# Time- and compartment-resolved proteome profiling of the extracellular niche in lung injury and repair

**DOI:** 10.15252/msb.20156123

**Published:** 2015-07-14

**Authors:** Herbert B Schiller, Isis E Fernandez, Gerald Burgstaller, Christoph Schaab, Richard A Scheltema, Thomas Schwarzmayr, Tim M Strom, Oliver Eickelberg, Matthias Mann

**Affiliations:** 1 Department of Proteomics and Signal Transduction, Max Planck Institute of BiochemistryMartinsried, Germany; 2 Comprehensive Pneumology Center, University Hospital of the Ludwig-Maximilians-University Munich and Helmholtz Zentrum München, Member of the German Center for Lung Research (DZL)Munich, Germany; 3 Institute of Human Genetics, Helmholtz Zentrum MünchenNeuherberg, Germany

**Keywords:** extracellular matrix, fibrosis, proteomics, regeneration, secretome

## Abstract

The extracellular matrix (ECM) is a key regulator of tissue morphogenesis and repair. However, its composition and architecture are not well characterized. Here, we monitor remodeling of the extracellular niche in tissue repair in the bleomycin-induced lung injury mouse model. Mass spectrometry quantified 8,366 proteins from total tissue and bronchoalveolar lavage fluid (BALF) over the course of 8 weeks, surveying tissue composition from the onset of inflammation and fibrosis to its full recovery. Combined analysis of proteome, secretome, and transcriptome highlighted post-transcriptional events during tissue fibrogenesis and defined the composition of airway epithelial lining fluid. To comprehensively characterize the ECM, we developed a quantitative detergent solubility profiling (QDSP) method, which identified Emilin-2 and collagen-XXVIII as novel constituents of the provisional repair matrix. QDSP revealed which secreted proteins interact with the ECM, and showed drastically altered association of morphogens to the insoluble matrix upon injury. Thus, our proteomic systems biology study assigns proteins to tissue compartments and uncovers their dynamic regulation upon lung injury and repair, potentially contributing to the development of anti-fibrotic strategies.

## Introduction

The lung is constantly subjected to harmful exposures, such as inhaled toxic substances, particulate matter, autoimmune reactions, and viral or bacterial infections that cause injury to the airway and alveolar epithelium. The epithelial lining of the airway lumen has a relatively slow turnover in homeostasis. Upon injury, however, rapid mobilization of several multipotent progenitor cell and stem cell lineages can regenerate the epithelial barrier (Kumar *et al*, 2011; Desai *et al*, 2014; Hogan *et al*, 2014; Kotton & Morrisey, 2014; Lee *et al*, 2014; Vaughan *et al*, 2015; Zuo *et al*, 2015). Lung regeneration is mediated by the reactivation of developmental programs, where the crosstalk between mesenchyme and epithelium via secreted proteins is essential. In a process called fibrogenesis, several mesenchymal cell populations secrete and assemble a specialized provisional extracellular matrix (ECM), which acts as a scaffold and master regulator of developmental programs in concert with extracellular morphogens, such as growth factors, cytokines, and chemokines (Gurtner *et al*, 2008). Morphogens can interact specifically with ECM proteins and glycosaminoglycans, which alter biological activity by affecting their signaling capacity (Chen *et al*, 2010), relative positioning to other receptor ligands (Hynes, 2009), or tissue residence time and spatial positioning (Weber *et al*, 2013). Bioinformatic analysis of protein domain architecture, together with literature mining, has defined an ECM component list (the “matrisome”) by classifying secreted proteins into structural constituents of the ECM (“core matrisome”) and ECM-interacting proteins (“matrisome-associated”) (Cromar *et al*, 2012; Naba *et al*, 2012a,b). Many of these annotations, however, are not based on direct experimental observations or have not been comprehensively tested *in vivo*. Developmental signaling pathways active in tissue repair, such as the TGF-β, Wnt, Shh, or Bmp pathways, which emanate from secreted morphogens and are regulated by interacting ECM components (Kleinman *et al*, 2003), are often deregulated in chronic lung diseases, potentially causing persistent pulmonary fibrosis (Fernandez & Eickelberg, 2012). Bleomycin-induced lung injury, which induces robust fibrogenesis 2 weeks after injury, is the most frequently used animal model of pulmonary fibrosis (Mouratis & Aidinis, 2011; Bauer *et al*, 2015). In contrast to progressive and irreversible fibrosis in many chronic lung diseases, bleomycin-induced fibrogenesis is a transient physiological reaction that largely resolves within 4–8 weeks, leading to almost complete regeneration of functional alveolar organization (Rock *et al*, 2011; Hecker *et al*, 2014).

Here, we employed the bleomycin lung injury mouse model and recent advances in mass spectrometry (MS)-based proteomics to generate a proteomic systems biology view on tissue injury, fibrosis, and repair. In particular, a next-generation Quadrupole–Orbitrap mass spectrometer (Q Exactive) with improvements in scan speed and sensitivity (Michalski *et al*, 2011; Altelaar & Heck, 2012; Mann *et al*, 2013) was combined with improved chromatography (Kocher *et al*, 2011; Thakur *et al*, 2011), biochemical sample processing (Kulak *et al*, 2014), and data analysis software for accurate intensity-based label-free quantification (Cox *et al*, 2014). The development of streamlined proteomic workflows, including a novel quantitative detergent solubility profiling (QDSP) method, resolved the pulmonary proteome into the interstitial proteome, its ECM components, and the epithelial lining fluid proteome of the alveolar and airway lumen. We directly measured the interactions of morphogens and other secreted proteins with the ECM in an unbiased way, revealing those that are bound to the matrix, signaling from that location or awaiting release by specific activating events. The time-resolved proteomic signatures revealed candidate molecular players for both the mobilization of multipotent epithelial progenitor cells early after injury and the resolution of fibrosis later in regeneration.

## Results

### Quantitative detergent solubility profiling (QDSP) defines matrisome composition *in vivo*

**Figure 1:**
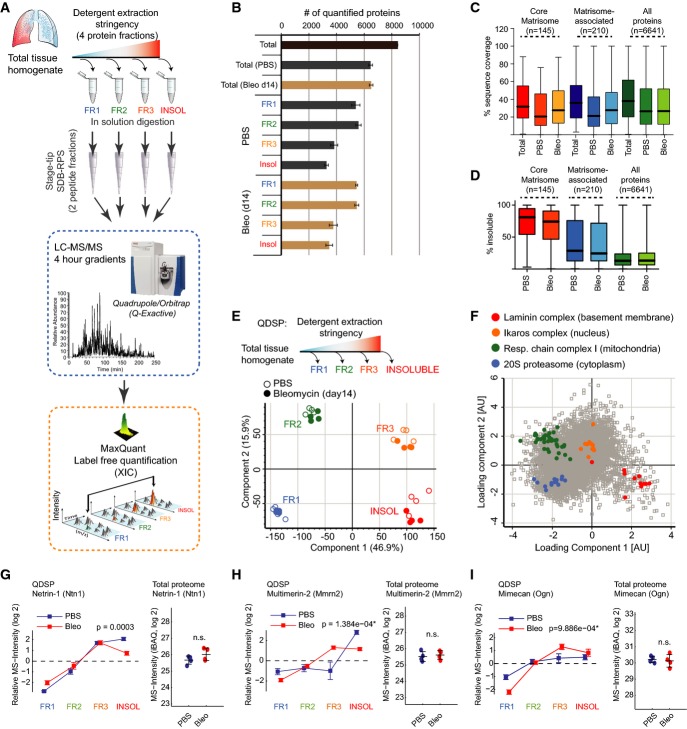
Quantitative detergent solubility profiling (QDSP) enables in-depth analysis of matrisome composition A Experimental workflow.

Morphogen solubility differences and gradients are critical in development and tissue repair (Martino *et al*, 2014). The ECM is highly insoluble, which allows its relative enrichment by extraction and depletion of more soluble proteins (Naba *et al*, 2012a). Interactions of secreted proteins with the ECM niche decrease their solubility, and thus, correlation analysis of the characteristic protein solubility profiles would enable the assignment of “matrisome association” *in vivo*. We therefore developed a protein correlation profiling method (quantitative detergent solubility profiling, QDSP), in which we extracted four distinct fractions from total lung tissue homogenates by gradually increasing the stringency of the detergents (see Materials and Methods), and analyzed the fractions separately by LC-MS/MS. Using label-free protein quantification in the MaxQuant software environment (Cox *et al*, 2014), we compared the relative abundance of proteins in the four solubility fractions and between experimental conditions. To increase throughput, we analyzed each protein solubility fraction with minimal peptide separation into two fractions. Thus, measurement time was lower than approaches based on extensive peptide fractionation, while yielding an extra dimension of spatial information together with a deep total proteome (Fig1A).

B Number of quantified proteins in the indicated protein fractions and experimental conditions. The mean and standard deviation are shown (PBS, *n*  = 4; Bleo d14, *n* = 4).

C, D The box and whisker plots depict the distribution of protein sequence coverage (coverage of possible tryptic peptides per protein in %) (C) or the percentage of MS intensity in the most insoluble protein fraction (D) for the indicated matrisome categories (Naba *et al*, 2012a) and experimental conditions.

E Principal component analysis (PCA) separates protein fractions derived from sequential detergent extraction. The first two components of data variability of 6,641 proteins, from four replicates of PBS control (open circles) and four replicates of bleomycin-treated lungs at day 14 after injury (closed circles), are shown.

F The scatter plot depicts the protein feature loadings of component 1 and component 2 of the PCA in (E) for the four indicated multiprotein complexes.

G–I Normalized QDSP MS intensity profiles (left panel) and total protein abundance (right panel) are shown for the indicated example proteins Netrin-1 (G), Multimerin-2 (H), and Mimecan (I). Error bars depict the standard error of the mean, and the indicated *P*-values are derived from an ANOVA test (all fractions: PBS, *n*  = 4; Bleo d14, *n* = 4).

**Figure EV1:**
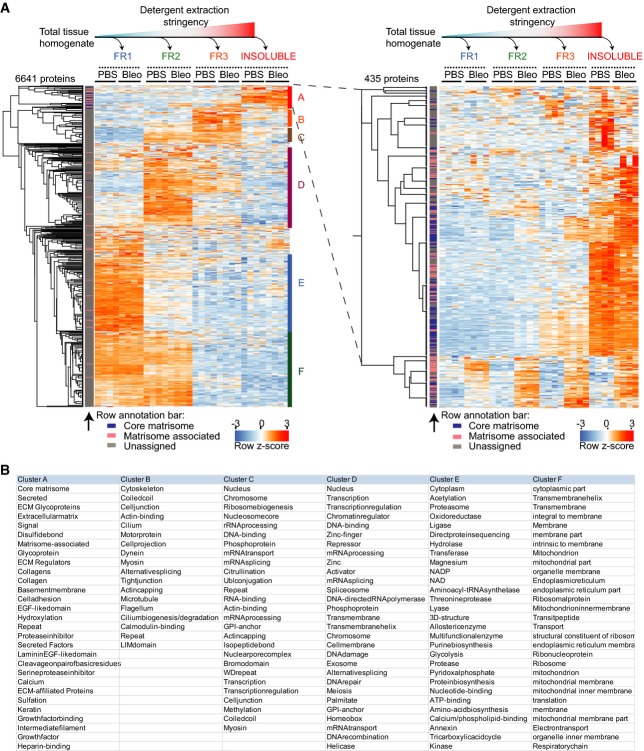
Efficient separation of subcellular and tissue compartments using QDSP Non-supervised hierarchical clustering of *z*-scored MS intensities from the indicated protein fractions and conditions (PBS, *n*  = 4; Bleo day 14, *n* = 4). The left panel depicts all 6,641 proteins, while the right panel shows a zoom in on the 435 most detergent-insoluble proteins in cluster A. The row annotation bar assigns proteins into the indicated matrisome categories.

We quantified 8,366 proteins including 435 matrisome proteins (171 core matrisome and 264 matrisome-associated) from healthy mouse lungs (PBS; *n* = 4) and lungs 14 days after a single intratracheal instillation of bleomycin (Bleo; 3 U/kg; *n* = 4) (Fig1B; Table EV1). We did not observe a strong bias against matrisome proteins (Naba *et al*, 2012a) in the proteomic analysis, evidenced by the only slightly reduced median sequence coverage of core matrisome proteins compared to all other proteins (Fig1C). As expected, most core matrisome proteins were strongly enriched in the detergent-insoluble protein fraction, while putative matrisome-associated proteins displayed a more heterogeneous enrichment (Fig1D). Principal component analysis (PCA) (Fig1E) and unsupervised hierarchical cluster analysis (Fig EV1A), together with annotation enrichment of the observed clusters (FigEV1B; Table EV2), clearly confirmed the successful quantitative separation of cytosolic, membrane, nuclear, cytoskeletal, and ECM proteins by their respective detergent solubility profiles. Examination of the proteins driving the separation (the “loadings” of the multidimensional PCA) showed that members of various multiprotein complexes were consistently grouped together by QDSP. For instance, the cytosolic 20S proteasome complex was most soluble, followed by the members of the mitochondrial respiratory chain complex, the nuclear Ikaros complex, and the basement membrane-associated laminins, which represented the most insoluble complexes (Fig1F). Lung injury could change protein abundance or protein localization or both, and these scenarios can be distinguished by QDSP. We first determined changes in the total abundance of proteins based on the summed peptide MS intensity values across the four solubility fractions. A *t*-test between total protein abundance of PBS-treated lungs and lungs 14 days after bleomycin revealed 1,125 significantly regulated proteins (FDR < 5%; Table EV1). Next, we normalized the data to remove differences in total protein abundance and investigated changes in solubility profiles between bleomycin-treated lungs and PBS controls. FDR-controlled ANOVA testing on the normalized solubility profiles identified 283 proteins with altered QDSP behavior (FigEV2A; Table EV1). Annotation term enrichment identified the two ECM categories “basal lamina” and “fibrinogen complex” with significant changes upon bleomycin injury (Table EV3). The fibrinogen complex, which is soluble in blood plasma and insoluble upon blood coagulation, significantly shifted into the insoluble compartment upon injury (FigEV2B). In contrast, the core structural constituents of the basement membrane (collagen-IV chains, laminins, nidogens, perlecan; *n* = 21) became significantly more soluble (FigEV2C). Matrisome proteins (Naba *et al*, 2012a) whose abundance was unchanged but whose solubility profiles were significantly altered included the glycoproteins Netrin-1 (Ntn1) (Fig1G) and Multimerin-2 (Mmrn2) (Fig1H), which were less strongly associated with the ECM, and the proteoglycan Mimecan (Ogn), whose association with the ECM increased after injury (Fig1I).

The table shows UniProt keywords that were significantly enriched (FDR < 2%) in the indicated protein clusters of the clustering analysis in (A).

**Figure EV2:**
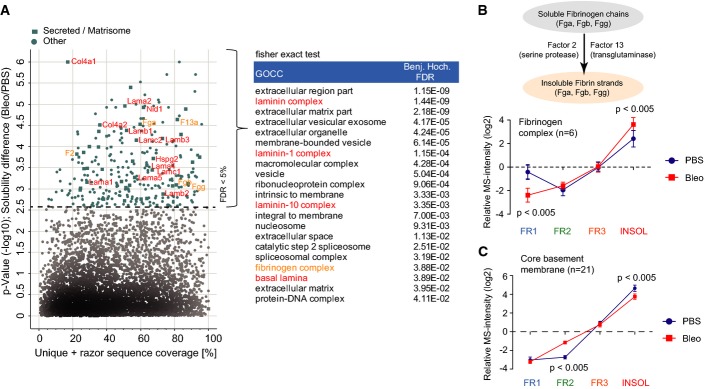
Differential solubility of matrisome complexes in tissue fibrogenesis A Proteins (*n* = 283) with significantly altered normalized MS intensity profiles (Benjamini–Hochberg FDR < 0.05) between PBS controls (*n* = 4) and bleomycin-treated (day 14; *n* = 4) samples are highlighted in a scatter plot depicting the percent coverage of identified tryptic peptides across the protein sequence and the *P*-value of the solubility difference. Significantly enriched GOCC terms (Fisher’s exact test, FDR < 2%) in the 283 protein group with altered QDSP profiles are shown in the right panel.

B, C The mean normalized QDSP MS intensity profiles of the fibrinogen complex (B; *n* = 6) and all core basement membrane proteins (C; *n* = 21) from PBS controls (*n* = 4) in blue and bleomycin (day 14; *n* = 4) in red are shown. The mean is shown and the error bars depict the standard error of the mean. The indicated *P*-values are derived from a *t*-test.

**Figure 2:**
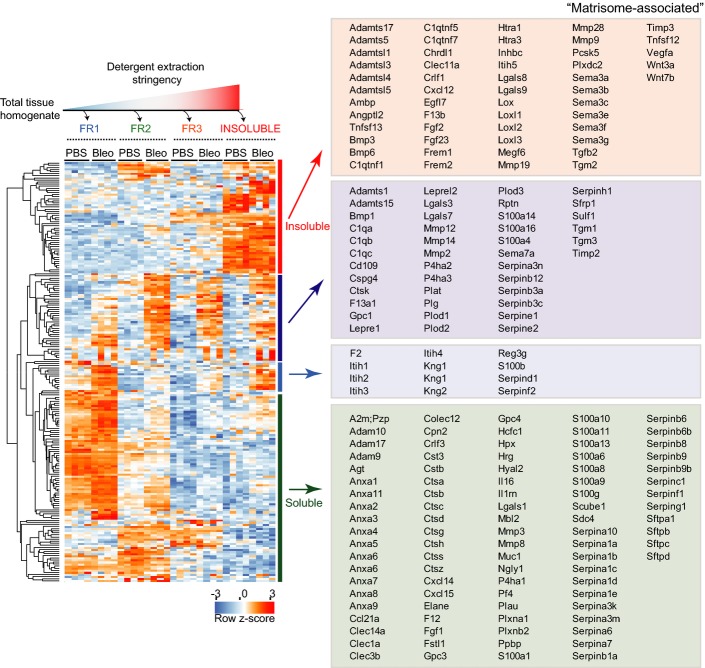
Assignment of secreted proteins to the extracellular matrix niche *in vivo* Proteins annotated with the term “matrisome-associated” (Naba *et al*, 2012a) were grouped using unsupervised hierarchical clustering of the *z*-scored MS intensities across the indicated experimental groups and protein fractions. Gene names within individual clusters are shown in boxes in the right panel.

Next, we analyzed putative matrisome-associated proteins by unsupervised hierarchical clustering (Fig2), which separated highly soluble proteins from insoluble proteins, while simultaneously showing regulation by injury. Notably, the most insoluble cluster contained important secreted morphogens of the Wnt, Bmp/Tgfb, and Fgf families, indicating strong interactions of these proteins with matrisome constituents. Another cluster contained proteins, including members of the Mmp, S100, and Serpin families, which were highly upregulated after bleomycin treatment. These proteins were spread over all four solubility fractions, indicating that they still partially reside in the endoplasmic reticulum and Golgi compartments or were just secreted and not yet incorporated into the ECM. Almost half of the putative matrisome-associated proteins (Naba *et al*, 2012a), including many members of the Annexin, Cathepsin, S100, and Serpin family of proteins, clustered together as highly soluble proteins, demonstrating that they were not significantly bound to the ECM (Fig2).

### Transcriptional and post-transcriptional regulation of tissue fibrogenesis

**Figure 3:**
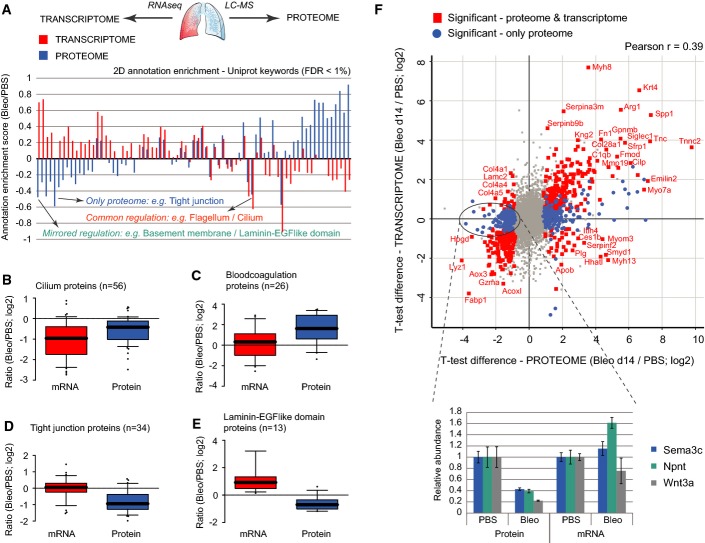
Combined proteomic and transcriptomic analyses uncouple transcriptional and post-transcriptional events upon lung injury A The bar graph shows the normalized annotation enrichment score (−1 min to +1 max) of UniProt keyword annotations that were significantly regulated (FDR < 1%) in the proteome (blue bars) and/or the transcriptome (red bars) dataset.

To investigate the nature and extent of post-transcriptional events upon lung injury, we analyzed identical tissue homogenates with both RNA-seq and mass spectrometry (*n* = 8; 4 × PBS, 4 × Bleo). The RNA FPKM values from RNA-seq (Table EV4) could be matched with protein MS intensities for 6,672 genes (FigEV3A). Using a cutoff of FPKM values at −1 (log2), we determined coverage of gene categories in the proteome relative to the transcriptome. Almost all categories, including the matrisome, were equally covered, demonstrating that the proteomic analysis was largely unbiased (FigEV3B). To systematically compare the proteomic and transcriptomic datasets in terms of gene categories, we used the statistically controlled 2D annotation enrichment algorithm (Cox & Mann, 2012), which detects correlated and uncorrelated changes between two data dimensions (Fig3A; Table EV5). For instance, Cilium proteins were downregulated in both datasets (Fig3B), whereas only the proteome data showed highly significant upregulation of blood coagulation proteins (Fig3C) and downregulation of tight junction proteins (Fig3D). Interestingly, the basement membrane proteins were reduced even though their transcripts were upregulated (Fig3E). The correlation of RNA abundance ratios with protein abundance ratios (Bleo/PBS) was rather moderate (Pearson *r* = 0.39), indicating post-transcriptional regulation of many biological processes in lung injury repair. Plotting individual RNA and protein ratios (Bleo/PBS) revealed the significant outlier proteins with the highest magnitude changes on both transcript and protein levels. Interestingly, several matrisome-associated morphogens and known regulators of lung morphogenesis (Dean *et al*, 2005; Vadivel *et al*, 2013), including semaphorin-3C (Sema3c), nephronectin (Npnt), and Wnt3a, were only regulated at the protein level (Fig3F). Thus, combined transcriptomic and proteomic analyses uncovered the level of regulation of proteins and processes important in lung repair, pointing to important post-transcriptional regulation of the extracellular niche.

**Figure EV3:**
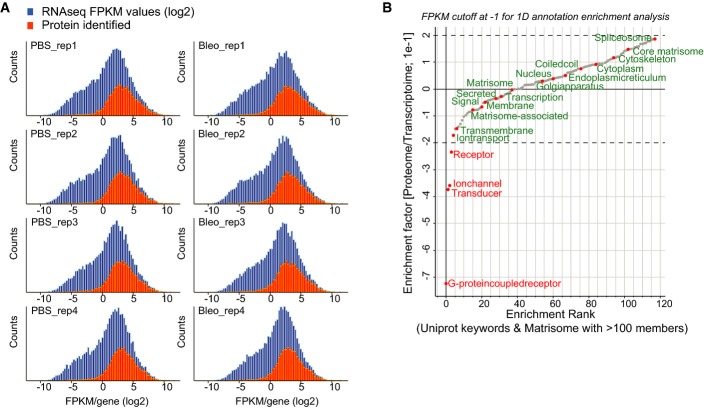
Combined proteomic and transcriptomic analyses of tissue fibrogenesis The histogram shows the distribution of the FPKM values from the RNA-seq experiment in log2 space (blue bars). The orange bars show the distribution of FPKM values for a total of 6,672 genes for which we also quantified the corresponding protein.

Relative enrichment (coverage) of the indicated gene categories (UniProt keywords) in the proteome and transcriptome was calculated using the 1D annotation enrichment algorithm embedded in the Perseus software suite. The enrichment is normalized to a score spanning from −1 to 1 as indicated. Gene categories are ranked according to their relative enrichment in the transcriptome or proteome (PBS, *n*  = 4; Bleo day 14, *n* = 4).

B–E The box and whisker plots depict the distribution of median log2 ratios from the transcriptome experiment (red boxes) and the proteome experiment (blue boxes) for the indicated UniProt keyword gene categories.

F The scatter plot shows the median log2 ratios of MS intensities and FPKM values for individual genes (*n* = 6,672). Genes that were significantly regulated in both RNA and protein are highlighted in red (FDR < 5%). Three example proteins that were only significantly regulated in the proteome but not in the transcriptome are shown in the bar graph inset. Error bars depict the standard error of the mean (PBS, *n*  = 4; Bleo day 14, *n* = 4).

### Time-resolved analysis of tissue proteome remodeling upon lung injury and repair

**Figure 4:**
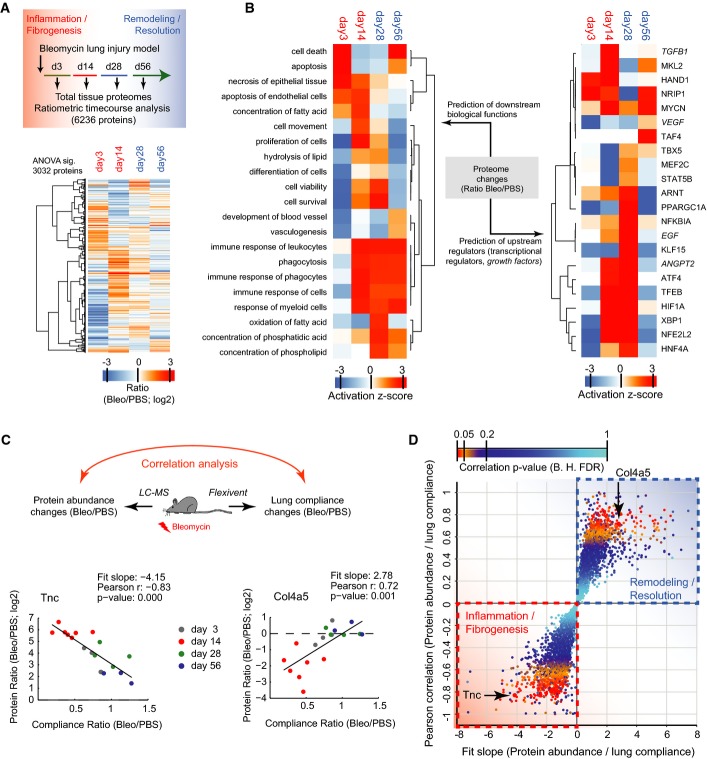
Tissue proteome time course analysis reveals lung injury repair protein signatures Schematic of experimental design and hierarchical clustering analysis of 3,032 MS intensity ratios (Bleo/PBS; log2) that were significant in ANOVA (day 3, *n* = 3; day 14, *n* = 7; day 28, *n* = 4; day 56, *n* = 3).

The bleomycin-mediated injury of the alveolar epithelium leads to an inflammatory response, which leads to maximal fibrogenesis 2 weeks after injury. Subsequently, the provisional ECM gets remodeled and repair is resolved within 8 weeks after injury (Bakowska & Adamson, 1998; Hecker *et al*, 2014). We characterized the dynamics of tissue repair upon bleomycin treatment using H&E (FigEV4A) and collagen type I stainings (FigEV4B) at different time points after bleomycin injury and confirmed the almost complete resolution of tissue repair within 8 weeks post-bleomycin treatment. To characterize the proteome changes after injury associated with inflammation (day 3), fibrogenesis (day 14), remodeling (day 28), and resolution (day 54), we homogenized total lung lobes from eight mice for each time point after a single intratracheal instillation of bleomycin (*n* = 18; Bleo 3 U/kg) or control saline (*n* = 16; PBS) (Fig4A). We quantified 8,019 protein groups with a median number of 5,020 identified proteins per single replicate sample (*n* = 34), and generated abundance ratios (Bleo/PBS) by dividing individual replicates by the median value of all PBS control samples (*n* = 16). In case of only missing intensity values (protein not identified) in one of the experimental conditions, we used data imputation (see Materials and Methods) at the low end of the intensity dynamic range if we obtained at least 50% valid intensity values in the other condition. In this way, the MS intensities of a total number of 6,236 protein groups (with protein quantification for at least three replicates in one of the experimental conditions) were finally used for the ratiometric time course analysis (Table EV6). Remarkably, a total of 3,032 of these proteins, including 154 matrisome components, changed significantly in at least one of the time points (ANOVA; FDR < 5%) (Fig4A). We assigned biological processes, and their upstream transcriptional regulators and growth factors to the consecutive phases of tissue repair (Fig4B; Table EV7) (see “Ingenuity pathway analysis” in Materials and Methods). For instance, abundance changes in 365 known targets of TGF-β signaling indicated that the activity of this master regulator of tissue fibrogenesis was highly upregulated at day 14 after injury and that it was back to baseline at day 28 (Fig4B). This revealed the temporal dynamics of transcriptional networks, allowing the identification of transcriptional regulators with putative novel functions in lung repair.

**Figure EV4:**
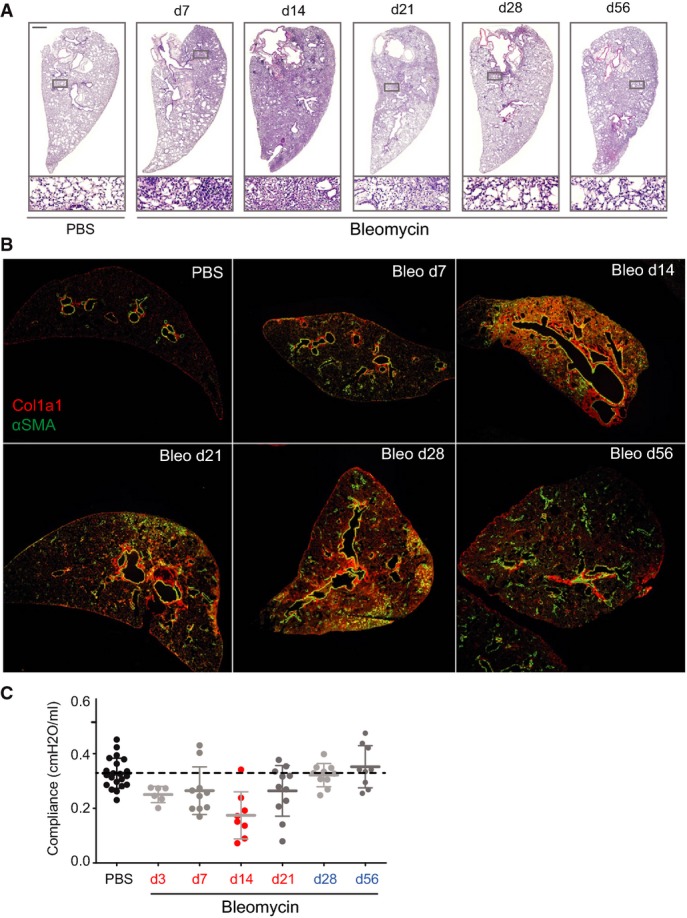
Transient fibrogenesis in the bleomycin model Representative tissue sections of the indicated experimental conditions and time points were analyzed using hematoxylin and eosin stain (H&E).

Tissue sections of the indicated experimental conditions and time points were stained for α-smooth muscle actin (α-SMA in green) and collagen type I (Col1a1 in red).

Lung compliance was measured in the indicated experimental conditions and time points using the forced oscillation technique from FlexiVent systems (Scireq, Montreal, Canada). The values for mean and standard deviation are shown.

Hierarchical clustering of the activity score of downstream biological functions (left panel) and the upstream transcriptional regulators and growth factors (right panel) for the indicated time points after injury as determined by Ingenuity pathway analysis using the 3,032 significant protein ratios.

Correlation of protein abundance changes with lung compliance changes in individual mouse lungs at the indicated time points after bleomycin instillation into the airways. The ECM glycoprotein Tnc serves as an example for proteins that have a negative slope of the correlation fit. The basement membrane protein Col4a5 serves as an example for proteins that have a positive slope of the correlation fit.

The scatter plot depicts the Pearson correlation coefficient and the correlation fit slope of all 6,236 proteins that were used in the ratiometric analysis of protein abundance versus lung compliance. The statistical significance of the correlation coefficient is color-coded as indicated.

Individual differences in severity of bleomycin-induced lung injury and dynamics of the repair response lead to variations in the degree of fibrogenesis, which could be correlated with the proteome of individual mice. We employed a test of lung compliance to capture the degree of tissue fibrogenesis. In clinical practice, measurement of pulmonary compliance captures the lung’s ability to stretch and expand, which is reduced in fibrosis and increased in lung emphysema. In the bleomycin model, we observed that the median lung compliance was slightly reduced at day 3 and severely reduced at day 14 after injury, after which it returned back to the level of PBS-instilled control mice at days 28 and 56 (FigEV4C). For each protein, we determined how their temporal abundance profiles correlated with lung compliance changes (Table EV6). For instance, the ECM glycoprotein tenascin-C (Tnc) correlated negatively with the compliance ratio, meaning that larger amounts of the protein were associated with stiffer lung tissue. The opposite was the case for the collagen-IV triple helical subunit Col4a5, a key constituent of the alveolar basement membrane (Fig4C; *n* = 34). We plotted the correlation fit slope of all quantified proteins against the Pearson r as a measure of the tightness of the correlation. Those with negative slope can be associated with a possible function in the inflammatory and fibrogenic phase and those with a positive slope with the remodeling and resolution phase. A total of 507 proteins (54 matrisome proteins) had a significant correlation with lung compliance (FDR < 5%) (Fig4D). A statistical test (1D annotation enrichment) revealed gene categories that were enriched with negative and positive slopes, respectively (Table EV8).

**Figure 5:**
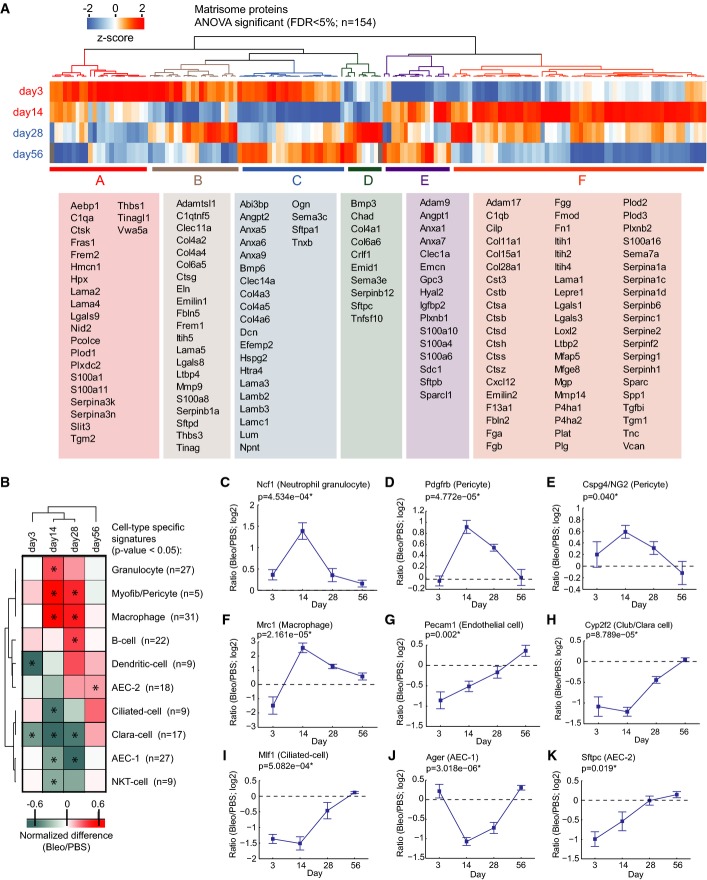
Matrisome dynamics in tissue repair and extraction of individual cell-type dynamics from the proteomic data A The *z*-scored MS intensity ratios of the indicated 154 matrisome proteins (ANOVA FDR < 0.05) were grouped by correlation using unsupervised hierarchical clustering. The table shows the gene names of the proteins associated with the indicated clusters.

**Figure EV5:**
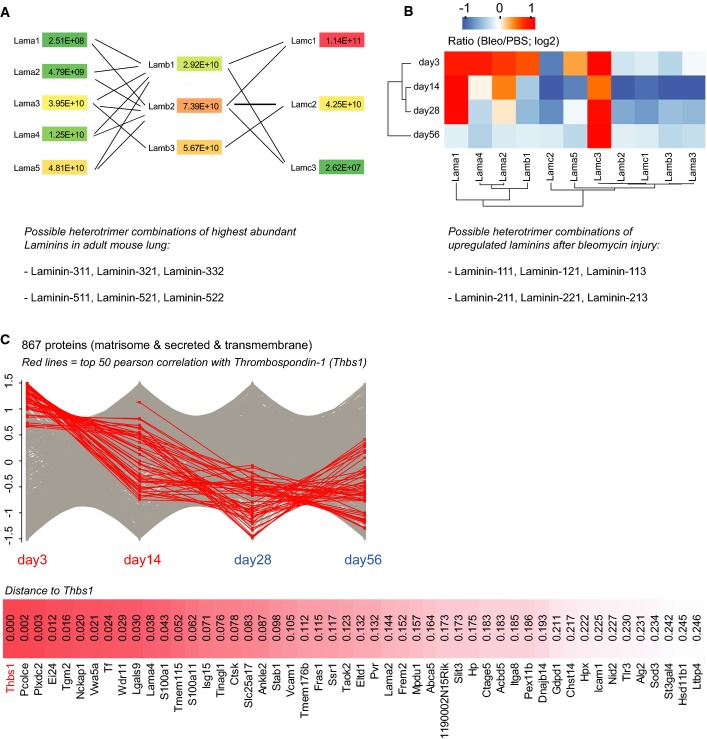
Pulmonary basement membrane laminins and immediate early extracellular niche factors upon injury The median MS intensity of all laminin chains was normalized for the theoretical number of tryptic peptides (iBAQ), yielding a relative stoichiometry of laminin chains, which is shown in the network graph for healthy control lungs (PBS,*n* = 16). The lower panel depicts the possible laminin heterotrimer combinations of the highly abundant α3- and α5-laminins, based on previous literature.

Next, we *z*-scored MS intensity ratios of the 154 matrisome proteins that were significantly regulated (FDR < 0.05) and grouped them by correlation using unsupervised hierarchical clustering into early and late factors in repair (Fig5A). This analysis revealed interesting heterogeneous regulation of interacting proteins, such as for instance the laminin heterotrimers of the basement membrane, which exist in 16 possible combinations and have a tissue- and developmental stage-specific distribution and differential functions in lung development and homeostasis (Domogatskaya *et al*, 2012). We quantified all existing laminin chains (five α-, three β-, and three γ-chains) and predicted the possible heterotrimer combinations based on their MS intensity (Fig EV5A). The α3- and α5-laminins were most abundant in adult mouse lung, followed by α4-laminins. The α1- and α2-laminins, which are mainly restricted to embryonic development (Nguyen & Senior, 2006), were at least ten-fold lower expressed than all other laminins. Upon injury, the high-abundance laminin chains were downregulated at day 3 and day 14 together with the collagen type IV chains. In contrast, the laminin α1-, α2-, and α4-chains were upregulated early upon injury, suggesting a potential role in initiation of tissue repair. The α1-laminins had their peak of expression at day 14, while the α2-laminins were upregulated very early upon bleomycin injury, and peaked already at day 3 (FigEV5B). We identified a signature of 50 other secreted and transmembrane proteins with a peak of expression at day 3 (FigEV5C). Interestingly, this signature also contained the ECM protein thrombospondin-1 (Thbs1), recently reported to be a key factor for alveolar differentiation of bronchoalveolar stem cells upon bleomycin injury (Lee *et al*, 2014).

B The normalized enrichment score (−1 to +1; 1D annotation enrichment in the Perseus software) of the indicated cell-type-specific protein signatures and time points after injury was grouped based on non-supervised hierarchical clustering. Conditions with significant enrichment or depletion of a cell-type-specific signature are marked with an asterisk (*P*-value < 0.05).

C–K The line plots show dynamic changes in relative abundance (log2 ratio bleomycin/PBS) of the indicated examples of cell-type-specific proteins over the tissue repair progression timeline. The mean and standard error of the mean and a *P*-value from ANOVA are shown (day 3, *n* = 3; day 14, *n* = 7; day 28, *n* = 4; day 56, *n* = 3).

The median *z*-scored MS intensity ratios of the indicated laminin chains were grouped by correlation using unsupervised hierarchical clustering (day 3, *n* = 3; day 14, *n* = 7; day 28, *n* = 4; day 56, *n* = 3). Possible heterotrimer combinations of the upregulated α1- and α2-laminins are shown in the lower panel.

The median *z*-scored MS intensity ratios of 867 extracellular proteins were grouped by correlation analysis of their temporal profiles. The 50 proteins with highest correlation to thrombospondin-1 (Thbs1) are shown (day 3, *n* = 3; day 14, *n* = 7; day 28, *n* = 4; day 56, *n* = 3).

The relative proportion of different cell populations after injury is affected by differential proliferation, immigration, and cell death. To extract individual cell-type dynamics from the proteomic data, we employed cell-type-specific gene expression signatures derived from single-cell RNA-seq profiling of lung-resident epithelial cell types (Treutlein *et al*, 2014), or microarray-based profiling of highly purified leukocyte populations (Gautier *et al*, 2012; Miller *et al*, 2012; Jojic *et al*, 2013) (Table EV9). This identified ten different cell types, whose abundance changed significantly during lung injury (Fig5B). For instance, the set of 27 proteins quantified from the signature of alveolar type 1 epithelial cells (AEC-1), indicated that AEC-1 cells were unchanged at day 3, significantly downregulated at day 14 and day 28 and recovered at day 56. Overall, one group of cell types, including granulocytes, myofibroblasts, pericytes, macrophages, and B cells, were increased in the fibrogenic or remodeling phase. At the same stage, all major epithelial cell types, including type 1 and type 2 (AEC-1 and AEC-2), ciliated, and Club/Clara cells were downregulated and, interestingly, recovered with different kinetics (Fig5B–K).

### Novel ECM proteins upregulated upon lung injury

**Figure 6:**
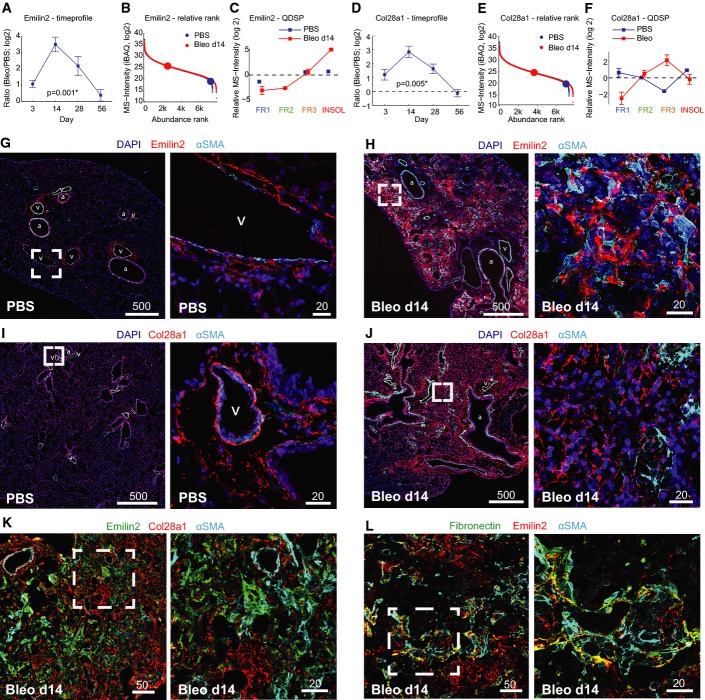
Validation of novel ECM proteins strongly upregulated upon lung injury A–F Quantitative proteome data depict protein abundance changes over time relative to healthy control mice (A, D), the abundance rank relative to all other quantified proteins (b, e), and the QDSP profiles indicative for the detergent solubility (C, F). The mean and standard error of the mean are shown (day 3, *n* = 3; day 14, *n* = 7; day 28, *n* = 4; day 56, *n* = 3).

Among the most highly regulated proteins, we unexpectedly found the ECM proteins Emilin-2 and collagen-XXVIII, which have never been described in the context of tissue injury repair or fibrosis. They showed similar temporal profiles of expression with significant upregulation already at day 3 and a peak at day 14 (Fig6A and D). Both proteins ranked in the lowest abundance quartile of the proteome in steady-state conditions and increased to median abundance range at day 14 (Fig6B and 6E). Furthermore, the QDSP profiles of both proteins showed that they became more insoluble in the bleomycin-treated lungs, thus confirming their incorporation into the ECM (Fig6C and 6F). We validated these findings using immunofluorescence on tissue sections from PBS- and bleomycin-treated animals (day 14). Emilin-2 localized to perivascular and peribronchiolar regions and was absent from the alveolar regions in the lungs of healthy control mice (Fig6G). In the bleomycin-treated lungs, it also occupied alveolar regions and was localized around α-SMA-positive myofibroblasts (Fig6H). Staining Emilin-2 together with a fibronectin antibody revealed the co-localization of these two proteins in a fibrillar network around myofibroblasts (Fig6L). Collagen-XXVIII localized in thin sheets around vessels, airways, and alveoli, which were reminiscent of basement membranes in healthy control mice (Fig6I). Upon bleomycin injury, we observed strong staining with a patchy appearance in fibrotic foci that, however, did not co-localize with α-SMA-positive myofibroblasts (Fig6J). Co-immunostaining of Emilin-2 with collagen-XXVIII confirmed their mutually exclusive localization and suggests that these ECM proteins are secreted and assembled by distinct cell lineages even though their temporal expression profile upon injury is highly similar (Fig6K). Thus, immunofluorescence-based validation of novel ECM proteins identified by our proteomic analysis of lung injury reveals the complexity of the provisional wound ECM and its spatiotemporal distribution into distinct zones of differential ECM composition.

G–L Lung tissue sections from control mice (PBS) and bleomycin-treated mice 2 weeks after injury (Bleo d14) were stained as indicated. Scale bars are in μm. Vessels are labeled with (v) and airways with (a).

### *In vivo* secretome dynamics in tissue repair

The airway lumen is covered by the epithelial lining fluid (ELF) into which epithelial cells and alveolar macrophages secrete proteins and peptides with a multitude of functions, including innate immunity, mucociliary clearance, and antioxidant defense. ELF and non-adherent cells in the airways can be sampled from lungs using bronchoalveolar lavage (BAL) (Reynolds, 2011), which is also a frequently used clinical procedure for diagnosis and monitoring of chronic lung diseases.

**Figure 7:**
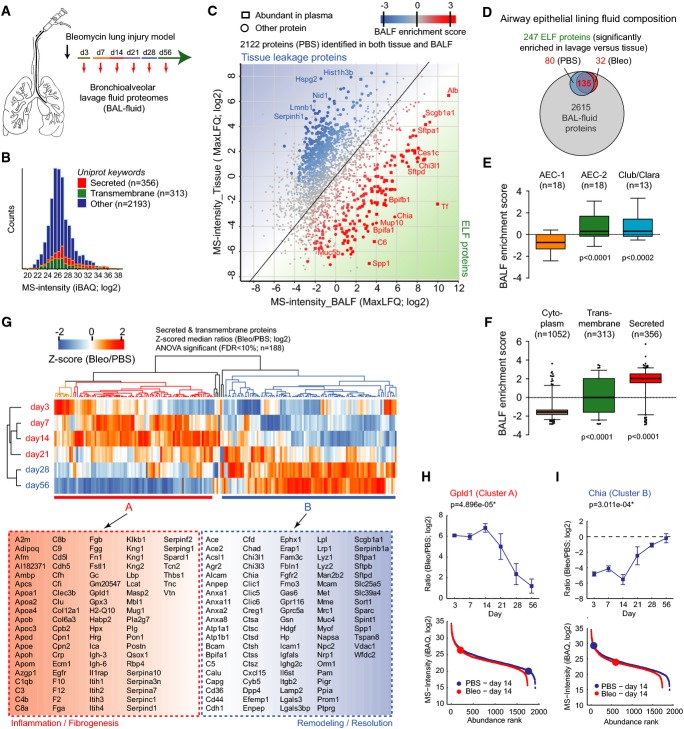
Epithelial lining fluid proteome composition and dynamic changes upon injury and repair A Experimental design.

**Figure EV6:**
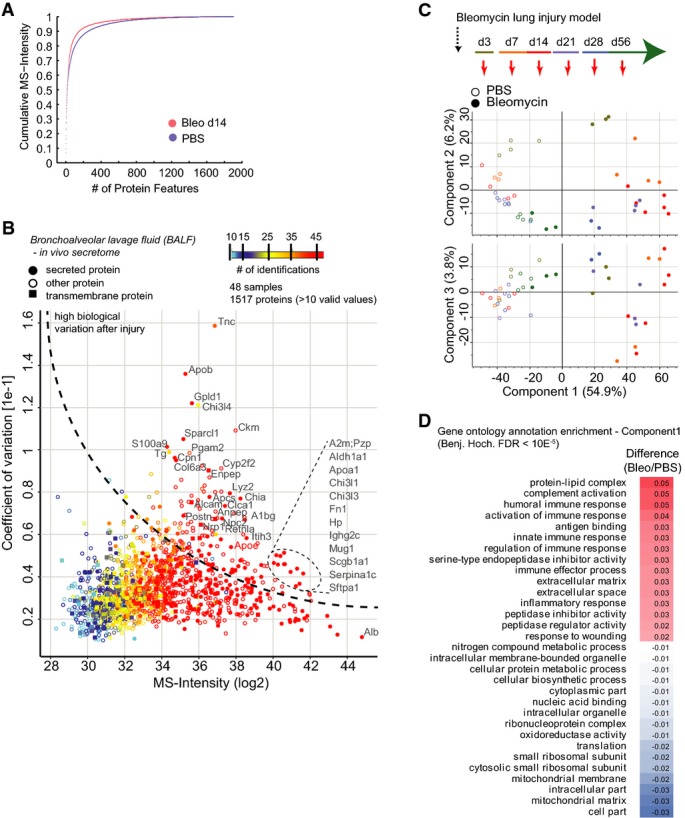
Proteomic analysis of bronchoalveolar lavage fluid upon bleomycin injury The mean cumulative MS intensity of the indicated conditions demonstrates differences in dynamic range of protein copy numbers (PBS, *n*  = 24; Bleo day 14, *n* = 7). The lower number after bleomycin treatment was likely due to the leakage of plasma through the endothelial barrier upon injury, which makes MS analysis more challenging.

We used MS-based secretome analysis (Meissner *et al*, 2013) to profile BALF of the mice from which tissue samples were taken and of additional mice at day 7 and day 21, for a total of six time points (four mice each for bleomycin treatment and control, *n* = 48; Fig7A; Table EV10). Single-shot LC-MS/MS quantified a remarkable median of 1,363 proteins per replicate in the PBS control and 823 after bleomycin application (FigEV6A). Of the more than 2,000 identified proteins, 356 were previously categorized as “secreted”. Interestingly, 313 proteins were annotated as “transmembrane,” including aminopeptidase A (ENPEP), aminopeptidase N (ANPEP), CD166 (Alcam), neuropillin-1 (NRP1), and epidermal growth factor receptor (EGFR), which were highly regulated in BALF (Figs7B and EV6B). Proteins identified in BALF with high-sensitivity MS methods could also originate from tissue leakage or cell lysis artifacts. By quantifying the tissue proteome against the BALF proteome, we constructed a *t*-test-based enrichment score, which unequivocally assigned ELF-specific proteins (Fig7C). This analysis distinguished obvious contaminants such as nuclear lamins (Lmnb1), histones (Hist1h3b), or basement membrane proteins (Hspg2, Nid1), from dedicated ELF proteins such as the Clara cell secretory protein (Scgb1a1) or the pulmonary surfactants (Sftpa, Sftpd). In total, we identified 247 proteins significantly enriched in ELF (Fig7D). Proteins specific for the non-secretory AEC-1 had significantly lower BALF enrichment scores than the proteins specific for the secretory AEC-2 and Club/Clara cells (Fig7E). Further validating the analysis, proteins annotated with the term “cytoplasm” showed a negative BALF enrichment score, while most “secreted” and many “transmembrane” proteins had a significant positive score (Fig7F).

B The histogram depicts MS intensity distributions of the indicated categories.

C The scatter plot shows the relative enrichment of proteins in either tissue or bronchoalveolar lavage fluid proteome. The enrichment score is color-coded as indicated, and proteins that are highly abundant in plasma are shown as squares.

D The Venn diagram depicts relative proportions of the number of proteins in the indicated categories and experimental conditions.

E, F The box plots depict the distribution of BALF enrichment scores in the indicated gene categories.

G The *z*-scored MS intensity ratios of the 189 secreted and transmembrane proteins that were significantly regulated (Benjamini–Hochberg FDR < 0.05) were grouped by correlation using unsupervised hierarchical clustering.

H, I Protein abundance changes over time of the indicated example proteins are shown in the upper panels. The mean log2 MS intensity ratio of bleomycin-treated to the PBS controls with the standard error of the mean and the *P*-value of ANOVA are shown. The lower panels depict the protein abundance rank relative to the complete BALF proteomes. The error bars depict the standard error of the mean.

The scatter plot depicts the median MS intensity versus the coefficient of variation across the injury time course and all replicates, which highlights proteins with high biological variation upon injury. The number of protein identifications across replicates and time points (*n* = 48) is color-coded, and secreted and transmembrane proteins are marked with the indicated symbols.

Principal component analysis of BALF proteomes from the indicated experimental conditions and time points separates experimental groups.

The table shows significantly regulated gene ontology terms (FDR < 2%) that were found to be enriched in either the negative (PBS) or positive (Bleo) data dimension of component 1 in the principal component analysis shown in (C).

Having determined the composition and origin of proteins in BALF, we applied ANOVA to the total time course (Table EV10) and *z*-scored the 188 significant secreted or transmembrane proteins. The temporal behaviors of most proteins were either consistent with a possible function in inflammation or fibrogenesis (cluster A) or remodeling and resolution (cluster B) (Fig7G). Representative for cluster A, the abundance of the glycosylphosphatidylinositol (GPI)-specific phospholipase D (Gpld1), which is abundant in serum (LeBoeuf *et al*, 1998), was drastically increased within the first 2 weeks after injury and thereafter gradually declined back to baseline, concomitant with repair of the blood barrier in the lung (Fig7H). Cluster B is exemplified by the immunomodulatory secreted protein acidic mammalian chitinase (Chia) (Lee *et al*, 2011), which was strongly downregulated the first 2 weeks after injury and recovered thereafter (Fig7I).

## Discussion

**Figure 8:**
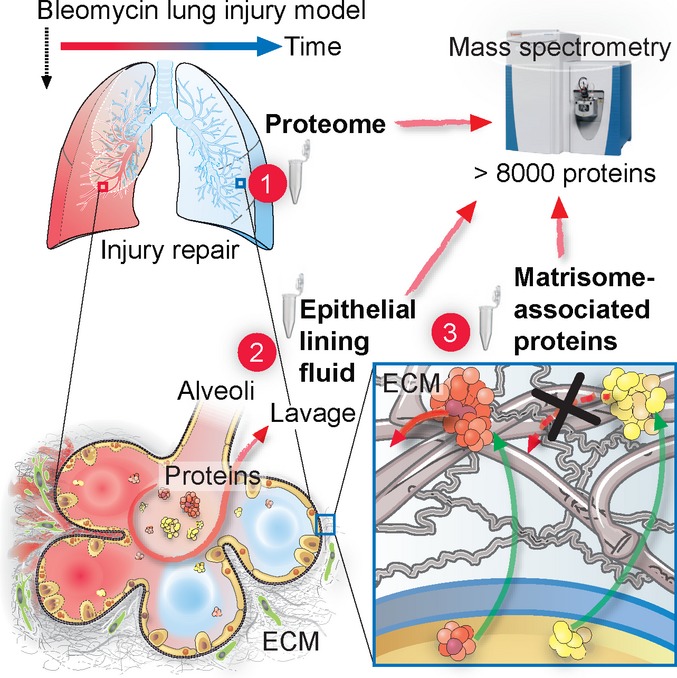
Proteomic systems biology view of tissue remodeling upon injury and repair and protein compartmentalization in the lung Tissue repair upon injury involves drastic remodeling of the proteome and the reactivation of developmental programs. Using state of the art quantitative mass spectrometry, we quantified more than 8,000 proteins and resolved their association with cell and tissue compartments as well as their relative abundance in the inflammation, fibrosis, remodeling, and resolution phases of tissue repair: (i) The tissue proteome data reflected the dynamics of biological processes and the transcriptional networks driving them and determined the relative proportions of various cell lineages along the tissue repair progression timeline; (ii) in-depth proteomic analysis of BALF and whole tissue discriminated true components of epithelial lining fluid from tissue leakage proteins and untangled the temporal regulation of *in vivo* protein secretion and receptor ectodomain shedding. This highlighted a multitude of unexpected proteins in ELF that may have important autocrine and paracrine functions in the different phases of lung repair and lung homeostasis; and (iii) our solubility profiling method (QDSP) enabled unbiased analysis of protein interactions in the ECM *in vivo* and quantified gain and loss of protein interactions within the ECM upon bleomycin-induced injury.

We developed advanced proteomics technologies and applied them to study fibrosis and regeneration in the lung. This shed light on the interactions of morphogens and other secreted proteins with the ECM, quantified protein secretion, and receptor shedding into the airway lumen and uncovered the temporal dynamics of a large fraction of the proteome upon lung injury and repair (summarized in Fig8). The proteomic datasets and data plots can be viewed and mined for analysis via a unified resource at the MaxQB proteomics database (Schaab *et al*, 2012), and in the Tables EV1, EV6 and EV10.

Proteomic studies of the ECM typically analyze the insoluble material left after application of different decellularization protocols (Didangelos *et al*, 2010; Barallobre-Barreiro *et al*, 2012; Naba *et al*, 2012a, 2014; Rashid *et al*, 2012; Zanivan *et al*, 2013). These workflows, however, do not distinguish true ECM constituents from contaminant non-ECM proteins. Some recent studies explored the possibility of measuring and comparing the protein pools after differential extraction from tissue (Barallobre-Barreiro *et al*, 2012; Decaris *et al*, 2014). The latter study for instance quantified the turnover of ECM proteins and compared the insoluble compartment with the guanidinium hydrochloride-soluble compartment (Decaris *et al*, 2014). We further explored the idea of a quantitative comparison of sequential protein extracts to characterize ECM proteins based on their solubility profiles, which we also used to directly measure the association of secreted proteins with ECM filaments *in vivo*. The proteome-wide QDSP data presented here can serve as confirmation of some of the available affinity measurements for ECM interactions and as a starting point for new investigations. For instance, our *in vivo* QDSP data validated a recent study, which showed that fibroblast growth factor 2 (Fgf2) strongly bound ECM proteins in ELISA-based experiments *in vitro*, while its family member Fgf1 did not (Martino *et al*, 2014). In agreement with this data, we found that Fgf2 was highly insoluble in contrast to Fgf1. Thus, we believe that QDSP will be useful for future proteomic investigations of matrisome composition in diverse biological contexts.

Secreted lung proteins can be present in the interstitial space, where they are exposed to ECM filaments and might be associated with them, or in the ELF of the airway lumen, where they are in solution. We were able to distinguish these two scenarios using QDSP and the BALF score, which measures relative enrichment in ELF compared to the tissue compartment. This can be illustrated by the ECM glycoprotein osteopontin (Spp1), a known marker of progressive fibrotic disease in IPF patients (Pardo *et al*, 2005). Spp1 had a soluble QDSP profile (no or weak ECM interaction) but also a high BALF enrichment score in both PBS controls and bleomycin-treated animals. Thus, we conclude that surprisingly the pulmonary Spp1 is mainly derived from epithelial cells and secreted apically into the airway lumen. Similarly, our global analysis identified many unexpected proteins as true constituents of ELF and distinguished apical from basolateral secretion of epithelium-derived proteins. Temporal profiling of ELF proteins uncovered a host of novel candidate proteins with putative functions in early or late phases of tissue repair, providing a rich resource for functional validation. Since BAL is a frequent diagnostic procedure for patients with chronic lung diseases, the highly streamlined nature of our LC-MS-based proteomic workflow makes the clinical translation of BALF proteomics an attractive direction for both patient stratification and drug target discovery (Blackwell *et al*, 2014).

Mobilization of tissue-resident progenitor cell and stem cell populations needed for regeneration of the correct bronchoalveolar epithelial organization is initiated in the early inflammatory and fibrogenic phase after injury (up to day 14 in our model), in which the perivascular niche plays a key role by contributing paracrine factors (Ding *et al*, 2011; Lee *et al*, 2014; Rafii *et al*, 2015; Ramasamy *et al*, 2015). The basement membrane underneath the epithelial and endothelial cell layers, in particular, is strategically located to impact on stem cell behavior. Here, we identified an interesting group of extracellular niche proteins, which was upregulated early after injury (days 3 and 14). It contained a specific set of basement membrane laminins that is normally restricted to embryonic development (Nguyen & Senior, 2006), and several basement membrane-associated proteins, including Frem-1 and Slit-3, which genetically interact in the regulation of lung development (Petrou *et al*, 2005; Beck *et al*, 2013). Moreover, QDSP uncovered increased solubility of basement membrane proteins, which could be due to proteolytic processing, as previously shown in lung injury and repair (Davey *et al*, 2011), and branching morphogenesis of the developing lung (Harunaga *et al*, 2014). Proteolytic processing might also explain the observed release of basement membrane-associated Netrins from the insoluble compartment. Netrins inhibit the outgrowth of ectopic epithelial buds during branching morphogenesis in lung development (Liu *et al*, 2004), and it will be thus interesting to determine the functional relevance of their release upon injury. Validation of the newly discovered provisional ECM proteins Emilin-2 and collagen-XXVIII interestingly suggests that different mesenchymal cell populations contribute a distinct set of ECM proteins to the provisional matrix. Indeed, lineage tracing studies in different organs have recently revealed a previously unexpected heterogeneity of mesenchymal cell populations during wound repair (Driskell *et al*, 2013; Rinkevich *et al*, 2015). For instance, the expansion of α-SMA-positive myofibroblasts in injured lungs is accompanied by the expansion of a set of Pdgfr-β-/Ng2-positive pericyte-like cells in alveolar regions upon bleomycin-induced injury (Rock *et al*, 2011). Emilin-2 is believed to associate with elastic microfibrils (Doliana *et al*, 2001), was recently shown to bind Wnt1 in the extracellular space (Marastoni *et al*, 2014), and can directly activate the extrinsic apoptosis pathway (Mongiat *et al*, 2007). Collagen-XXVIII (Col28a1) is a beaded-filament-forming collagen of unclear function, which associates with basement membranes (Veit *et al*, 2006). It will now be interesting to determine the specific molecular function of these ECM proteins in the context of tissue repair processes.

Our quantification of the cell-type-specific protein signatures is in agreement with lineage tracing studies, demonstrating that damaged AEC-1 cells are replaced by transdifferentiation of Club/Clara and AEC-2 cells (Rock *et al*, 2011). The differentiation of AEC-2 into AEC-1 can be recapitulated to some extend *in vitro*, and such studies have recently identified the transcription factor hepatocyte nuclear factor 4 alpha (HNF4a) as a putative regulator of this process (Marconett *et al*, 2013). We performed bioinformatics analysis of transcriptional networks and found that HNF4A activity coincided with recovery of the AEC-1 signature, supporting a function of HNF4a in AEC-2 to AEC-1 differentiation *in vivo*. At days 14 and 28, we observed increased activity of the transcription factor Nrf2 (Nfe2l2), which is activated by reactive oxygen species (ROS) and known to protect against oxidative damage (Ma, 2013). Nrf2 activity is essential for successful resolution of fibrosis upon bleomycin-induced injury (Hecker *et al*, 2014), and conditional deletion of Nrf2 in airway epithelium causes chronic inflammation upon injury (Reddy *et al*, 2011). Furthermore, aged mice and IPF patients, both of which have a defect in fibrosis resolution, have an imbalance in Nrf2-dependent redox homeostasis (Hecker *et al*, 2014). Thus, our data reemphasize the importance of the Nrf2 module for physiologic fibrogenesis and wound repair on the level of its target proteins. We also identified novel putative transcriptional regulators of lung repair. For instance, the activity of the transcription factor Tbx5 was in contrast to Nrf2 significantly downregulated in fibrogenesis and then upregulated in remodeling and resolution. Tbx5 is expressed in the lung mesenchyme and together with Fgf10 promotes the growth and branching of the epithelium in the developing lung (Arora *et al*, 2012). Interestingly, upregulation of Tbx5 was observed in axolotl limb regeneration (Khan *et al*, 2002), which supports a potential role of this factor in lung regeneration.

In conclusion, we provide an extensive resource of methods, novel molecules, and hypotheses for the regenerative medicine community and the field of ECM research. Clearly, proteomics is now capable of tackling highly complex tissue environments, providing very deep proteome coverage. Integration of the various datasets enabled us to add an additional dimension of spatial resolution to the pulmonary proteome, which for the first time revealed epithelial lining fluid composition and association of secreted proteins with the ECM *in vivo*. With the time-resolved data, we were able to predict functions of individual proteins in the early stages of repair, such as stem cell mobilization, or late stages, such as the resolution of fibrosis. We also showed that higher-level systems information can be extracted, such as the activity of transcriptional regulators and signaling pathways along the tissue repair progression timeline. While we here studied the lung, the physiological process of the onset, progression, and resolution of fibrosis, and thus the dynamics of protein expression described here, are likely to have many similarities in the regeneration of other organ systems.

## Materials and Methods

### Animal experiments

#### Bleomycin-induced lung injury

Pathogen-free female C57BL/6 mice (10–12 weeks old) were obtained from Charles River and housed in rooms maintained at constant temperature and humidity with a 12-h light cycle. Animals were allowed food and water *ad libitum*. All animal experiments were conducted under strict governmental and international guidelines. Bleomycin lung injury was induced according to the published protocols (Konigshoff *et al*, 2009). Briefly, a single intratracheal instillation of 50 μl of bleomycin (3 U/kg; Sigma-Aldrich, Taufkirchen, Germany), dissolved in sterile saline, was applied using the MicroSprayer Aerosolizer, Model IA-1C (Penn-Century, Wyndmoor, PA). Control mice were instilled with 50 μl of saline. After instillation, mice were kept for 3, 7, 14, 21, 28, and 56 days, respectively. For analysis, mice were anesthetized with ketamine (135 mg/kg) and xylazine (7 mg/kg) intraperitoneally, and lung function measurements and bronchoalveolar lavage were performed as described below. Finally, lungs were perfused through the heart with saline to remove remaining blood, and snap-frozen in liquid nitrogen for proteomic and transcriptomic analyses.

#### Lung function measurements and bronchoalveolar lavage

To investigate alterations of lung function after bleomycin application, we used the forced oscillation technique from FlexiVent systems (Scireq, Montreal, Canada). We used this system to measure standard and maximal pressure–volume curves resulting in clinically relevant parameters such as total lung capacity (TLC), resistance (R), compliance (C), elastance (E), tissue damping (resistance) (G), and tissue elasticity (H) of the whole respiratory system. Anesthetized and relaxed mice were tracheotomized, and an 18-G cannula placed intratracheally to connect the FlexiVent system. Lungs were ventilated in volume-driven mode corrected for mouse whole-body weight, with a positive end-expiratory pressure [PEEP] between three and five and a frequency between 160 and 180 breath/min. After lung function measurements, mice were exsanguinated, and bronchoalveolar lavage (BAL) was performed by applying three times 500 μl of PBS with proteinase inhibitor (Roche, Penzberg, Germany), through the intratracheal cannula. A return of 1.3 ml approximately was obtained. BAL was centrifuged, and supernatant was snap-frozen in liquid nitrogen for further analysis.

### Antibodies, immunohistochemistry, and microscopy

For immunofluorescence microscopy, the following primary (i) and secondary (ii) antibodies (Ab) were used: (i) goat polyclonal Ab to collagen 28a1(N-12) (Santa Cruz, sc-104151, 1:100), mouse monoclonal Ab to smooth muscle actin (clone 1A4) (Sigma-Aldrich, 06-920, 1:100), rabbit monoclonal Ab to tenascin-C (Abcam, ab108930, 1:100), rabbit and guinea pig Emelin-2 antisera (generous gift of Prof. Raimund Wagener (University of Cologne) and Prof. Paolo Bonaldo (University of Padua) (1:500) and (ii) donkey anti-goat IgG Alexa Fluor 568, donkey anti-rabbit IgG Alexa Fluor 488, goat anti-mouse Alexa Fluor 488, goat anti-rabbit Alexa Fluor 568 (all from Invitrogen, 1:250). Cell nuclei were stained with DAPI (4′,6-diamidino-2-phenylindole, Sigma-Aldrich, 1:2,000). For immunohistochemistry, mouse lungs were perfused with PBS, fixed in 4% paraformaldehyde (pH 7.0), and embedded in paraffin. The paraffin sections (2 μm) were deparaffinized and rehydrated, and the antigen retrieval was accomplished by pressure-cooking (30 s at 125°C and 10 s at 90°C) in citrate buffer (10 mM, pH 6.0). Inhibition of endogenous peroxidase activity was achieved by blocking in 3% H_2_O_2_ in dH_2_O for 15 min. The lung sections were incubated with the primary antibodies over night at 4°C, washed three times with PBS, and subsequently incubated with the secondary antibodies and DAPI (4′,6-diamidino-2-phenylindole, Sigma-Aldrich, 1:2,000) for 1 h at room temperature. Finally, the sections were washed three times with PBS and mounted in fluorescent mounting medium (Dako). Images were acquired with an LSM 710 (Zeiss) operated in multitrack mode using the following objectives: Plan-Apochromat 20×/0.8 M27 and Plan-Apochromat 63×/1.4 M27. The automated microscopy system was driven by ZEN2009 (Zeiss) software. In order to get an overview image of whole lung paraffin sections, a tile image was acquired (8 × 8 images) with a Plan-Apochromat 20×/0.8 M27 objective. The final images were cropped and adjusted for contrast and brightness by using the ZEN2012 (Zeiss) software. High-resolution images were taken with a Plan-Apochromat 63×/1.4 M27 as a *z*-stack. Subsequently, the *z*-stacks were processed as a maximum intensity projection and adjusted for contrast and brightness by using the ZEN2009 software.

### Sample preparation procedures for proteome analysis—QDSP

Lungs were perfused with PBS through the heart to remove blood. Then, ∼100 mg of total lung tissue (wet weight) was homogenized in 500 μl PBS (with protease inhibitor cocktail and EDTA) using an Ultra-turrax homogenizer. After centrifugation, the soluble proteins were collected and proteins were extracted from the insoluble pellet in three steps using buffers with increasing stringency [buffer 1: 150 mM NaCl, 50 mM Tris–HCl (pH 7.5), 5% glycerol, 1% IGEPAL® CA-630 (Sigma, #I8896), 1 mM MgCl_2_, 1× protease inhibitors (+EDTA), 1% benzonase (Merck, #70746-3), 1× phosphatase inhibitors (Roche, #04906837001); buffer 2: 50 mM Tris–HCl (pH 7.5), 5% glycerol, 150 mM NaCl, fresh protease inhibitor tablet (+EDTA), 1.0% IGEPAL® CA-630, 0.5% sodium deoxycholate, 0.1% SDS, 1% benzonase (Merck, #70746-3); and buffer 3: 50 mM Tris–HCl (pH 7.5), 5% glycerol, 500 mM NaCl, protease inhibitor tablet (+EDTA), 1.0% IGEPAL® CA-630, 2% sodium deoxycholate, 1% SDS, 1% benzonase (Merck, #70746-3)]. Insoluble pellets were resuspended in detergent-containing buffers and incubated for 20 min on ice (except for buffer 3, which was used at room temperature), followed by separation of soluble and insoluble material using centrifugation for 20 min at 16,000 *g*. The PBS from the tissue homogenate and the NP40-soluble fraction (buffer 1) was pooled, which together with the two fractions derived from ionic detergent extraction (buffers 2 and 3) resulted in a total of three soluble fractions and one insoluble pellet that were subjected to LC-MS/MS analysis. Soluble proteins were precipitated with 80% acetone and subjected to in-solution digestion using a modified published protocol (Kulak *et al*, 2014). In brief, protein reduction (10 mM TCEP) and alkylation (50 mM CAA) were performed at once in 6 M guanidinium hydrochloride (100 mM Tris–HCl pH 8.5) at 99°C for 15 min. Subsequent protein digestion was done in two steps. The first digestion was done at 37°C for 2 h with LysC (1:50 enzyme to protein ratio) in 10 mM Tris–HCl (pH 8.5) containing 2 M guanidinium hydrochloride (Gdm), 2.7 M urea, and 3% acetonitrile. The second digestion step was done using fresh LysC (1:50 enzyme to protein ratio) and trypsin (1:20 enzyme to protein ratio) in 600 mM Gdm, 800 mM urea, and 3% acetonitrile at 37°C overnight. For the insoluble protein pellet, which is strongly enriched for insoluble ECM proteins, we optimized the in-solution digestion protocol with additional steps involving extensive mechanical disintegration and ultrasonication-aided digestion. The insoluble material was cooked, reduced, and alkylated in 6 M Gdm for 15 min and then subjected to 200 strokes in a micro-Dounce device, which reduced the particle size of the insoluble protein meshwork. We then proceeded with the two-step digestion protocol described above, which was additionally aided by 15-min ultrasonication (Bioruptor, Diagenode) in the presence of the enzymes in both digestion steps.

For tissue proteome time course analysis, a similar sequential extraction procedure as described above was used. However, in these experiments, we employed slightly different buffers for extraction following a commercially available protein extraction kit (Compartment Protein Extraction Kit, Millipore). We collected three protein fractions for LC-MS analysis (two soluble fractions and one insoluble fraction). The first fraction measured was derived from proteins soluble in buffer M of the extraction kit [HEPES (pH 7.9), MgCl_2_, KCl, EDTA, sucrose, glycerol, sodium deoxycholate, NP-40, sodium orthovanadate]; the second fraction was derived from proteins soluble in buffer CS [PIPES (pH 6.8), MgCl_2_, NaCl, EDTA, sucrose, SDS, sodium orthovanadate], and finally, we also analyzed the proteins insoluble in buffer CS as described above for QDSP. To perform relative quantification of full proteomes in the various conditions, we summed up the peptide intensities of the three protein fractions in MaxQuant (Cox & Mann, 2008).

Peptides were purified using stage tips containing a poly-styrene-divinylbenzene copolymer modified with sulfonic acid groups (SDB-RPS) material (3M, St. Paul, MN, USA) as previously described (Kulak *et al*, 2014). For the QDSP experiments, we separated peptides in two fractions by sequentially eluting from the SDB-RPS stage tip material (buffer 1: 150 mM NH_4_HCO_2_, 60% acetonitrile, 0.5% FA; buffer 2: 5% ammonia and 80% acetonitrile).

### LC-MS/MS analysis

Approximately 2 μg of peptides was separated in a 4-h gradient on a 50-cm long (75 μm inner diameter) column packed in-house with ReproSil-Pur C18-AQ 1.9-μm resin (Dr. Maisch GmbH). Reverse-phase chromatography was performed with an EASY-nLC 1000 ultra-high-pressure system (Thermo Fisher Scientific), which was coupled to a Q Exactive mass spectrometer (Thermo Scientific). Peptides were loaded with buffer A (0.1% (v/v) formic acid) and eluted with a nonlinear 240-min gradient of 5–60% buffer B (0.1% (v/v) formic acid, 80% (v/v) acetonitrile) at a flow rate of 250 nl/min. After each gradient, the column was washed with 95% buffer B and re-equilibrated with buffer A. Column temperature was kept at 50°C by an in-house designed oven with a Peltier element (Thakur *et al*, 2011), and operational parameters were monitored in real time by the SprayQc software (Scheltema & Mann, 2012). MS data were acquired with a shotgun proteomics method, where in each cycle a full scan, providing an overview of the full complement of isotope patterns visible at that particular time point, is followed by up to ten data-dependent MS/MS scans on the most abundant not yet sequenced isotopes (top 10 method) (Michalski *et al*, 2011). Target value for the full scan MS spectra was 3 × 10^6^ charges in the 300–1,650 *m/z* range with a maximum injection time of 20 ms and a resolution of 70,000 at *m/z* 400. Isolation of precursors was performed with the quadrupole at a window of 3 Th. Precursors were fragmented by higher-energy collisional dissociation (HCD) with normalized collision energy of 25% (the appropriate energy is calculated using this percentage, and *m/z* and charge state of the precursor). MS/MS scans were acquired at a resolution of 17,500 at *m/z* 400 with an ion target value of 1 × 10^5^, a maximum injection time of 120 ms, and fixed first mass of 100 Th. Repeat sequencing of peptides was minimized by excluding the selected peptide candidates for 40 s.

### Computational MS data analysis

MS raw files were analyzed by the MaxQuant software (Cox & Mann, 2008) (version 1.4.1.12), and peak lists were searched against the mouse UniProt FASTA database (version May 2013), and a common contaminants database (247 entries) by the Andromeda search engine (Cox *et al*, 2011). As fixed modification cysteine carbamidomethylation and as variable modifications, hydroxylation of proline and methionine oxidation was used. False discovery rate was set to 0.01 for proteins and peptides (minimum length of seven amino acids) and was determined by searching a reverse database. Enzyme specificity was set as C-terminal to arginine and lysine, and a maximum of two missed cleavages were allowed in the database search. Peptide identification was performed with an allowed precursor mass deviation up to 4.5 ppm after time-dependent mass calibration and an allowed fragment mass deviation of 20 ppm. For label-free quantification in MaxQuant [MaxLFQ (Cox *et al*, 2014)], the minimum ratio count was set to two. For matching between runs, the retention time alignment window was set to 30 min and the match time window was 1 min.

### RNA-seq

Liquid nitrogen-frozen tissue was homogenized using a micro-dismembrator (Sartorius, Göttingen, Germany). RNA extraction from mouse tissue was performed using the Roti Quick kit (Carl Roth, Karlsruhe, Germany) followed by RNA purification with the peqGold RNA isolation kit (Peqlab, Erlangen, Germany), according to the manufacturers’ instructions. For RNA quality assessment, RNA integrity numbers (RIN) were measured via Agilent 2100 Bioanalyzer (Agilent Technologies, Santa Clara, CA, USA), and samples with RIN number above 6.5 were used for sequencing. RNA library preparation was done using the Illumina TruSeq RNA Sample Prep kit v2 following the manufacturers’ protocol. RNA sequencing was performed as 100-bp paired-end runs on a HiSeq2500 (Illumina). Pools of 16 indexed libraries were sequenced on four lanes. Image analysis and base calling were performed using Illumina Real Time Analysis. CASAVA 1.8 was used for demultiplexing.

### Bioinformatic analysis and statistics

#### RNA-seq data analysis

The GEM mapper (v 1.7.1) (Marco-Sola *et al*, 2012) with modified parameter settings (mismatches = 0.04, min-decoded-strata = 2) was used for split-read alignment against the mouse genome assembly mm9 (NCBI37) and UCSC knownGene annotation. To quantify the number of reads mapping to the annotated genes, we used HTseq-count (v 0.6.0) (Anders *et al*, 2015). FPKM (fragments per kilobase of exon per million fragments mapped) values were calculated using custom scripts.

#### Data imputation for ratiometric time course analysis

MaxLFQ intensity values were taken from the MaxQuant protein Groups table, which represent the values after inter-experiment normalization. From these values, the median intensity from replicates for each of the time points was calculated. Missing values in these median values were imputed by random selection from a normal distribution generated at 1.8 standard deviations, subtracted from the mean, of the total intensity distribution and a width of 0.3 standard deviations. This places the imputed values at the lower limit of the intensity scale, which represents detection limit of the used instrumentation. The median values were used to calculate the ratio between bleomycin-treated and control mice for each replicate at each acquired time point for those cases where the bleomycin-treated intensity value was based on at least one valid value. If this was not the case, data imputation and ratio calculation were done only where the control had at least 50% valid values (the other values were marked as missing). The resulting data matrix was exported for further statistical treatment and visualization.

#### QDSP—quantitative detergent solubility profiling

The total abundance of proteins in the bleomycin- and PBS-treated samples were calculated by summing the linear iBAQ values across the four solubility fractions. The abundance rank plots show the rank of the mean log2 intensity of the selected protein compared to the mean log2 intensities of all other proteins. Furthermore, a two-sample, two-sided *t*-test is performed comparing the mean log2 intensities between bleomycin- and PBS-treated samples for each protein.

The solubility profiles across the four fractions are compared by first normalizing the intensities such that the mean log2 intensities of the bleomycin- and the PBS-treated samples are zero, respectively. Using the normalized intensities, a two-way ANOVA with the two-factor treatment (bleomycin/PBS) and solubility fraction (FR1, FR2, FR3, INSOL) and the corresponding interaction term was performed. Proteins significant in the interaction term correspond to proteins for which the solubility profile changes upon bleomycin treatment. Therefore, the corresponding *P*-value was used for filtering the significantly changed profiles after FDR correction.

#### Tissue proteome time course analysis

For each protein, the ratio between the protein intensity in bleomycin-treated samples, and the mean intensity of the protein in the PBS-treated samples was calculated and log2-transformed. To identify proteins whose expression changes over time, one-way ANOVA was applied to these log2 ratios. In addition, a linear regression analysis was performed with the independent variable compliance ratio of the corresponding sample and the dependent variable protein ratio. In addition, the Pearson correlation coefficient and the correlation *P*-value were calculated for each protein.

#### BALF proteome analysis

Similar as for the tissue proteome, the expression ratio of bleomycin- and PBS-treated samples was calculated for further analysis, where the PBS expression value was averaged across all replicates and time points. To identify proteins, whose expression changes upon treatment with bleomycin, a two-sample, two-sided *t*-test was performed for data obtained at day 14. To identify proteins, whose expression ratio changes over time, a one-way ANOVA was performed on the expression ratios.

The *t*-test-based BALF enrichment score was calculated as in formula one in the paper Tusher *et al* (2001). The statistic is based on the ratio of change in gene expression to standard deviation in the data for that gene. The “gene-specific scatter” s(i) is the standard deviation of the replicates.

#### FDR correction

All *P*-values were corrected for multiple hypothesis testing using the Benjamini–Hochberg method (Benjamini & Hochberg, 1995), and only proteins significant at the specified FDR were reported. *P*-values are marked by an asterisk in the plots, if they are smaller than 0.05. All profile plots show the mean intensity or ratio, respectively; the error bars correspond to the standard error of the mean. All scripts performing the statistical analysis and generating the plots were implemented in MATLAB (Mathworks).

#### Ingenuity pathway analysis

To predict the activity of downstream biological processes and upstream transcriptional regulators and growth factors based on the observed protein abundance ratios, we used the Ingenuity® Pathway Analysis platform (IPA®, QIAGEN Redwood City, www.qiagen.com/ingenuity). We used a suite of algorithms and tools embedded in IPA for inferring and scoring regulator networks upstream of gene expression data based on a large-scale causal network derived from the Ingenuity Knowledge Base. The analytics tool “Upstream Regulator Analysis” (Kramer *et al*, 2014) was used to compare the known effect (transcriptional activation or repression) of a transcriptional regulator on its target genes to the observed changes in protein abundance to assign an activation *Z-*score. Since it is *a priori* unknown which causal edges in the master network are applicable to the experimental context, the “Upstream Regulator Analysis” tool uses a statistical approach to determine and score those regulators whose network connections to dataset genes as well as associated regulation directions are unlikely to occur in a random model (Kramer *et al*, 2014). In particular, the tool defines an overlap *P*-value measuring enrichment of network-regulated genes in the proteomic dataset, as well as an activation *Z*-score which can be used to find likely regulating molecules based on a statistically significant pattern match of up- and downregulation, and also to predict the activation state (either activated or inhibited) of a putative regulator. In our analysis, we restricted the “Upstream Regulator Analysis” to the categories “transcriptional regulator” and “growth factor” in the IPA filter settings. We considered proteins with an overlap *P*-value of > 3 (log10) that had an activation *Z*-score > 2.5 as activated and those with an activation *Z-*score <−2.5 as inhibited. Using the “Downstream Effects Analysis” (Kramer *et al*, 2014) embedded in IPA, we aimed at identifying those biological processes and functions that are likely to be causally affected by up- and downregulated proteins in the proteomic dataset. The approach is very similar to that of “Upstream Regulator Analysis,” except that the direction of edges connecting the dataset genes with the predicted entity (here, the biological process) is reversed. We confined our analysis to “Molecular and Cellular Functions & Physiological System Development and Function” and accepted an overlap *P*-value of > 3 (log10). Biological processes with an activation *Z*-score > 2 were considered as activated and those with an activation *Z-*score <−2 as inhibited.

#### Software packages for statistics and data visualization

For basic data handling, normalization, statistics (if not stated otherwise), and annotation enrichment analysis, we used the freely available open-source bioinformatics platform Perseus (http://141.61.102.17/perseus_doku/doku.php?id=start). Perseus was used to visualize data from principal component analysis, non-supervised hierarchical clustering, and scatter plots. Furthermore, the 1D and 2D annotation algorithms and Fisher’s exact tests implemented in Perseus (Cox & Mann, 2012) were used for annotation enrichment analysis. Bar graphs and box plots were generated in the GraphPad PRISM software (http://www.graphpad.com/scientific-software/prism/).

### Data access via the MaxQB database and proteomics raw data repository

Protein centric data from proteomic experiments are available via the MaxQB database (Schaab *et al*, 2012) via the following URL: http://maxqb.biochem.mpg.de/mxdb/project/show/P013

The site offers detailed information on protein identification data as well as data visualization plots, which can be accessed via the protein groups tab.

The mass spectrometry proteomics data have been deposited to the ProteomeXchange Consortium (Vizcaino *et al*, 2014) via the PRIDE partner repository with the dataset identifier PXD001765.

## Supporting Information

**Expanded View Figures PDF**

**Table EV1**

**Table EV2**

**Table EV3**

**Table EV4**

**Table EV5**

**Table EV6**

**Table EV7**

**Table EV8**

**Table EV9**

**Table EV10**

**Table Legends**

**Review Process File**
