# Supplementary material for: Time- and compartment-resolved proteome profiling of the extracellular niche in lung injury and repair
Source: Mol Syst Biol. 2015 Jul 14;11(7):819. doi: 10.15252/msb.20156123 (PMC4547847; doi:10.15252/msb.20156123)
Supplement: Supplementary file 1 [file msb0011-0819-sd1.pdf]

Expanded View Figures

A

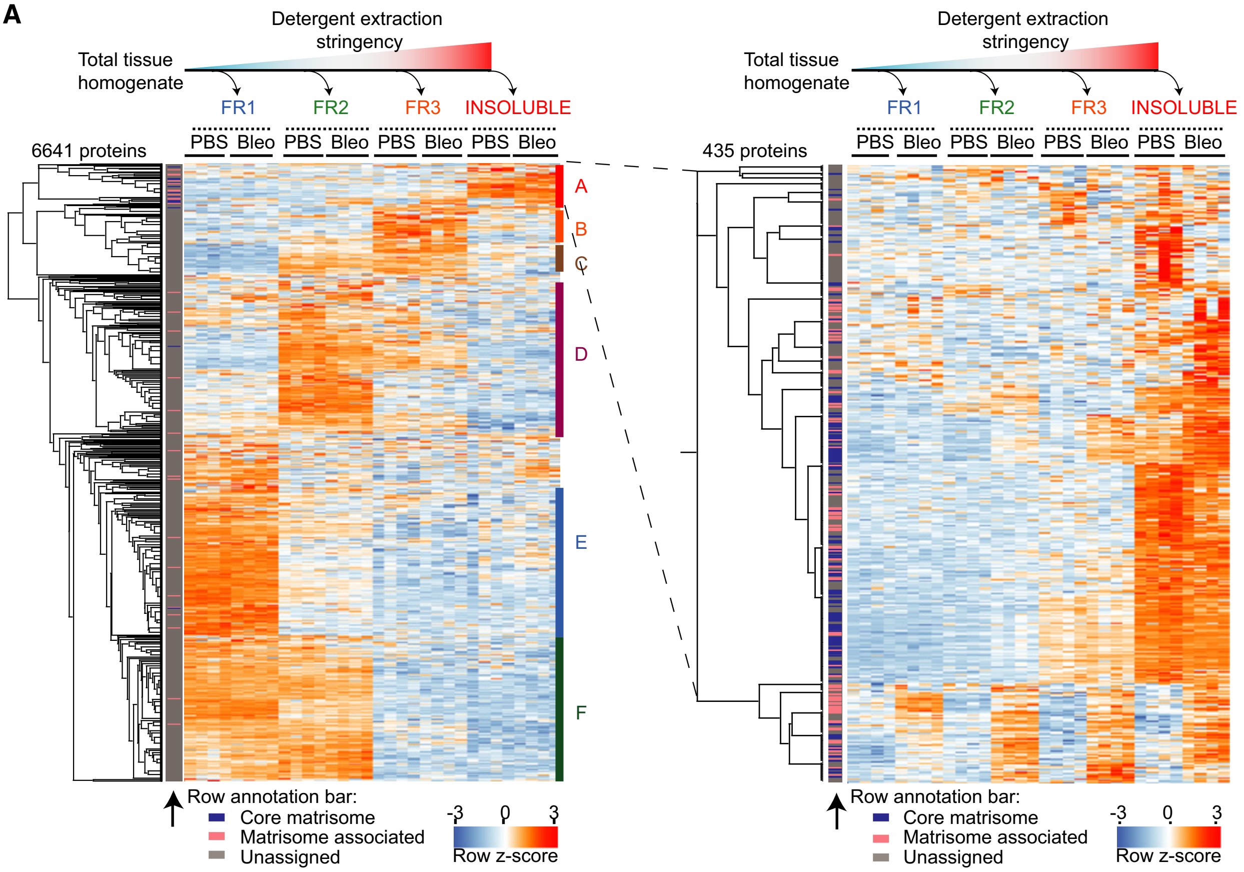

B

Cluster A	Cluster B	Cluster C	Cluster D	Cluster E	Cluster F
Core matrisome	Cytoskeleton	Nucleus	Nucleus	Cytoplasm	cytoplasmic part
Secreted	Coiledcoil	Chromosome	Transcription	Acetylation	Transmembranehelix
ECM Glycoproteins	Celljunction	Ribosomebiogenesis	Transcriptionregulation	Proteasome	Transmembrane
Extracellularmatrix	Actin-binding	Nucleosomecore	Chromatinregulator	Oxidoreductase	integral to membrane
Signal	Cilium	rRNAprocessing	DNA-binding	Ligase	Membrane
Disulfidebond	Motorprotein	DNA-binding	Zinc-finger	Directproteinsequencing	membrane part
Matrisome-associated	Cellprojection	Phosphoprotein	Repressor	Hydrolase	intrinsic to membrane
Glycoprotein	Dynein	mRNAtransport	mRNAprocessing	Transferase	Mitochondrion
ECM Regulators	Myosin	mRNAsplicing	Zinc	Magnesium	mitochondrial part
Collagens	Alternativesplicing	Citrullination	Activator	NADP	organelle membrane
Collagen	Tightjunction	Ublconjugation	mRNAsplicing	NAD	Endoplasmicreticulum
Basementmembrane	Actincapping	Repeat	Spliceosome	Aminoacyl-tRNASynthetase	endoplasmic reticulum part
Celladhesion	Microtubule	RNA-binding	DNA-directedRNApolymerase	Threonineprotease	Ribosomalprotein
EGF-likedomain	Flagellum	Actin-binding	Phosphoprotein	Lyase	Mitochondrioninnermembrane
Hydroxylation	Ciliumbiogenesis/degradation	mRNAprocessing	Transmembrane	3D-structure	Transitpeptide
Repeat	Calmodulin-binding	GPI-anchor	Transmembranehelix	Allostericenzyme	Transport
Proteaseinhibitor	Repeat	Actincapping	Chromosome	Multifunctionalenzyme	structural constituent of ribosom
Secreted Factors	LIMdomain	Isopeptidebond	Cellmembrane	Purinebiosynthesis	endoplasmic reticulum membra
LamininEGF-likedomain		Nuclearporecomplex	DNAdamage	Glycolysis	Ribonucleoprotein
Cleavageonpairofbasicresidues		Bromodomain	Exosome	Protease	Ribosome
Serineproteaseinhibitor		WDrepeat	Alternativesplicing	Pyridoxalphosphate	mitochondrion
Calcium		Transcription	DNArepair	Proteinbiosynthesis	mitochondrial membrane
ECM-affiliated Proteins		Transcriptionregulation	Meiosis	Nucleotide-binding	mitochondrial inner membrane
Sulfation		Celljunction	Palmitate	ATP-binding	translation
Keratin		Methylation	GPI-anchor	Amino-acidbiosynthesis	membrane
Growthfactorbinding		Coiledcoil	Homeobox	Calcium/phospholipid-binding	mitochondrial membrane part
Intermediatefilament		Myosin	mRNAtransport	Annexin	Electrontransport
Growthfactor			DNArecombination	Tricarboxylicacidcycle	organelle inner membrane
Heparin-binding			Helicase	Kinase	Respiratorychain

Figure EV1. Efficient separation of subcellular and tissue compartments using QDSP.

A Non-supervised hierarchical clustering of *z*-scored MS intensities from the indicated protein fractions and conditions (PBS, $n = 4$; Bleo day 14, $n = 4$). The left panel depicts all 6,641 proteins, while the right panel shows a zoom in on the 435 most detergent-insoluble proteins in cluster A. The row annotation bar assigns proteins into the indicated matrisome categories.

B The table shows UniProt keywords that were significantly enriched (FDR < 2%) in the indicated protein clusters of the clustering analysis in (A).

 © 2015 The Authors

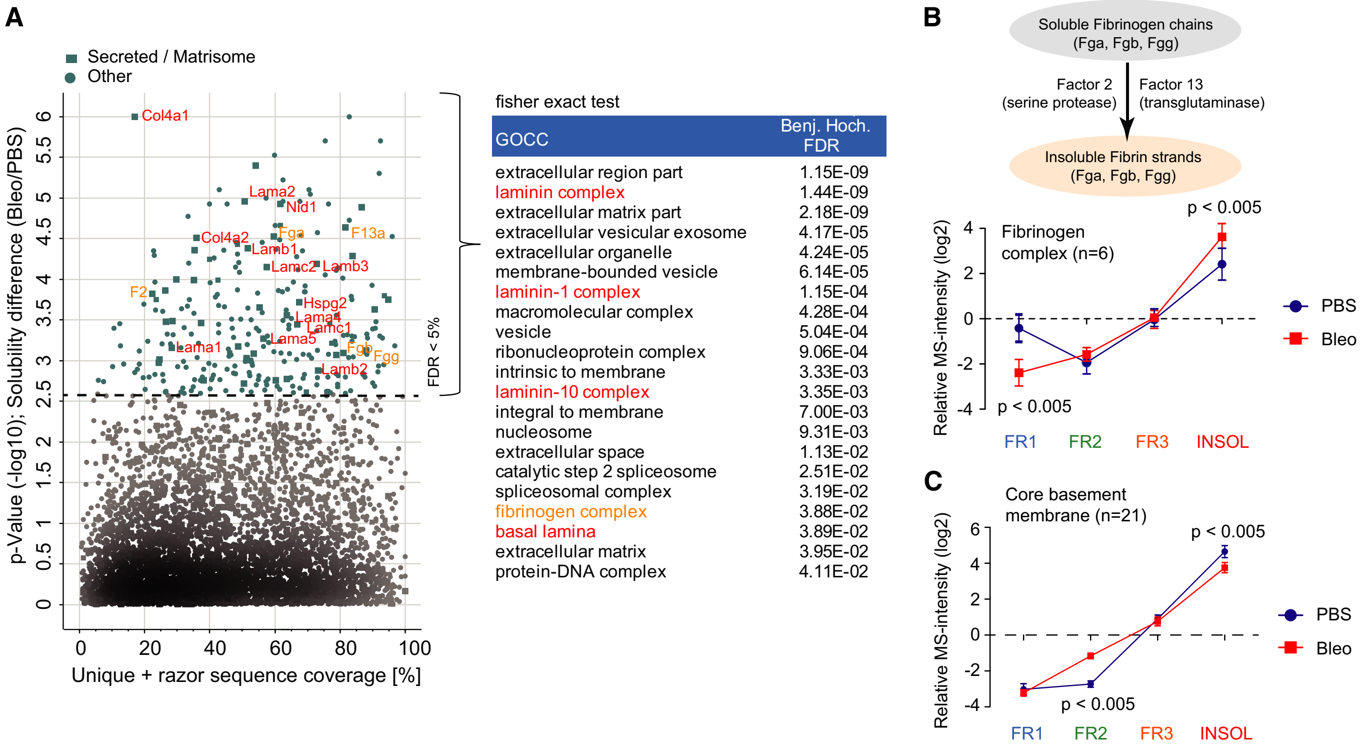

GOCC	Benj. Hoch. FDR
extracellular region part	1.15E-09
laminin complex	1.44E-09
extracellular matrix part	2.18E-09
extracellular vesicular exosome	4.17E-05
extracellular organelle	4.24E-05
membrane-bounded vesicle	6.14E-05
laminin-1 complex	1.15E-04
macromolecular complex	4.28E-04
vesicle	5.04E-04
ribonucleoprotein complex	9.06E-04
intrinsic to membrane	3.33E-03
laminin-10 complex	3.35E-03
integral to membrane	7.00E-03
nucleosome	9.31E-03
extracellular space	1.13E-02
catalytic step 2 spliceosome	2.51E-02
spliceosomal complex	3.19E-02
fibrinogen complex	3.88E-02
basal lamina	3.89E-02
extracellular matrix	3.95E-02
protein-DNA complex	4.11E-02

Figure EV2. Differential solubility of matrisome complexes in tissue fibrogenesis.

A Proteins (n = 283) with significantly altered normalized MS intensity profiles (Benjamini–Hochberg FDR < 0.05) between PBS controls (n = 4) and bleomycin-treated (day 14; n = 4) samples are highlighted in a scatter plot depicting the percent coverage of identified tryptic peptides across the protein sequence and the *P*-value of the solubility difference. Significantly enriched GOCC terms (Fisher's exact test, FDR < 2%) in the 283 protein group with altered QDSP profiles are shown in the right panel.

B, C The mean normalized QDSP MS intensity profiles of the fibrinogen complex (B; n = 6) and all core basement membrane proteins (C; n = 21) from PBS controls (n = 4) in blue and bleomycin (day 14; n = 4) in red are shown. The mean is shown and the error bars depict the standard error of the mean. The indicated *P*-values are derived from a *t*-test.

© 2015 The Authors

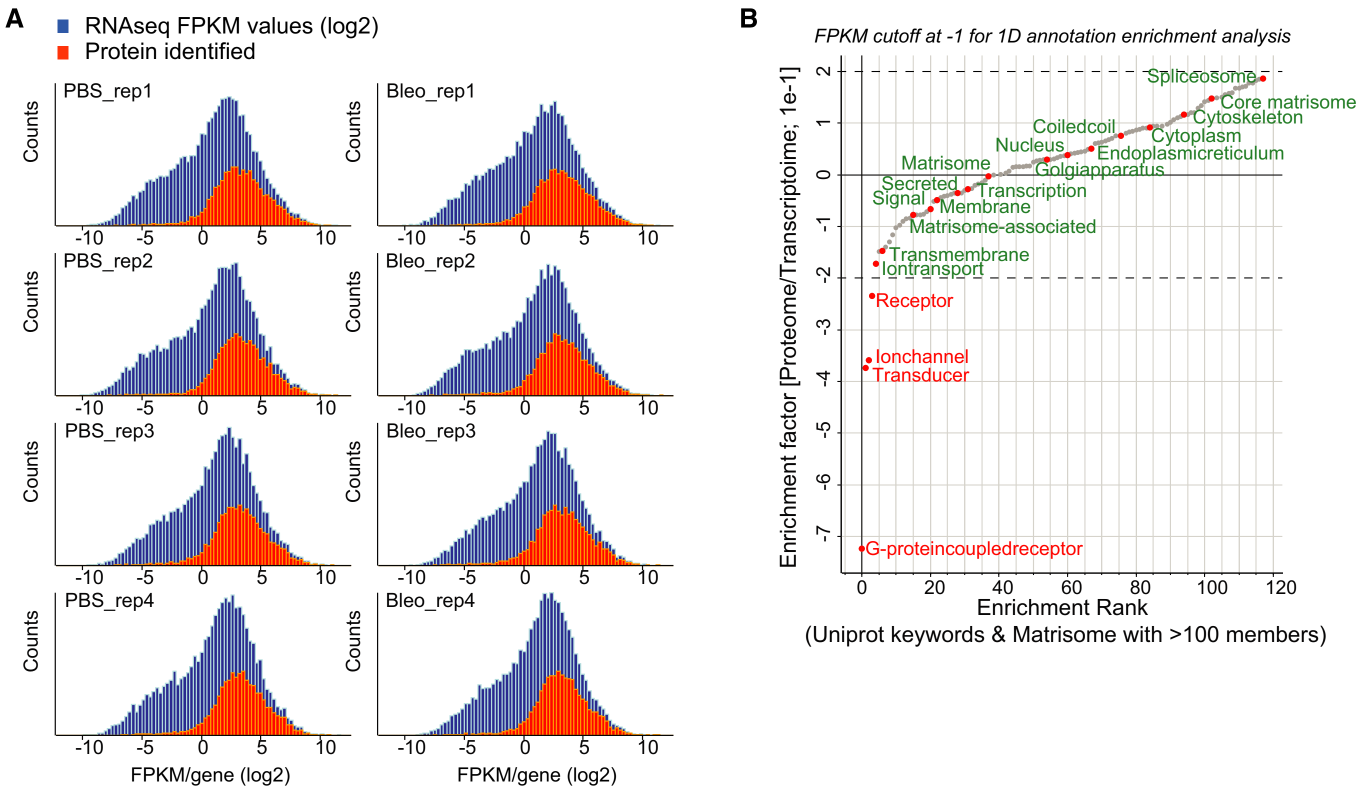

Figure EV3. Combined proteomic and transcriptomic analyses of tissue fibrogenesis.

A The histogram shows the distribution of the FPKM values from the RNA-seq experiment in log2 space (blue bars). The orange bars show the distribution of FPKM values for a total of 6,672 genes for which we also quantified the corresponding protein.

B Relative enrichment (coverage) of the indicated gene categories (UniProt keywords) in the proteome and transcriptome was calculated using the 1D annotation enrichment algorithm embedded in the Perseus software suite. The enrichment is normalized to a score spanning from −1 to 1 as indicated. Gene categories are ranked according to their relative enrichment in the transcriptome or proteome (PBS, *n* = 4; Bleo day 14, *n* = 4).

 © 2015 The Authors

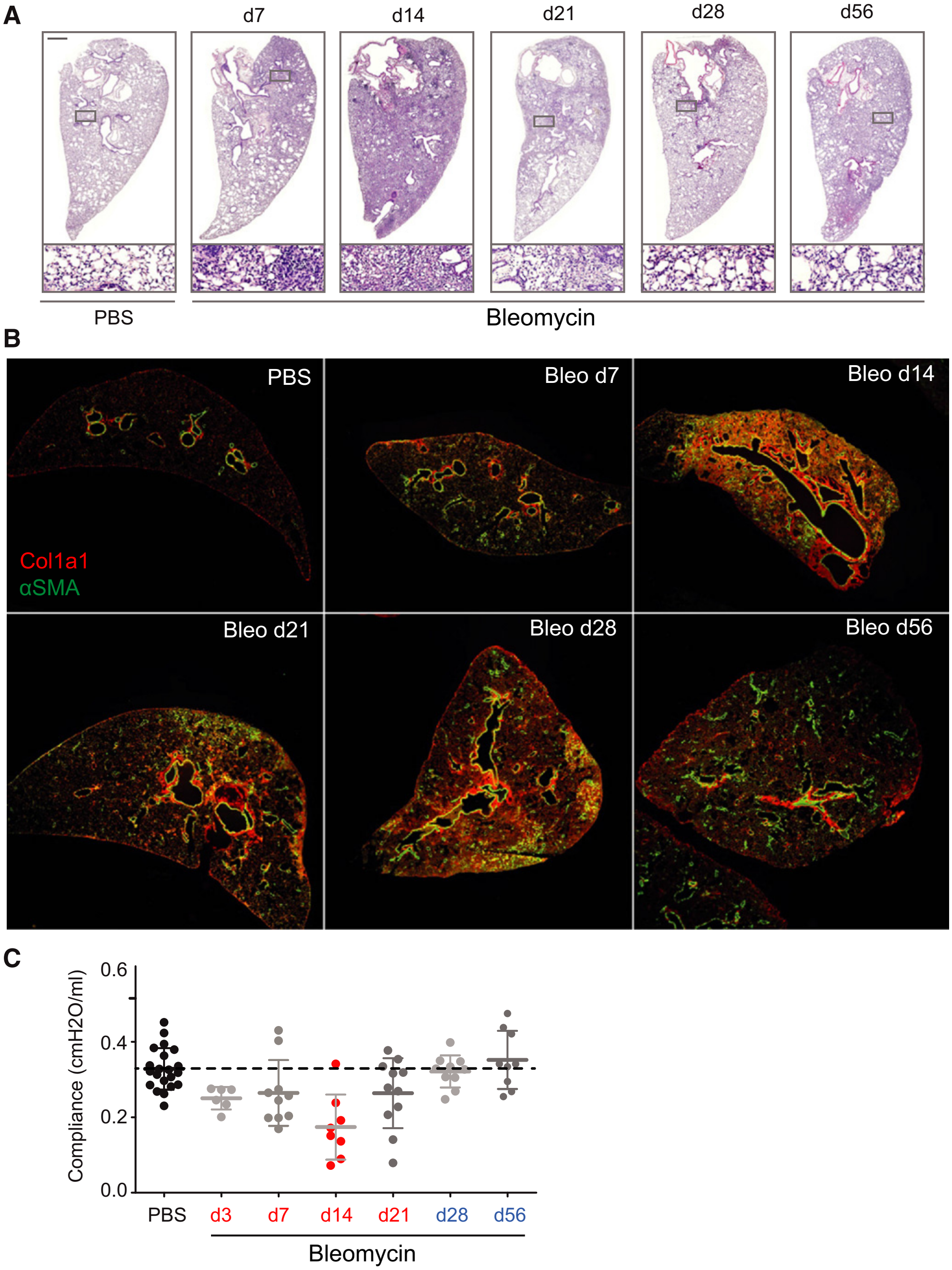

Figure EV4. Transient fibrogenesis in the bleomycin model.

A Representative tissue sections of the indicated experimental conditions and time points were analyzed using hematoxylin and eosin stain (H&E).

B Tissue sections of the indicated experimental conditions and time points were stained for α-smooth muscle actin (α-SMA in green) and collagen type I (Col1a1 in red).

C Lung compliance was measured in the indicated experimental conditions and time points using the forced oscillation technique from FlexiVent systems (Scireq, Montreal, Canada). The values for mean and standard deviation are shown.

© 2015 The Authors

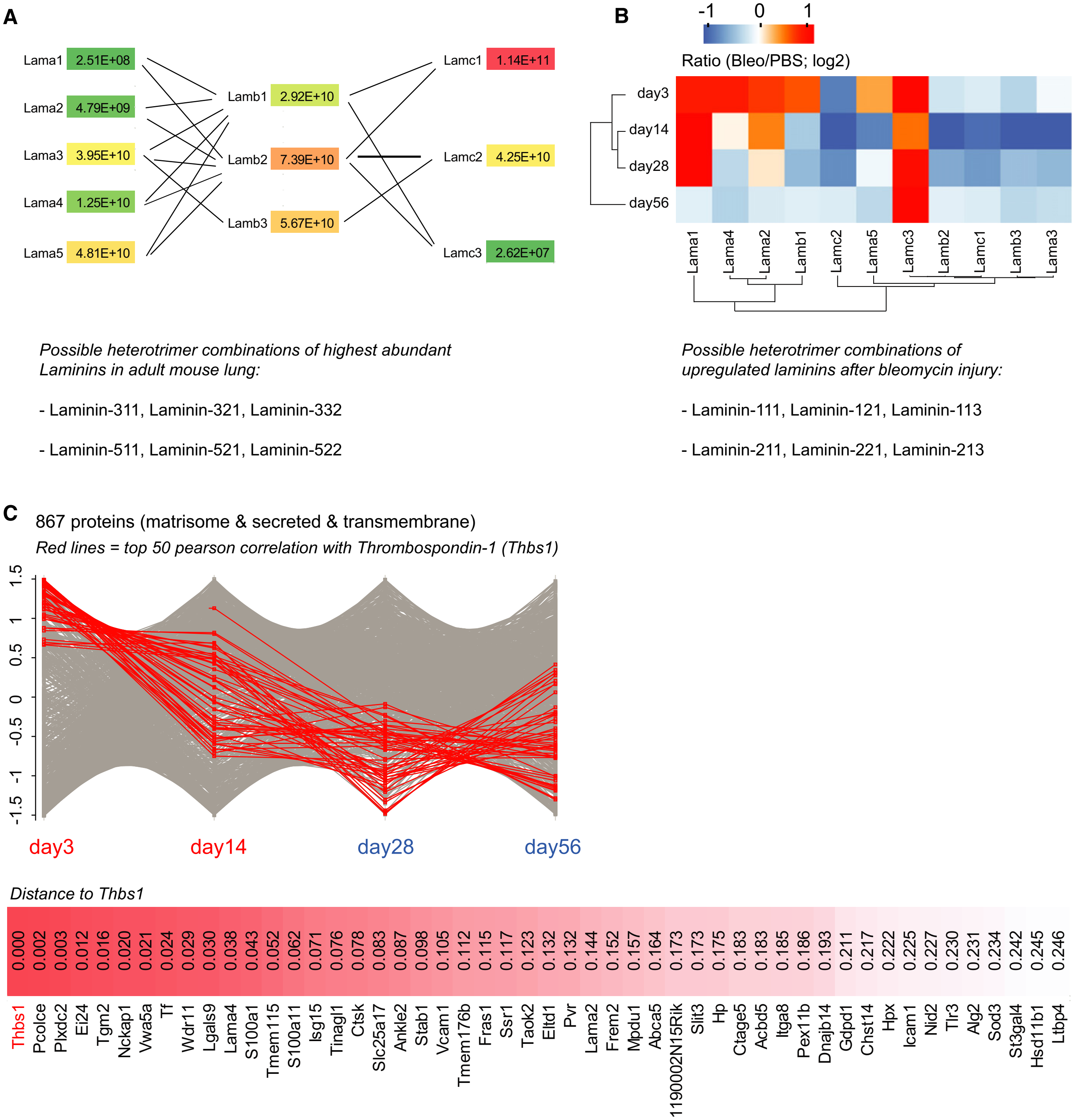

Figure EV5. Pulmonary basement membrane laminins and immediate early extracellular niche factors upon injury.

A The median MS intensity of all laminin chains was normalized for the theoretical number of tryptic peptides (iBAQ), yielding a relative stoichiometry of laminin chains, which is shown in the network graph for healthy control lungs (PBS, $n = 16$). The lower panel depicts the possible laminin heterotrimer combinations of the highly abundant α3- and α5-laminins, based on previous literature.

B The median z-scored MS intensity ratios of the indicated laminin chains were grouped by correlation using unsupervised hierarchical clustering (day 3, $n = 3$; day 14, $n = 7$; day 28, $n = 4$; day 56, $n = 3$). Possible heterotrimer combinations of the upregulated α1- and α2-laminins are shown in the lower panel.

C The median z-scored MS intensity ratios of 867 extracellular proteins were grouped by correlation analysis of their temporal profiles. The 50 proteins with highest correlation to thrombospondin-1 (Thbs1) are shown (day 3, $n = 3$; day 14, $n = 7$; day 28, $n = 4$; day 56, $n = 3$).

 © 2015 The Authors

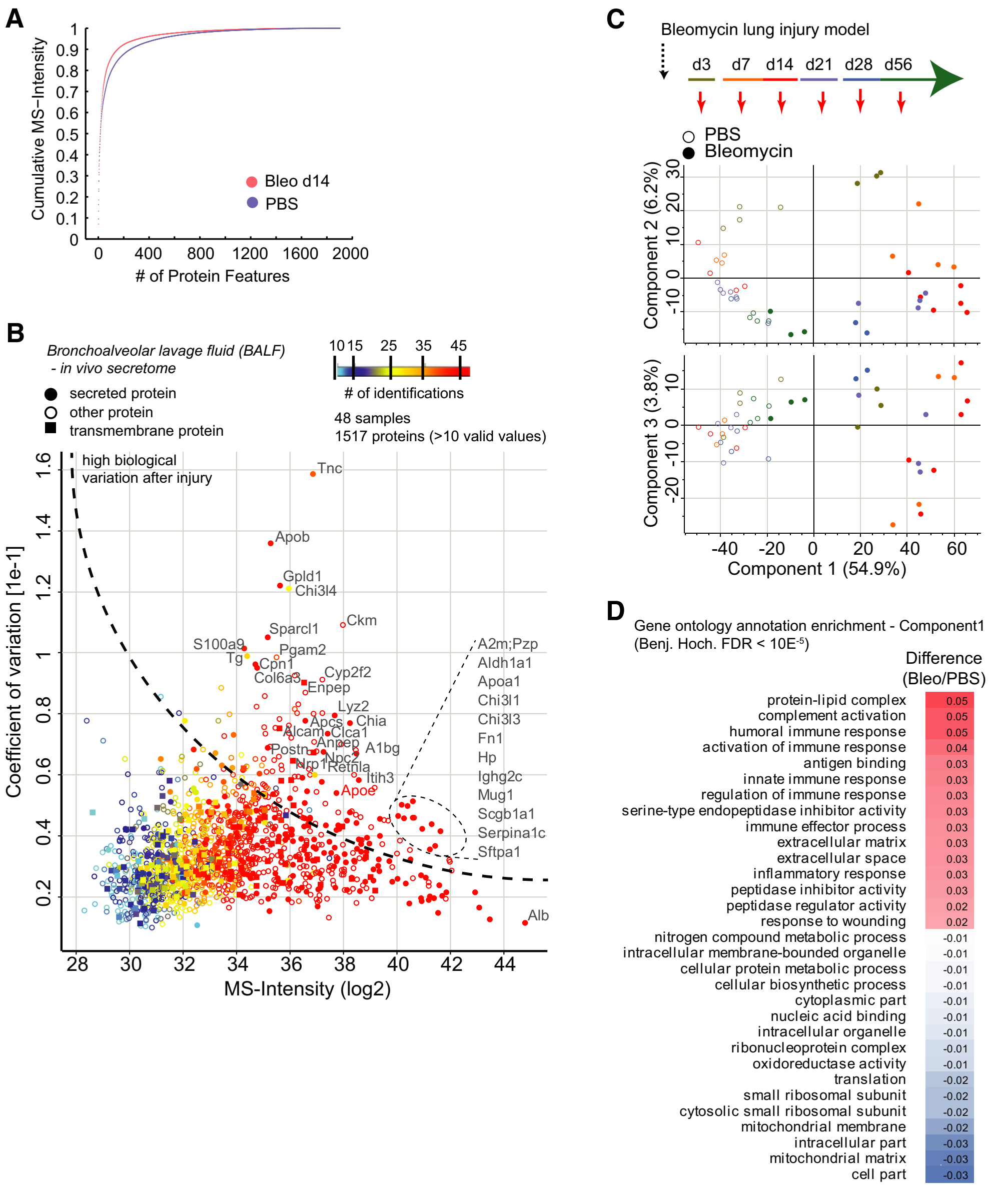

Figure EV6. Proteomic analysis of bronchoalveolar lavage fluid upon bleomycin injury.

A The mean cumulative MS intensity of the indicated conditions demonstrates differences in dynamic range of protein copy numbers (PBS, n = 24; Bleo day 14, n = 7). The lower number after bleomycin treatment was likely due to the leakage of plasma through the endothelial barrier upon injury, which makes MS analysis more challenging.

B The scatter plot depicts the median MS intensity versus the coefficient of variation across the injury time course and all replicates, which highlights proteins with high biological variation upon injury. The number of protein identifications across replicates and time points (n = 48) is color-coded, and secreted and transmembrane proteins are marked with the indicated symbols.

C Principal component analysis of BALF proteomes from the indicated experimental conditions and time points separates experimental groups.

D The table shows significantly regulated gene ontology terms (FDR < 2%) that were found to be enriched in either the negative (PBS) or positive (Bleo) data dimension of component 1 in the principal component analysis shown in (C).
