# Supplementary material for: Time- and compartment-resolved proteome profiling of the extracellular niche in lung injury and repair
Source: Mol Syst Biol. 2015 Jul 14;11(7):819. doi: 10.15252/msb.20156123 (PMC4547847; doi:10.15252/msb.20156123)

A2A547 − Rpl19 (id: 2)

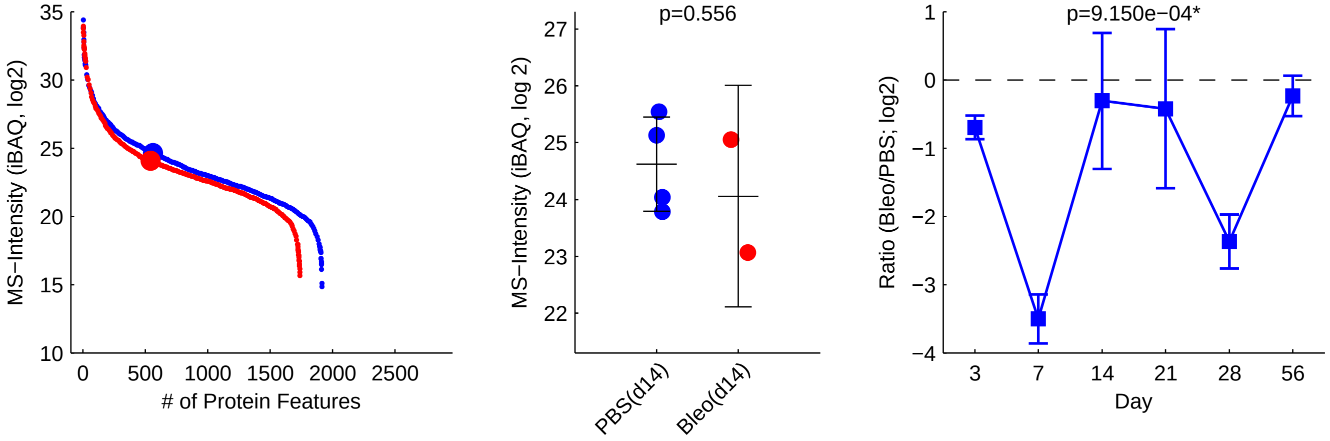

A2A6U3 − Sept9 (id: 6)

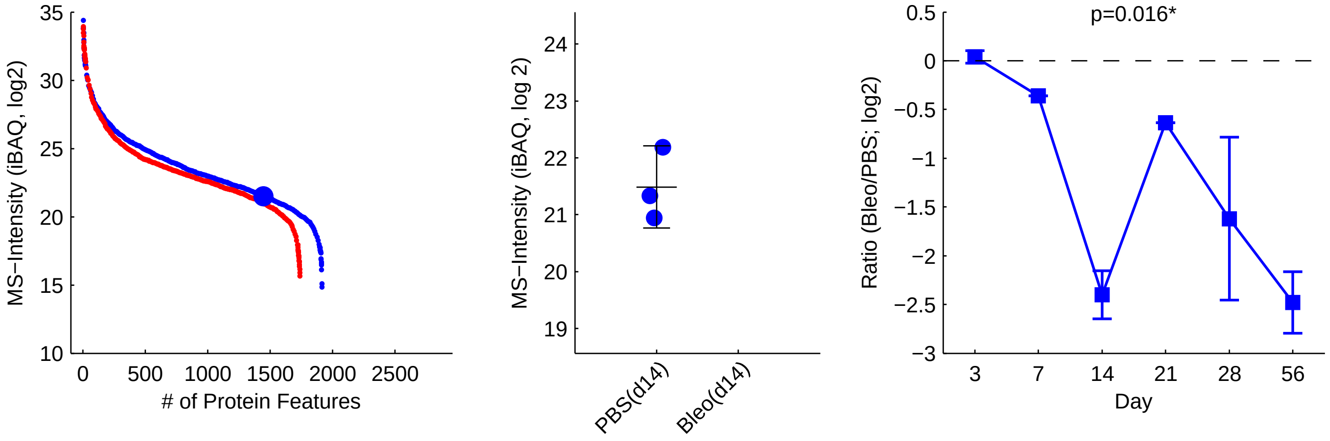

Q91WQ3 − Yars (id: 9)

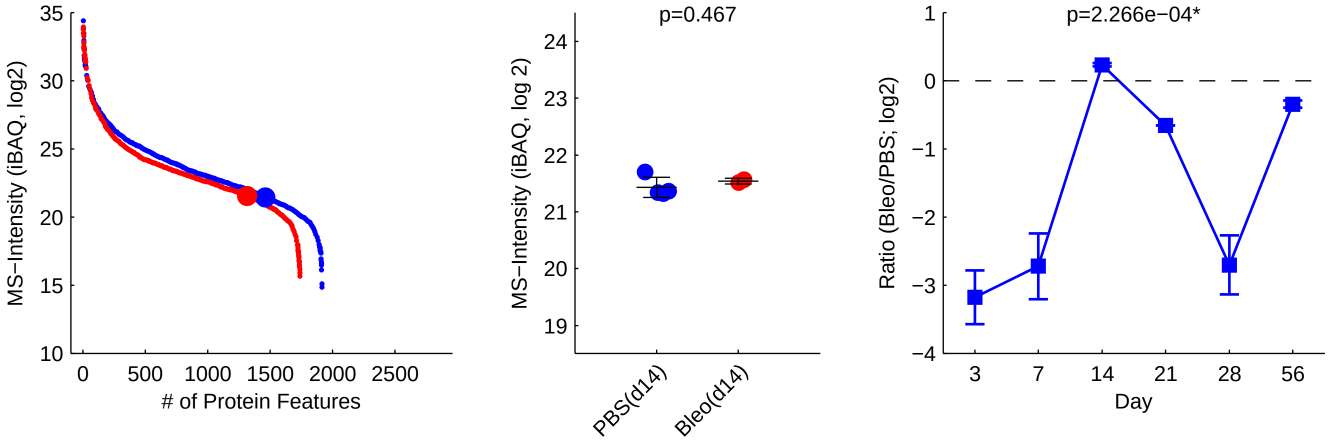

A2AIH8 − Pir (id: 35)

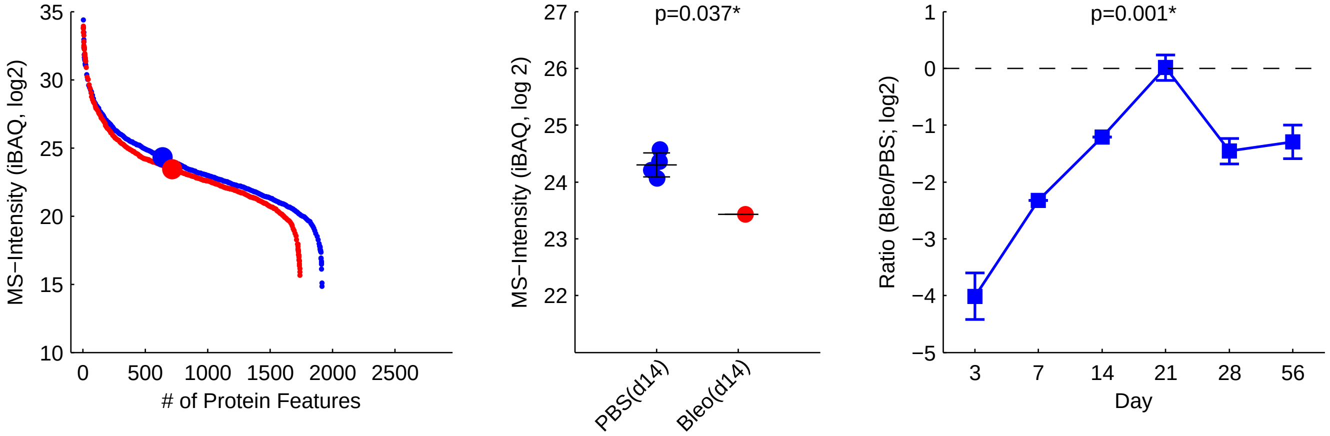

P15379−2 − Cd44 (id: 48)

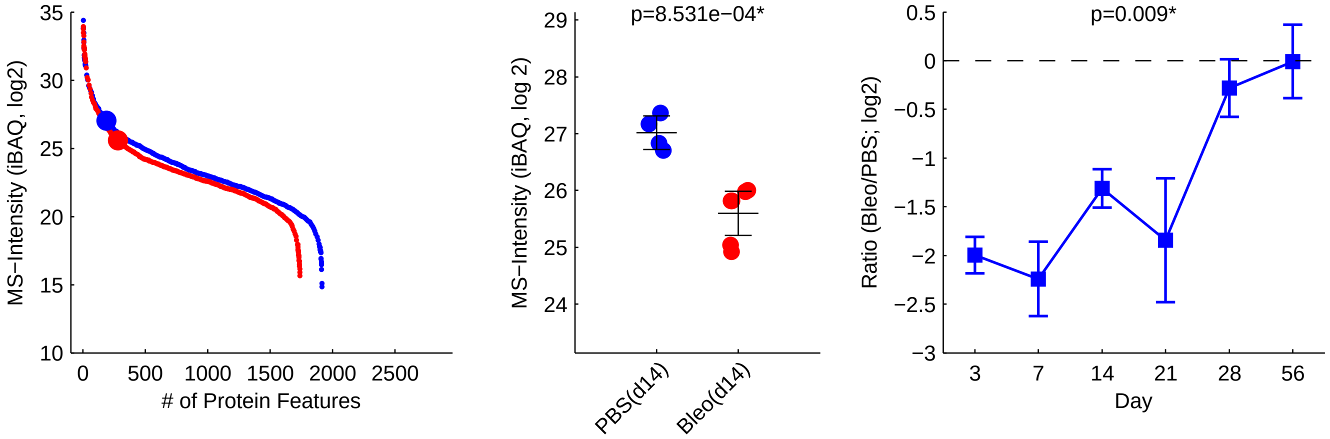

A2ARV4 − Lrp2 (id: 52)

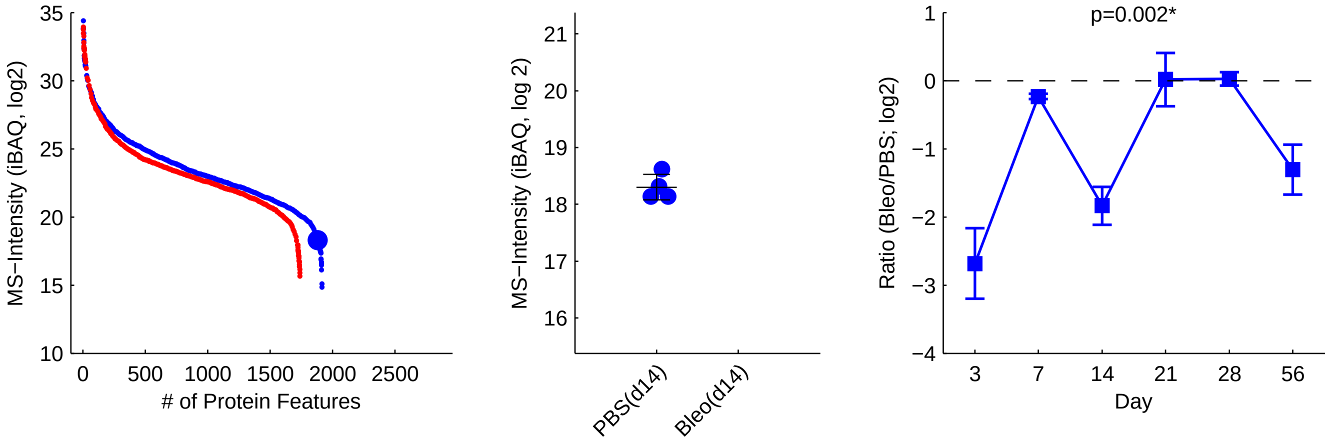

Q8BWN9 − AI182371 (id: 53)

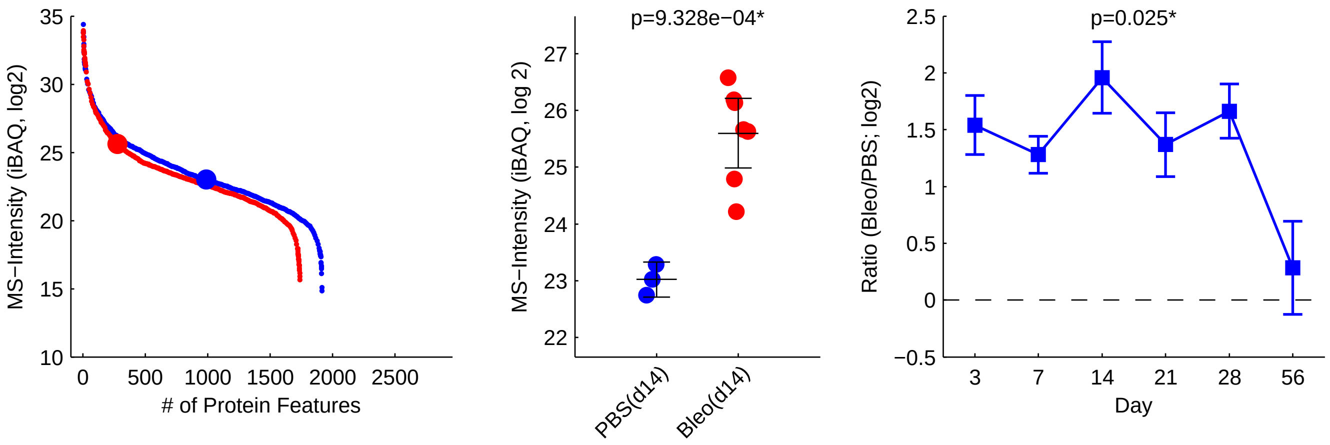

Q3TAI4 − Tpd52l2 (id: 58)

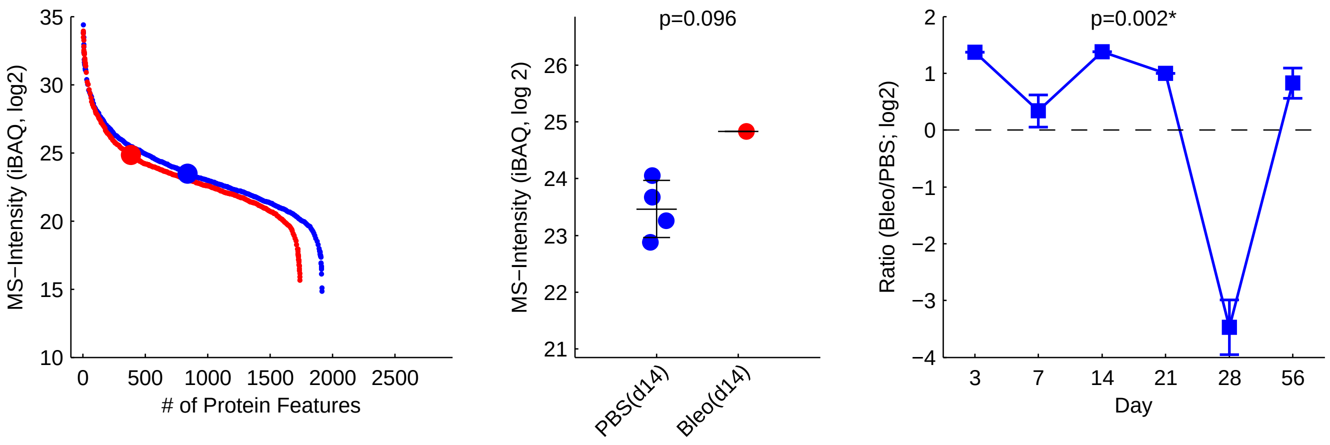

A2AVJ7 − Rrbp1 (id: 63)

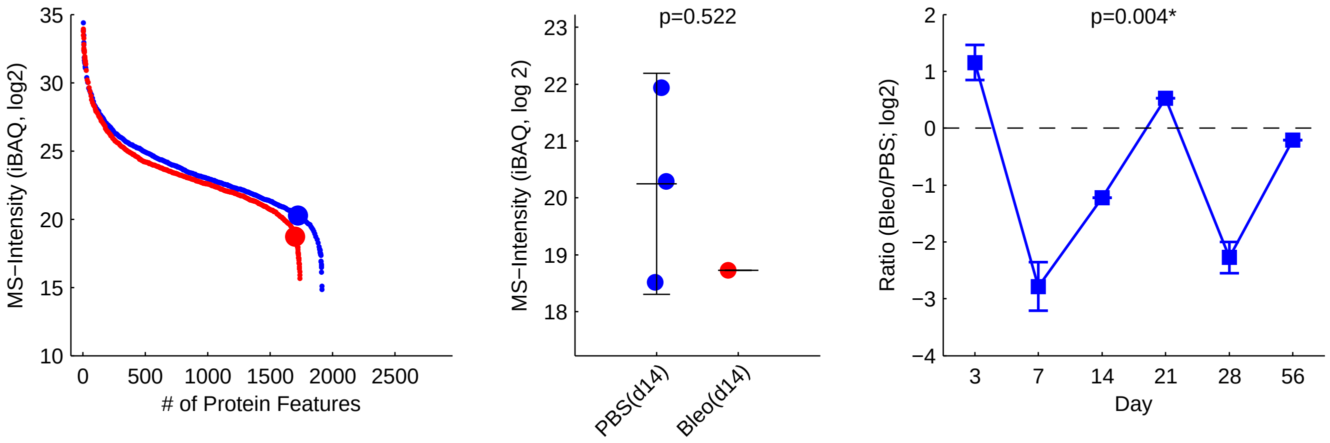

P62627 − Dynlrb1 (id: 64)

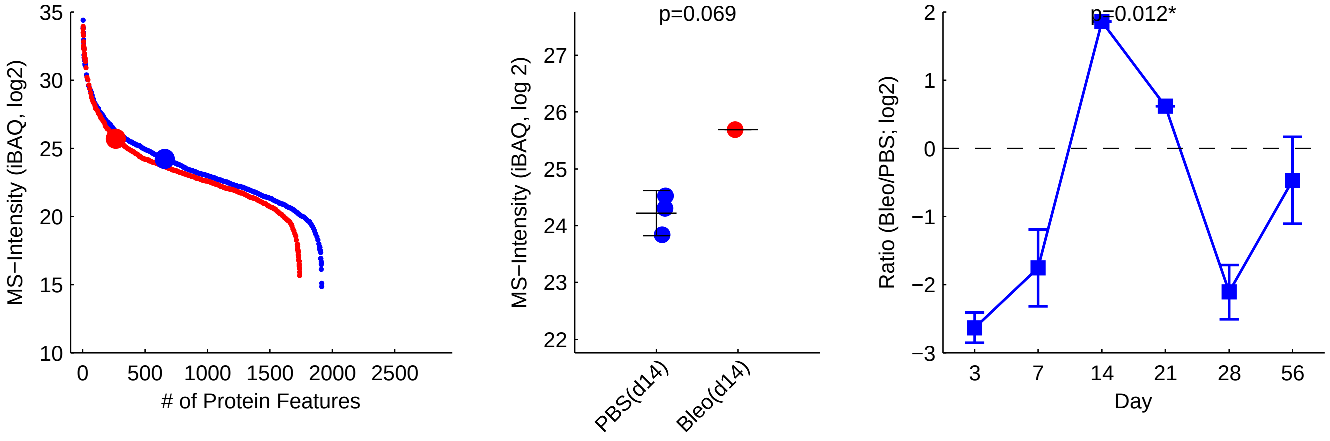

A2BFF8 − Dync1i2 (id: 67)

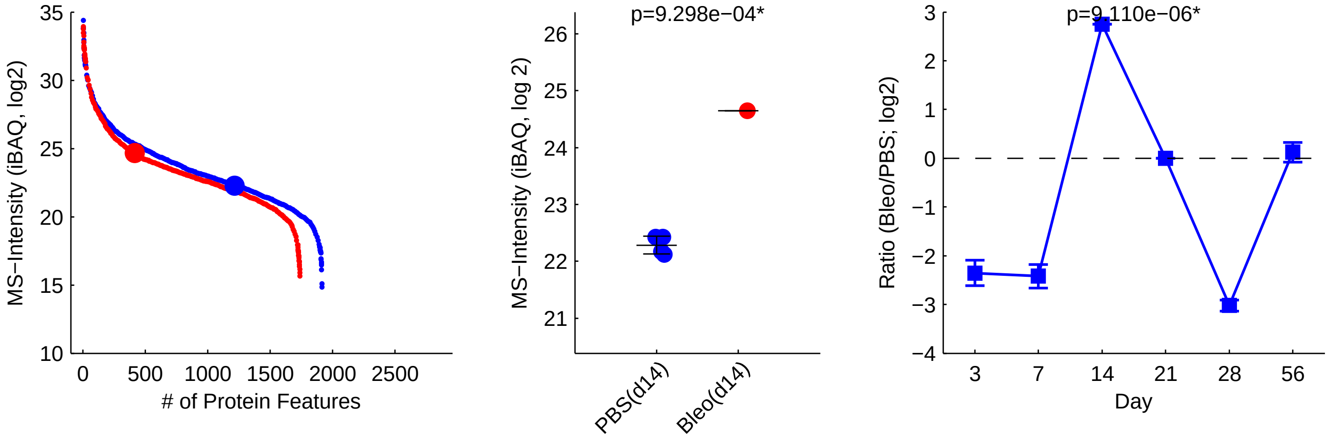

A3KGU7 − Sptan1 (id: 77)

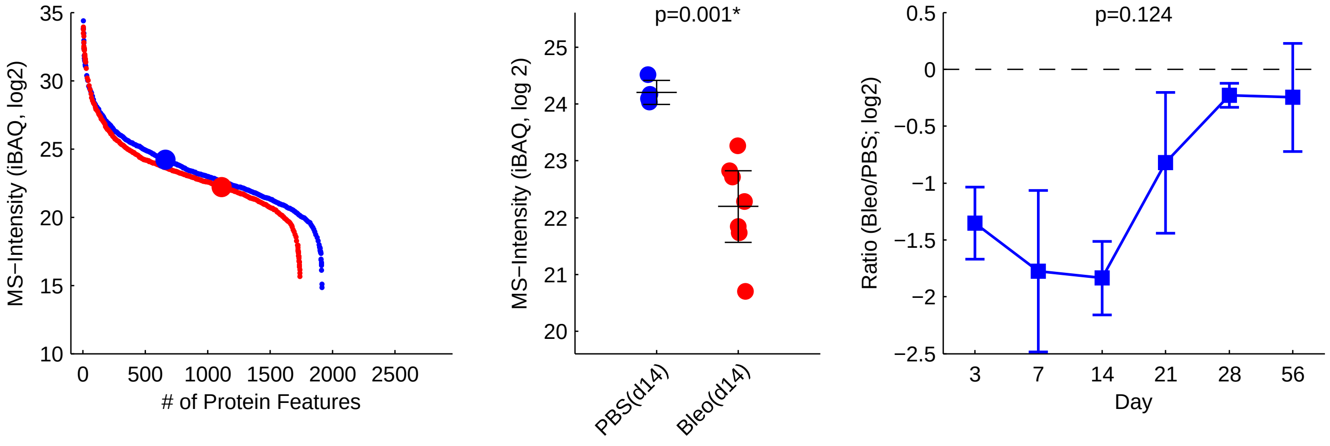

B1ASE2 − Atp5h (id: 104)

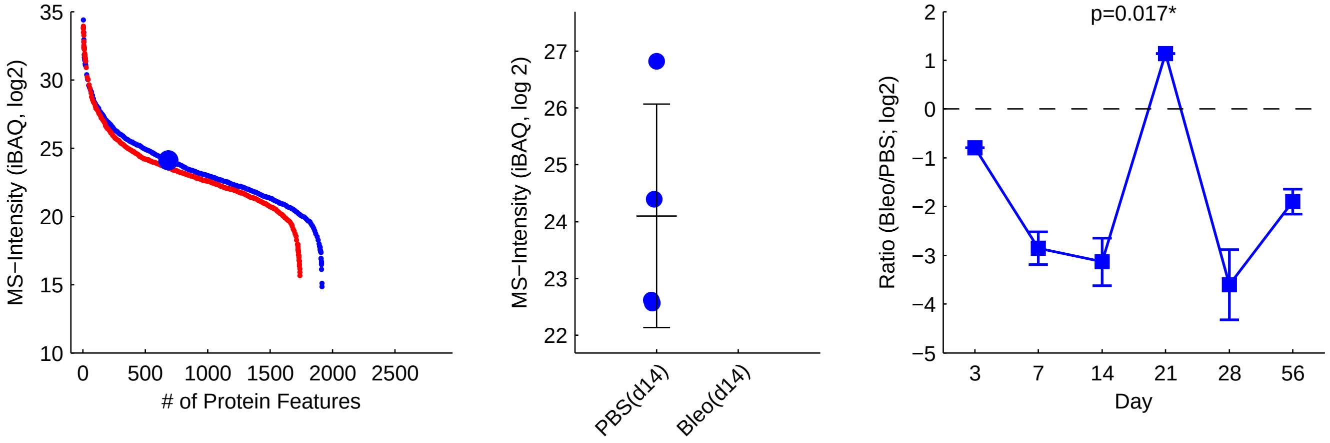

Q99LD4 − Gps1 (id: 110)

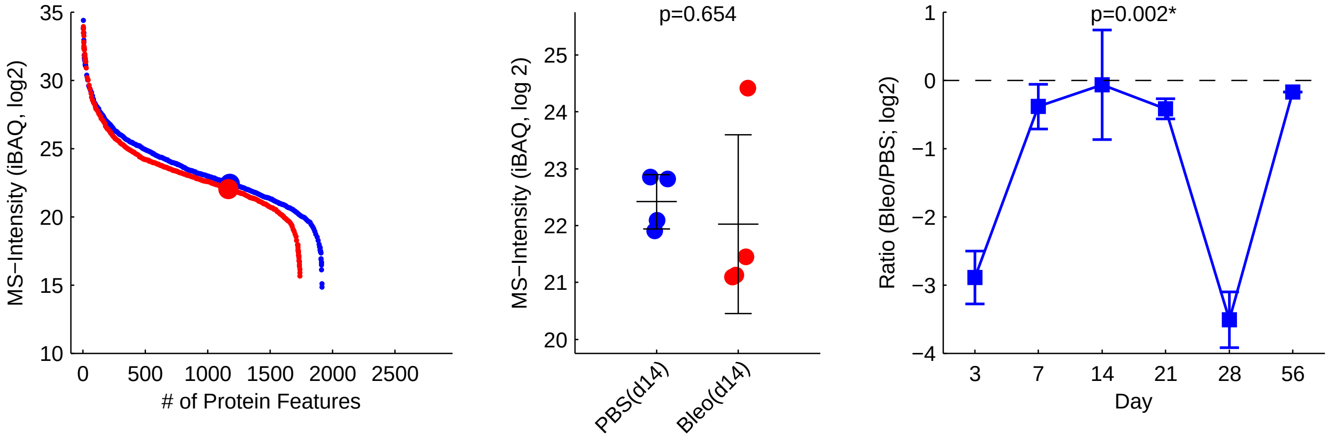

B1AWE0 − Clta (id: 122)

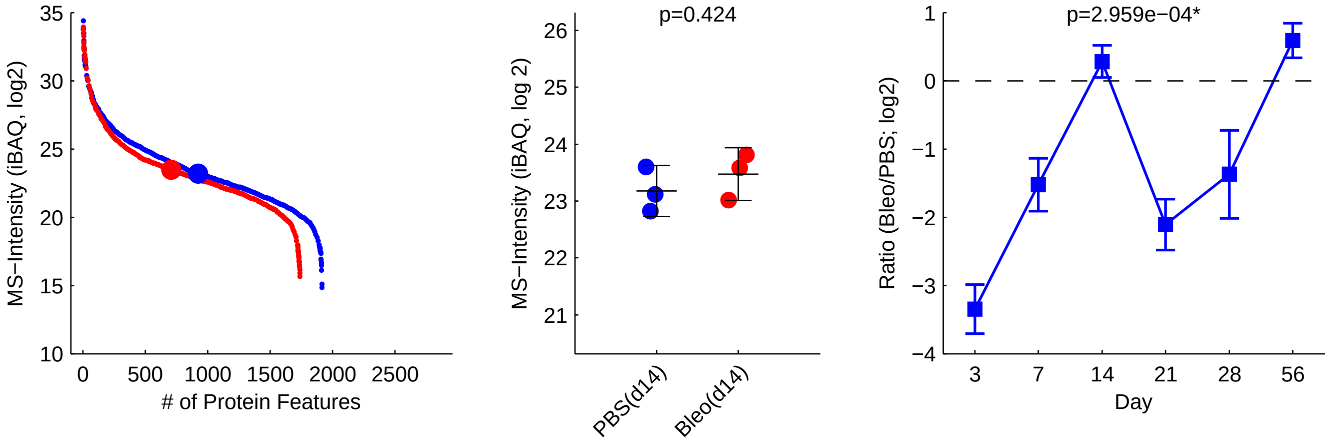

Q8BKX1−3 − Baiap2 (id: 128)

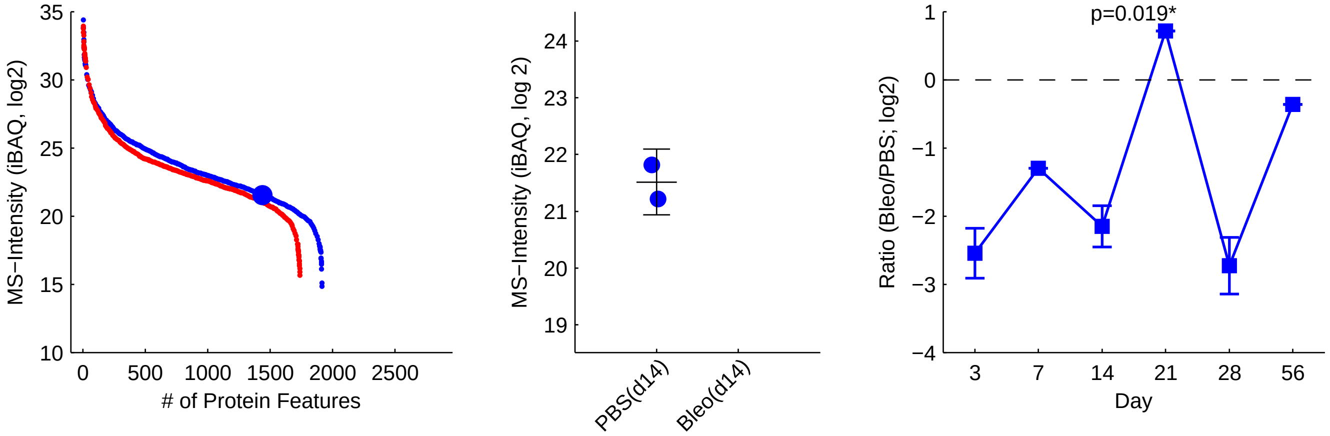

B1AZS9 − Prdx4 (id: 130)

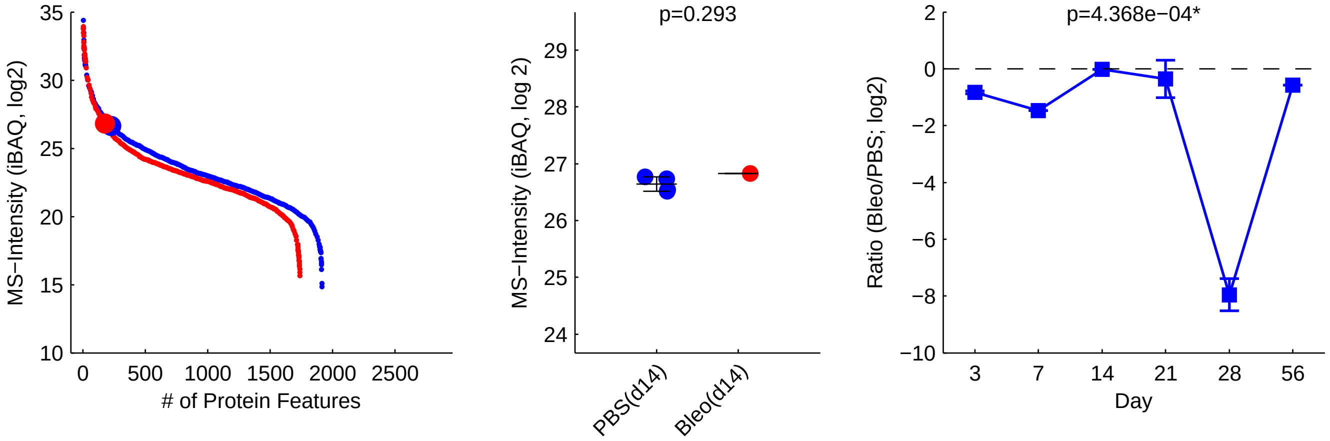

P61979−3 − Hnrnpk (id: 136)

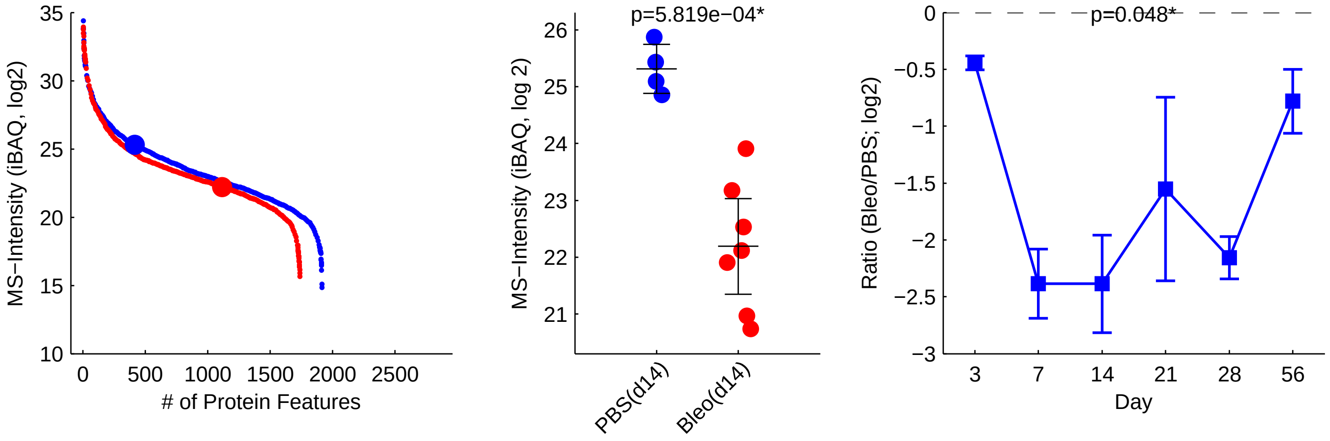

B2RXS4 − Plxnb2 (id: 142)

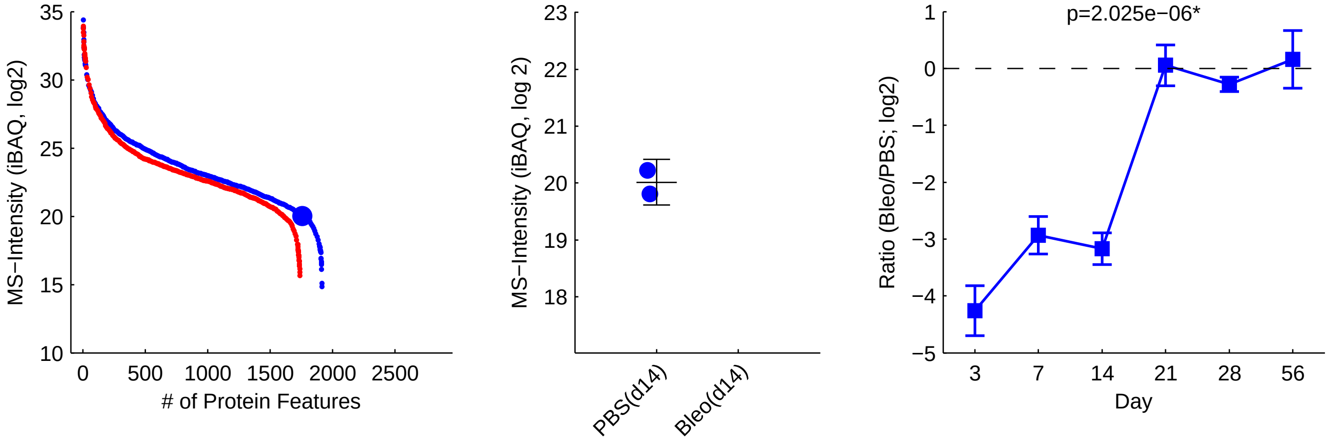

B5THE2 − Mgam (id: 145)

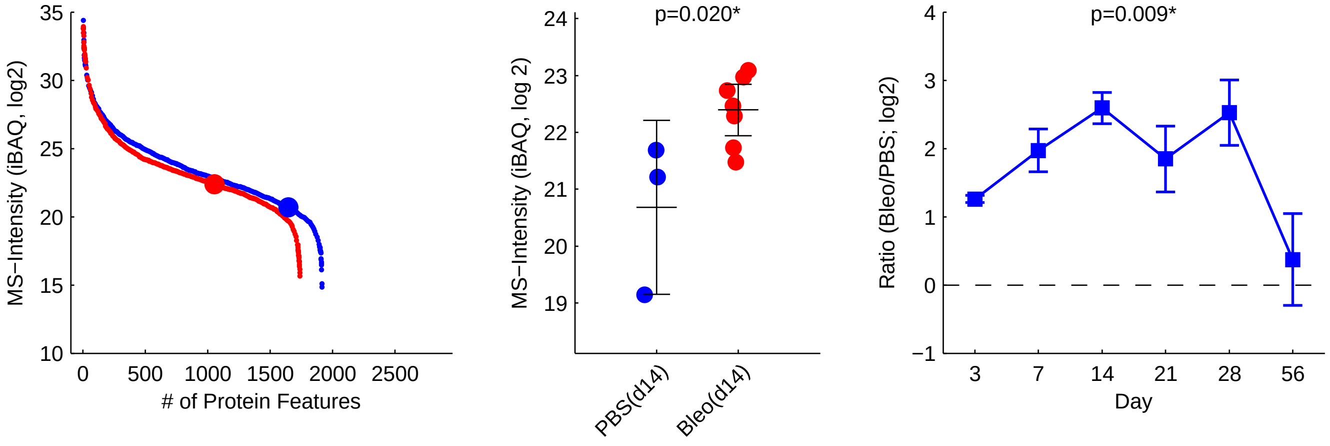

B8JJN0 − Gm20547 (id: 158)

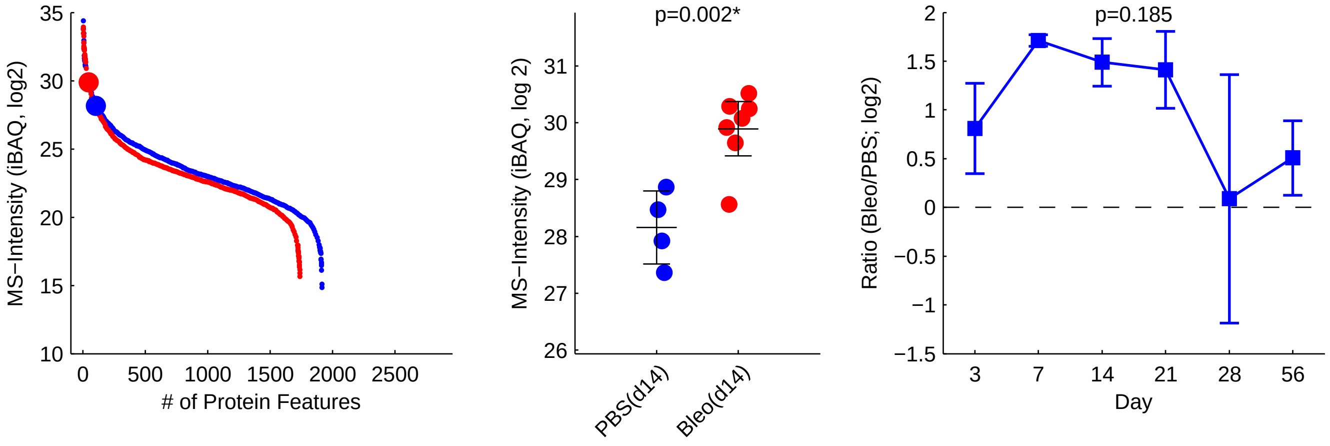

B8JK33 − Hnrnpm (id: 159)

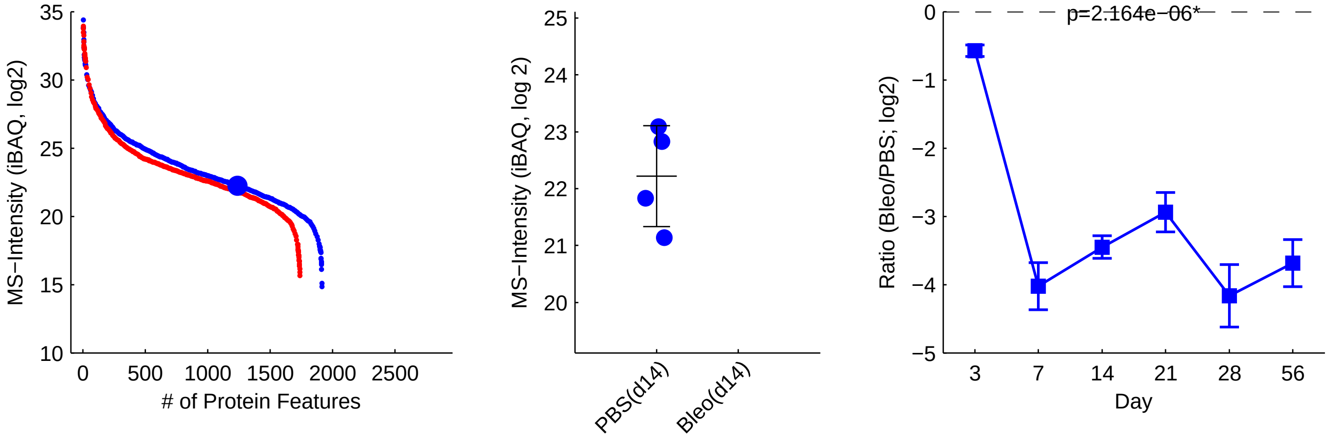

CON__P07477 − Tmprss13 (id: 177)

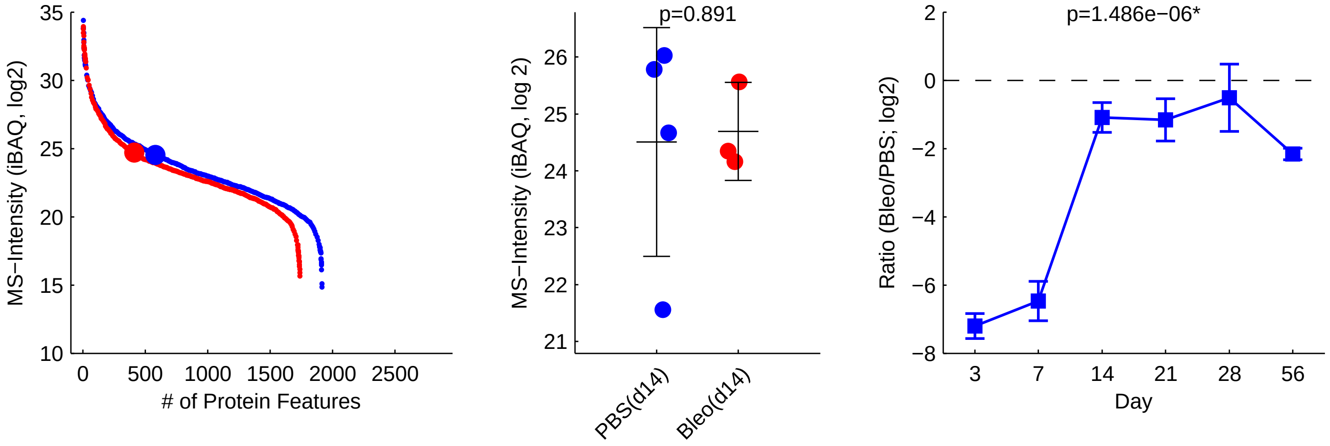

CON__P15636 − (id: 184)

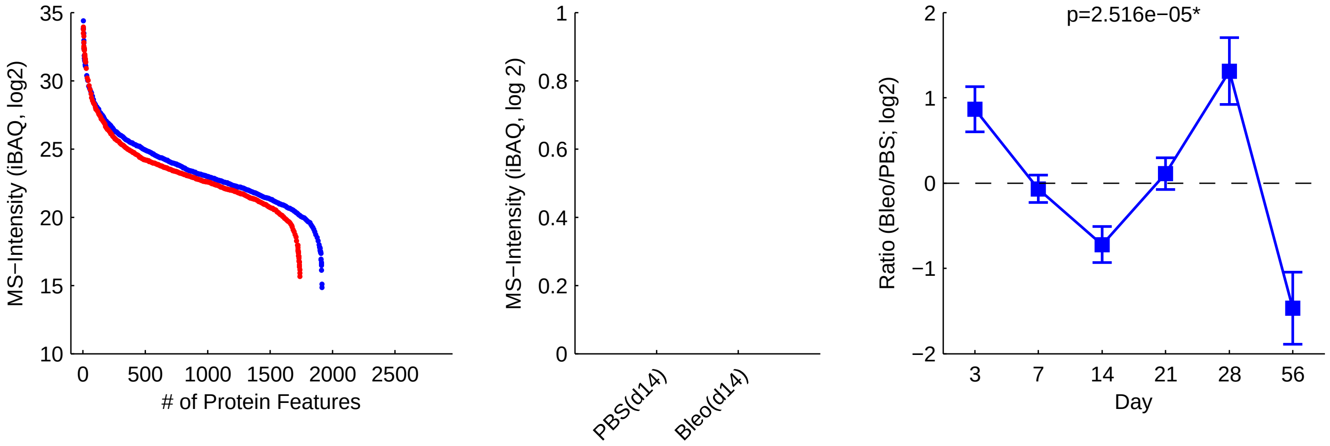

P60710 − Actb (id: 190)

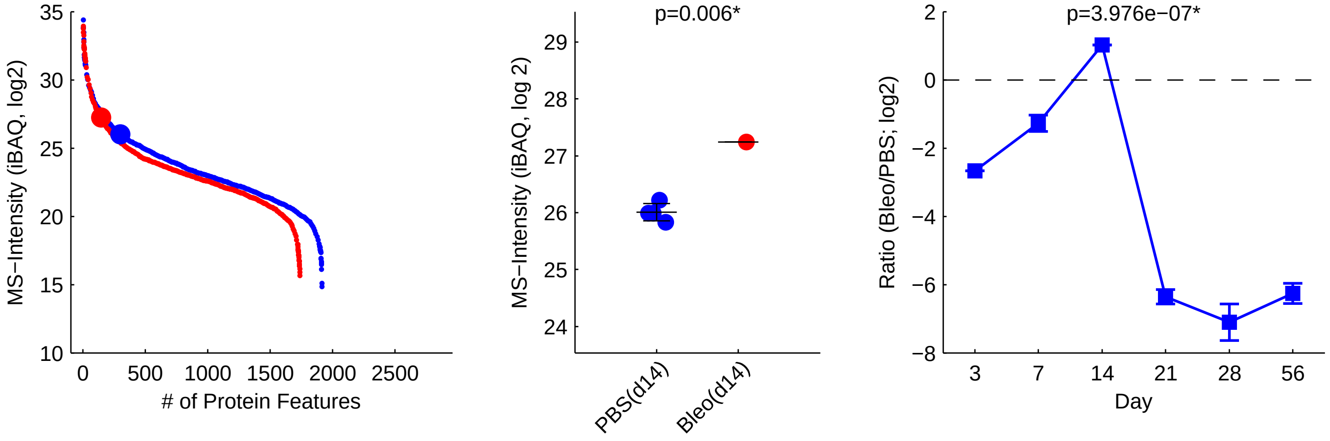

Q3UH68 − Limch1 (id: 211)

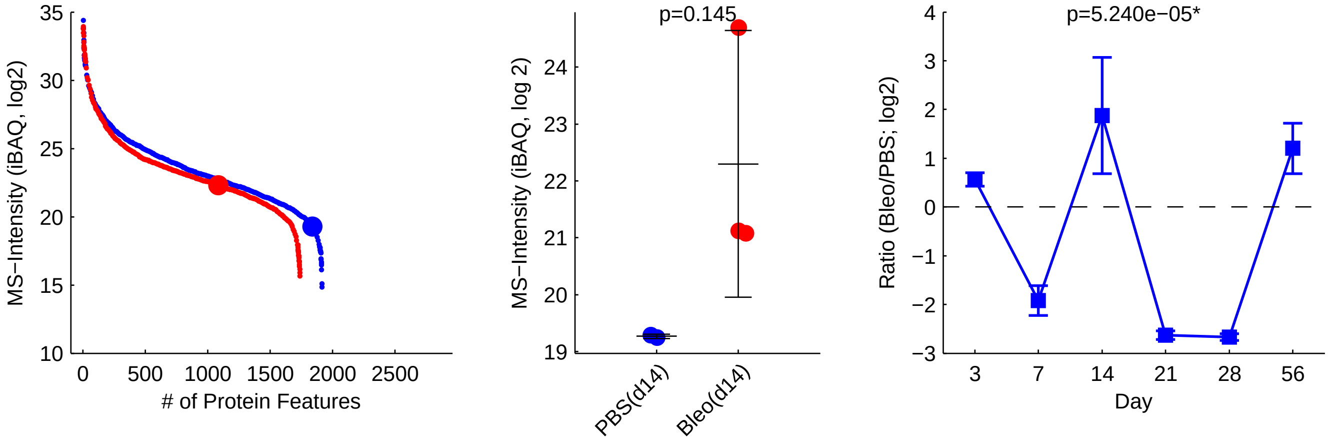

D3YU60 − Mgst1 (id: 212)

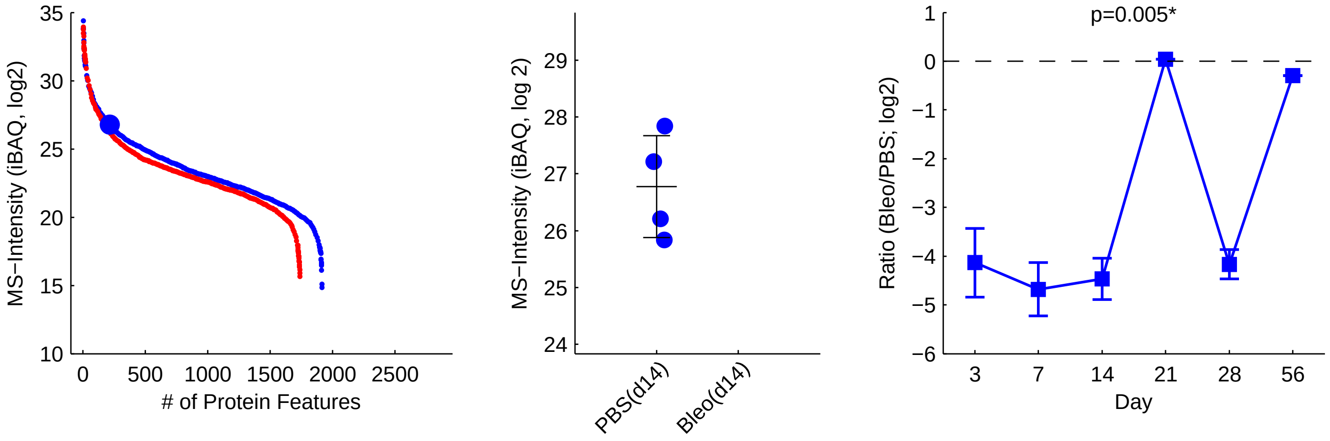

P12970 − Rpl7a (id: 219)

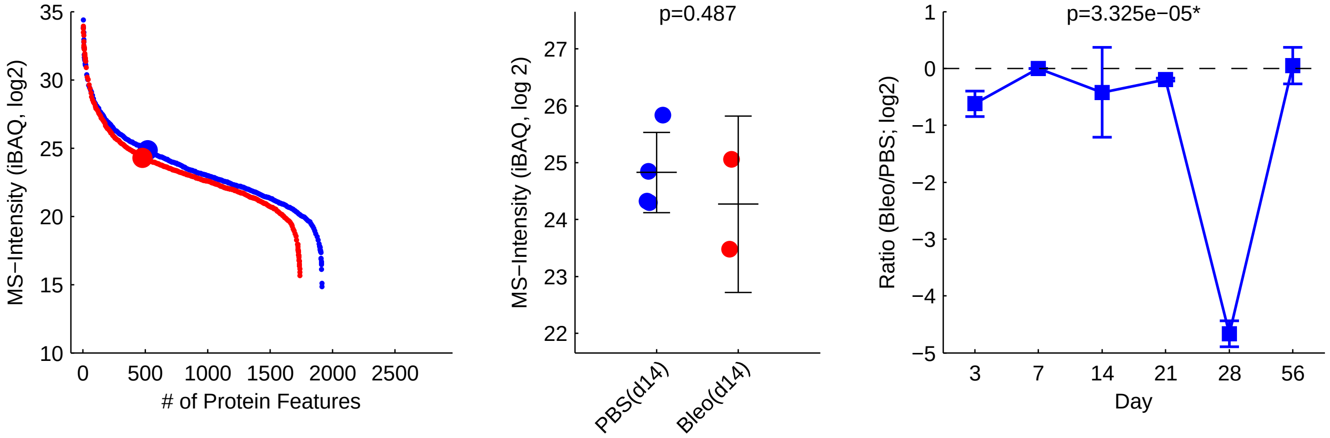

Q8BFR5 − Tufm (id: 224)

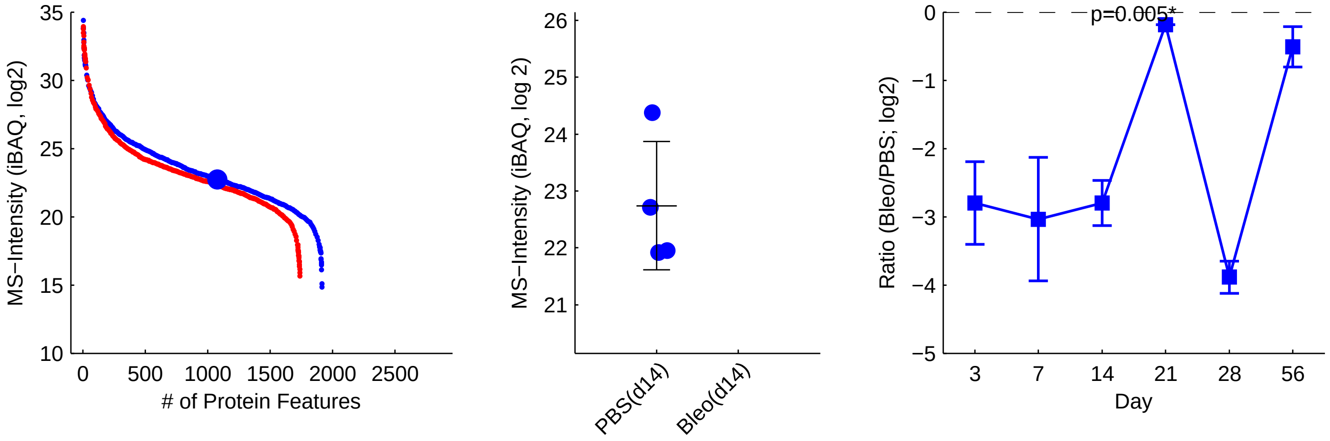

D3YWF6 − Otub1 (id: 230)

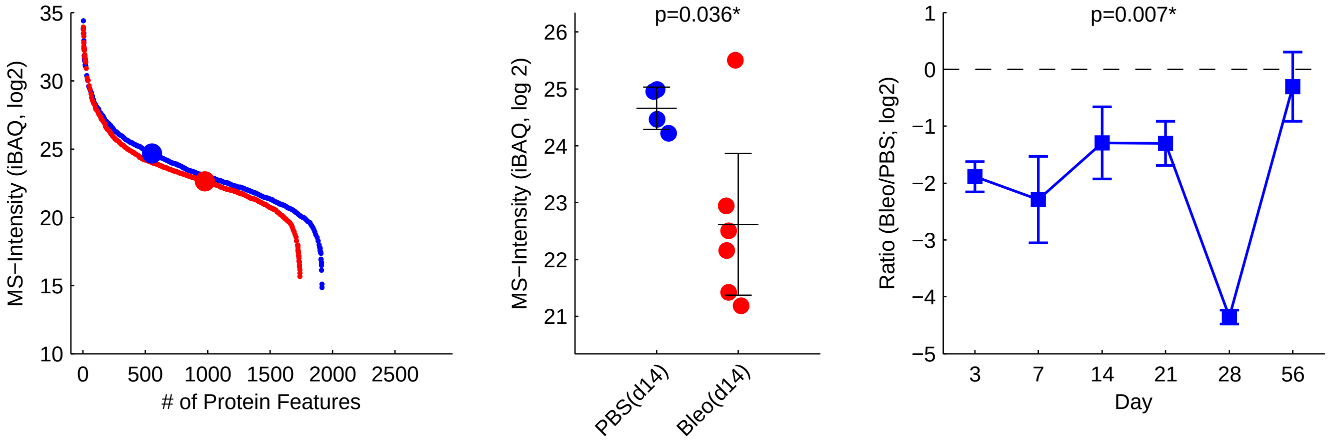

P62751 − Rpl23a (id: 232)

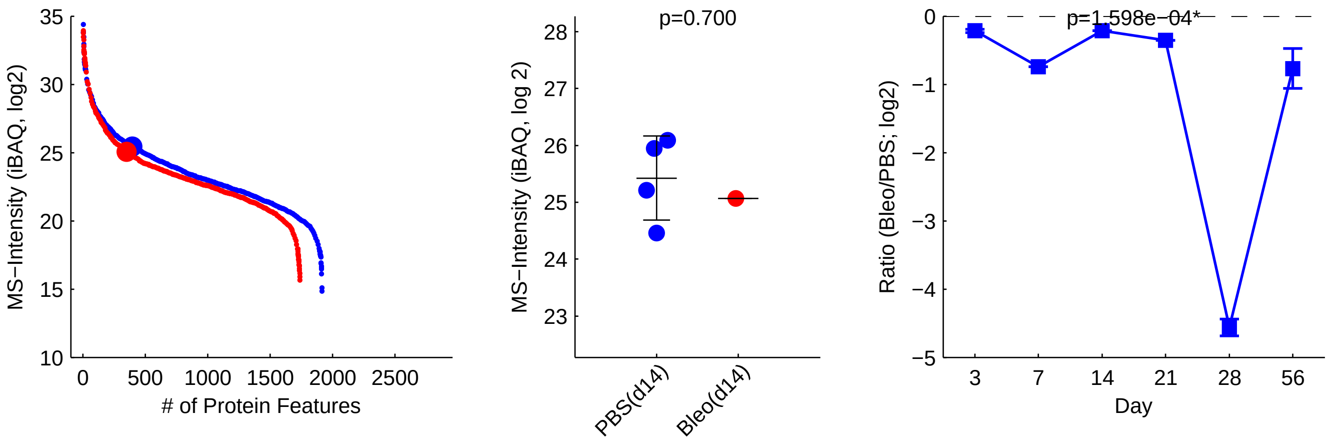

D3YX54 − Rpl13−ps3 (id: 234)

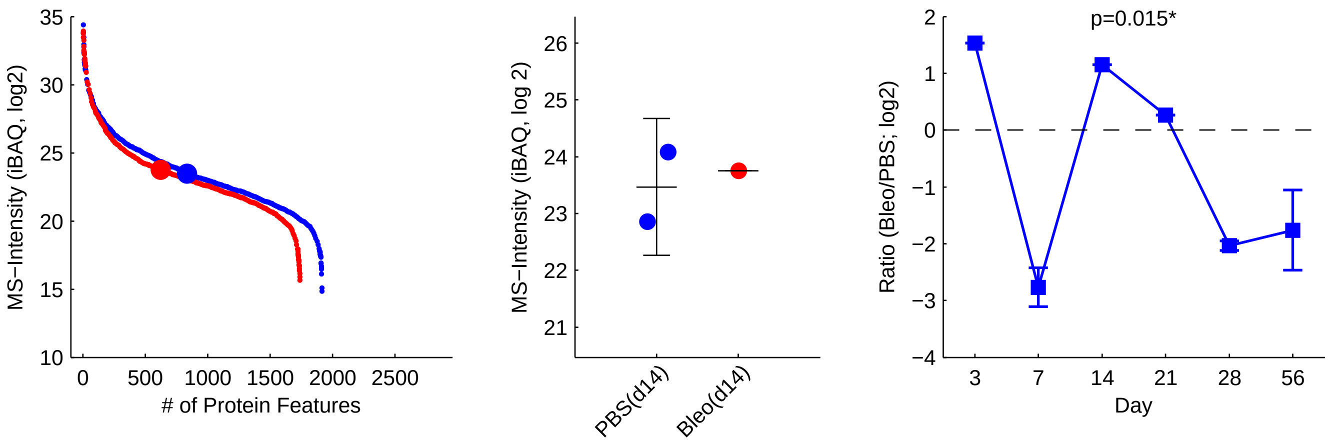

D3YYE1 − Anp32a (id: 246)

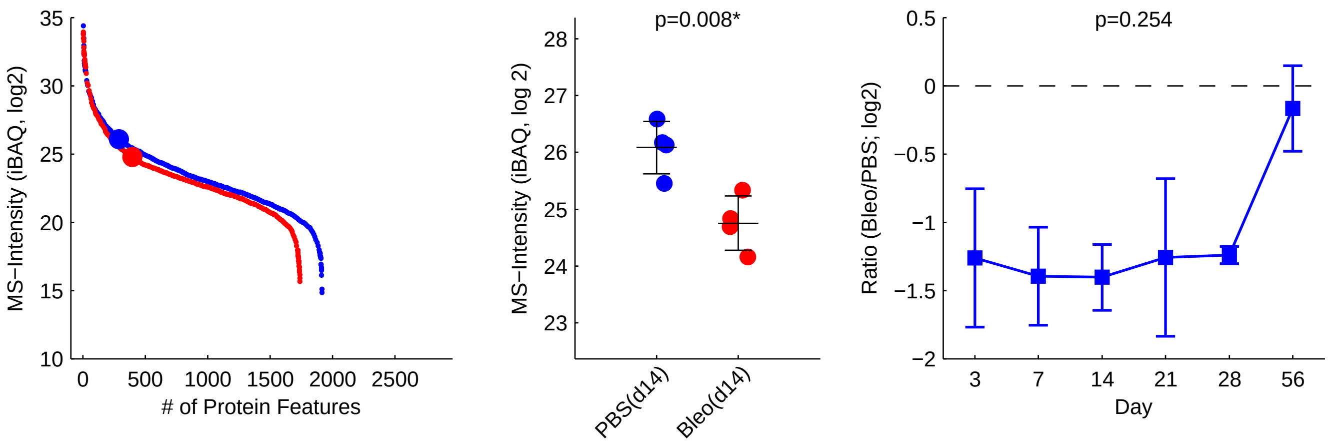

D3Z0J2 − Cav1 (id: 259)

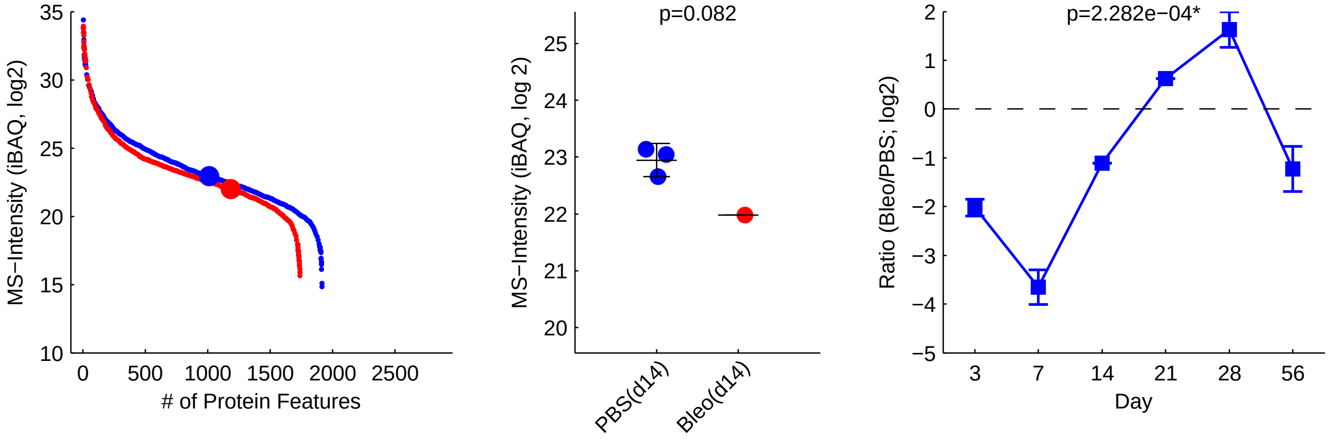

Q924U4 − Cav2 (id: 266)

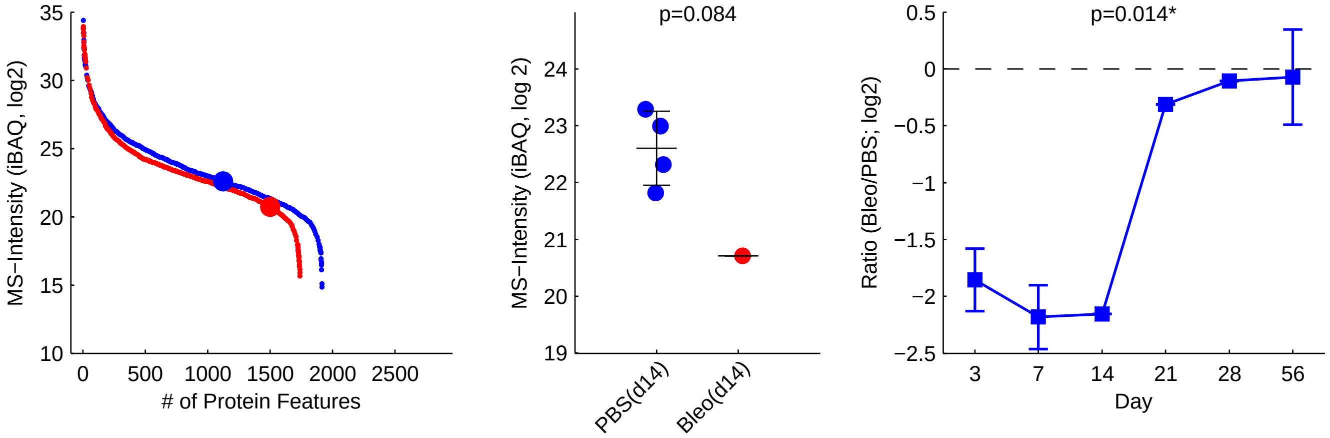

D3Z2P2 − Chi3l1 (id: 273)

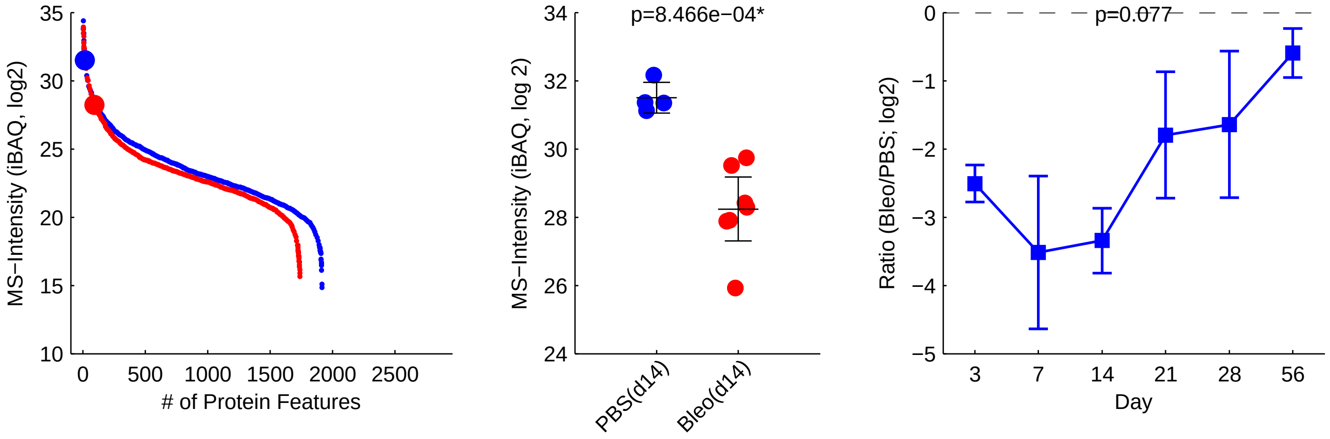

D3Z4D6 − Mnf1 (id: 282)

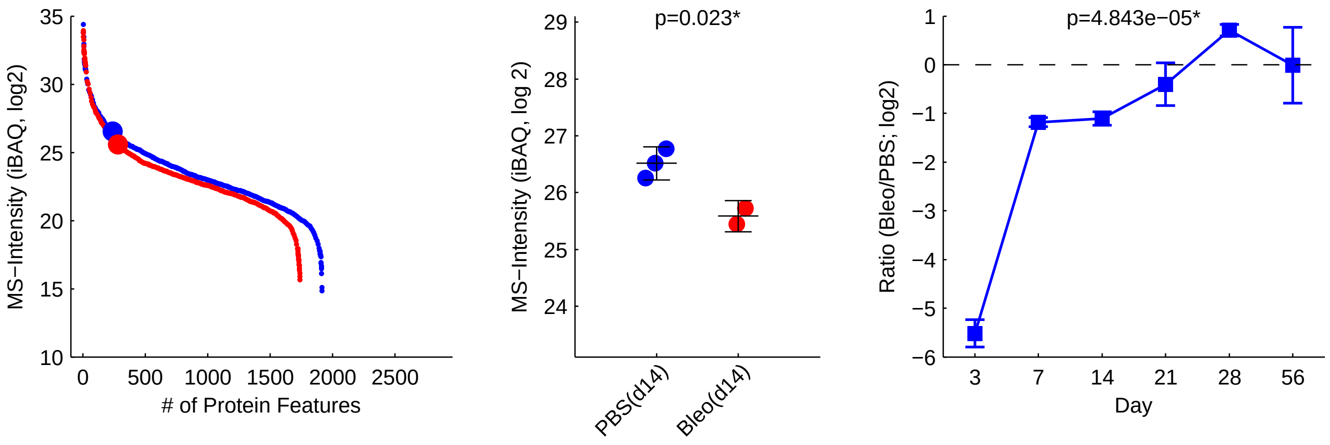

D3Z5G7 − Ces1b (id: 285)

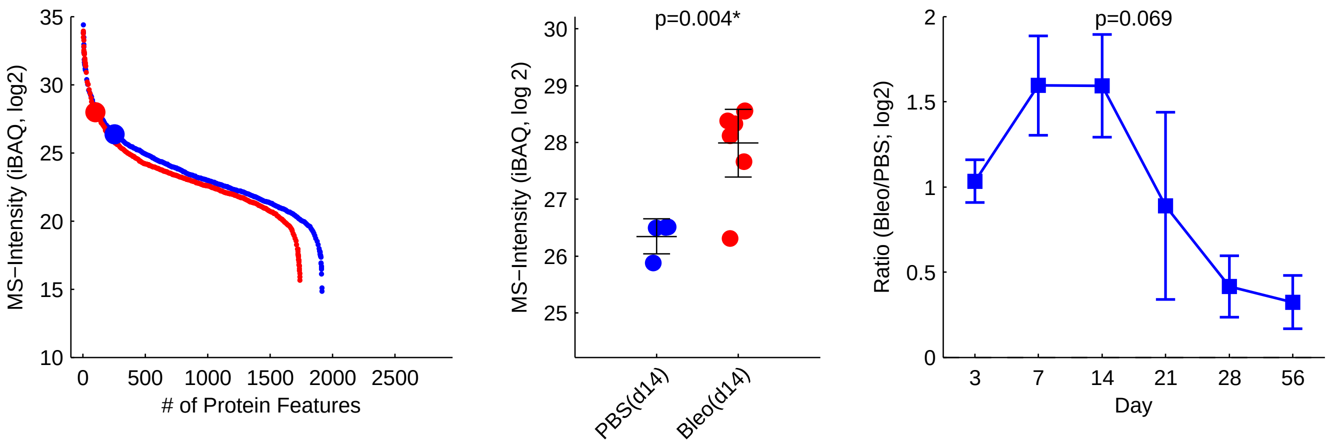

P97351 − Rps3a (id: 289)

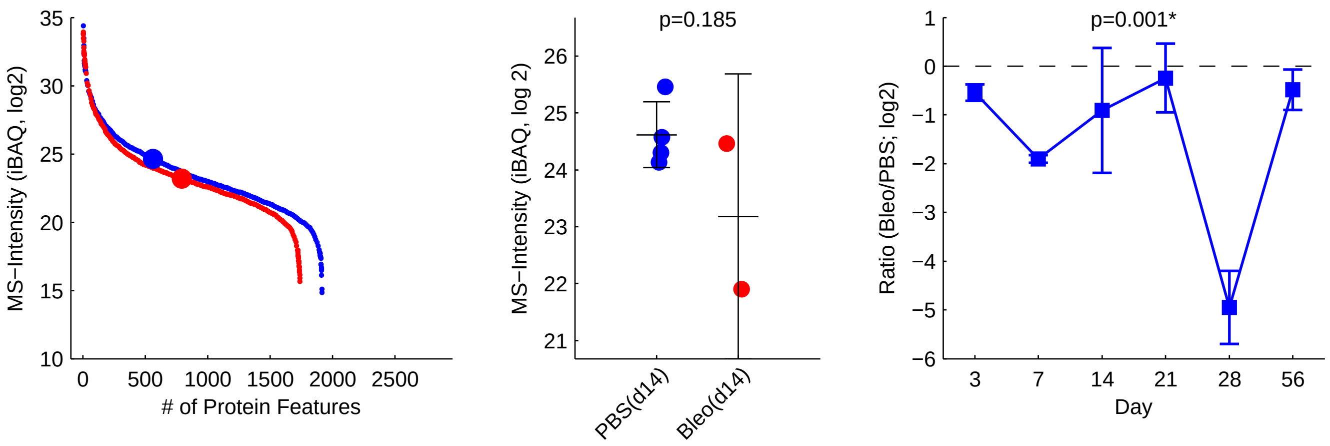

H7BWX9 − Sumo2 (id: 298)

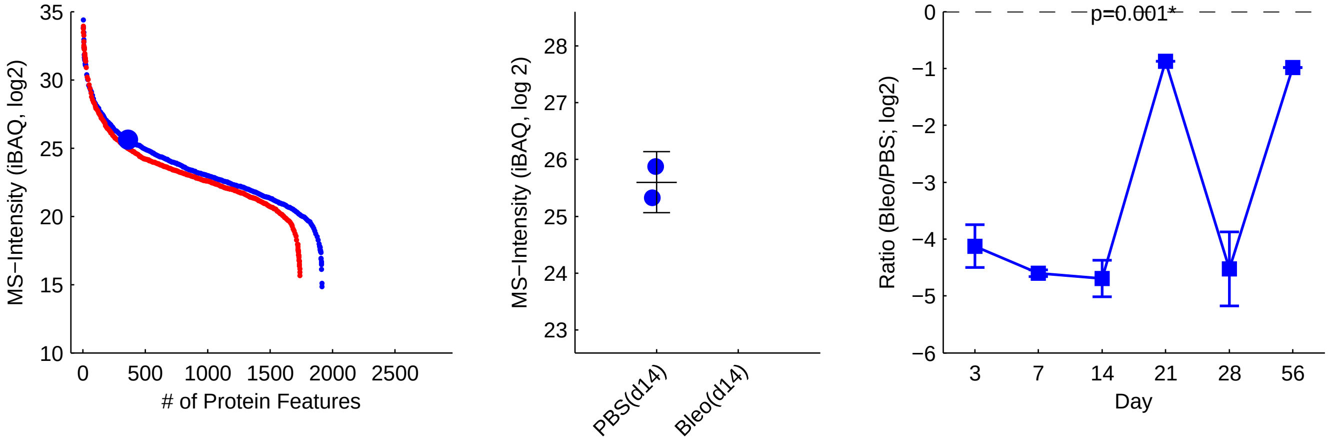

D3Z7E6 − Pafah1b3 (id: 301)

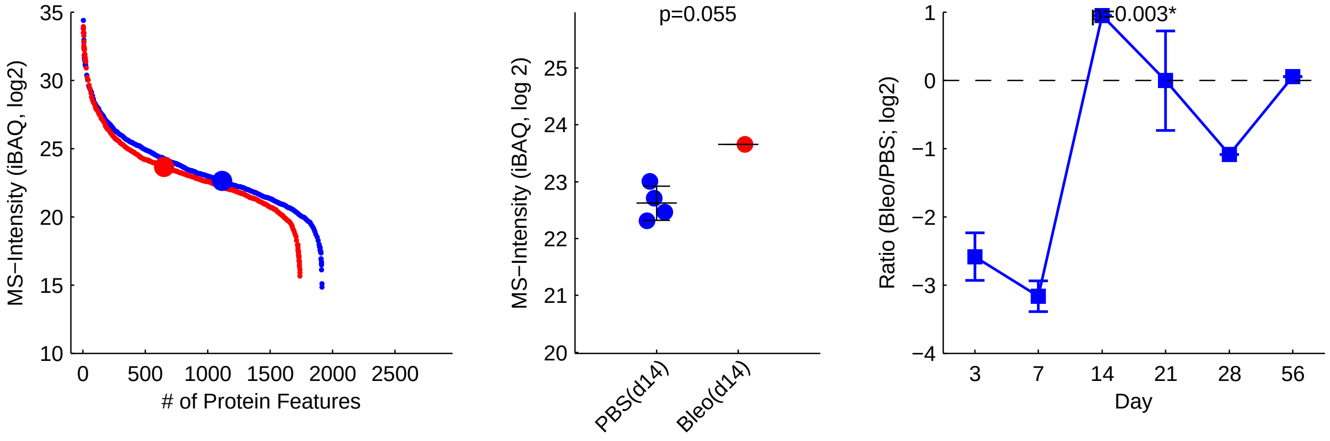

D5MCW4 − Cuta (id: 306)

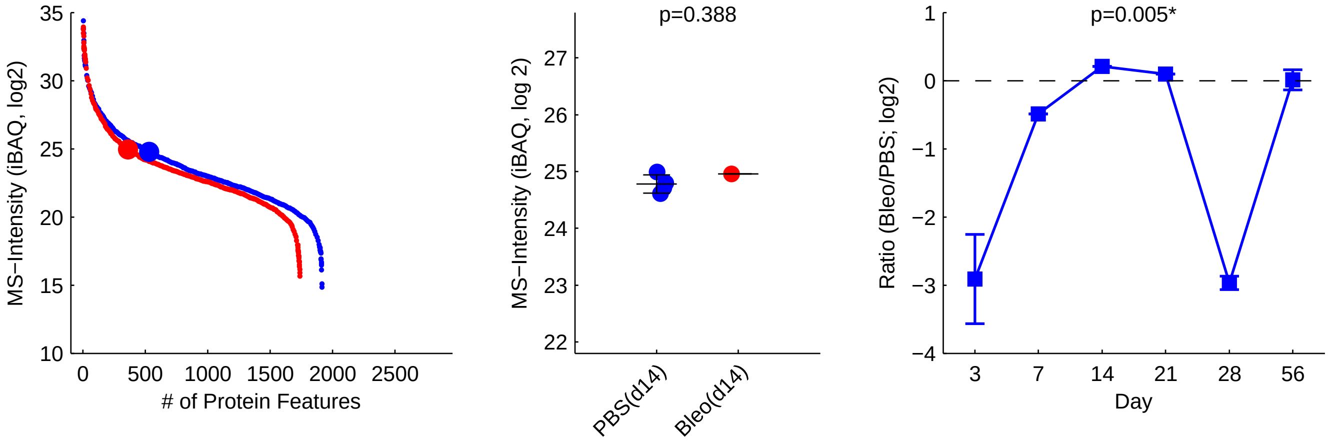

D6RJK5 − Fgfr2 (id: 312)

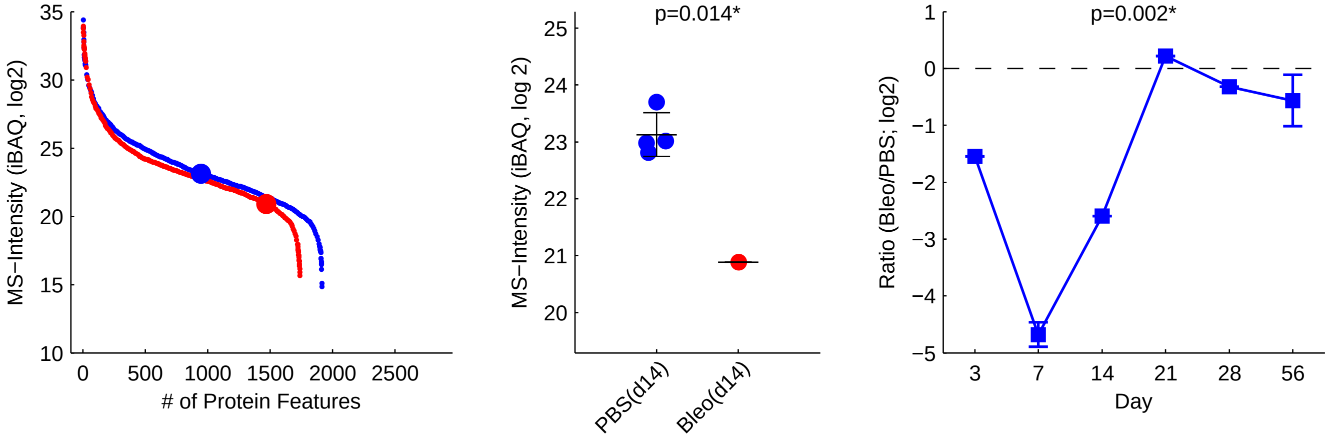

Q8R5M8−5 − Cadm1 (id: 320)

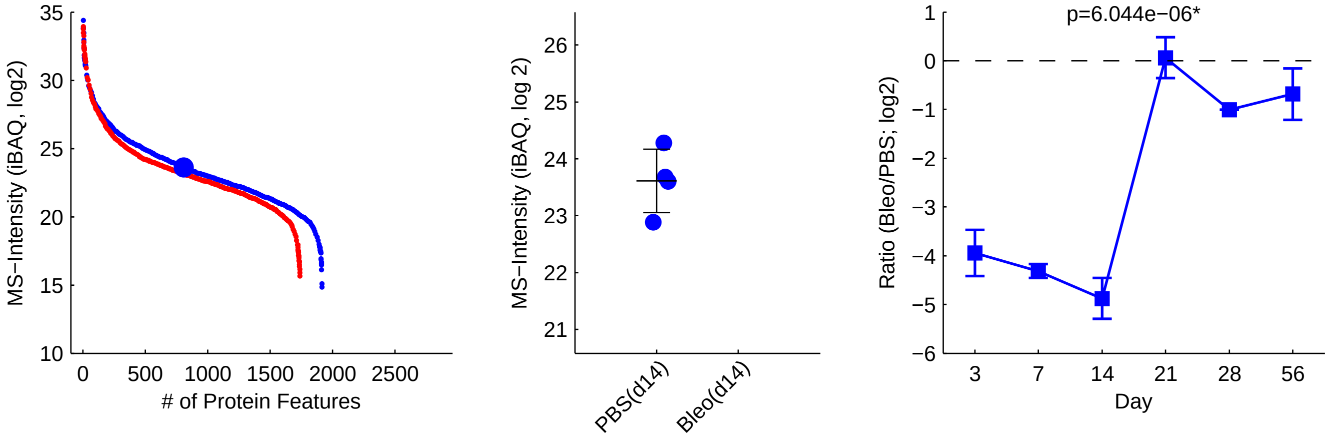

Q62393−2 − Tpd52 (id: 327)

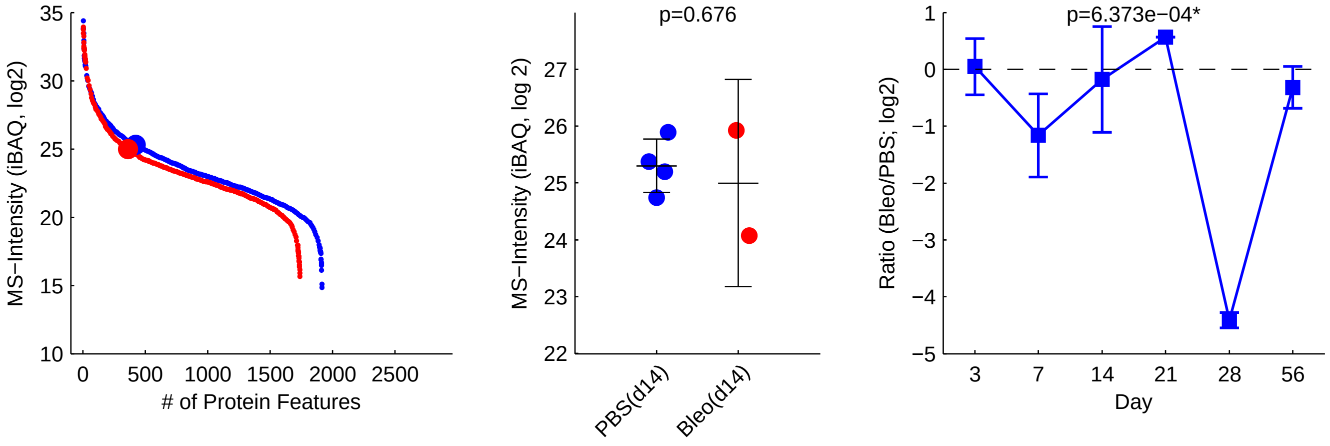

Q61210−3 − Arhgef1 (id: 332)

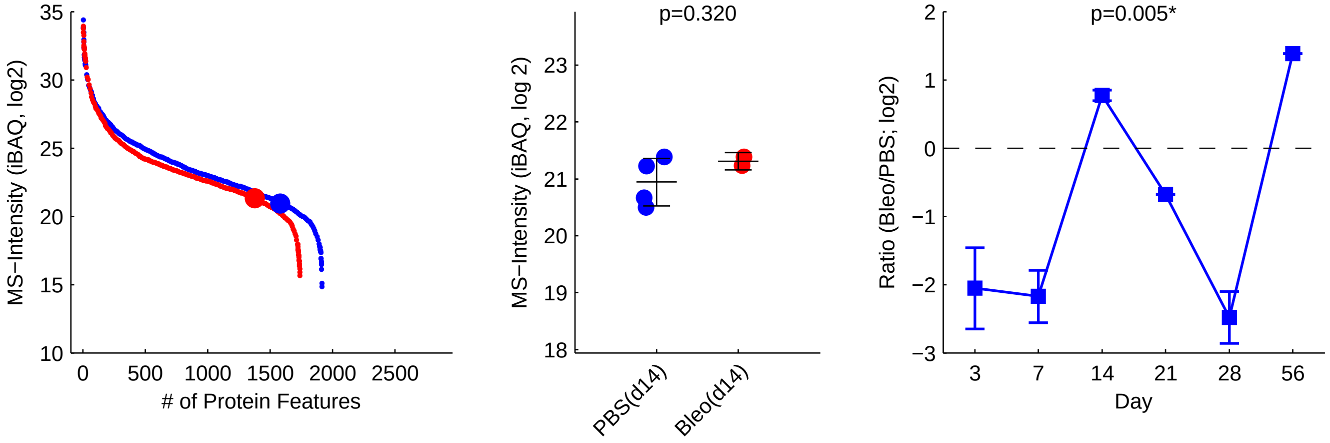

F8WGT1 − Ahcyl2 (id: 338)

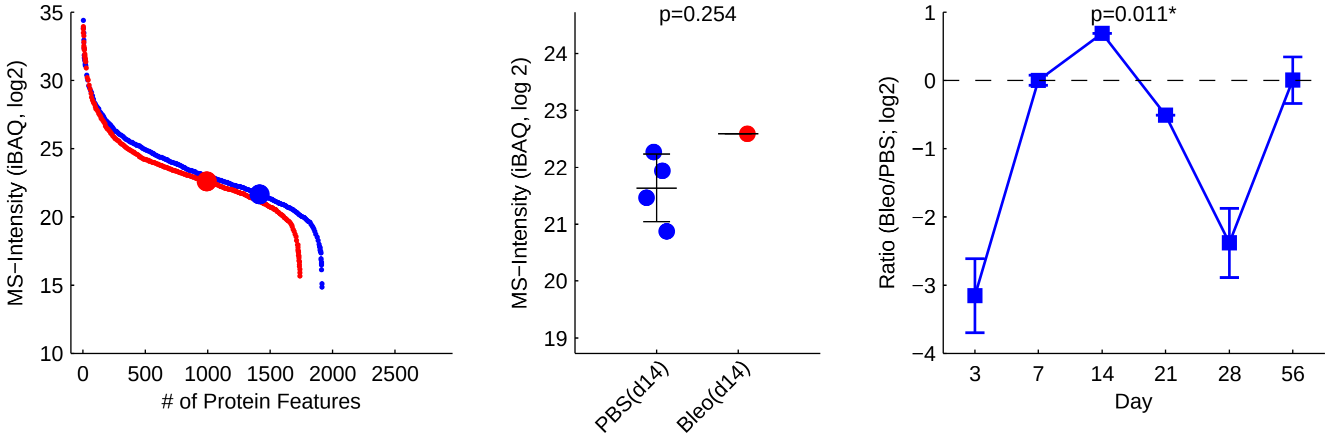

E9PV24 − Fga (id: 340)

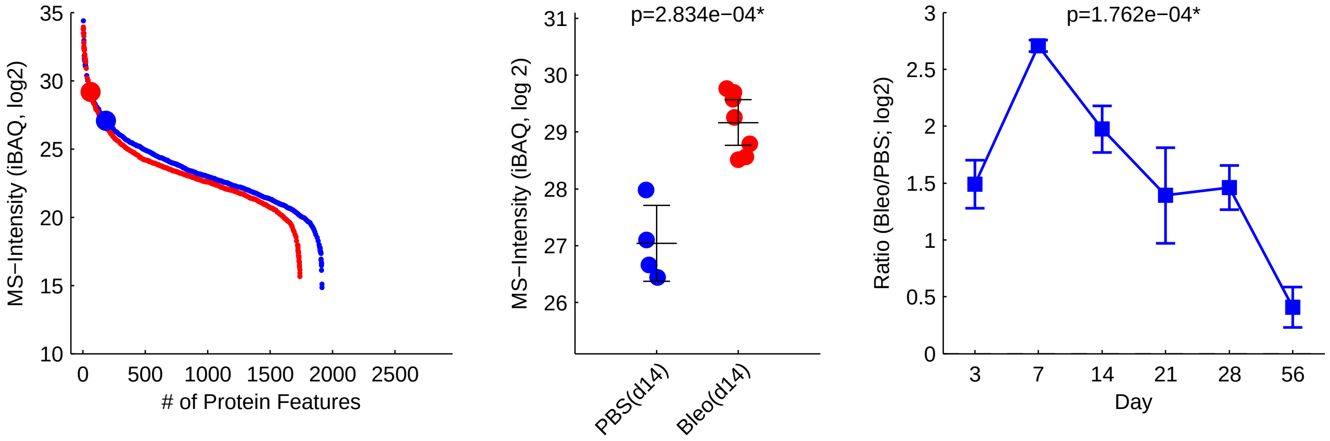

E9PVD2 − Itih4 (id: 344)

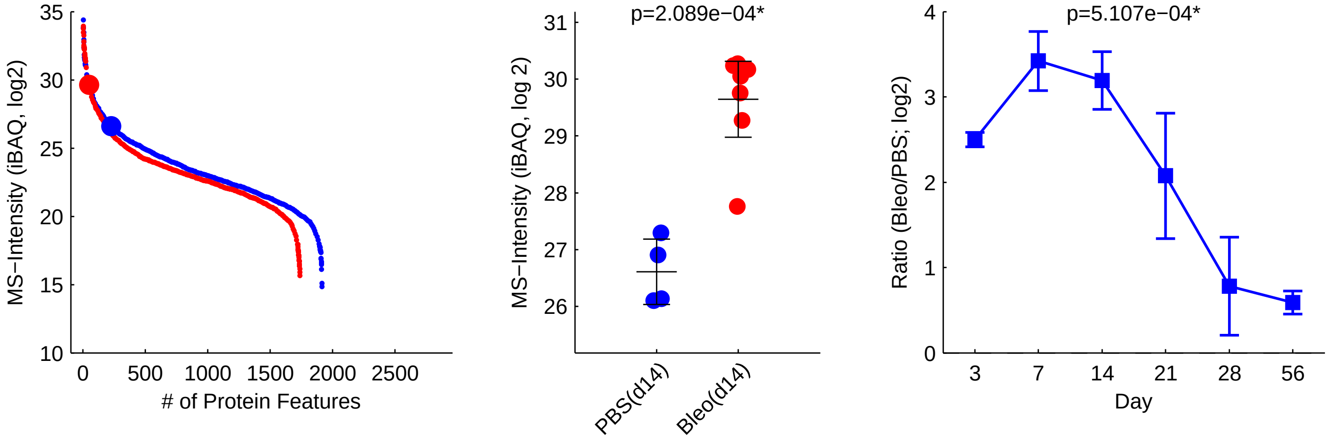

G3UW37 − Stard10 (id: 346)

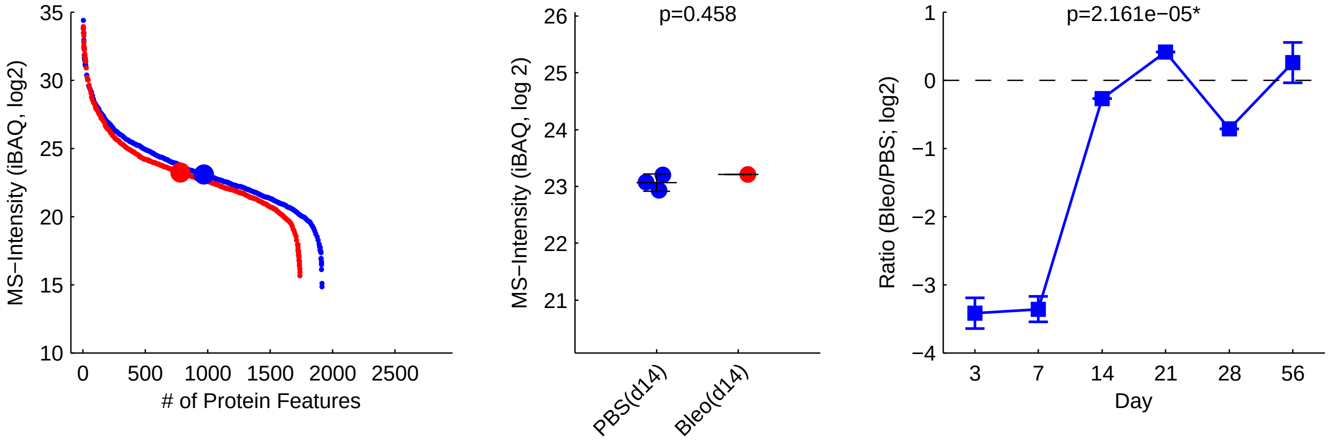

E9PWF0 − Thbs3 (id: 353)

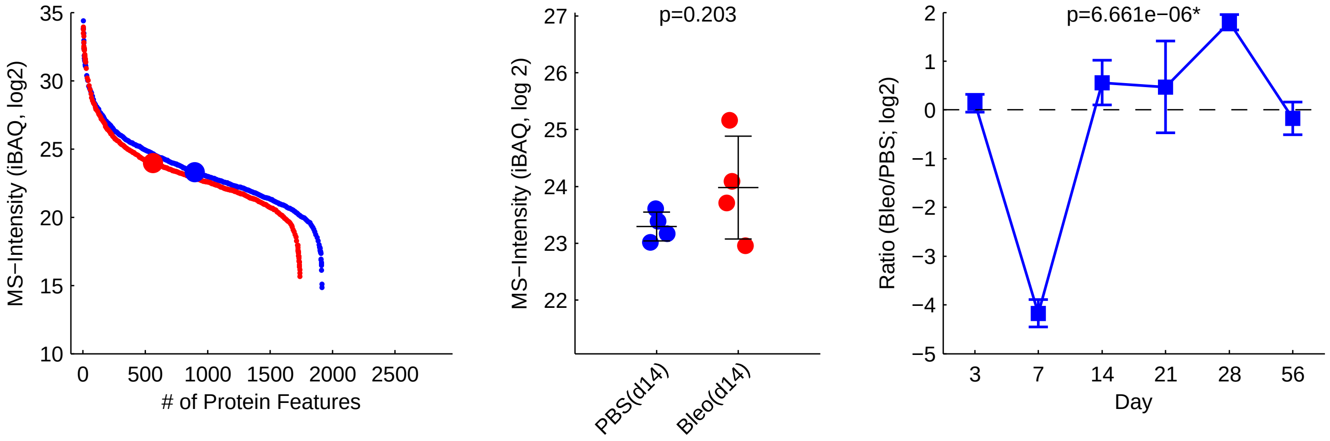

E9PWG9 − Map3k5 (id: 355)

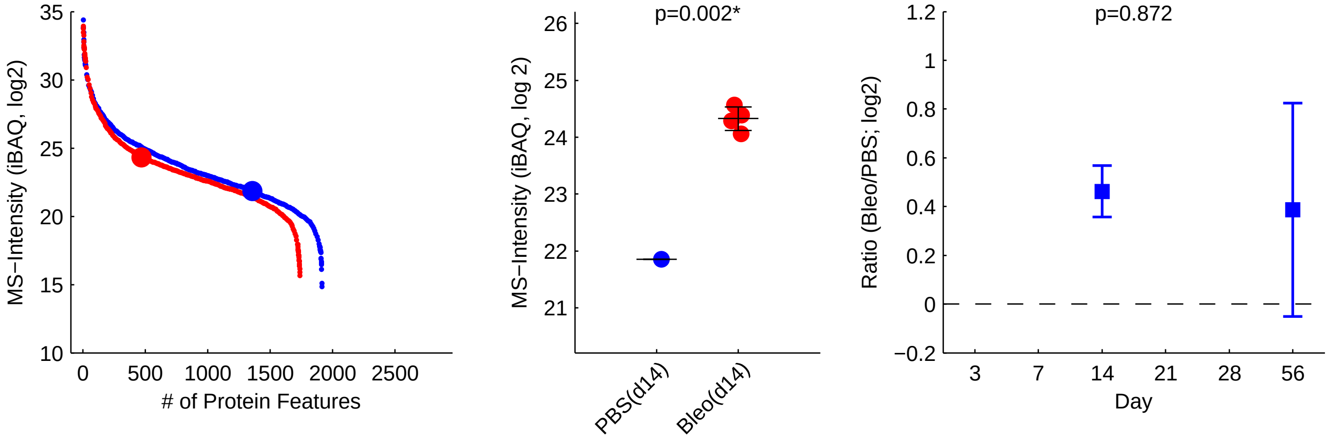

E9PWQ3 − Col6a3 (id: 357)

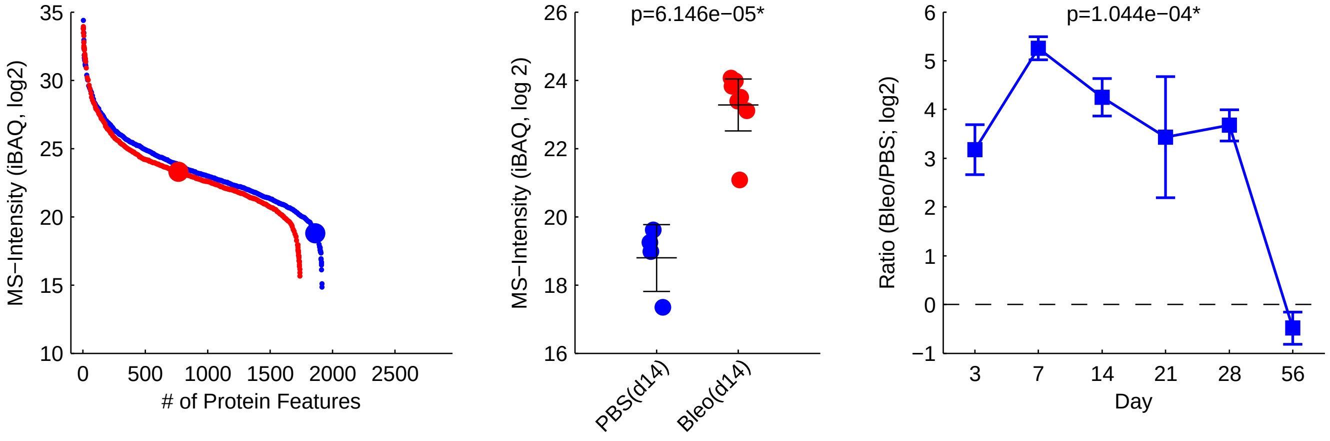

E9PX70 − Col12a1 (id: 362)

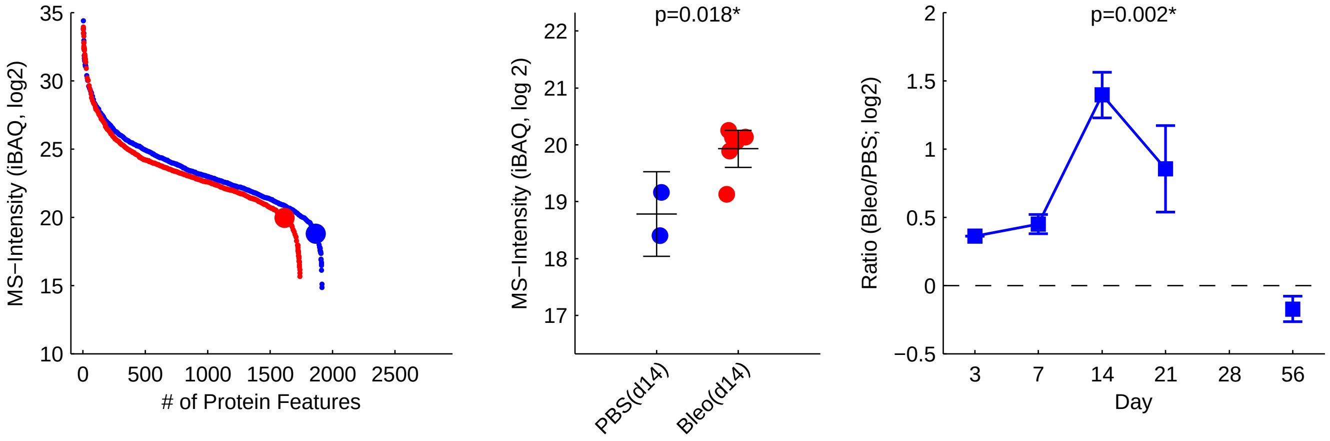

E9PXX7 − Txndc5 (id: 368)

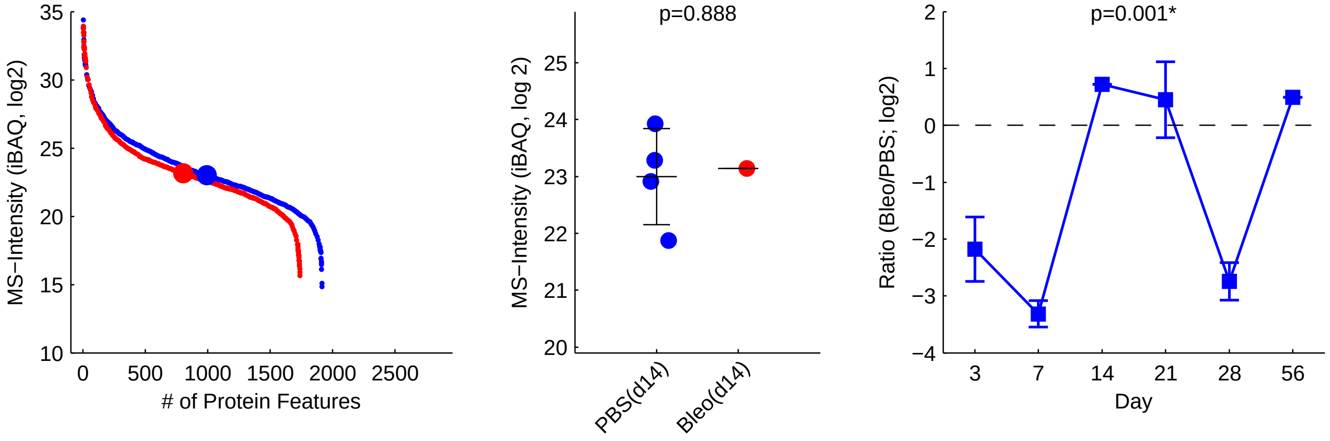

F7CVJ5 − Ahnak2 (id: 372)

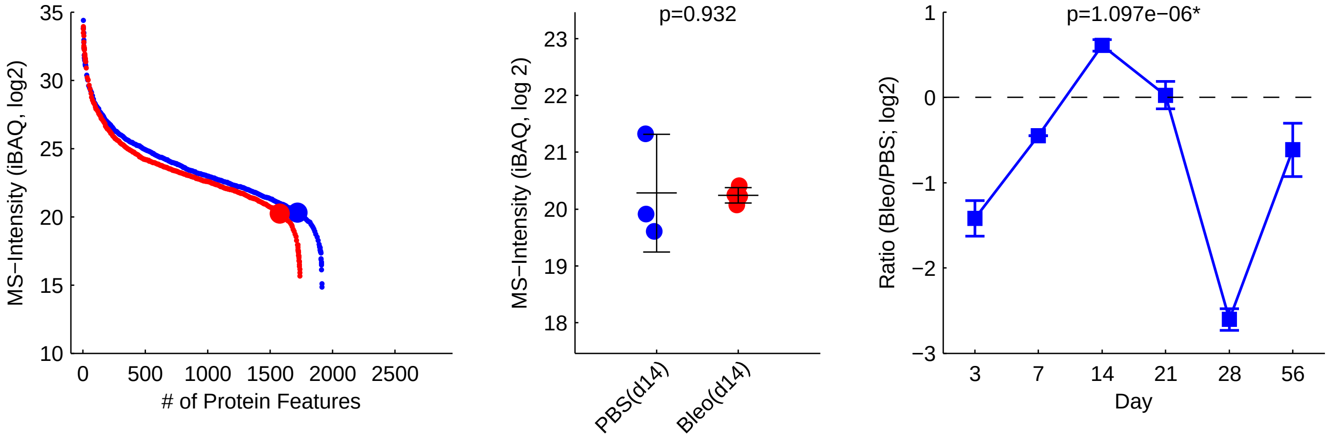

Q9CXW4 − Rpl11 (id: 375)

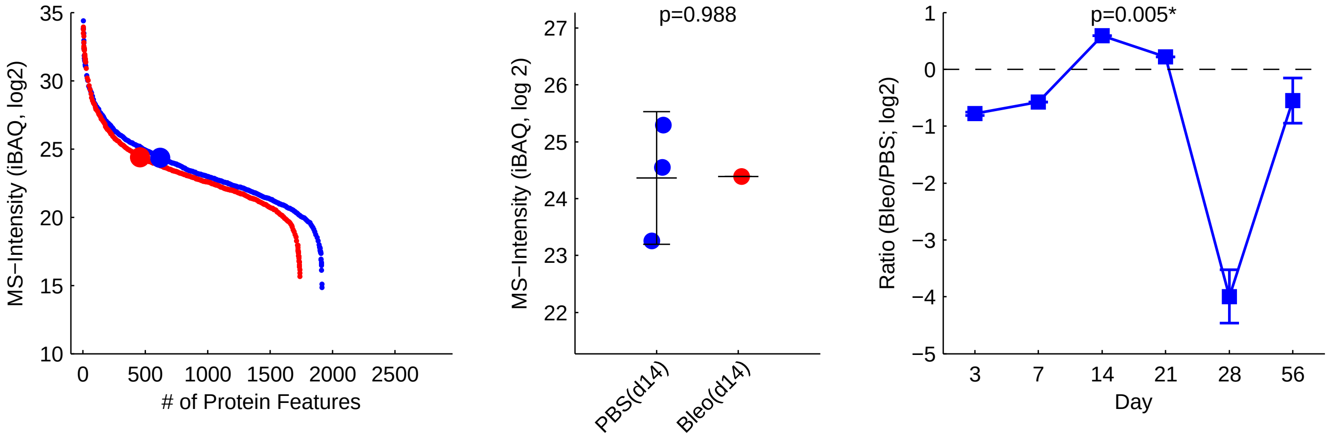

Q61147 − Cp (id: 381)

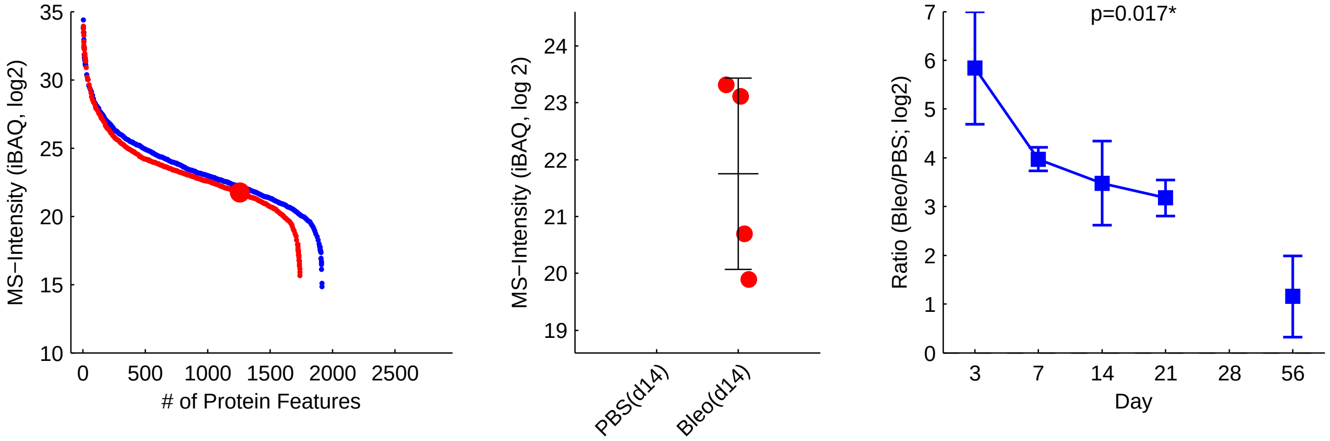

E9PZI9 − Cd200 (id: 383)

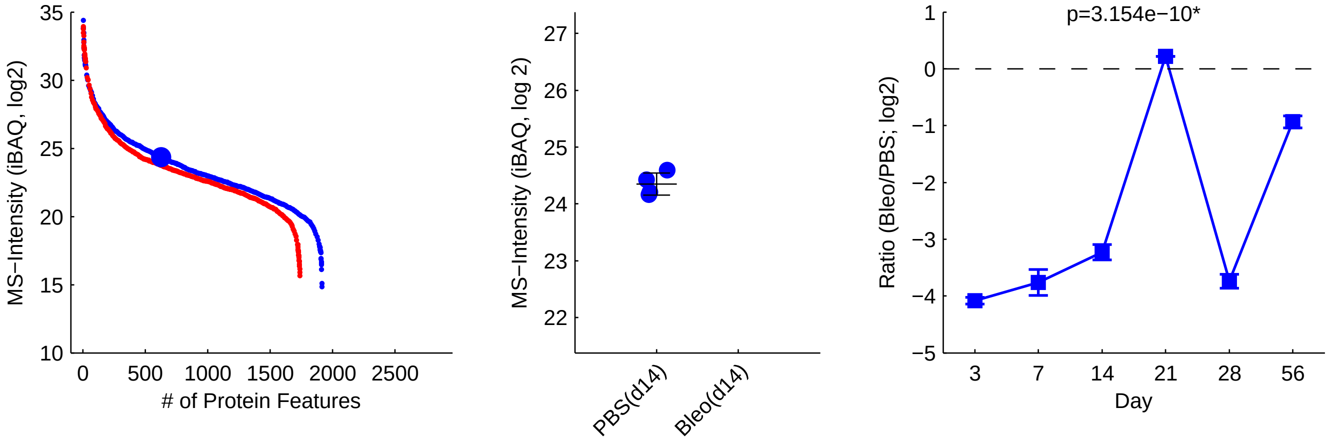

Q3V1J8 − Habp2 (id: 386)

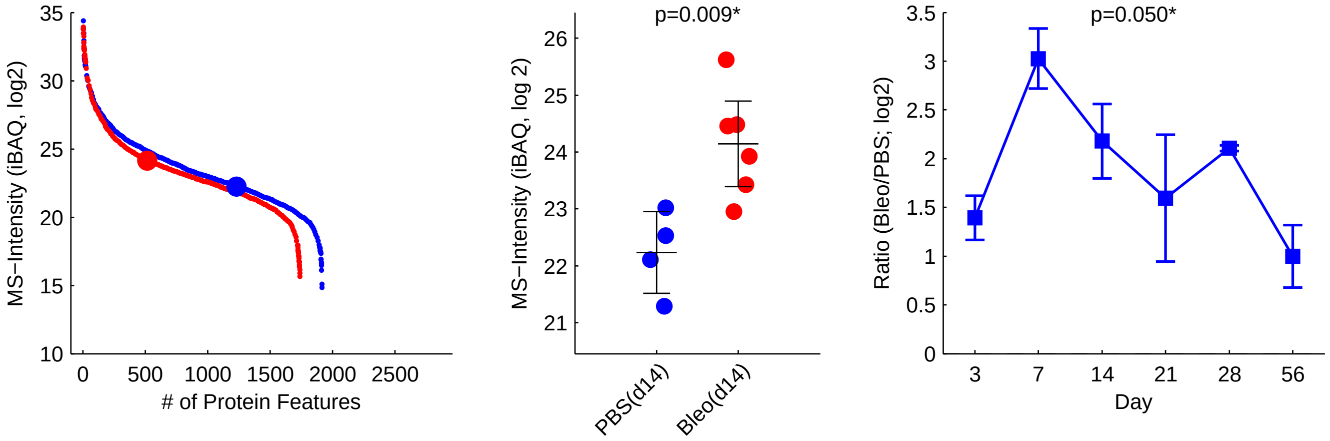

E9Q0W5 − Fam3c (id: 391)

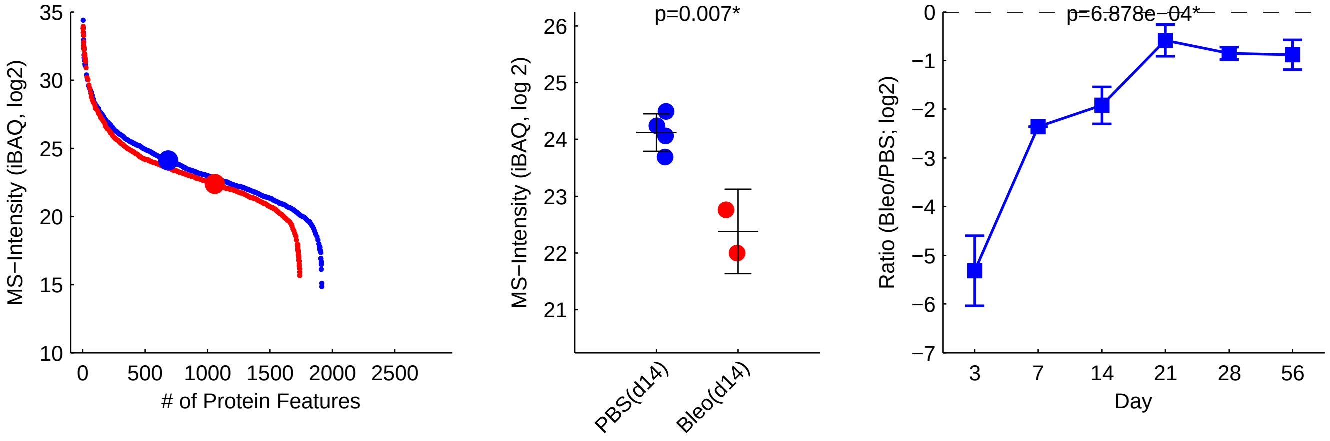

E9Q132 − Rpl24 (id: 392)

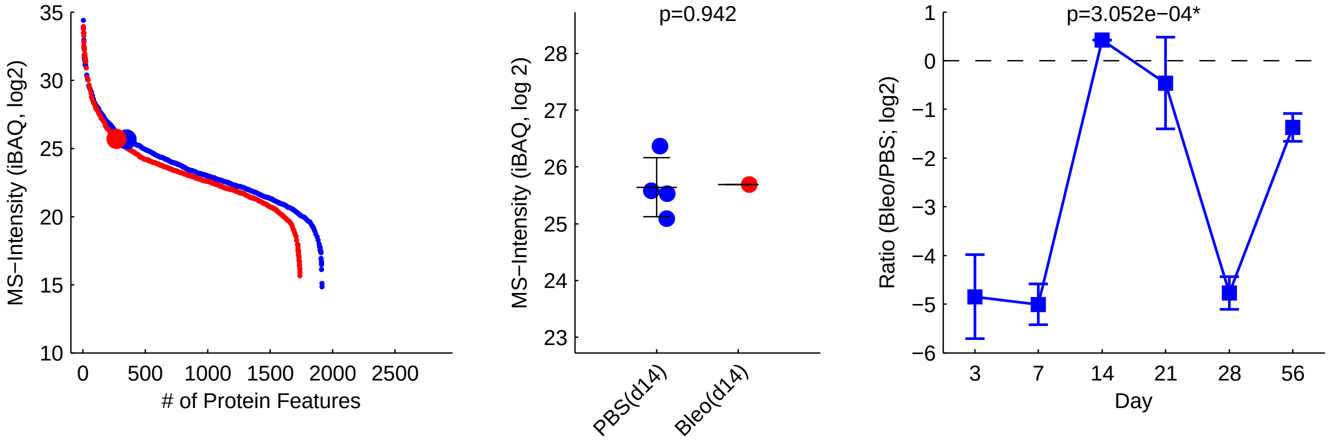

E9Q1F5 − Myo5c (id: 394)

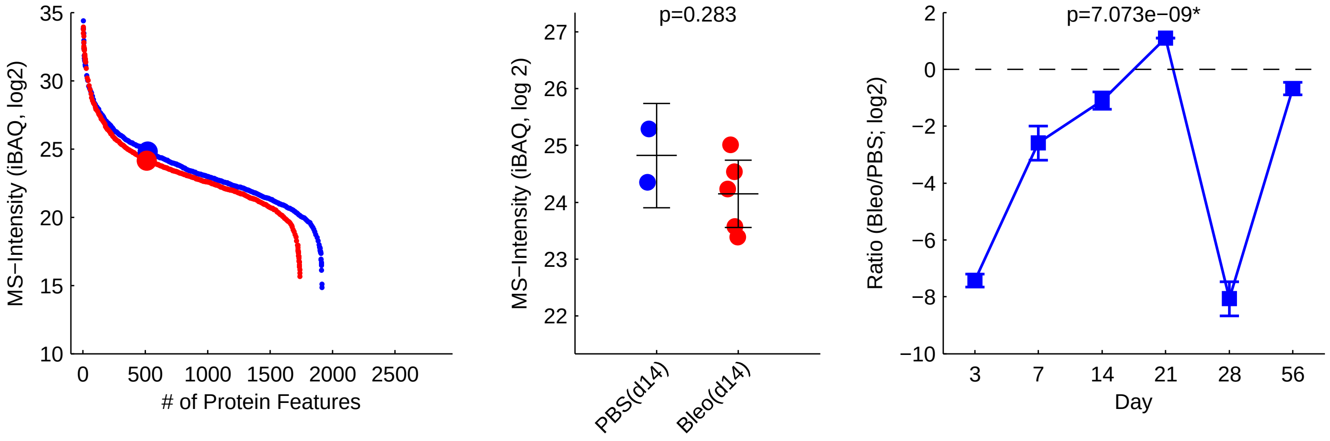

O55131 − Sept7 (id: 395)

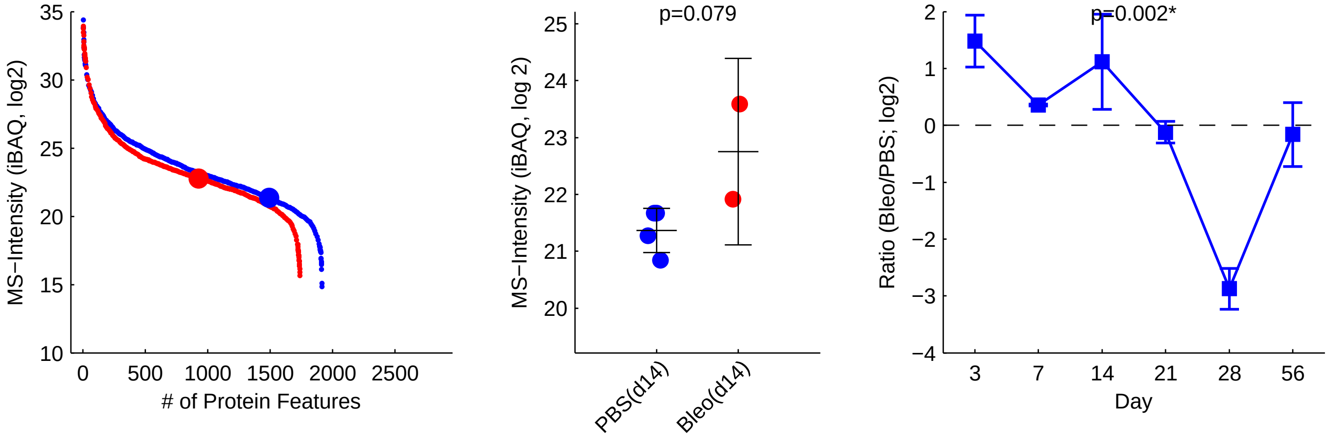

E9Q4G8 − Alcam (id: 405)

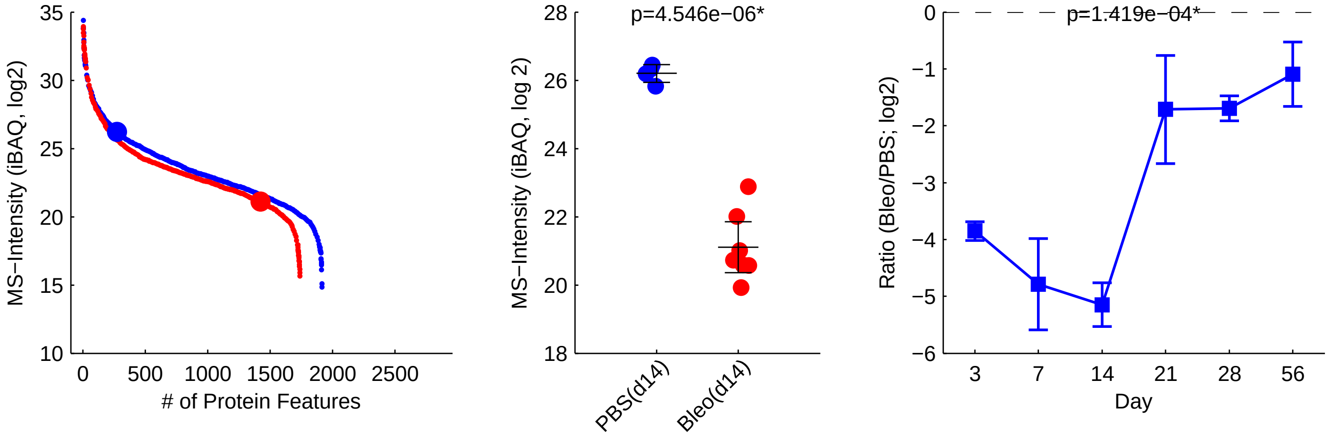

E9Q3V6 − Sept2 (id: 406)

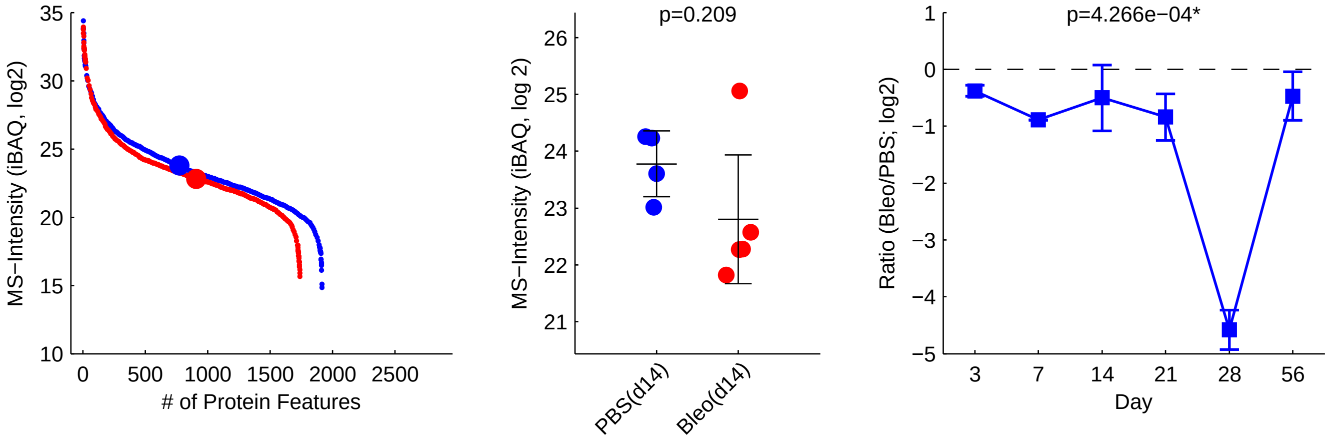

E9Q414 − Apob (id: 410)

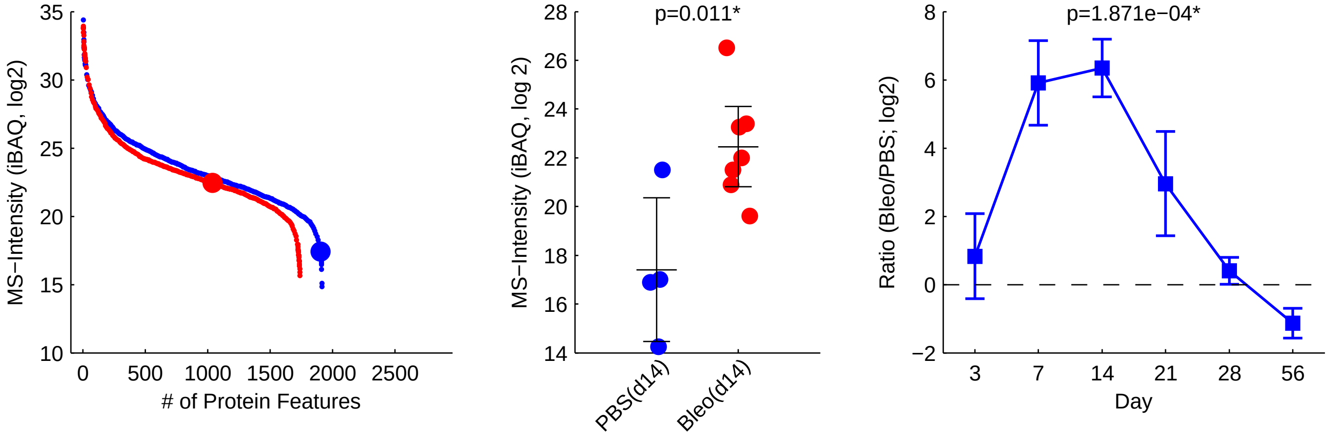

E9Q9J0 − Uba52 (id: 413)

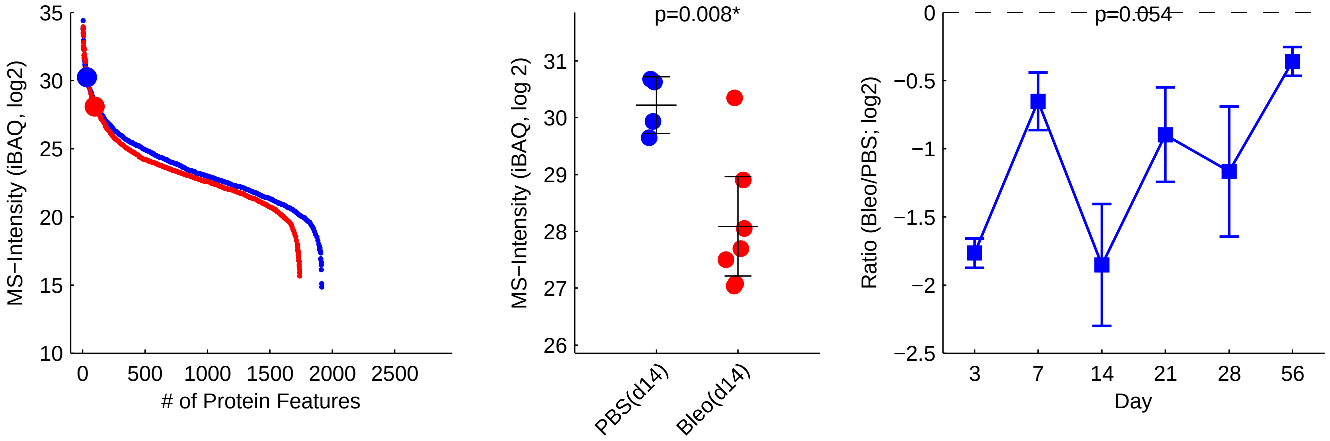

E9Q557 − Dsp (id: 417)

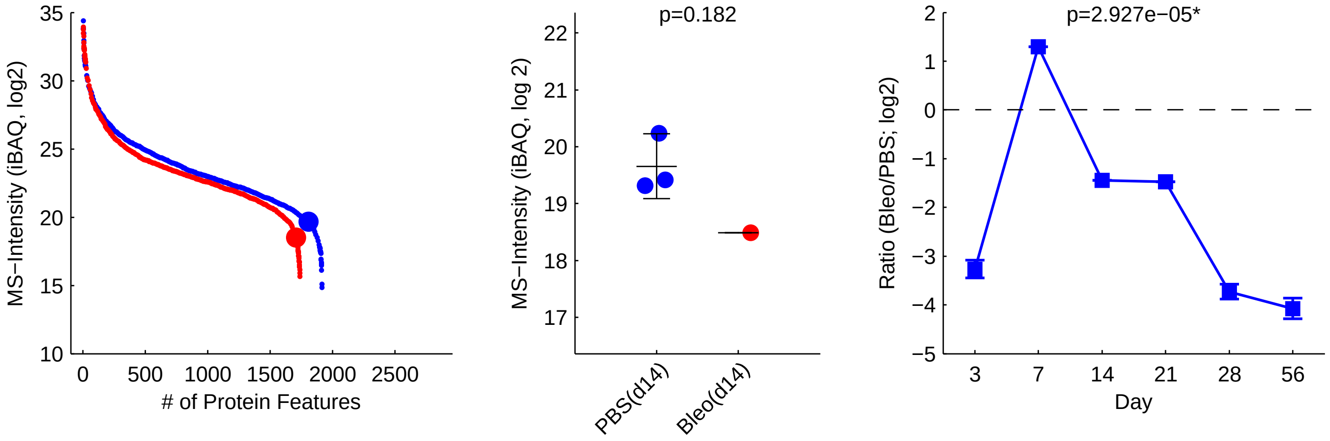

E9Q616 − Ahnak (id: 425)

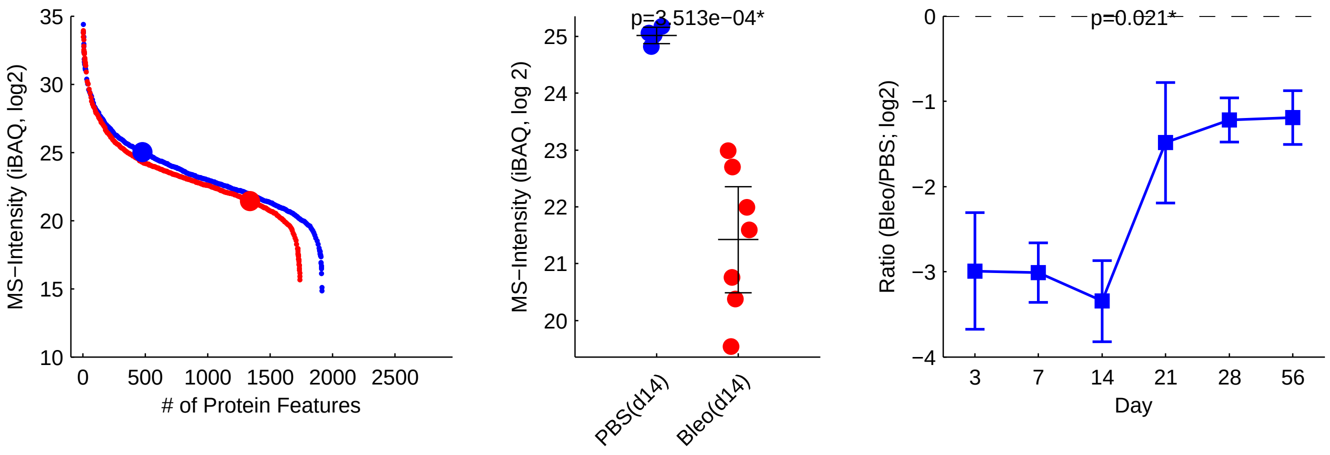

Q91X70 − C6 (id: 428)

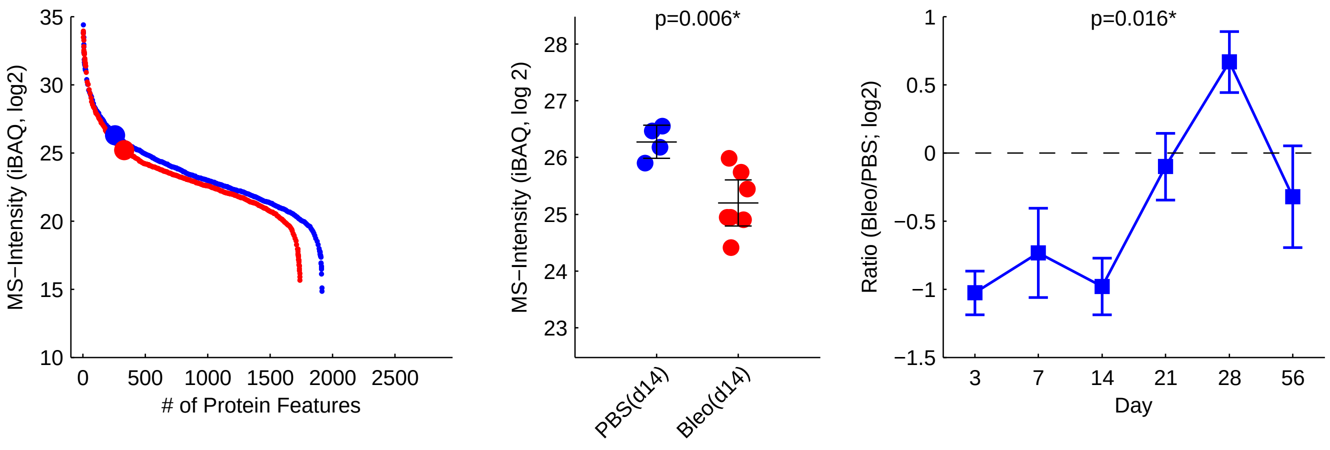

Q61730−2 − Il1rap (id: 429)

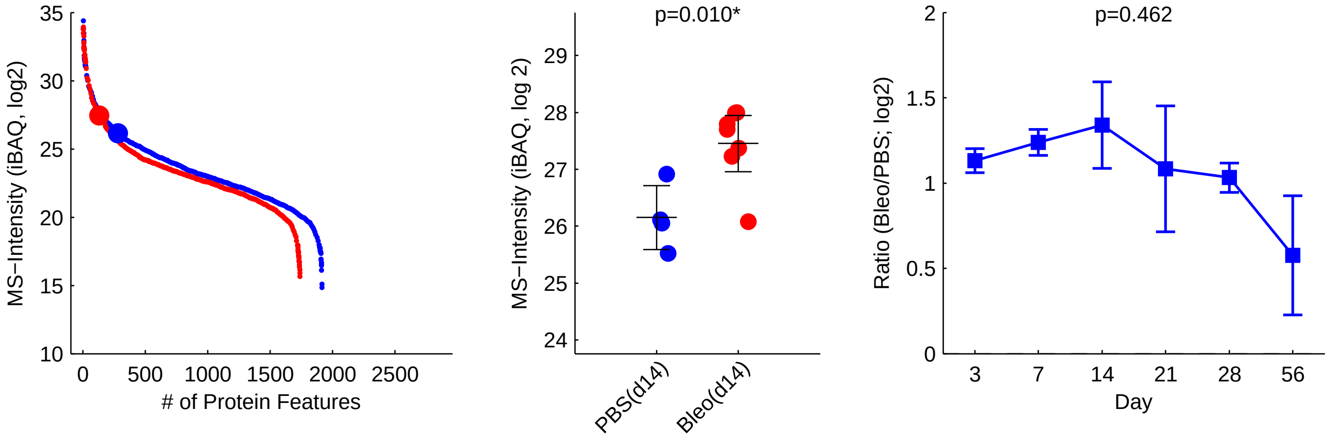

E9Q704 − Pam (id: 431)

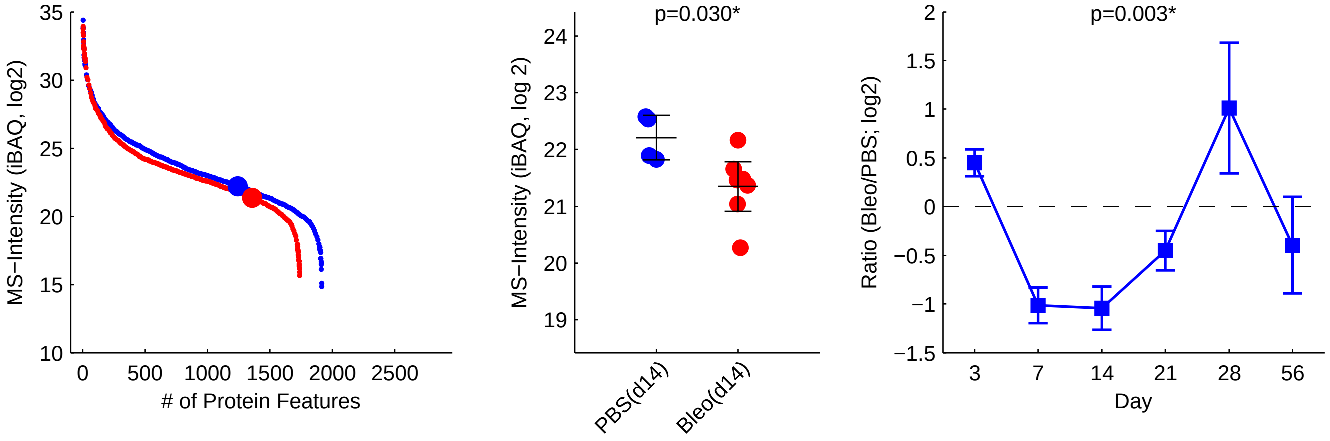

P06909 − Cfh (id: 441)

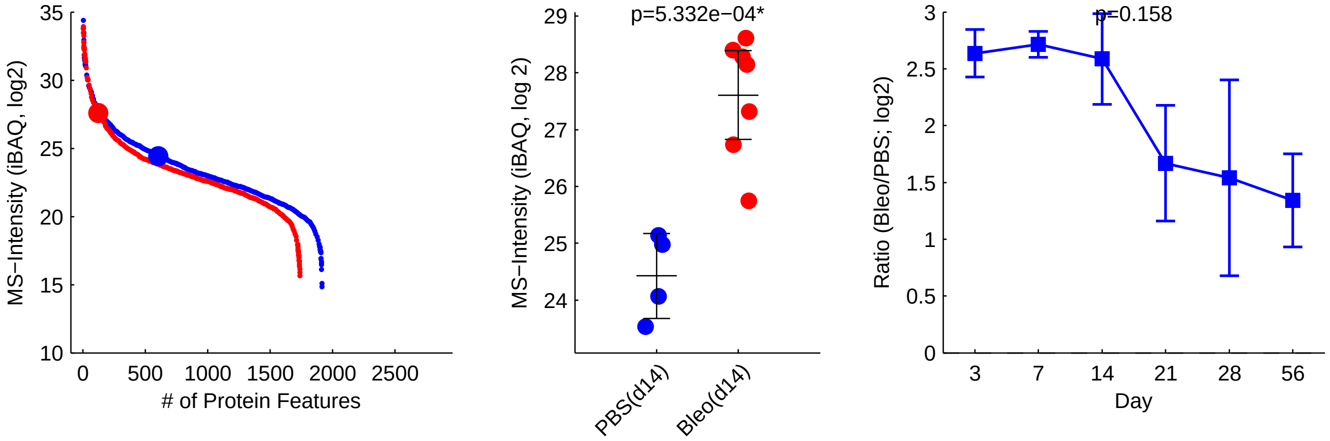

Q9CZM2 − Rpl15 (id: 453)

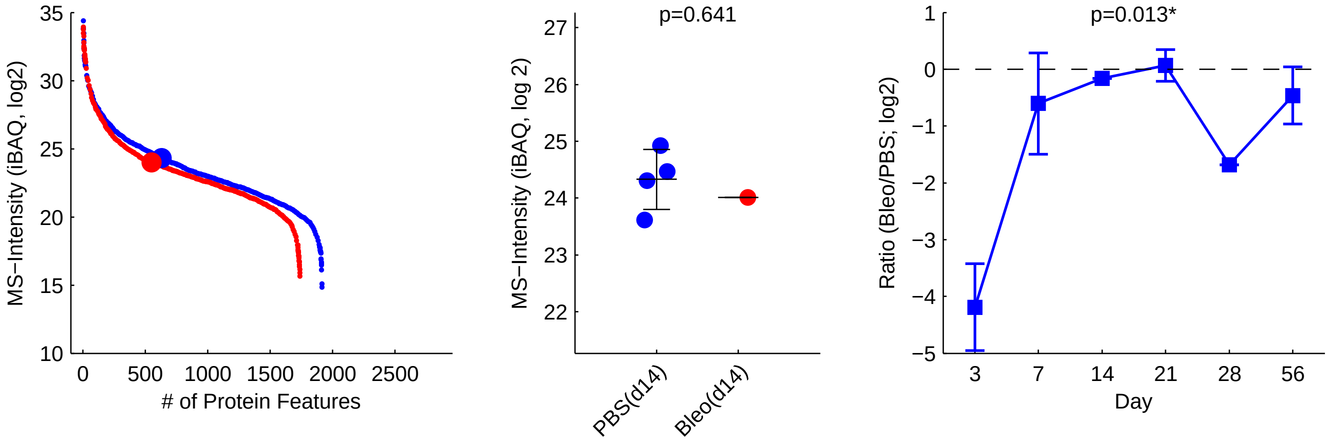

Q7TNG5 − Eml2 (id: 458)

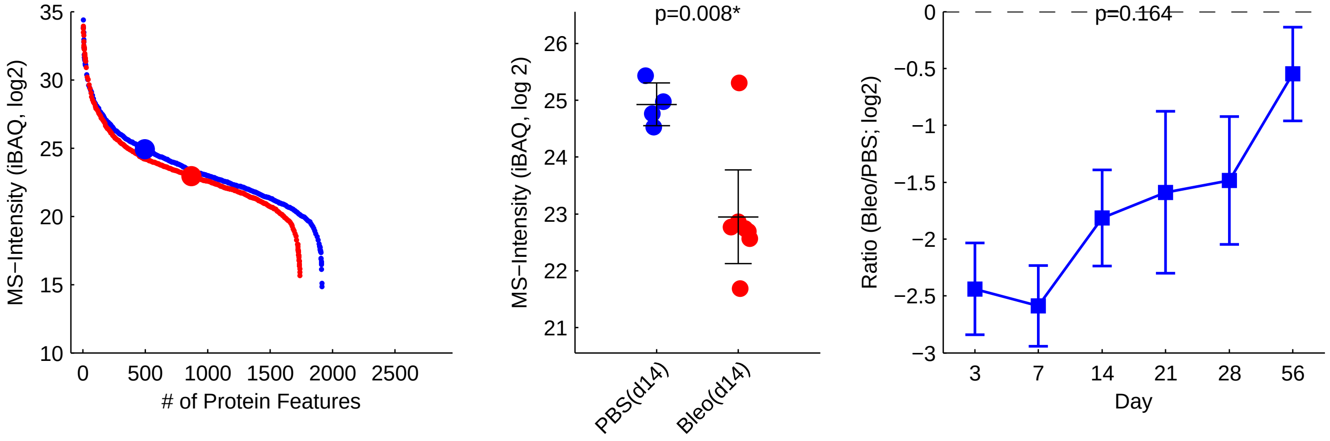

Q03142−2 − Fgfr3 (id: 467)

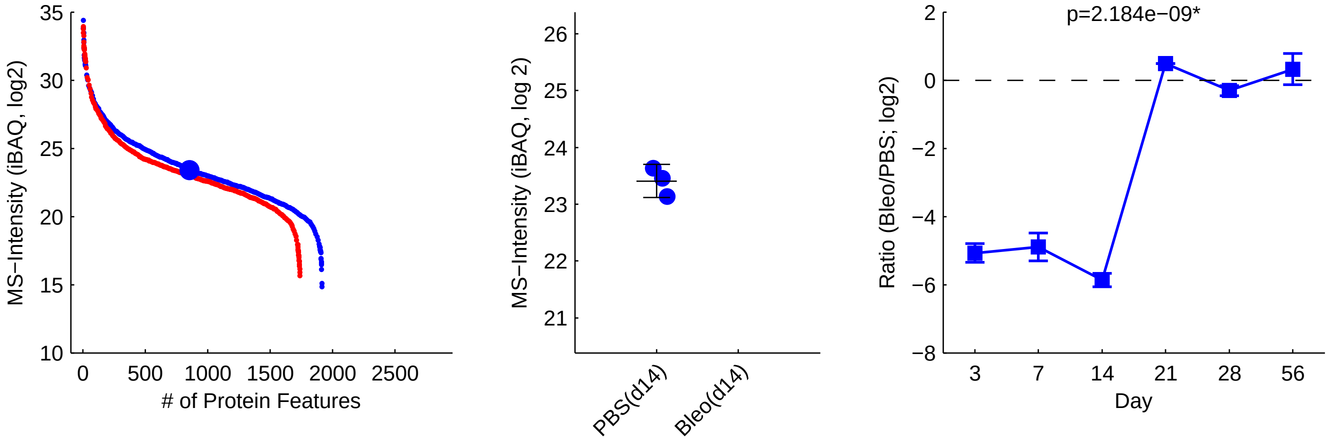

P33622 − Apoc3 (id: 469)

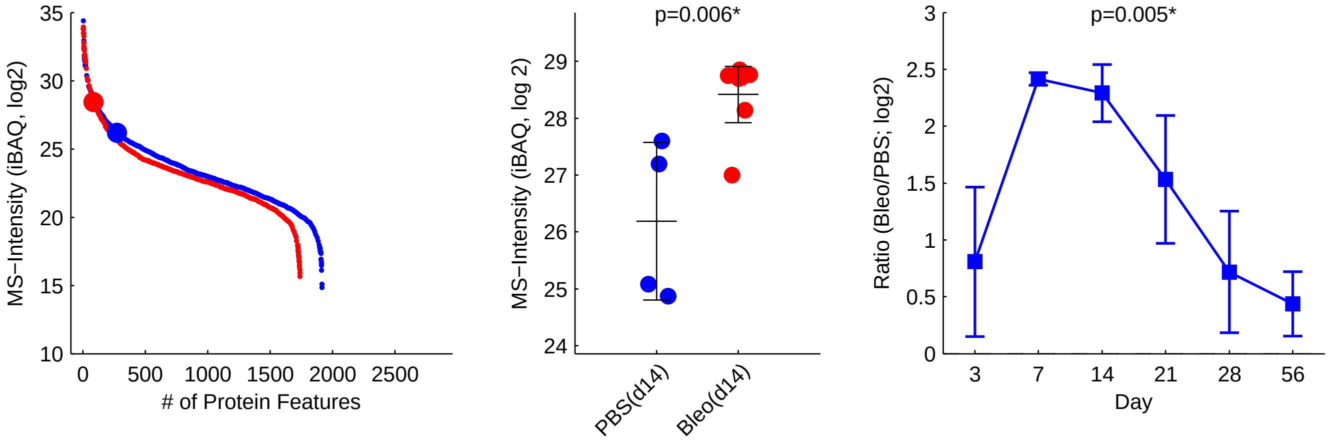

G5E8R3 − Pcx (id: 471)

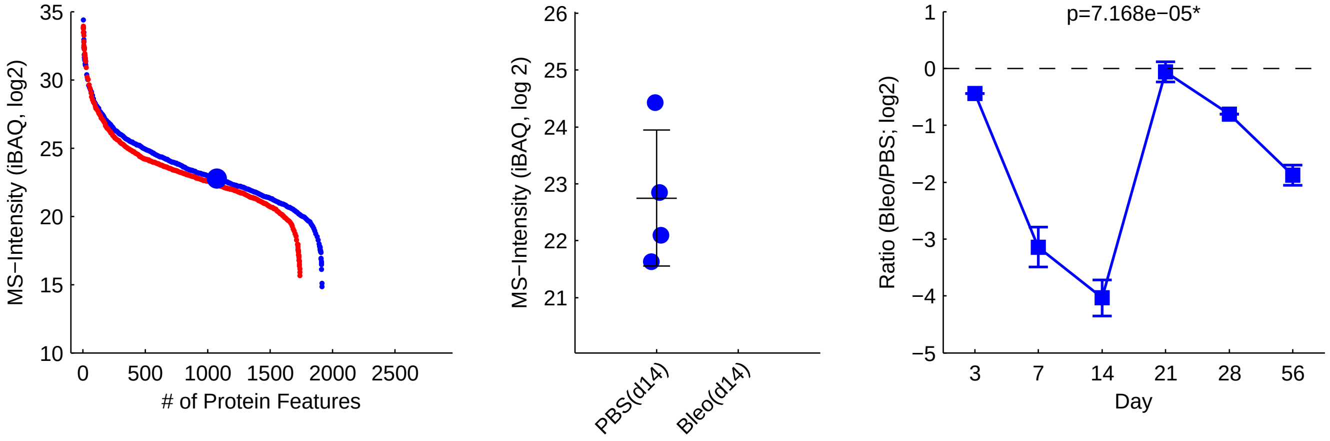

Q9DCN2−2 − Cyb5r3 (id: 478)

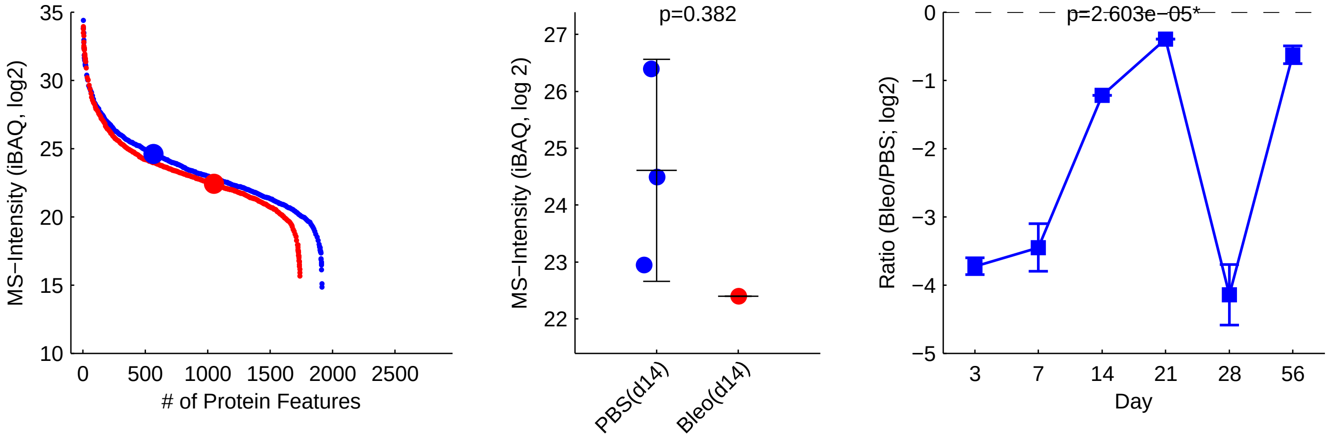

F6RPJ9 − Ide (id: 483)

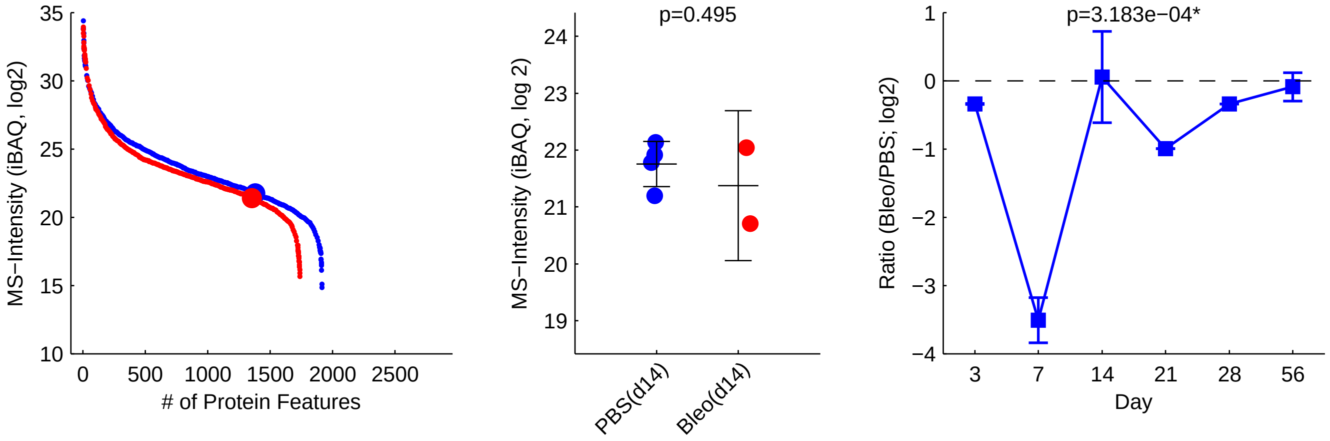

Q60739−2 − Bag1 (id: 486)

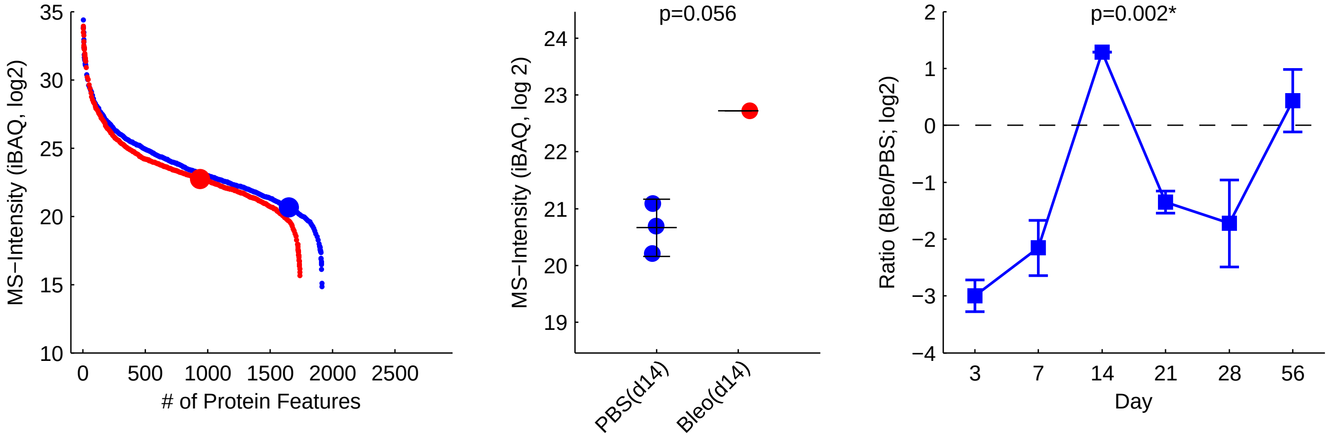

F6TFN2 − Lmo7 (id: 487)

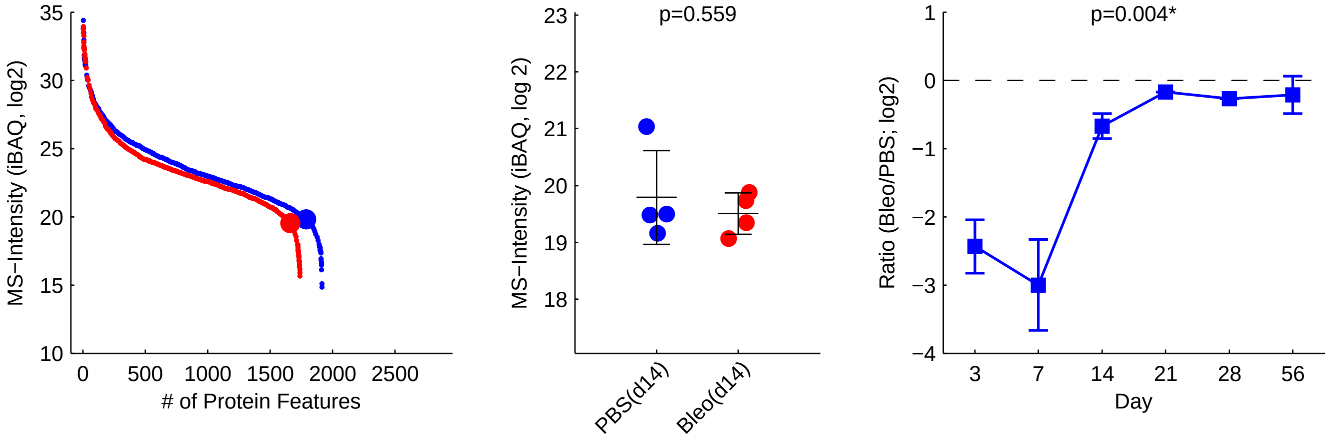

F6TQW2 − Ighg2c (id: 488)

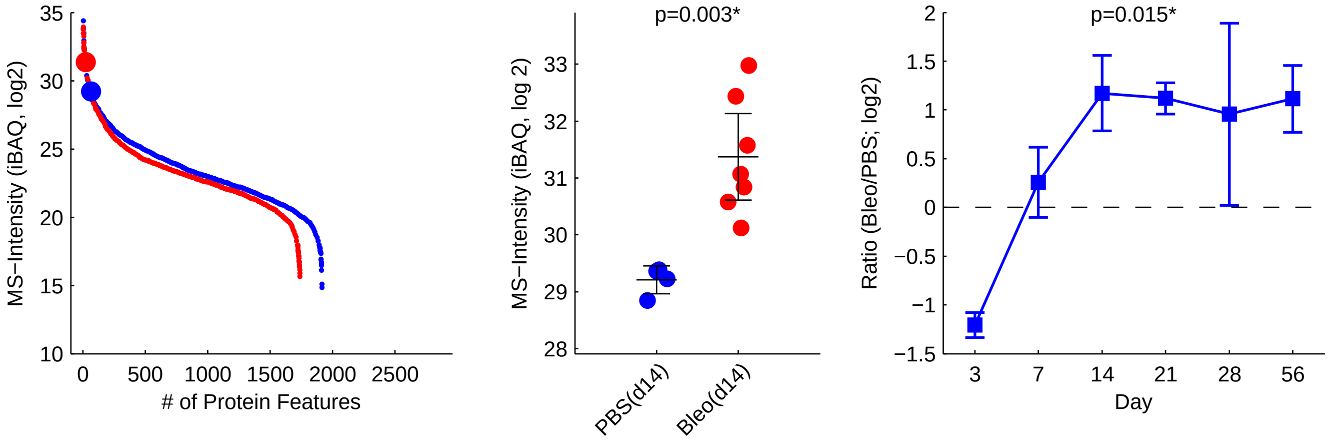

G3UW30 − Gbe1 (id: 501)

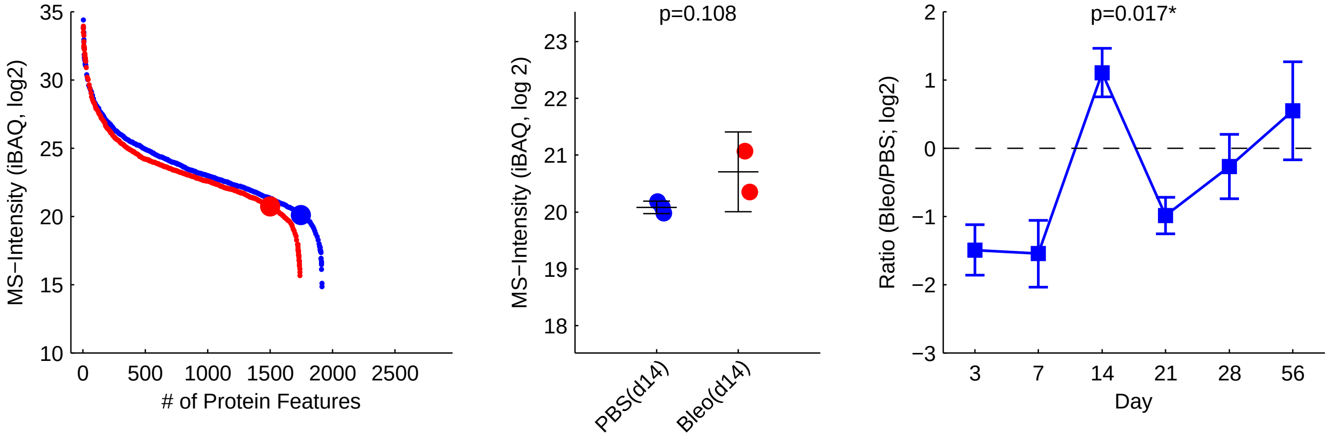

Q05909 − Ptprg (id: 517)

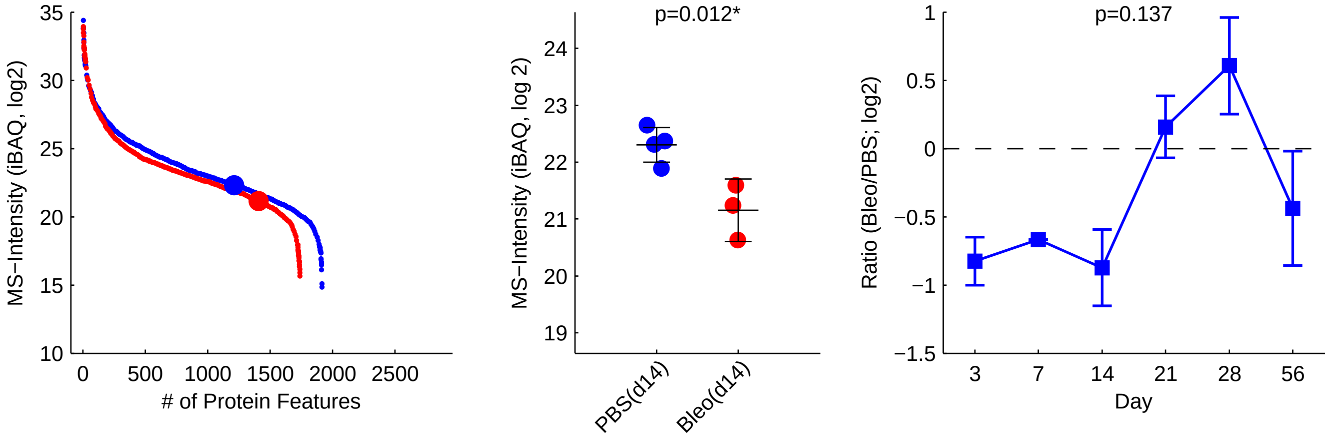

F8VQL0 − Met (id: 519)

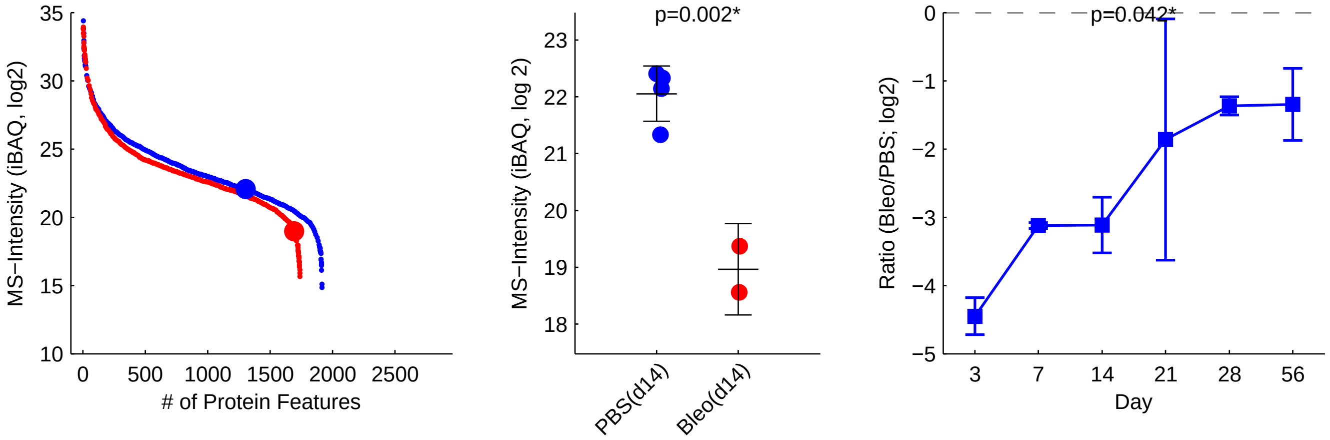

F8WGK6 − Sftpb (id: 520)

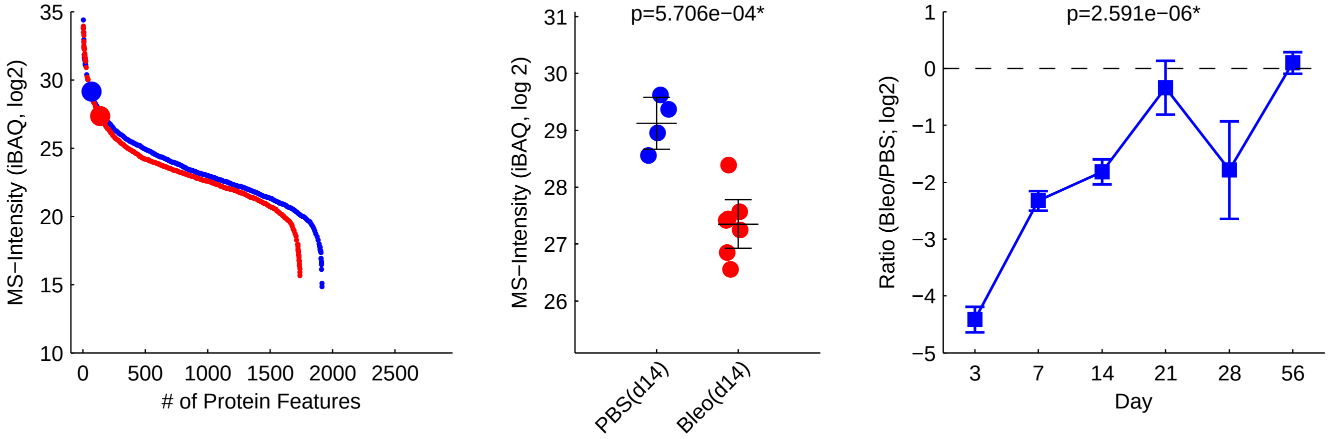

P10923 − Spp1 (id: 527)

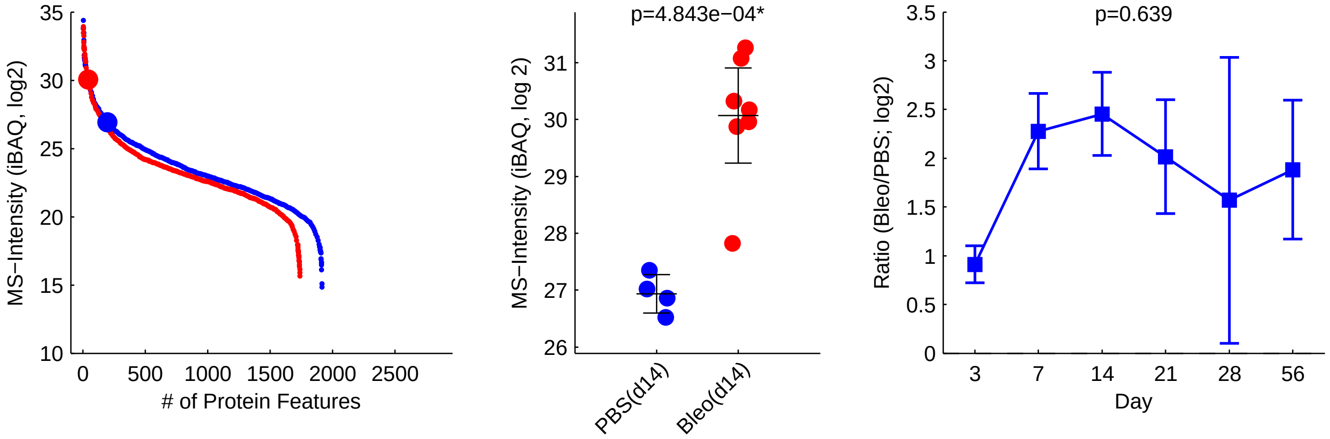

F8WIX8 − Hist1h2al (id: 530)

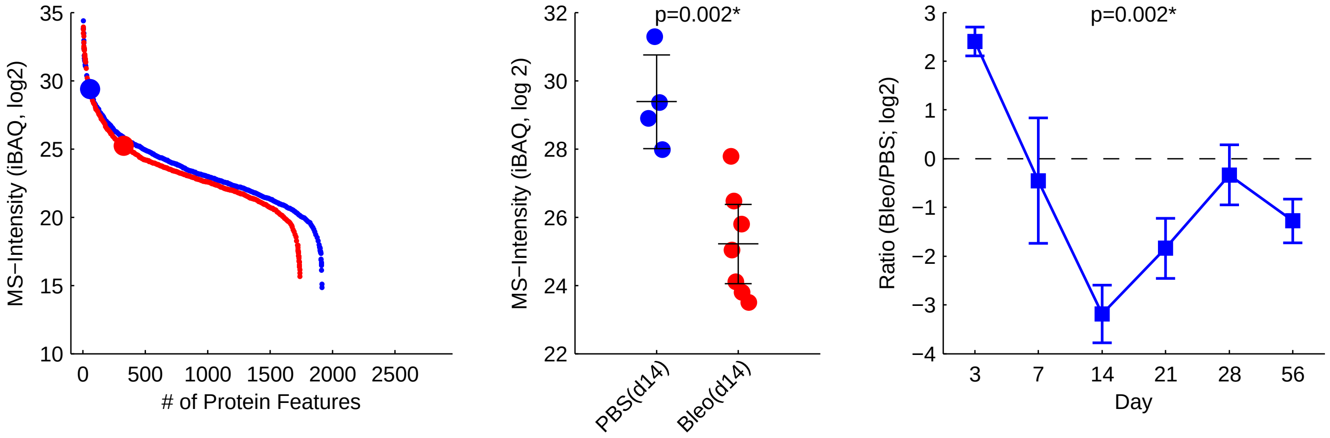

G3UX26 − Vdac2 (id: 539)

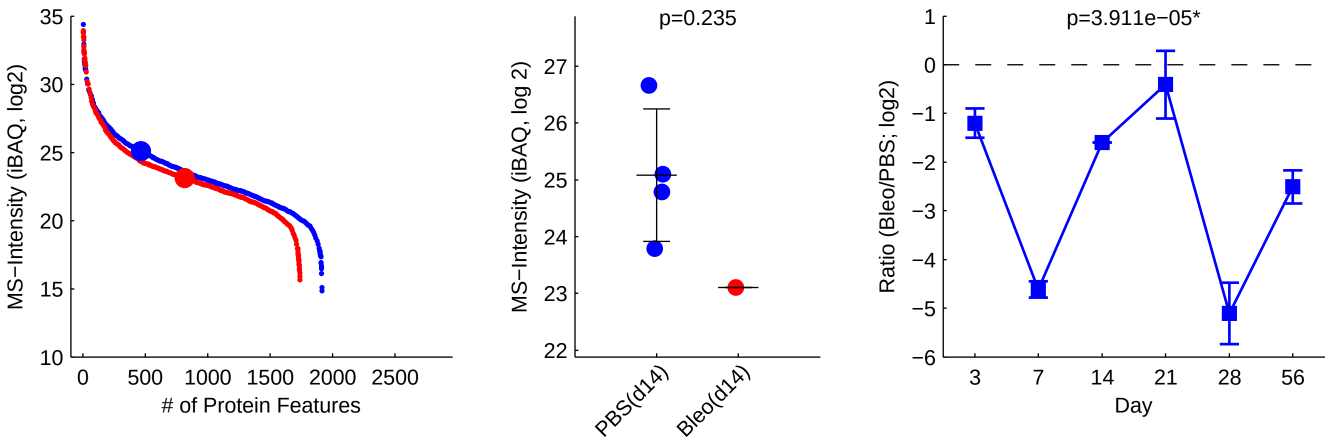

G3X9T8 − Cp (id: 552)

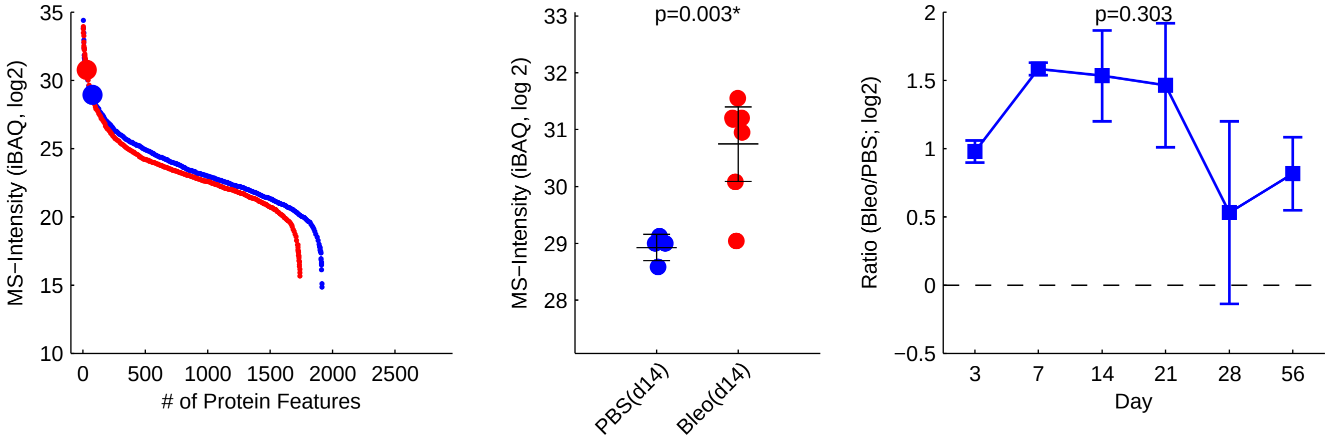

P16675 − Ctsa (id: 555)

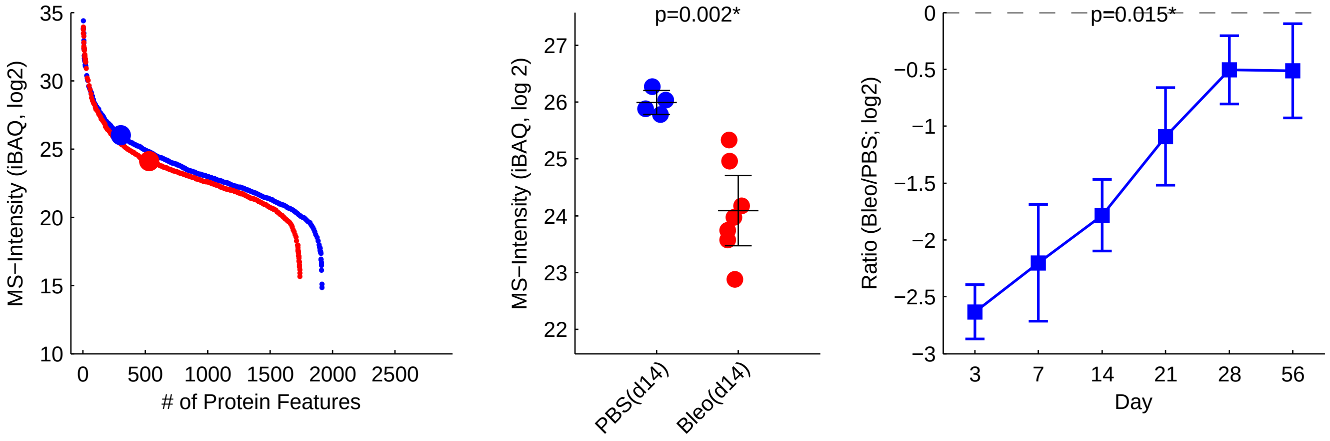

G3X8T9 − Serpina3n (id: 556)

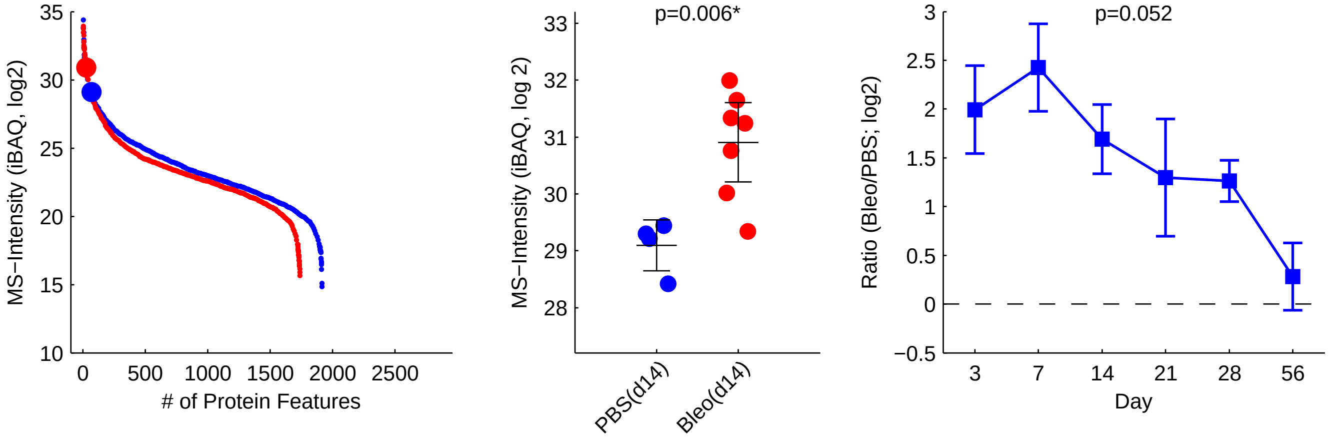

Q61703 − Itih2 (id: 567)

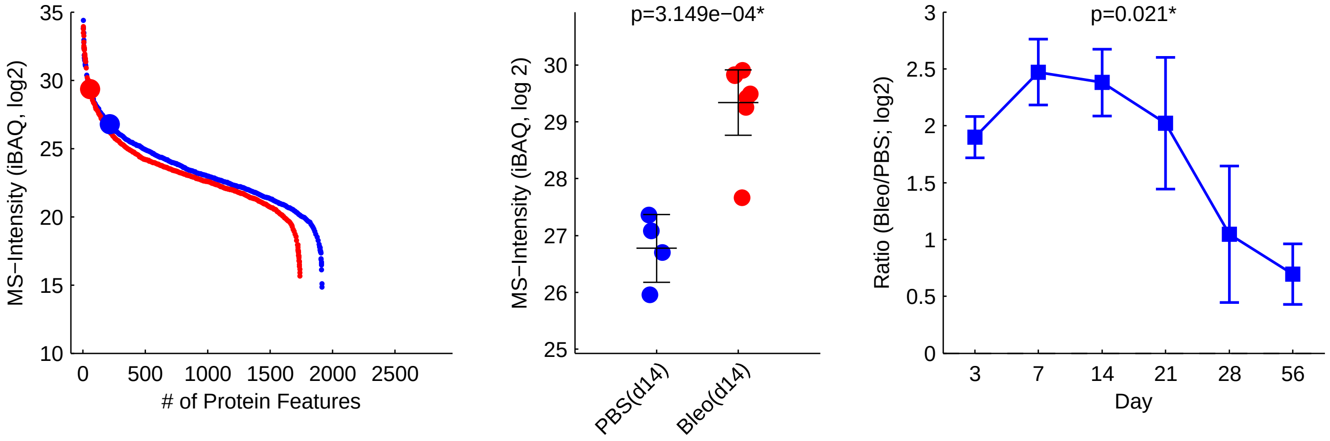

G3X982 − Aox3 (id: 568)

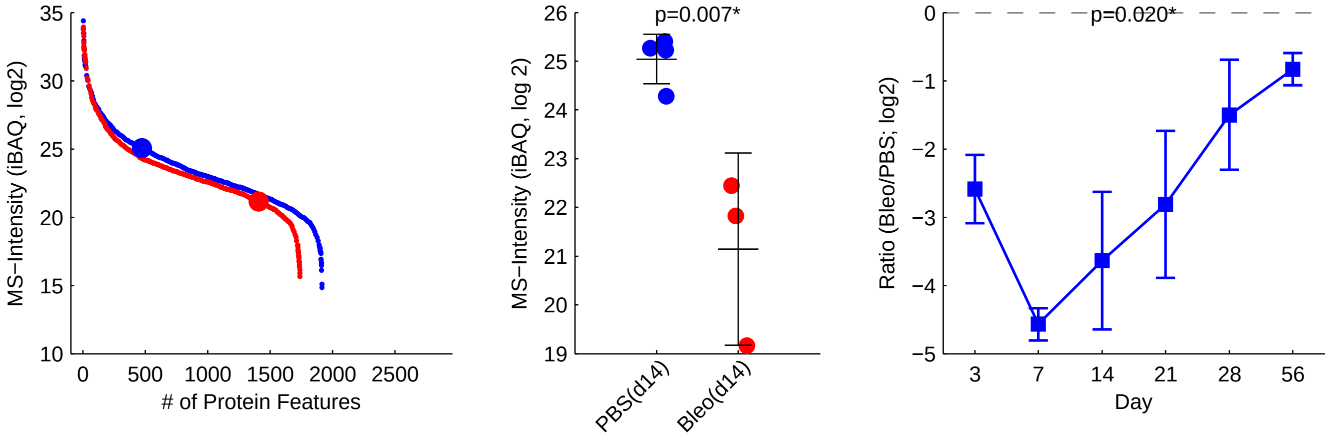

Q8CDK8 − Prom1 (id: 570)

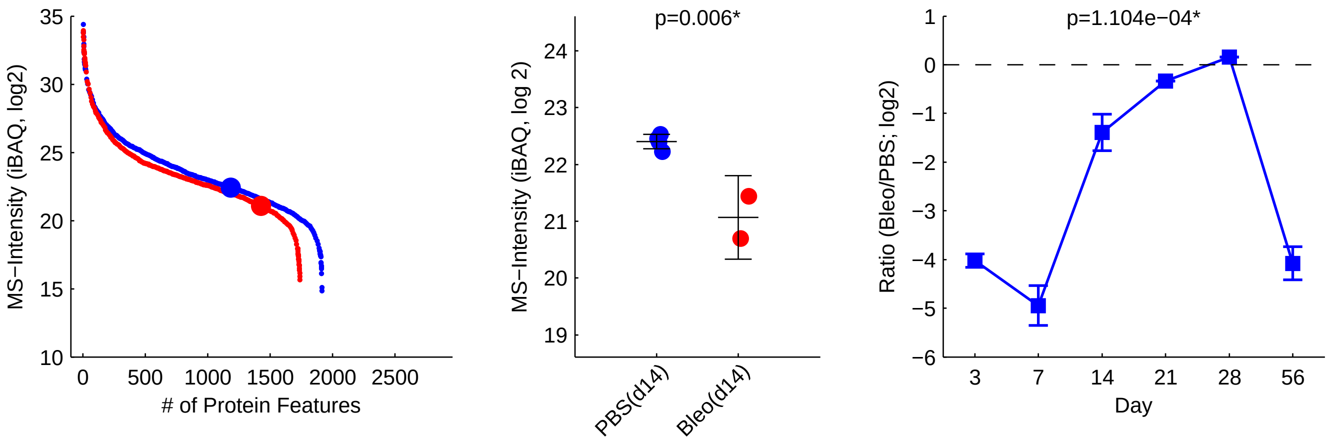

G5E850 − Cyb5 (id: 578)

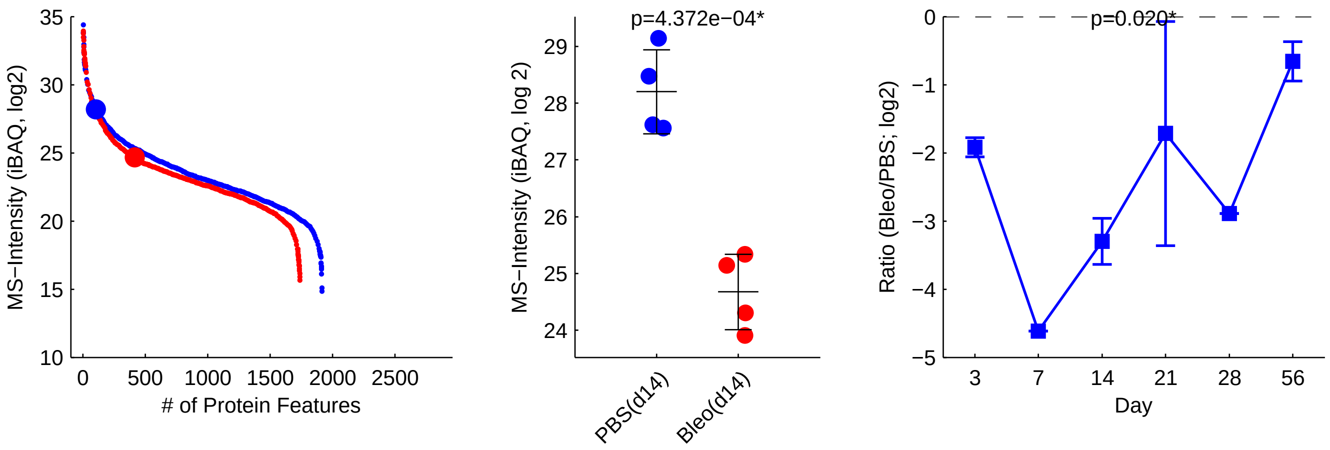

G5E8B5 − Scgb1c1 (id: 584)

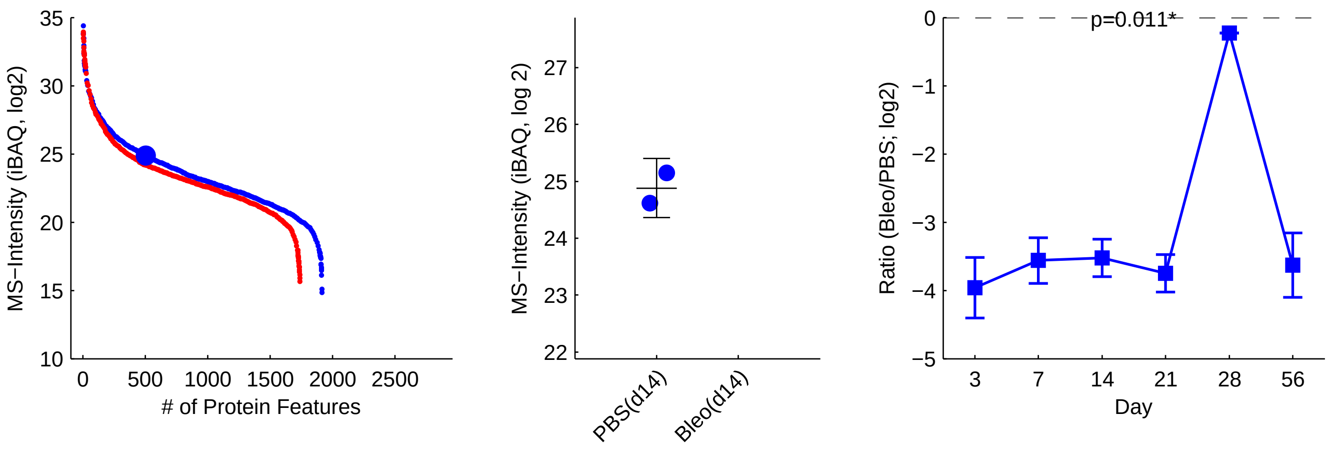

Q8BHL4 − Gprc5a (id: 585)

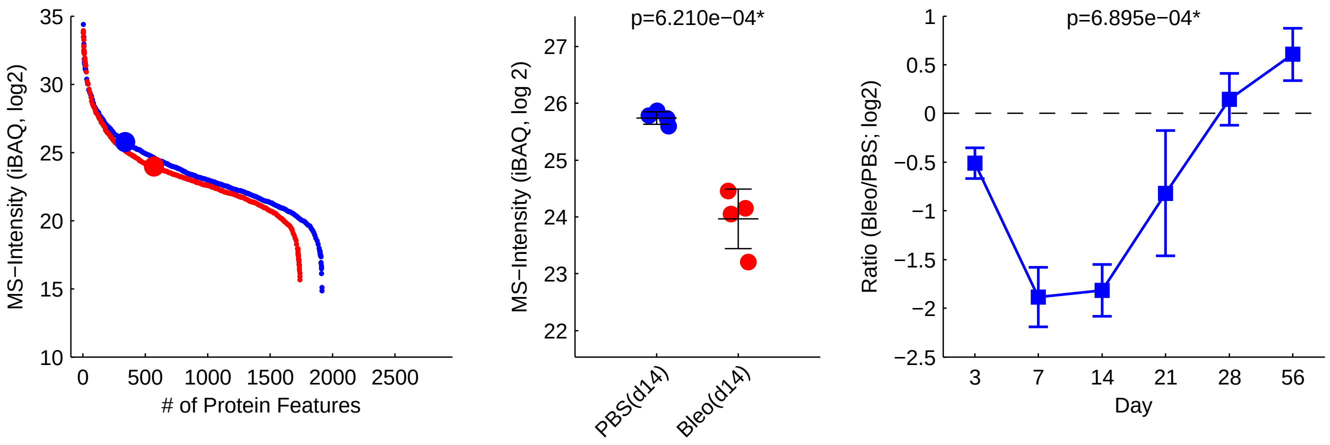

G5E8Q8 − Gpr116 (id: 589)

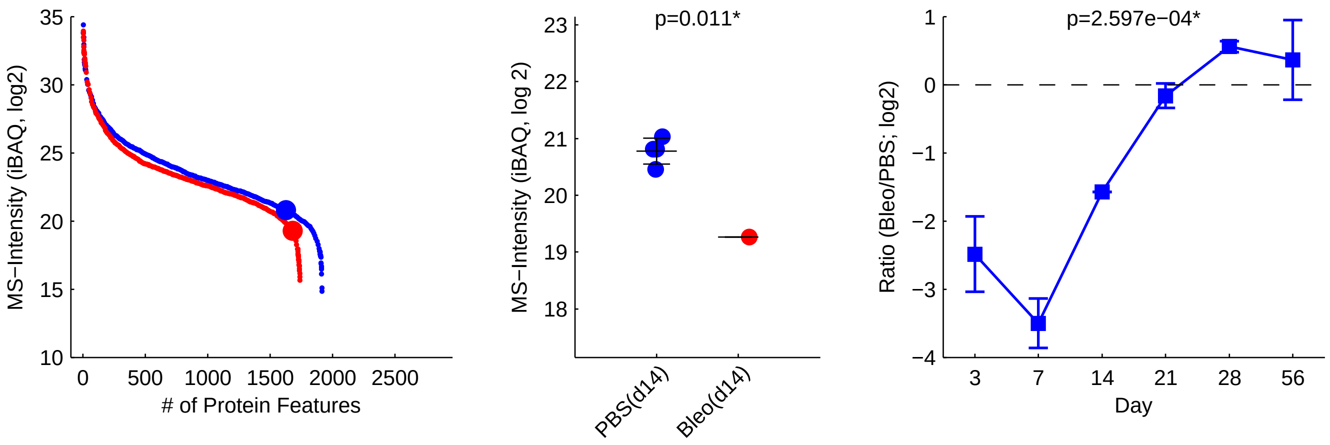

Q99KB8−2 − Hagh (id: 592)

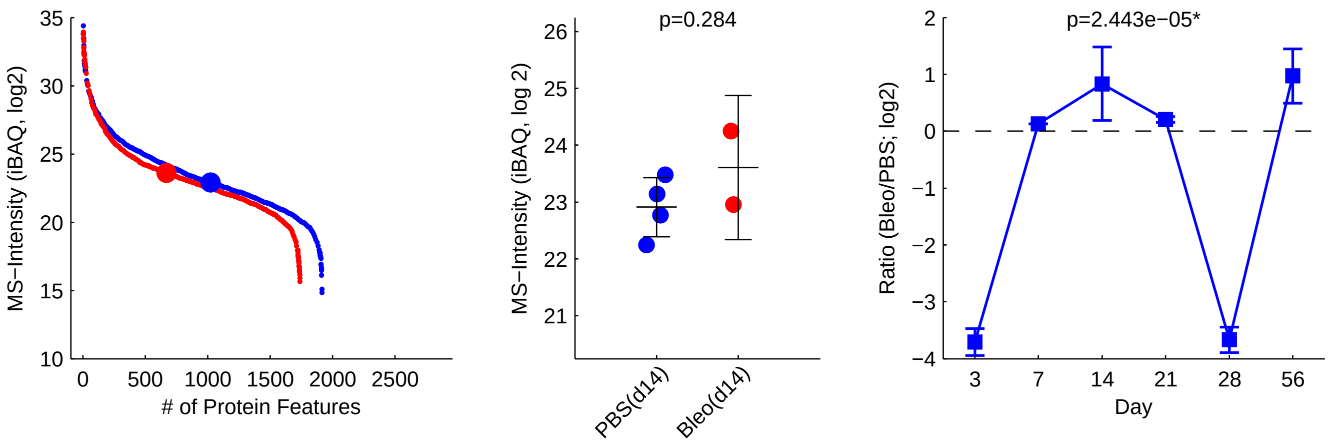

Q8R081 − Hnrnpl (id: 593)

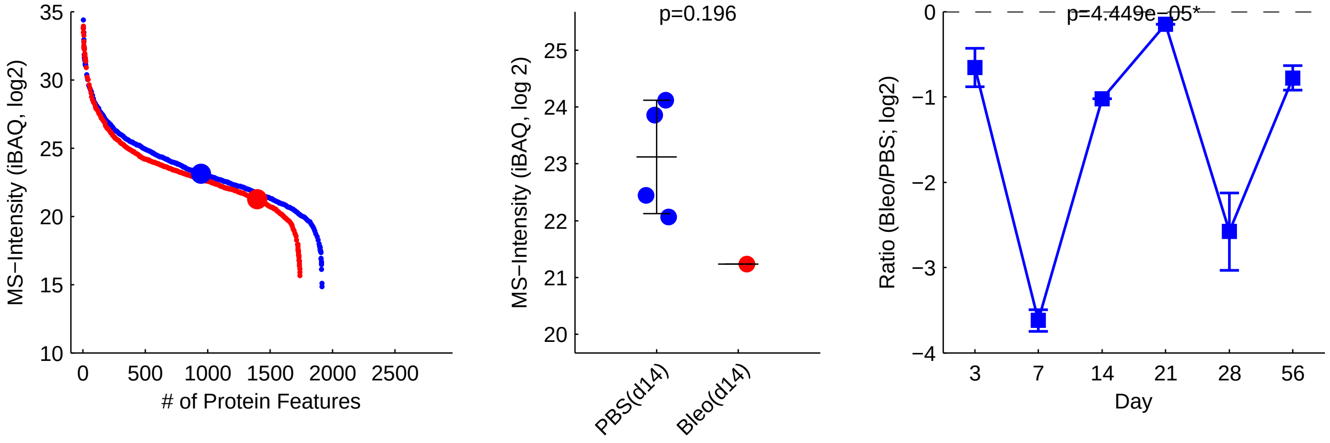

Q00724 − Rbp4 (id: 601)

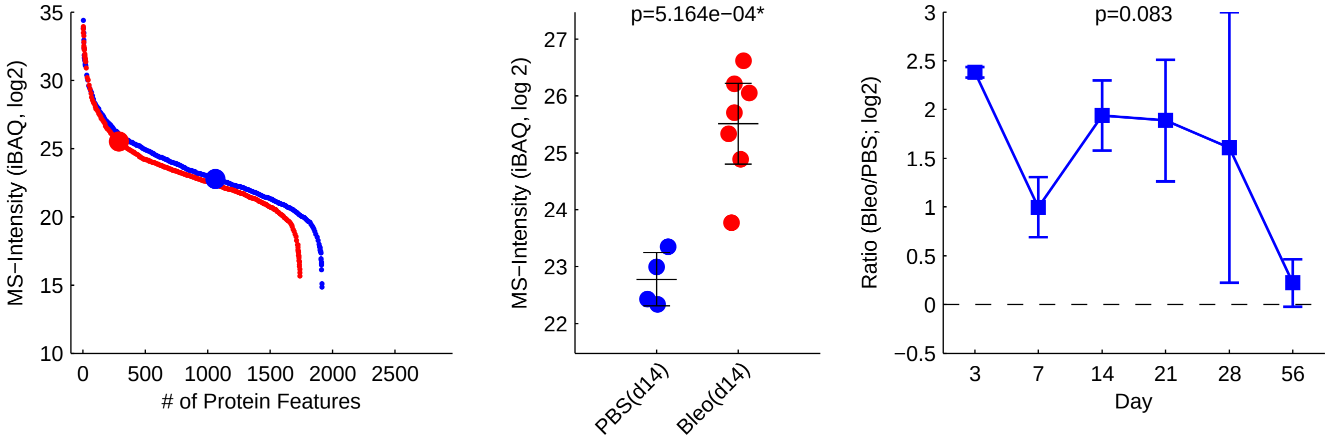

O55143−2 − Atp2a2 (id: 609)

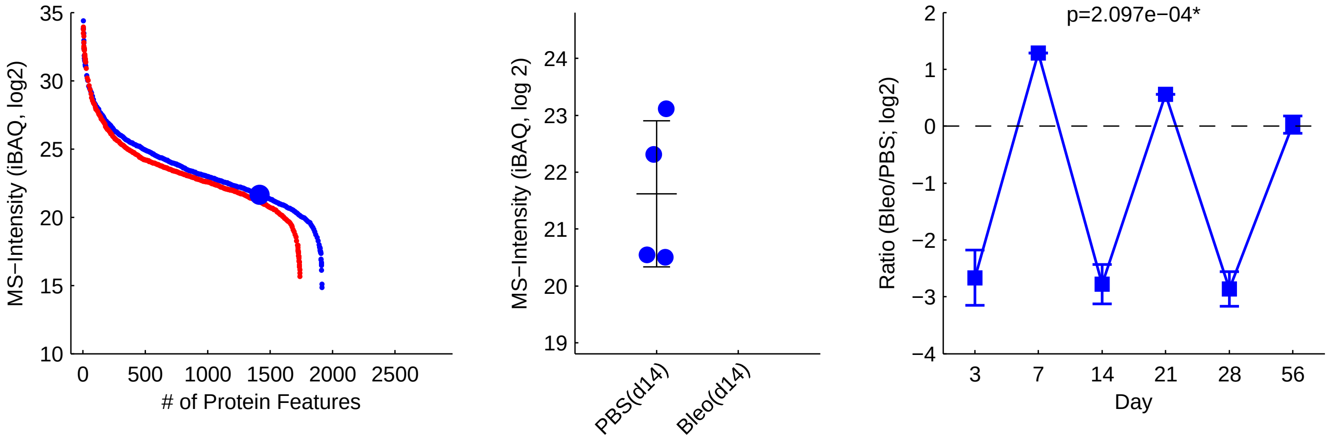

P11404 − Fabp3 (id: 613)

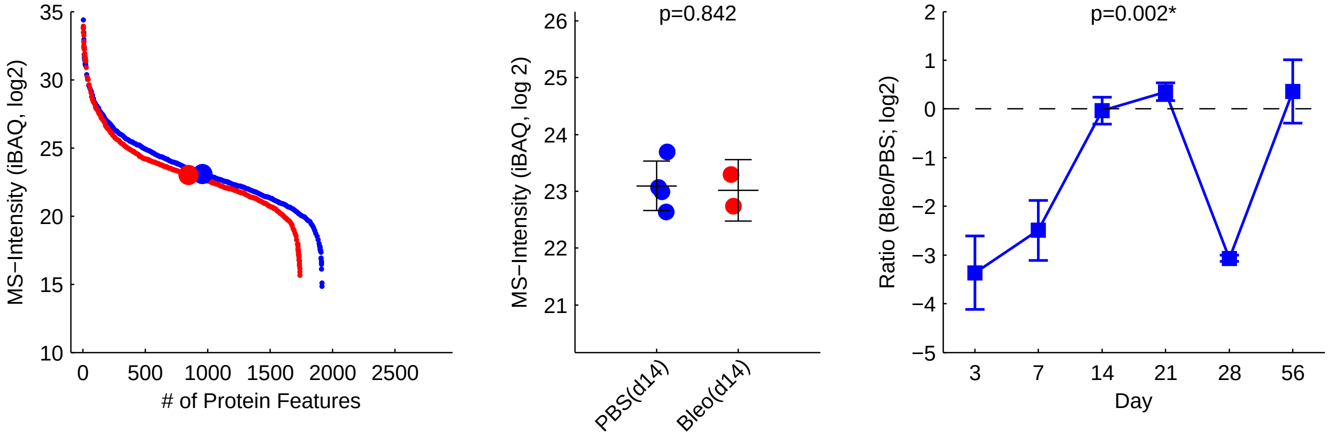

Q60931 − Vdac3 (id: 616)

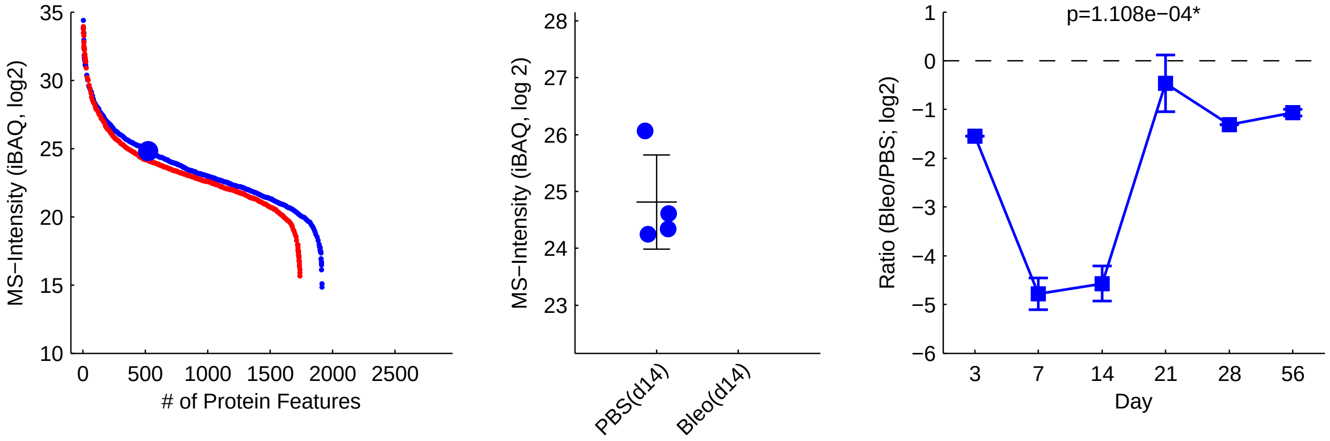

Q6URW6−2 − Myh14 (id: 619)

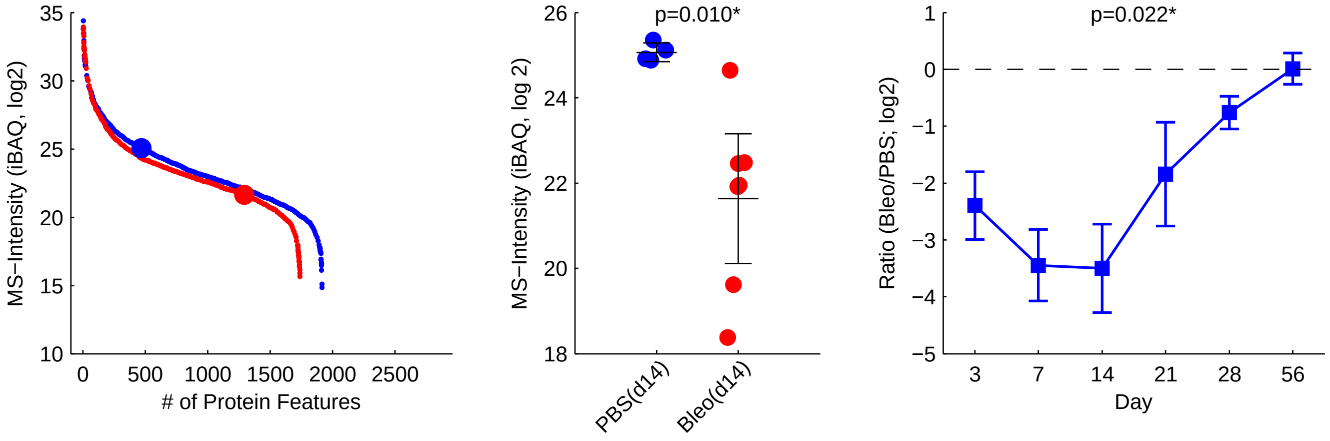

O08677 − Kng1 (id: 631)

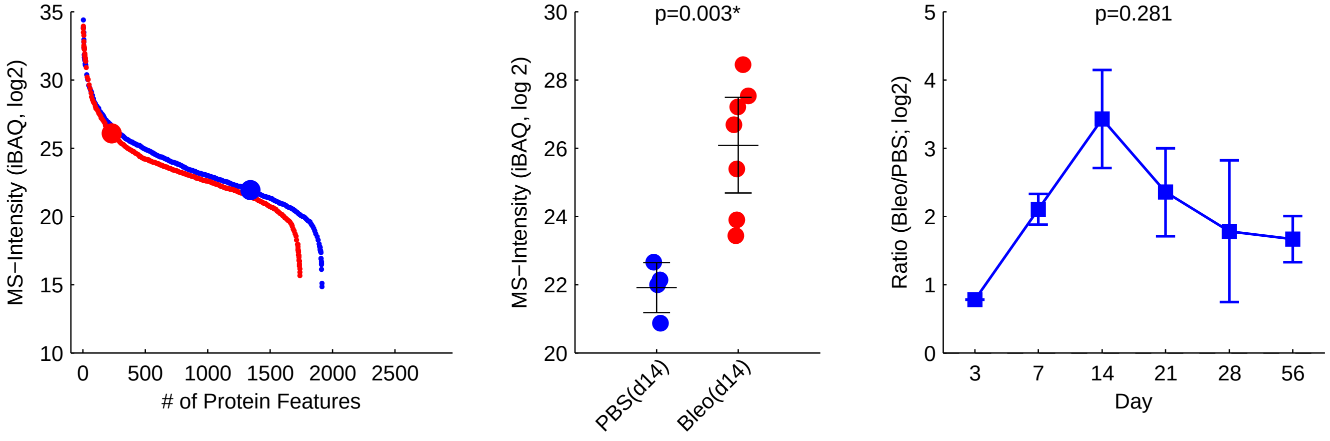

O08677−2 − (id: 632)

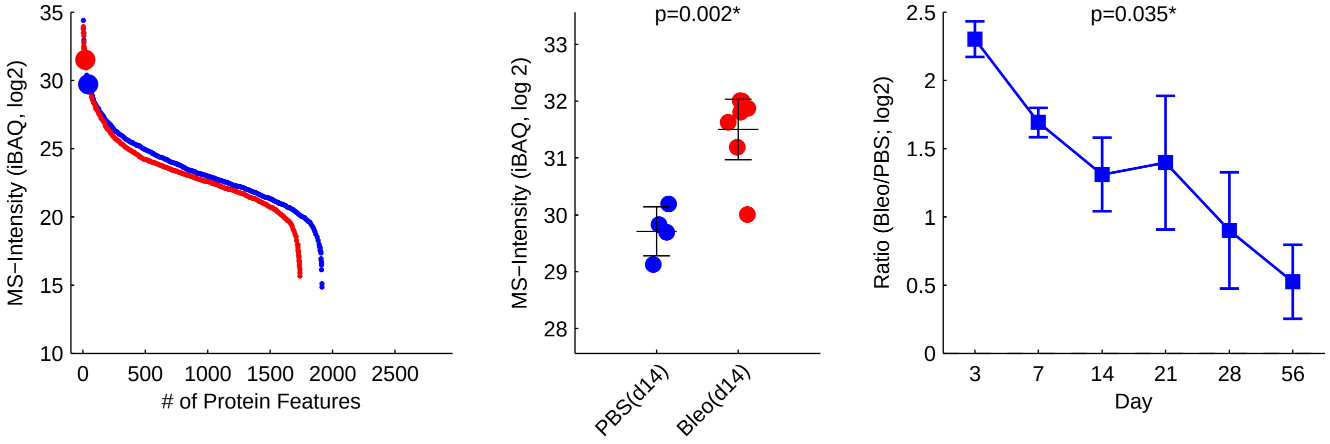

Q3TMX0 − Sdcbp (id: 645)

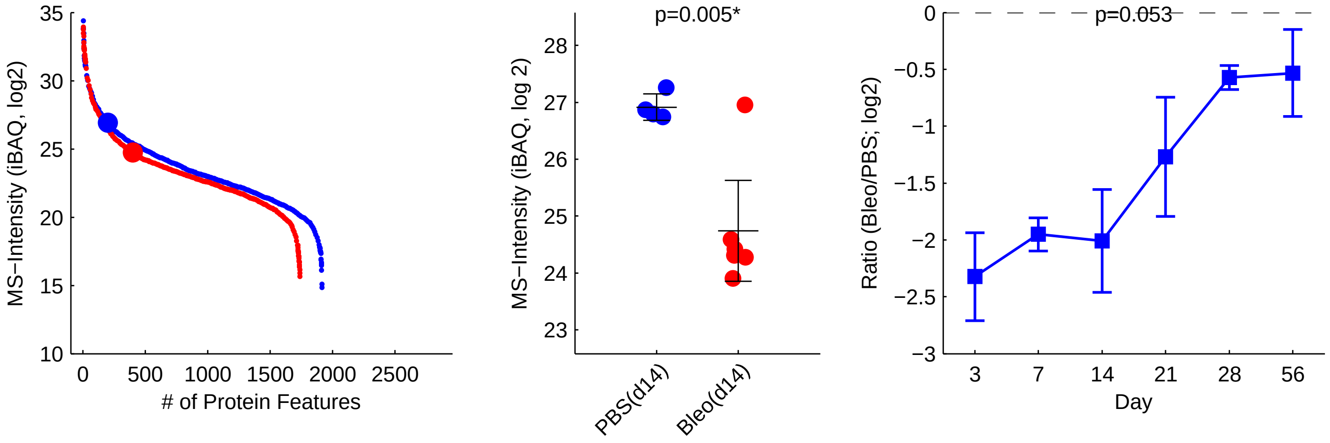

O09043 − Napsa (id: 647)

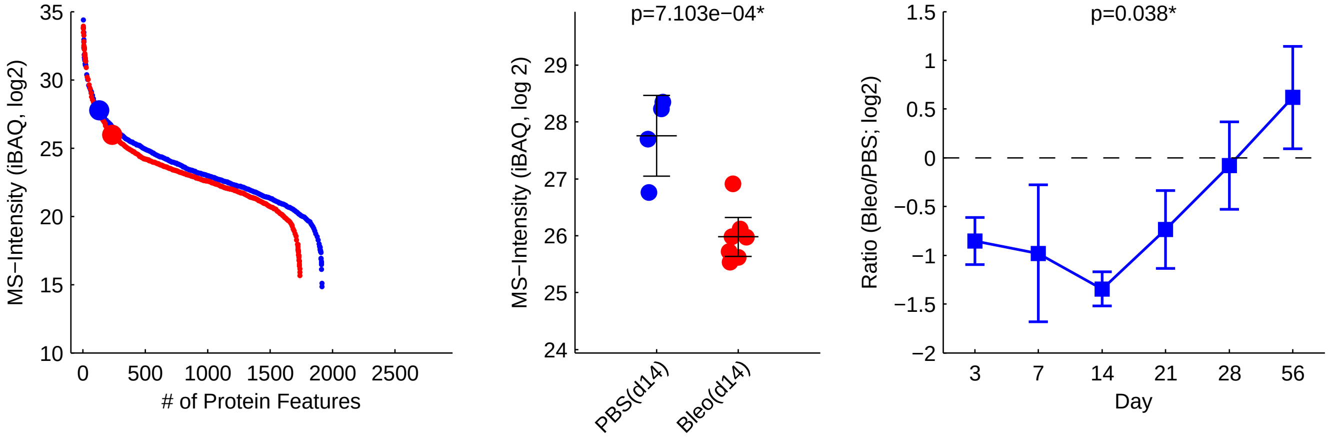

O09131 − Gsto1 (id: 652)

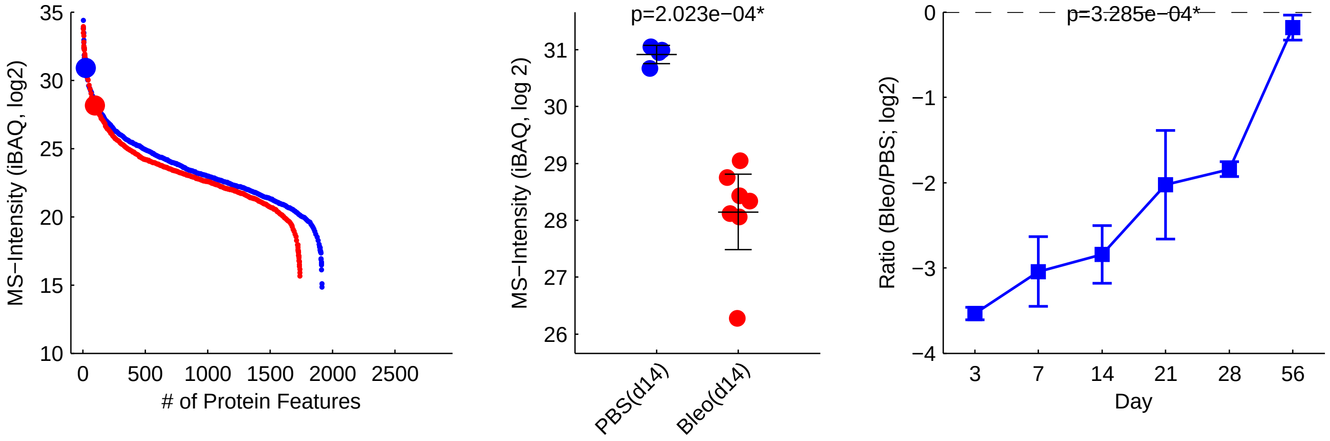

O09159 − Man2b1 (id: 653)

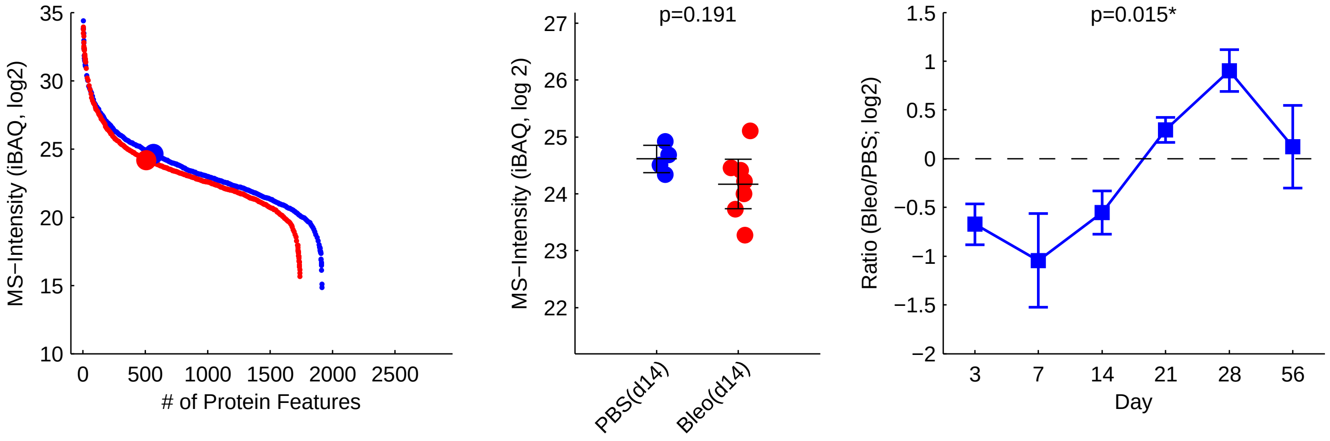

Q9CQM8 − Rpl21 (id: 655)

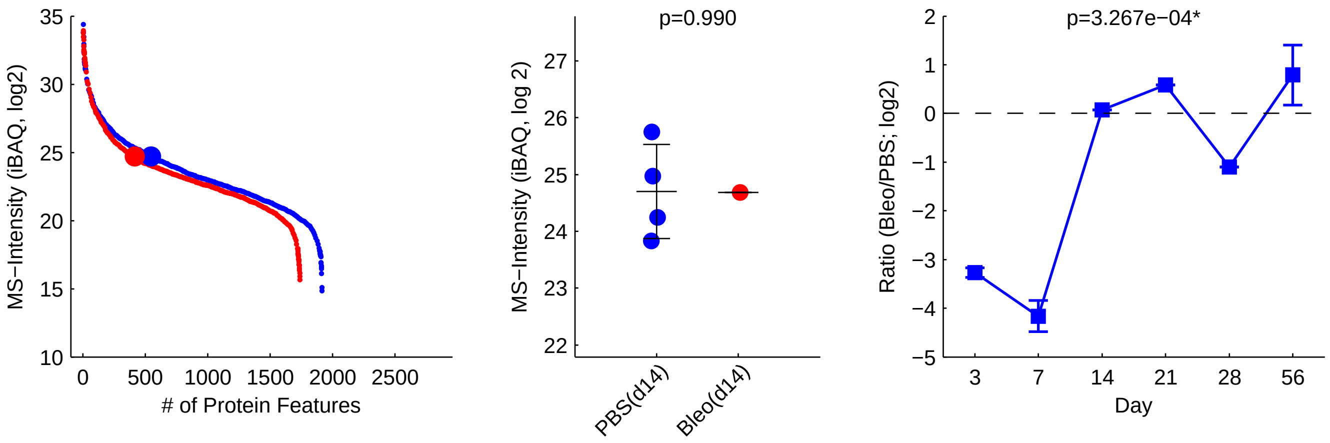

O35129 − Phb2 (id: 660)

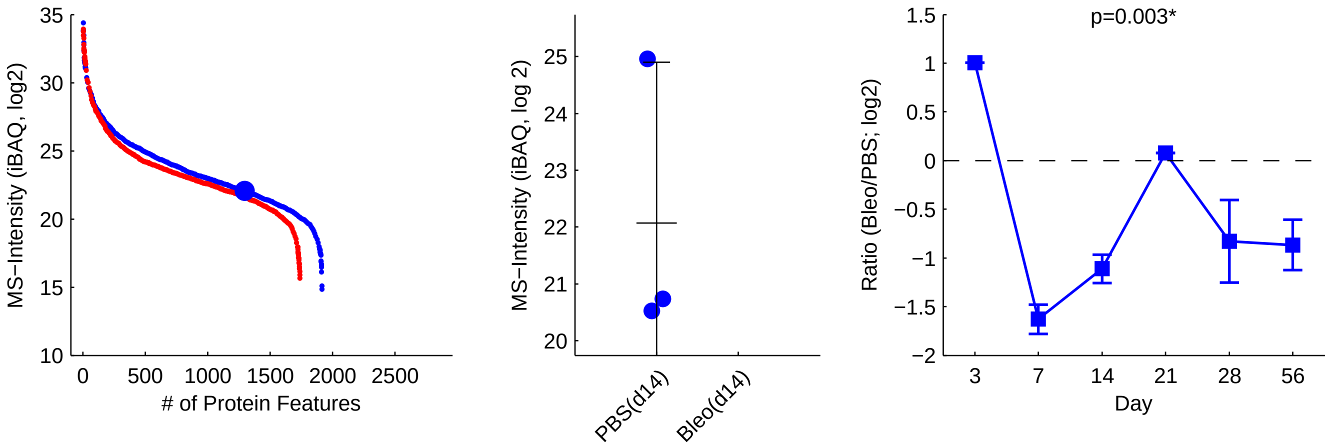

O35215 − Ddt (id: 661)

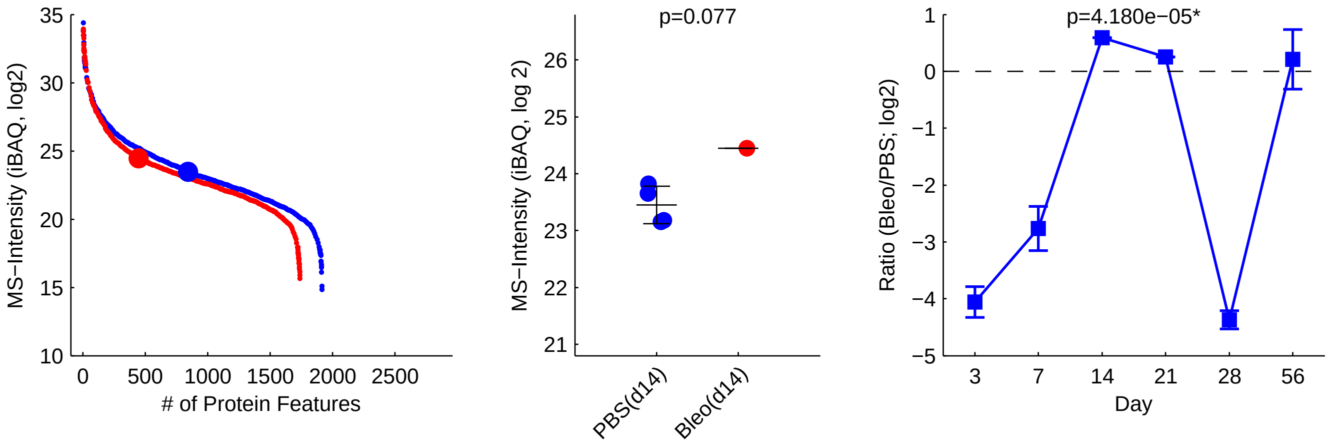

O35226 − Psmd4 (id: 662)

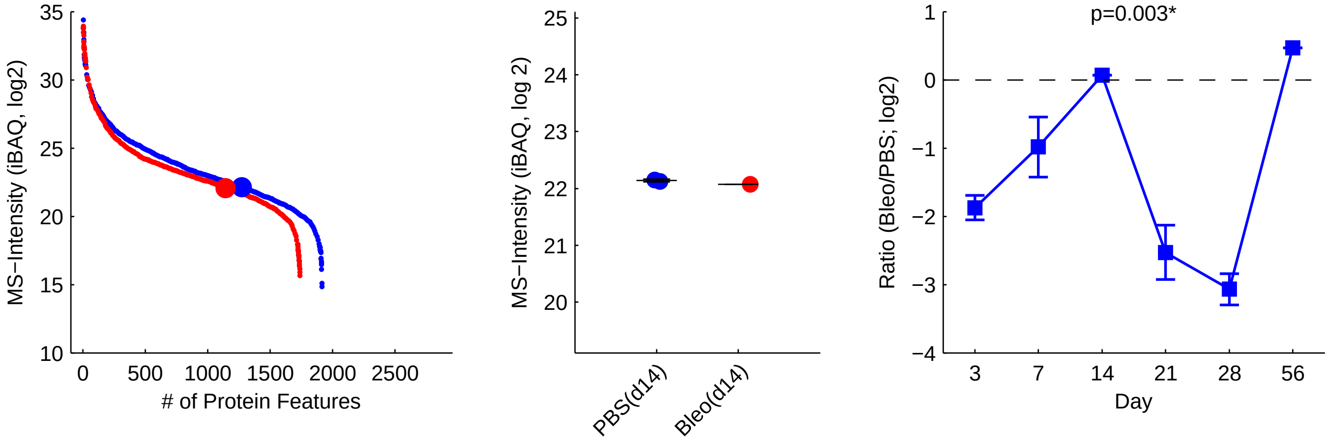

O35309 − Nmi (id: 664)

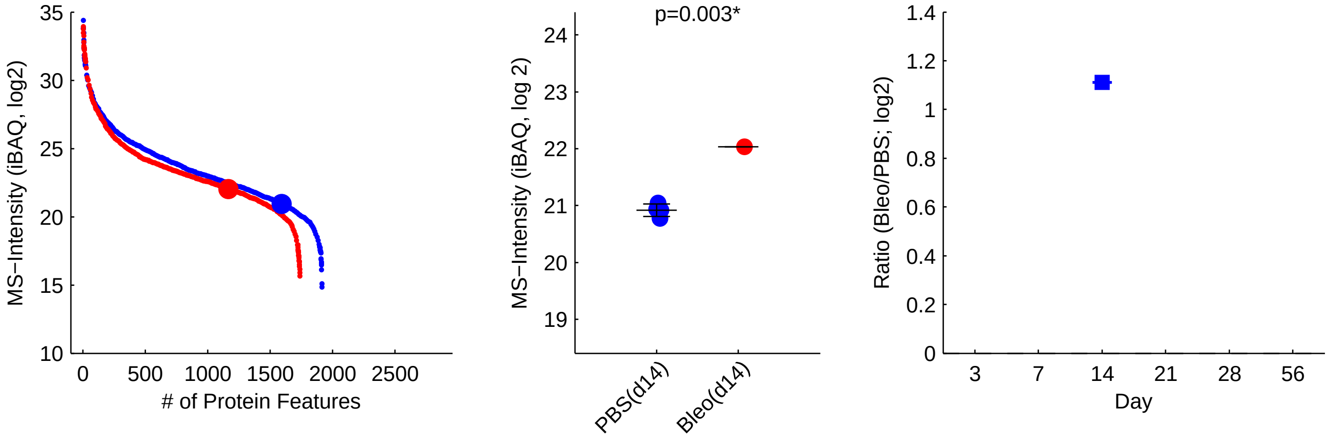

O35604 − Npc1 (id: 676)

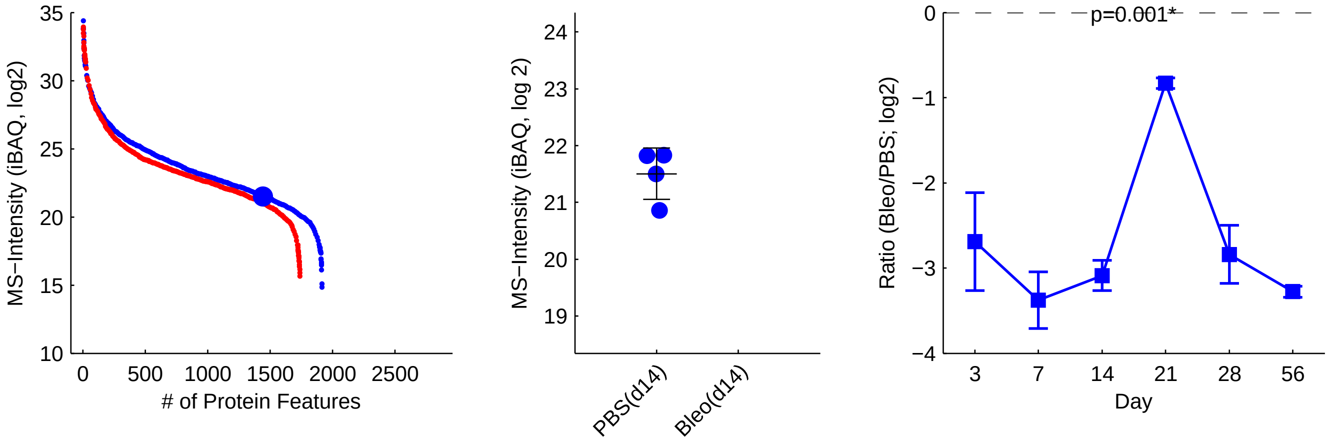

O35640 − Anxa8 (id: 678)

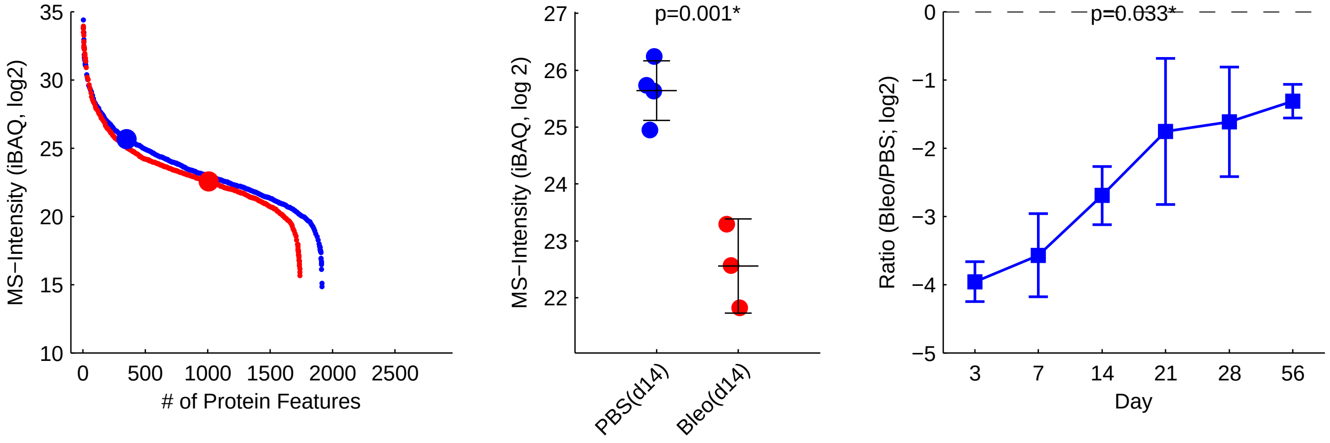

O35737 − Hnrnph1 (id: 683)

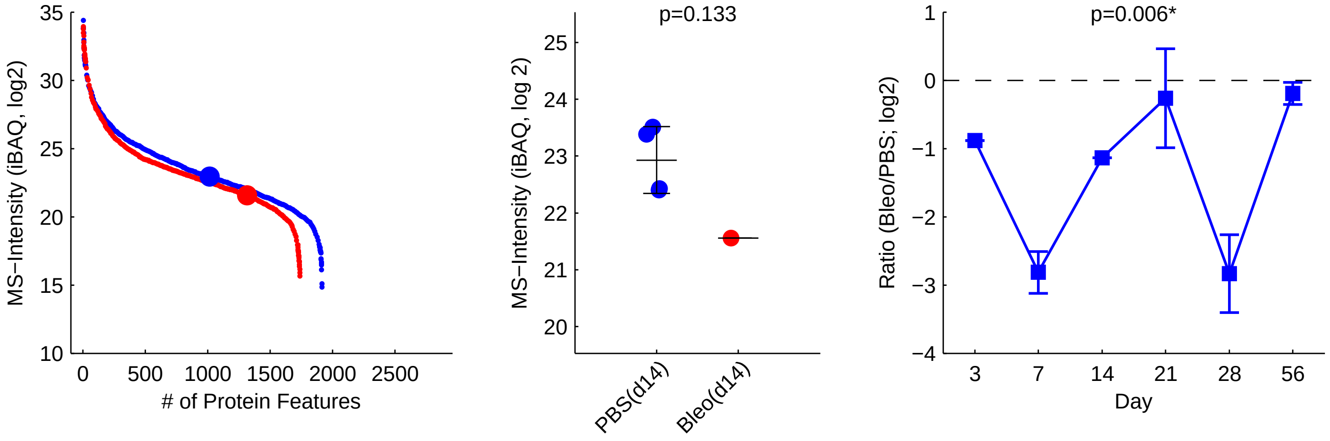

O35744 − Chi3l3 (id: 684)

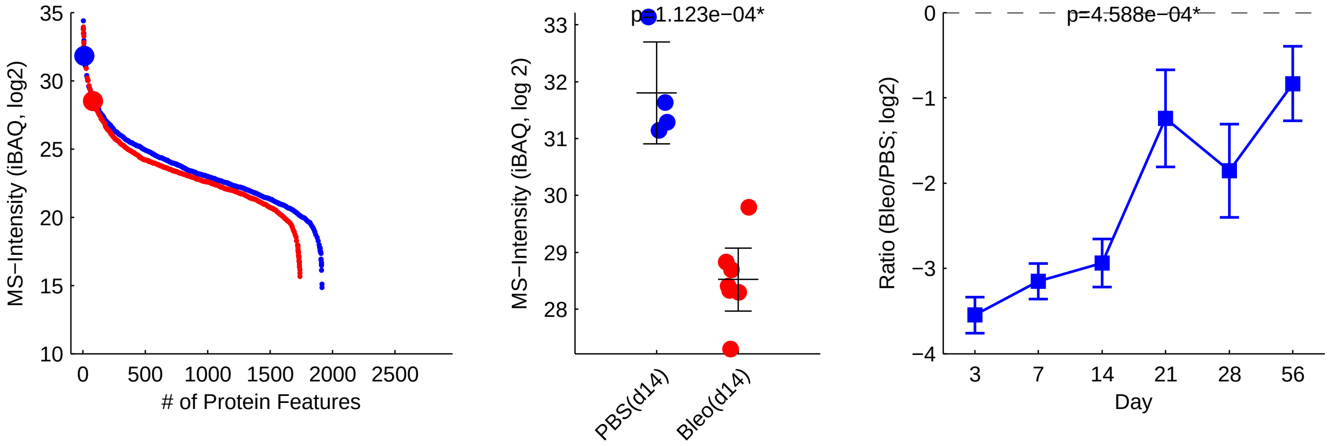

O35887 − Calu (id: 687)

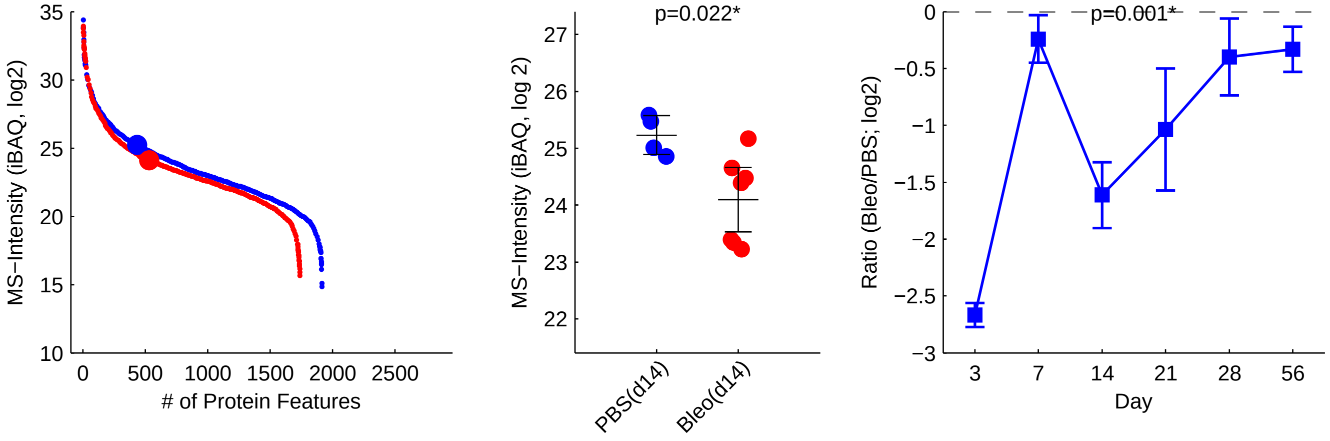

O35945 − Aldh1a7 (id: 691)

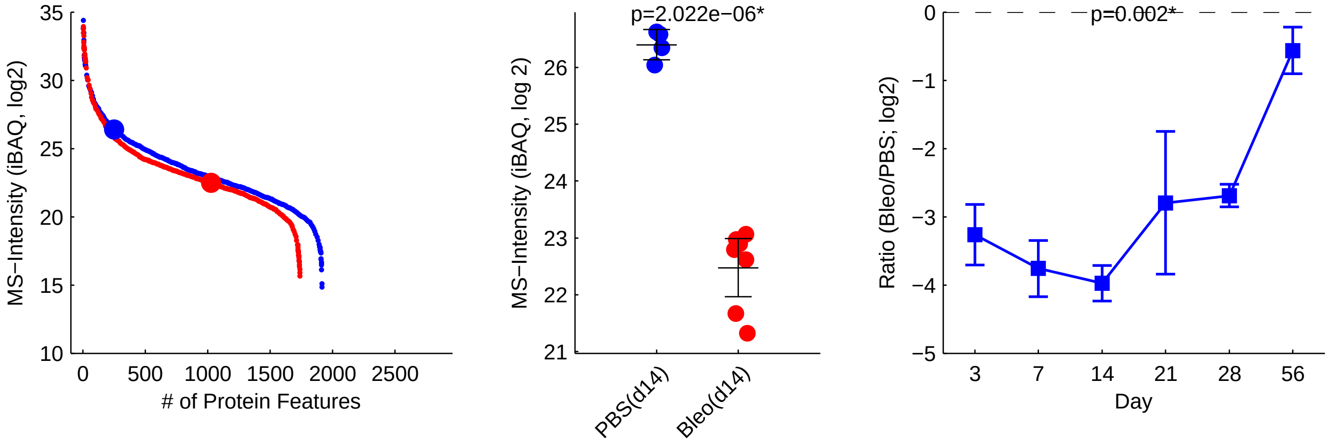

O54734 − Ddost (id: 695)

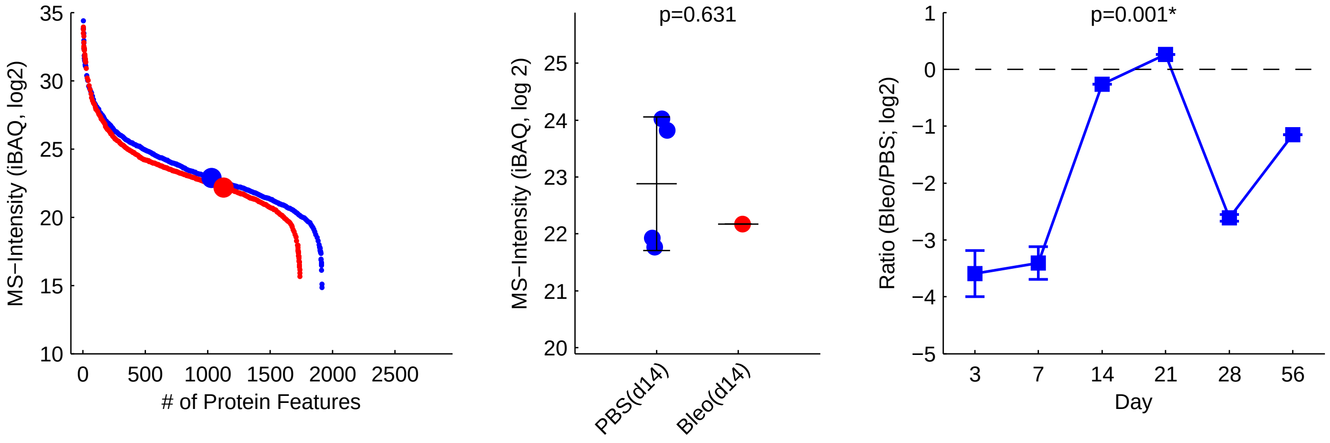

O54782 − Man2b2 (id: 697)

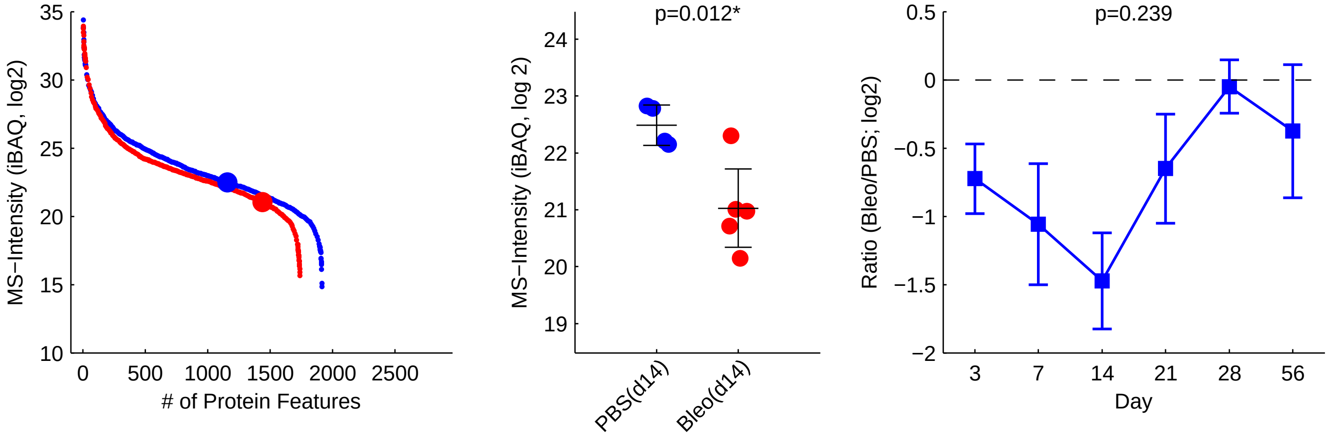

O54950 − Prkag1 (id: 699)

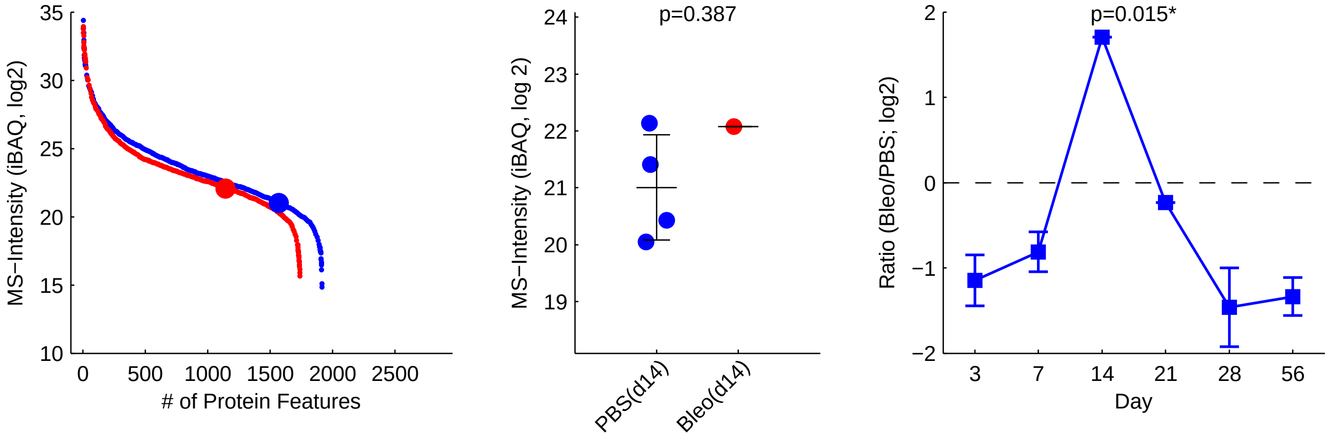

O54988 − Slk (id: 702)

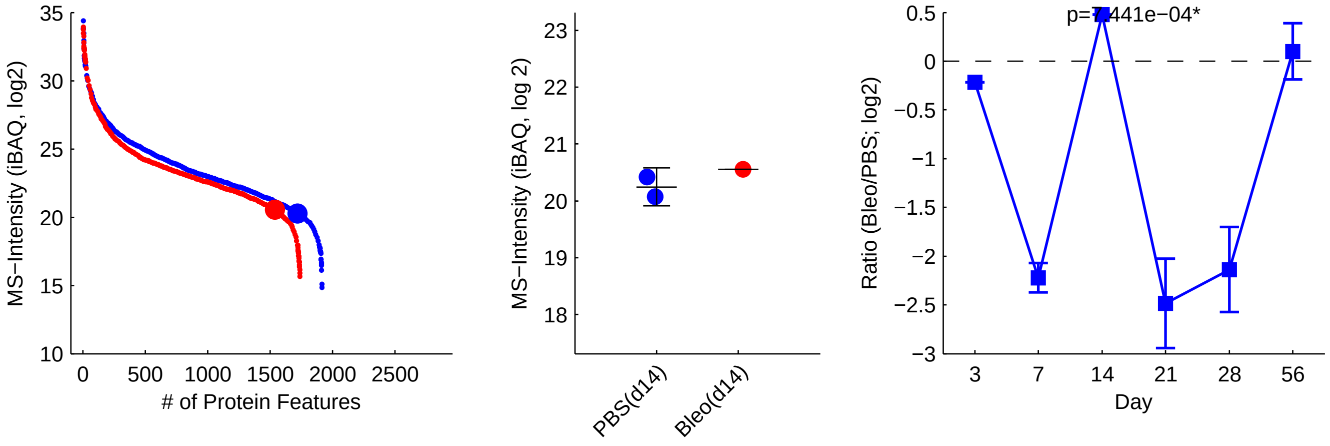

O55022 − Pgrmc1 (id: 703)

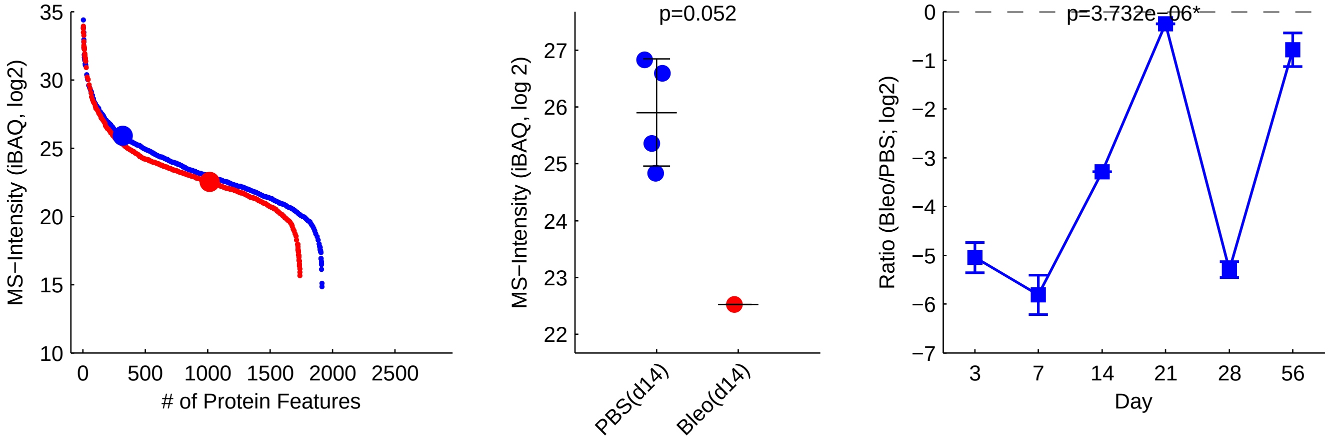

O55137 − Acot1 (id: 711)

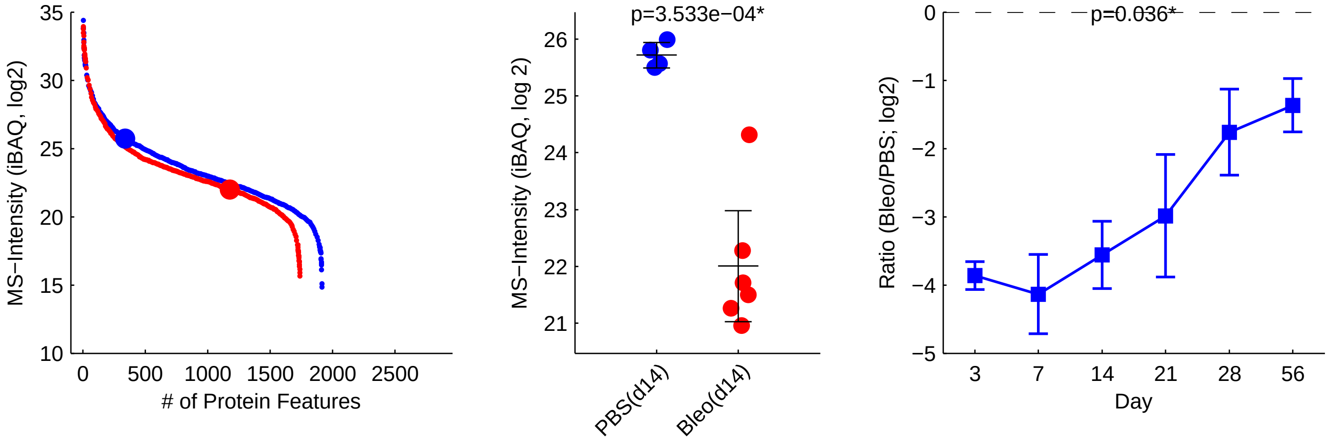

O55226 − Chad (id: 714)

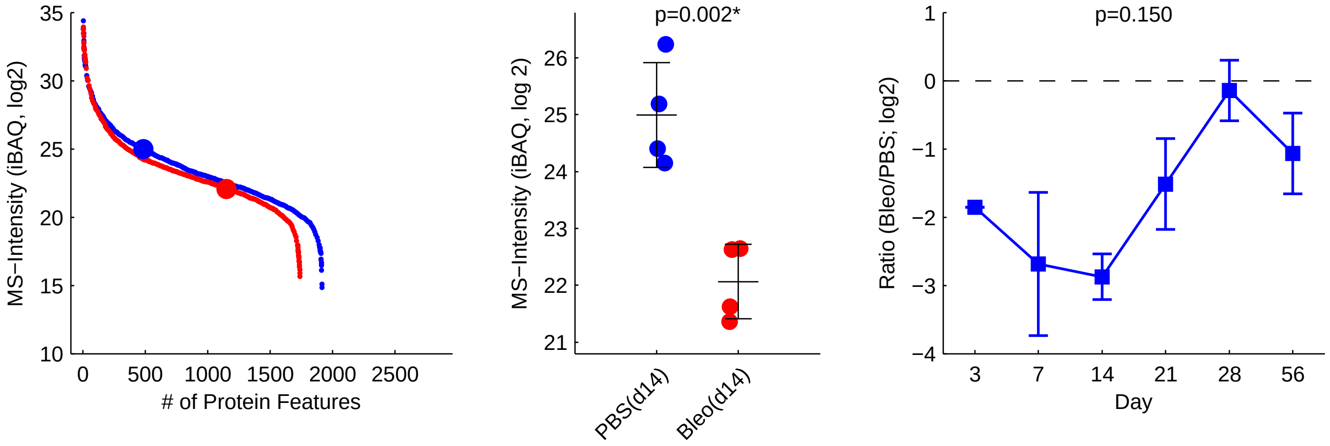

O70251 − Eef1b (id: 721)

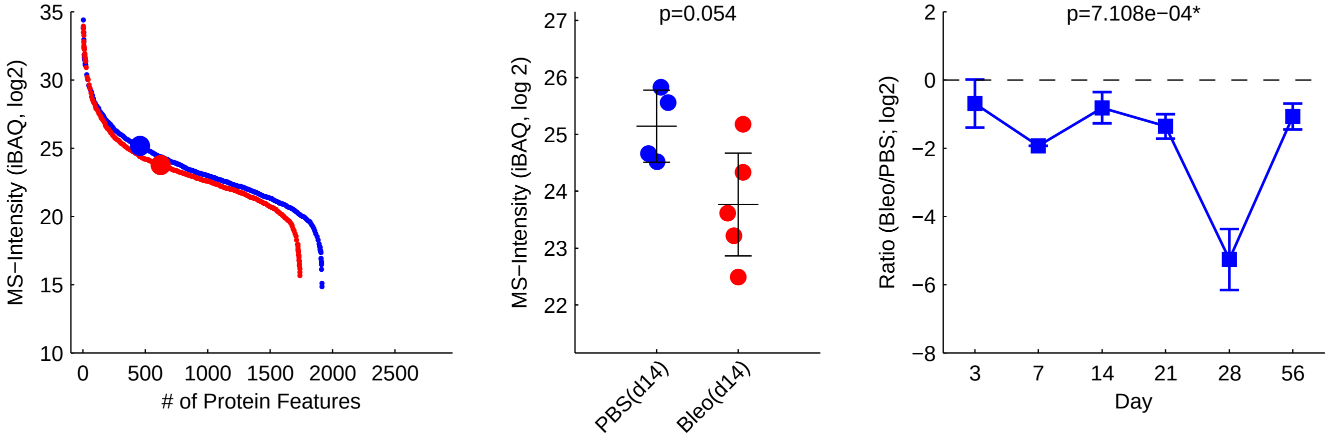

O70318 − Epb41l2 (id: 723)

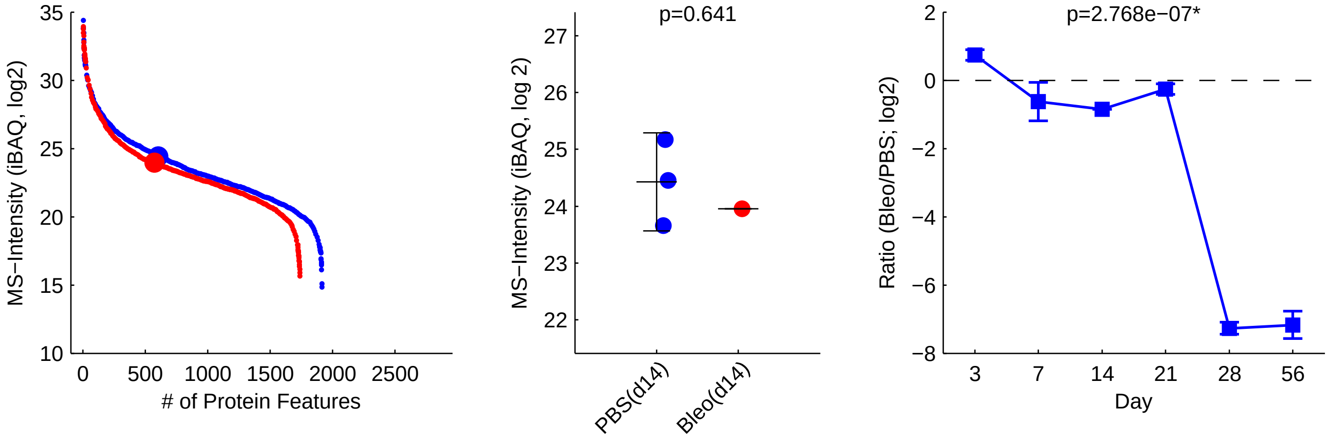

O70370 − Ctss (id: 725)

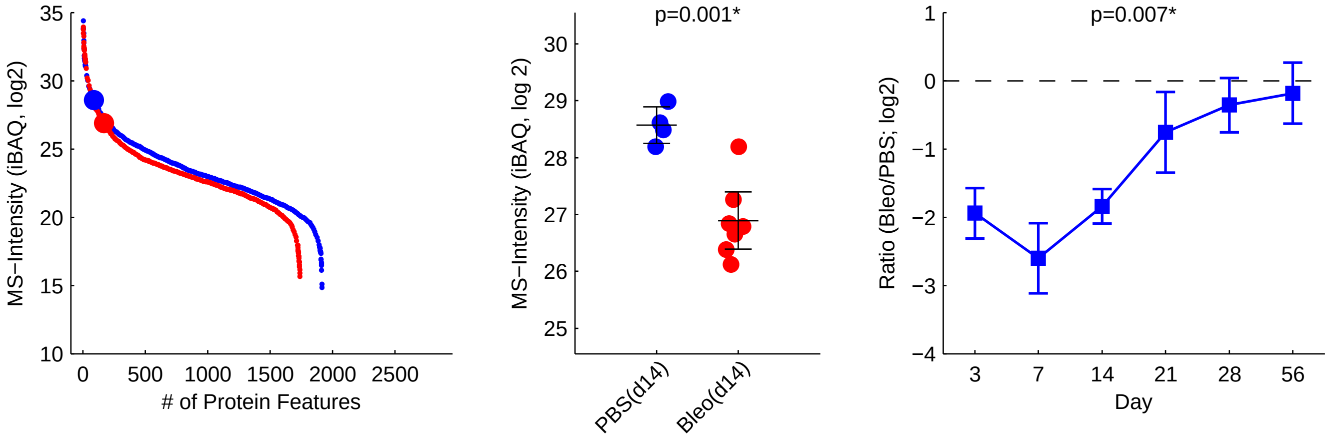

O70400 − Pdlim1 (id: 726)

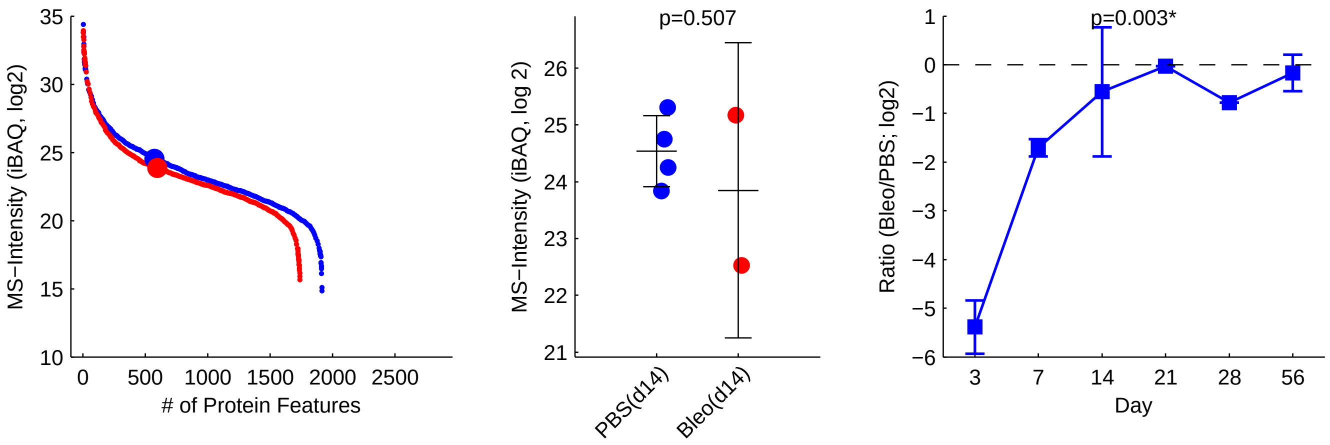

O70451 − Slc16a7 (id: 730)

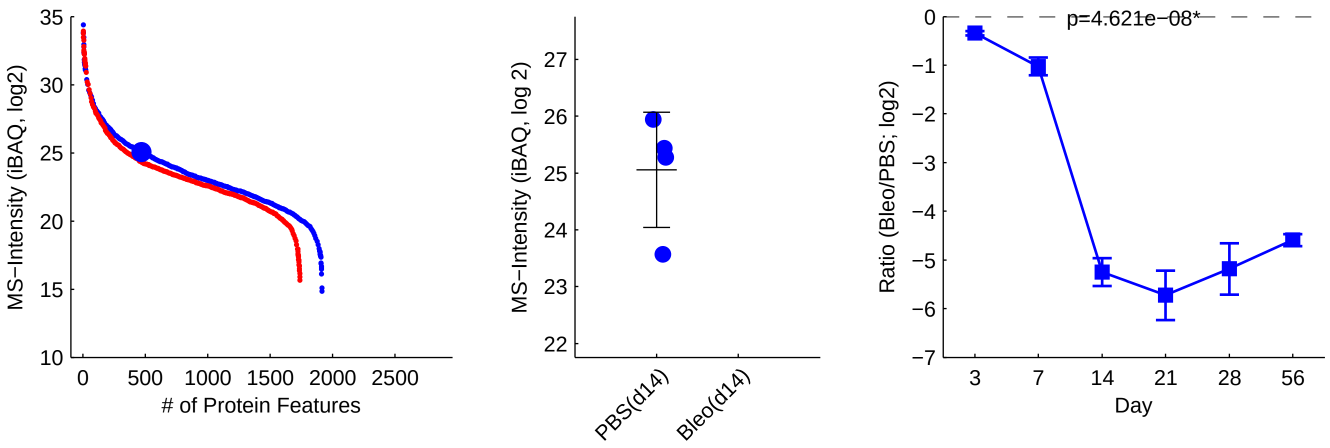

Q6ZWQ5 − Snx12 (id: 733)

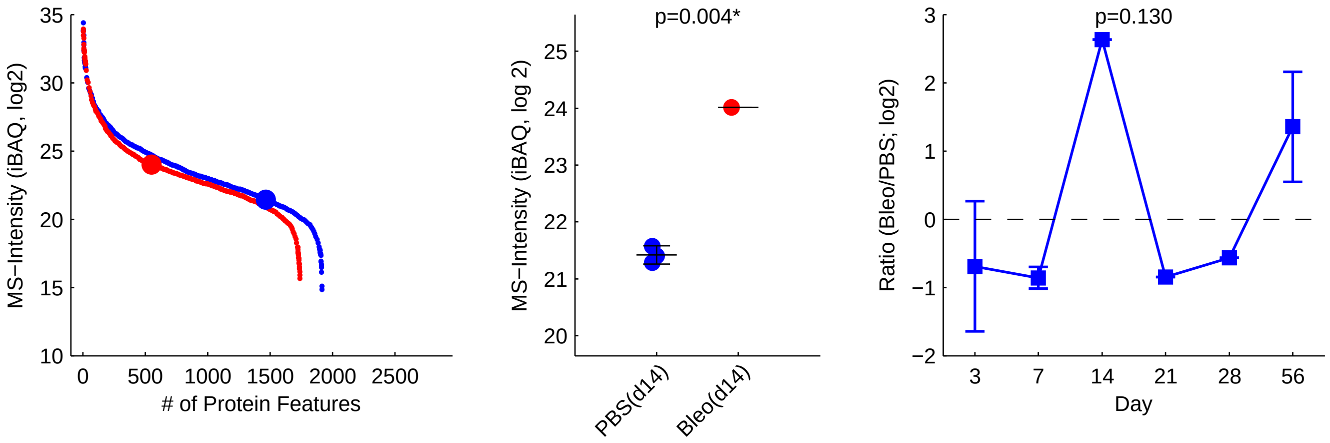

O70570 − Pigr (id: 735)

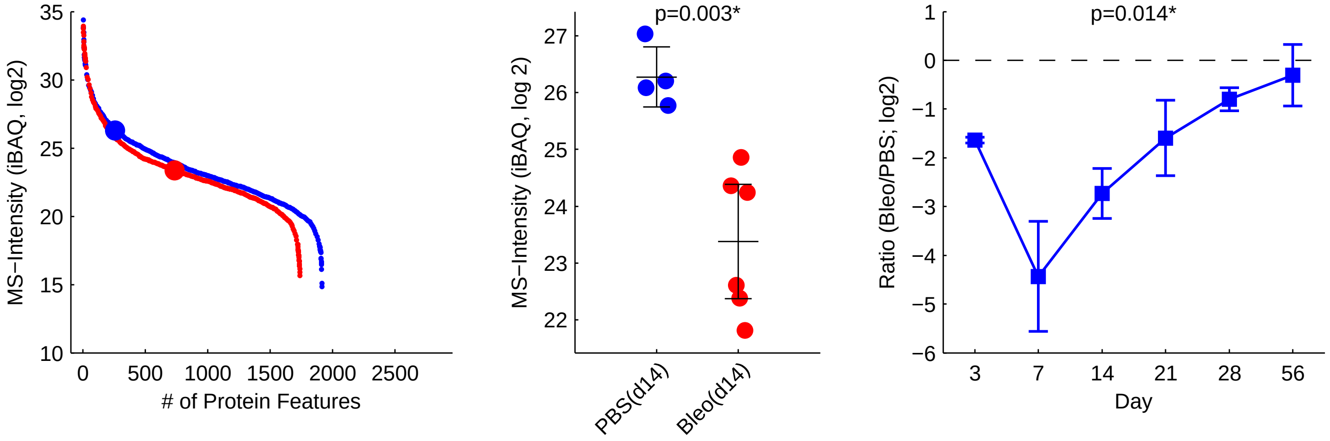

O88312 − Agr2 (id: 741)

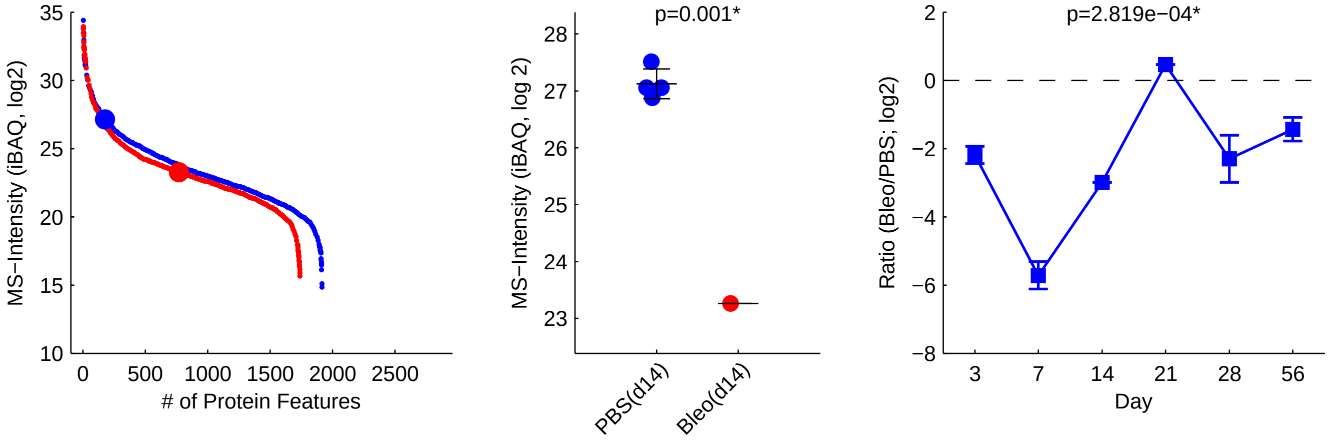

O88543 − Cops3 (id: 746)

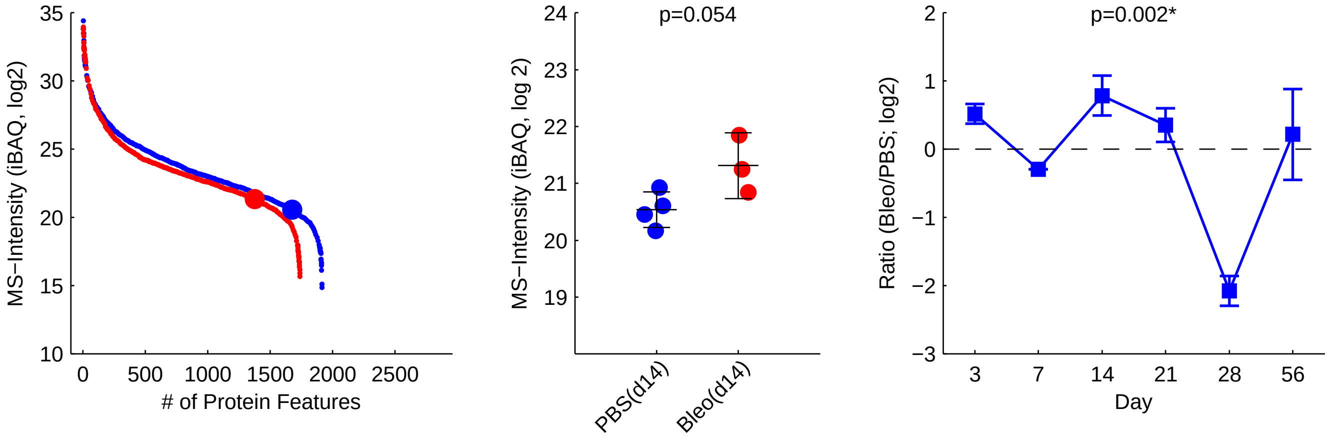

O88569 − Hnrnpa2b1 (id: 748)

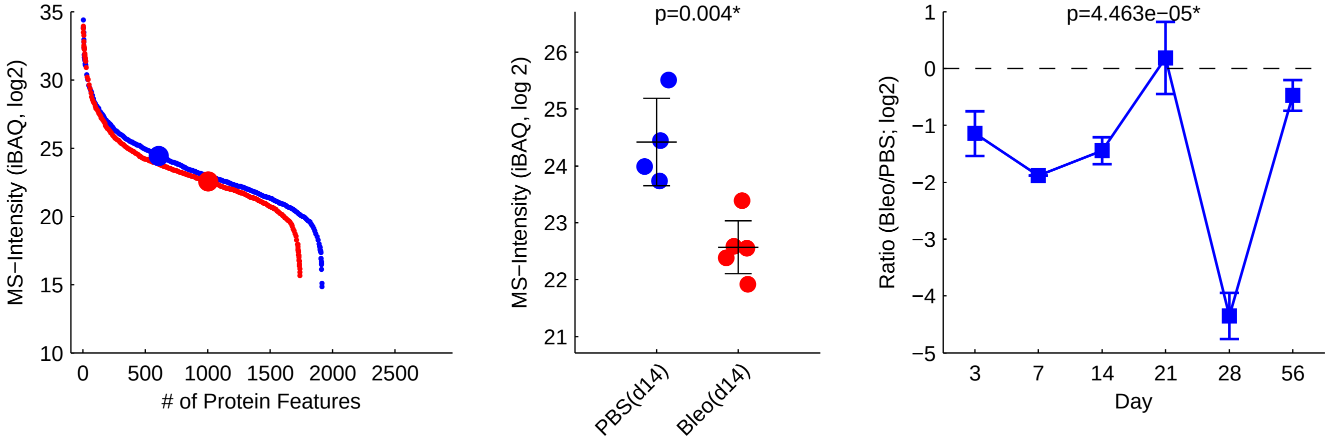

O88587−2 − Comt (id: 749)

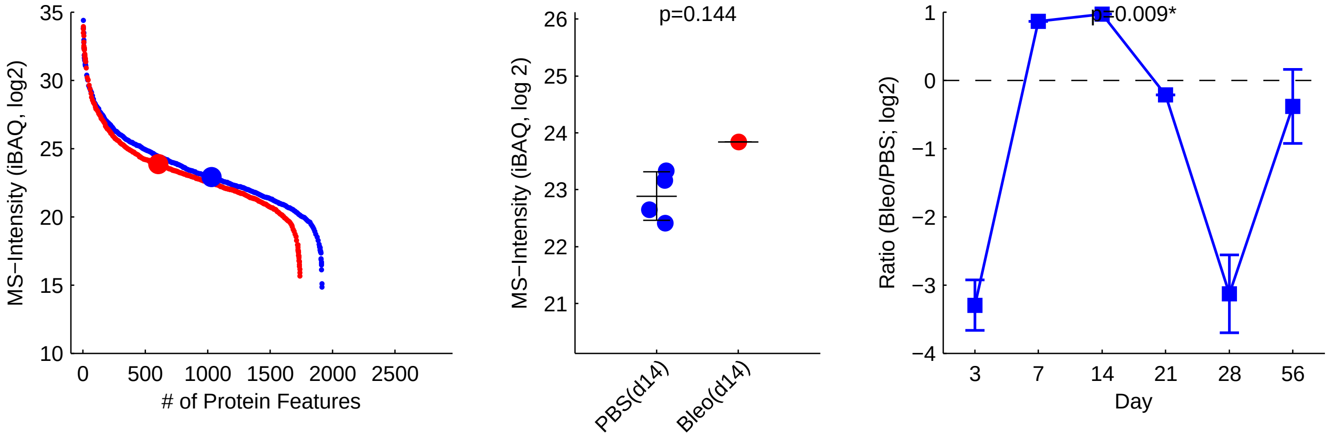

O88668 − Creg1 (id: 753)

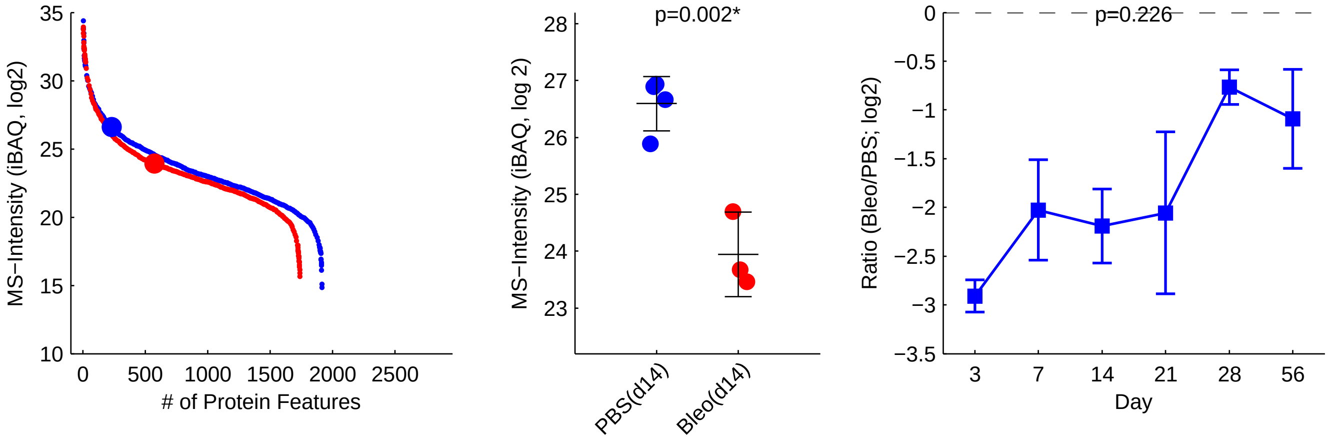

O88844 − Idh1 (id: 758)

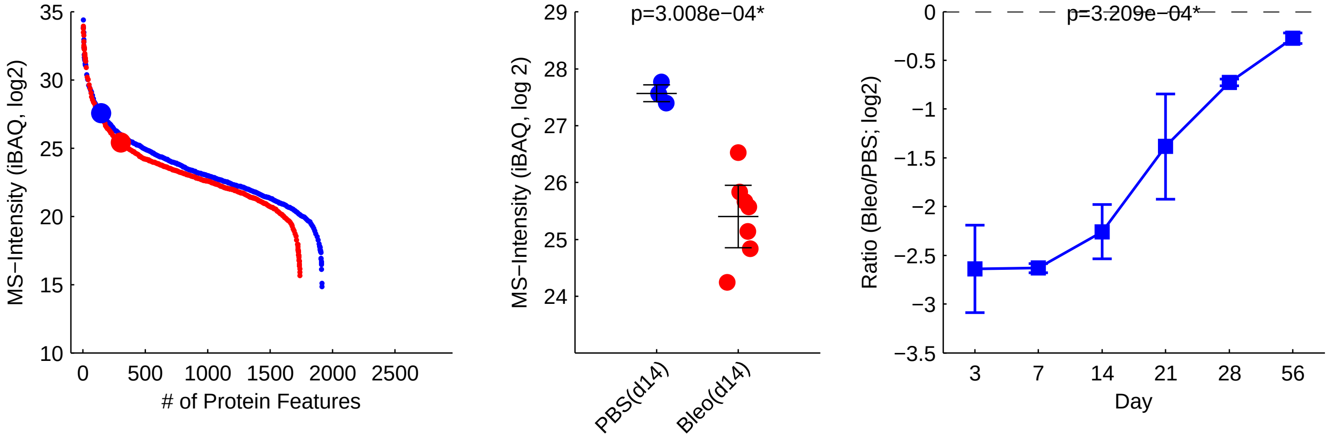

O88851 − Rbbp9 (id: 759)

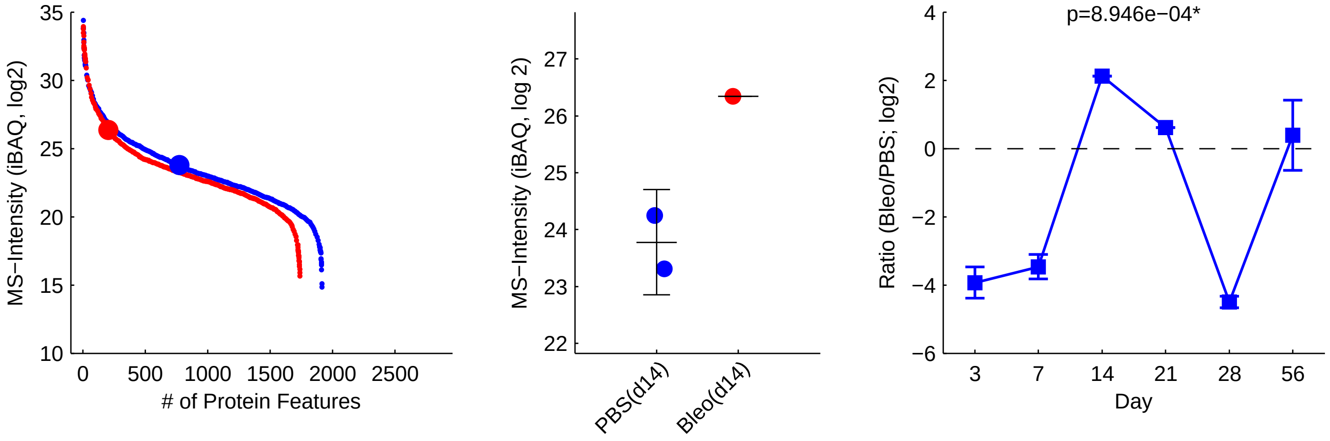

O88947 − F10 (id: 760)

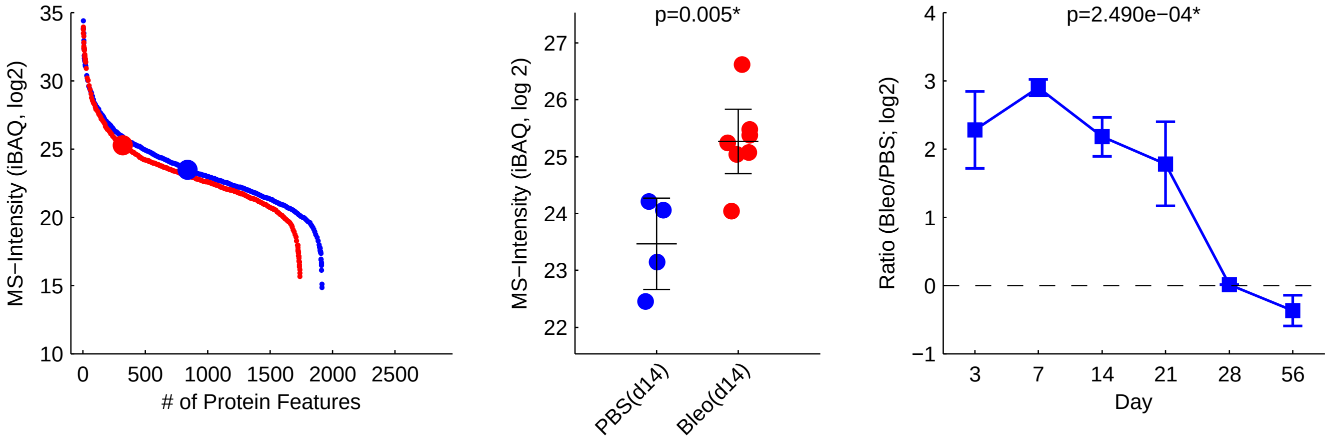

O88958 − Gnpda1 (id: 762)

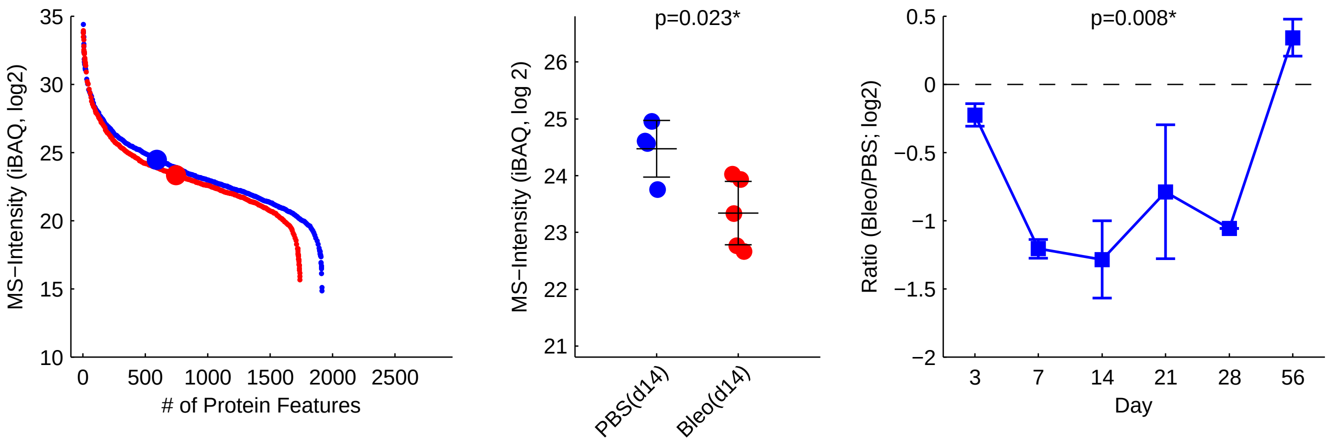

O88968 − Tcn2 (id: 763)

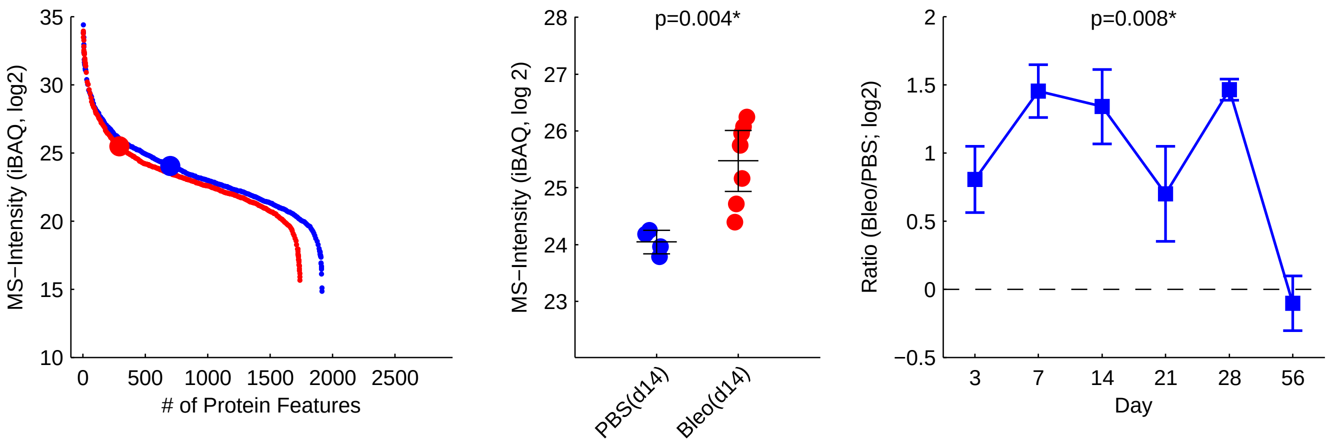

O89023 − Tpp1 (id: 767)

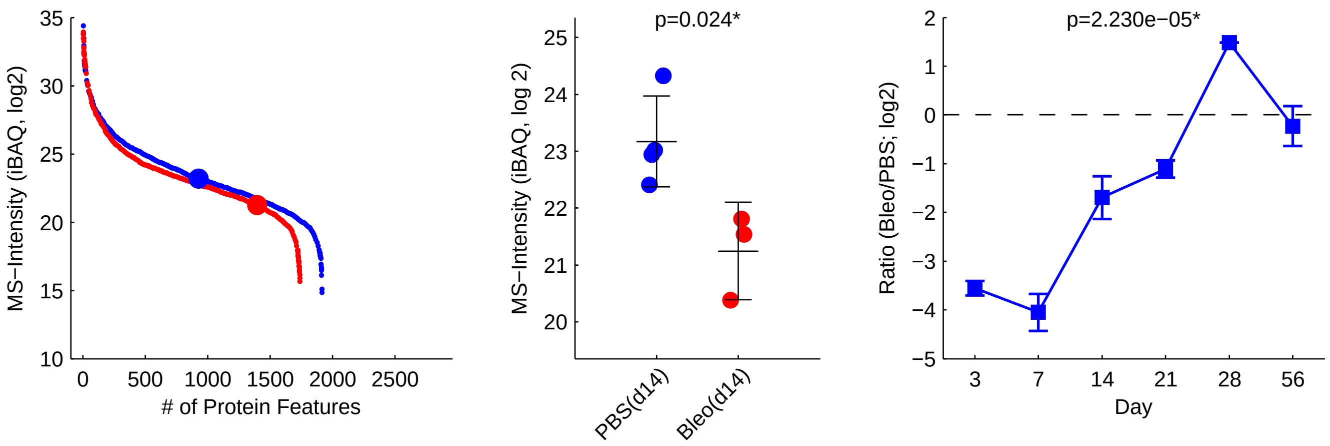

O89053 − Coro1a (id: 769)

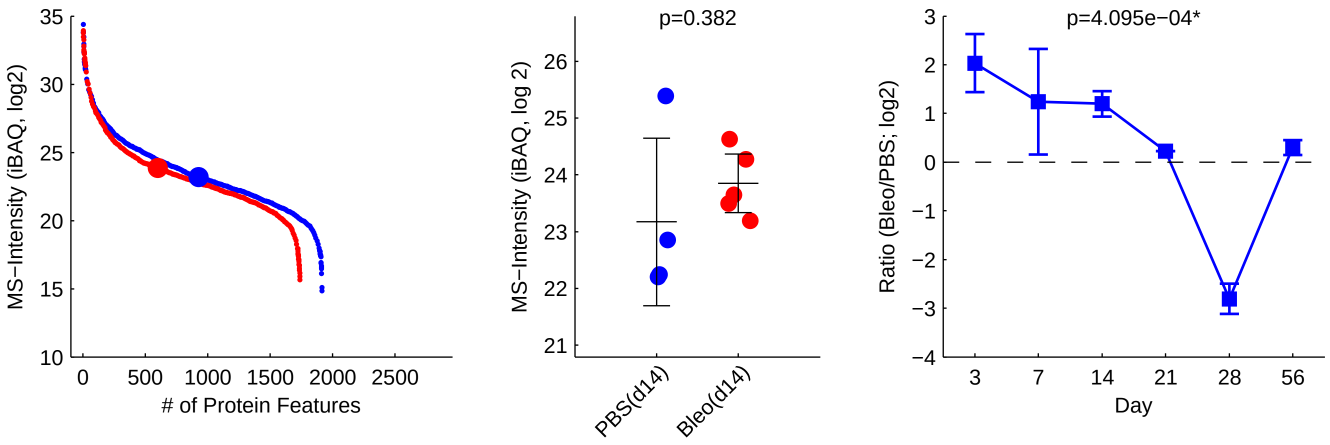

P00493 − Hprt1 (id: 776)

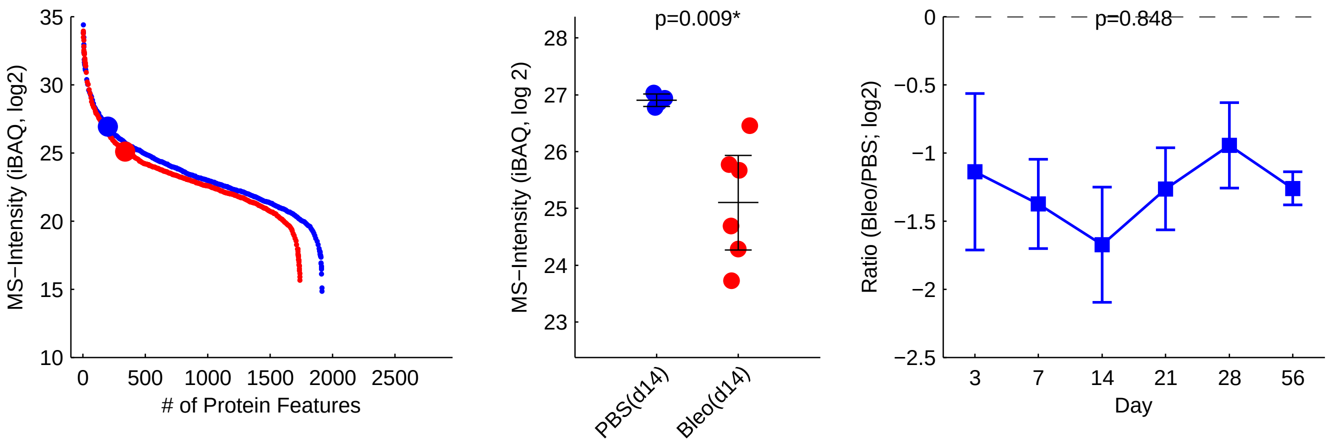

P00688 − Amy2 (id: 778)

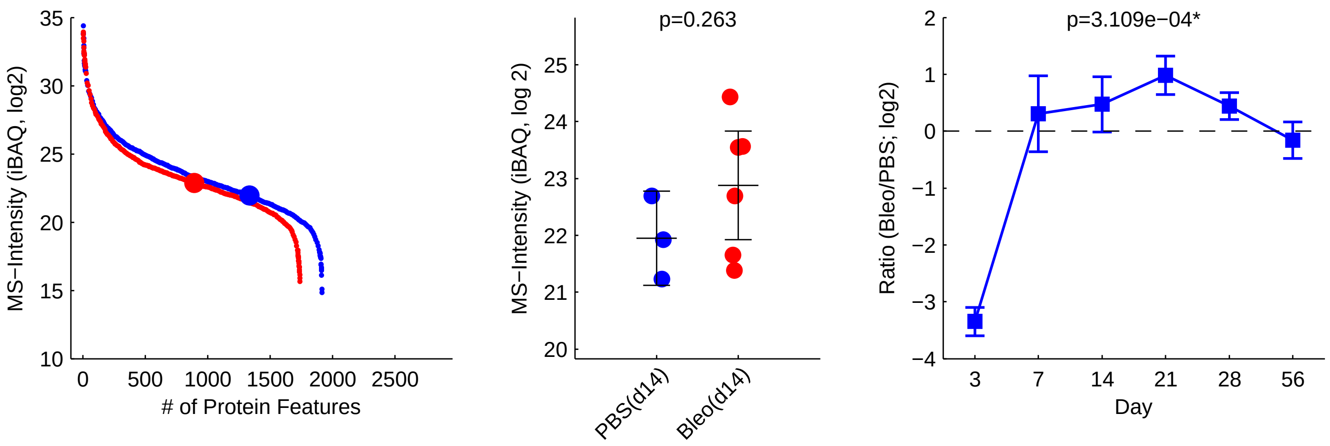

P01027 − C3 (id: 780)

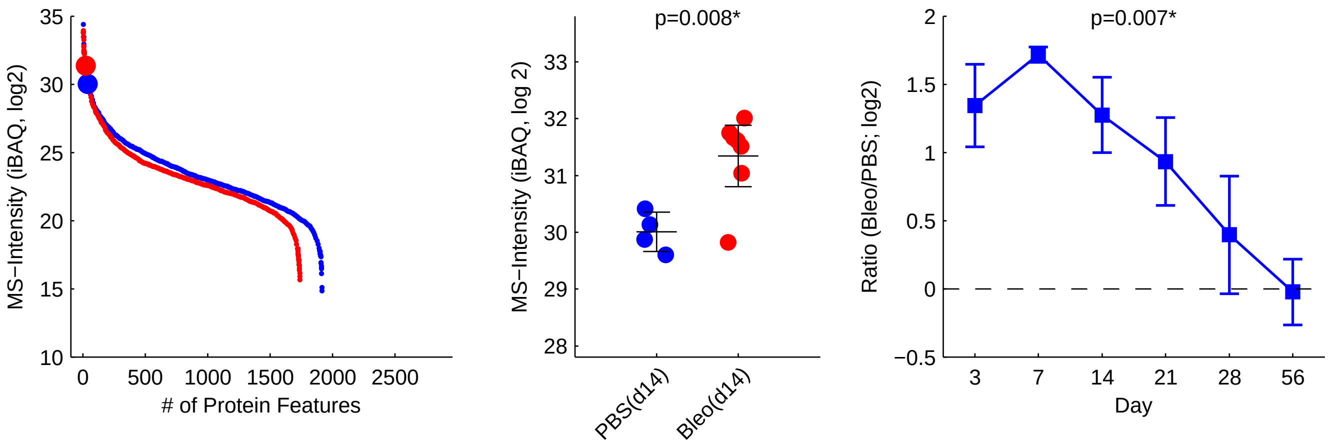

P01029 − C4b (id: 781)

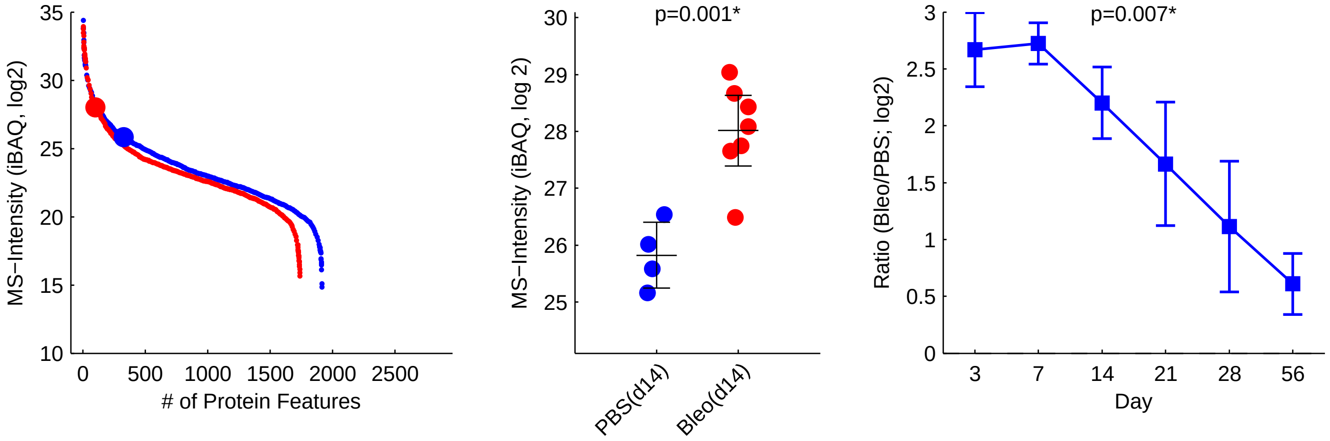

P01633 − Igk−V19−17 (id: 786)

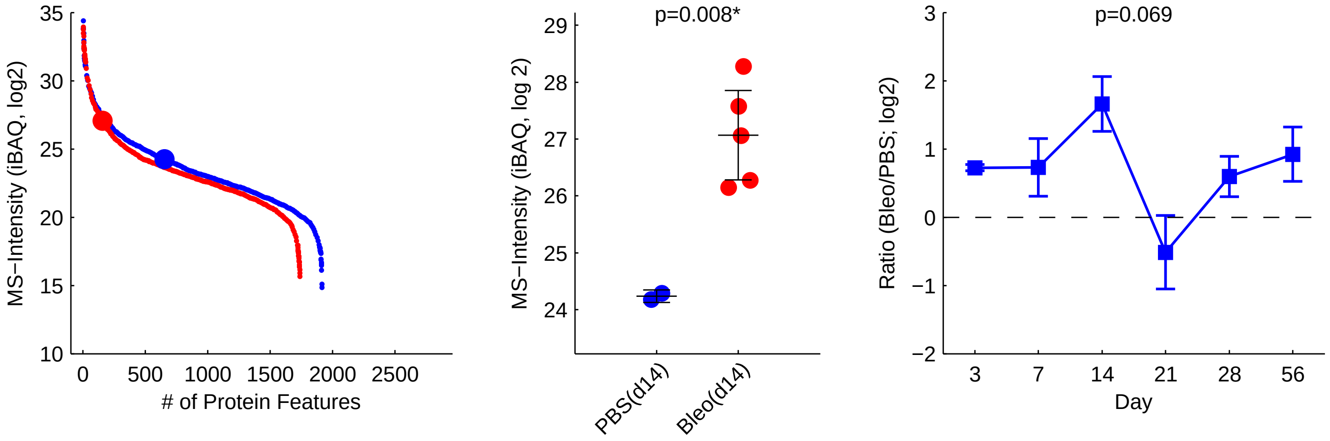

P01636 − (id: 788)

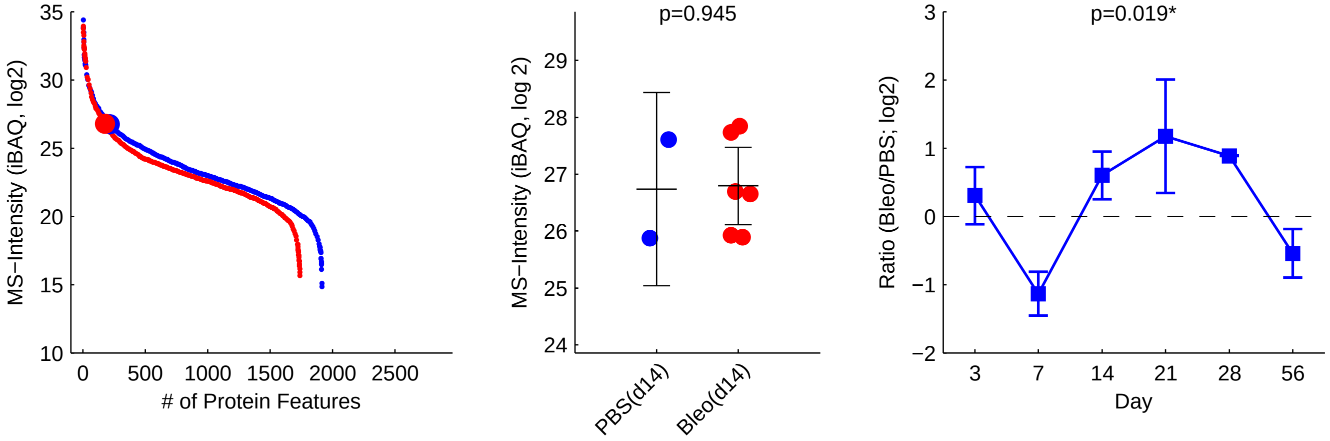

P01638 − (id: 789)

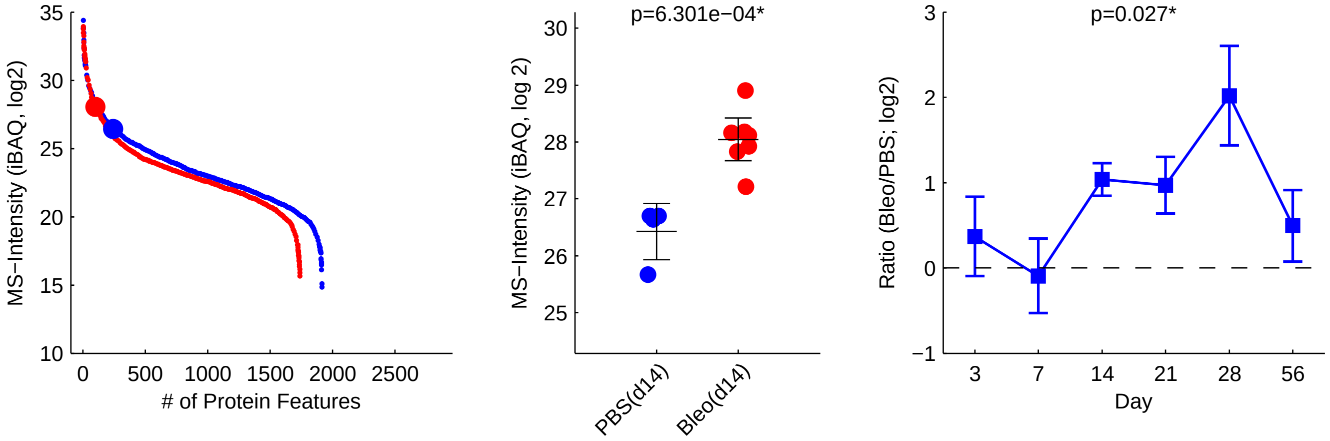

P01642 − Gm10881 (id: 791)

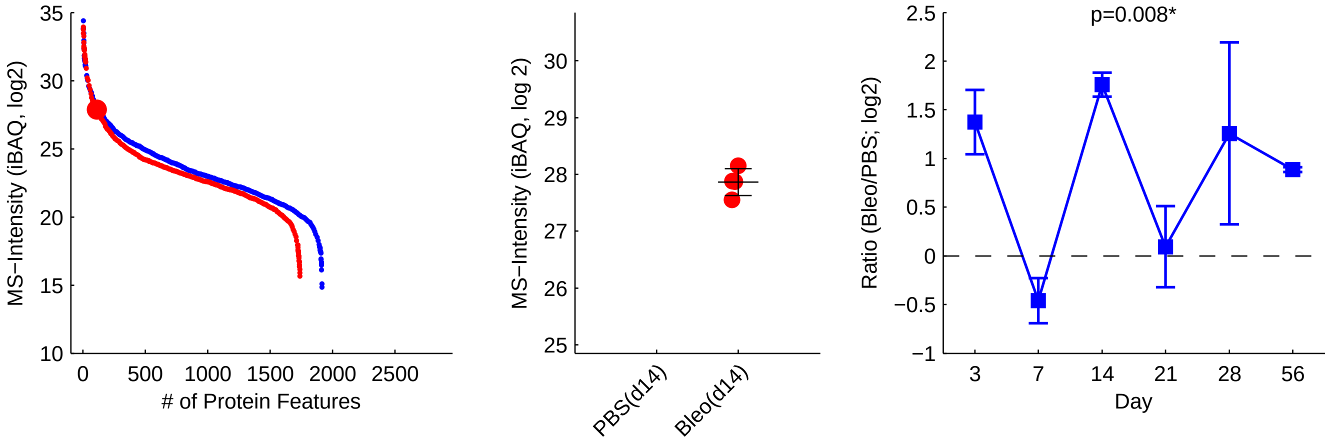

P01679 − (id: 804)

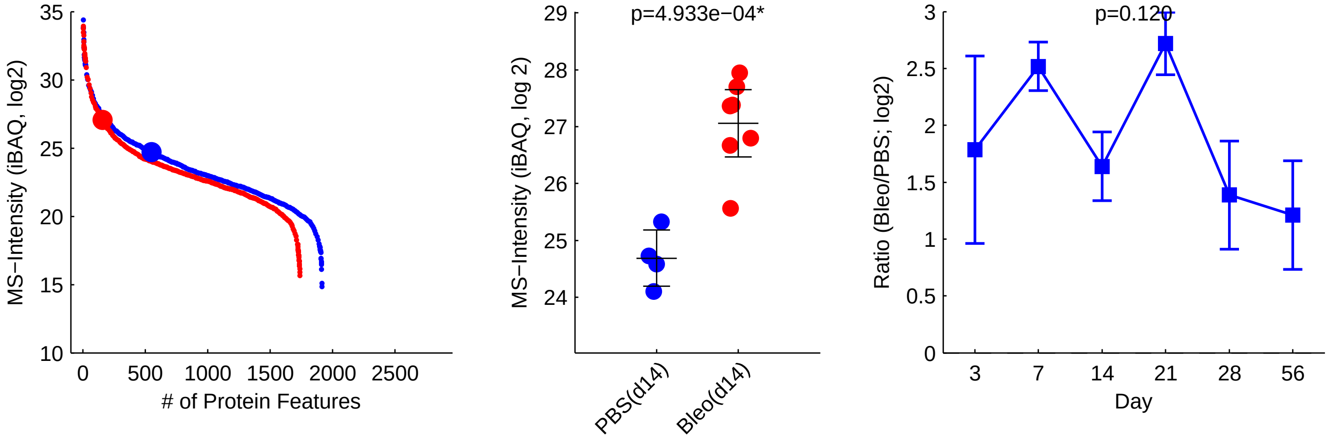

P01758 − Igh−VJ558 (id: 811)

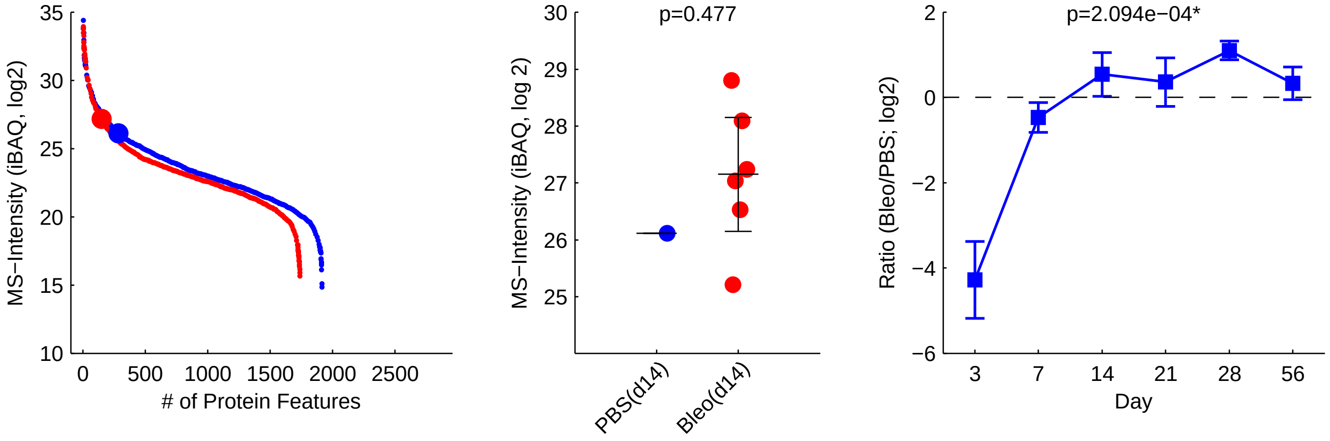

P01799 − (id: 815)

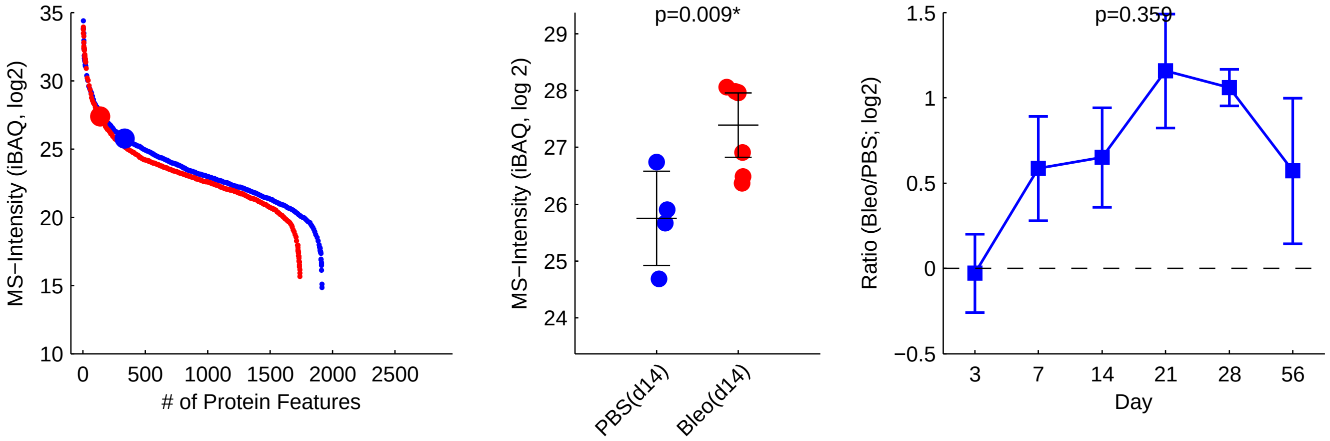

P01806 − (id: 816)

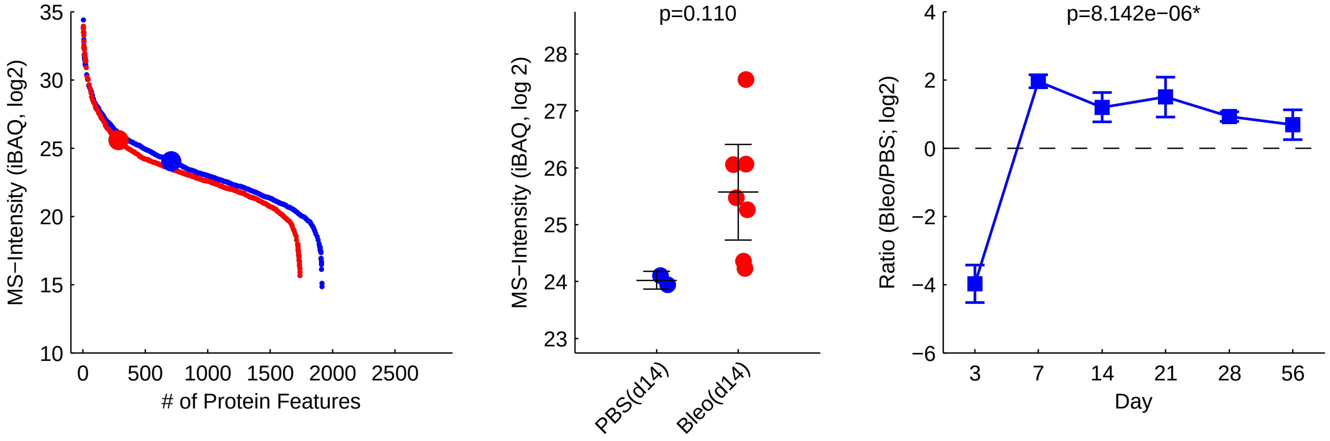

P01837 − (id: 819)

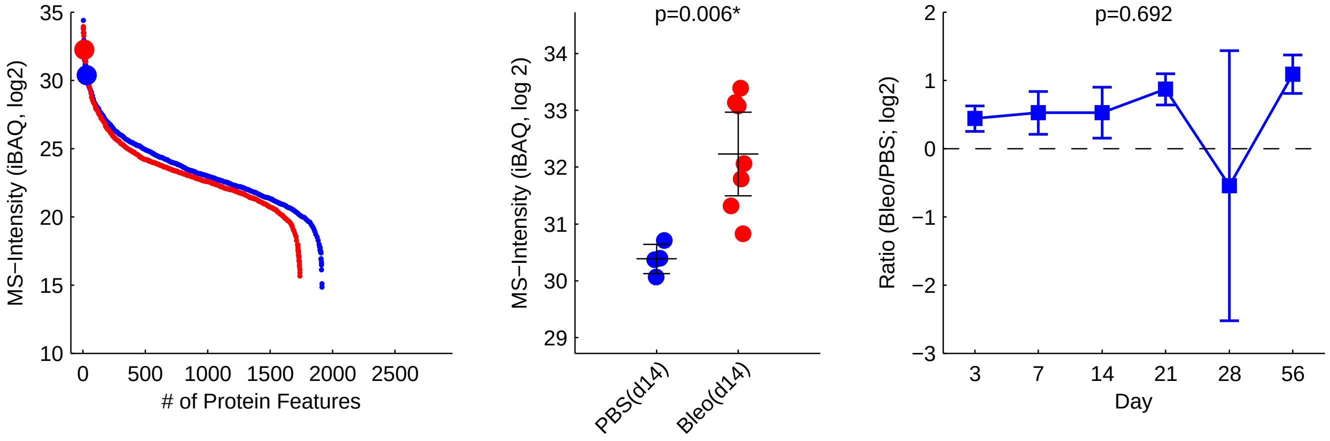

P01844 − Iglc2 (id: 821)

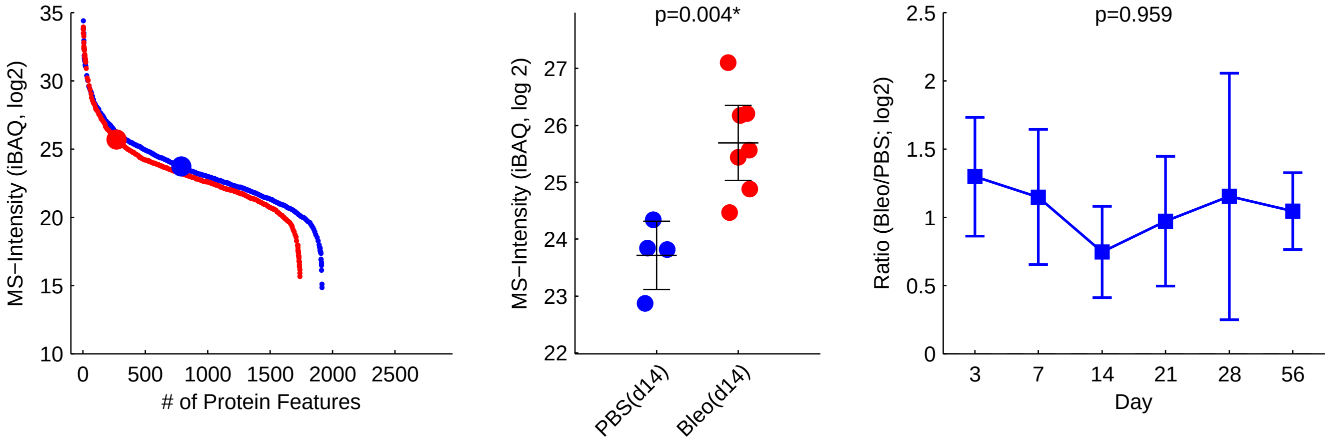

P01867−2 − Igh−3 (id: 823)

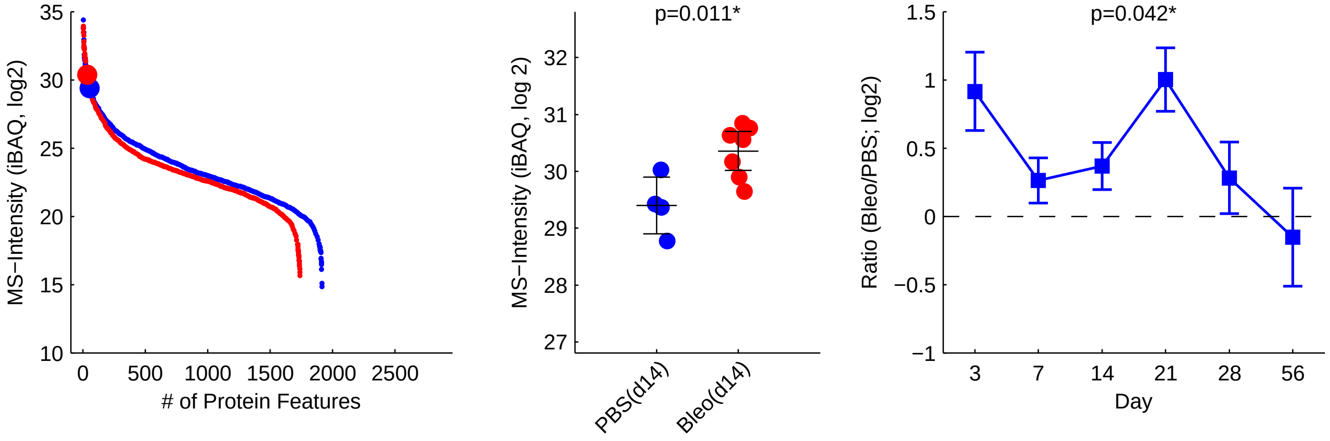

P01872 − Igh−6 (id: 825)

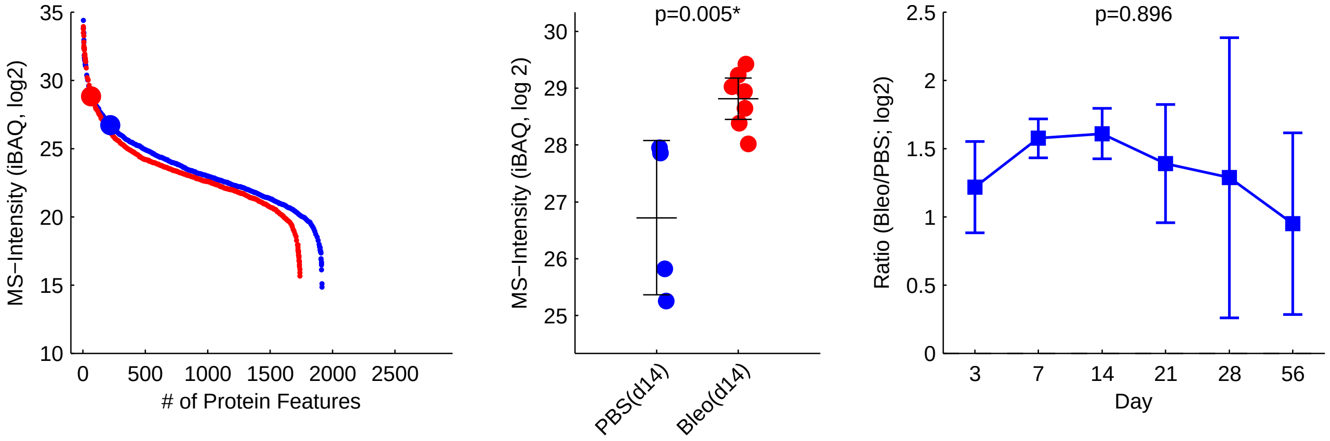

P01878 − (id: 826)

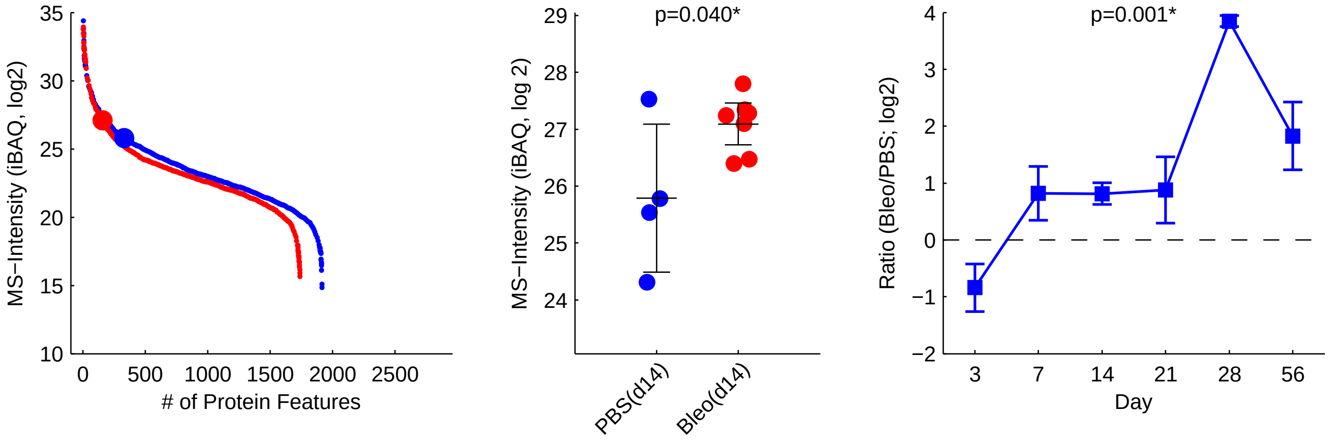

P01898 − H2−Q10 (id: 828)

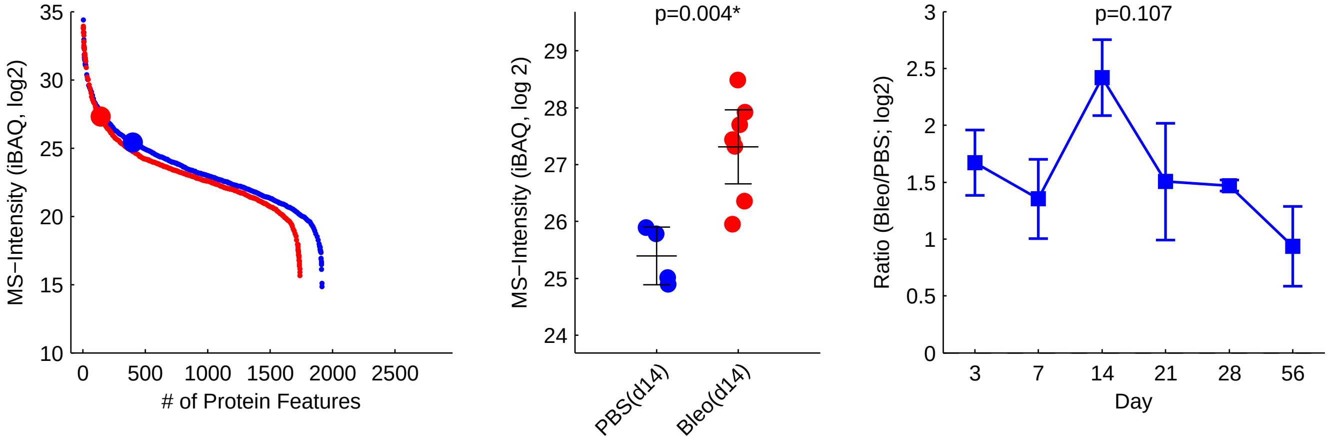

P01899 − H2−D1 (id: 829)

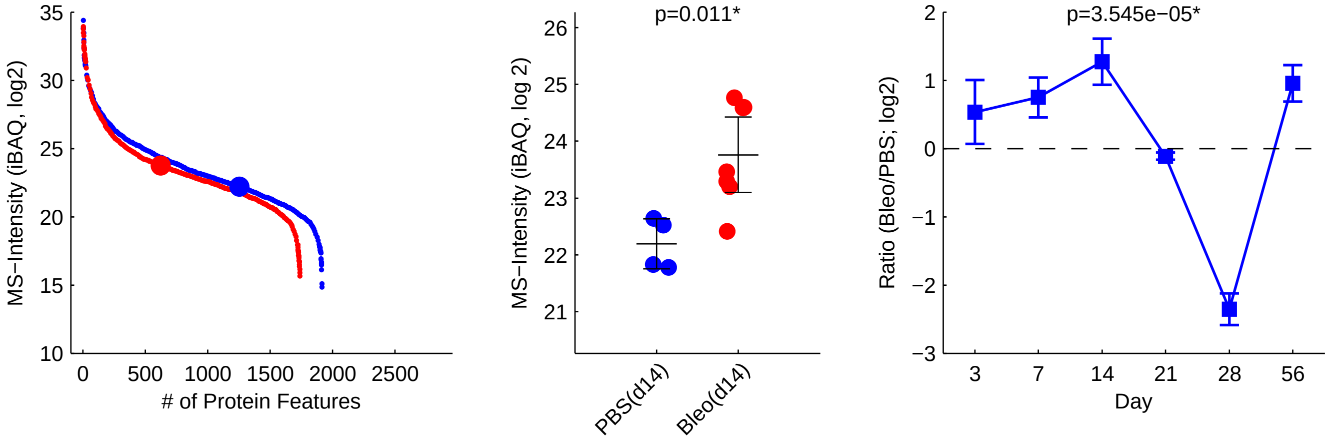

P03953−2 − Cfd (id: 835)

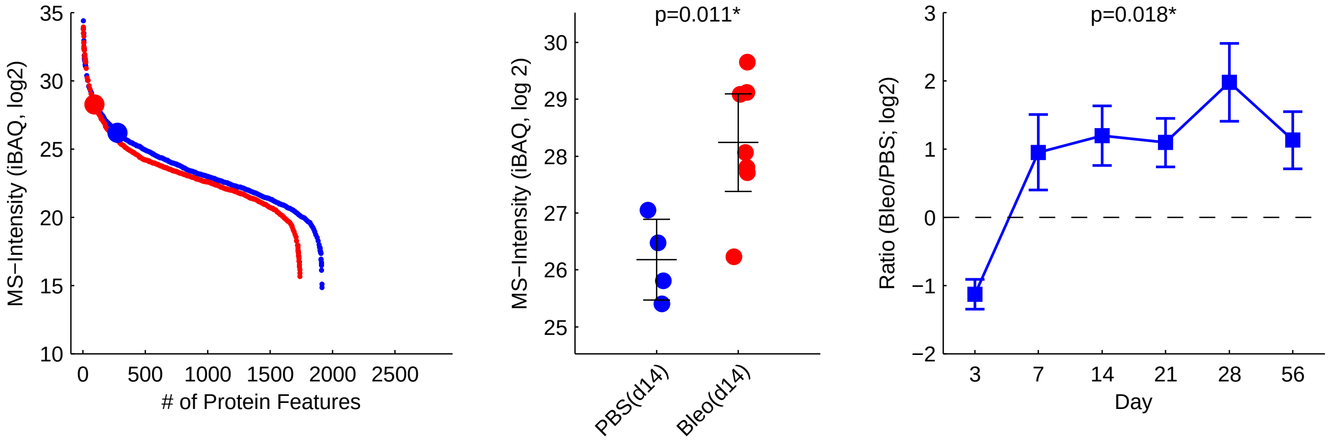

P04117 − Fabp4 (id: 839)

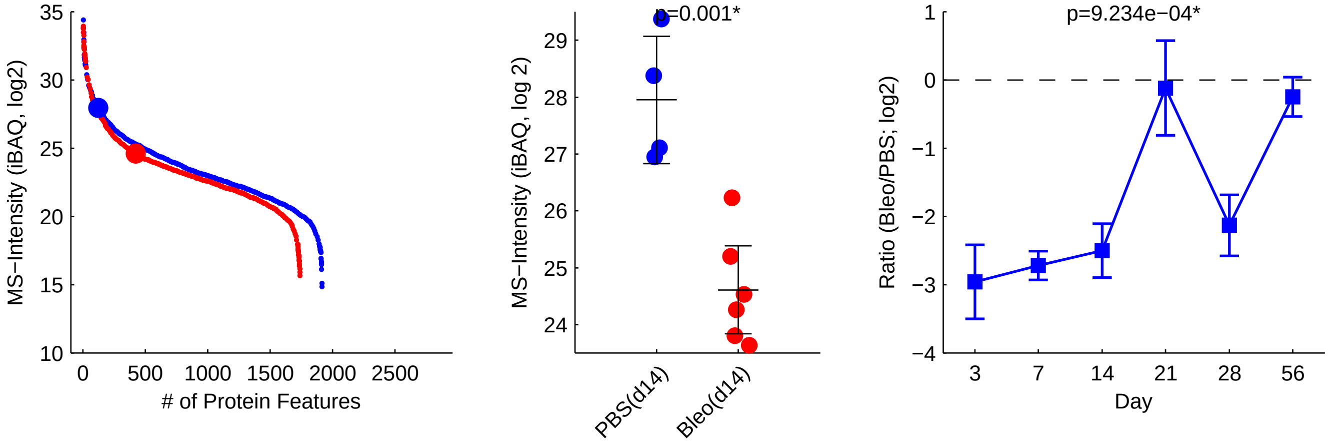

P04247 − Mb (id: 841)

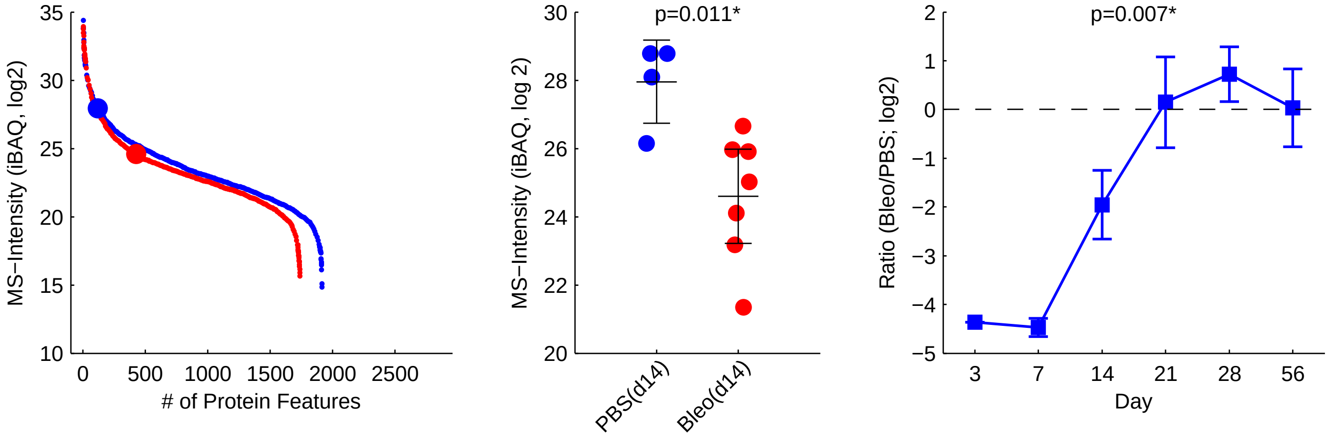

P05132−2 − Prkaca (id: 848)

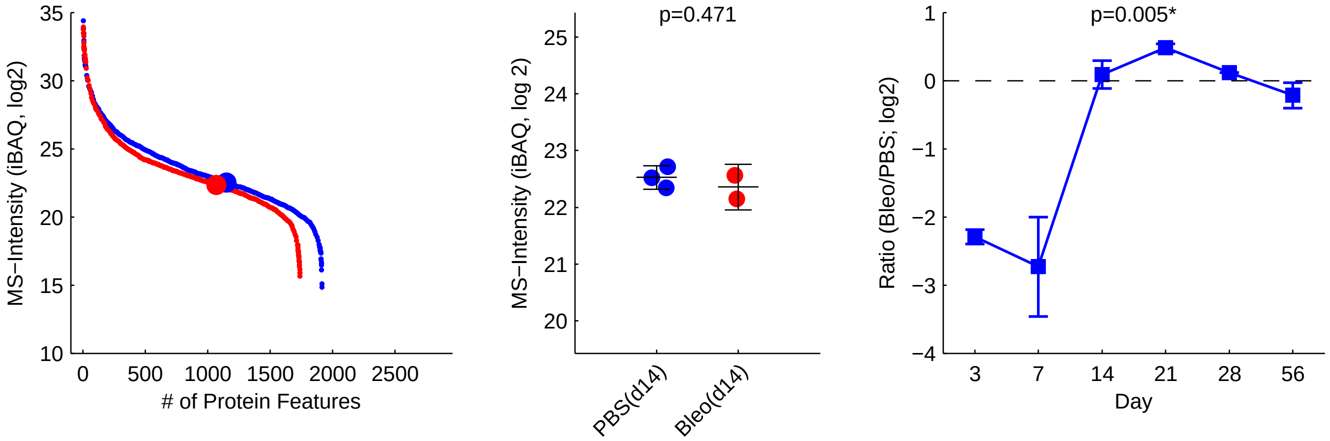

P05202 − Got2 (id: 850)

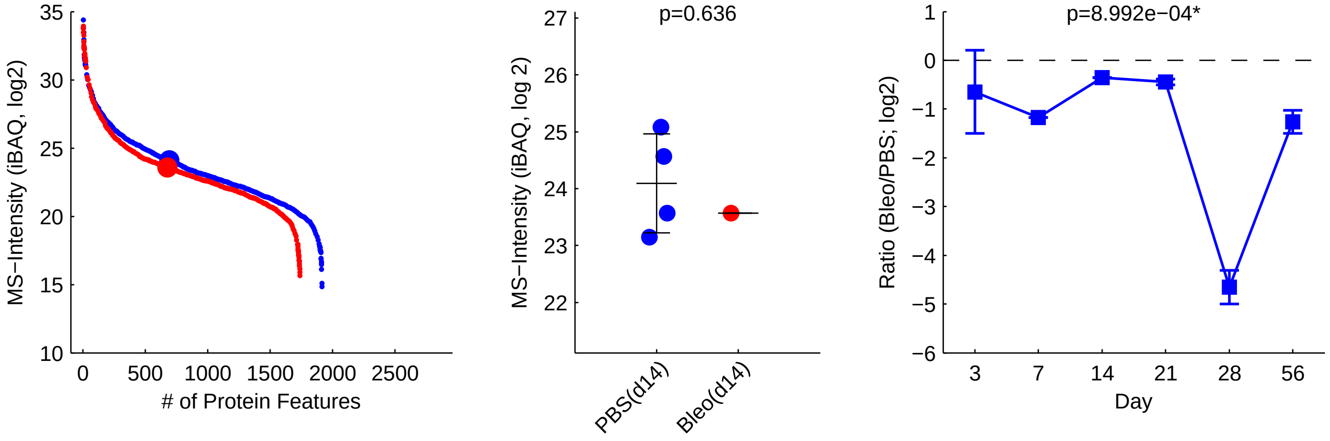

P06151 − Ldha (id: 856)

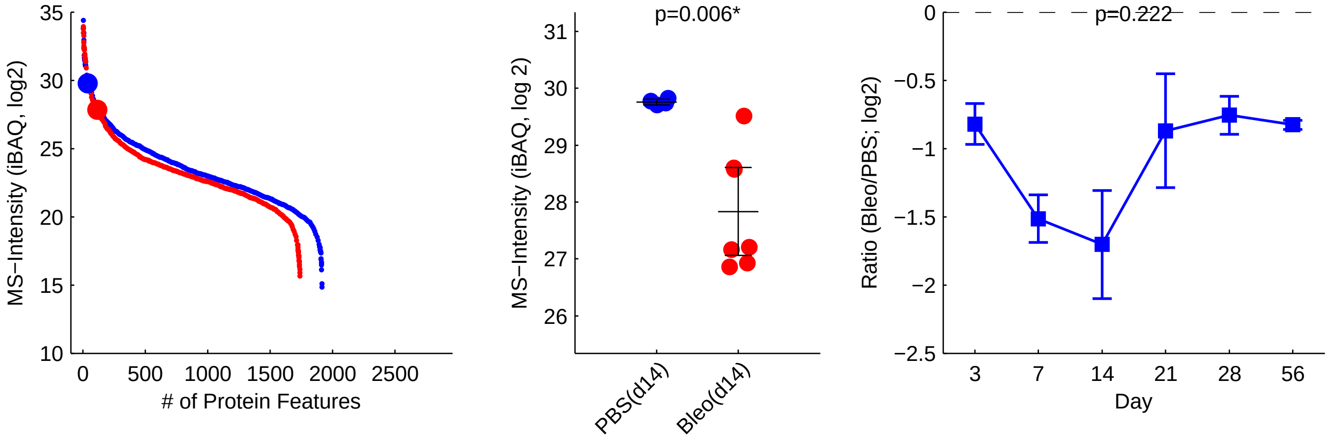

P06327 − Gm5629 (id: 857)

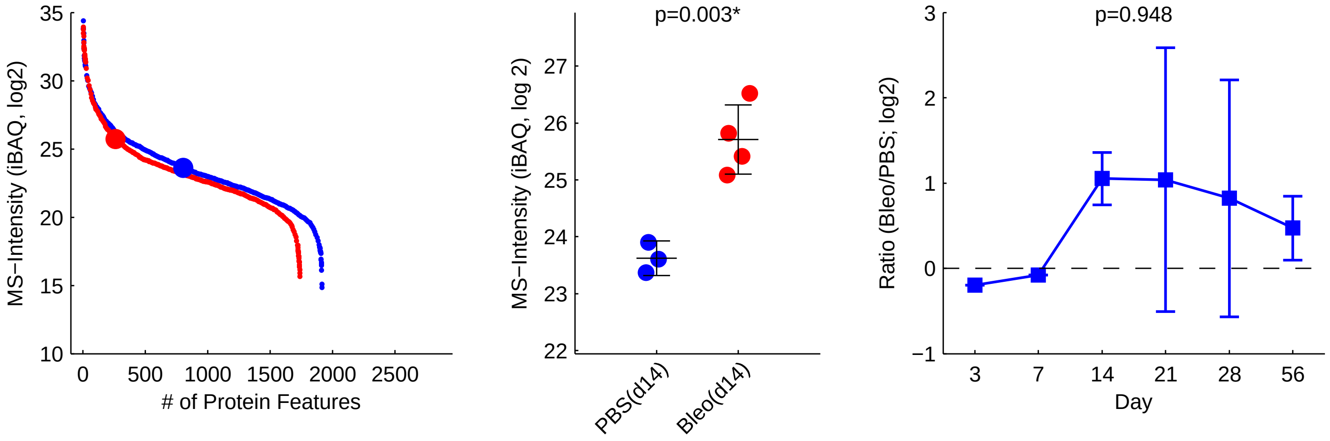

P06330 − (id: 858)

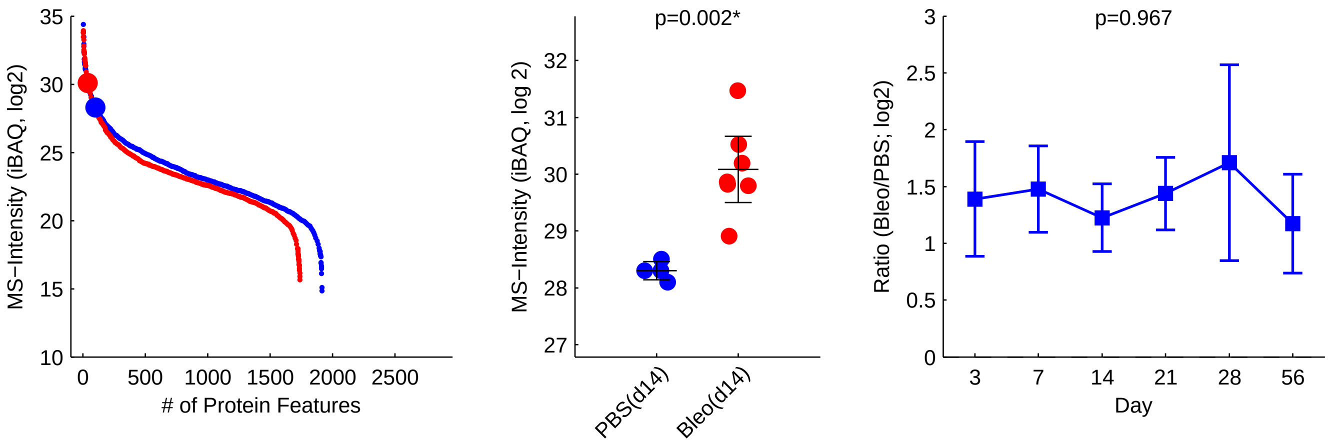

P06684 − C5 (id: 860)

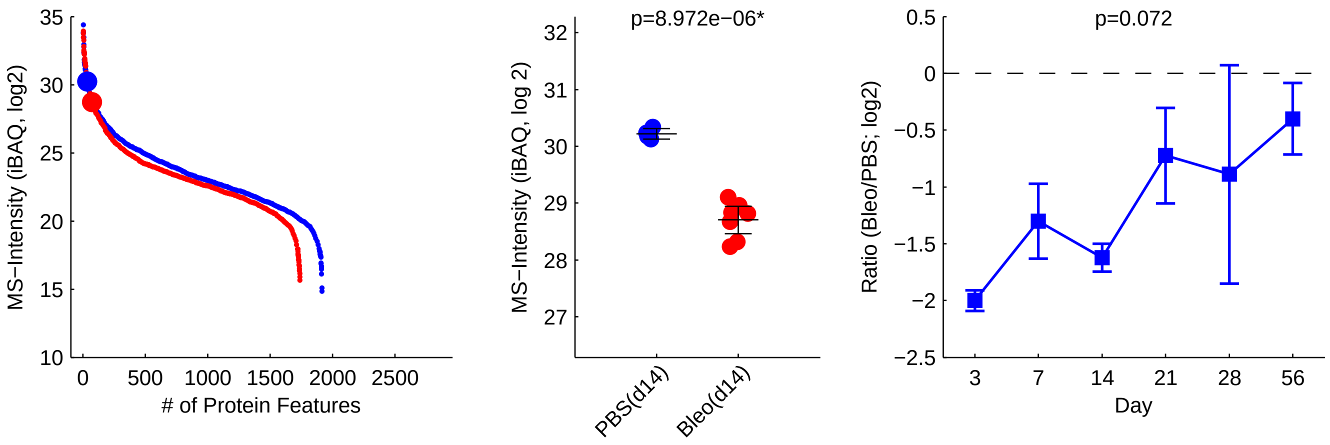

P06728 − Apoa4 (id: 861)

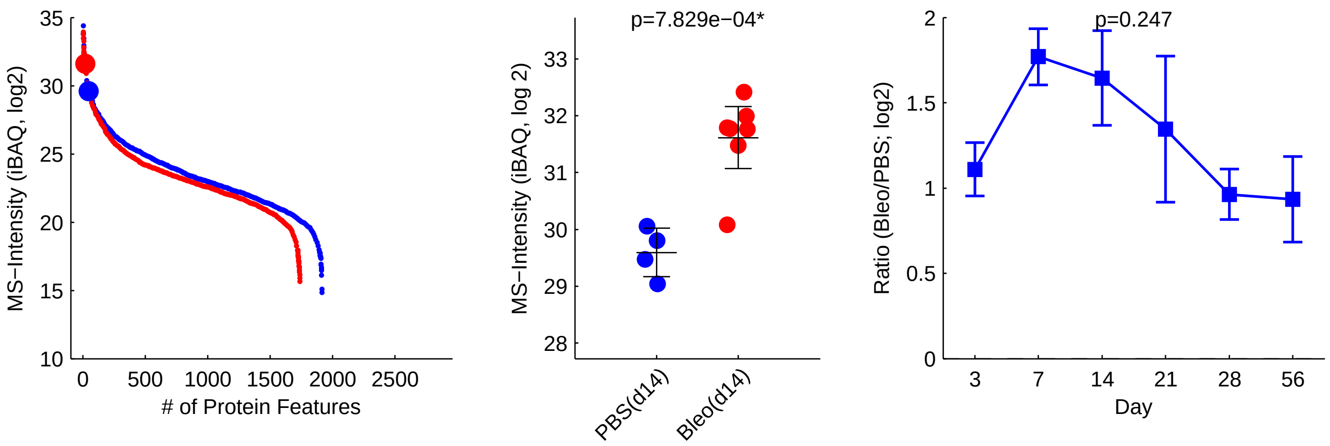

P06797 − Ctsl1 (id: 863)

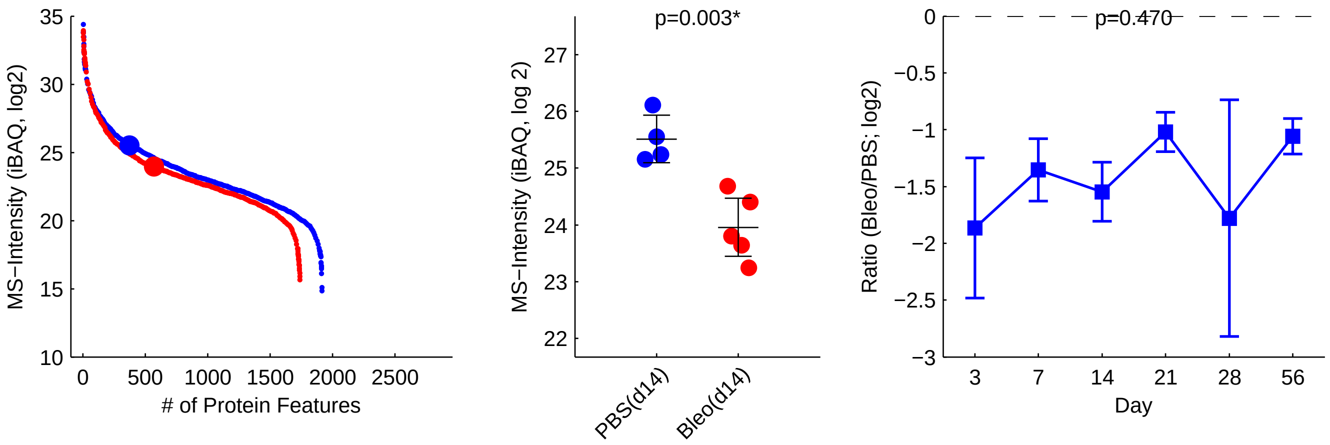

P06801 − Me1 (id: 865)

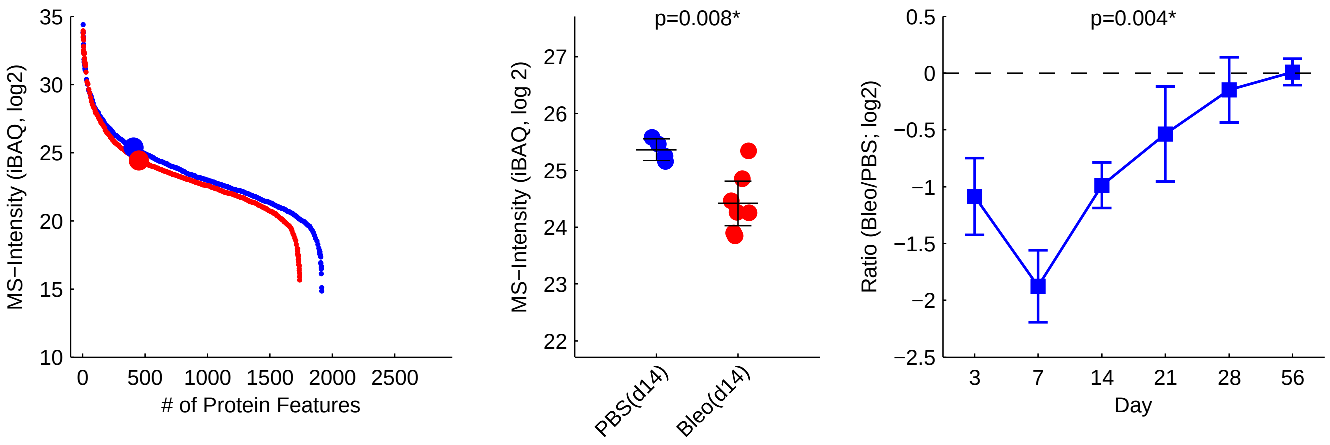

P07091 − S100a4 (id: 866)

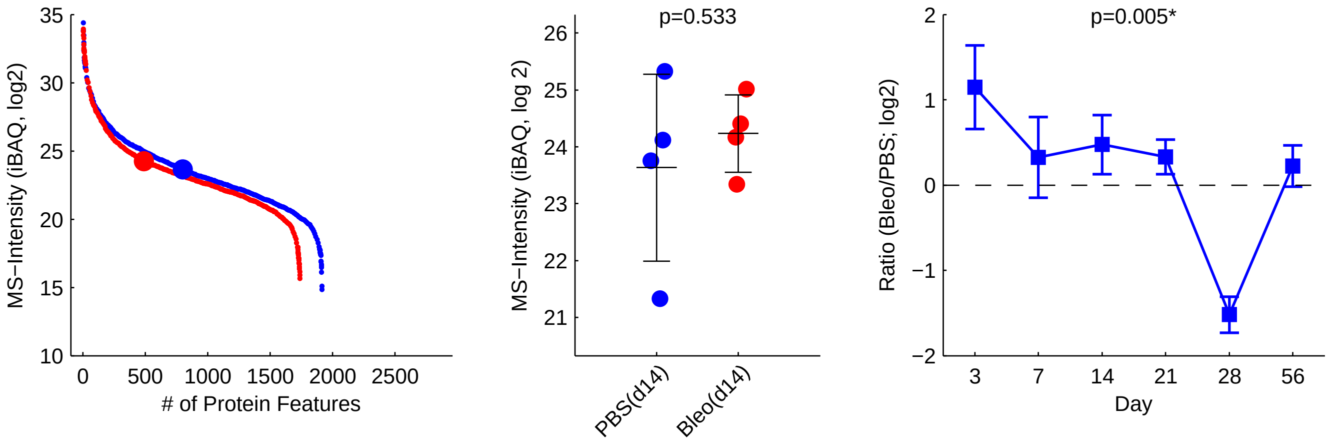

Q5NCU4 − Sparc (id: 869)

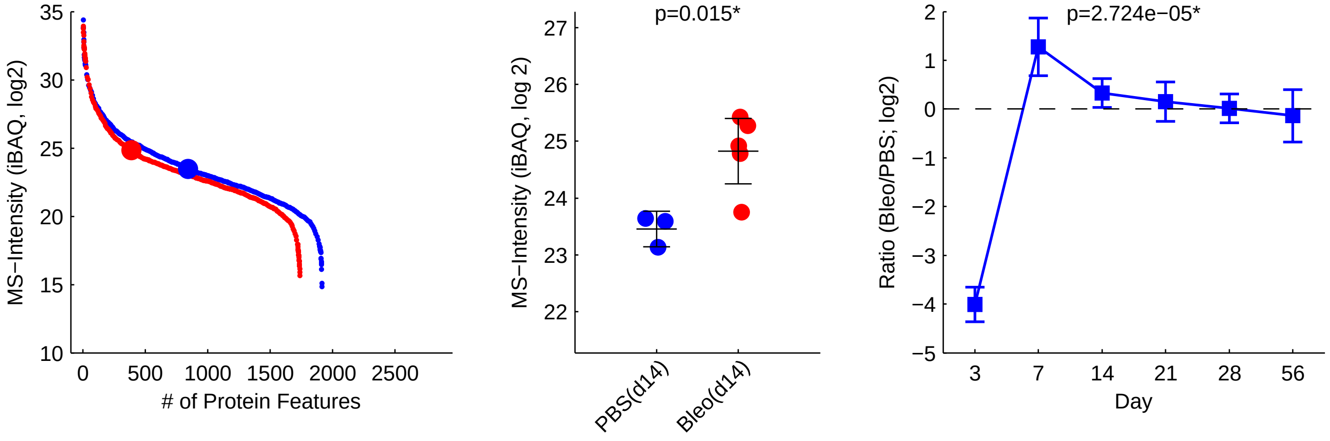

P07310 − Ckm (id: 871)

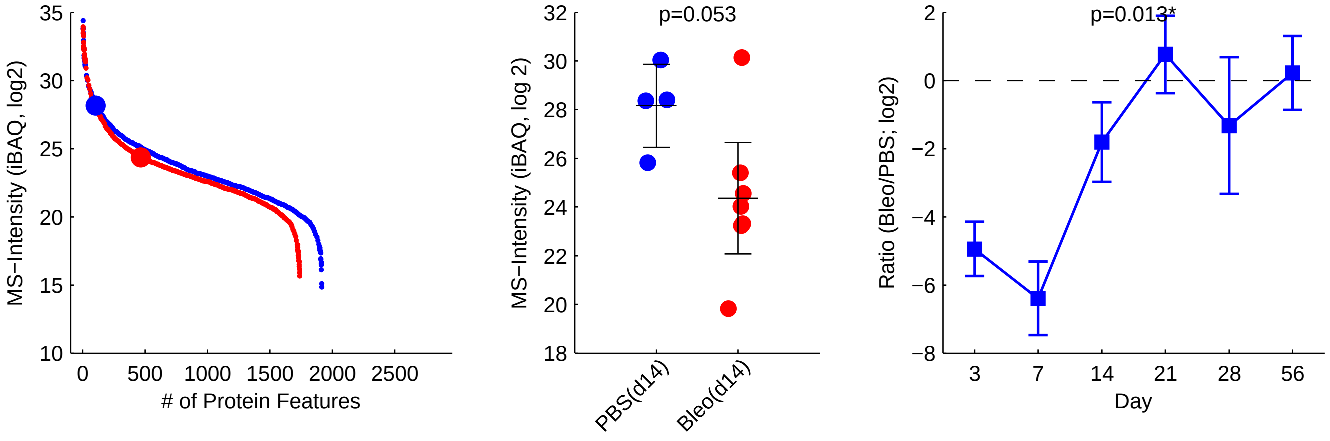

P07356 − Anxa2 (id: 872)

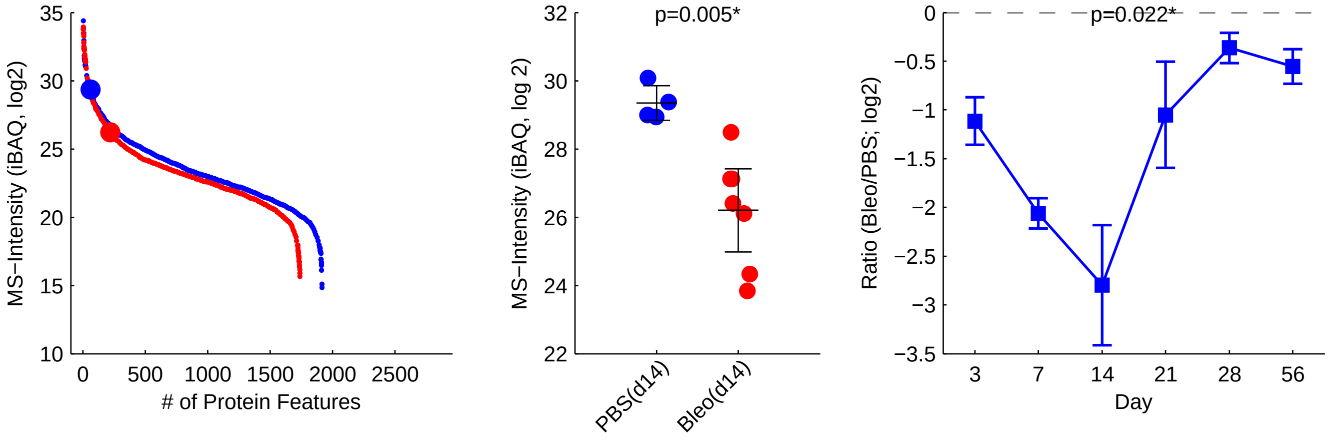

P07361 − Orm2 (id: 873)

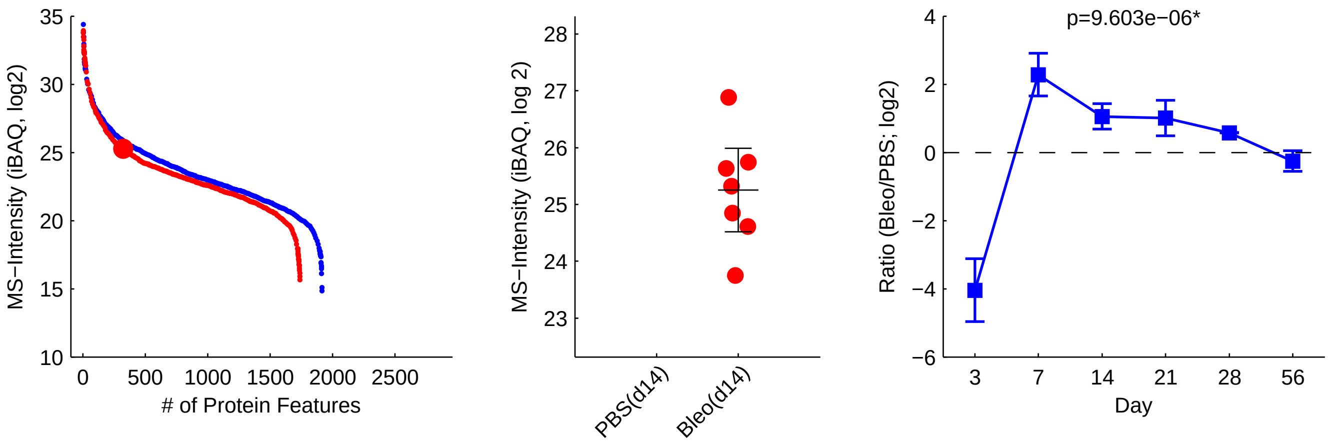

P07901 − Hsp90aa1 (id: 878)

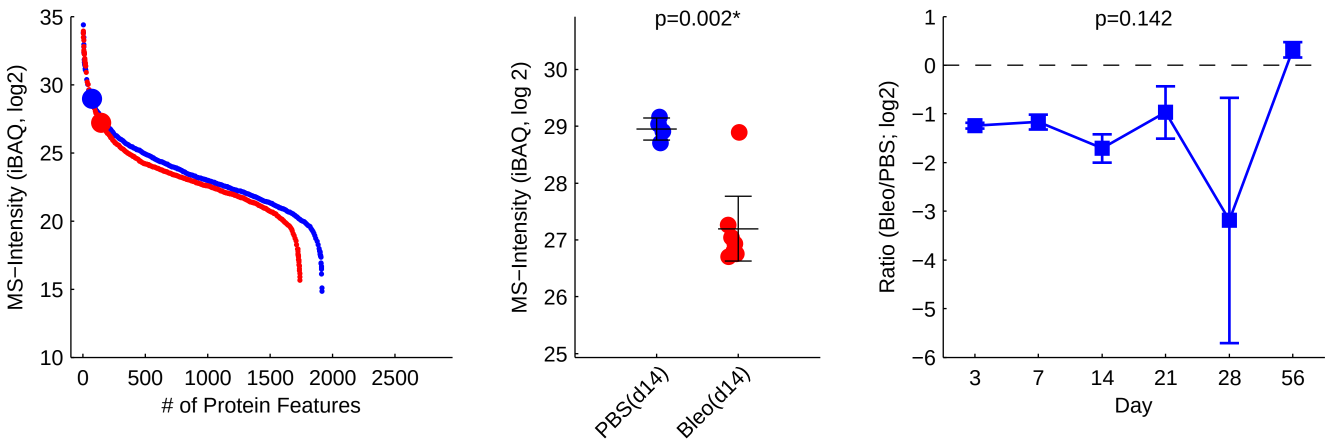

P08003 − Pdia4 (id: 879)

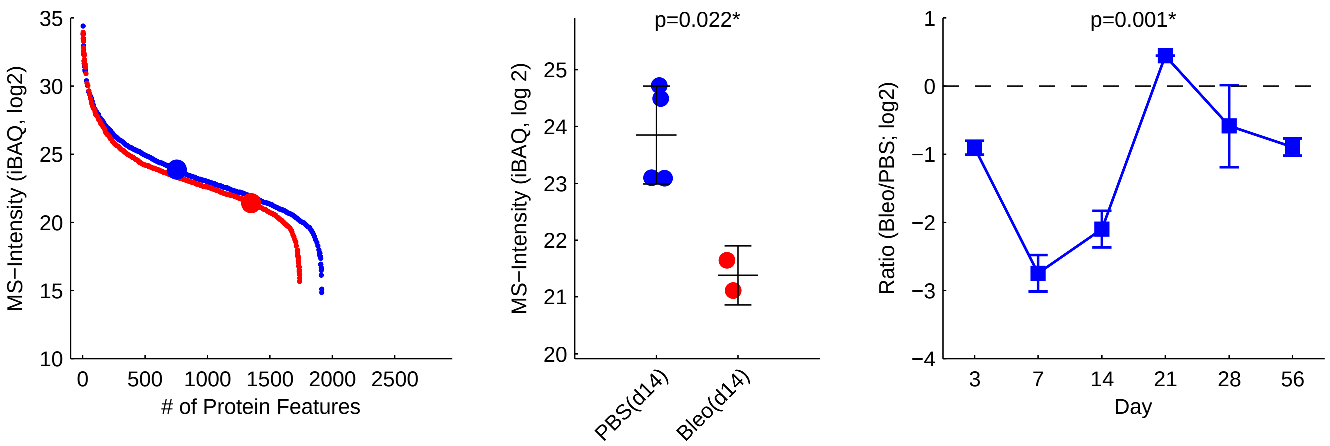

P08074 − Cbr2 (id: 882)

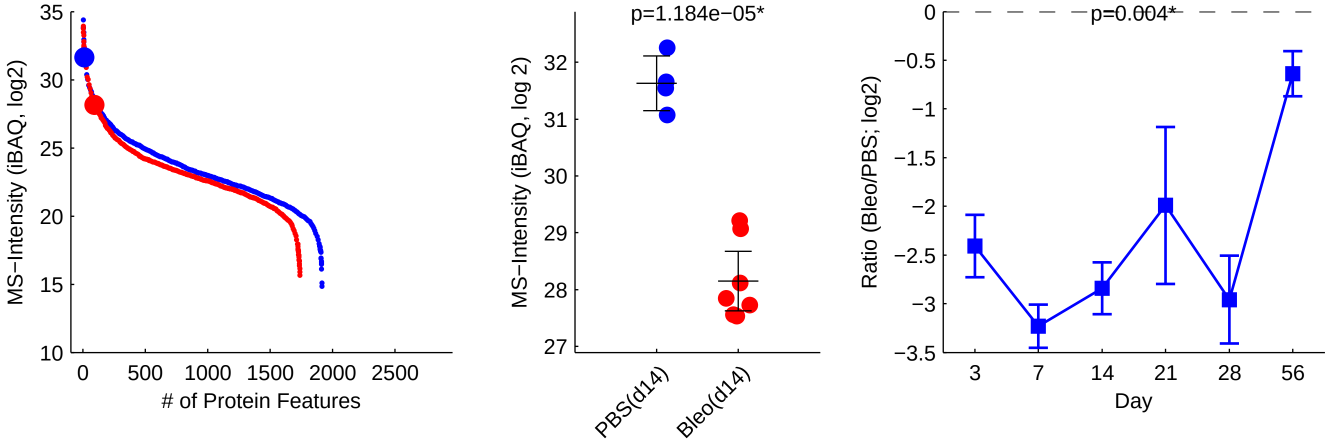

P08113 − Hsp90b1 (id: 883)

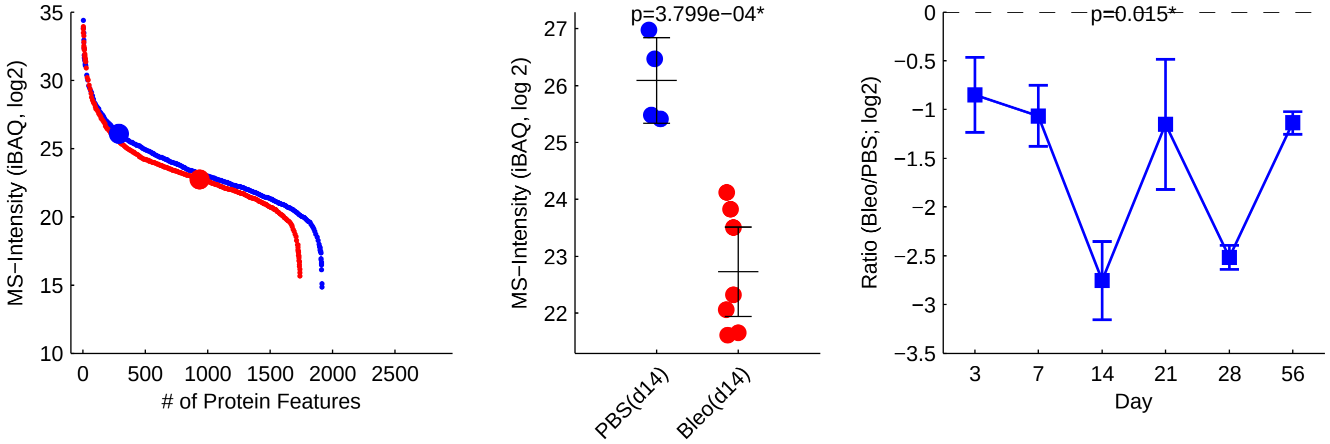

P08121 − Col3a1 (id: 884)

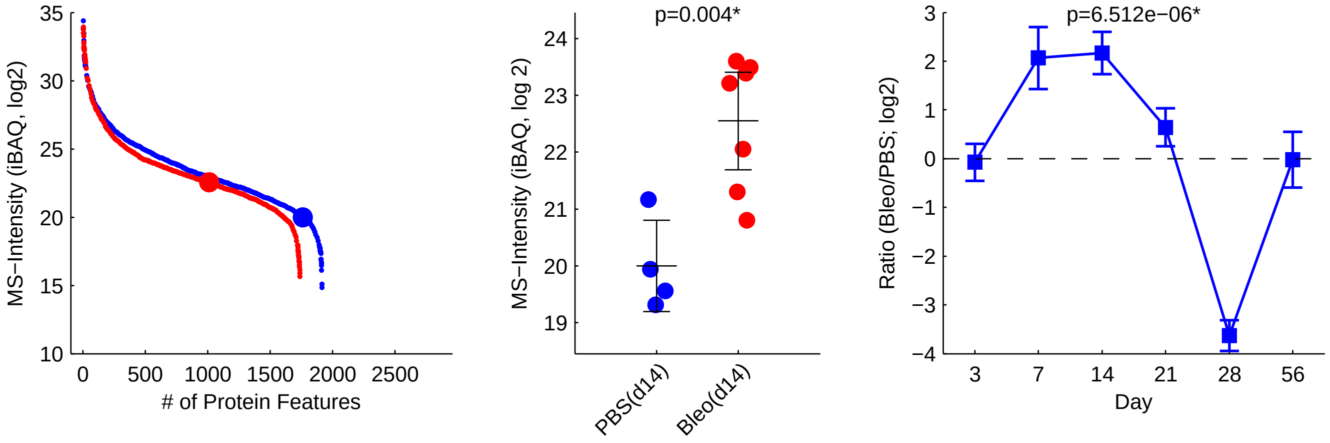

P08122 − Col4a2 (id: 885)

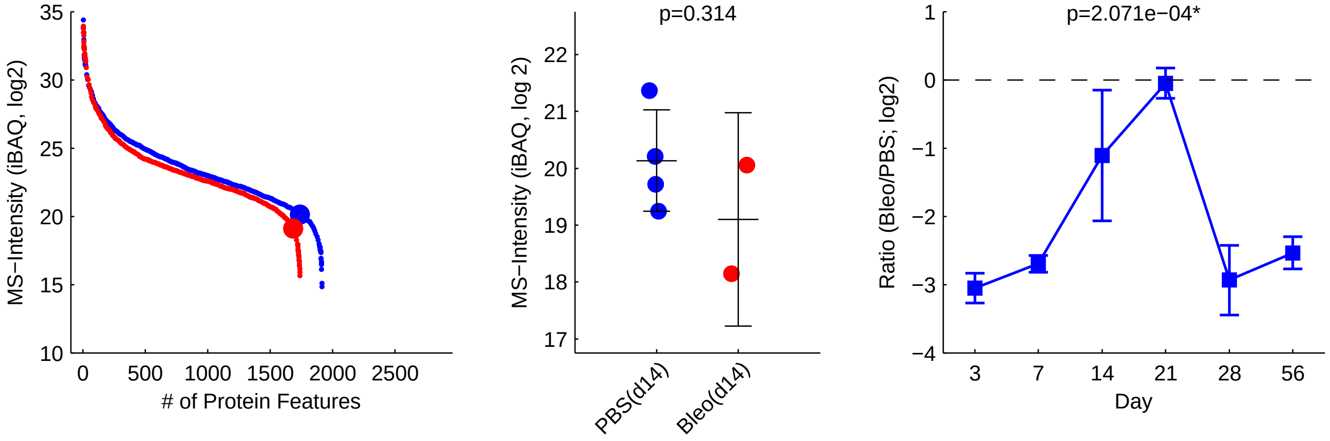

P08226 − Apoe (id: 887)

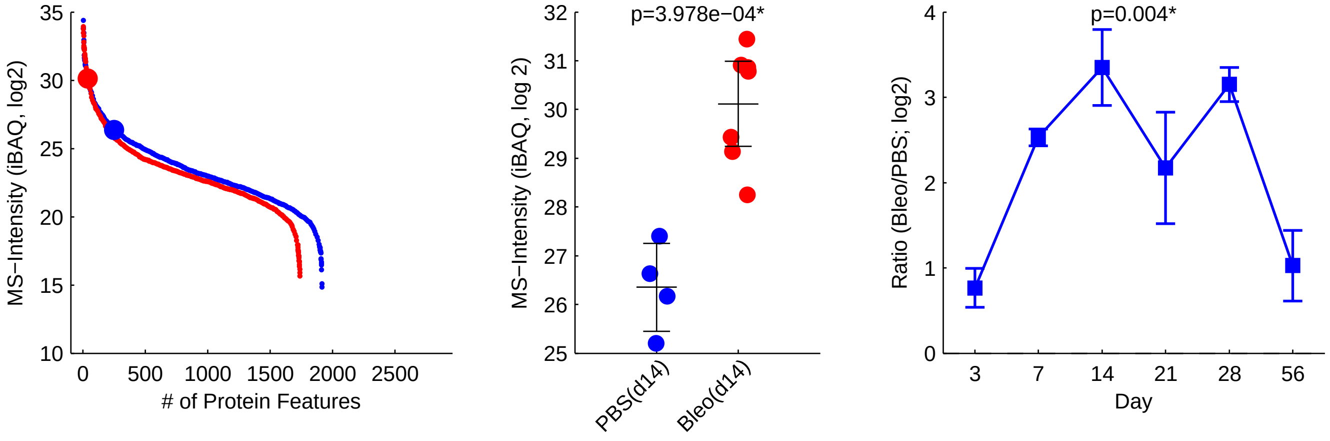

P08228 − Sod1 (id: 888)

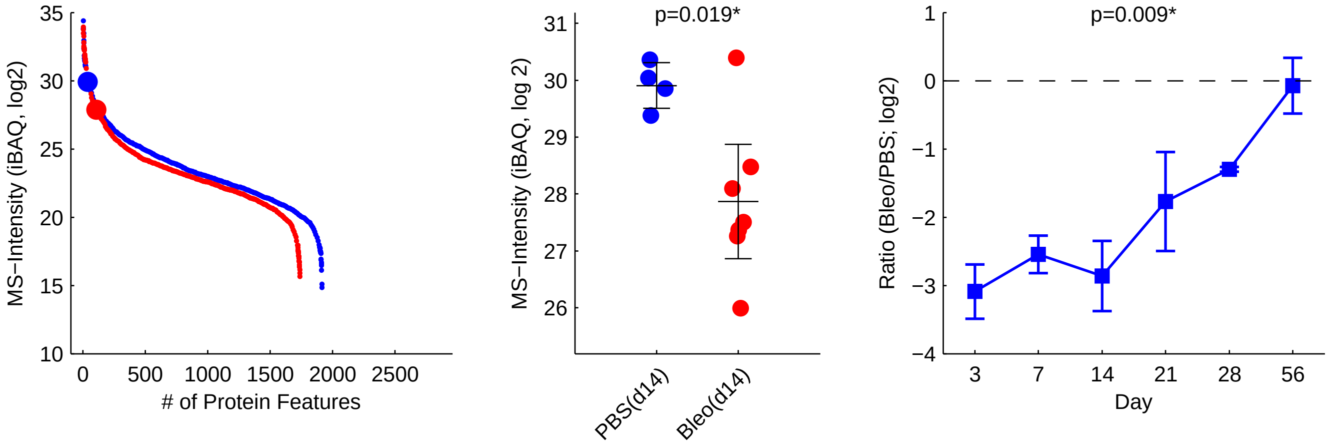

P08249 − Mdh2 (id: 889)

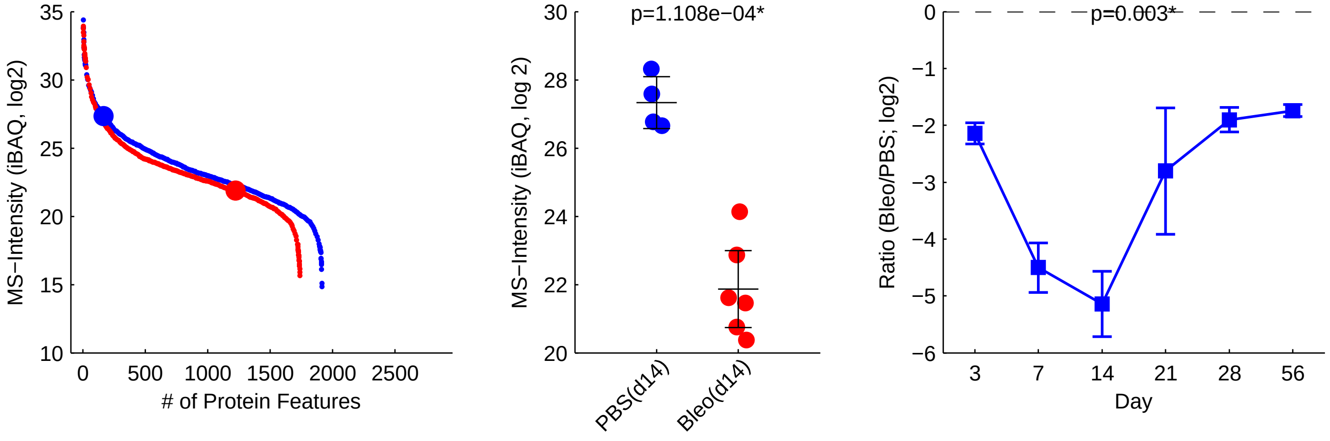

P08752 − Gnai2 (id: 891)

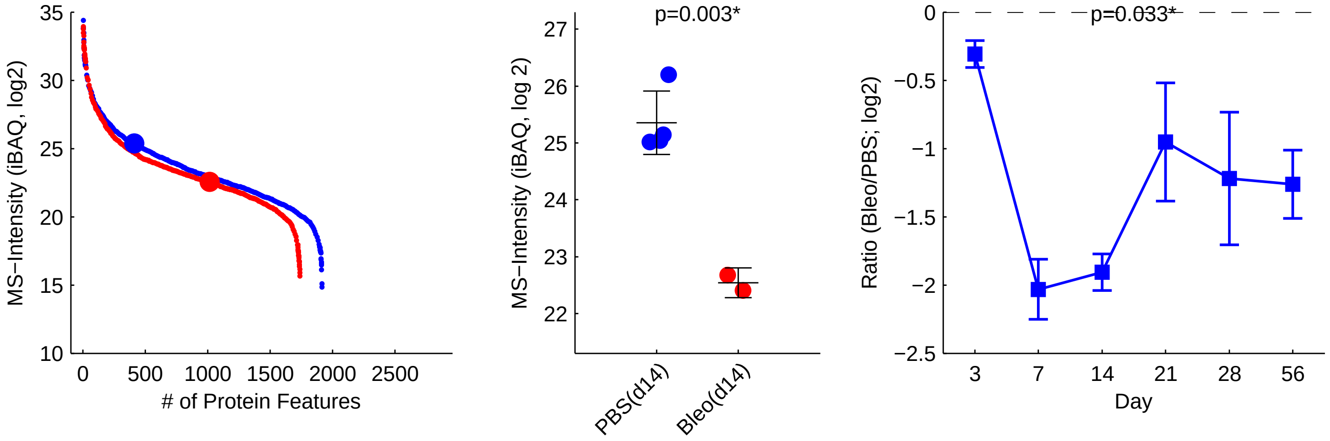

P08905 − Lyz2 (id: 892)

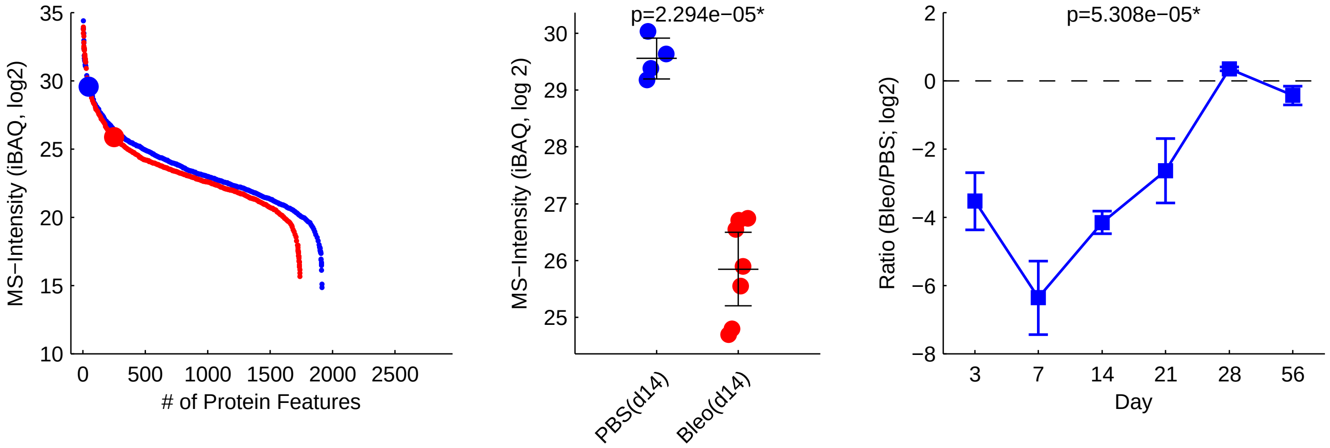

P09055 − Itgb1 (id: 893)

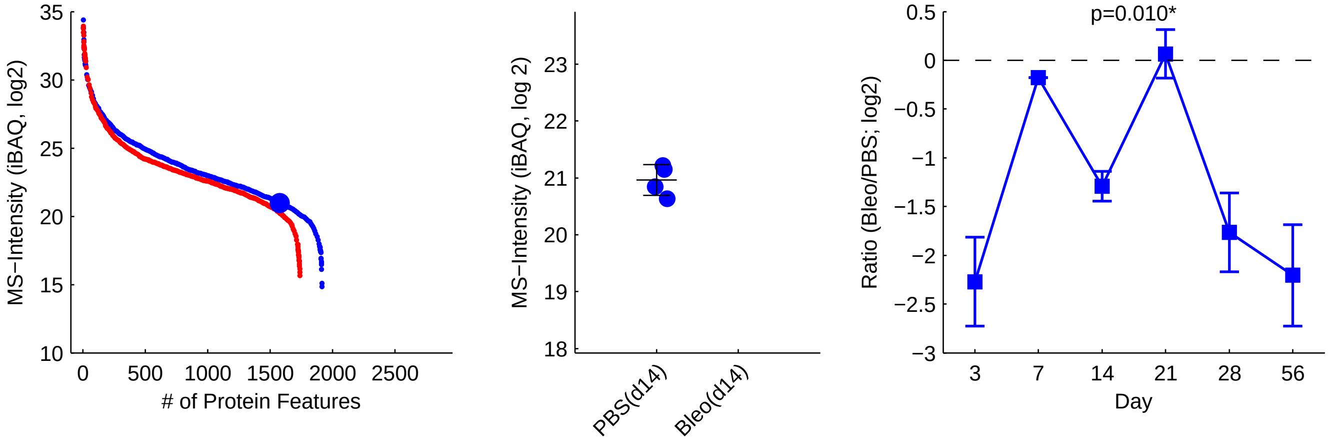

P09103 − P4hb (id: 894)

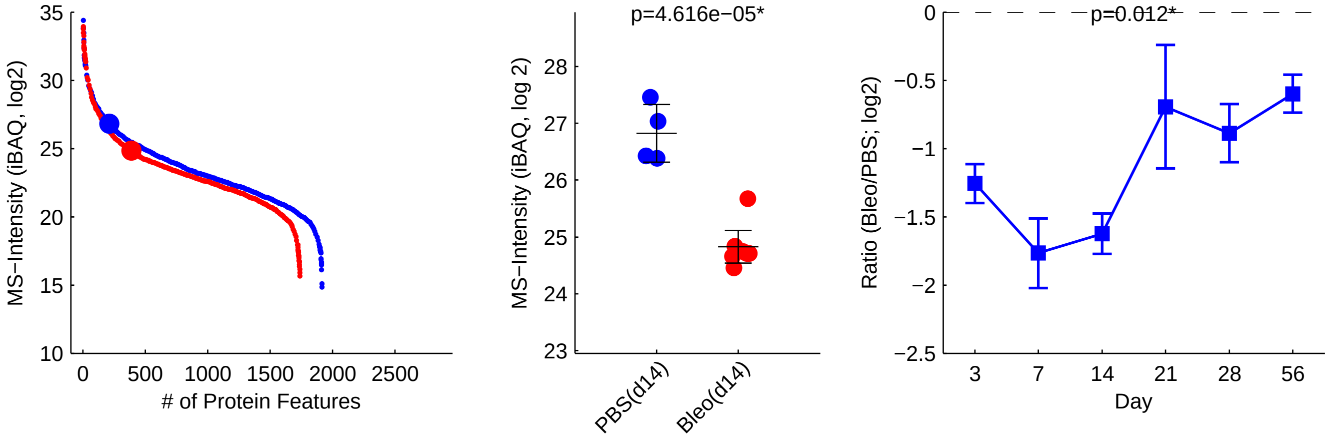

P09405 − Ncl (id: 895)

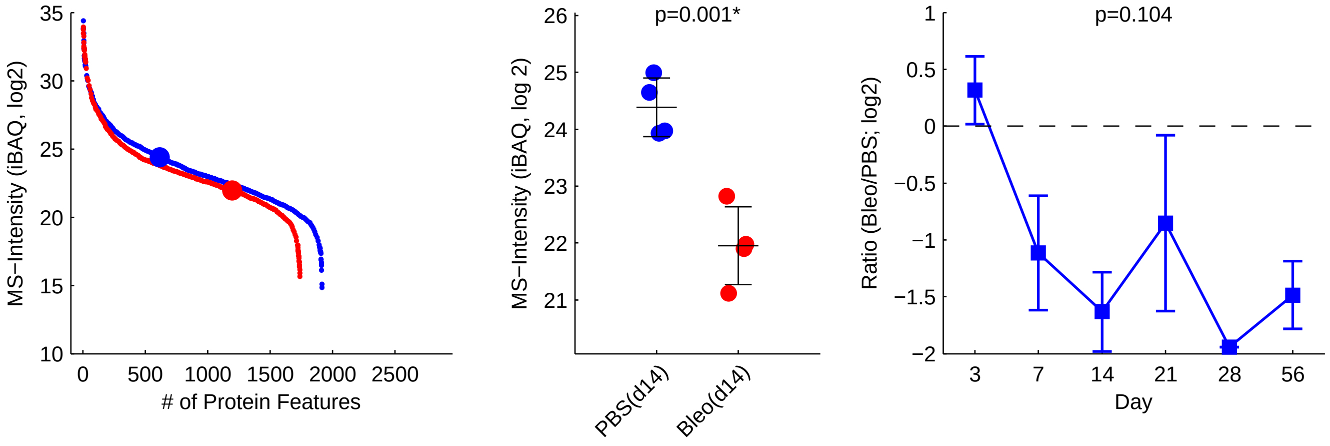

P09528 − Fth1 (id: 898)

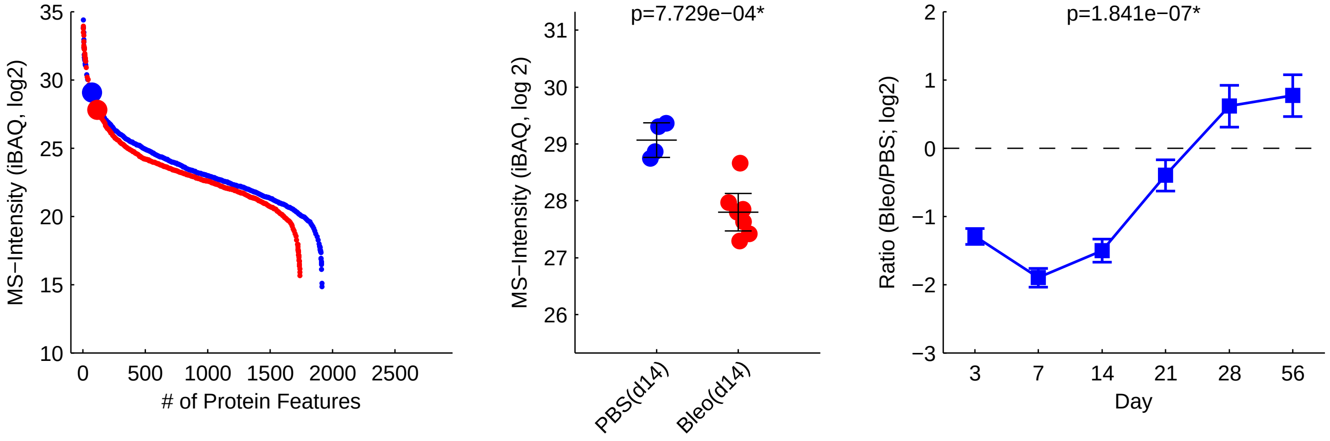

P09803 − Cdh1 (id: 901)

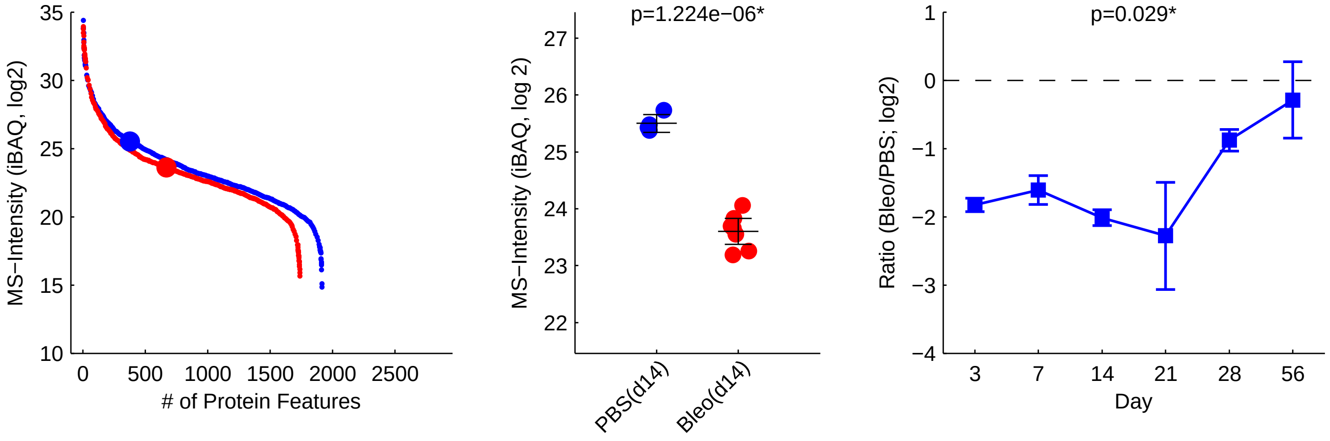

P09813 − Apoa2 (id: 902)

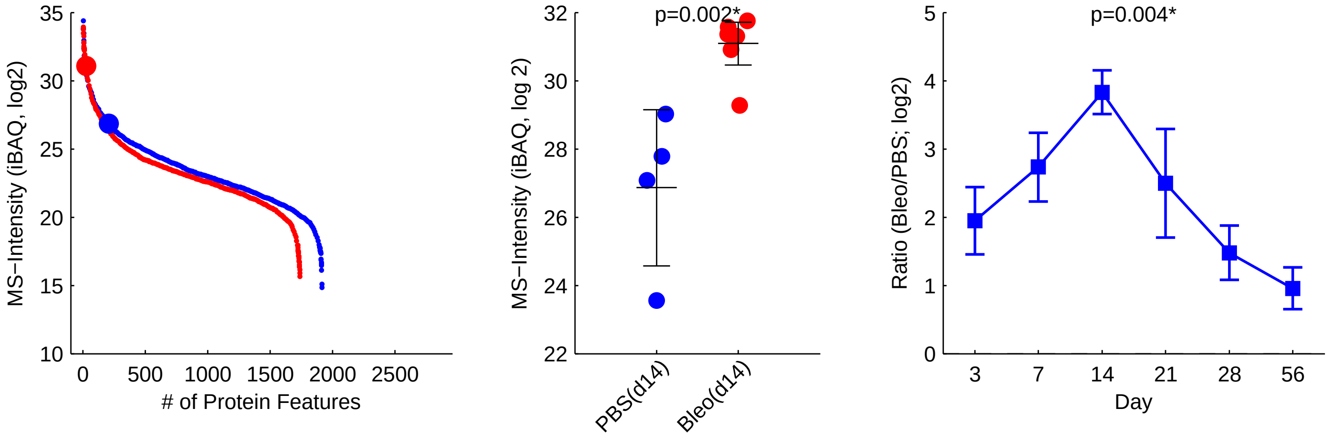

P10518 − Alad (id: 909)

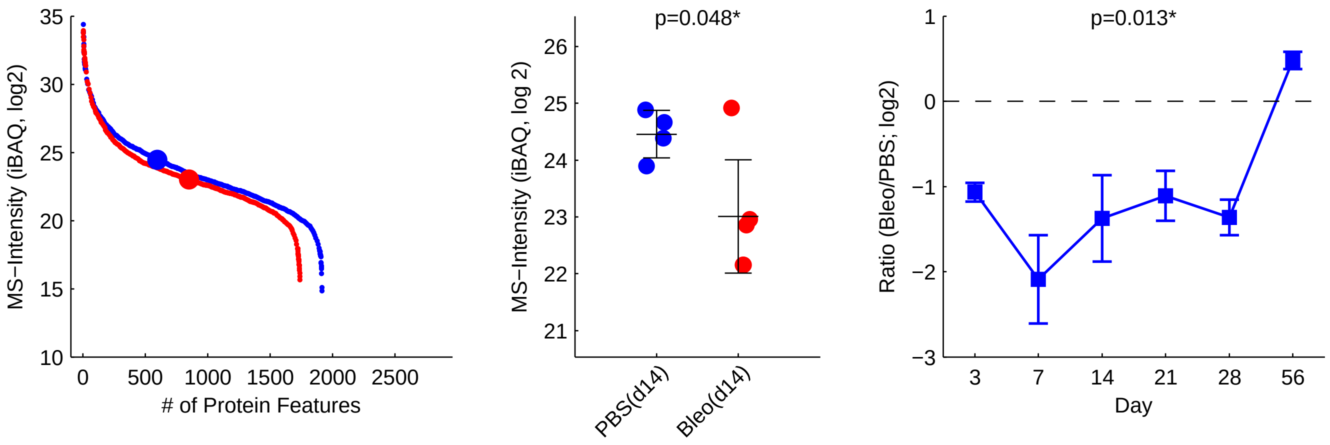

P10630 − Eif4a2 (id: 911)

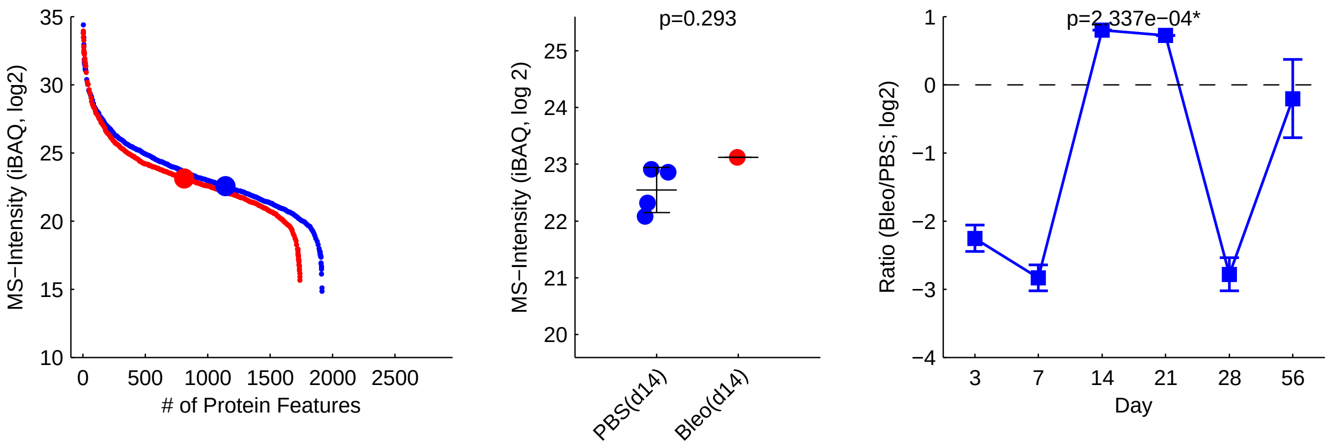

P10639 − Txn (id: 912)

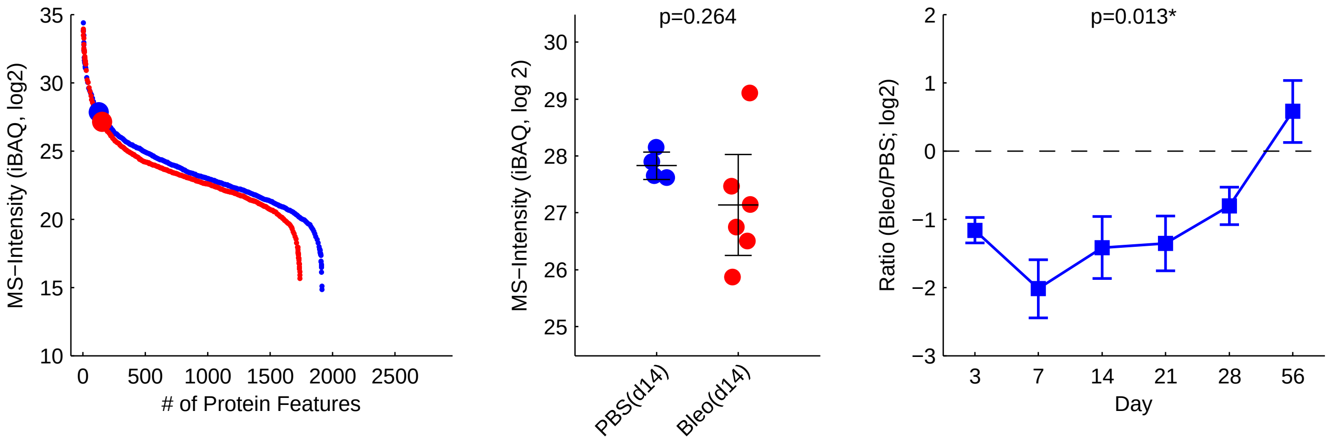

P10649 − Gstm1 (id: 913)

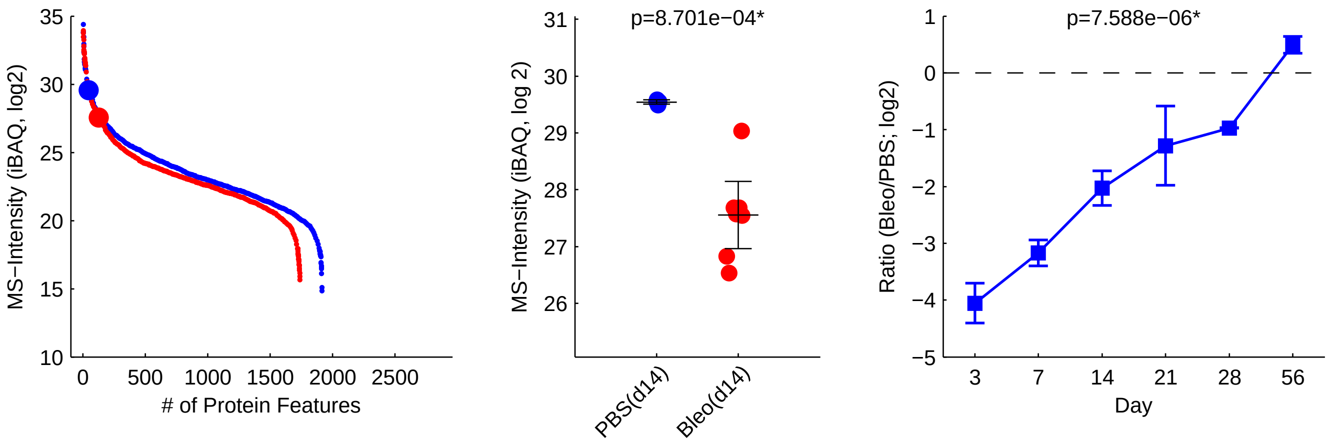

P10810 − Cd14 (id: 914)

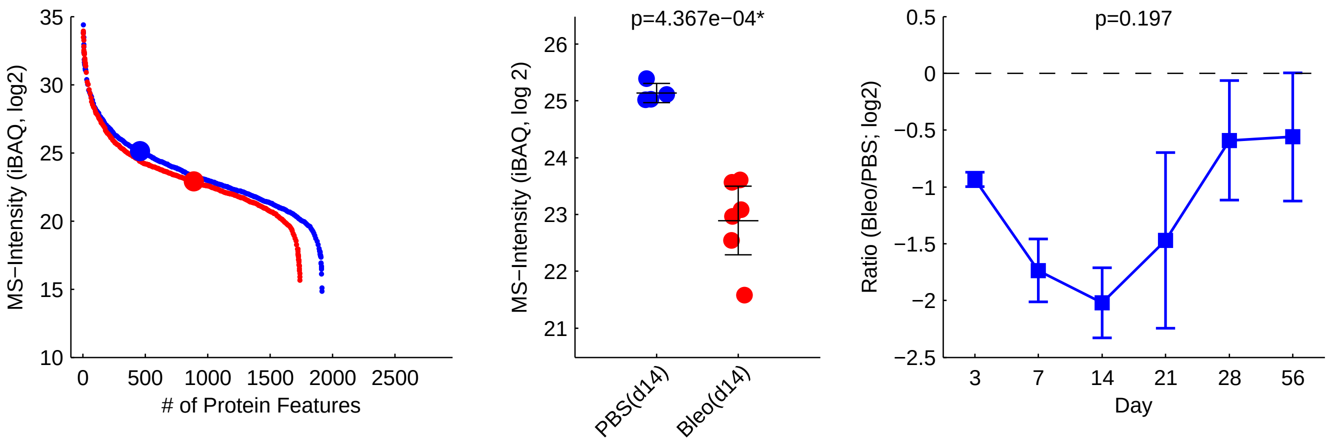

Q8CGP2 − Hist1h2bp (id: 916)

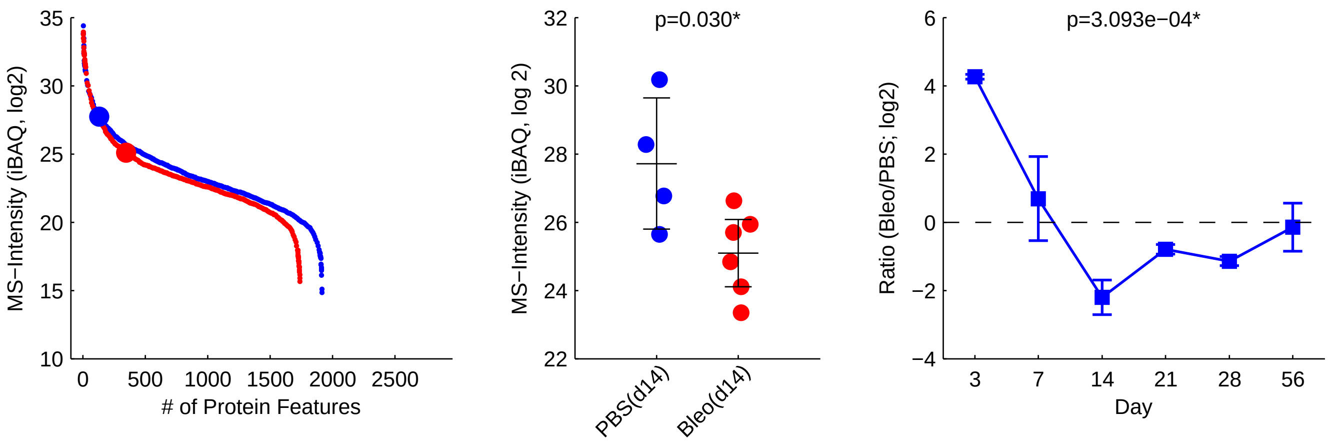

P11031 − Sub1 (id: 919)

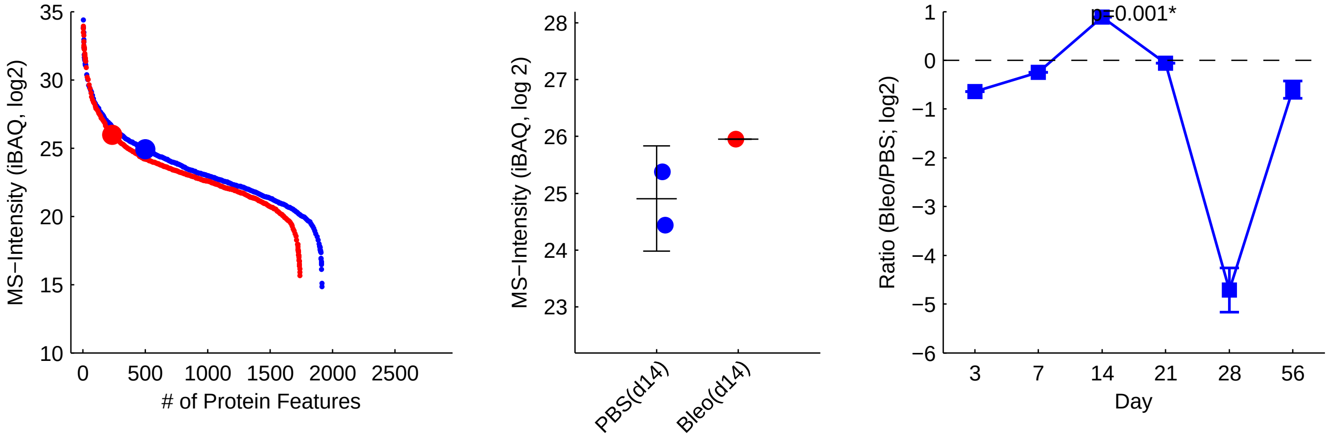

P11152 − Lpl (id: 921)

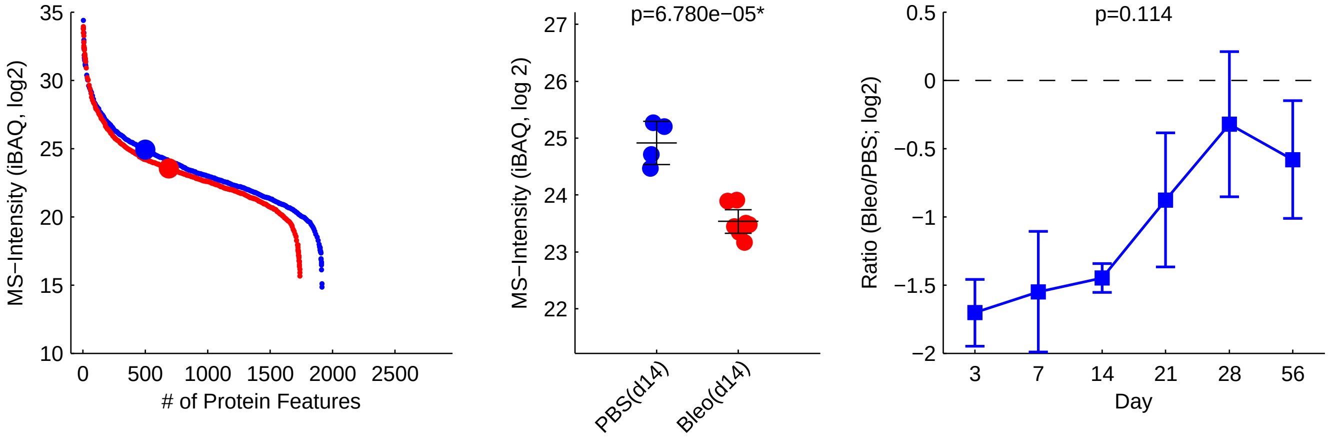

P11276 − Fn1 (id: 923)

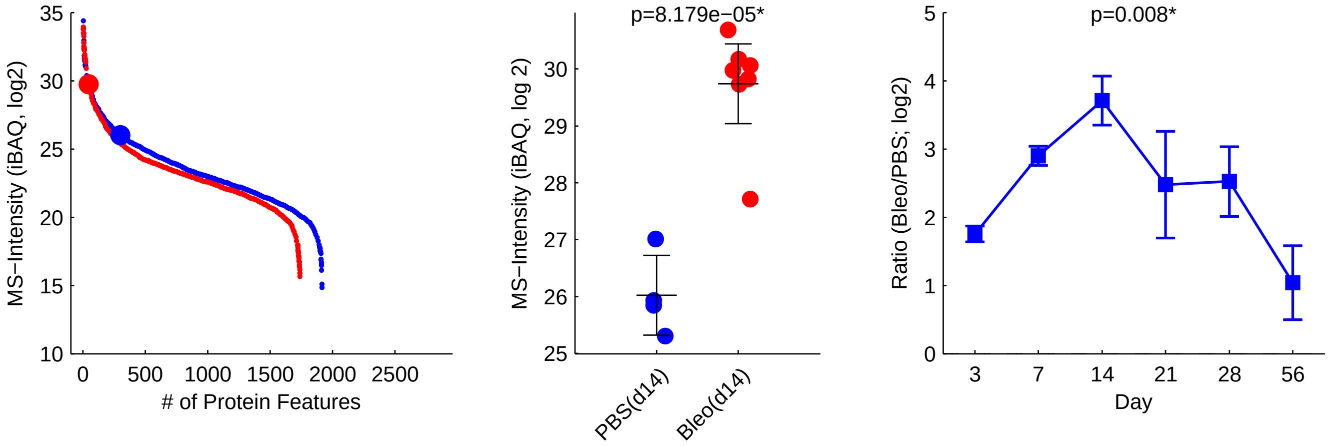

P11499 − Hsp90ab1 (id: 926)

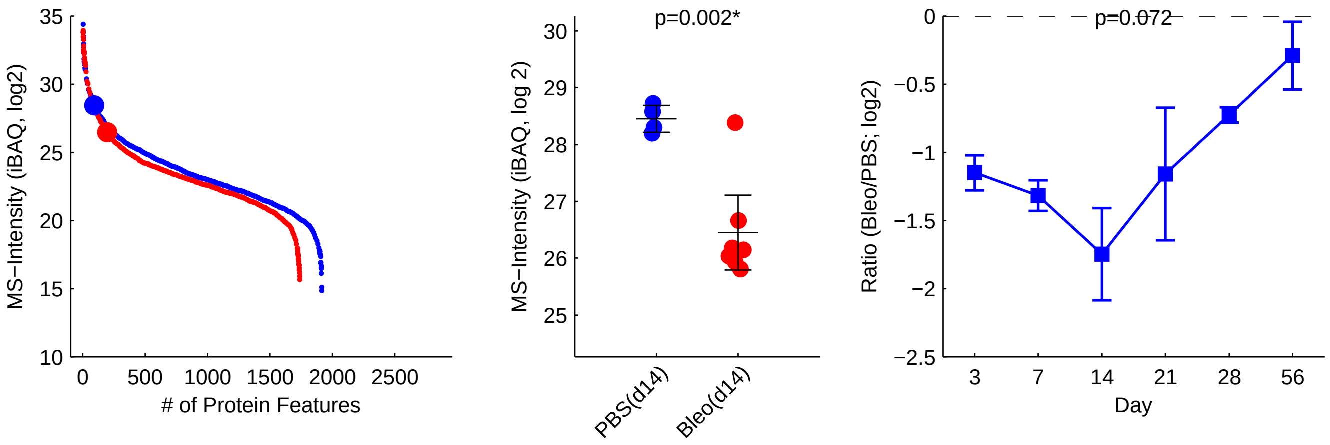

P11679 − Krt8 (id: 928)

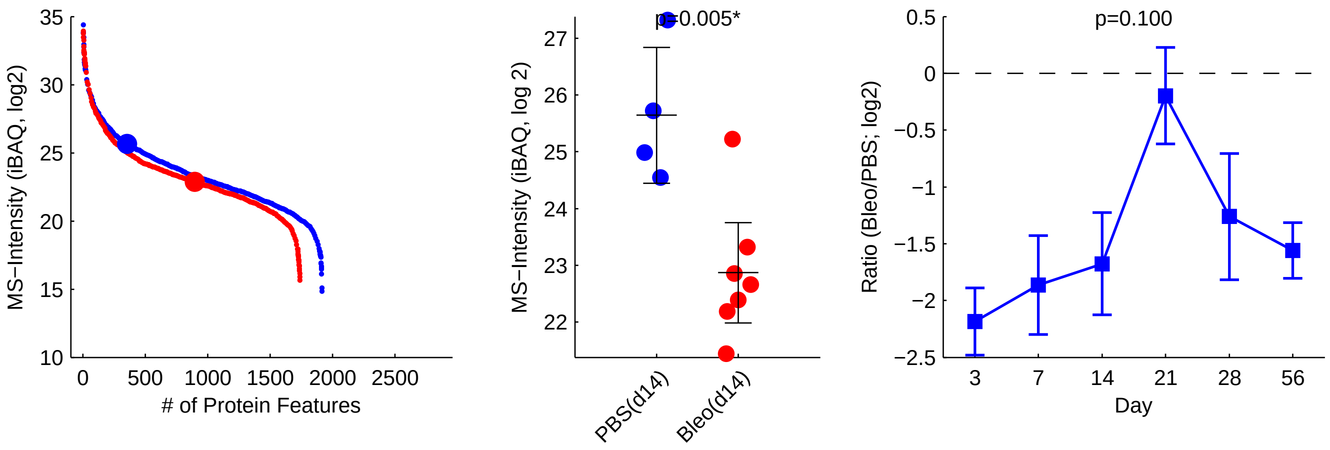

Q542I8 − Itgb2 (id: 930)

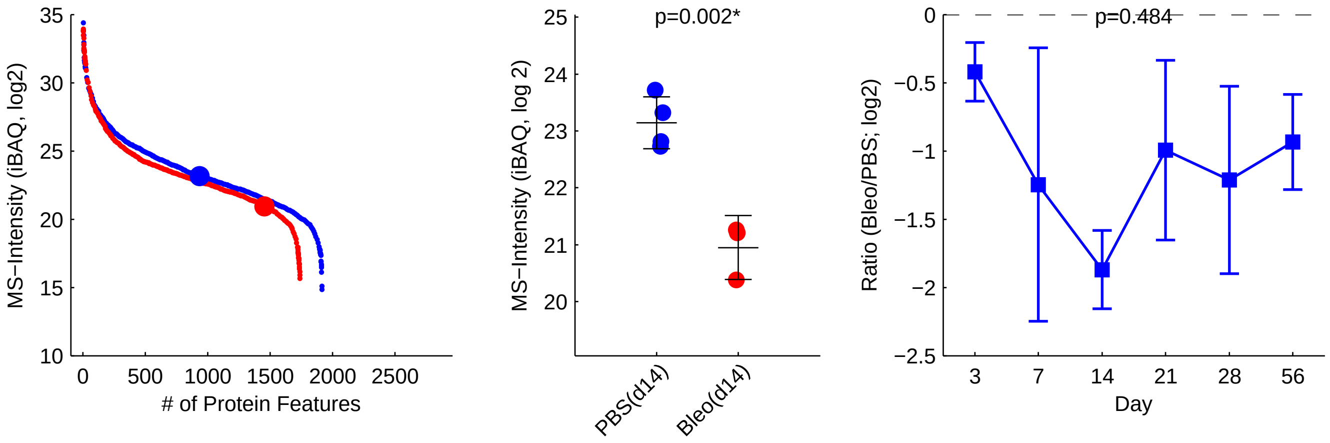

P12246 − Apcs (id: 935)

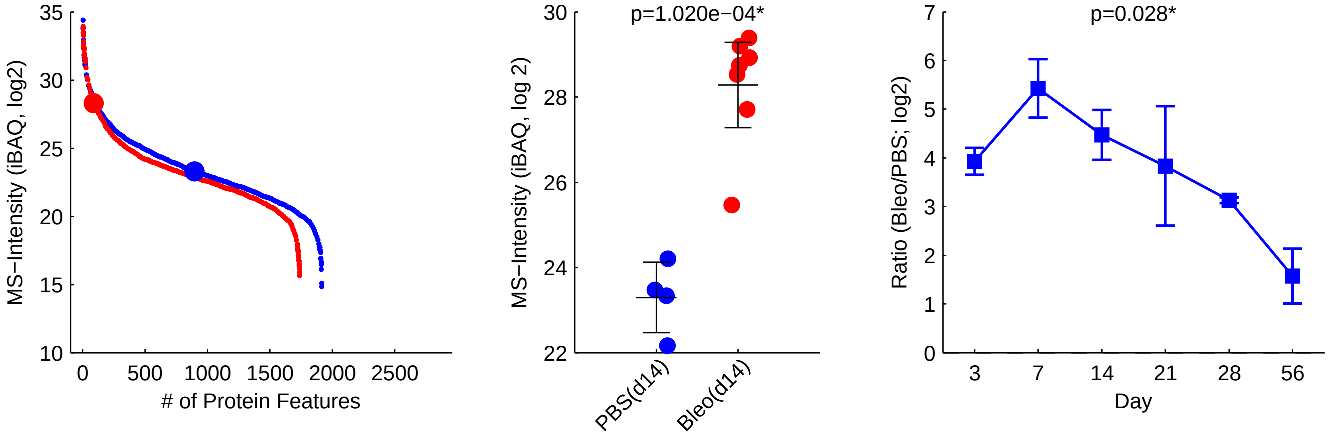

P12265 − Gusb (id: 936)

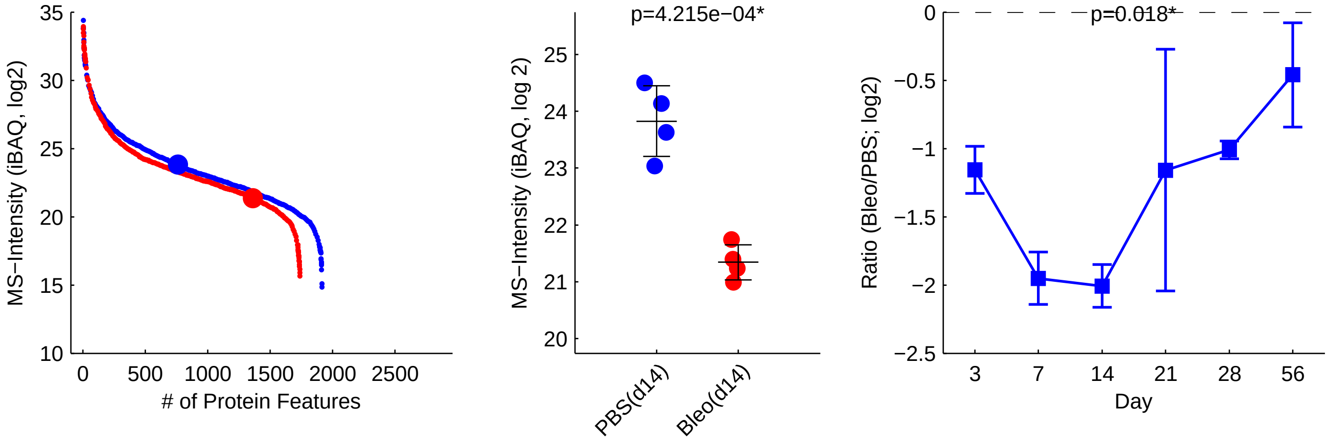

P12710 − Fabp1 (id: 939)

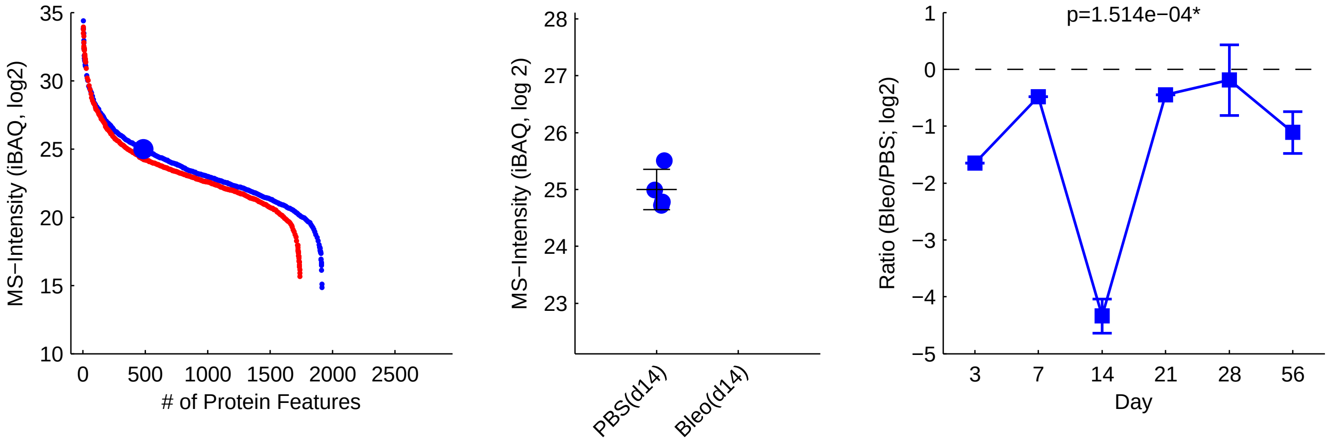

P13020 − Gsn (id: 942)

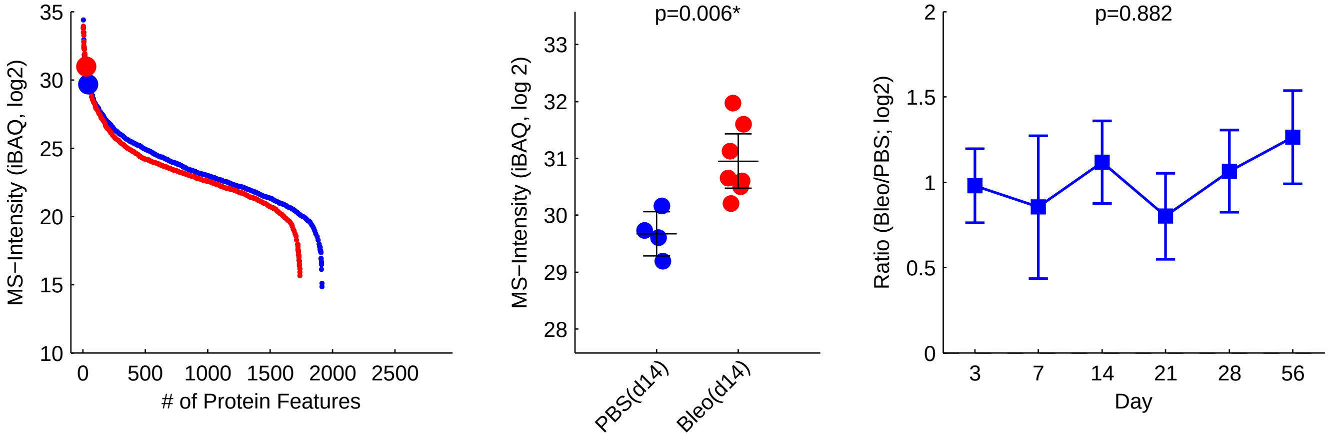

P13597−2 − Icam1 (id: 946)

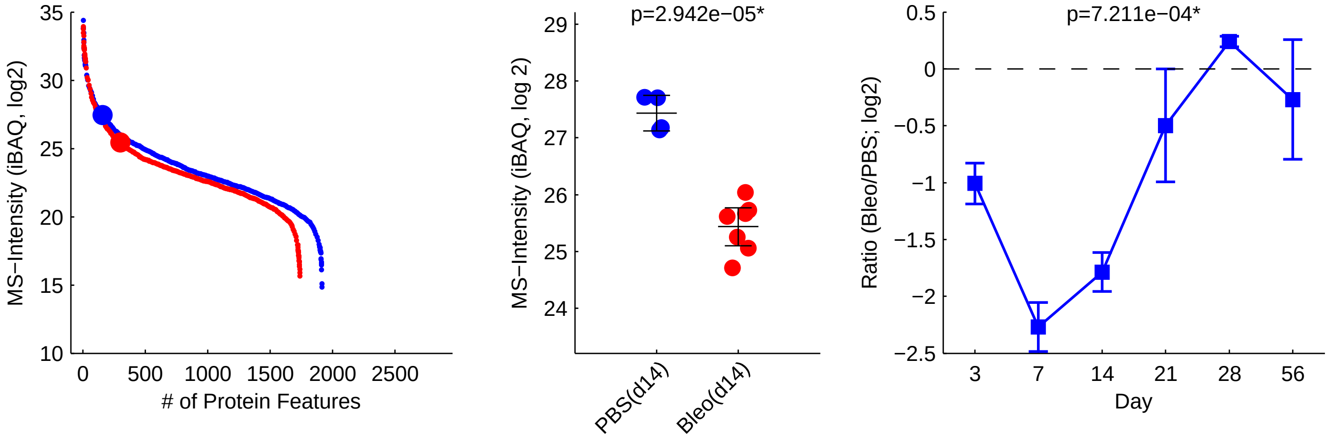

P14069 − S100a6 (id: 950)

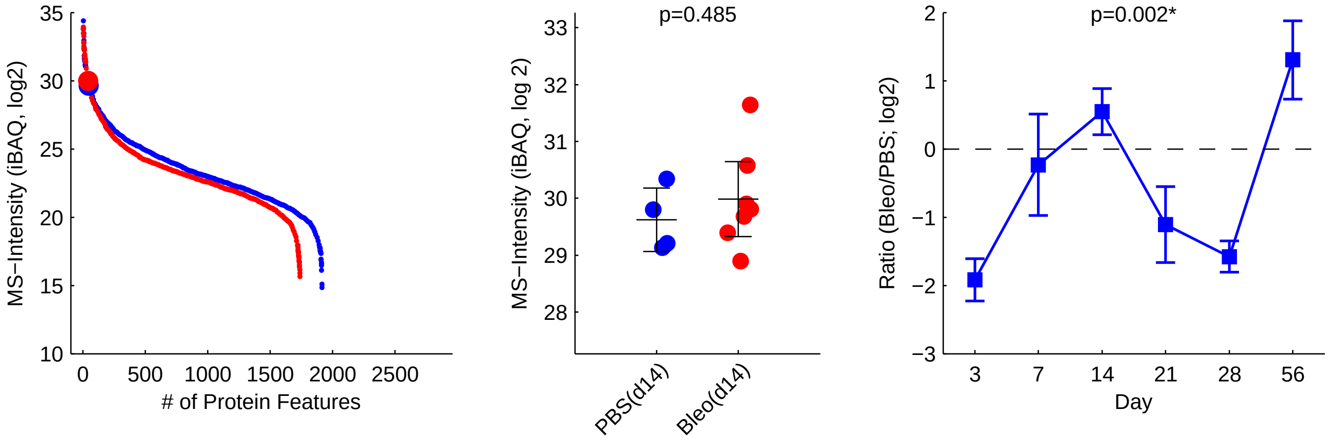

P14094 − Atp1b1 (id: 951)

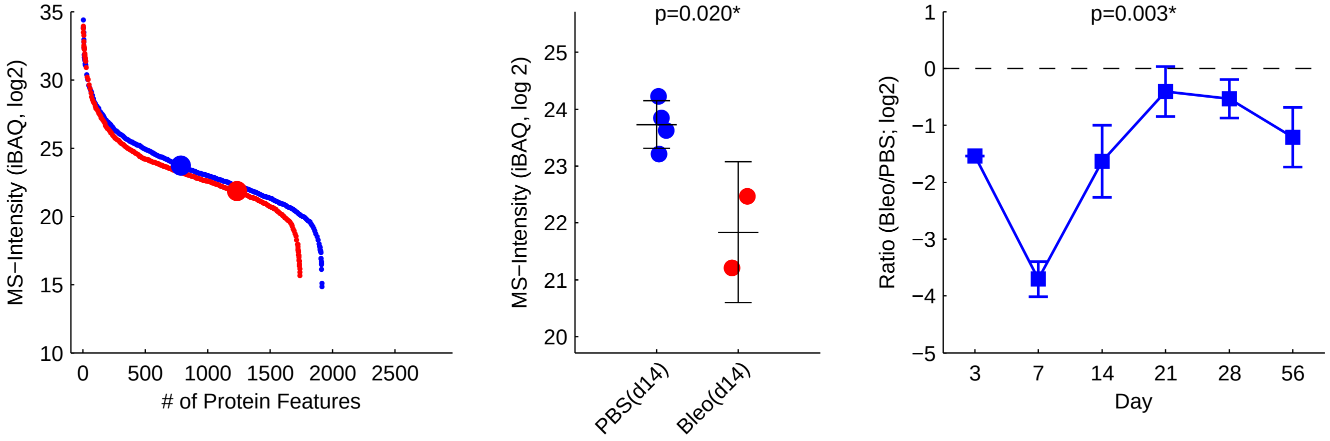

P14106 − C1qb (id: 952)

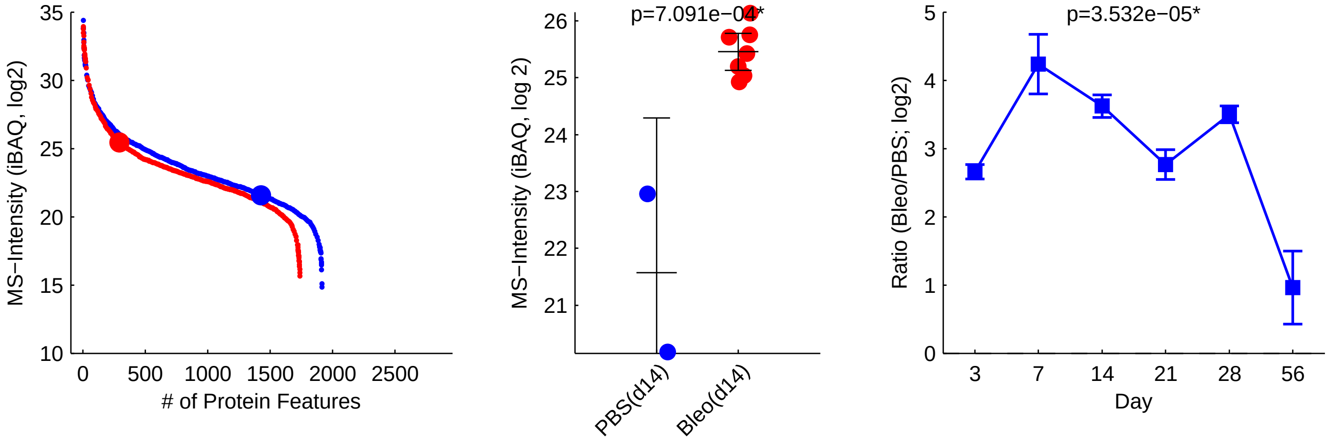

P14115 − Rpl27a (id: 953)

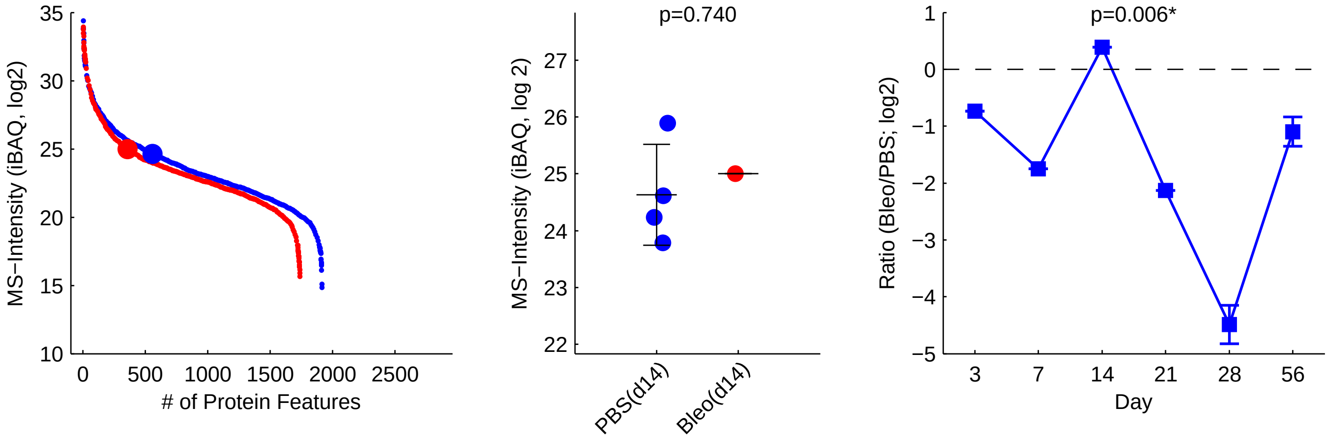

P14131 − Rps16 (id: 954)

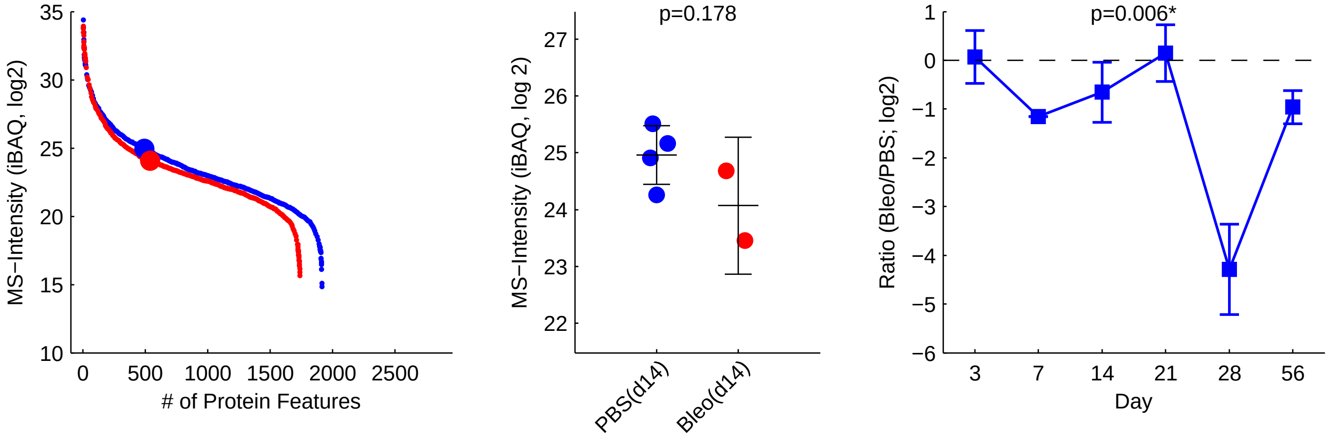

P14152 − Mdh1 (id: 956)

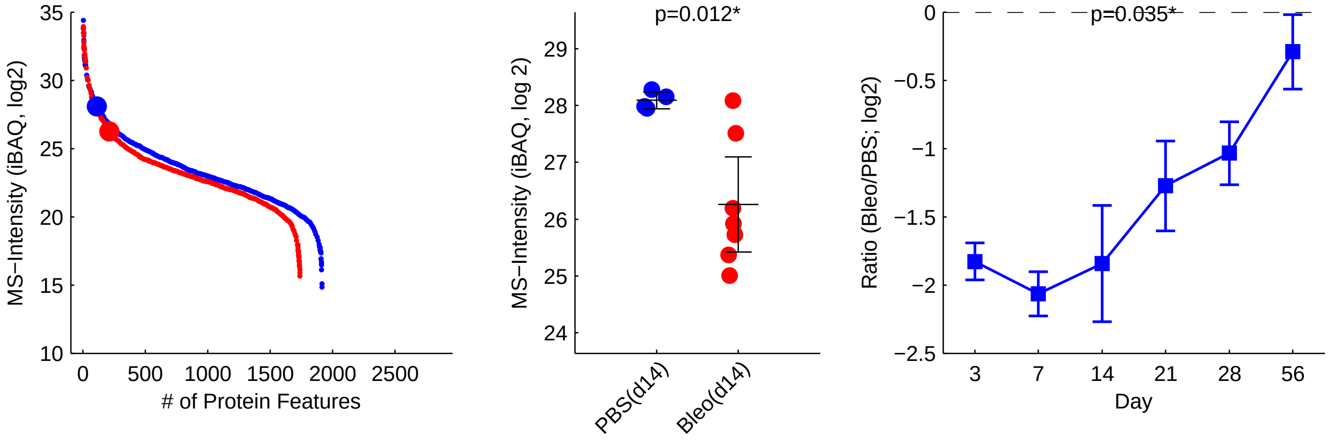

P14206 − Rpsa (id: 957)

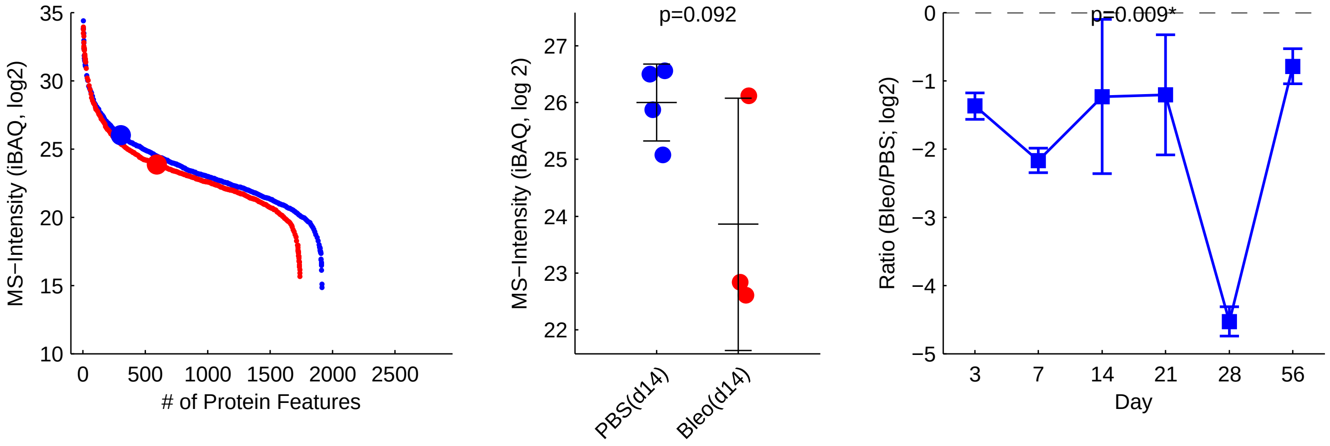

P14211 − Calr (id: 958)

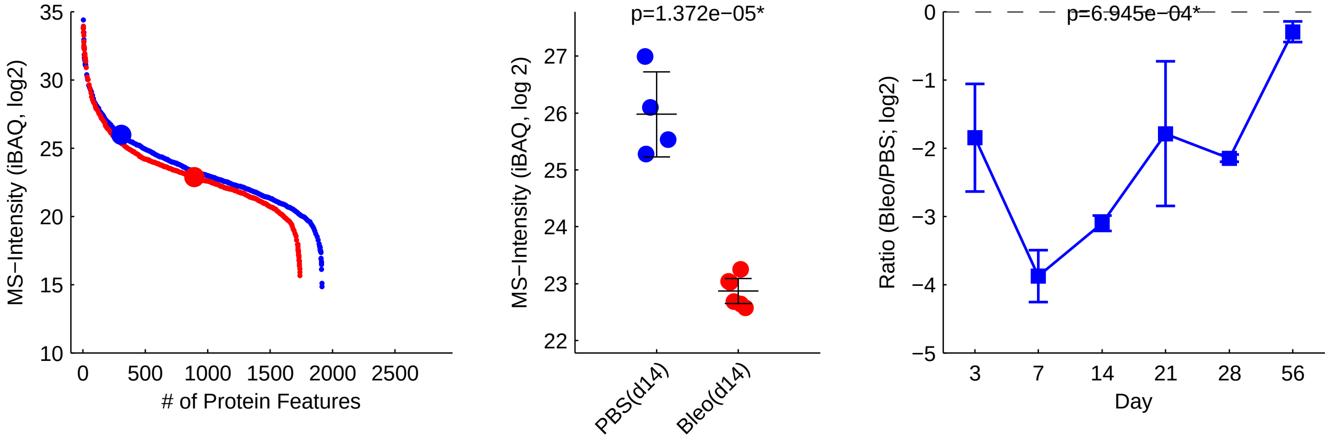

P14434 − H2−Aa (id: 961)

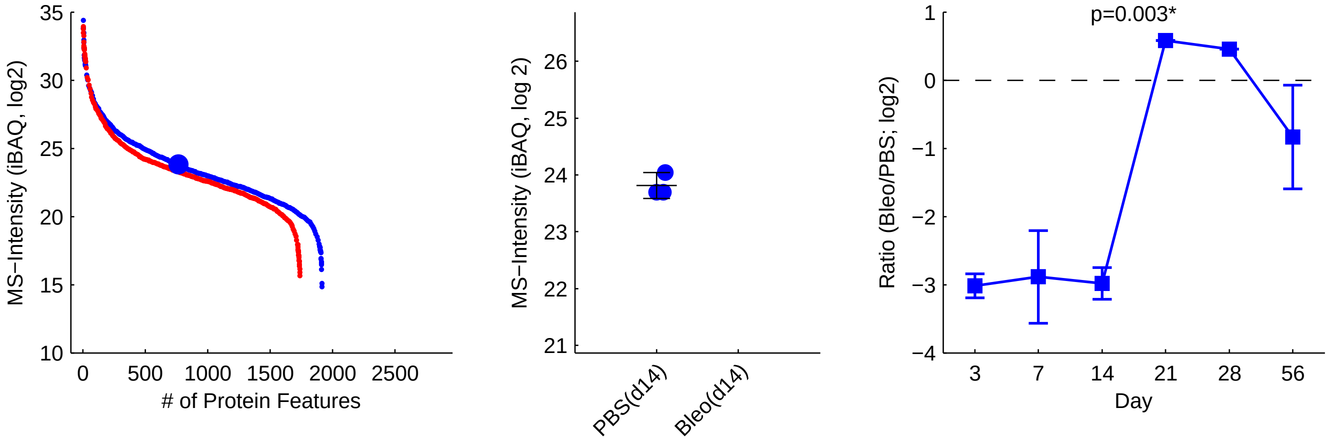

P14483 − H2−Ab1 (id: 962)

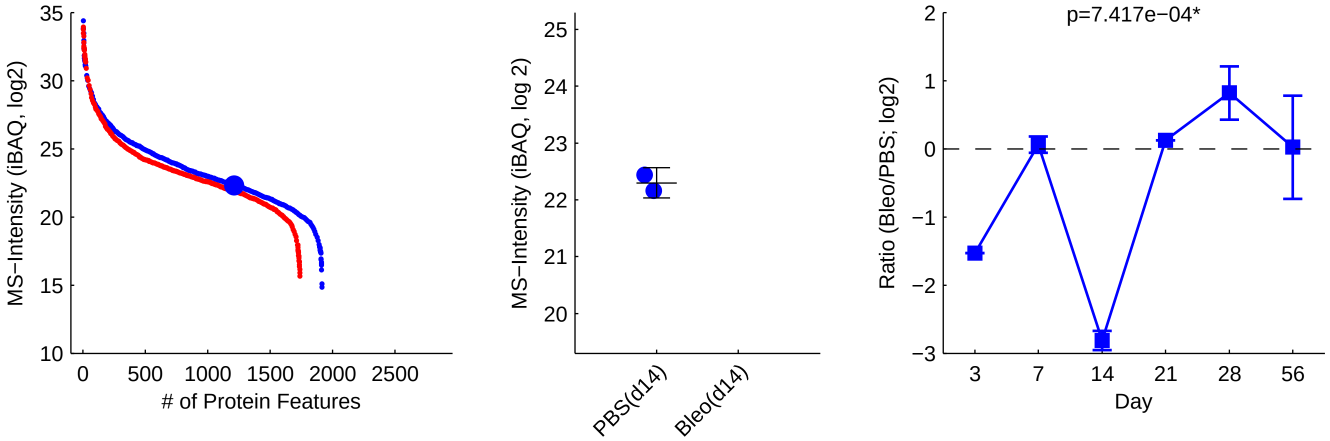

P14685 − Psmd3 (id: 964)

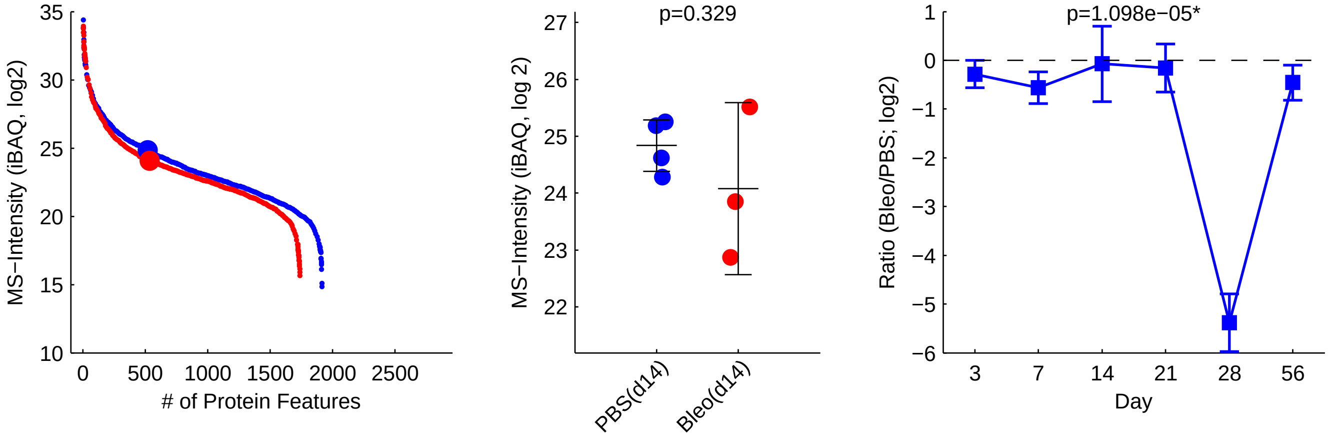

P14847 − Crp (id: 967)

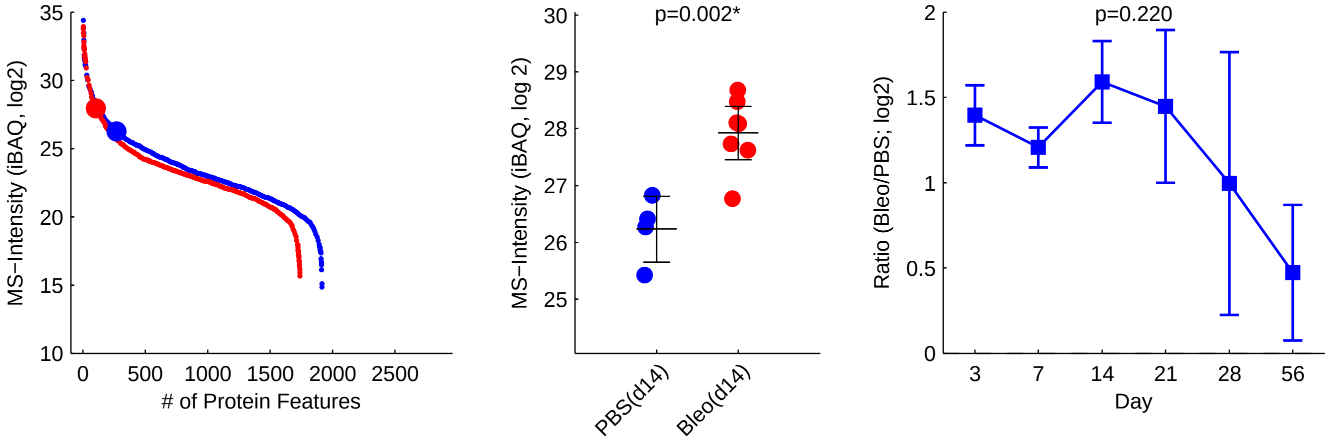

P15532 − Nme1 (id: 971)

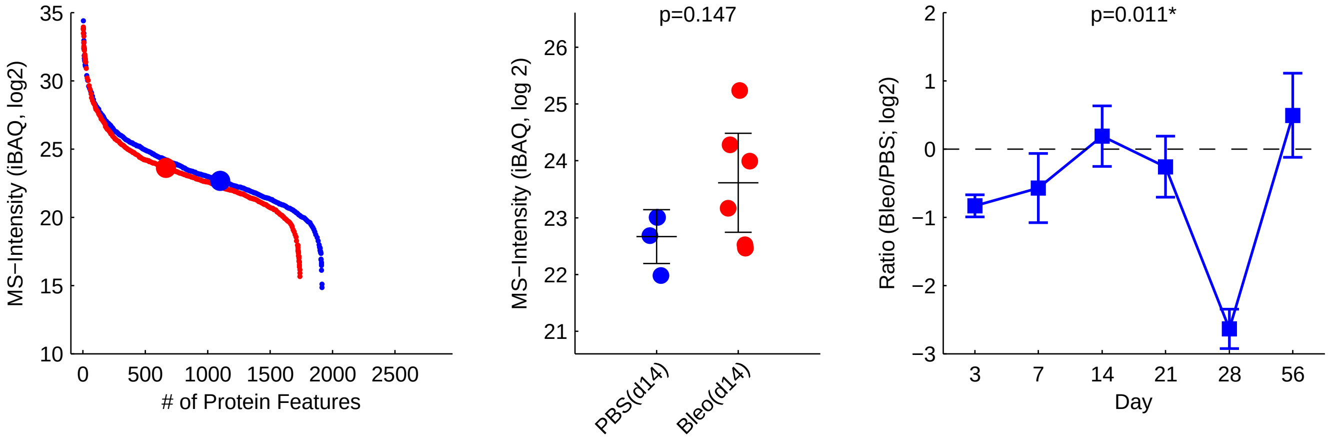

P15626 − Gstm2 (id: 973)

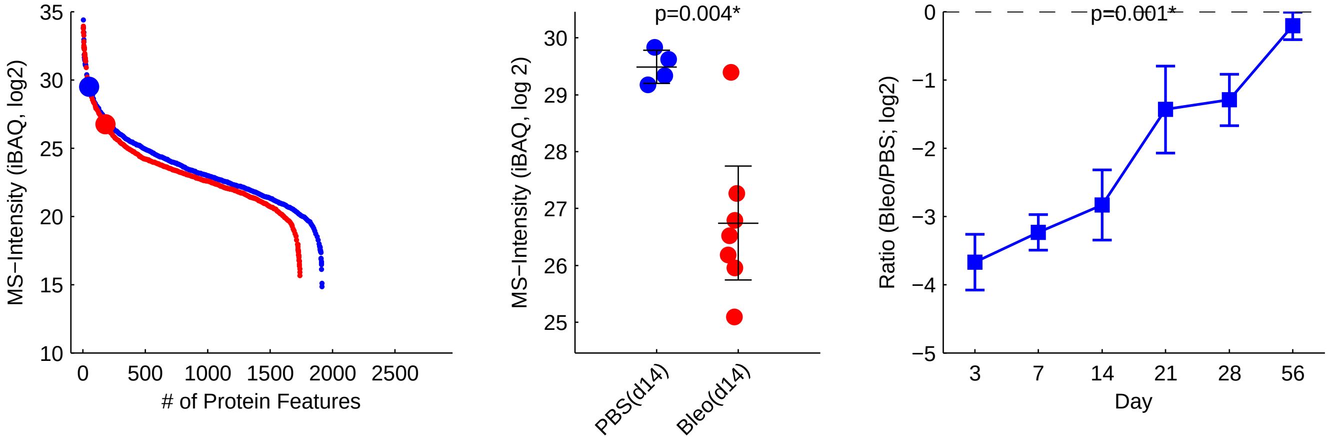

P15864 − Hist1h1c (id: 974)

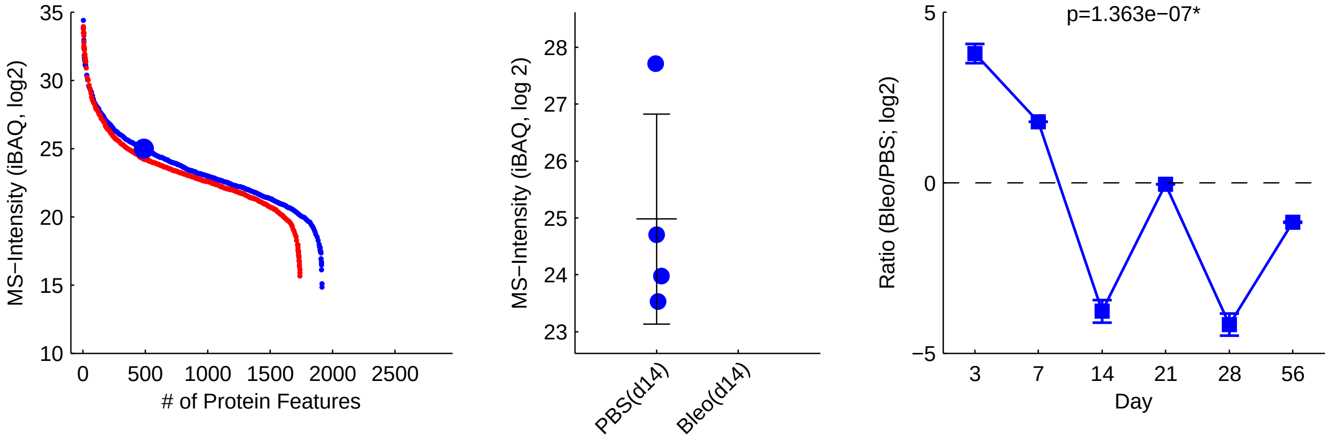

P16015 − Ca3 (id: 976)

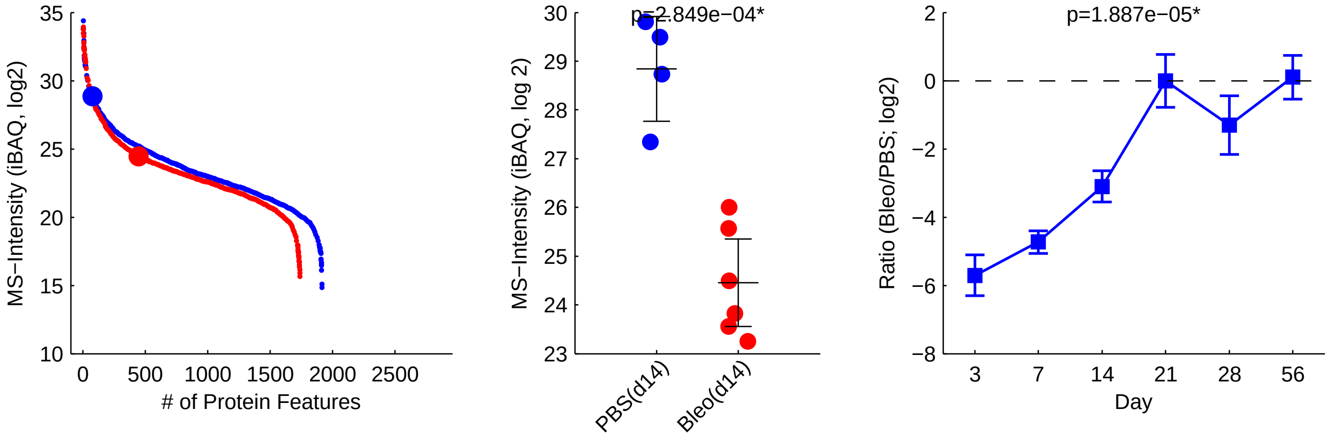

P16045 − Lgals1 (id: 977)

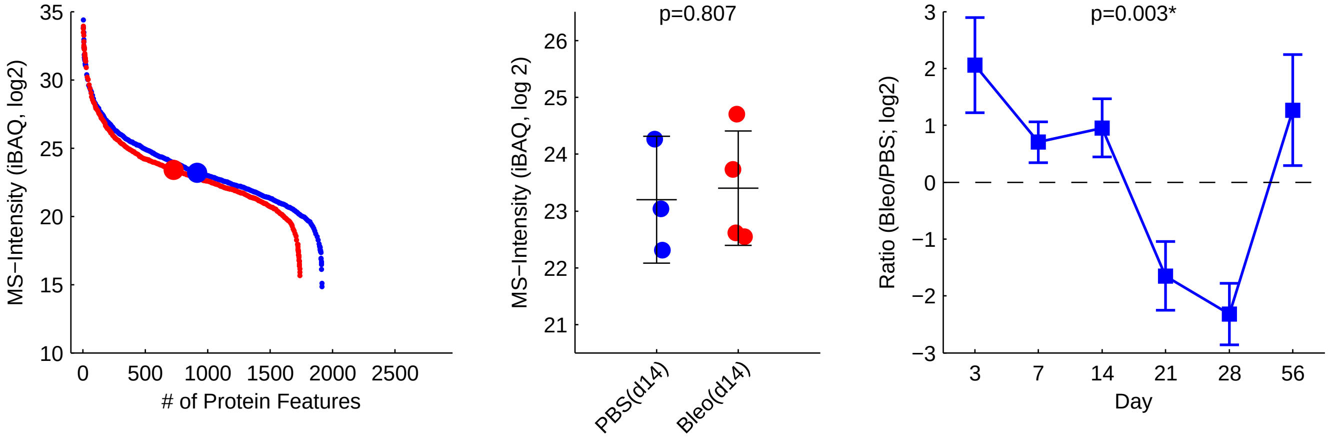

Q8C253 − Lgals3 (id: 978)

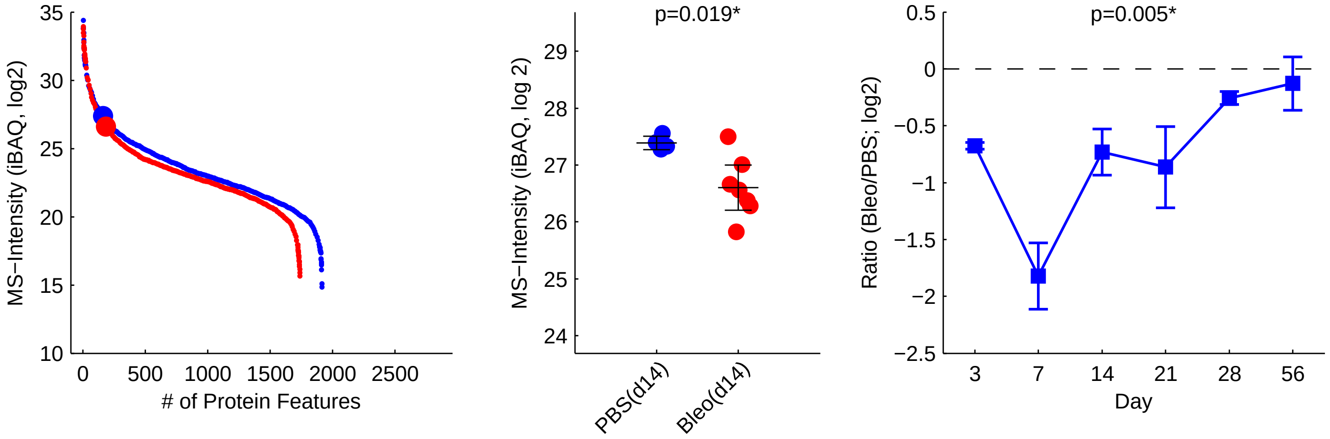

P16125 − Ldhb (id: 979)

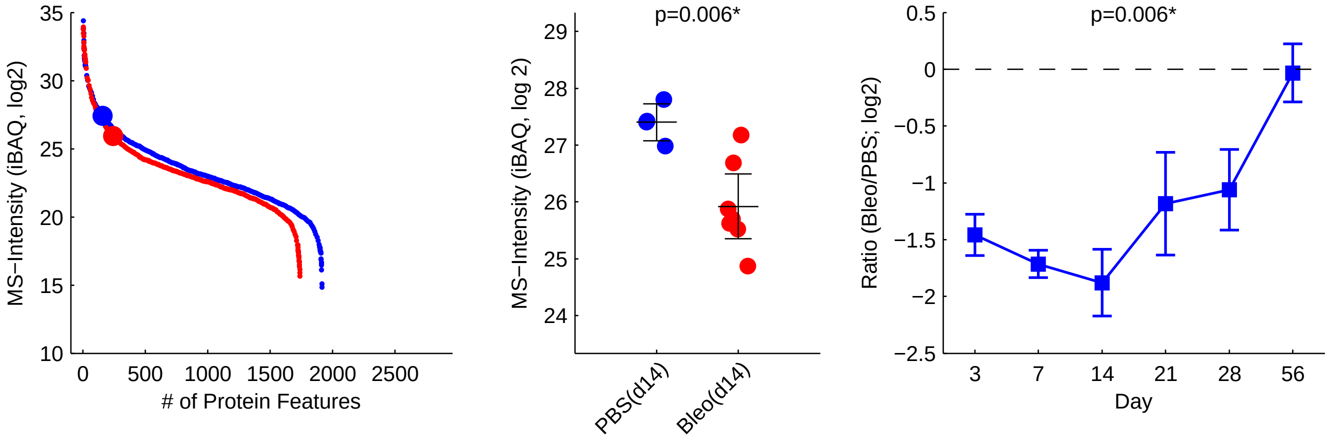

P16301 − Lcat (id: 981)

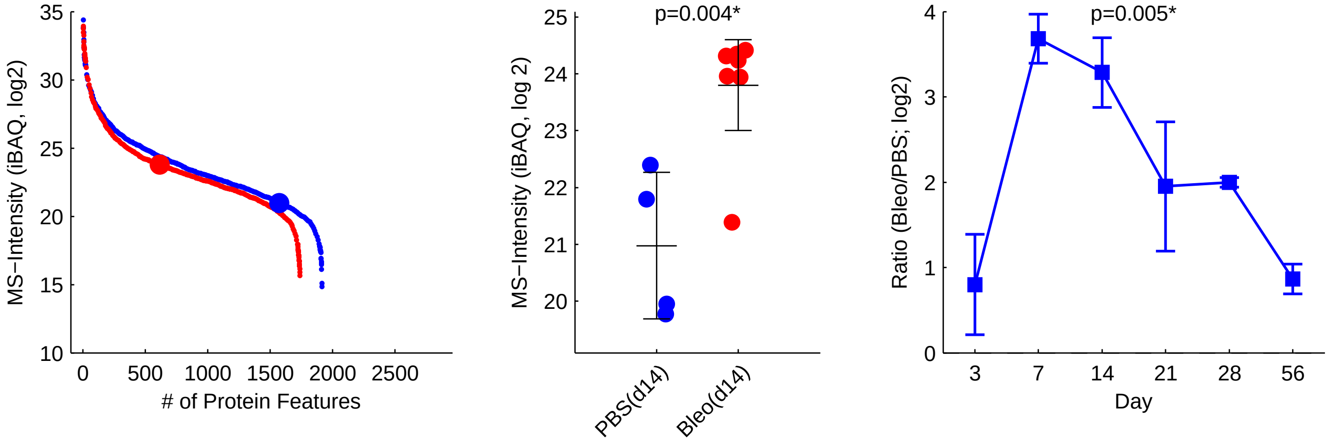

P16406 − Enpep (id: 984)

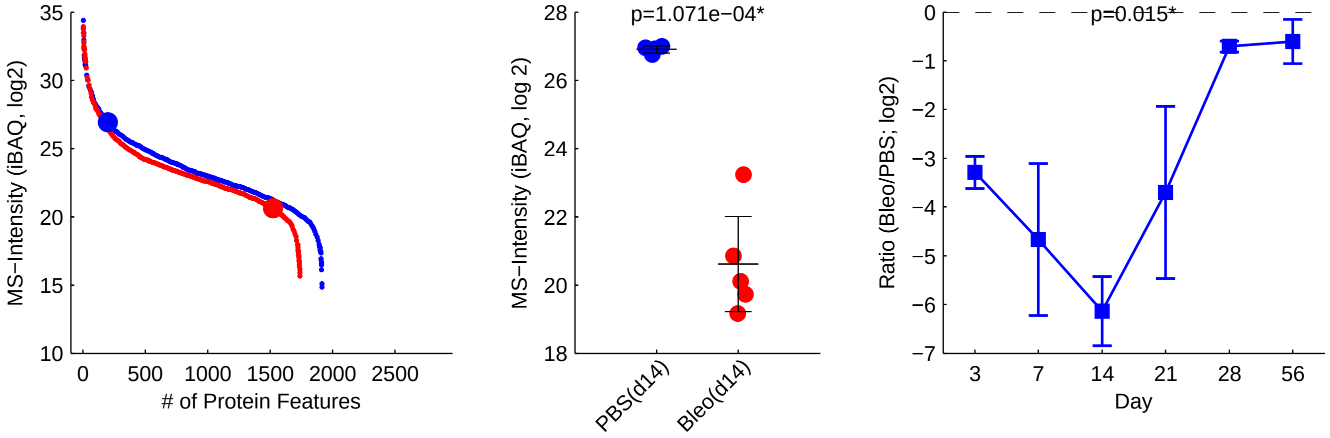

P16858 − Gapdh (id: 988)

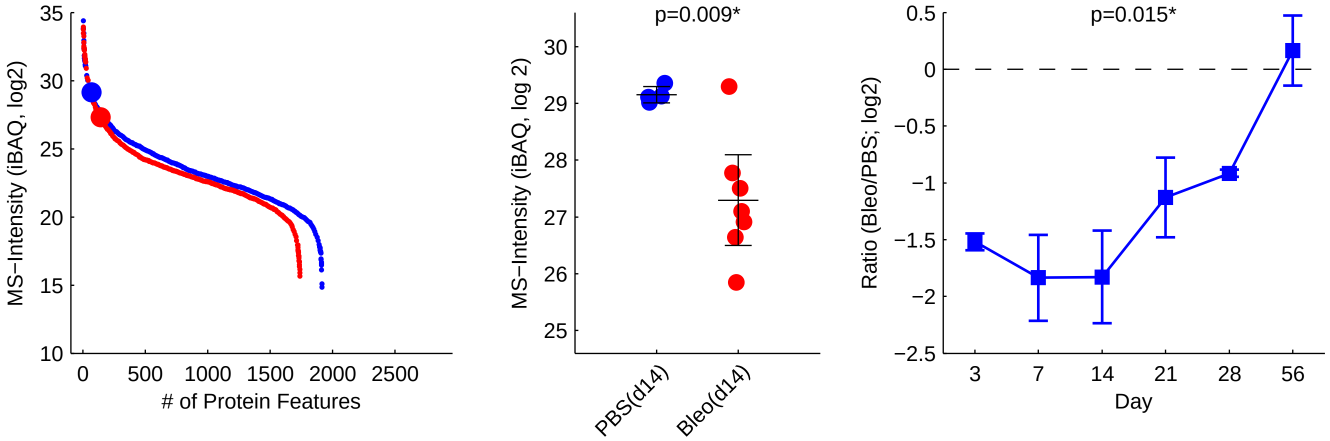

P17047 − Lamp2 (id: 989)

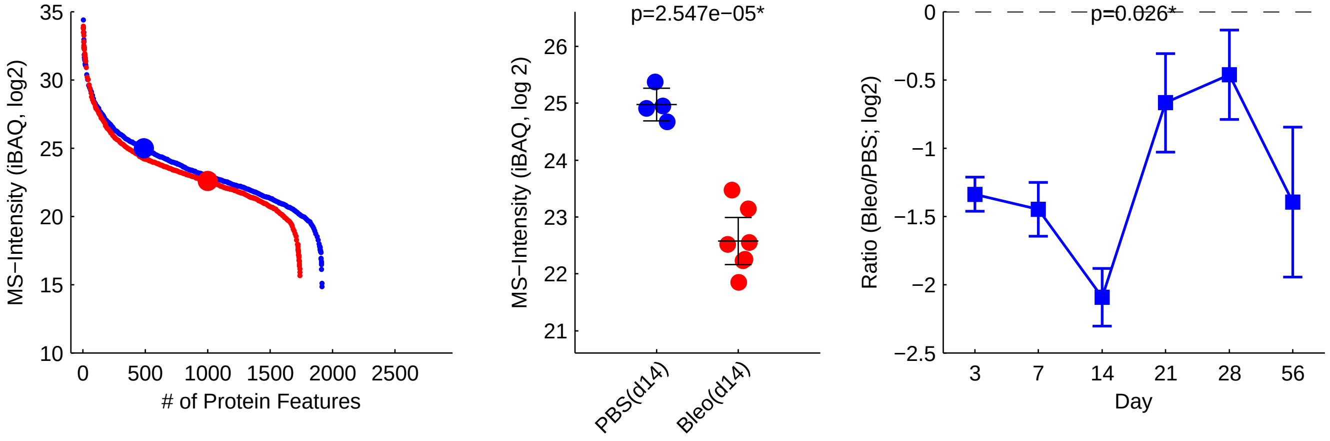

P17156 − Hspa2 (id: 990)

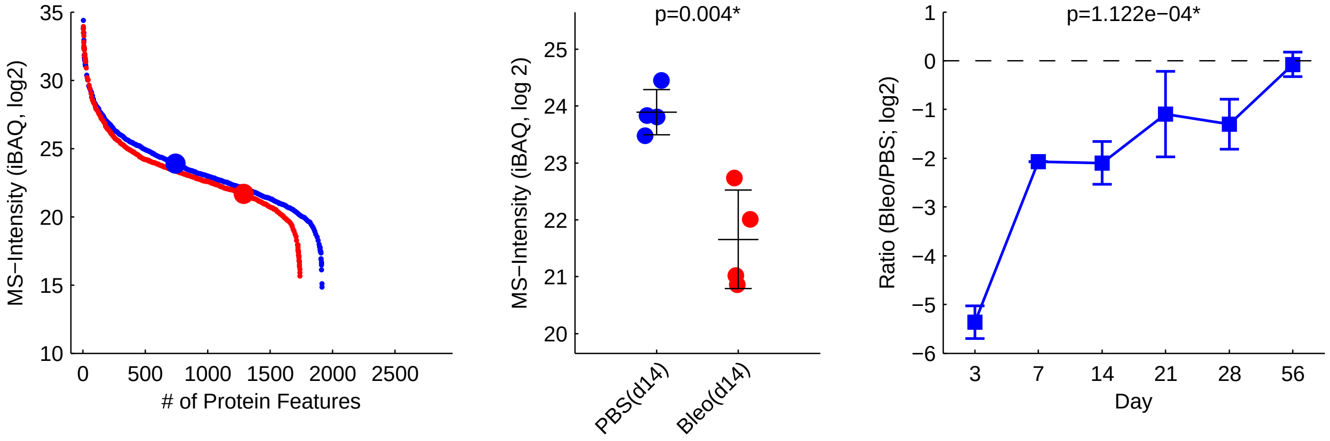

P17563 − Selenbp1 (id: 994)

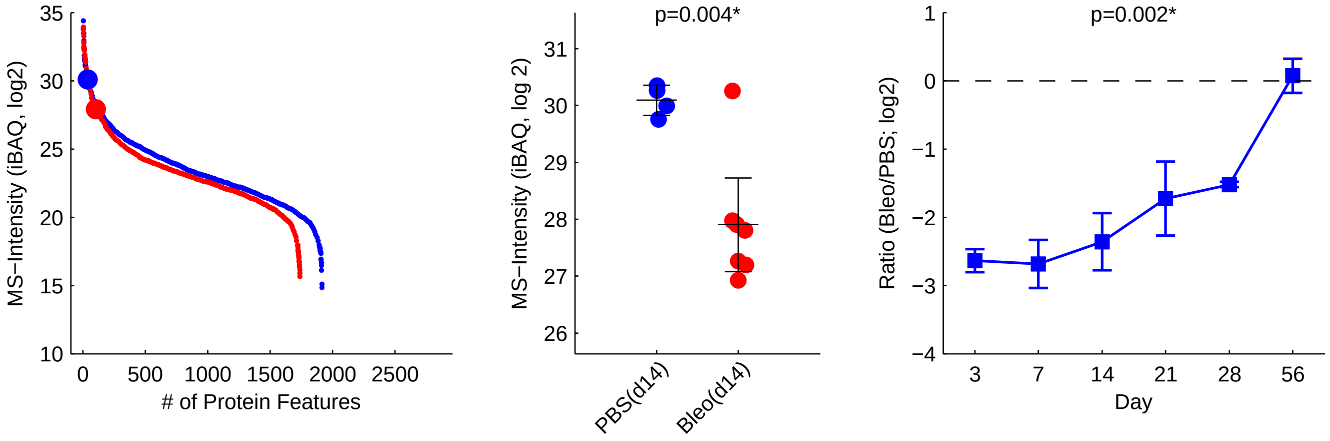

P17710−3 − Hk1 (id: 995)

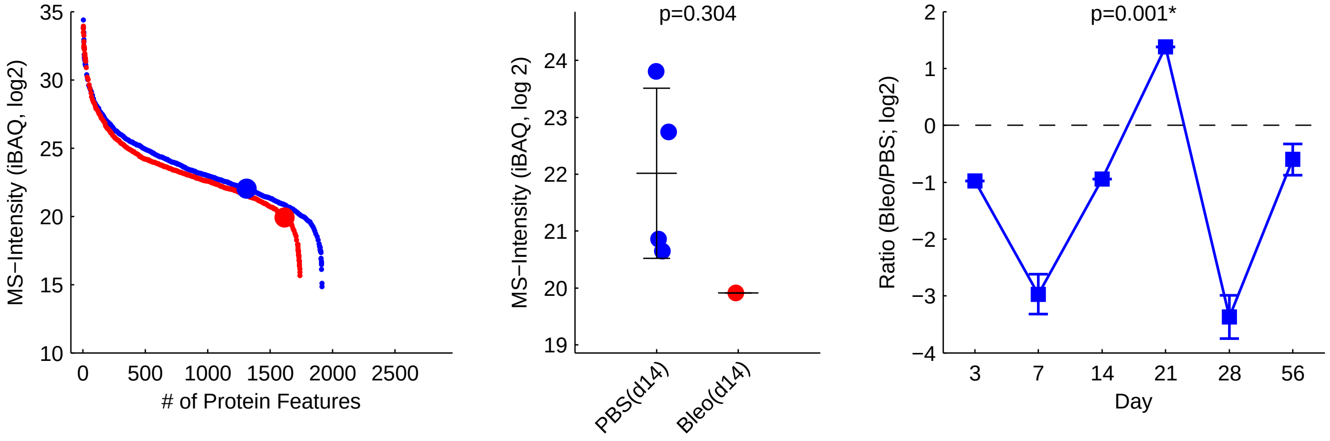

Q61696 − Hspa1a (id: 998)

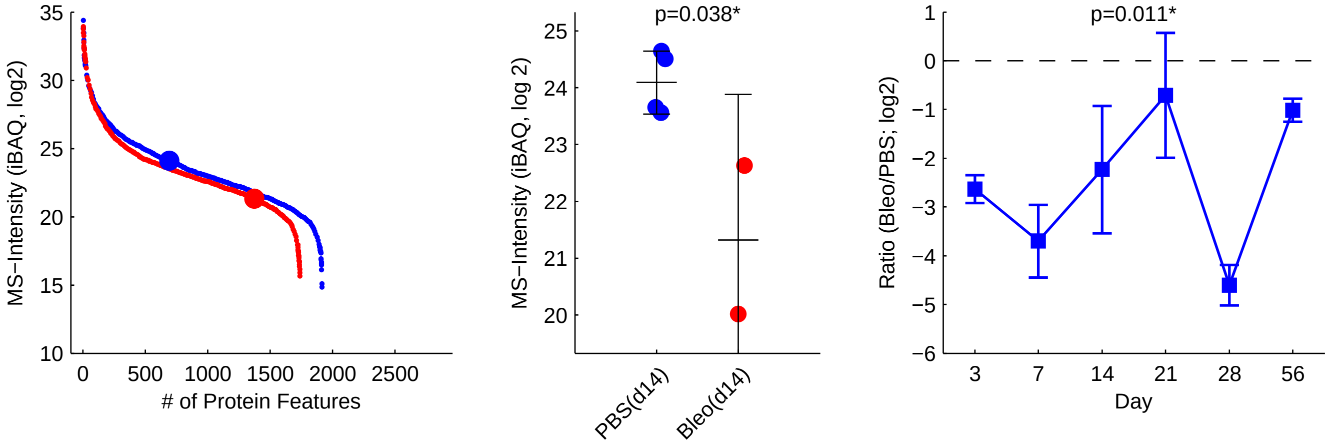

P18242 − Ctsd (id: 1001)

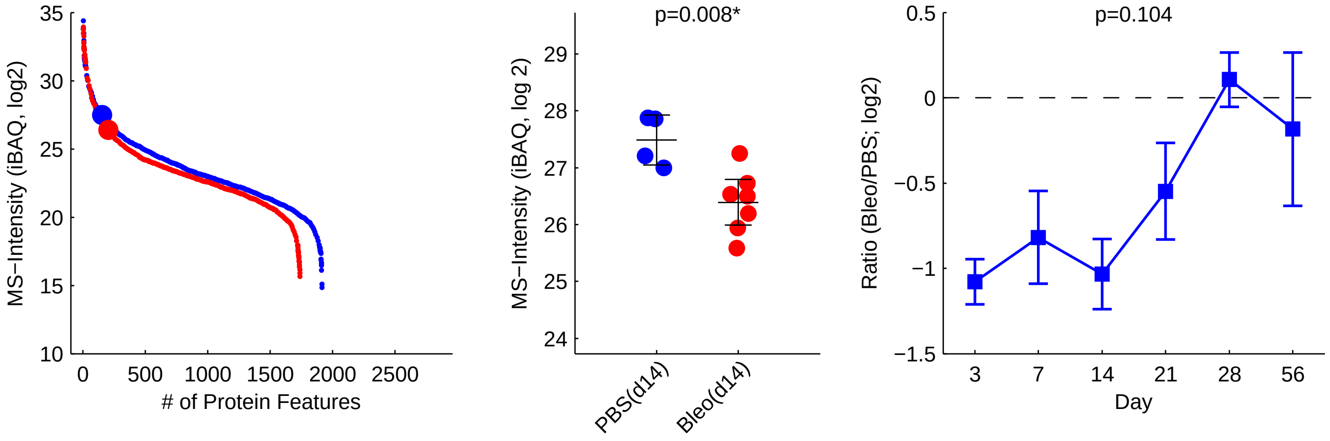

P19253 − Rpl13a (id: 1010)

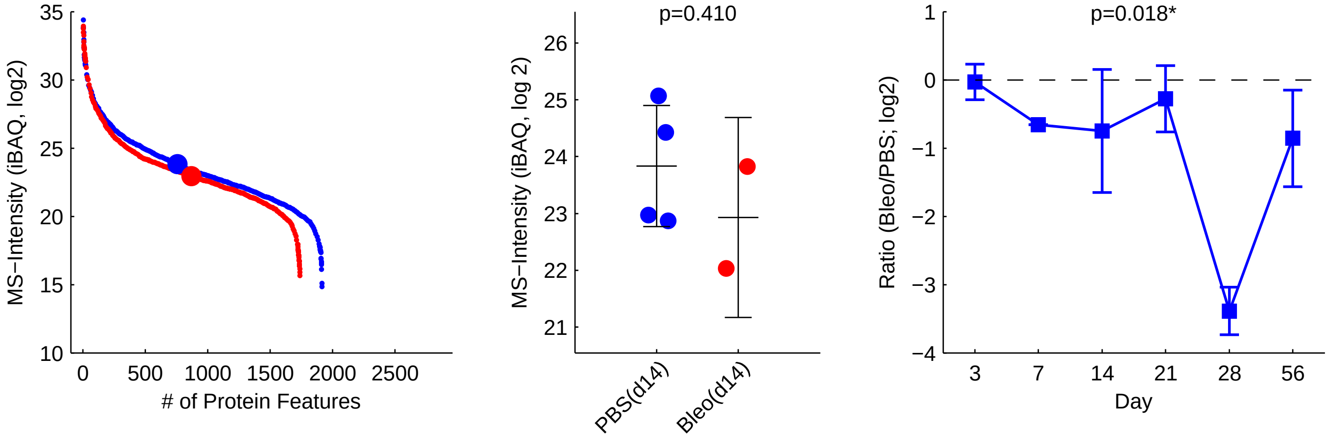

P19973−2 − Lsp1 (id: 1016)

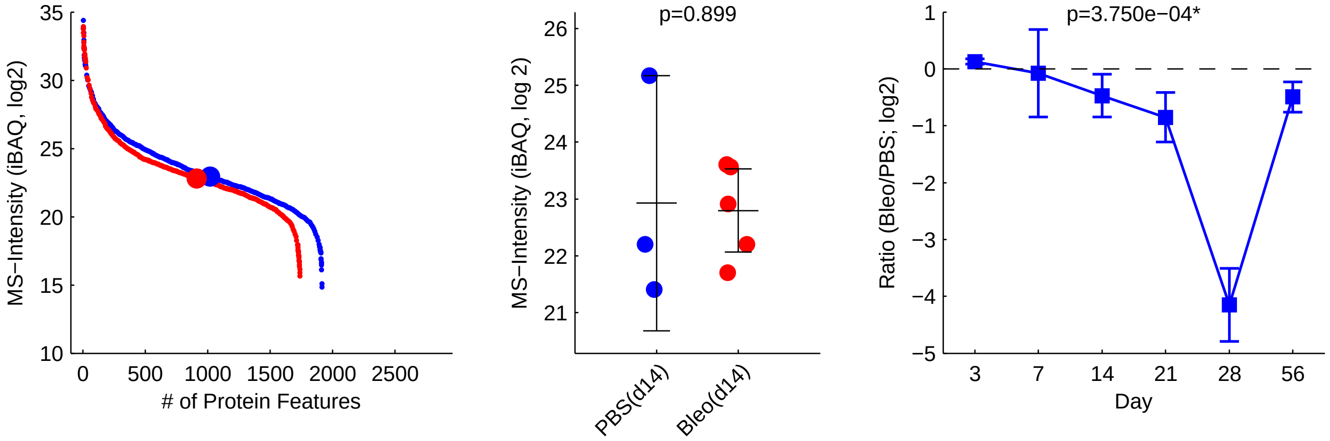

P20029 − Hspa5 (id: 1017)

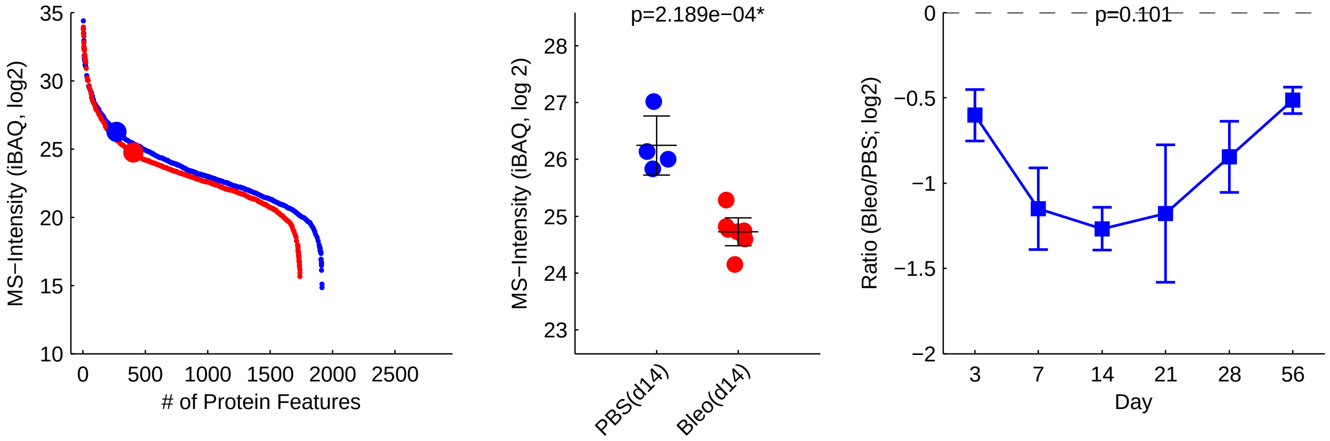

P20108 − Prdx3 (id: 1020)

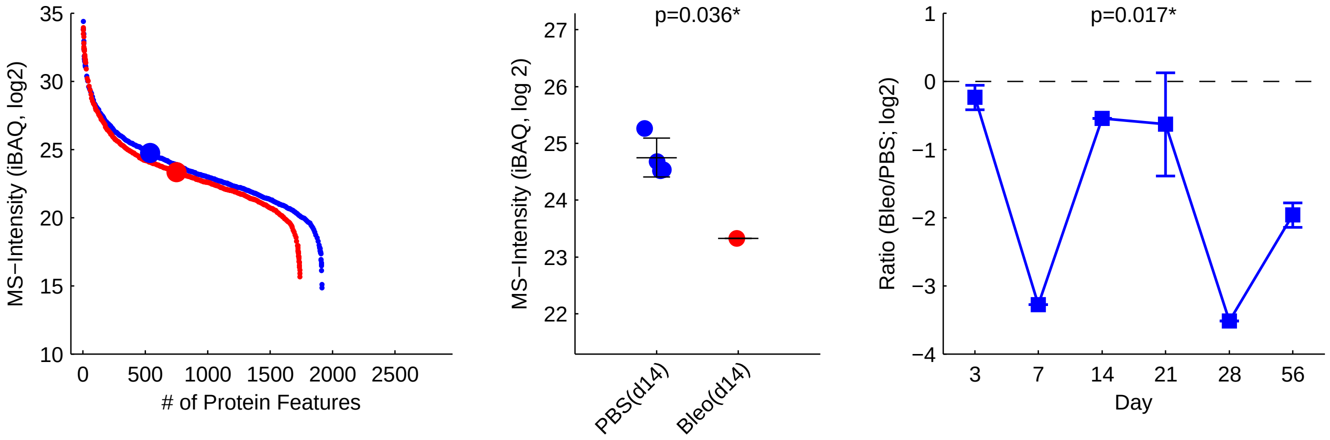

P20152 − Vim (id: 1021)

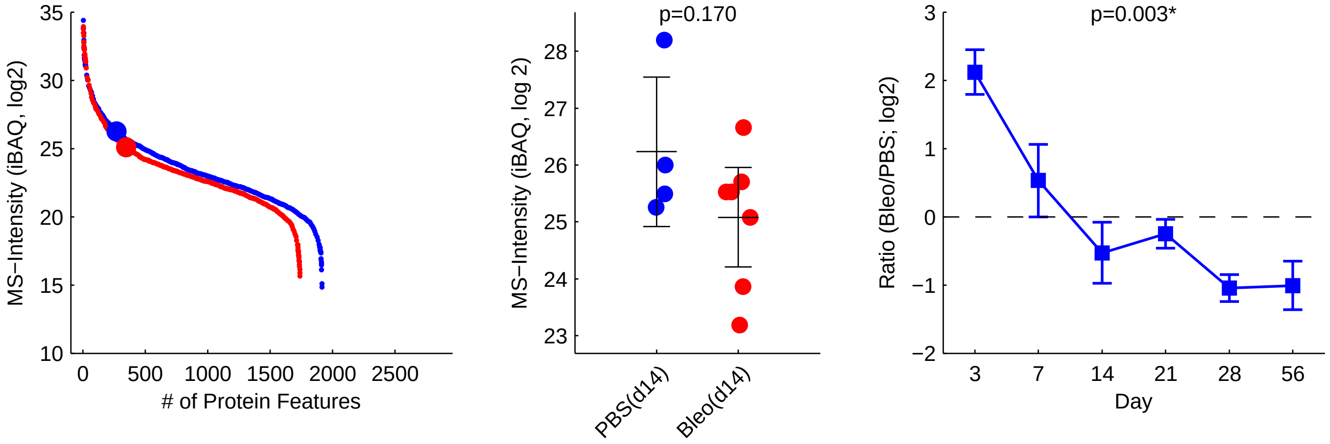

P20918 − Plg (id: 1024)

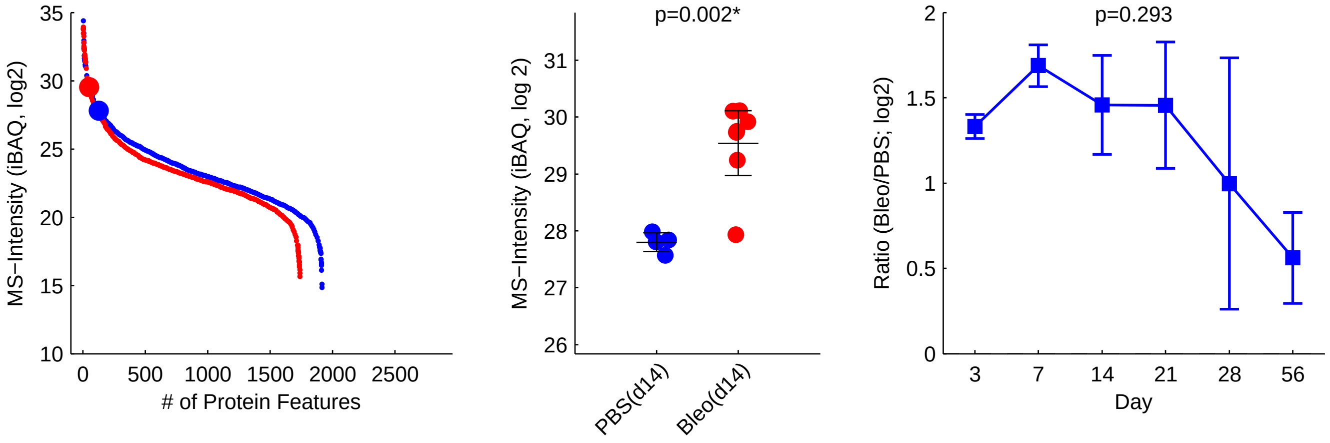

P21279 − Gnaq (id: 1028)

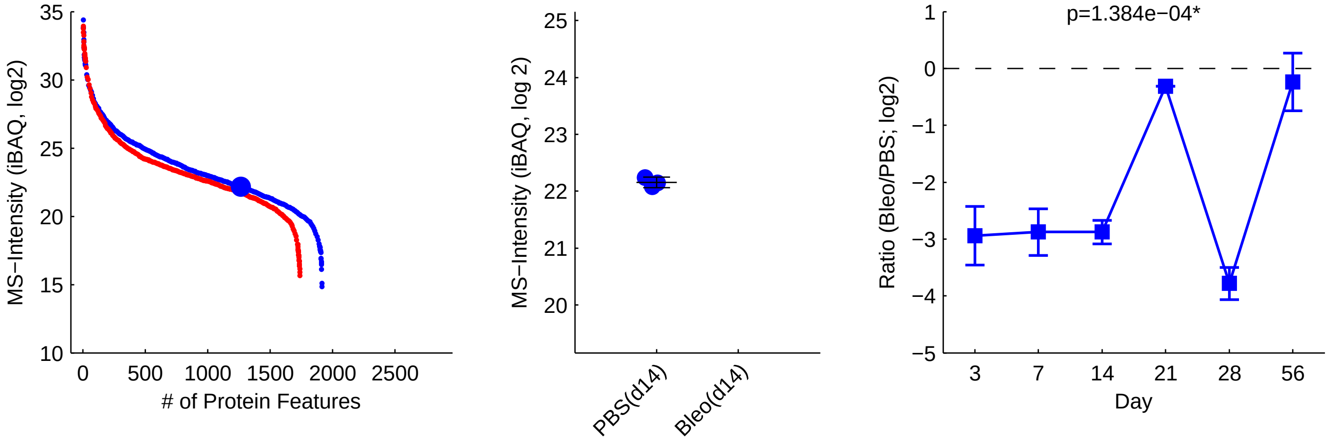

P21460 − Cst3 (id: 1030)

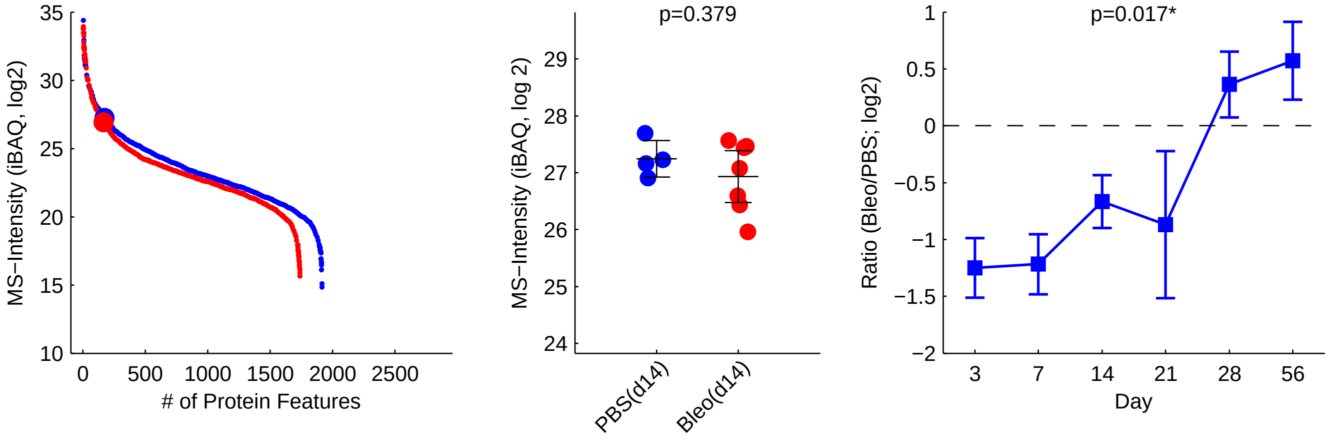

P21614 − Gc (id: 1033)

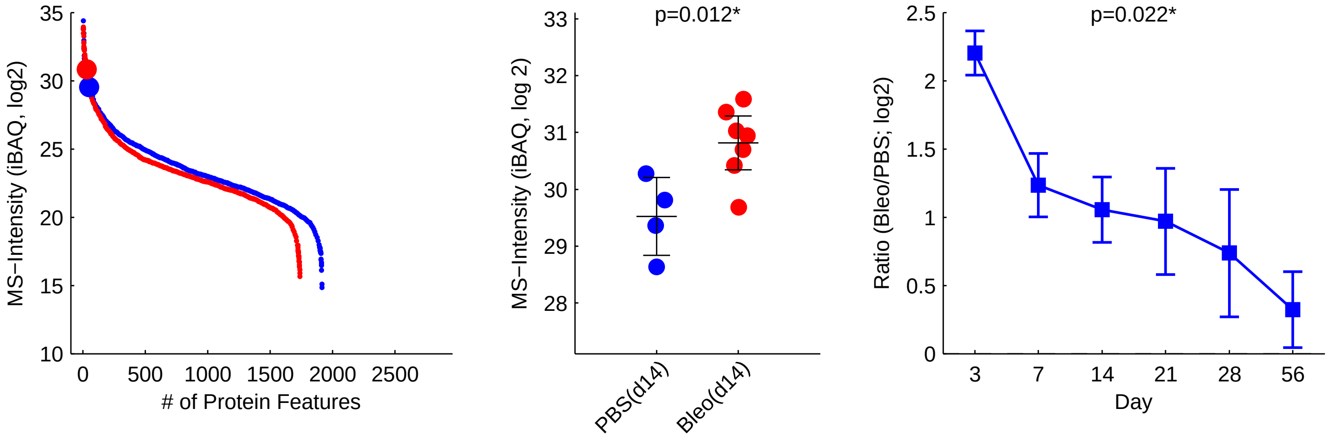

Q6P8P8 − Sftpc (id: 1035)

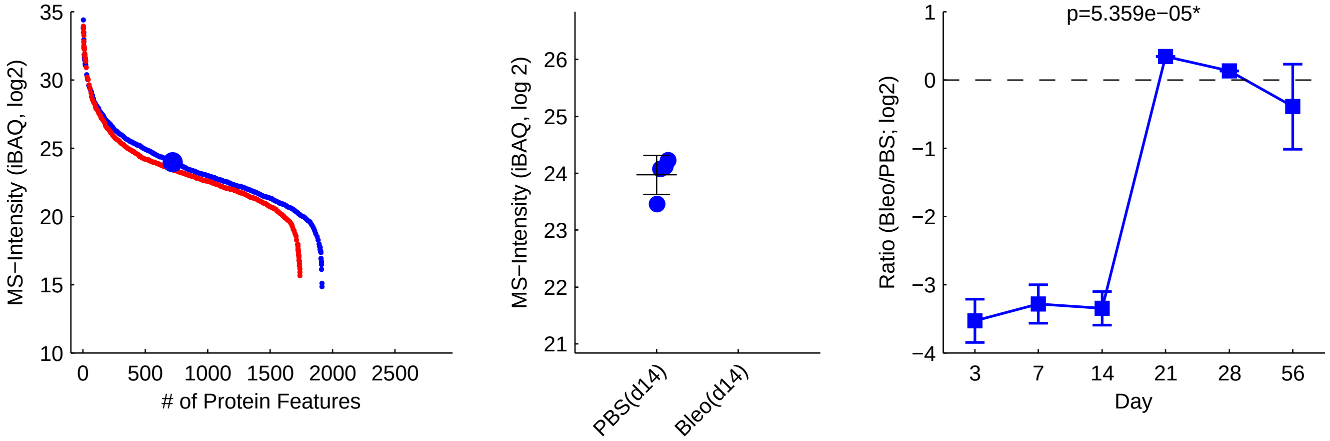

P21956−2 − Mfge8 (id: 1036)

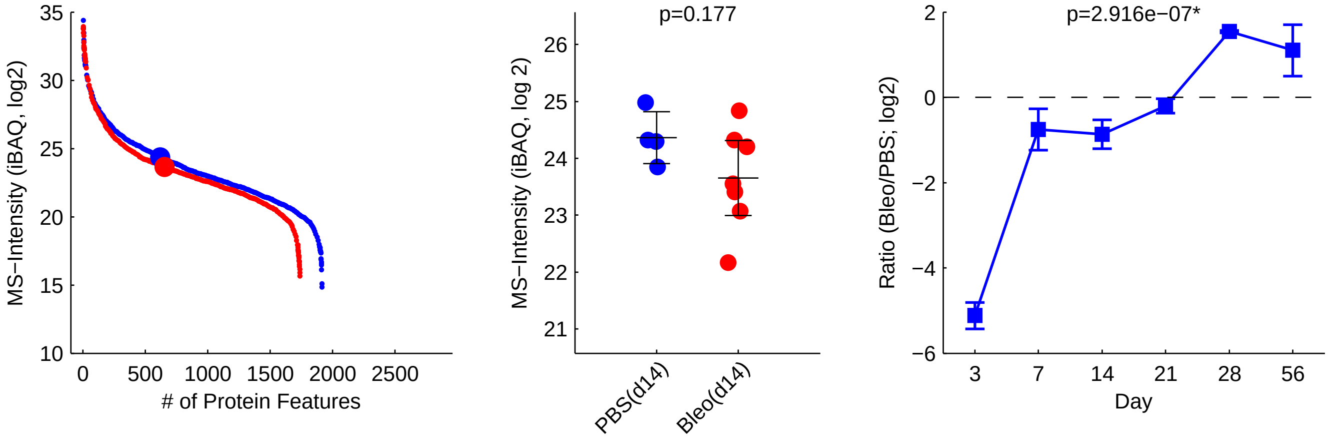

Q8CGP6 − Hist1h2ah (id: 1041)

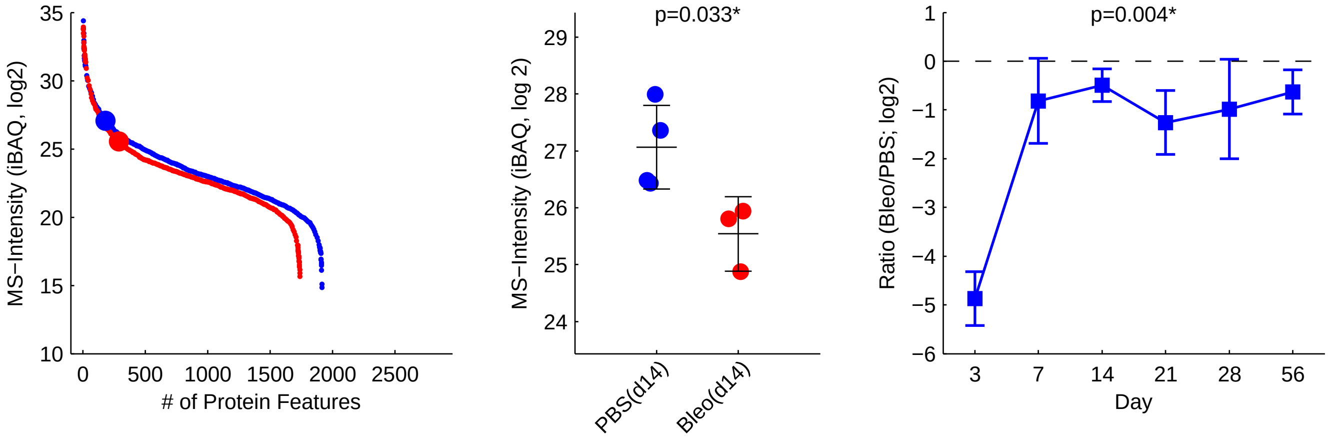

P22892 − Ap1g1 (id: 1042)

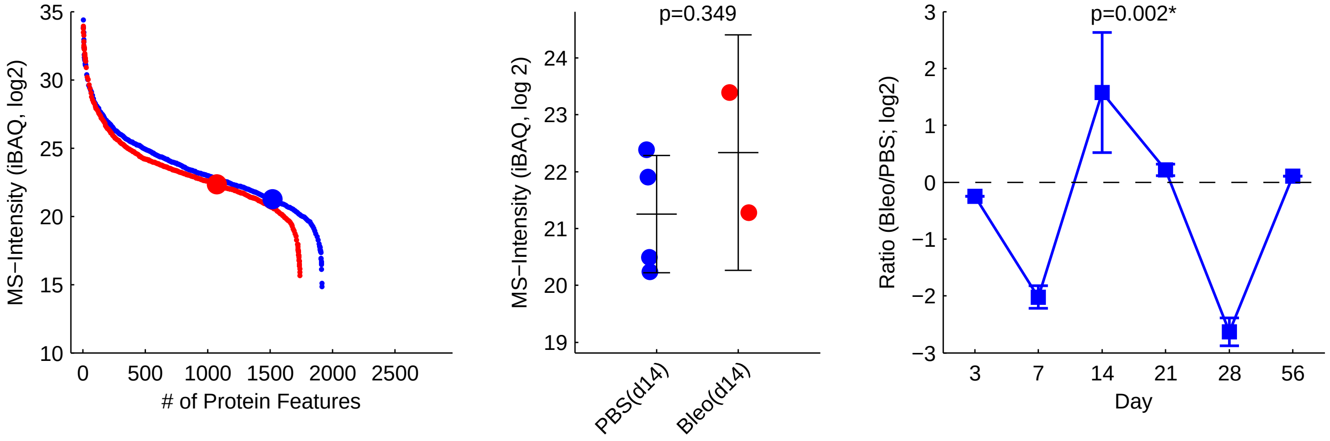

P23116 − Eif3a (id: 1044)

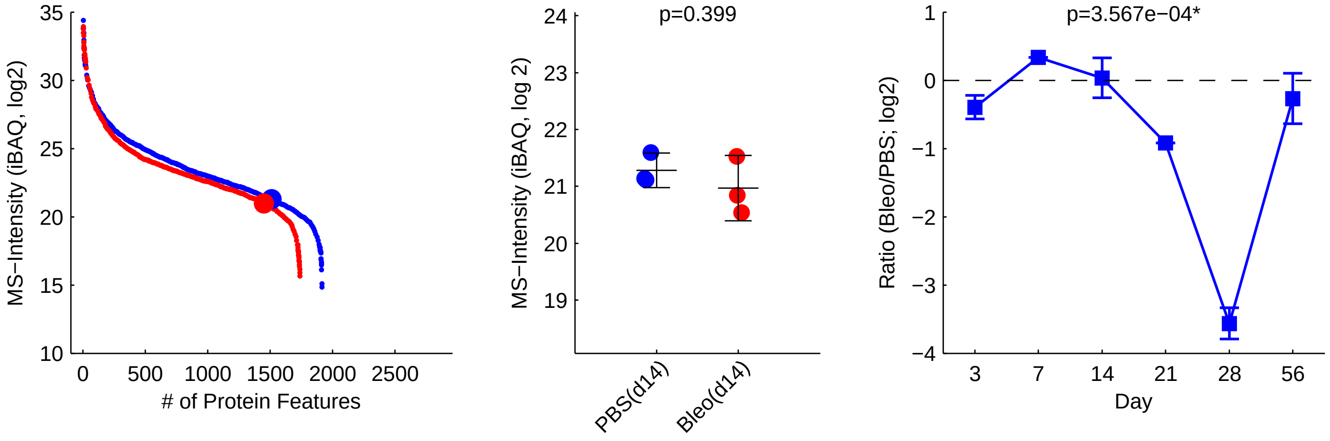

Q543K9 − Pnp (id: 1046)

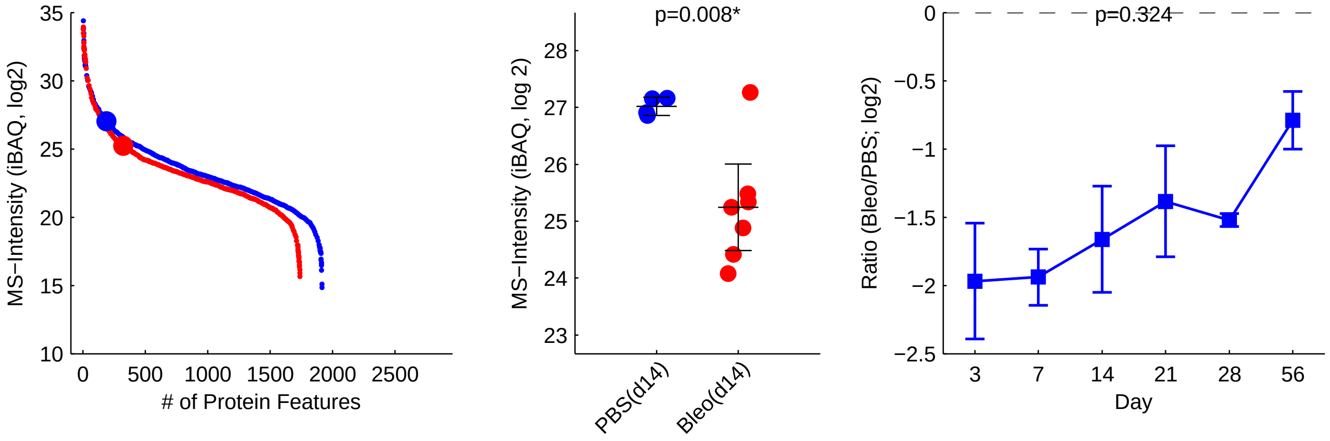

P23953 − Ces1c (id: 1050)

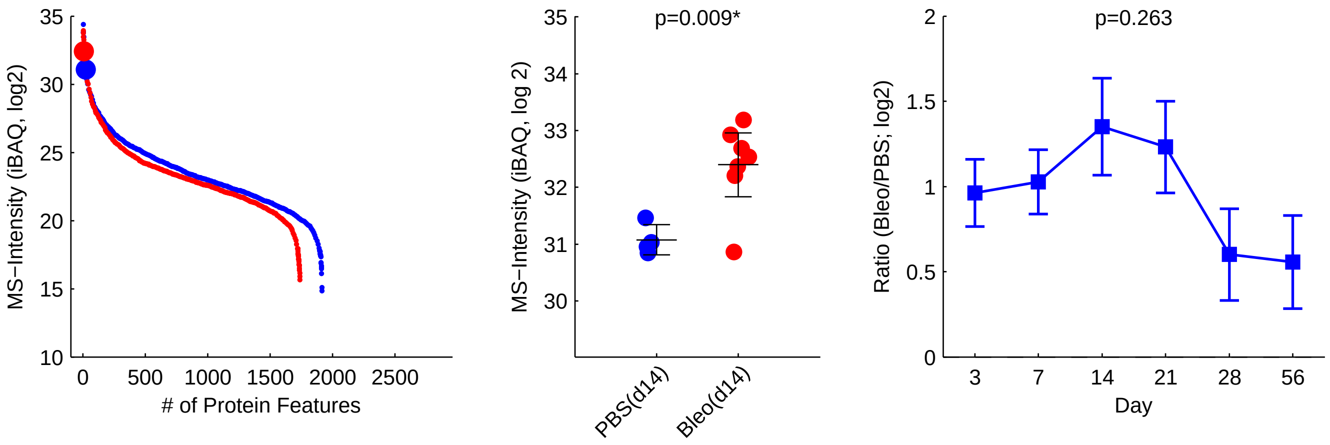

P24472 − Gsta4 (id: 1053)

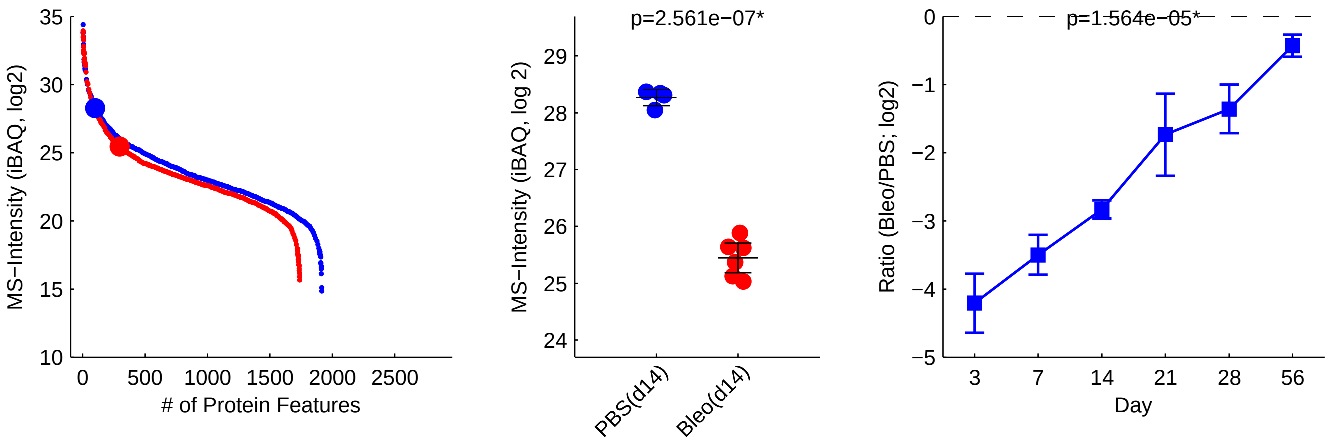

P24549 − Aldh1a1 (id: 1056)

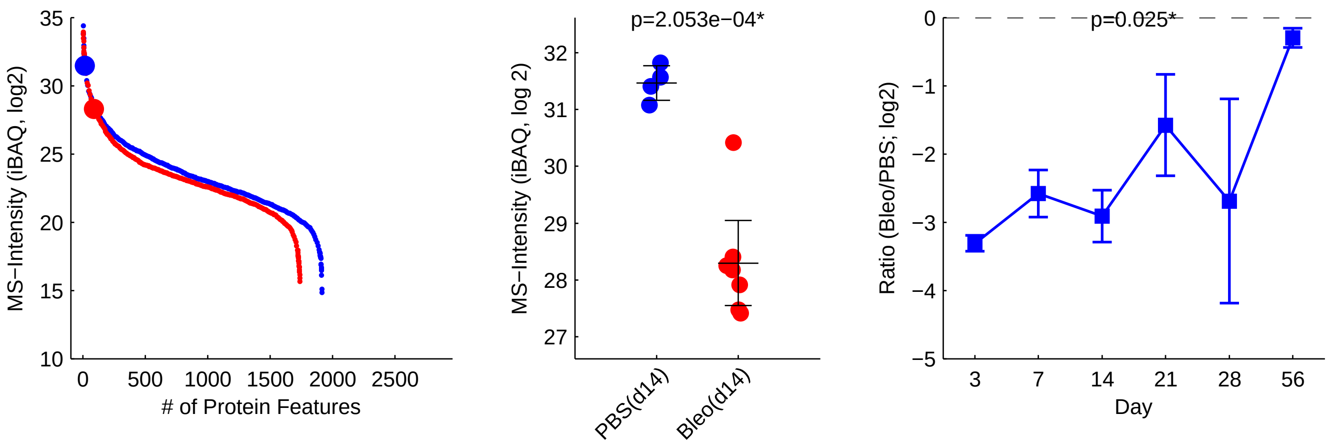

P26041 − Msn (id: 1063)

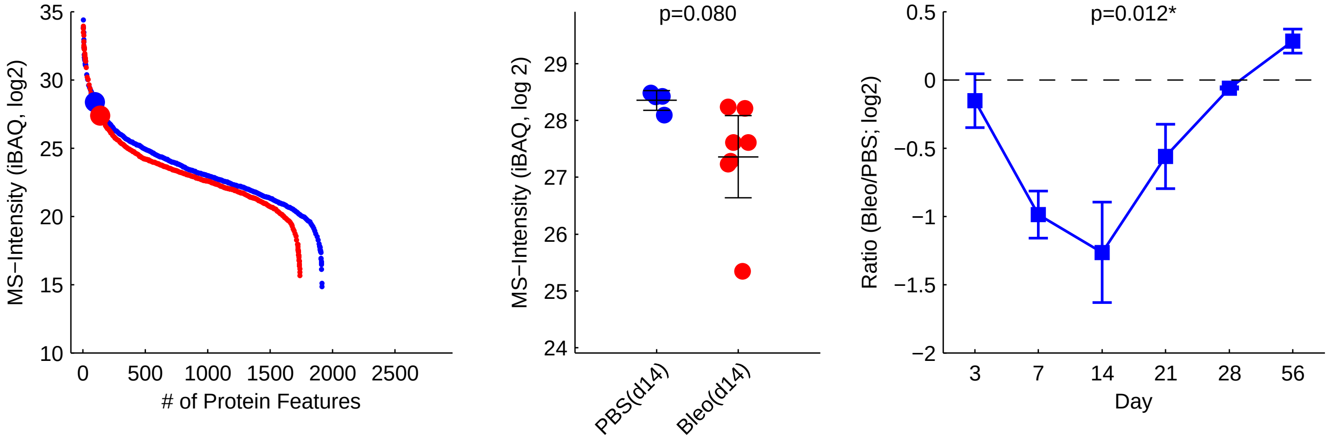

P26043 − Rdx (id: 1064)

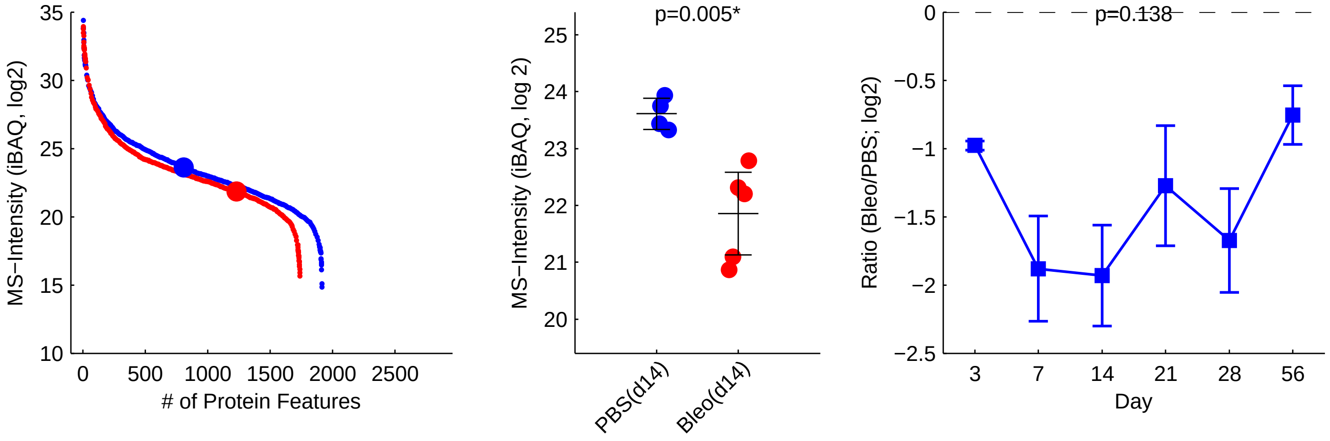

P26262 − Klkb1 (id: 1066)

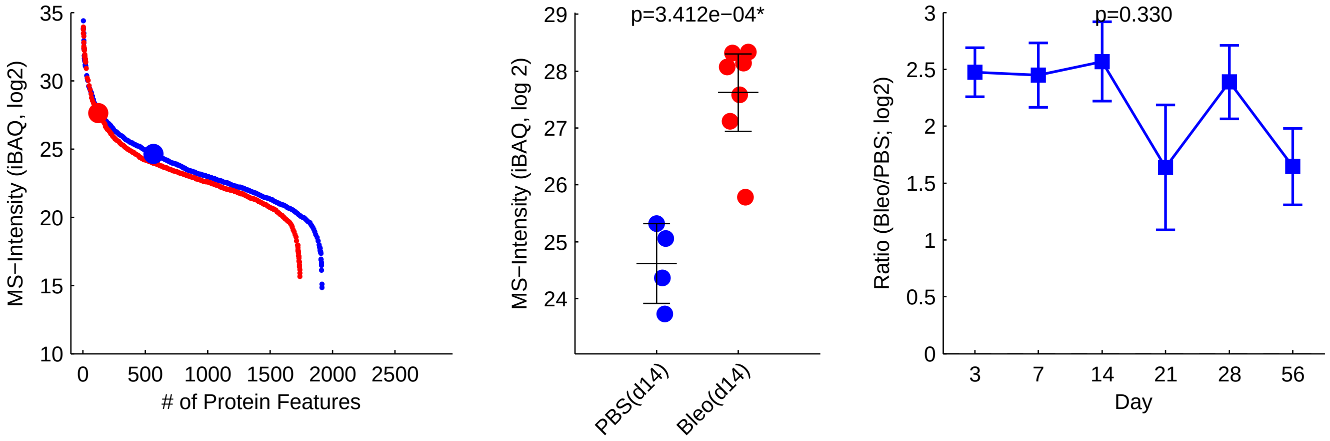

P26350 − Ptma (id: 1067)

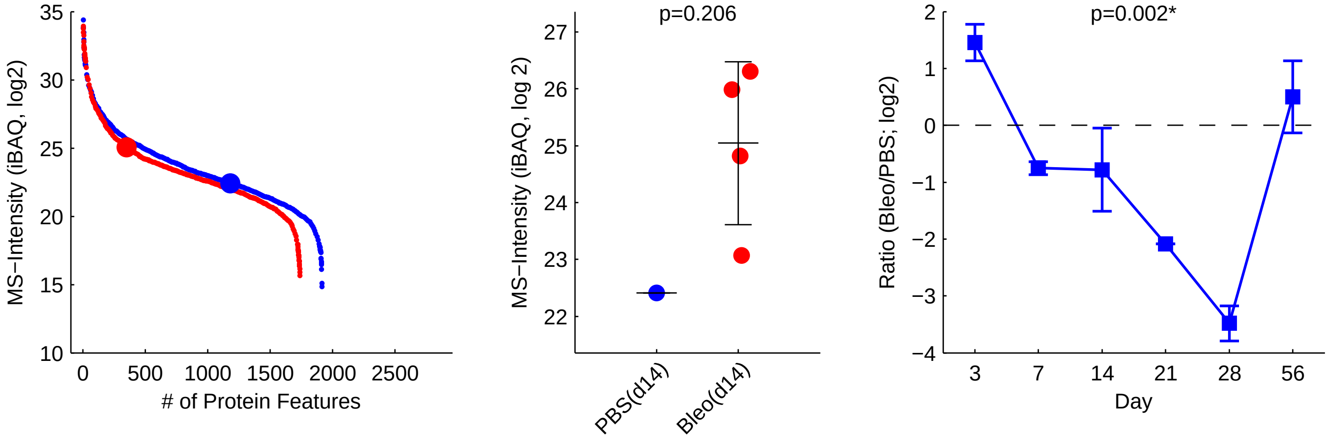

P26443 − Glud1 (id: 1069)

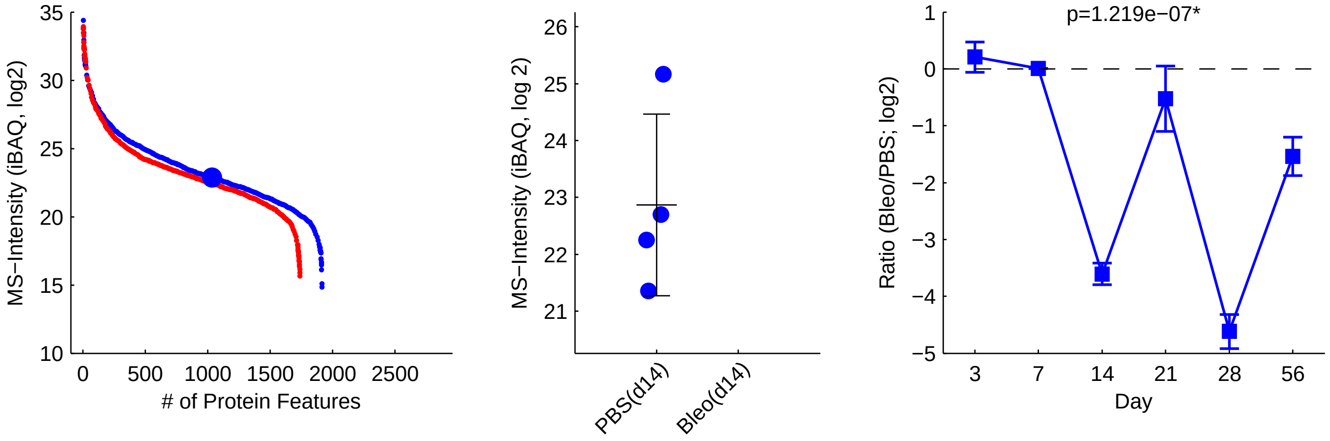

P27773 − Pdia3 (id: 1082)

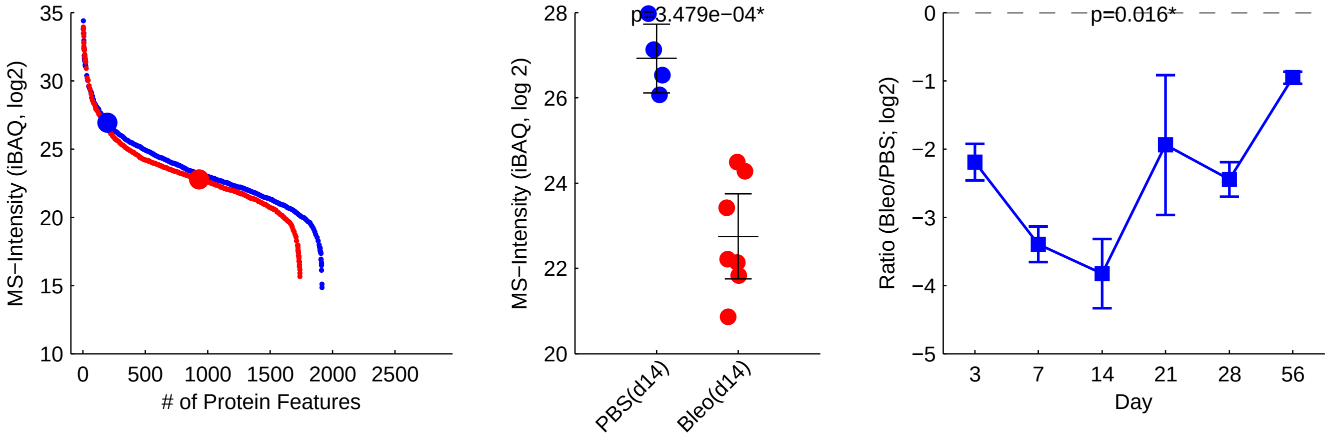

P28665 − Mug1 (id: 1092)

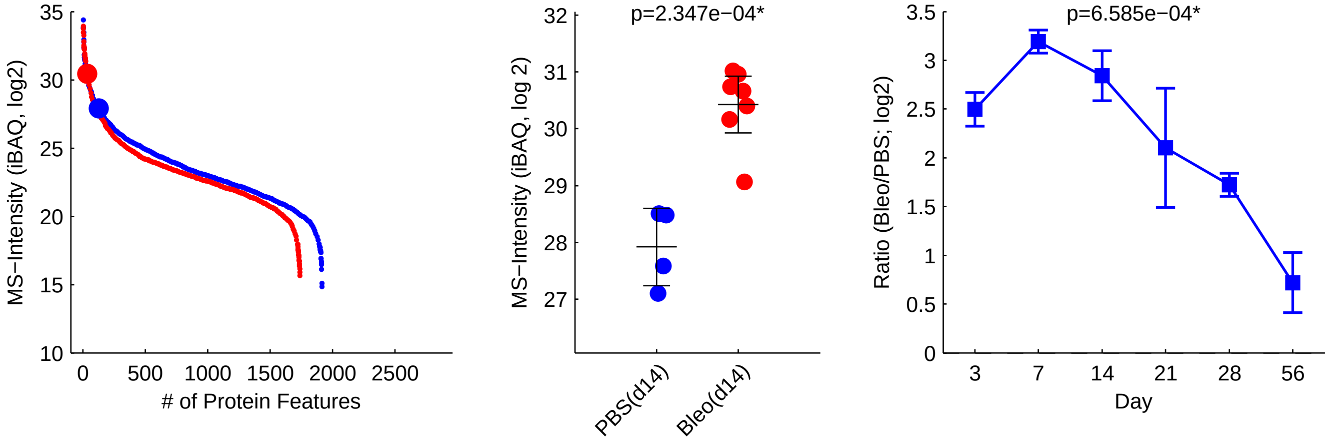

P28828 − Ptprm (id: 1094)

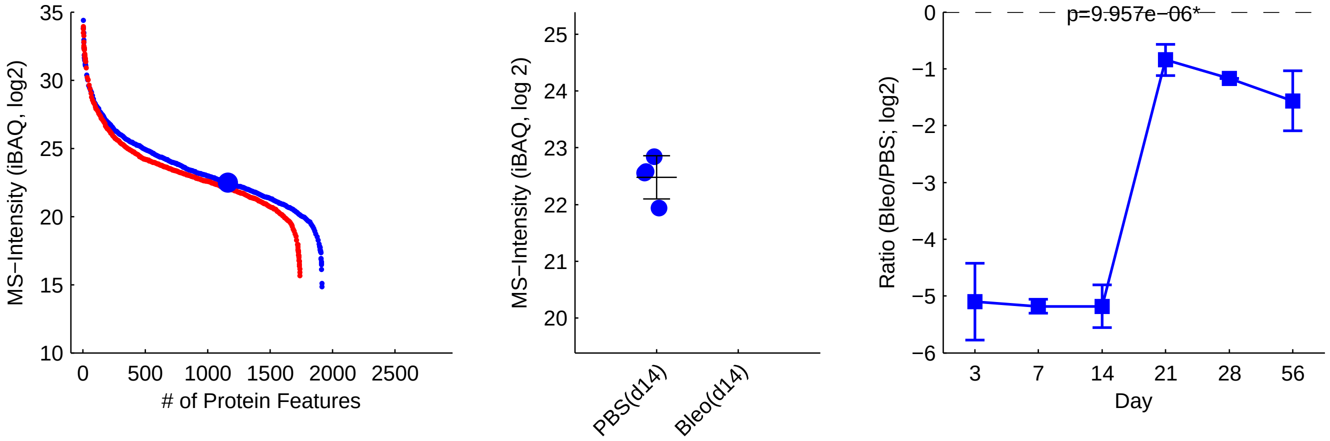

P28843 − Dpp4 (id: 1095)

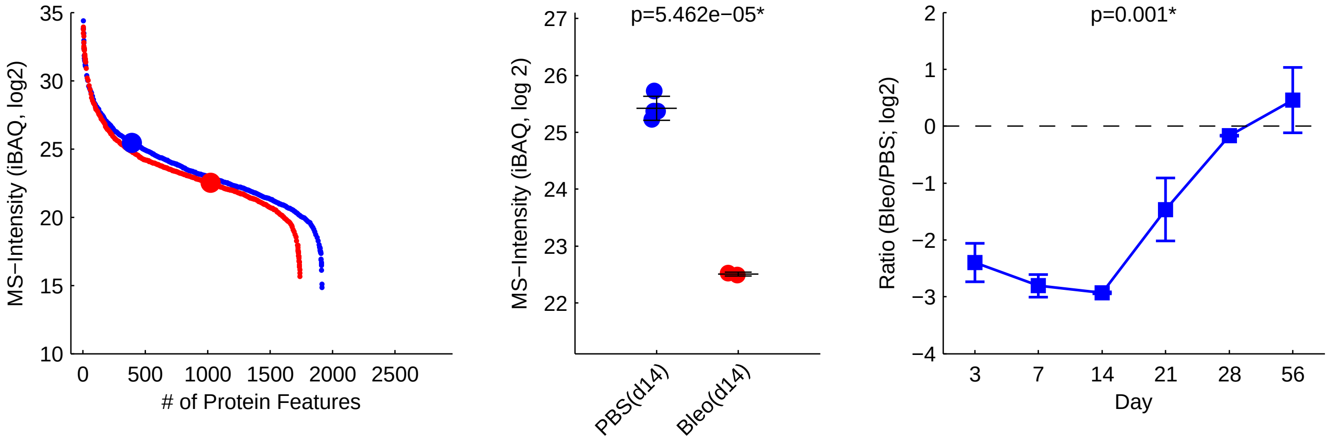

P29699 − Ahsg (id: 1104)

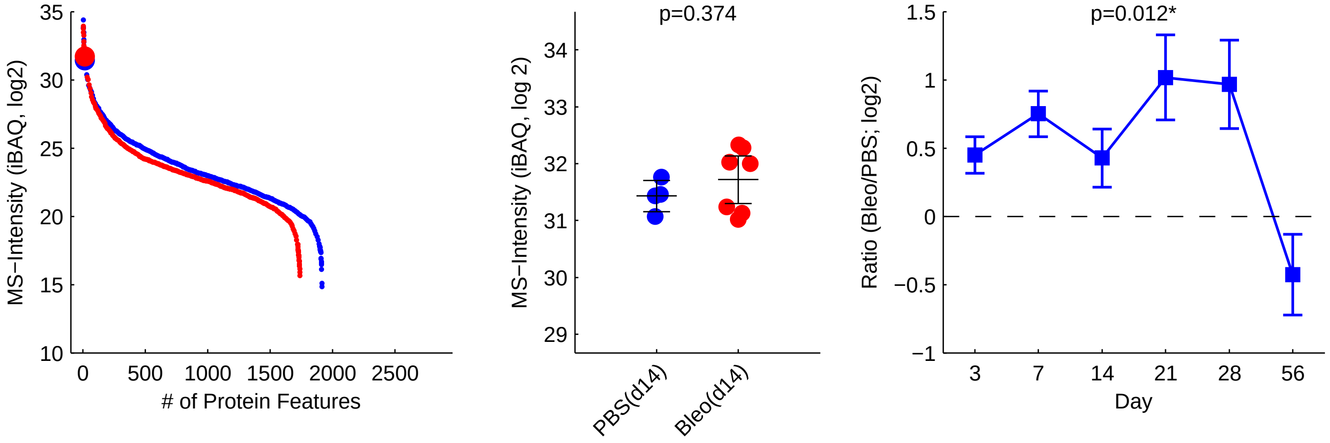

P29788 − Vtn (id: 1106)

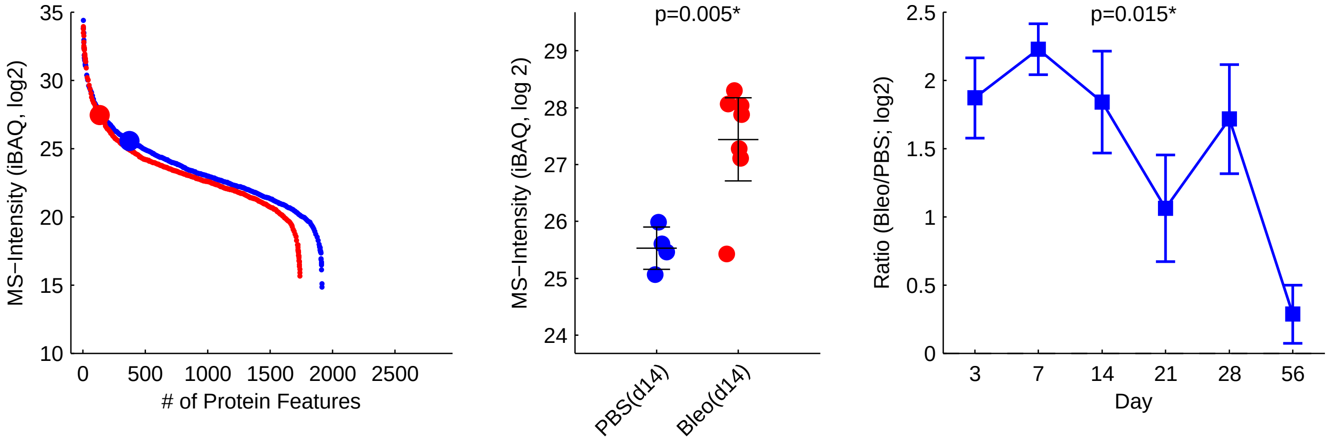

P30115 − Gsta3 (id: 1107)

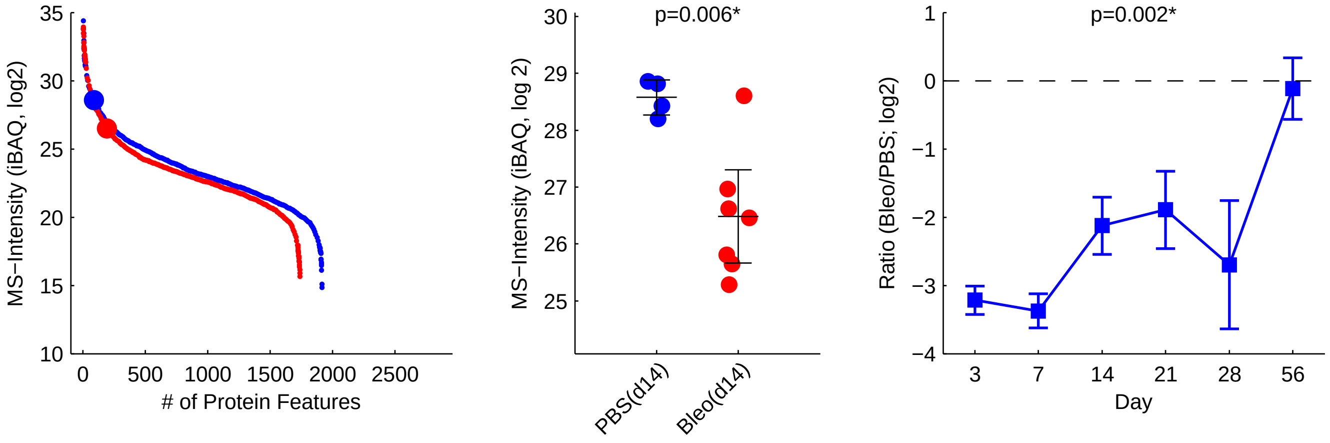

P30275 − Ckmt1 (id: 1108)

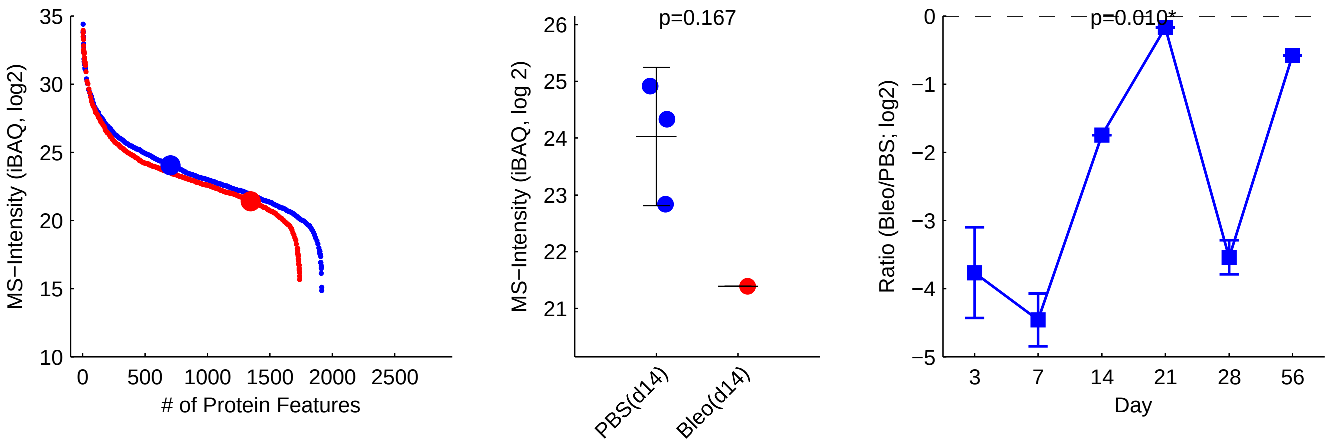

P30412 − Ppic (id: 1109)

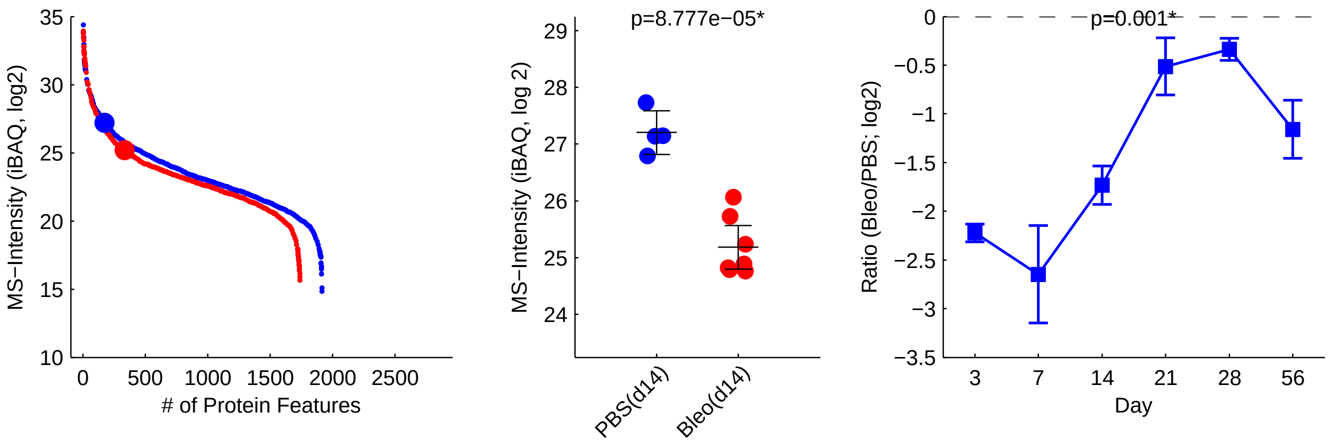

P30416 − Fkbp4 (id: 1110)

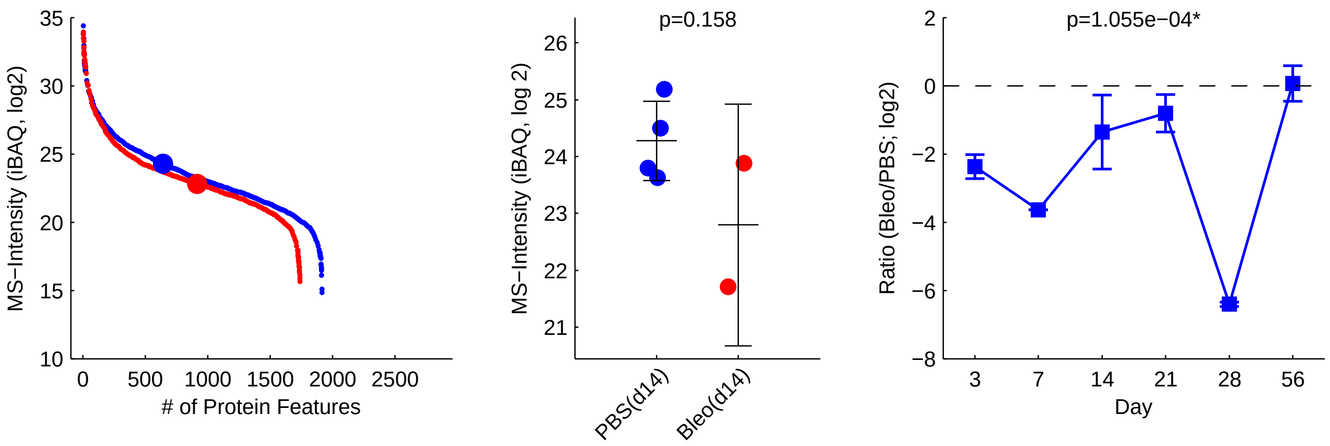

P30681 − Hmgb2 (id: 1111)

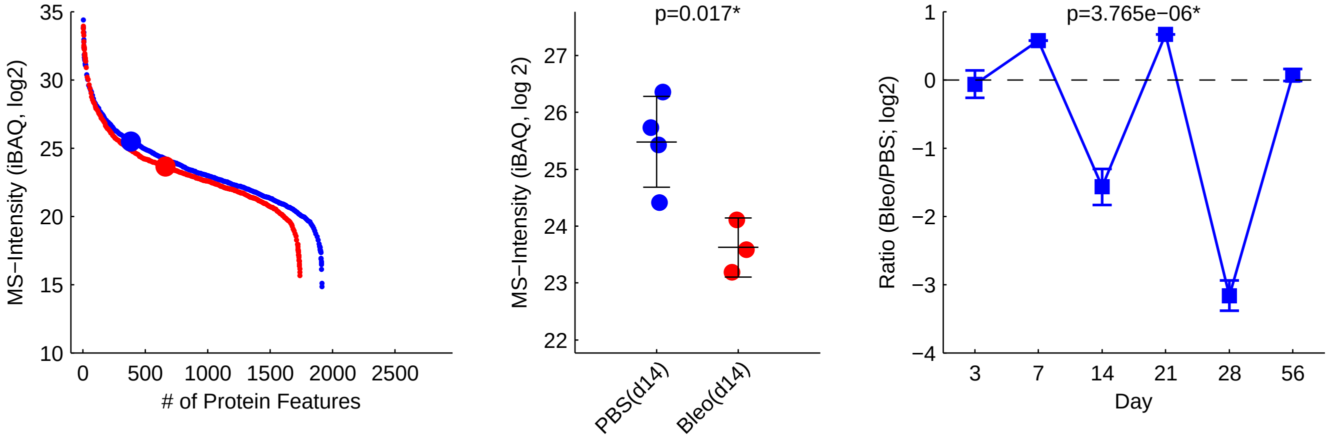

P31324 − Prkar2b (id: 1113)

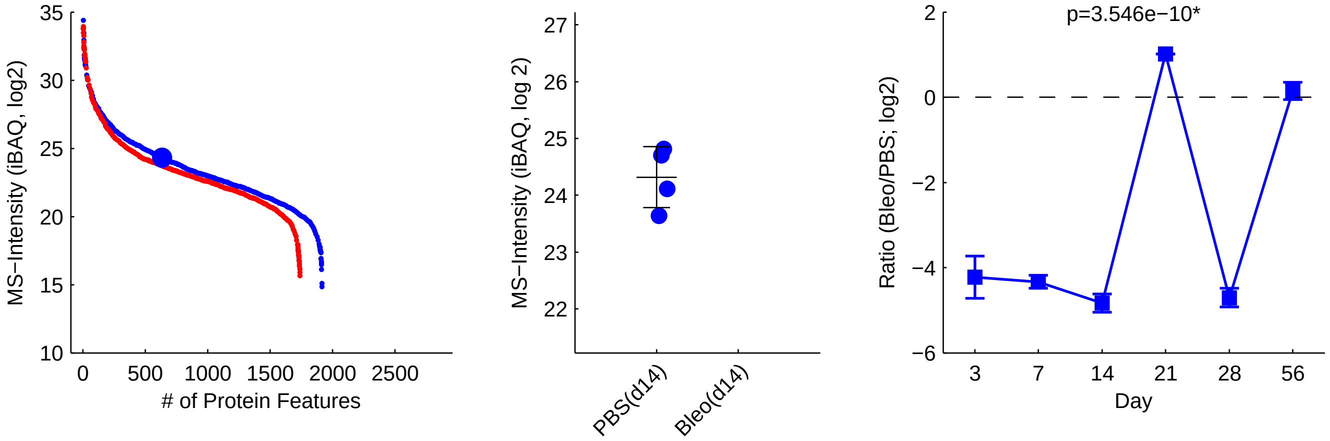

P31428 − Dpep1 (id: 1114)

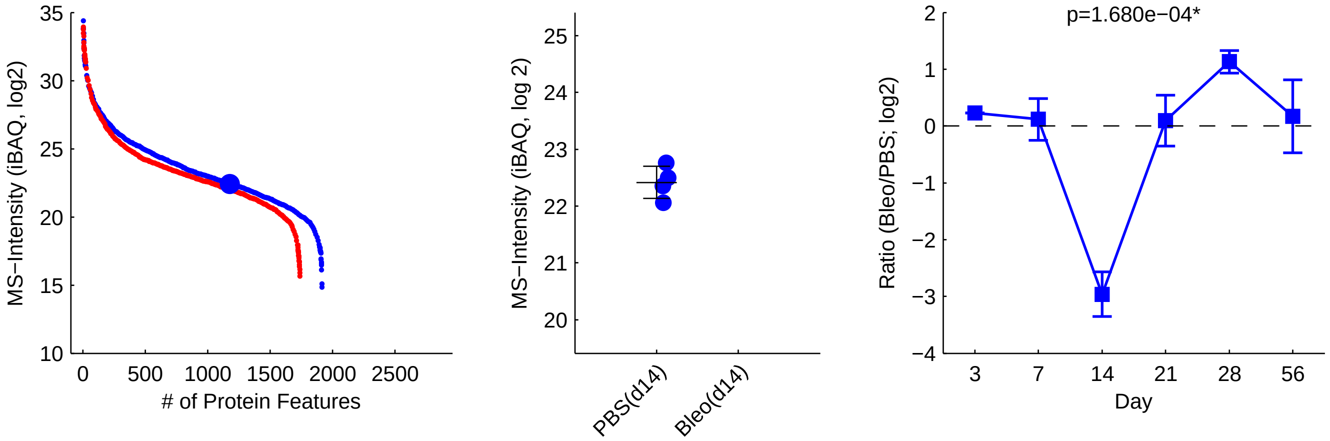

P32507−2 − Pvrl2 (id: 1122)

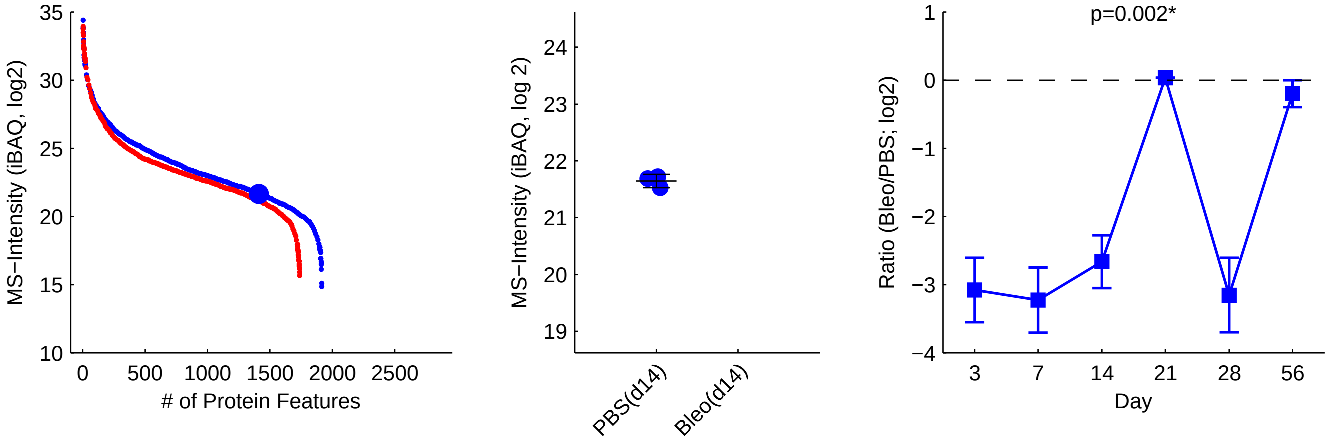

P32848 − Pvalb (id: 1123)

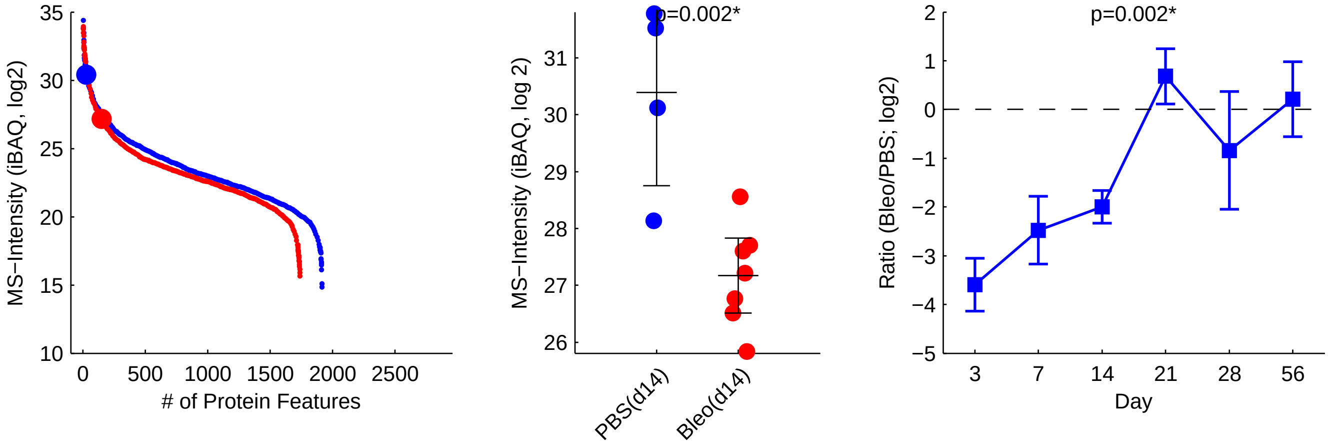

P32883−2 − Kras (id: 1124)

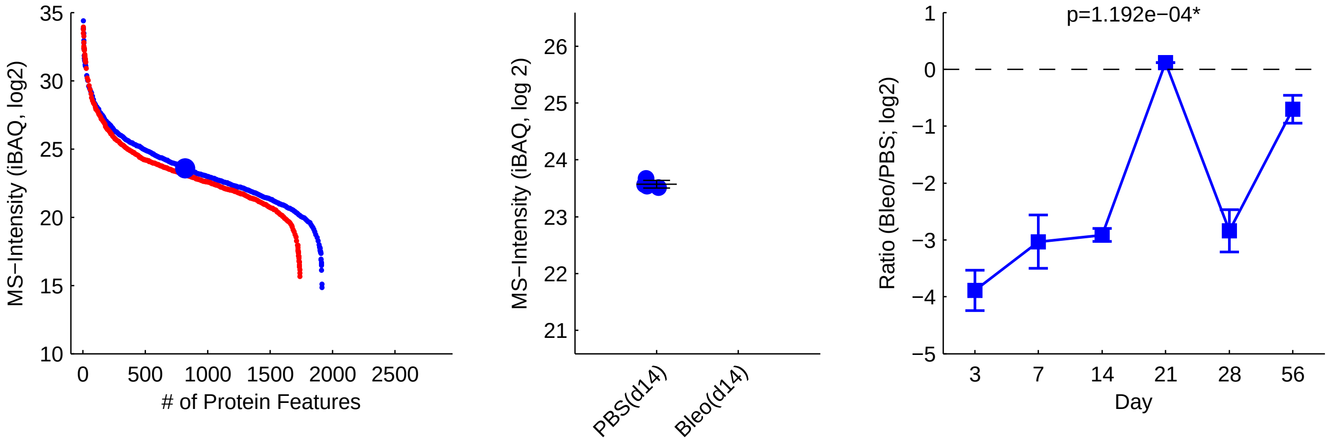

P33267 − Cyp2f2 (id: 1126)

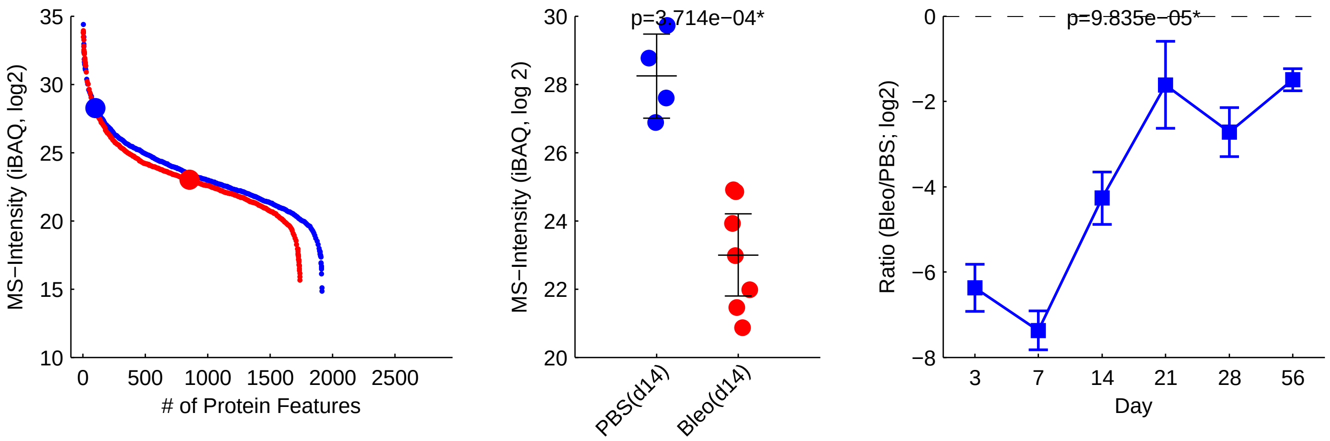

P33587 − Proc (id: 1128)

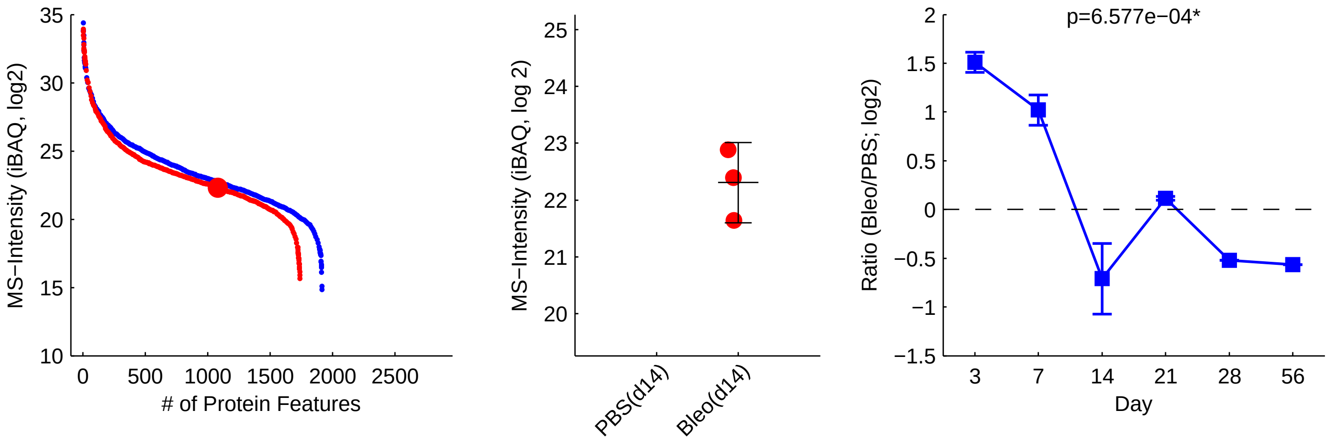

P34022 − Ranbp1 (id: 1129)

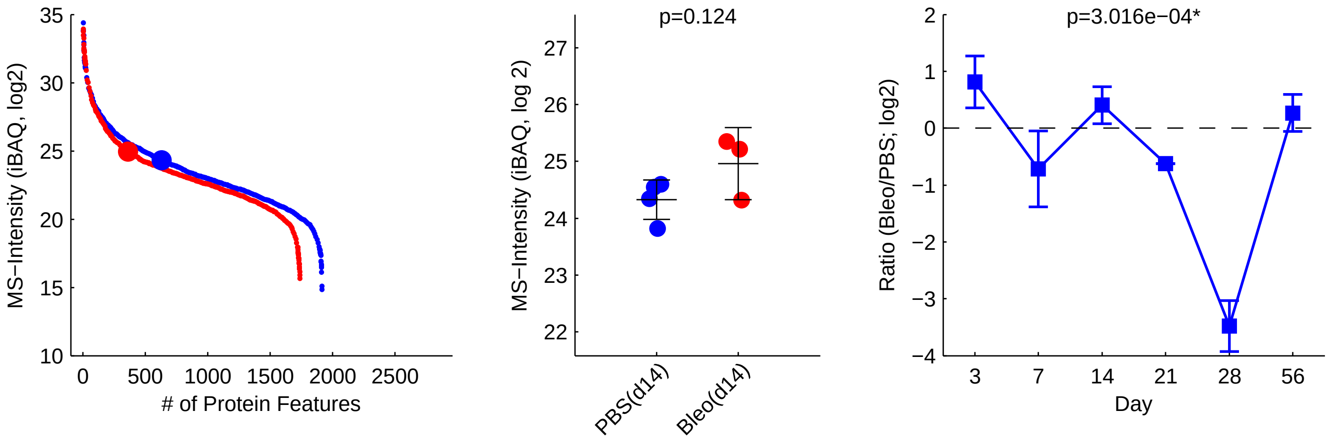

P34884 − Mif (id: 1130)

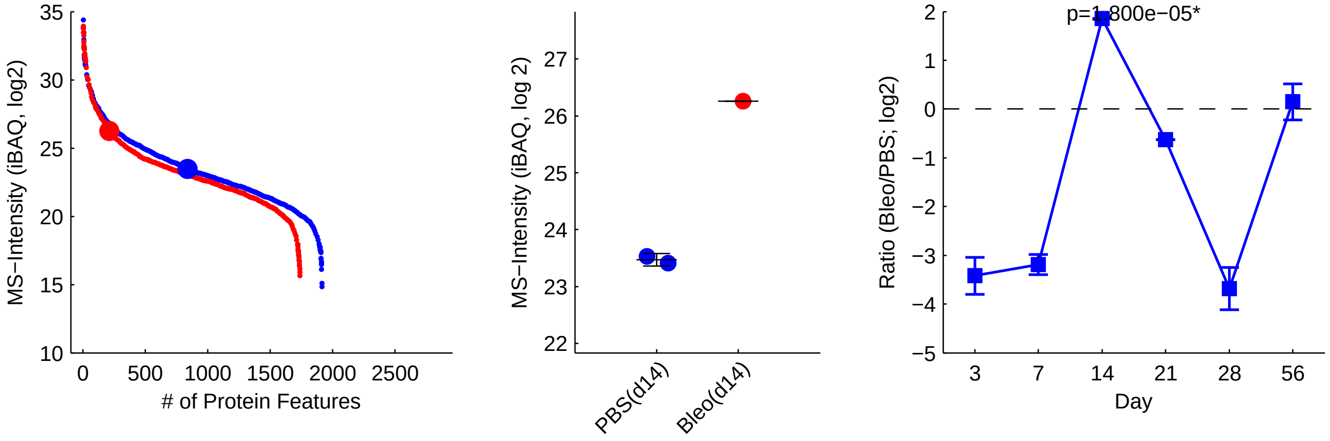

Q9CQI1 − Sftpa1 (id: 1134)

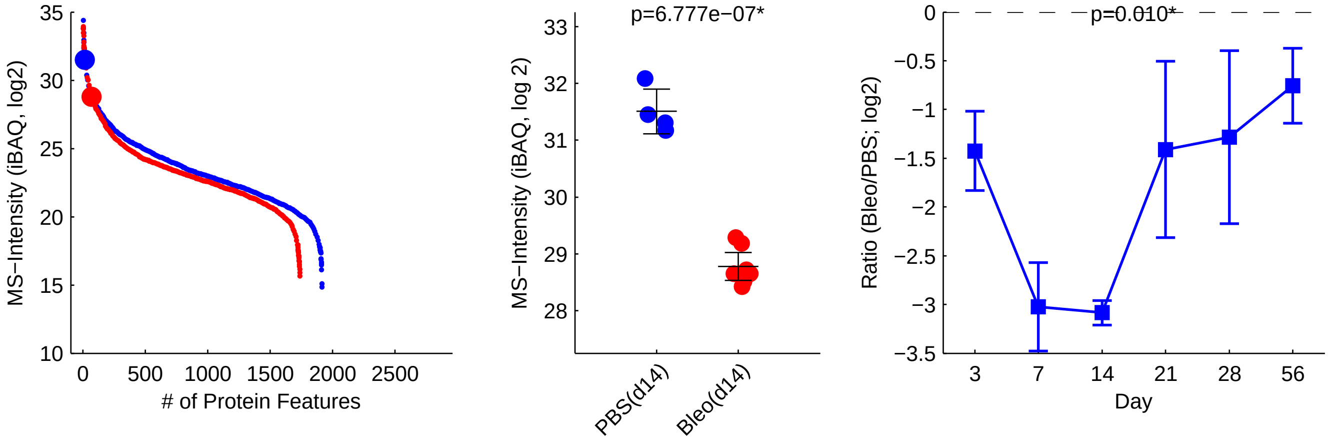

P35278 − Rab5c (id: 1136)

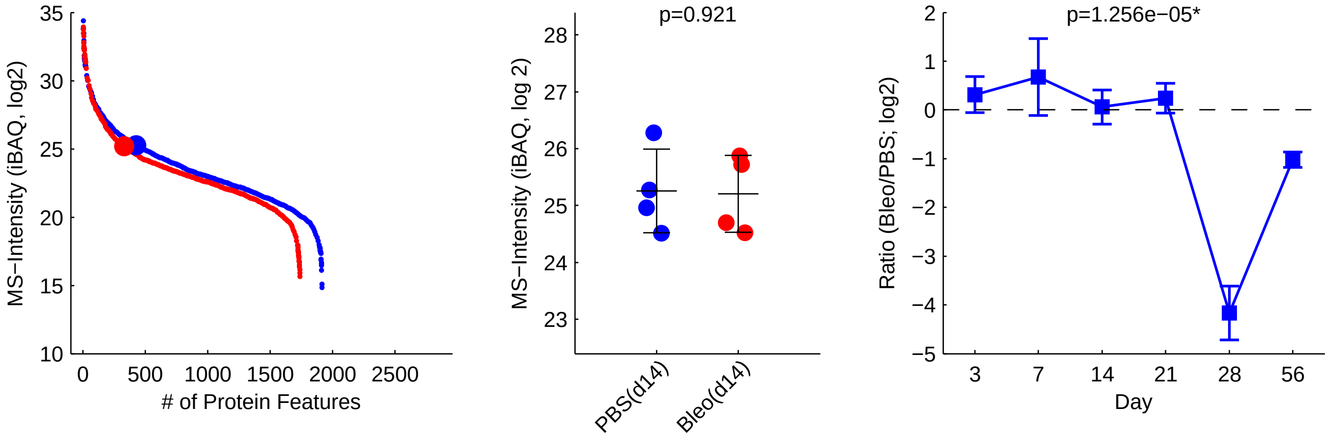

P35279−2 − Rab6a (id: 1137)

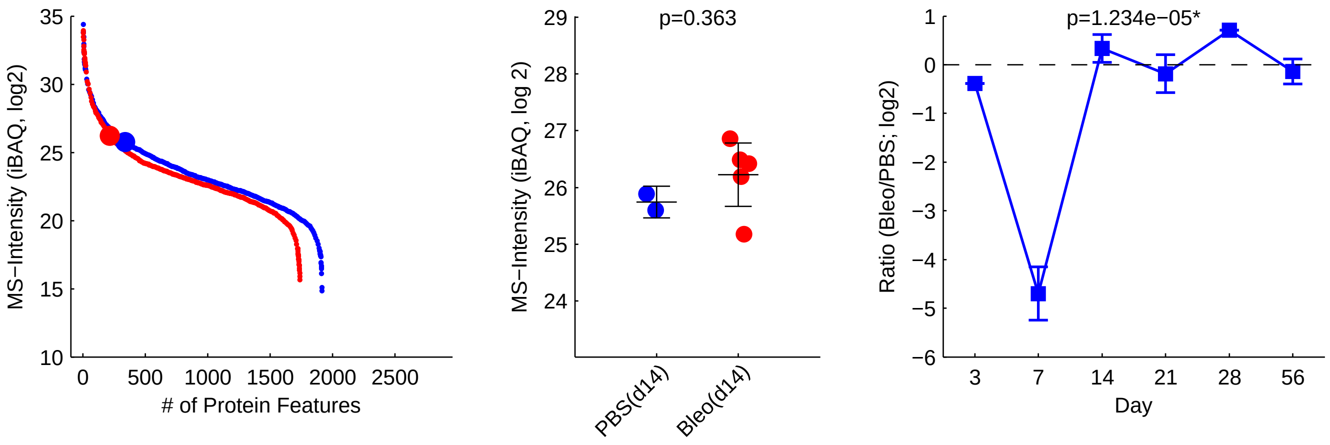

P35564 − Canx (id: 1142)

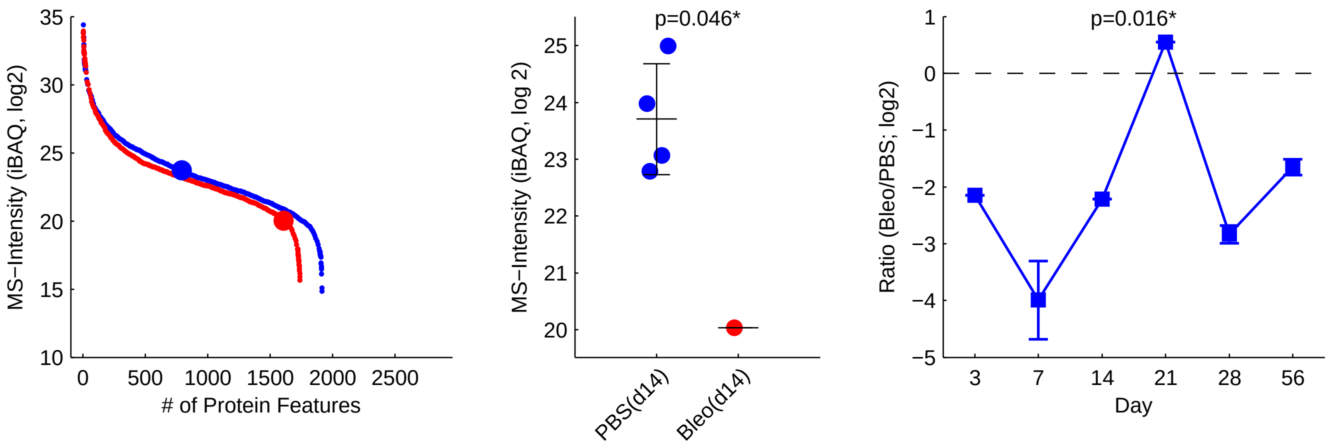

P37040 − Por (id: 1154)

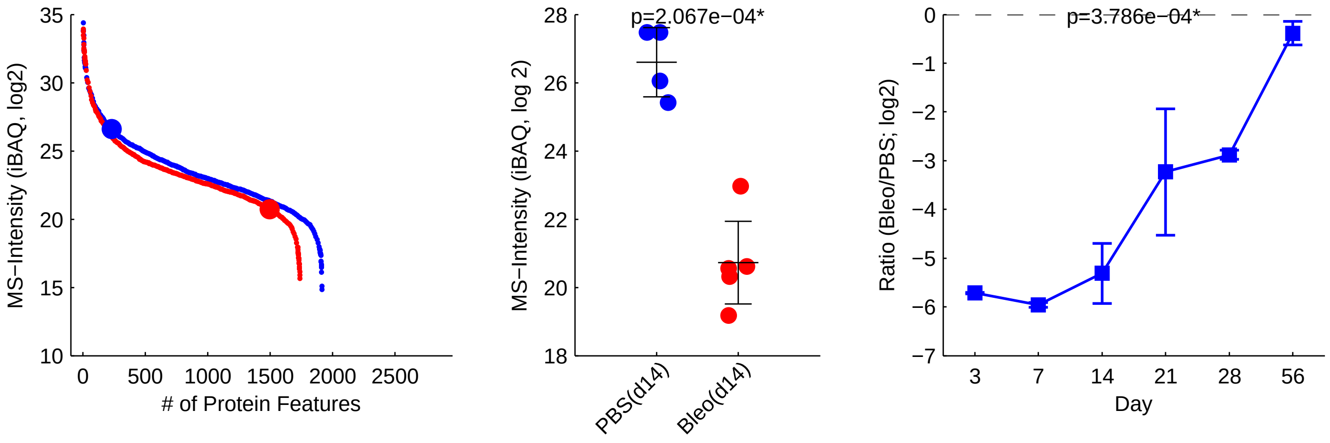

P37804 − Tagln (id: 1155)

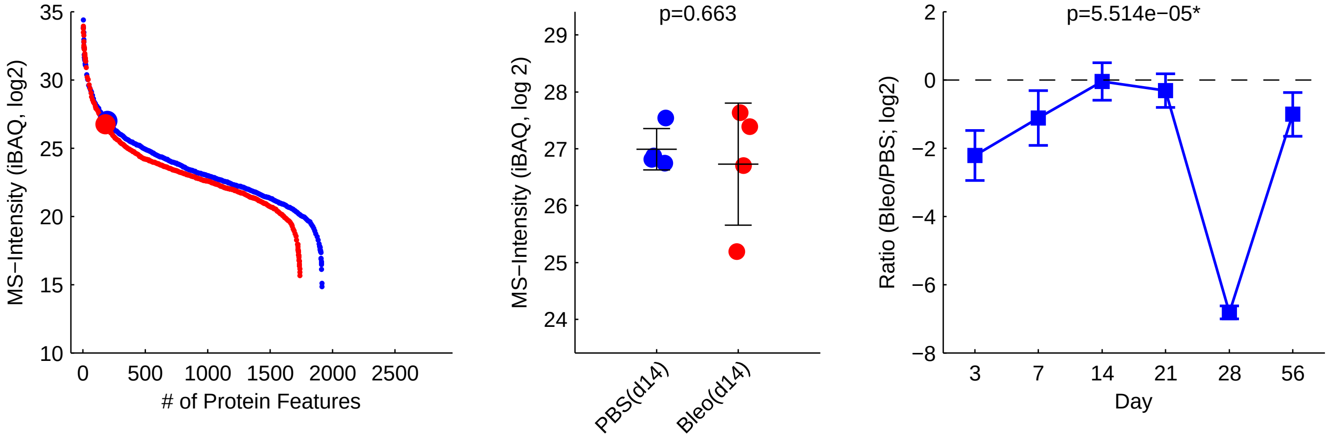

P38647 − Hspa9 (id: 1157)

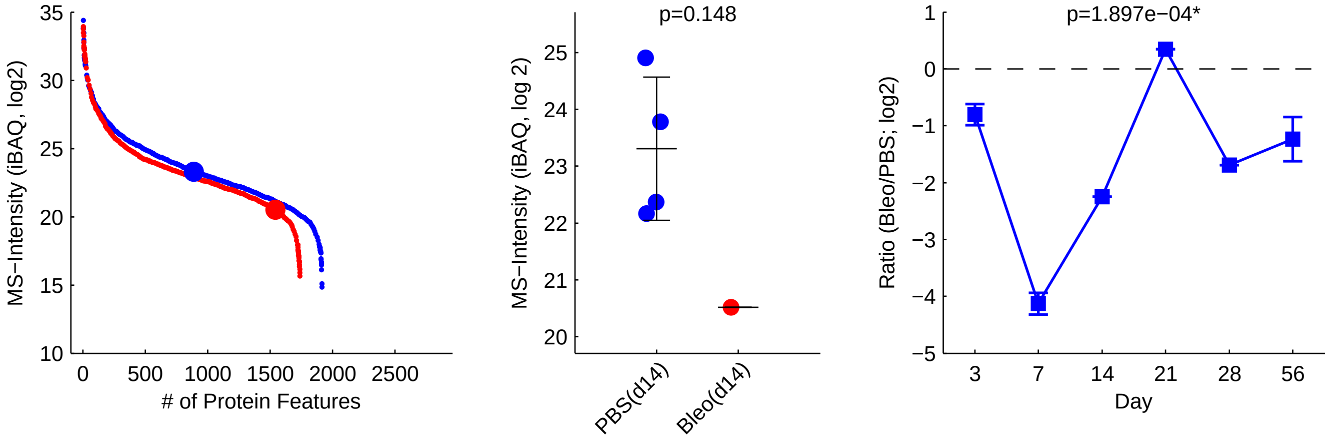

P39039 − Mbl1 (id: 1158)

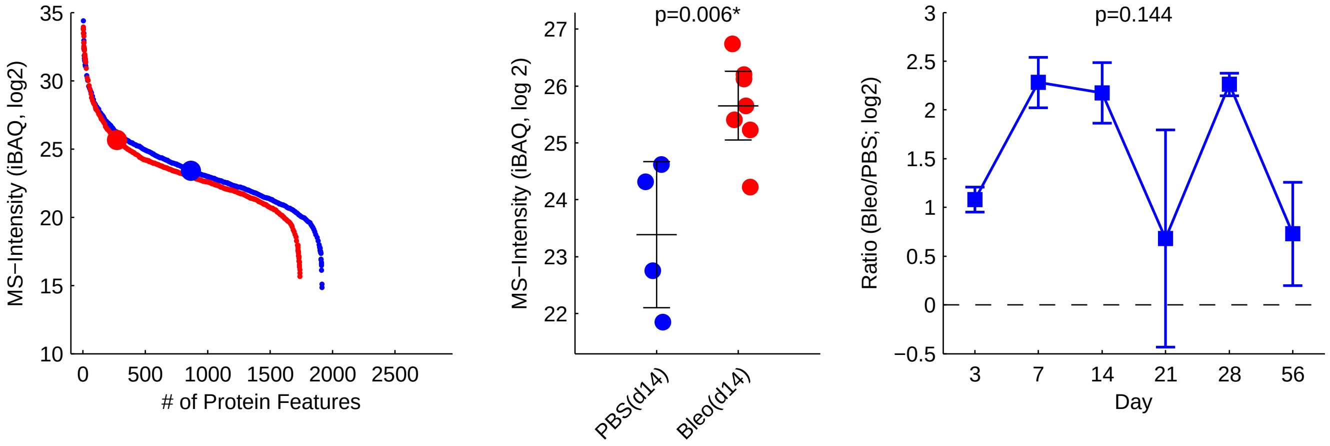

P40336 − Vps26a (id: 1164)

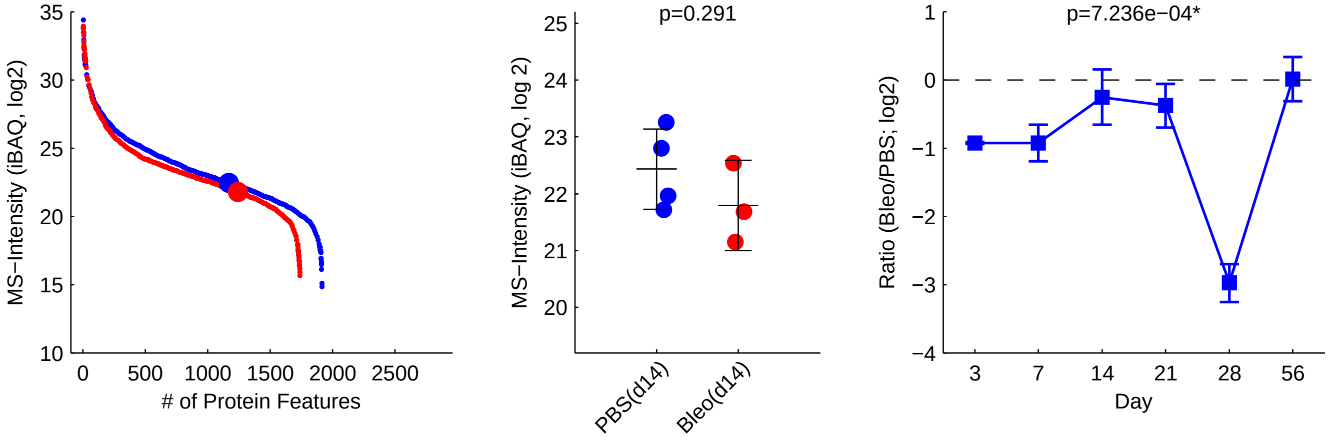

P41317 − Mbl2 (id: 1168)

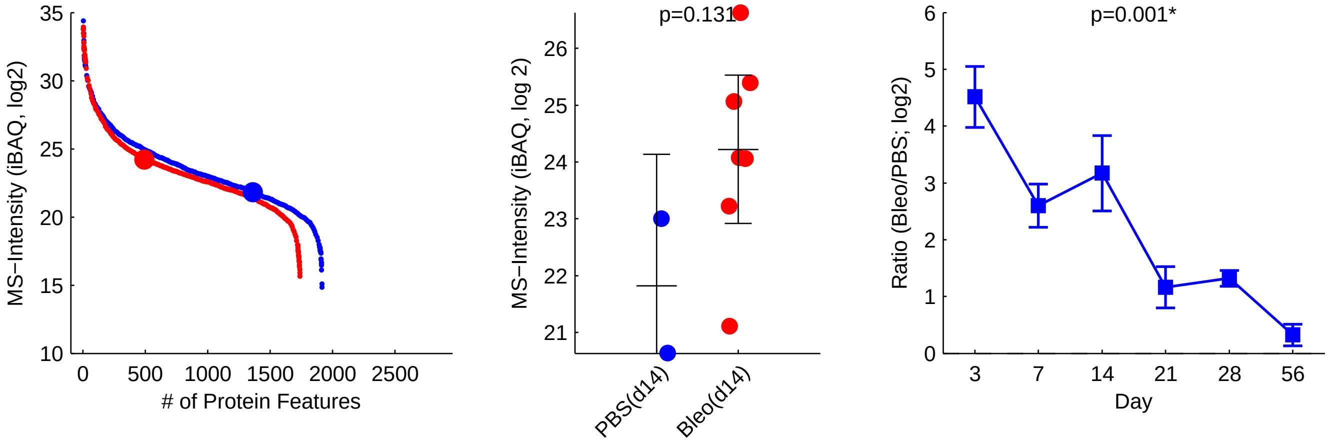

P42227−2 − Stat3 (id: 1170)

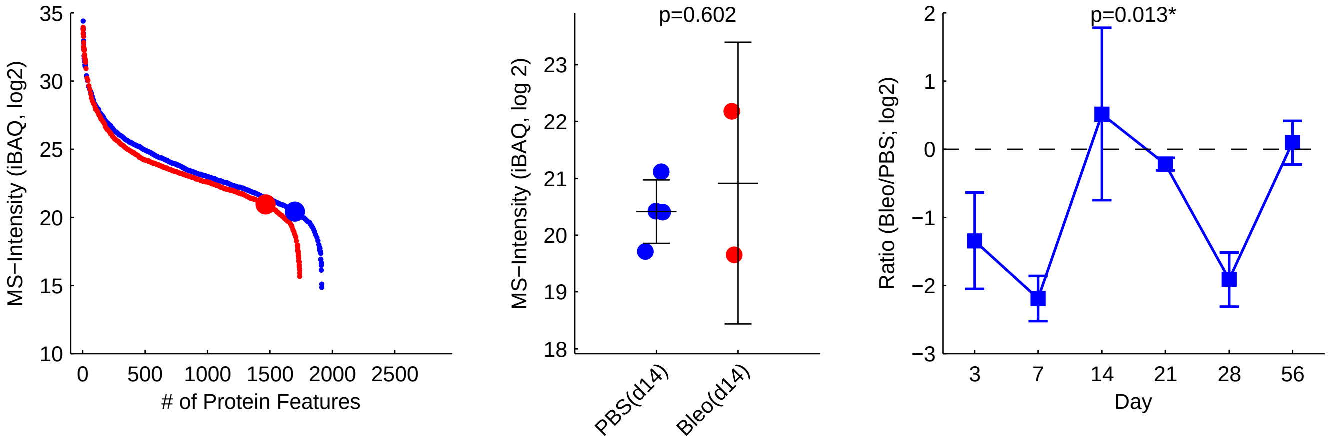

P45952 − Acadm (id: 1186)

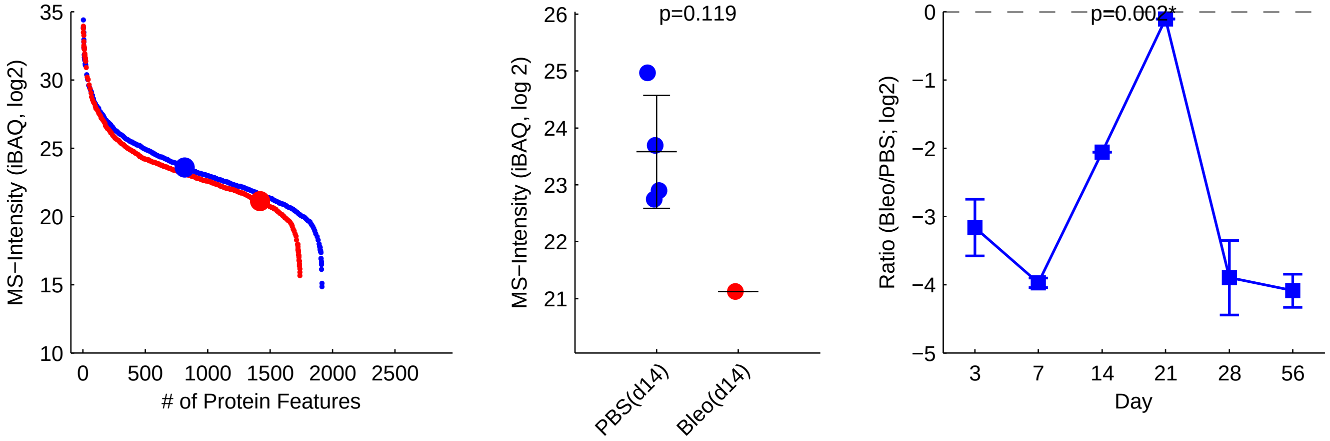

P46412 − Gpx3 (id: 1188)

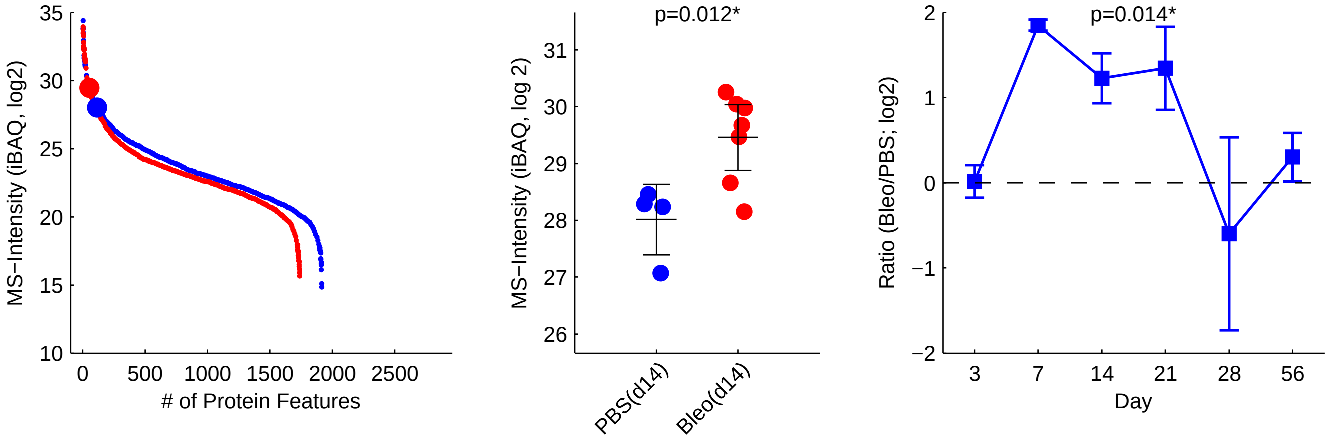

P46467 − Vps4b (id: 1190)

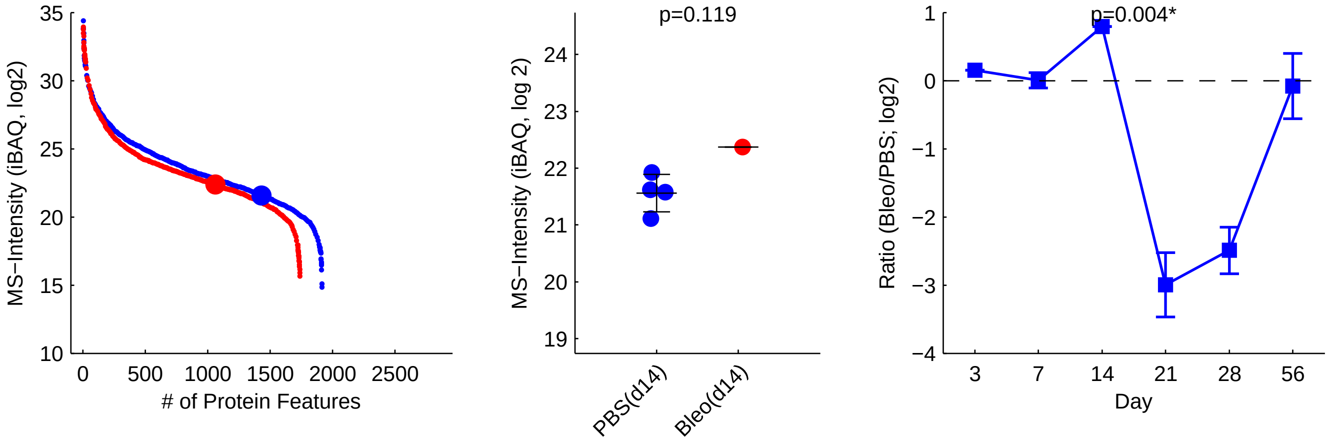

P46471 − Psmc2 (id: 1191)

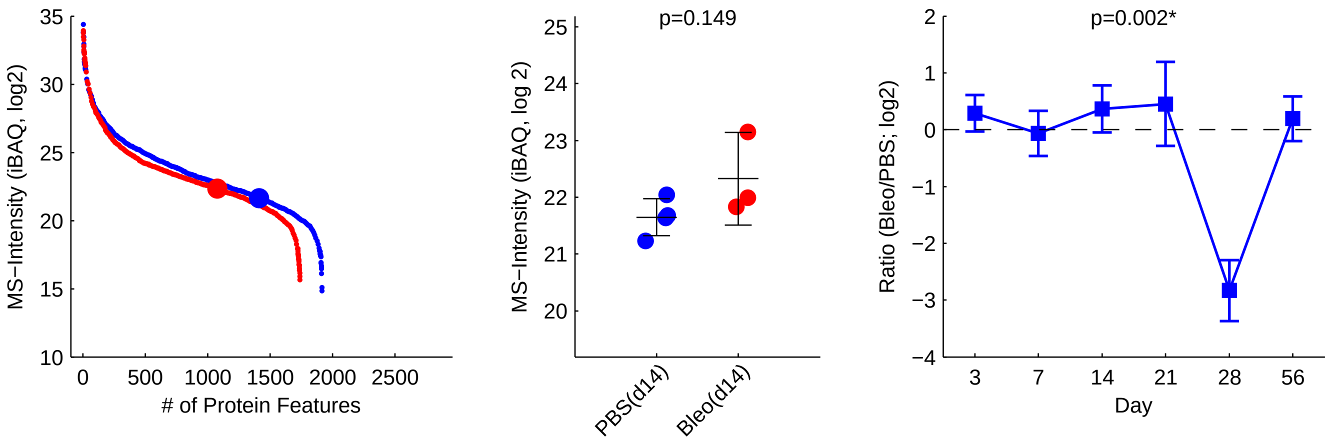

P46638 − Rab11b (id: 1192)

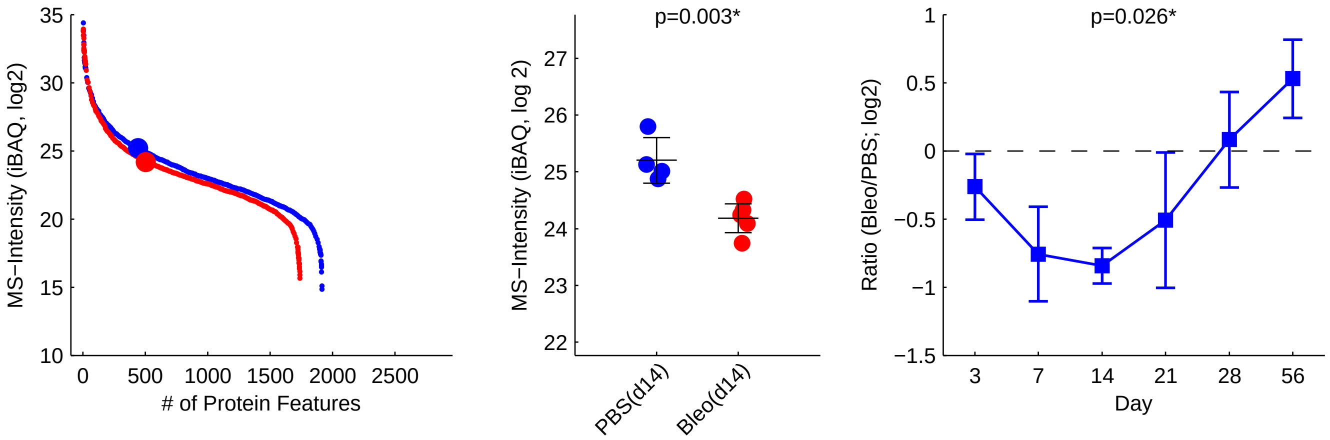

P47738 − Aldh2 (id: 1197)

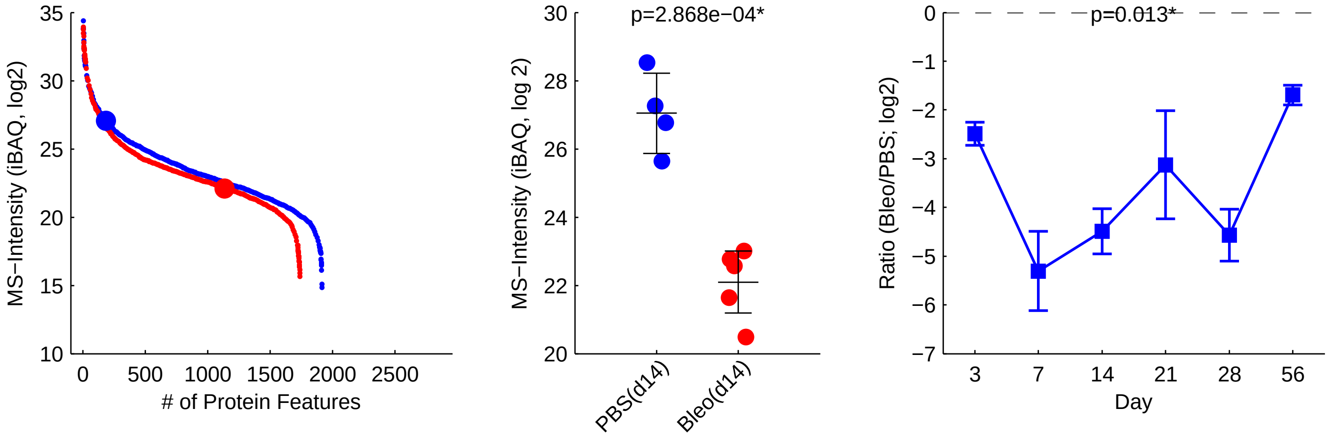

P47739 − Aldh3a1 (id: 1198)

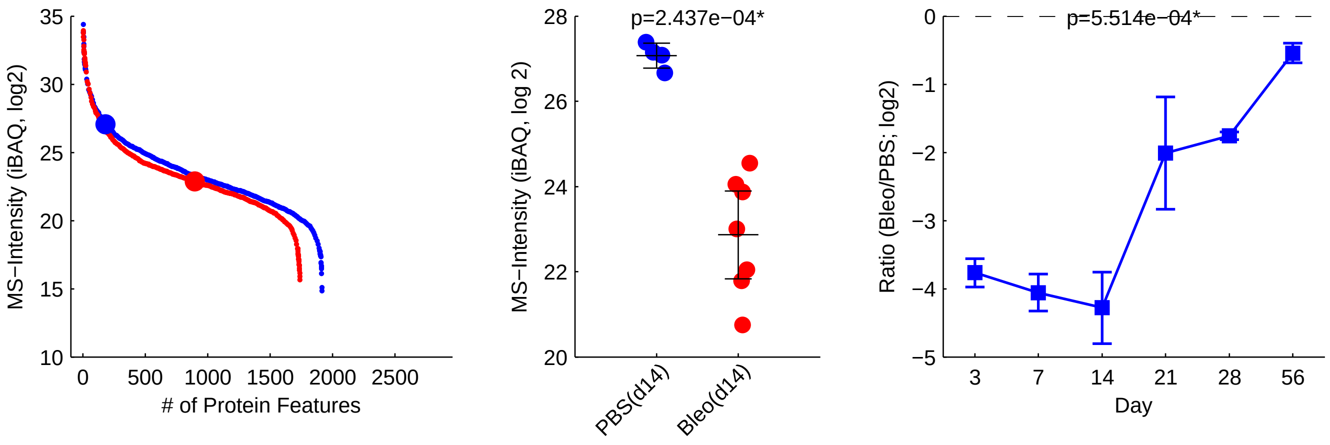

P47791−2 − Gsr (id: 1201)

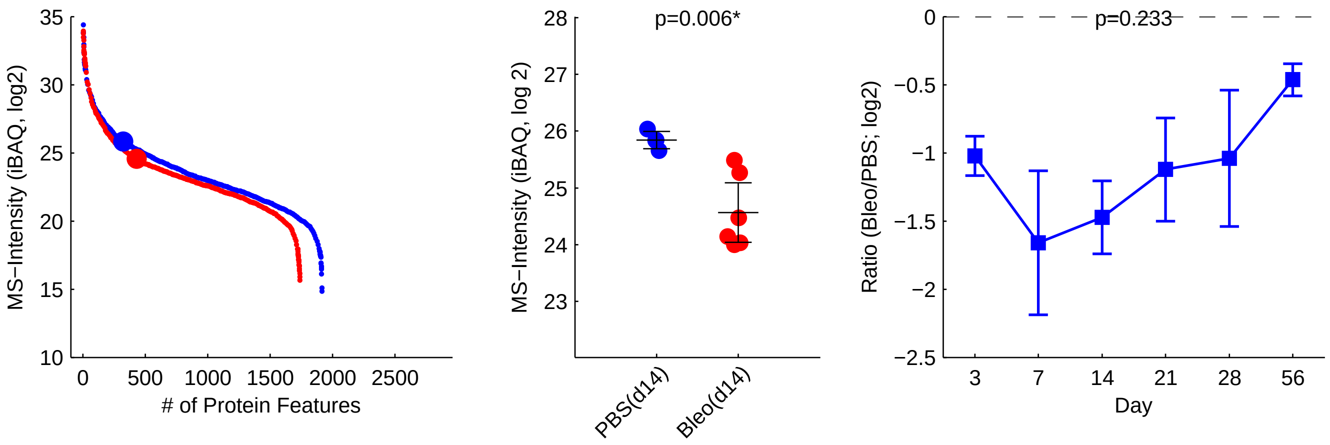

P47856−2 − Gfpt1 (id: 1203)

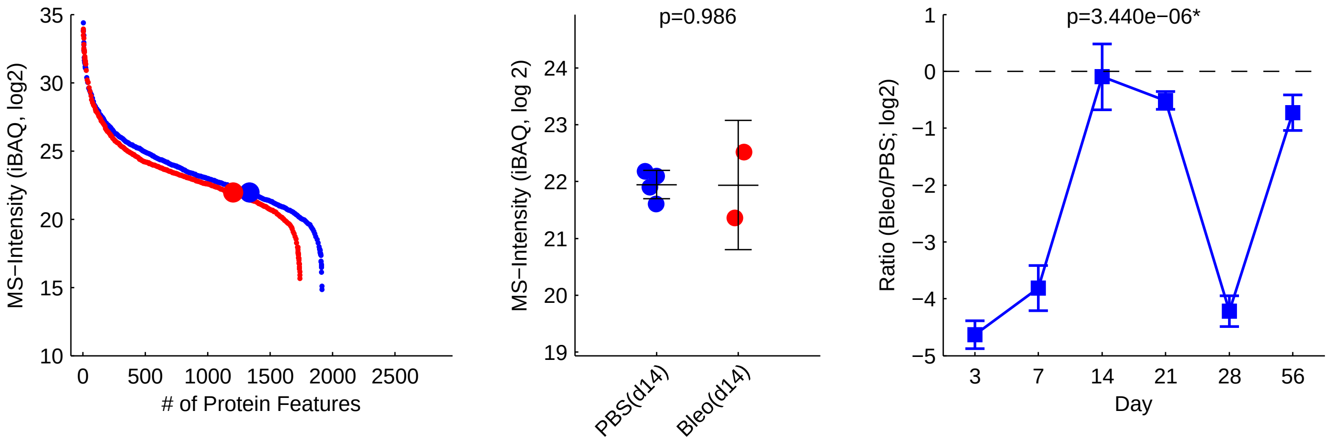

P47877 − Igfbp2 (id: 1205)

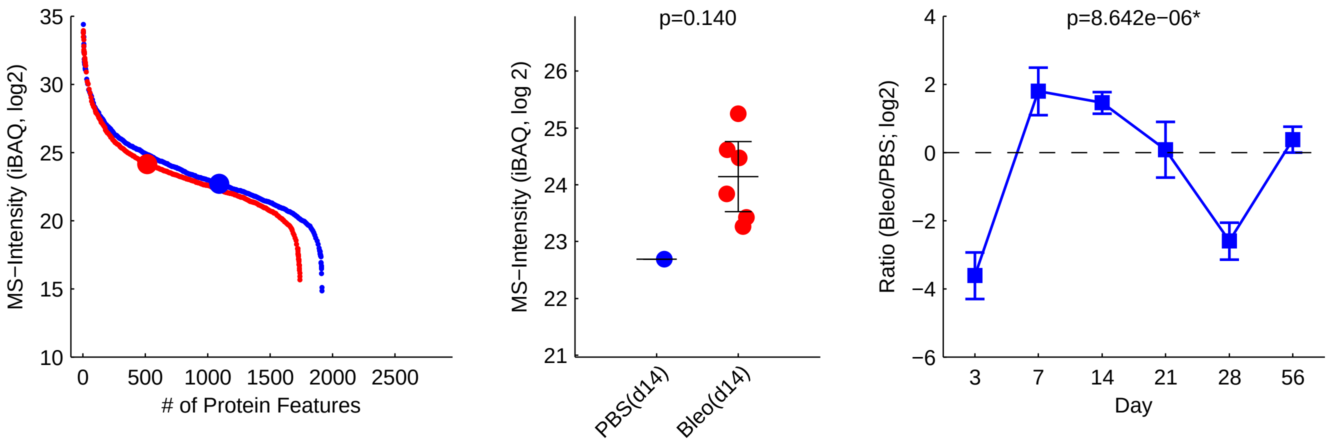

P47955 − Rplp1 (id: 1209)

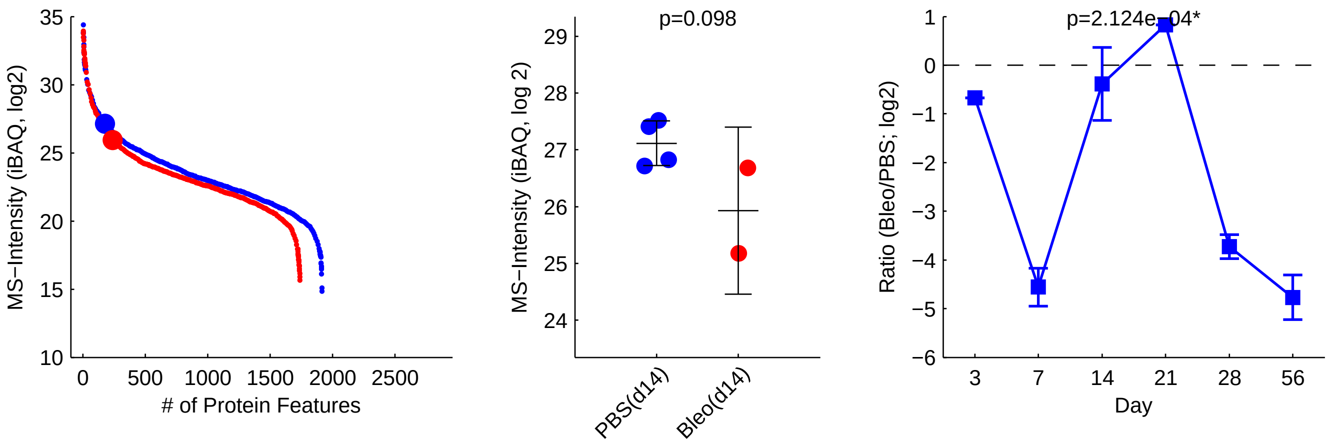

P48024 − Eif1 (id: 1212)

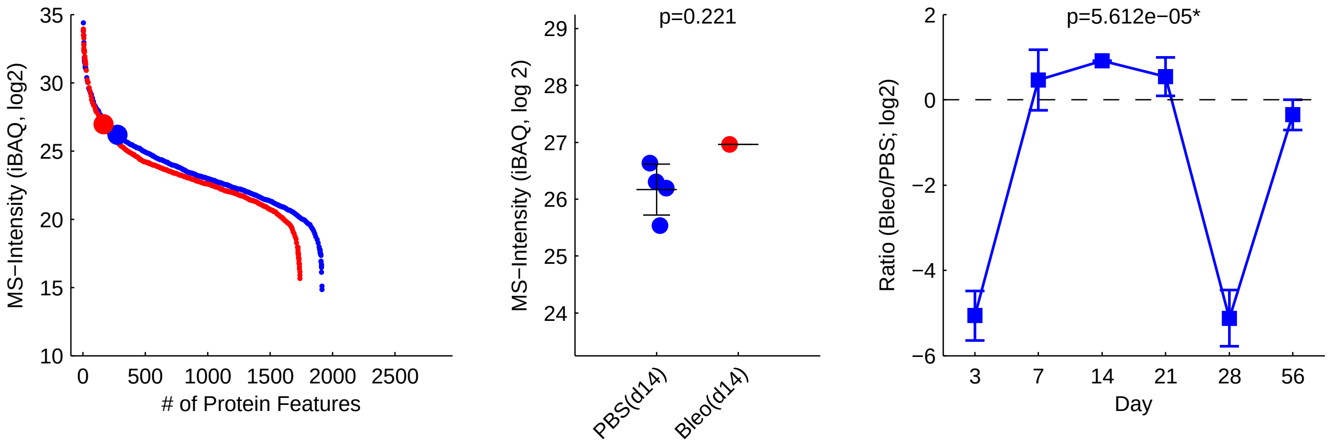

P48428 − Tbca (id: 1215)

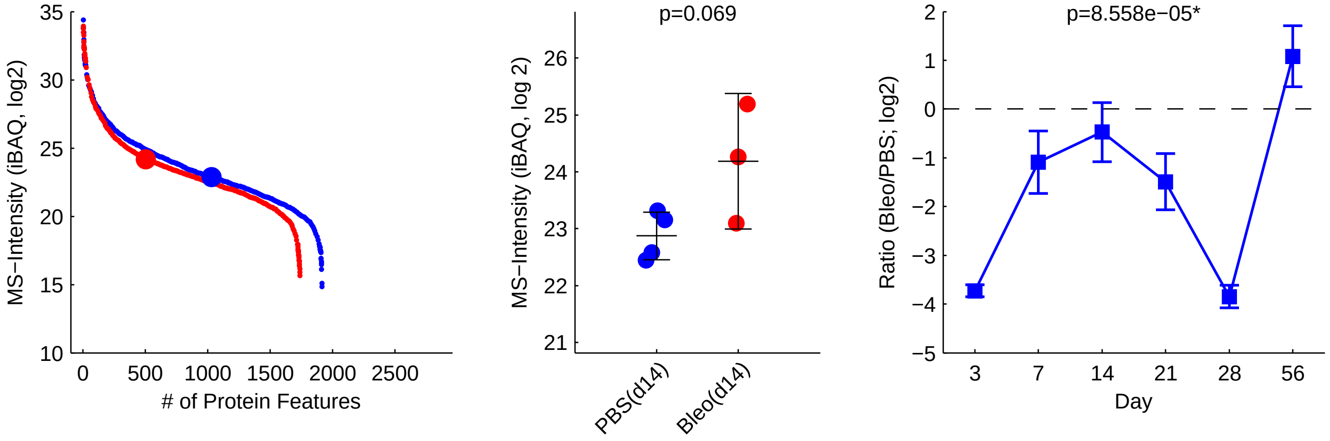

P48678 − Lmna (id: 1216)

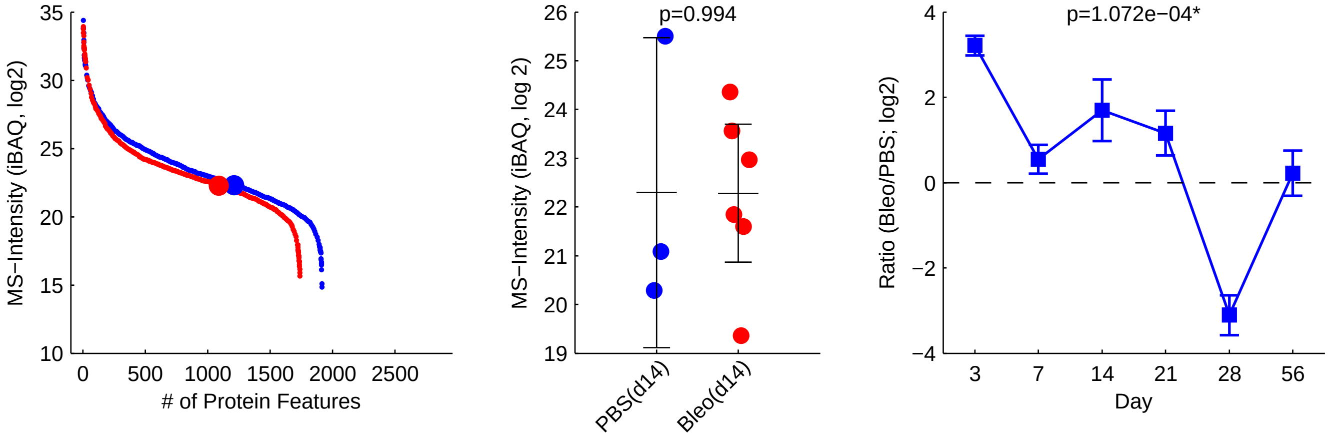

P48758 − Cbr1 (id: 1218)

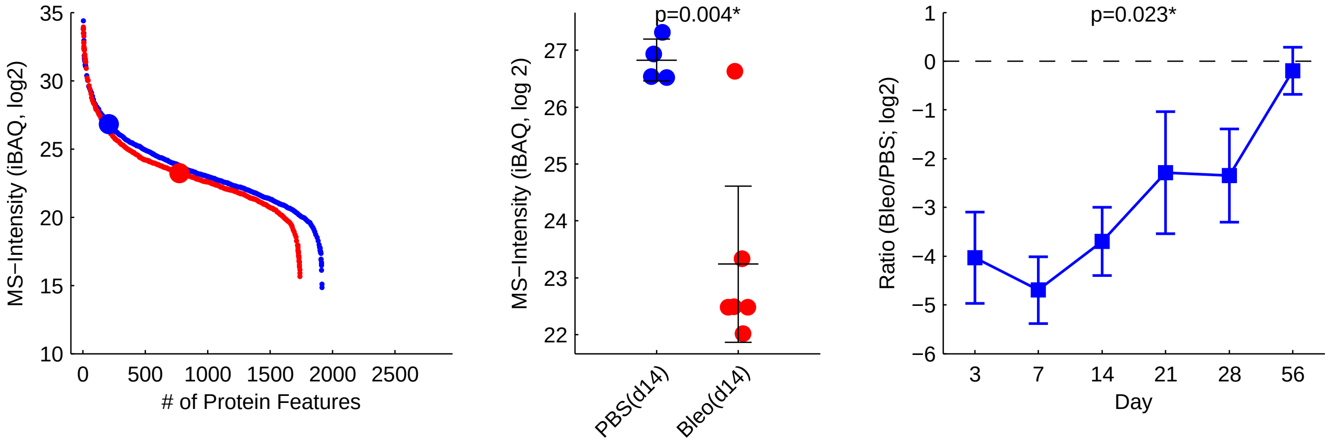

P49722 − Psma2 (id: 1225)

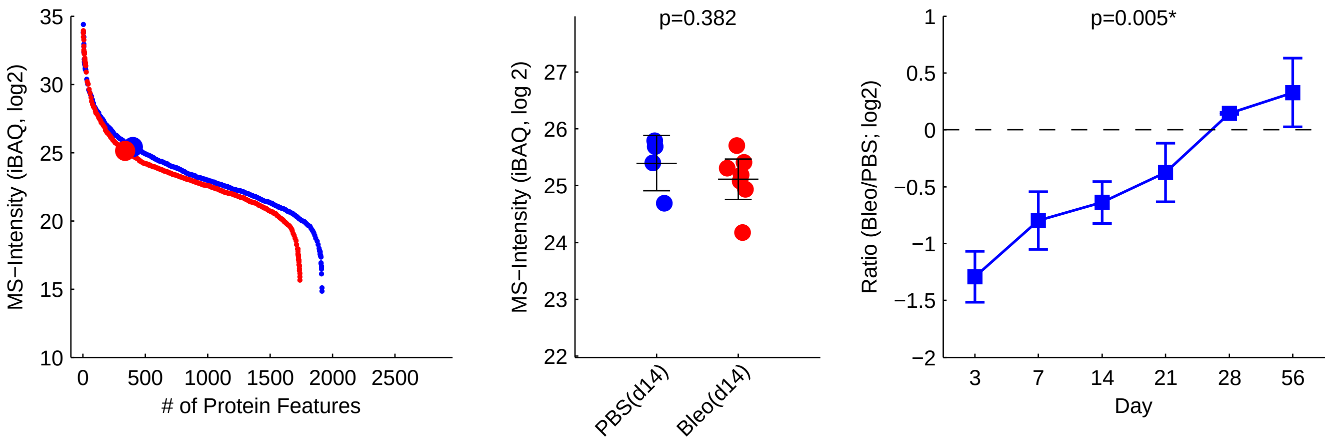

P49813 − Tmod1 (id: 1226)

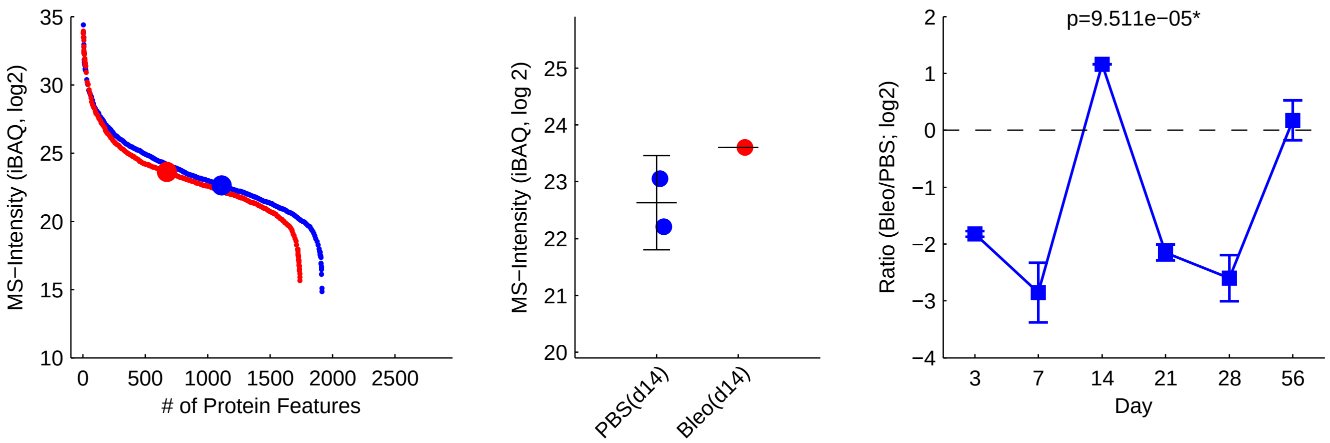

P49935 − Ctsh (id: 1227)

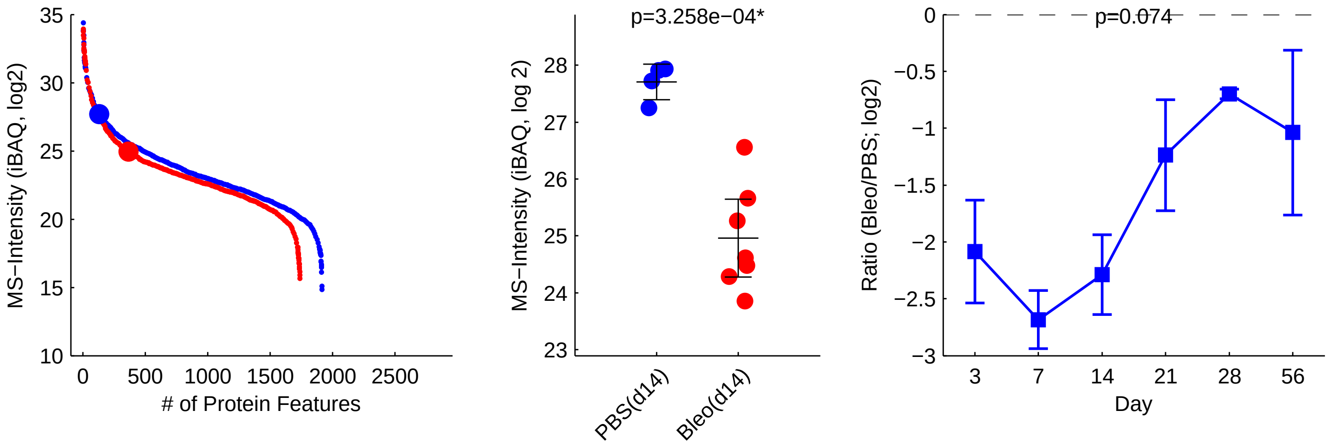

P50404 − Sftpd (id: 1233)

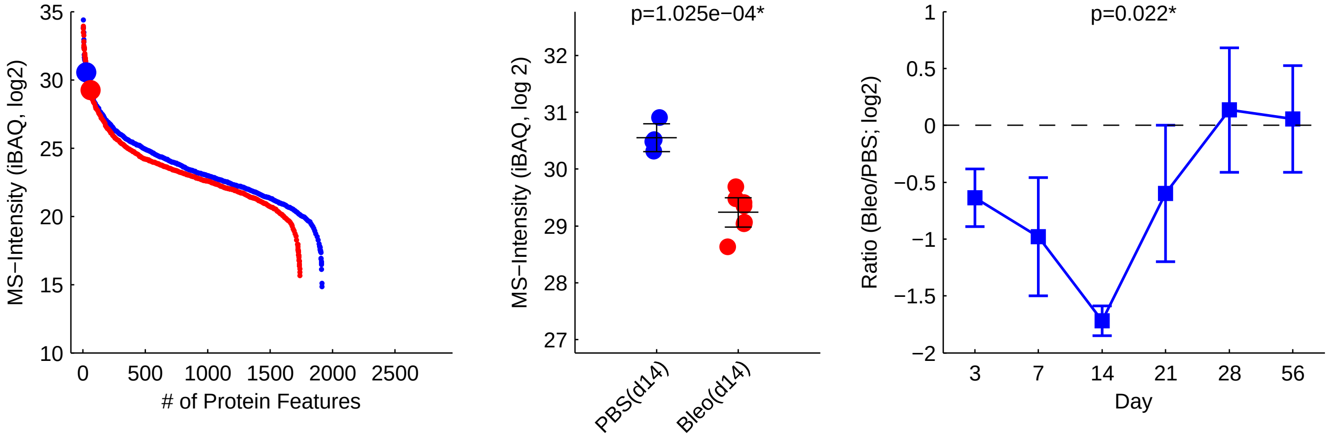

P50518 − Atp6v1e1 (id: 1238)

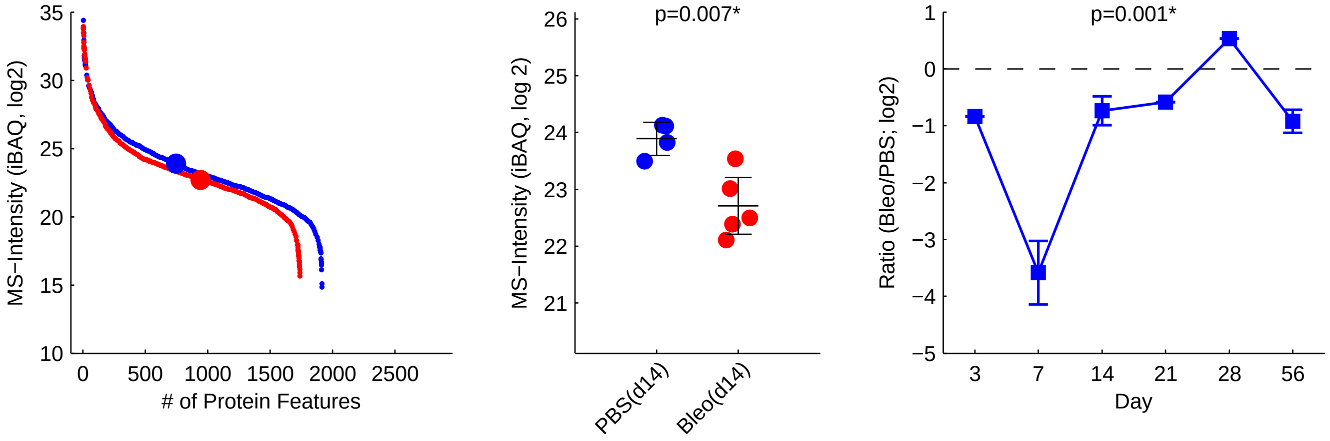

P50580 − Pa2g4 (id: 1240)

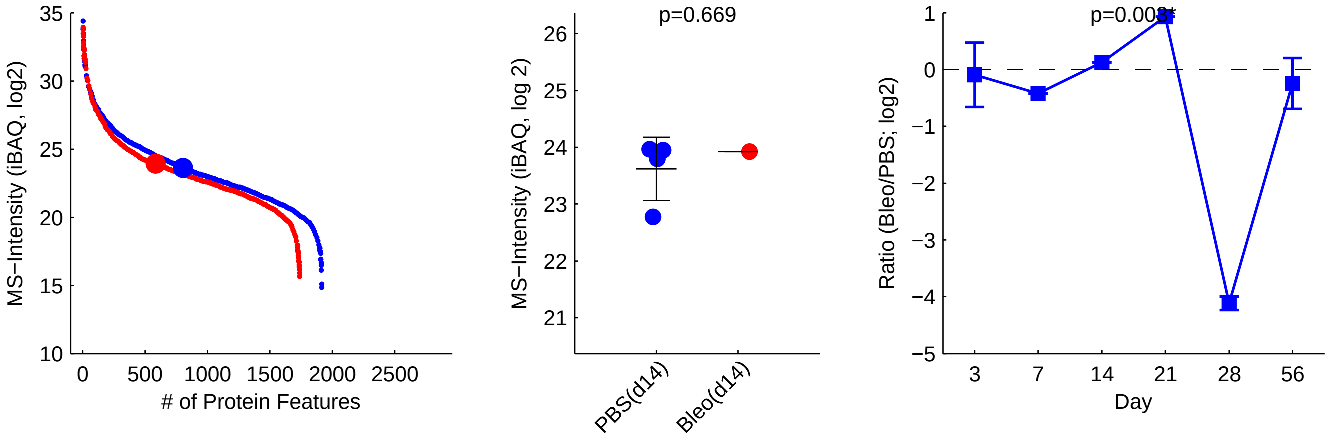

Q8CE80 − Cast (id: 1241)

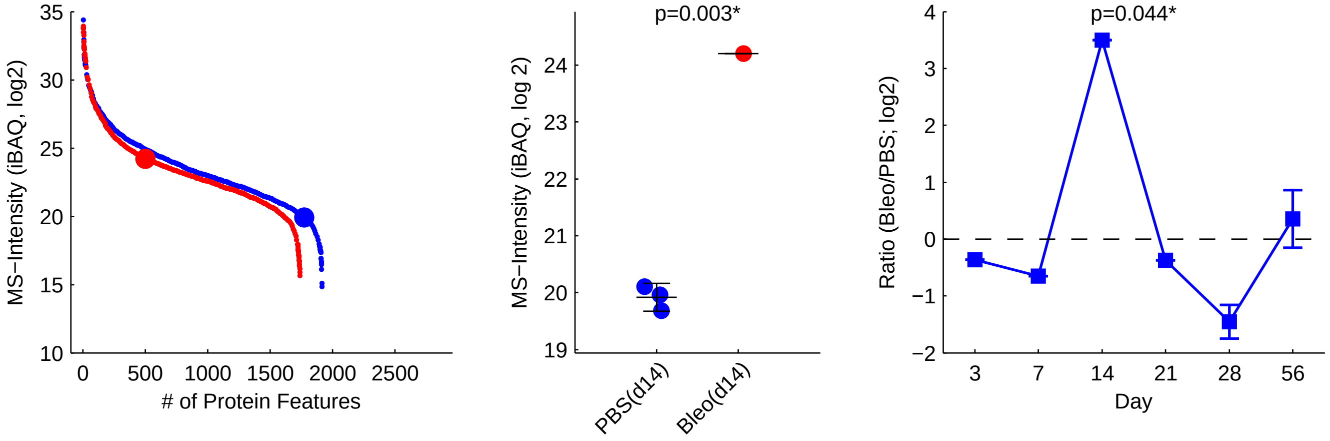

P51174 − Acadl (id: 1243)

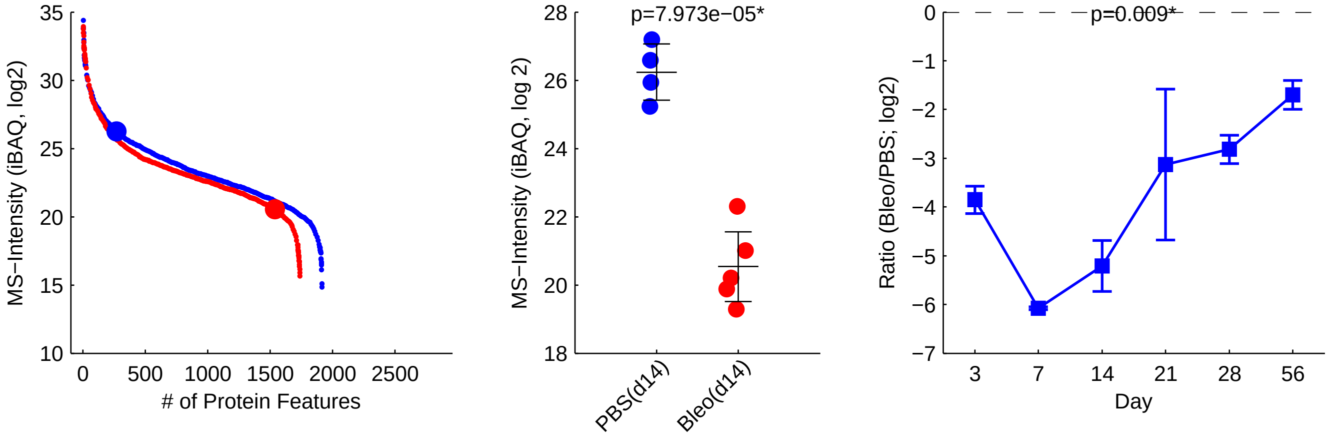

P51410 − Rpl9 (id: 1244)

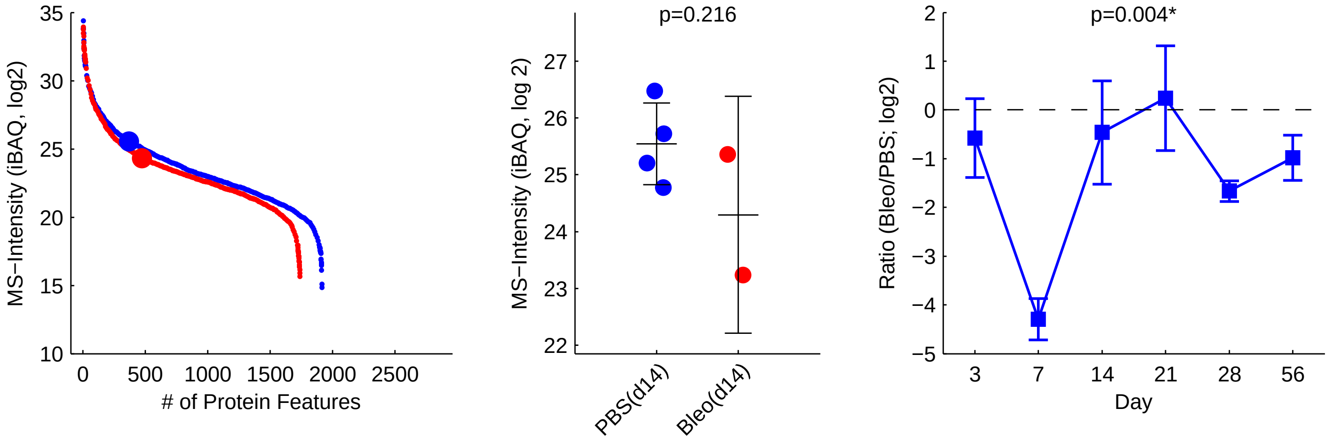

P51859 − Hdgf (id: 1251)

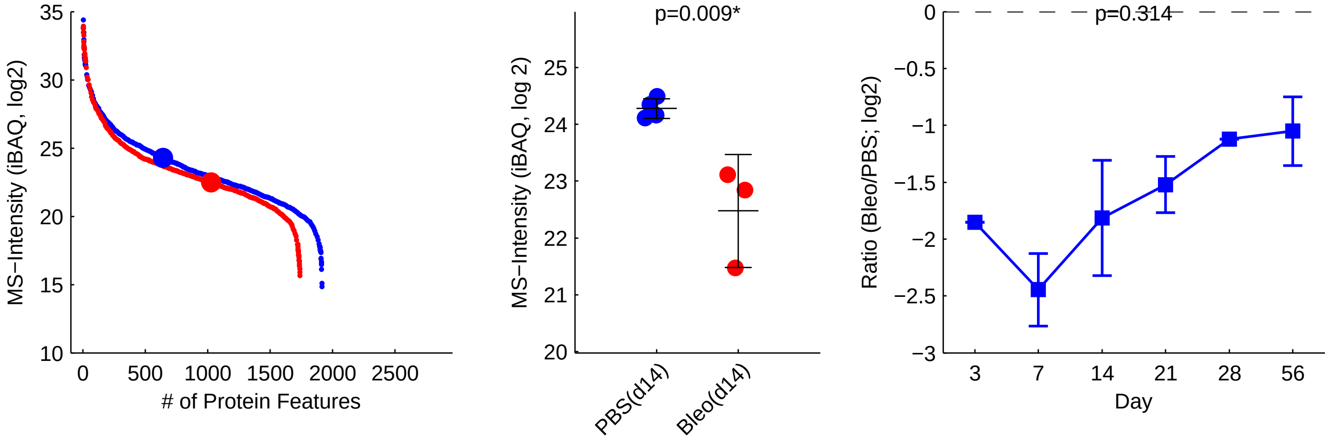

P51881 − Slc25a5 (id: 1253)

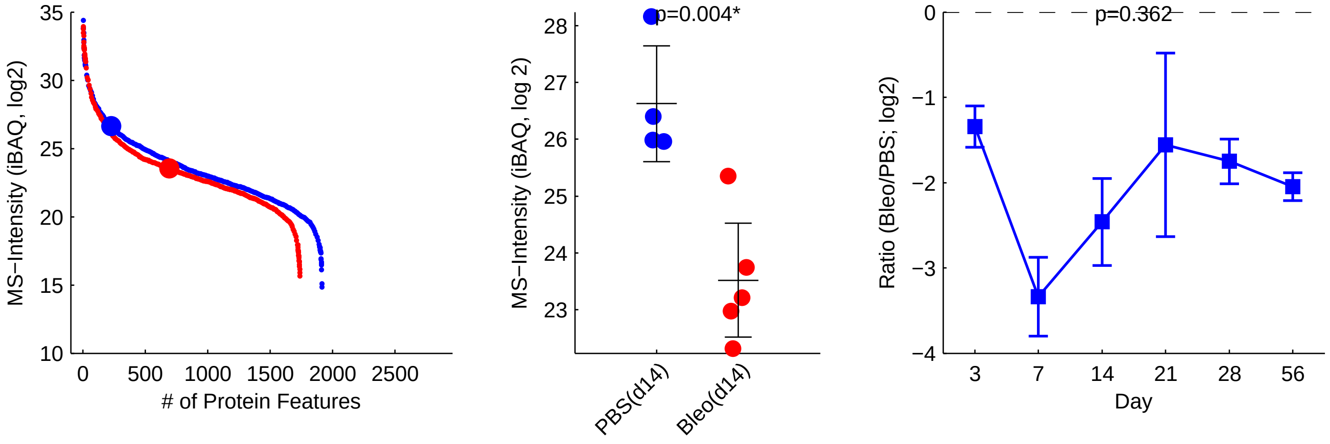

P51910 − Apod (id: 1255)

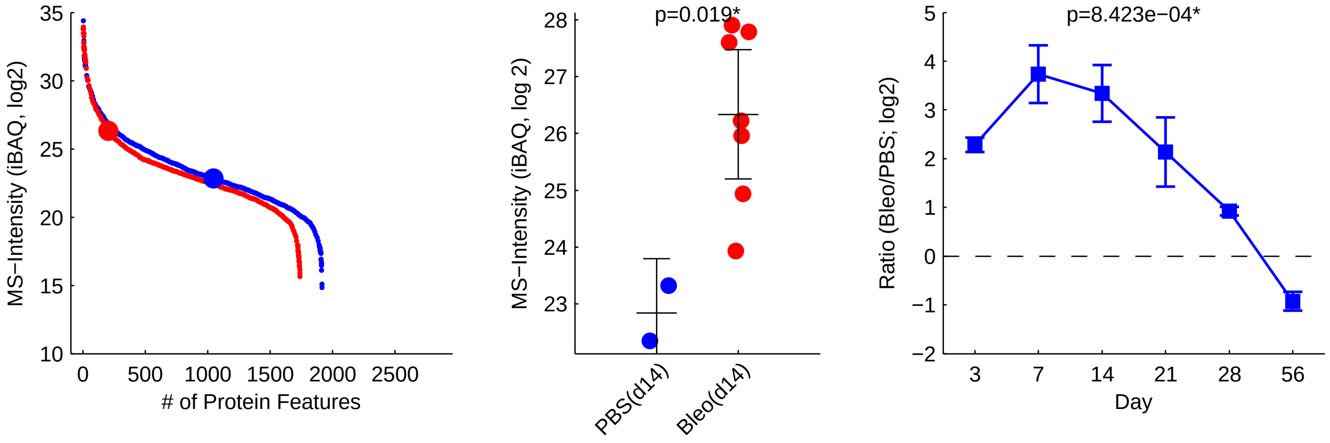

P52196 − Tst (id: 1256)

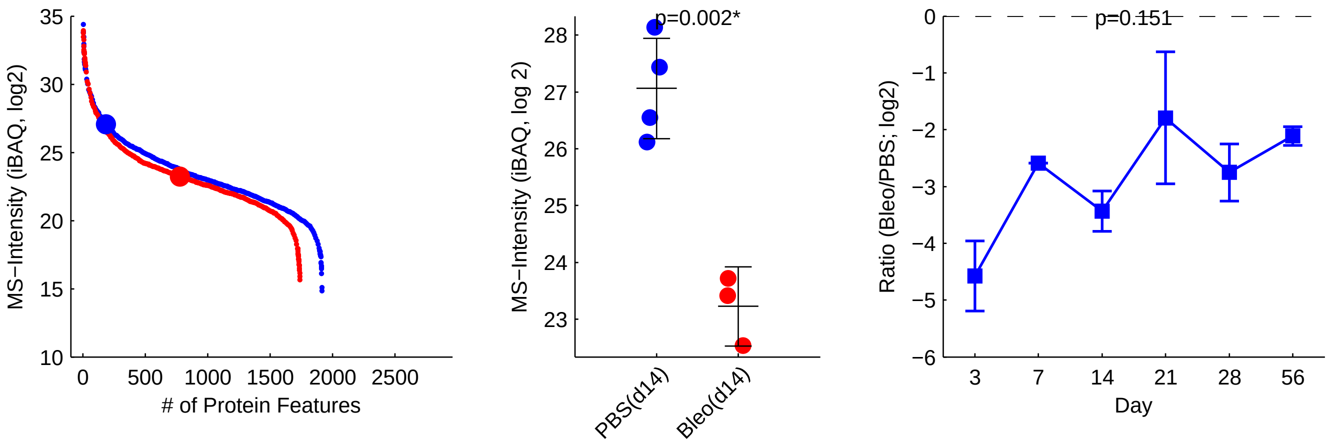

P52430 − Pon1 (id: 1257)

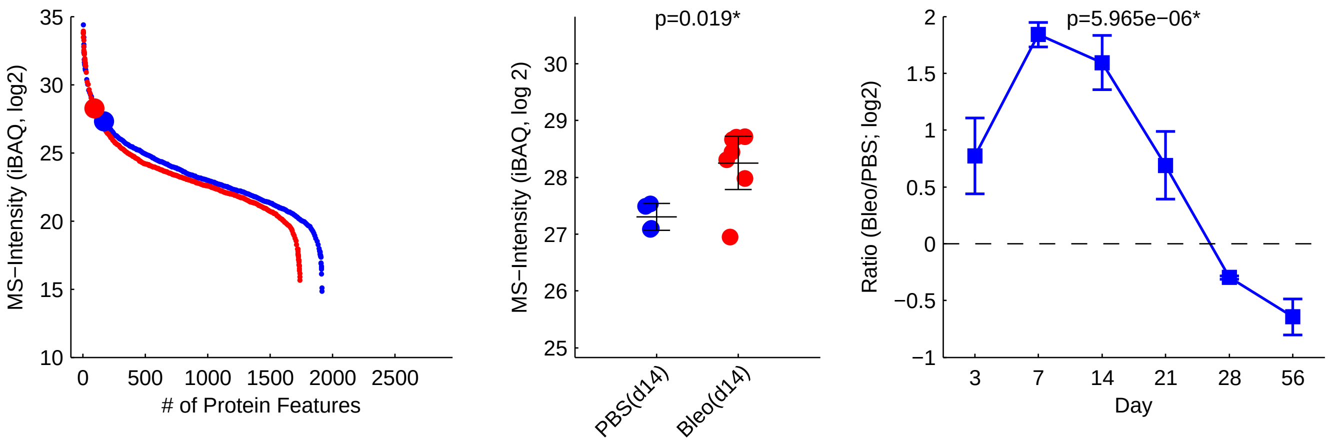

P52760 − Hrsp12 (id: 1260)

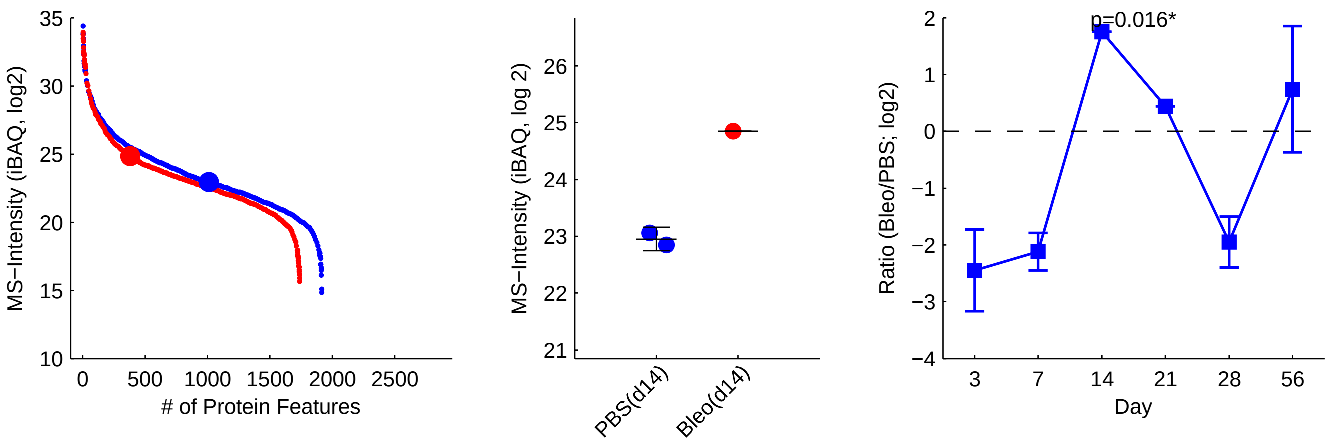

P53810 − Pitpna (id: 1263)

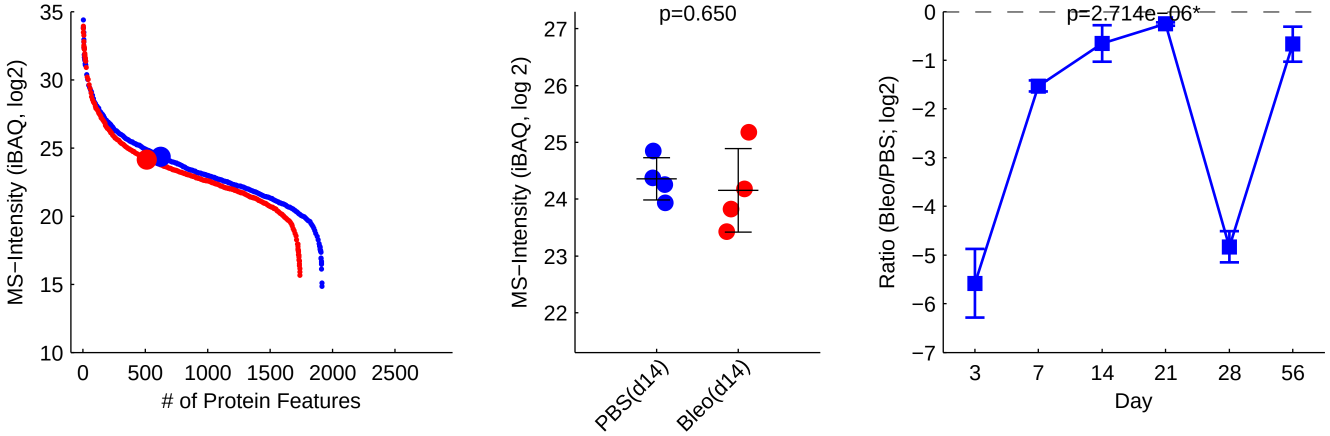

P54071 − Idh2 (id: 1266)

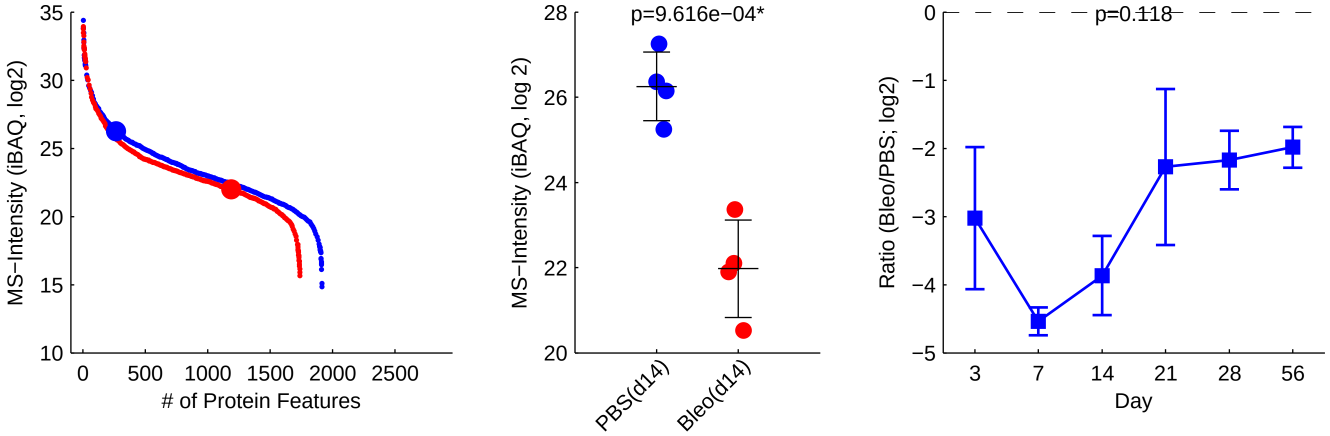

P54116 − Stom (id: 1267)

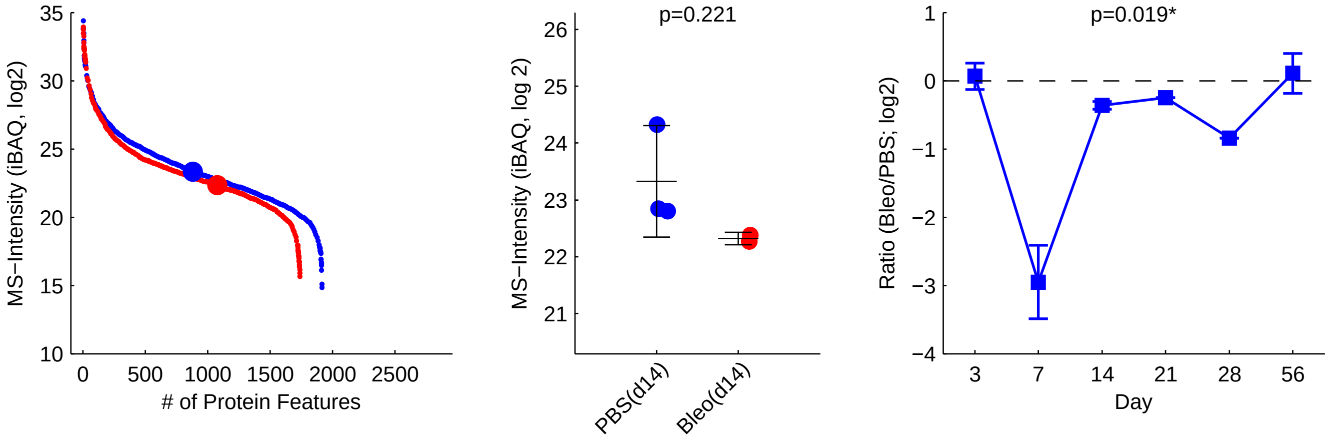

P54775 − Psmc4 (id: 1272)

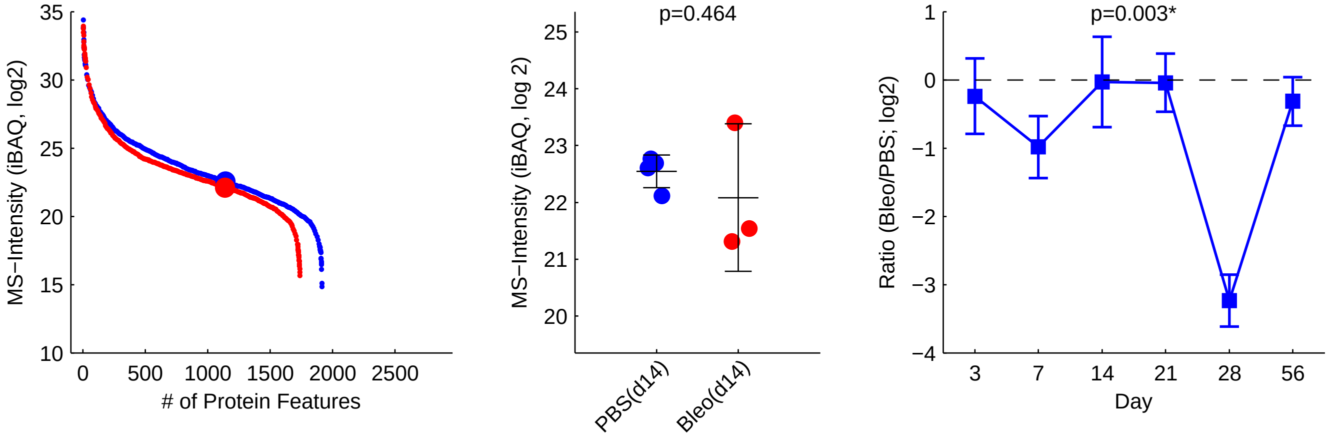

P55284 − Cdh5 (id: 1281)

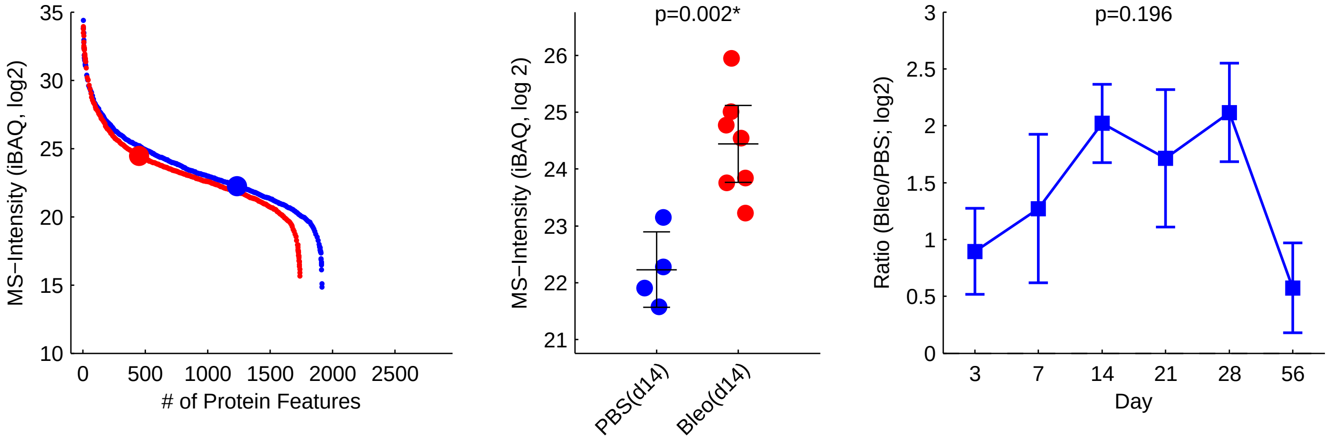

P56480 − Atp5b (id: 1288)

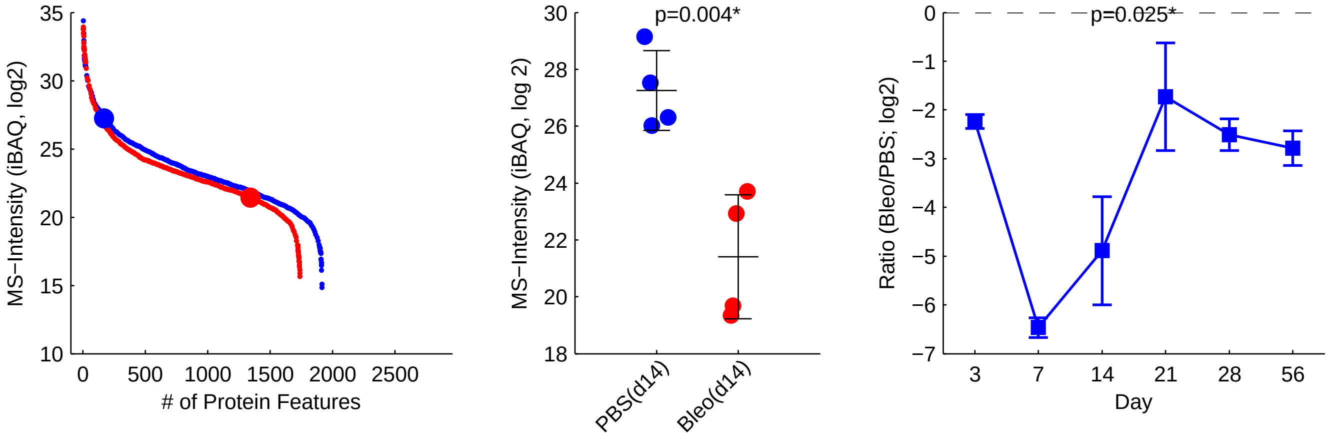

P56812 − Pdcd5 (id: 1291)

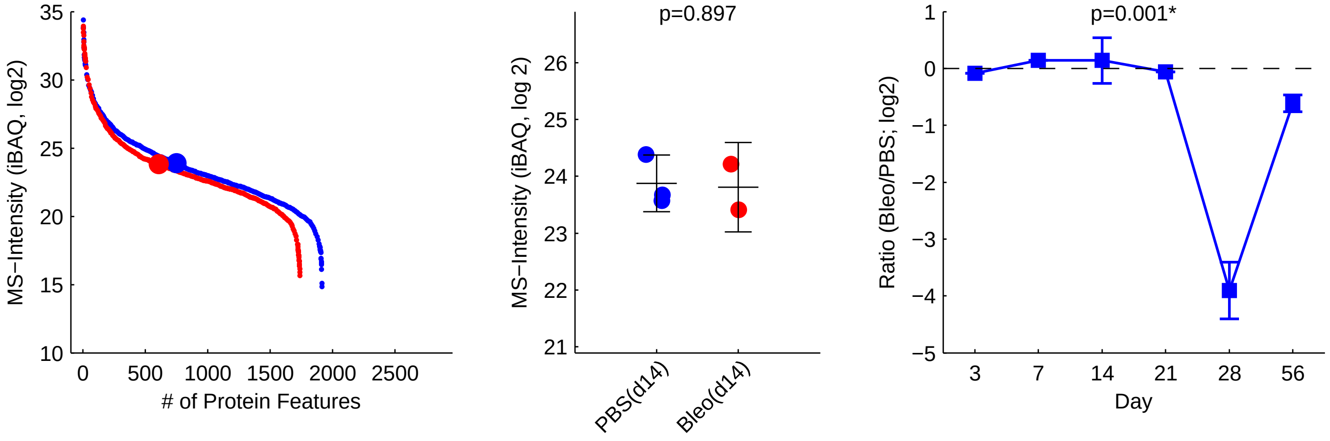

P57746 − Atp6v1d (id: 1293)

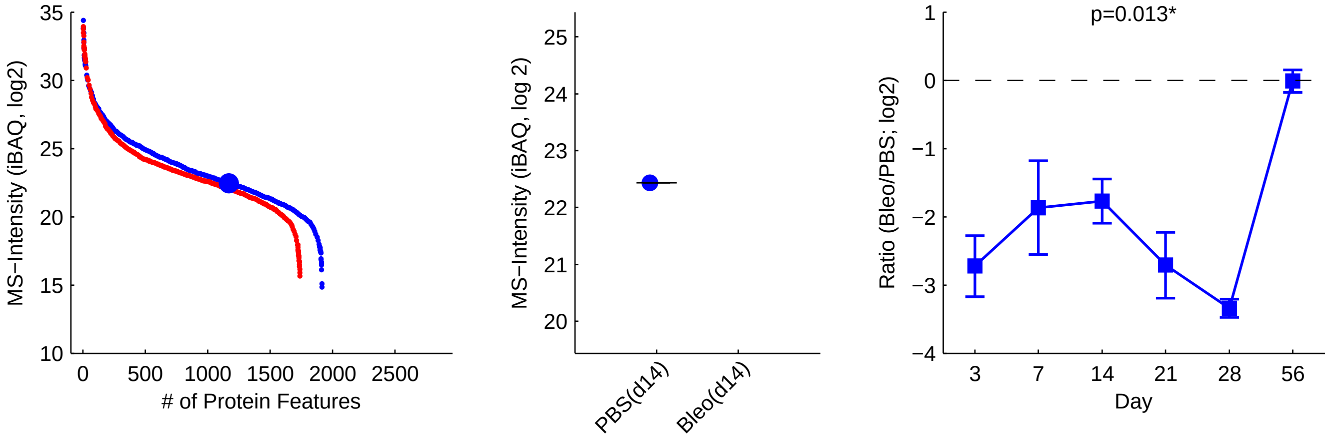

P57780 − Actn4 (id: 1295)

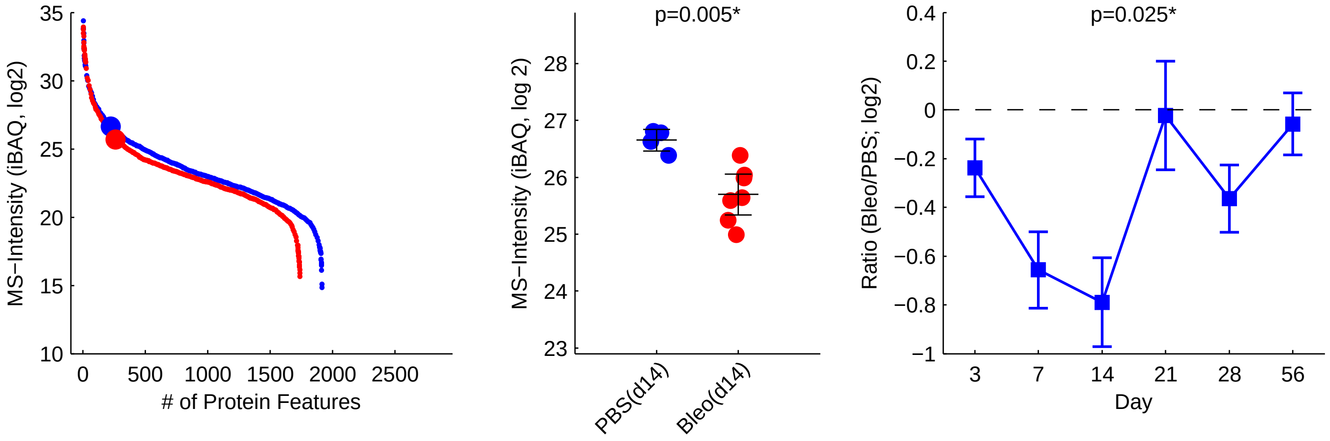

P58389 − Ppp2r4 (id: 1298)

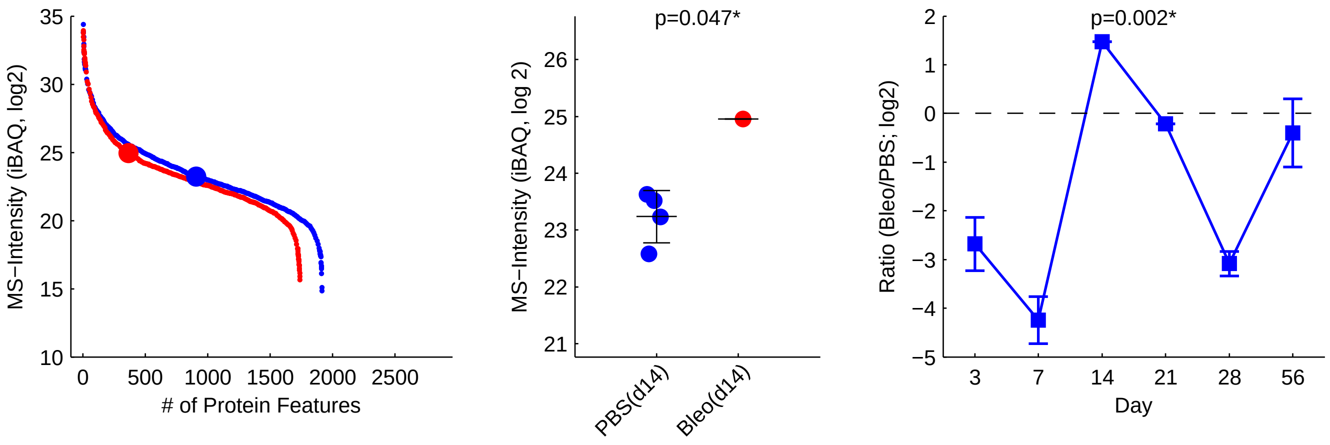

P60867 − Rps20 (id: 1310)

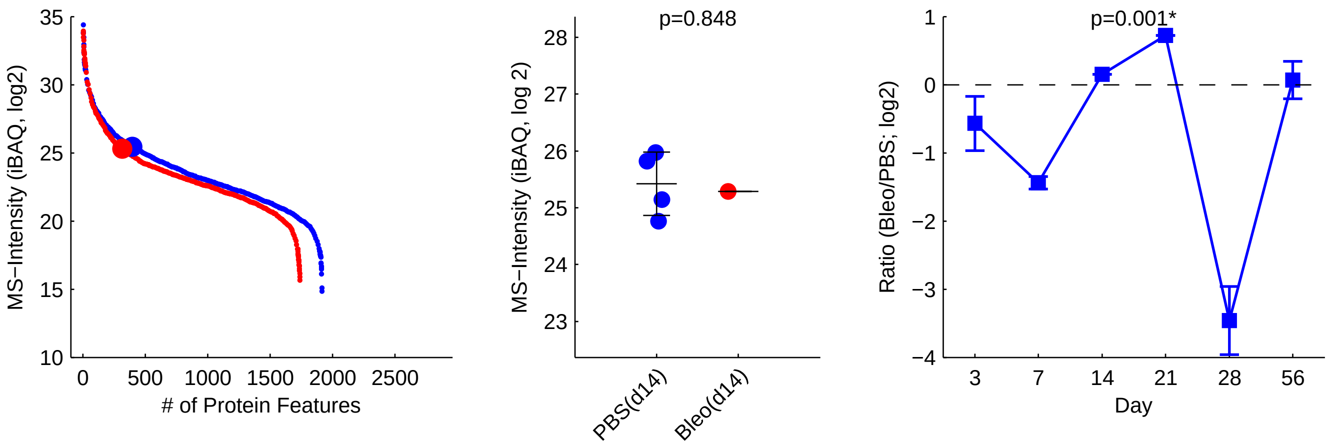

P62838 − Ube2d2 (id: 1314)

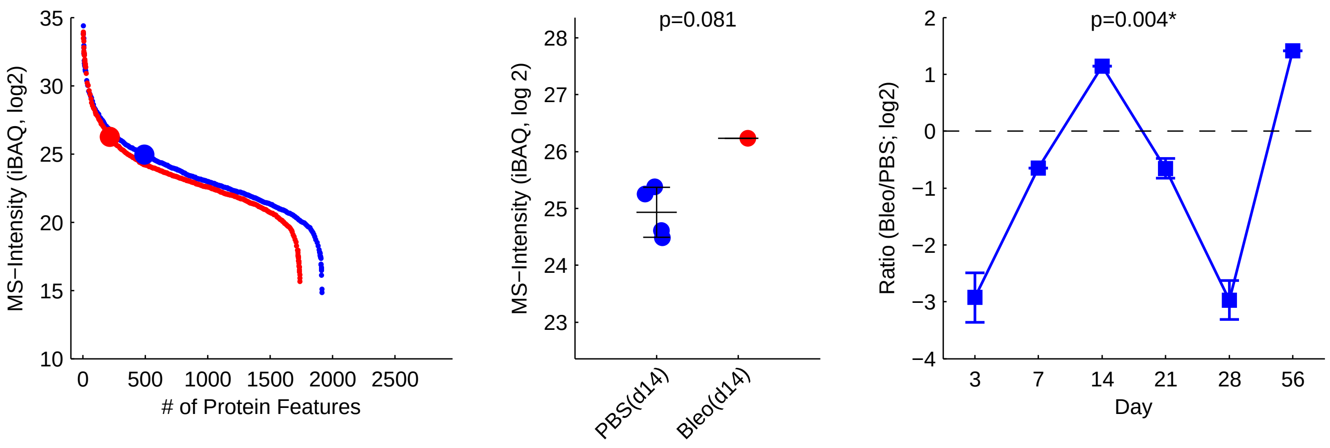

P61148 − Fgf1 (id: 1318)

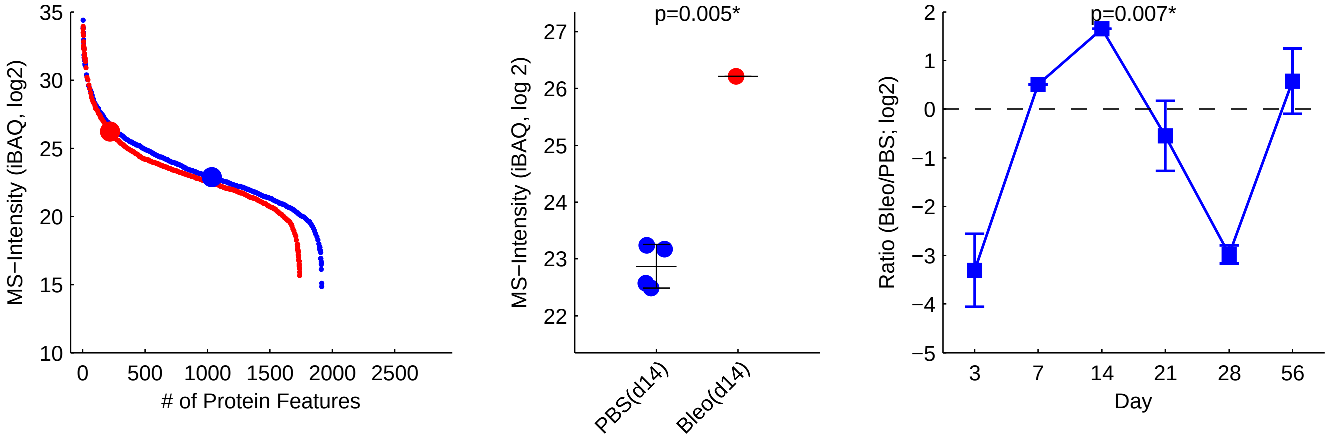

P61202 − Cops2 (id: 1321)

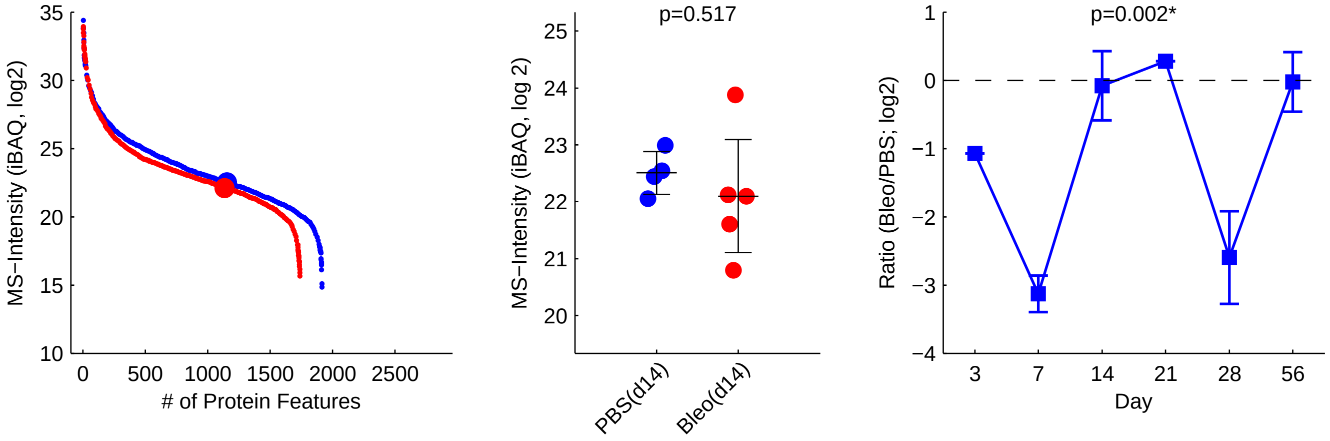

P61255 − Rpl26 (id: 1326)

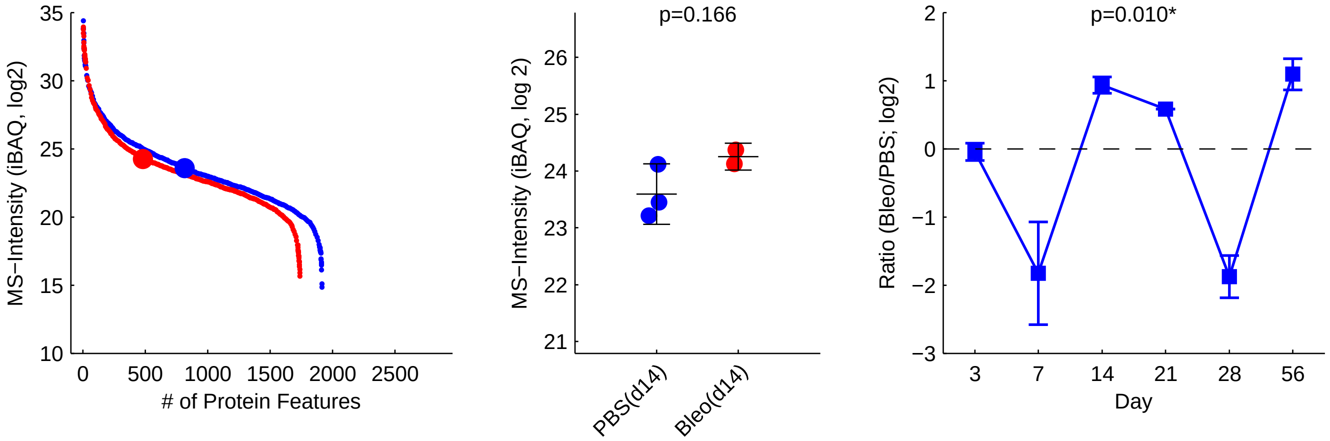

P61358 − Rpl27 (id: 1328)

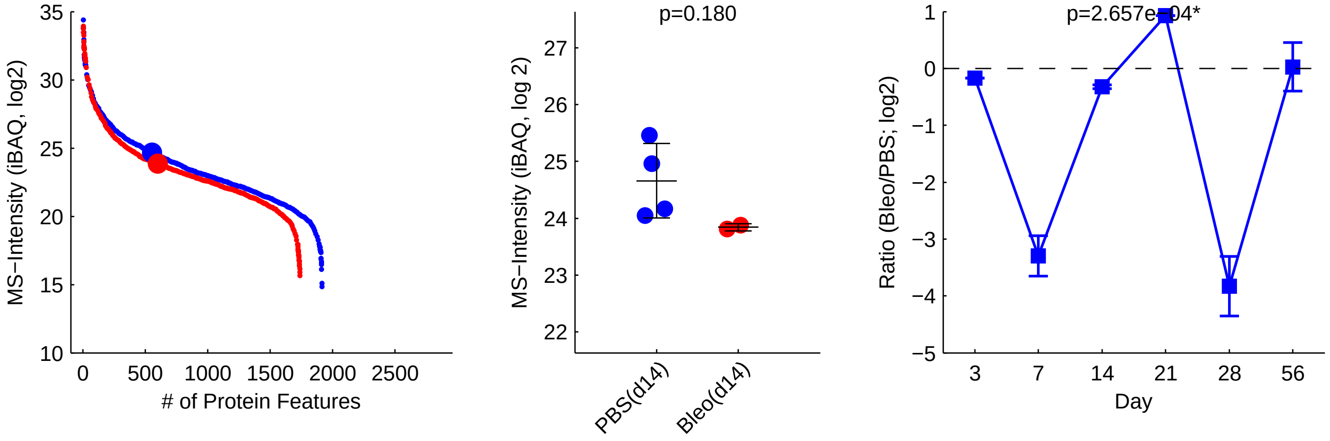

P61924 − Copz1 (id: 1333)

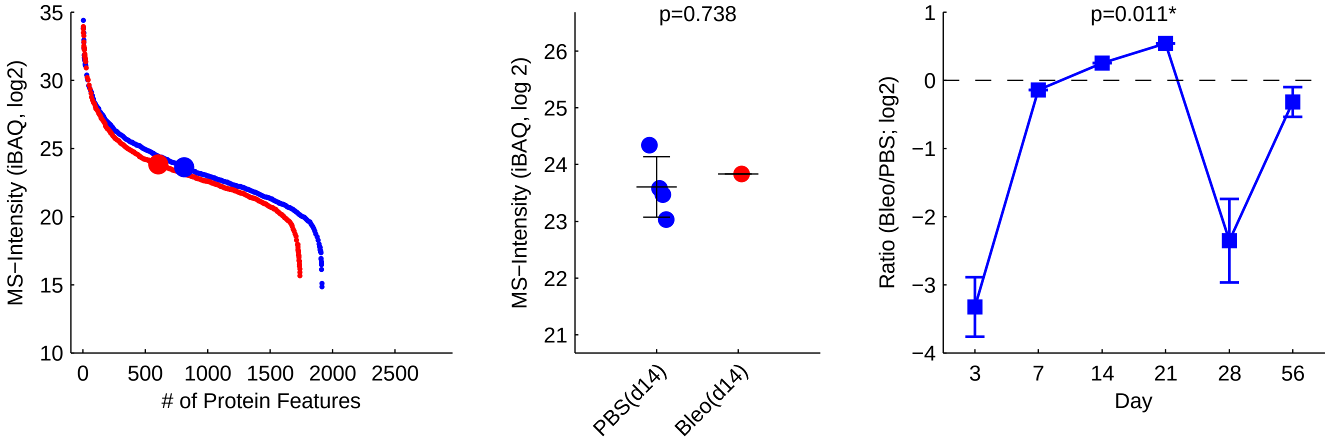

P61939 − Serpina7 (id: 1334)

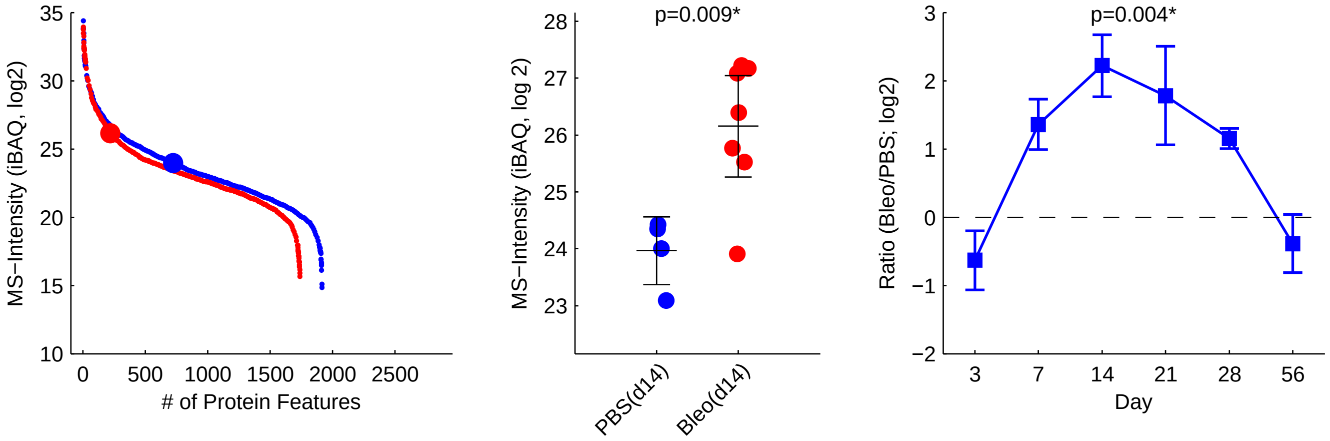

P61971 − Nutf2 (id: 1336)

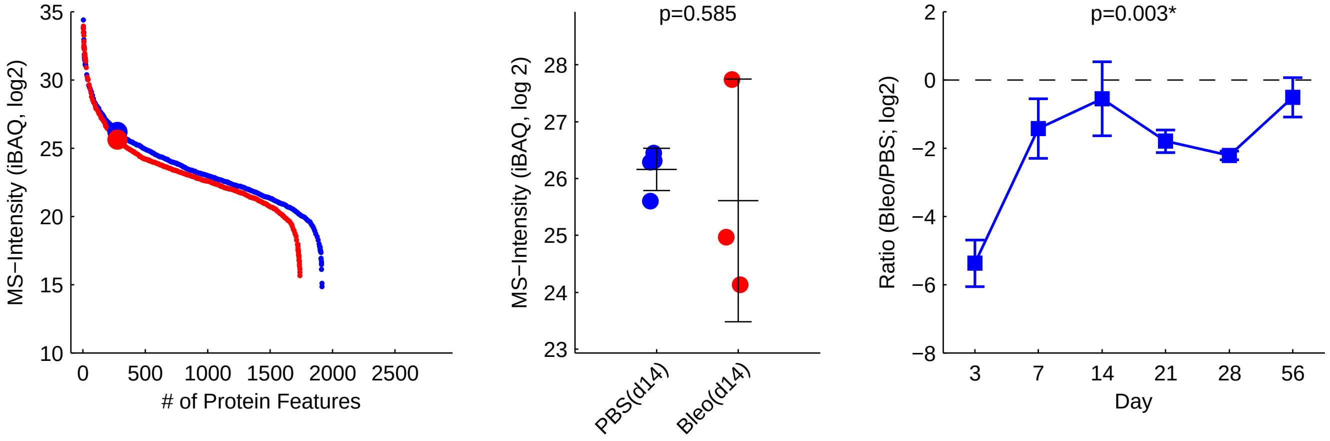

P62192 − Psmc1 (id: 1341)

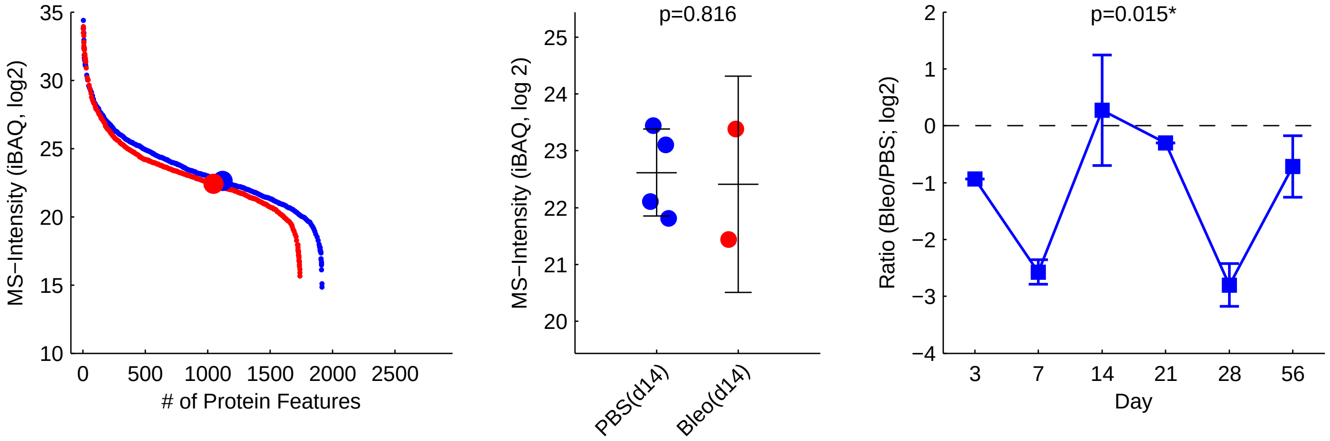

P62204 − Calm1 (id: 1343)

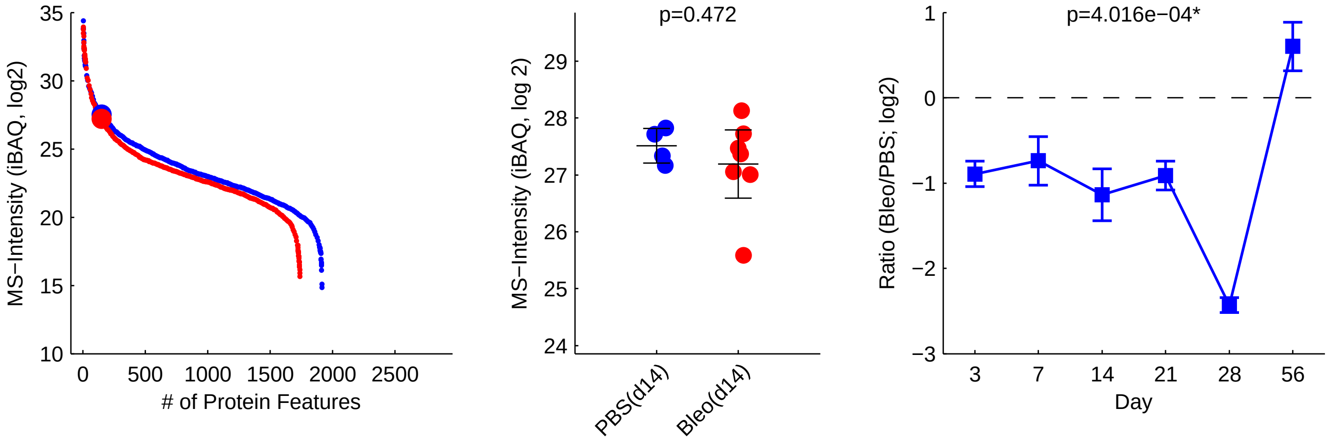

P62242 − Rps8 (id: 1344)

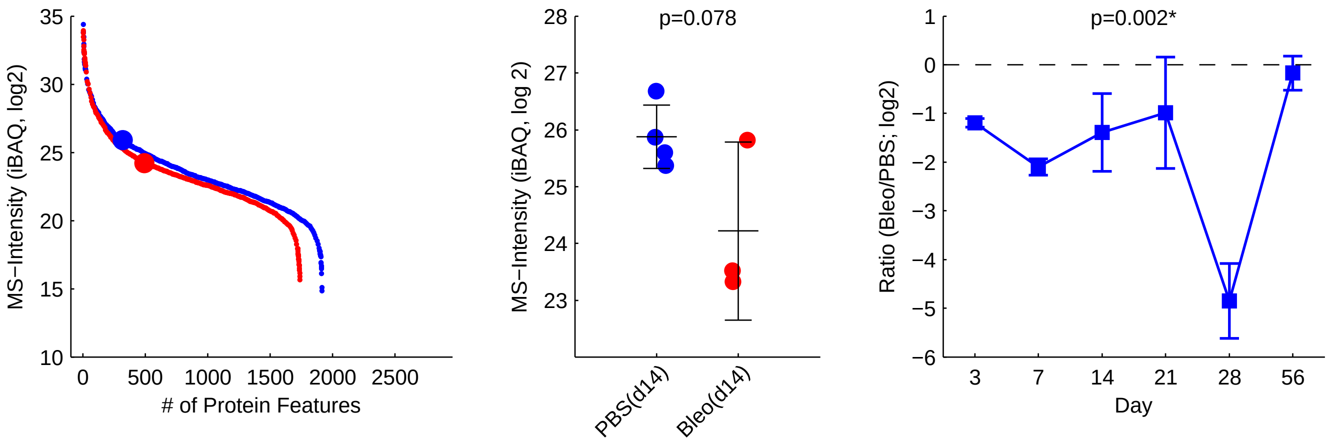

P62245 − Rps15a (id: 1345)

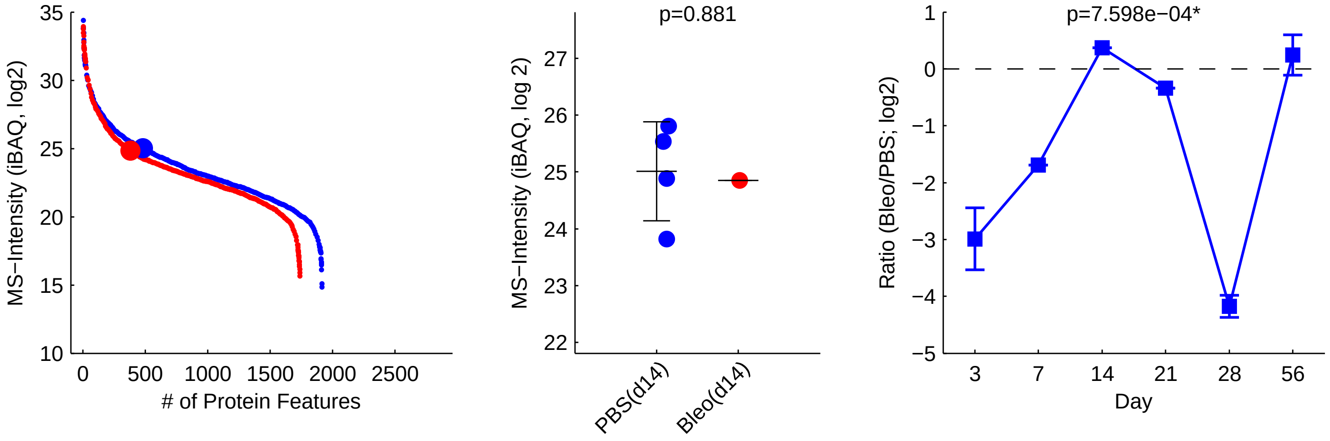

P62259 − Ywhae (id: 1348)

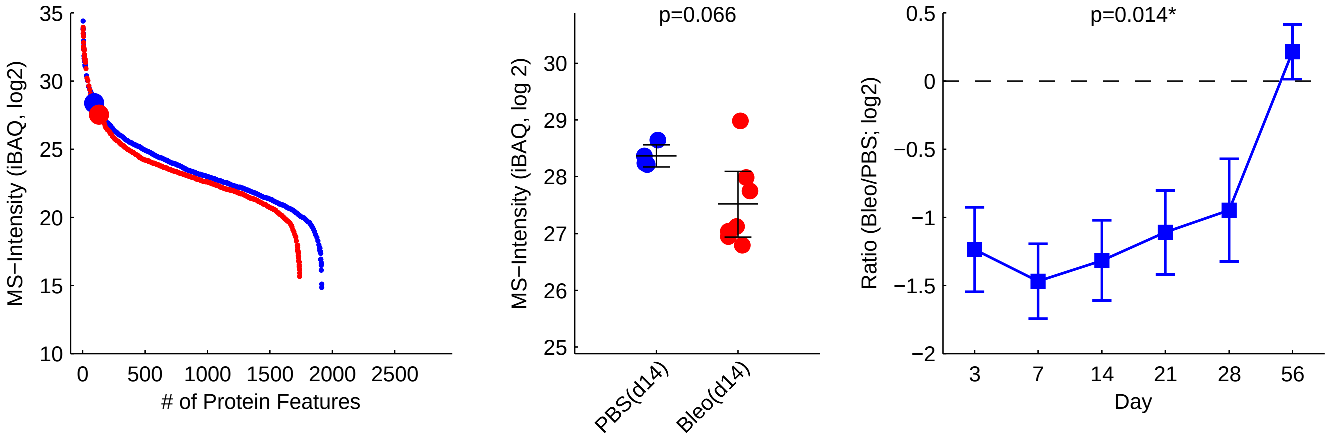

P62267 − Rps23 (id: 1350)

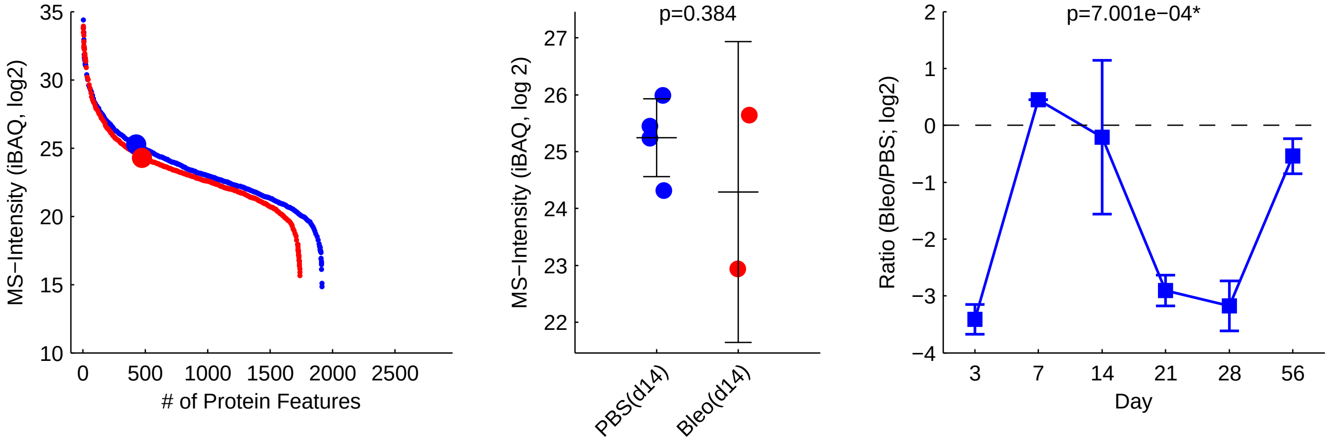

P62281 − Rps11 (id: 1352)

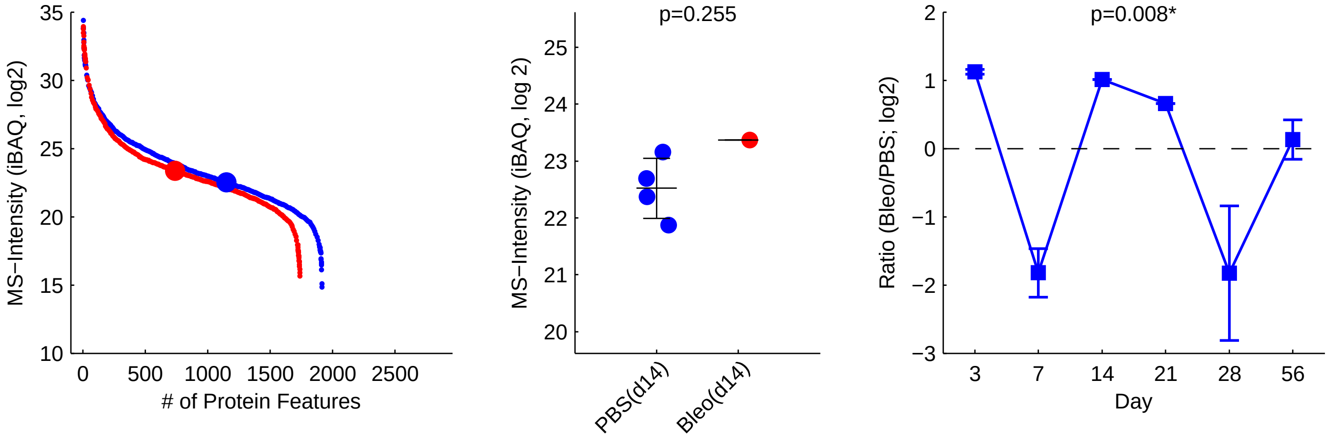

P62301 − Rps13 (id: 1353)

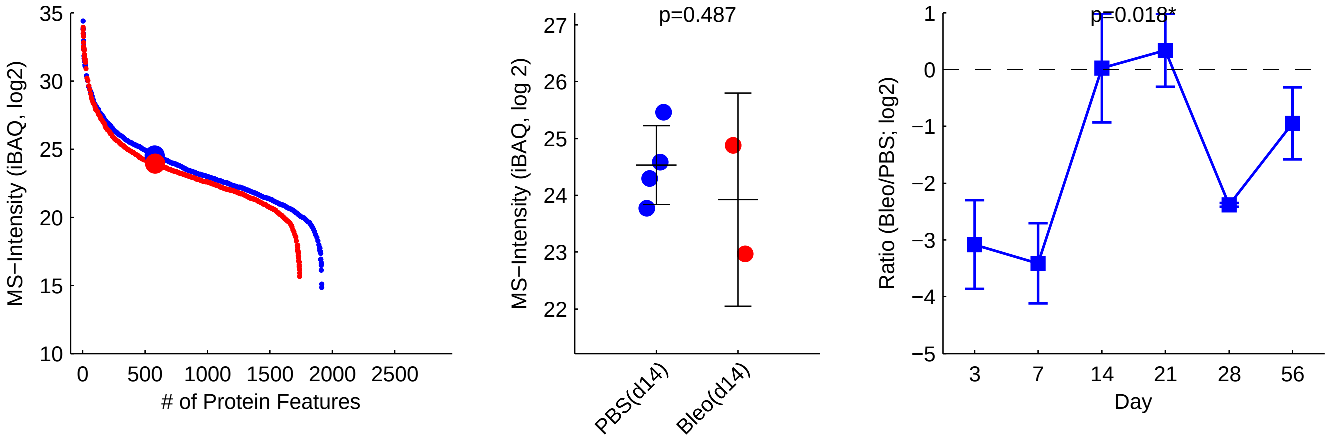

P62702 − Rps4x (id: 1360)

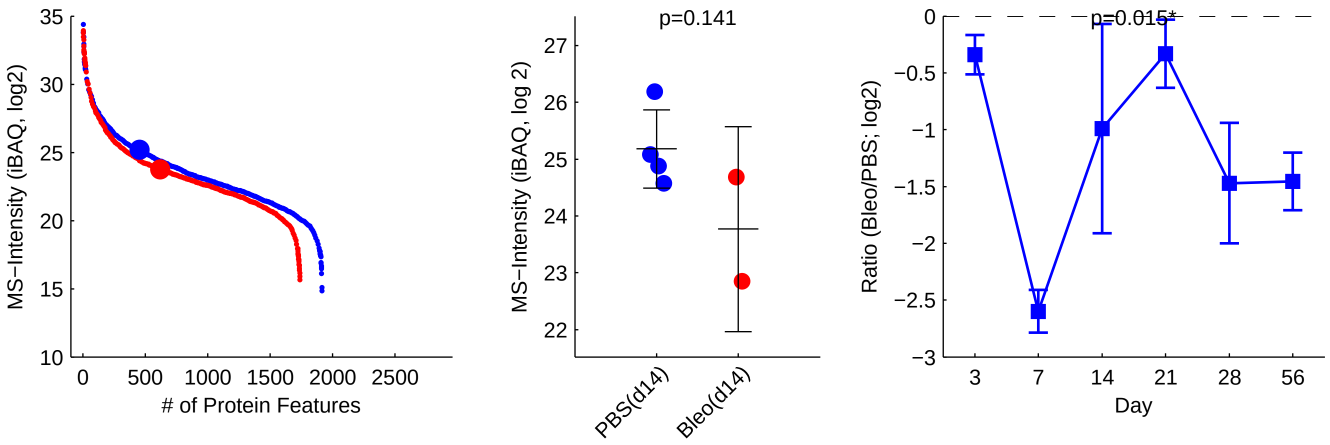

P62754 − Rps6 (id: 1365)

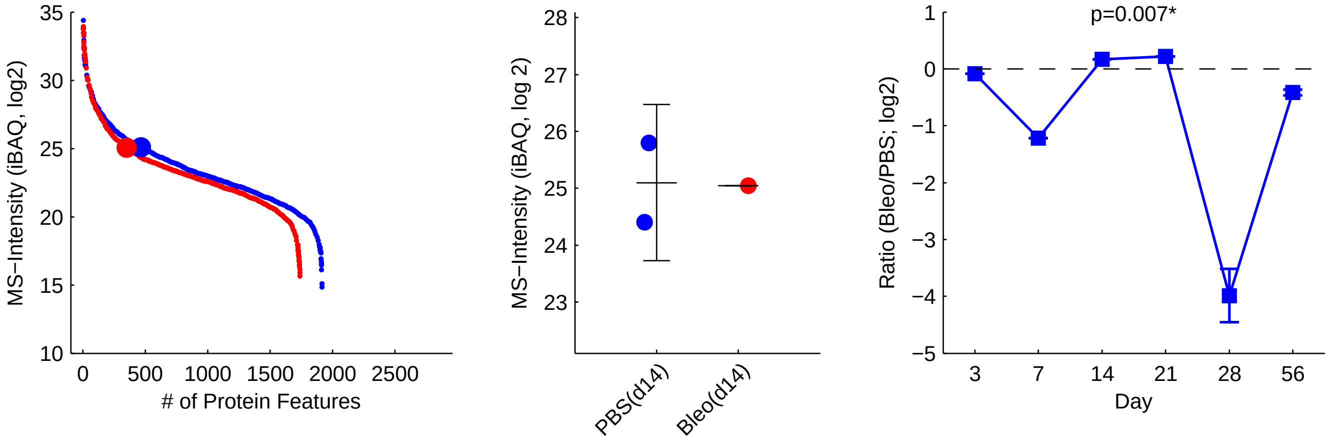

P62806 − Hist1h4a (id: 1367)

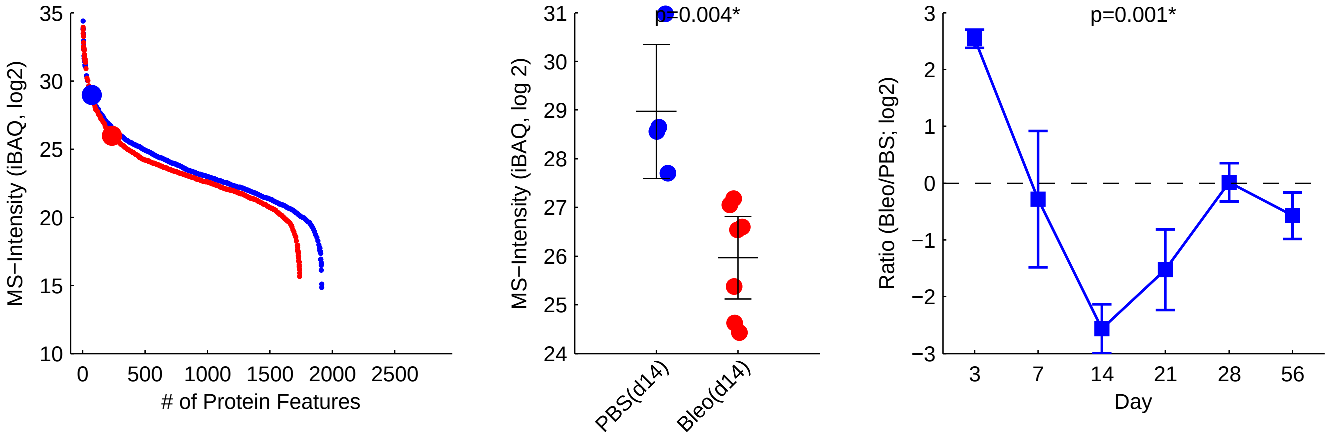

P62814 − Atp6v1b2 (id: 1368)

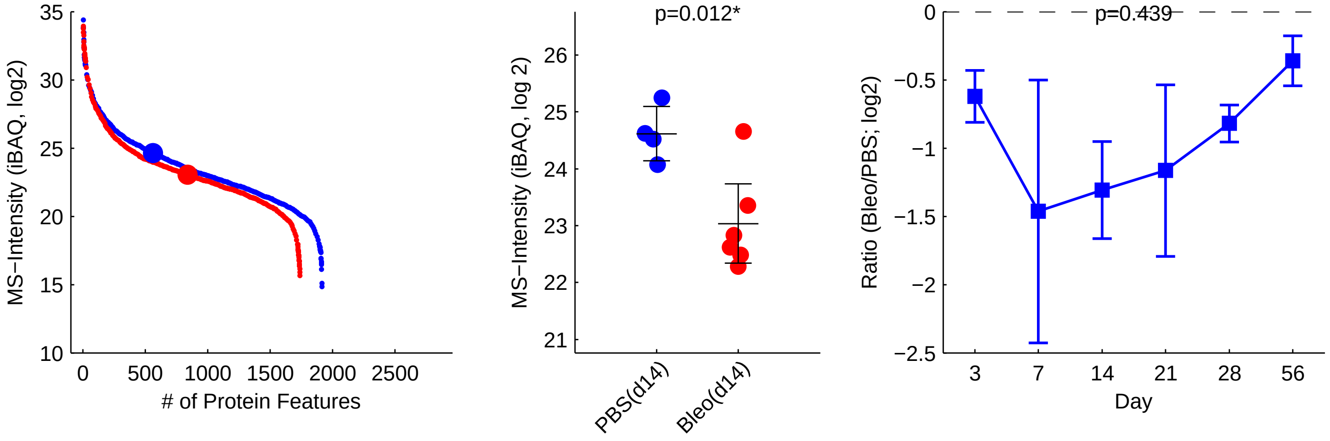

P62855 − Rps26 (id: 1375)

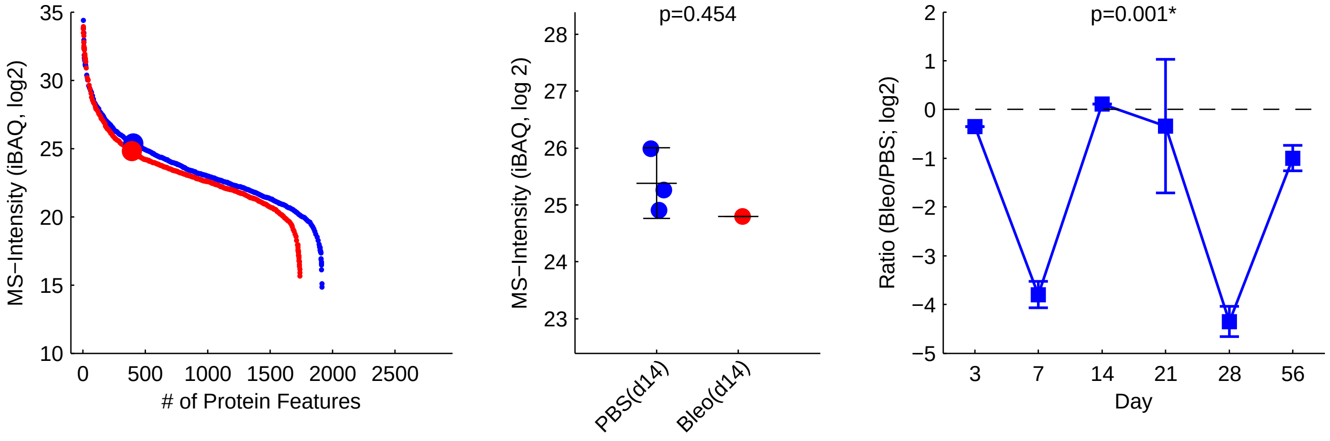

P62869 − Tceb2 (id: 1378)

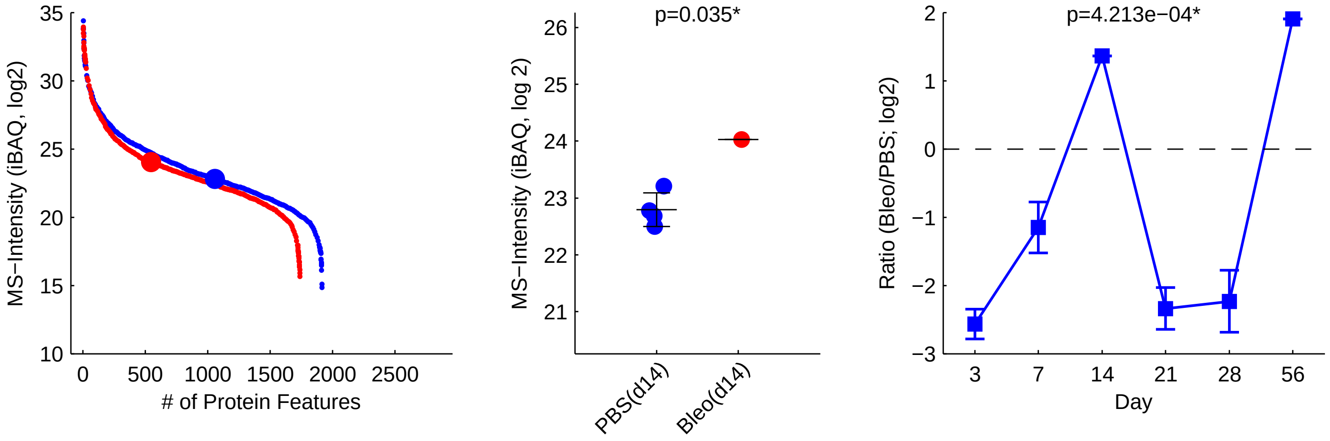

P62874 − Gnb1 (id: 1379)

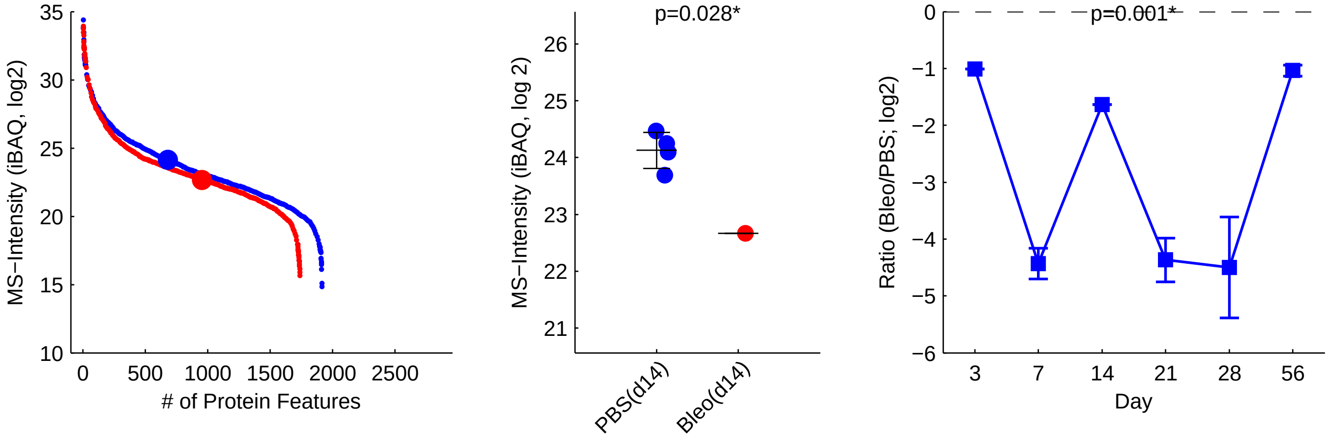

P62880 − Gnb2 (id: 1380)

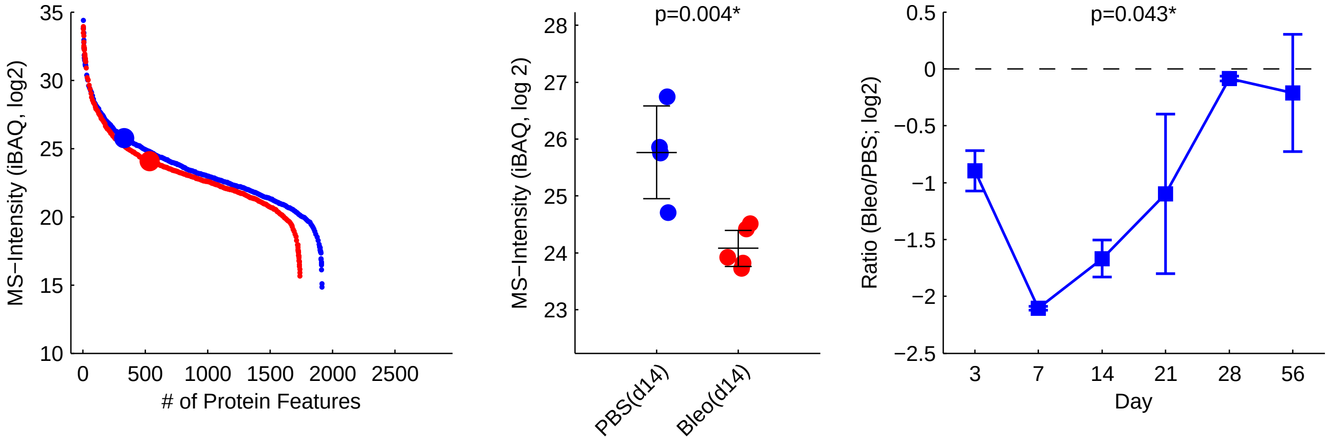

P62889 − Rpl30 (id: 1381)

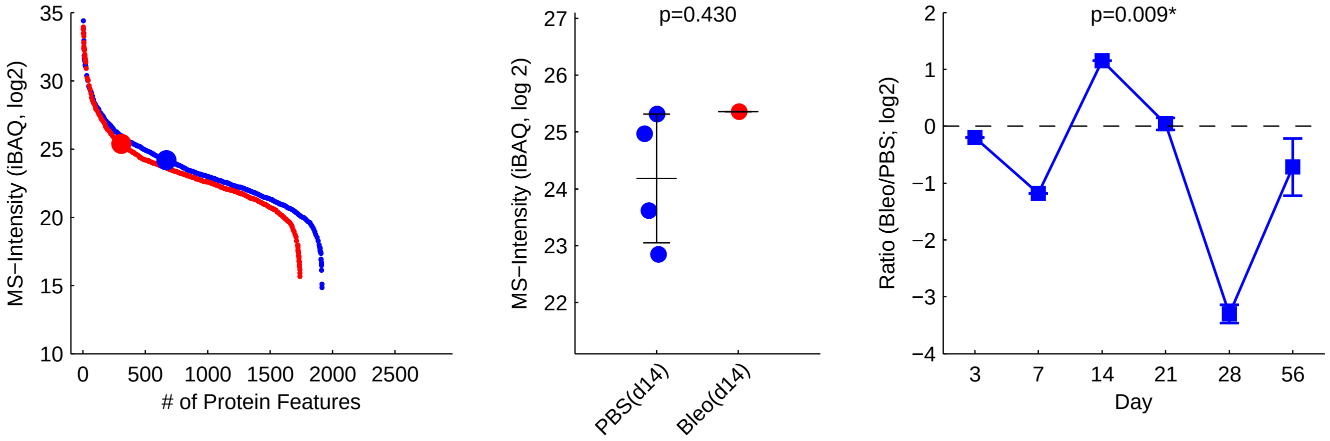

P62900 − Rpl31 (id: 1382)

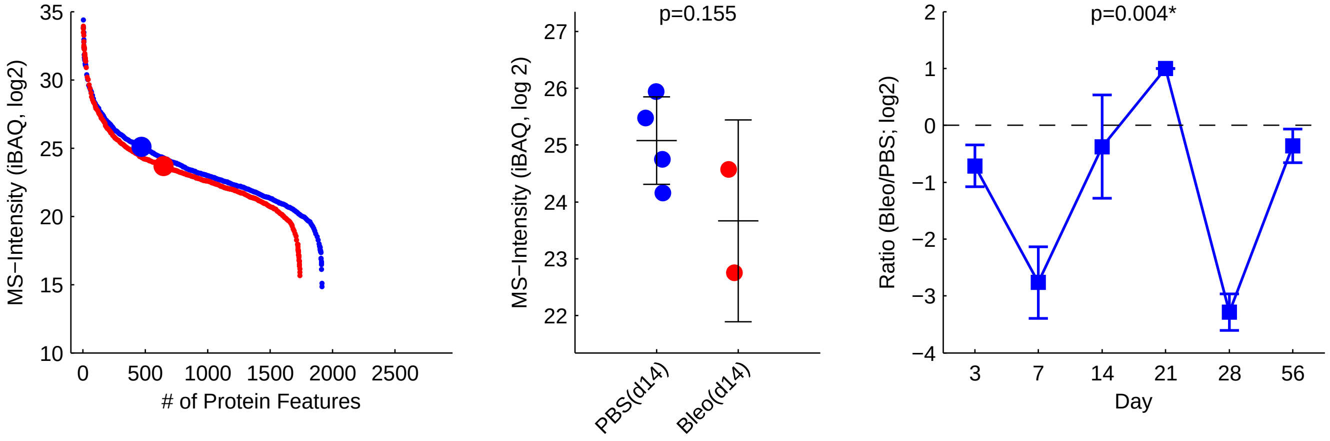

P62918 − Rpl8 (id: 1385)

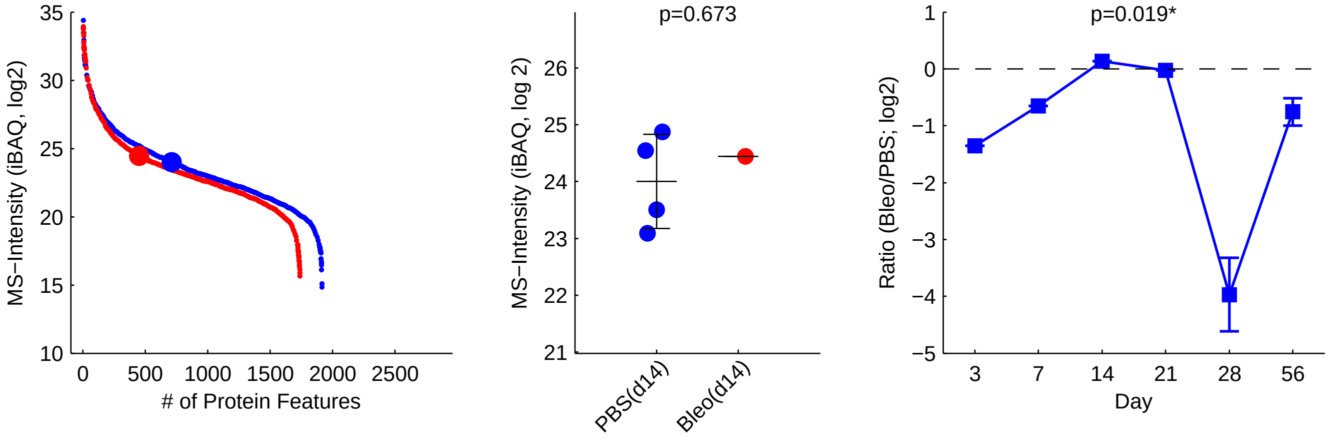

P62960 − Ybx1 (id: 1386)

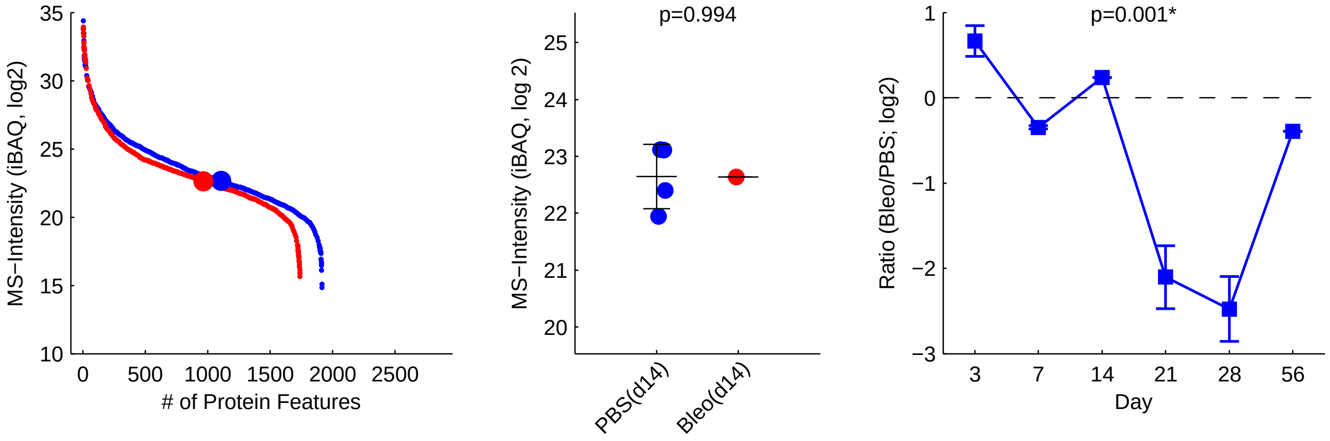

P63037 − Dnaja1 (id: 1393)

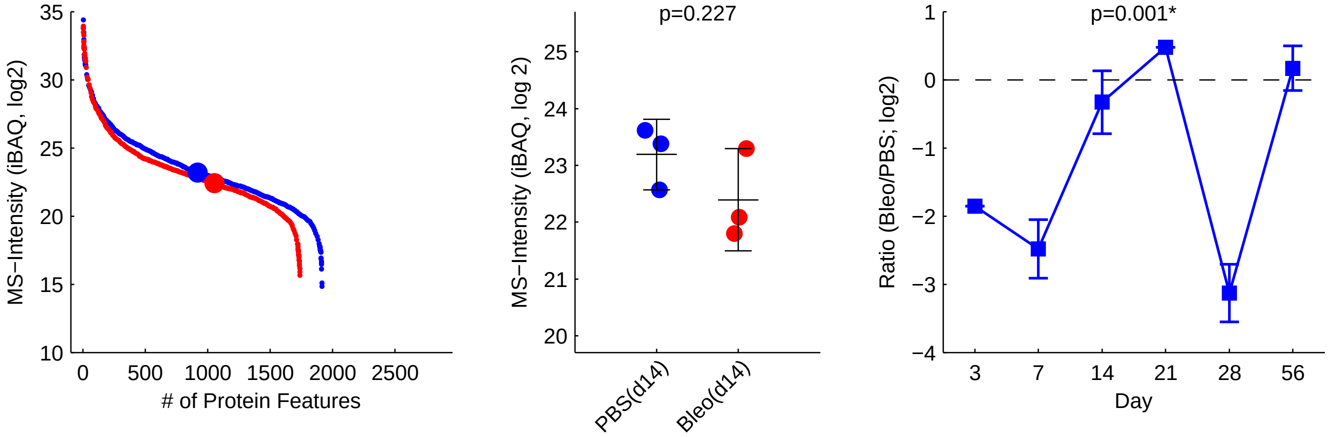

P63038 − Hspd1 (id: 1394)

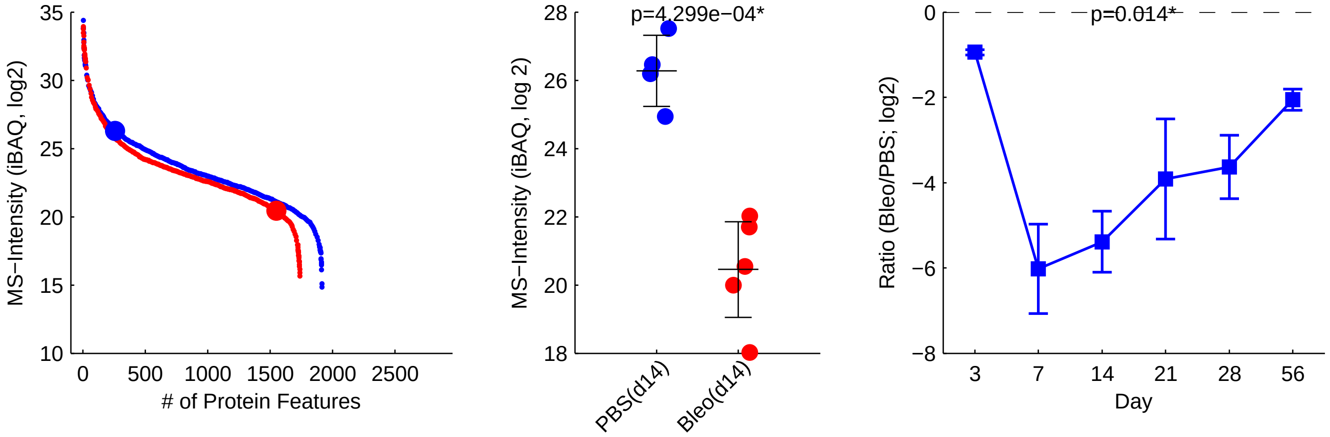

P63085 − Mapk1 (id: 1395)

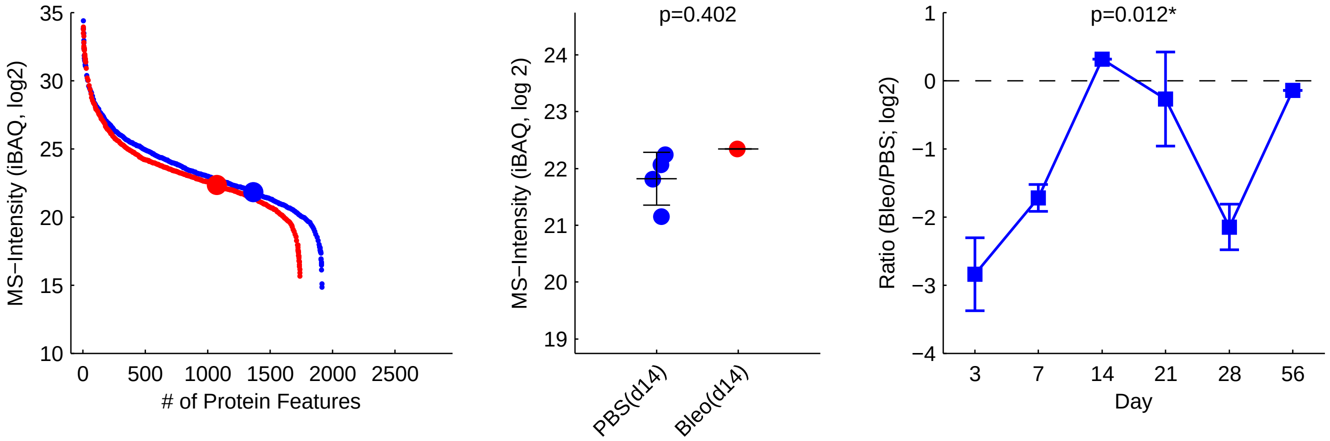

P63094−2 − Gnas (id: 1397)

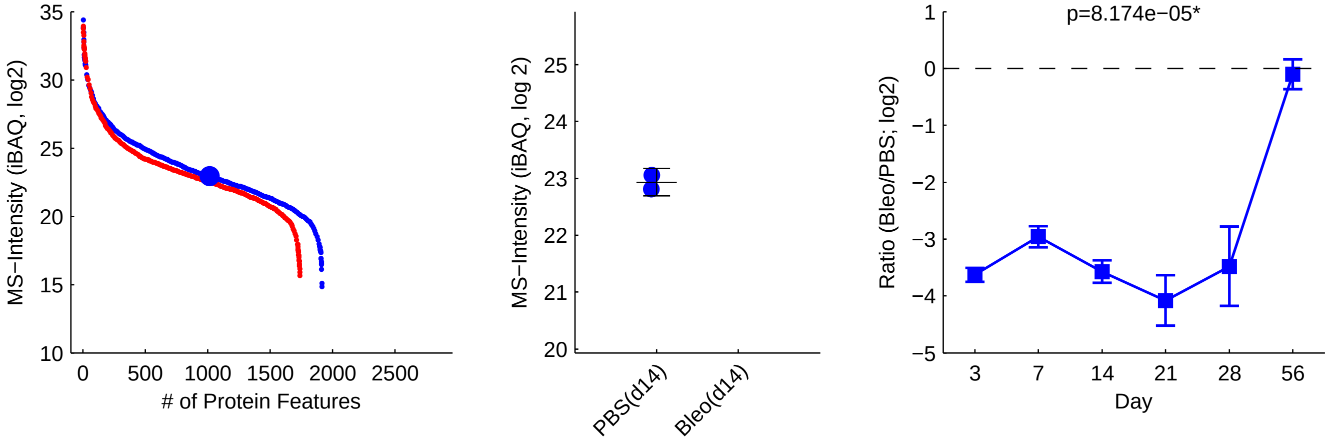

P63101 − Ywhaz (id: 1398)

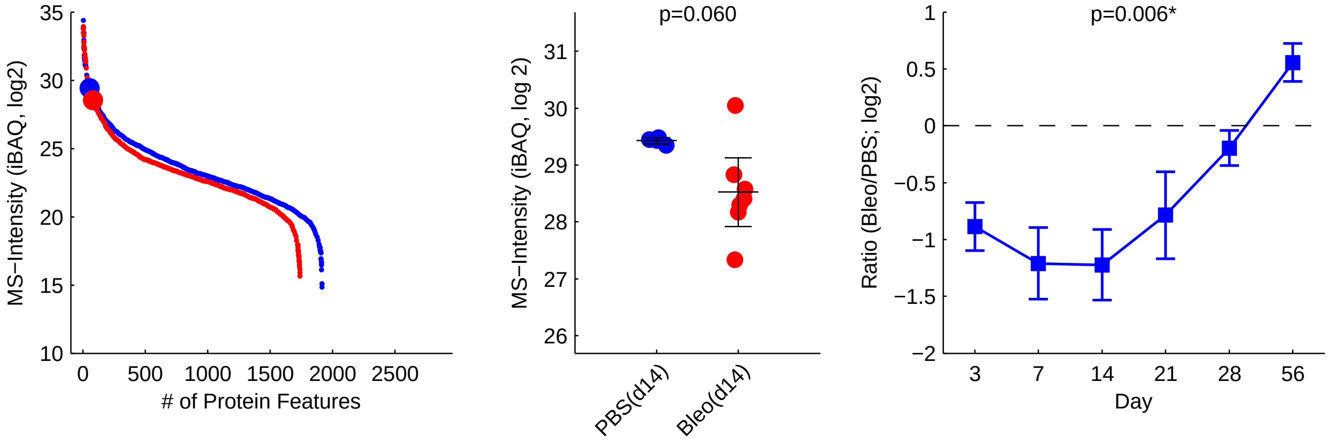

P63158 − Hmgb1 (id: 1399)

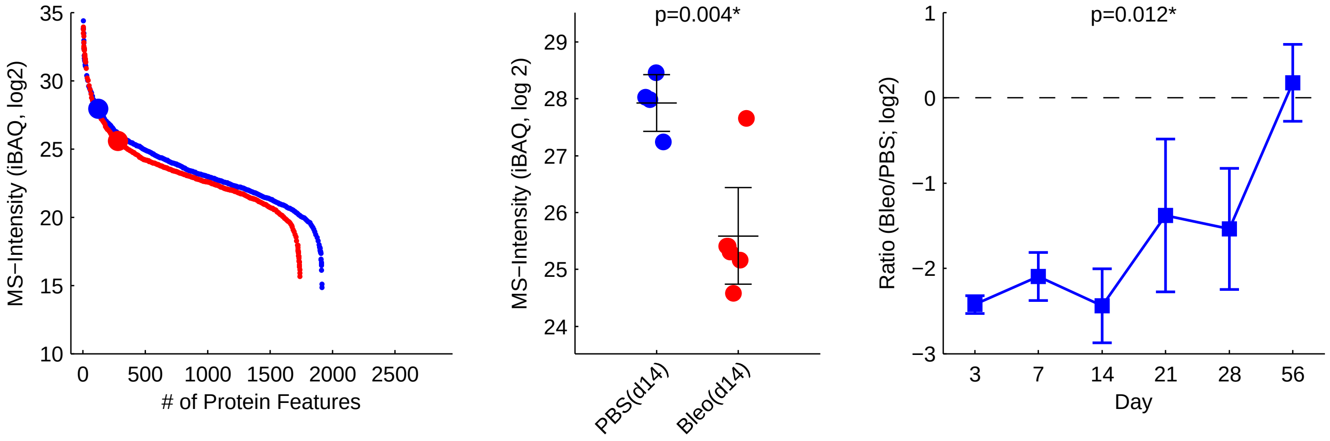

P63168 − Dynll1 (id: 1401)

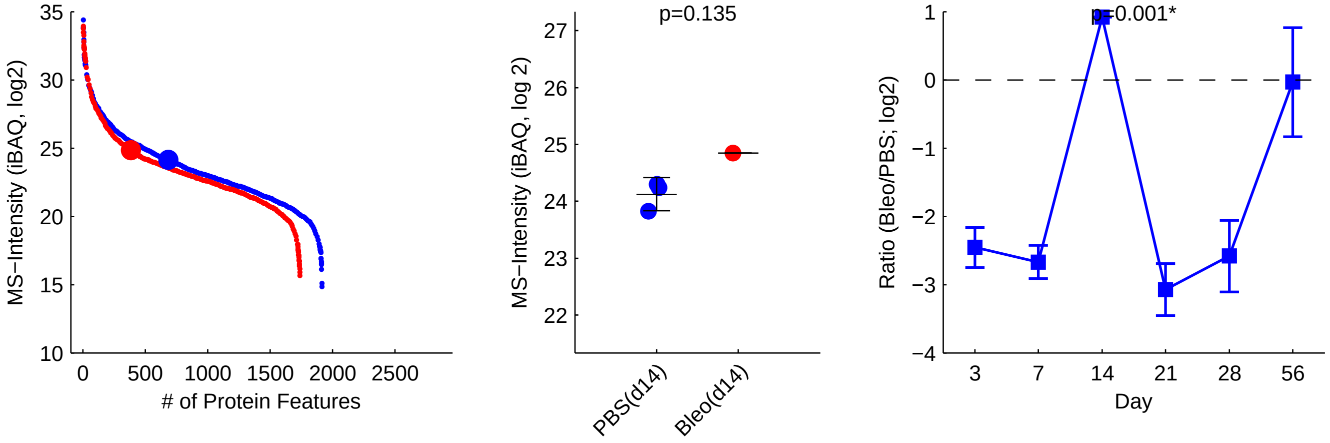

P63254 − Crip1 (id: 1403)

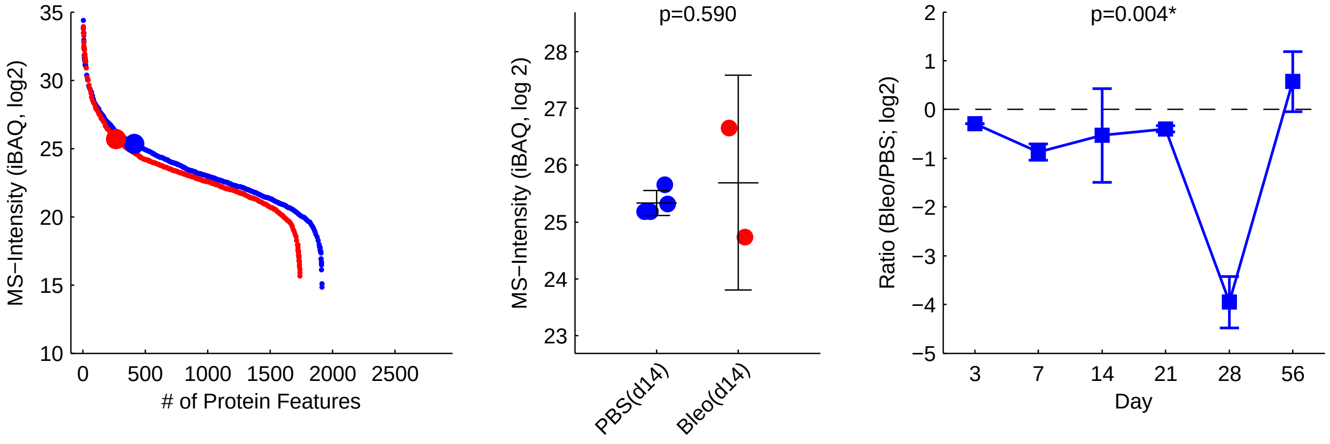

P63276 − Rps17 (id: 1405)

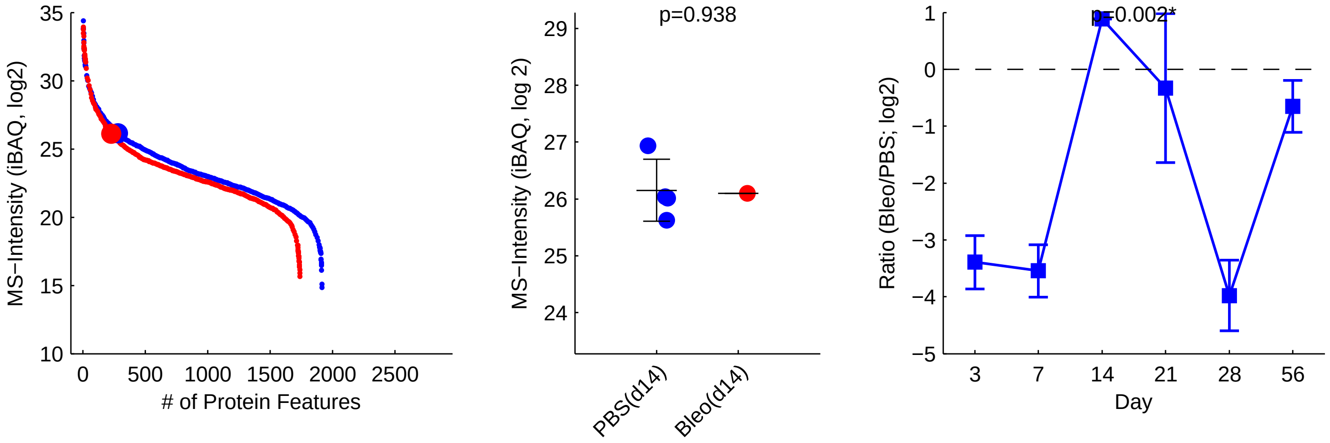

P67984 − Rpl22 (id: 1413)

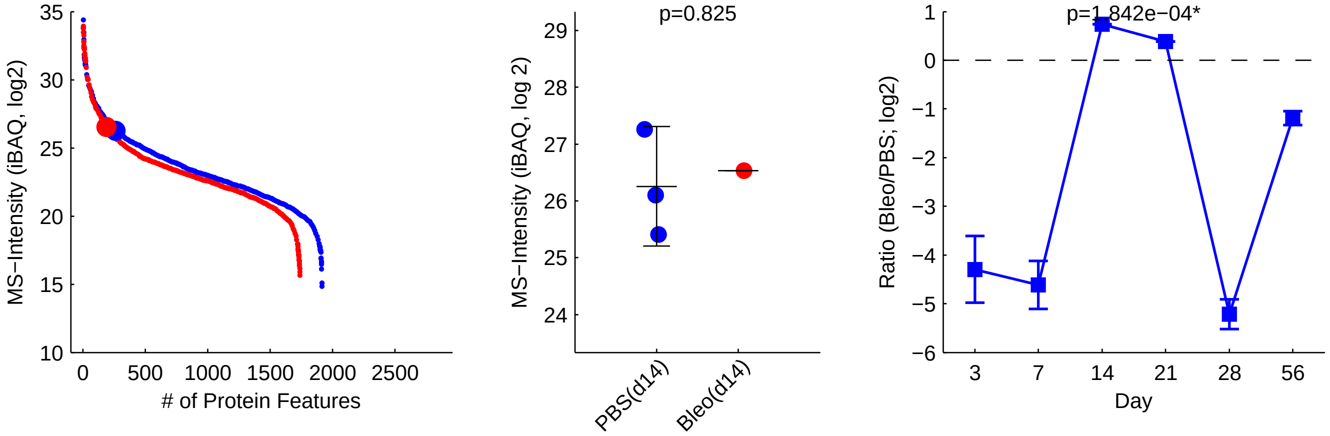

P68033 − Actc1 (id: 1414)

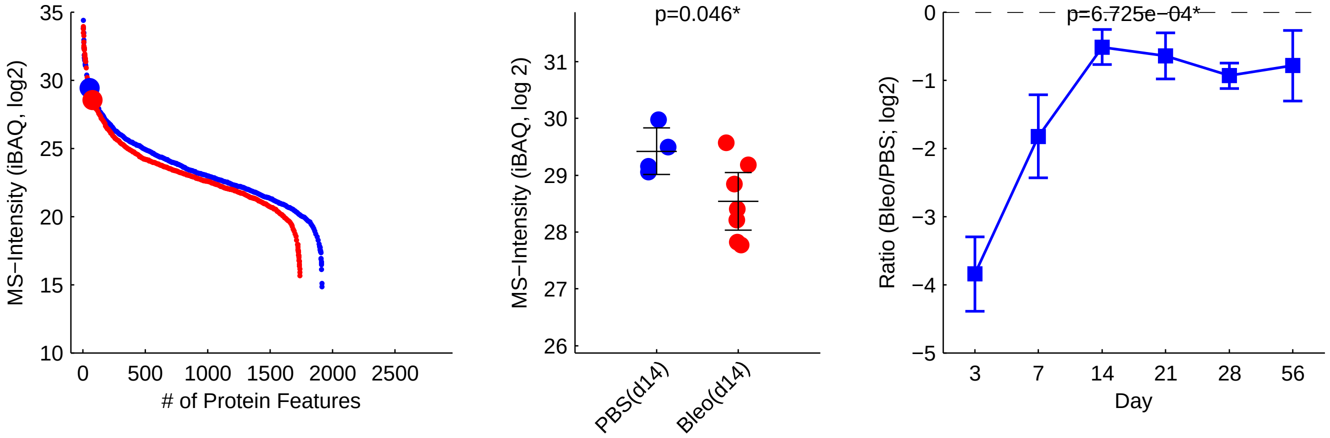

P68368 − Tuba4a (id: 1419)

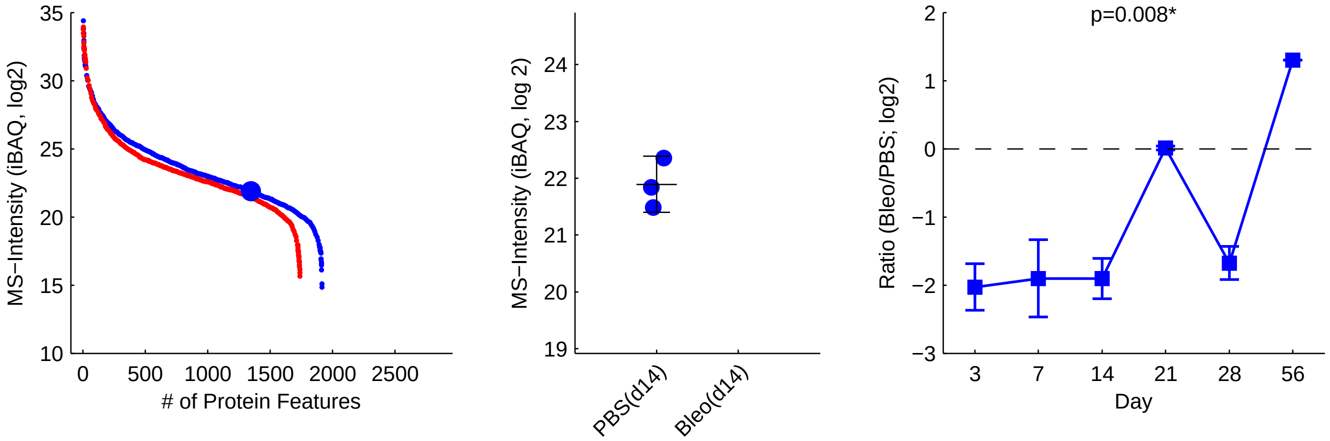

P68369 − Tuba1a (id: 1420)

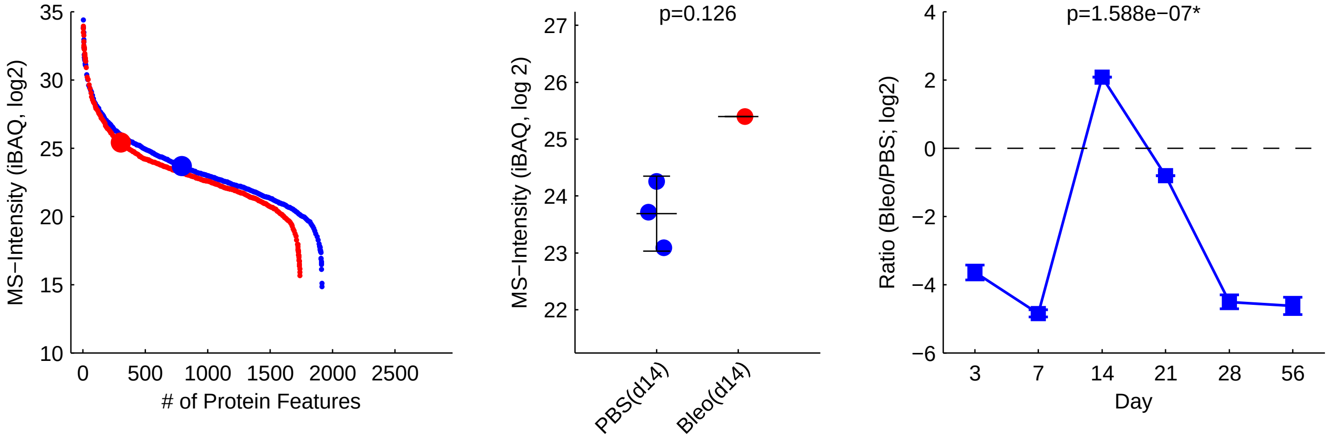

P68372 − Tubb4b (id: 1421)

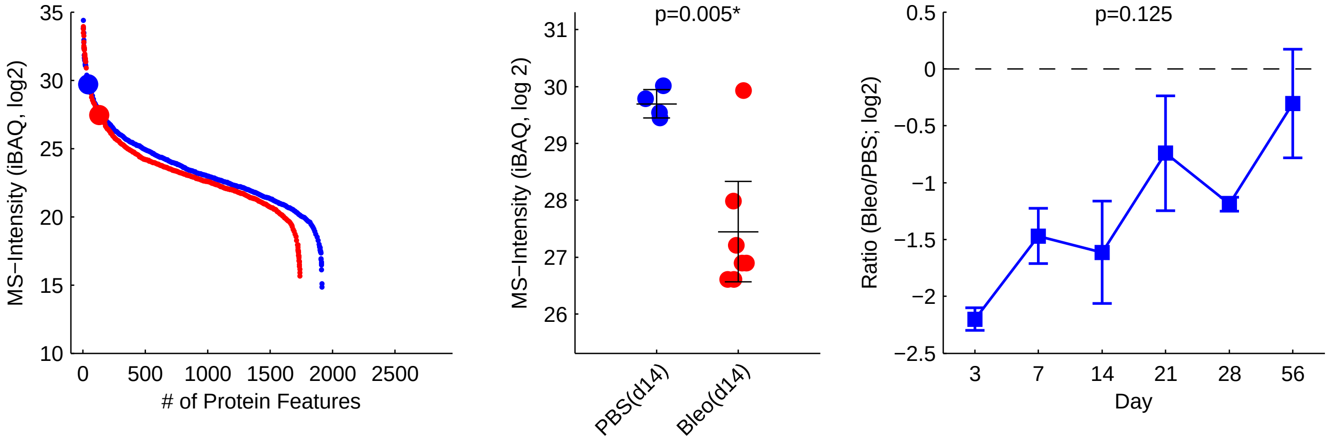

P70122 − Sbds (id: 1425)

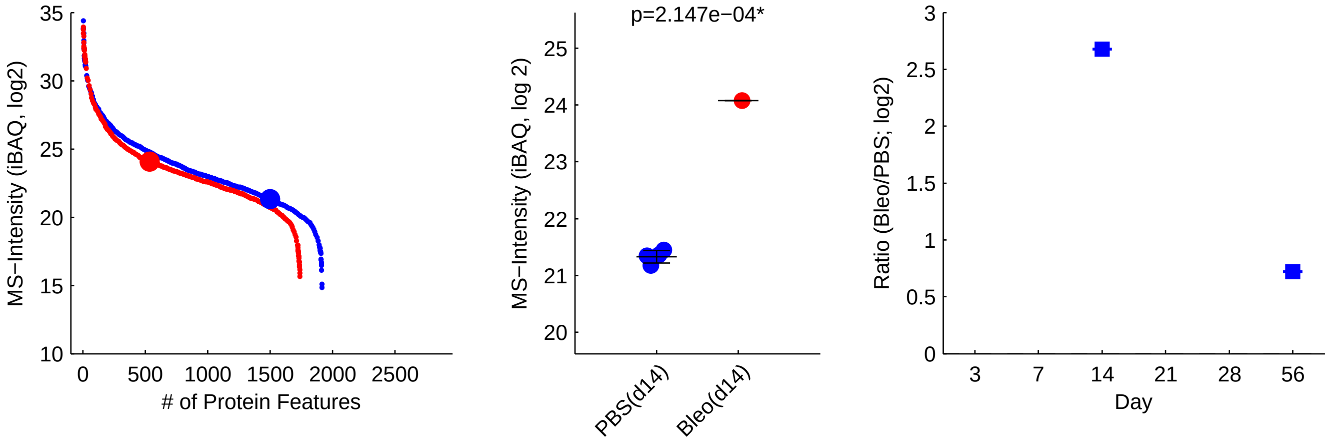

P70195 − Psmb7 (id: 1429)

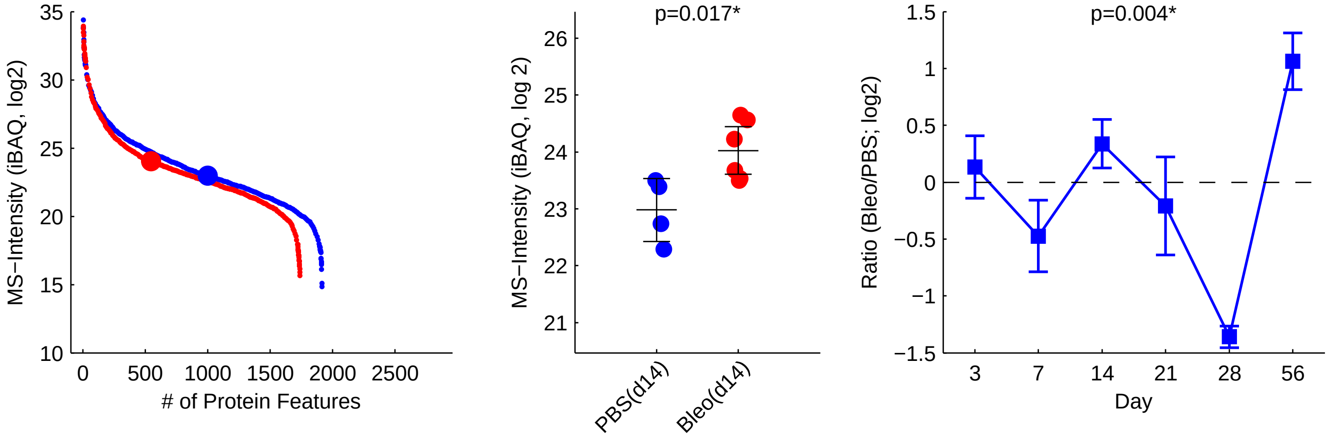

P70202 − Lxn (id: 1430)

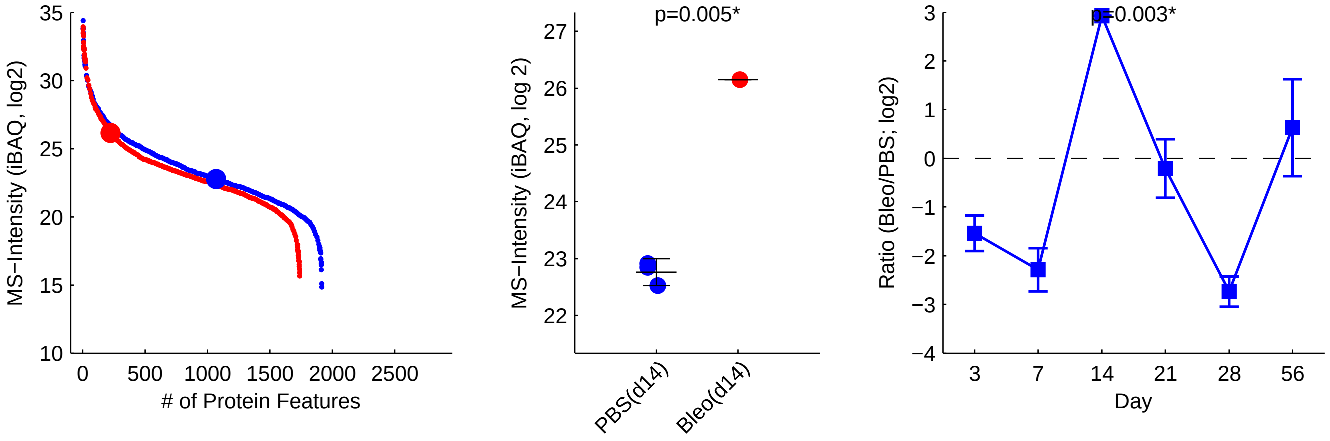

P70375 − F7 (id: 1440)

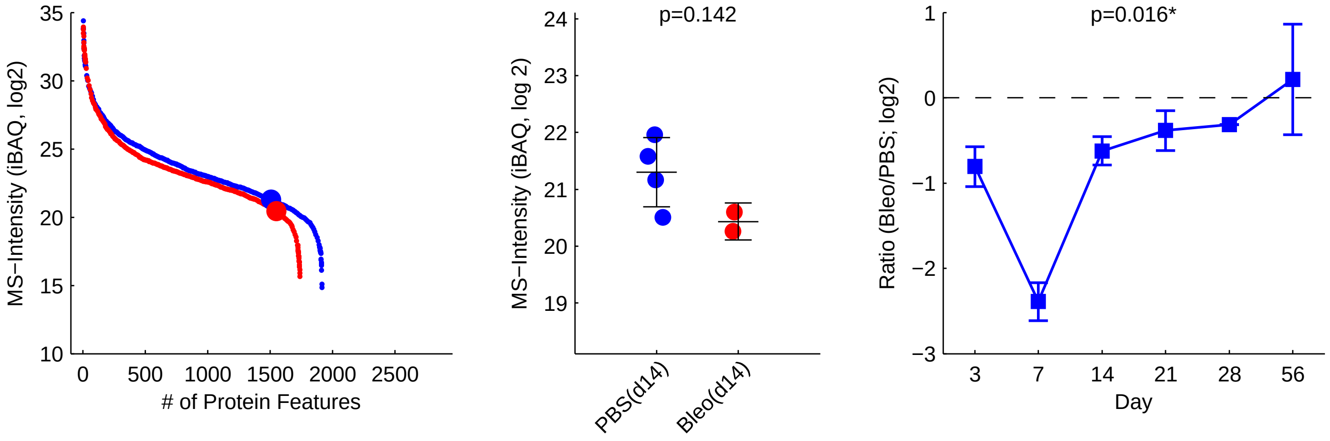

P70441 − Slc9a3r1 (id: 1445)

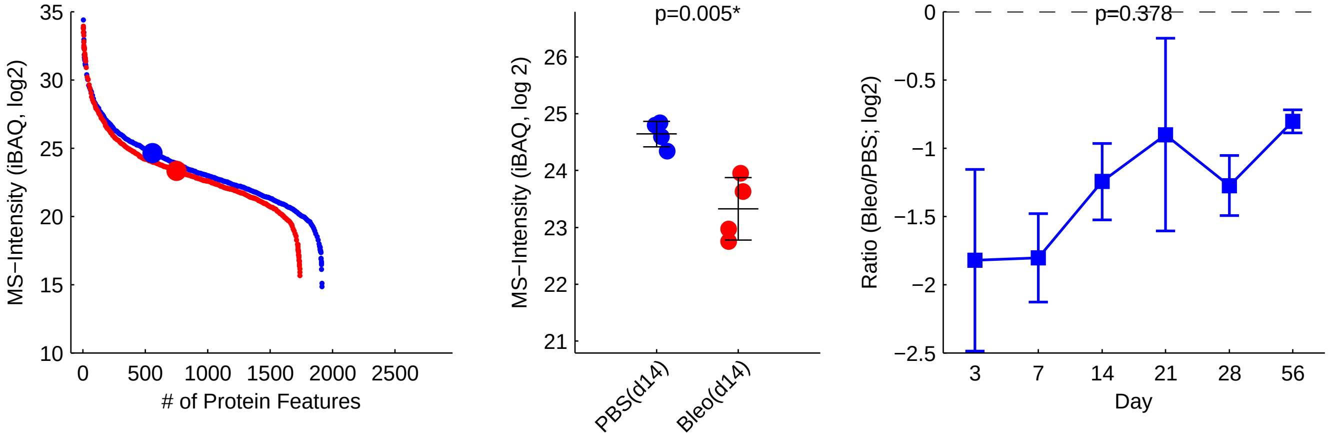

P70663 − Sparcl1 (id: 1447)

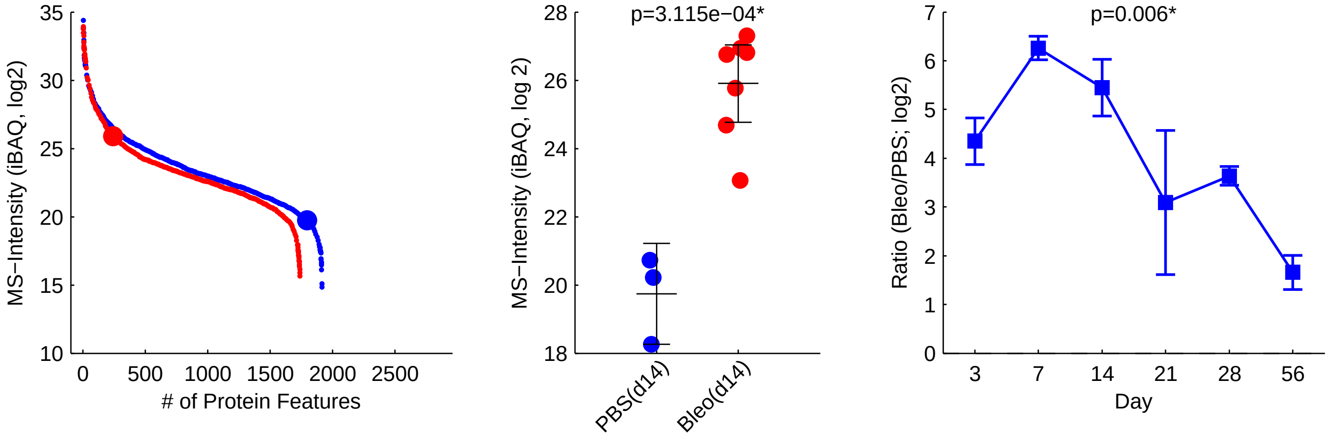

Q60817 − Naca (id: 1448)

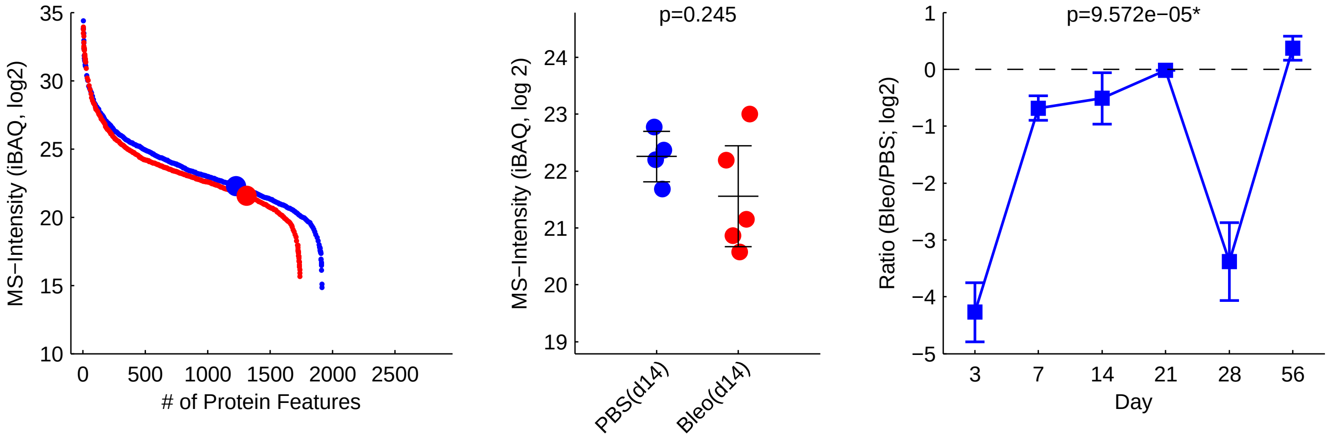

P70699 − Gaa (id: 1453)

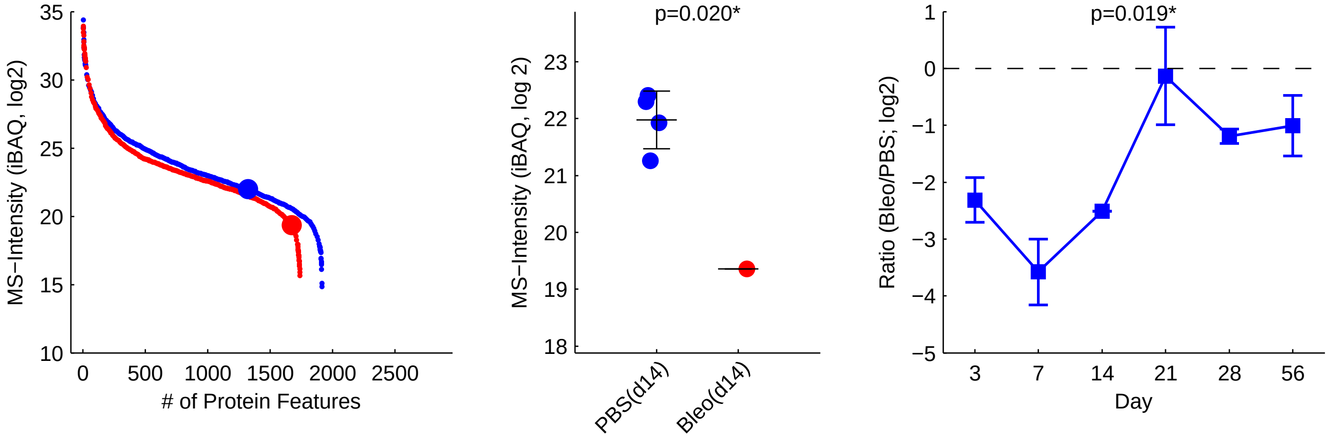

P80317 − Cct6a (id: 1458)

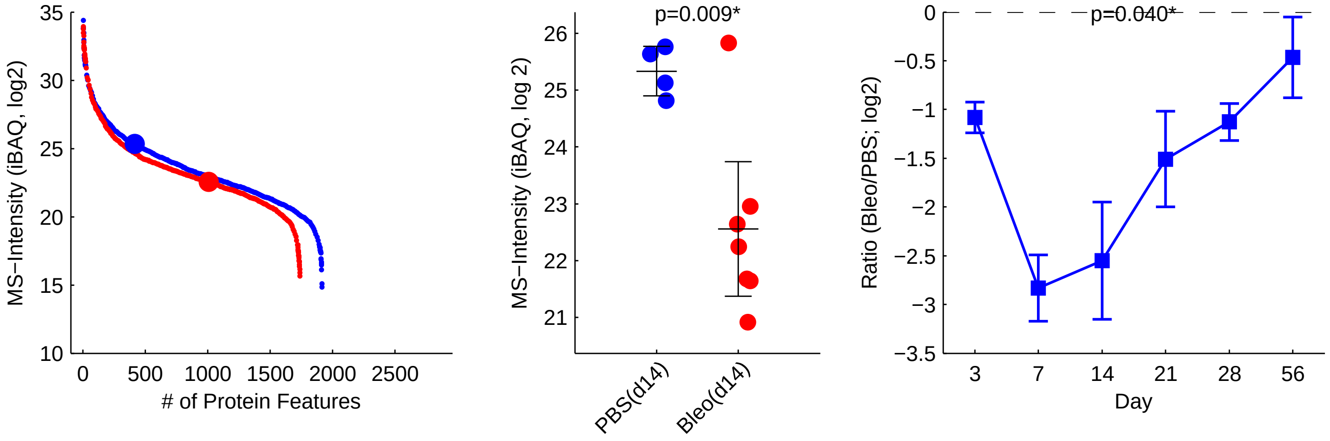

P84096 − Rhog (id: 1470)

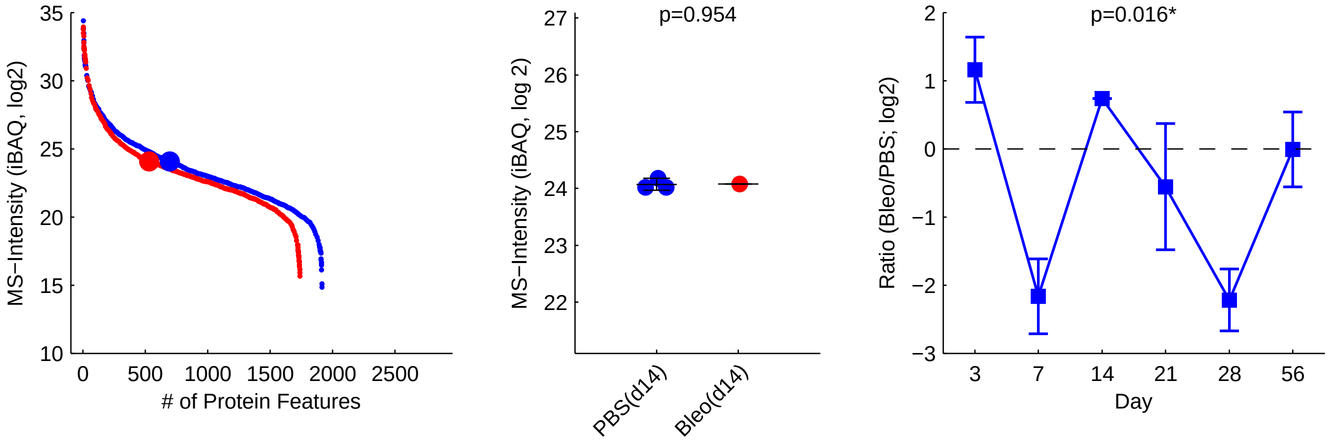

P84228 − Hist1h3b (id: 1472)

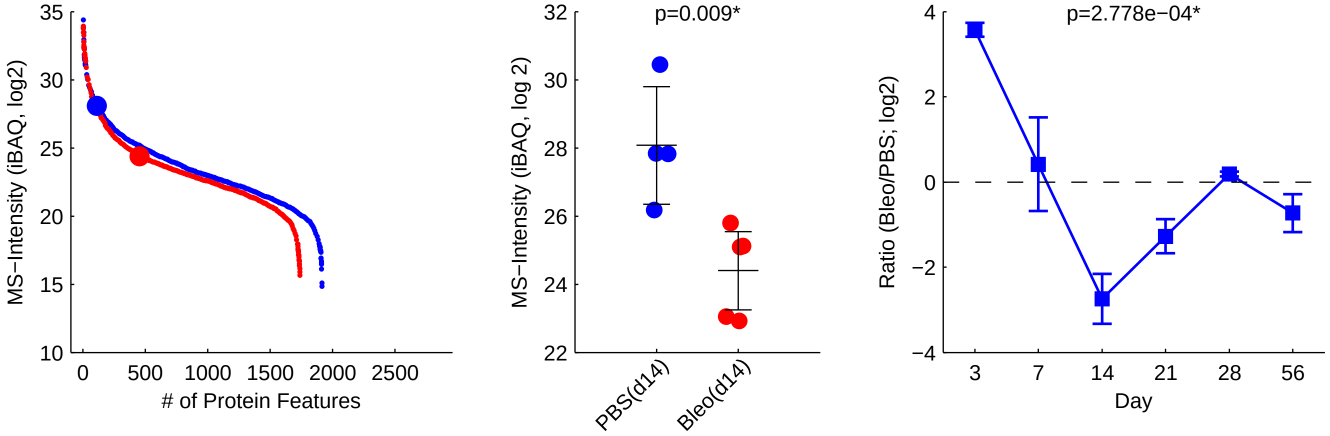

P97290 − Serping1 (id: 1473)

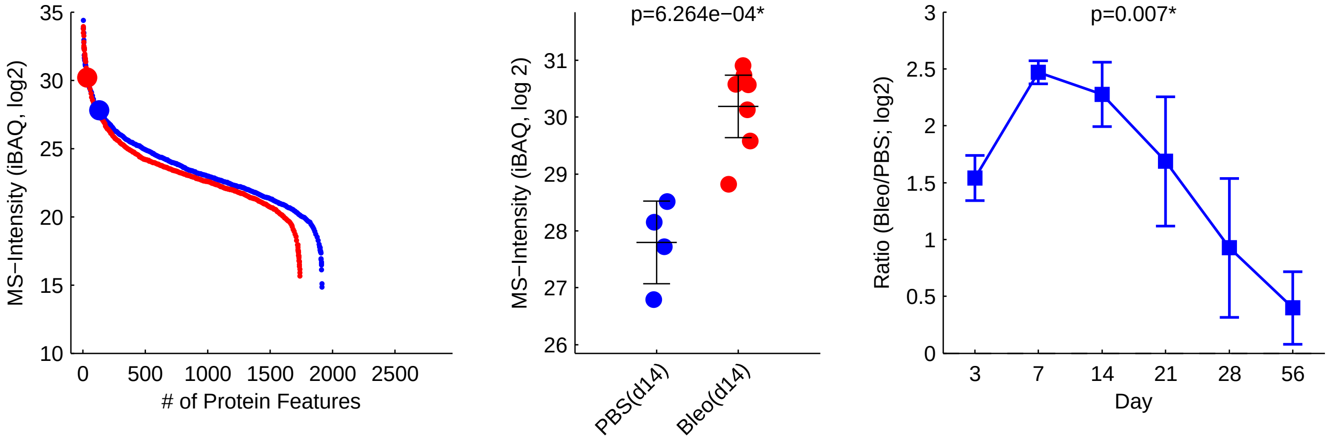

P97333 − Nrp1 (id: 1476)

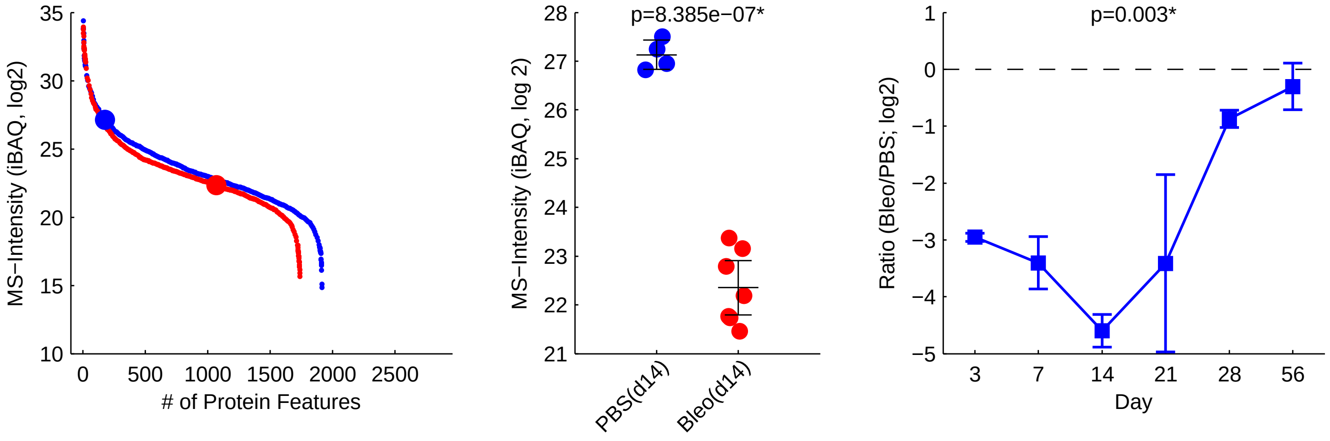

P97352 − S100a13 (id: 1477)

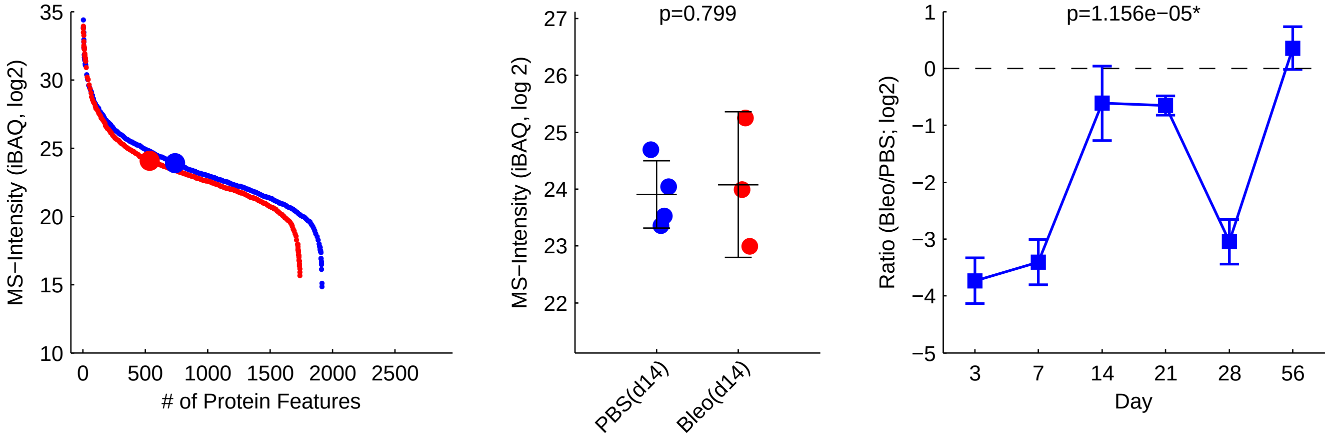

P97361 − Bpifa1 (id: 1479)

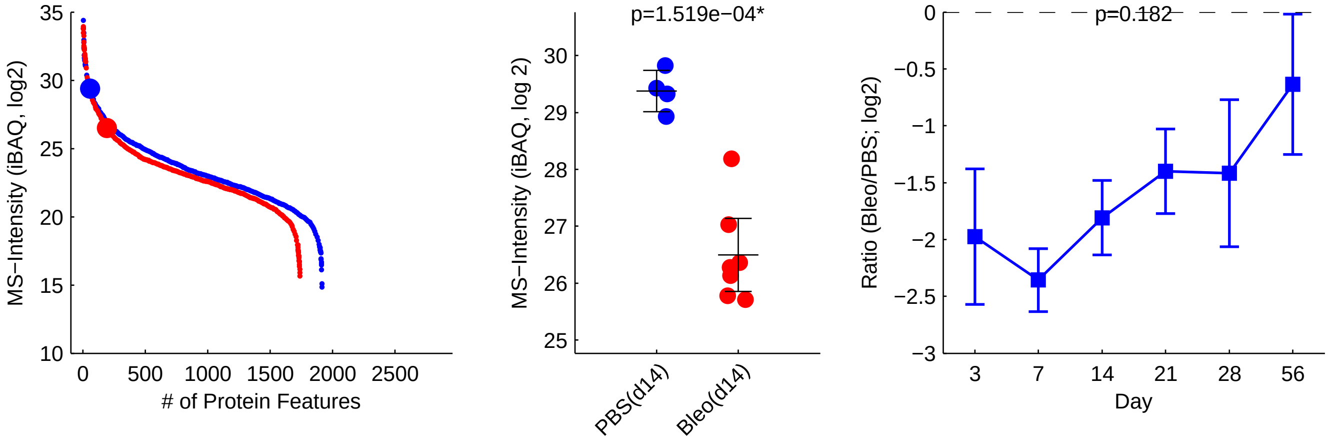

P97425 − Ear2 (id: 1484)

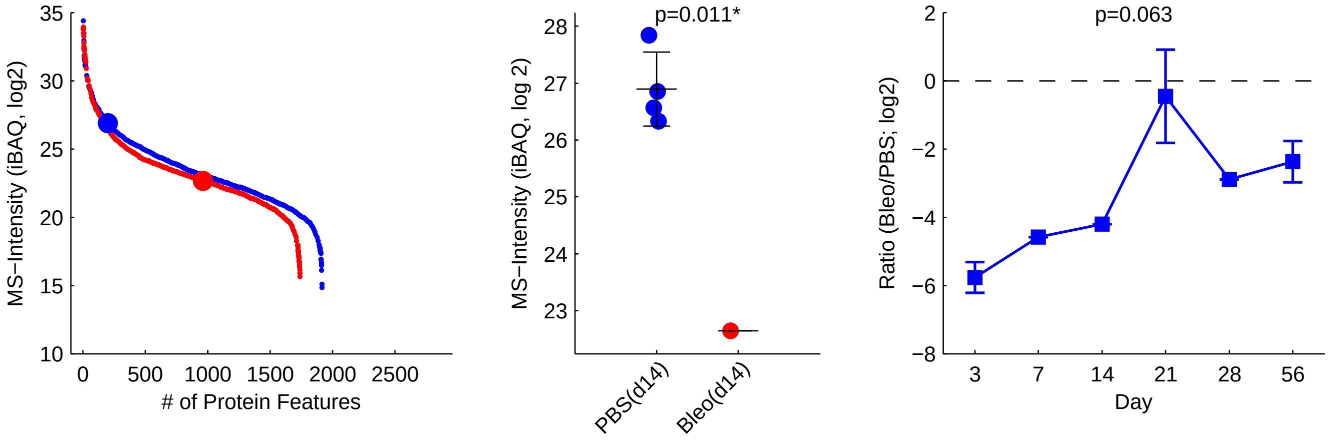

P97449 − Anpep (id: 1486)

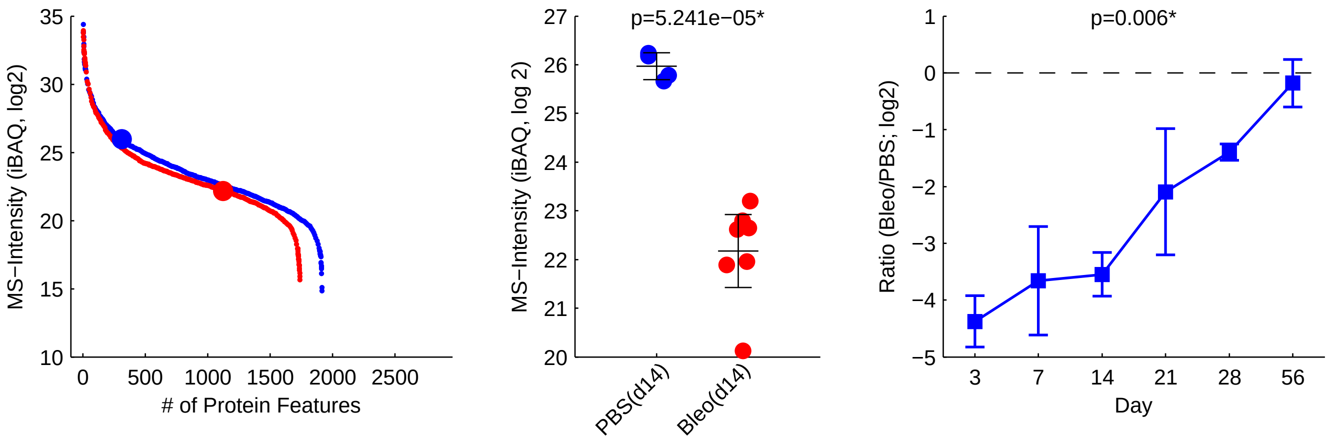

P97501 − Fmo3 (id: 1489)

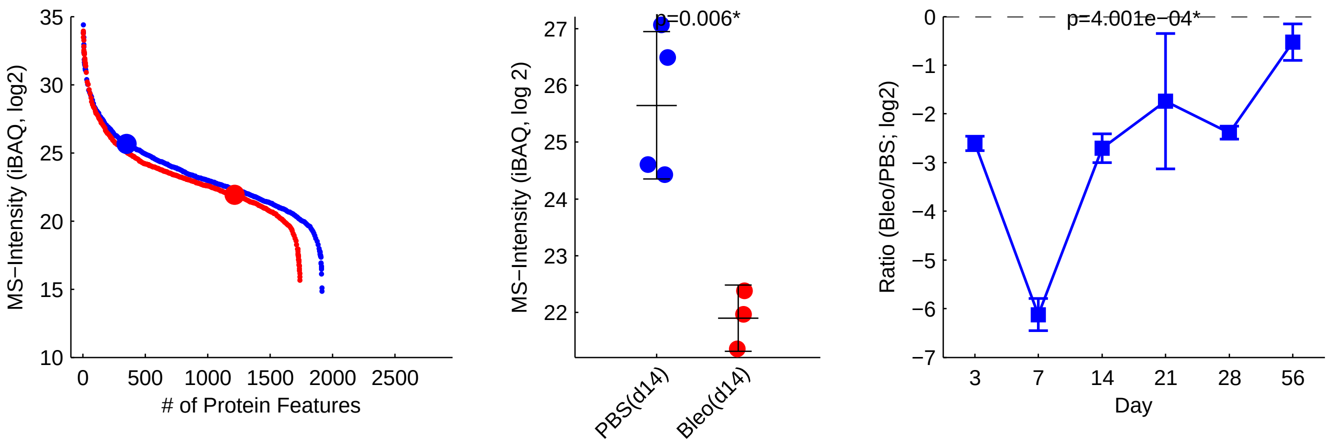

P97807−2 − Fh (id: 1492)

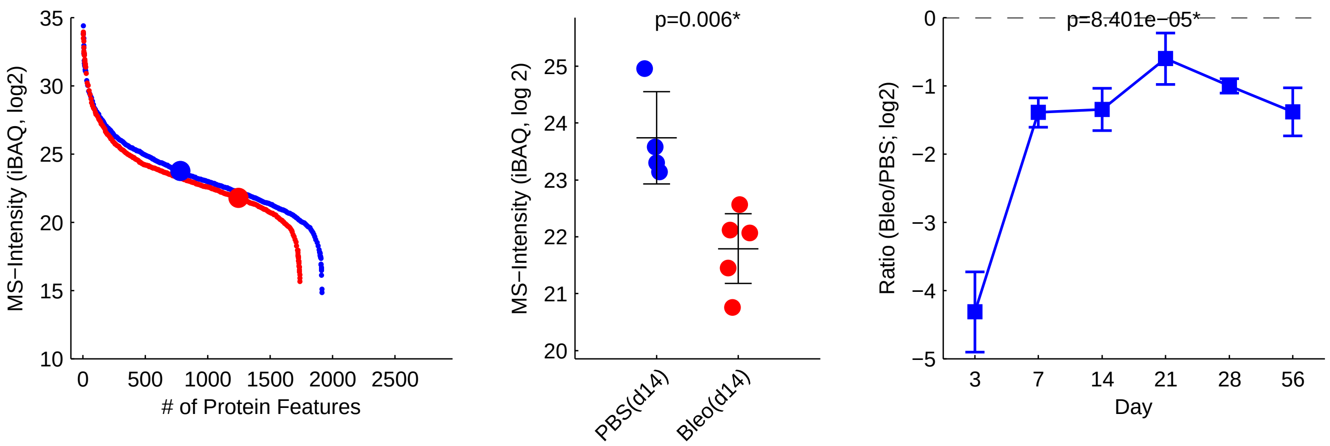

P97821 − Ctsc (id: 1494)

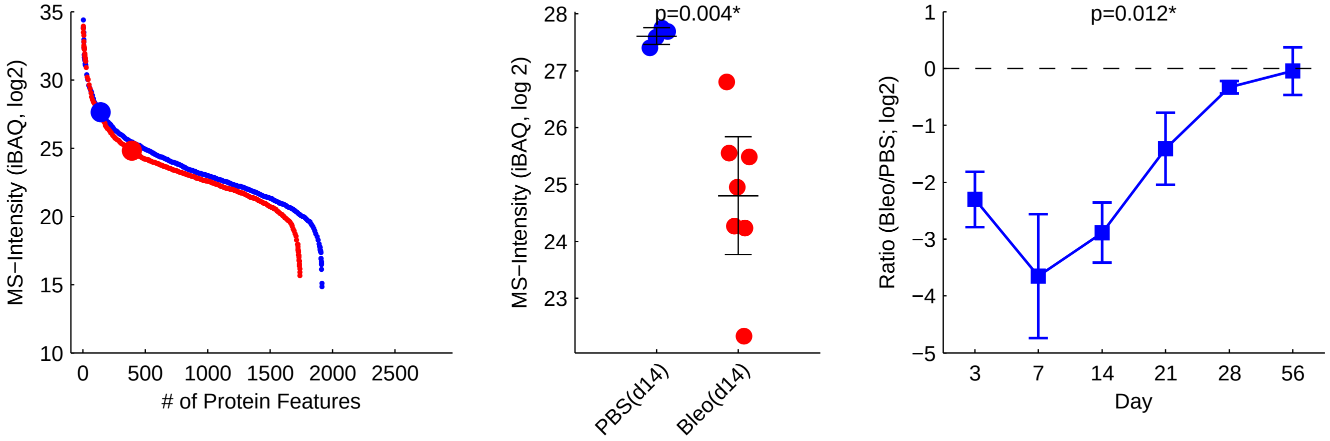

P98086 − C1qa (id: 1500)

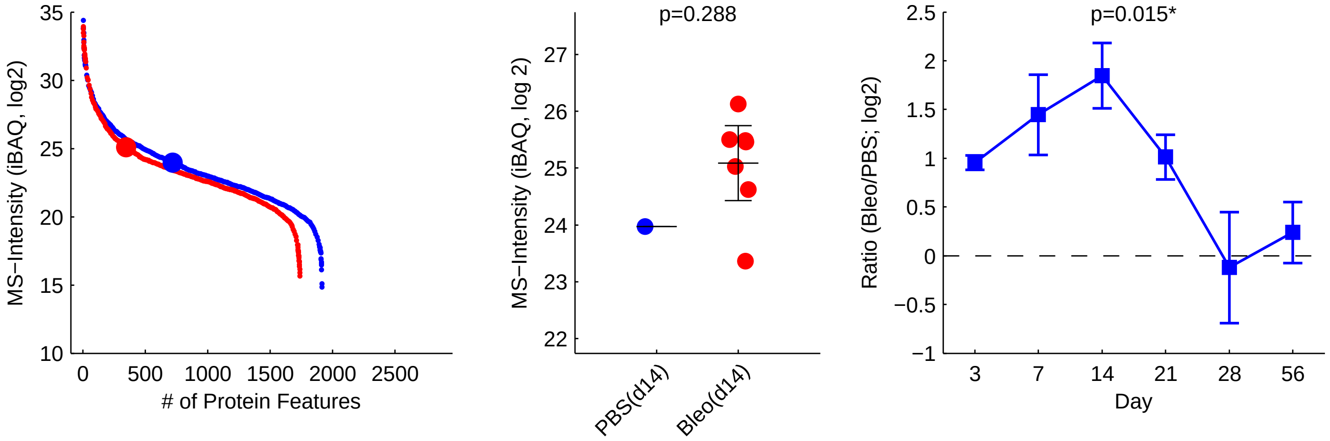

P99029−2 − Prdx5 (id: 1504)

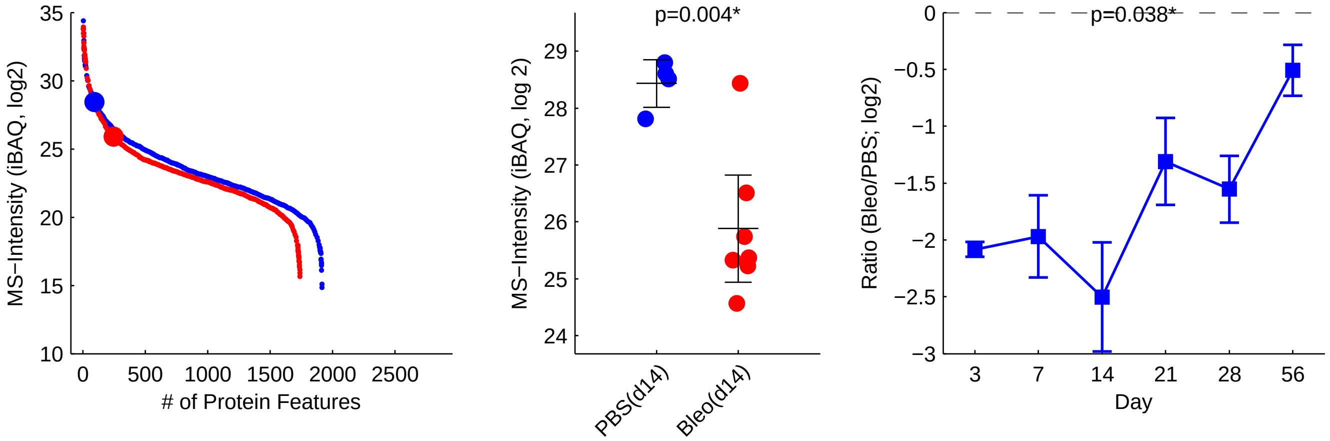

Q00519 − Xdh (id: 1505)

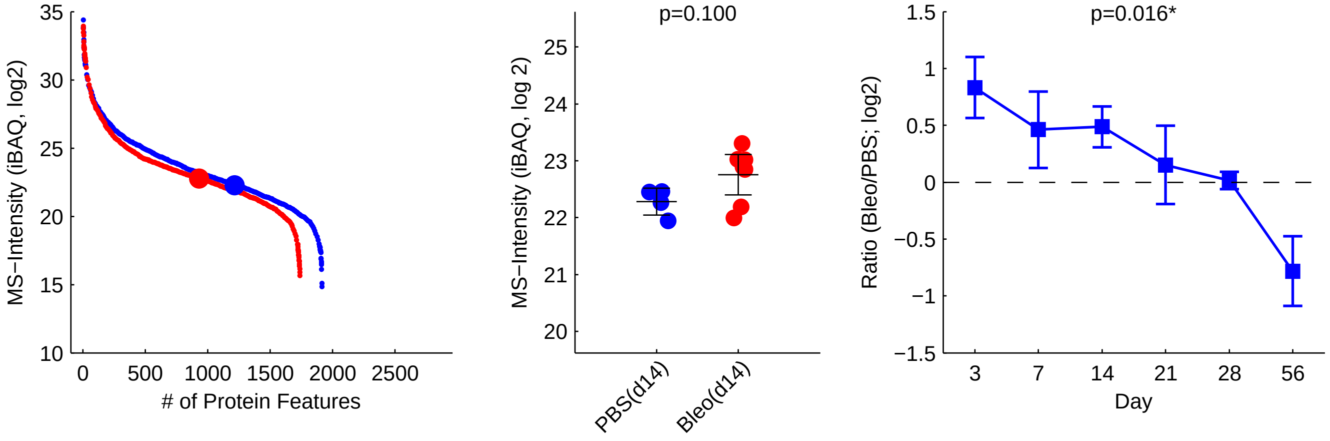

Q00560 − Il6st (id: 1506)

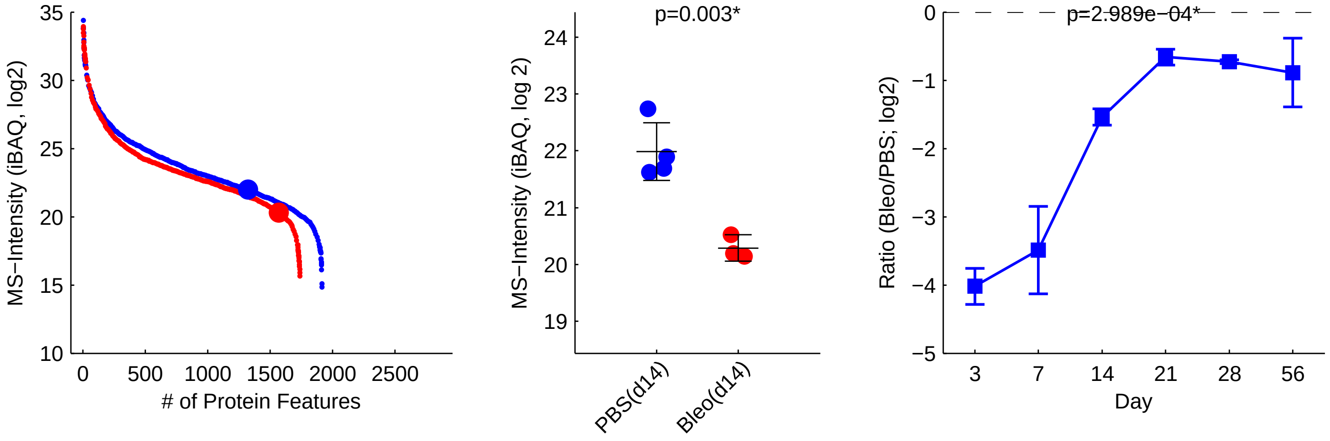

Q00623 − Apoa1 (id: 1508)

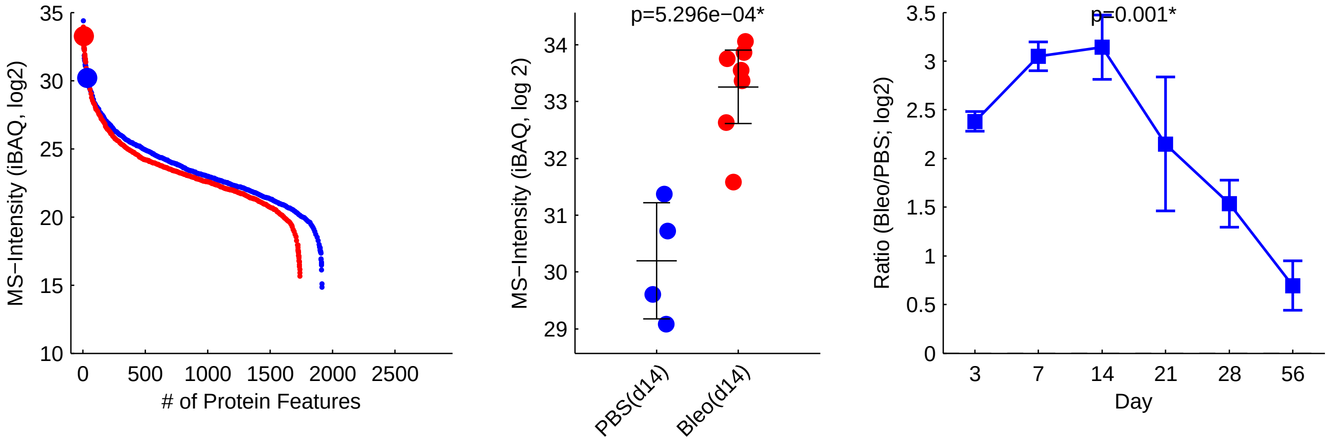

Q00896 − Serpina1c (id: 1509)

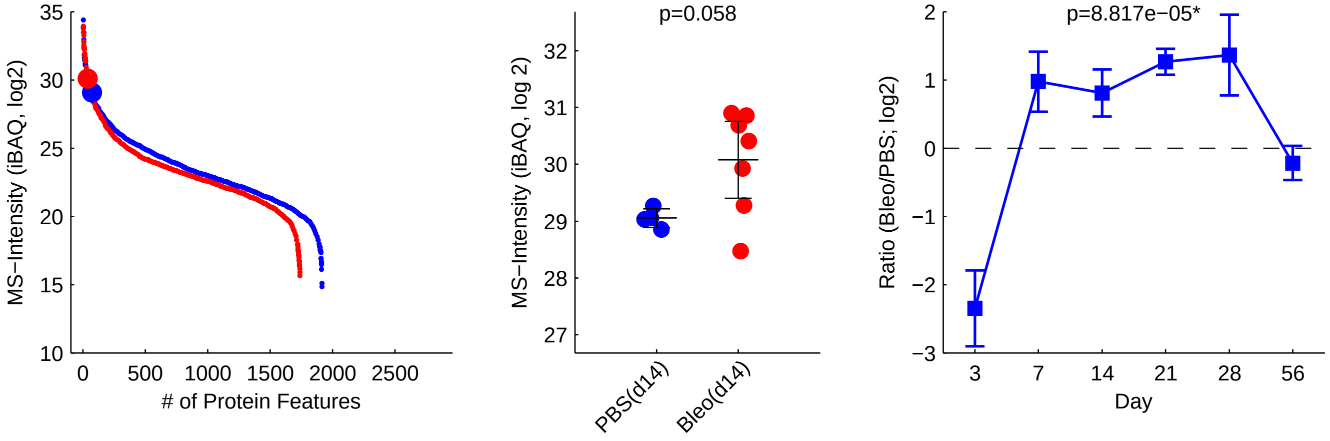

Q00898 − Serpina1e (id: 1511)

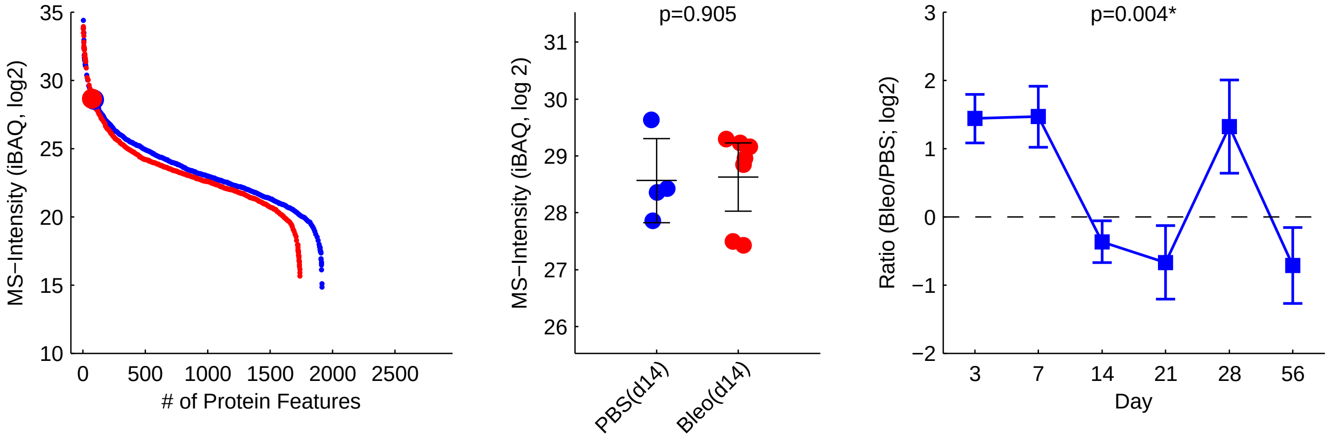

Q00915 − Rbp1 (id: 1512)

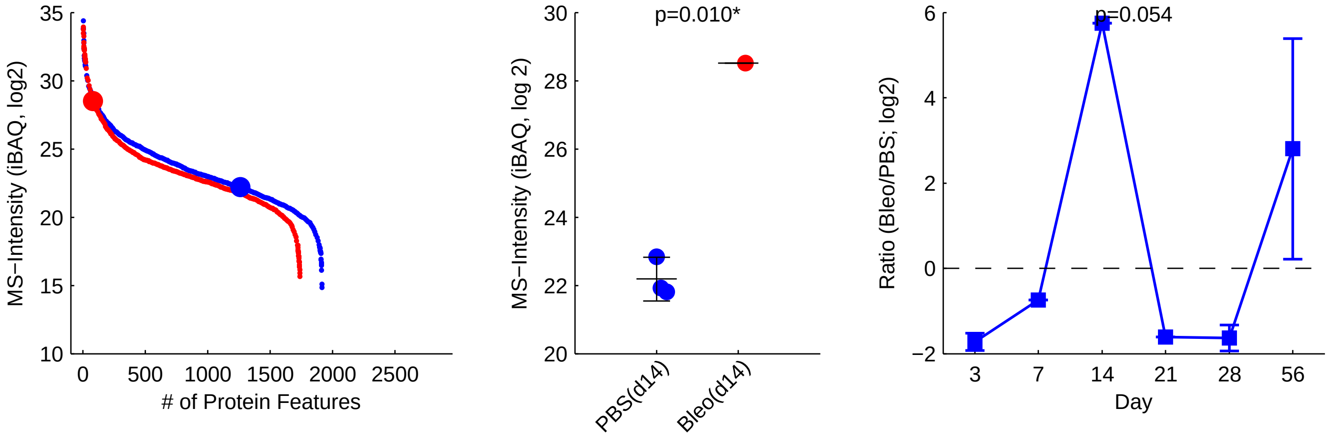

Q01149 − Col1a2 (id: 1515)

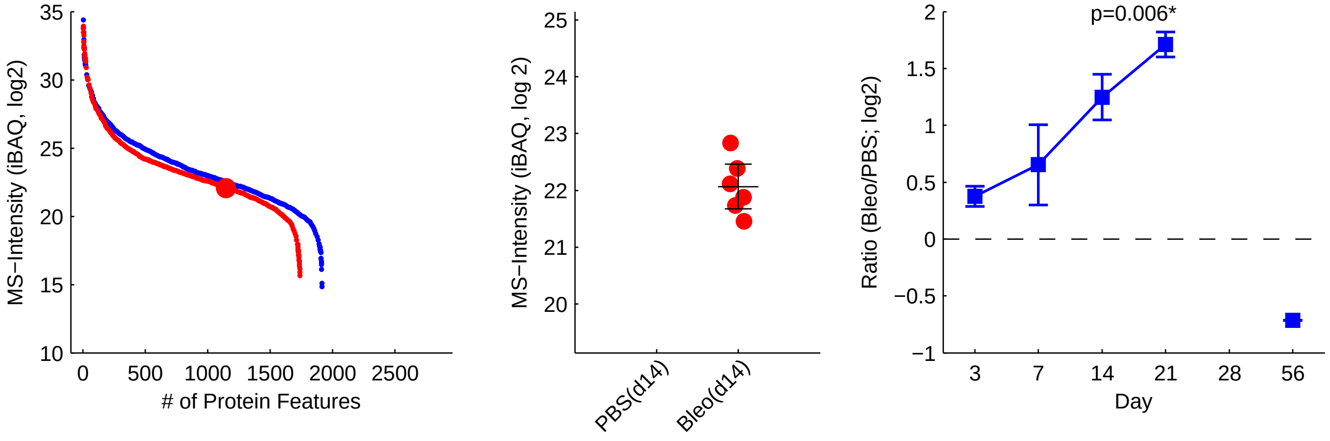

Q9WVF5 − Egfr (id: 1516)

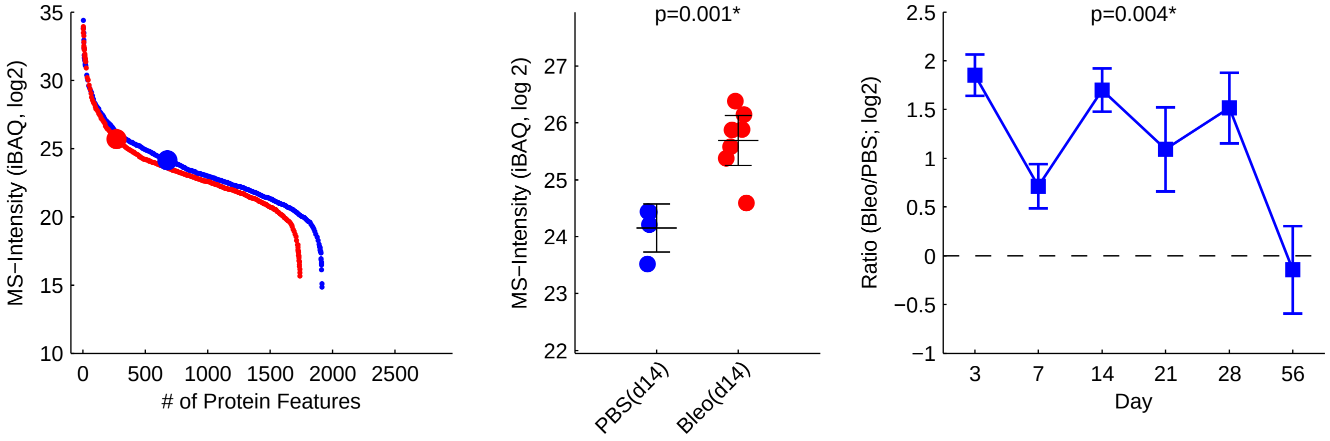

Q01339 − Apoh (id: 1517)

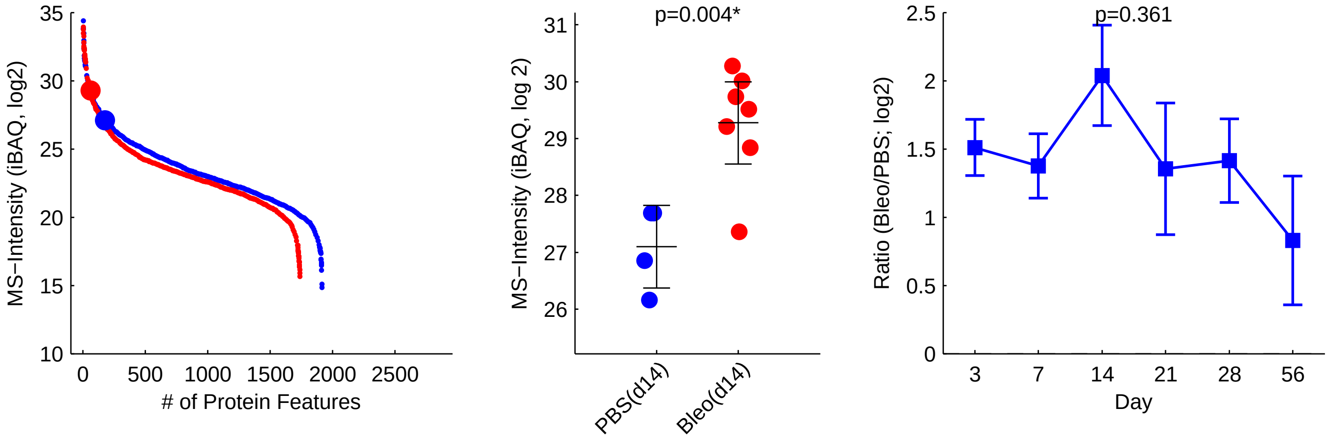

Q01853 − Vcp (id: 1518)

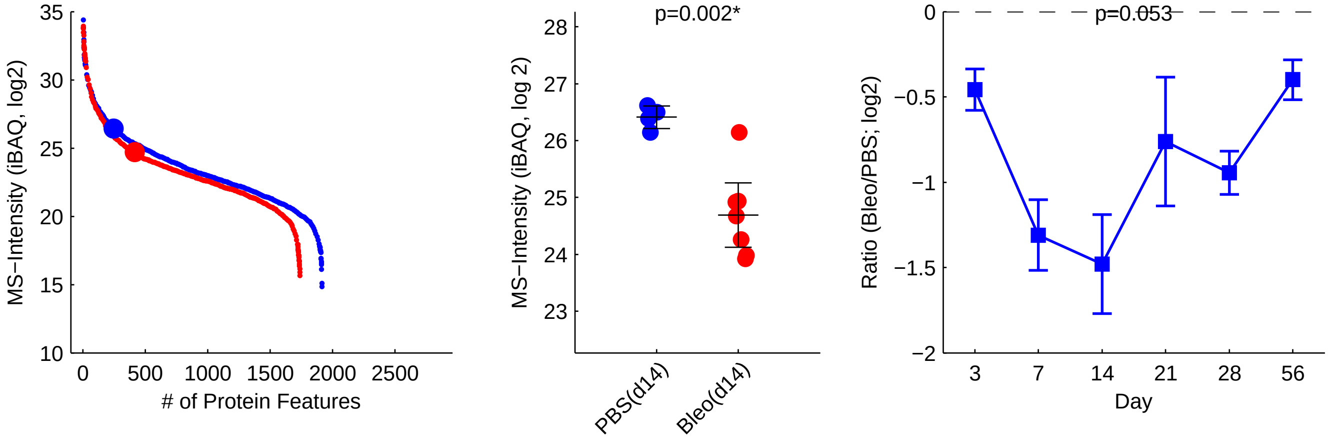

Q02105 − C1qc (id: 1520)

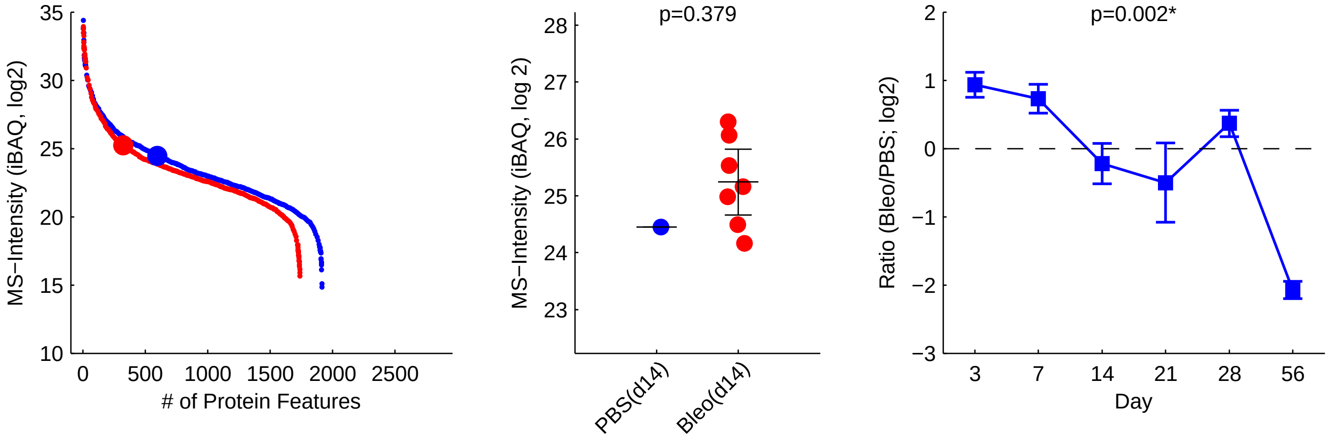

Q02788 − Col6a2 (id: 1525)

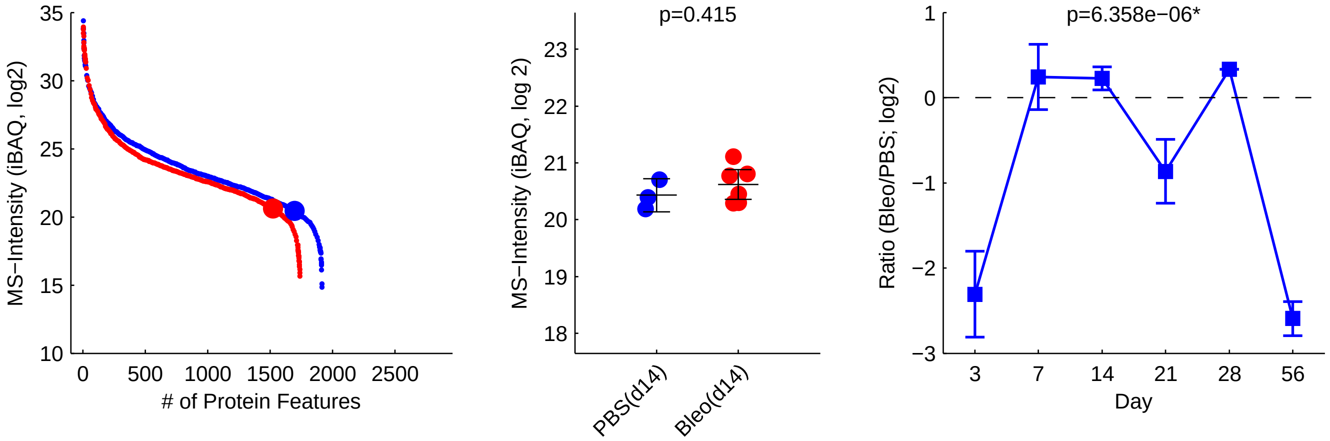

Q03265 − Atp5a1 (id: 1528)

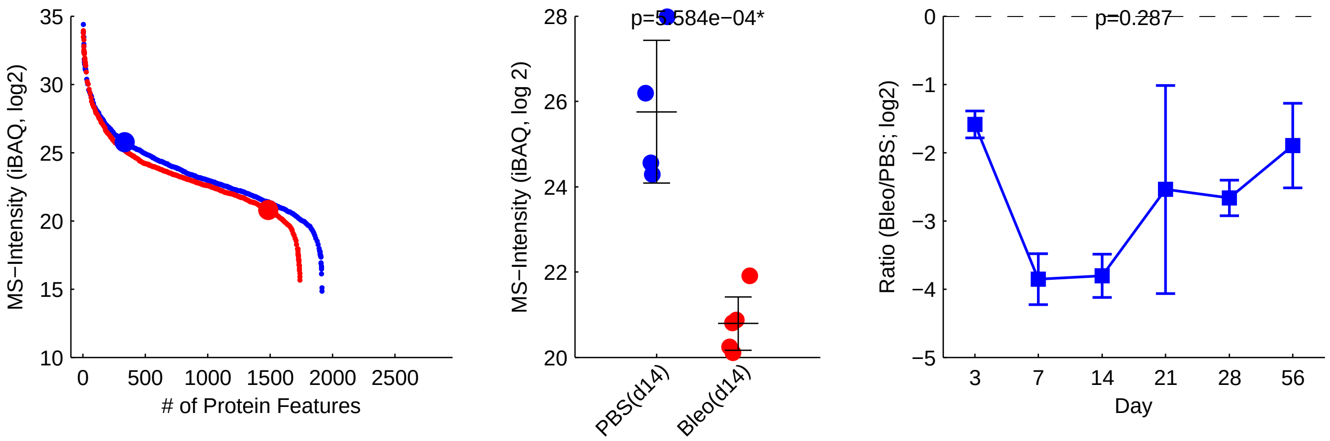

Q05816 − Fabp5 (id: 1539)

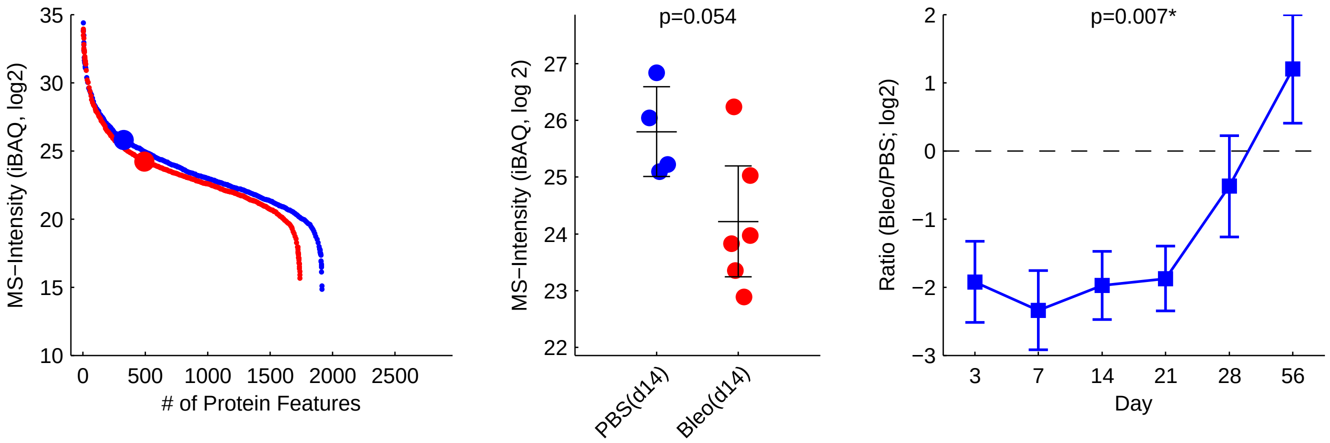

Q06318 − Scgb1a1 (id: 1543)

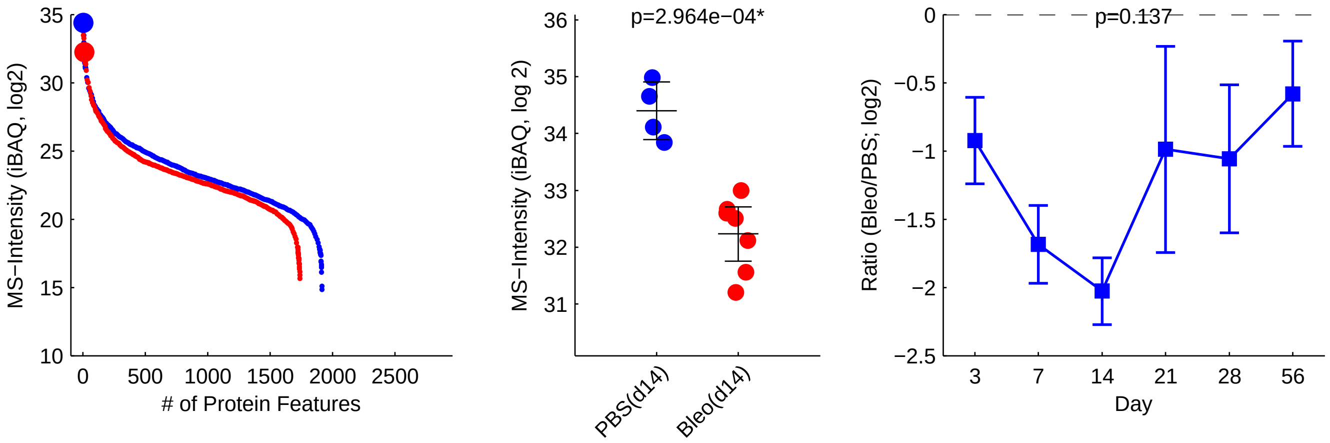

Q06335−2 − Aplp2 (id: 1544)

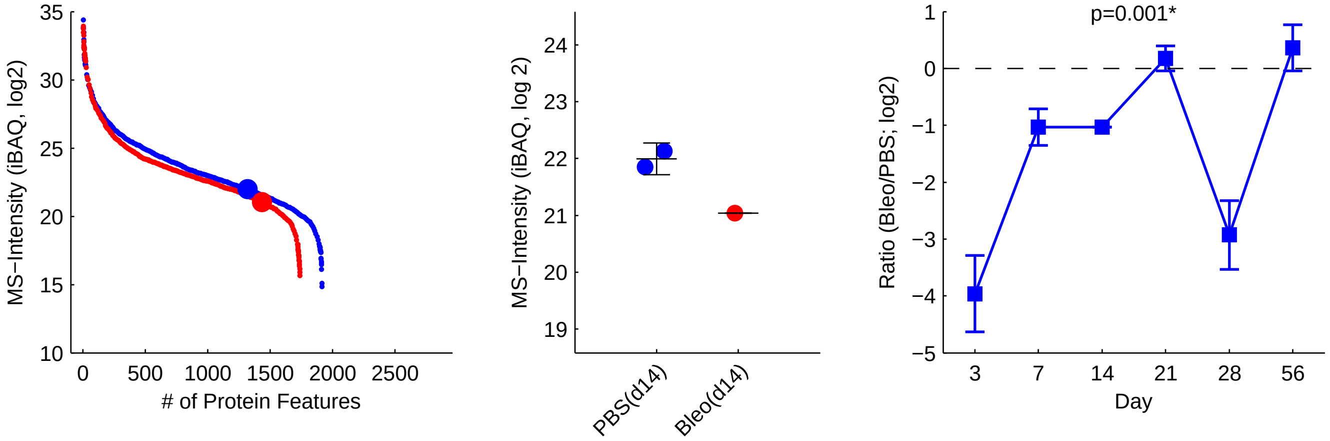

Q06890 − Clu (id: 1547)

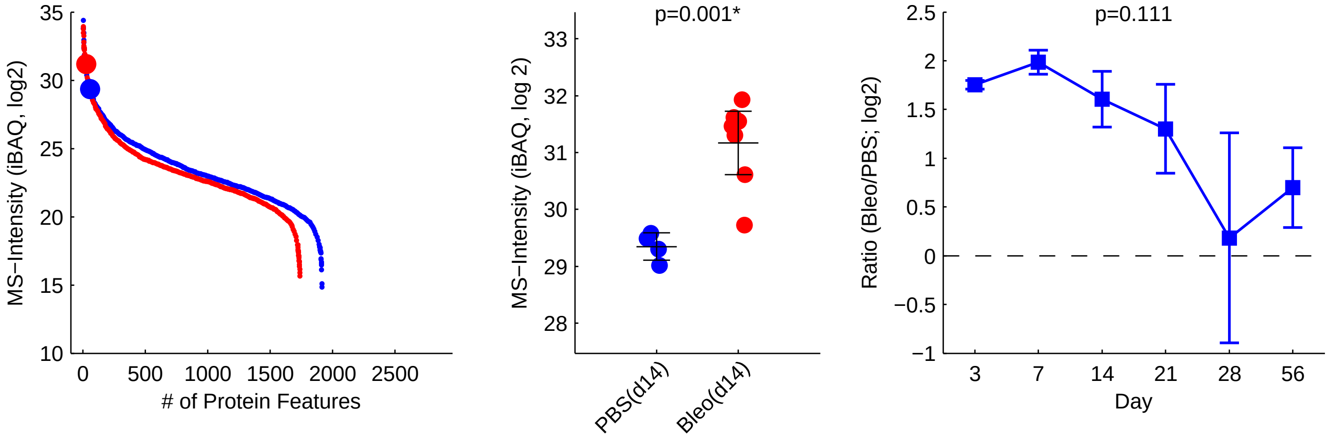

Q07113 − Igf2r (id: 1550)

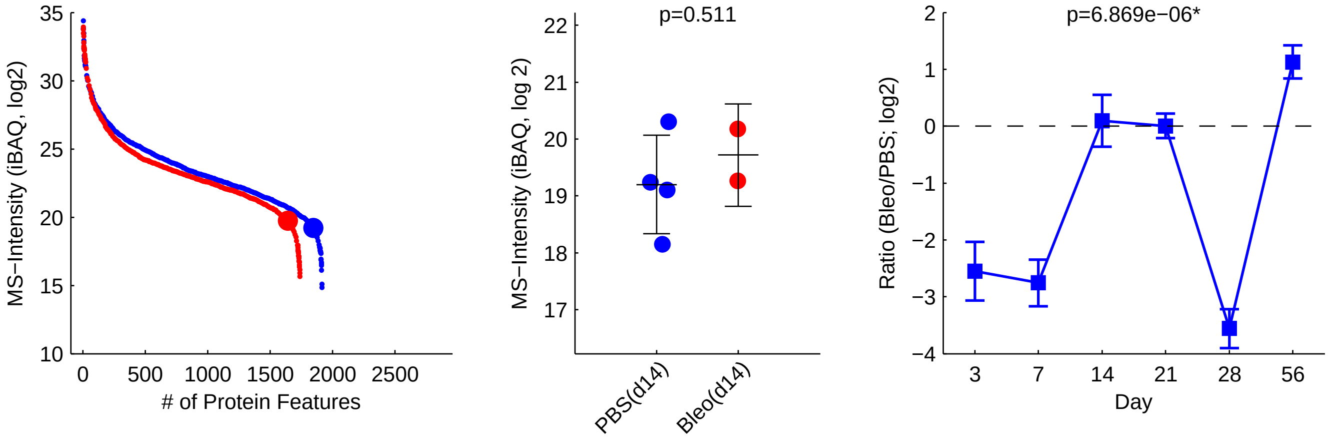

Q07456 − Ambp (id: 1553)

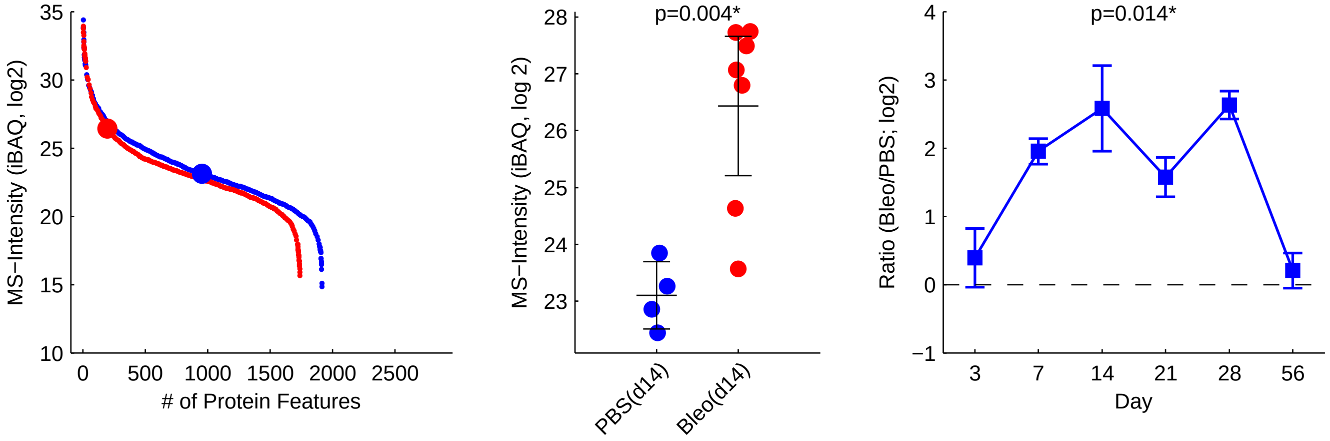

Q07797 − Lgals3bp (id: 1554)

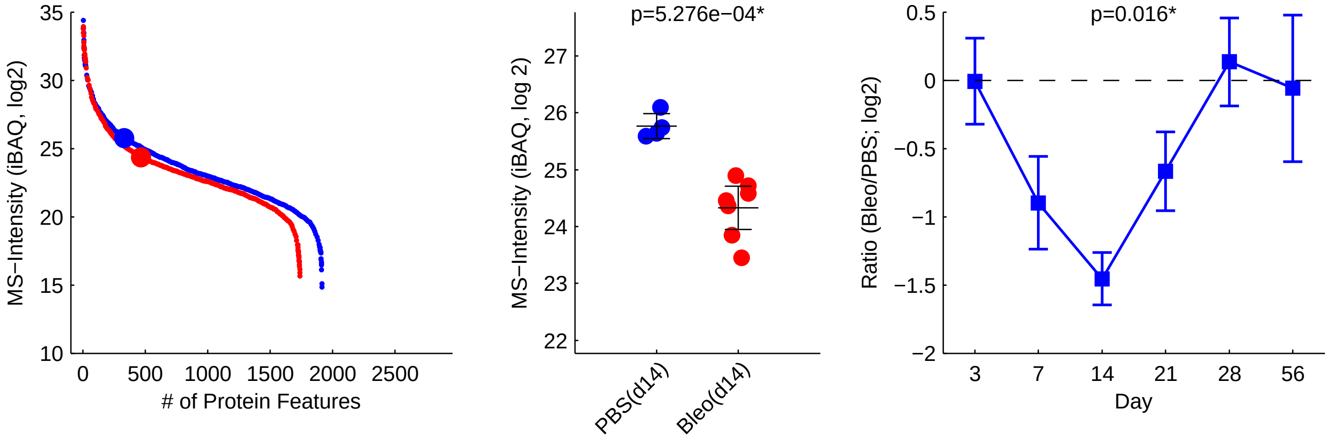

Q08857 − Cd36 (id: 1559)

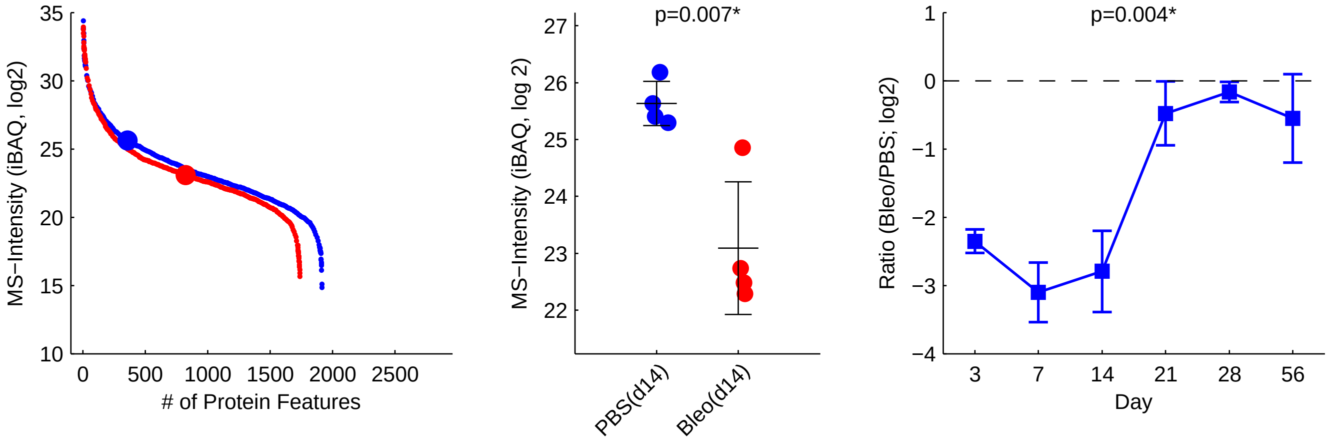

Q08879 − Fbln1 (id: 1560)

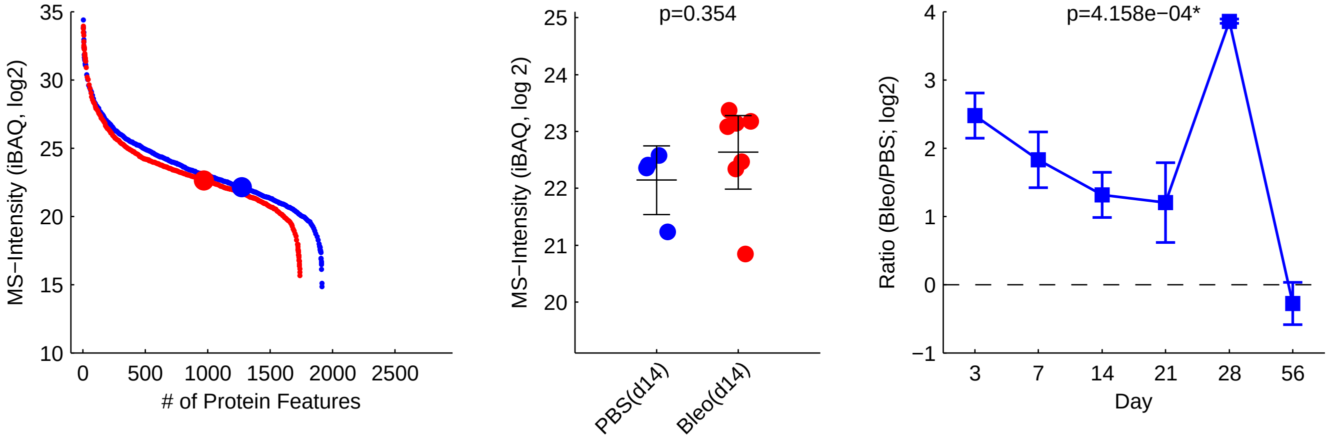

Q99020 − Hnrnpab (id: 1568)

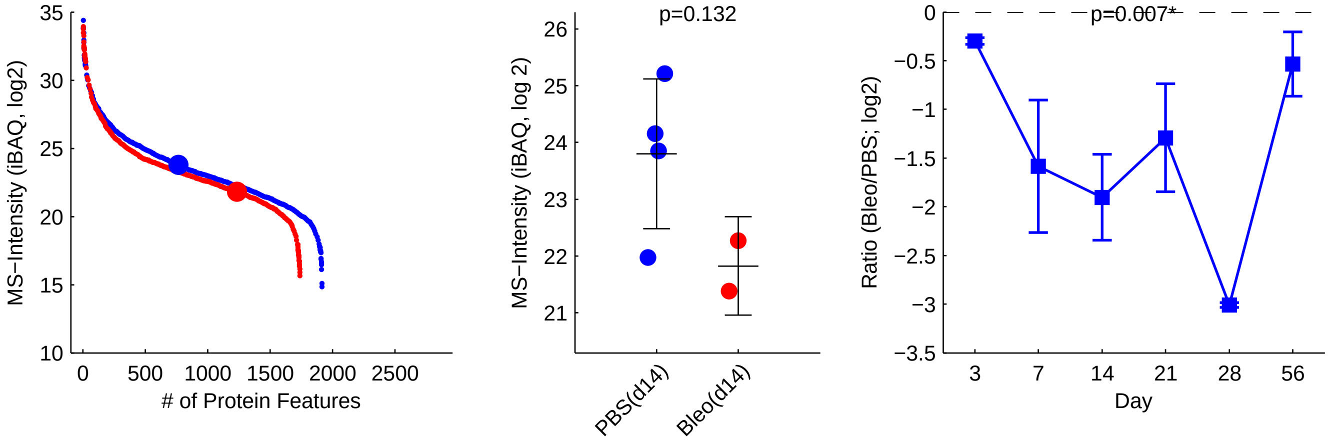

Q3B7Z2 − Osbp (id: 1571)

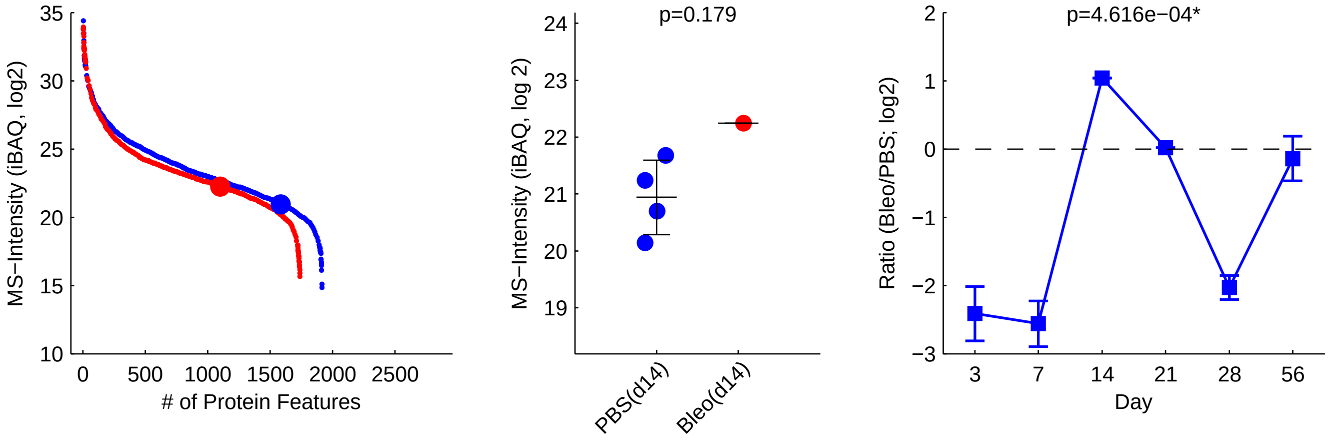

Q3THS6 − Mat2a (id: 1580)

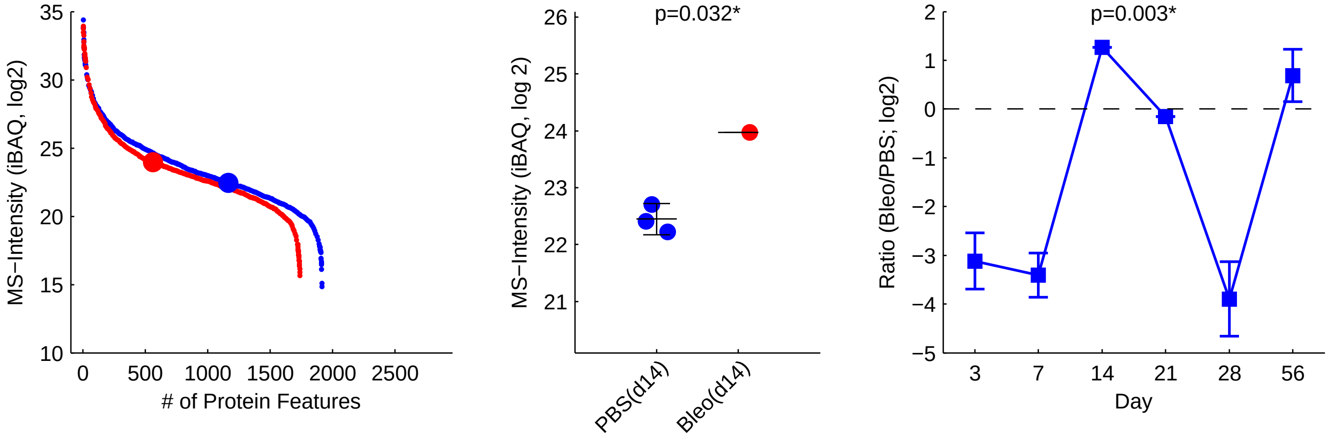

Q3TML0 − Pdia6 (id: 1584)

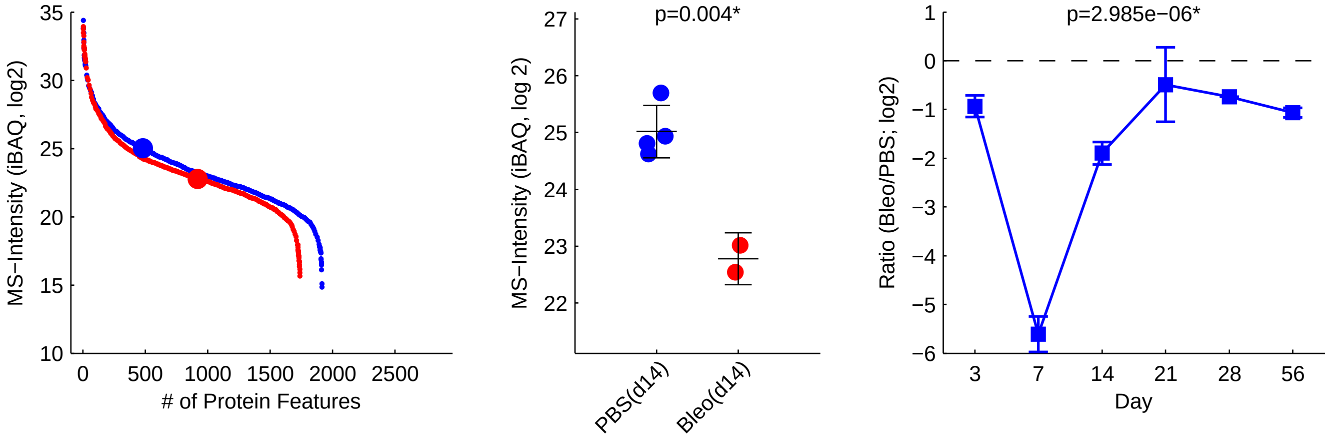

Q80ZP8 − Manf (id: 1585)

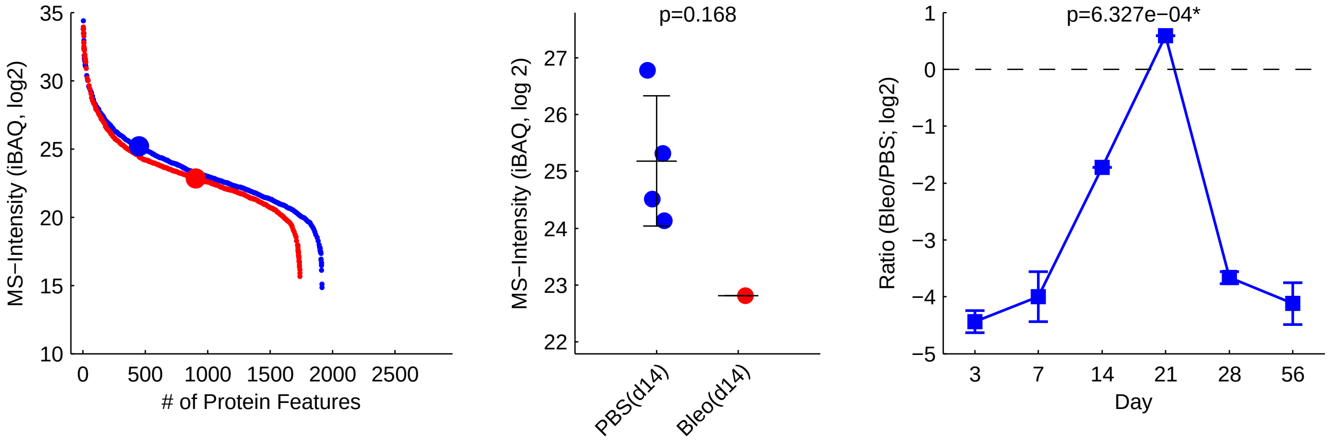

Q9WUZ9 − Entpd5 (id: 1587)

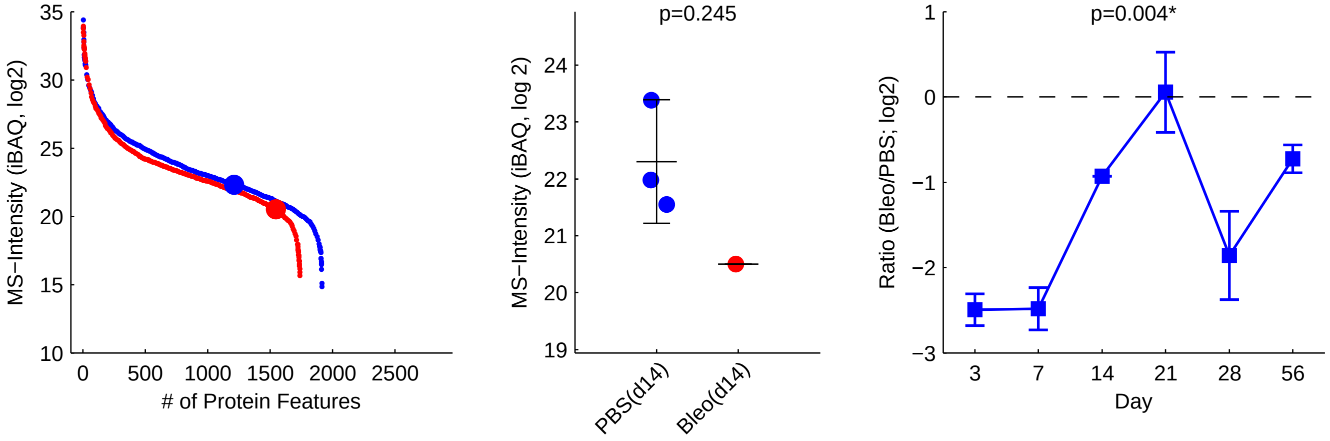

Q3TUY3 − Sbpl (id: 1589)

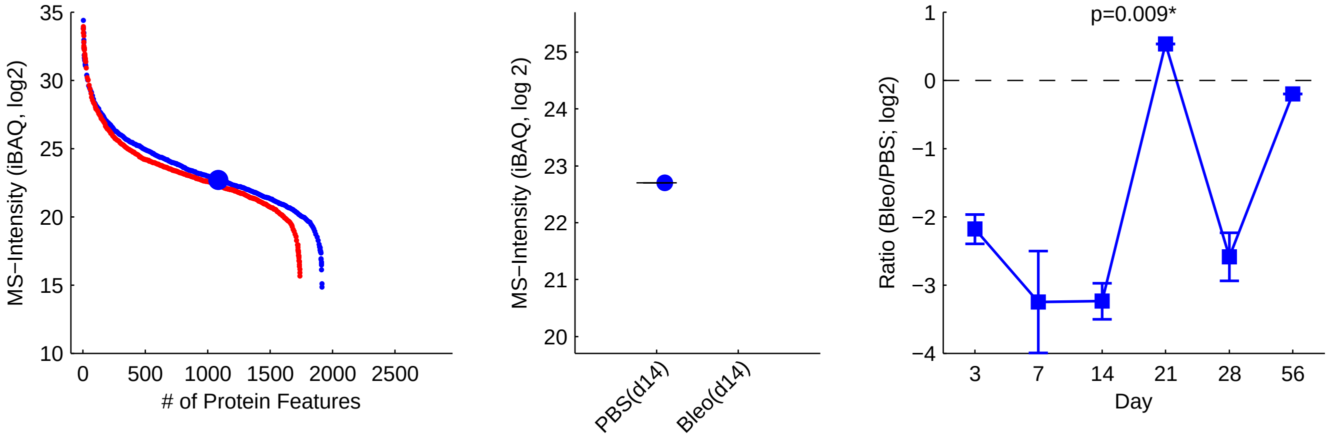

Q3TXS7 − Psmd1 (id: 1591)

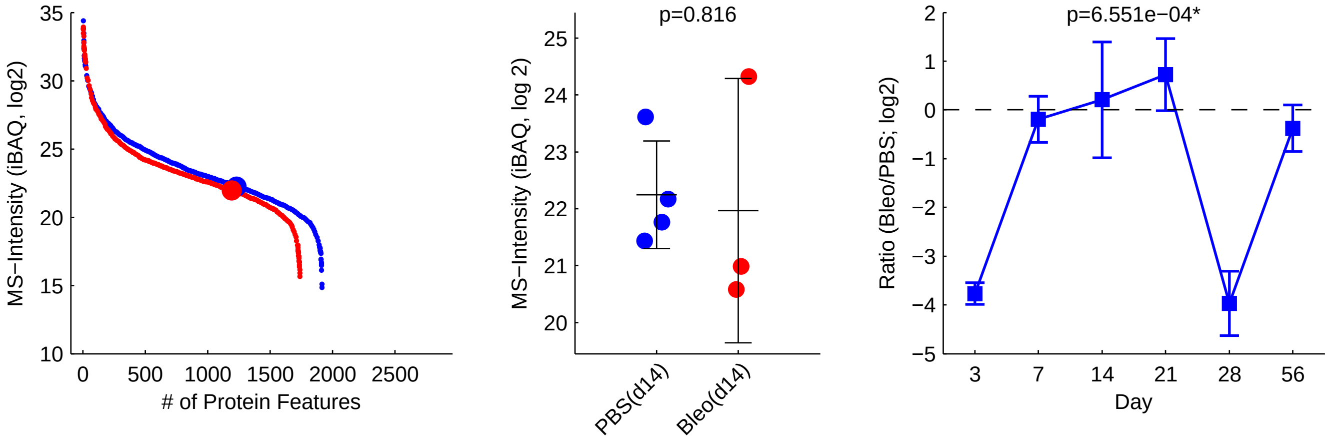

Q9JLJ2 − Aldh9a1 (id: 1600)

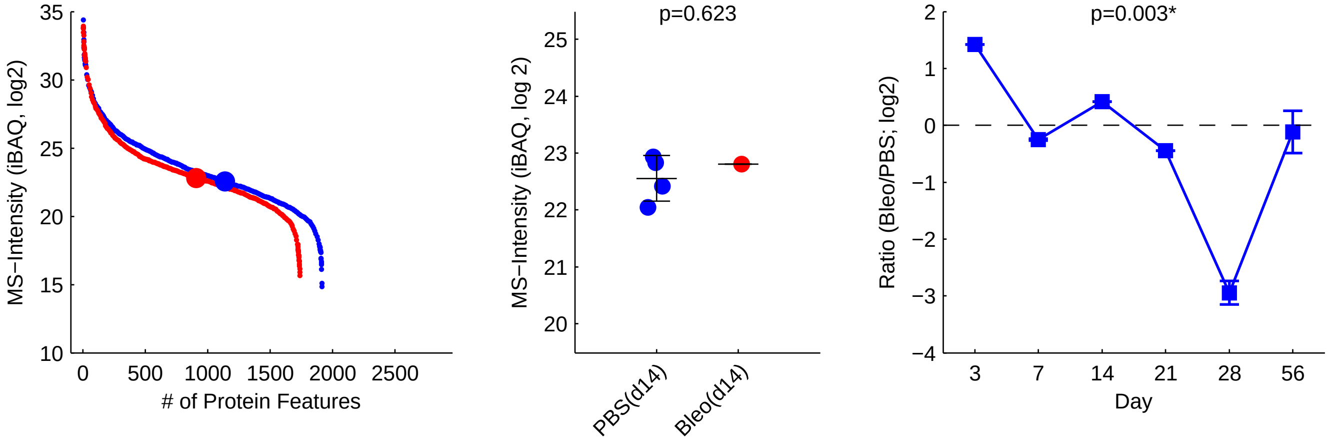

Q3U6K9 − Psat1 (id: 1604)

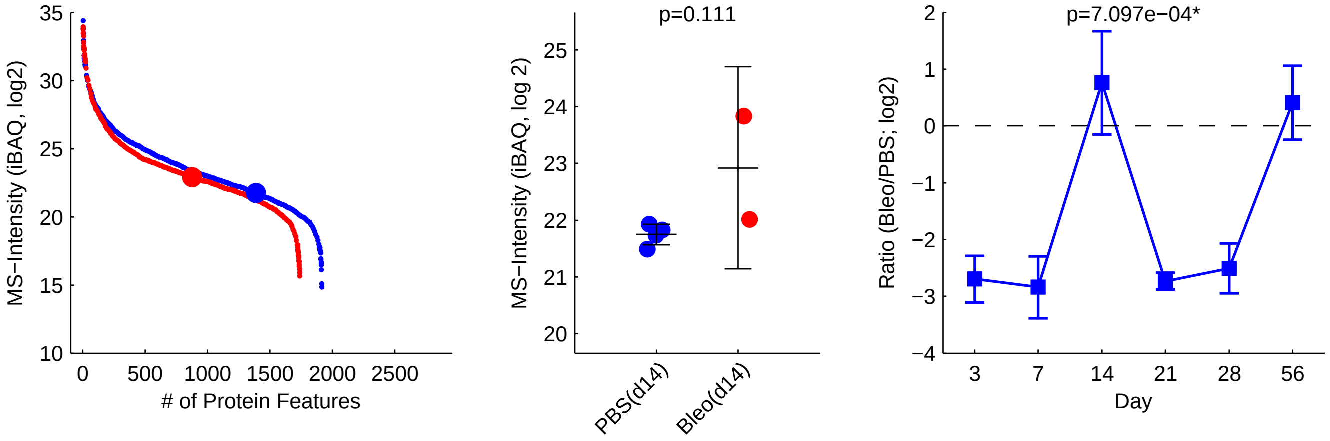

Q921Y0 − Mob1a (id: 1608)

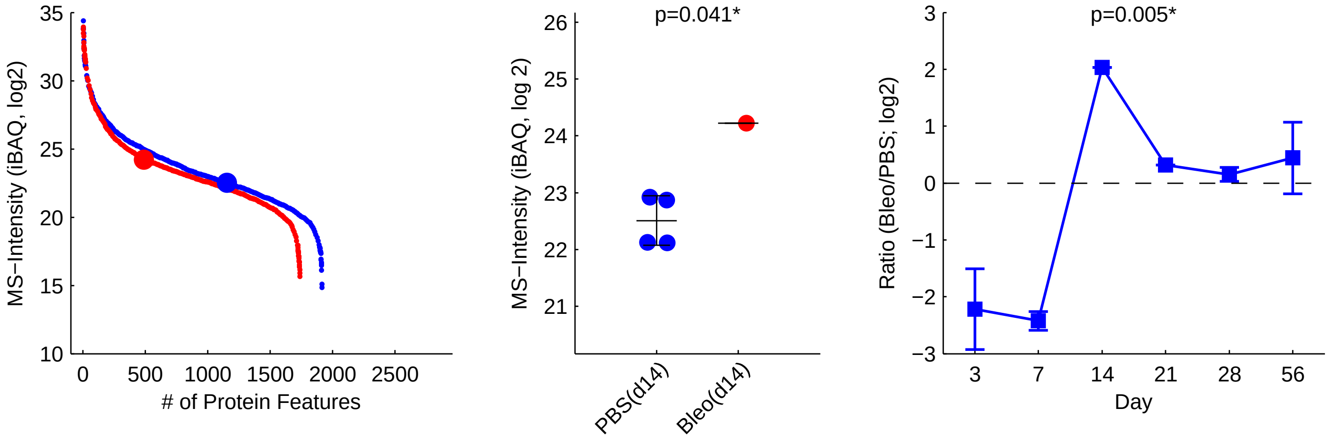

Q3UGR5 − Hdhd2 (id: 1615)

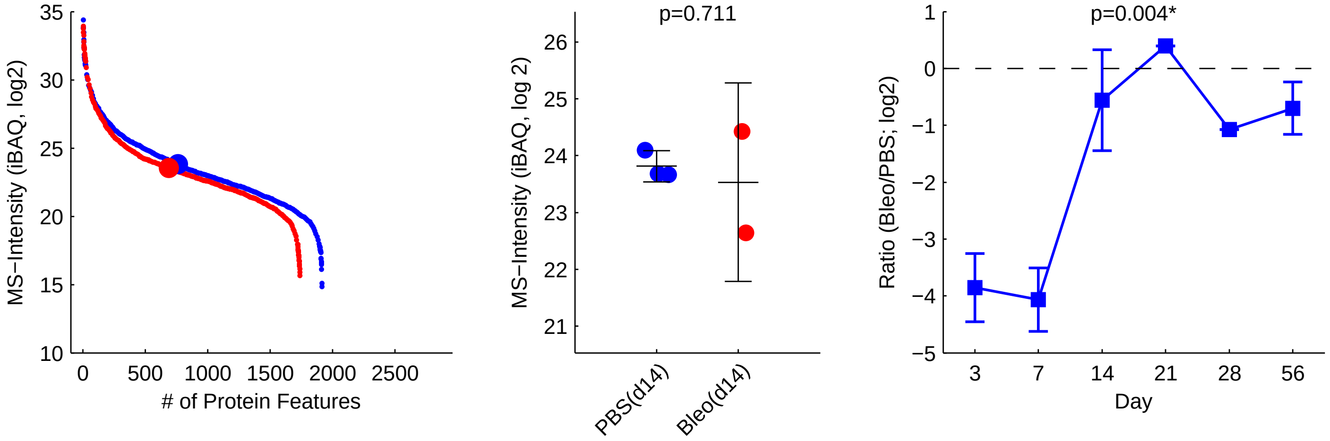

Q3UPL0 − Sec31a (id: 1628)

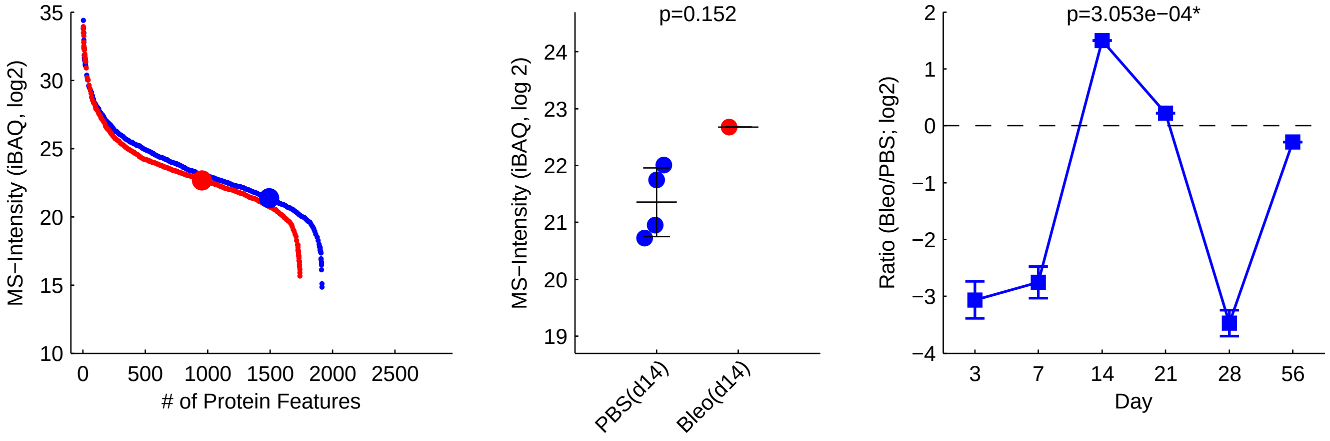

Q3UQ05 − Bpifb5 (id: 1629)

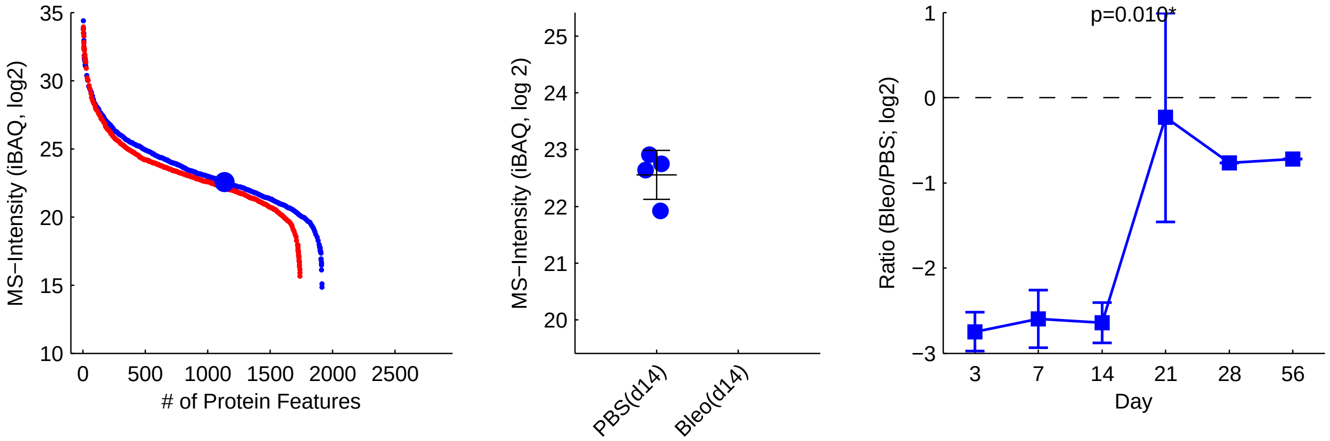

Q3UU35 − Ovos (id: 1635)

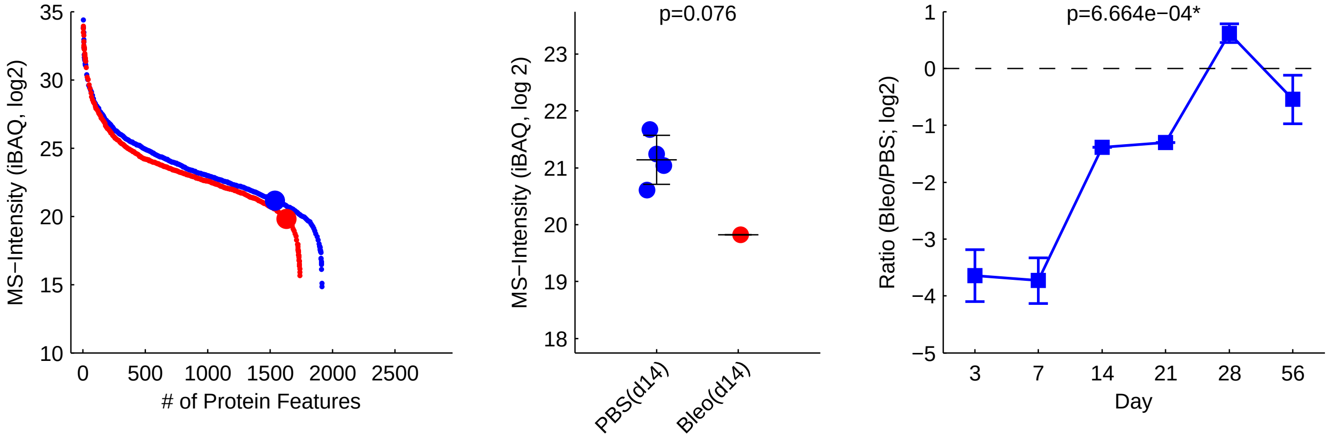

Q3UW53 − Fam129a (id: 1638)

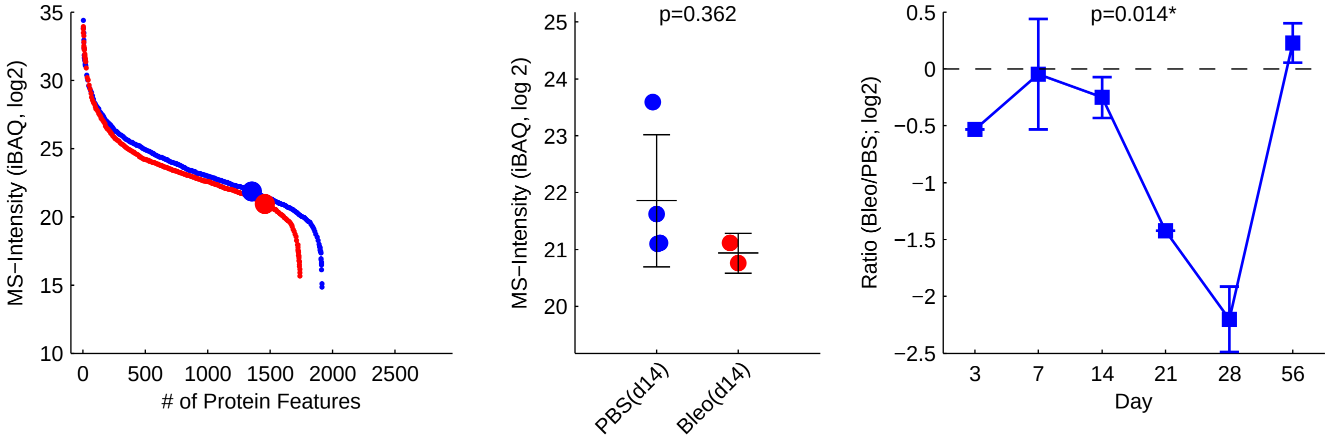

Q3UZZ6 − Sult1d1 (id: 1642)

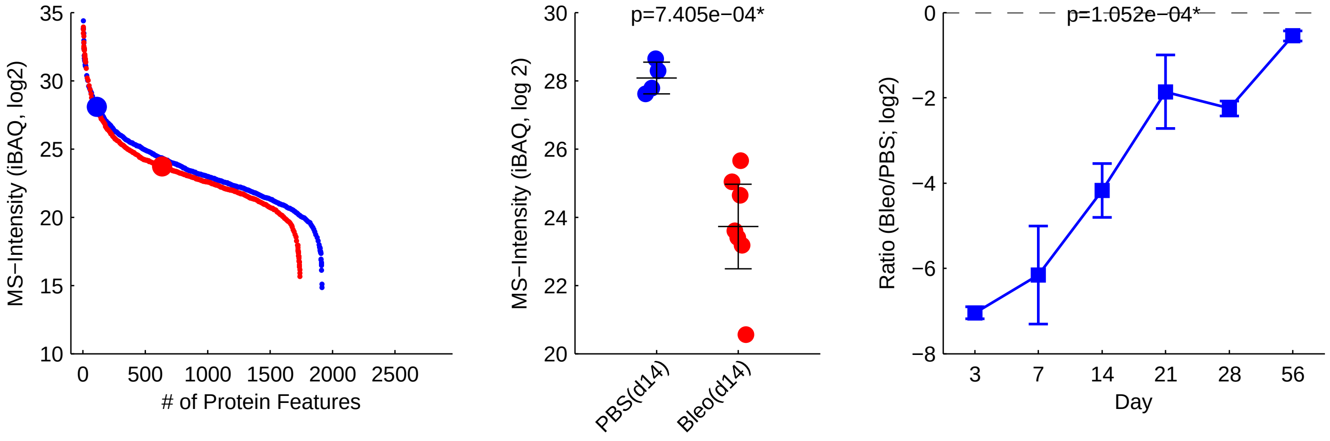

Q91V92 − Acly (id: 1644)

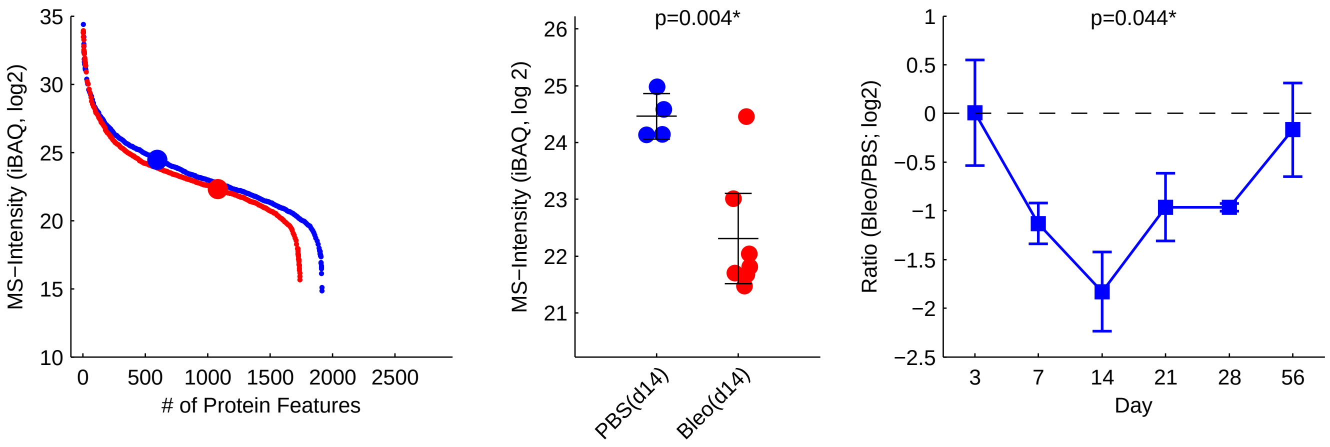

Q5RKN9 − Capza1 (id: 1665)

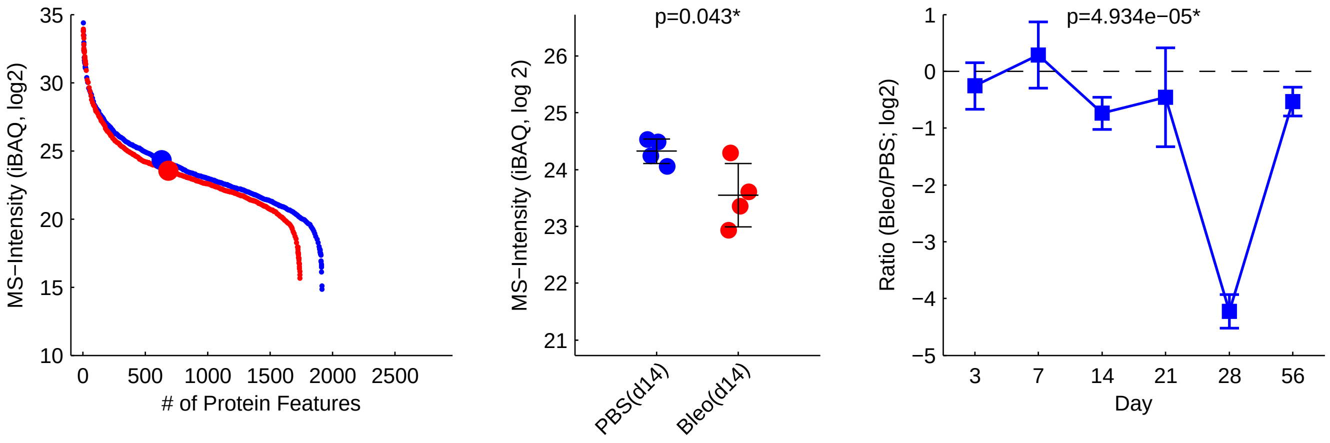

Q5SQ27 − Sec14l3 (id: 1666)

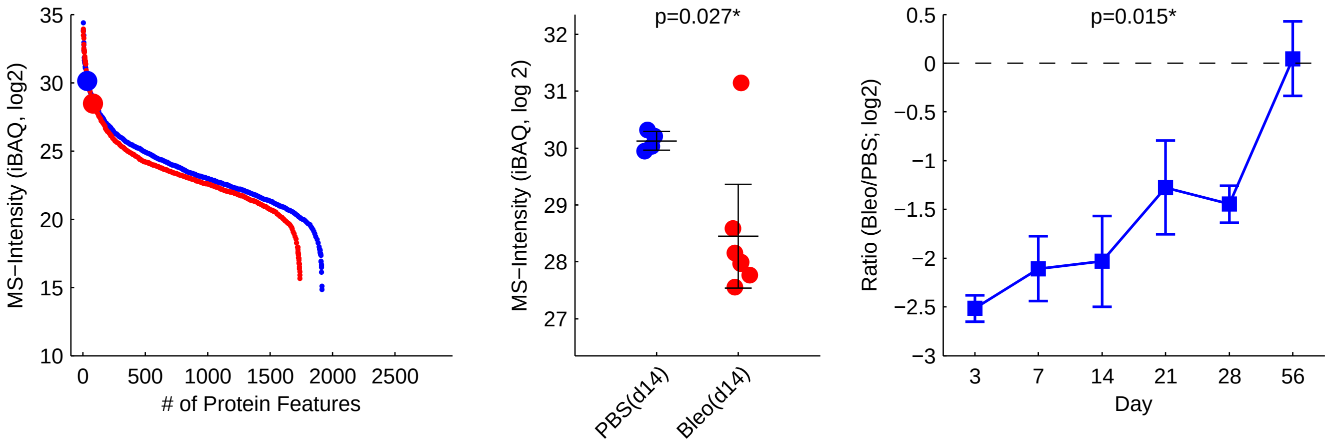

Q5SW88 − Rab1 (id: 1678)

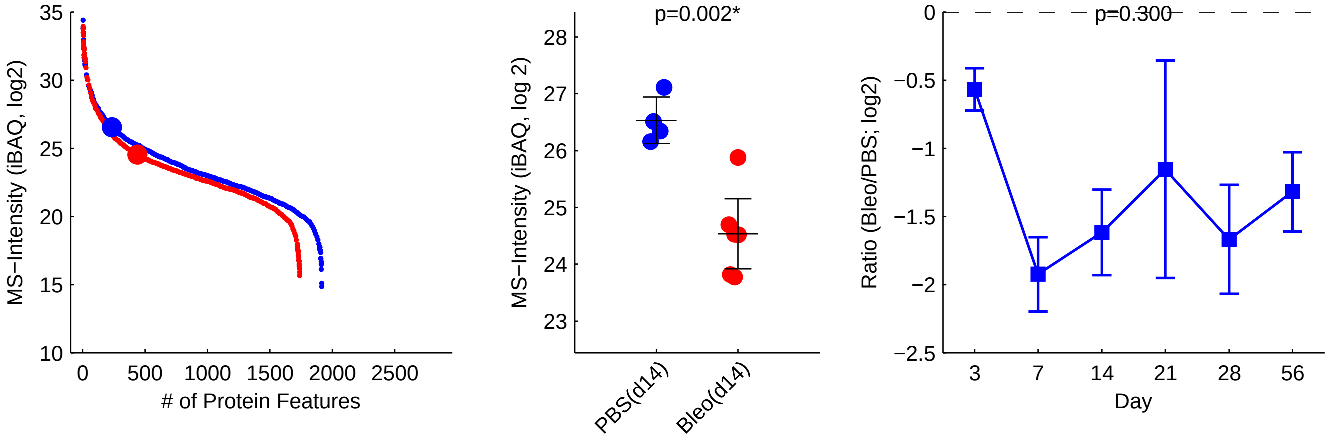

Q5SYD0 − Myo1d (id: 1683)

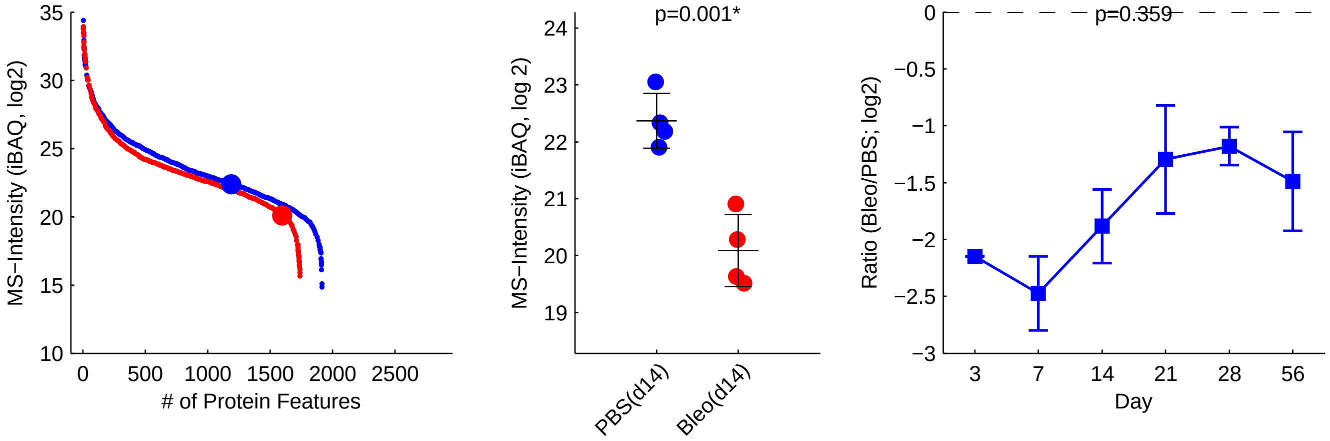

Q5XJY5 − Arcn1 (id: 1685)

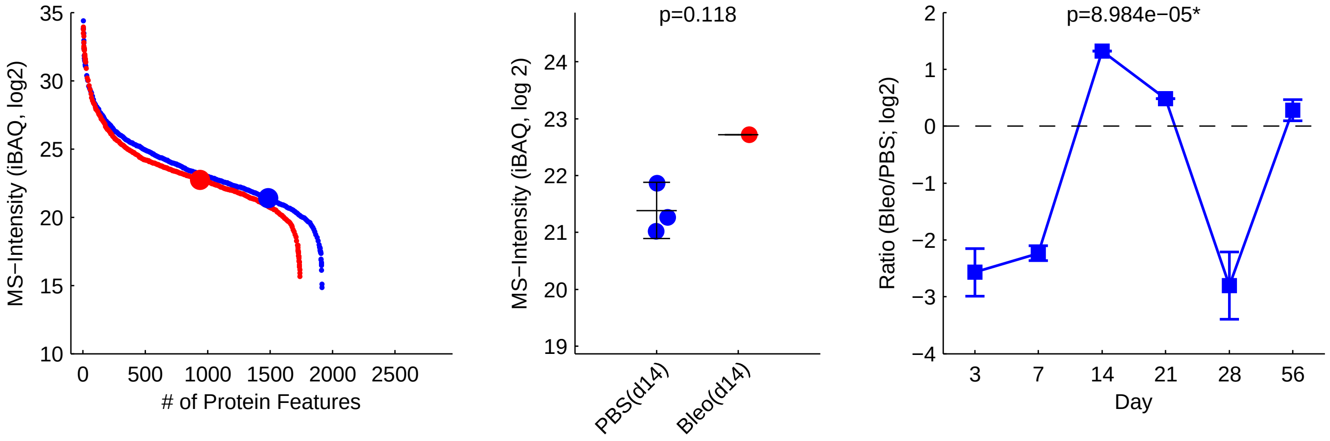

Q921L6 − Cttn (id: 1689)

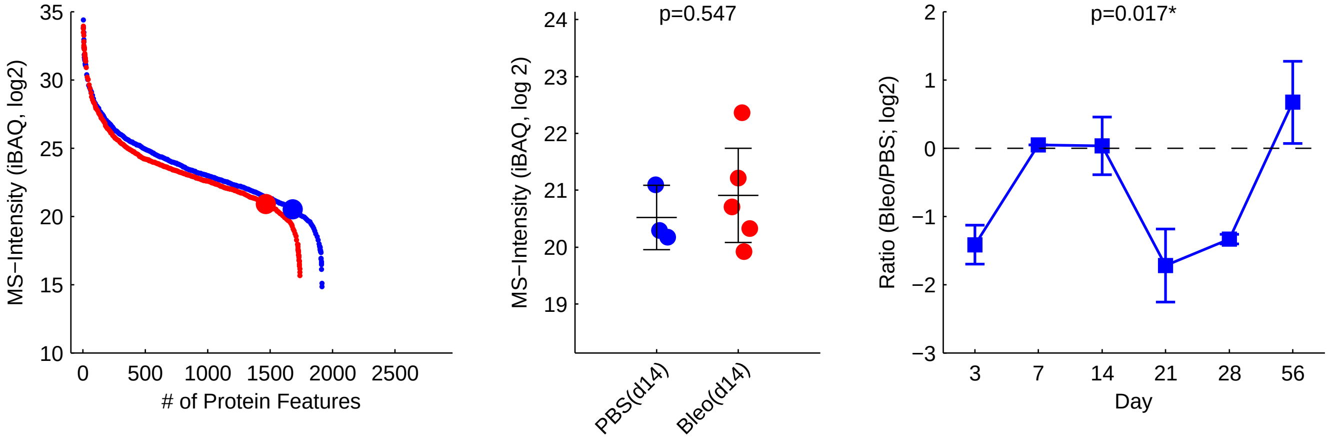

Q60648 − Gm2a (id: 1692)

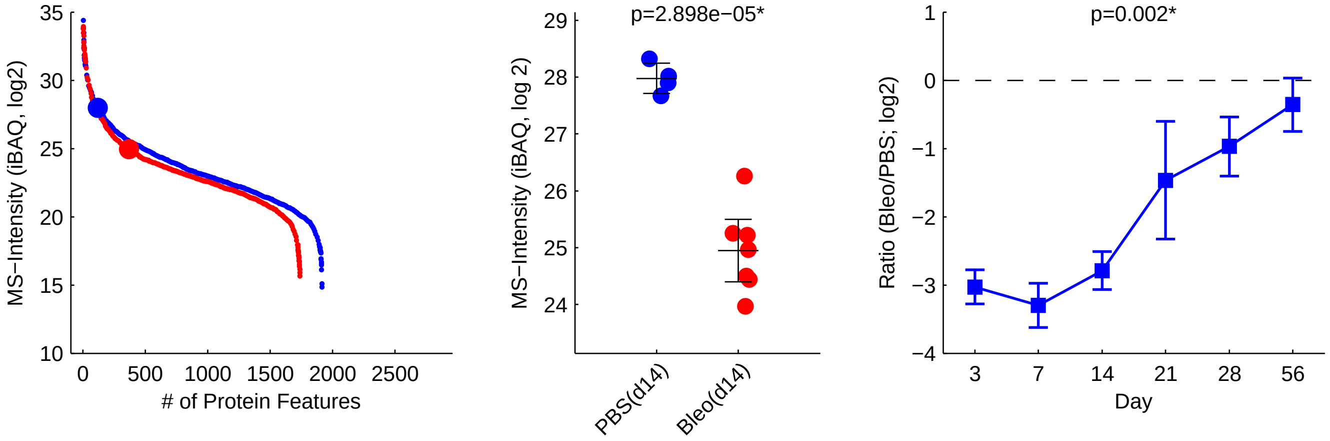

Q60668−3 − Hnrnpd (id: 1693)

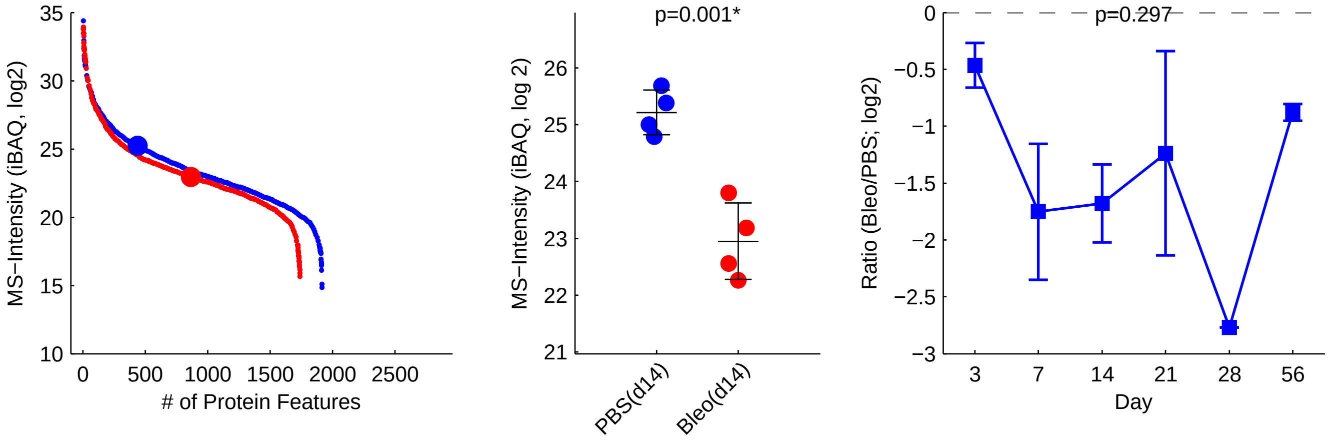

Q60805 − Mertk (id: 1699)

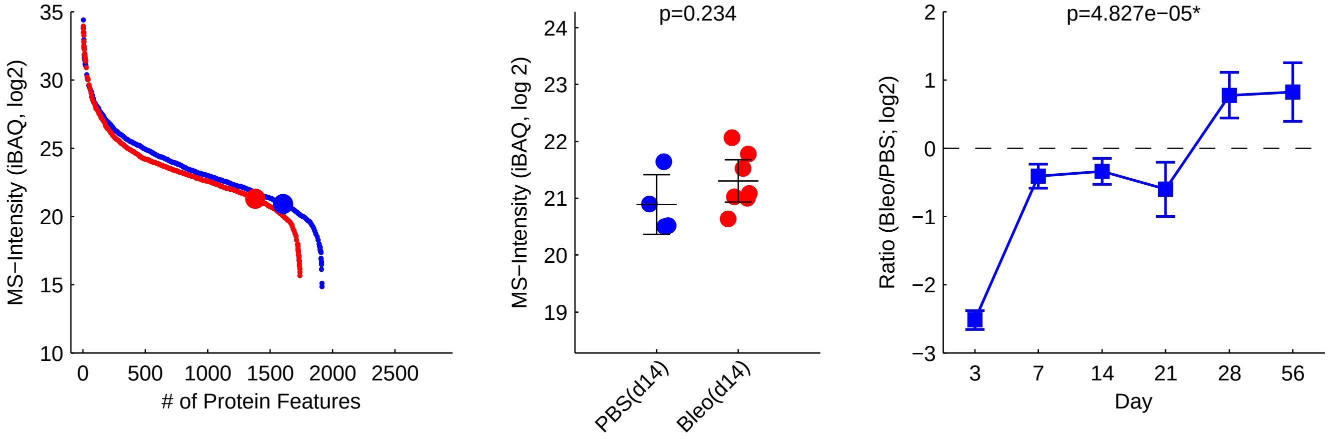

Q60864 − Stip1 (id: 1701)

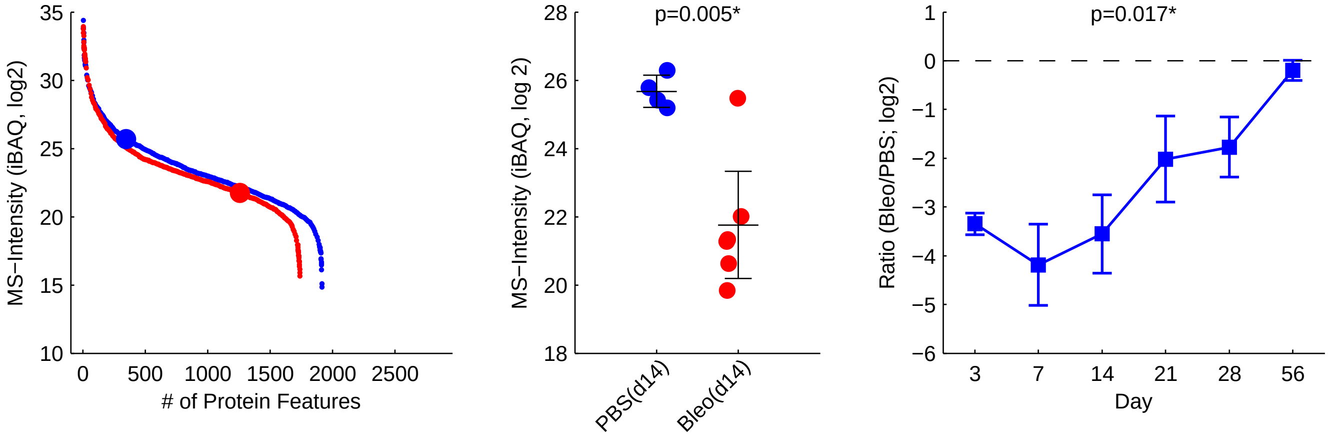

Q60932−2 − Vdac1 (id: 1705)

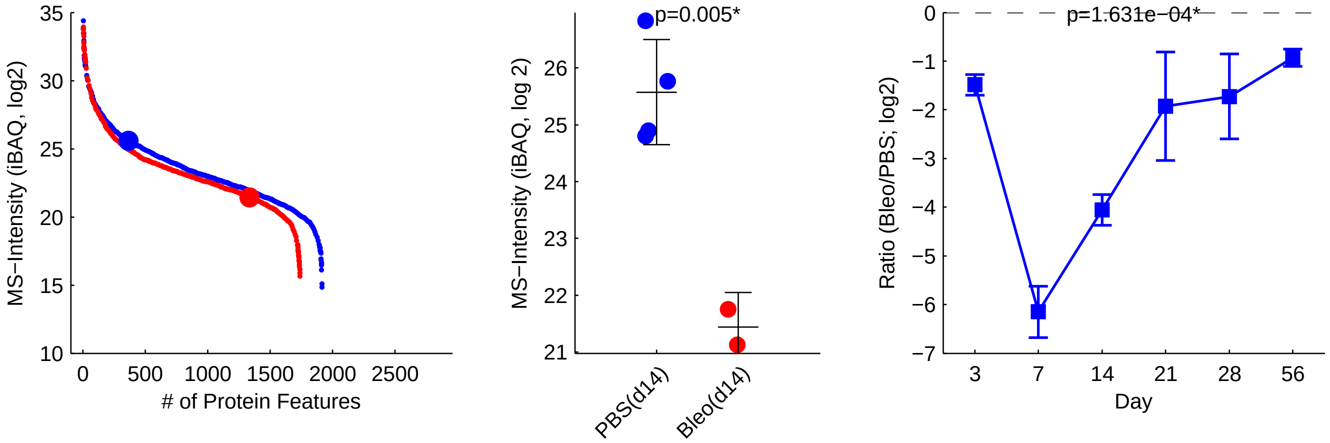

Q60963 − Pla2g7 (id: 1707)

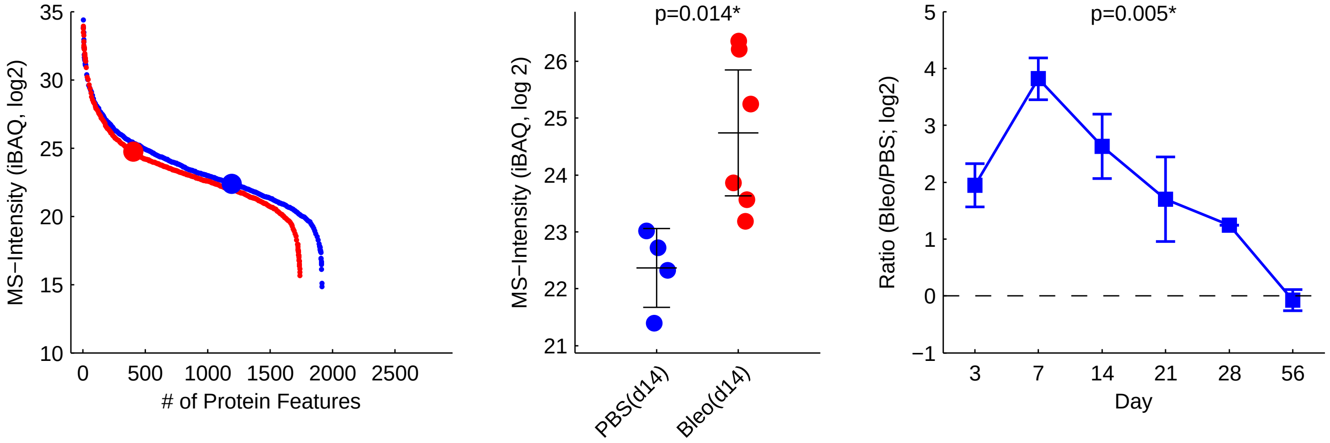

Q60994 − Adipoq (id: 1710)

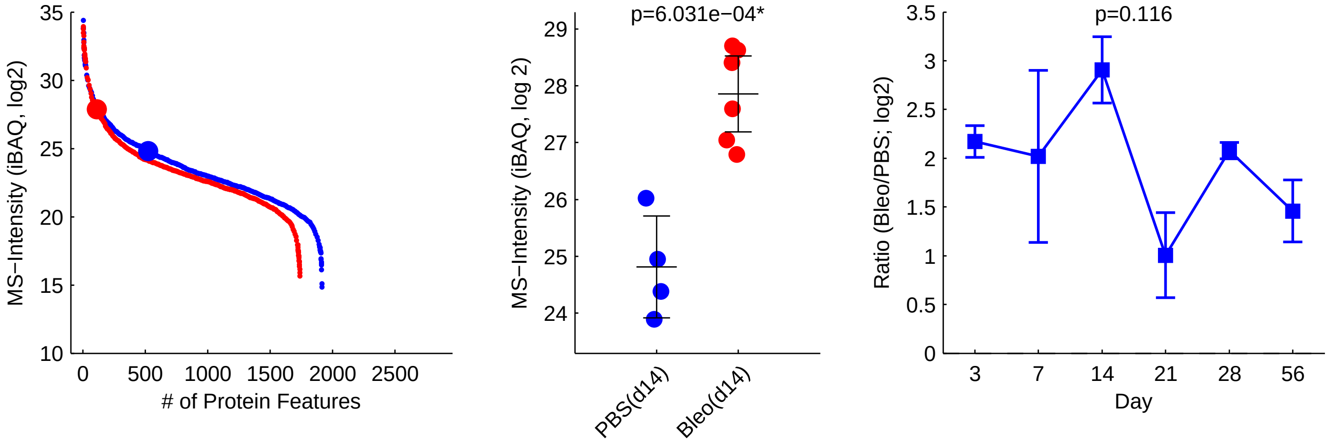

Q61035 − Hars (id: 1716)

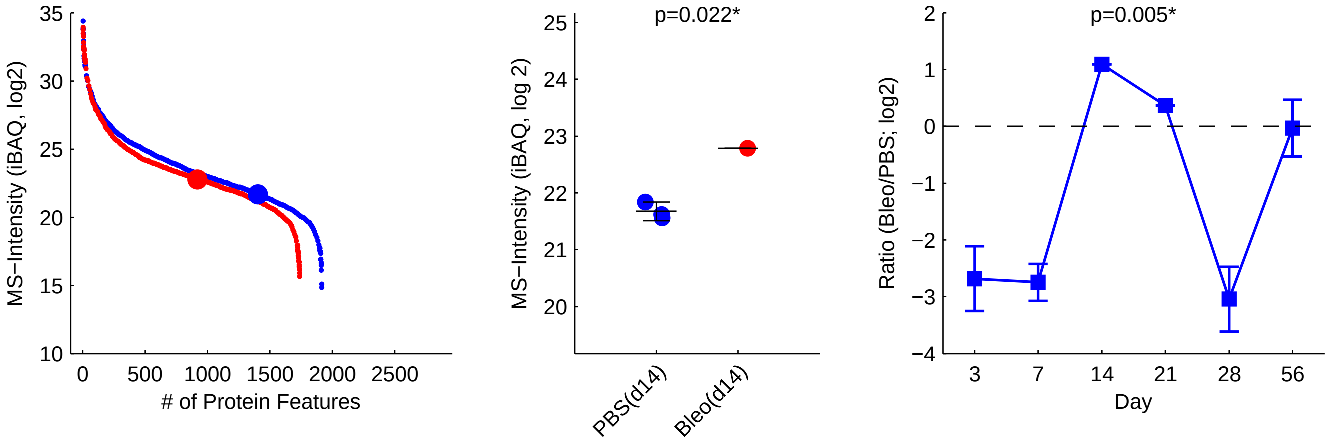

Q61081 − Cdc37 (id: 1718)

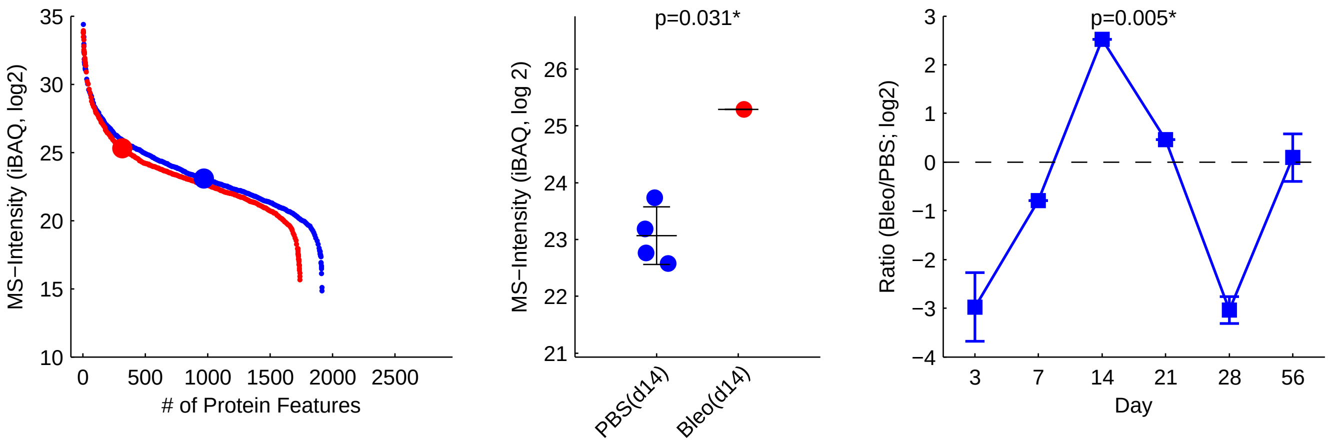

Q8C257 − Il18r1 (id: 1721)

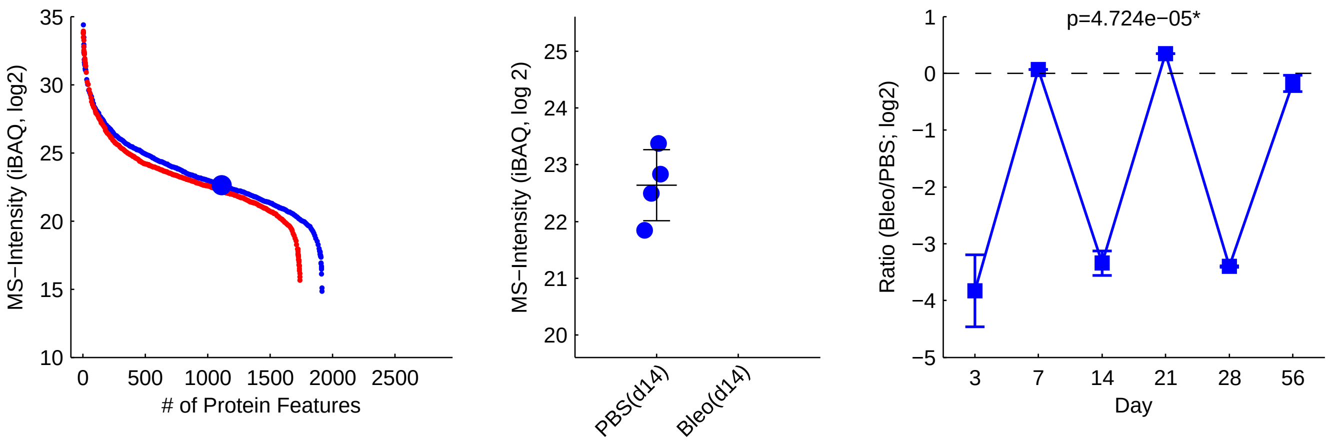

Q61129 − Cfi (id: 1724)

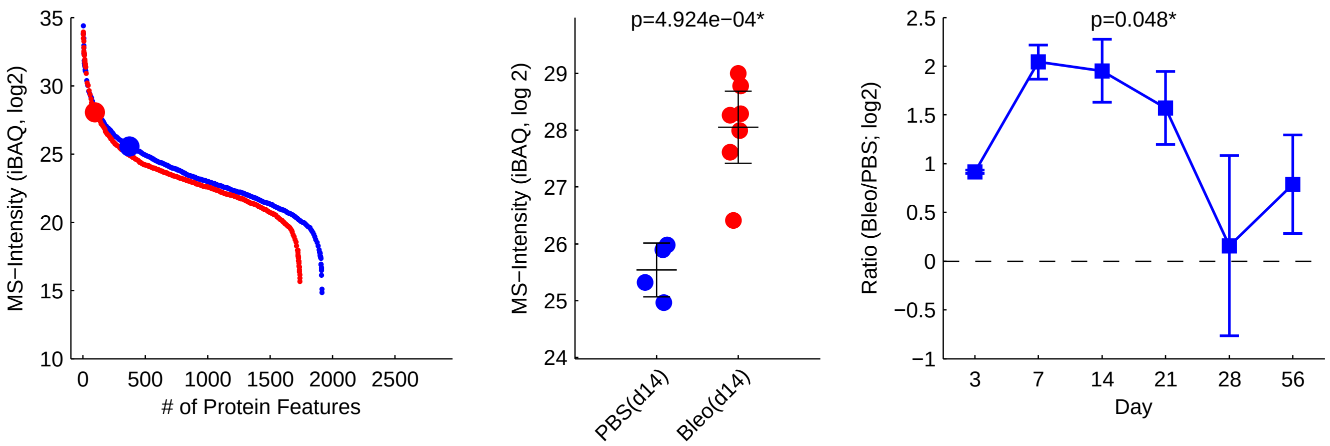

Q61133 − Gstt2 (id: 1725)

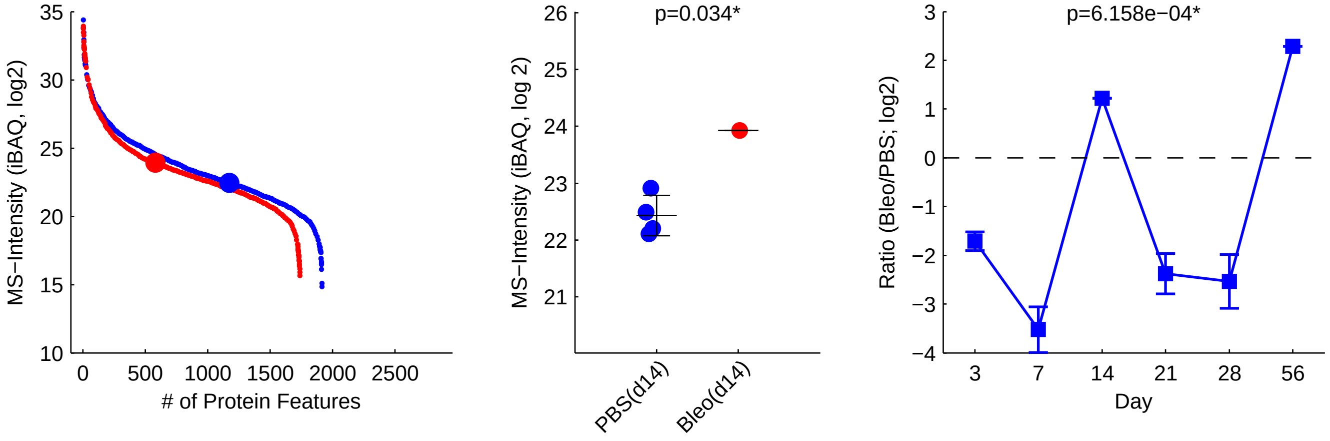

Q61206 − Pafah1b2 (id: 1731)

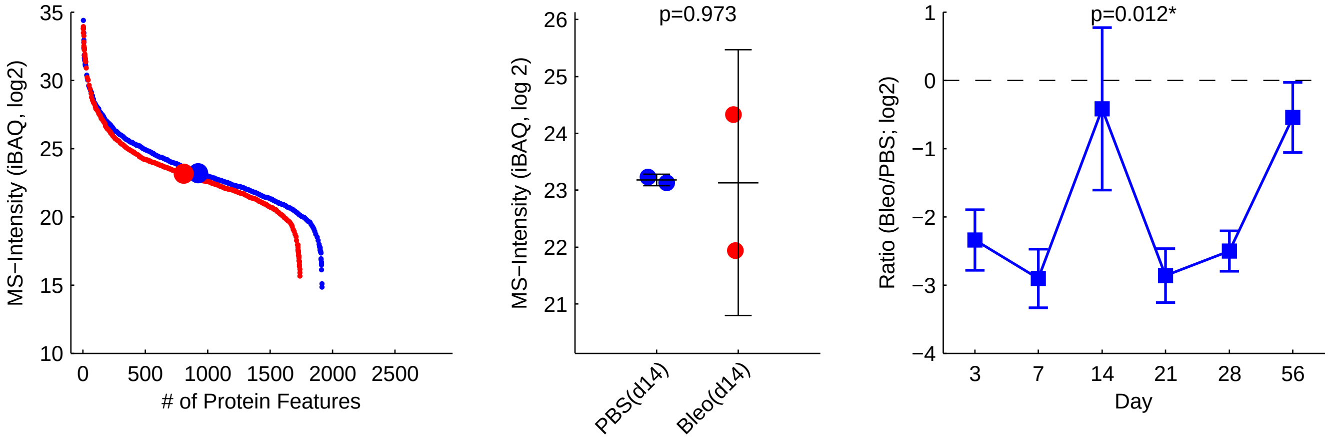

Q61233 − Lcp1 (id: 1732)

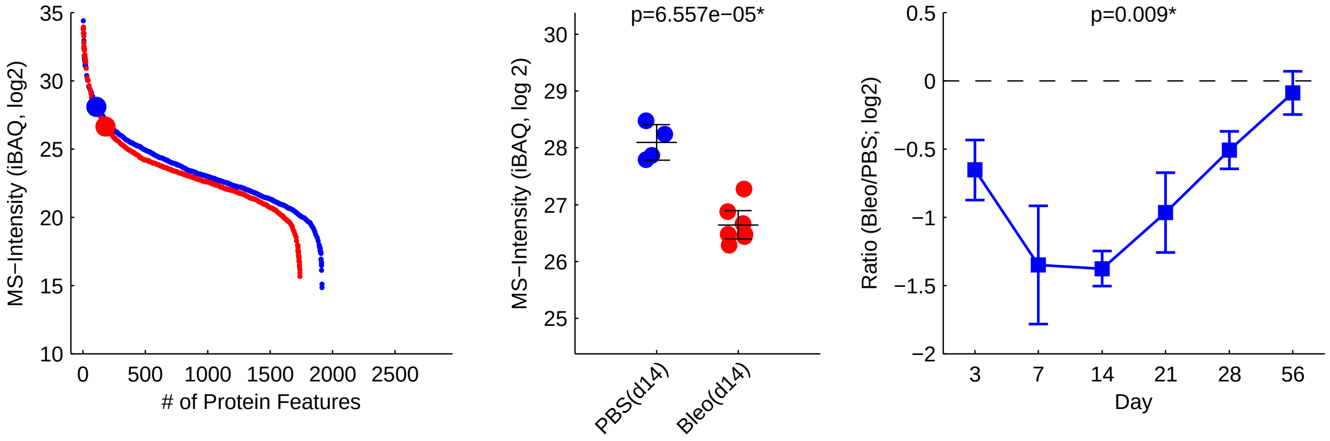

Q61247 − Serpinf2 (id: 1734)

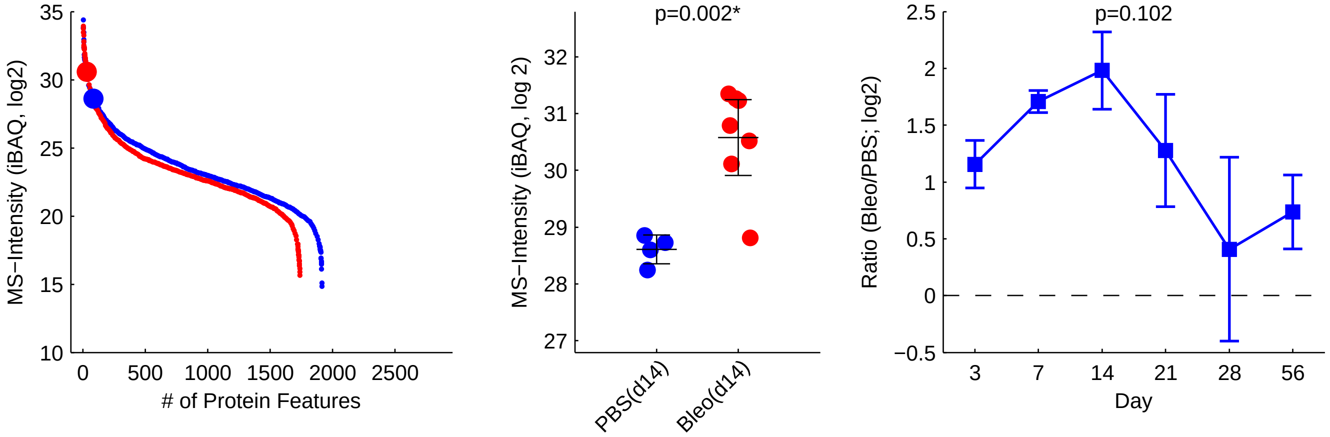

Q61425 − Hadh (id: 1741)

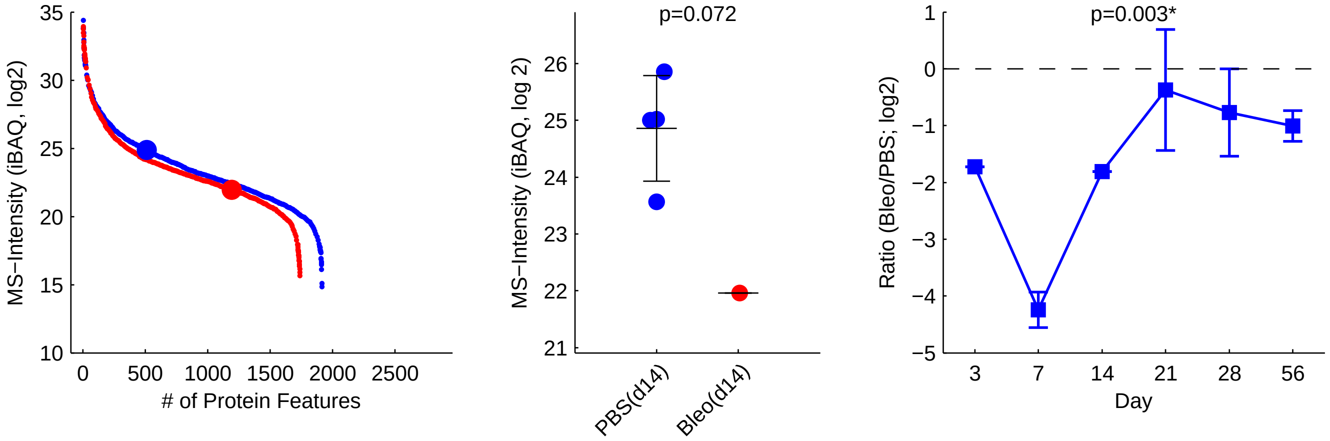

Q61503 − Nt5e (id: 1744)

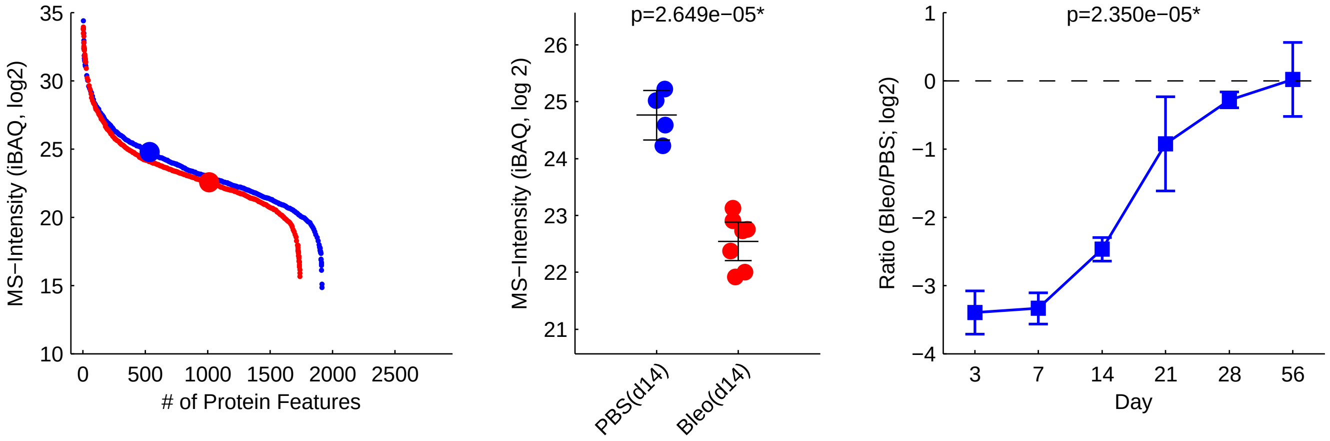

Q61508 − Ecm1 (id: 1745)

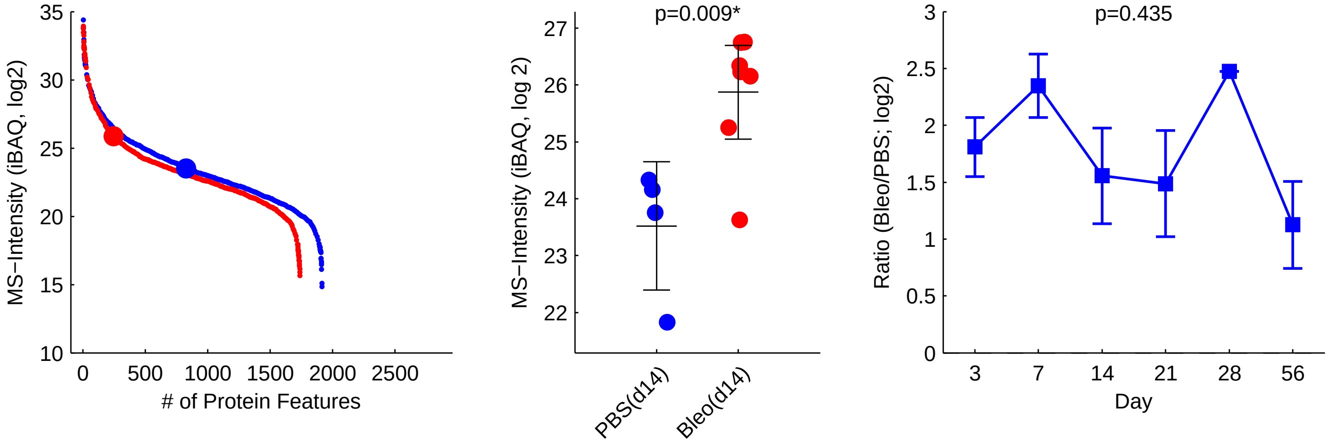

Q61592 − Gas6 (id: 1746)

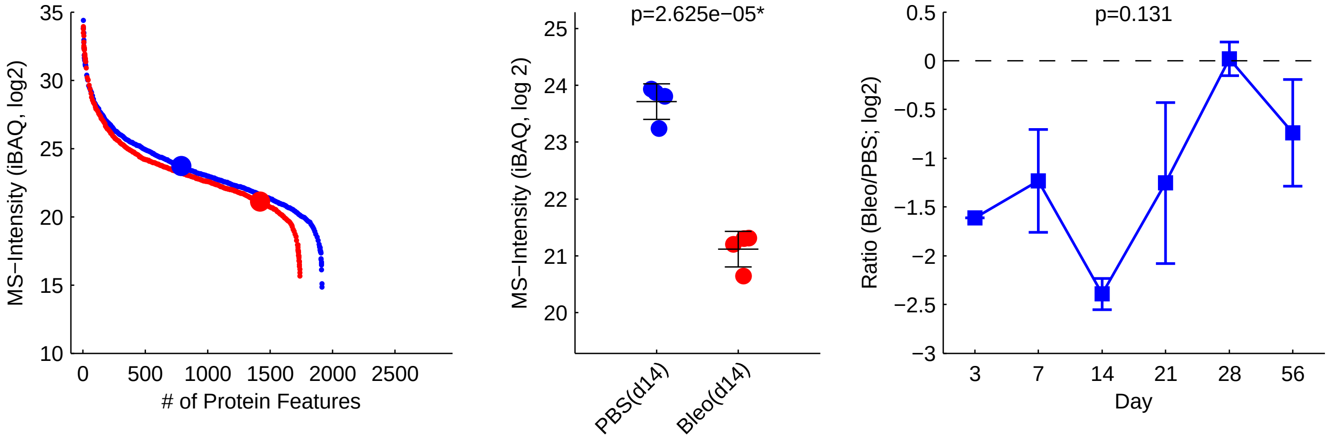

Q61599 − Arhgdib (id: 1748)

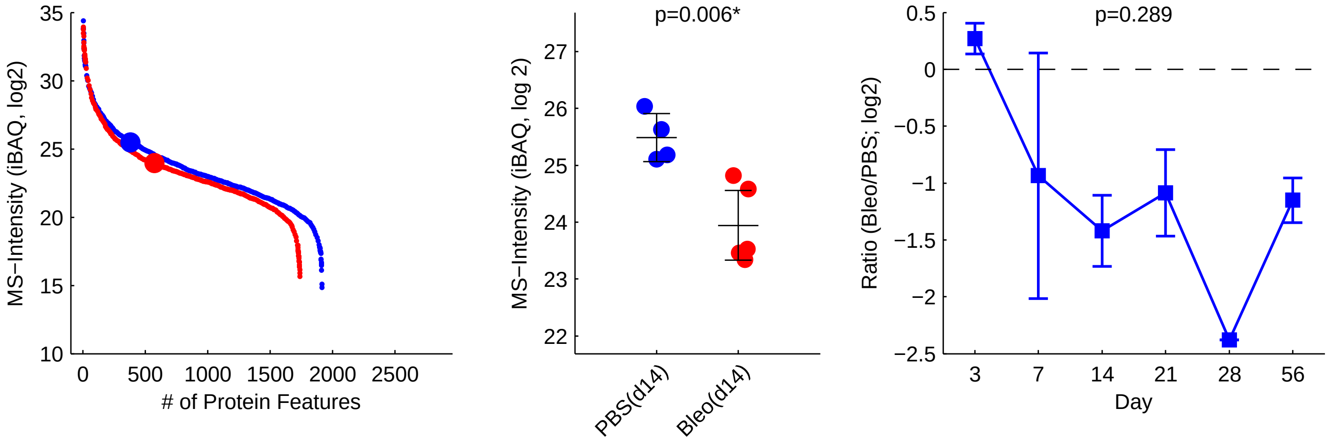

Q61646 − Hp (id: 1750)

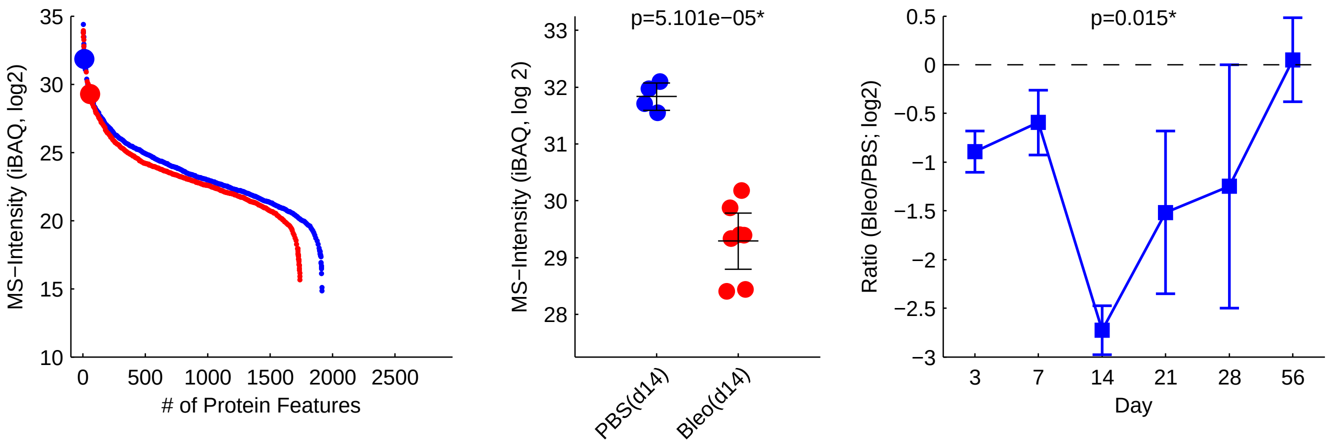

Q61699−2 − Hsph1 (id: 1753)

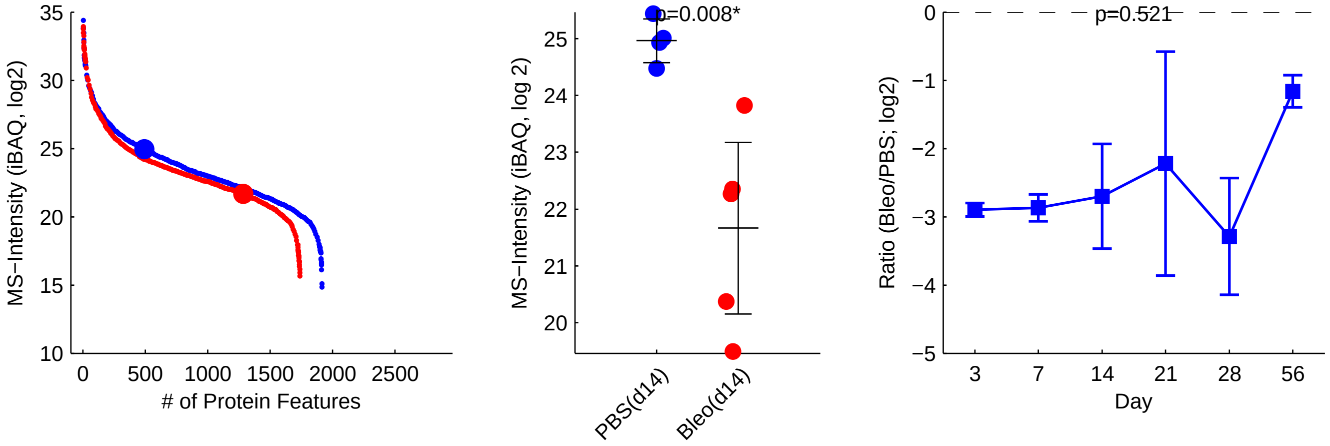

Q61702 − Itih1 (id: 1754)

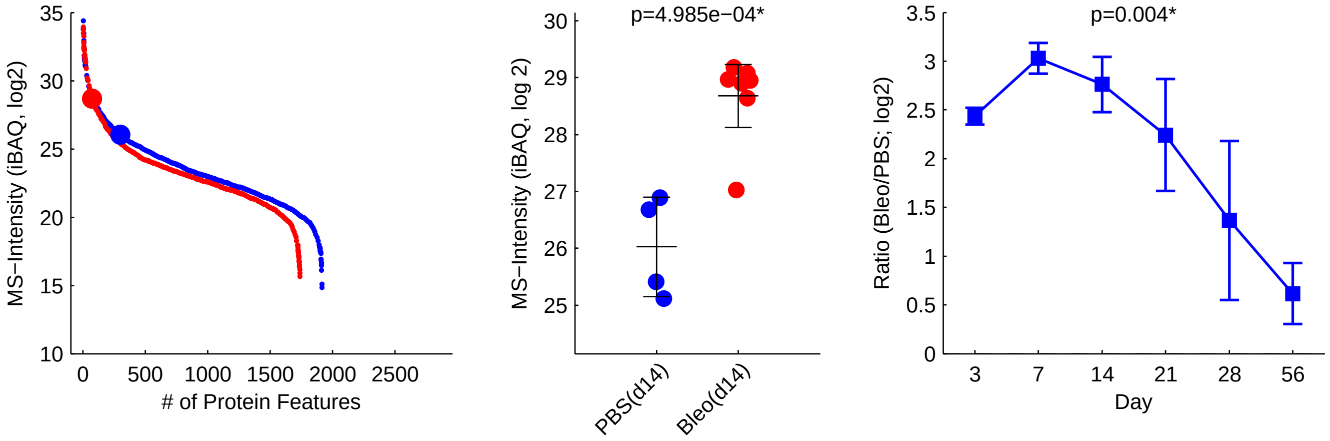

Q61704 − Itih3 (id: 1755)

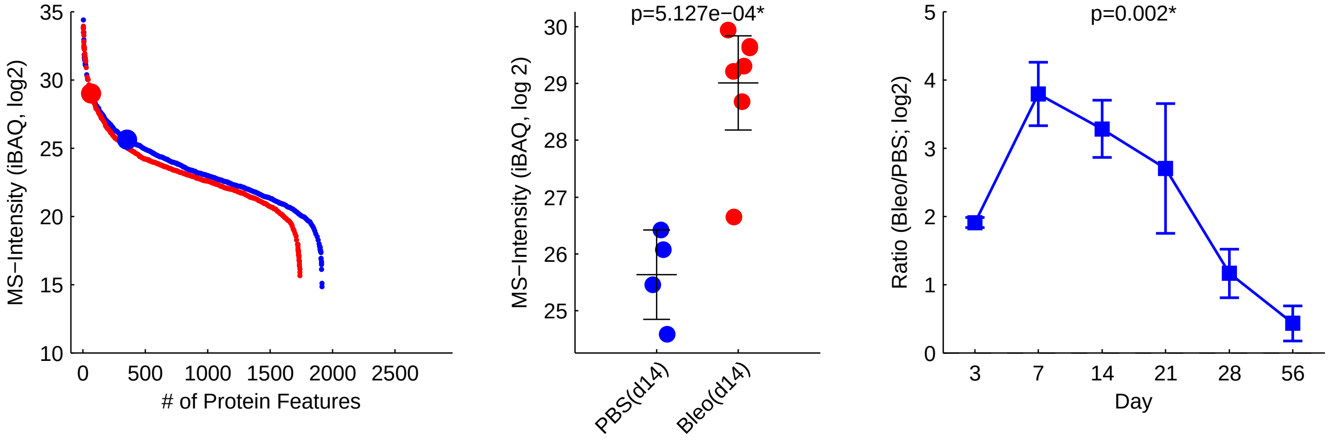

Q61735 − Cd47 (id: 1756)

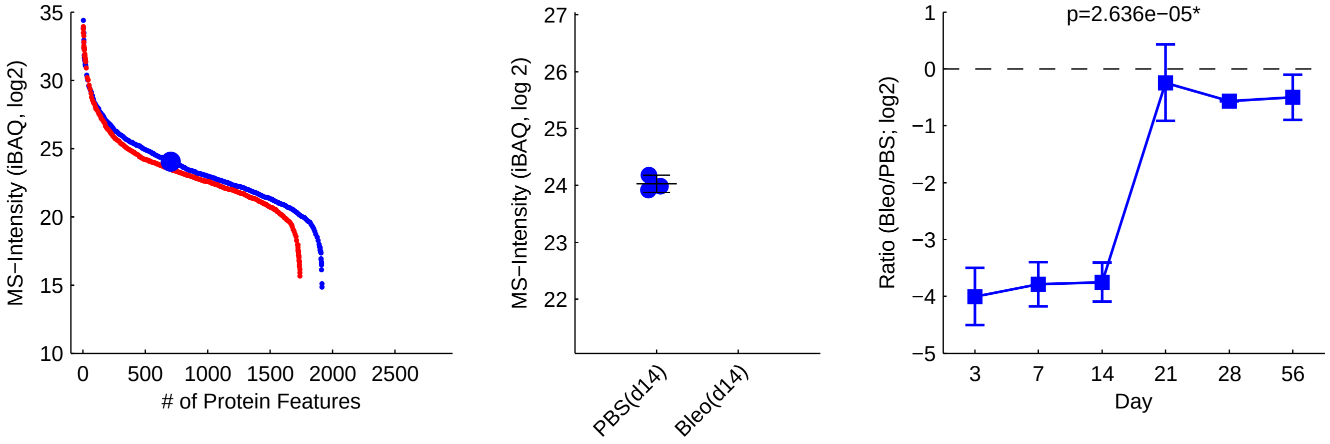

Q61805 − Lbp (id: 1761)

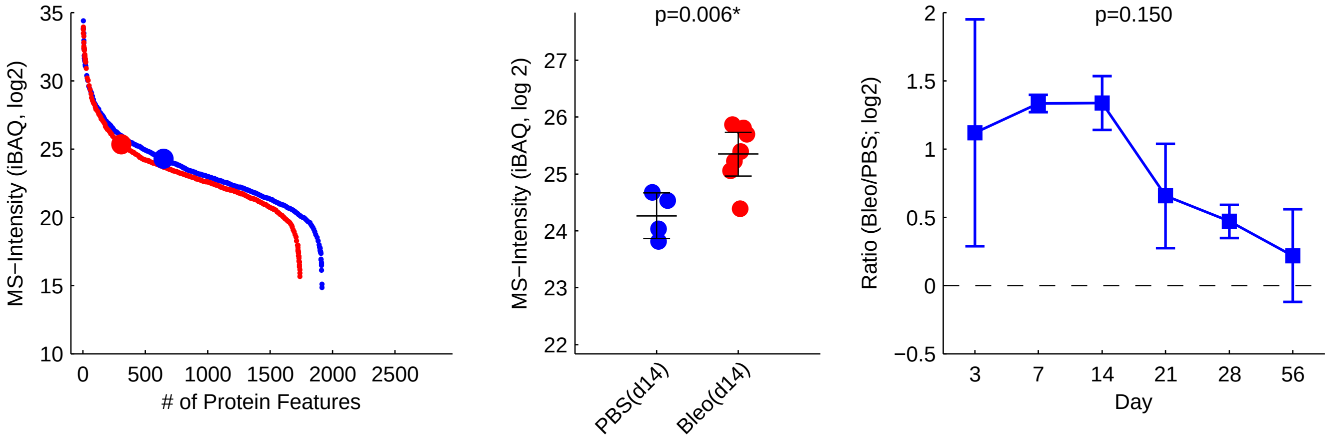

Q61830 − Mrc1 (id: 1762)

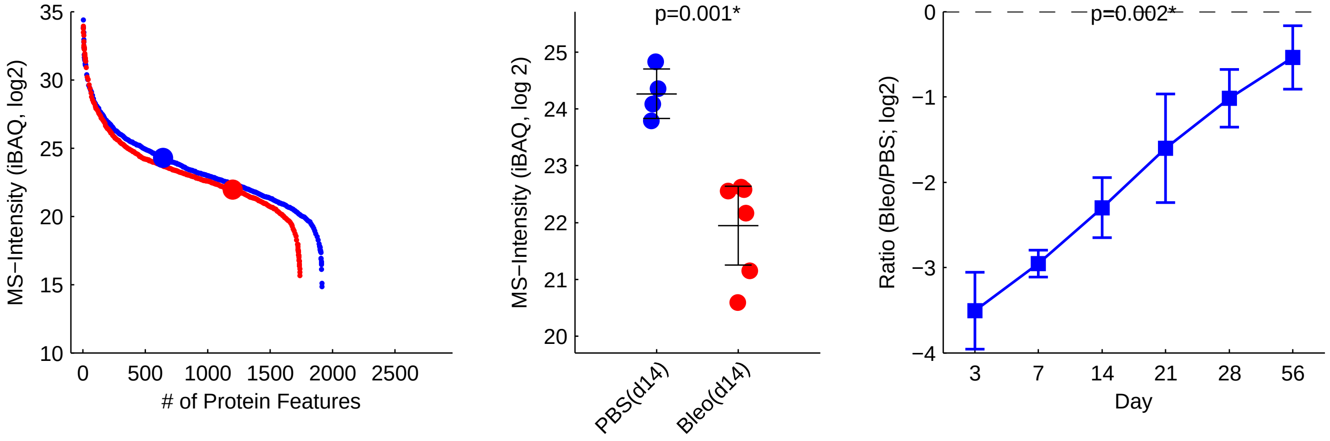

Q61838 − A2m (id: 1763)

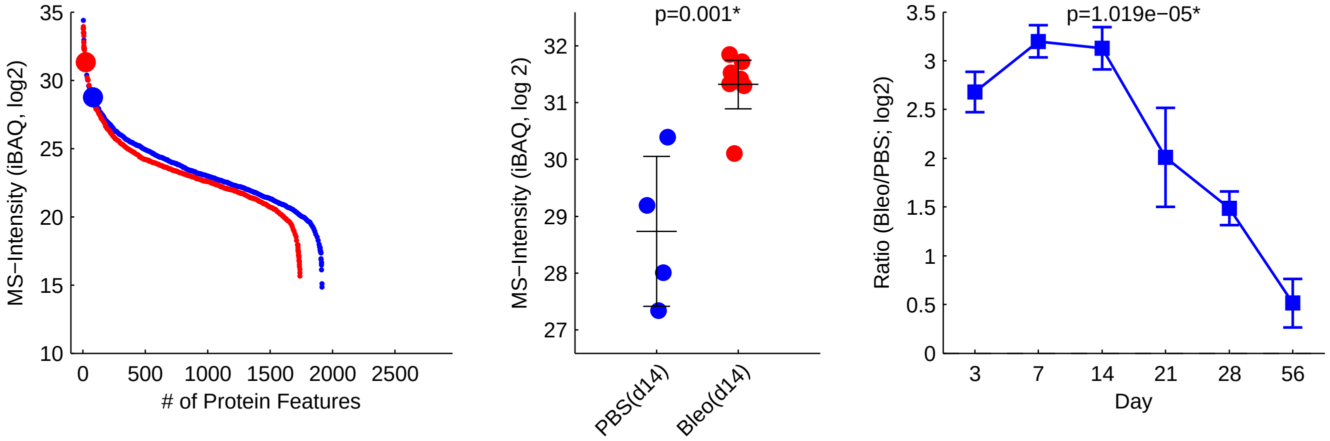

Q61937 − Npm1 (id: 1764)

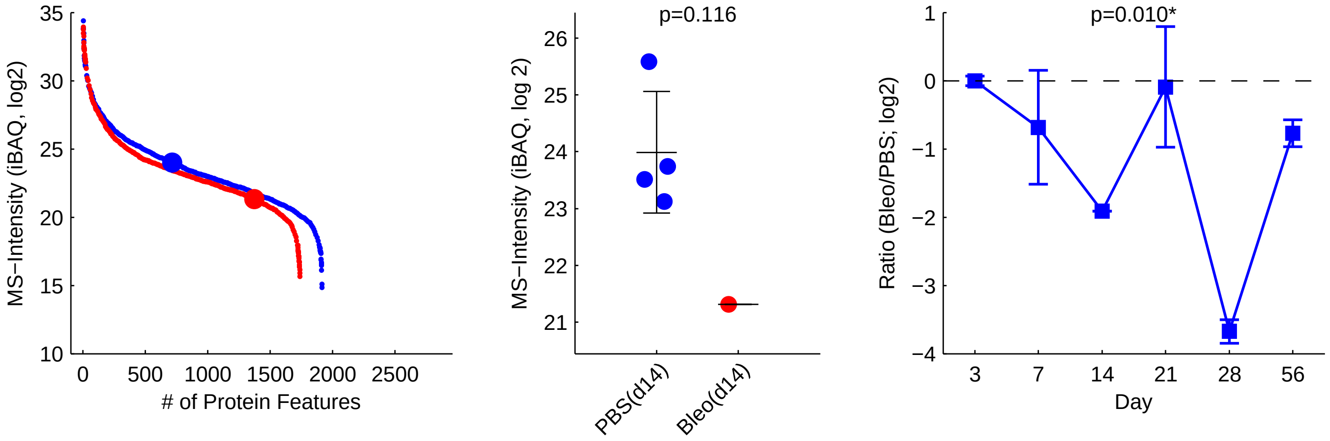

Q61990−2 − Pcbp2 (id: 1765)

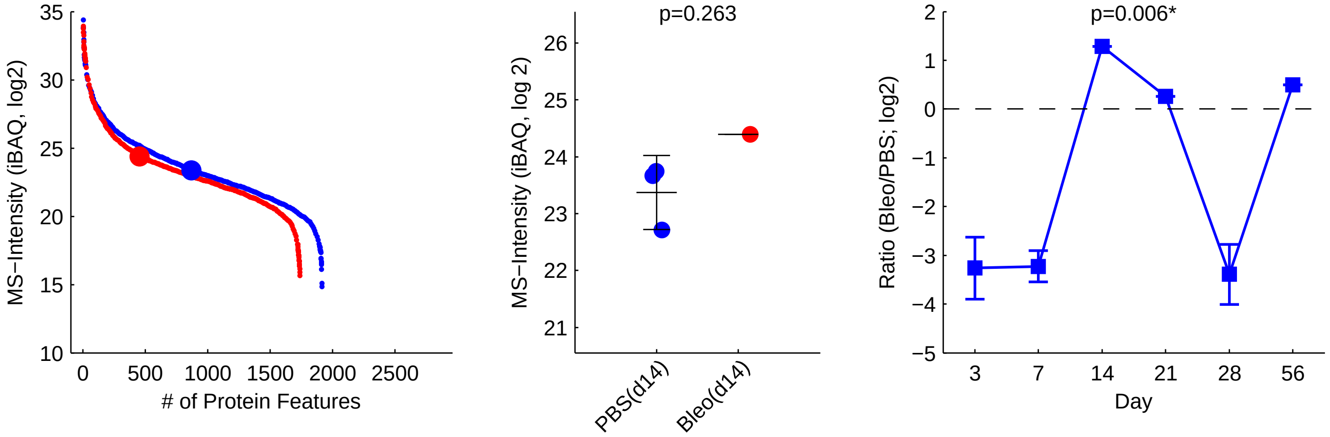

Q62009−5 − Postn (id: 1767)

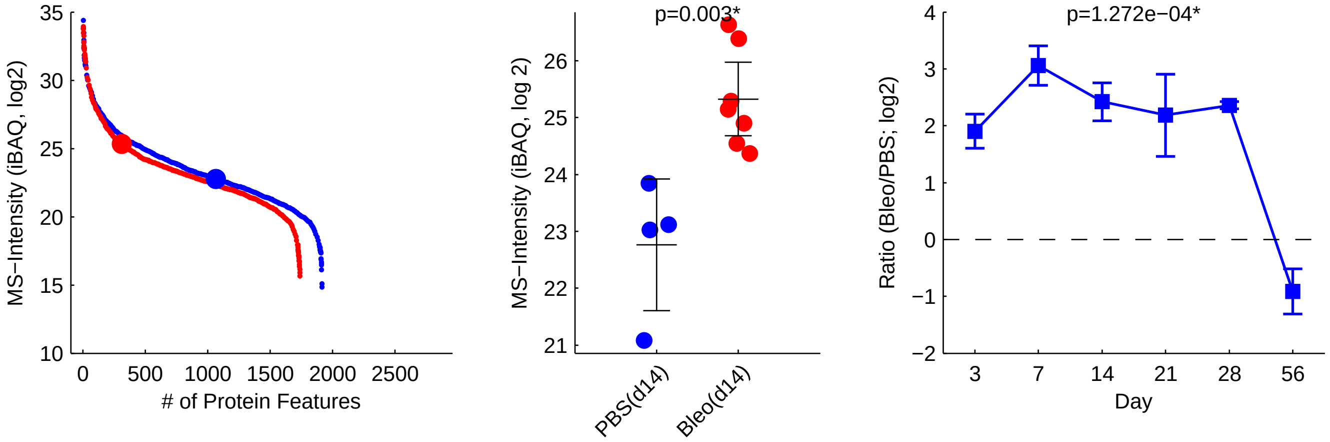

Q62048 − Pea15 (id: 1769)

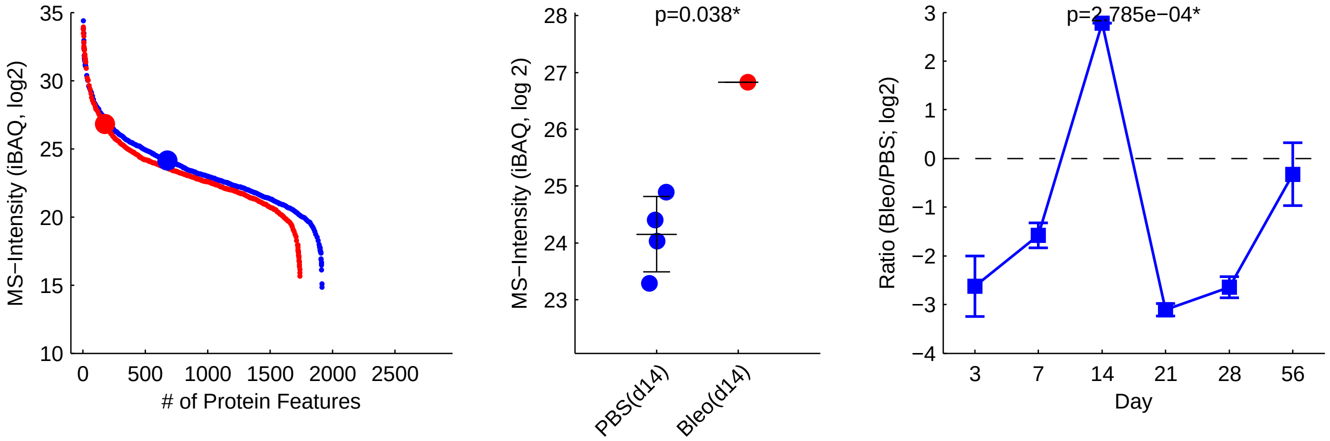

Q62087 − Pon3 (id: 1771)

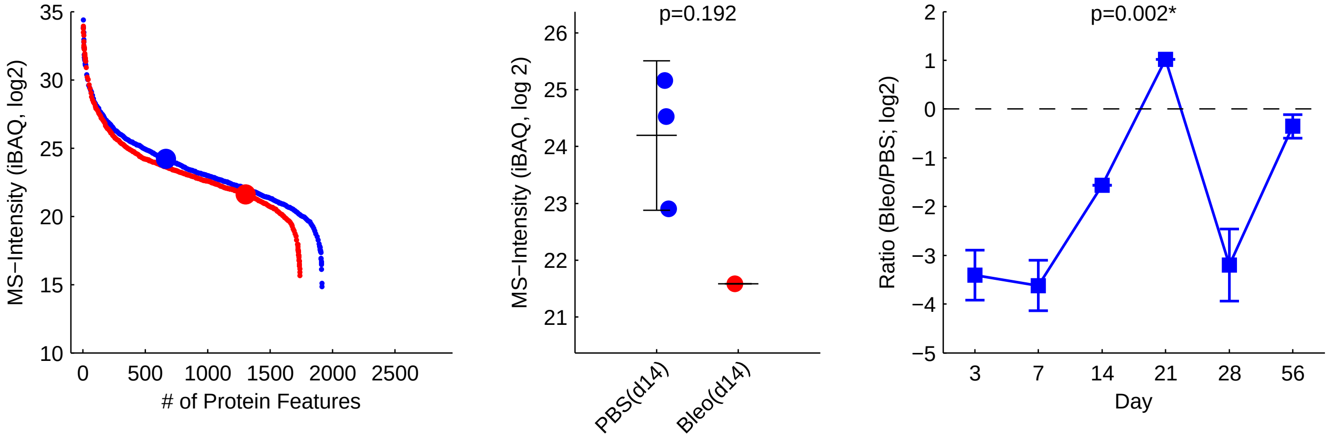

Q62188 − Dpysl3 (id: 1779)

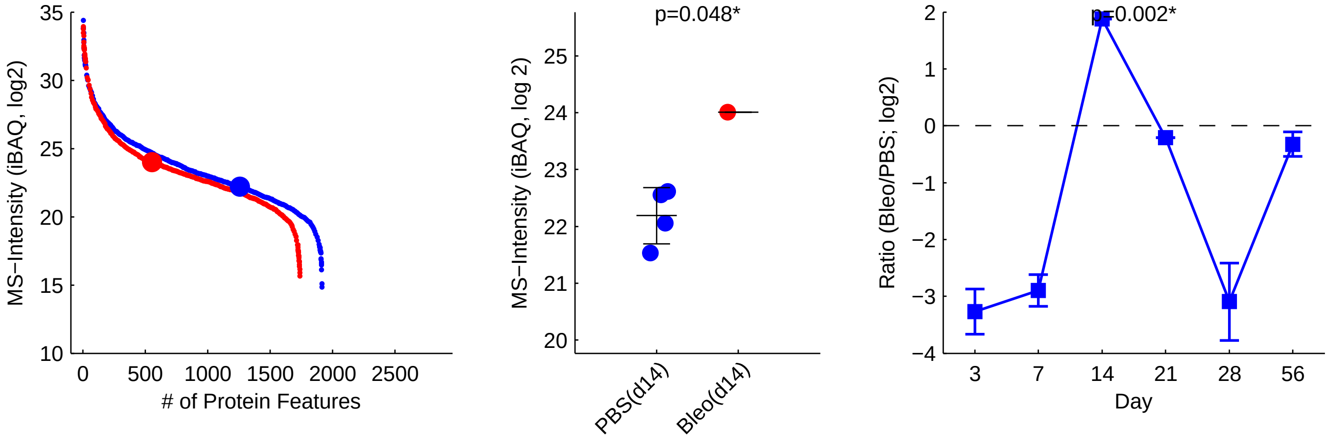

Q62261 − Sptbn1 (id: 1781)

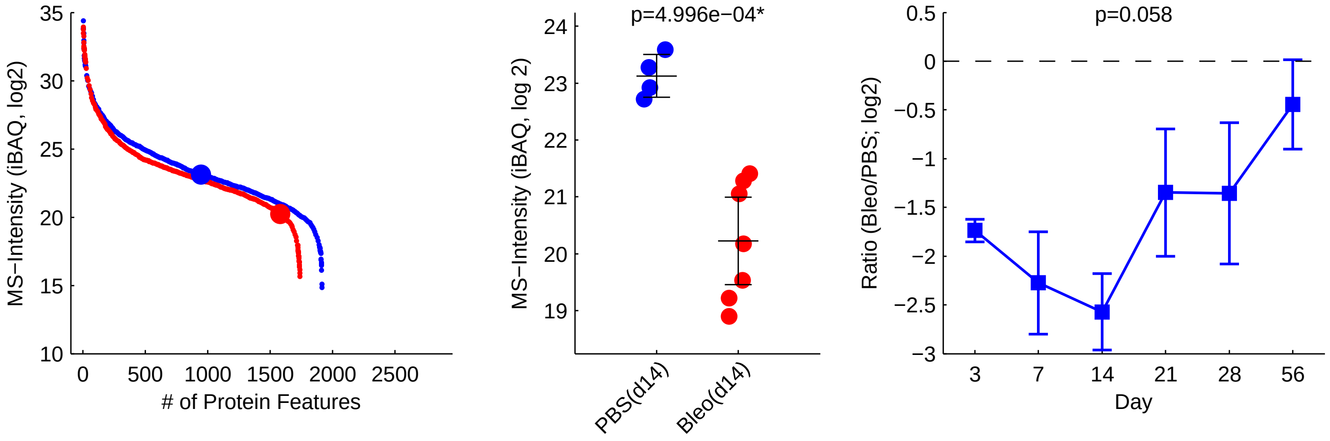

Q62348 − Tsn (id: 1784)

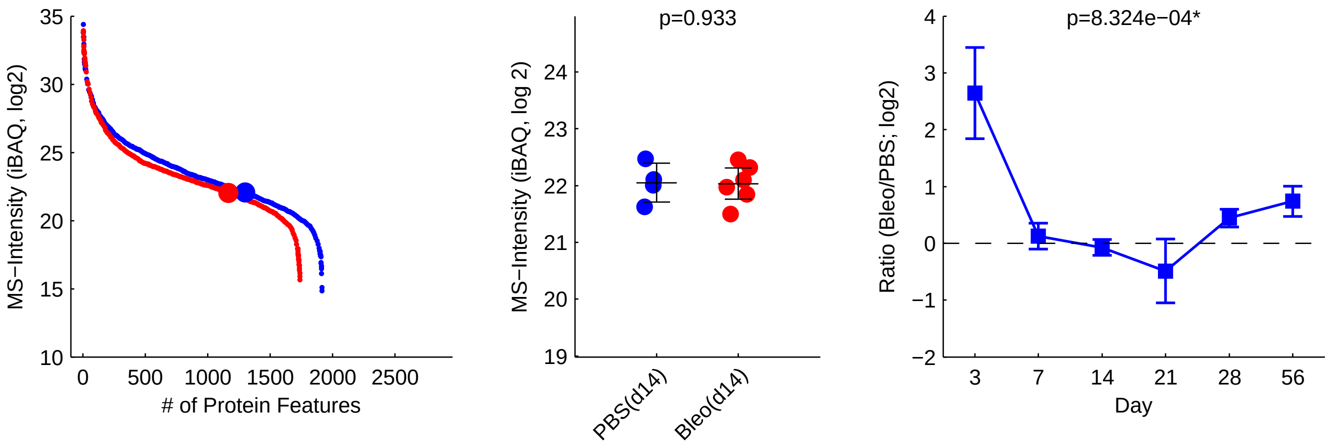

Q62426 − Cstb (id: 1792)

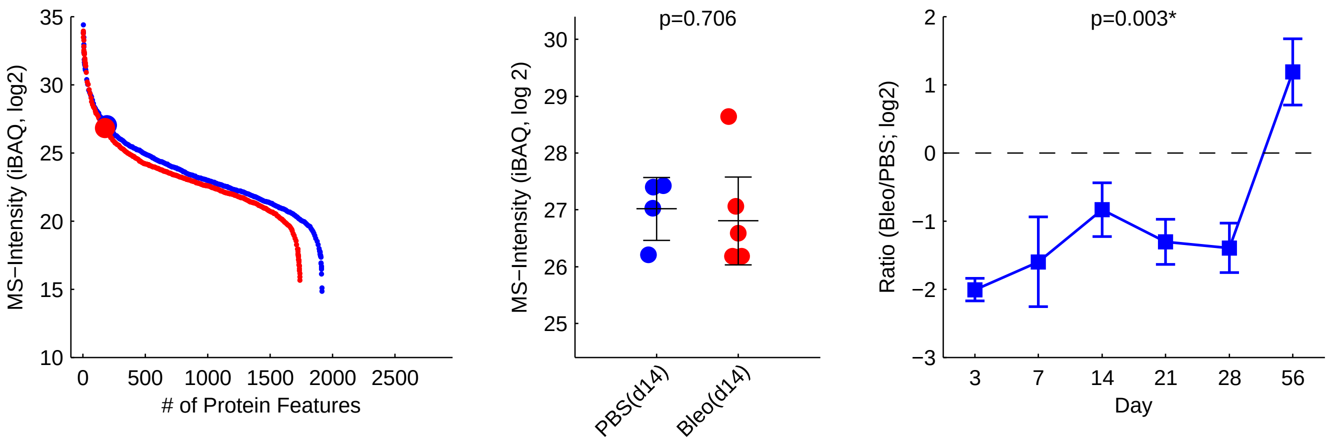

Q63810−2 − Ppp3r1 (id: 1796)

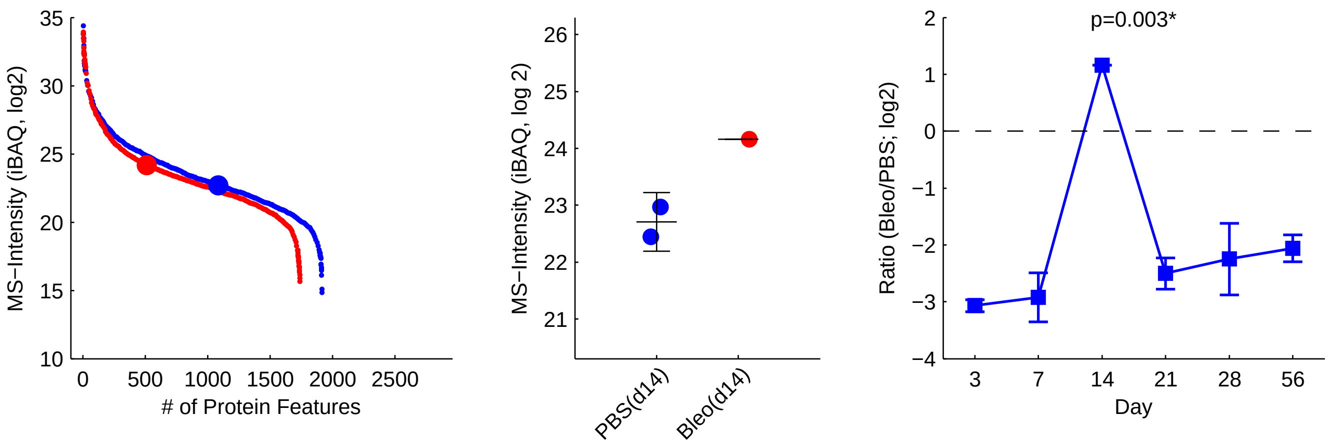

Q64282 − Ifit1 (id: 1806)

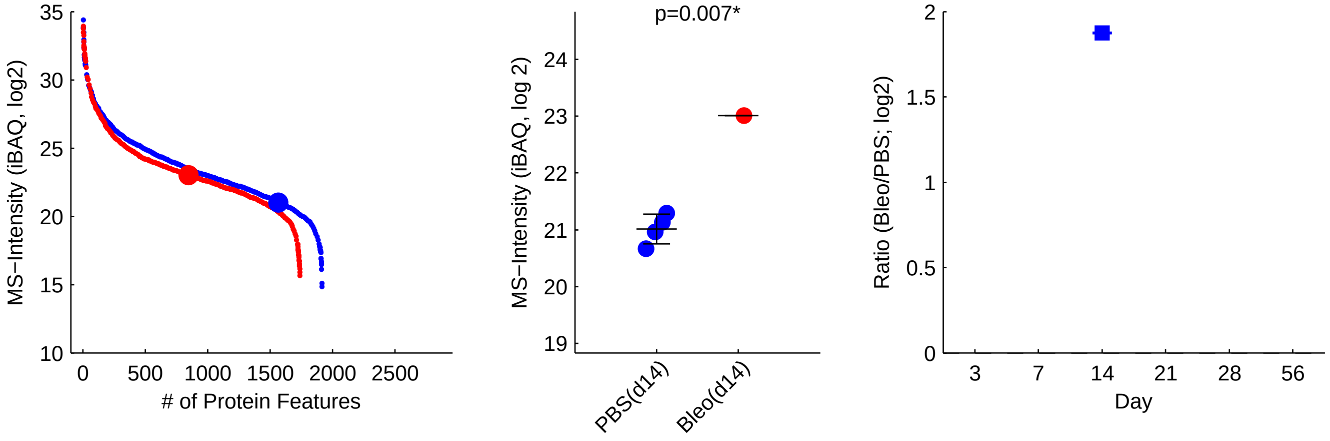

Q64339 − Isg15 (id: 1808)

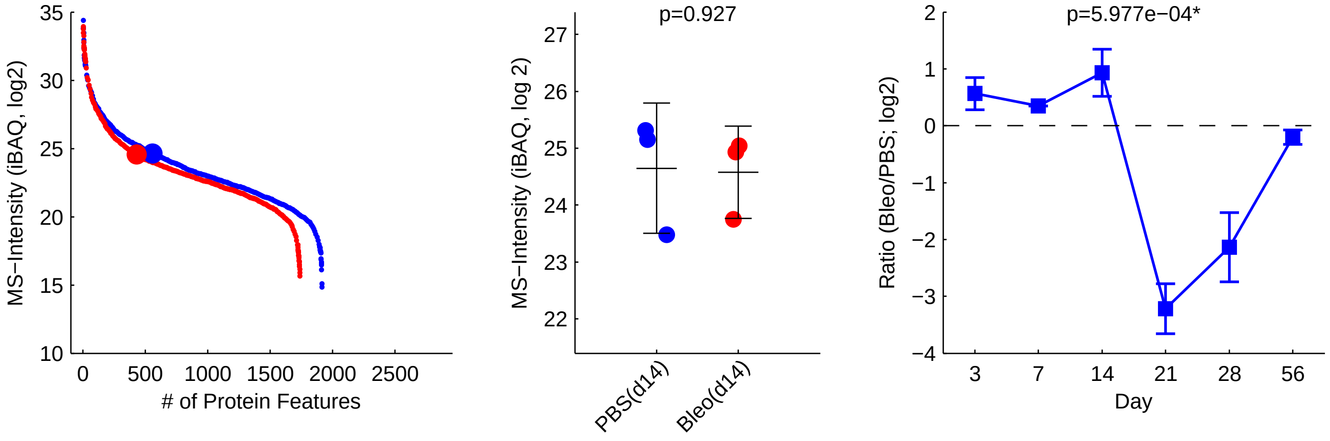

Q64435 − Ugt1a6 (id: 1811)

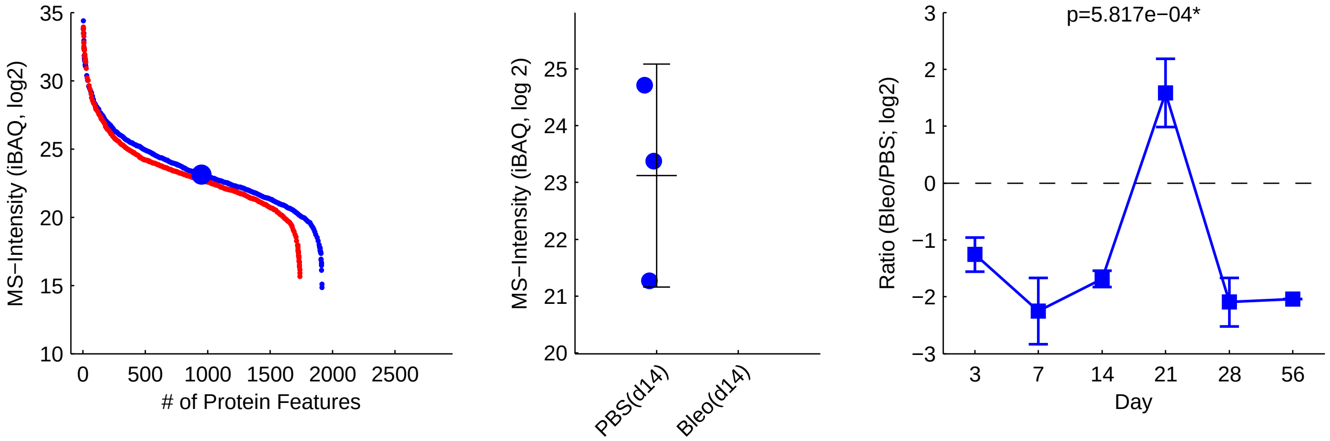

Q64437 − Adh7 (id: 1812)

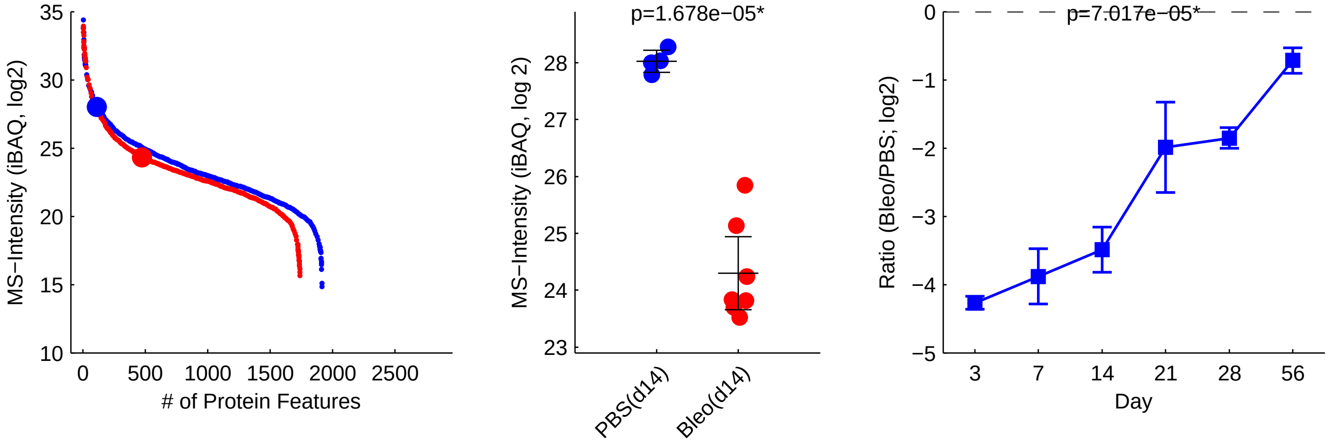

Q64442 − Sord (id: 1813)

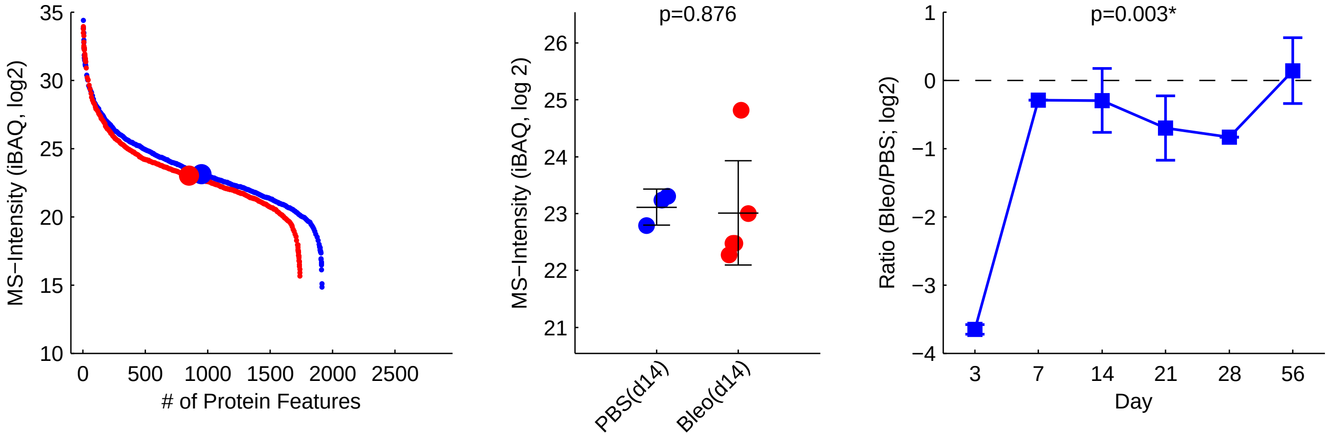

Q64462 − Cyp4b1 (id: 1815)

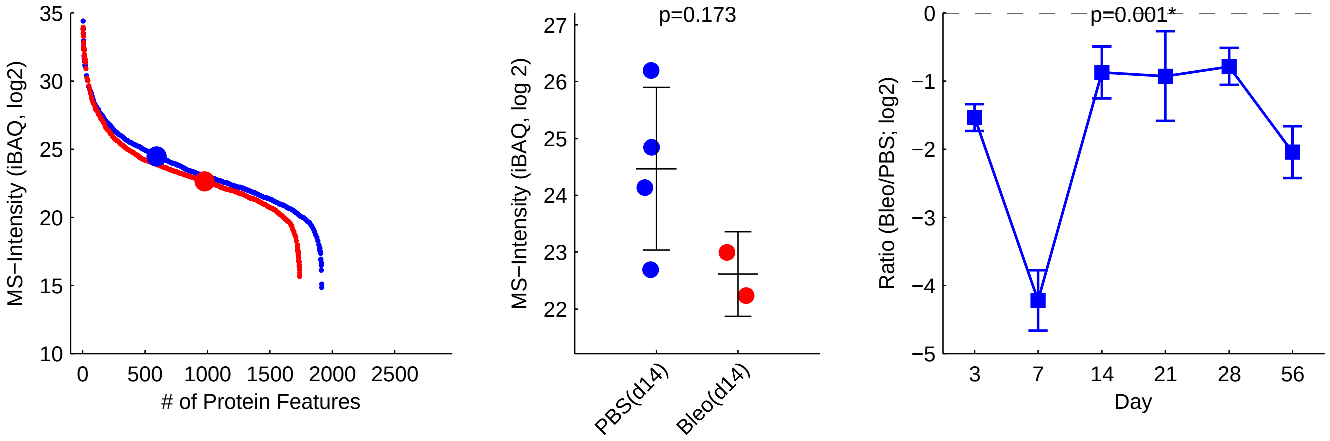

Q64521 − Gpd2 (id: 1818)

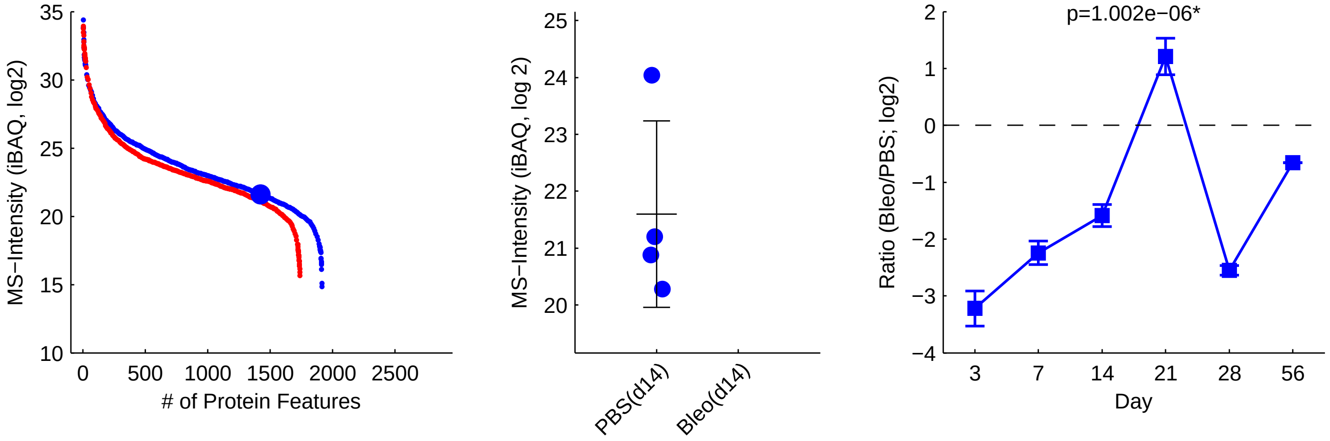

Q6P069−2 − Sri (id: 1839)

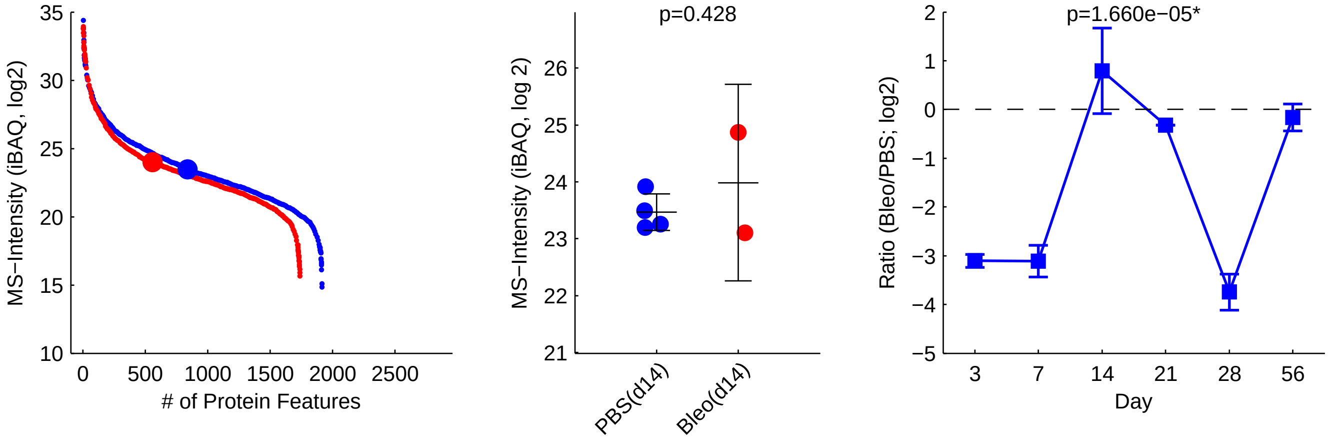

Q6P1B1 − Xpnpep1 (id: 1840)

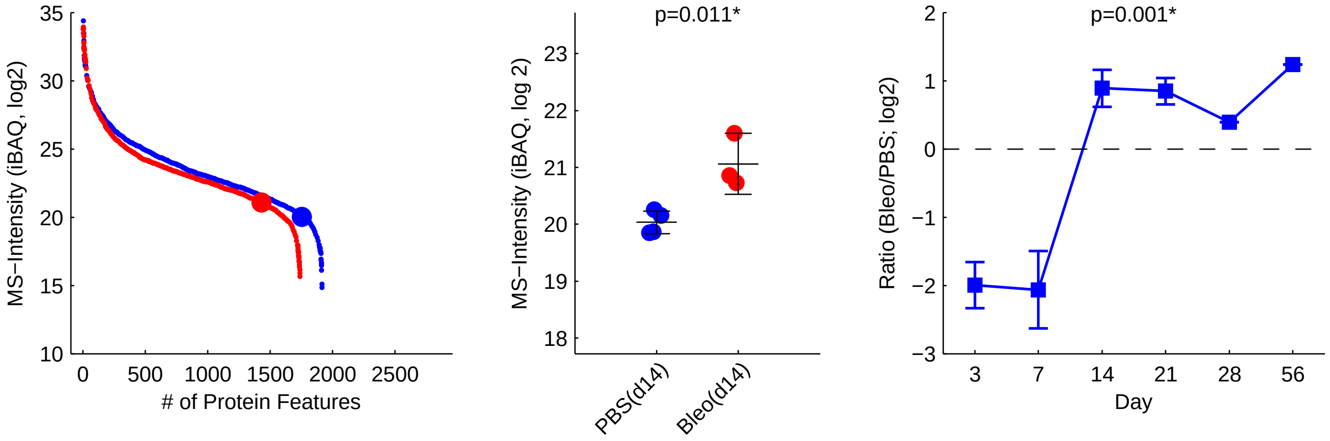

Q6P5E4 − Uggt1 (id: 1845)

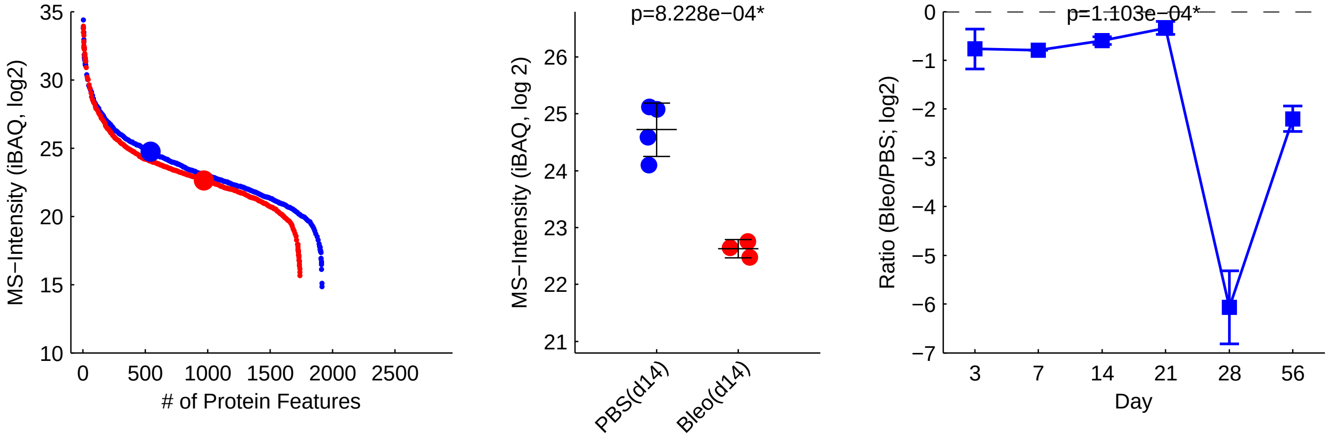

Q6P5F9 − Xpo1 (id: 1846)

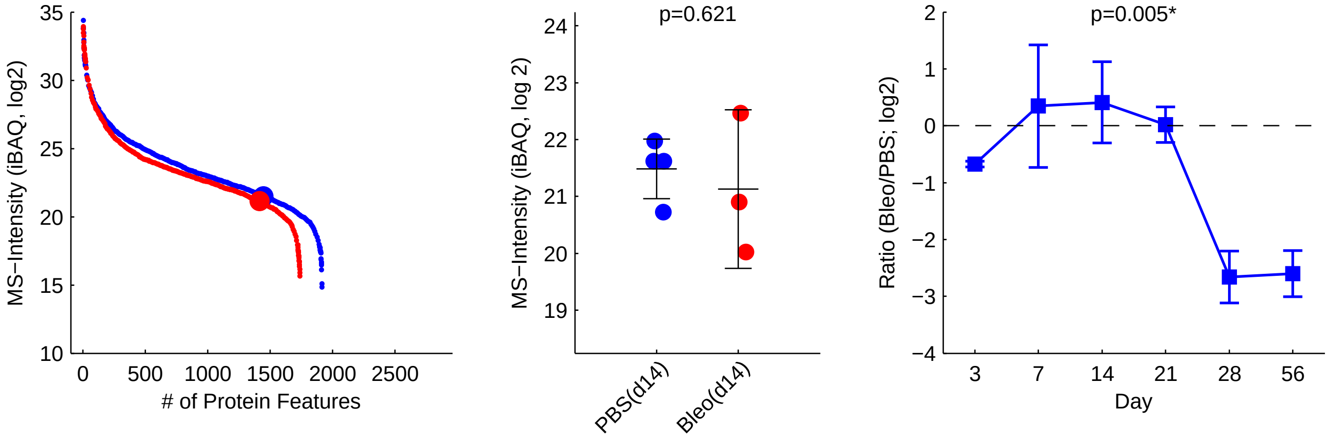

Q6P8X1 − Snx6 (id: 1849)

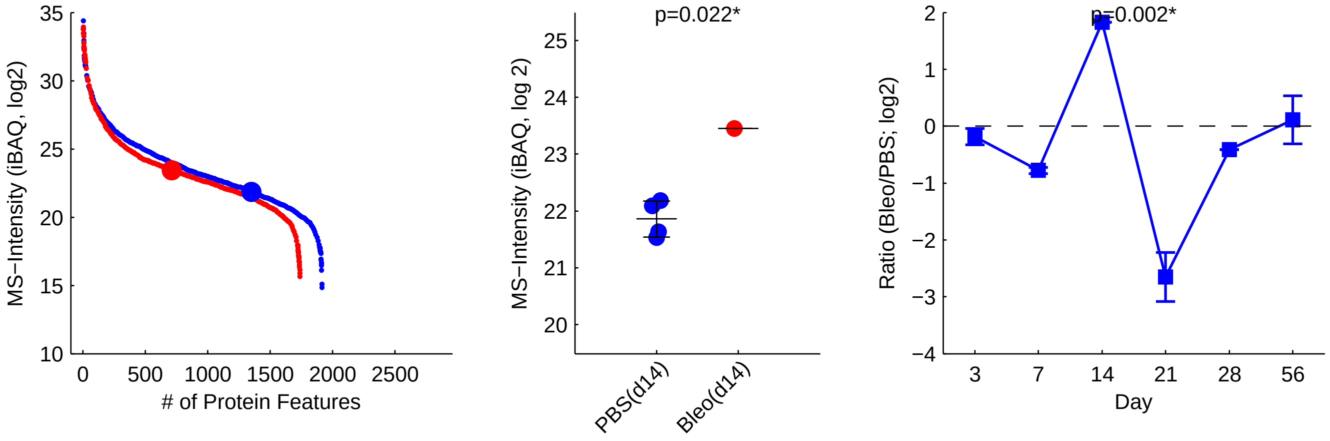

Q6PHU5 − Sort1 (id: 1869)

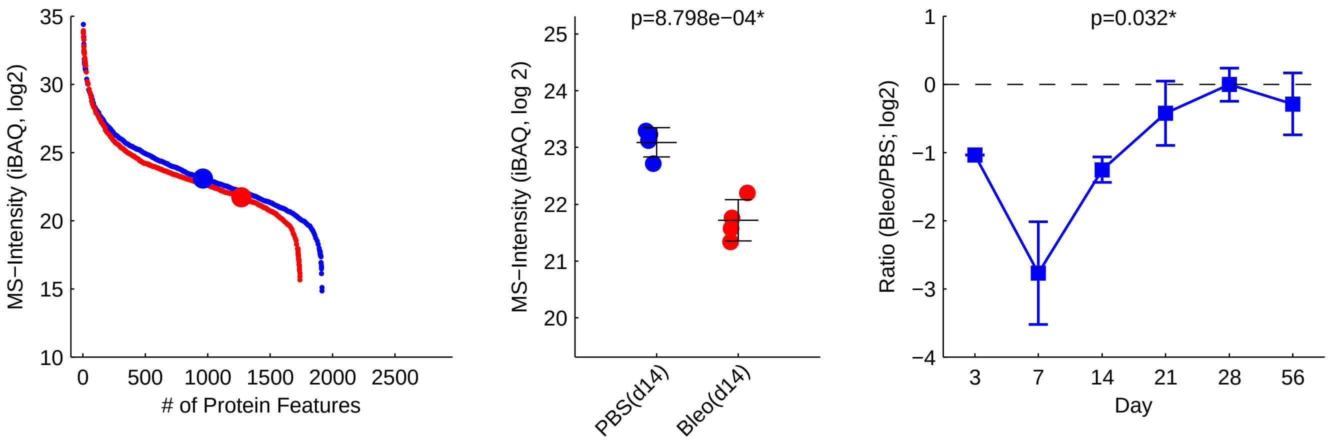

Q6Q899 − Ddx58 (id: 1871)

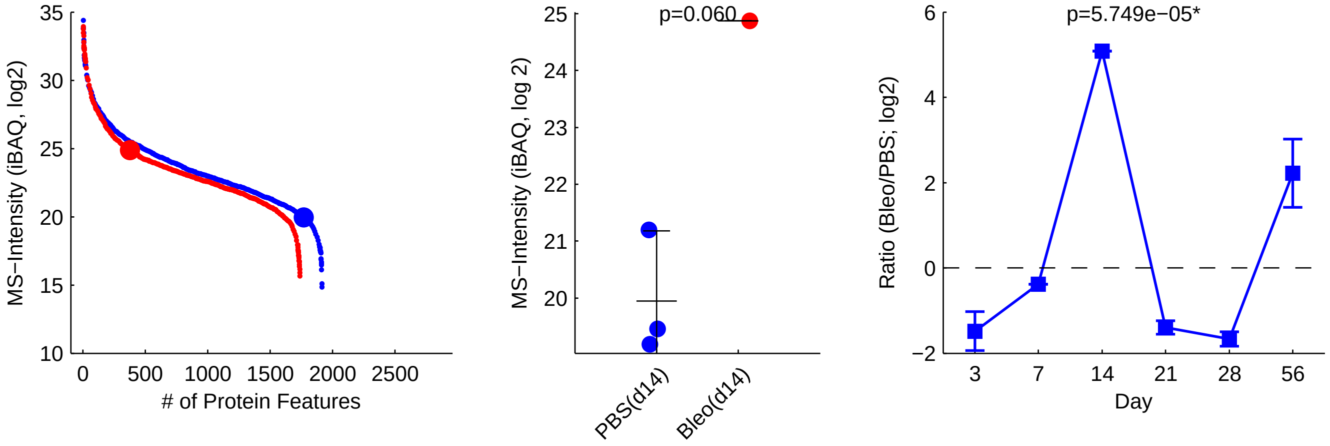

Q6S9I0 − Kng2 (id: 1872)

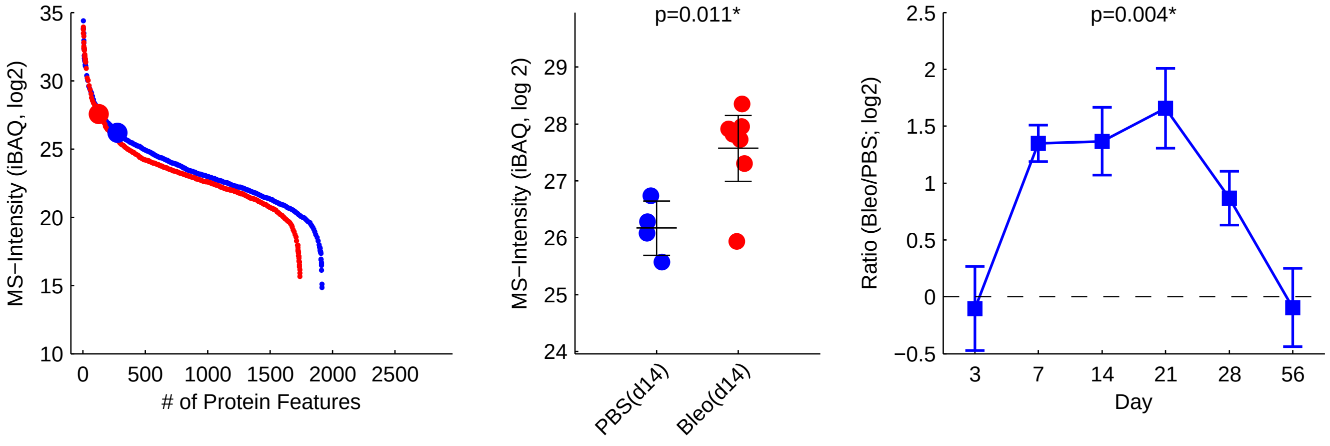

Q8R0B4 − Tardbp (id: 1875)

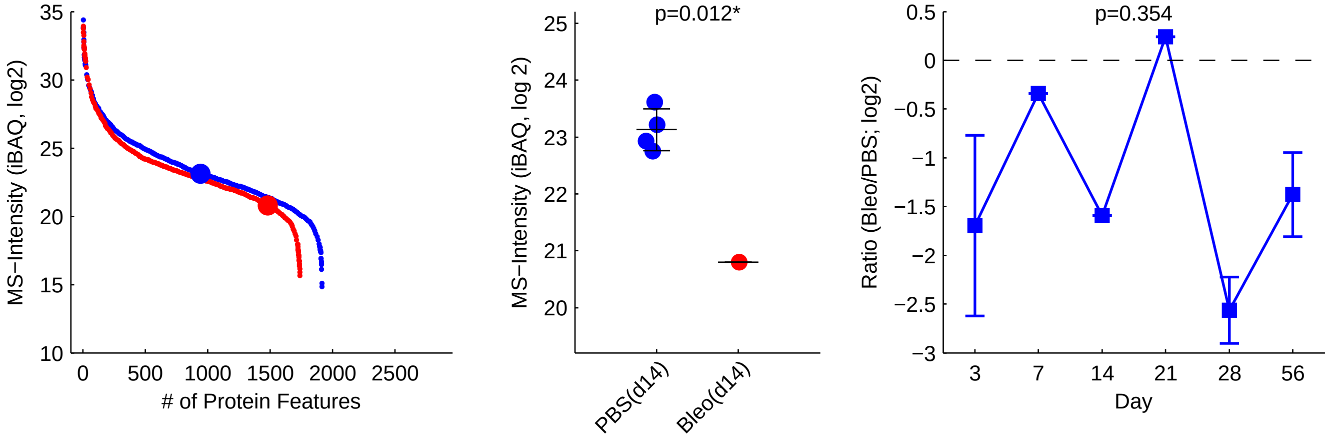

Q6WVG3 − Kctd12 (id: 1877)

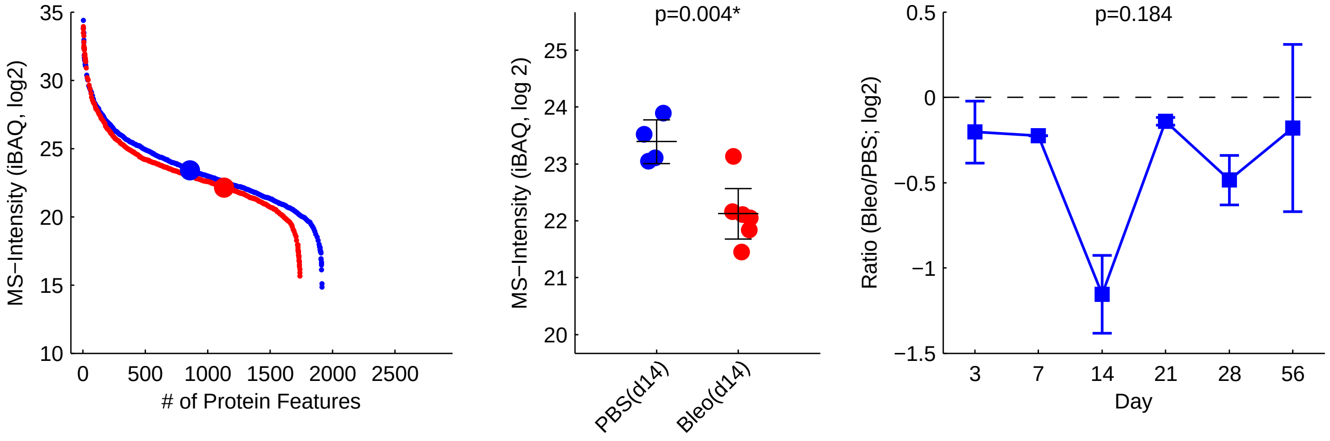

Q6ZWV3 − Rpl10 (id: 1886)

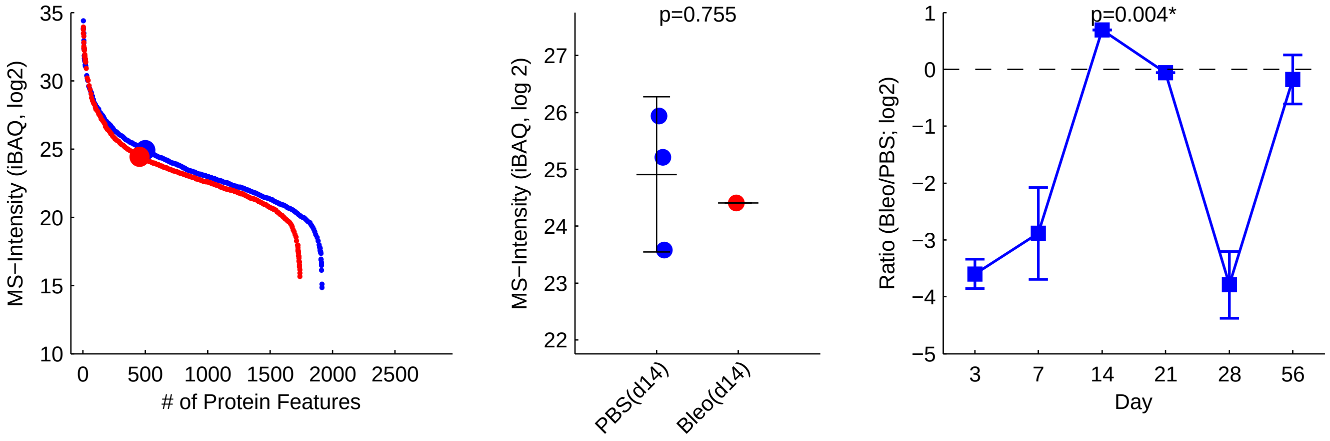

Q6ZWV7 − Rpl35 (id: 1887)

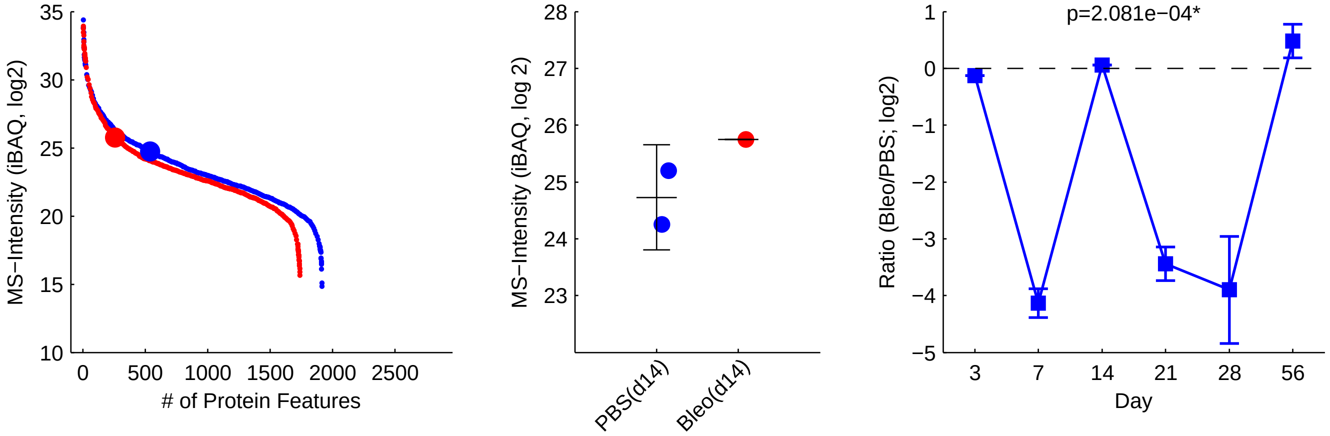

Q78IQ7 − Slc39a4 (id: 1894)

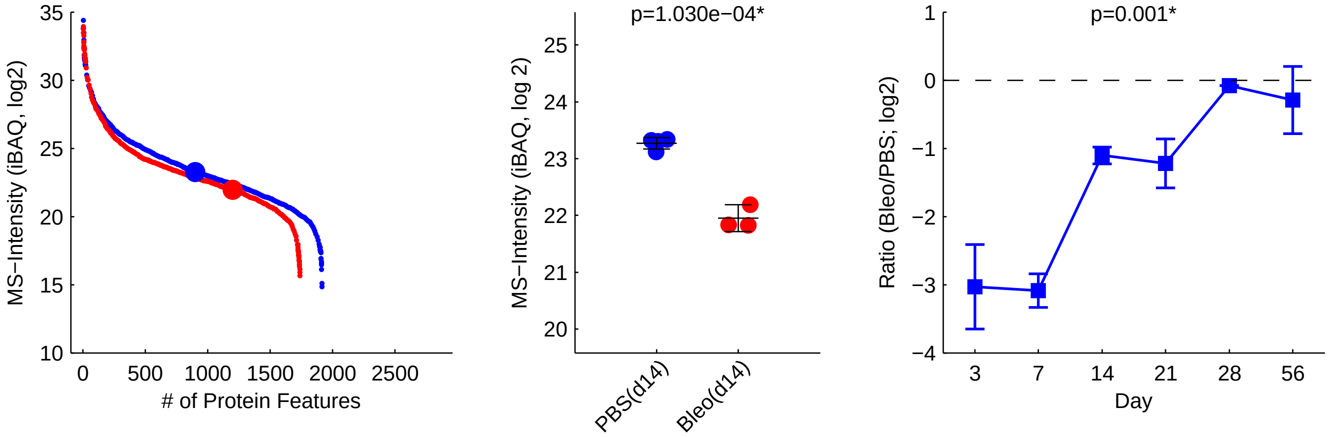

Q78PY7 − Snd1 (id: 1895)

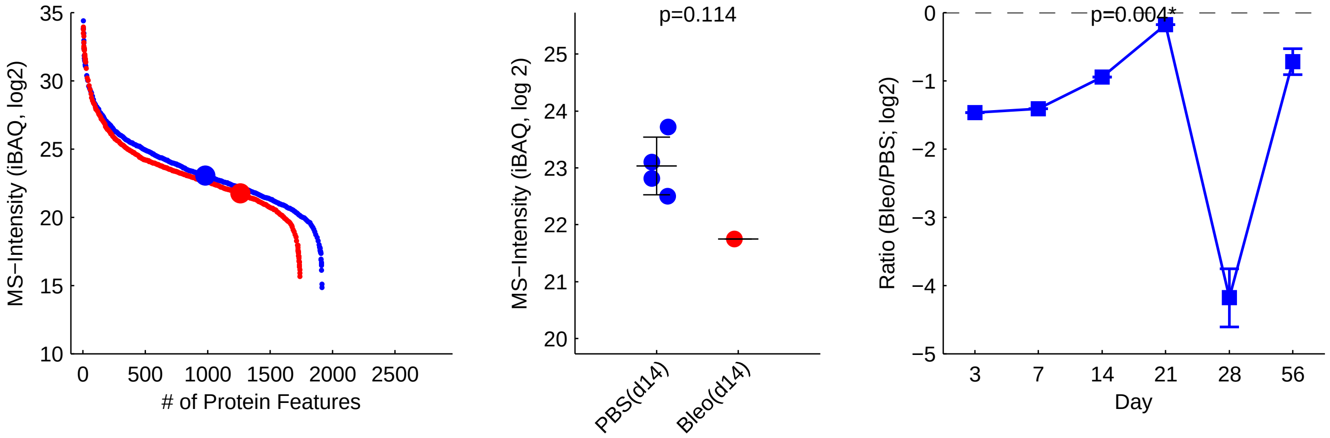

Q7TSV4 − Pgm2 (id: 1906)

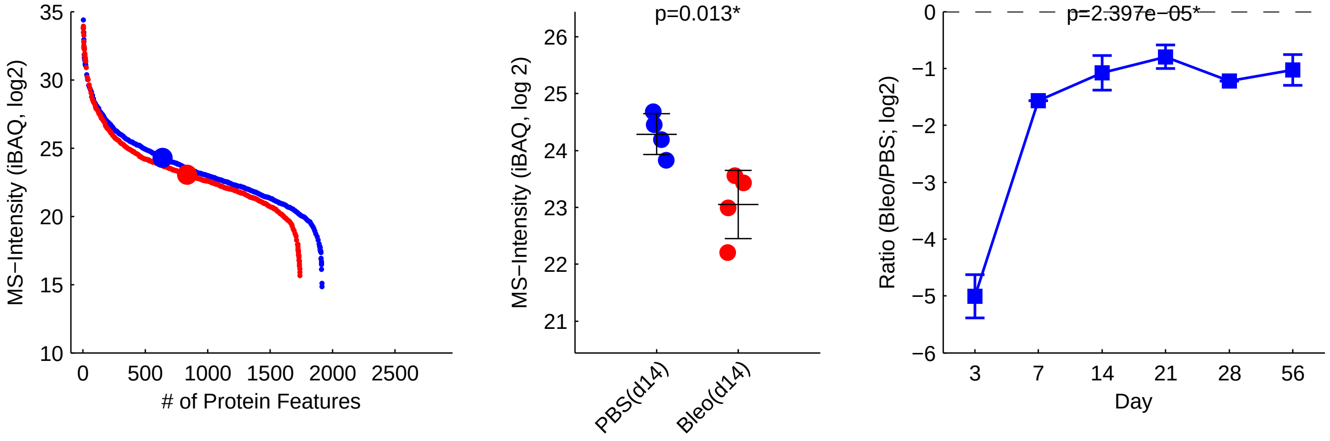

Q80V42 − Cpm (id: 1917)

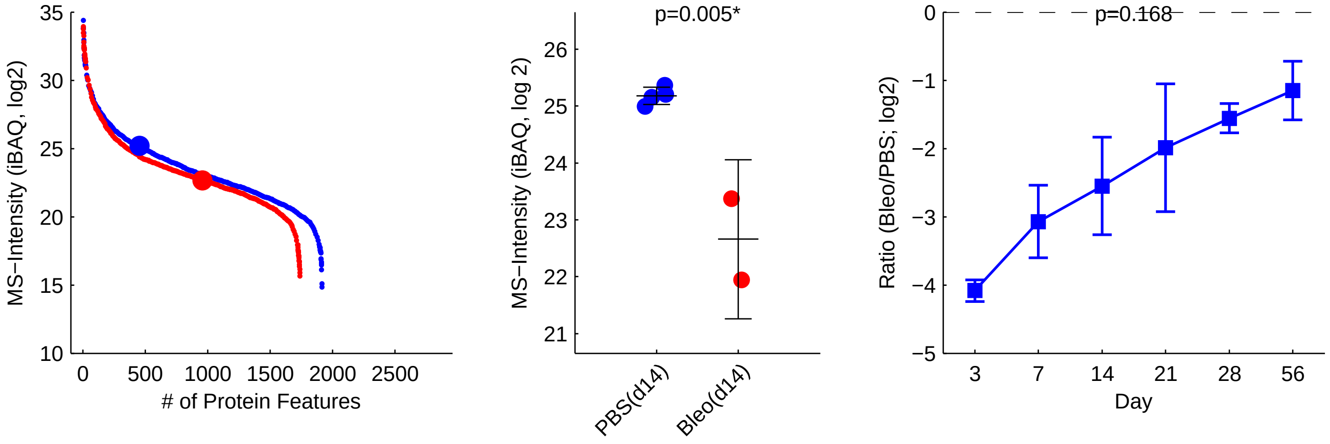

Q80VQ0 − Aldh3b1 (id: 1919)

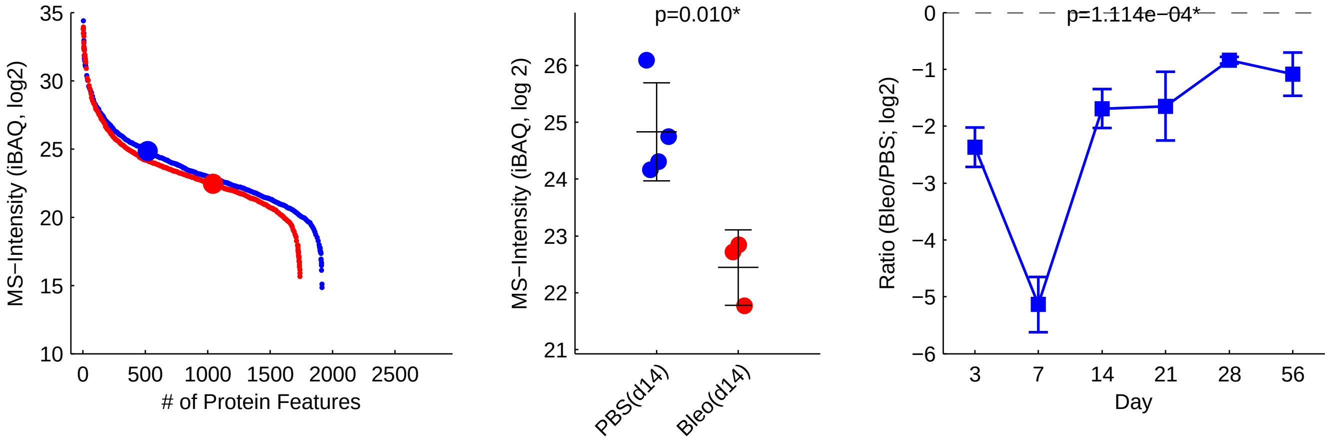

Q80X90 − Flnb (id: 1927)

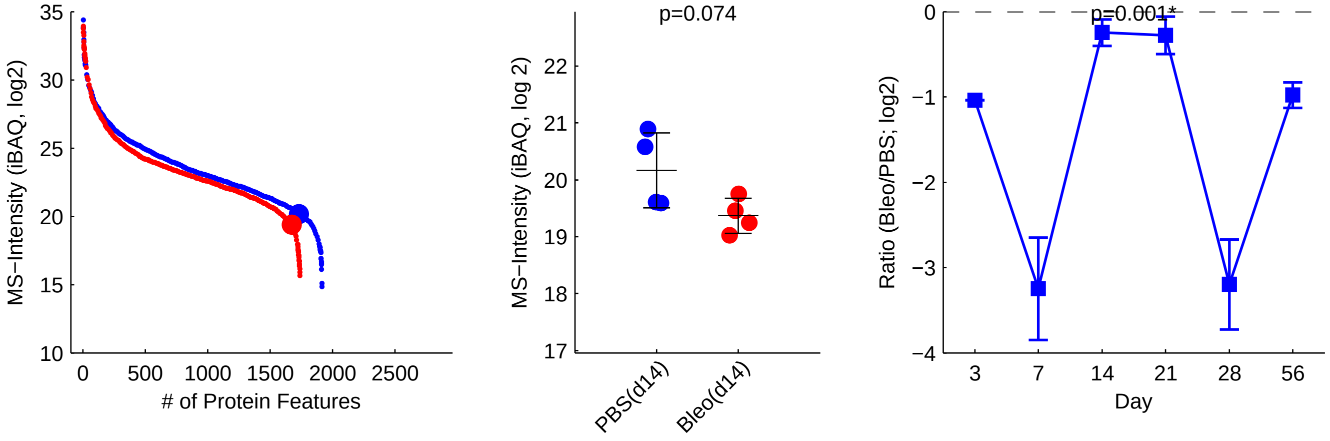

Q80Y75 − Dnajb13 (id: 1929)

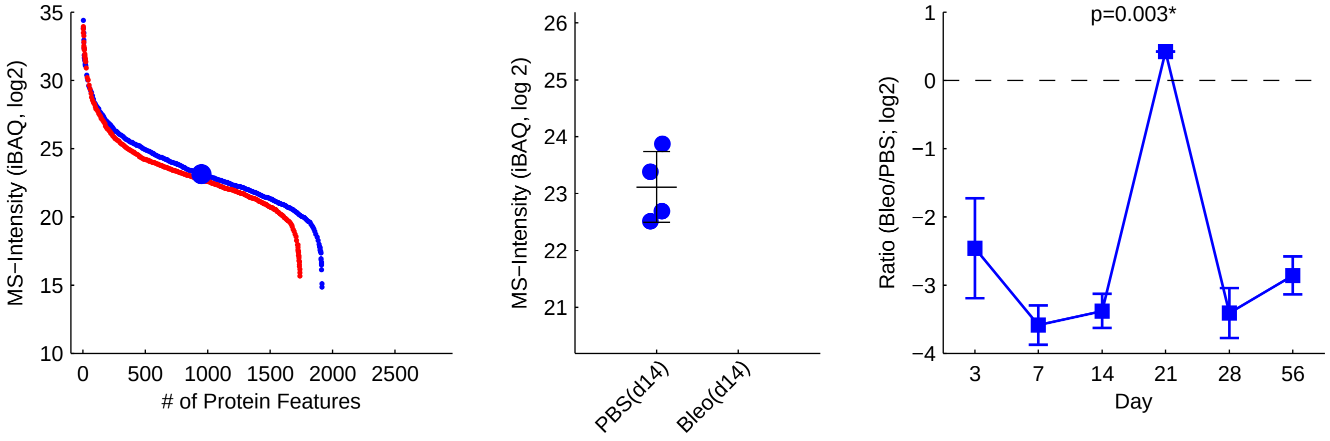

Q80YC5 − F12 (id: 1931)

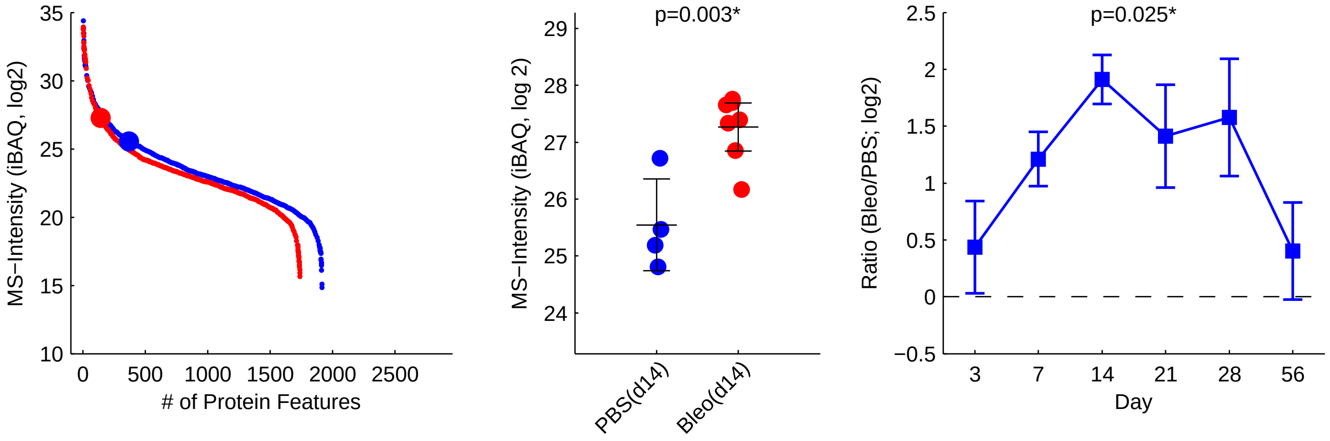

Q80YQ1 − Thbs1 (id: 1932)

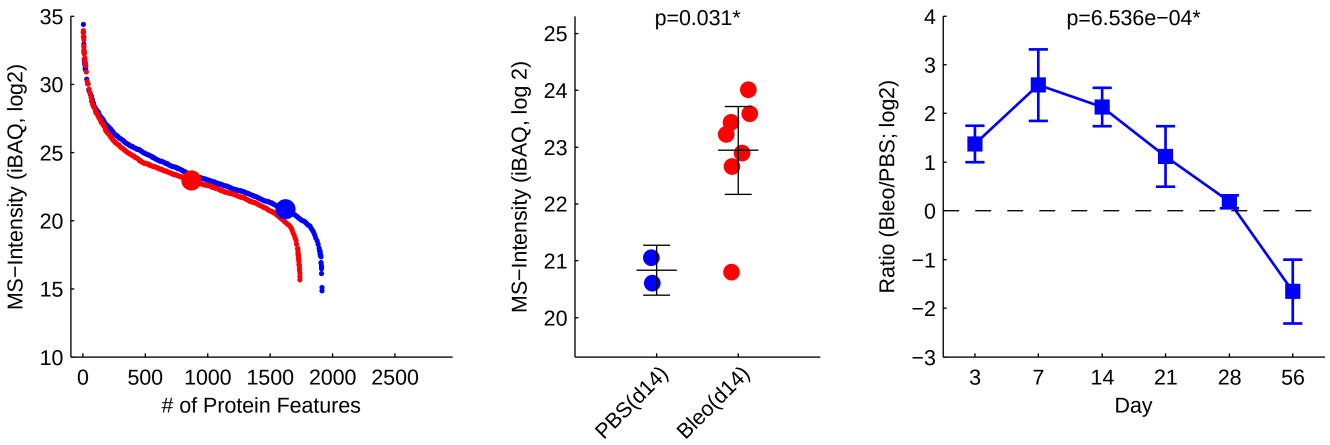

Q80YX1 − Tnc (id: 1933)

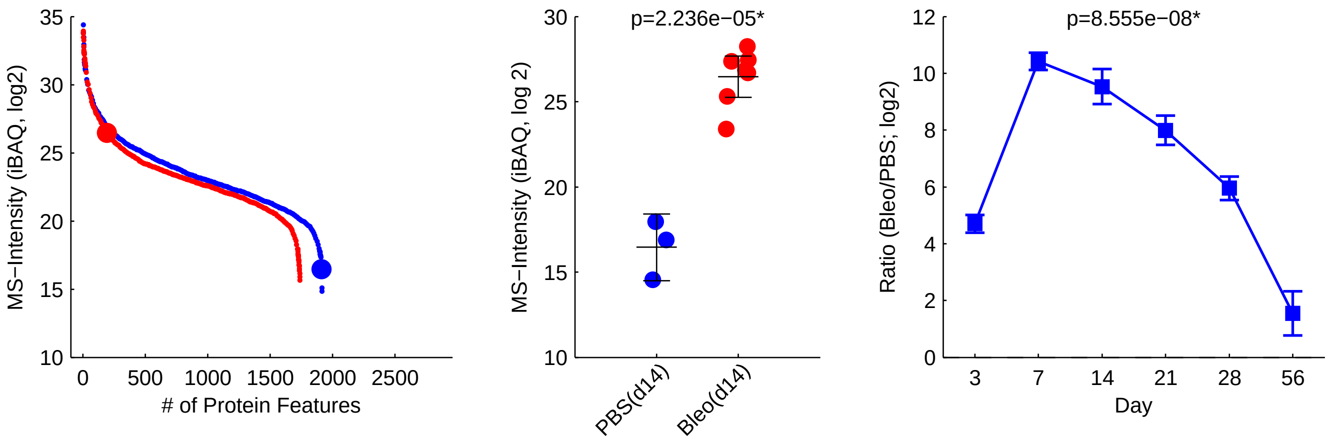

Q8BFR4 − Gns (id: 1938)

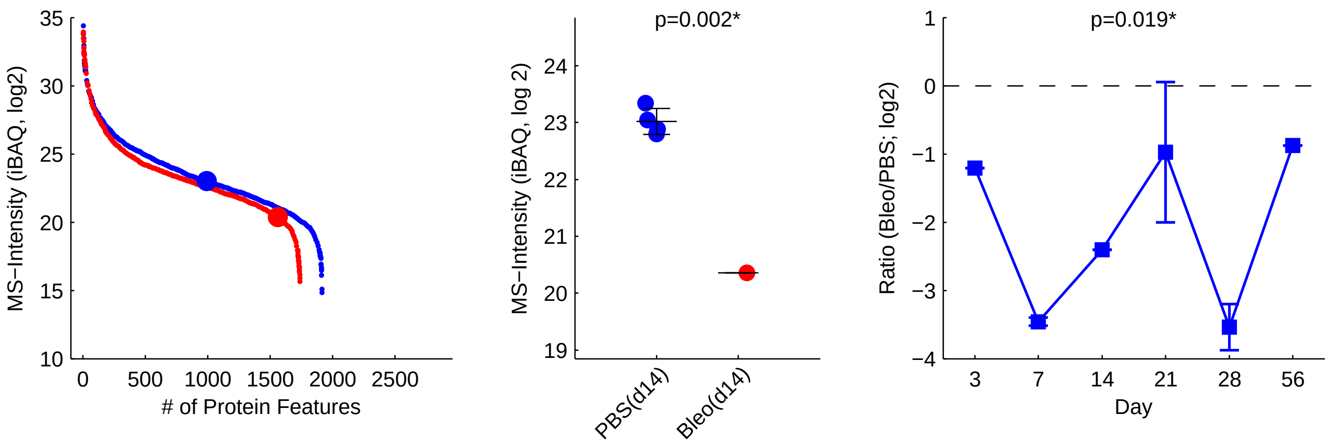

Q8BG05−2 − Gm9242 (id: 1945)

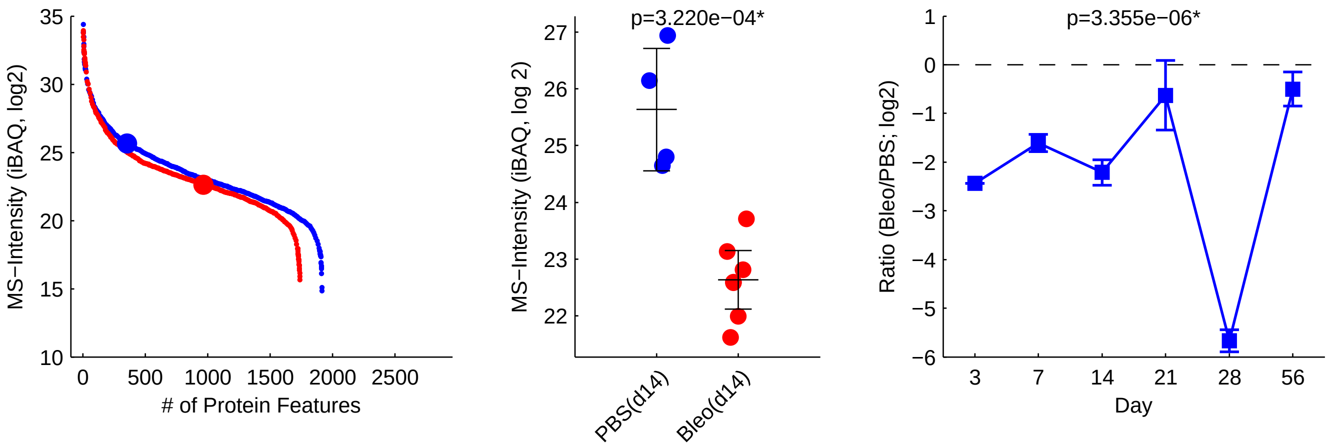

Q8BGB7 − Enoph1 (id: 1949)

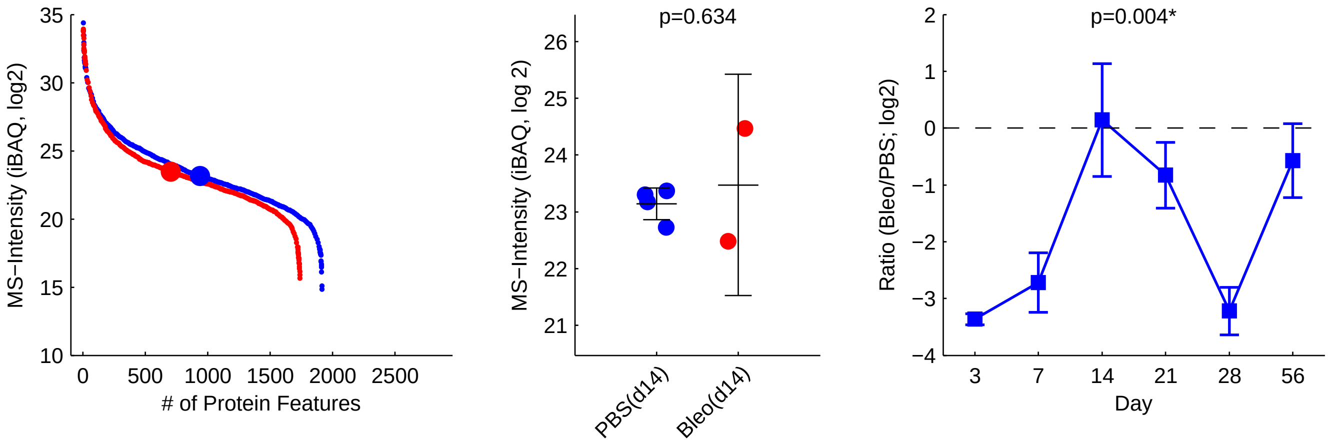

Q8BGJ5 − Ptbp1 (id: 1951)

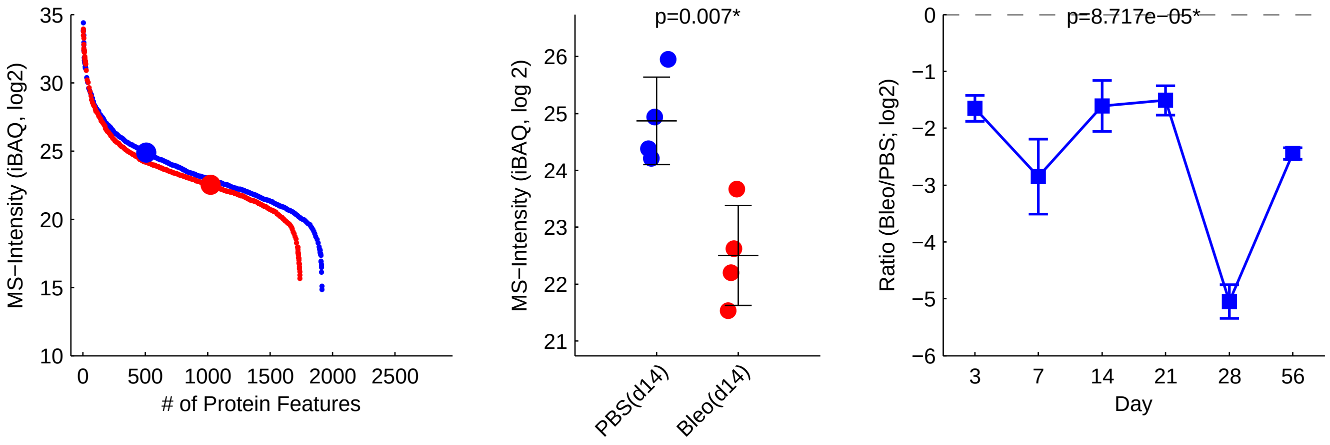

Q8BH35 − C8b (id: 1957)

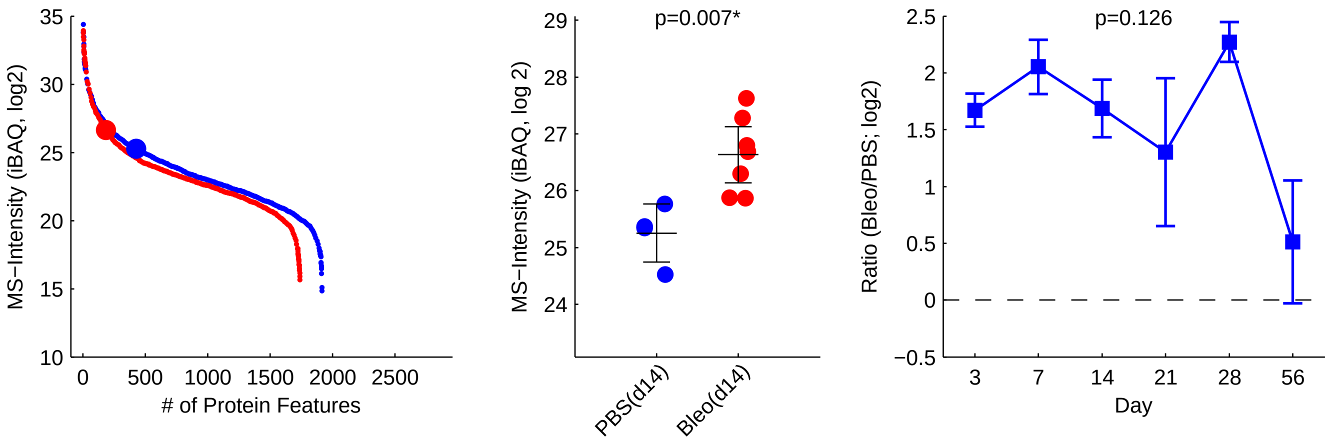

Q8BH95 − Echs1 (id: 1966)

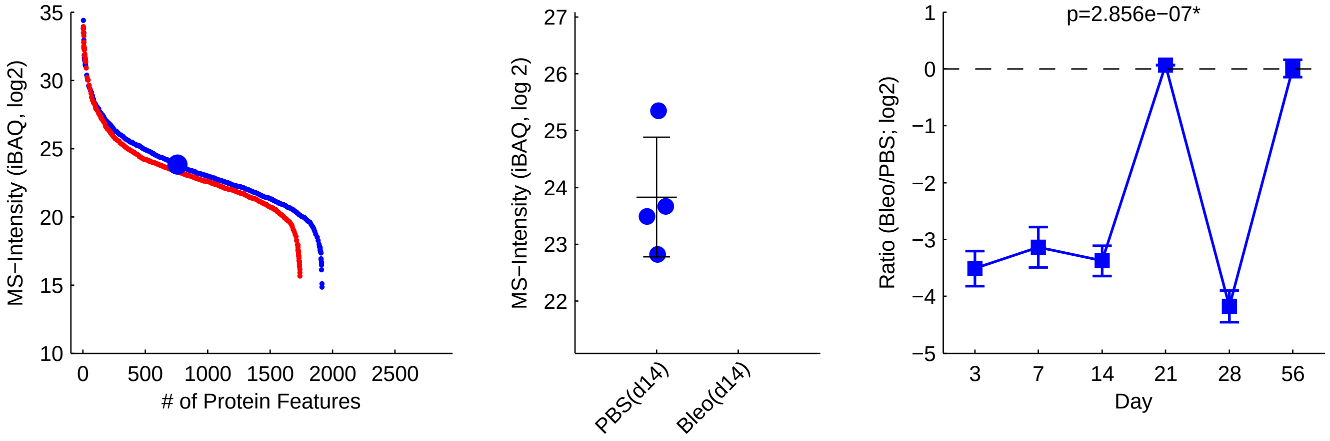

Q8BHB9 − Clic6 (id: 1967)

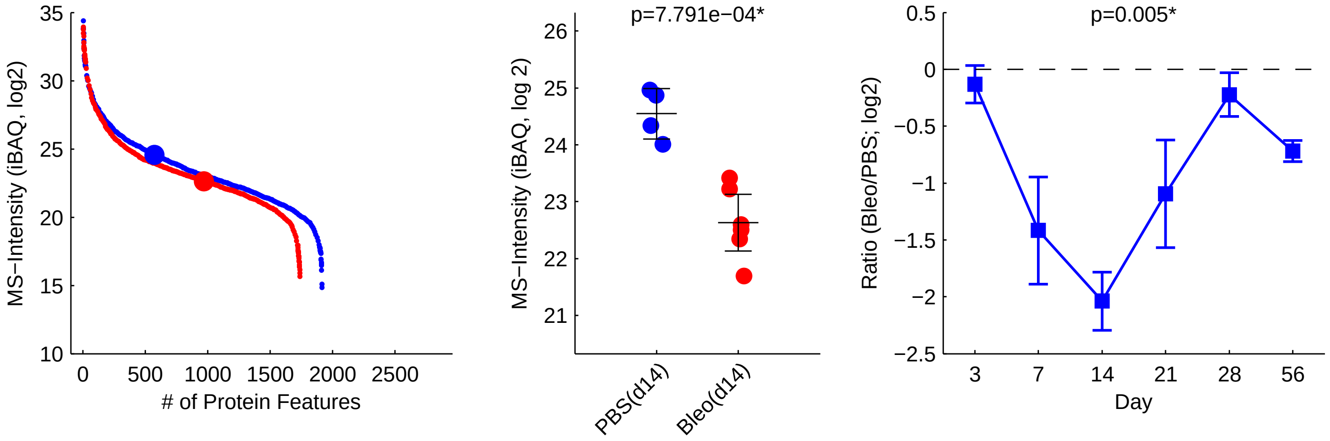

Q8BHN3 − Ganab (id: 1971)

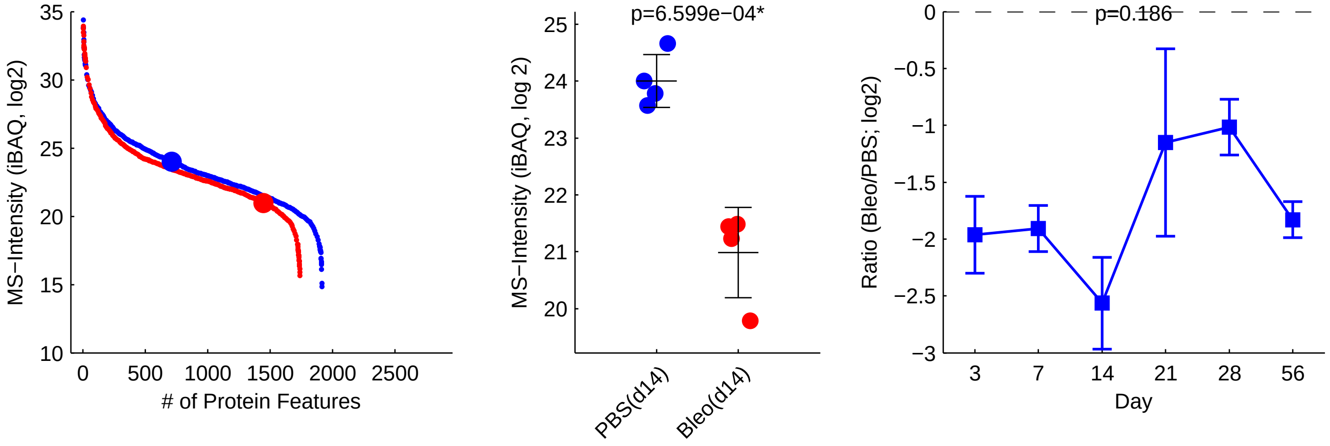

Q8BJU0−2 − Sgta (id: 1980)

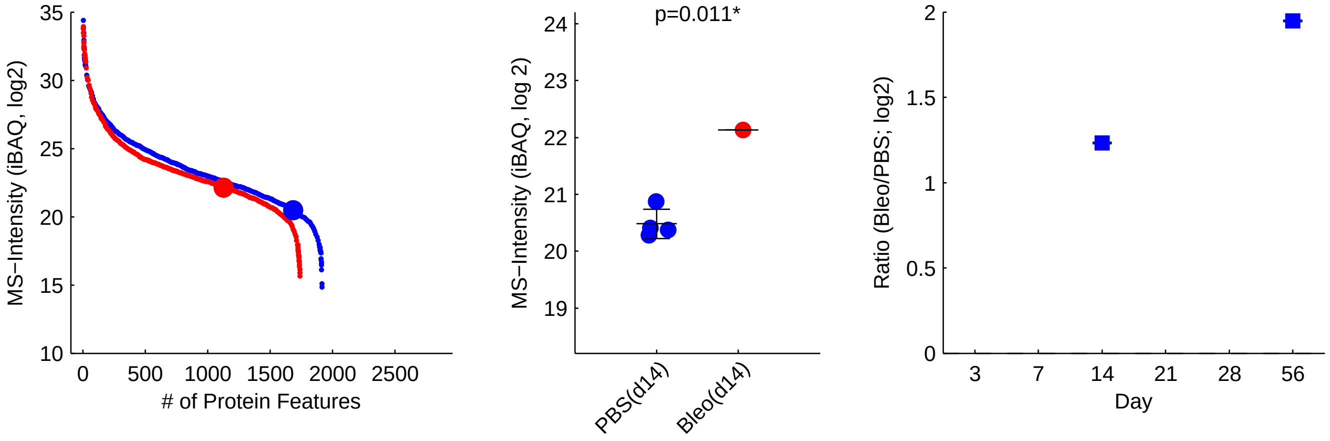

Q8BK48 − Ces2e (id: 1983)

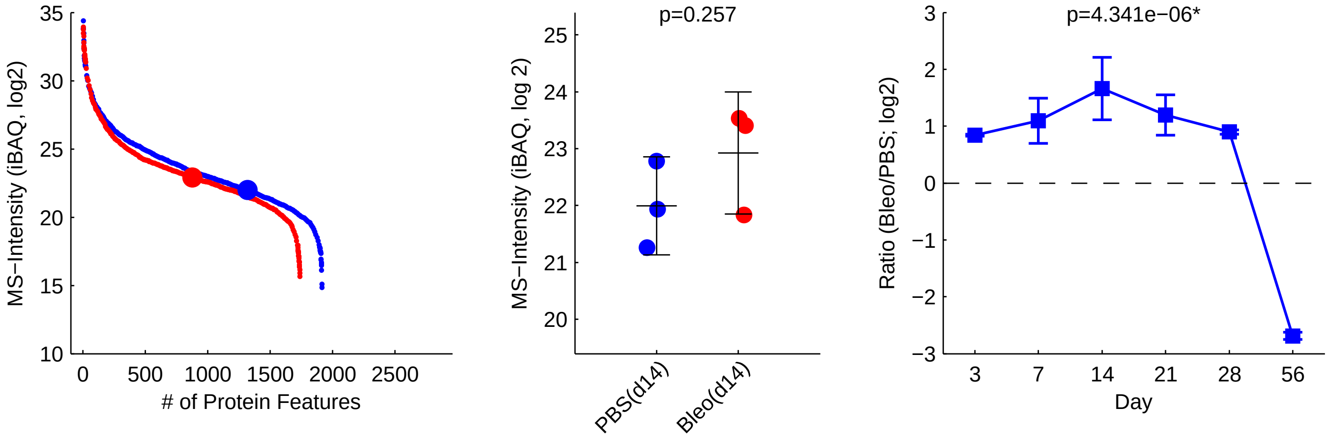

Q8BK62 − Olfml3 (id: 1984)

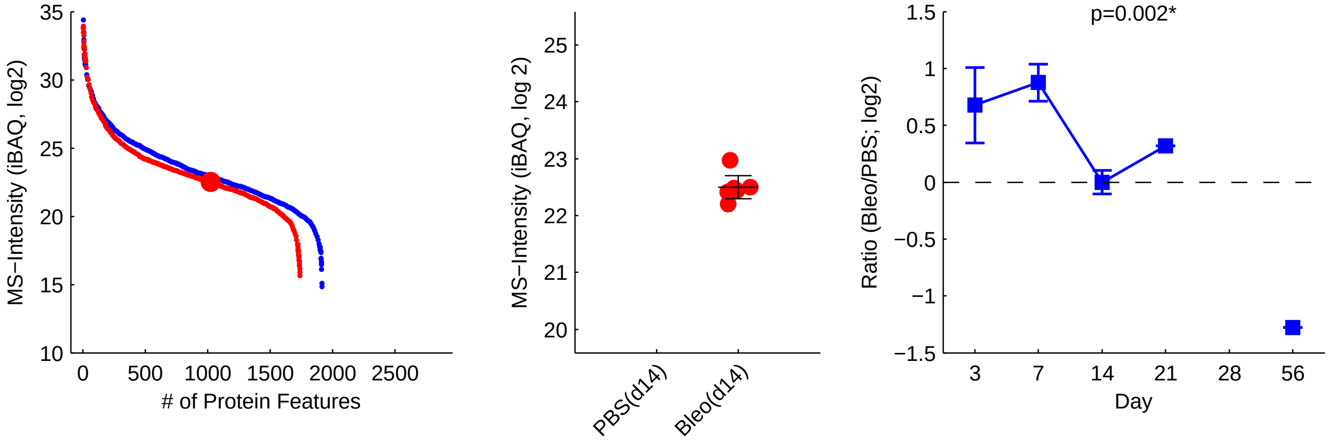

Q8BK64 − Ahsa1 (id: 1985)

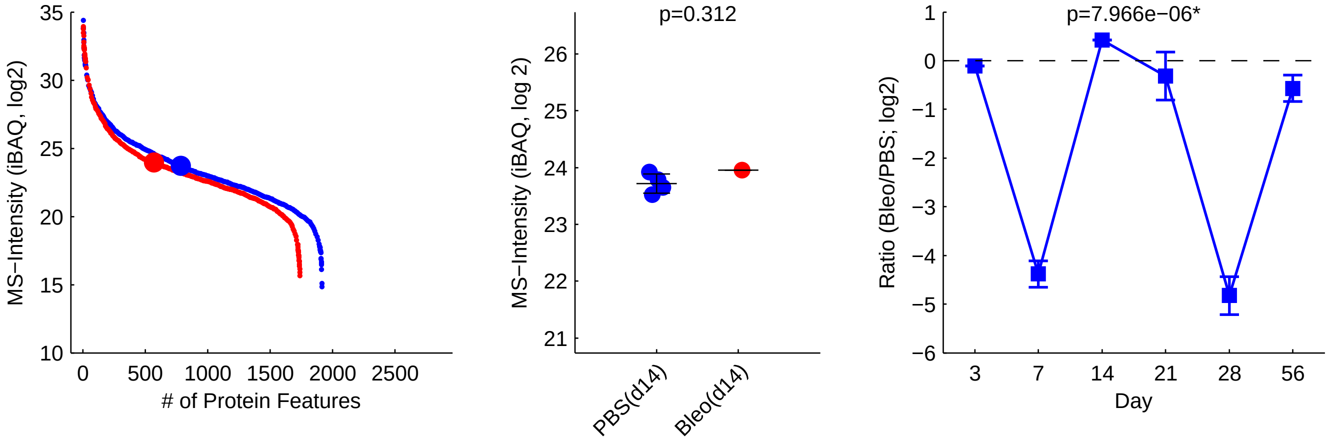

Q8BM88 − Ctso (id: 1993)

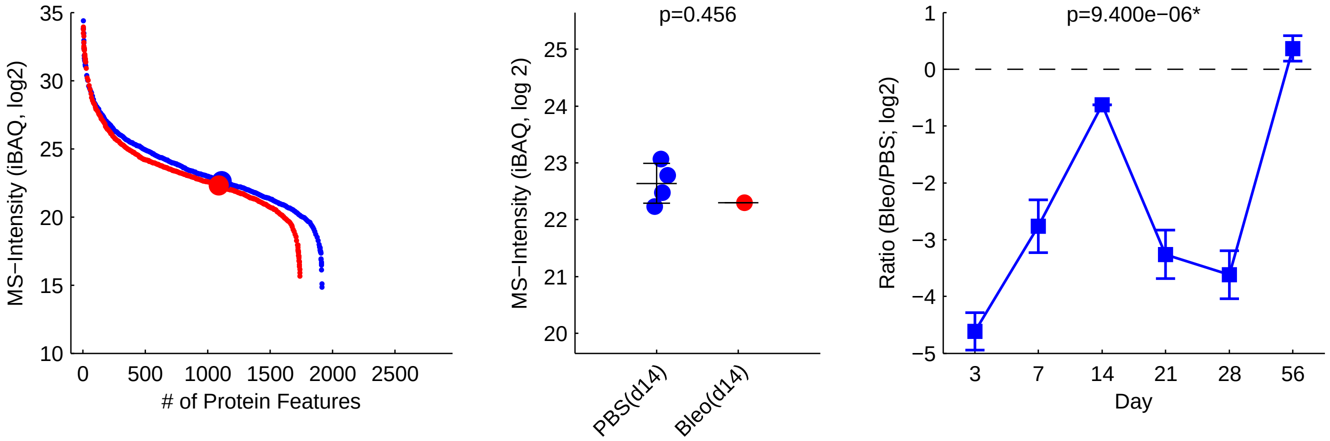

Q8BML9 − Qars (id: 1997)

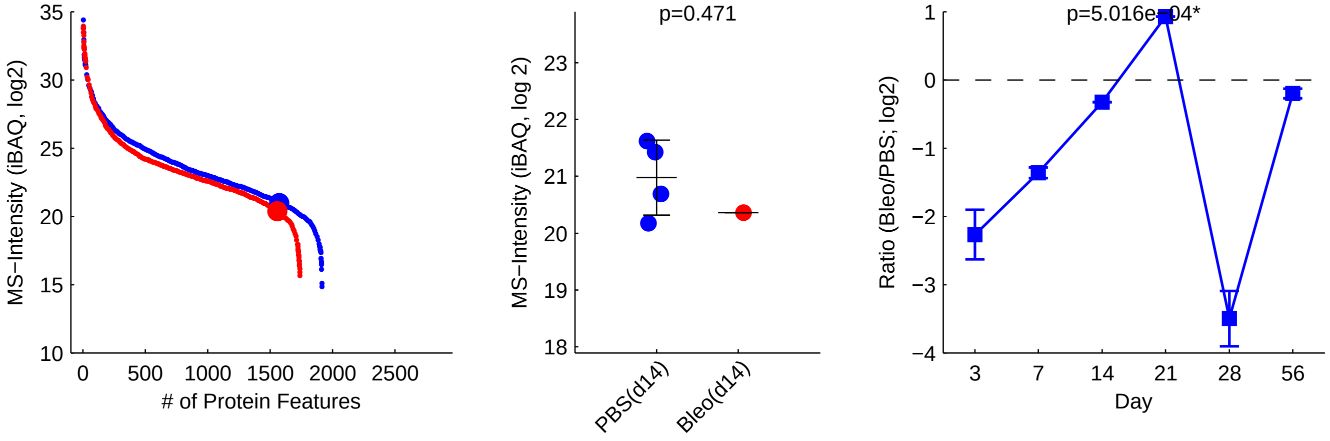

Q8BMS1 − Hadha (id: 1998)

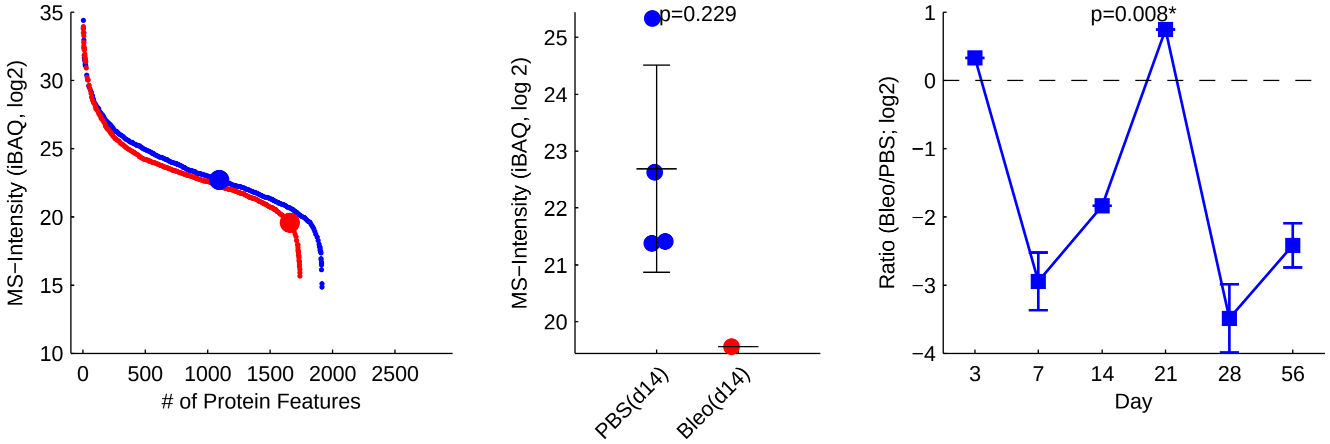

Q8BND5−2 − Qsox1 (id: 2000)

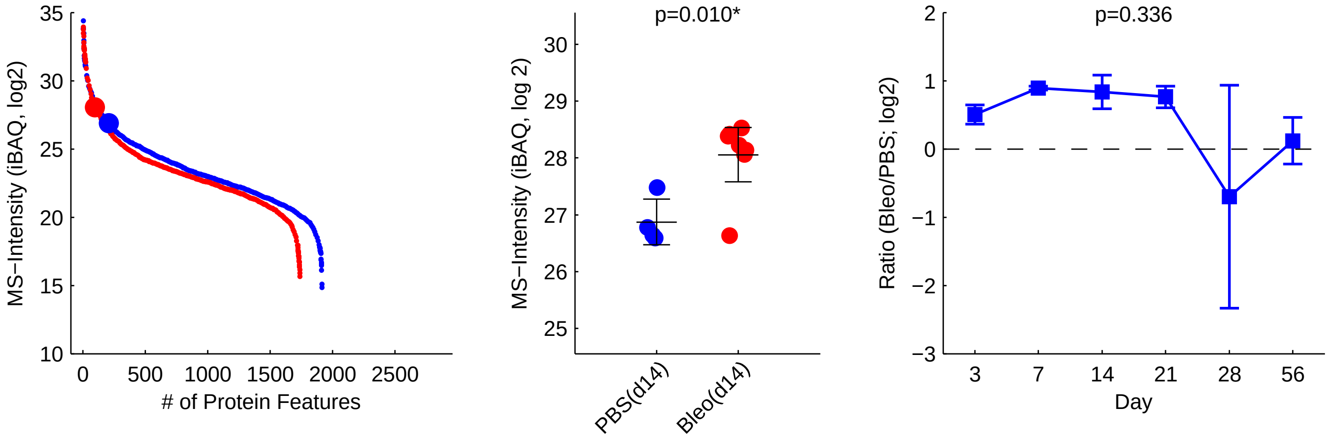

Q8BTZ7 − Gmppb (id: 2016)

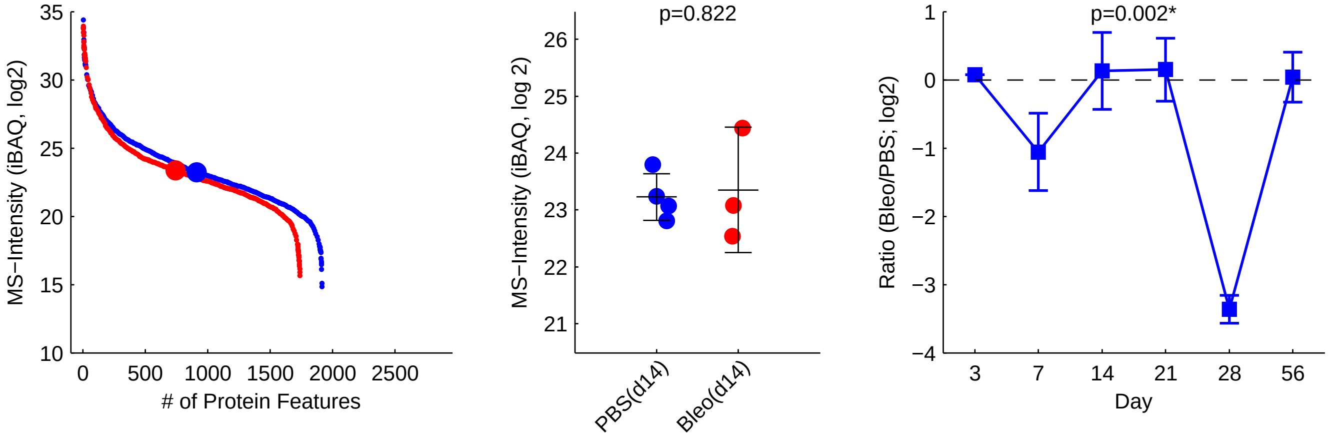

Q8BWT1 − Acaa2 (id: 2031)

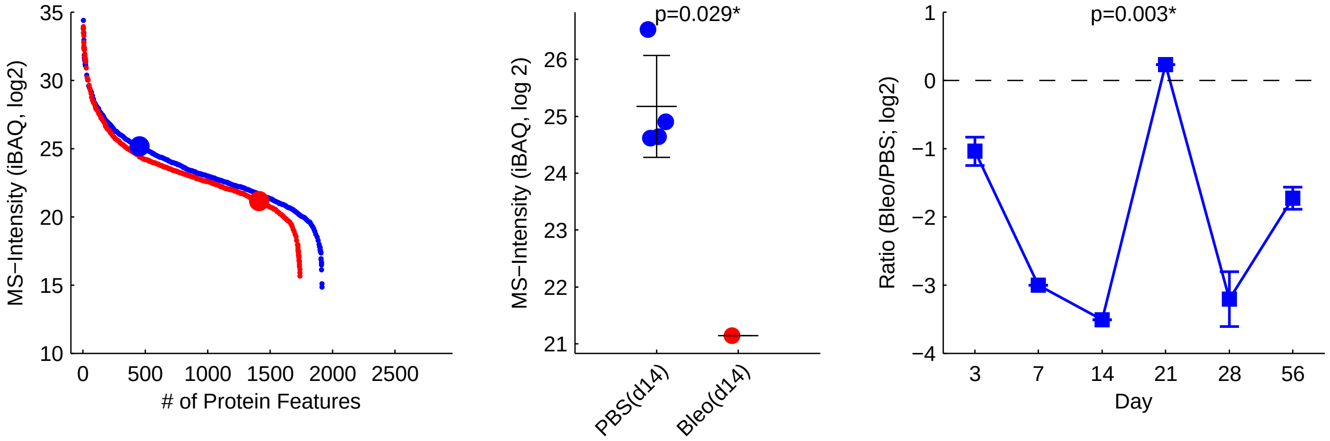

Q8BWY3 − Etf1 (id: 2032)

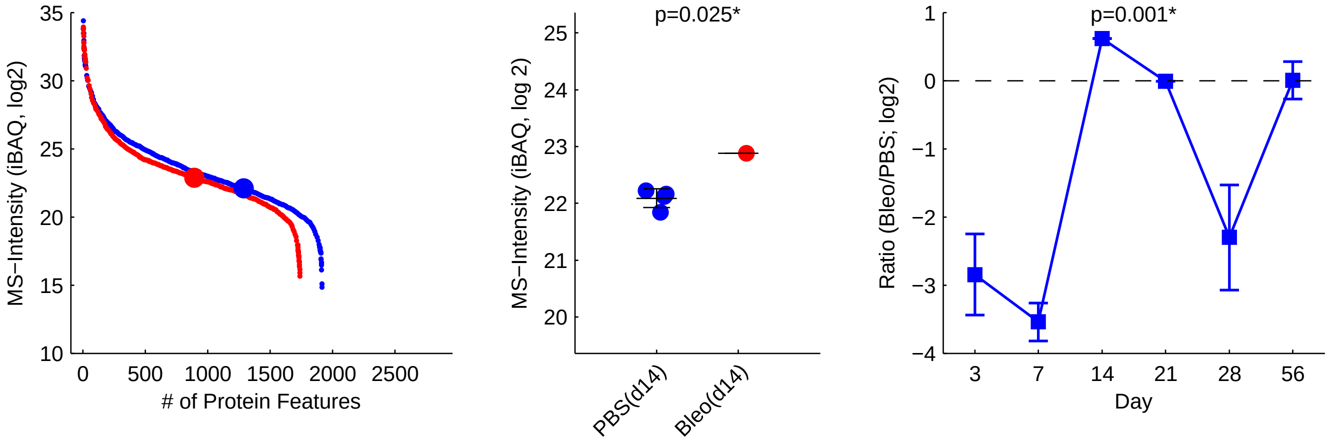

Q8BXK9 − Clic5 (id: 2034)

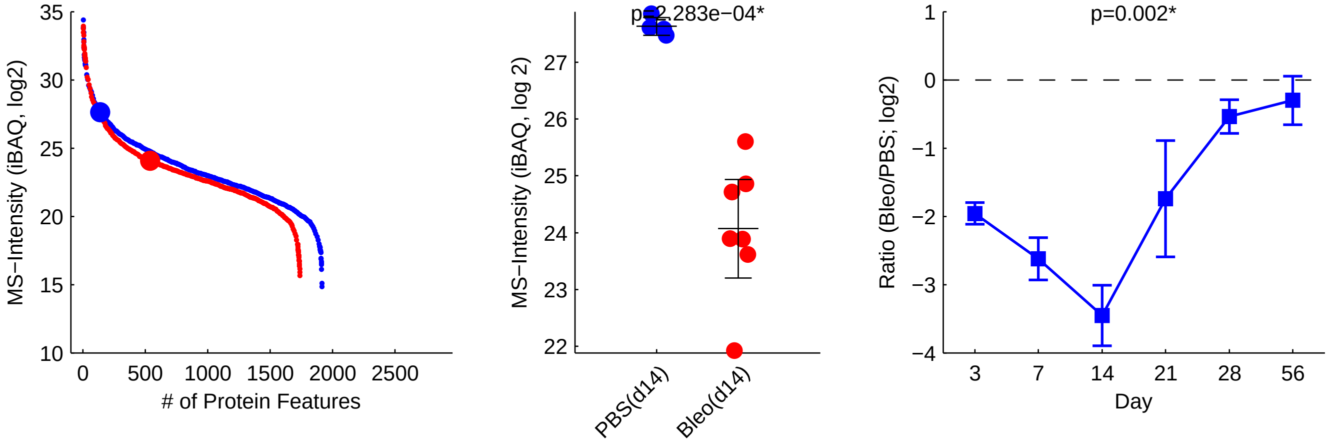

Q8BYM7 − Rsph4a (id: 2039)

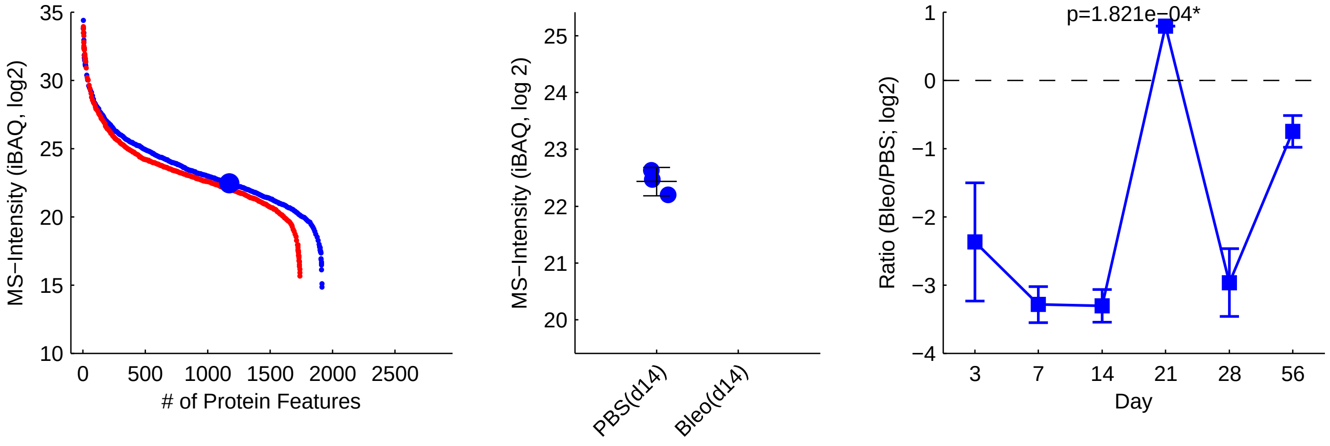

Q8C255 − Dpep2 (id: 2051)

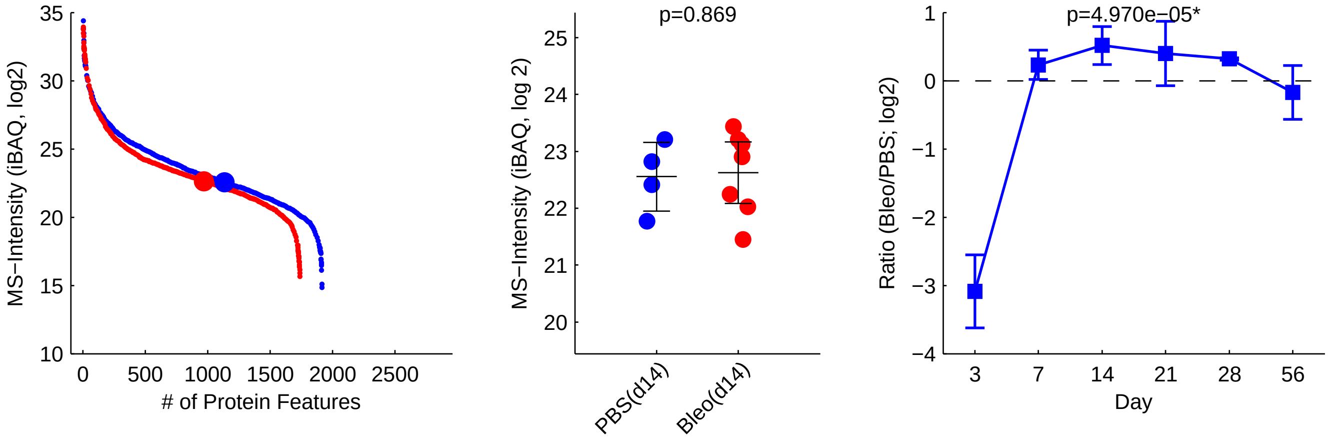

Q8C3V4 − Stat1 (id: 2053)

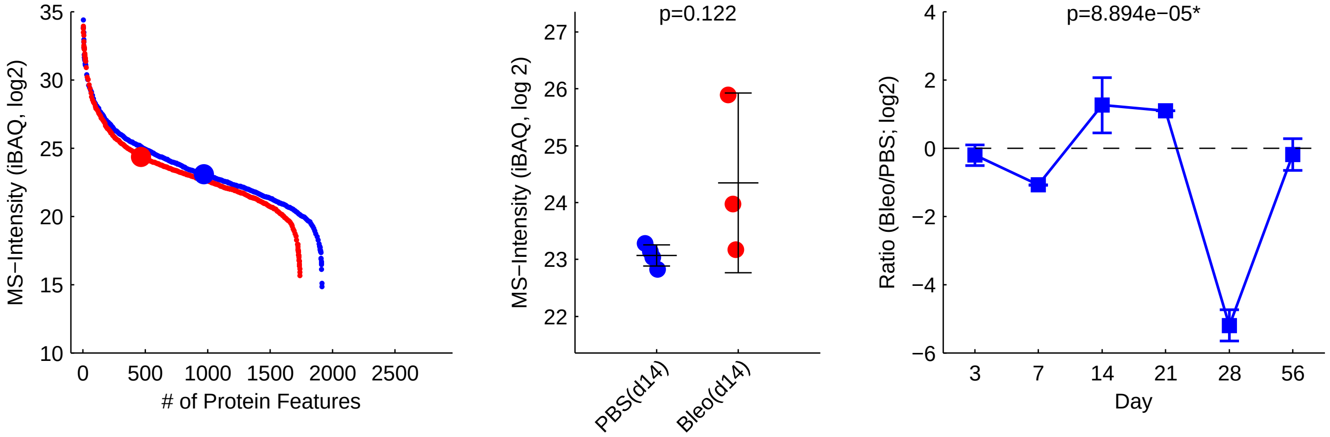

Q8C5R8 − Prps1l1 (id: 2057)

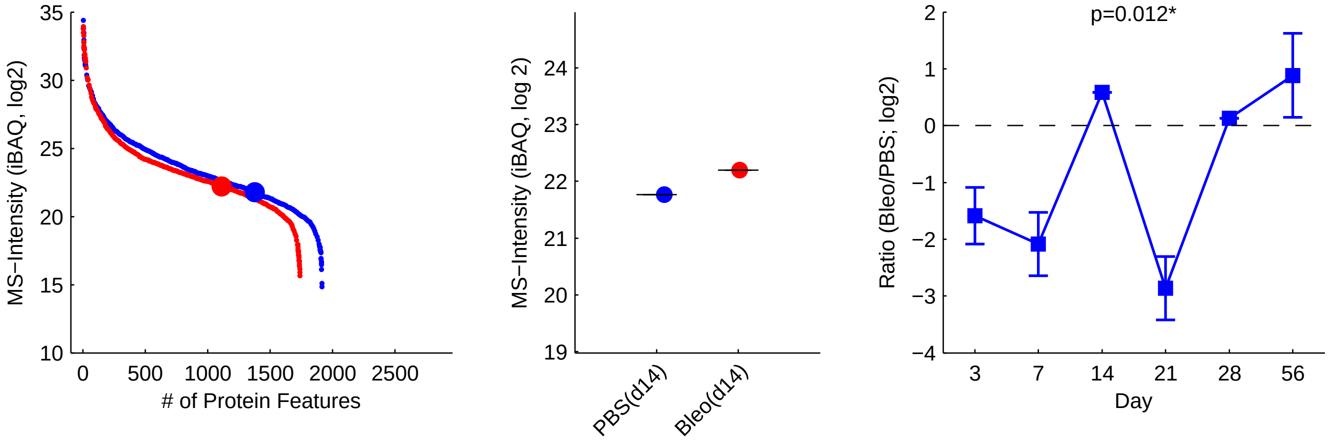

Q8C6B0 − Mettl7a1 (id: 2060)

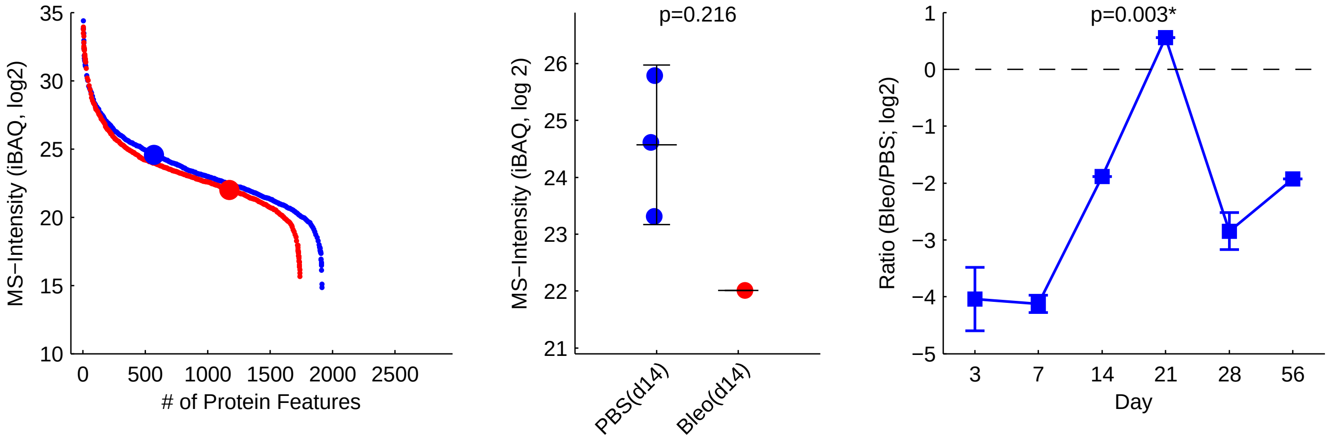

Q8C845 − Efhd2 (id: 2065)

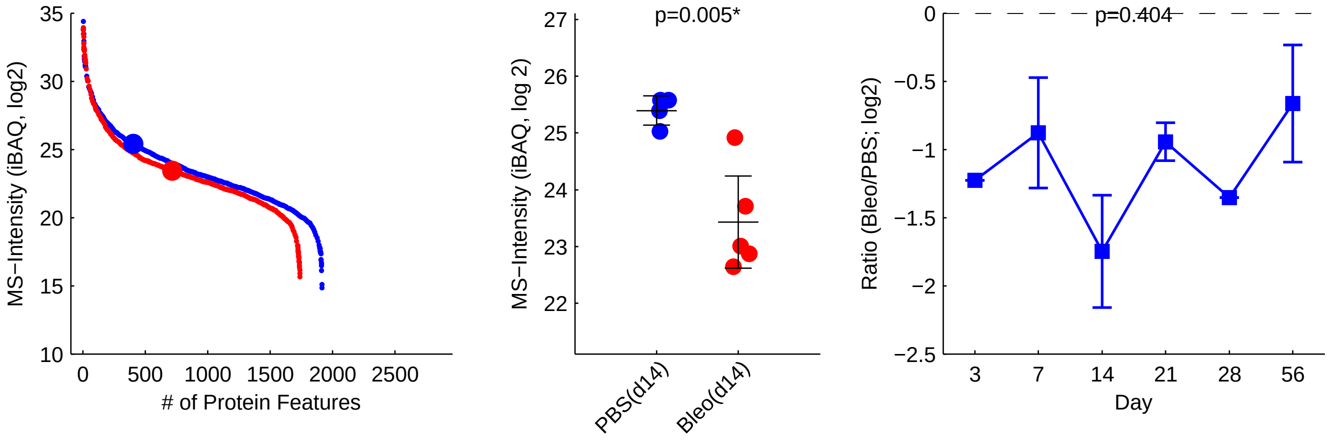

Q8CDN6 − Txnl1 (id: 2074)

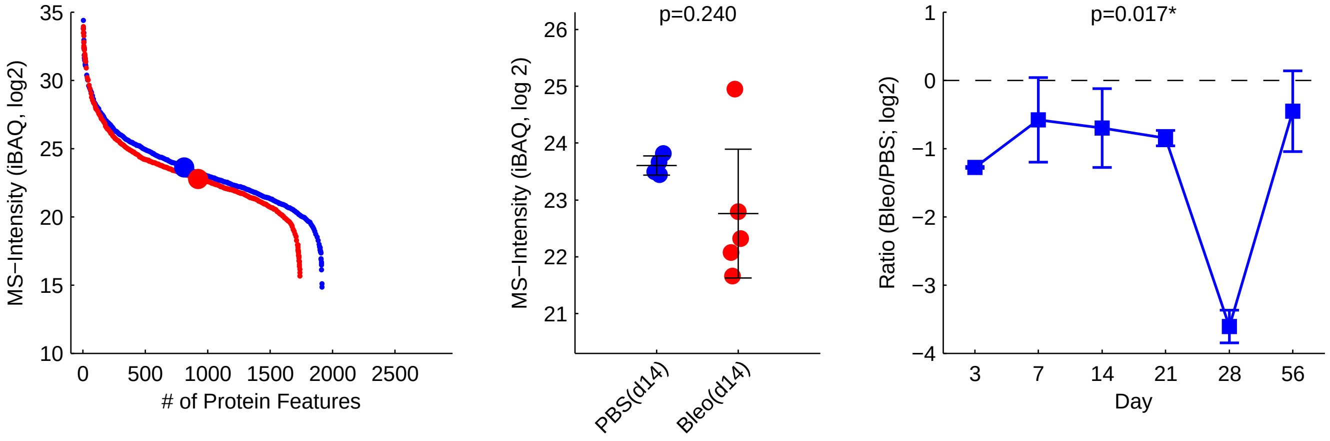

Q8CFV9 − Rfk (id: 2076)

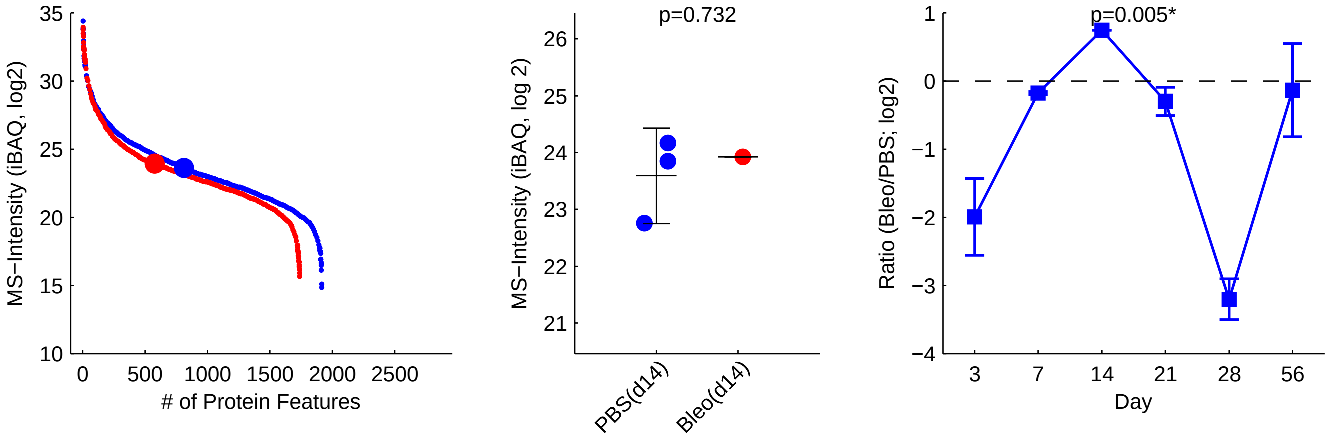

Q8CG16 − C1ra (id: 2078)

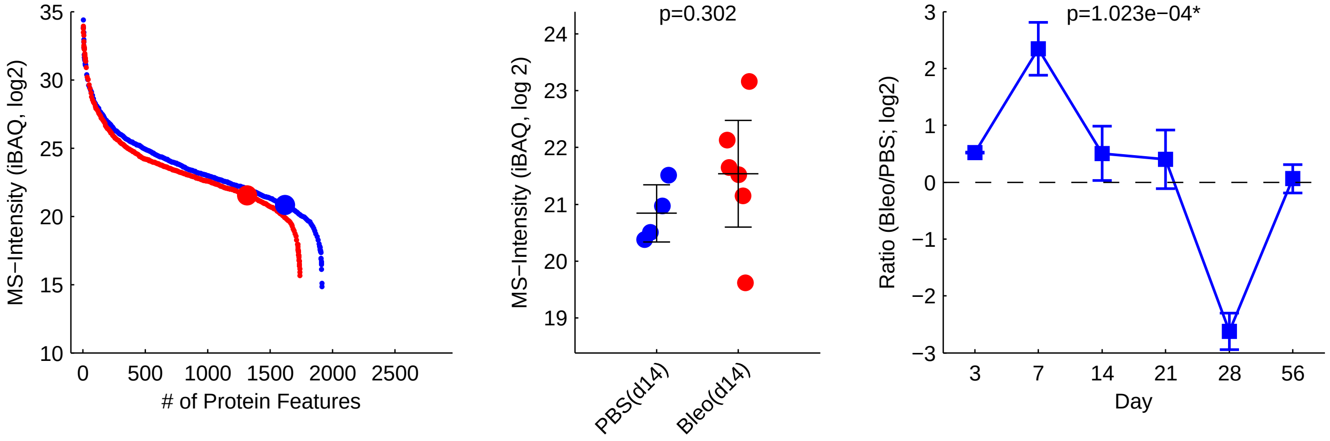

Q8CG76 − Akr7a2 (id: 2081)

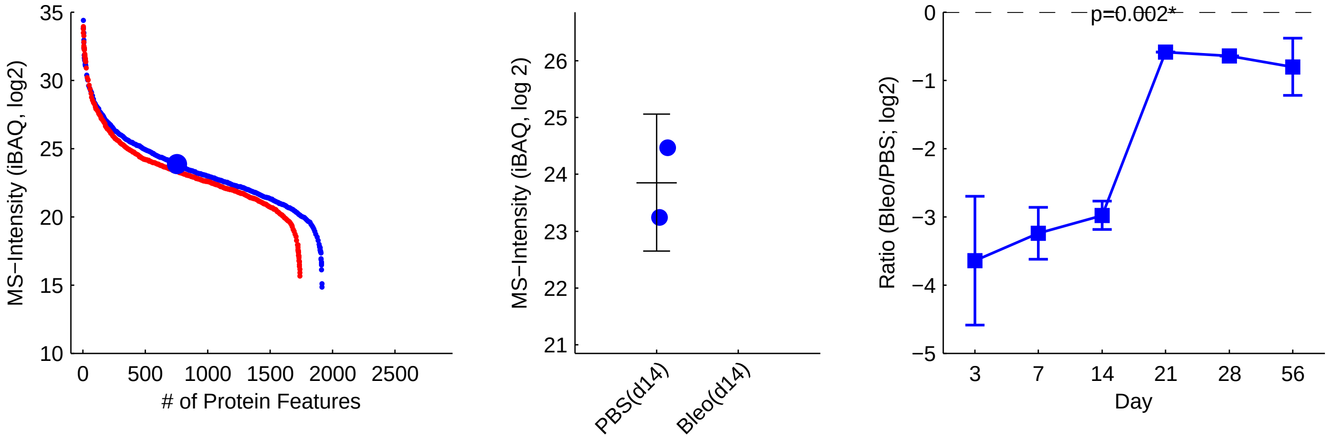

Q8CGC7 − Eprs (id: 2083)

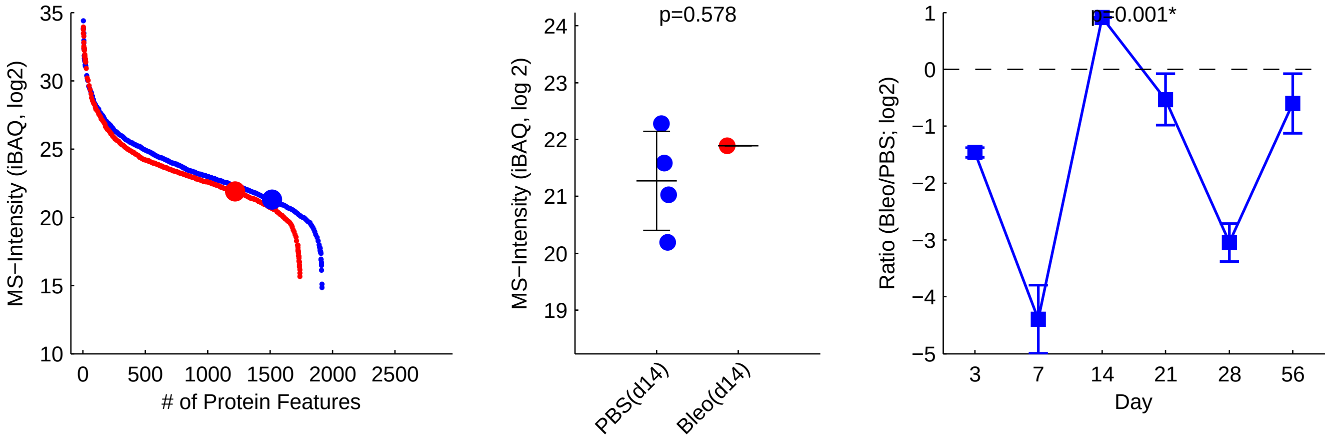

Q8CHP8 − Pgp (id: 2087)

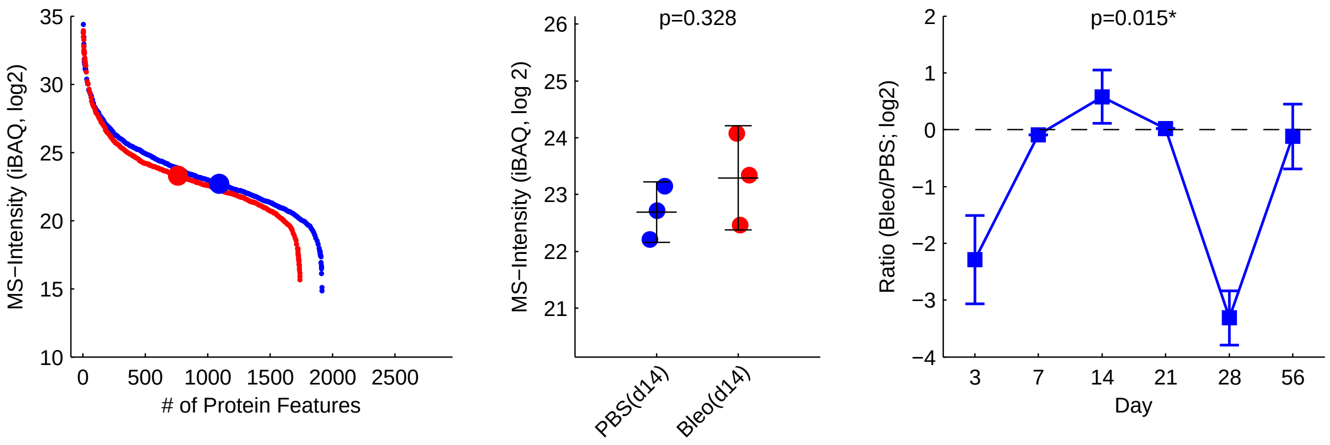

Q8CIF4 − Btd (id: 2092)

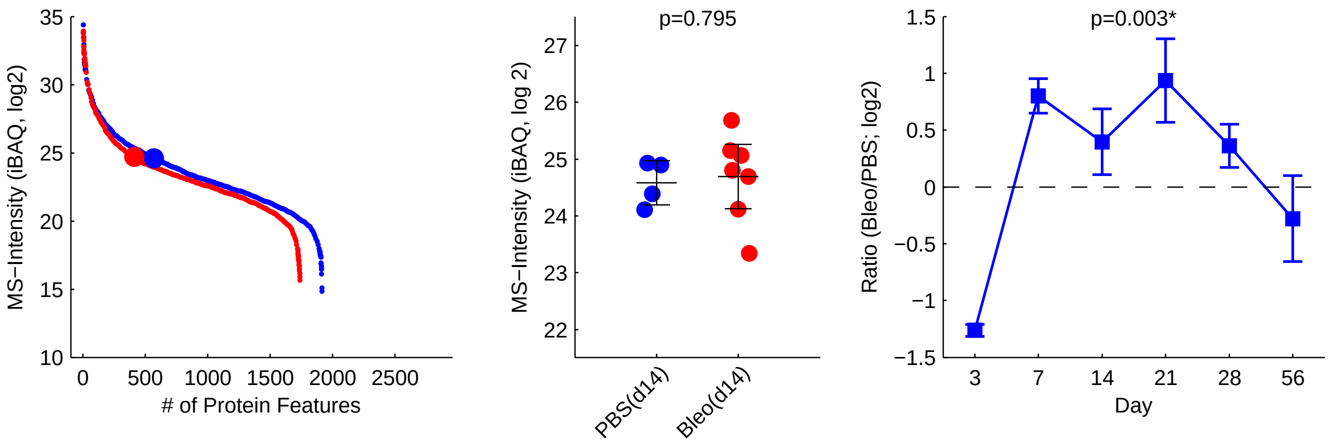

Q8JZQ9 − Eif3b (id: 2098)

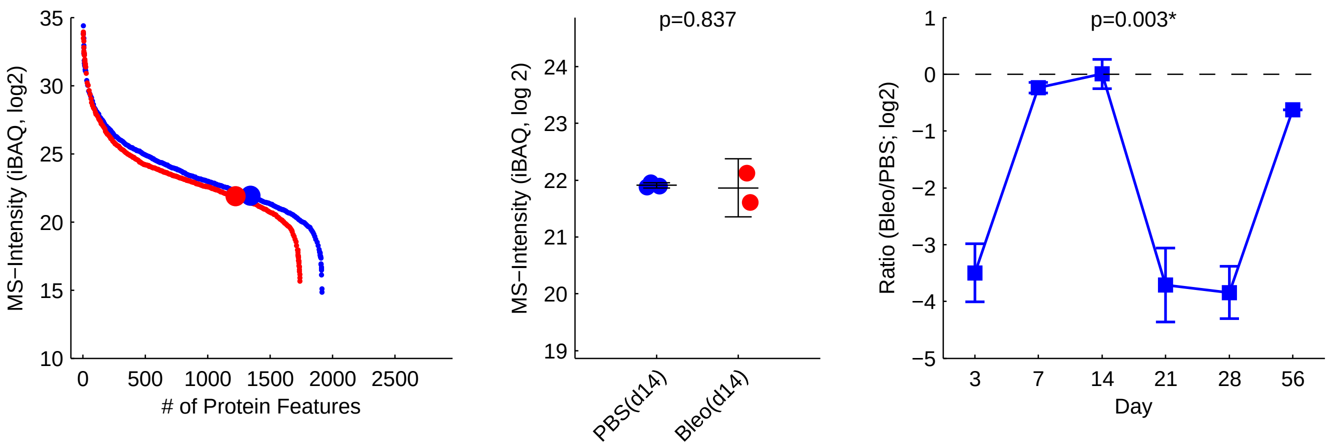

Q8K010 − Oplah (id: 2102)

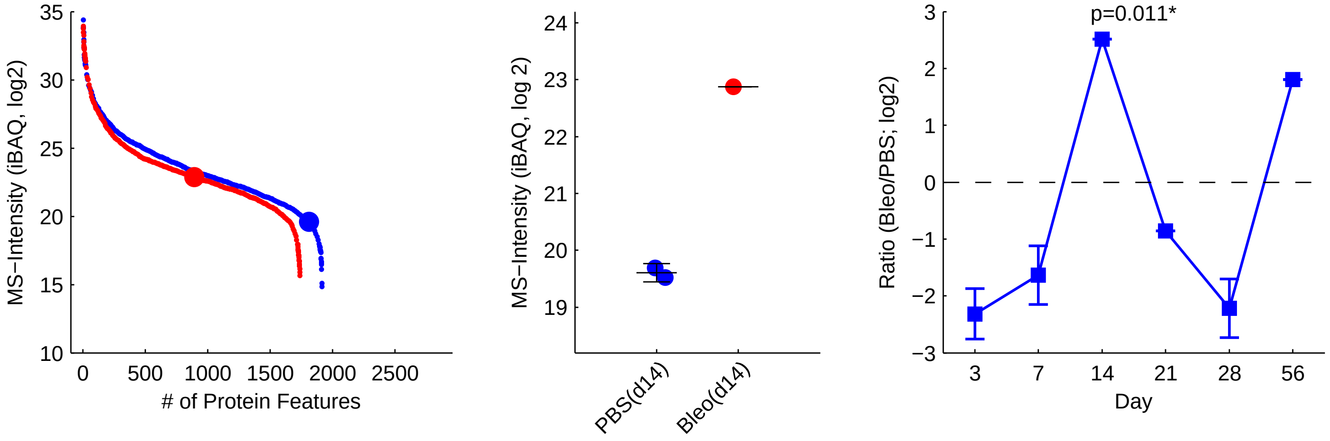

Q8K023 − Akr1c18 (id: 2104)

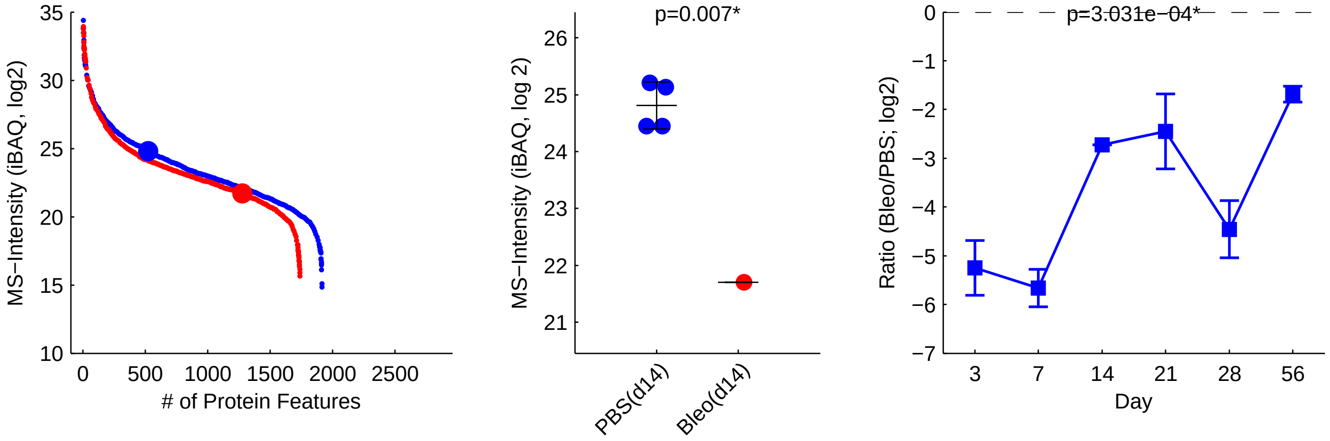

Q8K0C9 − Gmds (id: 2106)

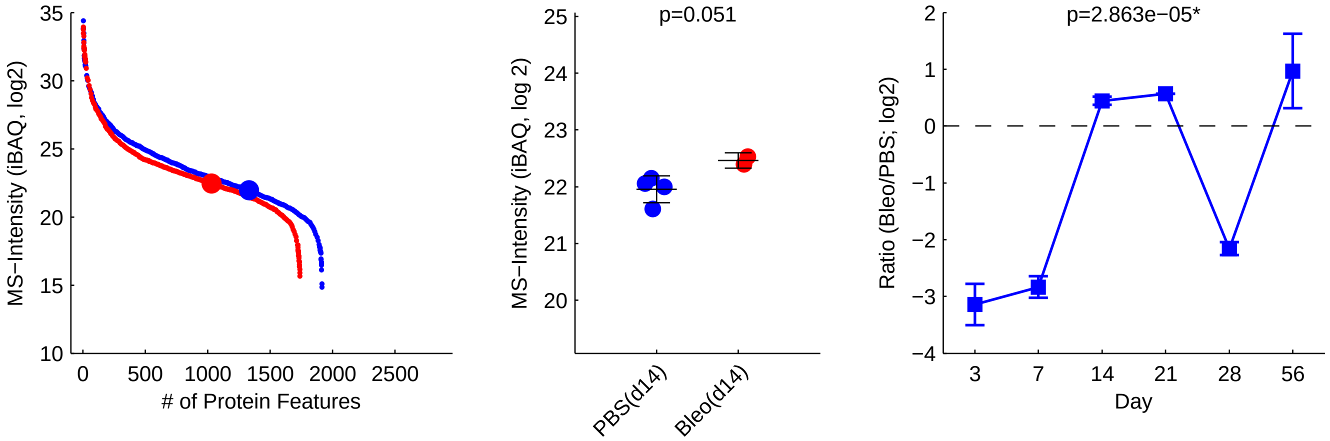

Q8K0E8 − Fgb (id: 2107)

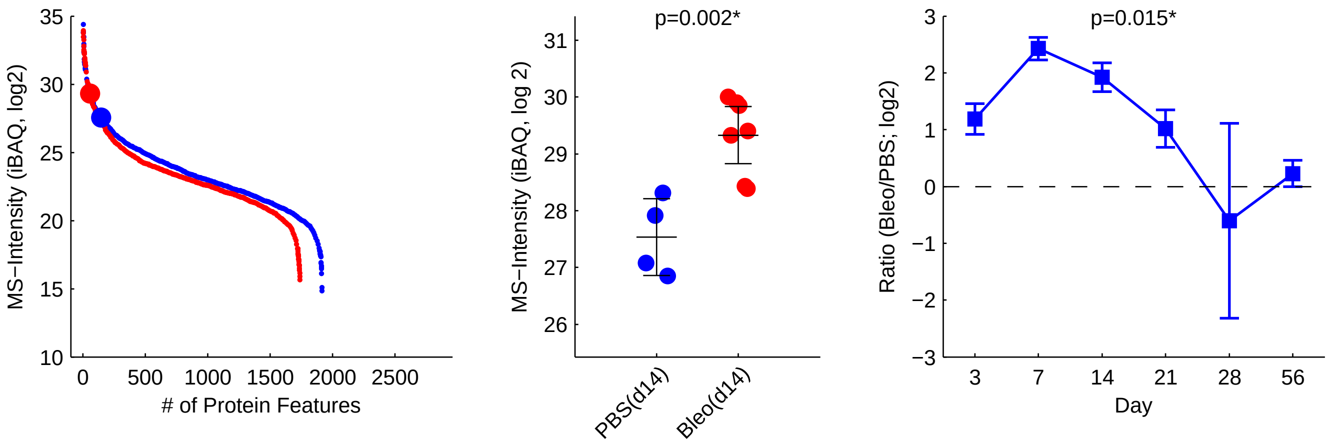

Q8K157 − Galm (id: 2112)

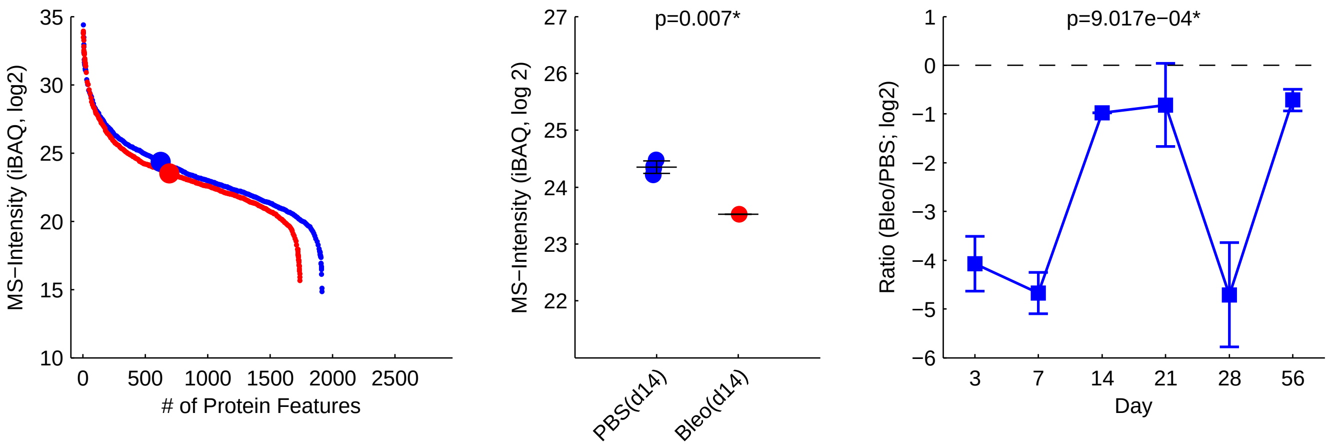

Q8K182 − C8a (id: 2113)

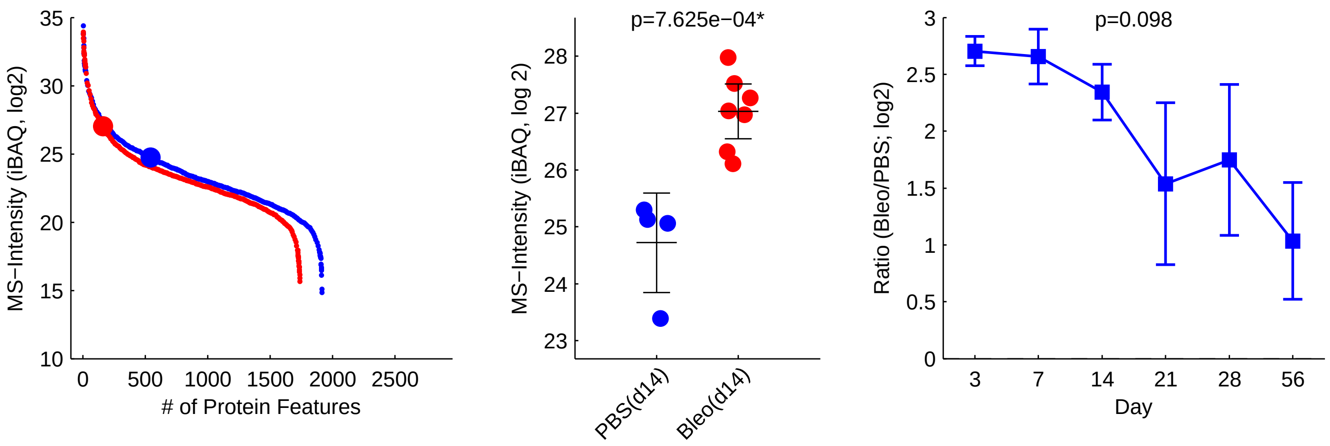

Q8K1M3 − Prkar2a (id: 2116)

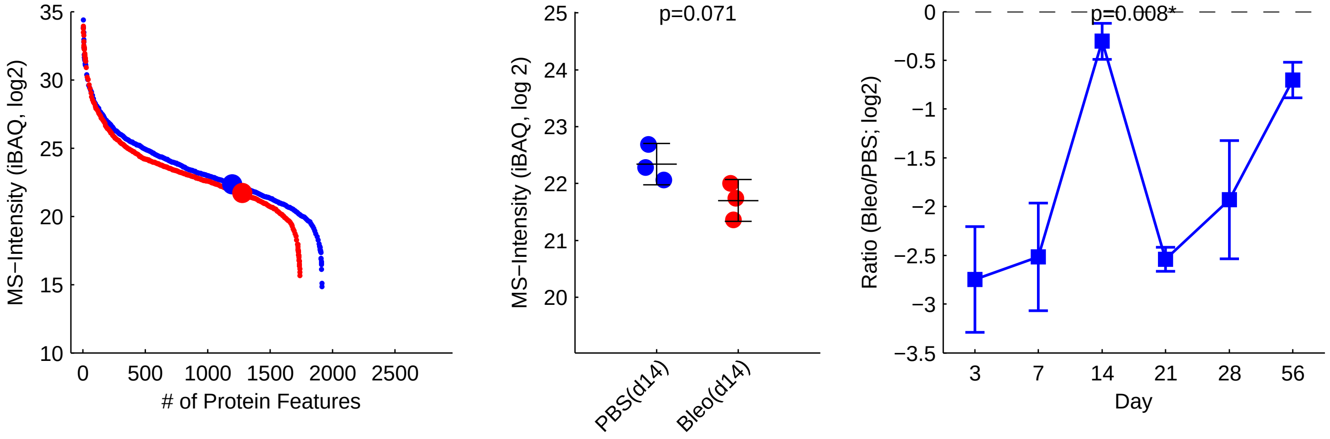

Q8K2B3 − Sdha (id: 2119)

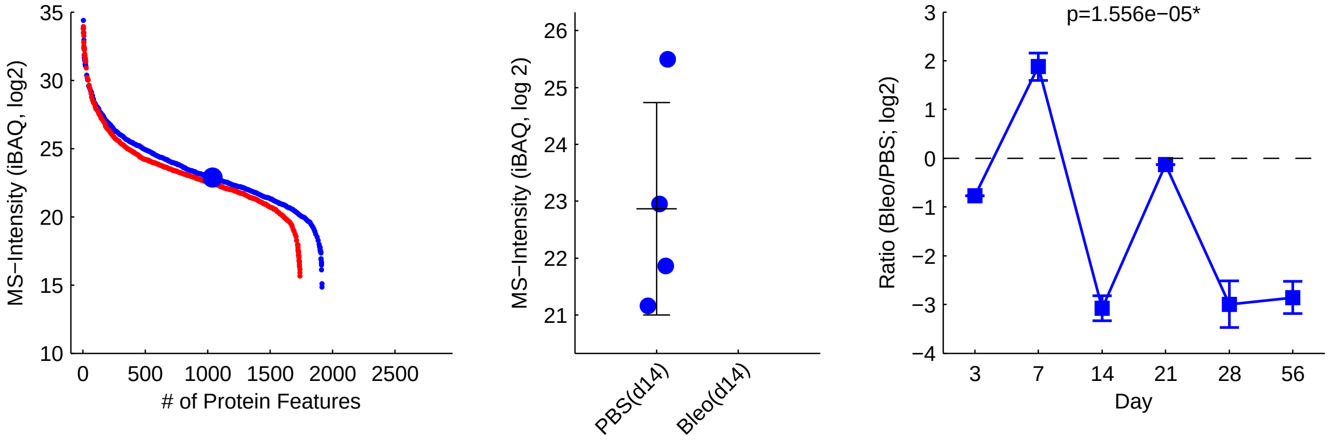

Q8K2I3 − Fmo2 (id: 2122)

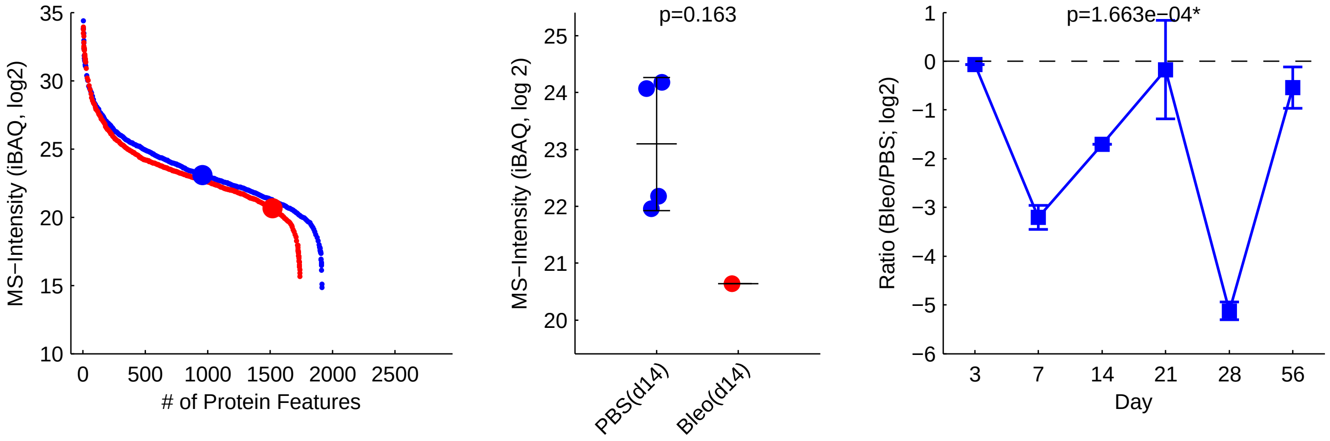

Q8K354 − Cbr3 (id: 2131)

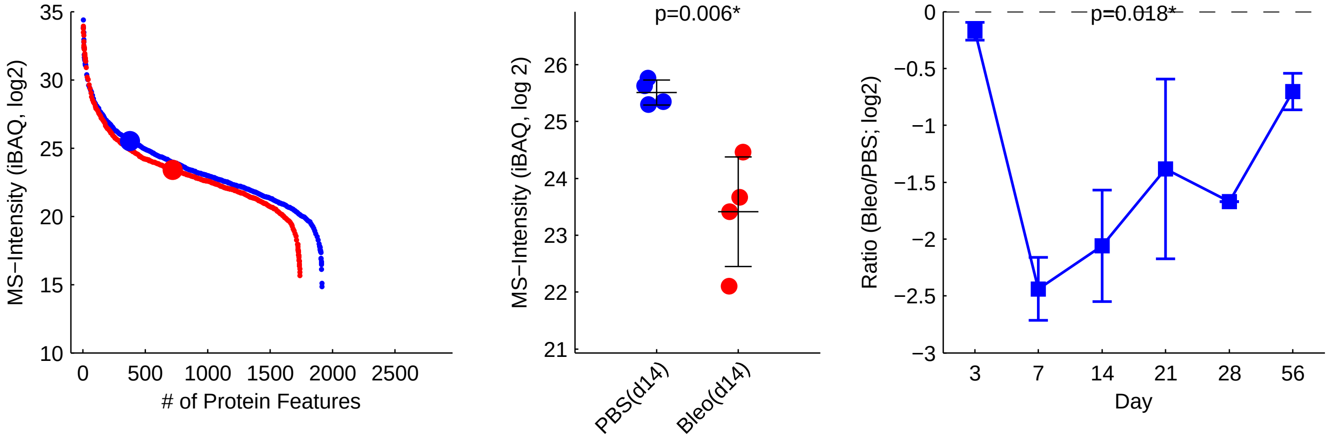

Q8QZR5 − Gpt (id: 2138)

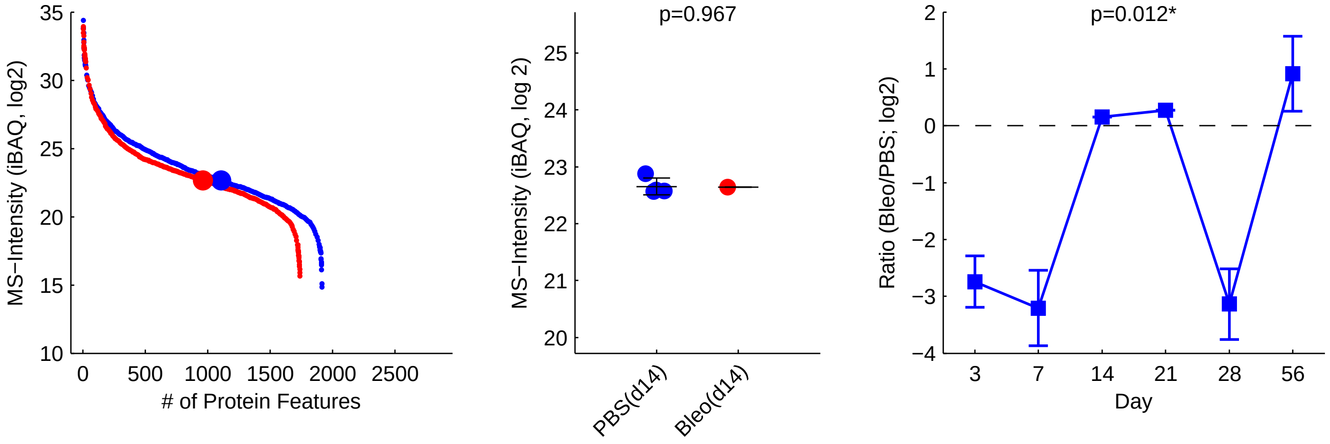

Q8QZS1 − Hibch (id: 2139)

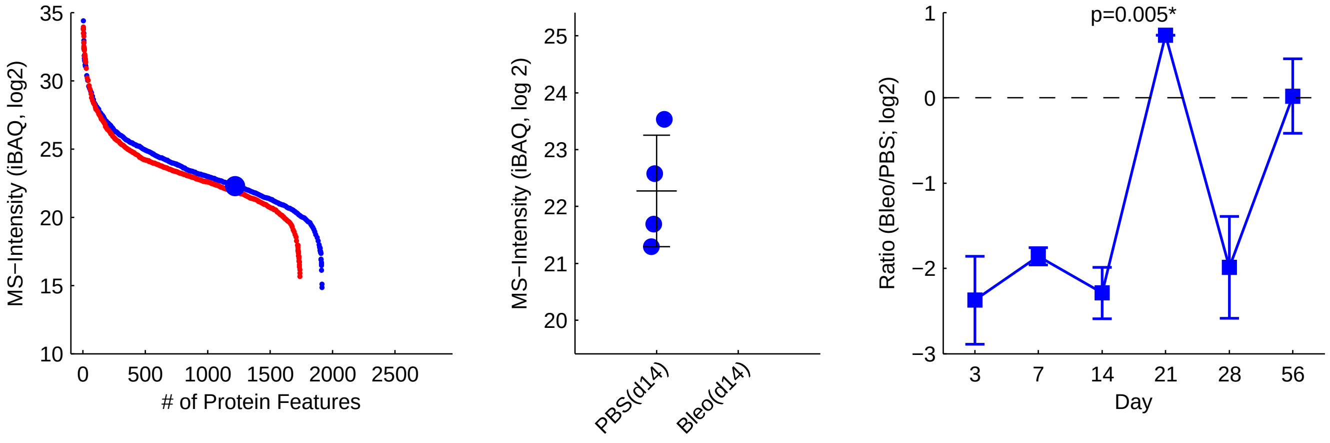

Q8QZT1 − Acat1 (id: 2140)

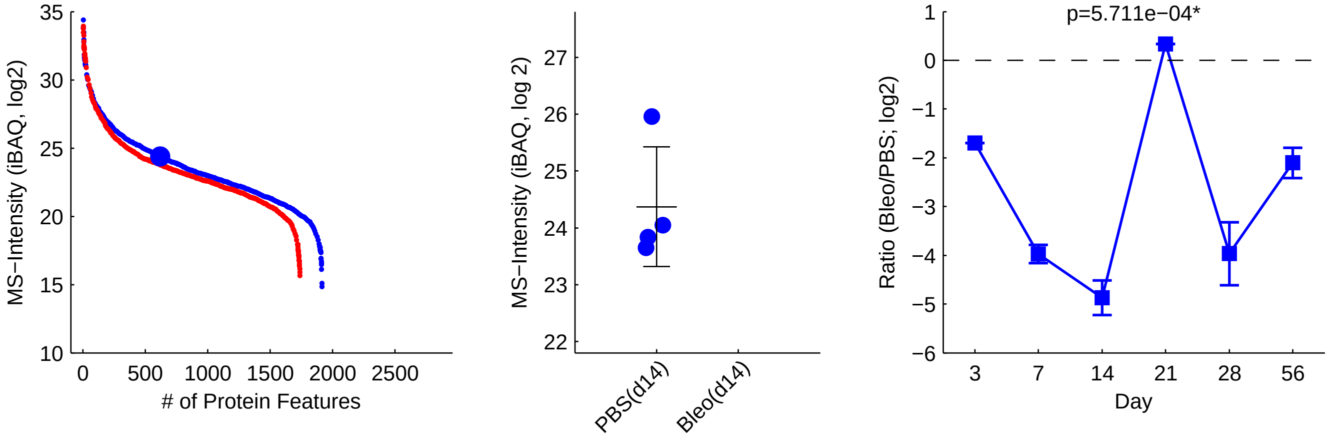

Q8R048 − Mycbp (id: 2144)

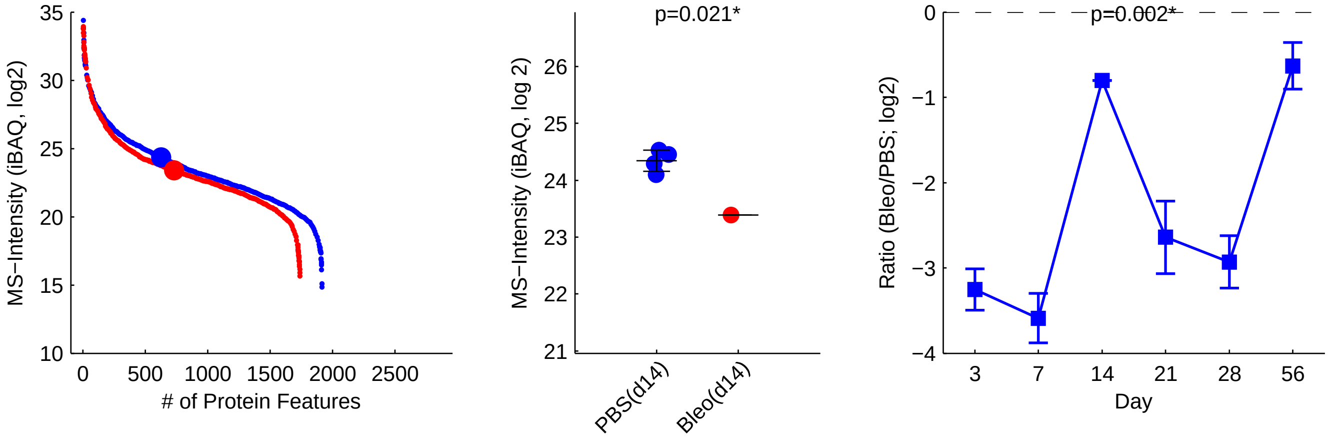

Q8R0I0 − Ace2 (id: 2149)

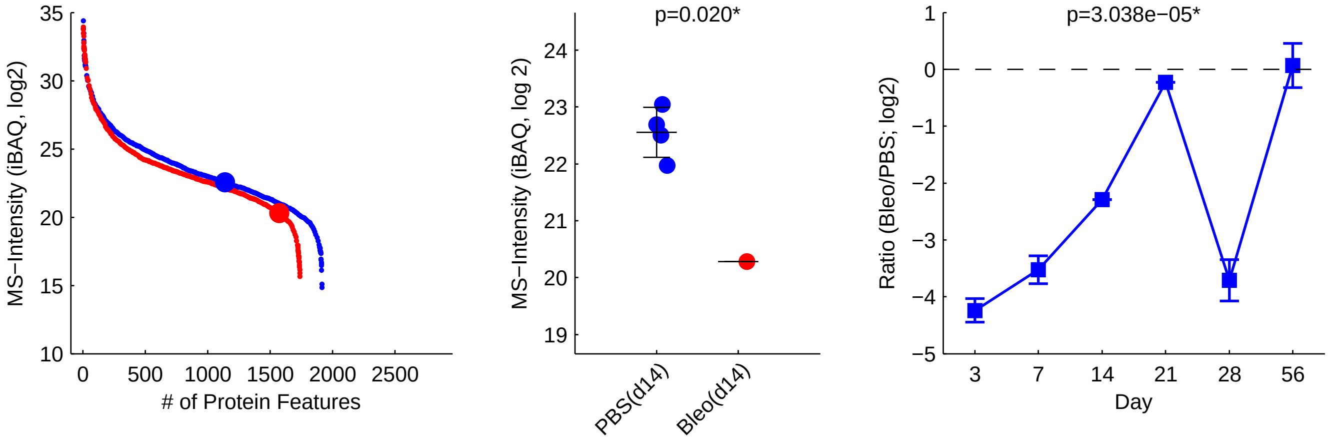

Q8R0Y6 − Aldh1l1 (id: 2151)

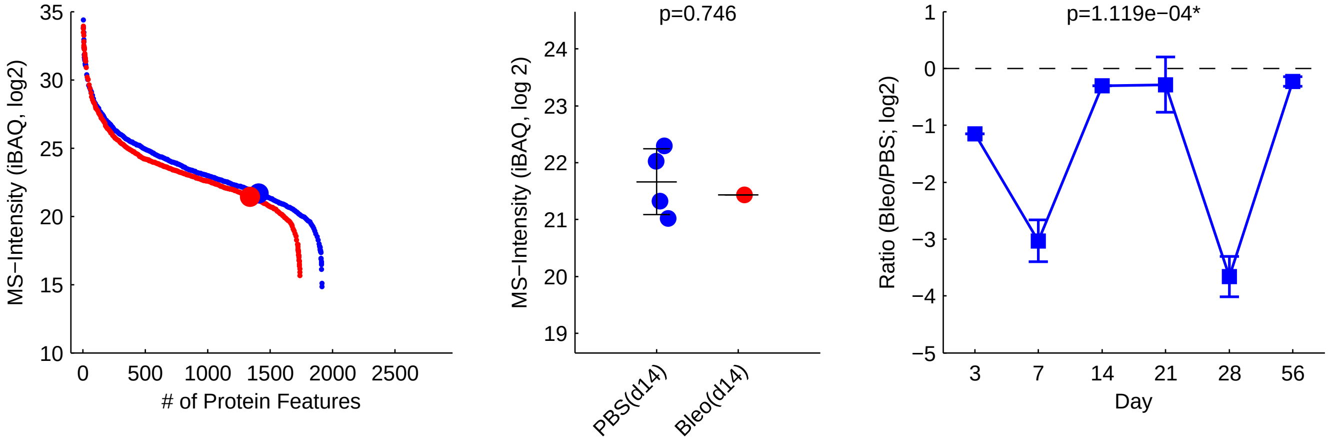

Q8R121 − Serpina10 (id: 2152)

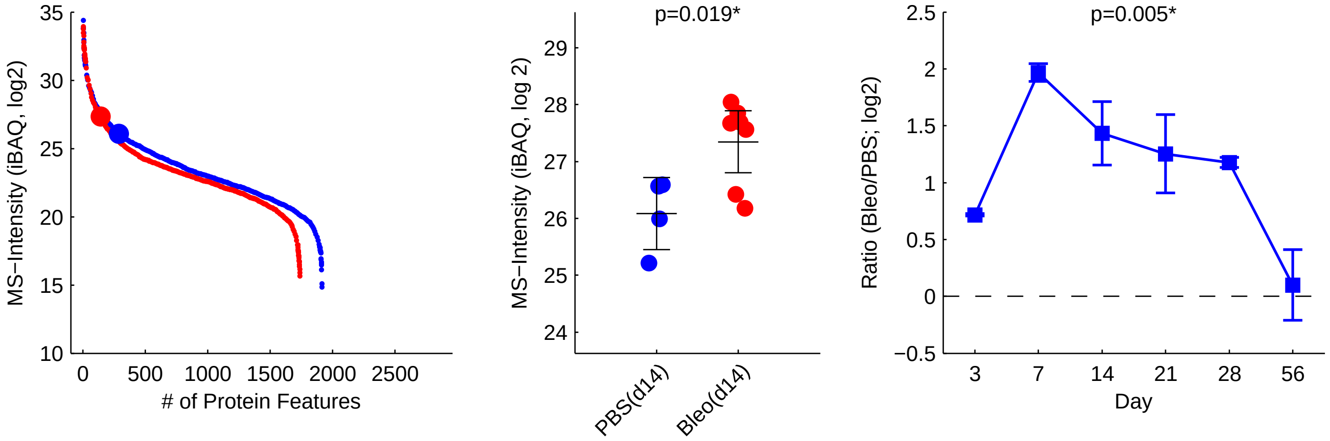

Q8R164 − Bphl (id: 2154)

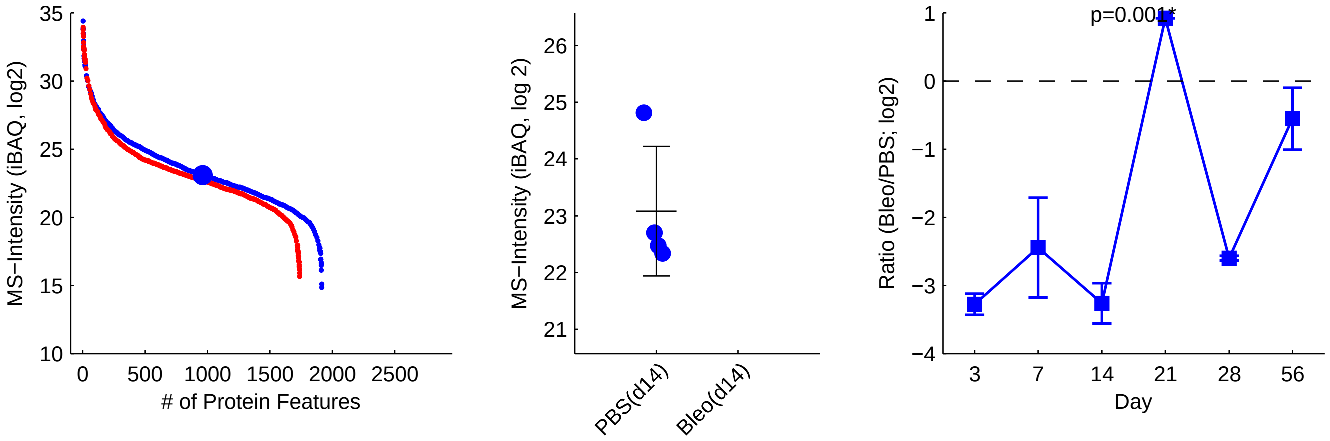

Q8R1B4 − Eif3c (id: 2155)

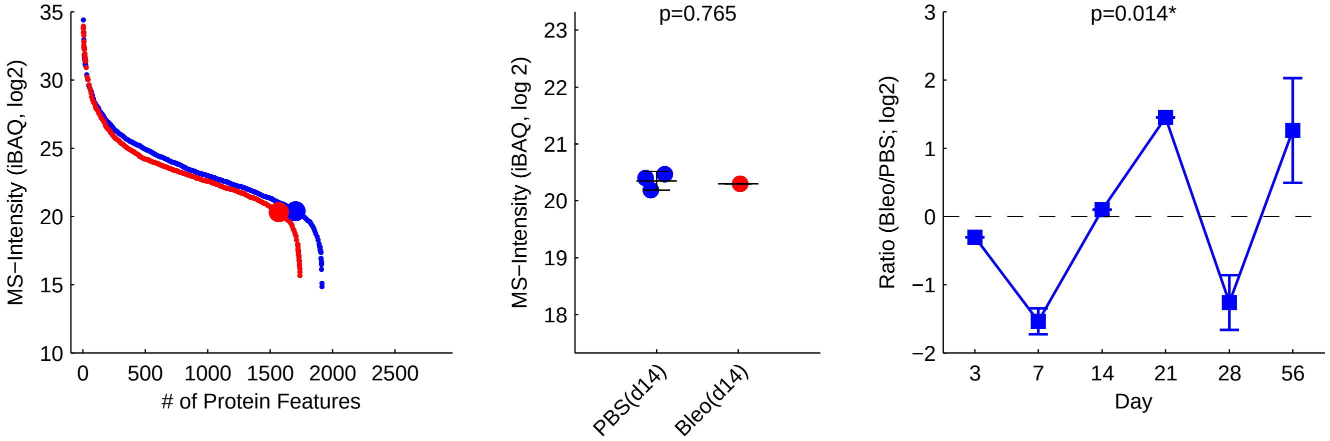

Q8R1G2 − Cmbl (id: 2158)

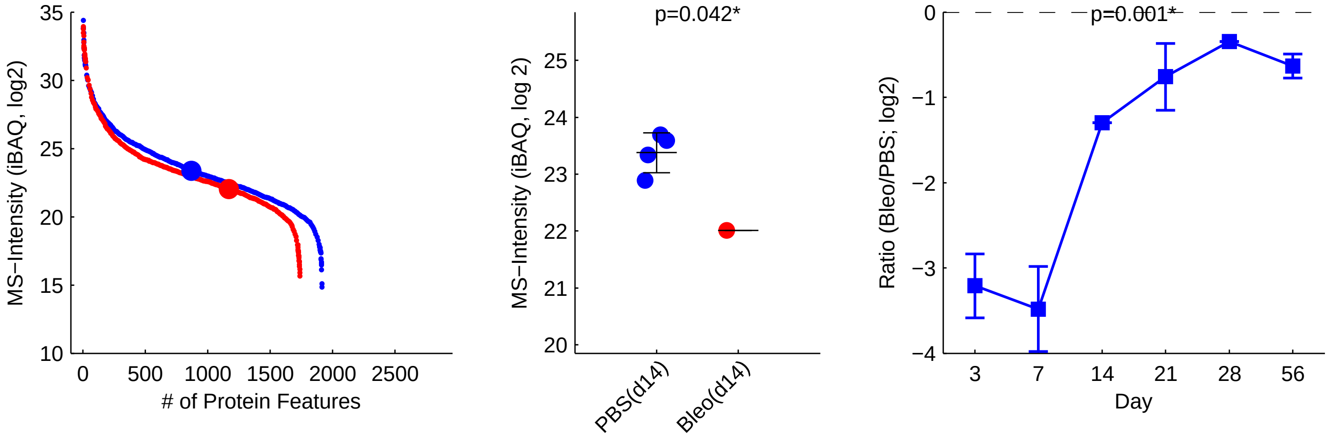

Q8R1H0 − Hopx (id: 2160)

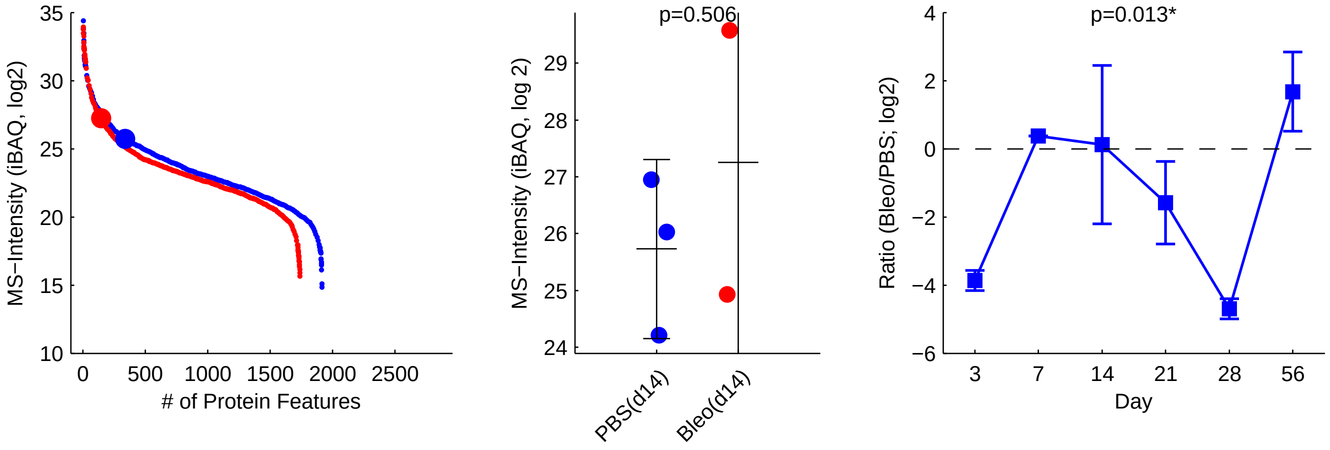

Q8R242 − Ctbs (id: 2164)

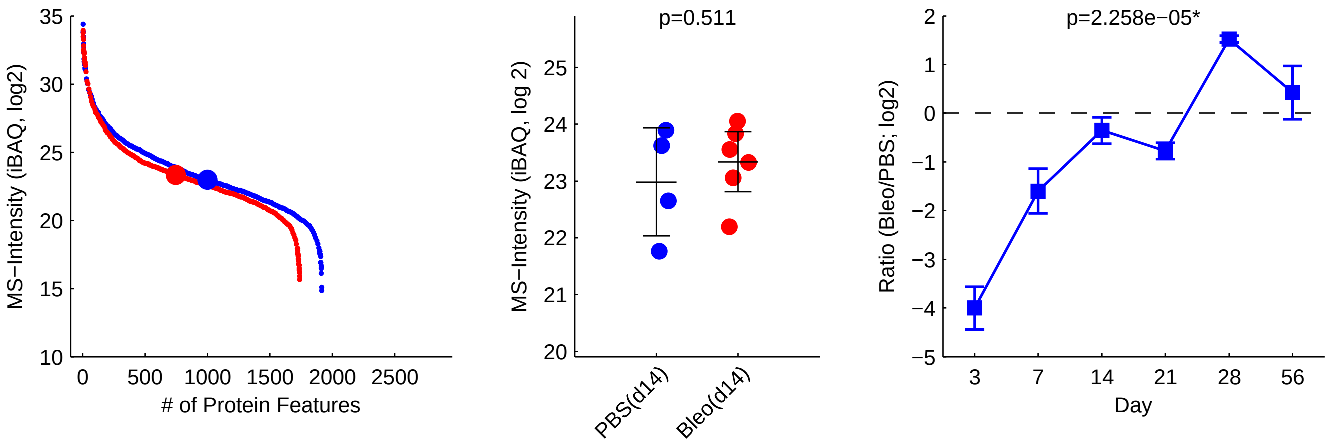

Q8R2Y2−2 − Mcam (id: 2168)

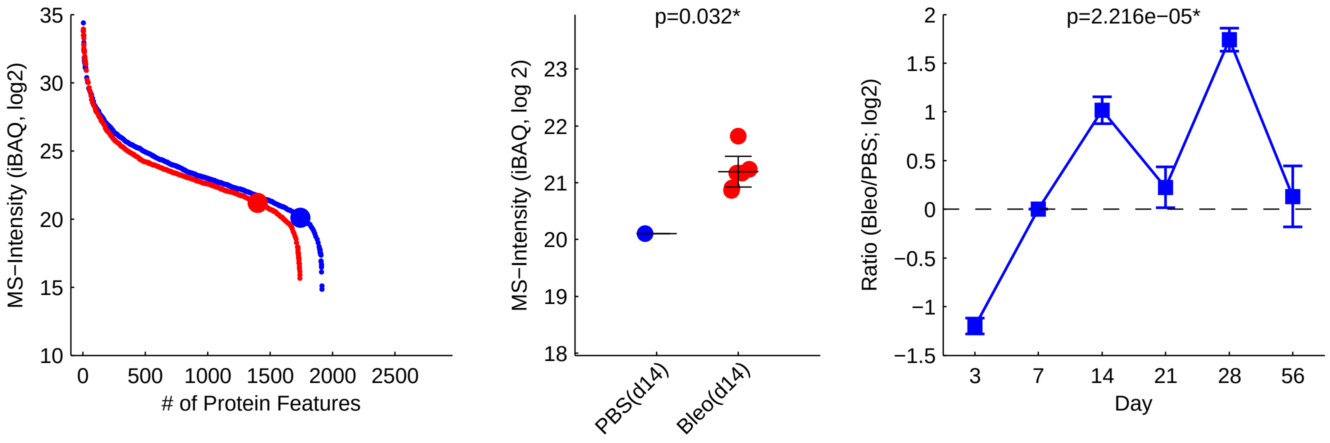

Q8R3G9 − Tspan8 (id: 2174)

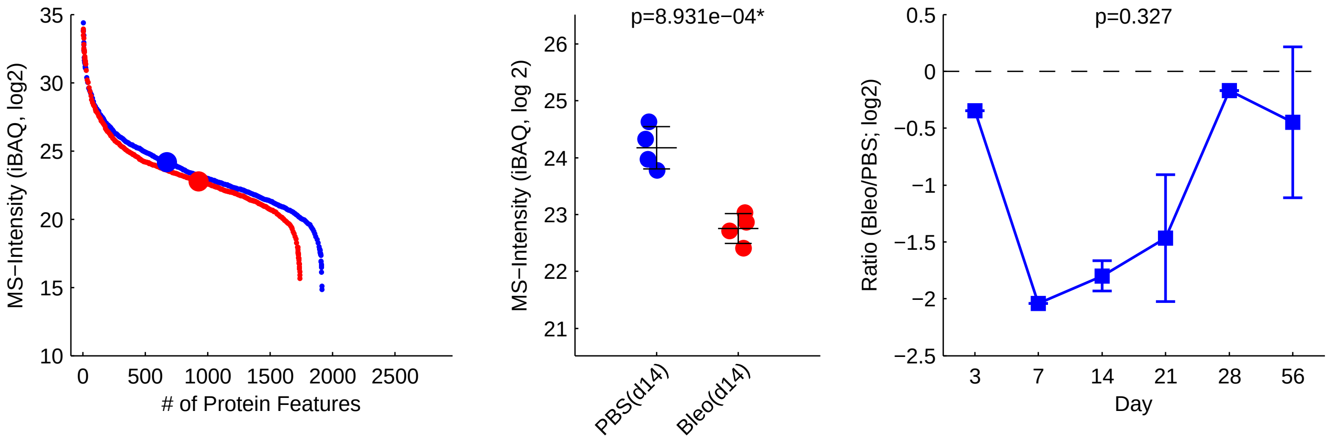

Q8R574 − Prpsap2 (id: 2178)

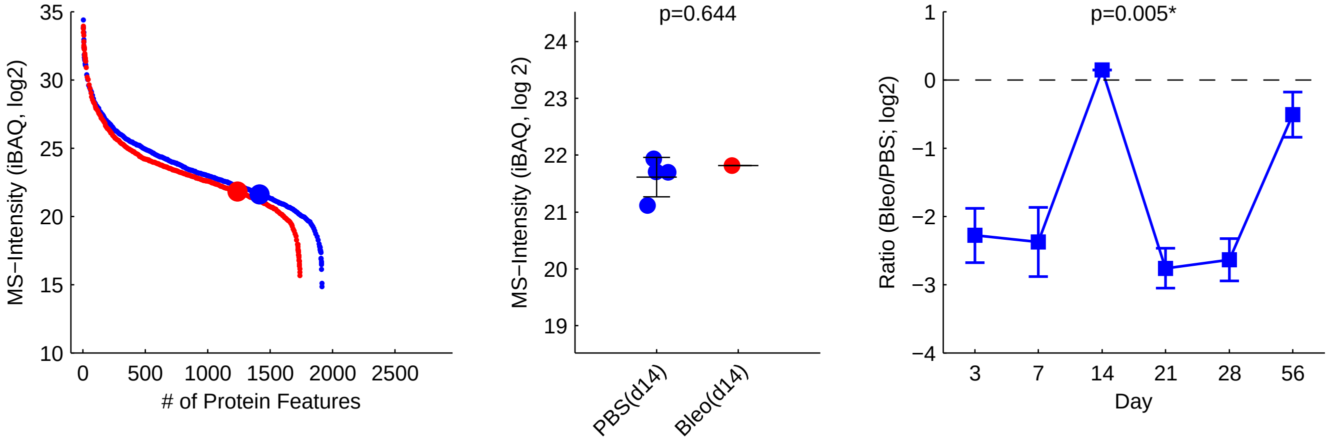

Q8VCG4 − C8g (id: 2192)

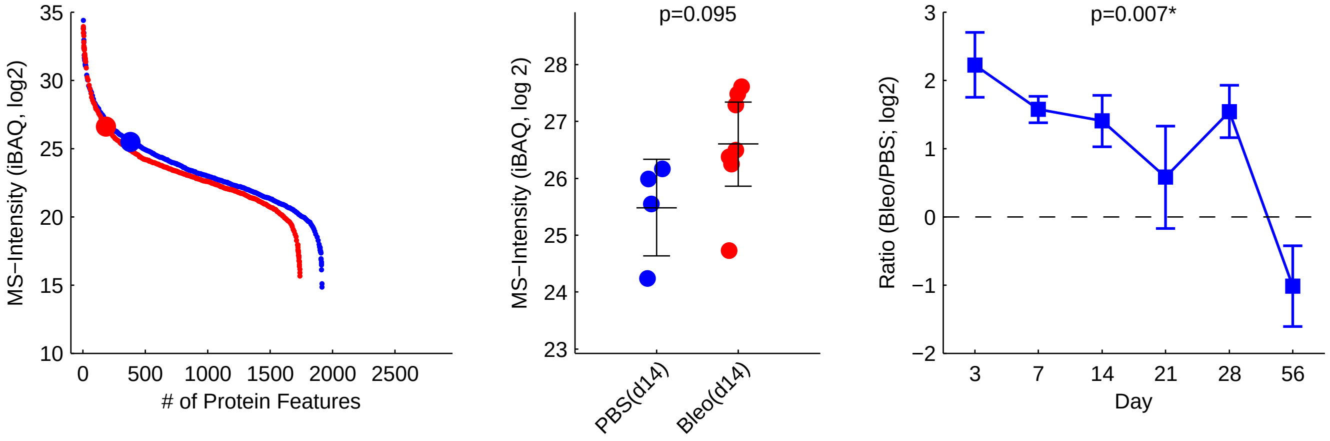

Q8VCM7 − Fgg (id: 2195)

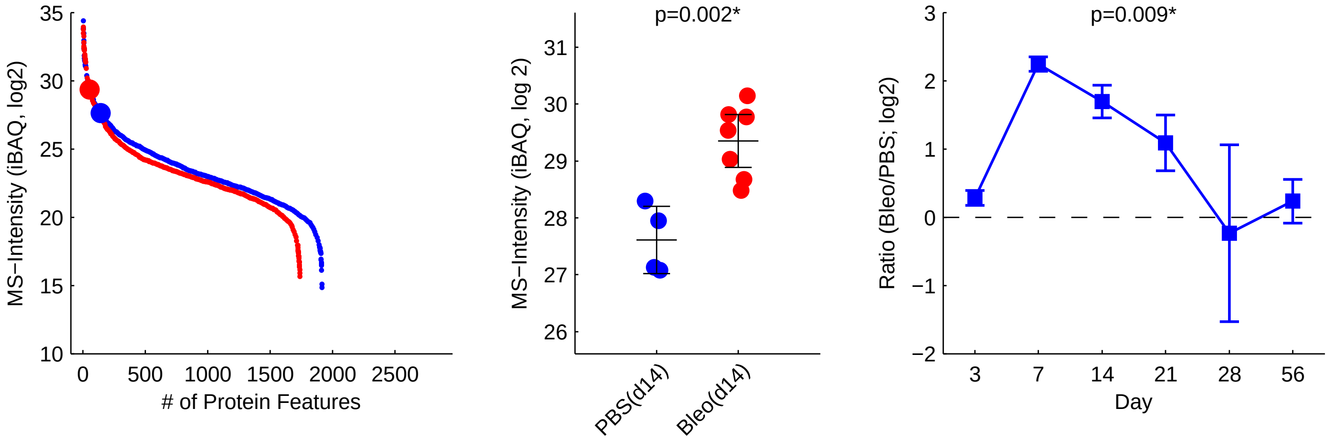

Q8VCS0 − Pglyrp2 (id: 2197)

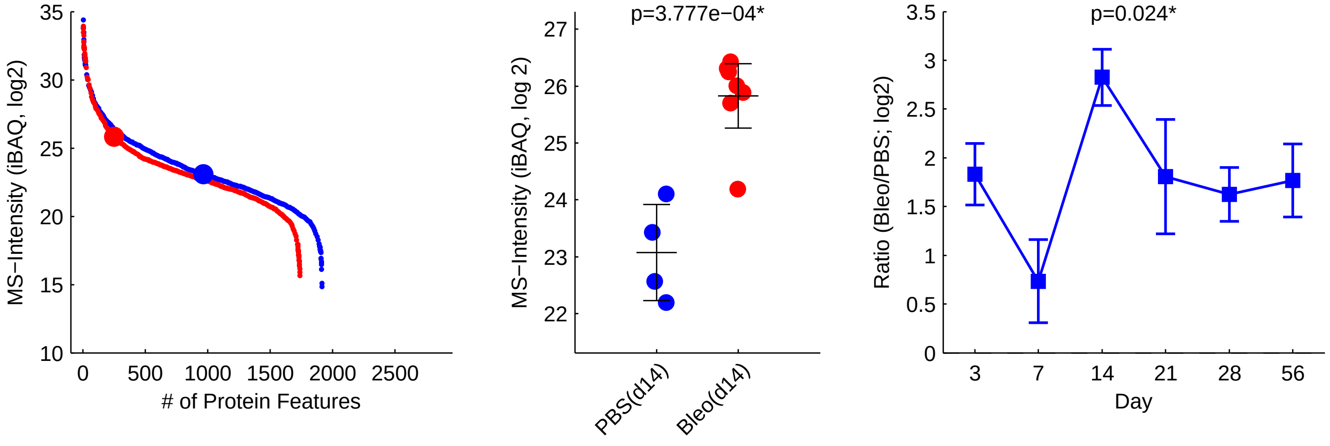

Q8VCT4 − Ces1d (id: 2199)

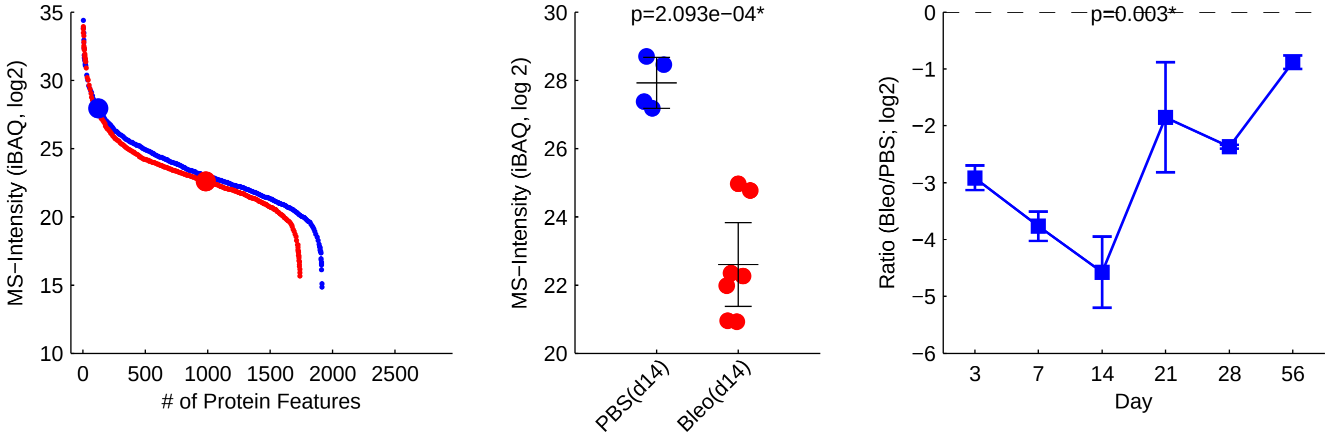

Q8VCU2 − Gpld1 (id: 2200)

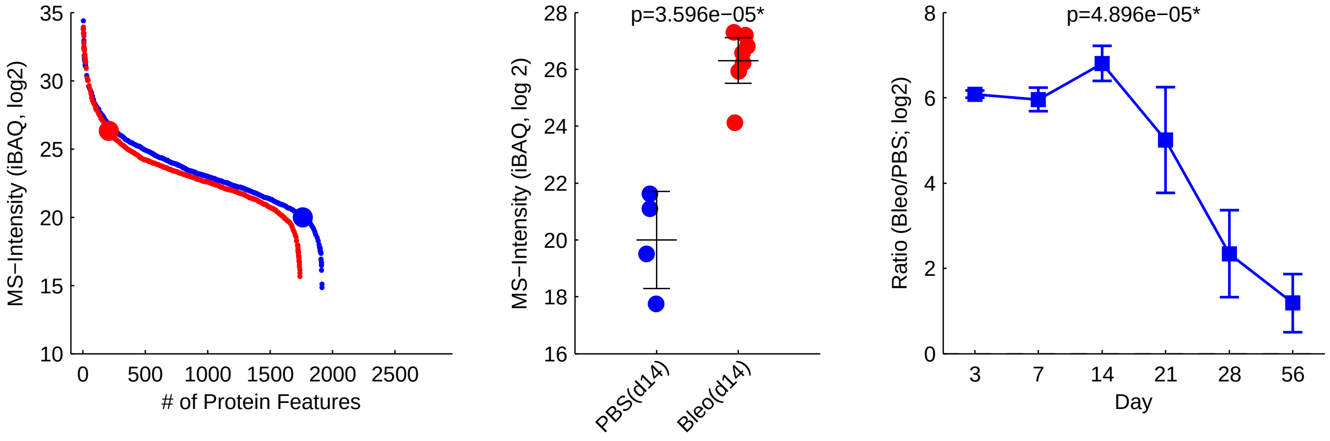

Q8VCW8 − Acsf2 (id: 2201)

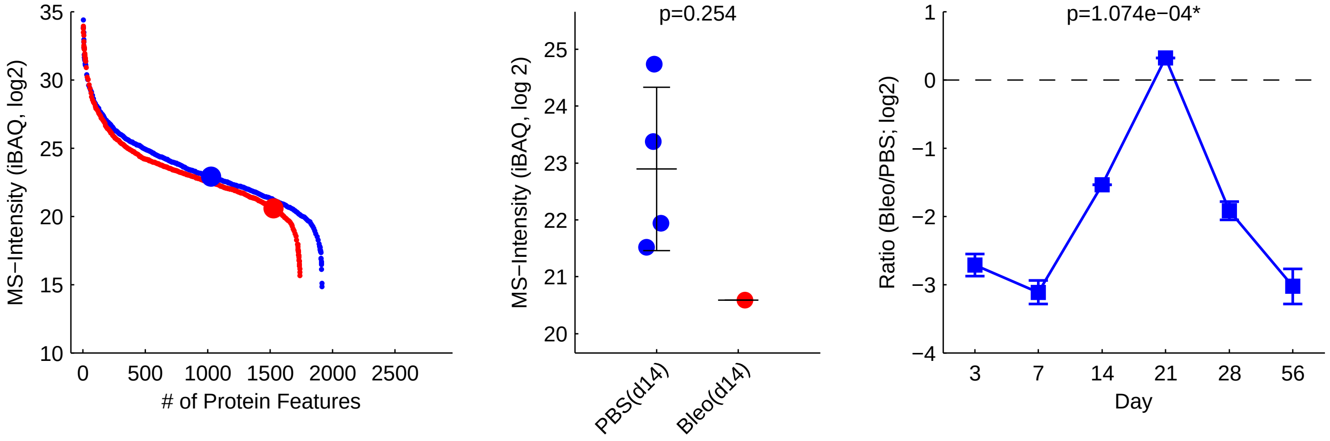

Q8VDJ3 − Hdlbp (id: 2205)

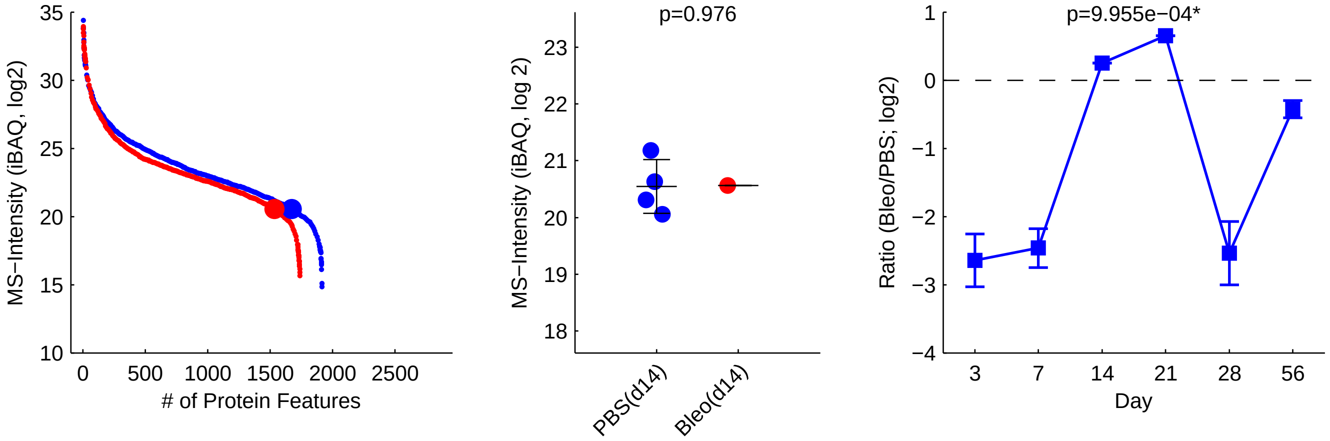

Q8VDN2 − Atp1a1 (id: 2209)

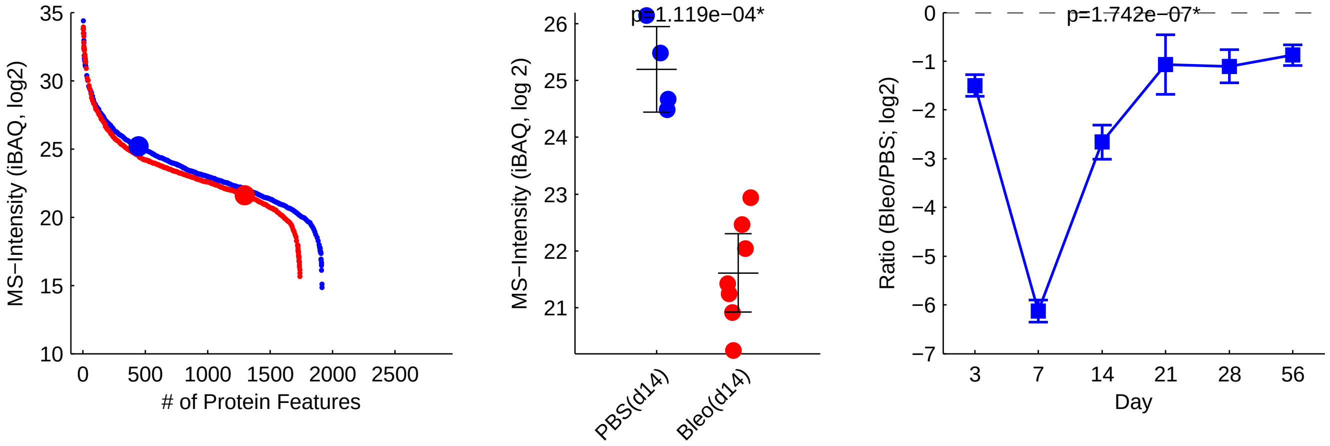

Q8VDQ1 − Ptgr2 (id: 2212)

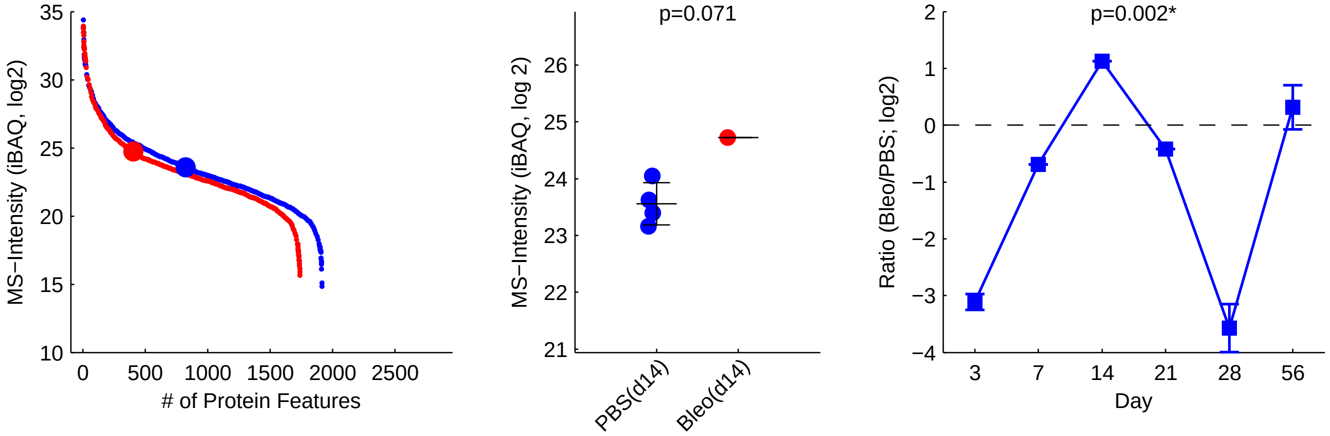

Q8VE70 − Pdcd10 (id: 2215)

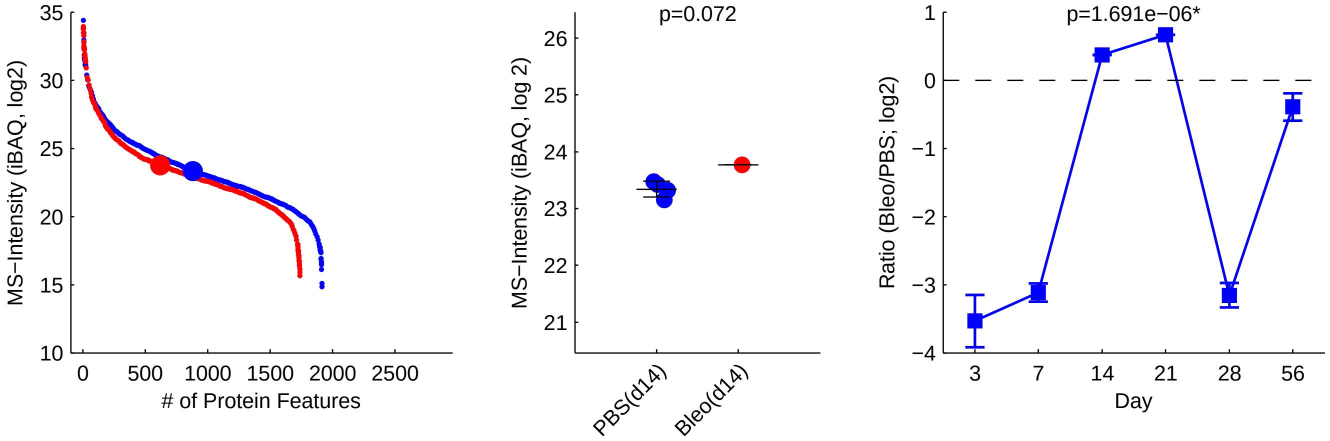

Q91V77 − S100a1 (id: 2238)

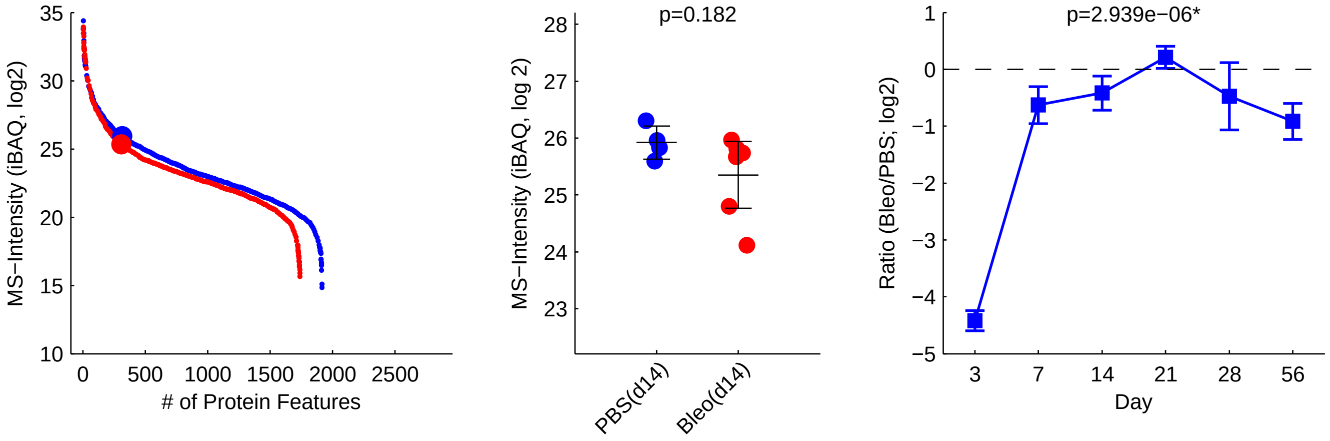

Q91VW3 − Sh3bgrl3 (id: 2255)

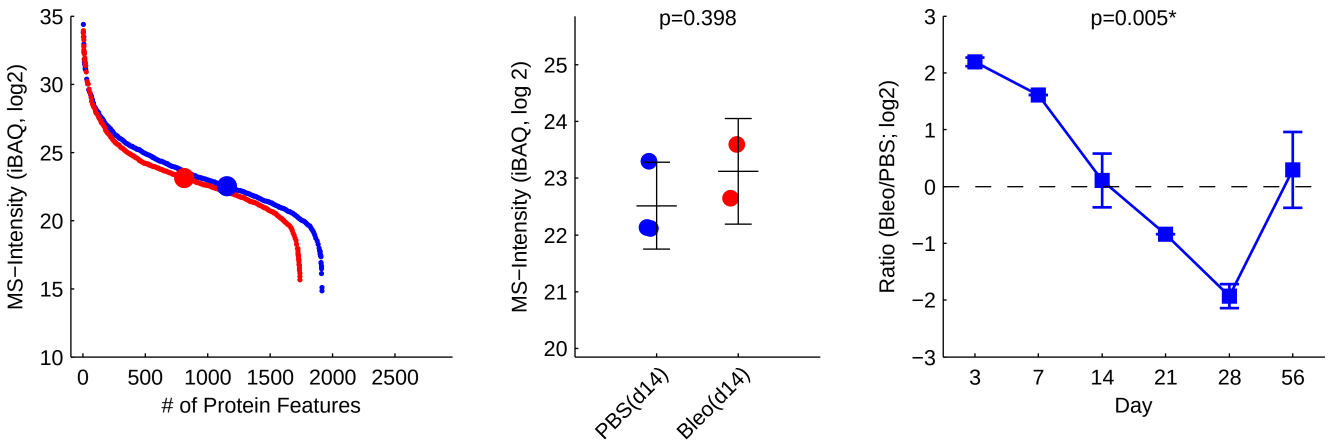

Q91WS0 − Cisd1 (id: 2265)

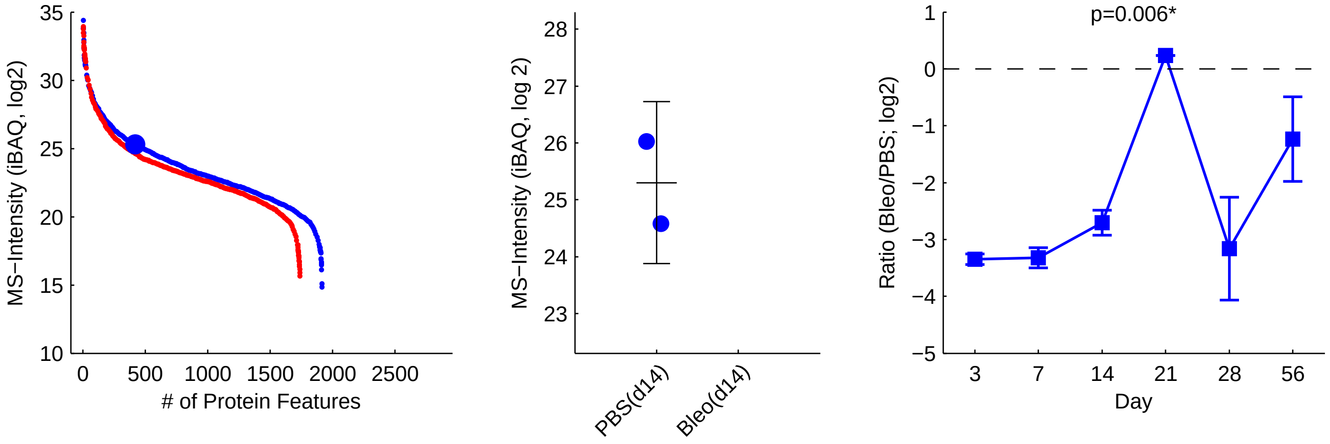

Q91X75 − Cyp2a5 (id: 2271)

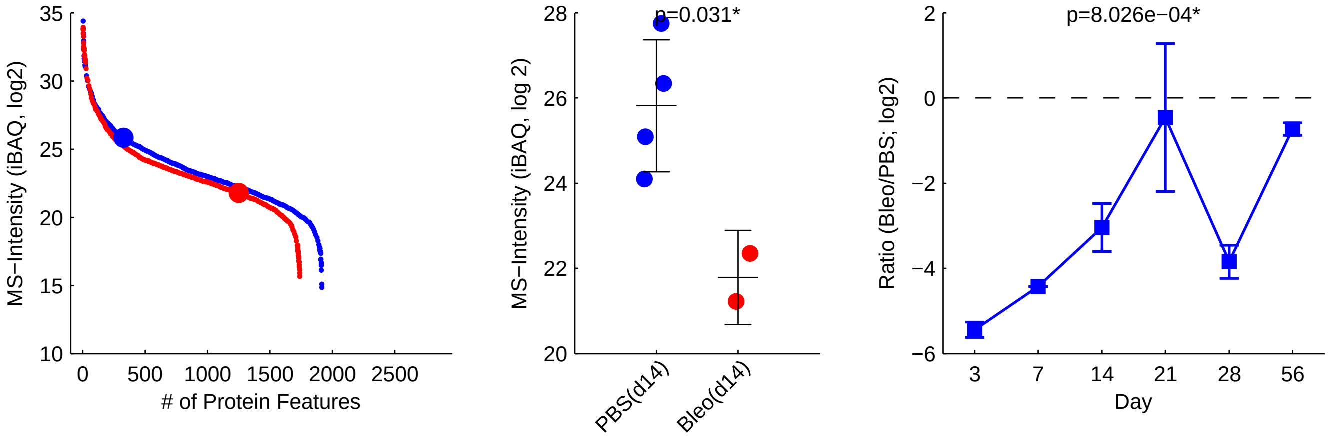

Q91X79 − Cela1 (id: 2272)

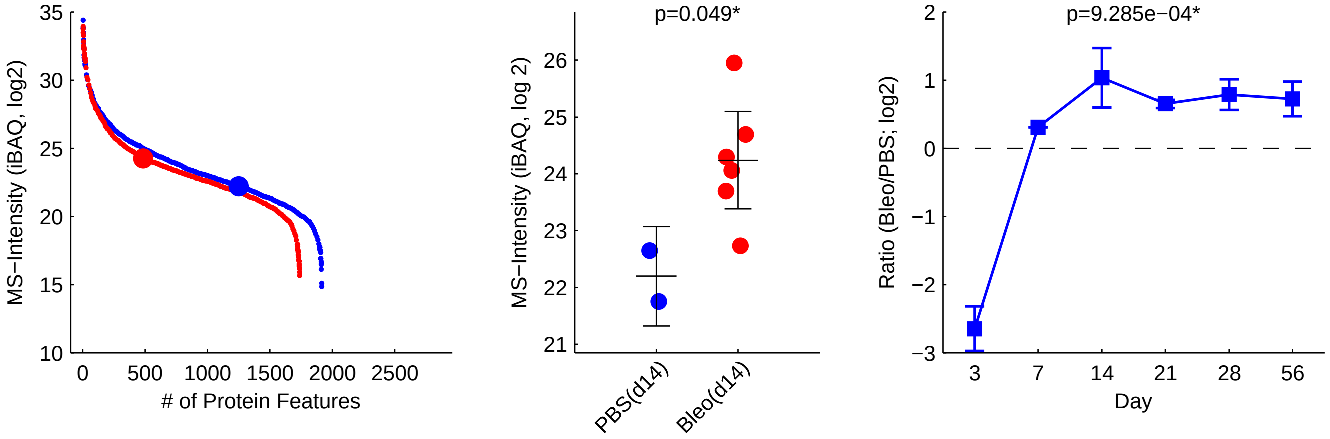

Q91XA2 − Golm1 (id: 2274)

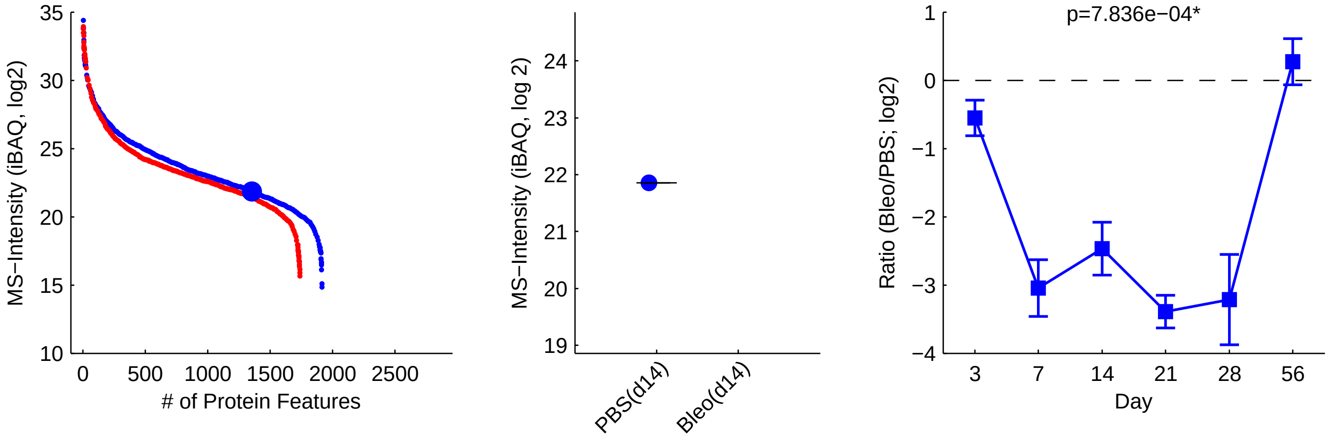

Q91XA9 − Chia (id: 2275)

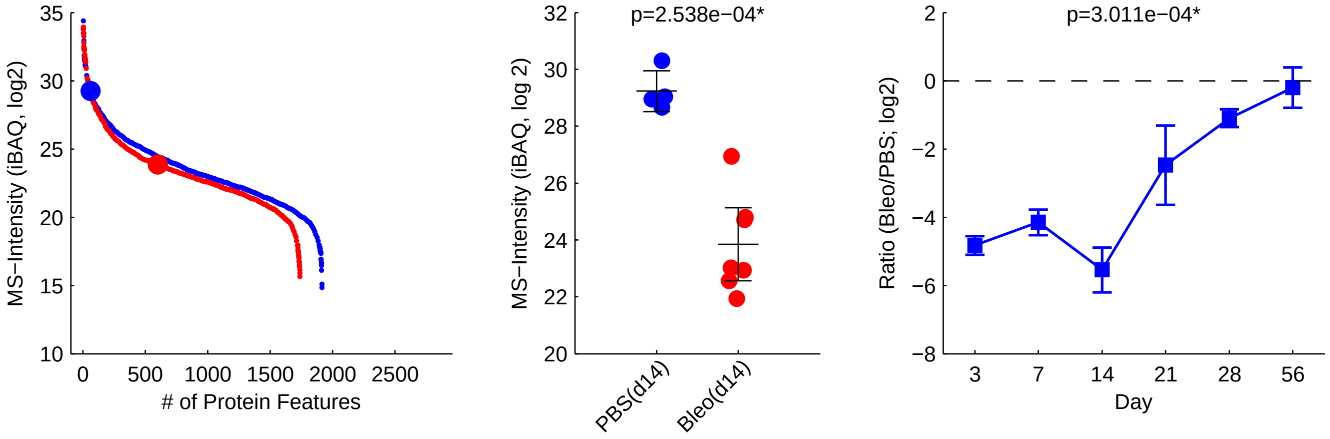

Q91XV3 − Basp1 (id: 2281)

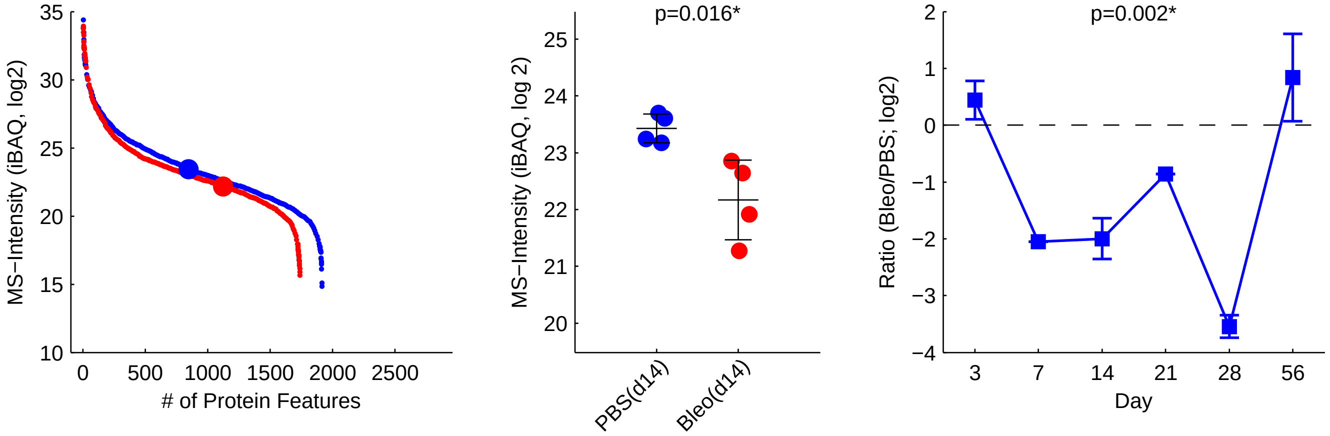

Q91YQ5 − Rpn1 (id: 2289)

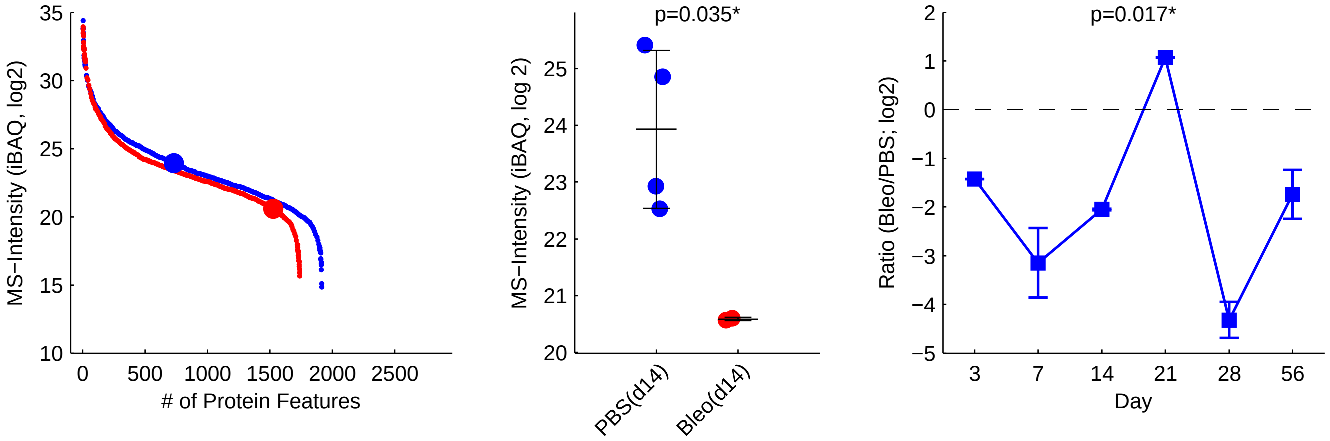

Q91Z53 − Grhpr (id: 2294)

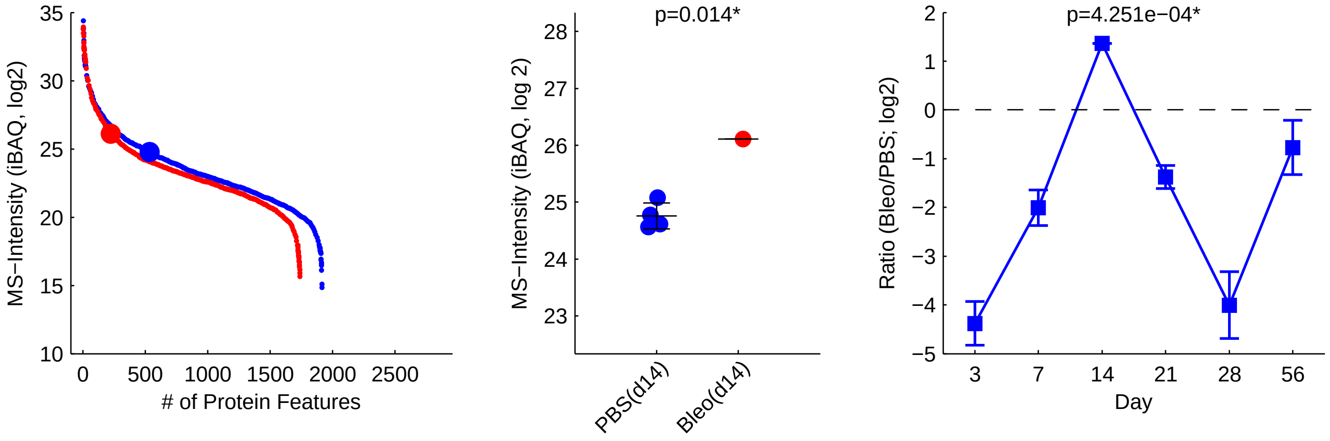

Q91ZX7 − Lrp1 (id: 2299)

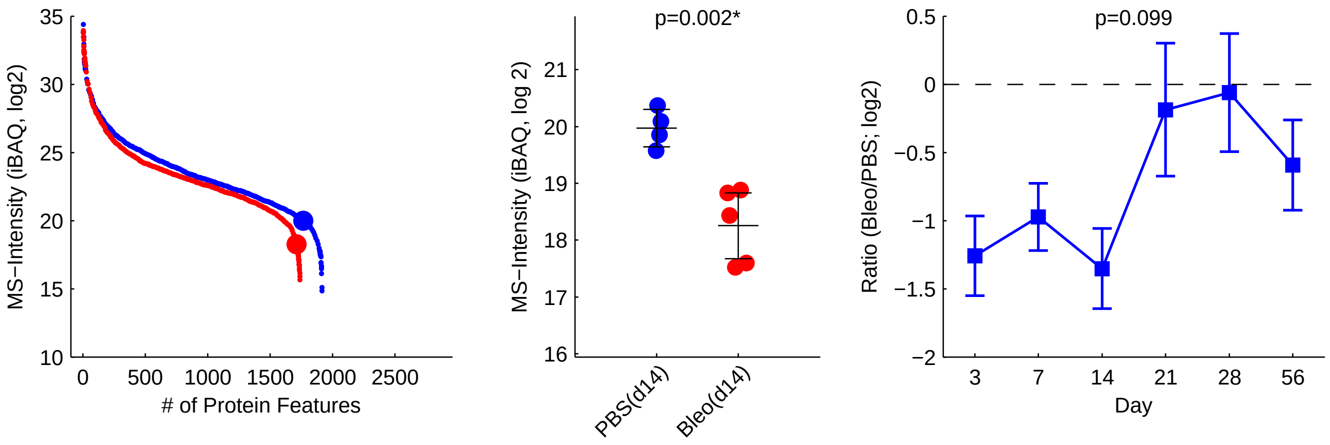

Q920A5 − Scpep1 (id: 2300)

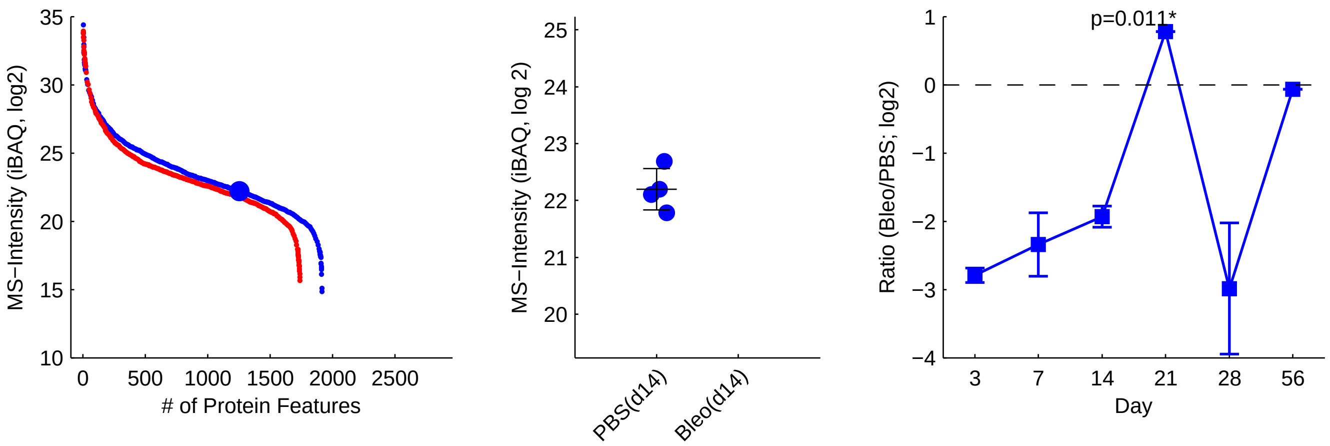

Q93092 − Taldo1 (id: 2324)

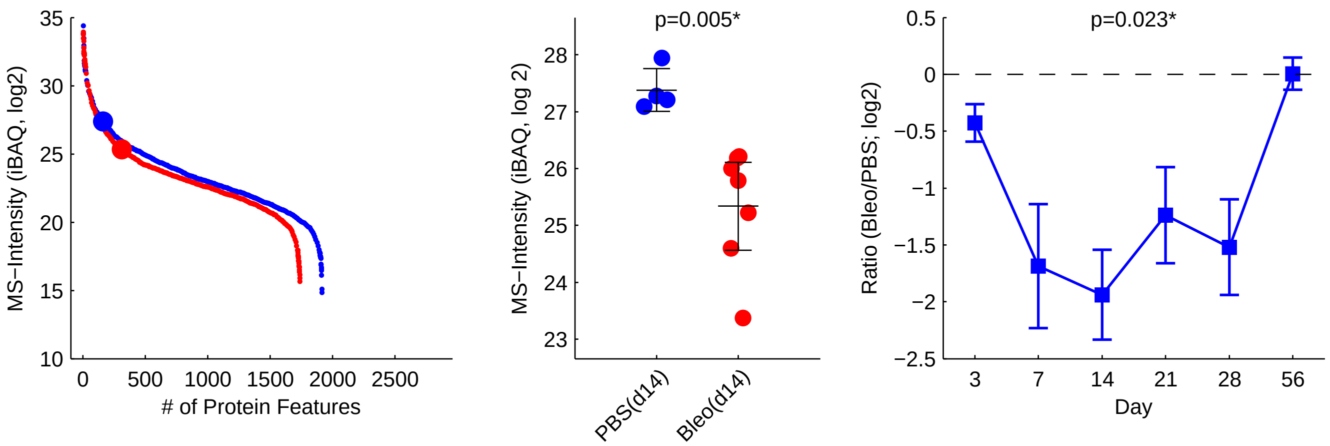

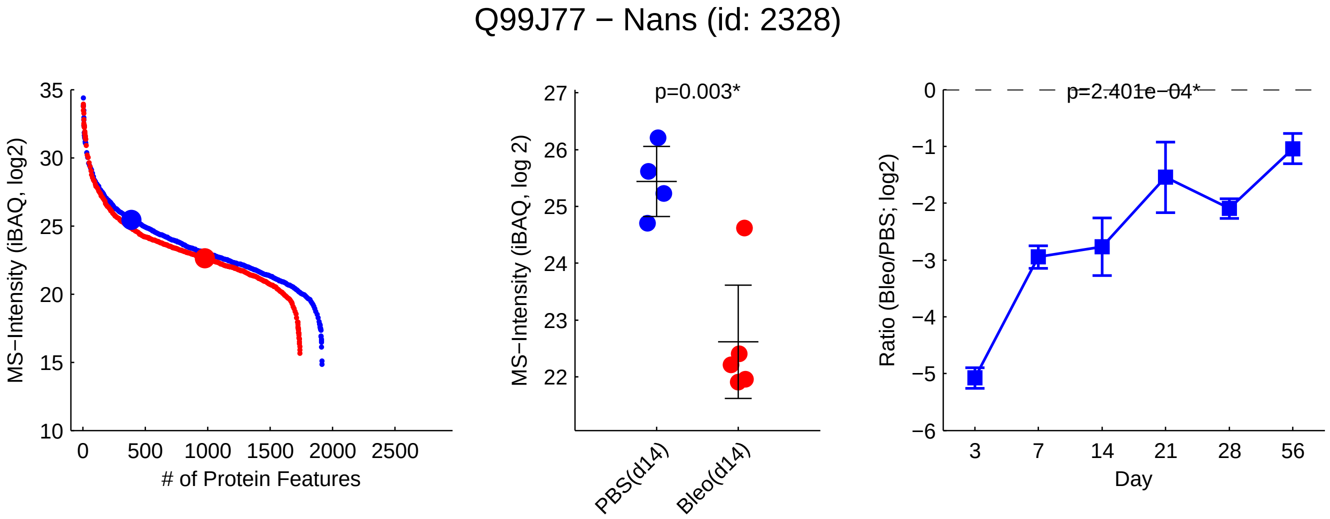
Q99J77 − Nans (id: 2328)
MS−Intensity (iBAQ, log2)
# of Protein Features
p=0.003*
MS−Intensity (iBAQ, log 2)
PBS(d14)
Bleo(d14)
p=2.401e−04*
Ratio (Bleo/PBS; log2)
Day

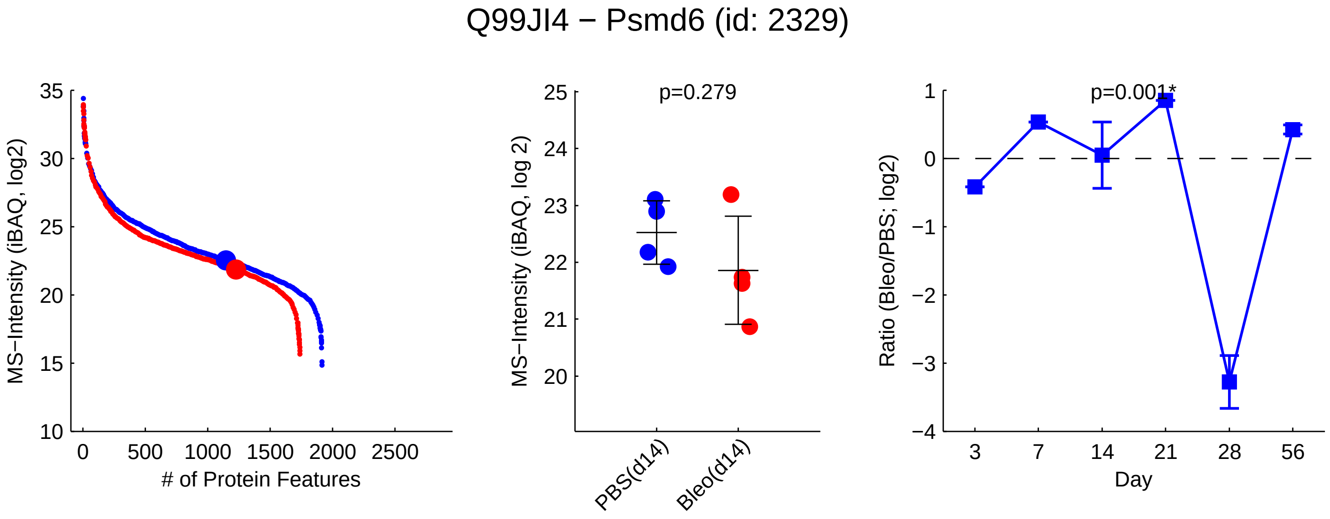
Q99JI4 − Psmd6 (id: 2329)
MS−Intensity (iBAQ, log2)
# of Protein Features
p=0.279
MS−Intensity (iBAQ, log 2)
PBS(d14)
Bleo(d14)
p=0.001*
Ratio (Bleo/PBS; log2)
Day

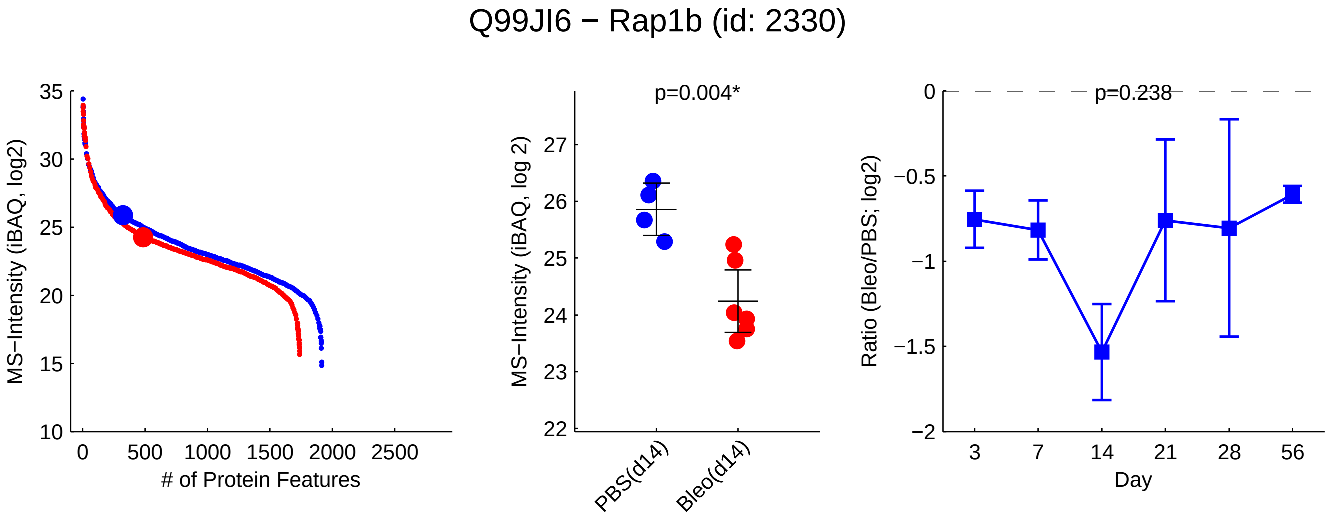
Q99JI6 − Rap1b (id: 2330)
MS−Intensity (iBAQ, log2)
# of Protein Features
p=0.004*
MS−Intensity (iBAQ, log 2)
PBS(d14)
Bleo(d14)
p=0.238
Ratio (Bleo/PBS; log2)
Day

Q99JX4 − Eif3m (id: 2332)

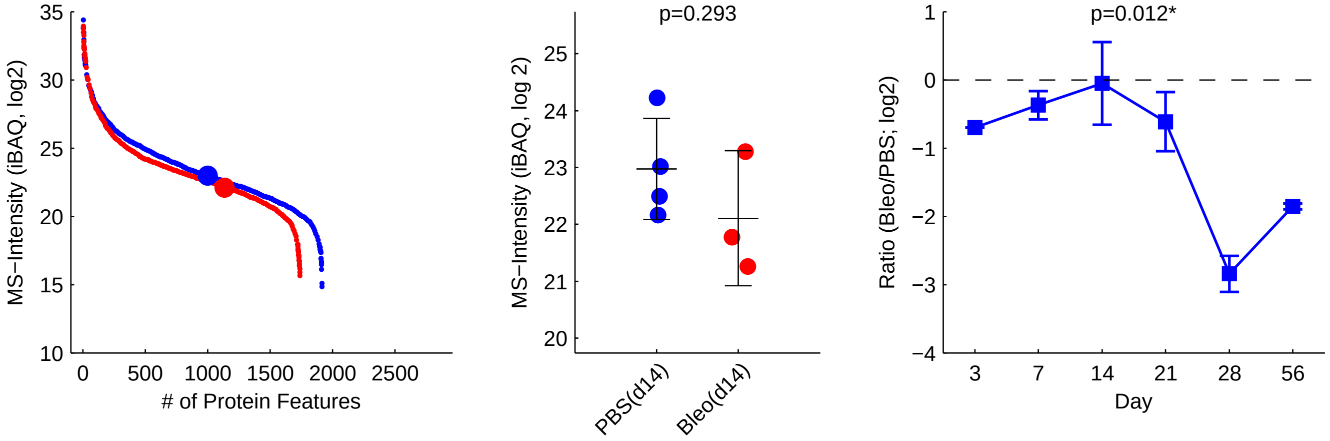

Q99JY9 − Actr3 (id: 2334)

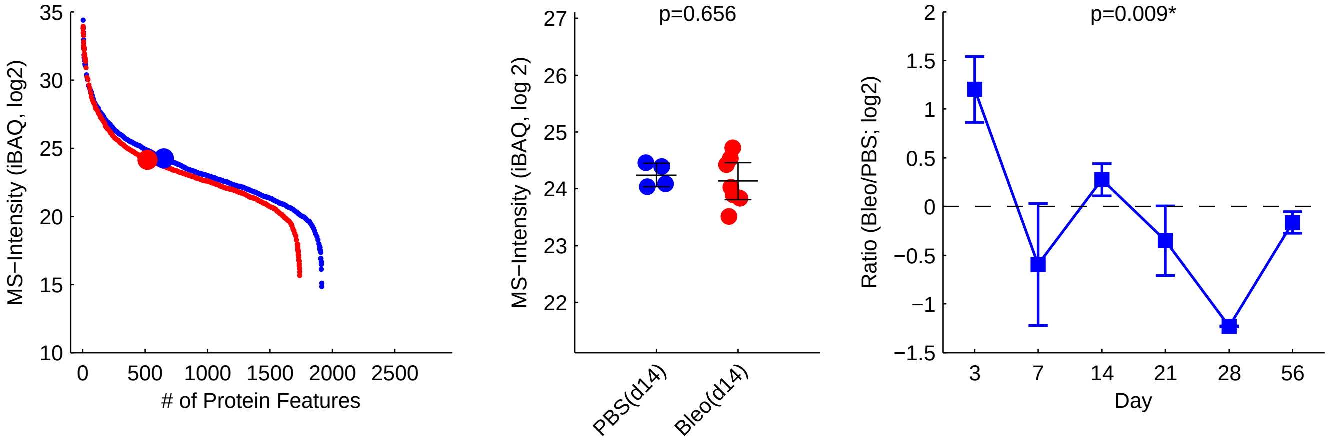

Q99KI0 − Aco2 (id: 2345)

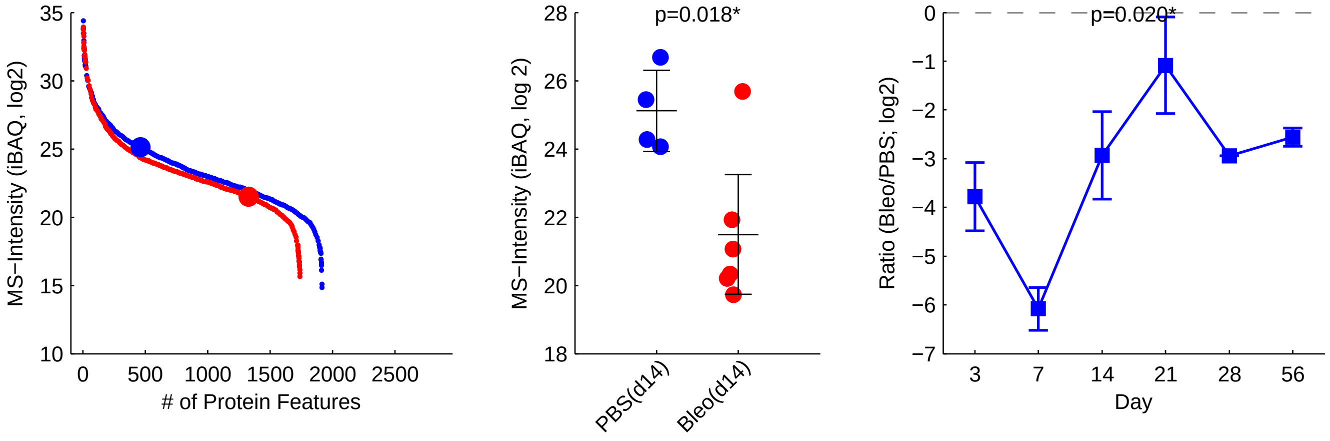

Q99KP3 − Cryl1 (id: 2349)

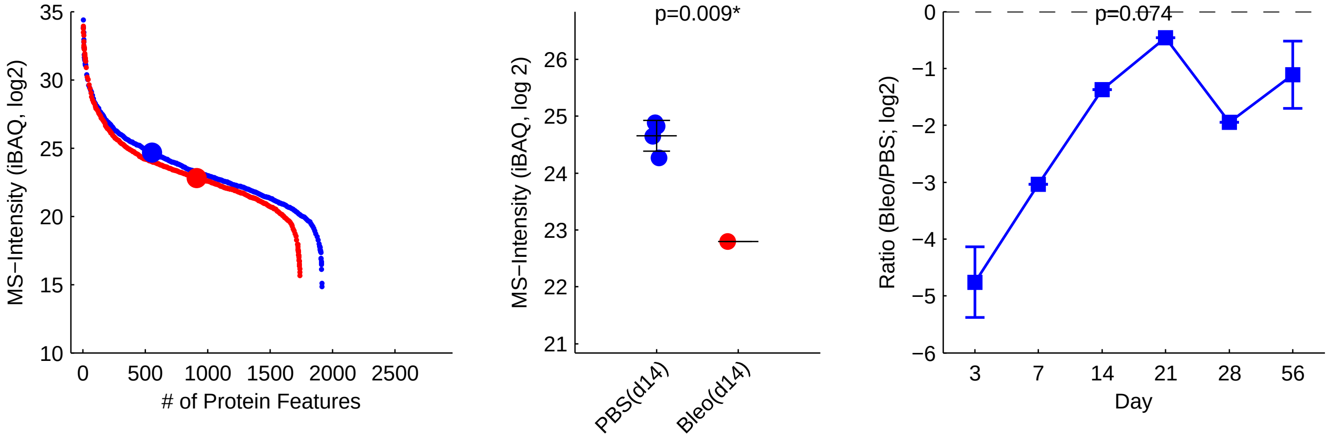

Q99KR3 − Lactb2 (id: 2352)

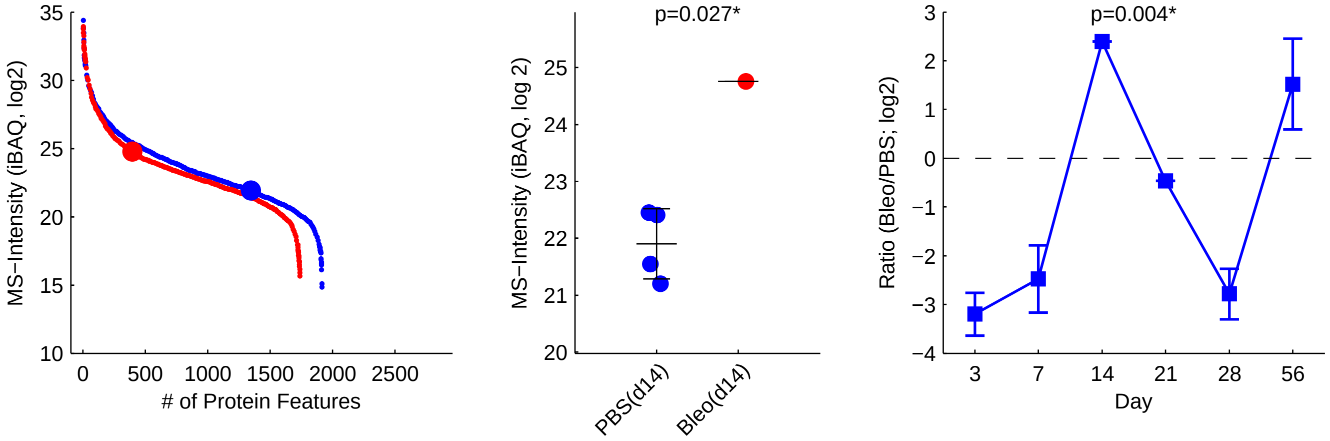

Q99LB6−2 − Mat2b (id: 2362)

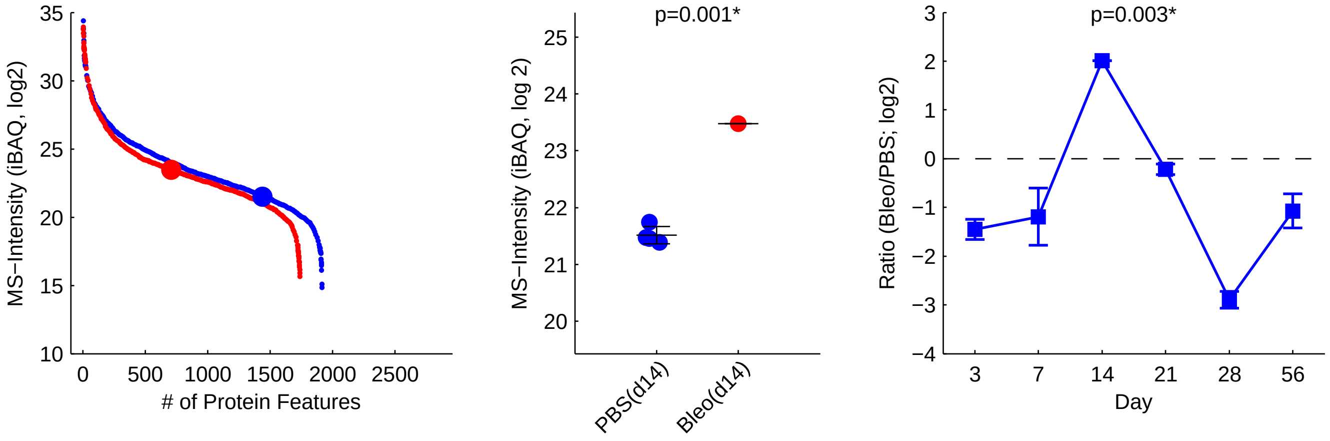

Q99LC5 − Etfa (id: 2364)

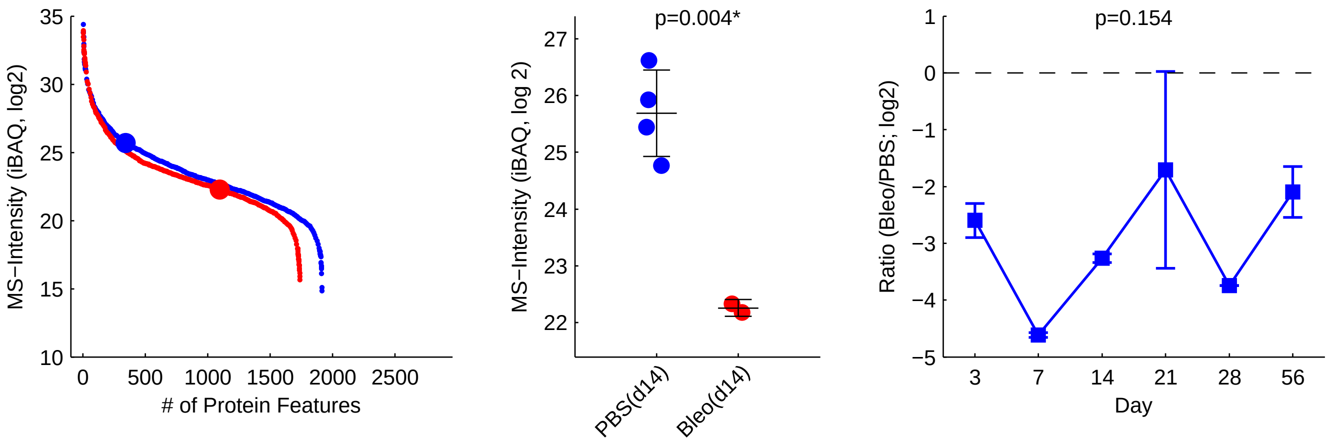

Q99LF4 − D10Wsu52e (id: 2367)

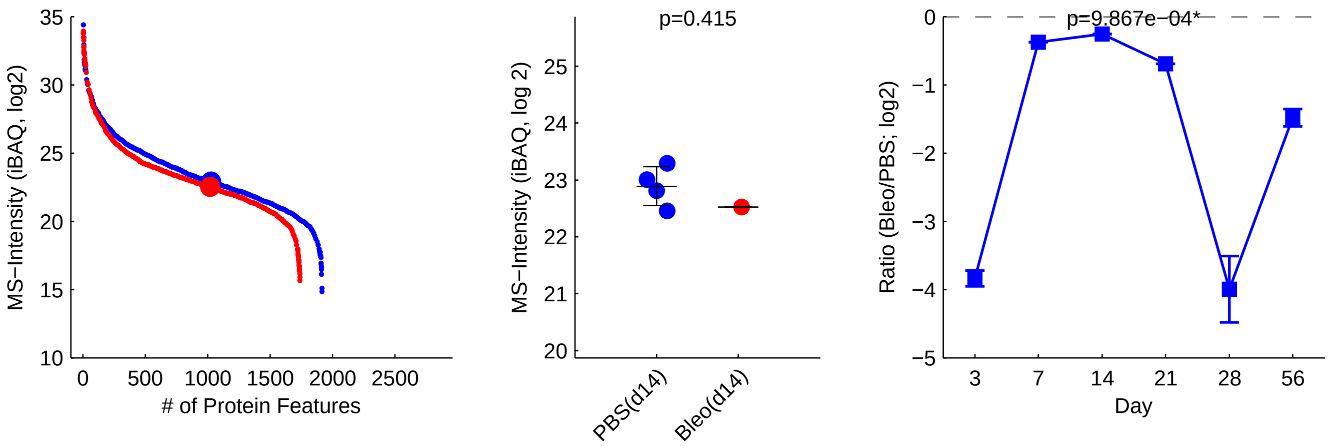

Q99LJ1 − Fuca1 (id: 2369)

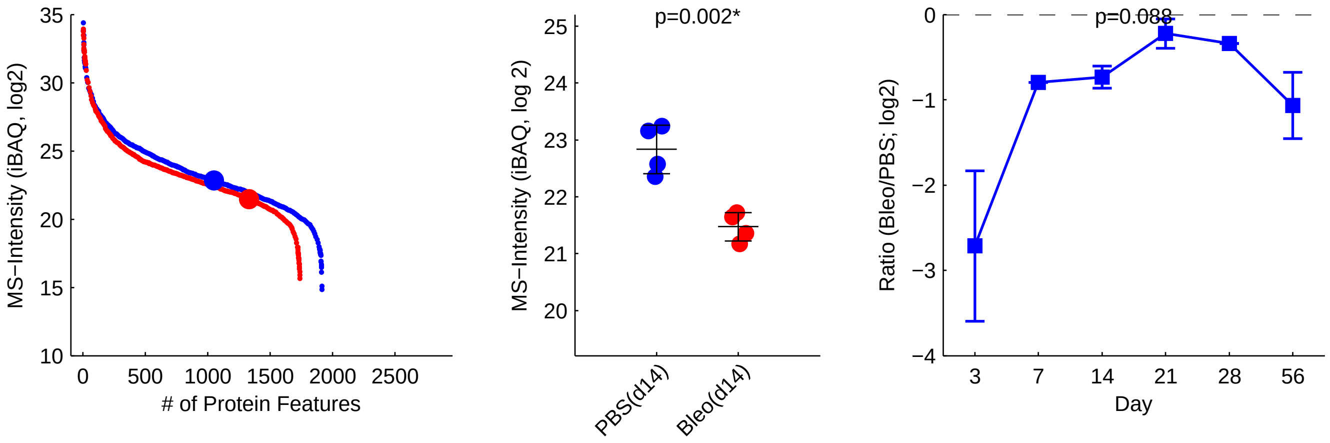

Q99MN9 − Pccb (id: 2378)

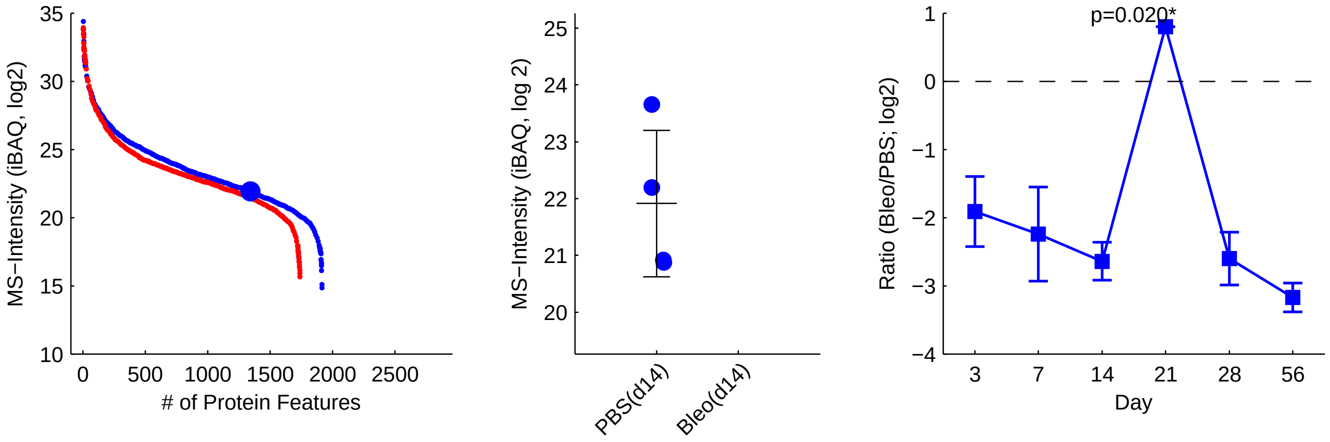

Q99N15 − Hsd17b10 (id: 2381)

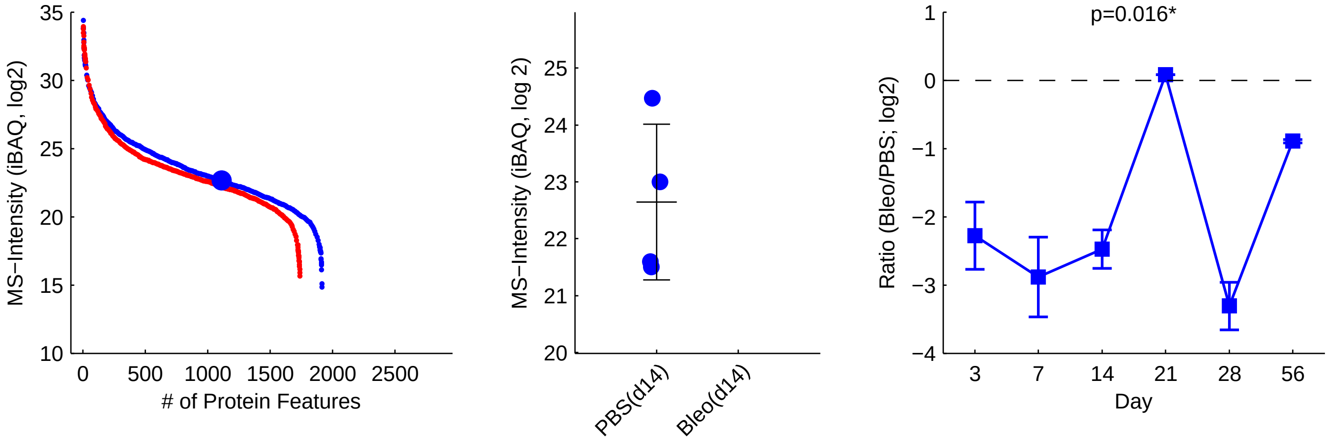

Q99NB1 − Acss1 (id: 2382)

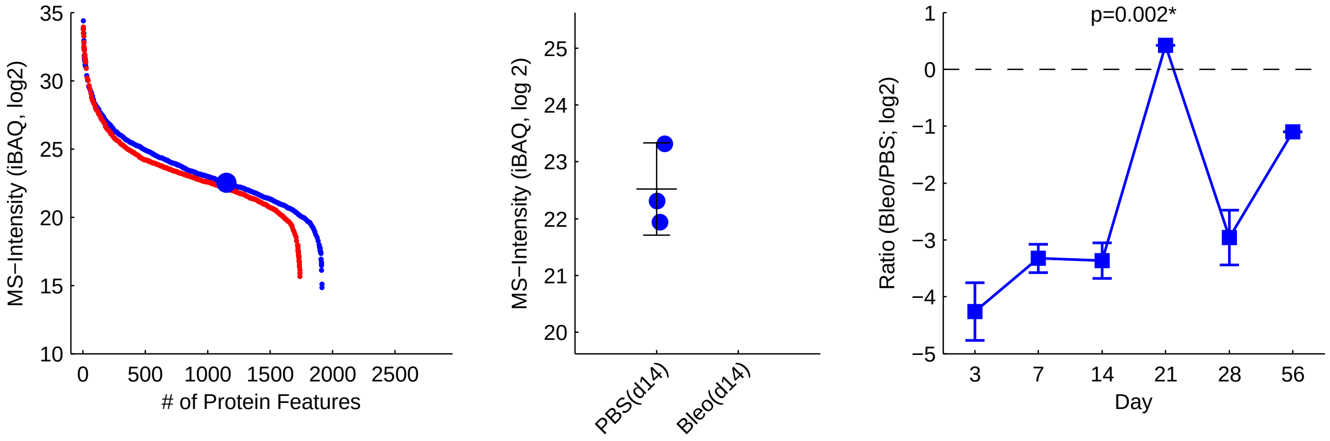

Q9CPP7 − Lipf (id: 2388)

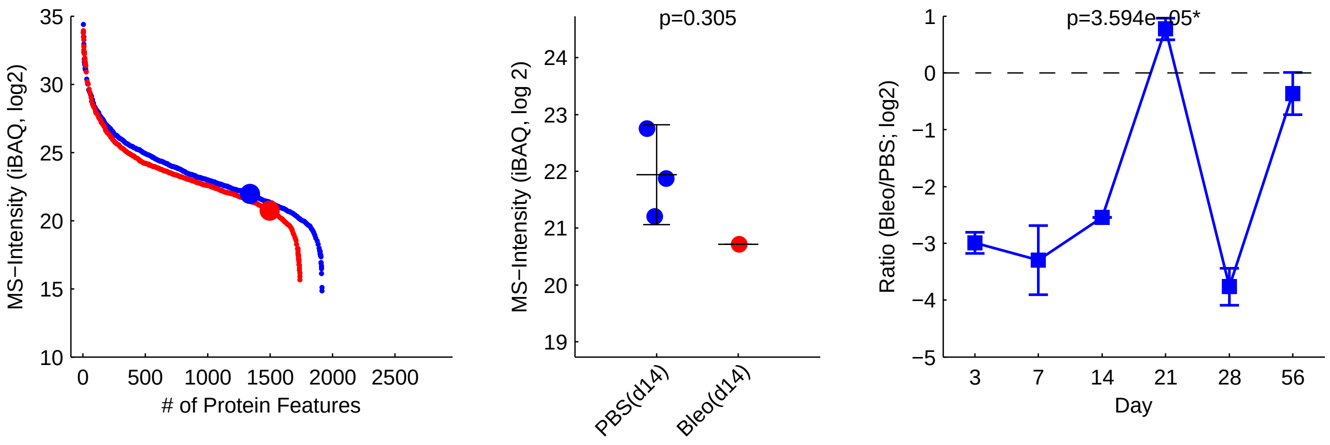

Q9CPV4 − Glod4 (id: 2392)

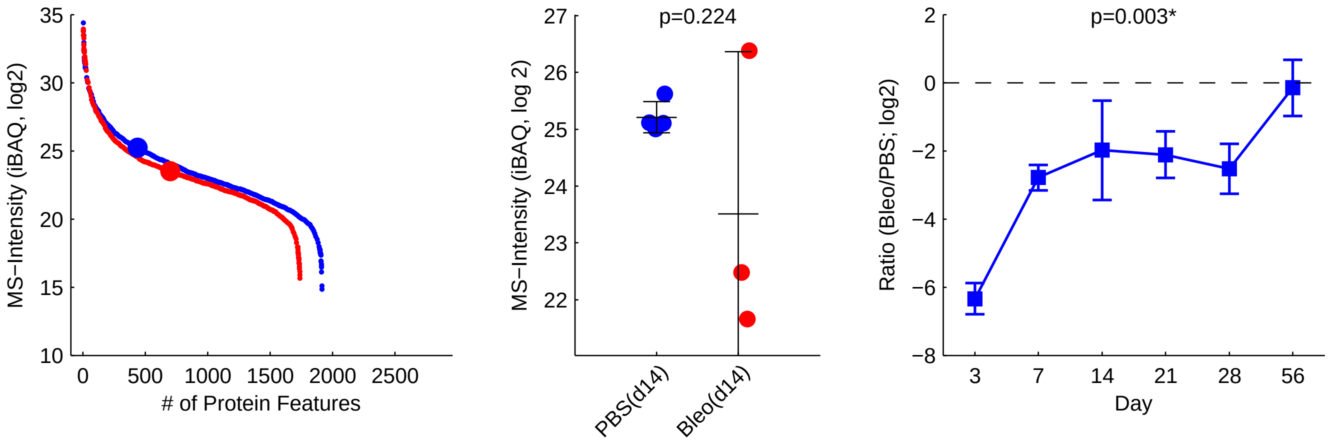

Q9CPX4 − Ftl1 (id: 2394)

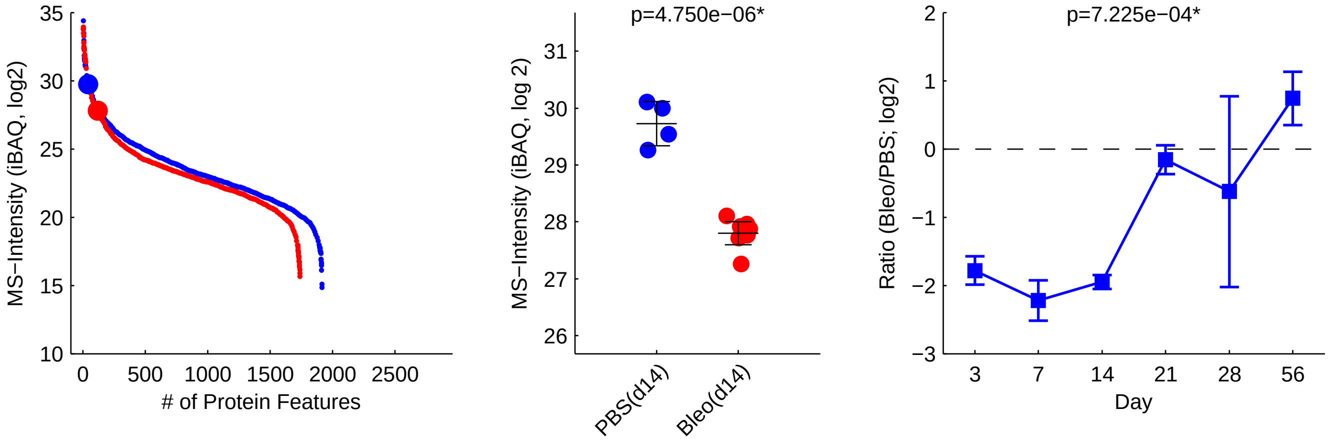

Q9CPX6 − Atg3 (id: 2395)

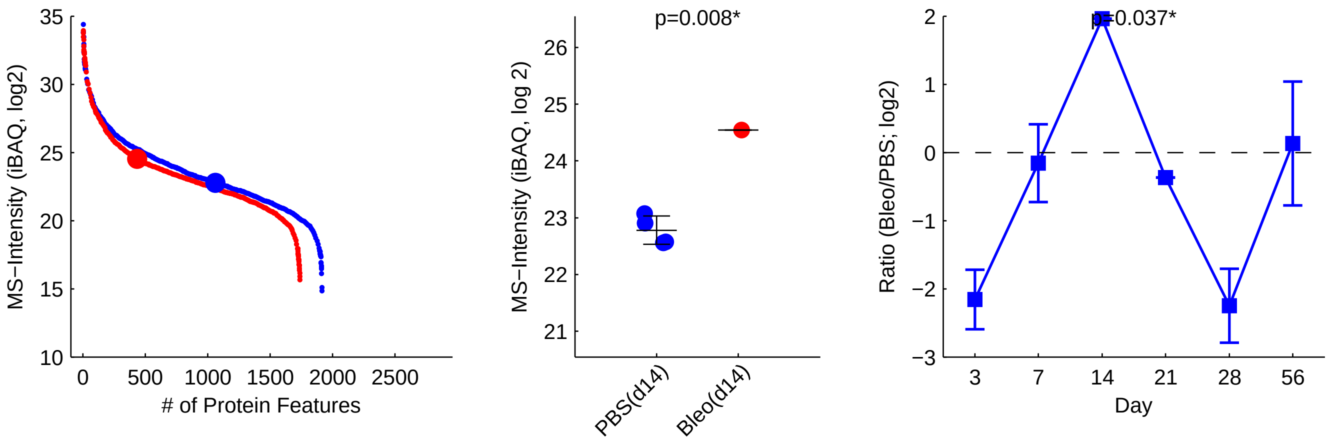

Q9CPY7−2 − Lap3 (id: 2396)

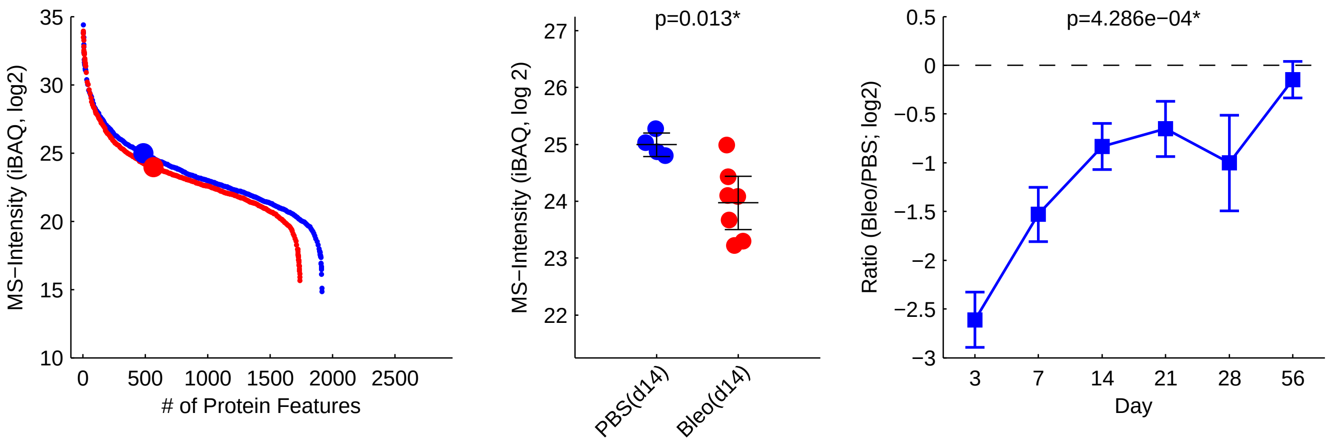

Q9CQ01 − Rnaset2 (id: 2397)

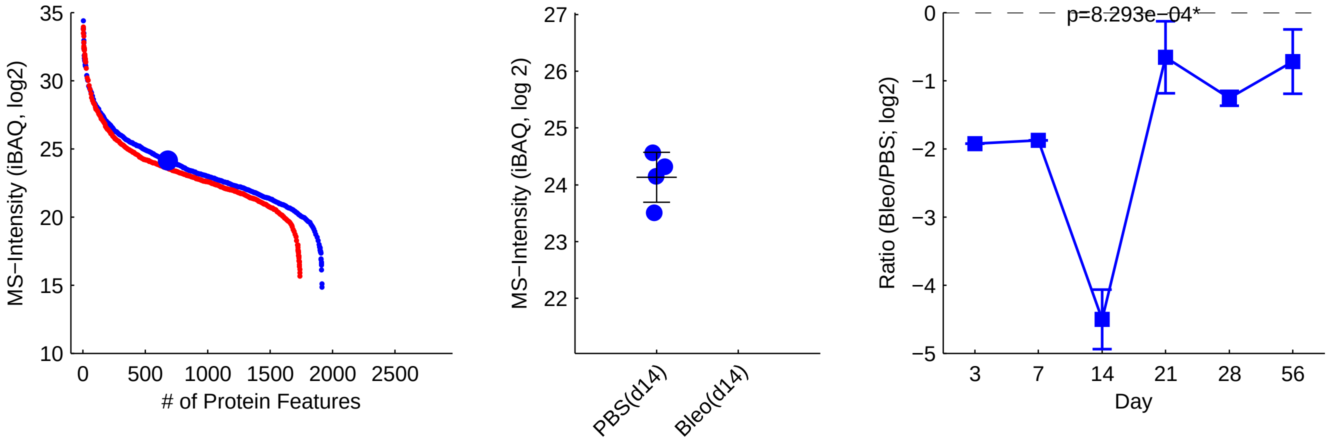

Q9CQI3 − Gmfb (id: 2418)

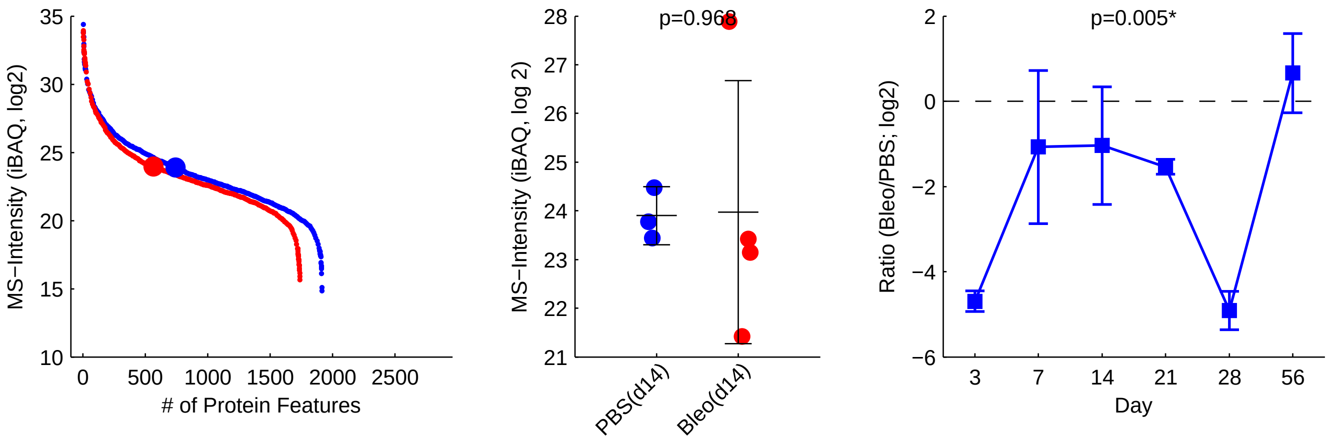

Q9CQI6 − Cotl1 (id: 2419)

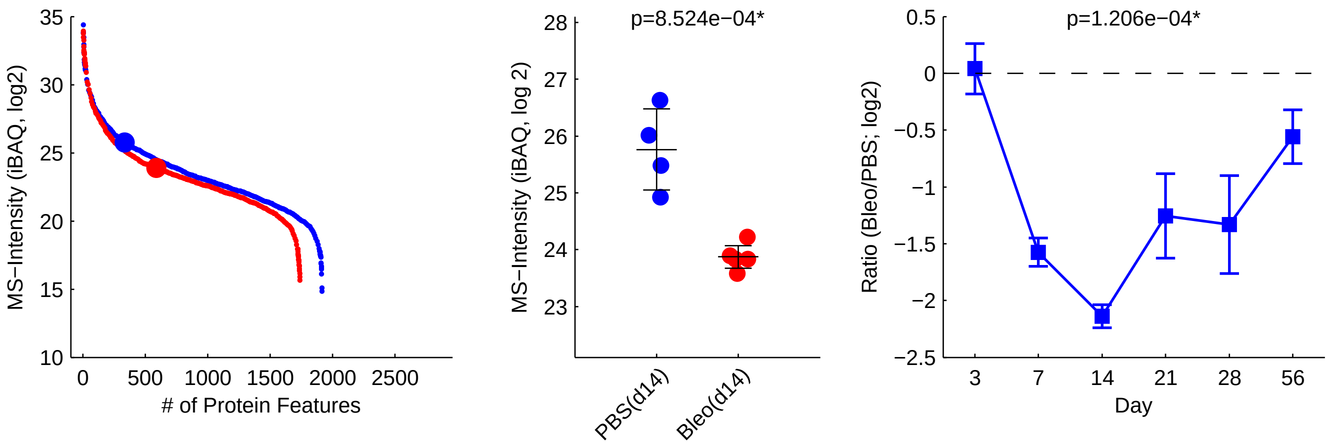

Q9CQM5 − Txndc17 (id: 2420)

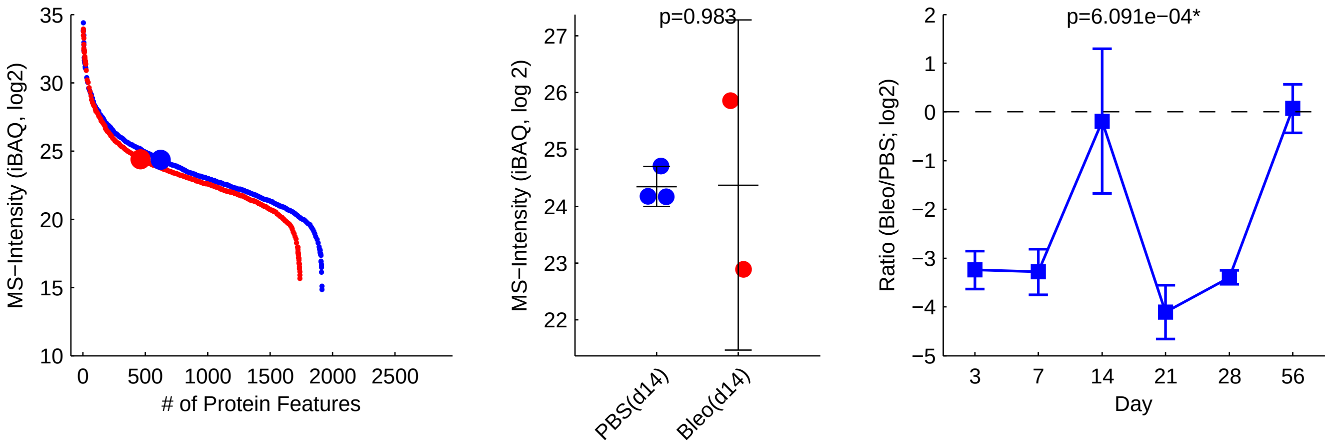

Q9CQR2 − Rps21 (id: 2426)

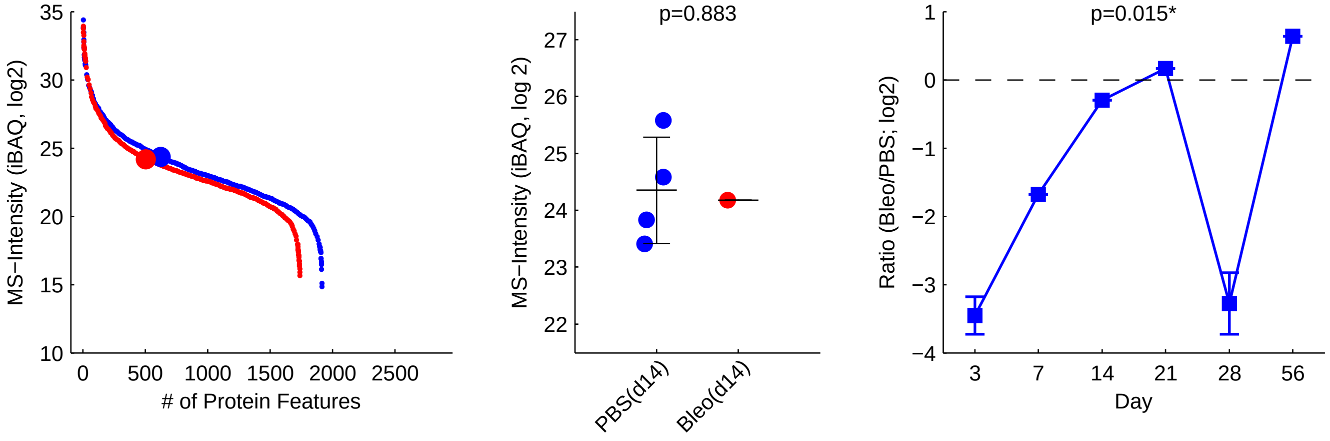

Q9CQT1 − Mri1 (id: 2429)

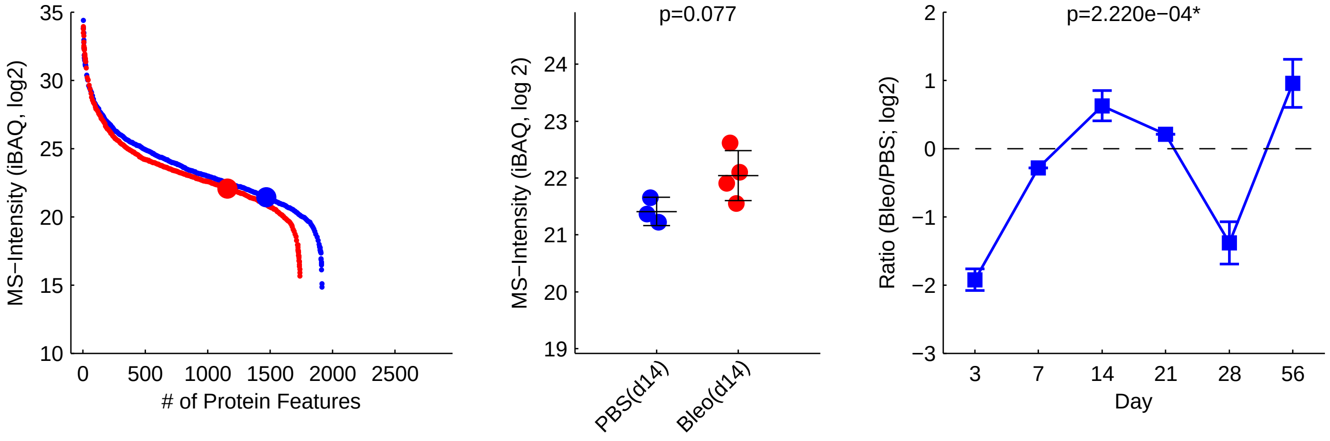

Q9CR51 − Atp6v1g1 (id: 2446)

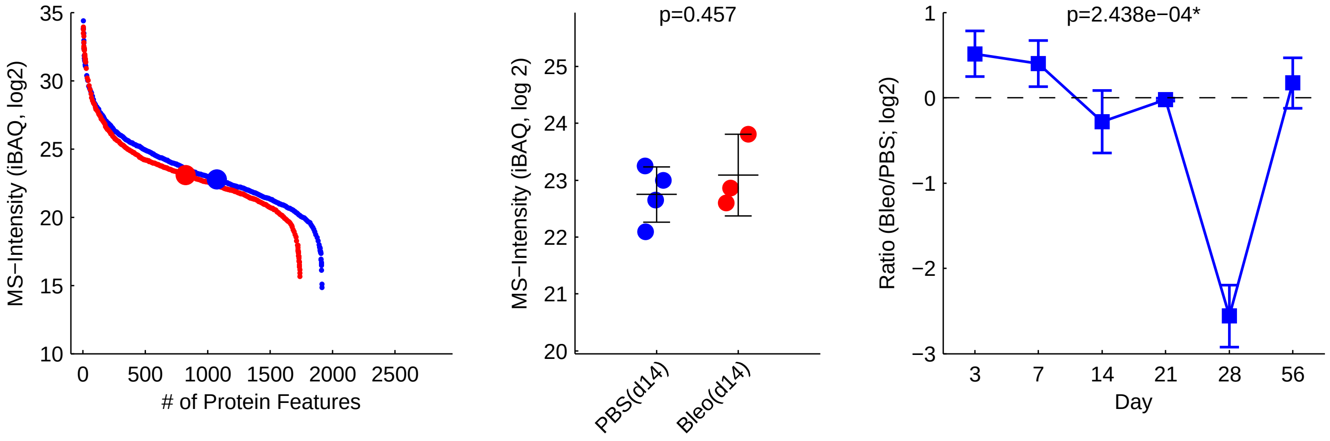

Q9CR57 − Rpl14 (id: 2447)

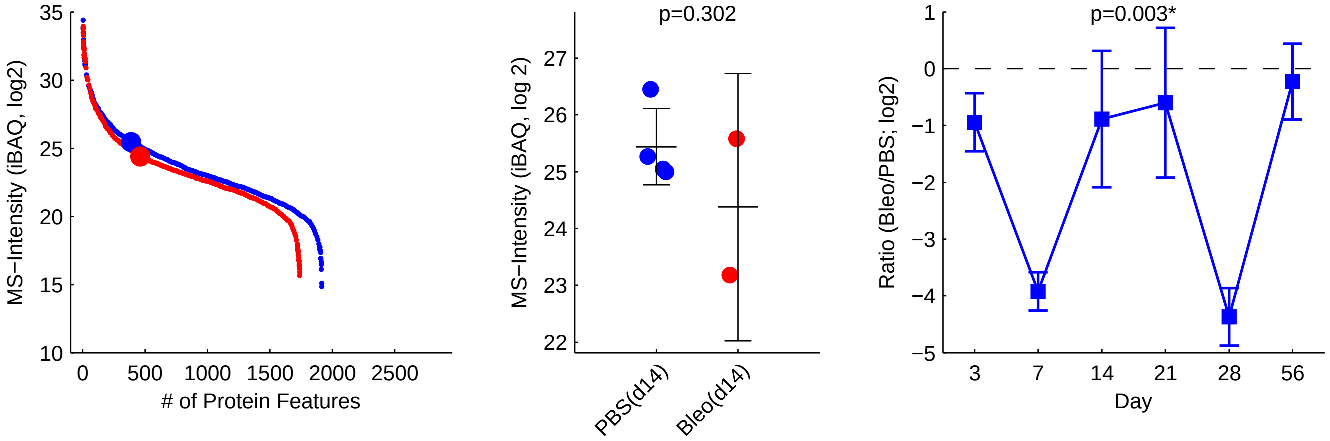

Q9CRB6 − Tppp3 (id: 2451)

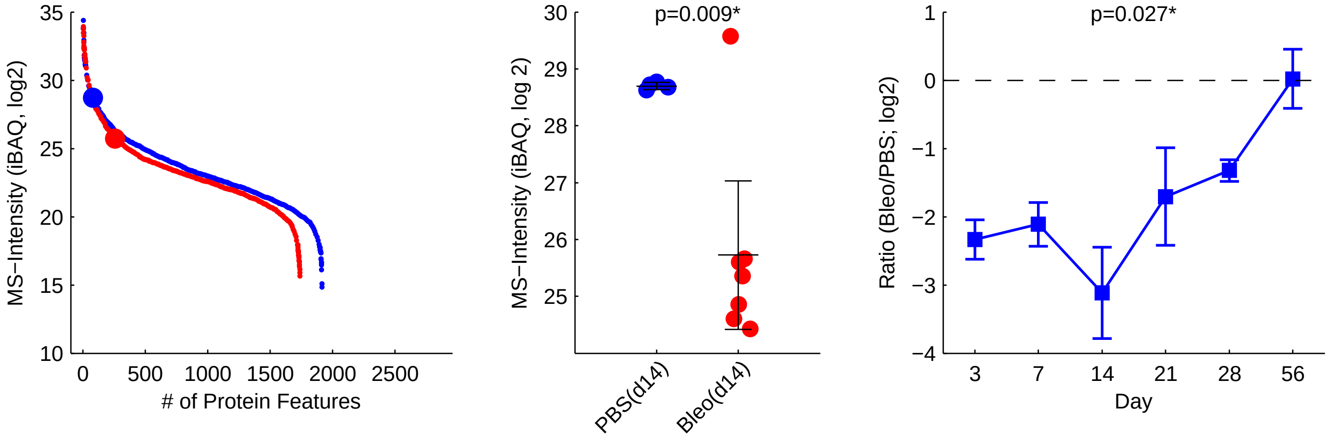

Q9CRC9 − Gnpda2 (id: 2452)

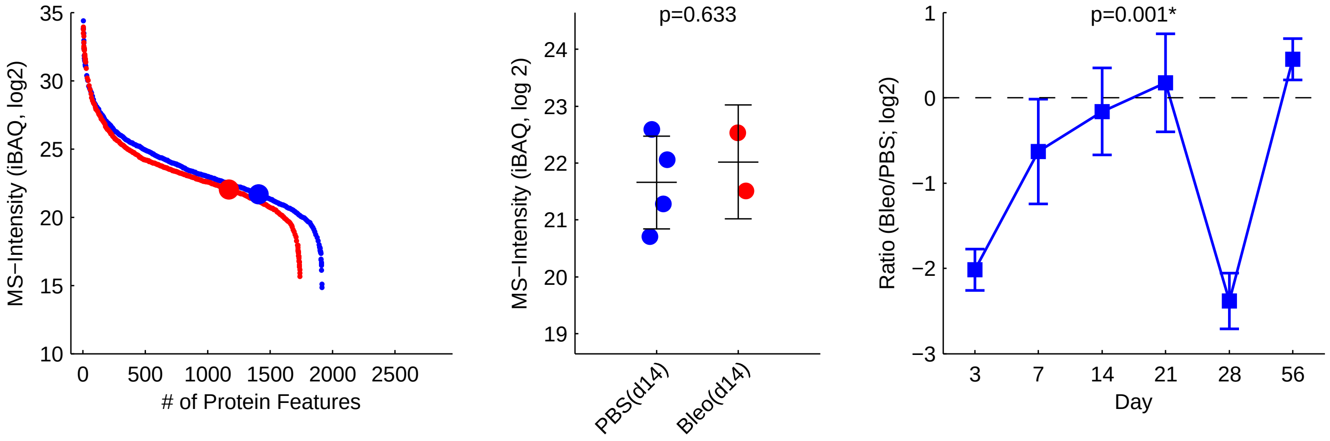

Q9CWF2 − Tubb2b (id: 2456)

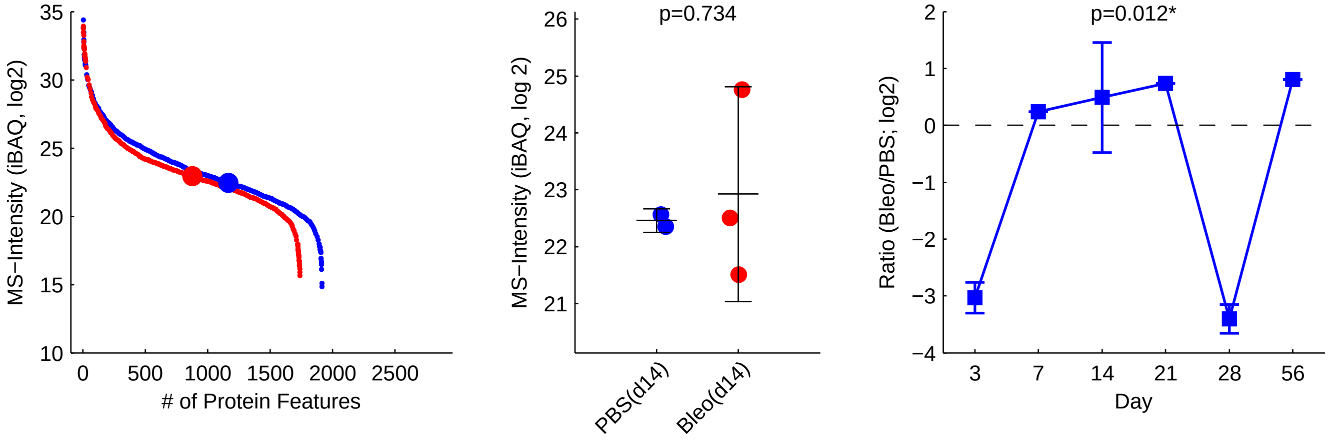

Q9CWK8 − Snx2 (id: 2458)

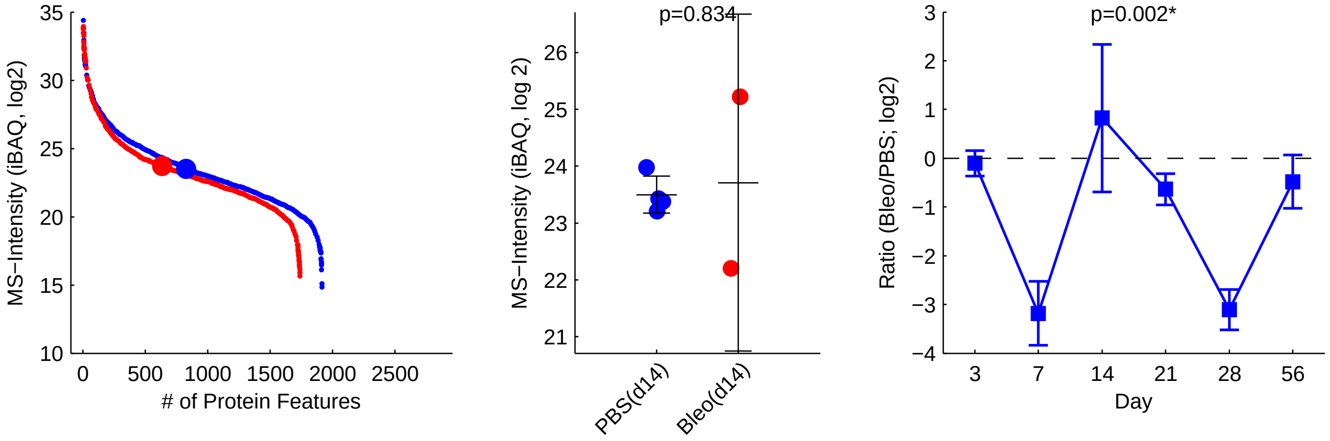

Q9CX00 − Ist1 (id: 2460)

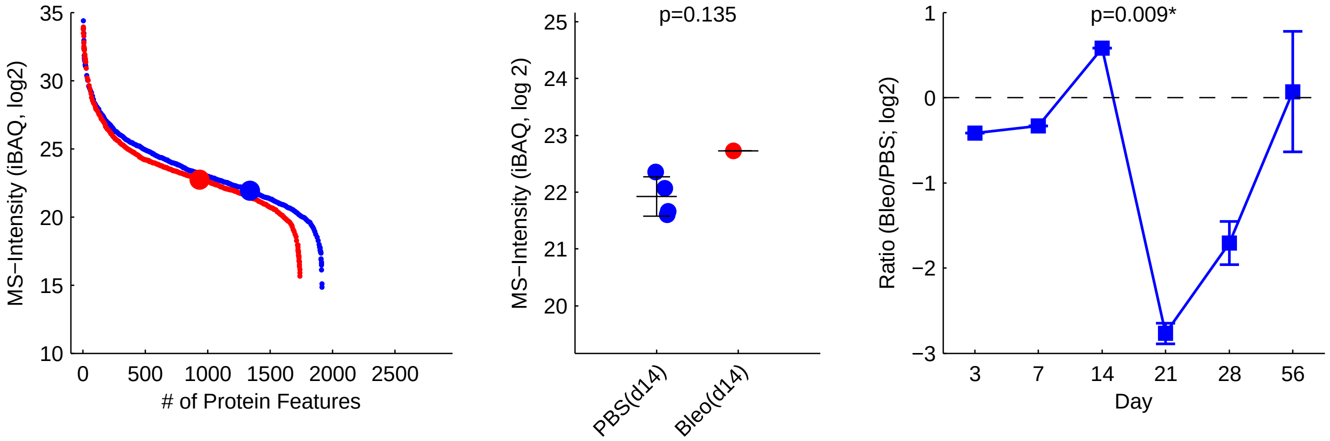

Q9CX56 − Psmd8 (id: 2462)

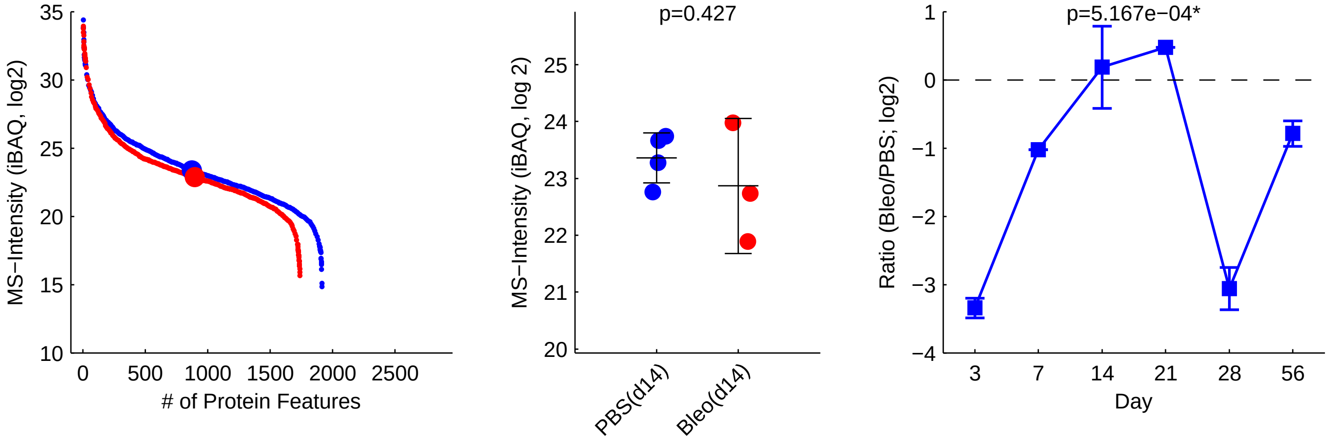

Q9CXW3 − Cacybp (id: 2467)

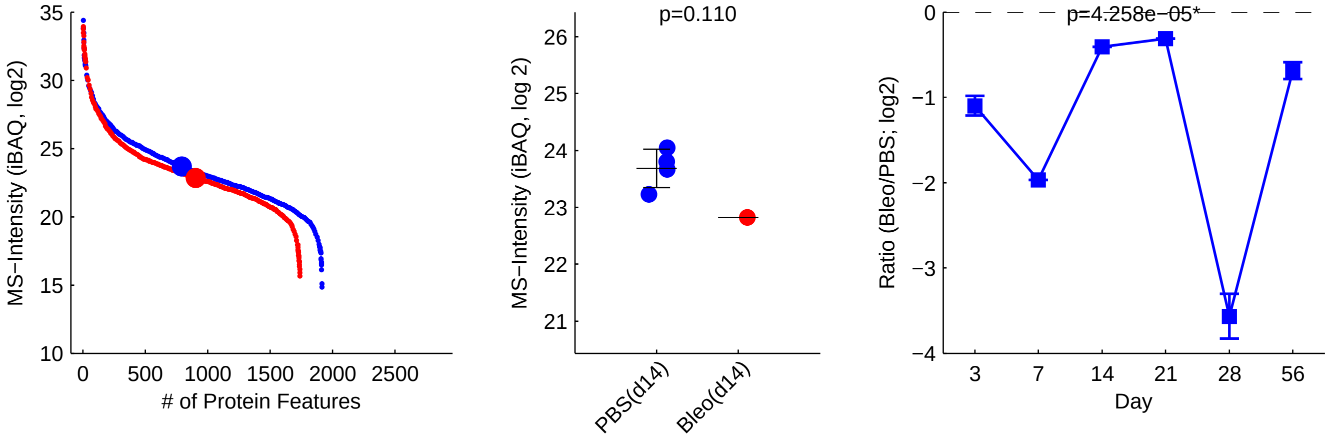

Q9CZ44 − Nsfl1c (id: 2483)

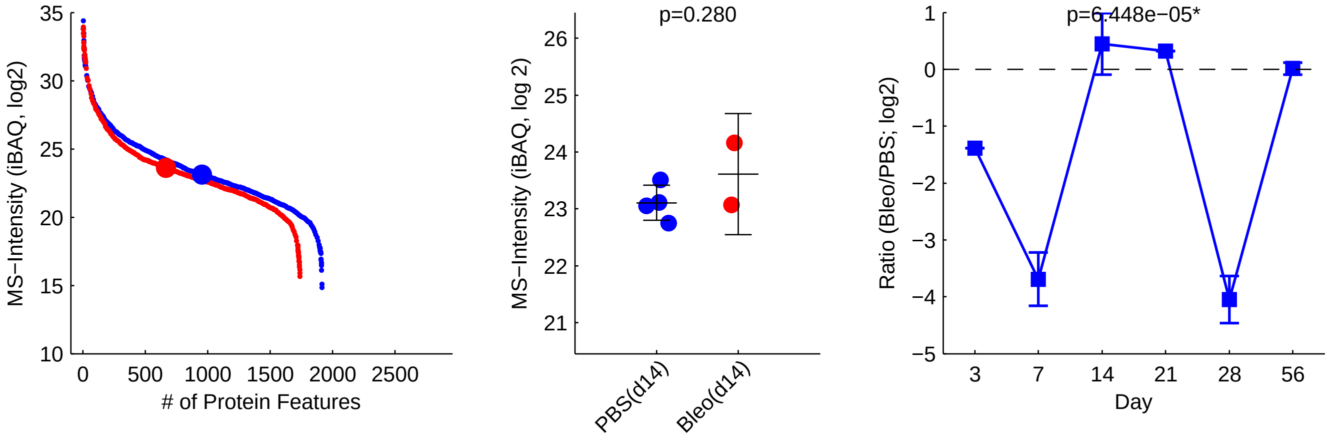

Q9CZU6 − Cs (id: 2487)

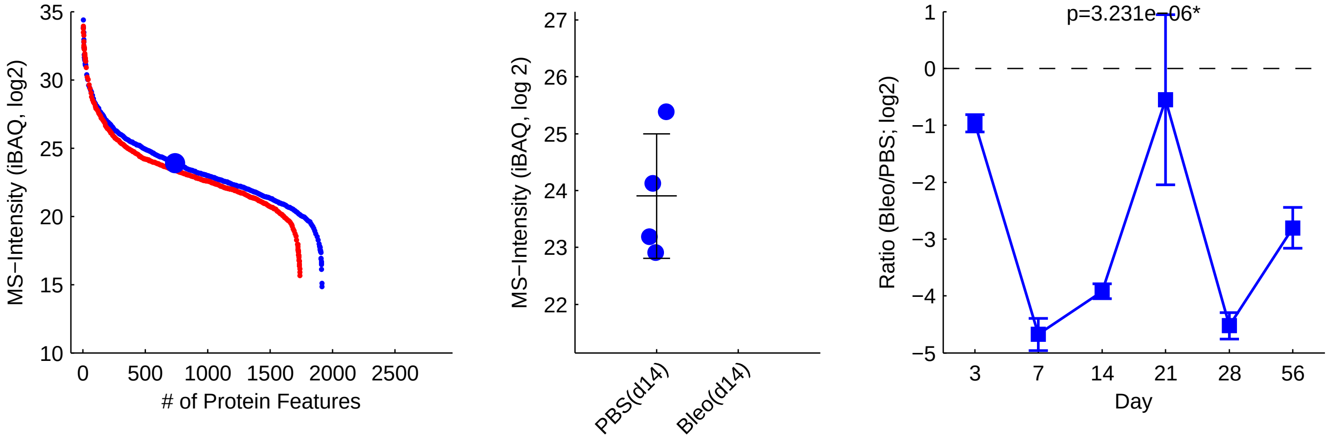

Q9CZX8 − Rps19 (id: 2488)

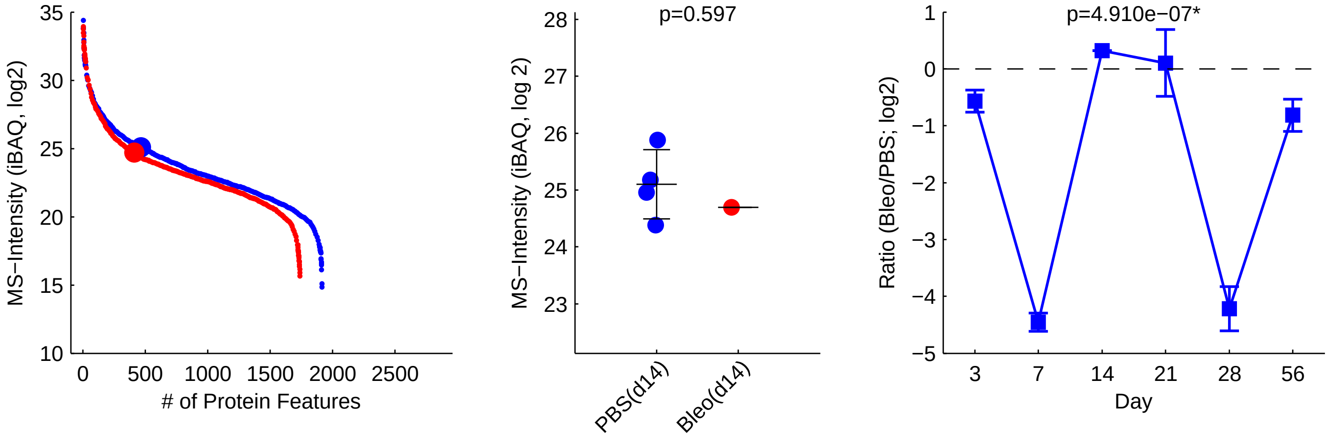

Q9D051 − Pdhb (id: 2493)

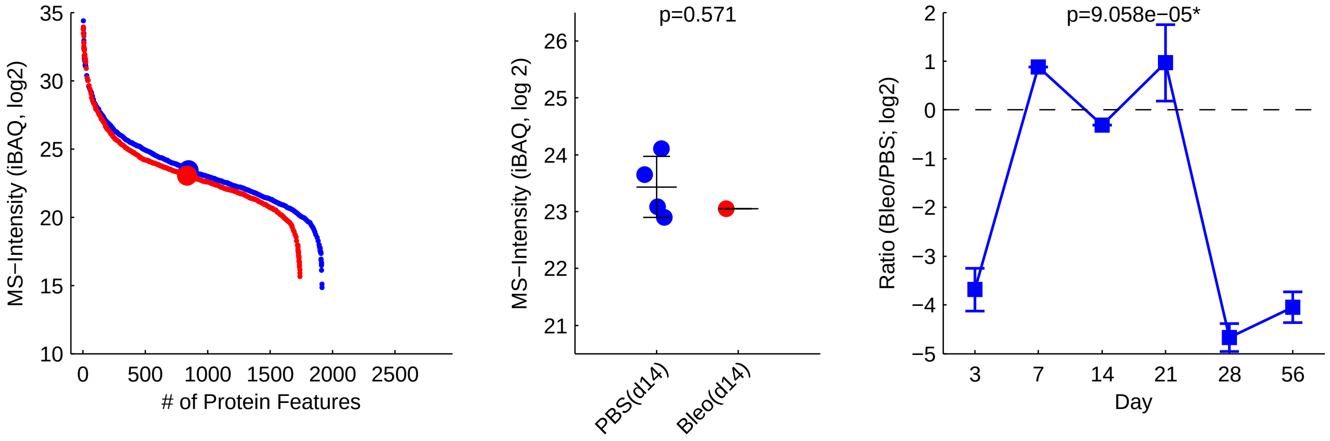

Q9D0J8 − Ptms (id: 2501)

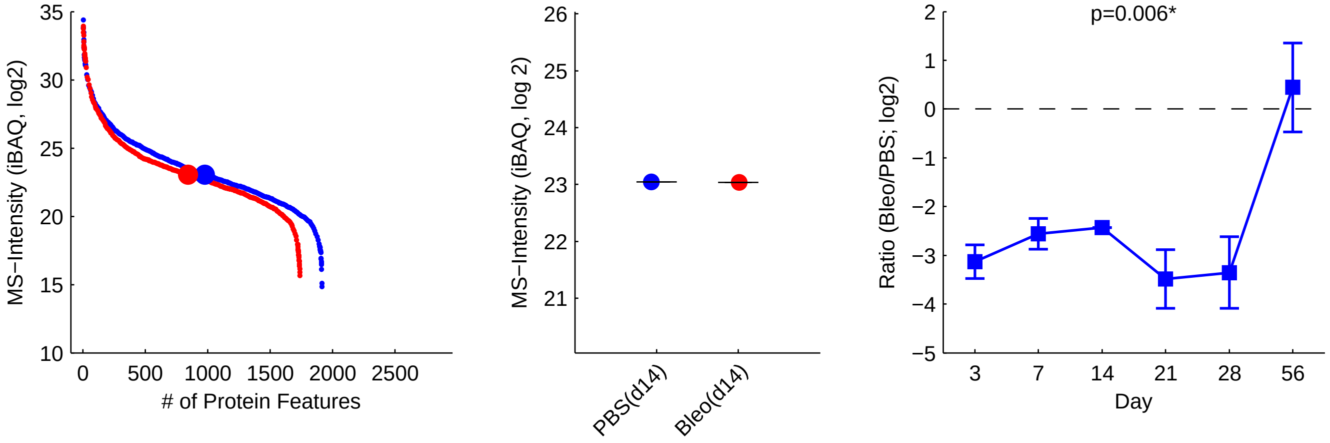

Q9D0M5 − Dynll2 (id: 2503)

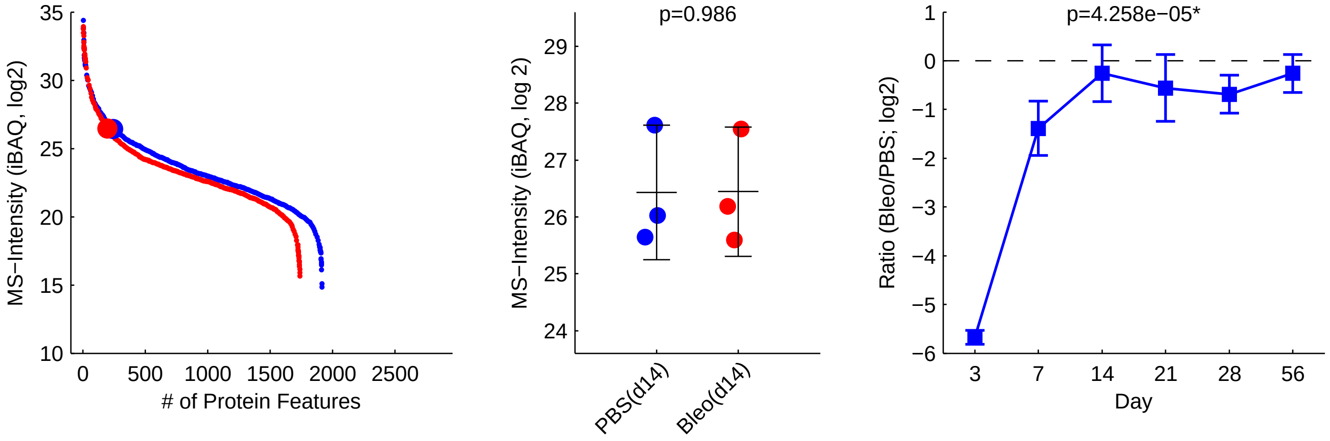

Q9D154 − Serpinb1a (id: 2510)

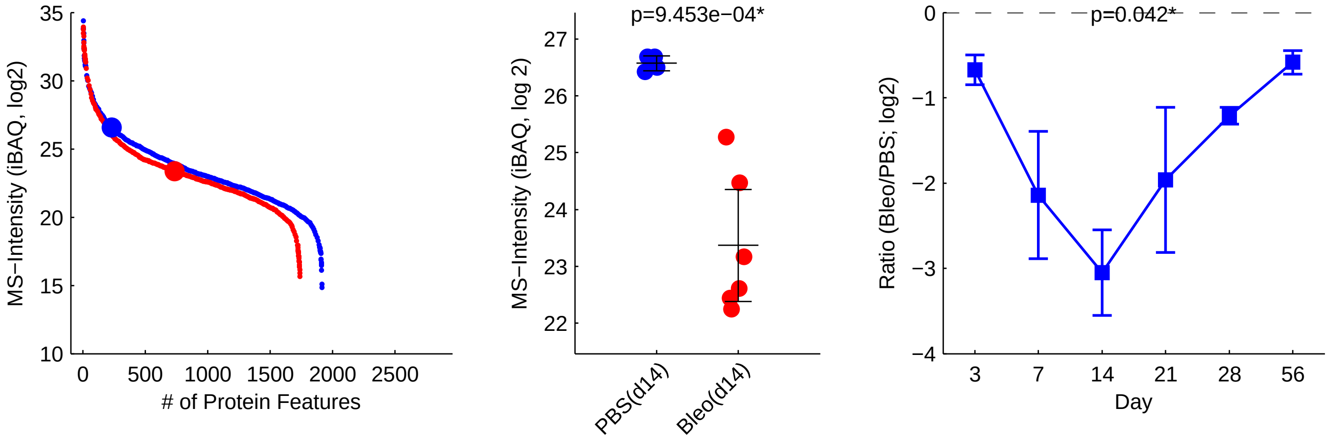

Q9D1G1 − Rab1b (id: 2518)

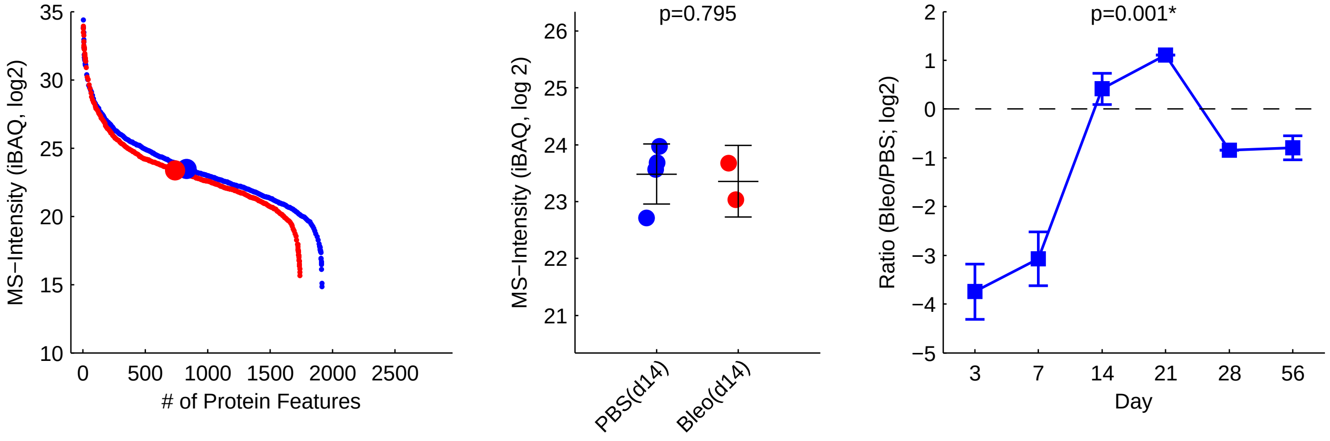

Q9D1P4 − Chordc1 (id: 2525)

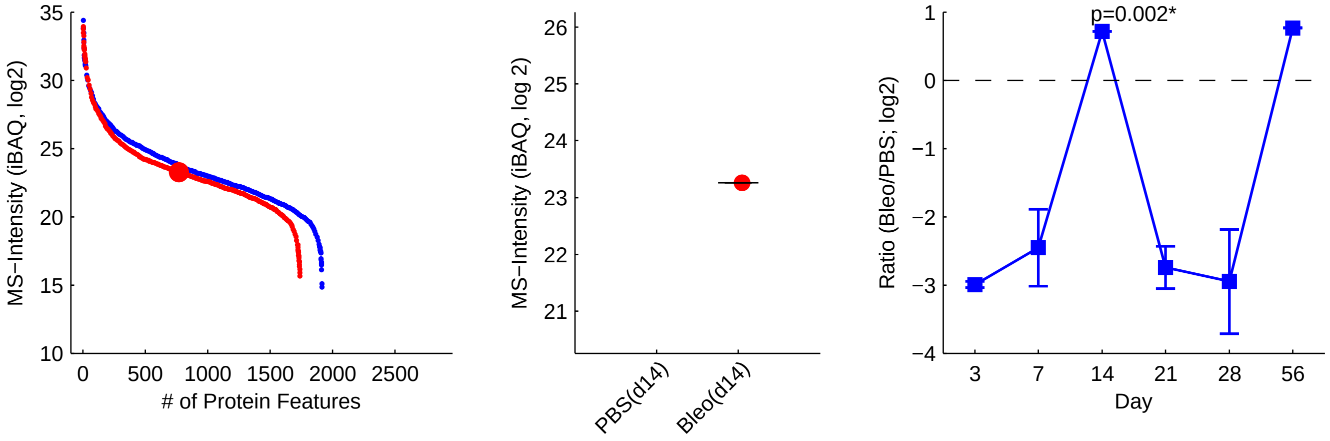

Q9D379 − Ephx1 (id: 2536)

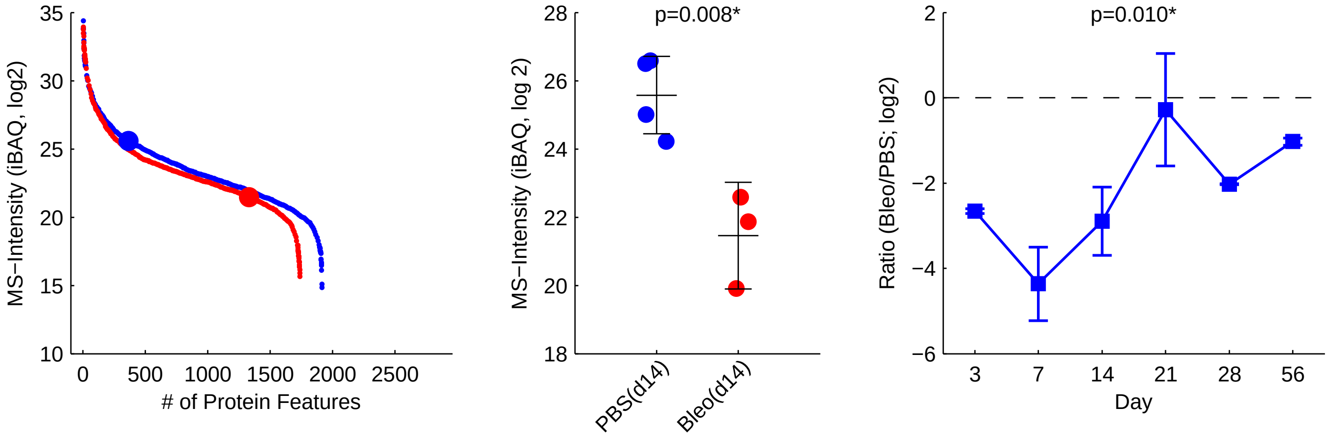

Q9D6Y7−3 − Msra (id: 2552)

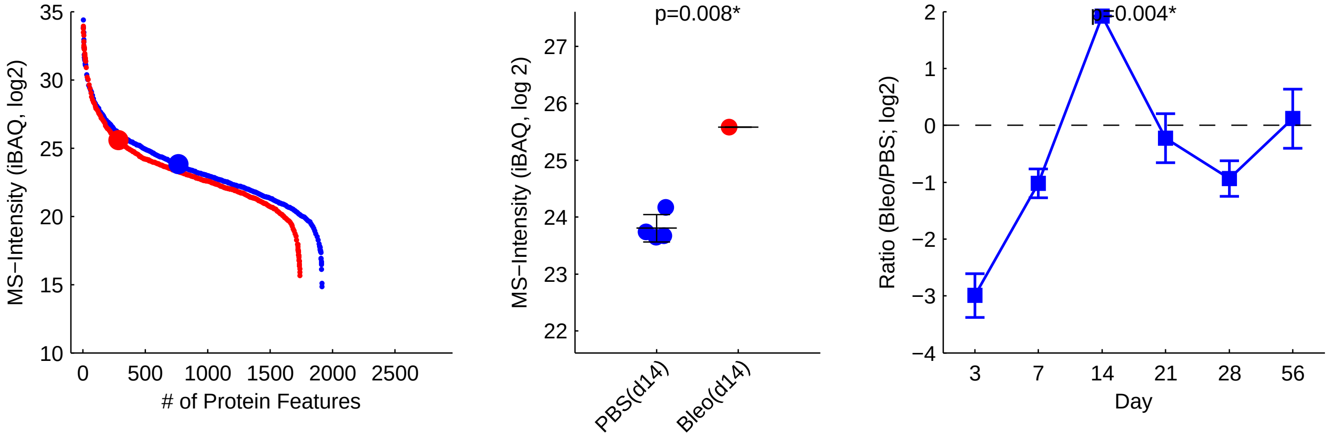

Q9D7P7 − Clic3 (id: 2557)

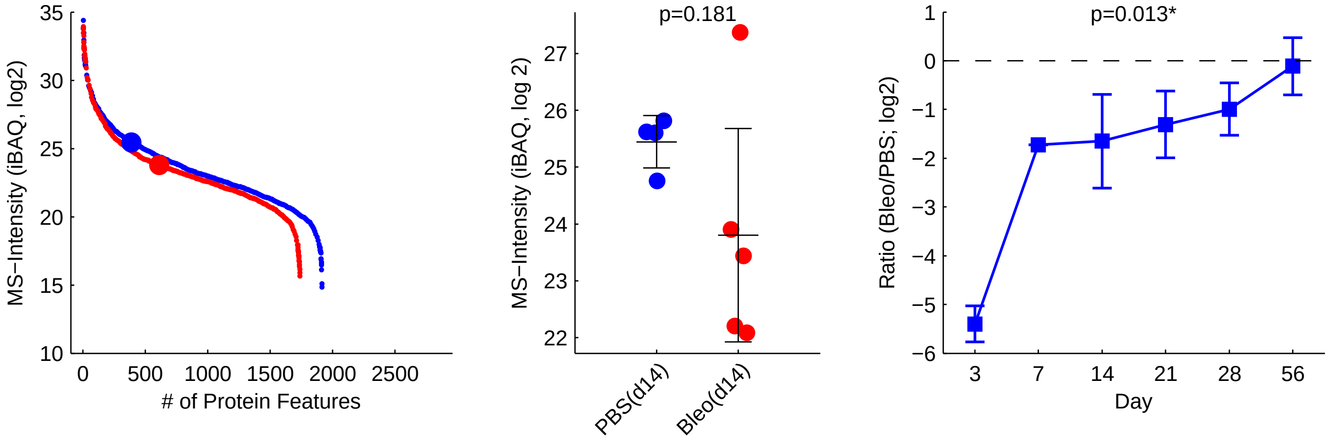

Q9D8B3 − Chmp4b (id: 2567)

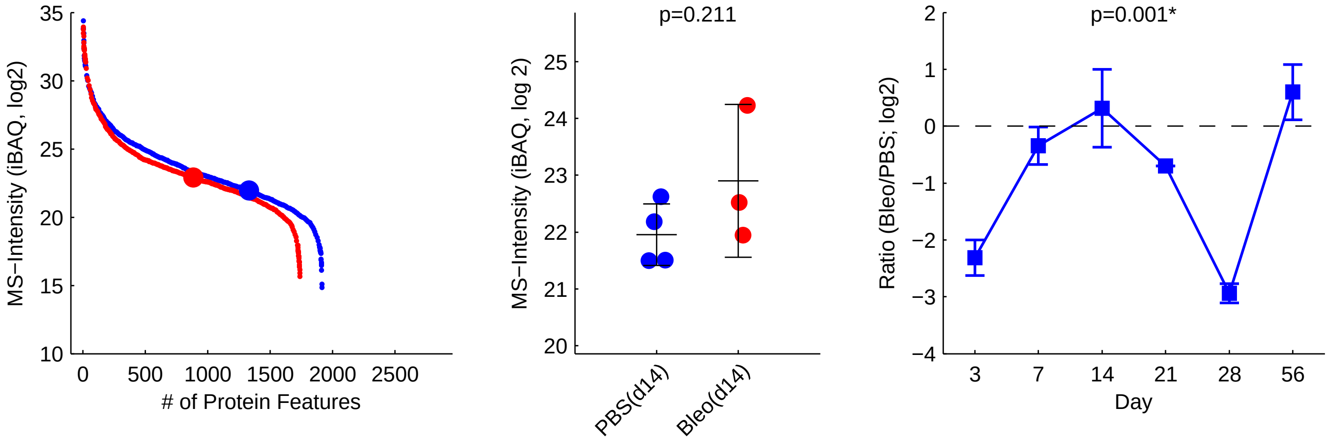

Q9D8E6 − Rpl4 (id: 2569)

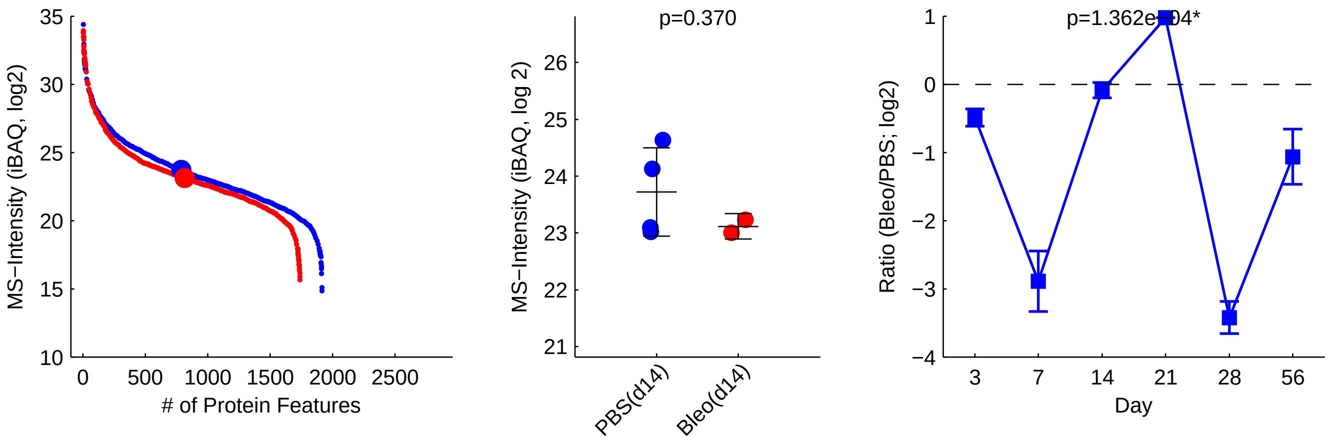

Q9D8W5 − Psmd12 (id: 2574)

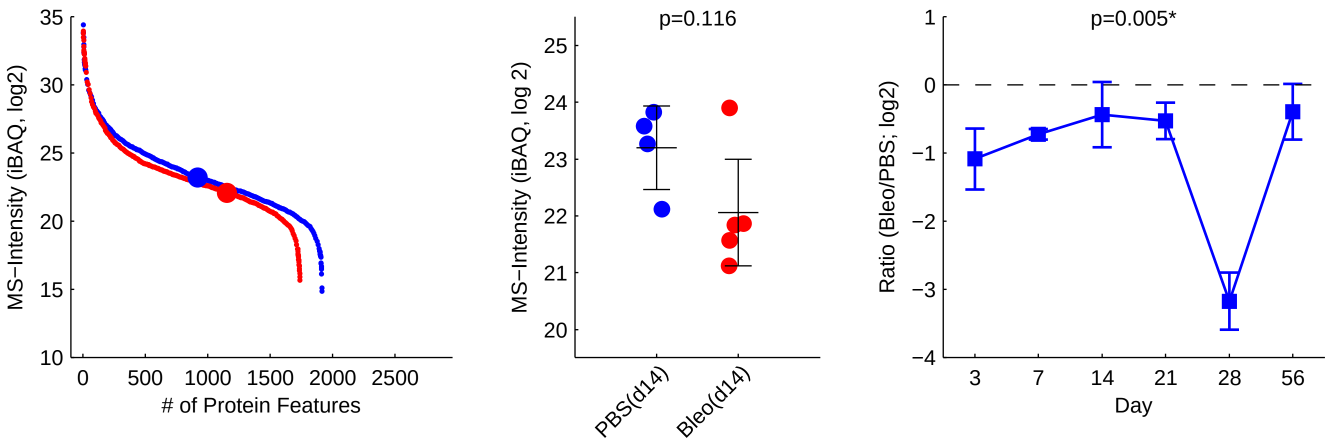

Q9QZ08 − Nagk (id: 2577)

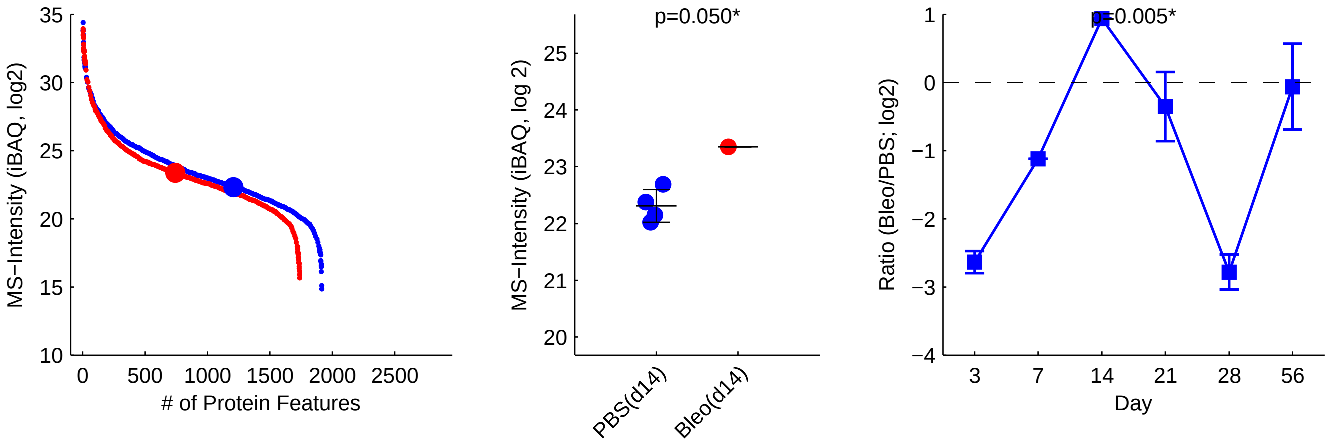

Q9D9V4 − Rsph9 (id: 2579)

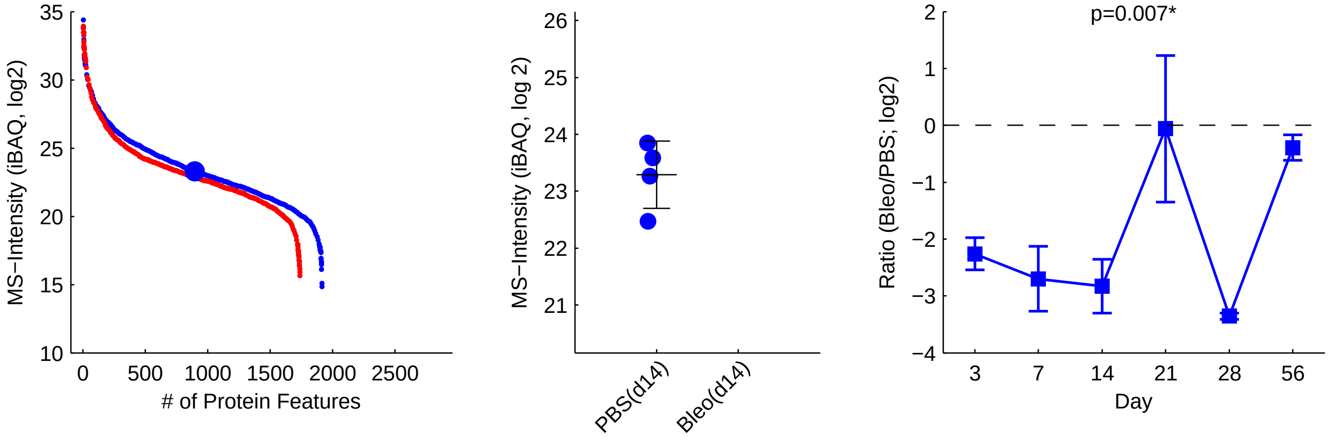

Q9DAI2 − Rabl5 (id: 2581)

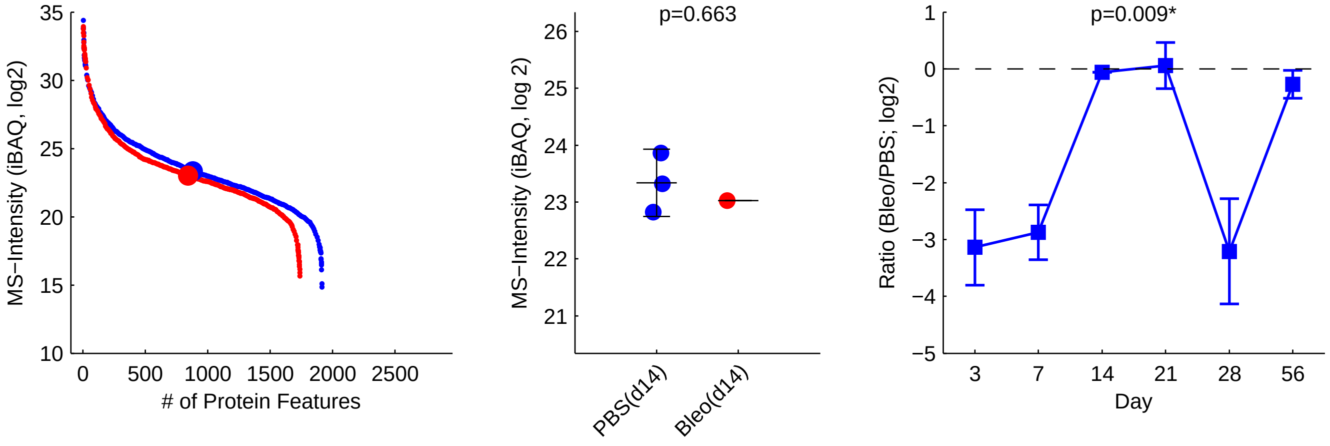

Q9DAJ5 − Dynlrb2 (id: 2583)

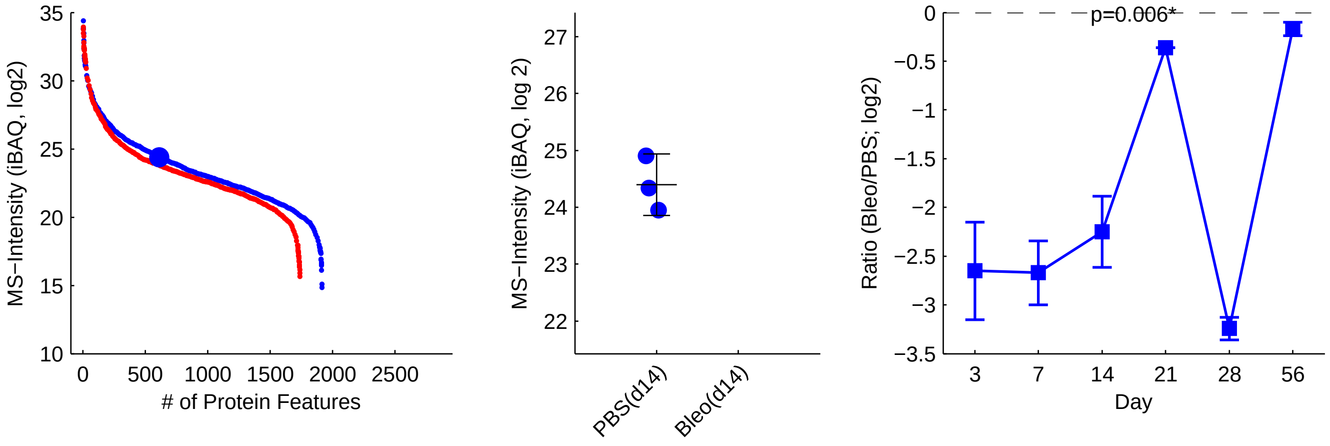

Q9DAK9 − Phpt1 (id: 2585)

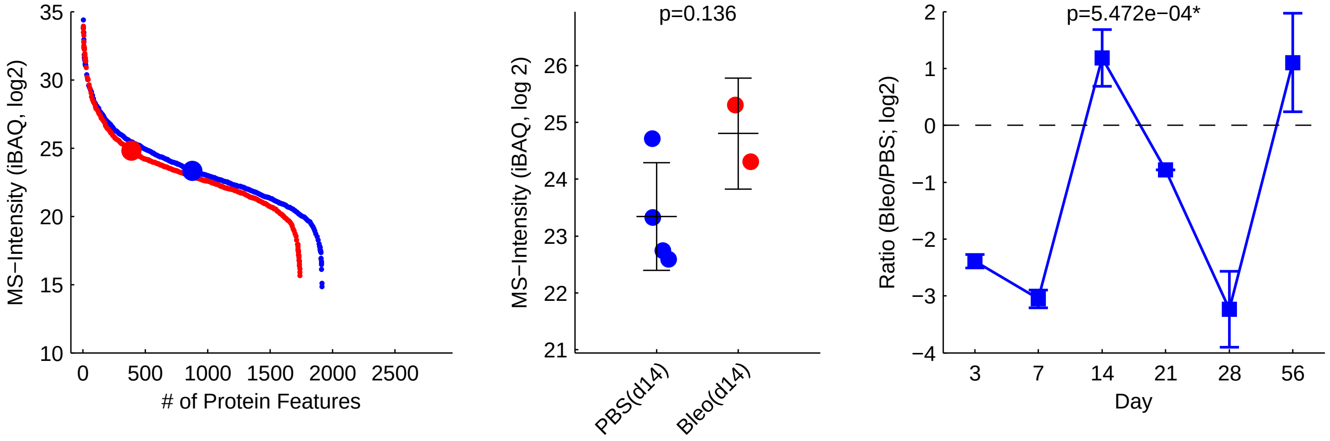

Q9DAR7 − Dcps (id: 2588)

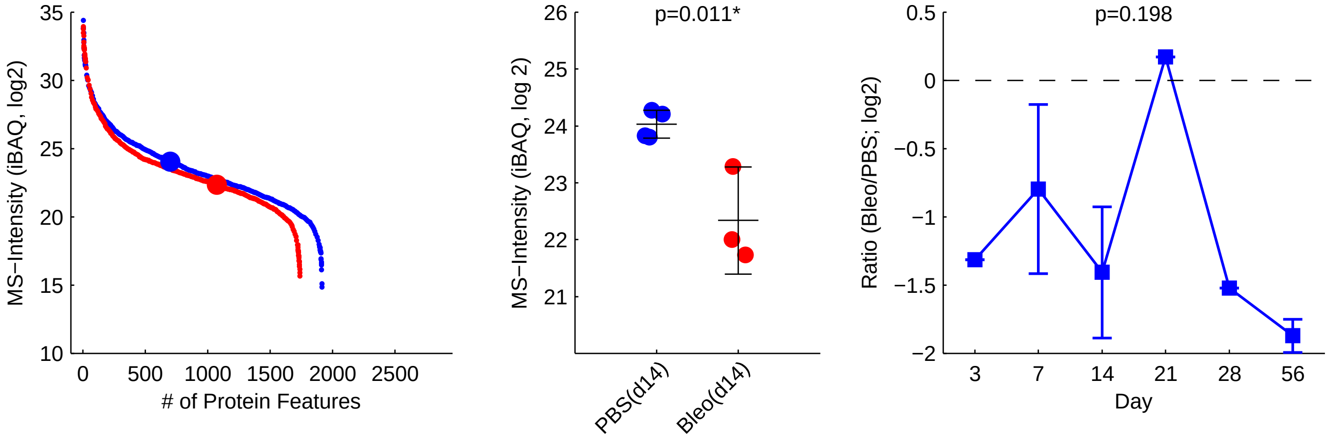

Q9DAV6 − Serpinb9b (id: 2591)

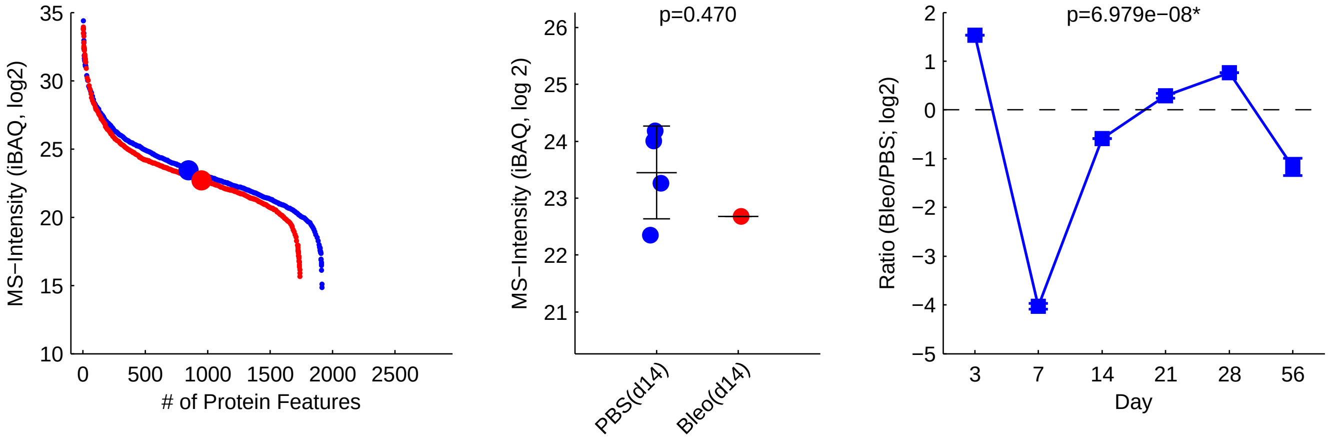

Q9DB16 − Cab39l (id: 2595)

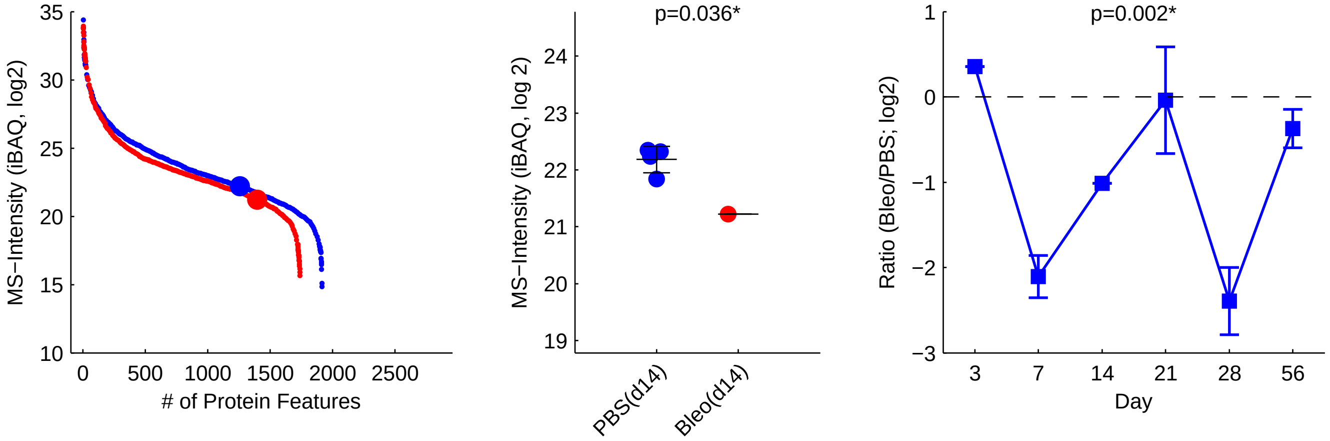

Q9DB20 − Atp5o (id: 2596)

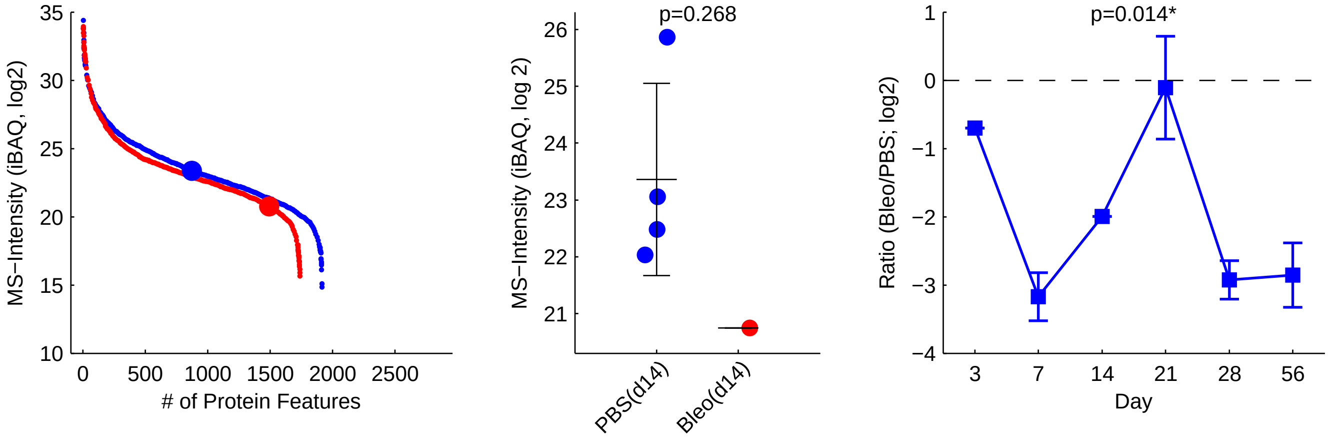

Q9DB77 − Uqcrc2 (id: 2600)

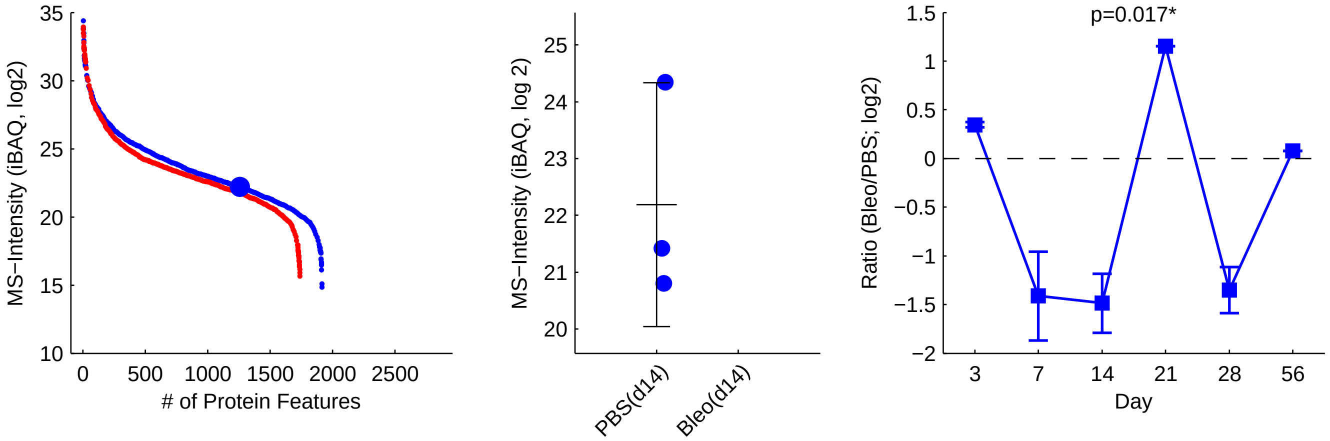

Q9DBB9 − Cpn2 (id: 2602)

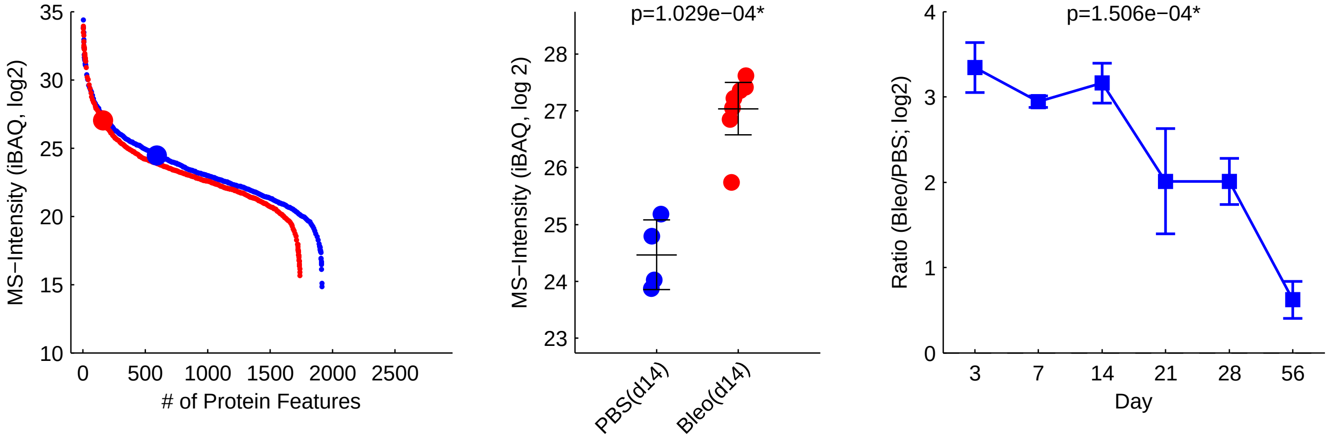

Q9DBD0 − Ica (id: 2604)

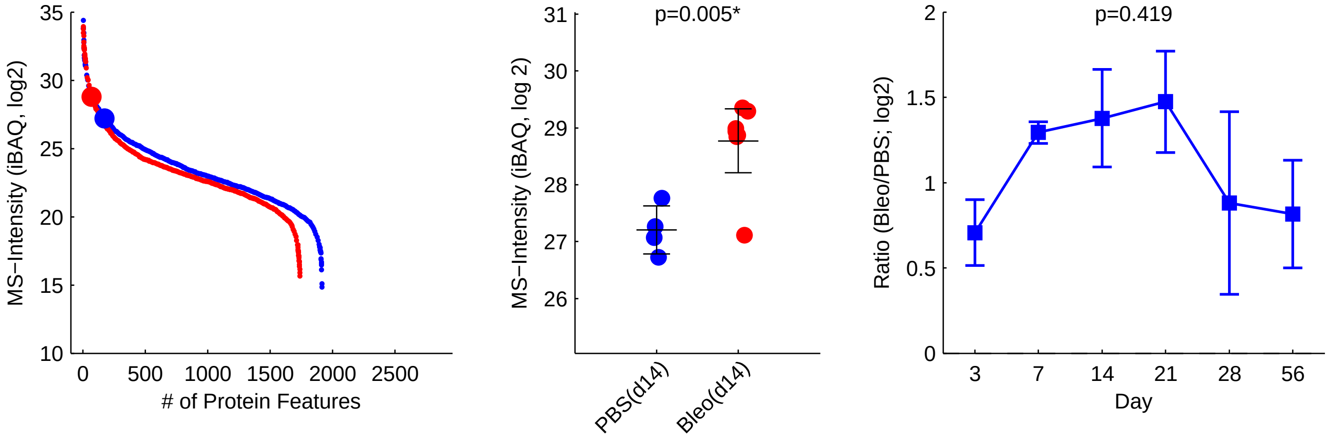

Q9DBE0 − Csad (id: 2605)

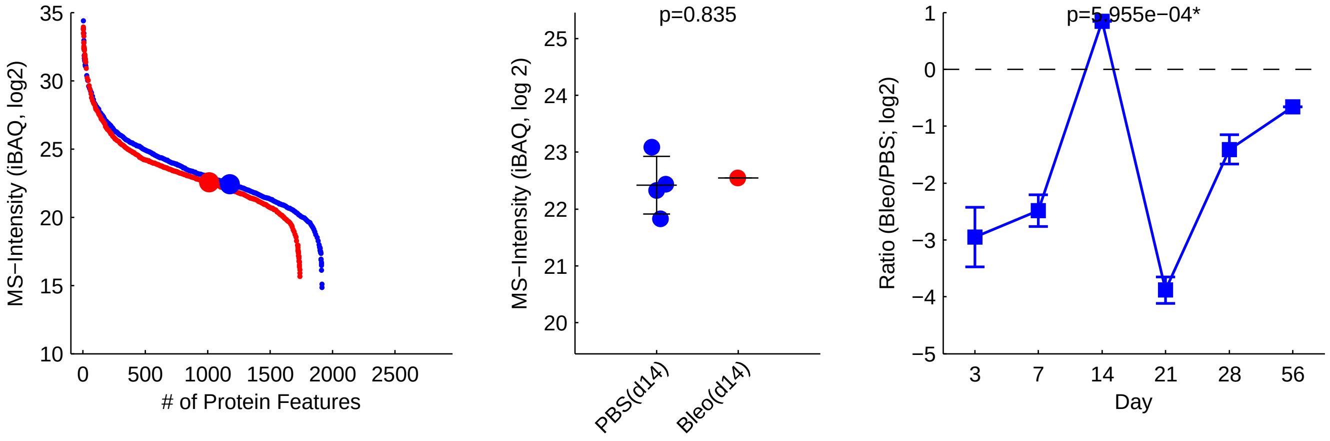

Q9DBG3 − Ap2b1 (id: 2607)

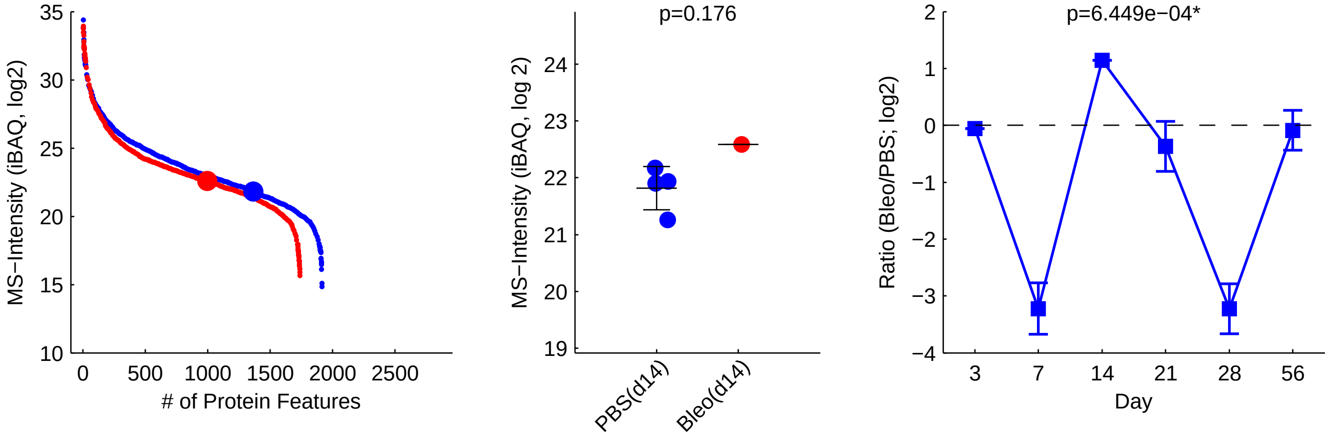

Q9DBG6 − Rpn2 (id: 2609)

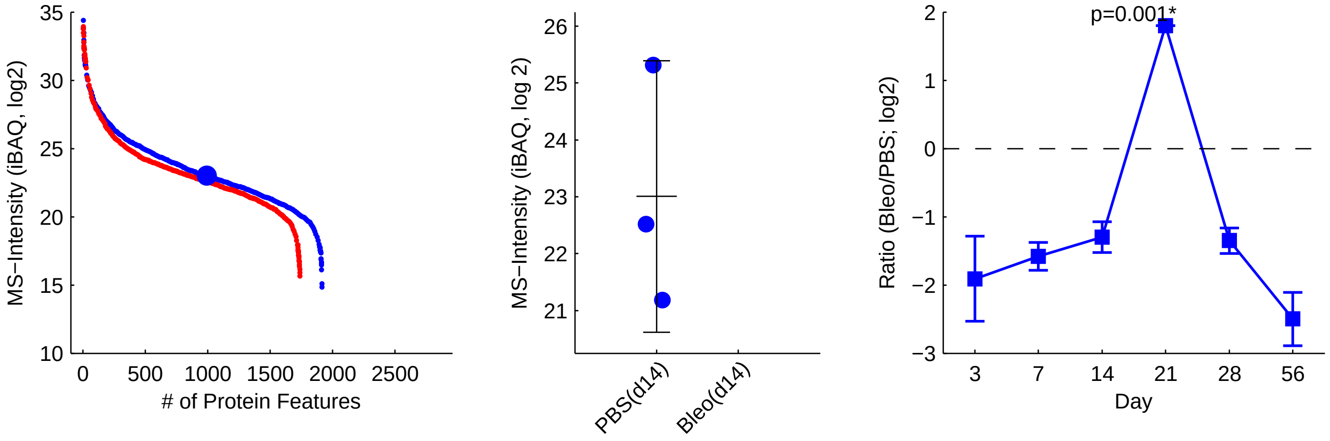

Q9DBJ1 − Pgam1 (id: 2612)

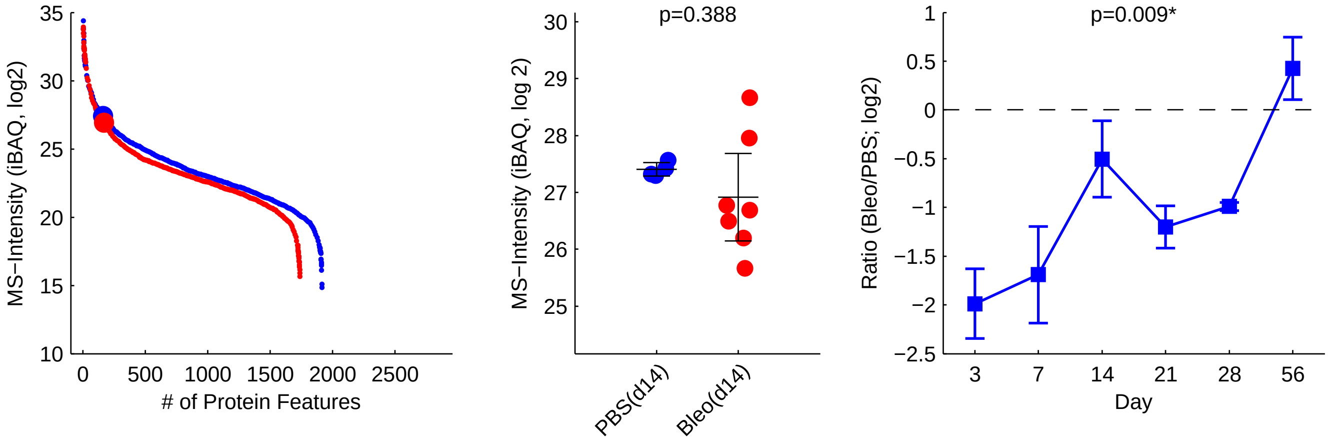

Q9DBJ3 − Baiap2l1 (id: 2613)

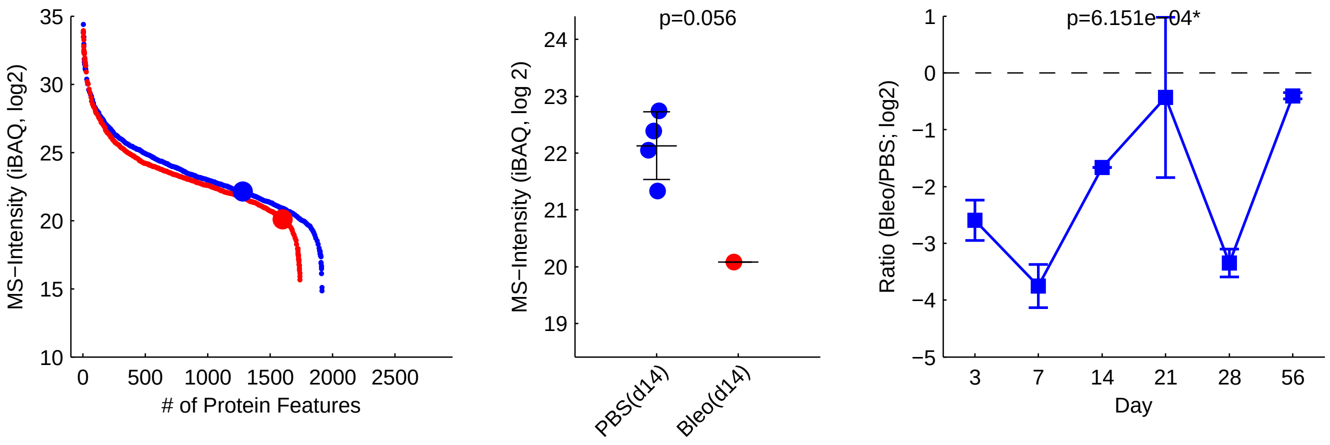

Q9DBK7 − Uba7 (id: 2614)

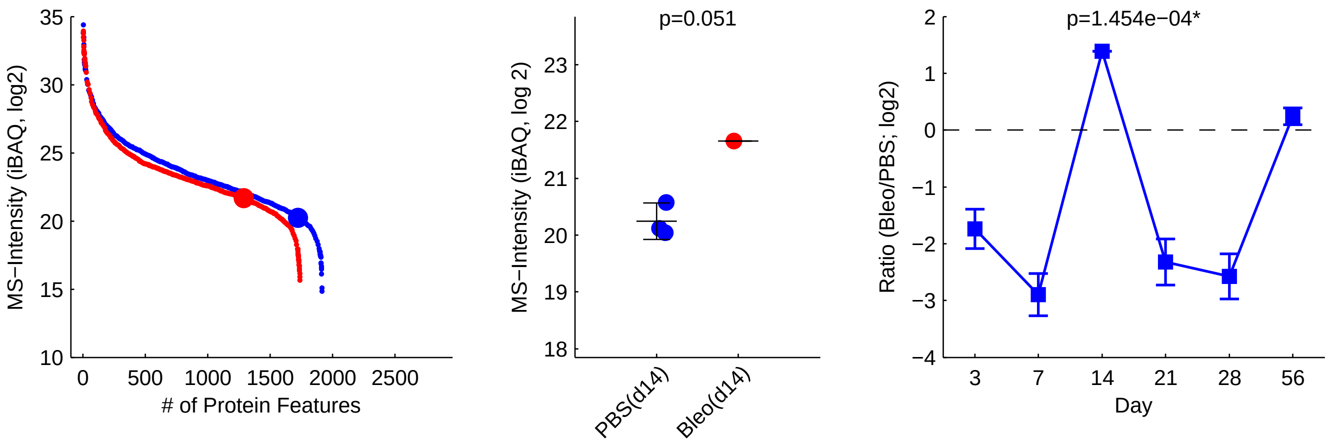

Q9DBP0 − Slc34a2 (id: 2616)

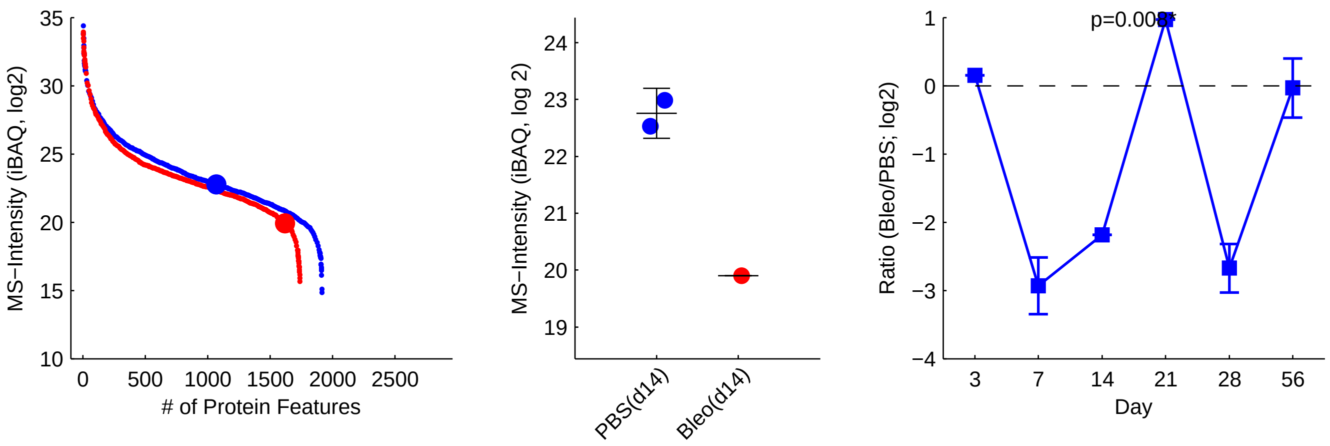

Q9DBX3 − Susd2 (id: 2621)

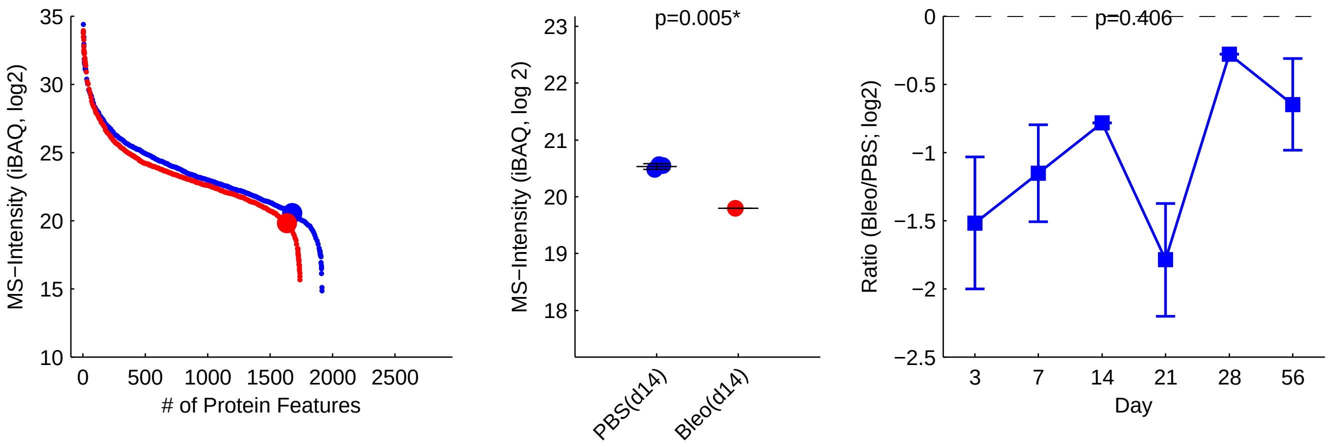

Q9DC07 − Nebl (id: 2623)

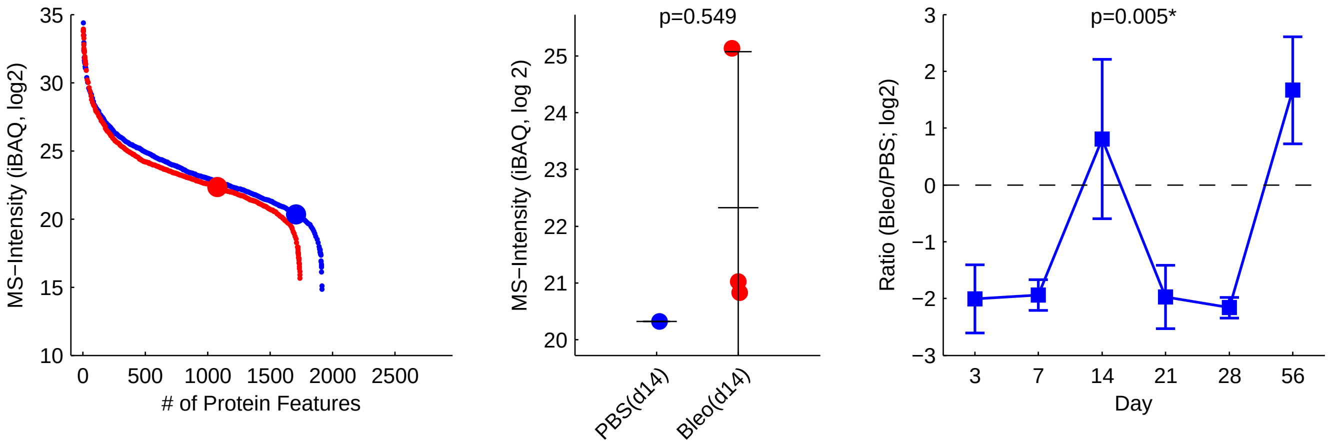

Q9DCL9 − Paics (id: 2635)

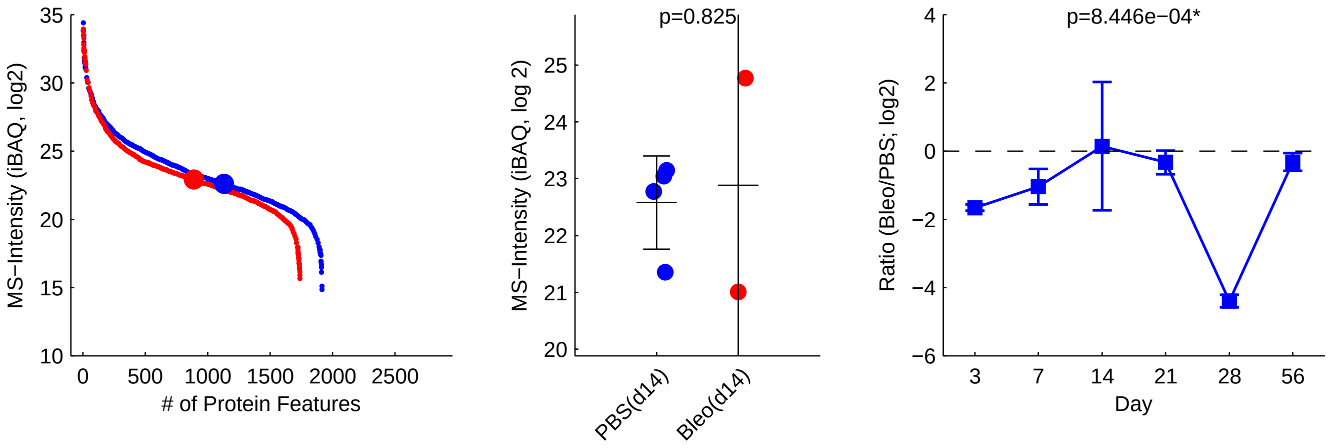

Q9DCT1 − Akr1e2 (id: 2638)

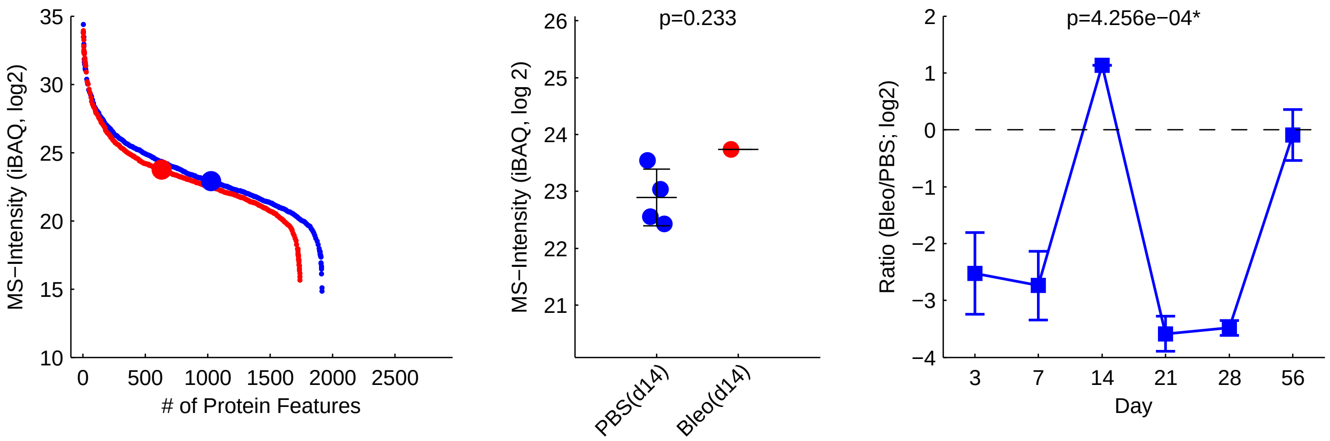

Q9DCW4 − Etfb (id: 2640)

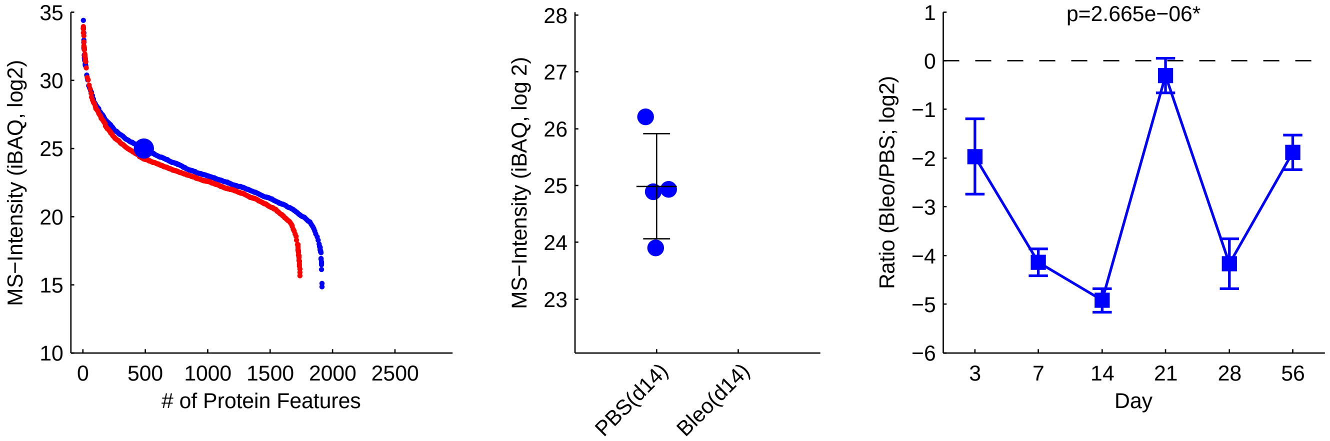

Q9DD23 − Lypd2 (id: 2643)

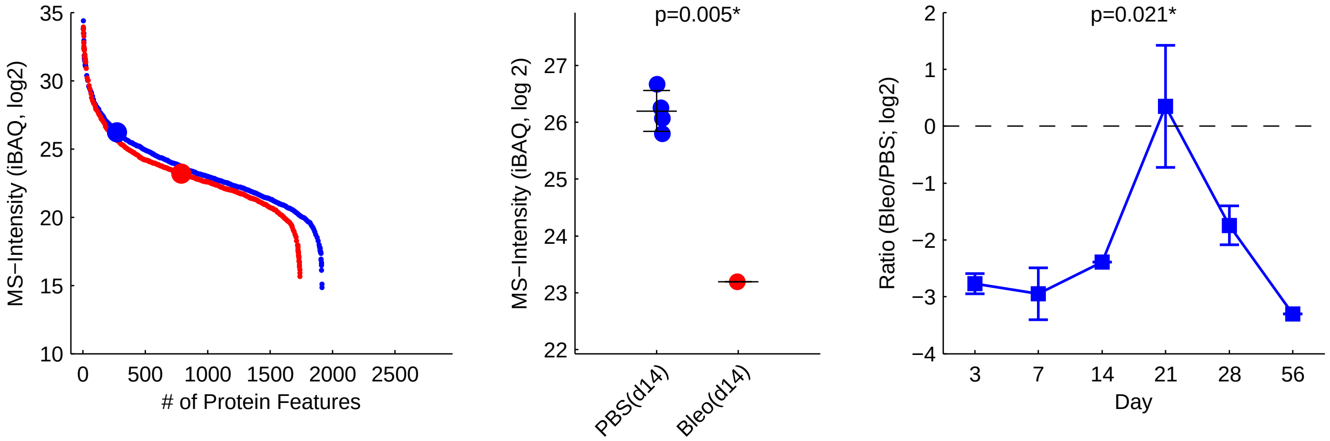

Q9EQ08 − Sgsh (id: 2654)

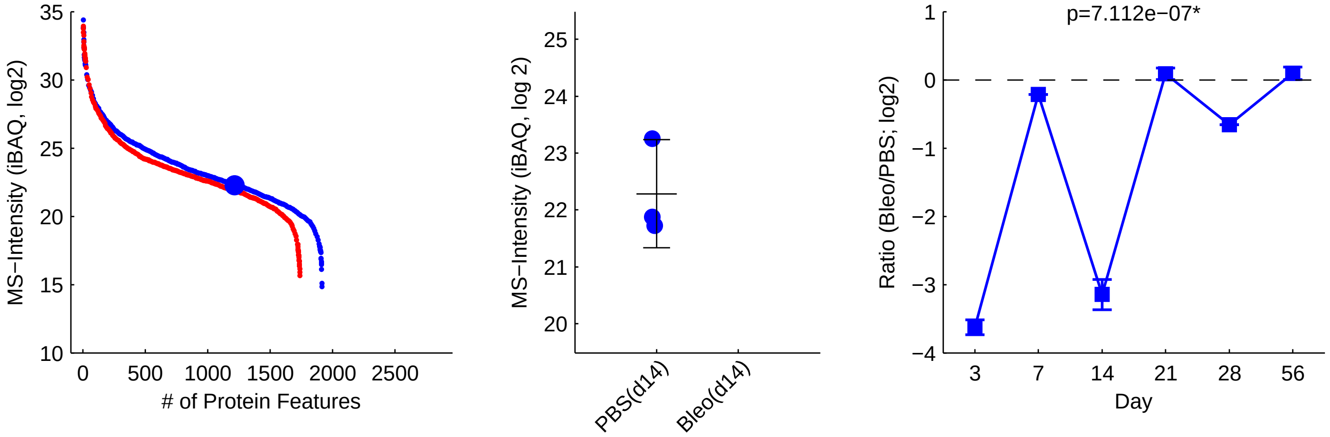

Q9EQ20 − Aldh6a1 (id: 2655)

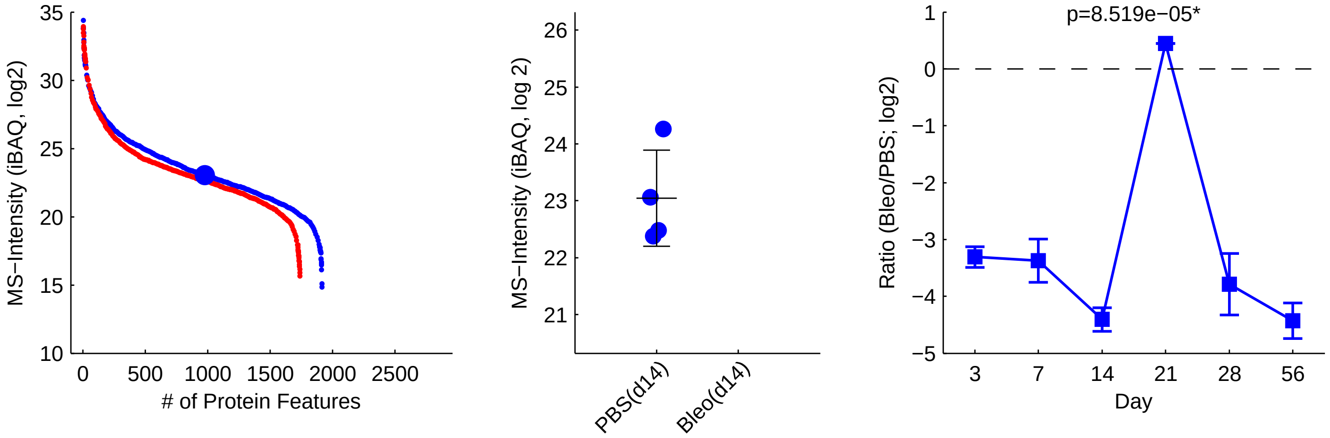

Q9EQU5−2 − Set (id: 2664)

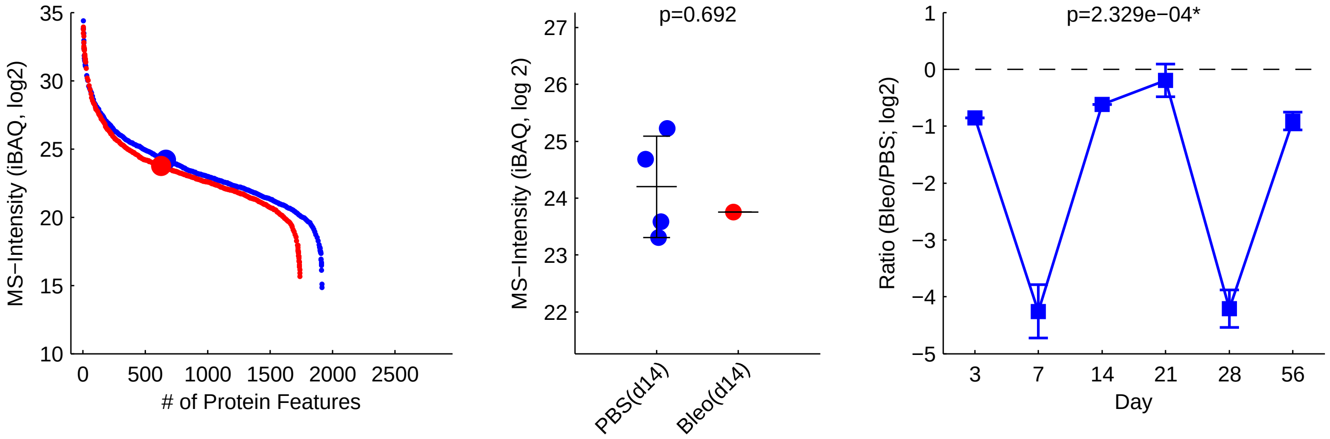

Q9ER72−2 − Cars (id: 2669)

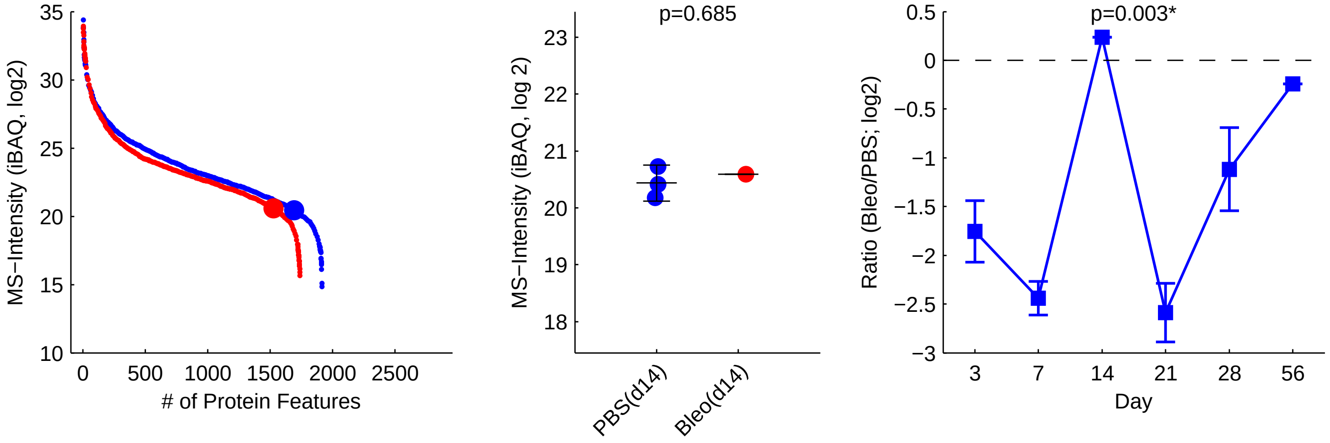

Q9ESB3 − Hrg (id: 2676)

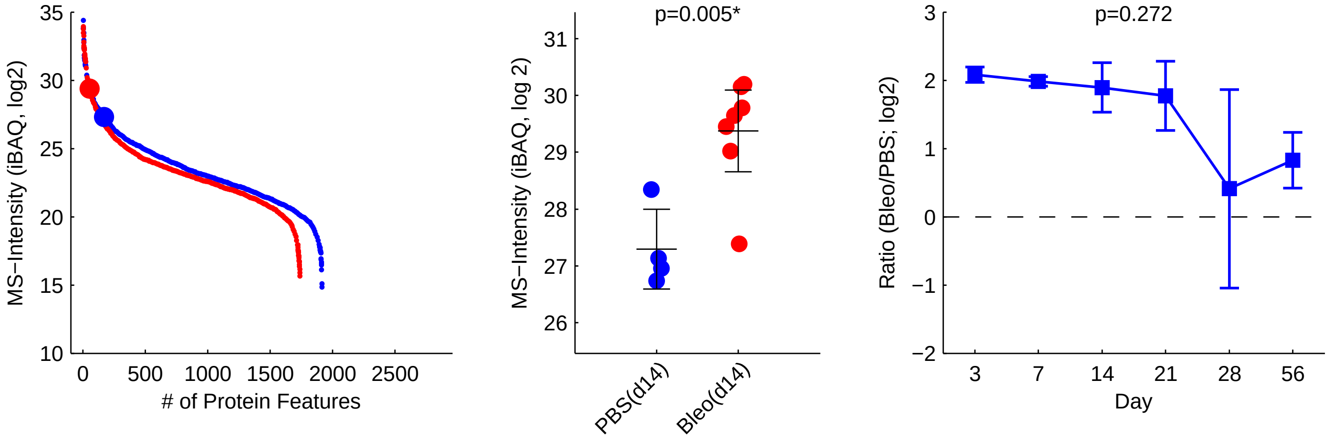

Q9ET01 − Pygl (id: 2682)

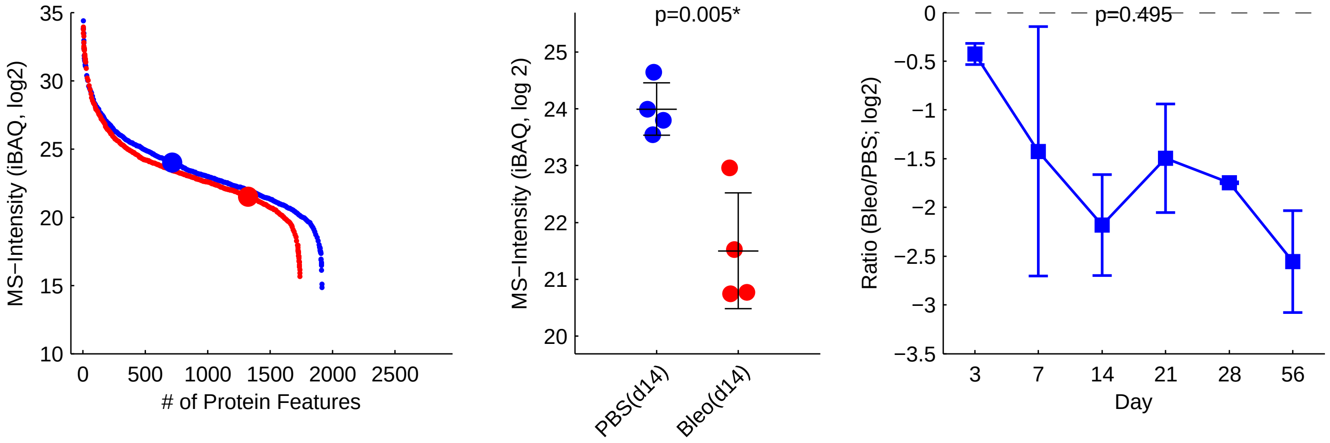

Q9JHH6 − Cpb2 (id: 2687)

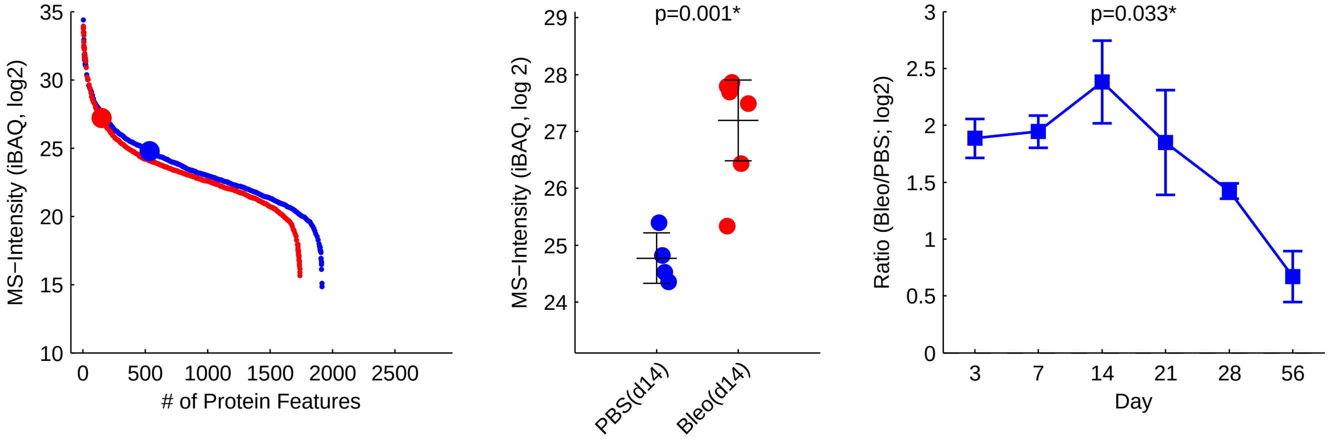

Q9JHQ5 − Lztfl1 (id: 2694)

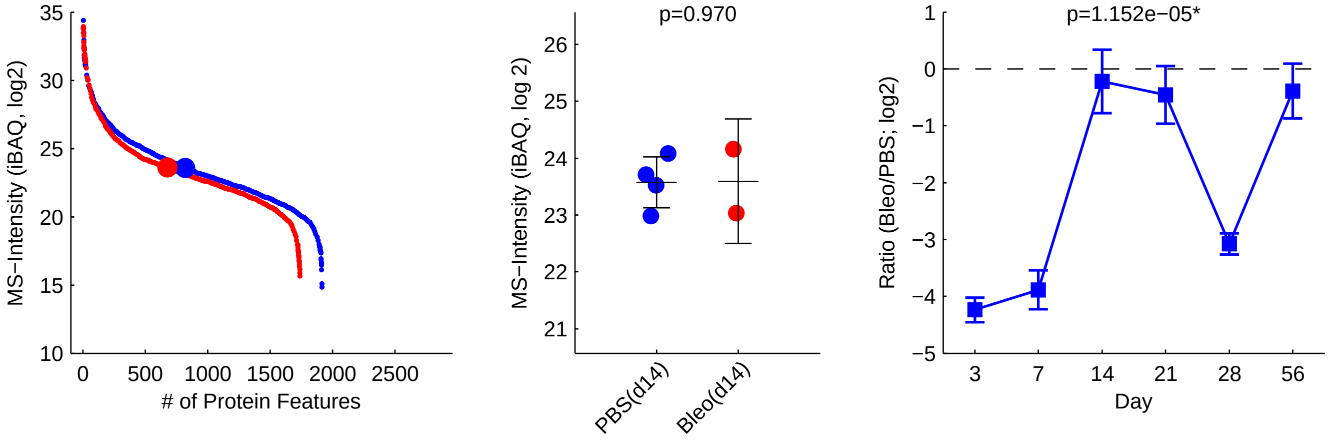

Q9JHW2 − Nit2 (id: 2697)

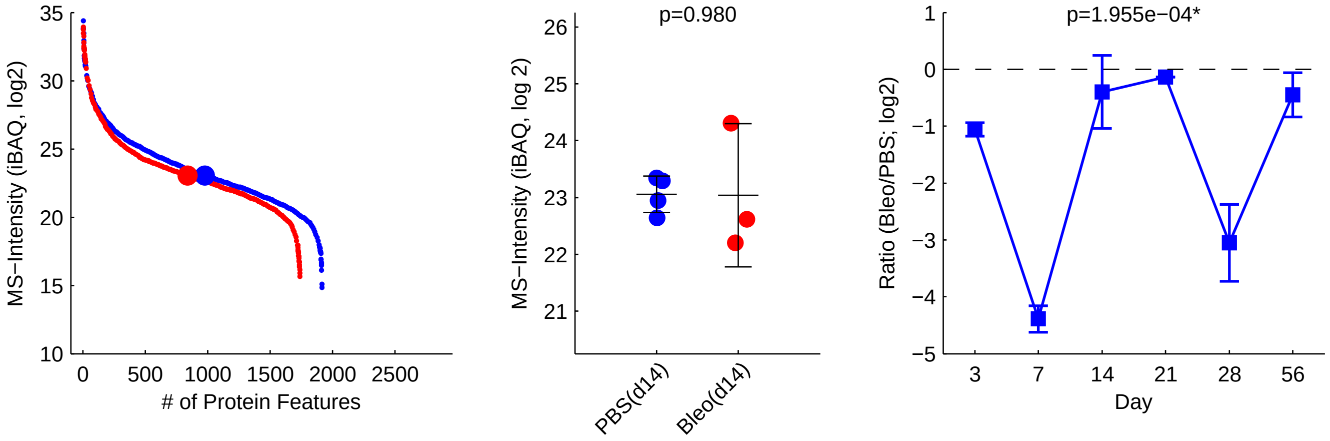

Q9JII6 − Akr1a1 (id: 2702)

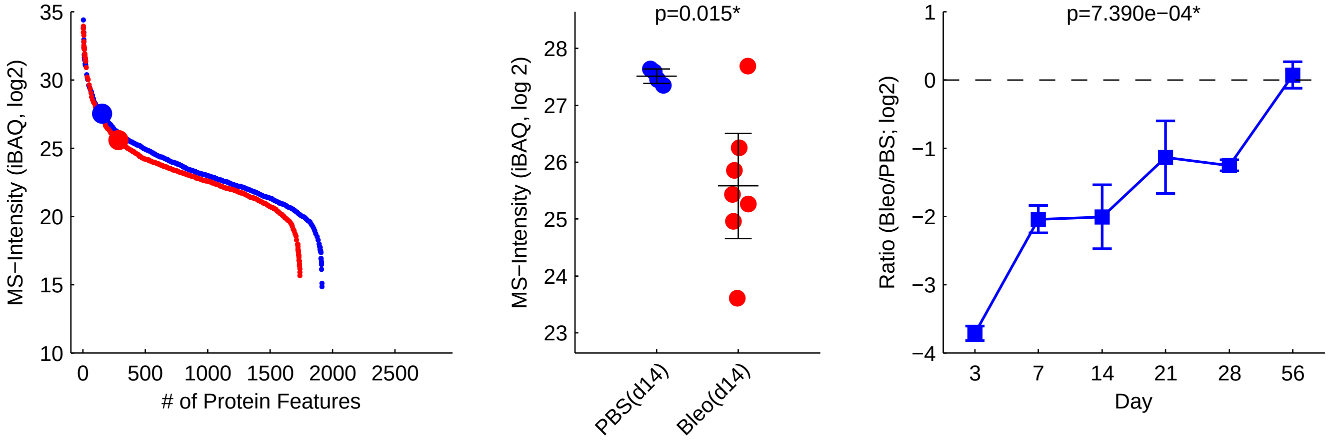

Q9JIW9 − Ralb (id: 2703)

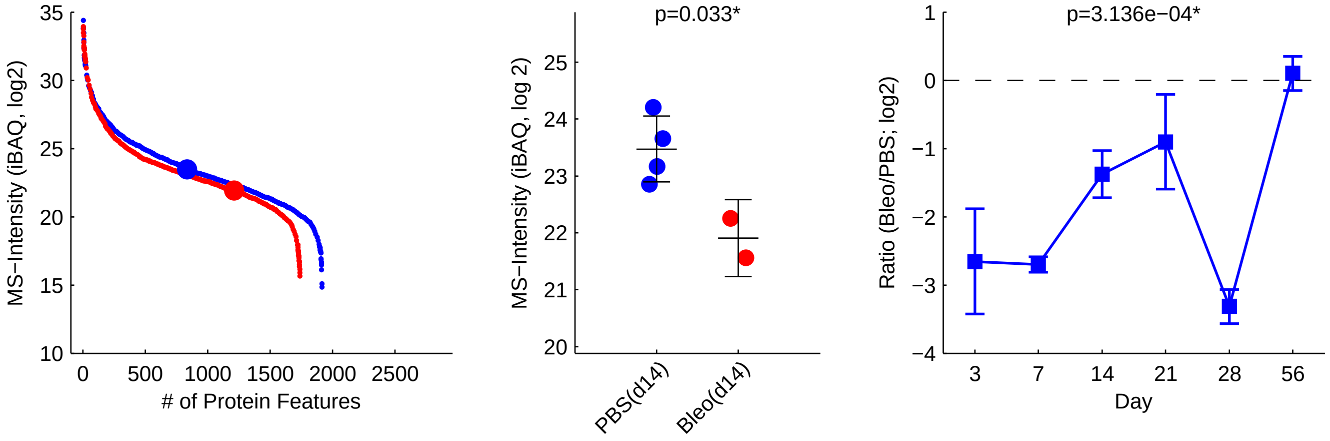

Q9JJI8 − Rpl38 (id: 2708)

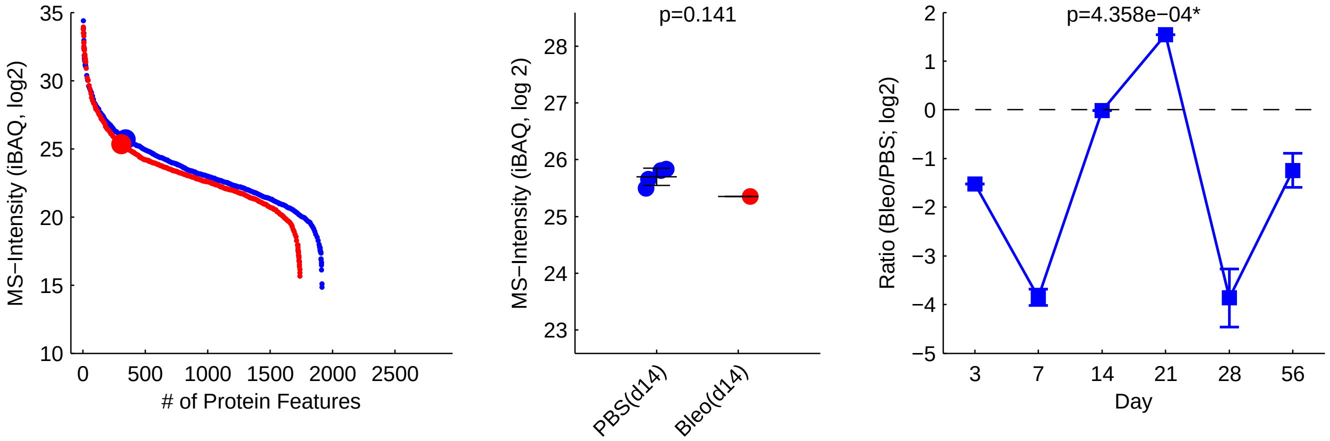

Q9JJN5 − Cpn1 (id: 2709)

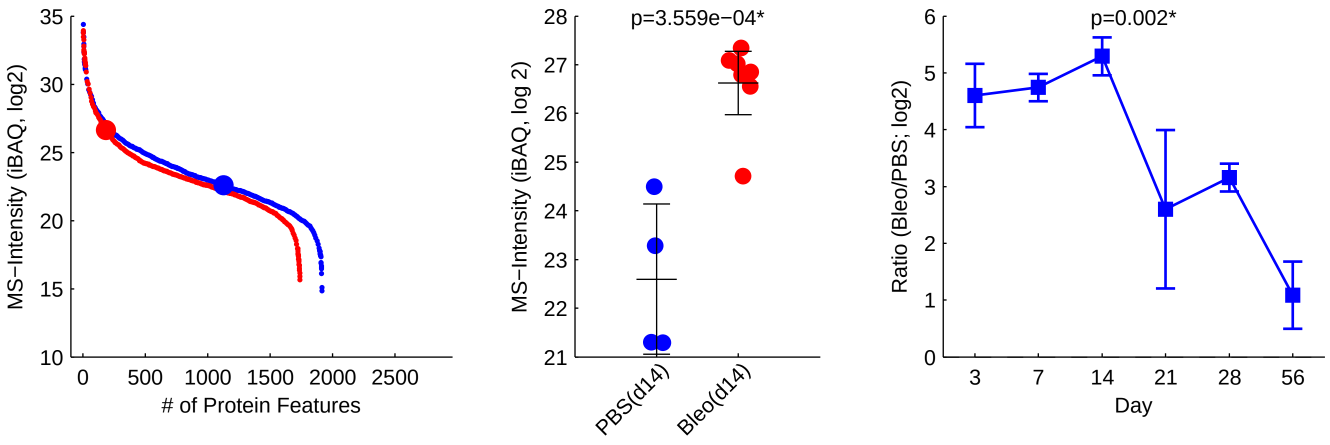

Q9JJV2 − Pfn2 (id: 2711)

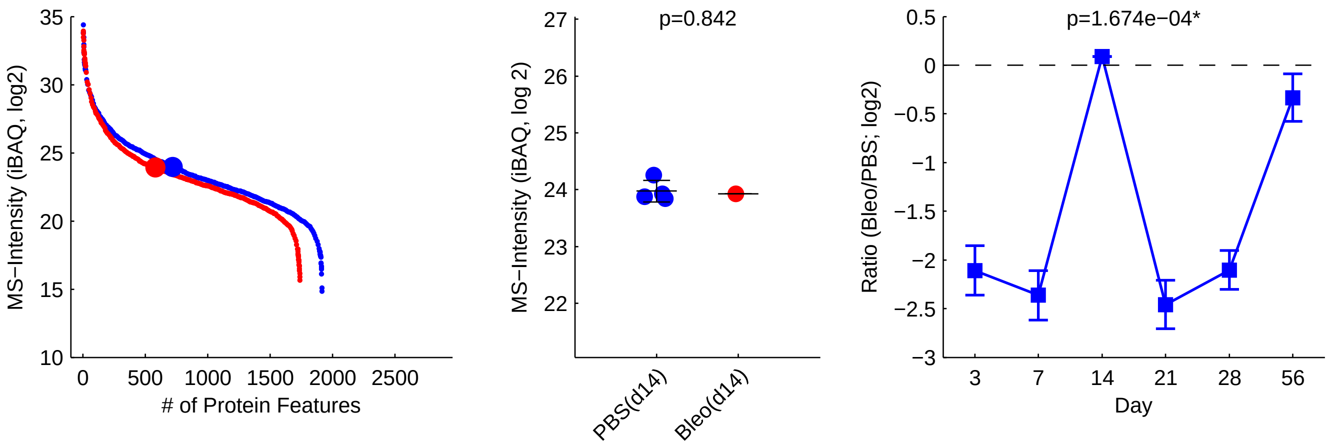

Q9JKB1 − Uchl3 (id: 2716)

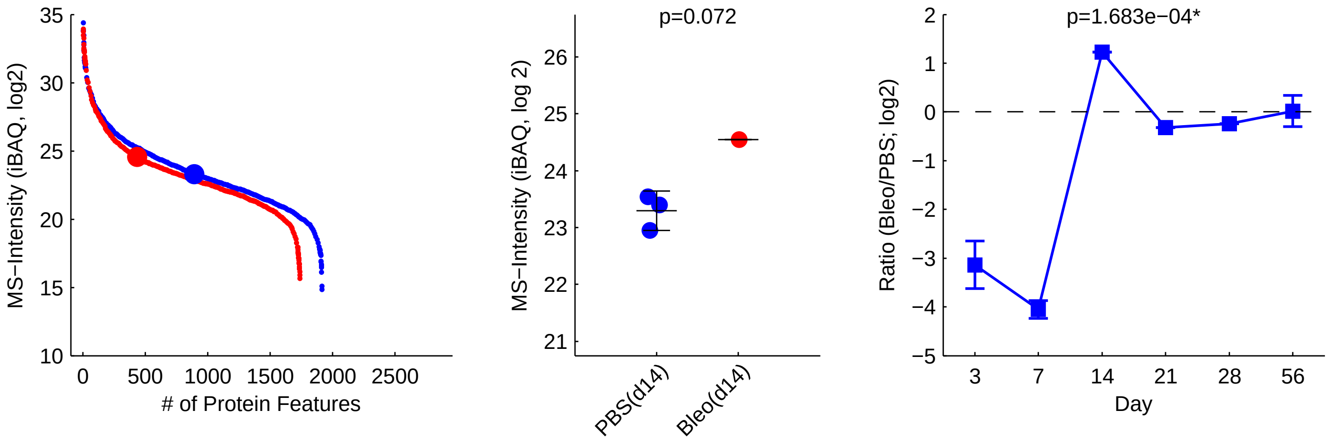

Q9JKX6 − Nudt5 (id: 2720)

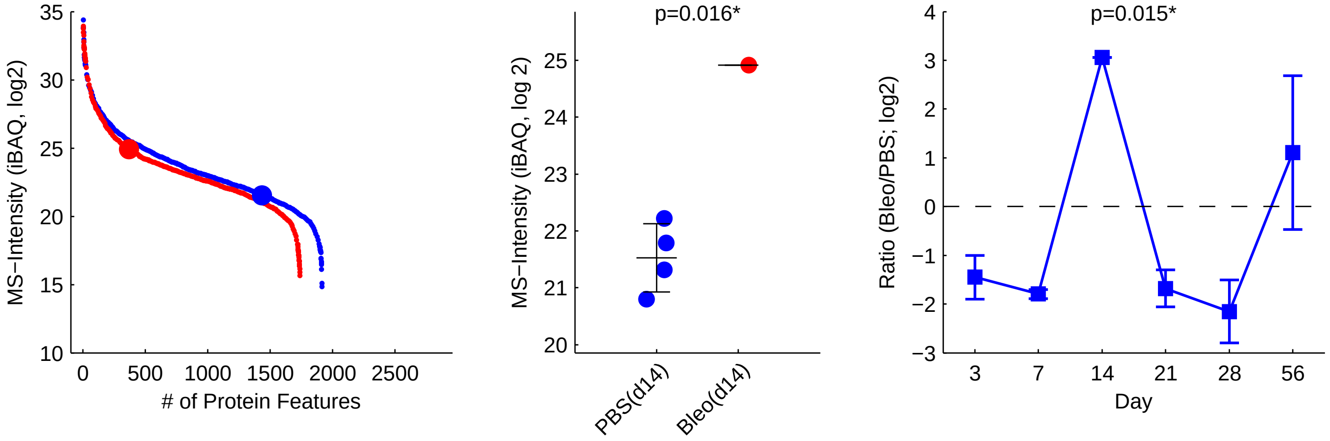

Q9JLB9−3 − Pvrl3 (id: 2725)

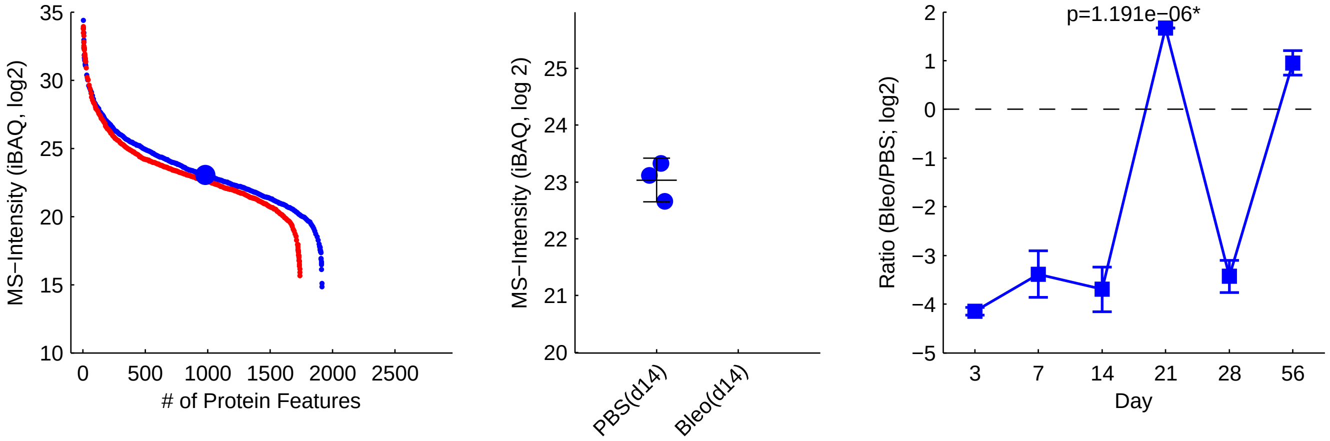

Q9JM14 − Nt5c (id: 2735)

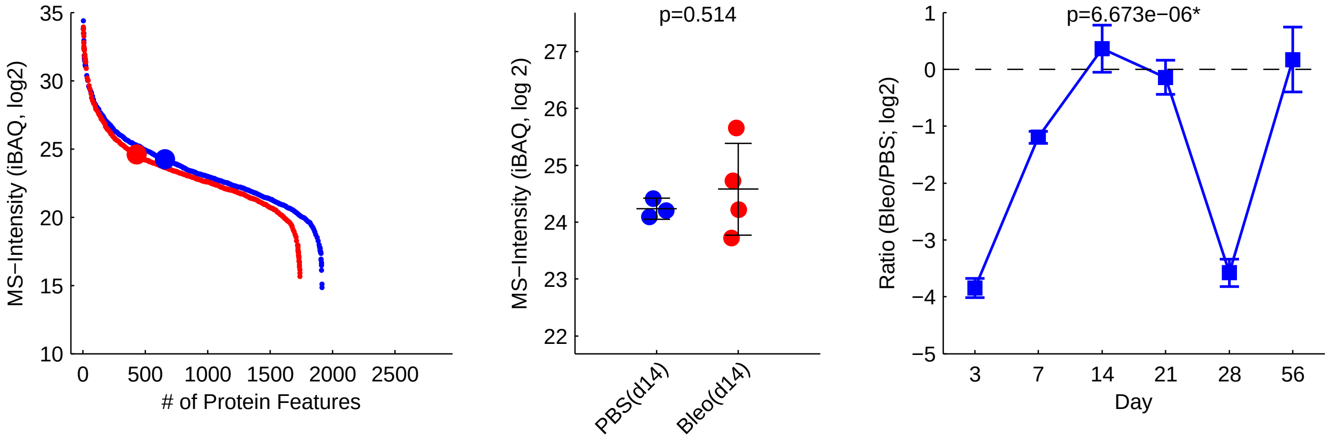

Q9JMC3 − Dnaja4 (id: 2739)

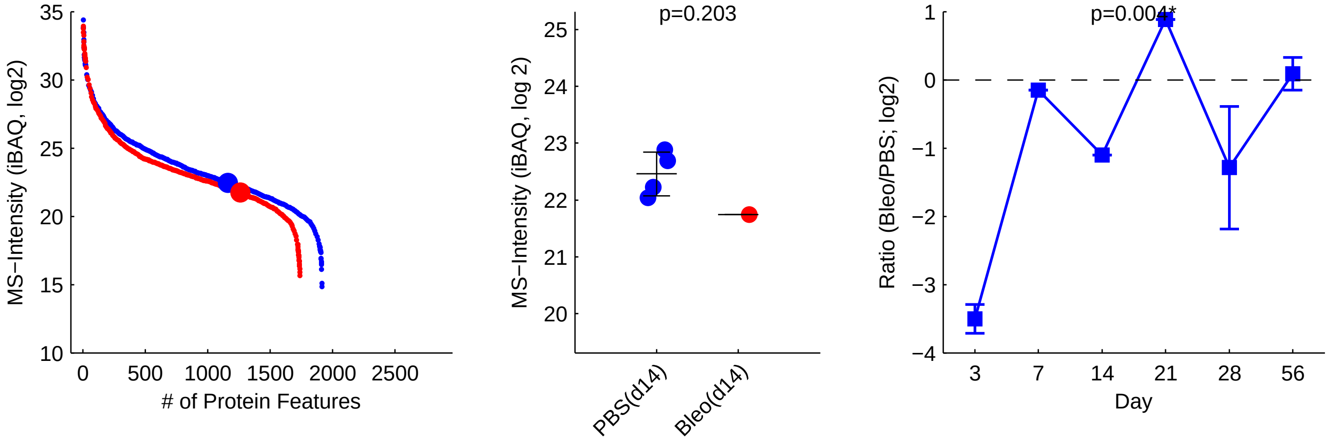

Q9QUM9 − Psma6 (id: 2746)

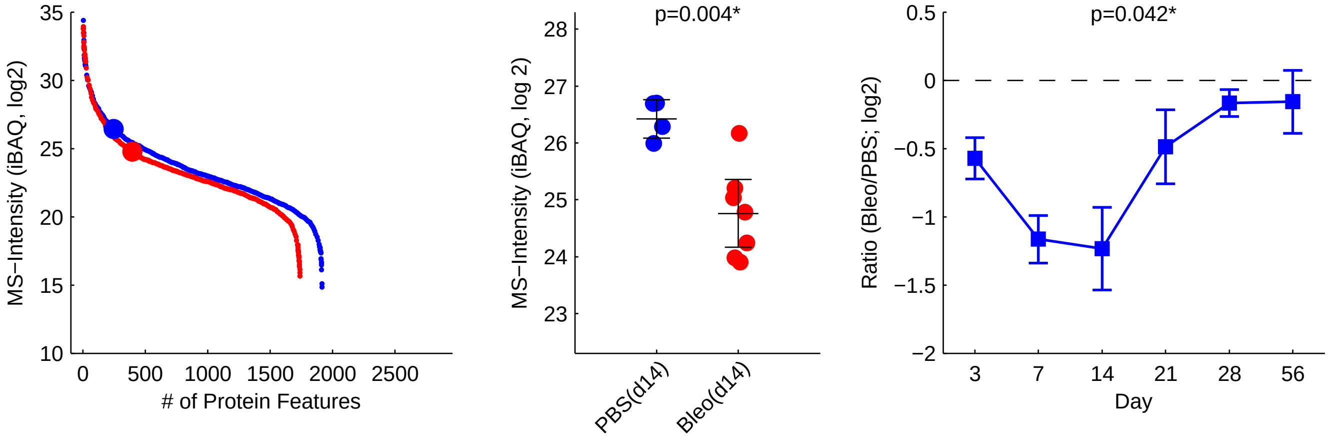

Q9QUN9 − Dkk3 (id: 2747)

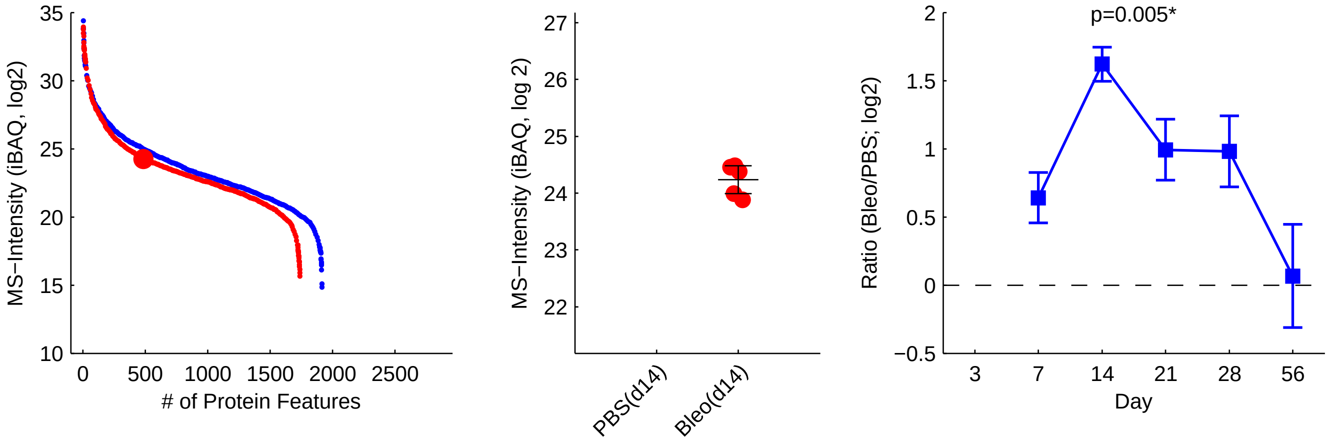

Q9QWK4 − Cd5l (id: 2752)

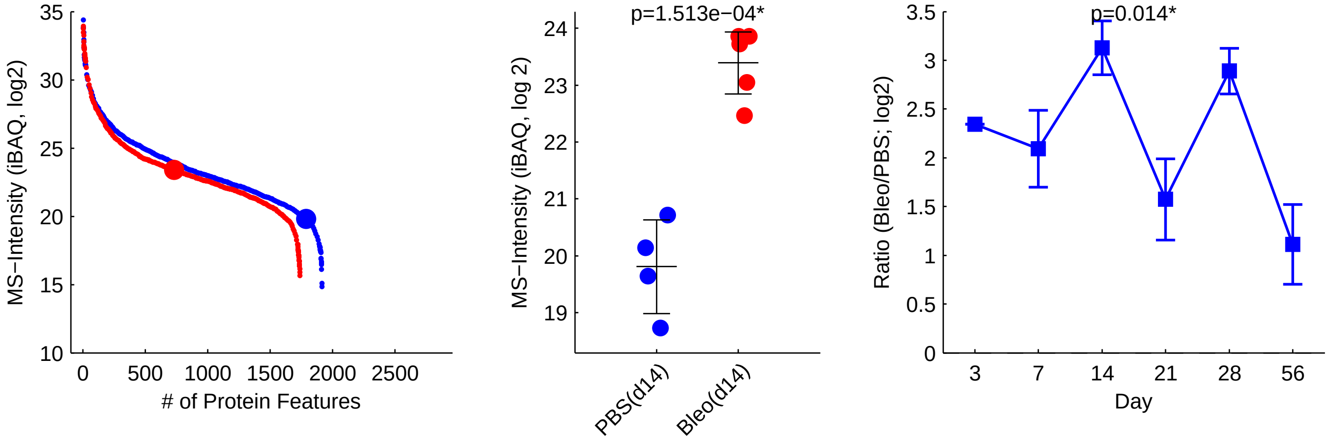

Q9QWR8 − Naga (id: 2753)

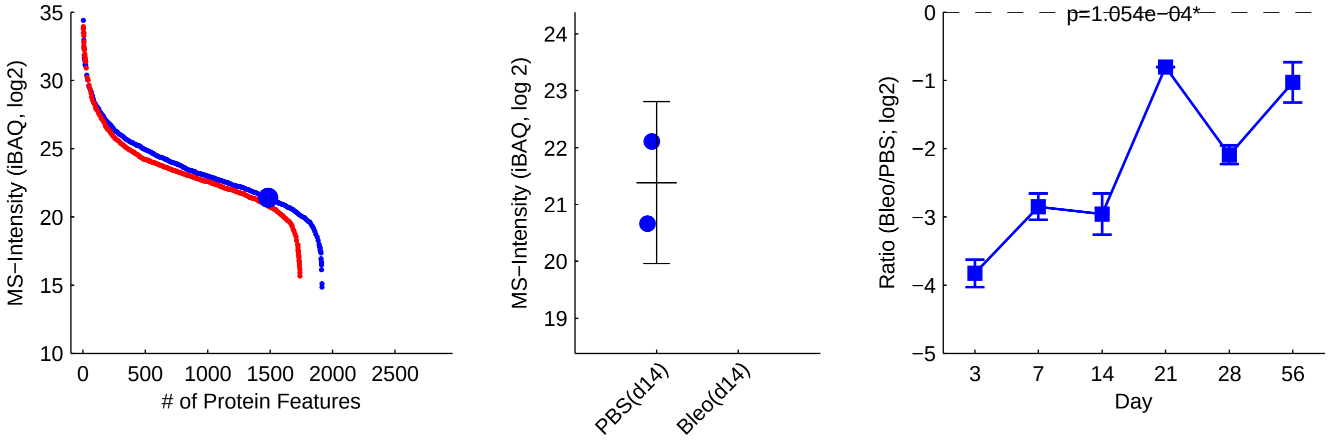

Q9QWV4 − Mlf1 (id: 2754)

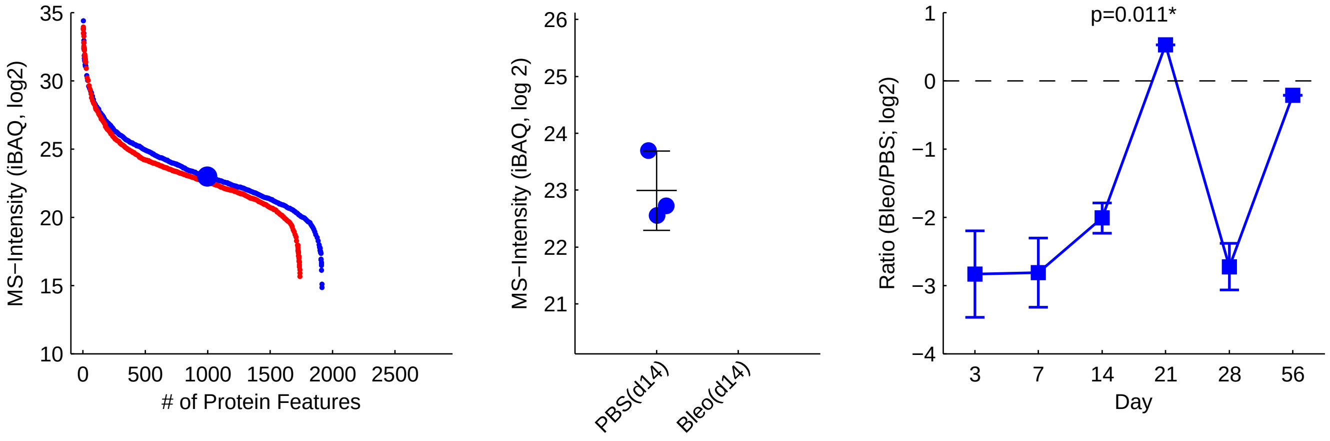

Q9QYJ0 − Dnaja2 (id: 2766)

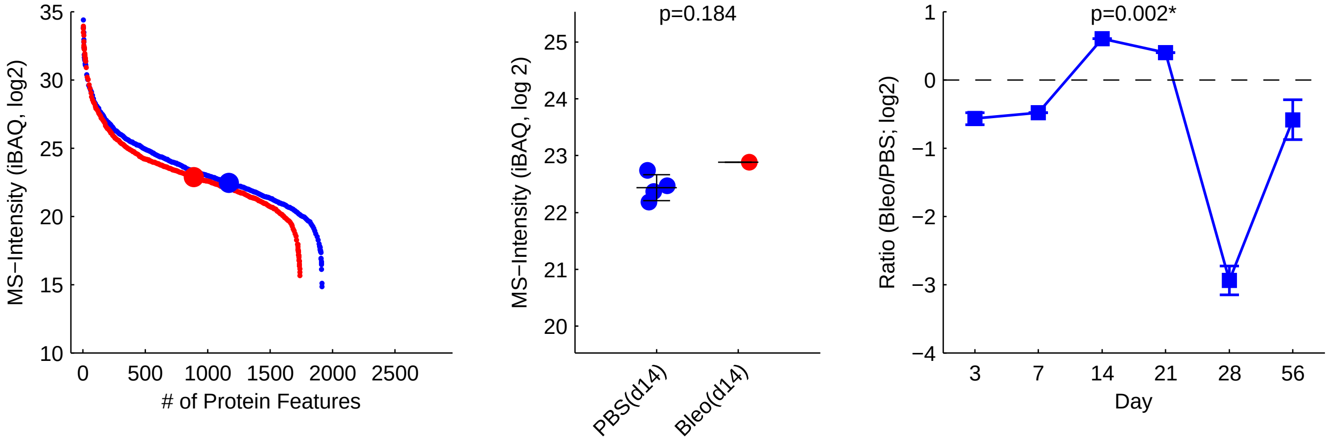

Q9QZE5 − Copg1 (id: 2773)

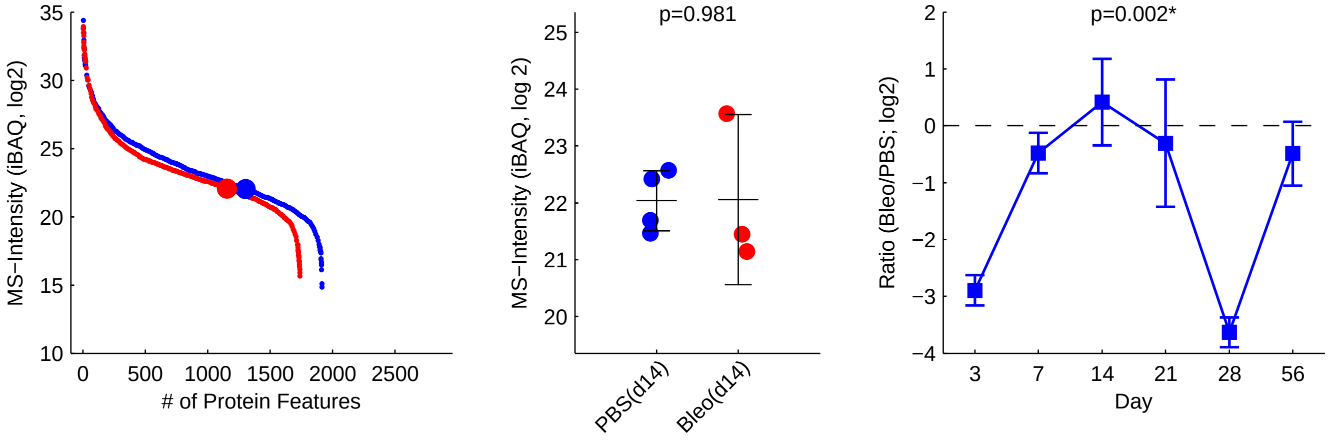

Q9QZE7 − Tsnax (id: 2774)

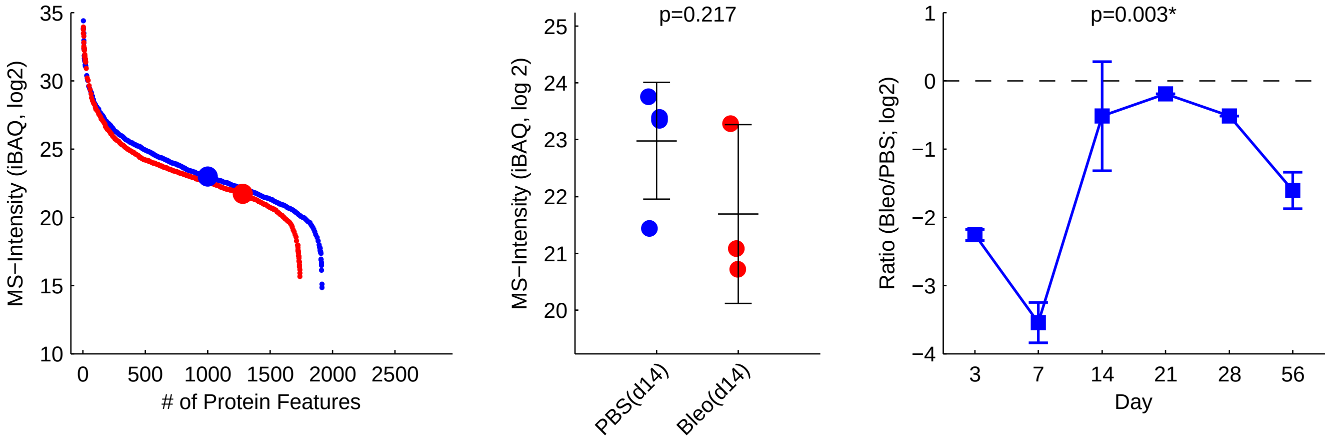

Q9QZQ8−2 − H2afy (id: 2776)

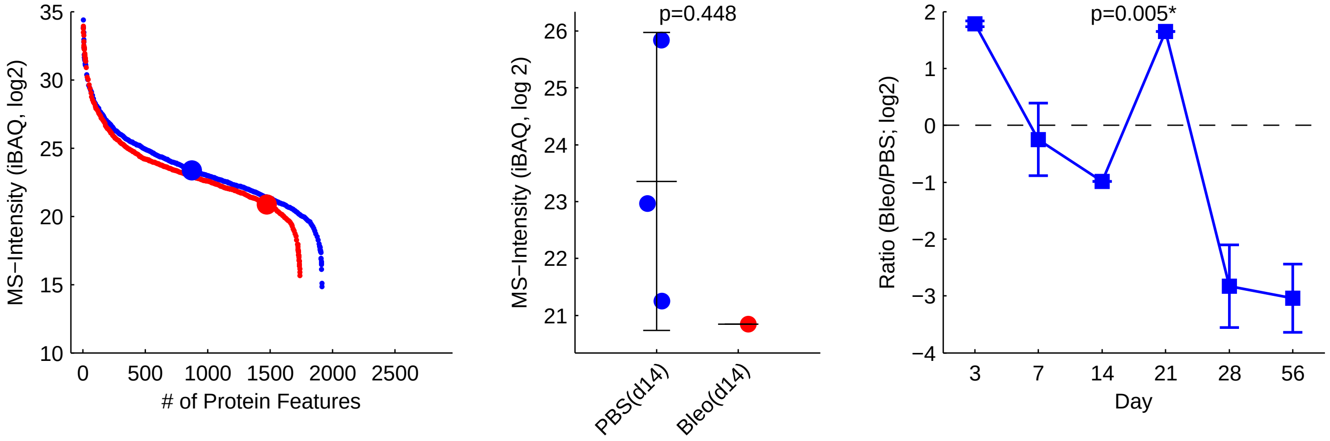

Q9R062 − Gyg1 (id: 2780)

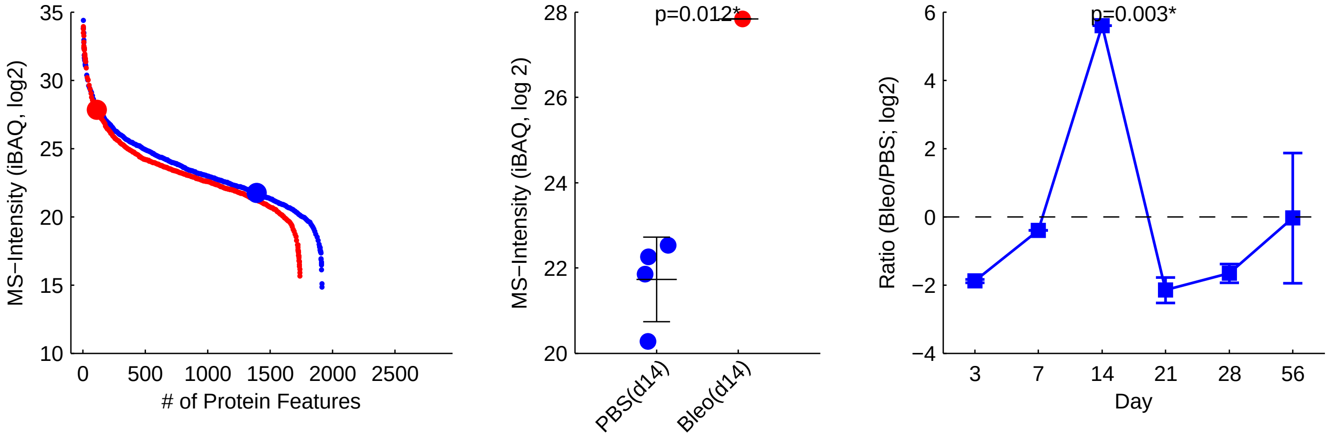

Q9R069 − Bcam (id: 2781)

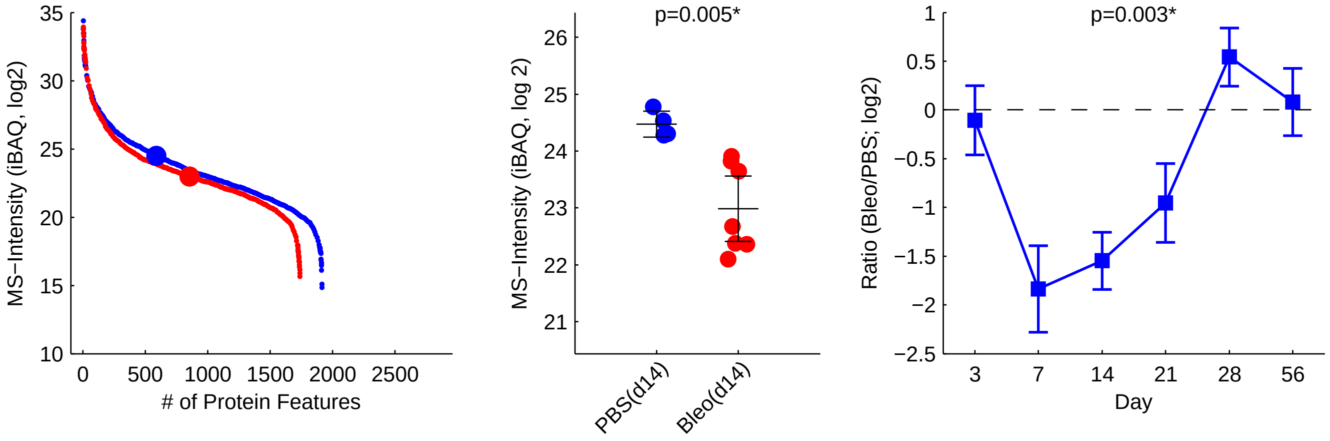

Q9R097 − Spint1 (id: 2783)

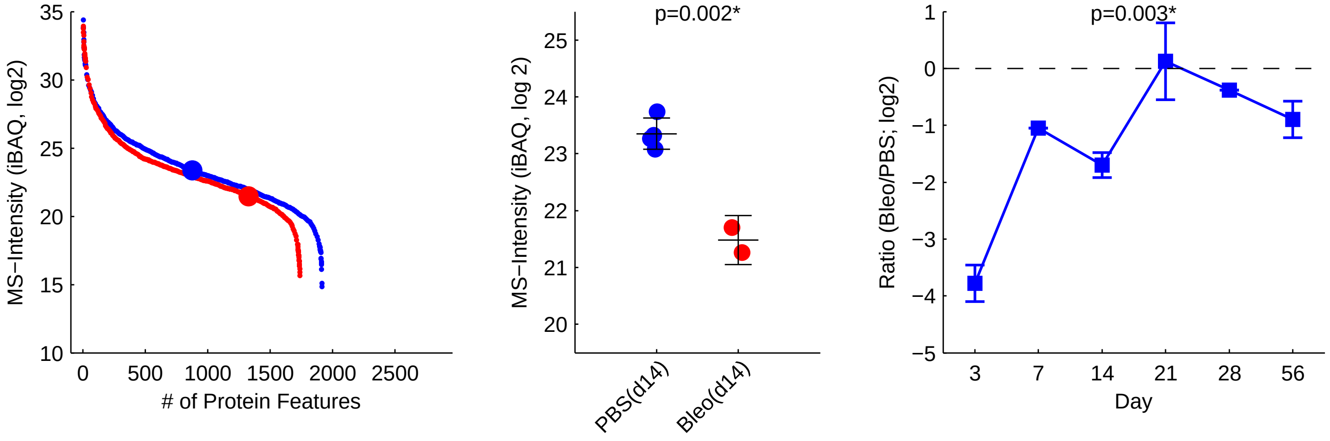

Q9R098 − Hgfac (id: 2784)

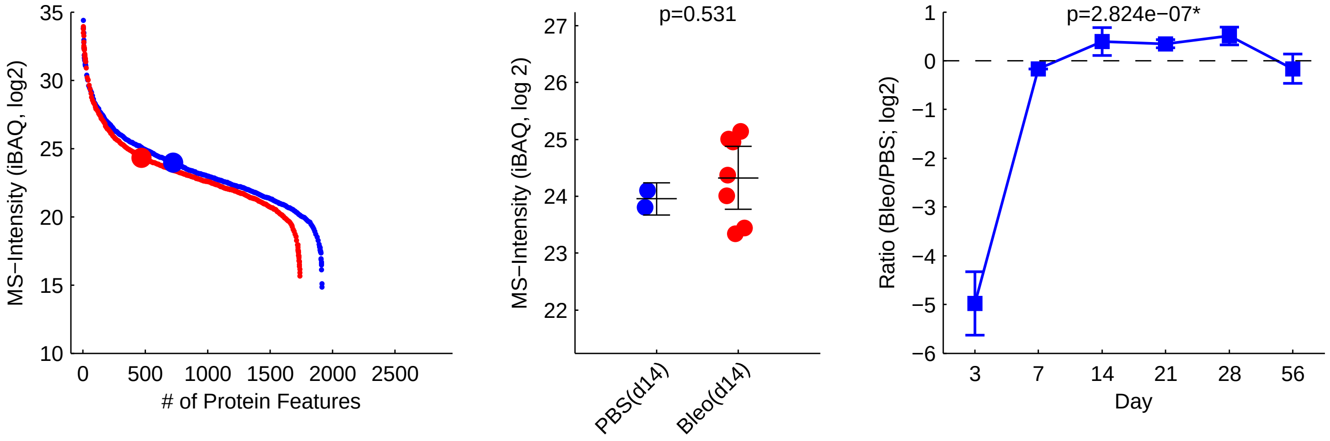

Q9R0E2 − Plod1 (id: 2787)

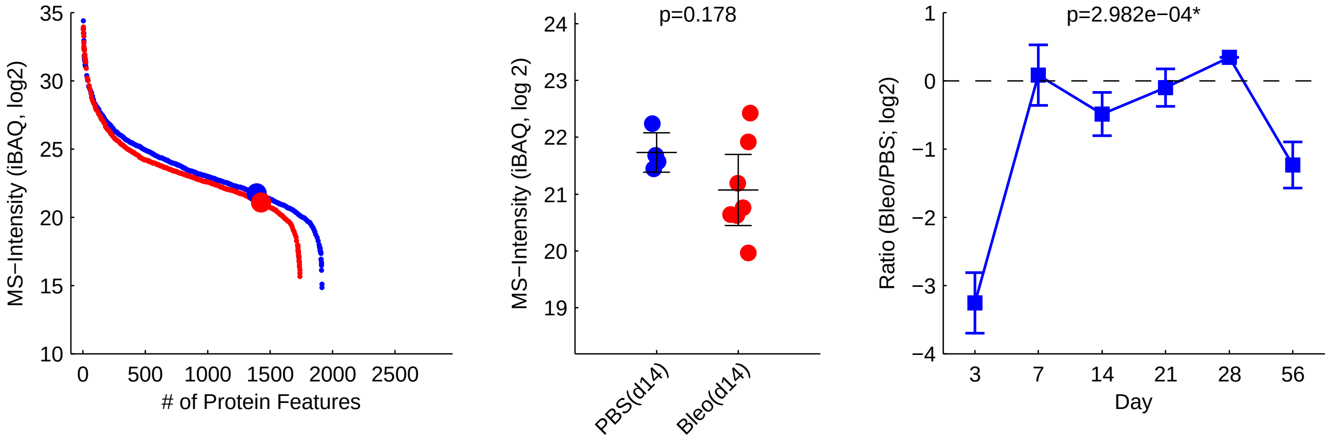

Q9R0Q7 − Ptges3 (id: 2790)

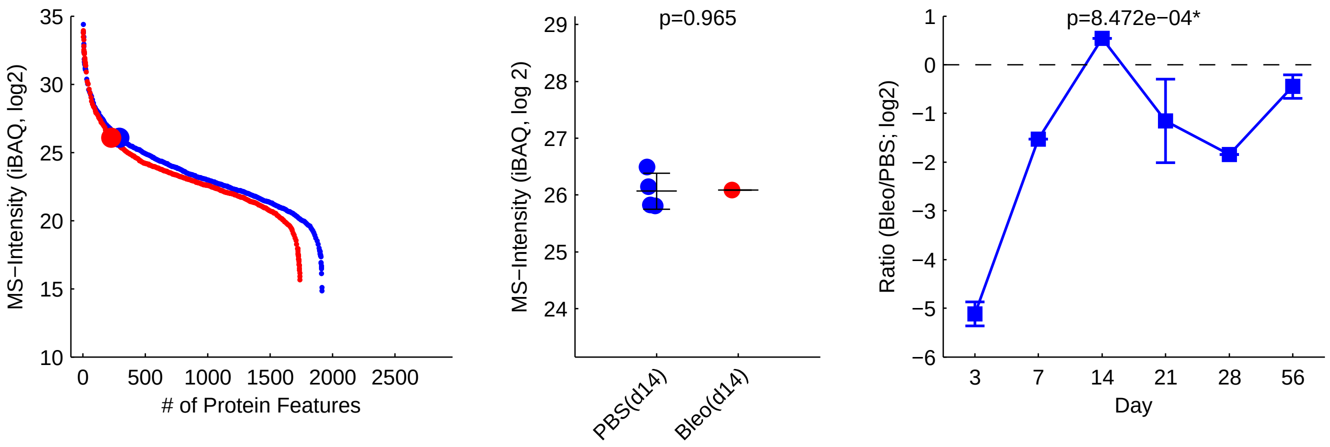

Q9R0Y5 − Ak1 (id: 2792)

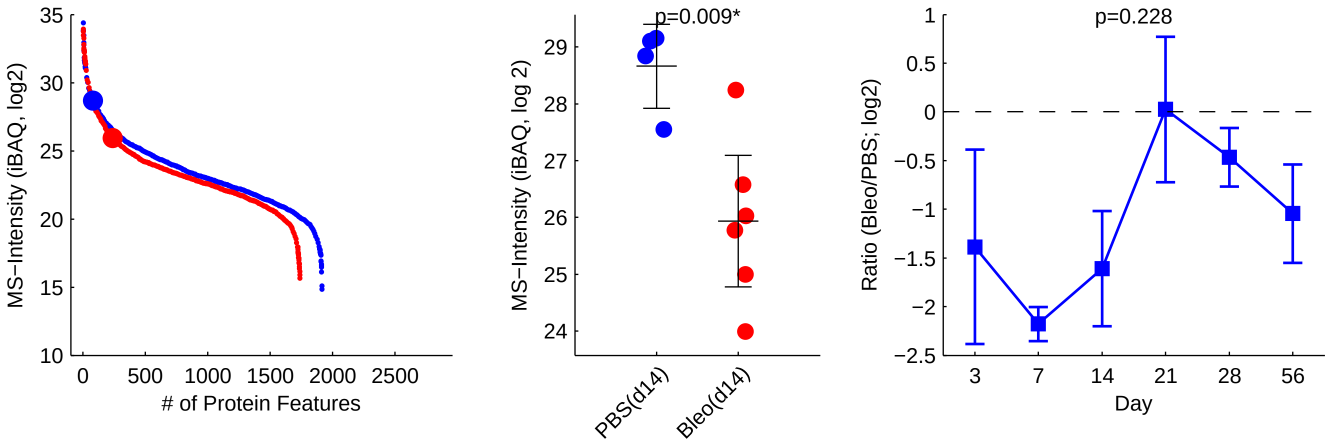

Q9R111 − Gda (id: 2793)

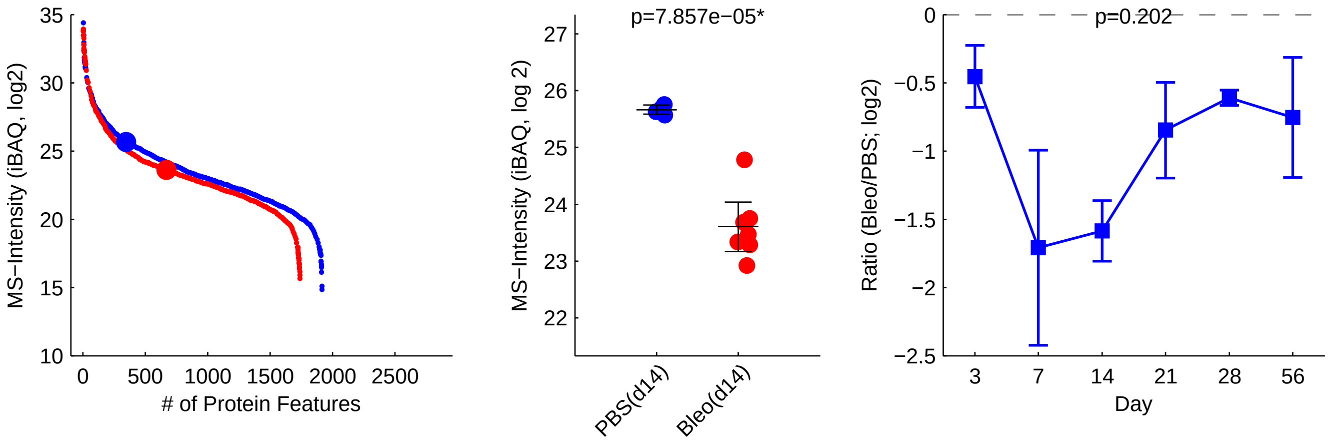

Q9R1P1 − Psmb3 (id: 2796)

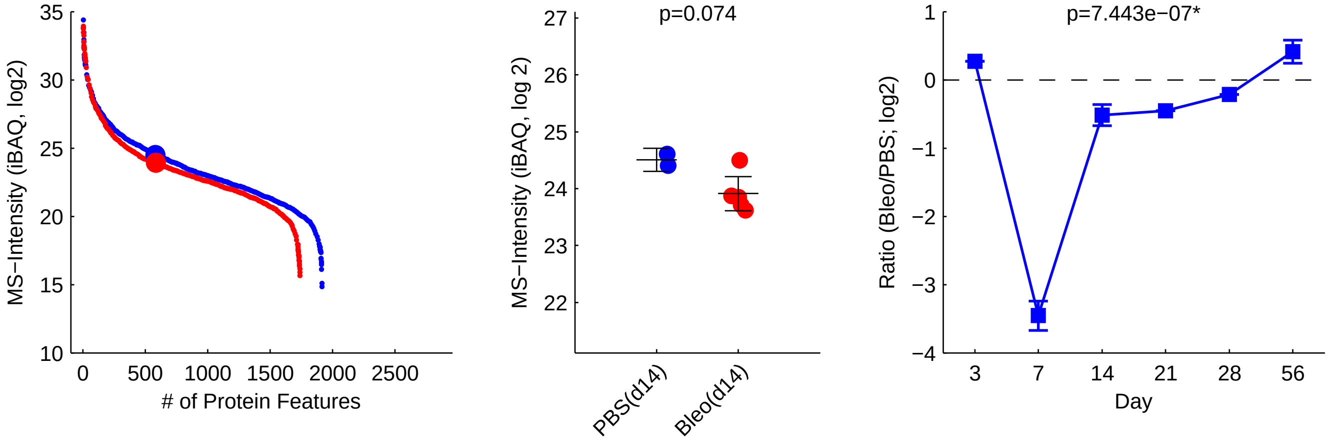

Q9R1P3 − Psmb2 (id: 2797)

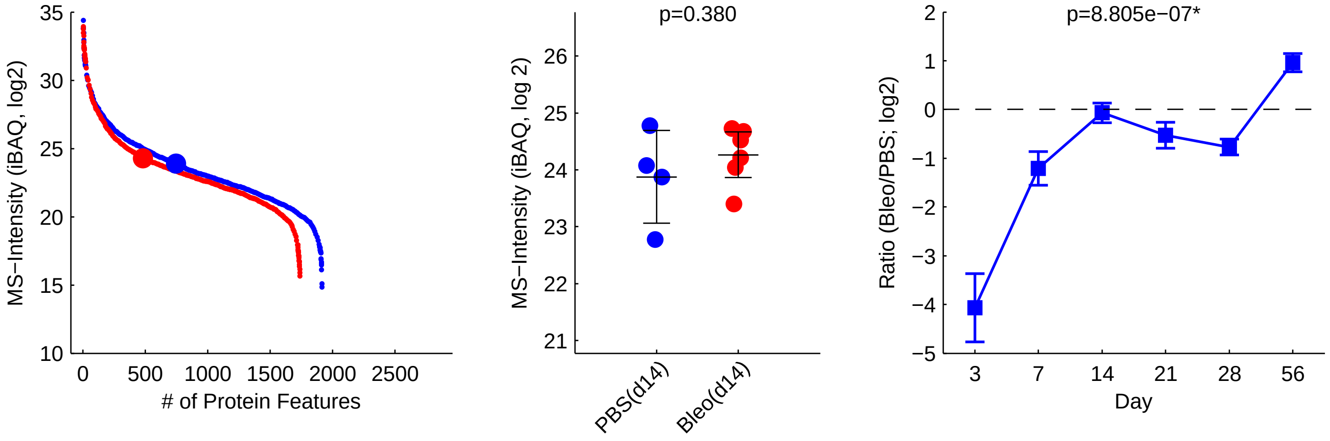

Q9R1Z7 − Pts (id: 2800)

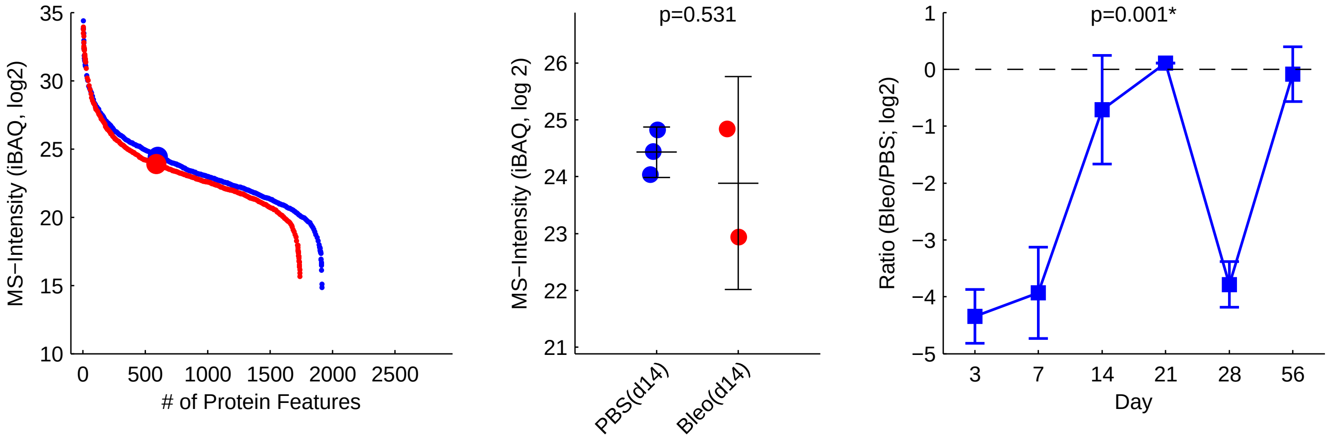

Q9WTL7 − Lypla2 (id: 2805)

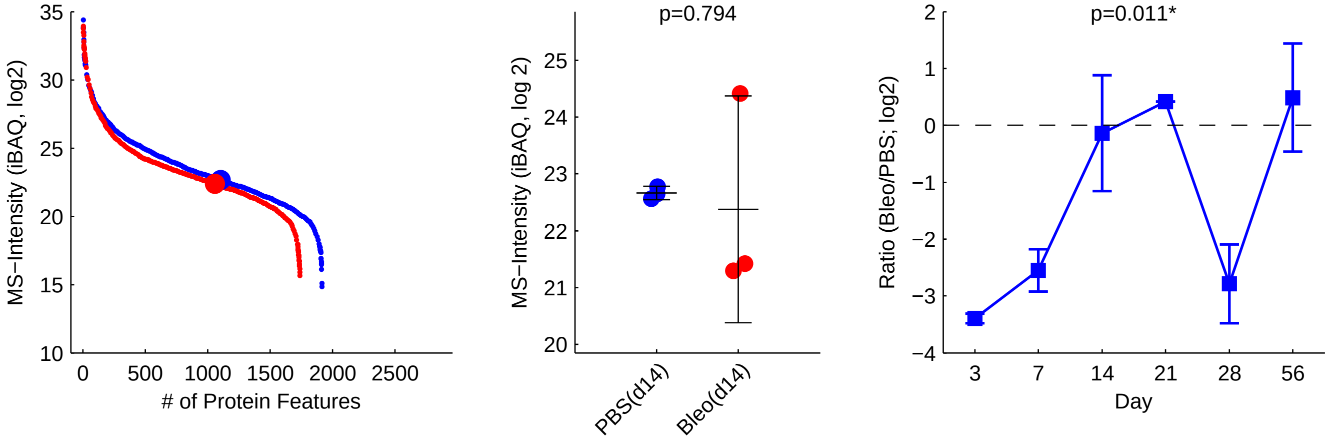

Q9WTR5 − Cdh13 (id: 2810)

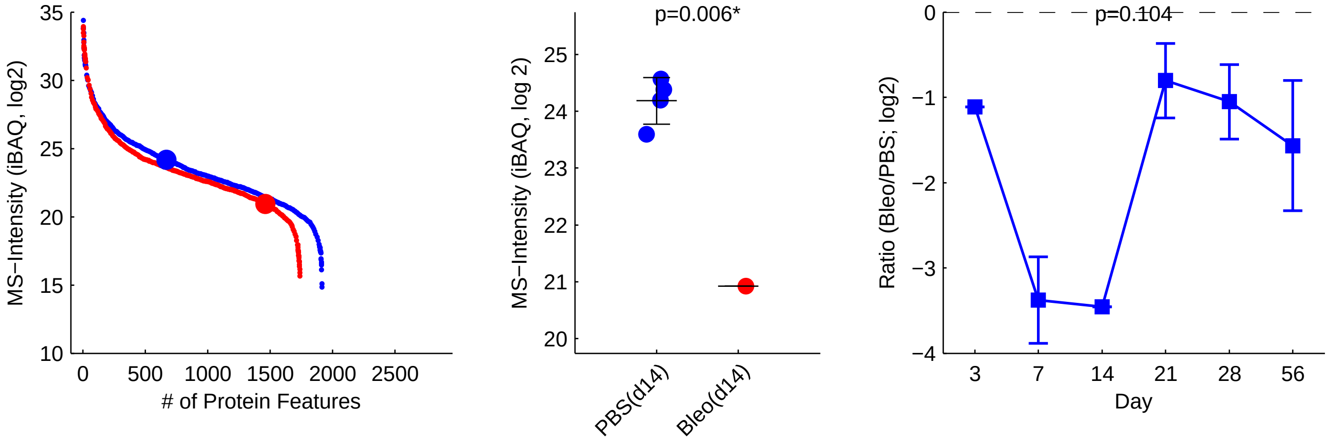

Q9WTY4 − Aqp5 (id: 2815)

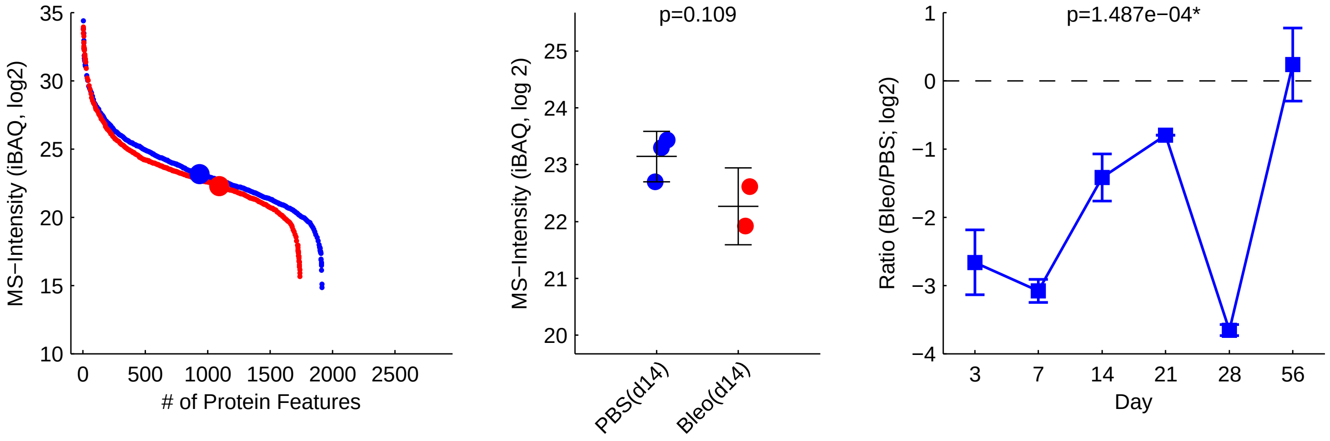

Q9WUA2 − Farsb (id: 2818)

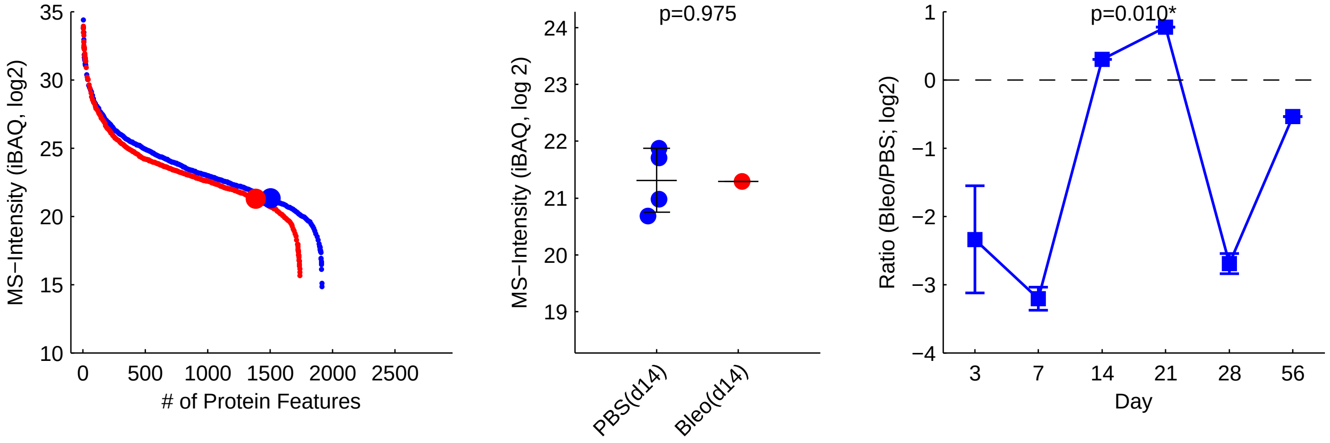

Q9WUD0 − Cyp2b10 (id: 2820)

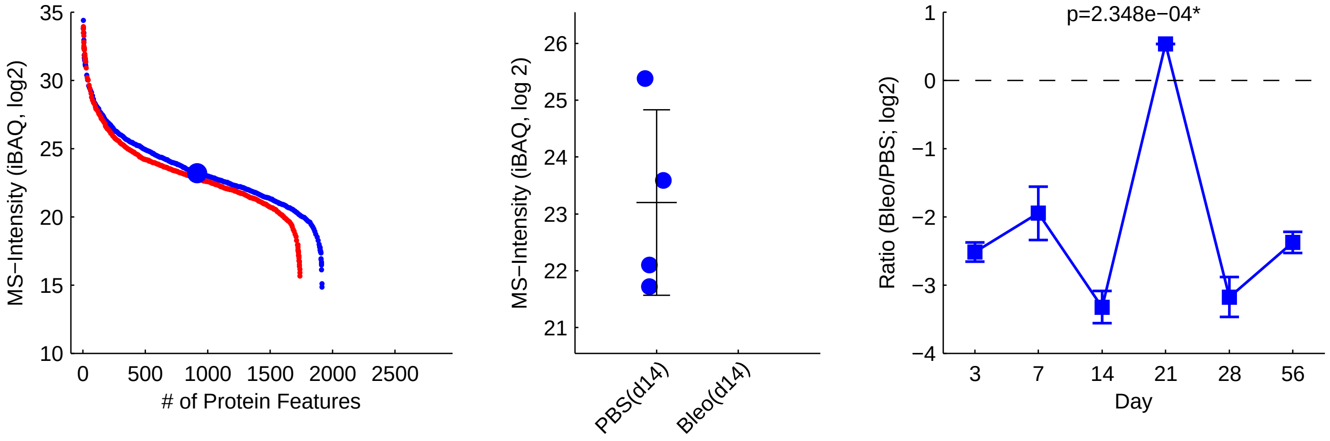

Q9WUK2−2 − Eif4h (id: 2821)

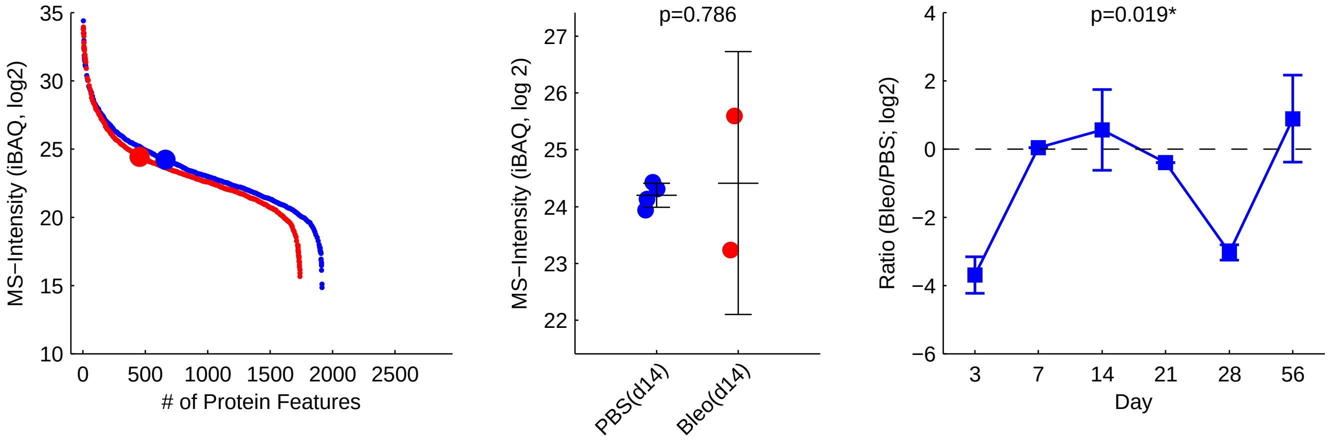

Q9WUM3 − Coro1b (id: 2823)

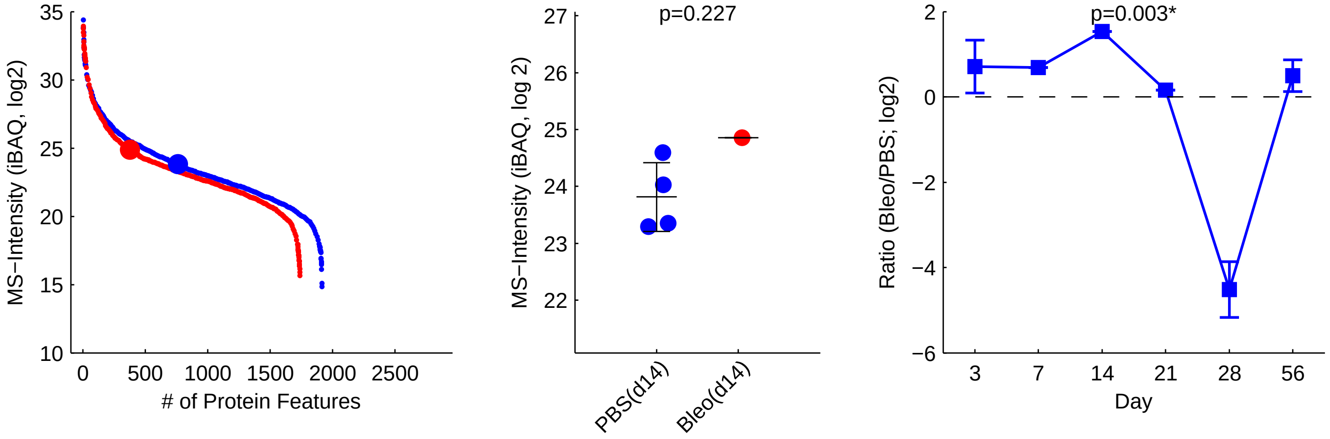

Q9WUM4 − Coro1c (id: 2824)

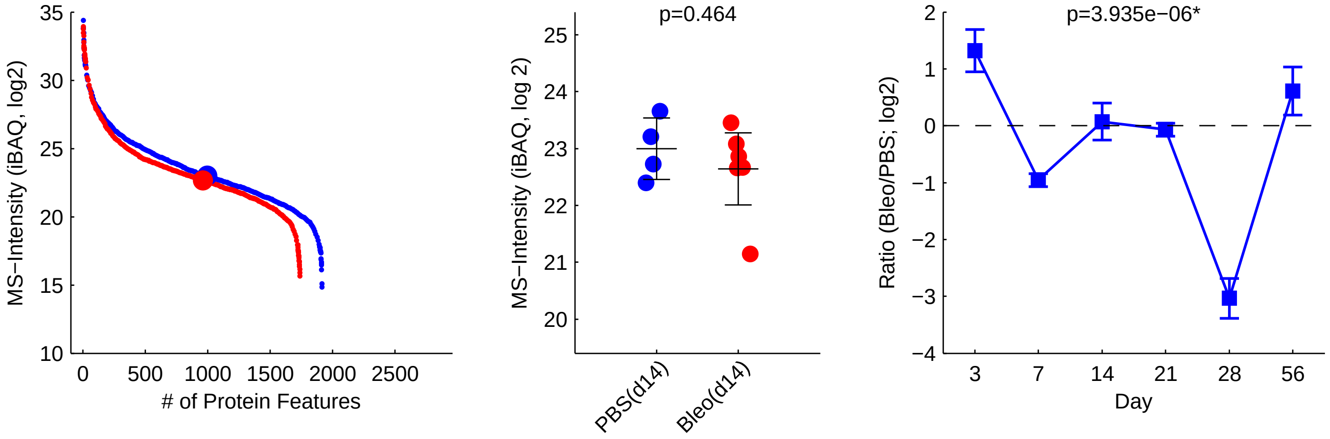

Q9WUM5 − Suclg1 (id: 2825)

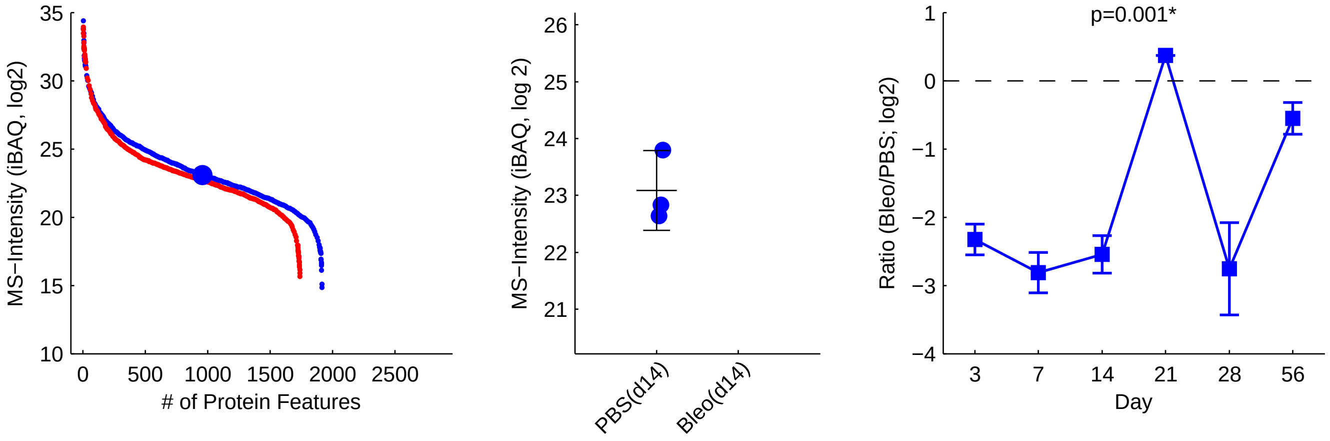

Q9WUU7 − Ctsz (id: 2827)

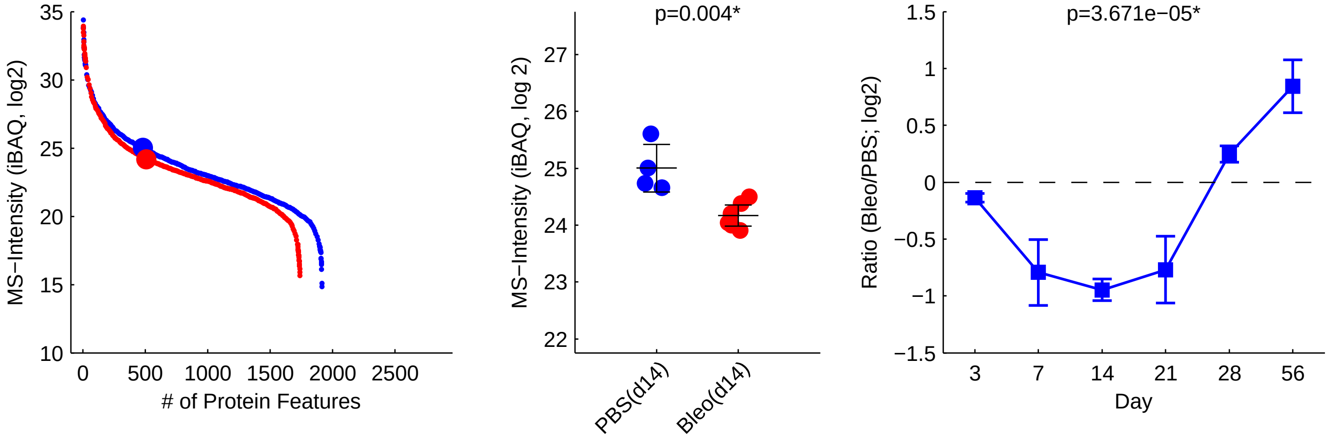

Q9WV54 − Asah1 (id: 2831)

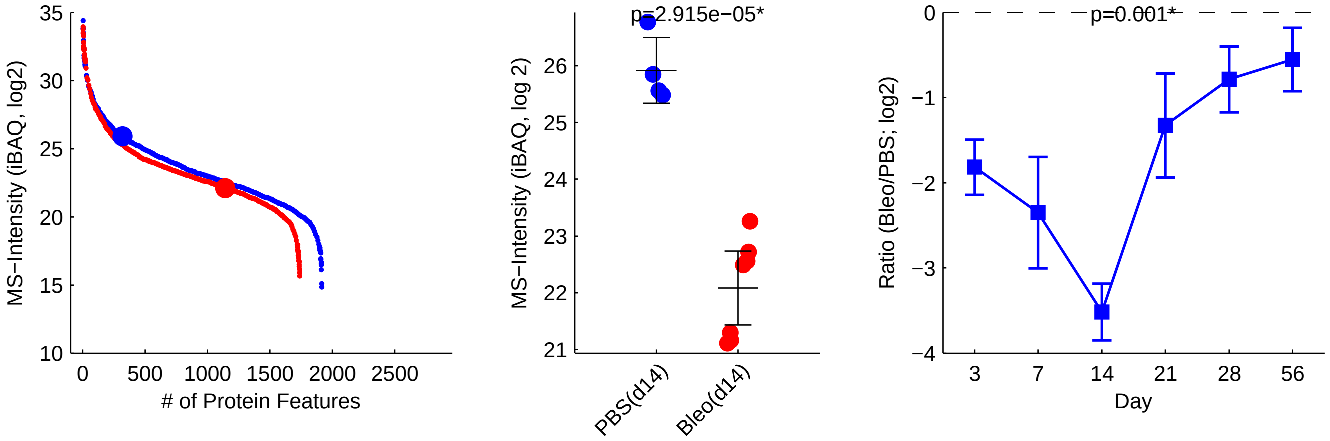

Q9WVE8 − Pacsin2 (id: 2835)

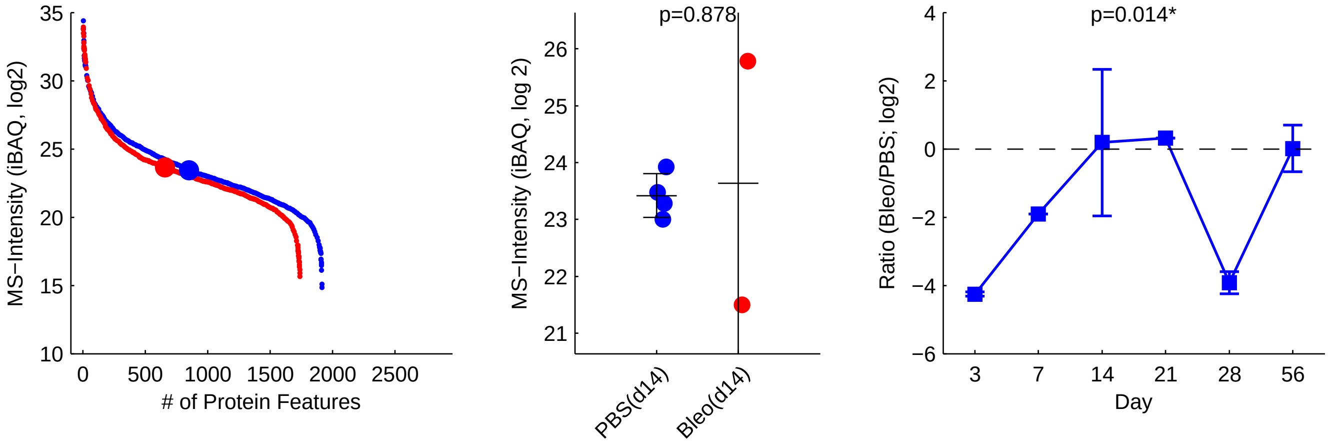

Q9WVL7 − Cxcl15 (id: 2841)

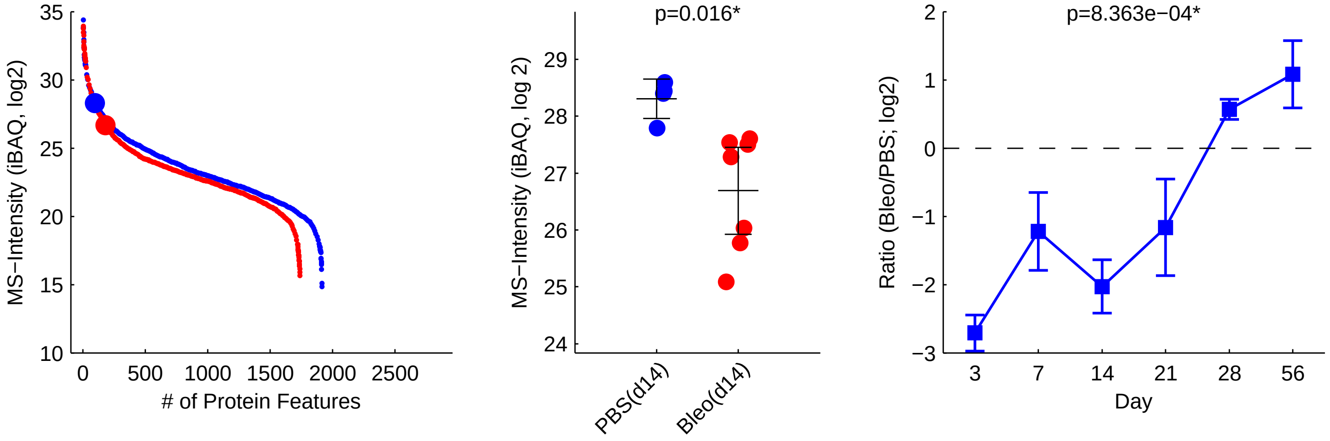

Q9Z0J0 − Npc2 (id: 2847)

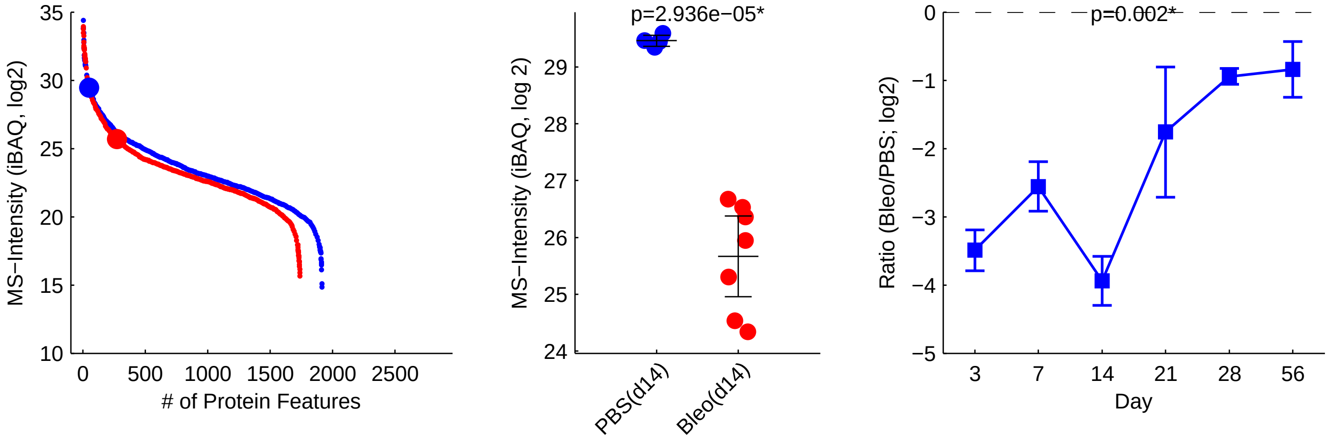

Q9Z0K8 − Vnn1 (id: 2849)

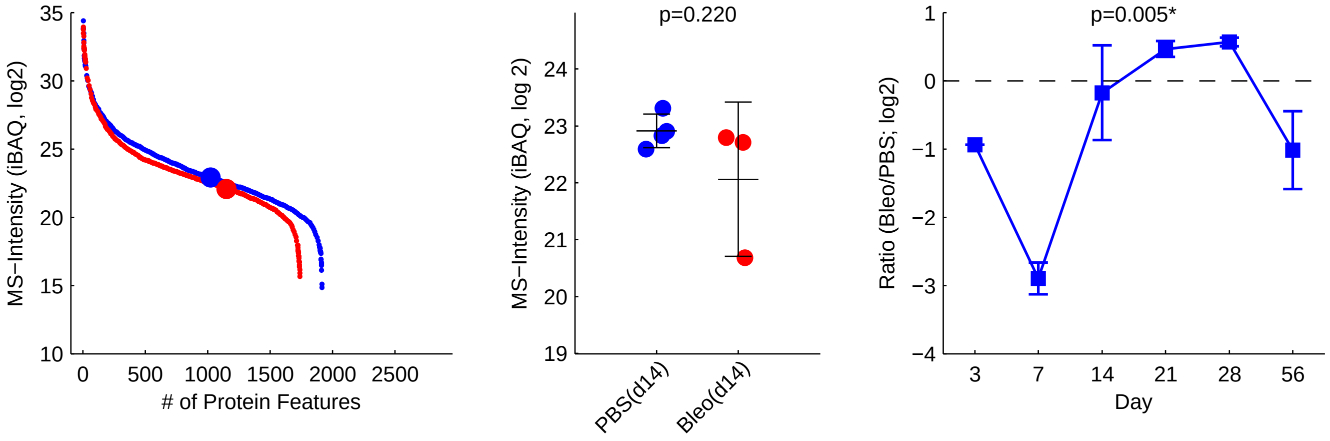

Q9Z0N1 − Eif2s3x (id: 2853)

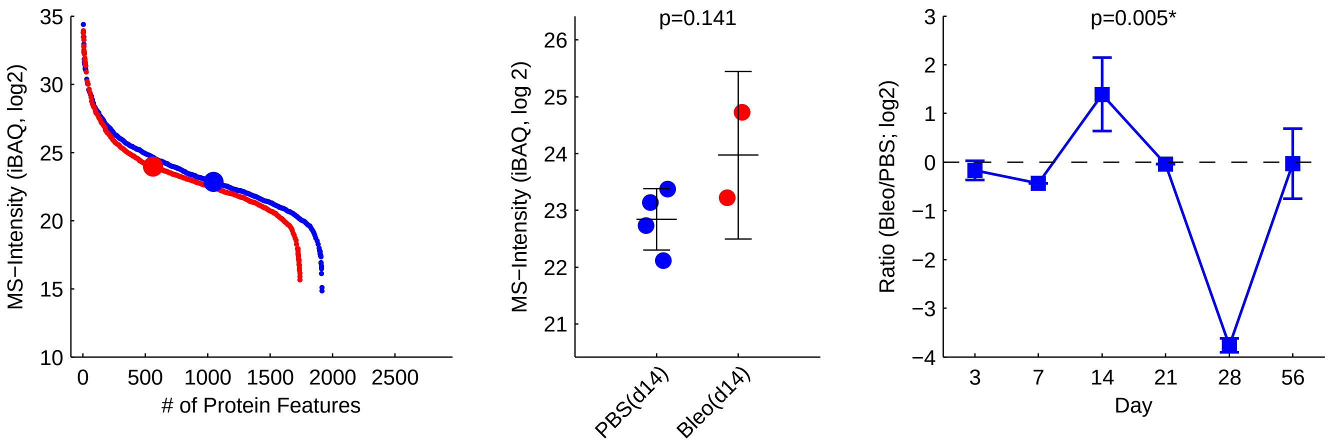

Q9Z0U1 − Tjp2 (id: 2857)

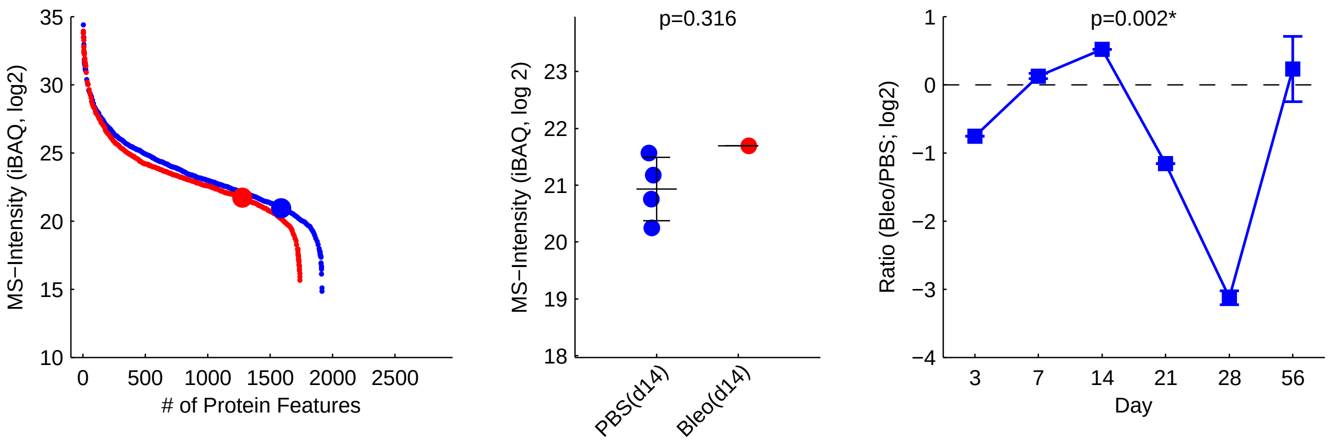

Q9Z1A1 − Tfg (id: 2858)

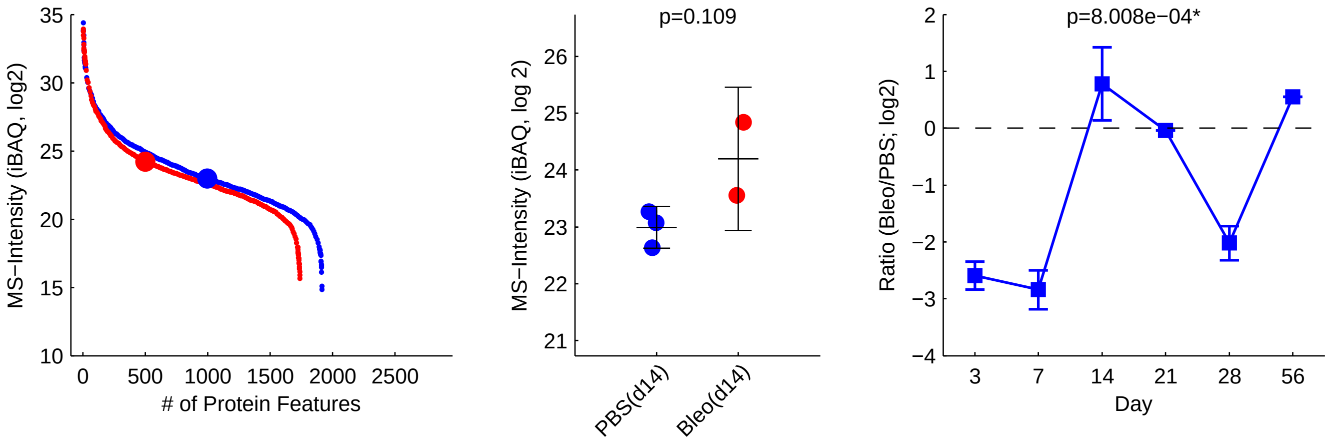

Q9Z1F9 − Uba2 (id: 2860)

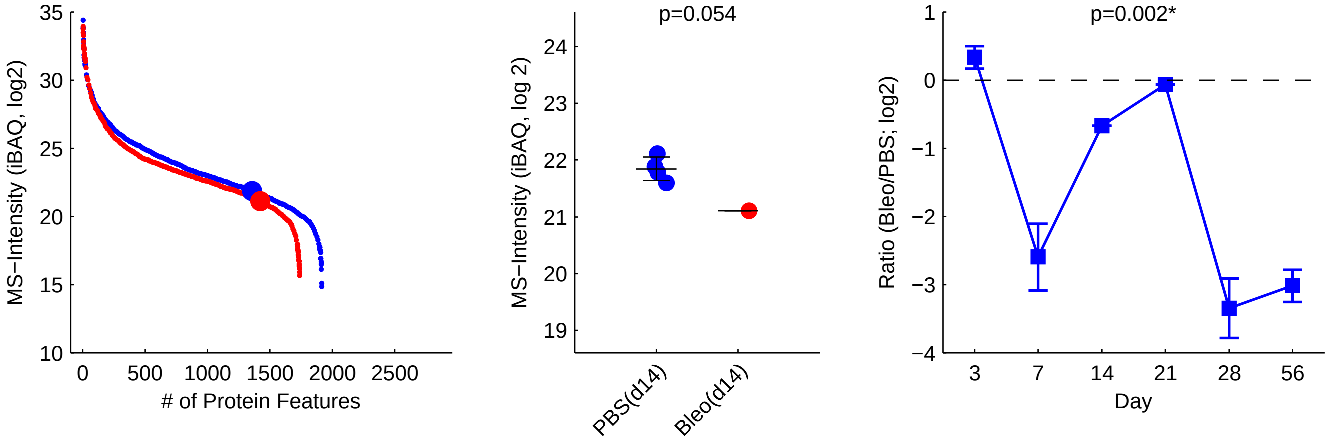

Q9Z1G3 − Atp6v1c1 (id: 2861)

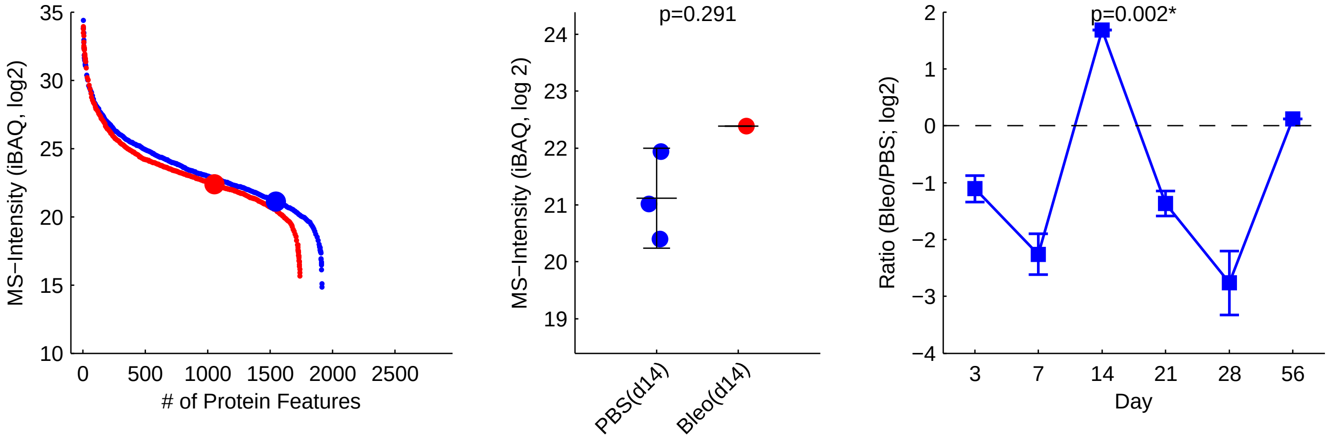

Q9Z1N5 − Ddx39b (id: 2862)

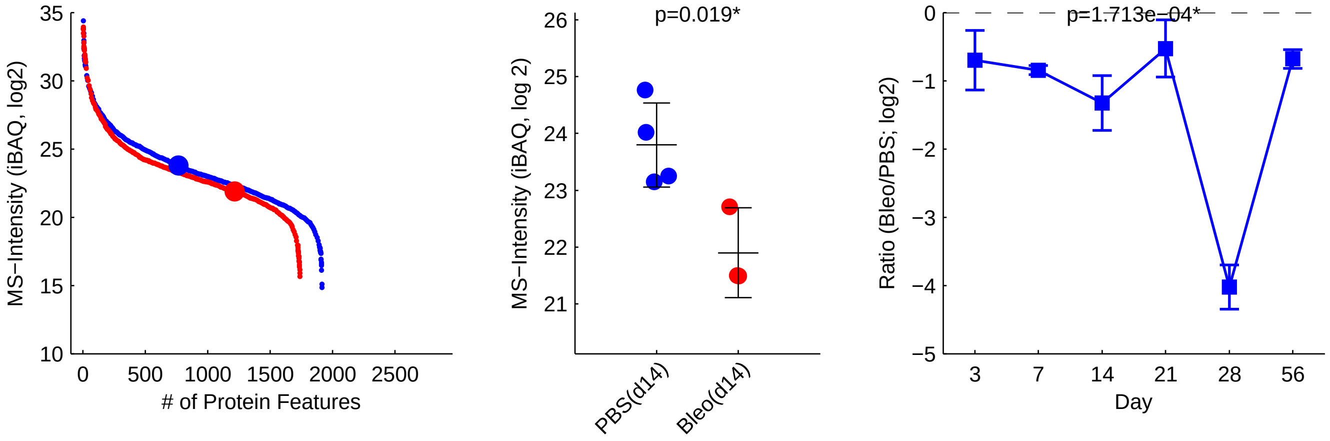

Q9Z1Q5 − Clic1 (id: 2863)

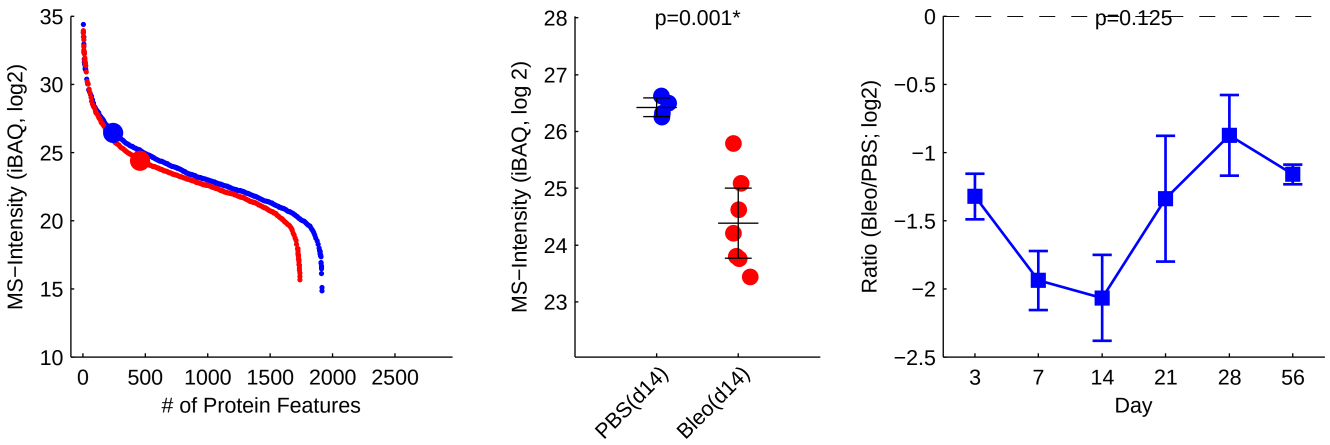

Q9Z1Q9 − Vars (id: 2864)

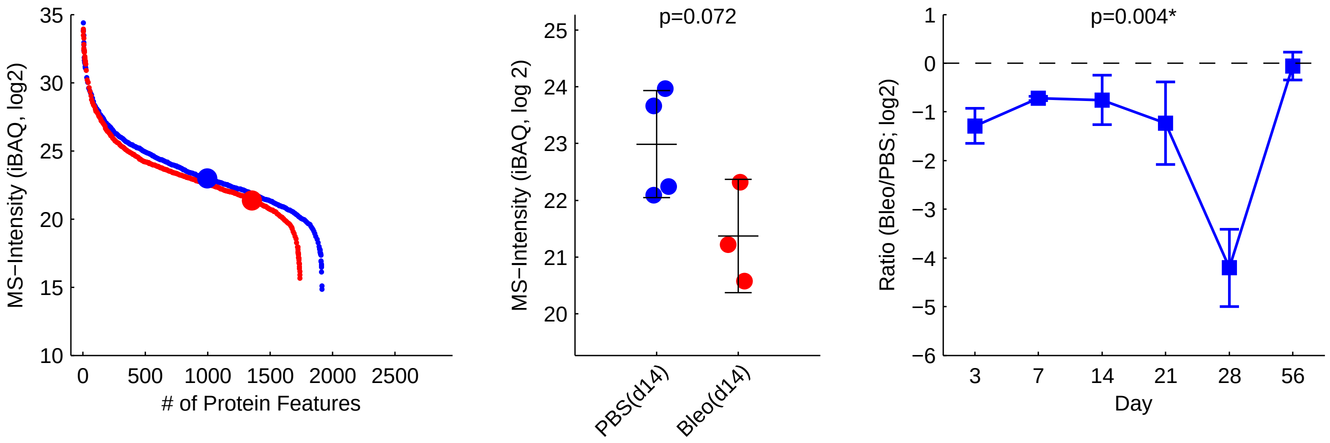

Q9Z1R3 − Apom (id: 2865)

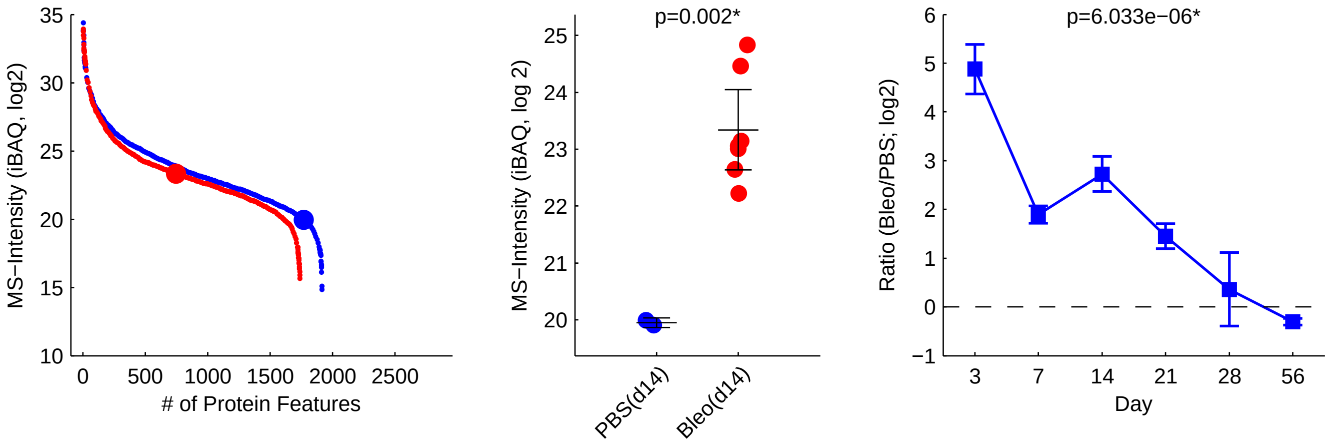

Q9Z1Z2 − Strap (id: 2870)

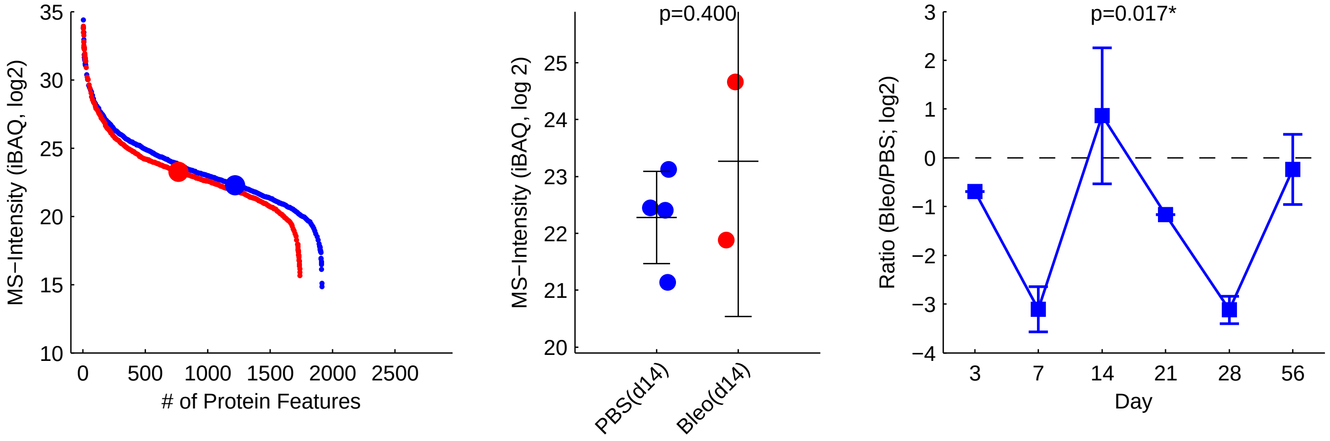

Q9Z204−2 − Hnrnpc (id: 2871)

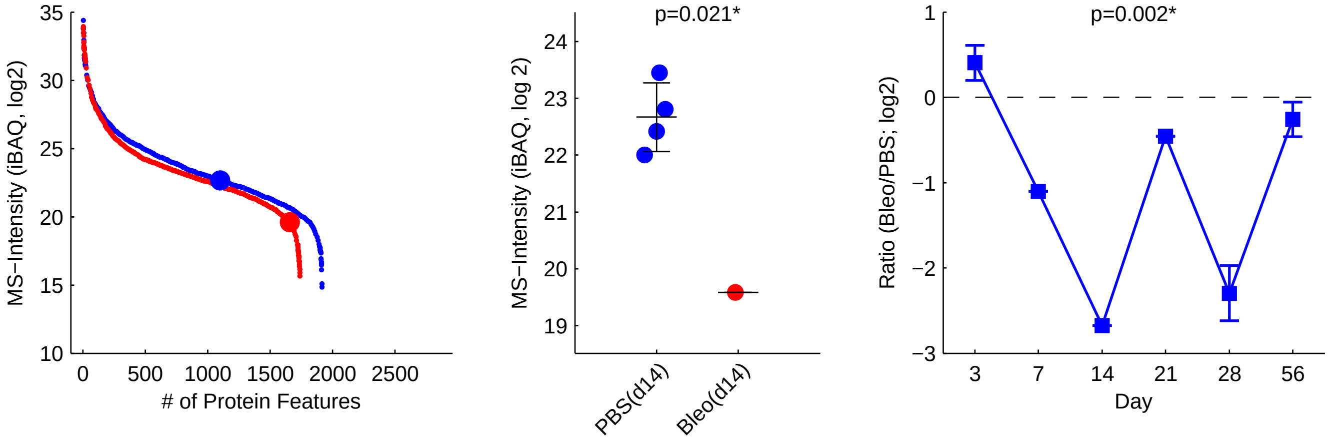

Q9Z2H7 − Gipc2 (id: 2872)

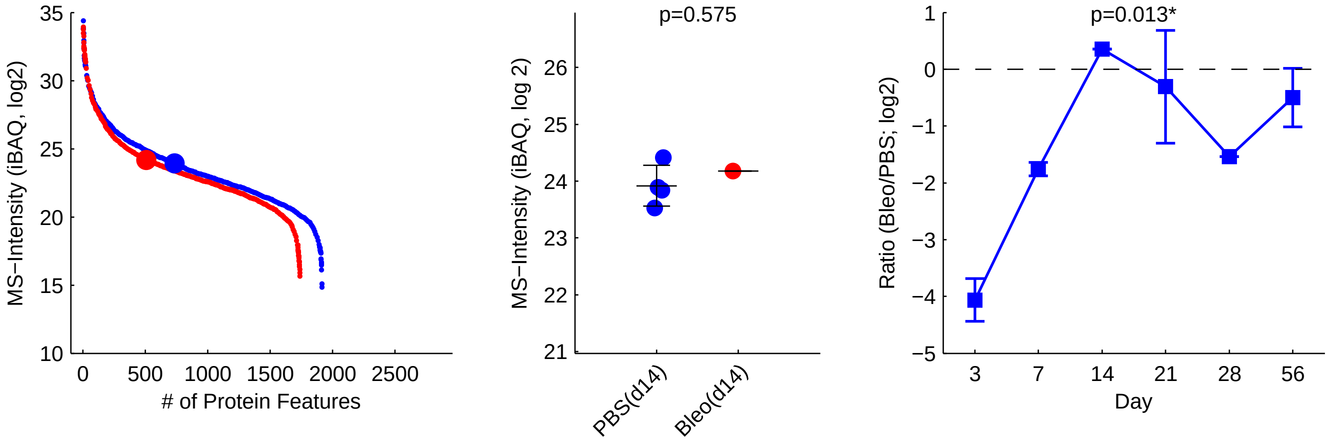

Q9Z2I9 − Sucla2 (id: 2875)

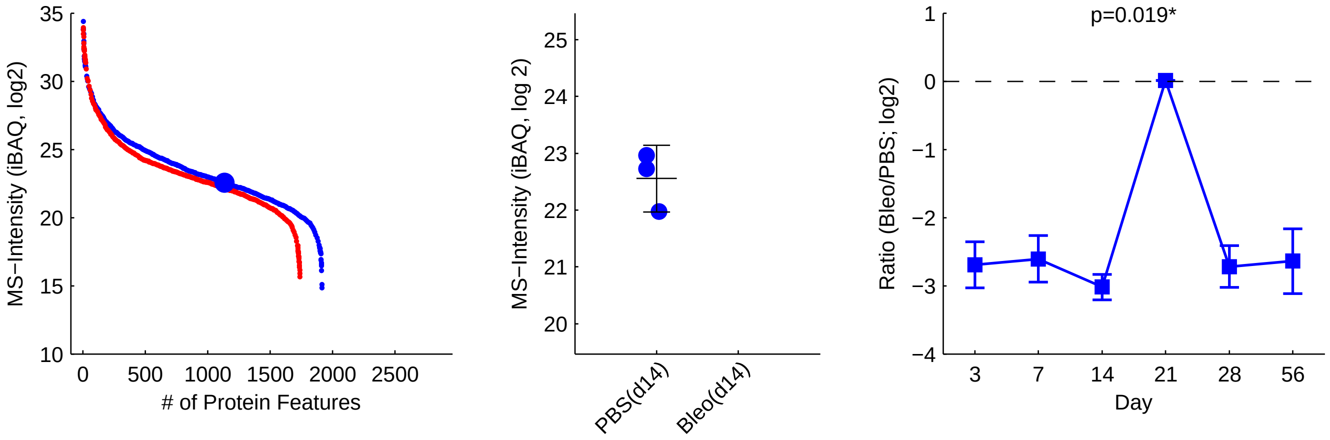

Q9Z2M7 − Pmm2 (id: 2878)

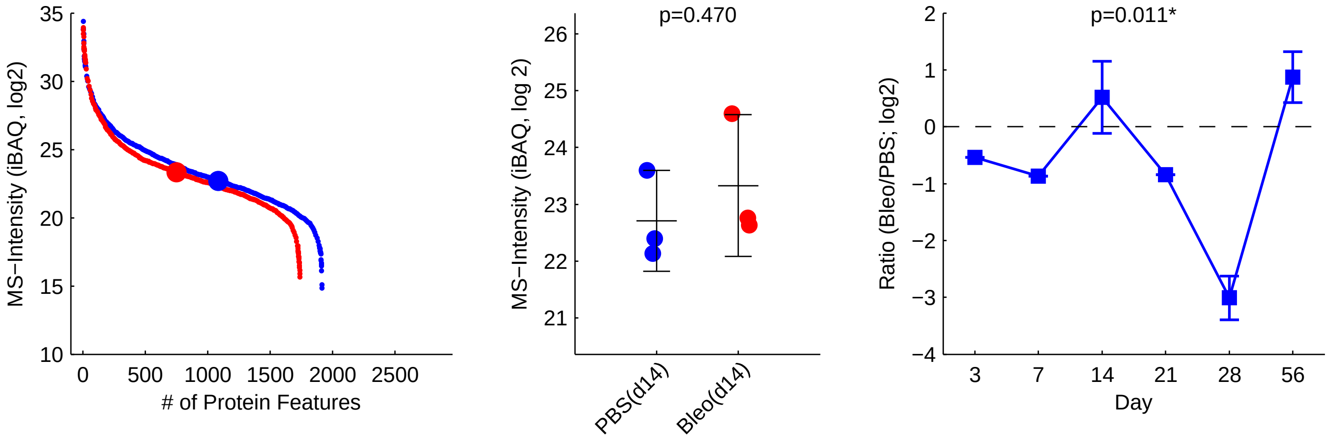

Q9Z2U0 − Psma7 (id: 2879)

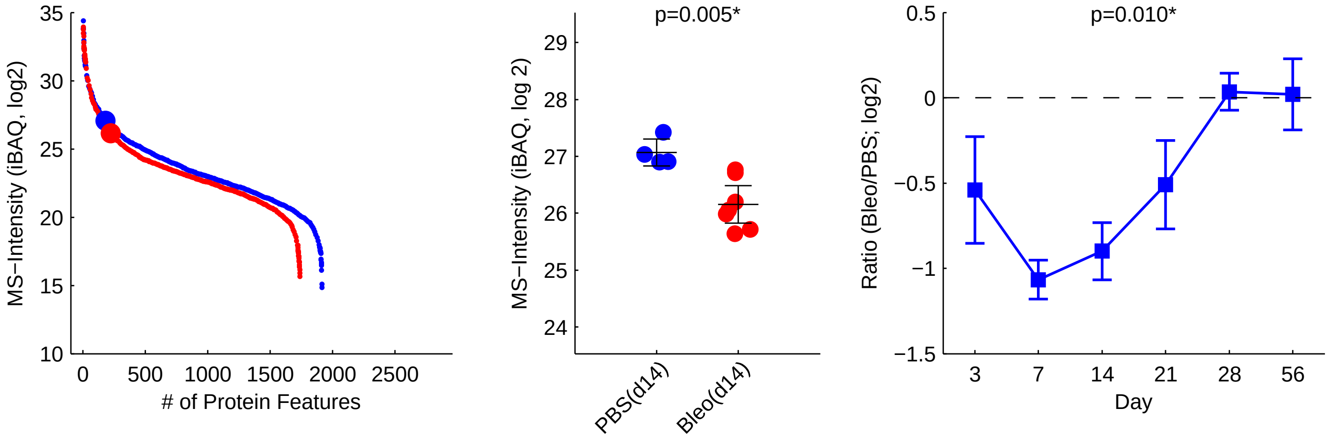

Q9Z2U1 − Psma5 (id: 2880)

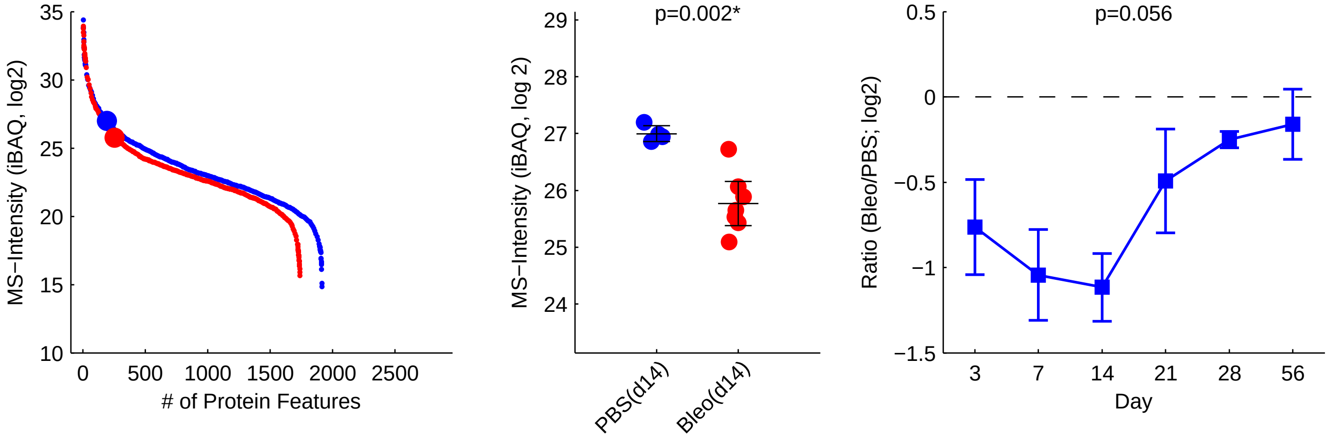

Q9Z2W0 − Dnpep (id: 2881)

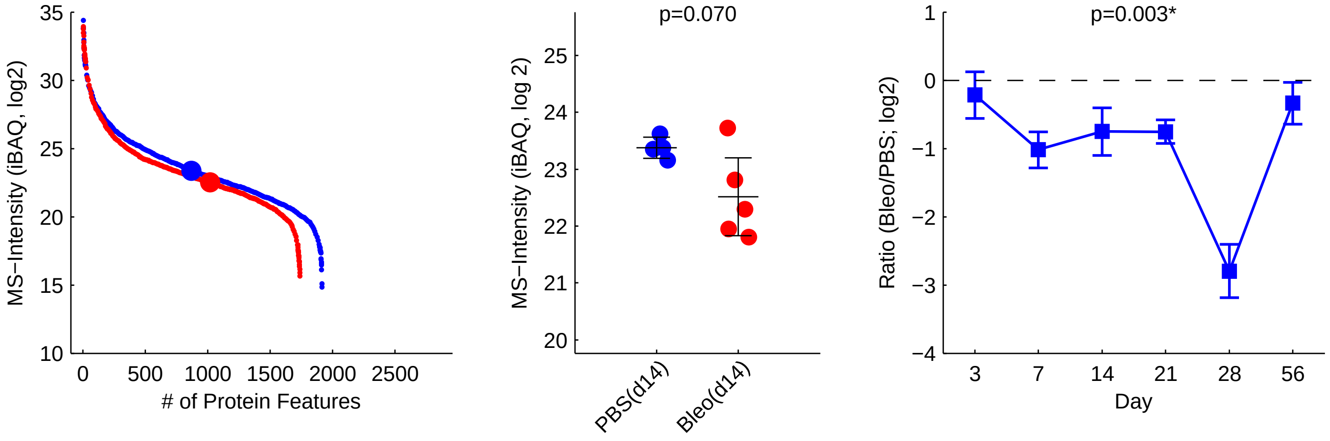

Q9Z2X1 − Hnrnpf (id: 2883)

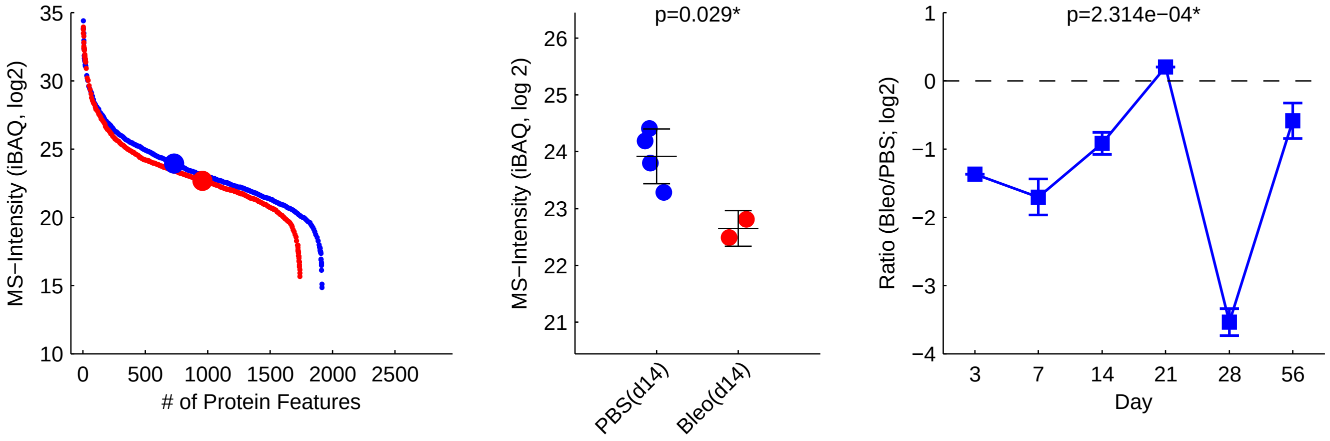

Q9Z2Y8 − Prosc (id: 2884)

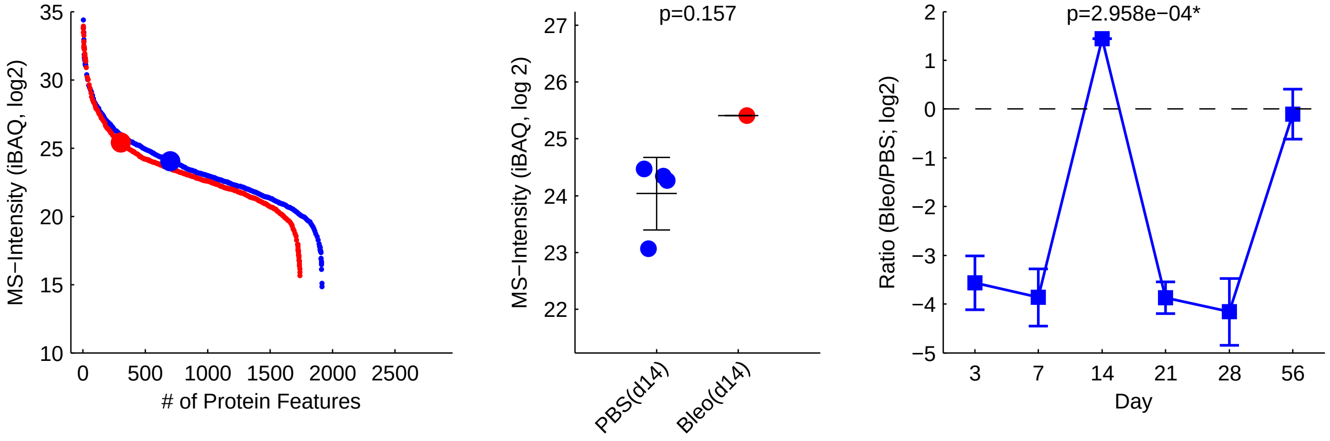

Supplement: Supplementary file 11 [file msb0011-0819-sd11.zip › Table EV10/BalfPlots_TableEV10.pdf]
