# Supplementary material for: Time- and compartment-resolved proteome profiling of the extracellular niche in lung injury and repair
Source: Mol Syst Biol. 2015 Jul 14;11(7):819. doi: 10.15252/msb.20156123 (PMC4547847; doi:10.15252/msb.20156123)
Supplement: Supplementary file 12 [file msb0011-0819-sd12.docx]

**Table Legends**

Table EV1 – QDSP data table – protein detergent solubility quantification

Table EV2 – Annotation enrichment analysis QDSP clustering

Table EV3 – Solubility differences (bleomycin versus PBS) – fisher exact annotation enrichment analysis

Table EV4 – RNA-seq data table (FPKM; bleomycin day 14, n=4; PBS, n=4)

Table EV5 – 2D annotation enrichment – proteome versus transcriptome

Table EV6 – Time course analysis – tissue proteome data (protein quantification)

Table EV7 – Activation Z-scores of the ‘Upstream Regulator Analysis’ and the ‘Downstream Effects Analysis’

Table EV8 – Annotation enrichment analysis – lung compliance correlation fit slope (negative versus positive slope)

Table EV9 – Cell type specific protein signatures

Table EV10 - Time course analysis – BAL-fluid proteome data (protein quantification)
