# Supplementary material for: Time- and compartment-resolved proteome profiling of the extracellular niche in lung injury and repair
Source: Mol Syst Biol. 2015 Jul 14;11(7):819. doi: 10.15252/msb.20156123 (PMC4547847; doi:10.15252/msb.20156123)

A1BN54 − Actn1 (id: 4)

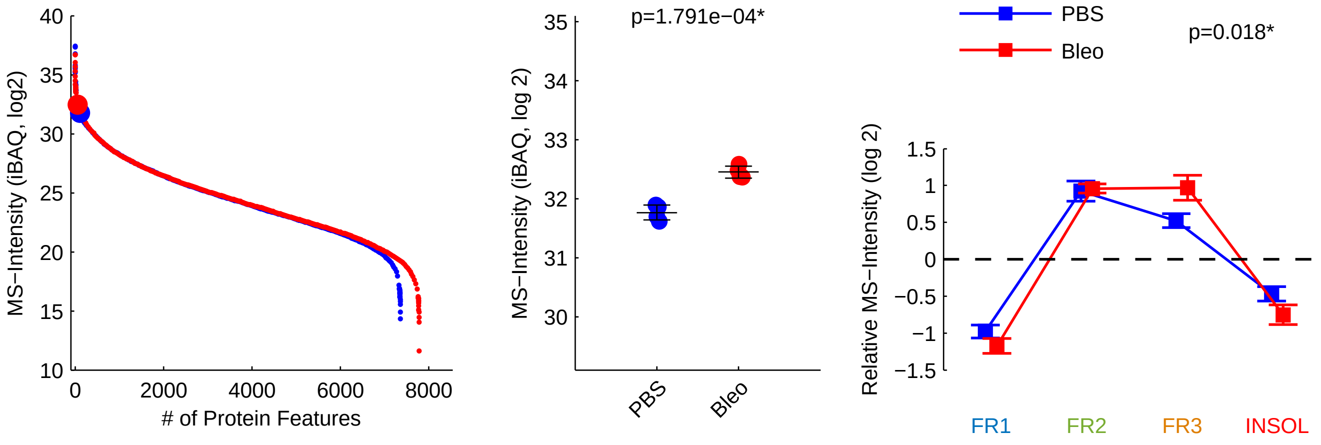

Q62077 − Plcg1 (id: 13)

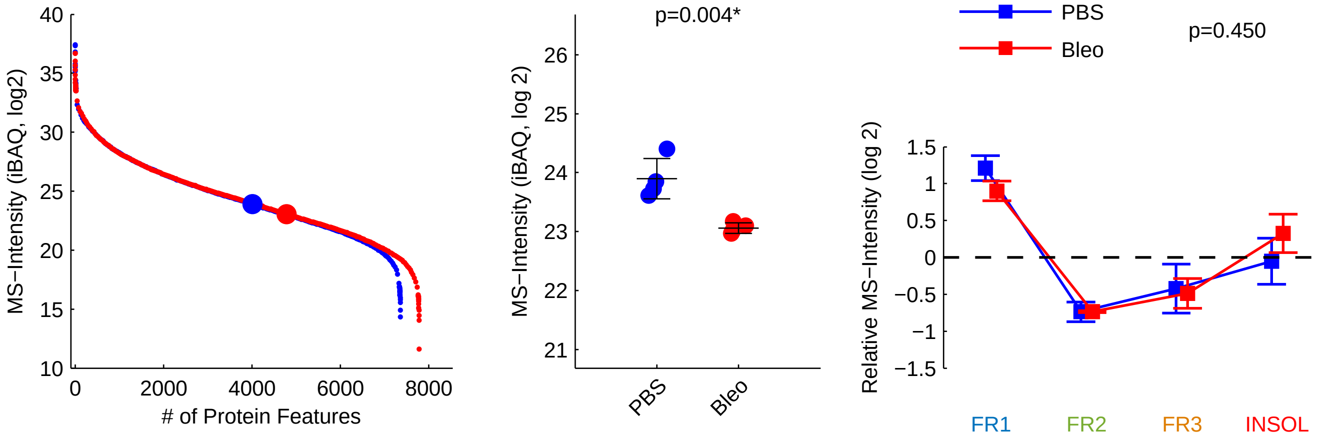

A2A4P0 − Dhx8 (id: 15)

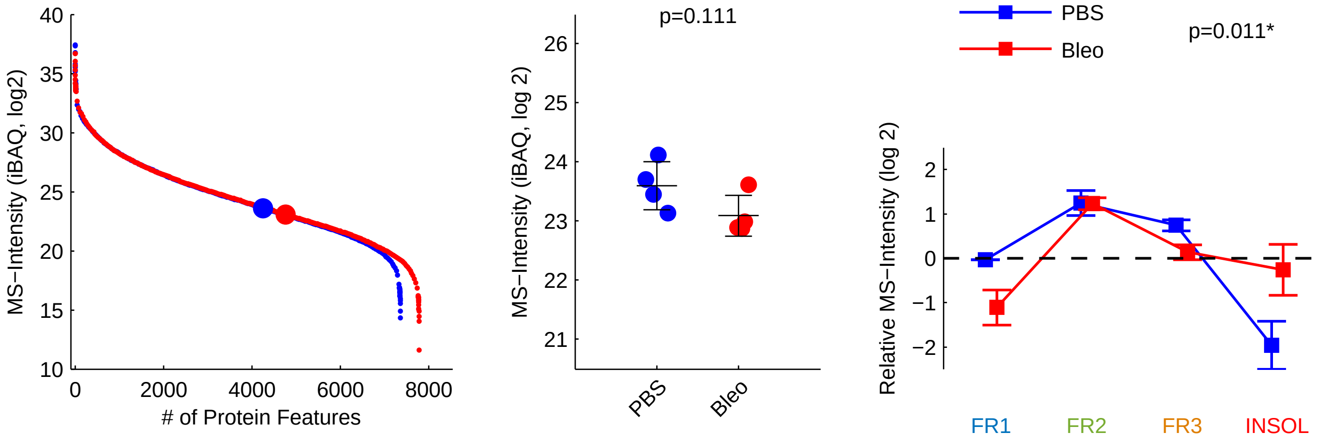

Q3THG9 − Aarsd1 (id: 16)

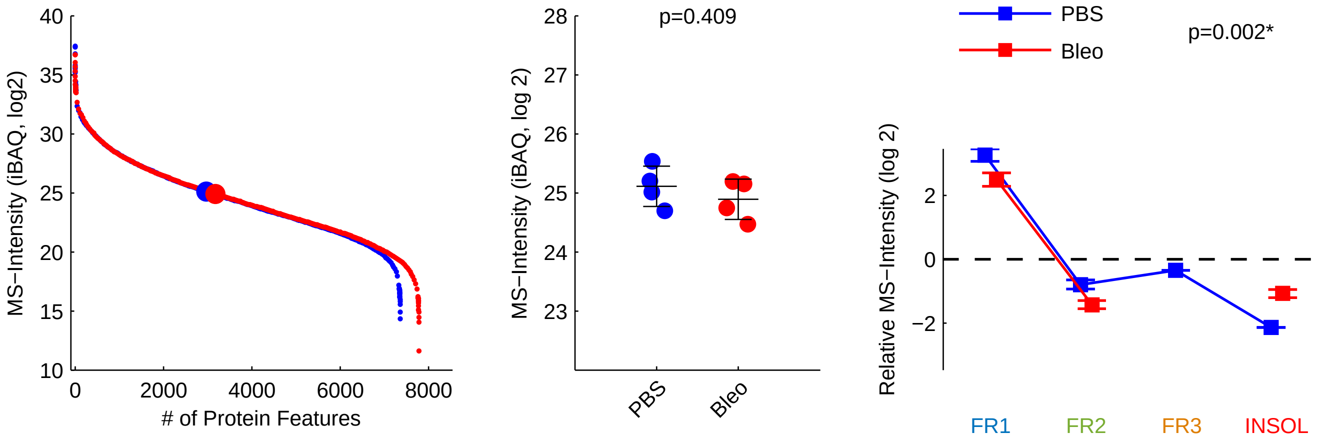

A2A5R2 − Arfgef2 (id: 21)

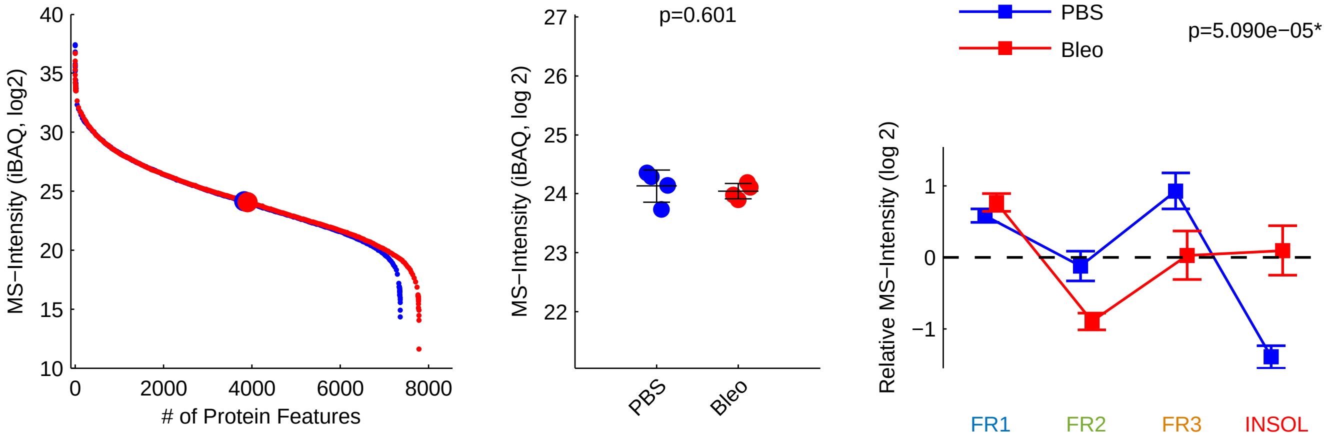

A2A5S3 − Stau1 (id: 22)

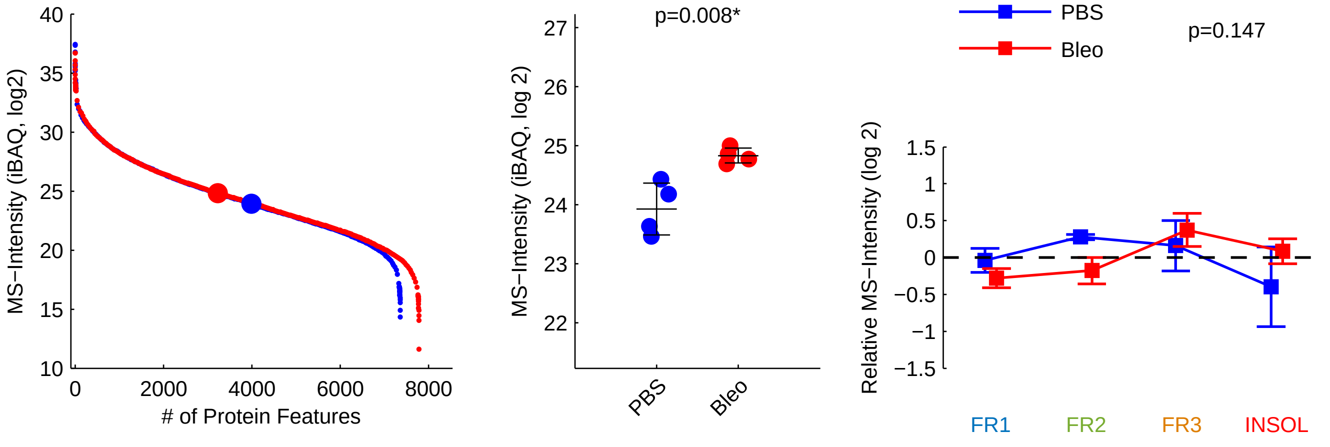

A2A6I0 − Tnnt3 (id: 29)

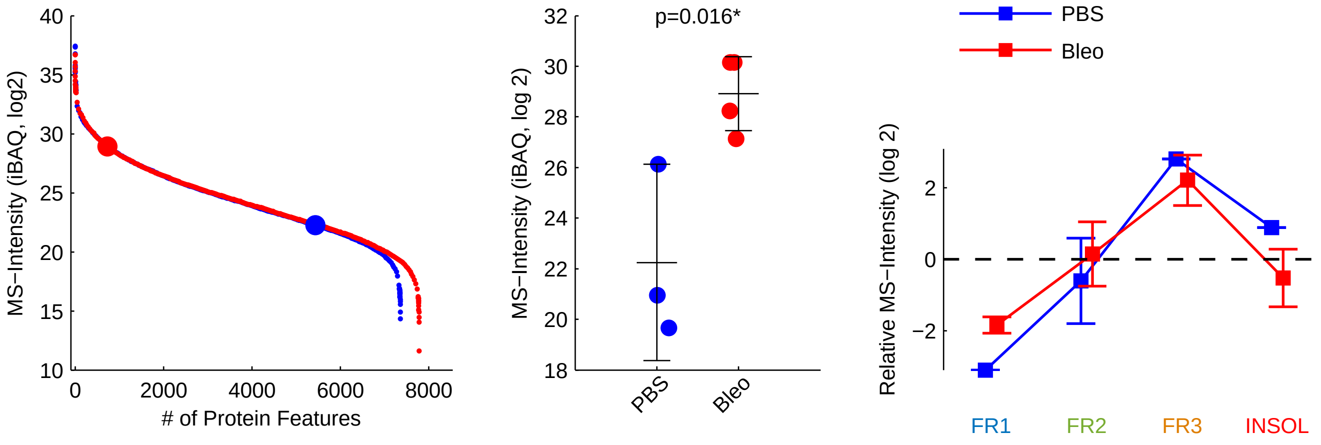

A8Y5G5 − Zfyve9 (id: 46)

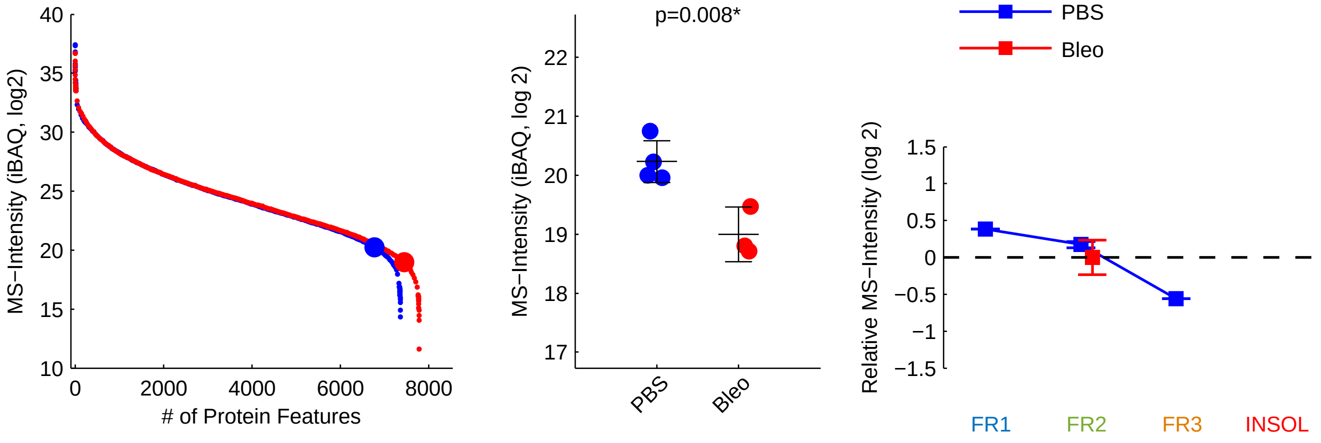

A2A8Z1 − Osbpl9 (id: 50)

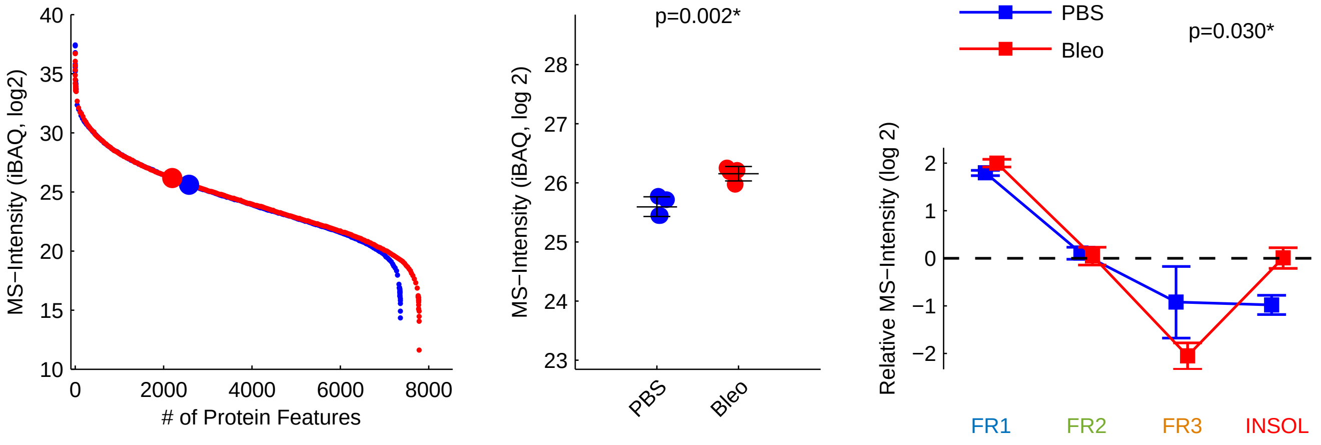

Q8K182 − C8a (id: 52)

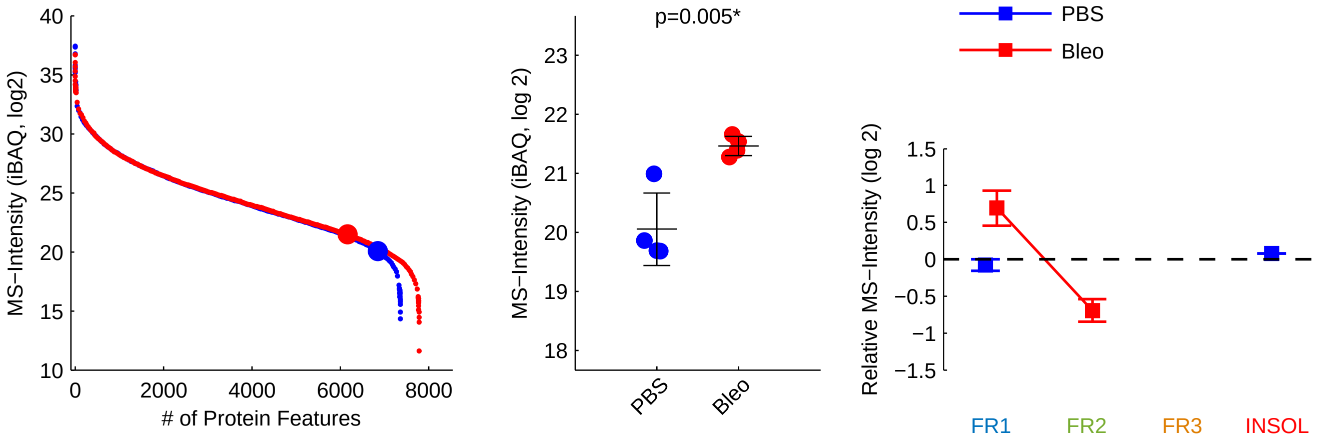

Q8R1A4 − Dock7 (id: 57)

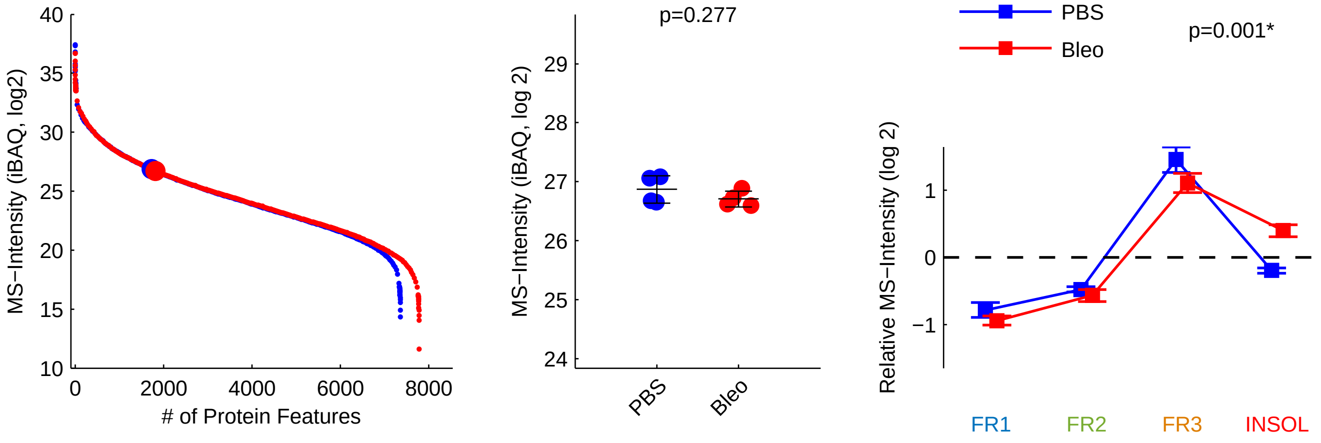

Q8BHG1 − Nrd1 (id: 59)

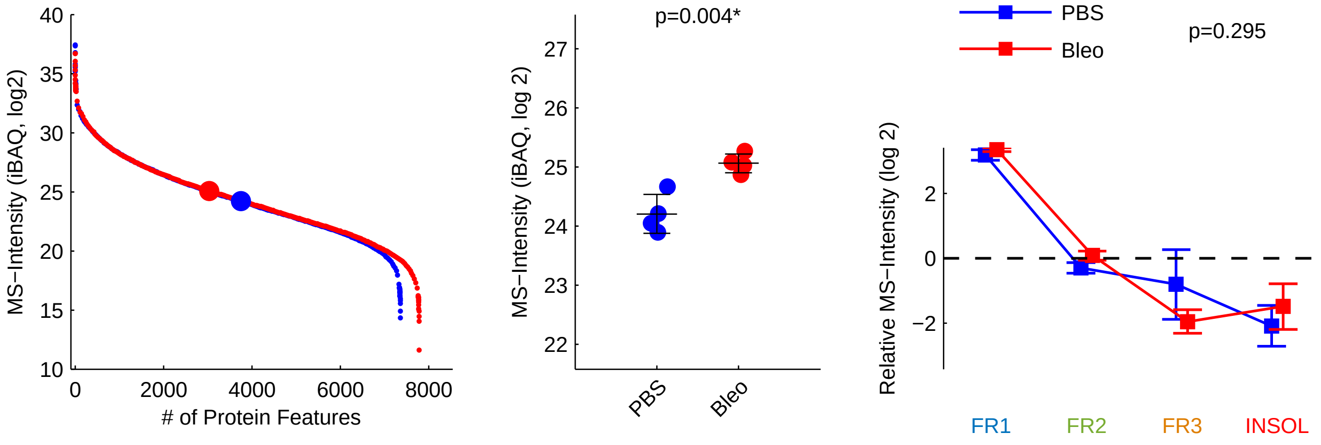

A2AAY5 − Sh3pxd2b (id: 66)

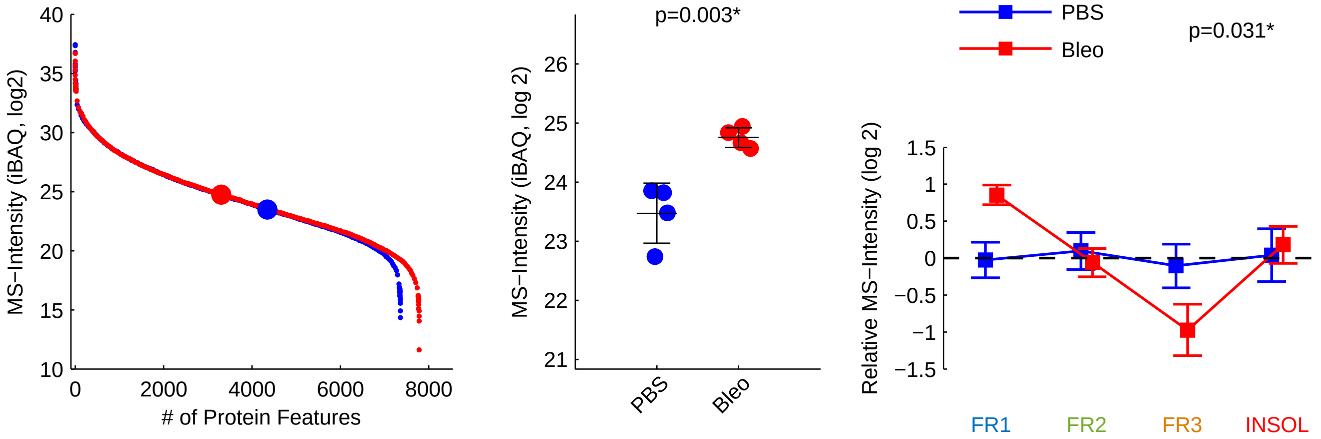

A2ADB1 − Spen (id: 82)

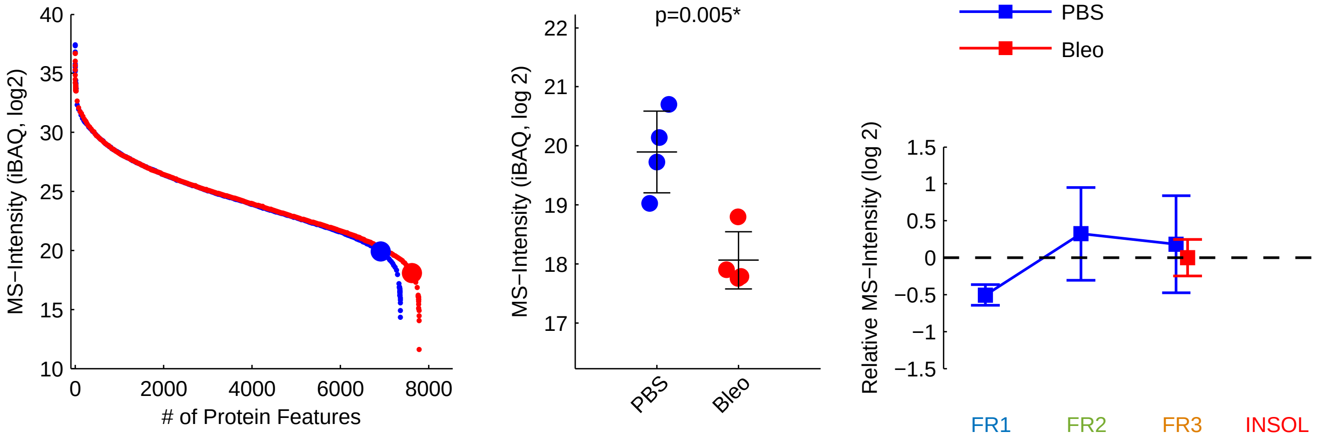

A2AF47 − Dock11 (id: 97)

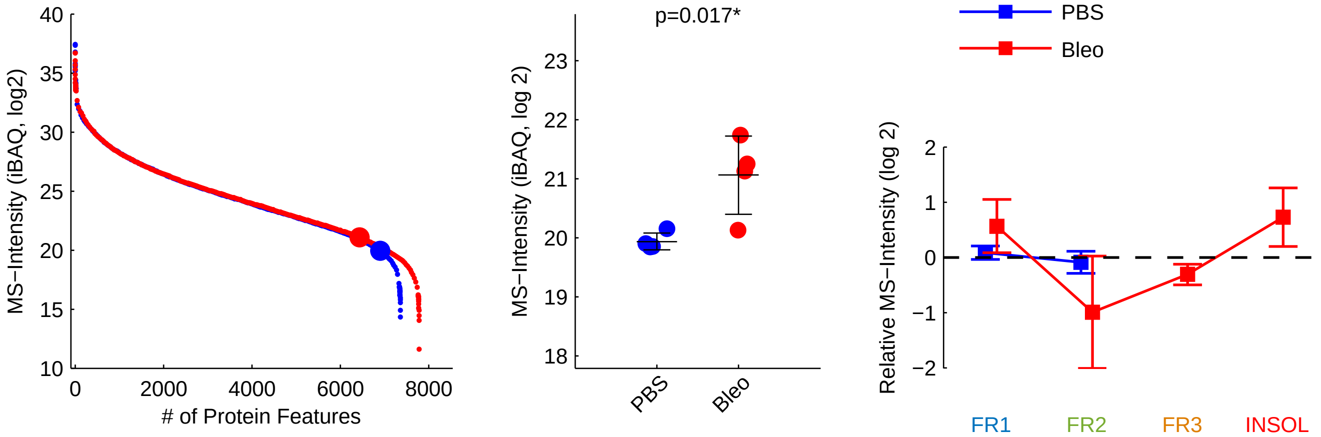

A2AFF6 − Stag2 (id: 98)

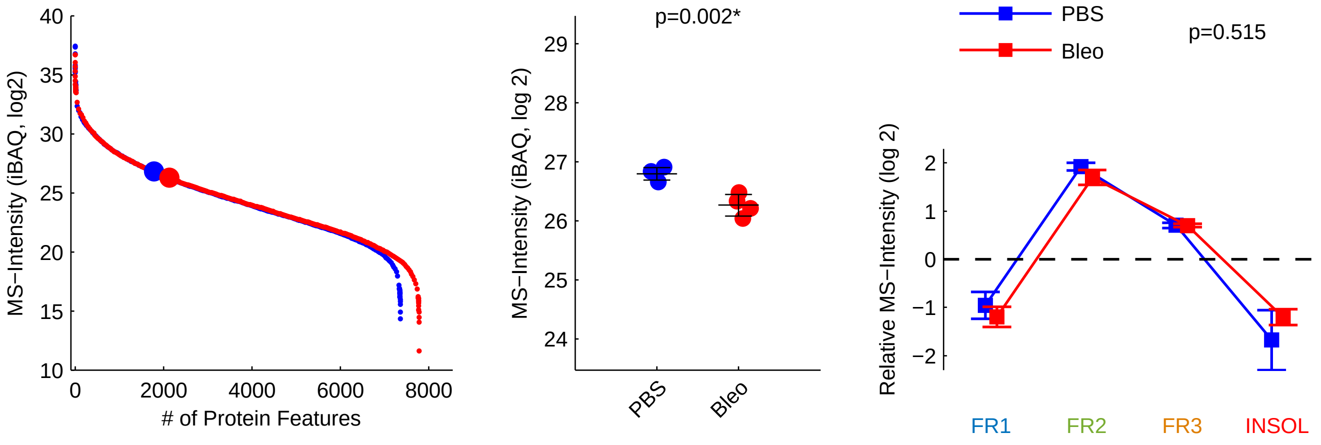

A2AFI8 − Reps2 (id: 99)

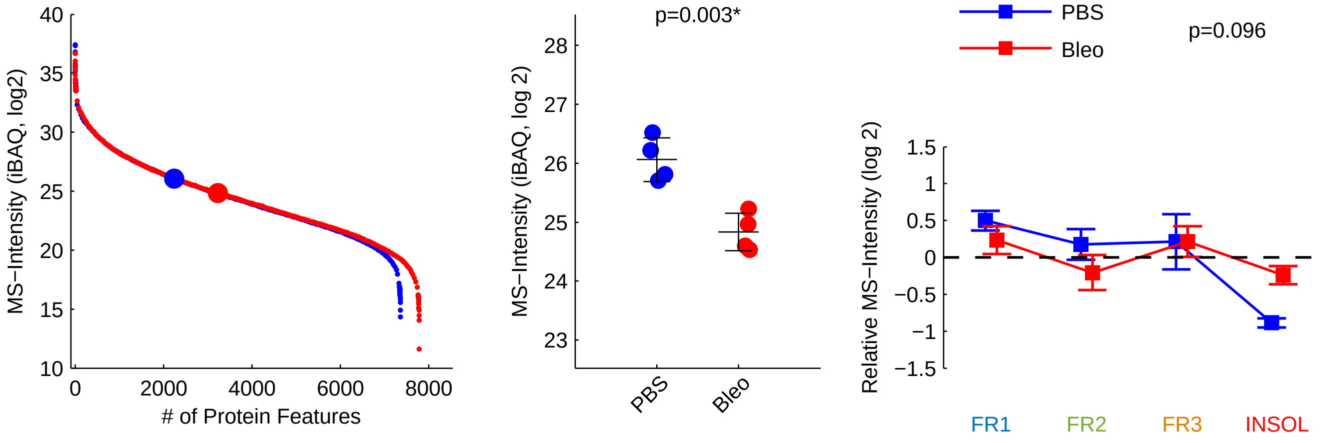

A2AGH6 − Med12 (id: 111)

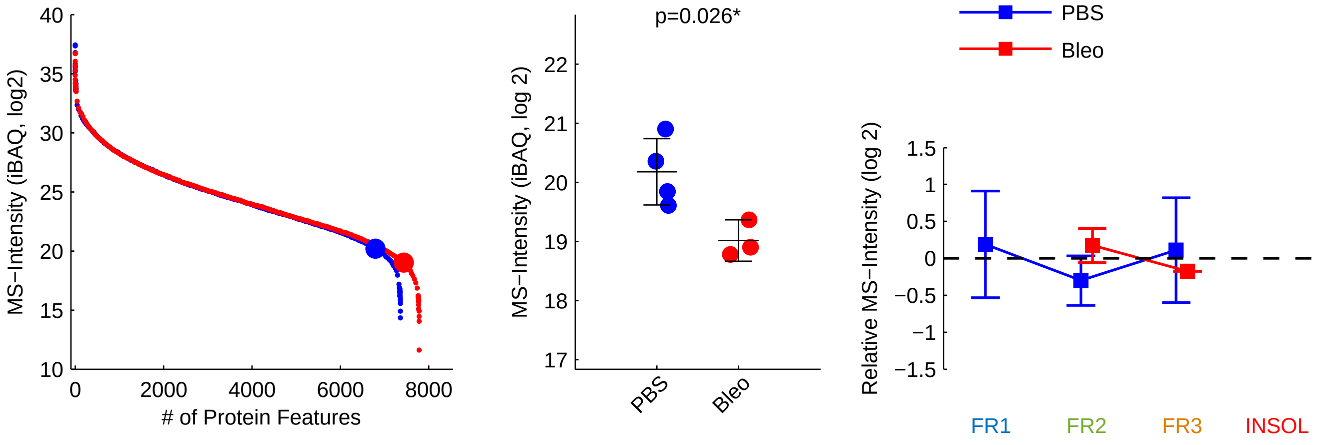

Q923J1 − Trpm7 (id: 125)

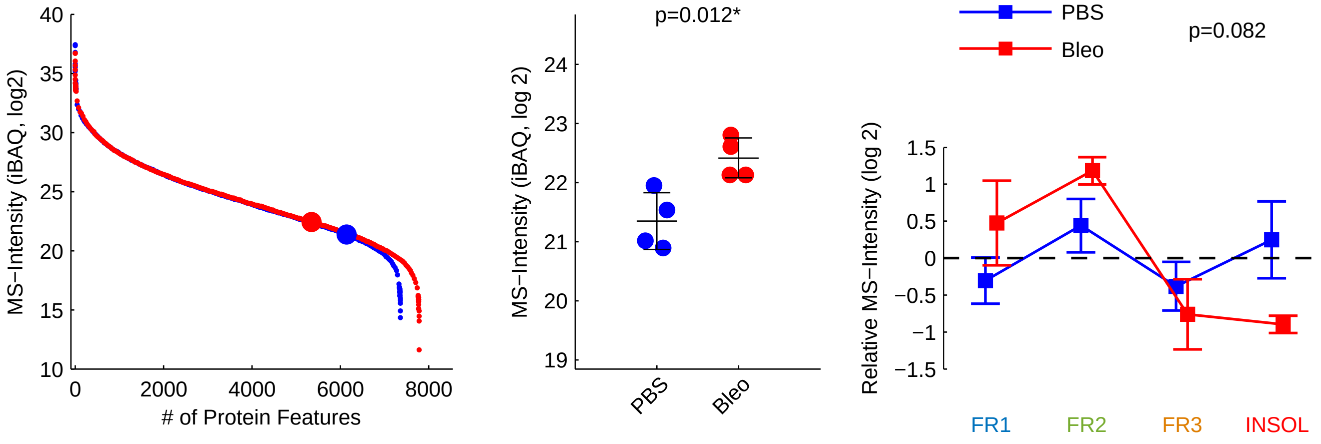

A2AIP0−2 − Fam166b (id: 130)

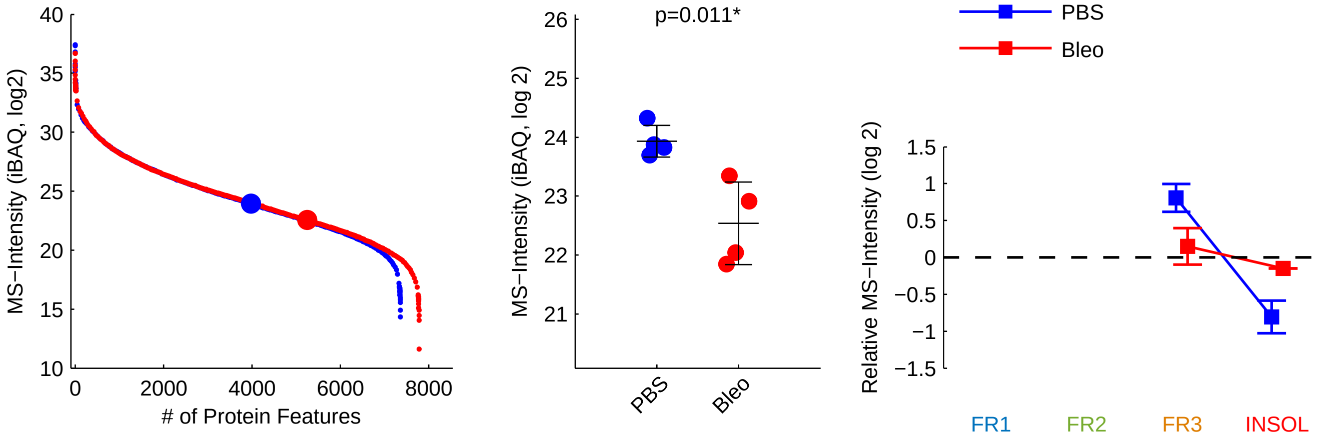

P25085−2 − Il1rn (id: 131)

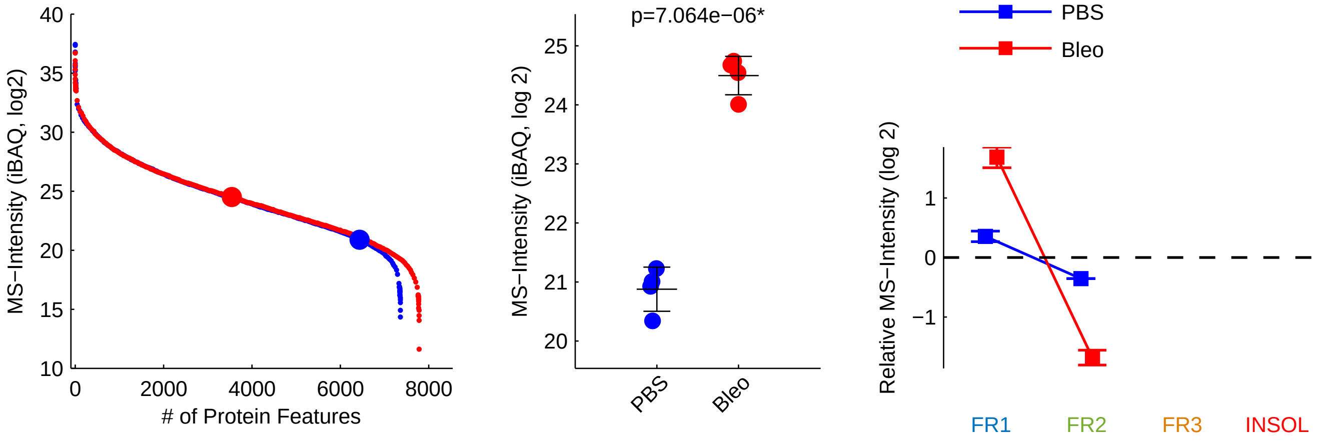

A2AJ15 − Man1b1 (id: 135)

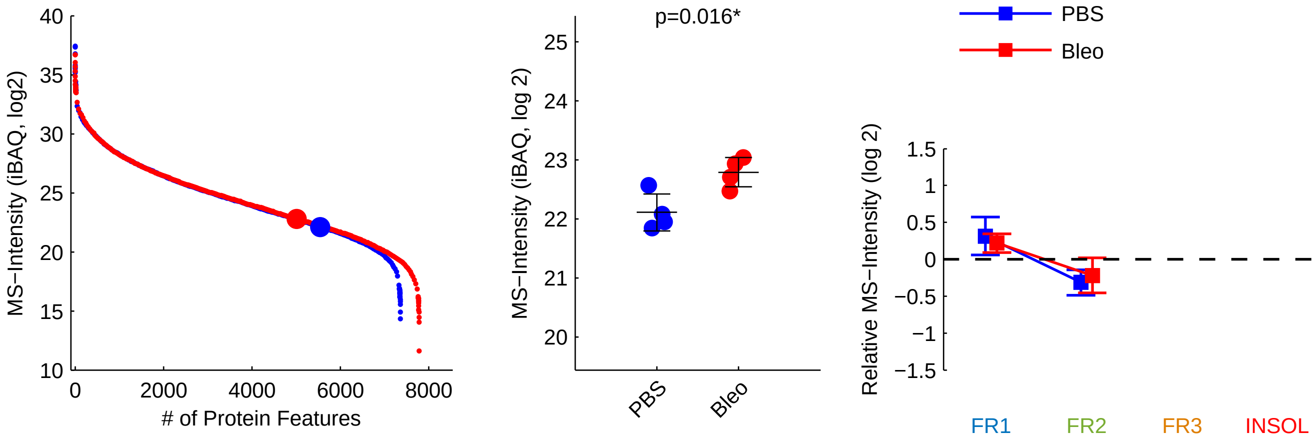

A2AJ88 − Pnpla7 (id: 139)

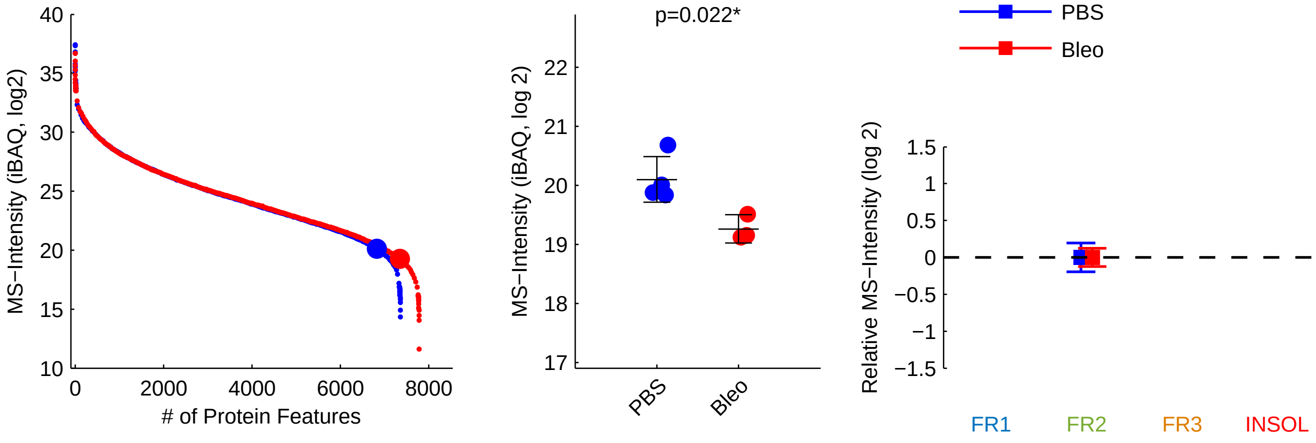

O35206 − Col15a1 (id: 150)

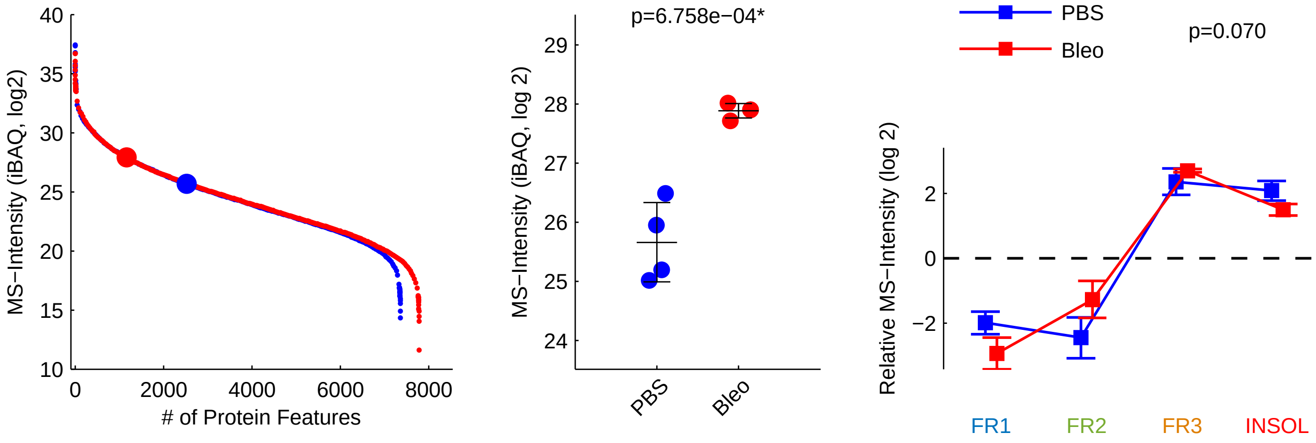

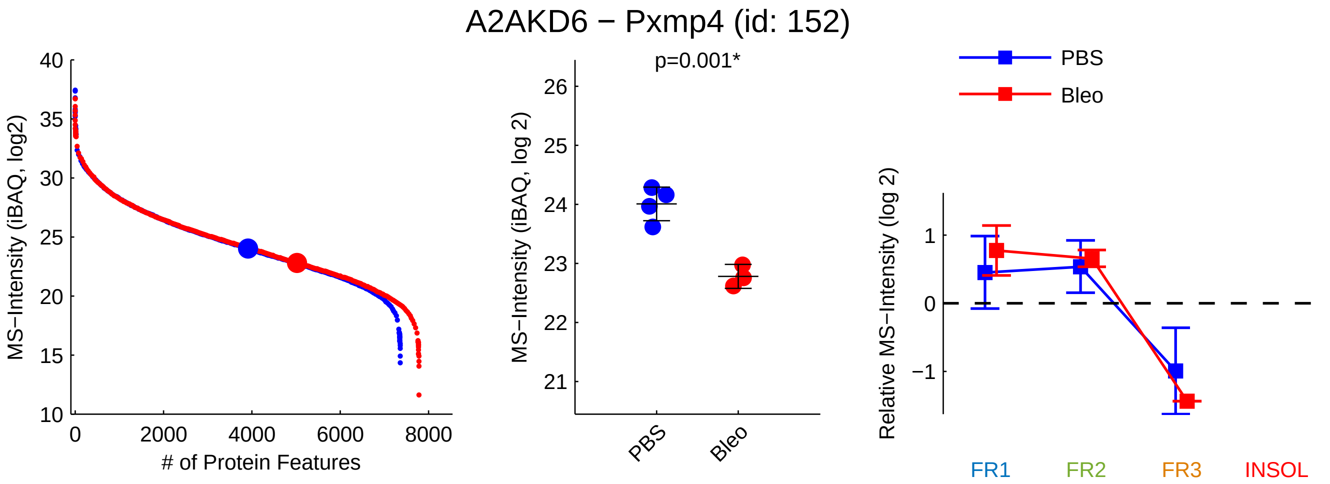
A2AKD6 − Pxmp4 (id: 152)
MS−Intensity (iBAQ, log2)
# of Protein Features
p=0.001*
MS−Intensity (iBAQ, log 2)
PBS
Bleo
Relative MS−Intensity (log 2)
FR1
FR2
FR3
INSOL

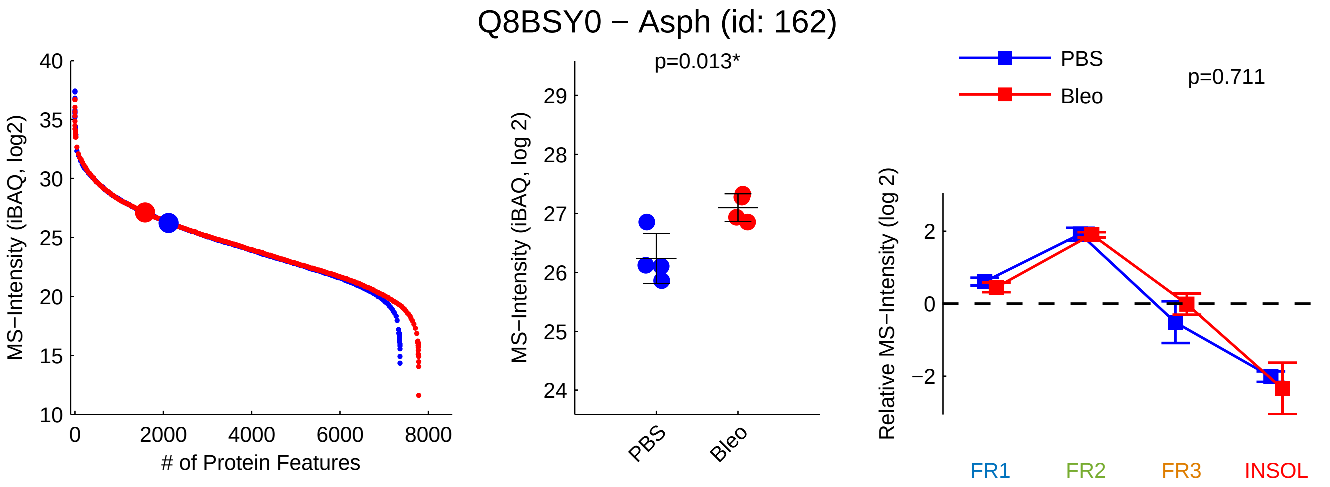
Q8BSY0 − Asph (id: 162)
MS−Intensity (iBAQ, log2)
# of Protein Features
p=0.013*
MS−Intensity (iBAQ, log 2)
PBS
Bleo
p=0.711
Relative MS−Intensity (log 2)
FR1
FR2
FR3
INSOL

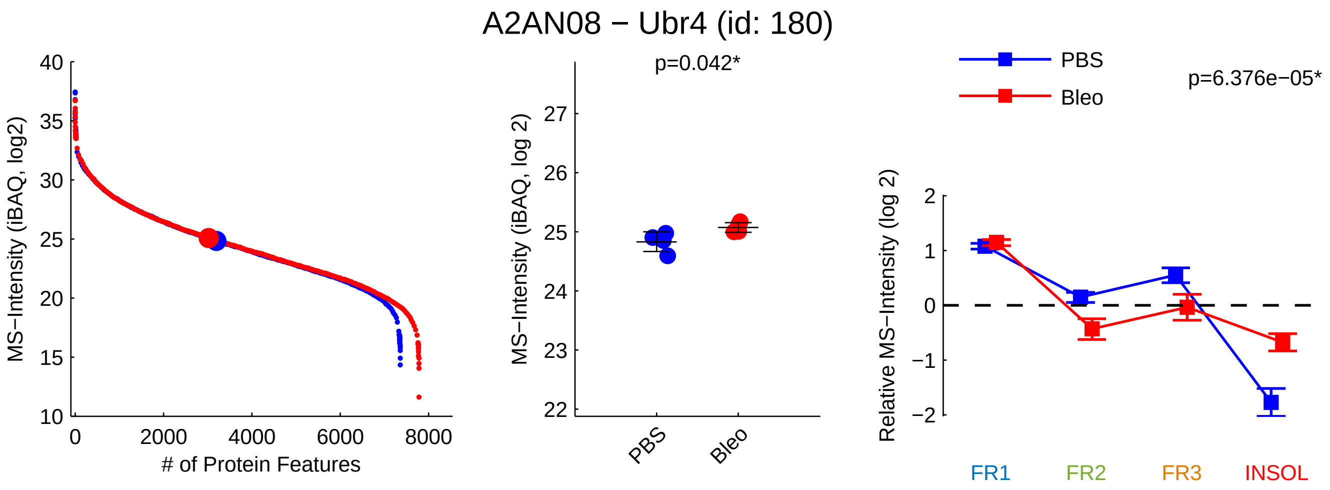
A2AN08 − Ubr4 (id: 180)
MS−Intensity (iBAQ, log2)
# of Protein Features
p=0.042*
MS−Intensity (iBAQ, log 2)
PBS
Bleo
p=6.376e−05*
Relative MS−Intensity (log 2)
FR1
FR2
FR3
INSOL

P15379 − Cd44 (id: 192)

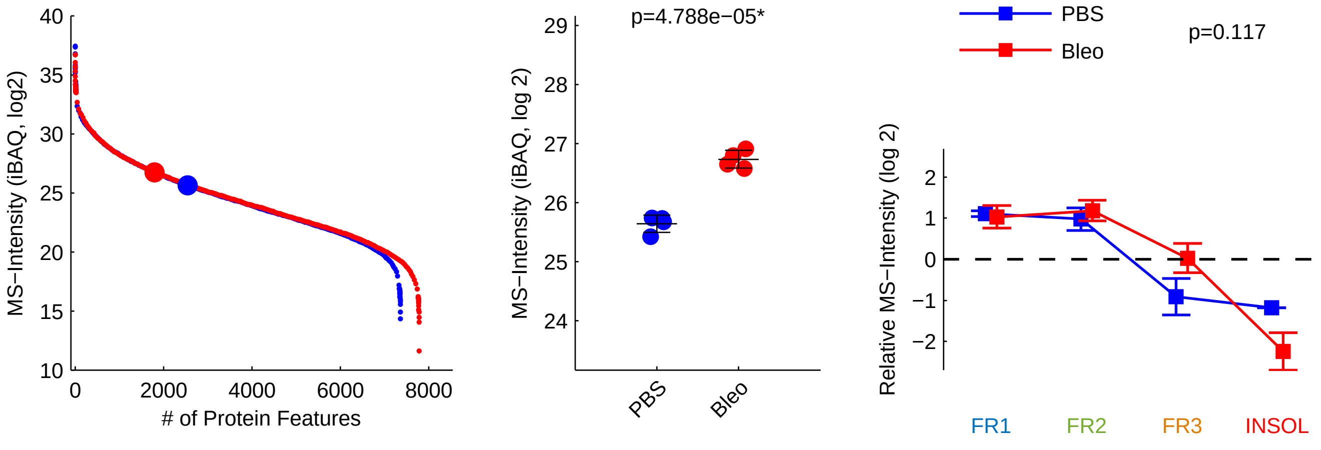

A2AQ07 − Tubb1 (id: 196)

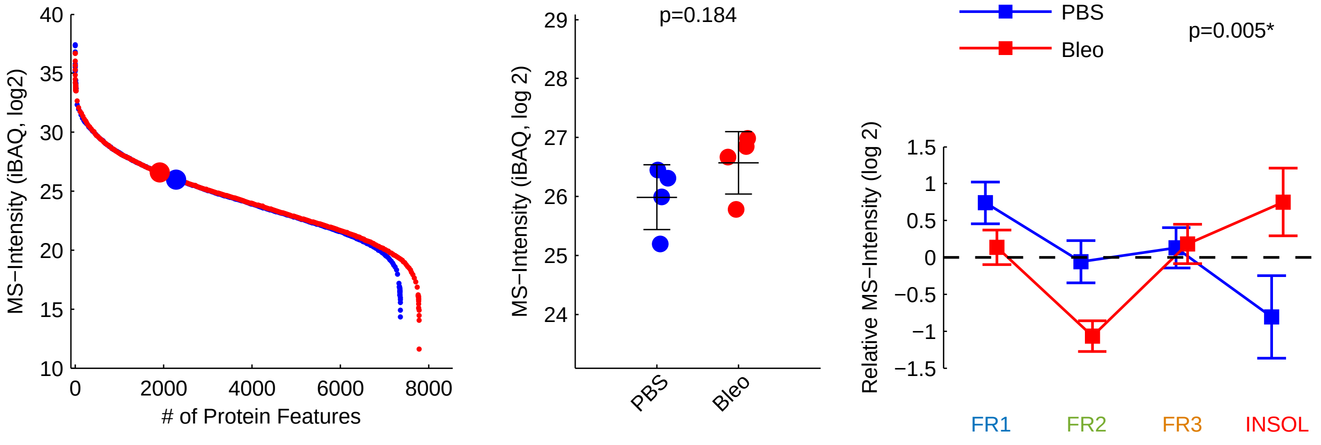

A2AQ53 − Fbn1 (id: 201)

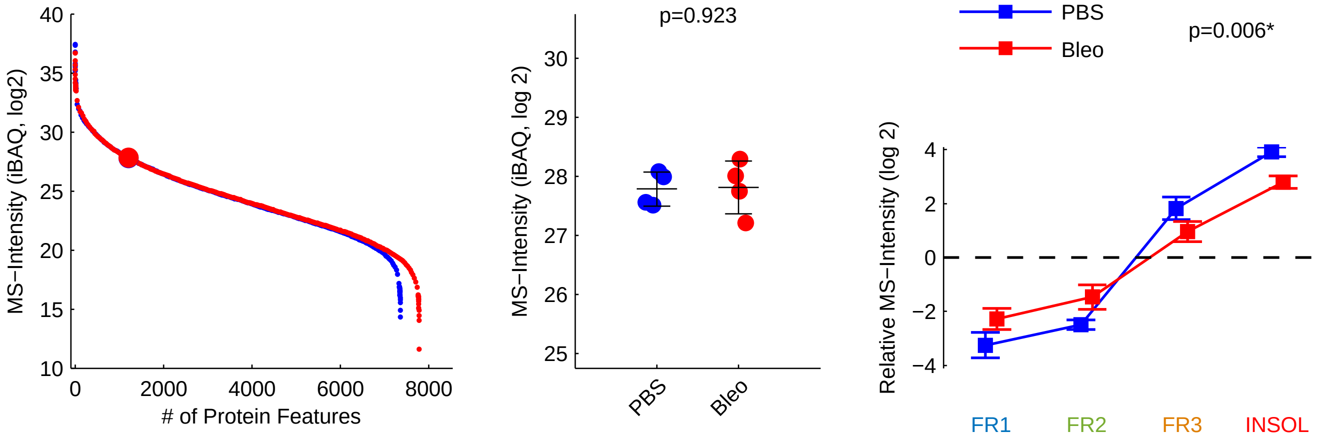

A2ARA8 − Itga8 (id: 219)

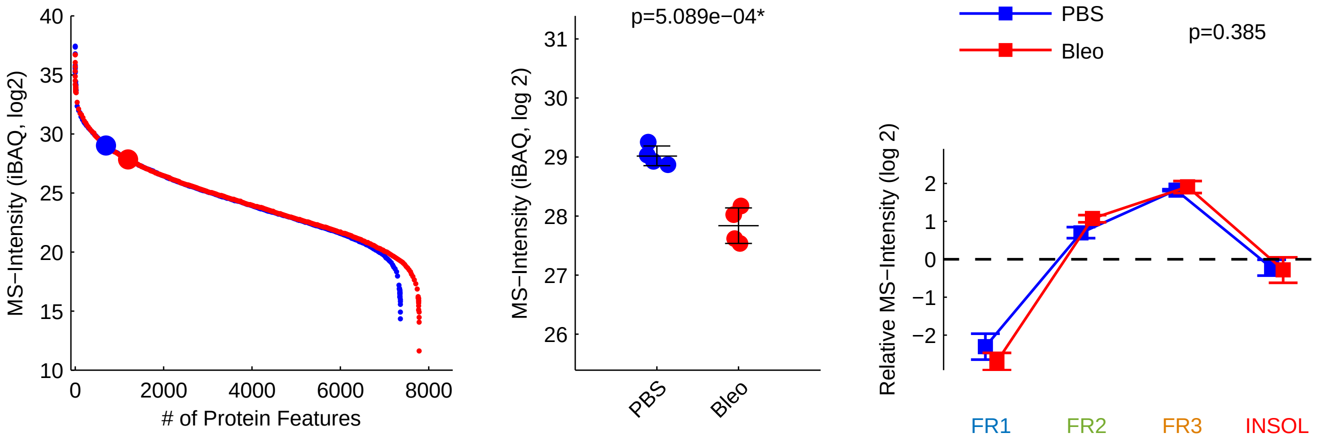

Q9QYR6 − Map1a (id: 222)

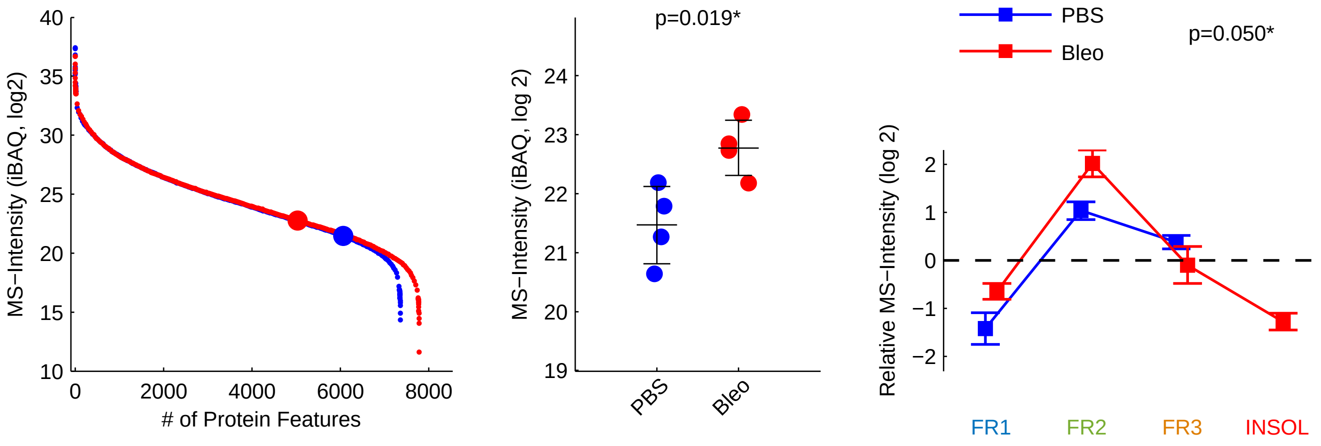

Q99JX3 − Gorasp2 (id: 236)

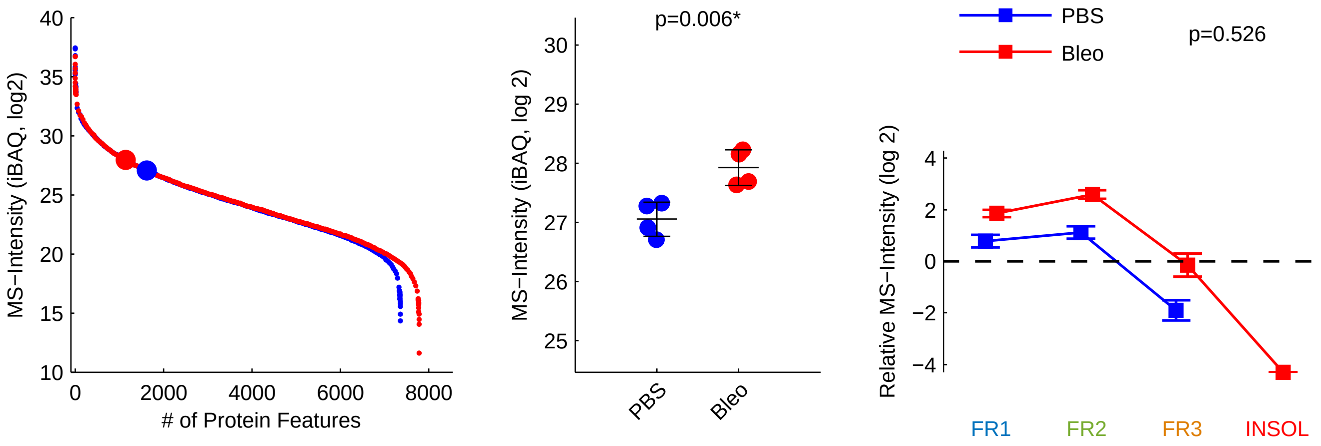

Q64012−2 − Raly (id: 242)

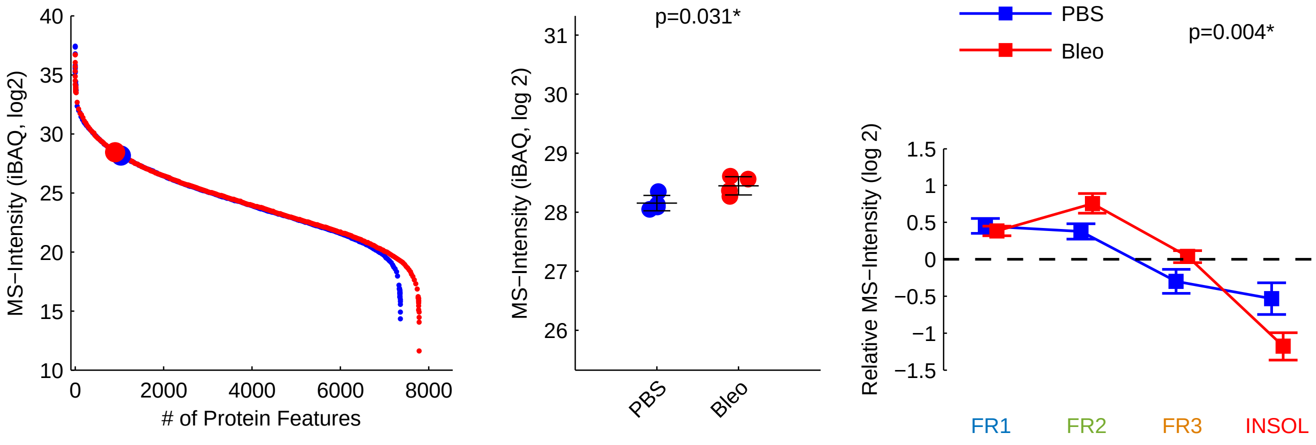

A2AUK7 − Epb4.1l1 (id: 248)

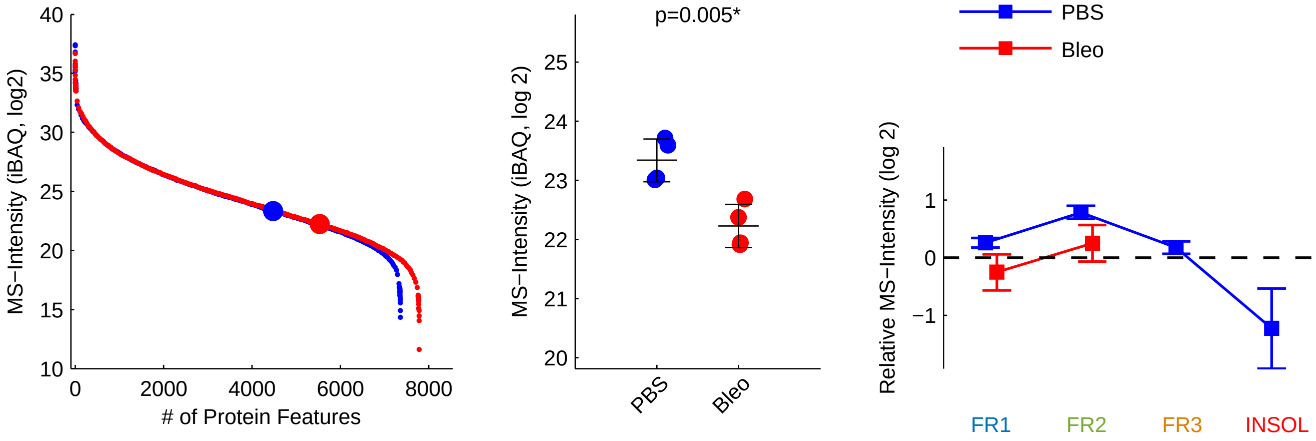

Q3UHC7 − Dab2ip (id: 253)

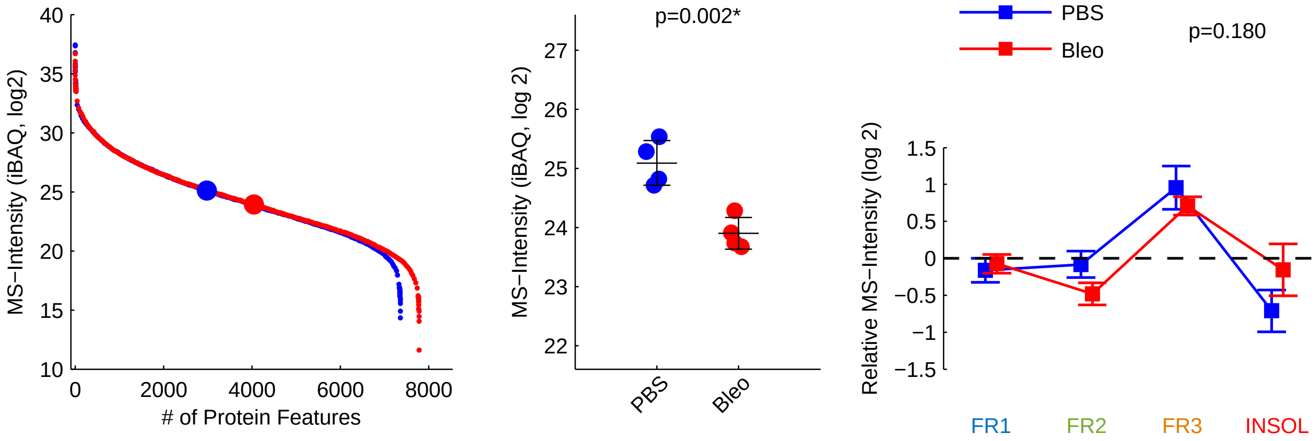

A2AVA0 − Svep1 (id: 256)

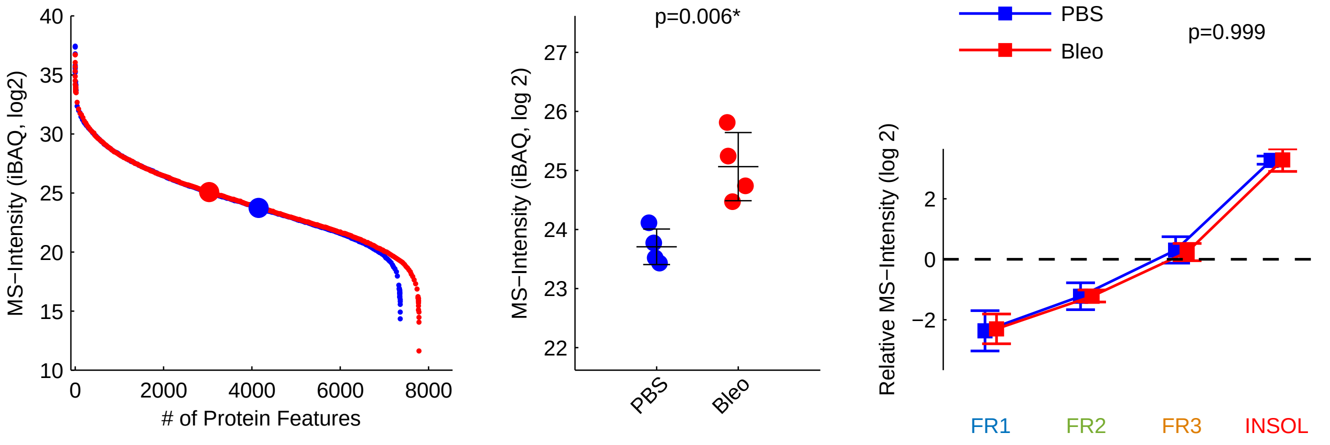

Q99PL5 − Rrbp1 (id: 258)

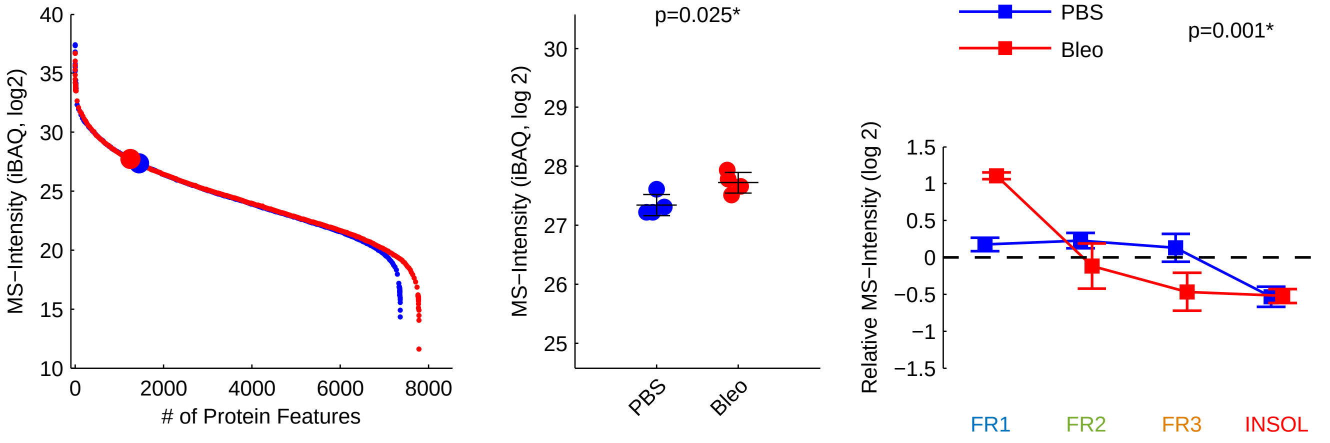

A2AWT5 − Ubtf (id: 267)

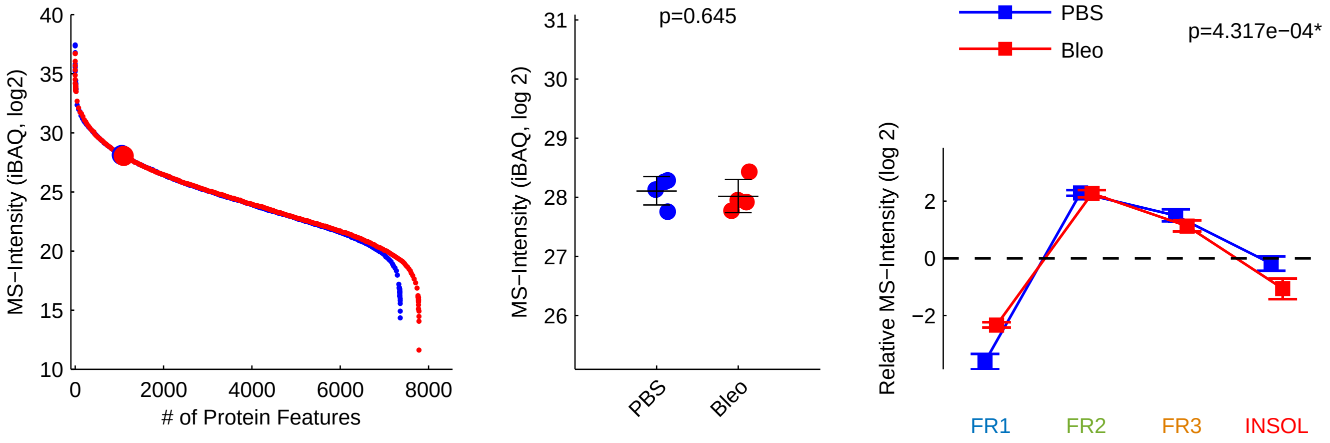

A2BDX0 − Adnp (id: 270)

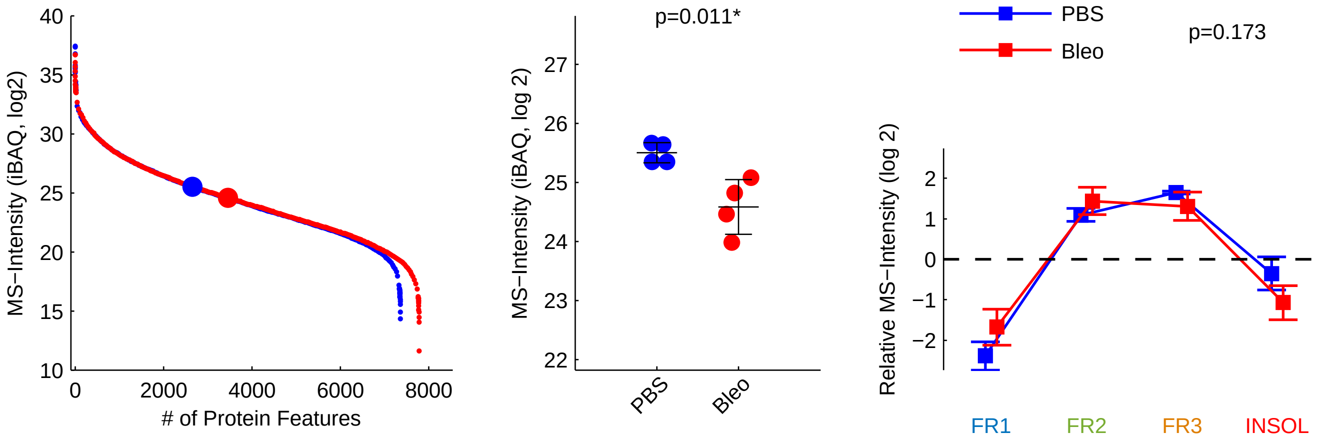

P70175 − Dlg3 (id: 276)

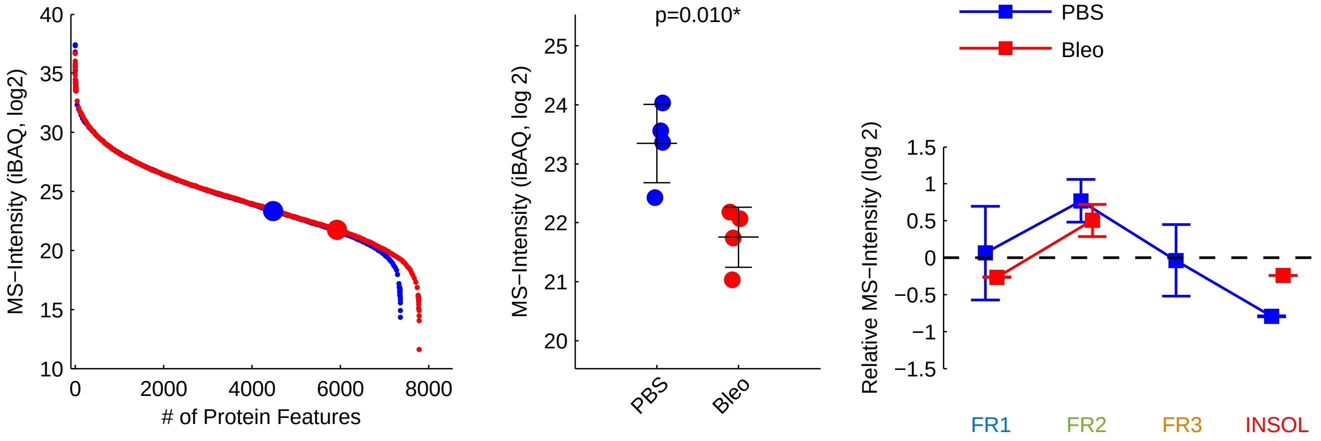

P35283 − Rab12 (id: 284)

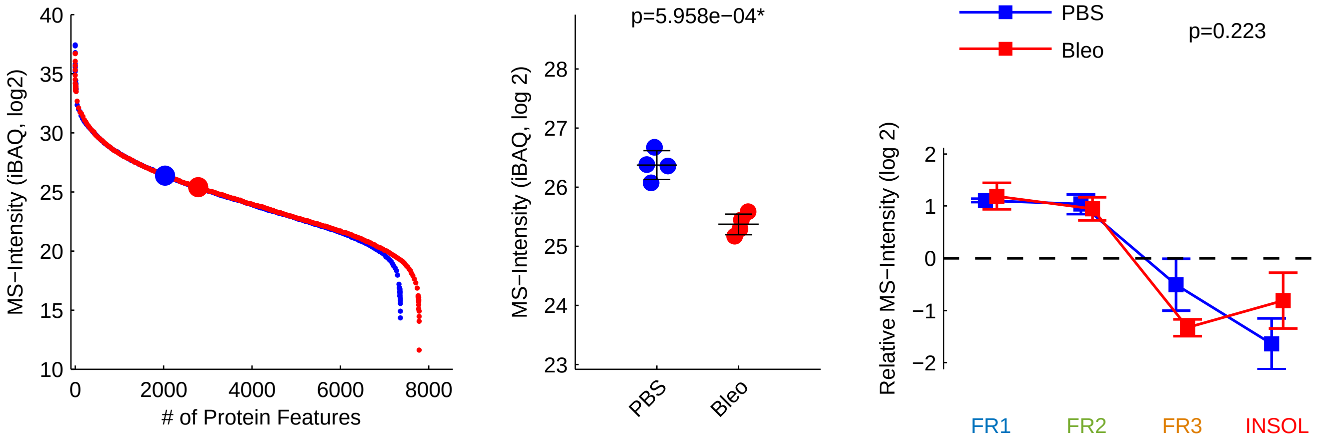

Q9D898 − Arpc5l (id: 307)

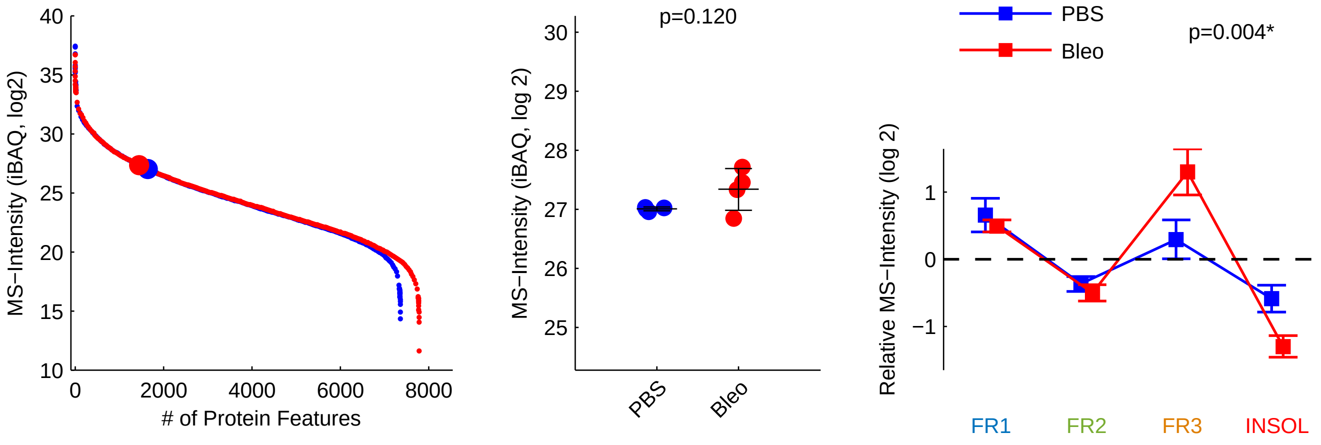

A3KGU7 − Sptan1 (id: 310)

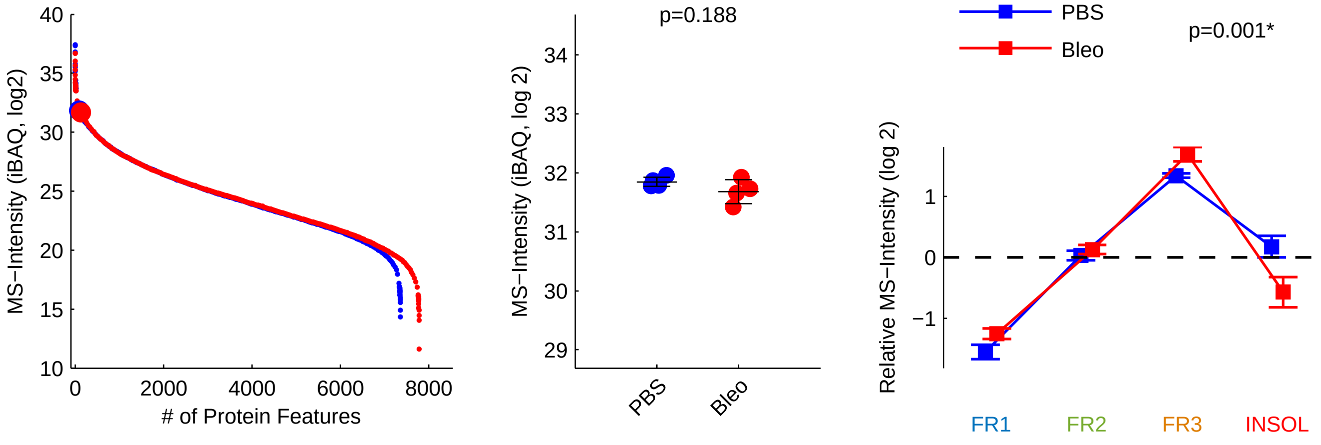

A3KMP2 − Ttc38 (id: 313)

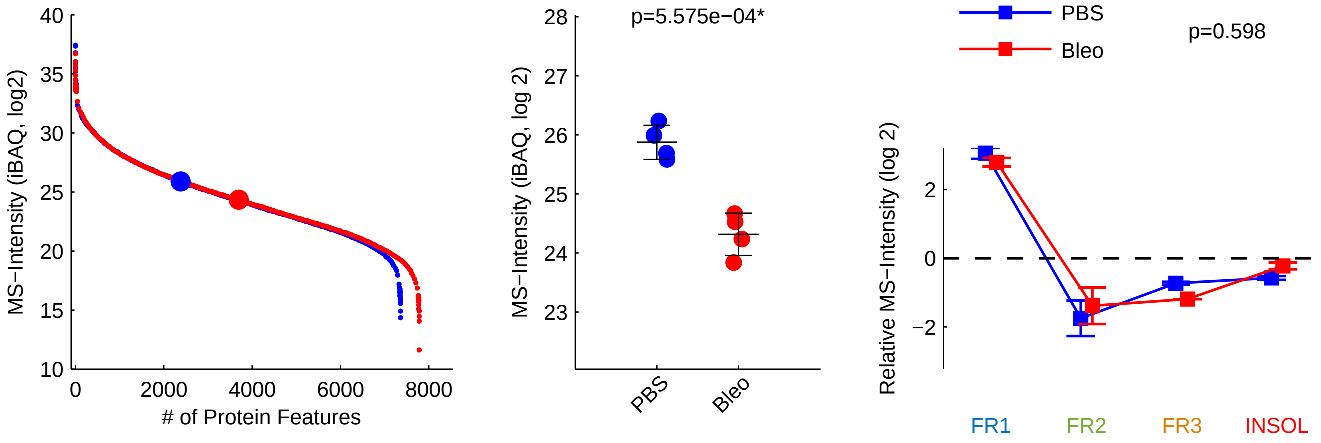

Q99NH2 − Pard3 (id: 317)

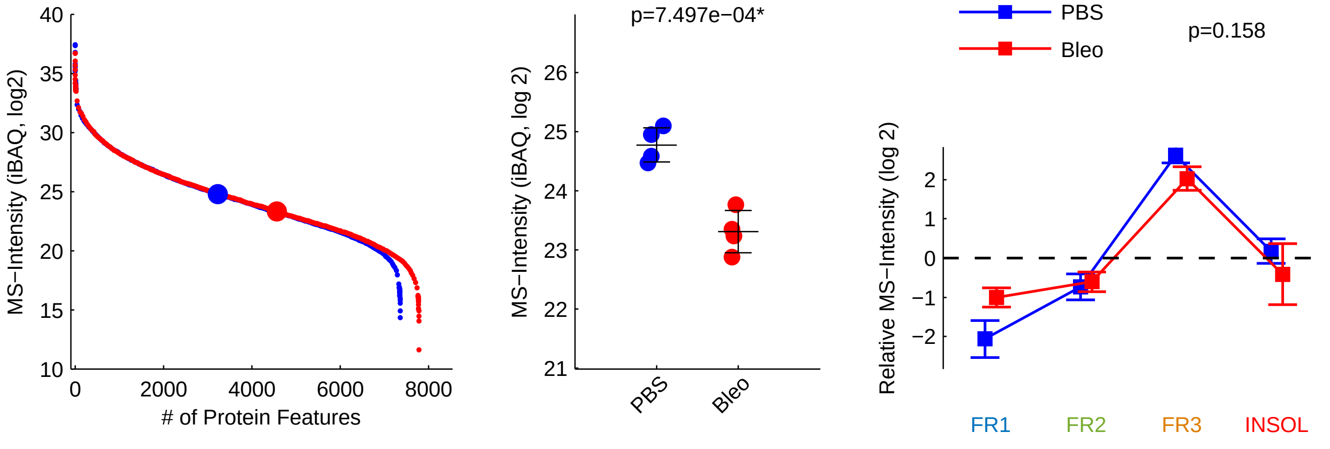

A6H5Z3 − Exoc6b (id: 322)

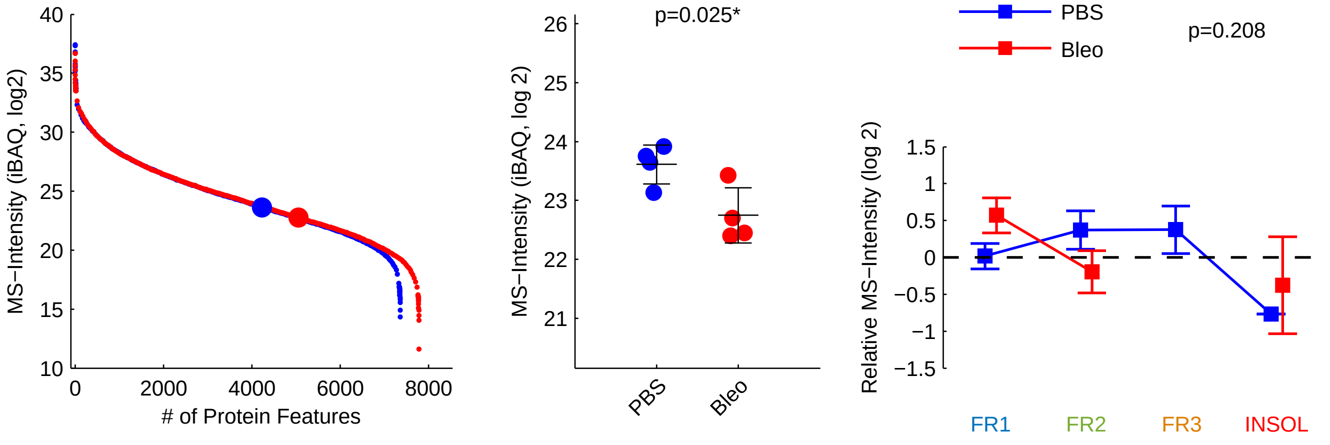

A6H6E2 − Mmrn2 (id: 329)

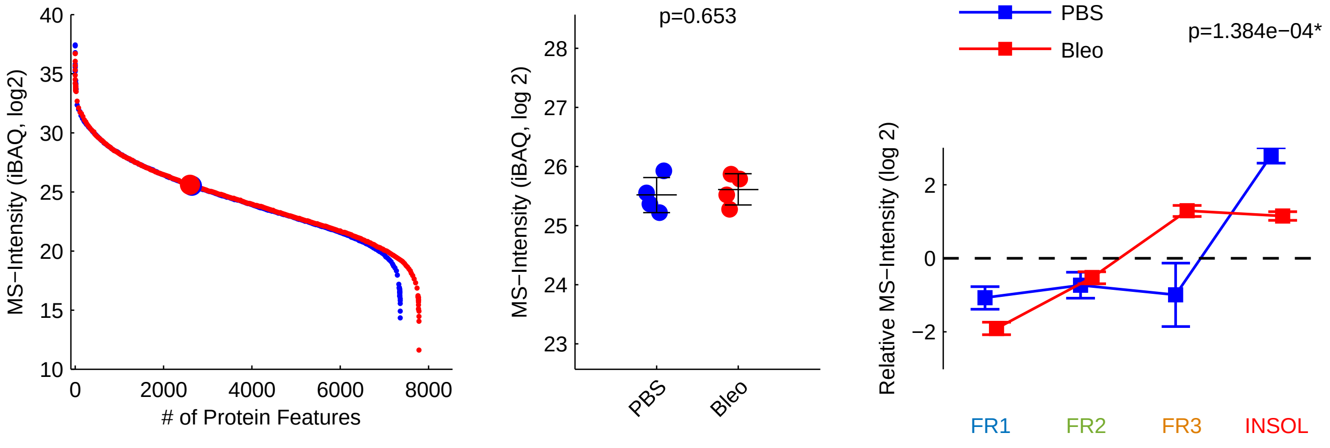

A6X8Z5 − Arhgap31 (id: 338)

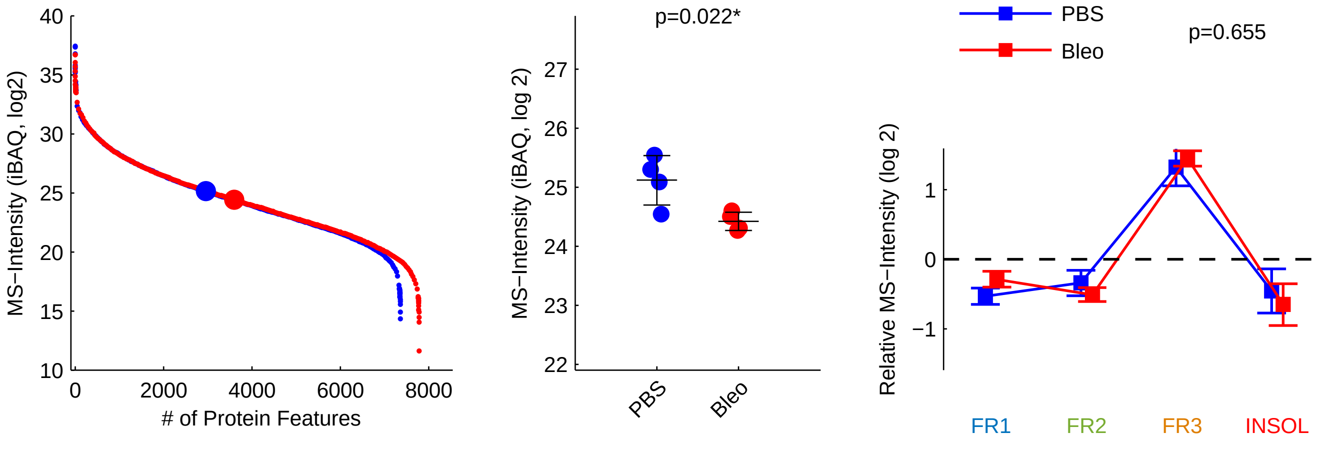

A6X919 − Dpy19l1 (id: 339)

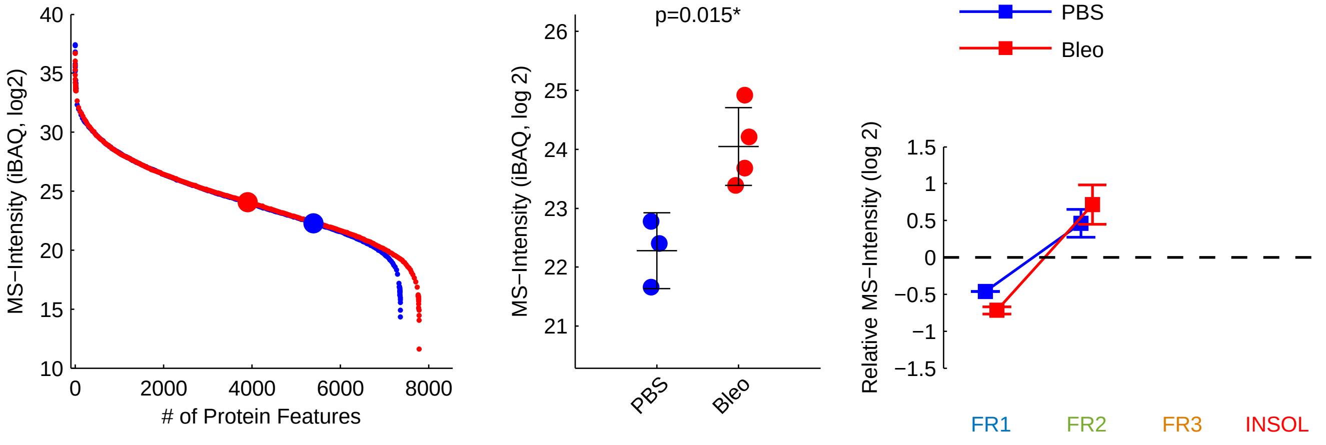

A6X935 − Itih4 (id: 340)

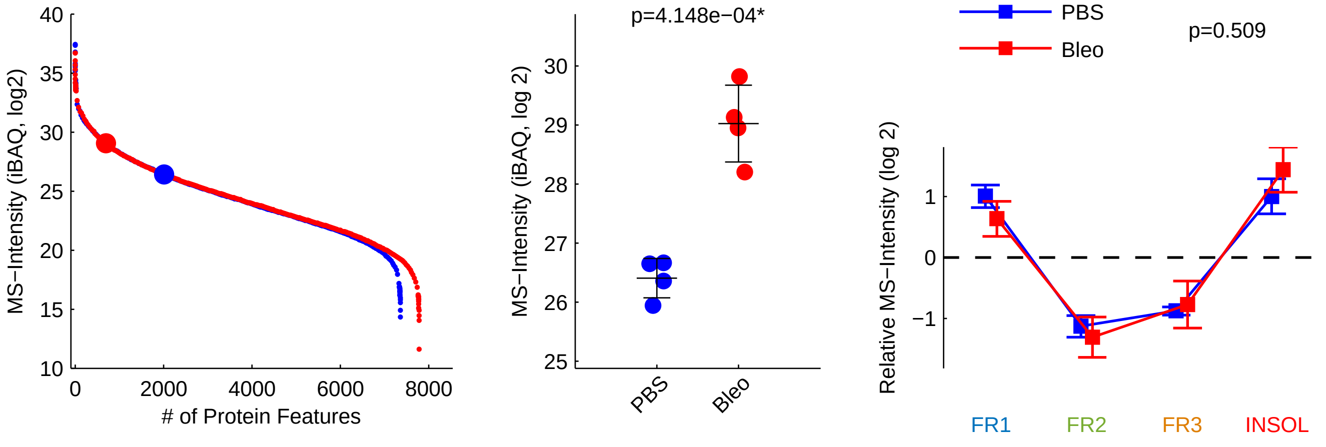

A6X940 − Fermt2 (id: 341)

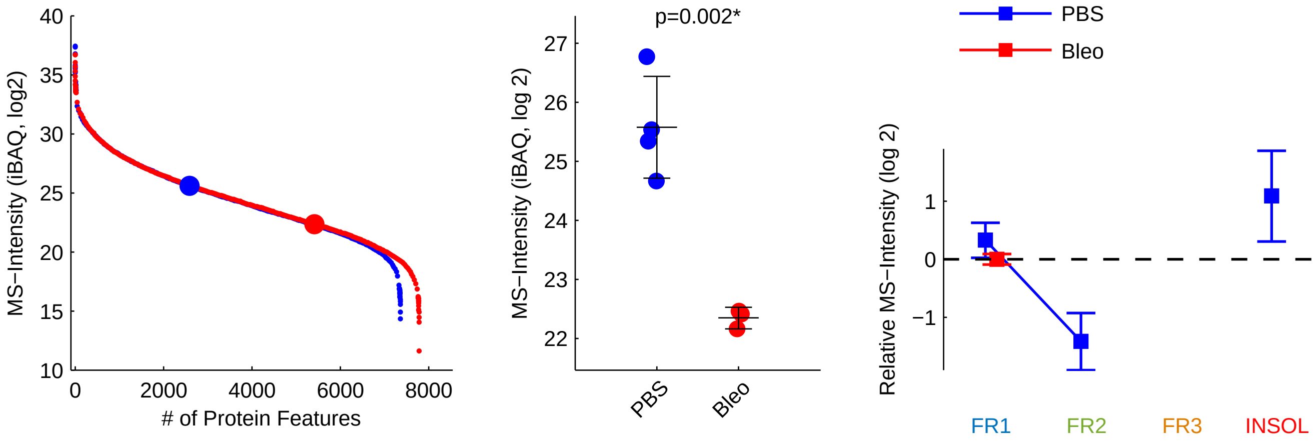

Q8CFI5 − Pars2 (id: 351)

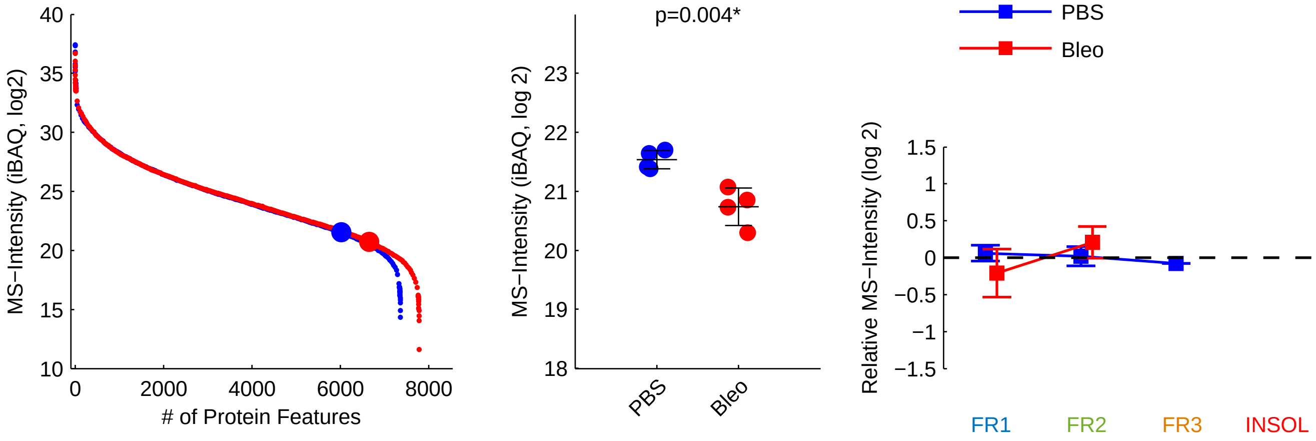

P63044 − Vamp2 (id: 357)

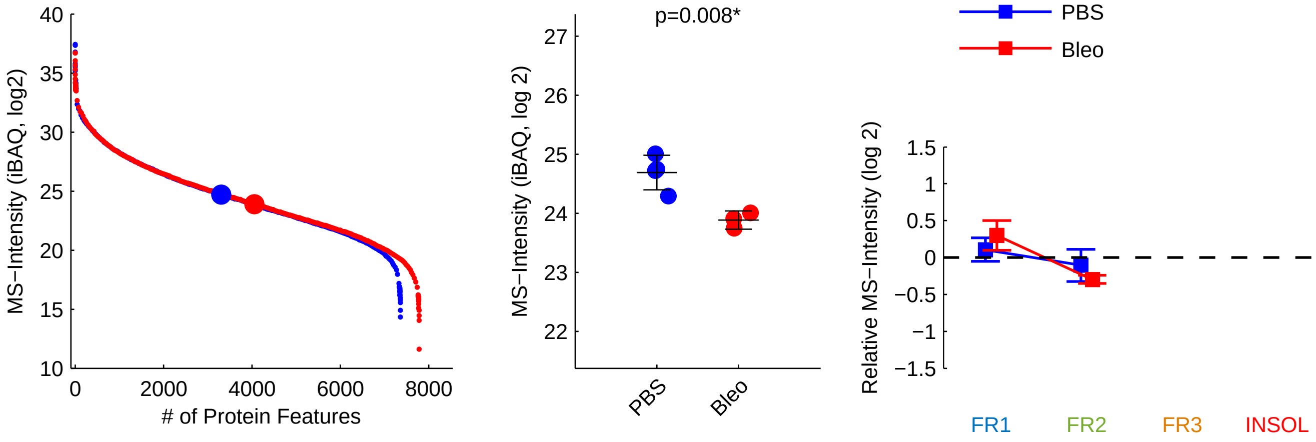

P47740 − Aldh3a2 (id: 390)

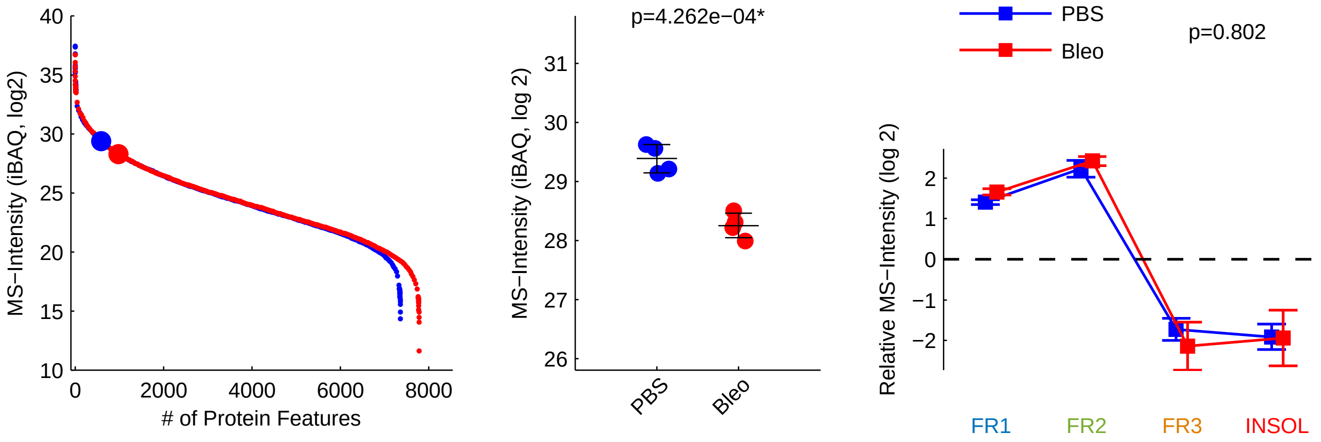

B1ATI9 − Gas7 (id: 391)

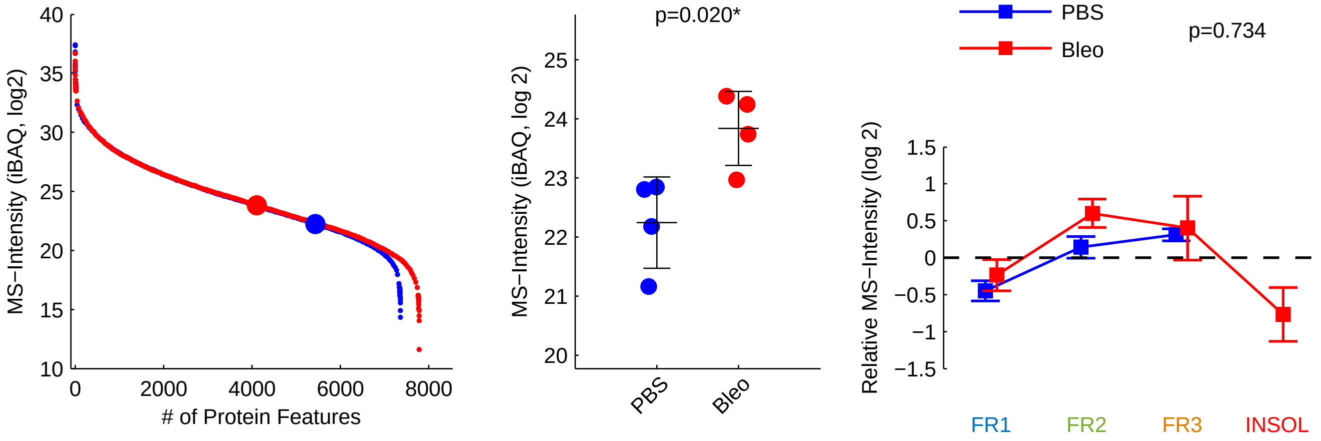

Q64518 − Atp2a3 (id: 393)

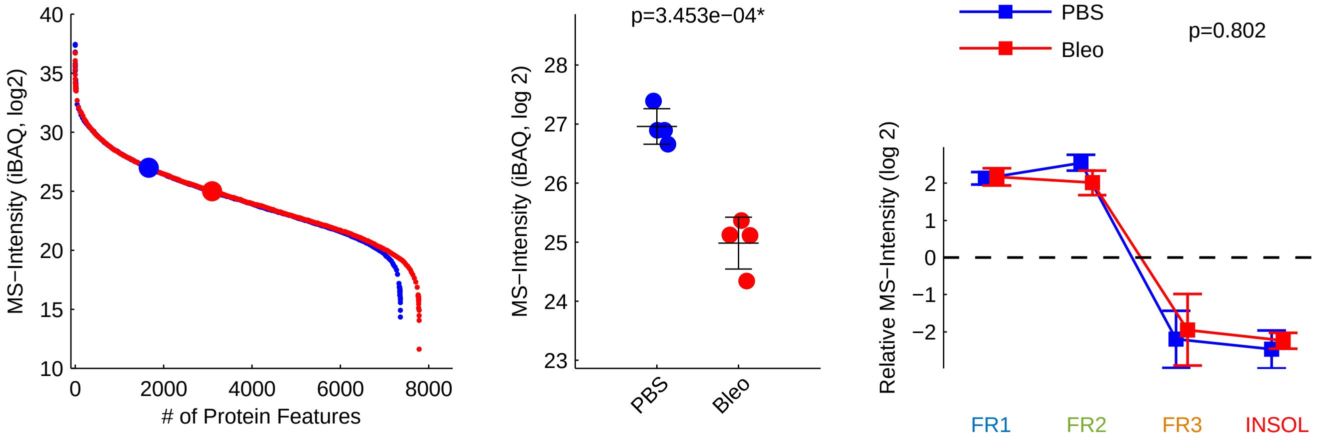

Q99LI8 − Hgs (id: 395)

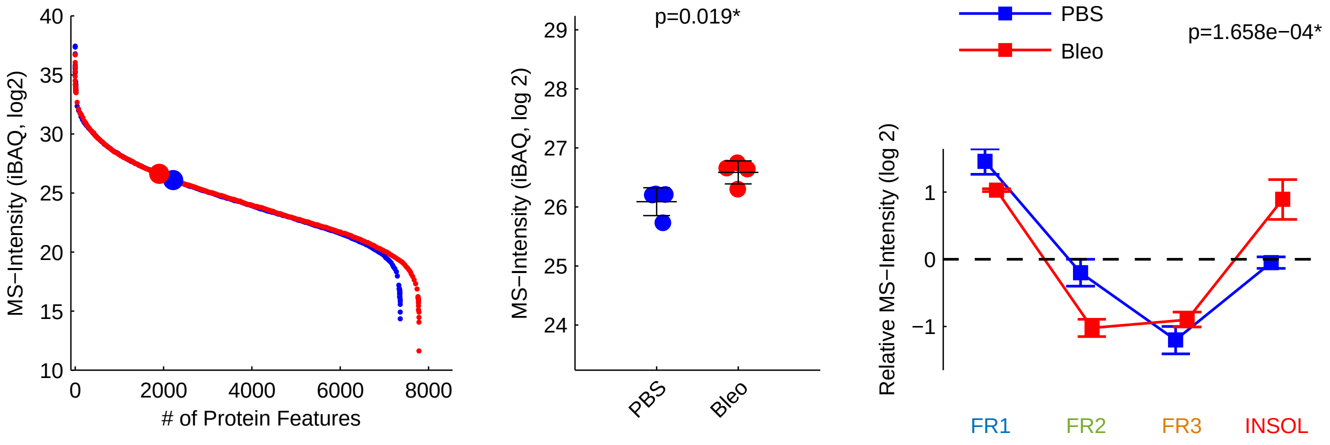

Q9CWP6 − Mospd2 (id: 396)

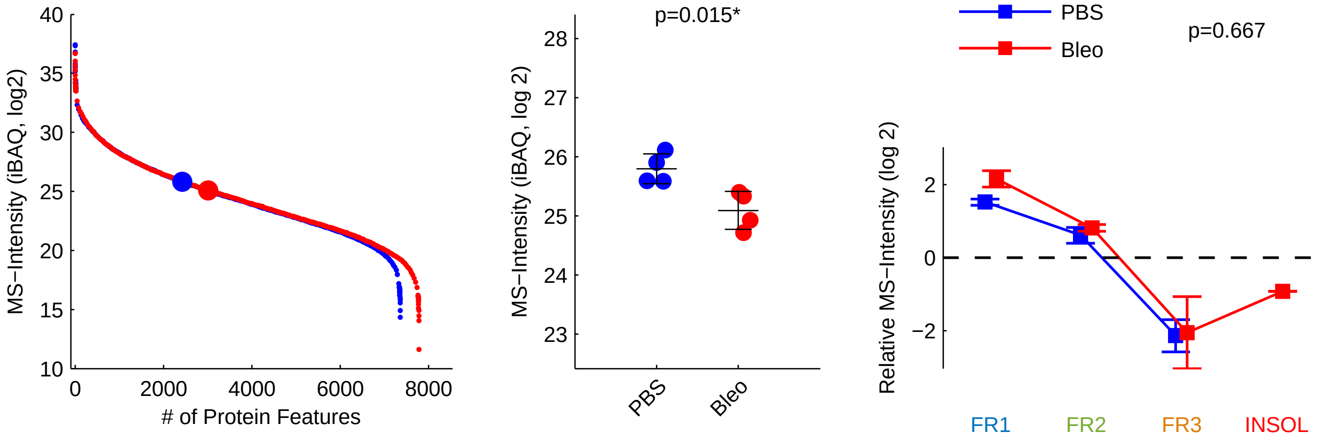

B1AVD1 − Xpnpep2 (id: 407)

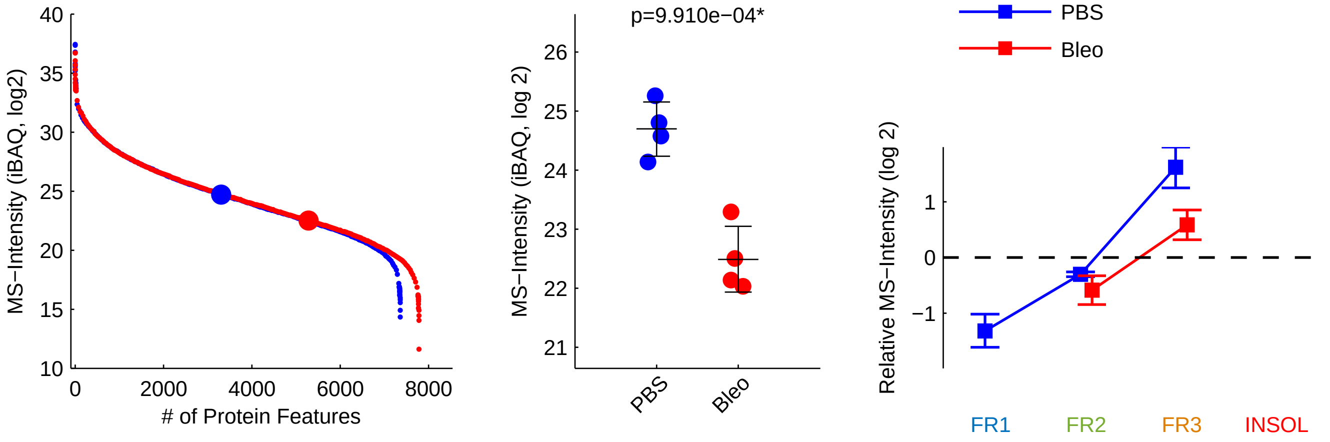

Q8C0P5 − Coro2a (id: 408)

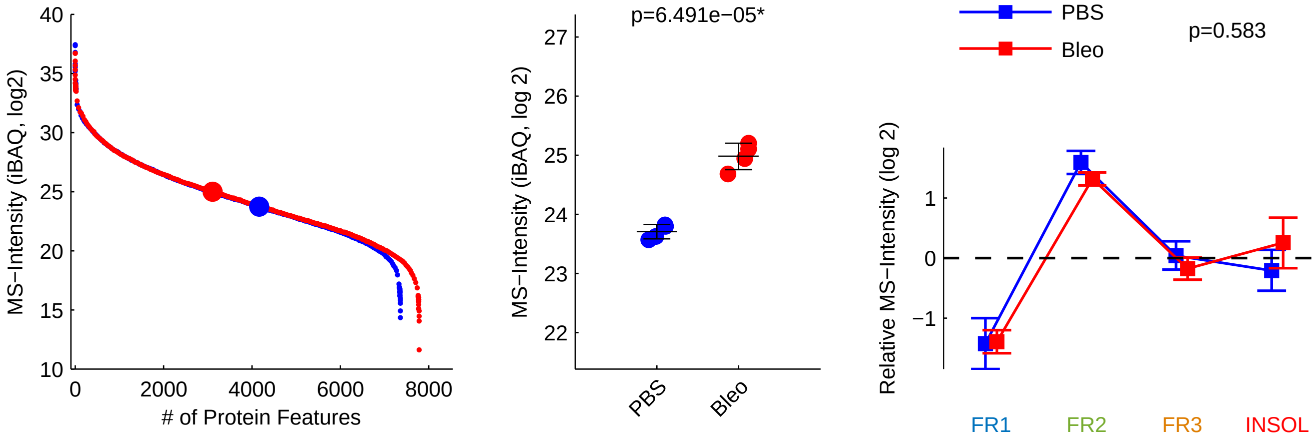

Q8R054 − Srpx2 (id: 410)

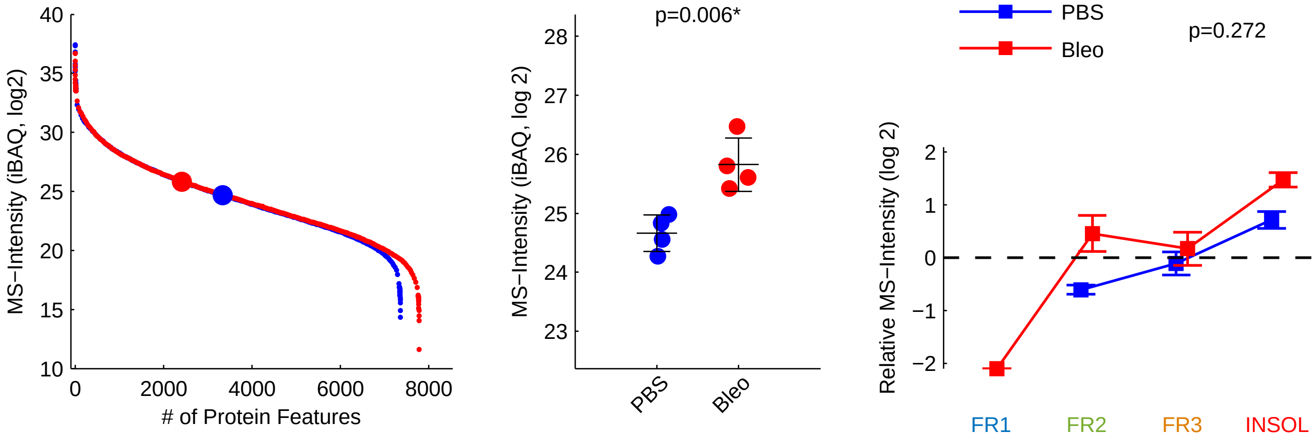

Q02858 − Tek (id: 419)

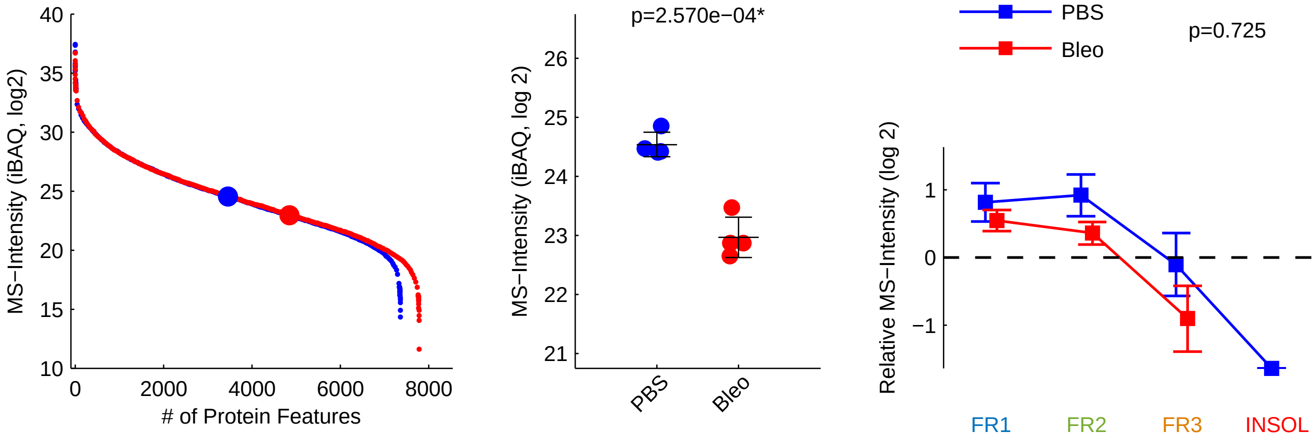

Q9DC11 − Plxdc2 (id: 425)

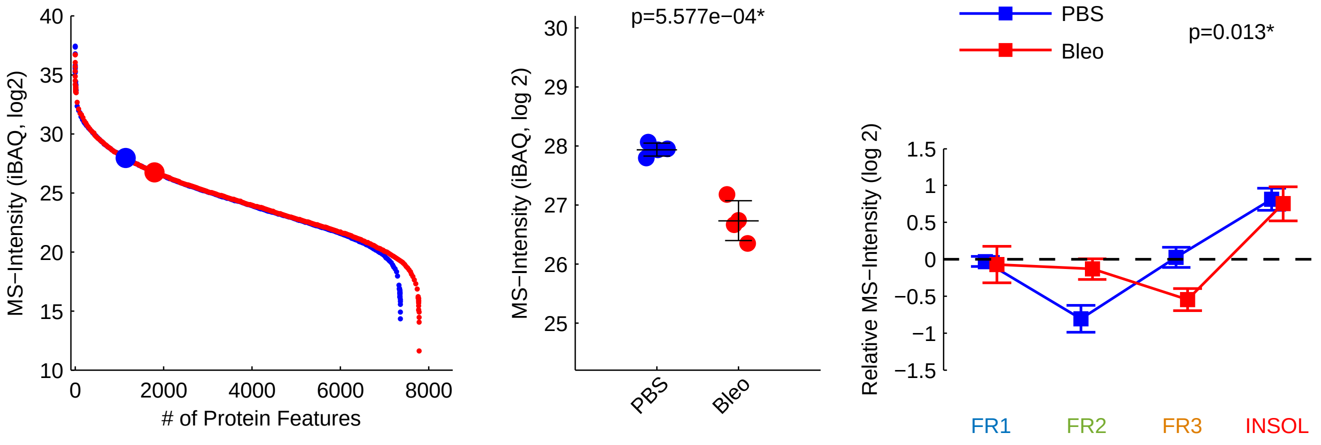

Q8C5N3 − Cwc22 (id: 429)

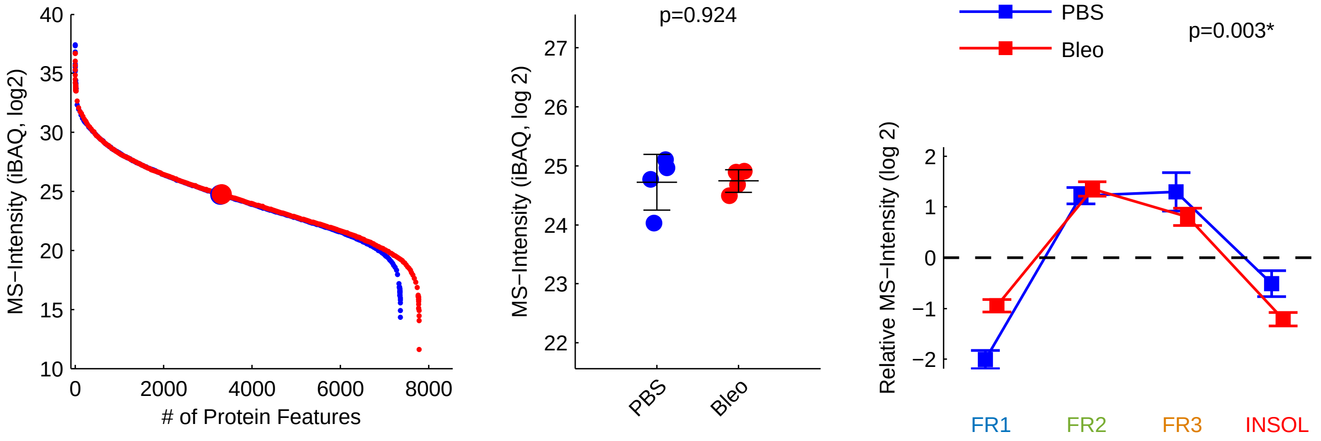

B1AZI6 − Thoc2 (id: 435)

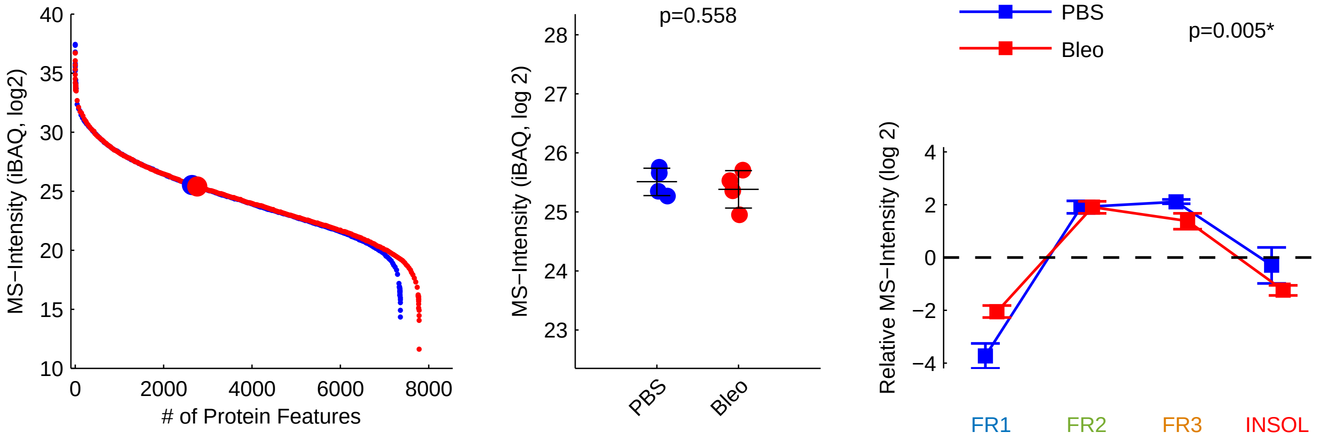

B1B1A8 − Mylk (id: 437)

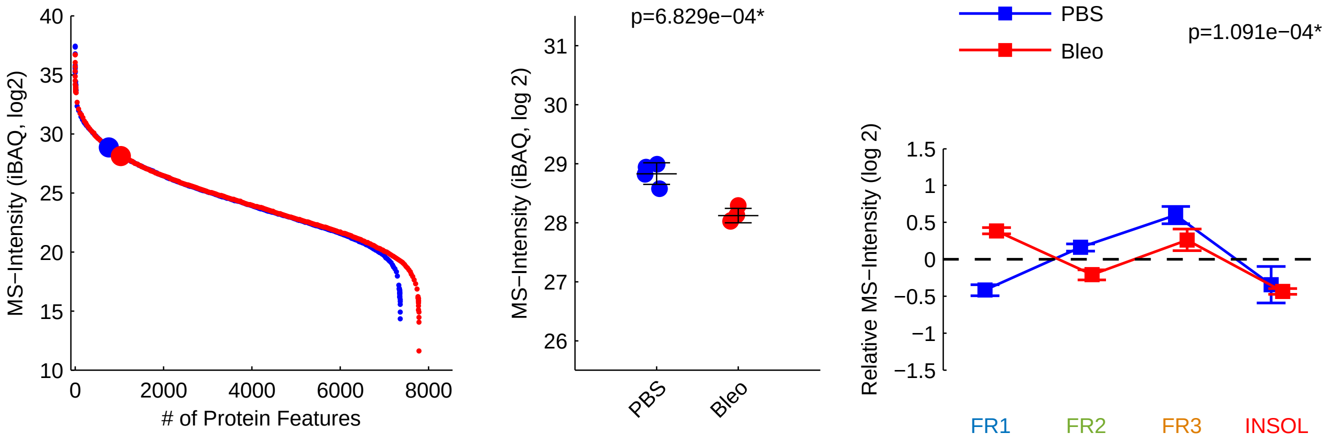

Q9D067 − Mdm1 (id: 449)

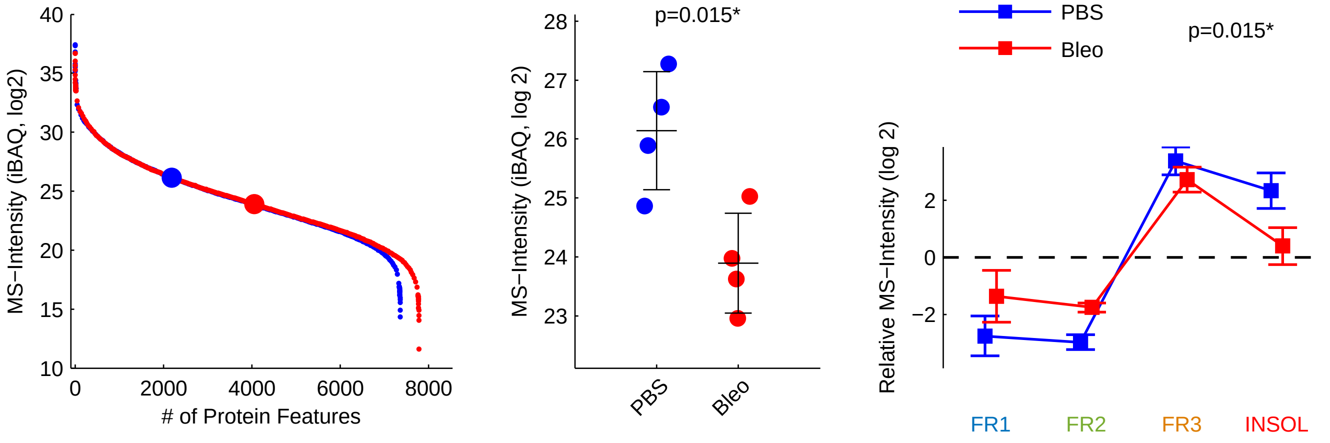

G3UVV6 − Mmrn1 (id: 450)

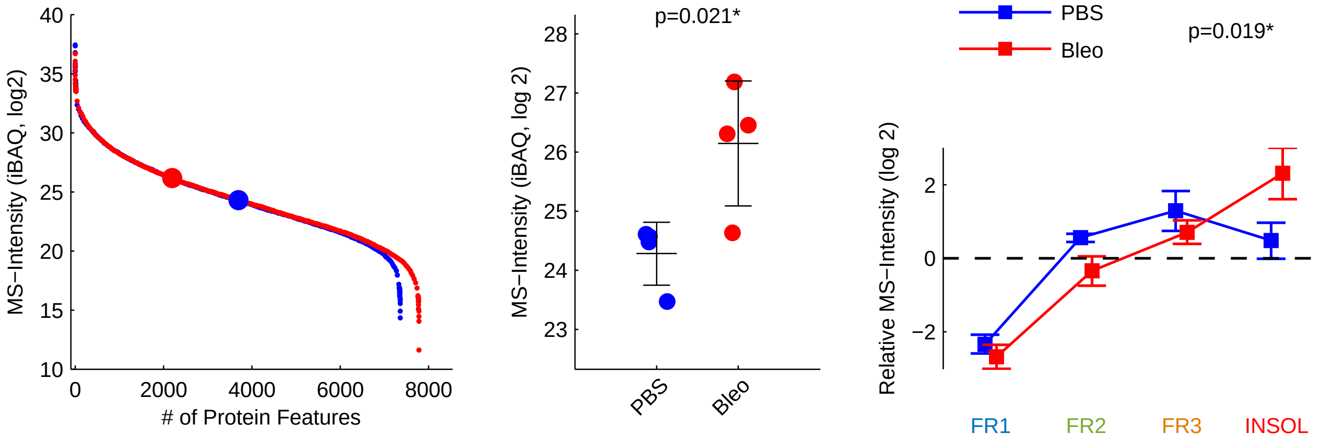

Q689Z5 − Sbno1 (id: 462)

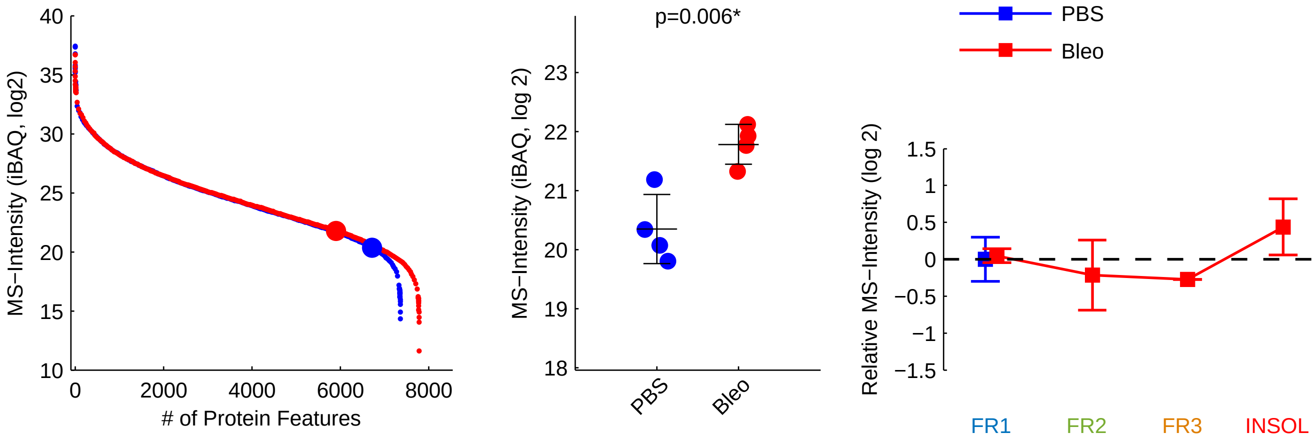

Q8CHG3 − Gcc2 (id: 469)

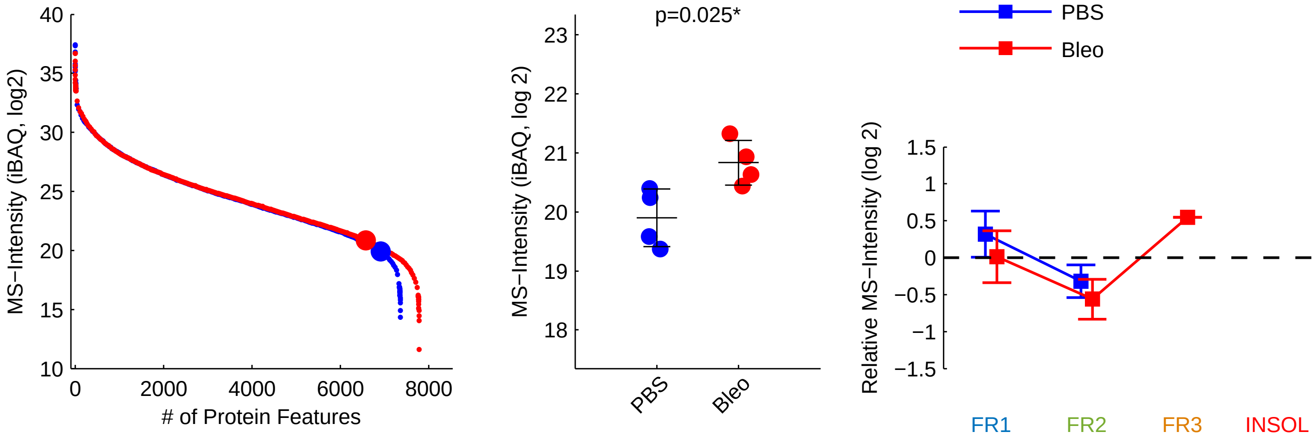

Q8C5L7 − Rbm34 (id: 475)

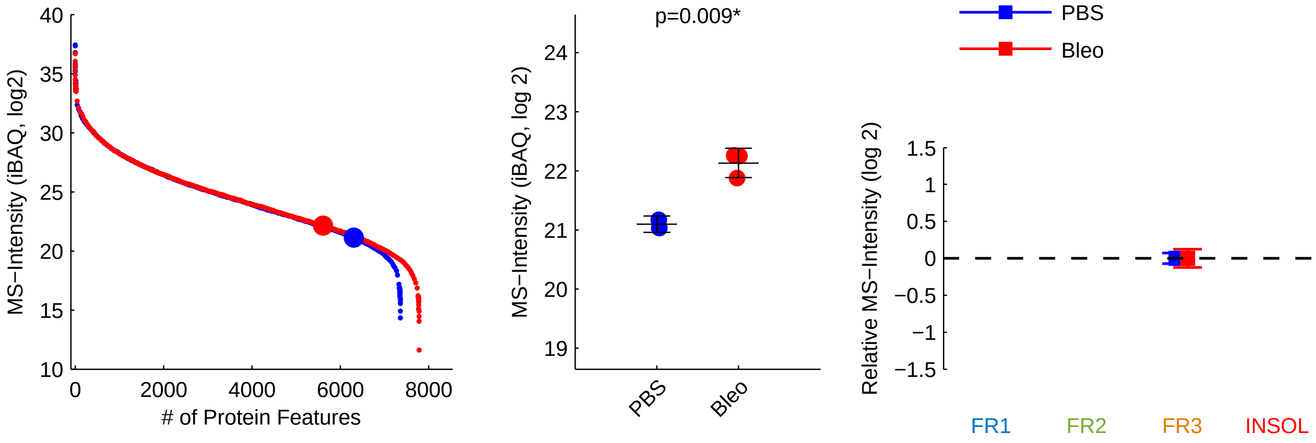

B2RUP2 − Unc13d (id: 476)

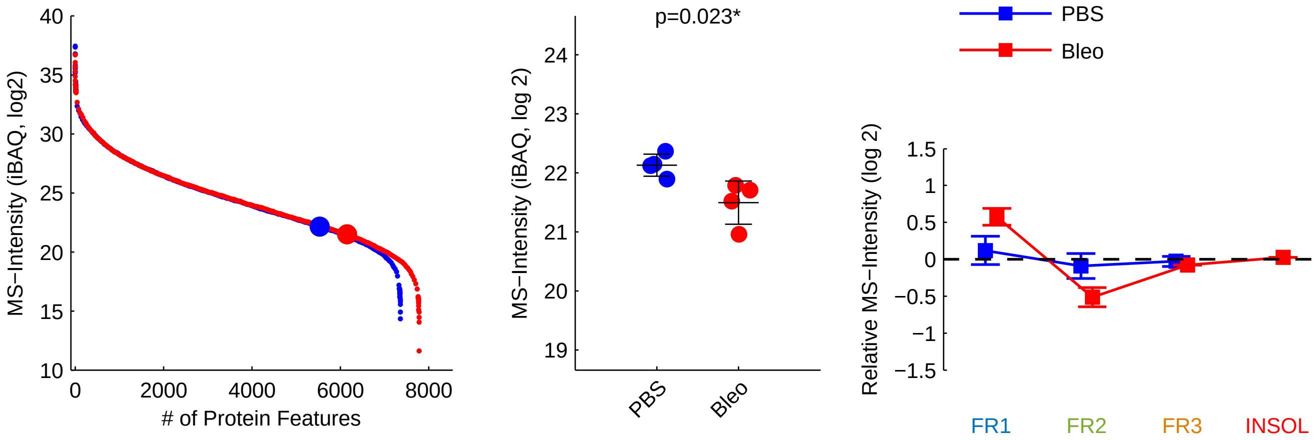

B2RX12 − Abcc3 (id: 482)

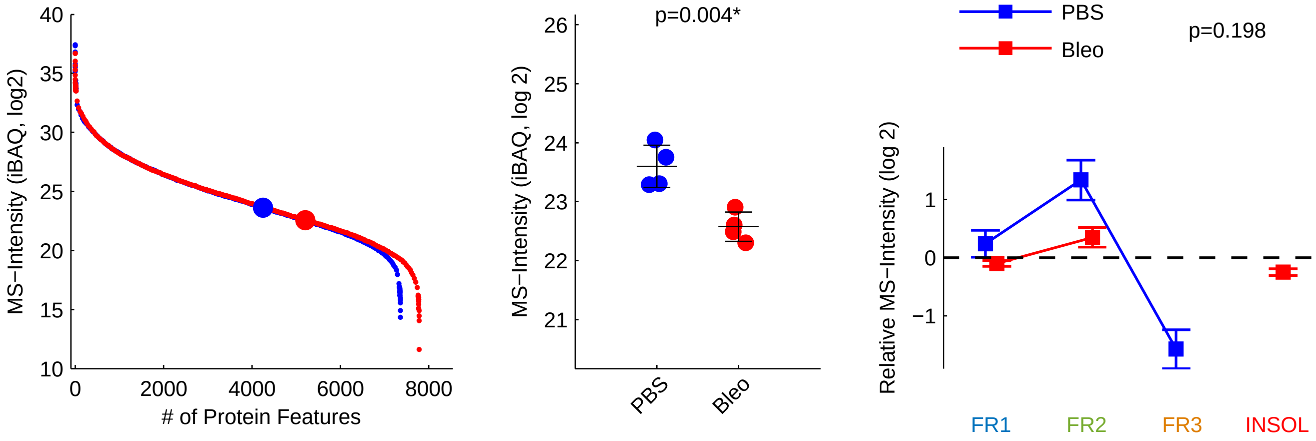

B2RXC2 − Itpkb (id: 485)

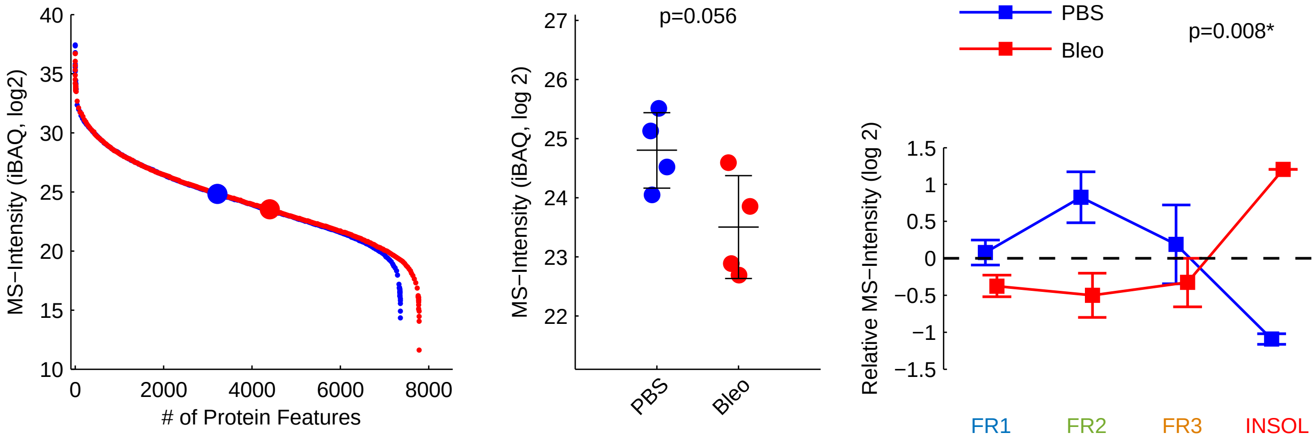

B2RXS4 − Plxnb2 (id: 489)

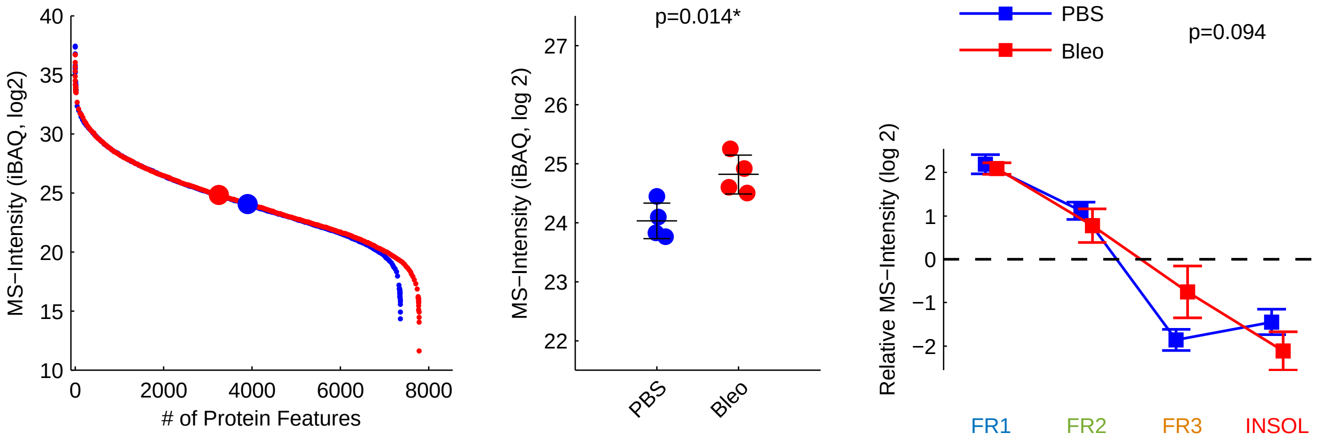

B7FAU9 − Flna (id: 498)

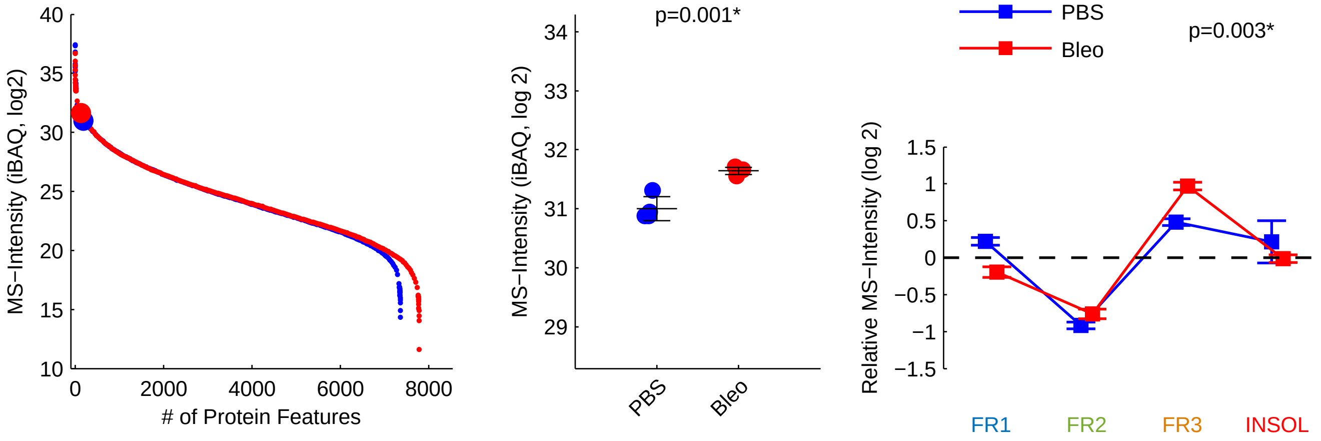

B7ZC22 − Pkig (id: 499)

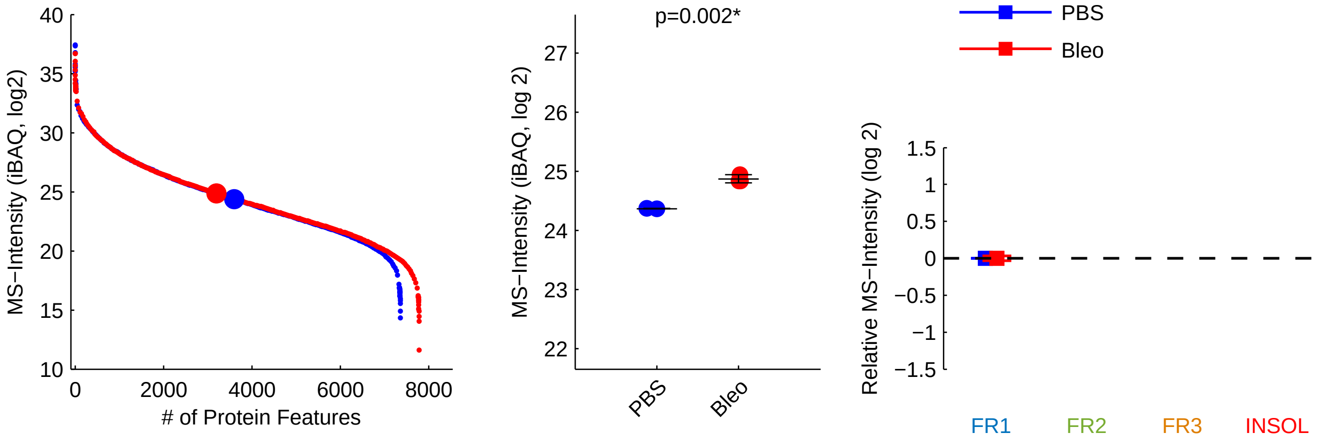

B7ZNH7 − Col14a1 (id: 515)

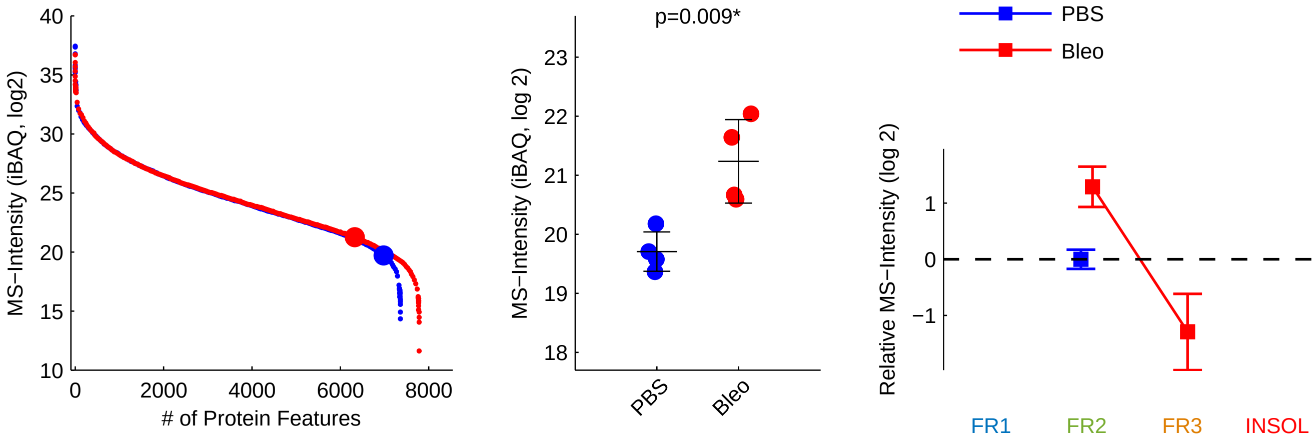

Q80TH2 − Erbb2ip (id: 516)

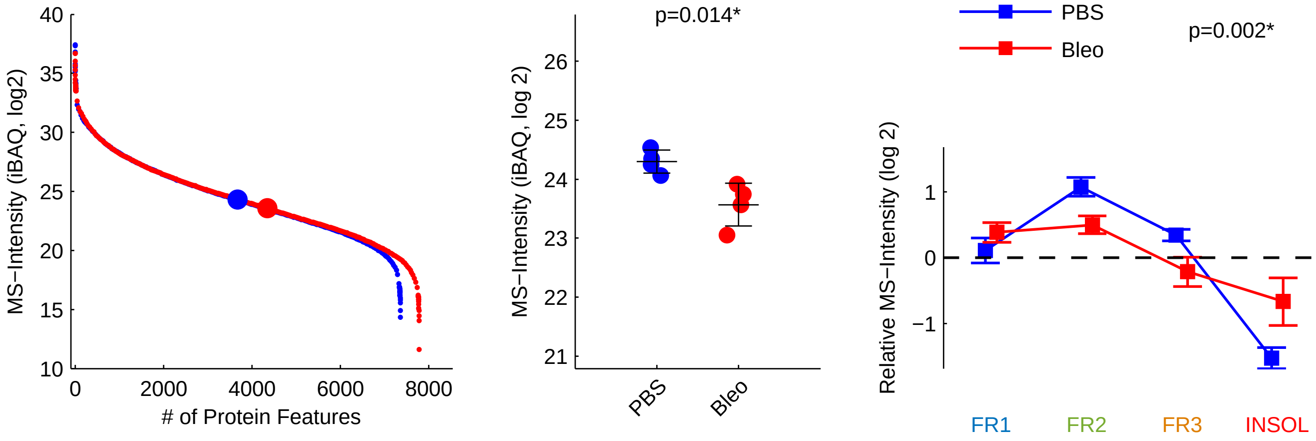

B8JJN0 − Gm20547 (id: 524)

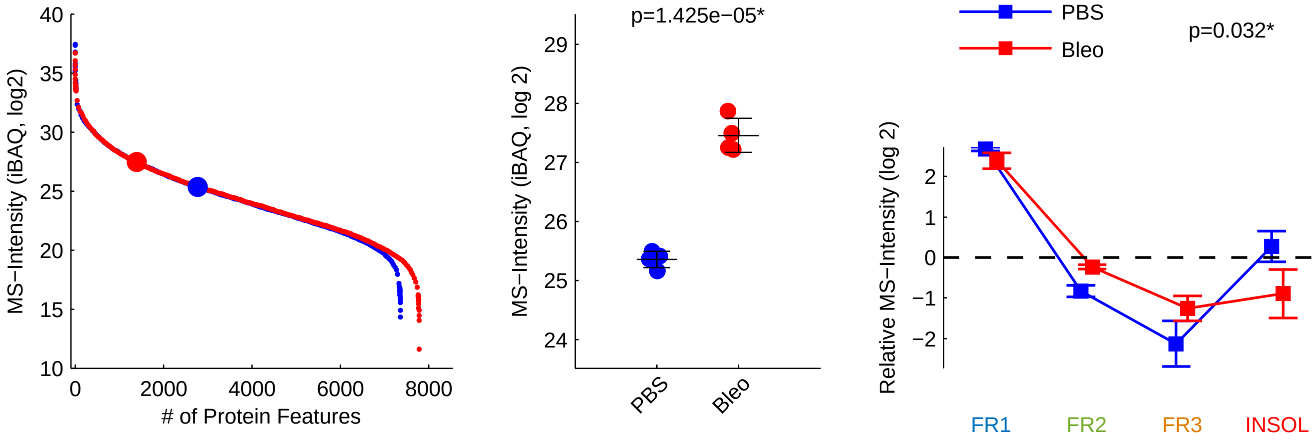

B9EHJ3 − Tjp1 (id: 532)

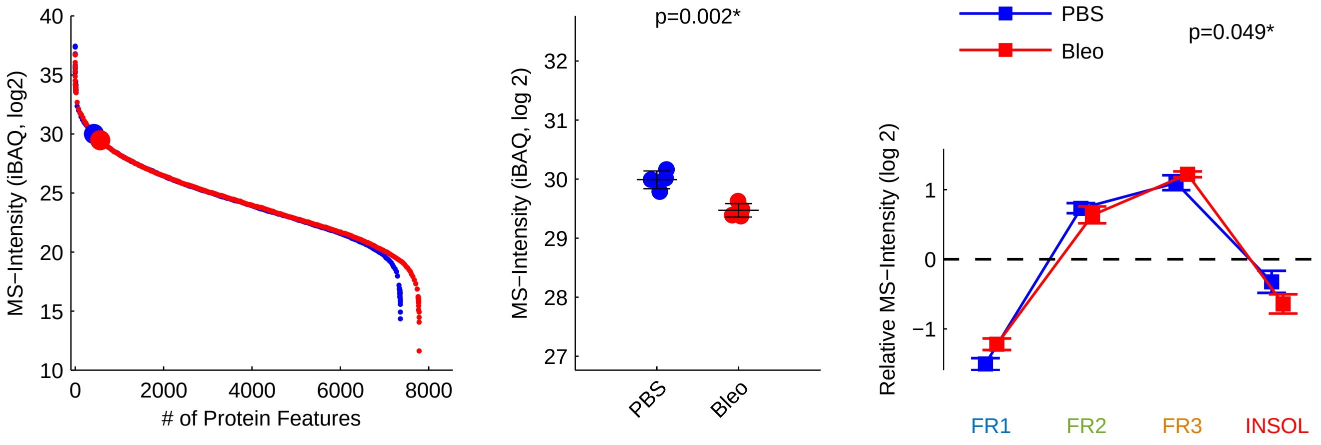

Q80TL7−2 − Mon2 (id: 546)

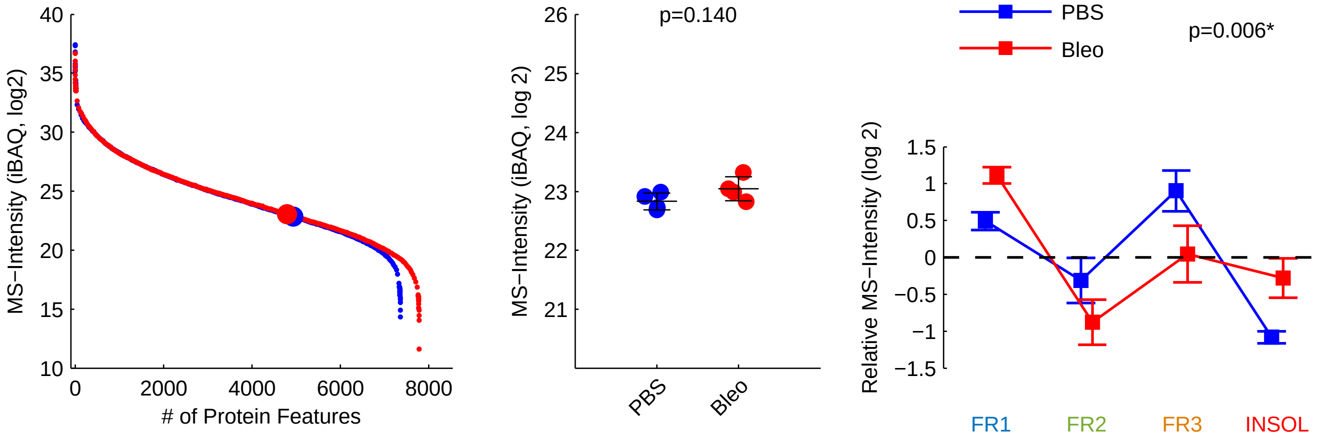

C5H3H7 − Ager (id: 549)

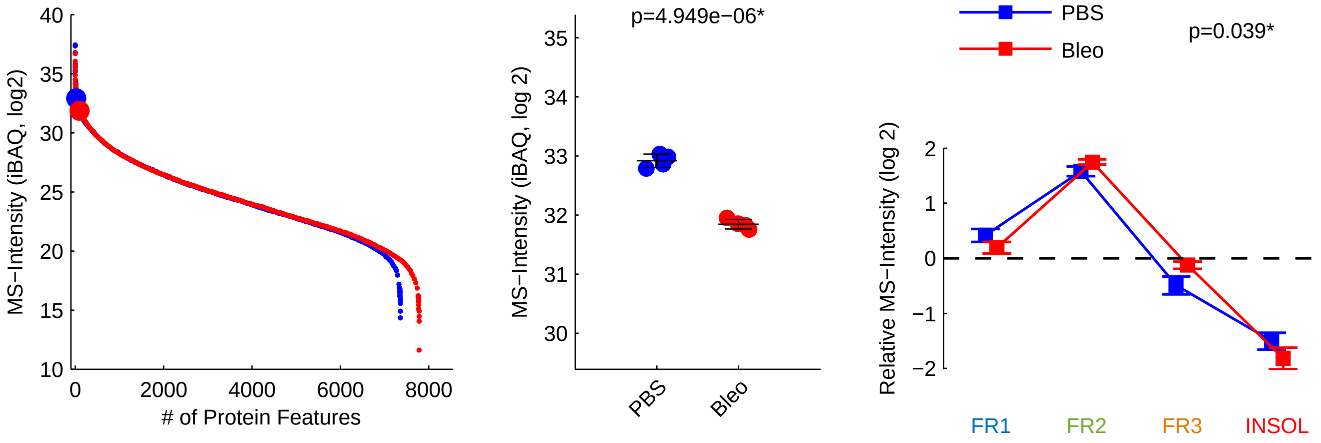

P19001 − Krt19 (id: 588)

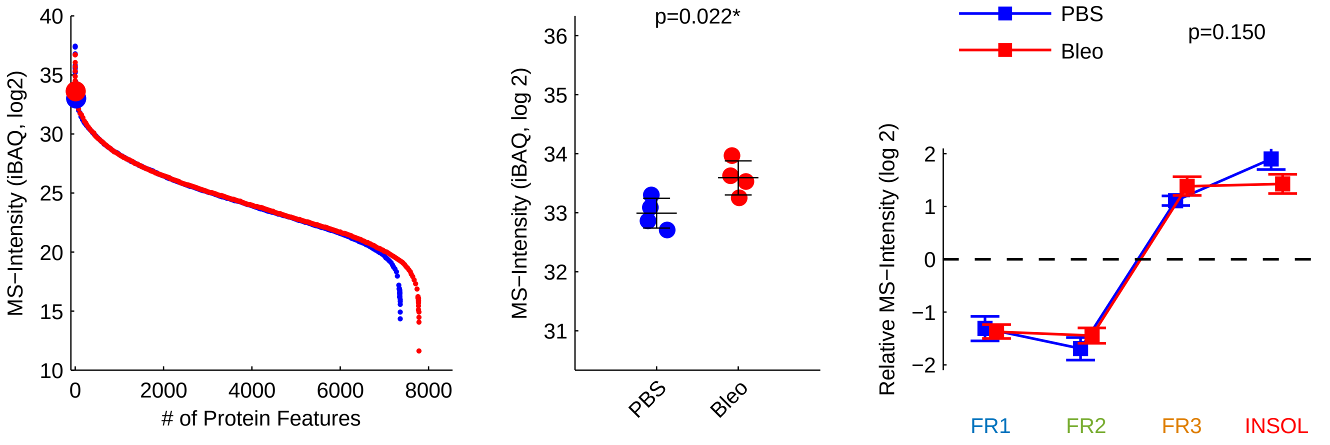

Q9DCV7 − Krt7 (id: 623)

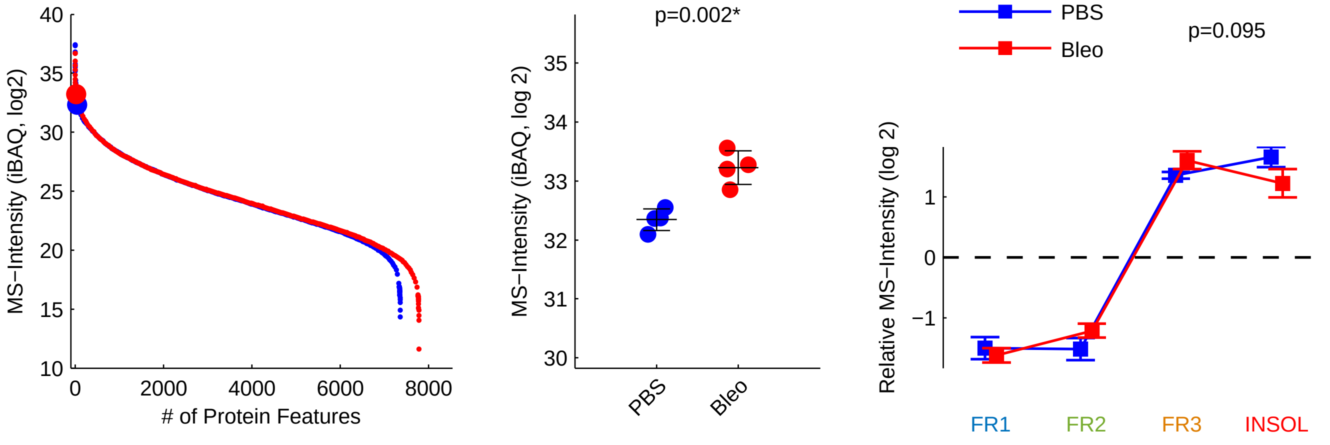

D0QMC3 − Mndal (id: 626)

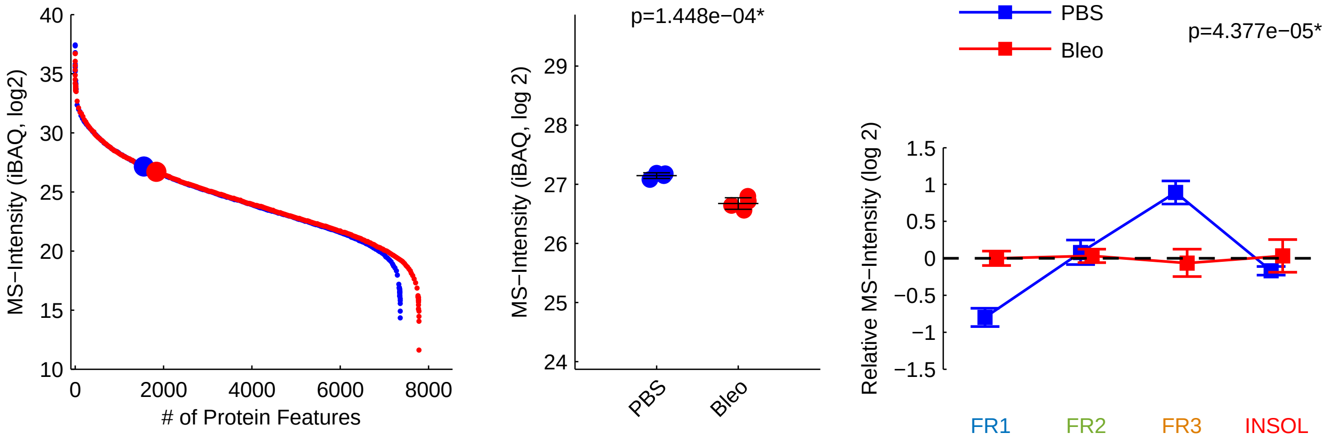

D3YTN4 − Gimap4 (id: 630)

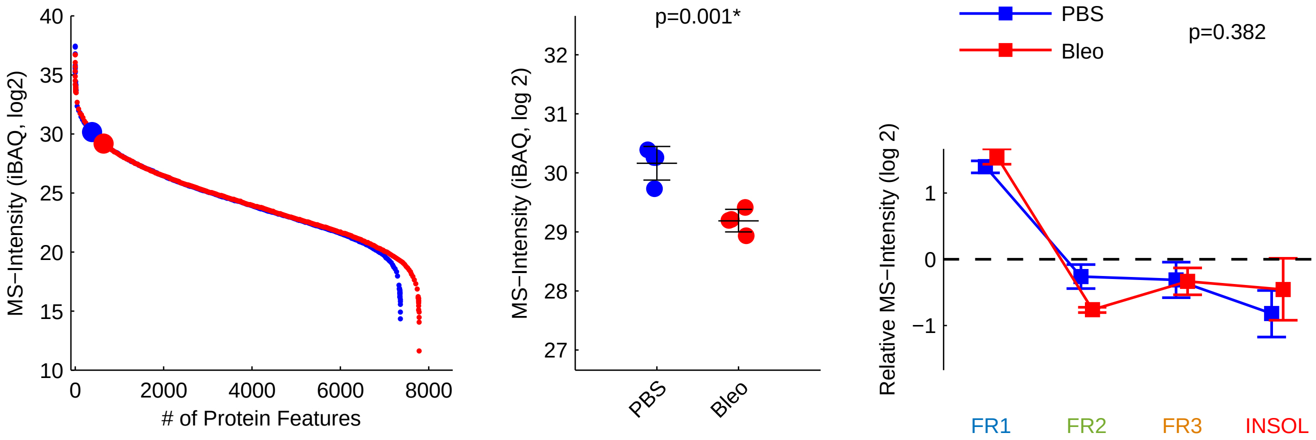

D3YTQ3 − Hnrpdl (id: 633)

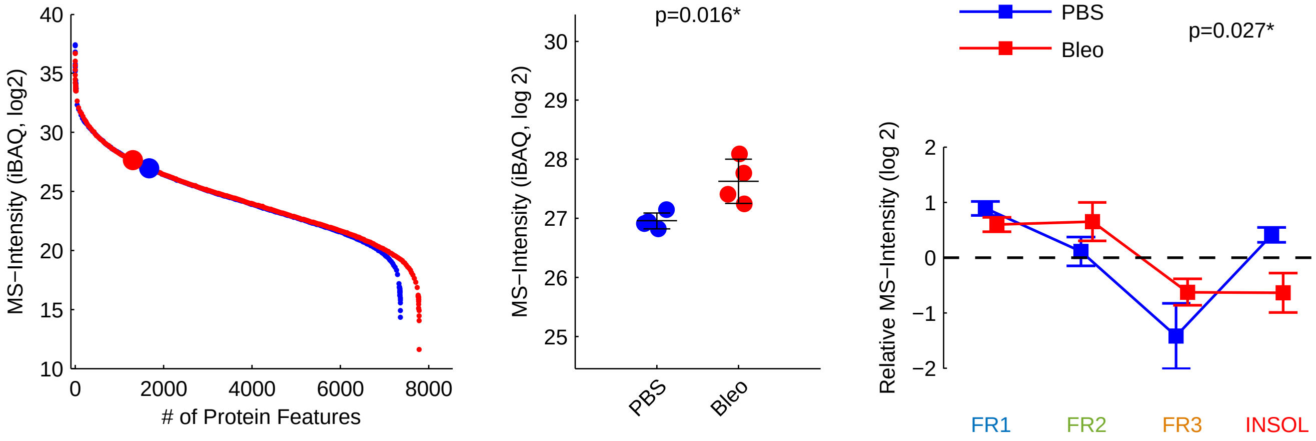

P52793 − Efna1 (id: 636)

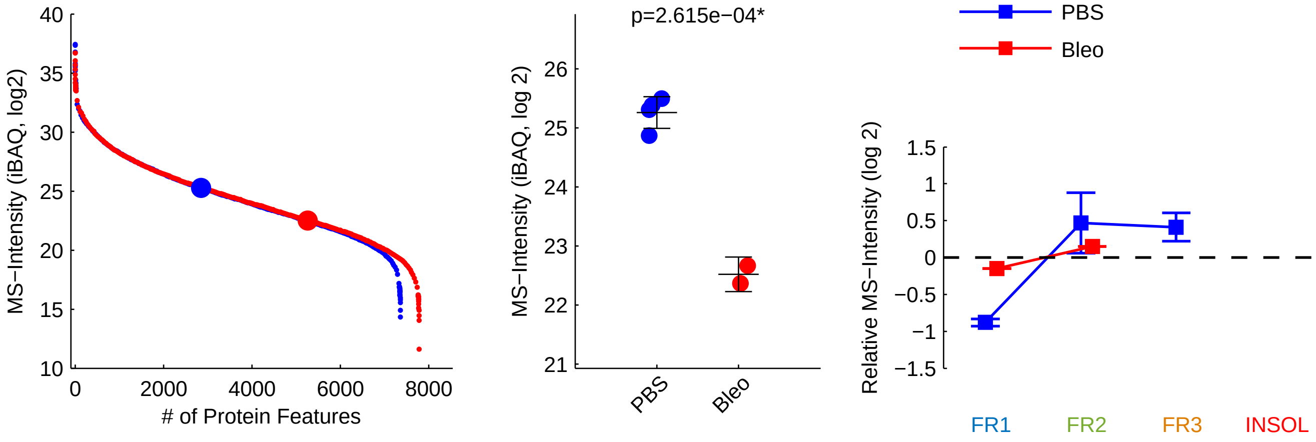

Q3UH68 − Limch1 (id: 649)

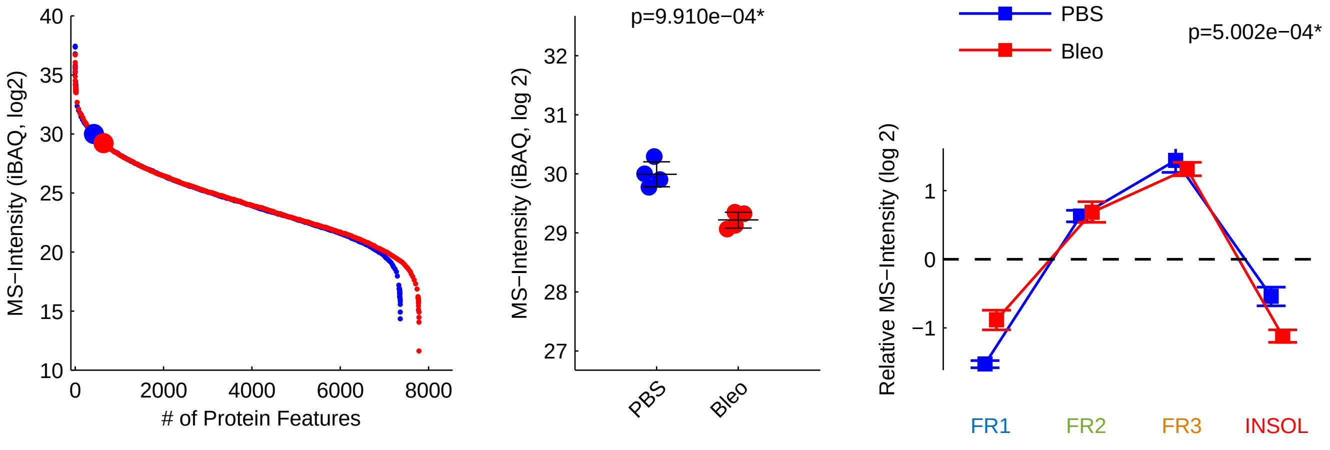

Q91VS7 − Mgst1 (id: 650)

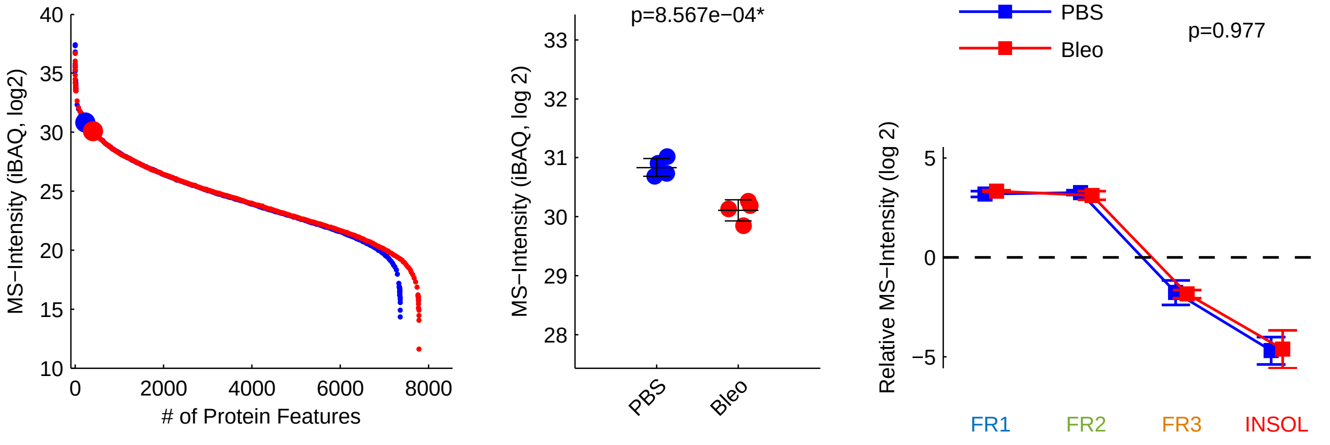

D3YVF0 − Akap5 (id: 674)

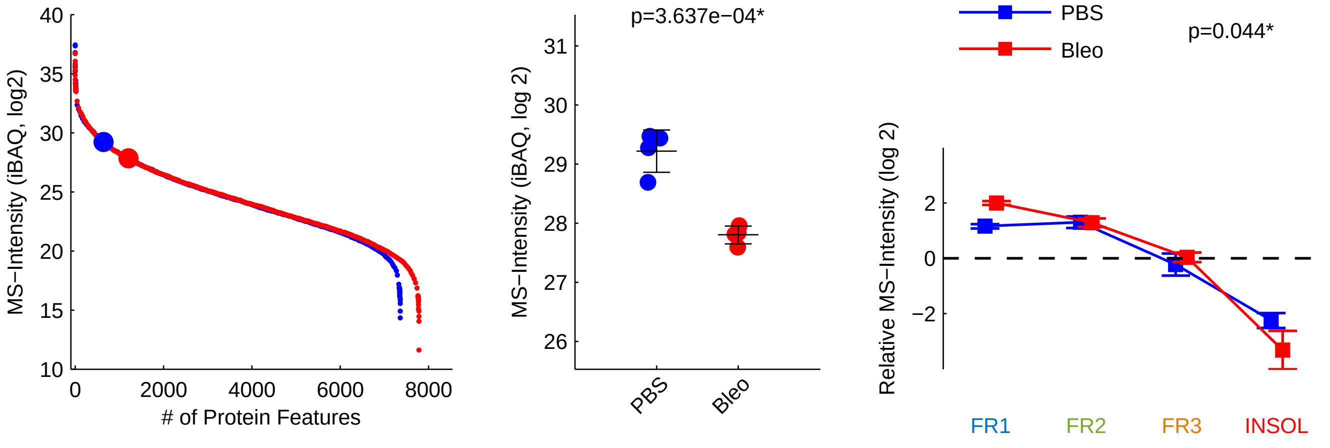

E9Q1U2 − Synpo2 (id: 689)

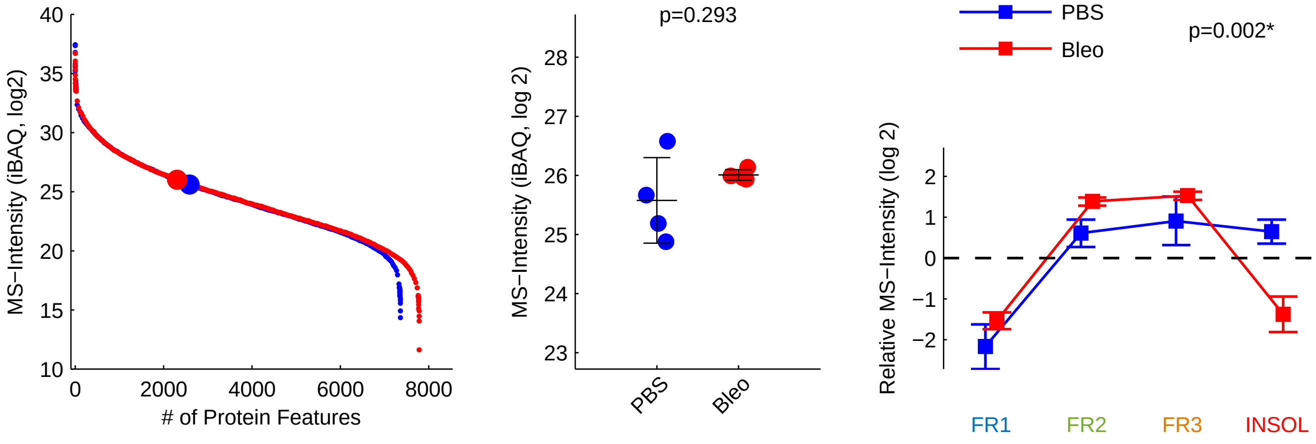

D3YVW2 − Golim4 (id: 690)

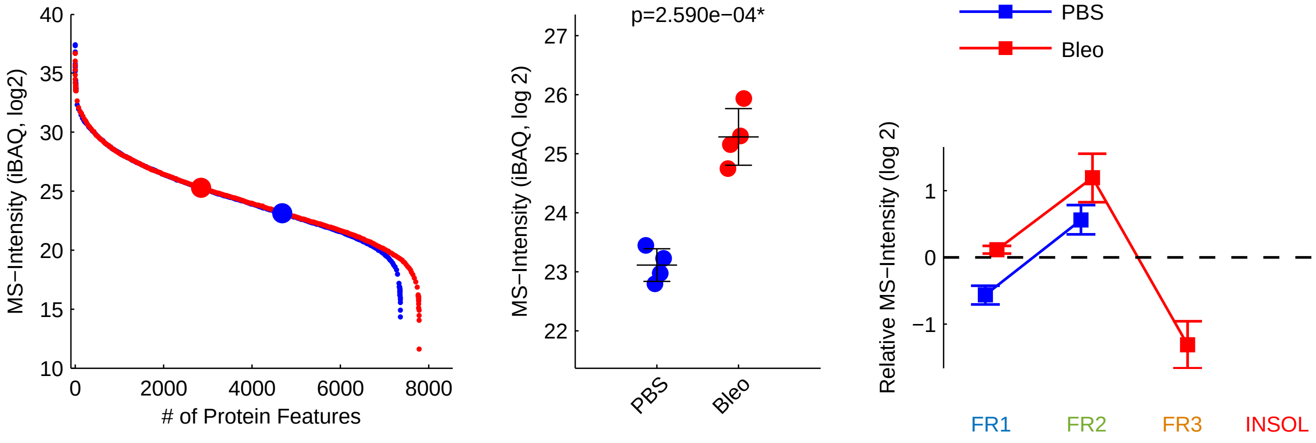

D6RFN5 − Ninj1 (id: 692)

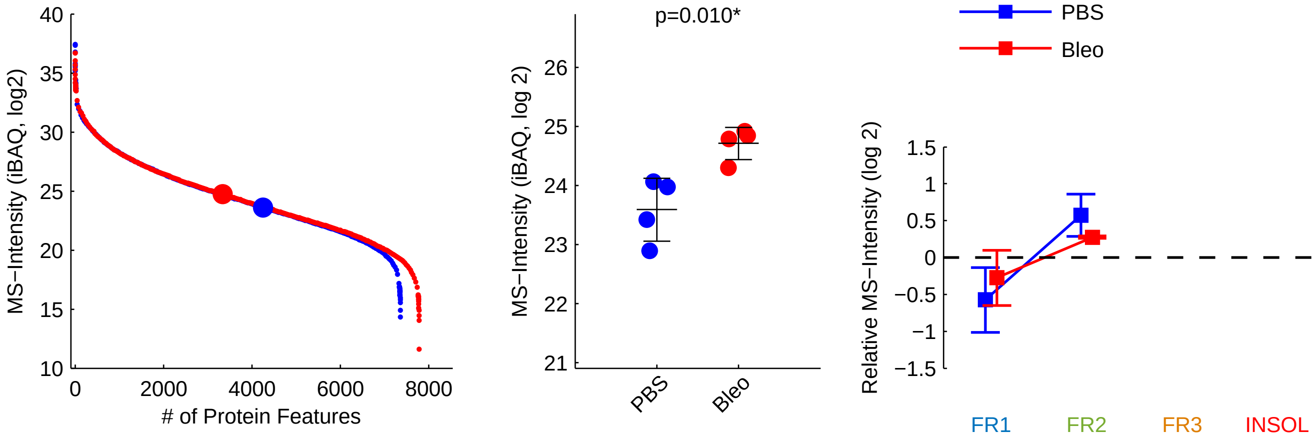

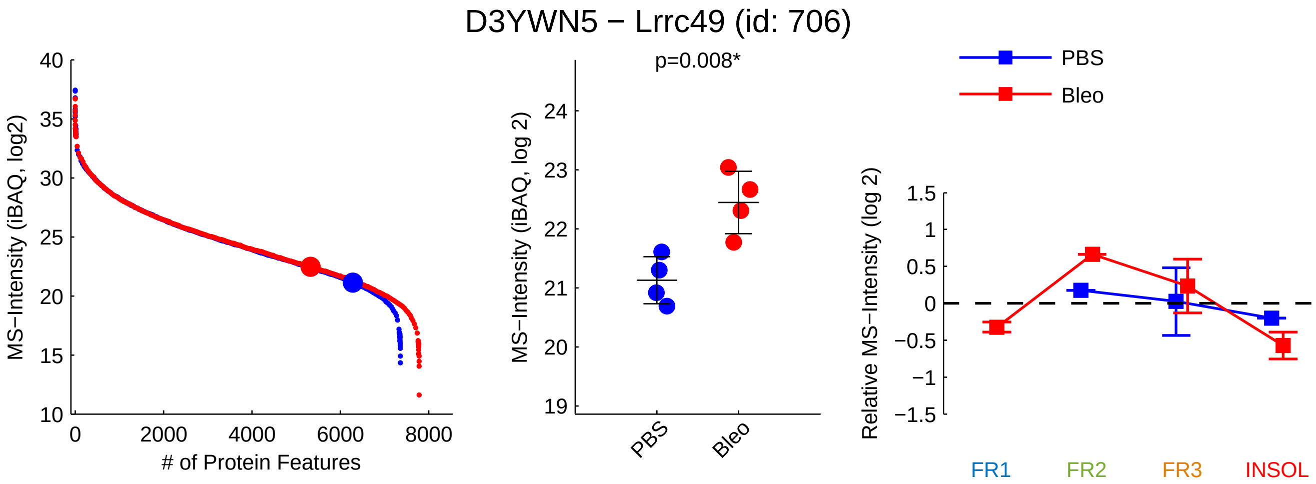
D3YWN5 − Lrrc49 (id: 706)
MS−Intensity (iBAQ, log2)
# of Protein Features
p=0.008*
MS−Intensity (iBAQ, log 2)
PBS
Bleo
Relative MS−Intensity (log 2)
FR1
FR2
FR3
INSOL

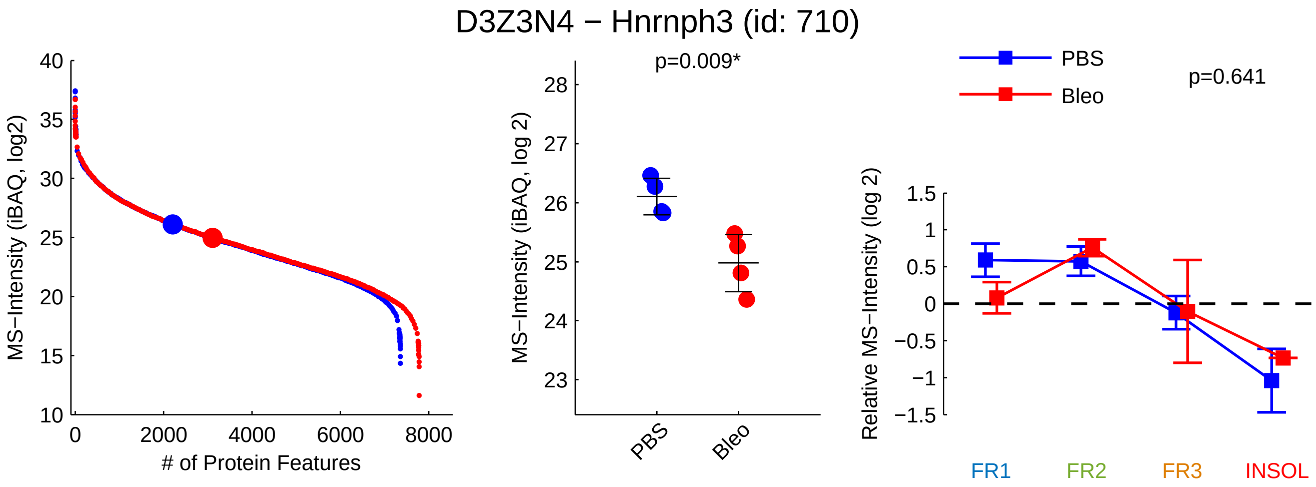
D3Z3N4 − Hnrnph3 (id: 710)
MS−Intensity (iBAQ, log2)
# of Protein Features
p=0.009*
MS−Intensity (iBAQ, log 2)
PBS
Bleo
p=0.641
Relative MS−Intensity (log 2)
FR1
FR2
FR3
INSOL

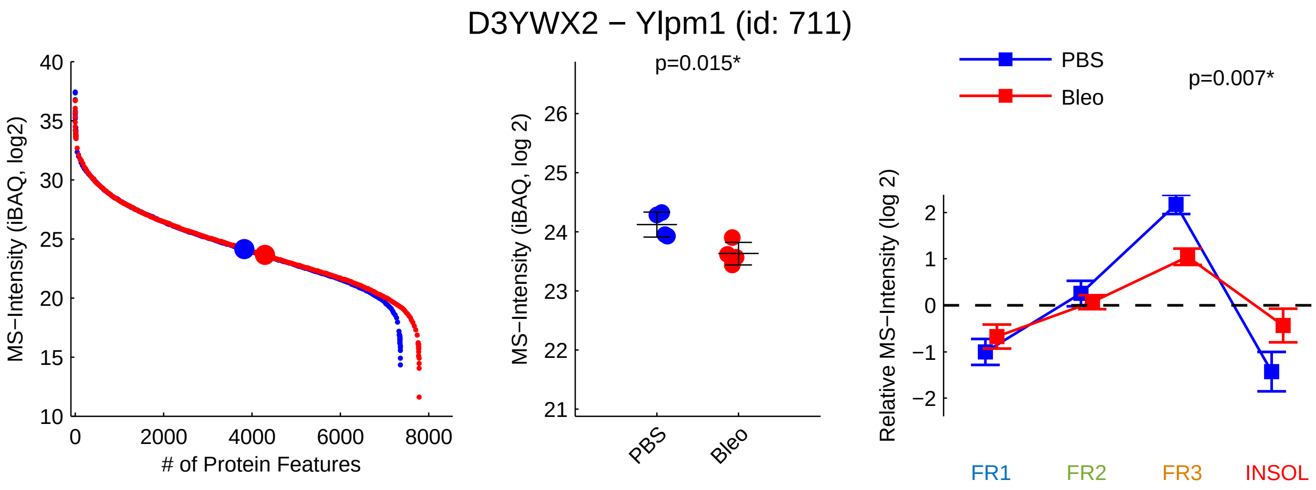
D3YWX2 − Ylpm1 (id: 711)
MS−Intensity (iBAQ, log2)
# of Protein Features
p=0.015*
MS−Intensity (iBAQ, log 2)
PBS
Bleo
p=0.007*
Relative MS−Intensity (log 2)
FR1
FR2
FR3
INSOL

Q7SIG6 − Asap2 (id: 712)

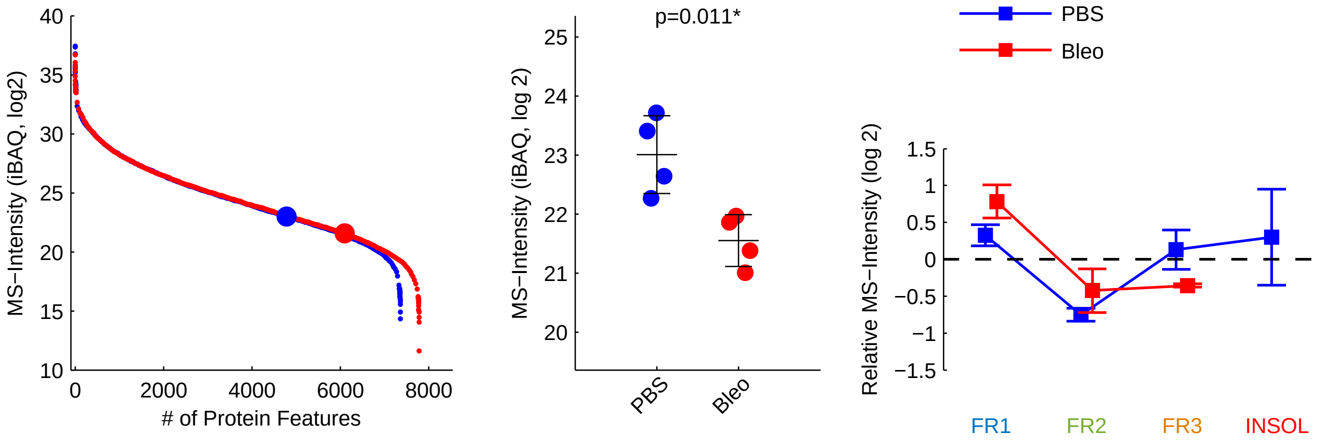

D3Z2Q7 − Hmcn1 (id: 716)

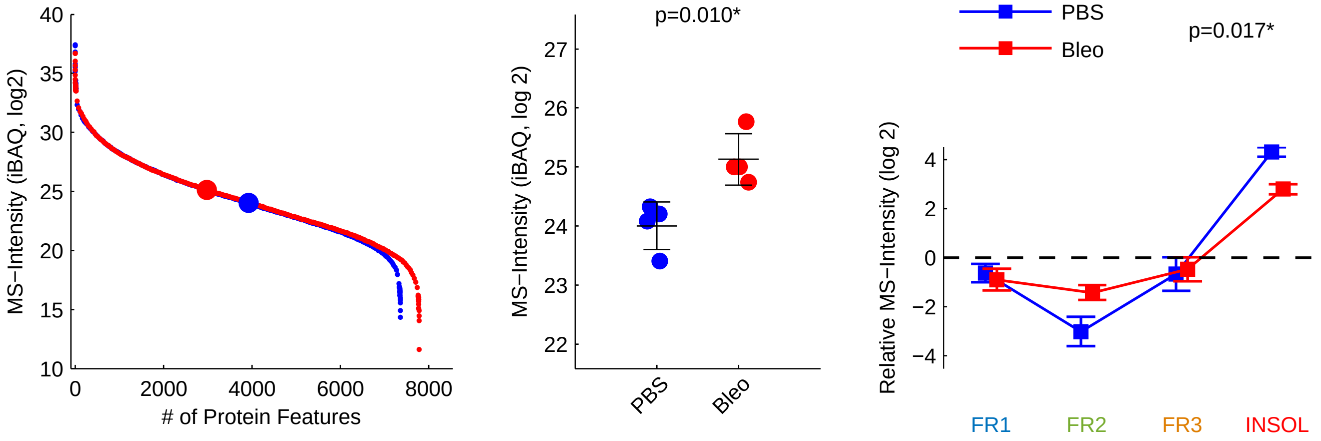

D3YXK2 − Safb (id: 721)

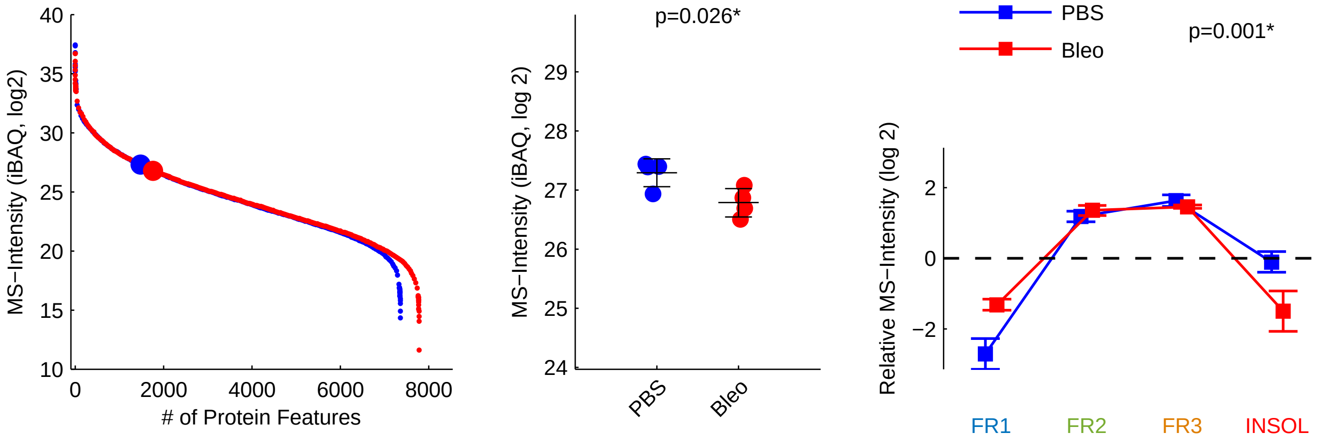

Q9D1G2 − Pmvk (id: 725)

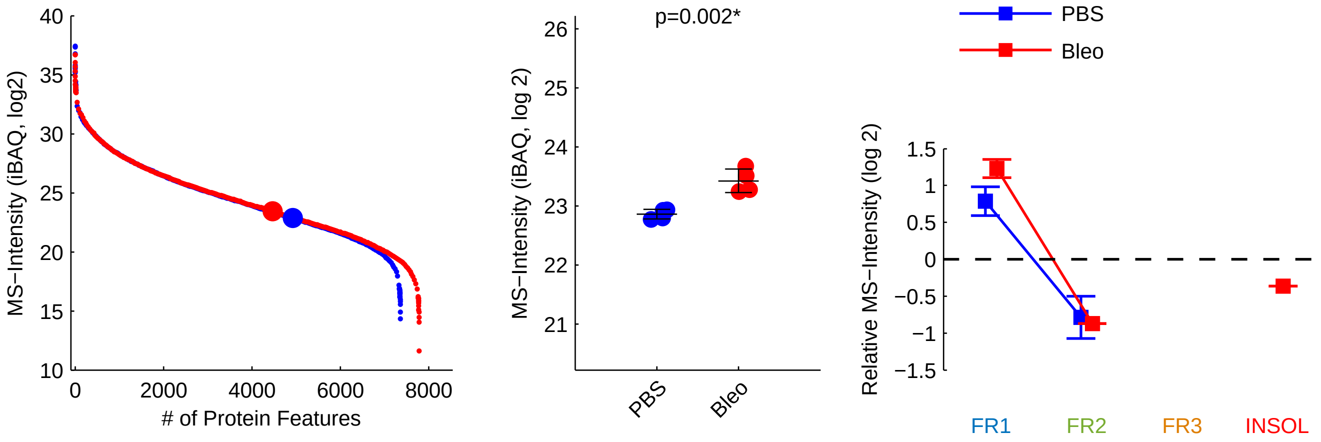

D3YYT1 − Glyr1 (id: 742)

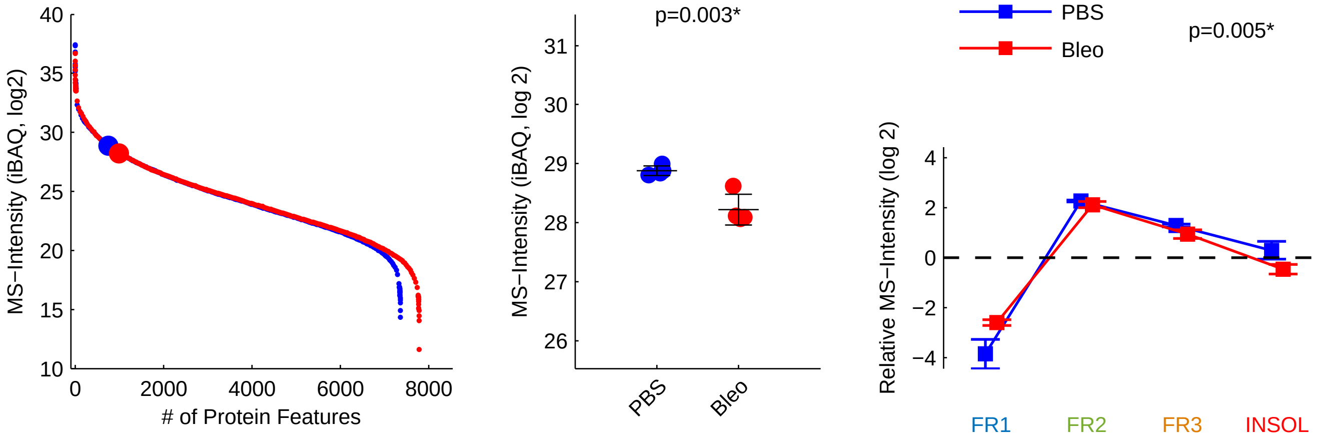

D3YYY8 − Arhgef26 (id: 747)

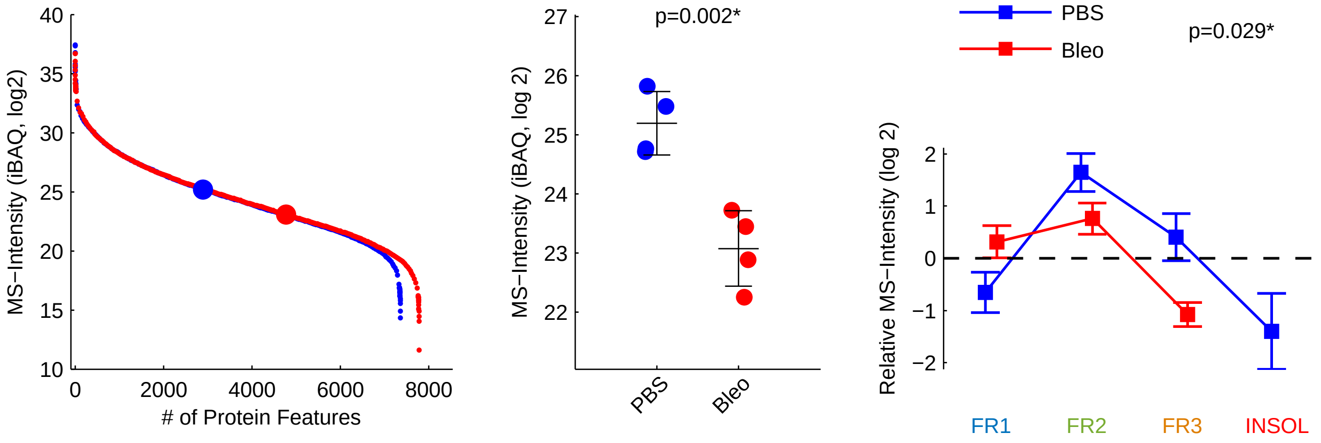

D3YZP9 − Ccdc6 (id: 759)

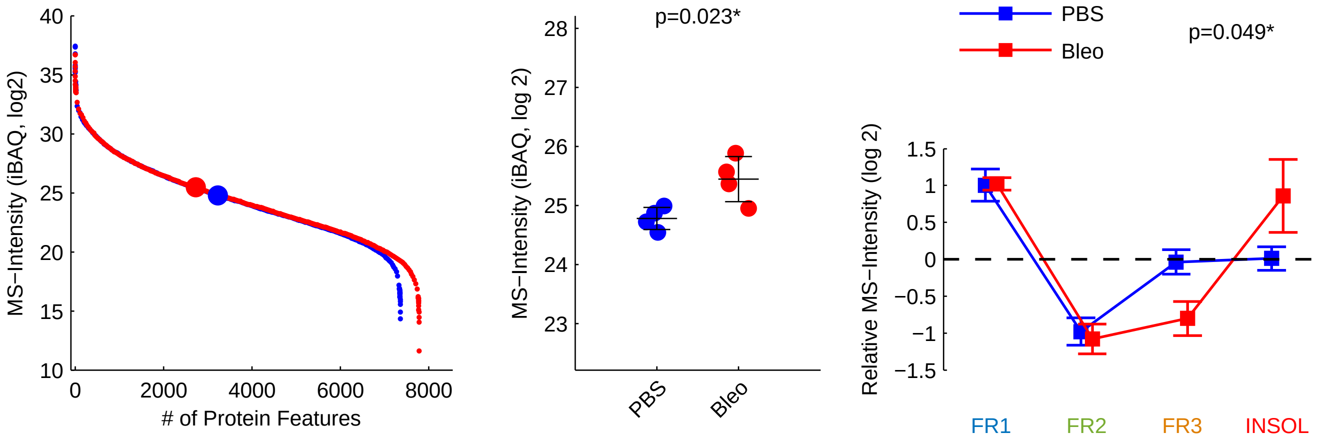

D3Z030 − Lrrc16a (id: 764)

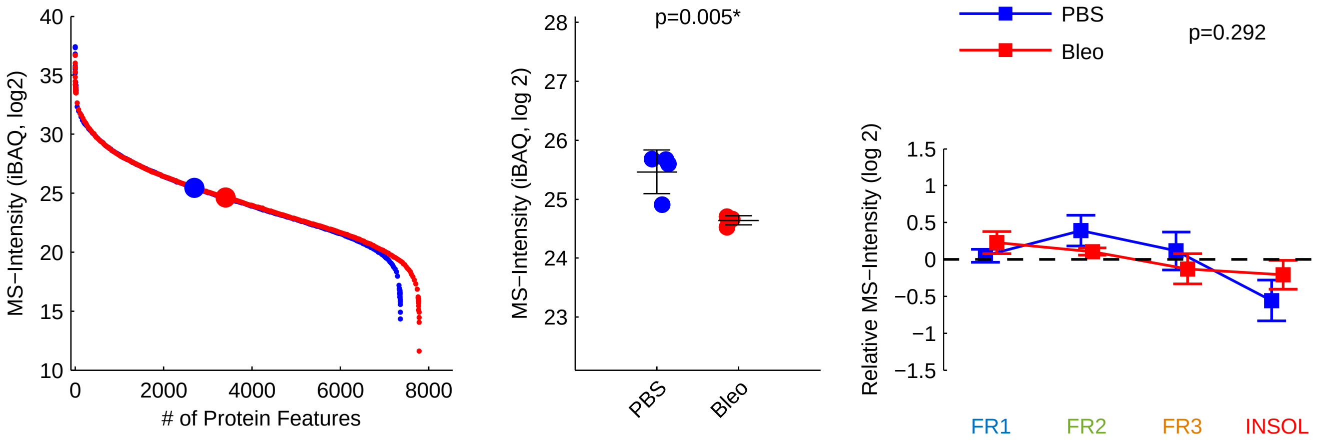

P41216 − Acsl1 (id: 766)

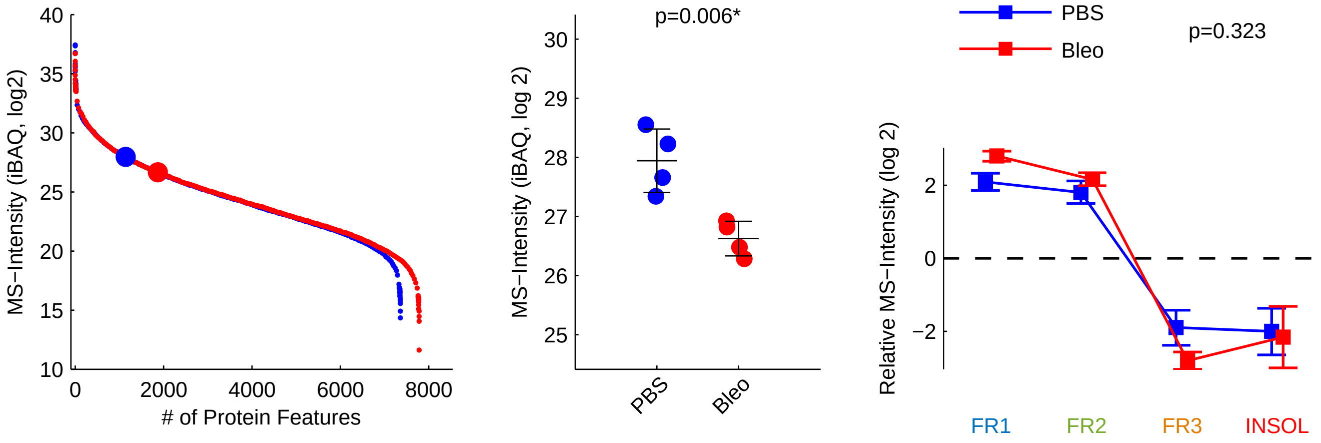

Q9JLT4 − Txnrd2 (id: 776)

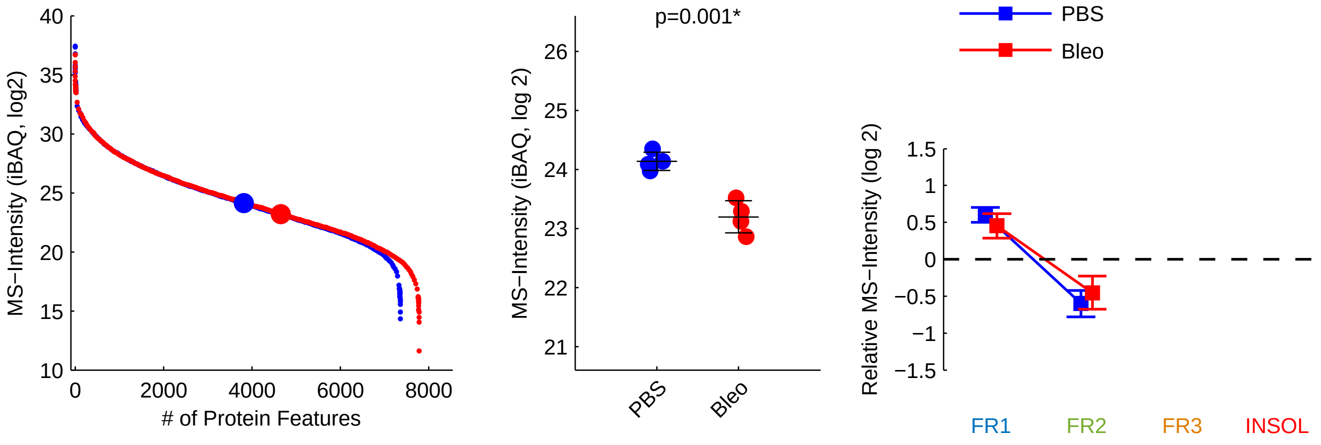

O54916 − Reps1 (id: 805)

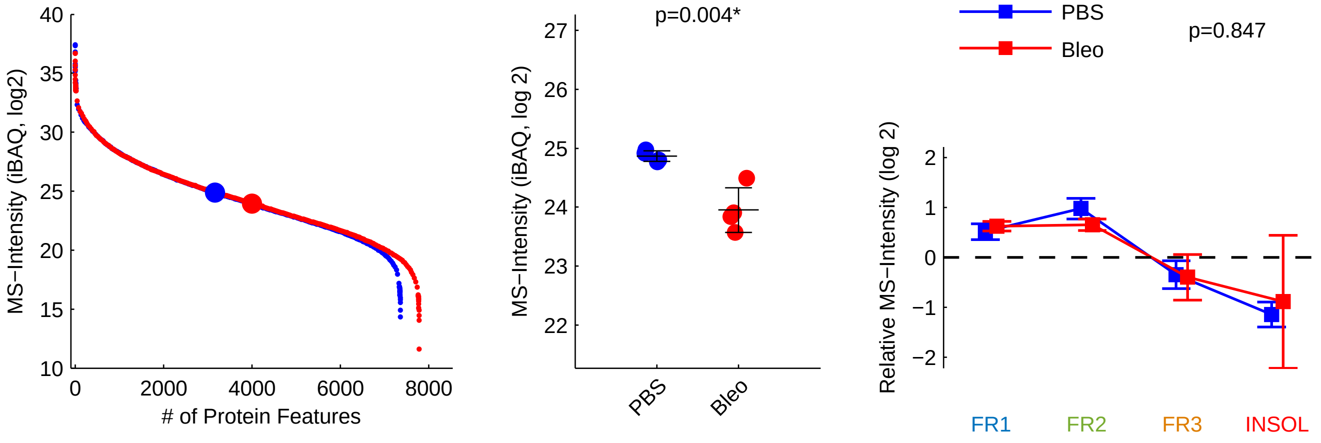

D3Z2P2 − Chi3l1 (id: 809)

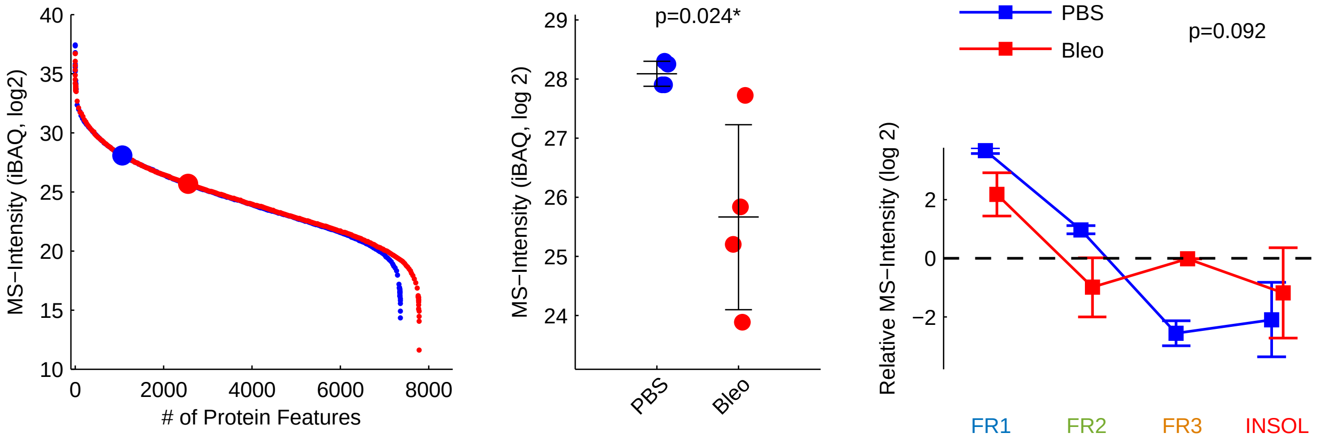

D3Z2R5 − Sepn1 (id: 810)

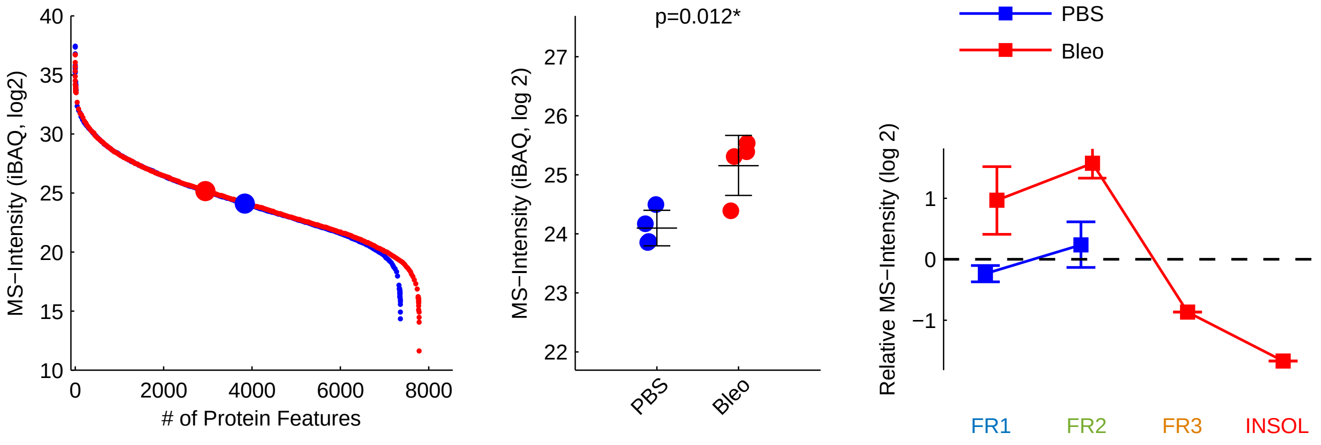

D3Z2Y6 − S100a16 (id: 811)

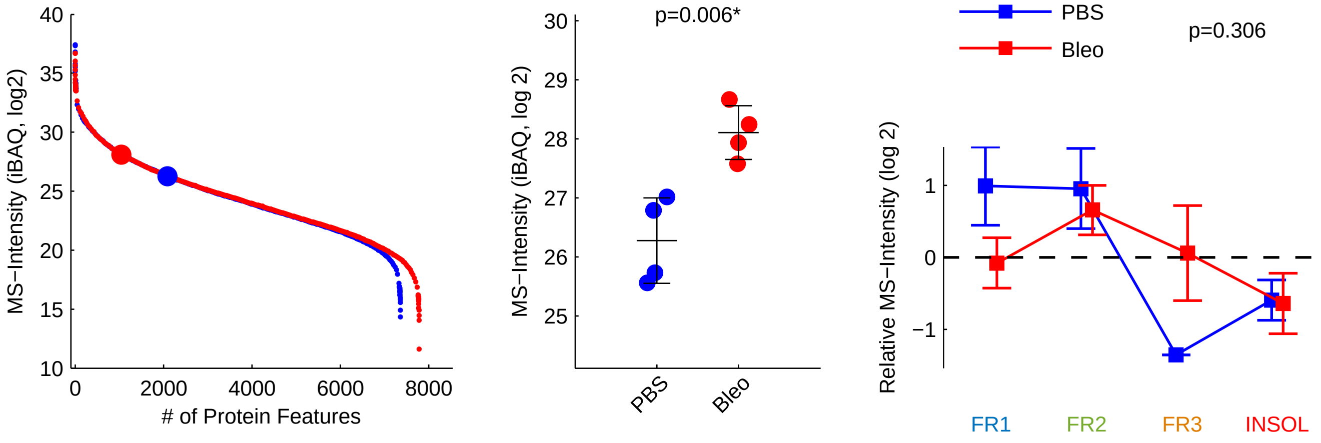

Q922J3 − Clip1 (id: 812)

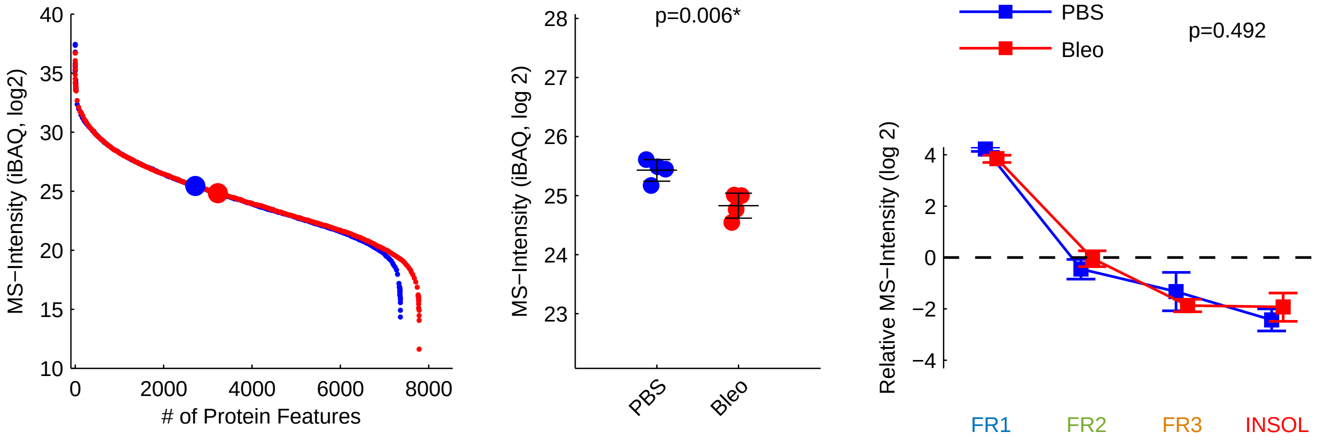

Q921U8 − Smtn (id: 821)

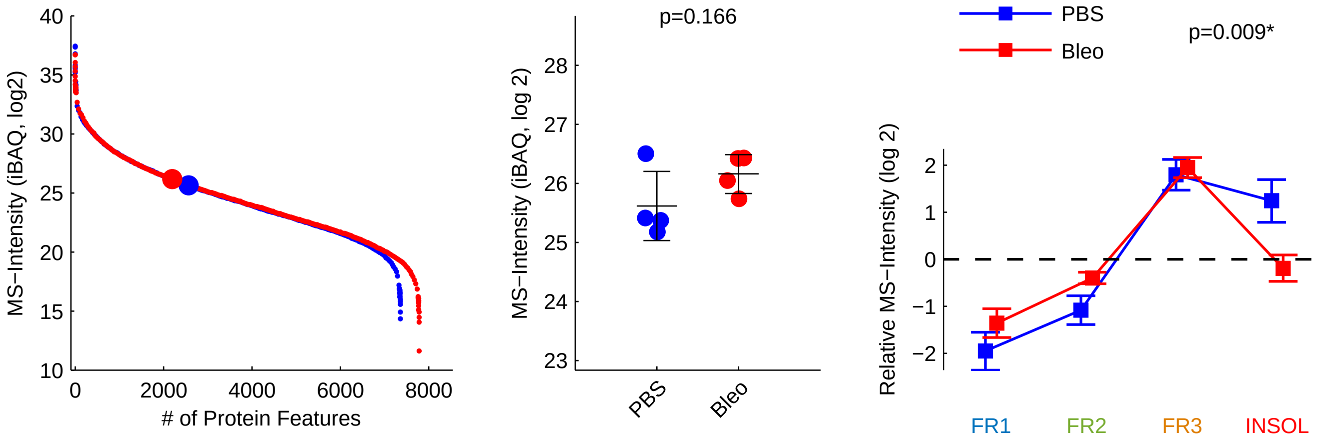

D3Z3R1 − Gm5745 (id: 822)

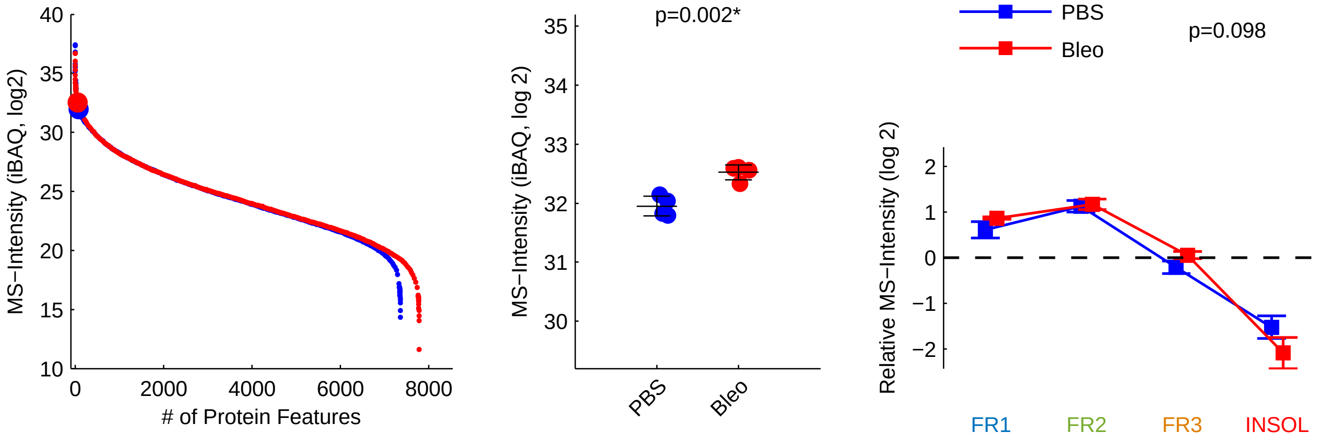

D3Z4C9 − Mnf1 (id: 832)

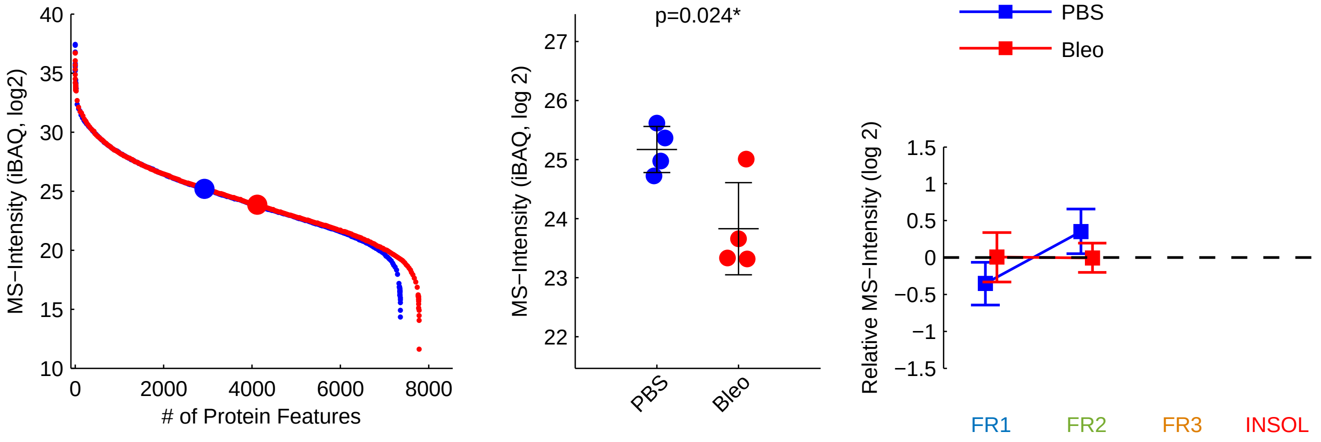

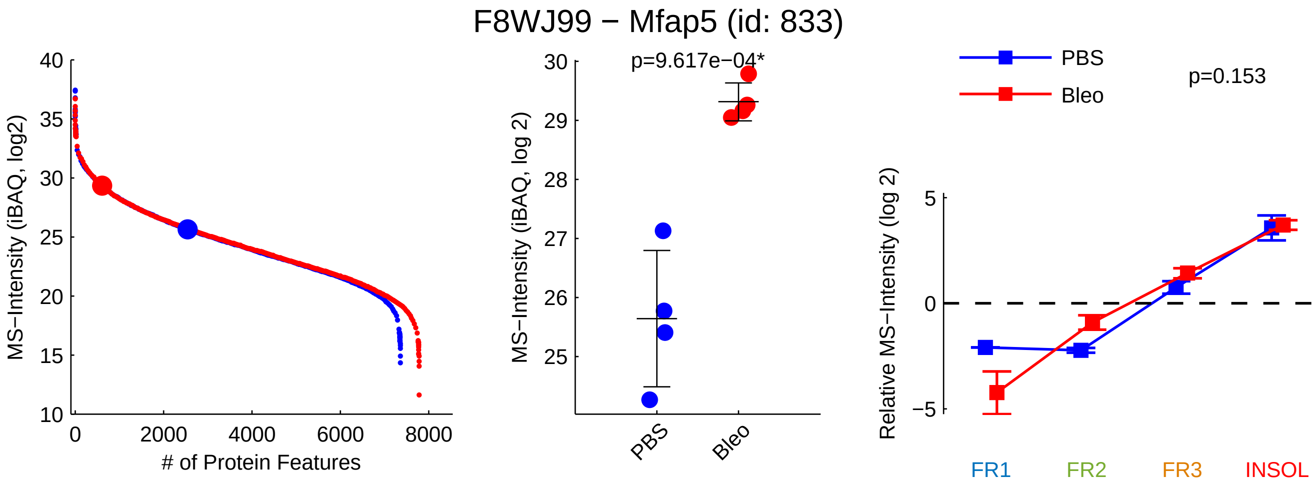
F8WJ99 − Mfap5 (id: 833)
MS−Intensity (iBAQ, log2)
# of Protein Features
MS−Intensity (iBAQ, log 2)
p=9.617e−04*
PBS
Bleo
Relative MS−Intensity (log 2)
p=0.153
FR1
FR2
FR3
INSOL

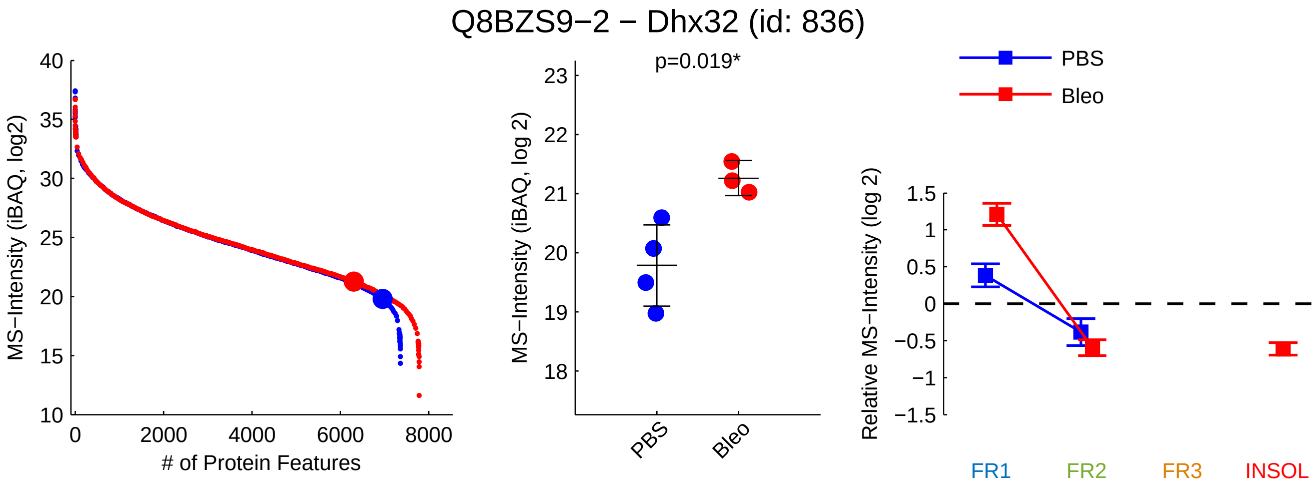
Q8BZS9−2 − Dhx32 (id: 836)
MS−Intensity (iBAQ, log2)
# of Protein Features
MS−Intensity (iBAQ, log 2)
p=0.019*
PBS
Bleo
Relative MS−Intensity (log 2)
FR1
FR2
FR3
INSOL

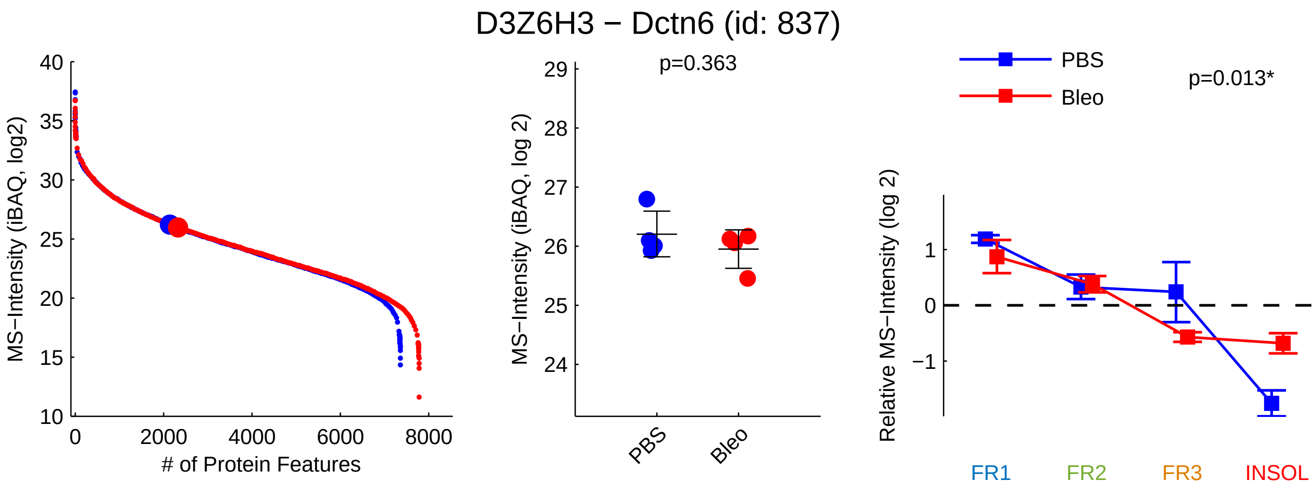
D3Z6H3 − Dctn6 (id: 837)
MS−Intensity (iBAQ, log2)
# of Protein Features
MS−Intensity (iBAQ, log 2)
p=0.363
PBS
Bleo
Relative MS−Intensity (log 2)
p=0.013*
FR1
FR2
FR3
INSOL

P56542 − Dnase2 (id: 849)

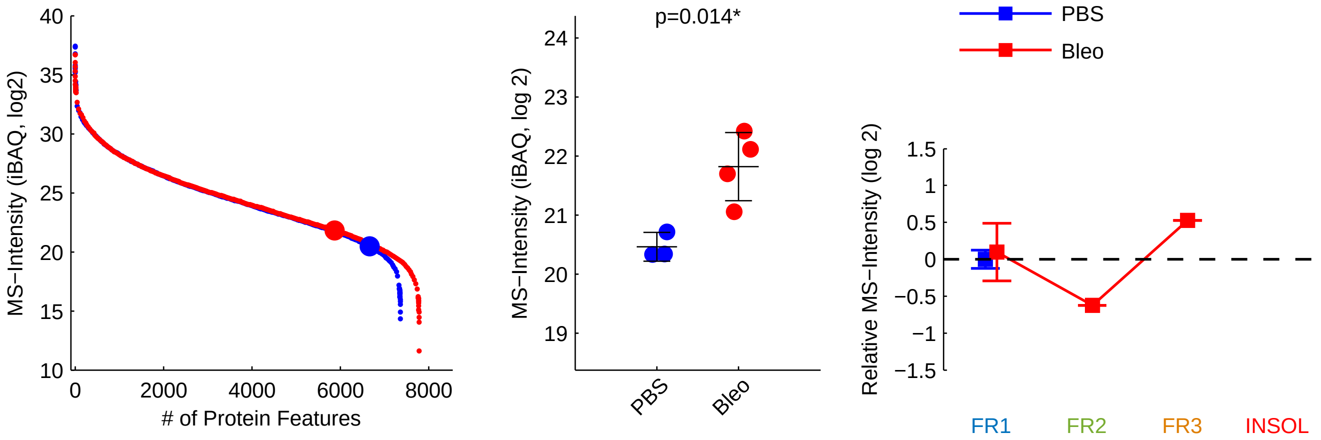

D3Z5G7 − Ces1b (id: 854)

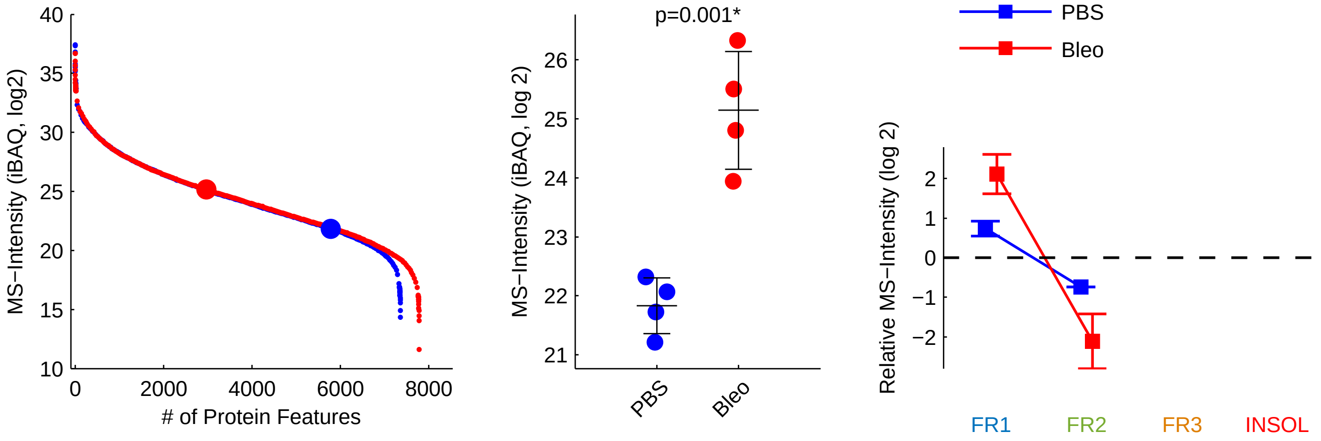

Q8CHC4 − Synj1 (id: 869)

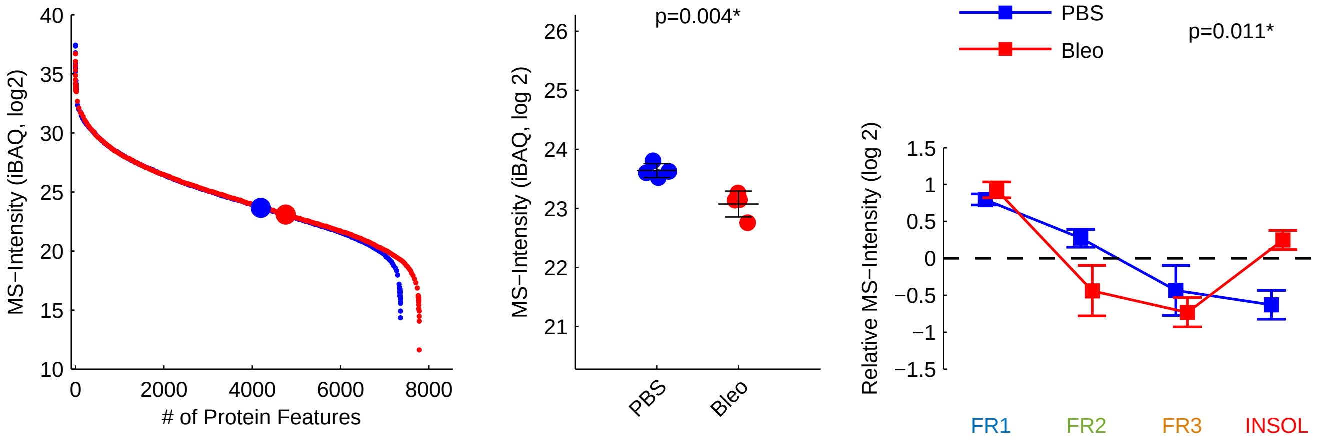

Q3UHH8 − Gxylt1 (id: 882)

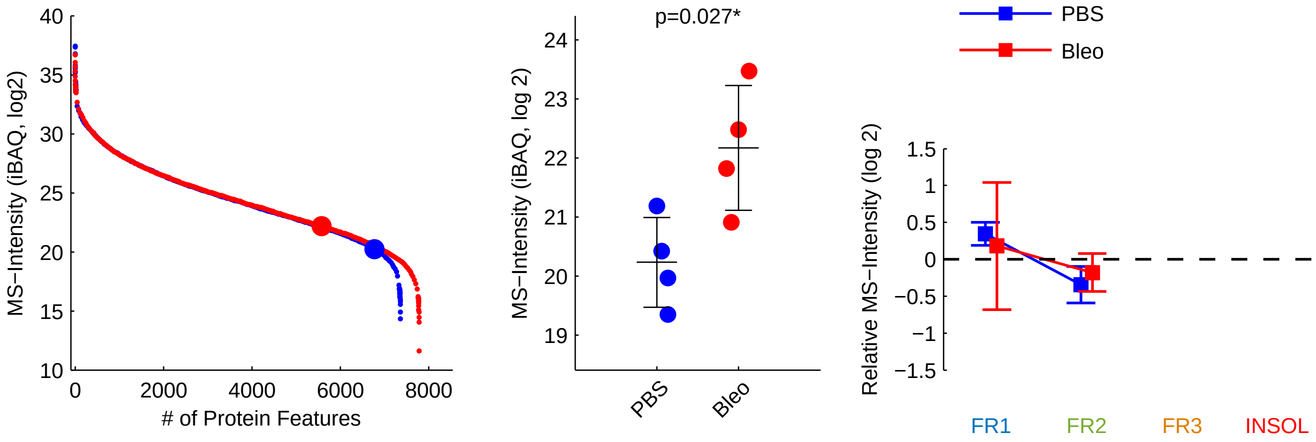

D3Z7X0 − Acad12 (id: 903)

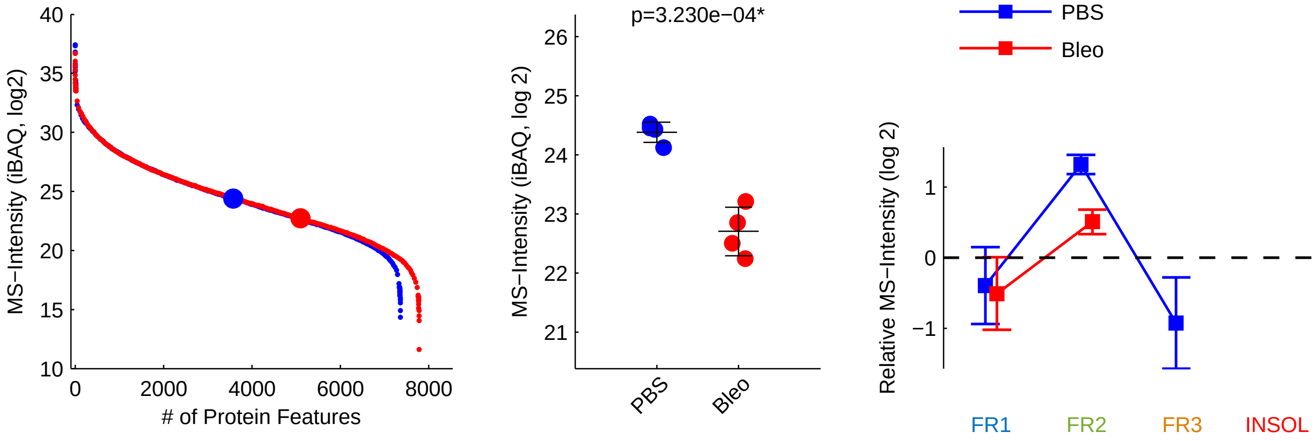

E0CX20 − Bud31 (id: 916)

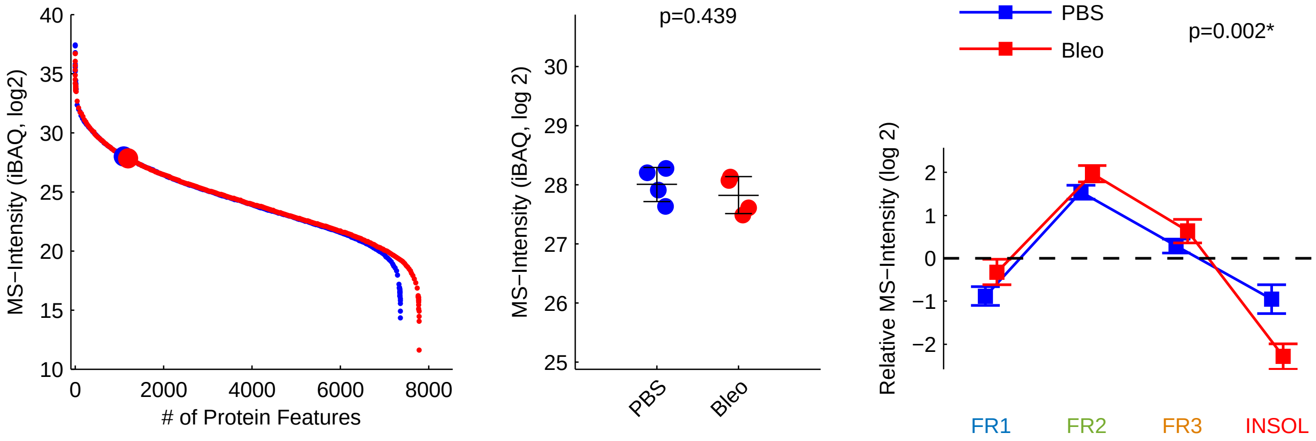

Q61301 − Ctnna2 (id: 922)

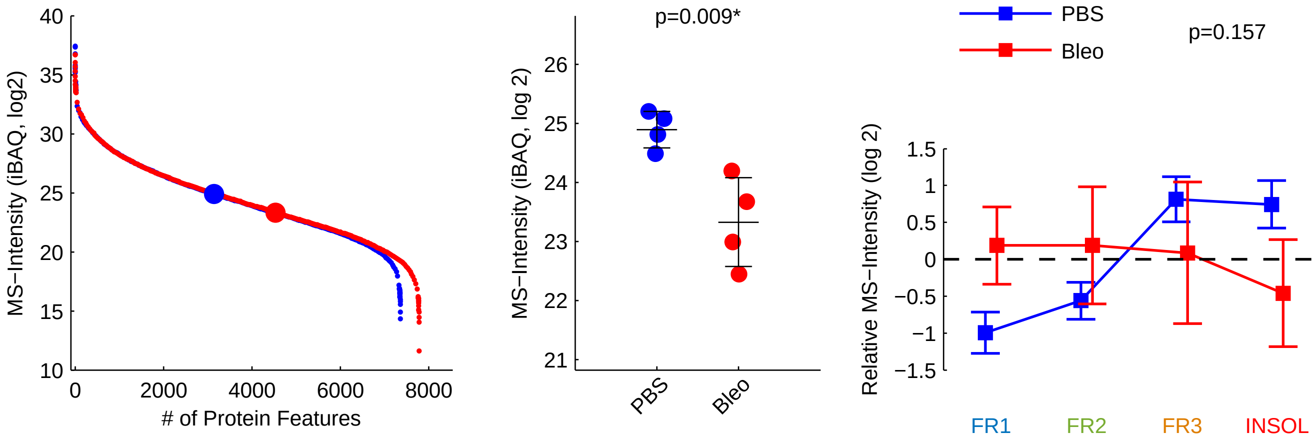

Q9ER69 − Wtap (id: 936)

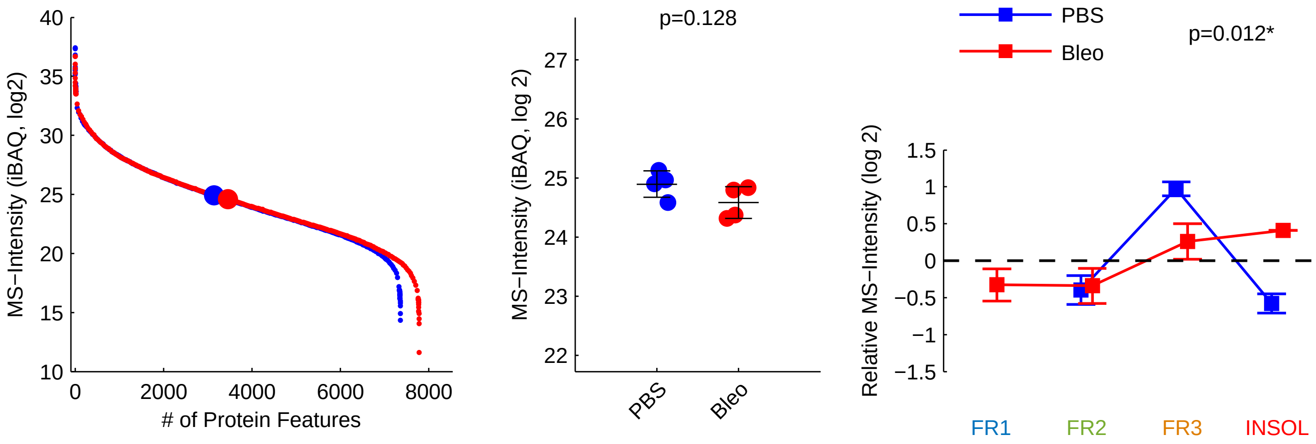

E0CYH4 − Wdr26 (id: 937)

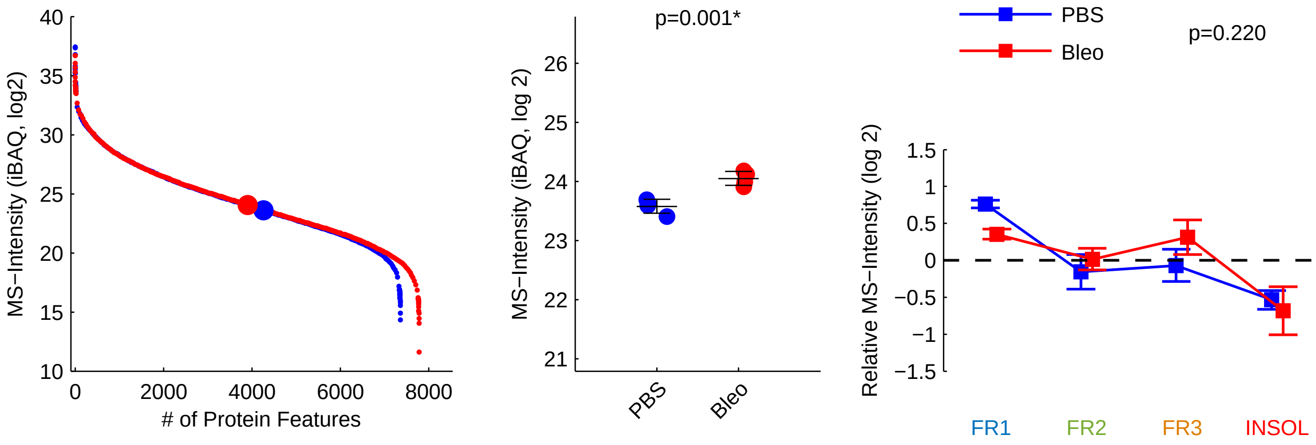

E0CYM1 − Filip1l (id: 938)

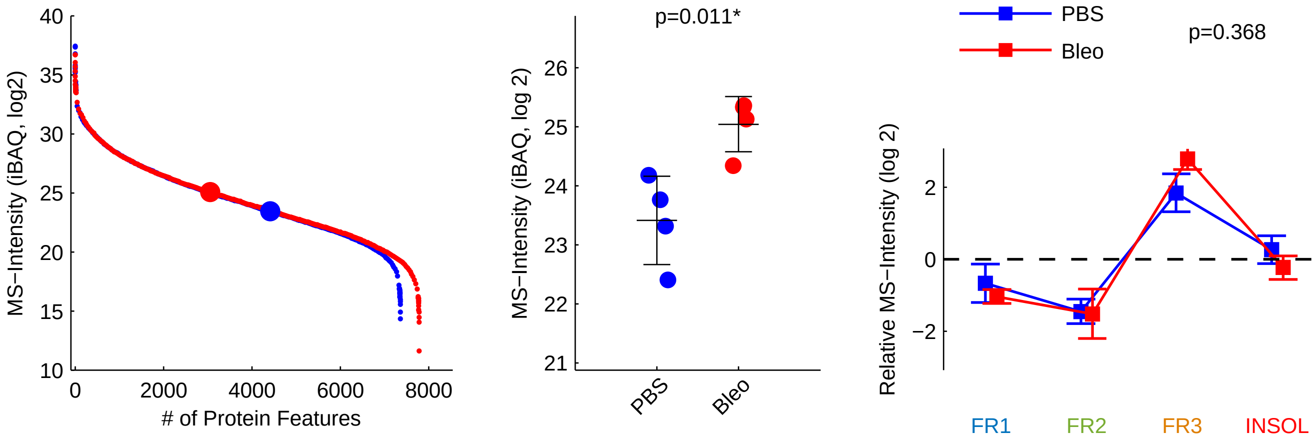

P23506 − Pcmt1 (id: 942)

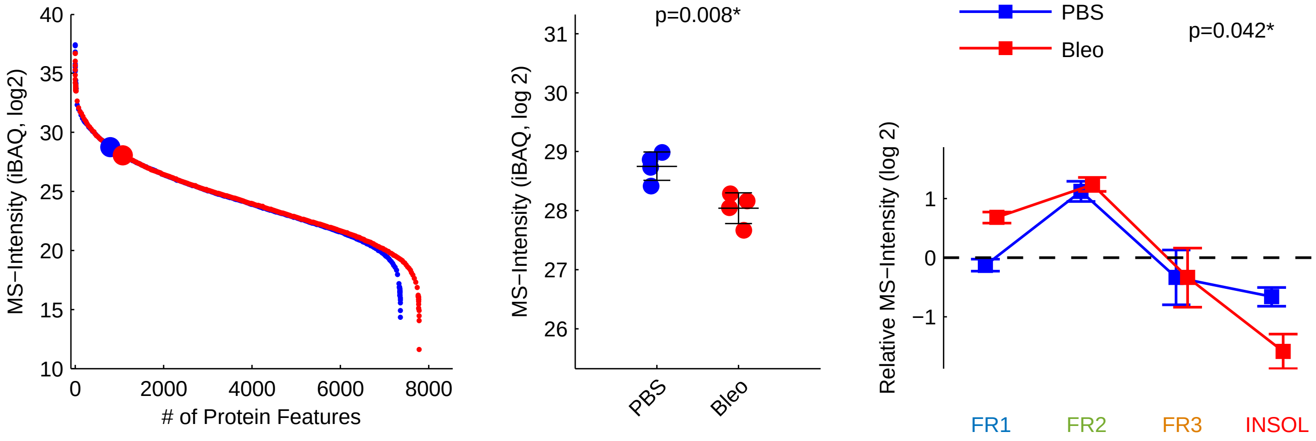

Q61146 − Ocln (id: 948)

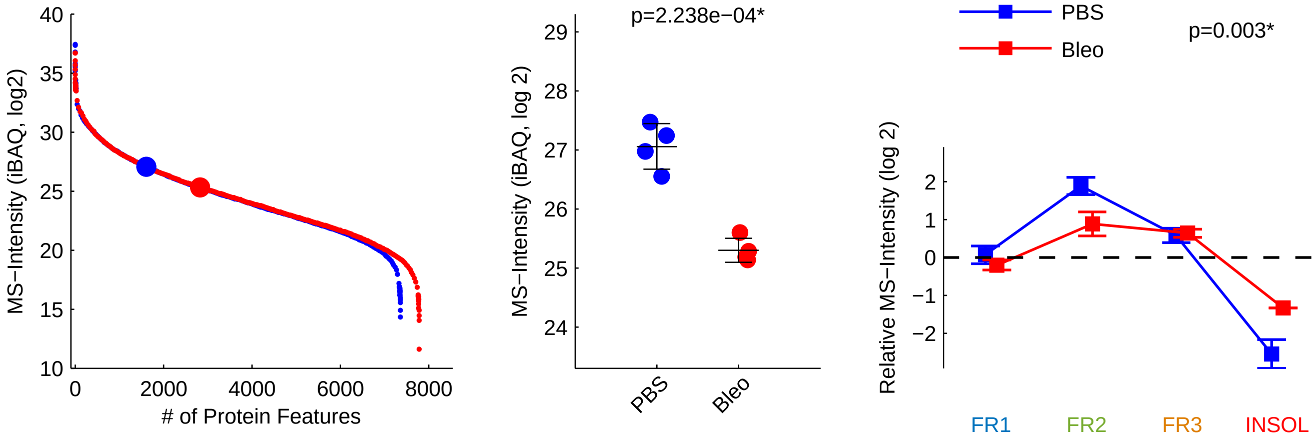

Q91X20 − Ash2l (id: 956)

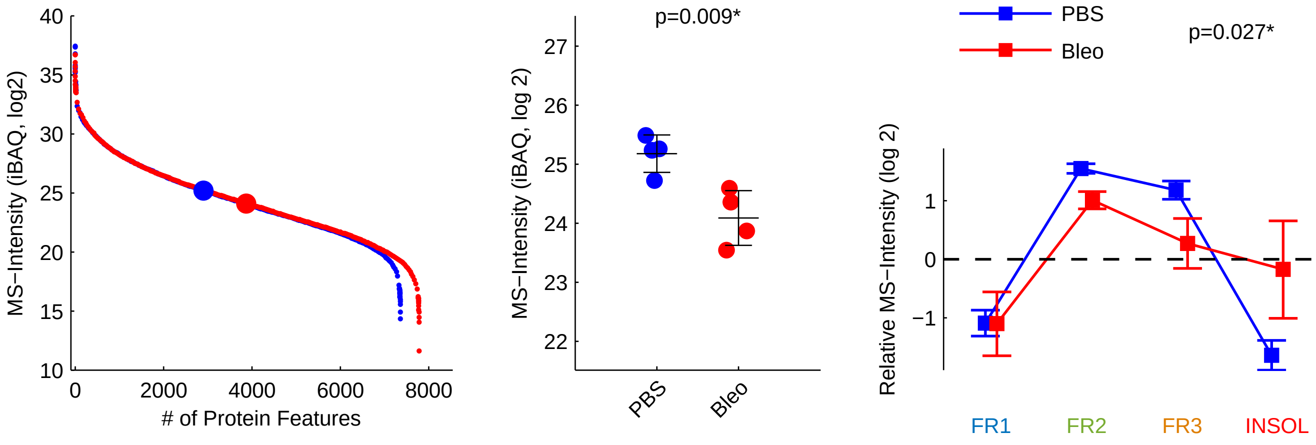

Q571H0 − Urb1 (id: 957)

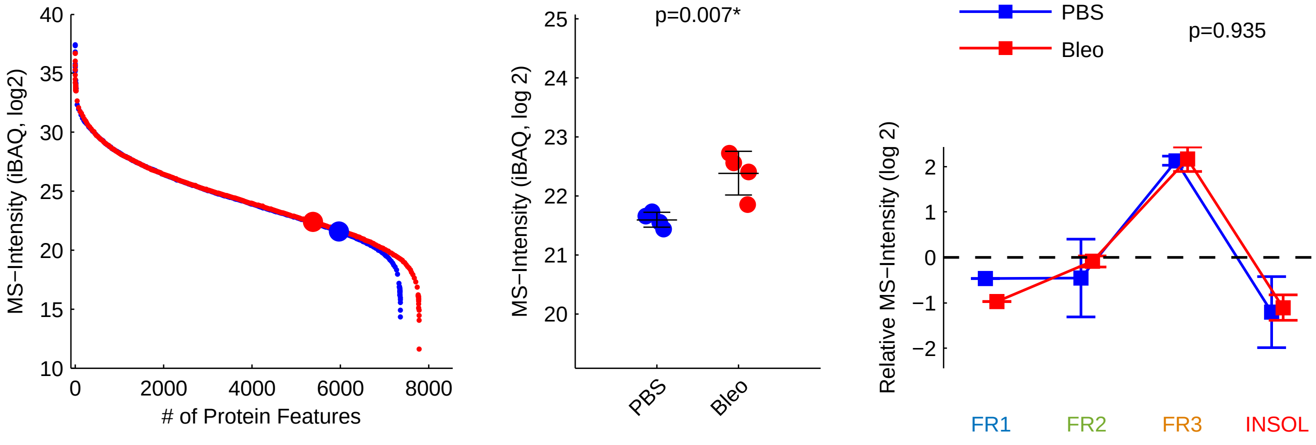

Q62393−2 − Tpd52 (id: 960)

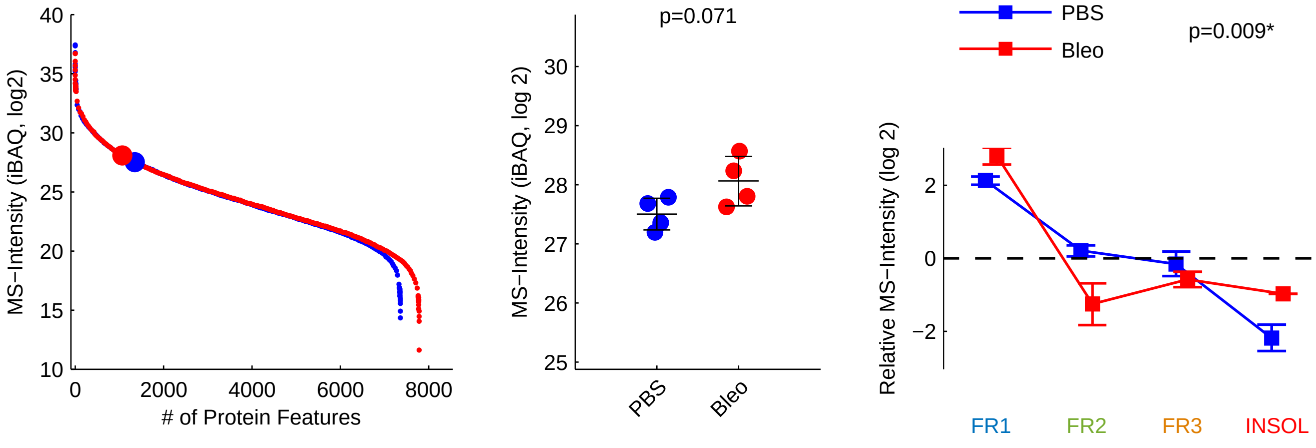

Q61210 − Arhgef1 (id: 965)

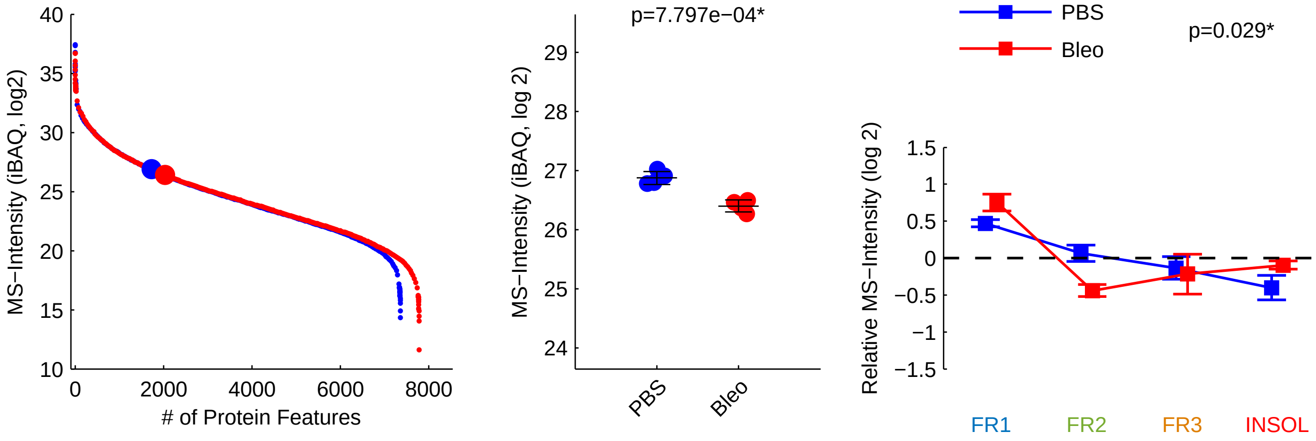

E9PUK3 − Fnbp1l (id: 966)

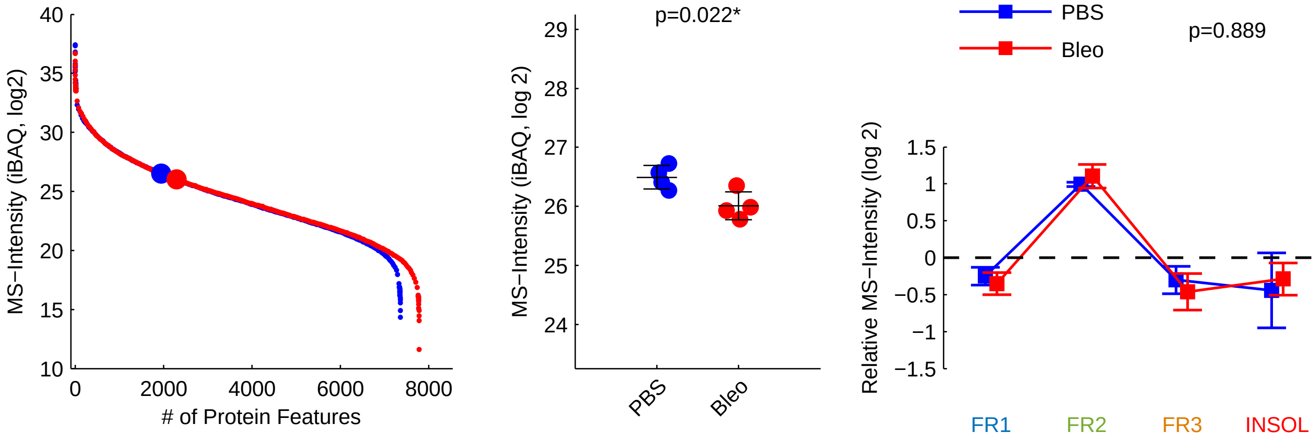

E9PUM4 − Tln2 (id: 967)

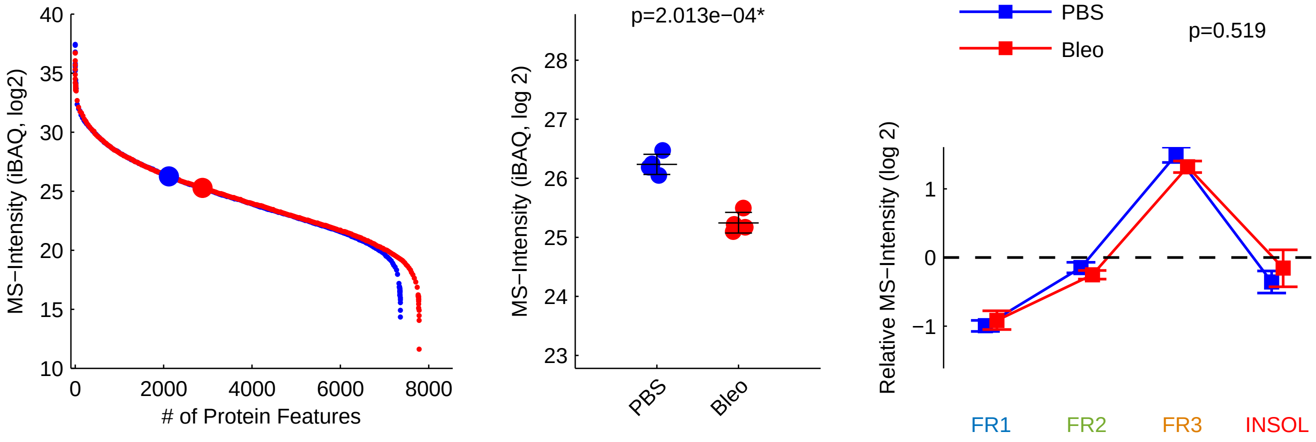

E9PUM5 − Gm4788 (id: 968)

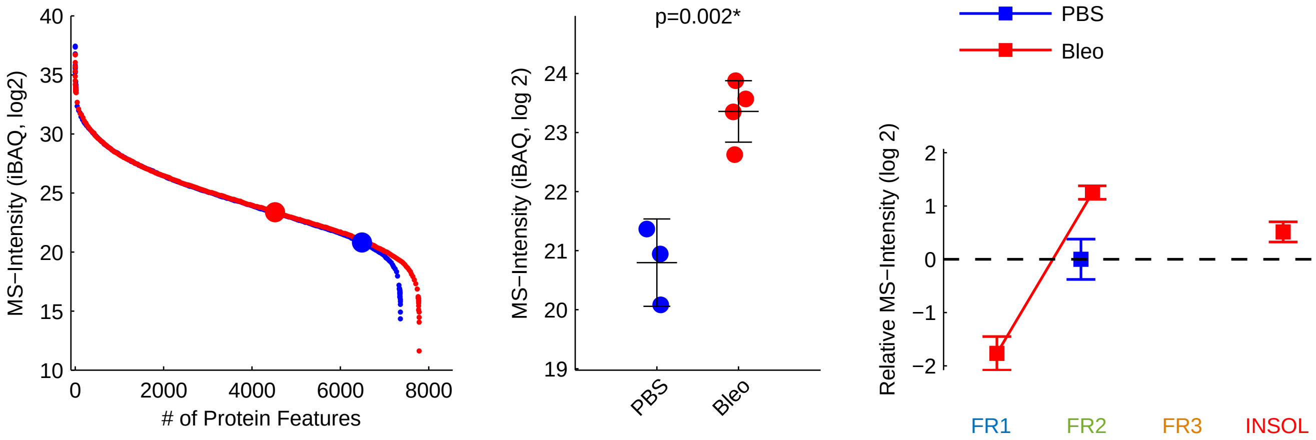

Q921M4−2 − Golga2 (id: 971)

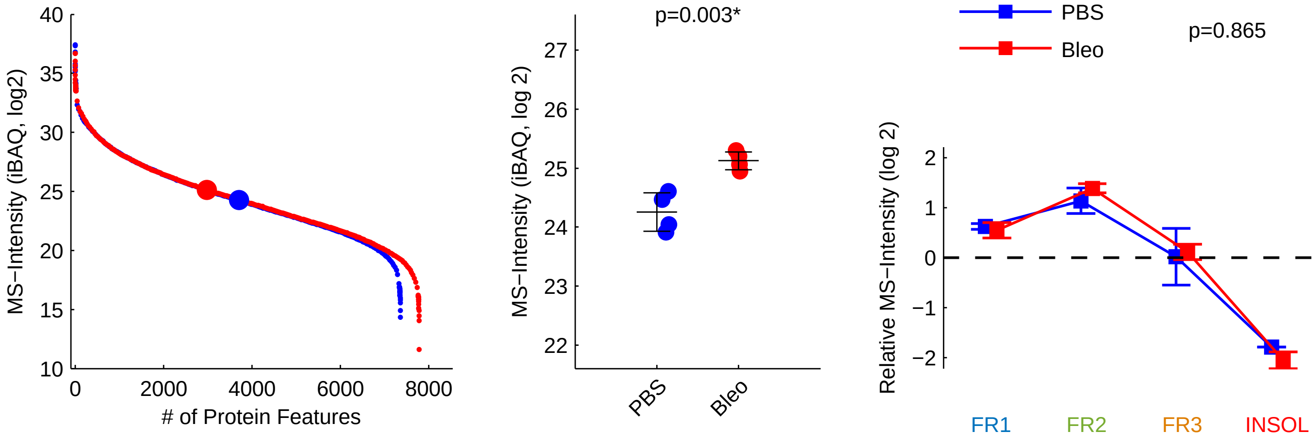

E9PUR4 − Lama3 (id: 974)

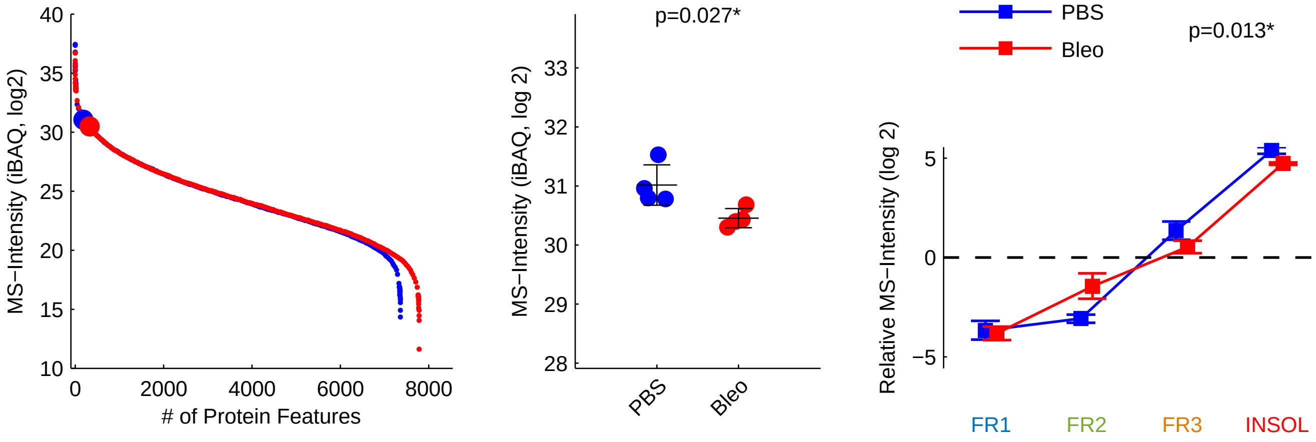

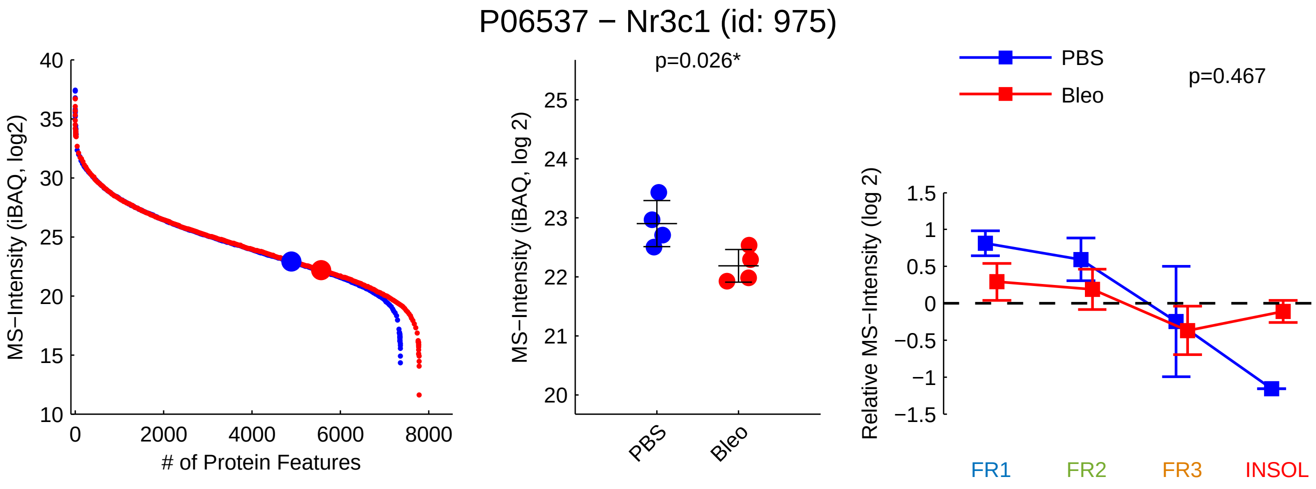
P06537 − Nr3c1 (id: 975)
MS−Intensity (iBAQ, log2)
# of Protein Features
p=0.026*
MS−Intensity (iBAQ, log 2)
PBS
Bleo
p=0.467
Relative MS−Intensity (log 2)
FR1
FR2
FR3
INSOL

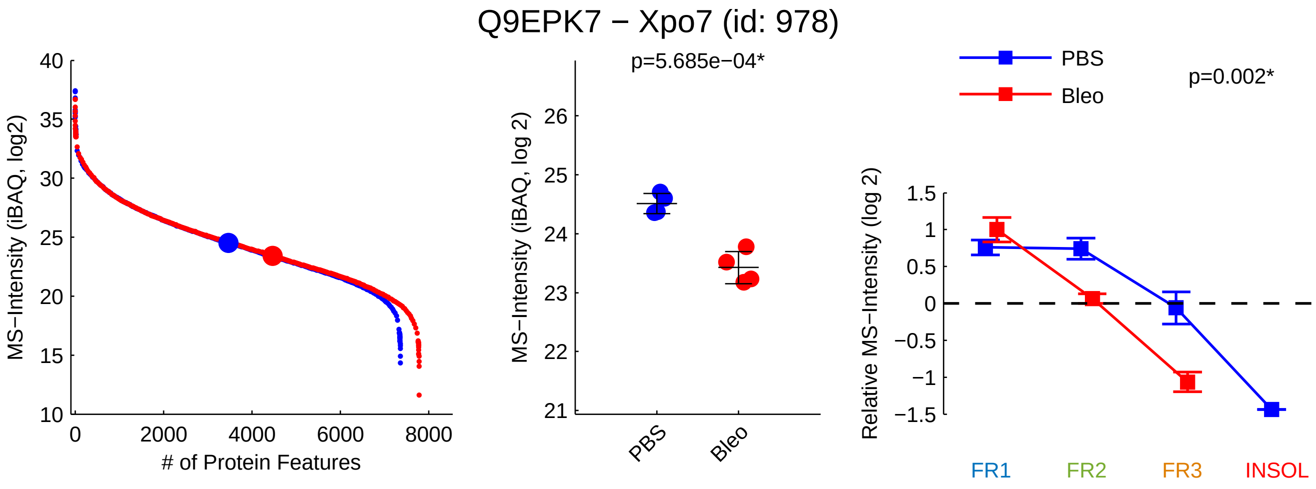
Q9EPK7 − Xpo7 (id: 978)
MS−Intensity (iBAQ, log2)
# of Protein Features
p=5.685e−04*
MS−Intensity (iBAQ, log 2)
PBS
Bleo
p=0.002*
Relative MS−Intensity (log 2)
FR1
FR2
FR3
INSOL

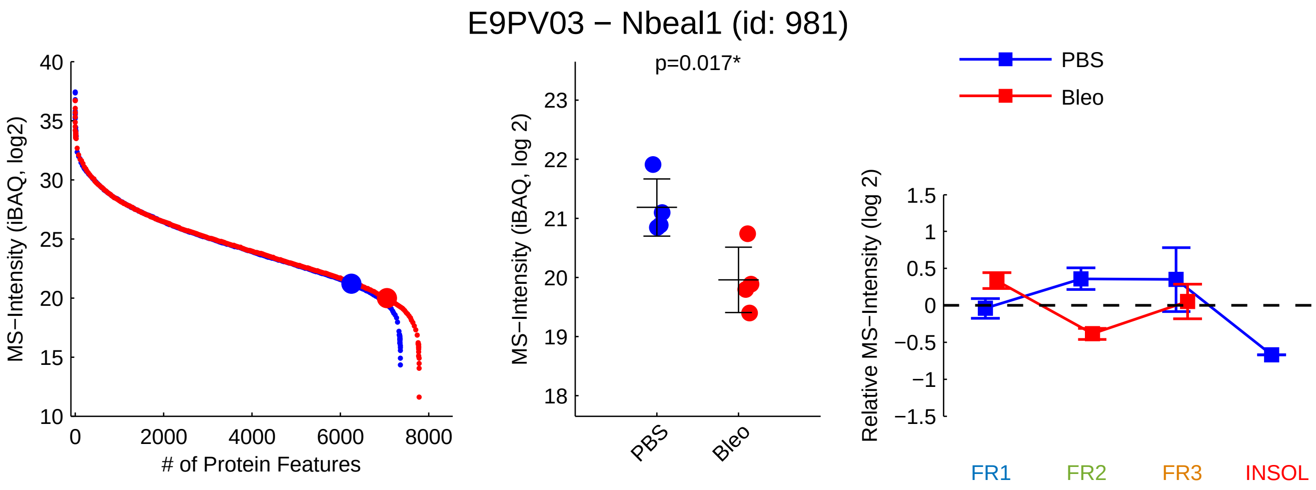
E9PV03 − Nbeal1 (id: 981)
MS−Intensity (iBAQ, log2)
# of Protein Features
p=0.017*
MS−Intensity (iBAQ, log 2)
PBS
Bleo
Relative MS−Intensity (log 2)
FR1
FR2
FR3
INSOL

E9PV24 − Fga (id: 982)

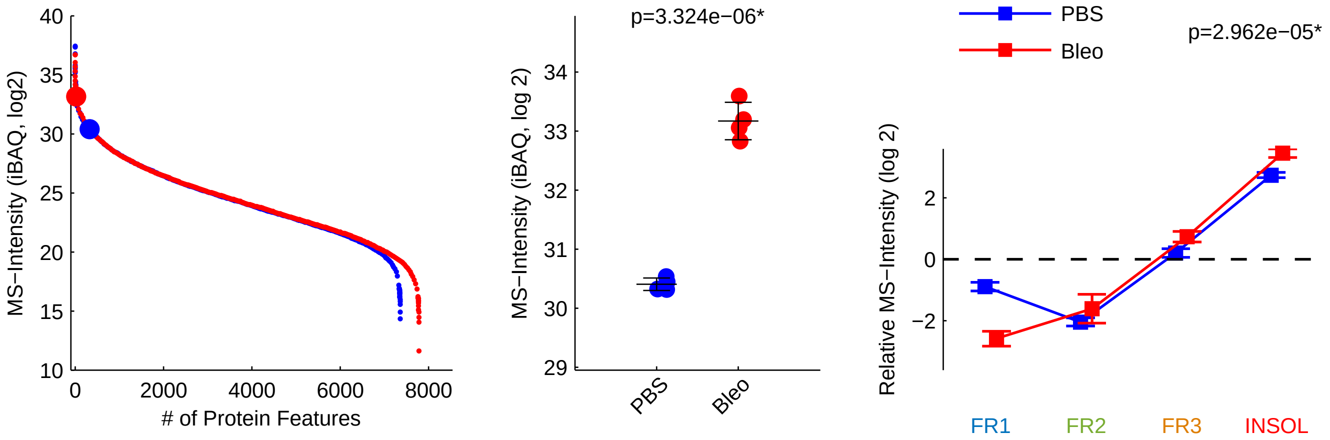

E9PV38 − Ces2g (id: 983)

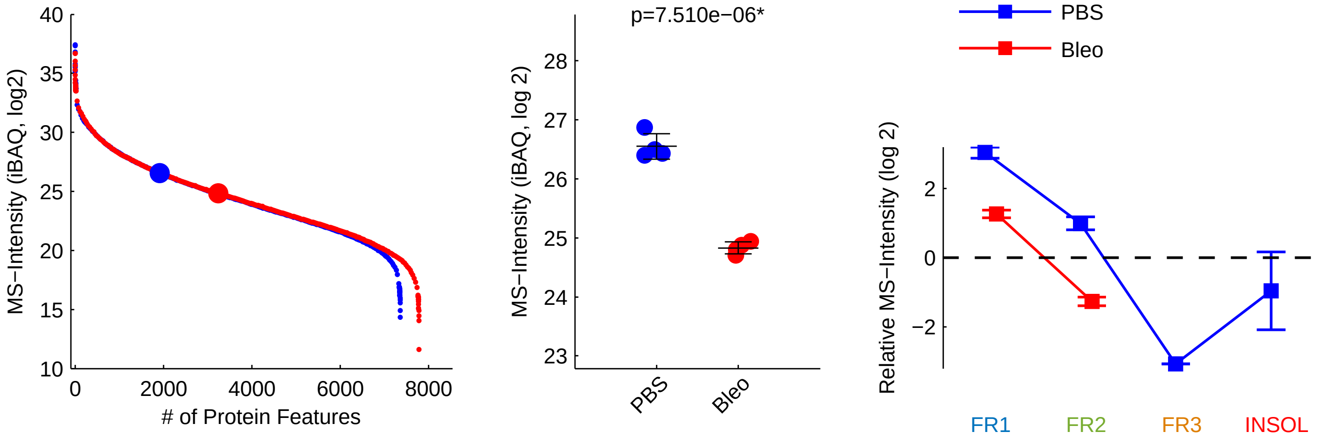

E9PV41 − Diap1 (id: 984)

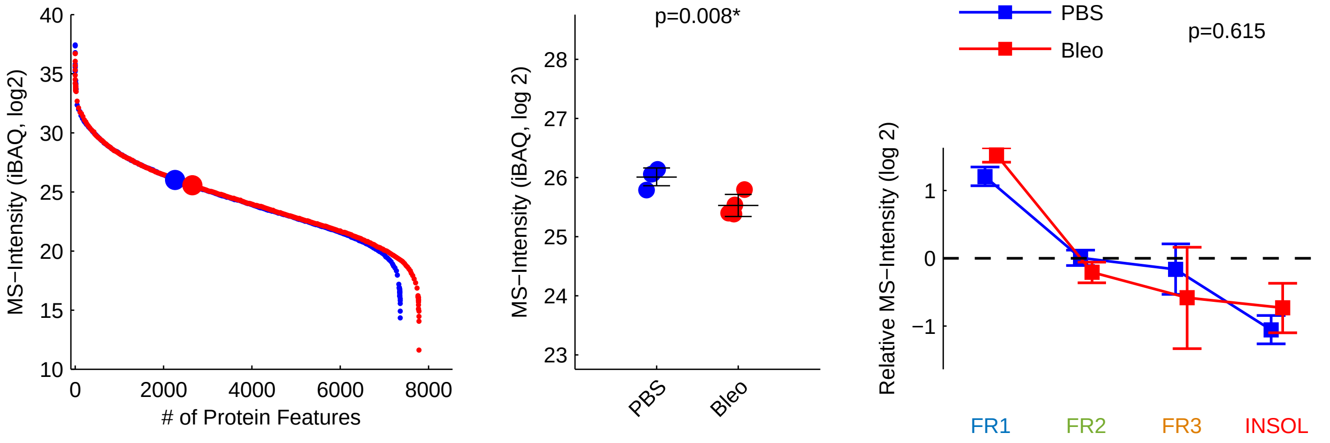

E9PV44 − Atpif1 (id: 985)

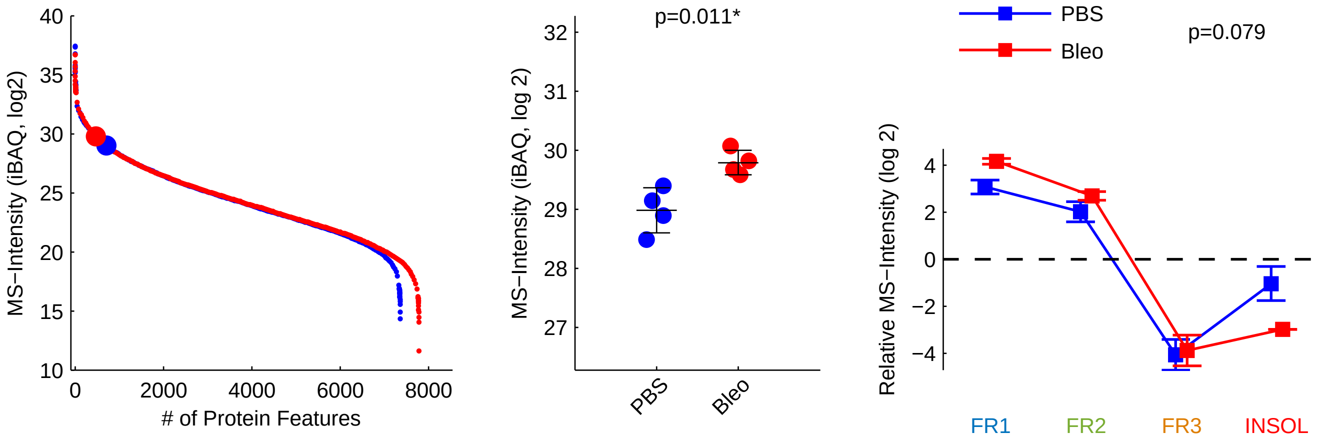

E9PVA8 − Gcn1l1 (id: 991)

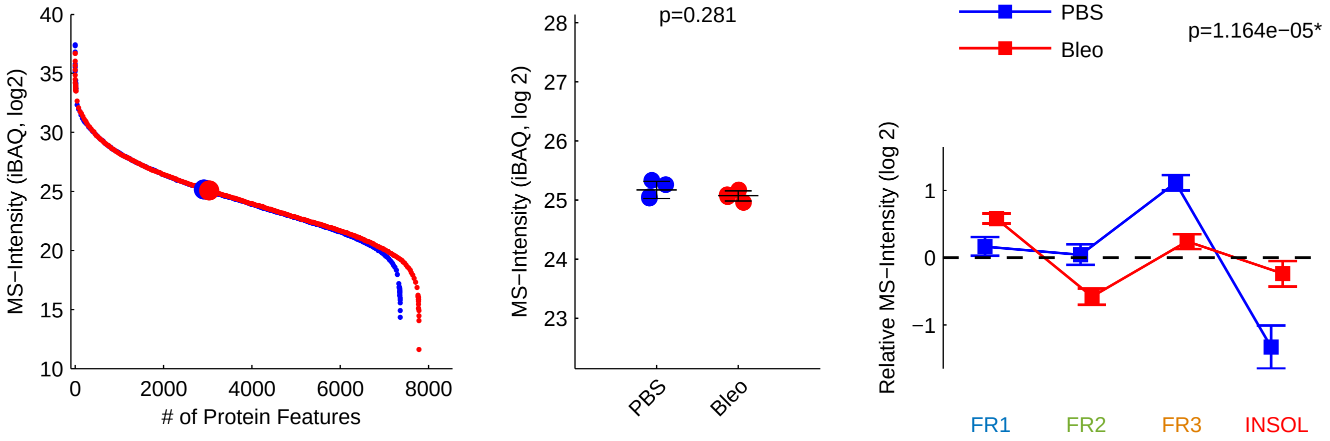

E9PVC6 − Eif4g1 (id: 993)

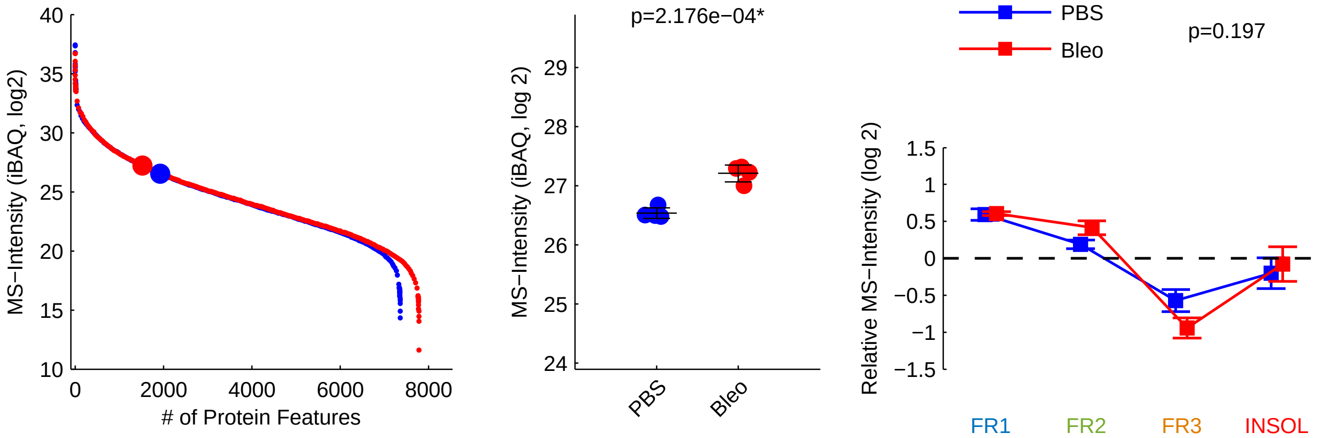

E9QAH1 − Golgb1 (id: 1007)

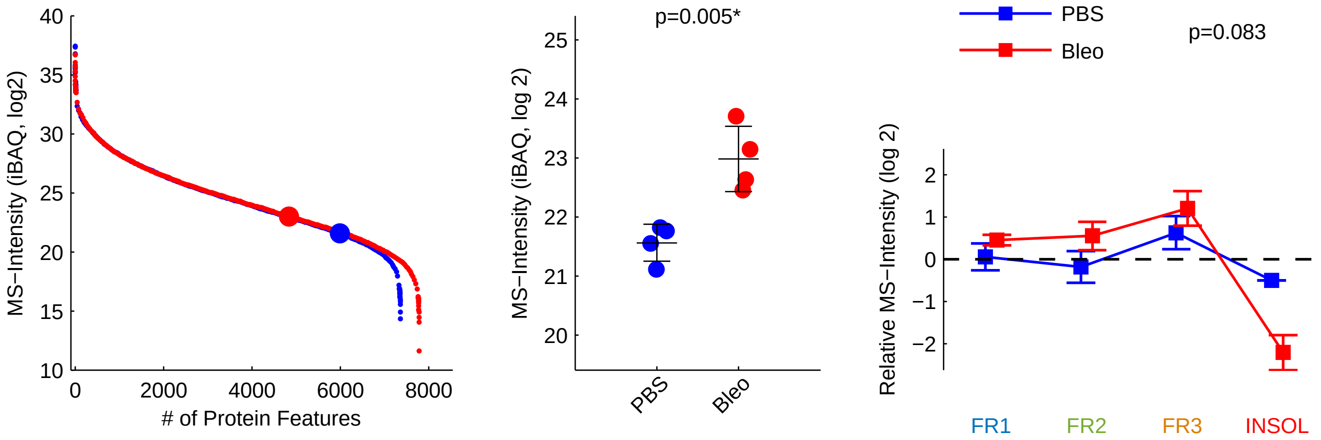

O35099 − Map3k5 (id: 1019)

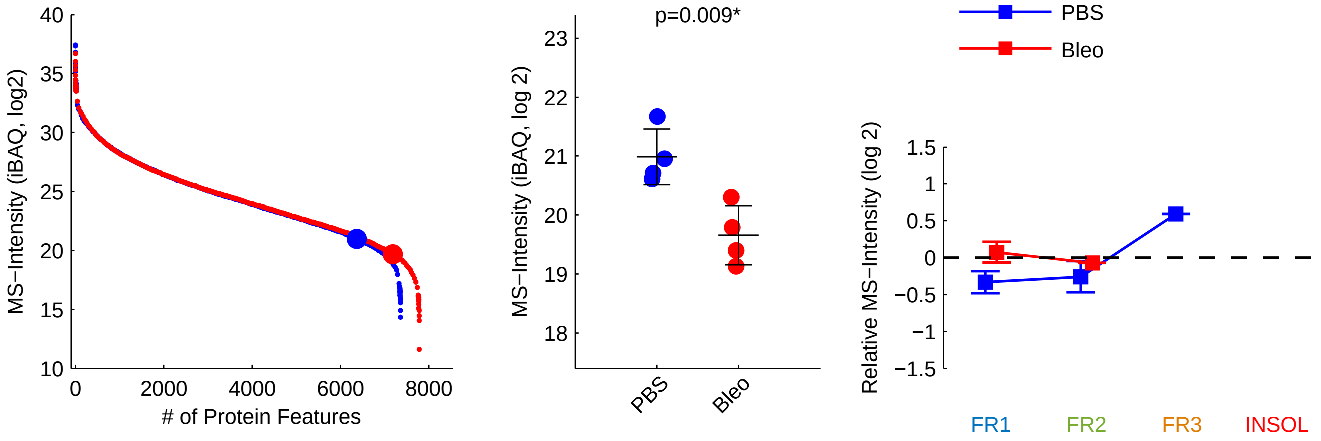

E9PWQ3 − Col6a3 (id: 1027)

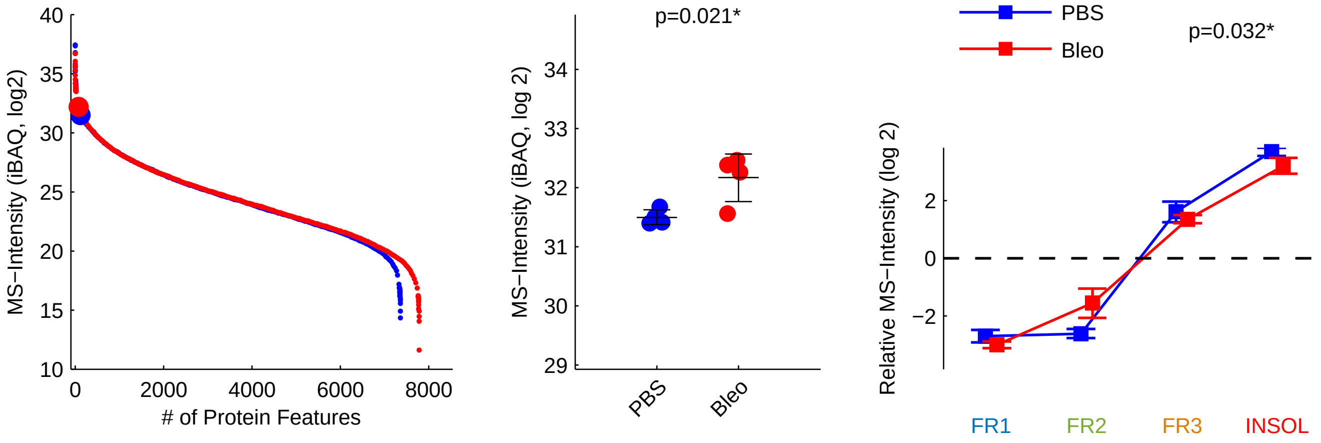

E9PWT4 − H2−Q7 (id: 1030)

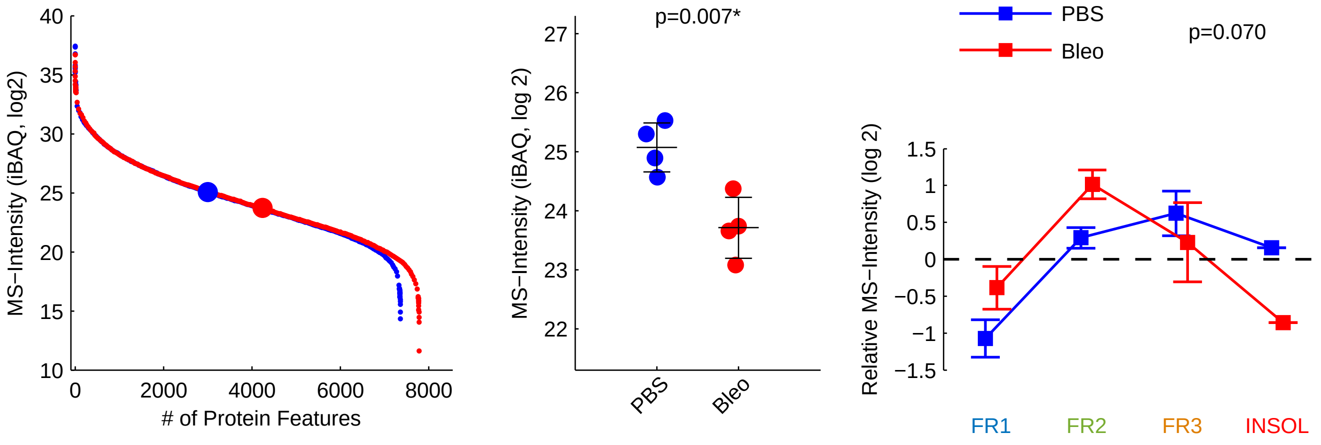

Q69Z37 − Samd9l (id: 1045)

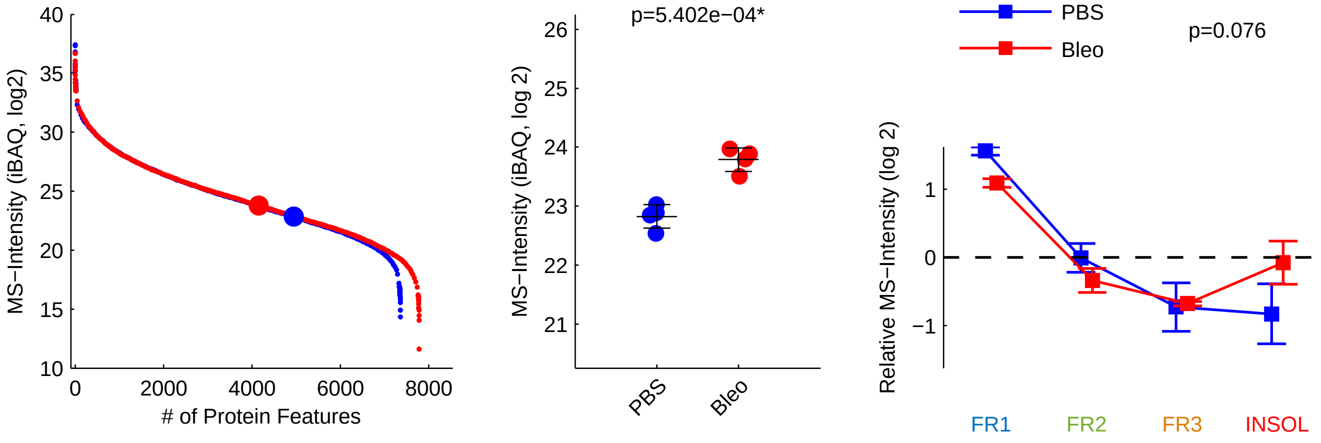

Q60847 − Col12a1 (id: 1049)

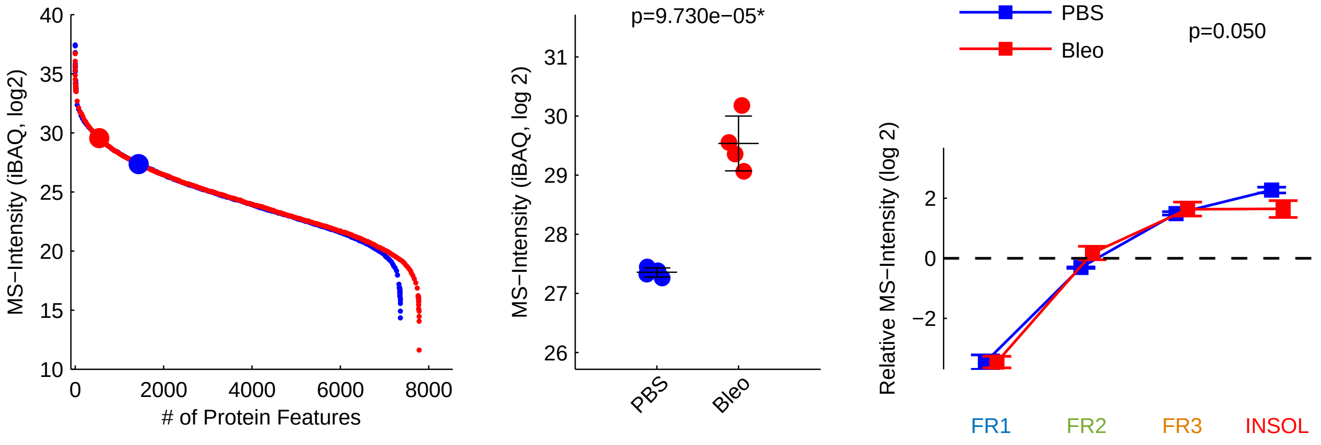

P27870 − Vav1 (id: 1056)

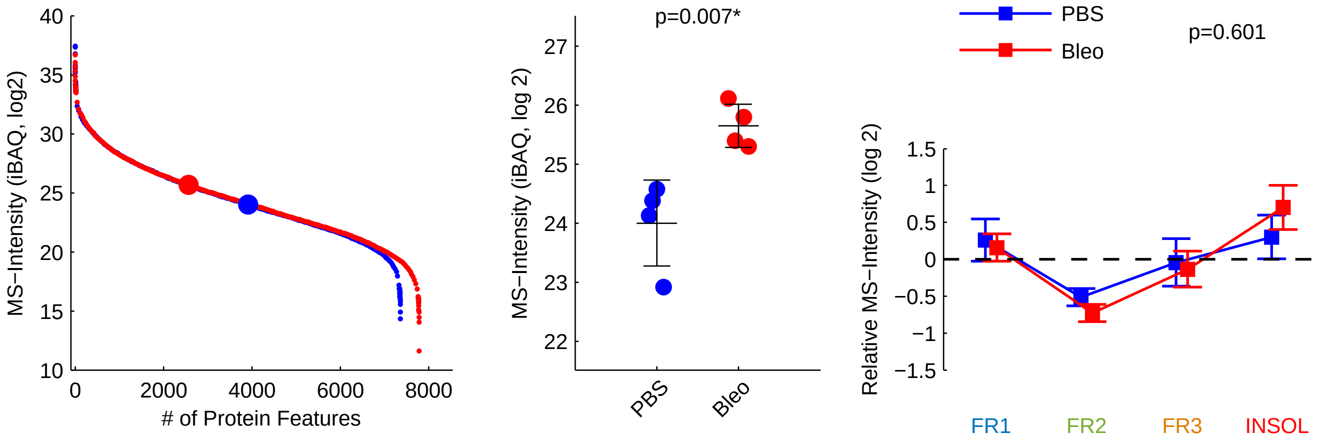

E9PY08 − Adamts1 (id: 1061)

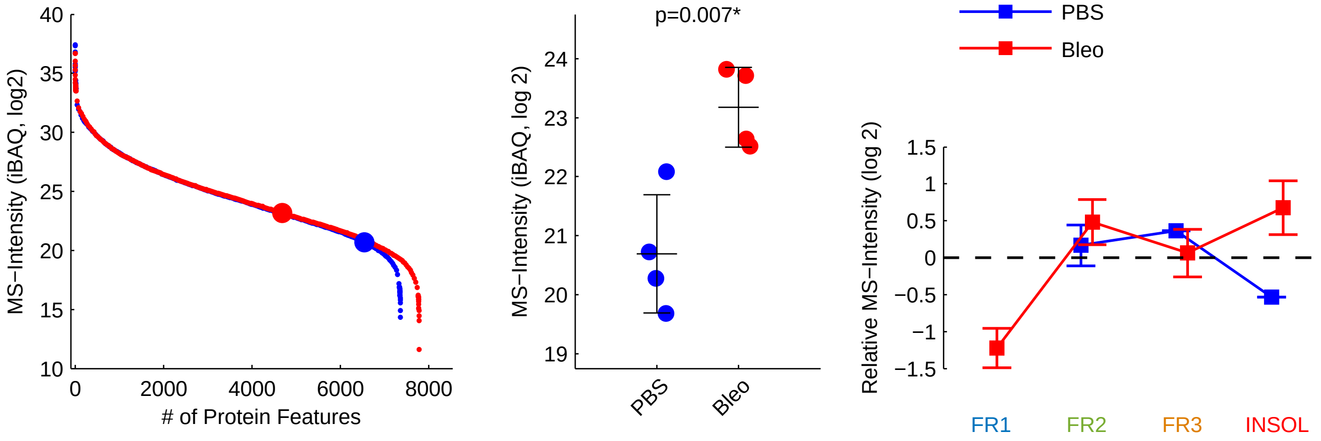

G3XA35 − Vcan (id: 1072)

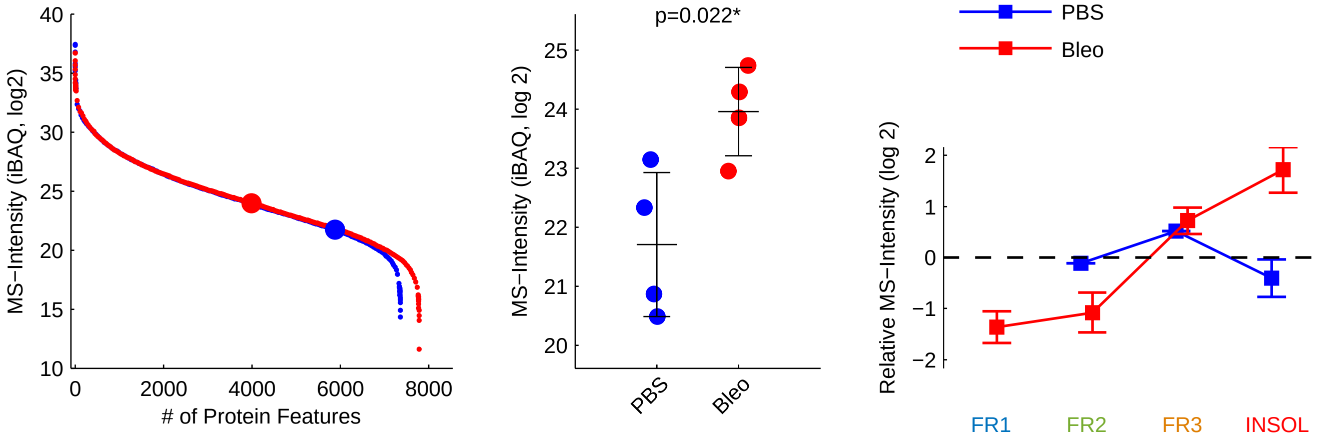

E9PYK3 − Parp4 (id: 1077)

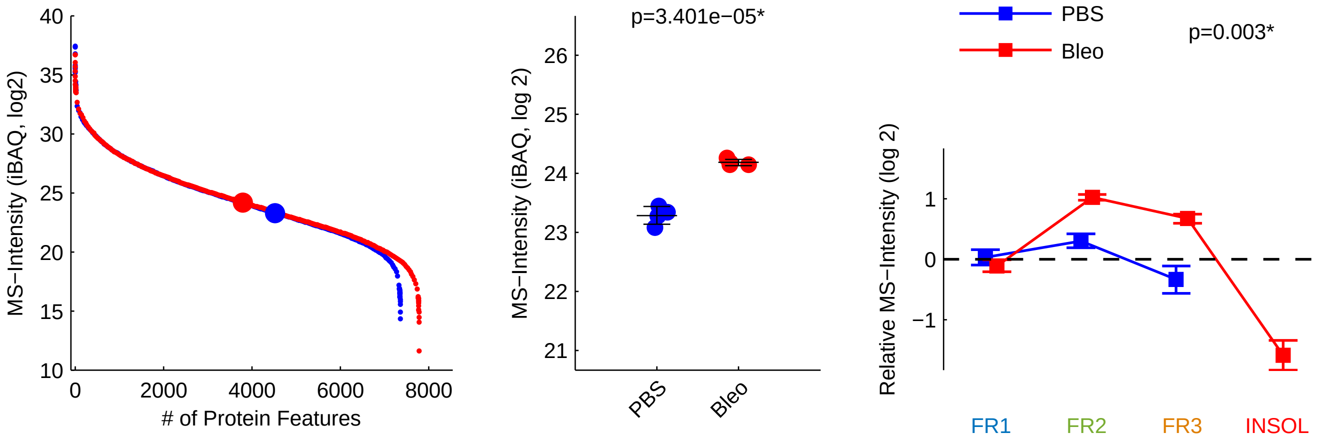

Q9CXW4 − Rpl11 (id: 1081)

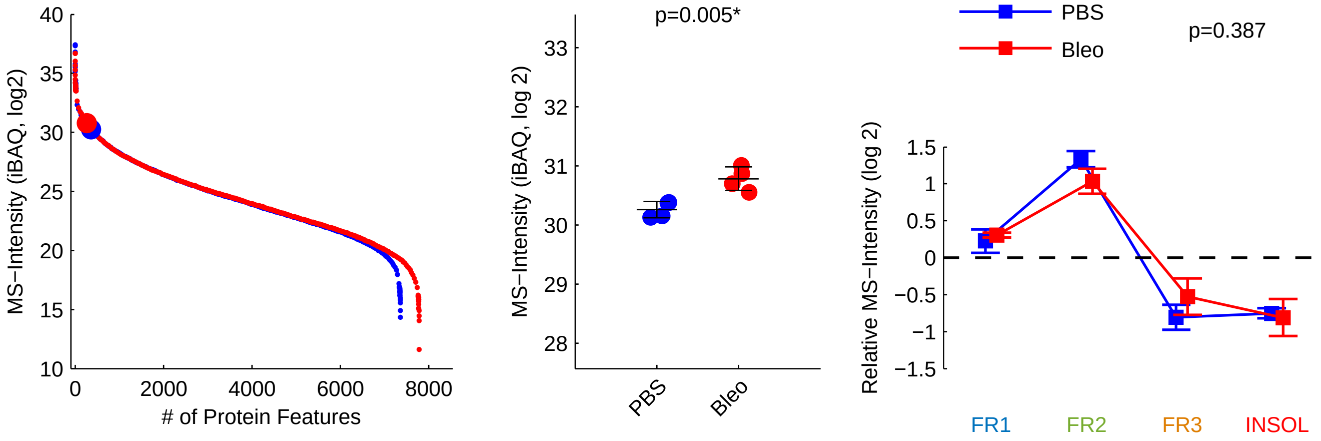

Q8R5M8 − Cadm1 (id: 1082)

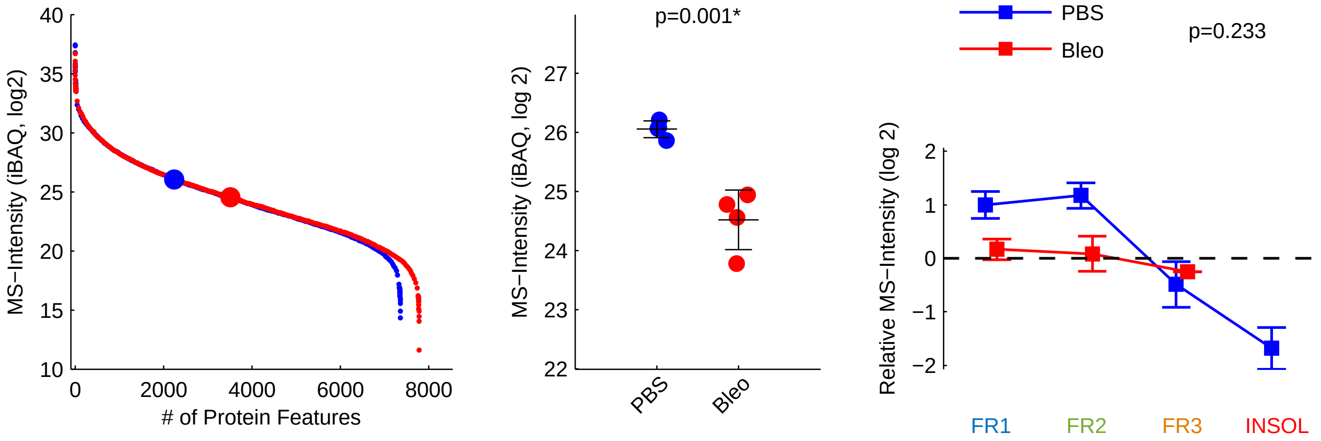

E9PZ00 − Psap (id: 1087)

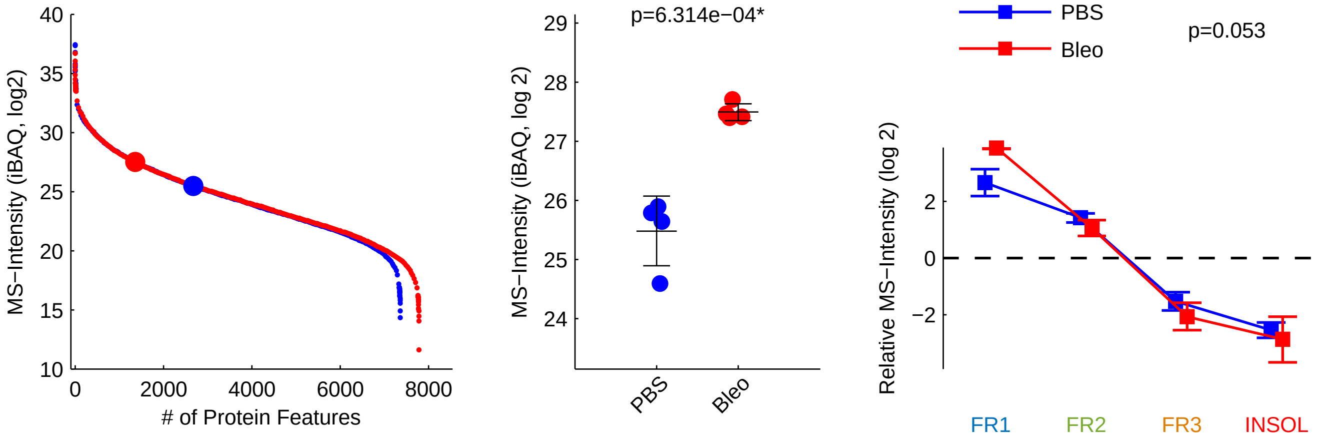

E9PZ16 − Hspg2 (id: 1089)

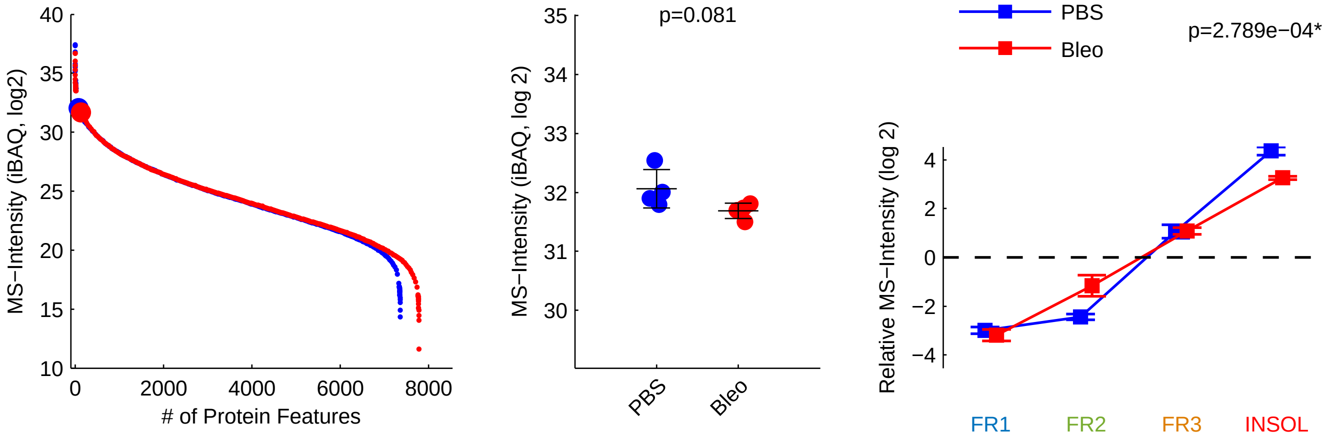

G5E8P1 − Brd1 (id: 1091)

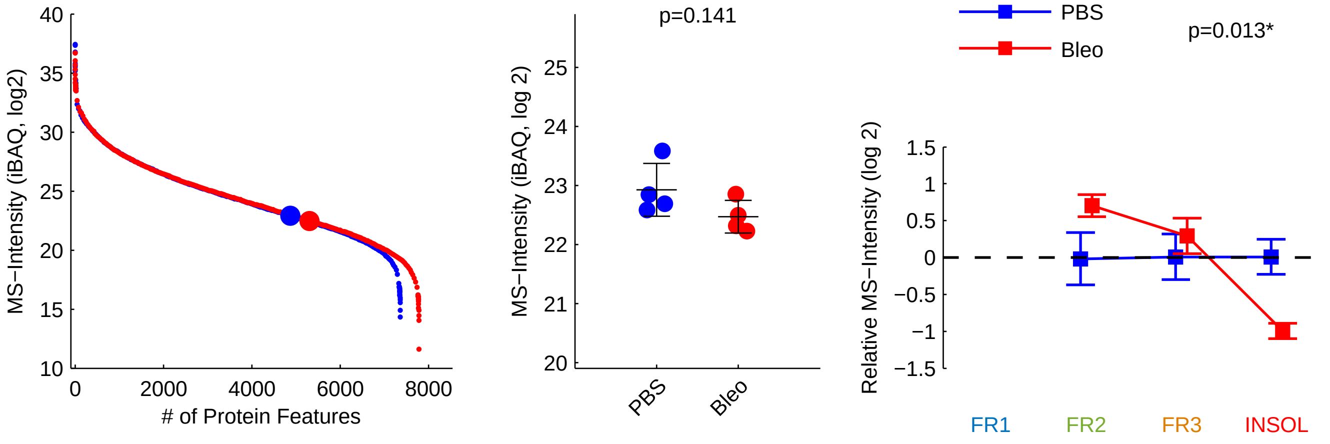

E9PZF0 − Gm20390 (id: 1097)

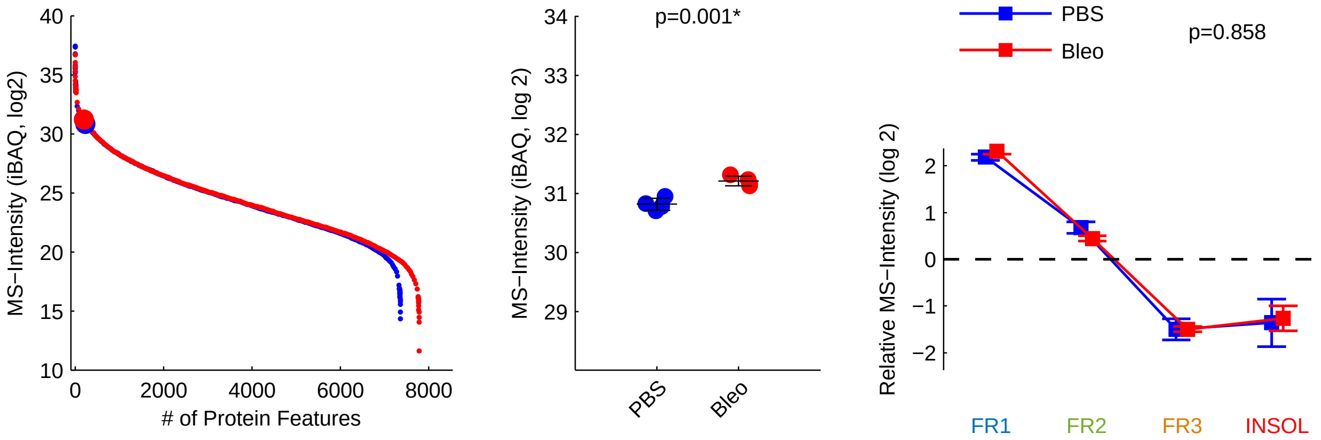

E9PZI9 − Cd200 (id: 1098)

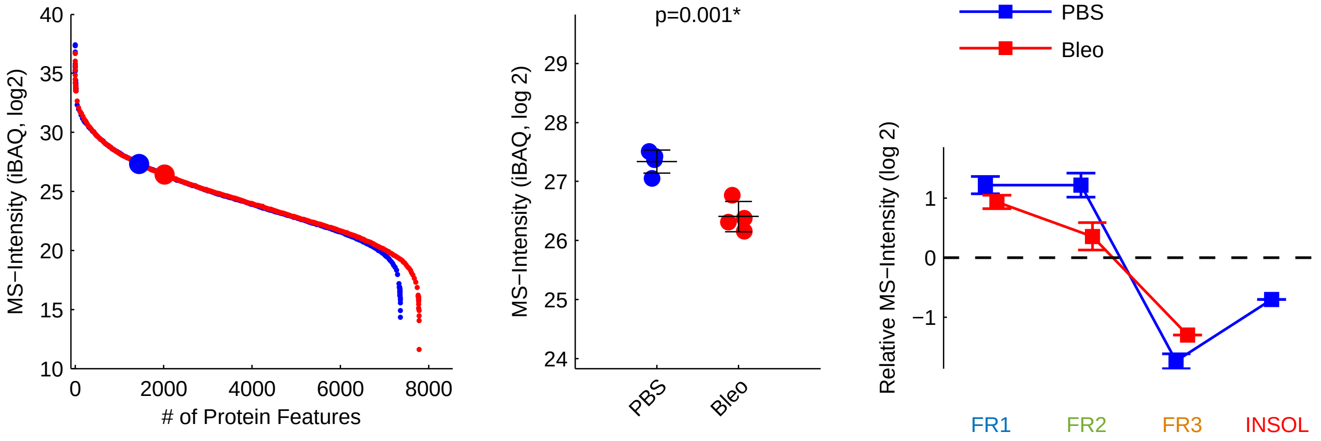

E9PZJ8 − Ascc3 (id: 1100)

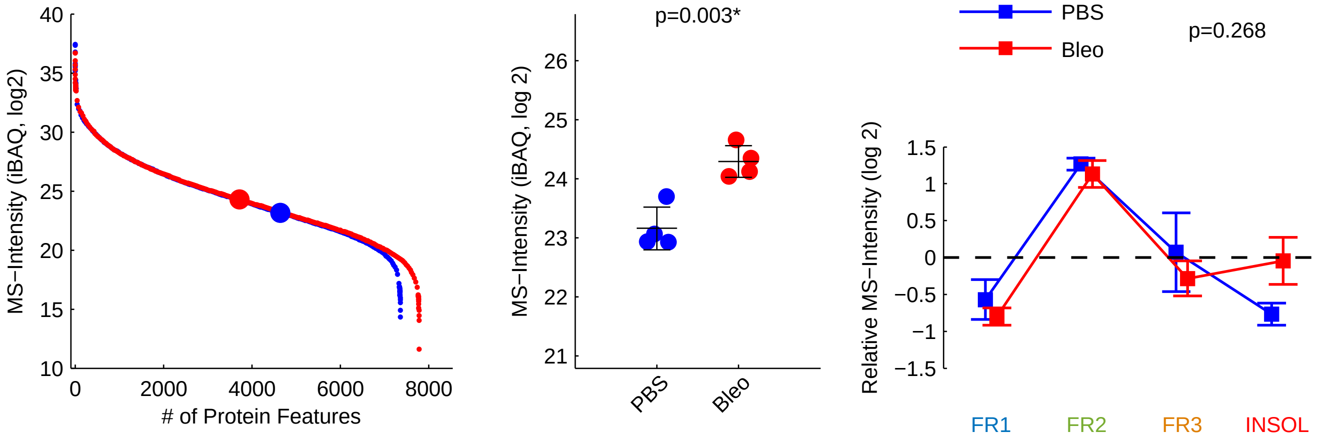

E9PZM7 − Scaf11 (id: 1102)

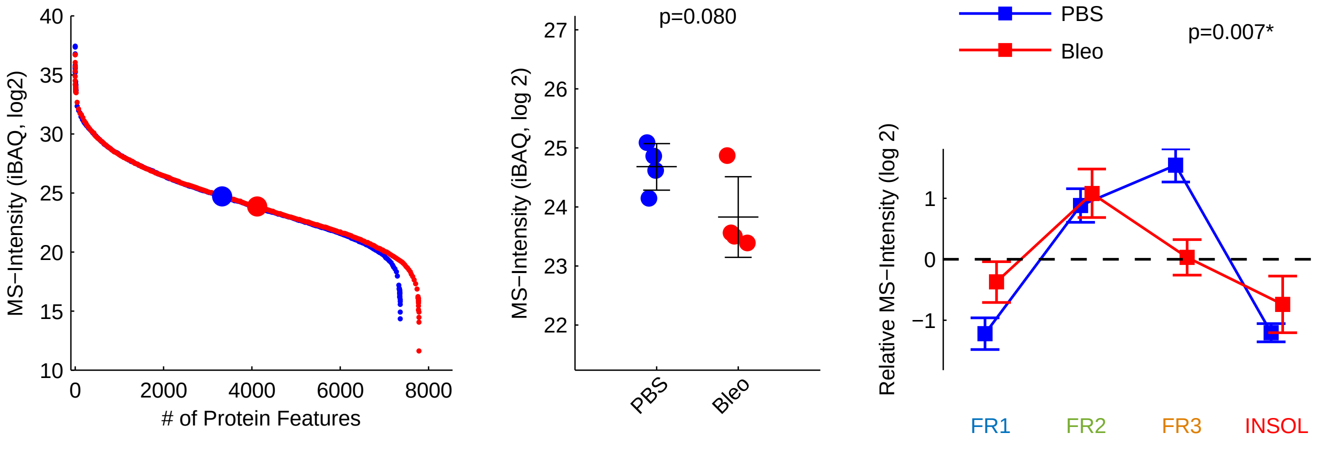

E9PZW8 − Myo9b (id: 1108)

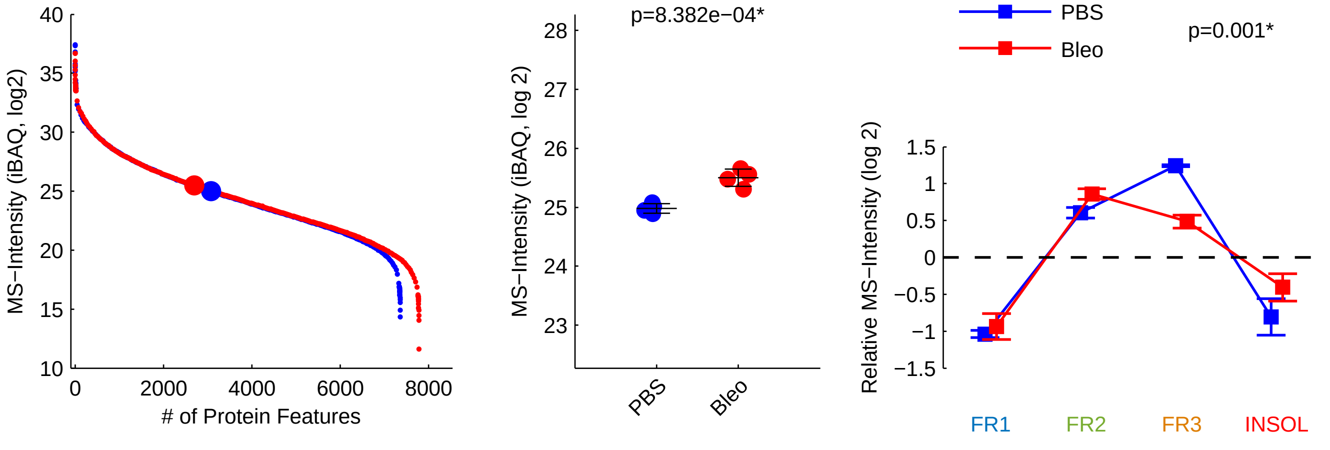

E9Q035 − Gm20425 (id: 1111)

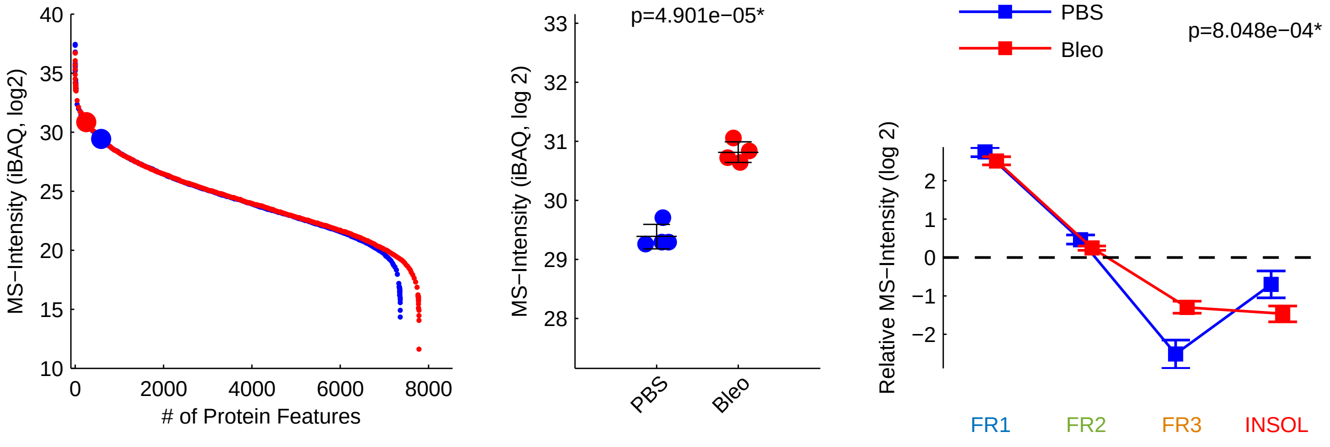

Q68FM7 − Arhgef11 (id: 1116)

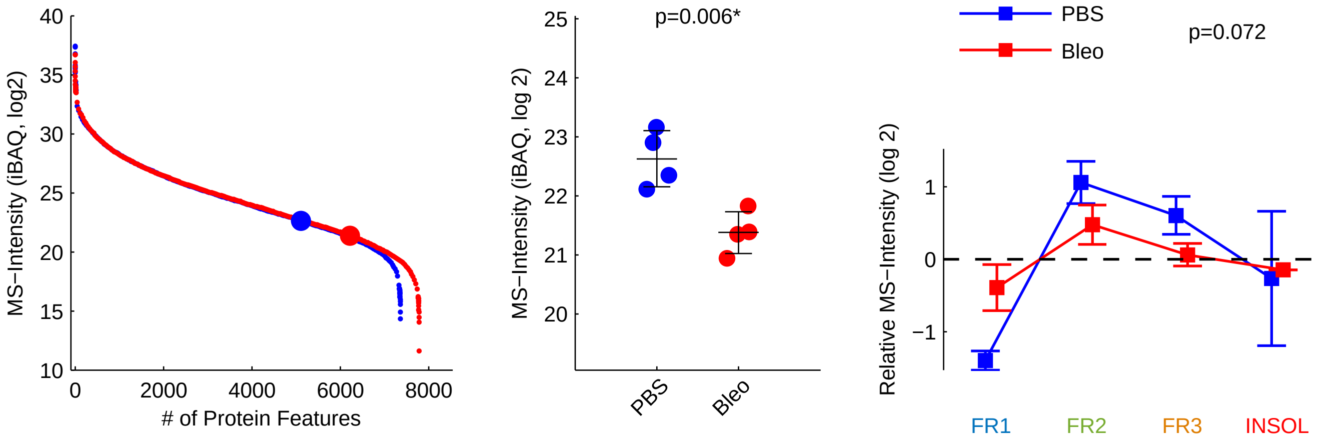

E9Q0S6 − Tns1 (id: 1124)

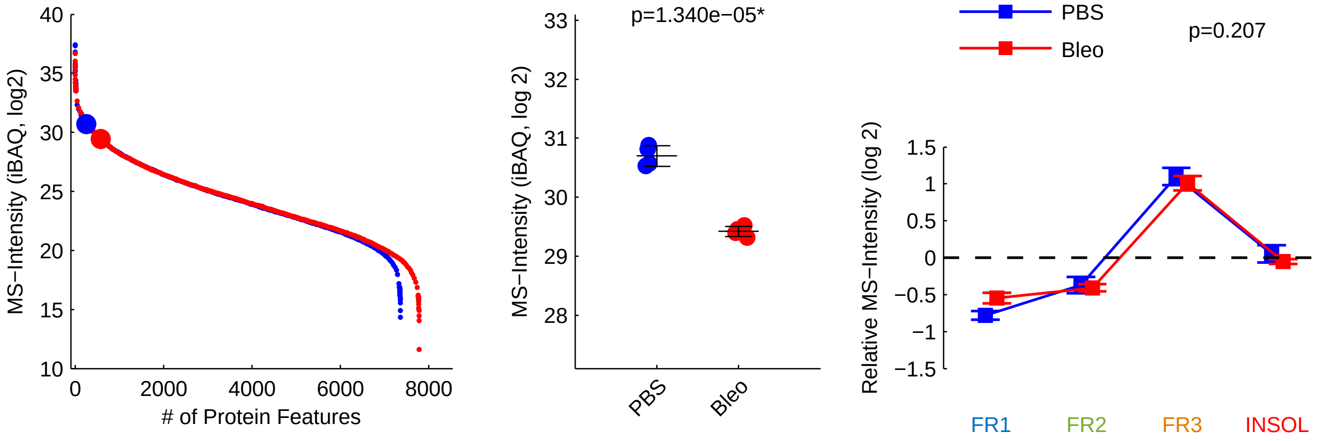

E9Q0Y4 − Sipa1 (id: 1129)

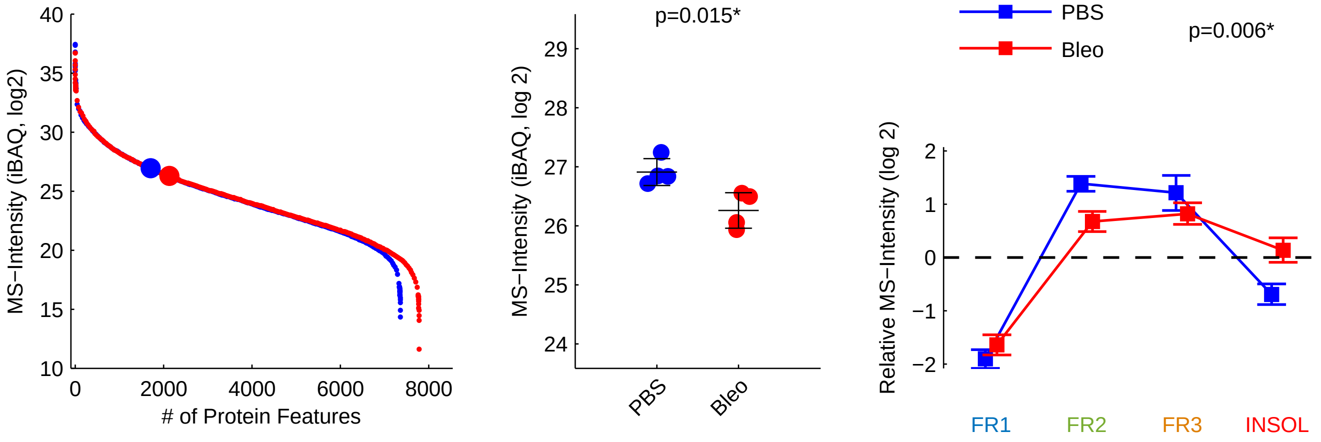

Q8BP67 − Rpl24 (id: 1130)

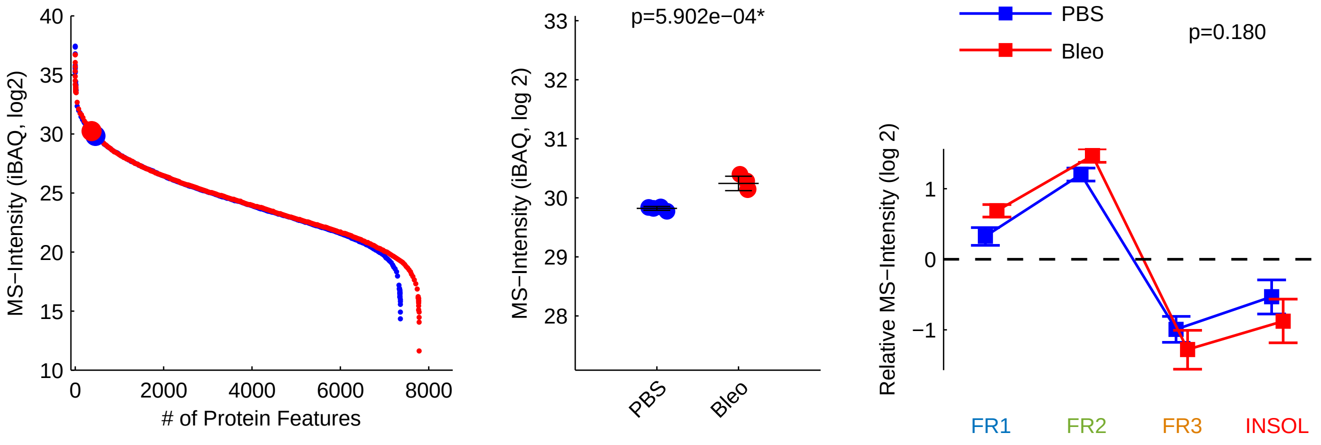

E9Q175 − Myo6 (id: 1135)

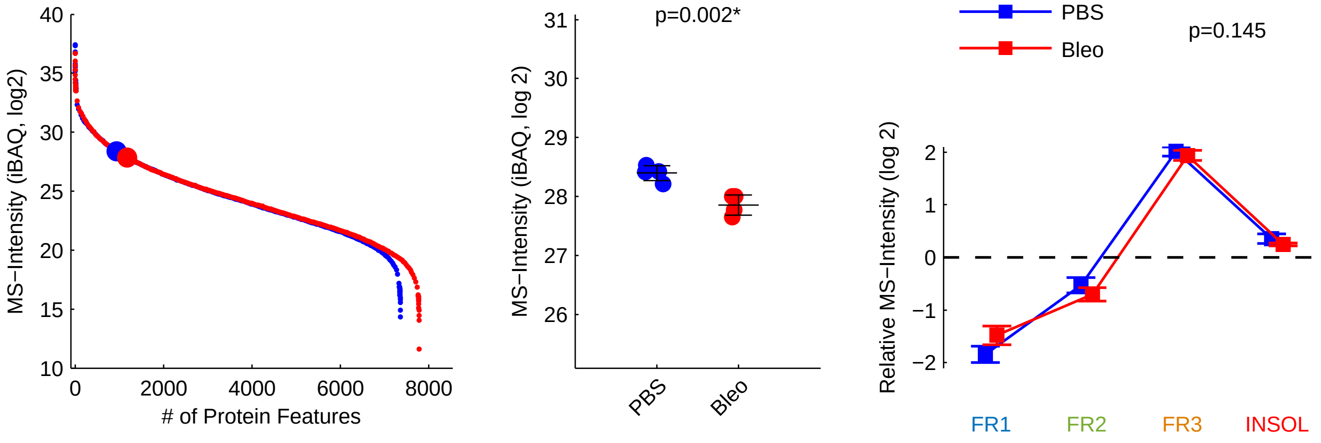

E9Q1D6 − Ltbp2 (id: 1140)

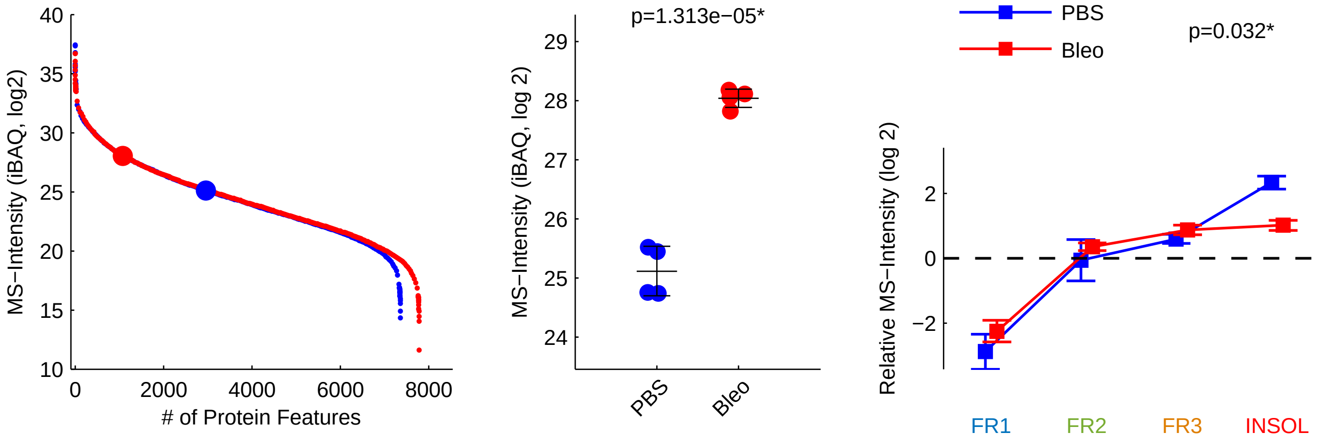

E9Q1M1 − Pdzd2 (id: 1146)

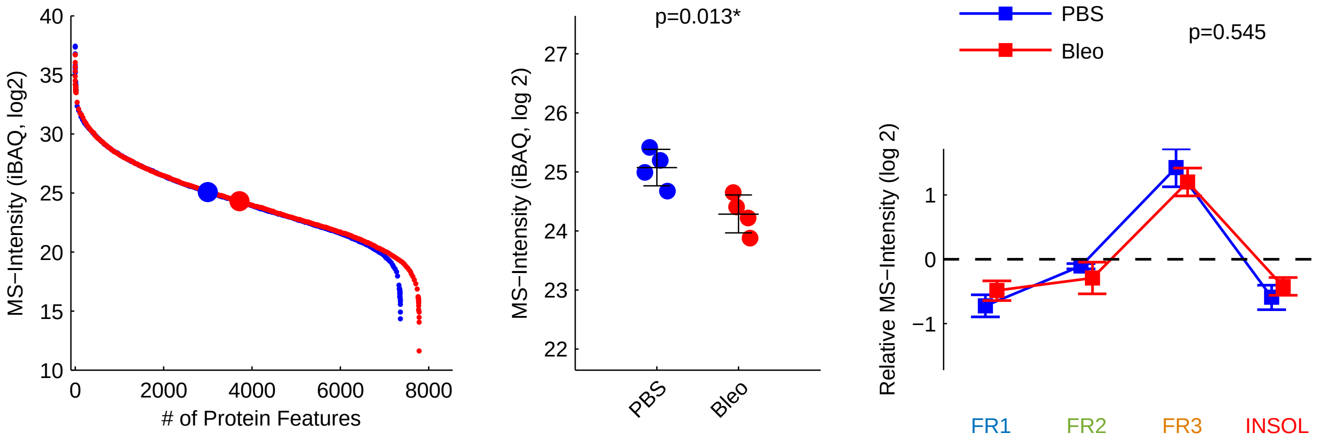

Q01405 − Sec23a (id: 1150)

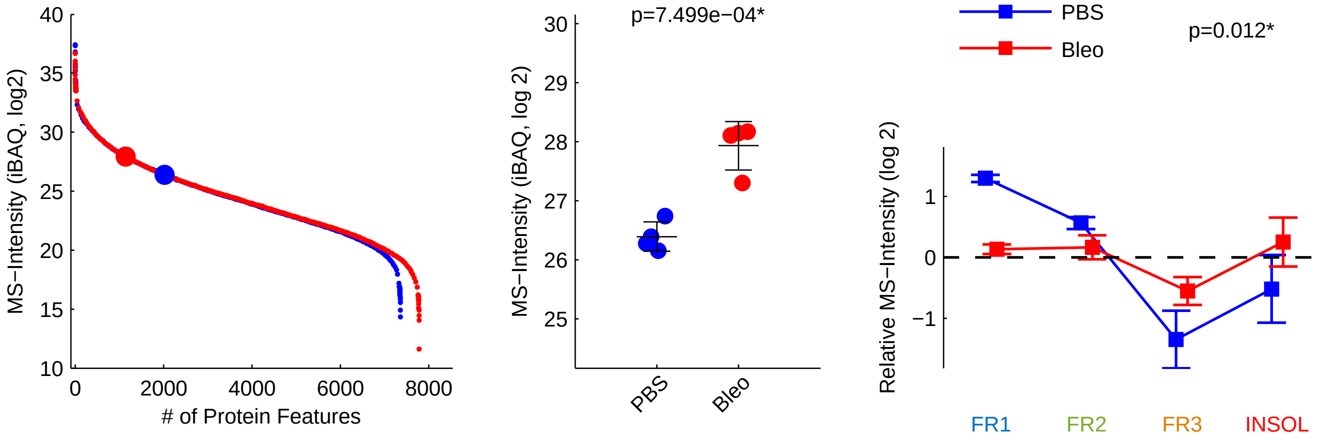

E9Q2I4 − Elmsan1 (id: 1168)

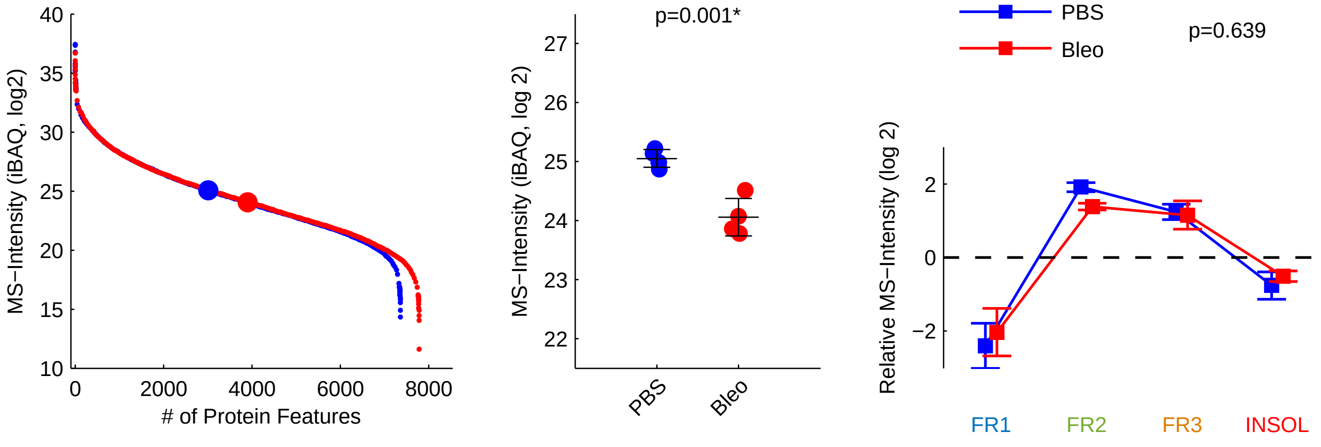

E9Q2M9 − Wdfy4 (id: 1169)

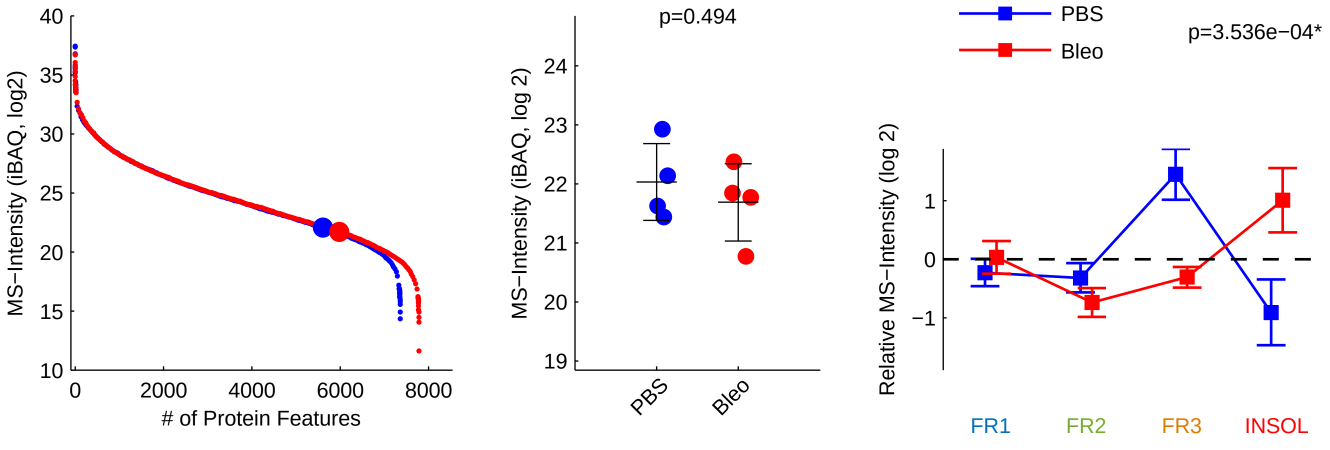

E9Q317 − Gm10094 (id: 1180)

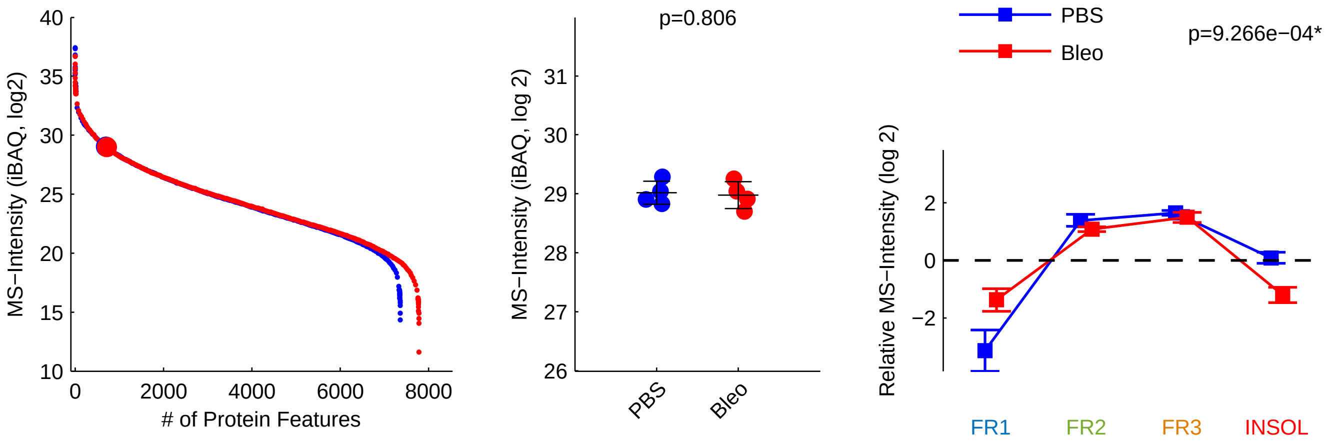

E9Q3C1 − C2cd2 (id: 1183)

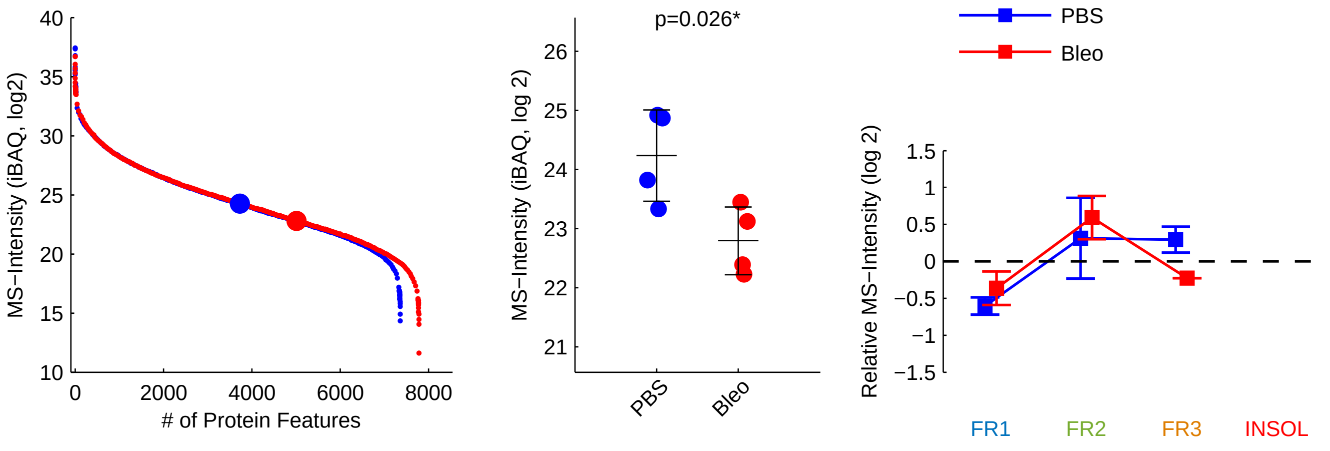

E9Q3E2 − Synpo (id: 1185)

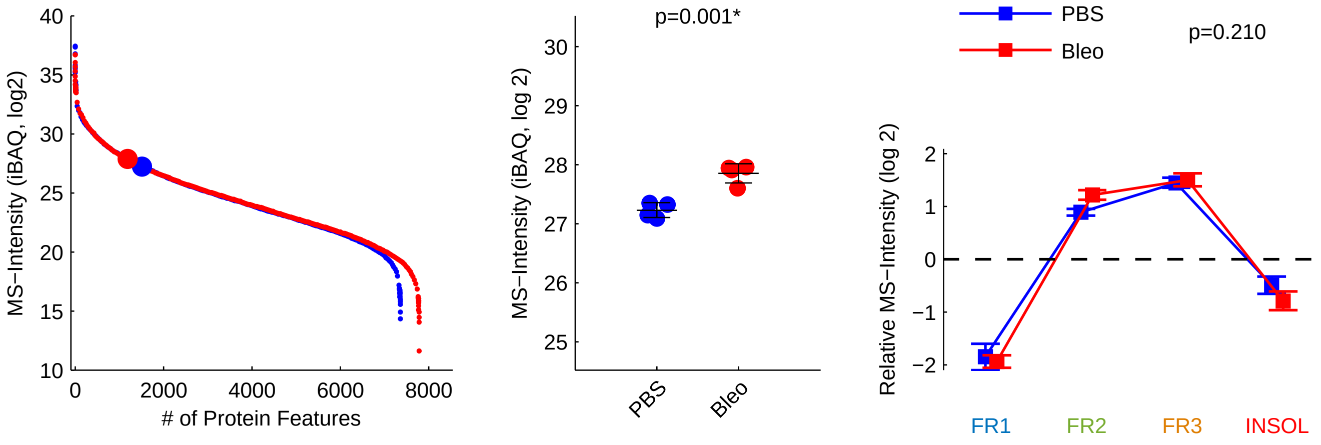

Q8BXK8 − Agap1 (id: 1189)

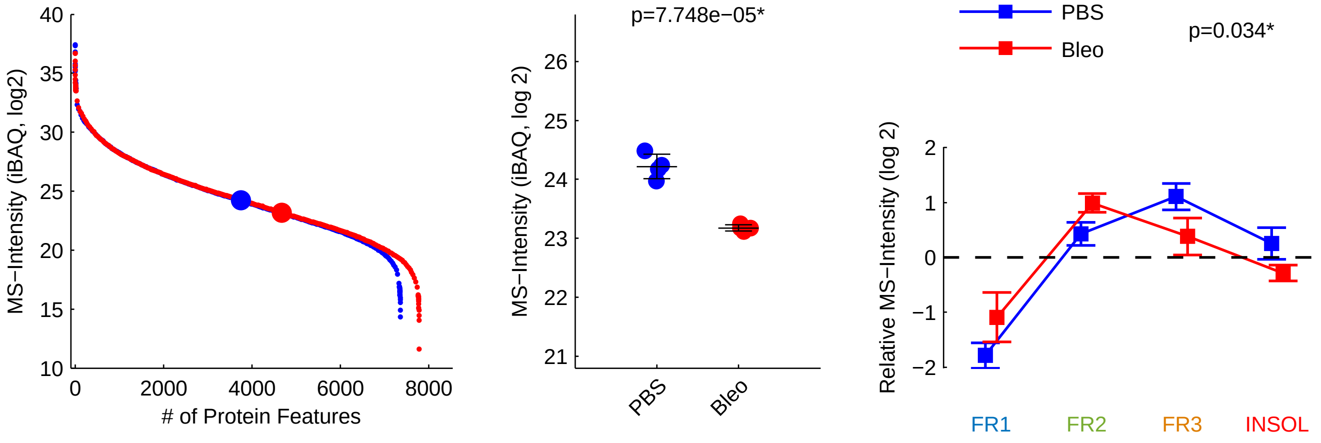

E9Q3L2 − Pi4ka (id: 1191)

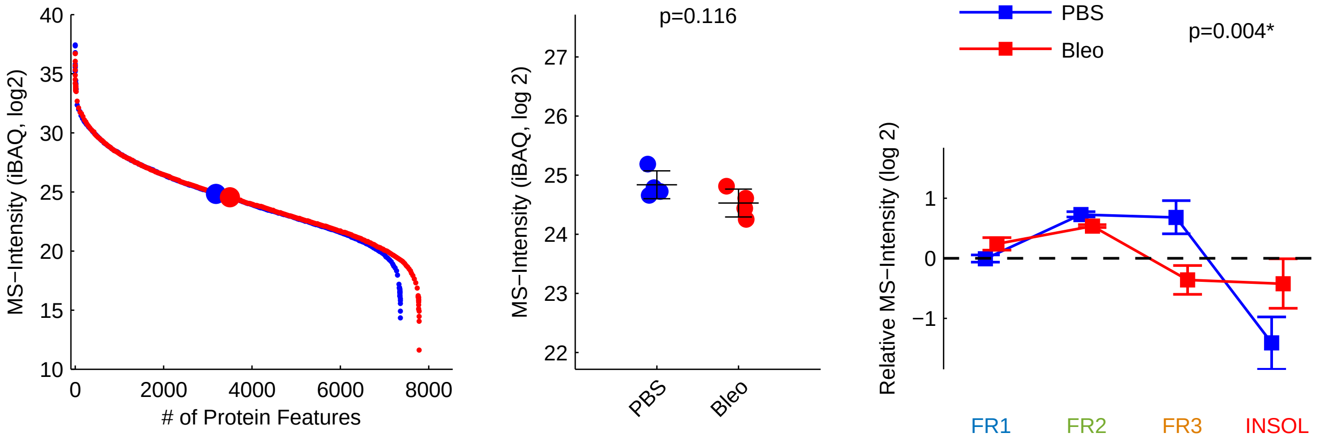

E9Q586 − Dctn1 (id: 1192)

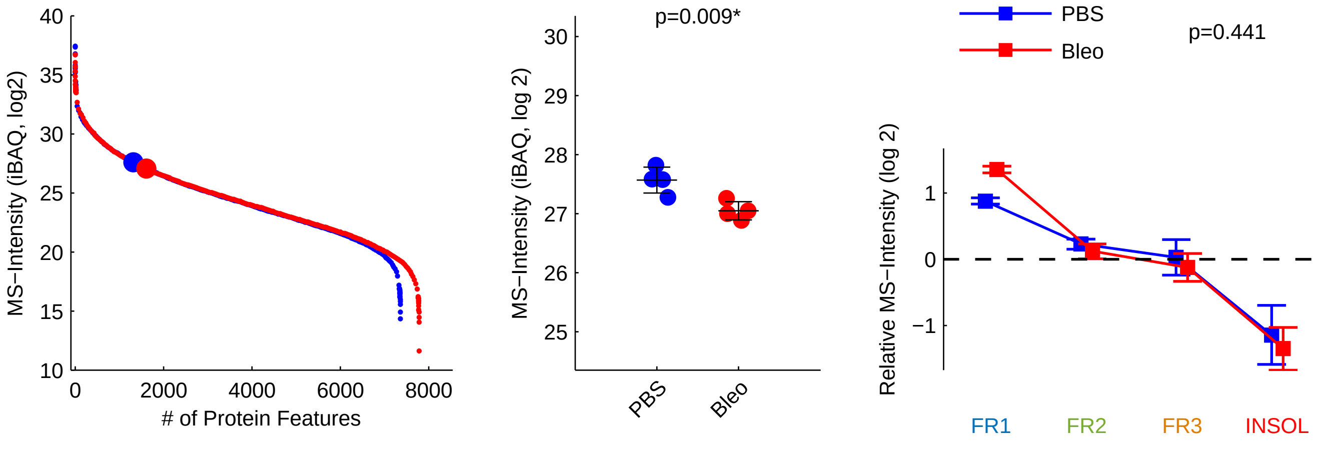

E9Q3N9 − Efemp2 (id: 1195)

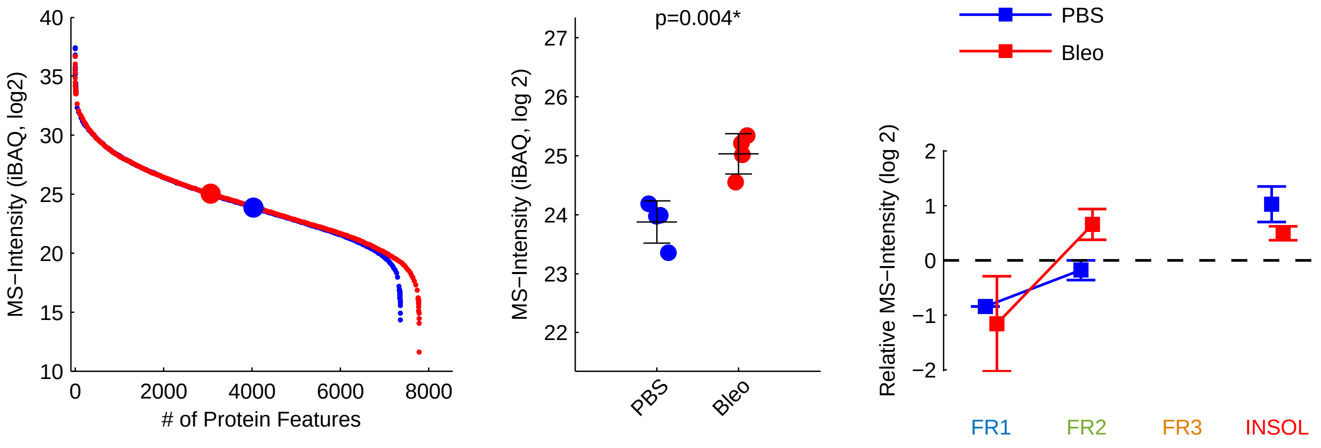

Q61490 − Alcam (id: 1196)

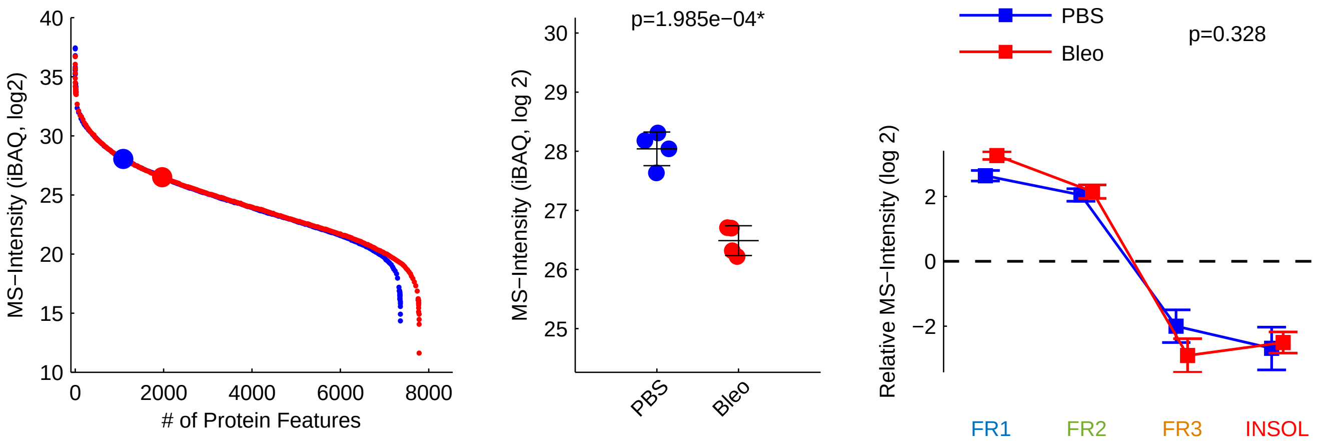

E9Q4M2 − Lipe (id: 1216)

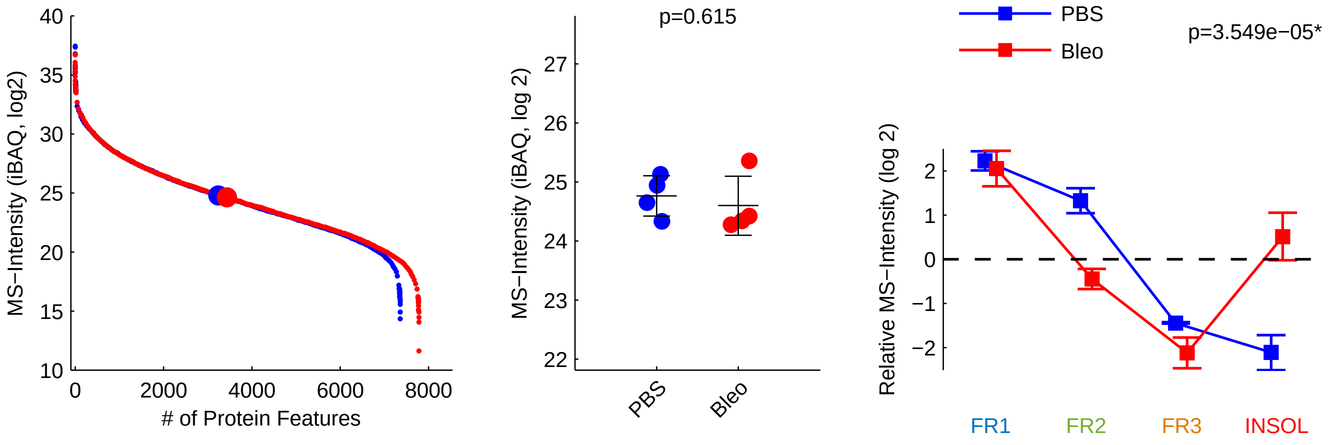

E9Q4X2 − Uggt2 (id: 1223)

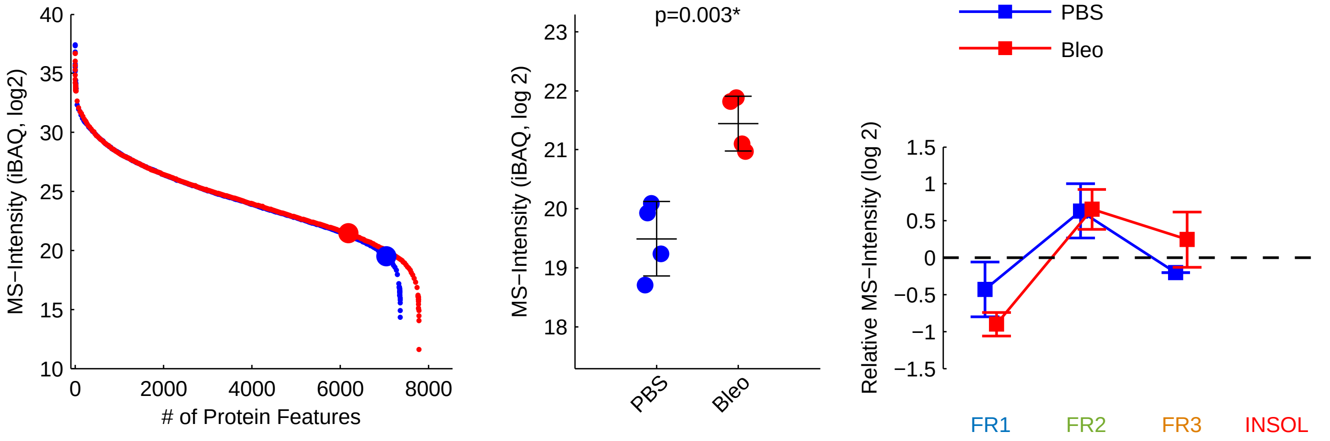

E9Q4Y4 − Cep192 (id: 1225)

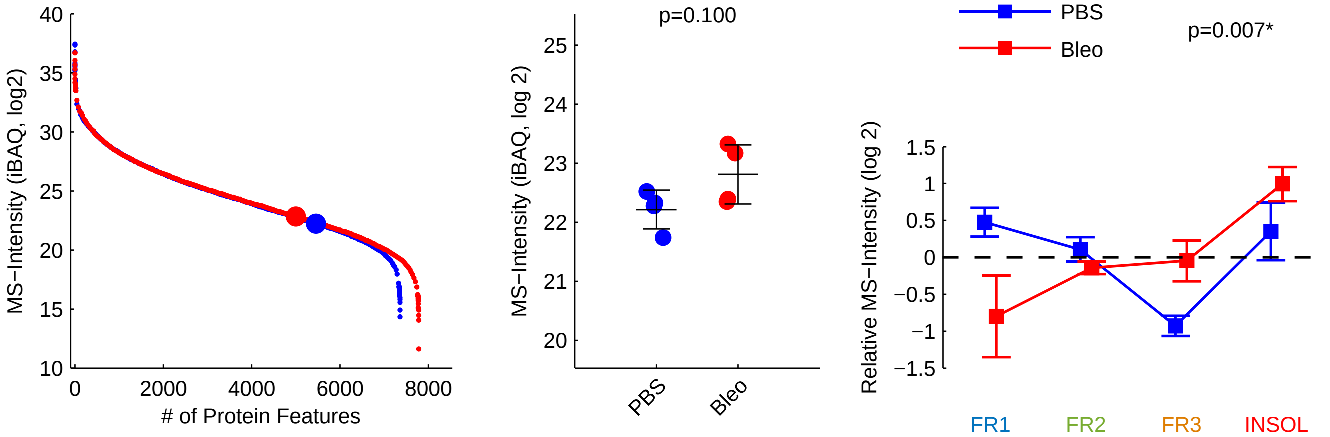

Q8C008 − Dzank1 (id: 1226)

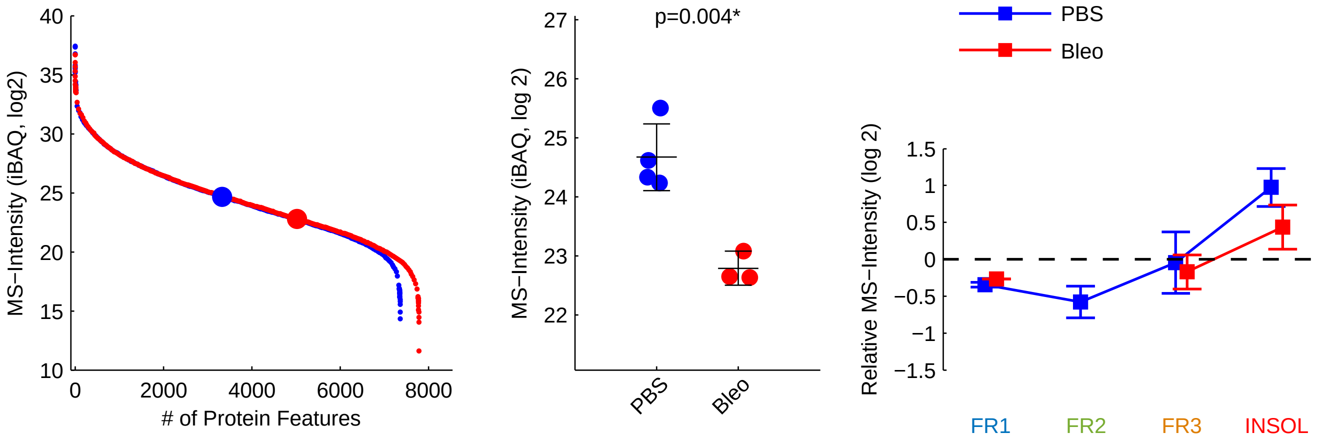

E9Q555 − Rnf213 (id: 1228)

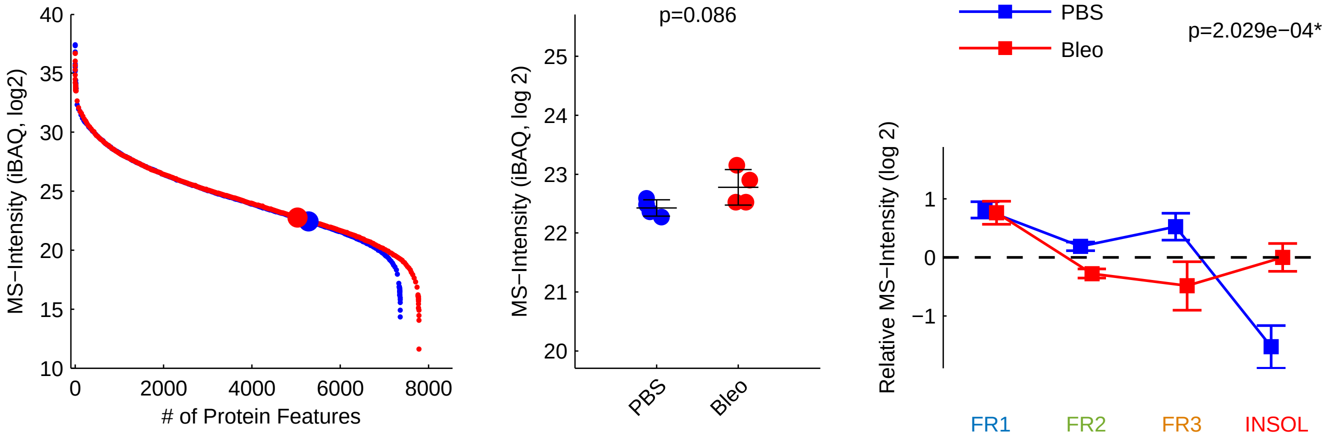

E9QNH6 − Myo1b (id: 1230)

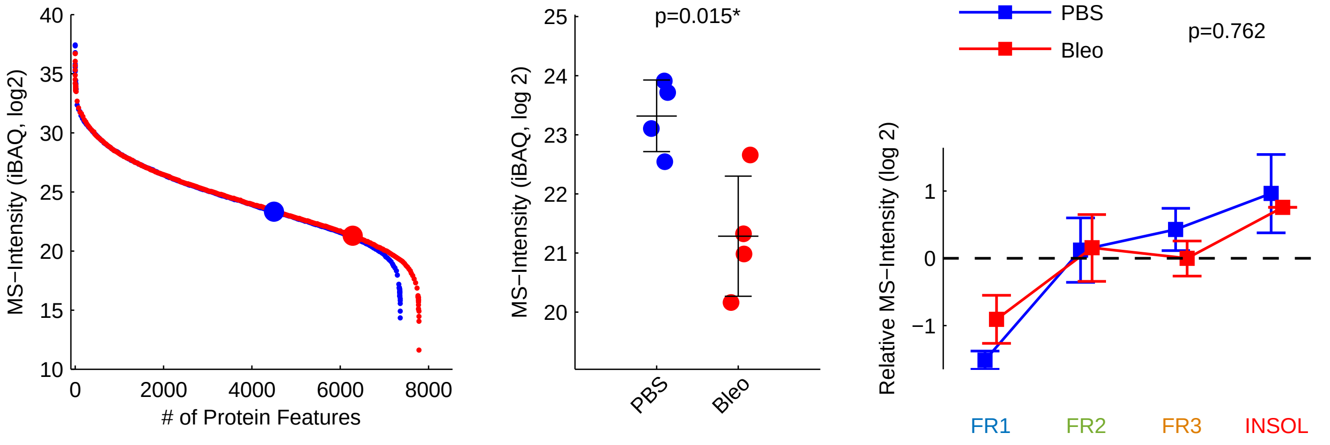

E9Q5C9 − Nolc1 (id: 1234)

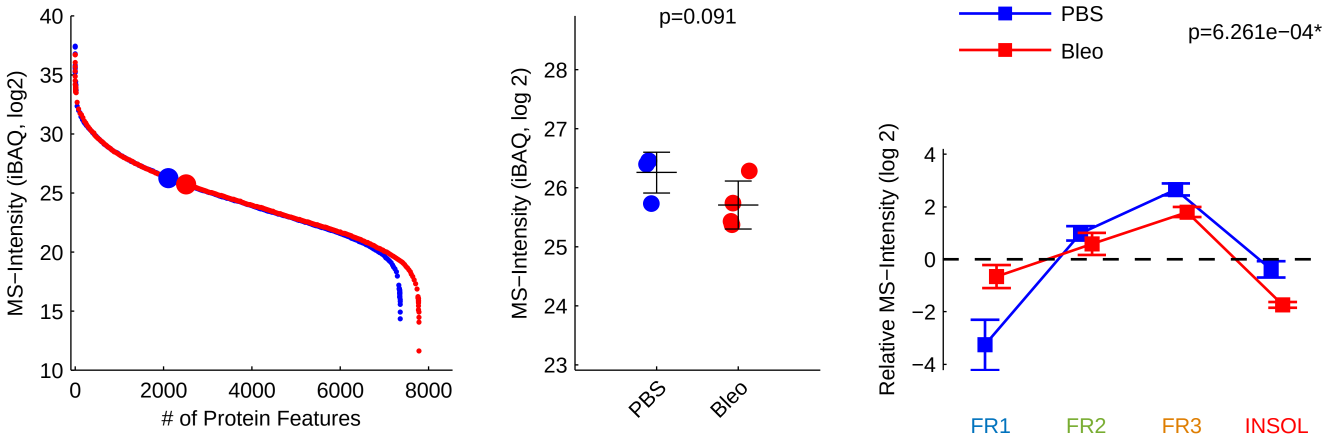

E9Q5I7 − Eng (id: 1242)

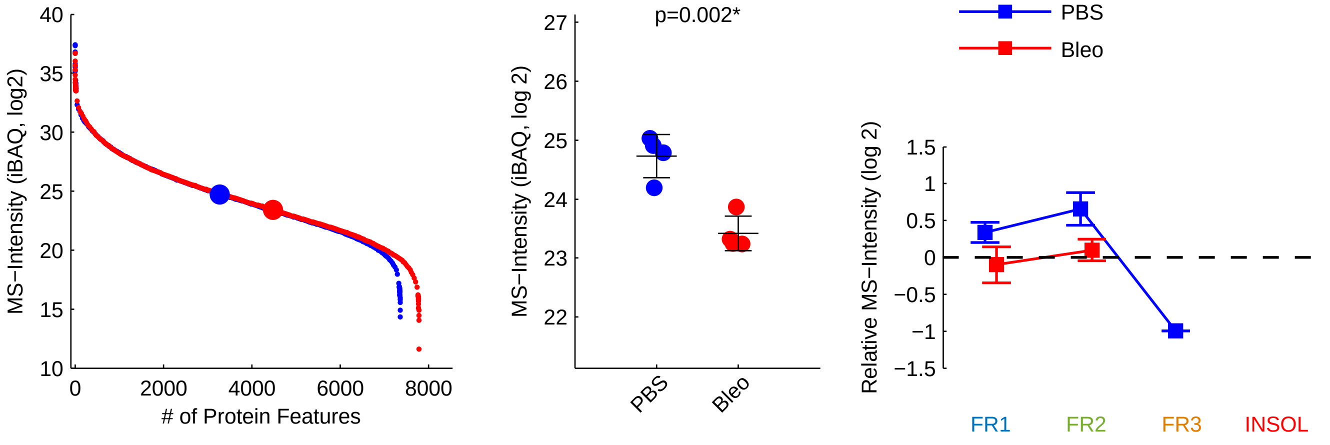

Q8K0Z5 − Tpm3 (id: 1243)

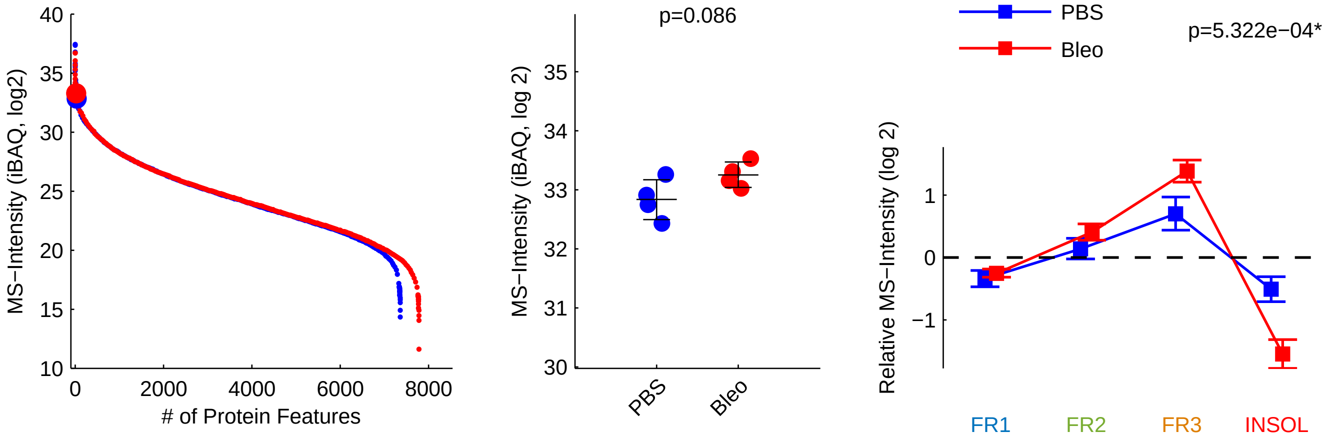

E9Q616 − Ahnak (id: 1251)

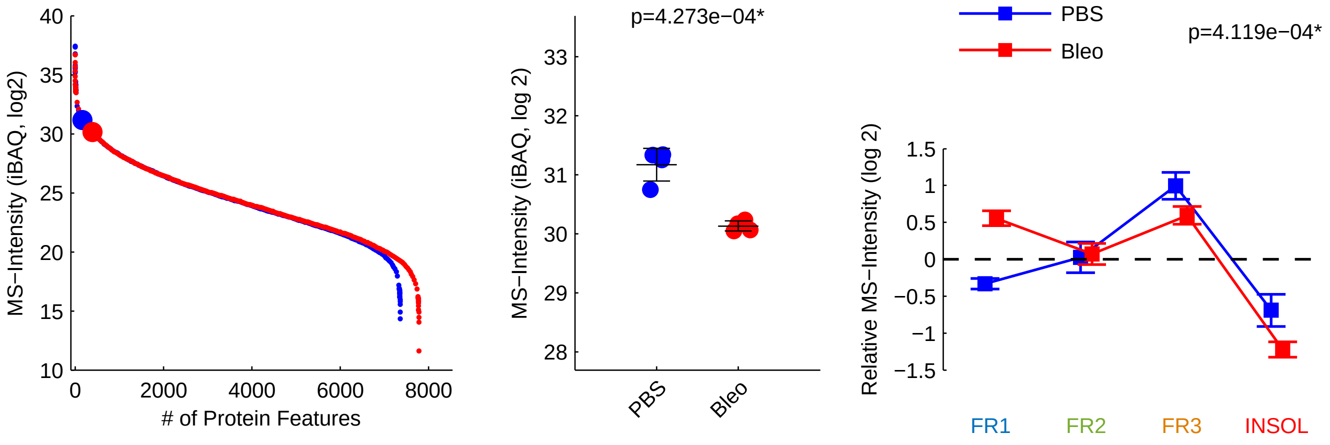

E9Q634 − Myo1e (id: 1253)

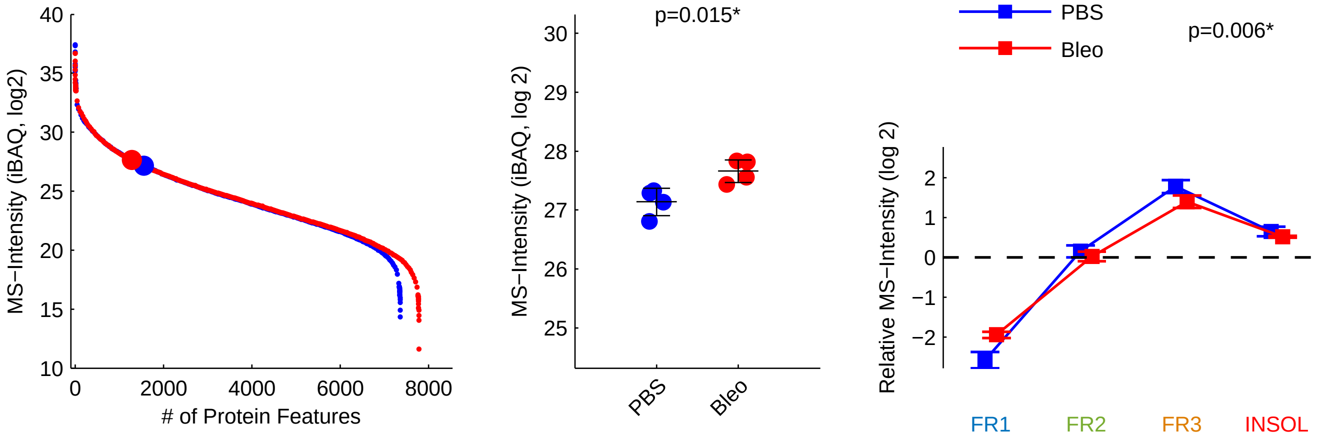

E9Q6C7 − Lphn2 (id: 1262)

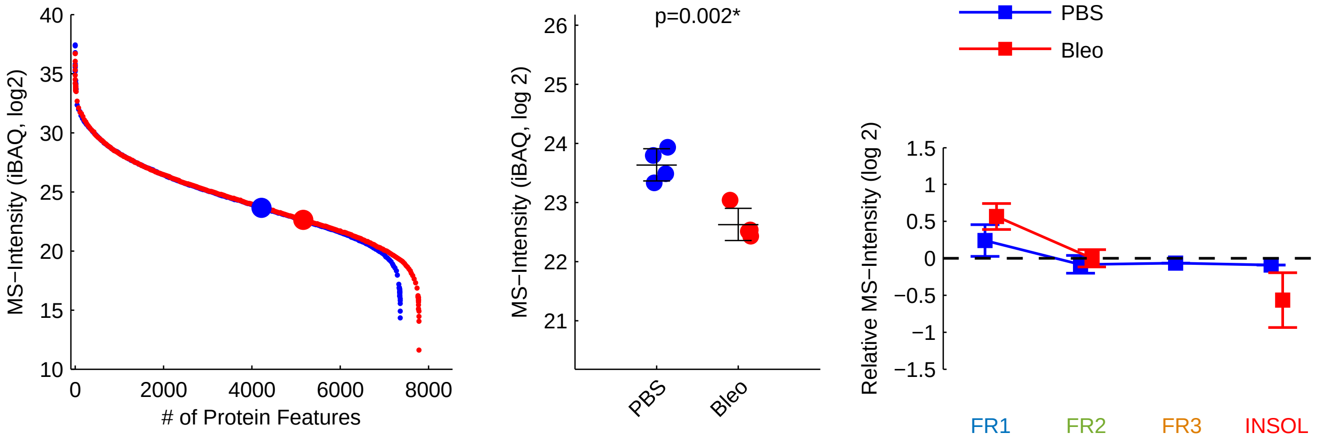

Q91X70 − C6 (id: 1263)

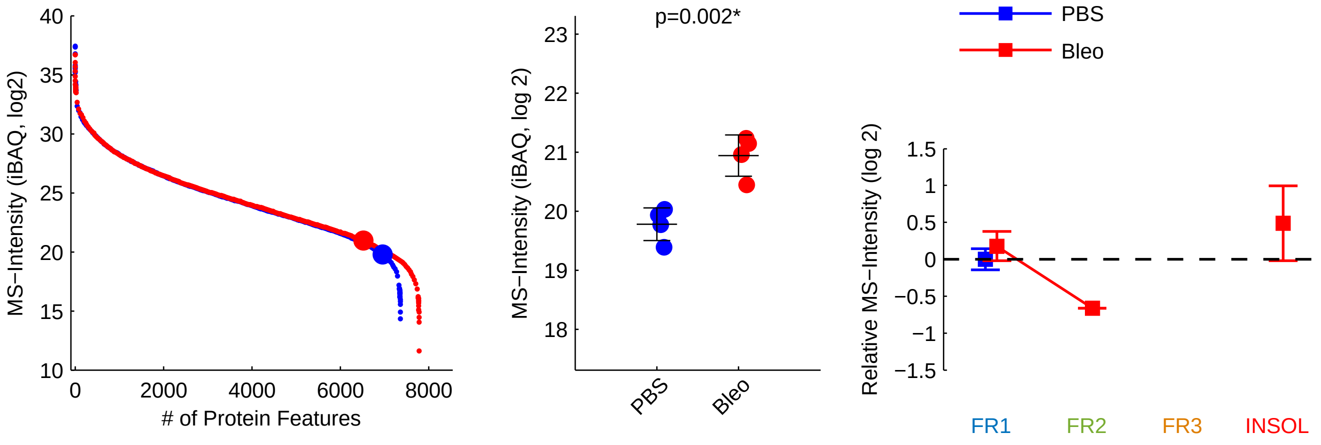

E9Q6H8 − Plekha5 (id: 1266)

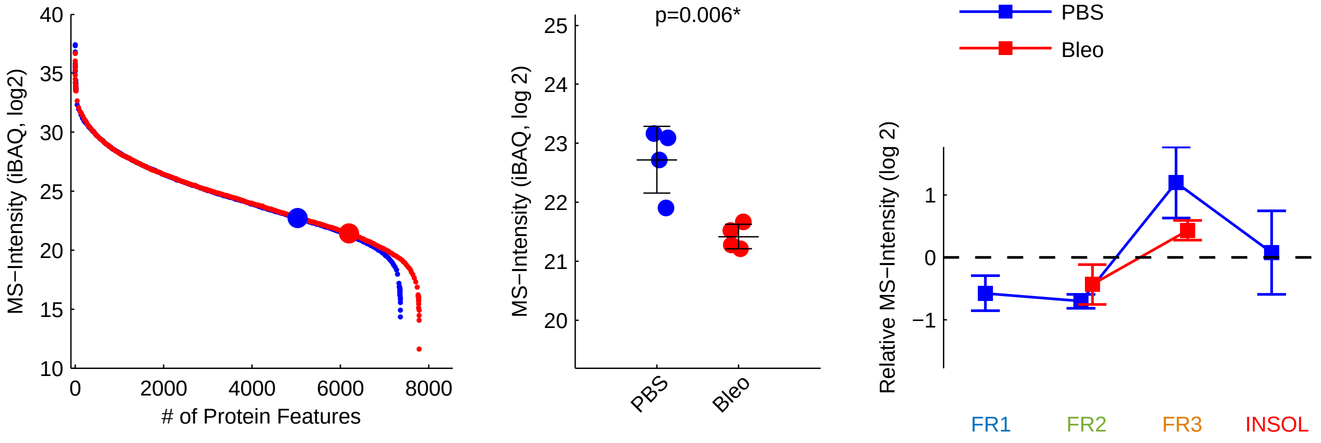

E9Q6L4 − Tbc1d1 (id: 1271)

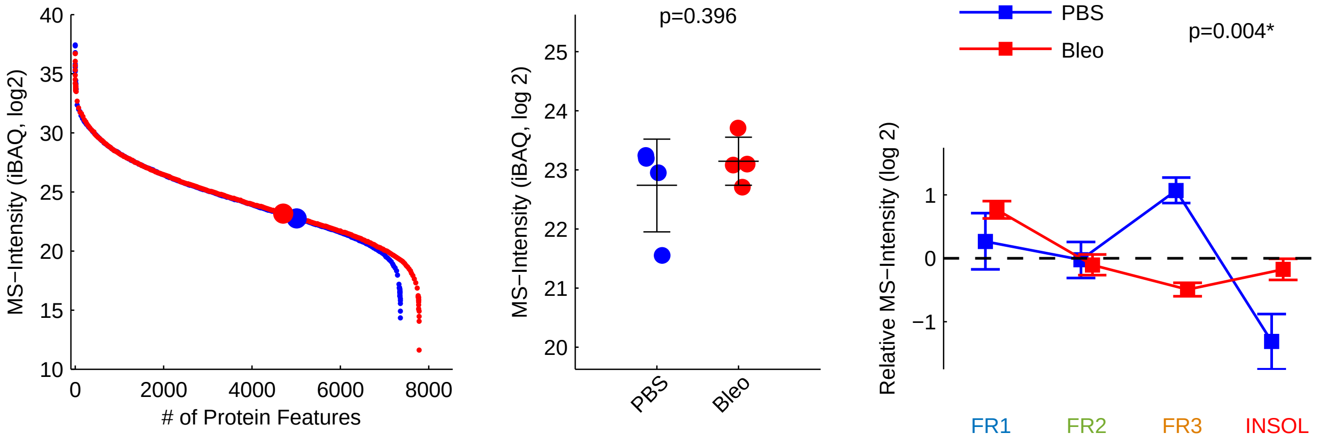

E9Q6R7 − Utrn (id: 1275)

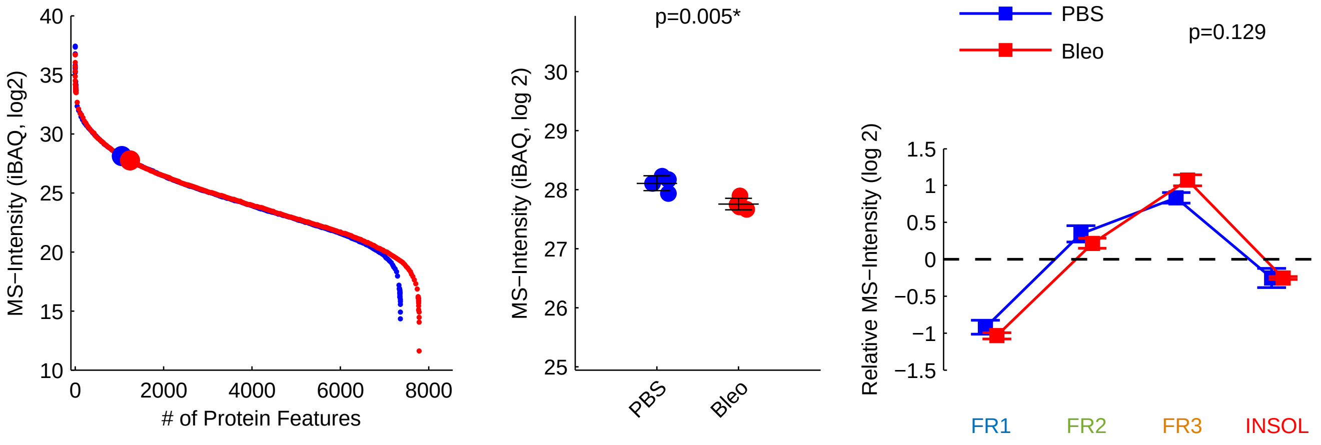

E9Q718 − Plod2 (id: 1279)

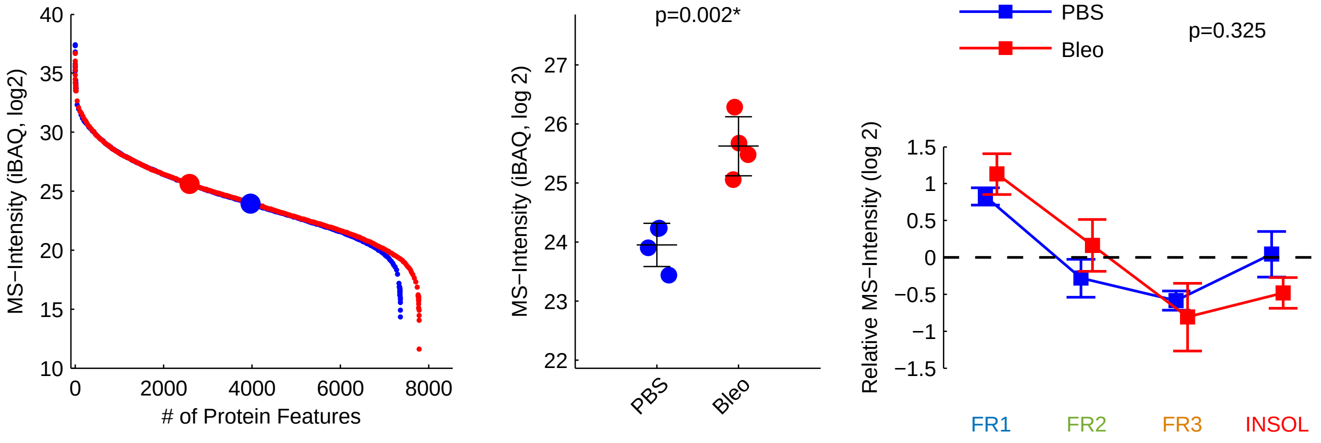

E9Q777 − Akap11 (id: 1283)

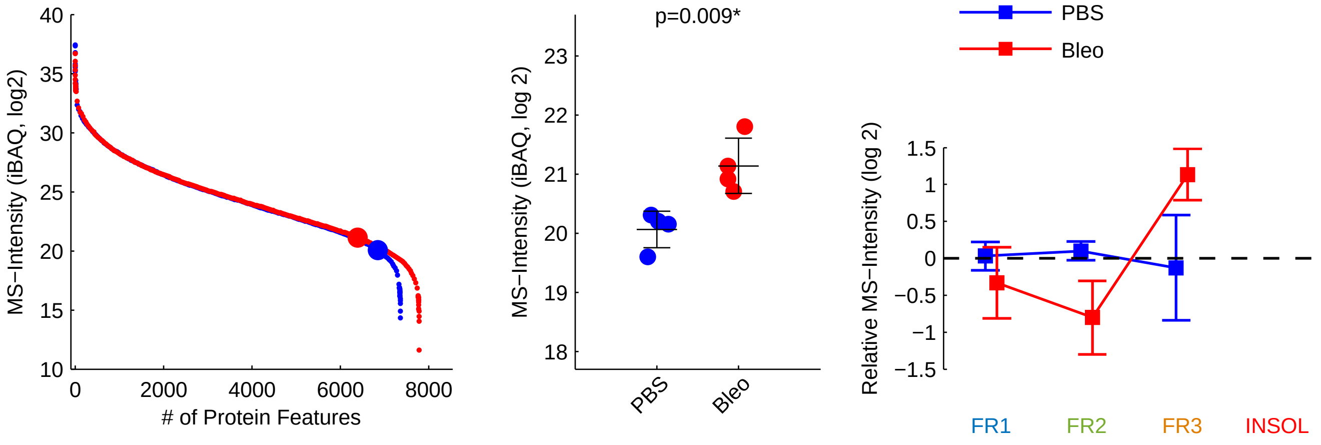

E9Q7D5 − Arhgef5 (id: 1288)

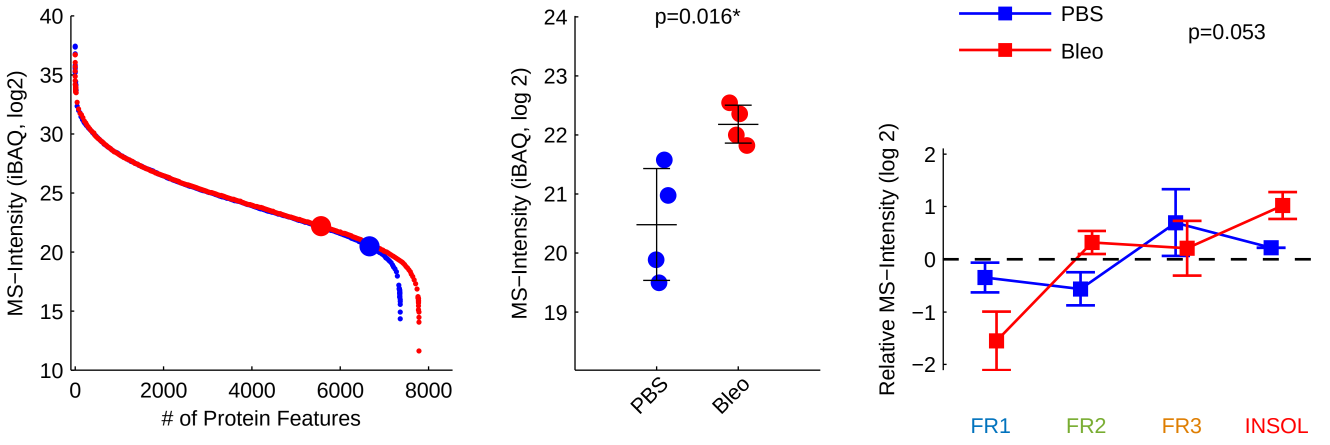

E9Q7G0 − Numa1 (id: 1292)

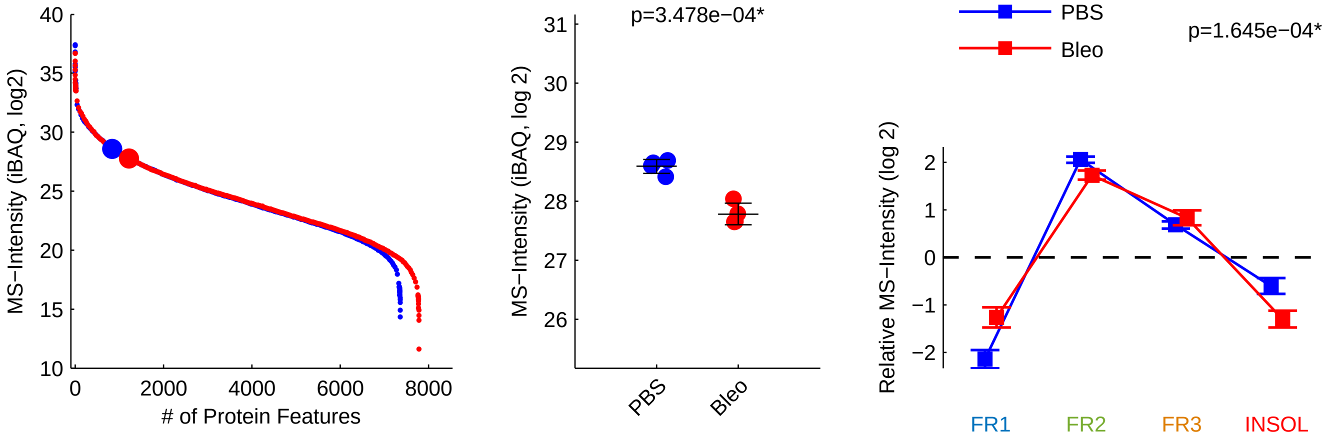

Q59IW6 − Abi3bp (id: 1312)

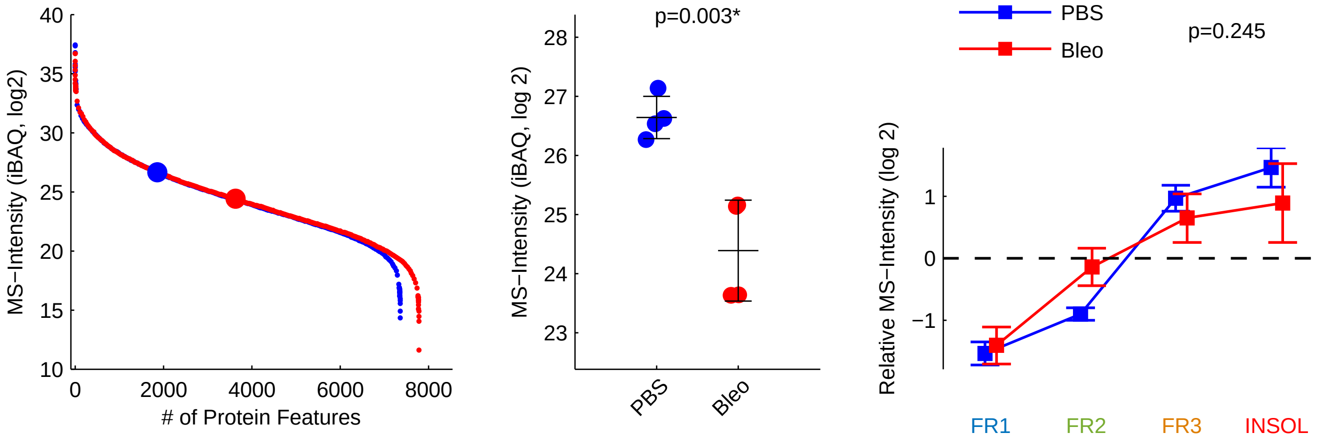

P06909 − Cfh (id: 1315)

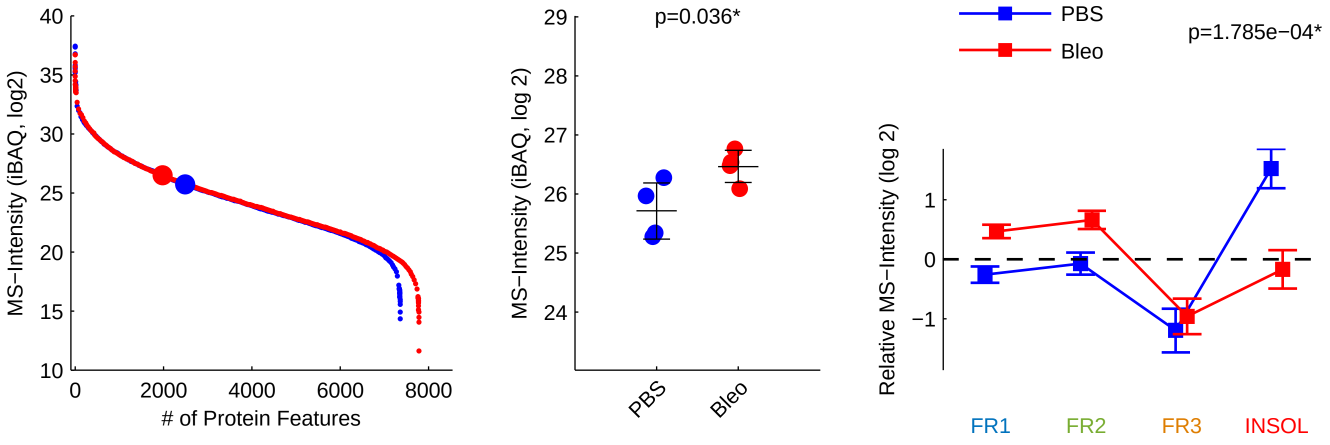

E9Q8L9 − Rab11fip1 (id: 1321)

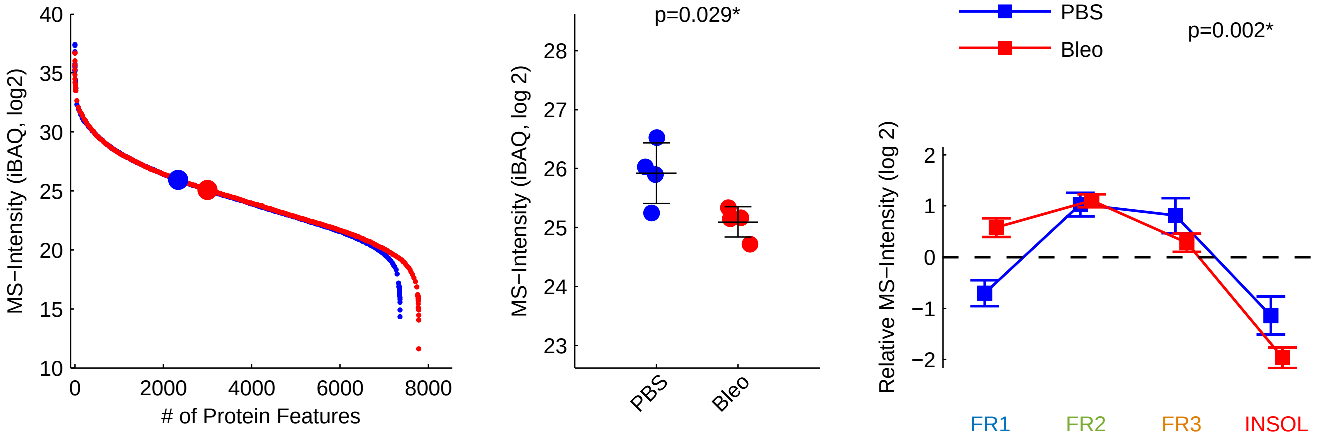

Q8BRT1 − Clasp2 (id: 1324)

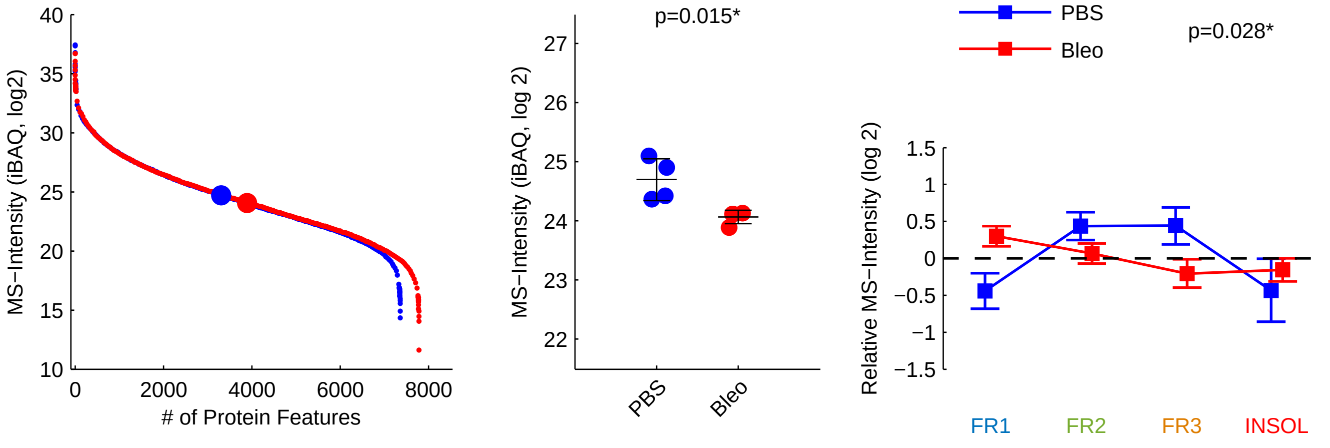

E9Q8Q6 − Ccdc141 (id: 1326)

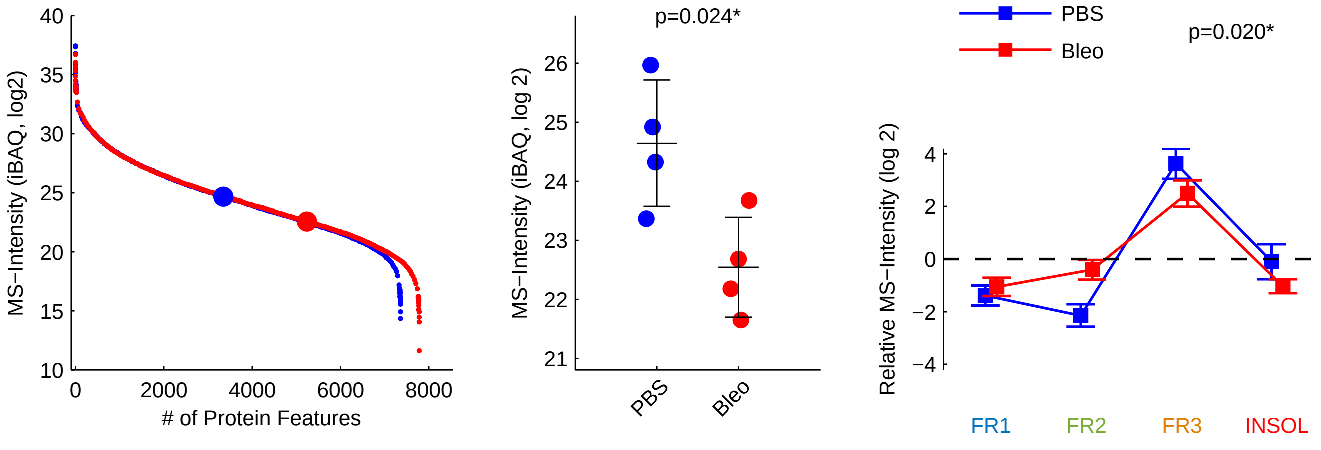

Q3TRM8 − Hk3 (id: 1327)

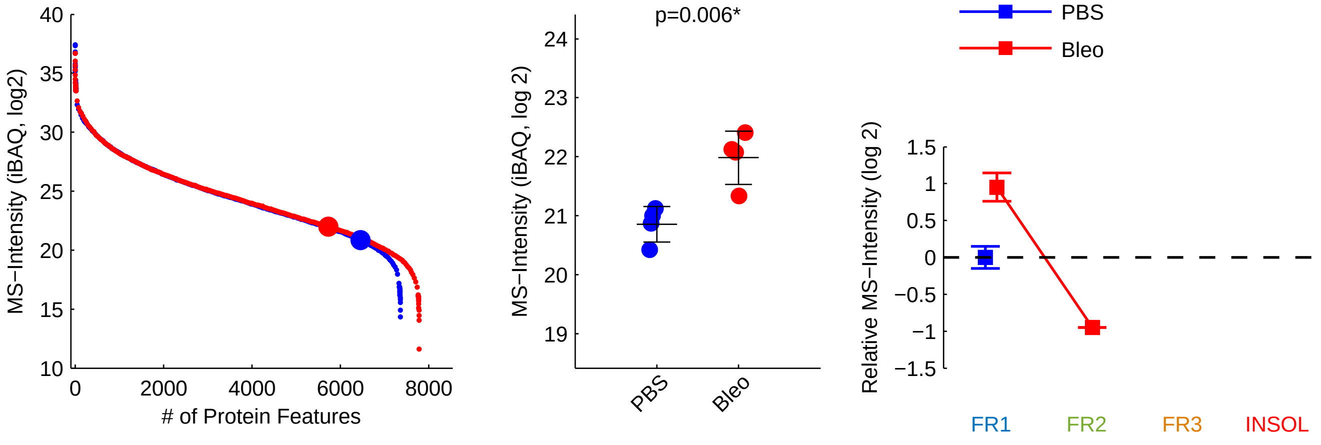

E9Q8V6 − Dennd4a (id: 1330)

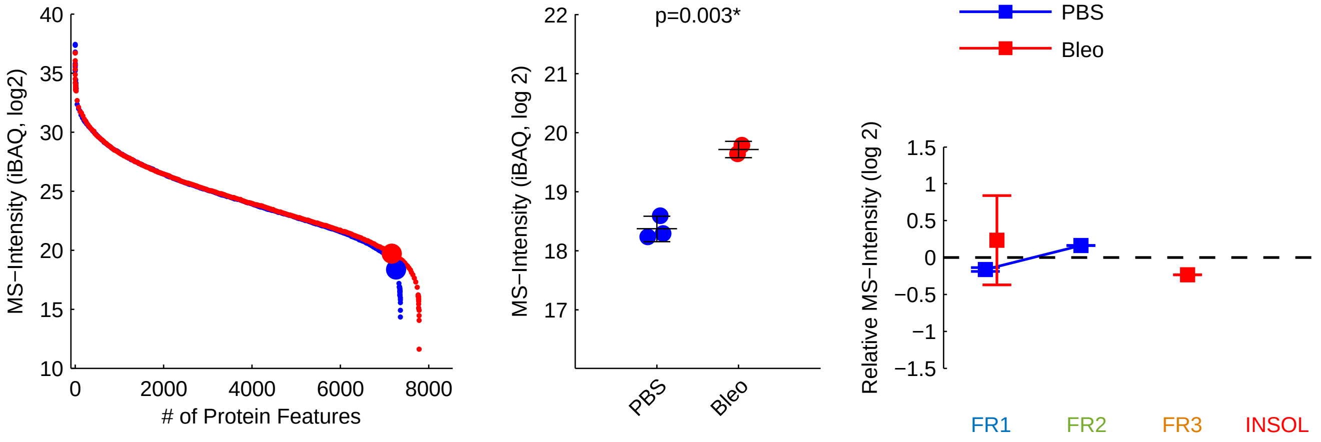

E9Q9B7 − Kidins220 (id: 1337)

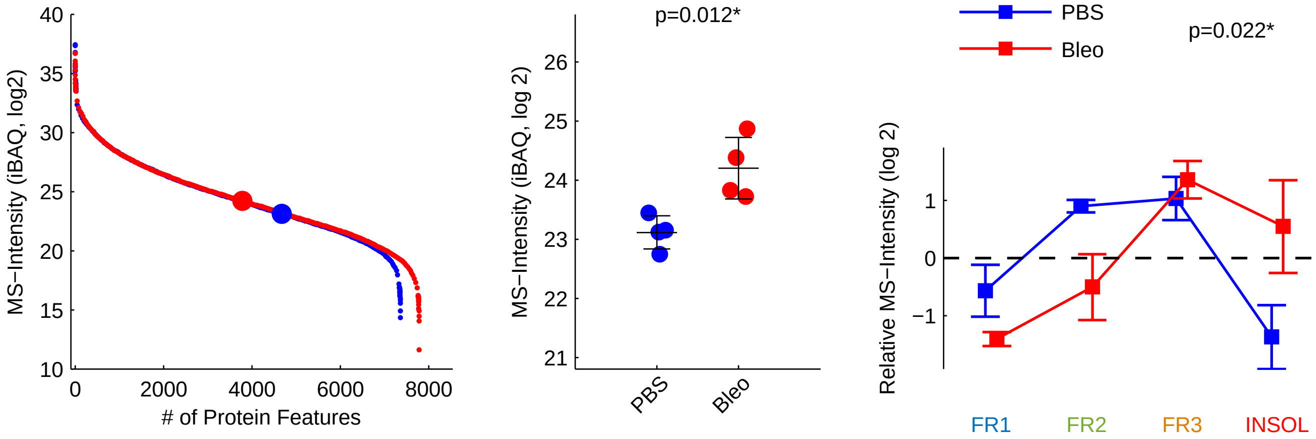

E9Q9C3 − Mllt4 (id: 1340)

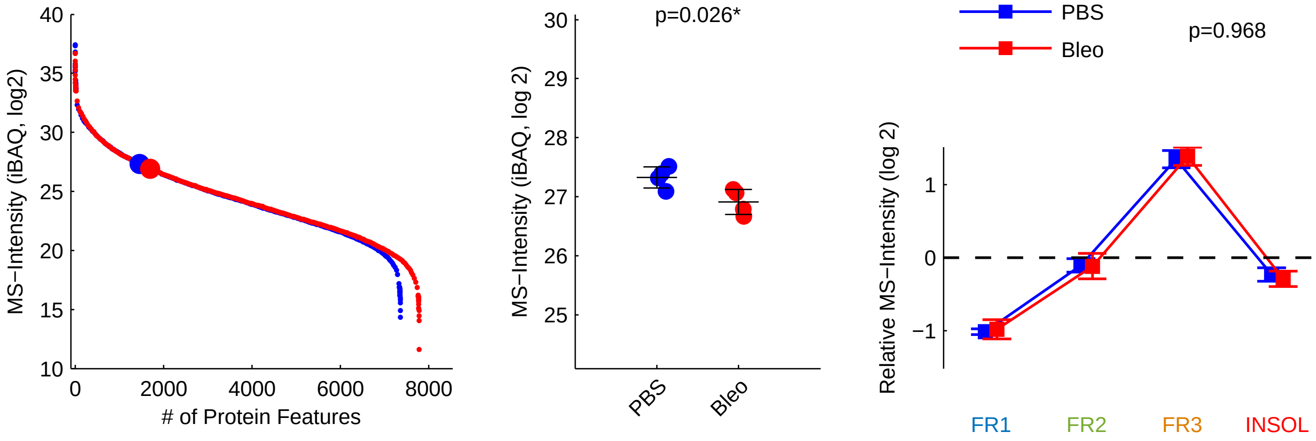

E9Q9C5 − Atp6v0c (id: 1341)

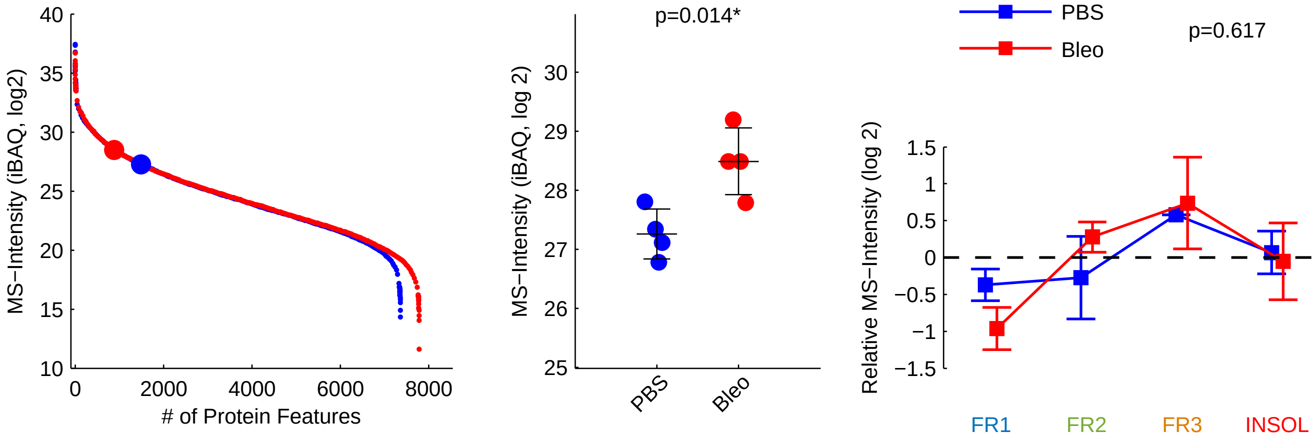

Q3UJD6 − Usp19 (id: 1354)

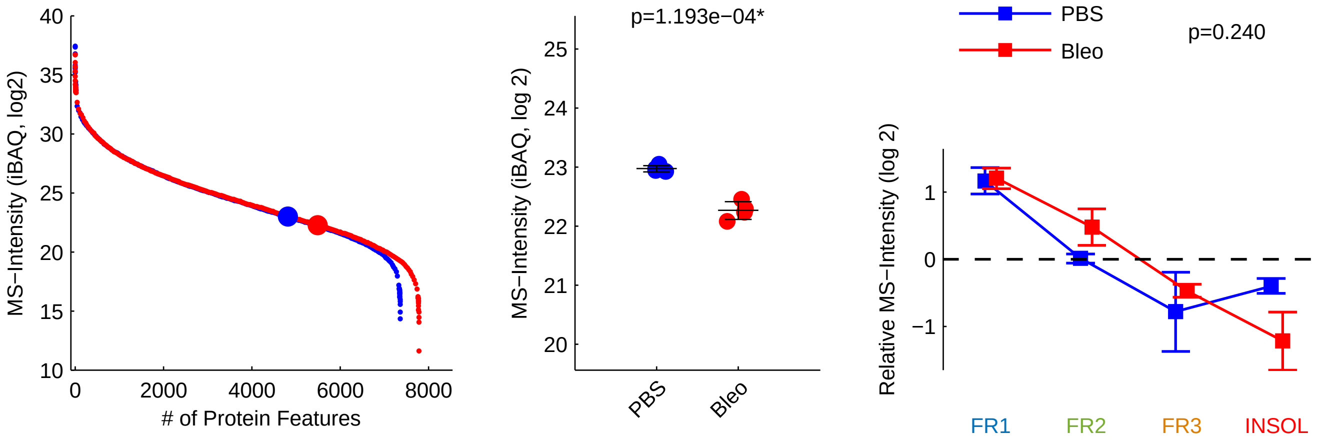

E9QA15 − Cald1 (id: 1360)

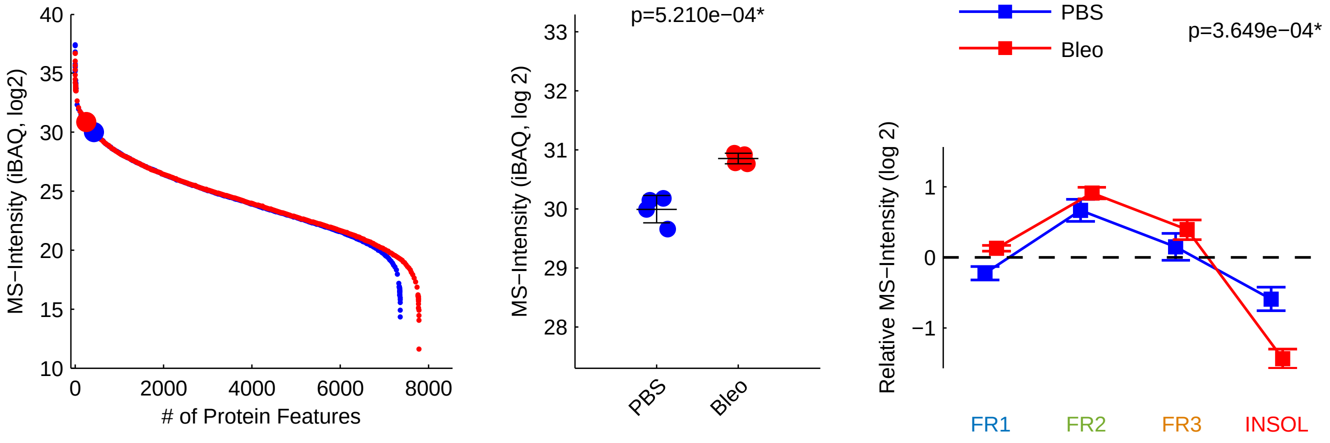

E9QA16 − Cald1 (id: 1361)

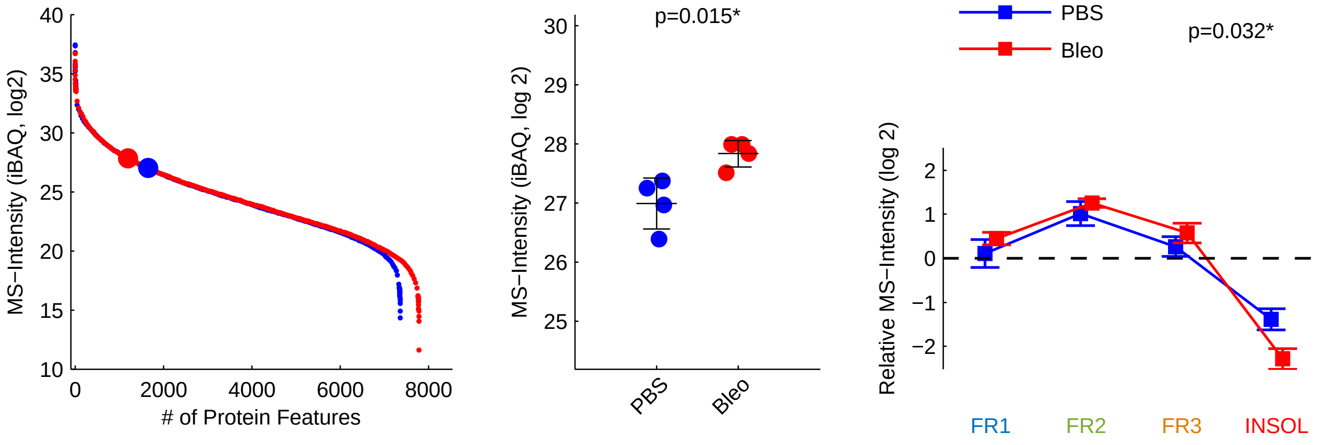

Q6NZK2 − Nckap5 (id: 1366)

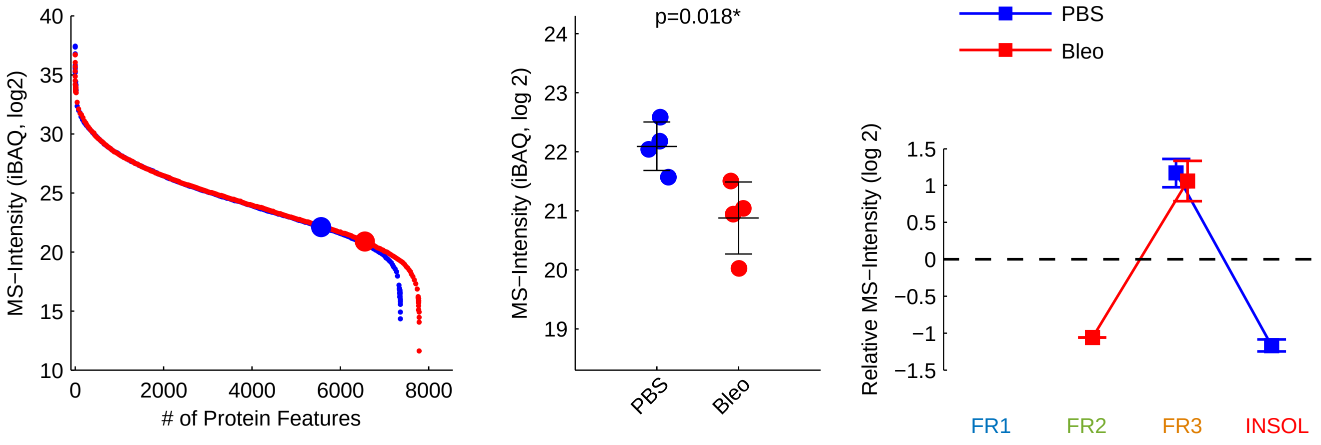

E9QAE3 − Btaf1 (id: 1367)

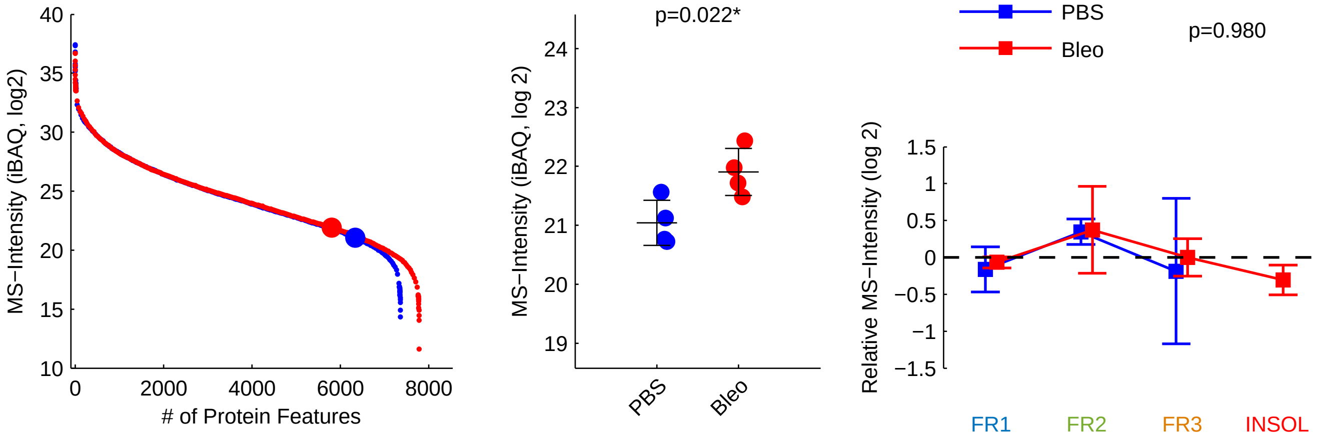

E9QAS5 − Chd4 (id: 1376)

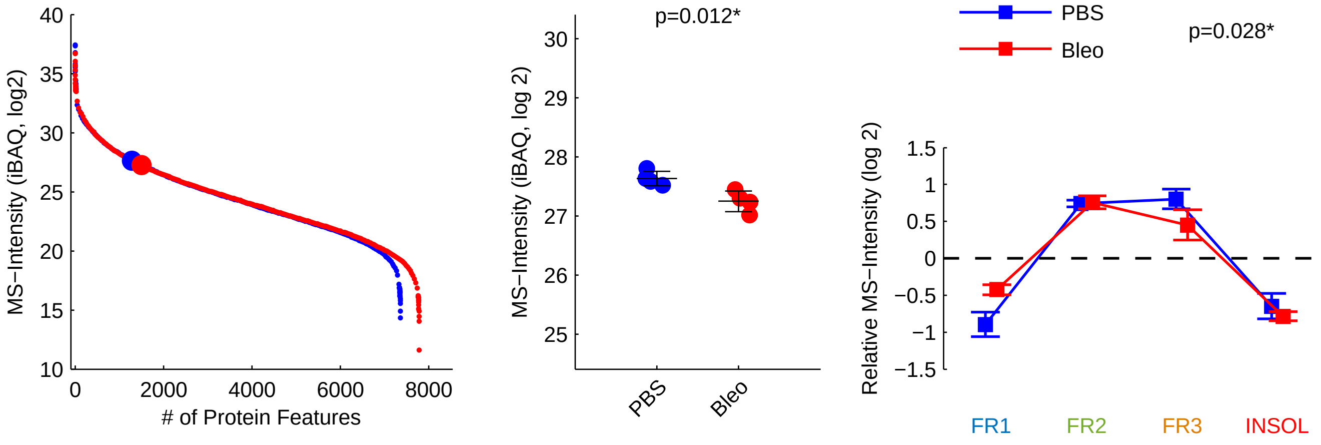

Q9DC42 − Cd97 (id: 1382)

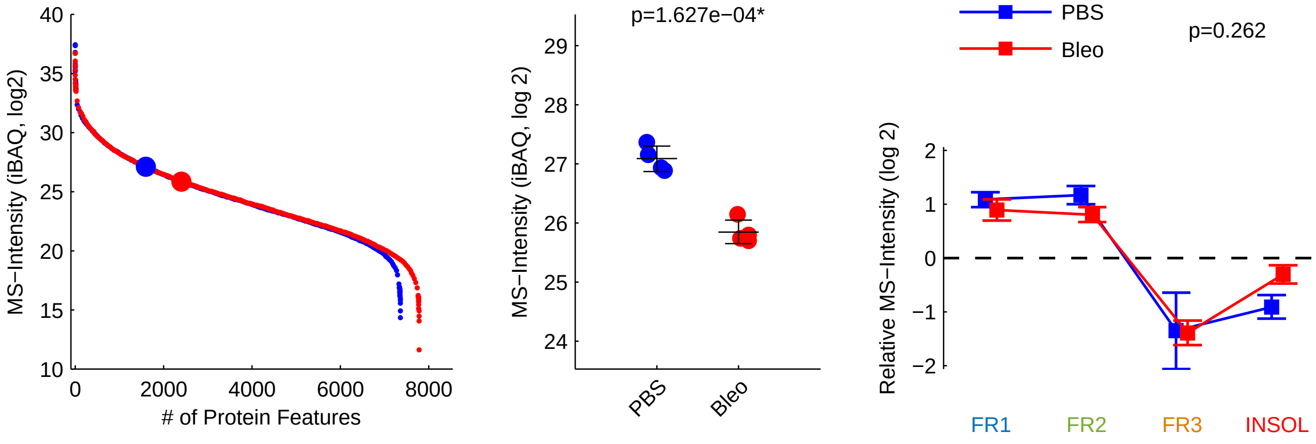

Q8CG79 − Tp53bp2 (id: 1385)

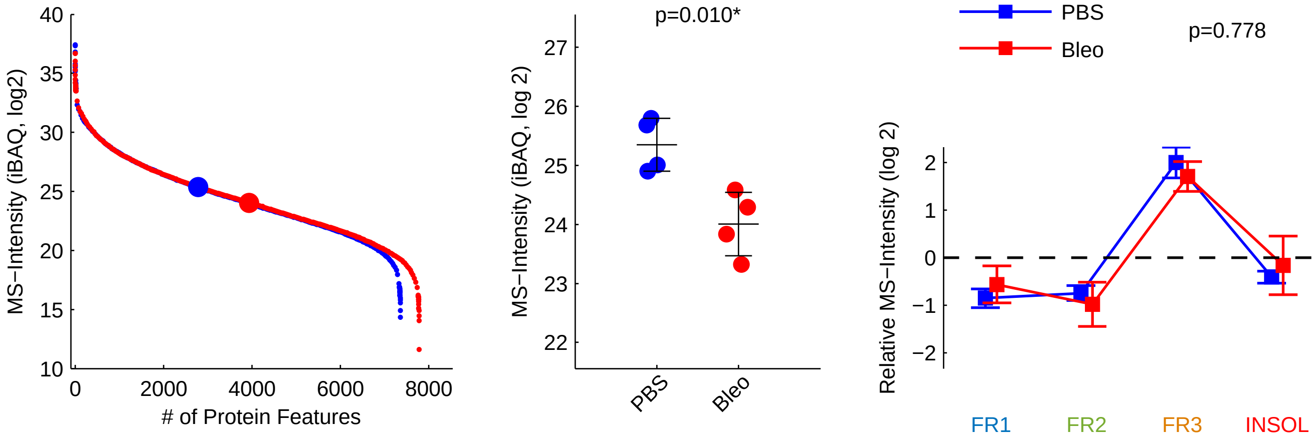

Q5XG71 − Utp20 (id: 1390)

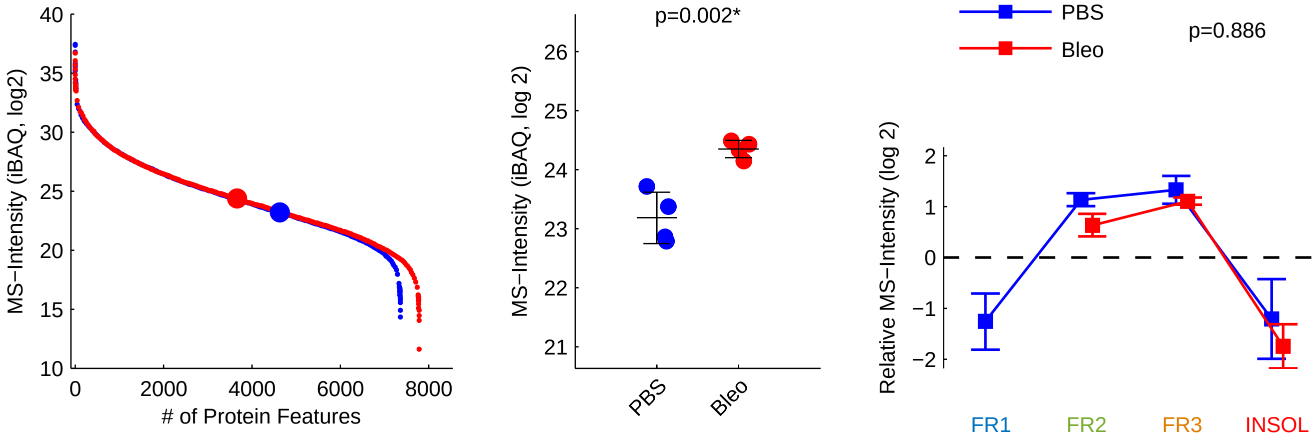

Q91YE6 − Ipo9 (id: 1405)

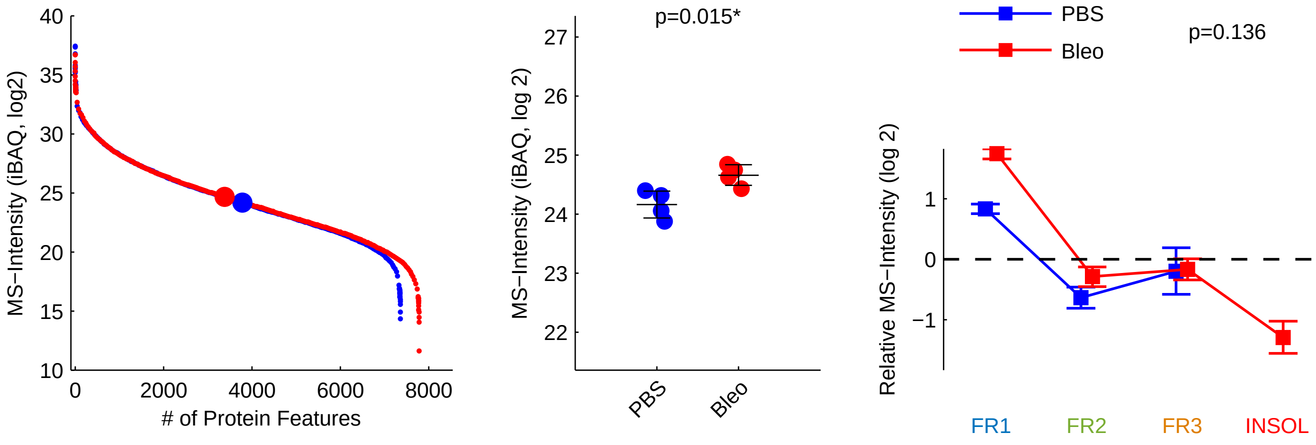

P98078 − Dab2 (id: 1406)

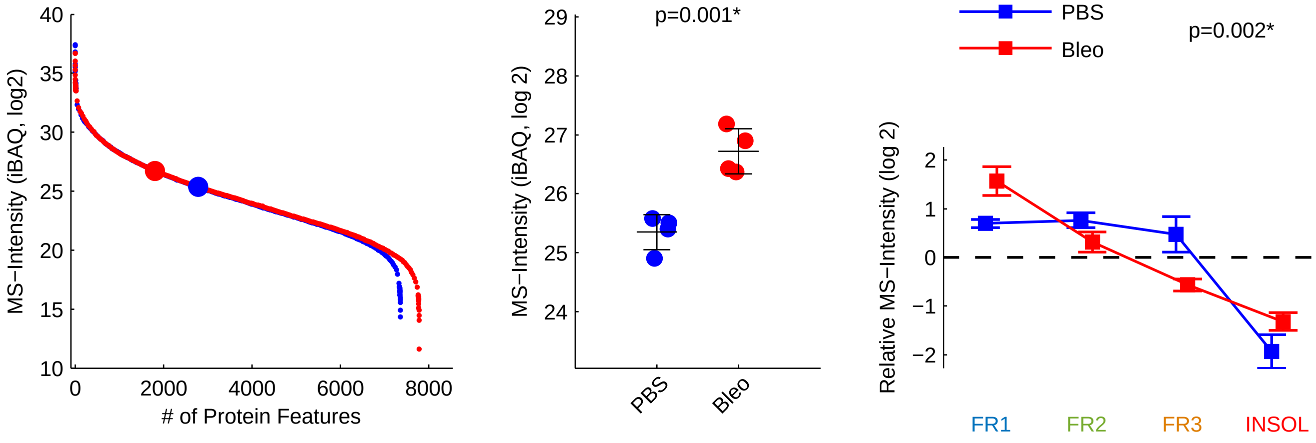

Q0GNC1 − Inf2 (id: 1409)

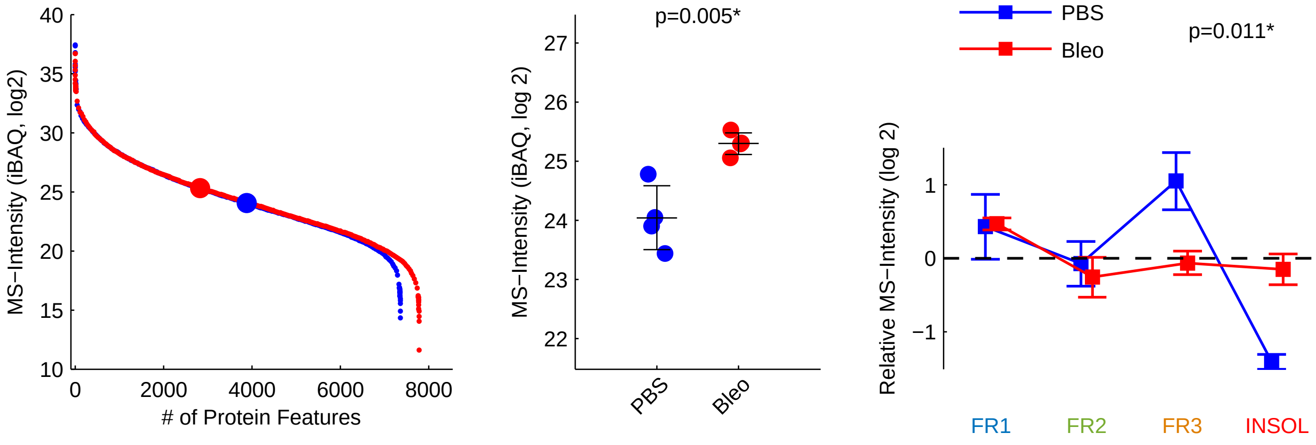

Q566J8 − Adck4 (id: 1411)

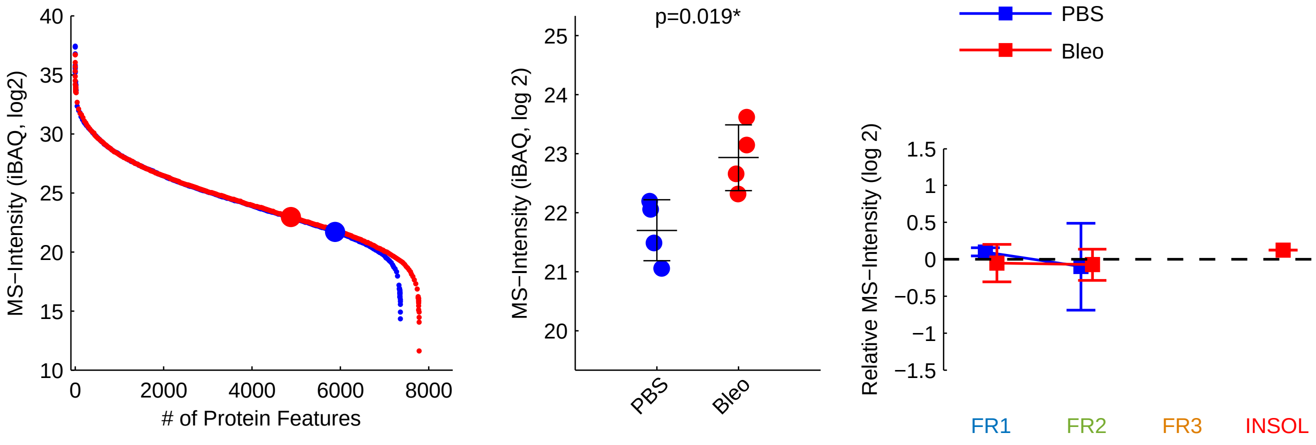

O70305 − Atxn2 (id: 1420)

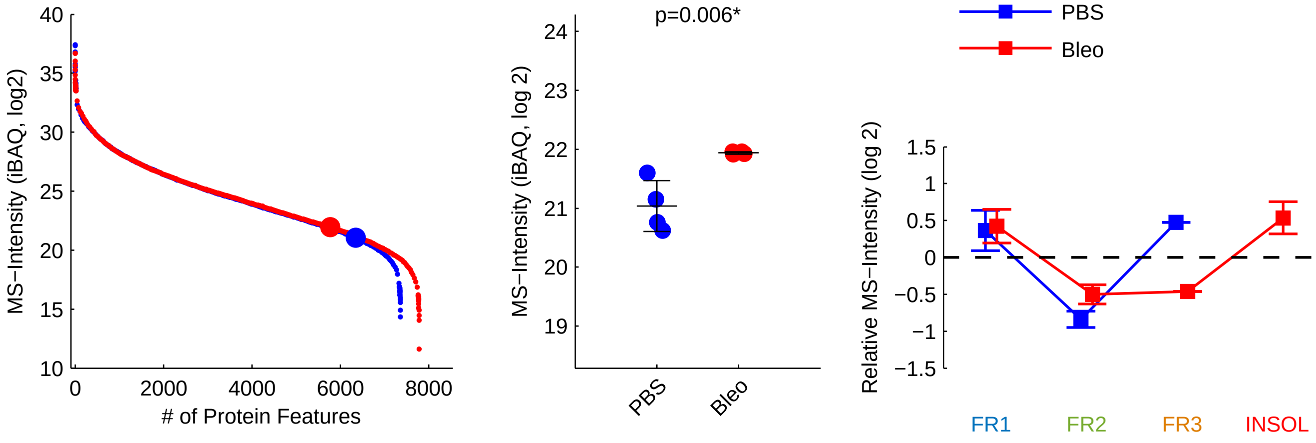

E9QMC1 − Cgn (id: 1424)

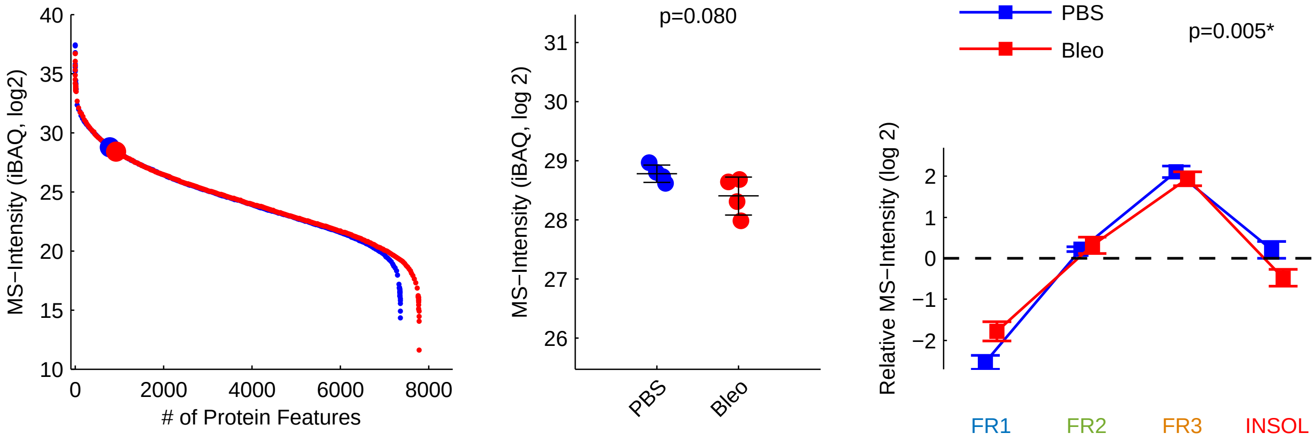

Q99J72 − Apobec3 (id: 1427)

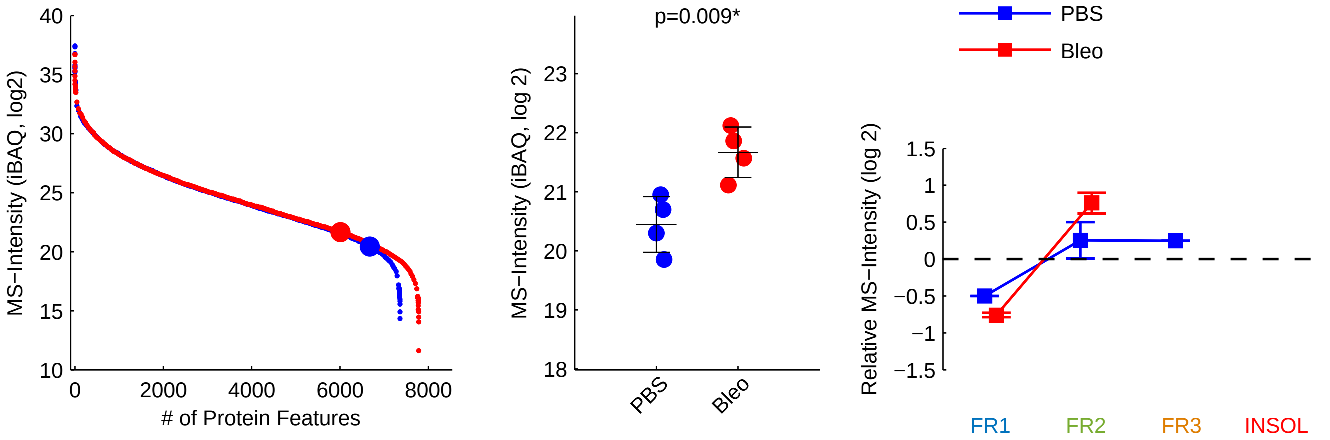

E9QMH7 − Ikbip (id: 1428)

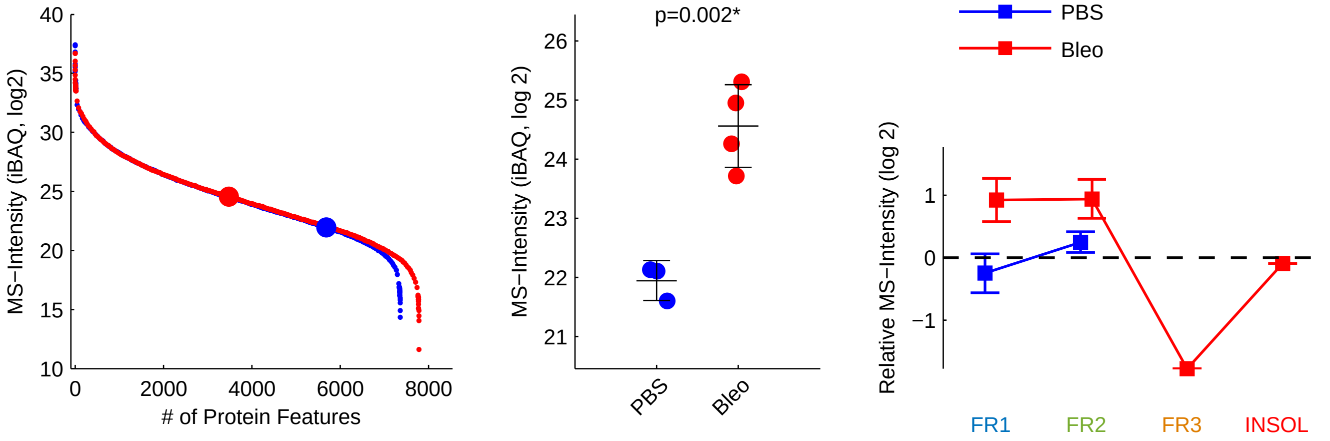

E9QMP6 − Mark2 (id: 1433)

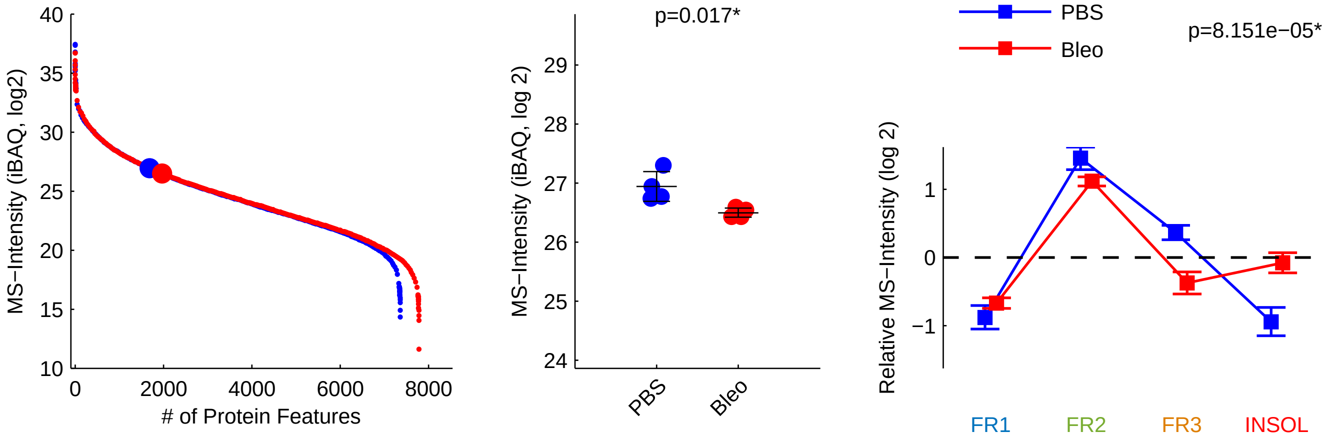

E9QN52 − Lrrfip2 (id: 1444)

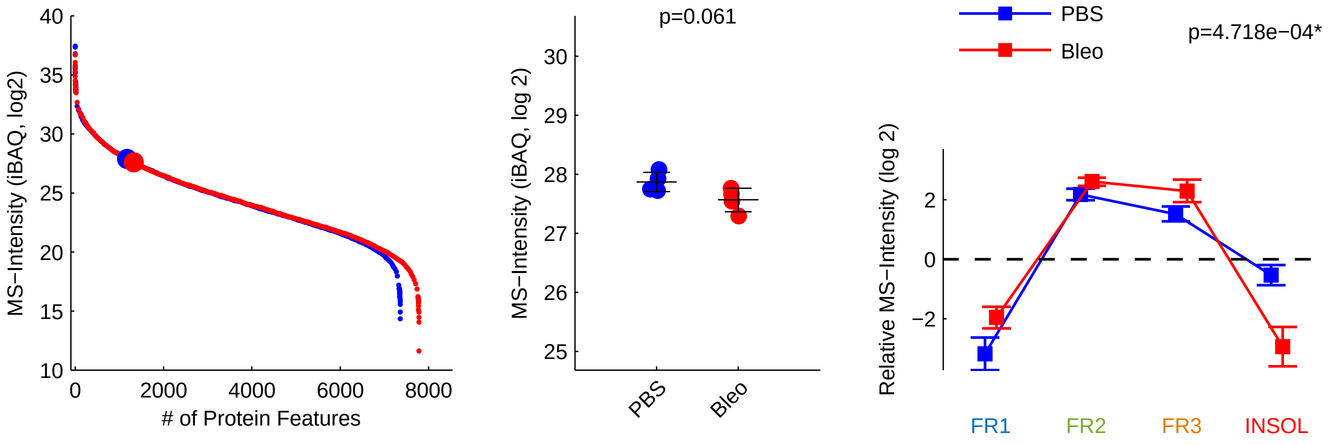

P02469 − Lamb1 (id: 1445)

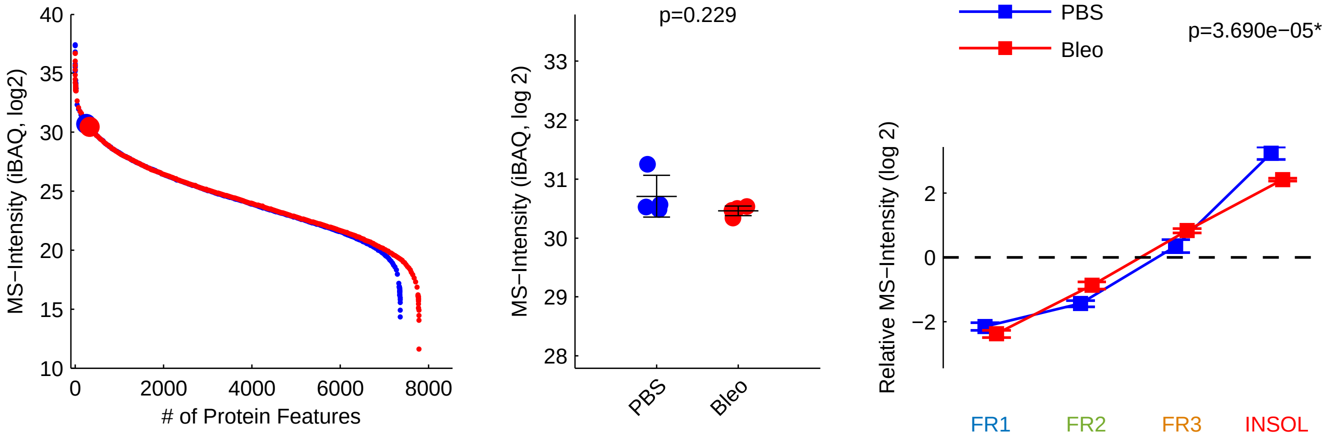

E9QN87 − Plec (id: 1446)

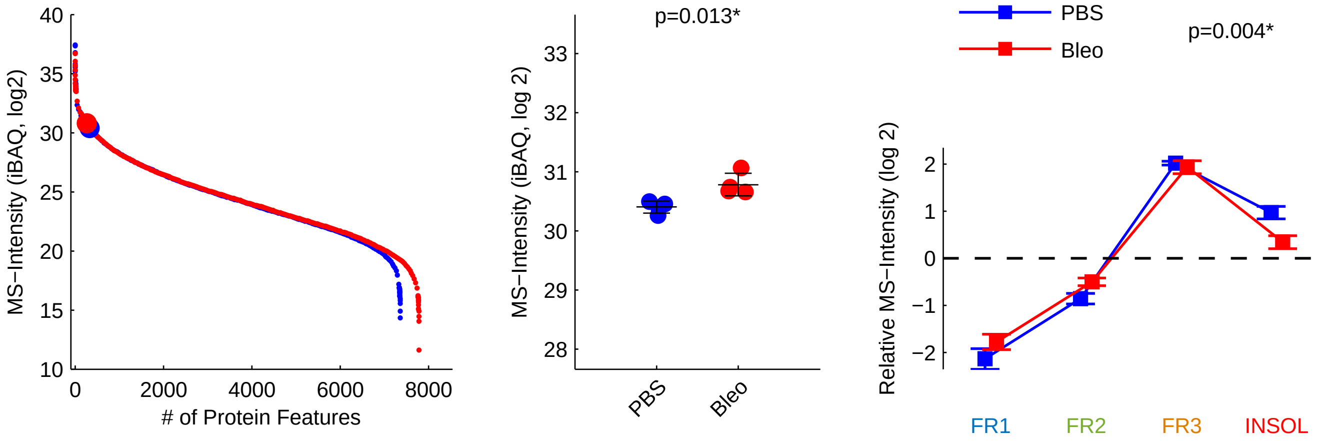

E9QN99 − Abhd14b (id: 1447)

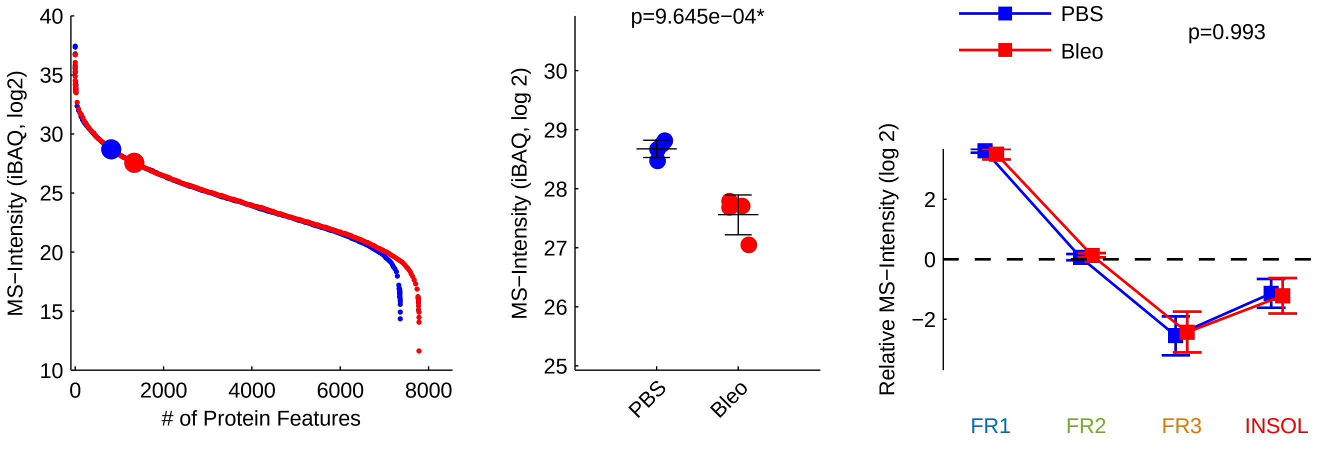

E9QNA7 − Sorbs1 (id: 1448)

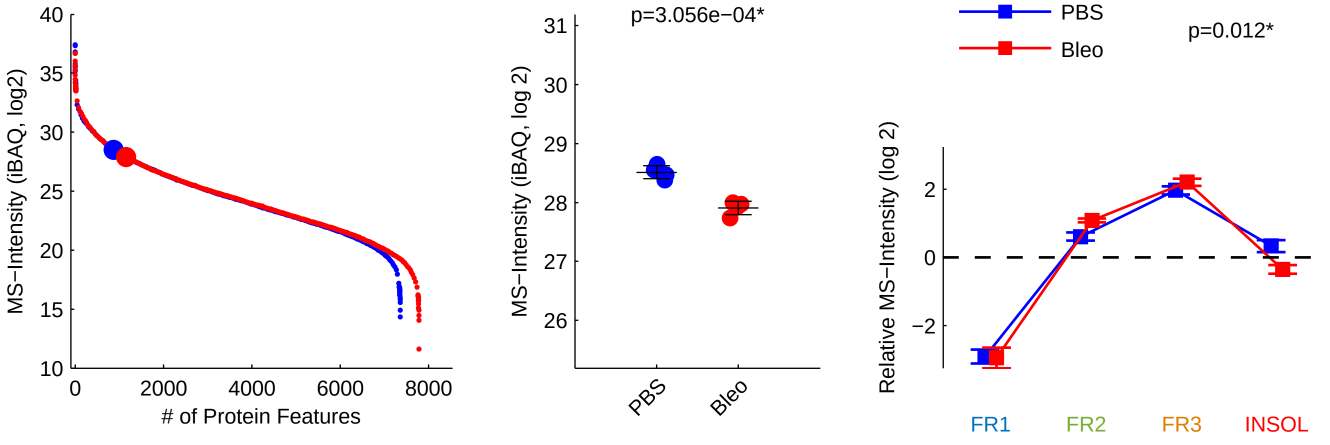

E9QNN1 − Dhx9 (id: 1454)

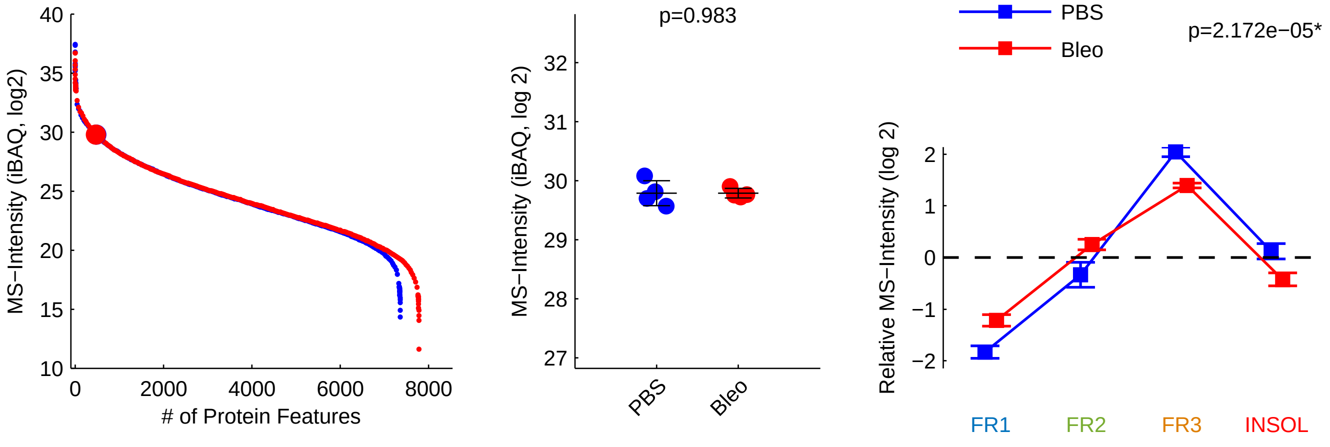

Q8VDG7 − Pafah2 (id: 1462)

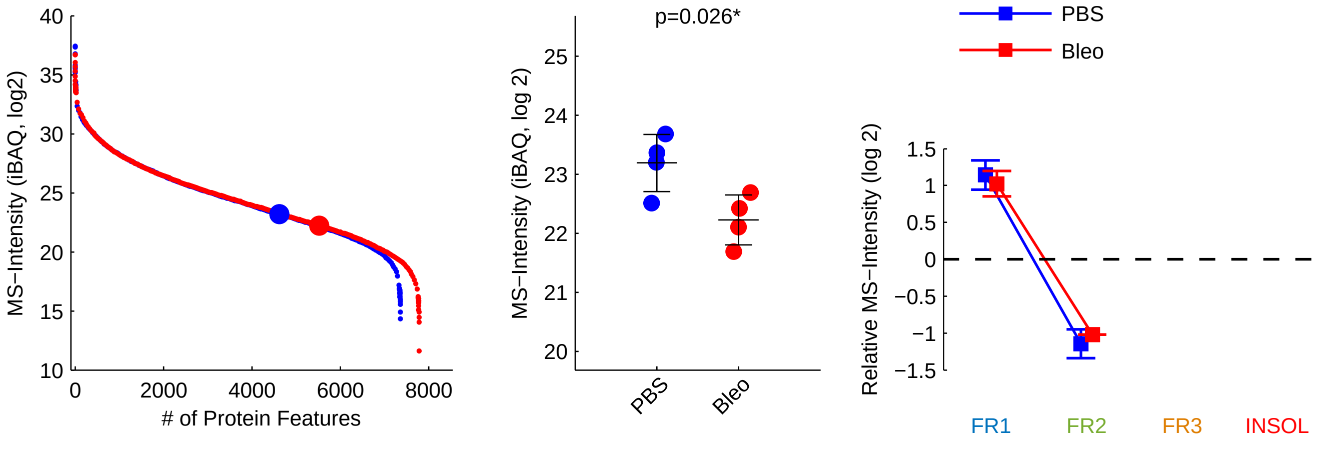

Q9JLC8 − Sacs (id: 1465)

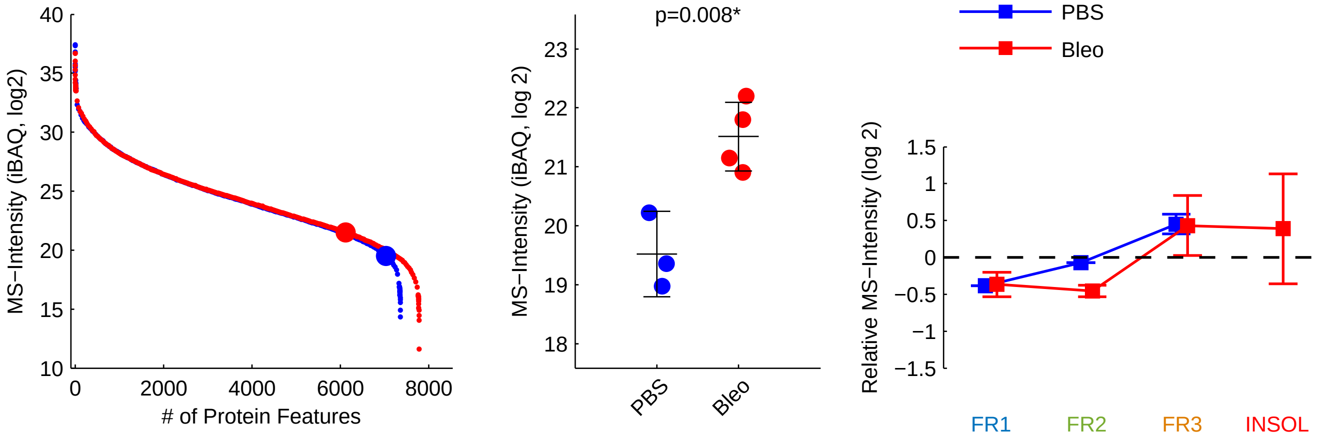

E9QP46 − Syne2 (id: 1468)

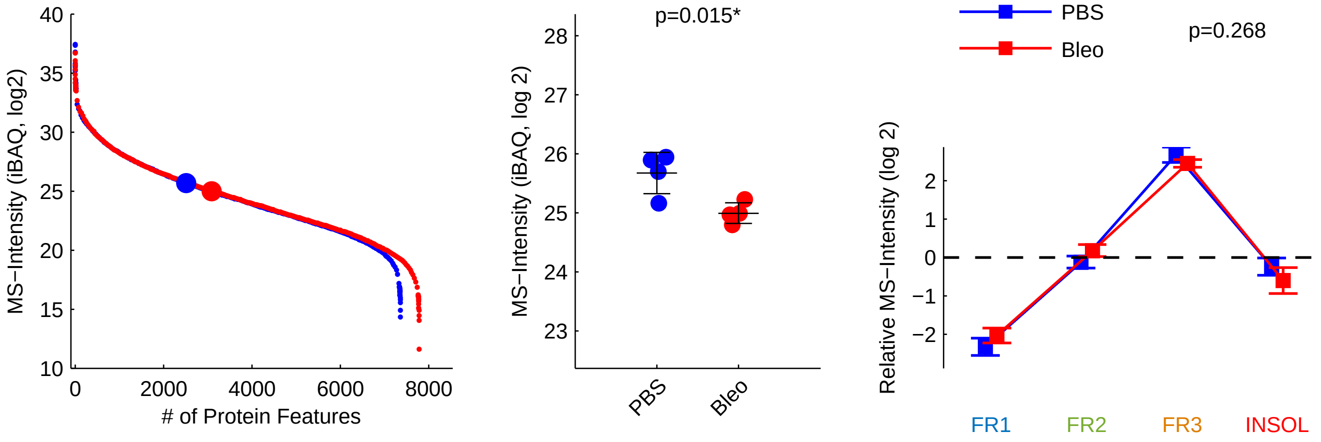

E9QP62 − Lims1 (id: 1471)

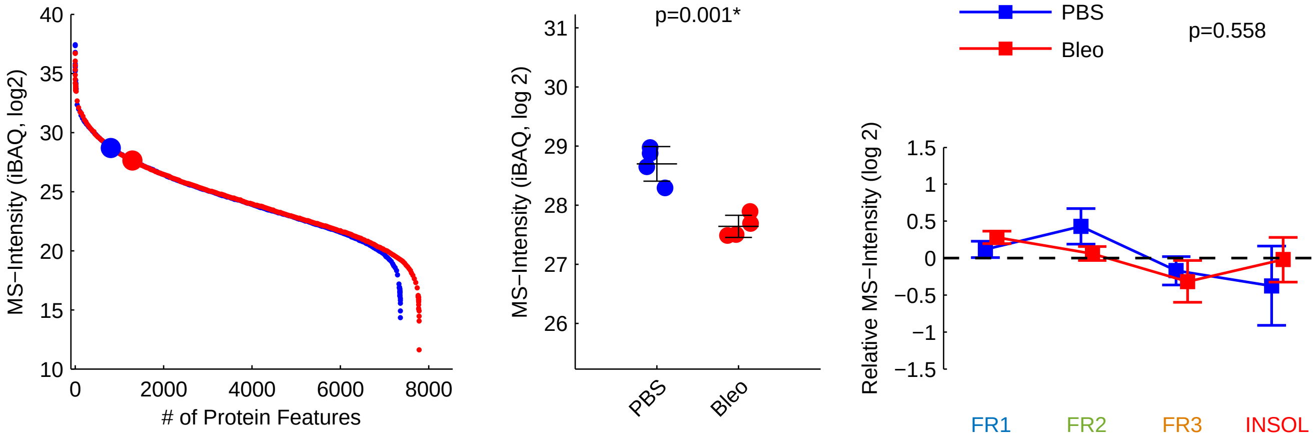

G5E8R3 − Pcx (id: 1479)

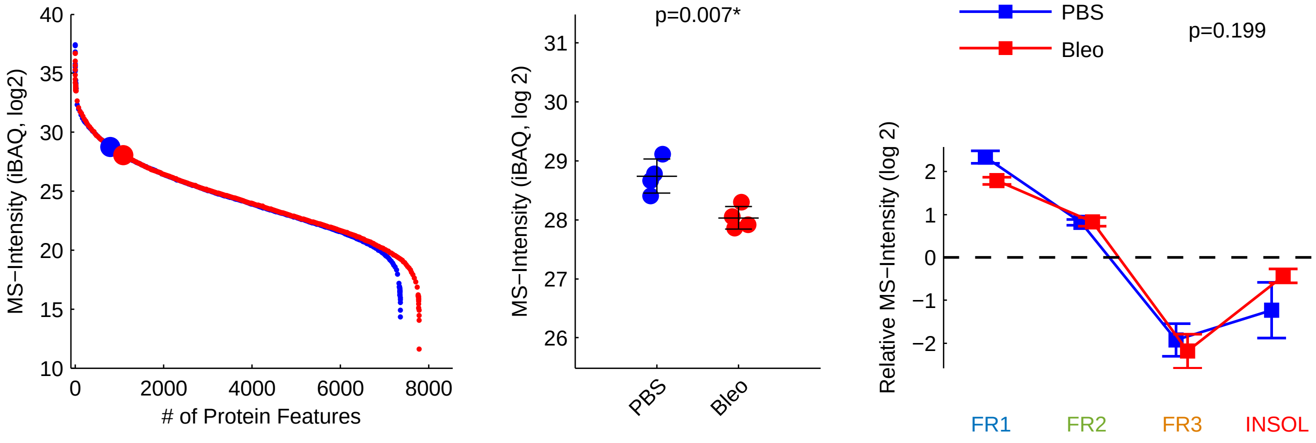

E9QPE7 − Myh11 (id: 1480)

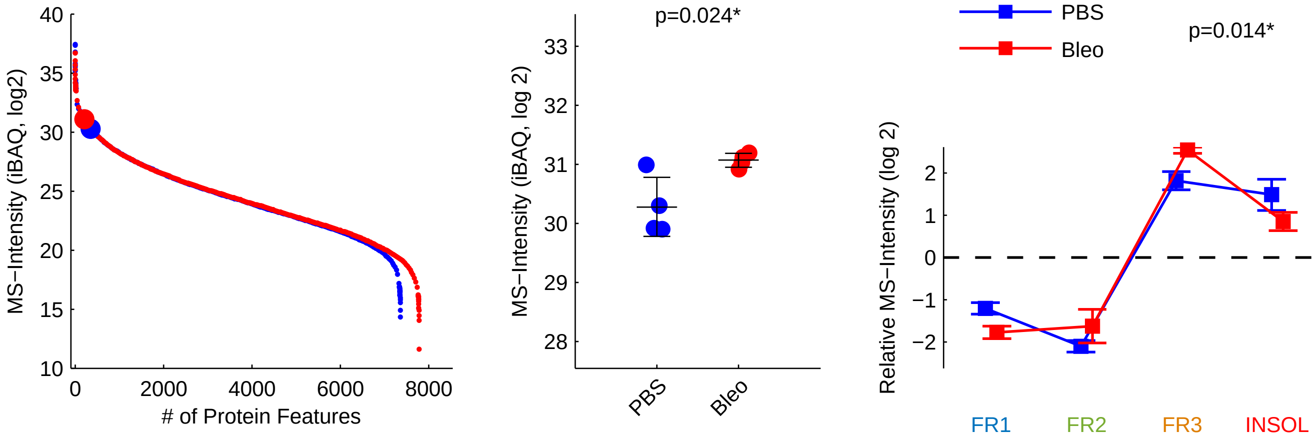

Q8CIZ8 − Vwf (id: 1485)

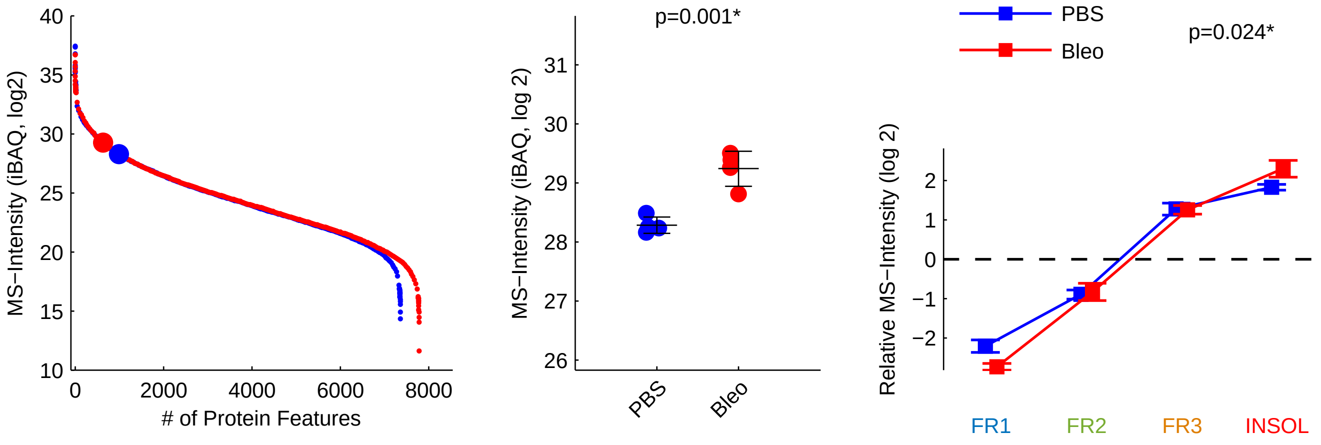

Q9DBR4 − Apbb2 (id: 1487)

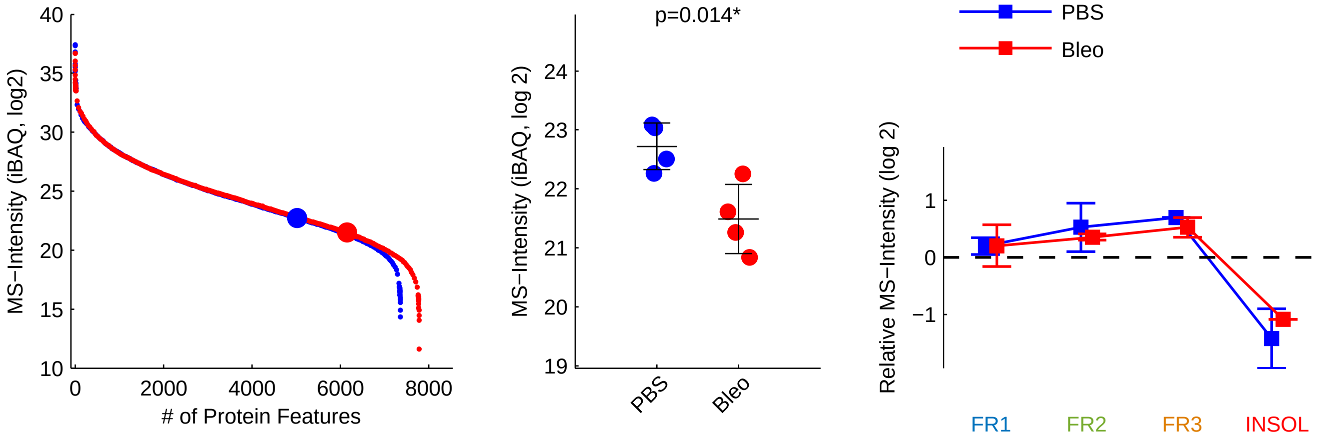

P39061−1 − Col18a1 (id: 1488)

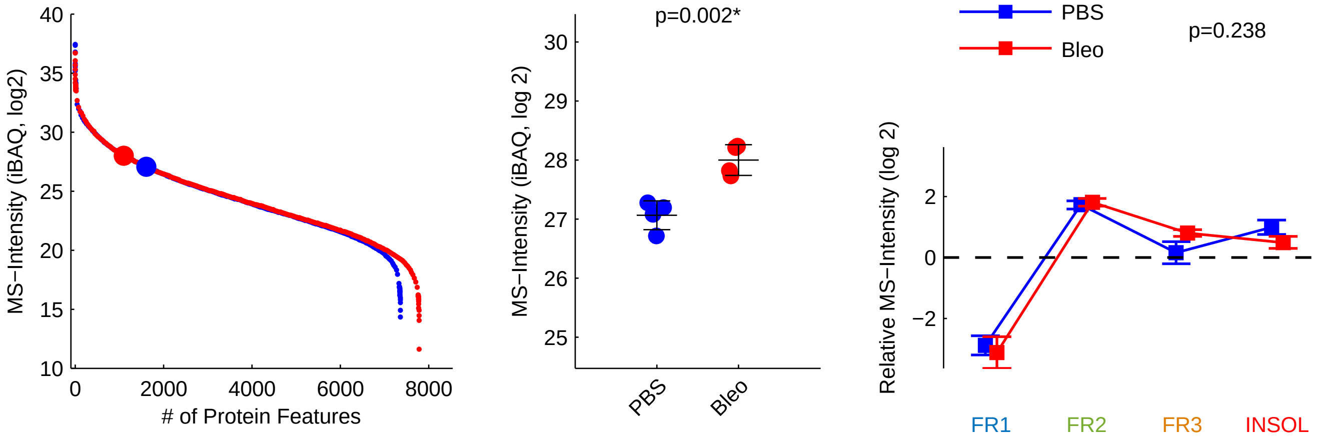

E9QQ57 − Prx (id: 1494)

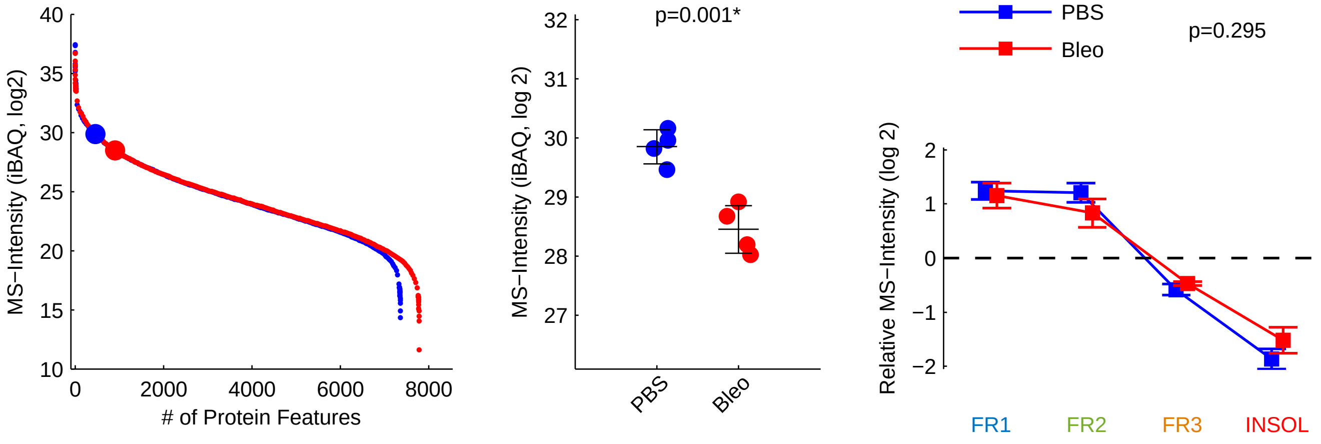

F2Z468 − Matn4 (id: 1498)

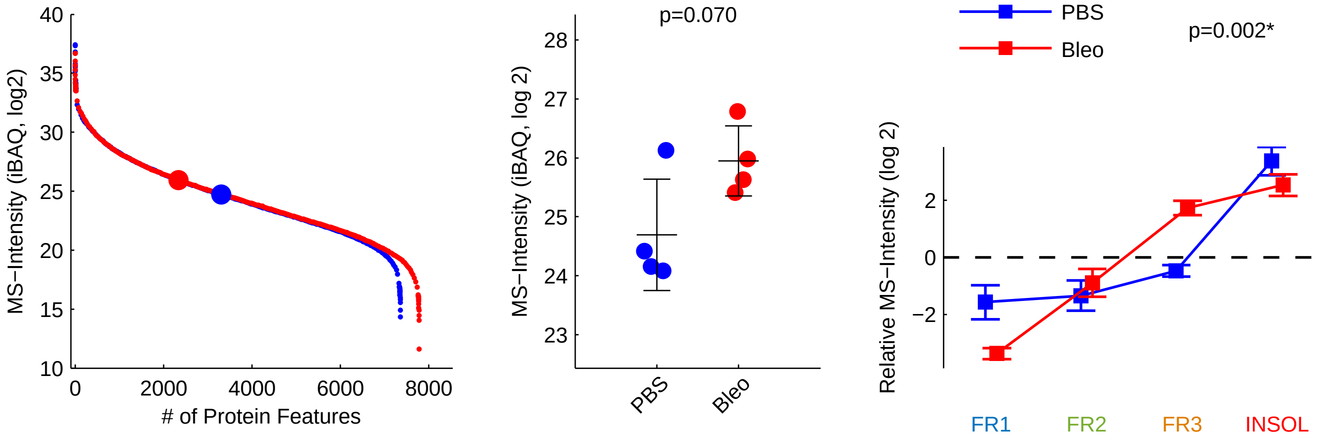

F2Z4A3 − Fat1 (id: 1504)

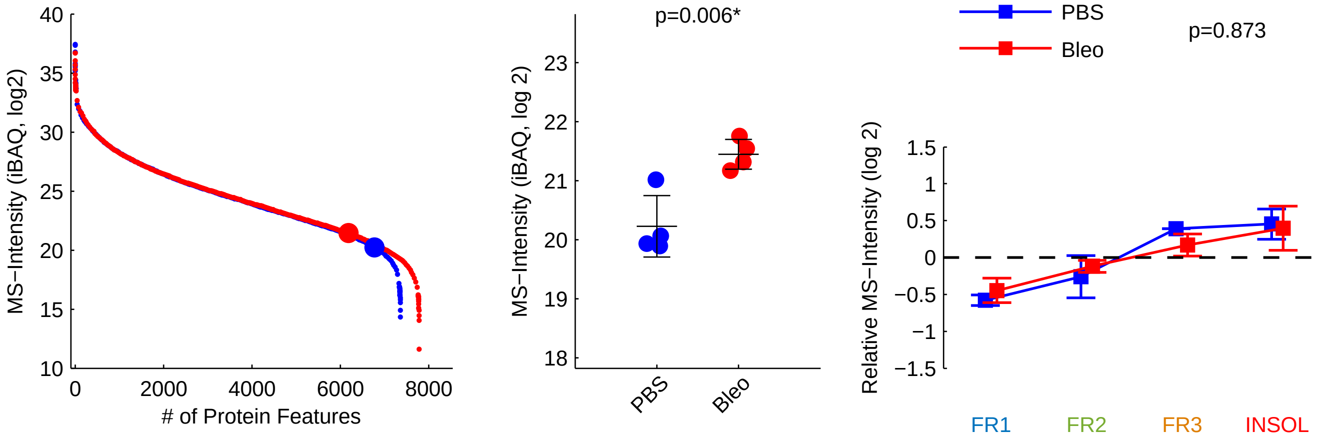

Q6DIC0 − Smarca2 (id: 1505)

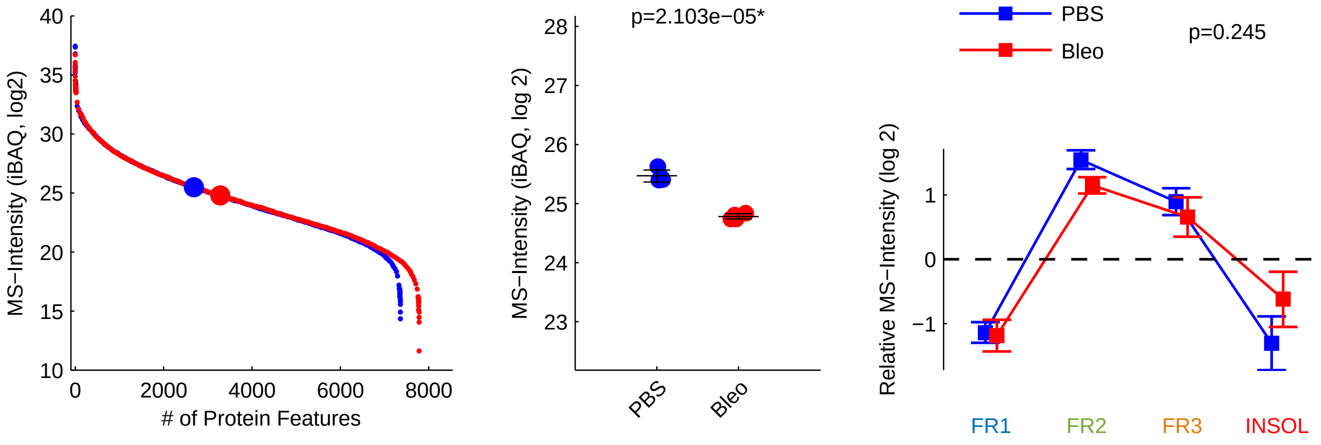

F6SMY7 − Mycbp2 (id: 1533)

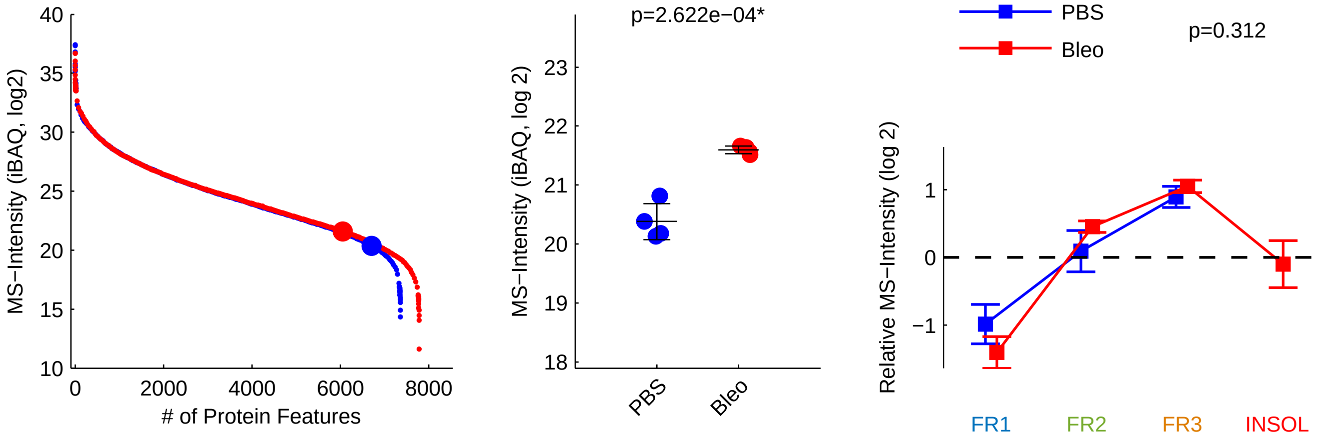

F6TQW2 − Ighg2c (id: 1542)

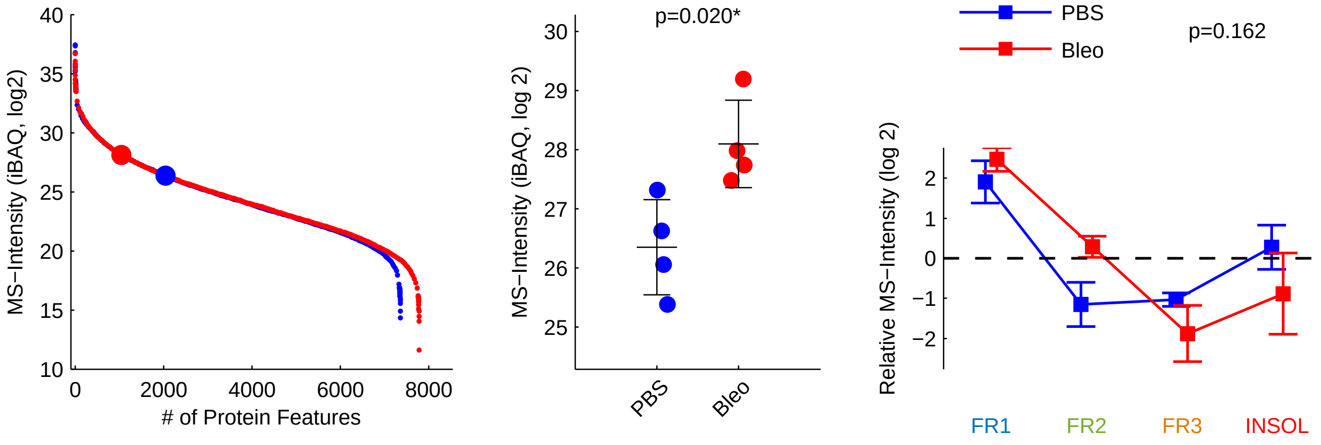

Q9R0Q3 − Tmed2 (id: 1548)

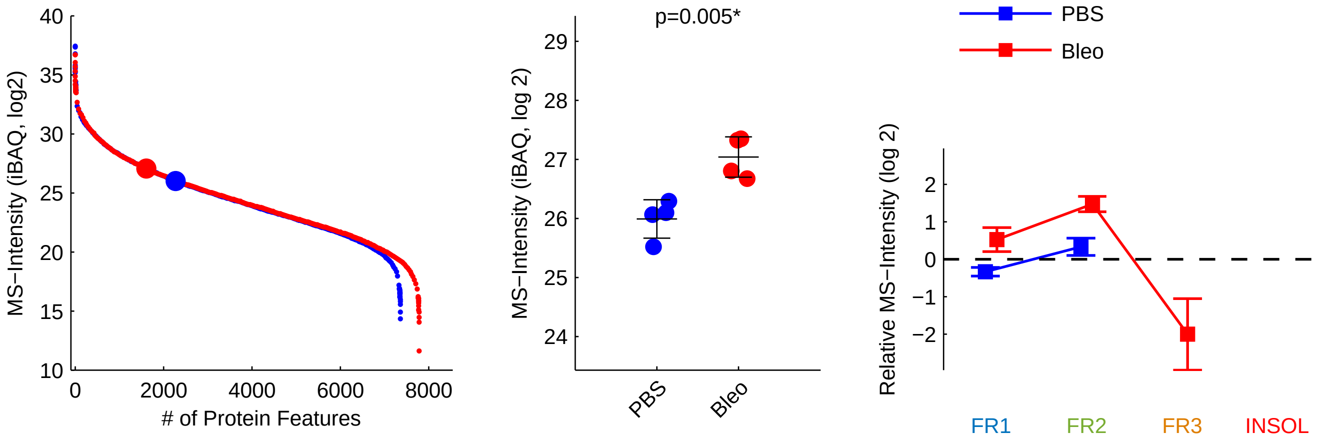

F6VRP8 − Lgals3bp (id: 1553)

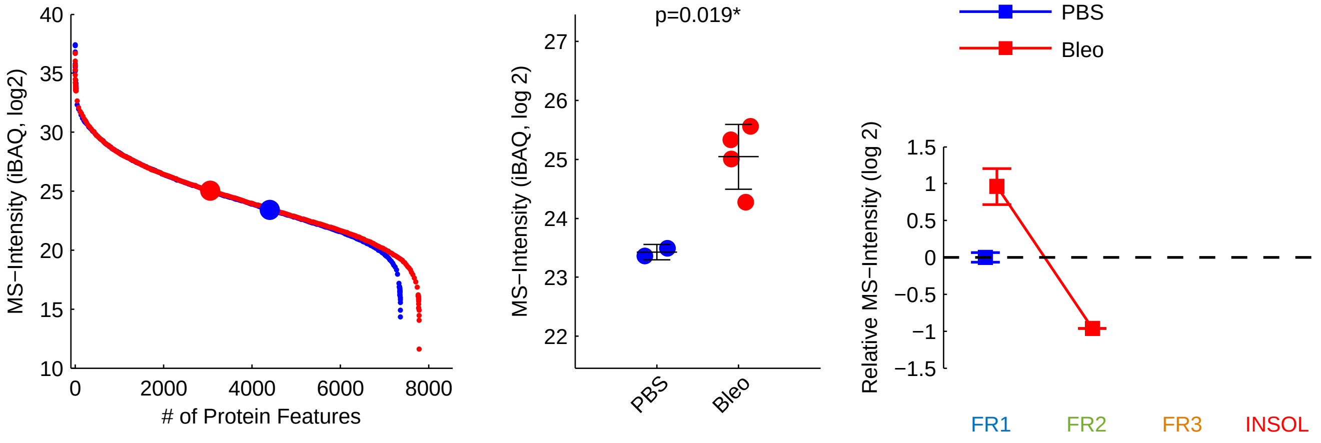

F6WBA1 − 2210011C24Rik (id: 1557)

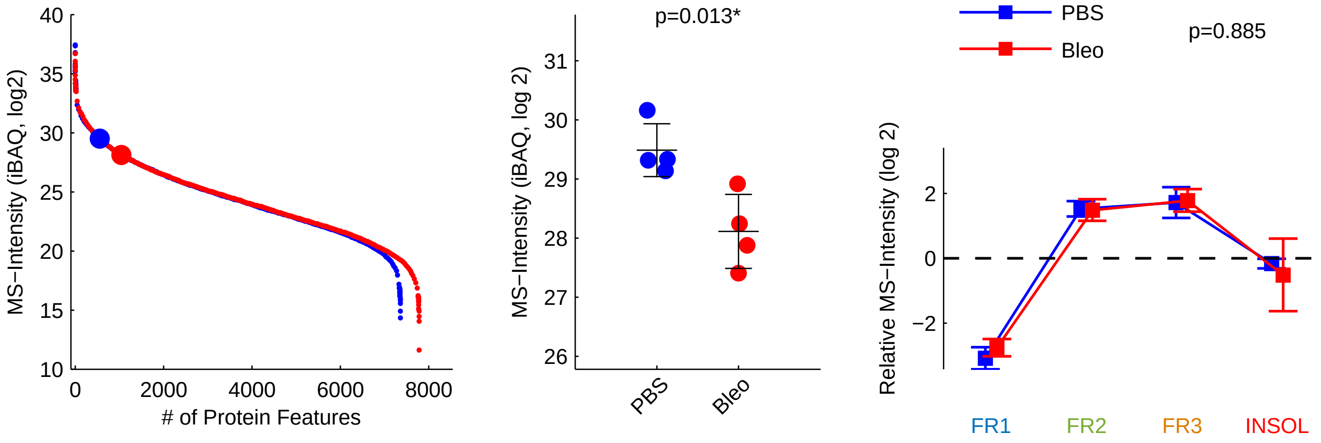

P61961 − Ufm1 (id: 1568)

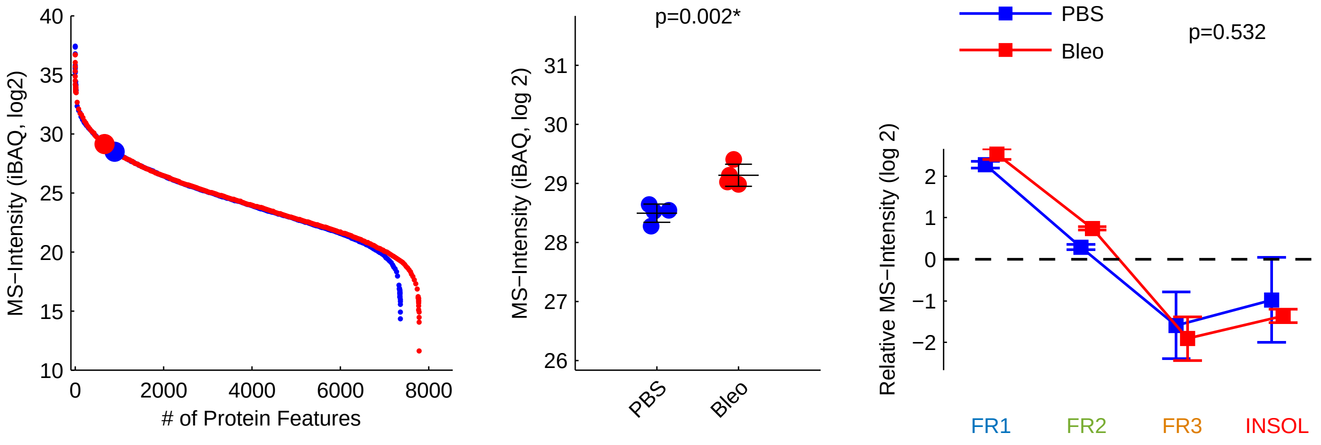

P56477 − Irf5 (id: 1578)

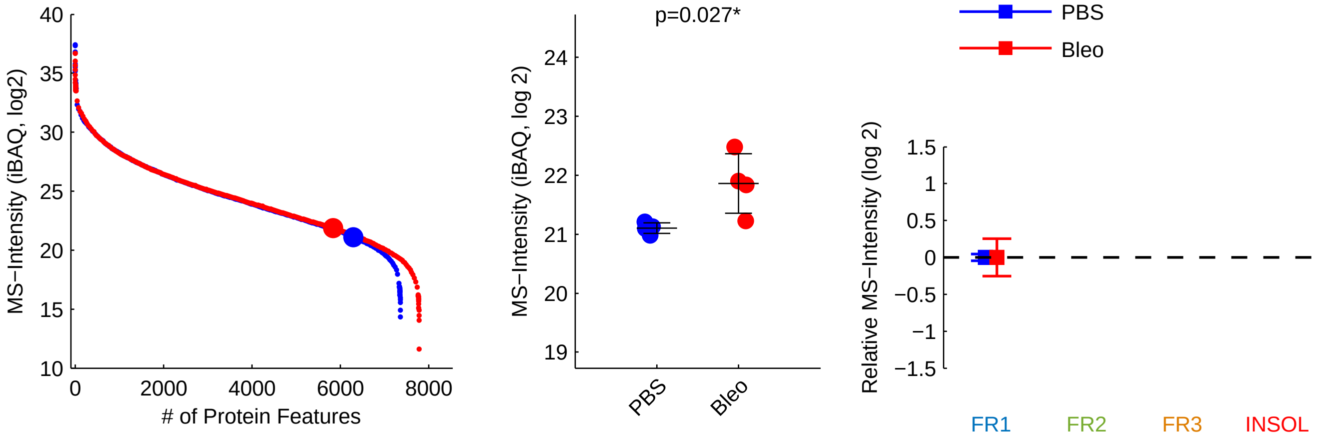

F7AA26 − Gm20459 (id: 1591)

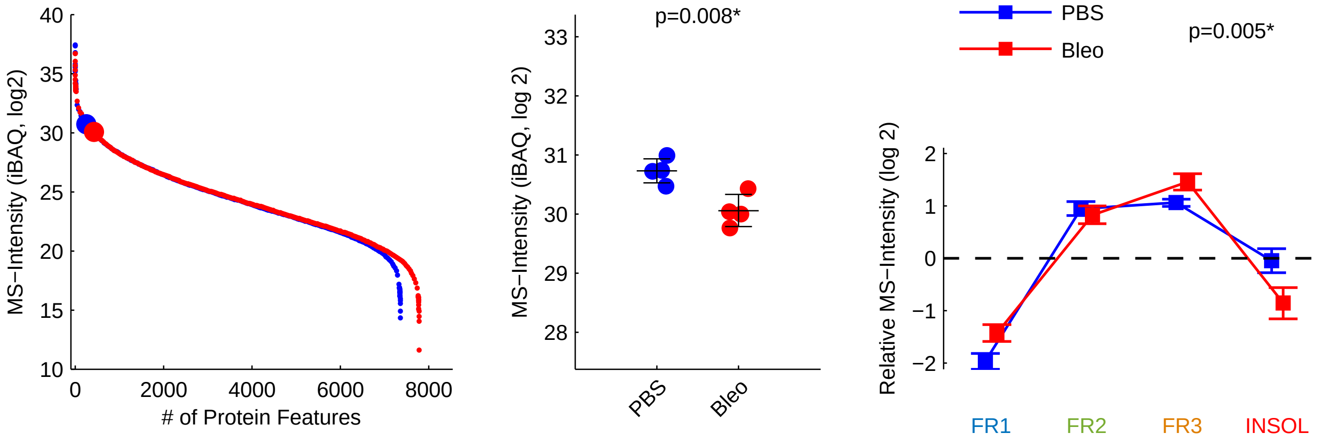

F7BJK1 − Pcdh1 (id: 1602)

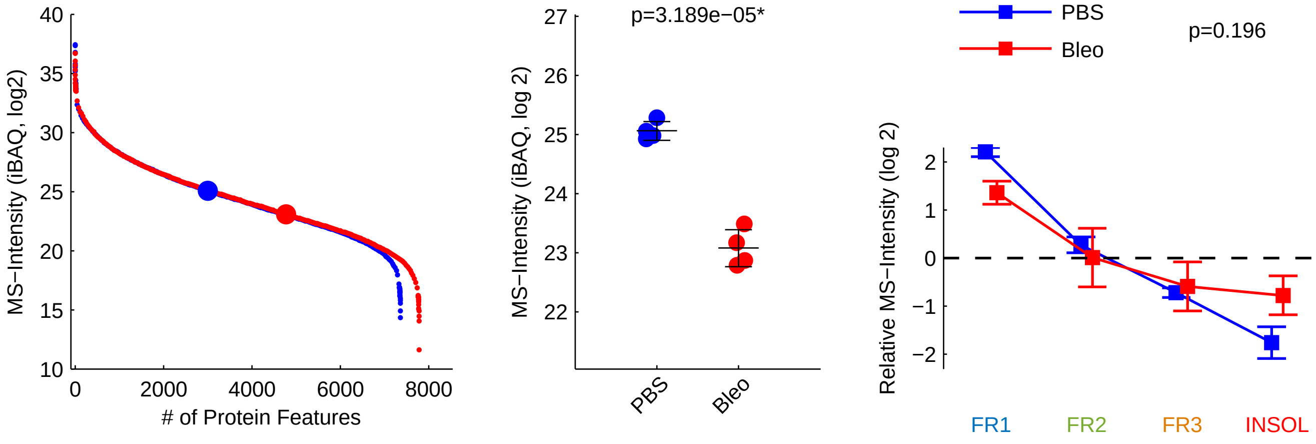

F7DBB3 − Ahnak2 (id: 1615)

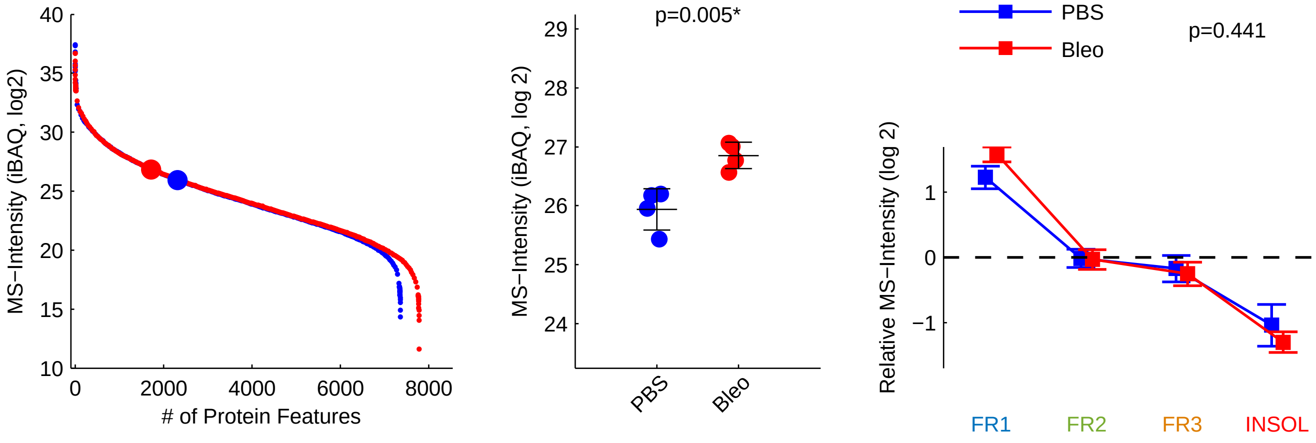

P05532 − Kit (id: 1618)

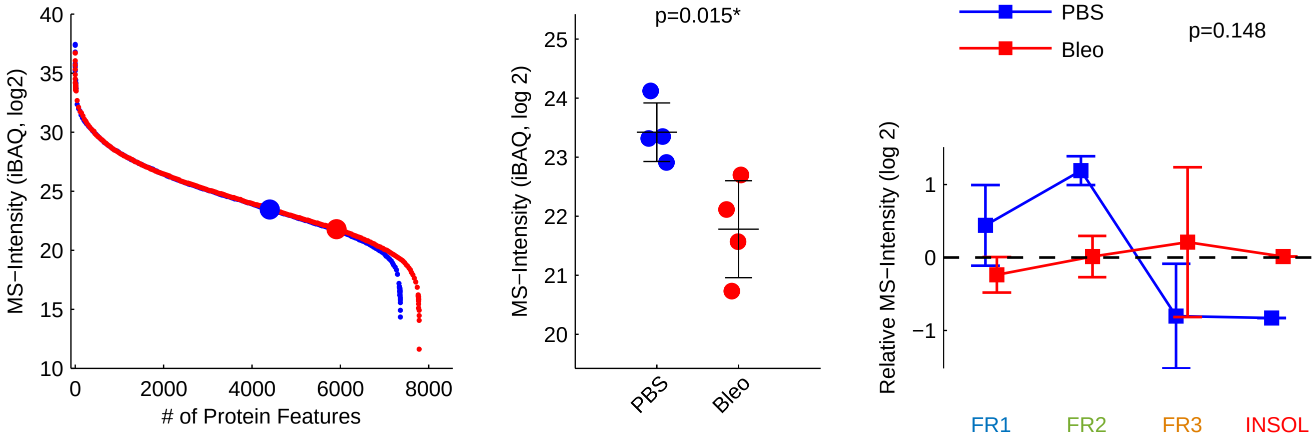

F7DEU6 − Impdh1 (id: 1619)

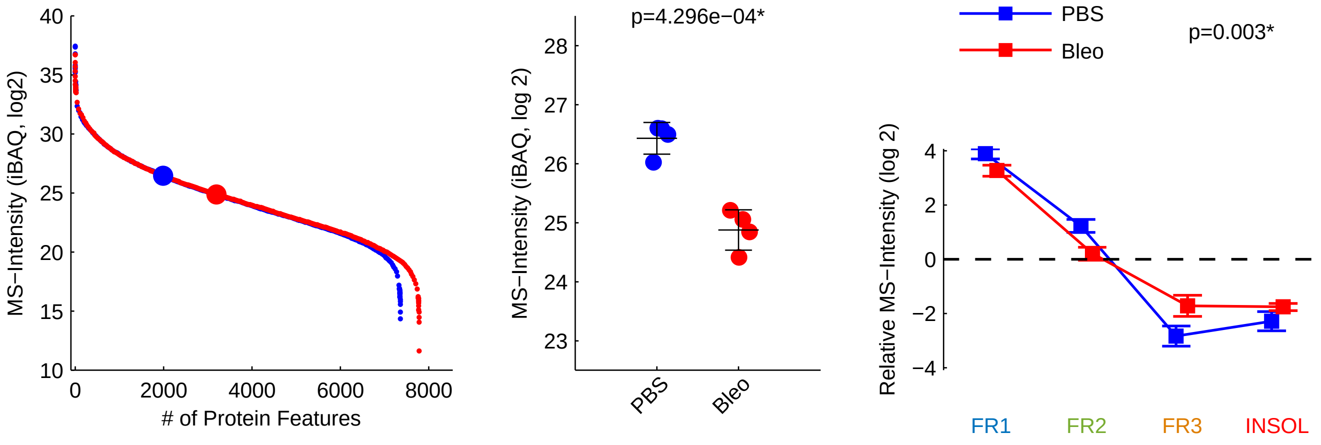

F8VPK0 − Ttc37 (id: 1622)

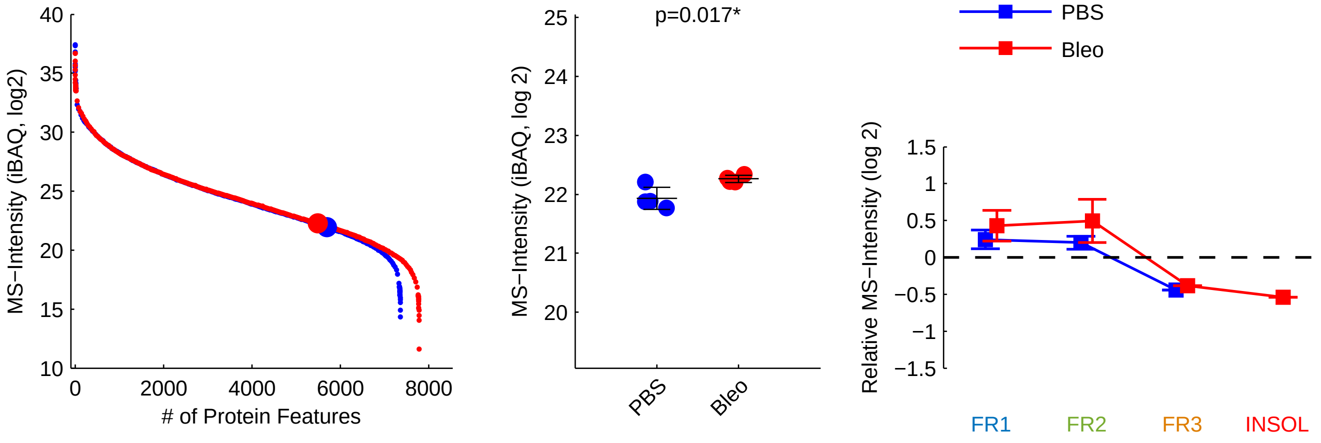

F8VPL5 − Map4k4 (id: 1626)

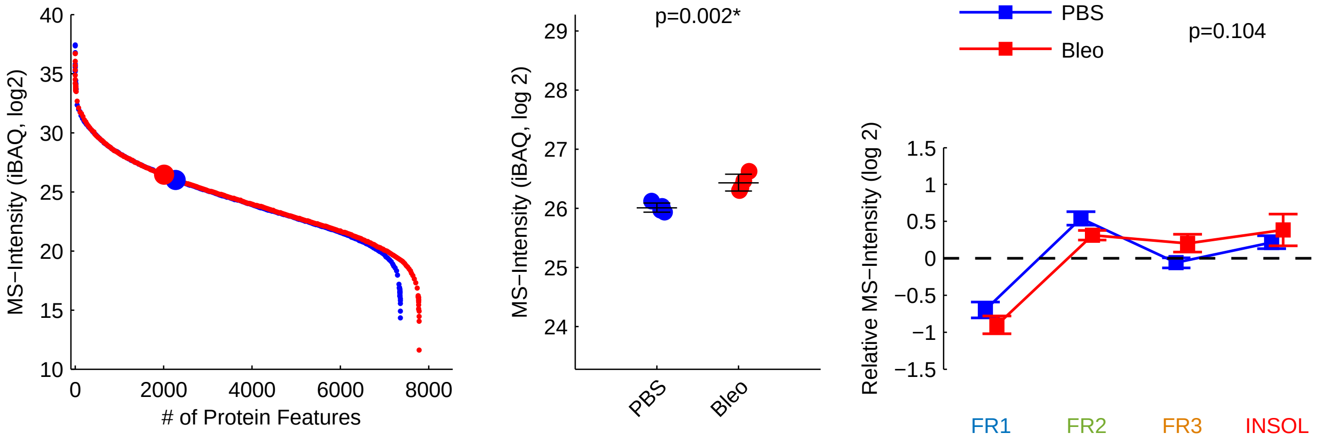

F8VPY2 − Taf5 (id: 1636)

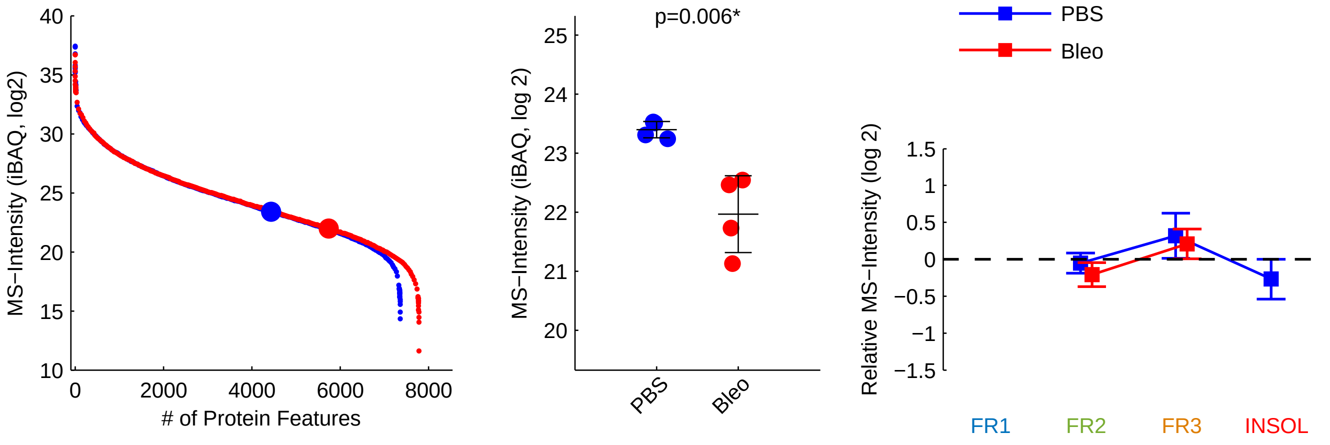

F8VQ40 − Lama1 (id: 1645)

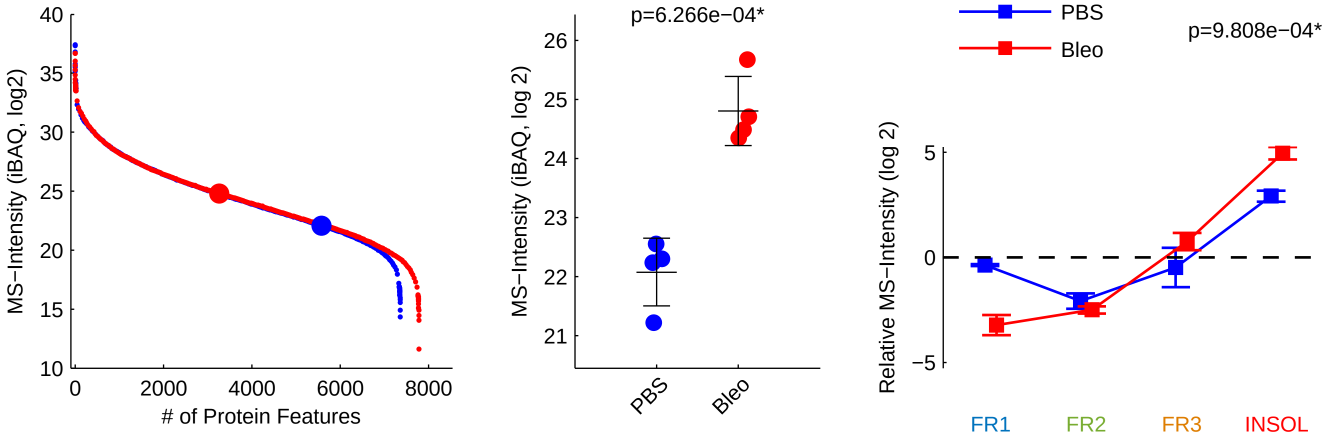

F8VQ43 − Lama2 (id: 1646)

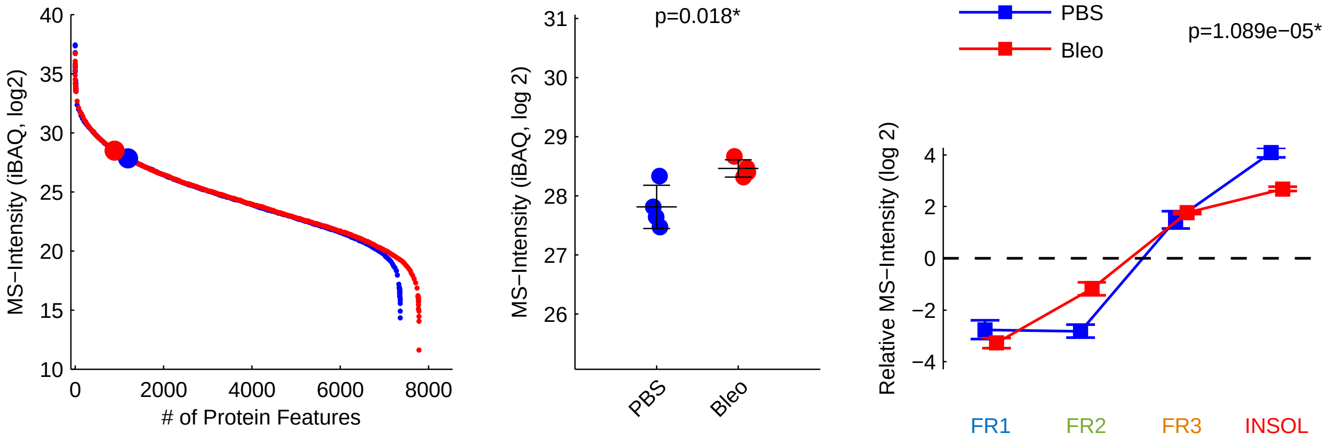

Q9EQW7 − Kif13a (id: 1651)

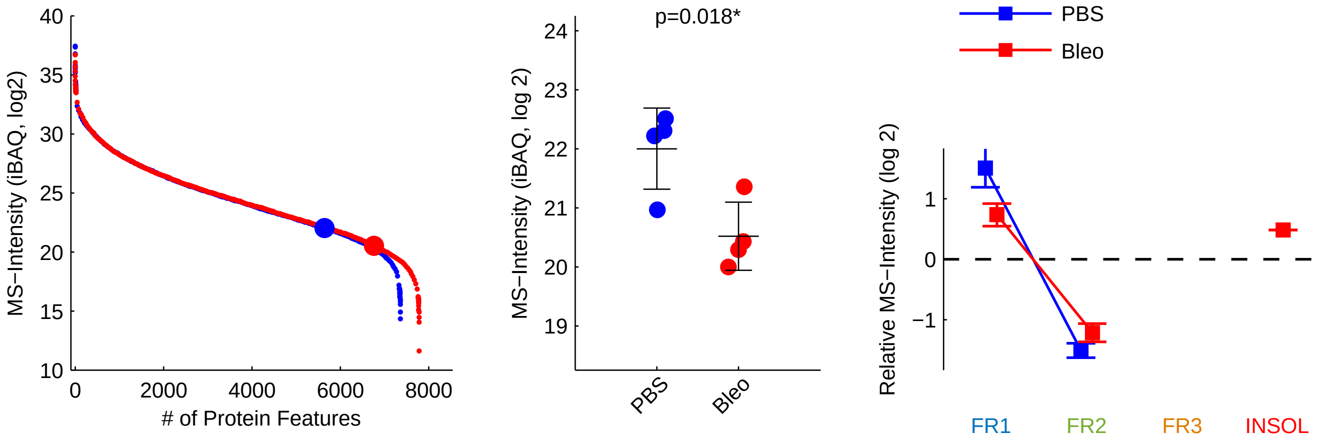

F8VQ87 − Xrn1 (id: 1652)

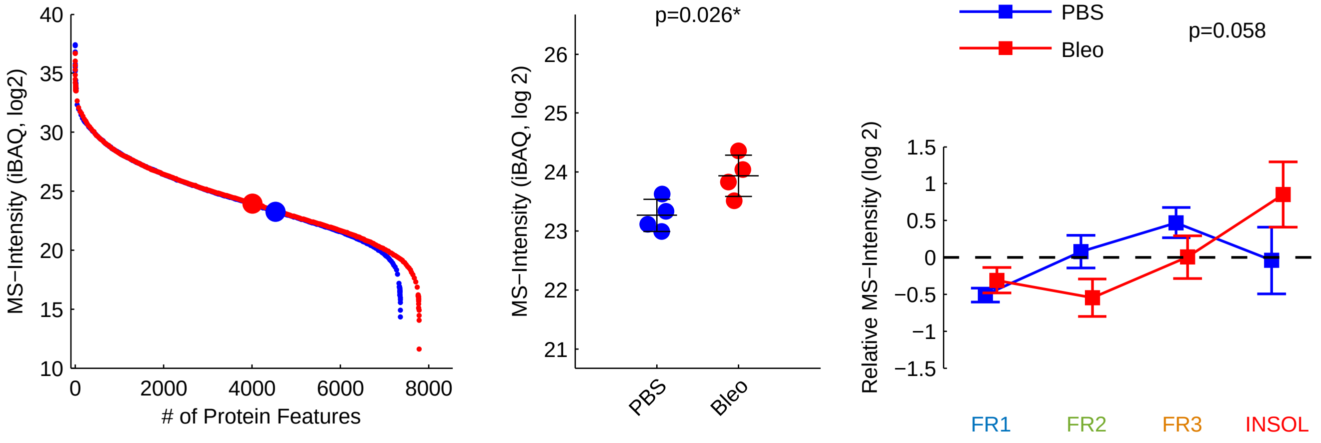

F8VQ93 − Phip (id: 1653)

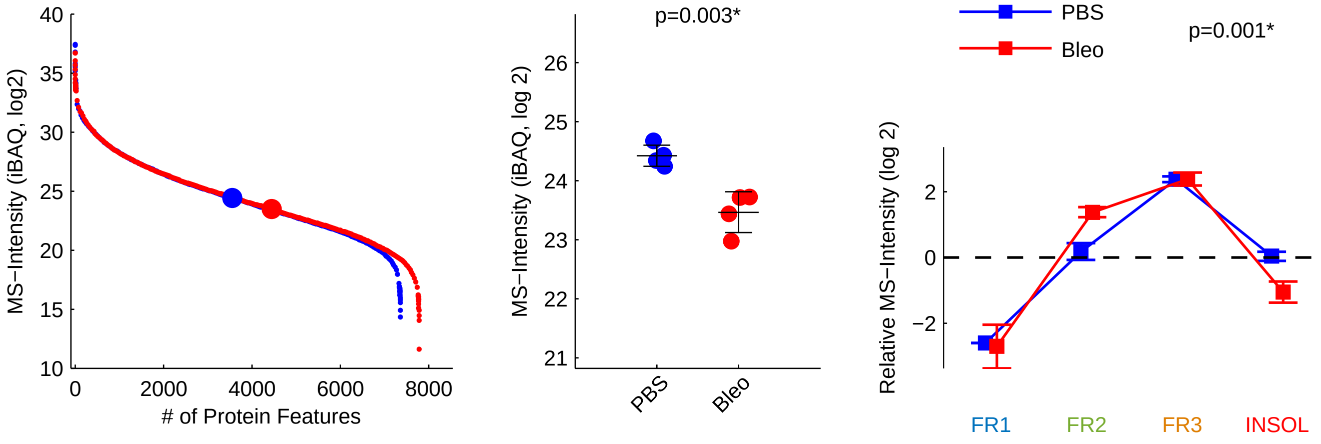

Q6Y685 − Tacc1 (id: 1654)

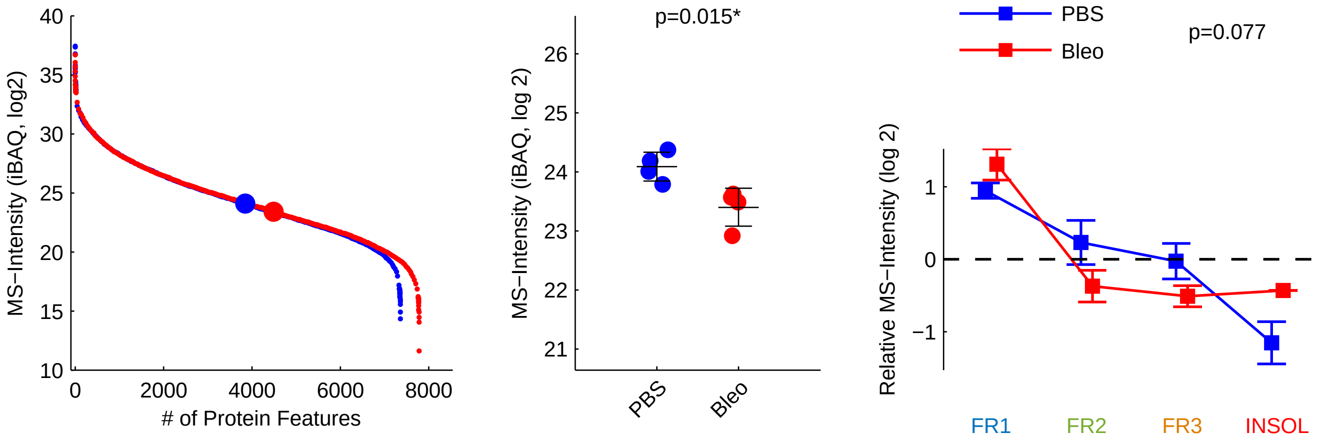

F8VQB6 − Myo10 (id: 1655)

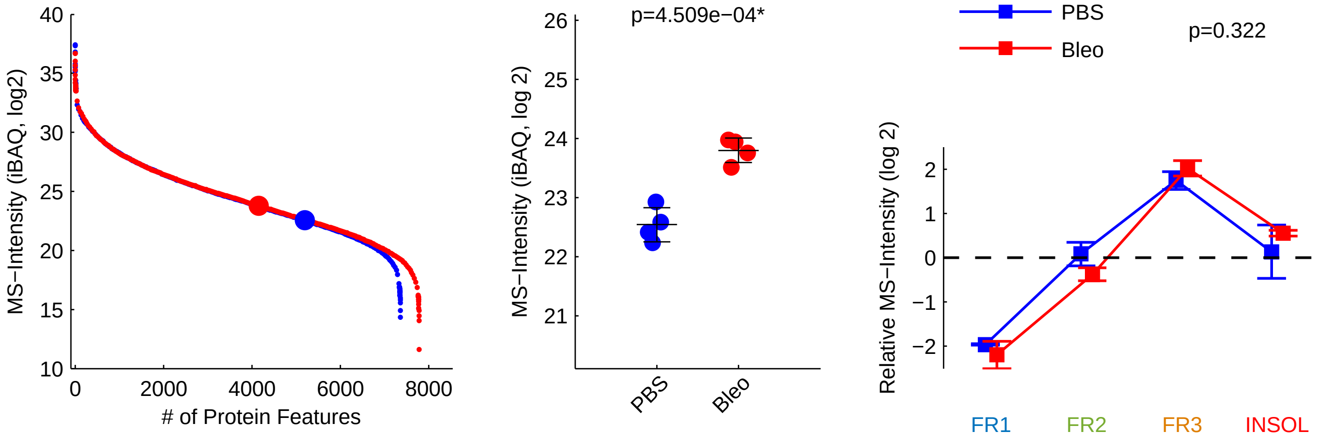

Q8VHH5 − Agap3 (id: 1660)

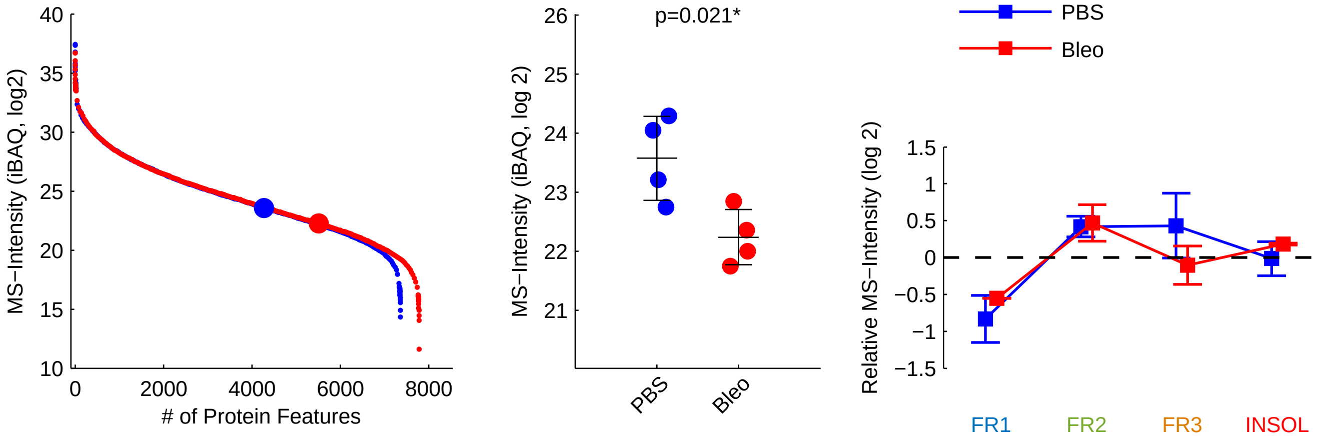

F8VQJ3 − Lamc1 (id: 1662)

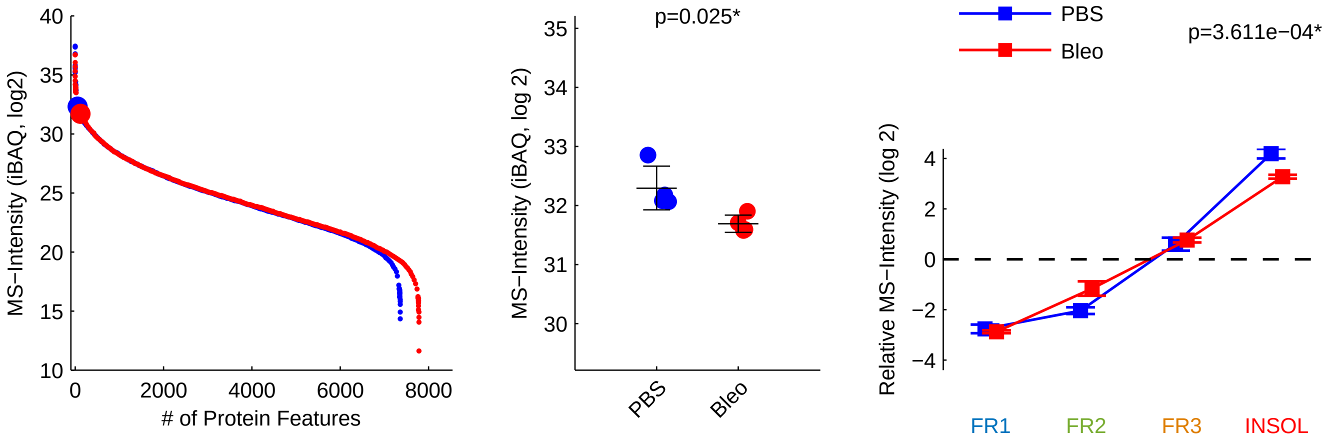

F8VQK3 − Gucy1a2 (id: 1663)

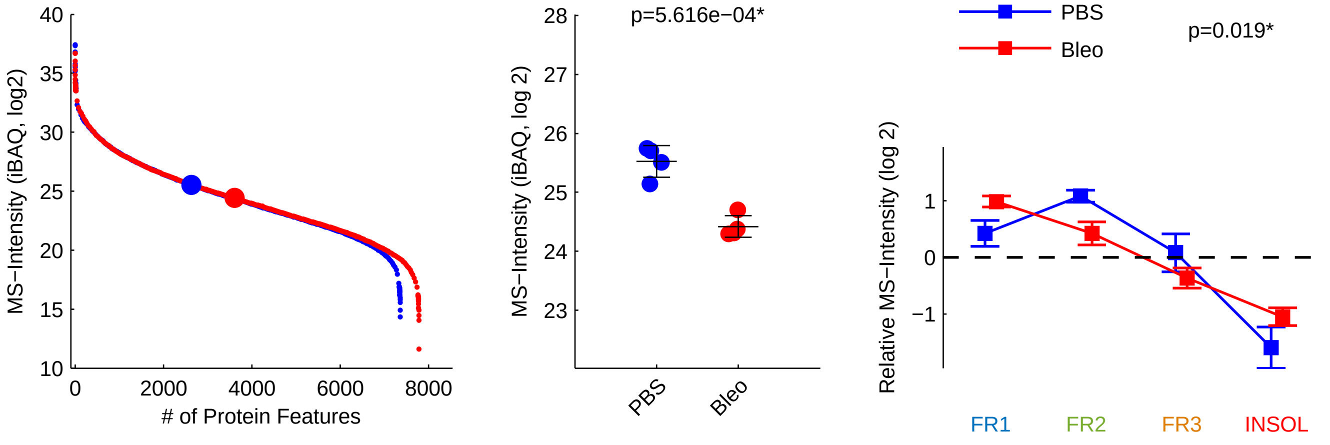

F8VQN6 − Arhgef12 (id: 1666)

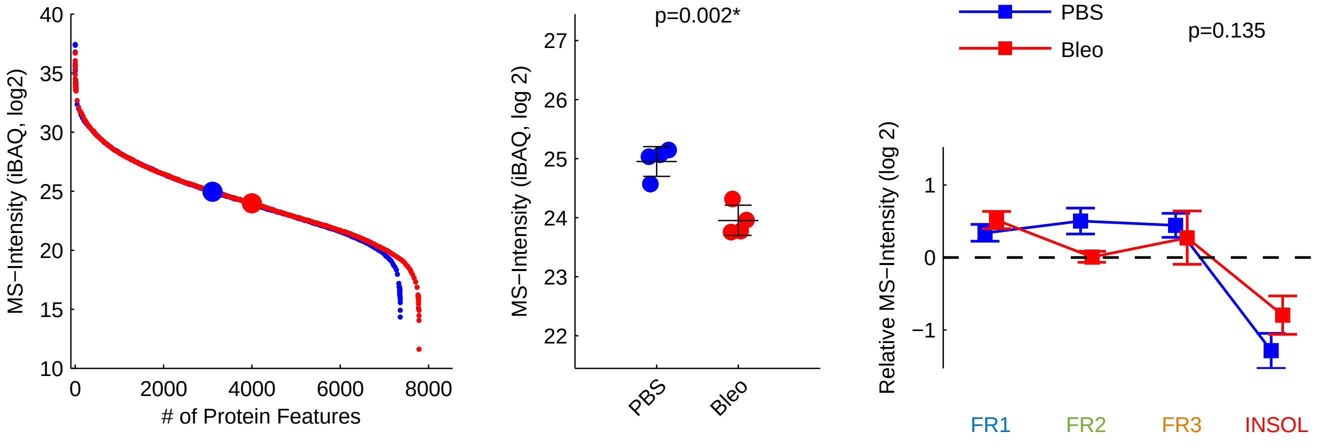

Q68FL4 − Ahcyl2 (id: 1669)

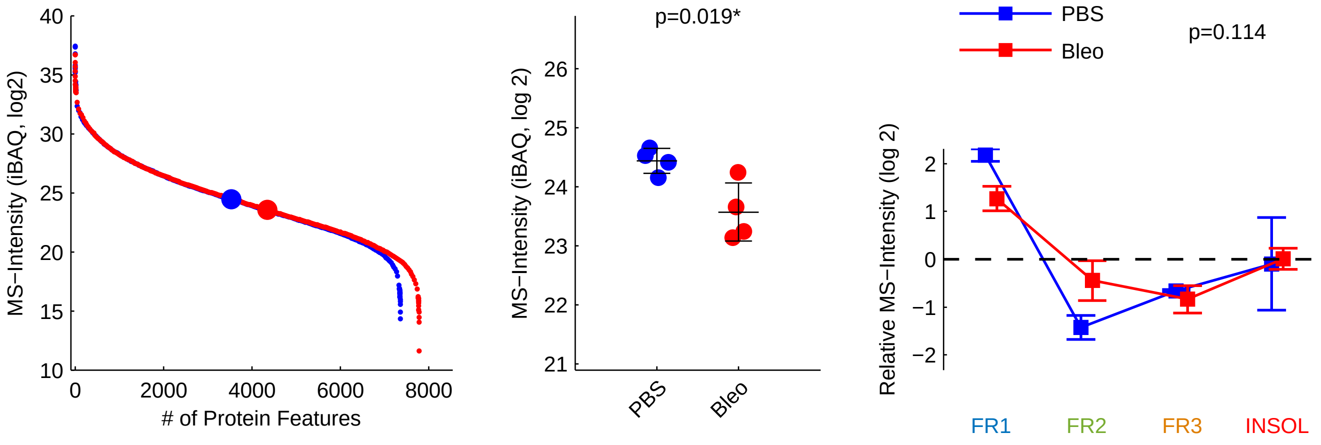

Q61581 − Igfbp7 (id: 1673)

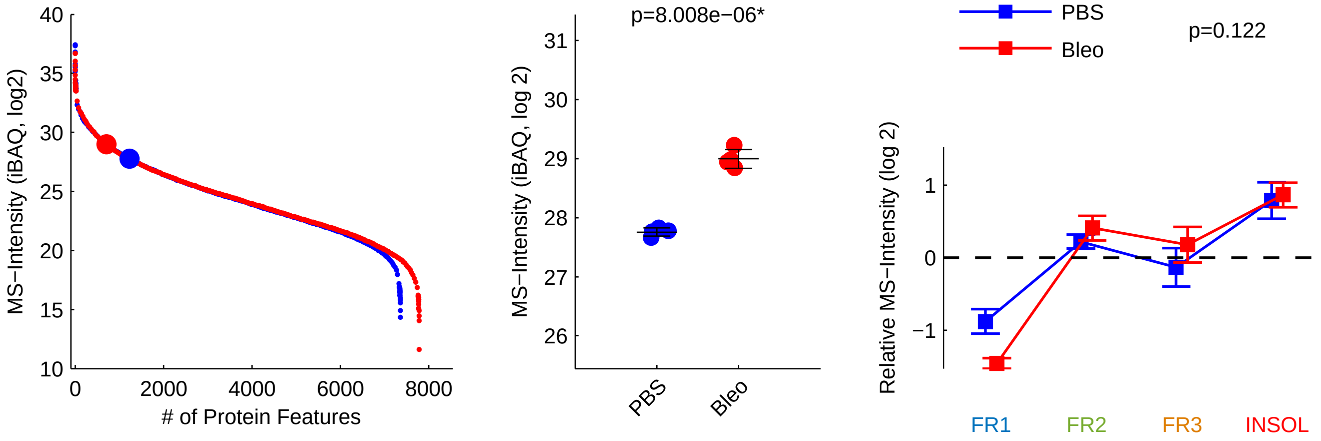

Q09014 − Ncf1 (id: 1674)

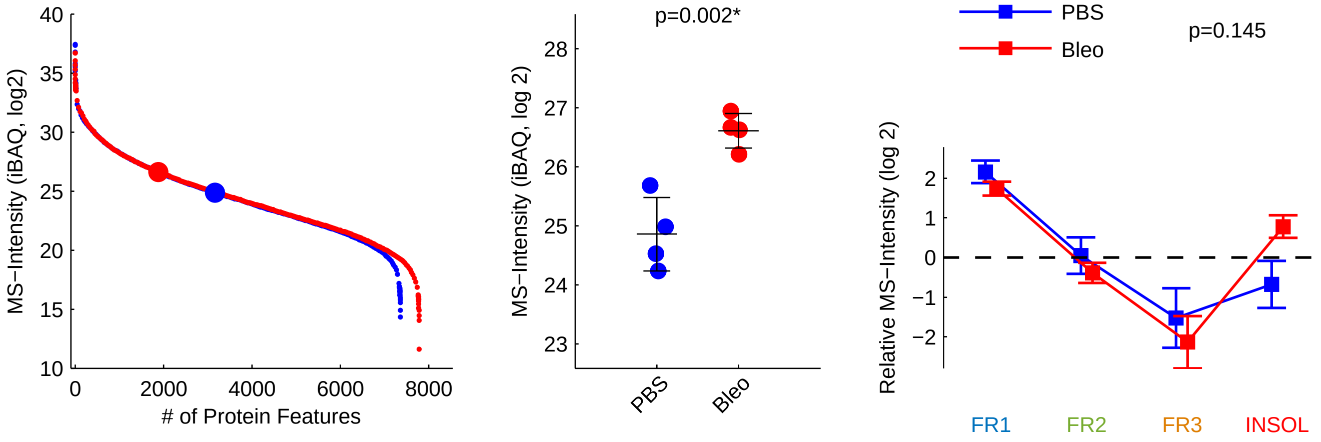

Q61543 − Glg1 (id: 1678)

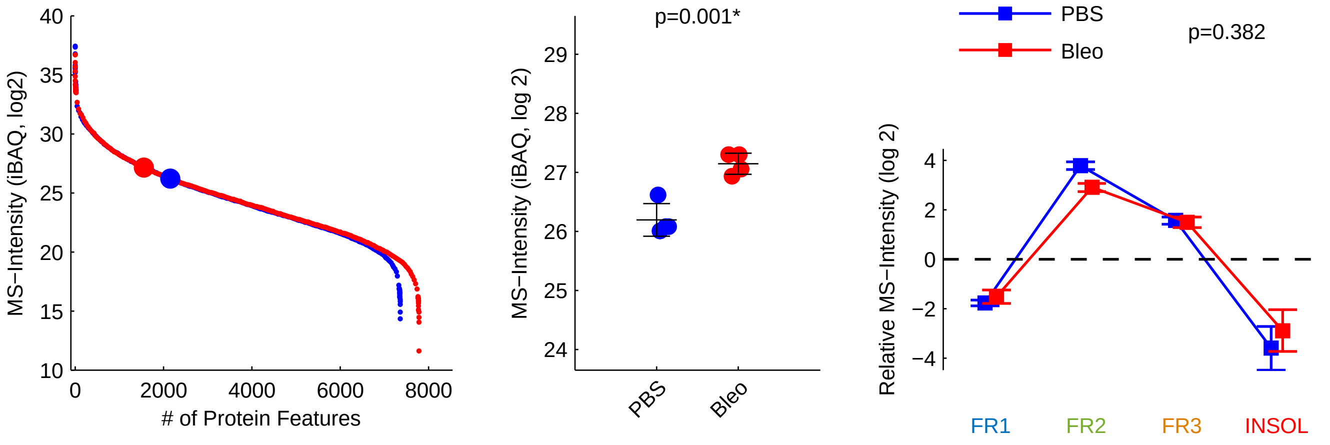

F8WHY8 − Mta1 (id: 1687)

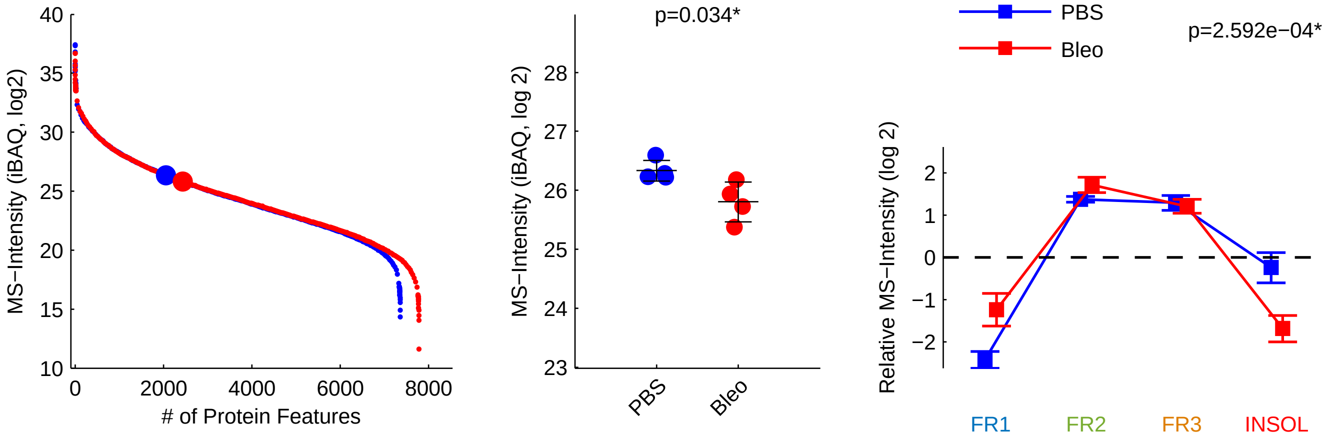

F8WID5 − Tpm1 (id: 1697)

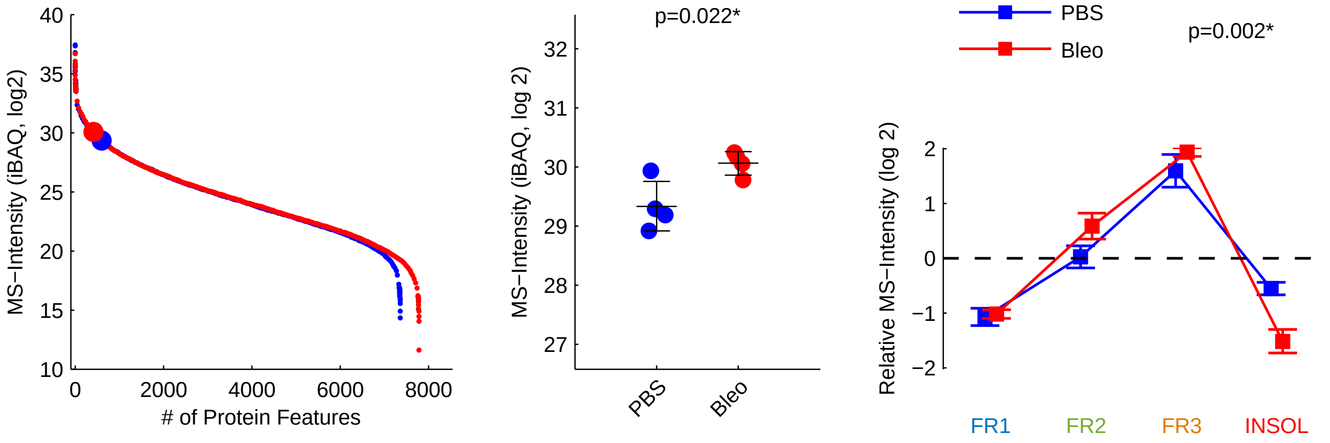

F8WIE5 − Hectd1 (id: 1698)

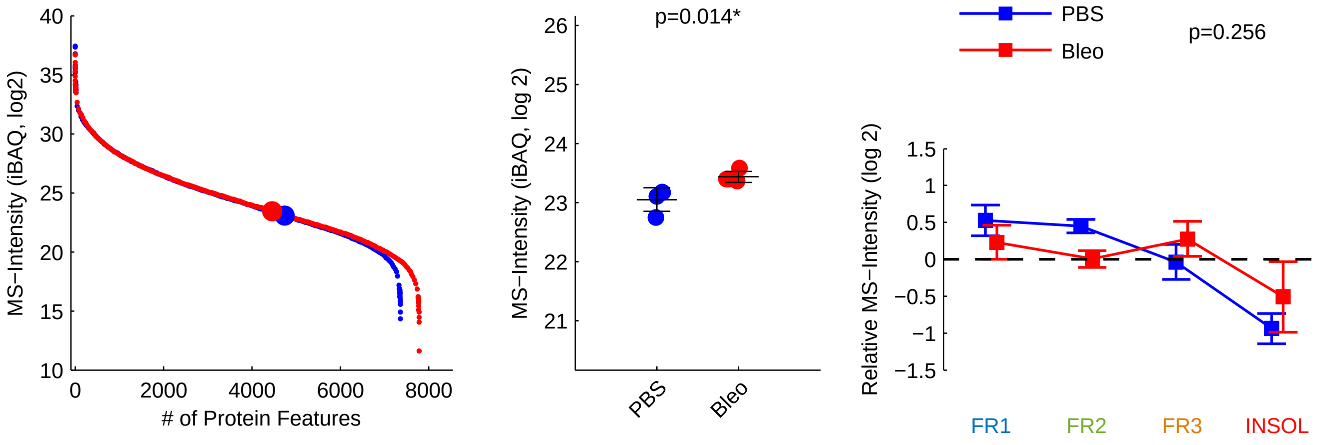

P10923 − Spp1 (id: 1701)

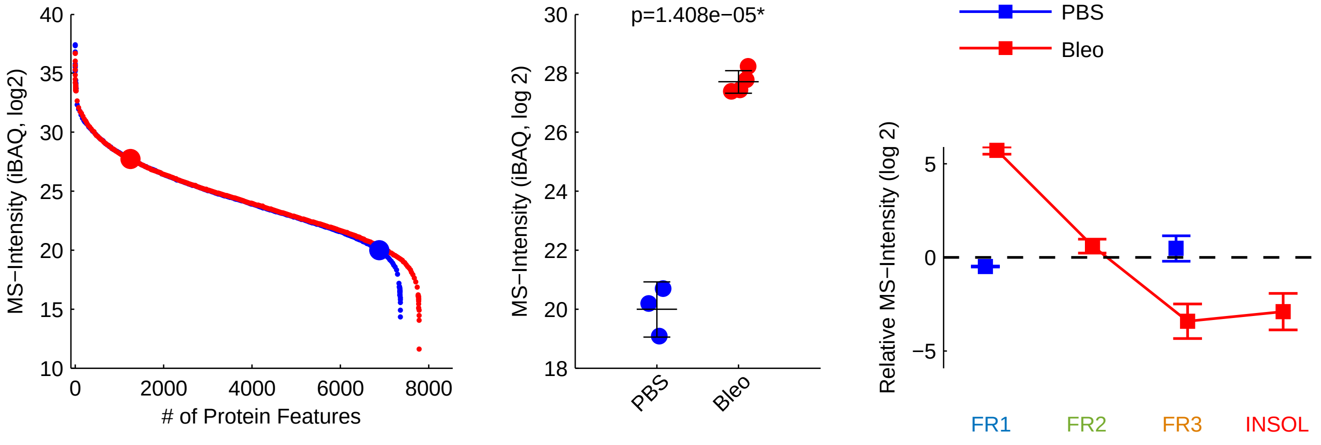

Q923Q2 − Stard13 (id: 1707)

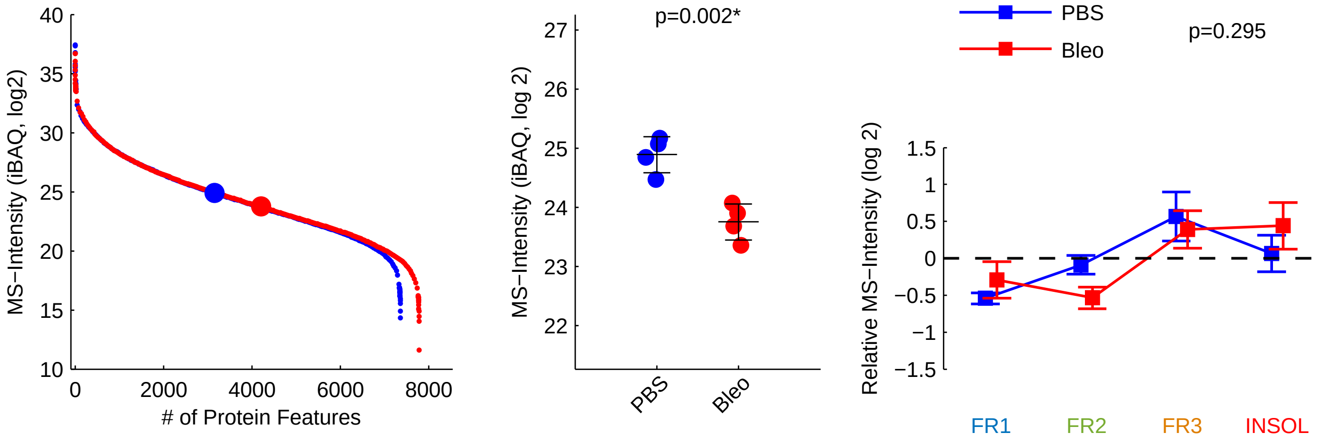

O08550 − Kmt2b (id: 1709)

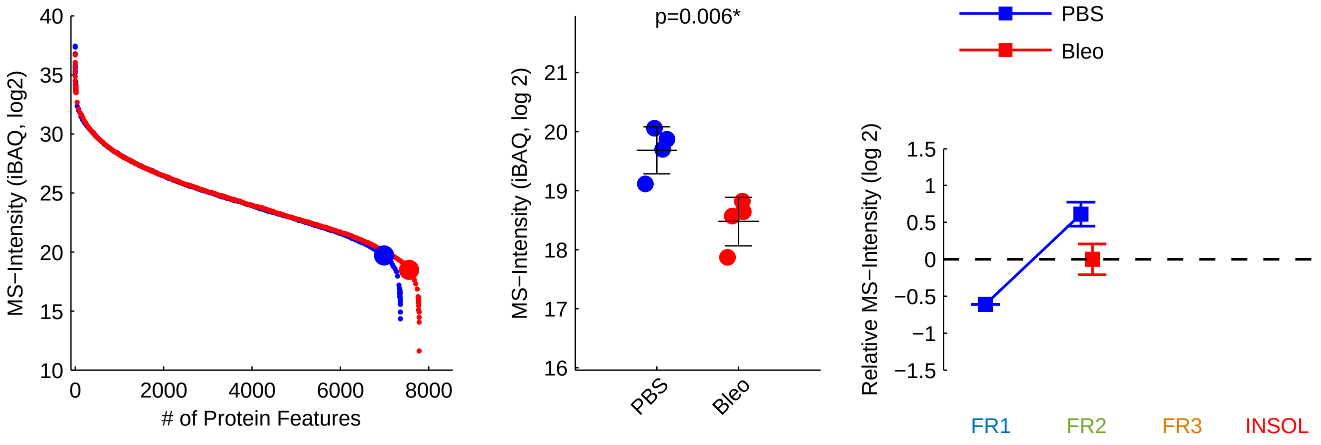

Q80X82 − Sympk (id: 1712)

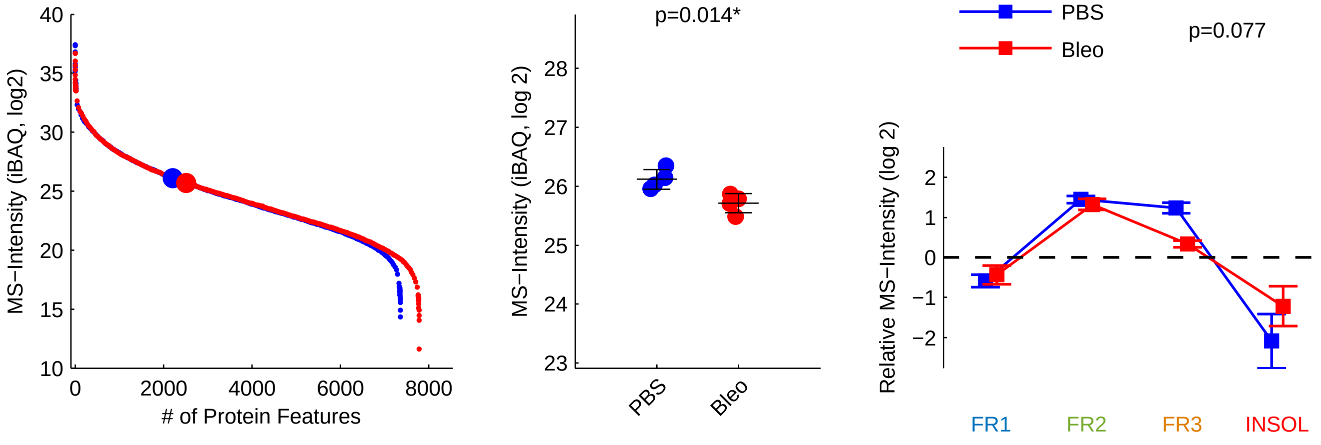

P62897 − Cycs (id: 1729)

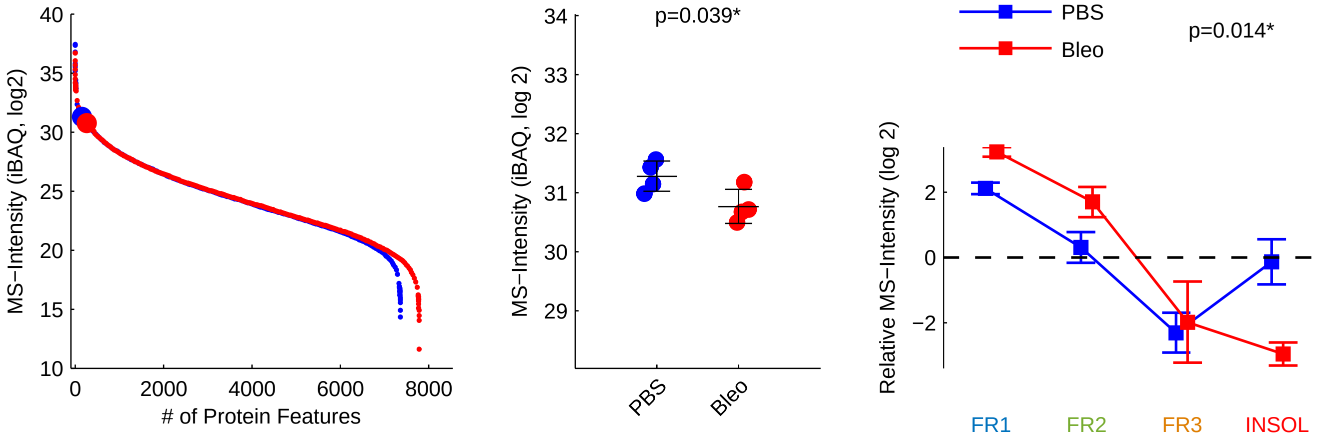

Q9D7G0 − Prps1 (id: 1741)

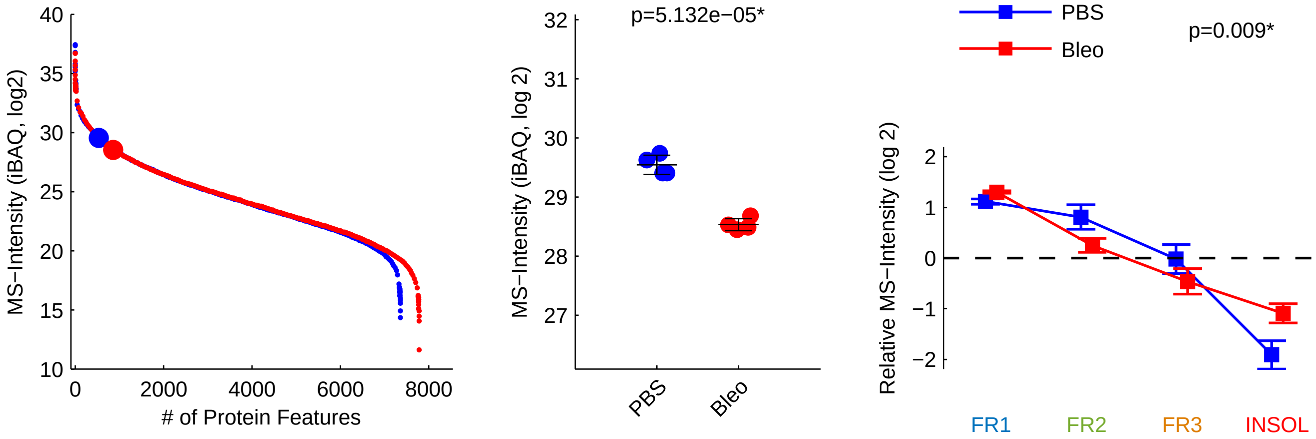

Q64487 − Ptprd (id: 1748)

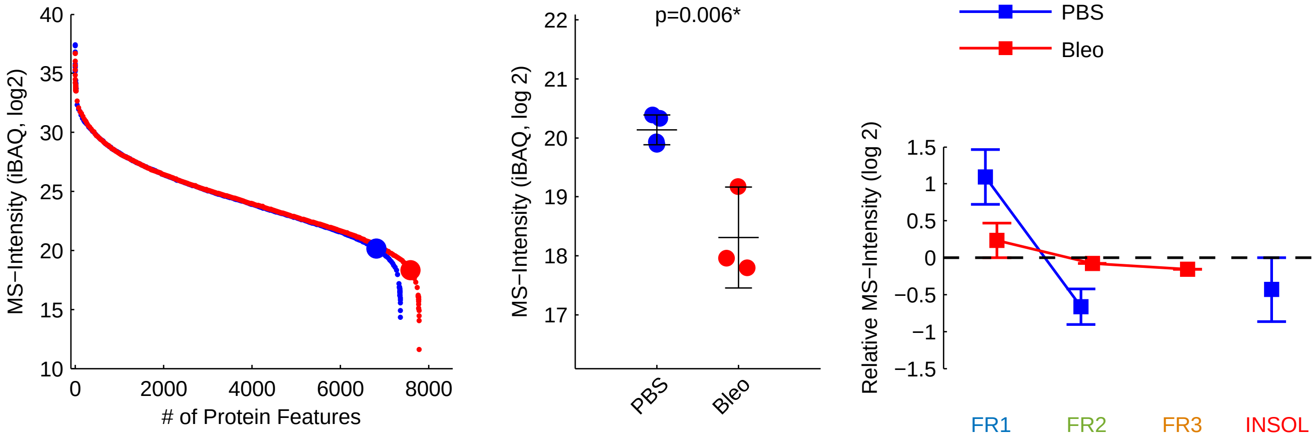

G3UYJ7 − Gm20441 (id: 1752)

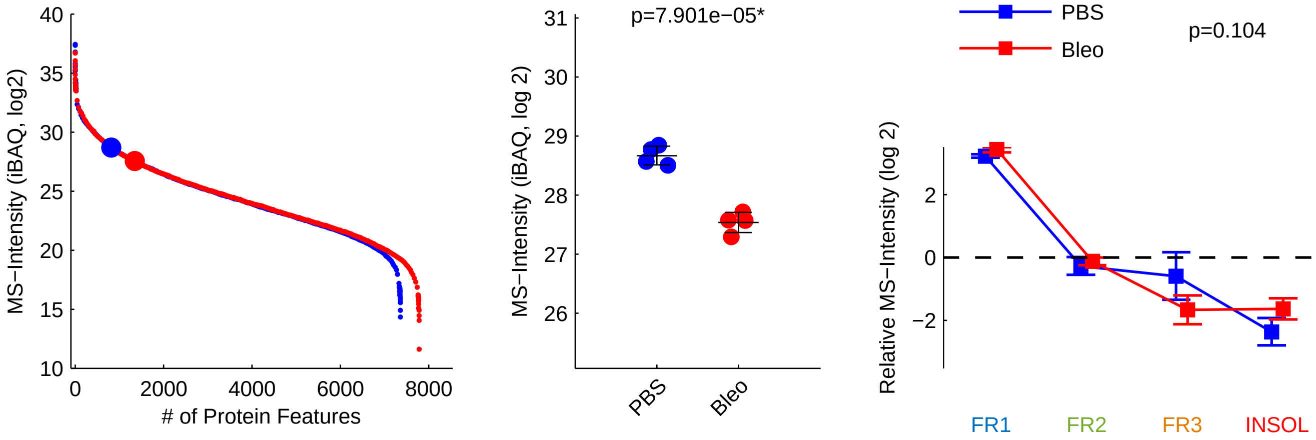

Q8BWW9 − Pkn2 (id: 1769)

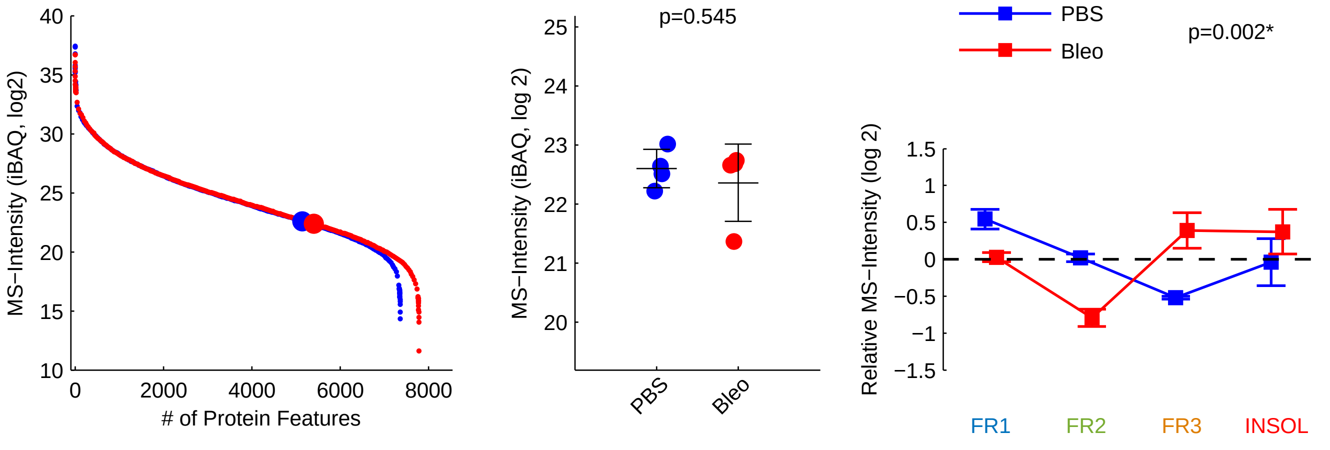

O54754 − Aox1 (id: 1773)

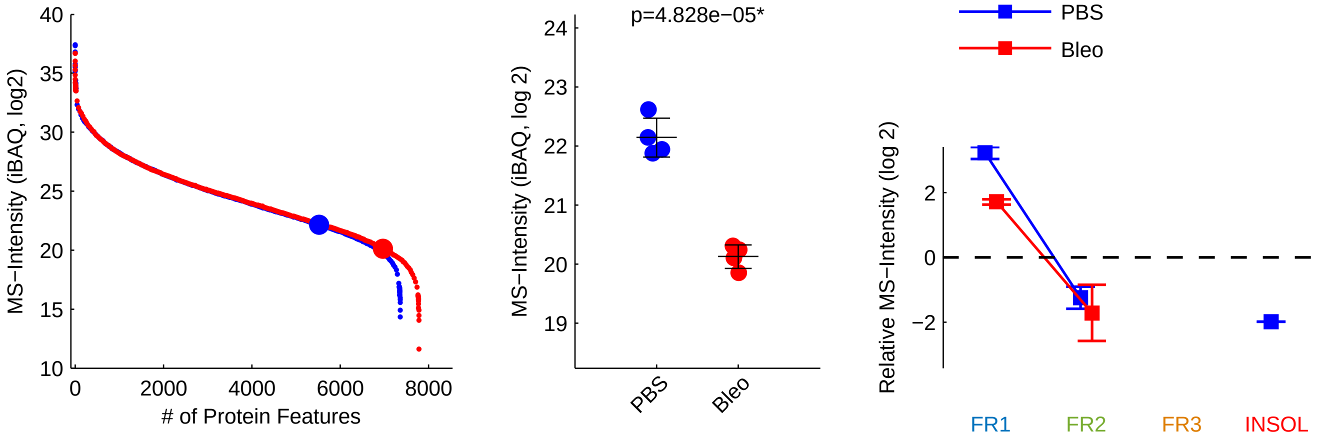

G3X9T8 − Cp (id: 1776)

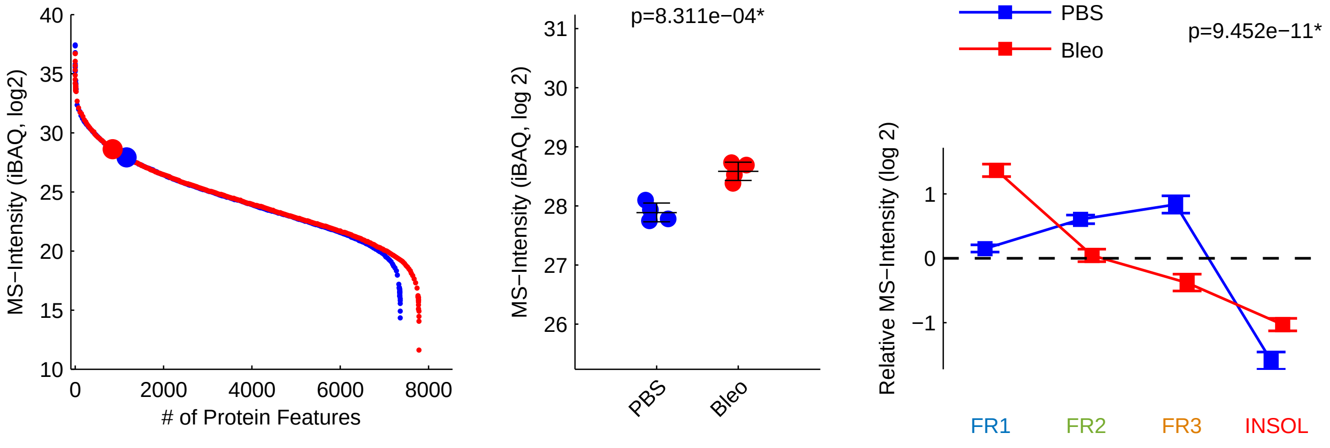

P16675 − Ctsa (id: 1784)

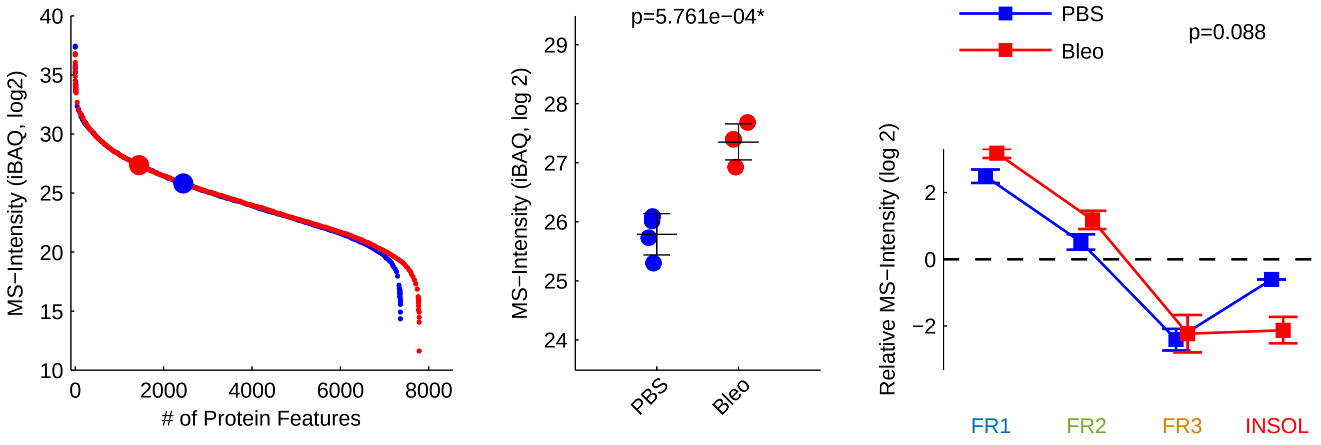

G3X8T9 − Serpina3n (id: 1785)

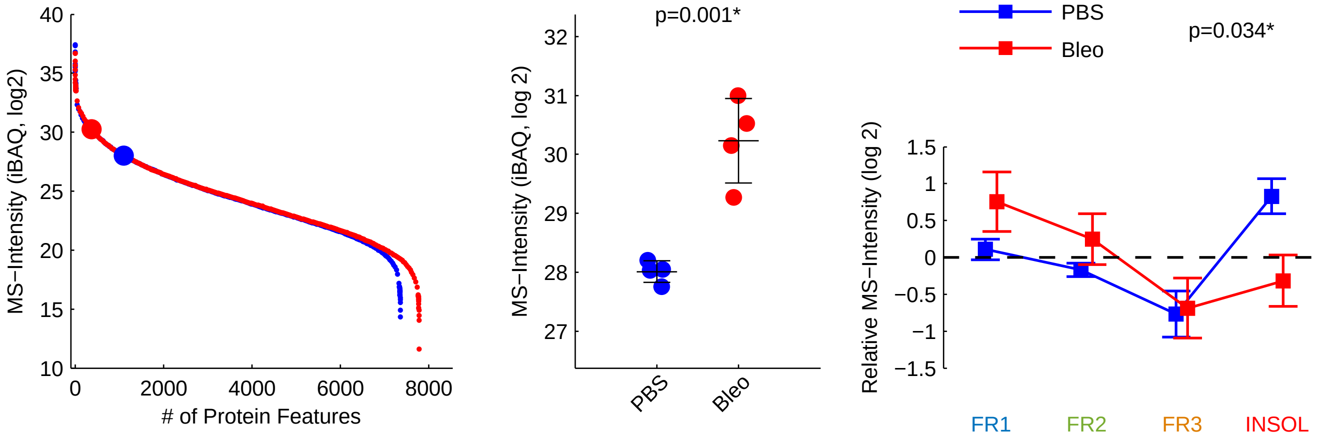

G3X8V8 − Ripk3 (id: 1789)

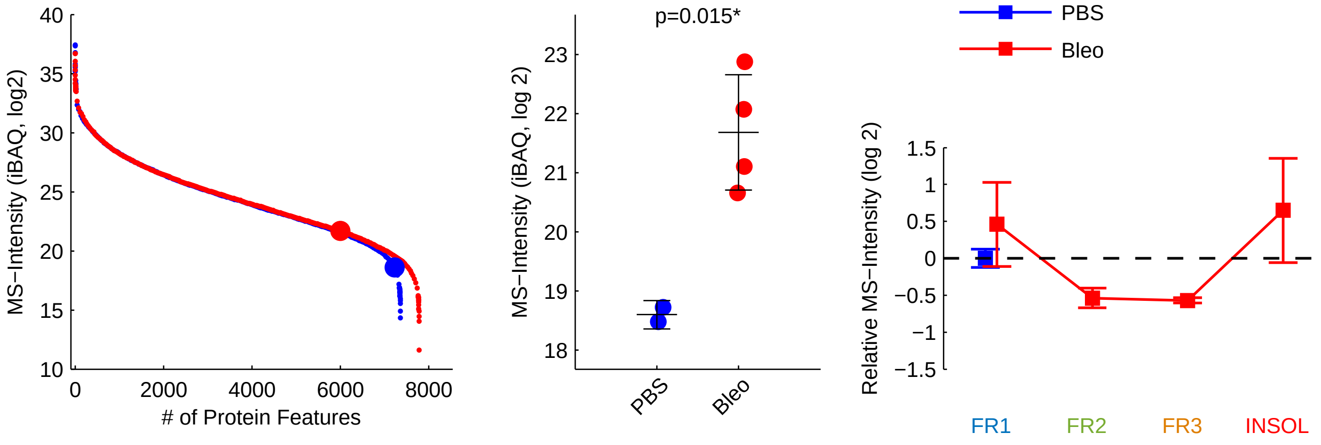

Q62230 − Siglec1 (id: 1792)

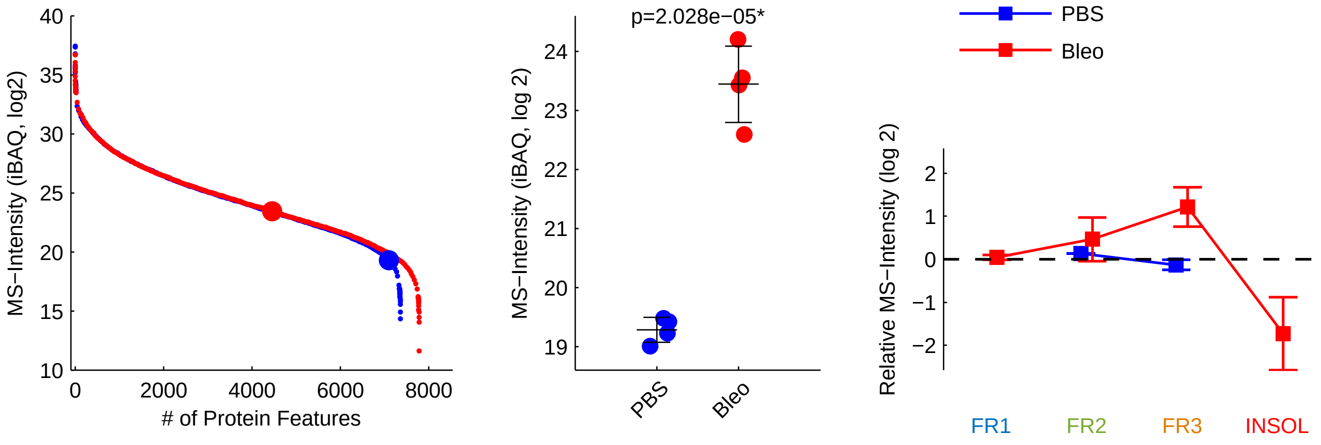

G3X922 − Dnajc13 (id: 1799)

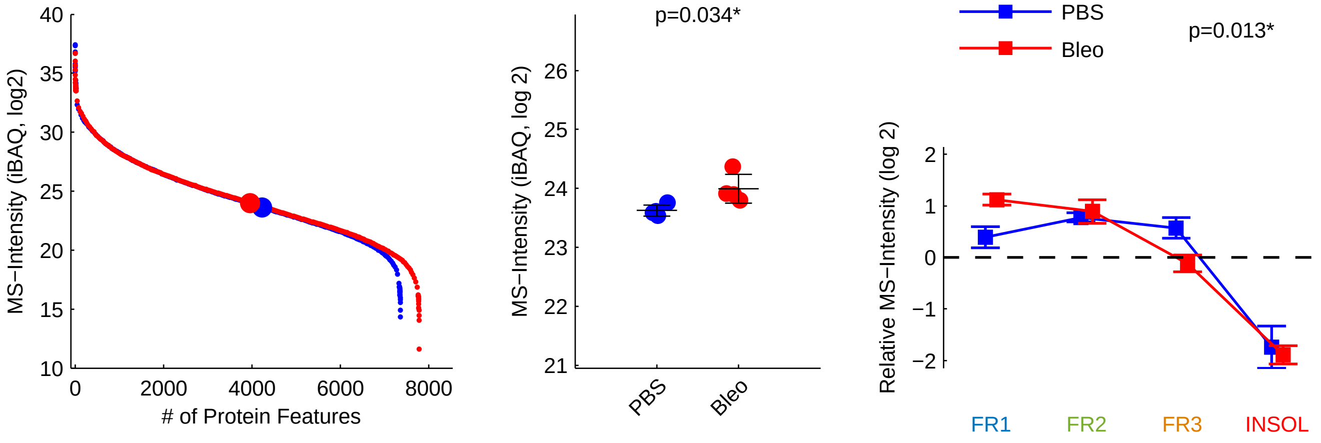

G3X924 − Plvap (id: 1800)

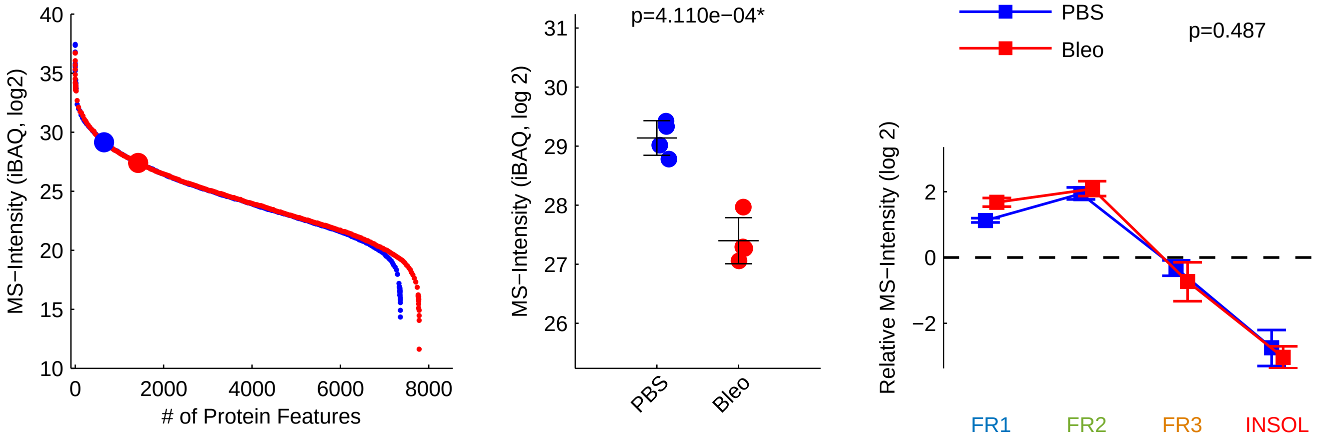

G3X926 − Rpf2 (id: 1802)

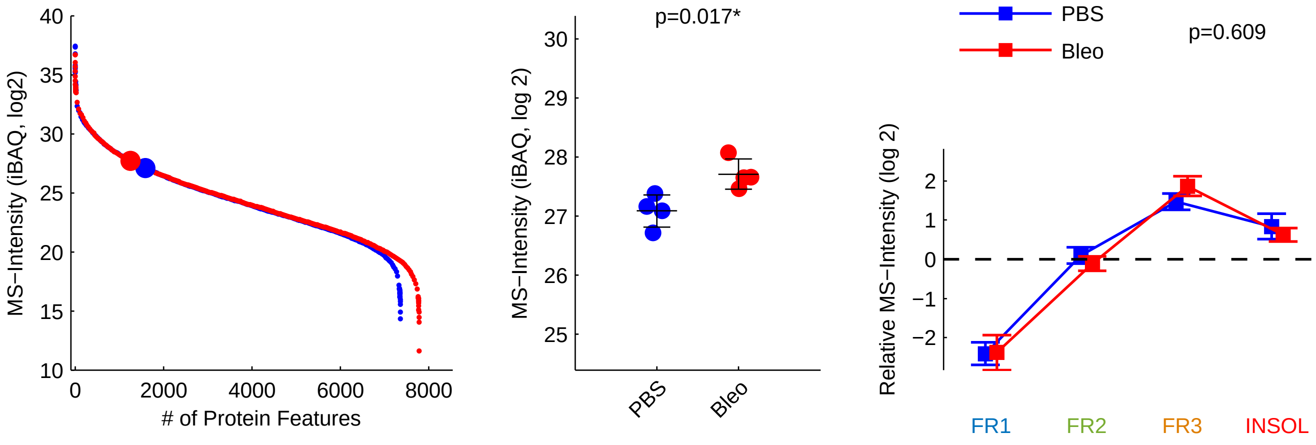

G3X928 − Sec23ip (id: 1803)

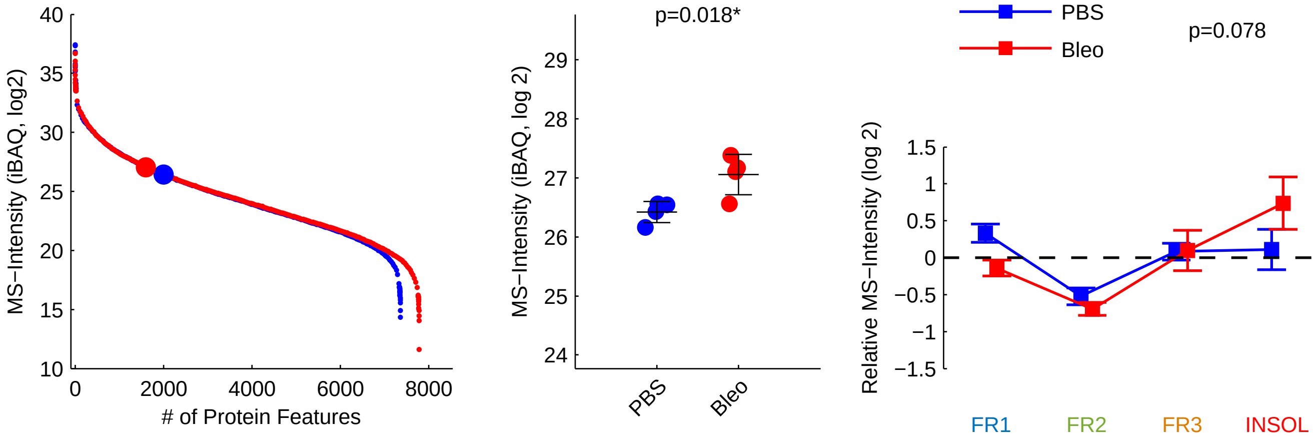

Q8BSN3 − Ccdc151 (id: 1807)

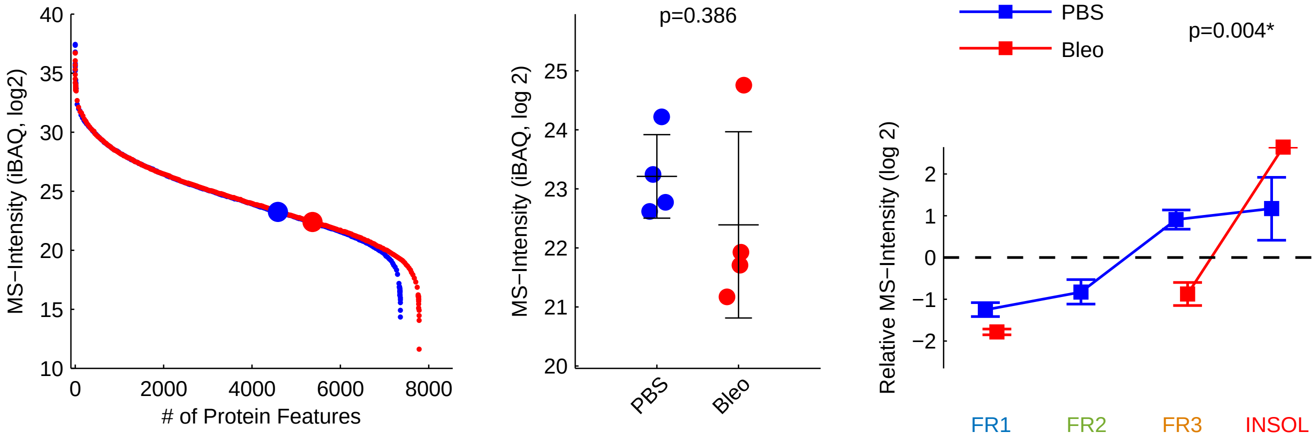

O35711 − Ppfibp2 (id: 1809)

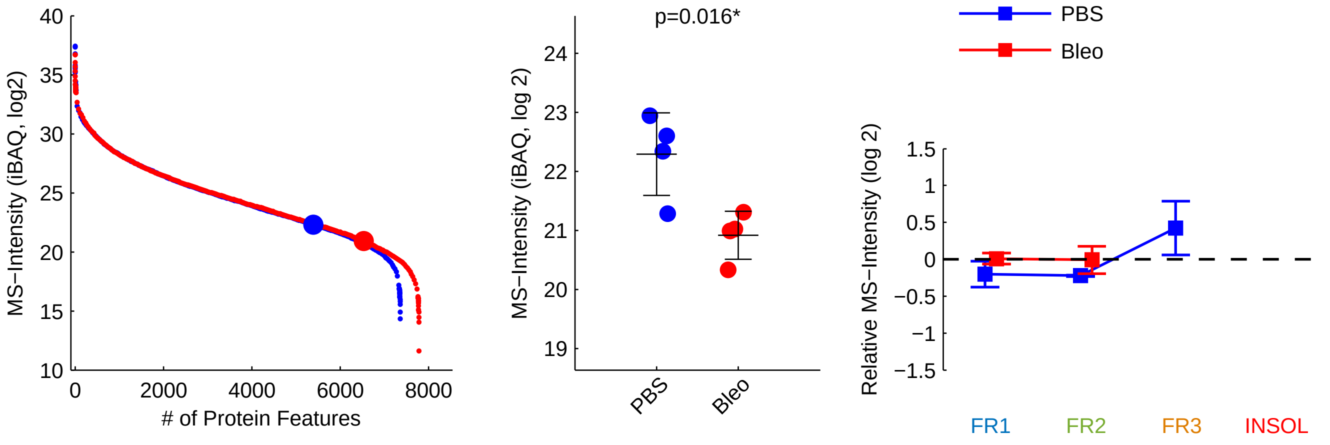

G3X972 − Sec24c (id: 1813)

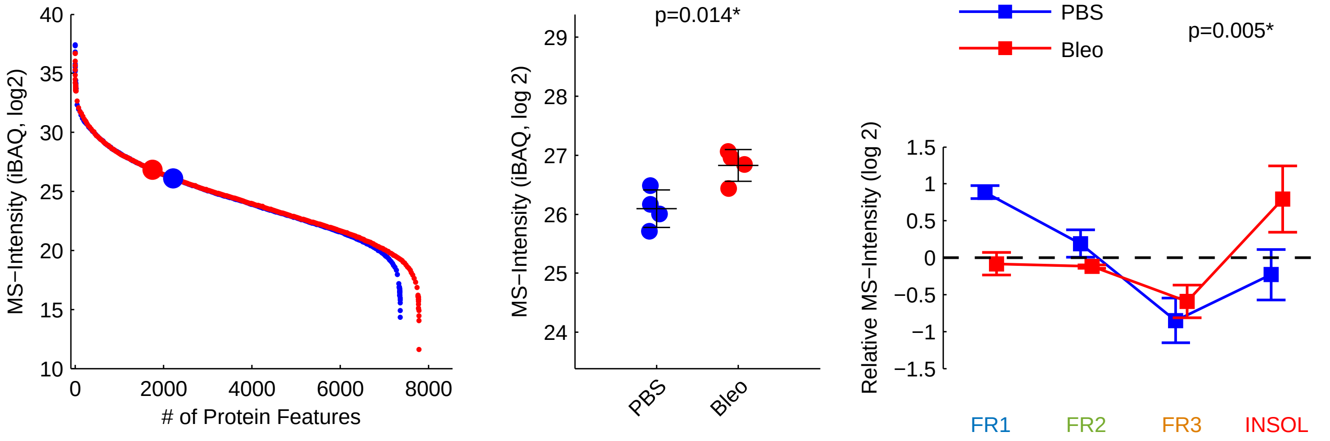

G3X973 − Stab1 (id: 1814)

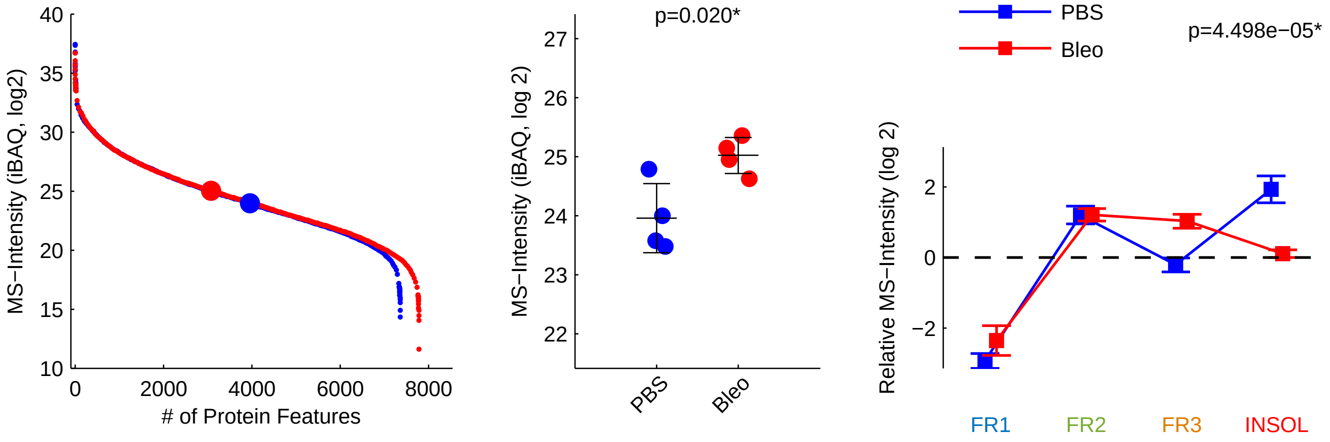

Q61703 − Itih2 (id: 1815)

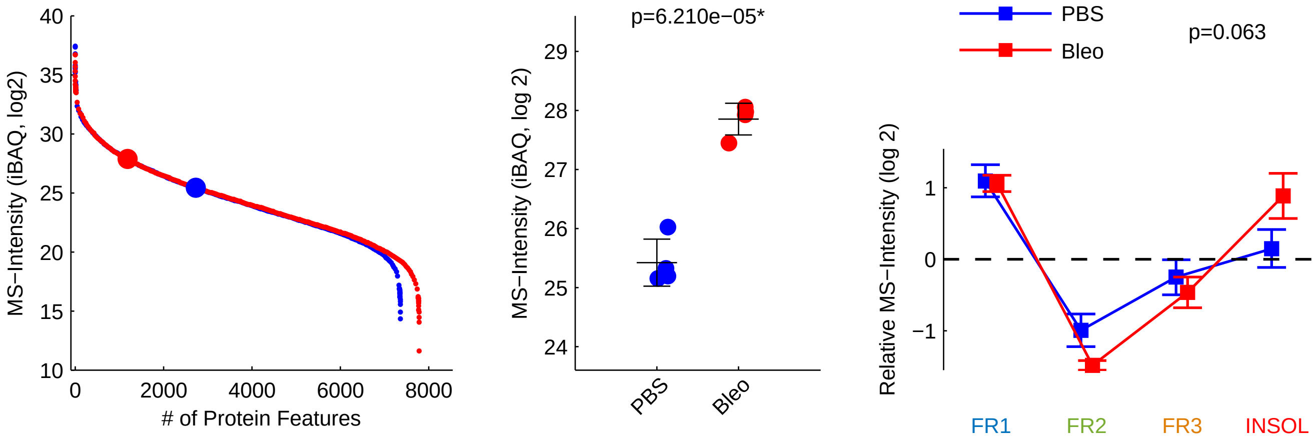

G3X982 − Aox3 (id: 1816)

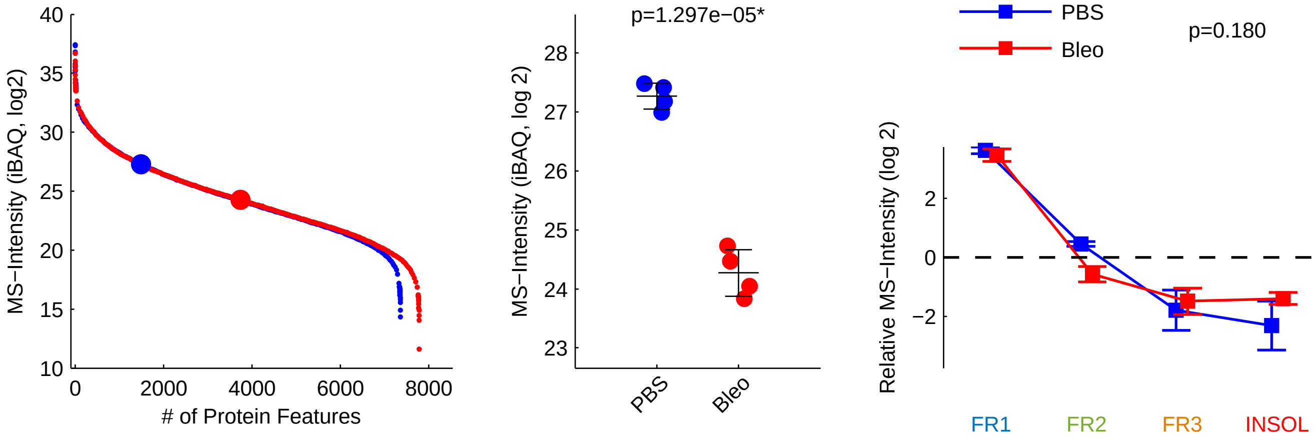

G3X9B1 − Heatr1 (id: 1819)

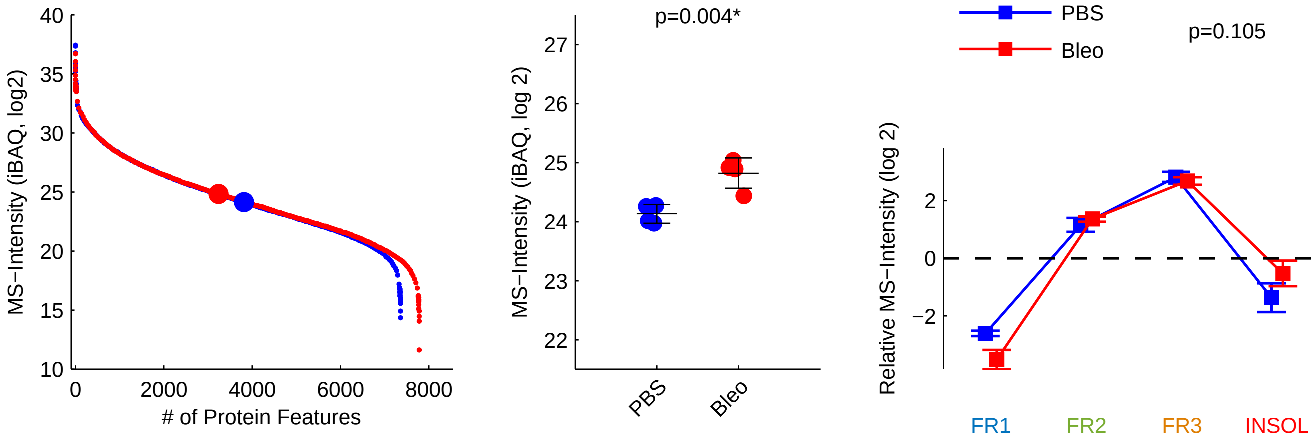

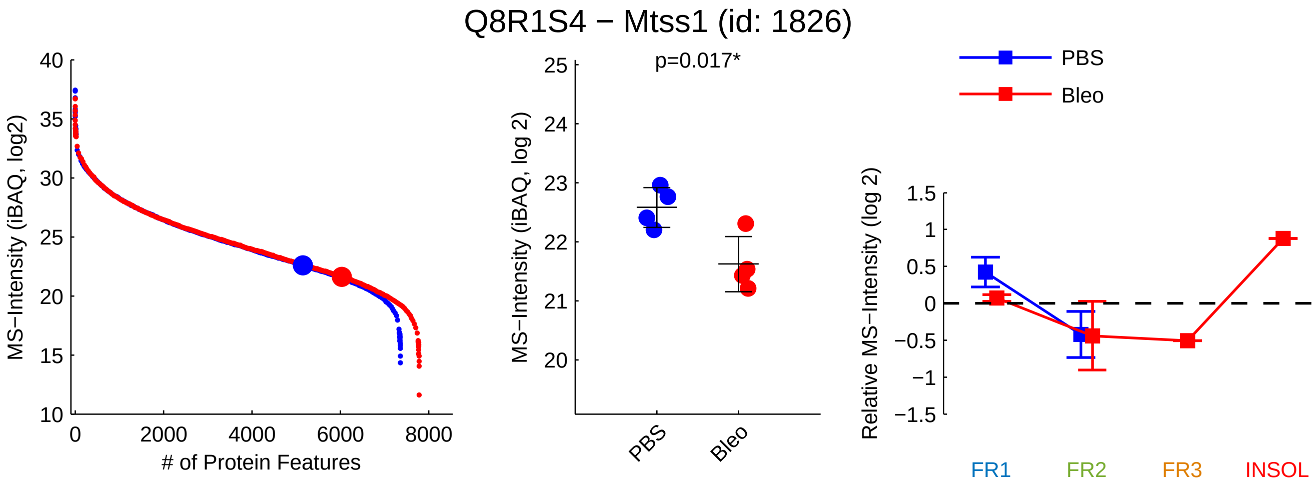
Q8R1S4 − Mtss1 (id: 1826)
MS−Intensity (iBAQ, log2)
# of Protein Features
p=0.017*
MS−Intensity (iBAQ, log 2)
PBS
Bleo
Relative MS−Intensity (log 2)
FR1
FR2
FR3
INSOL

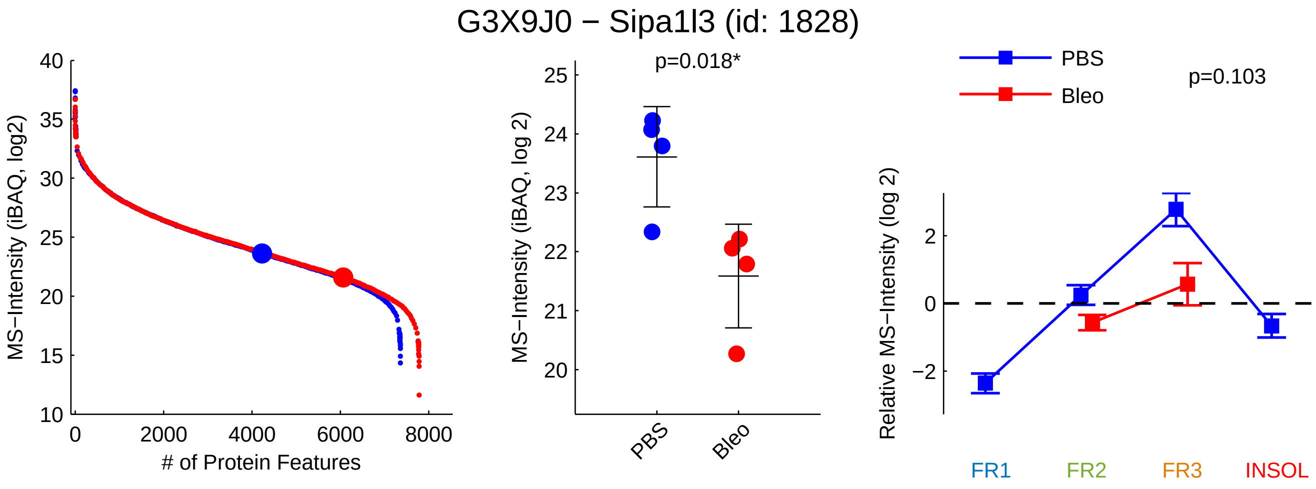
G3X9J0 − Sipa1l3 (id: 1828)
MS−Intensity (iBAQ, log2)
# of Protein Features
p=0.018*
MS−Intensity (iBAQ, log 2)
PBS
Bleo
p=0.103
Relative MS−Intensity (log 2)
FR1
FR2
FR3
INSOL

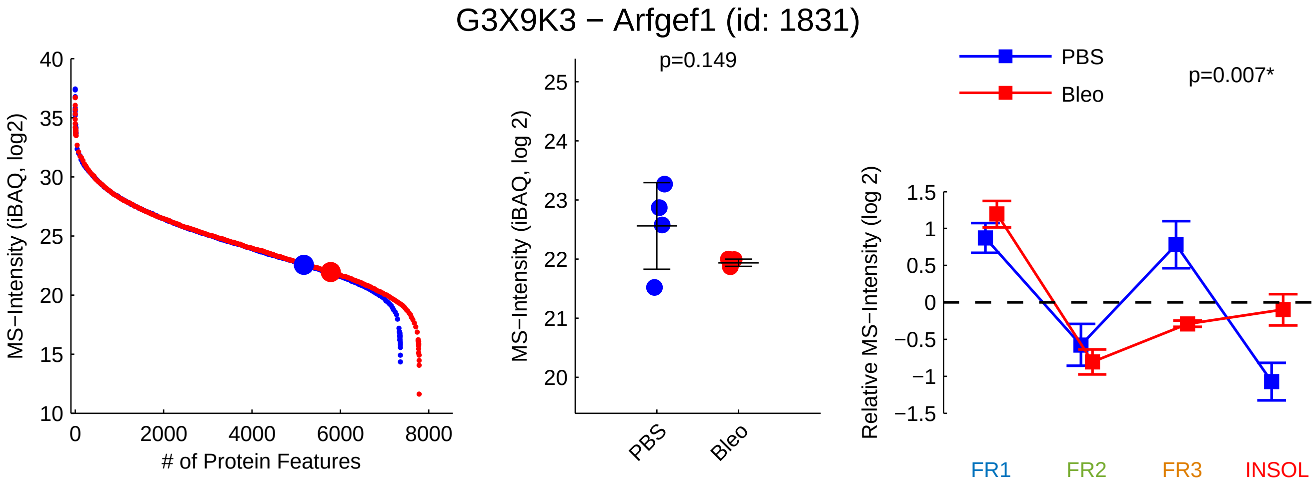
G3X9K3 − Arfgef1 (id: 1831)
MS−Intensity (iBAQ, log2)
# of Protein Features
p=0.149
MS−Intensity (iBAQ, log 2)
PBS
Bleo
p=0.007*
Relative MS−Intensity (log 2)
FR1
FR2
FR3
INSOL

Q9JKP5 − Mbnl1 (id: 1834)

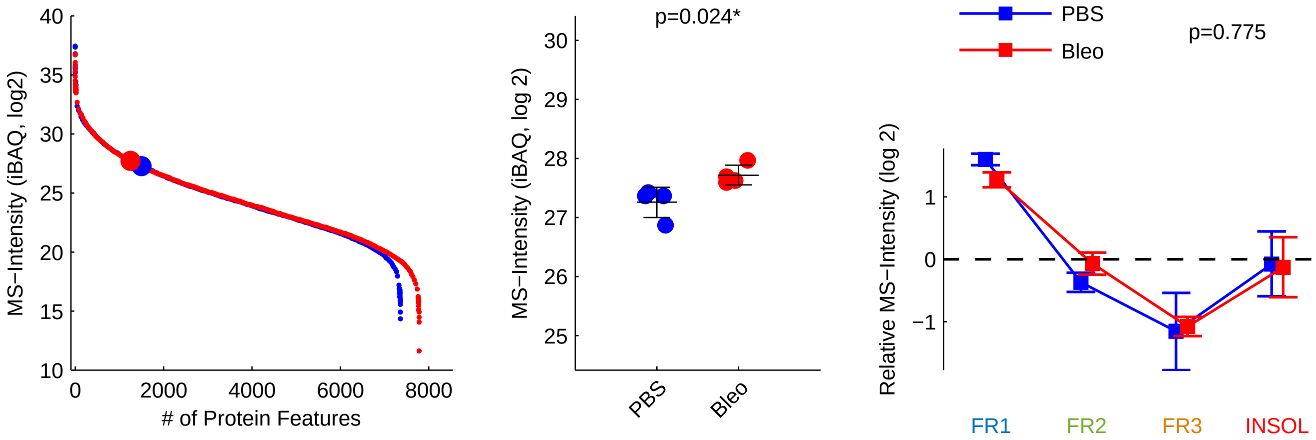

G3X9Q3 − Hmha1 (id: 1837)

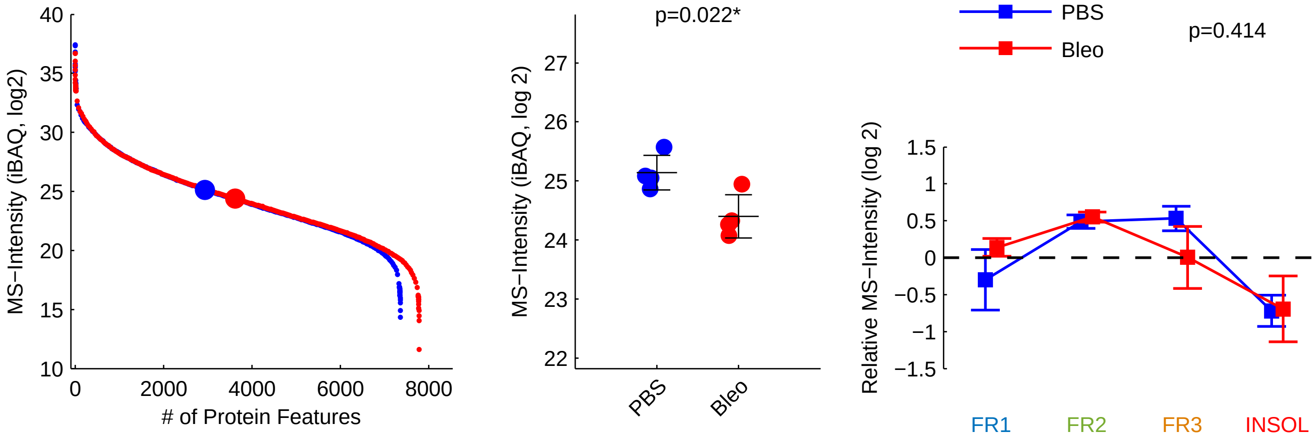

G3X9T7 − Lgals9 (id: 1839)

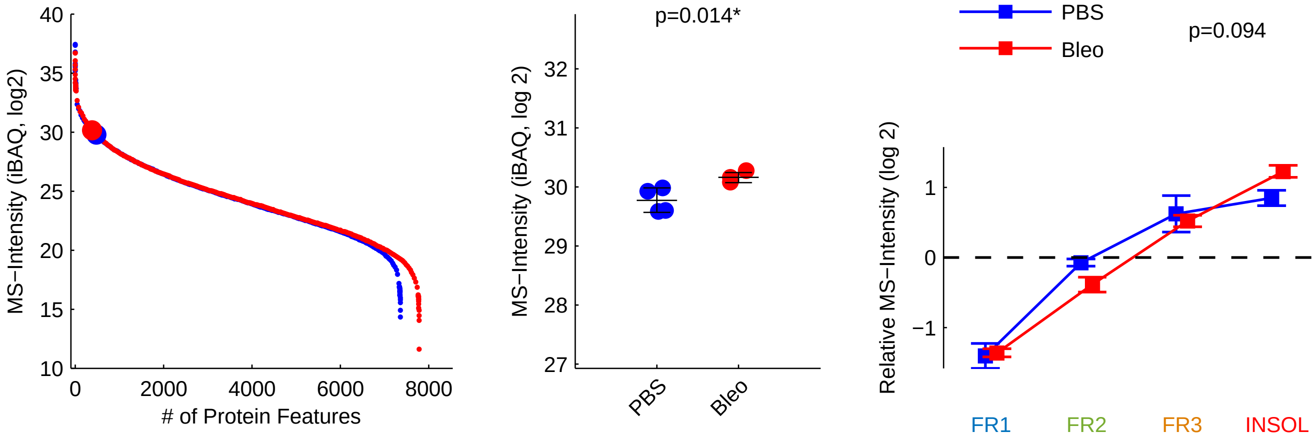

P49117 − Nr2c2 (id: 1842)

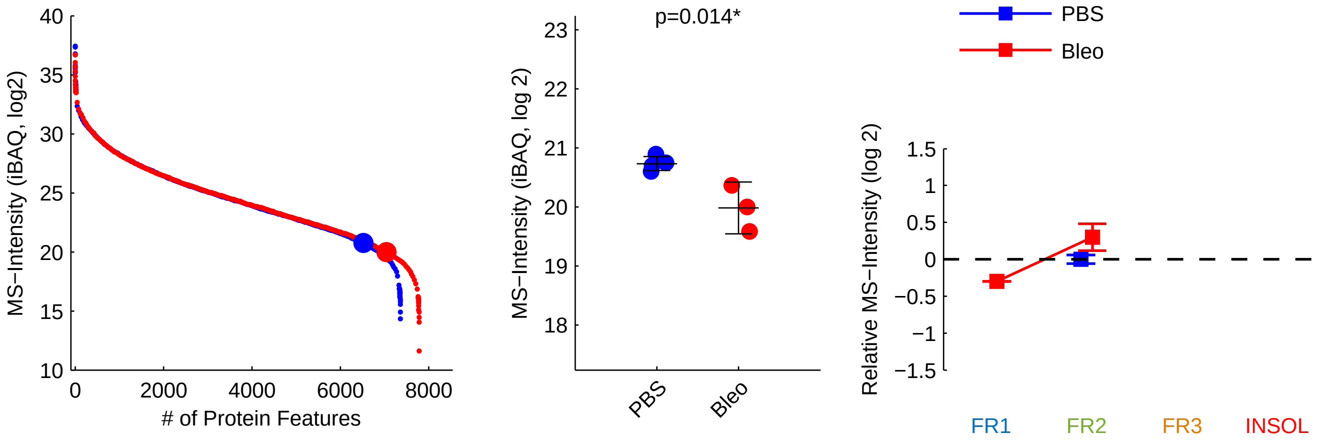

G3XA17 − Eif4g2 (id: 1847)

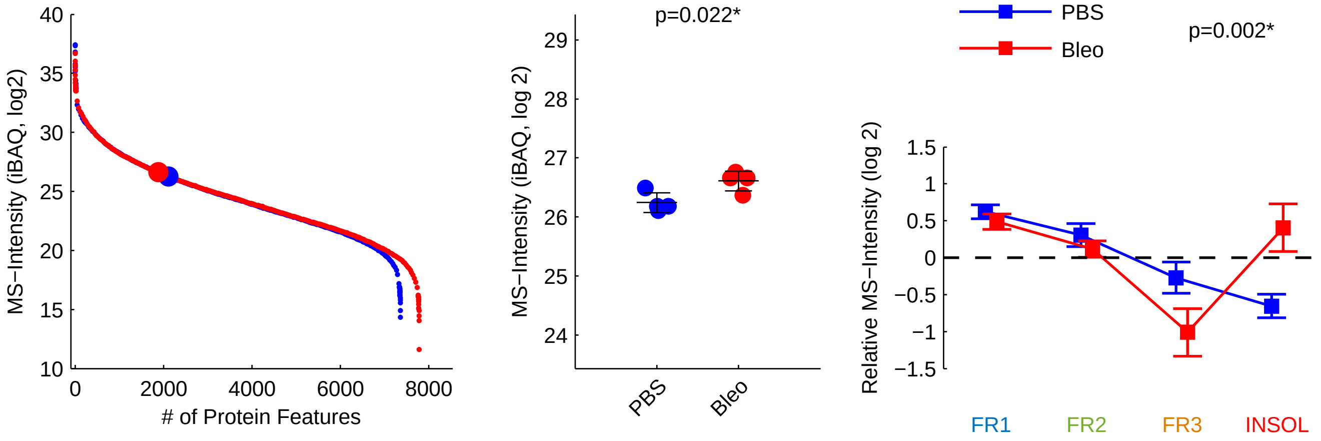

G5E829 − Atp2b1 (id: 1853)

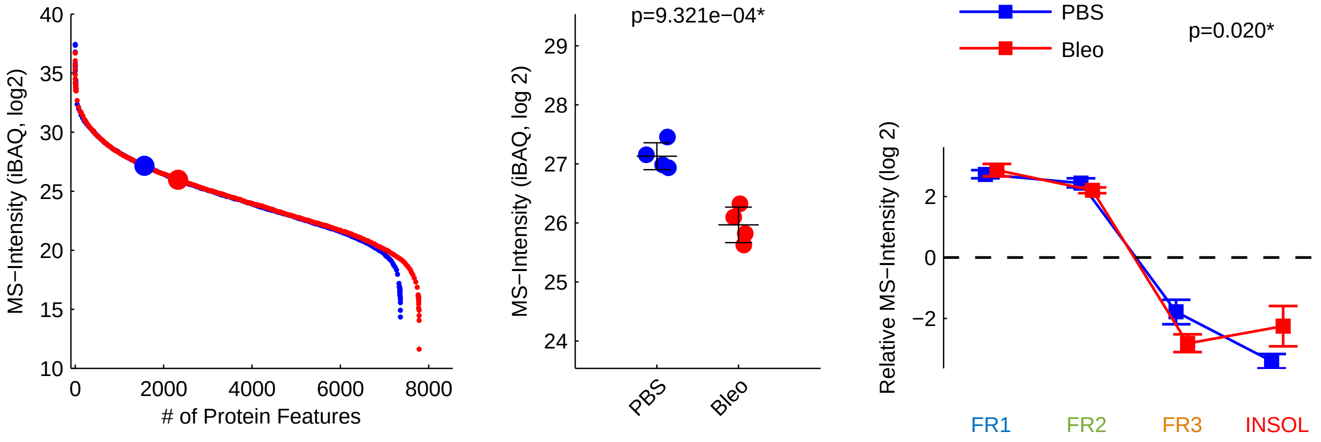

G5E843 − Robo1 (id: 1856)

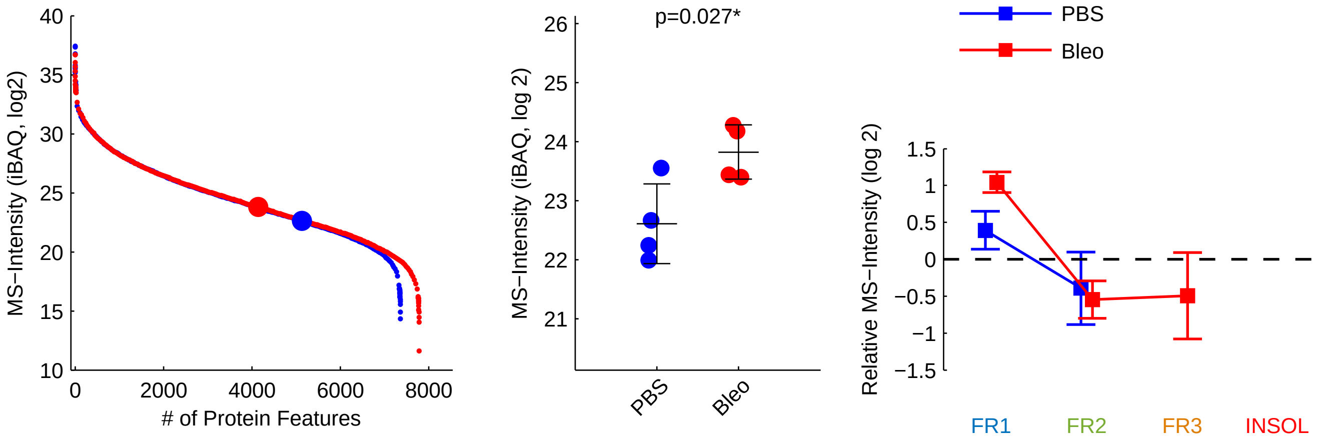

P15331 − Prph (id: 1857)

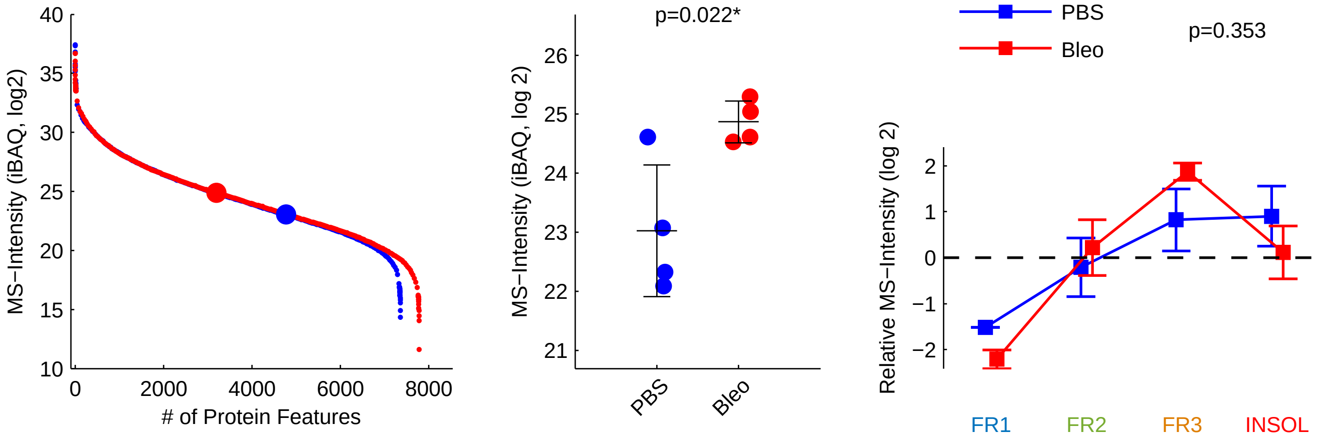

P56395 − Cyb5a (id: 1859)

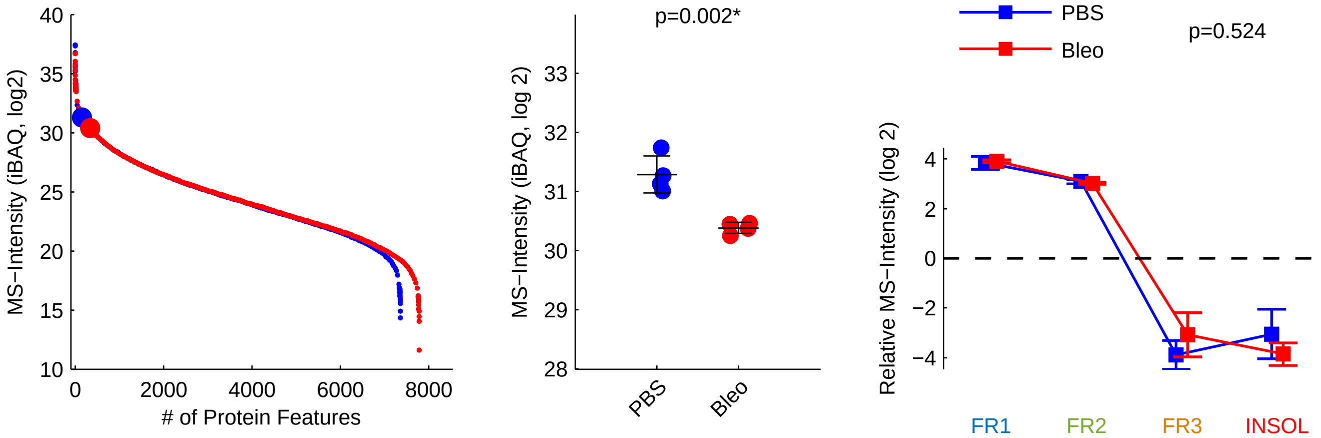

G5E866 − Sf3b1 (id: 1862)

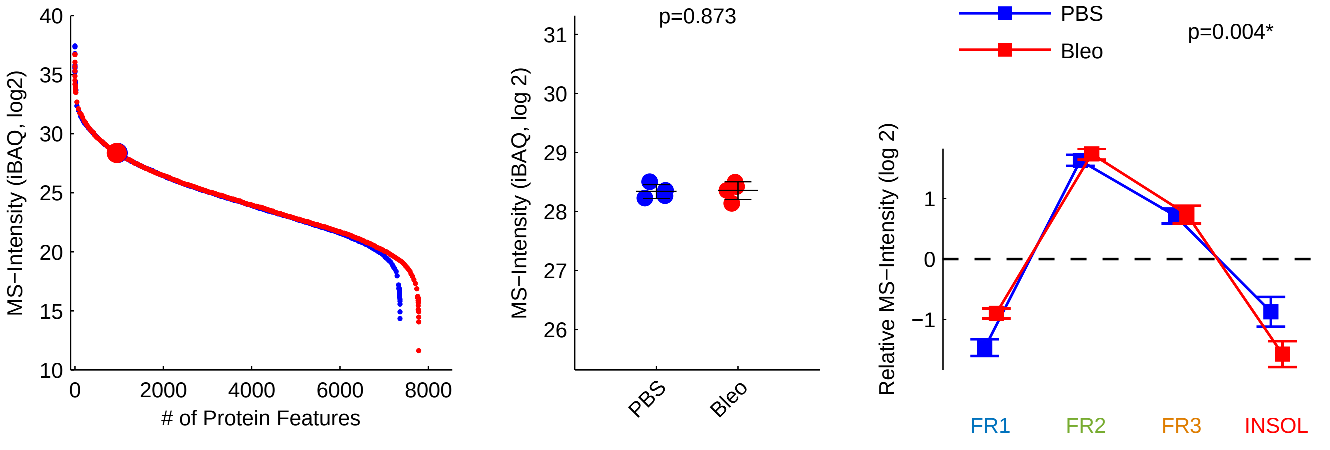

G5E874 − Lamc2 (id: 1864)

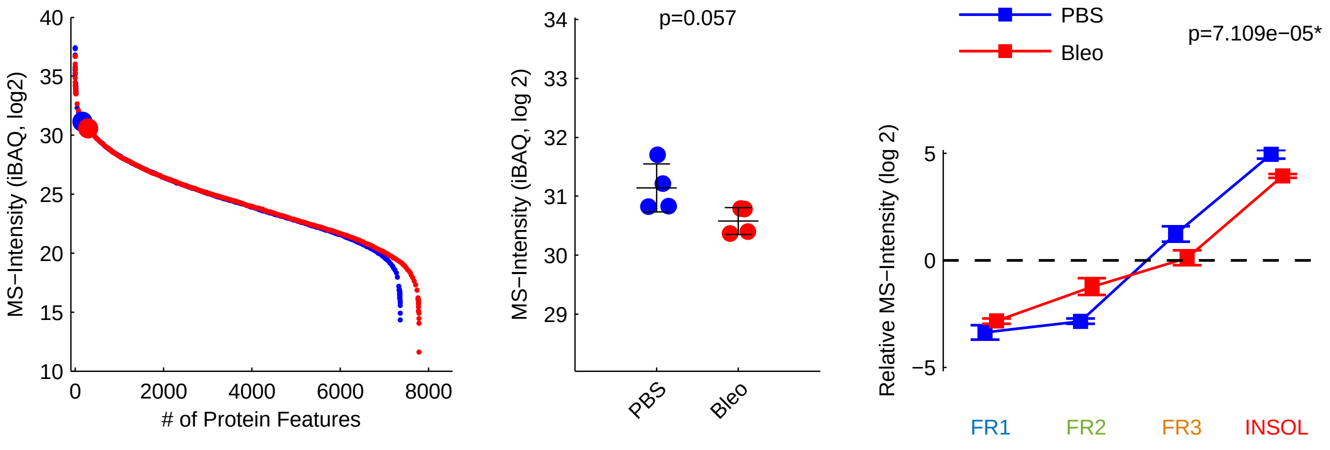

G5E895 − Akr1b10 (id: 1870)

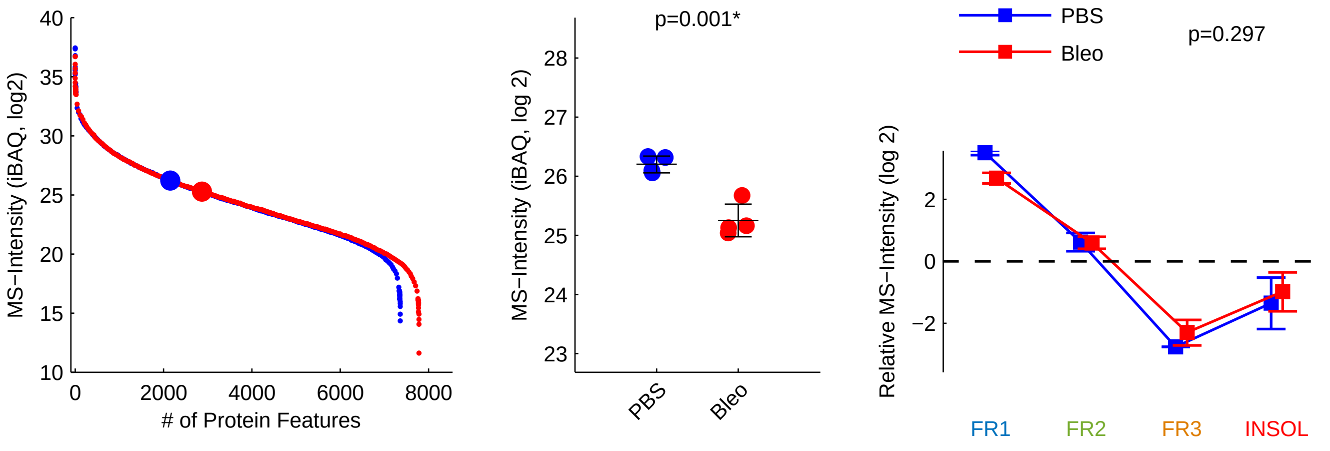

G5E899 − Serpine1 (id: 1874)

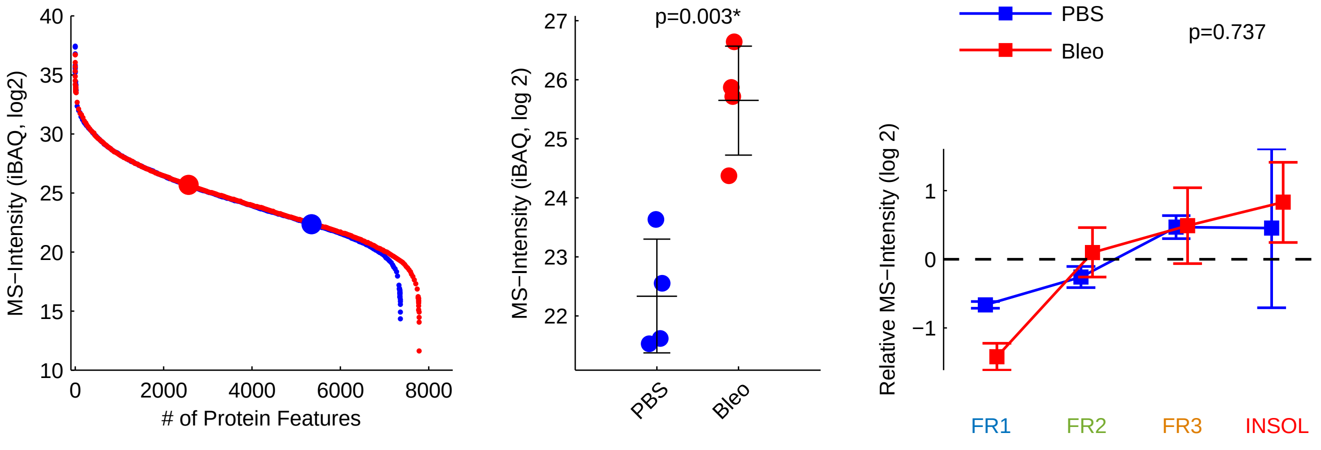

G5E8B1 − Ptpn13 (id: 1877)

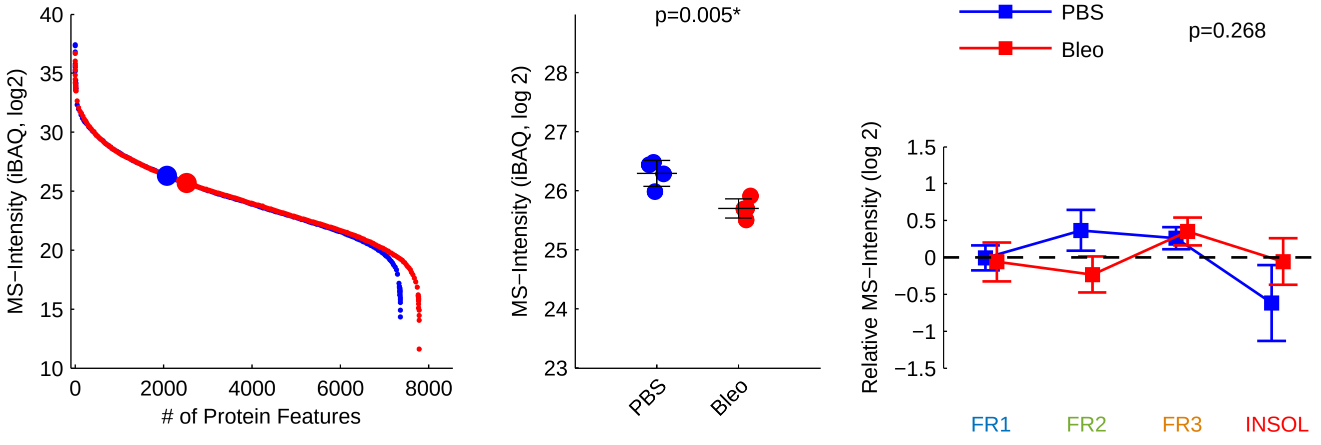

Q8BRH0 − Tmtc3 (id: 1881)

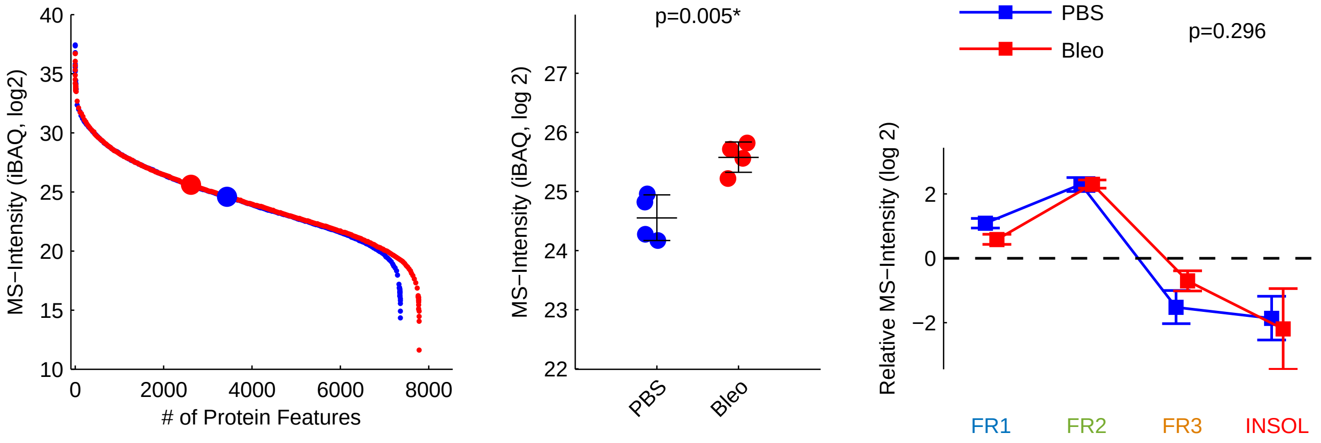

G5E8C6 − Vill (id: 1882)

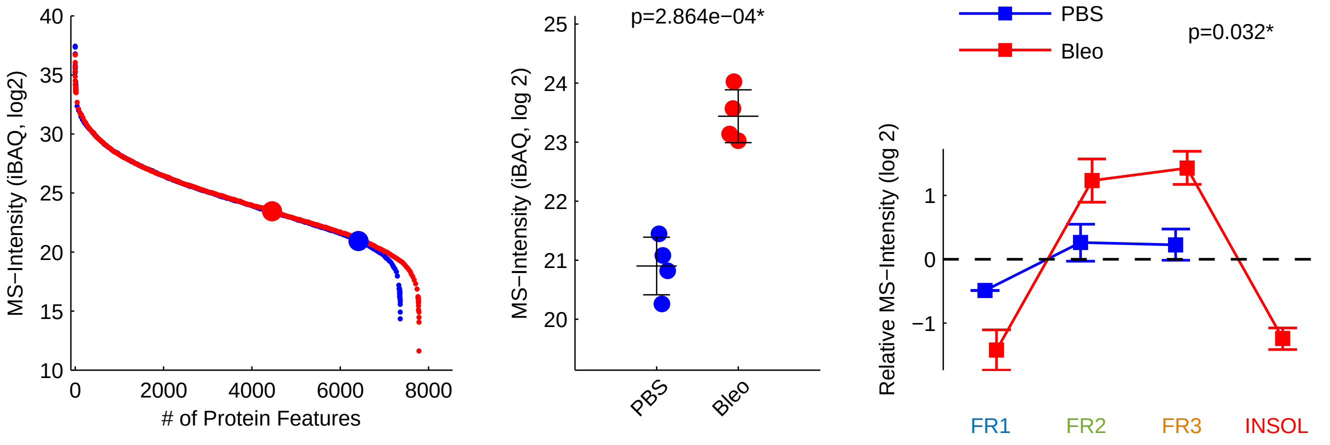

G5E8E1 − Lrrfip1 (id: 1883)

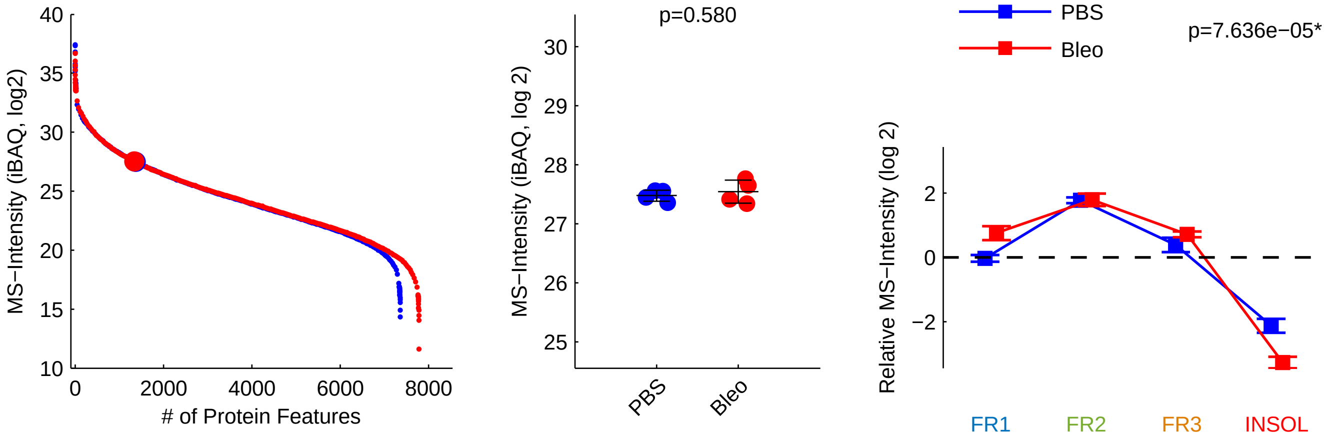

G5E8E3 − 2310022A10Rik (id: 1884)

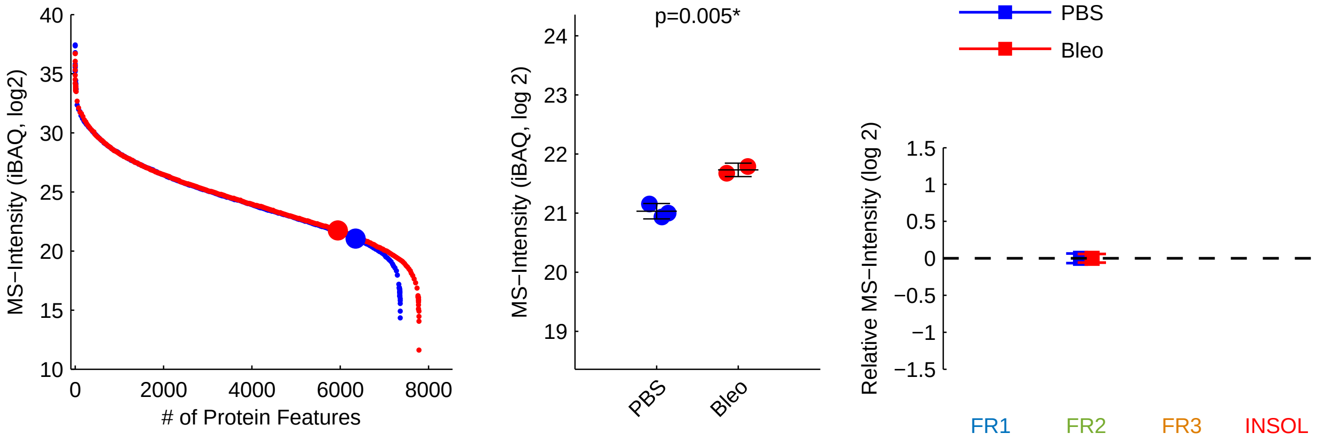

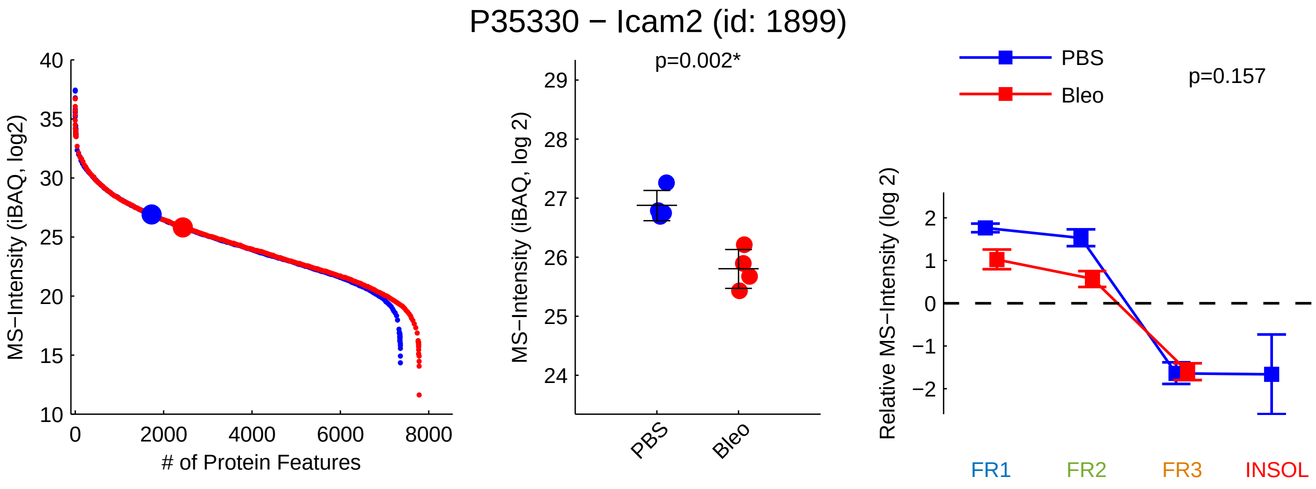
P35330 − Icam2 (id: 1899)
MS−Intensity (iBAQ, log2)
# of Protein Features
p=0.002*
MS−Intensity (iBAQ, log 2)
PBS
Bleo
p=0.157
Relative MS−Intensity (log 2)
FR1
FR2
FR3
INSOL

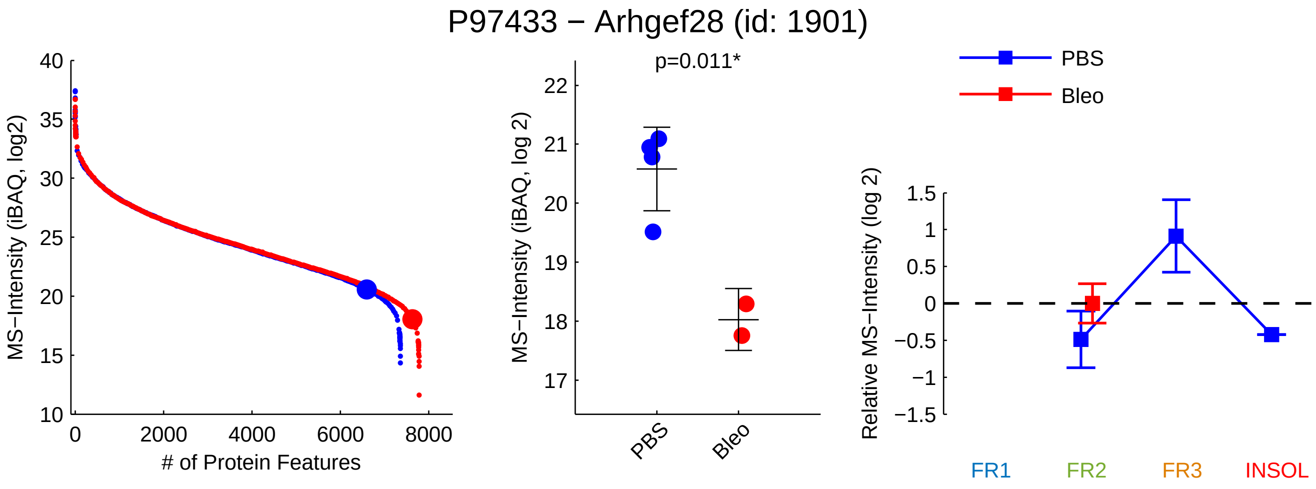
P97433 − Arhgef28 (id: 1901)
MS−Intensity (iBAQ, log2)
# of Protein Features
p=0.011*
MS−Intensity (iBAQ, log 2)
PBS
Bleo
Relative MS−Intensity (log 2)
FR1
FR2
FR3
INSOL

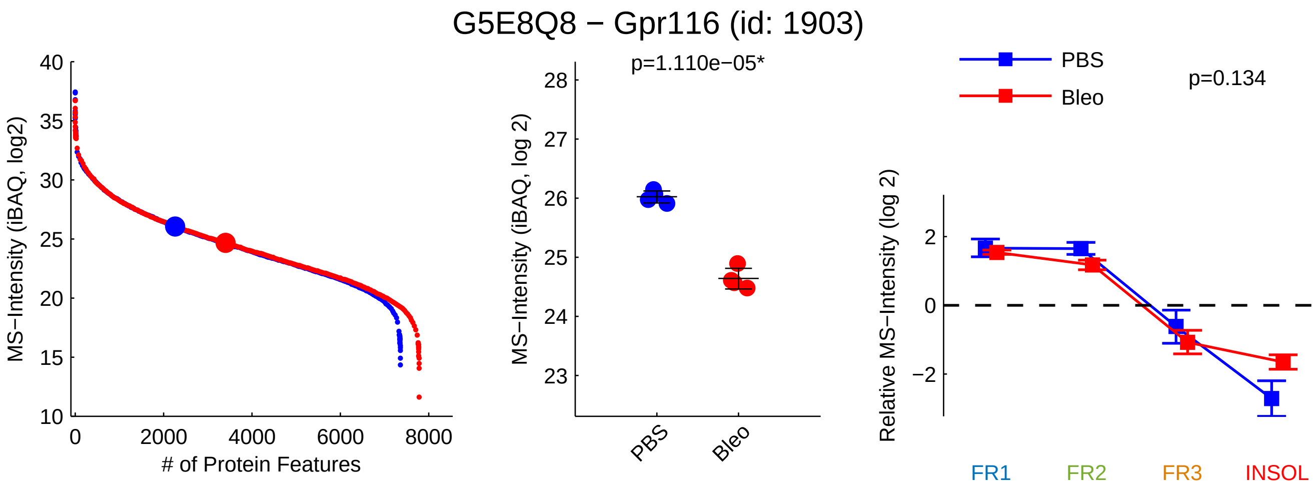
G5E8Q8 − Gpr116 (id: 1903)
MS−Intensity (iBAQ, log2)
# of Protein Features
p=1.110e−05*
MS−Intensity (iBAQ, log 2)
PBS
Bleo
p=0.134
Relative MS−Intensity (log 2)
FR1
FR2
FR3
INSOL

G5E8S8 − Mpp7 (id: 1907)

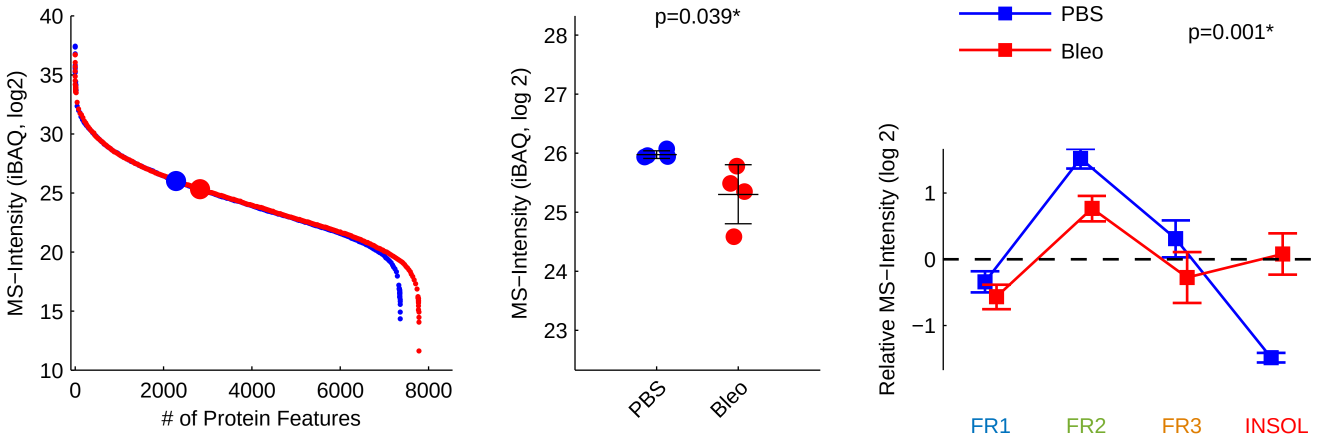

Q9EQJ9 − Magi3 (id: 1908)

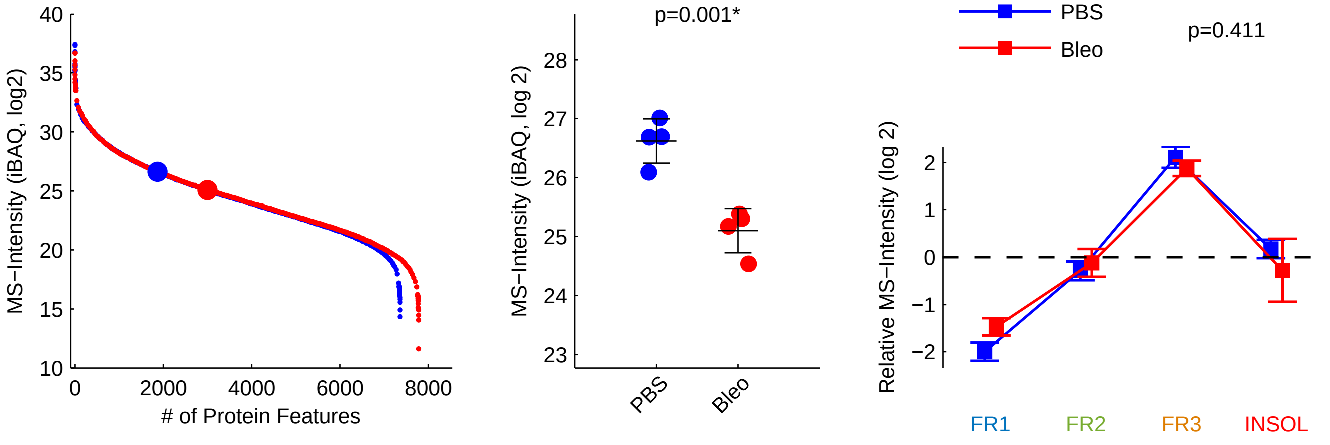

G5E8V9 − Arfip1 (id: 1912)

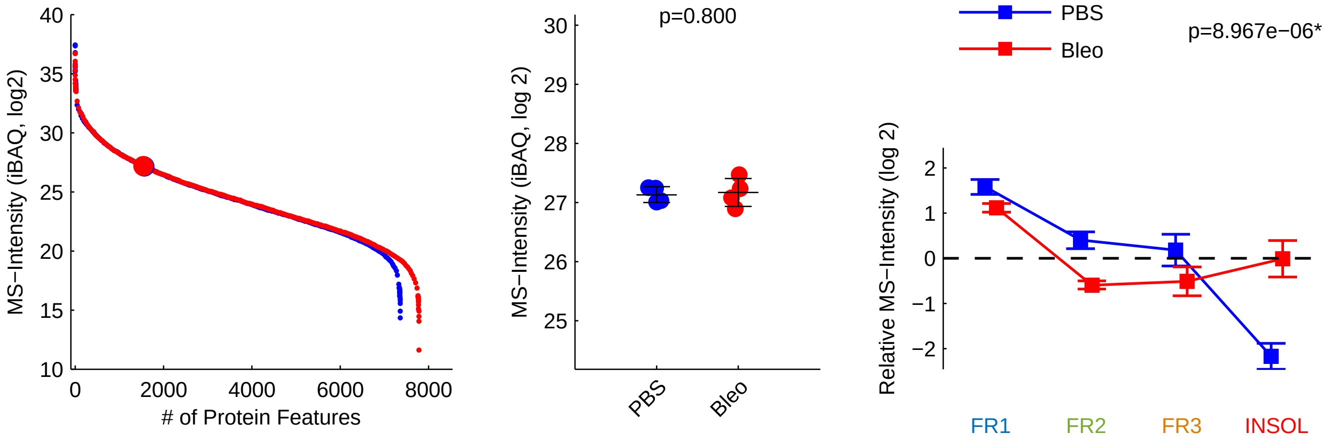

G5E911 − Fam3c (id: 1916)

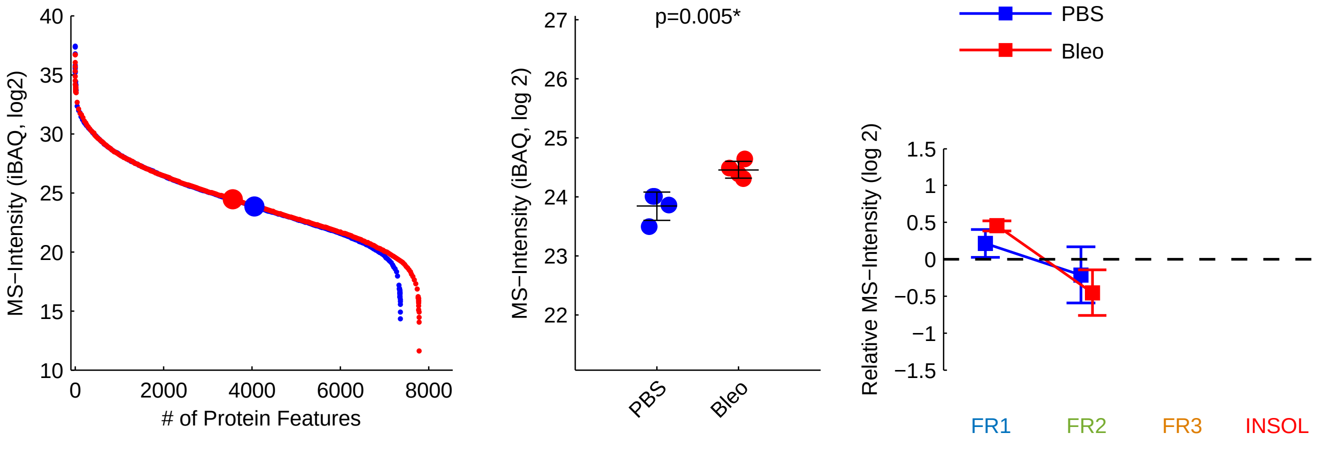

G5E924 − Hnrnpl (id: 1917)

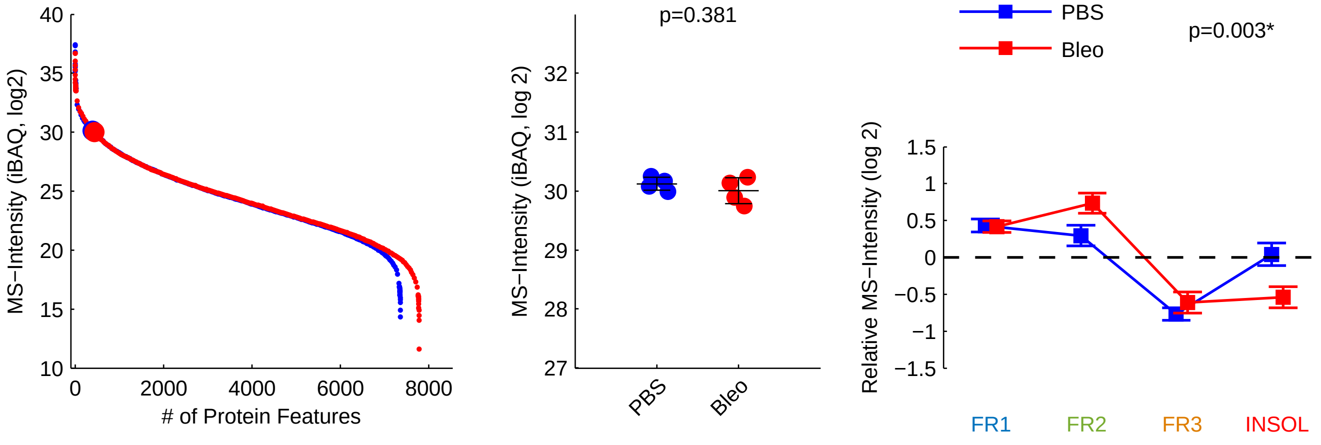

Q6NVF9 − Cpsf6 (id: 1925)

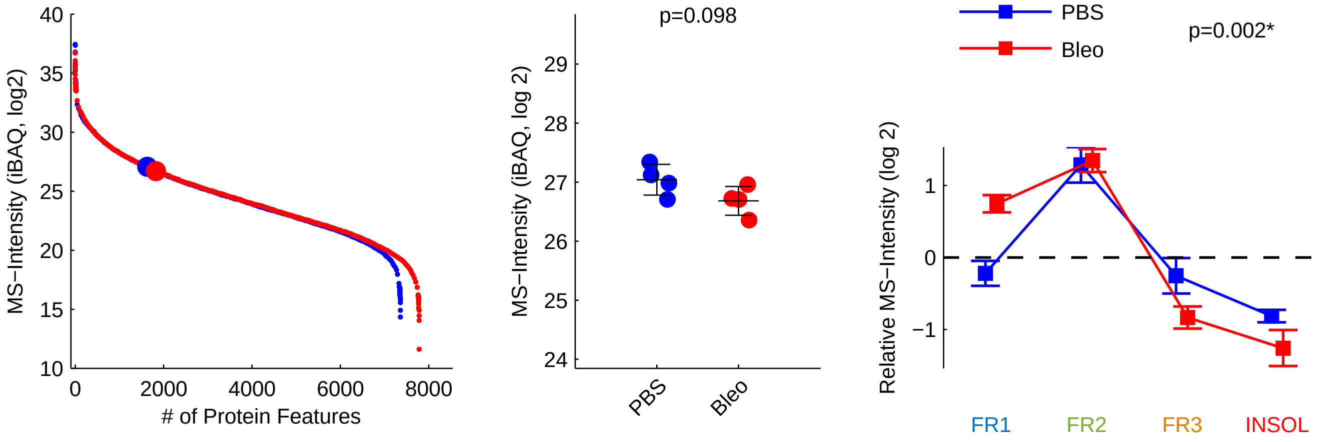

H3BJD6 − Ppp1r9a (id: 1931)

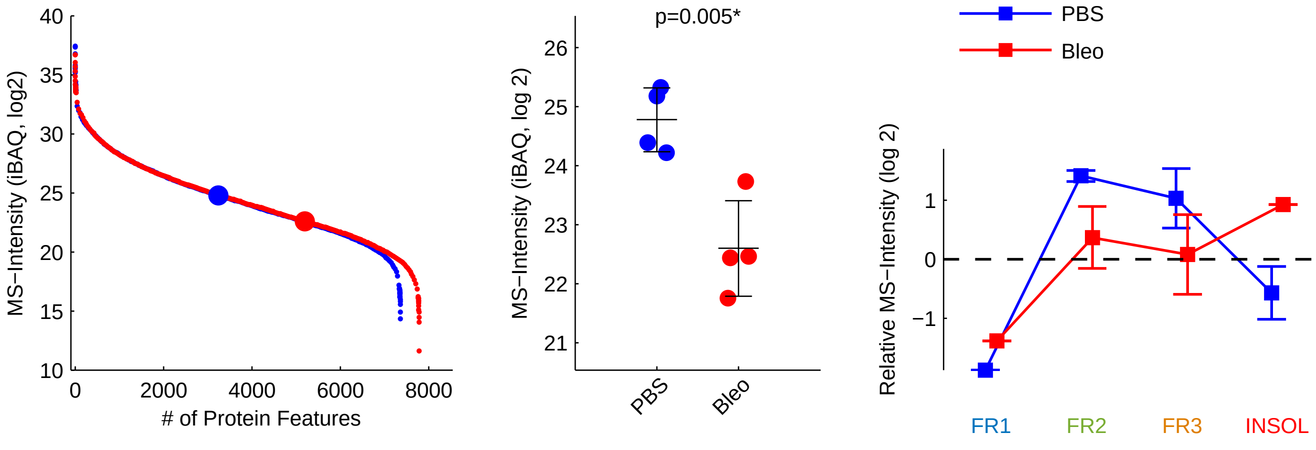

O08784 − Tcof1 (id: 1949)

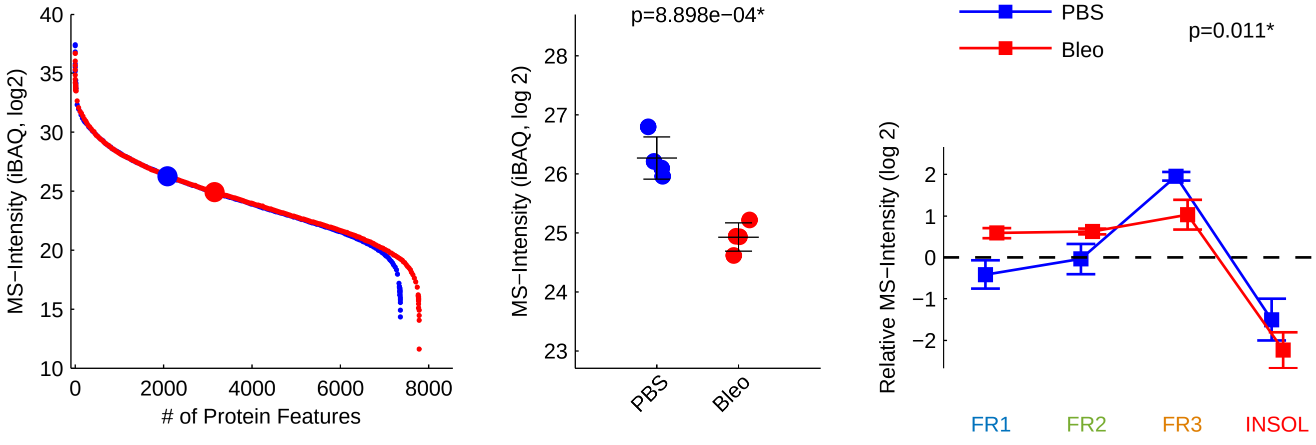

P40224−1 − Cxcl12 (id: 1961)

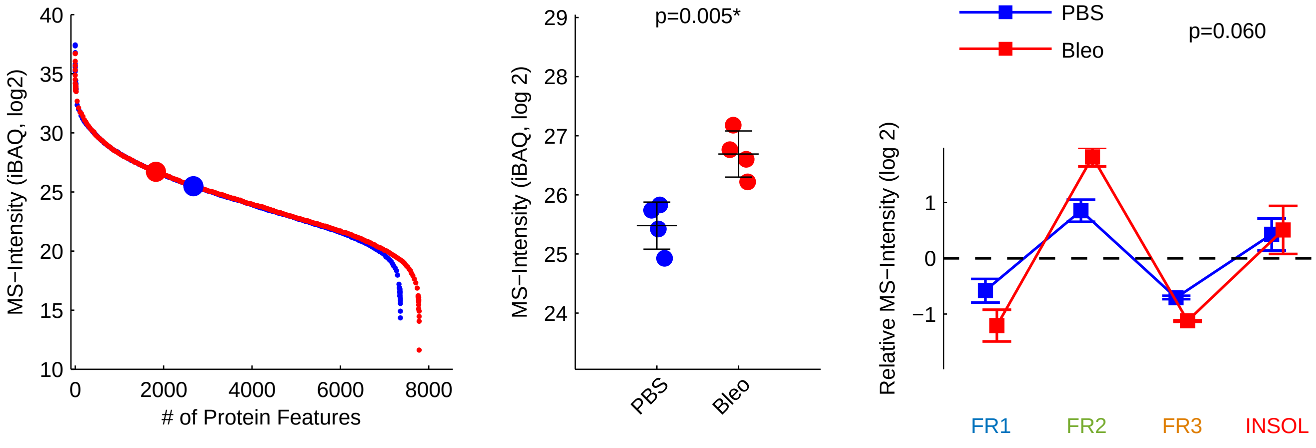

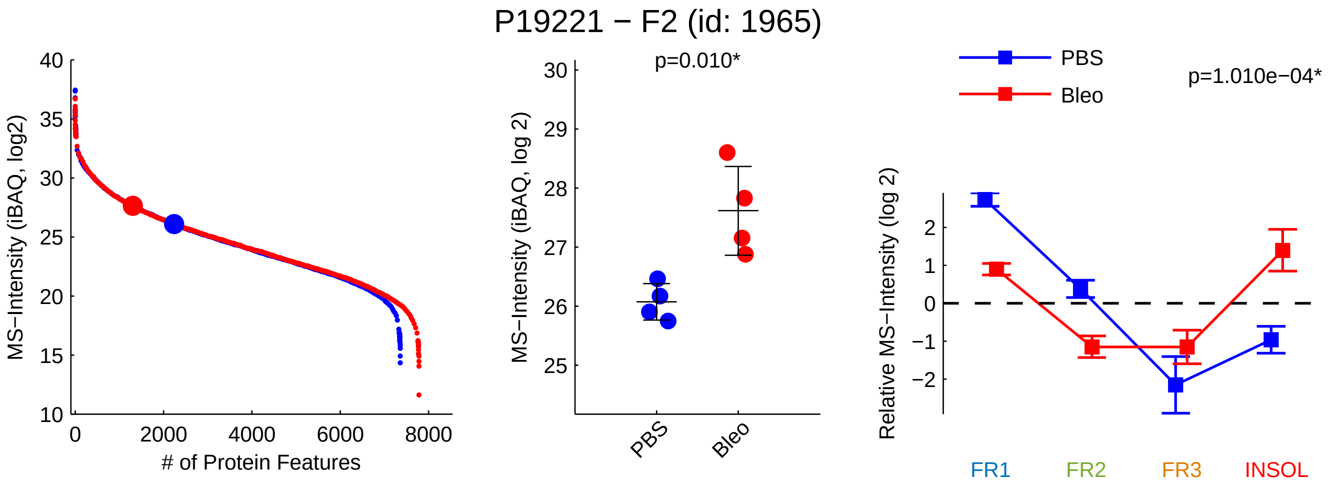
P19221 − F2 (id: 1965)
MS−Intensity (iBAQ, log2)
# of Protein Features
p=0.010*
MS−Intensity (iBAQ, log 2)
PBS
Bleo
p=1.010e−04*
Relative MS−Intensity (log 2)
FR1
FR2
FR3
INSOL

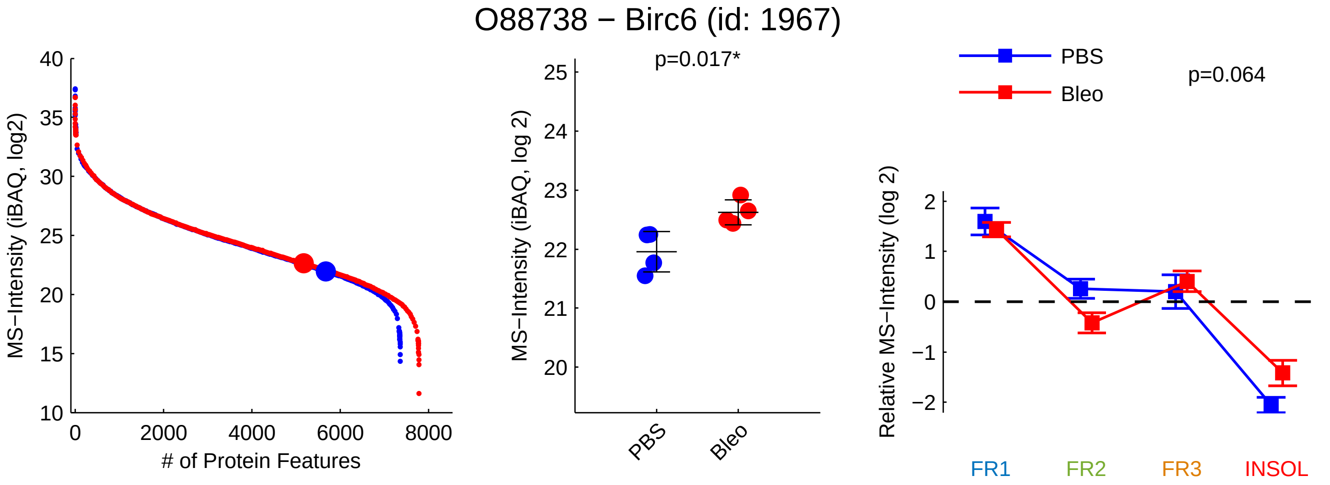
O88738 − Birc6 (id: 1967)
MS−Intensity (iBAQ, log2)
# of Protein Features
p=0.017*
MS−Intensity (iBAQ, log 2)
PBS
Bleo
p=0.064
Relative MS−Intensity (log 2)
FR1
FR2
FR3
INSOL

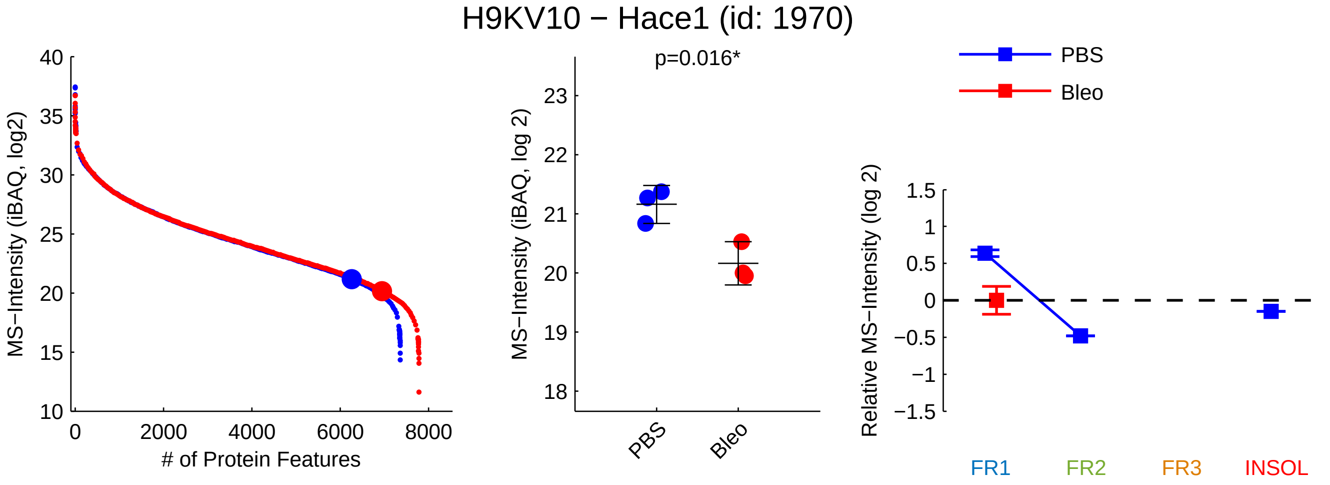
H9KV10 − Hace1 (id: 1970)
MS−Intensity (iBAQ, log2)
# of Protein Features
p=0.016*
MS−Intensity (iBAQ, log 2)
PBS
Bleo
Relative MS−Intensity (log 2)
FR1
FR2
FR3
INSOL

Q8C827 − Zfp62 (id: 1980)

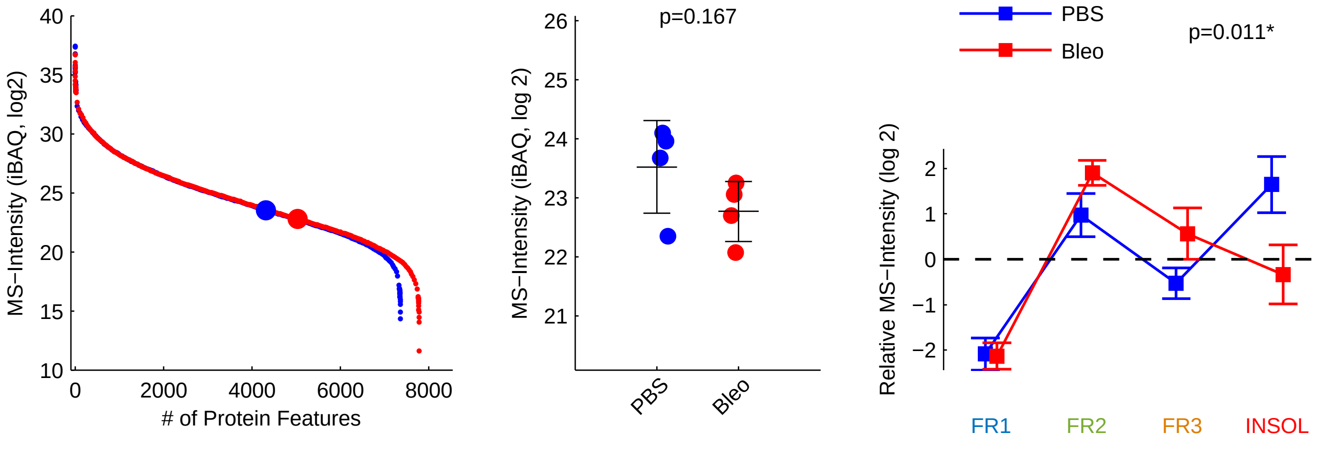

J3QK52 − Noc2l (id: 1988)

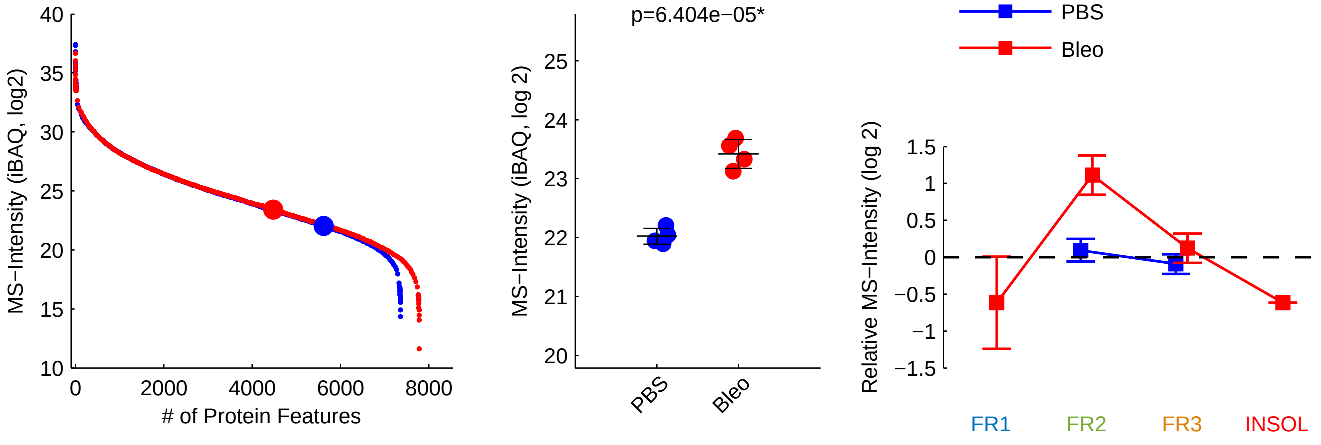

Q9CZ42 − Carkd (id: 1992)

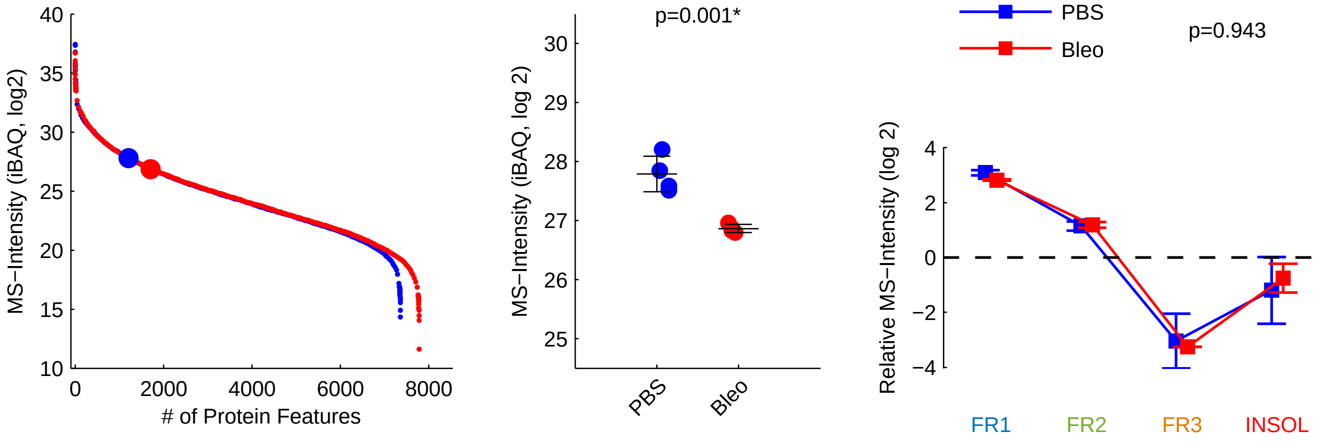

Q0P557−3 − Spata18 (id: 2007)

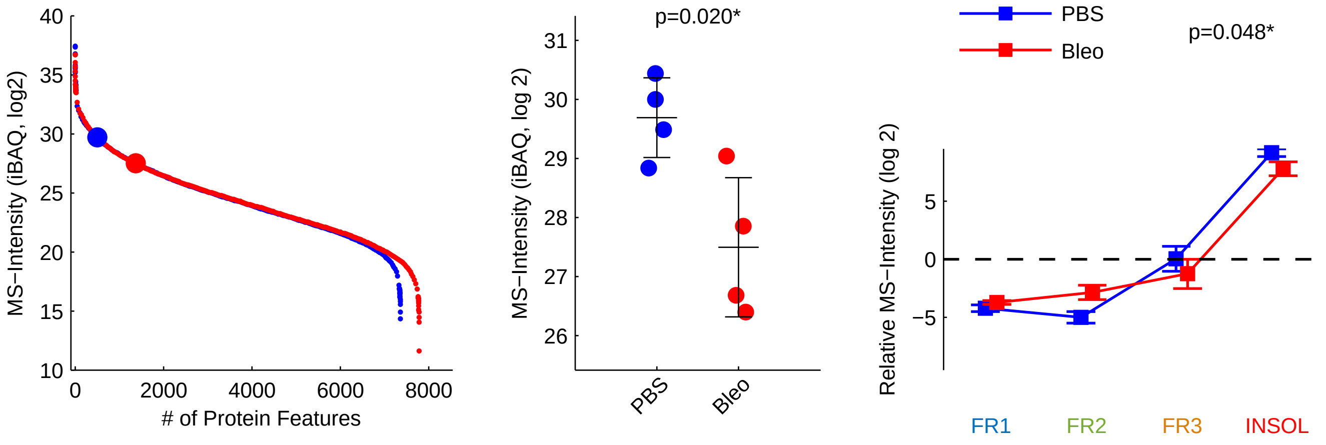

Q8BH60 − Gopc (id: 2017)

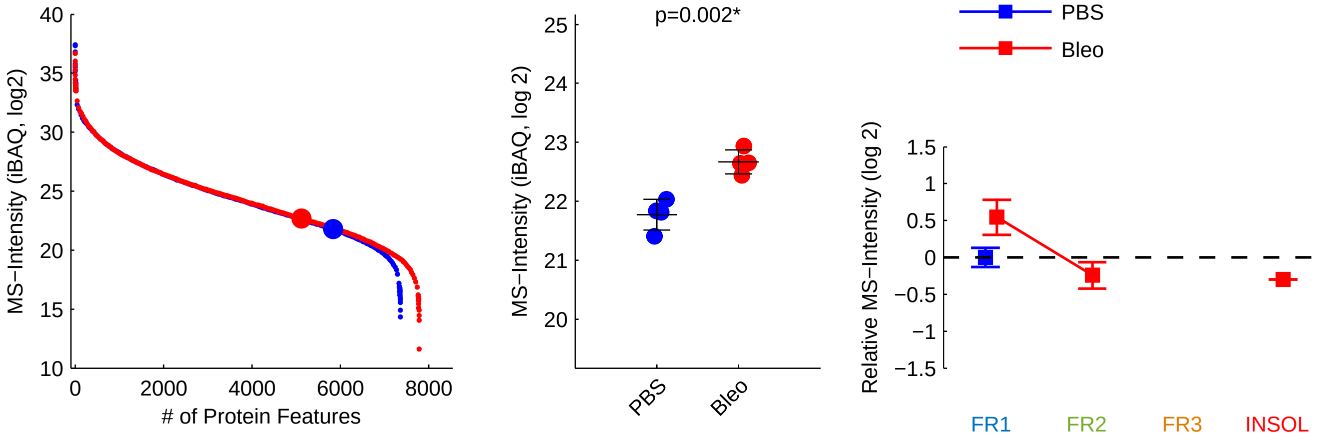

L7N1X8 − Gbp6 (id: 2024)

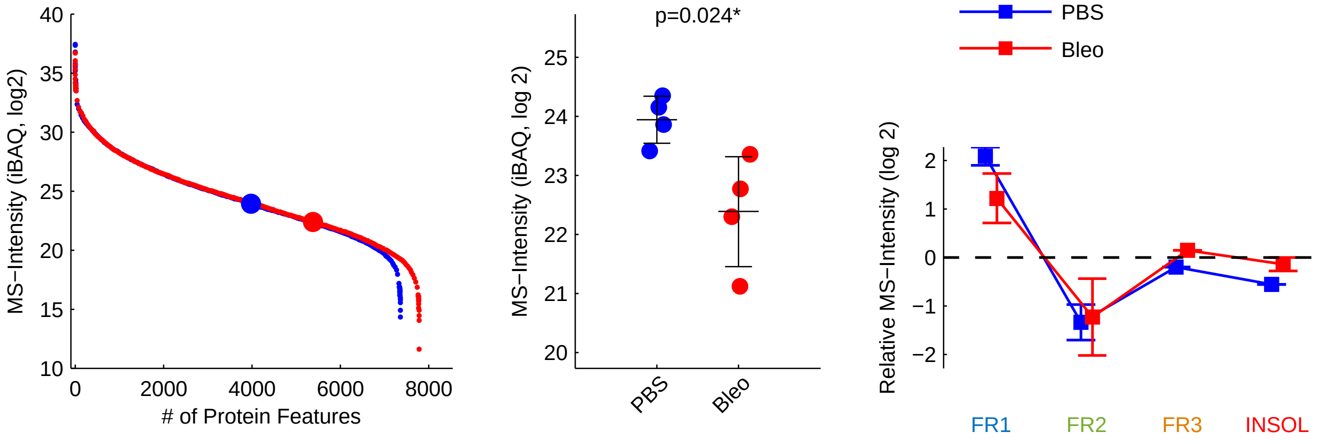

Q80SU7 − Gvin1 (id: 2028)

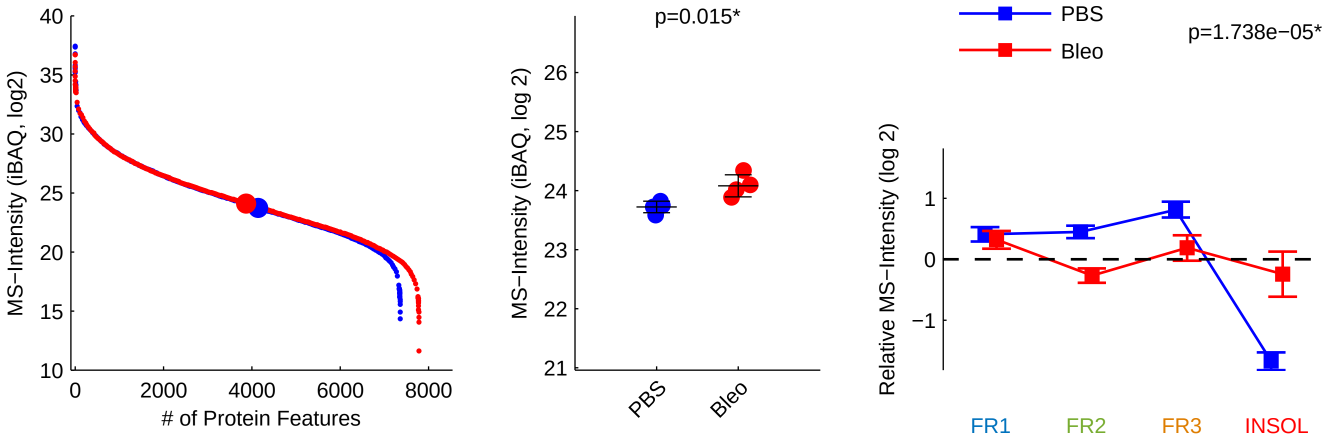

M0QWP1 − Agrn (id: 2031)

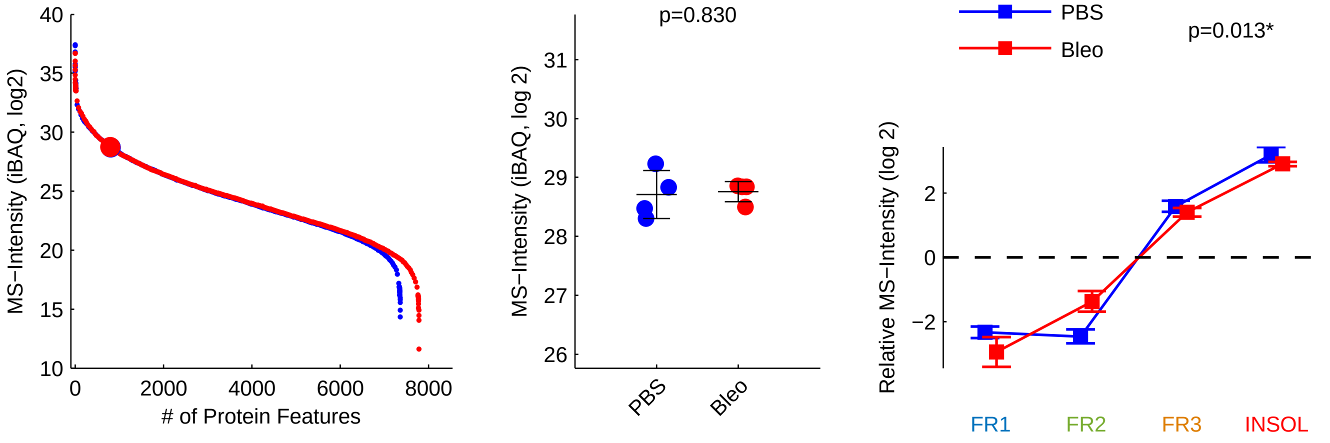

O08528 − Hk2 (id: 2034)

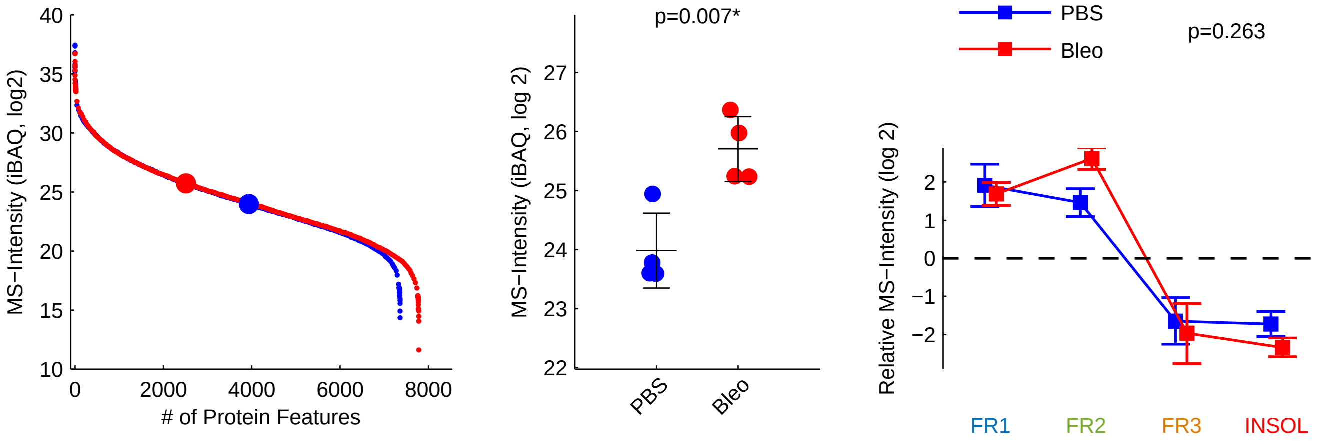

O08532 − Cacna2d1 (id: 2037)

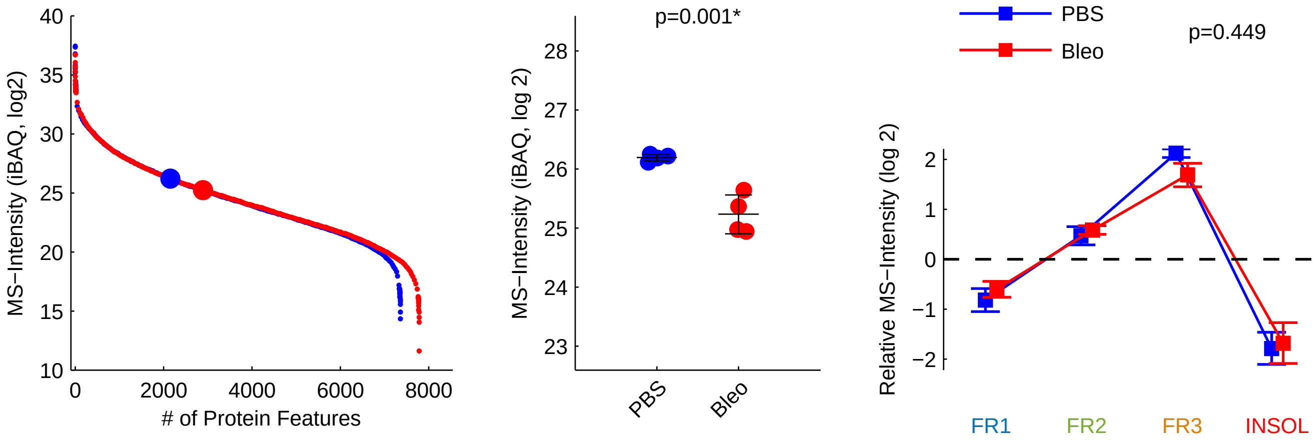

O08553 − Dpysl2 (id: 2040)

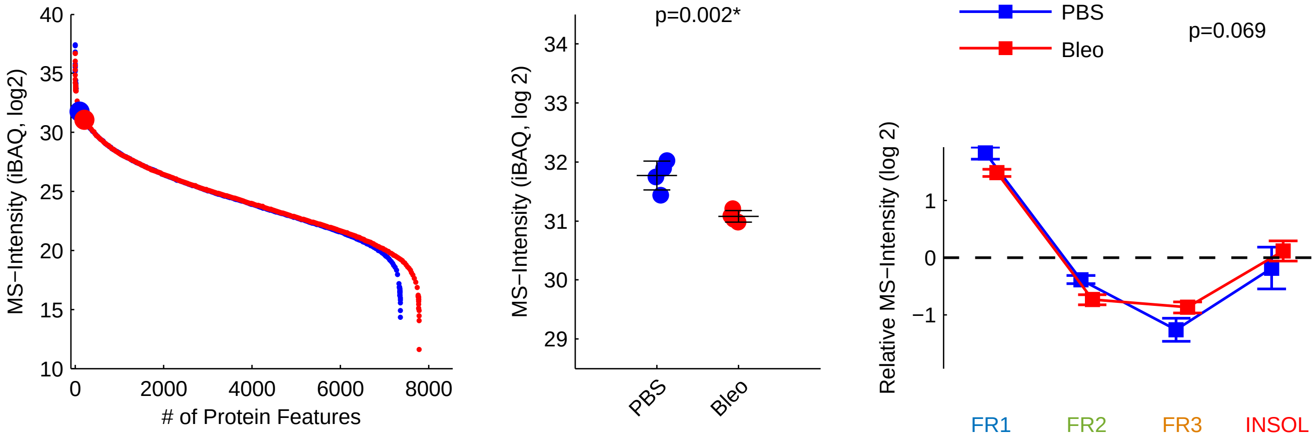

O08599 − Stxbp1 (id: 2046)

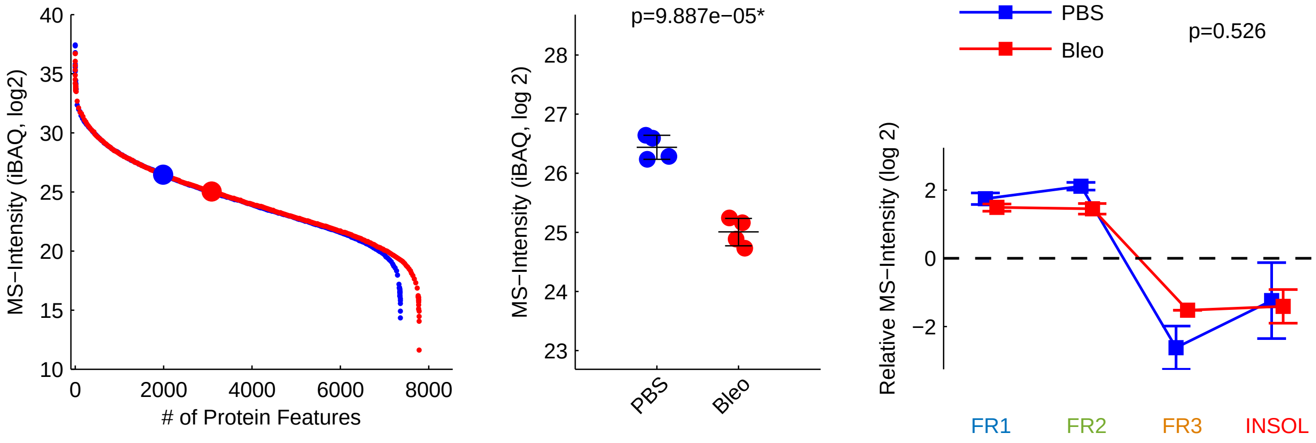

O08638 − Myh11 (id: 2048)

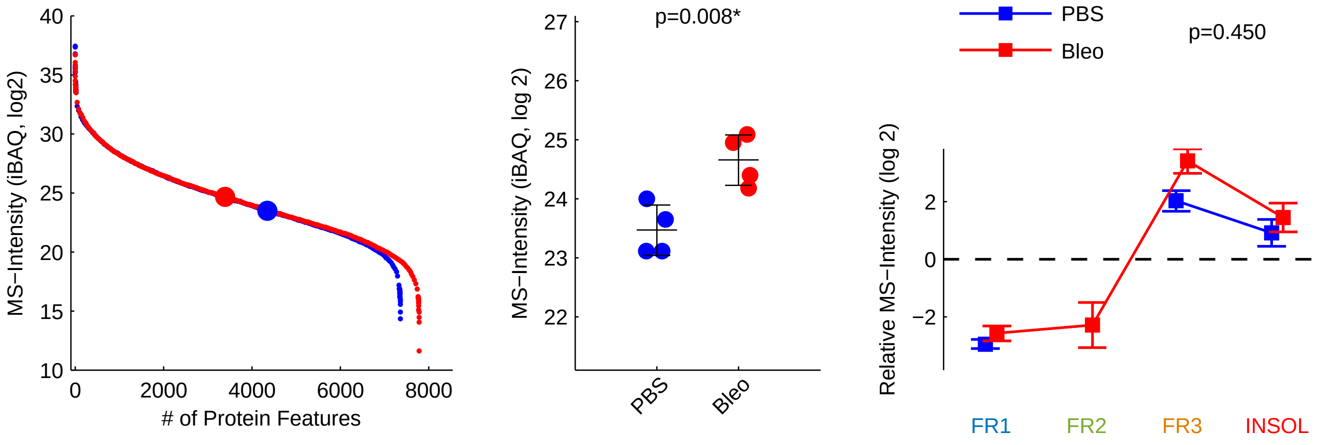

O08663 − Metap2 (id: 2050)

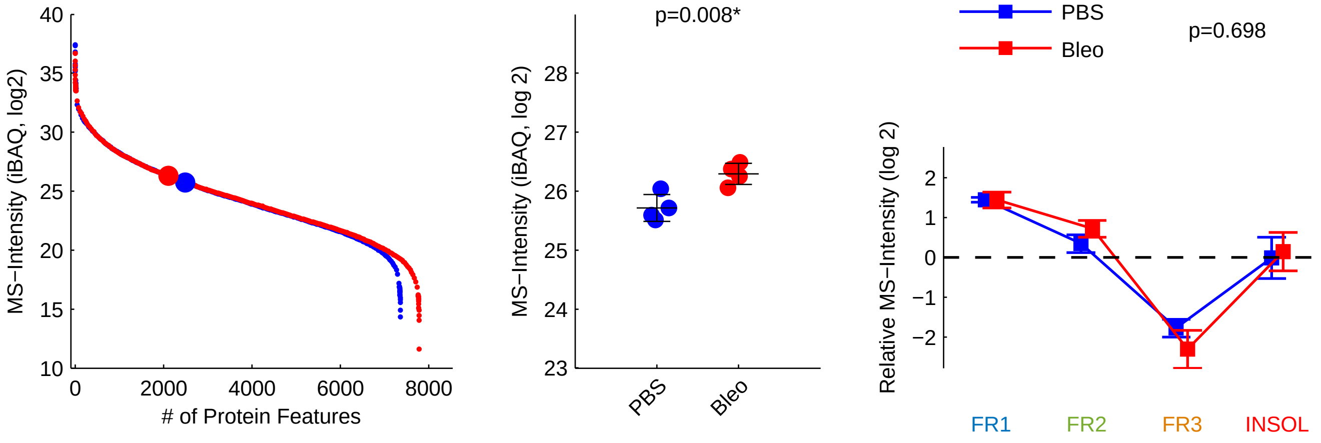

O08677−2 − Kng1 (id: 2053)

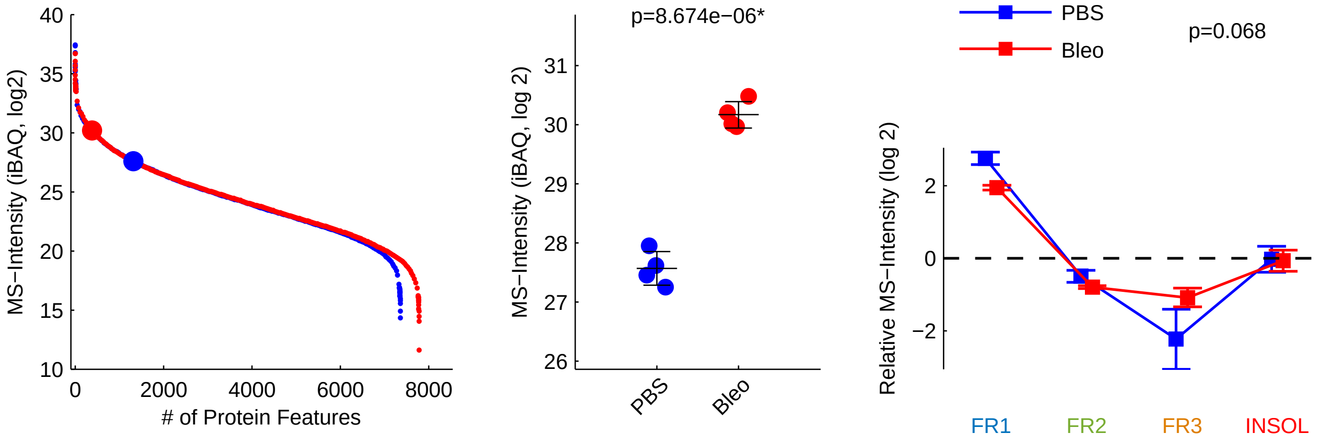

O08691 − Arg2 (id: 2055)

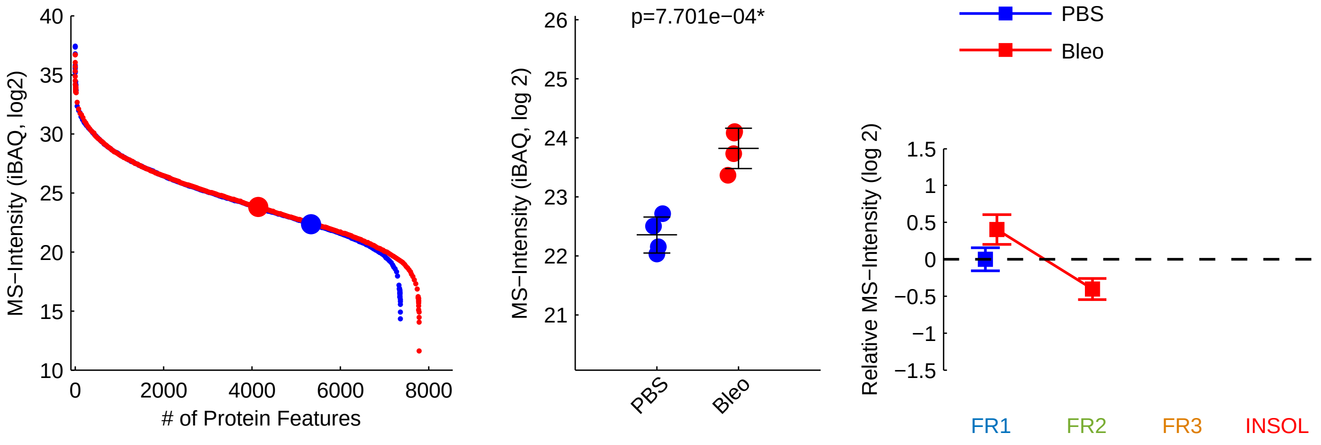

O08746 − Matn2 (id: 2063)

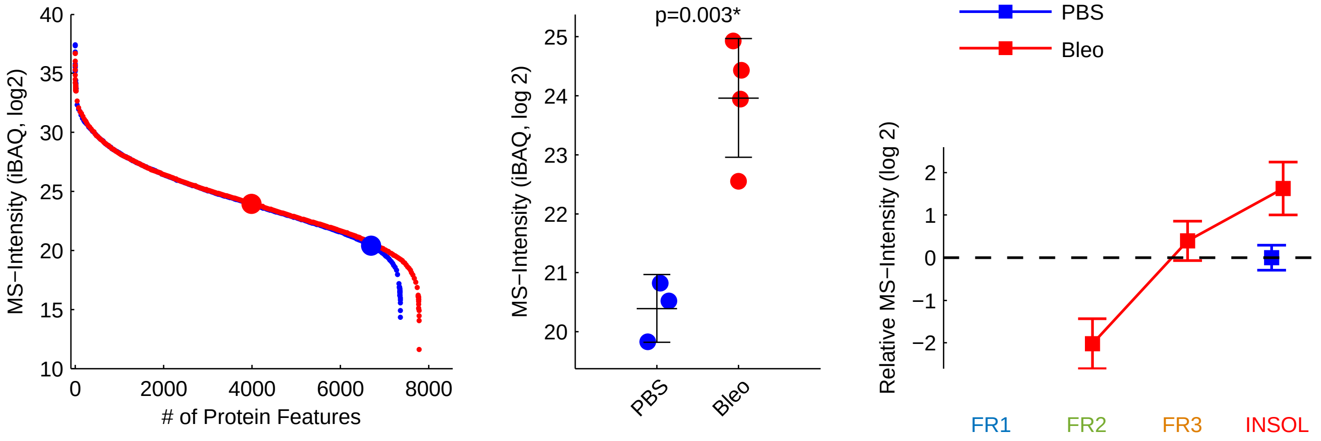

O08807 − Prdx4 (id: 2071)

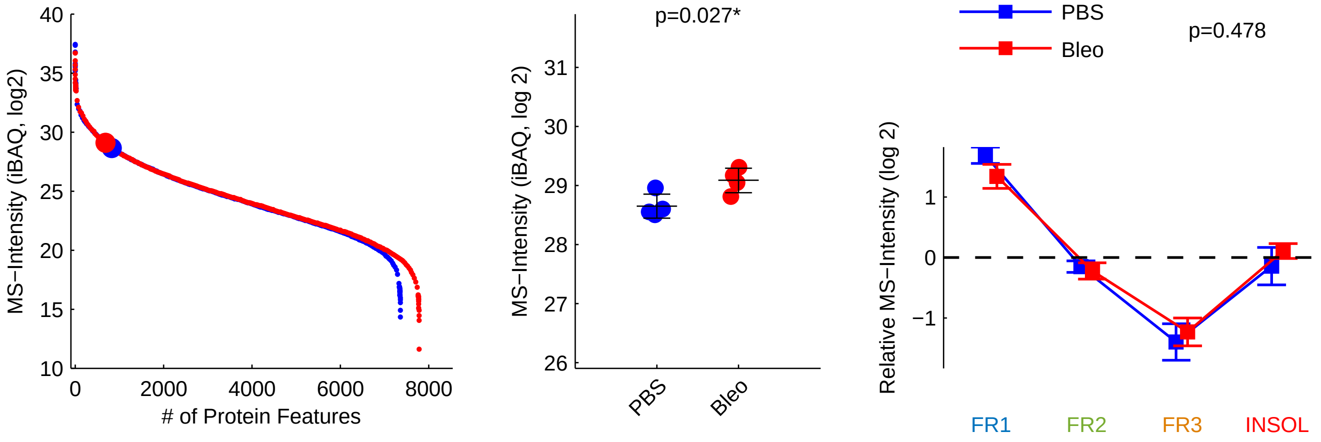

O08842 − Gfra2 (id: 2072)

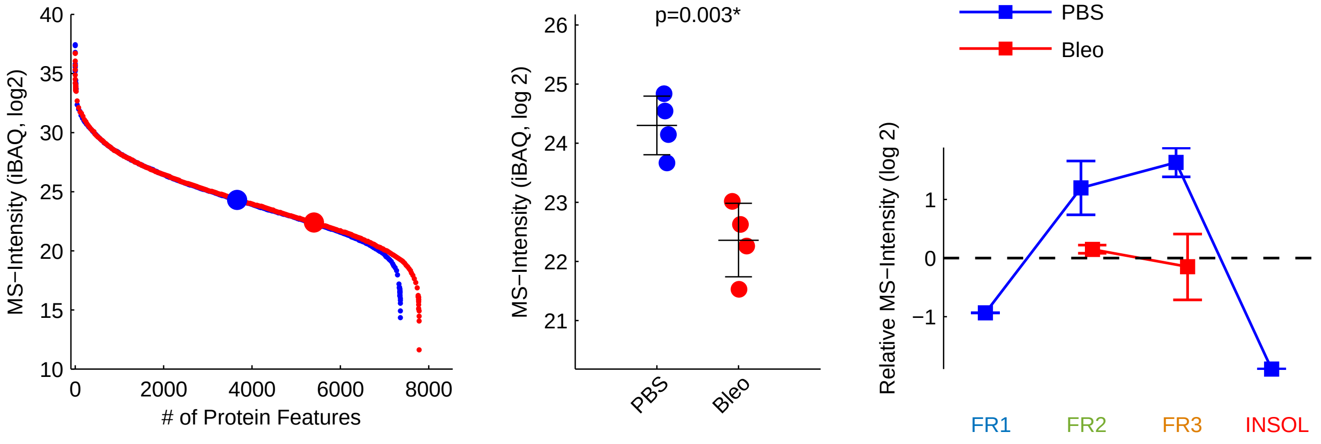

O08911 − Mapk12 (id: 2077)

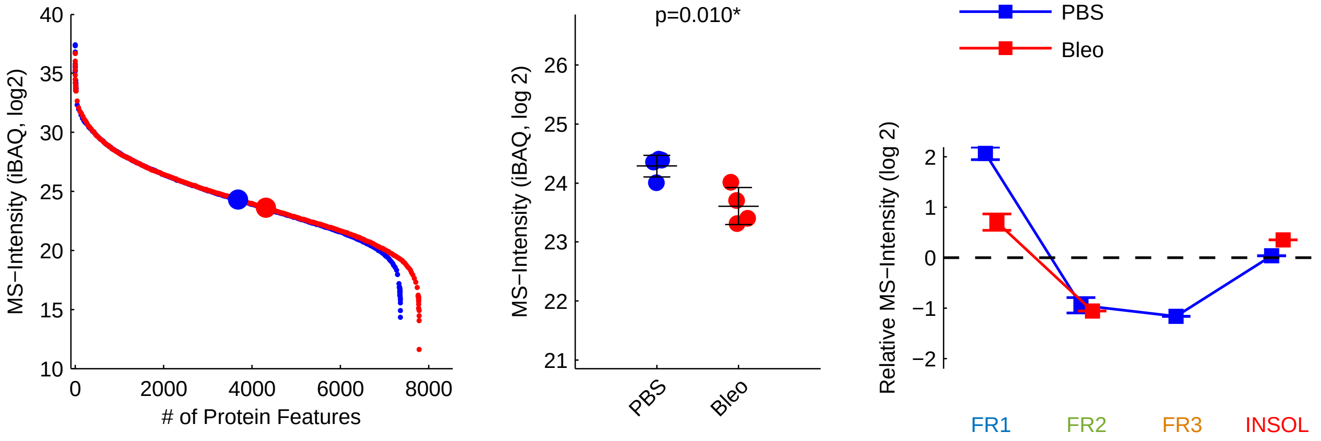

O08912 − Galnt1 (id: 2078)

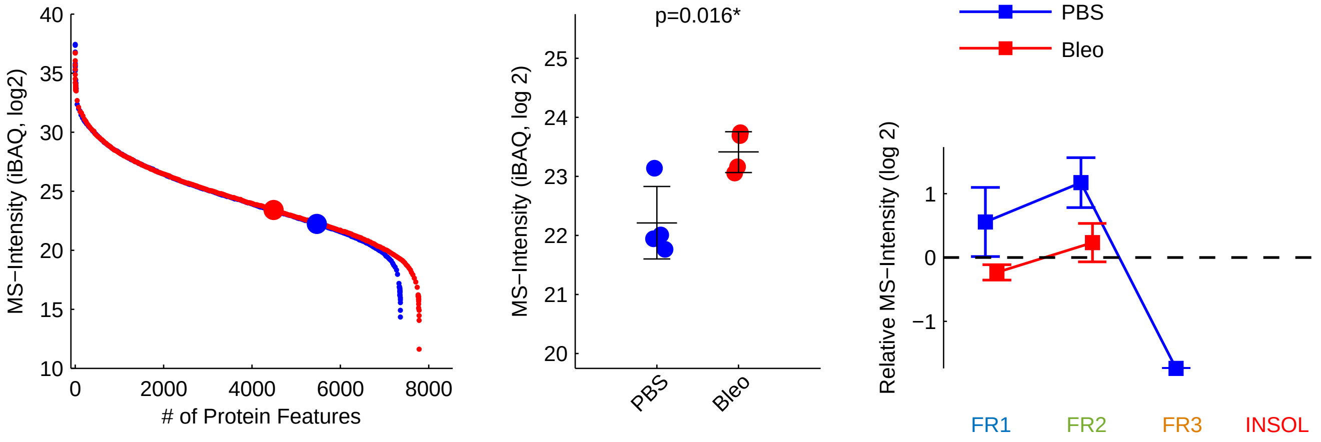

O08914 − Faah (id: 2079)

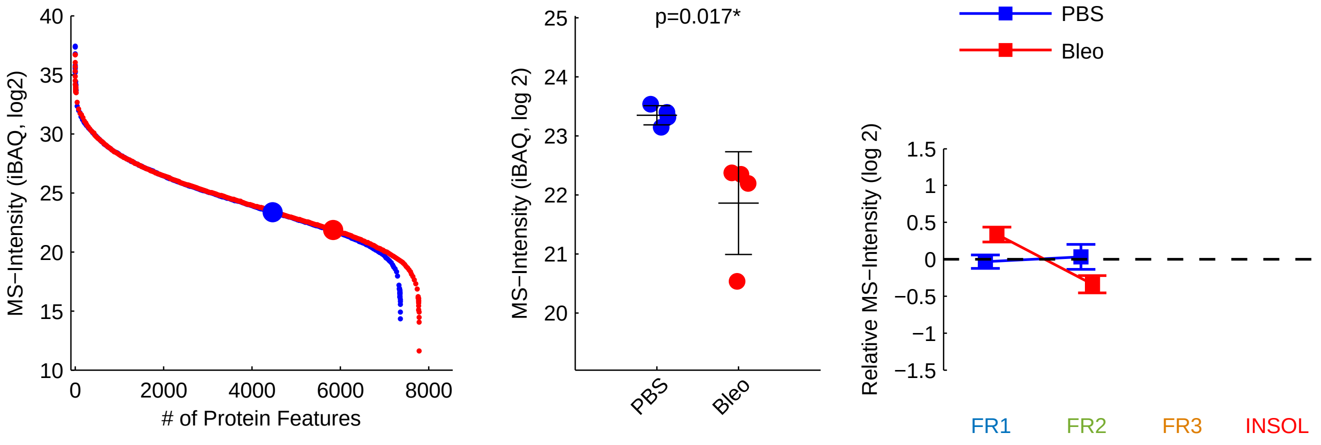

O08915 − Aip (id: 2080)

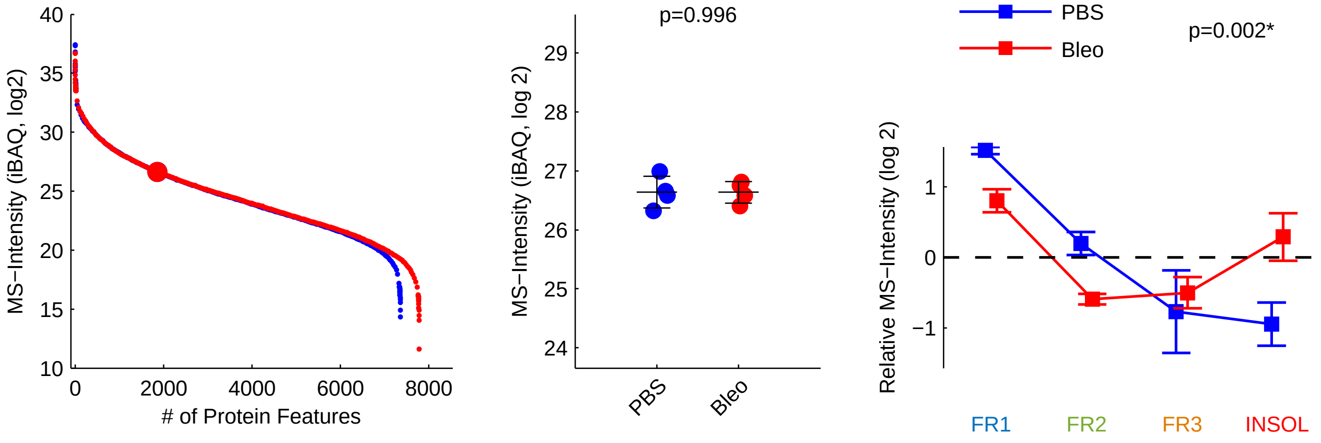

O08919 − Numbl (id: 2082)

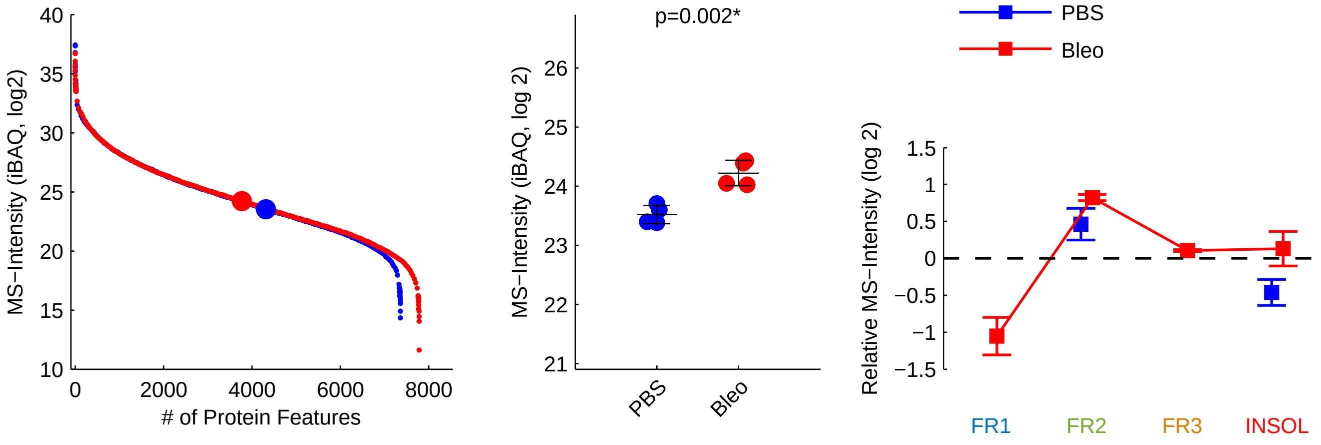

O08992 − Sdcbp (id: 2085)

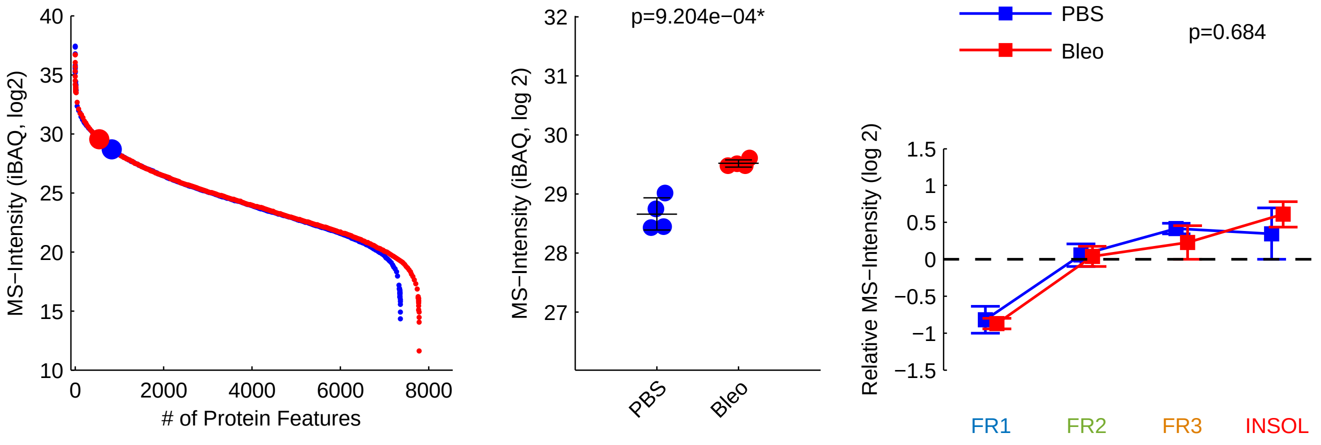

O09043 − Napsa (id: 2090)

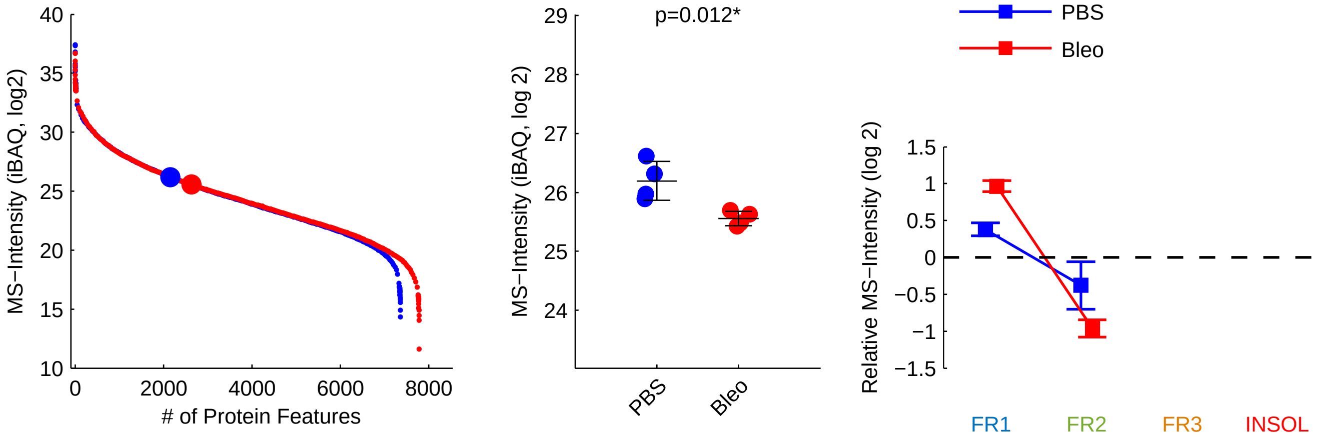

O09117−2 − Sypl1 (id: 2098)

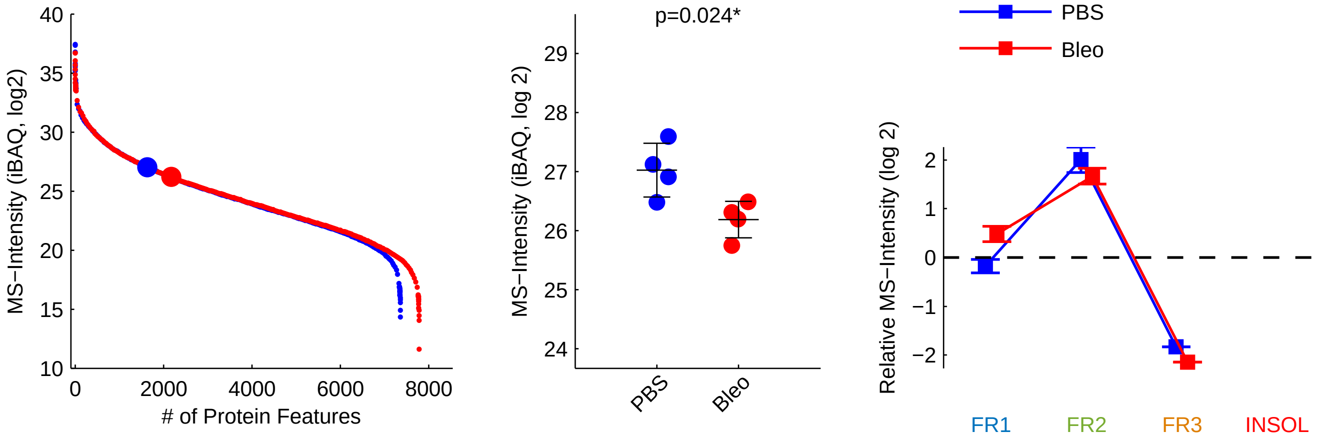

O09118 − Ntn1 (id: 2099)

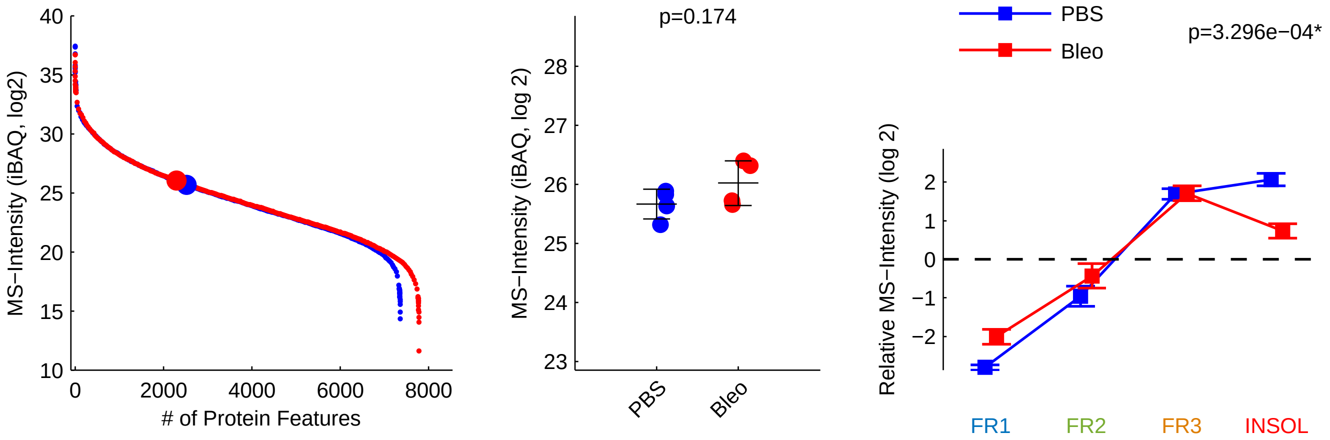

O09159 − Man2b1 (id: 2101)

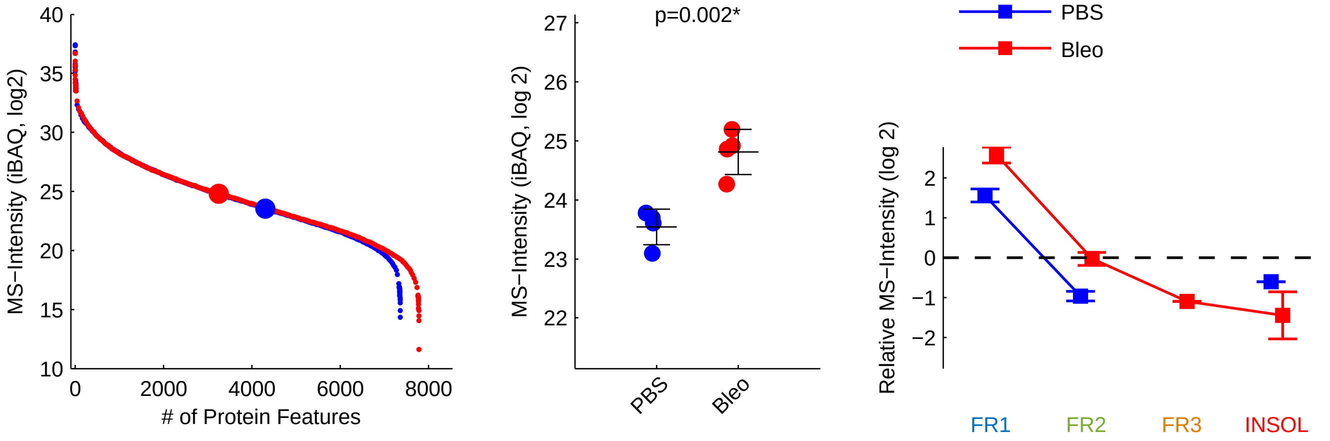

O09164 − Sod3 (id: 2103)

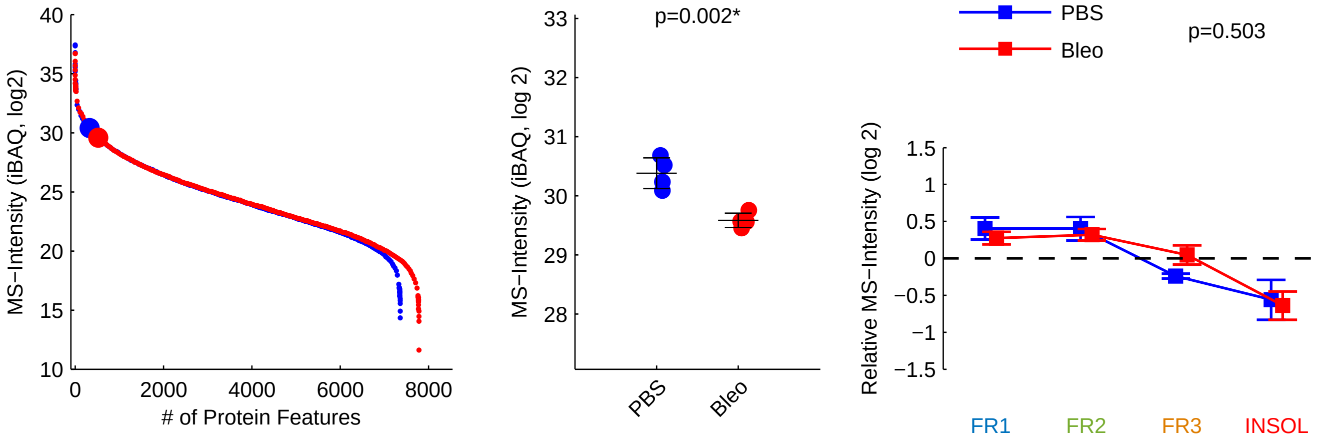

O35215 − Ddt (id: 2124)

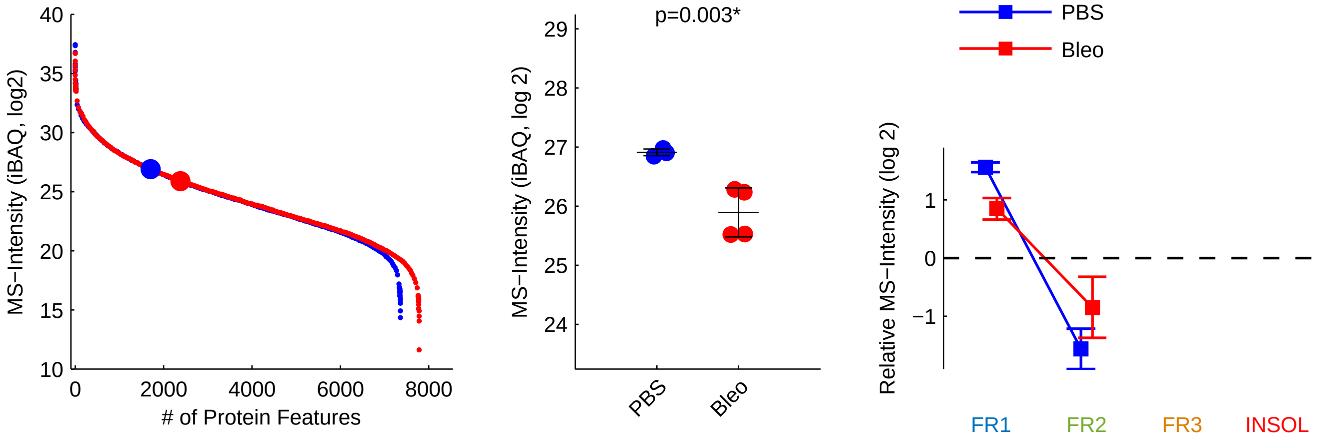

O35405 − Pld3 (id: 2145)

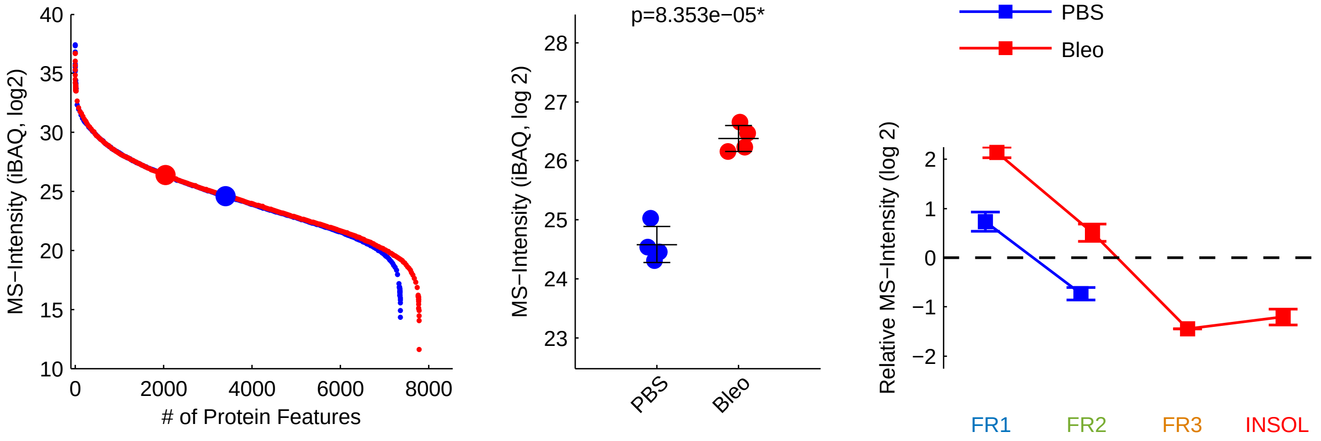

O35593 − Psmd14 (id: 2157)

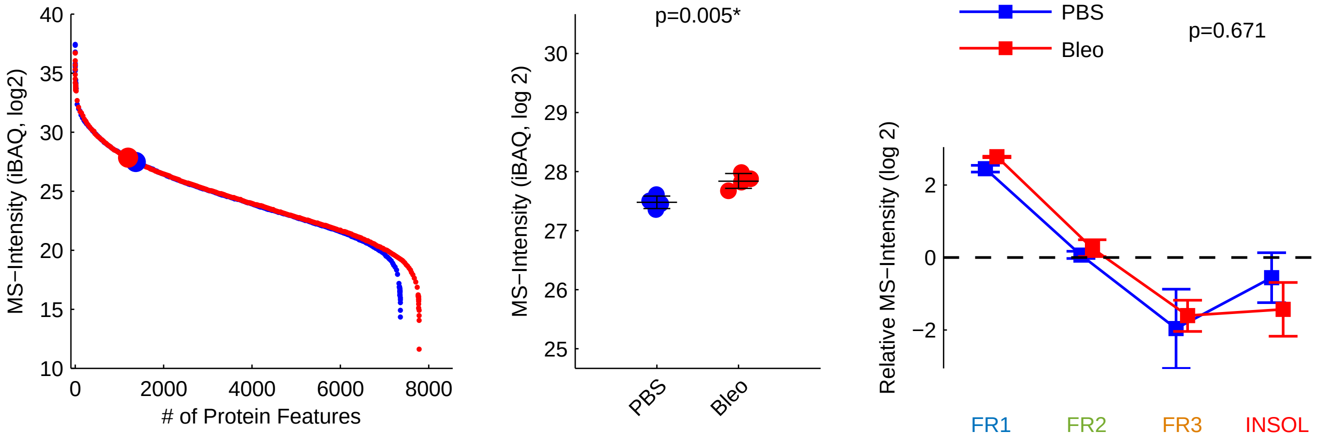

O35604 − Npc1 (id: 2161)

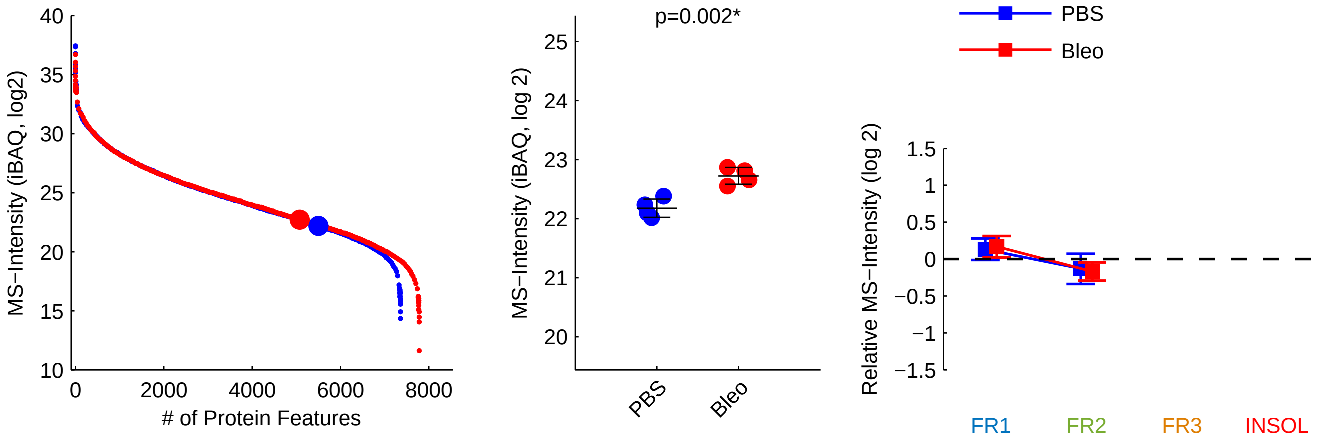

O35607 − Bmpr2 (id: 2162)

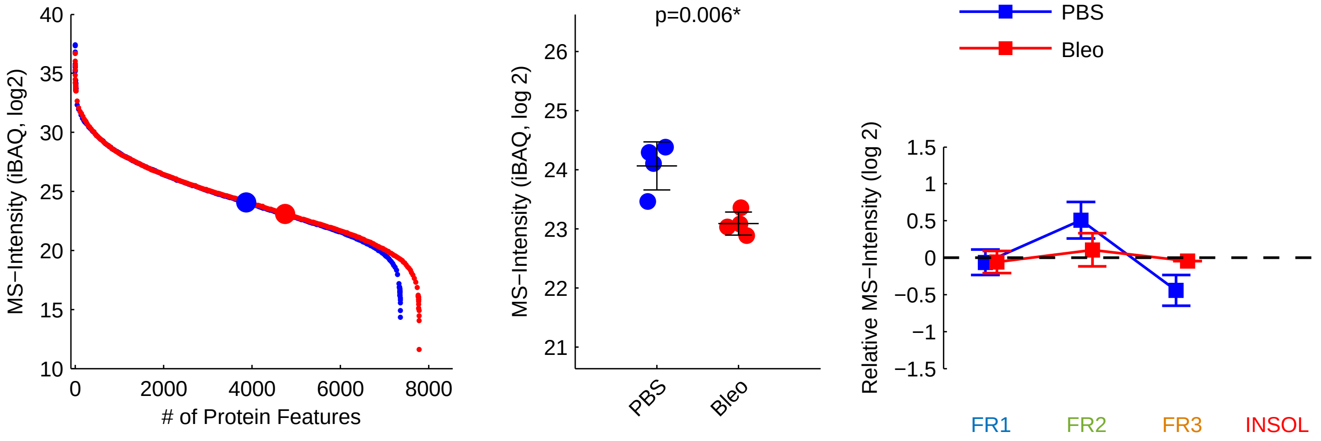

O35632 − Hyal2 (id: 2165)

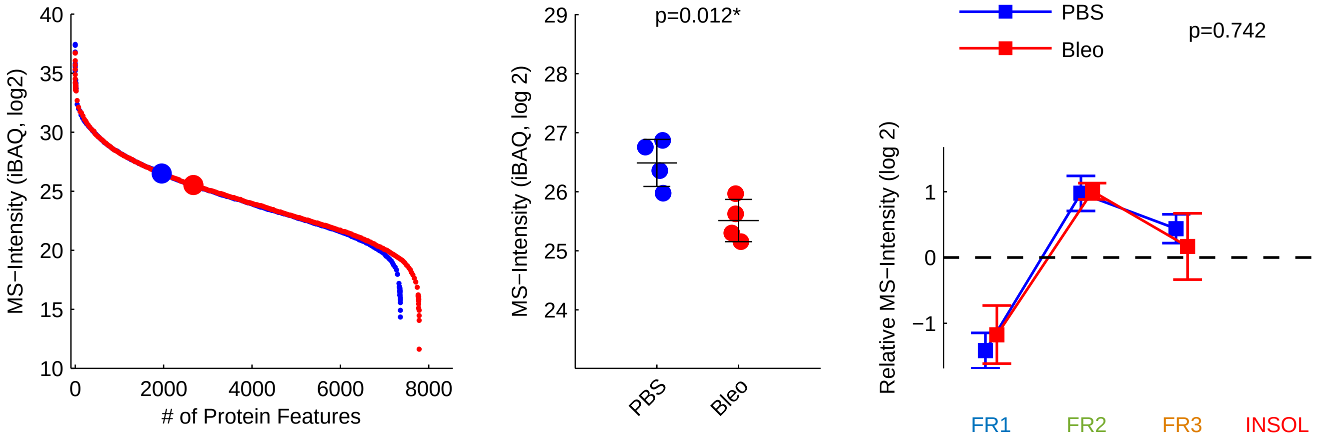

O35682 − Myadm (id: 2174)

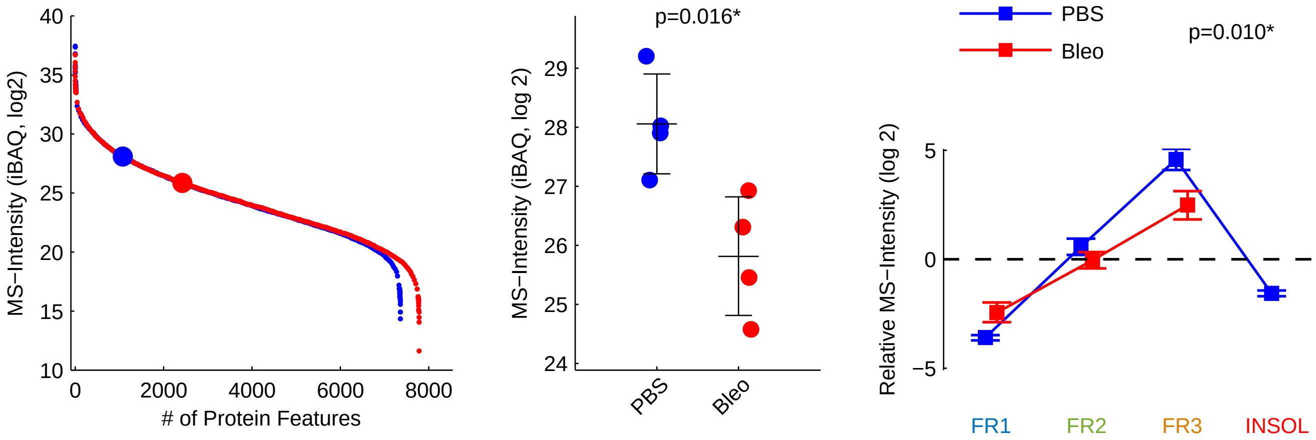

O35691 − Pnn (id: 2177)

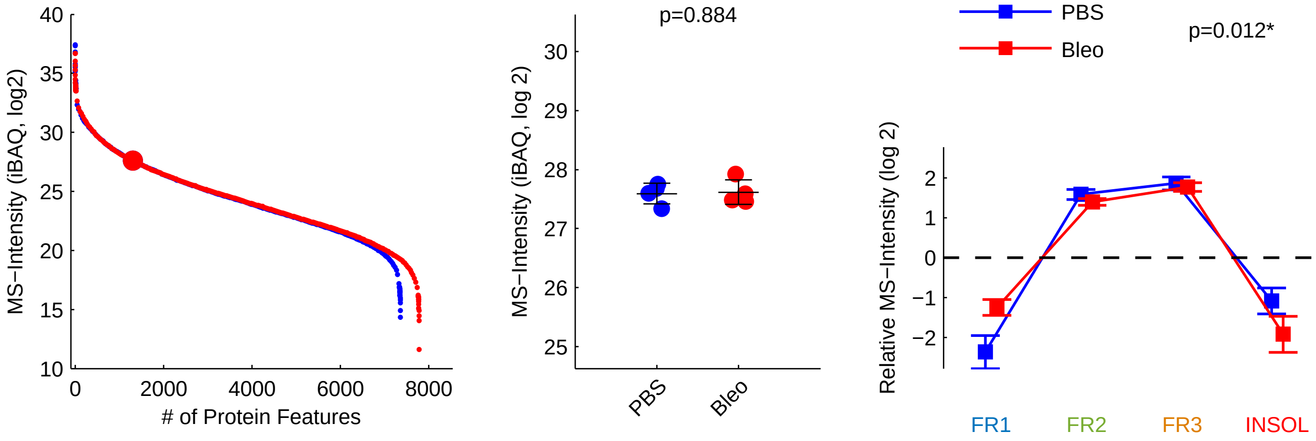

O35704 − Sptlc1 (id: 2178)

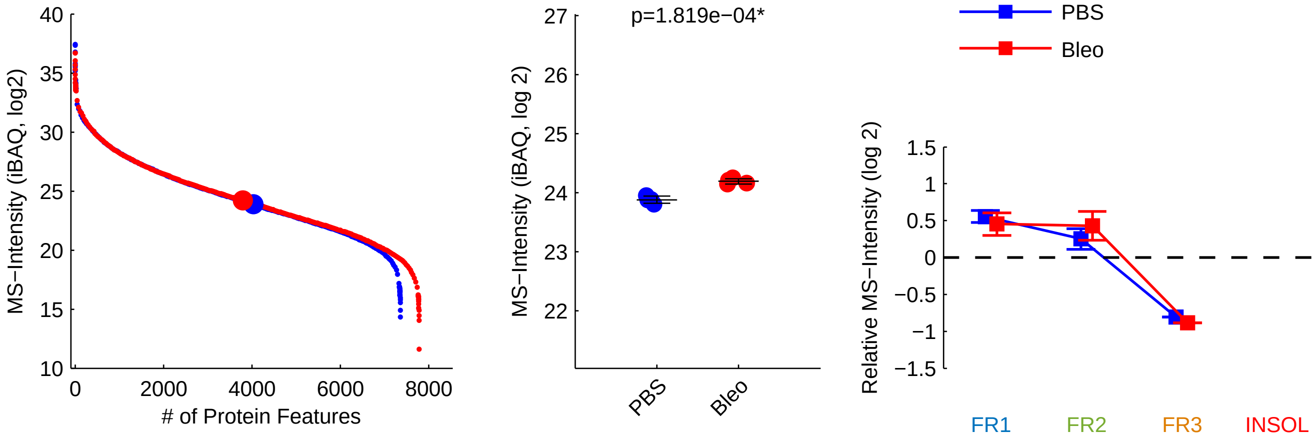

O35737 − Hnrnph1 (id: 2180)

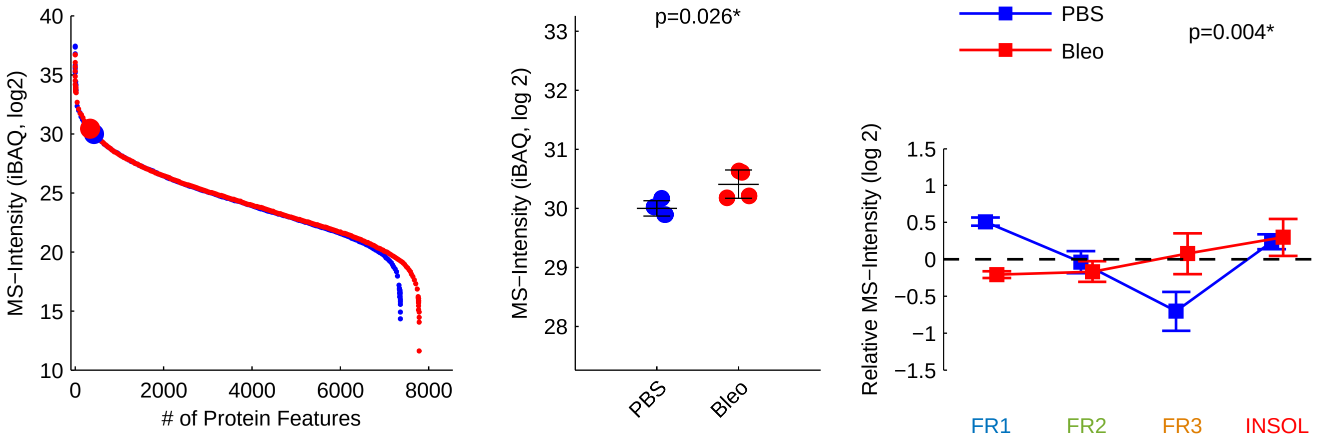

O35744 − Chi3l3 (id: 2181)

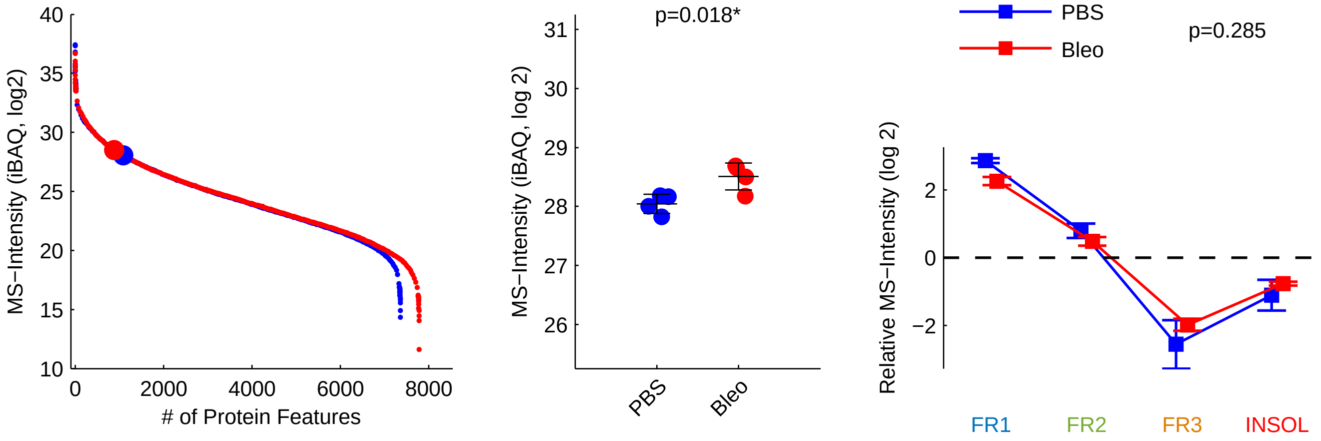

O35887 − Calu (id: 2185)

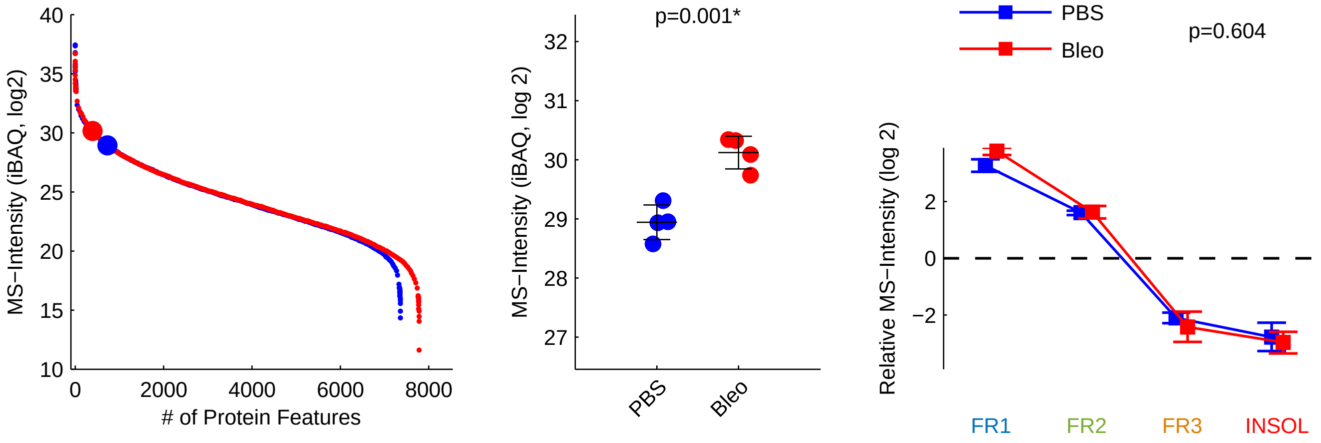

O35945 − Aldh1a7 (id: 2191)

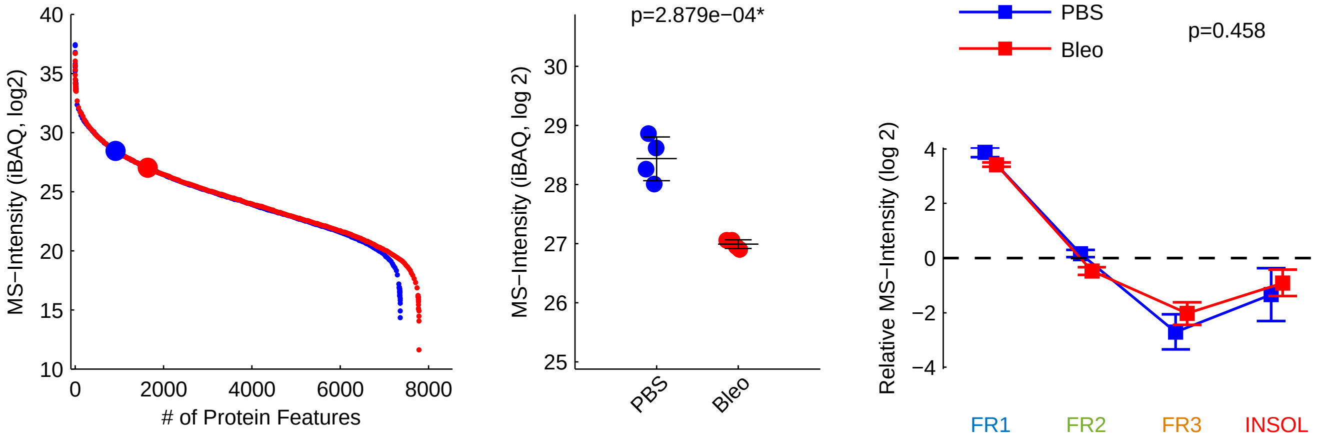

O54724 − Ptrf (id: 2198)

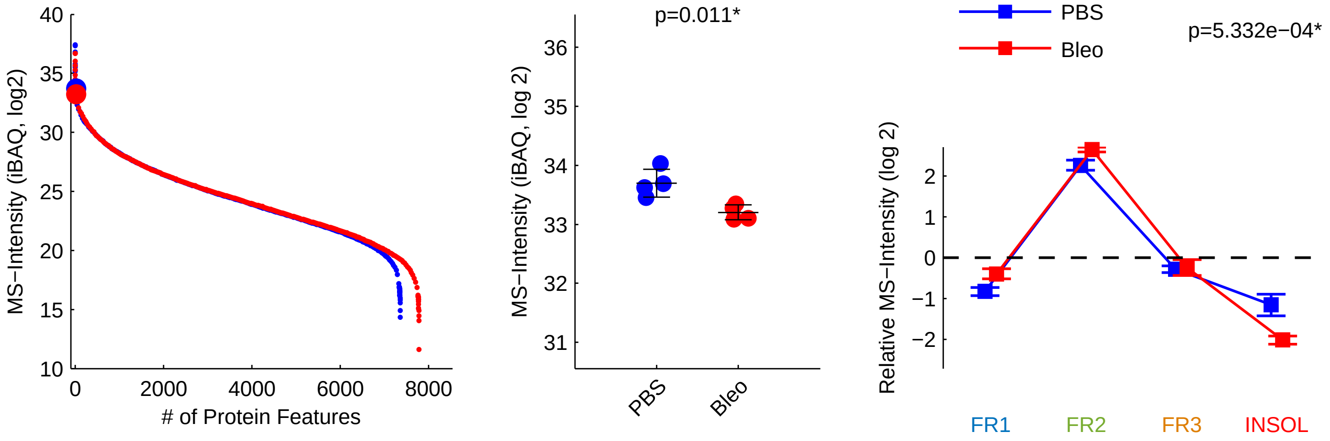

O54734 − Ddost (id: 2200)

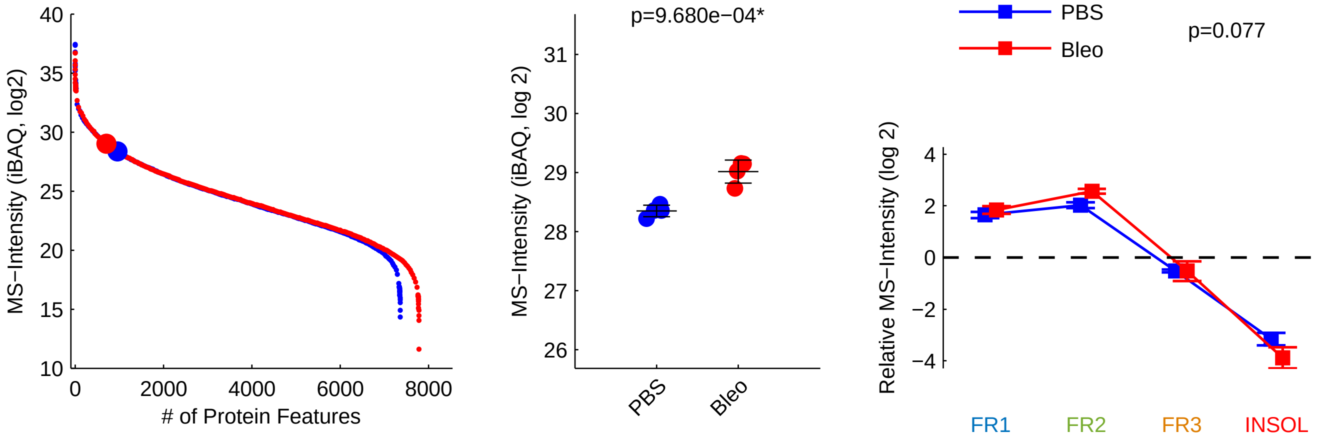

O54774 − Ap3d1 (id: 2201)

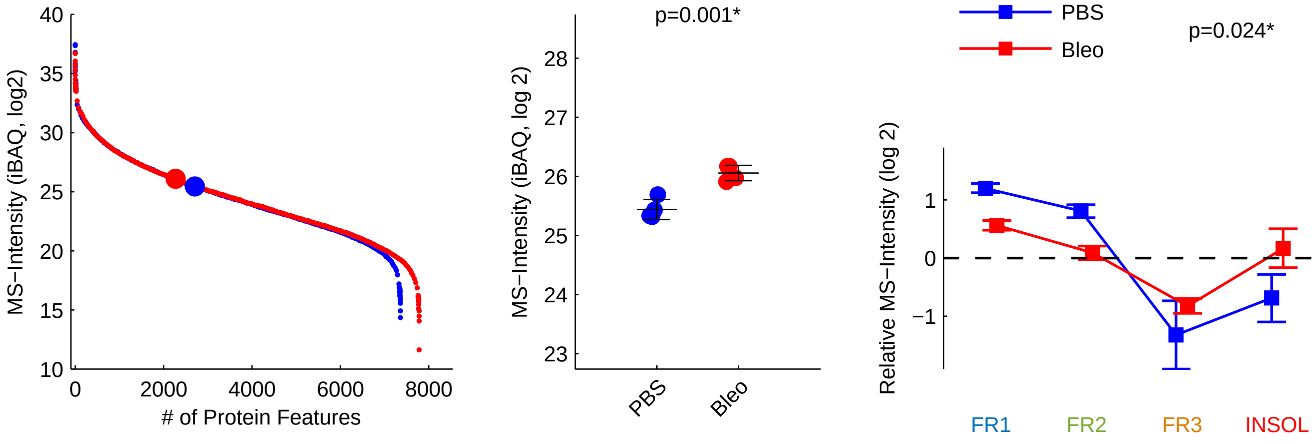

O54782 − Man2b2 (id: 2203)

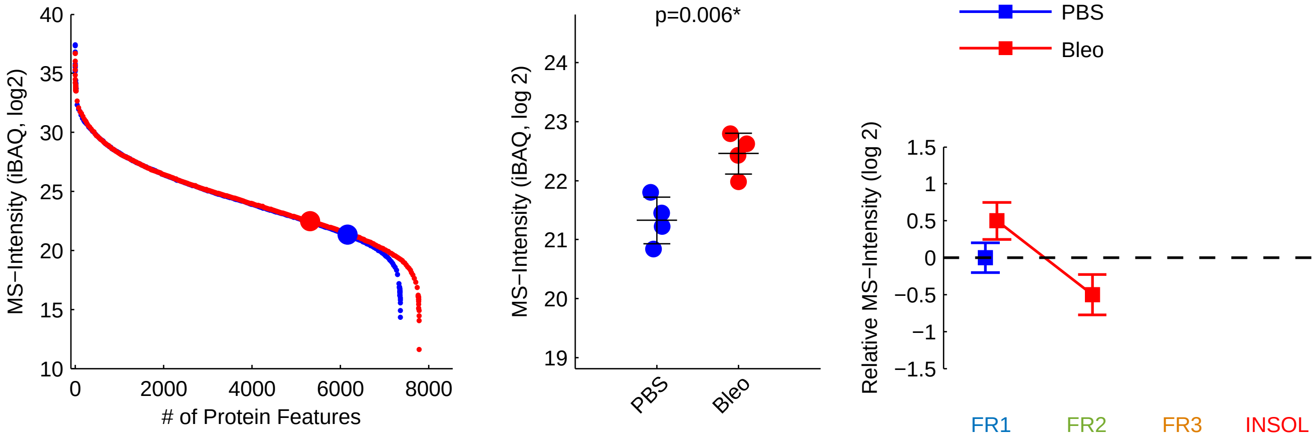

O54824 − Il16 (id: 2210)

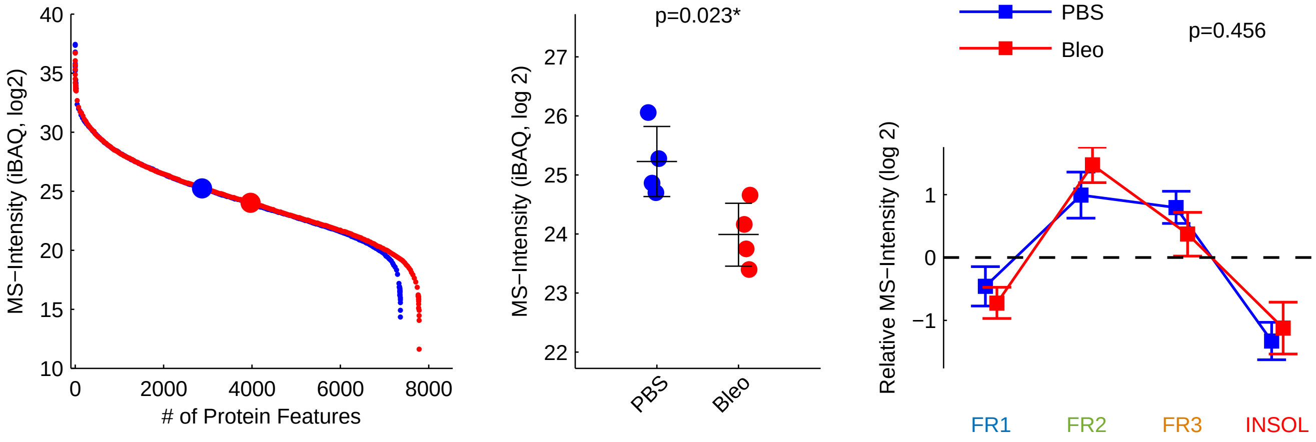

O54833 − Csnk2a2 (id: 2212)

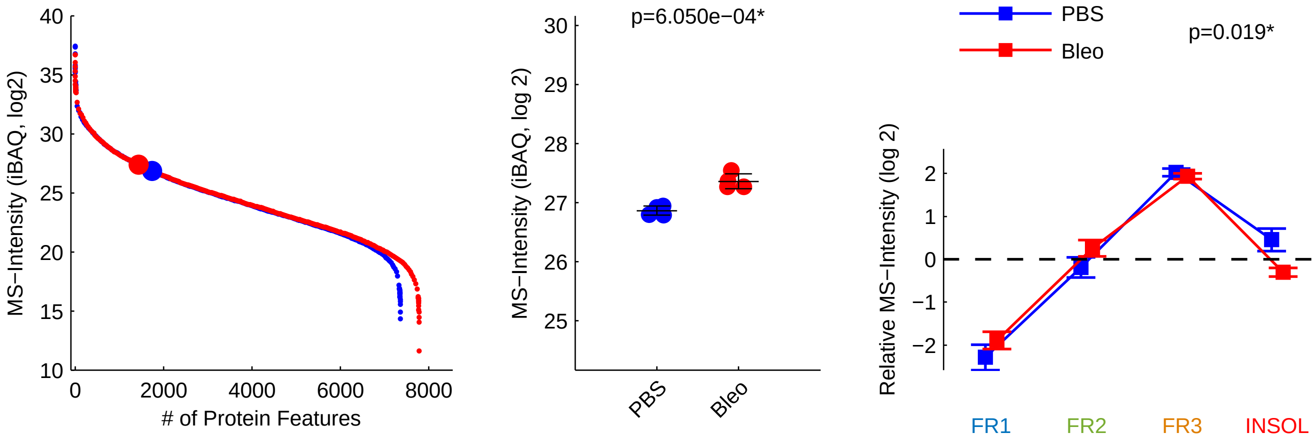

O54865 − Gucy1b3 (id: 2214)

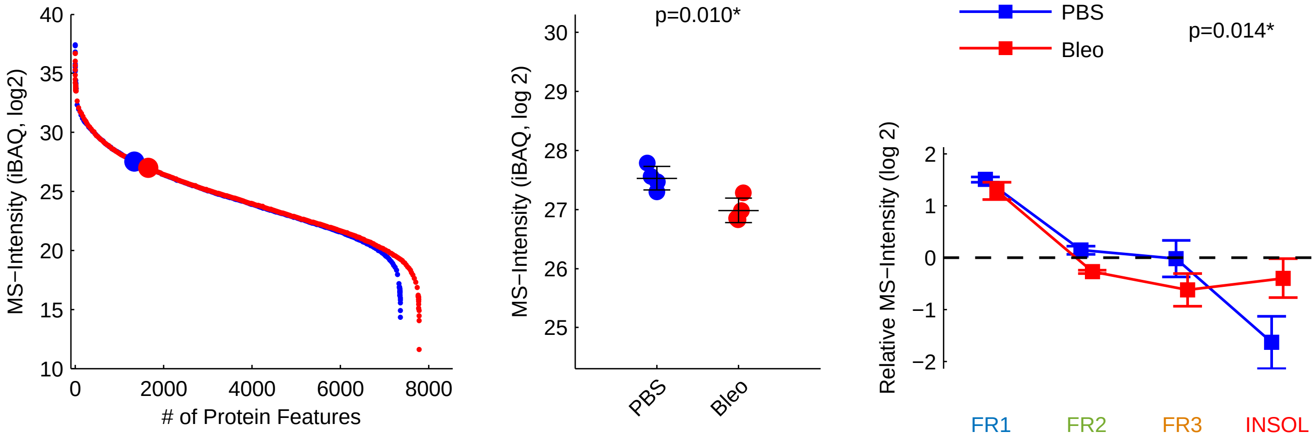

O54907 − Tnfsf12 (id: 2216)

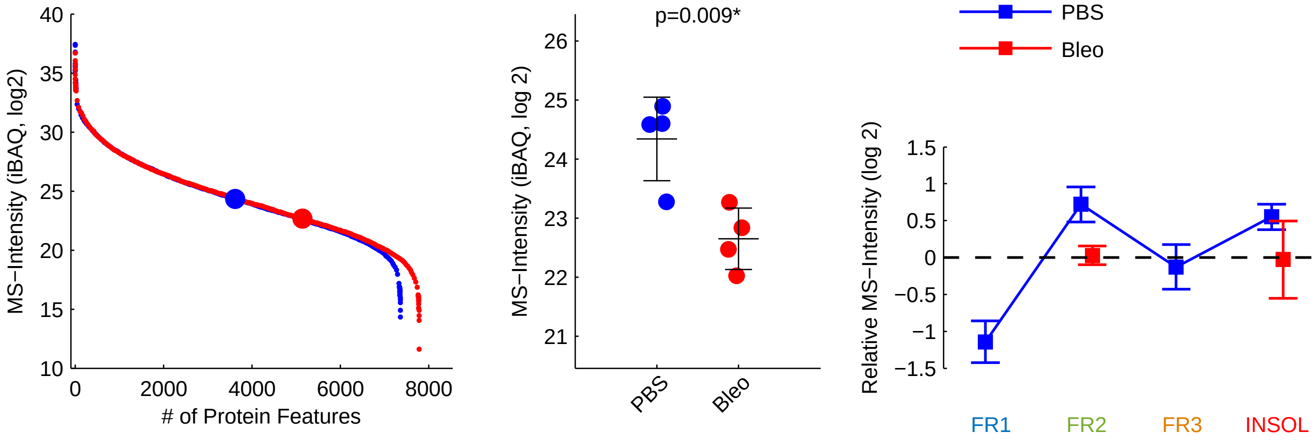

O54983 − Crym (id: 2227)

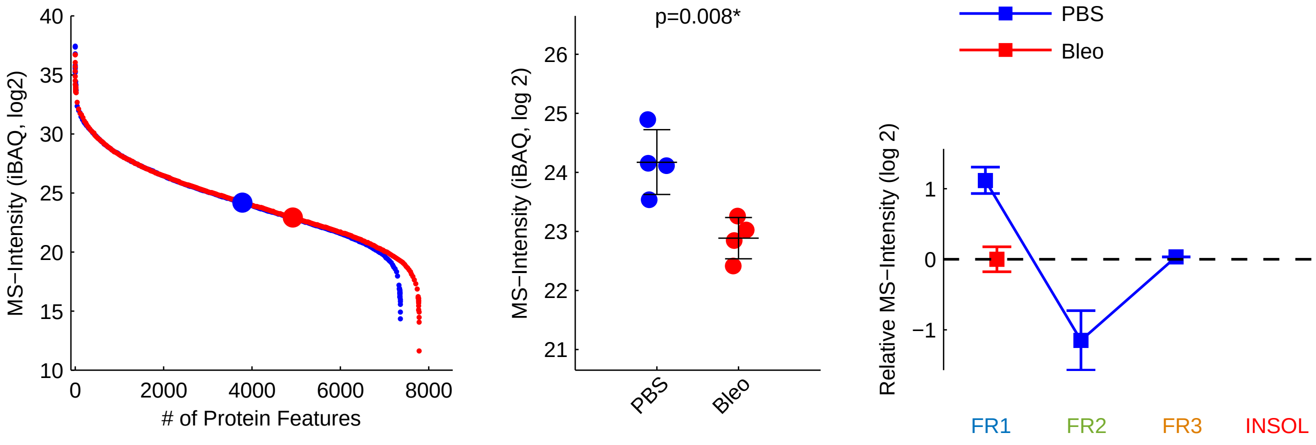

O54998 − Fkbp7 (id: 2230)

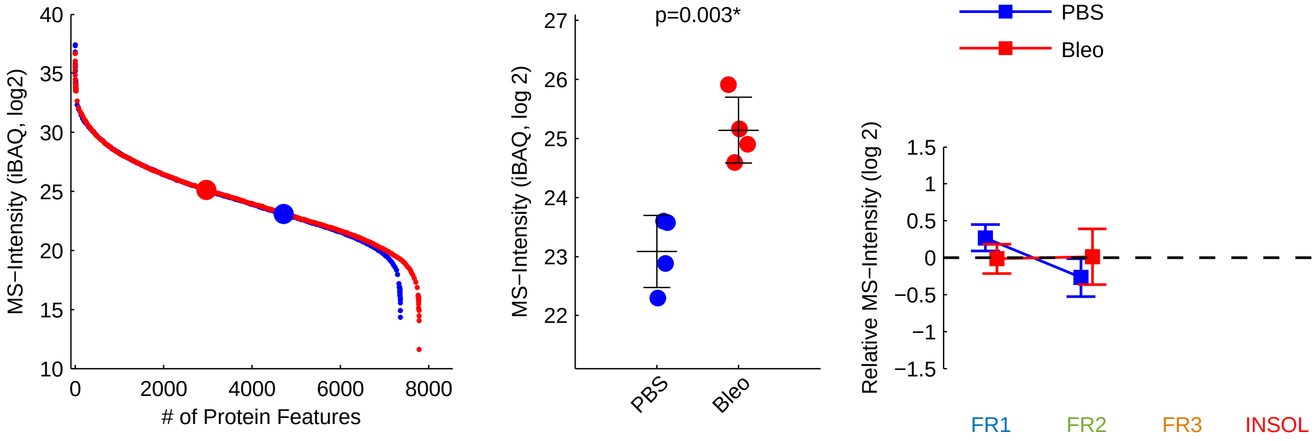

O55022 − Pgrmc1 (id: 2232)

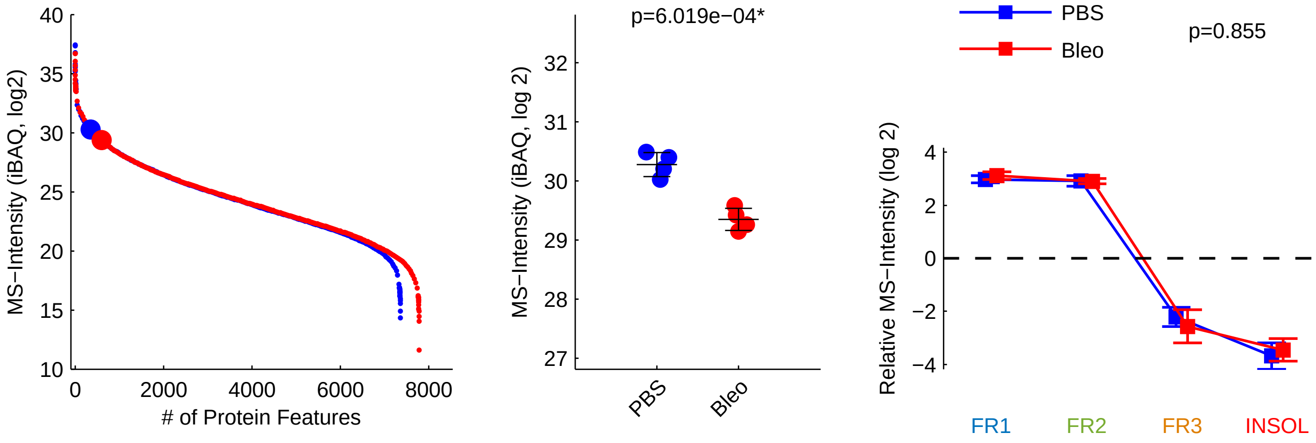

O55028 − Bckdk (id: 2234)

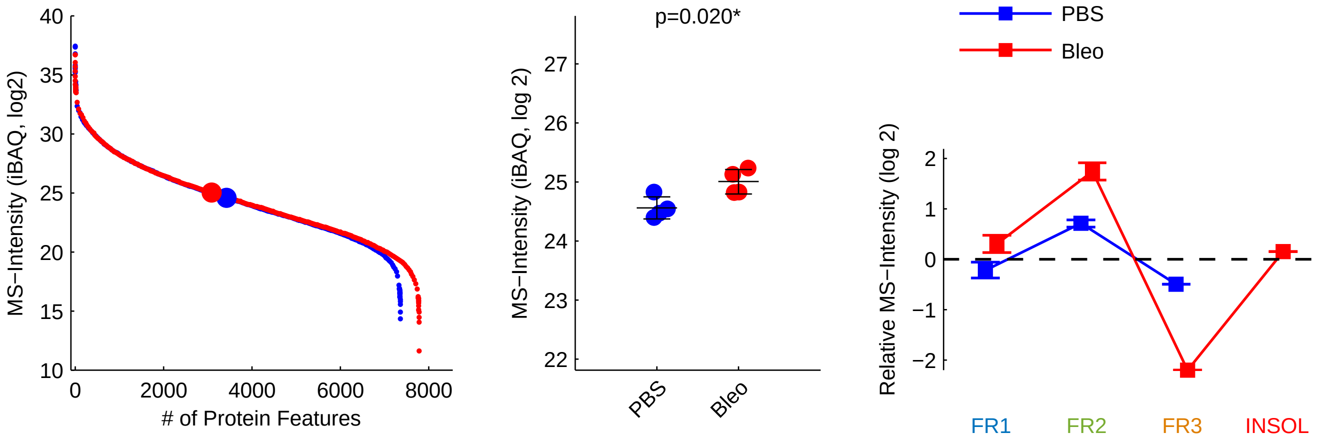

O55029 − Copb2 (id: 2235)

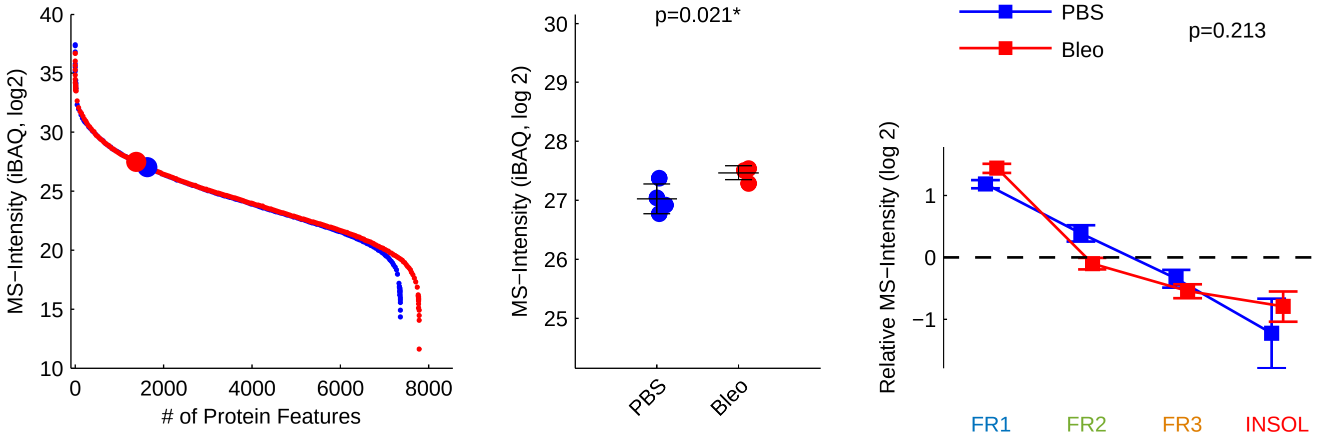

O55098 − Stk10 (id: 2242)

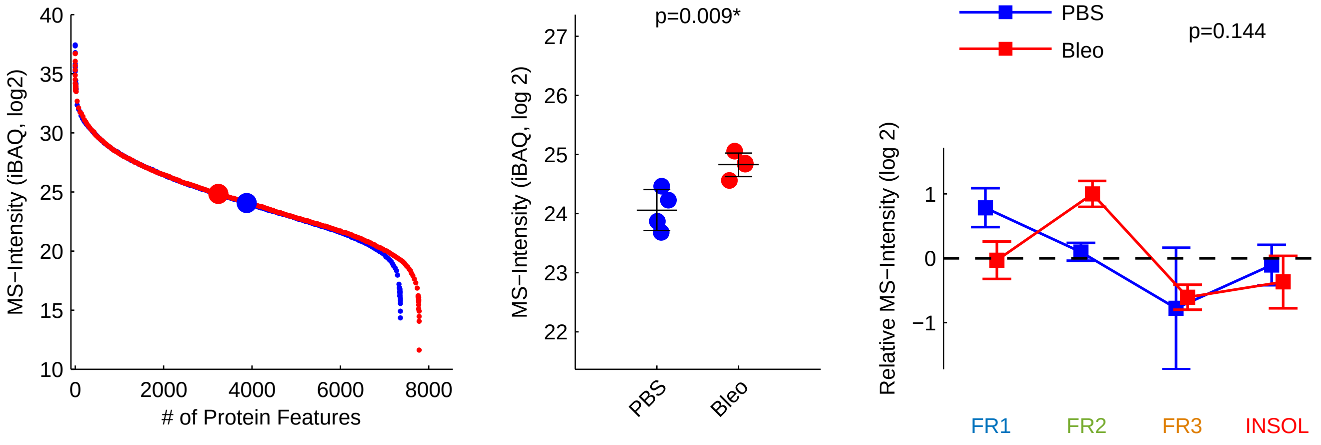

O55106 − Strn (id: 2244)

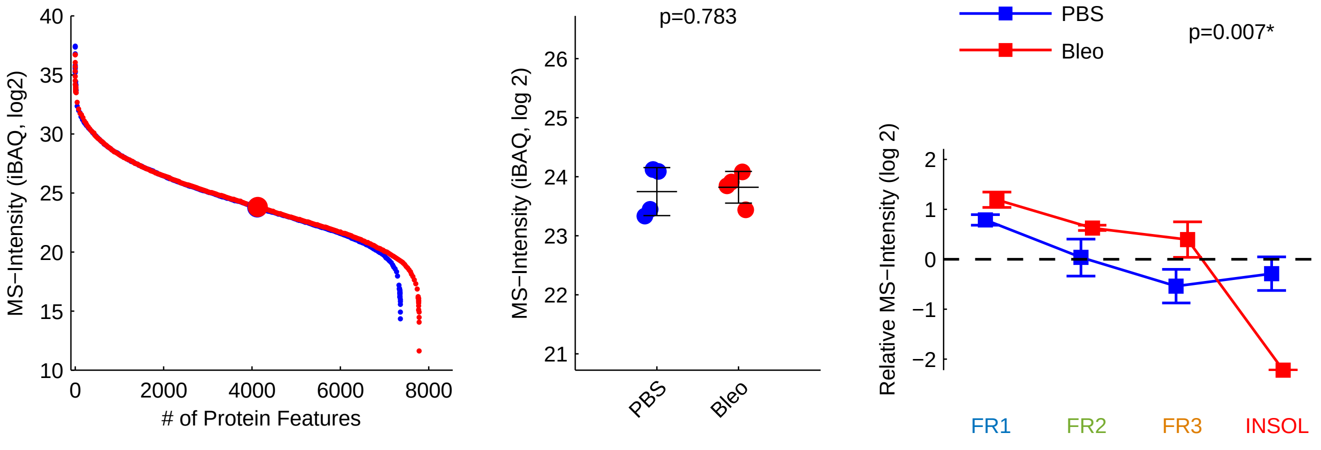

O55125 − Nipsnap1 (id: 2246)

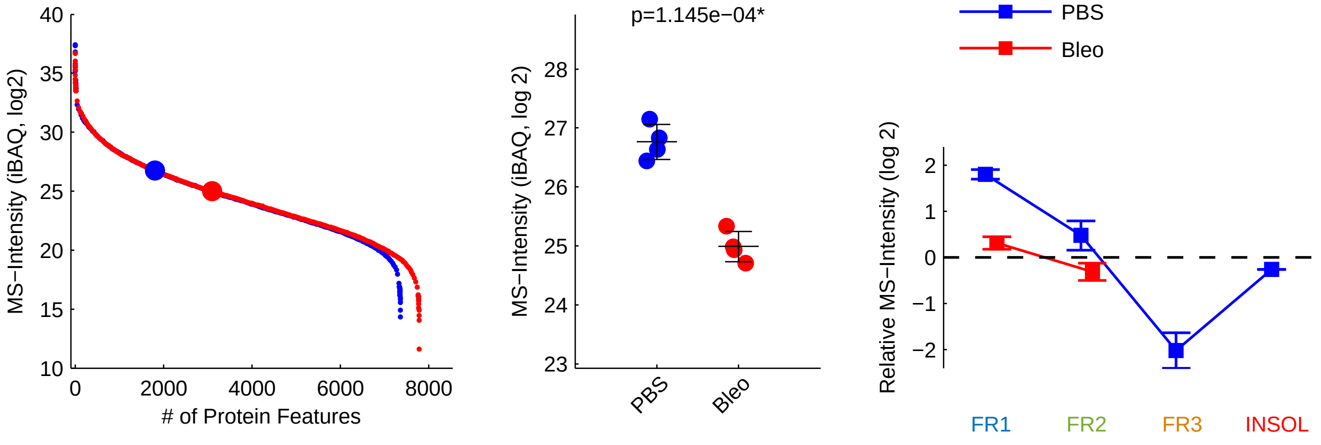

O55137 − Acot1 (id: 2249)

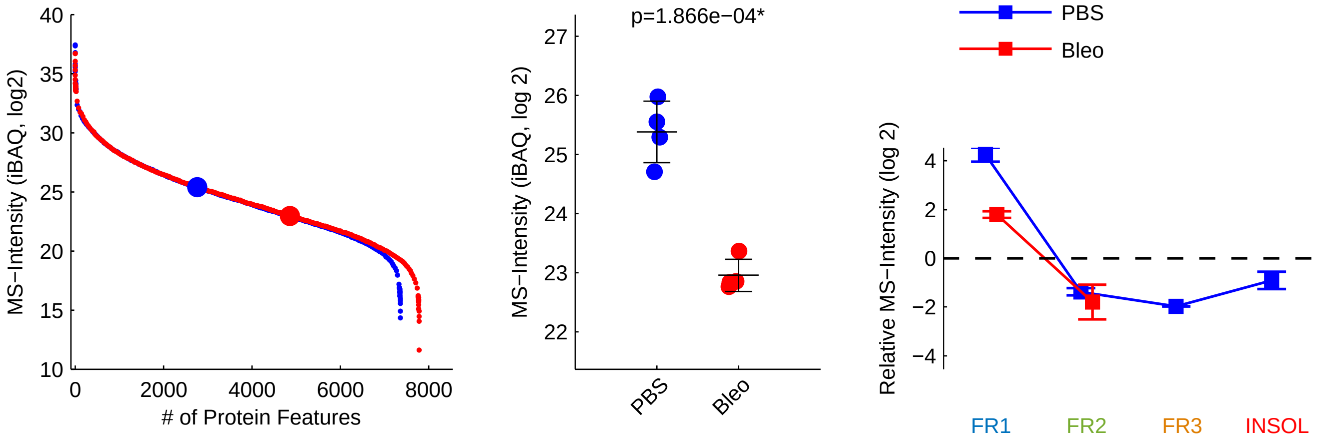

O55142 − Rpl35a (id: 2250)

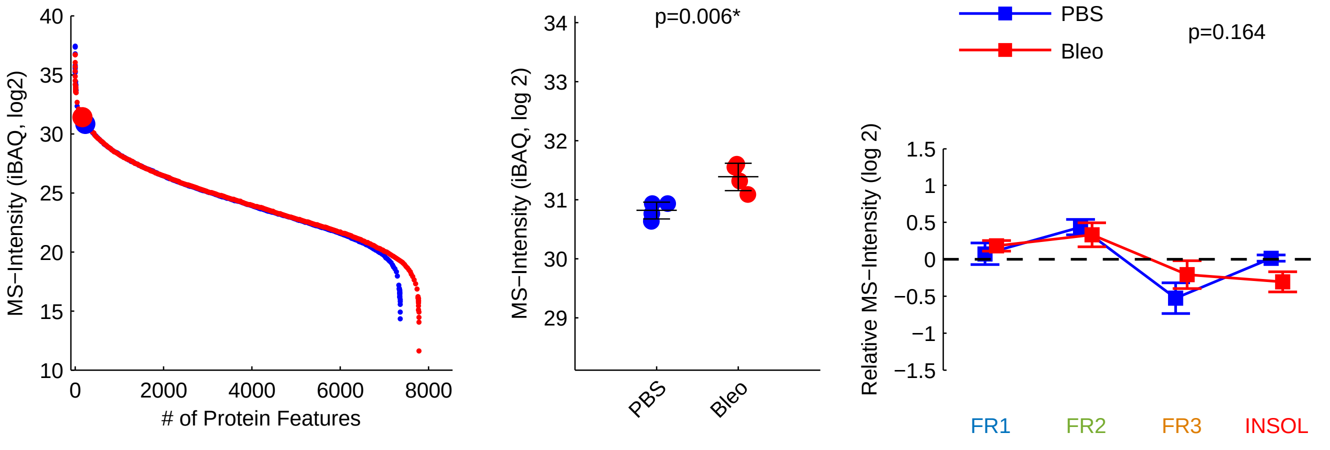

O55201 − Supt5h (id: 2255)

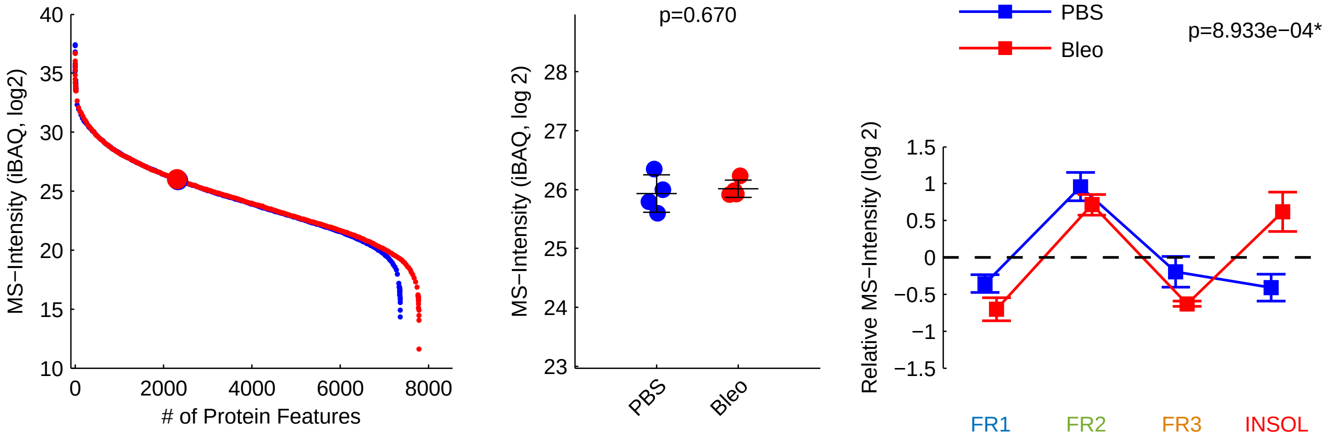

O55222 − Ilk (id: 2256)

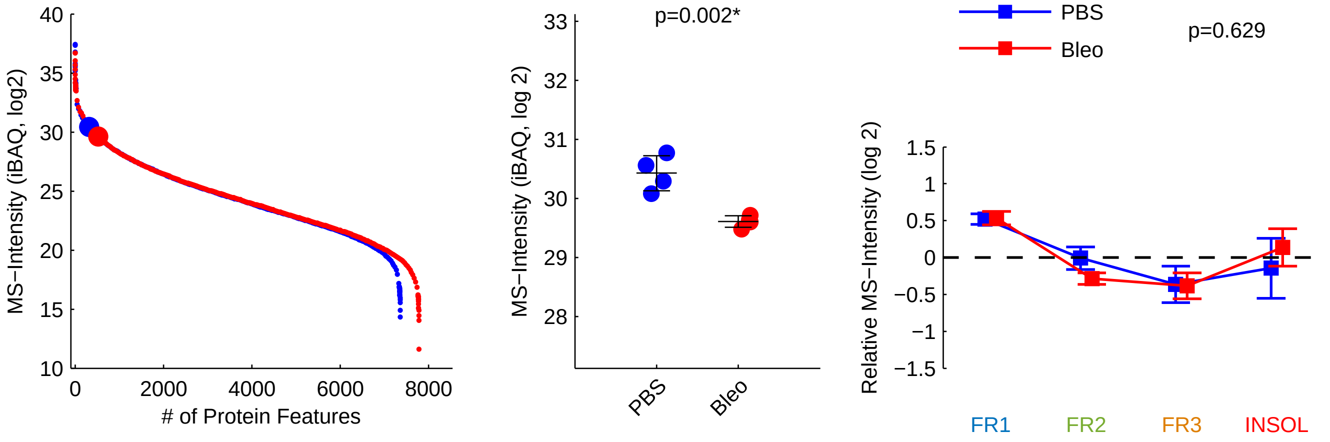

O70145 − Ncf2 (id: 2265)

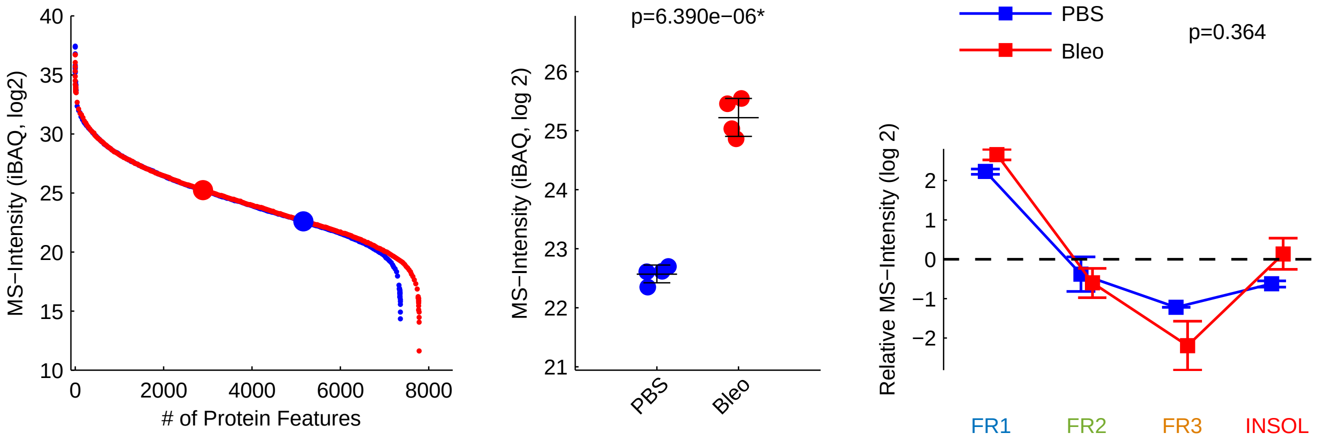

O70194 − Eif3d (id: 2268)

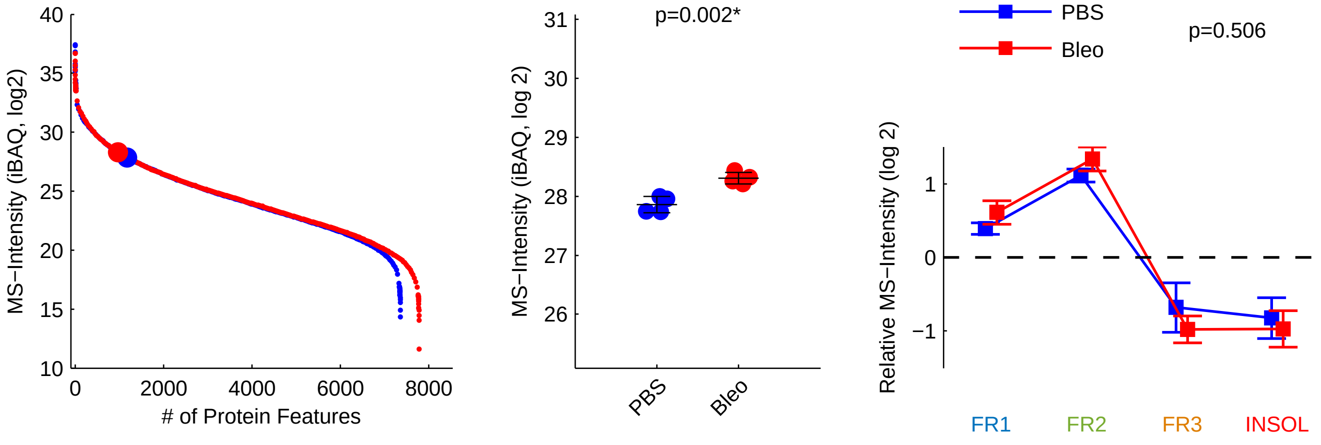

O70200 − Aif1 (id: 2269)

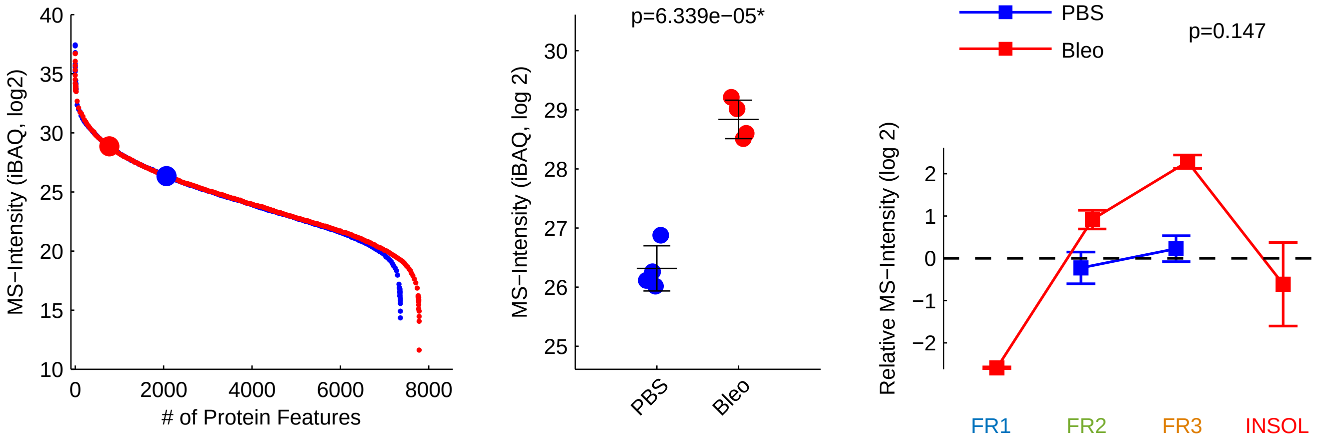

O70251 − Eef1b (id: 2274)

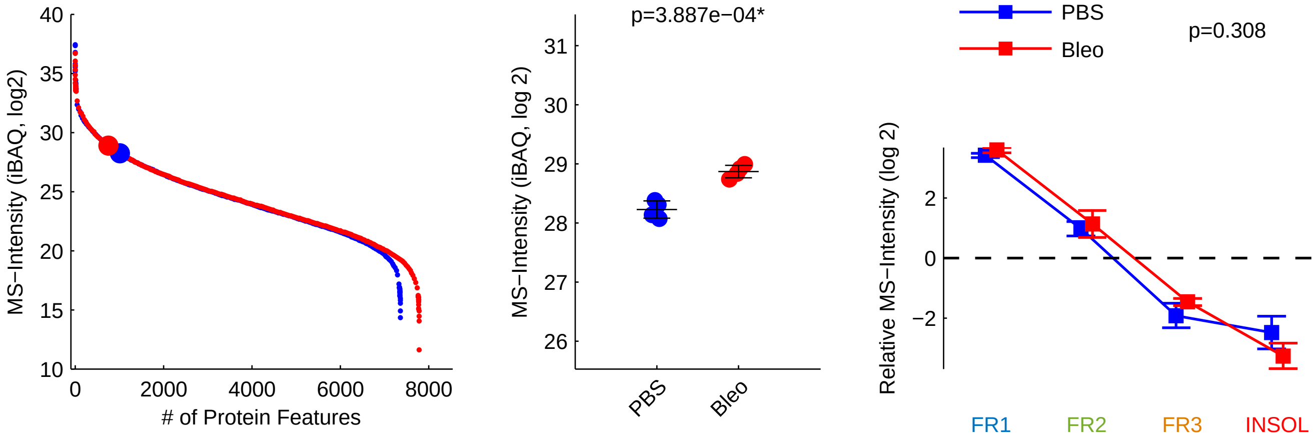

O70310 − Nmt1 (id: 2280)

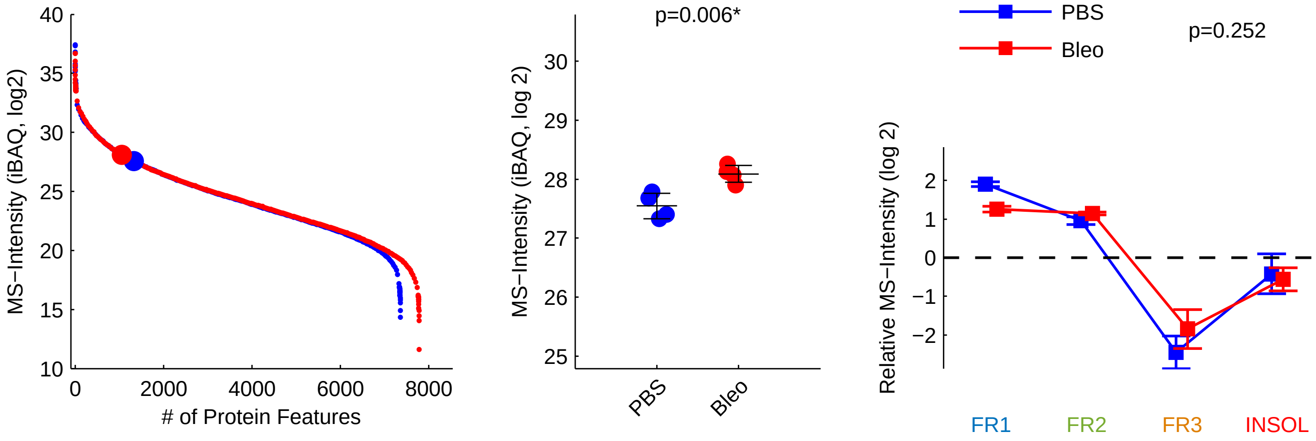

O70318 − Epb41l2 (id: 2281)

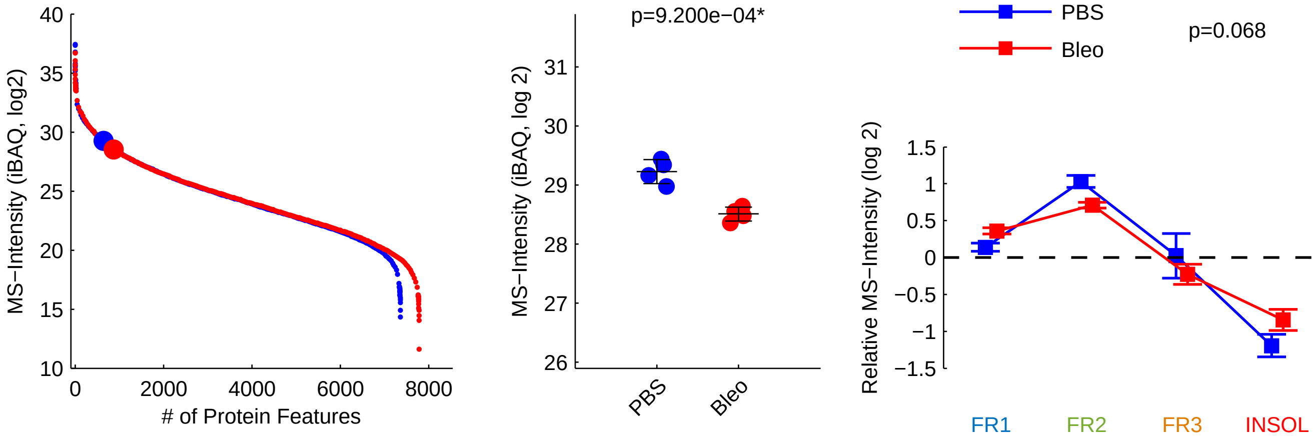

O70370 − Ctss (id: 2283)

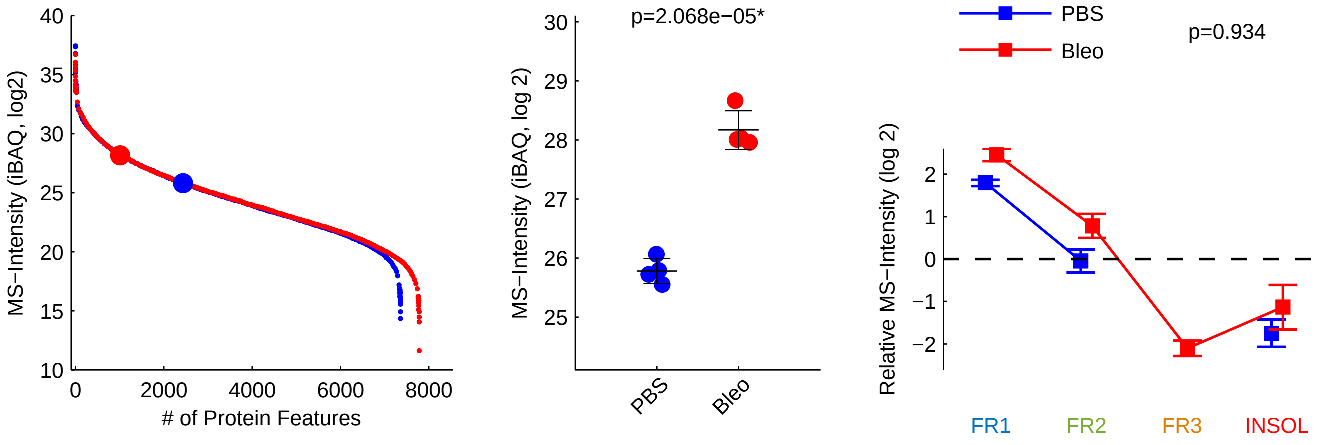

O70400 − Pdlim1 (id: 2285)

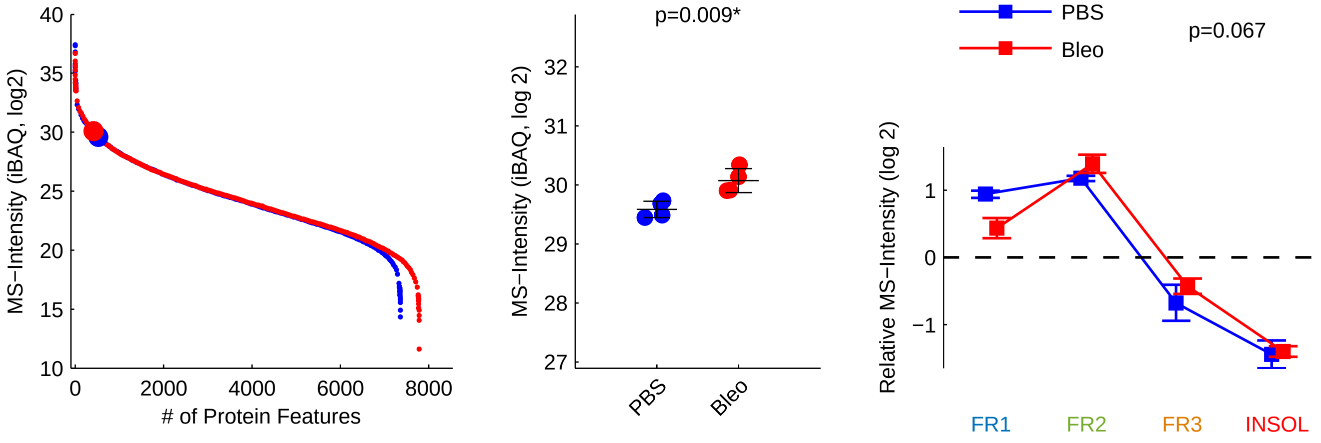

O70404 − Vamp8 (id: 2286)

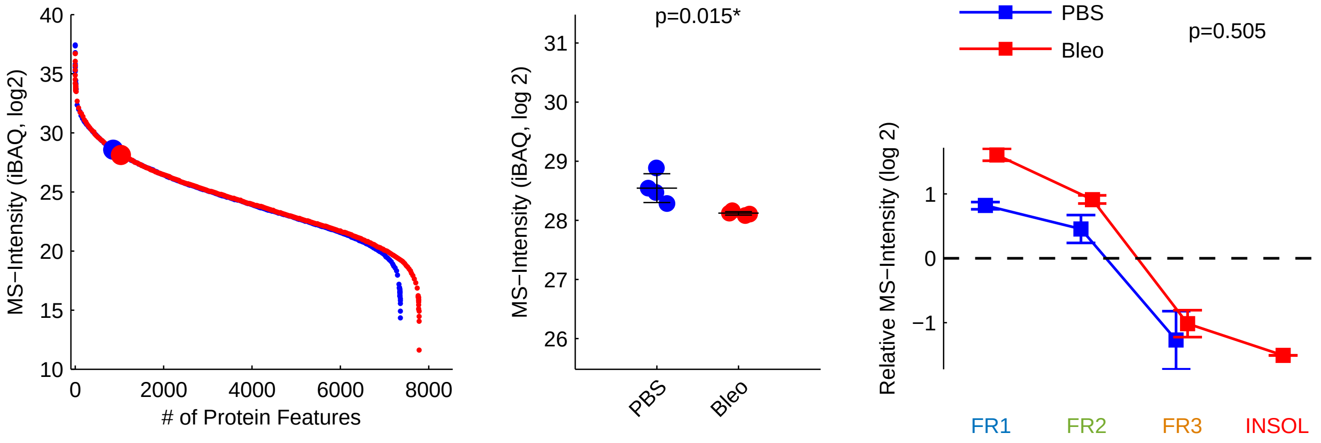

O70456 − Sfn (id: 2292)

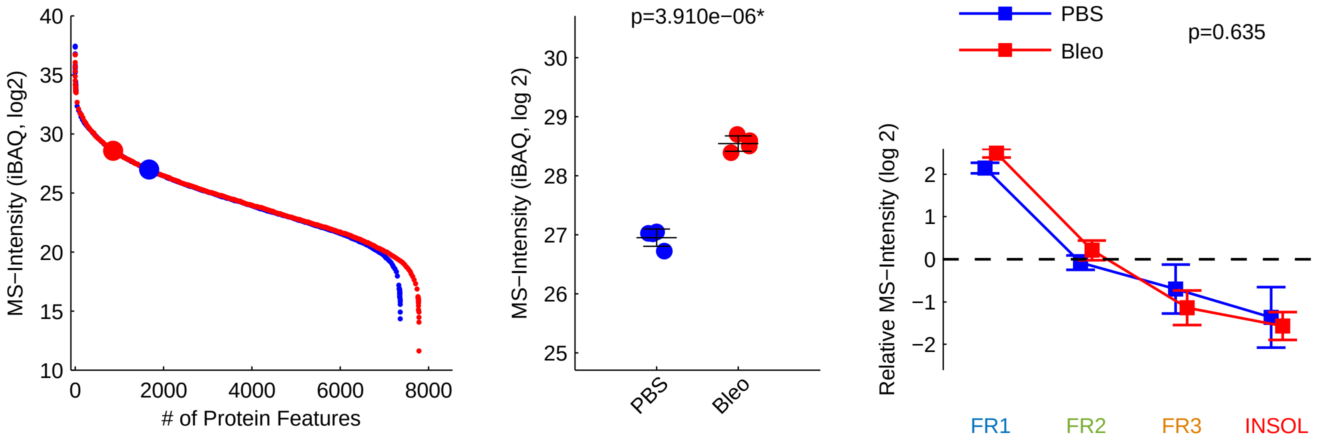

O70475 − Ugdh (id: 2293)

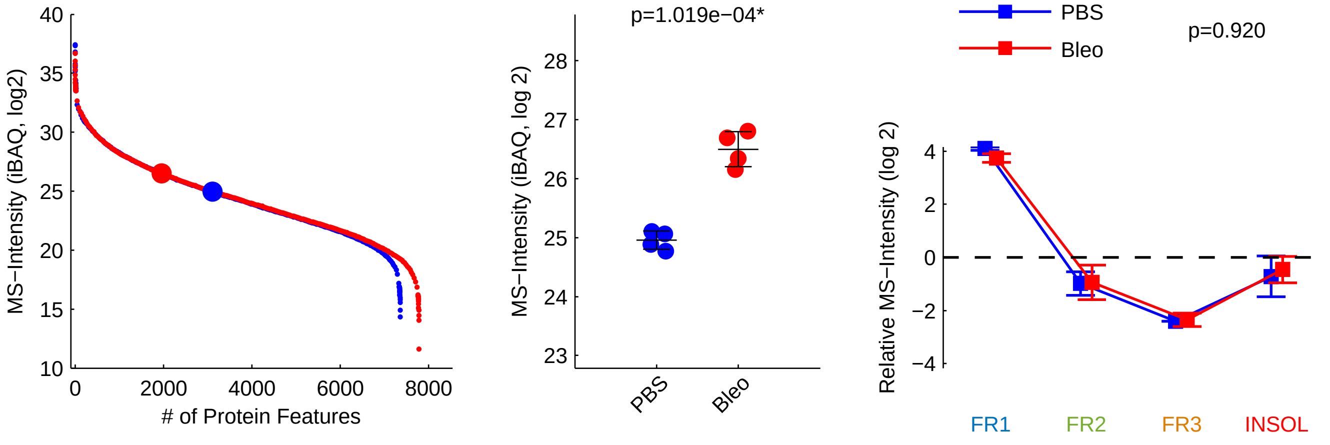

O70503 − Hsd17b12 (id: 2298)

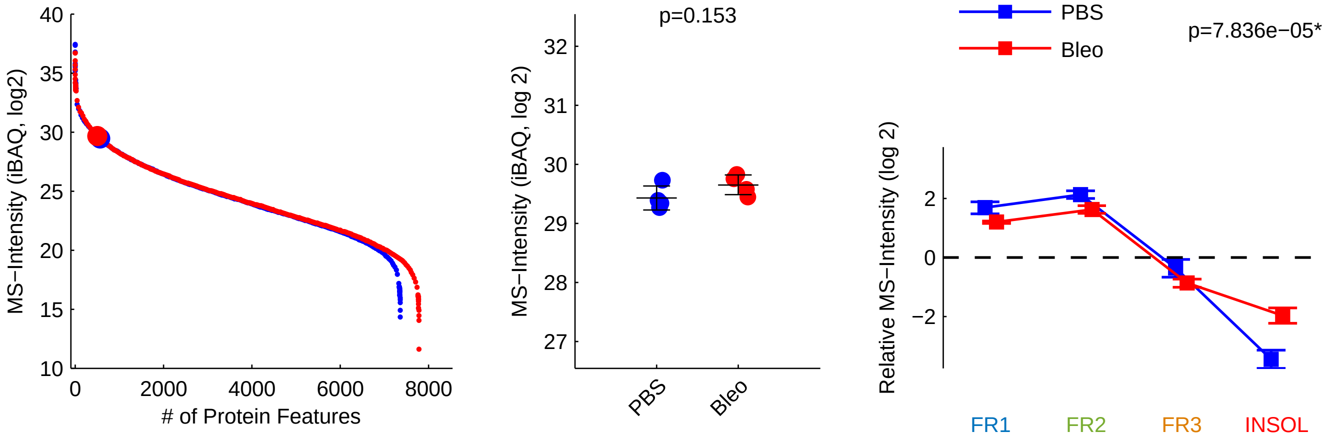

O70551 − Srpk1 (id: 2301)

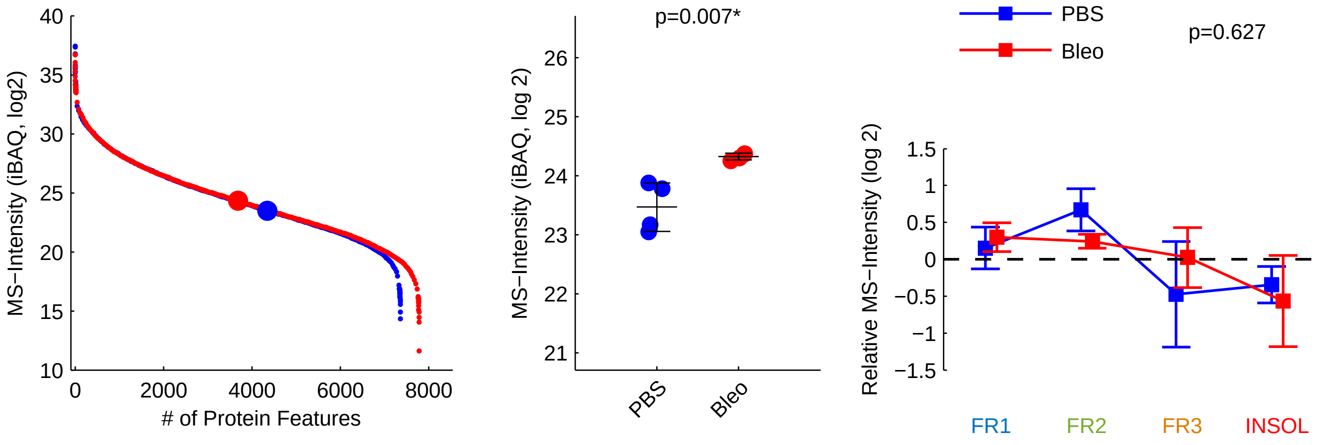

O70589 − Cask (id: 2309)

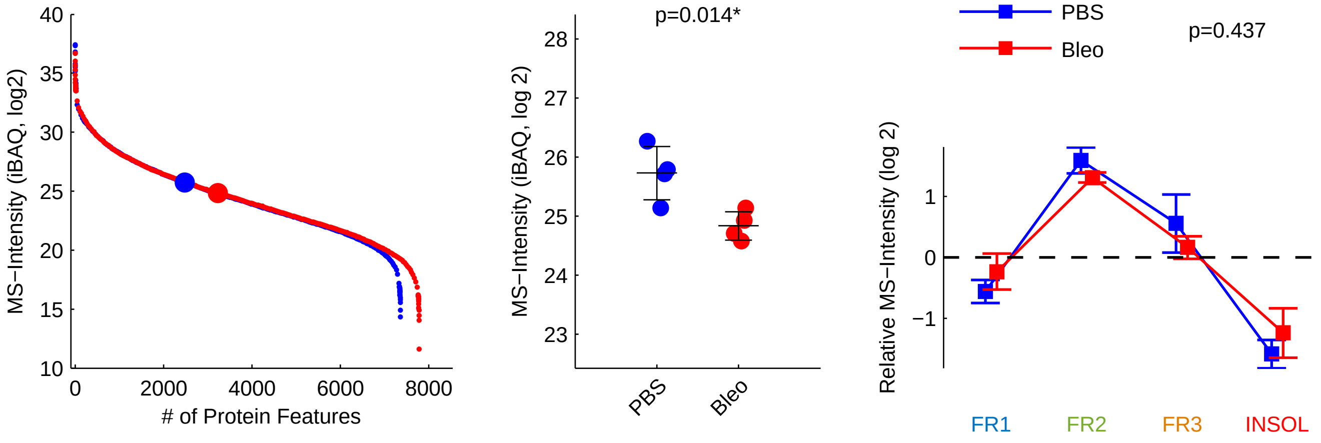

O88207 − Col5a1 (id: 2314)

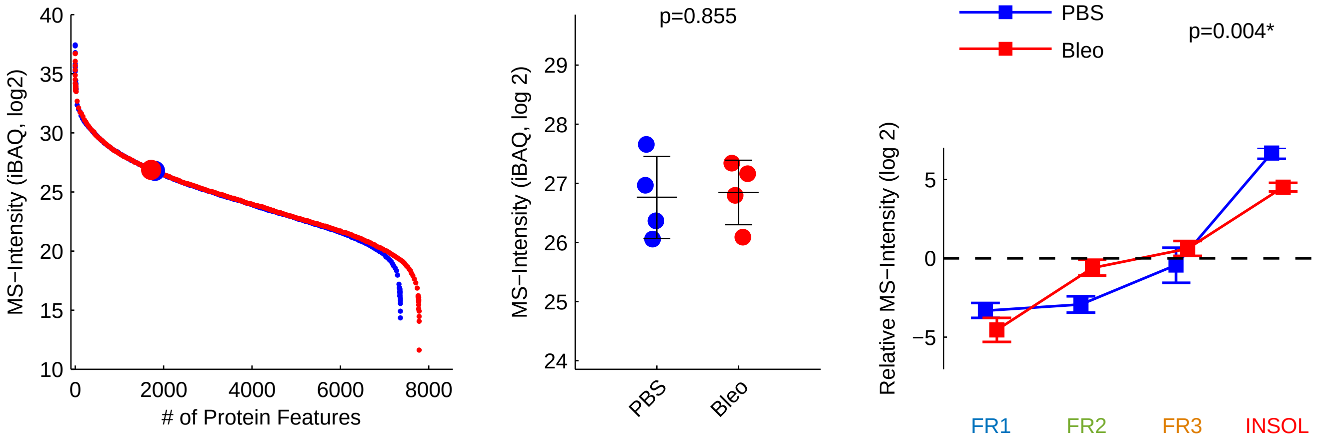

O88325 − Naglu (id: 2321)

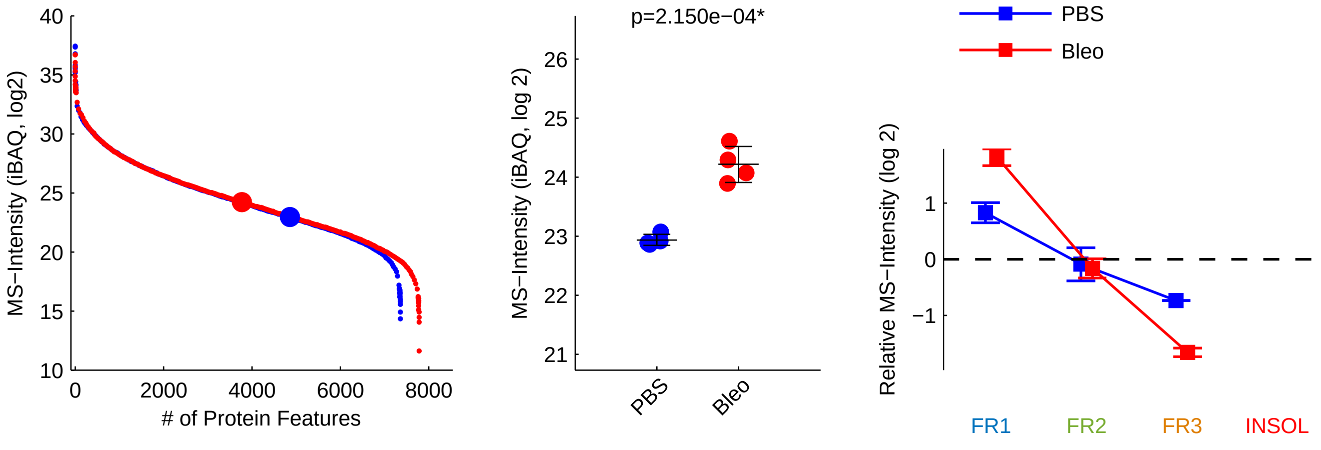

O88327 − Ctnnal1 (id: 2322)

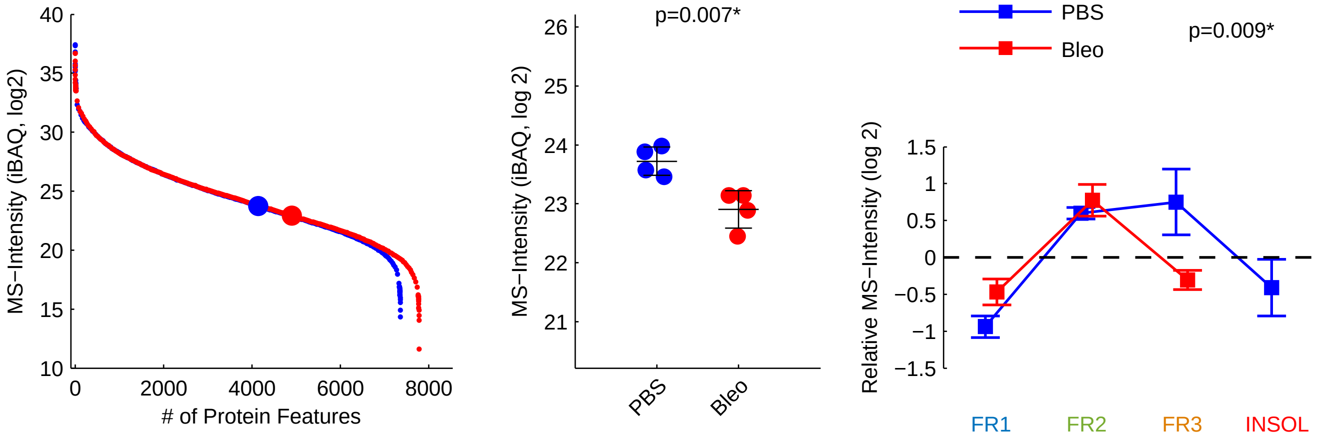

O88487 − Dync1i2 (id: 2333)

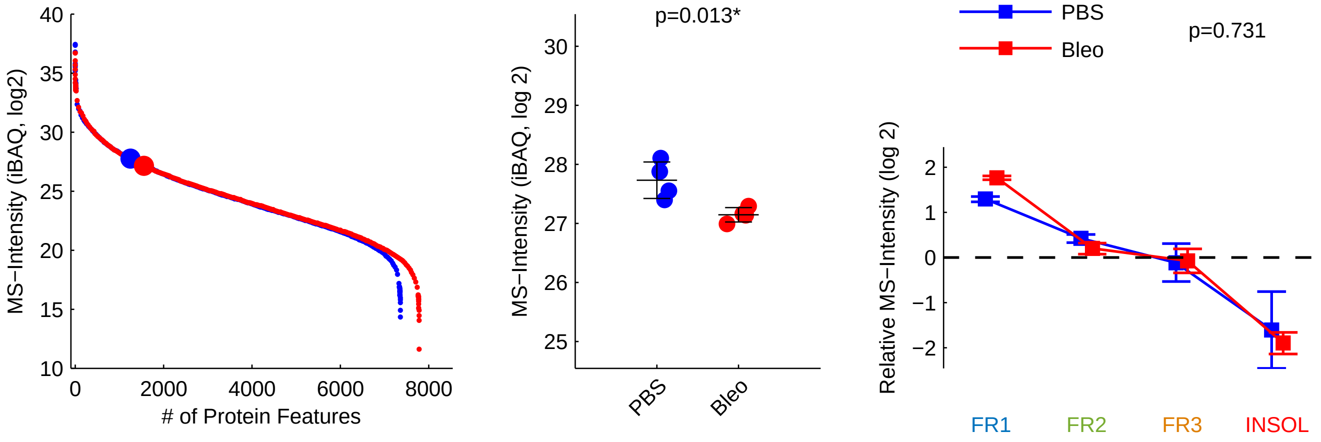

O88520 − Shoc2 (id: 2339)

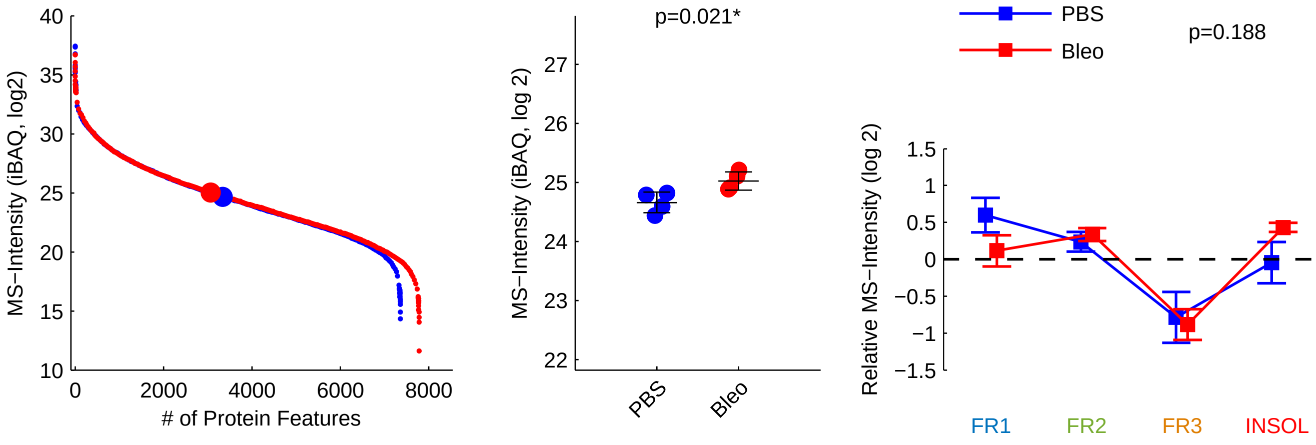

O88531 − Ppt1 (id: 2340)

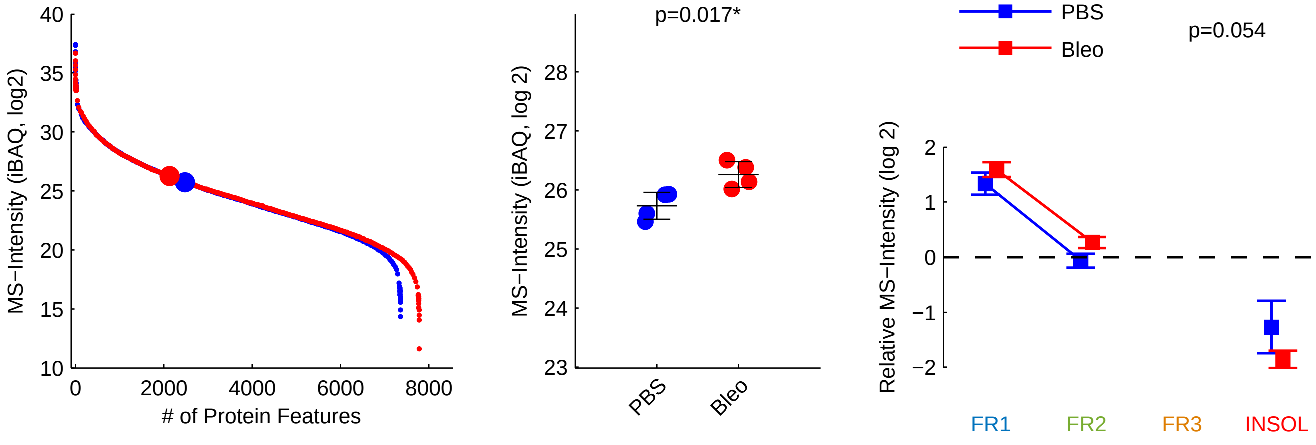

O88569 − Hnrnpa2b1 (id: 2346)

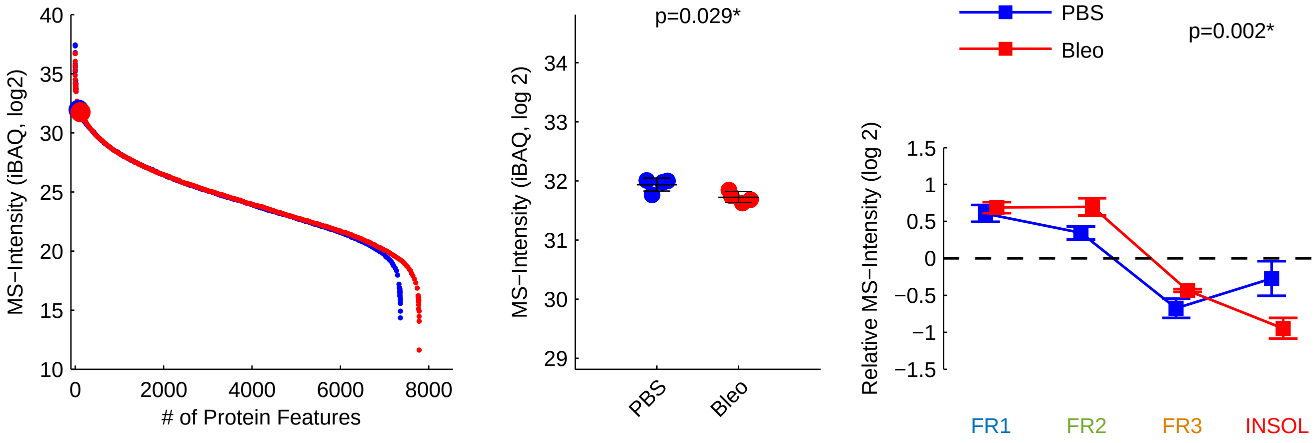

O88597 − Becn1 (id: 2350)

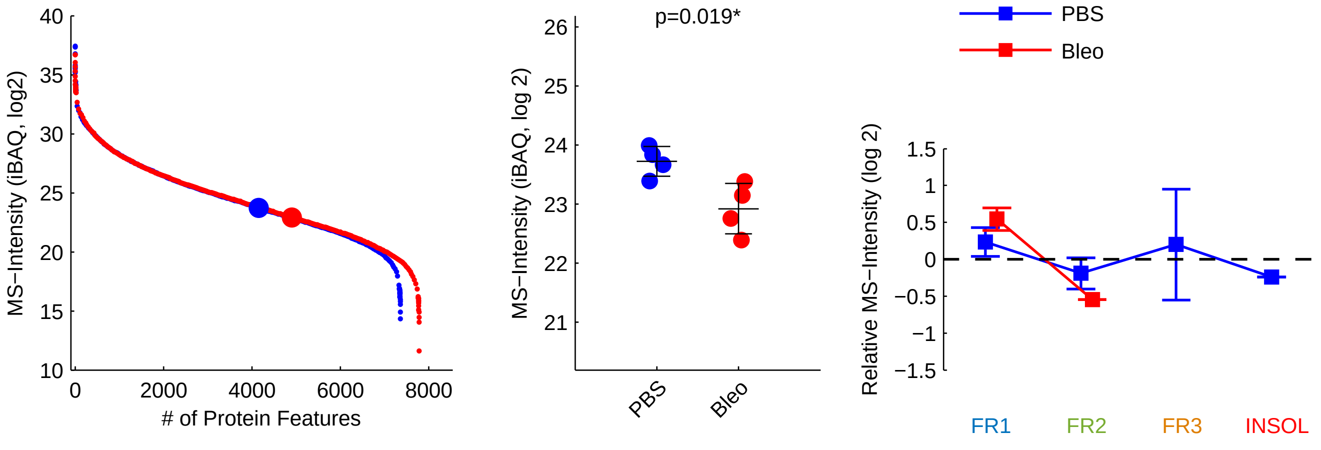

O88685 − Psmc3 (id: 2359)

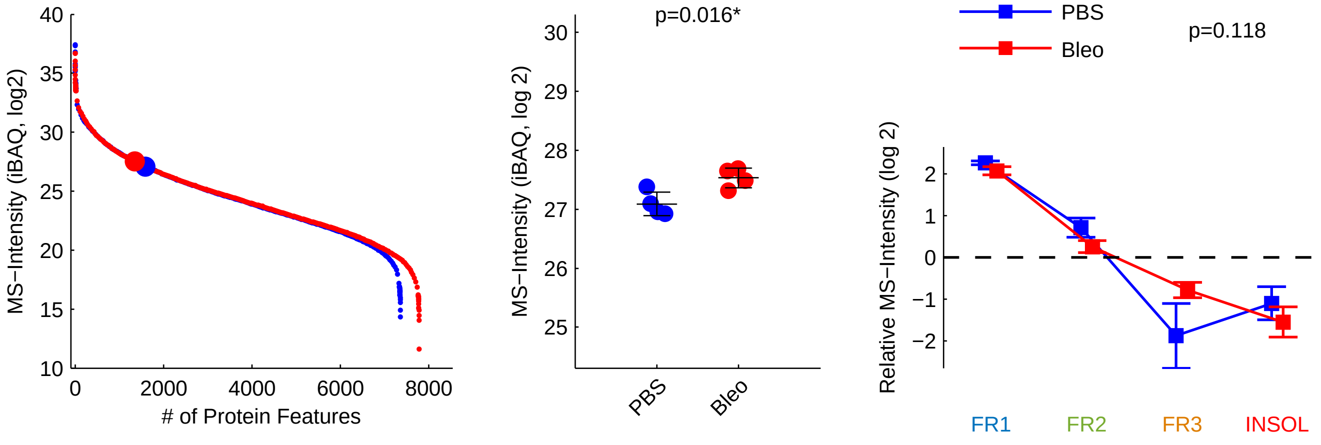

O88696 − Clpp (id: 2360)

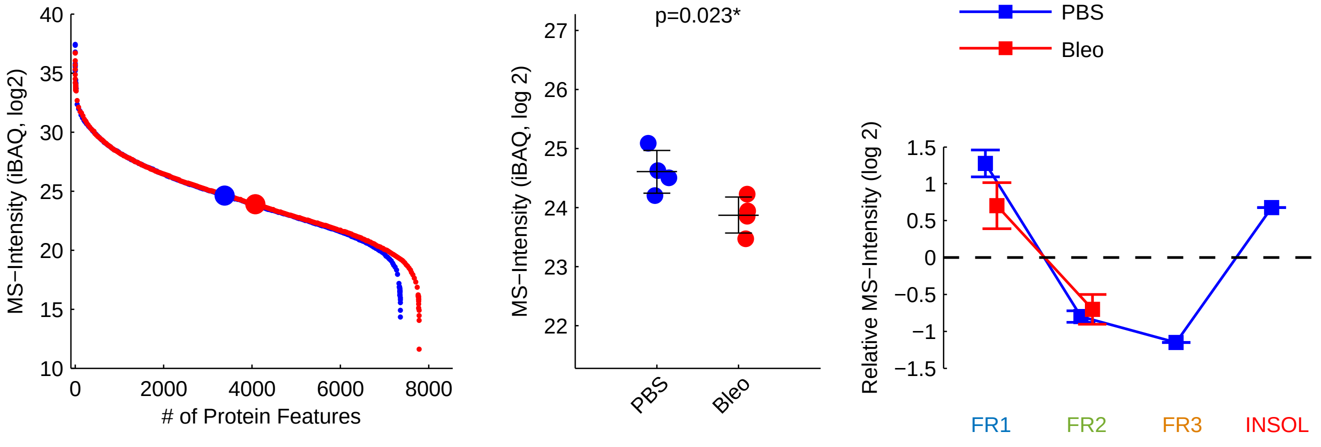

O88792 − F11r (id: 2367)

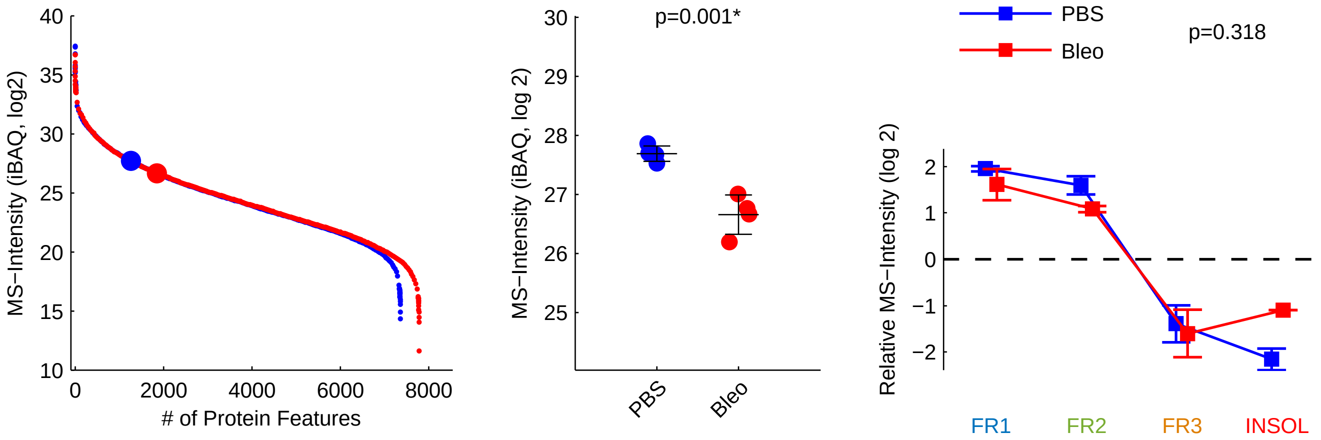

O88845 − Akap10 (id: 2373)

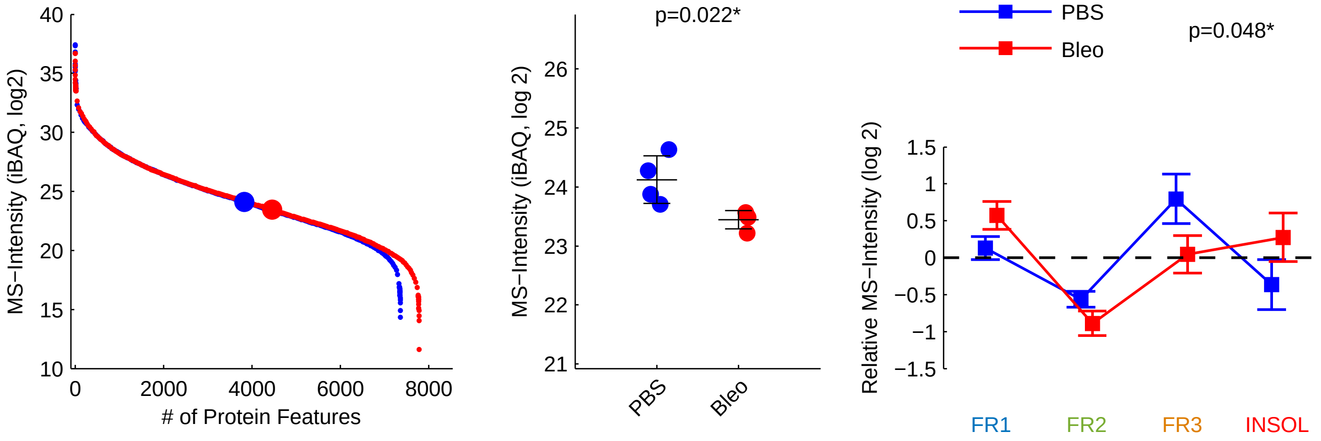

O88851 − Rbbp9 (id: 2374)

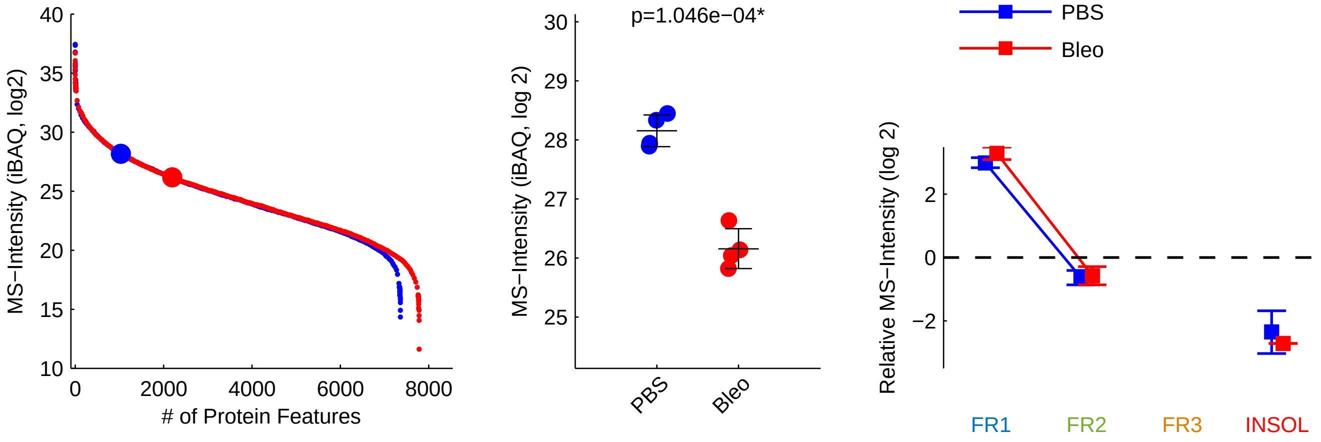

O88856 − Tpst2 (id: 2376)

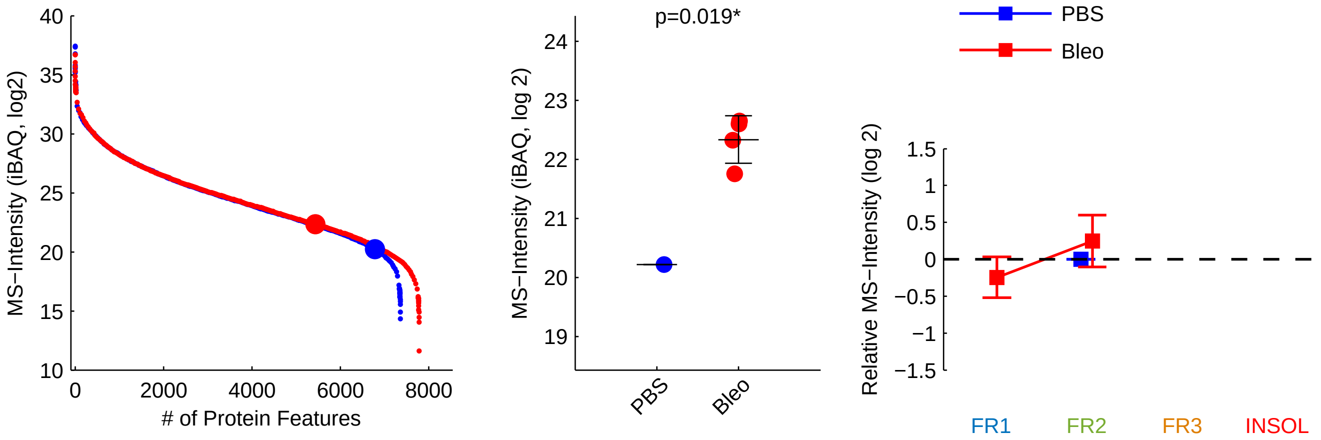

O88952 − Lin7c (id: 2386)

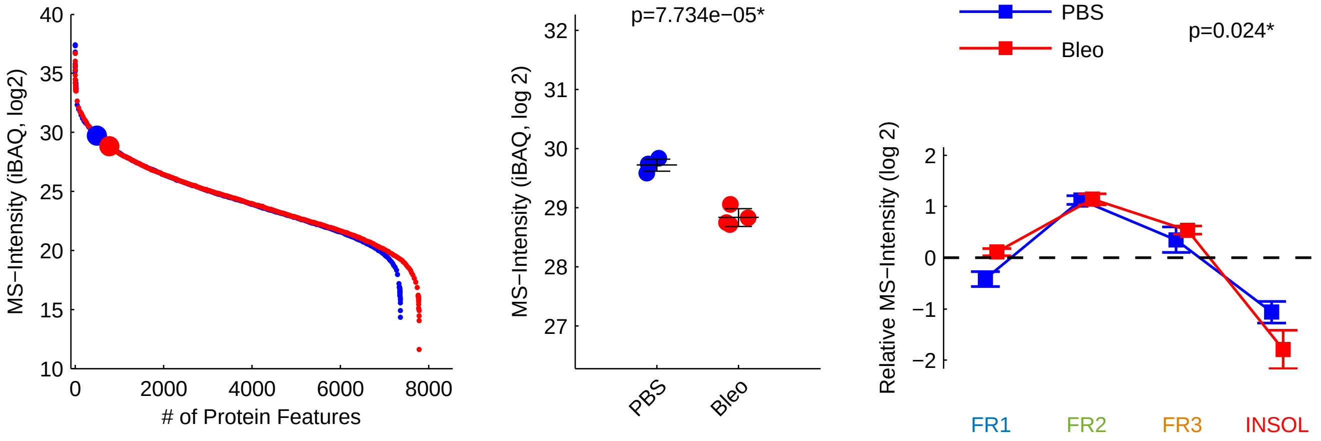

O88958 − Gnpda1 (id: 2387)

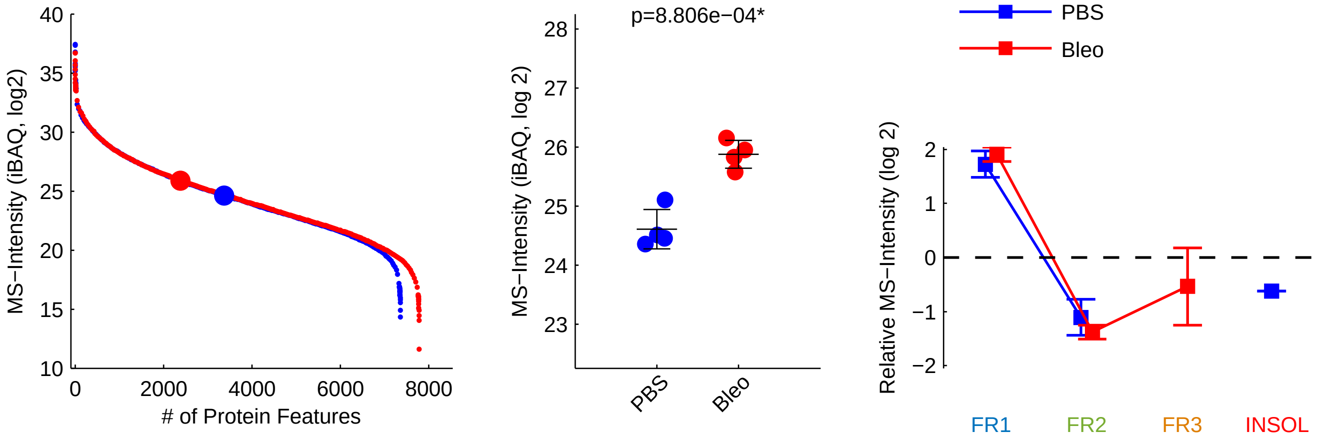

O88986 − Gcat (id: 2391)

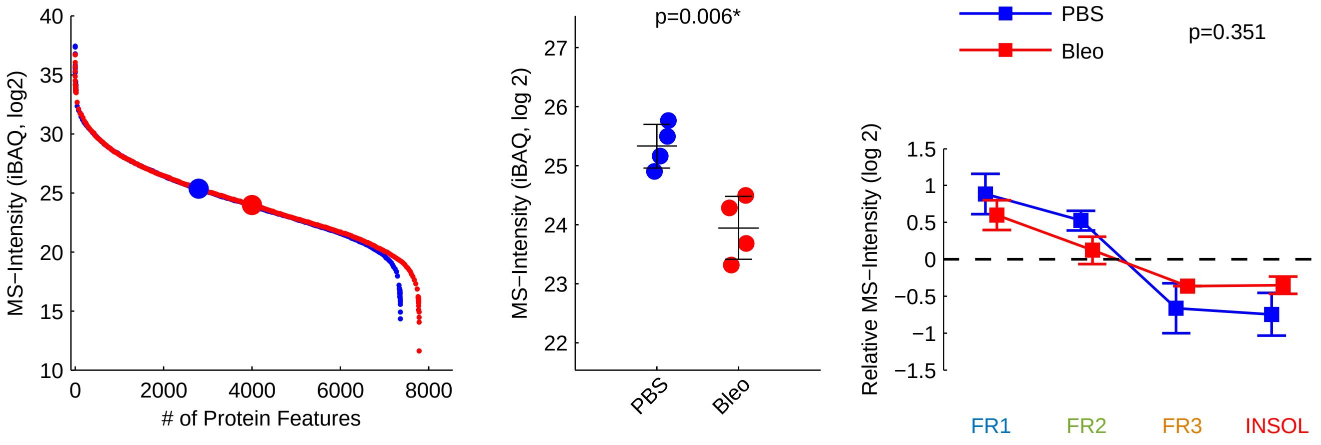

O89017 − Lgmn (id: 2394)

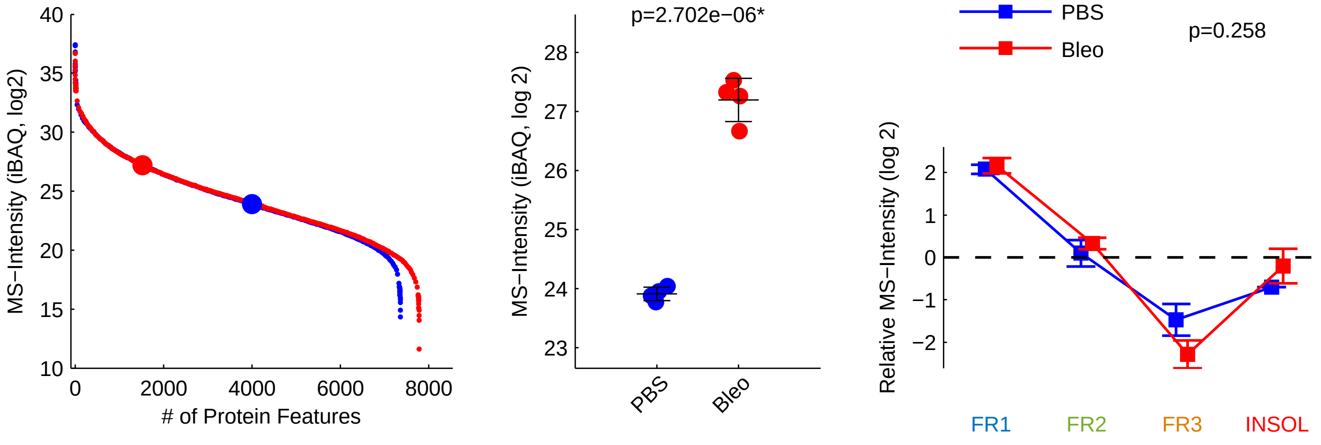

O89020 − Afm (id: 2395)

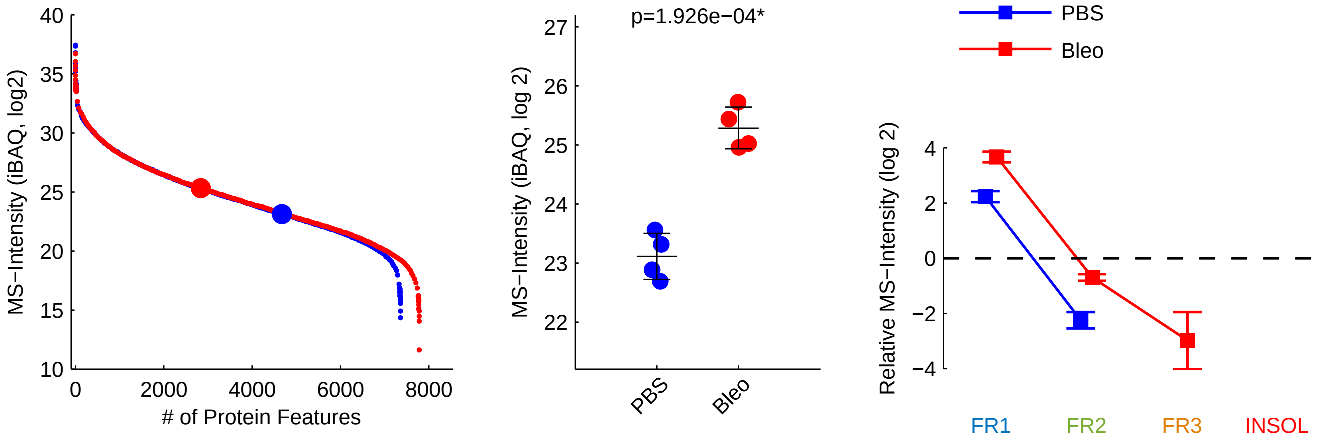

O89023 − Tpp1 (id: 2396)

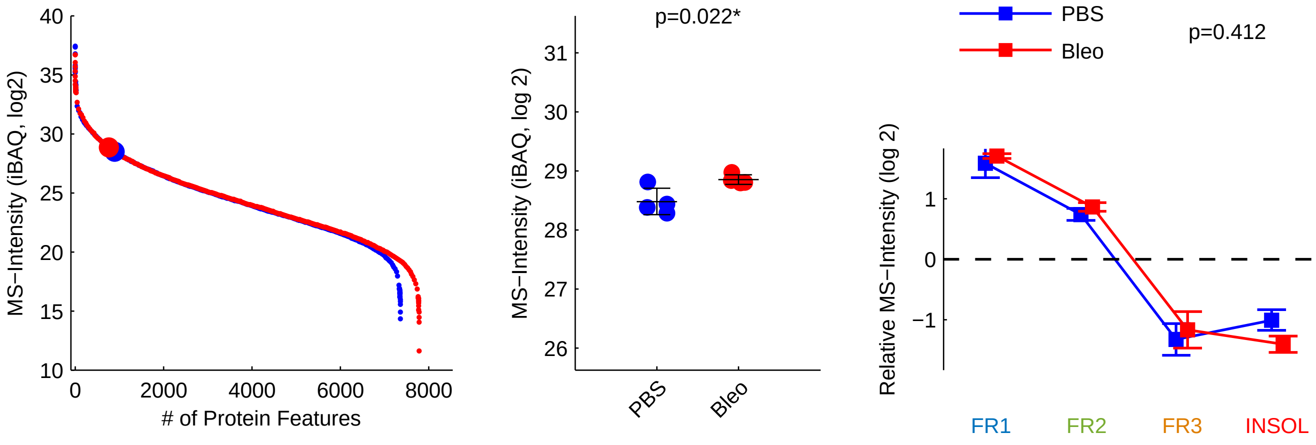

O89051 − Itm2b (id: 2399)

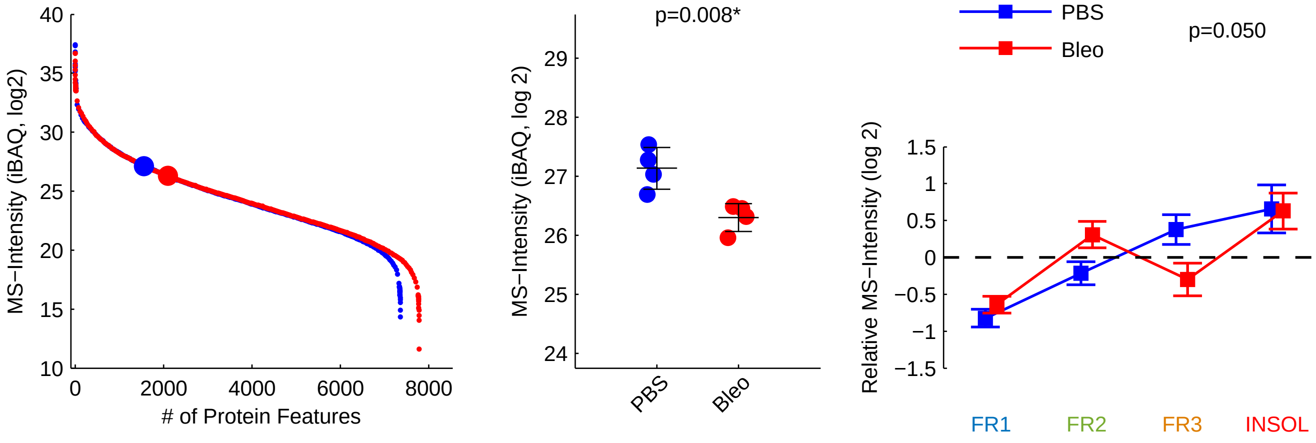

O89086 − Rbm3 (id: 2402)

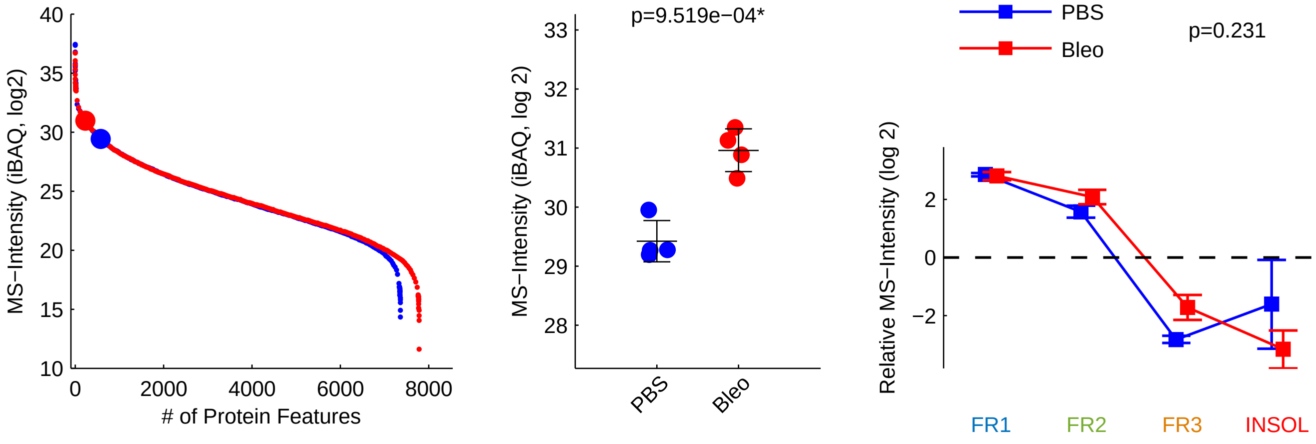

O89103 − Cd93 (id: 2404)

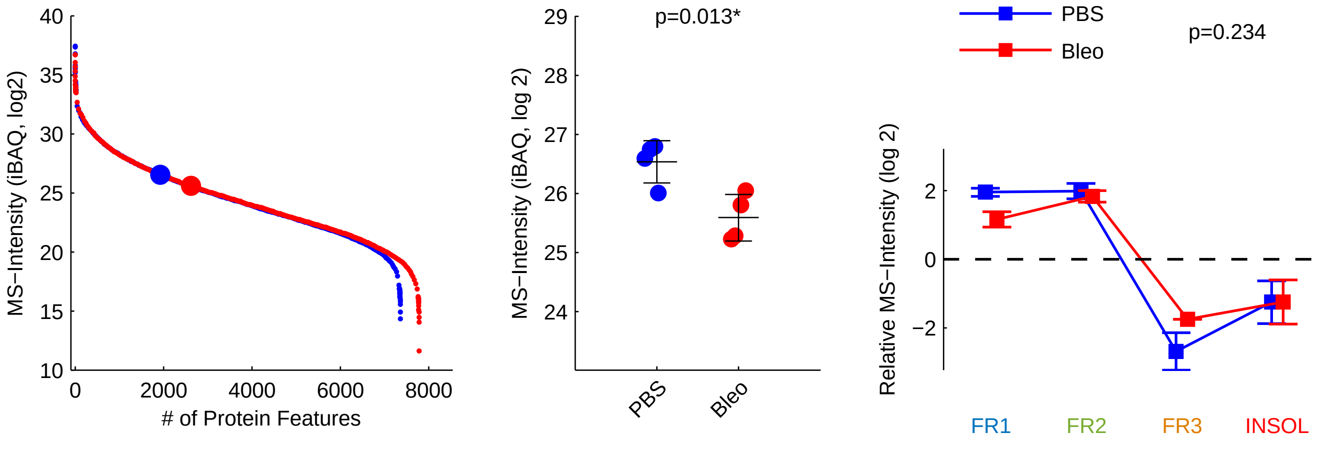

O89112 − Lancl1 (id: 2406)

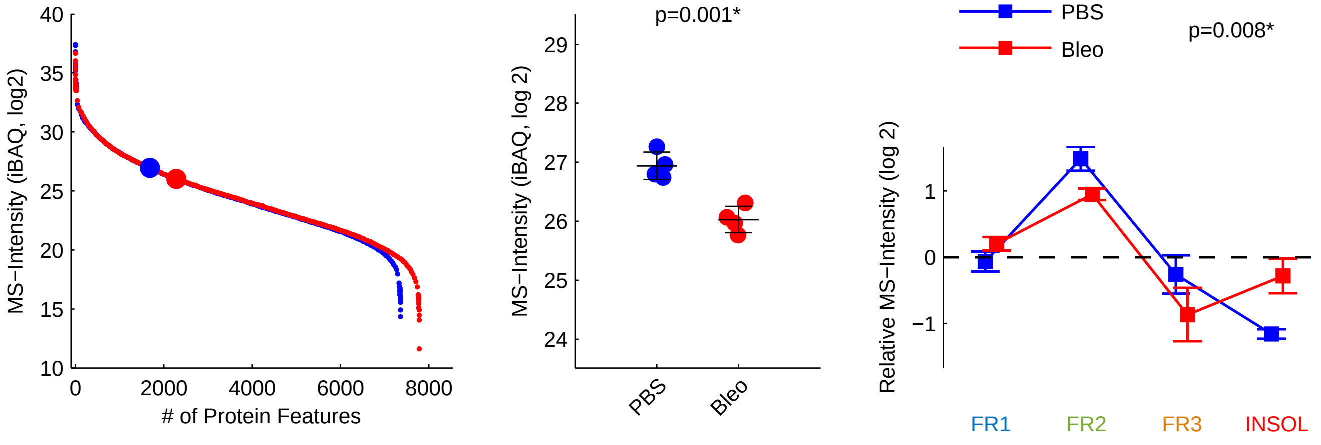

P00493 − Hprt1 (id: 2412)

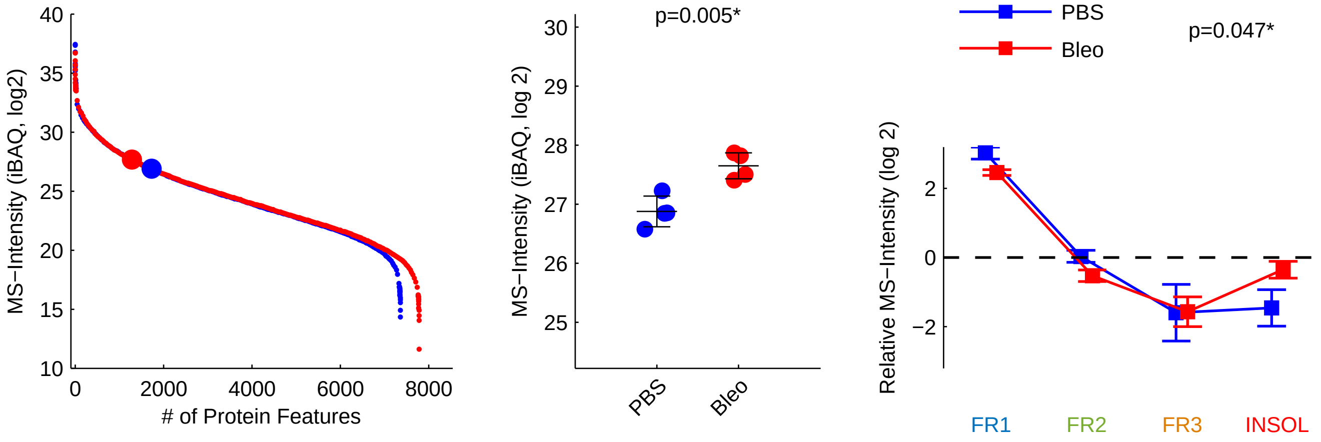

P01027 − C3 (id: 2417)

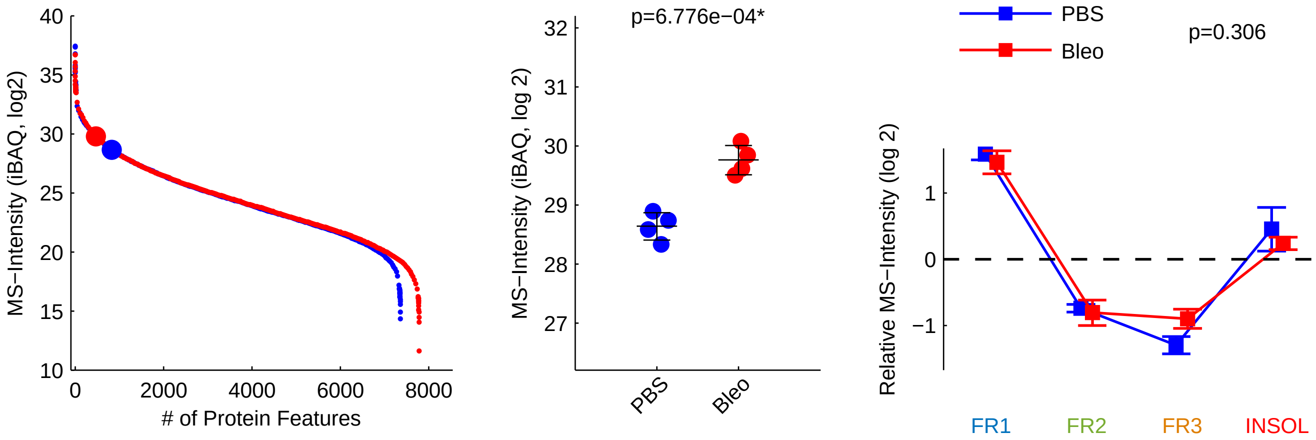

P01029 − C4b (id: 2418)

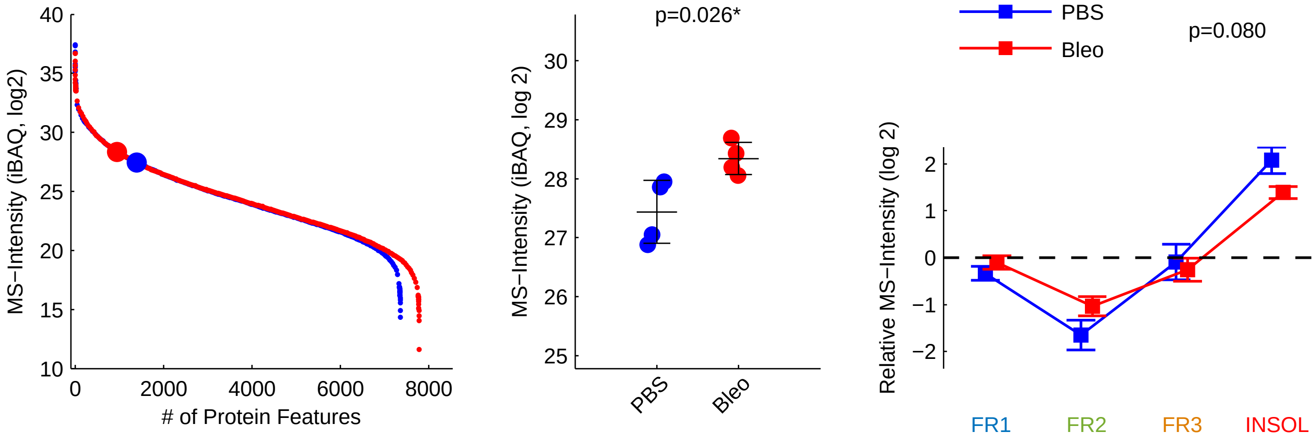

P01646 − (id: 2430)

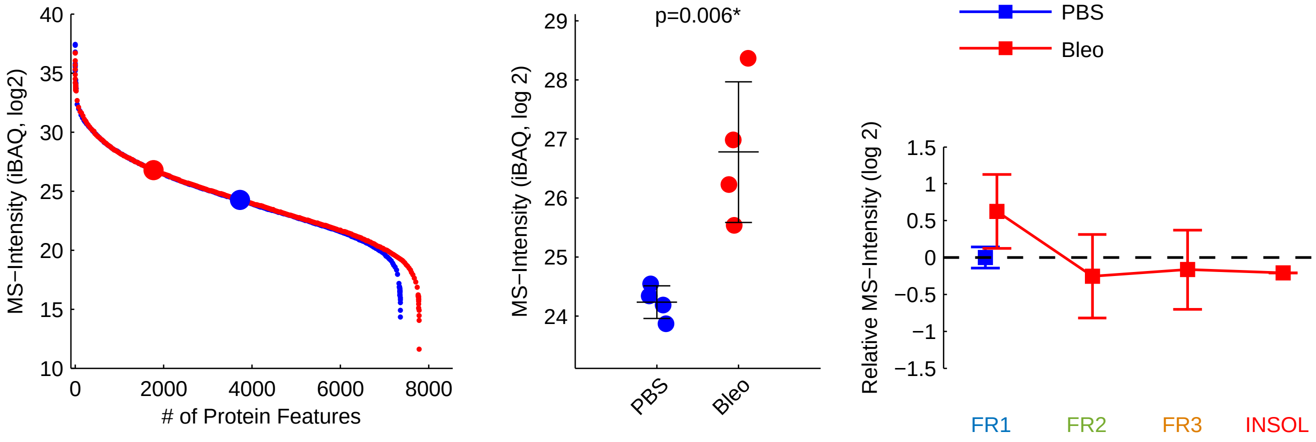

P01654 − (id: 2432)

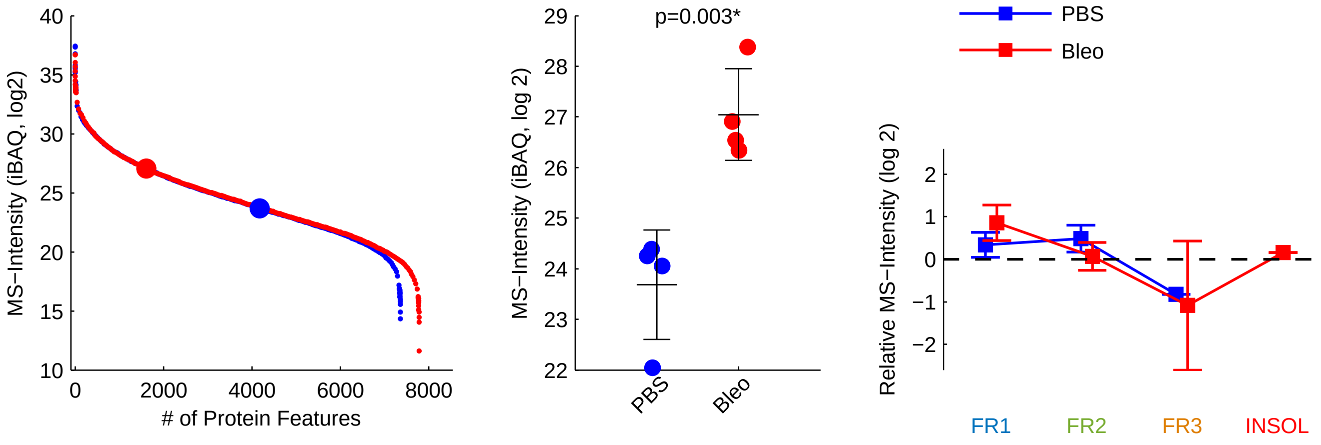

P01898 − H2−Q10 (id: 2458)

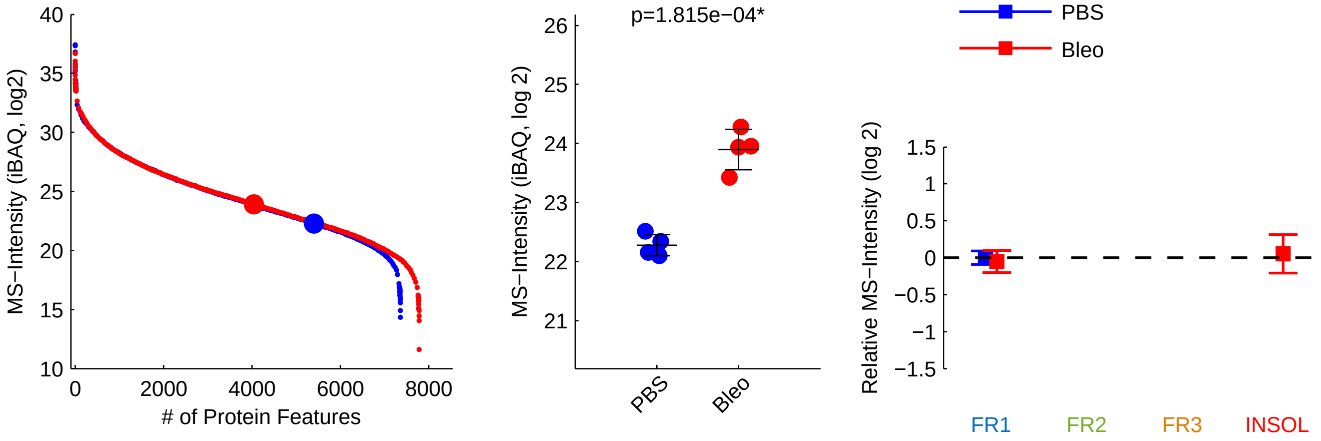

P01899 − H2−D1 (id: 2459)

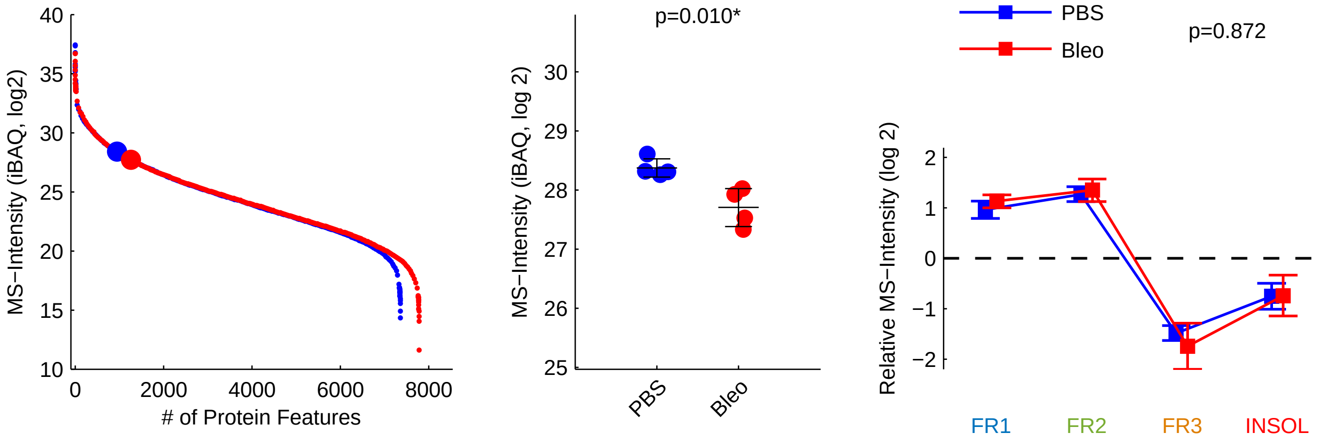

P02463 − Col4a1 (id: 2464)

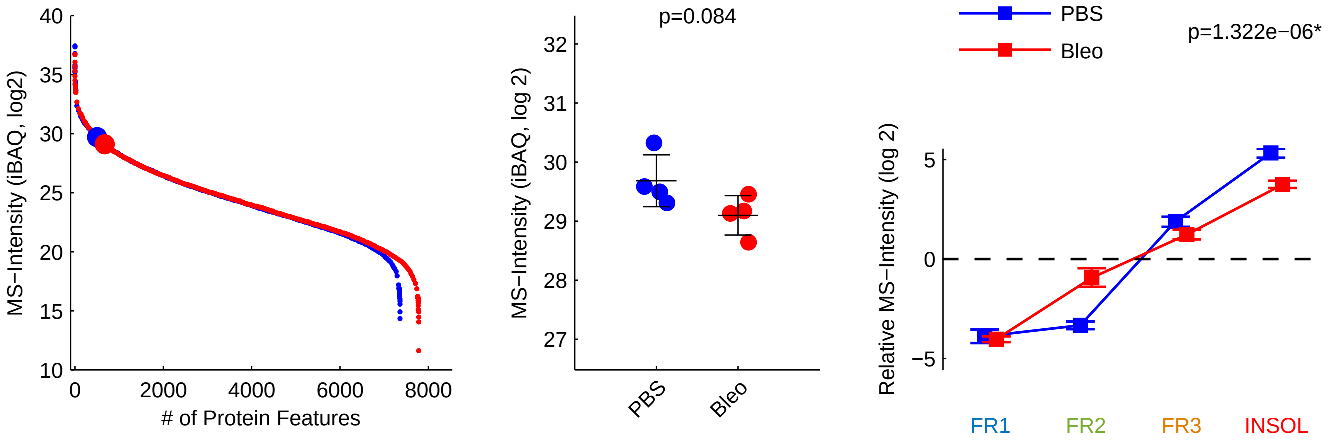

P03987−2 − (id: 2474)

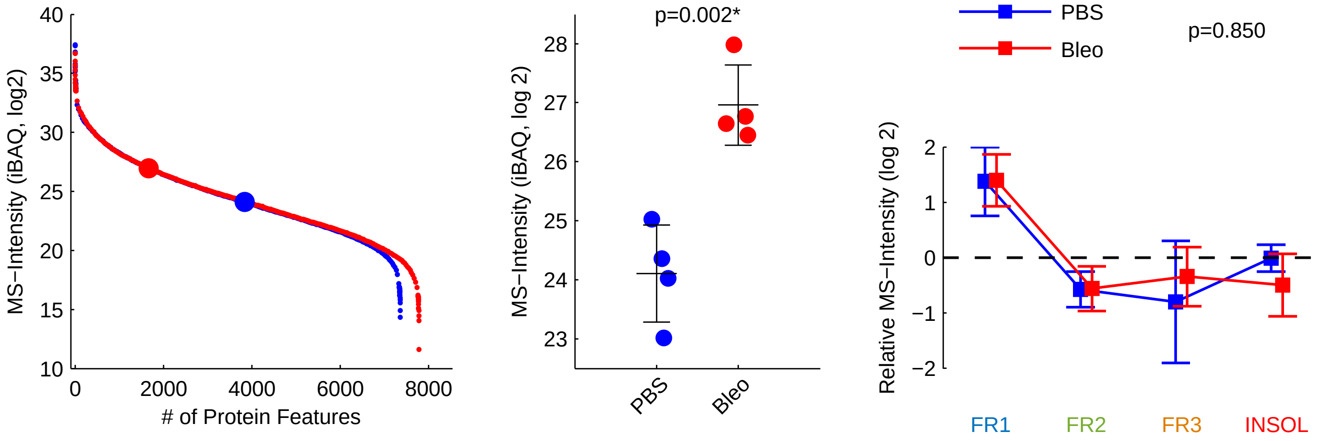

P04627 − Araf (id: 2487)

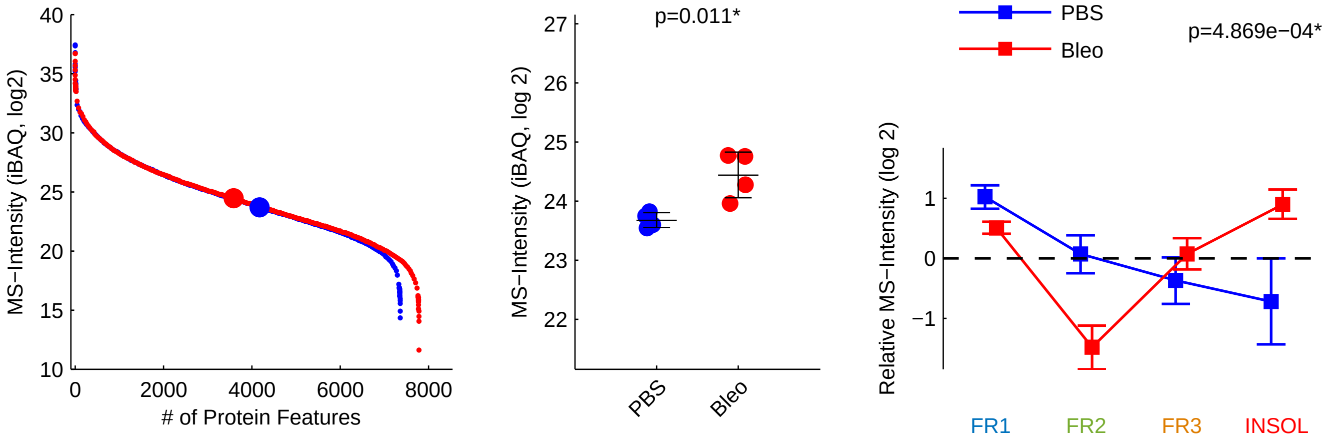

P05480 − Src (id: 2502)

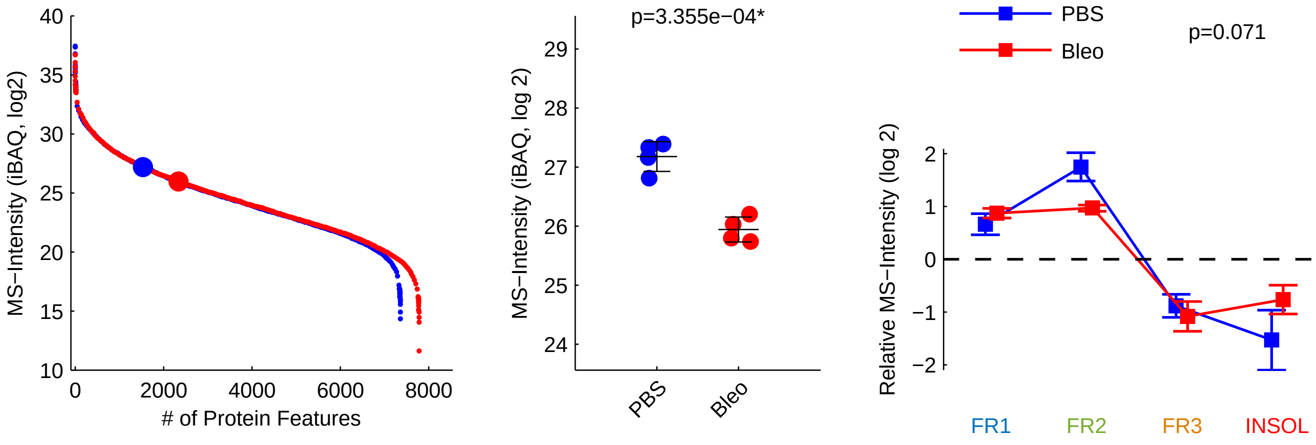

P06330 − (id: 2508)

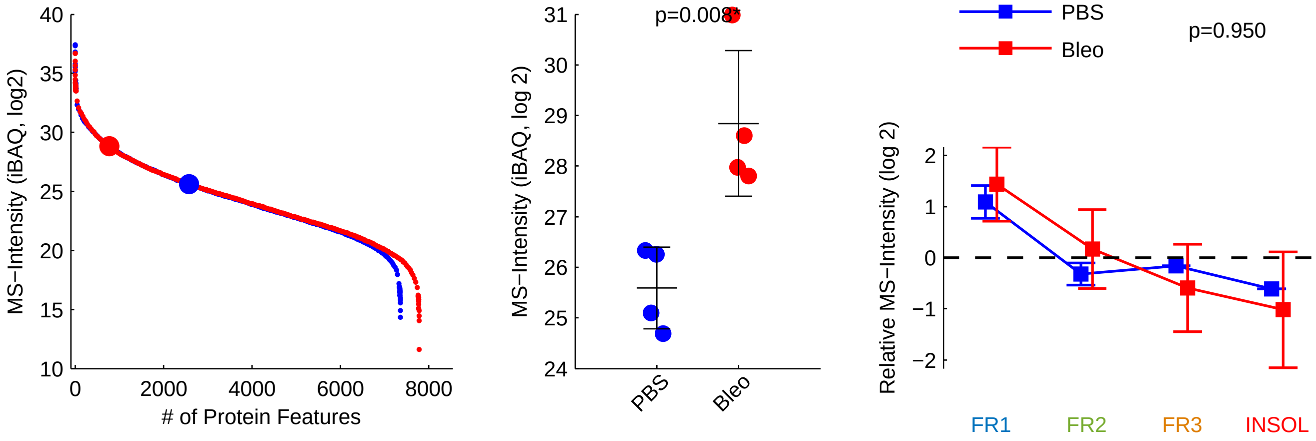

P06728 − Apoa4 (id: 2511)

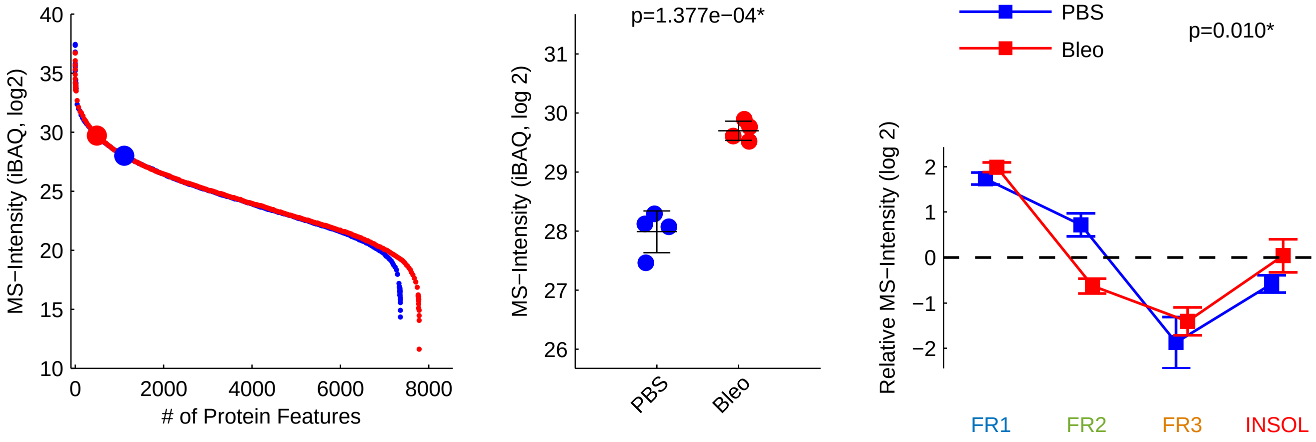

P07091 − S100a4 (id: 2519)

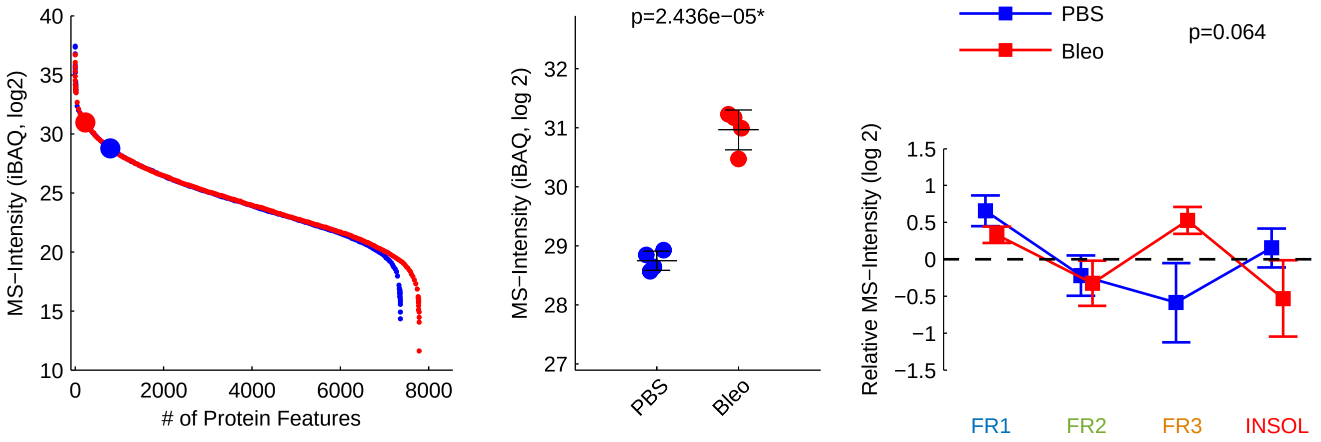

Q5NCU4 − Sparc (id: 2521)

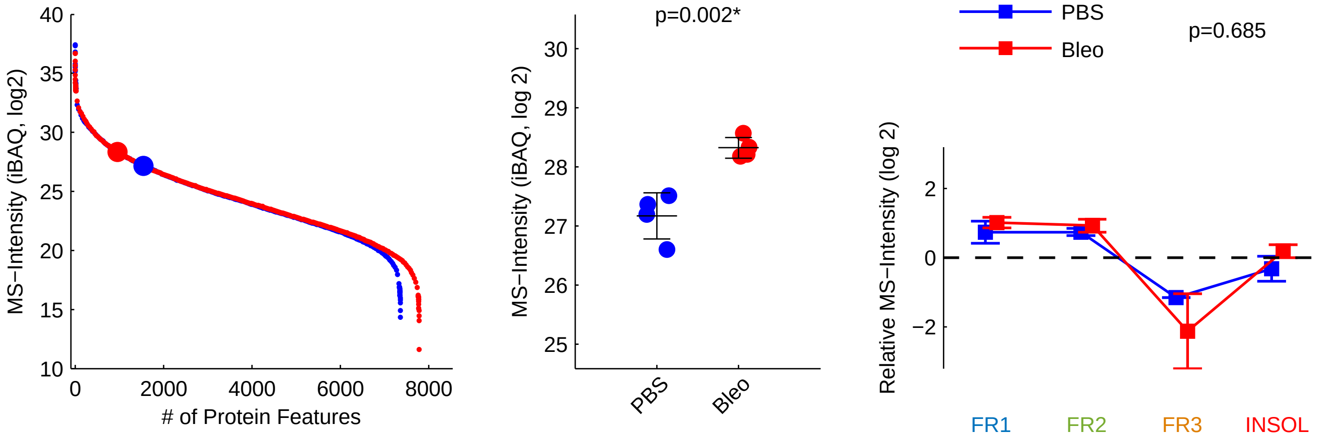

P07309 − Ttr (id: 2522)

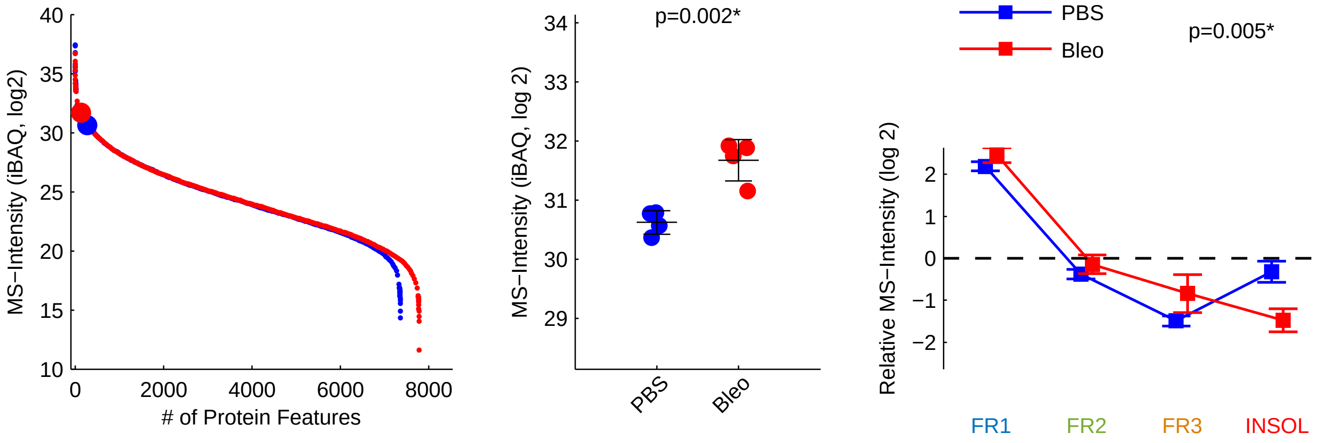

P07724 − Alb (id: 2527)

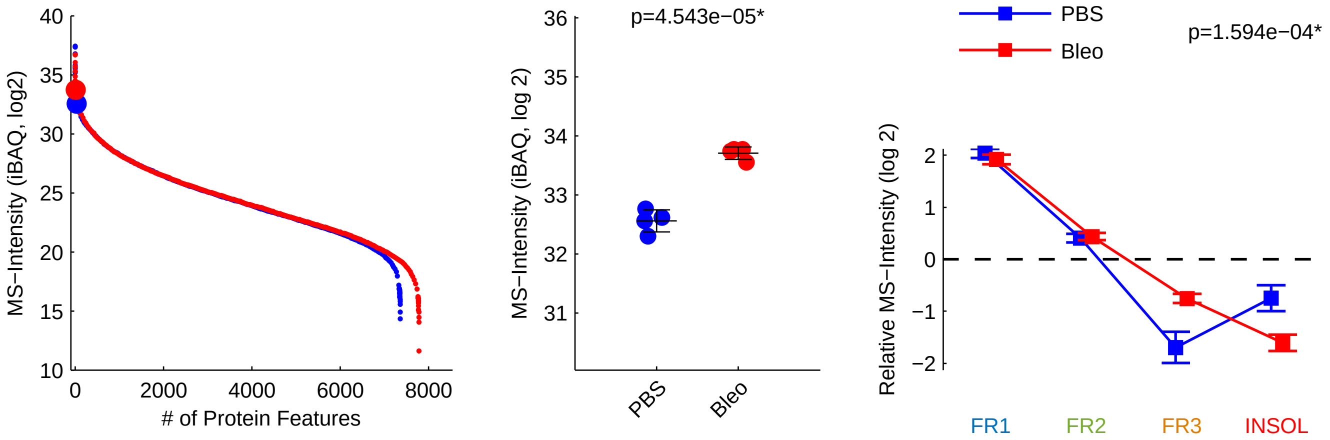

P07758 − Serpina1a (id: 2529)

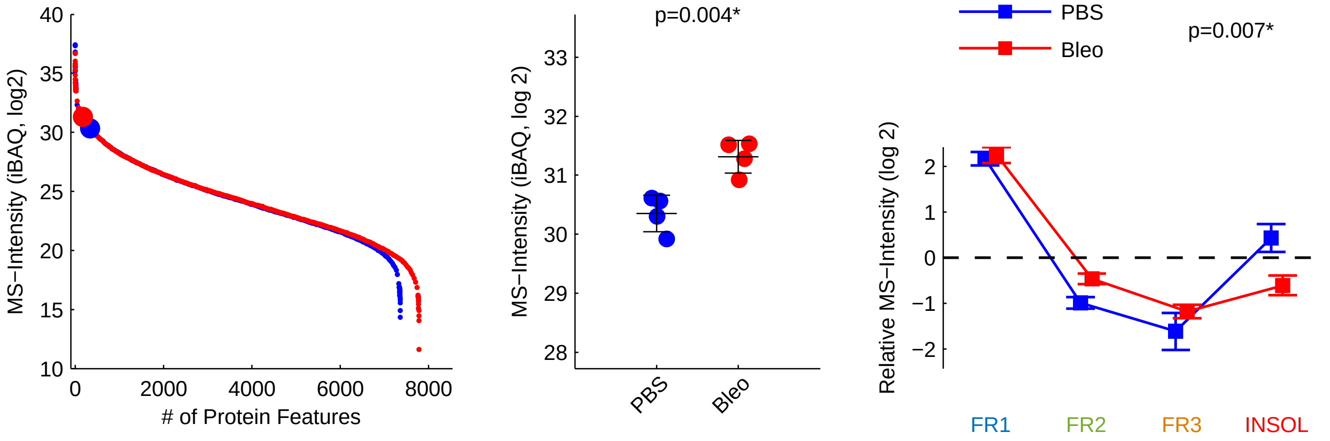

P07759 − Serpina3k (id: 2530)

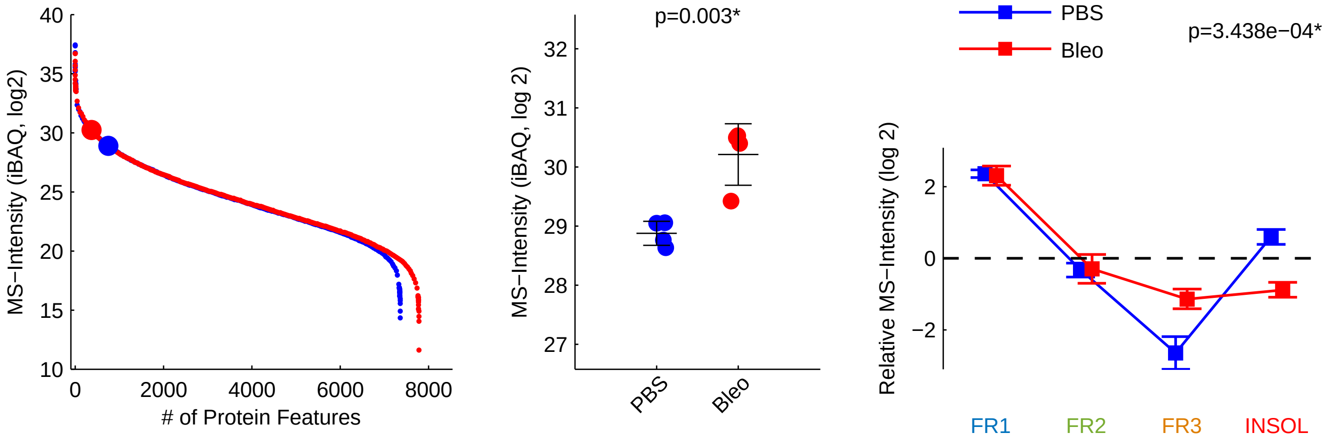

P07901 − Hsp90aa1 (id: 2531)

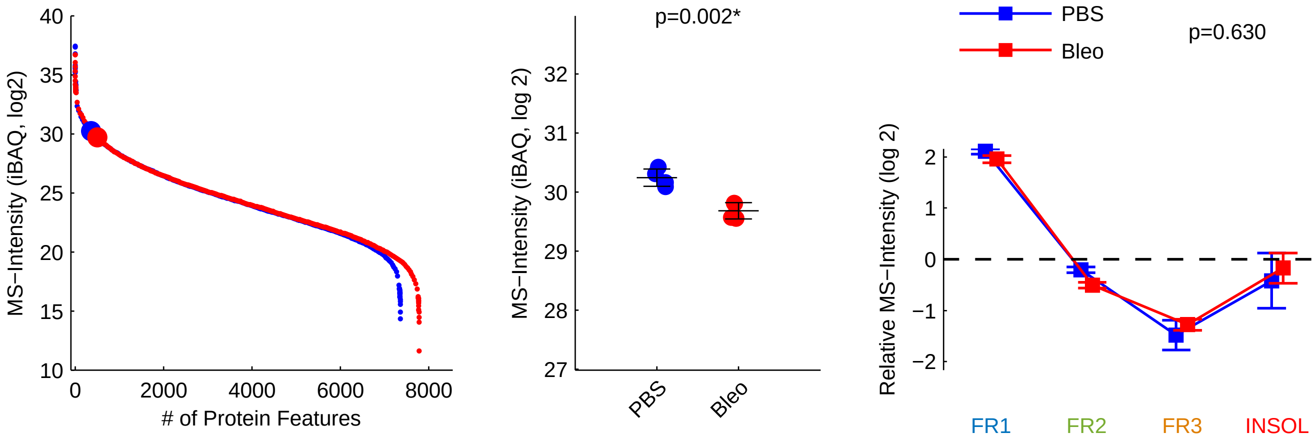

P08030 − Aprt (id: 2534)

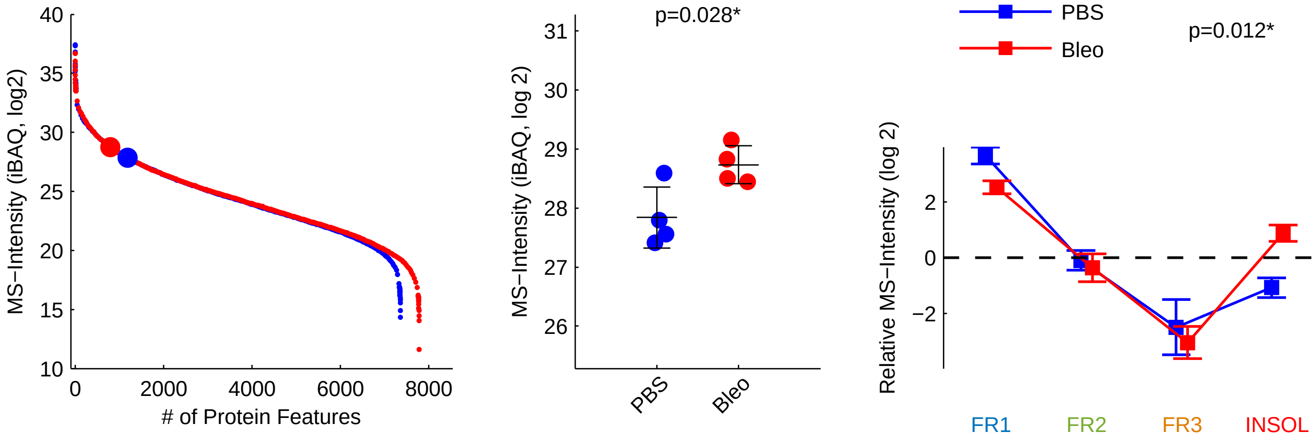

P08032 − Spta1 (id: 2535)

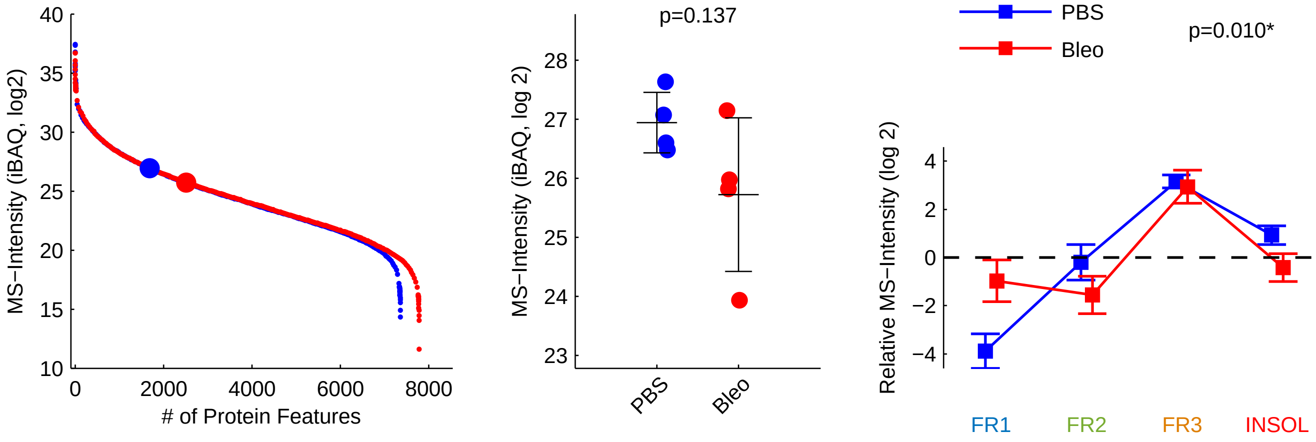

P08074 − Cbr2 (id: 2537)

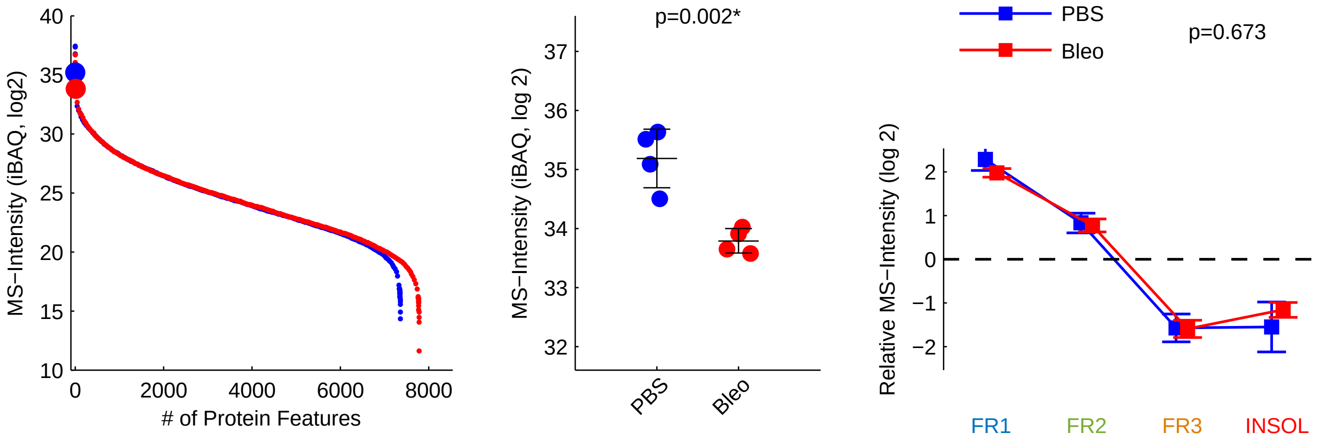

P08101−2 − Fcgr2 (id: 2538)

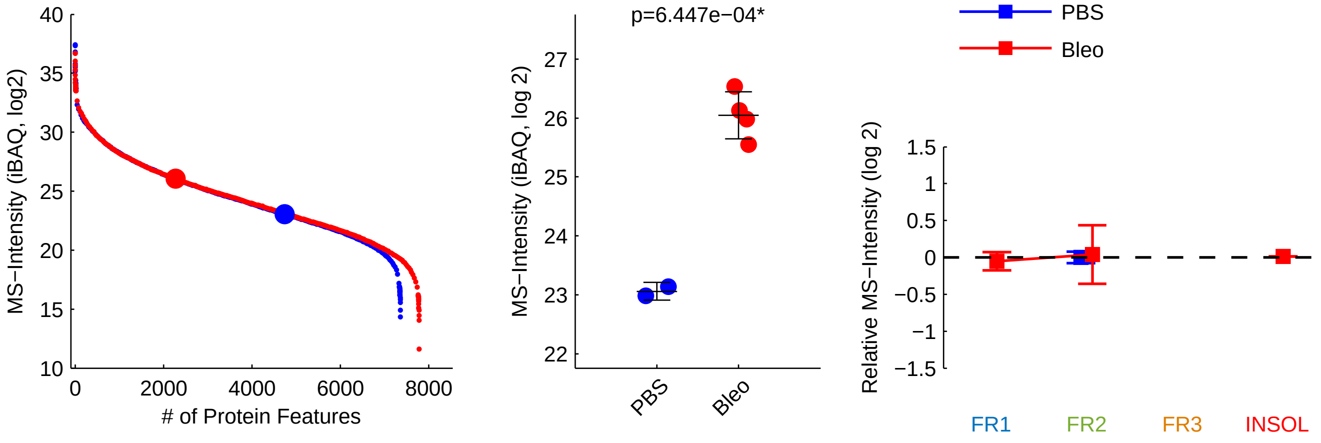

P08121 − Col3a1 (id: 2540)

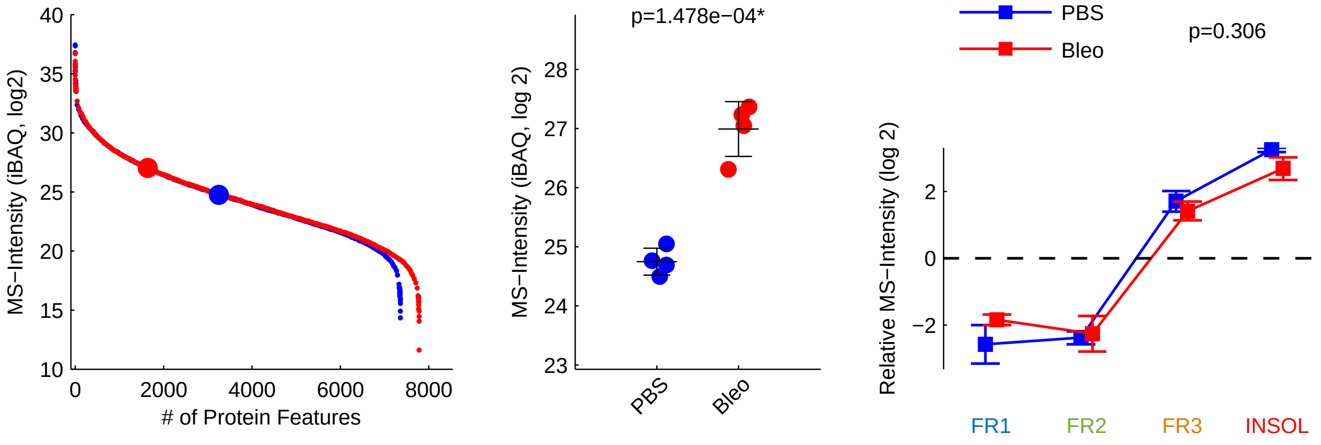

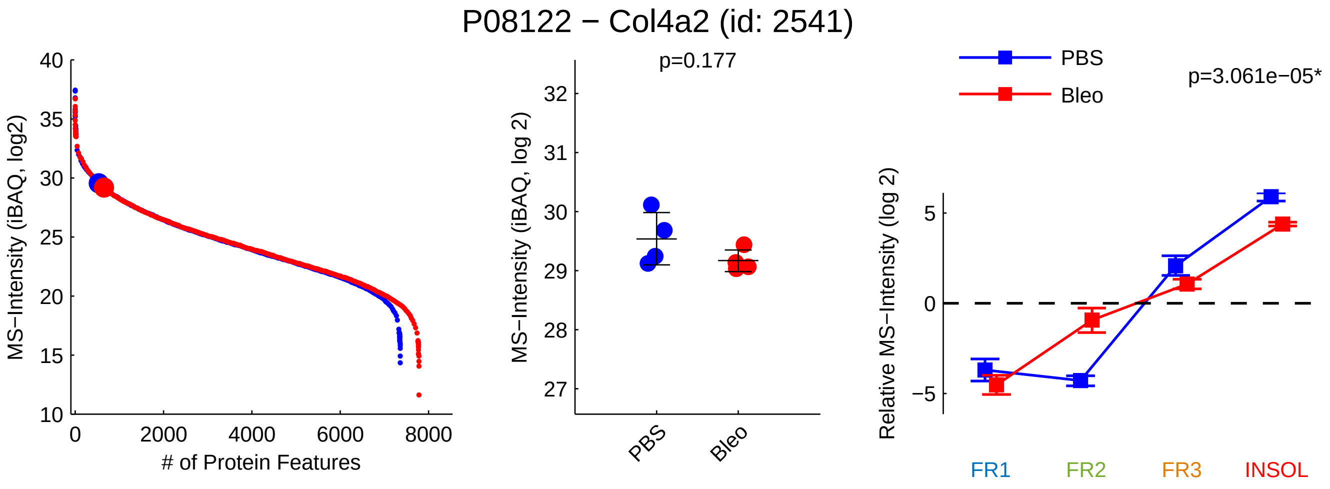

P08122 − Col4a2 (id: 2541)
MS−Intensity (iBAQ, log2)
# of Protein Features
p=0.177
MS−Intensity (iBAQ, log 2)
PBS
Bleo
p=3.061e−05*
Relative MS−Intensity (log 2)
FR1
FR2
FR3
INSOL

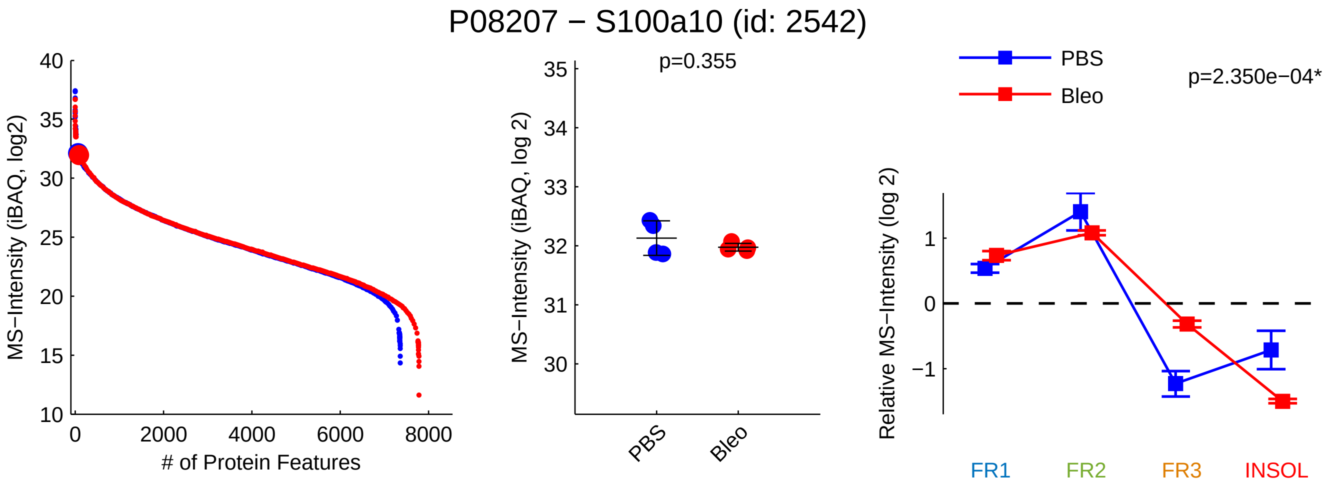

P08207 − S100a10 (id: 2542)
MS−Intensity (iBAQ, log2)
# of Protein Features
p=0.355
MS−Intensity (iBAQ, log 2)
PBS
Bleo
p=2.350e−04*
Relative MS−Intensity (log 2)
FR1
FR2
FR3
INSOL

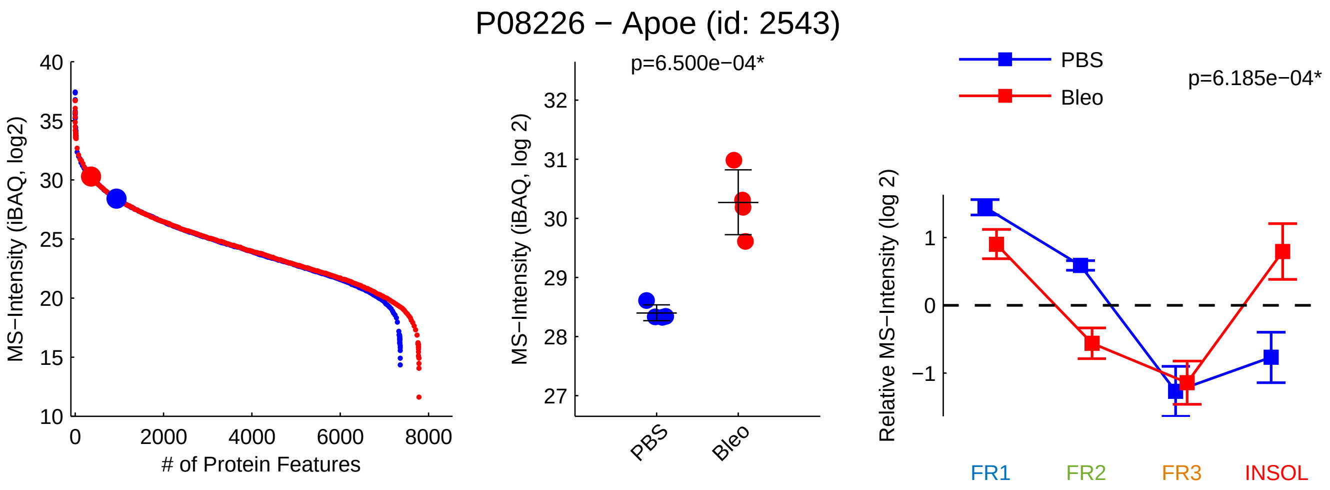

P08226 − Apoe (id: 2543)
MS−Intensity (iBAQ, log2)
# of Protein Features
p=6.500e−04*
MS−Intensity (iBAQ, log 2)
PBS
Bleo
p=6.185e−04*
Relative MS−Intensity (log 2)
FR1
FR2
FR3
INSOL

P08228 − Sod1 (id: 2544)

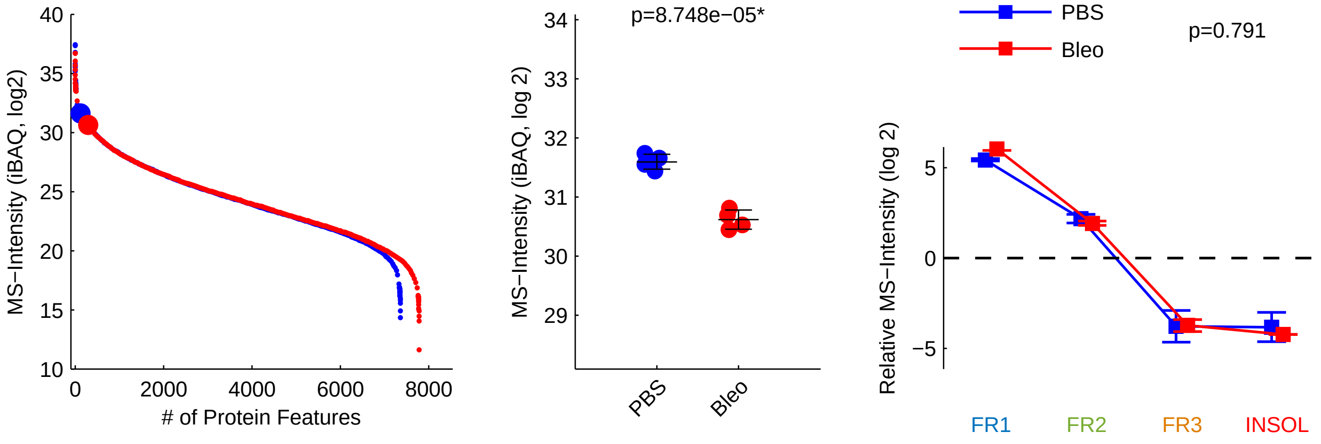

P08249 − Mdh2 (id: 2545)

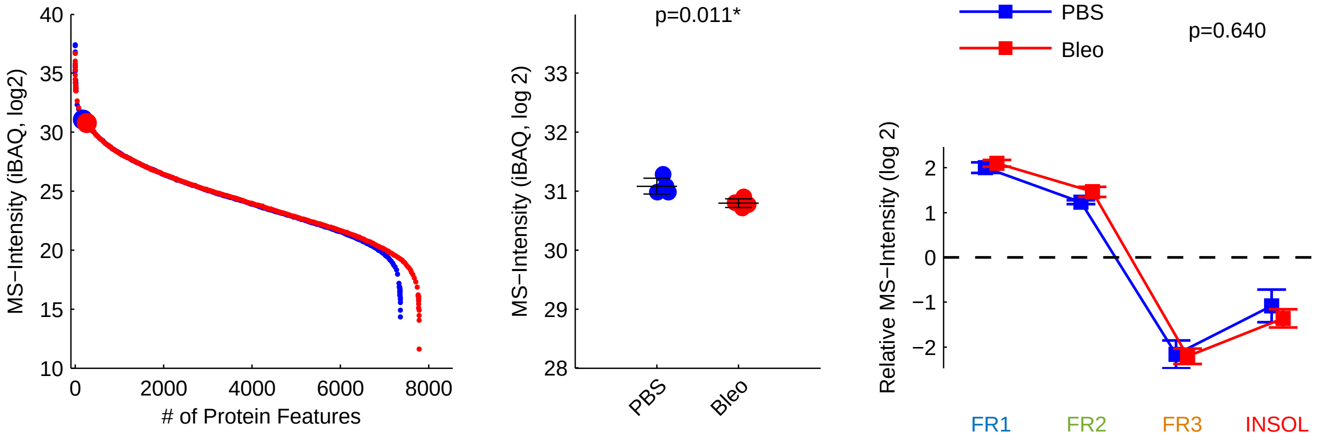

P08752 − Gnai2 (id: 2550)

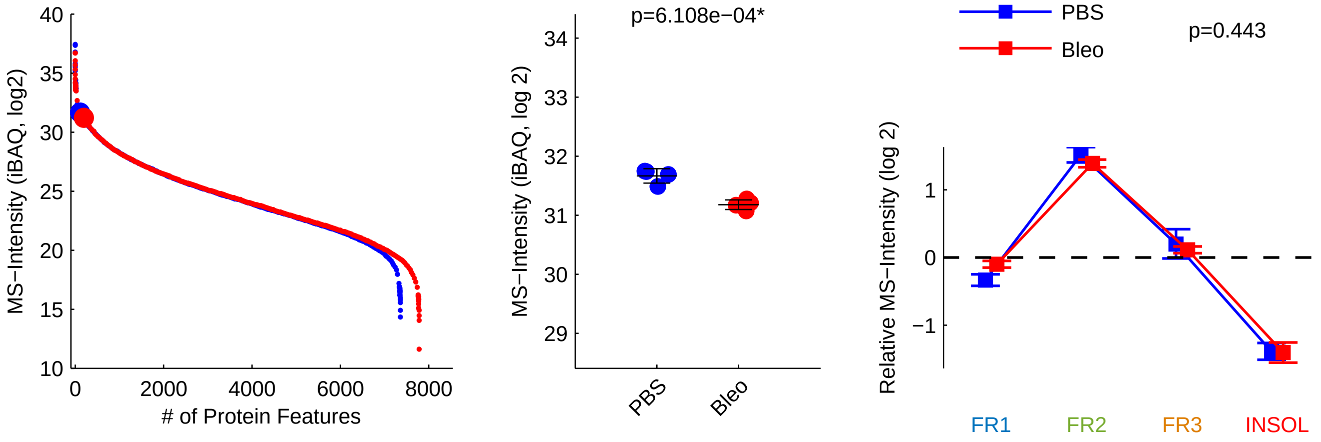

P08775 − Polr2a (id: 2551)

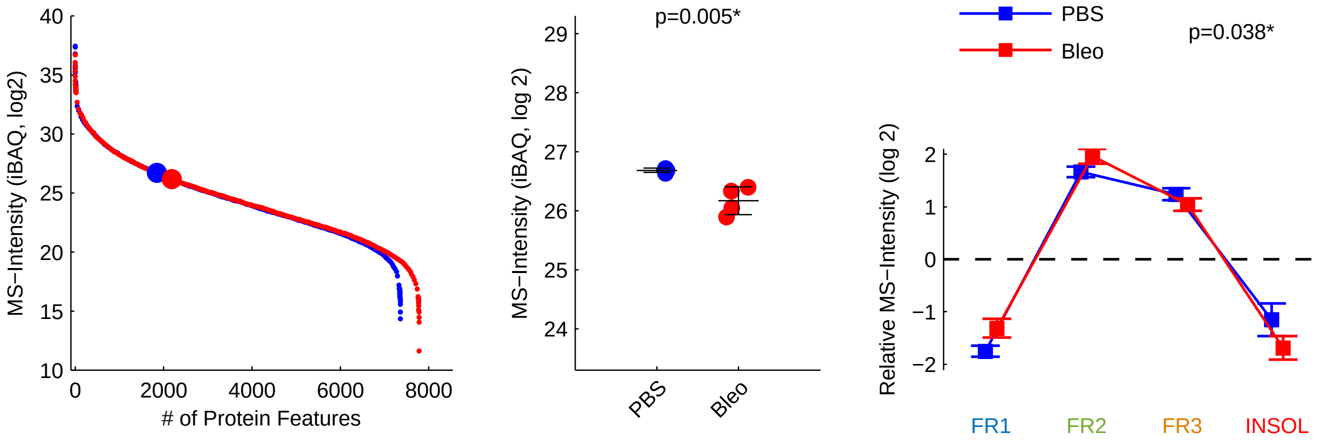

P09055 − Itgb1 (id: 2555)

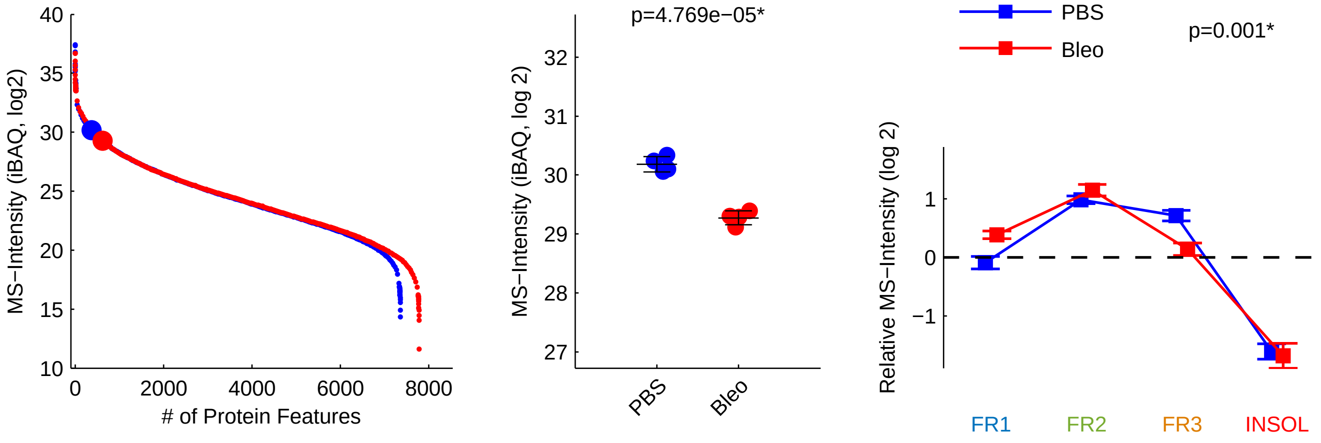

P09103 − P4hb (id: 2556)

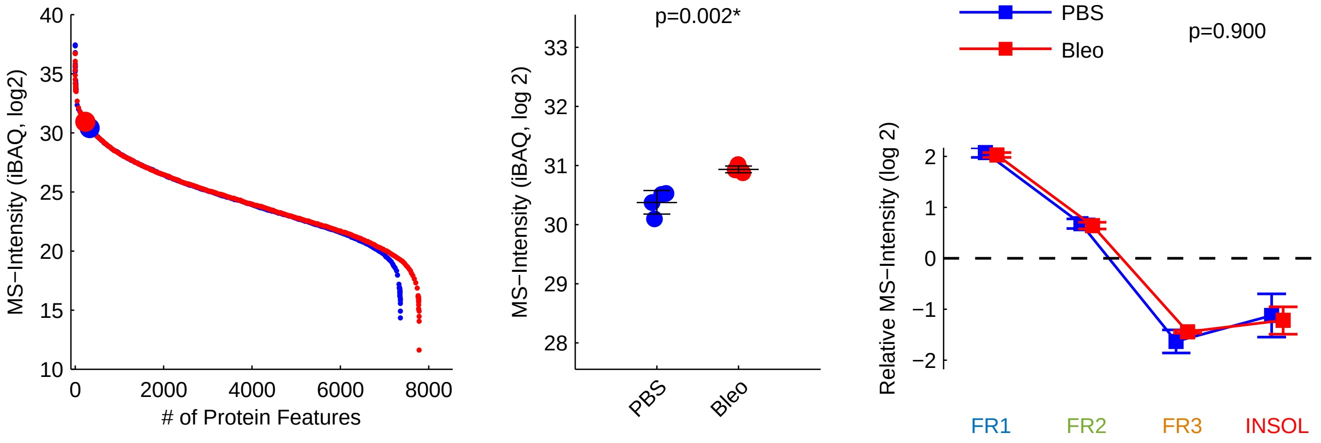

P09470 − Ace (id: 2560)

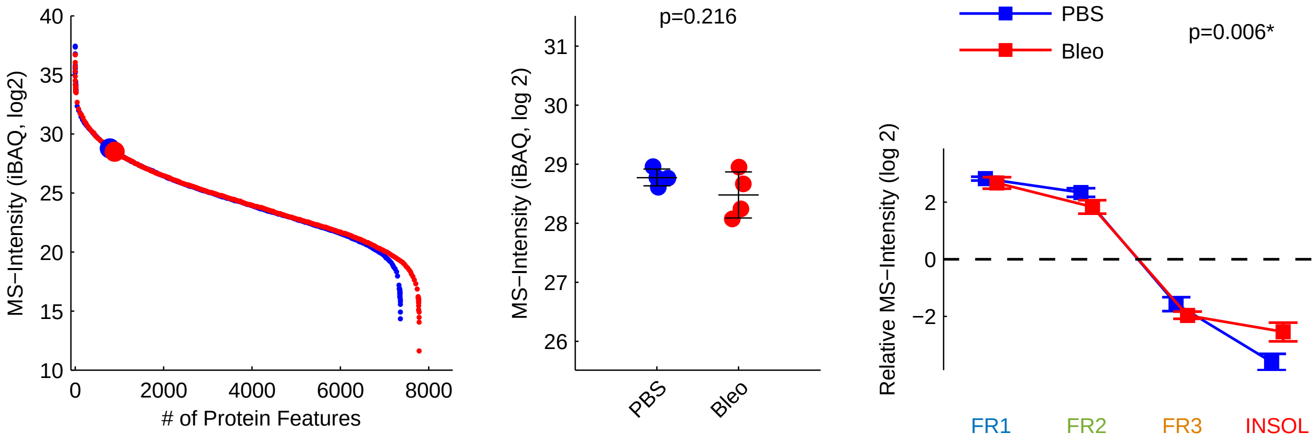

P09671 − Sod2 (id: 2564)

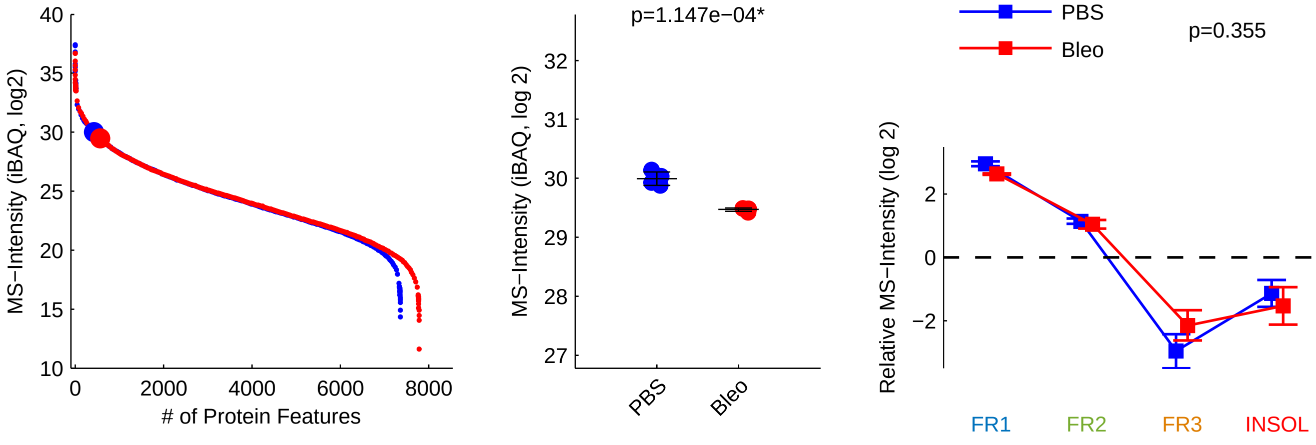

P09803 − Cdh1 (id: 2565)

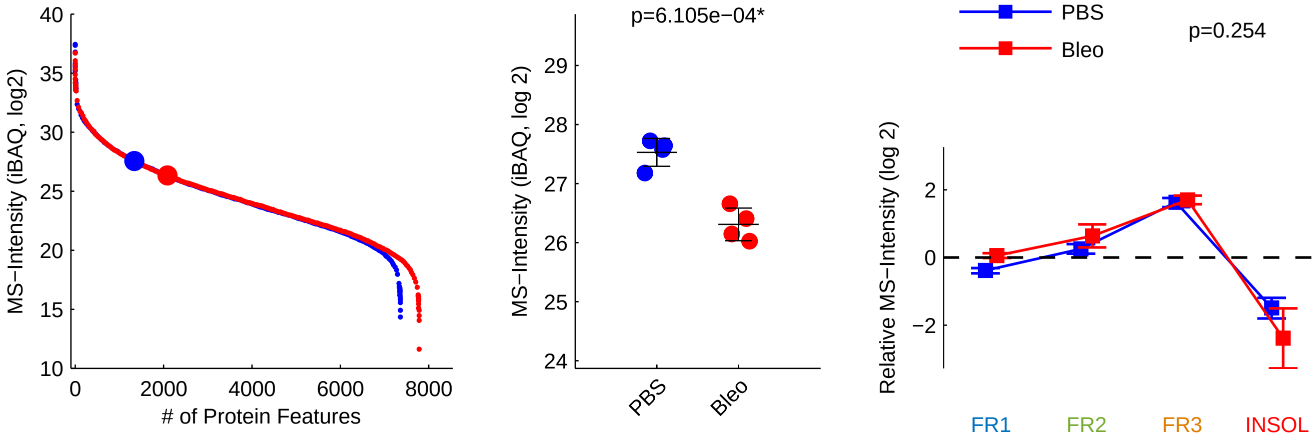

P09813 − Apoa2 (id: 2566)

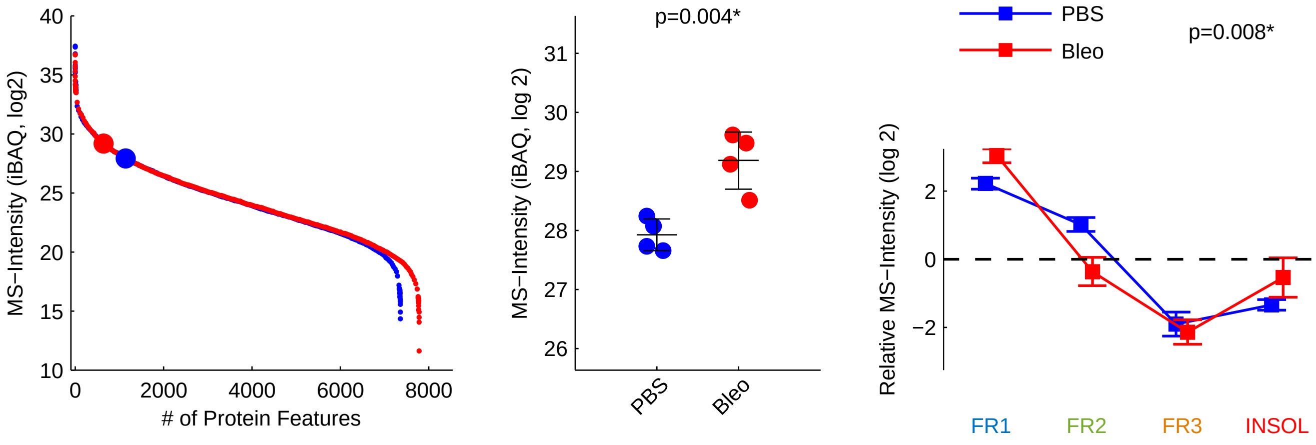

P0C605 − Prkg1 (id: 2571)

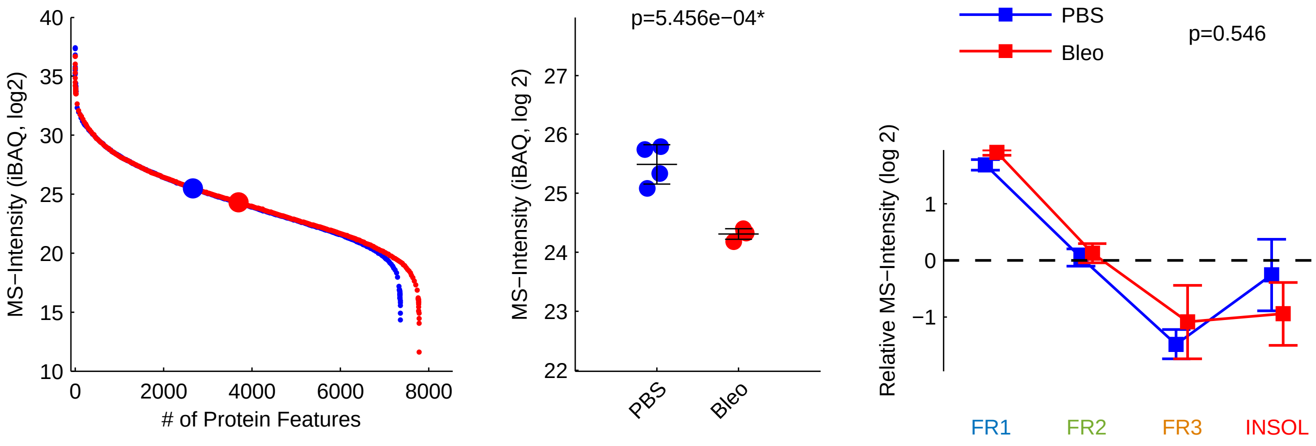

P10107 − Anxa1 (id: 2579)

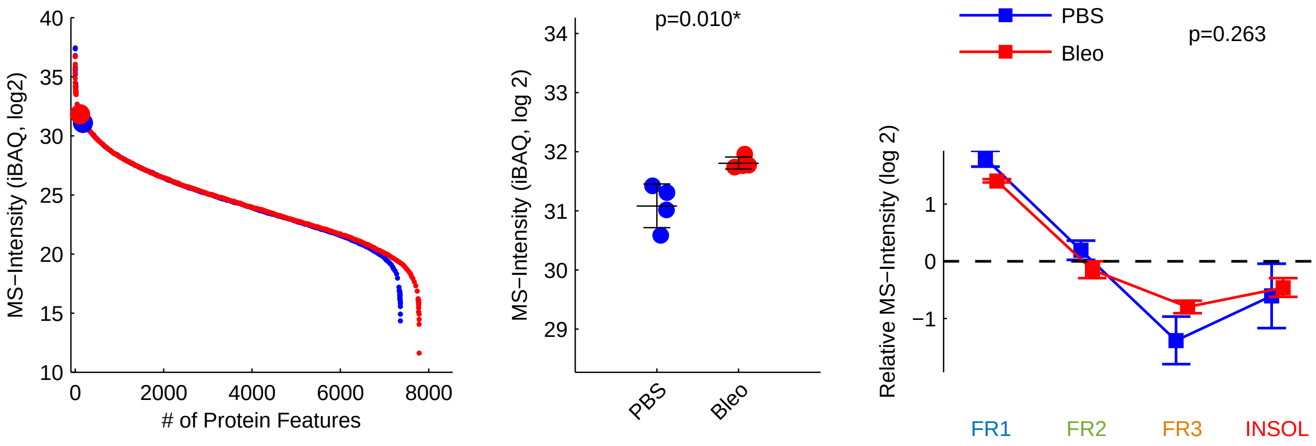

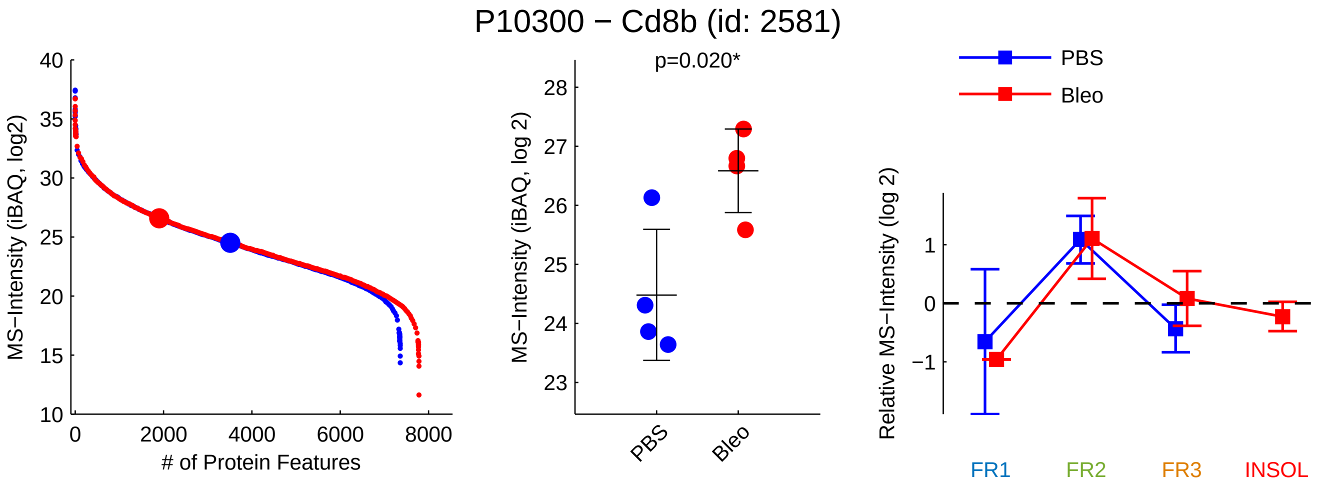
P10300 − Cd8b (id: 2581)
MS−Intensity (iBAQ, log2)
# of Protein Features
p=0.020*
MS−Intensity (iBAQ, log 2)
PBS
Bleo
Relative MS−Intensity (log 2)
FR1
FR2
FR3
INSOL

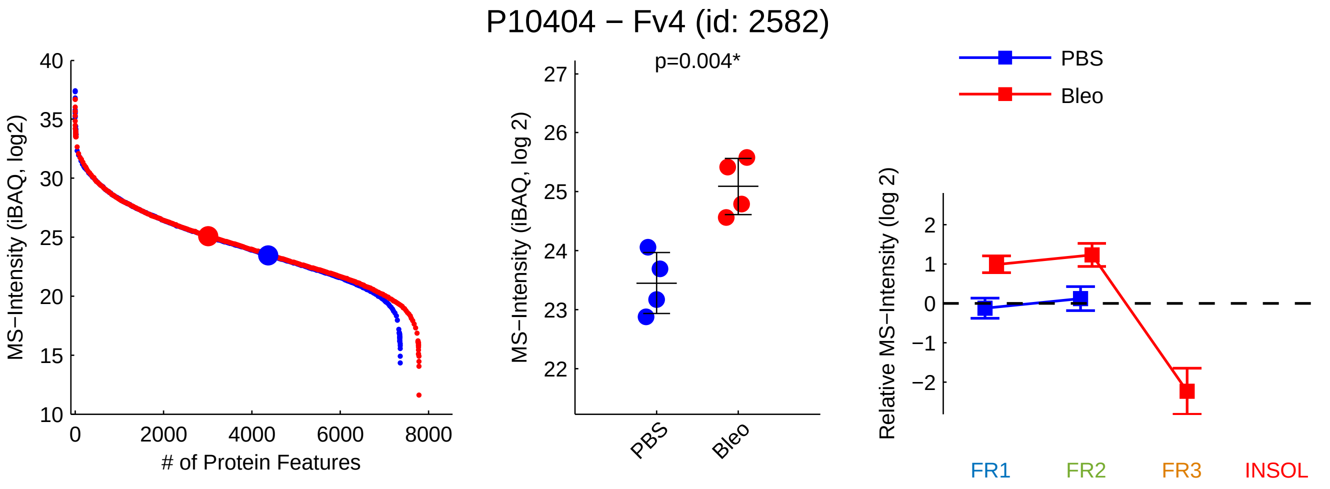
P10404 − Fv4 (id: 2582)
MS−Intensity (iBAQ, log2)
# of Protein Features
p=0.004*
MS−Intensity (iBAQ, log 2)
PBS
Bleo
Relative MS−Intensity (log 2)
FR1
FR2
FR3
INSOL

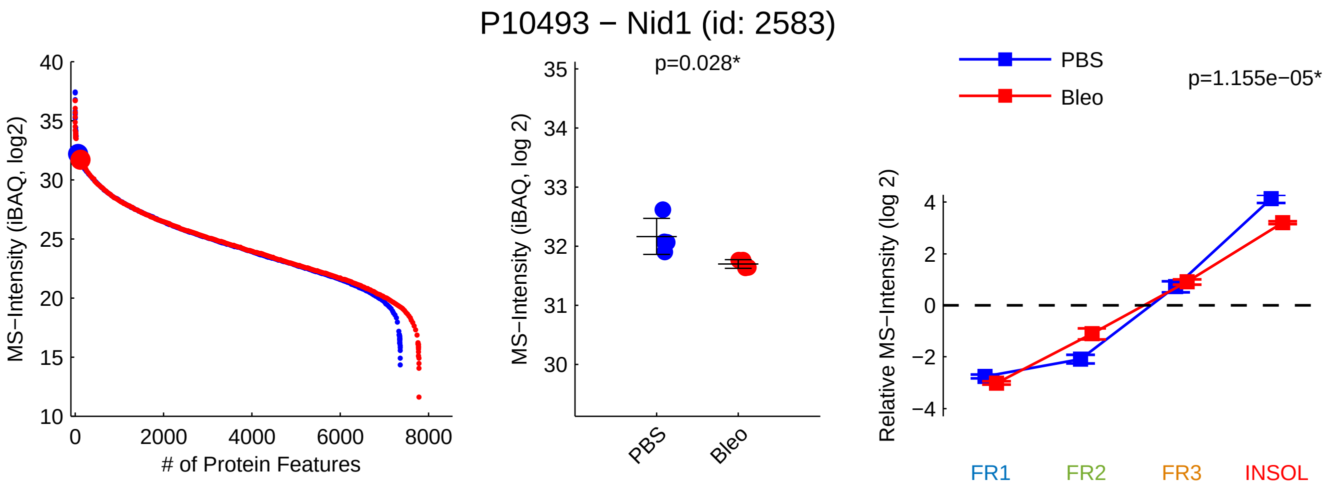
P10493 − Nid1 (id: 2583)
MS−Intensity (iBAQ, log2)
# of Protein Features
p=0.028*
MS−Intensity (iBAQ, log 2)
PBS
Bleo
p=1.155e−05*
Relative MS−Intensity (log 2)
FR1
FR2
FR3
INSOL

P10605 − Ctsb (id: 2585)

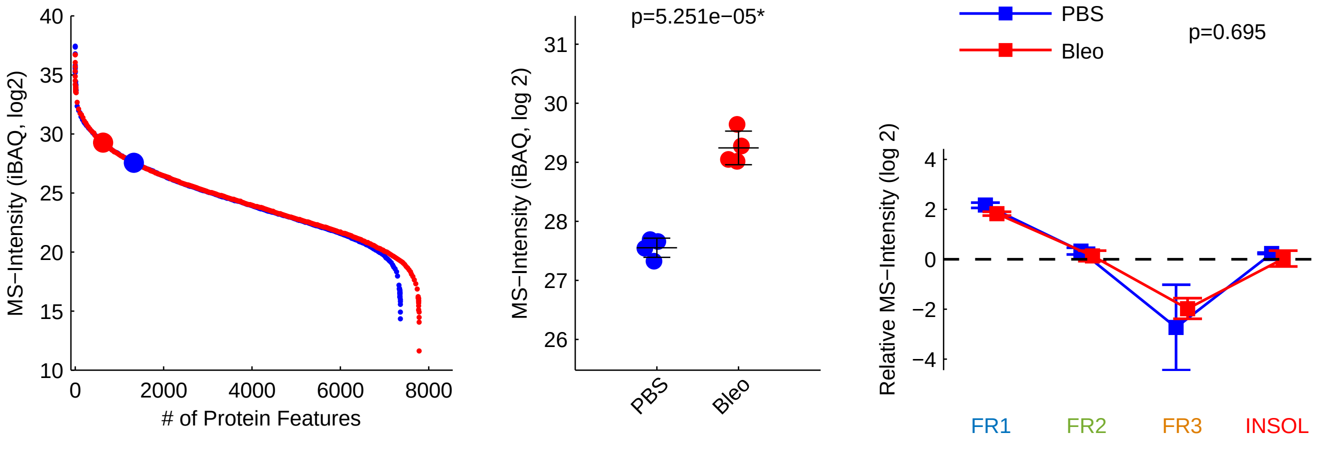

P10630 − Eif4a2 (id: 2586)

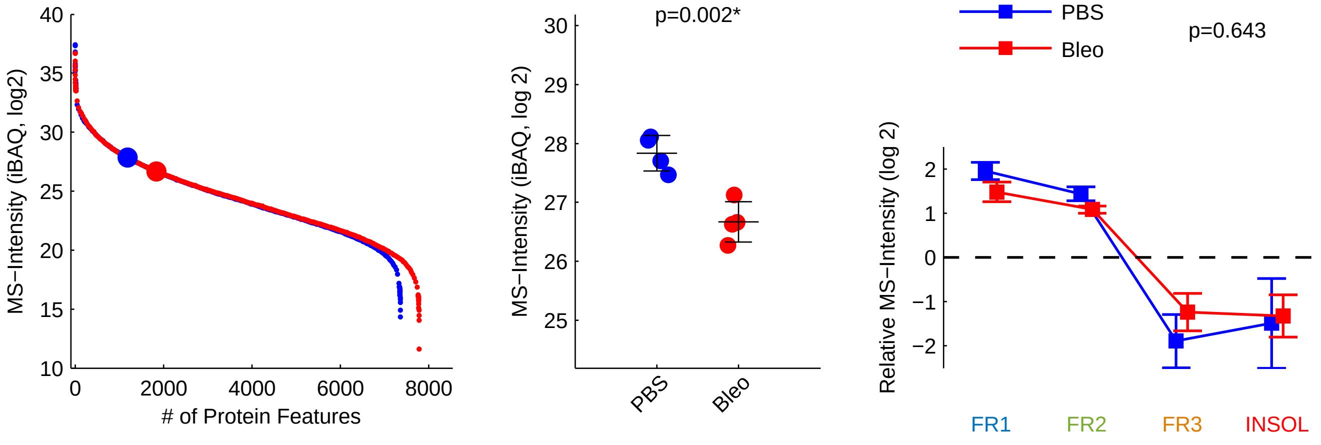

P10637 − Mapt (id: 2587)

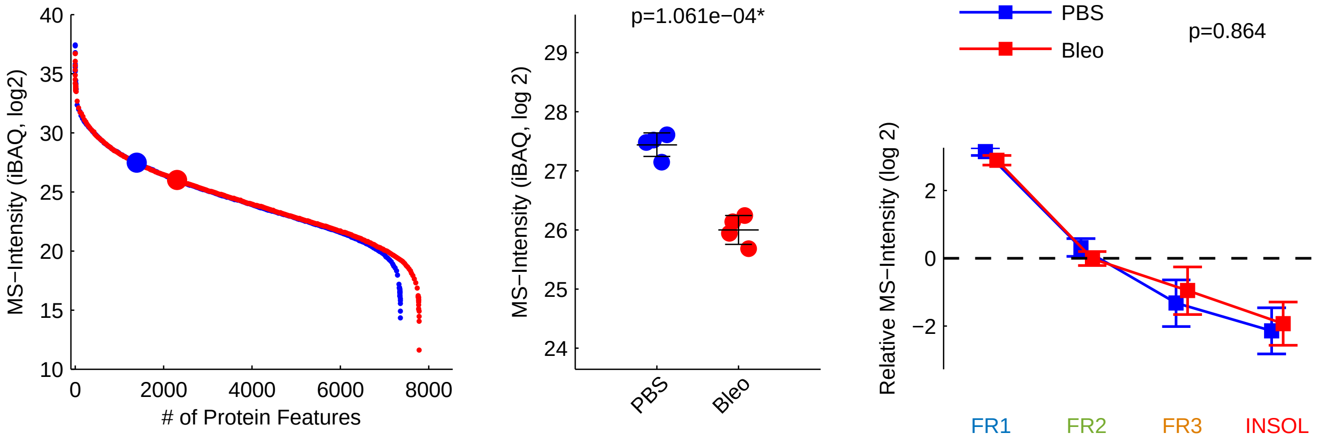

P10649 − Gstm1 (id: 2589)

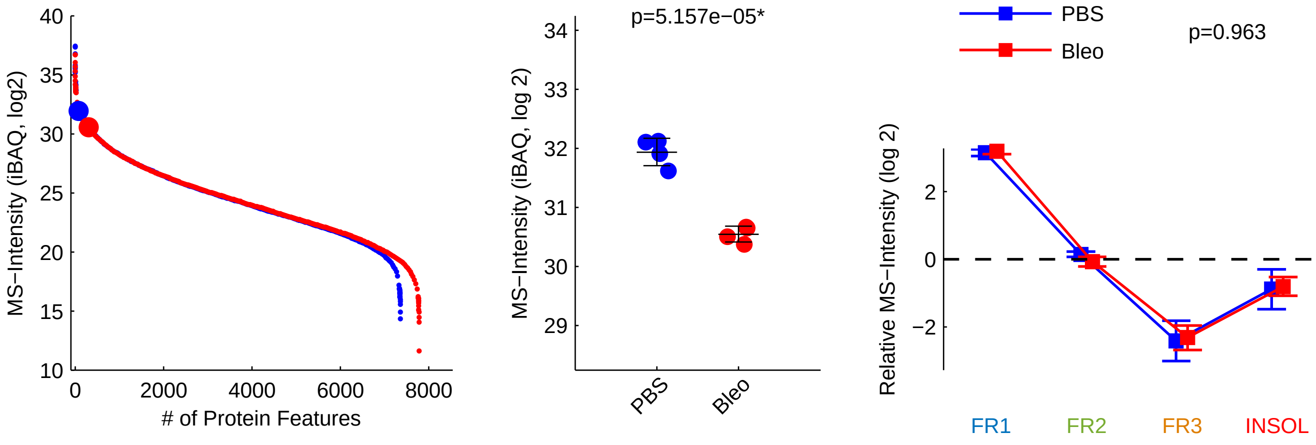

P10810 − Cd14 (id: 2592)

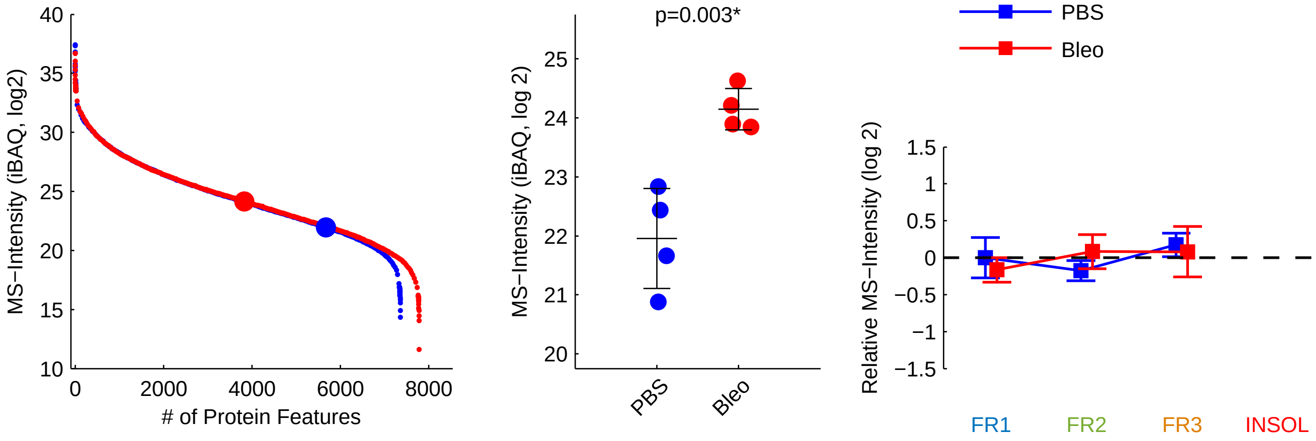

P10833 − Rras (id: 2594)

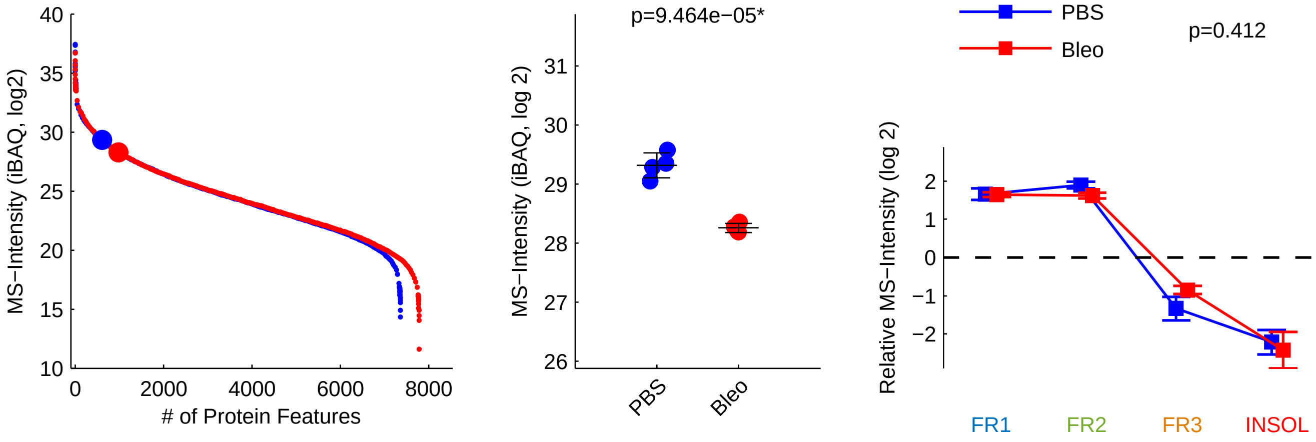

P10922 − H1f0 (id: 2597)

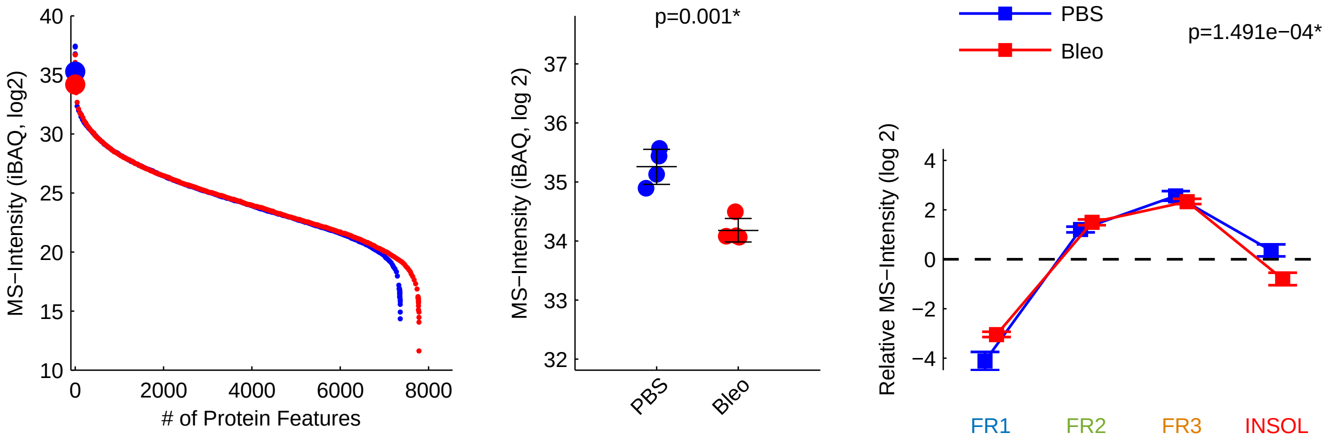

P11032−2 − Gzma (id: 2599)

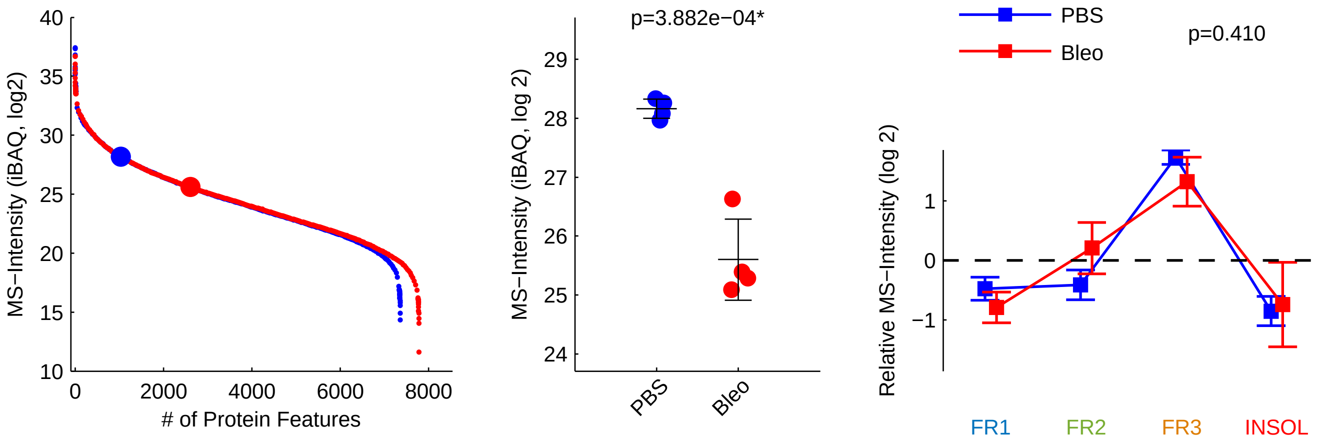

P11087 − Col1a1 (id: 2600)

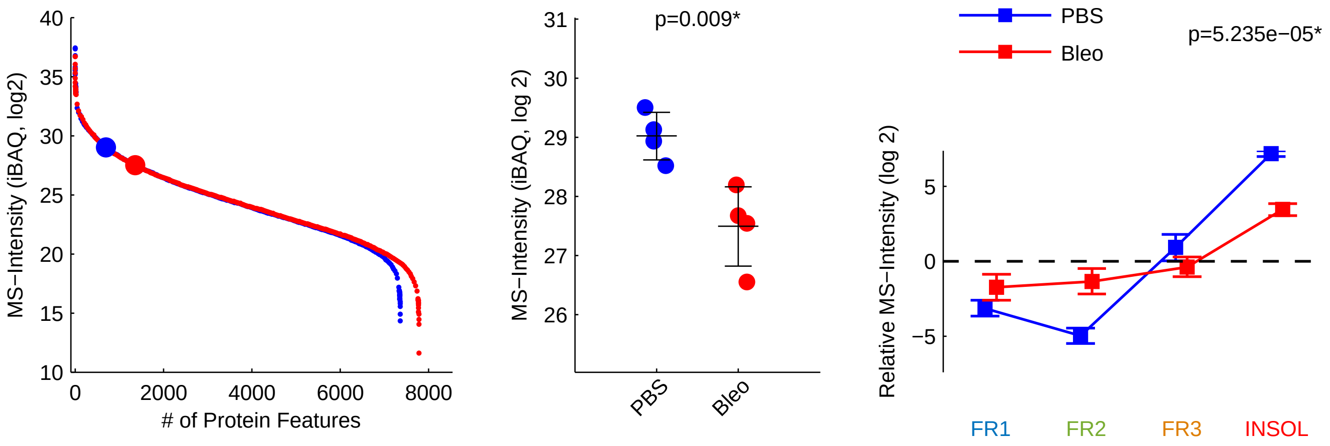

P11152 − Lpl (id: 2602)

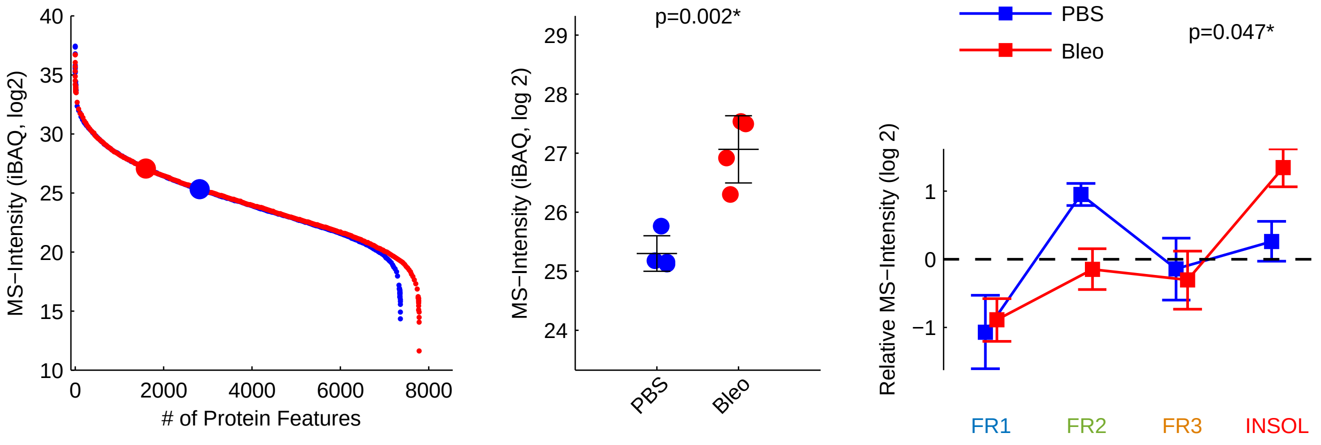

P11214 − Plat (id: 2604)

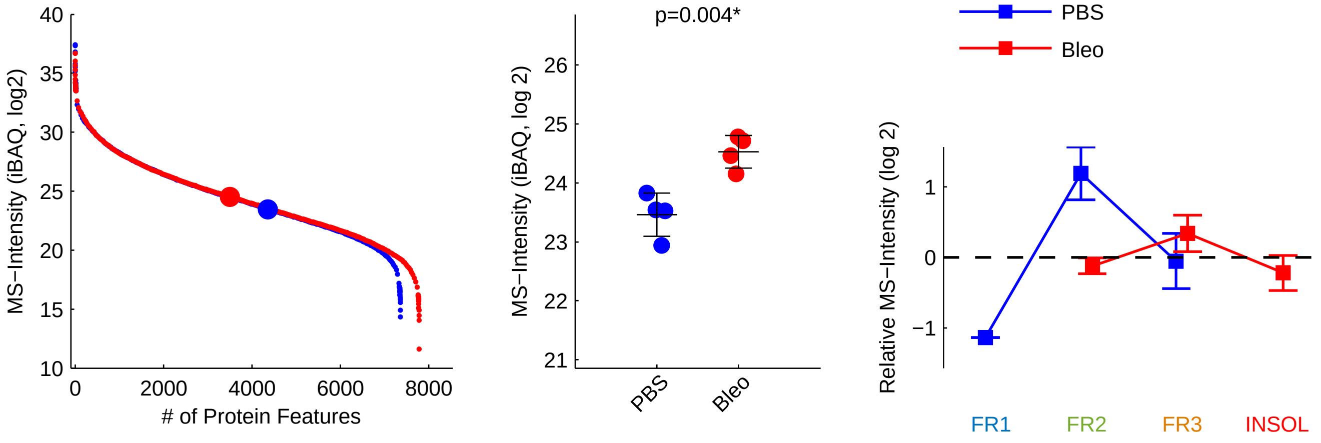

P11276 − Fn1 (id: 2606)

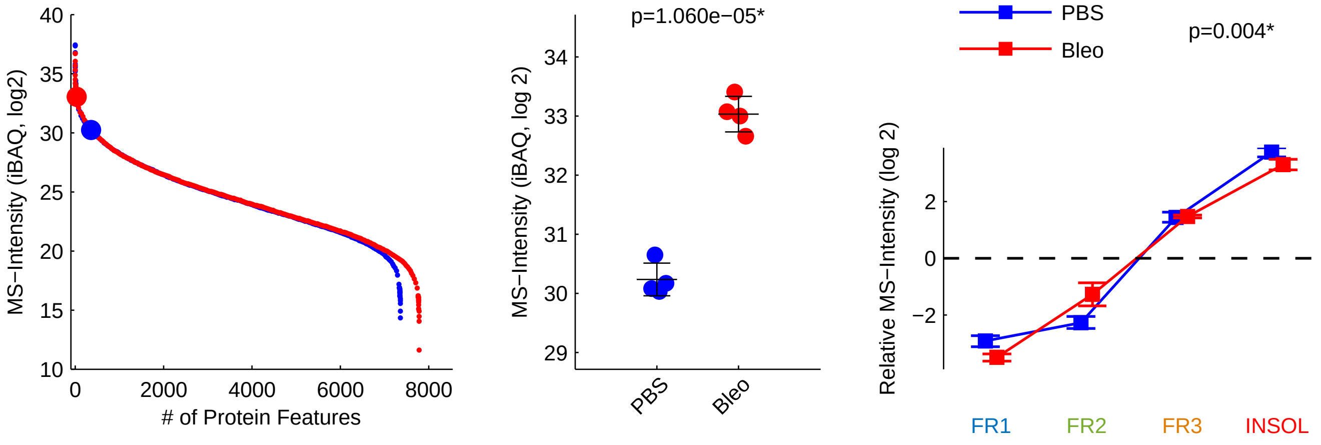

P11499 − Hsp90ab1 (id: 2611)

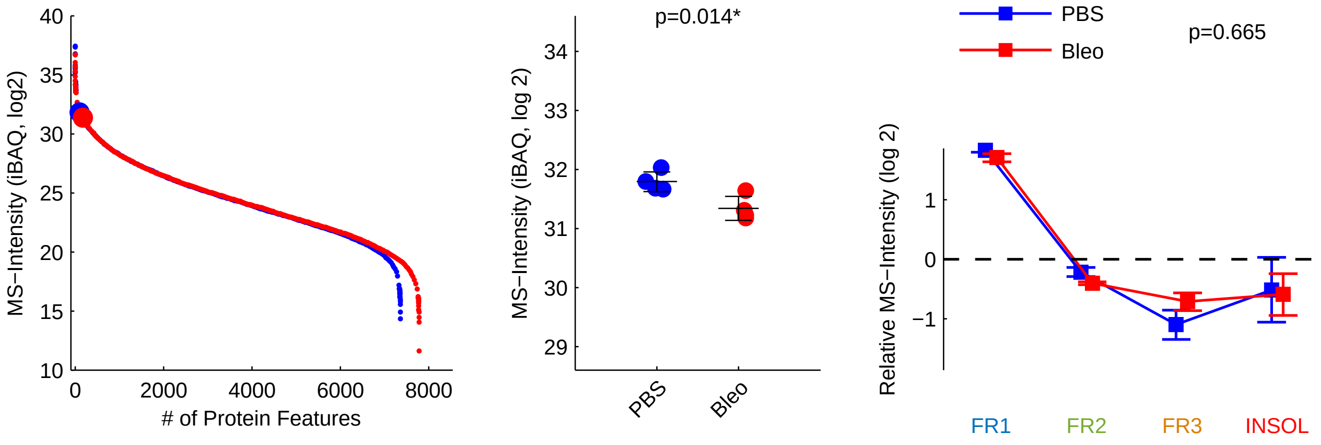

P11688 − Itga5 (id: 2615)

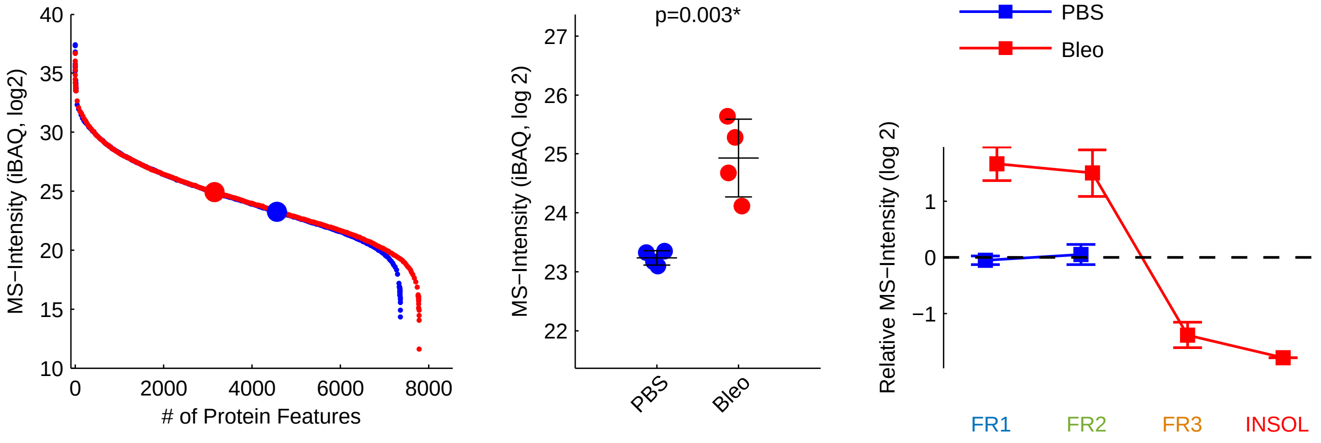

P11835 − Itgb2 (id: 2616)

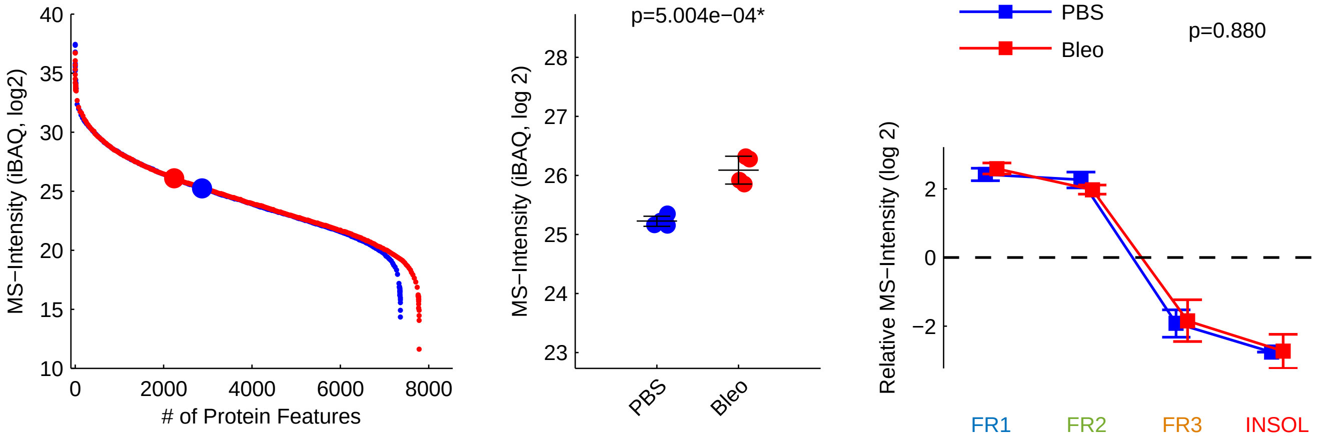

P11881 − Itpr1 (id: 2617)

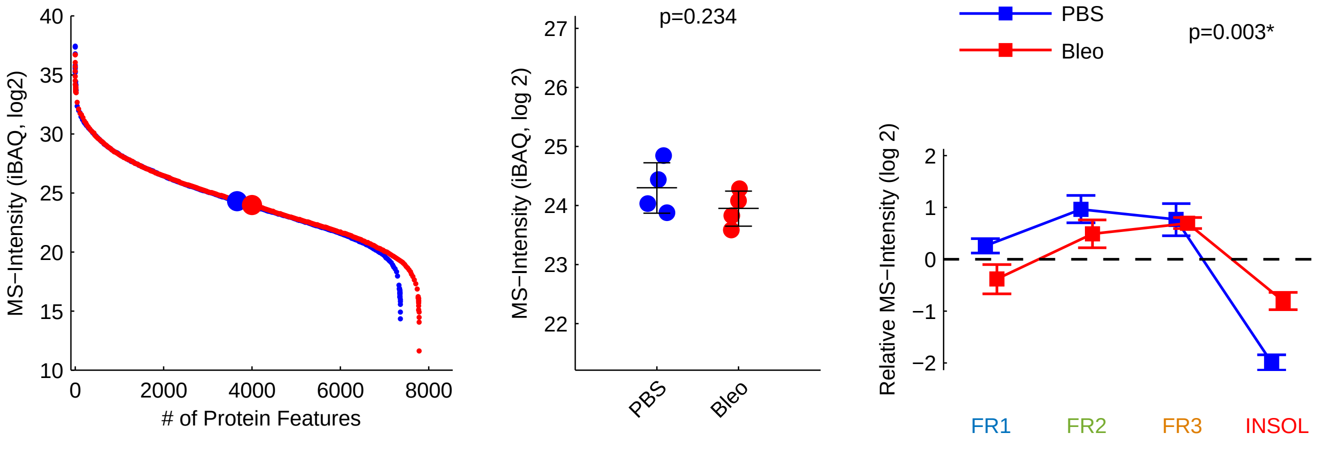

P11983 − Tcp1 (id: 2621)

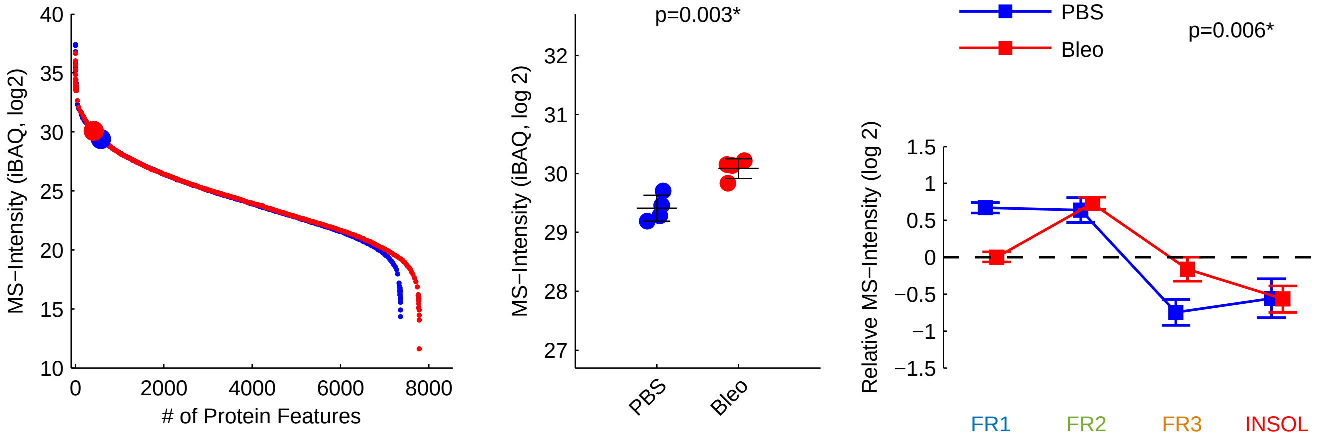

P12246 − Apcs (id: 2624)

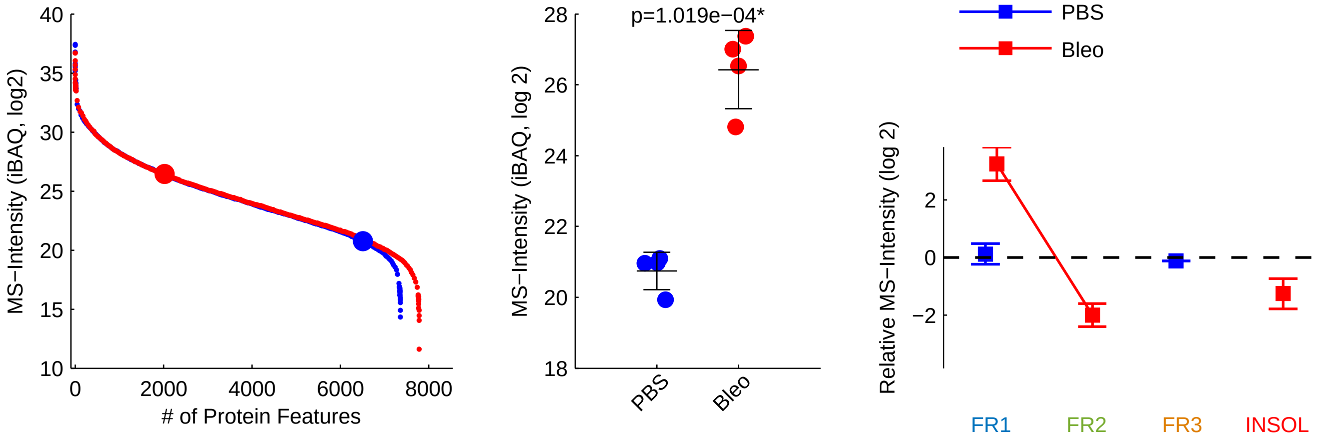

P12265 − Gusb (id: 2625)

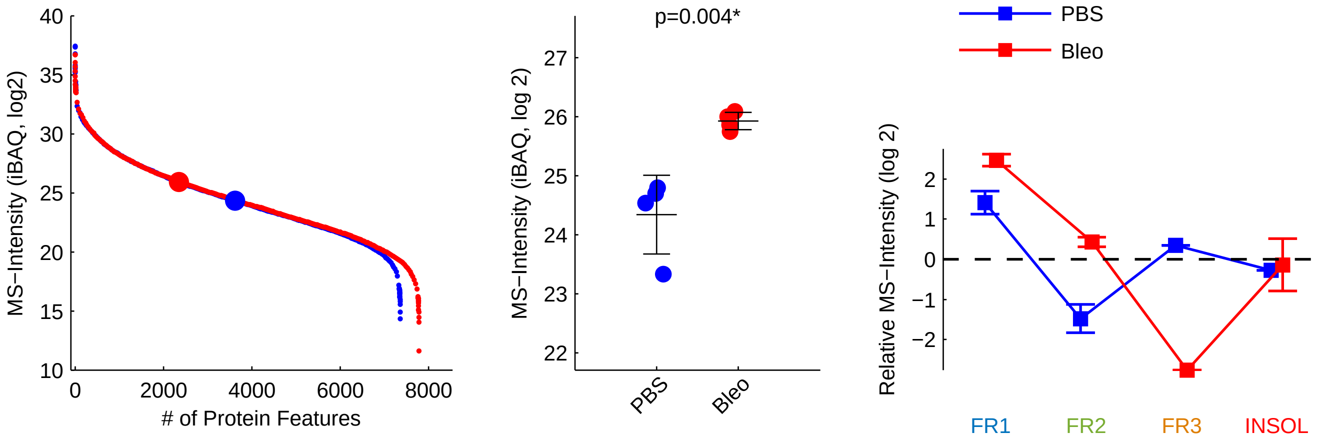

P12787 − Cox5a (id: 2631)

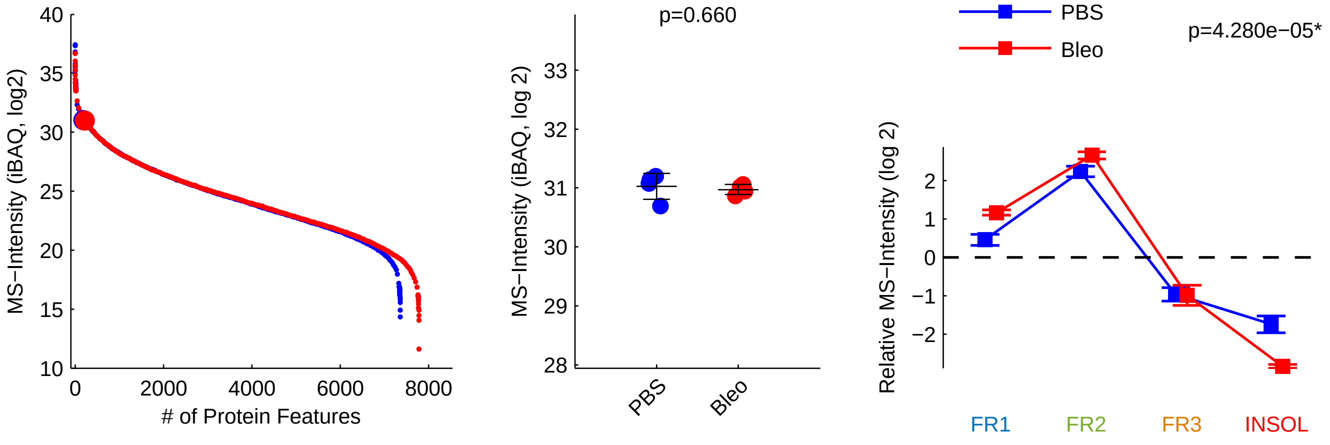

P12970 − Rpl7a (id: 2635)

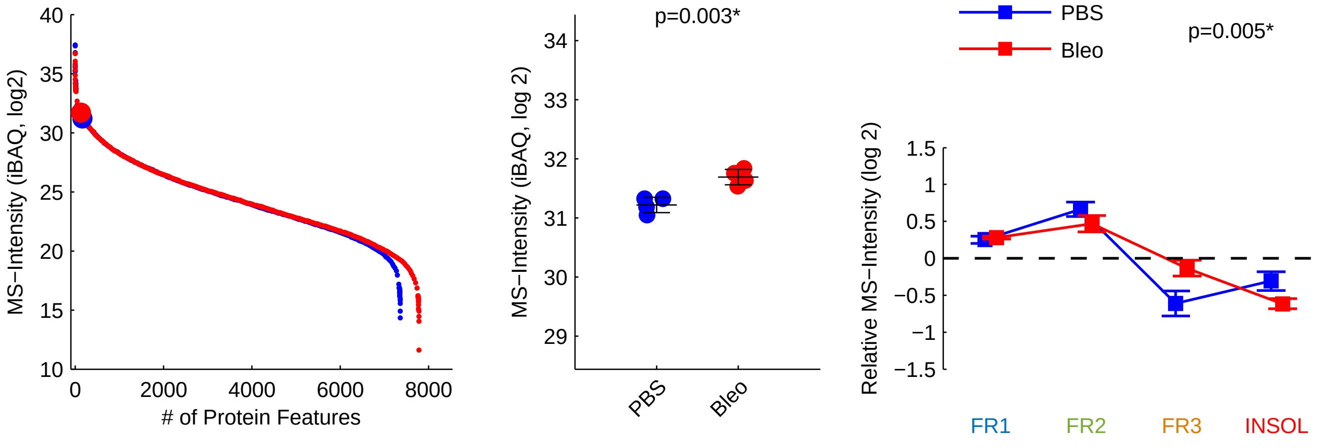

P13439 − Umps (id: 2640)

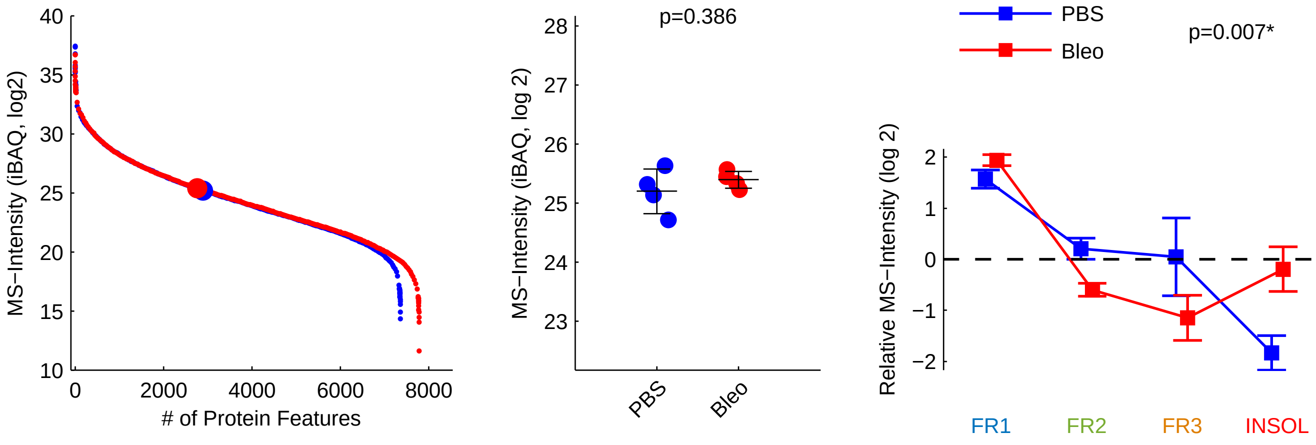

P13745 − Gsta1 (id: 2647)

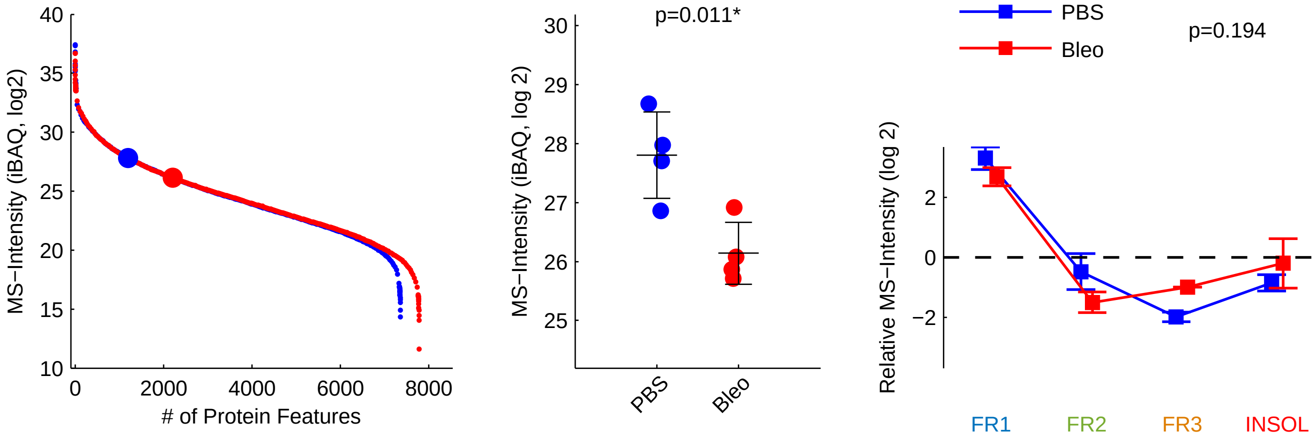

P13808 − Slc4a2 (id: 2648)

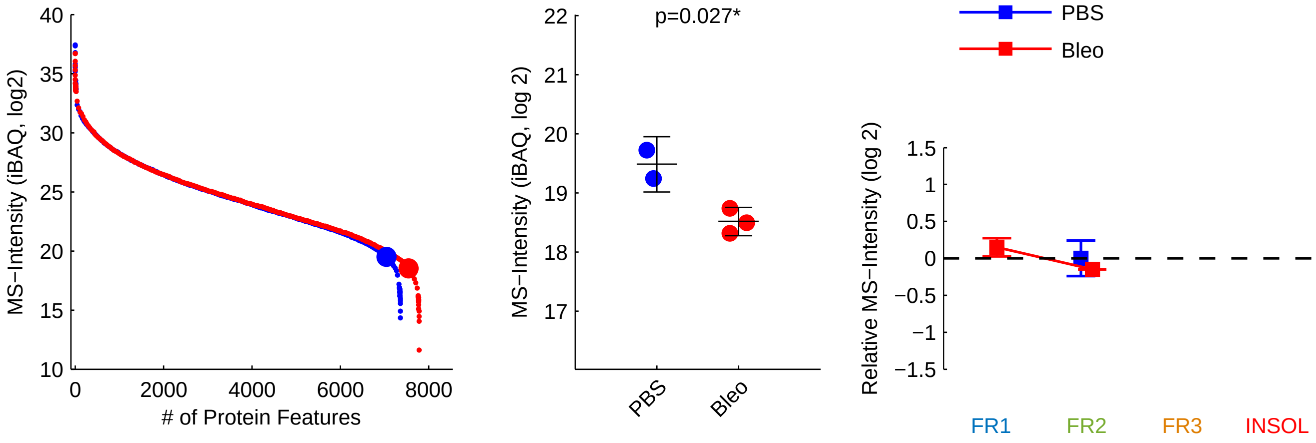

P14106 − C1qb (id: 2652)

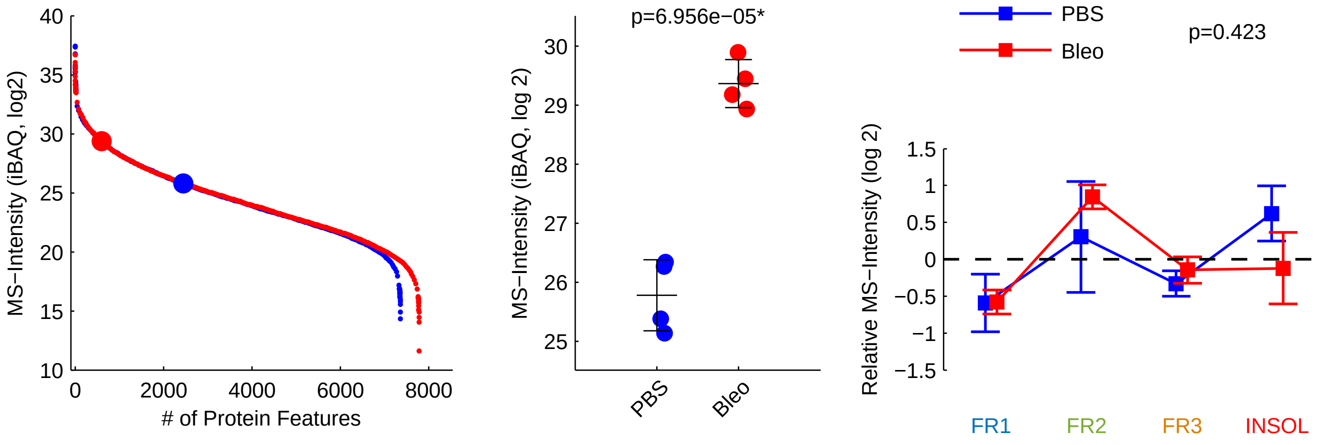

P14115 − Rpl27a (id: 2653)

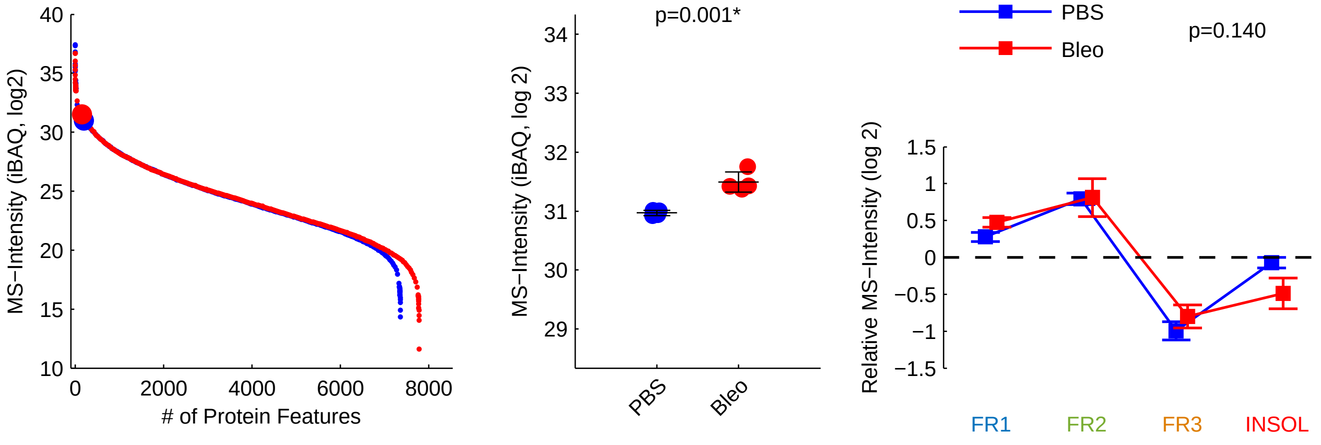

P14131 − Rps16 (id: 2654)

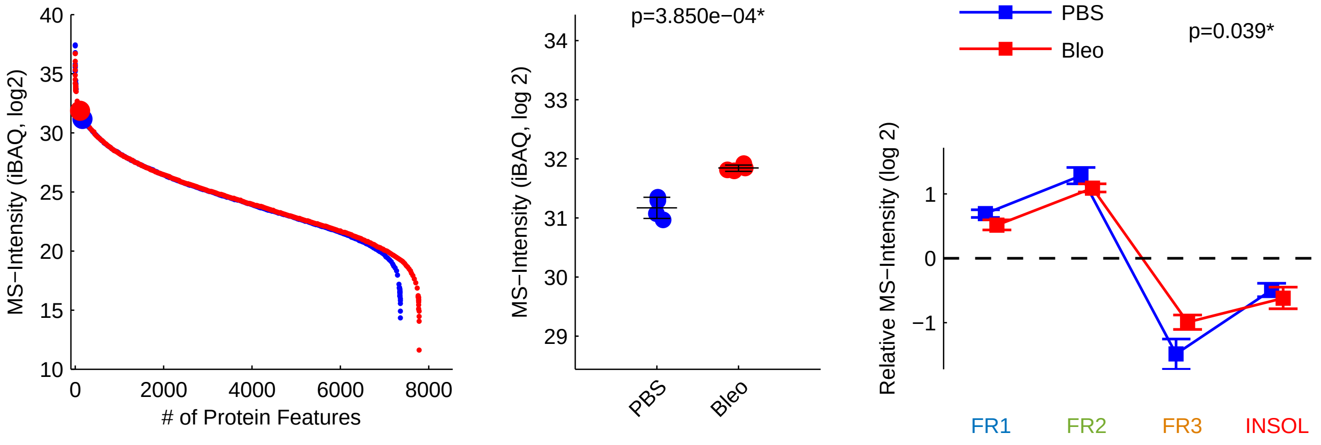

P14148 − Rpl7 (id: 2655)

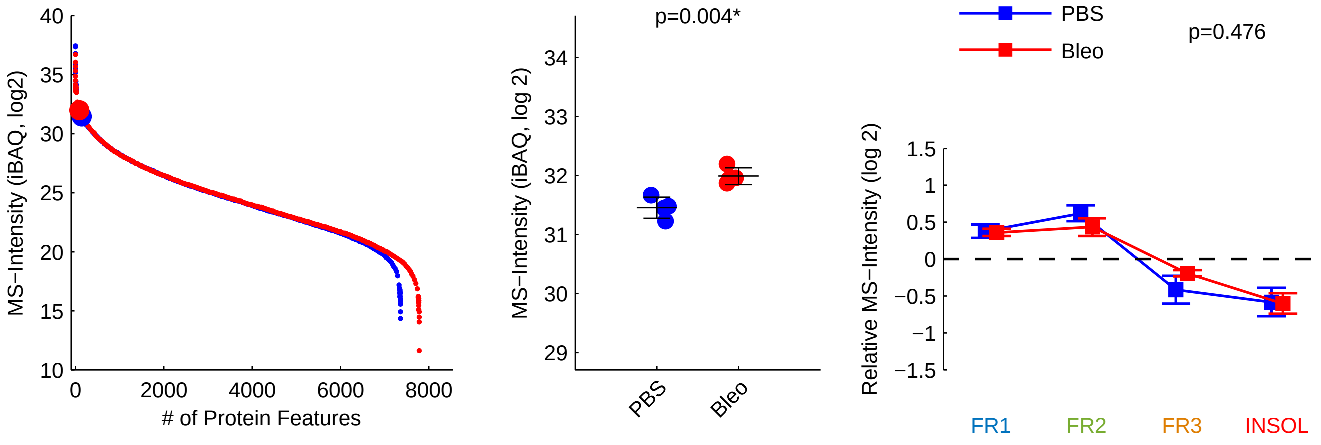

P14152 − Mdh1 (id: 2656)

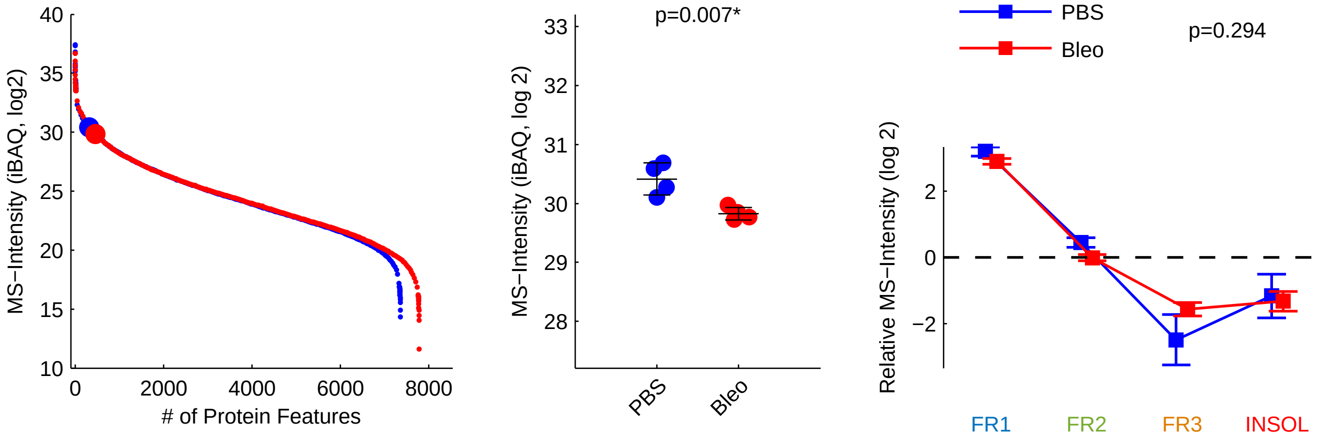

P14206 − Rpsa (id: 2657)

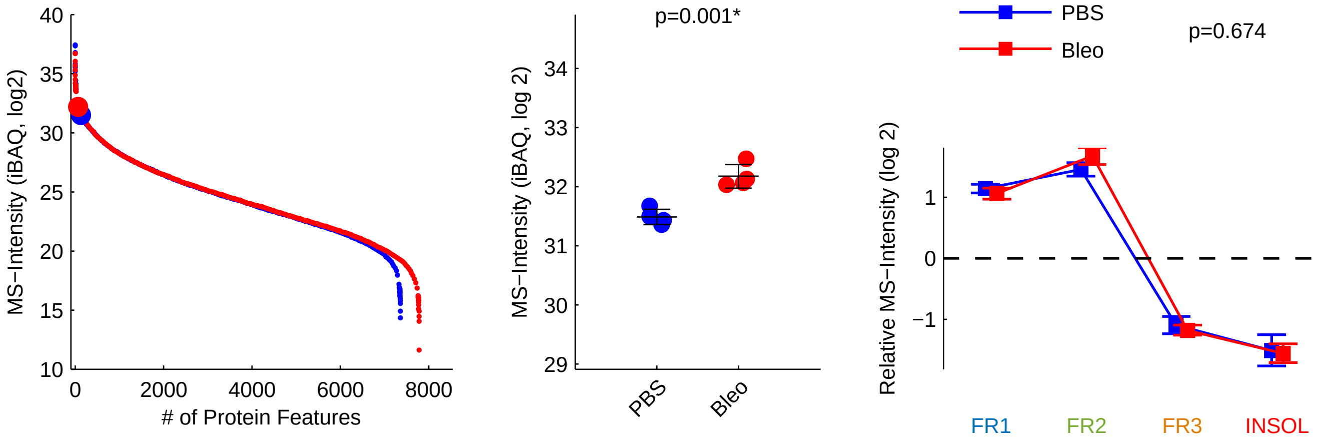

P14234 − Fgr (id: 2659)

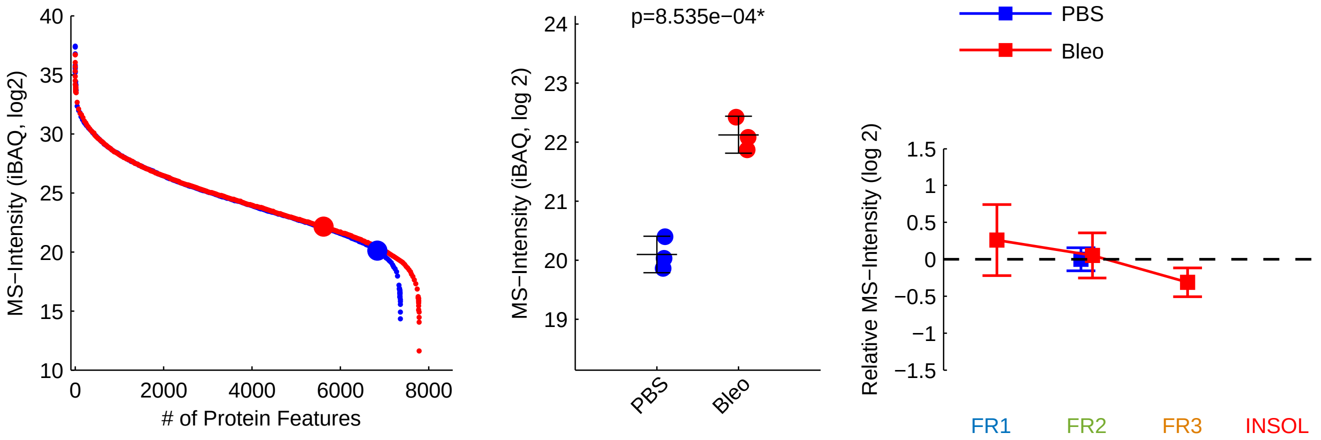

P14733 − Lmnb1 (id: 2666)

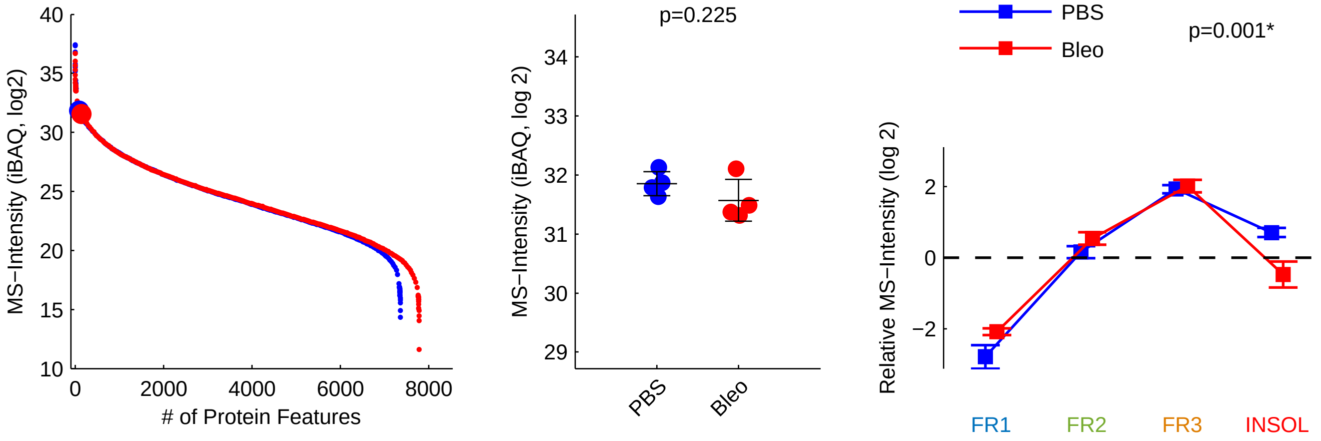

P14847 − Crp (id: 2668)

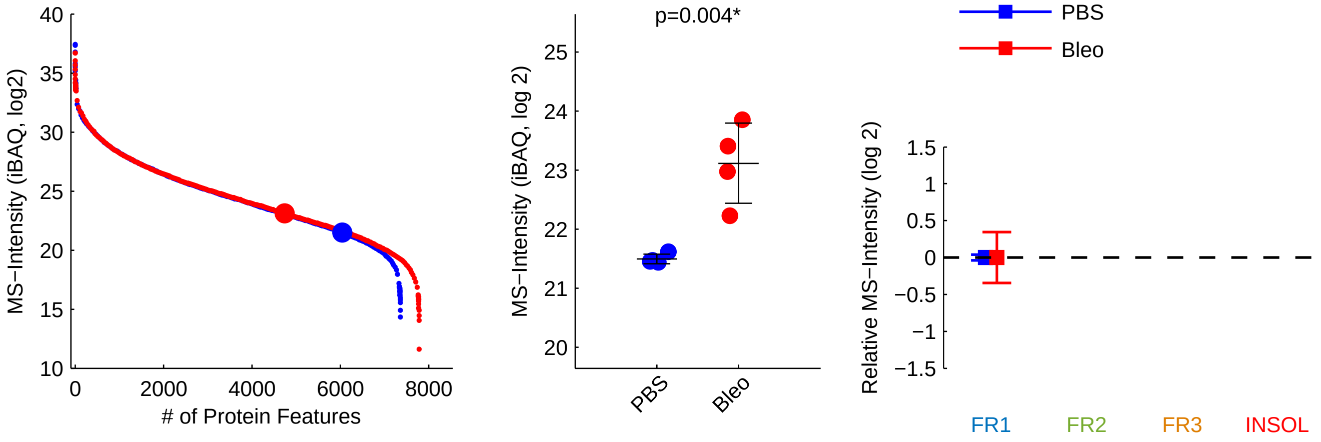

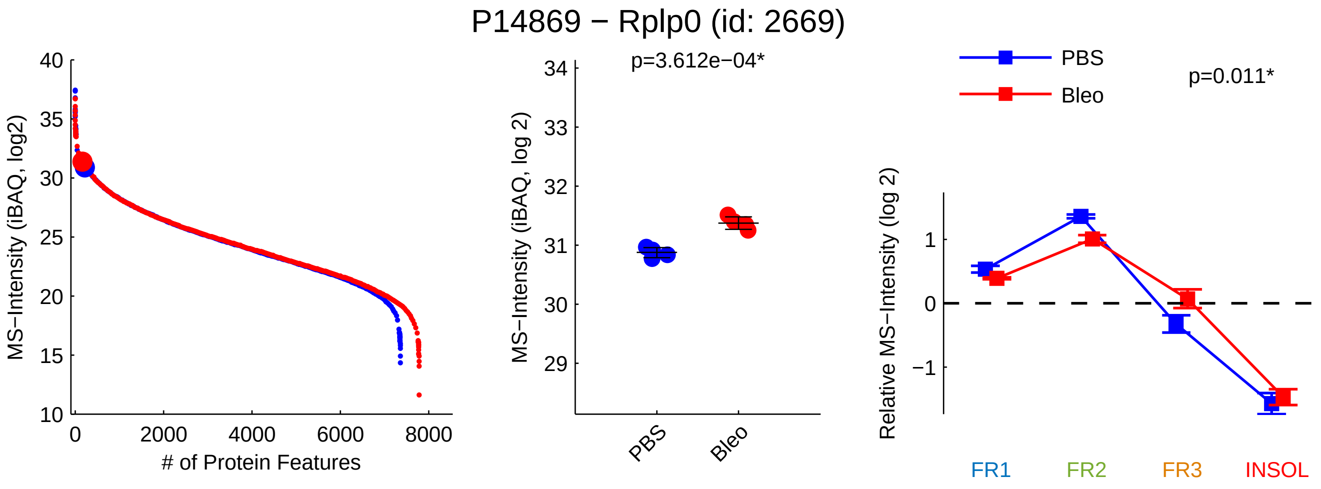
P14869 − Rplp0 (id: 2669)
MS−Intensity (iBAQ, log2)
# of Protein Features
p=3.612e−04*
MS−Intensity (iBAQ, log 2)
PBS
Bleo
p=0.011*
Relative MS−Intensity (log 2)
FR1
FR2
FR3
INSOL

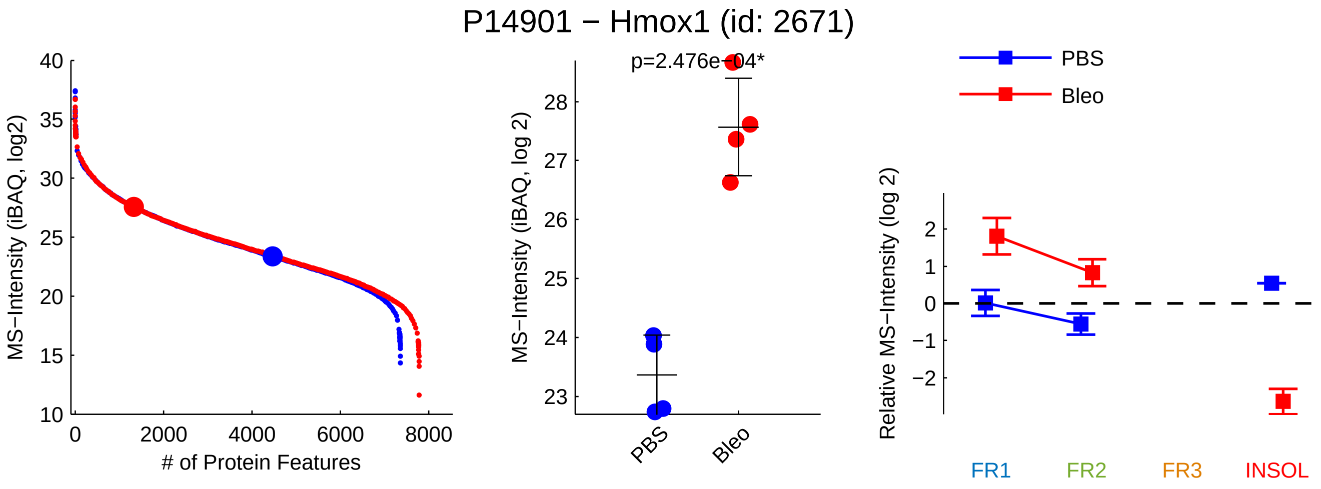
P14901 − Hmox1 (id: 2671)
MS−Intensity (iBAQ, log2)
# of Protein Features
p=2.476e−04*
MS−Intensity (iBAQ, log 2)
PBS
Bleo
Relative MS−Intensity (log 2)
FR1
FR2
FR3
INSOL

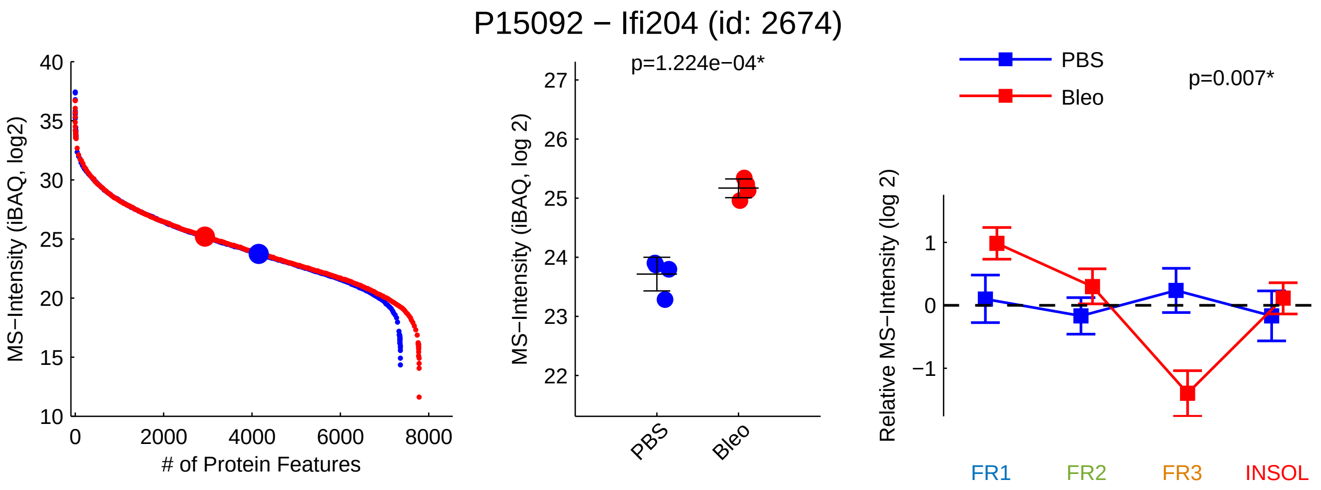
P15092 − Ifi204 (id: 2674)
MS−Intensity (iBAQ, log2)
# of Protein Features
p=1.224e−04*
MS−Intensity (iBAQ, log 2)
PBS
Bleo
p=0.007*
Relative MS−Intensity (log 2)
FR1
FR2
FR3
INSOL

P15306 − Thbd (id: 2677)

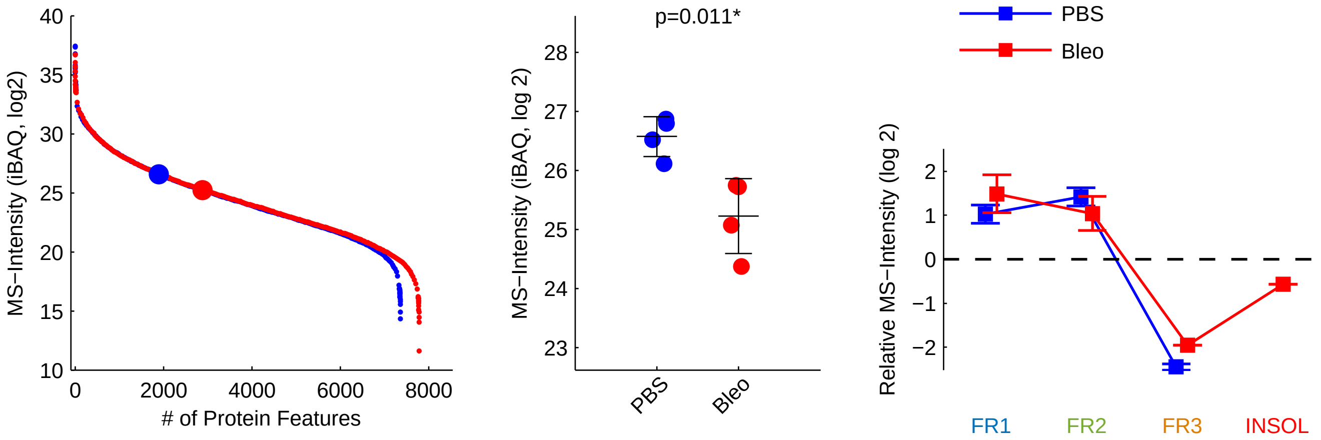

P15535−2 − B4galt1 (id: 2681)

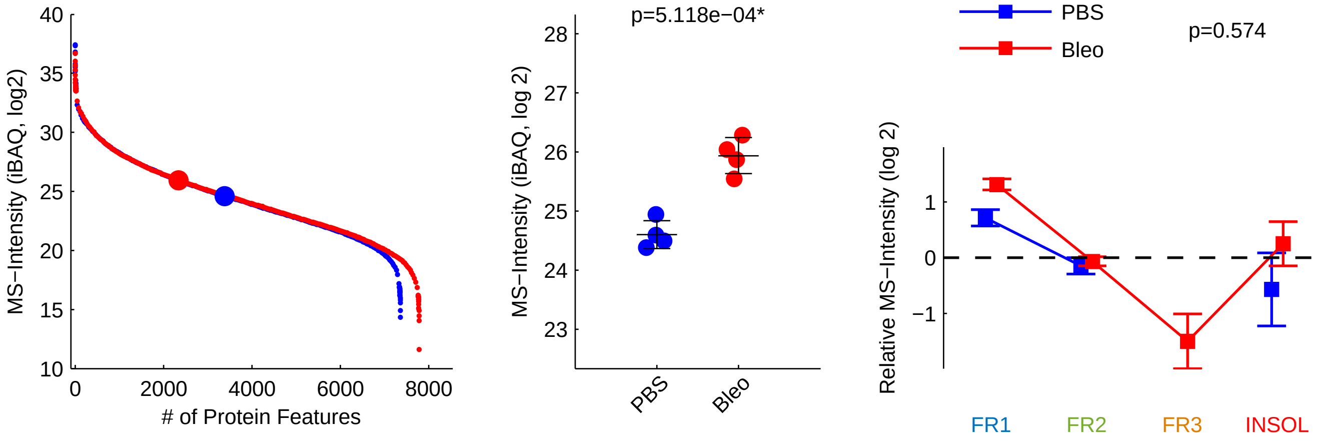

P15626 − Gstm2 (id: 2682)

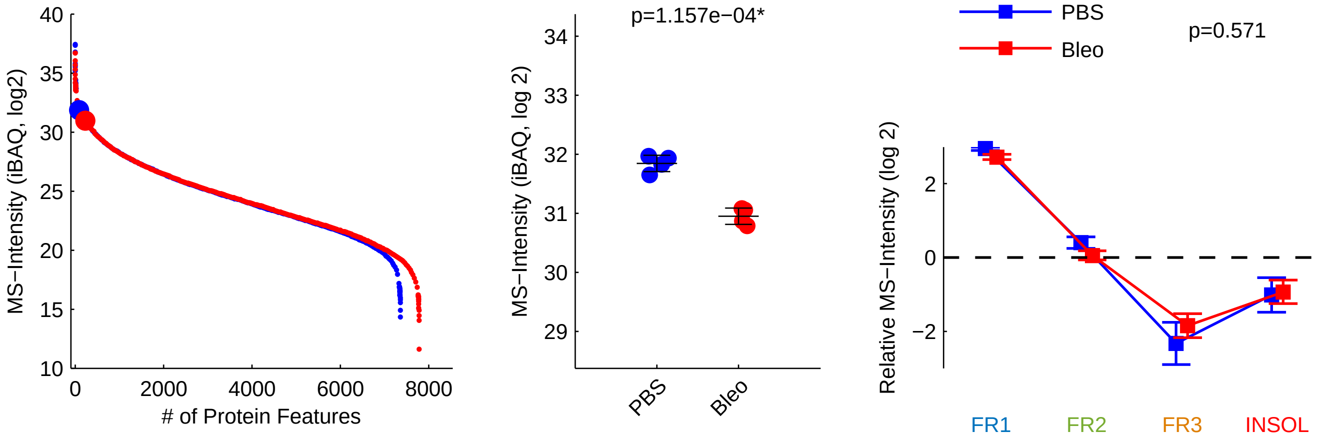

P15864 − Hist1h1c (id: 2685)

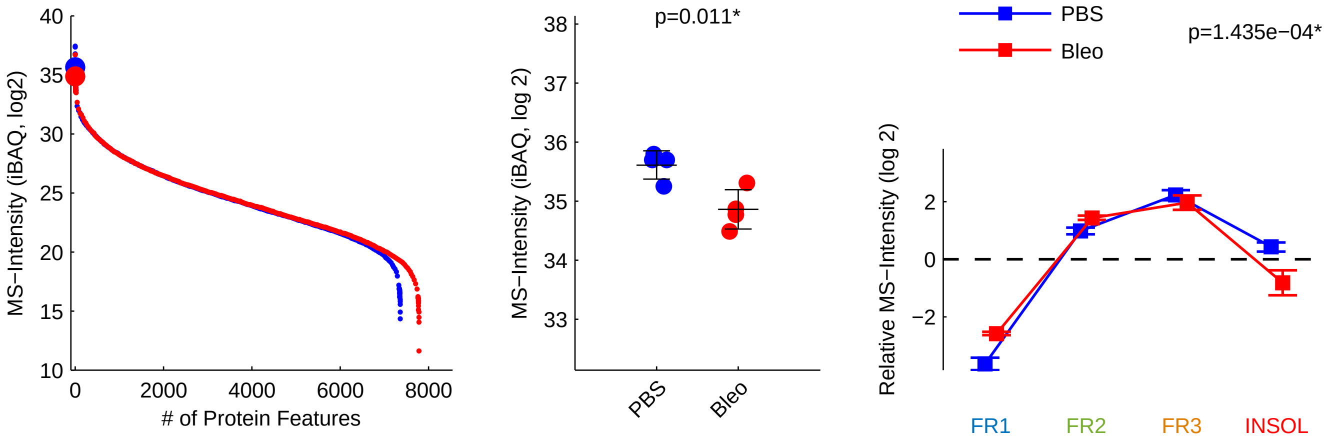

P16045 − Lgals1 (id: 2689)

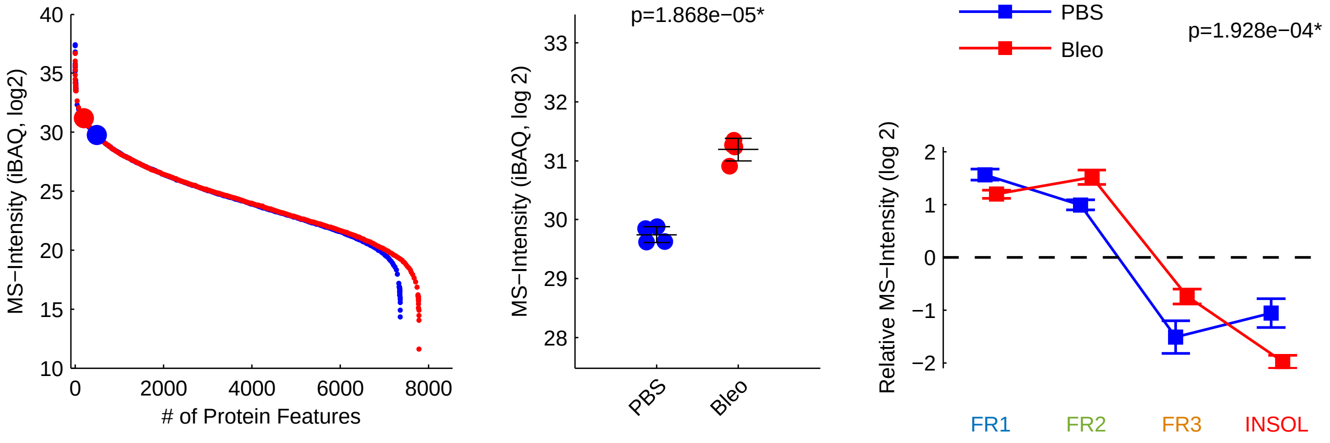

P16110 − Lgals3 (id: 2691)

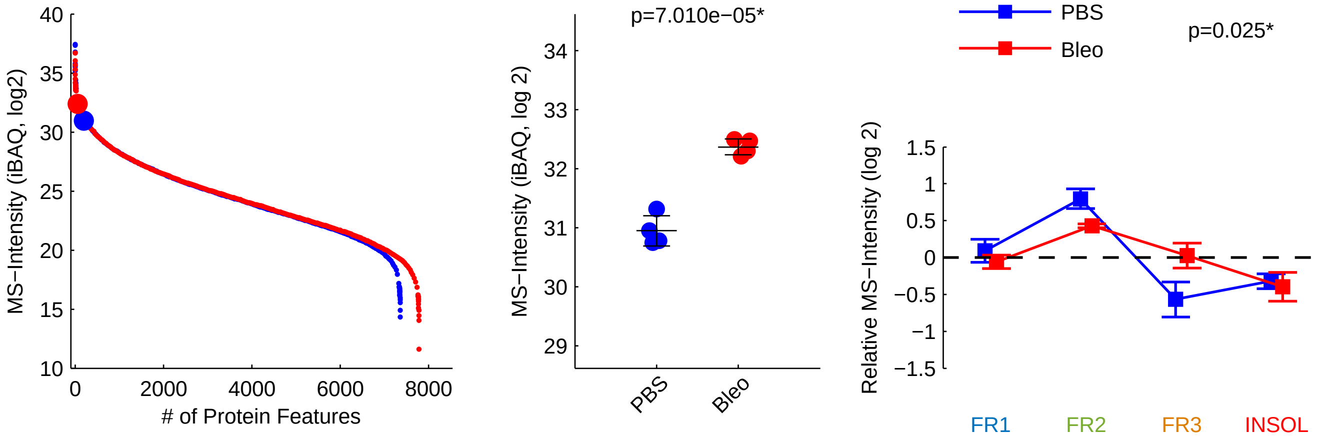

P16125 − Ldhb (id: 2692)

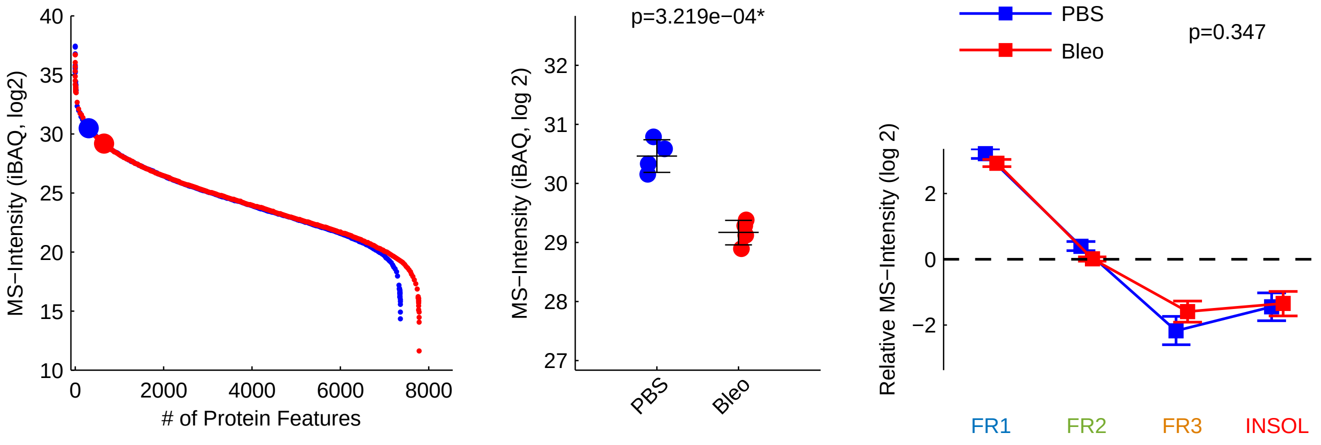

P16330 − Cnp (id: 2696)

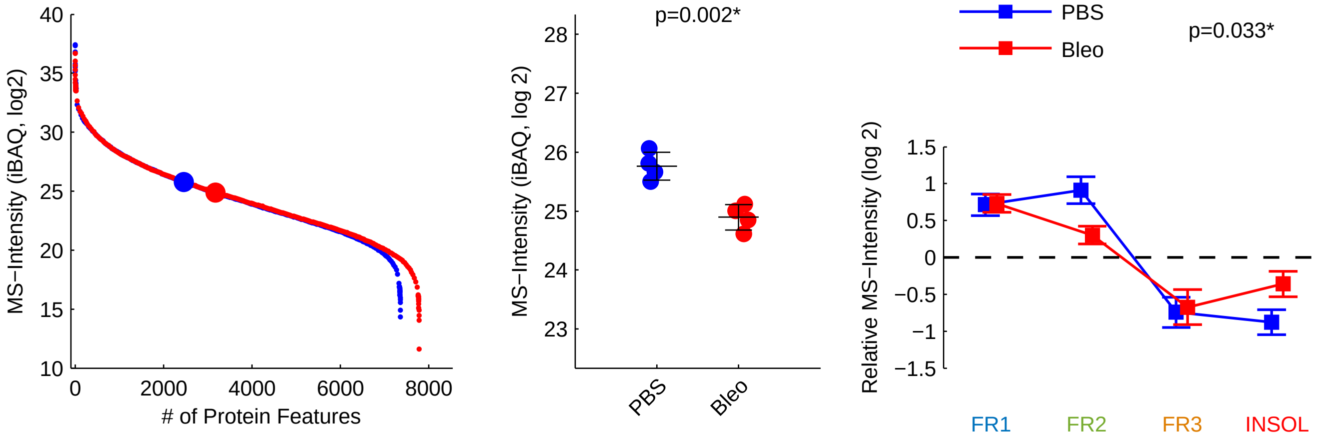

P16332 − Mut (id: 2697)

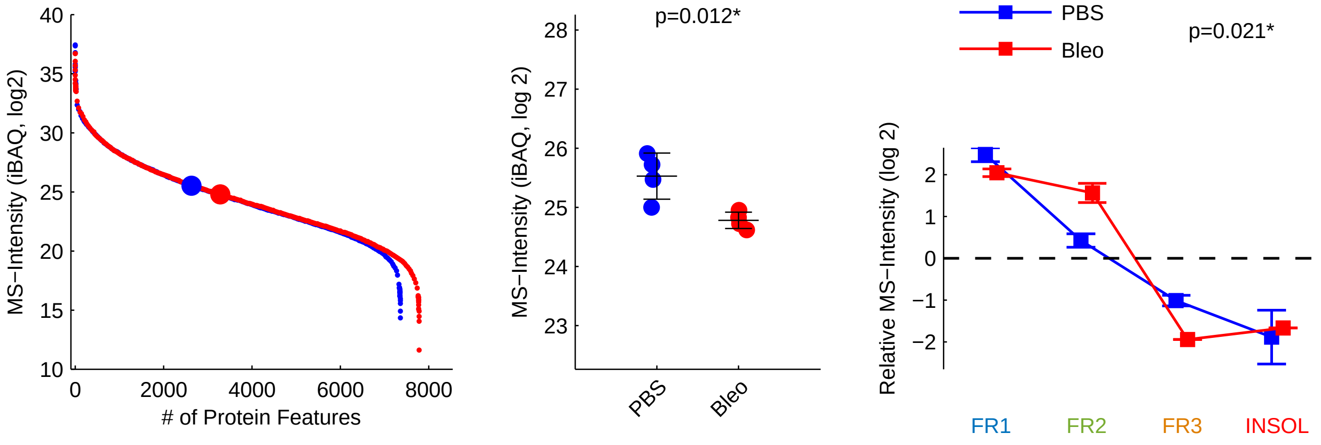

P16406 − Enpep (id: 2699)

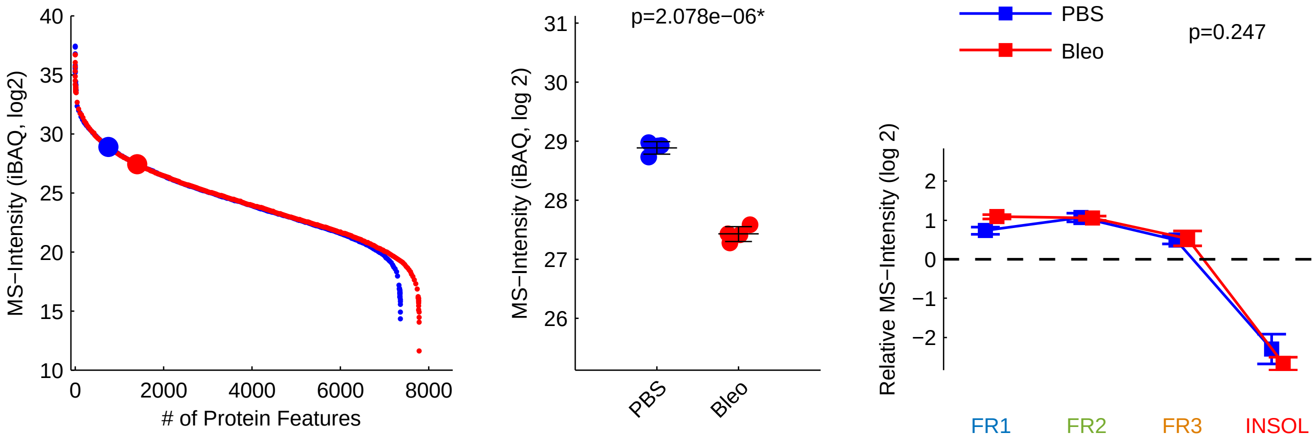

P17047−3 − Lamp2 (id: 2707)

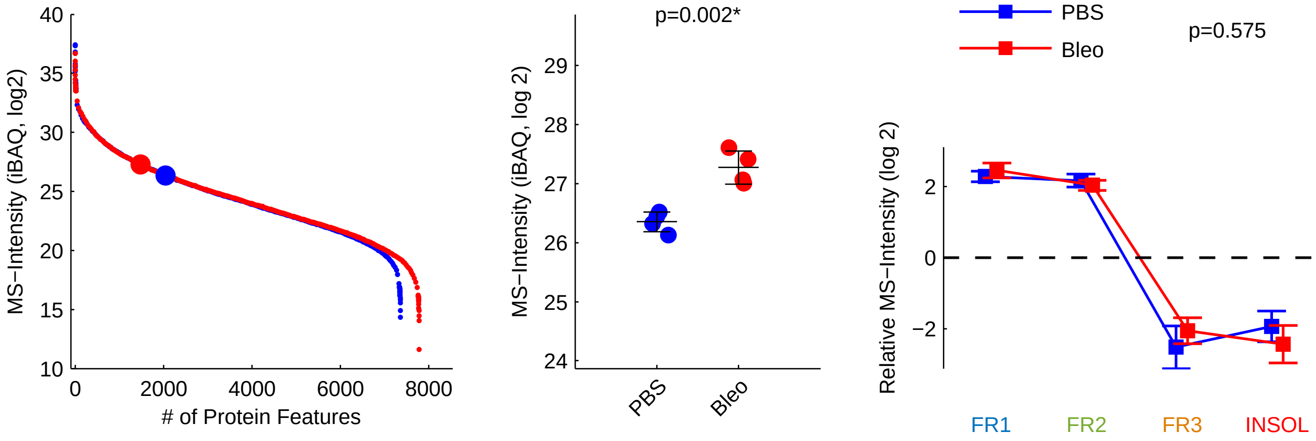

P17182 − Eno1 (id: 2712)

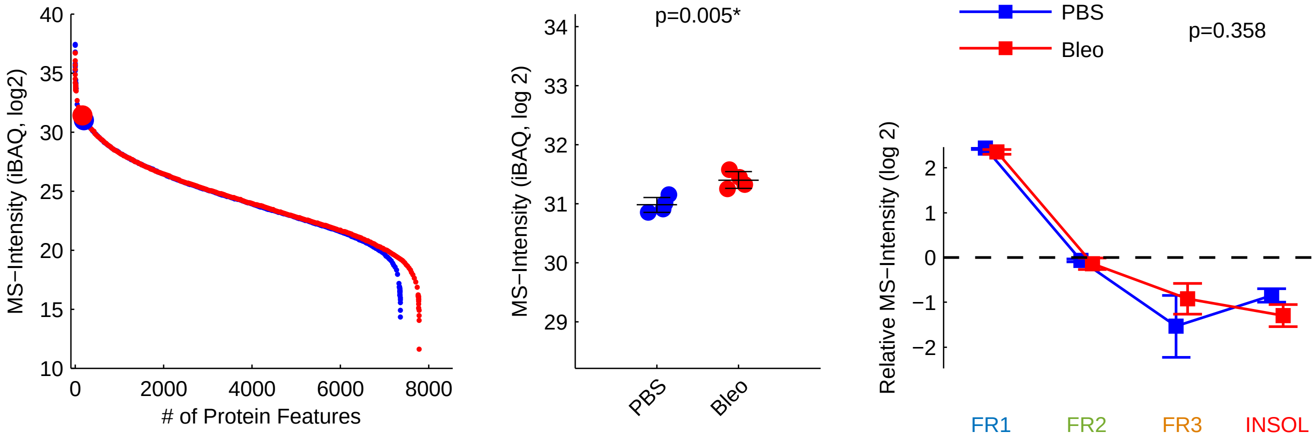

P17427 − Ap2a2 (id: 2715)

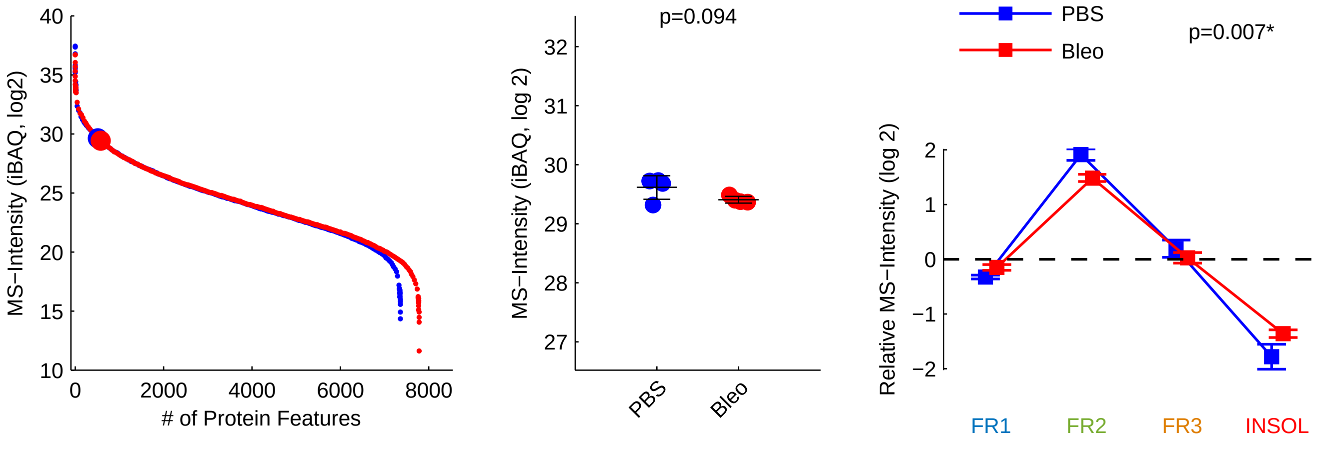

P17439 − Gba (id: 2717)

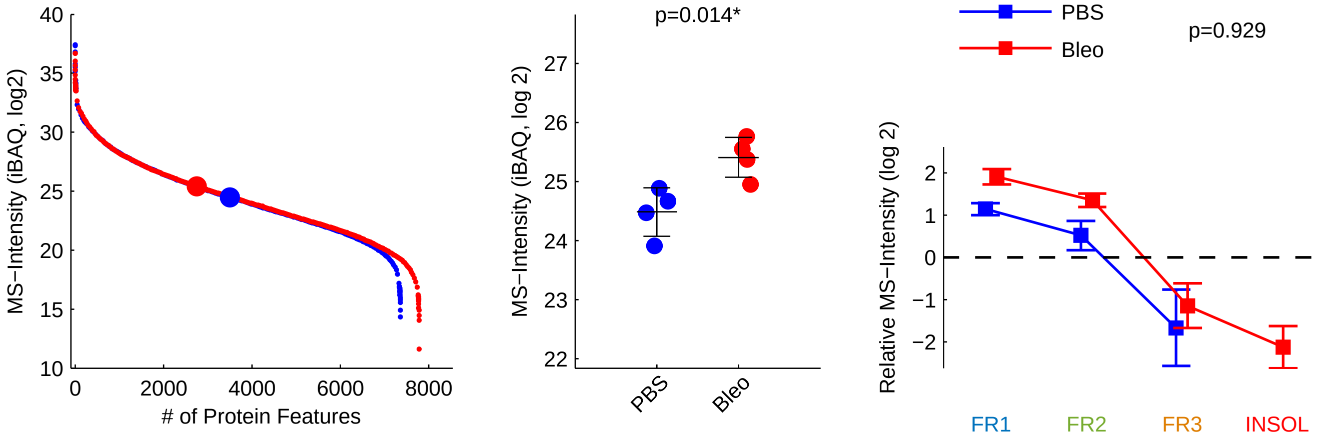

P17563 − Selenbp1 (id: 2718)

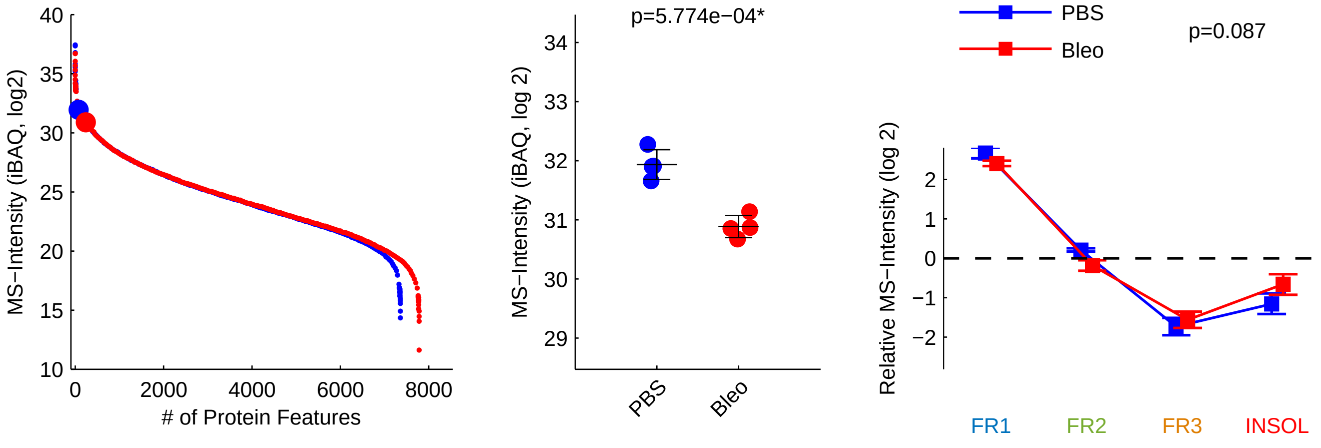

P17897 − Lyz1 (id: 2723)

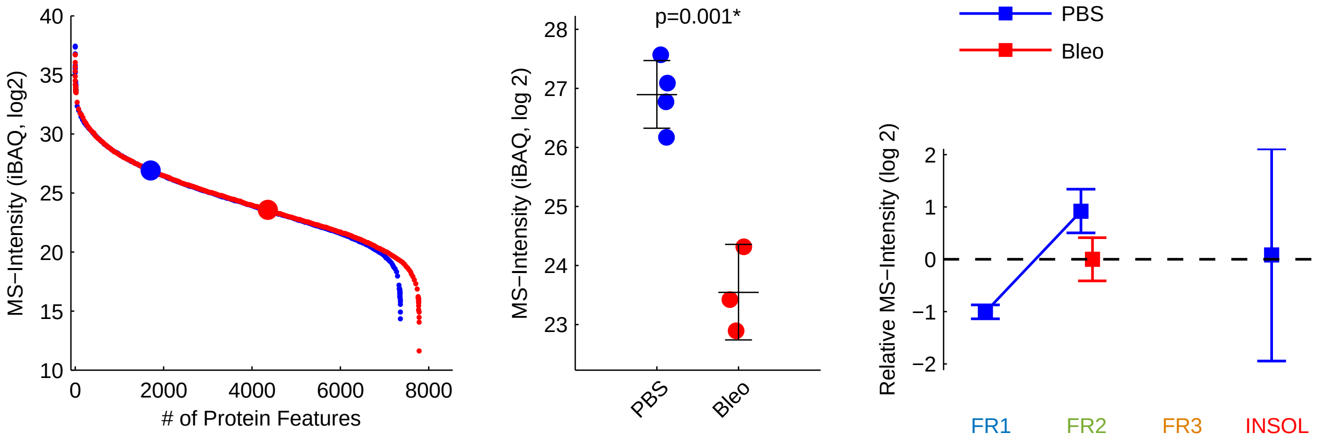

P18242 − Ctsd (id: 2726)

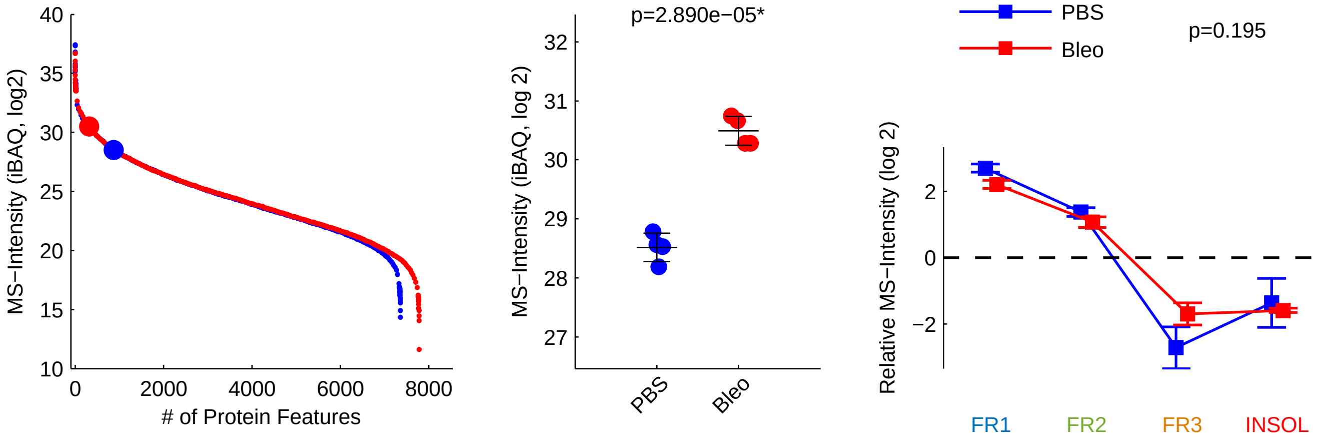

P18293 − Npr1 (id: 2727)

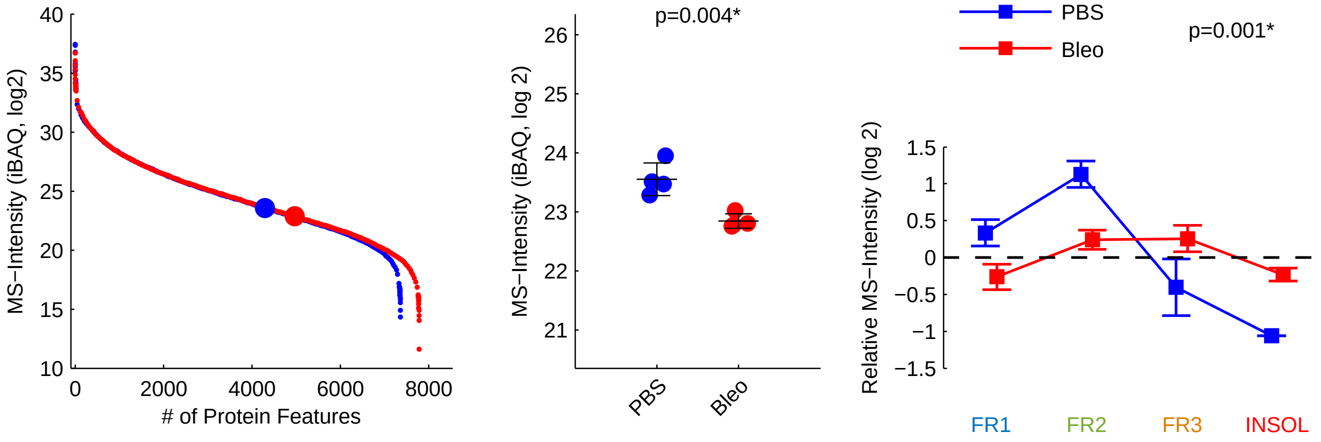

P18760 − Cfl1 (id: 2736)

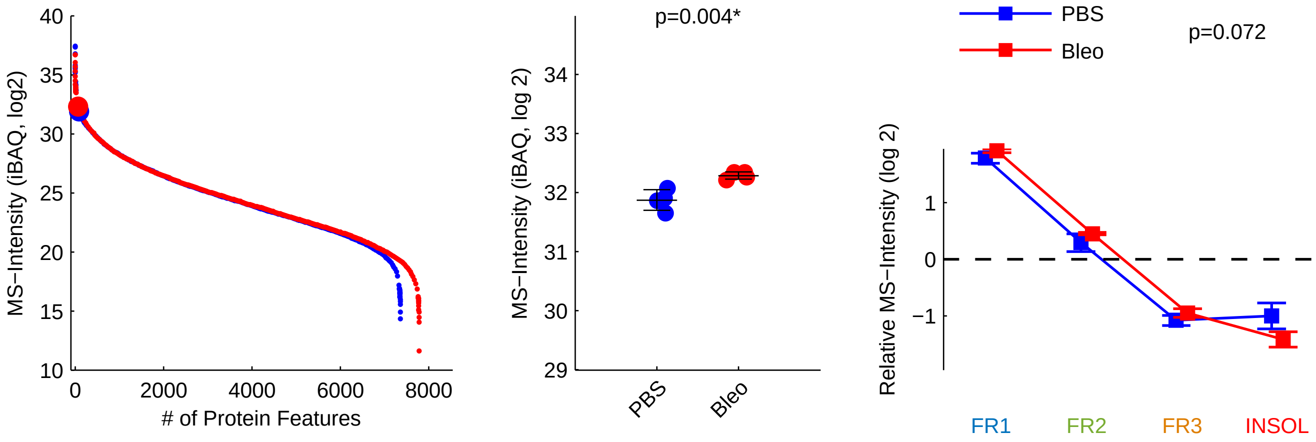

P18872−2 − Gnao1 (id: 2739)

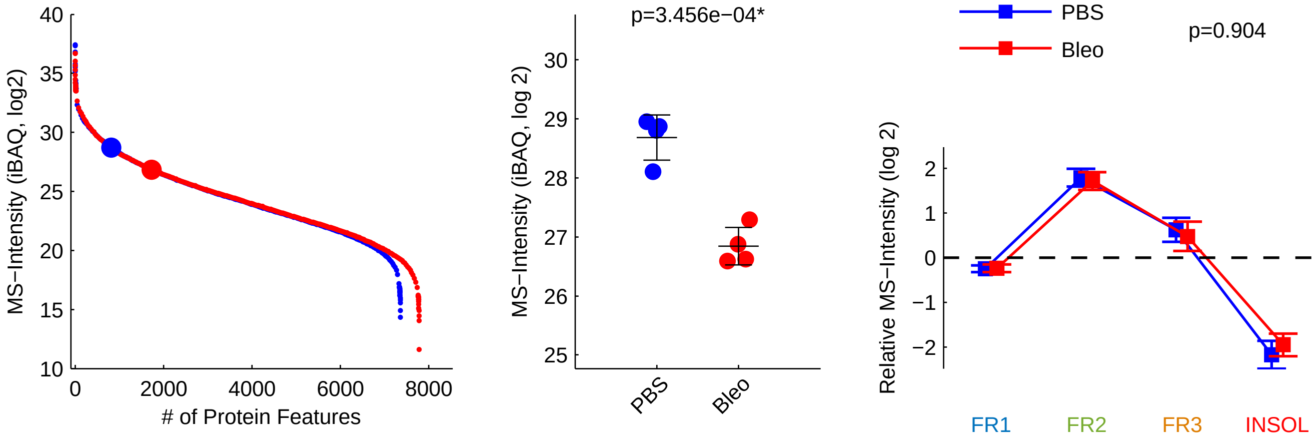

P19253 − Rpl13a (id: 2746)

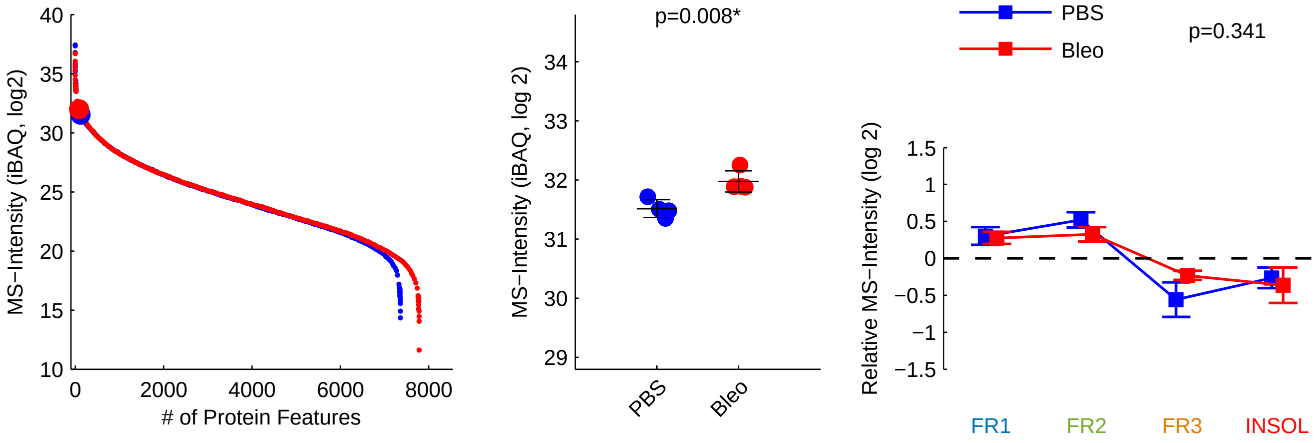

P19324 − Serpinh1 (id: 2747)

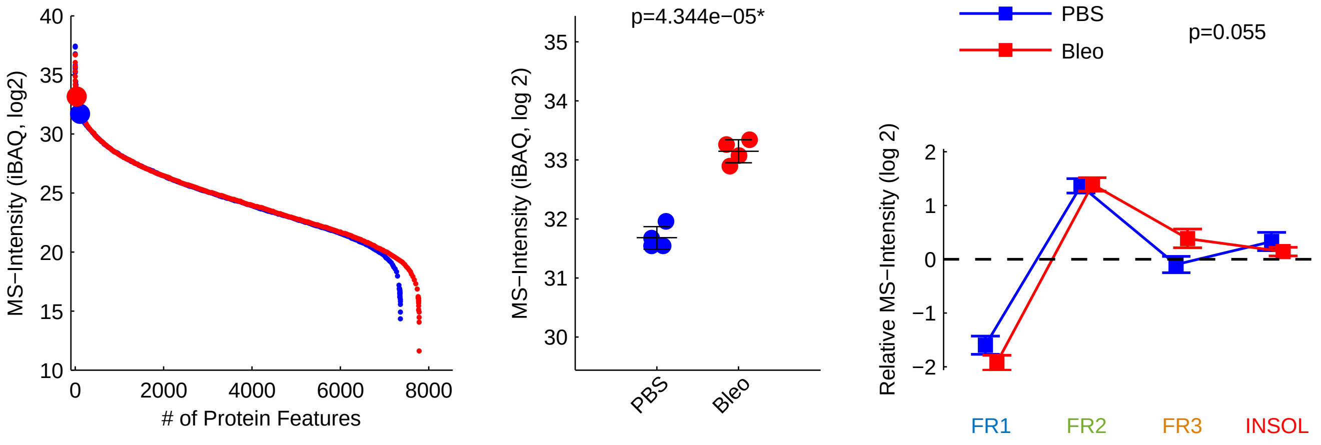

P19536 − Cox5b (id: 2750)

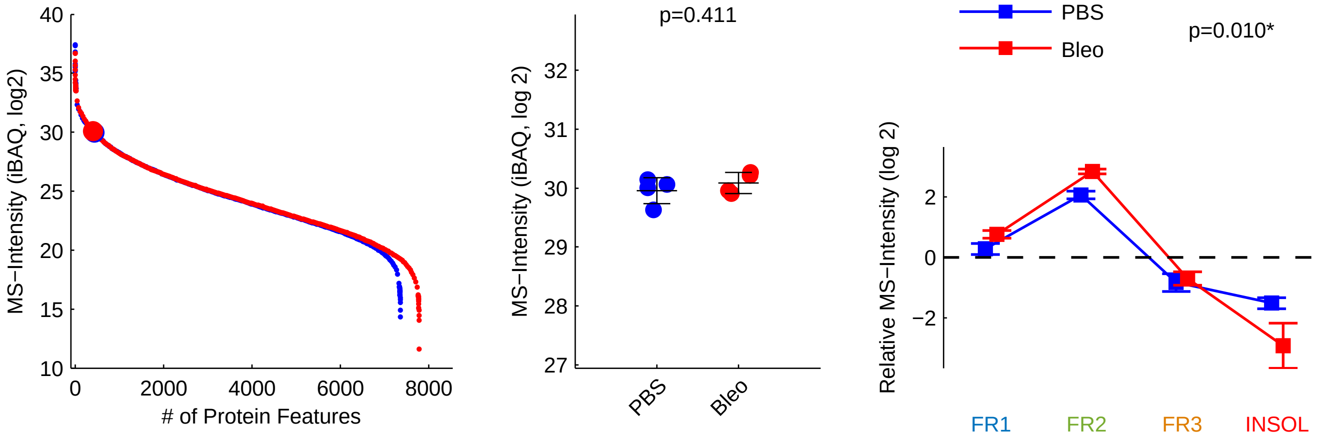

P19783 − Cox4i1 (id: 2752)

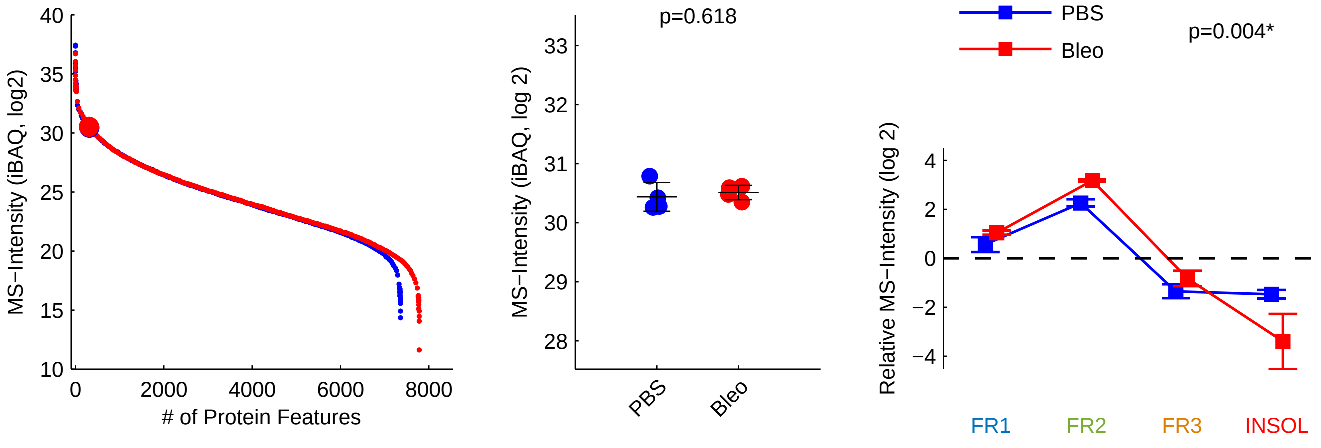

P19788 − Mgp (id: 2753)

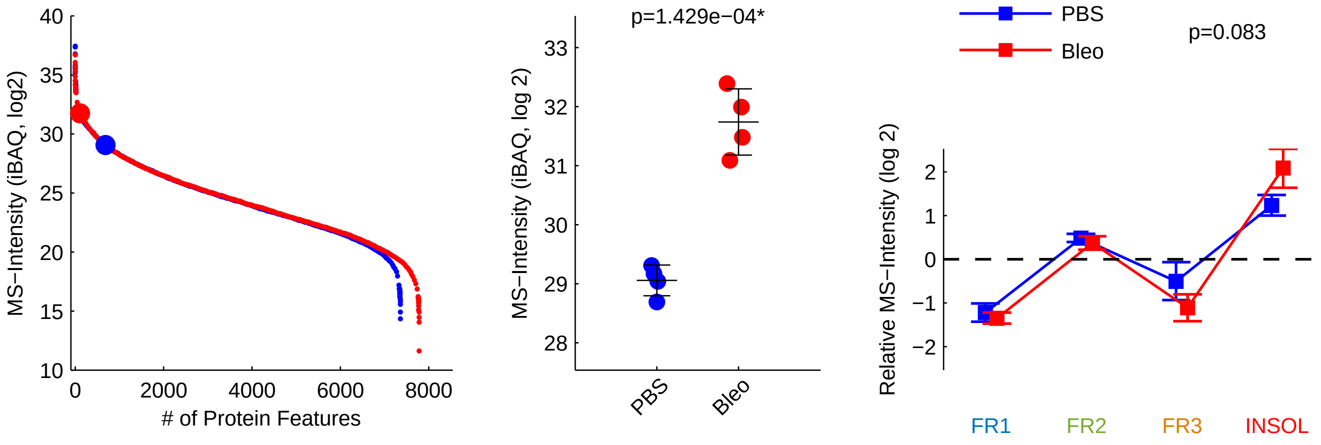

P20029 − Hspa5 (id: 2755)

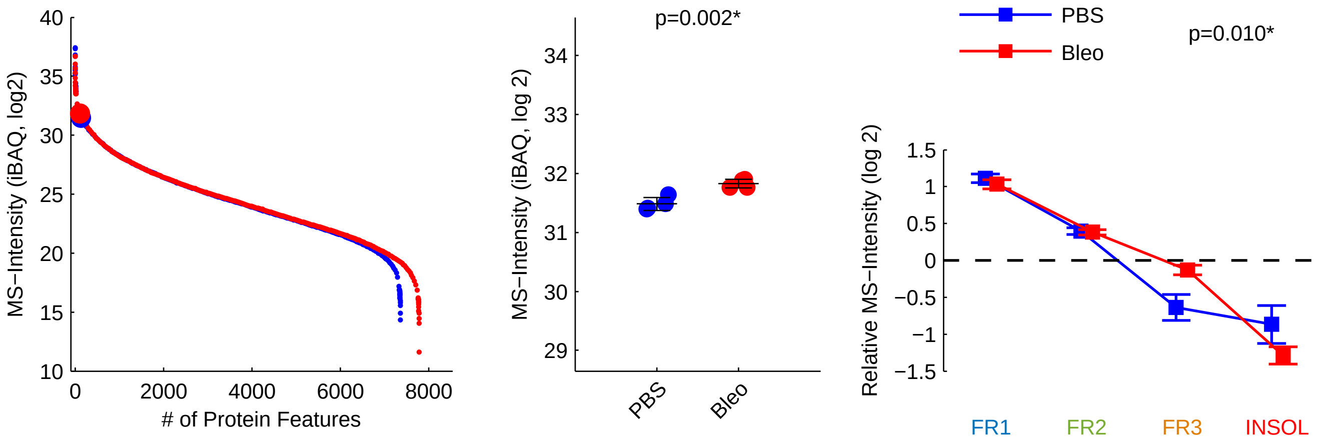

P20060 − Hexb (id: 2756)

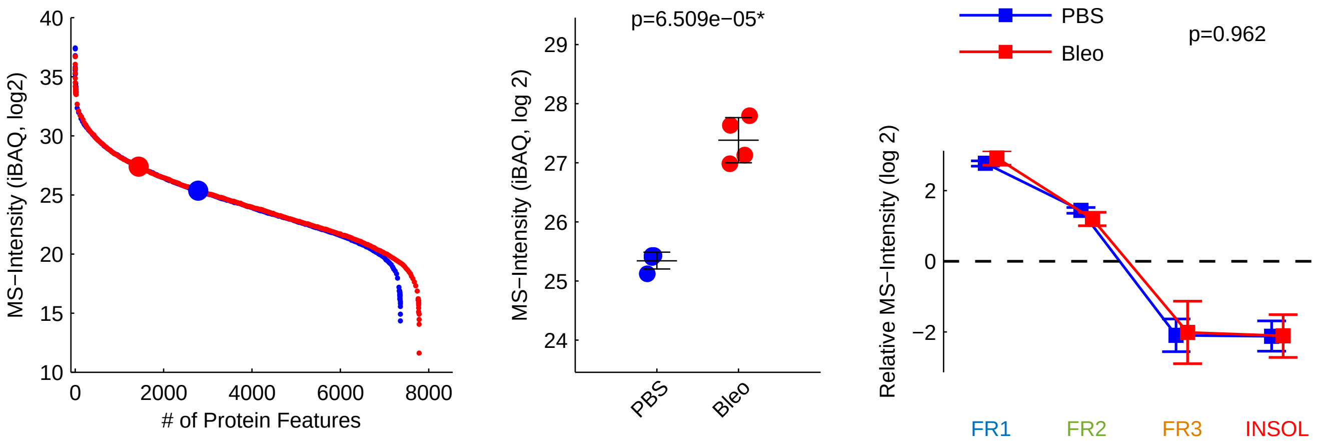

P20152 − Vim (id: 2759)

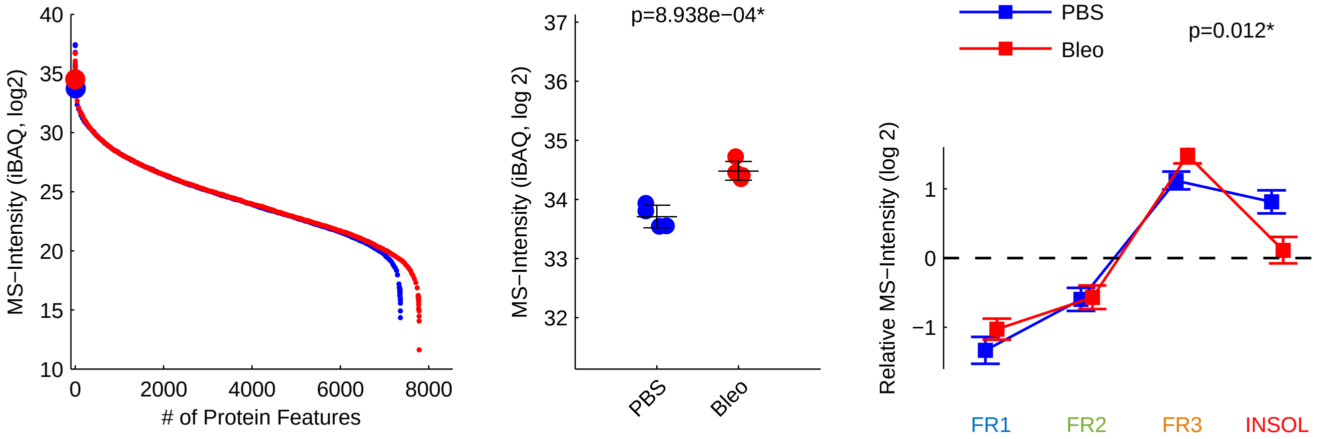

P20357 − Map2 (id: 2761)

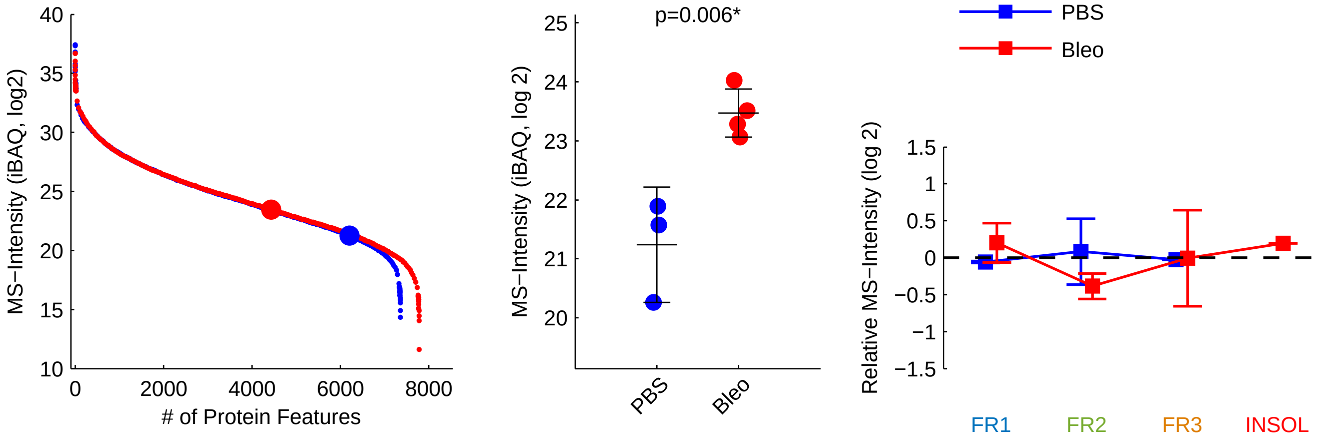

P20918 − Plg (id: 2766)

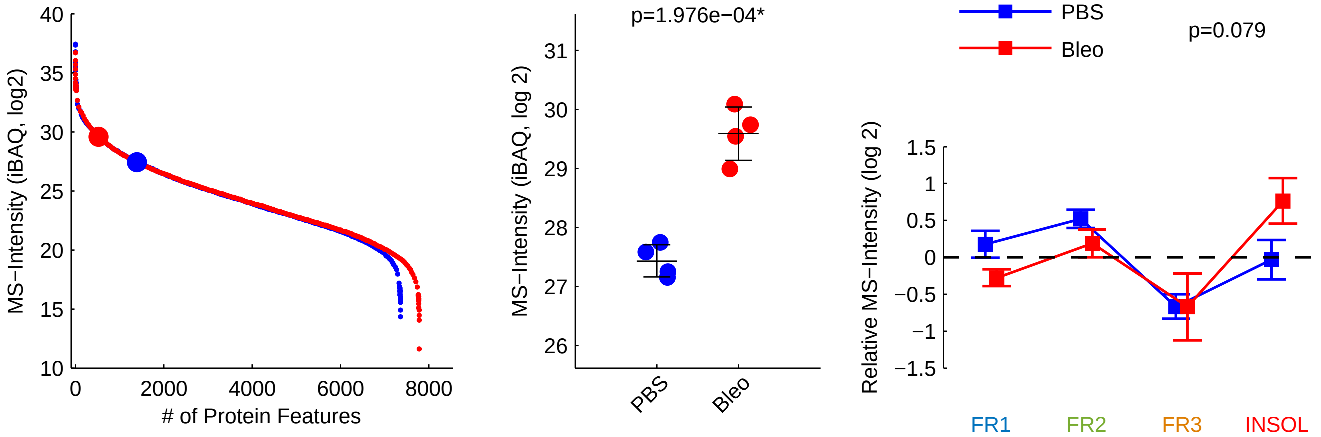

P21107 − Tpm3 (id: 2767)

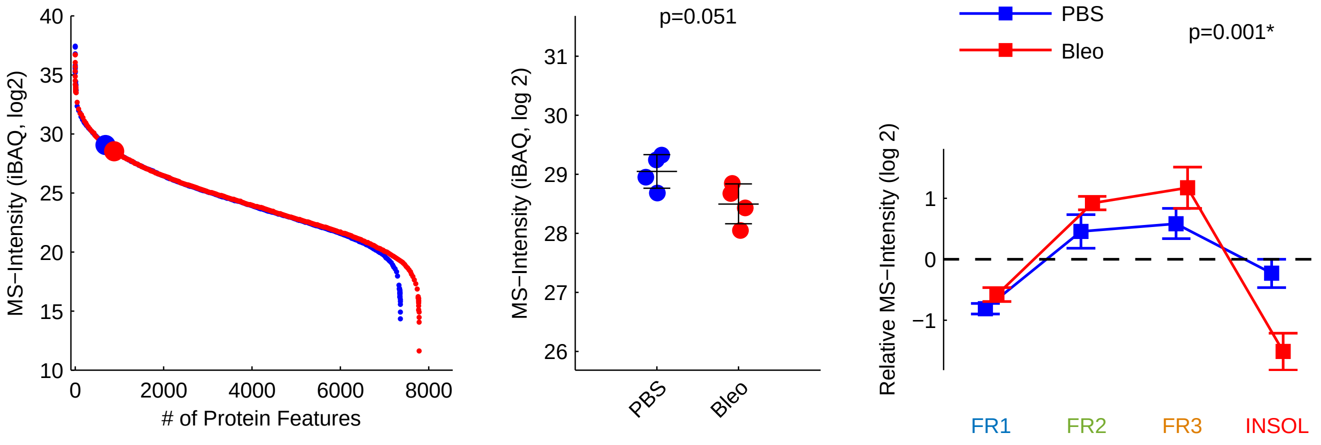

P21278 − Gna11 (id: 2770)

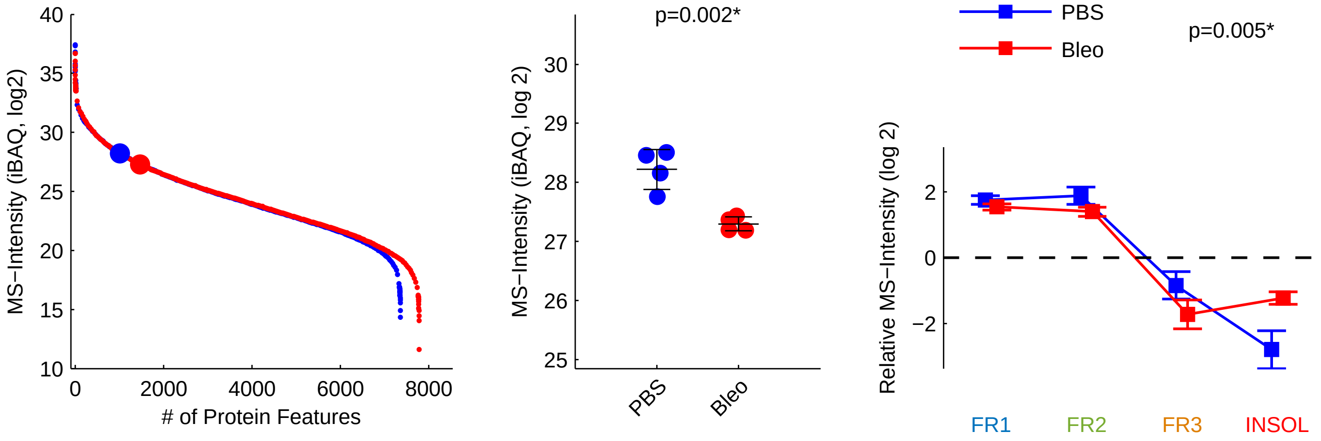

P21279 − Gnaq (id: 2771)

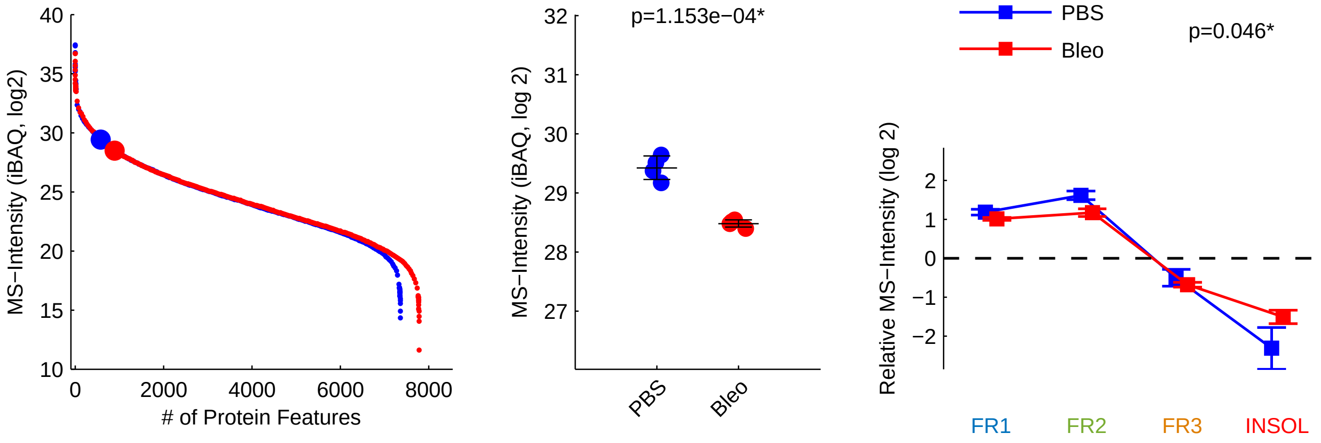

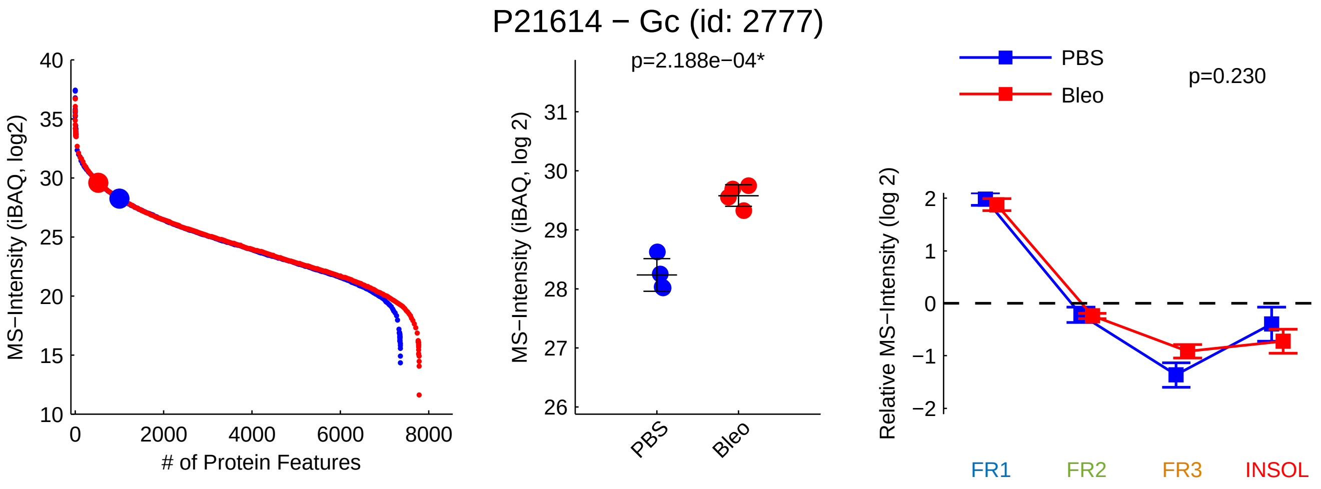
P21614 − Gc (id: 2777)
MS−Intensity (iBAQ, log2)
# of Protein Features
p=2.188e−04*
MS−Intensity (iBAQ, log 2)
PBS
Bleo
PBS
Bleo
p=0.230
Relative MS−Intensity (log 2)
FR1
FR2
FR3
INSOL

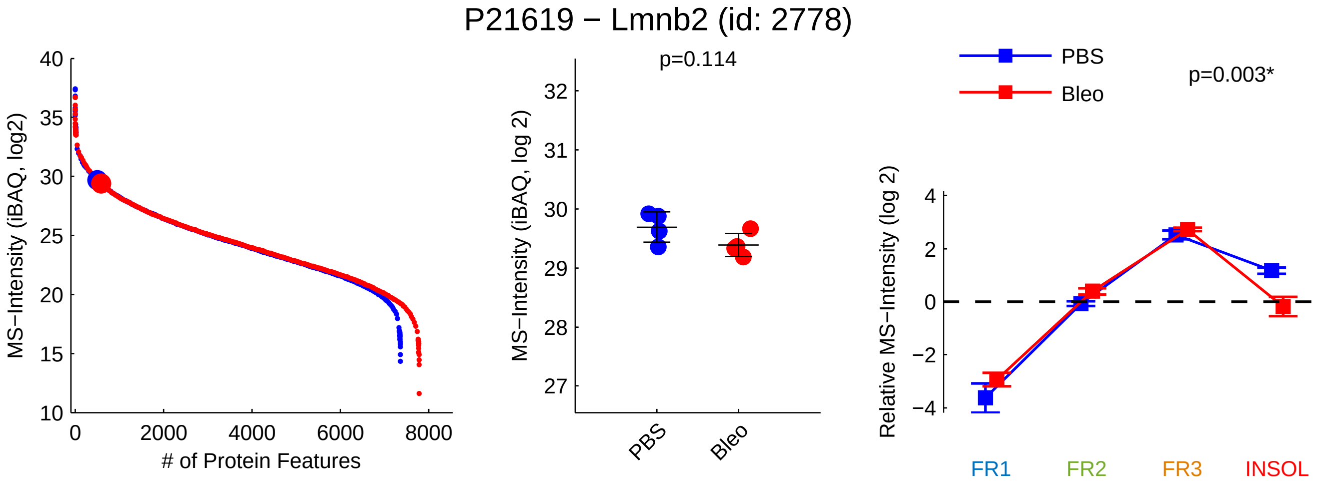
P21619 − Lmnb2 (id: 2778)
MS−Intensity (iBAQ, log2)
# of Protein Features
p=0.114
MS−Intensity (iBAQ, log 2)
PBS
Bleo
PBS
Bleo
p=0.003*
Relative MS−Intensity (log 2)
FR1
FR2
FR3
INSOL

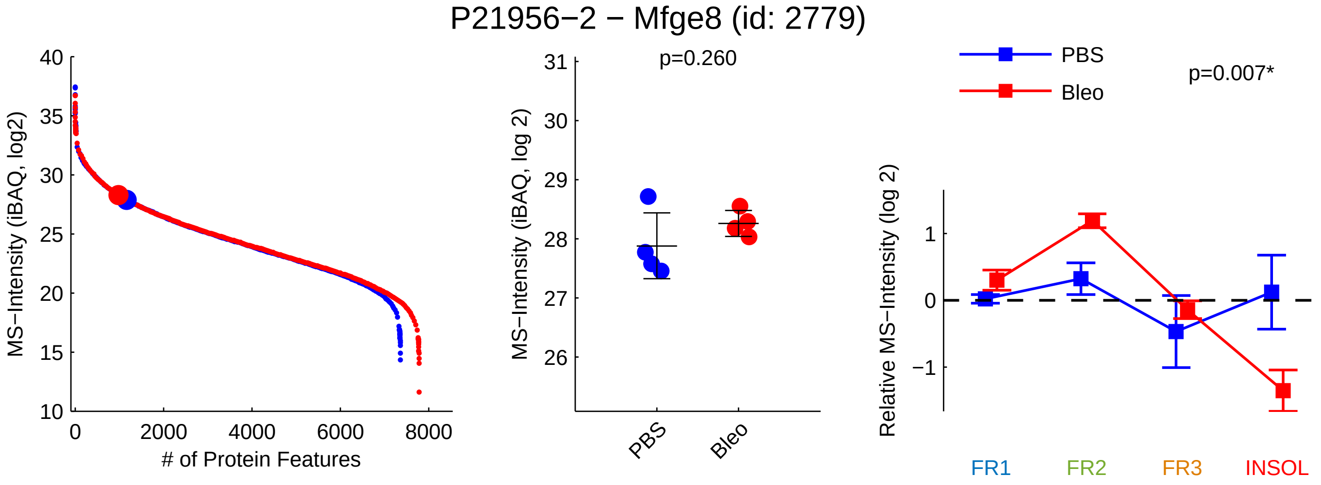
P21956−2 − Mfge8 (id: 2779)
MS−Intensity (iBAQ, log2)
# of Protein Features
p=0.260
MS−Intensity (iBAQ, log 2)
PBS
Bleo
PBS
Bleo
p=0.007*
Relative MS−Intensity (log 2)
FR1
FR2
FR3
INSOL

P21981 − Tgm2 (id: 2781)

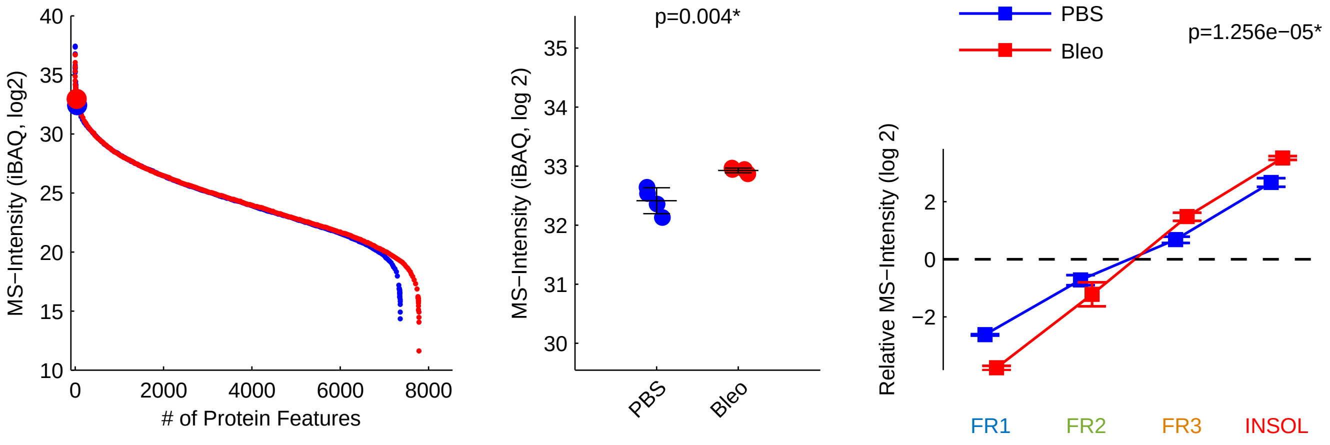

P21995 − Emb (id: 2782)

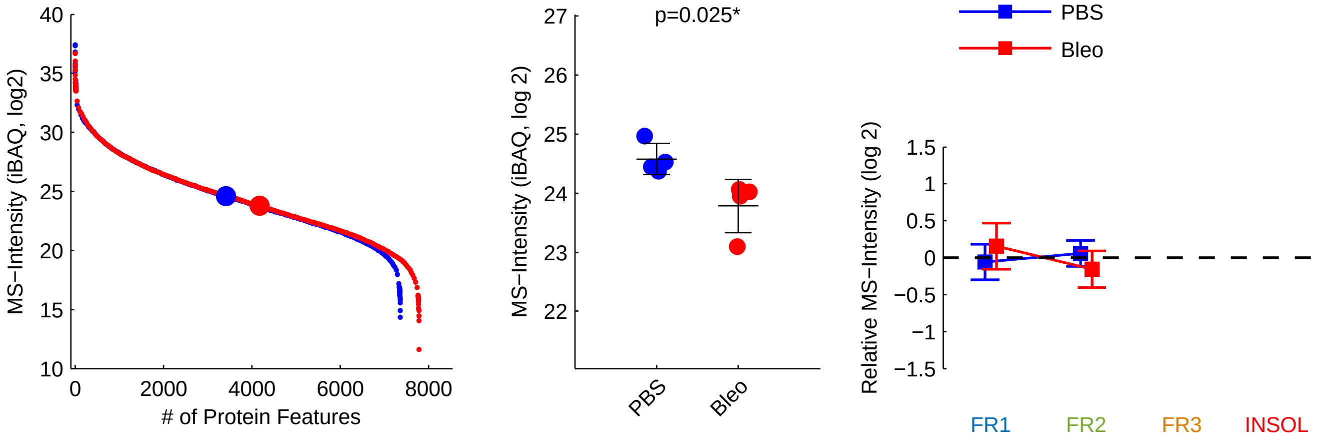

P22599 − Serpina1b (id: 2787)

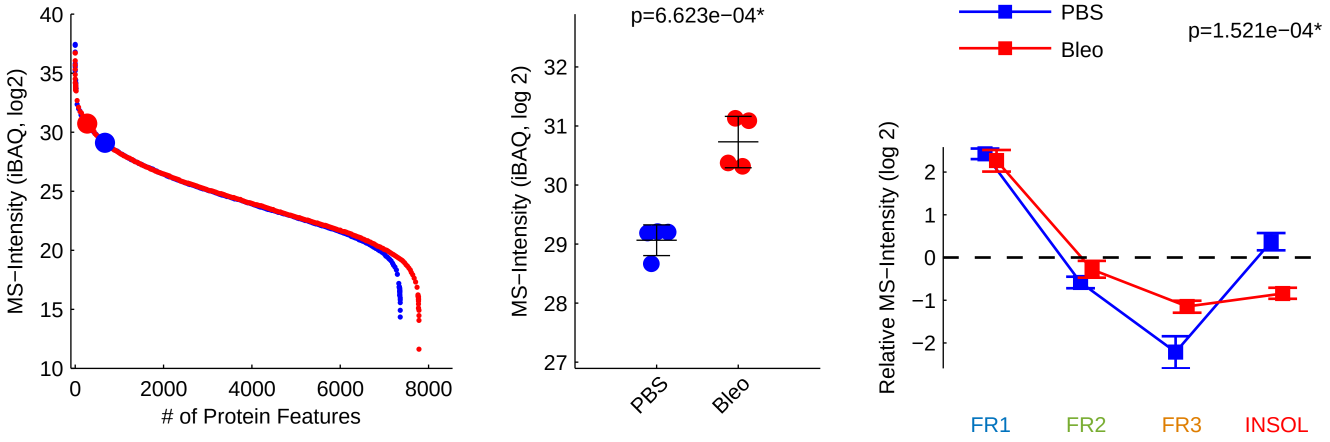

P22682 − Cbl (id: 2789)

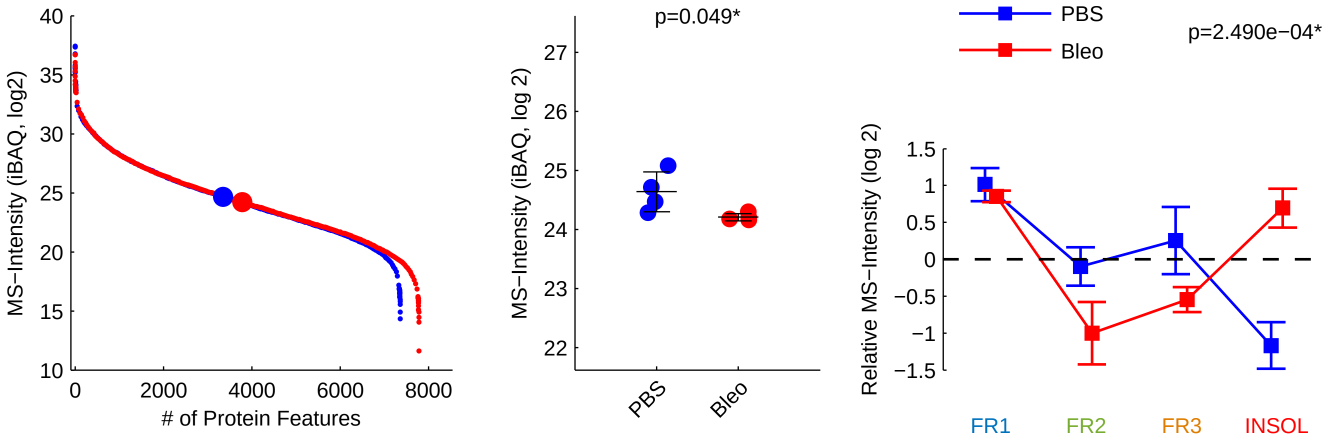

P22892 − Ap1g1 (id: 2790)

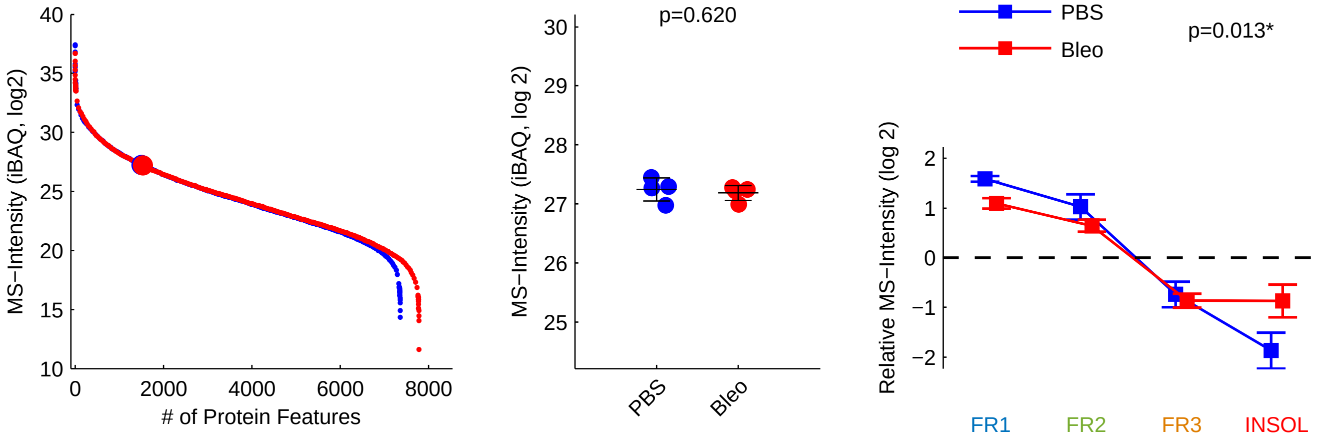

P23116 − Eif3a (id: 2792)

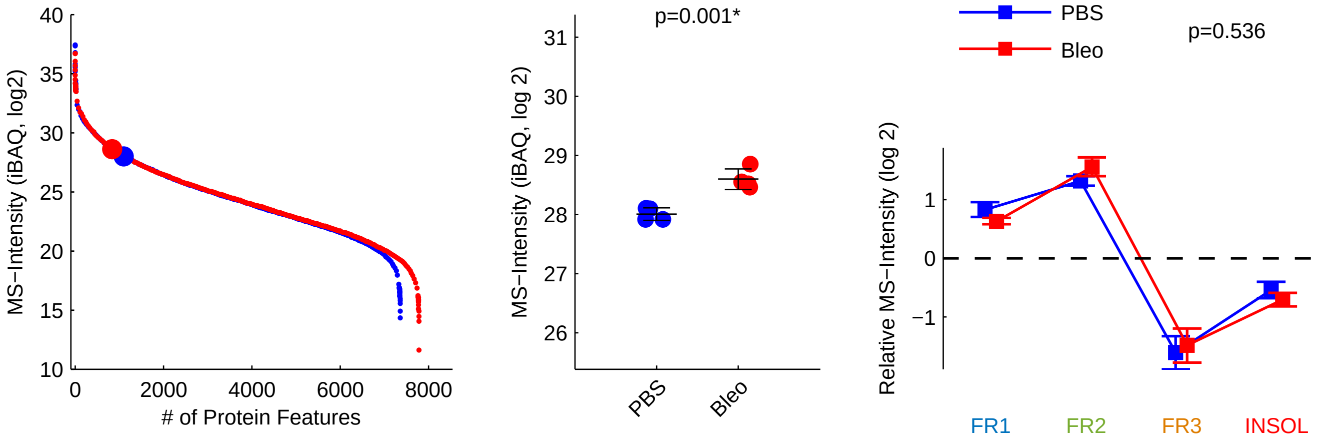

P23298 − Prkch (id: 2794)

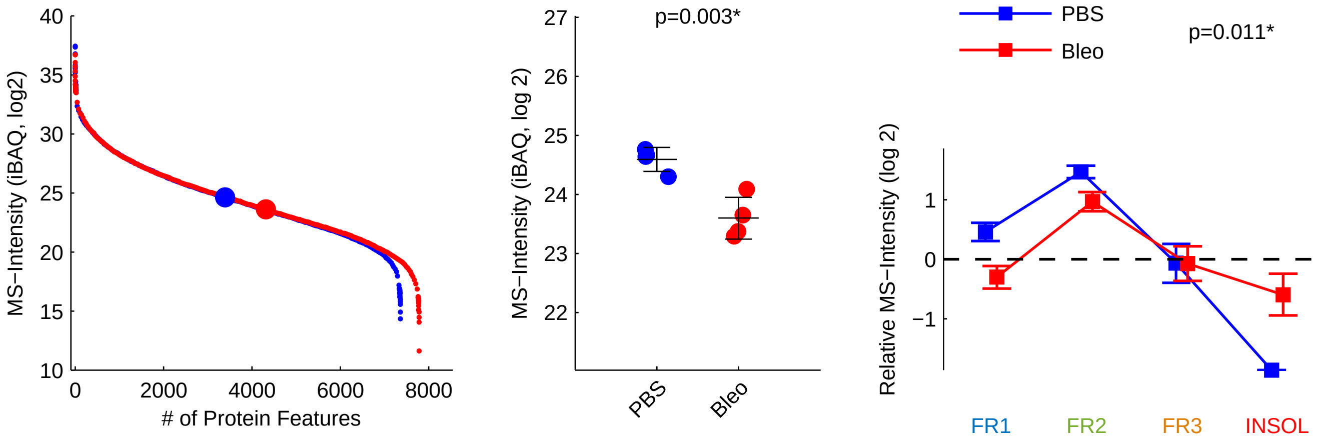

P23953 − Ces1c (id: 2805)

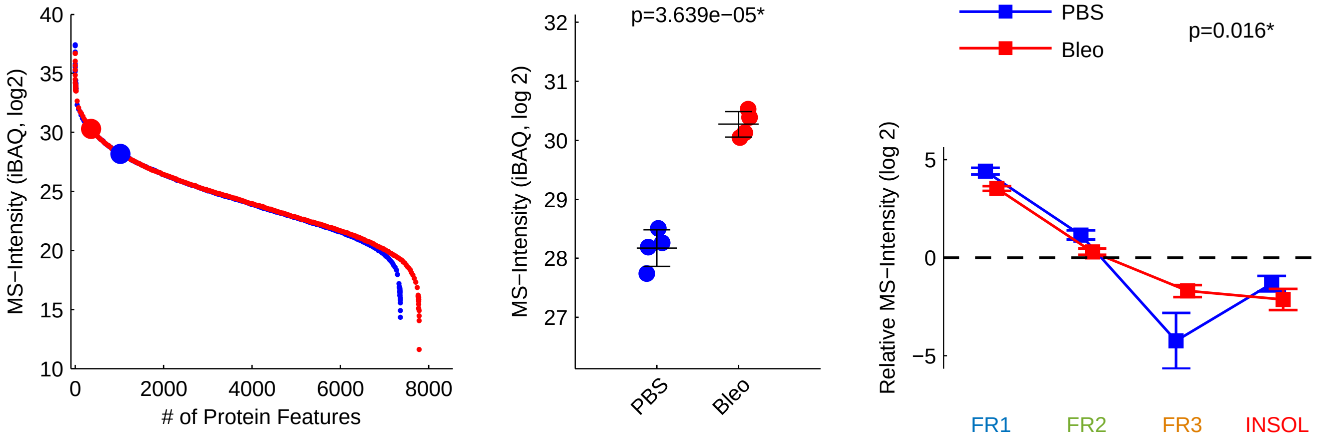

P24270 − Cat (id: 2806)

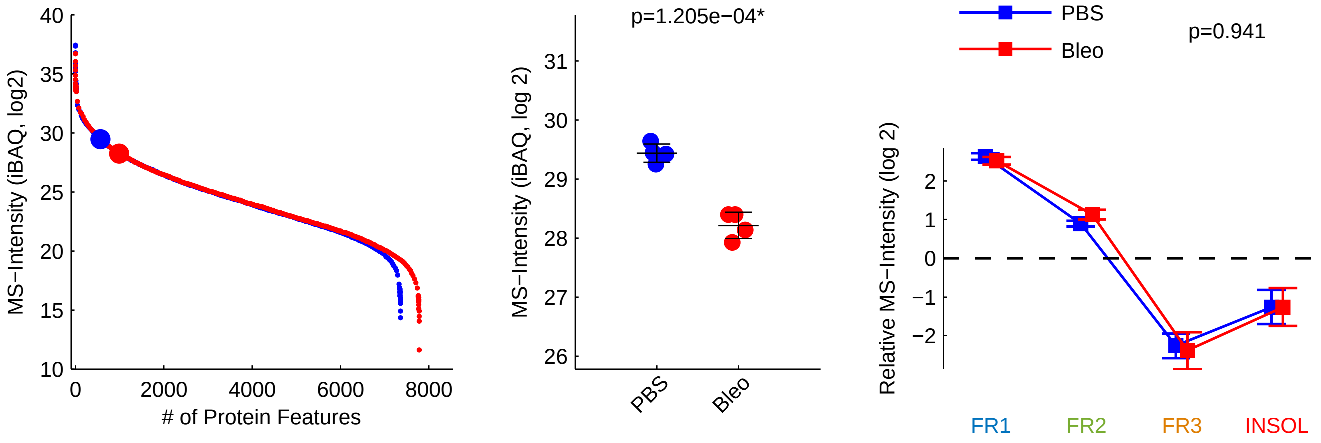

P24369 − Ppib (id: 2807)

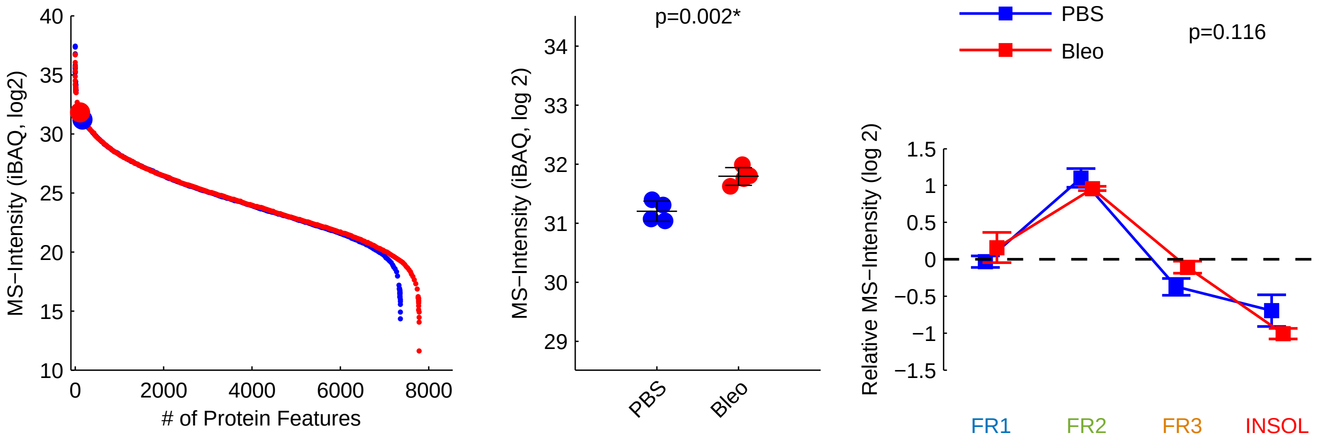

P24527 − Lta4h (id: 2809)

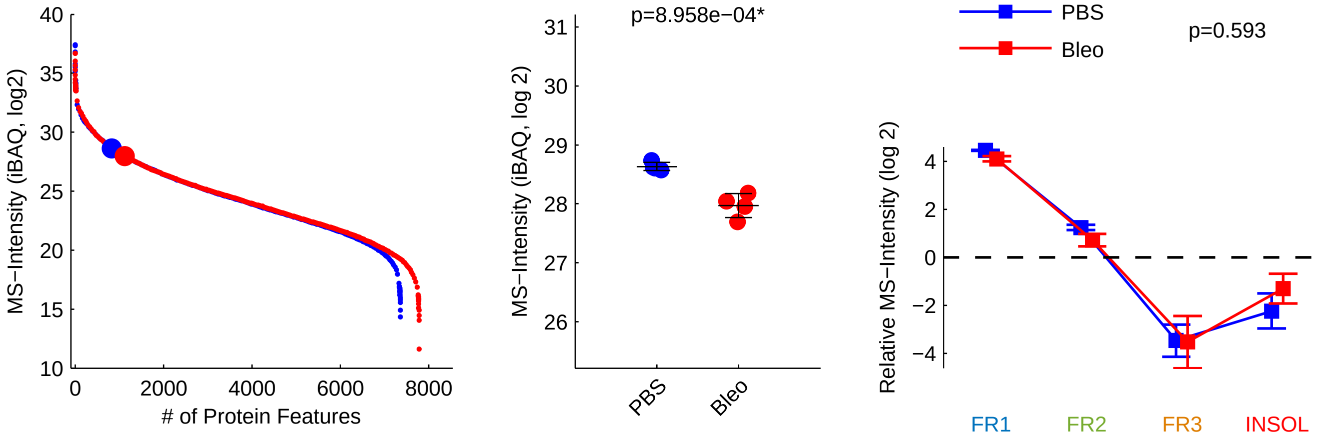

P24549 − Aldh1a1 (id: 2811)

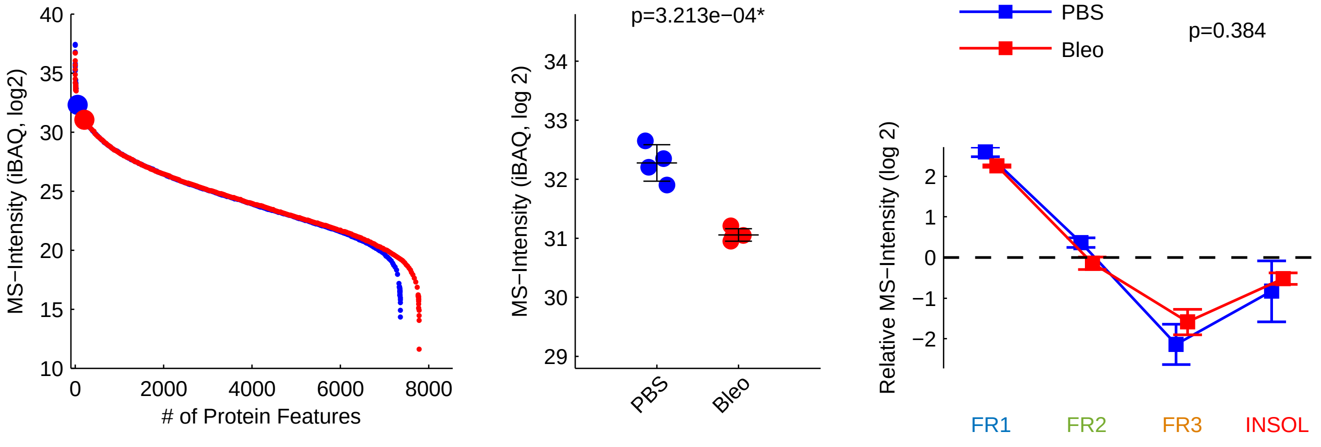

P24638 − Acp2 (id: 2812)

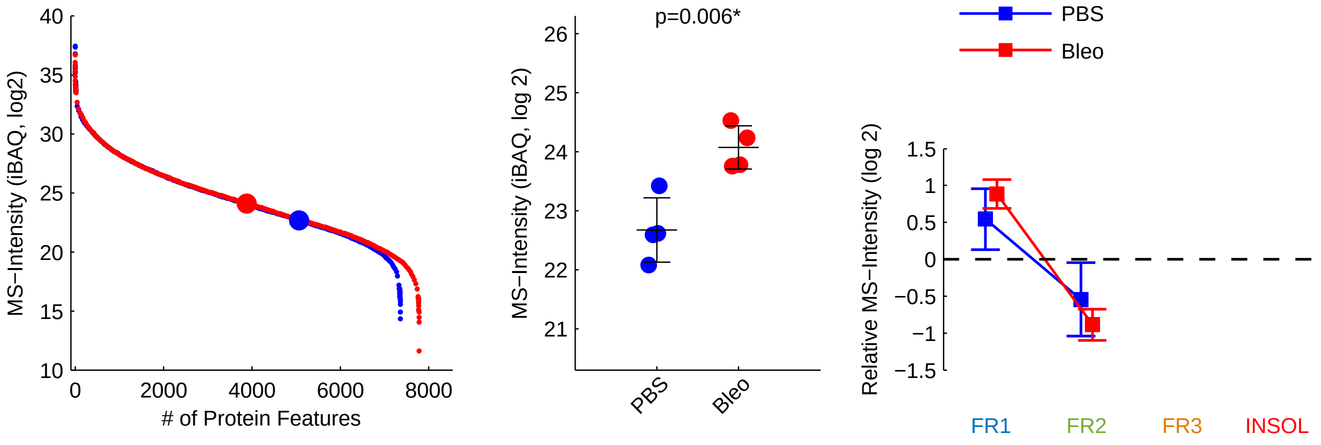

P25444 − Rps2 (id: 2816)

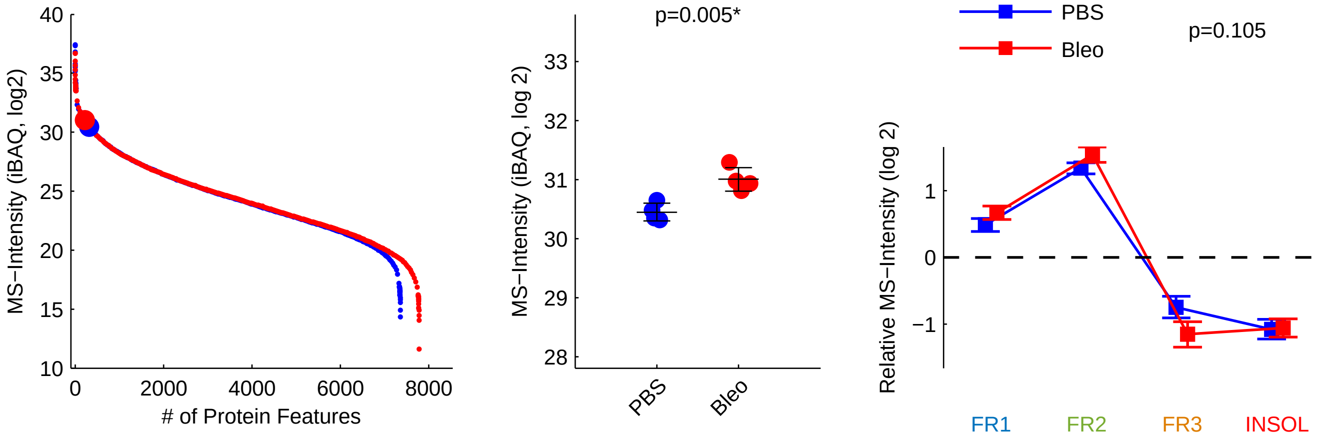

Q6PI17 − Timp2 (id: 2818)

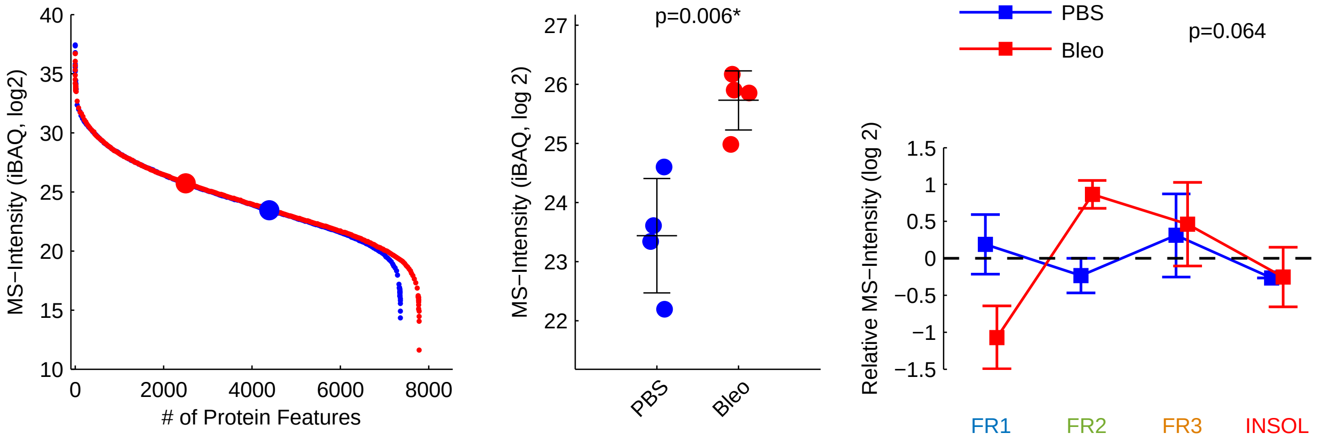

P25911 − Lyn (id: 2820)

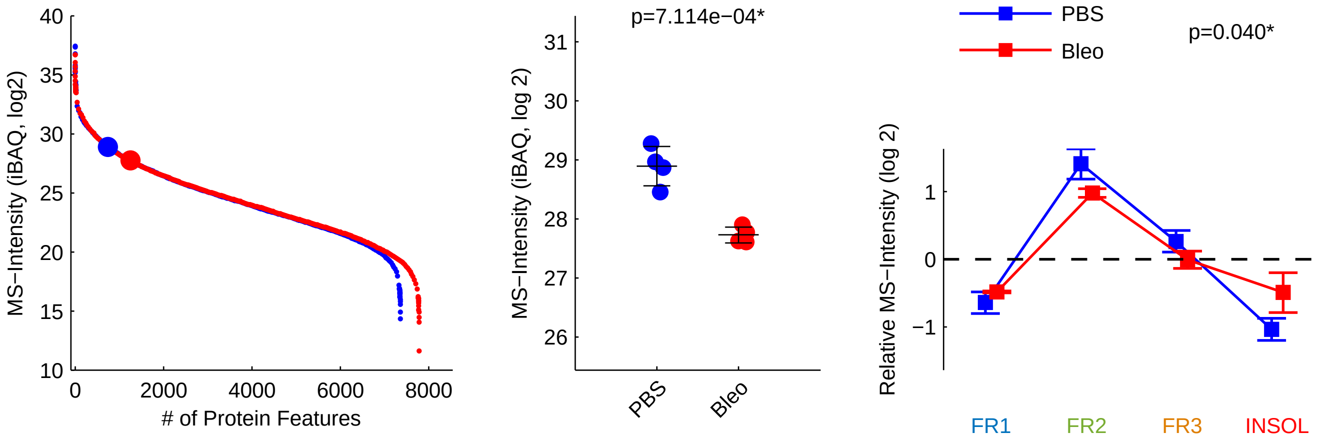

P26039 − Tln1 (id: 2824)

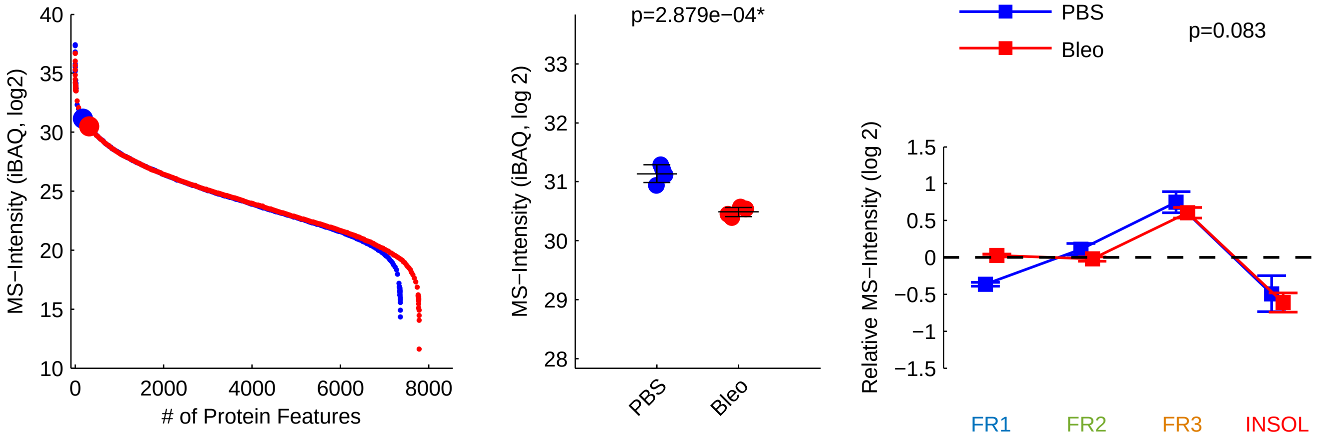

P26041 − Msn (id: 2826)

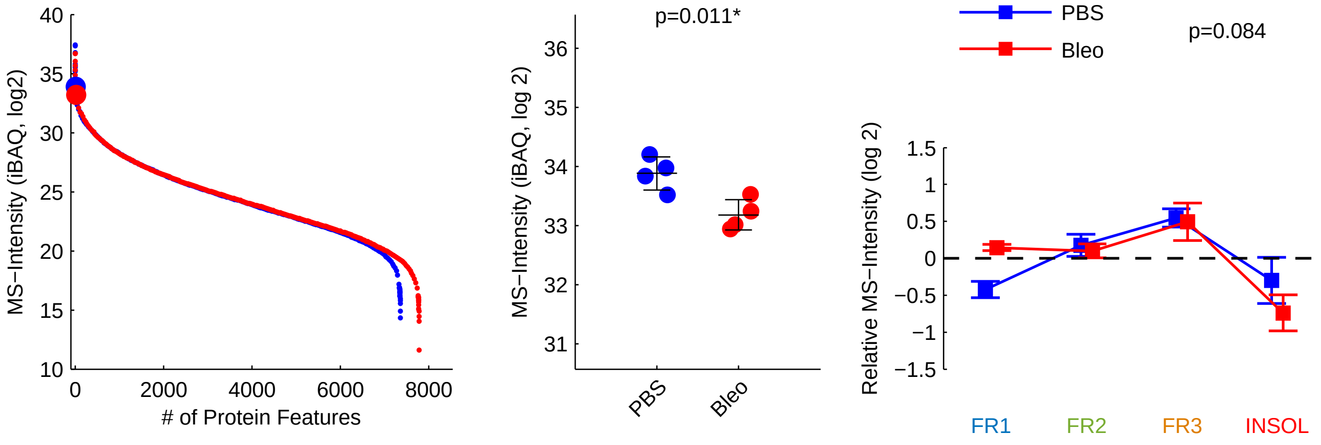

P26043 − Rdx (id: 2827)

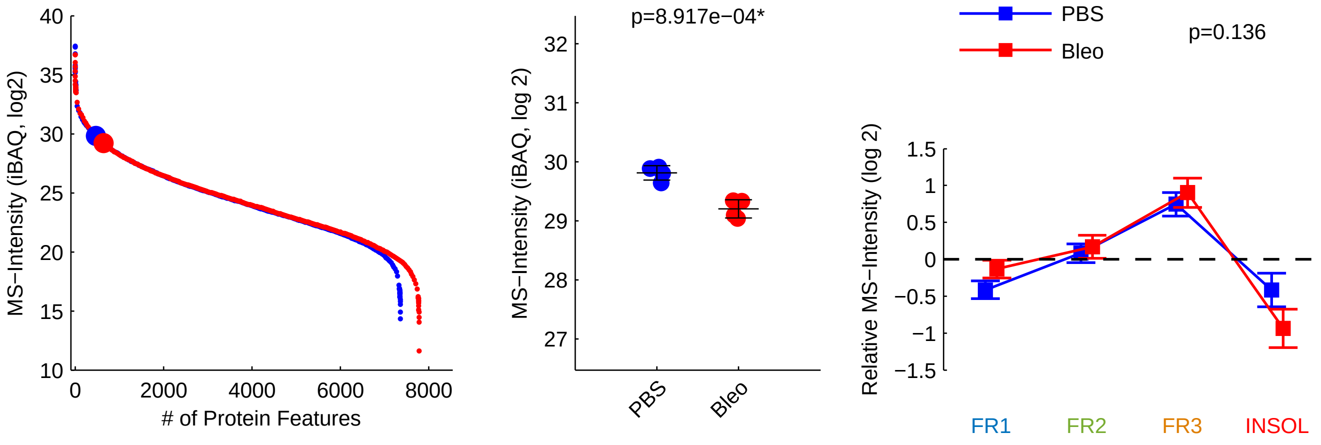

P26231 − Ctnna1 (id: 2829)

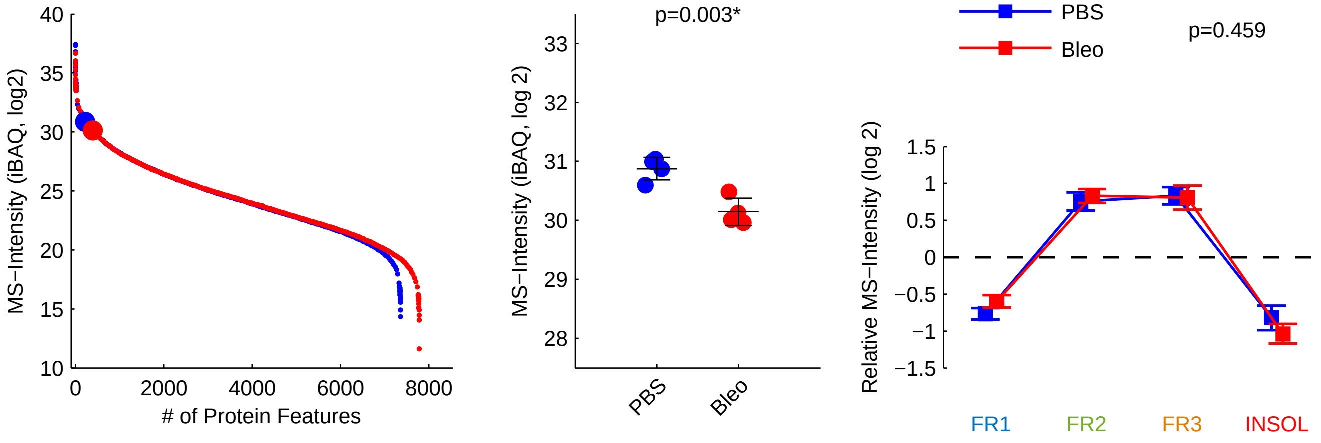

P26262 − Klkb1 (id: 2830)

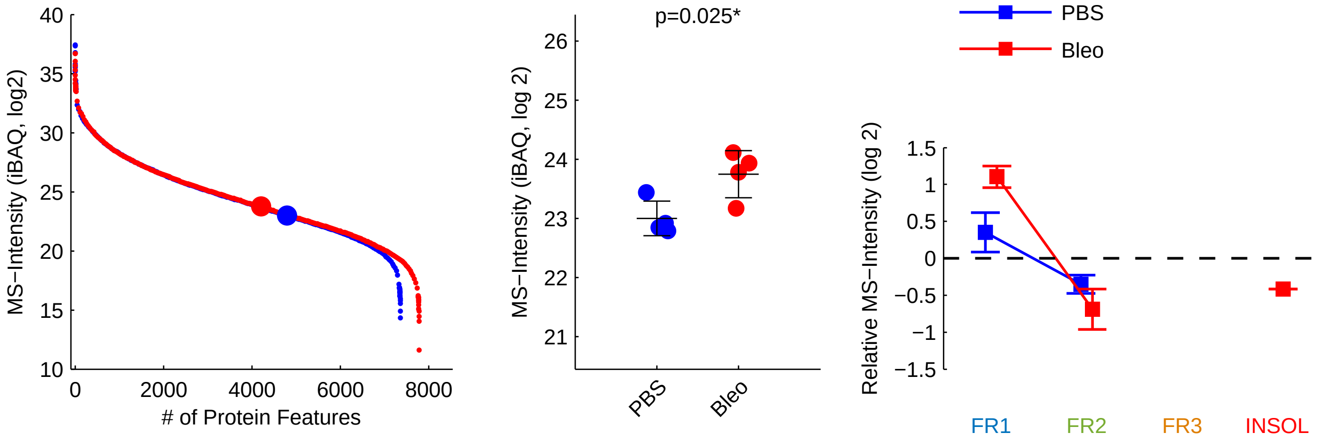

P26450 − Pik3r1 (id: 2835)

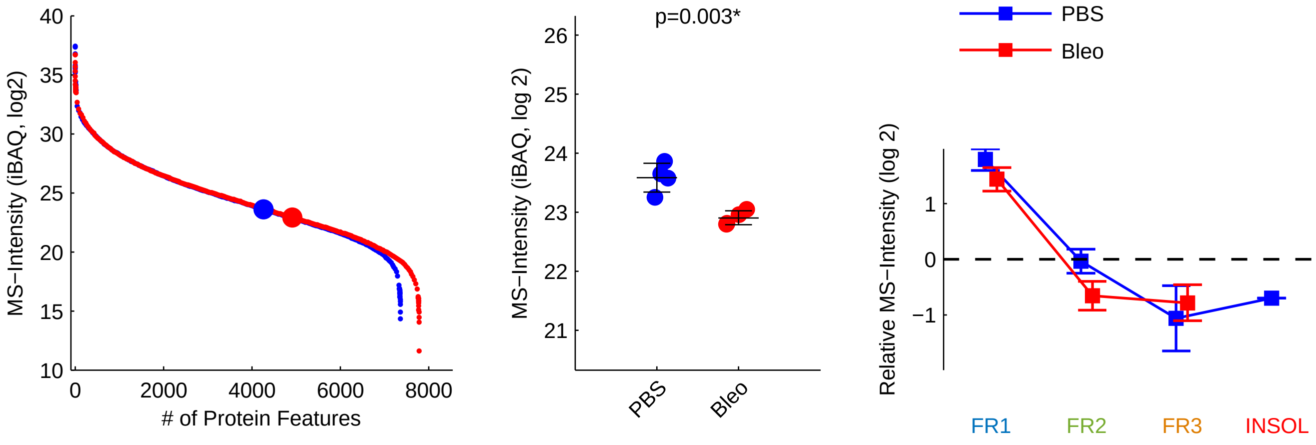

P27046 − Man2a1 (id: 2842)

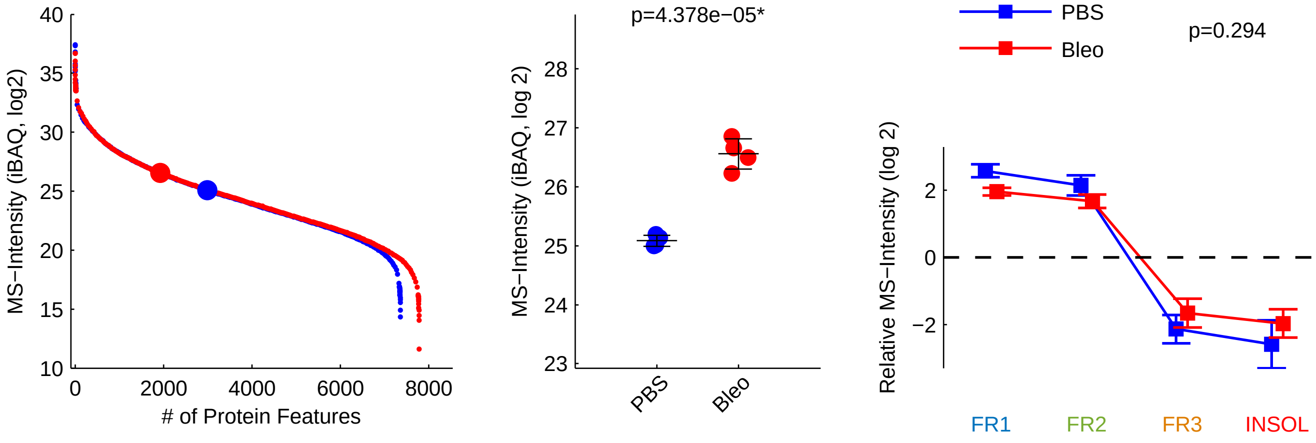

P27090 − Tgfb2 (id: 2844)

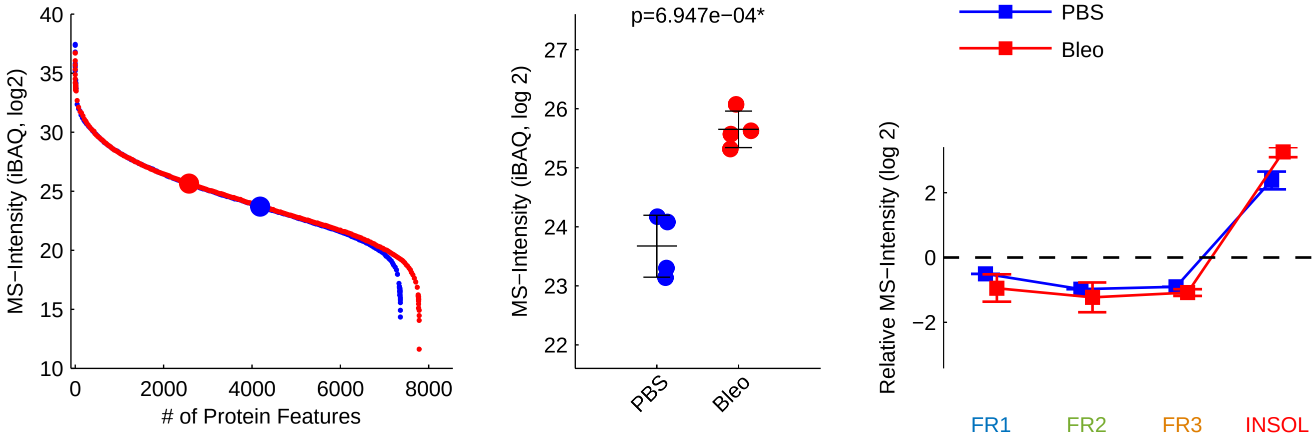

P27467 − Wnt3a (id: 2845)

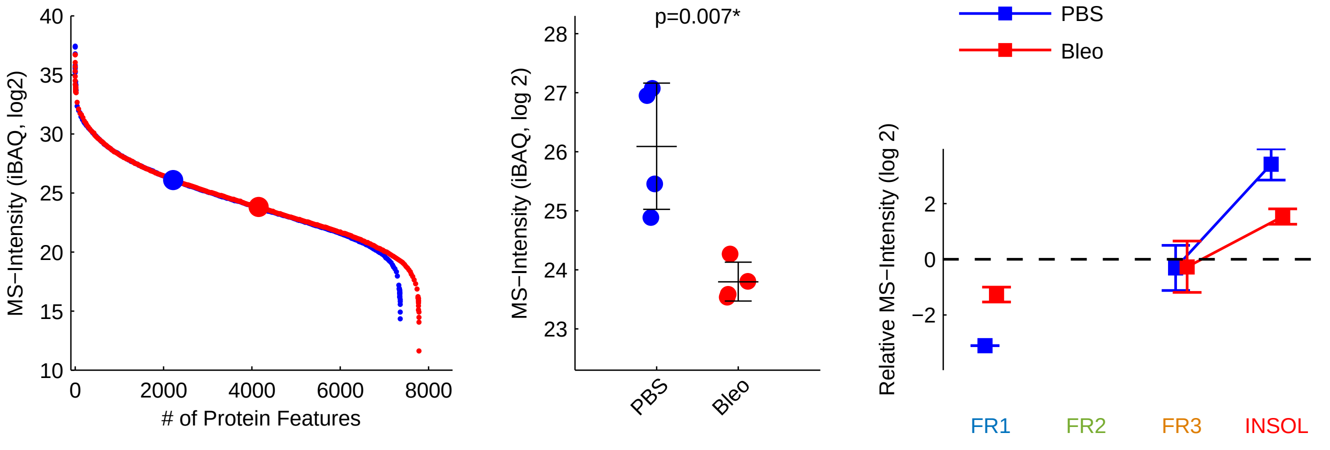

P27600 − Gna12 (id: 2848)

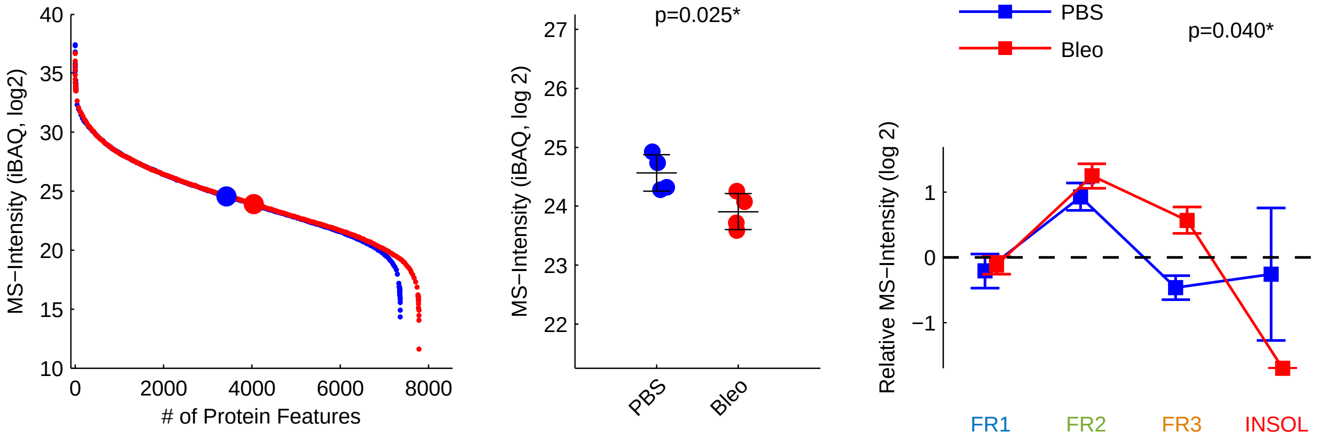

P27601 − Gna13 (id: 2849)

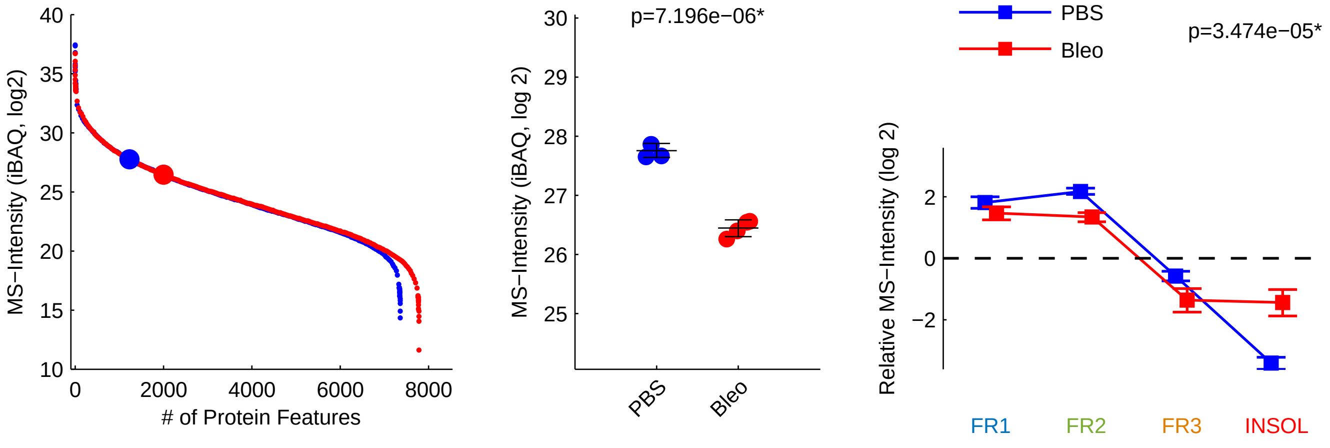

P27659 − Rpl3 (id: 2852)

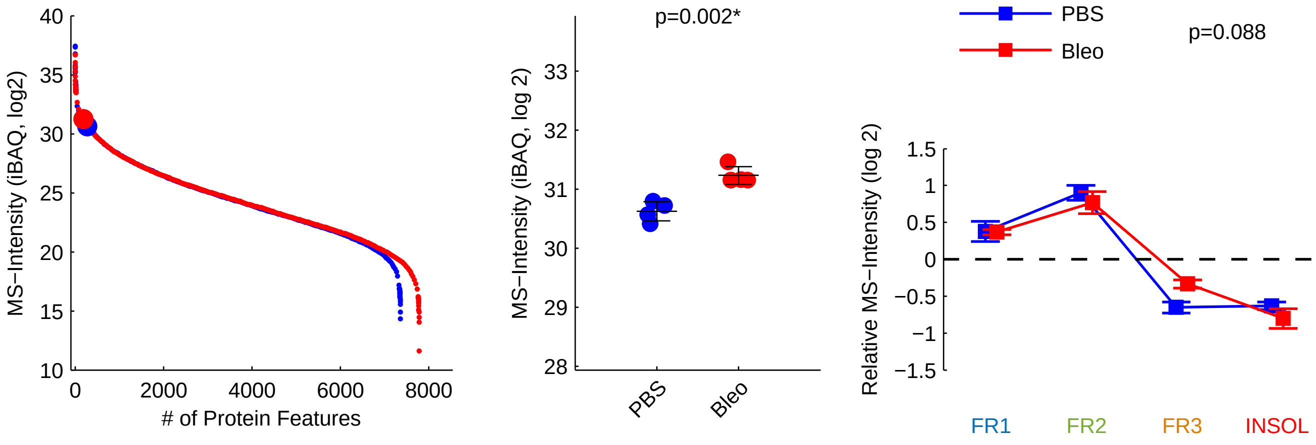

P27773 − Pdia3 (id: 2854)

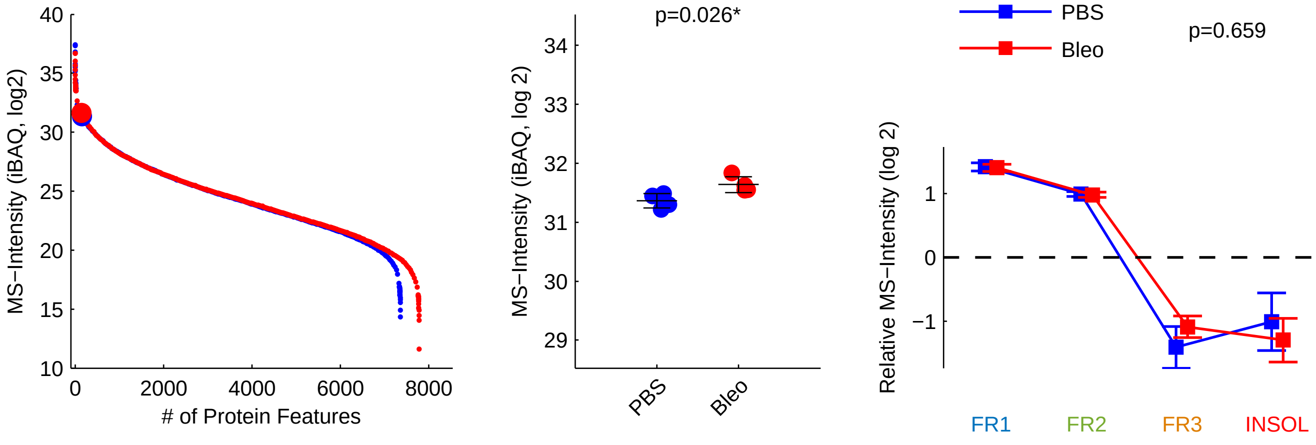

P28271 − Aco1 (id: 2861)

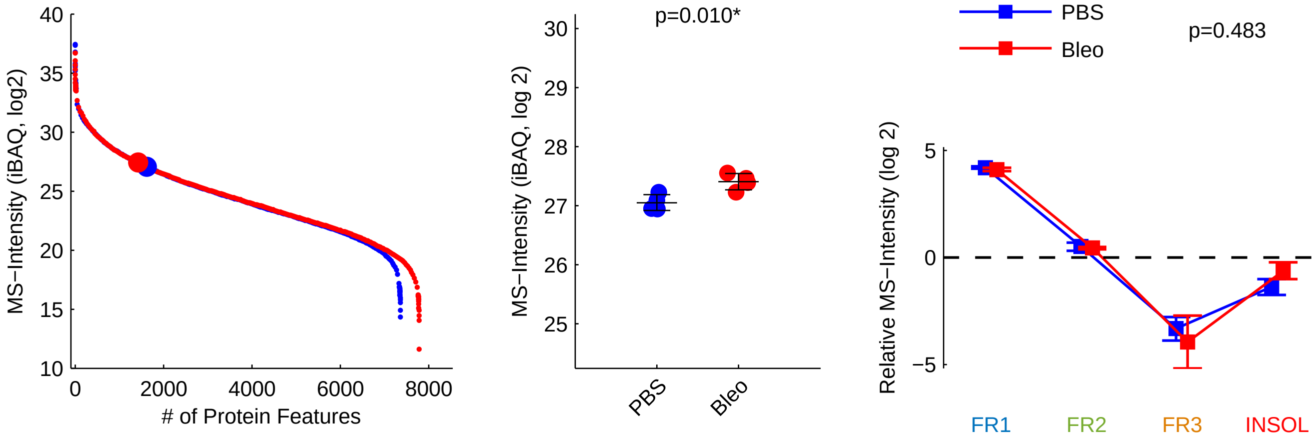

P28301 − Lox (id: 2863)

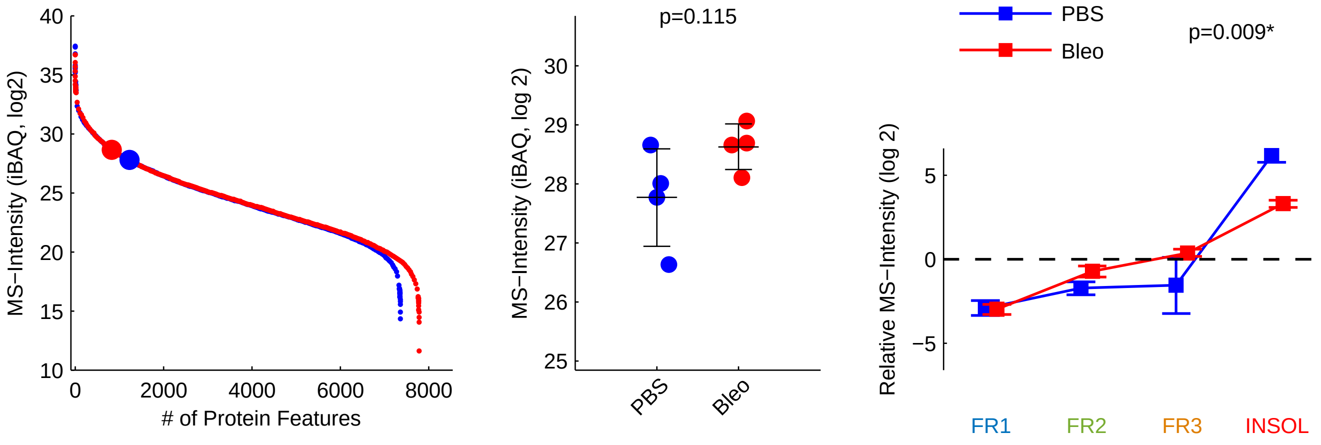

P28352 − Apex1 (id: 2864)

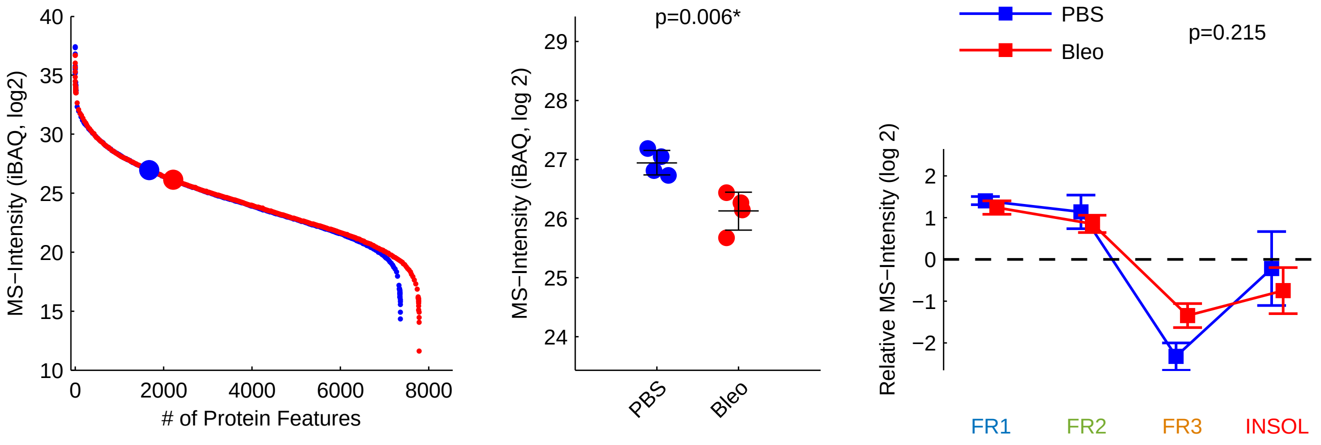

P28474 − Adh5 (id: 2865)

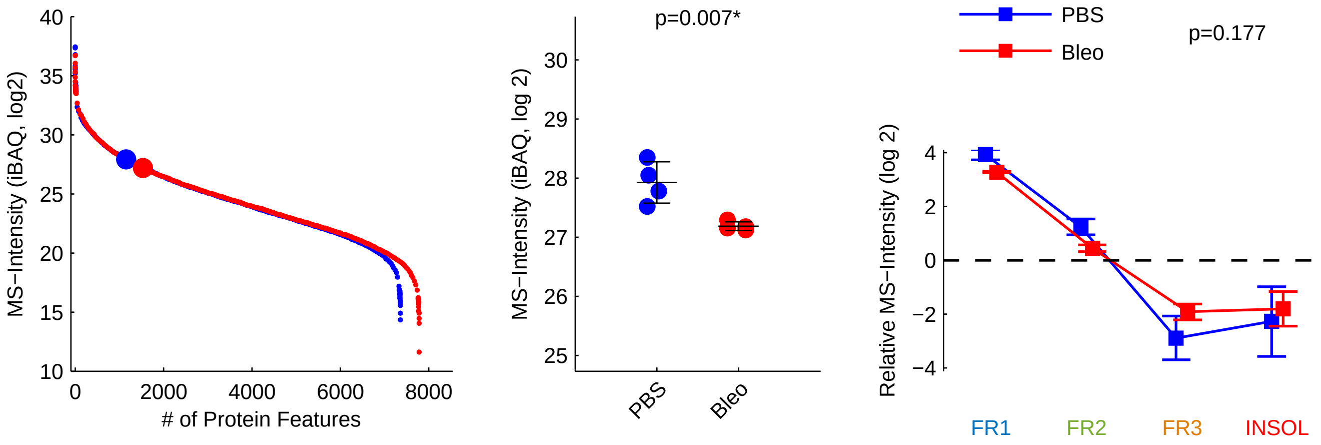

P28651 − Ca8 (id: 2868)

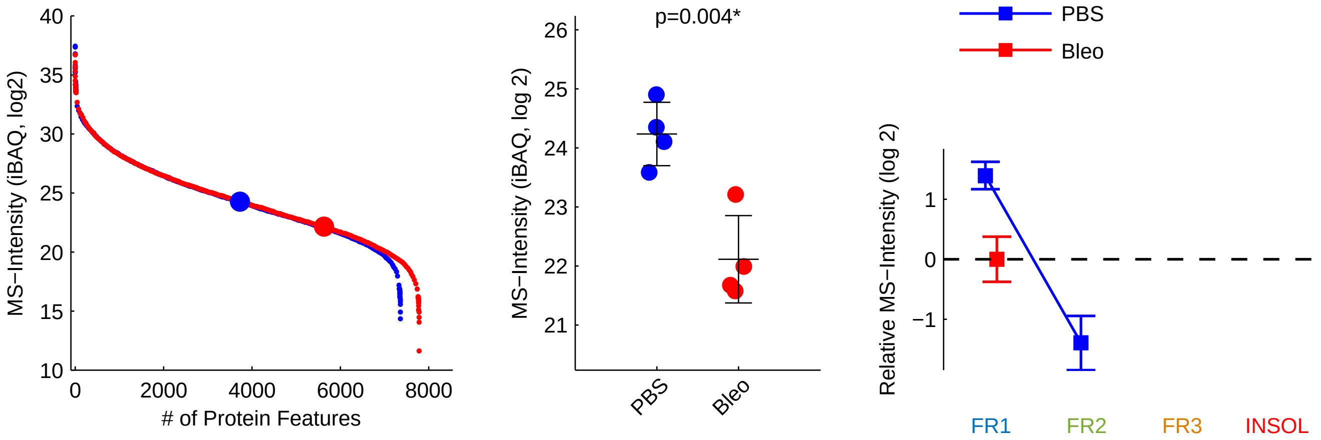

P28653 − Bgn (id: 2869)

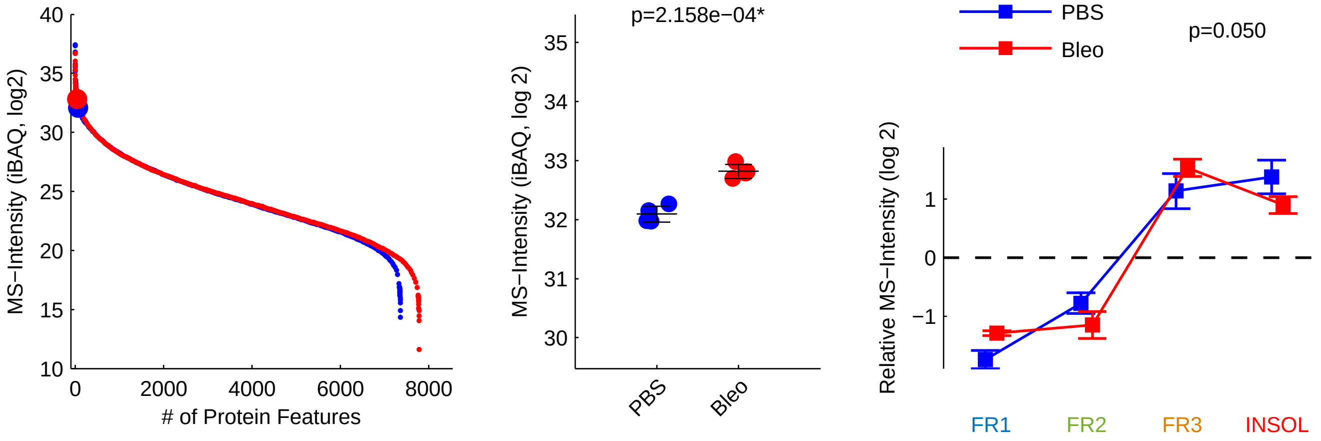

P28661 − Sept4 (id: 2873)

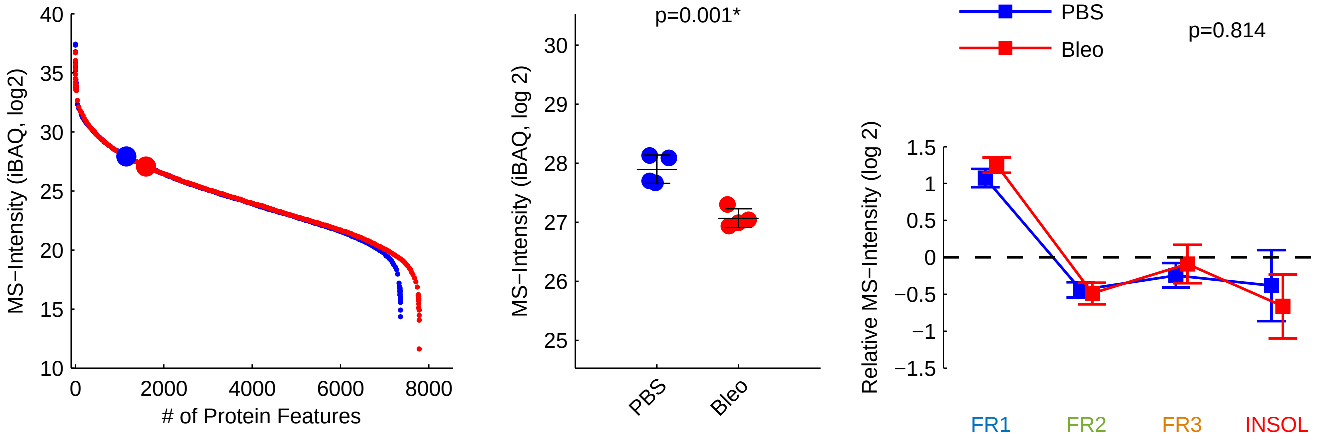

P28665 − Mug1 (id: 2874)

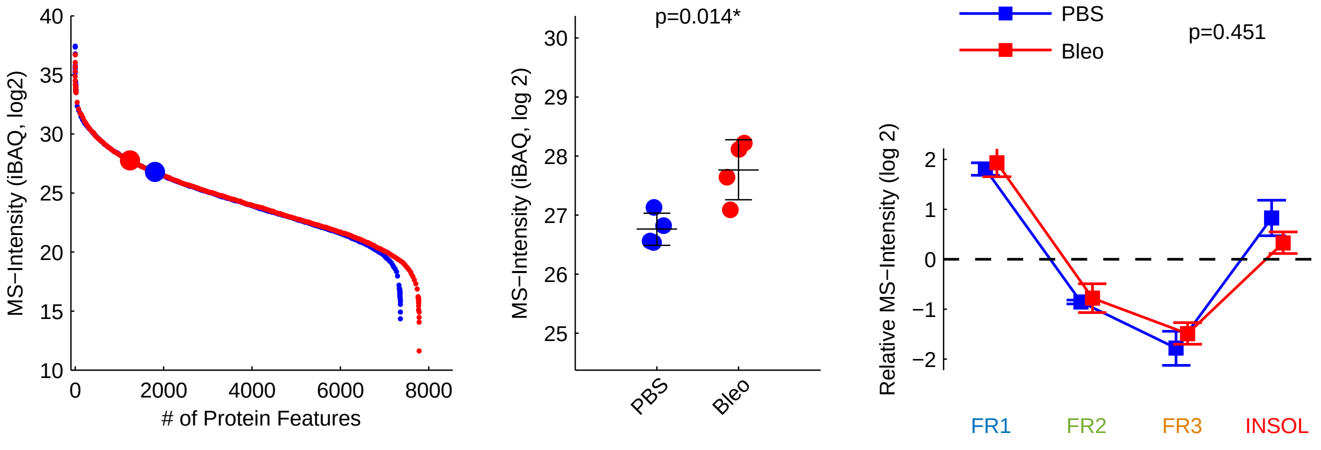

P28828 − Ptprm (id: 2880)

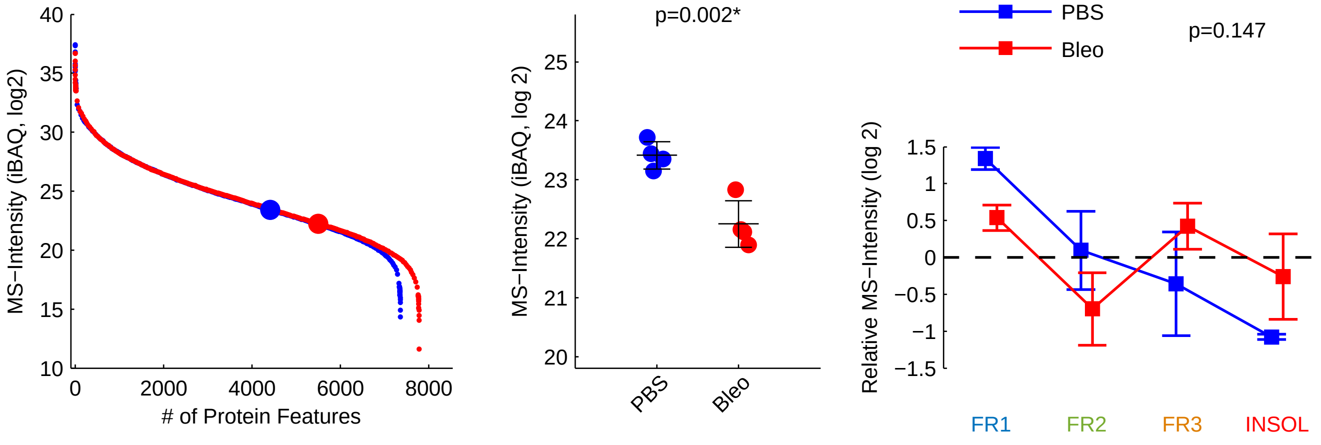

P28843 − Dpp4 (id: 2881)

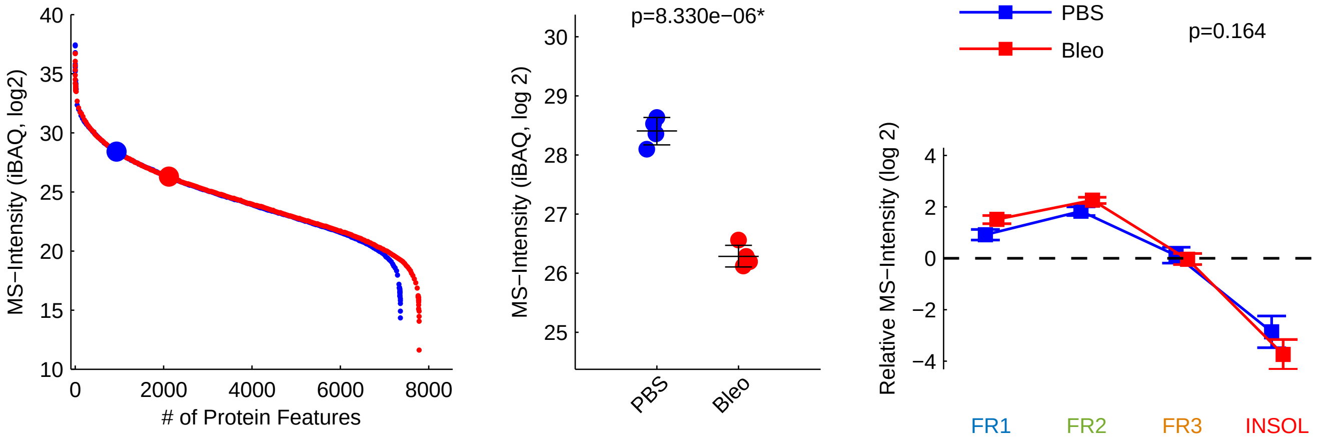

P28867 − Prkcd (id: 2883)

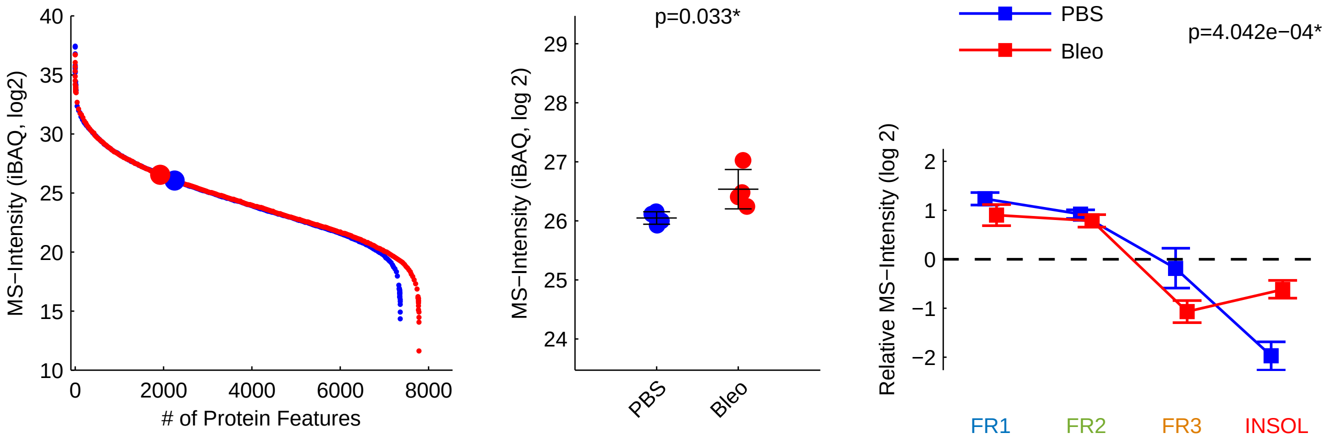

P29268 − Ctgf (id: 2884)

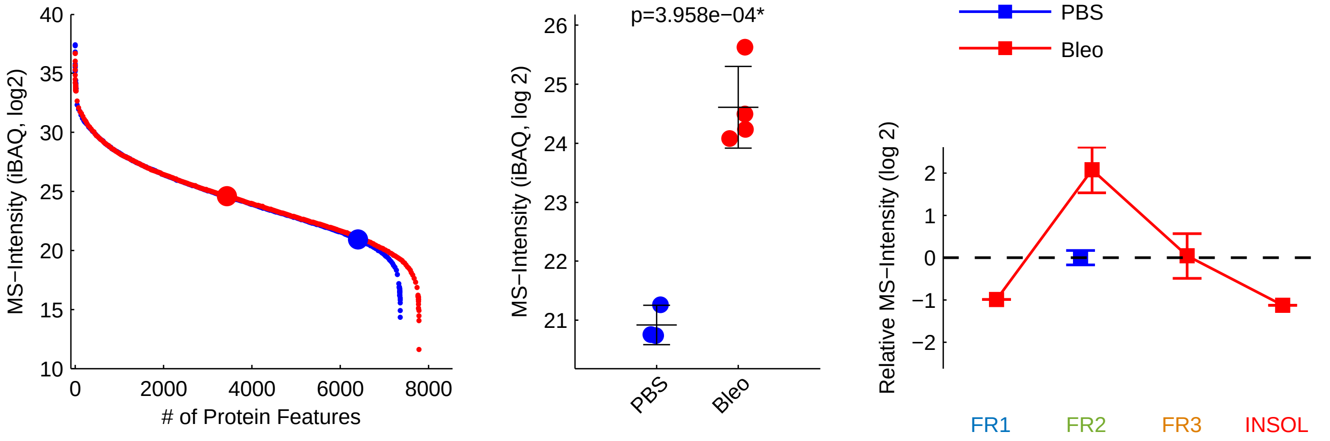

P29341 − Pabpc1 (id: 2885)

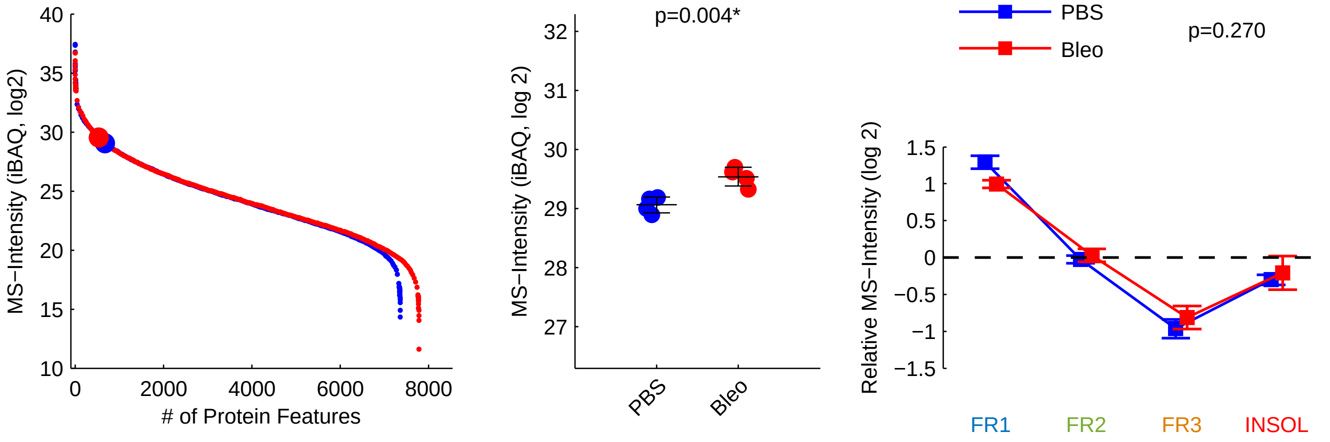

P29387 − Gnb4 (id: 2887)

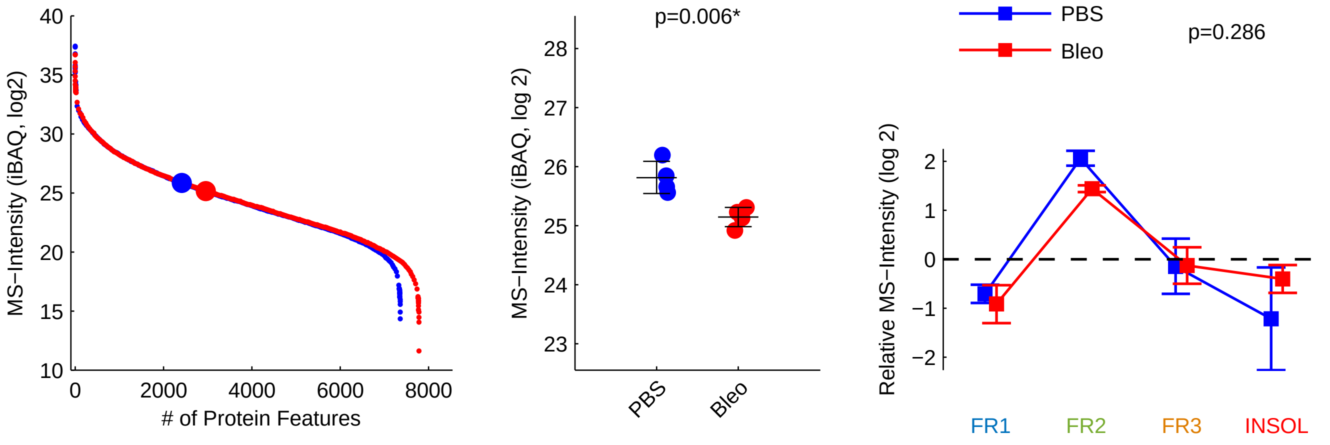

P29416 − Hexa (id: 2888)

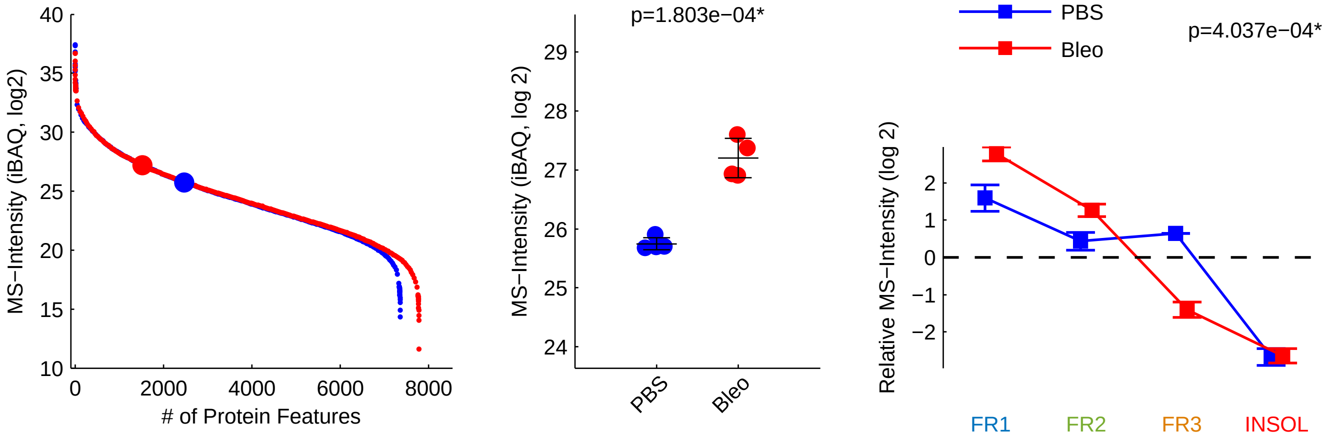

P29699 − Ahsg (id: 2893)

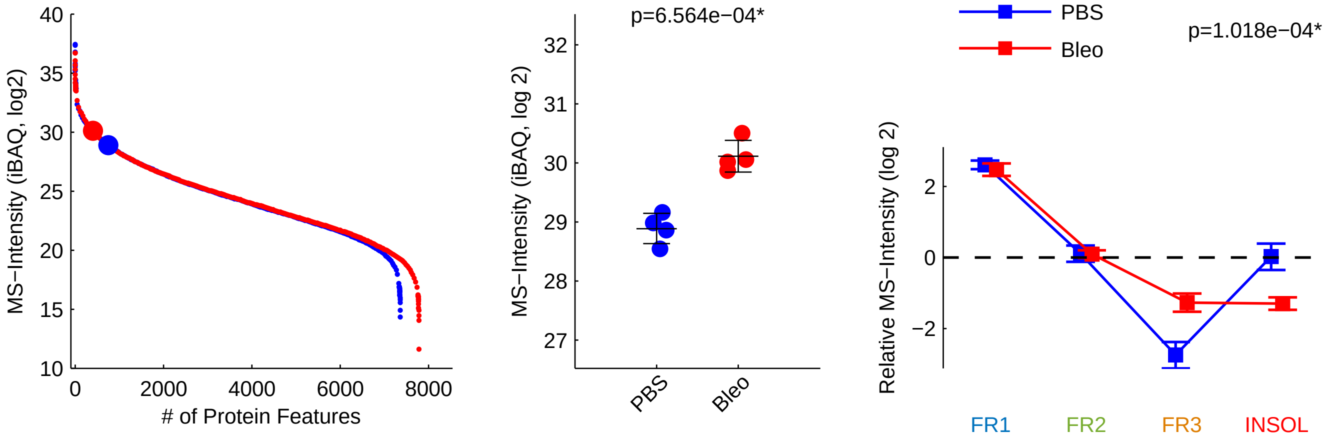

P29788 − Vtn (id: 2895)

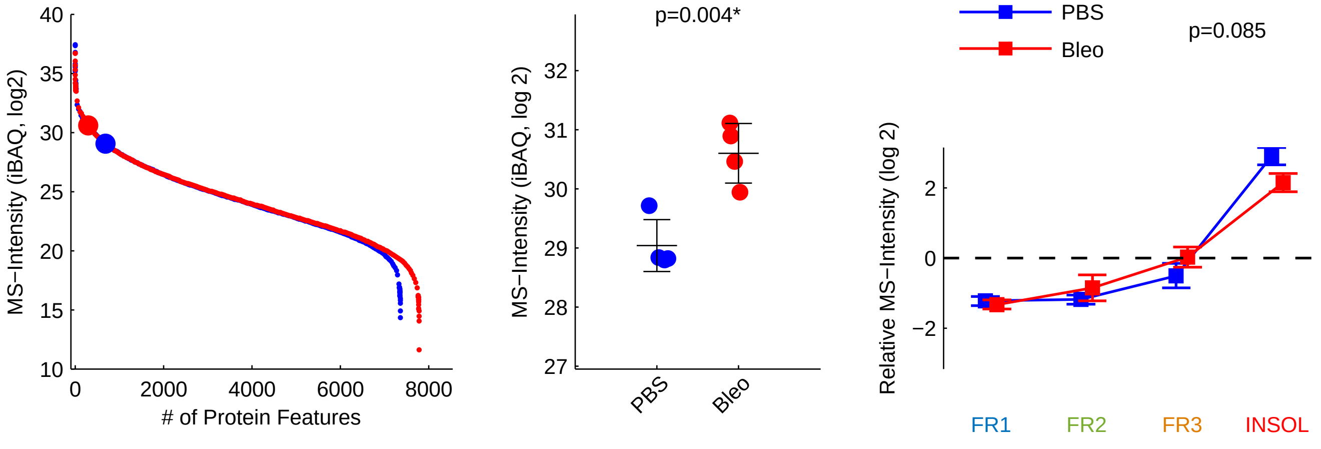

P30115 − Gsta3 (id: 2896)

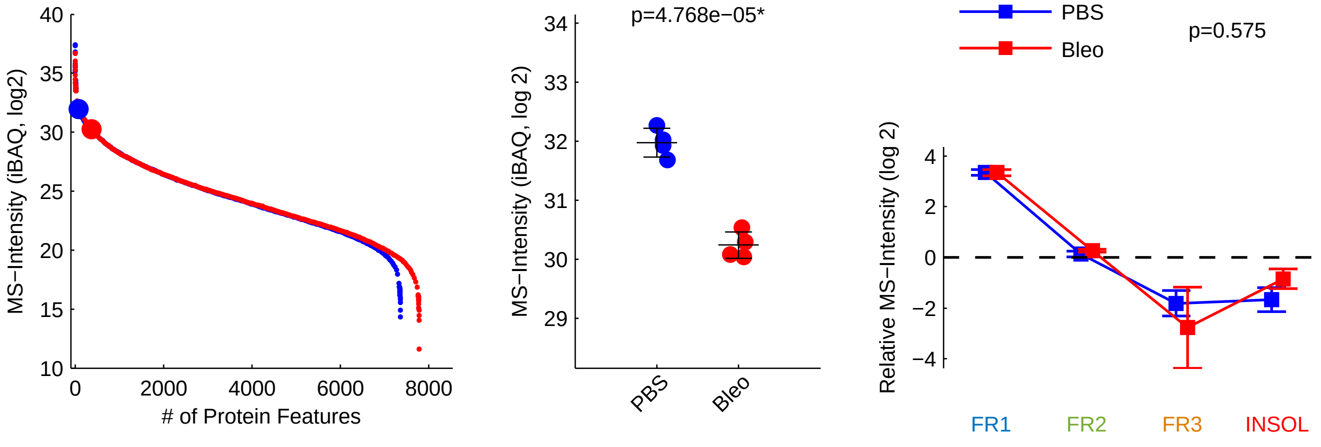

P30275 − Ckmt1 (id: 2898)

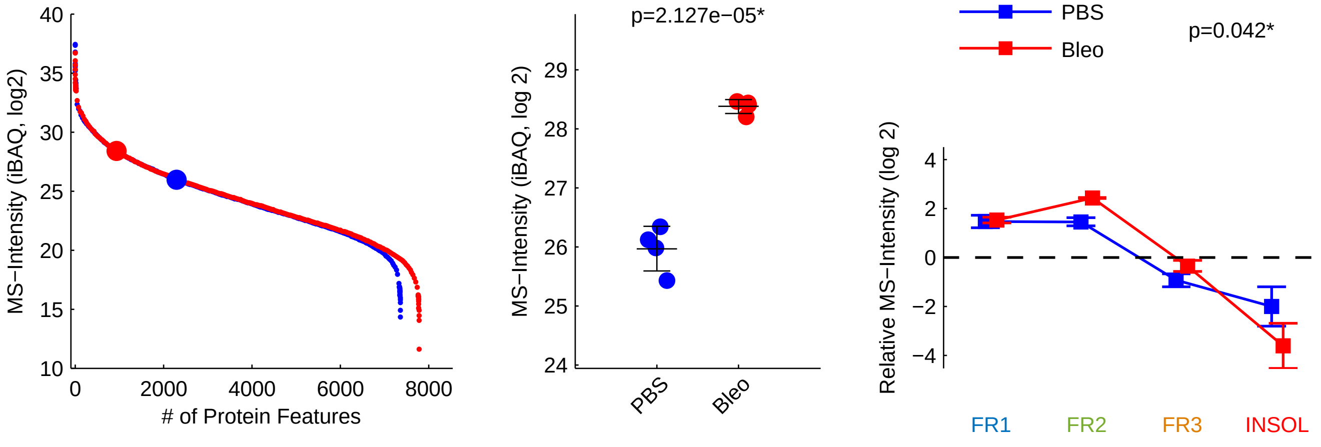

P30412 − Ppic (id: 2901)

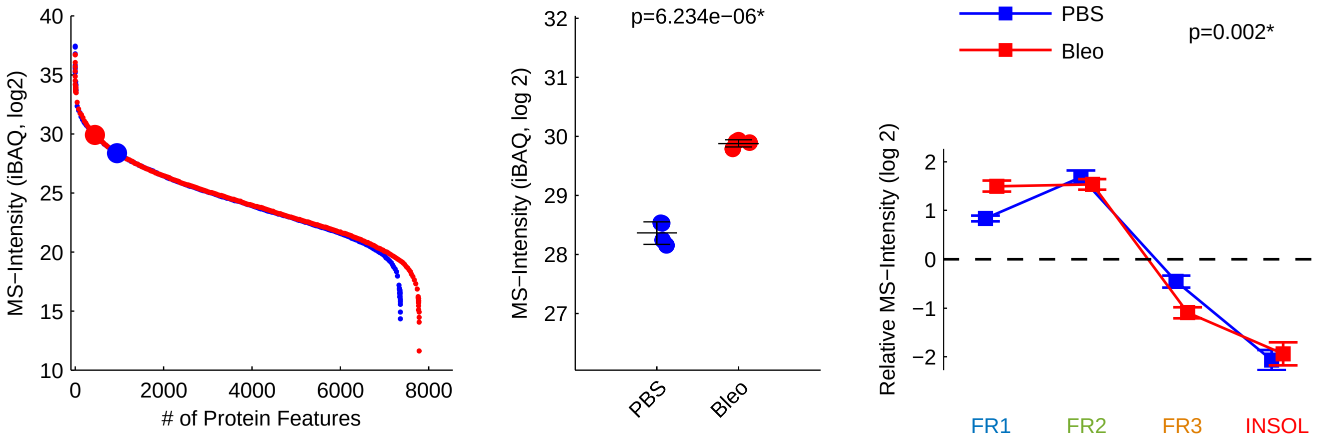

P30416 − Fkbp4 (id: 2903)

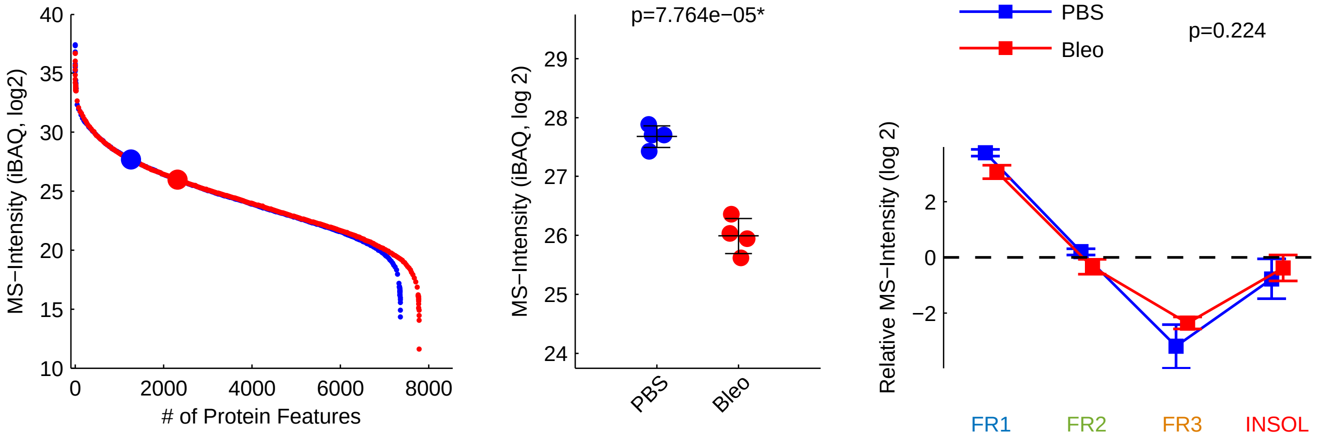

P30999 − Ctnnd1 (id: 2907)

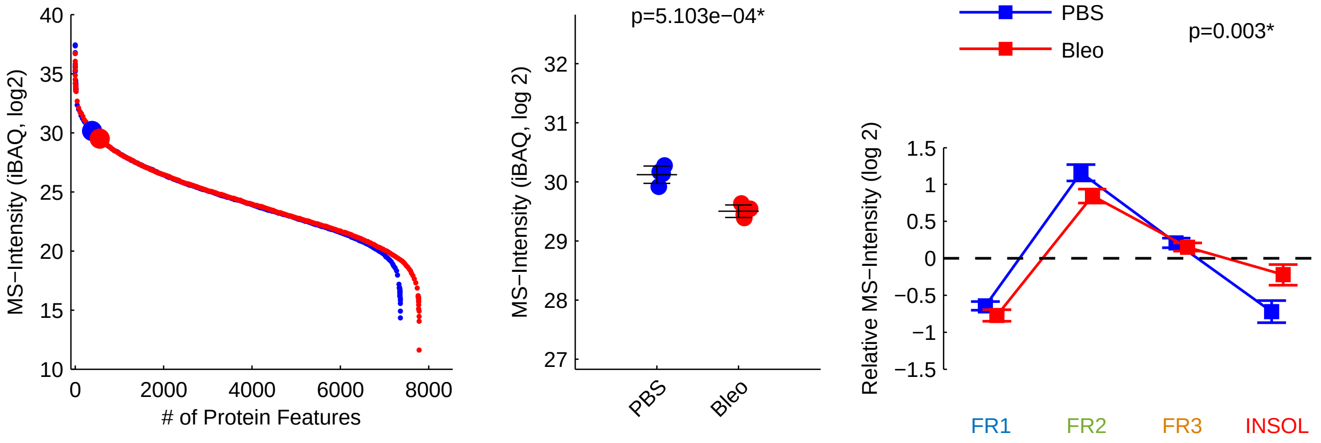

P30999−3 − Ctnnd1 (id: 2908)

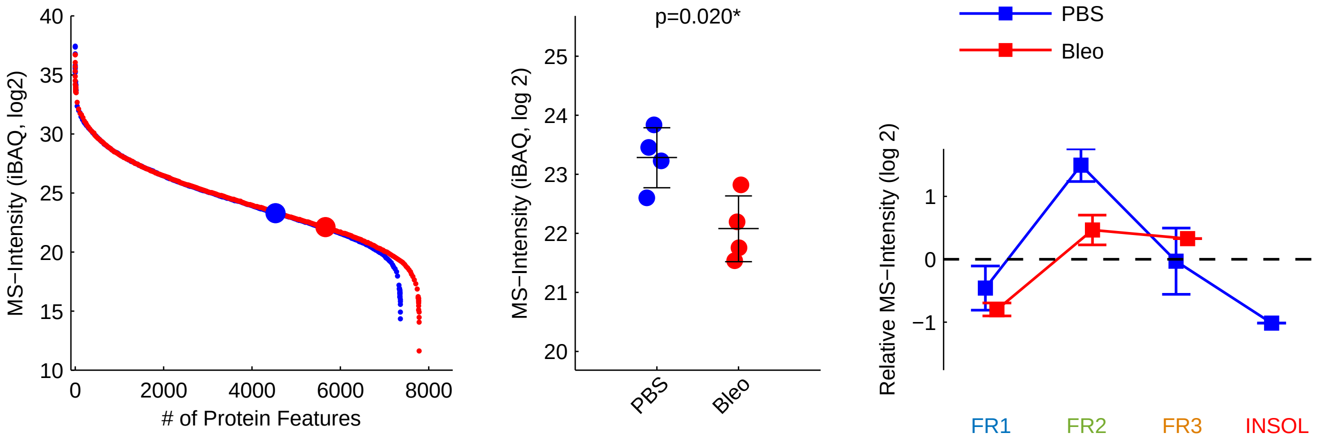

P31001 − Des (id: 2909)

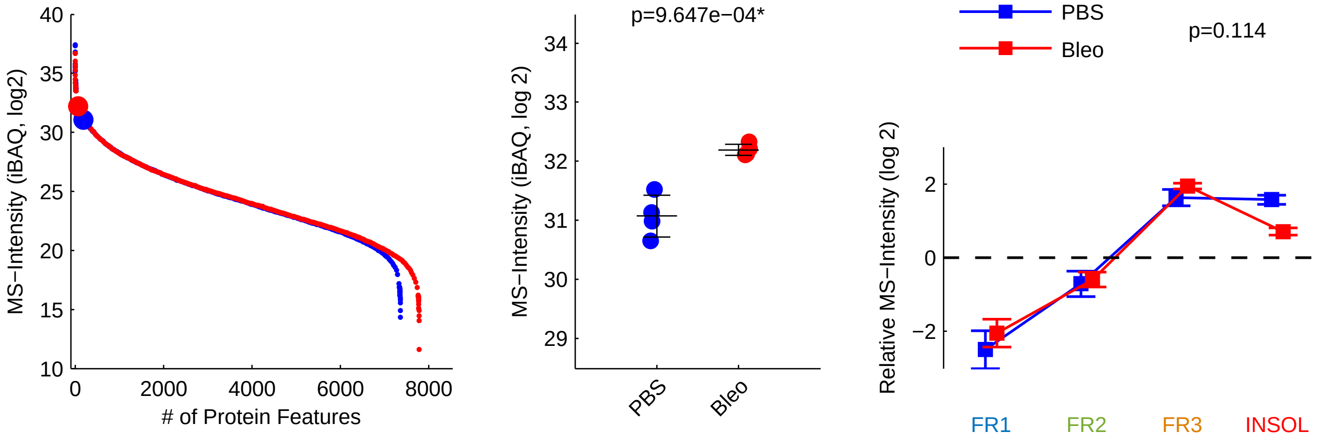

P31428 − Dpep1 (id: 2913)

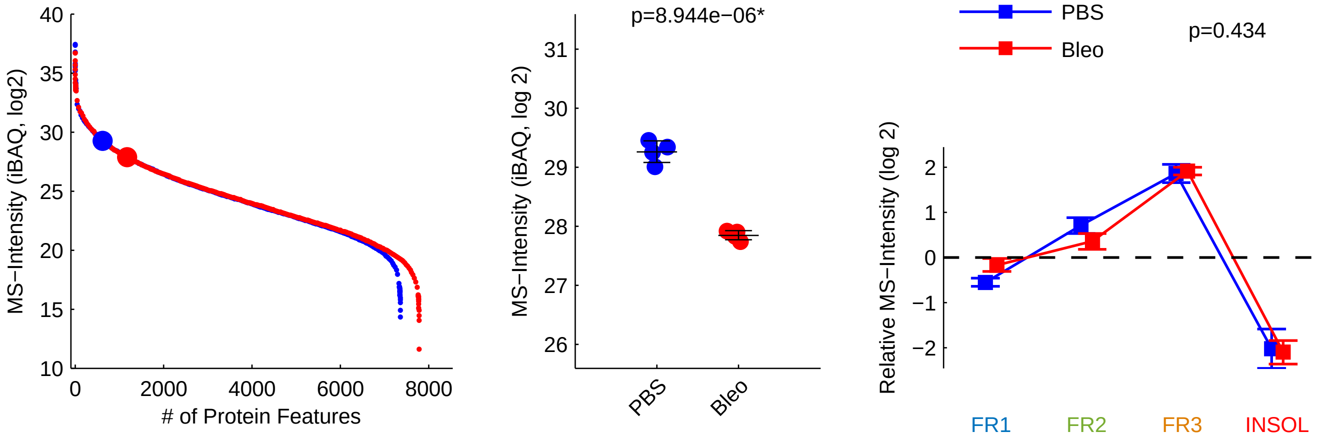

P31809 − Ceacam1 (id: 2918)

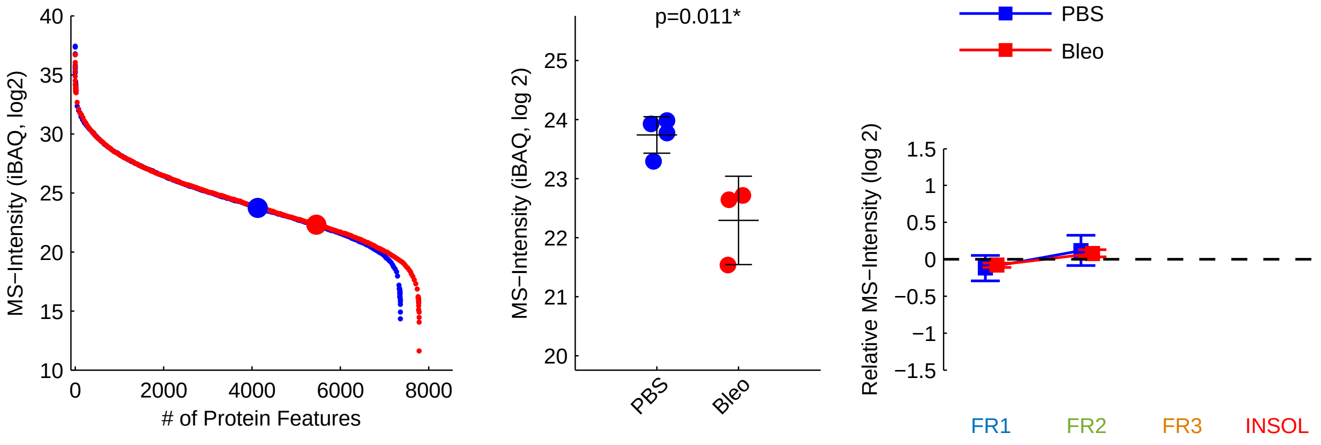

P31996 − Cd68 (id: 2920)

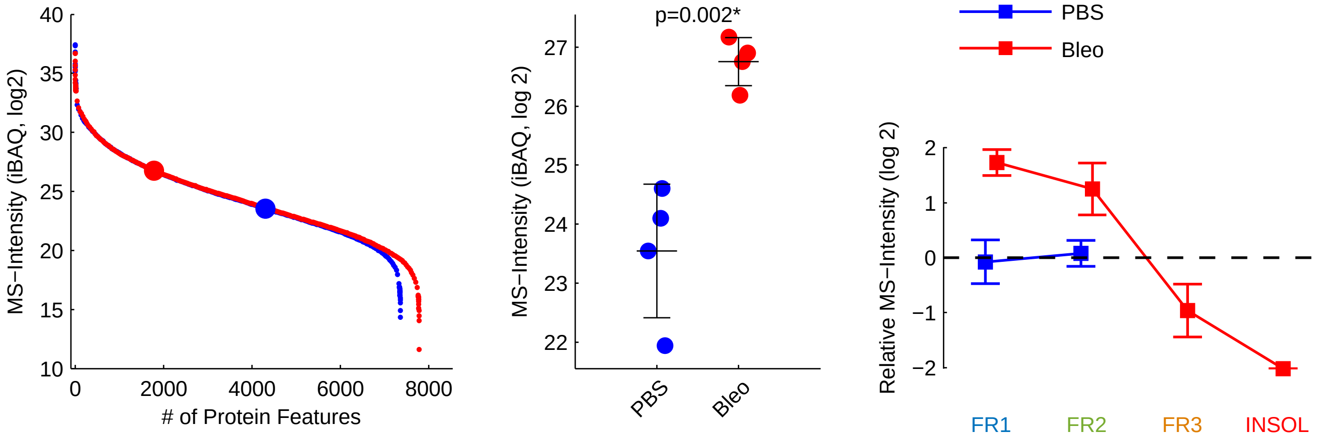

P32020 − Scp2 (id: 2921)

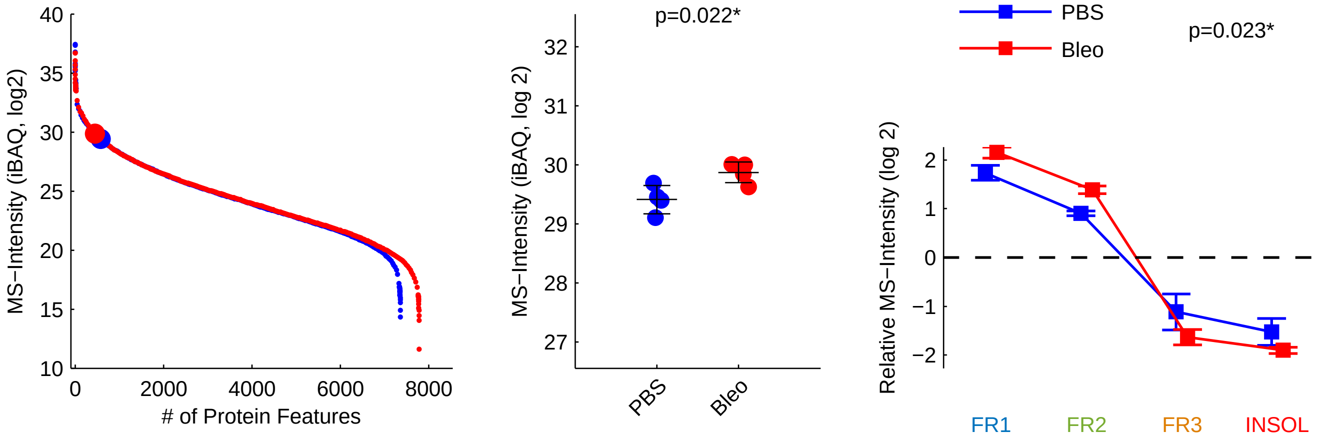

P32067 − Ssb (id: 2922)

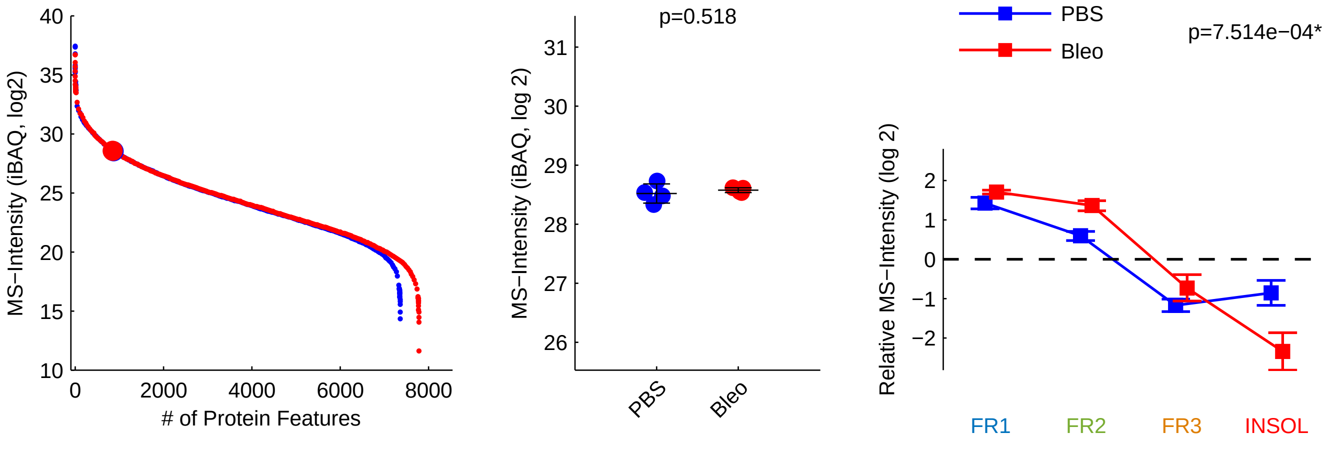

P32261 − Serpinc1 (id: 2924)

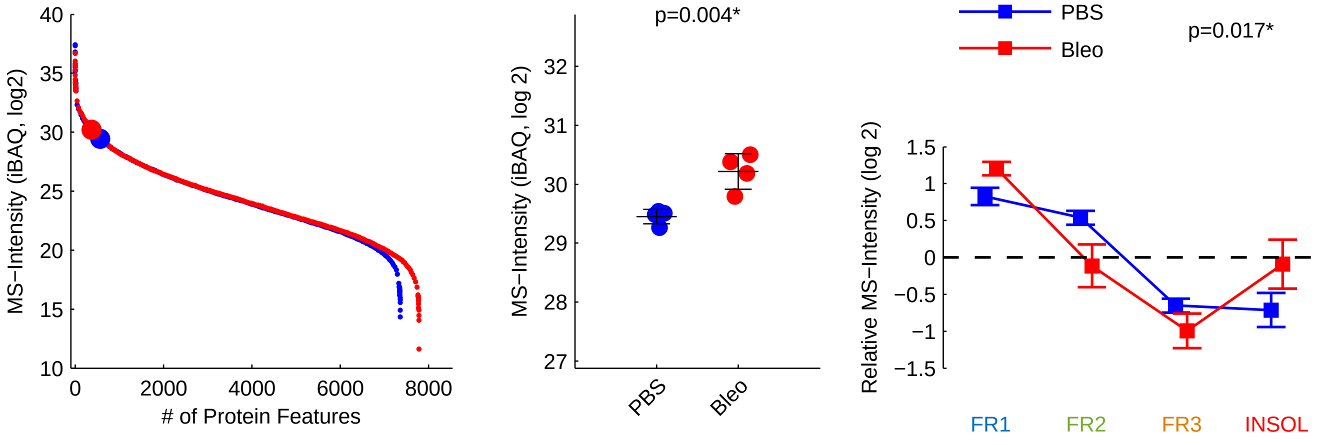

P32883−2 − Kras (id: 2927)

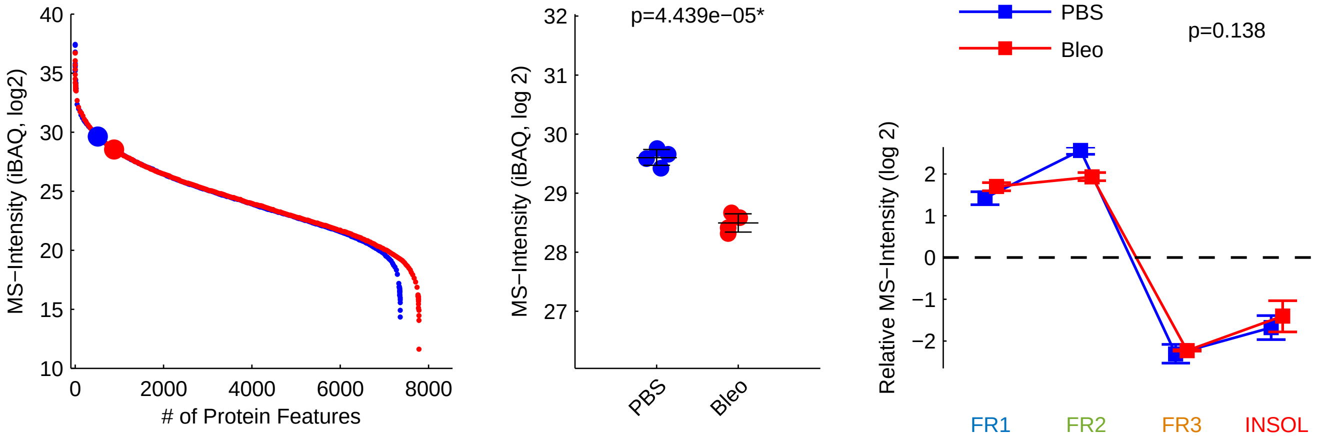

P33267 − Cyp2f2 (id: 2931)

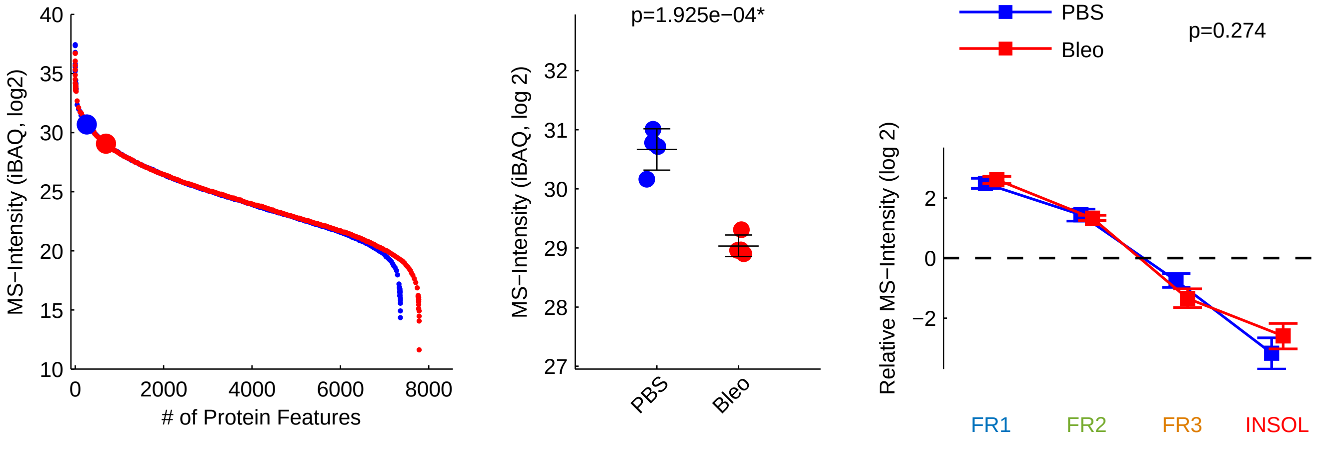

P33434 − Mmp2 (id: 2932)

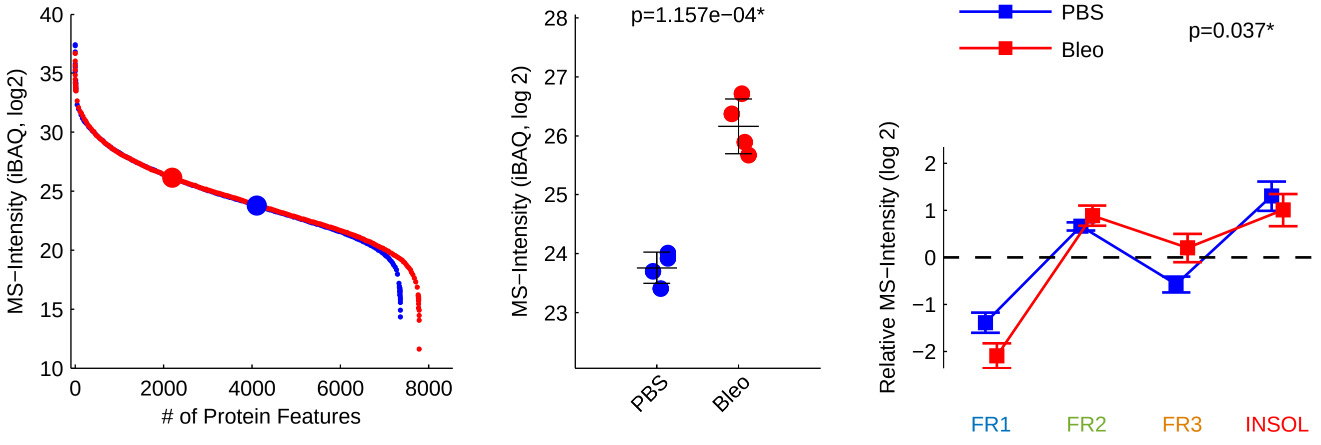

P34152 − Ptk2 (id: 2937)

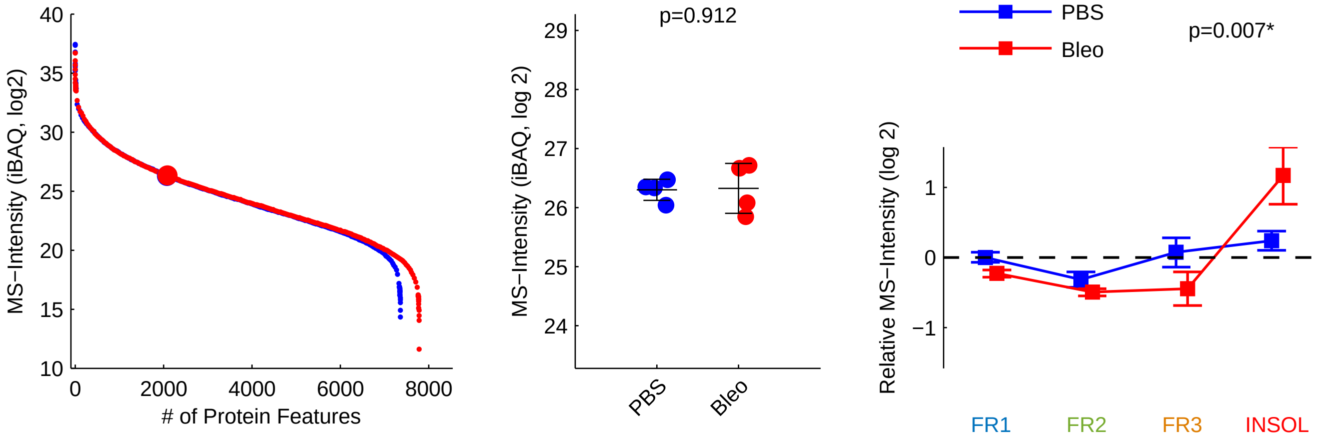

P34884 − Mif (id: 2938)

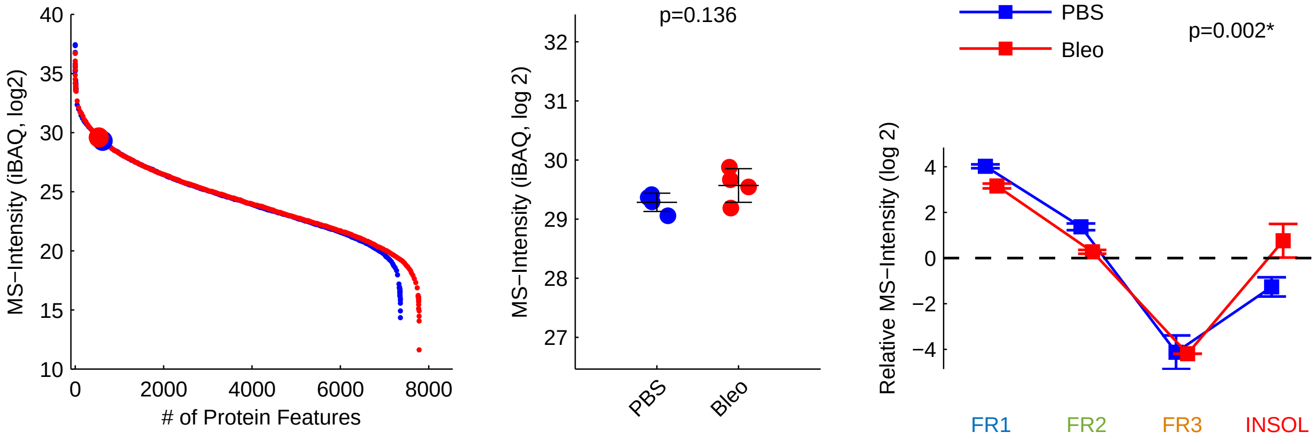

P35123 − Usp4 (id: 2942)

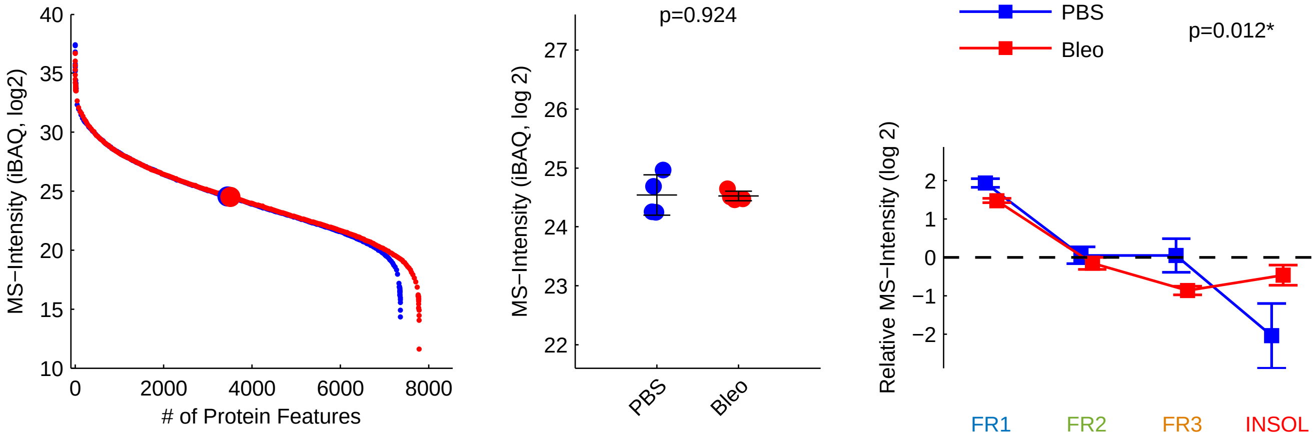

P35282 − Rab21 (id: 2948)

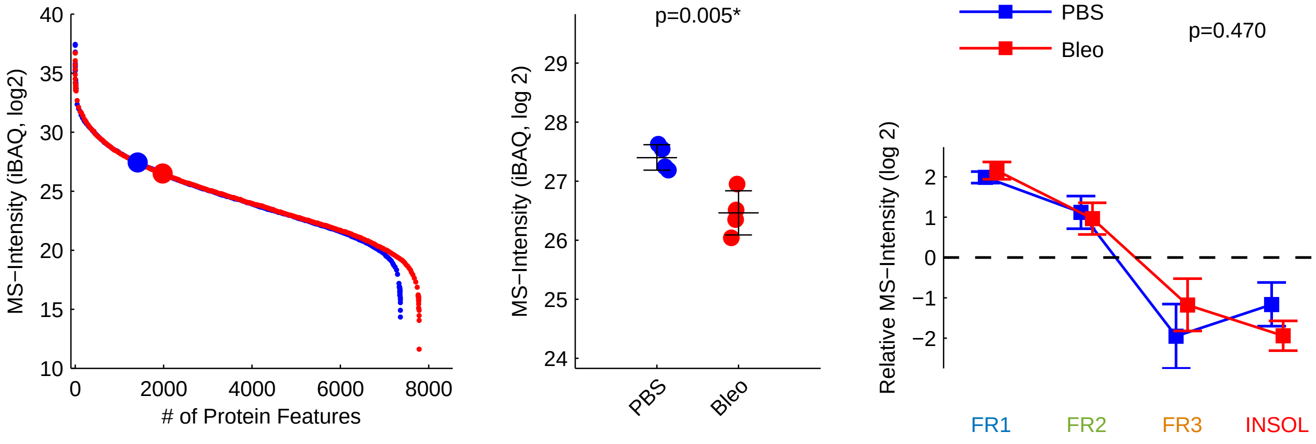

P35802 − Gpm6a (id: 2963)

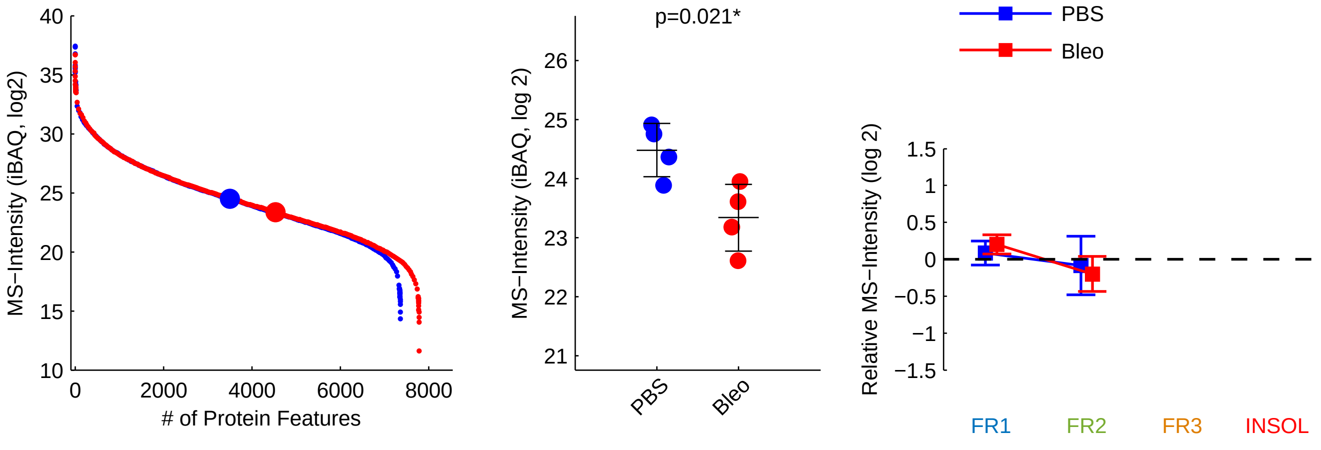

P35821 − Ptpn1 (id: 2964)

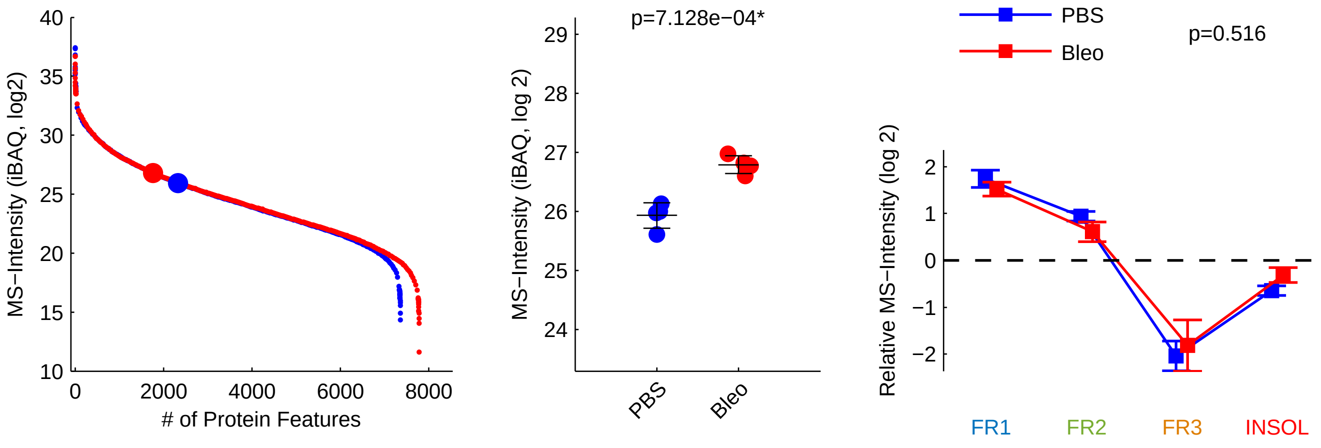

P35969 − Flt1 (id: 2970)

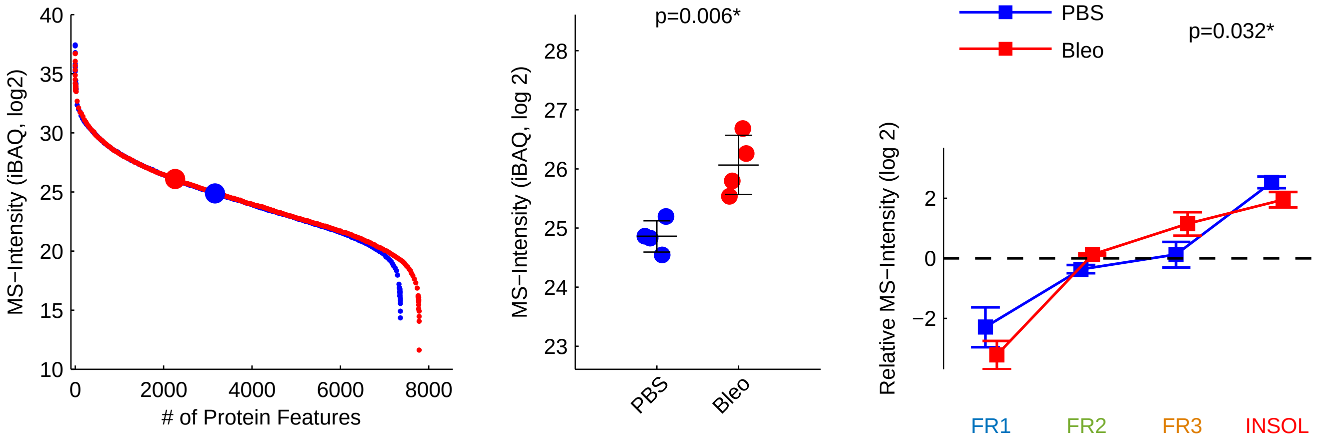

P35980 − Rpl18 (id: 2972)

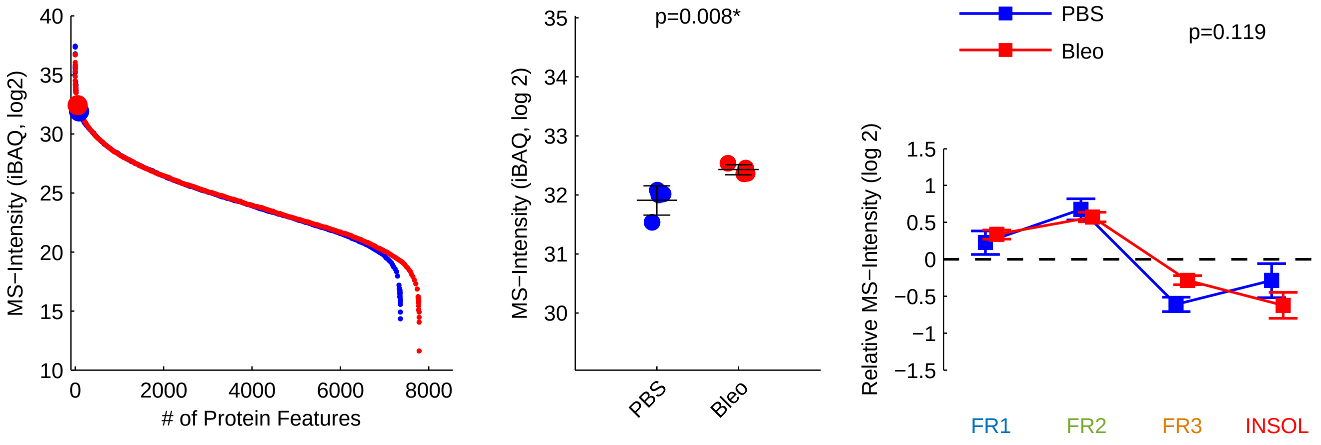

P35991 − Btk (id: 2973)

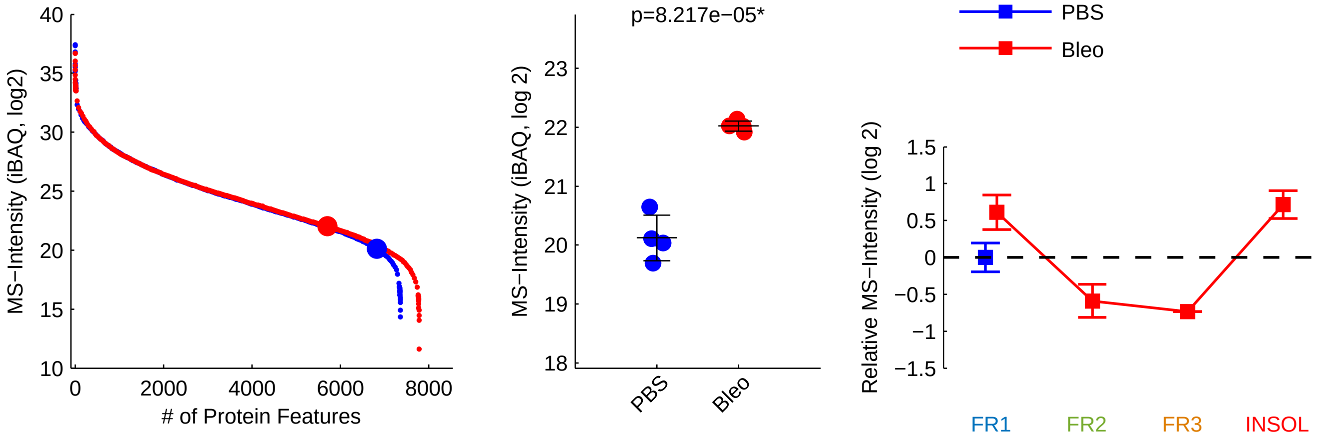

P37040 − Por (id: 2979)

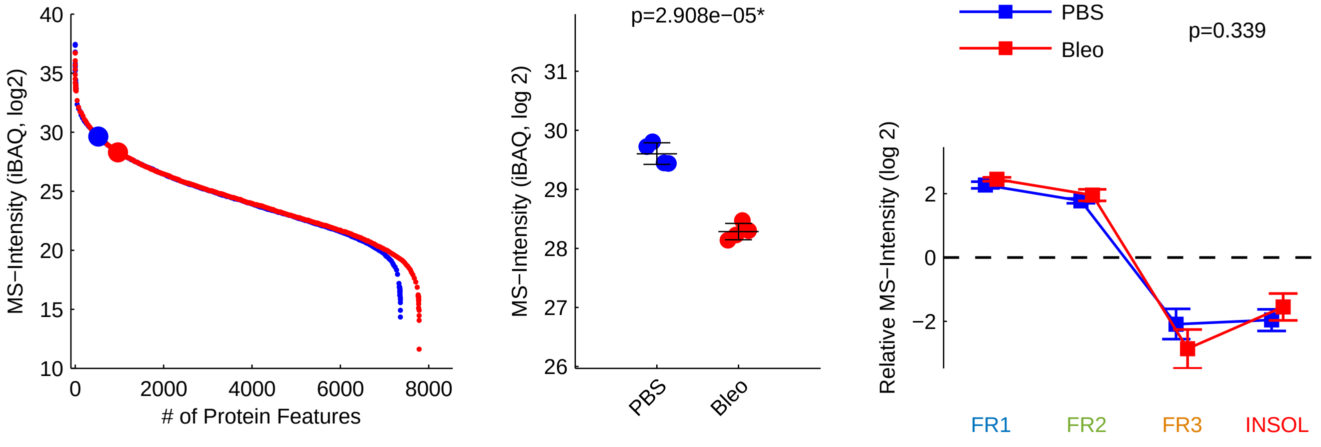

P37804 − Tagln (id: 2980)

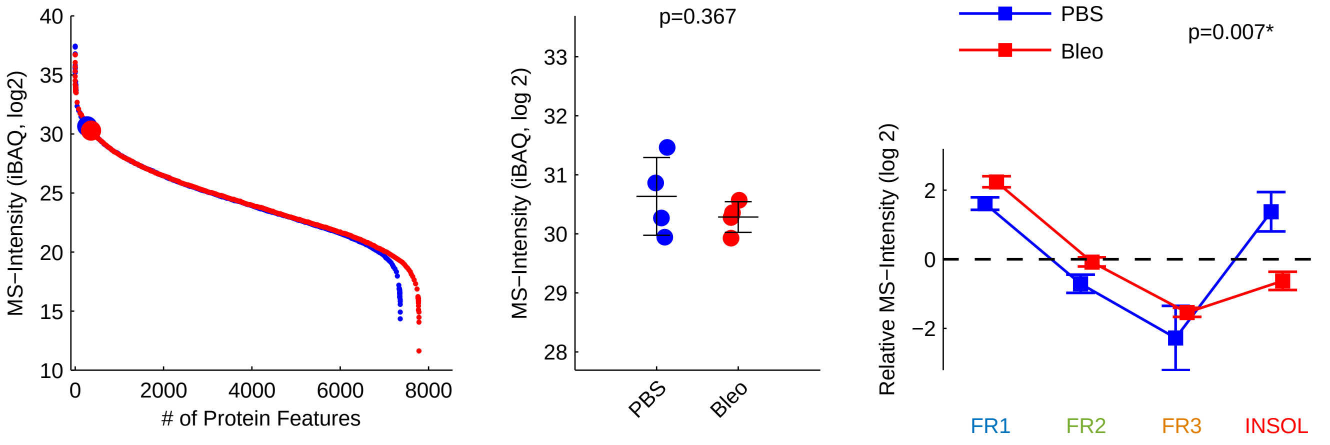

P37889 − Fbln2 (id: 2981)

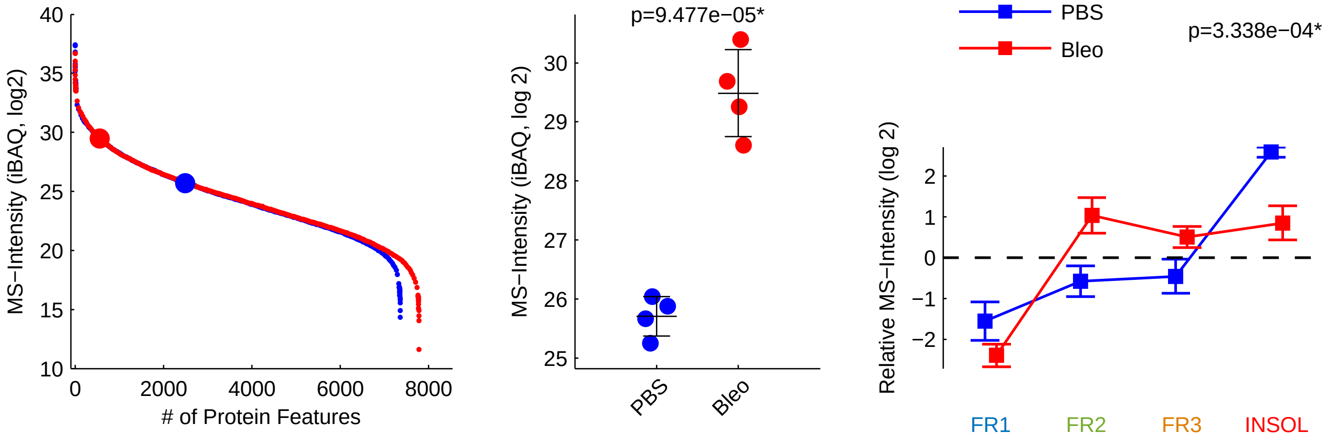

P39054 − Dnm2 (id: 2987)

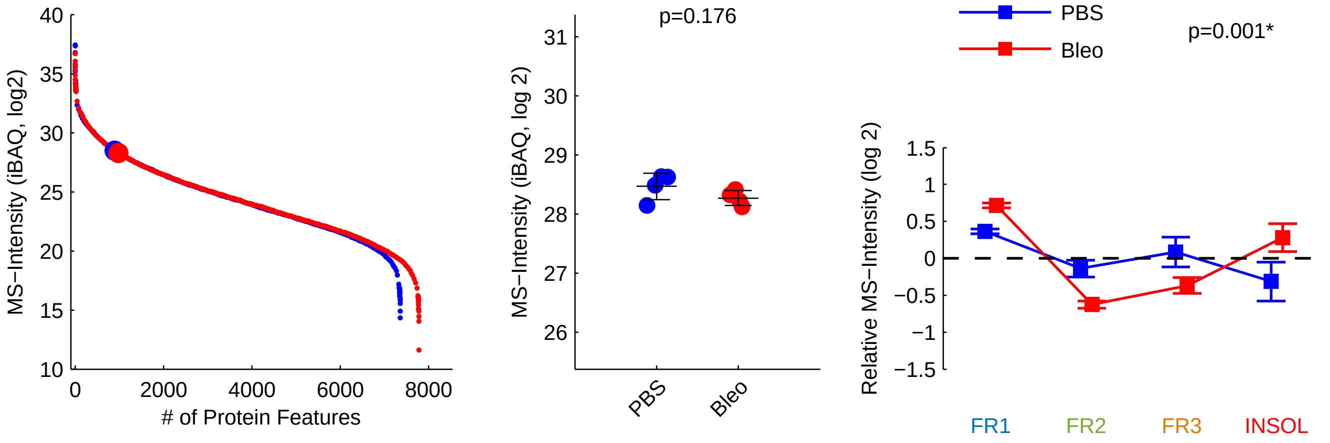

P39688 − Fyn (id: 2995)

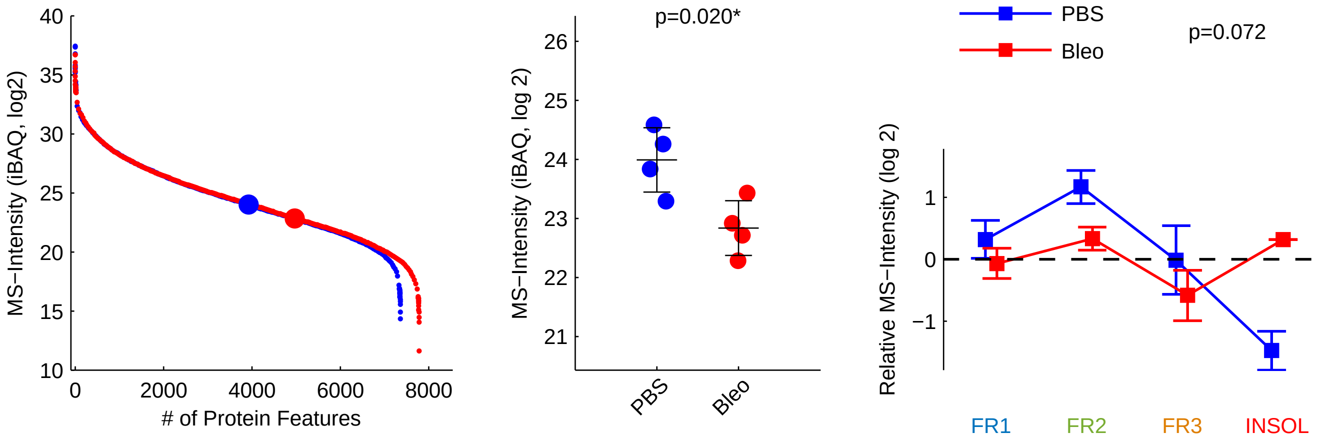

P40240 − Cd9 (id: 3002)

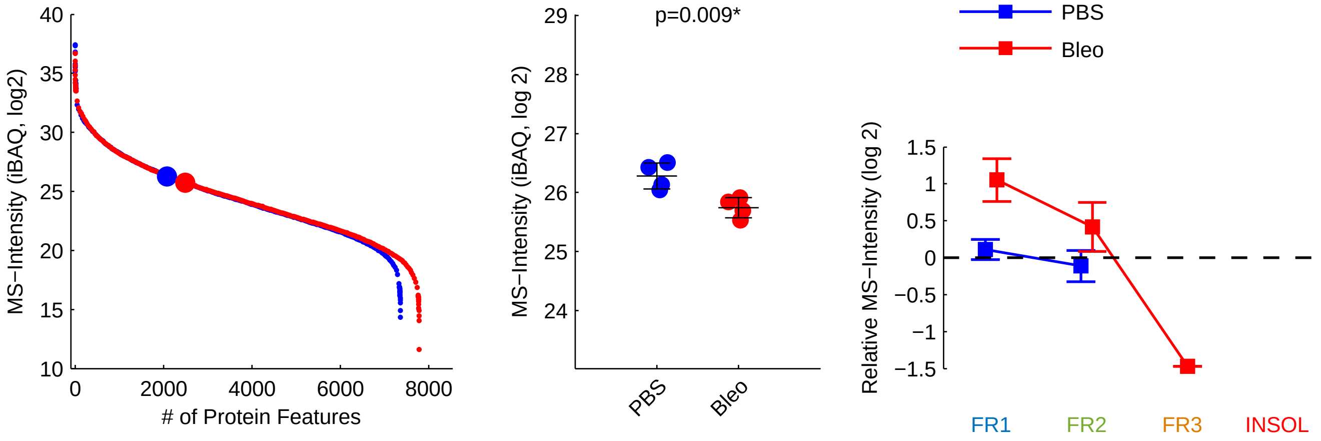

P40338 − Vhl (id: 3004)

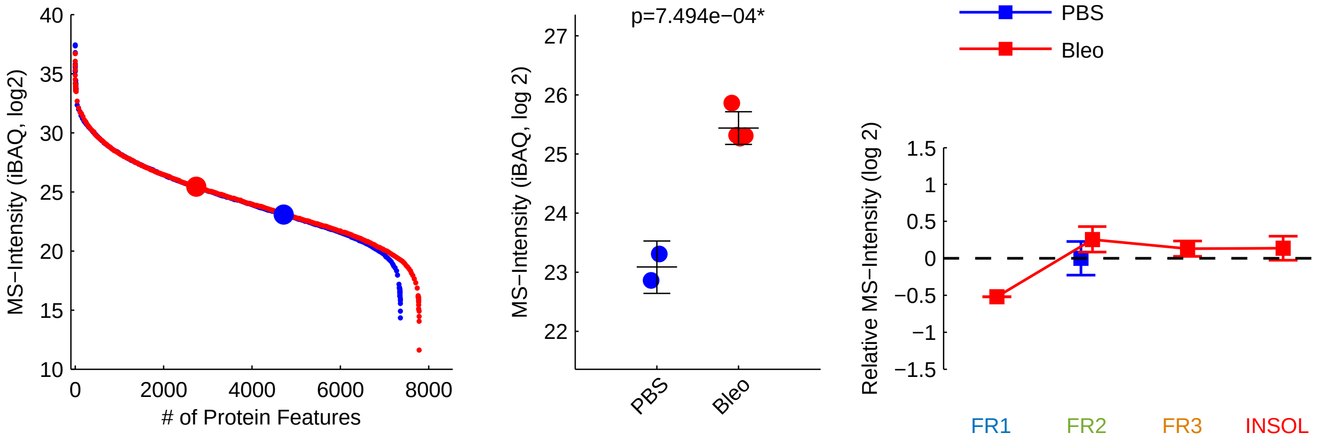

P40630 − Tfam (id: 3005)

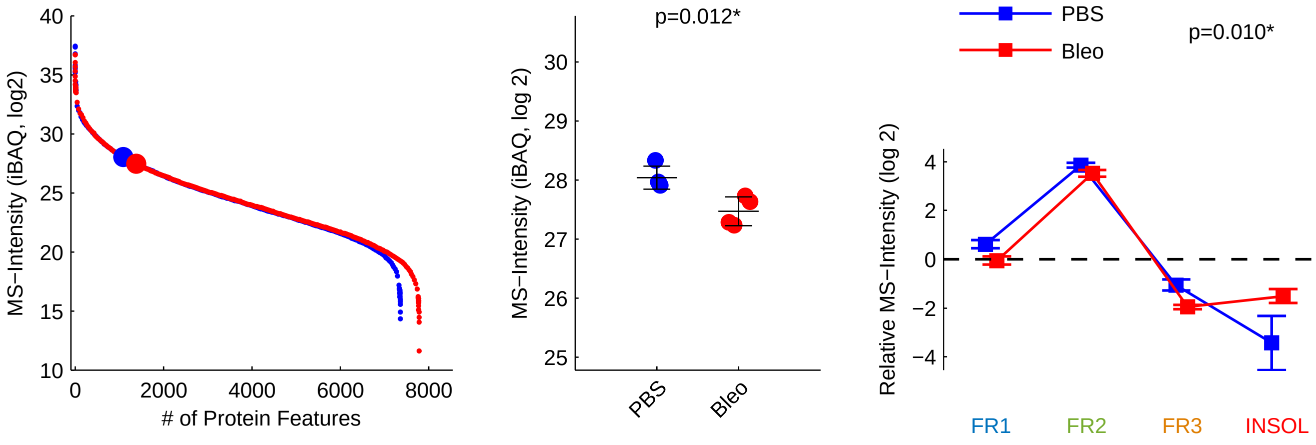

P40936 − Inmt (id: 3006)

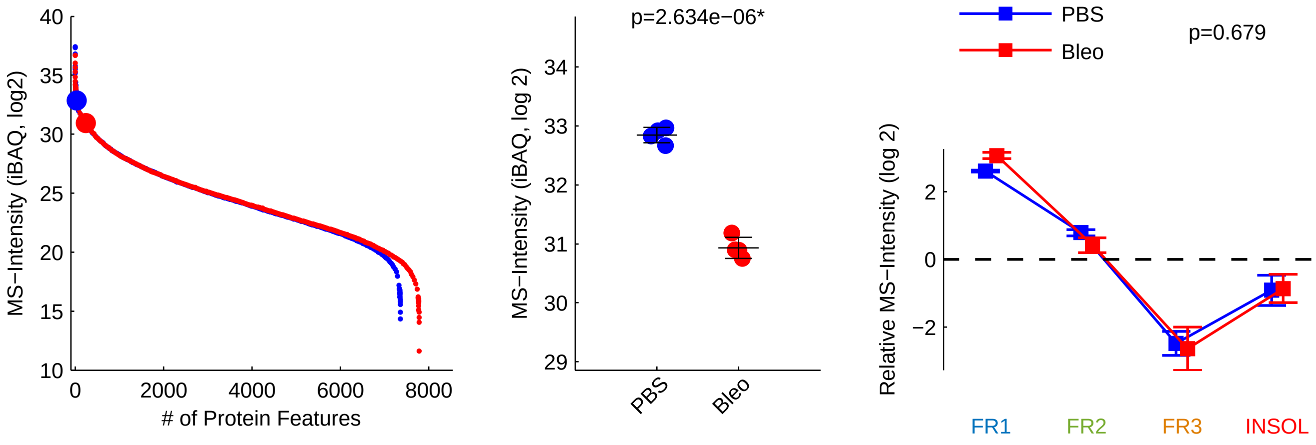

P41105 − Rpl28 (id: 3007)

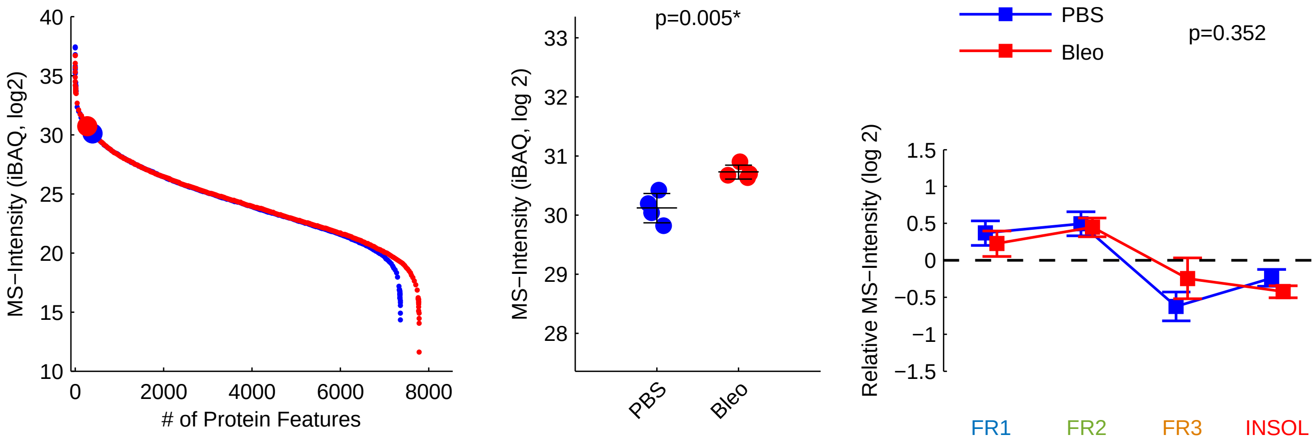

P42125 − Eci1 (id: 3014)

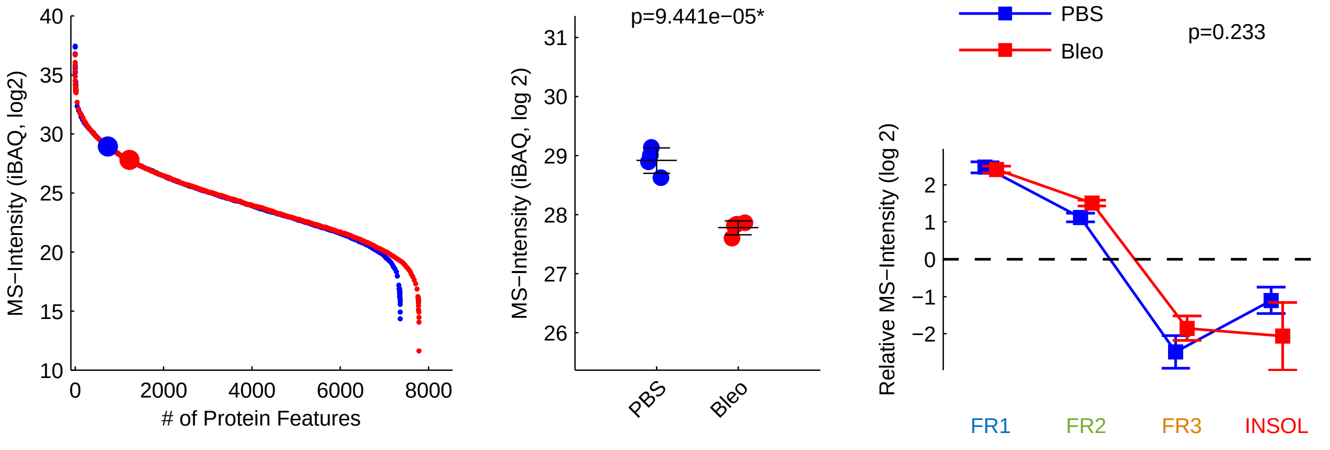

P42227 − Stat3 (id: 3019)

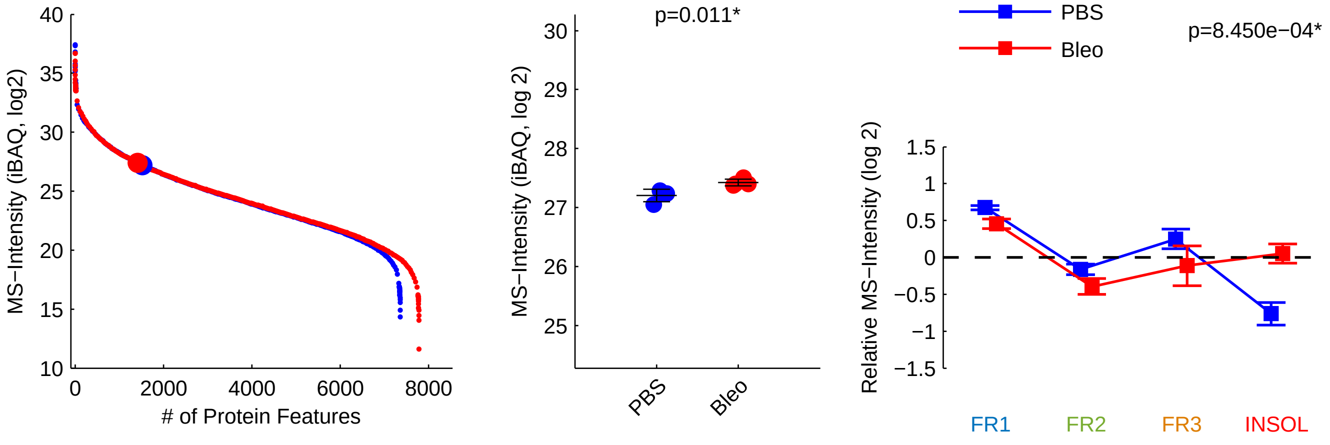

P42232 − Stat5b (id: 3020)

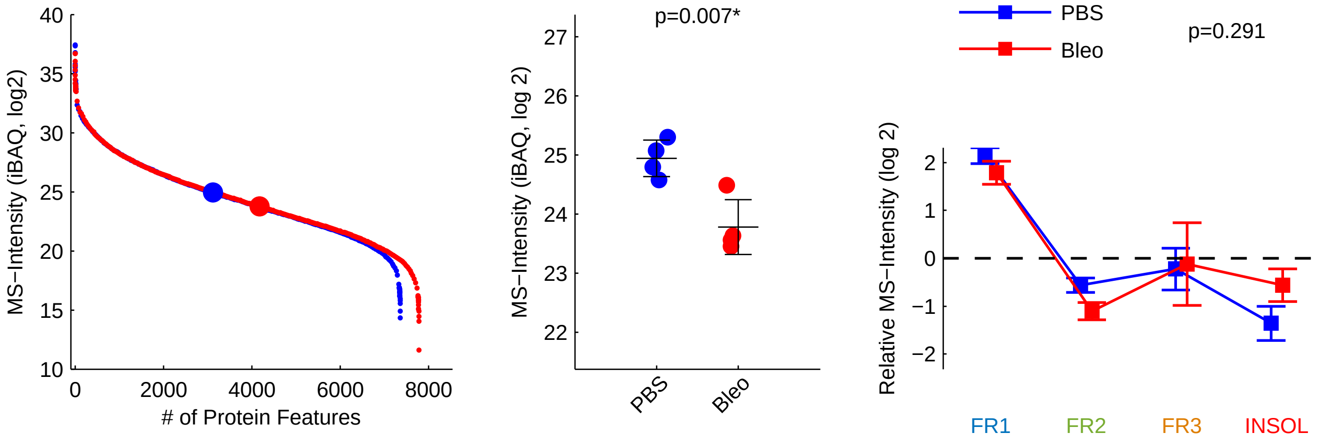

P42567 − Eps15 (id: 3022)

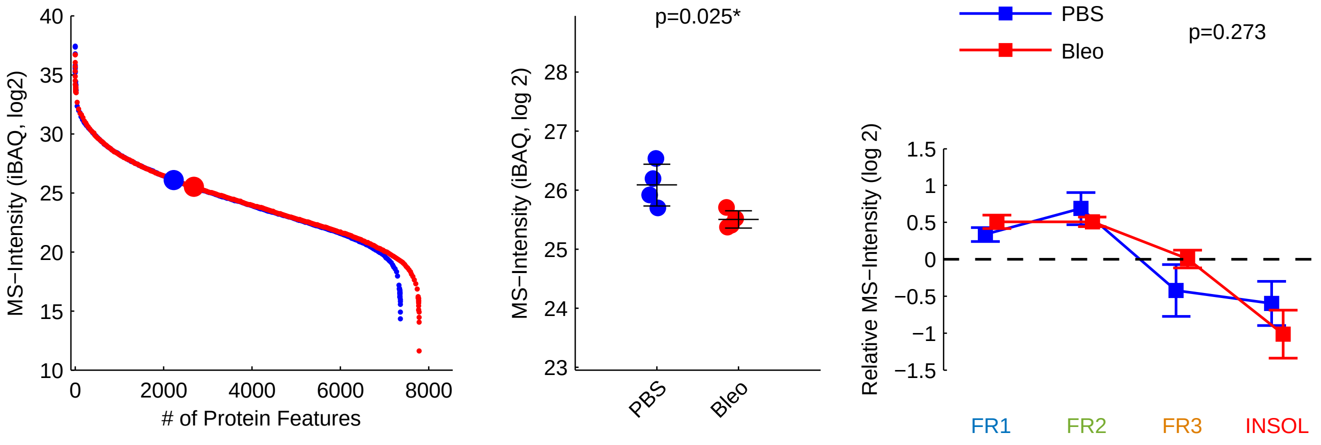

P42669 − Pura (id: 3023)

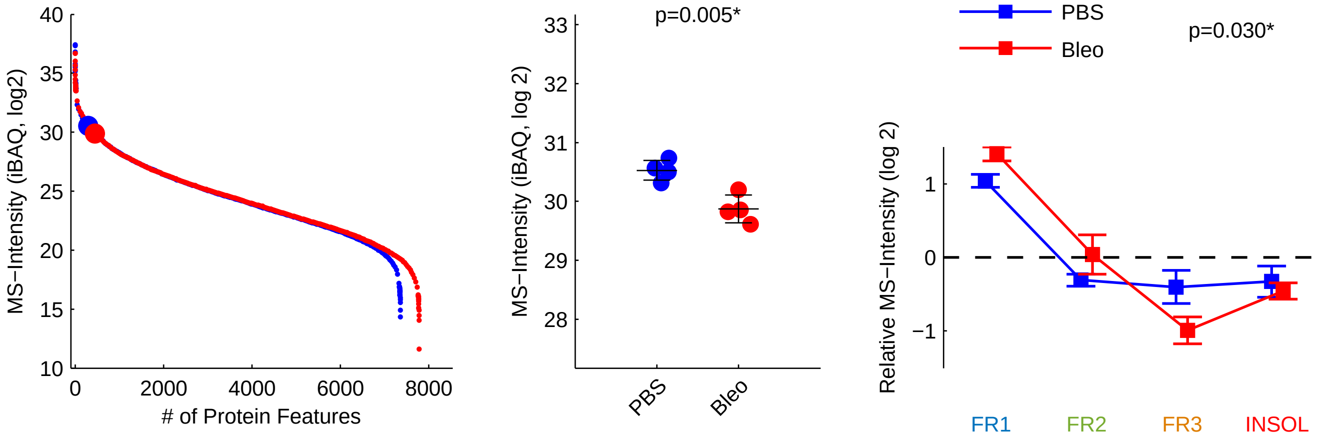

P42932 − Cct8 (id: 3026)

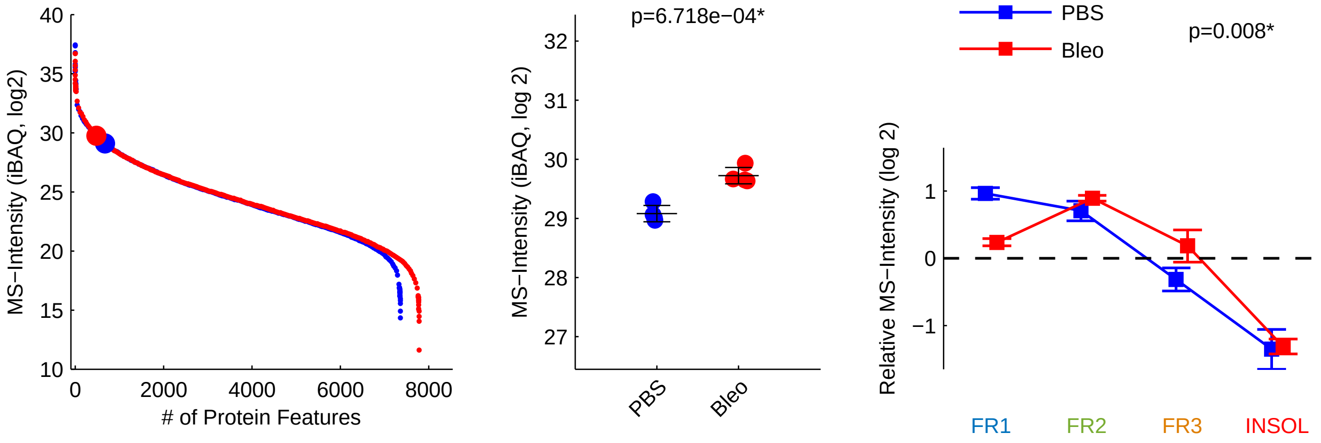

P43274 − Hist1h1e (id: 3033)

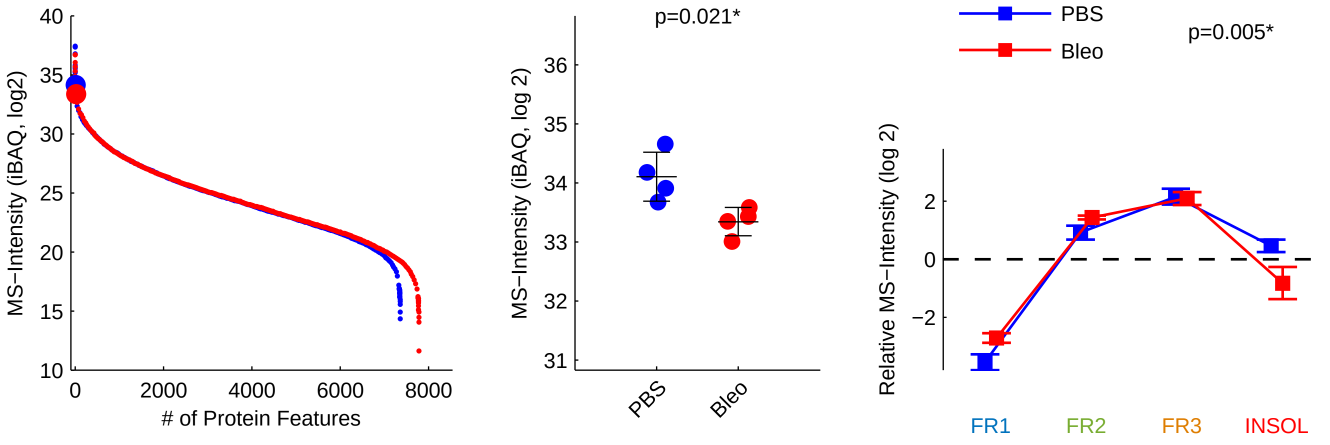

P43275 − Hist1h1a (id: 3034)

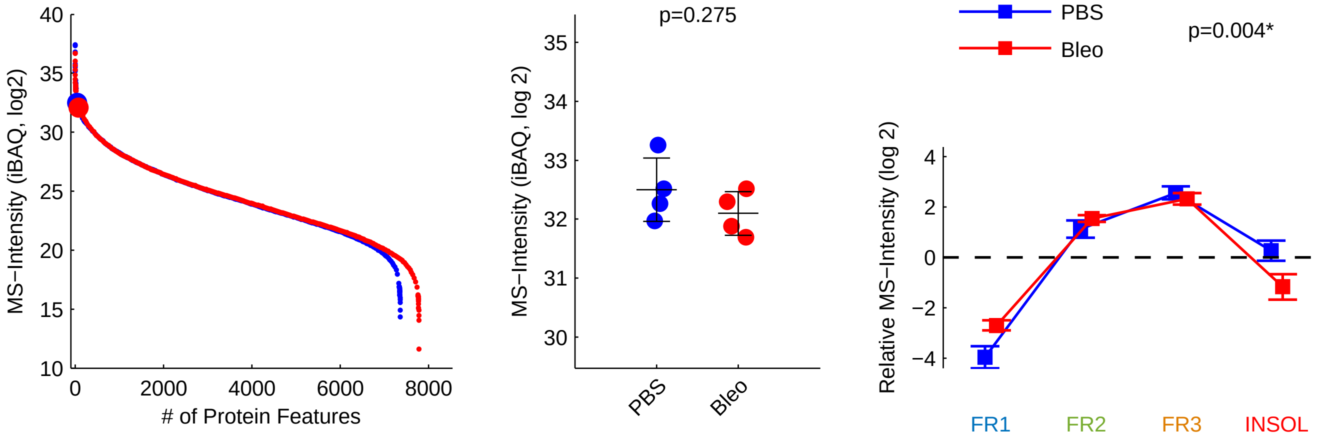

P43276 − Hist1h1b (id: 3035)

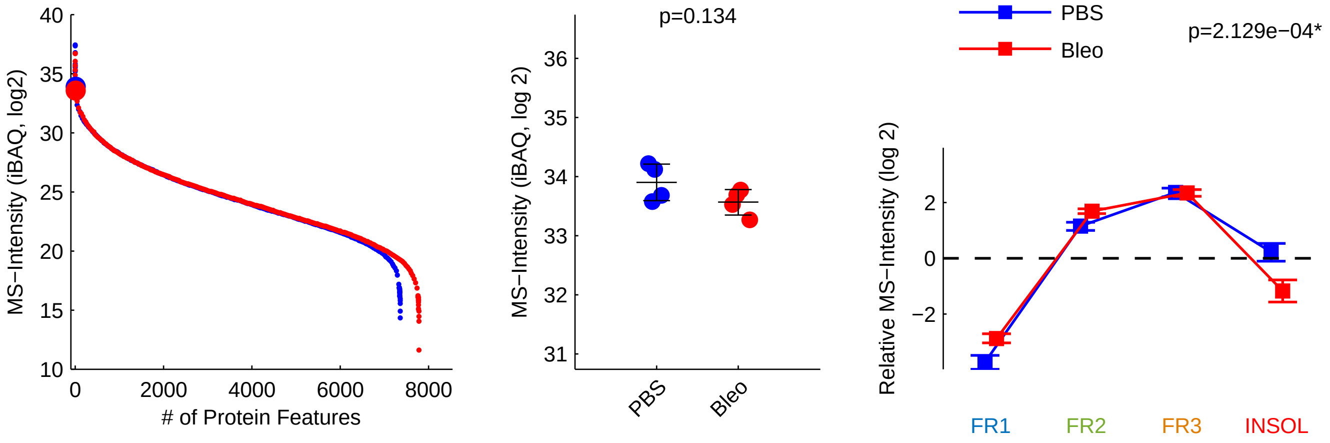

P43406 − Itgav (id: 3039)

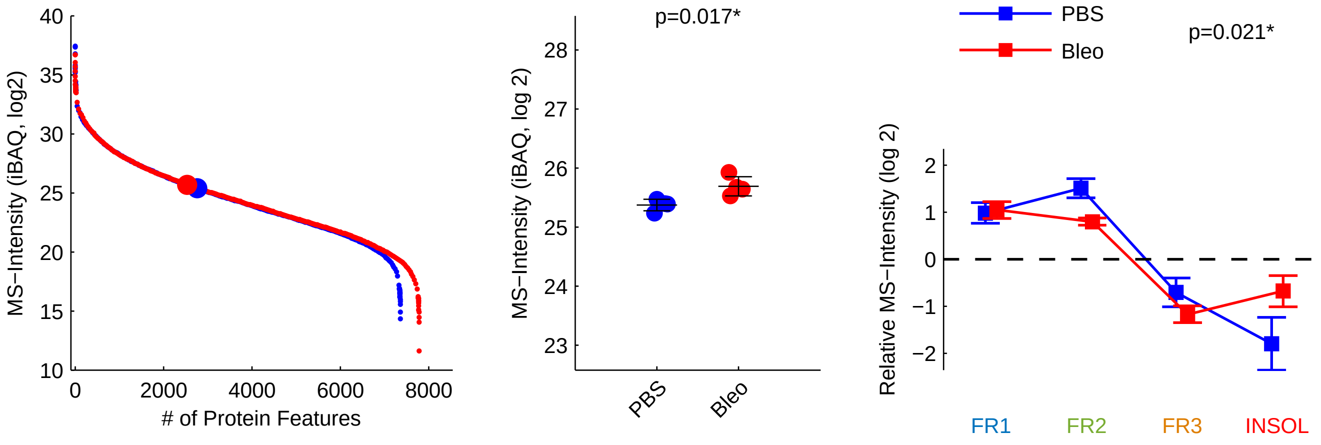

P45376 − Akr1b1 (id: 3042)

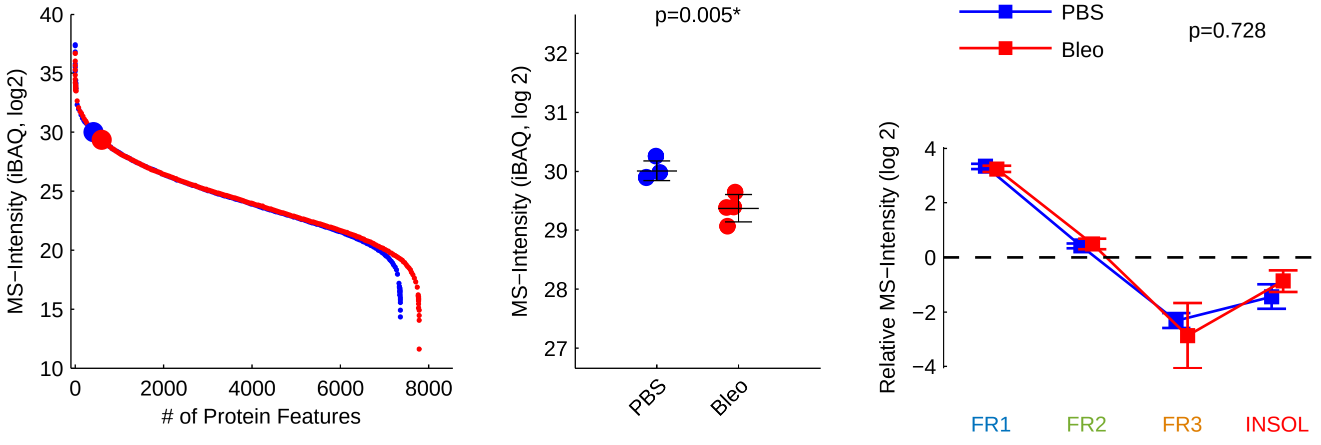

P45377 − Akr1b8 (id: 3043)

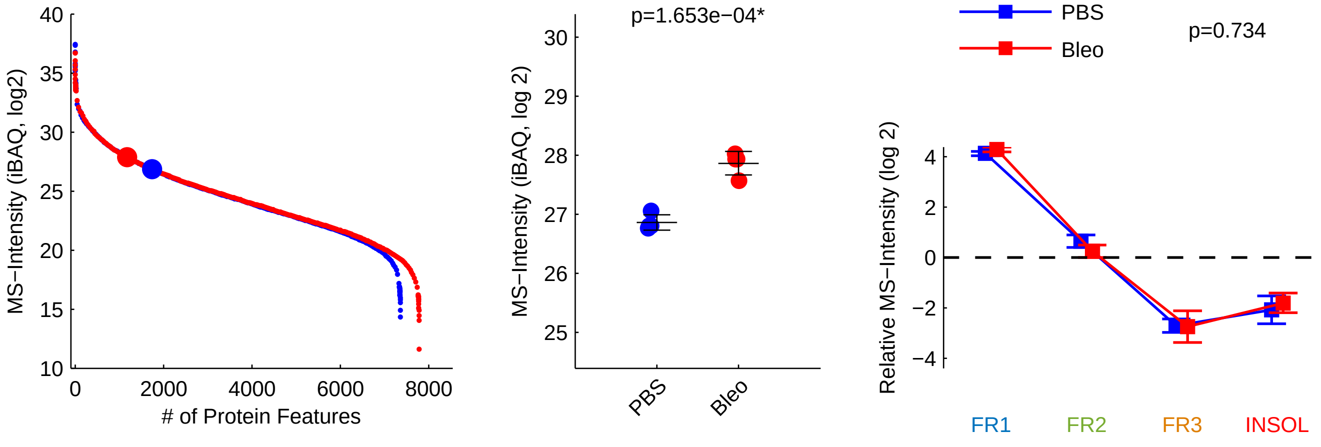

P45952 − Acadm (id: 3047)

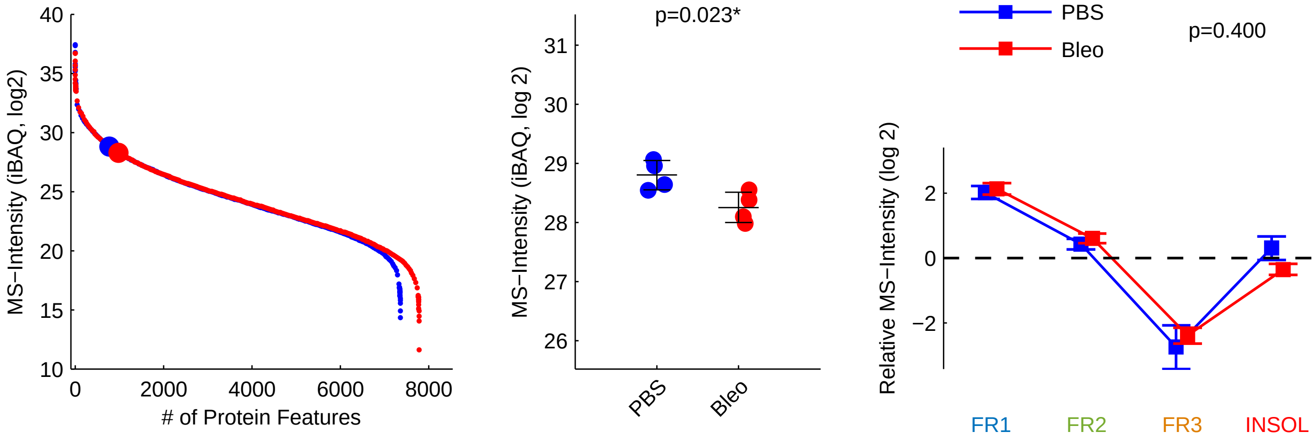

P46412 − Gpx3 (id: 3049)

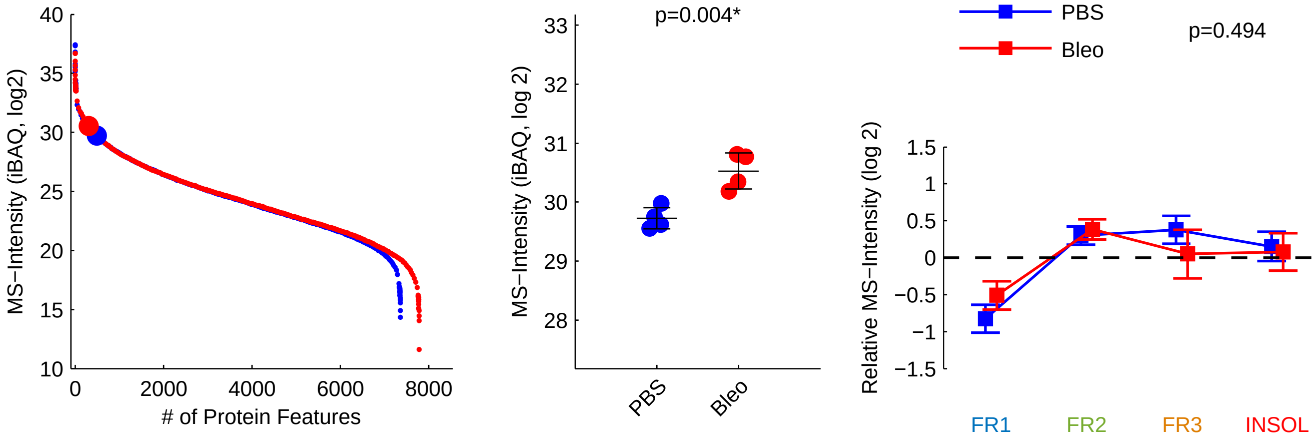

P46414 − Cdkn1b (id: 3050)

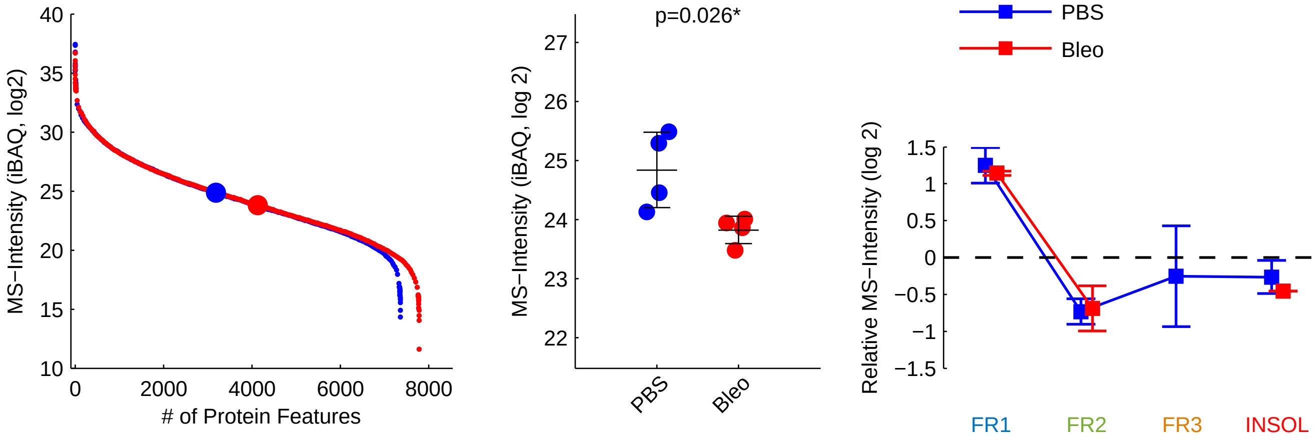

P46467 − Vps4b (id: 3052)

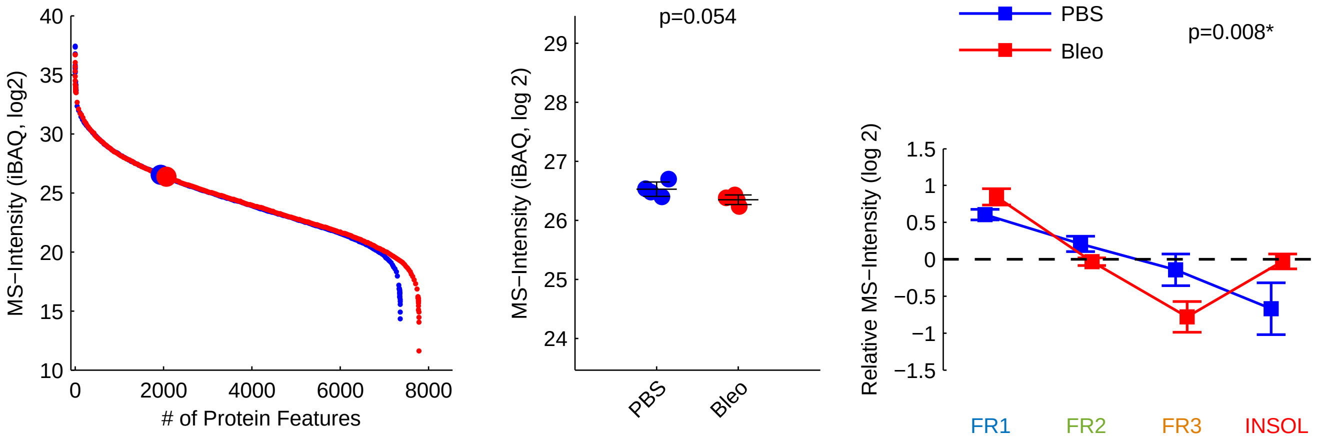

P46638 − Rab11b (id: 3054)

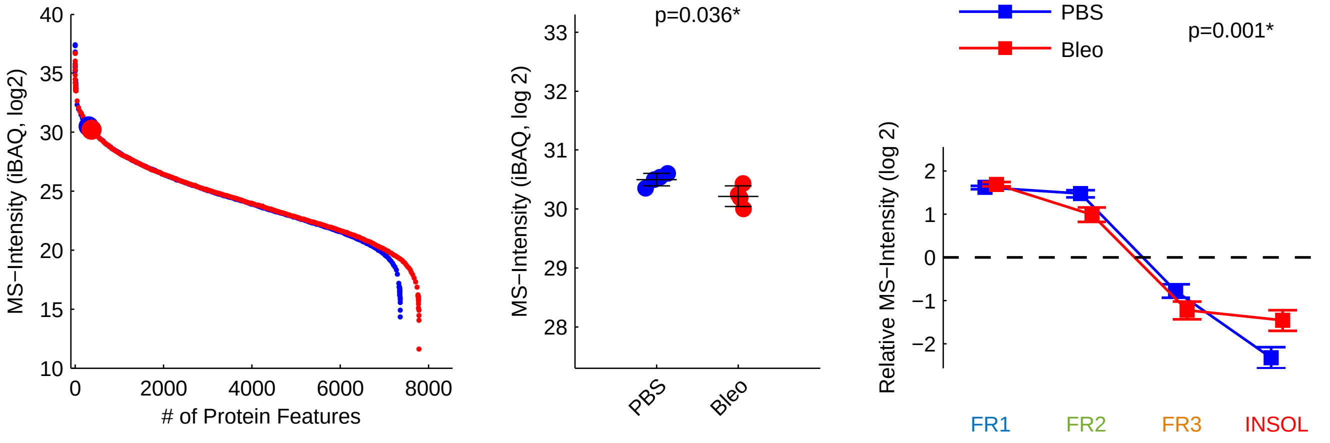

P46735 − Myo1b (id: 3060)

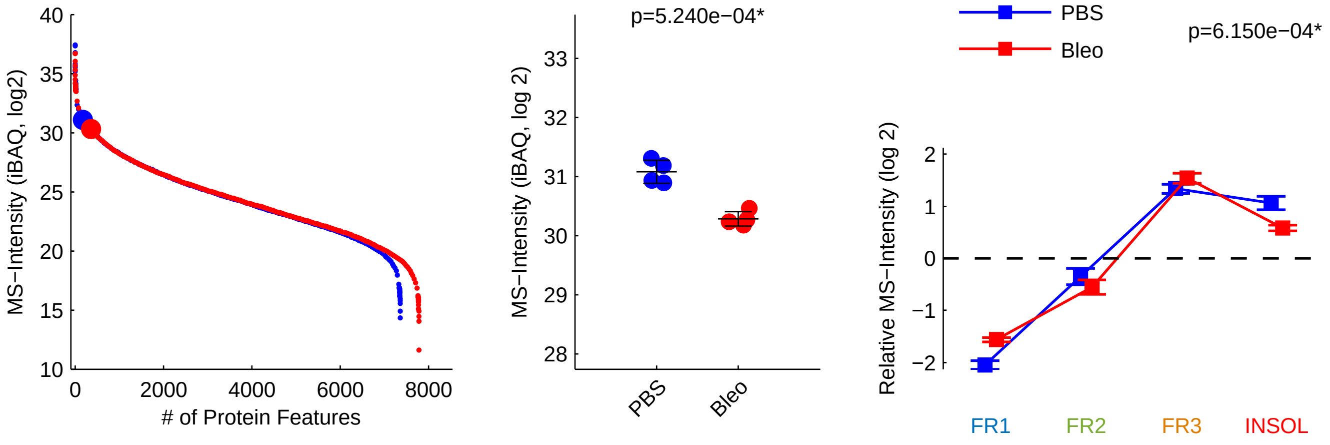

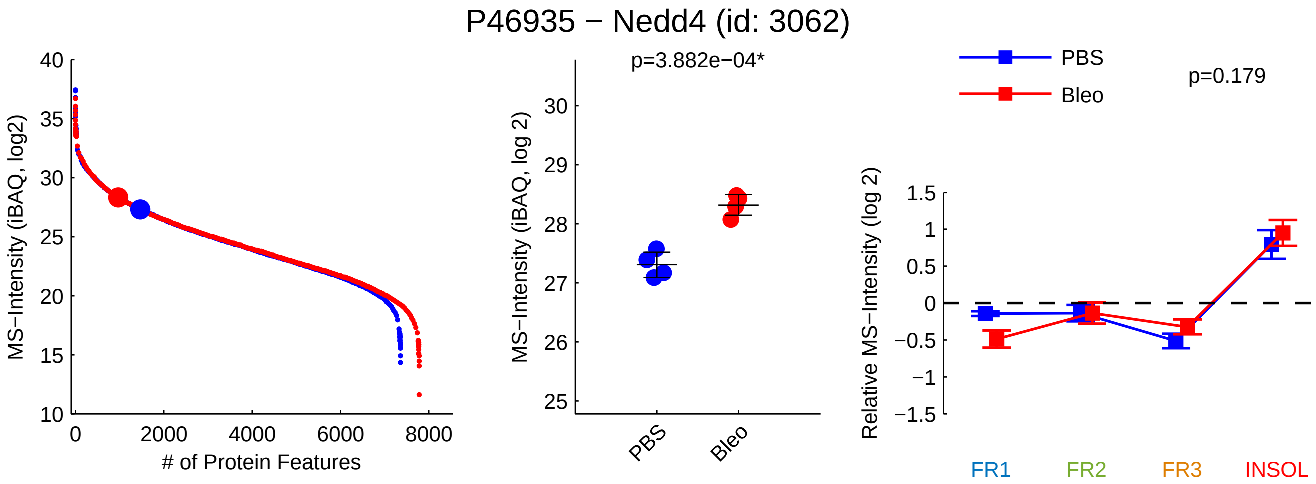
P46935 − Nedd4 (id: 3062)
MS−Intensity (iBAQ, log2)
# of Protein Features
p=3.882e−04*
MS−Intensity (iBAQ, log 2)
PBS
Bleo
p=0.179
Relative MS−Intensity (log 2)
FR1
FR2
FR3
INSOL

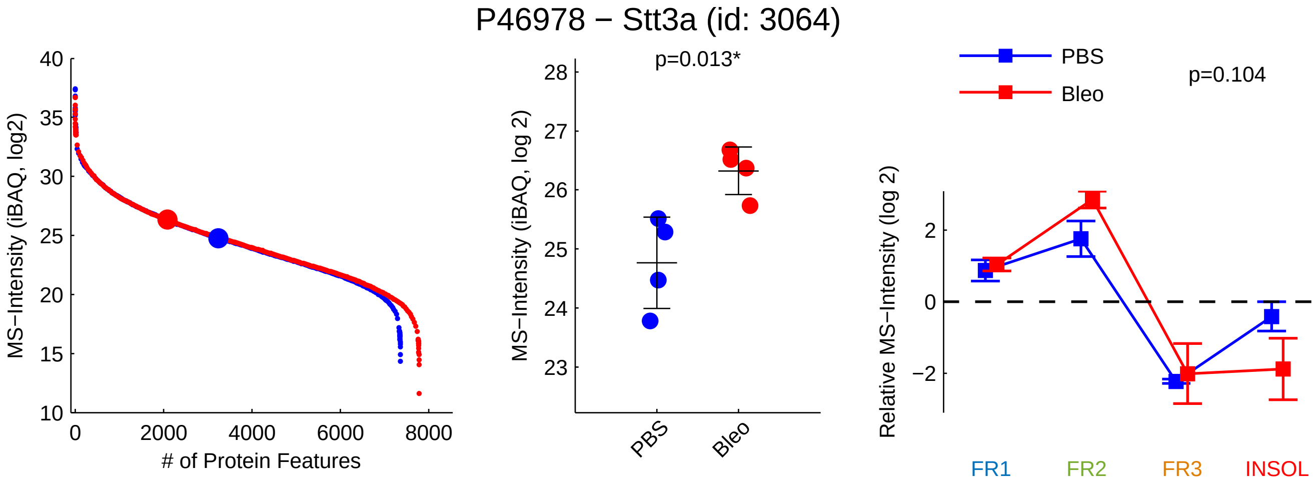
P46978 − Stt3a (id: 3064)
MS−Intensity (iBAQ, log2)
# of Protein Features
p=0.013*
MS−Intensity (iBAQ, log 2)
PBS
Bleo
p=0.104
Relative MS−Intensity (log 2)
FR1
FR2
FR3
INSOL

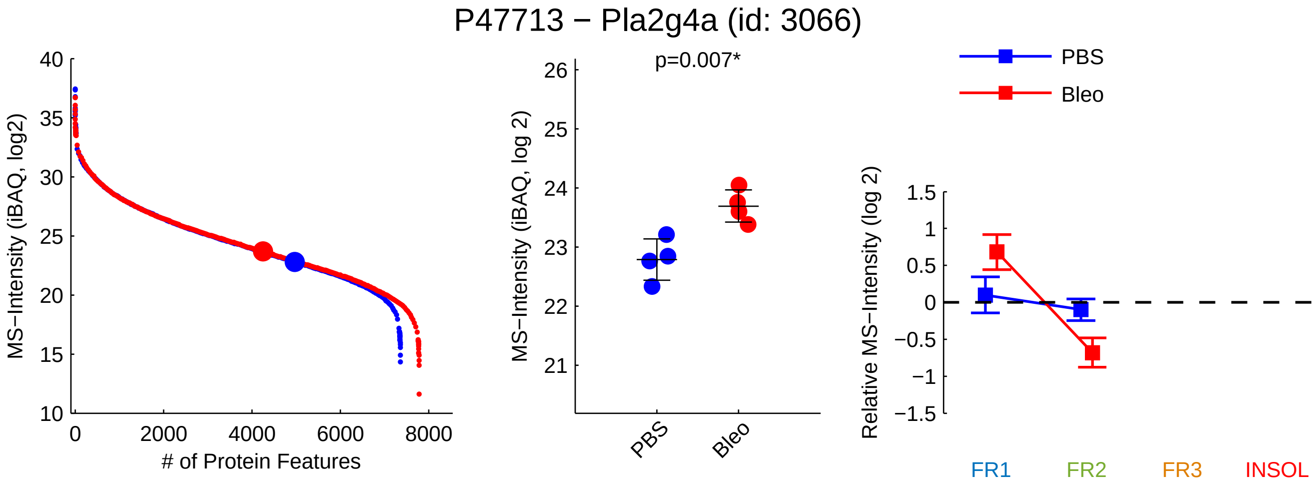
P47713 − Pla2g4a (id: 3066)
MS−Intensity (iBAQ, log2)
# of Protein Features
p=0.007*
MS−Intensity (iBAQ, log 2)
PBS
Bleo
Relative MS−Intensity (log 2)
FR1
FR2
FR3
INSOL

P47738 − Aldh2 (id: 3067)

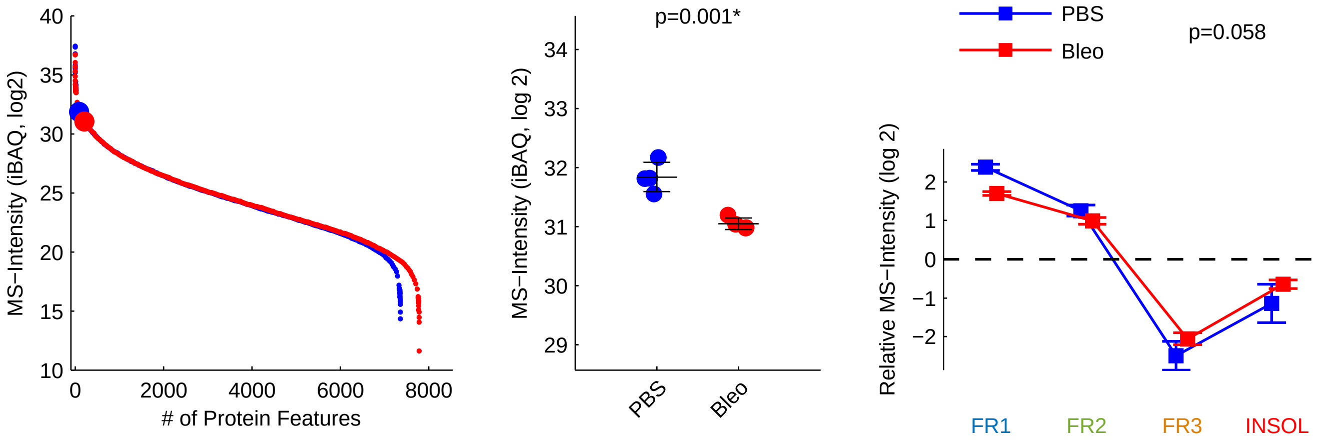

P47856 − Gfpt1 (id: 3075)

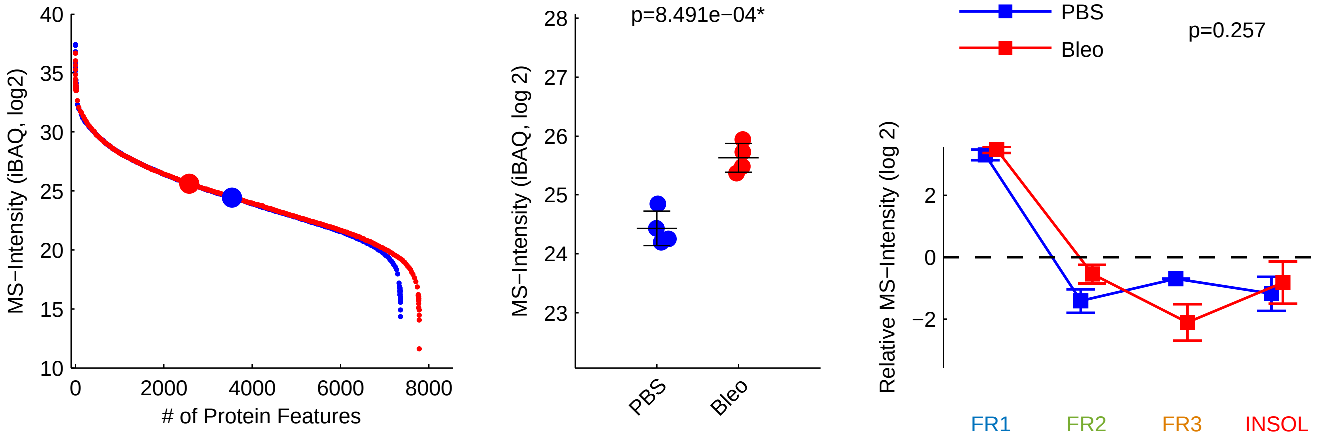

P47880 − Igfbp6 (id: 3077)

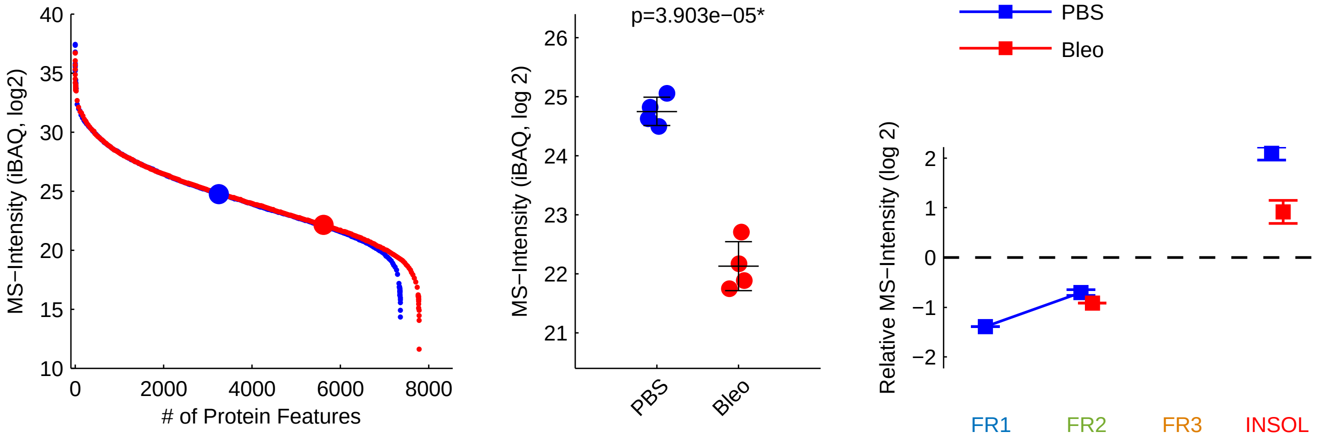

P47911 − Rpl6 (id: 3078)

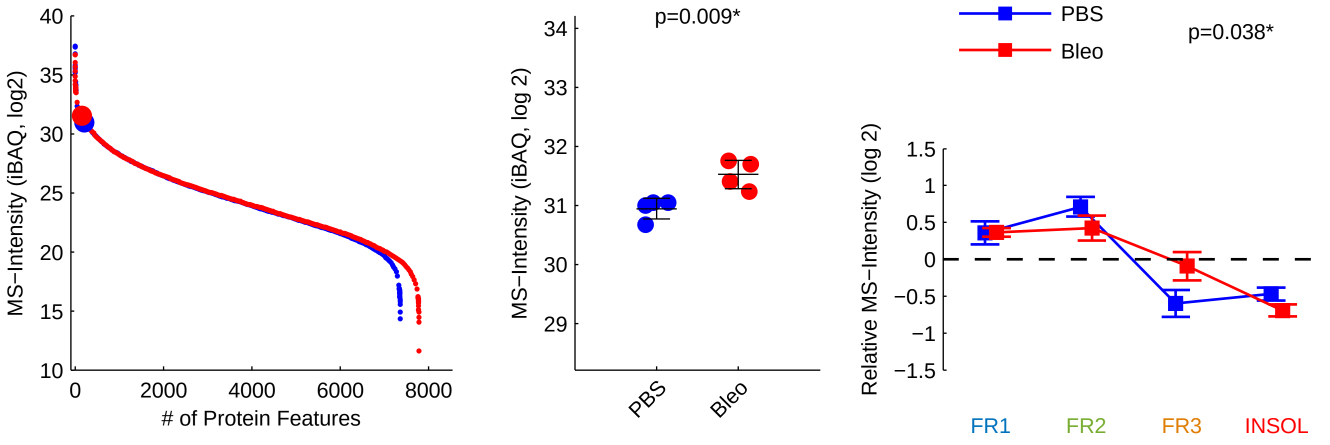

P47941 − Crkl (id: 3080)

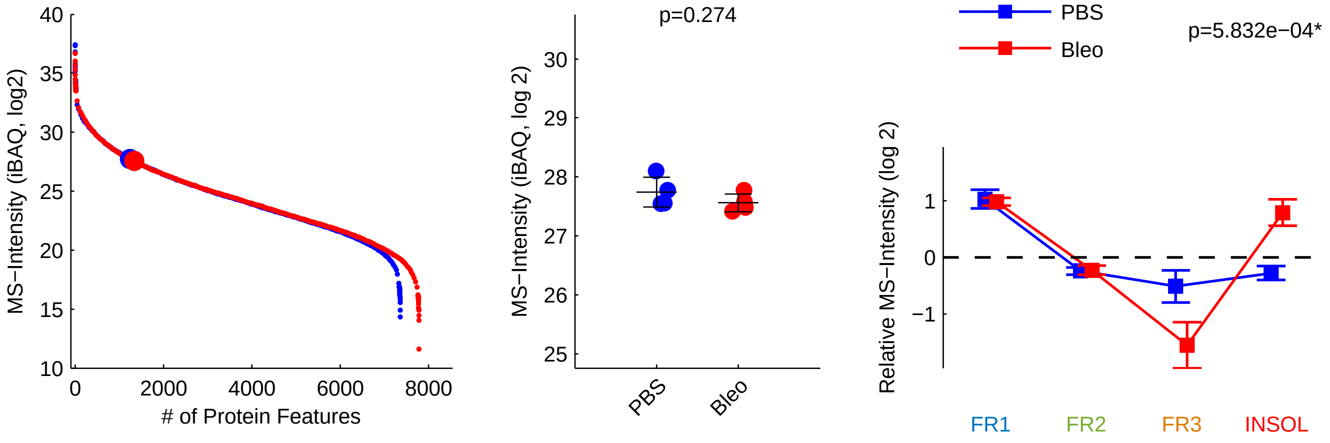

P47962 − Rpl5 (id: 3082)

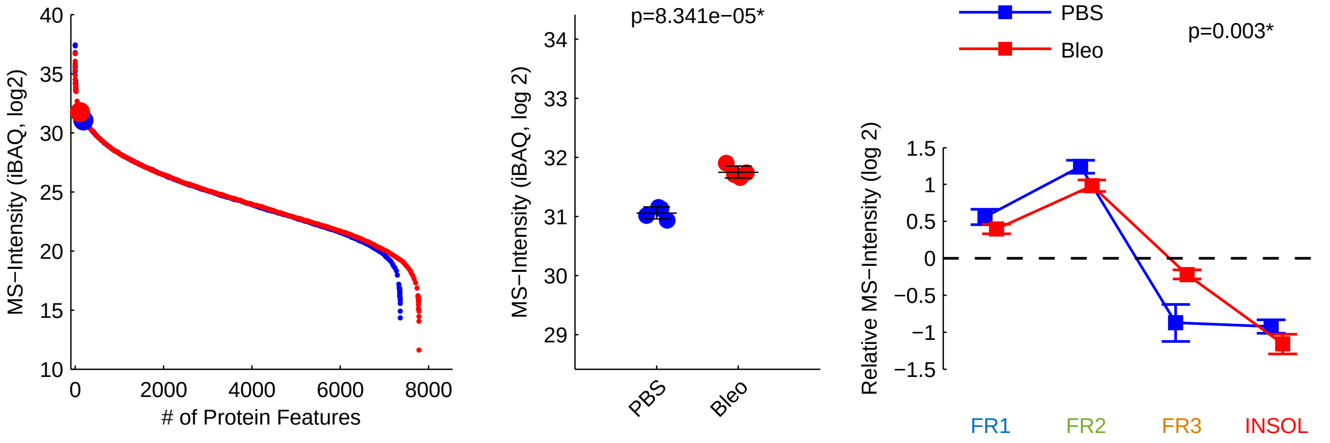

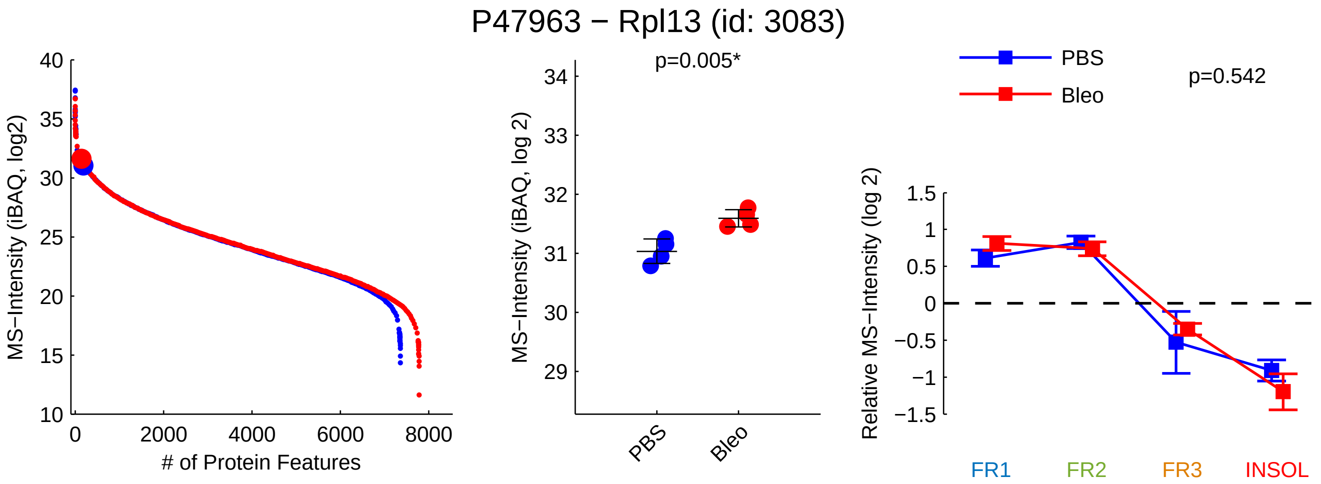
P47963 − Rpl13 (id: 3083)
MS−Intensity (iBAQ, log2)
# of Protein Features
p=0.005*
MS−Intensity (iBAQ, log 2)
PBS
Bleo
p=0.542
Relative MS−Intensity (log 2)
FR1
FR2
FR3
INSOL

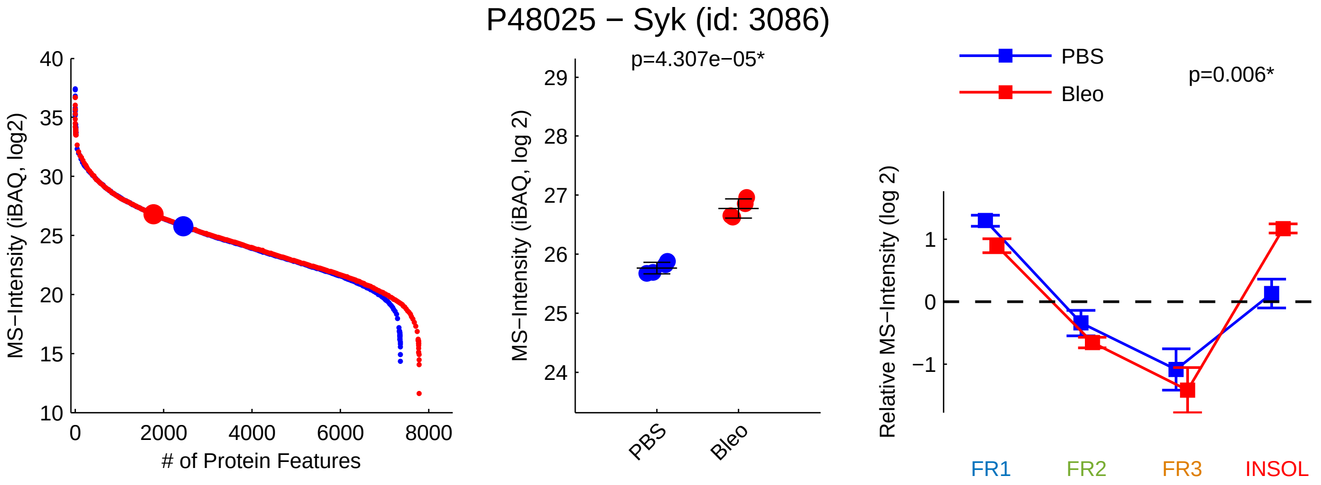
P48025 − Syk (id: 3086)
MS−Intensity (iBAQ, log2)
# of Protein Features
p=4.307e−05*
MS−Intensity (iBAQ, log 2)
PBS
Bleo
p=0.006*
Relative MS−Intensity (log 2)
FR1
FR2
FR3
INSOL

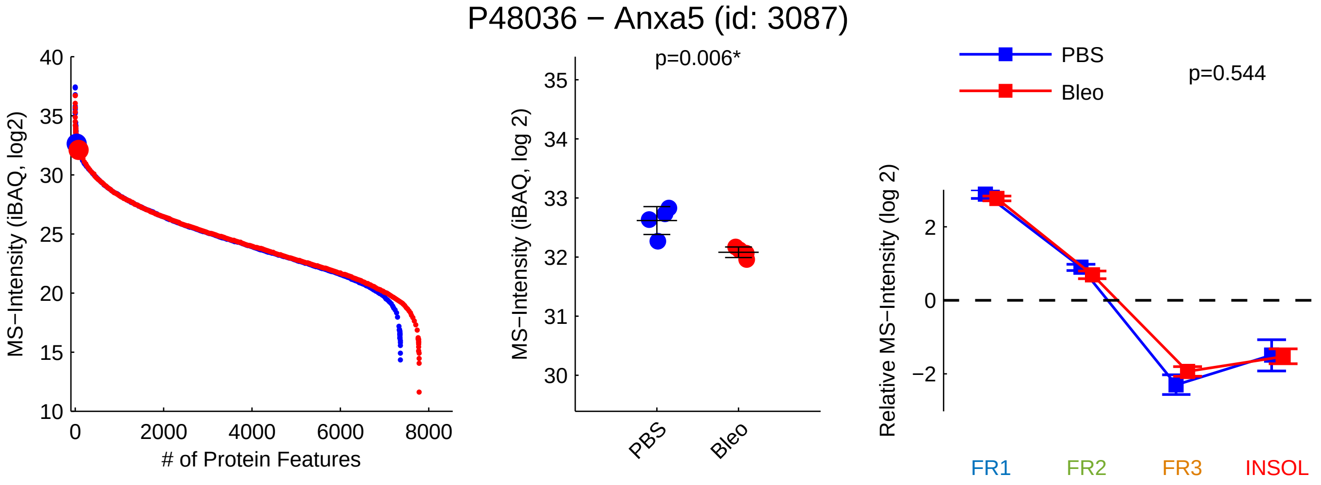
P48036 − Anxa5 (id: 3087)
MS−Intensity (iBAQ, log2)
# of Protein Features
p=0.006*
MS−Intensity (iBAQ, log 2)
PBS
Bleo
p=0.544
Relative MS−Intensity (log 2)
FR1
FR2
FR3
INSOL

P48193 − Epb41 (id: 3088)

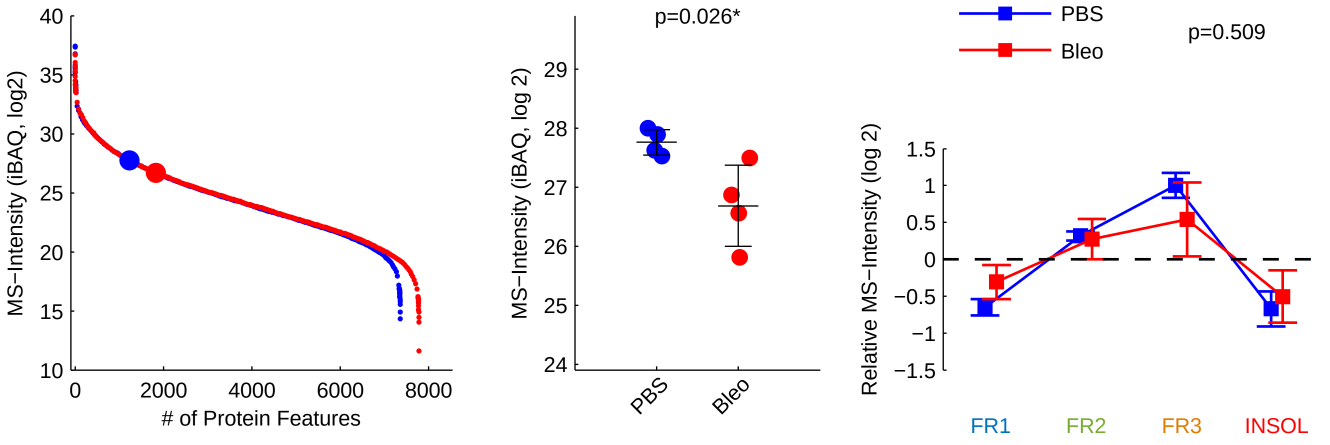

P48678 − Lmna (id: 3095)

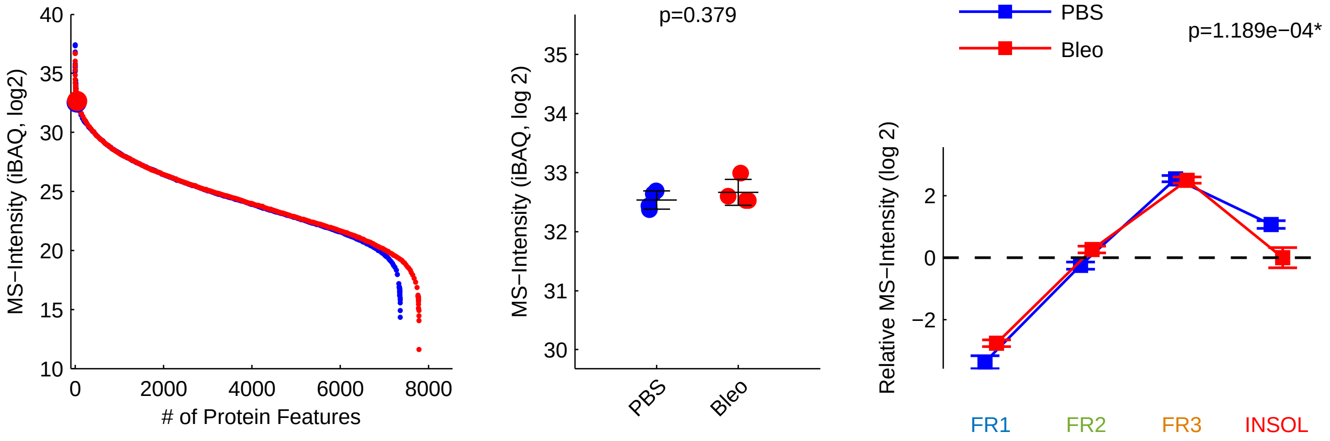

P48722 − Hspa4l (id: 3097)

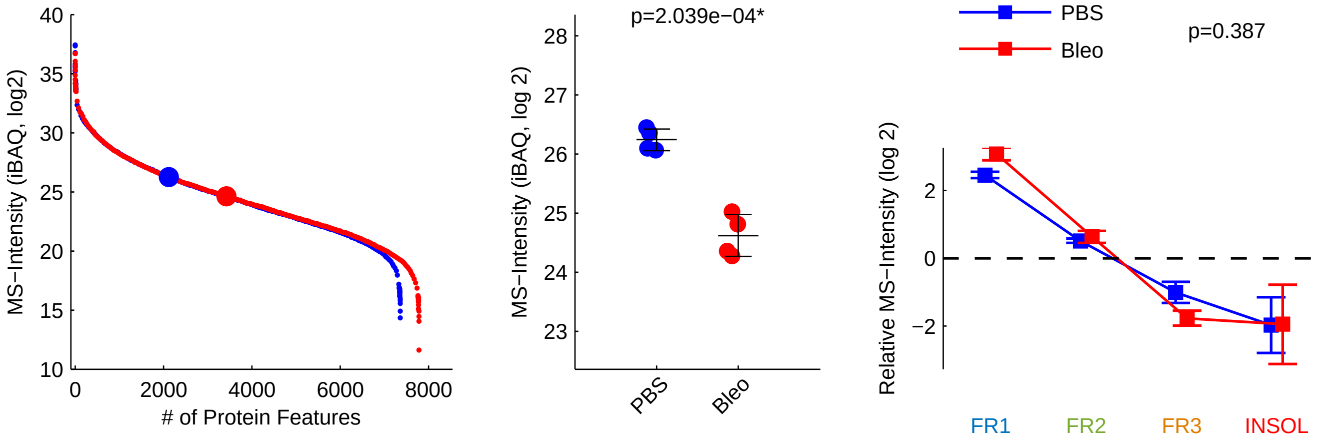

P48758 − Cbr1 (id: 3098)

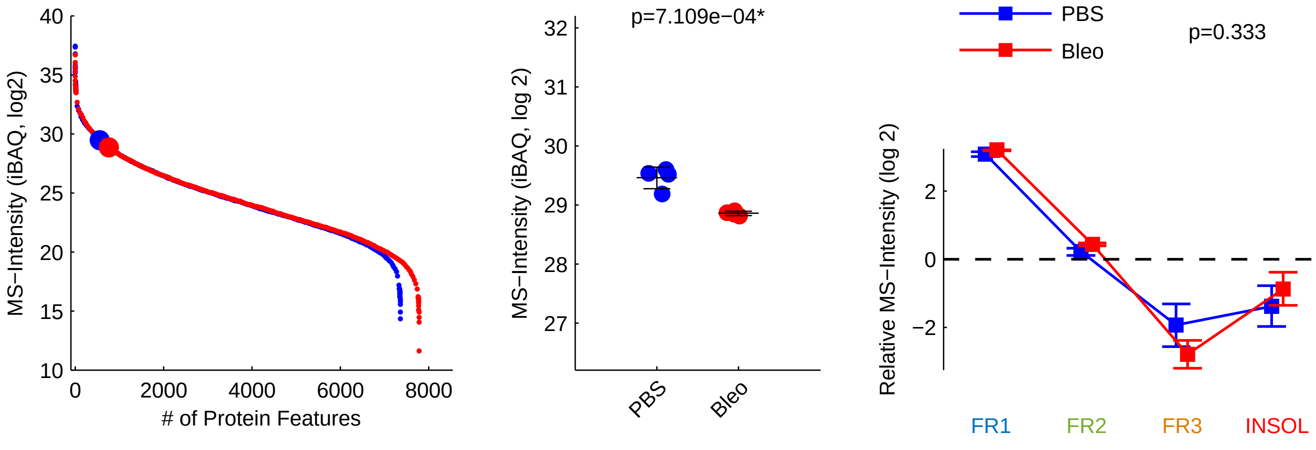

P49182 − Serpind1 (id: 3107)

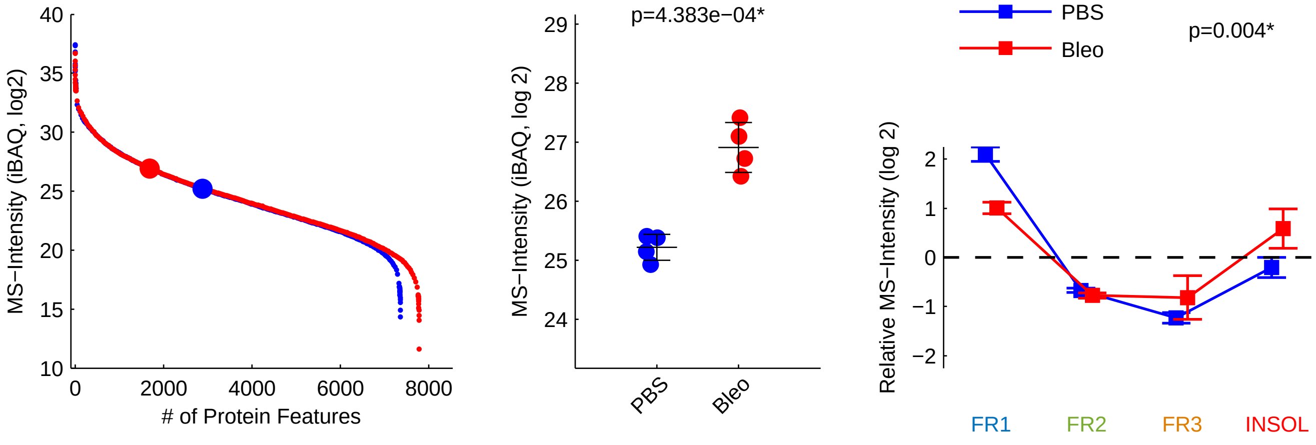

P49290 − Epx (id: 3109)

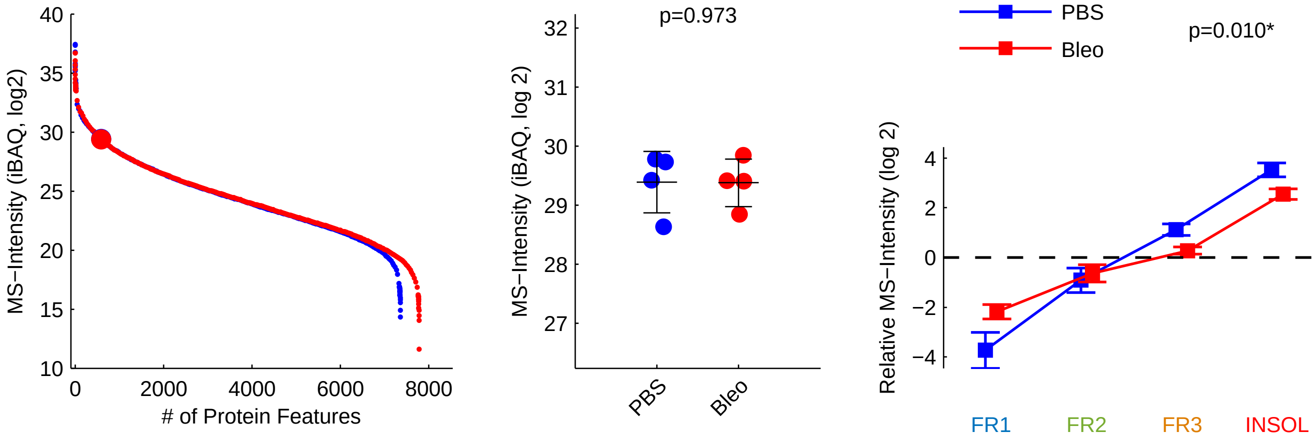

P49312 − Hnrnpa1 (id: 3110)

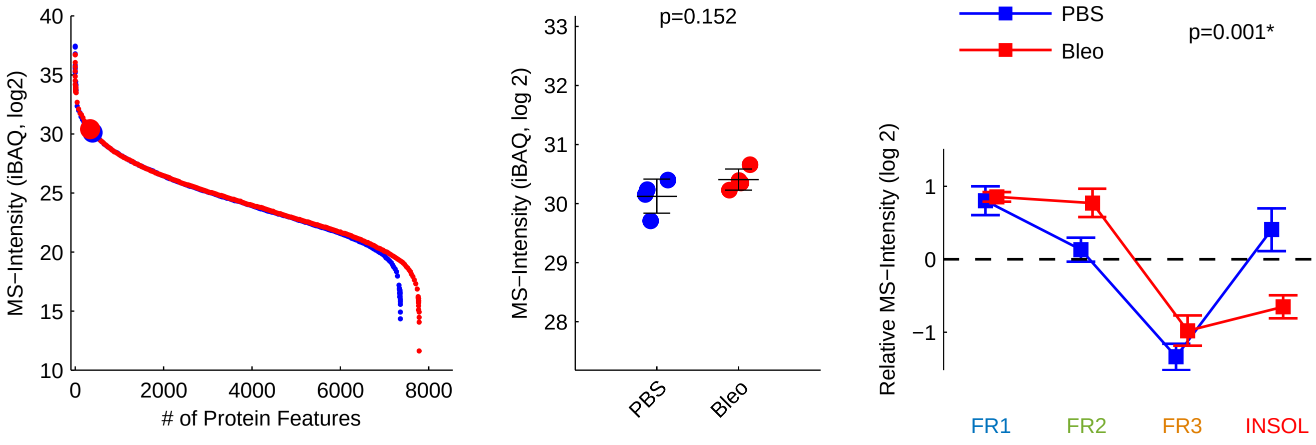

P49442 − Inpp1 (id: 3112)

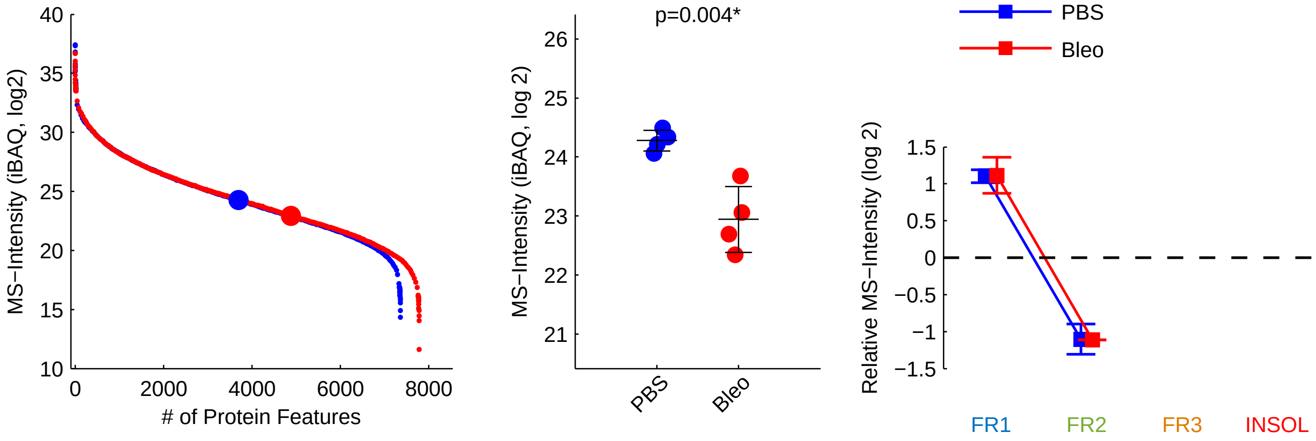

P49710 − Hcls1 (id: 3118)

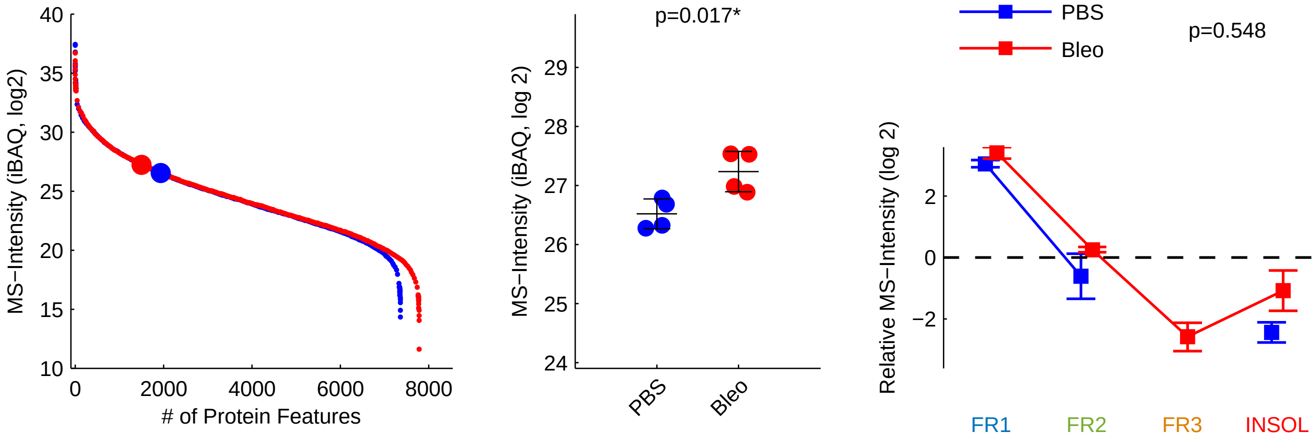

P49813 − Tmod1 (id: 3122)

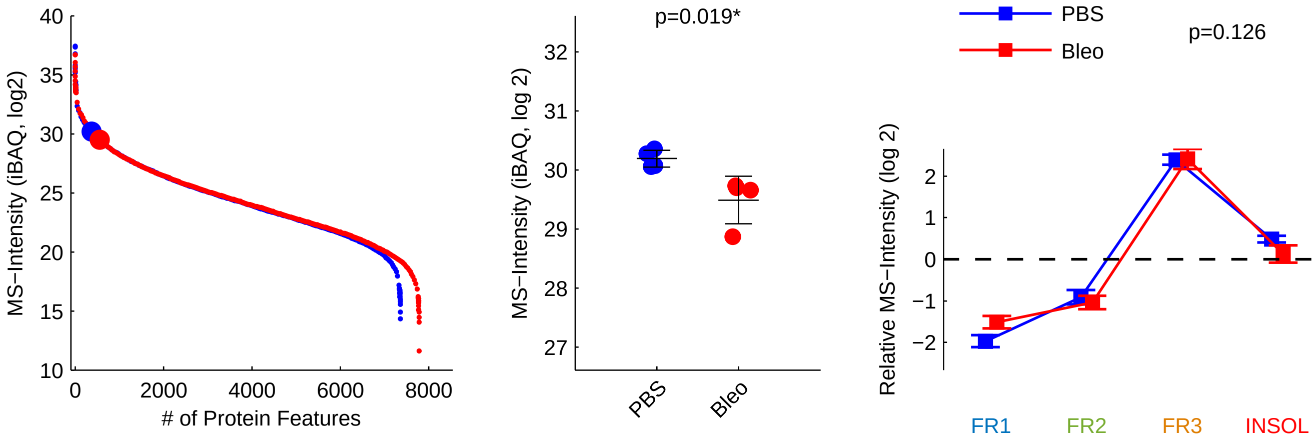

P49817 − Cav1 (id: 3123)

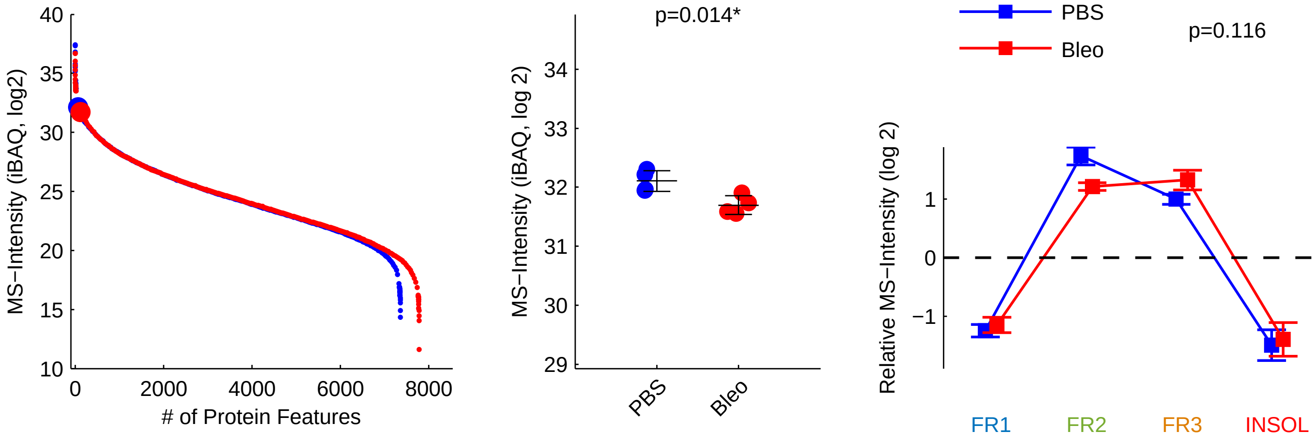

P49935 − Ctsh (id: 3125)

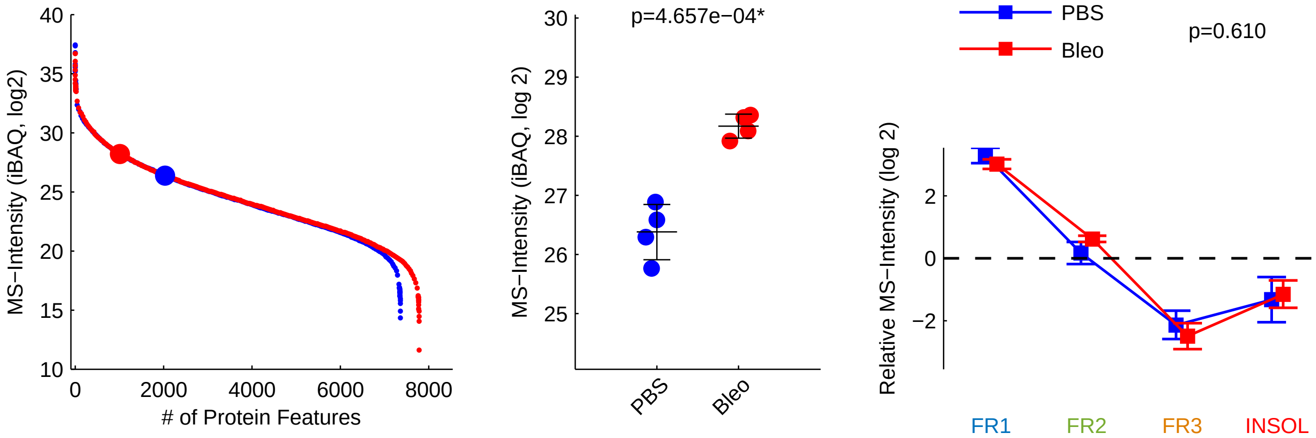

P50114 − S100b (id: 3127)

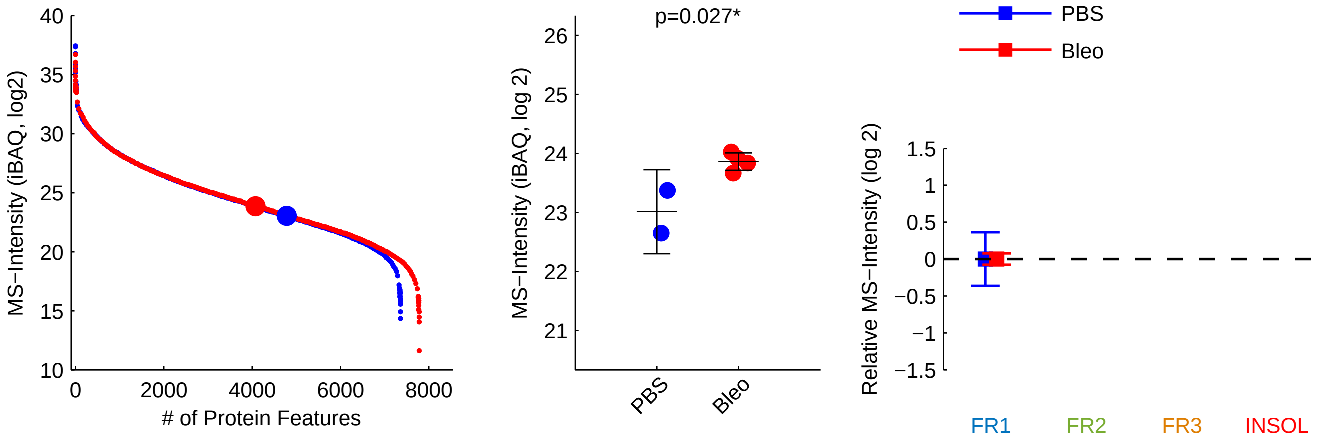

P50171 − Hsd17b8 (id: 3129)

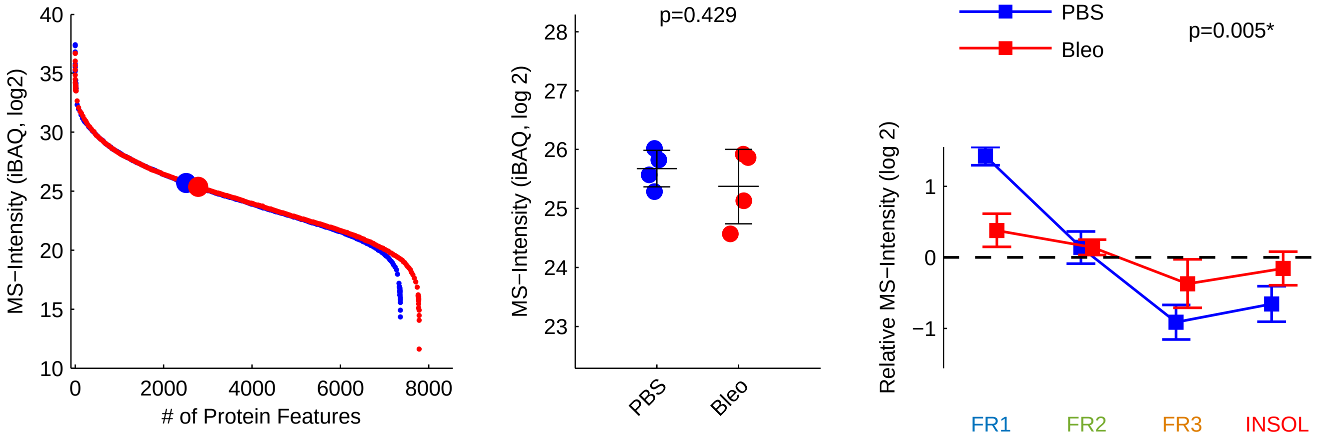

P50172 − Hsd11b1 (id: 3130)

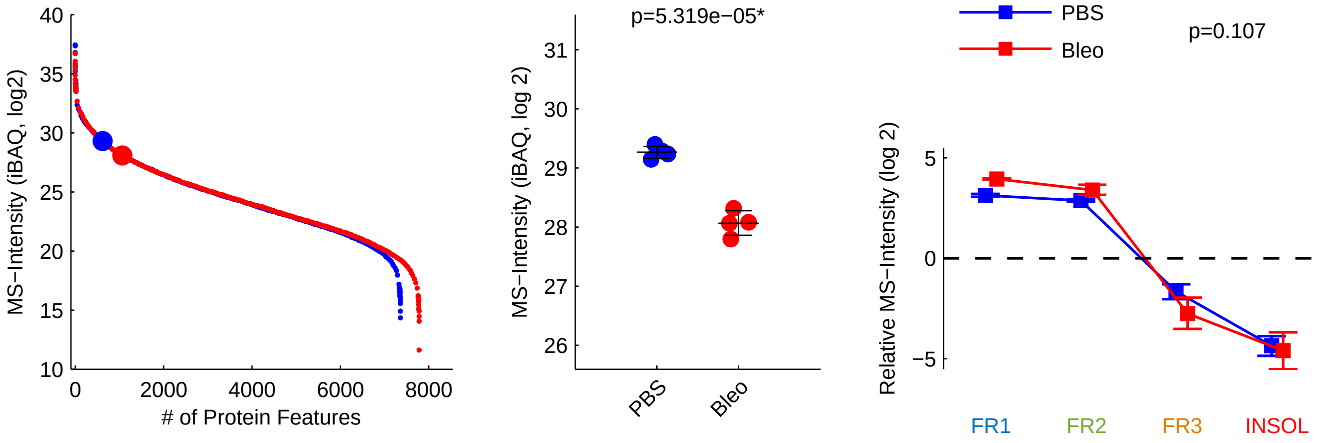

P50247 − Ahcy (id: 3132)

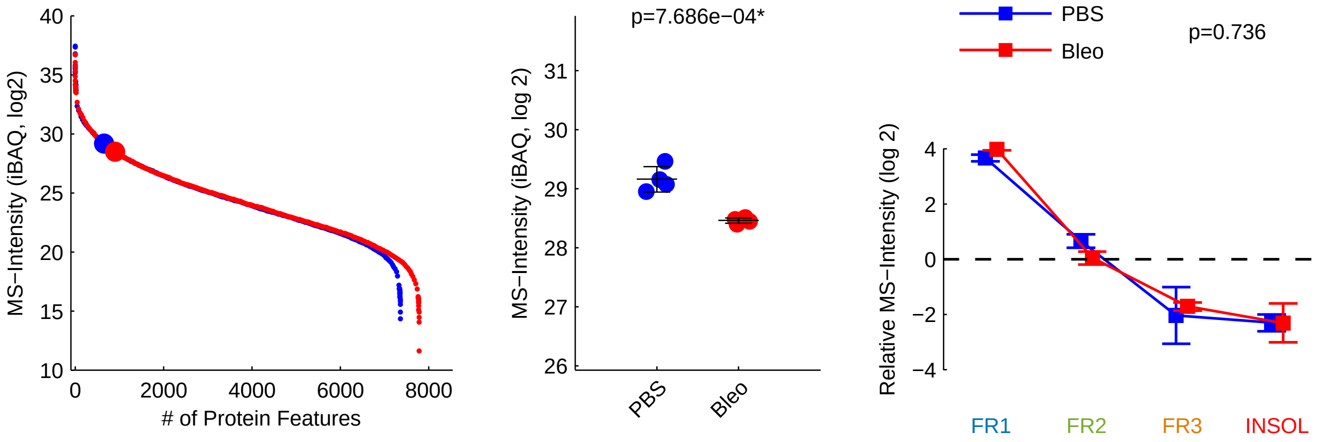

P50285 − Fmo1 (id: 3133)

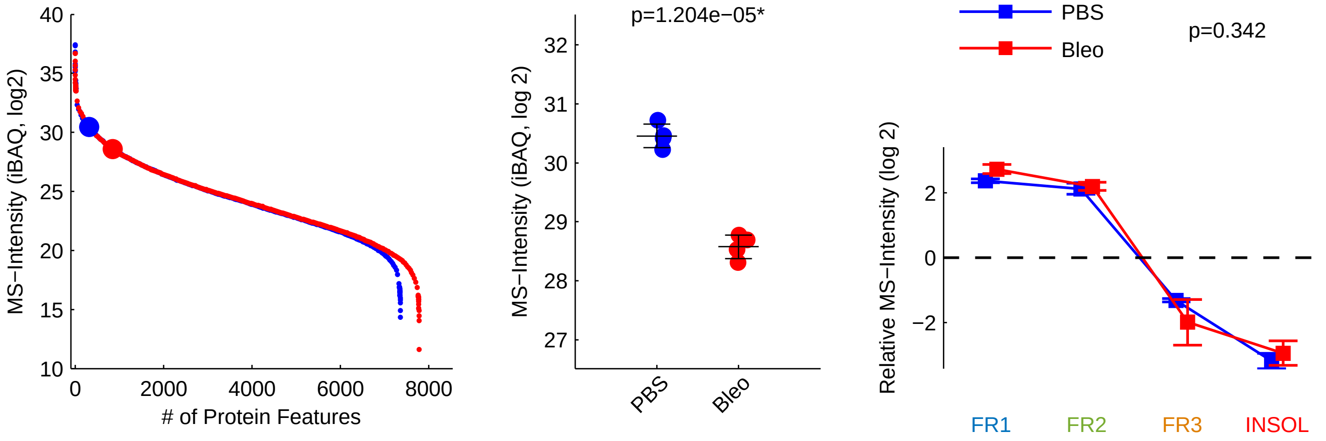

P50396 − Gdi1 (id: 3135)

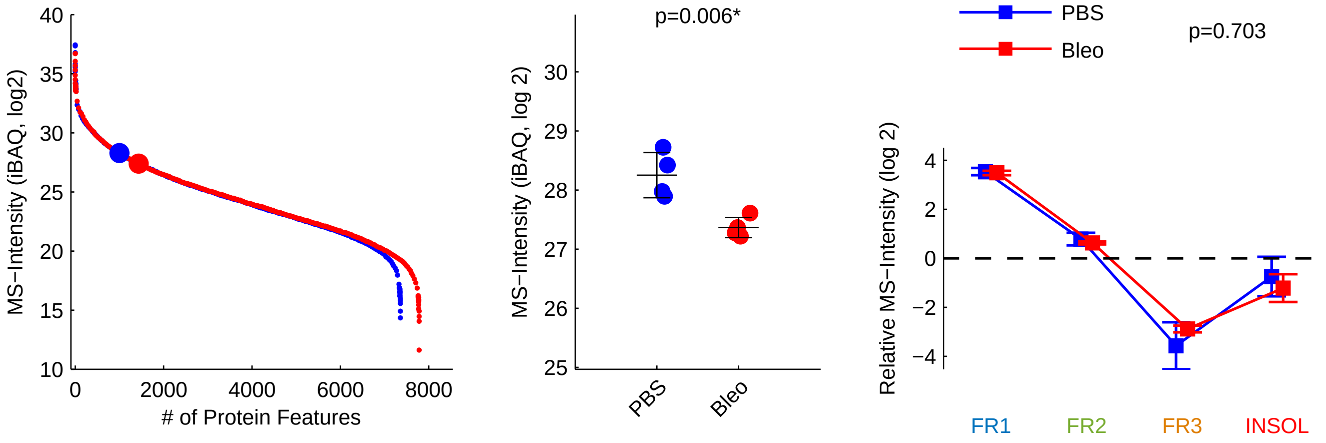

P50405 − Sftpb (id: 3137)

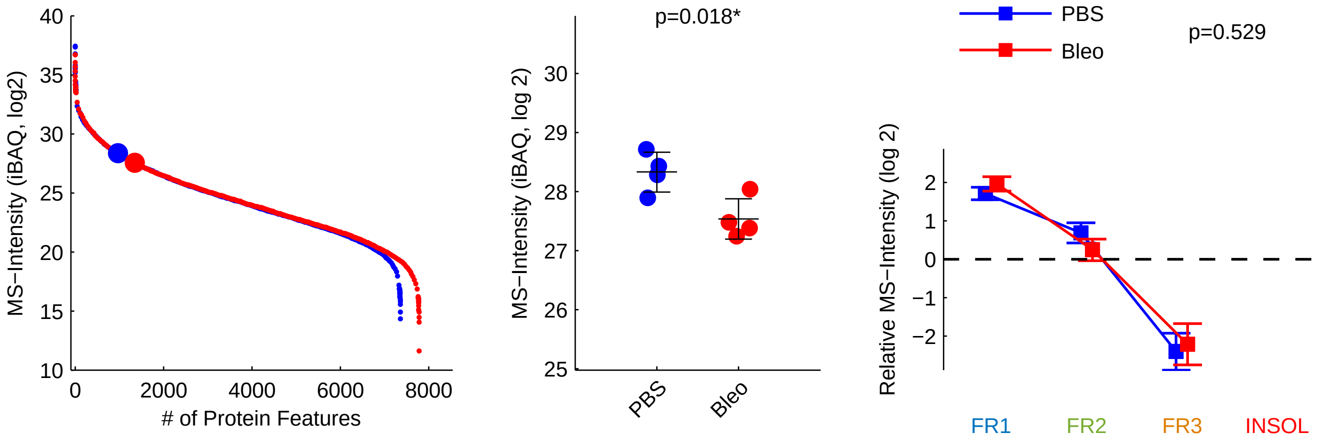

P50427 − Sts (id: 3138)

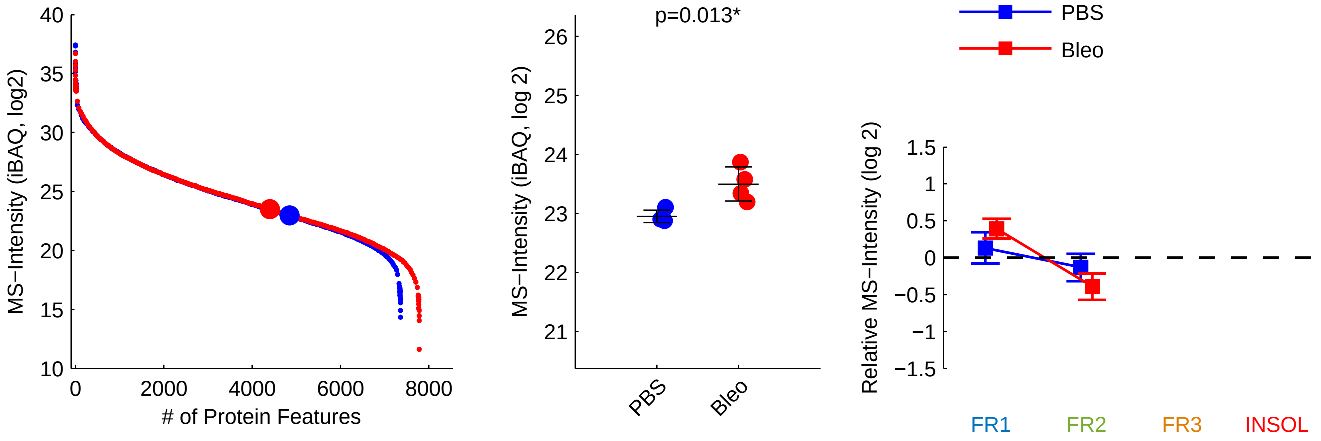

P50543 − S100a11 (id: 3145)

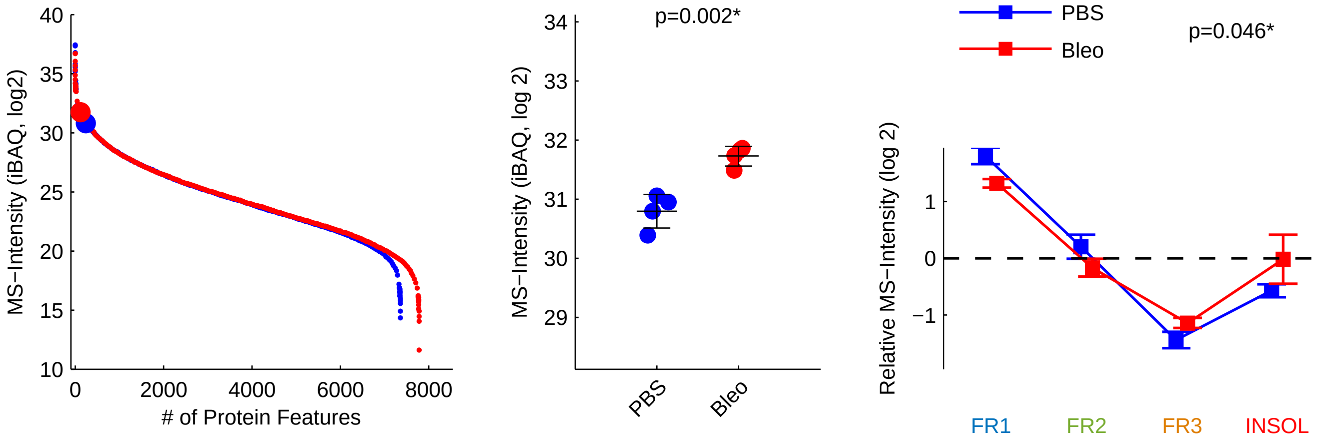

P50580 − Pa2g4 (id: 3147)

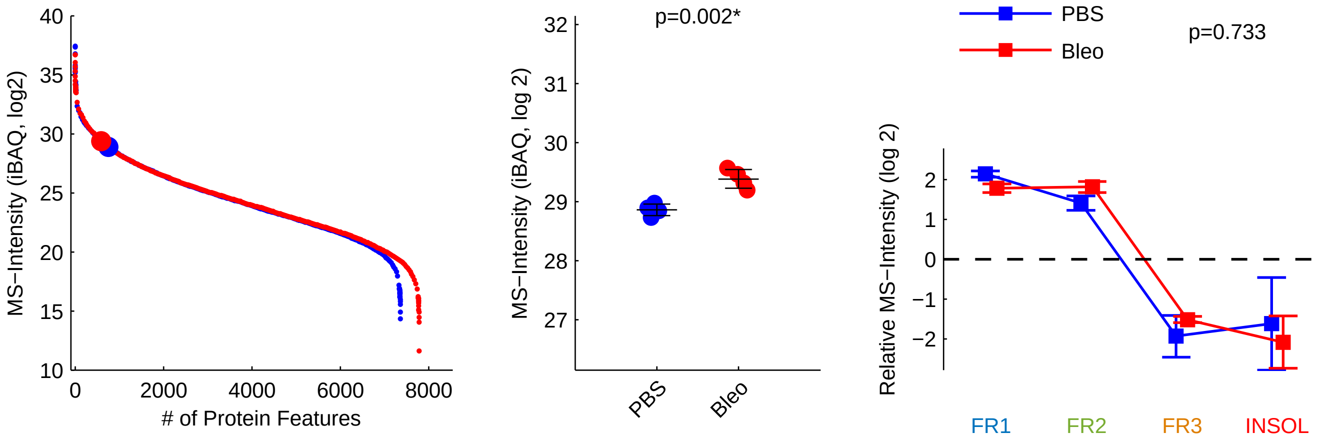

P50608 − Fmod (id: 3149)

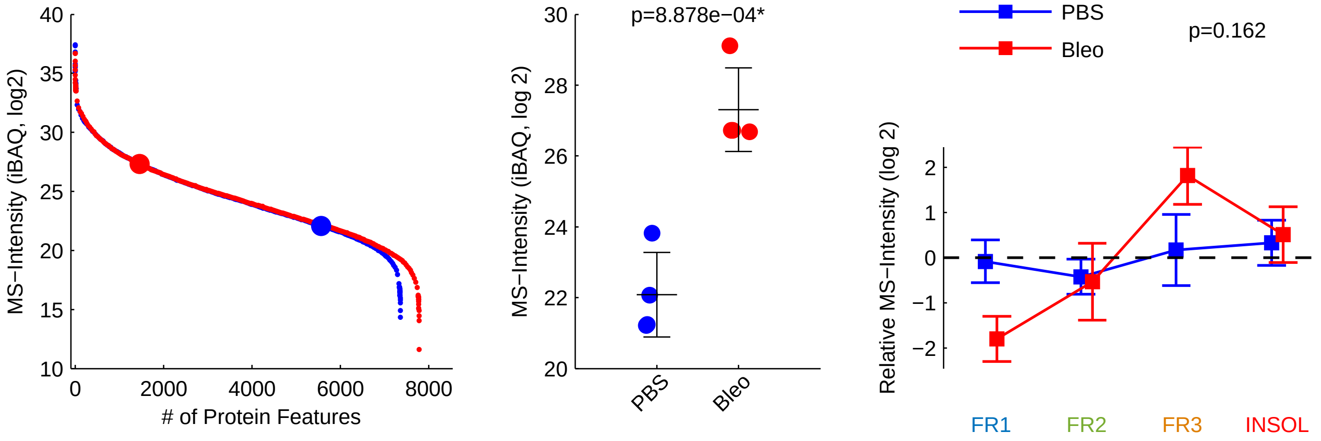

P51410 − Rpl9 (id: 3157)

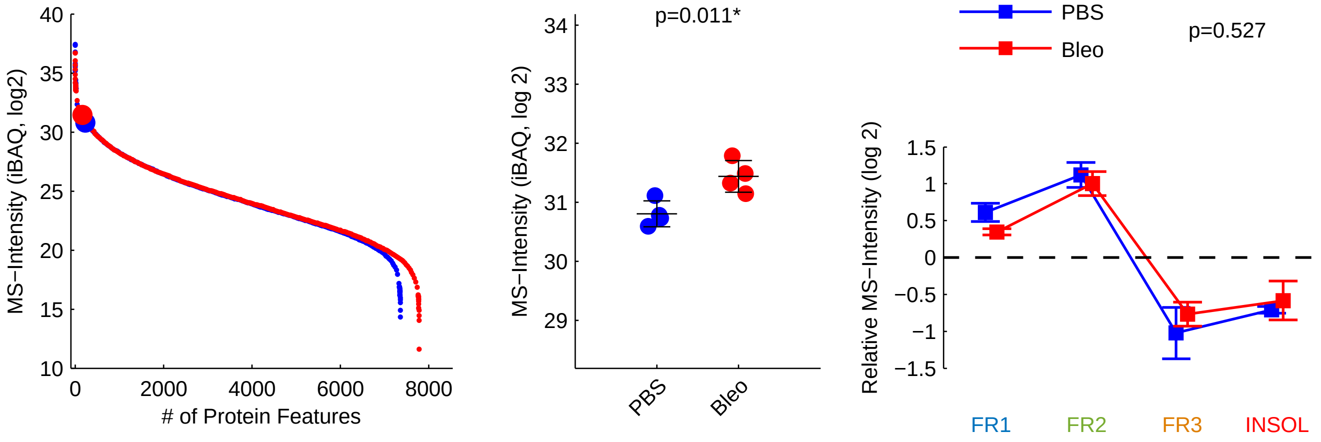

P51432 − Plcb3 (id: 3158)

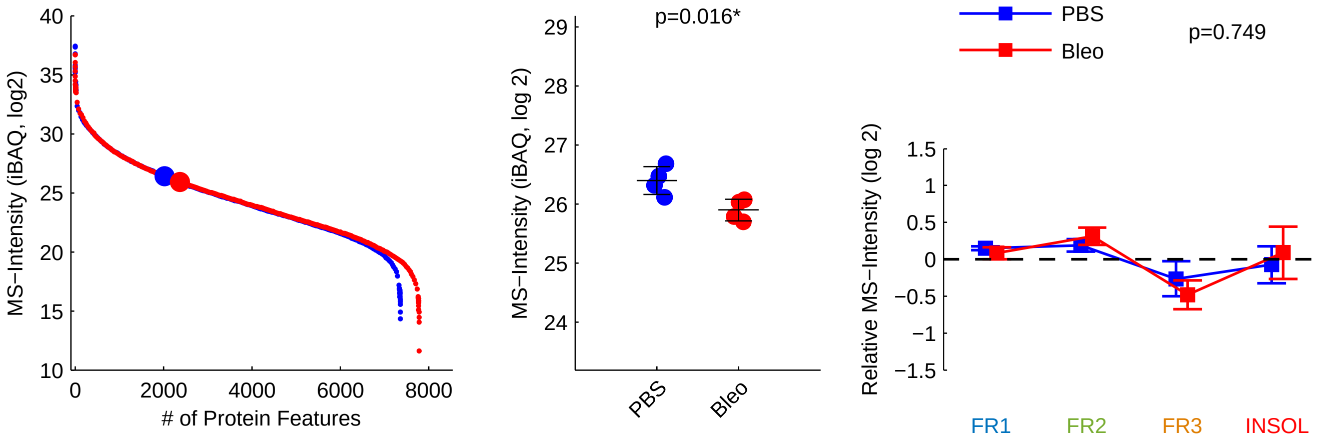

P51569 − Gla (id: 3160)

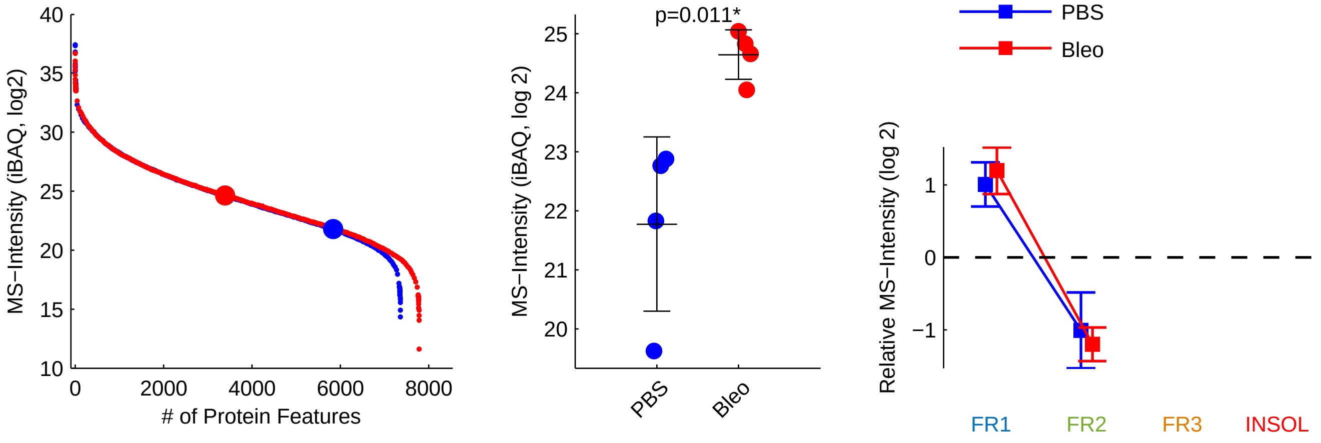

P51612 − Xpc (id: 3161)

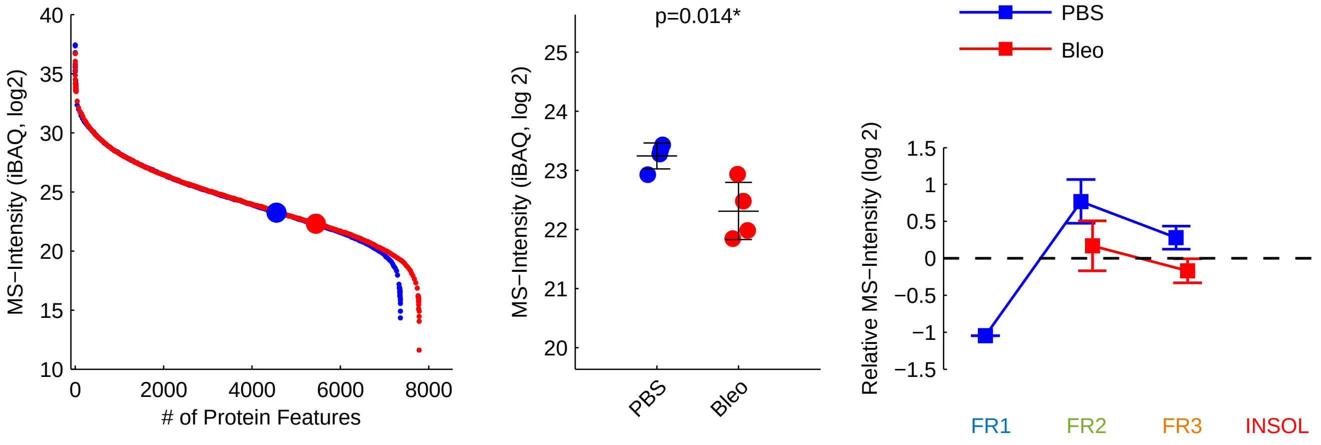

P51660 − Hsd17b4 (id: 3164)

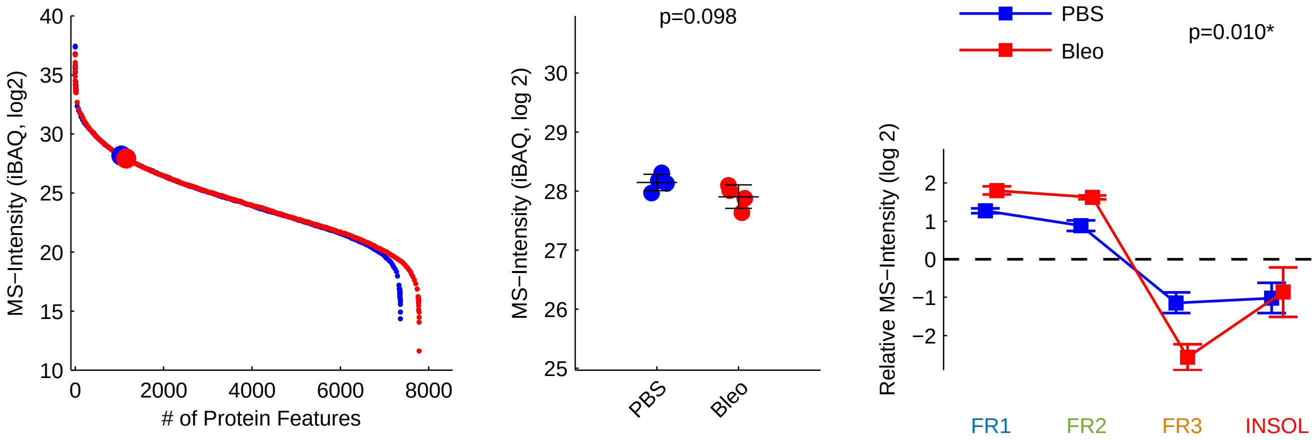

P51830 − Adcy9 (id: 3172)

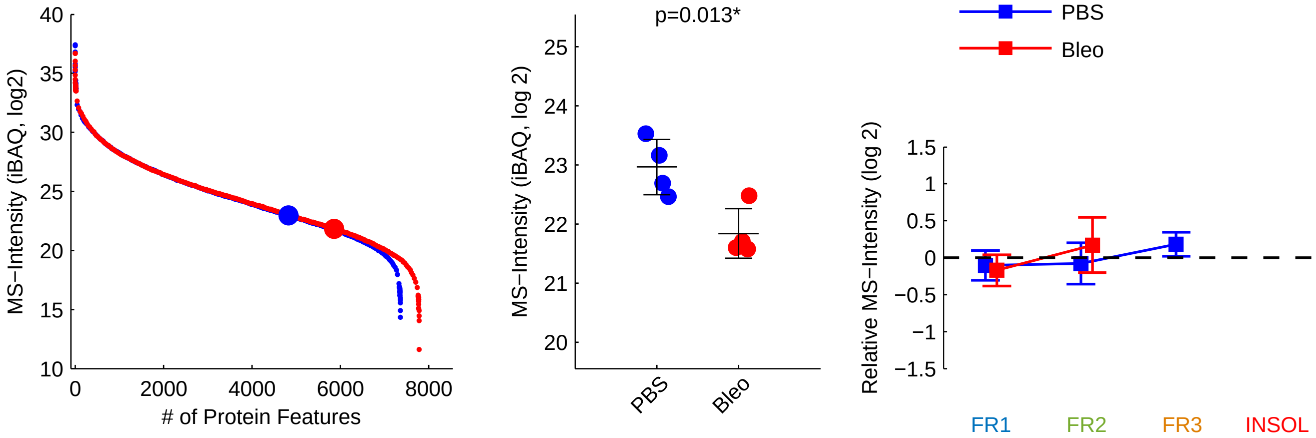

P51859 − Hdgf (id: 3174)

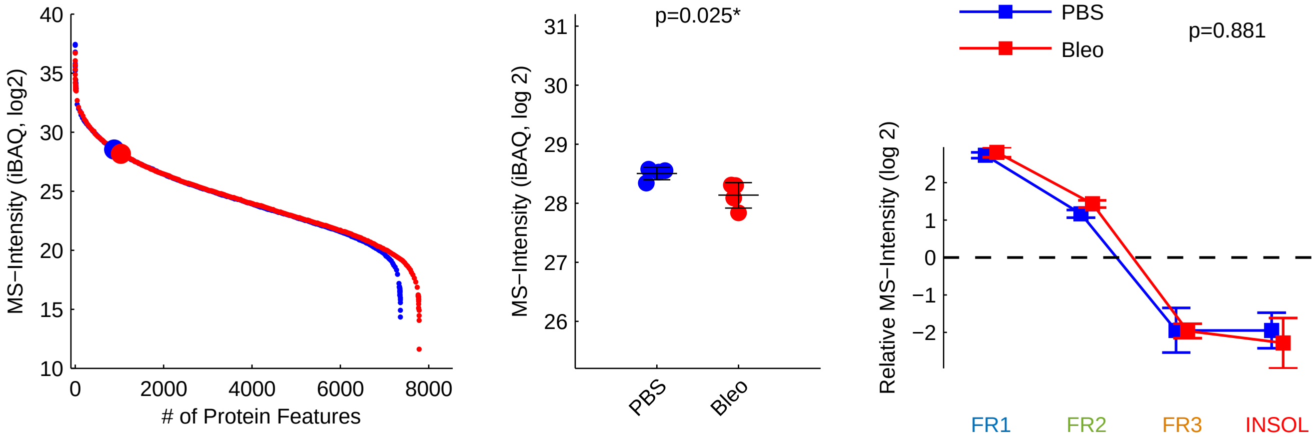

P51885 − Lum (id: 3177)

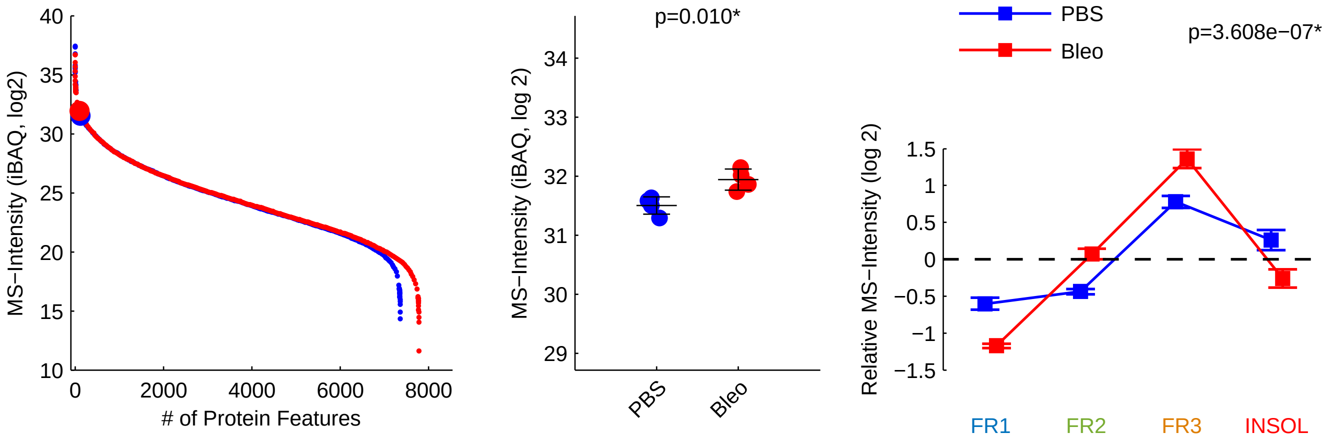

P52196 − Tst (id: 3180)

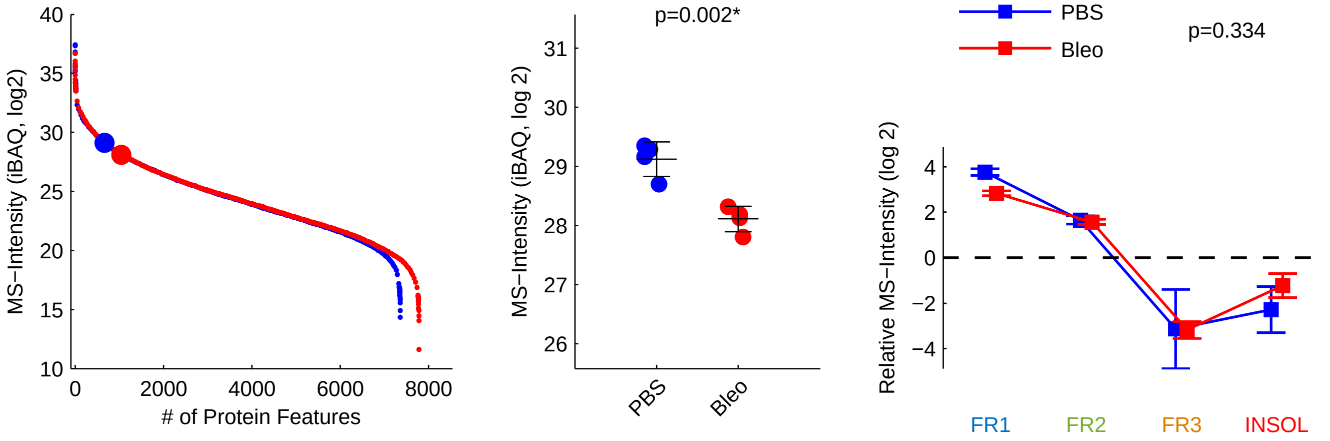

P52430 − Pon1 (id: 3182)

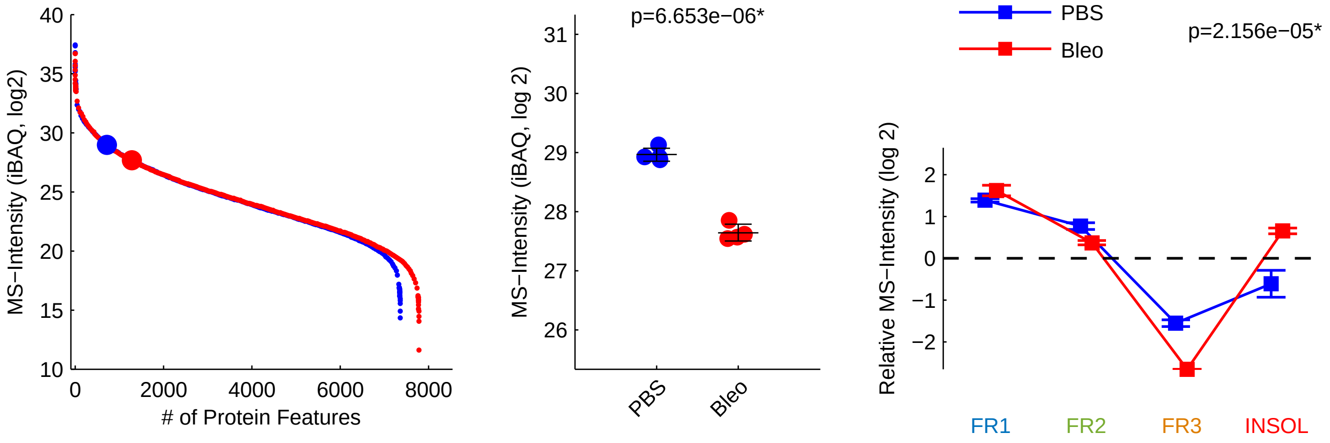

P52480 − Pkm (id: 3186)

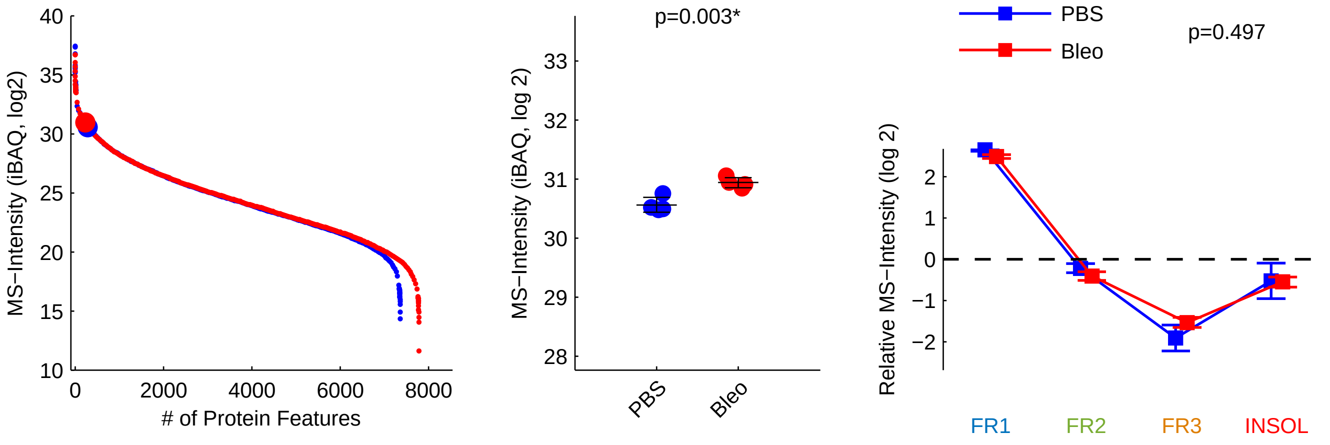

P52633 − Stat6 (id: 3191)

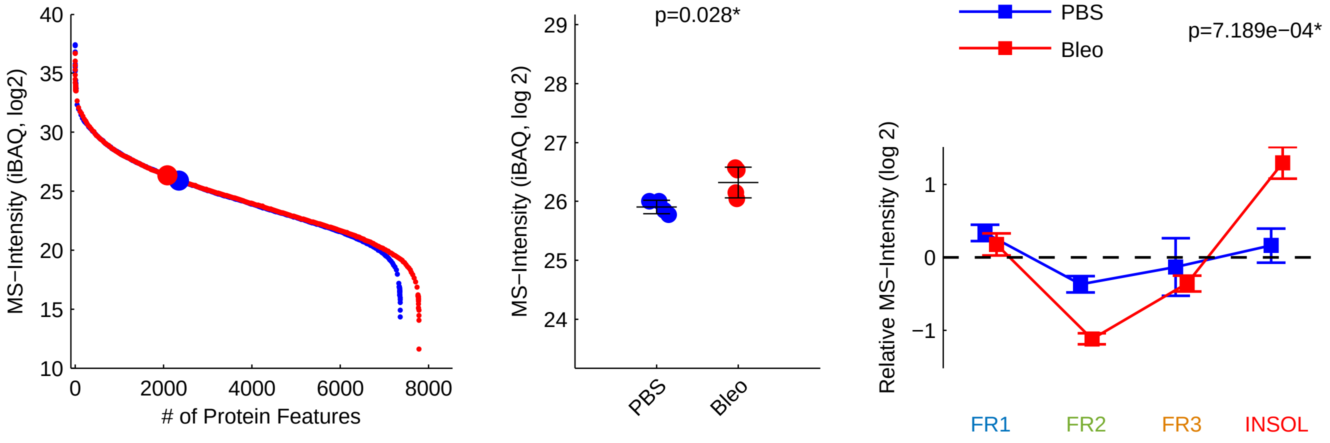

P53395 − Dbt (id: 3198)

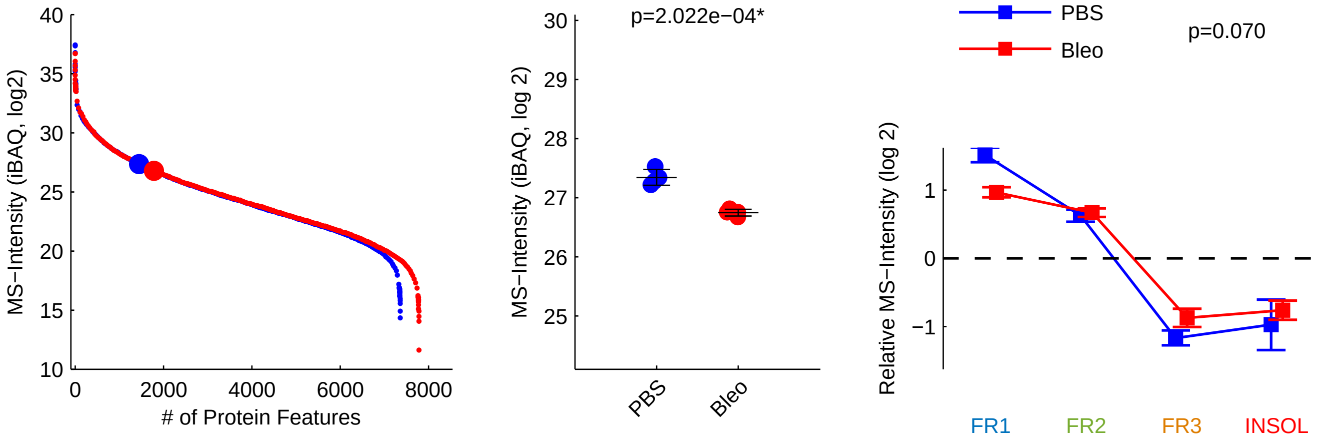

P53569 − Cebpz (id: 3201)

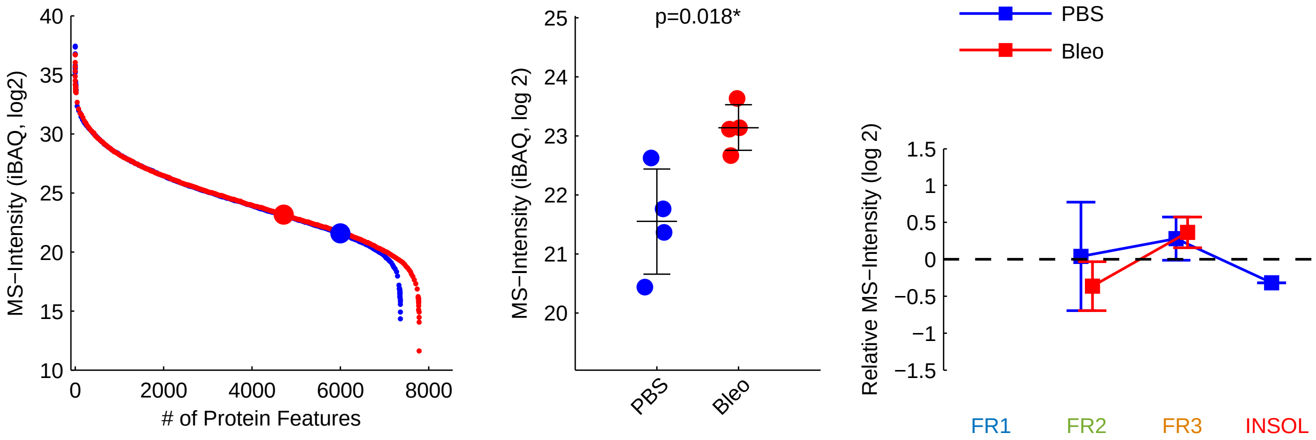

P53690 − Mmp14 (id: 3204)

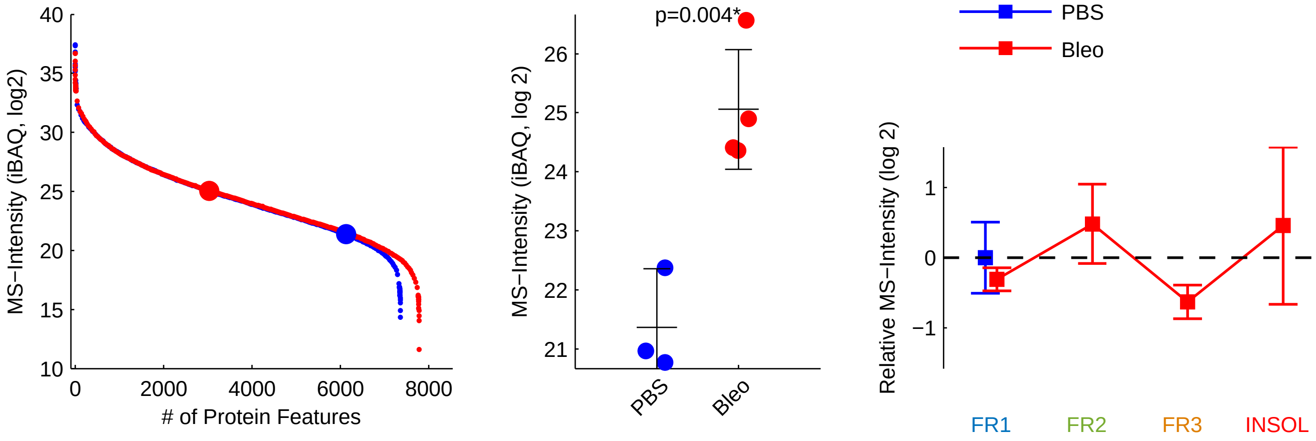

P53994 − Rab2a (id: 3209)

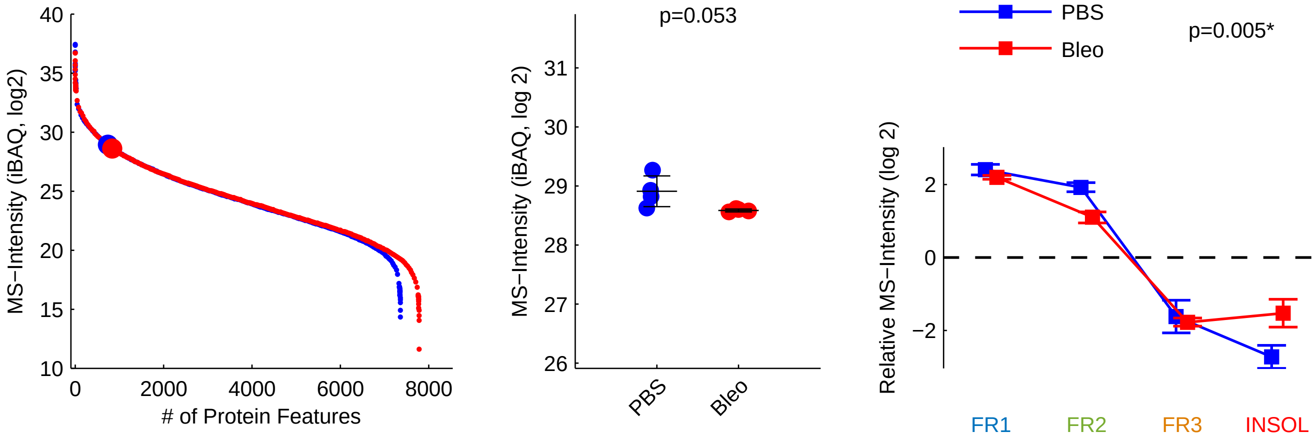

P54071 − Idh2 (id: 3212)

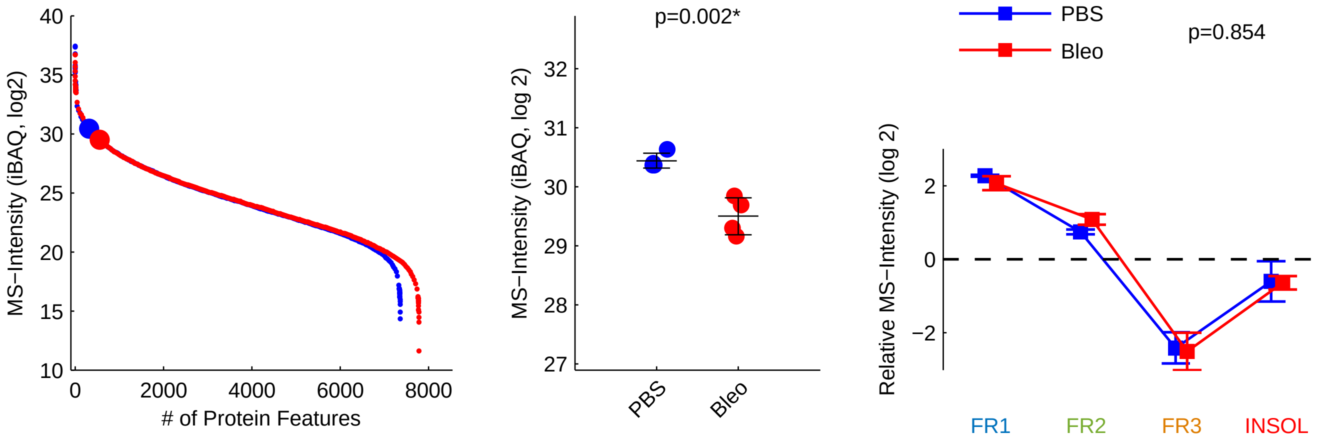

P54116 − Stom (id: 3213)

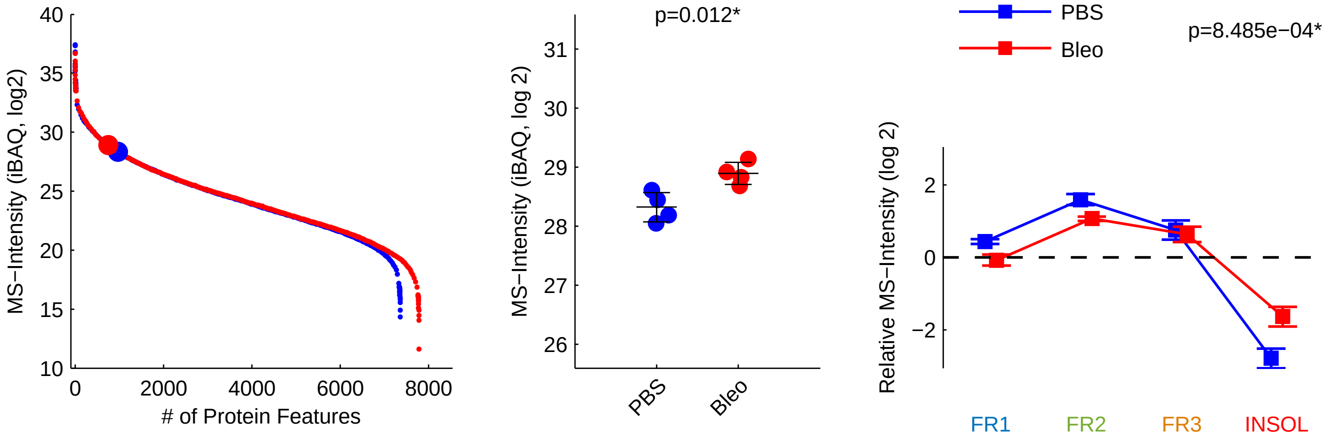

P54320 − Eln (id: 3216)

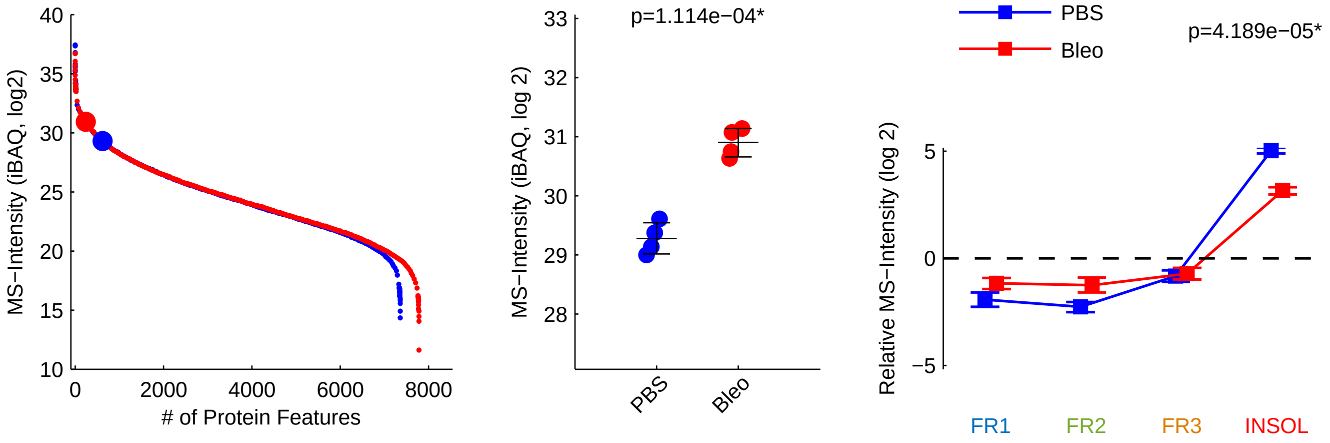

P54775 − Psmc4 (id: 3221)

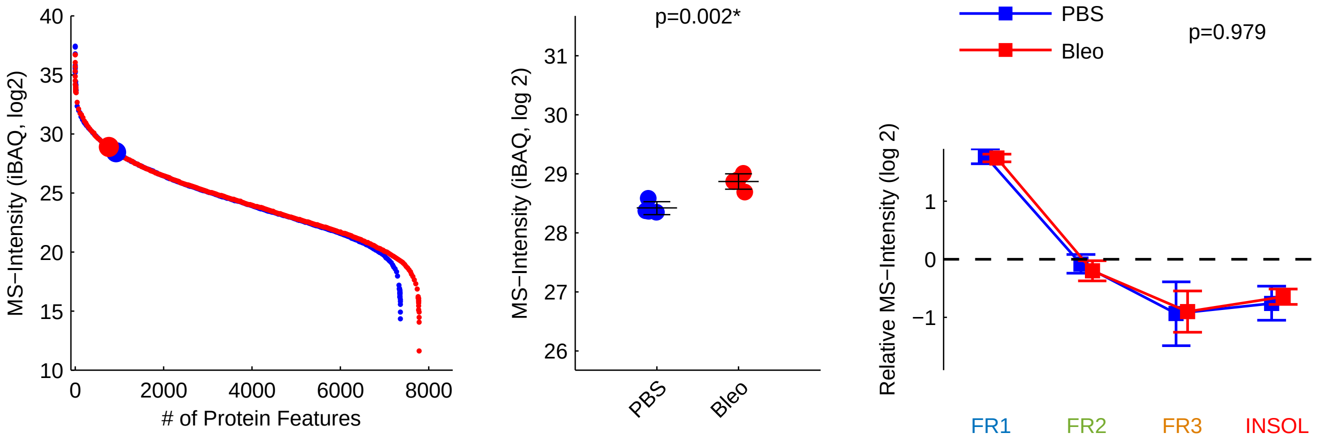

P54818 − Galc (id: 3223)

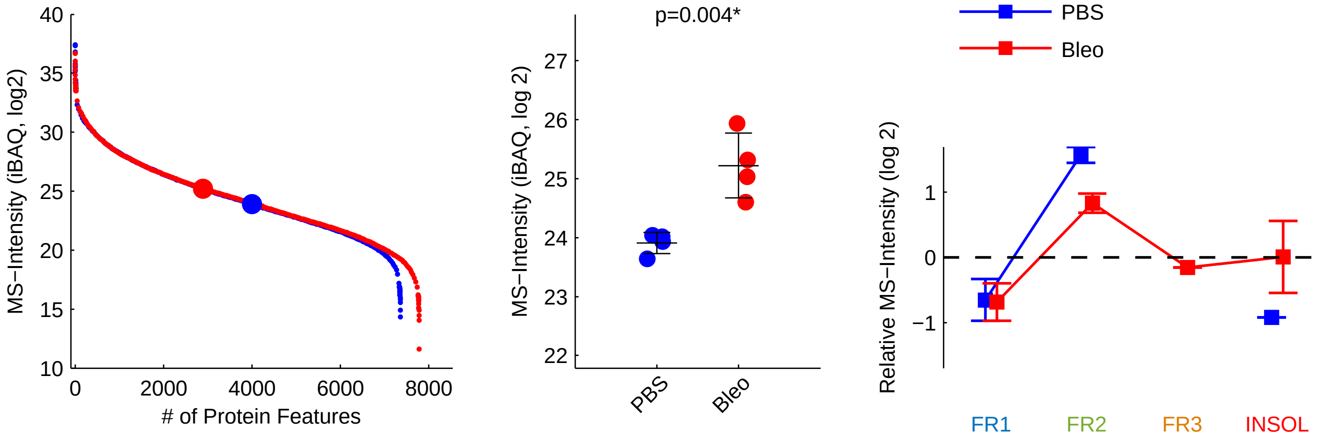

P54822 − Adsl (id: 3224)

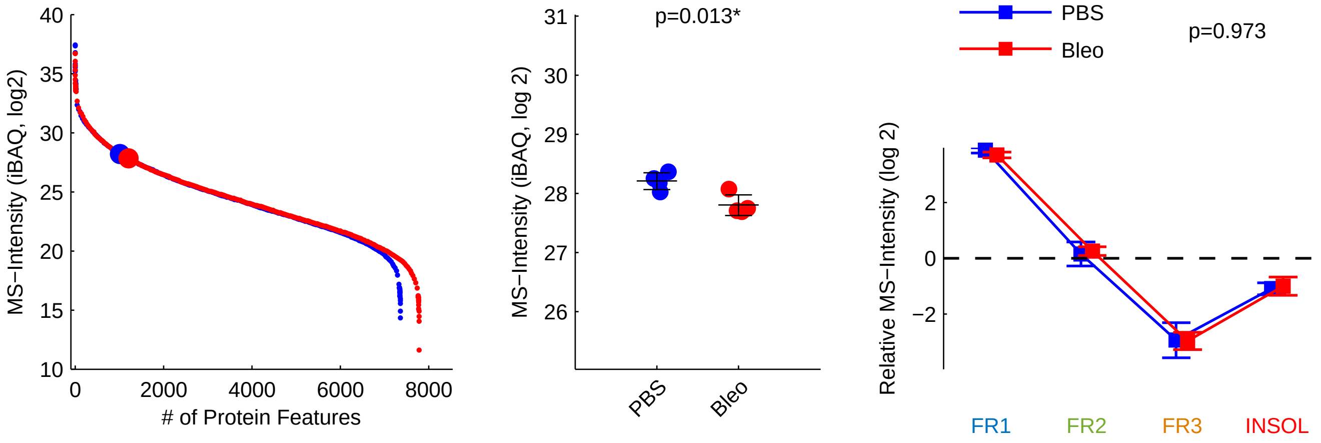

P54823 − Ddx6 (id: 3225)

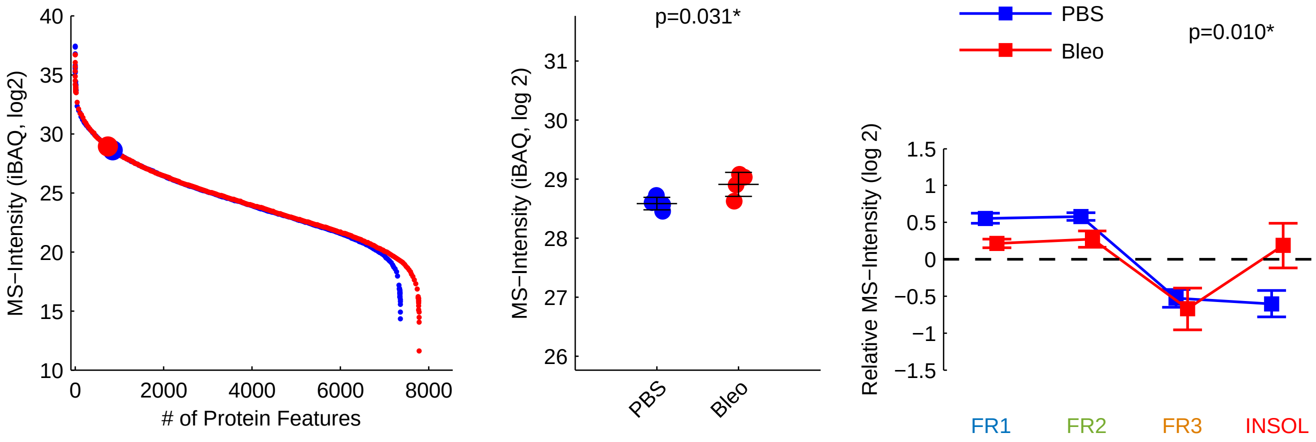

P54923 − Adprh (id: 3227)

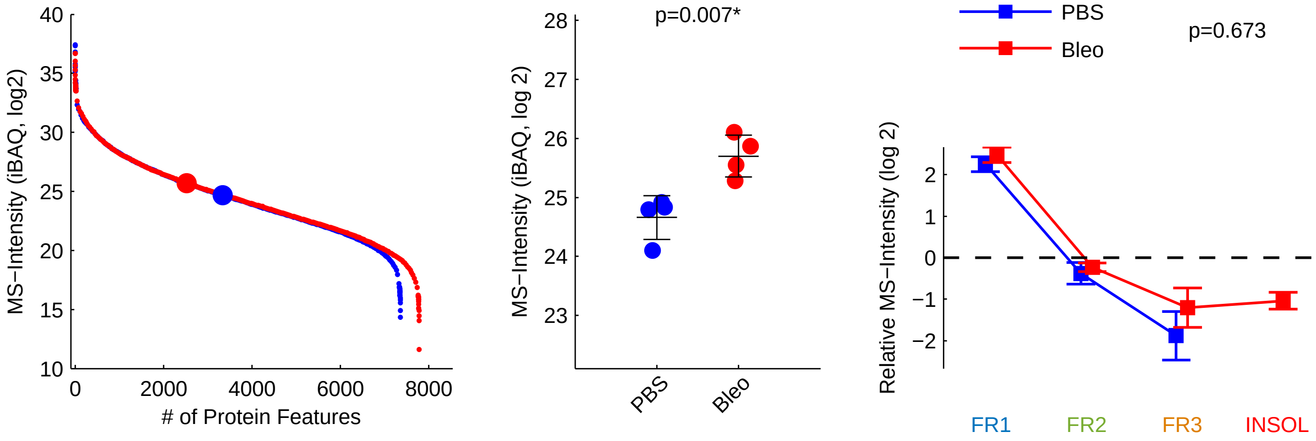

P55096 − Abcd3 (id: 3230)

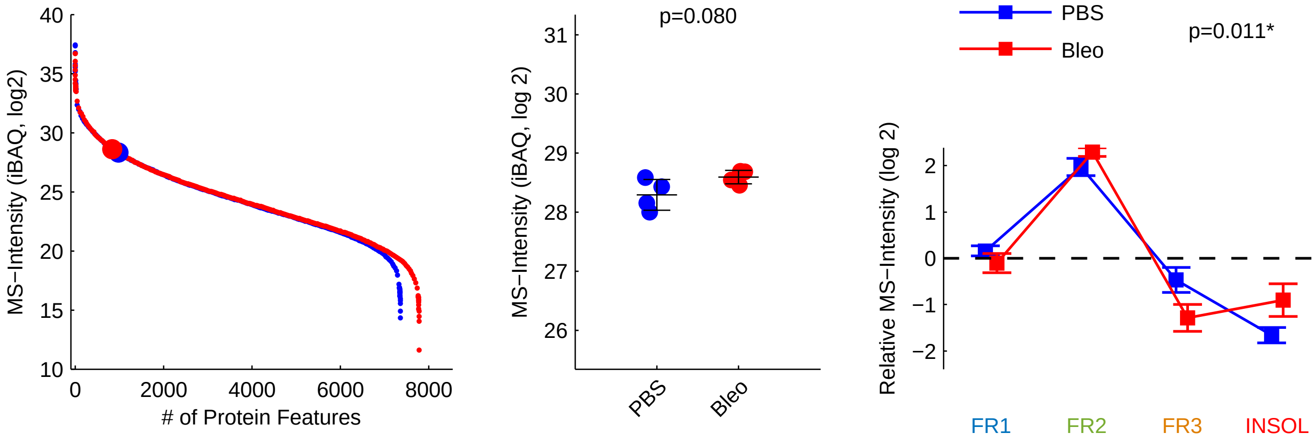

P55097 − Ctsk (id: 3231)

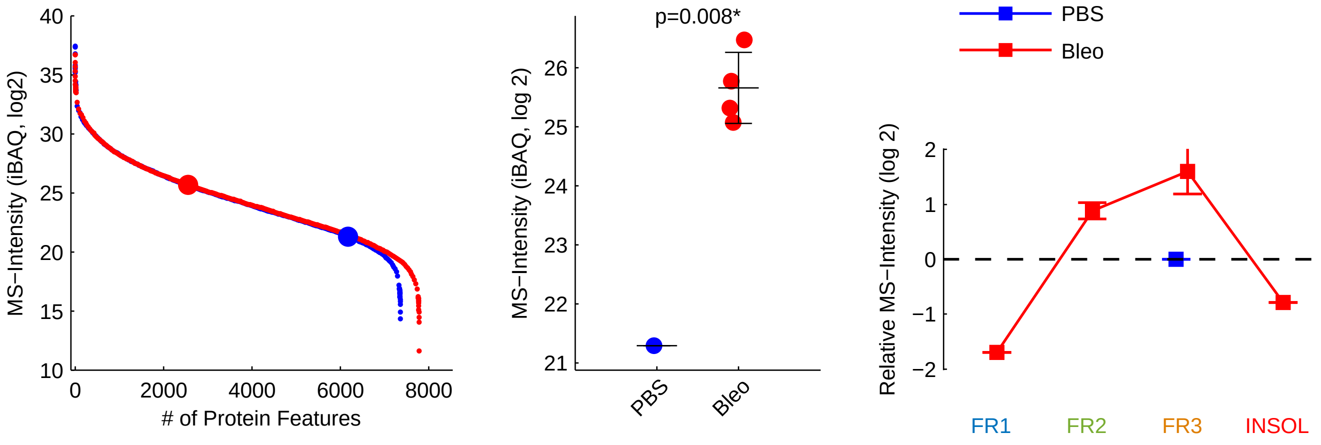

P55200 − Kmt2a (id: 3233)

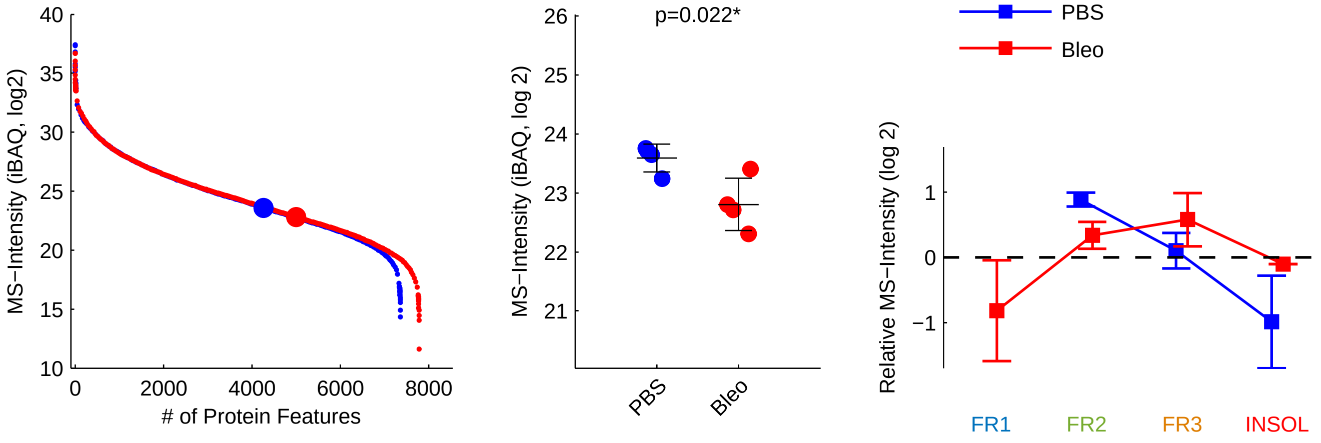

P55258 − Rab8a (id: 3234)

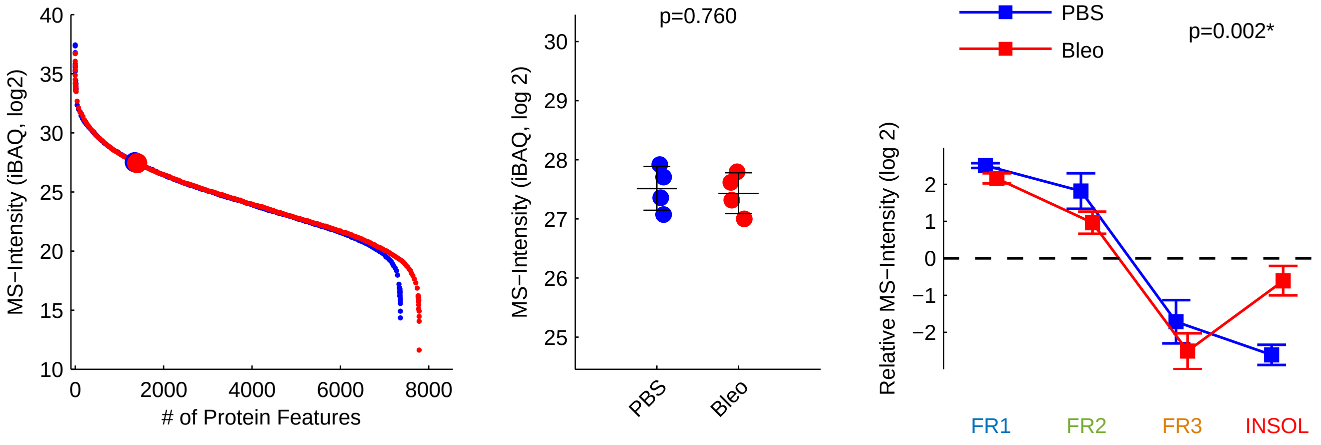

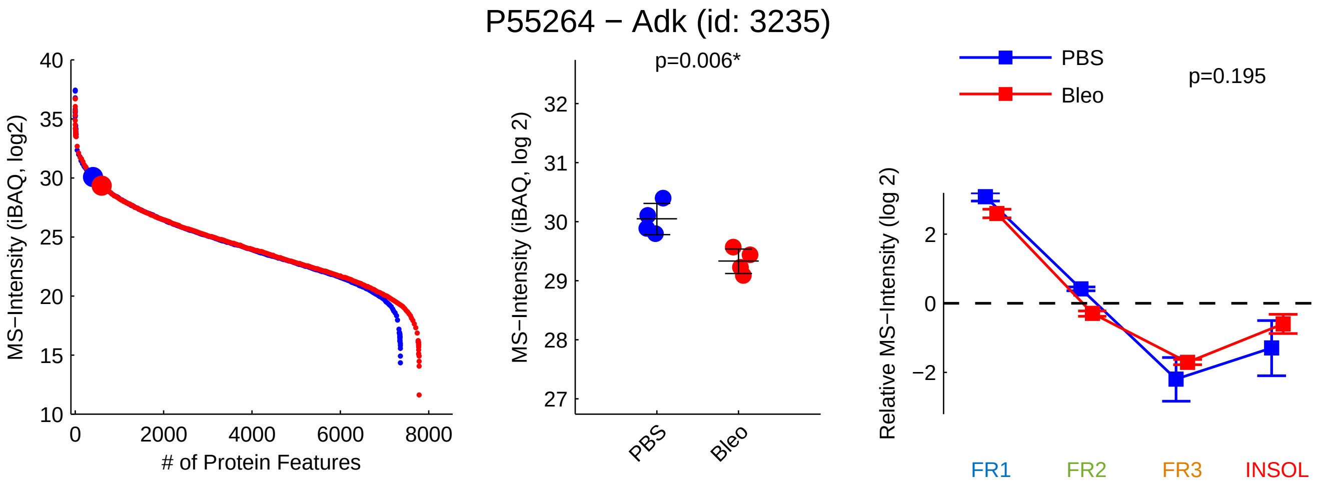
P55264 − Adk (id: 3235)
MS−Intensity (iBAQ, log2)
# of Protein Features
p=0.006*
MS−Intensity (iBAQ, log 2)
PBS
Bleo
p=0.195
Relative MS−Intensity (log 2)
FR1
FR2
FR3
INSOL

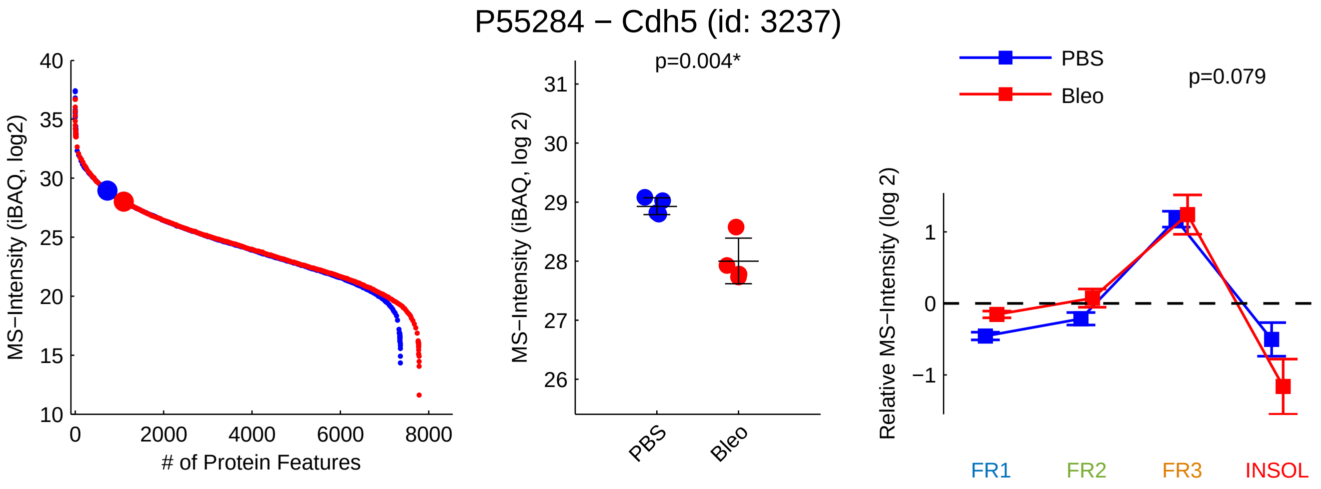
P55284 − Cdh5 (id: 3237)
MS−Intensity (iBAQ, log2)
# of Protein Features
p=0.004*
MS−Intensity (iBAQ, log 2)
PBS
Bleo
p=0.079
Relative MS−Intensity (log 2)
FR1
FR2
FR3
INSOL

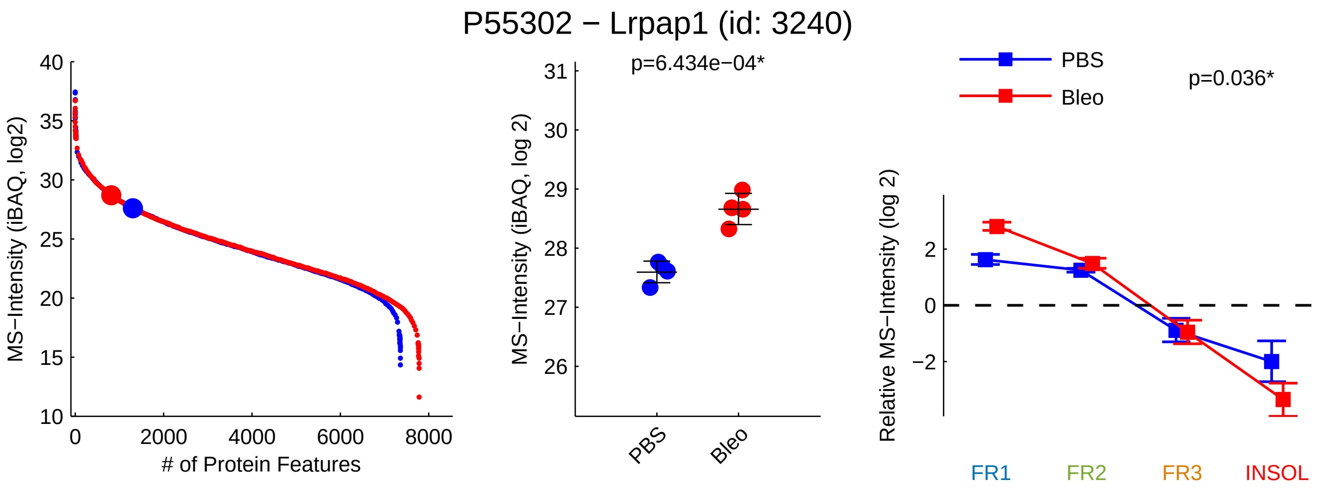
P55302 − Lrpap1 (id: 3240)
MS−Intensity (iBAQ, log2)
# of Protein Features
p=6.434e−04*
MS−Intensity (iBAQ, log 2)
PBS
Bleo
p=0.036*
Relative MS−Intensity (log 2)
FR1
FR2
FR3
INSOL

P56183 − Rrp1 (id: 3244)

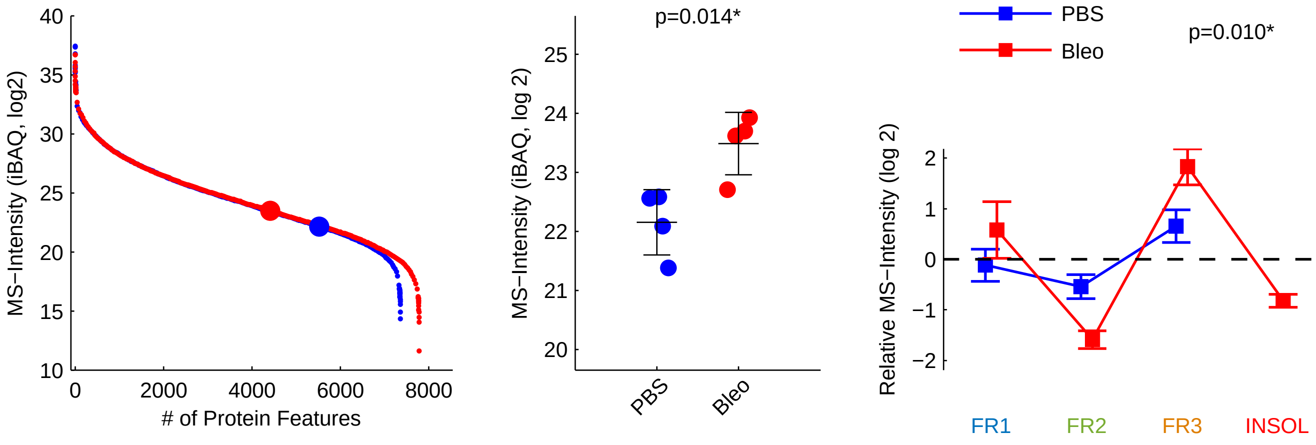

P56376 − Acyp1 (id: 3249)

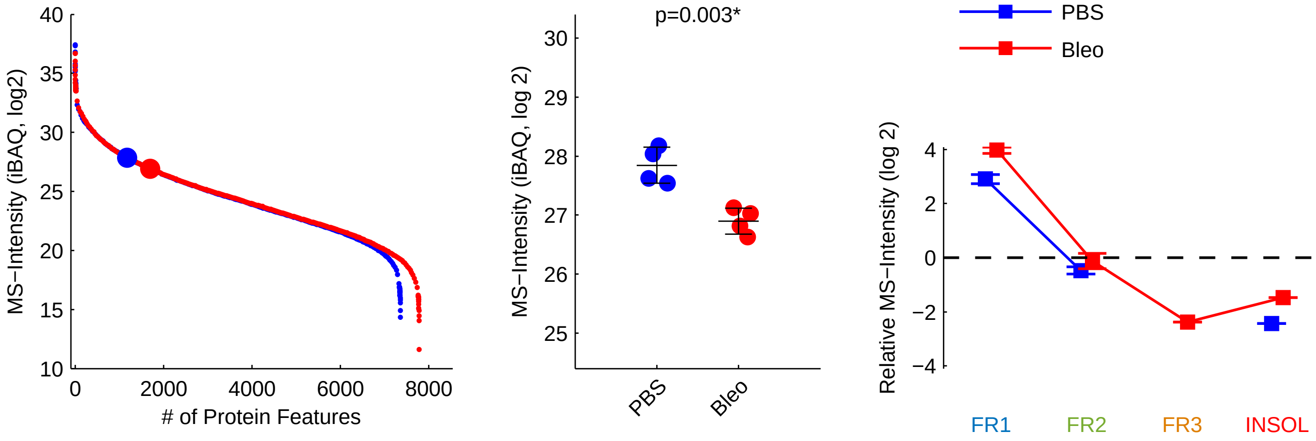

P56857 − Cldn18 (id: 3268)

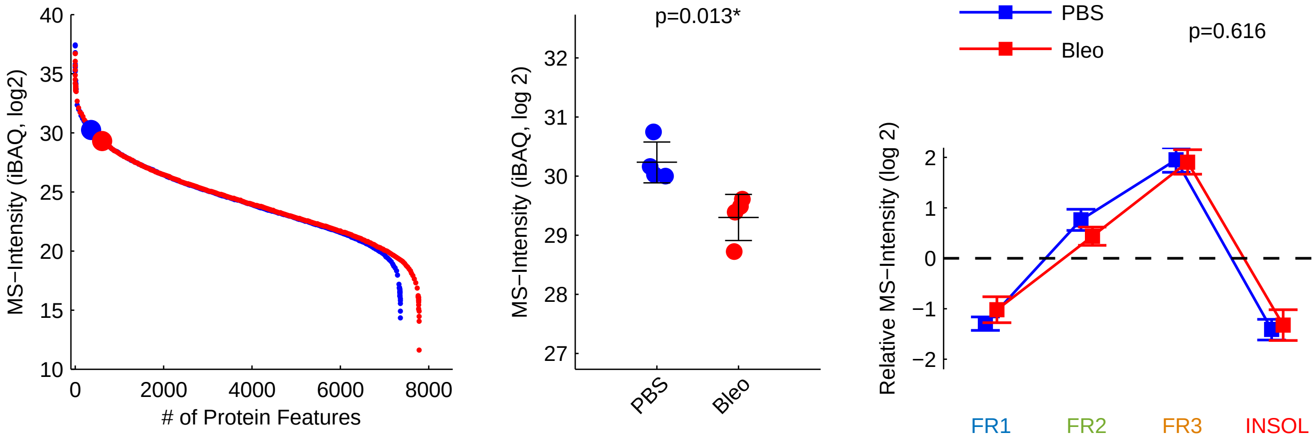

P57716 − Ncstn (id: 3273)

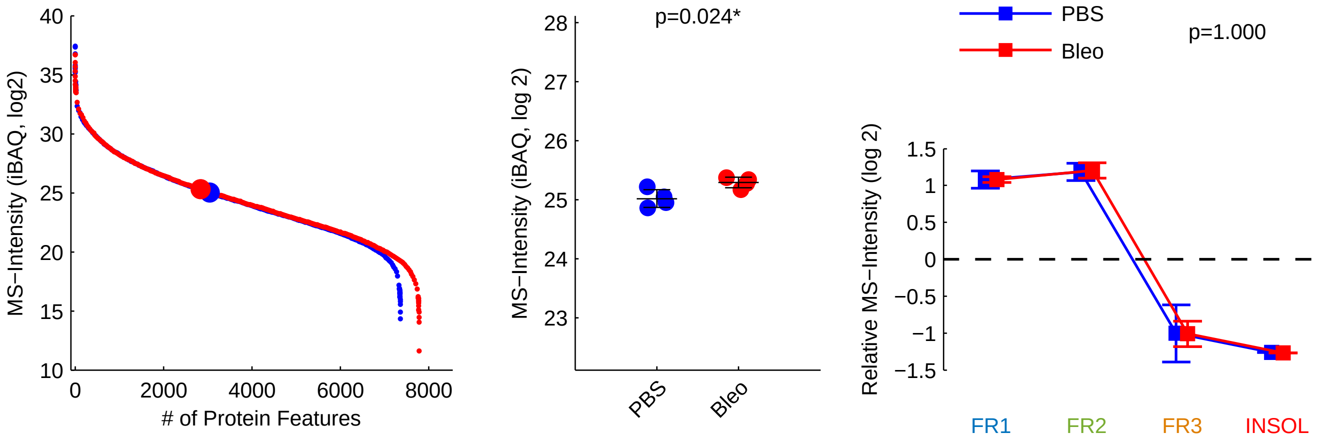

P57759 − Erp29 (id: 3277)

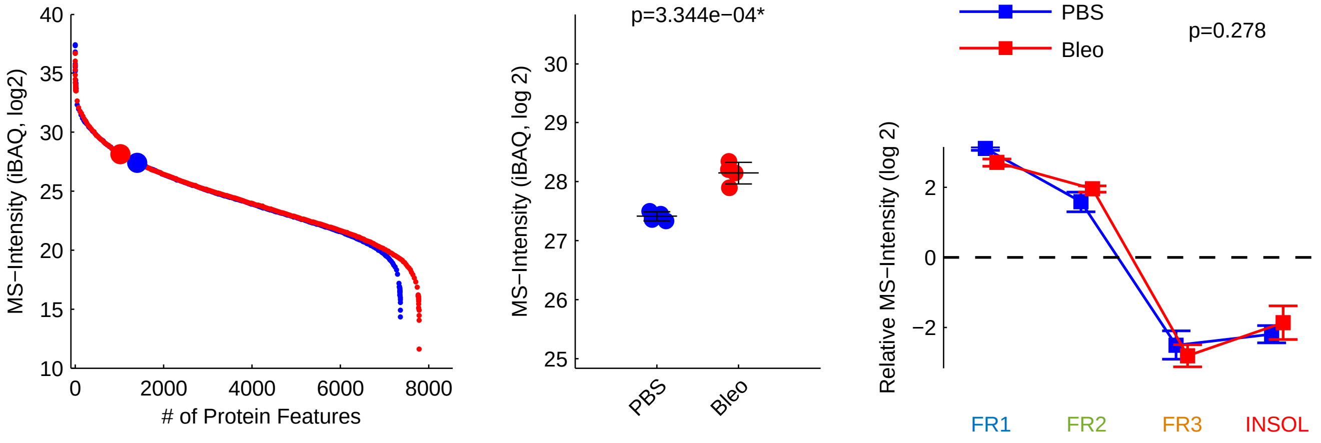

P57780 − Actn4 (id: 3279)

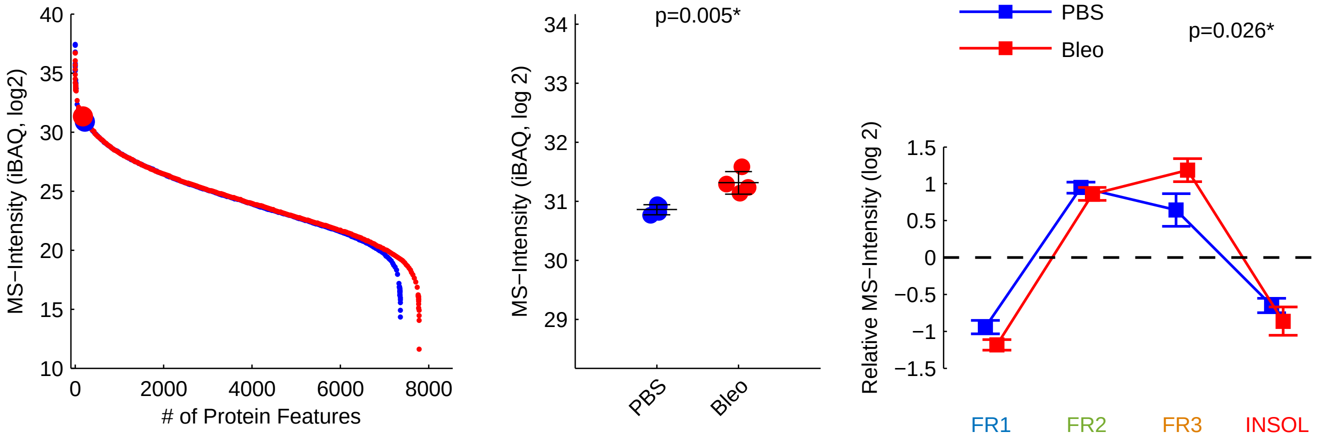

P58021 − Tm9sf2 (id: 3281)

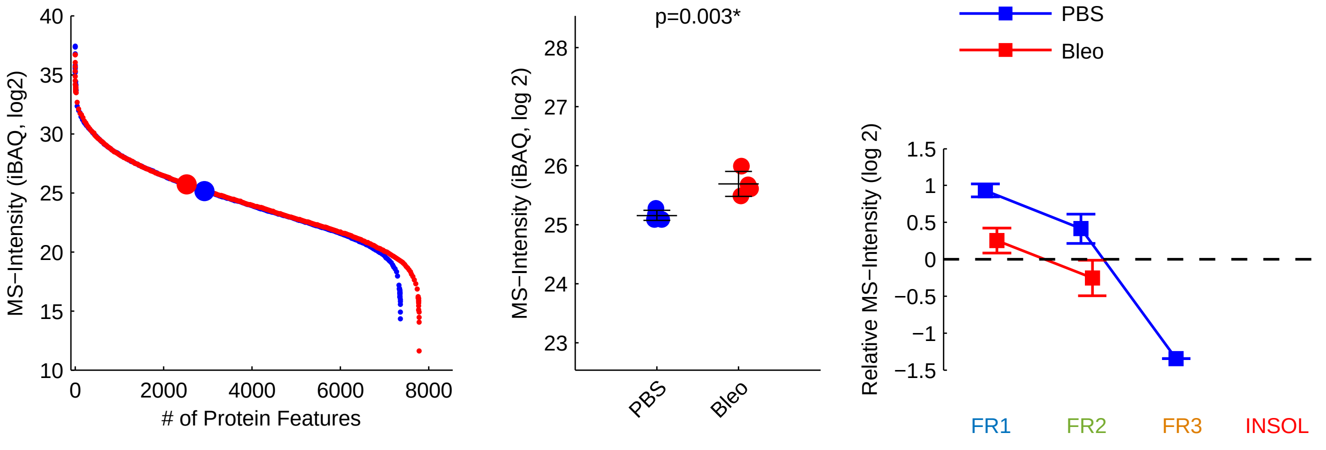

P58022 − Loxl2 (id: 3282)

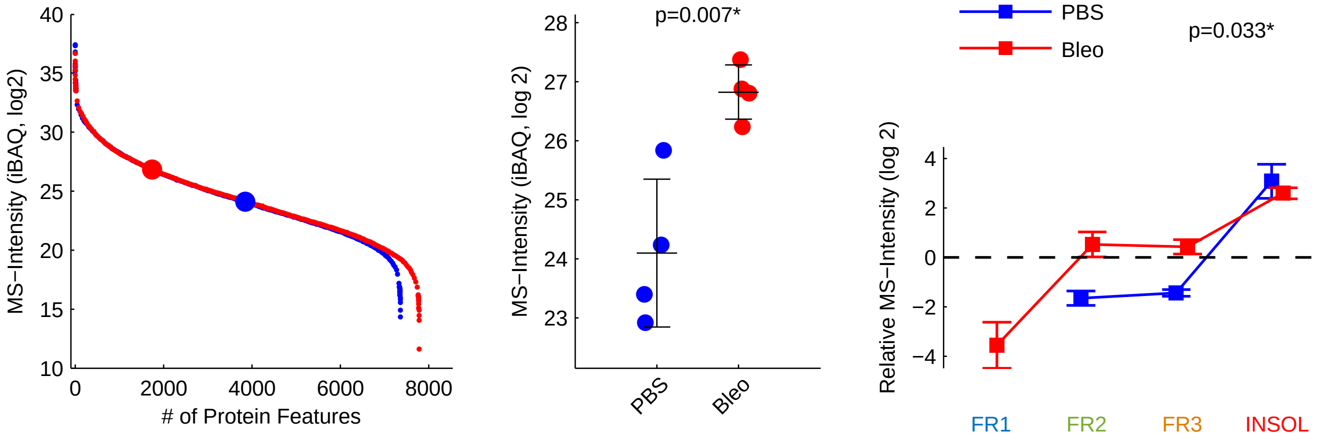

P58196 − Plscr4 (id: 3287)

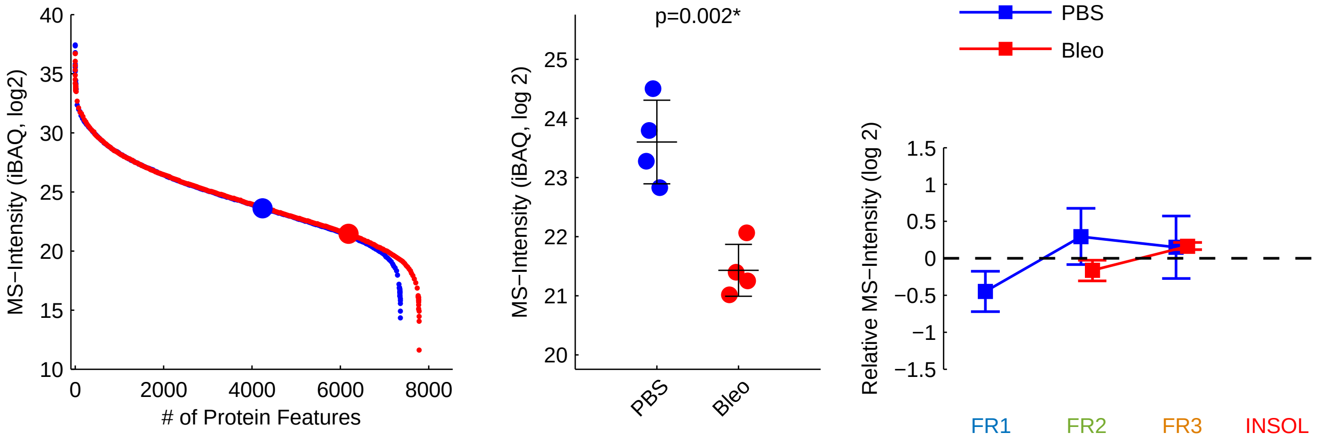

P58252 − Eef2 (id: 3288)

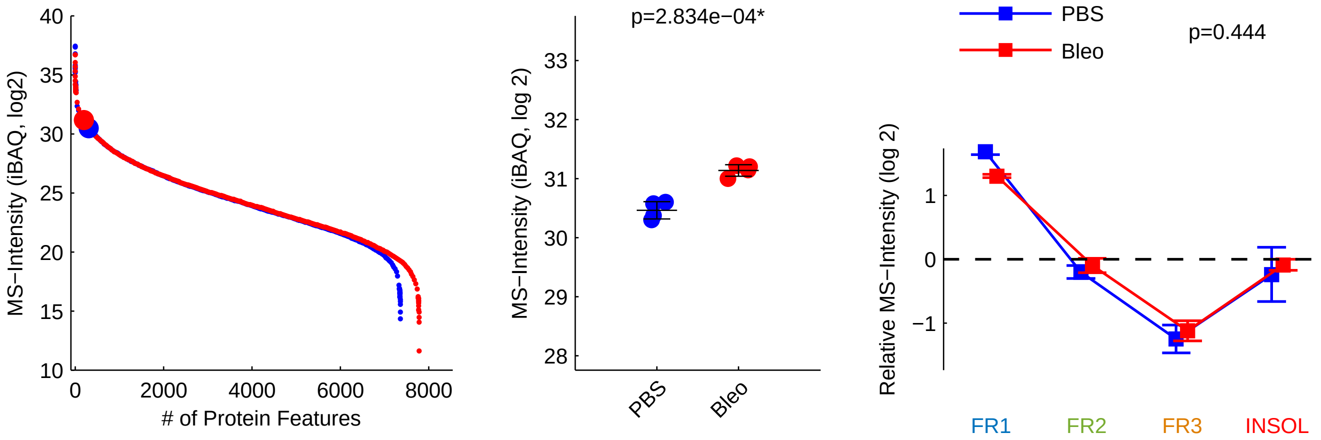

P58466 − Ctdsp1 (id: 3292)

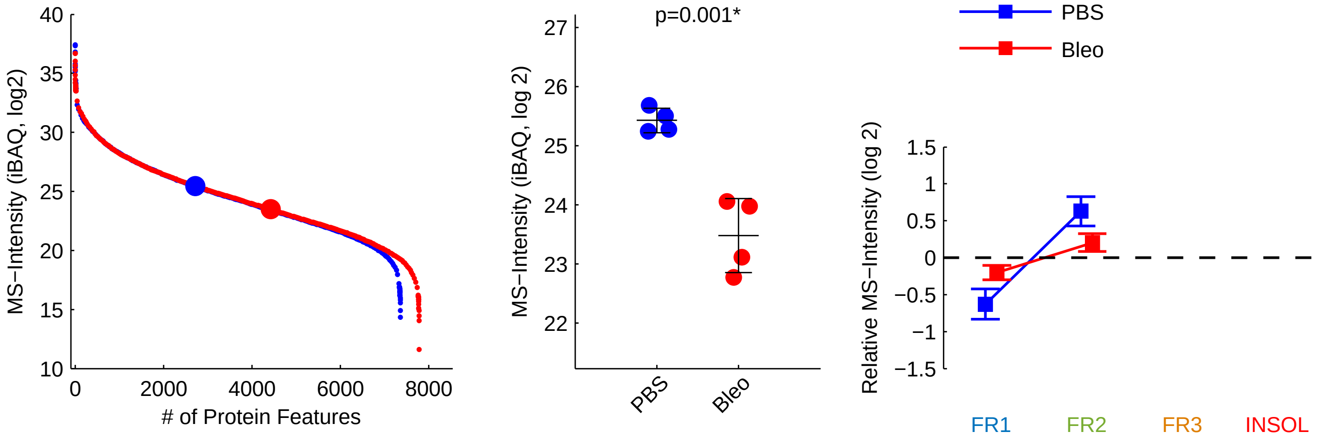

P58501 − Paxbp1 (id: 3294)

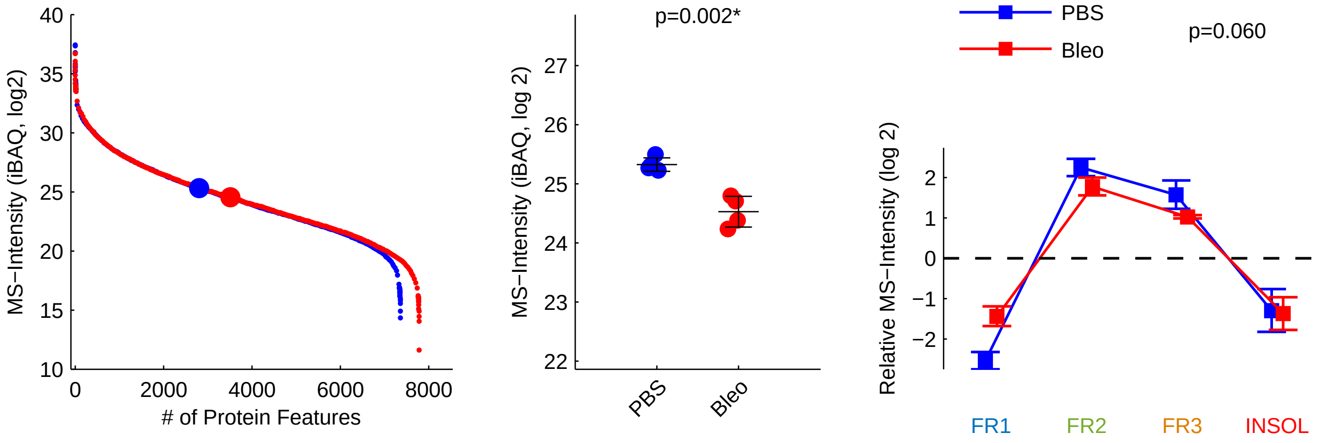

P58774−2 − Tpm2 (id: 3298)

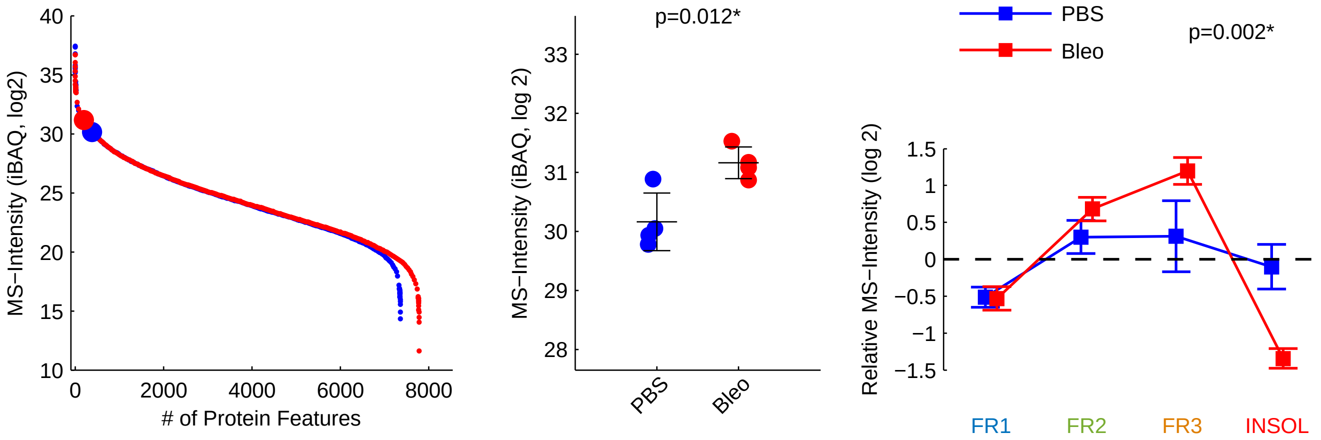

P58802 − Tbc1d10a (id: 3300)

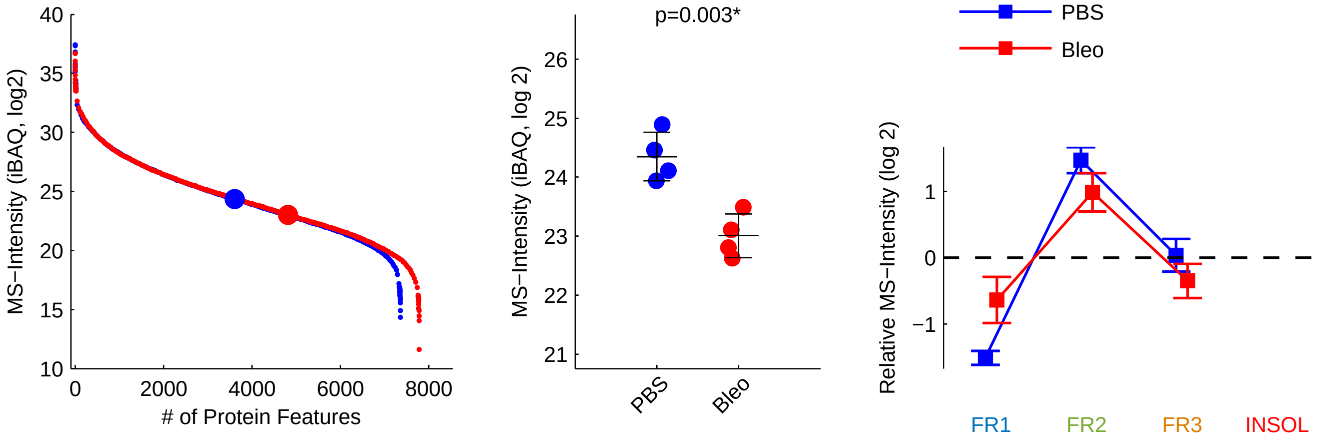

P59016 − Vps33b (id: 3304)

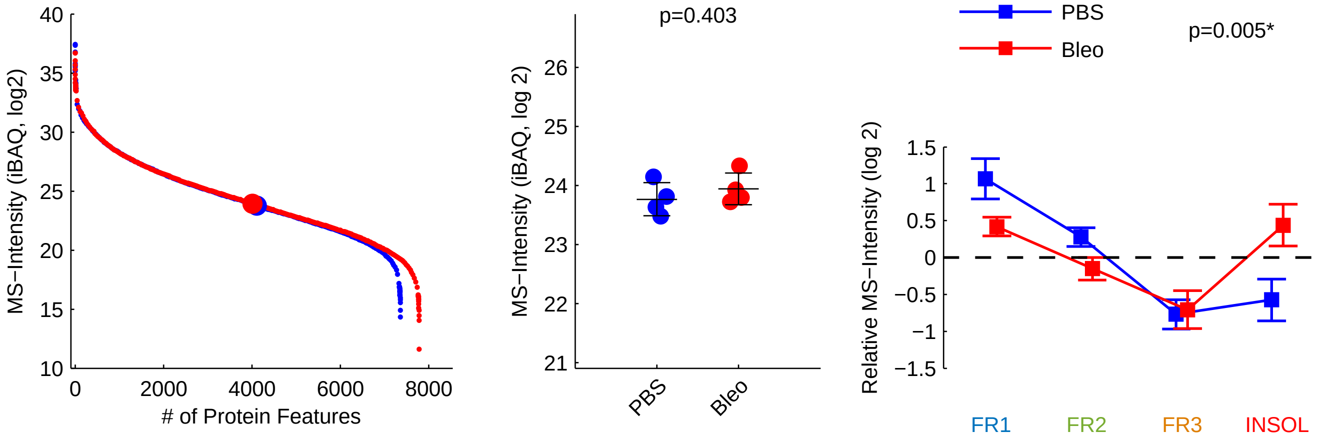

P59383 − Lrrn4 (id: 3316)

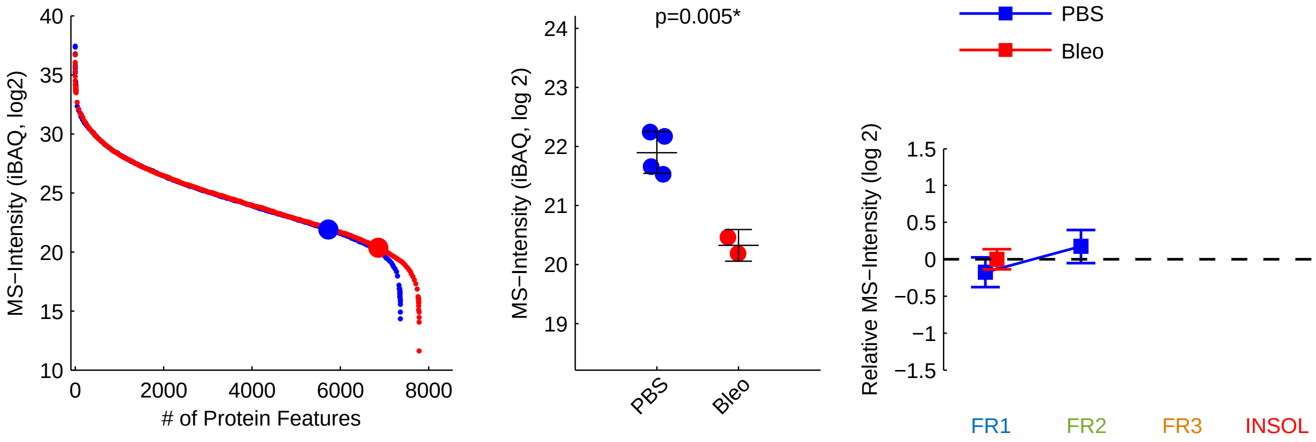

P59384 − Adamts15 (id: 3317)

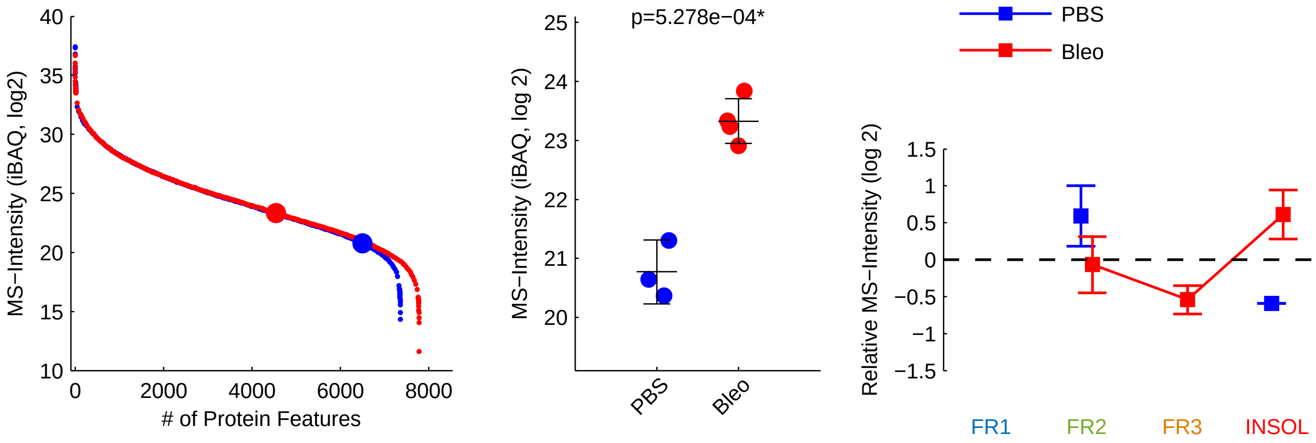

P59672 − Anks1a (id: 3319)

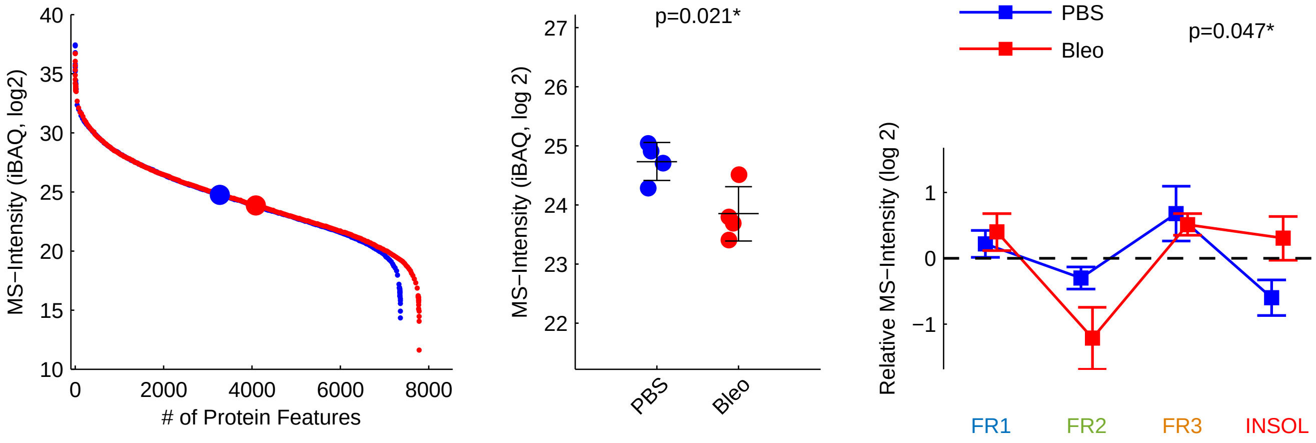

P59708 − Sf3b14 (id: 3320)

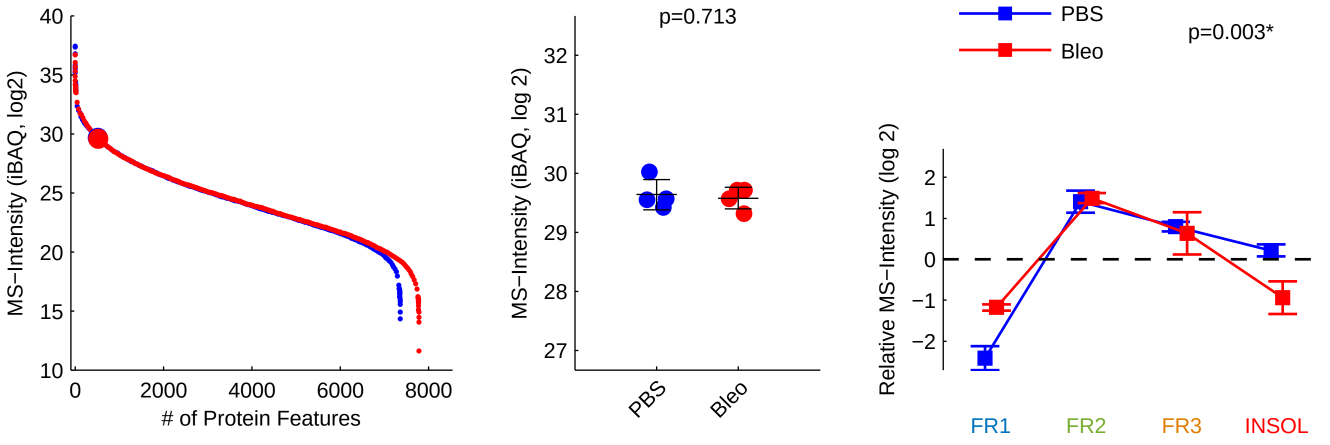

P59764 − Dock4 (id: 3322)

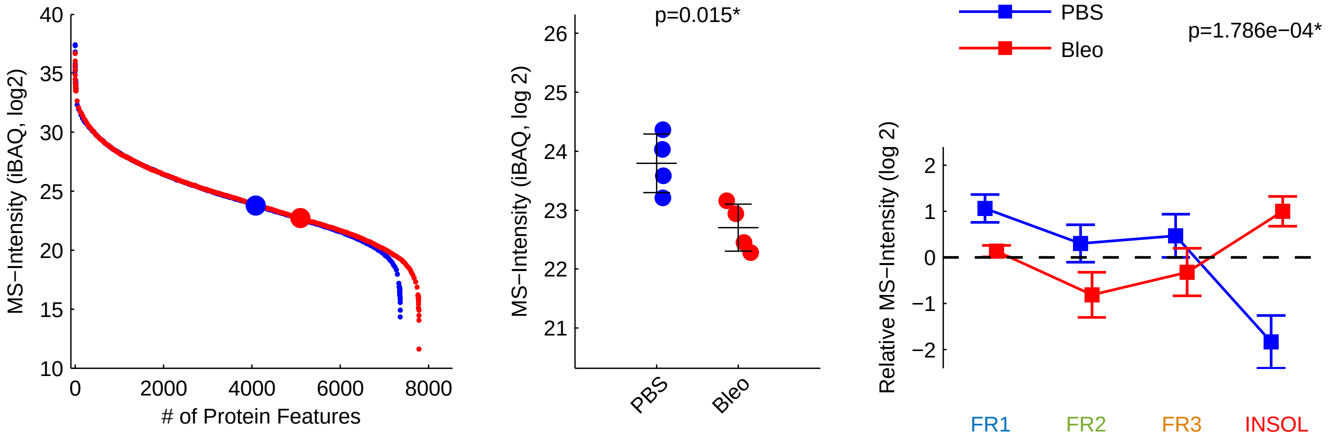

P59999 − Arpc4 (id: 3325)

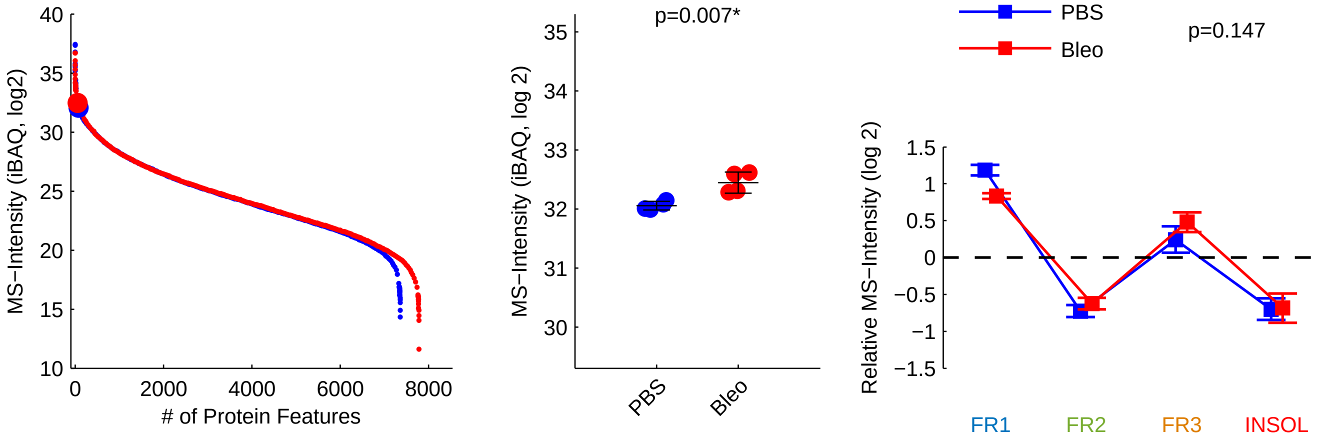

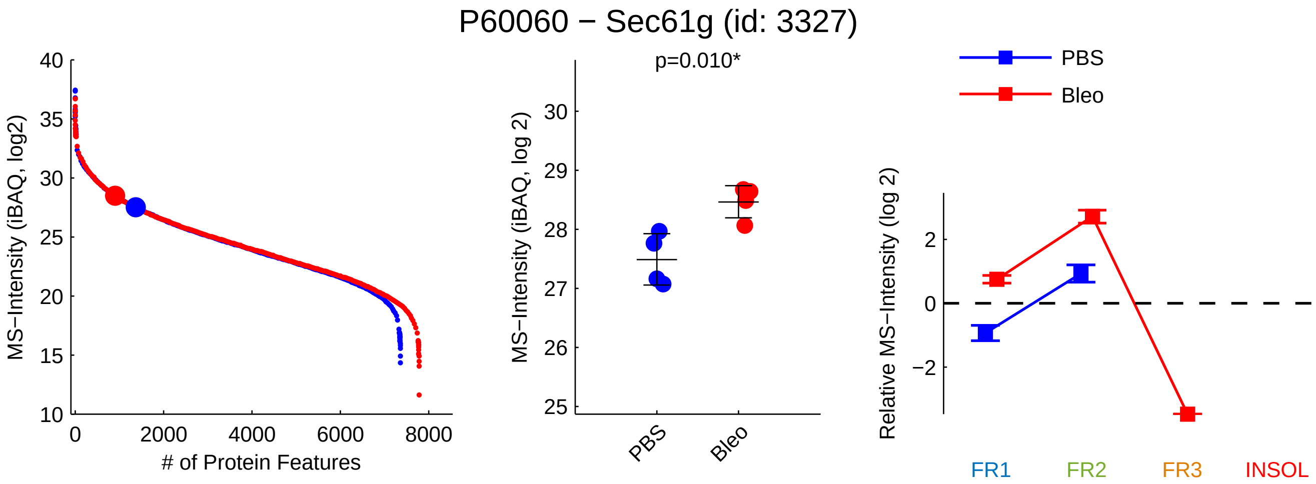
P60060 − Sec61g (id: 3327)
MS−Intensity (iBAQ, log2)
# of Protein Features
p=0.010*
MS−Intensity (iBAQ, log 2)
PBS
Bleo
Relative MS−Intensity (log 2)
FR1
FR2
FR3
INSOL

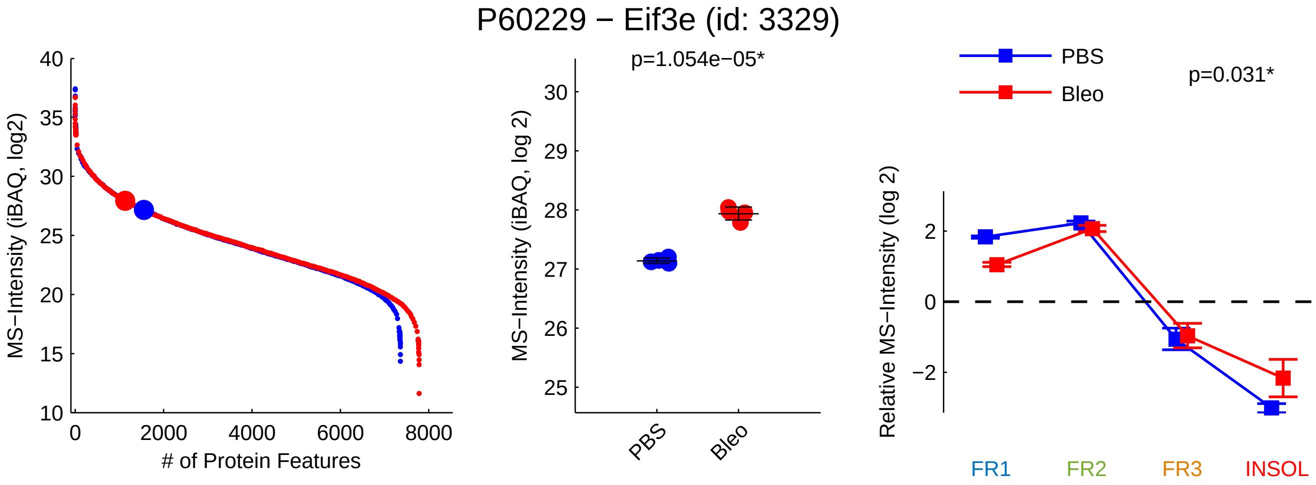
P60229 − Eif3e (id: 3329)
MS−Intensity (iBAQ, log2)
# of Protein Features
p=1.054e−05*
MS−Intensity (iBAQ, log 2)
PBS
Bleo
p=0.031*
Relative MS−Intensity (log 2)
FR1
FR2
FR3
INSOL

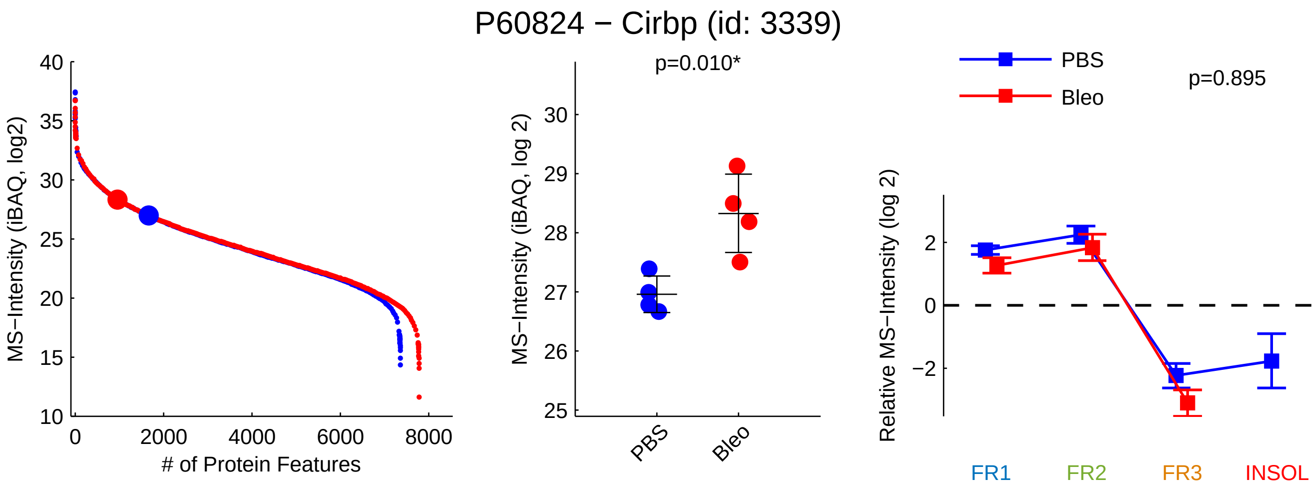
P60824 − Cirbp (id: 3339)
MS−Intensity (iBAQ, log2)
# of Protein Features
p=0.010*
MS−Intensity (iBAQ, log 2)
PBS
Bleo
p=0.895
Relative MS−Intensity (log 2)
FR1
FR2
FR3
INSOL

P60840 − Ensa (id: 3340)

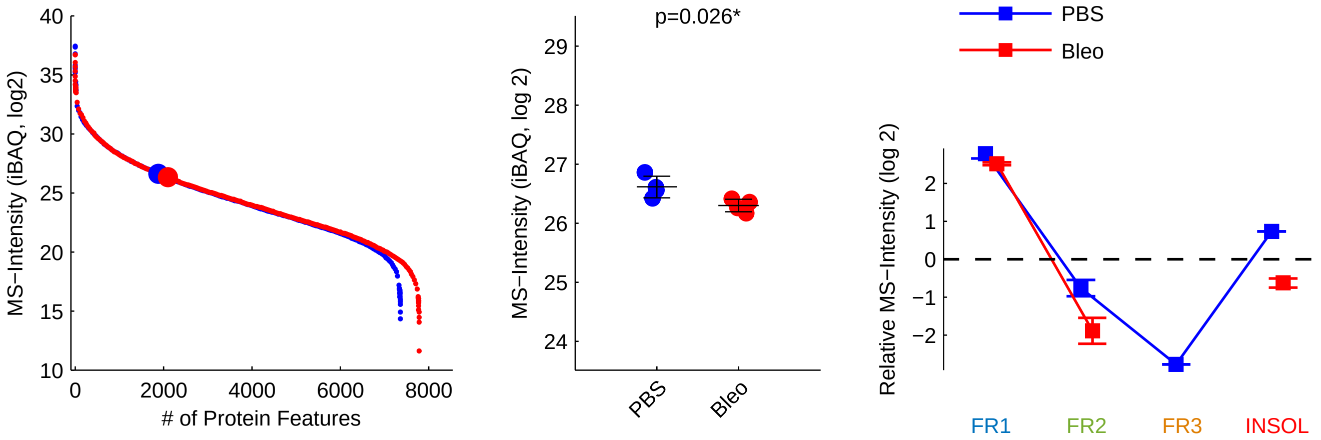

P60843 − Eif4a1 (id: 3341)

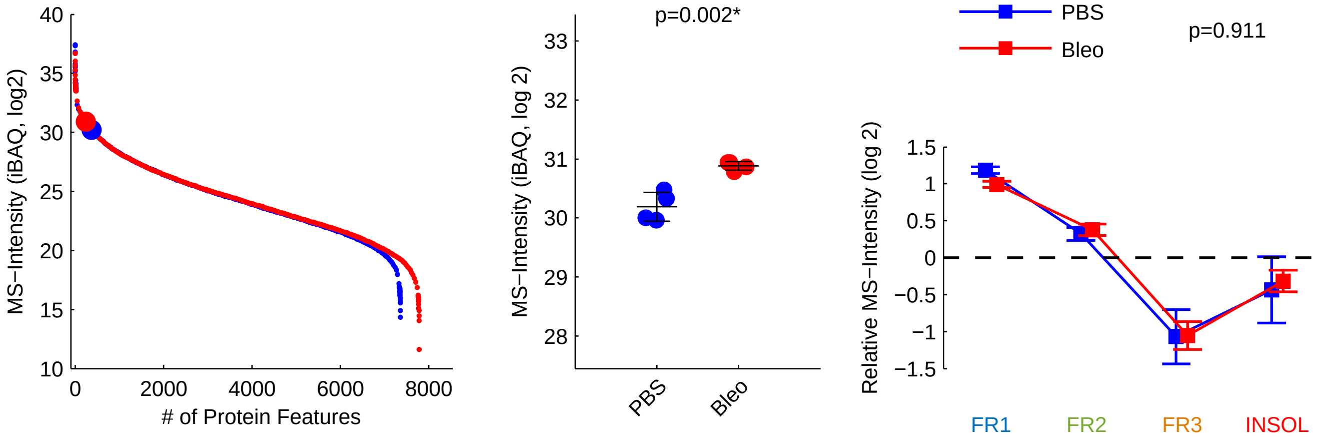

P60867 − Rps20 (id: 3342)

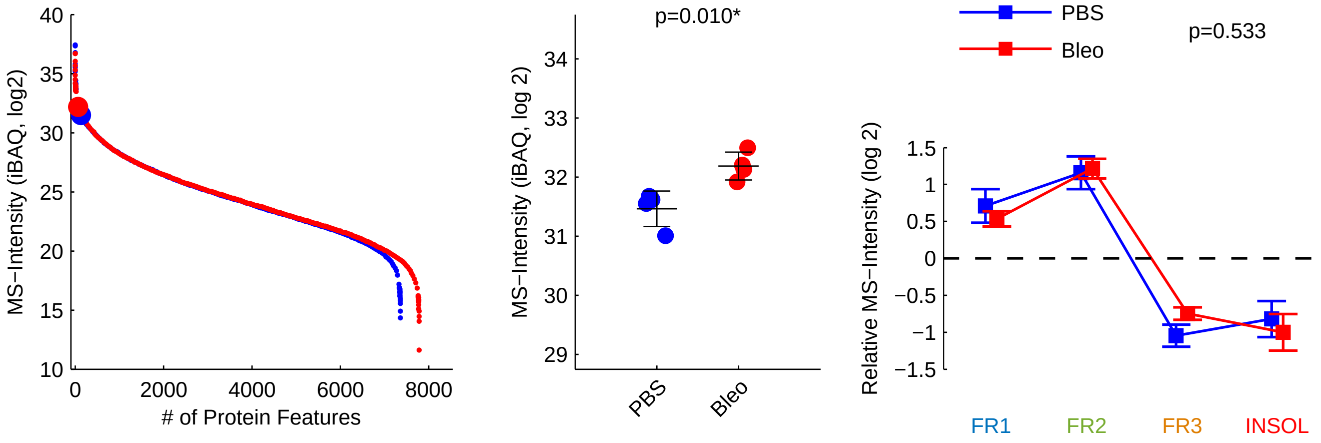

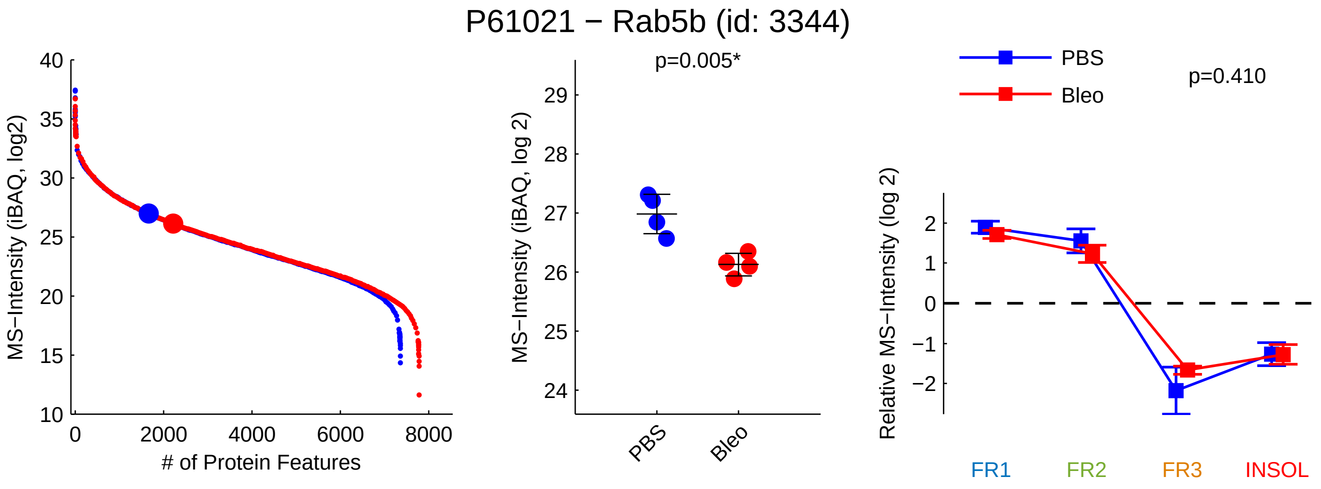

P61021 − Rab5b (id: 3344)
MS−Intensity (iBAQ, log2)
# of Protein Features
p=0.005*
MS−Intensity (iBAQ, log 2)
PBS
Bleo
p=0.410
Relative MS−Intensity (log 2)
FR1
FR2
FR3
INSOL

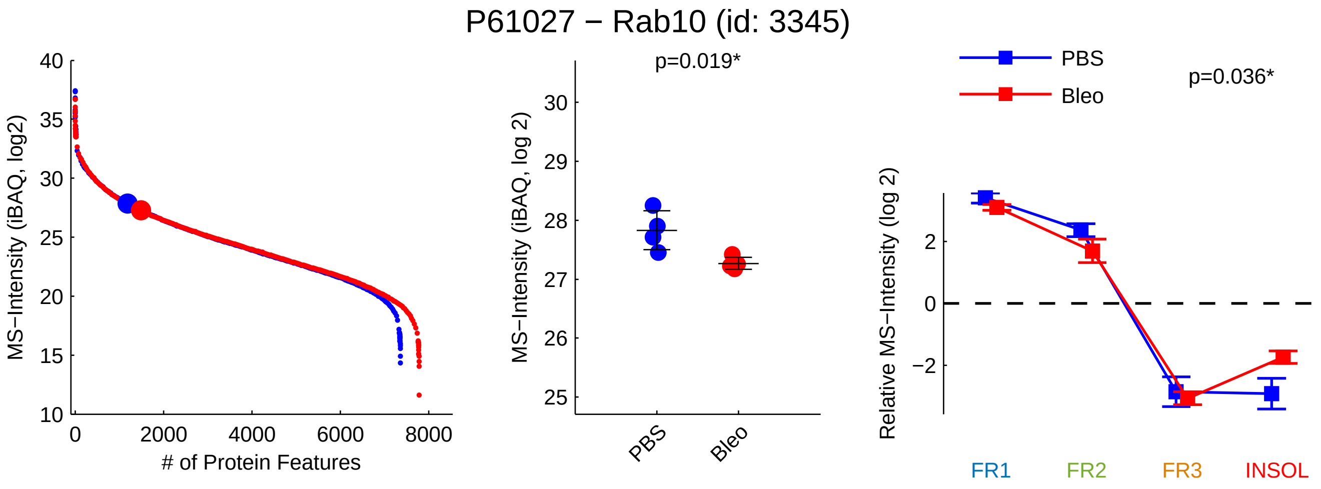

P61027 − Rab10 (id: 3345)
MS−Intensity (iBAQ, log2)
# of Protein Features
p=0.019*
MS−Intensity (iBAQ, log 2)
PBS
Bleo
p=0.036*
Relative MS−Intensity (log 2)
FR1
FR2
FR3
INSOL

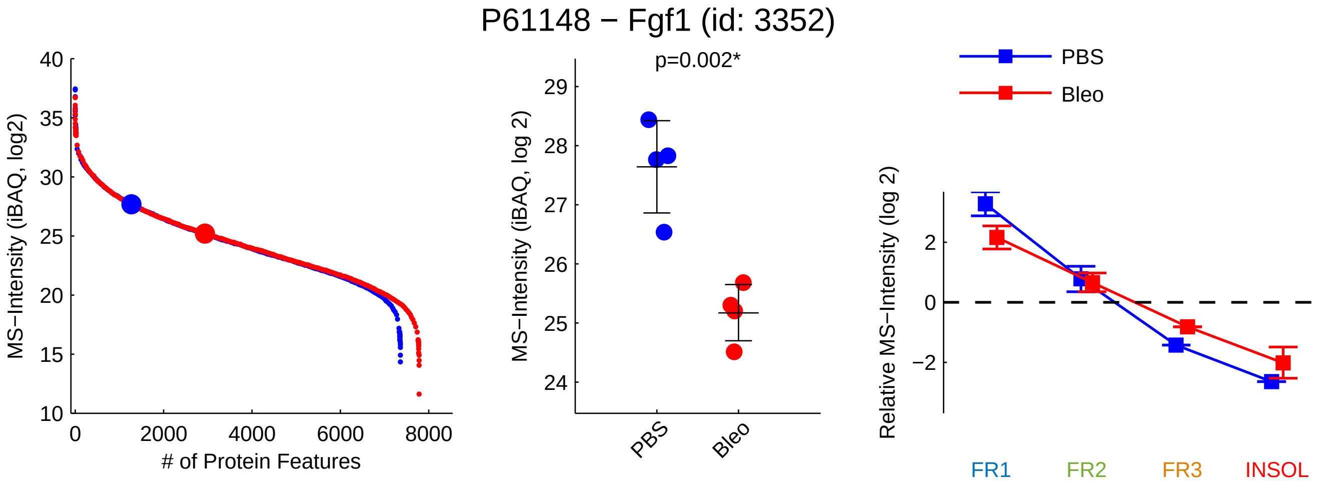

P61148 − Fgf1 (id: 3352)
MS−Intensity (iBAQ, log2)
# of Protein Features
p=0.002*
MS−Intensity (iBAQ, log 2)
PBS
Bleo
Relative MS−Intensity (log 2)
FR1
FR2
FR3
INSOL

P61255 − Rpl26 (id: 3361)

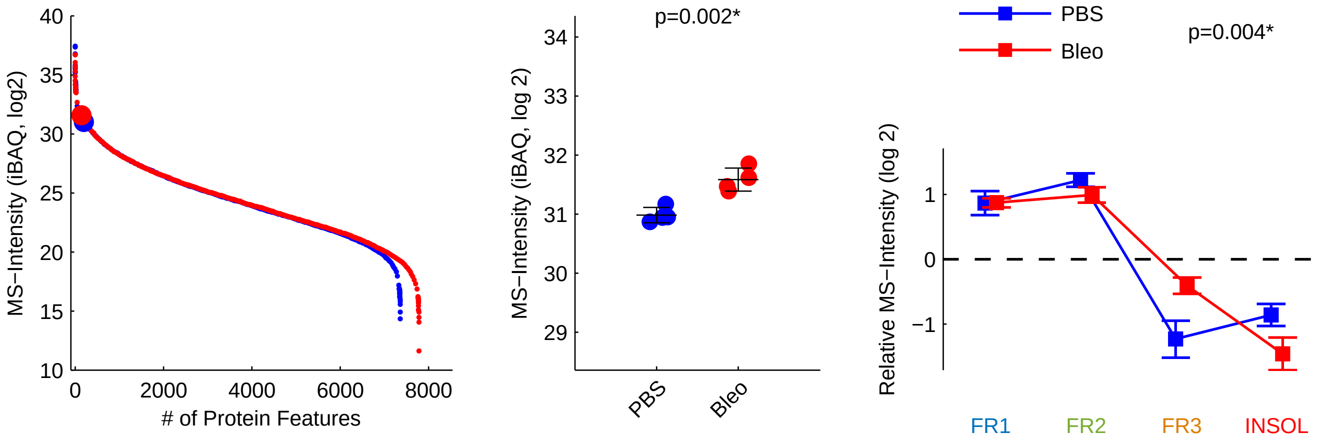

P61294 − Rab6b (id: 3363)

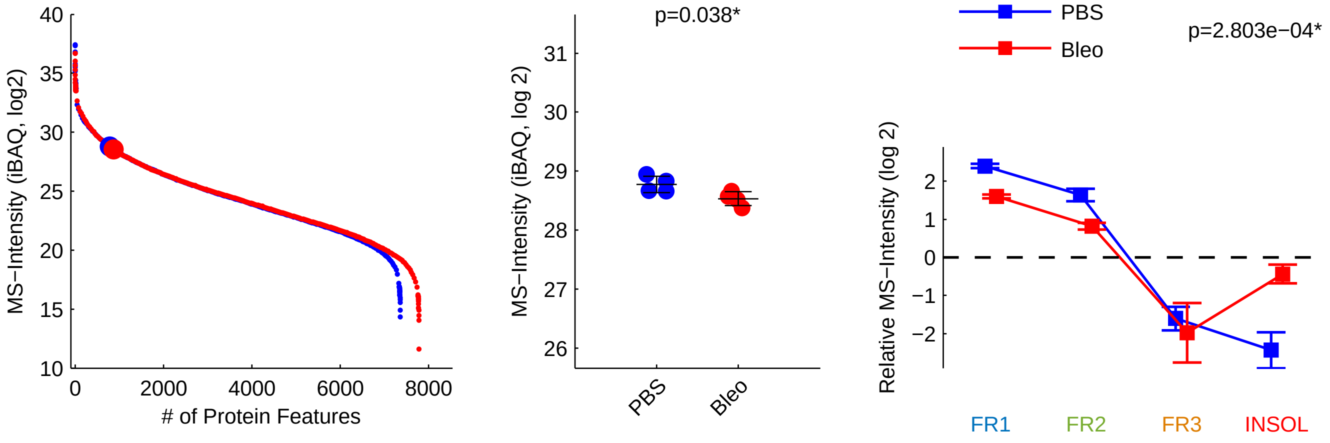

P61358 − Rpl27 (id: 3365)

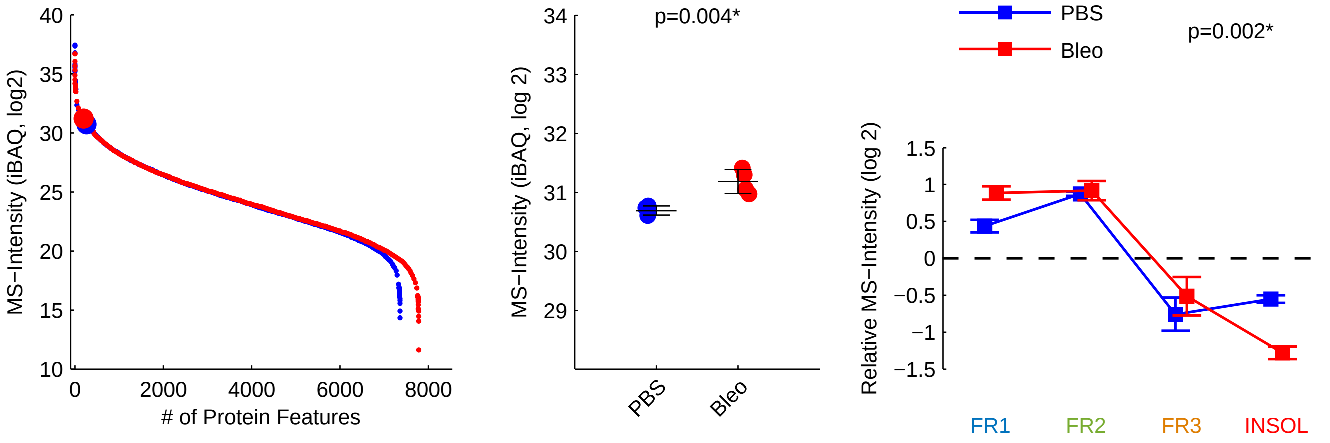

P61514 − Rpl37a (id: 3368)

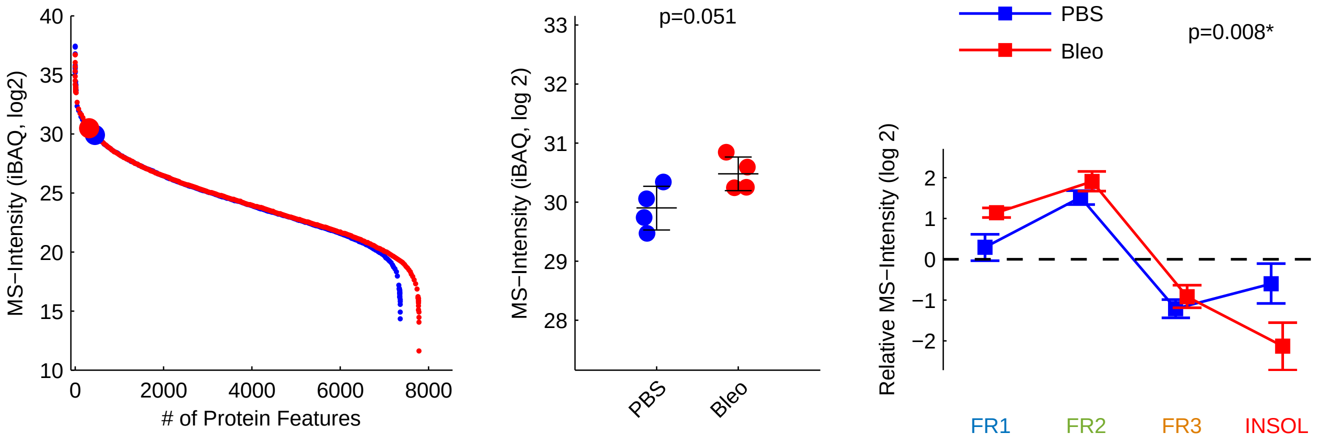

P61750 − Arf4 (id: 3371)

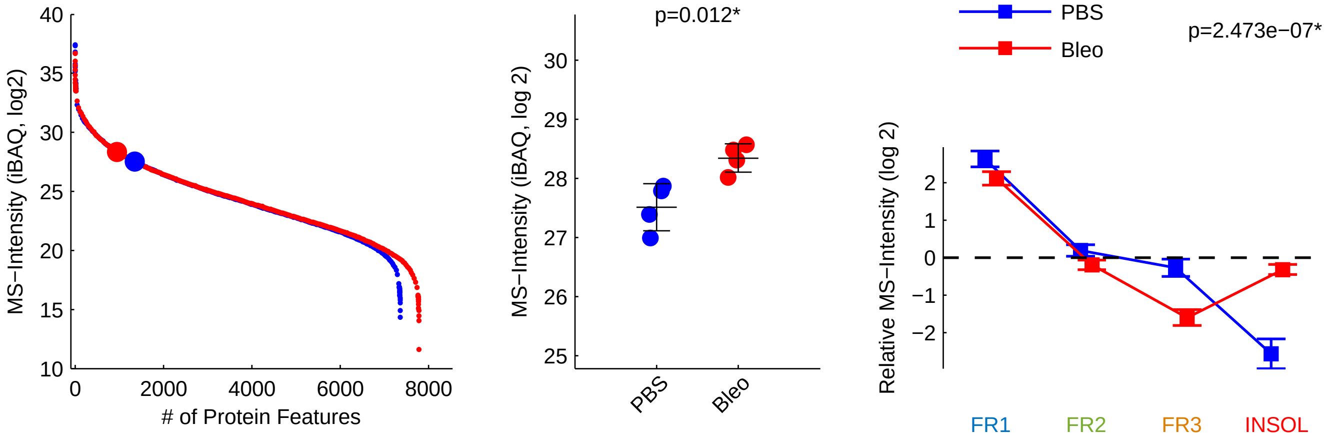

P61924 − Copz1 (id: 3375)

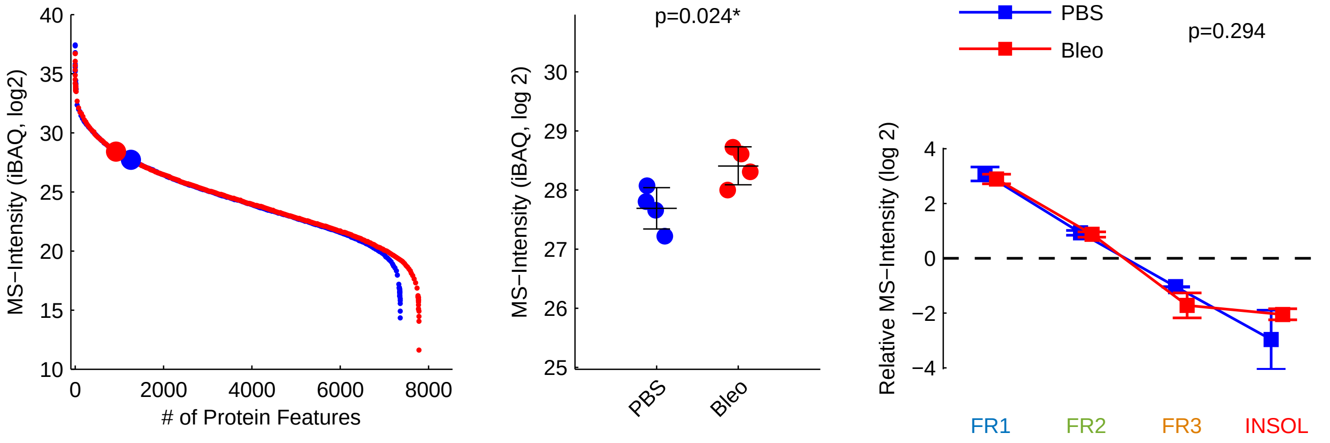

P61953 − Gng11 (id: 3377)

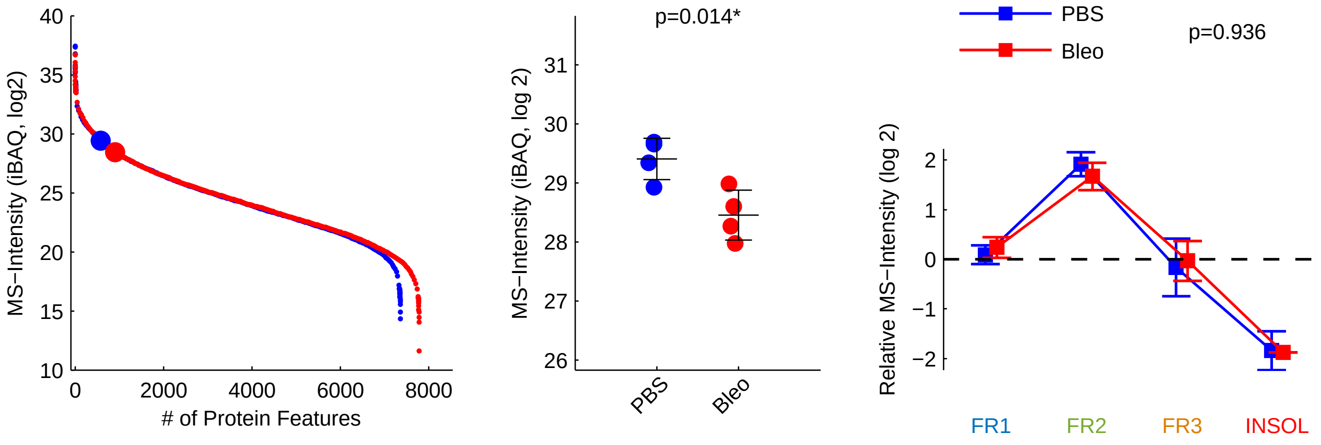

P61979−2 − Hnrnpk (id: 3383)

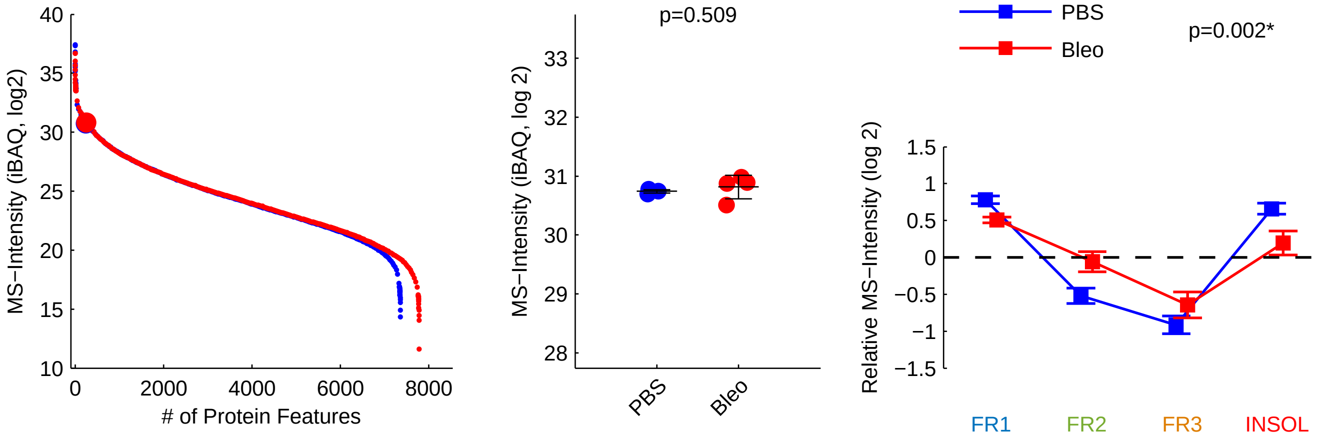

P62071 − Rras2 (id: 3386)

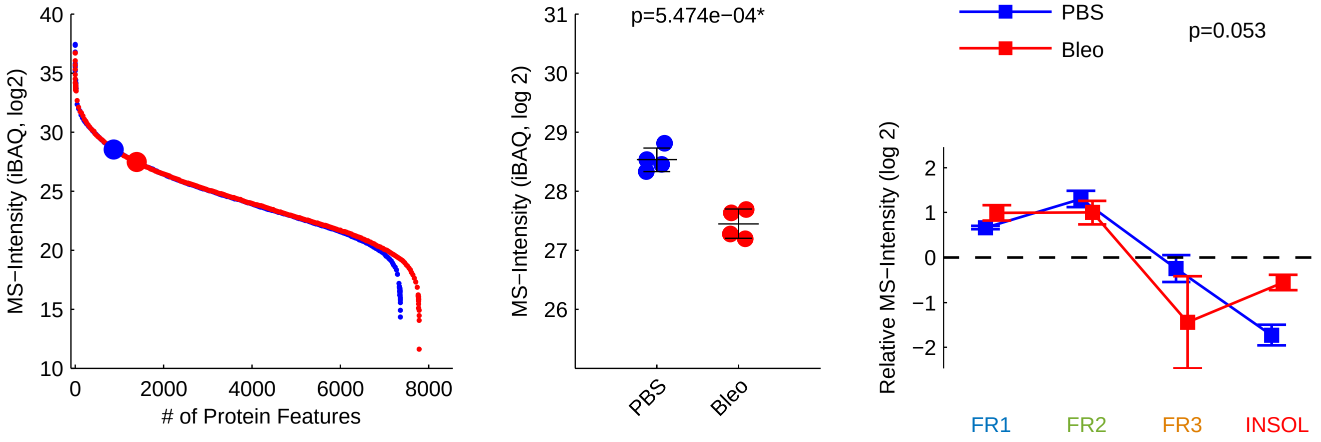

P62082 − Rps7 (id: 3390)

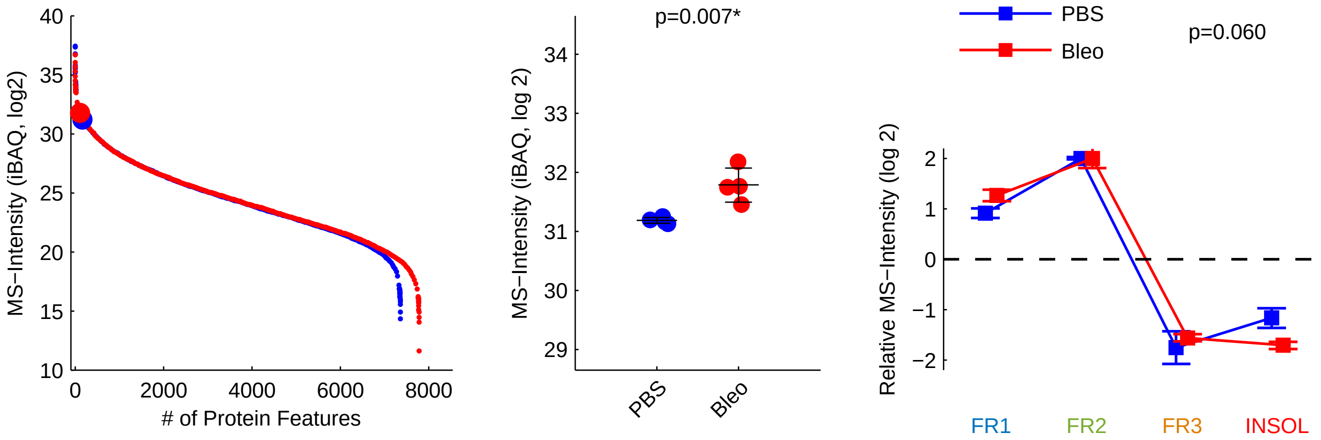

P62141 − Ppp1cb (id: 3392)

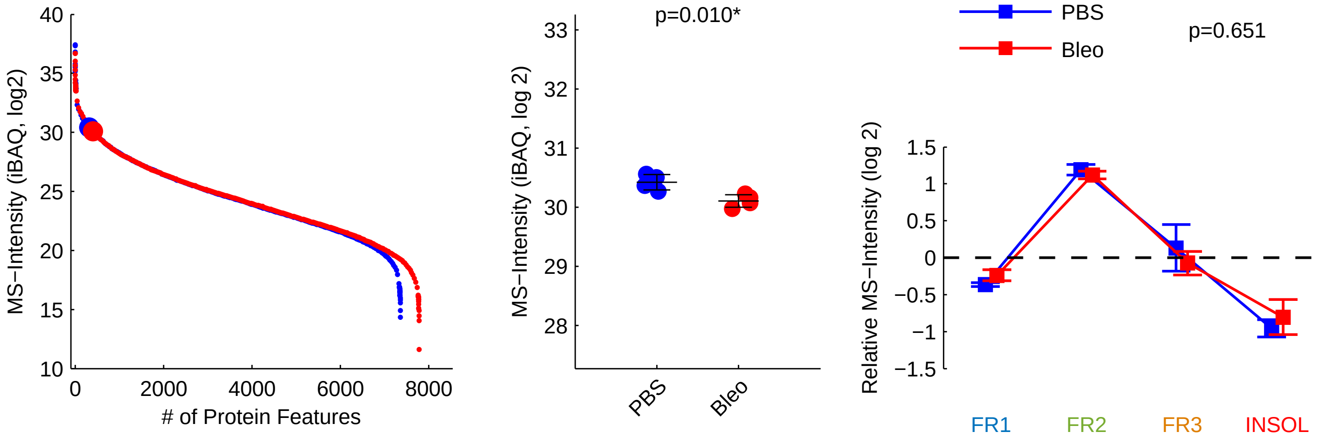

P62204 − Calm1 (id: 3395)

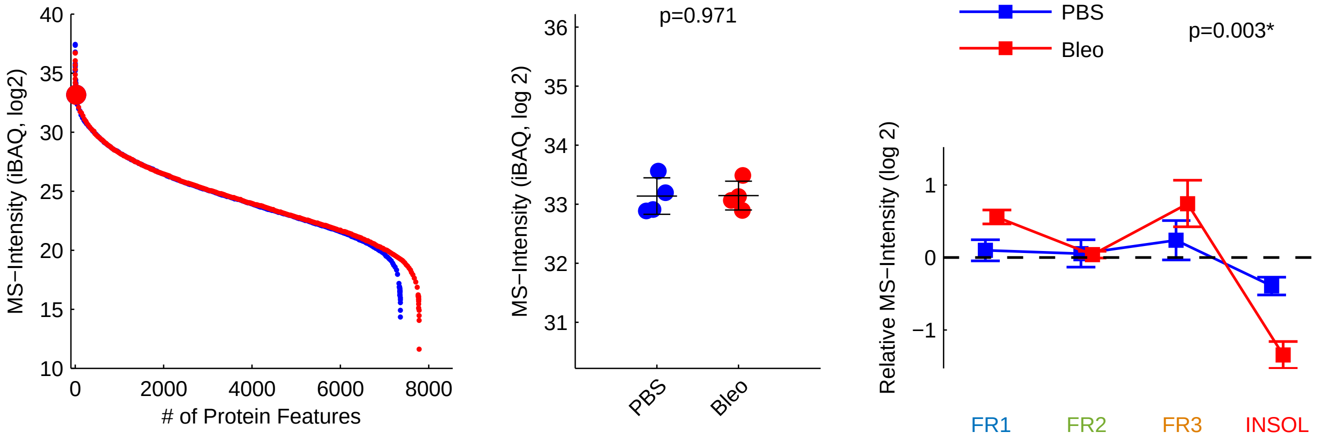

P62242 − Rps8 (id: 3396)

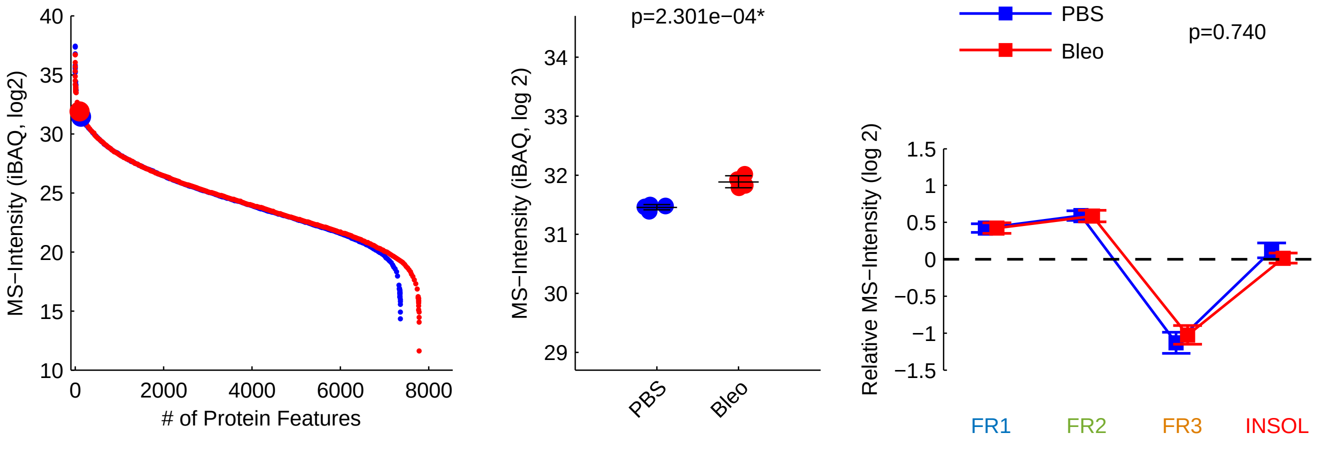

P62245 − Rps15a (id: 3397)

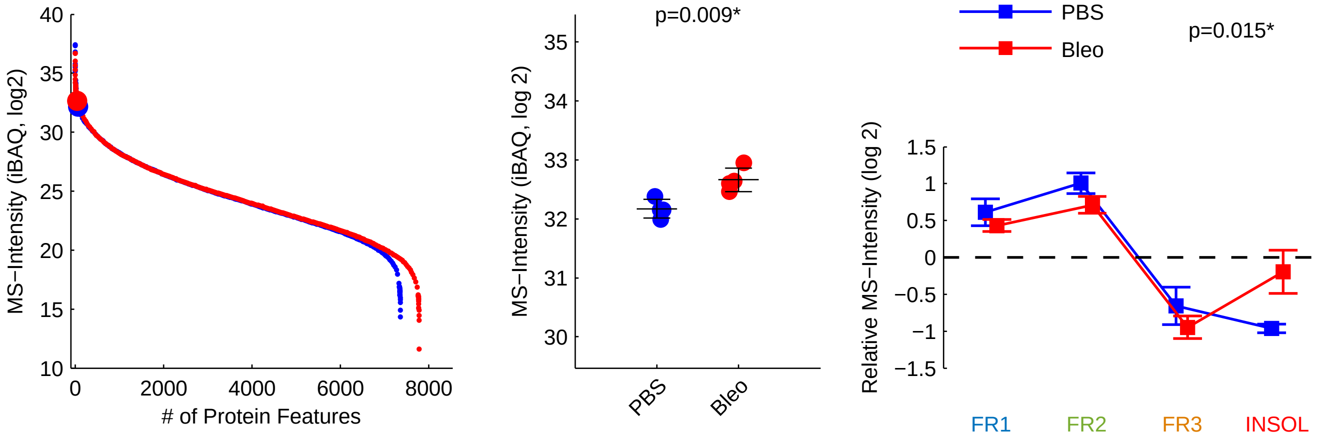

P62264 − Rps14 (id: 3401)

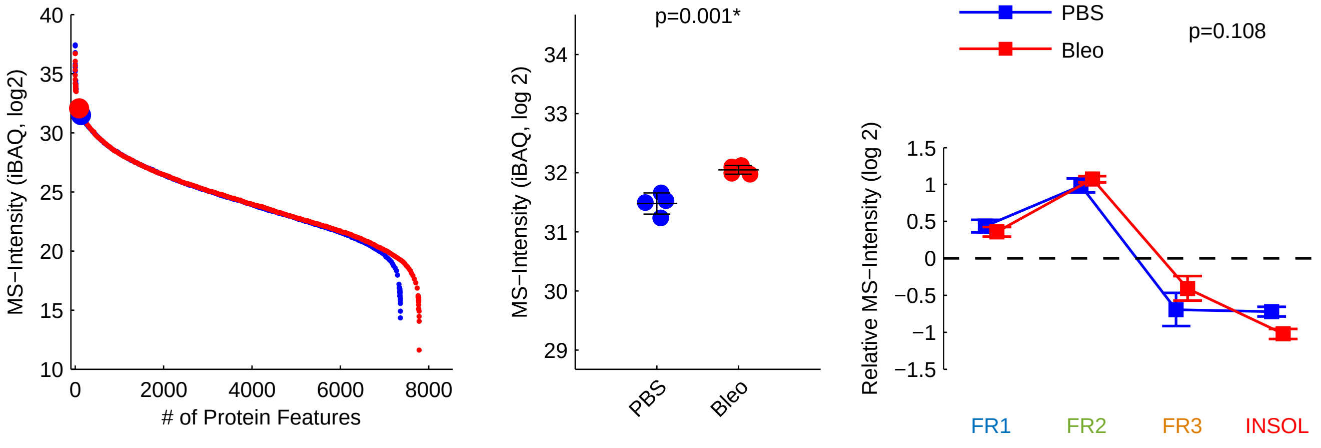

P62267 − Rps23 (id: 3402)

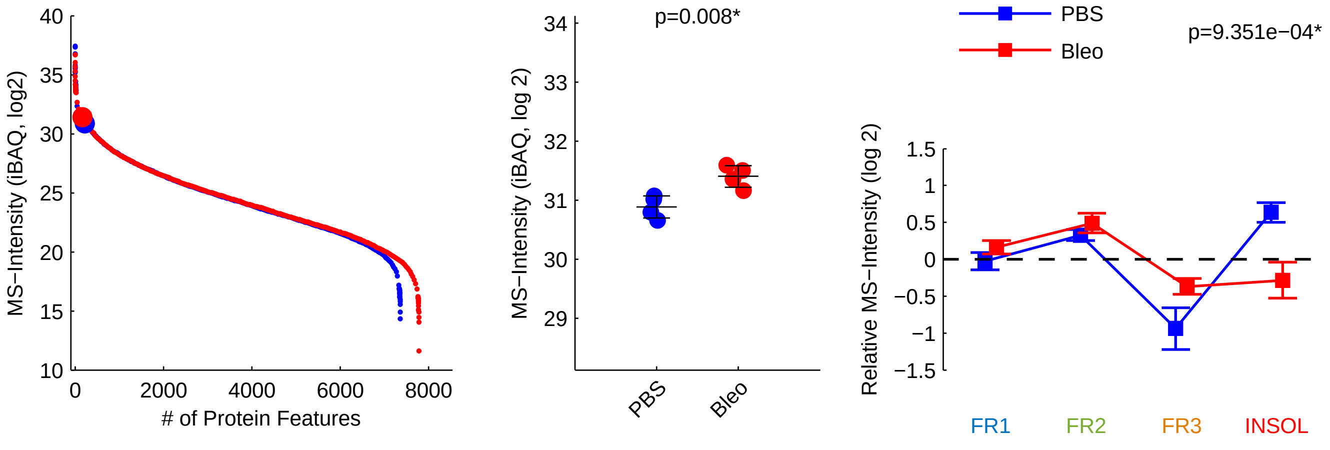

P62270 − Rps18 (id: 3403)

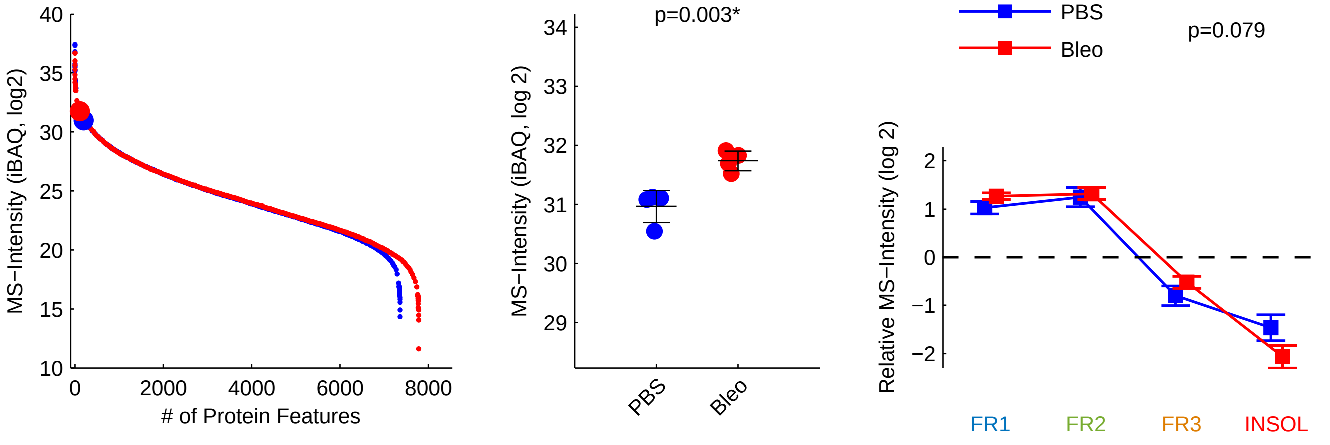

P62281 − Rps11 (id: 3405)

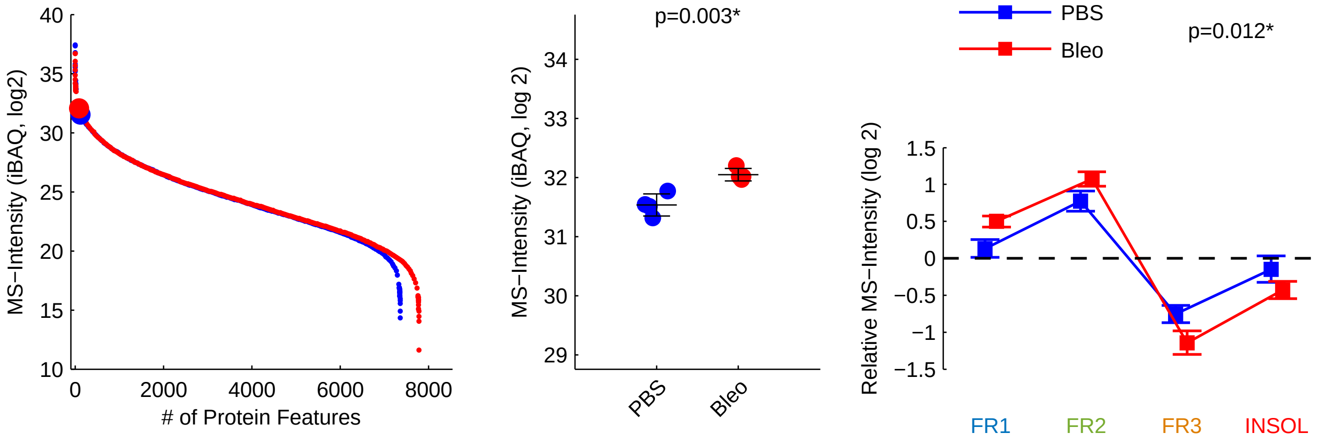

P62301 − Rps13 (id: 3406)

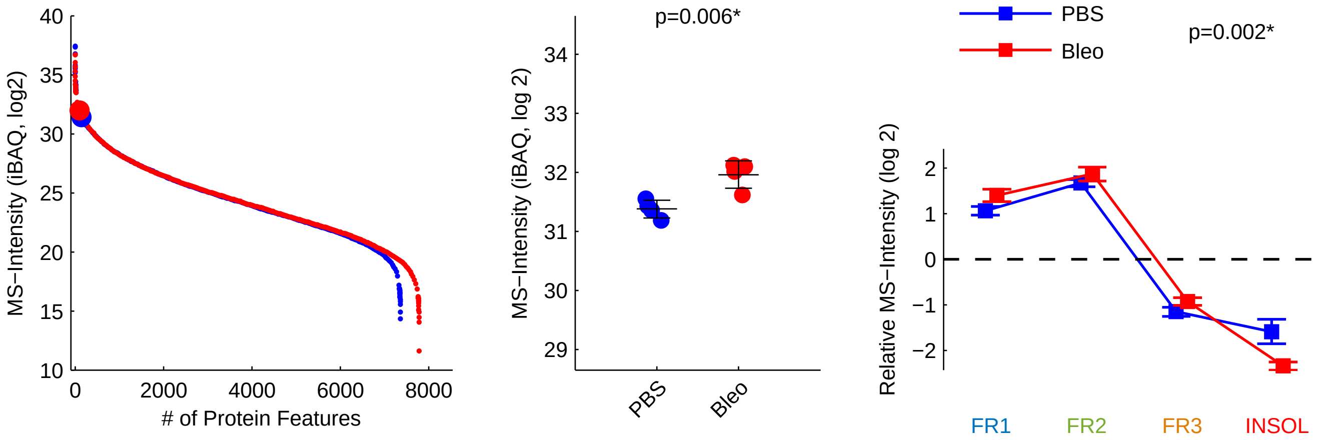

P62313 − Lsm6 (id: 3411)

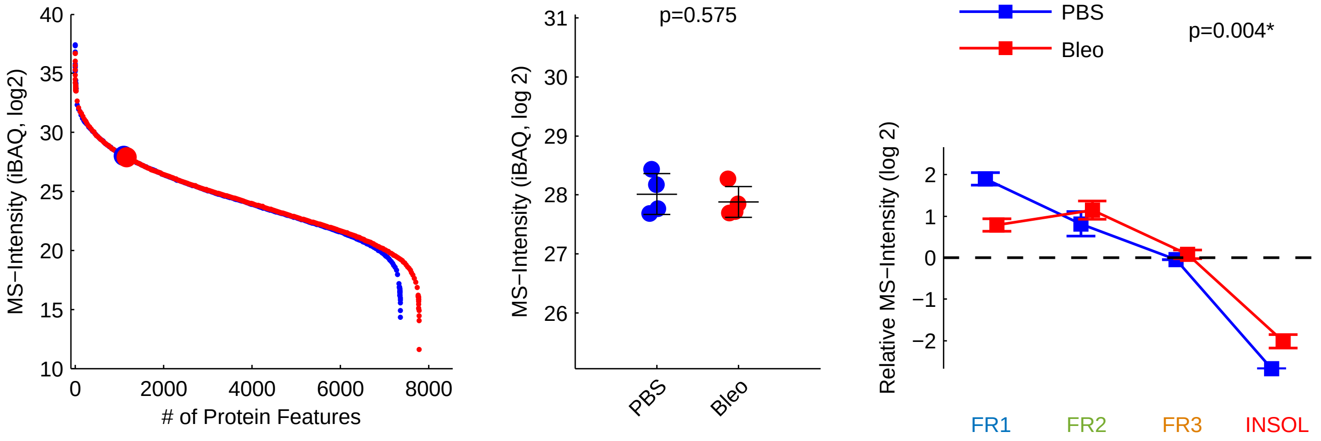

P62331 − Arf6 (id: 3416)

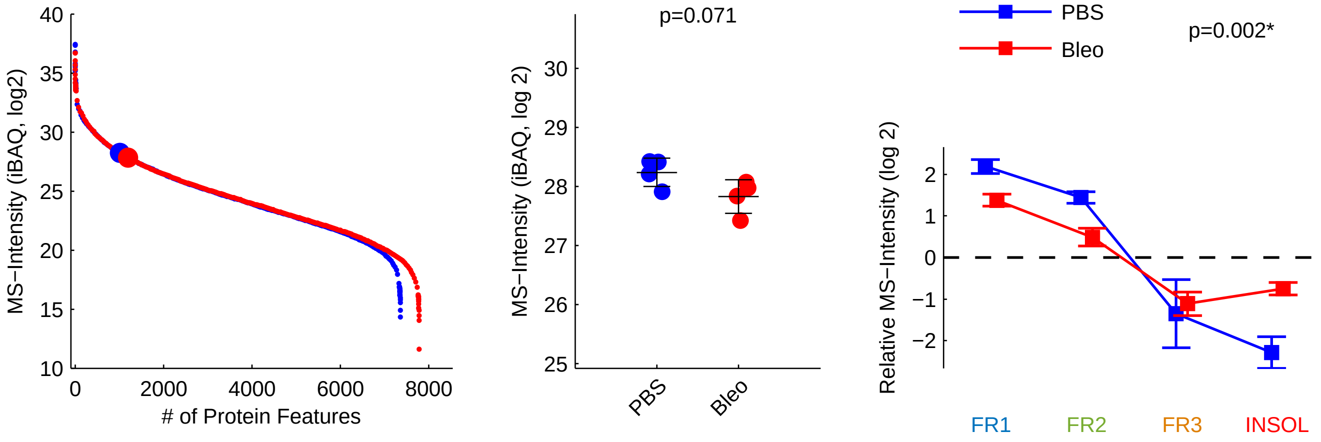

P62334 − Psmc6 (id: 3417)

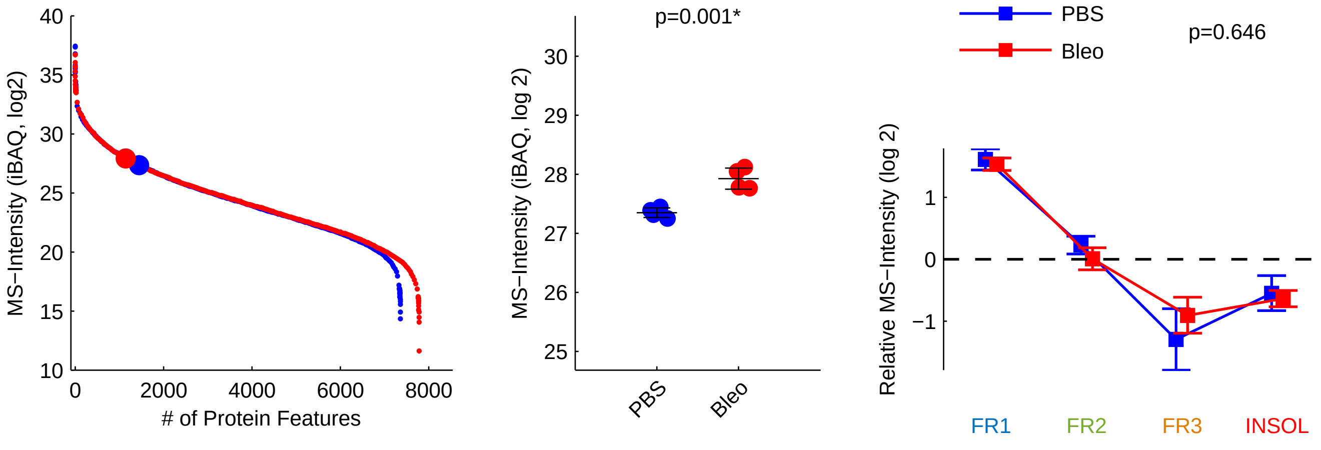

P62702 − Rps4x (id: 3425)

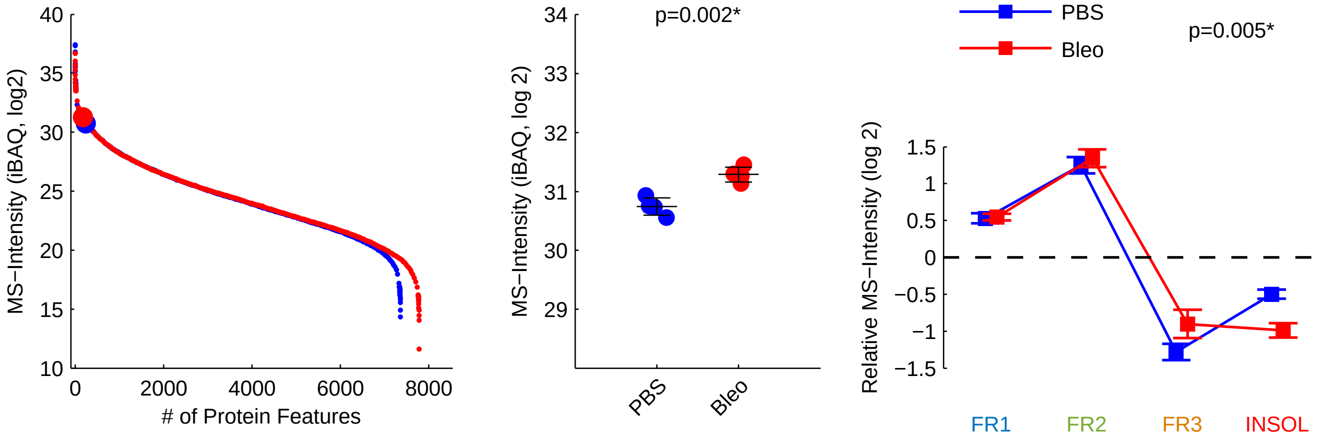

P62717 − Rpl18a (id: 3427)

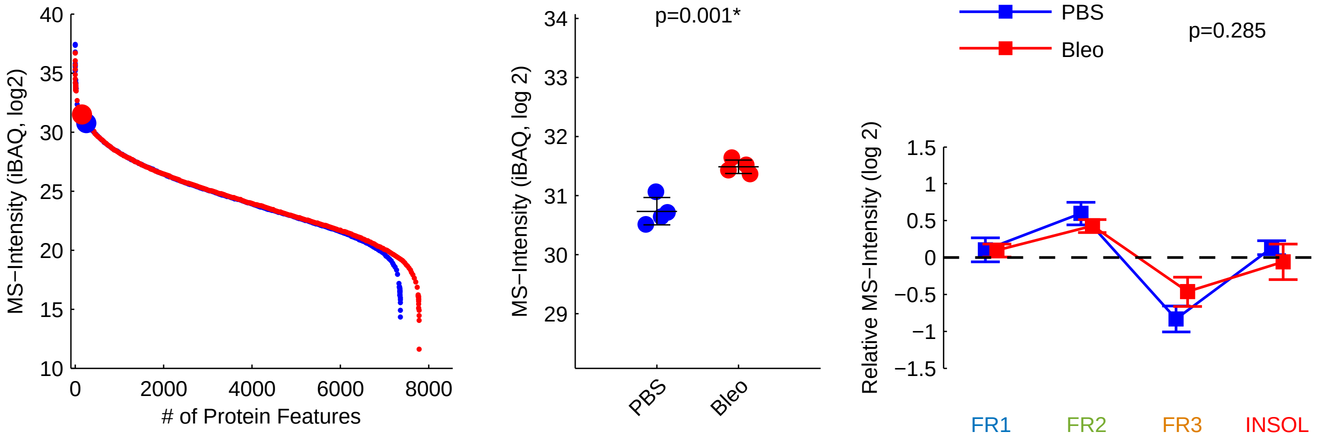

P62746 − Rhob (id: 3430)

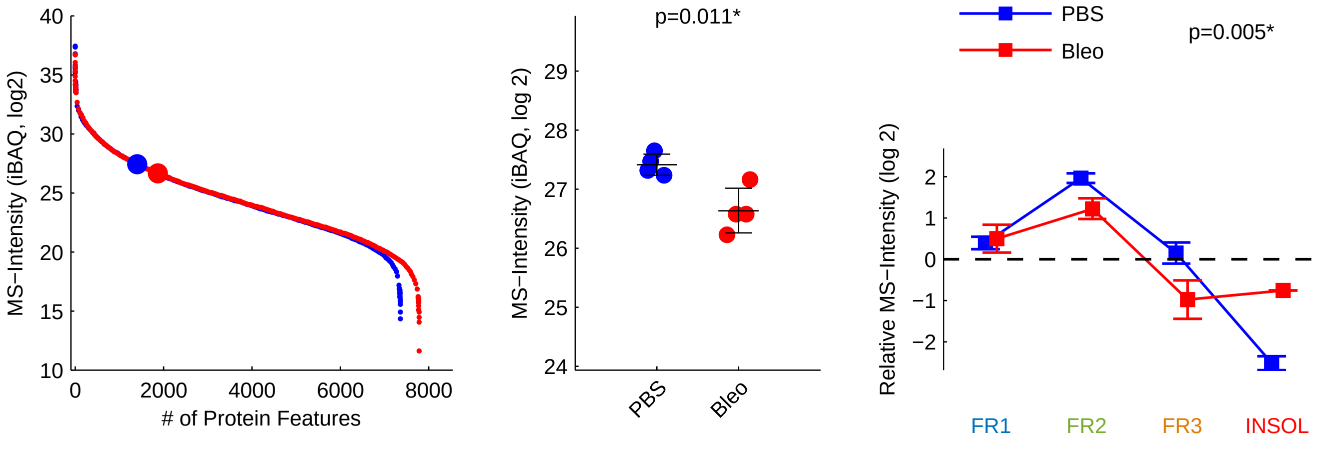

P62748 − Hpcal1 (id: 3431)

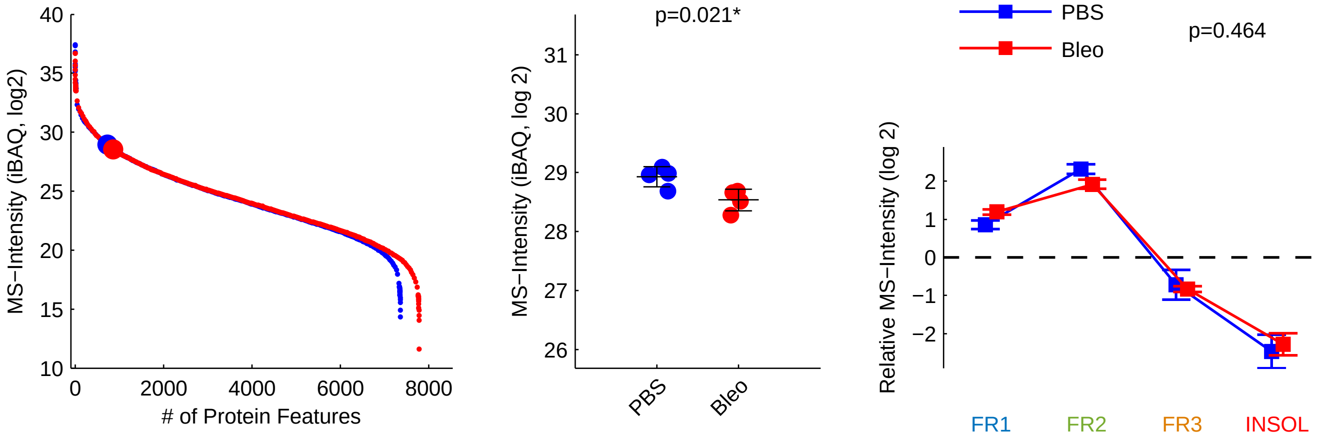

P62751 − Rpl23a (id: 3432)

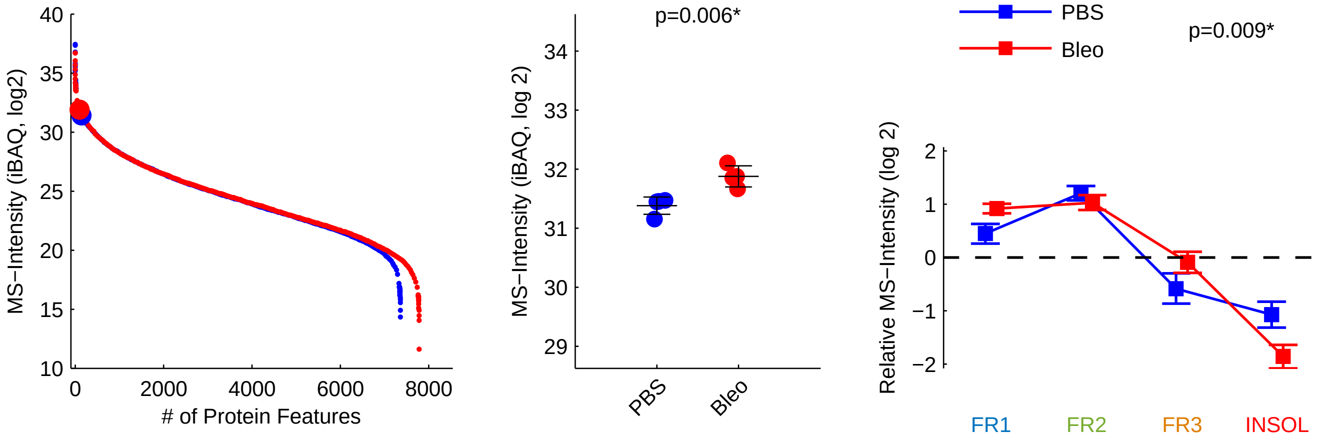

P62754 − Rps6 (id: 3433)

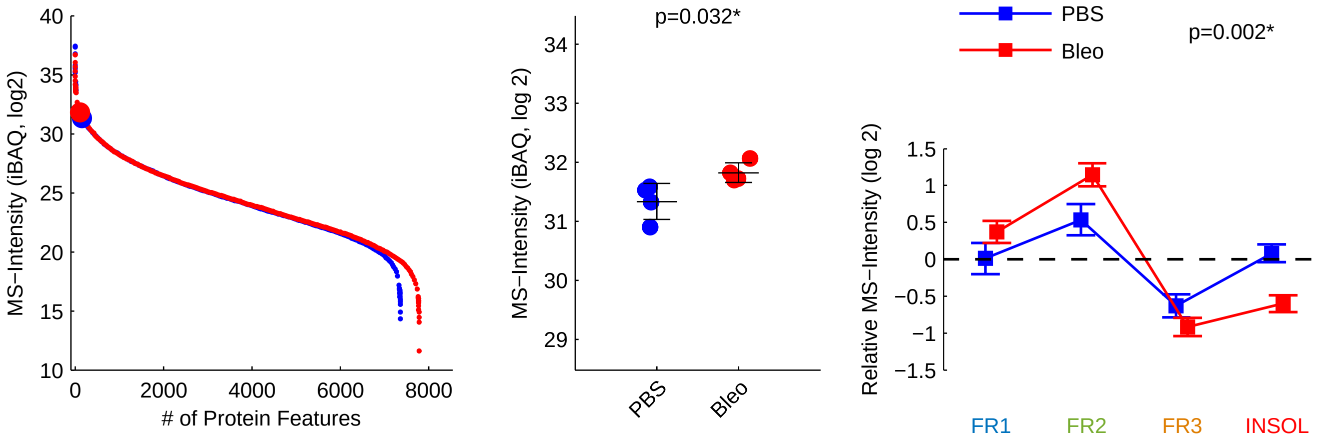

P62761 − Vsnl1 (id: 3434)

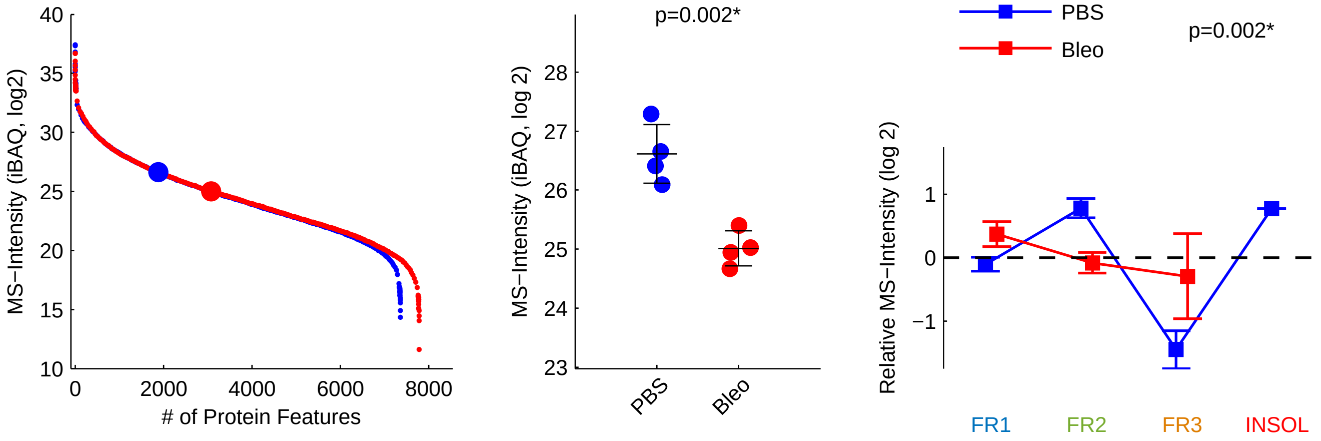

P62806 − Hist1h4a (id: 3436)

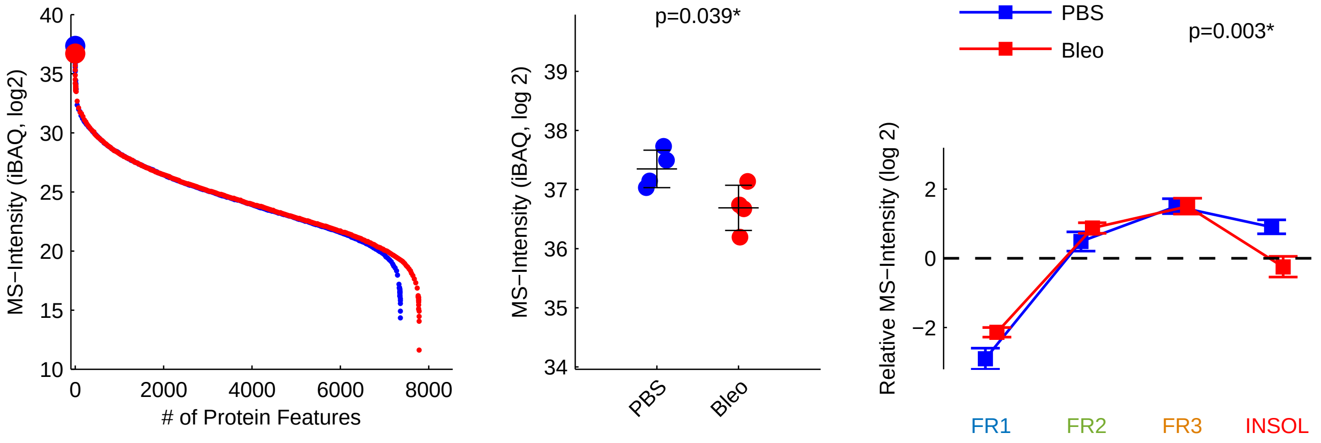

P62821 − Rab1A (id: 3438)

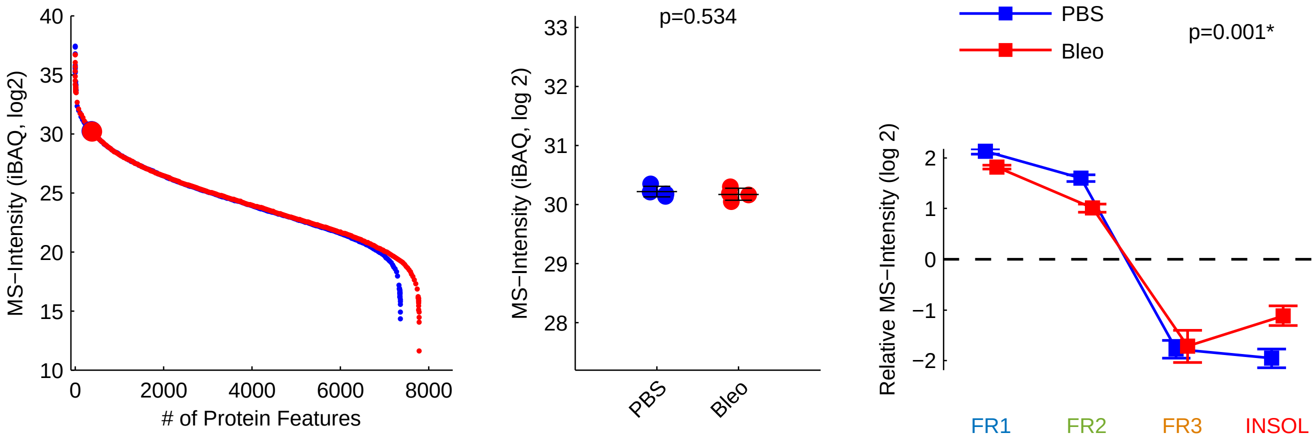

P62830 − Rpl23 (id: 3440)

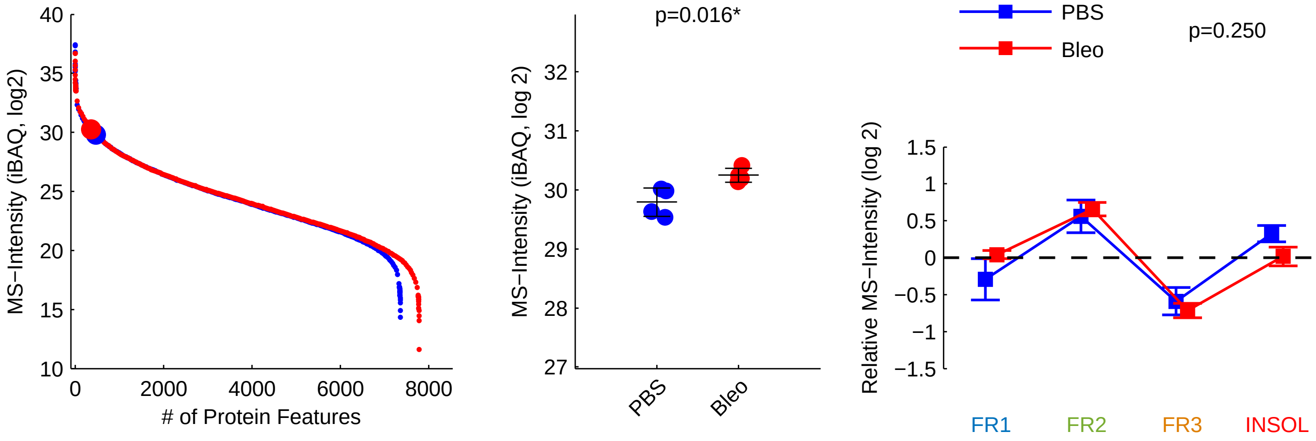

P62835 − Rap1a (id: 3441)

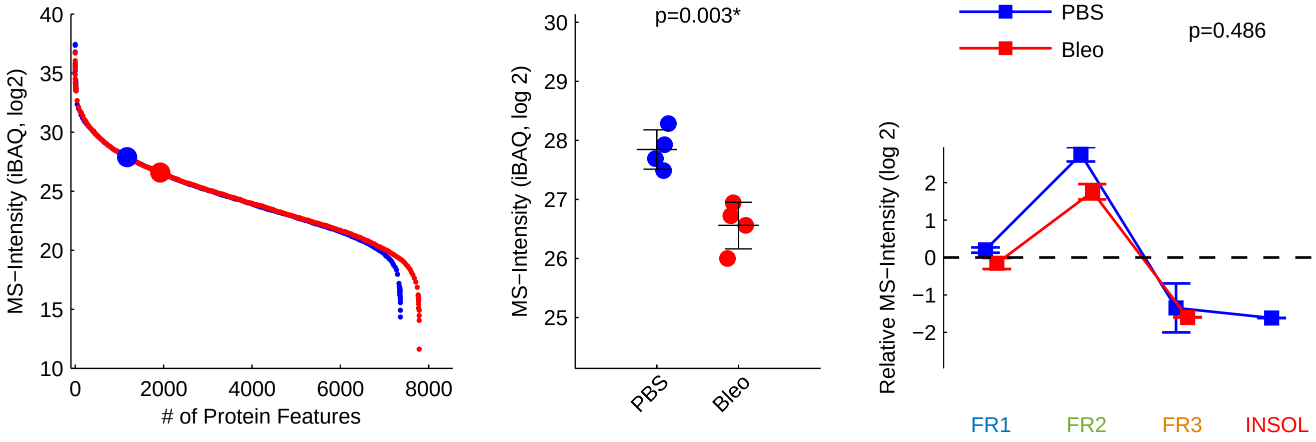

P62843 − Rps15 (id: 3443)

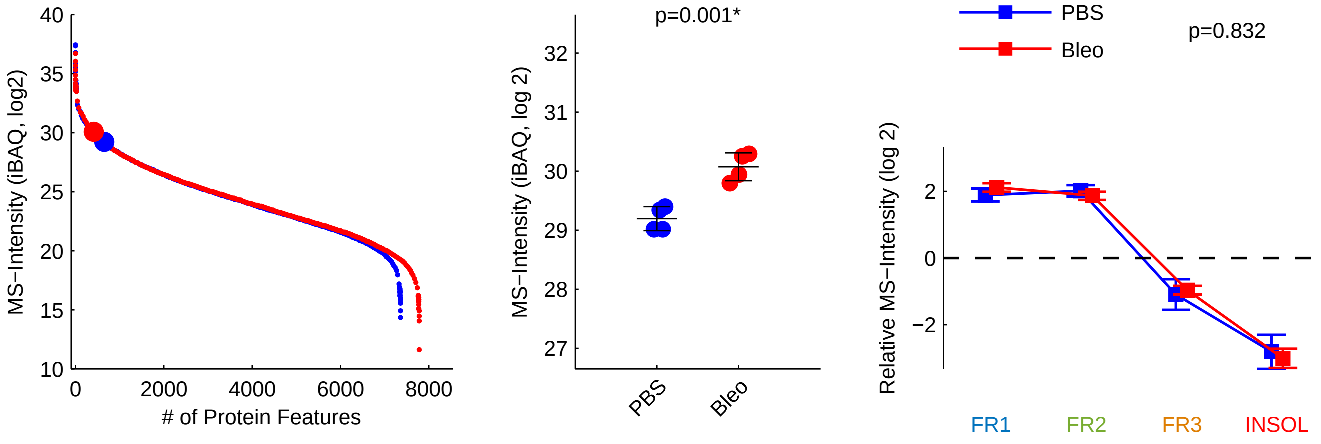

P62852 − Rps25 (id: 3445)

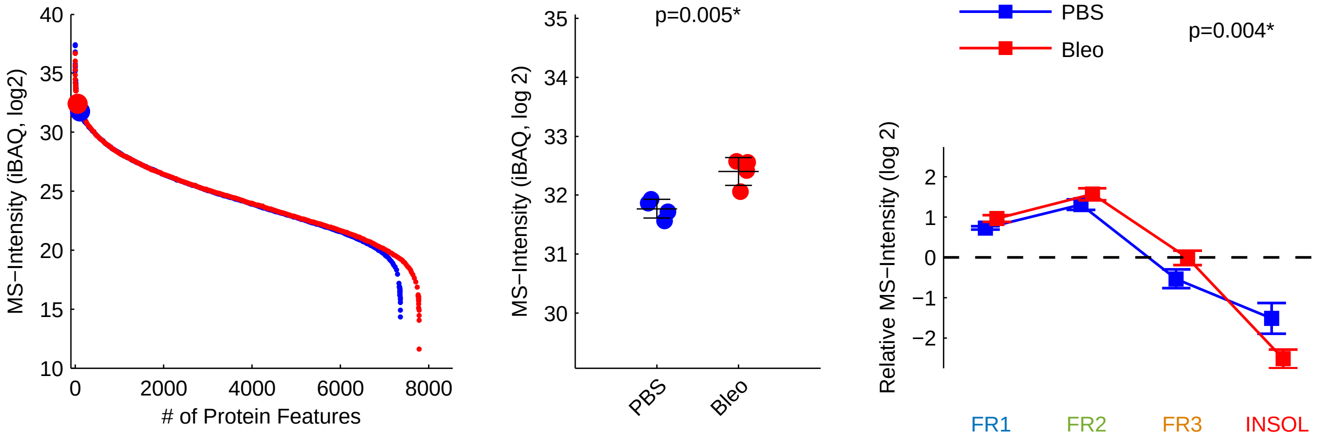

P62855 − Rps26 (id: 3446)

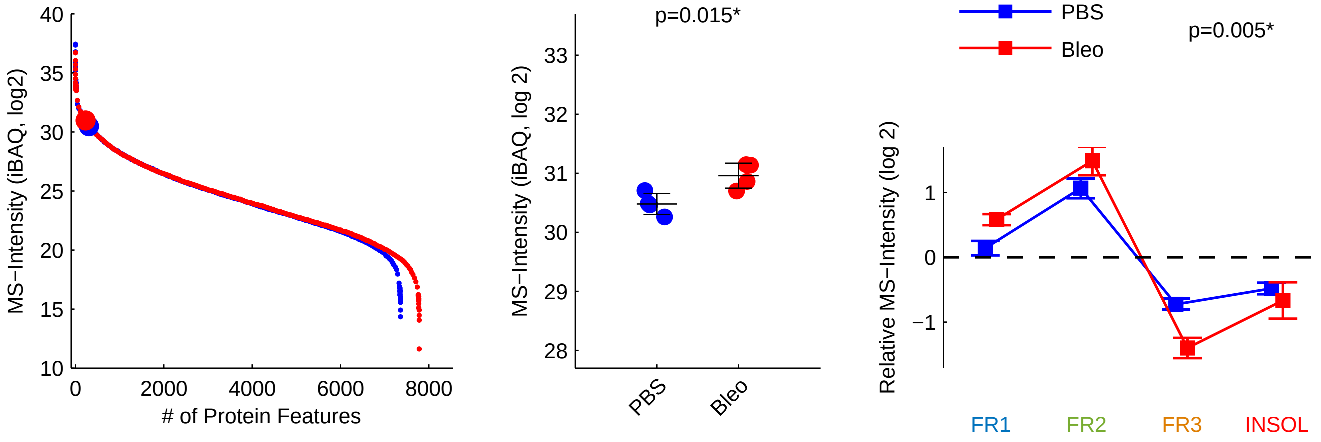

P62858 − Rps28 (id: 3447)

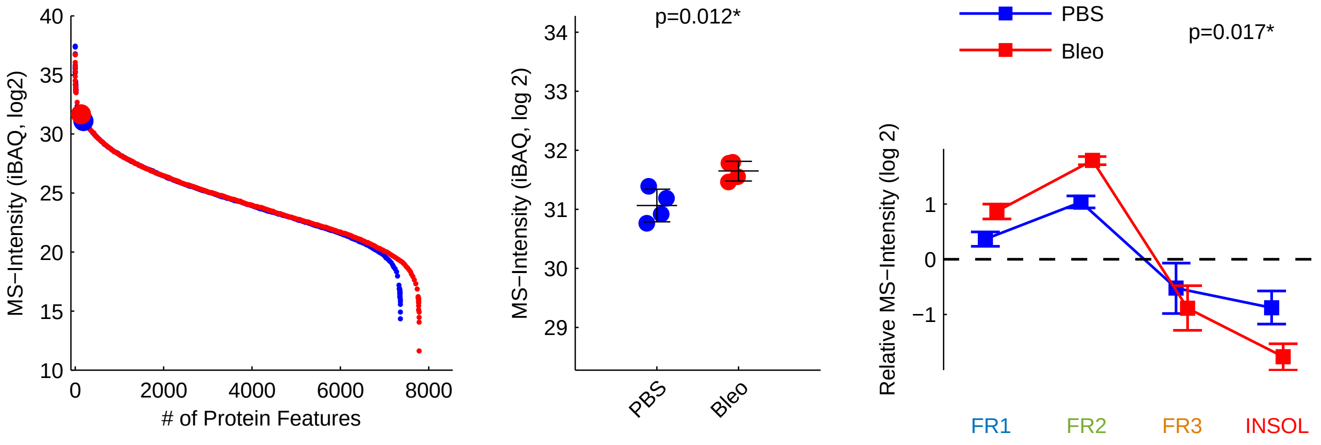

P62874 − Gnb1 (id: 3450)

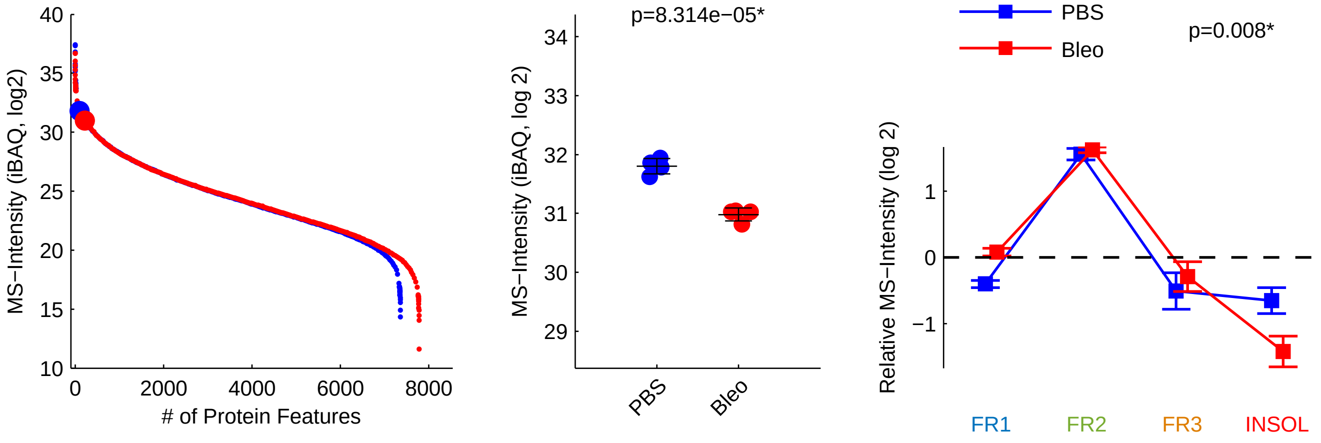

P62880 − Gnb2 (id: 3453)

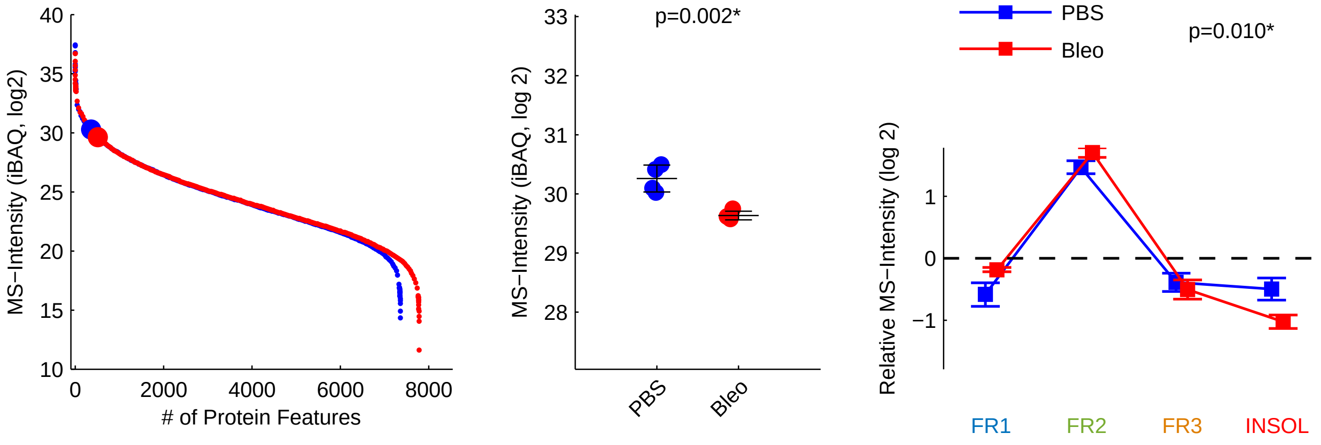

P62889 − Rpl30 (id: 3454)

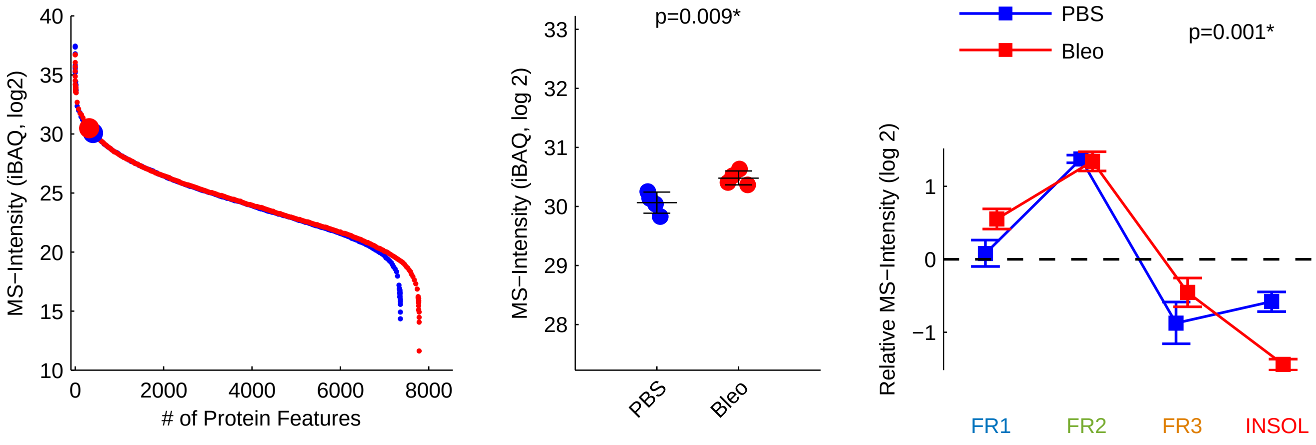

P62900 − Rpl31 (id: 3456)

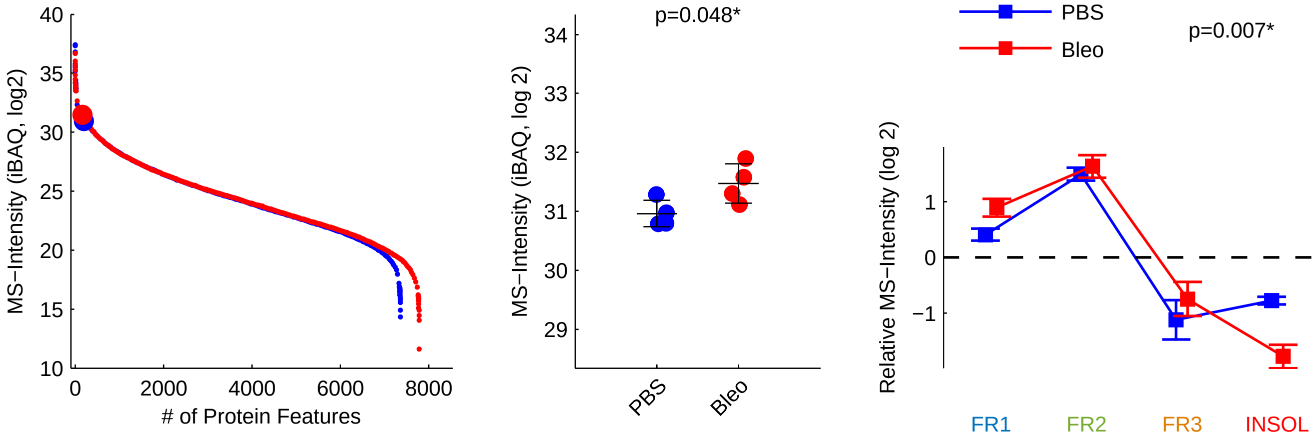

P62908 − Rps3 (id: 3457)

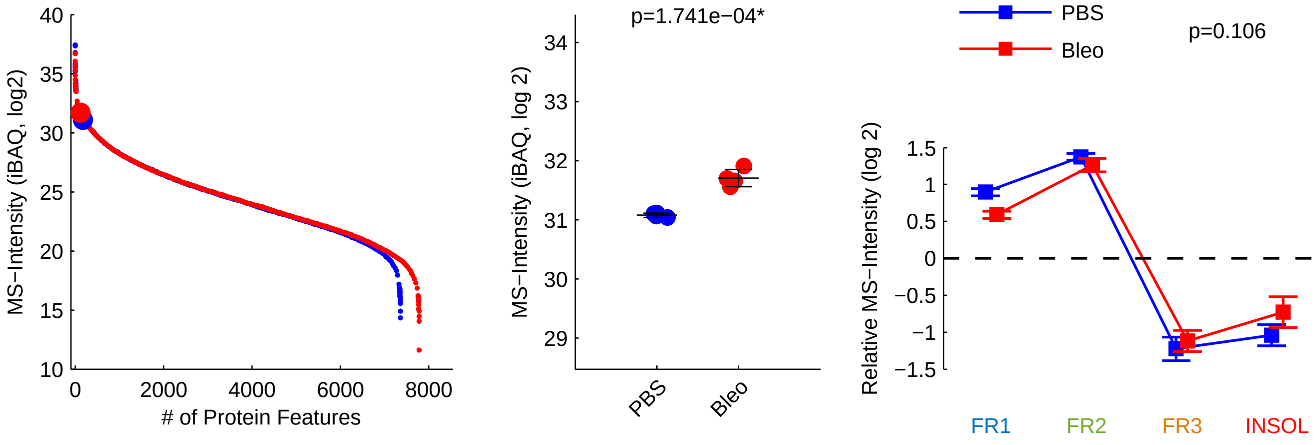

P62911 − Rpl32 (id: 3458)

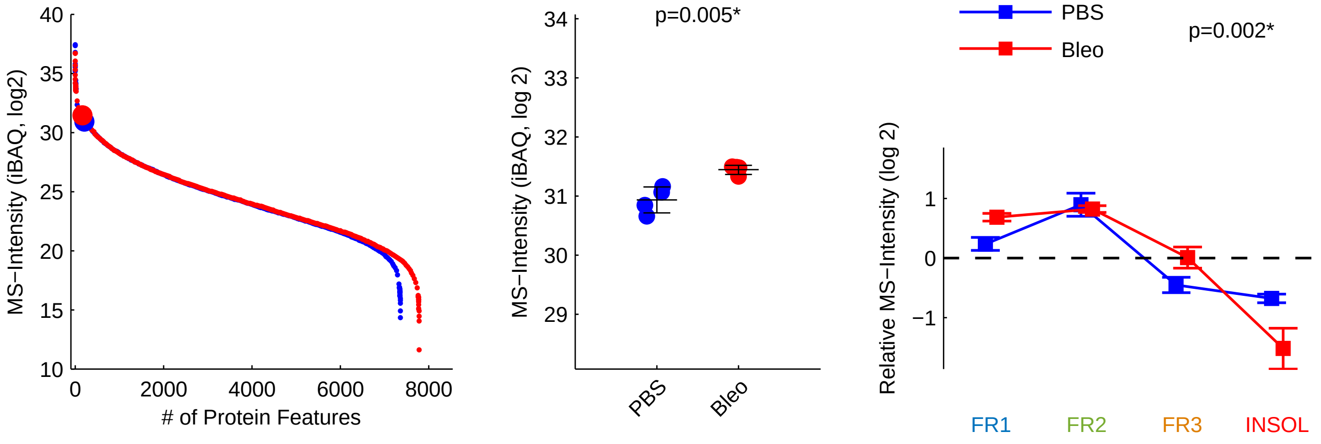

P62915 − Gtf2b (id: 3459)

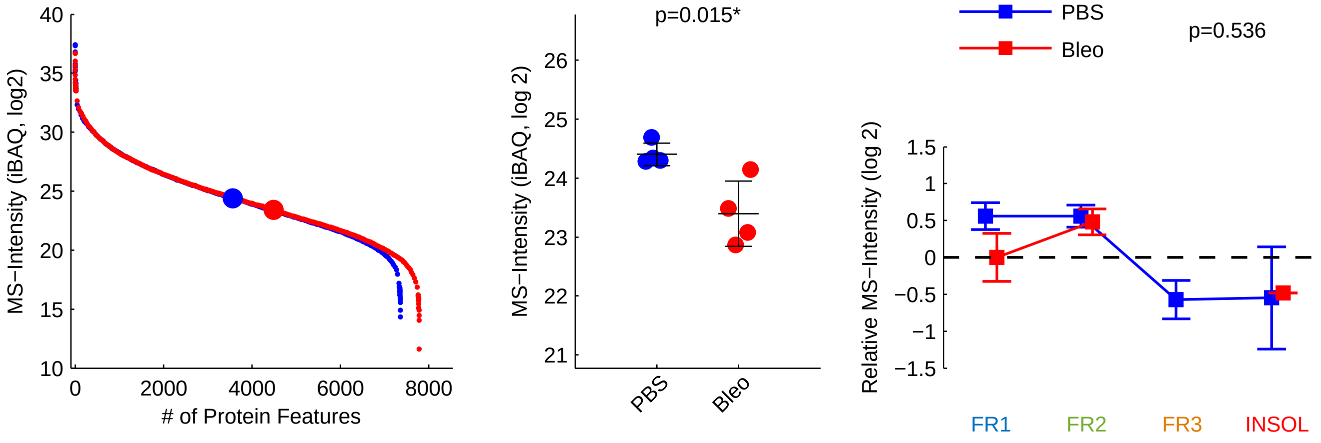

P62918 − Rpl8 (id: 3460)

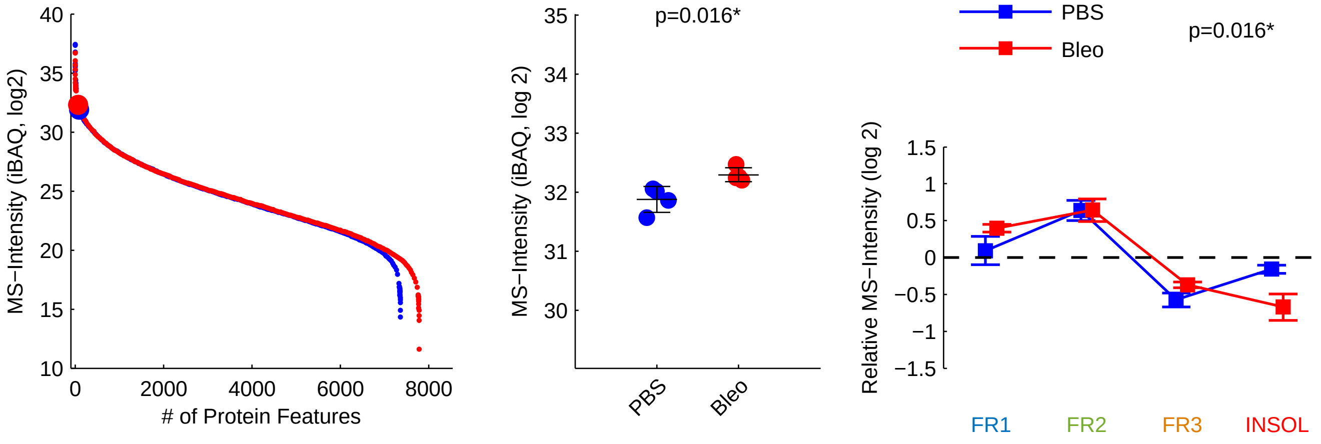

P62960 − Ybx1 (id: 3461)

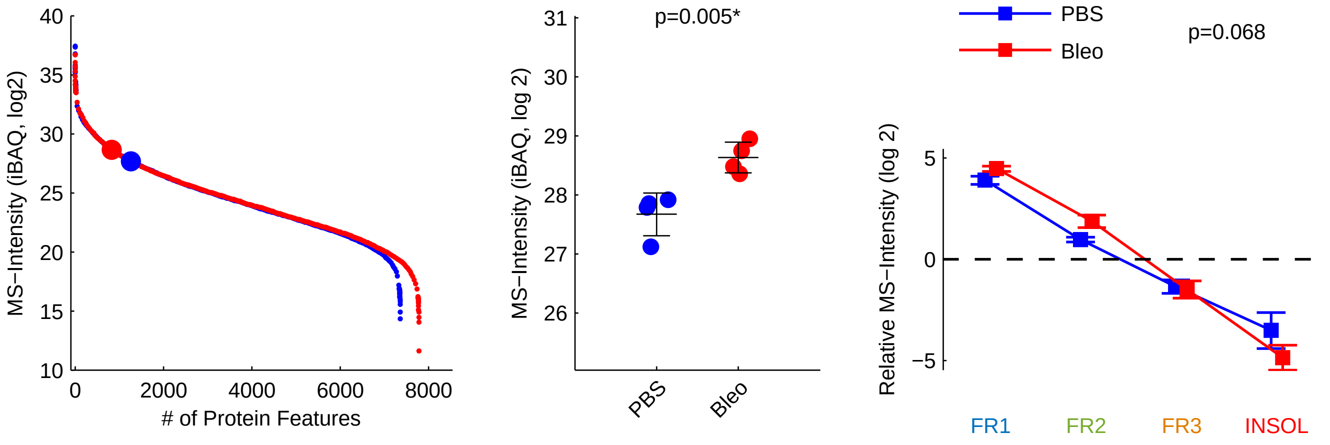

P62983 − Rps27a (id: 3463)

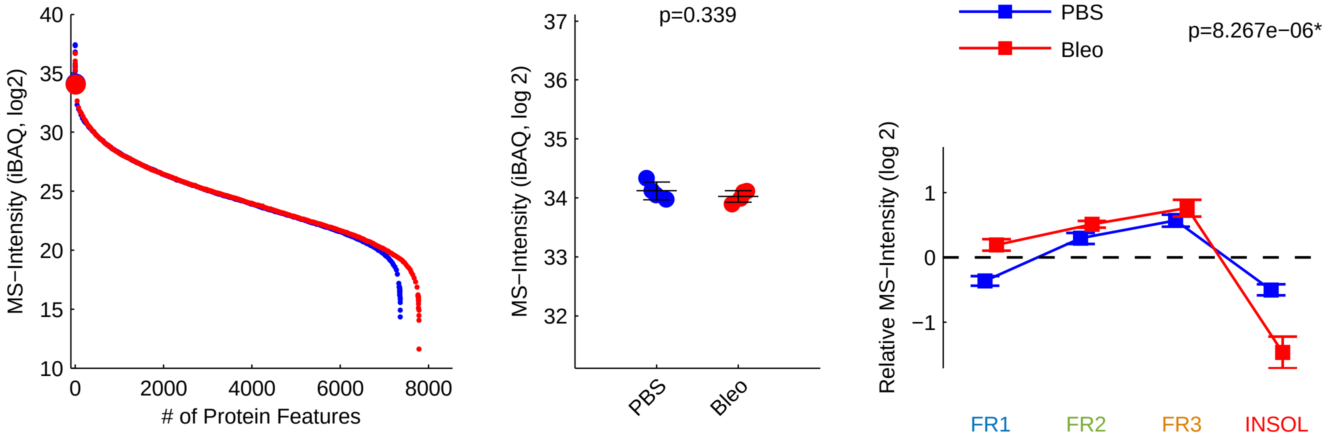

P62996 − Tra2b (id: 3464)

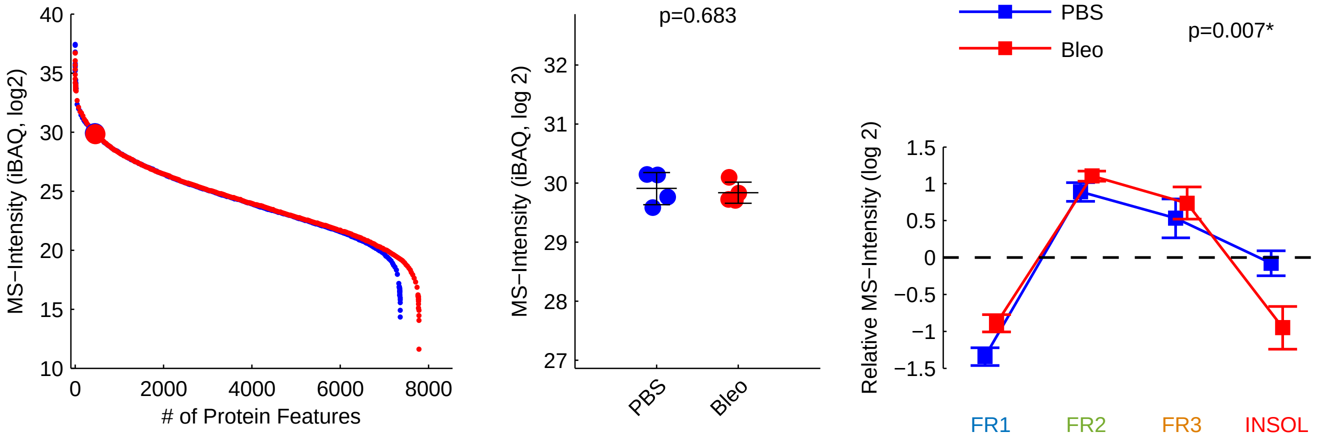

P63001 − Rac1 (id: 3465)

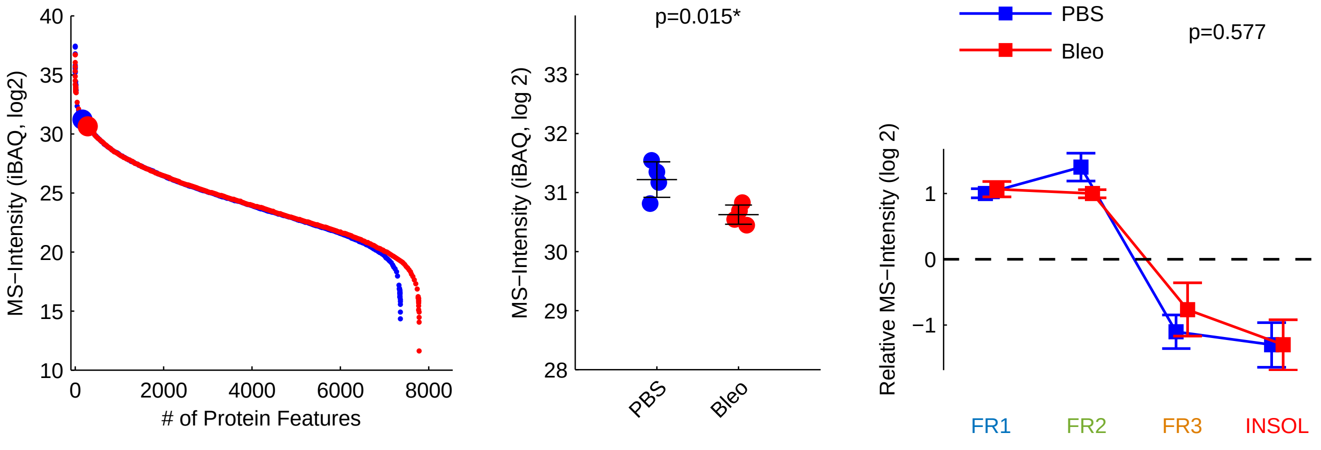

P63005 − Pafah1b1 (id: 3467)

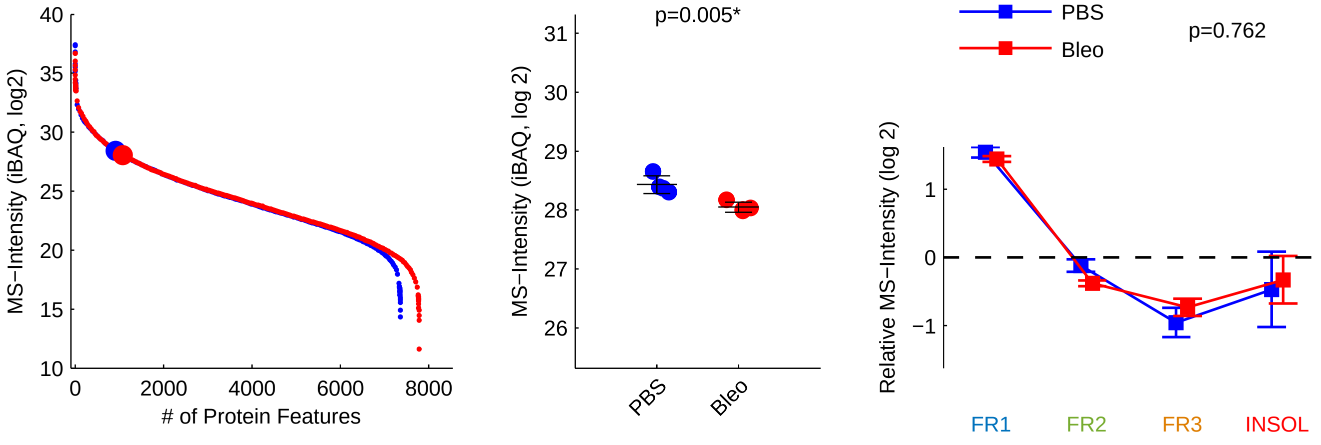

P63017 − Hspa8 (id: 3469)

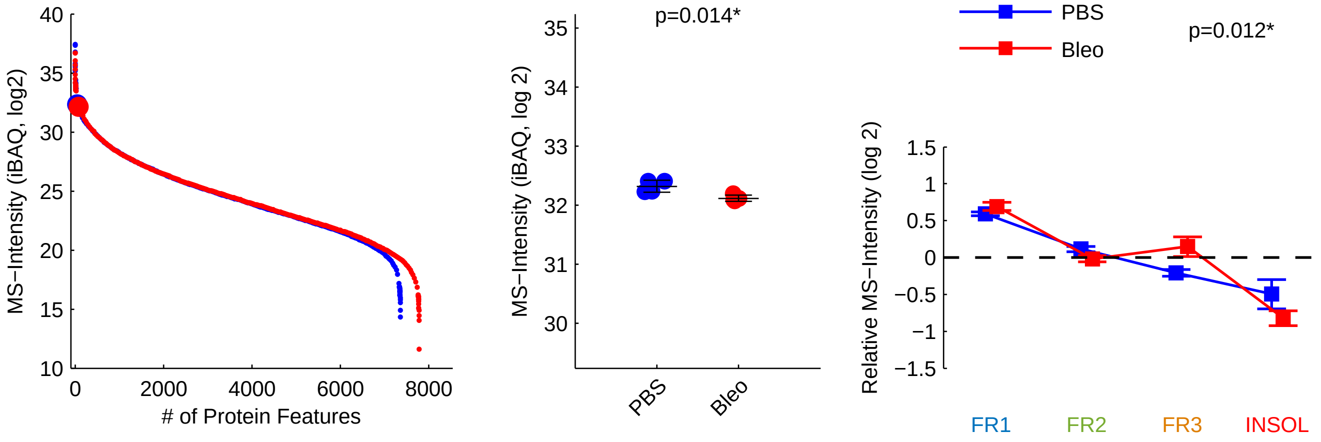

P63024 − Vamp3 (id: 3470)

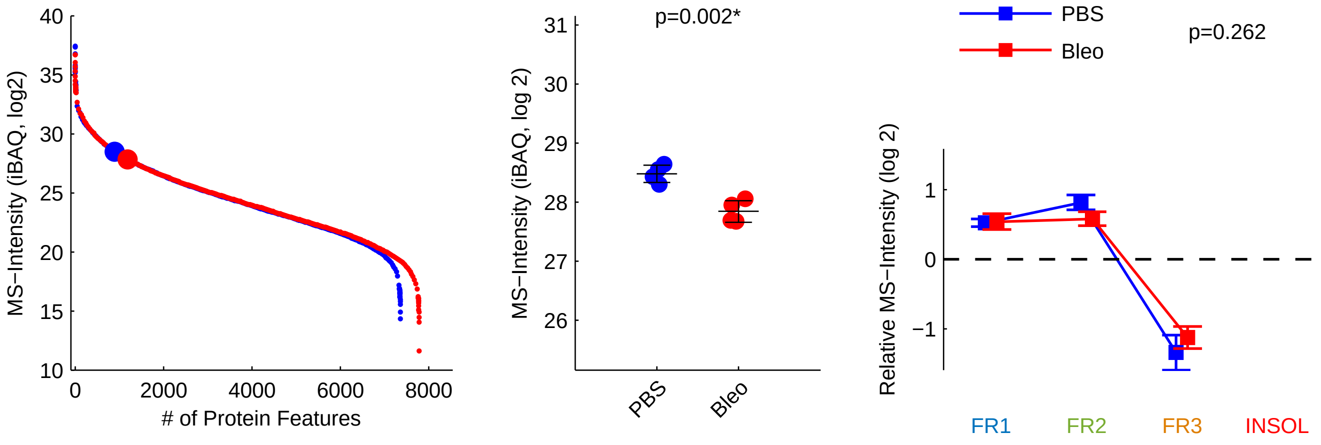

P63028 − Tpt1 (id: 3471)

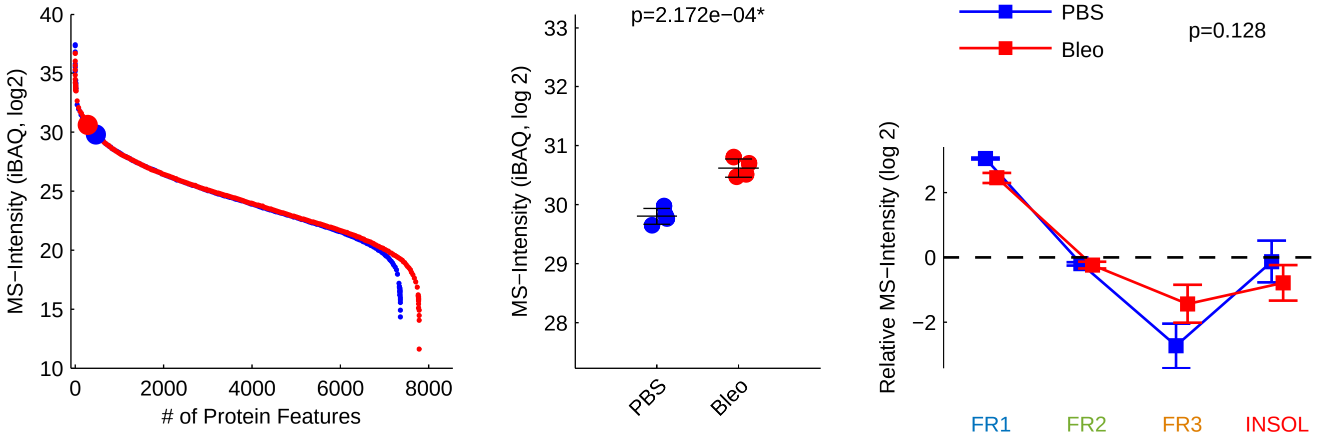

P63030 − Mpc1 (id: 3472)

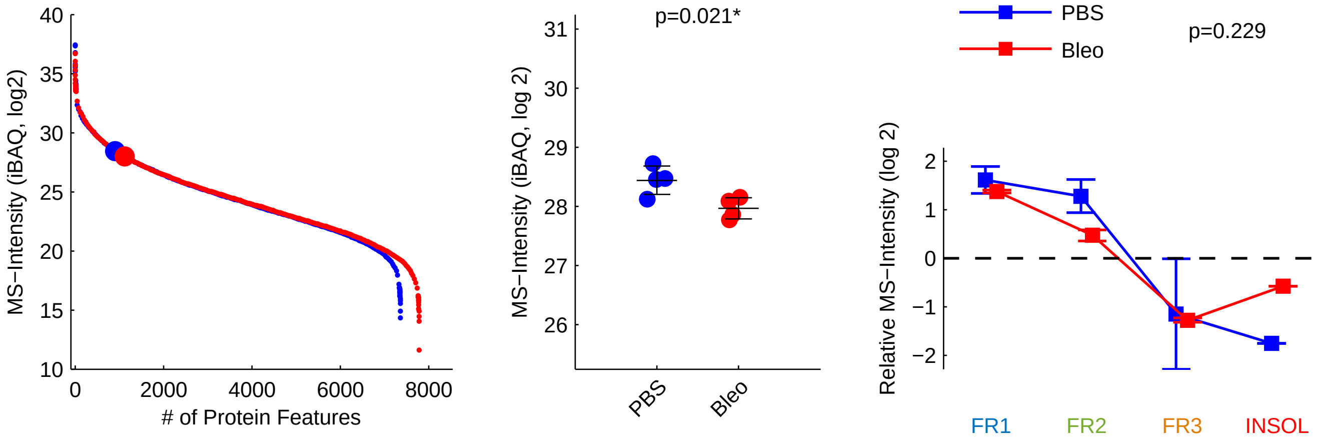

P63038 − Hspd1 (id: 3474)

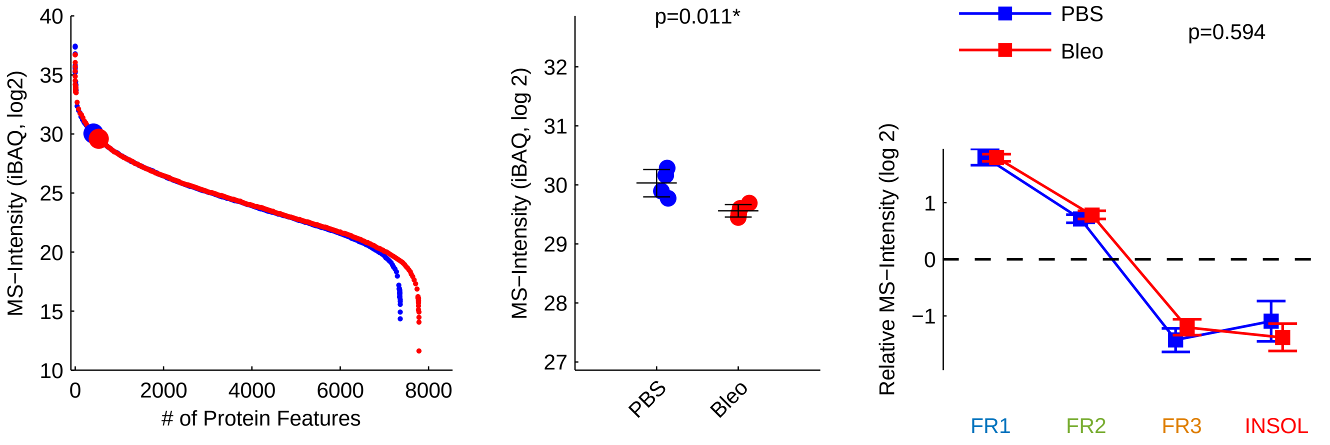

P63094 − Gnas (id: 3478)

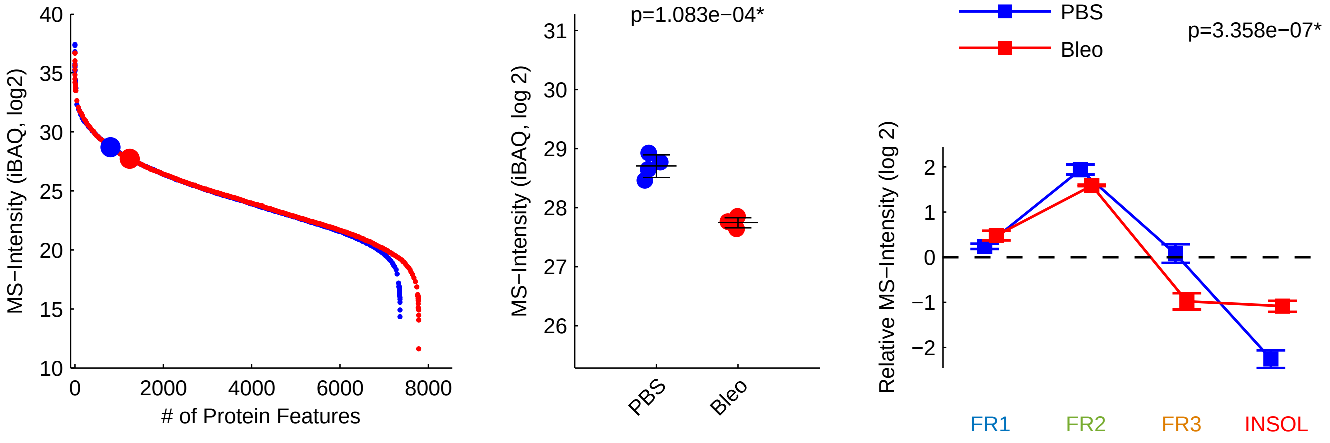

P63158 − Hmgb1 (id: 3483)

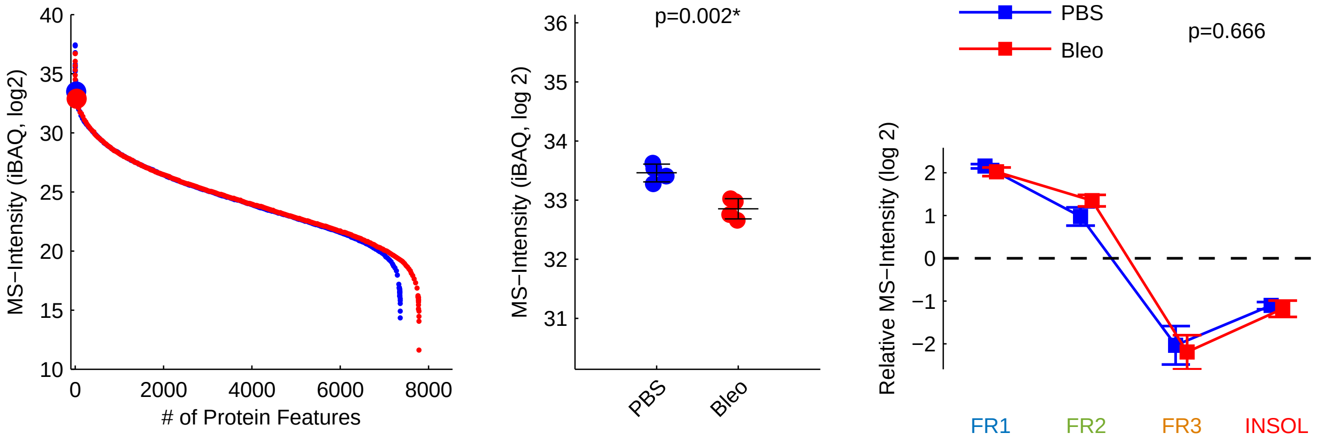

P63213 − Gng2 (id: 3486)

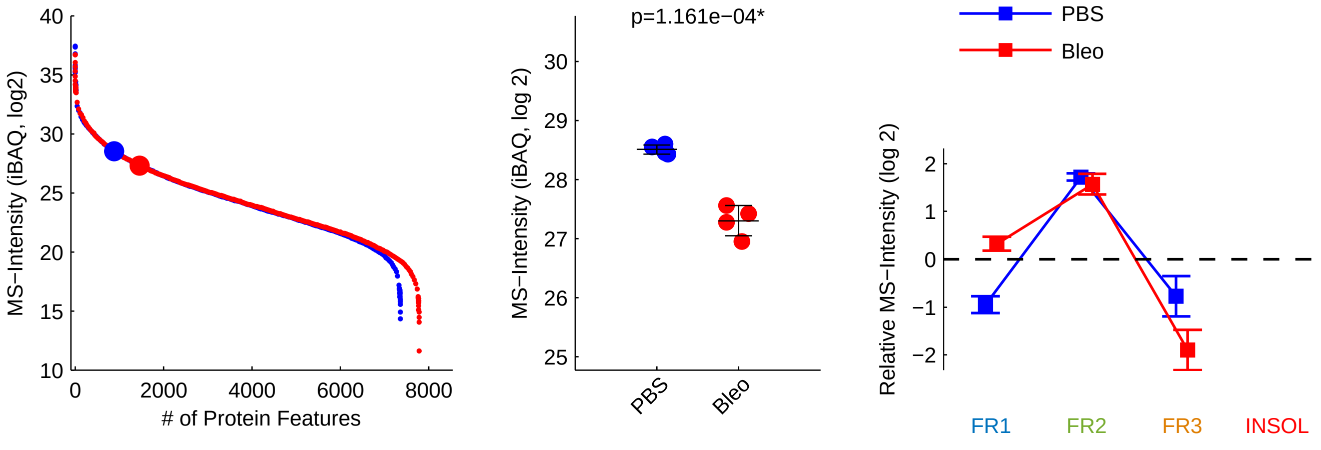

P63276 − Rps17 (id: 3492)

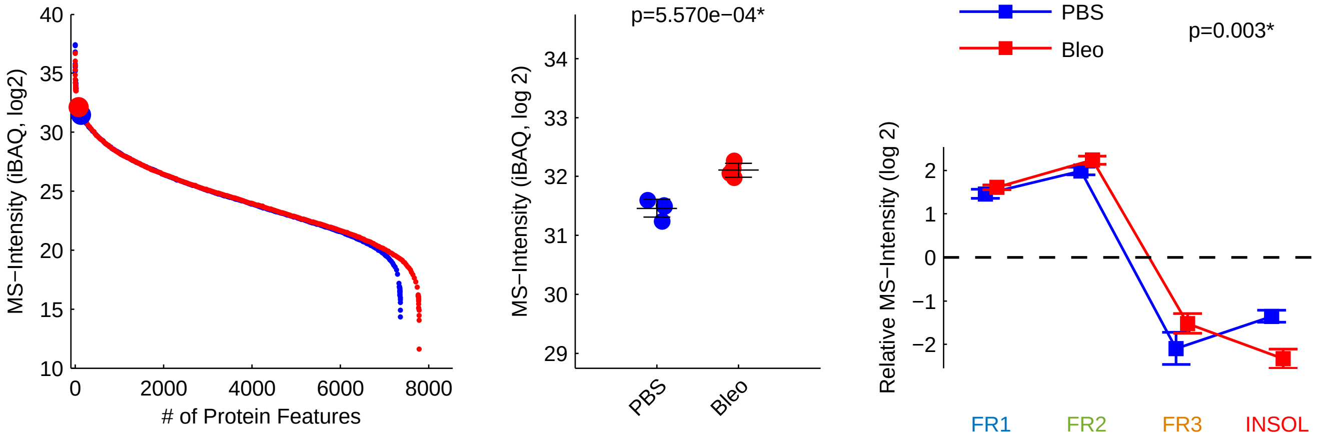

P63321 − Rala (id: 3495)

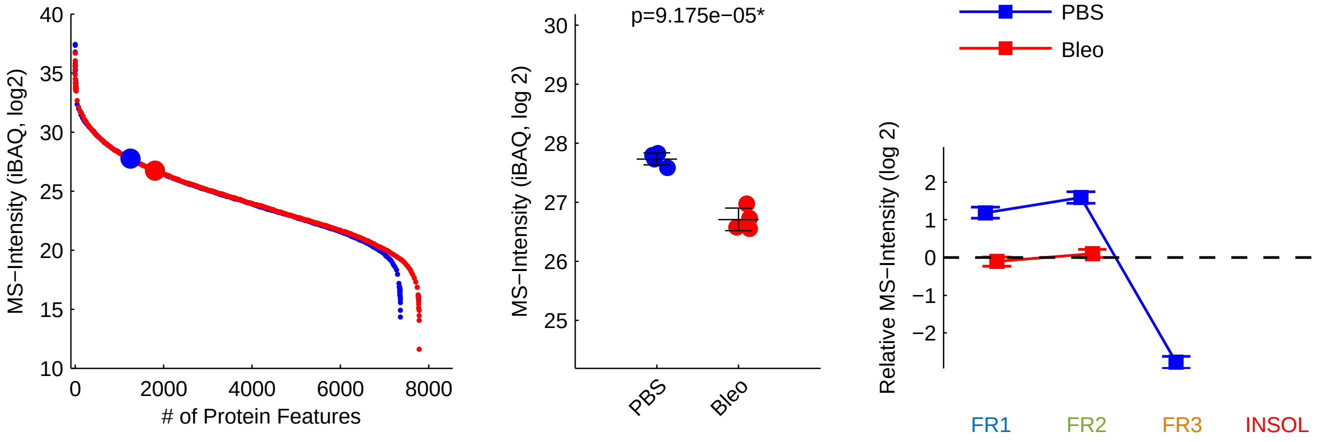

P63325 − Rps10 (id: 3496)

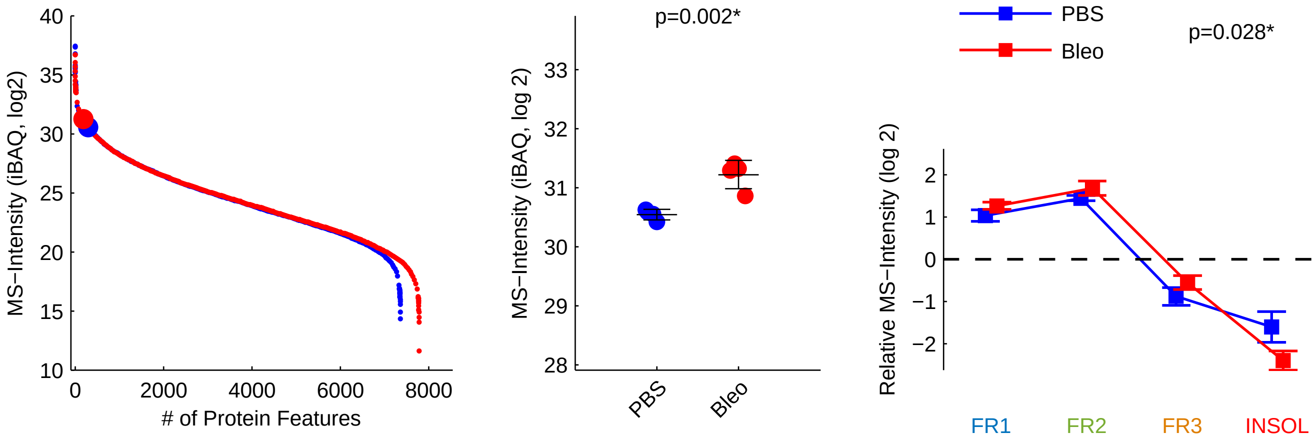

P63328−2 − Ppp3ca (id: 3497)

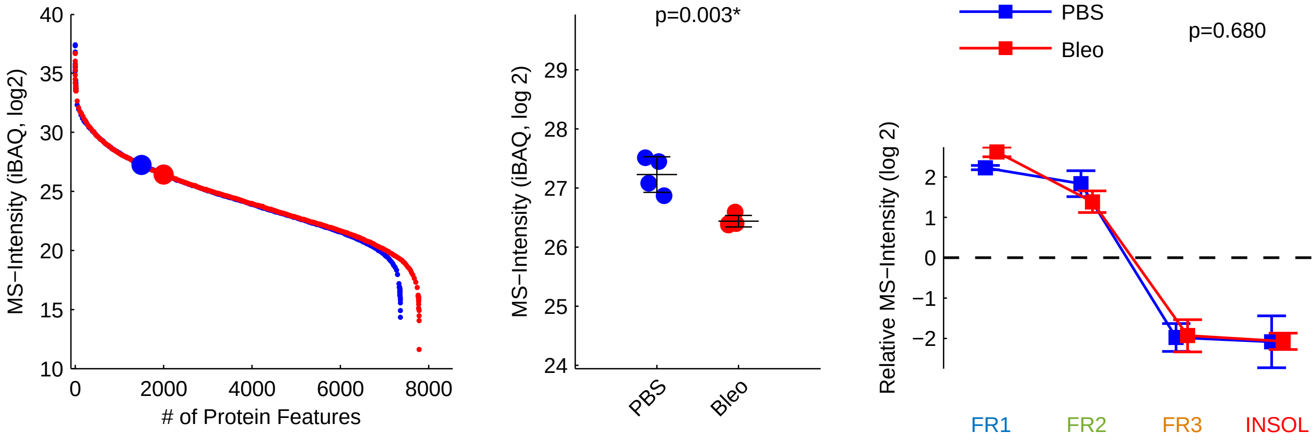

P67984 − Rpl22 (id: 3501)

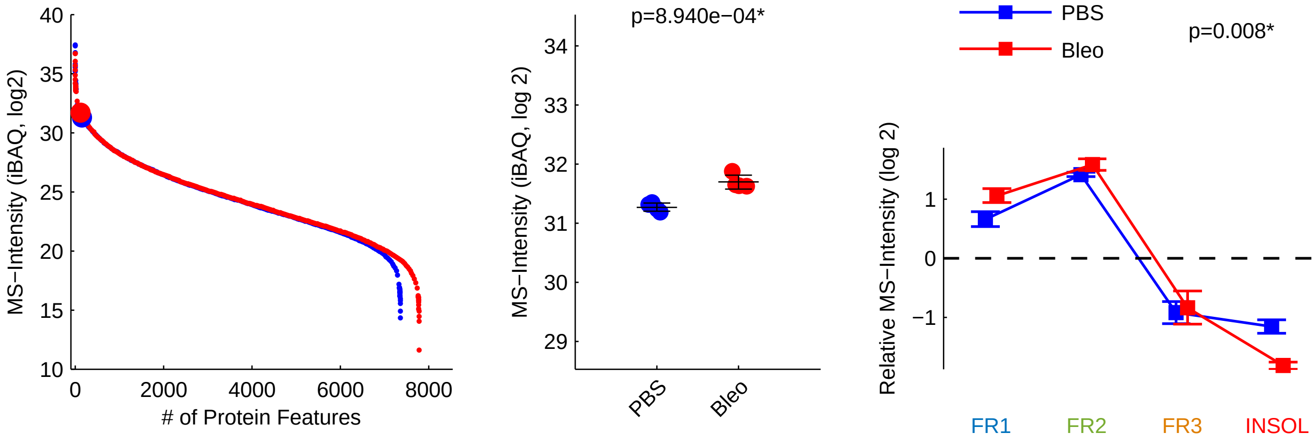

P68040 − Gnb2l1 (id: 3504)

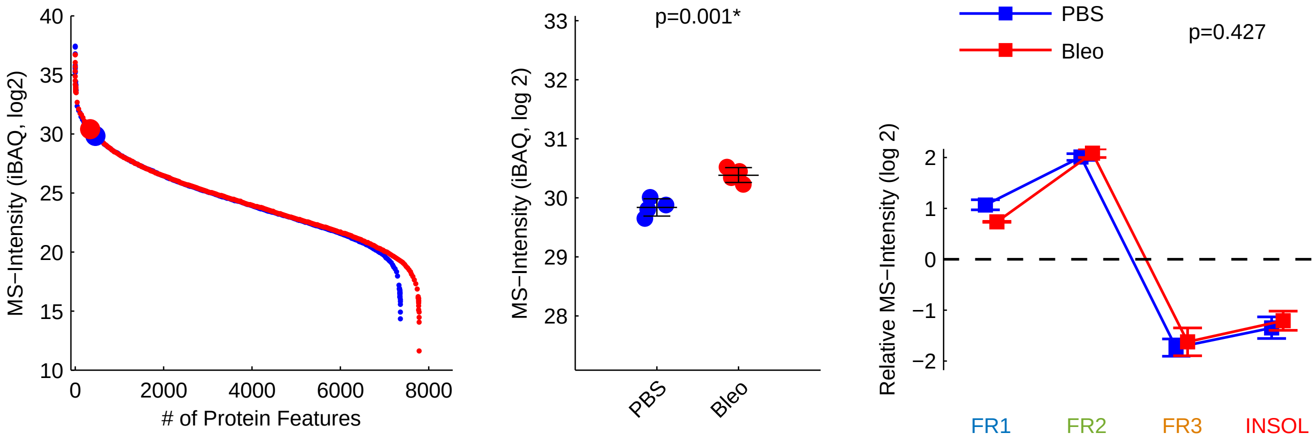

P68433 − Hist1h3a (id: 3512)

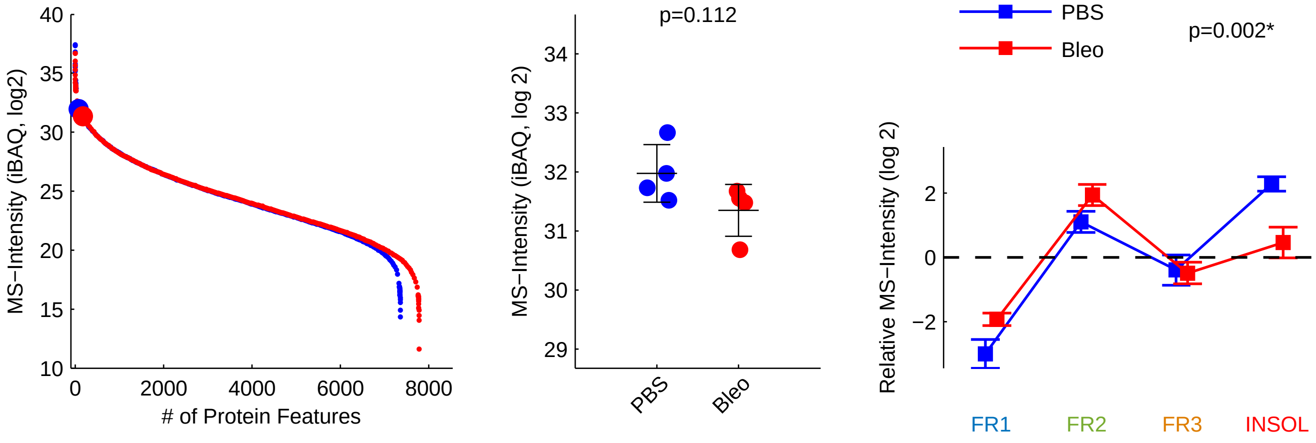

P70195 − Psmb7 (id: 3521)

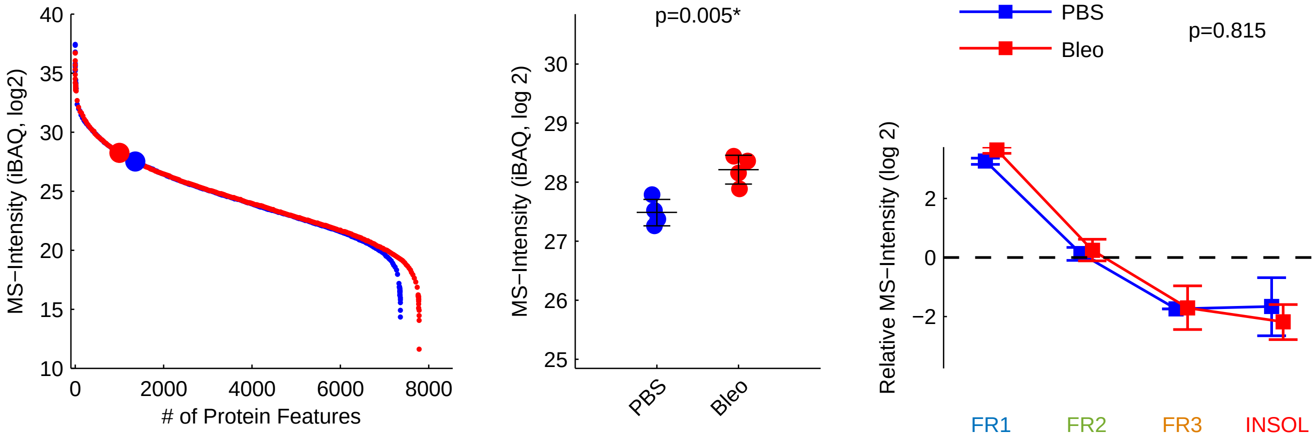

P70255−2 − Nfic (id: 3531)

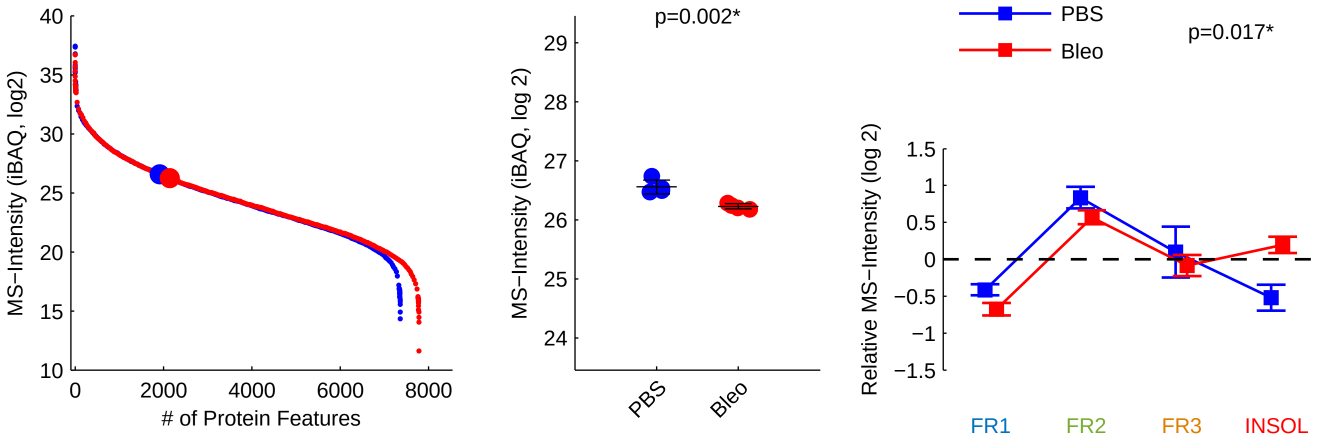

P70261 − Pald1 (id: 3533)

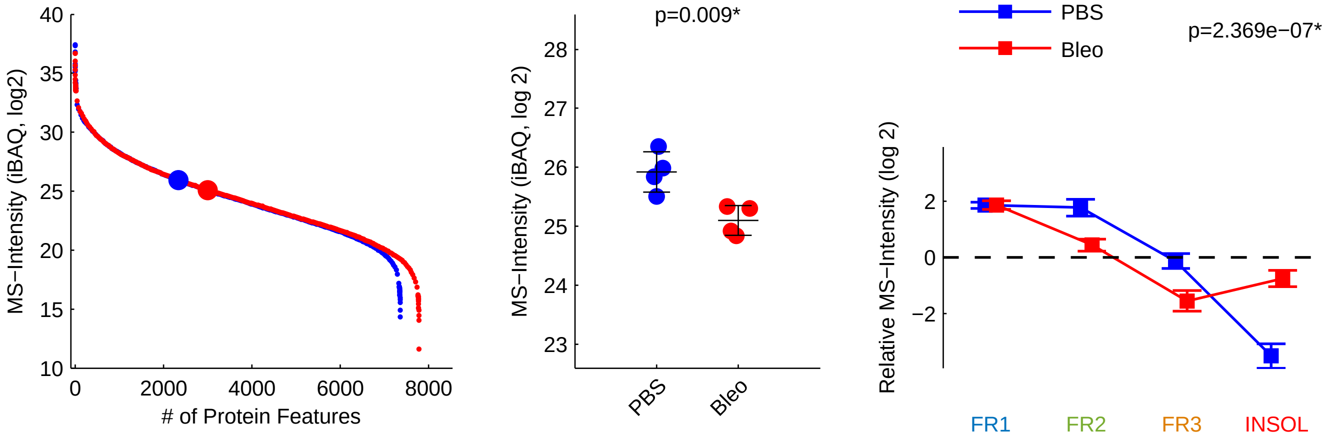

P70274 − Sepp1 (id: 3536)

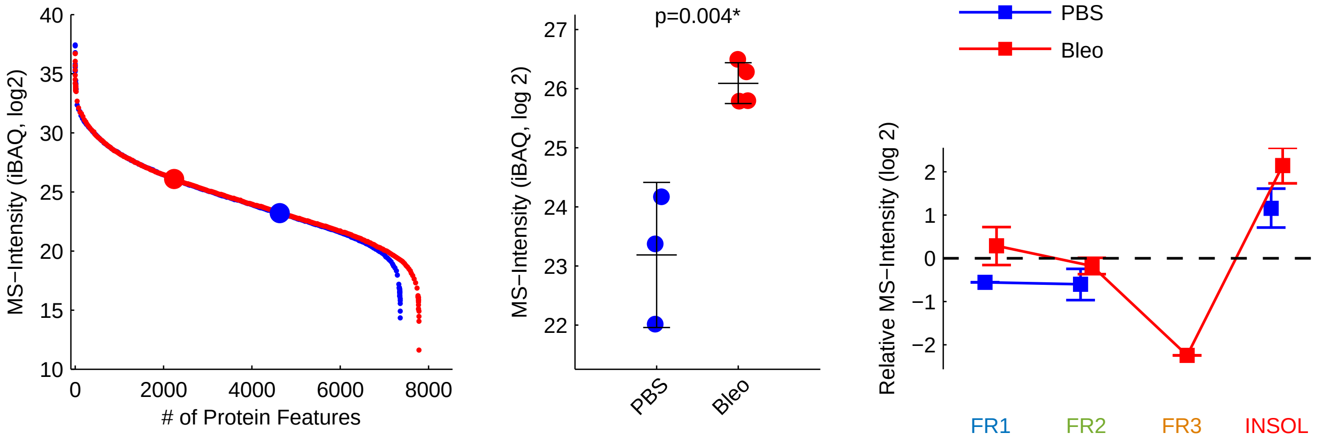

P70296 − Pebp1 (id: 3544)

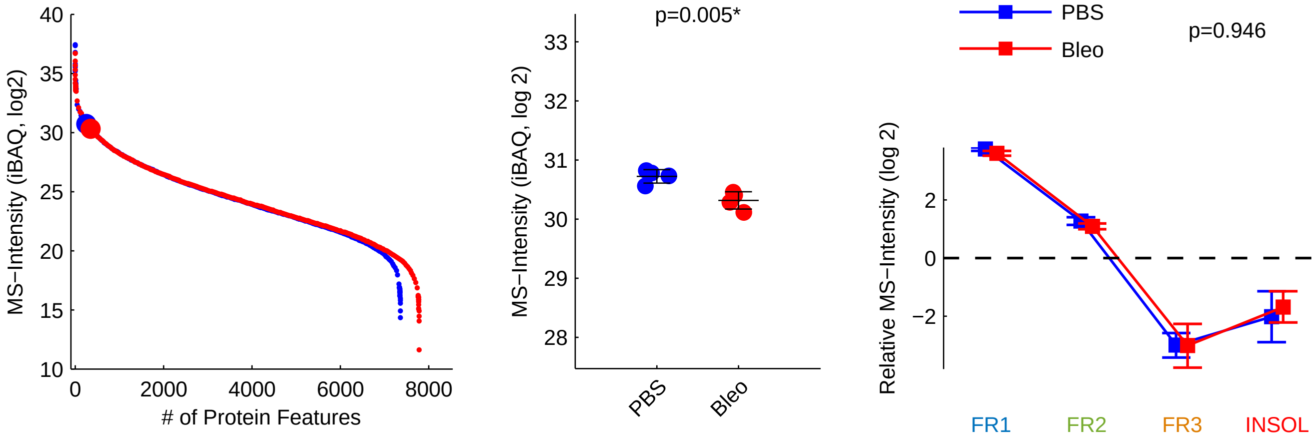

P70297 − Stam (id: 3545)

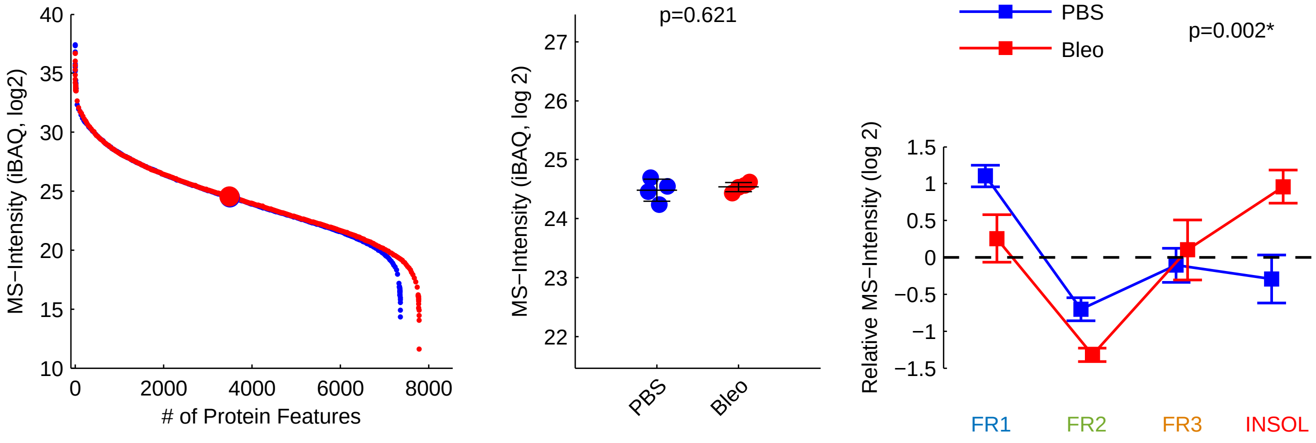

P70362 − Ufd1l (id: 3559)

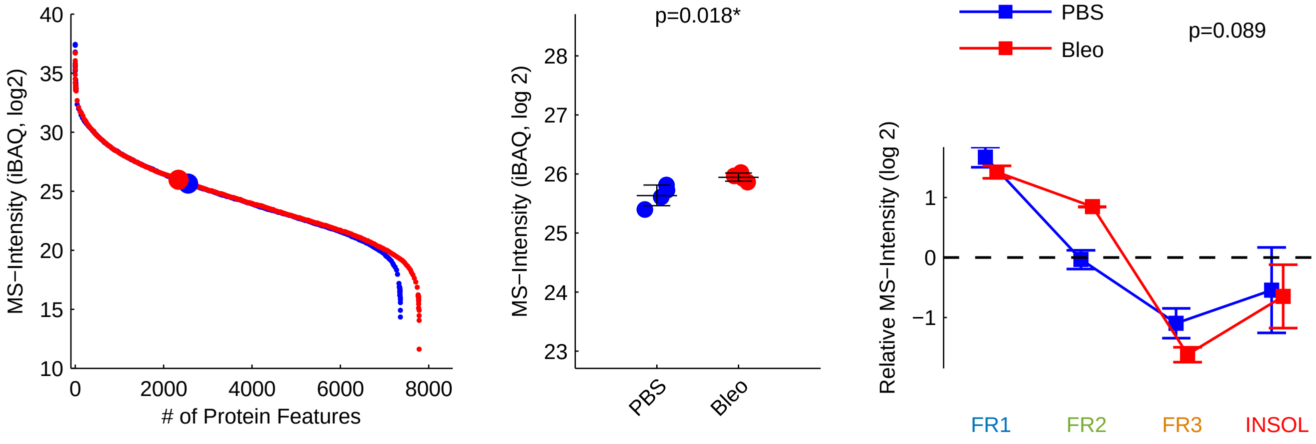

P70372 − Elavl1 (id: 3562)

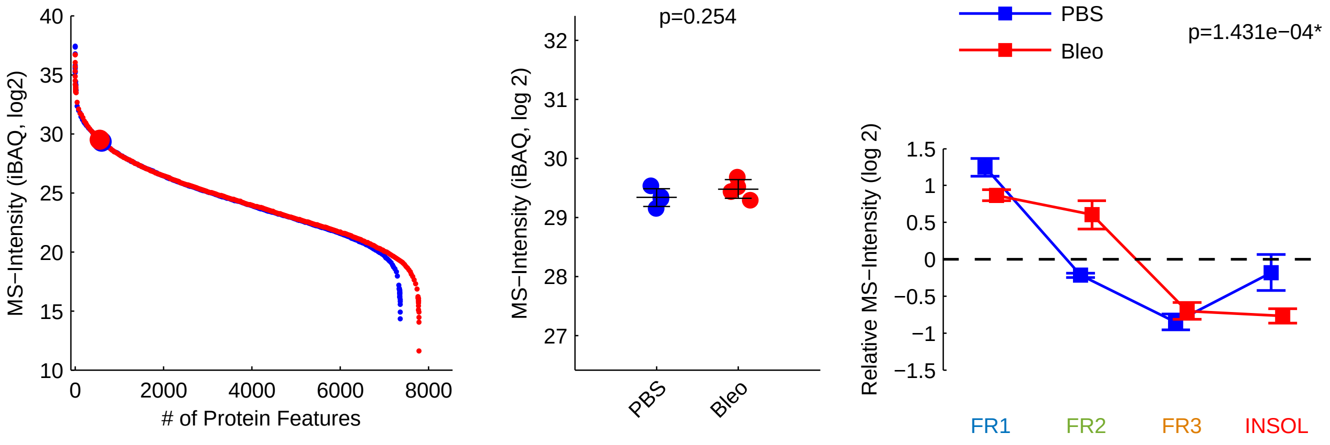

P70392 − Rasgrf2 (id: 3567)

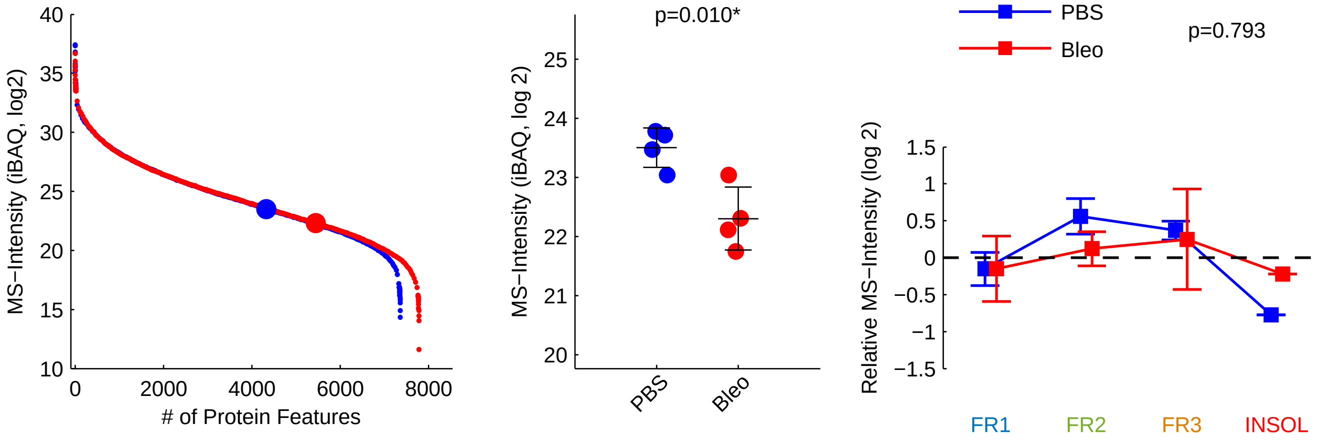

P70398 − Usp9x (id: 3568)

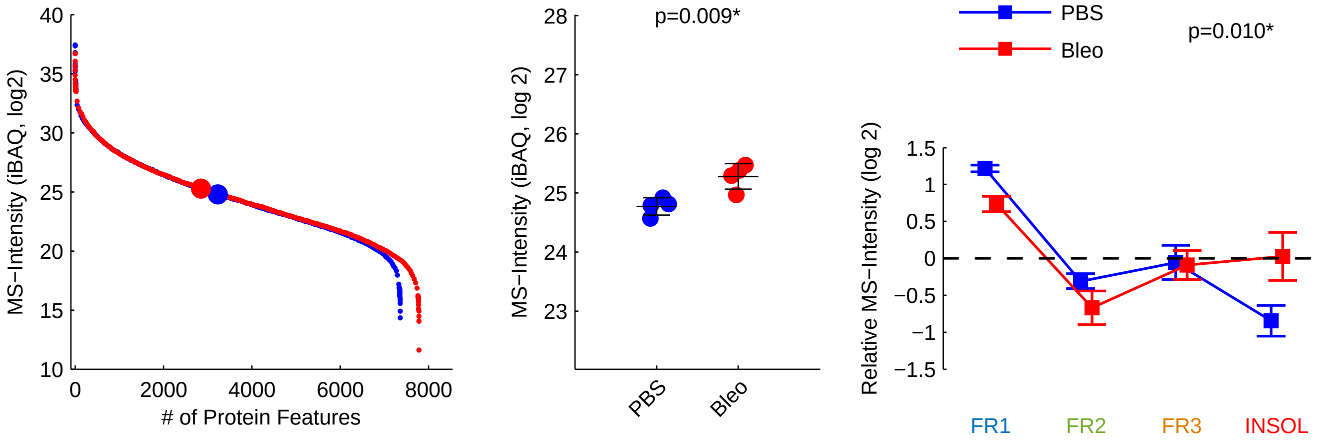

P70404 − Idh3g (id: 3570)

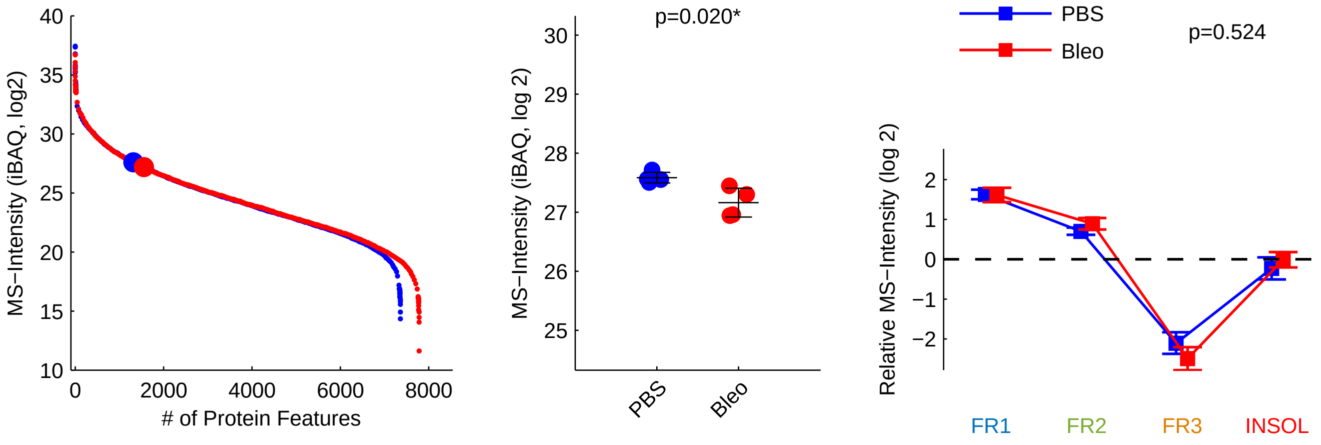

P70441 − Slc9a3r1 (id: 3574)

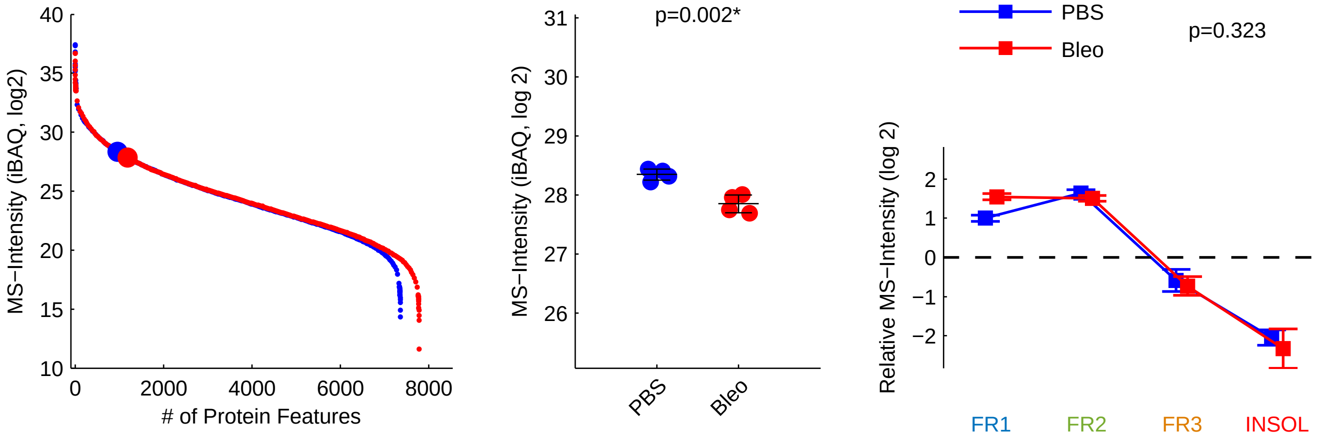

P70452 − Stx4 (id: 3578)

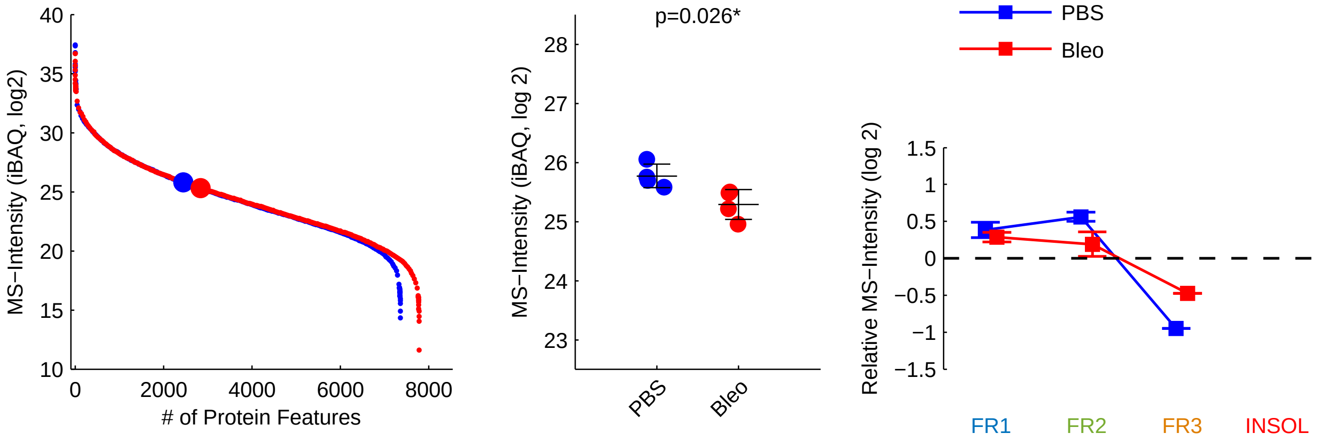

P70663 − Sparcl1 (id: 3582)

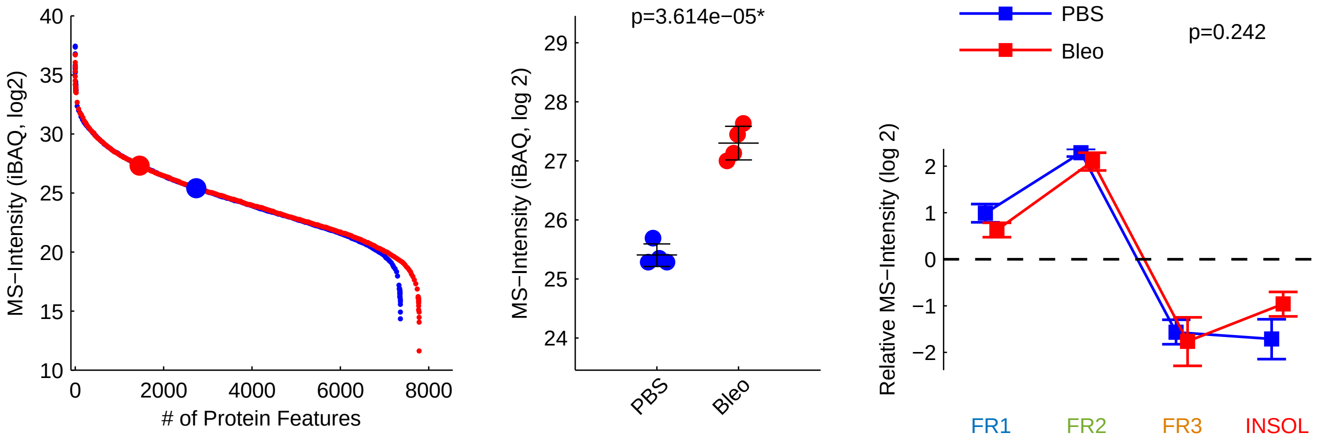

P70670 − Naca (id: 3583)

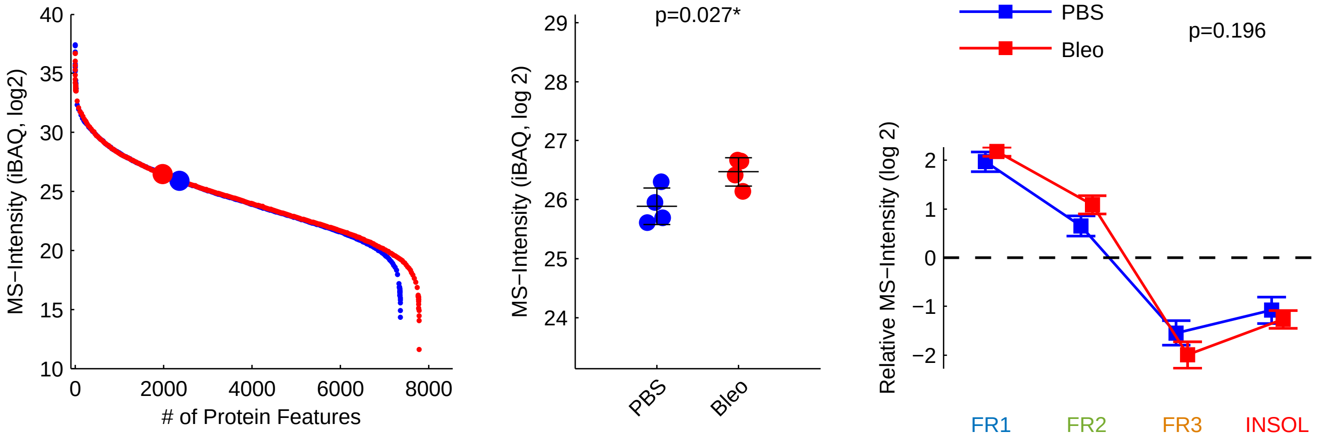

P70677 − Casp3 (id: 3585)

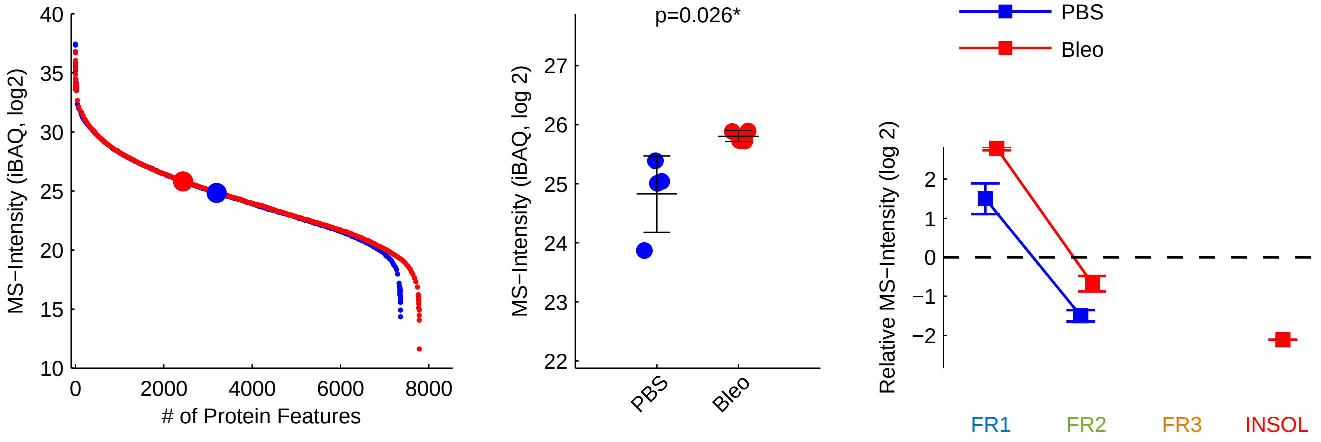

P70697 − Urod (id: 3588)

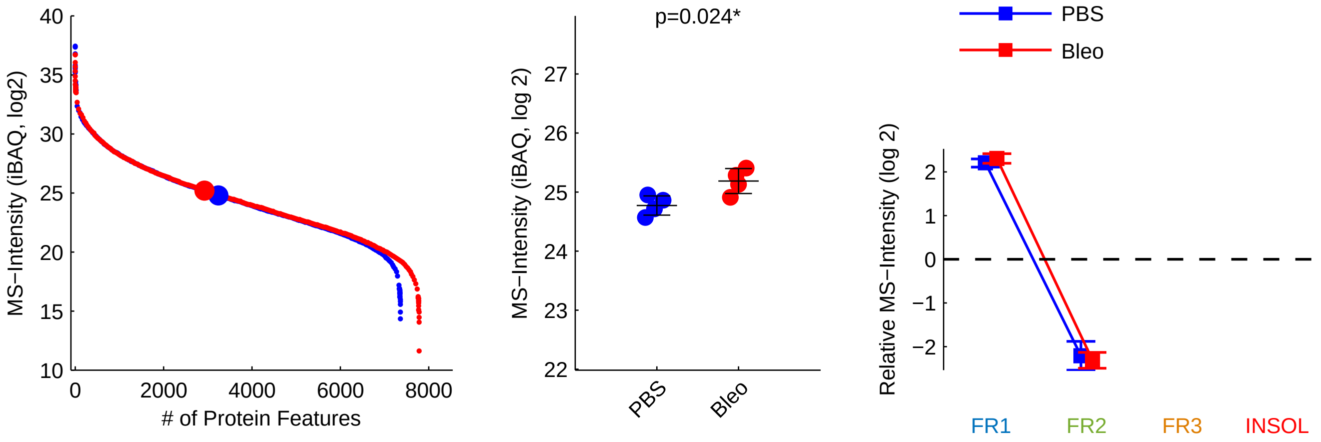

P70698 − Ctps1 (id: 3589)

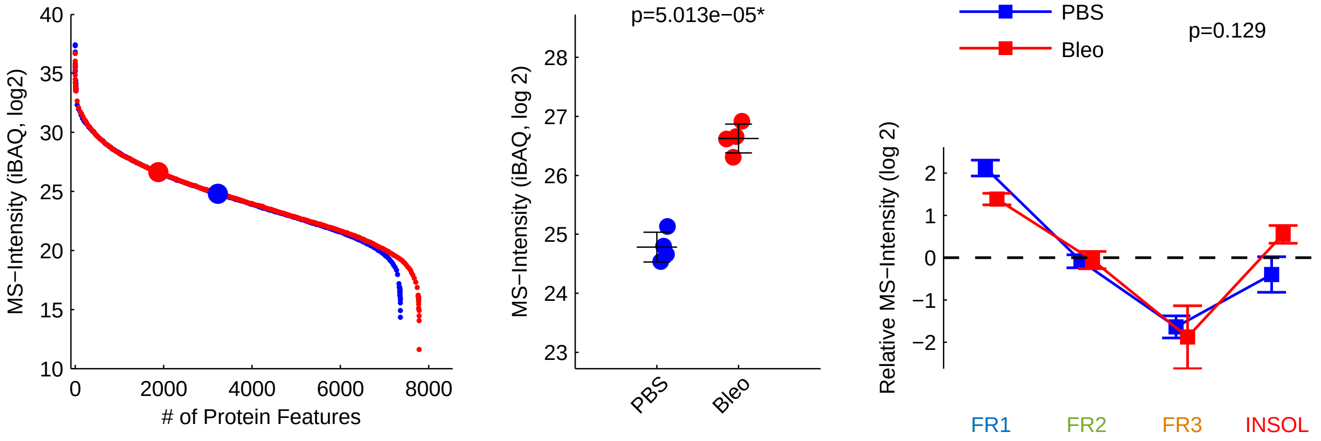

P70699 − Gaa (id: 3590)

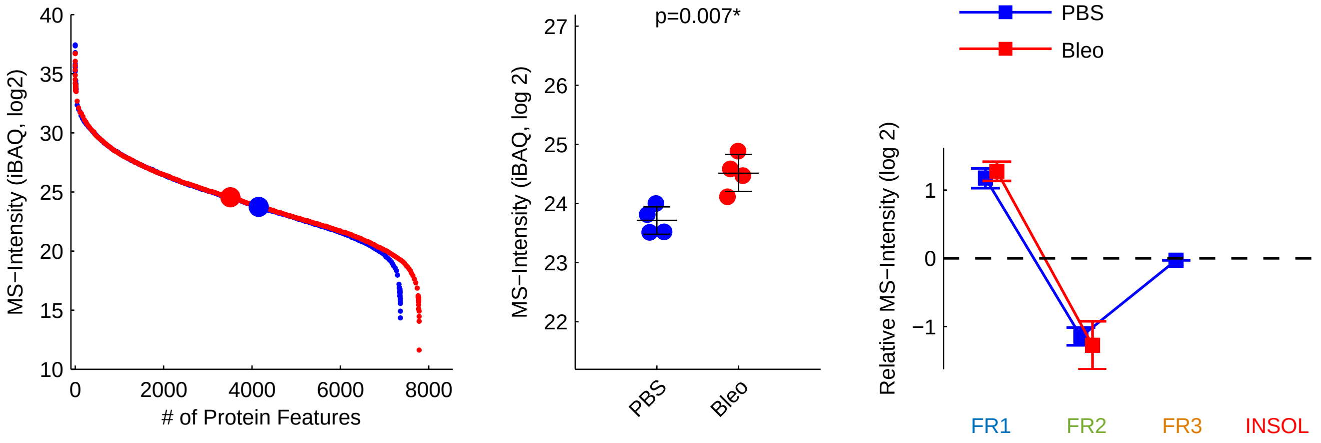

P80313 − Cct7 (id: 3592)

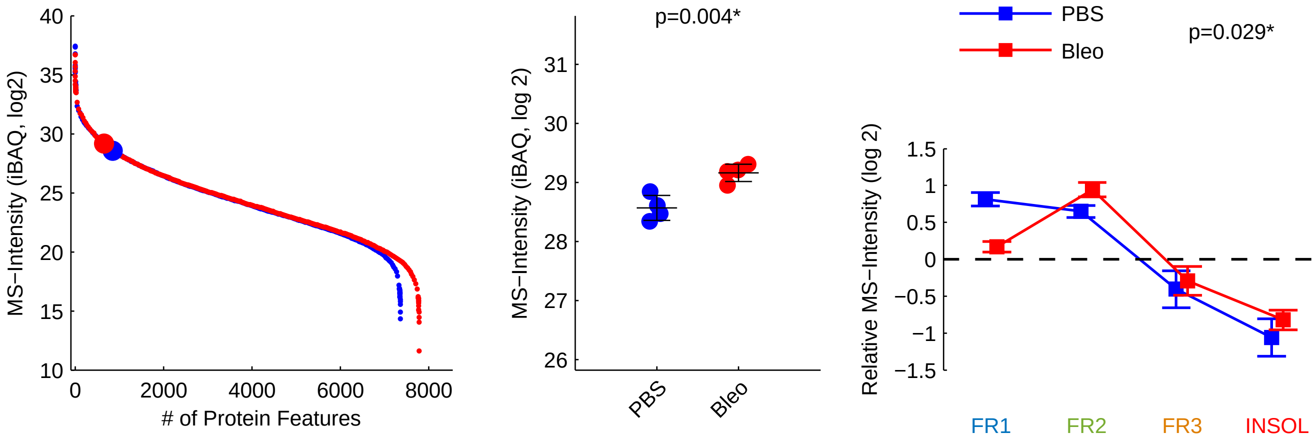

P80314 − Cct2 (id: 3593)

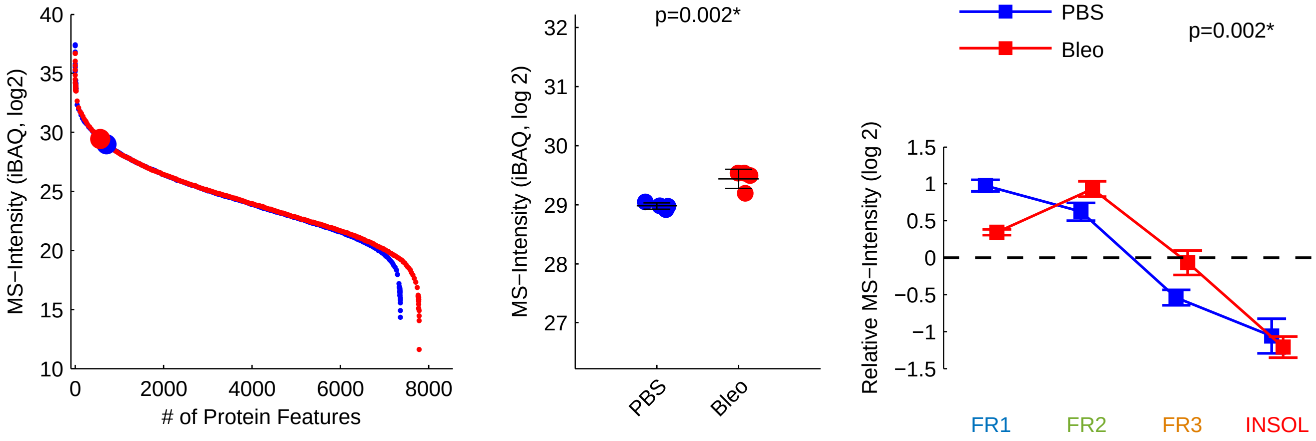

P80315 − Cct4 (id: 3594)

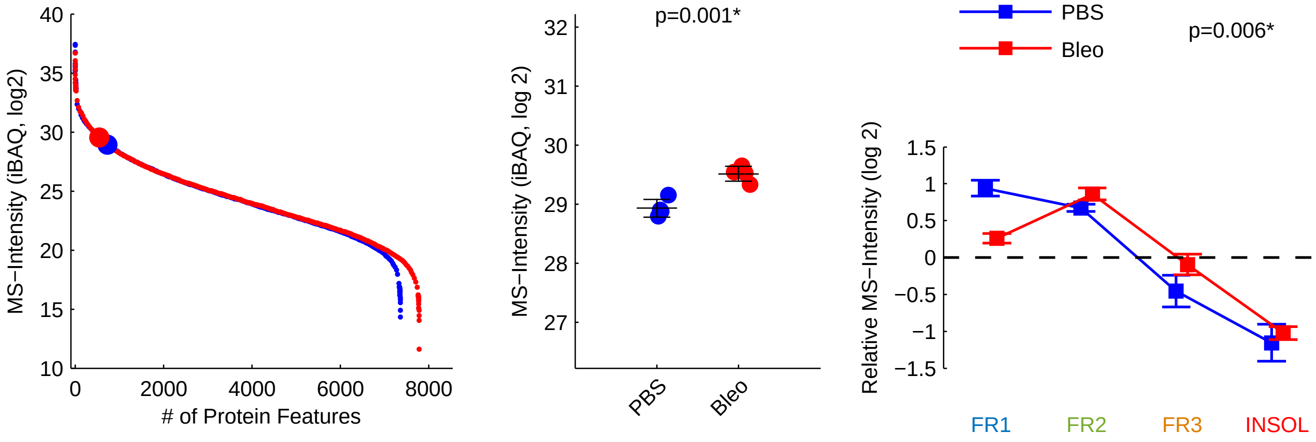

P80316 − Cct5 (id: 3595)

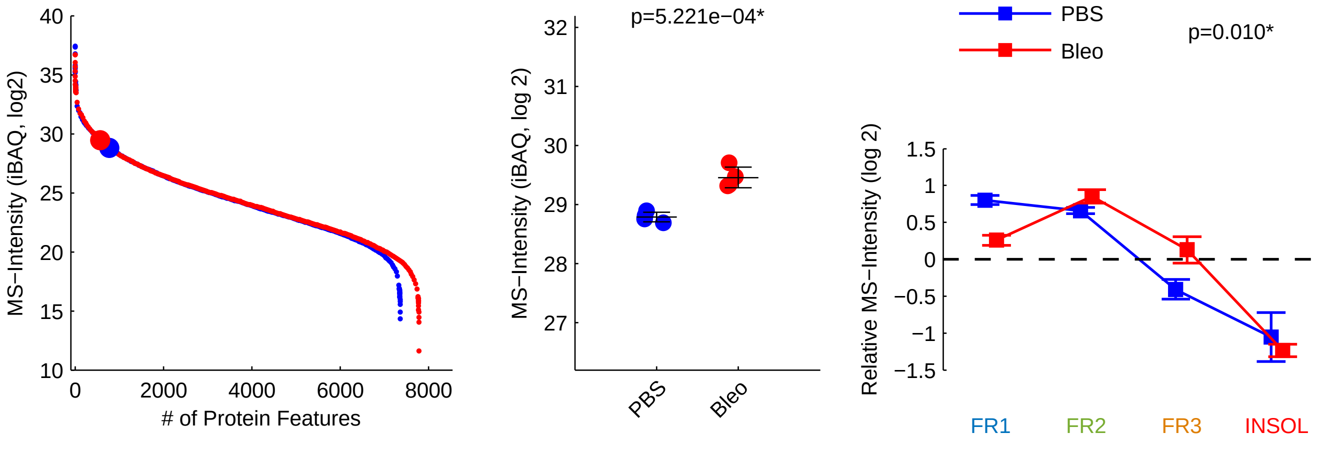

P80317 − Cct6a (id: 3596)

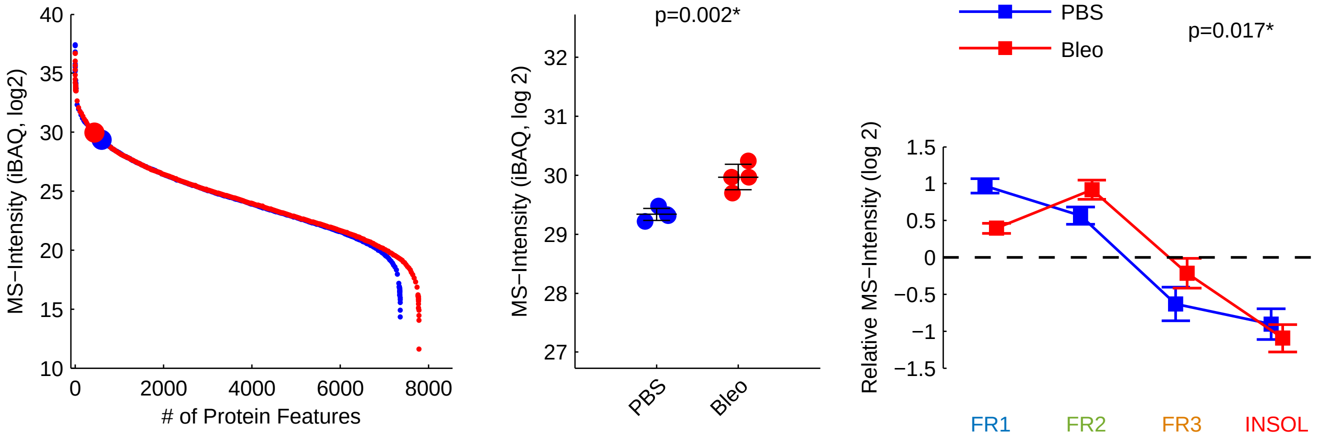

P80318 − Cct3 (id: 3597)

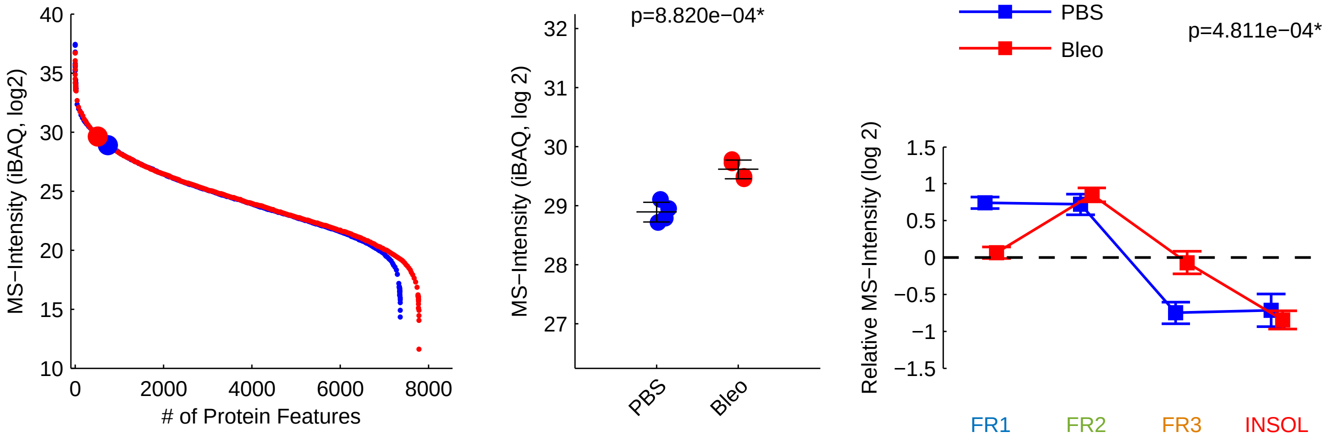

P81117 − Nucb2 (id: 3599)

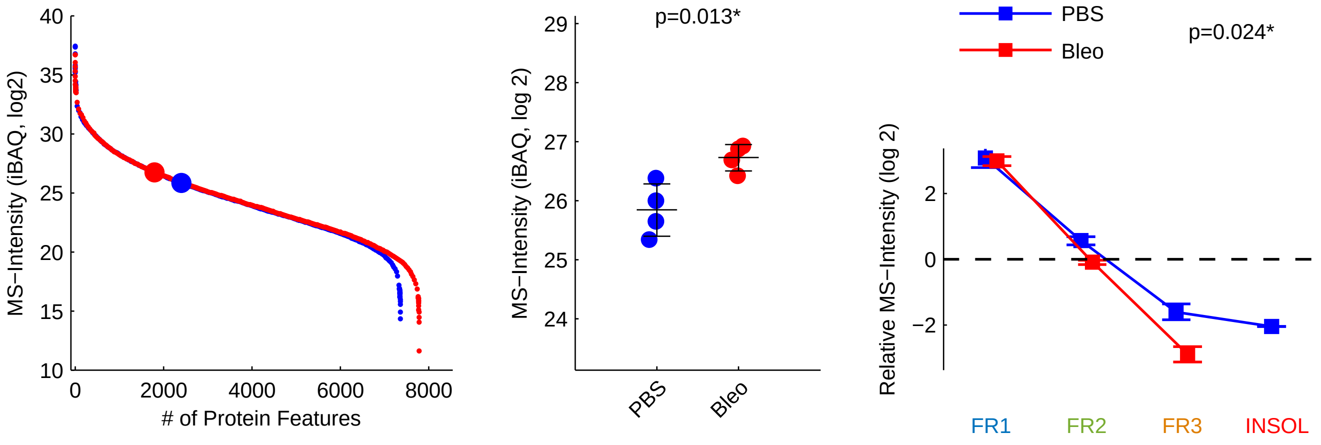

P82198 − Tgfbi (id: 3600)

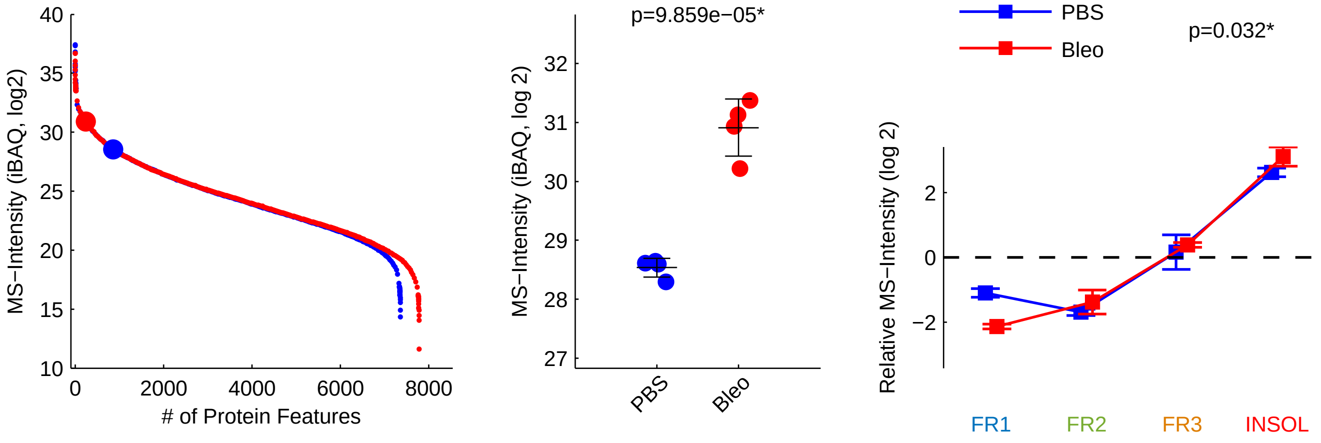

P82347 − Sgcd (id: 3602)

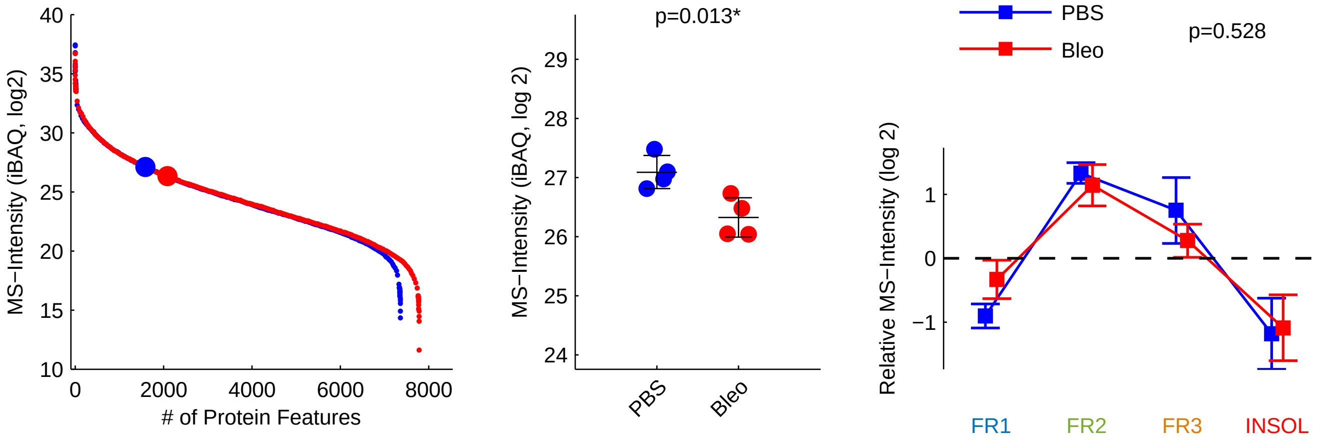

P82349 − Sgcb (id: 3603)

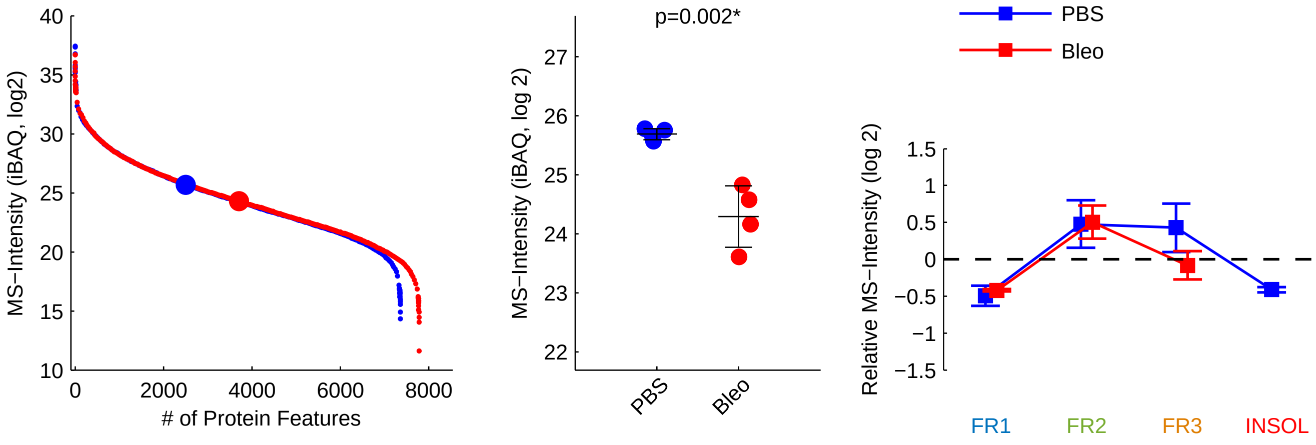

P83741 − Wnk1 (id: 3605)

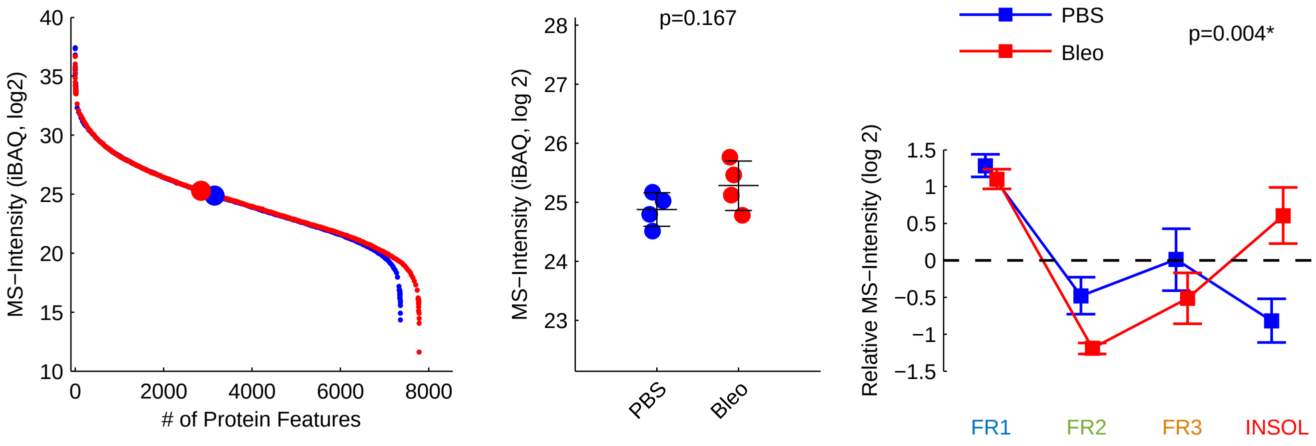

P83882 − Rpl36a (id: 3608)

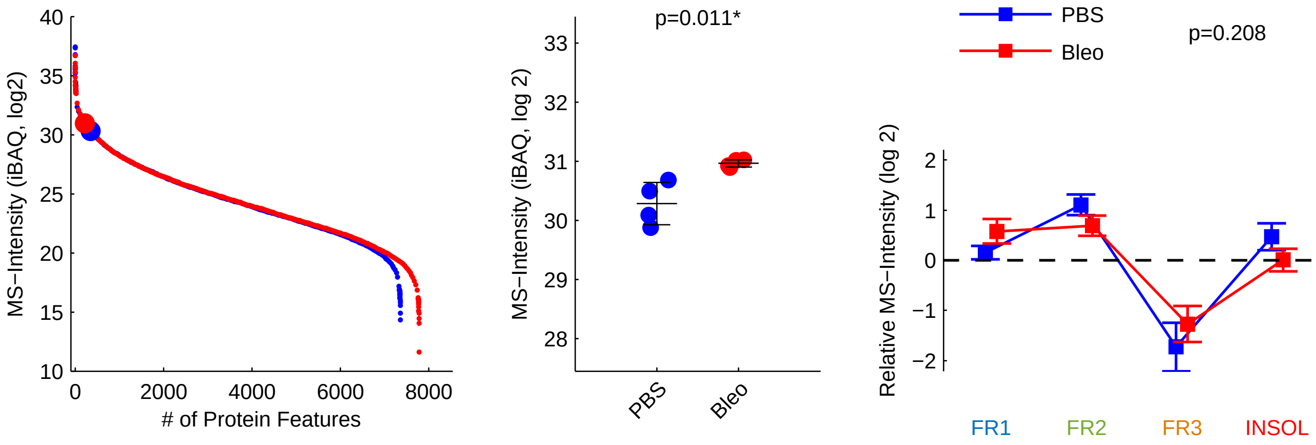

P84078 − Arf1 (id: 3612)

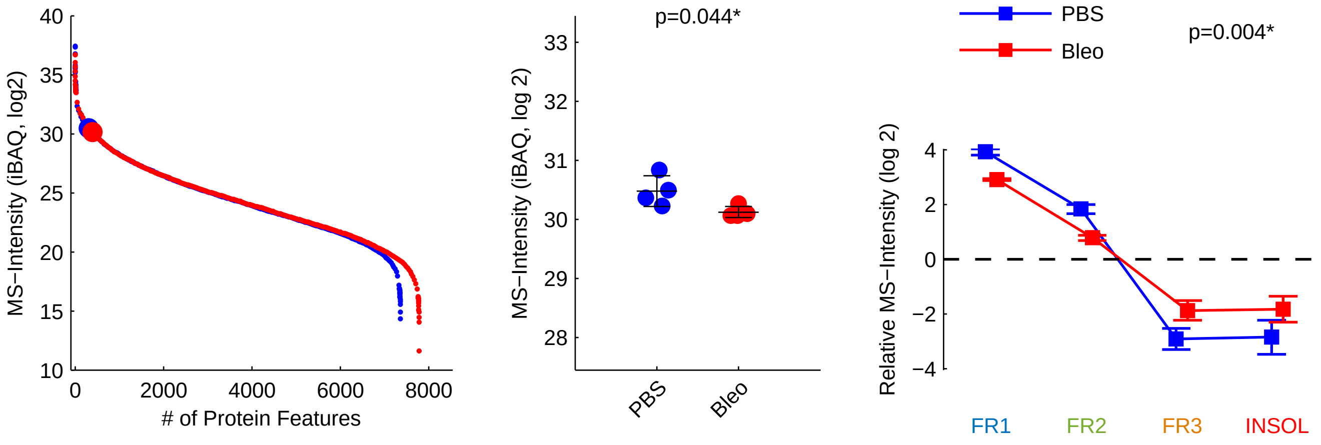

P84089 − Erh (id: 3614)

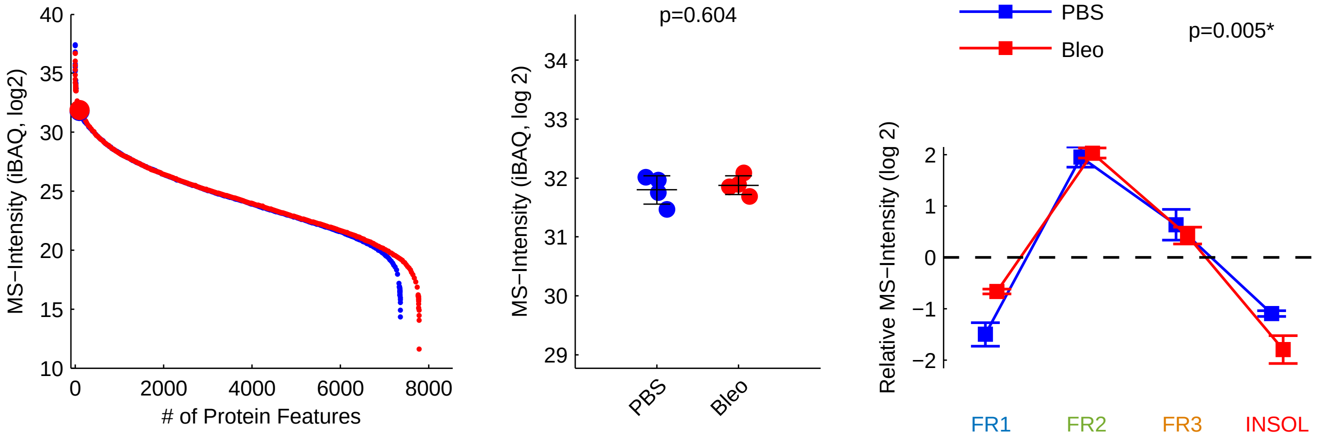

P84096 − Rhog (id: 3616)

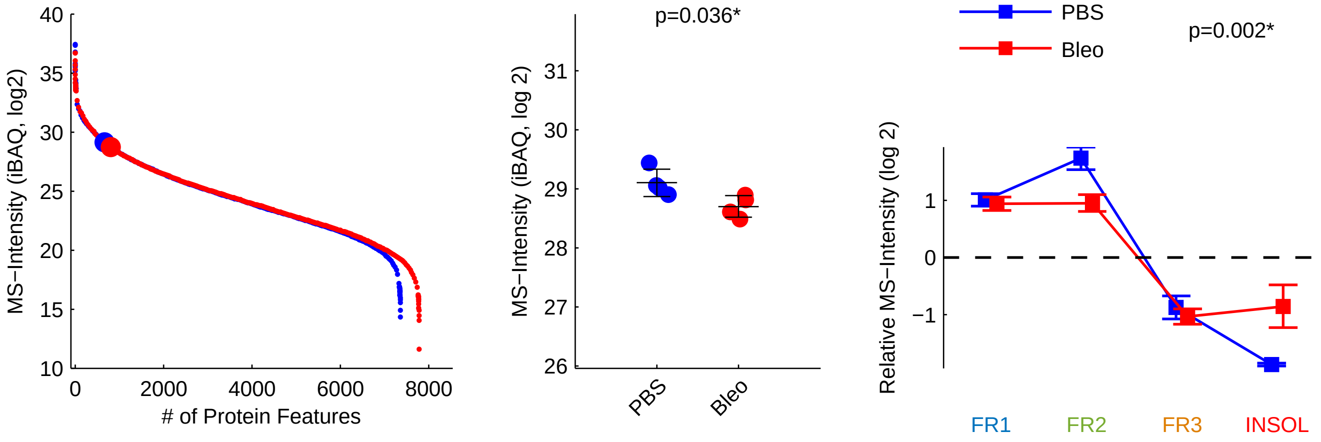

P84099 − Rpl19 (id: 3617)

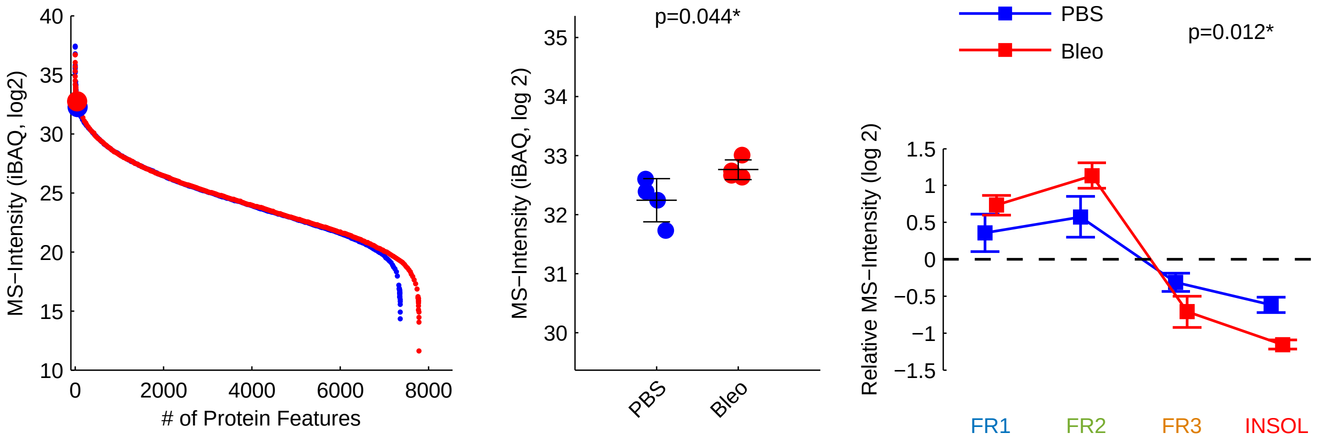

P84228 − Hist1h3b (id: 3620)

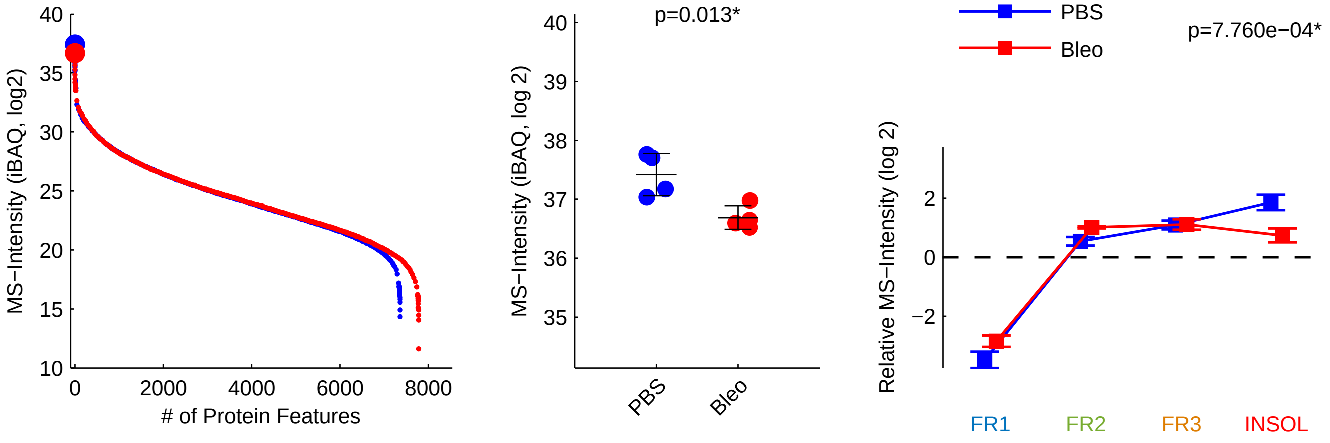

P84244 − H3f3a (id: 3621)

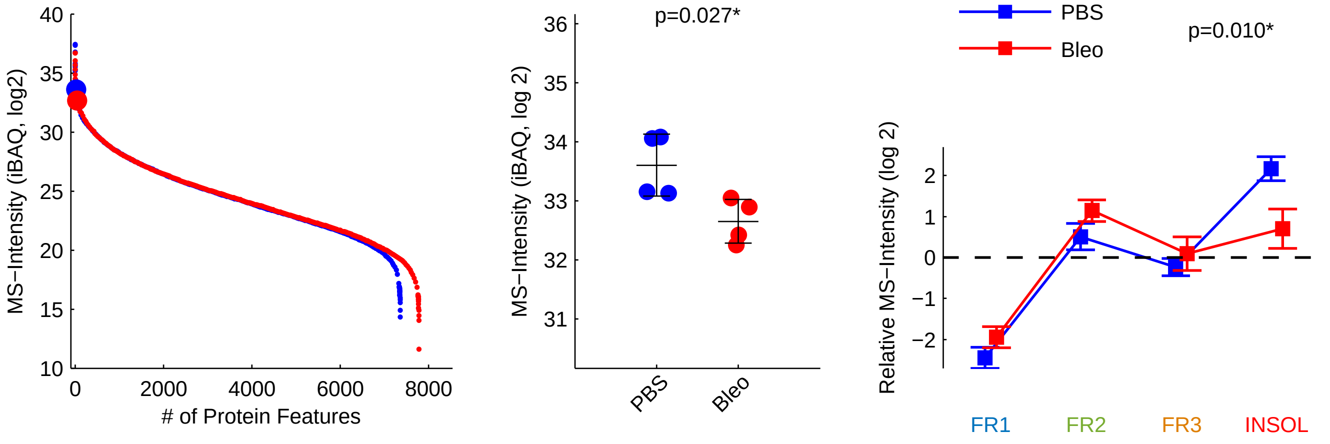

P97290 − Serping1 (id: 3625)

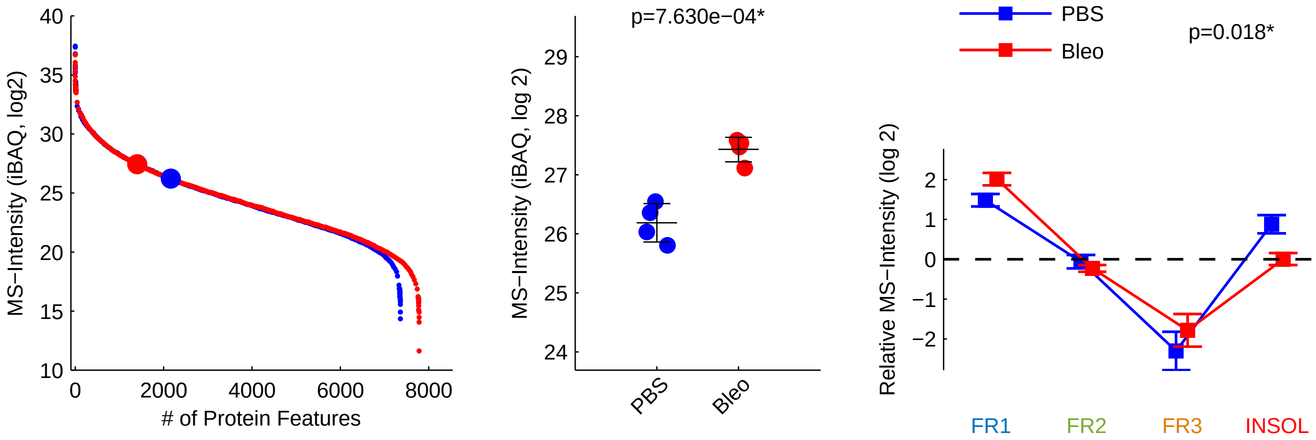

P97298 − Serpinf1 (id: 3626)

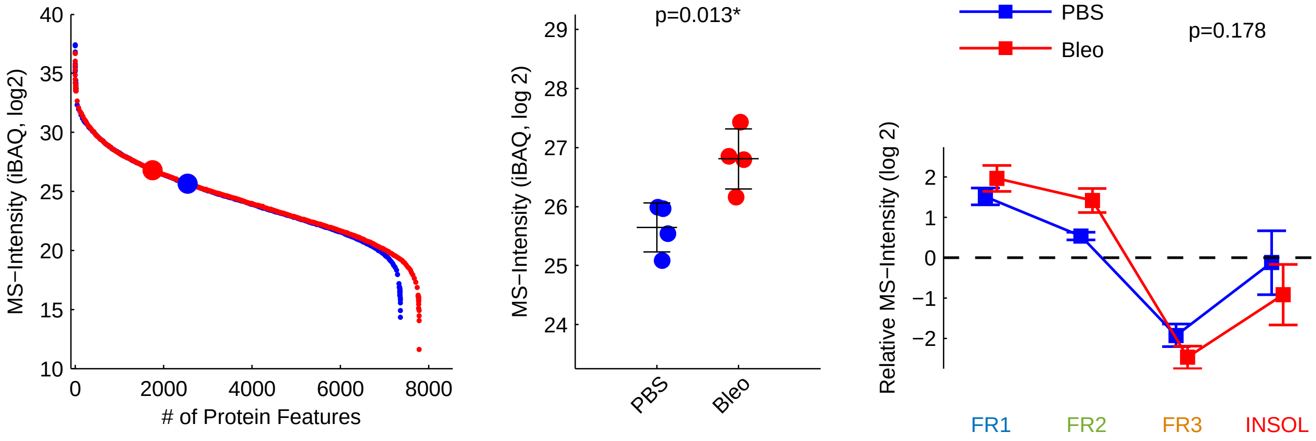

P97314 − Csrp2 (id: 3632)

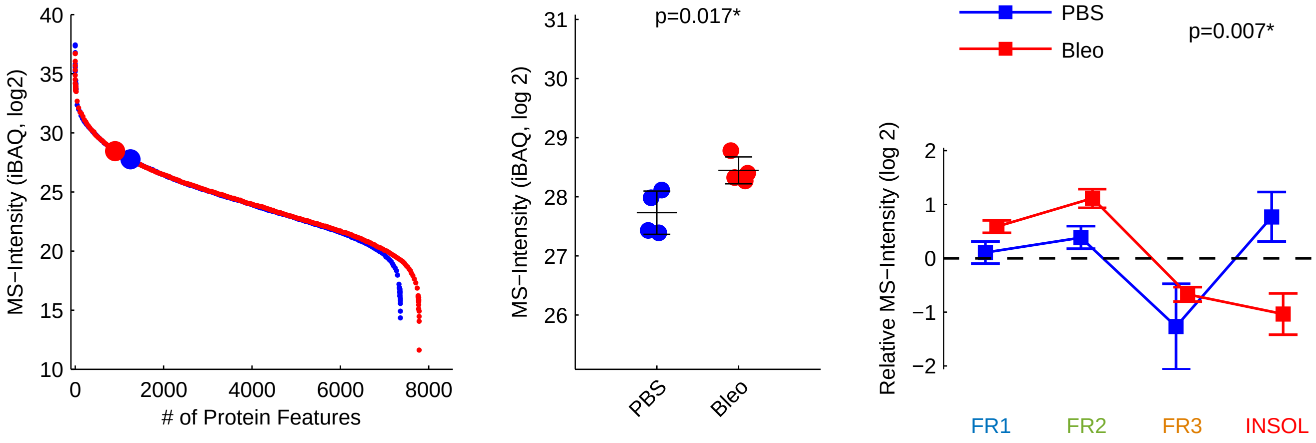

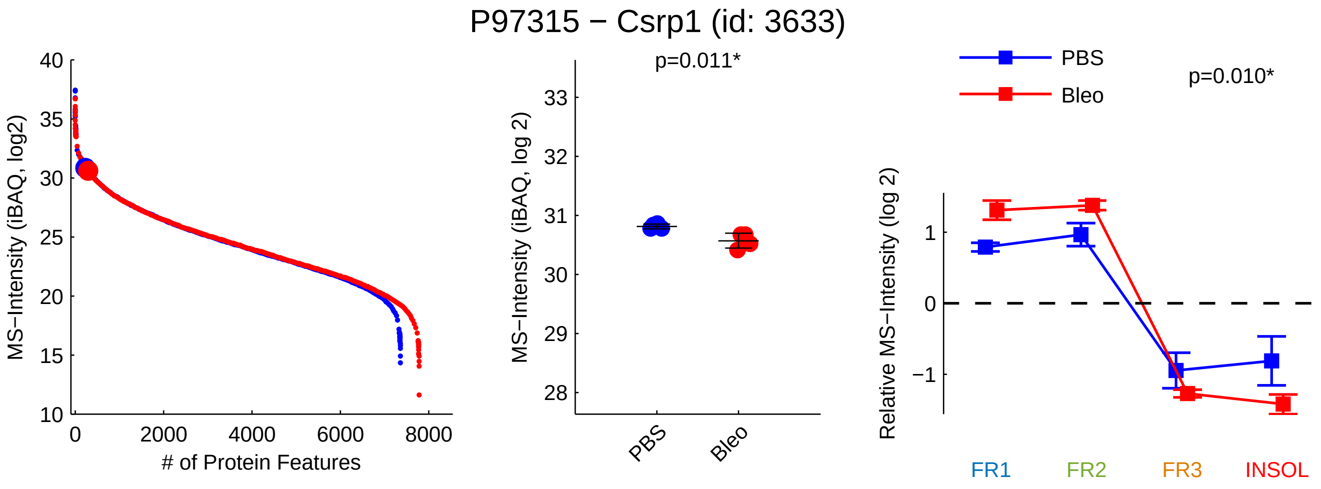
P97315 − Csrp1 (id: 3633)
MS−Intensity (iBAQ, log2)
# of Protein Features
p=0.011*
MS−Intensity (iBAQ, log 2)
PBS
Bleo
p=0.010*
Relative MS−Intensity (log 2)
FR1
FR2
FR3
INSOL

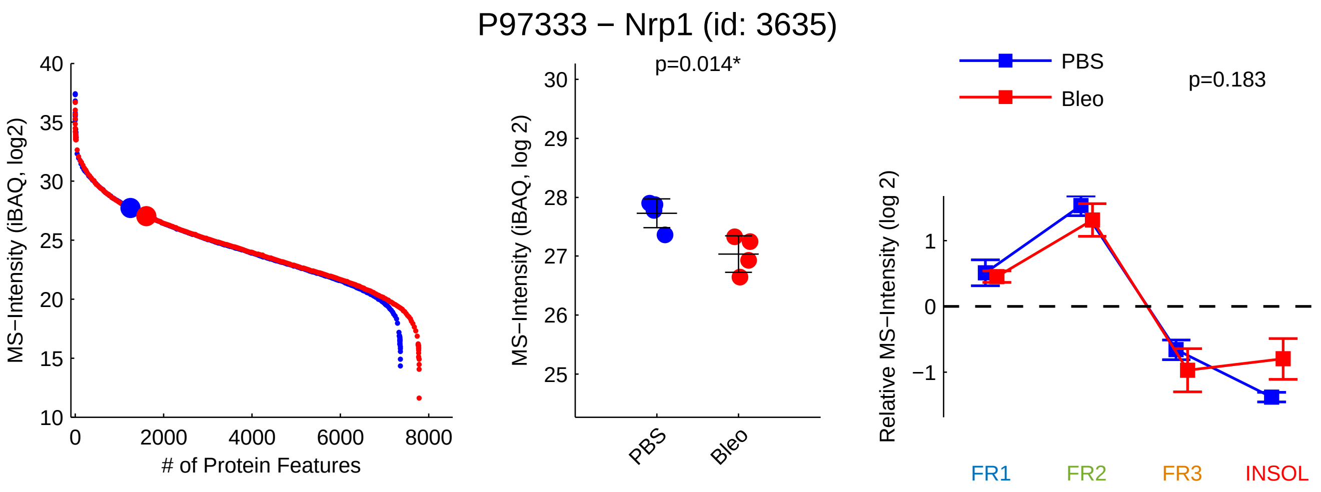
P97333 − Nrp1 (id: 3635)
MS−Intensity (iBAQ, log2)
# of Protein Features
p=0.014*
MS−Intensity (iBAQ, log 2)
PBS
Bleo
p=0.183
Relative MS−Intensity (log 2)
FR1
FR2
FR3
INSOL

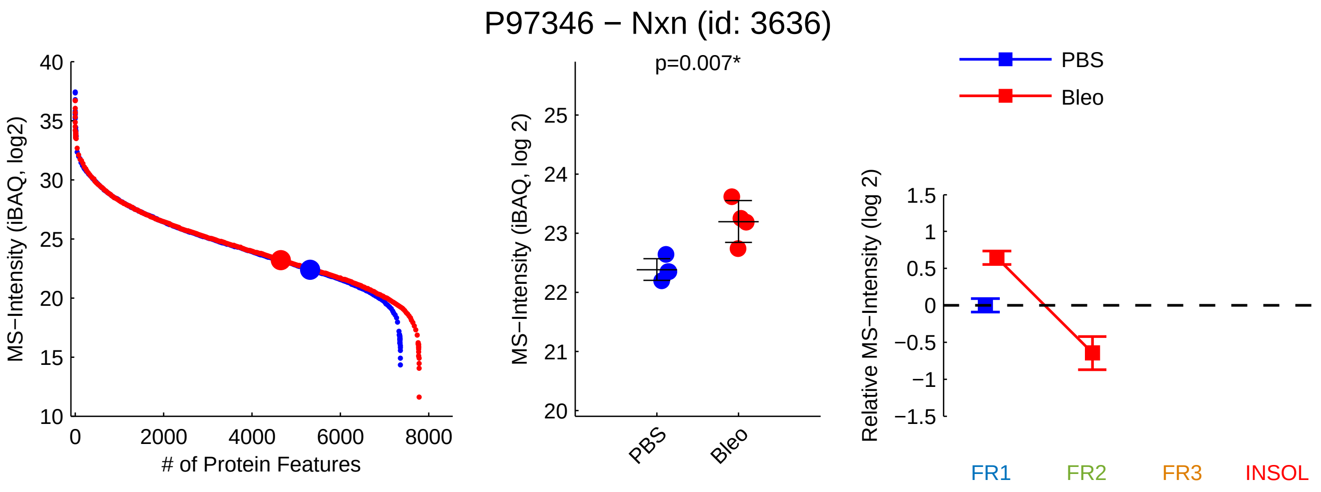
P97346 − Nxn (id: 3636)
MS−Intensity (iBAQ, log2)
# of Protein Features
p=0.007*
MS−Intensity (iBAQ, log 2)
PBS
Bleo
Relative MS−Intensity (log 2)
FR1
FR2
FR3
INSOL

P97351 − Rps3a (id: 3639)

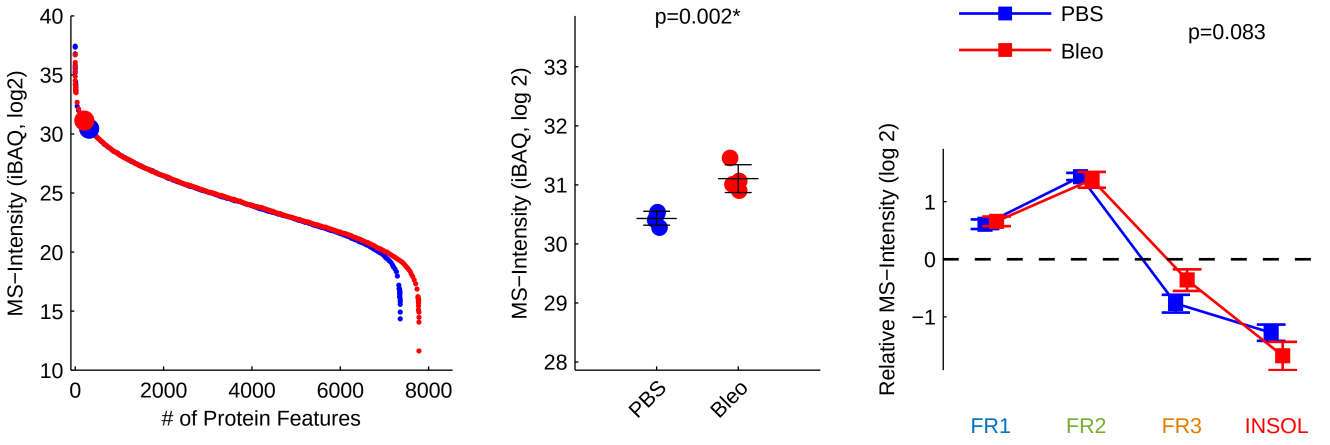

P97360 − Etv6 (id: 3642)

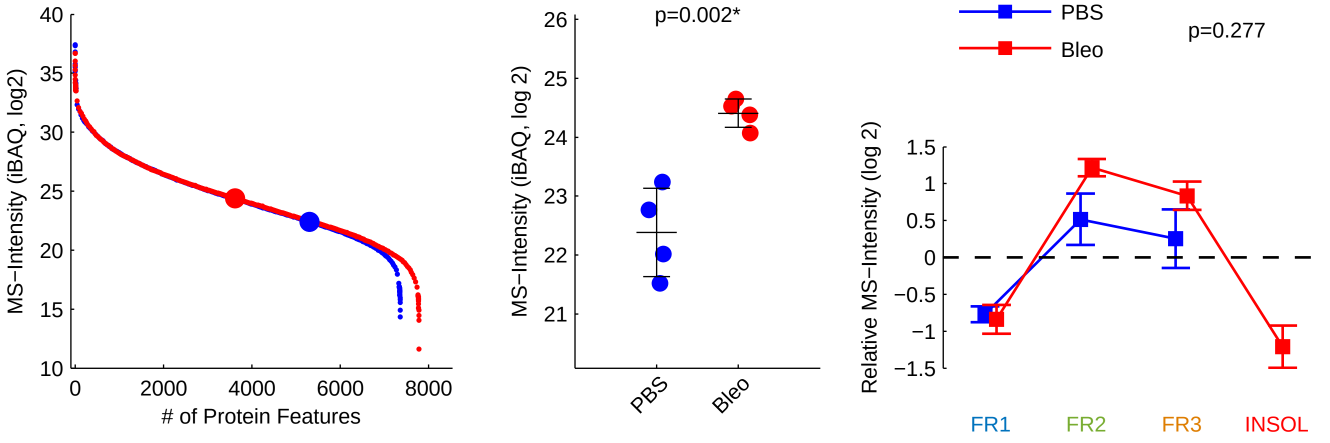

P97363 − Sptlc2 (id: 3644)

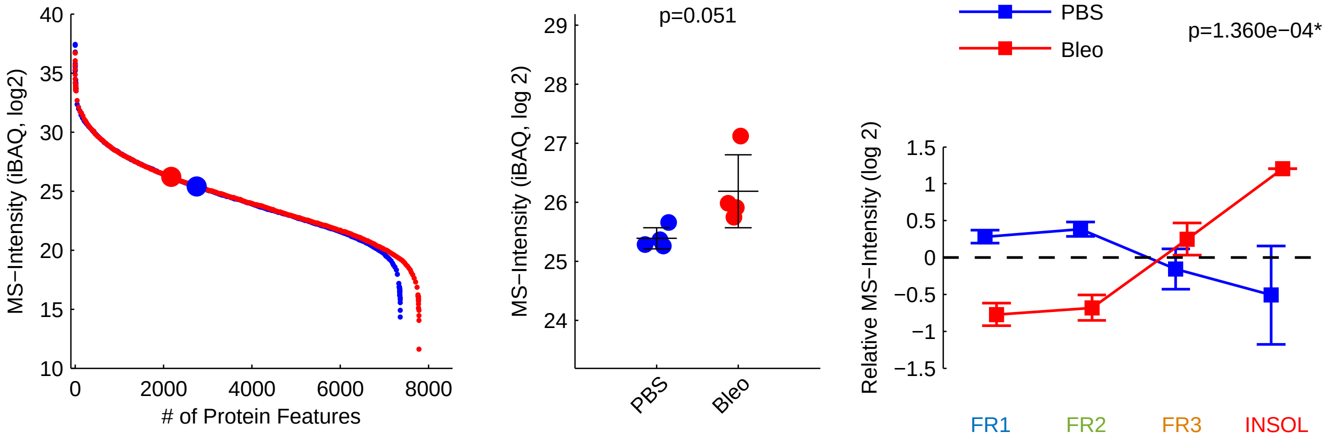

P97364 − Sephs2 (id: 3645)

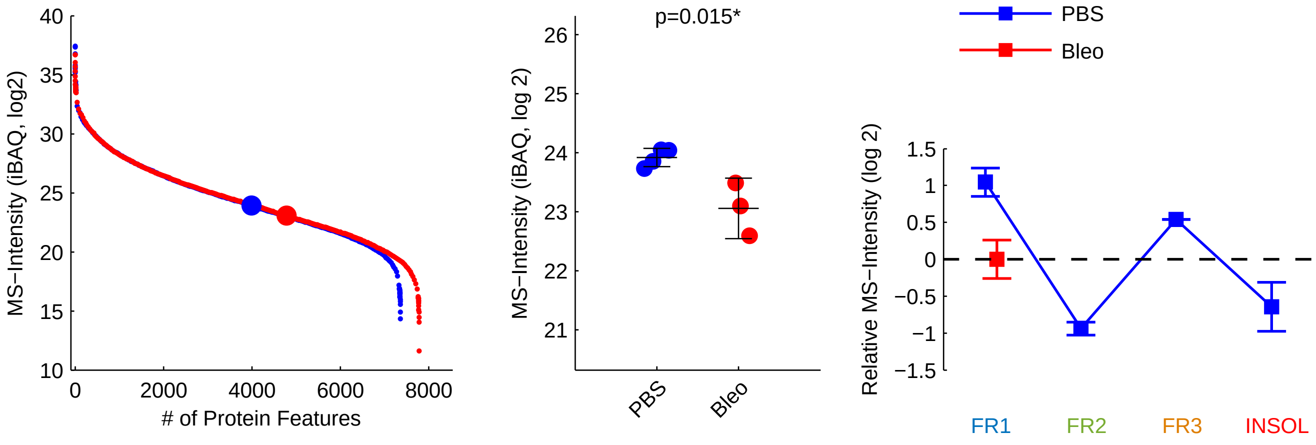

P97369 − Ncf4 (id: 3646)

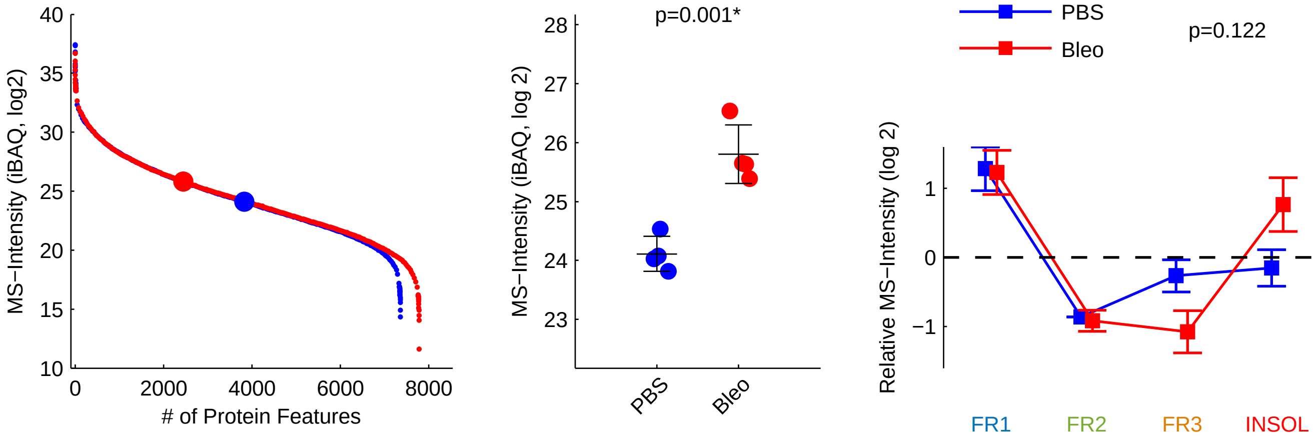

P97425 − Ear2 (id: 3654)

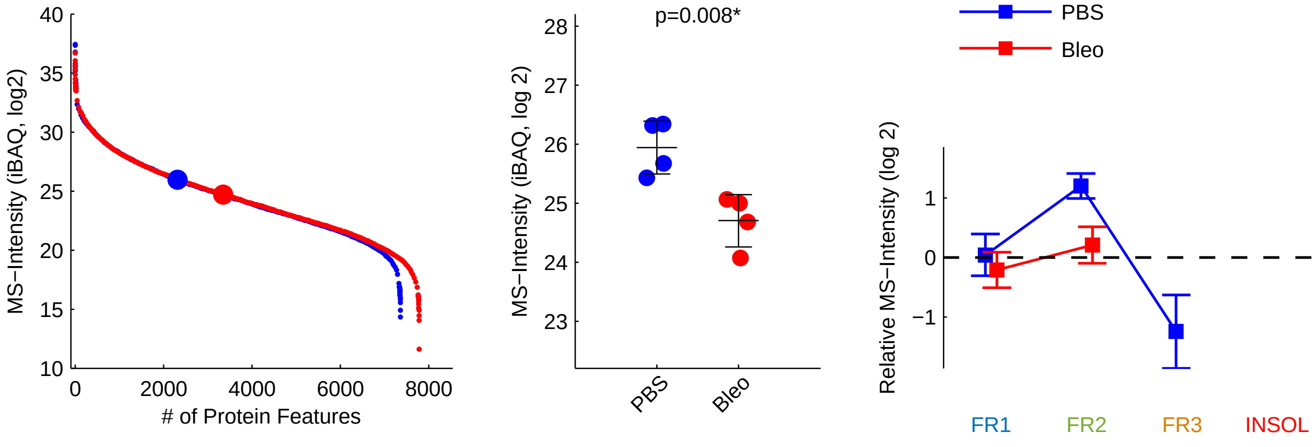

P97454 − Smad5 (id: 3664)

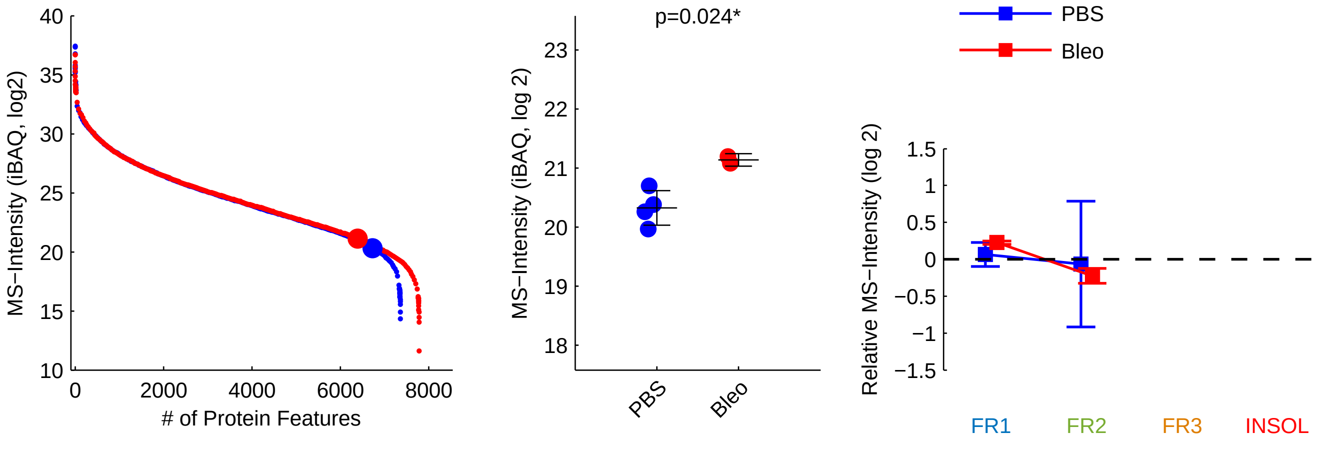

P97479 − Myo7a (id: 3672)

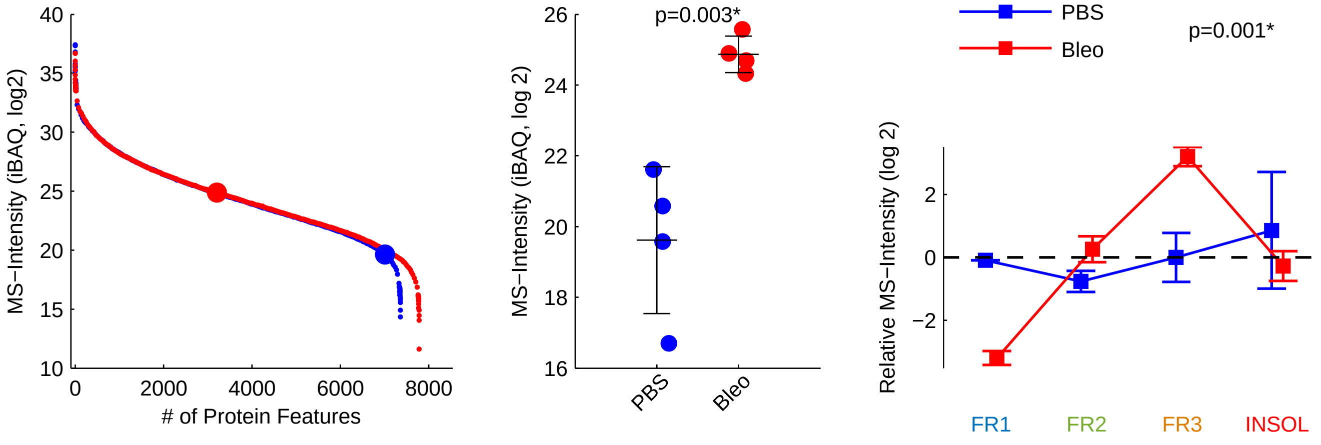

P97484 − Lilrb3 (id: 3673)

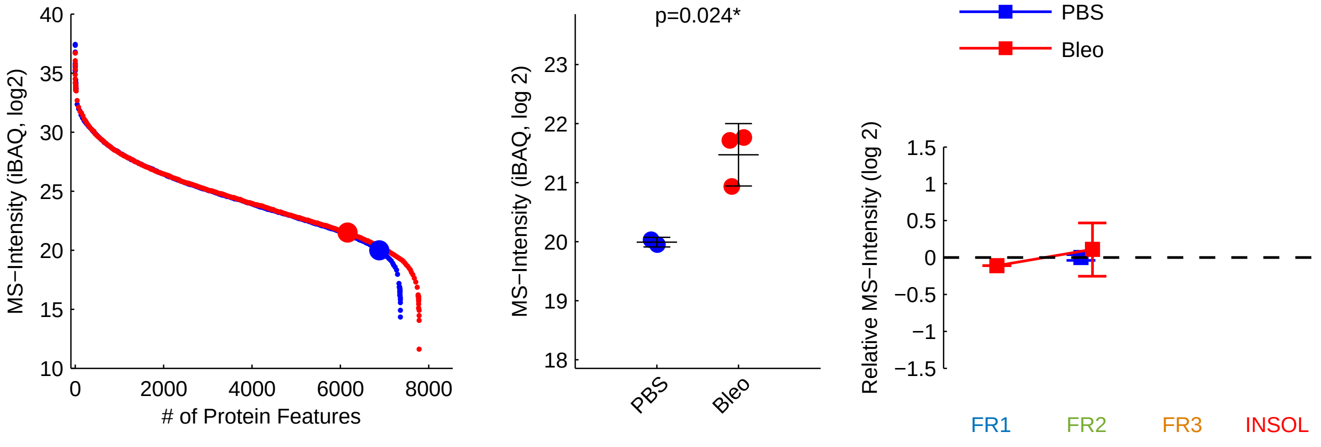

P97499 − Tep1 (id: 3675)

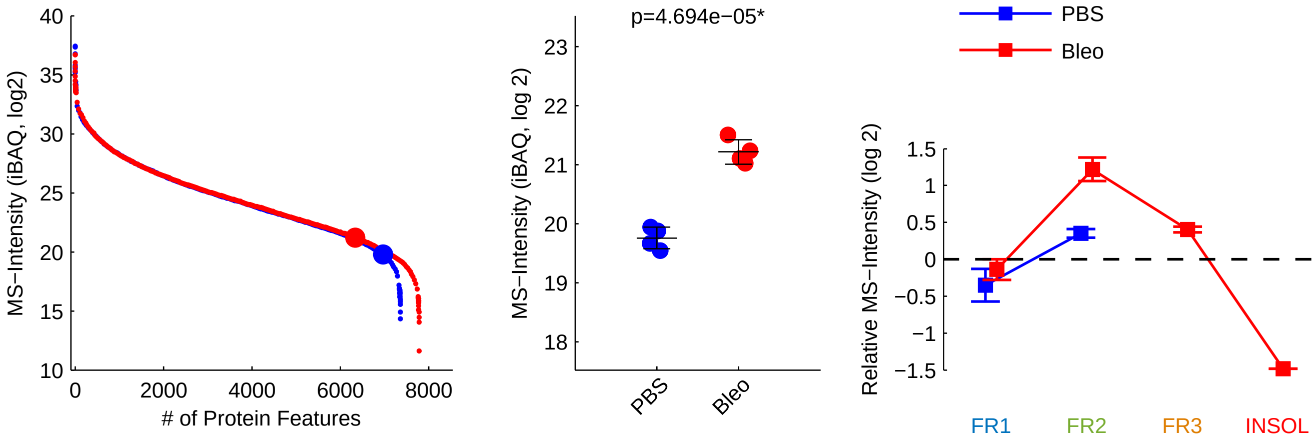

P97501 − Fmo3 (id: 3676)

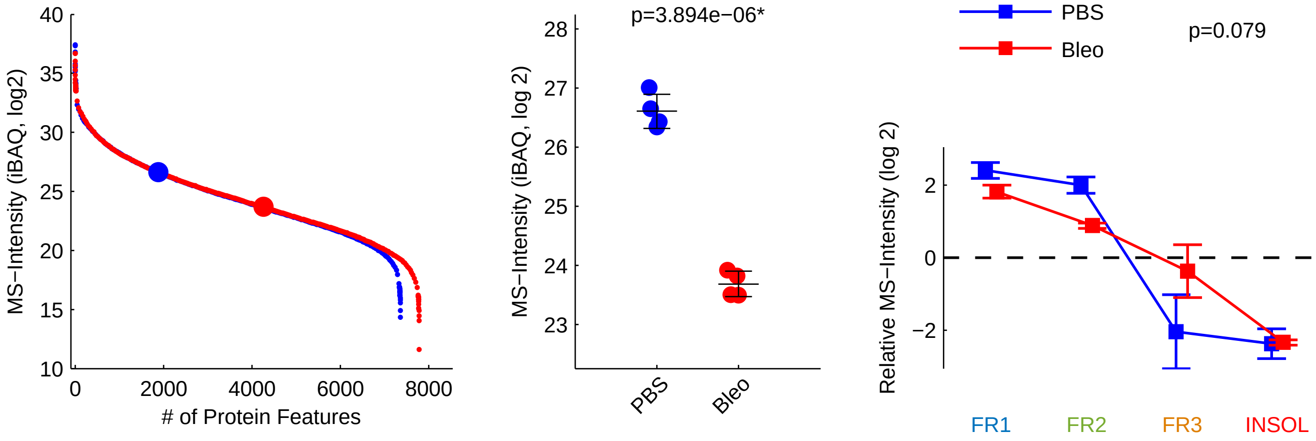

P97792 − Cxadr (id: 3681)

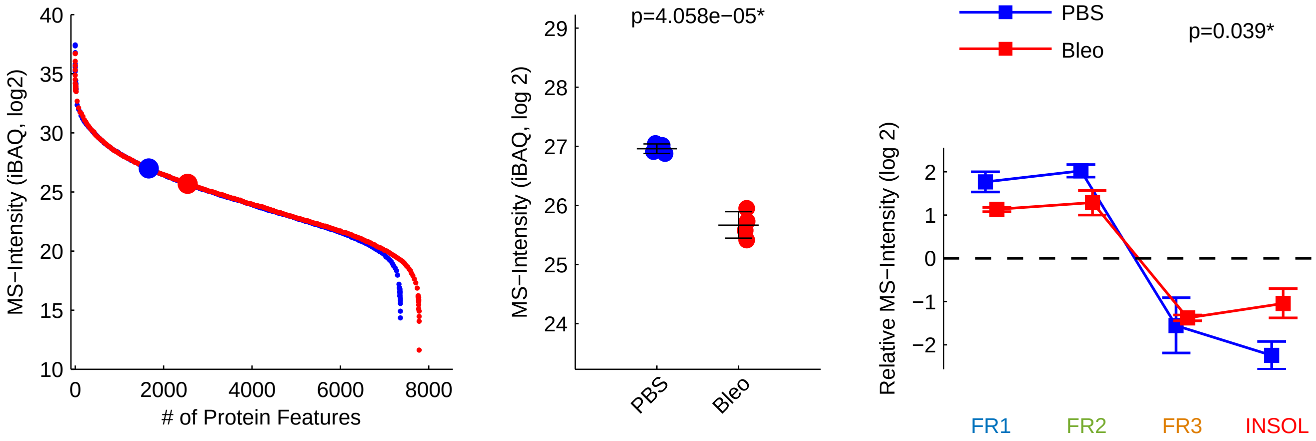

P97807−2 − Fh (id: 3682)

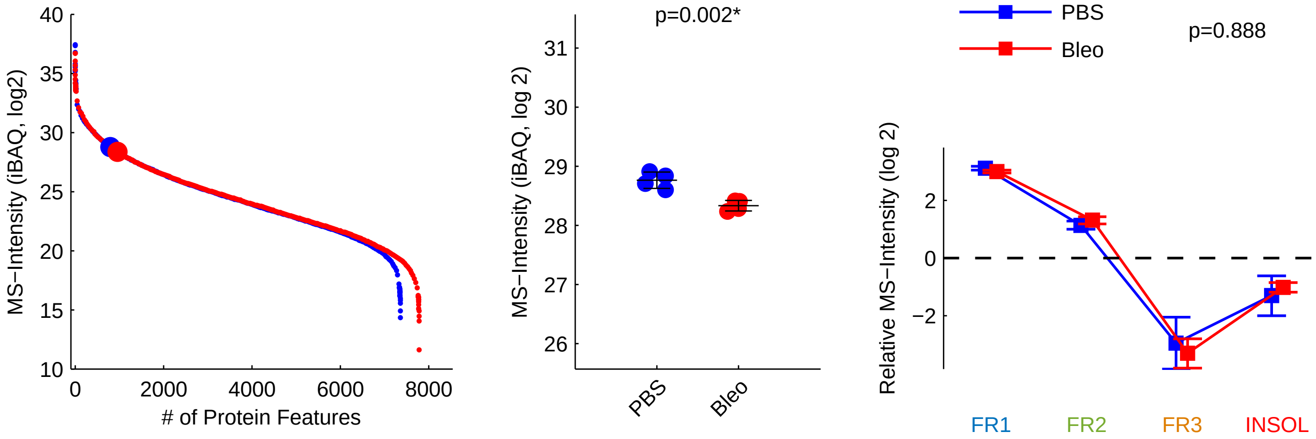

P97813 − Pld2 (id: 3683)

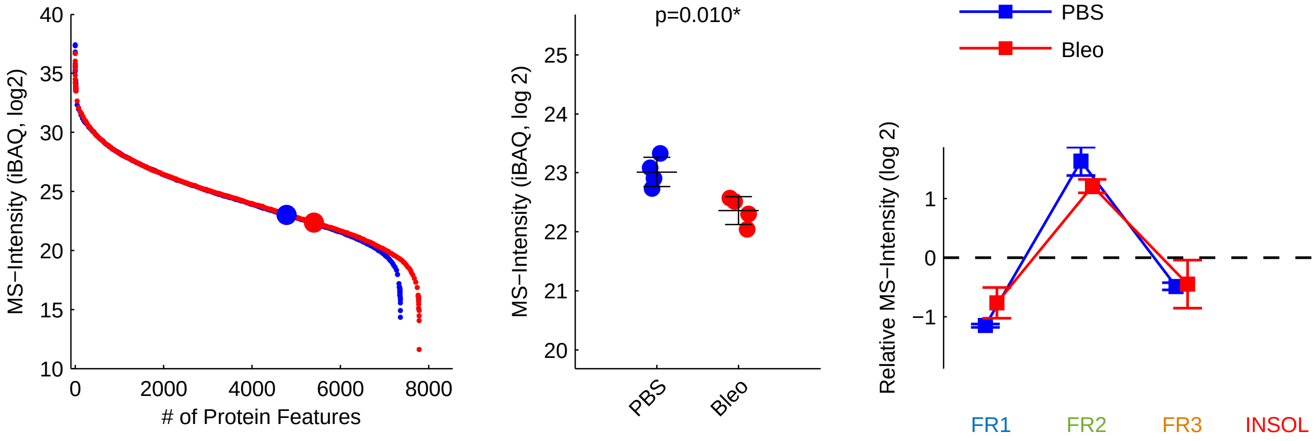

P97855 − G3bp1 (id: 3691)

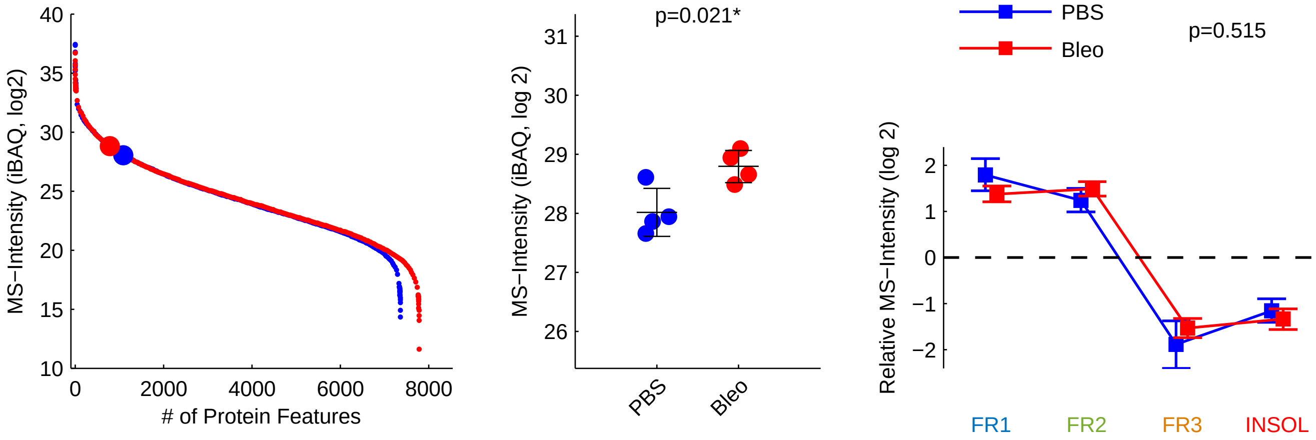

P97872 − Fmo5 (id: 3695)

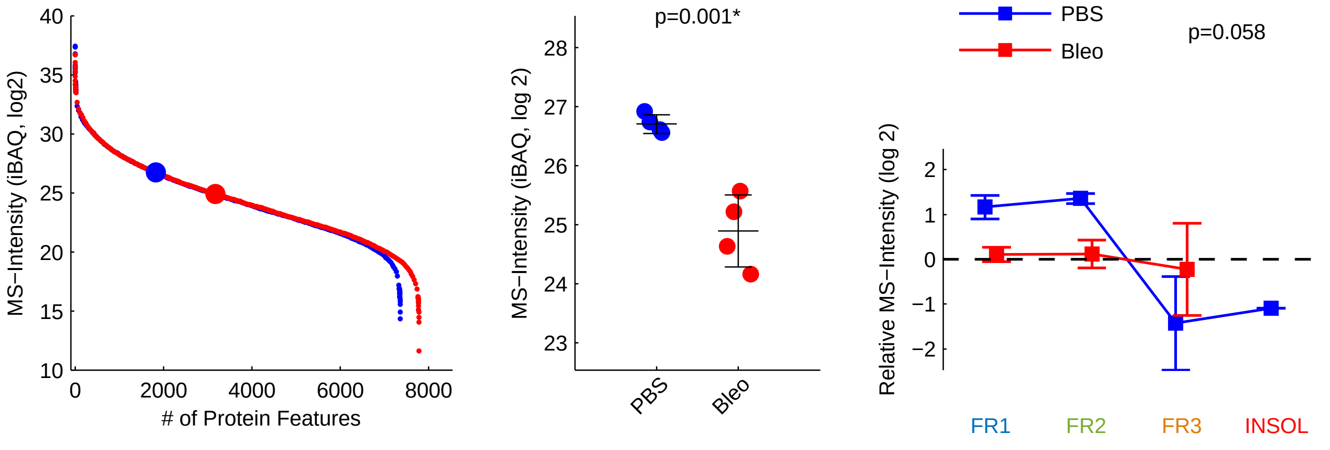

P97873 − Loxl1 (id: 3696)

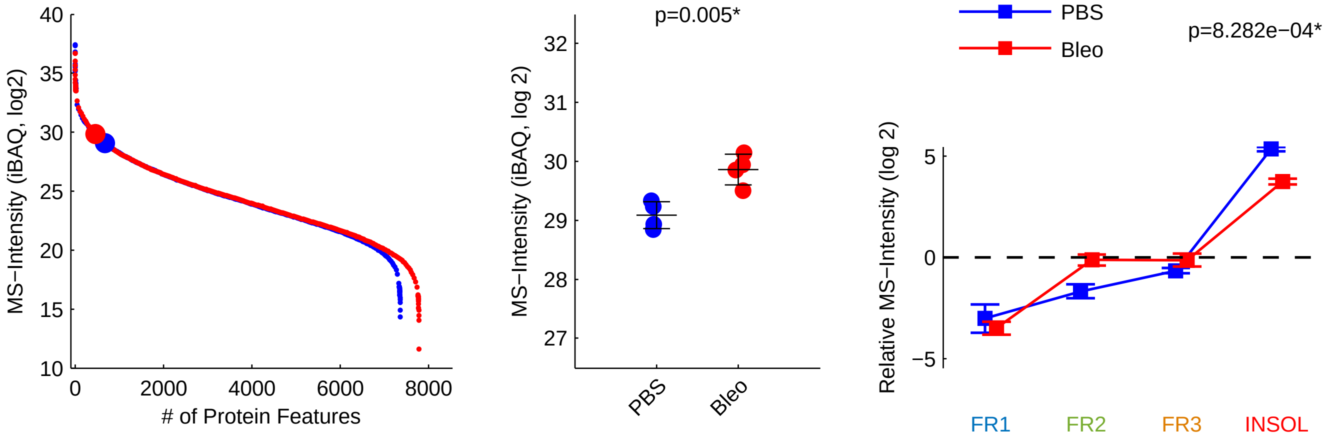

P97927 − Lama4 (id: 3697)

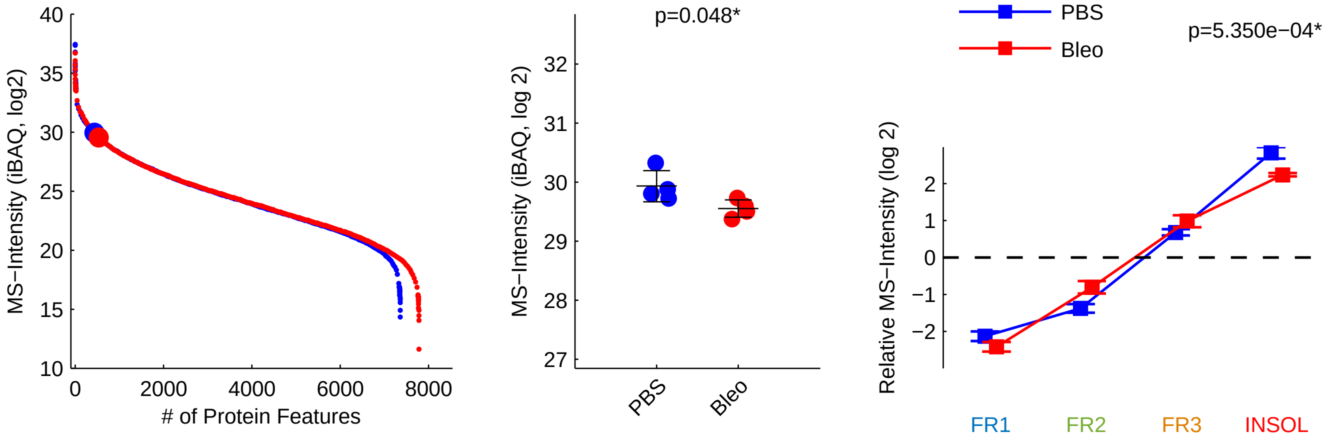

P98063 − Bmp1 (id: 3699)

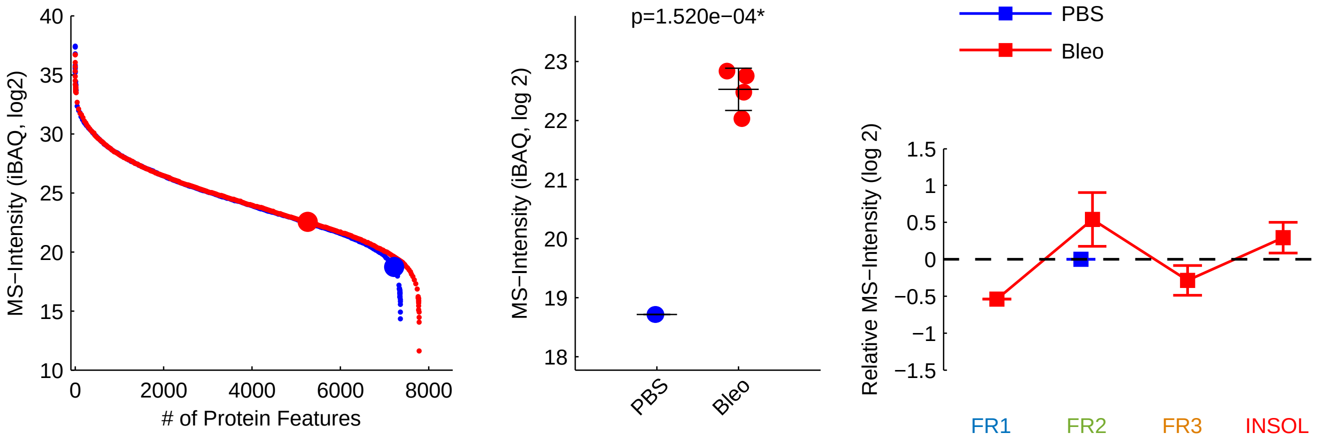

P98086 − C1qa (id: 3701)

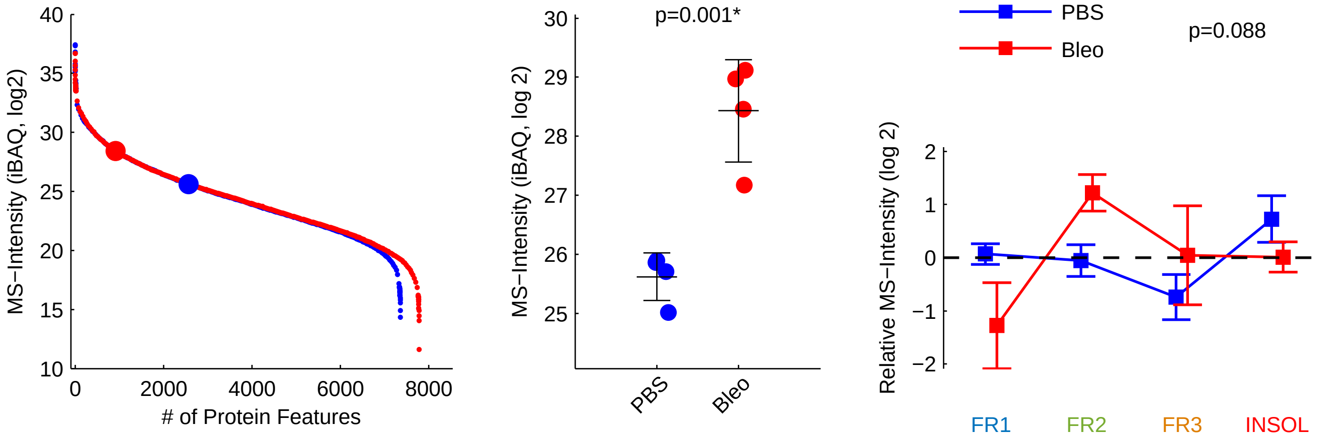

P98203 − Arvcf (id: 3703)

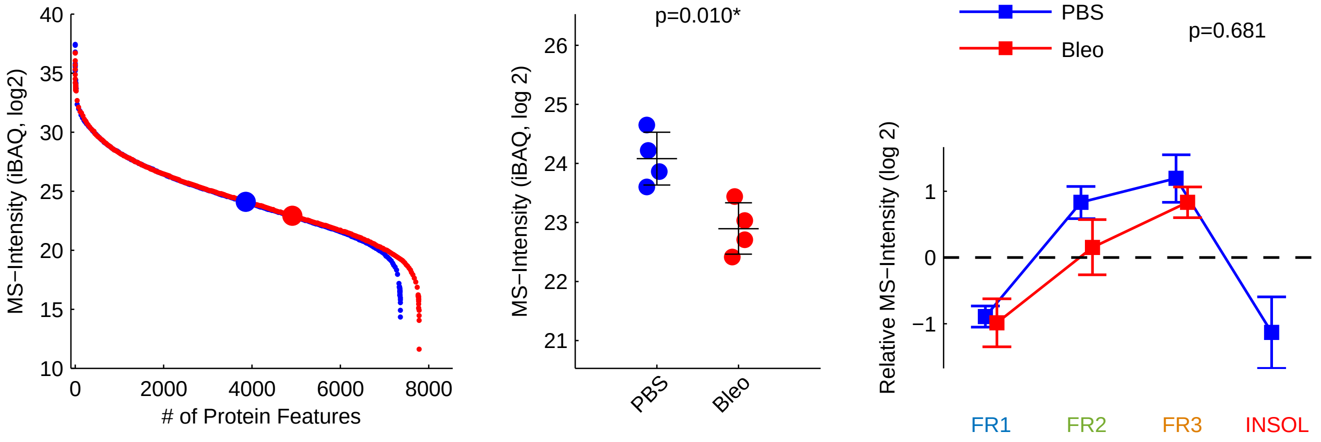

P99024 − Tubb5 (id: 3704)

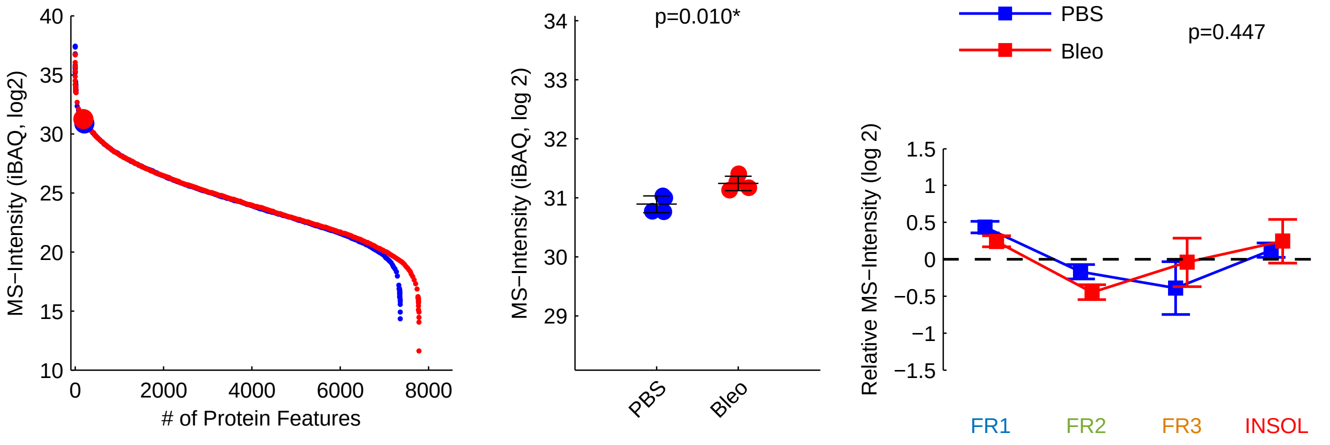

P99026 − Psmb4 (id: 3705)

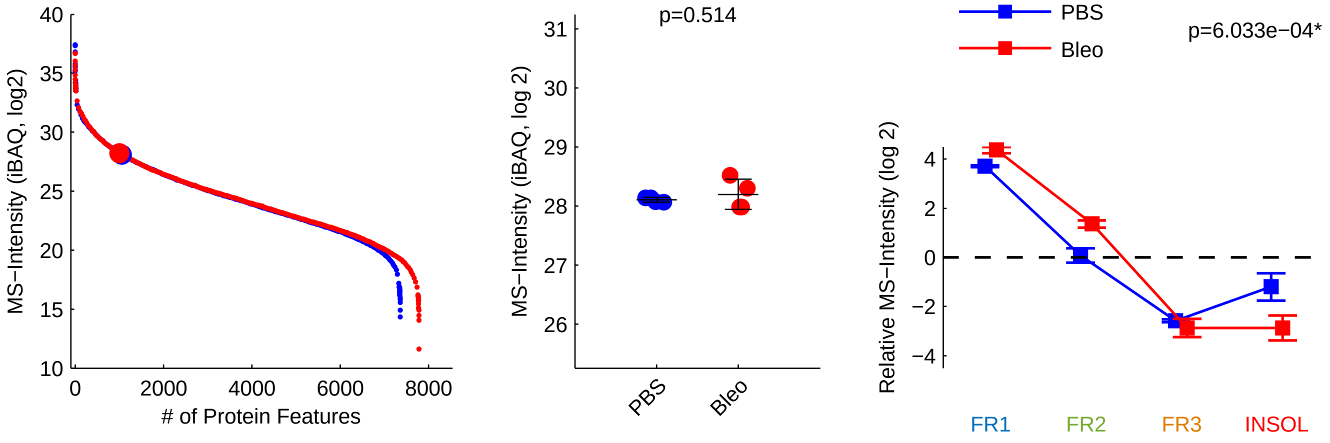

P99027 − Rplp2 (id: 3706)

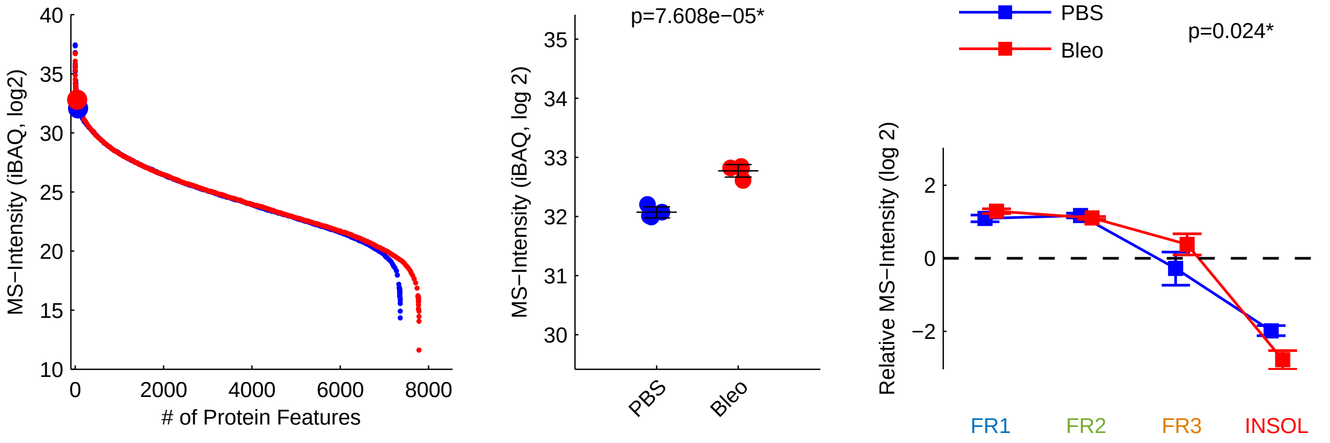

Q00519 − Xdh (id: 3712)

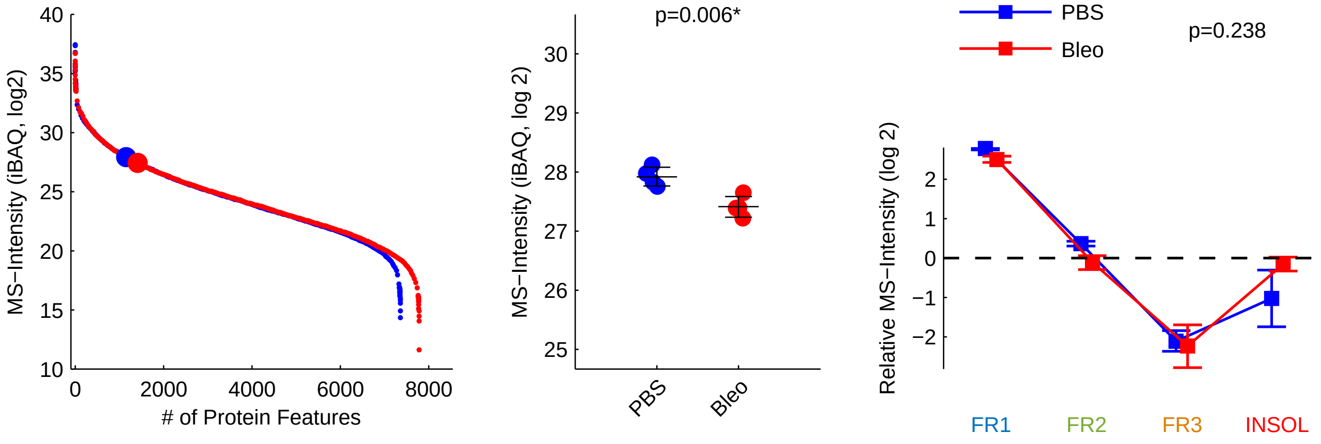

Q00612 − G6pdx (id: 3714)

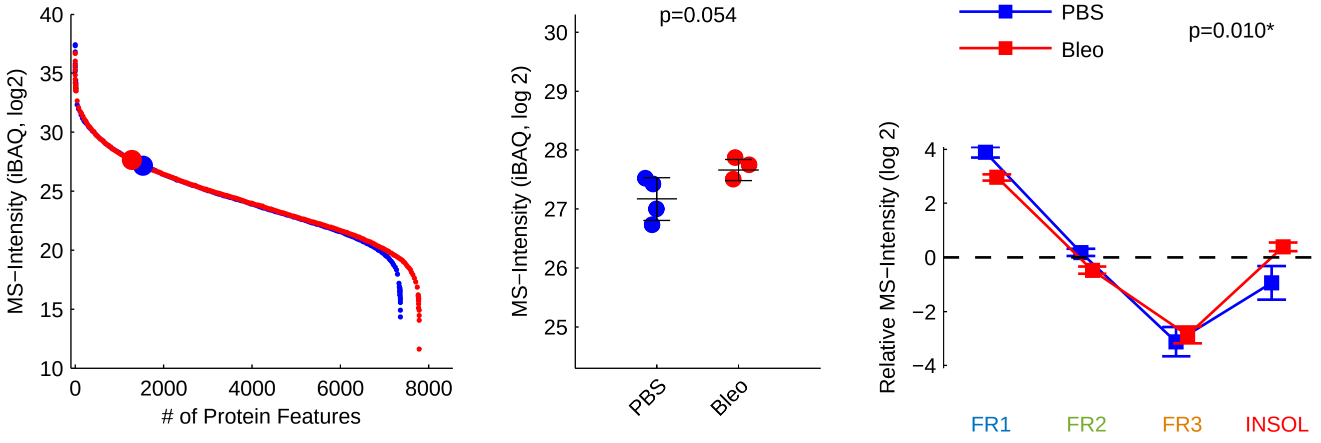

Q00623 − Apoa1 (id: 3715)

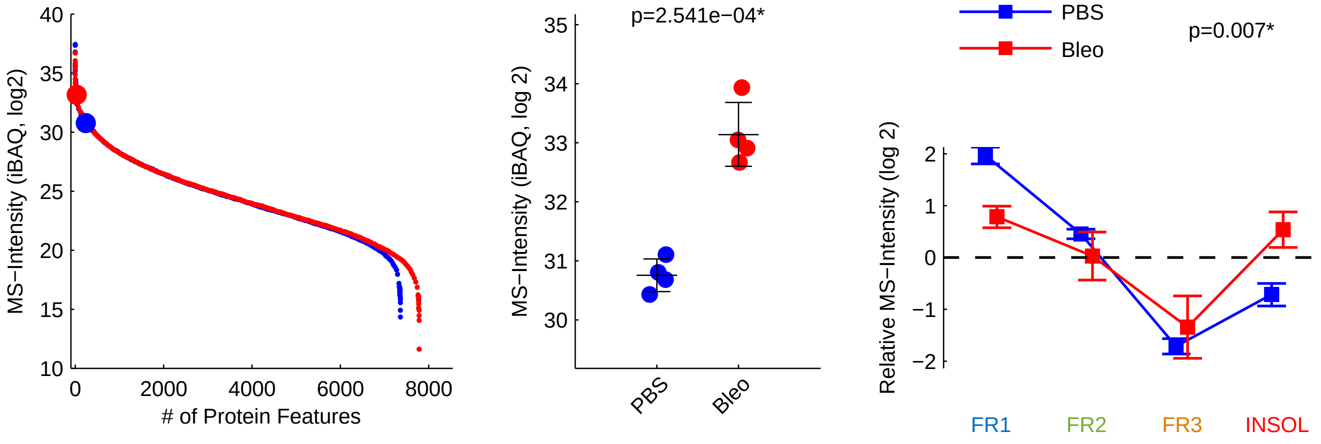

Q00780 − Col8a1 (id: 3716)

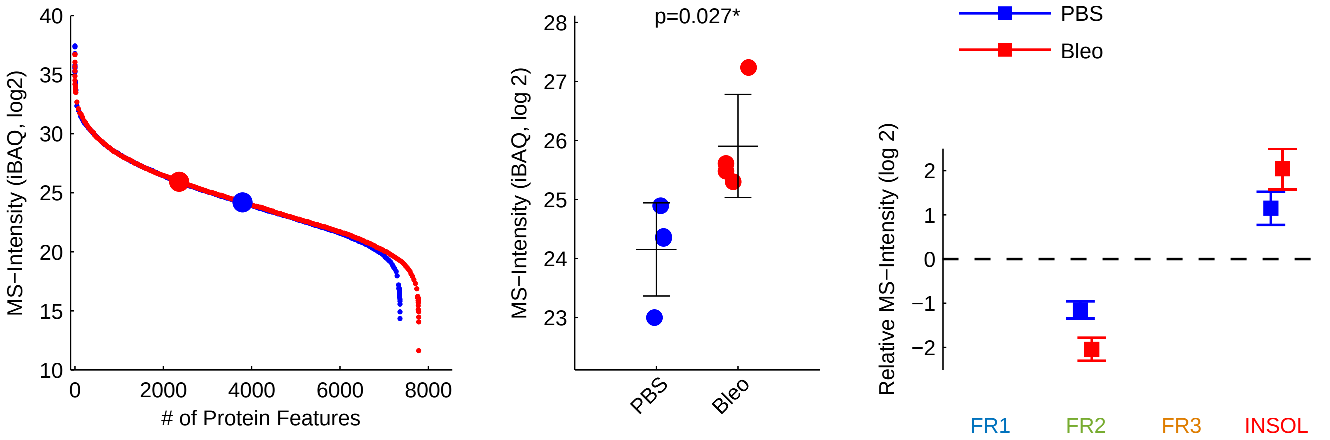

Q00897 − Serpina1d (id: 3718)

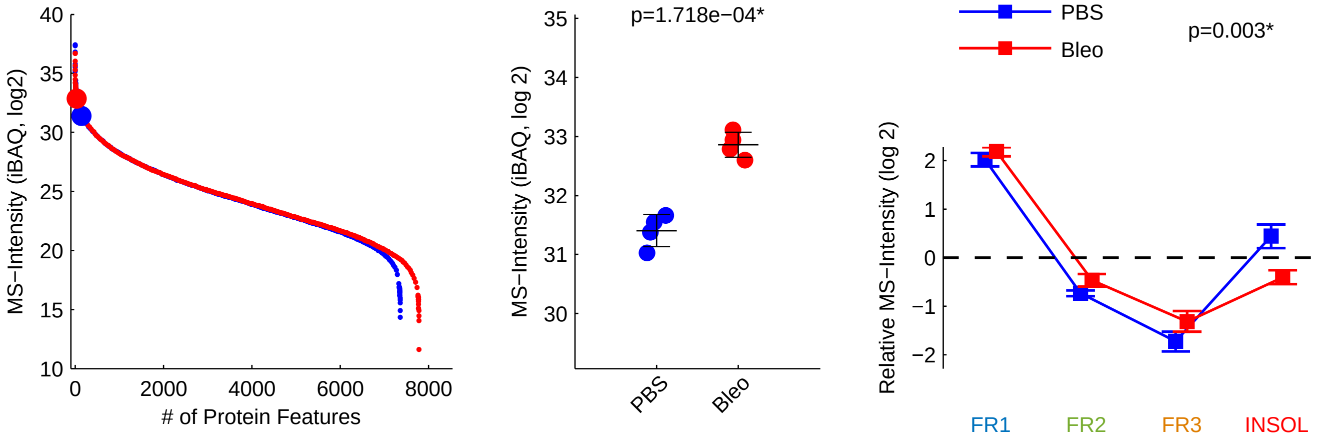

Q01149 − Col1a2 (id: 3725)

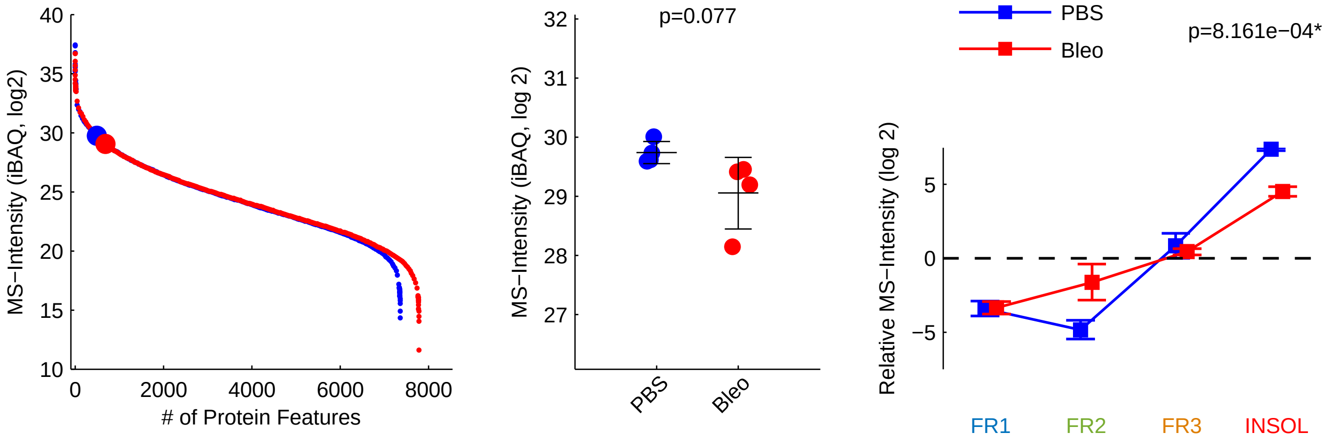

Q01279 − Egfr (id: 3727)

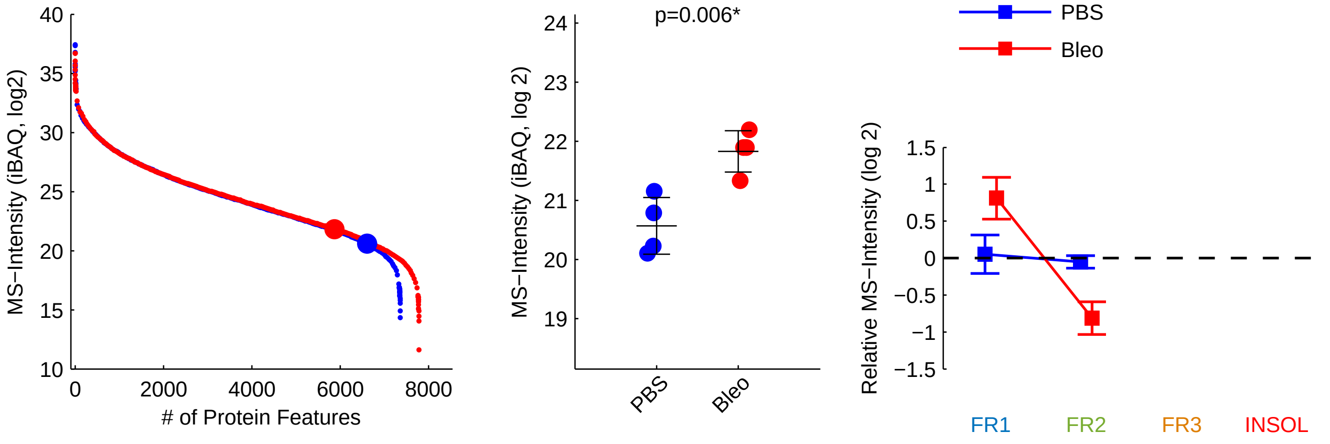

Q01339 − Apoh (id: 3729)

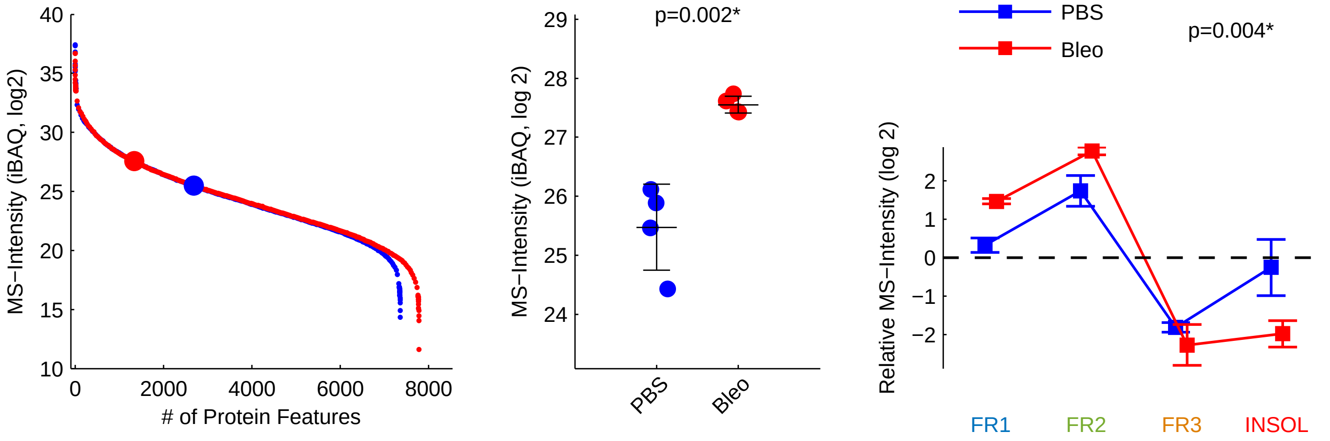

Q01853 − Vcp (id: 3730)

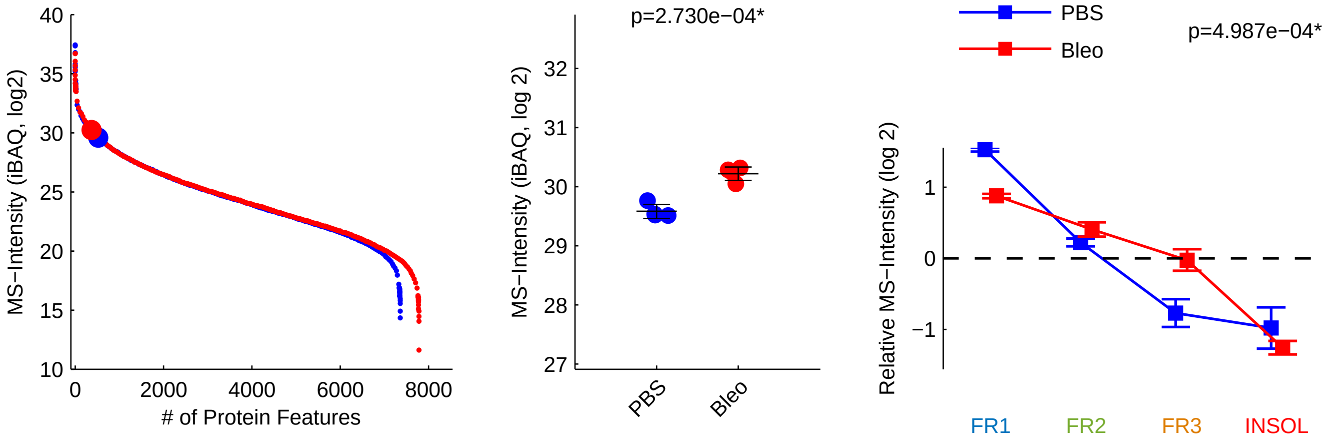

Q02013 − Aqp1 (id: 3731)

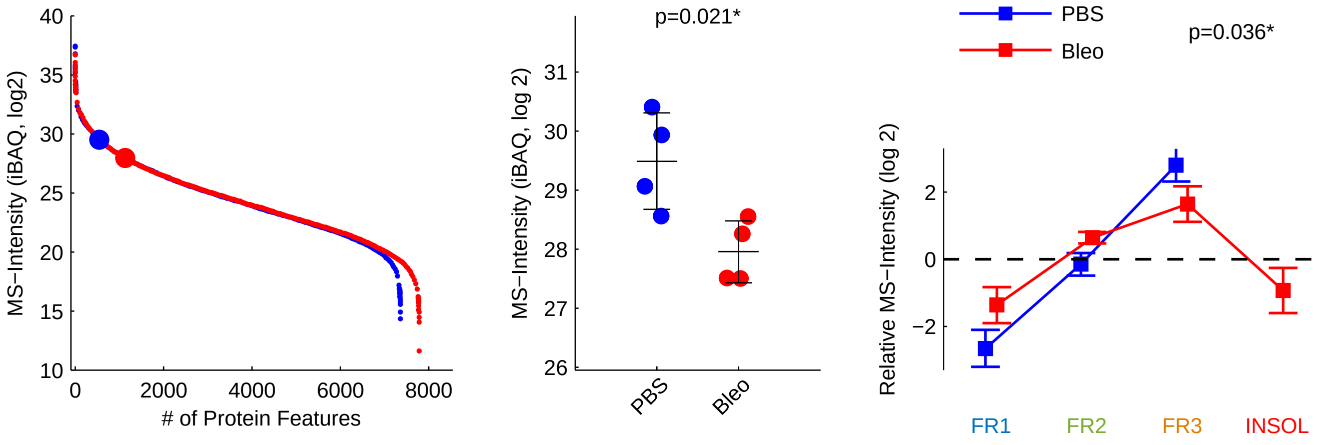

Q02105 − C1qc (id: 3733)

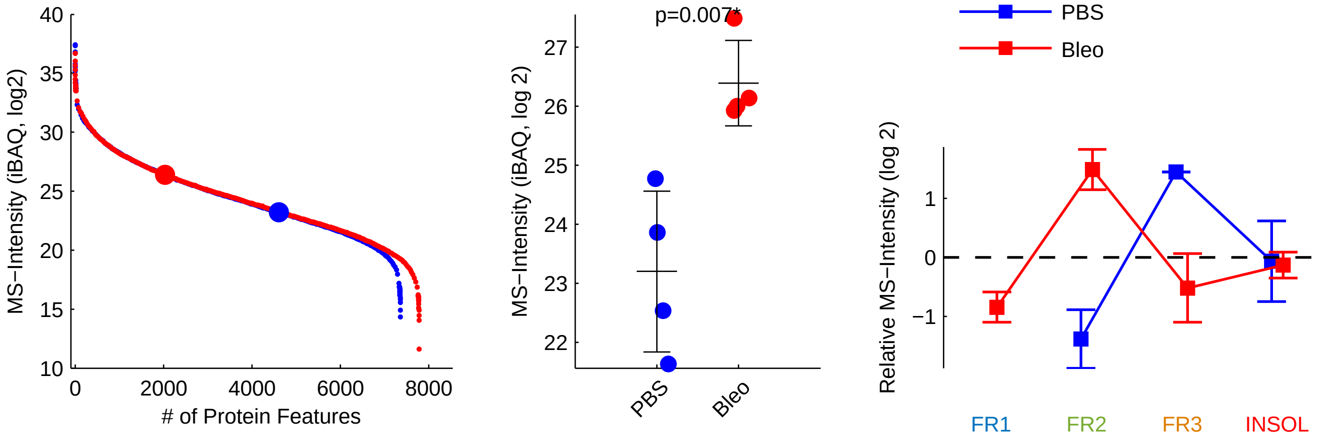

Q02257 − Jup (id: 3735)

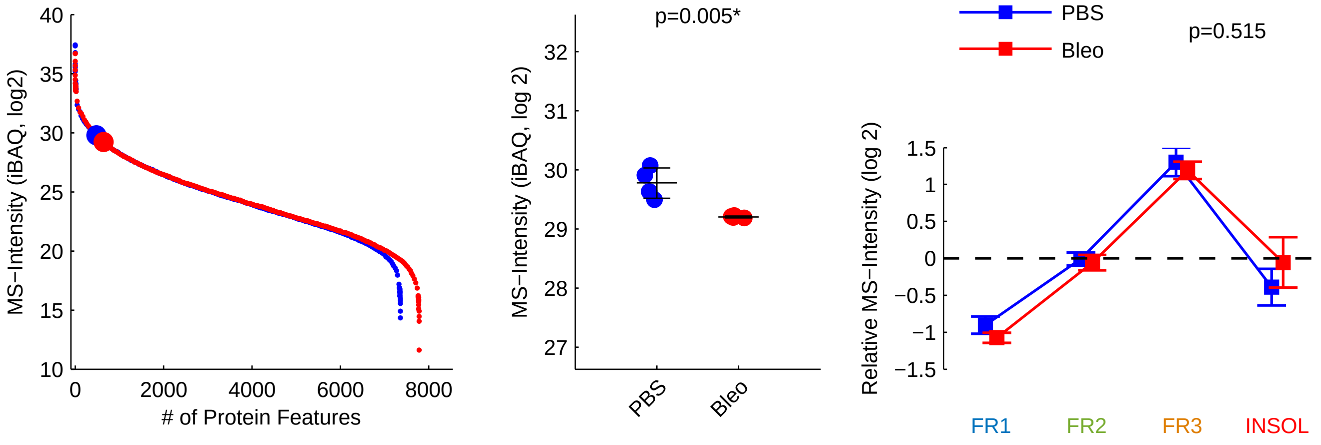

Q02526 − Zfp41 (id: 3738)

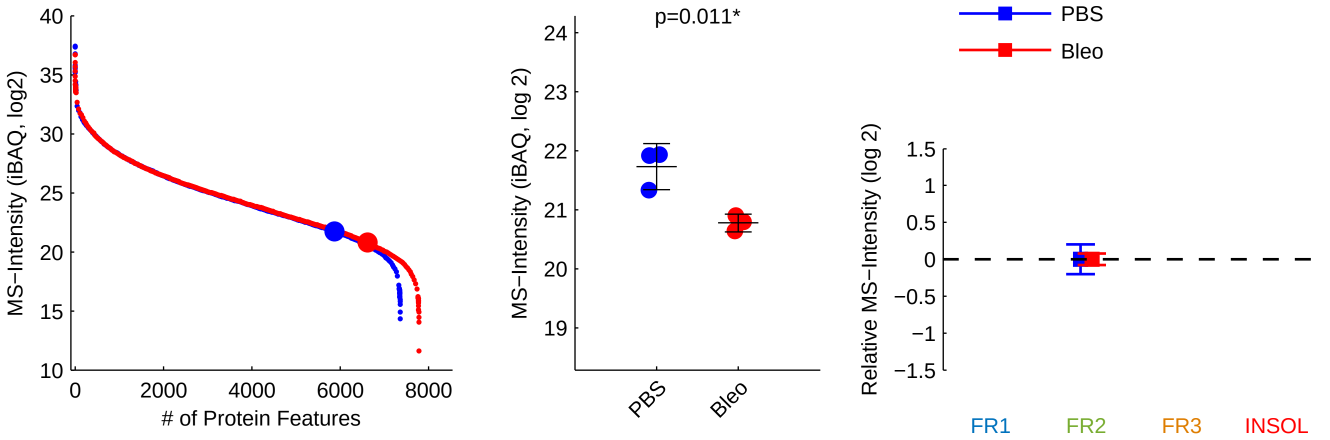

Q02819 − Nucb1 (id: 3743)

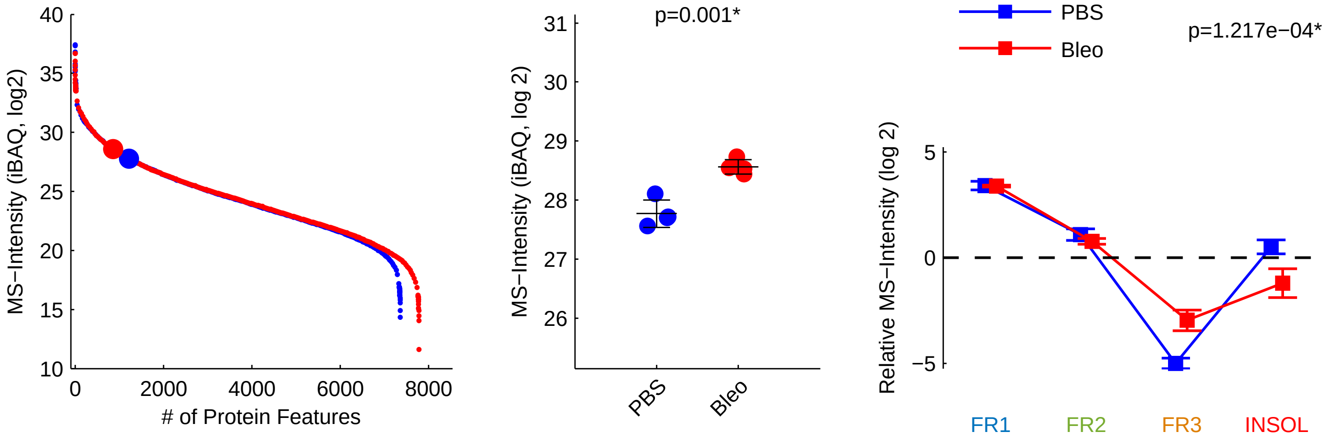

Q02956 − Prkcz (id: 3744)

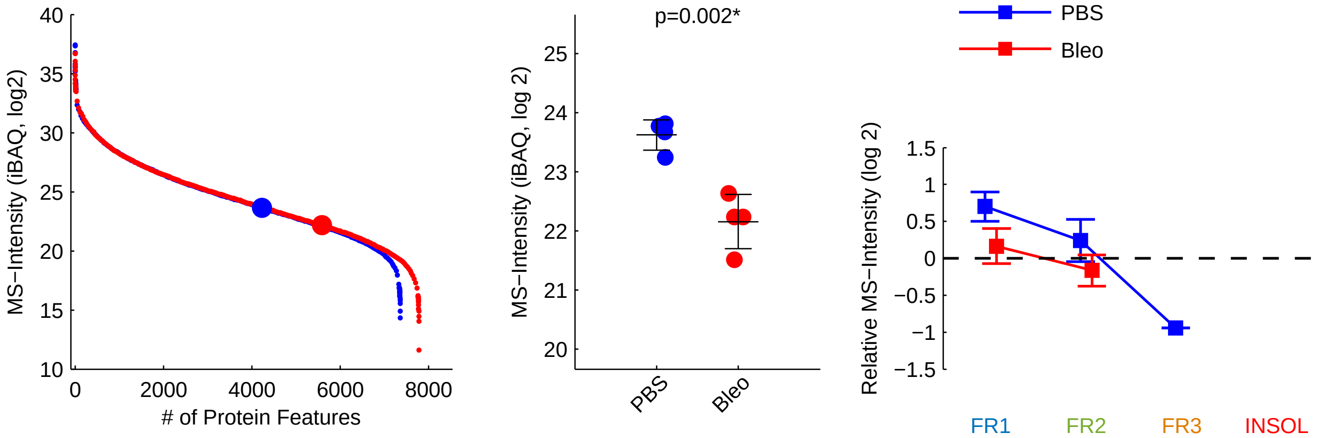

Q03145 − Epha2 (id: 3747)

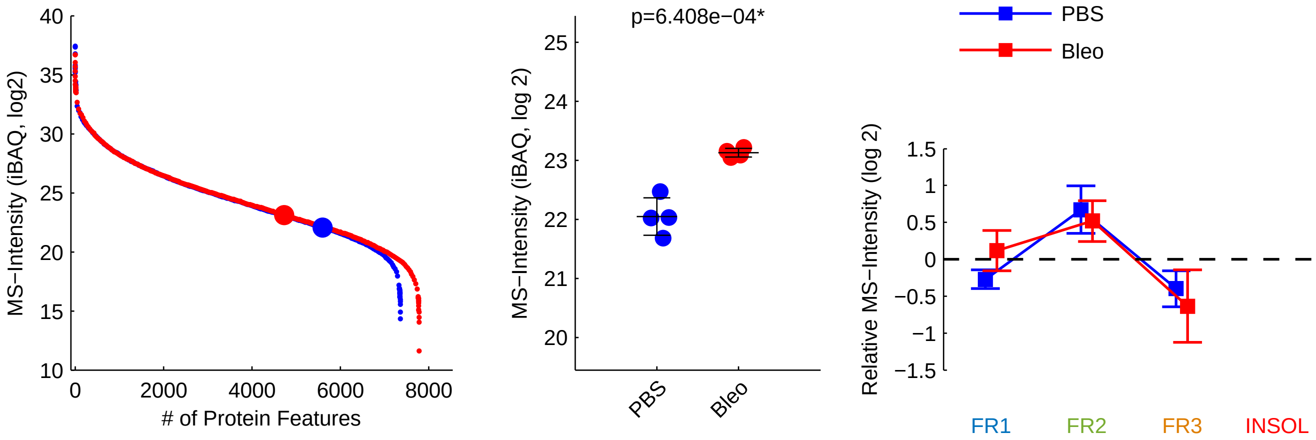

Q03734 − Serpina3m (id: 3752)

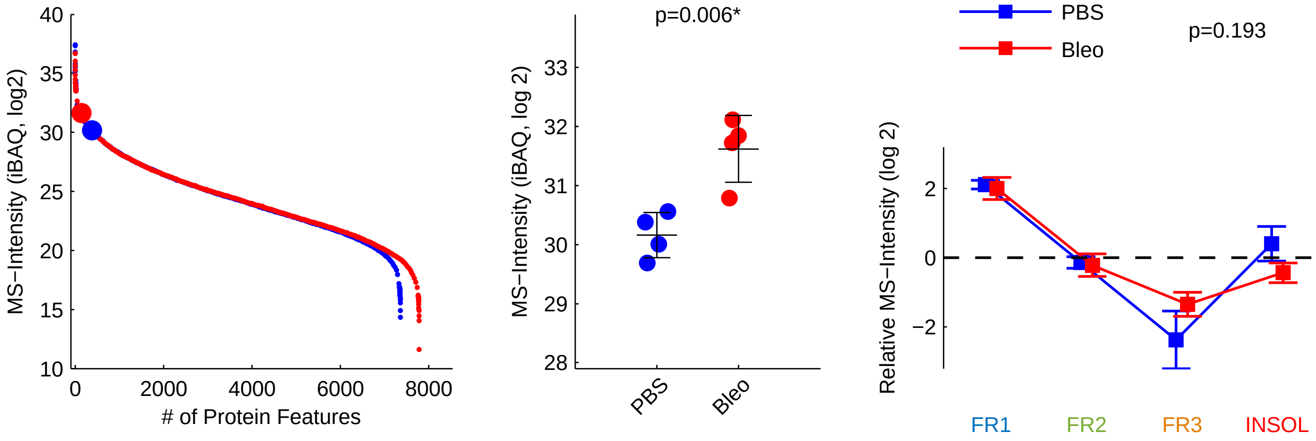

Q03963 − Eif2ak2 (id: 3754)

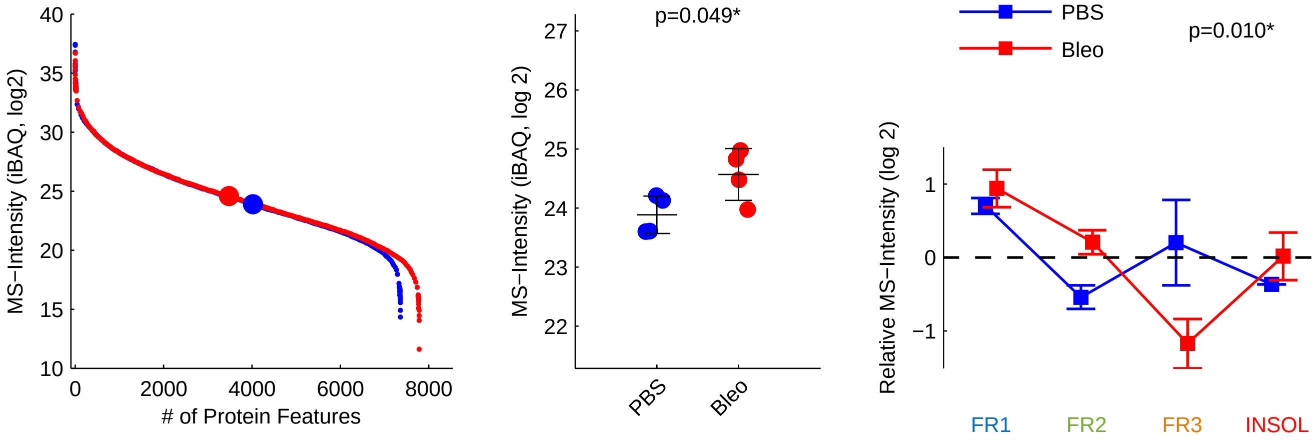

Q04447 − Ckb (id: 3756)

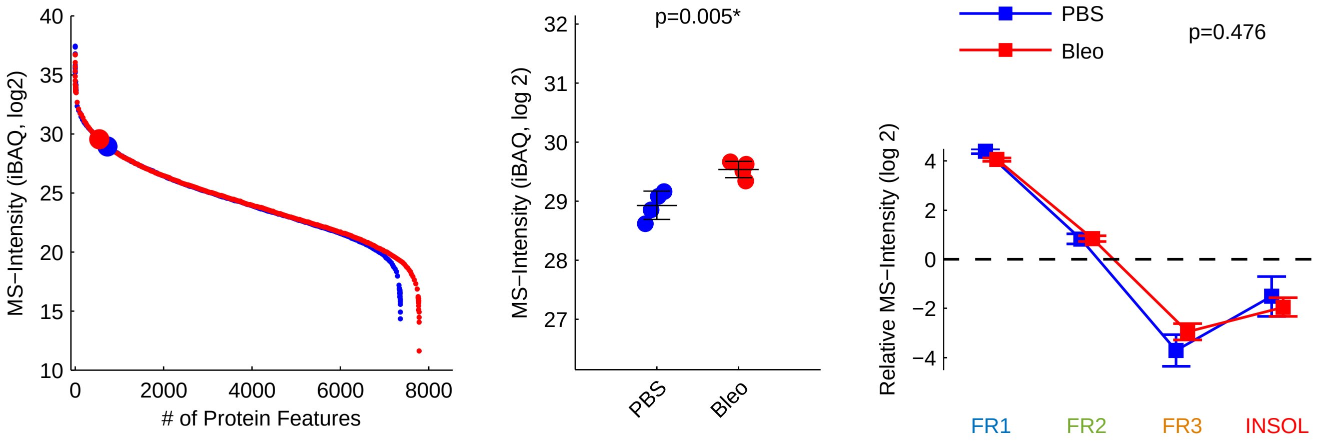

Q04690 − Nf1 (id: 3759)

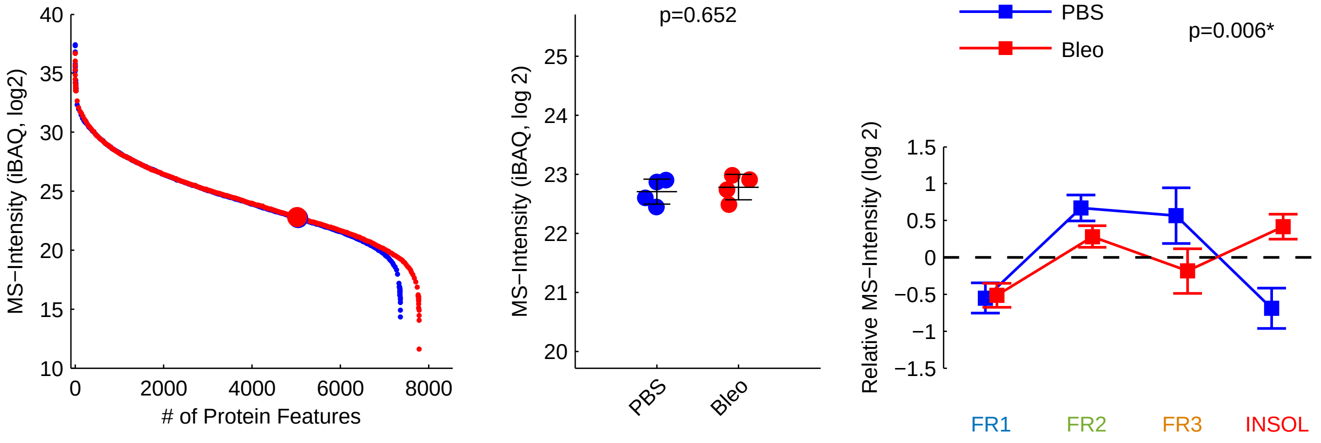

Q04736 − Yes1 (id: 3762)

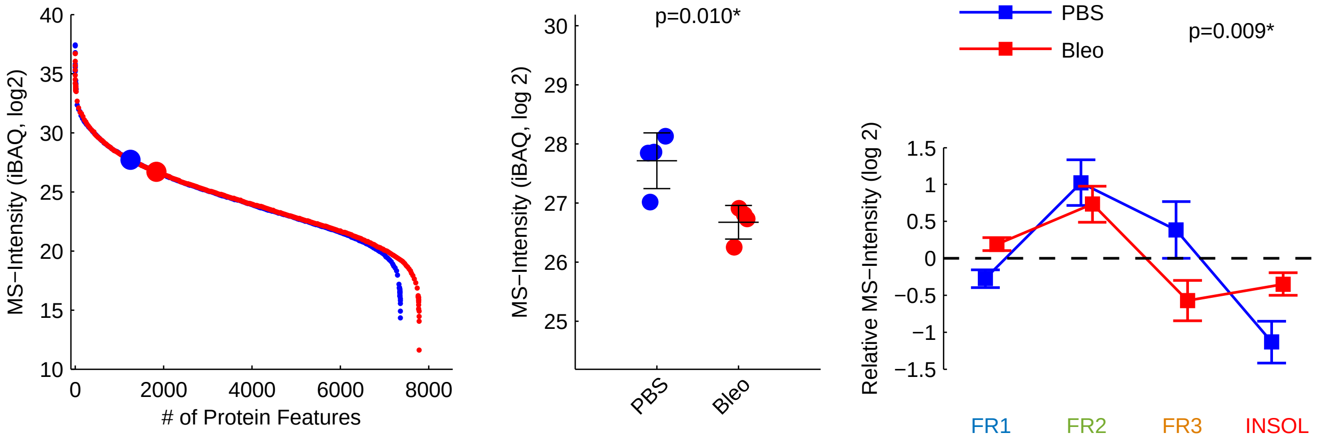

Q04857 − Col6a1 (id: 3764)

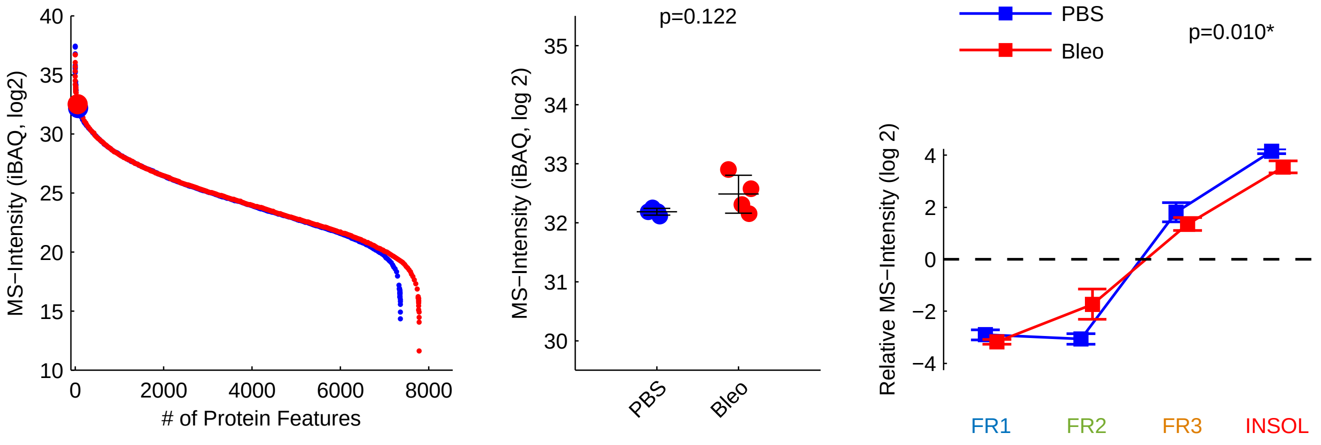

Q05117 − Acp5 (id: 3768)

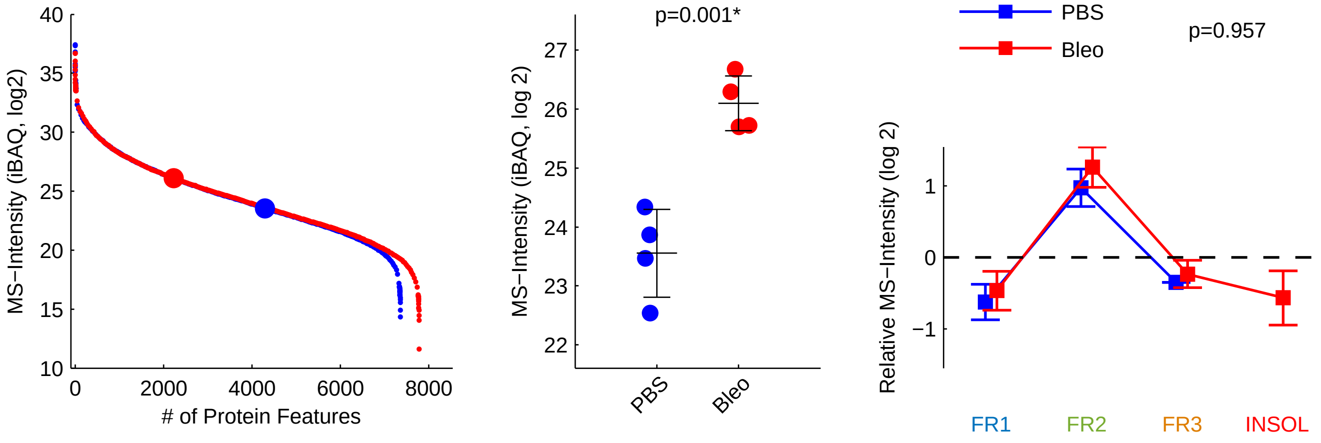

Q05BC3 − Eml1 (id: 3777)

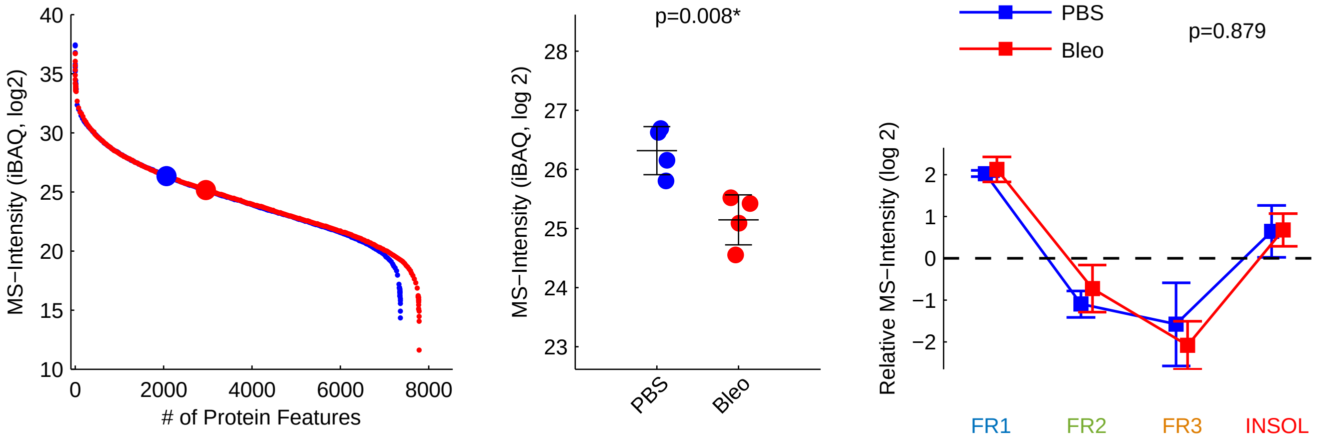

Q05D44 − Eif5b (id: 3780)

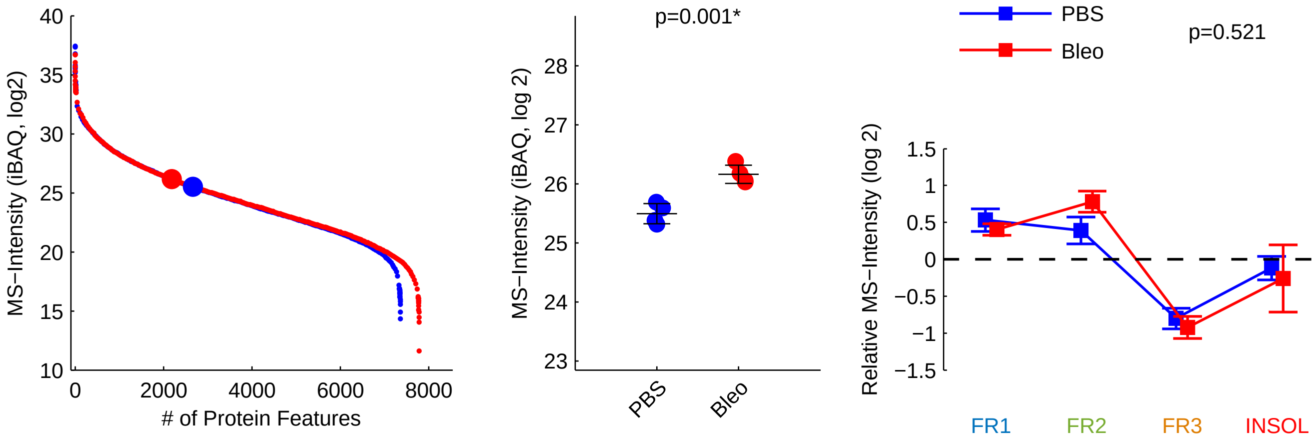

Q06138 − Cab39 (id: 3782)

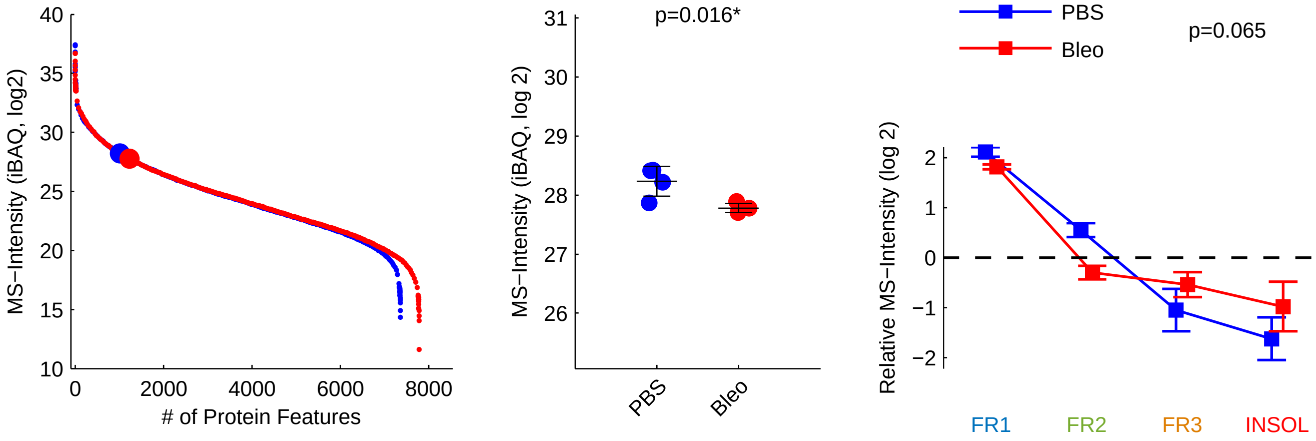

Q06318 − Scgb1a1 (id: 3784)

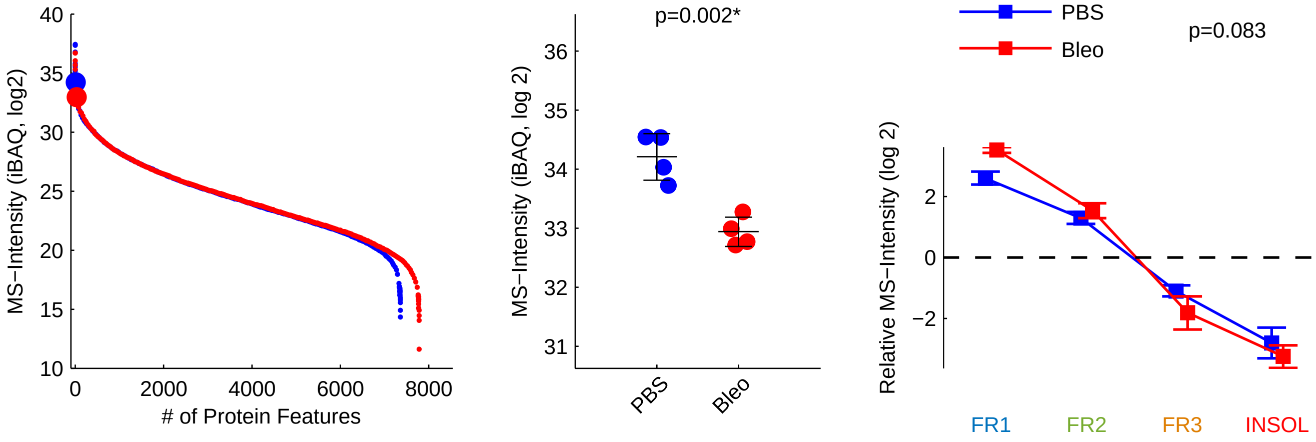

Q06770 − Serpina6 (id: 3785)

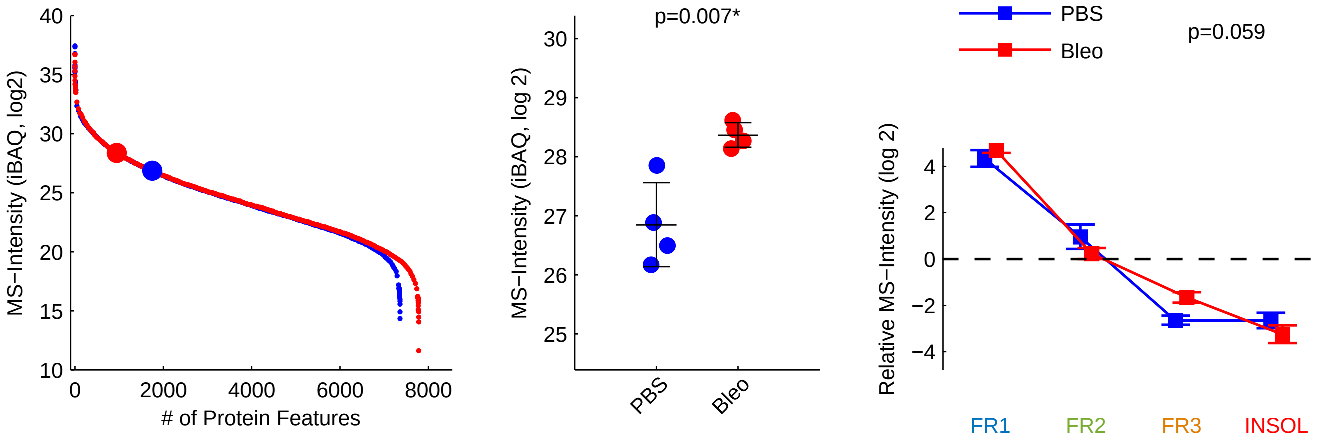

Q06890 − Clu (id: 3787)

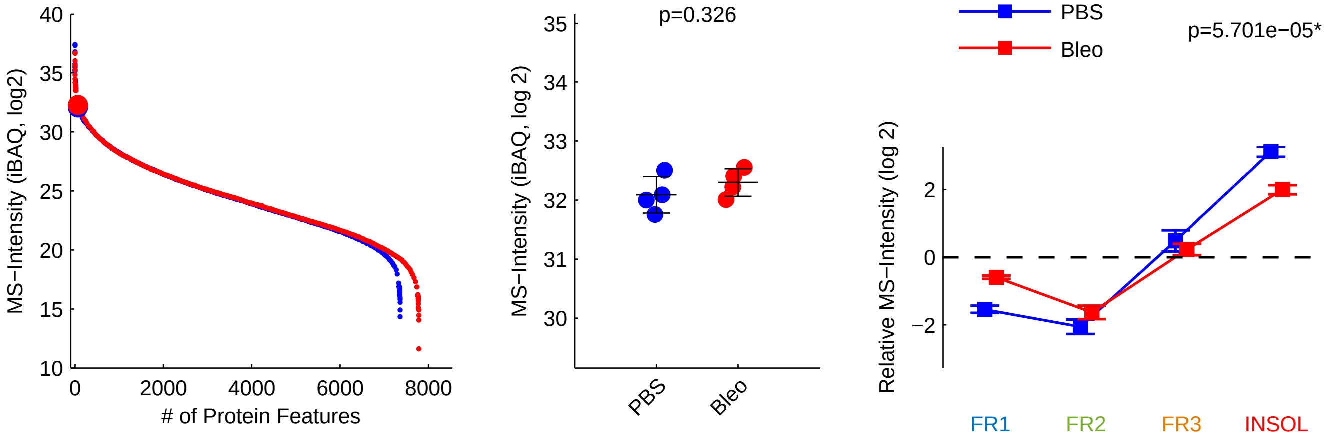

Q07113 − Igf2r (id: 3790)

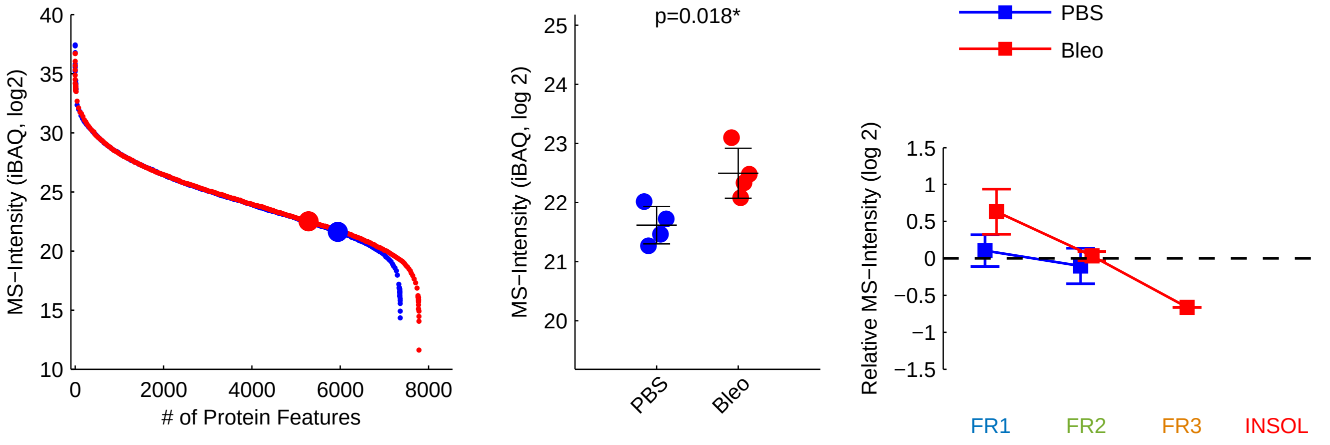

Q07235 − Serpine2 (id: 3791)

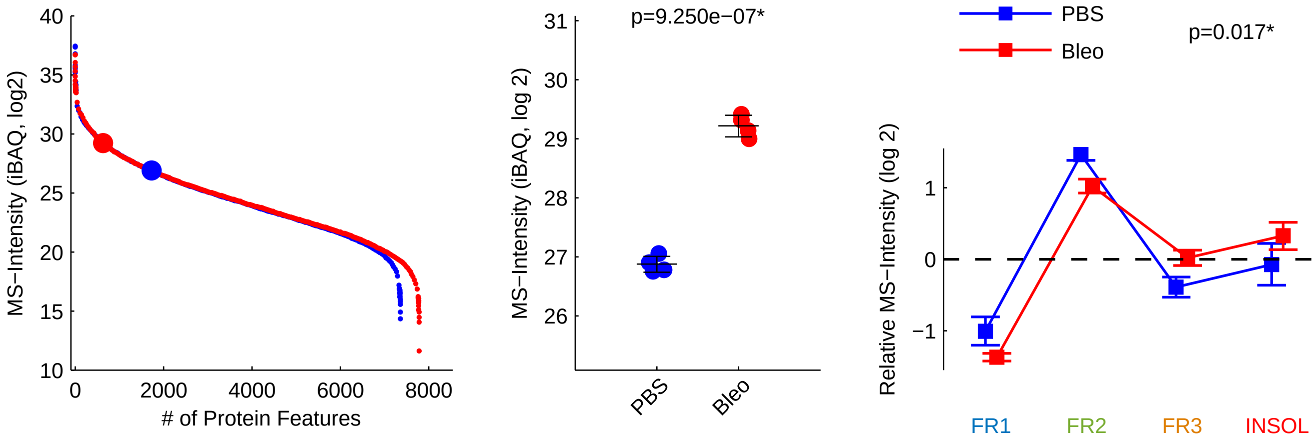

Q07646−2 − Mest (id: 3794)

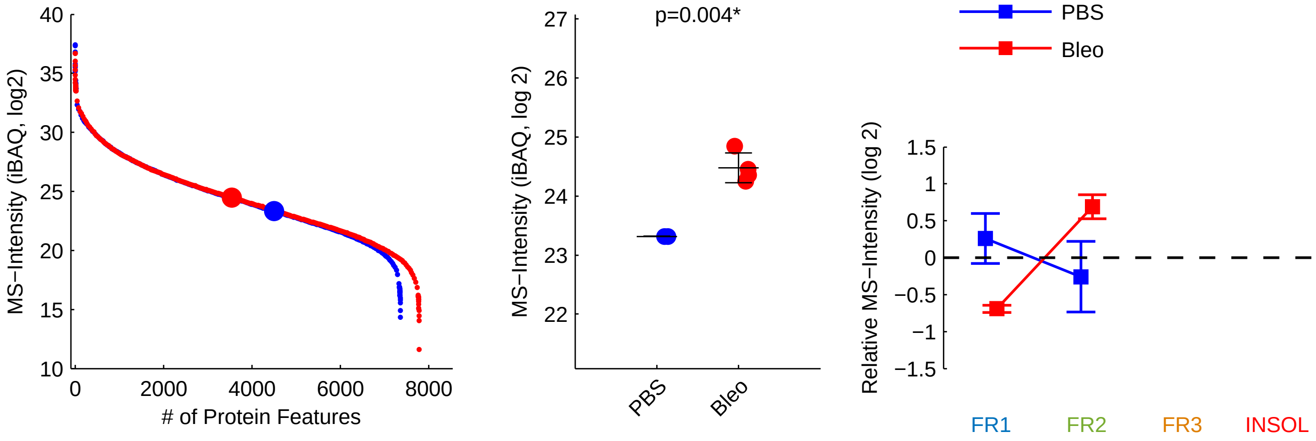

Q07797 − Lgals3bp (id: 3795)

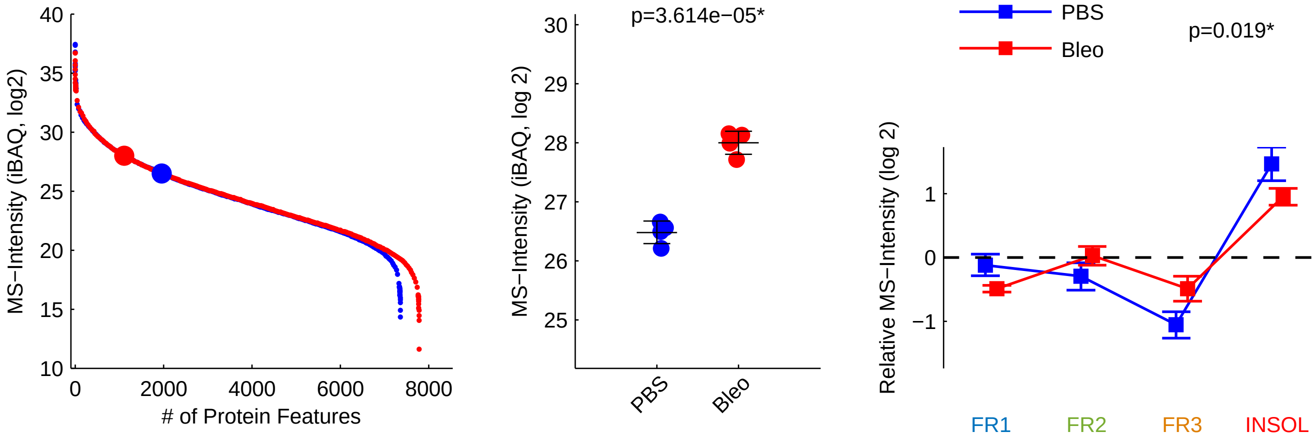

Q07968 − F13b (id: 3799)

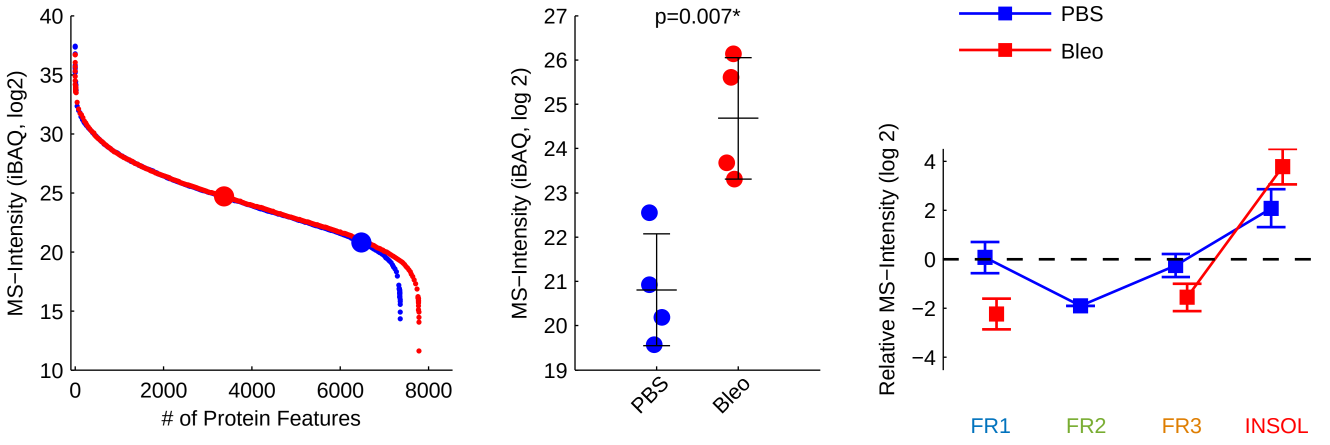

Q08481 − Pecam1 (id: 3807)

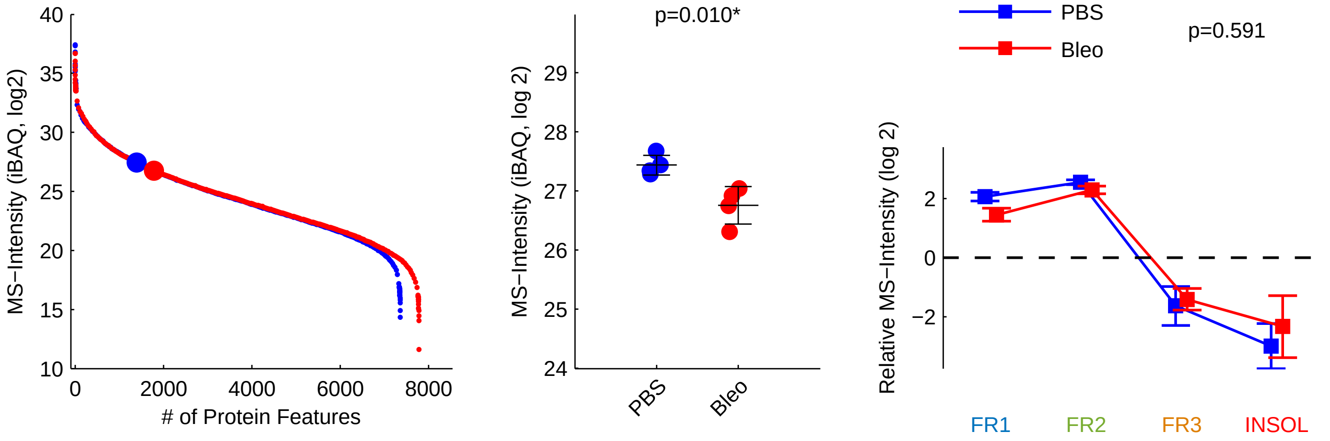

Q08619 − Ifi205b (id: 3809)

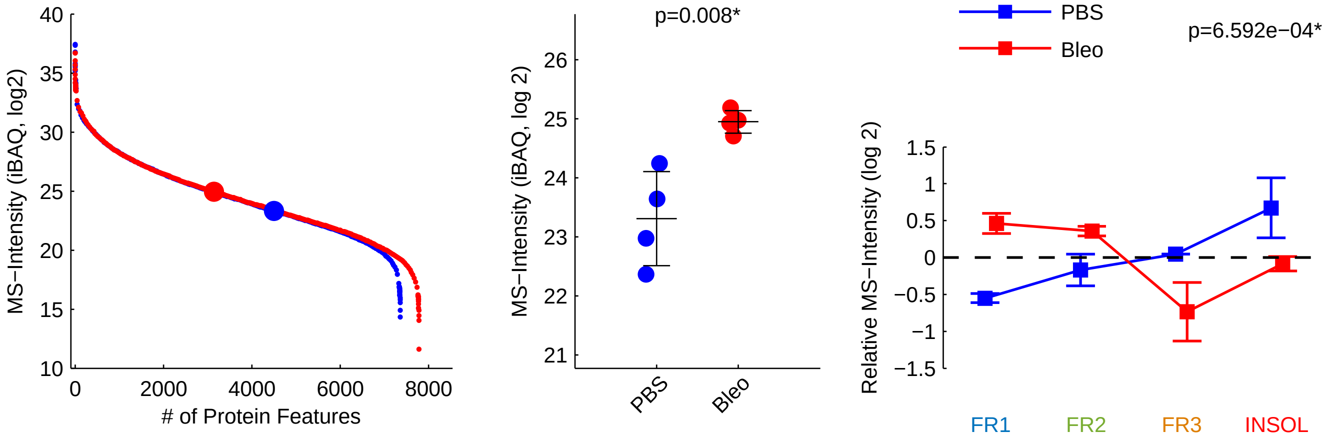

Q08761 − Pros1 (id: 3811)

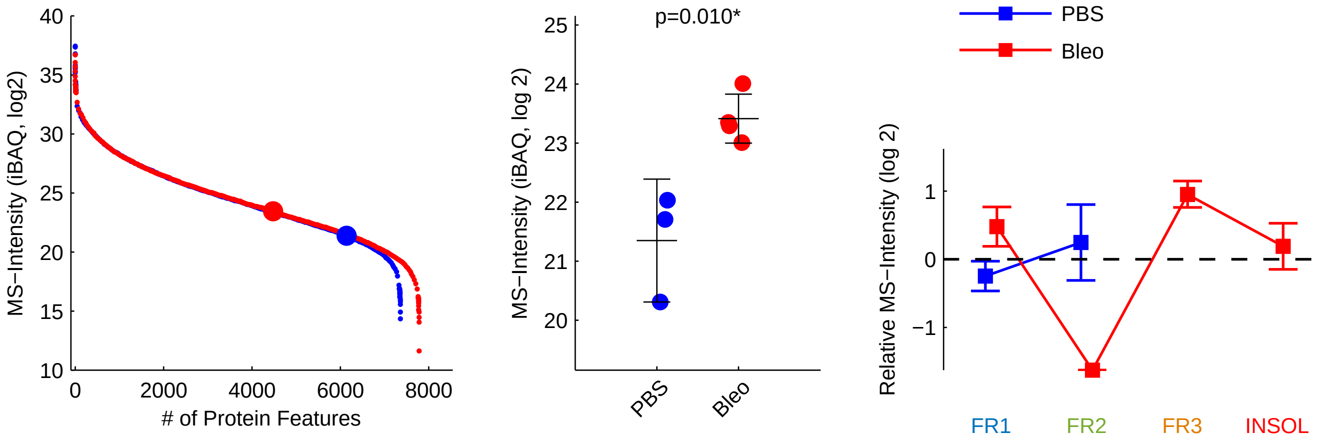

Q08857 − Cd36 (id: 3812)

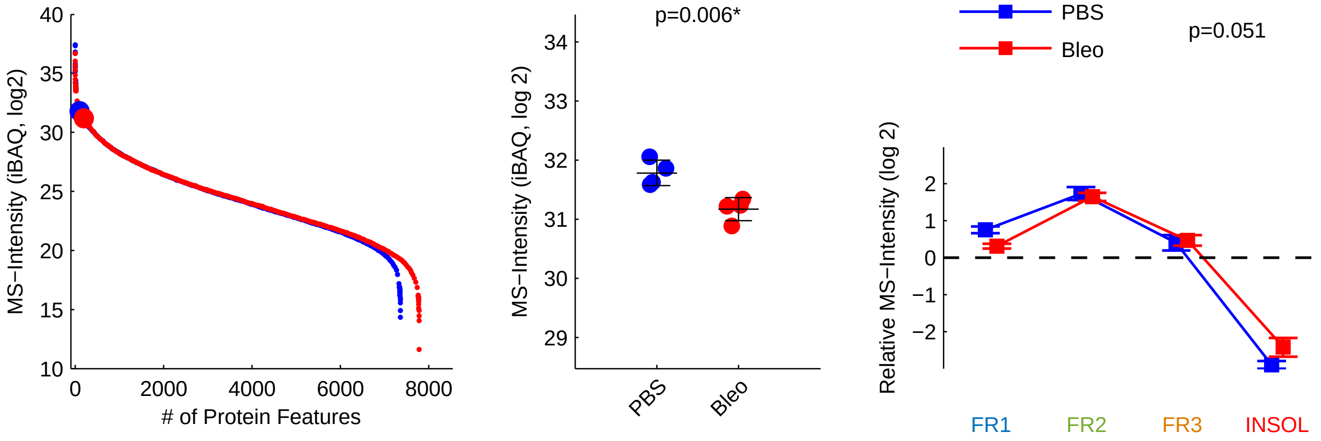

Q08AU9 − (id: 3818)

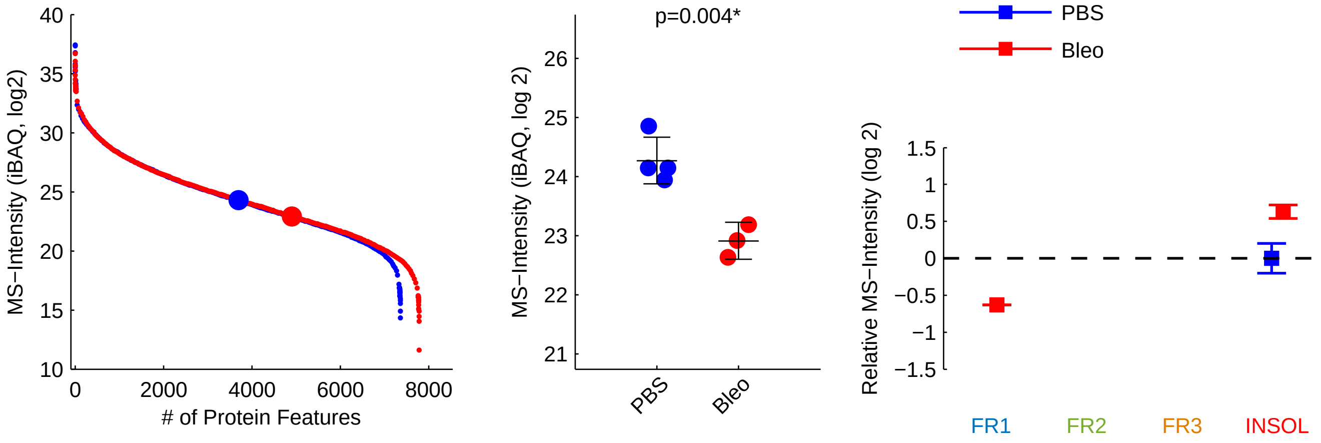

Q08EC4 − Cass4 (id: 3819)

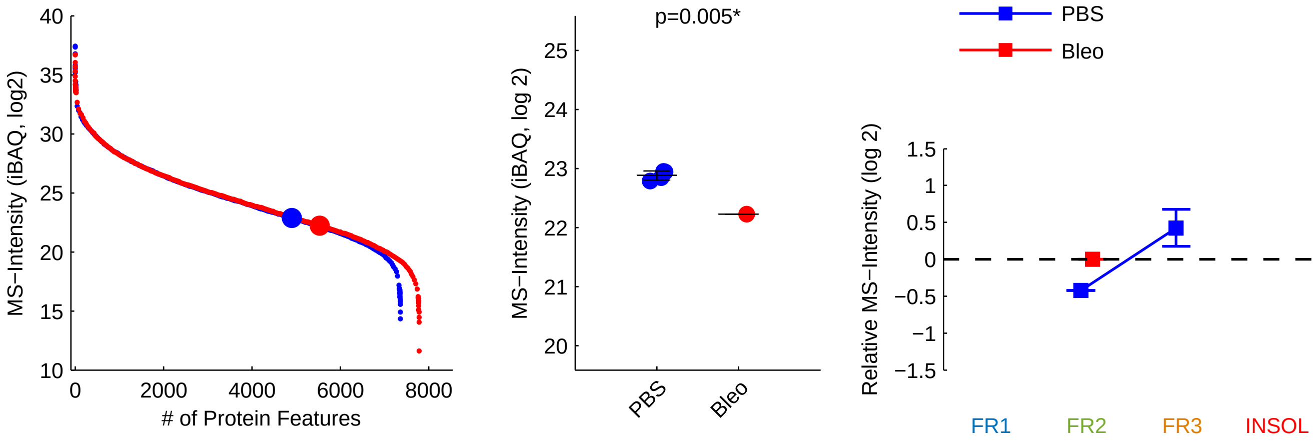

Q0KL02 − Trio (id: 3823)

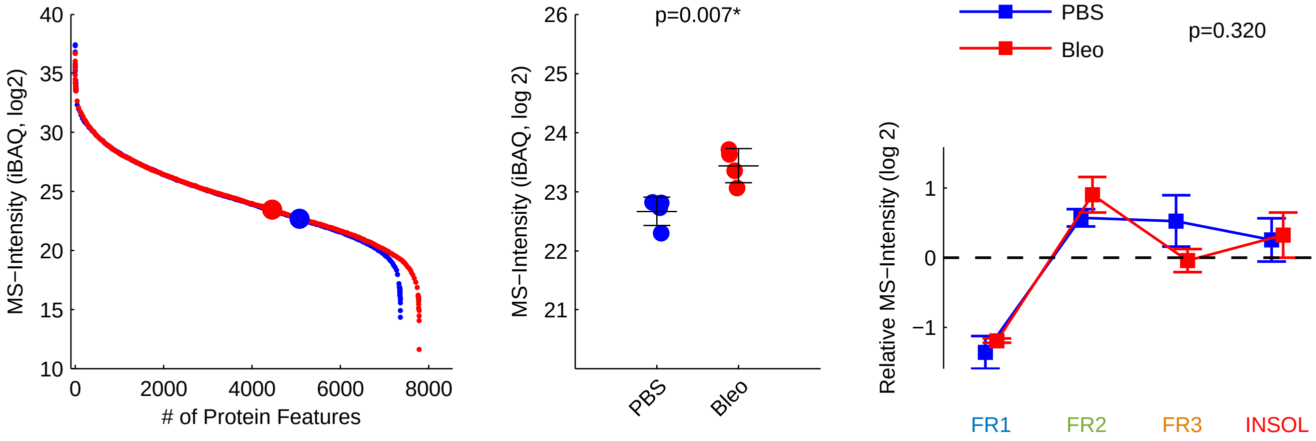

Q0VEE6 − Znf800 (id: 3831)

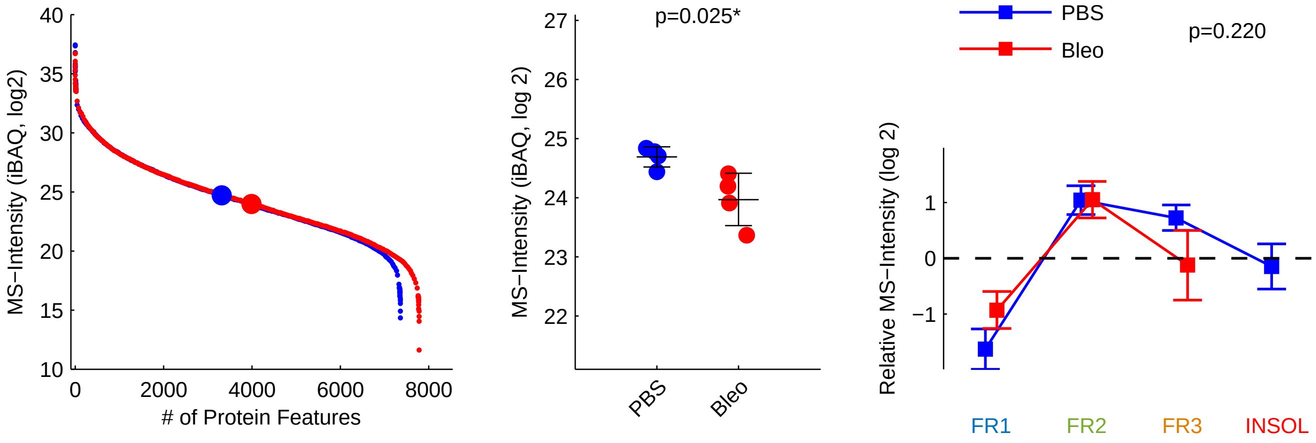

Q14B46 − Rtkn2 (id: 3844)

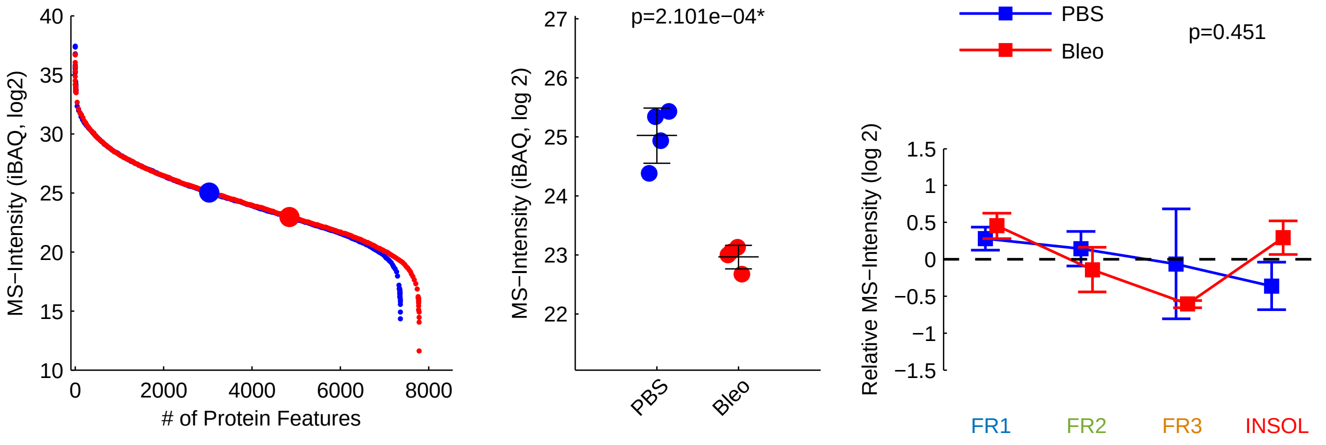

Q14C51 − Ptcd3 (id: 3847)

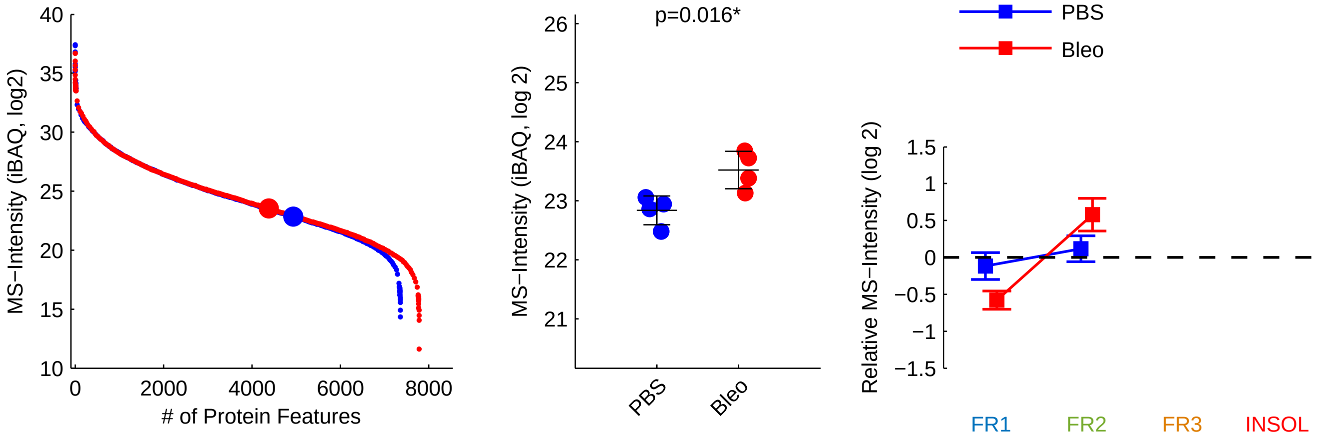

Q2KN98 − Specc1l (id: 3857)

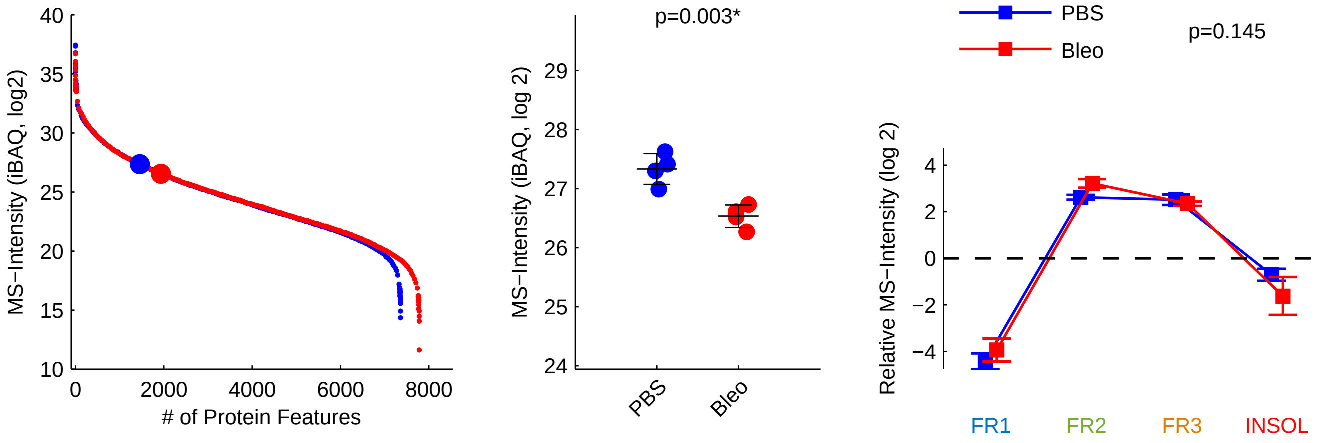

Q2TPA8 − Hsdl2 (id: 3860)

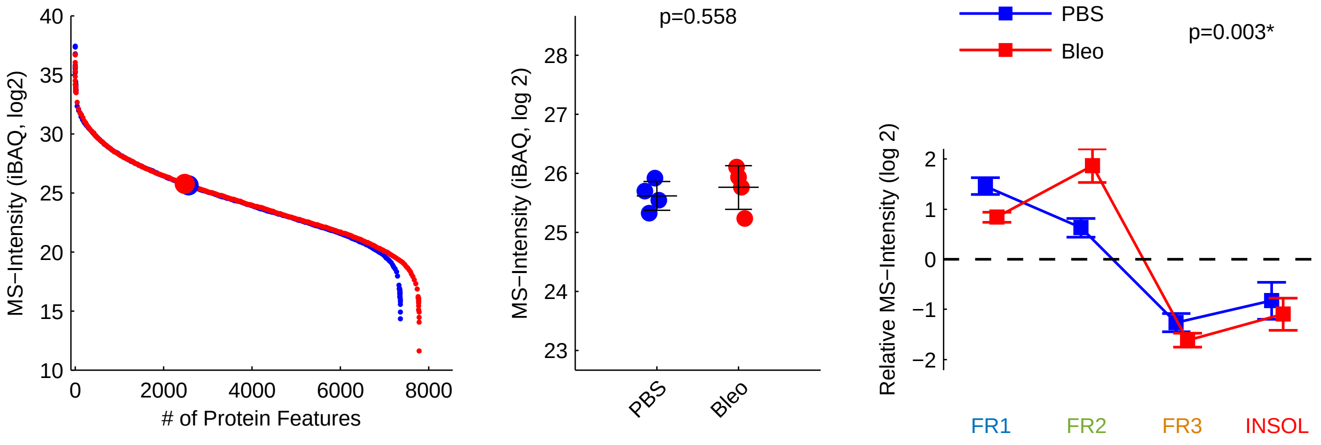

Q2UY11 − Col28a1 (id: 3862)

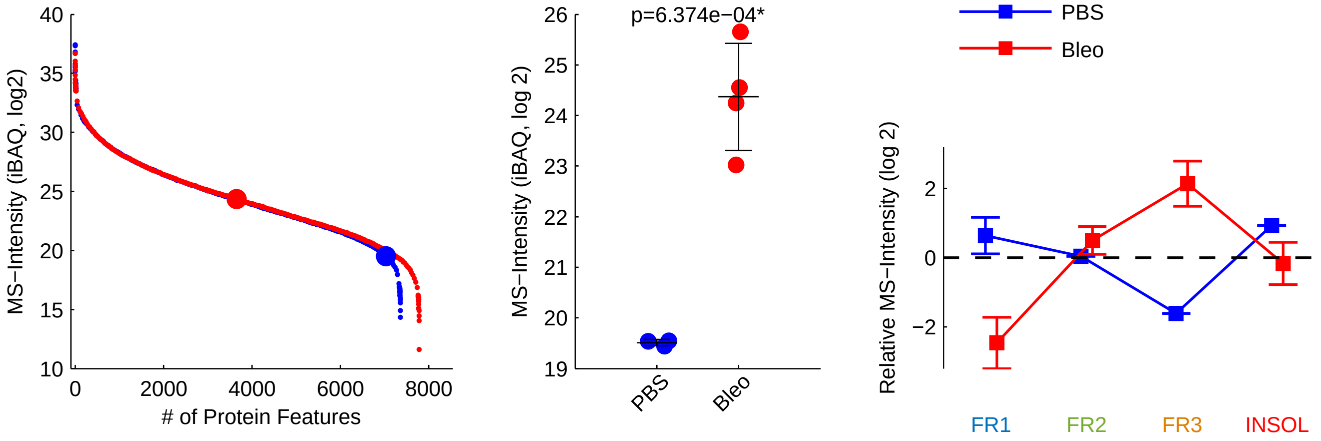

Q3LAC4 − Prex2 (id: 3871)

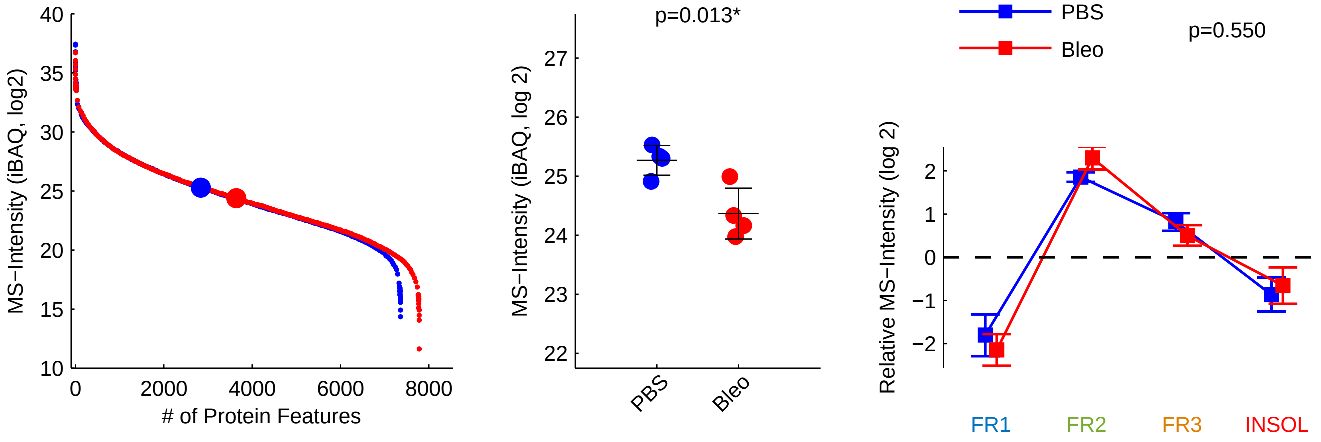

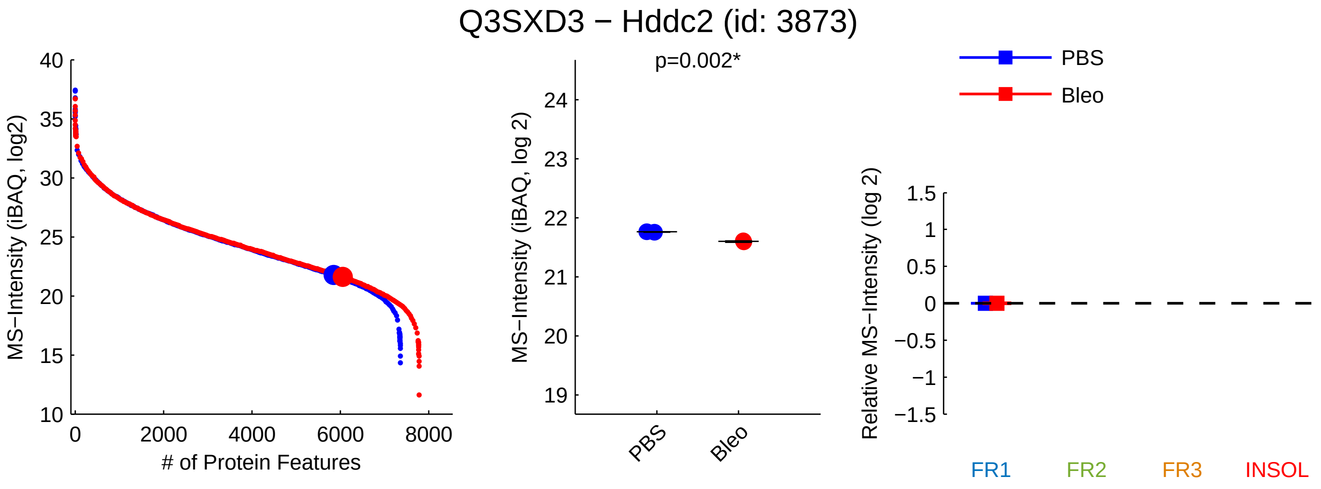
Q3SXD3 − Hddc2 (id: 3873)
MS−Intensity (iBAQ, log2)
# of Protein Features
p=0.002*
MS−Intensity (iBAQ, log 2)
PBS
Bleo
Relative MS−Intensity (log 2)
FR1
FR2
FR3
INSOL

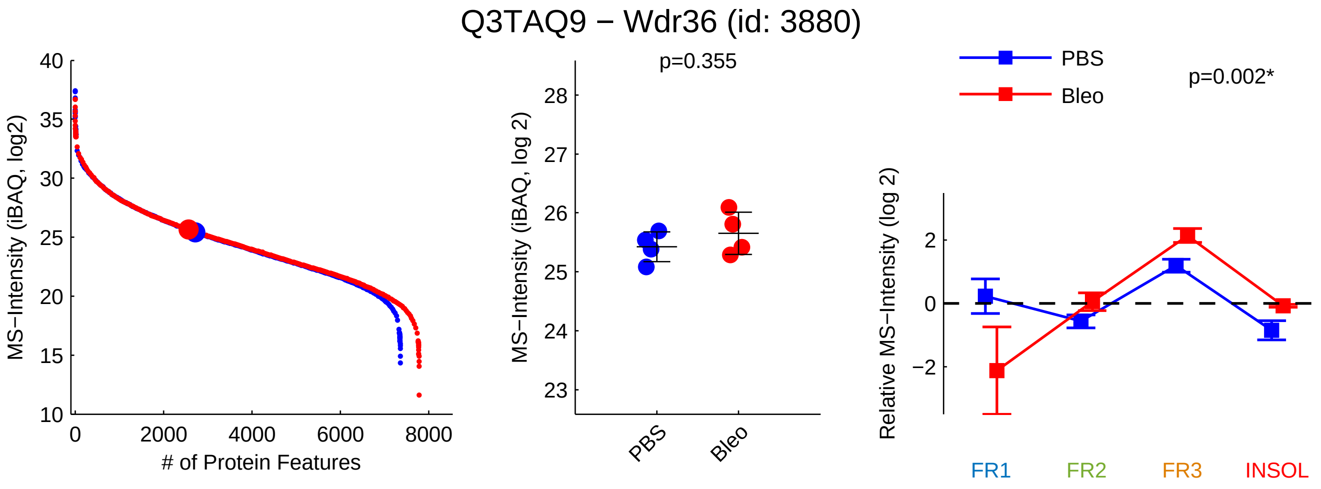
Q3TAQ9 − Wdr36 (id: 3880)
MS−Intensity (iBAQ, log2)
# of Protein Features
p=0.355
MS−Intensity (iBAQ, log 2)
PBS
Bleo
p=0.002*
Relative MS−Intensity (log 2)
FR1
FR2
FR3
INSOL

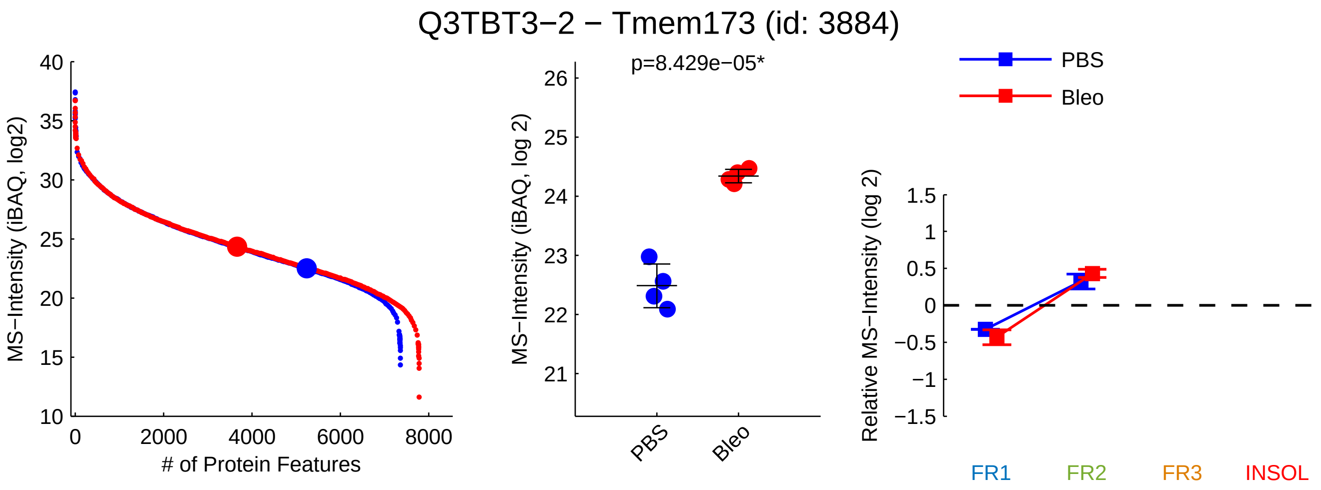
Q3TBT3−2 − Tmem173 (id: 3884)
MS−Intensity (iBAQ, log2)
# of Protein Features
p=8.429e−05*
MS−Intensity (iBAQ, log 2)
PBS
Bleo
Relative MS−Intensity (log 2)
FR1
FR2
FR3
INSOL

Q3TC72 − Fahd2 (id: 3887)

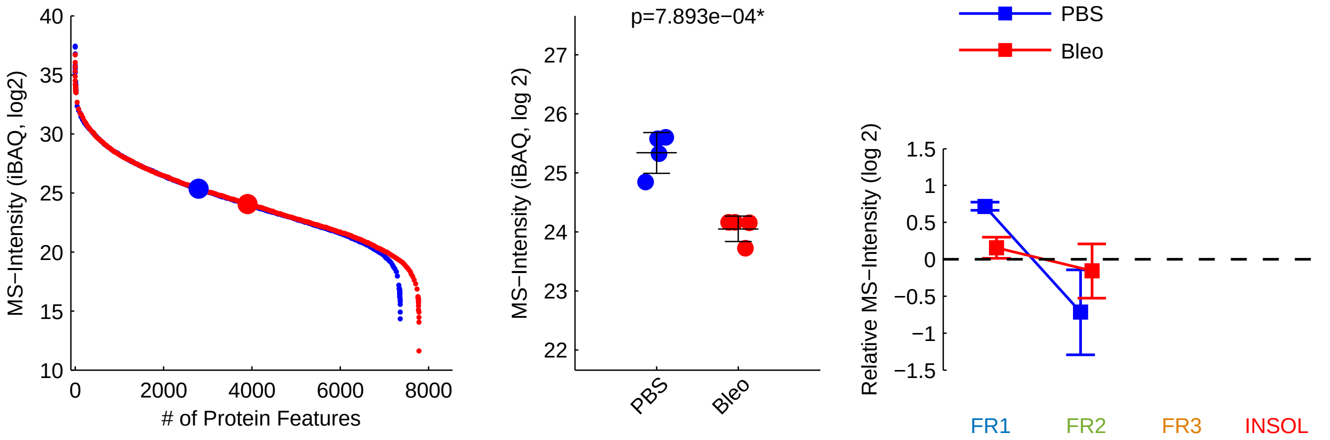

Q3TCN2 − Plbd2 (id: 3891)

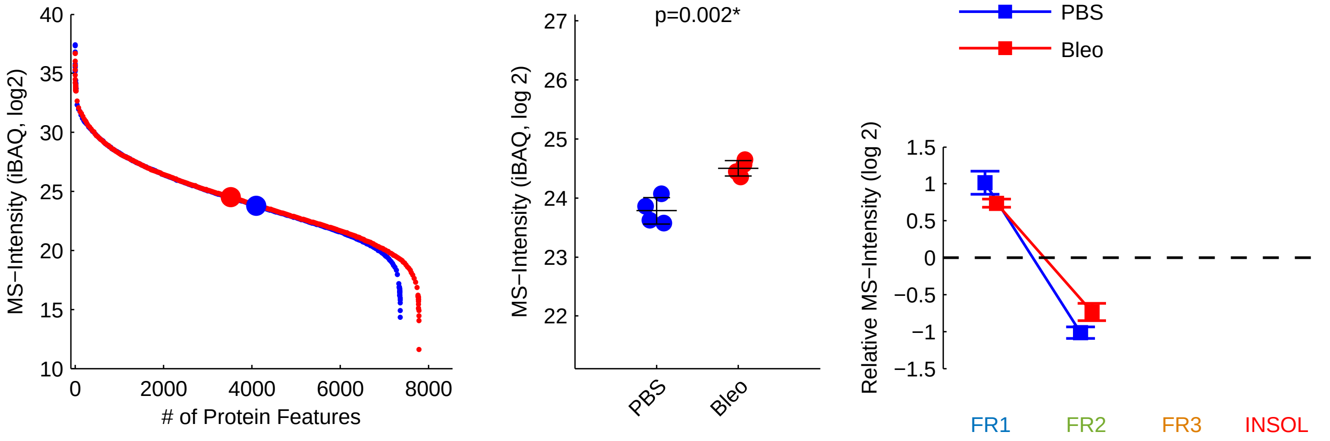

Q3TEA8−2 − Hp1bp3 (id: 3903)

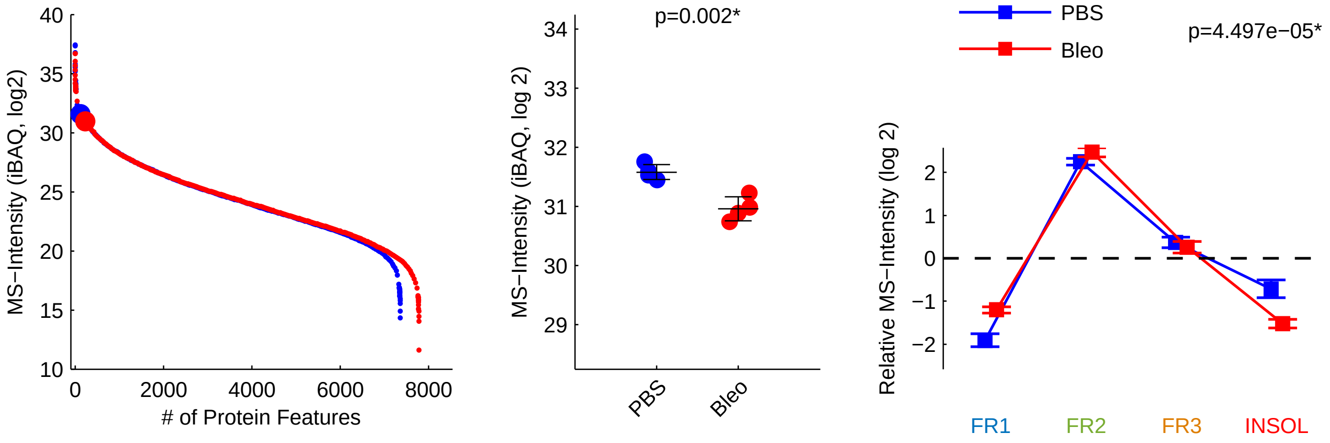

Q3TFD2 − Lpcat1 (id: 3904)

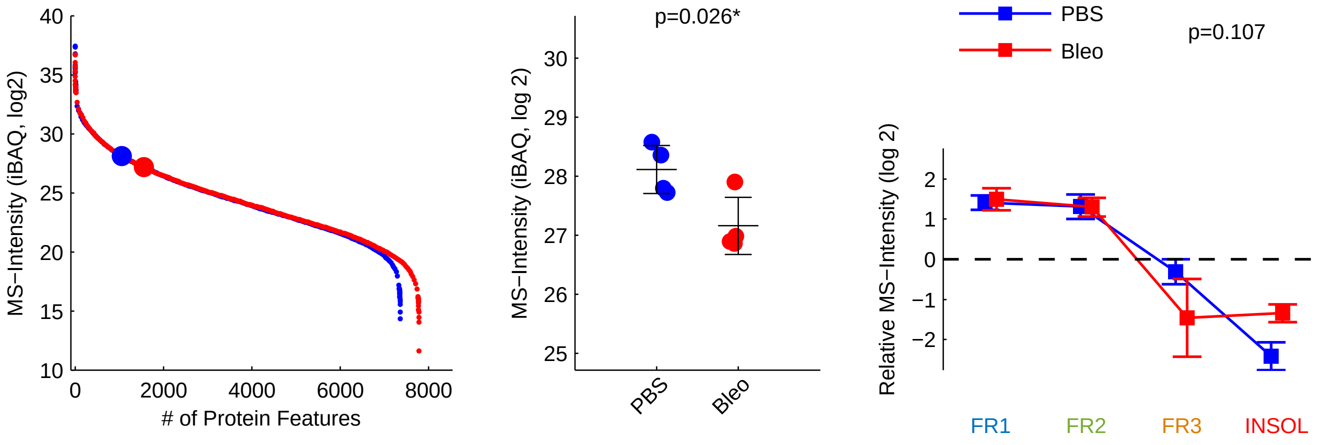

Q9R0U0 − Srsf10 (id: 3905)

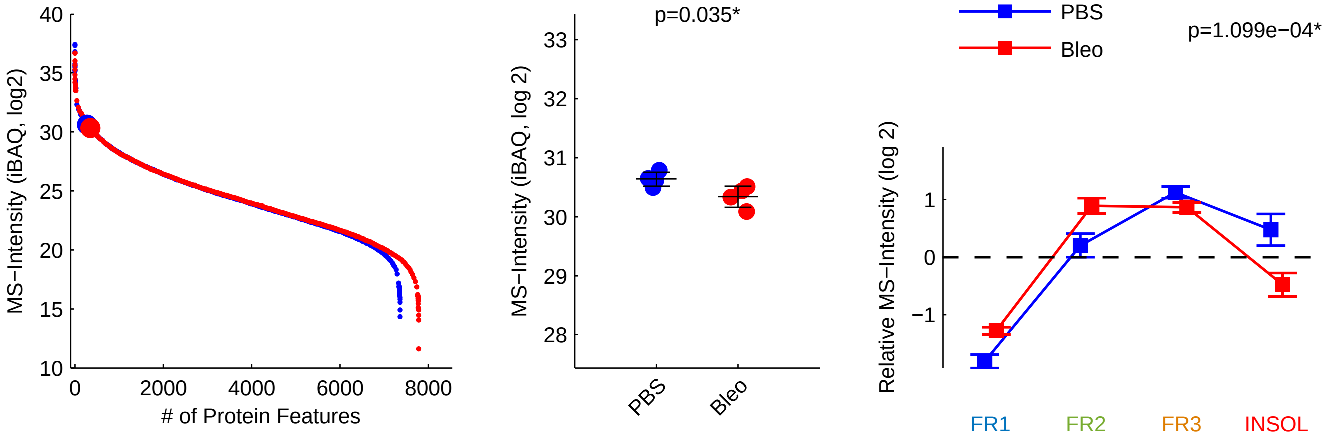

Q3TFQ1 − Spryd7 (id: 3906)

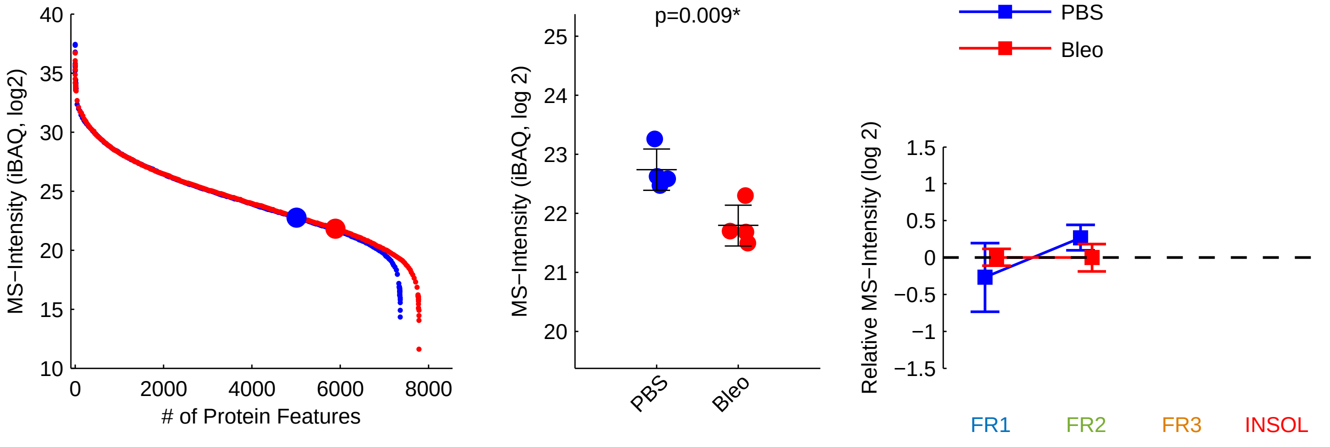

Q3TGW2 − Eepd1 (id: 3909)

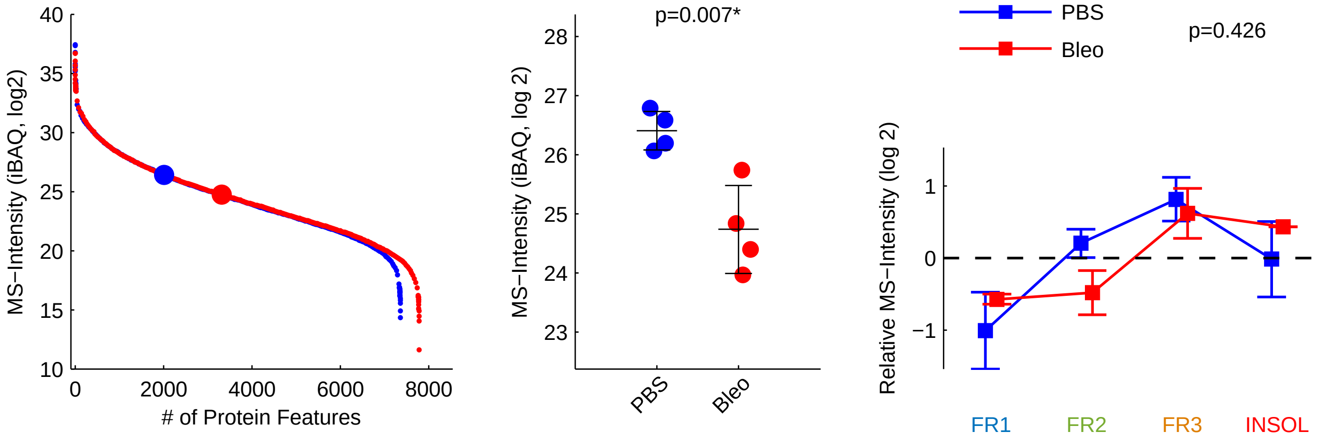

Q9DBU6 − Rsrc1 (id: 3910)

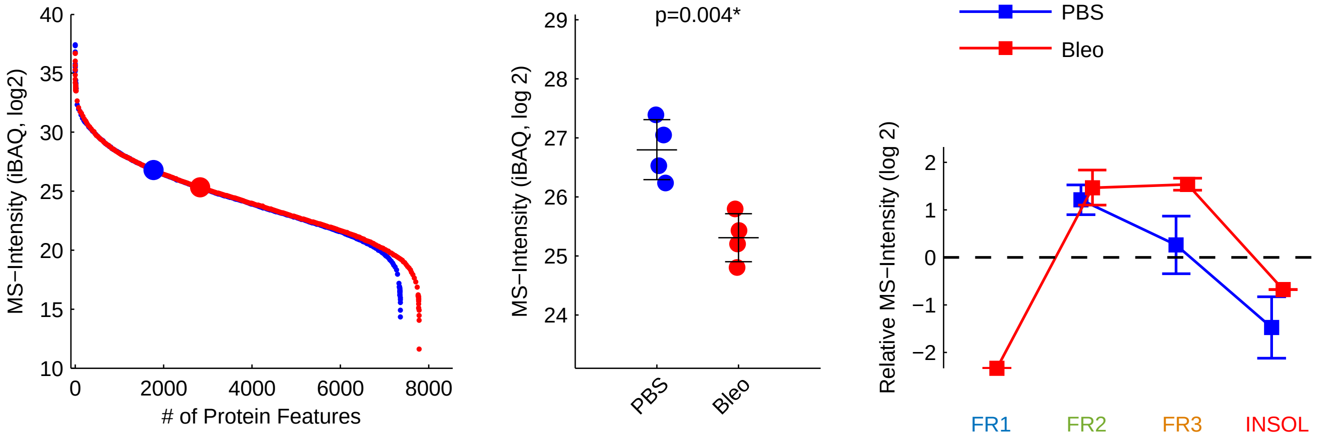

Q3THK7 − Gmps (id: 3914)

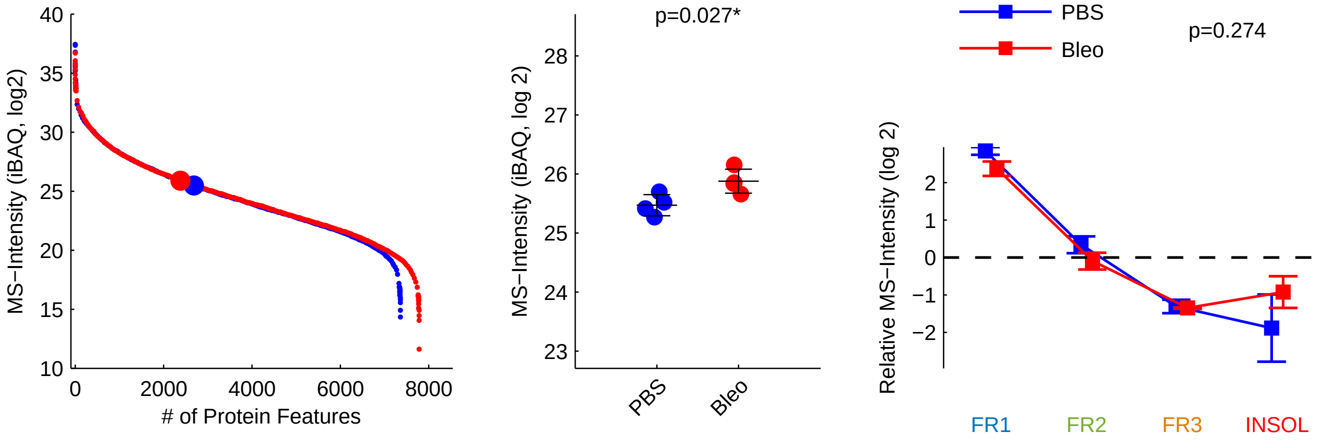

Q3THS6 − Mat2a (id: 3915)

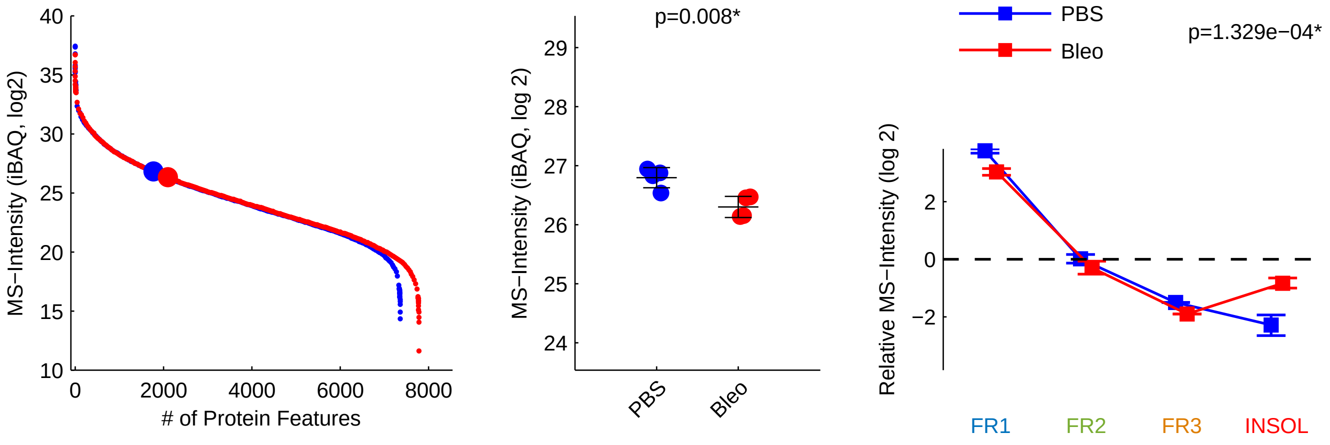

Q3TIX9 − Usp39 (id: 3921)

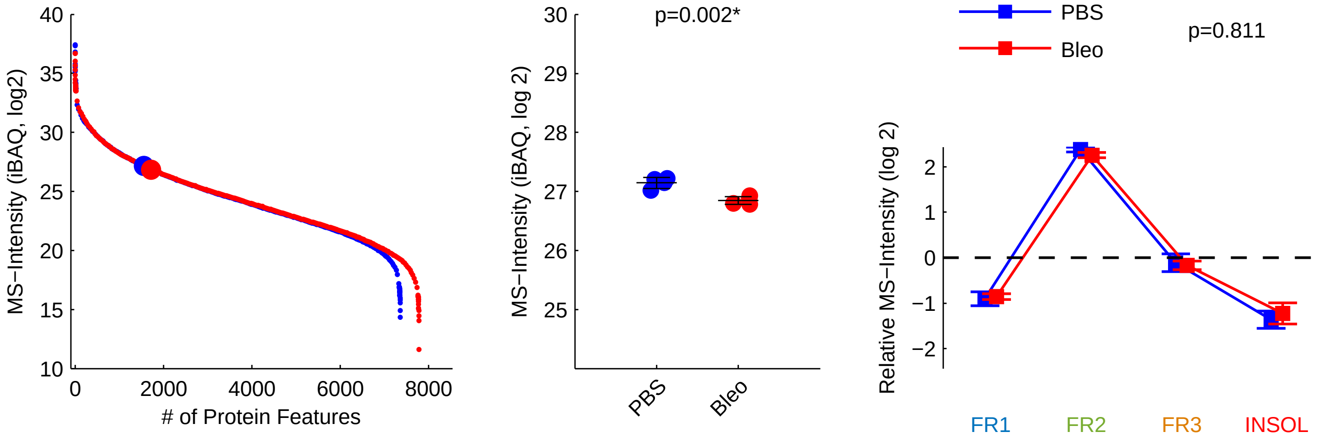

Q3TJD7 − Pdlim7 (id: 3924)

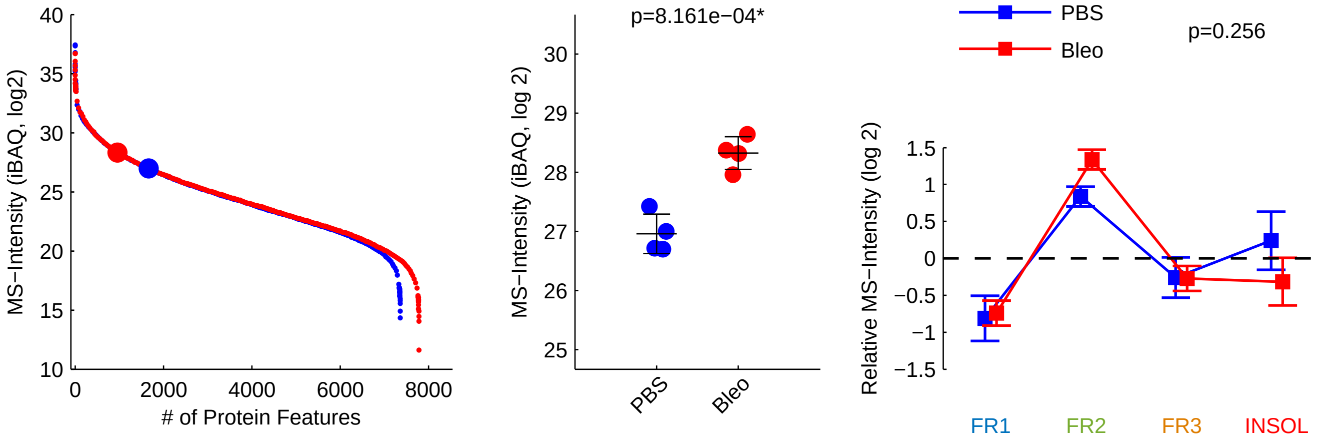

Q3TJZ6 − Fam98a (id: 3927)

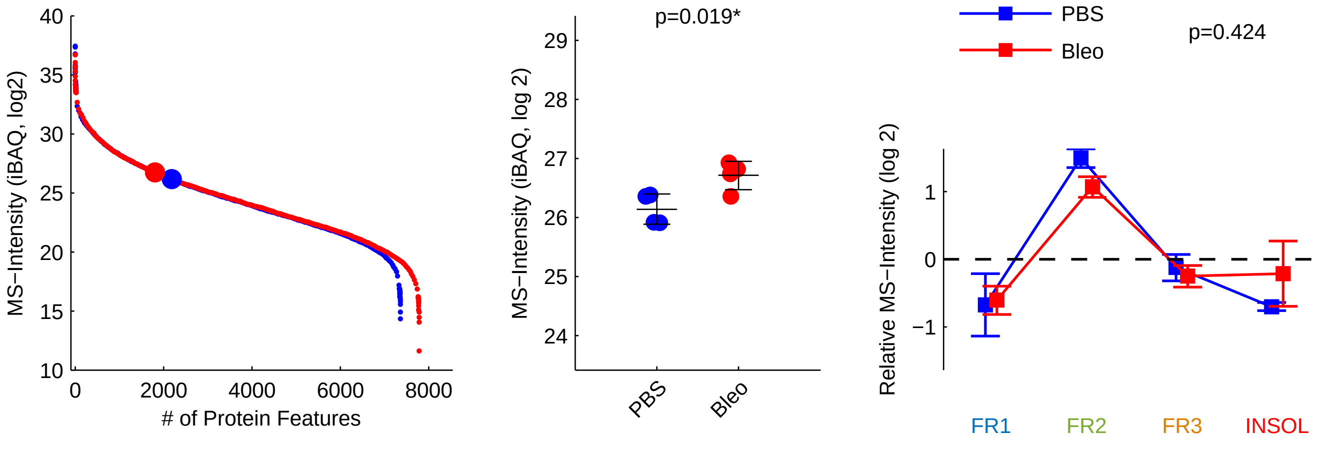

Q3TLH4−5 − Prrc2c (id: 3931)

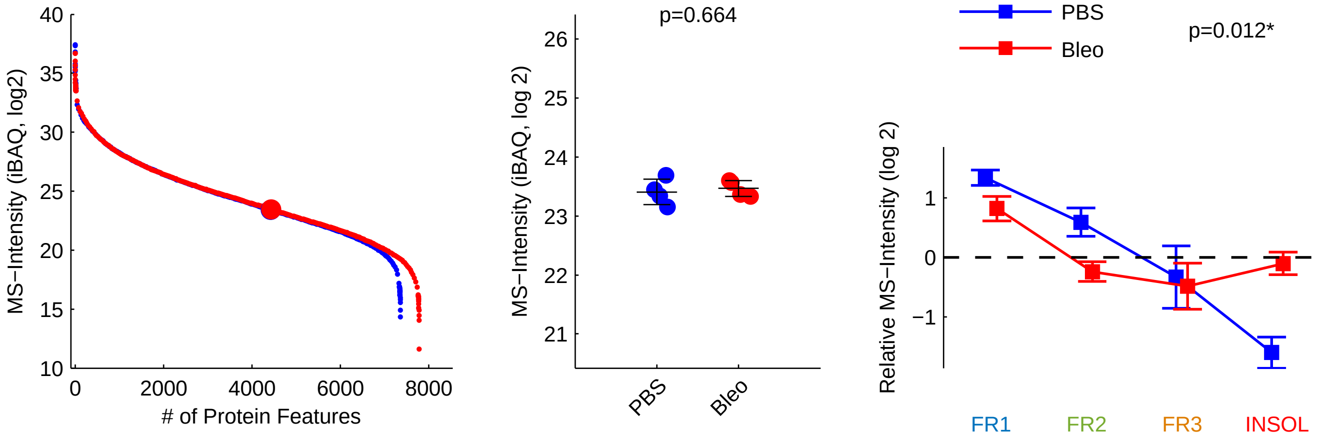

Q3TMH2 − Scrn3 (id: 3936)

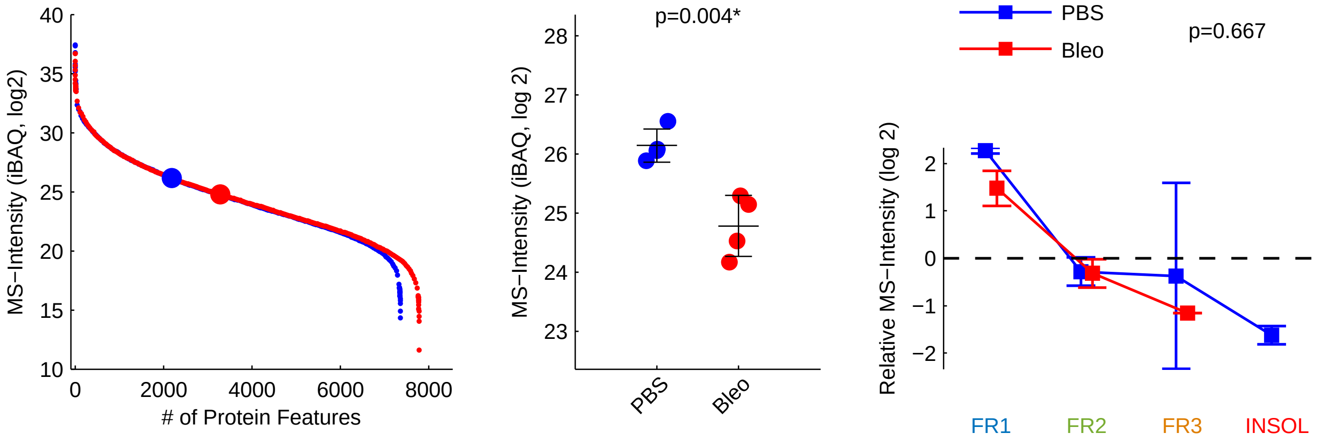

Q3TML0 − Pdia6 (id: 3937)

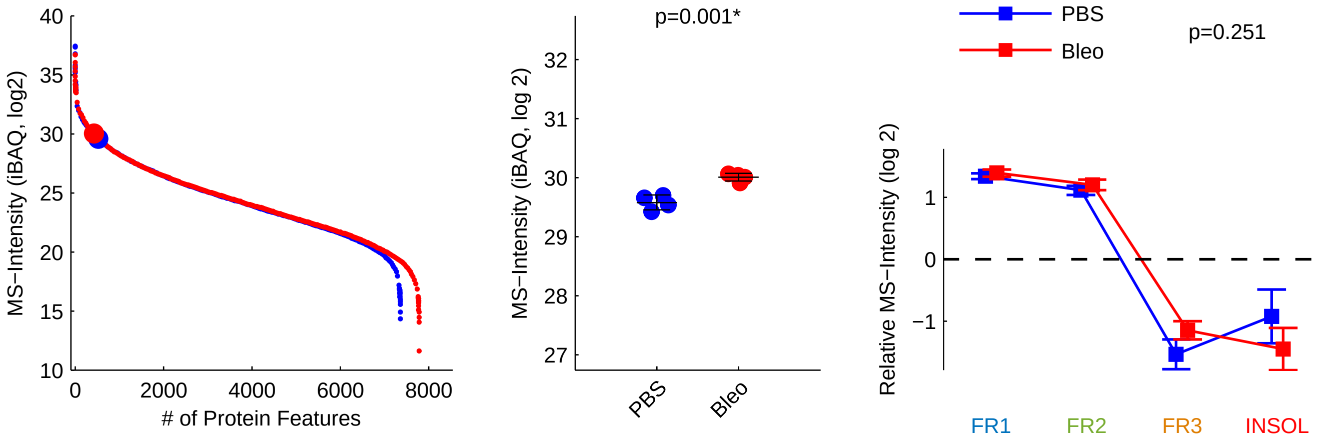

Q3TMP1 − Gtf3c3 (id: 3938)

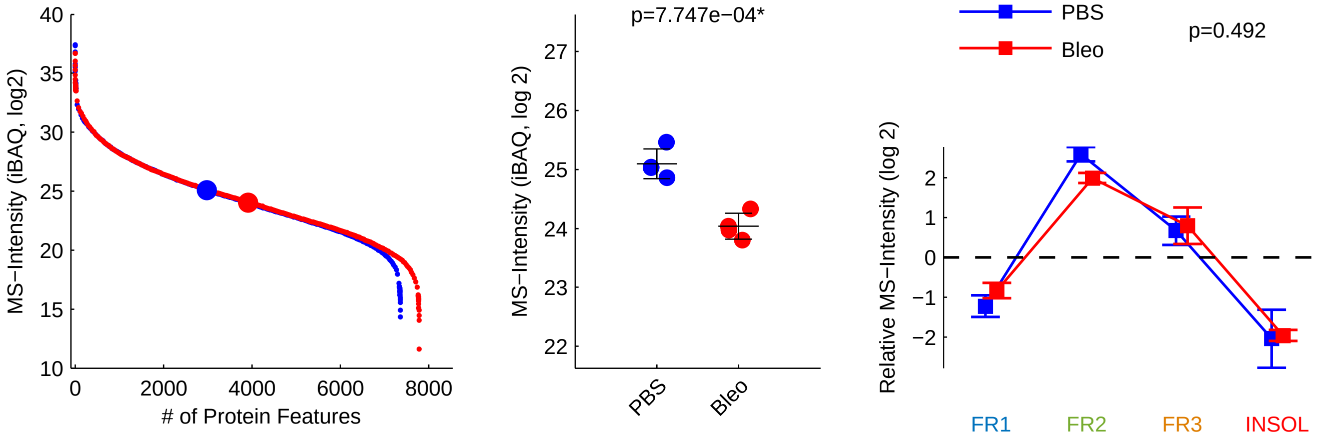

Q3TPX4 − Exoc5 (id: 3947)

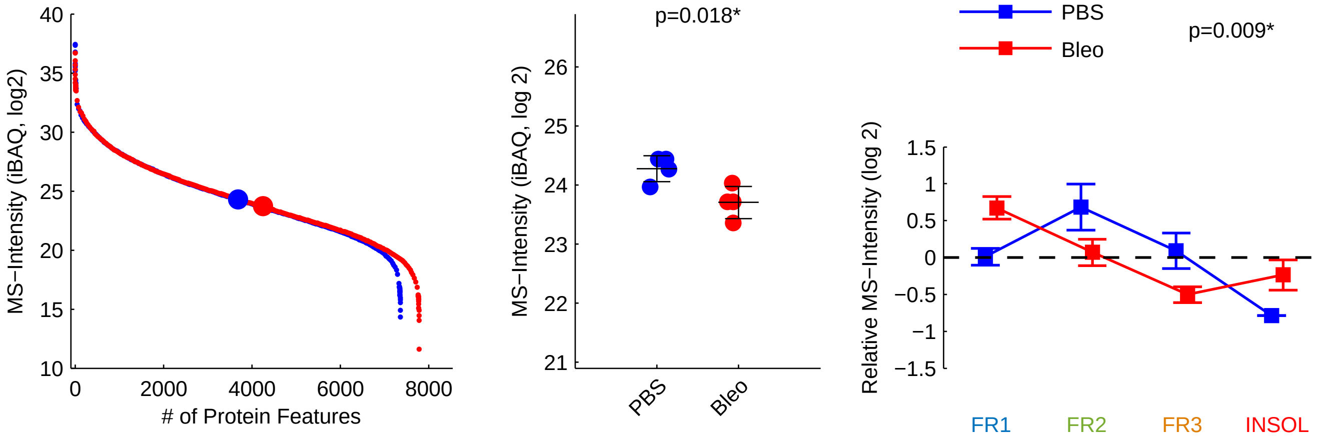

Q3TW96 − Uap1l1 (id: 3964)

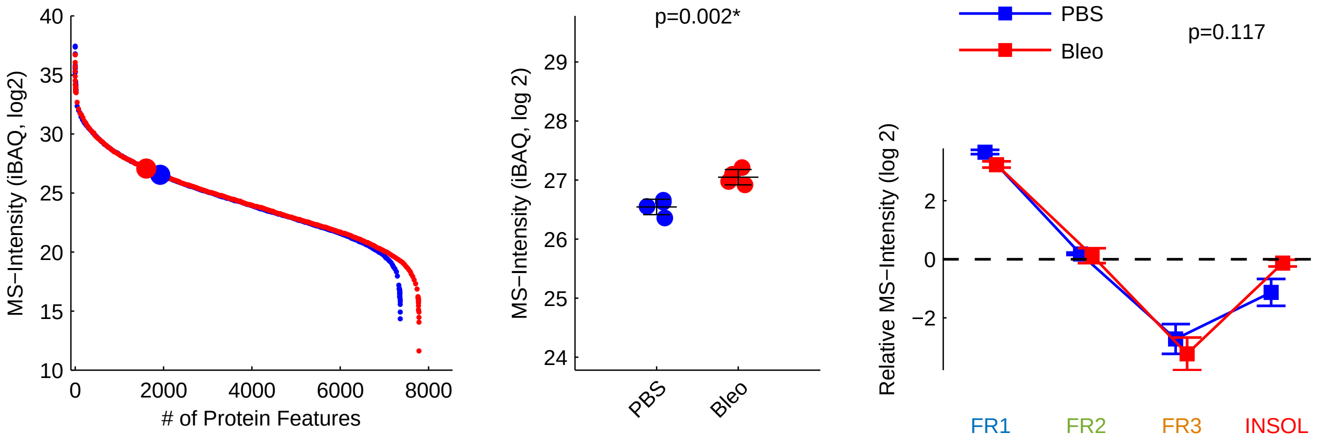

Q3TYA6 − Mphosph8 (id: 3976)

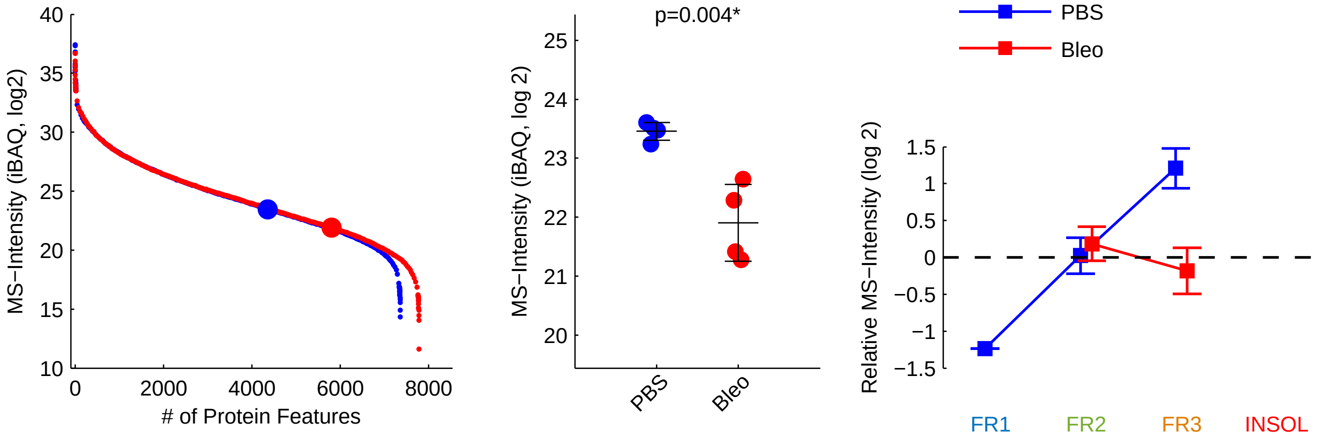

Q80W04 − Tmcc2 (id: 3986)

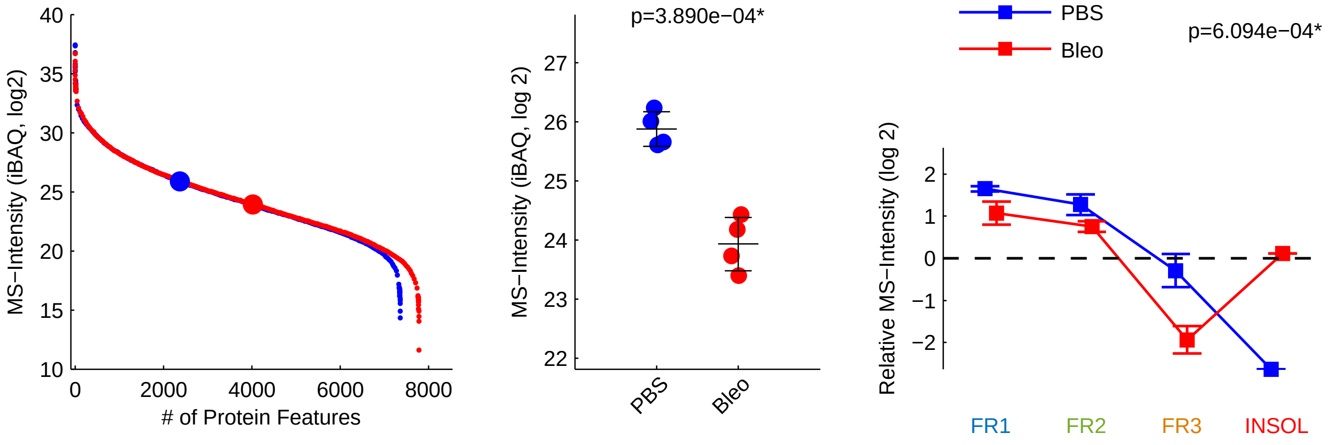

Q3TZZ7 − Esyt2 (id: 3987)

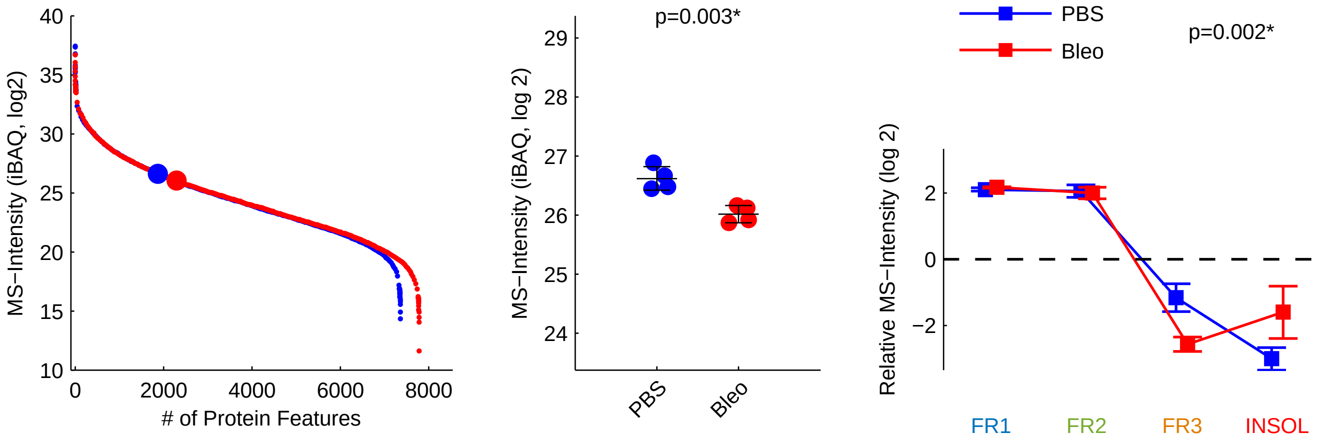

Q3U0S6 − Rasip1 (id: 3992)

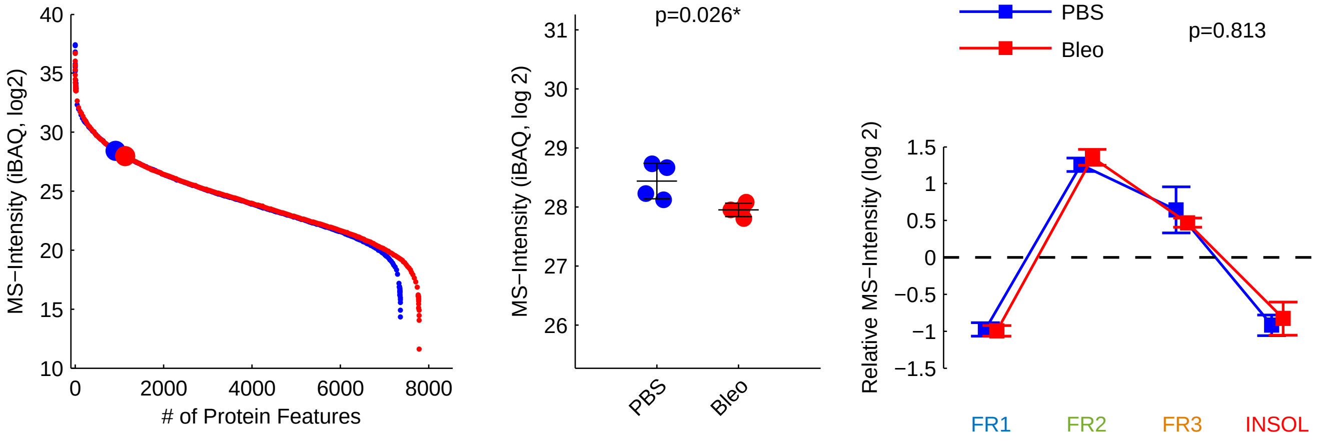

Q3U0V1 − Khsrp (id: 3993)

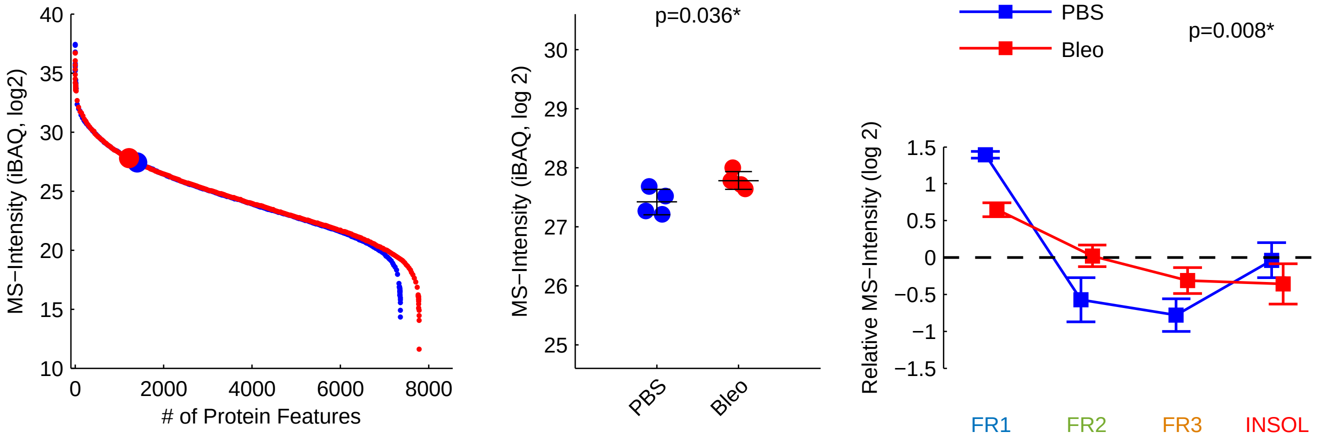

Q9CYH2 − Fam213a (id: 3995)

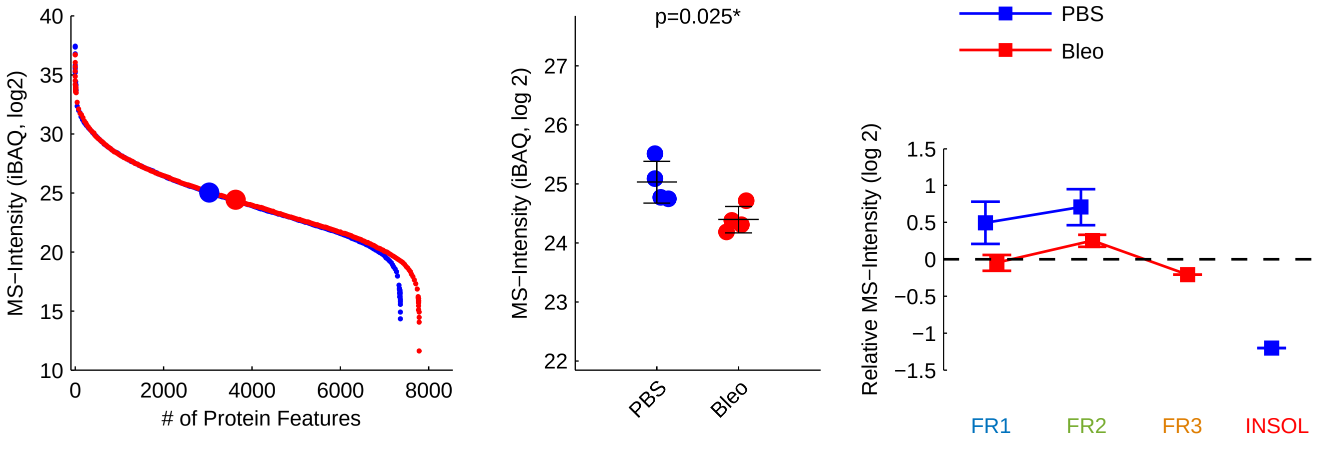

Q3U1U4 − Itgam (id: 4004)

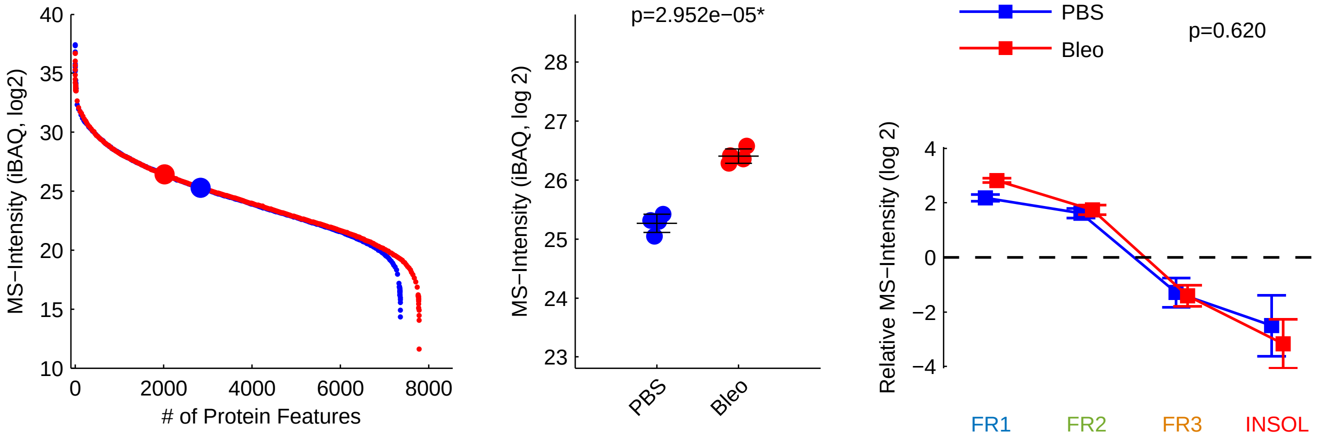

Q80WC7 − Agfg2 (id: 4009)

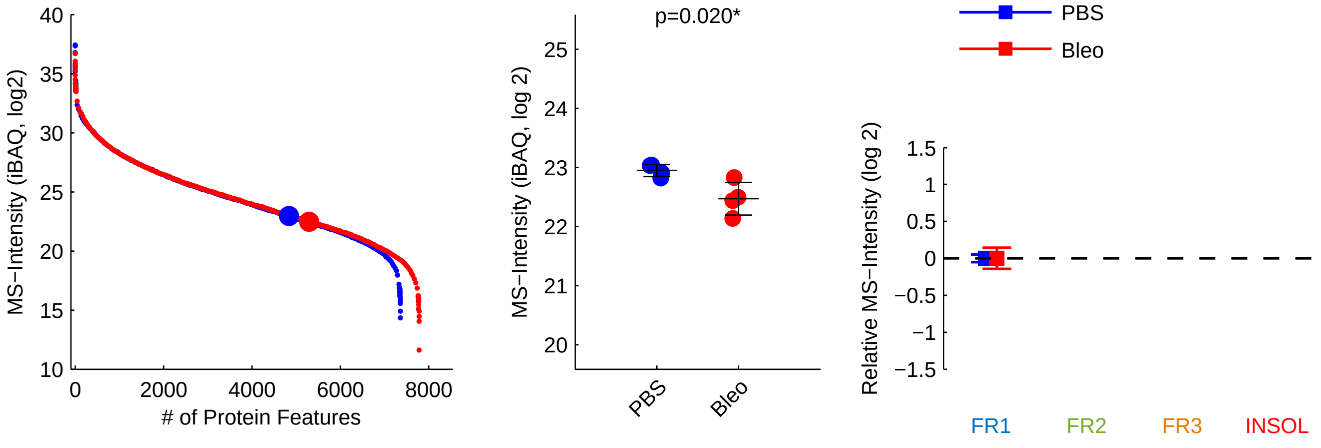

Q3U2P1 − Sec24a (id: 4010)

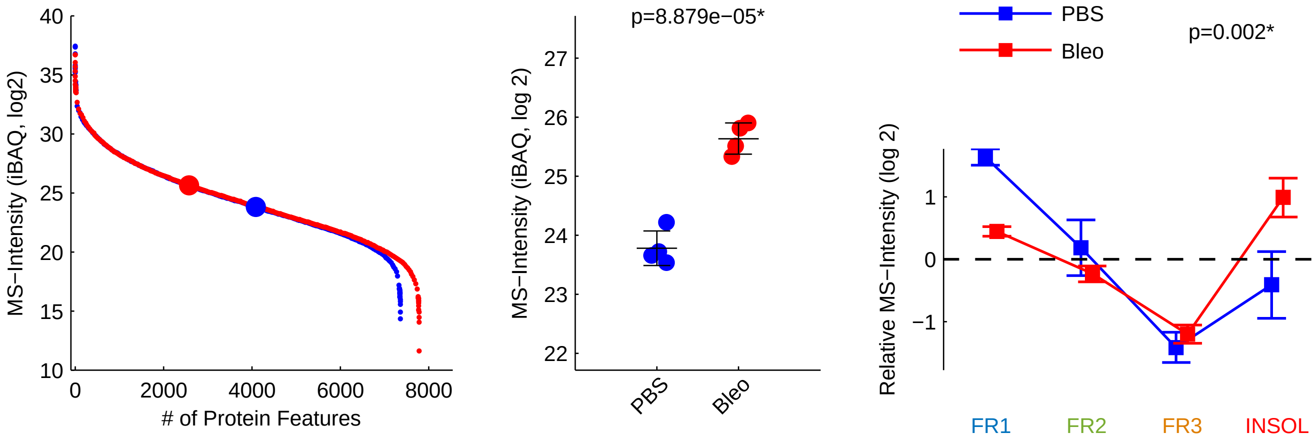

Q3U4S0 − Pank2 (id: 4026)

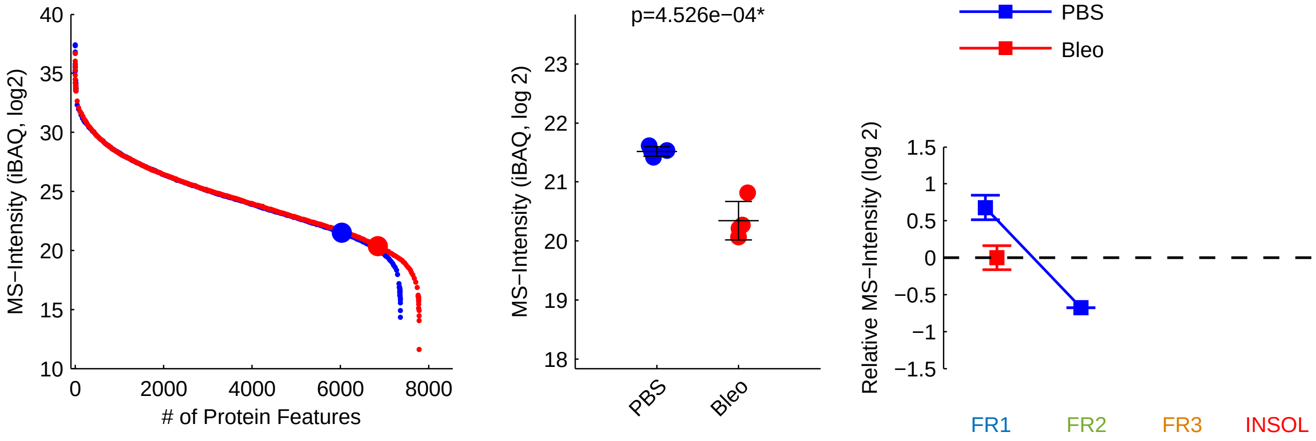

Q3U5C8 − Arhgef16 (id: 4029)

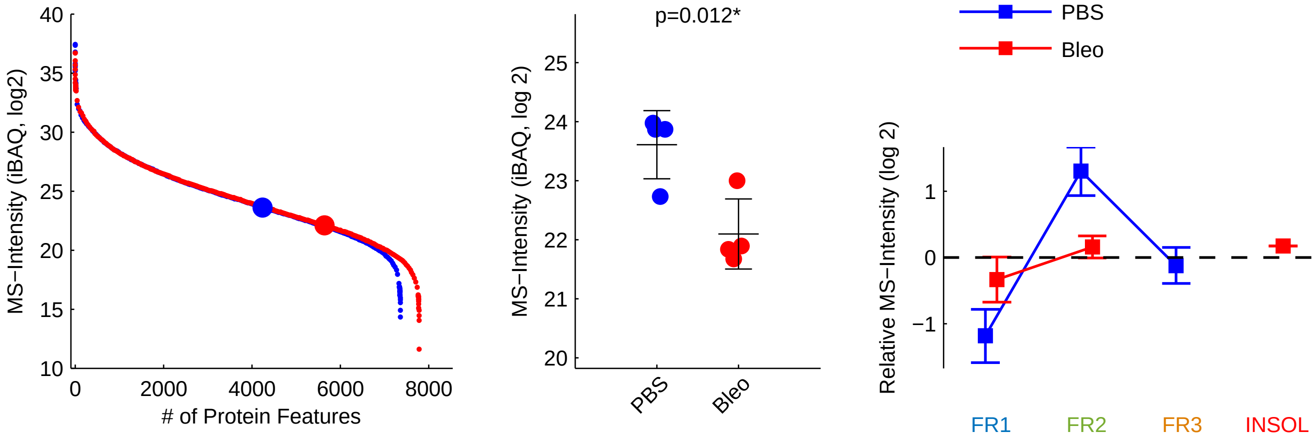

Q3U5Q7 − Cmpk2 (id: 4031)

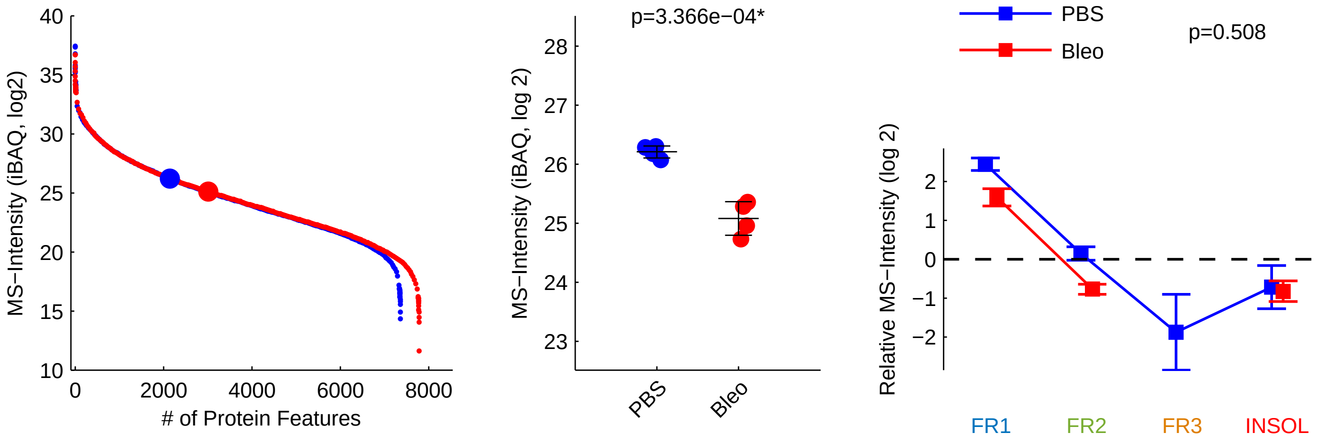

Q9D3G5 − Stx11 (id: 4032)

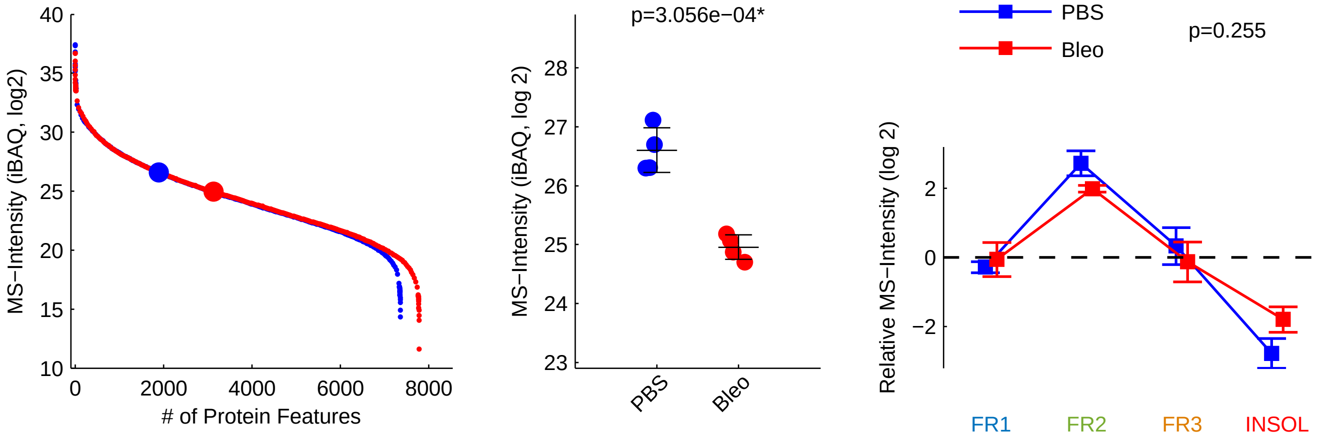

Q3U7R1 − Esyt1 (id: 4038)

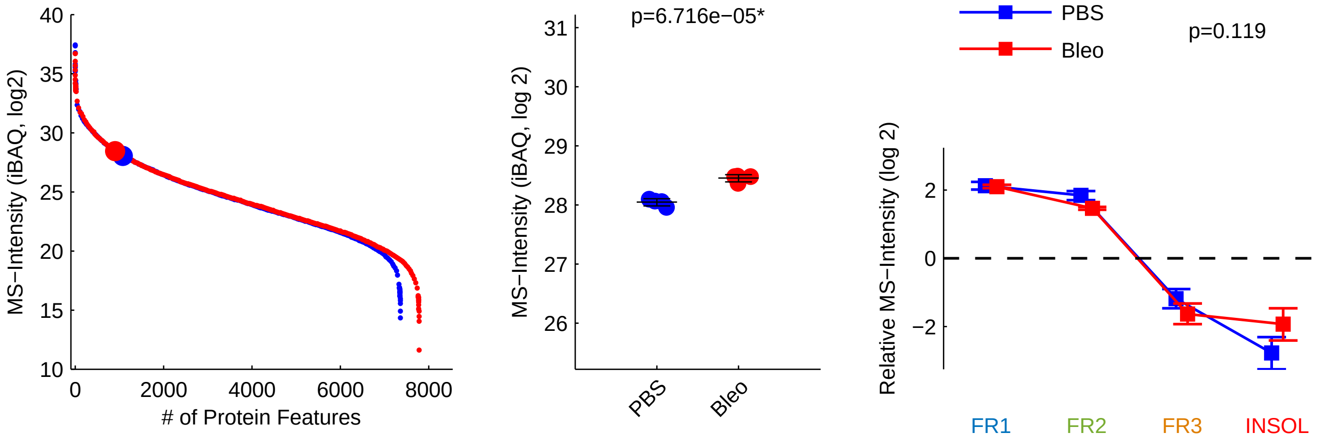

Q3UBX0 − Tmem109 (id: 4052)

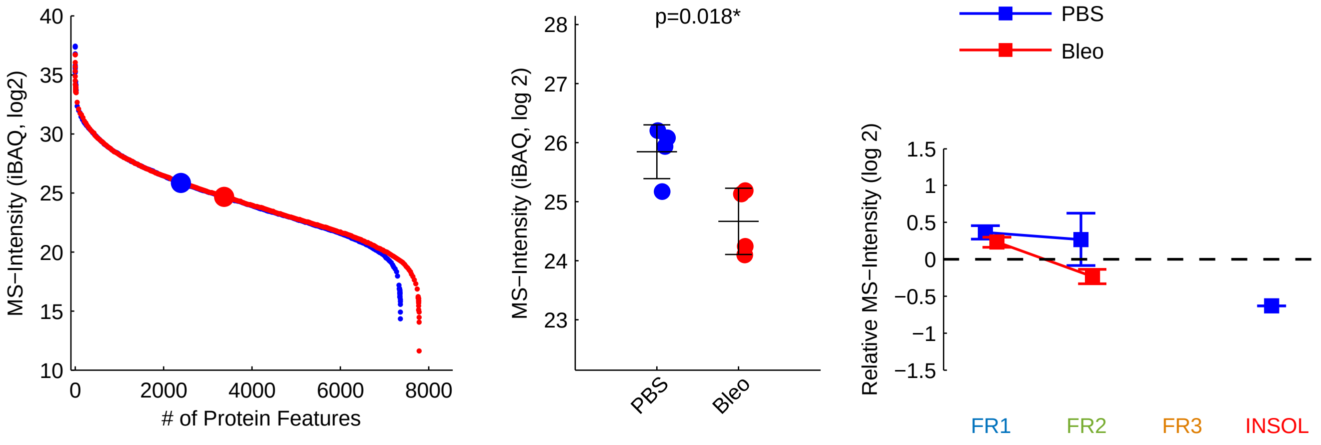

Q3UFK8 − Frmd8 (id: 4062)

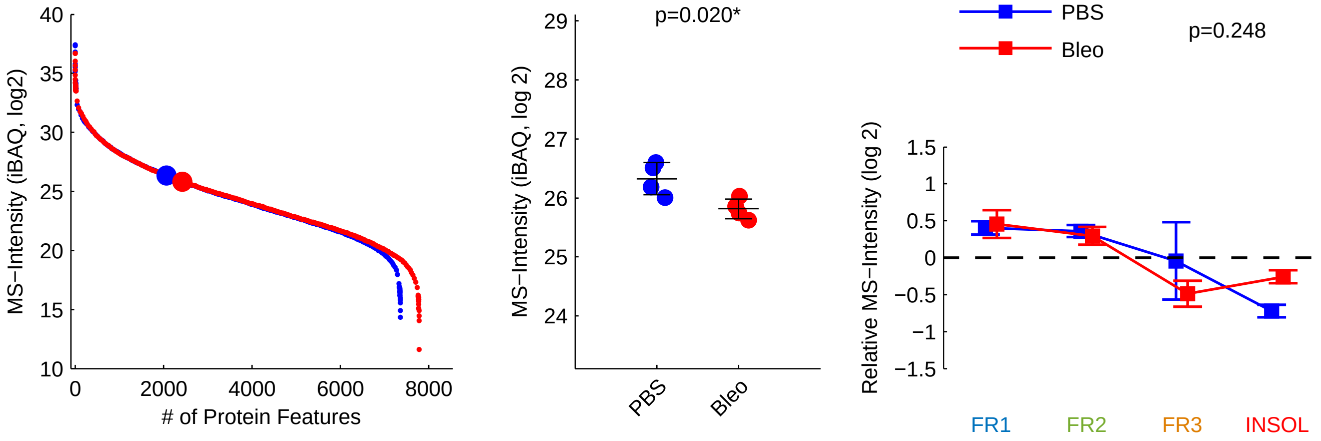

Q3UGC7 − Eif3j1 (id: 4070)

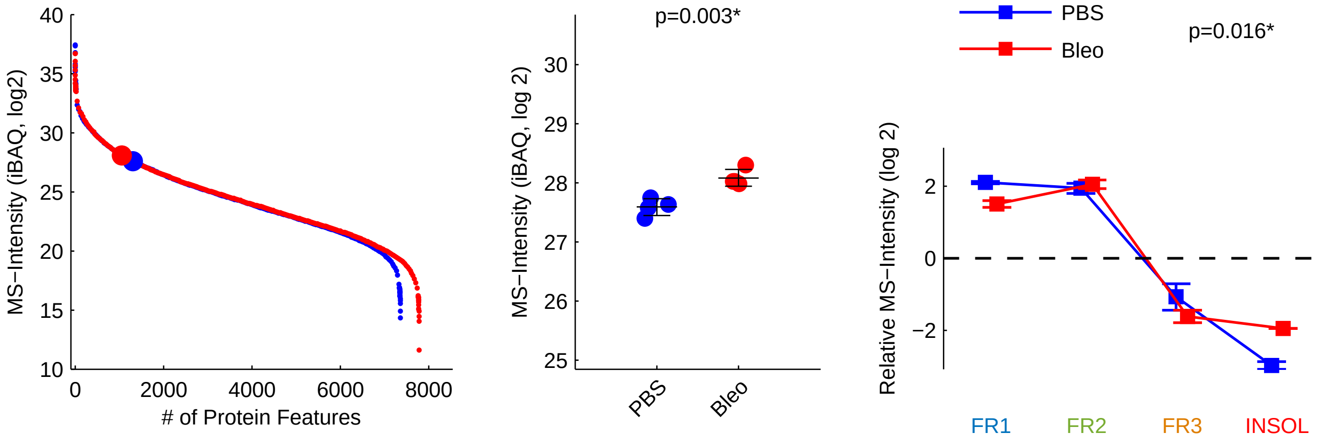

Q3UGR5 − Hdhd2 (id: 4074)

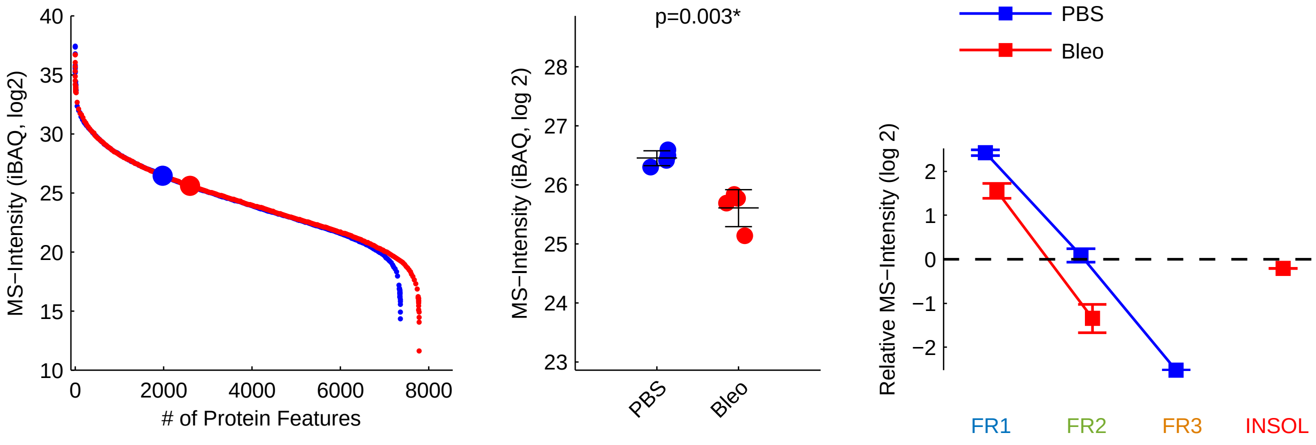

Q3UH06−3 − Rreb1 (id: 4079)

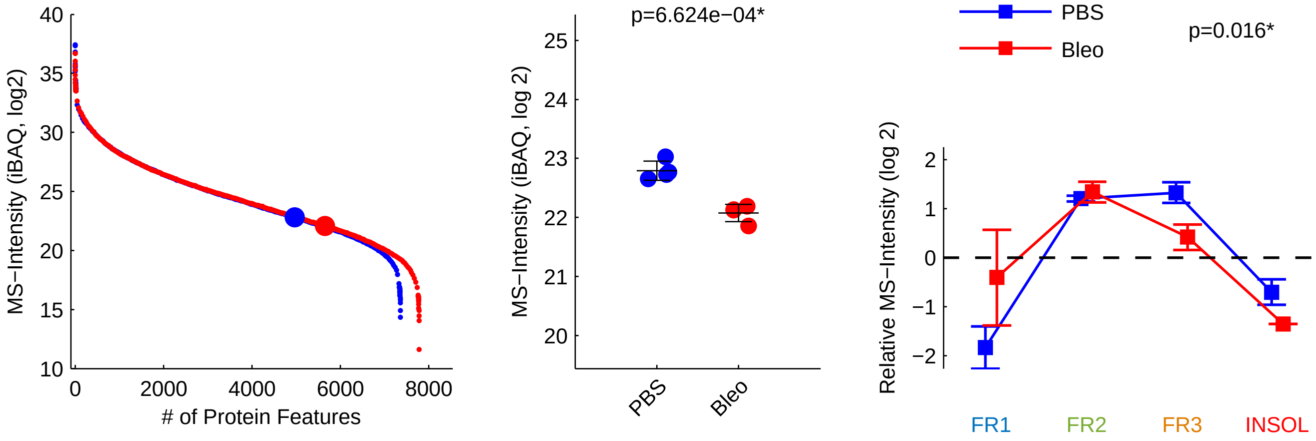

Q3UHH1 − Zswim8 (id: 4088)

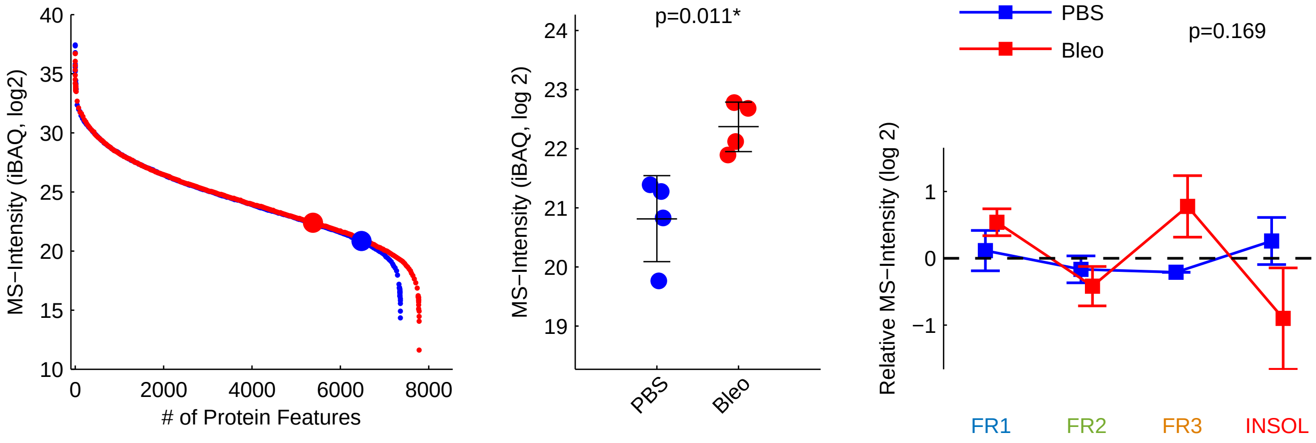

Q3UHU8 − Gtf2i (id: 4095)

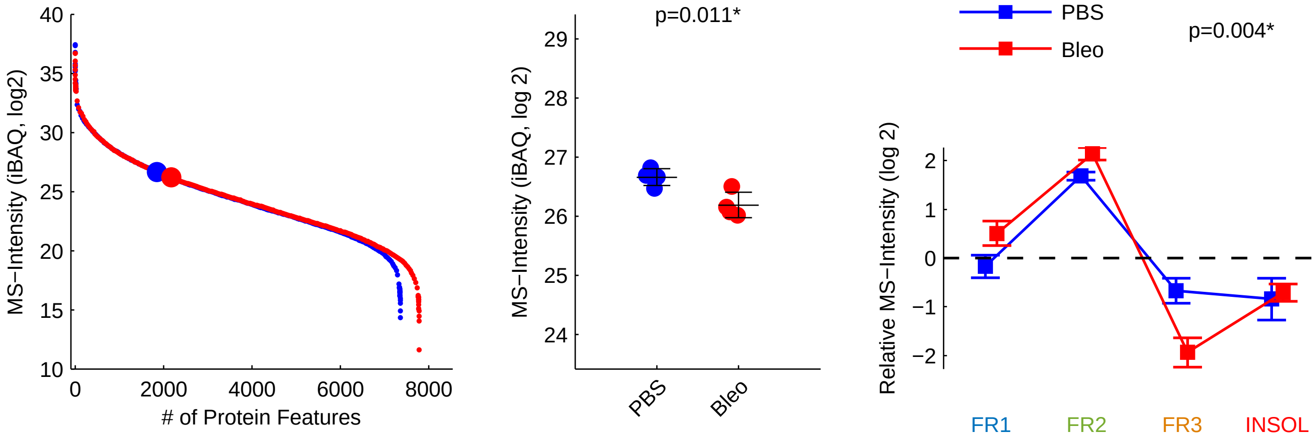

Q3UID0 − Smarcc2 (id: 4103)

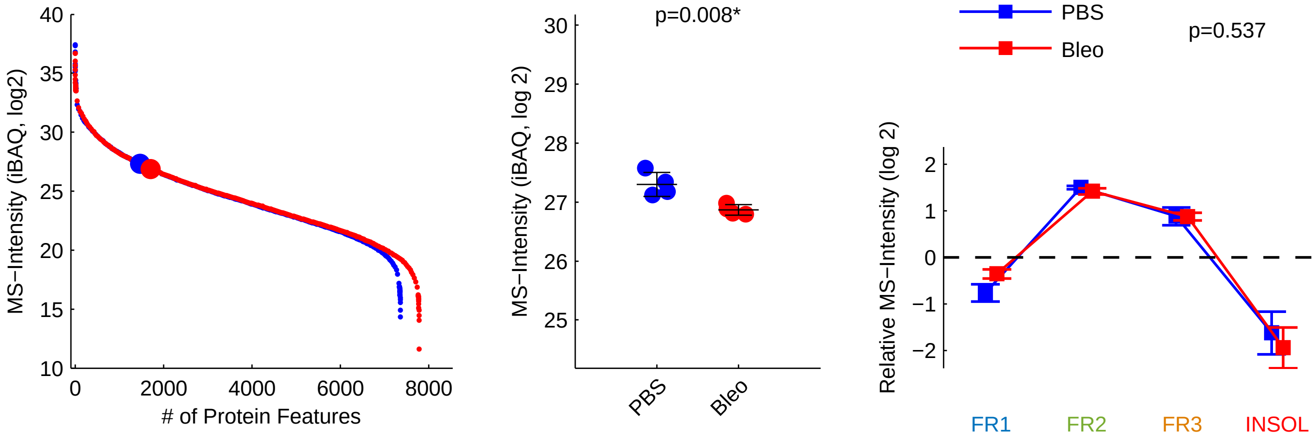

Q3UIJ9 − Myzap (id: 4104)

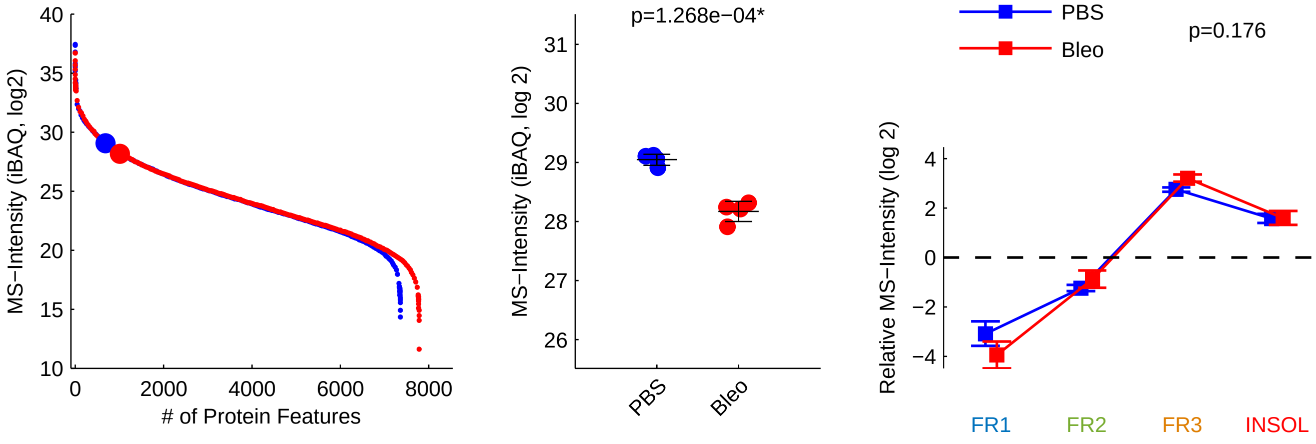

Q3UIL6−2 − Plekha7 (id: 4105)

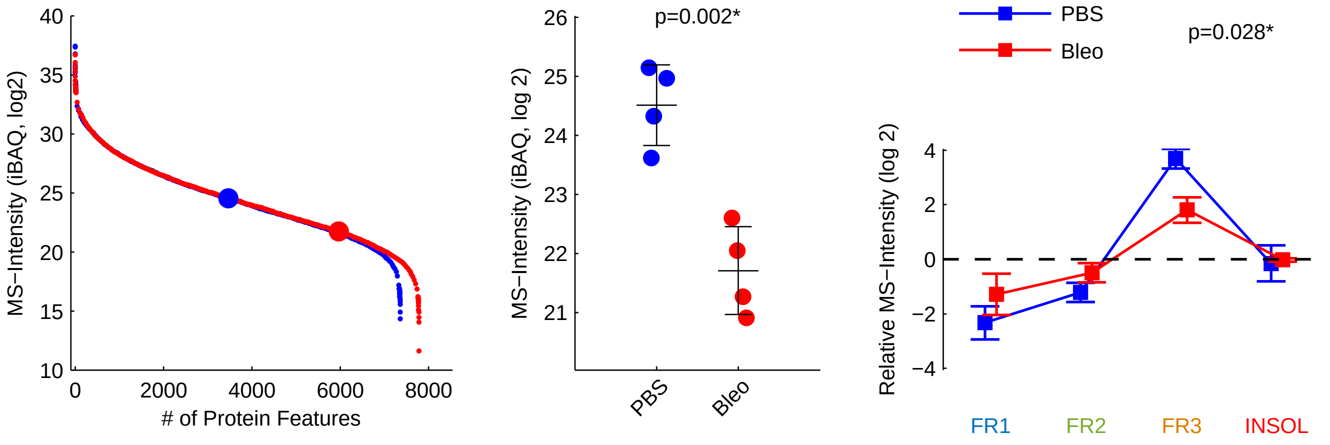

Q3UJB0 − Sf3b2 (id: 4108)

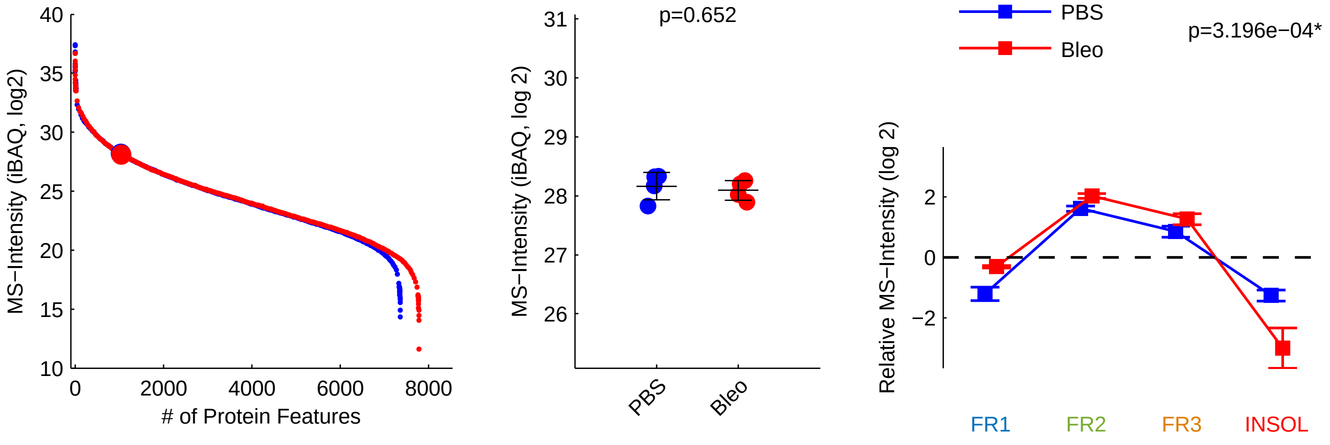

Q9R210 − Tfeb (id: 4115)

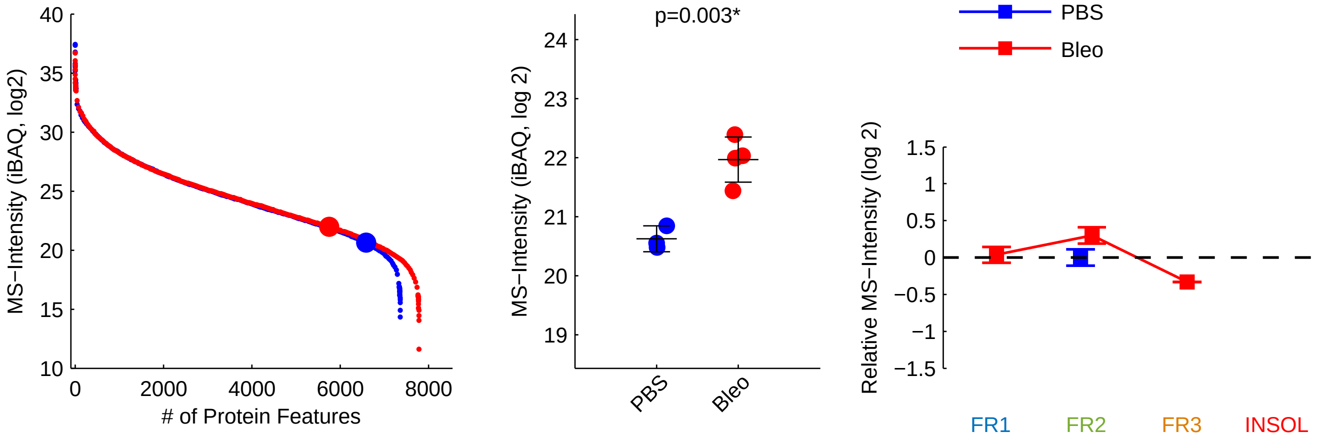

Q3ULD5 − Mccc2 (id: 4120)

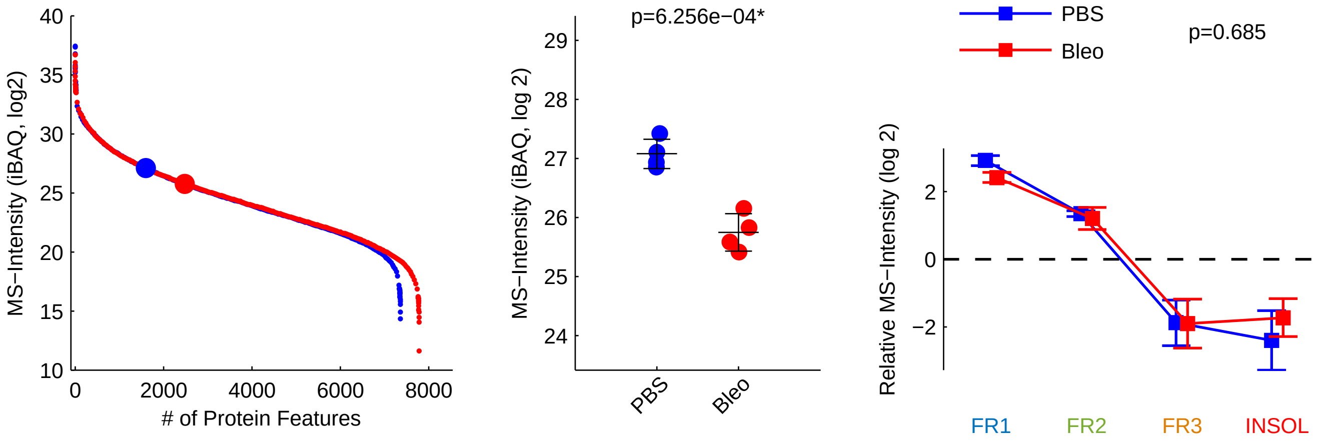

Q3ULJ0 − Gpd1l (id: 4121)

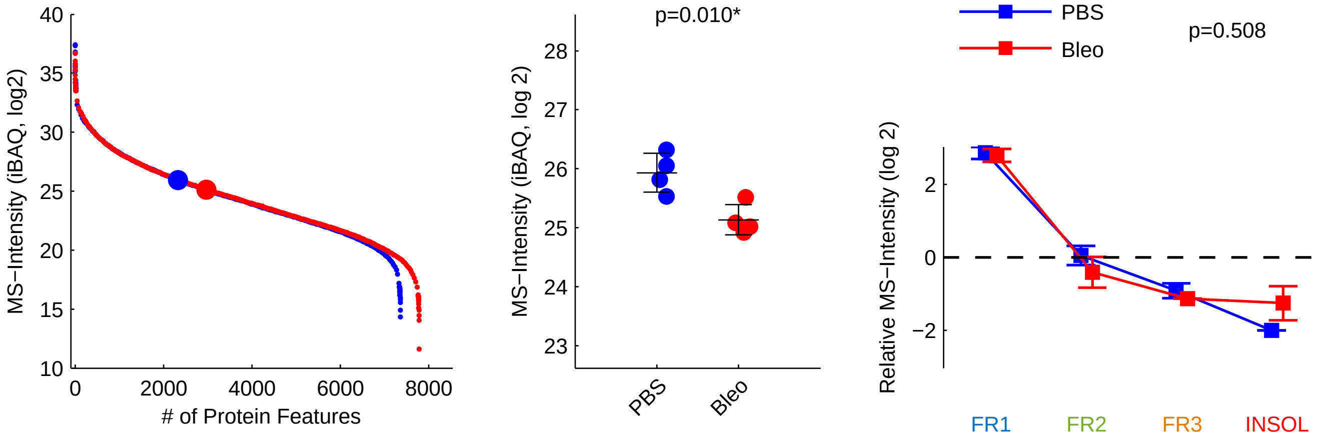

Q3ULL6 − Upf3b (id: 4122)

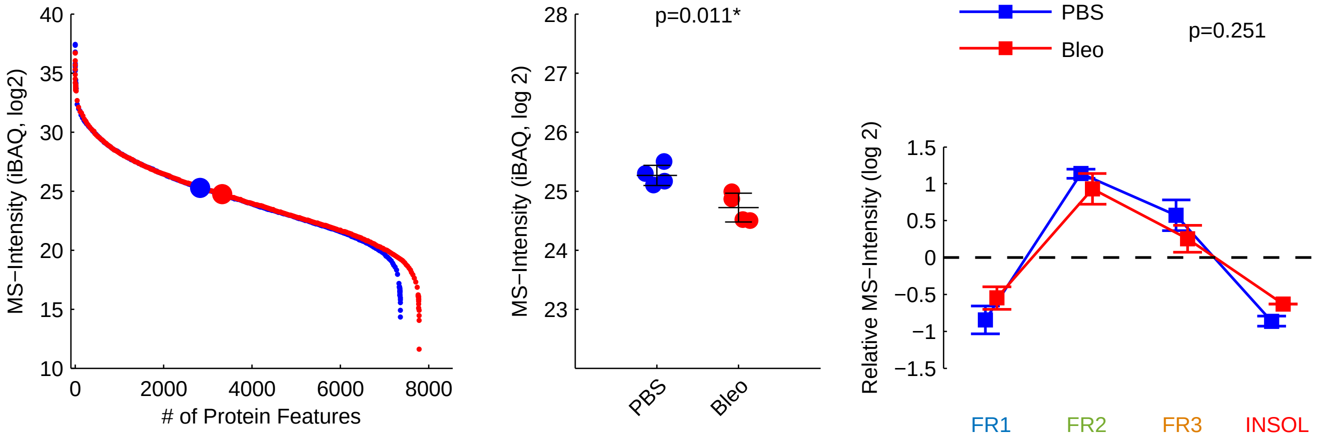

Q3UM29 − Cog7 (id: 4123)

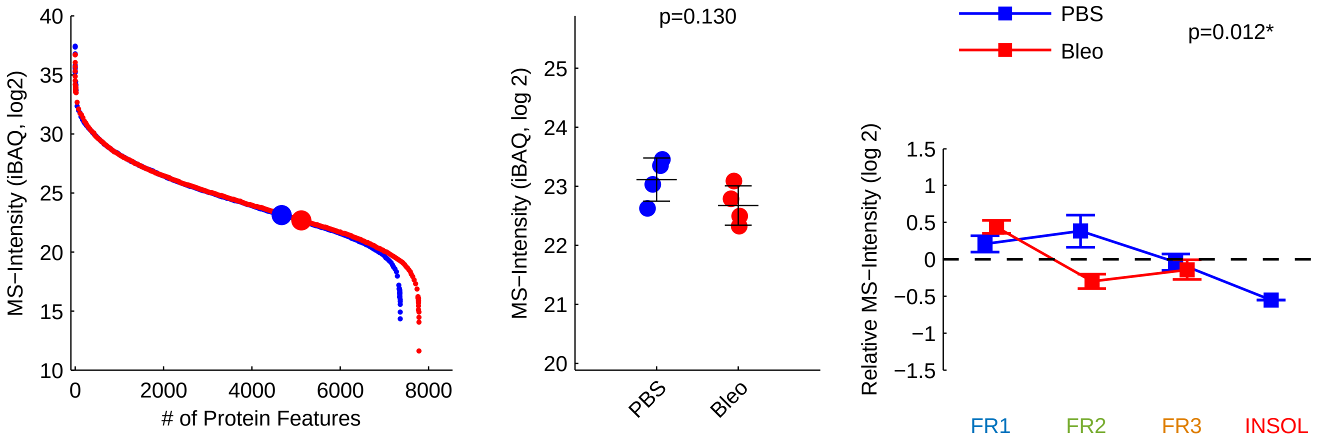

Q3UNZ8 − (id: 4139)

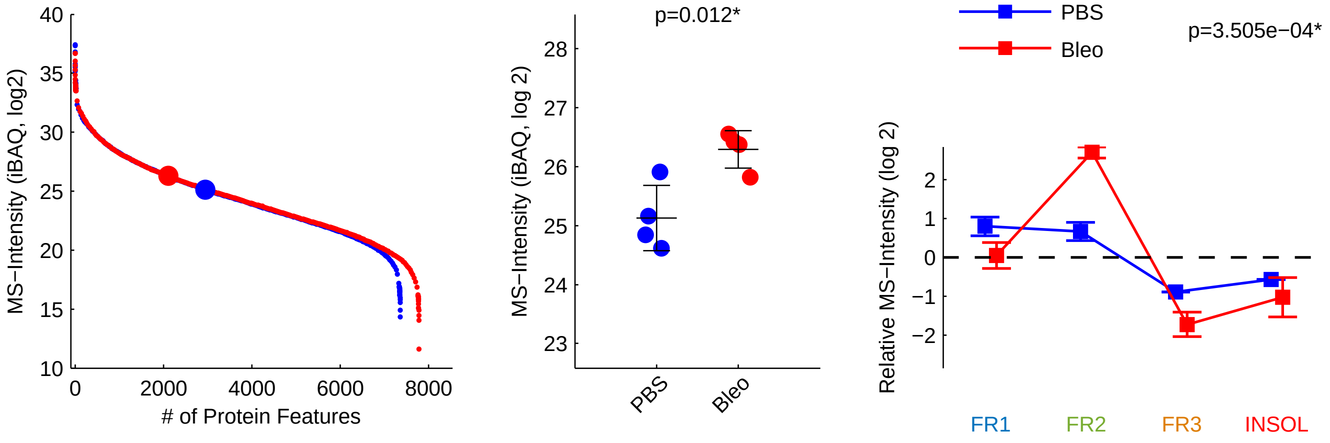

Q3UPH1 − Prrc1 (id: 4141)

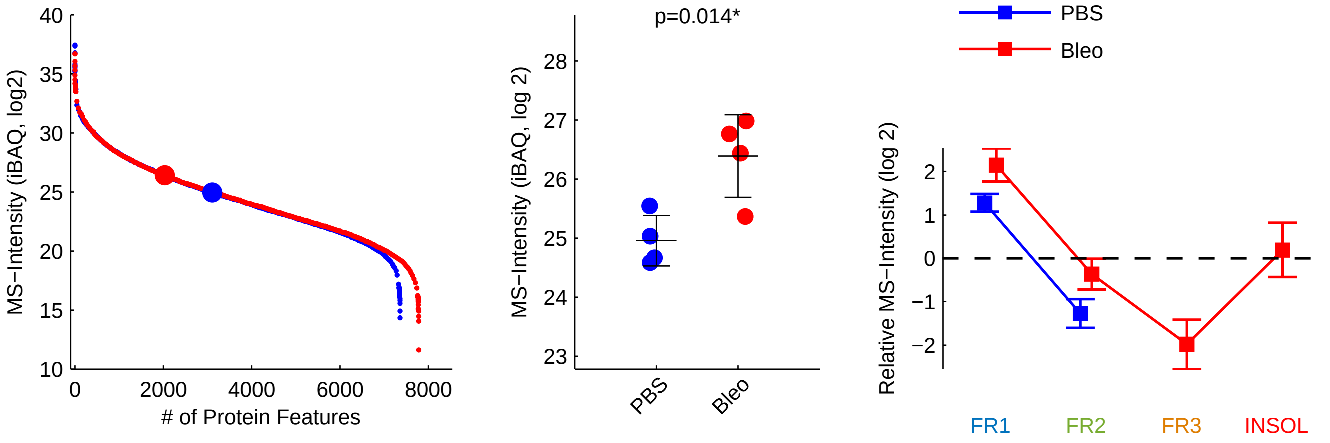

Q3UPL0 − Sec31a (id: 4144)

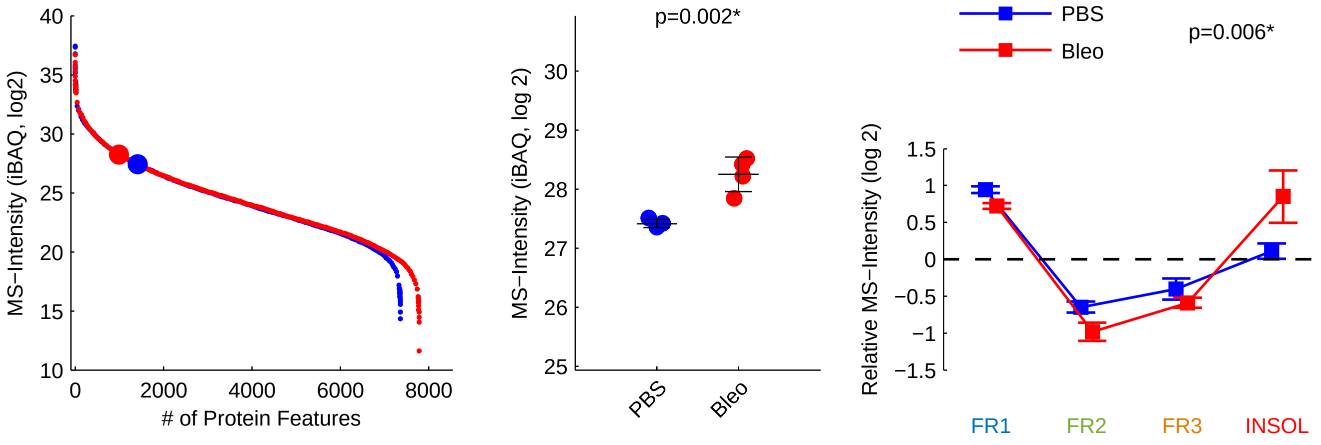

Q3UPV6 − Kcnab2 (id: 4148)

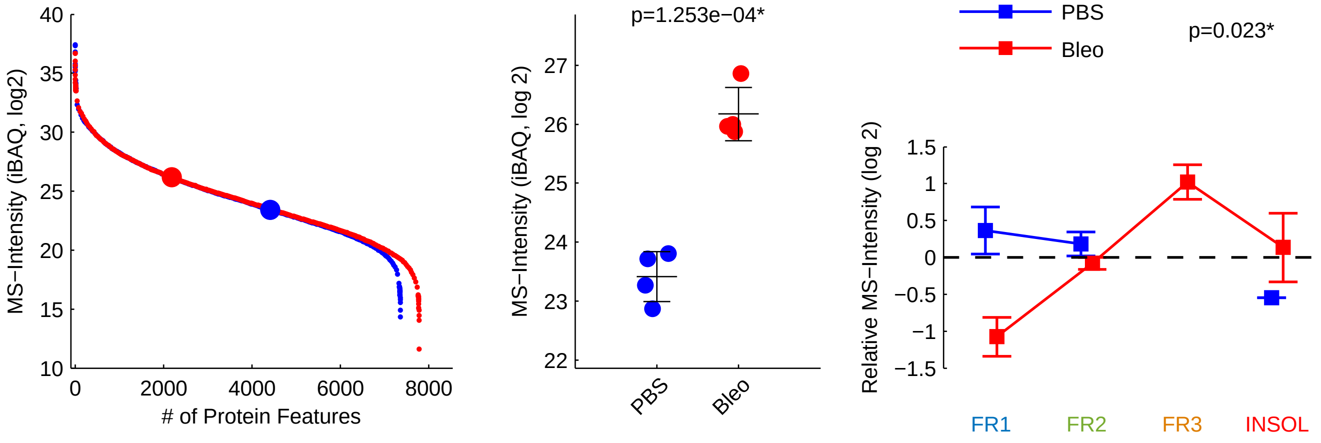

Q3UQ28 − Pxdn (id: 4150)

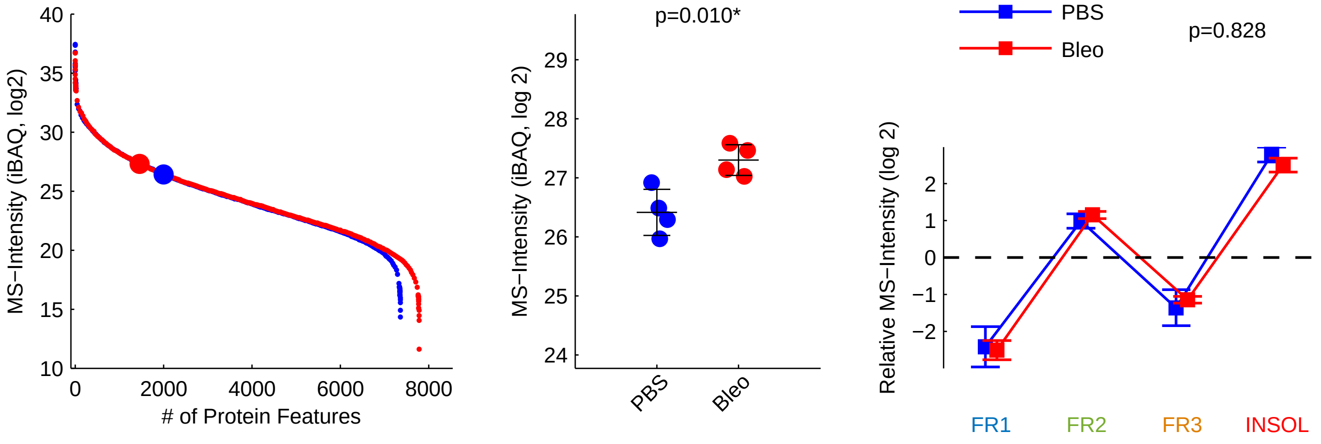

Q3UQ44 − Iqgap2 (id: 4151)

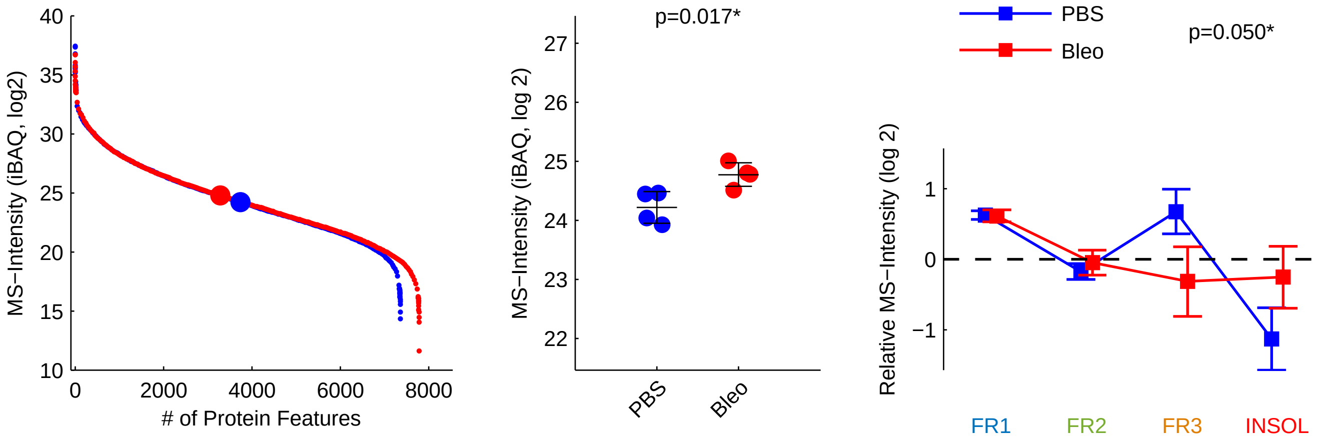

Q3UQU0 − Brd9 (id: 4154)

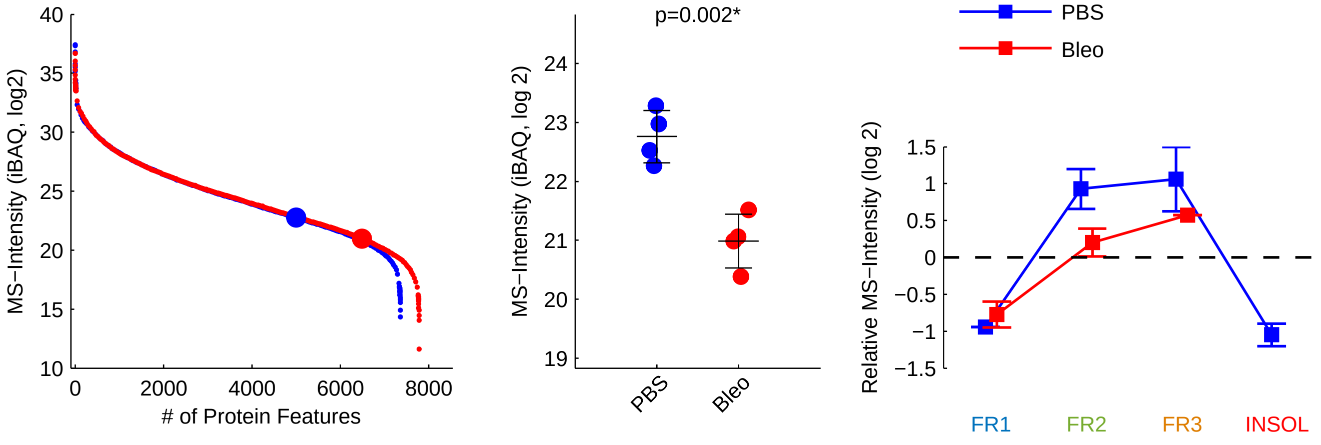

Q3US17 − Znf48 (id: 4161)

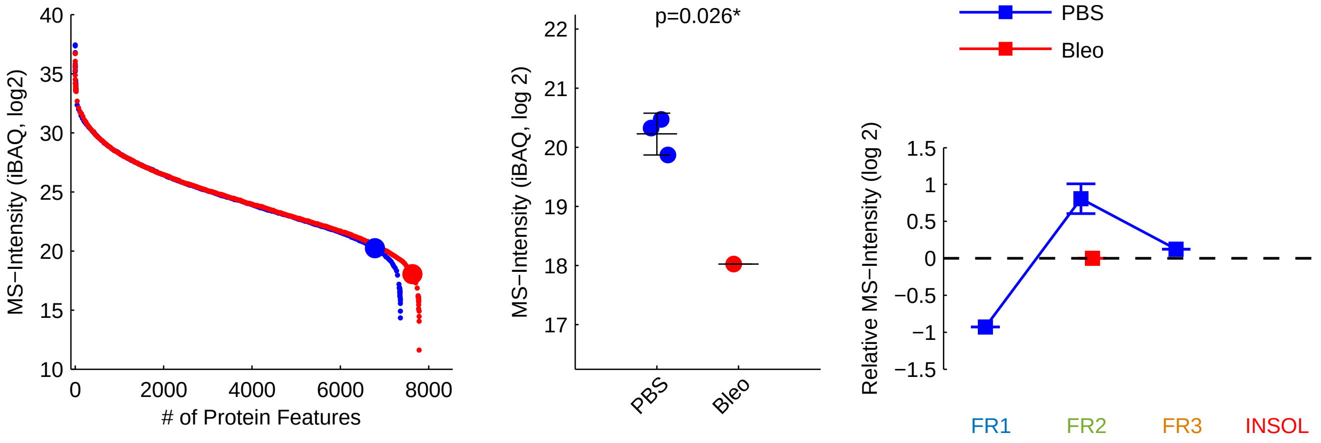

Q3USS3 − Drc1 (id: 4165)

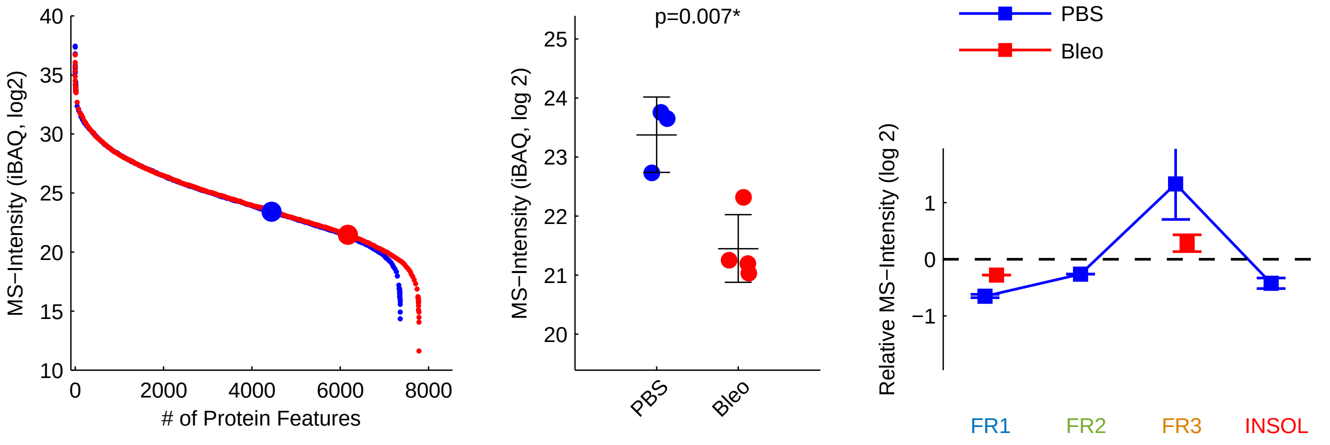

Q3UTJ2−4 − Sorbs2 (id: 4167)

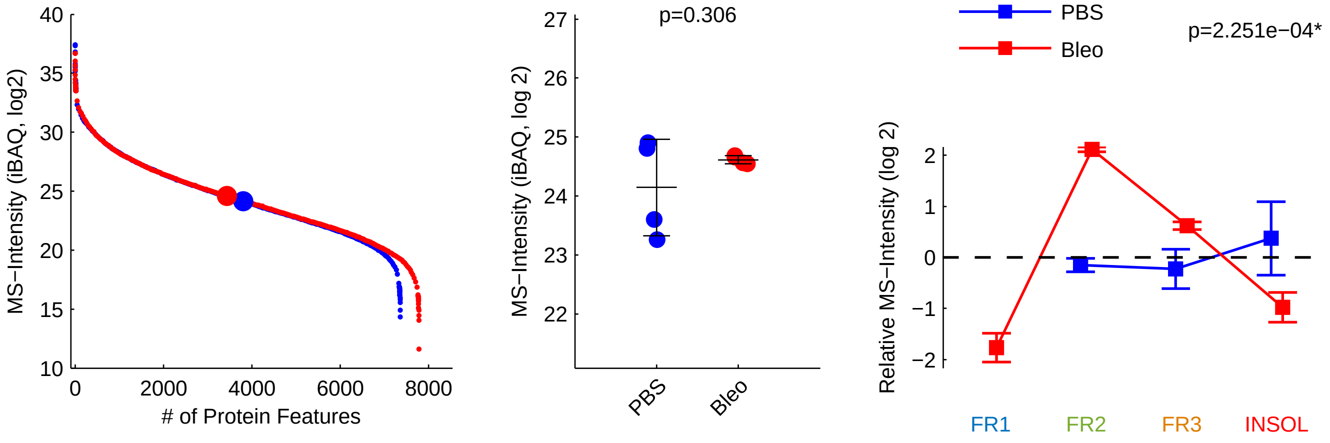

Q3UTR7 − Agt (id: 4170)

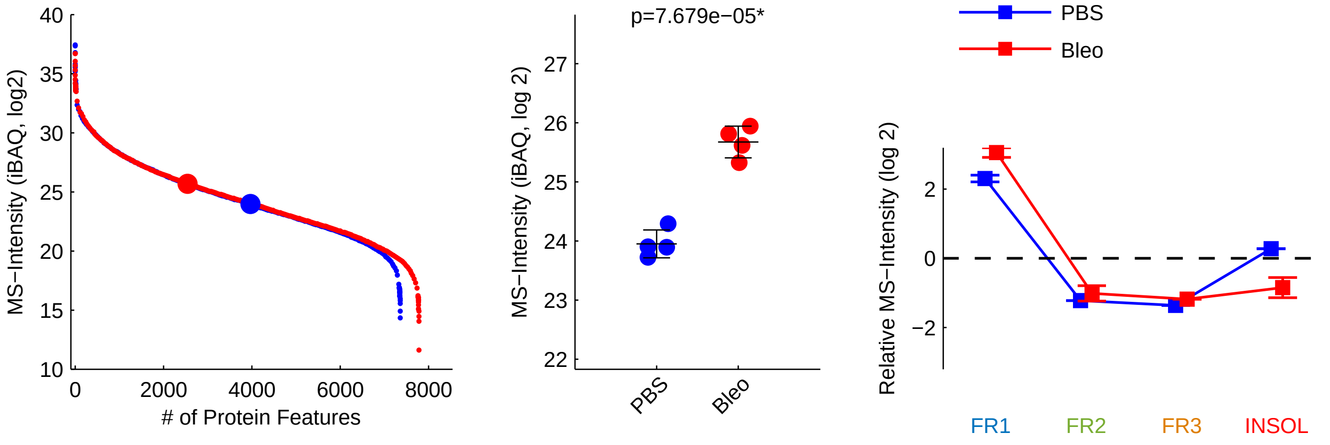

Q3UUQ7 − Pgap1 (id: 4175)

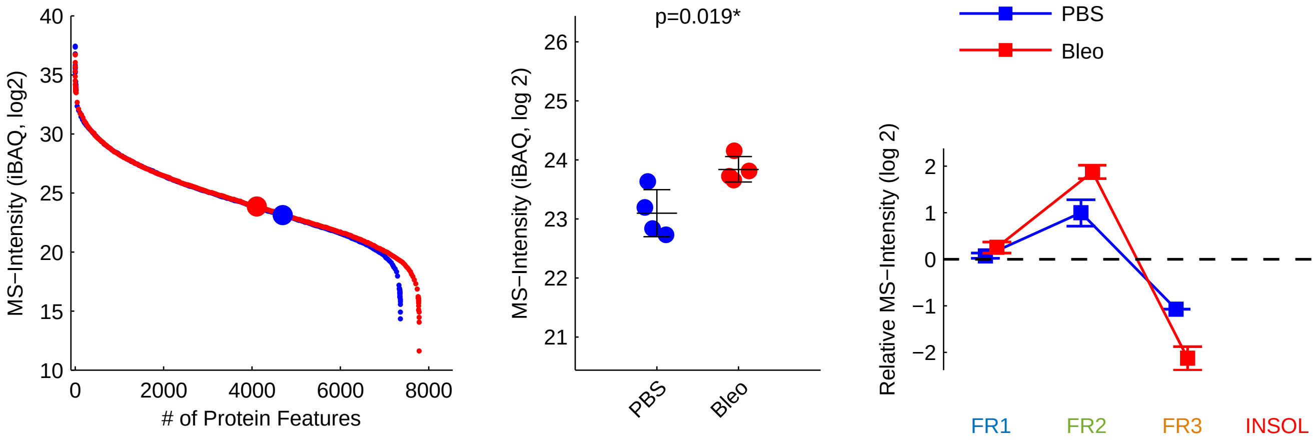

Q3UVL4 − Vps51 (id: 4182)

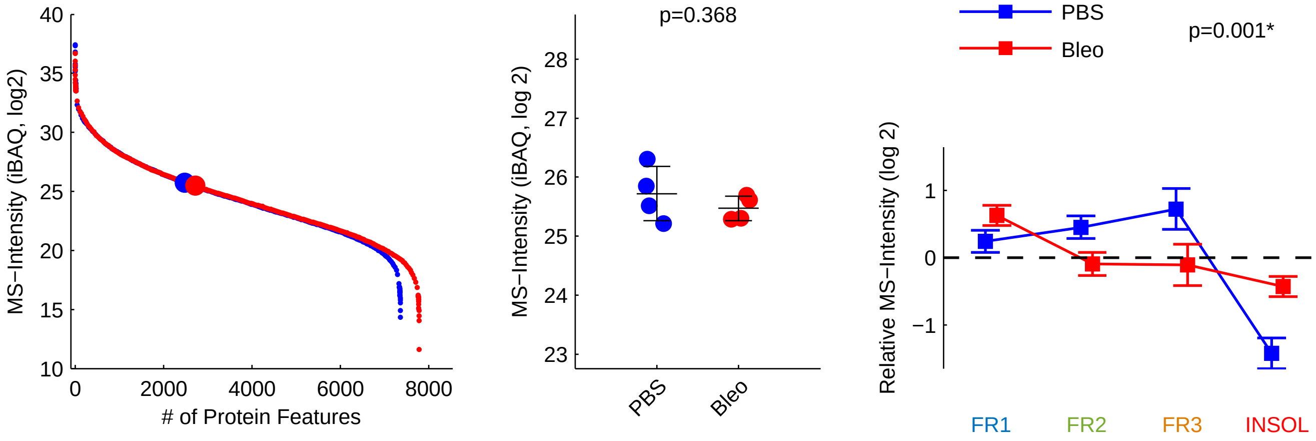

Q3UW53 − Fam129a (id: 4184)

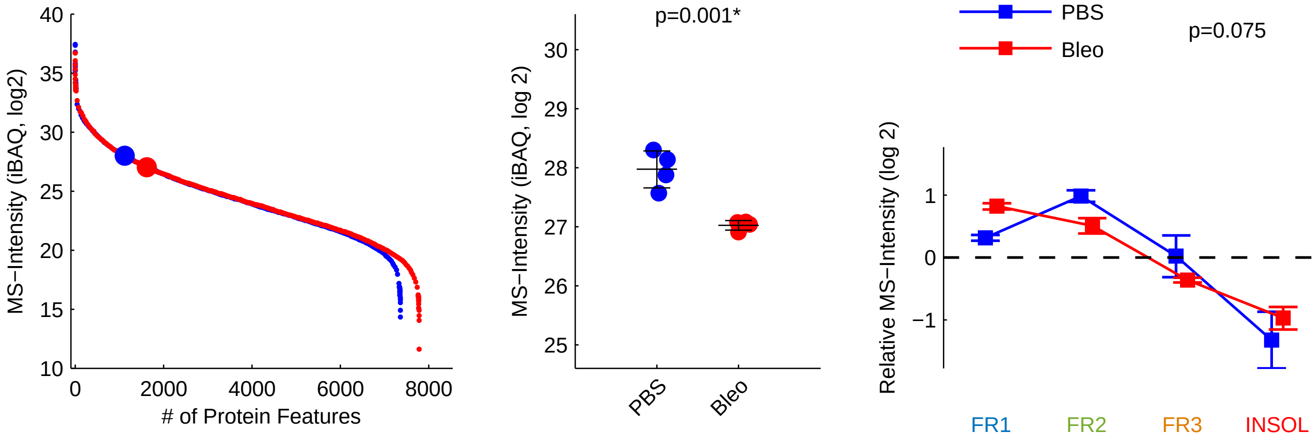

Q99J99 − Mpst (id: 4186)

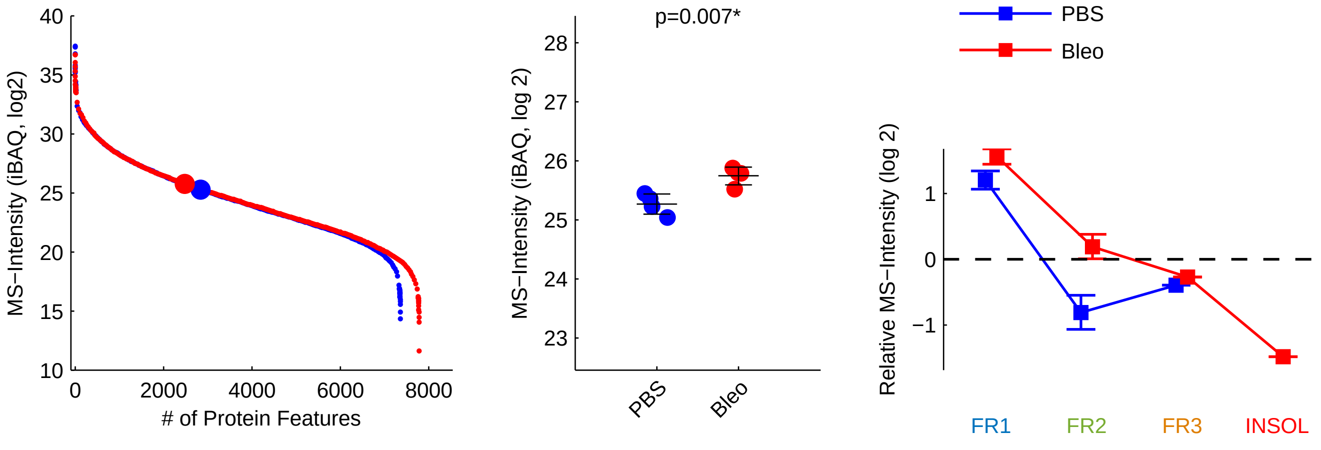

Q3UWE6 − Wdr20a (id: 4187)

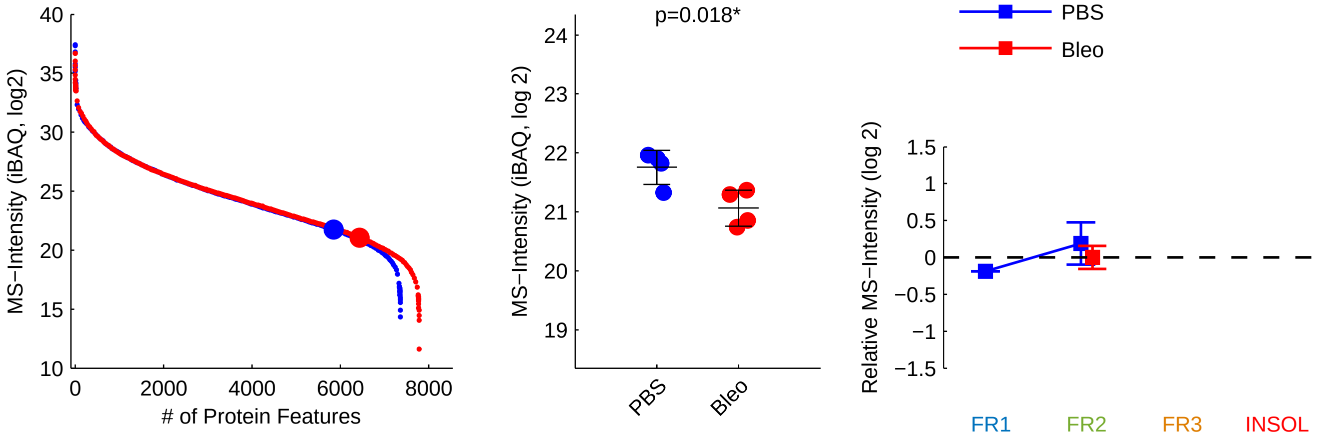

Q3UZA1 − Rcsd1 (id: 4204)

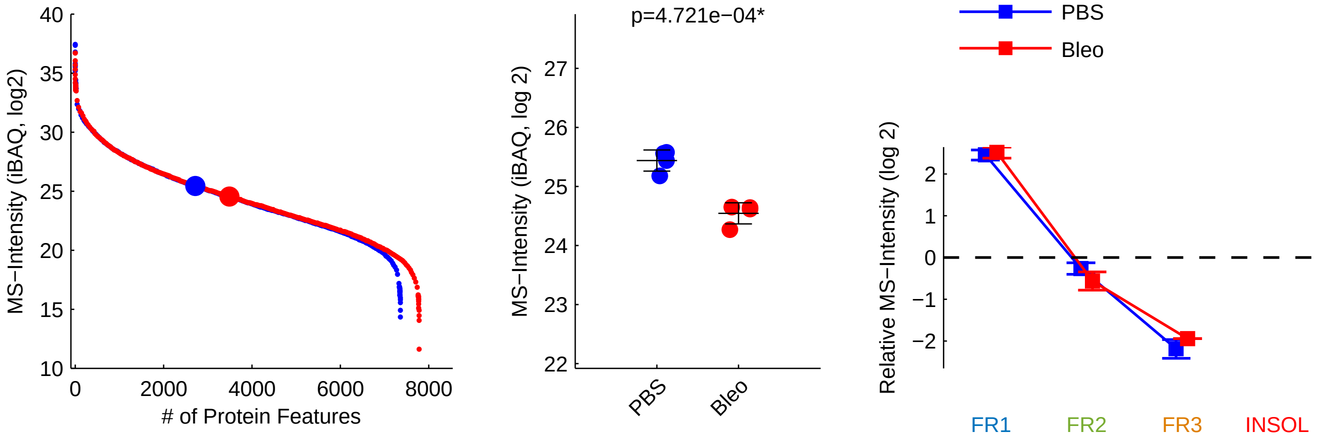

Q3V117 − Acly (id: 4212)

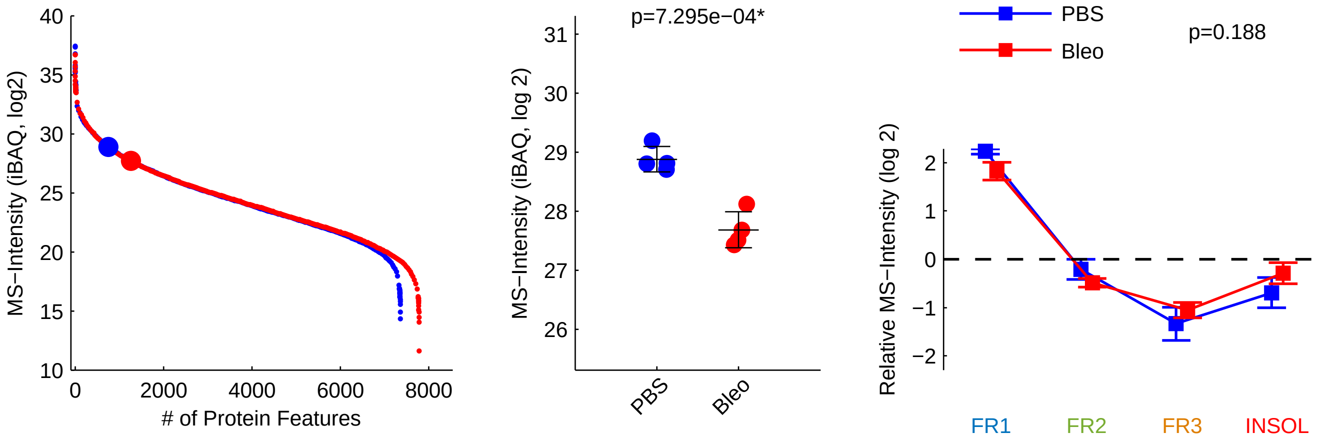

Q3V1L4 − Nt5c2 (id: 4217)

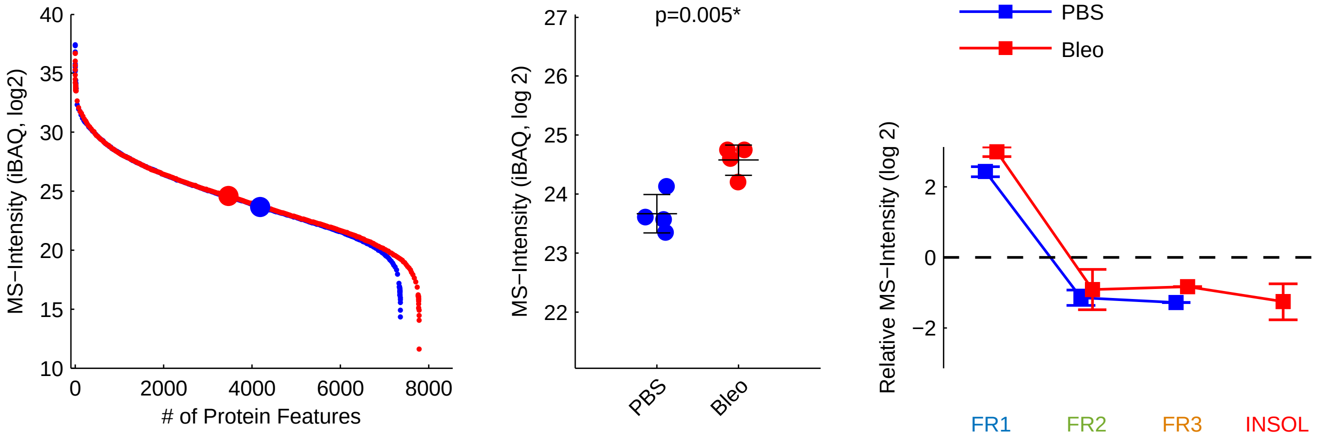

Q3V1M1 − Igsf10 (id: 4218)

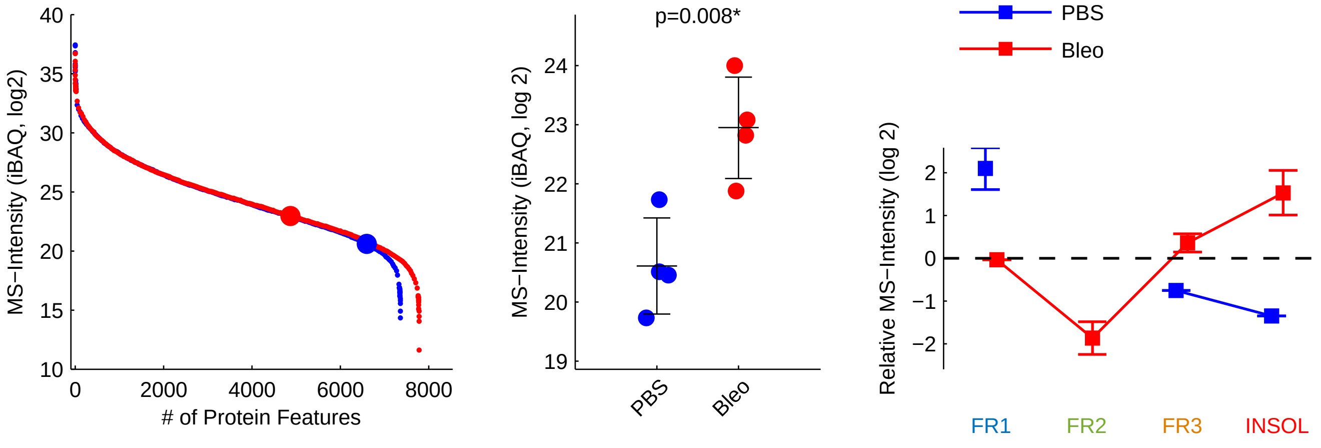

Q3V1T4 − Lepre1 (id: 4221)

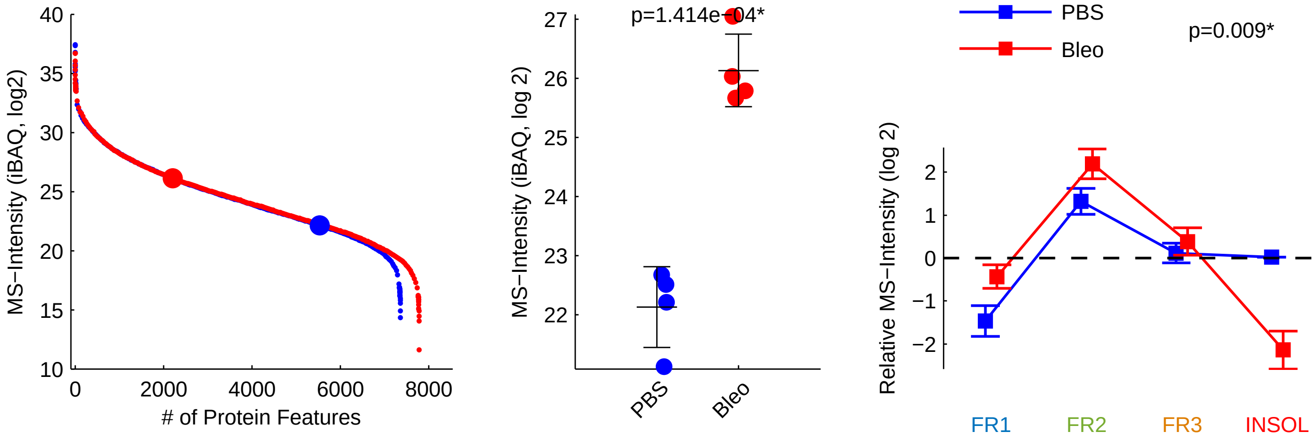

Q3V1Z5 − Rps4y2 (id: 4223)

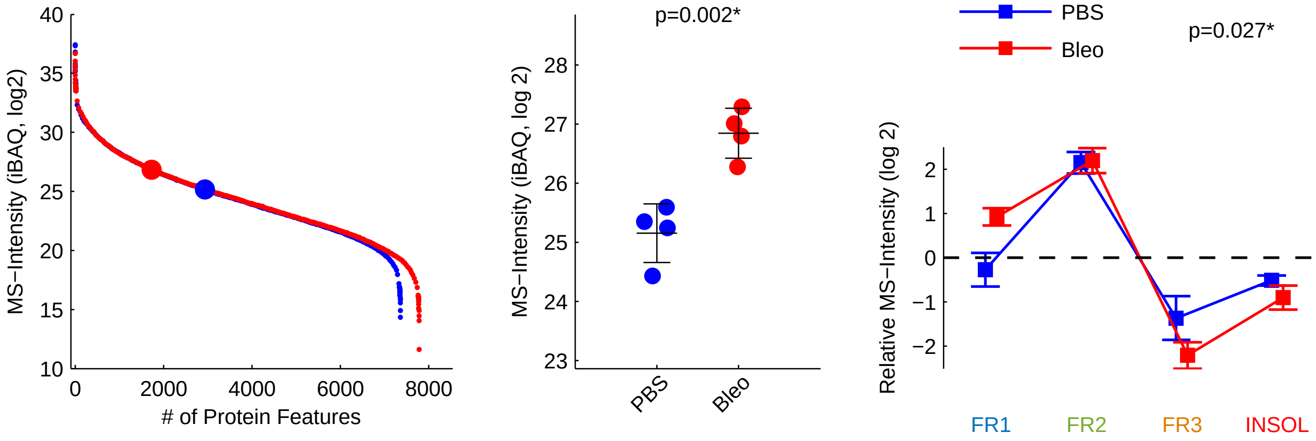

Q3V3R1 − Mthfd1l (id: 4229)

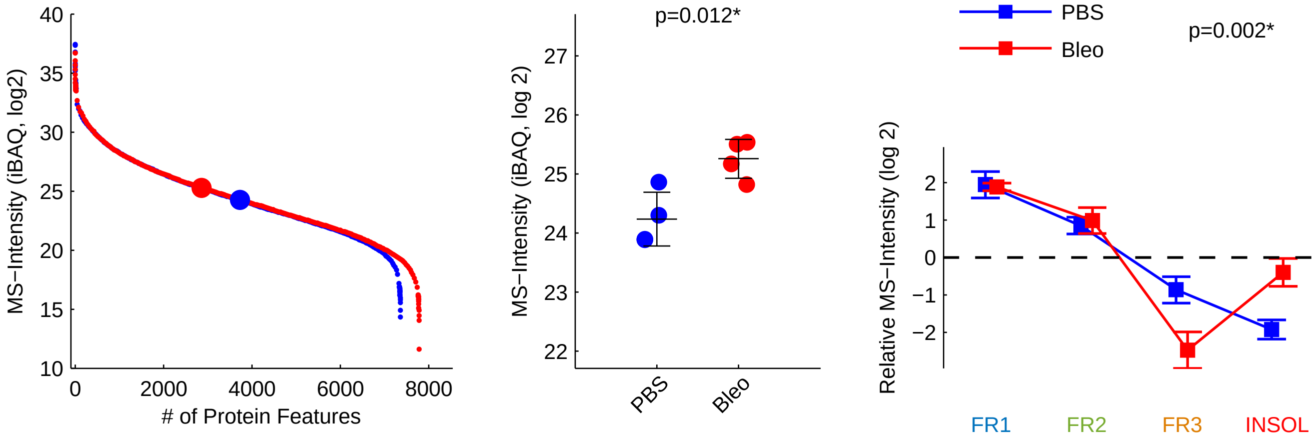

Q3V3R4 − Itga1 (id: 4230)

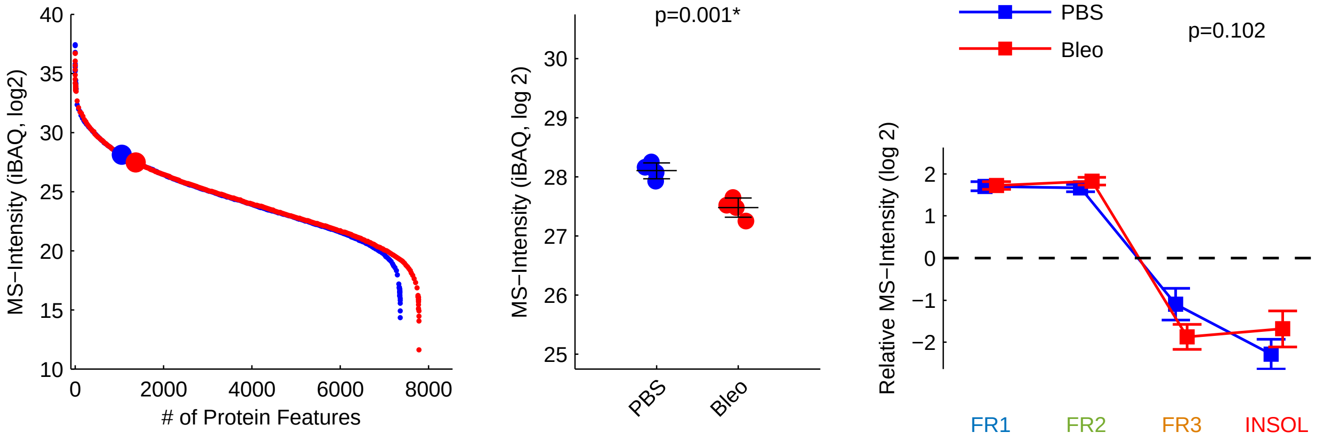

Q45VK7 − Dync2h1 (id: 4236)

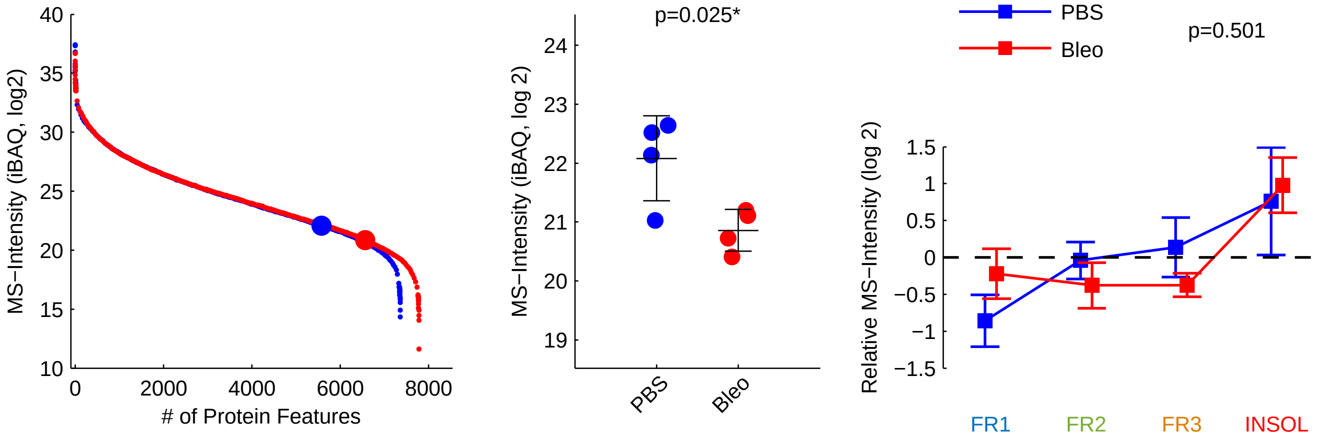

Q4PZA2−4 − Ece1 (id: 4253)

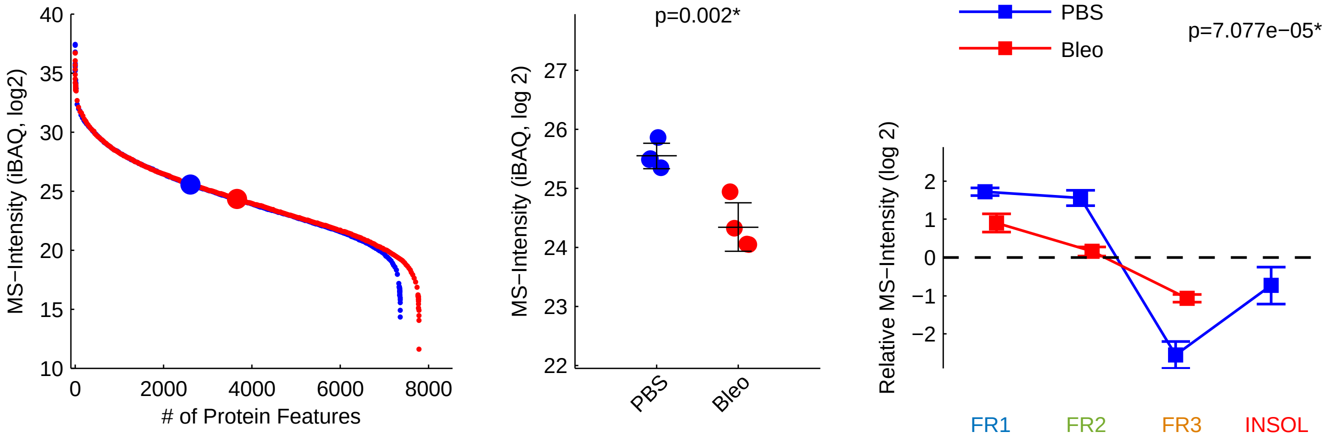

Q4VA53 − Pds5b (id: 4256)

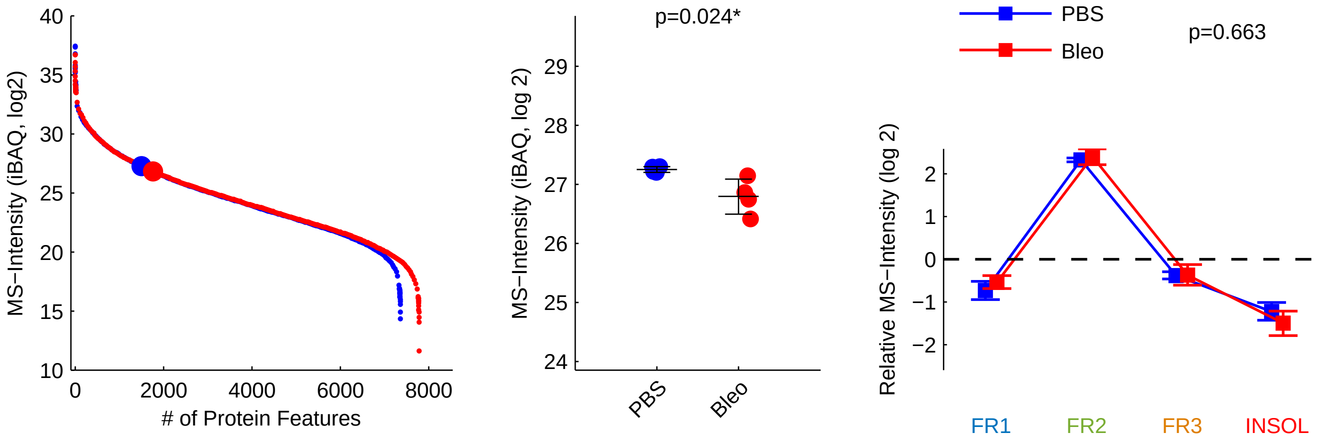

Q4VA93 − Prkca (id: 4258)

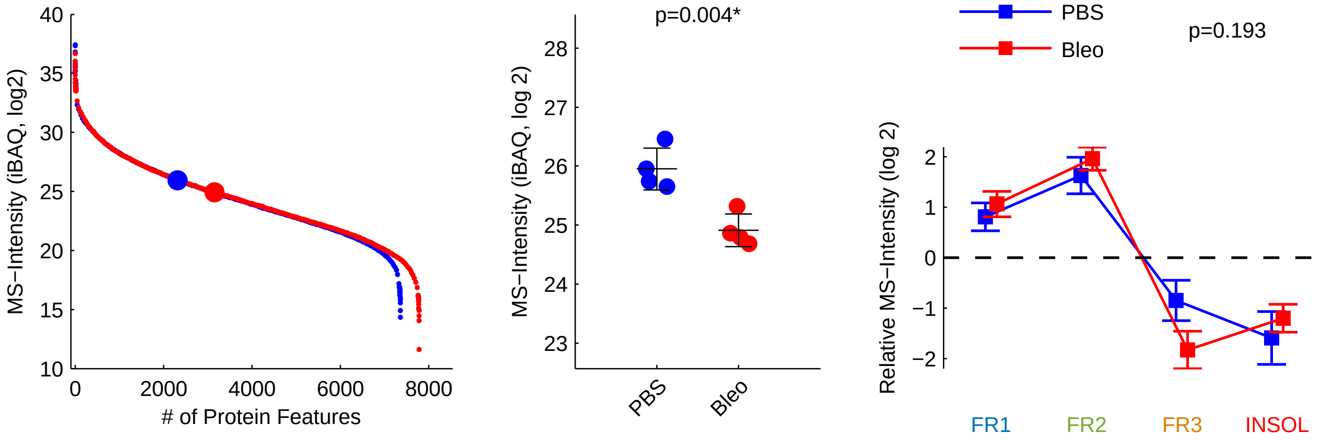

Q501J6 − Ddx17 (id: 4265)

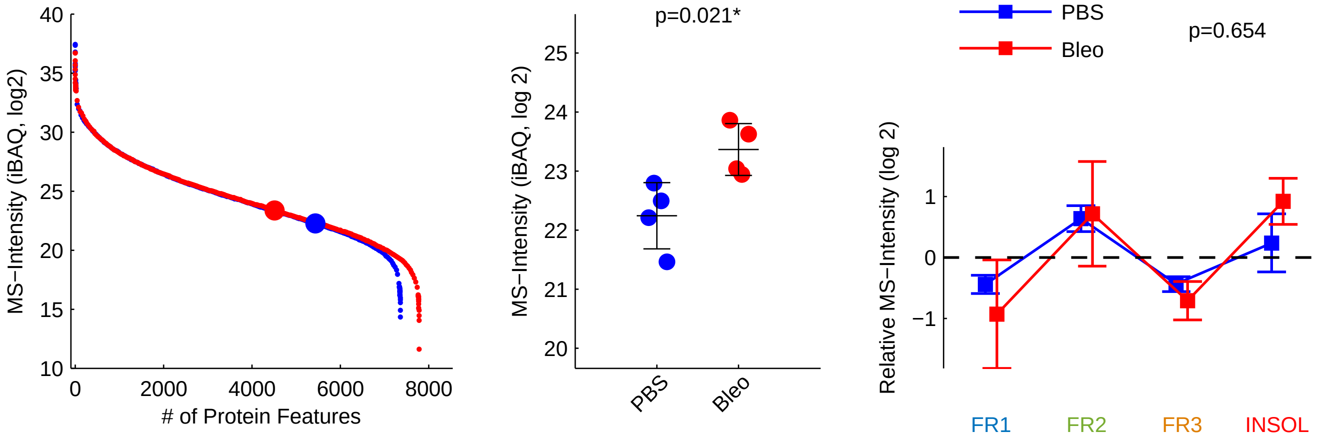

Q569Z5 − Ddx46 (id: 4279)

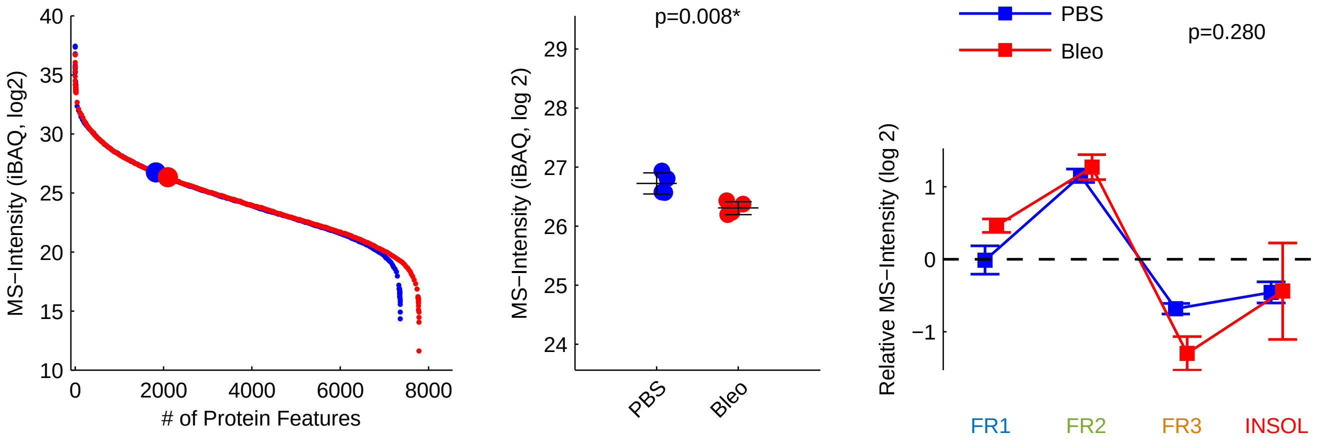

Q569Z6 − Thrap3 (id: 4280)

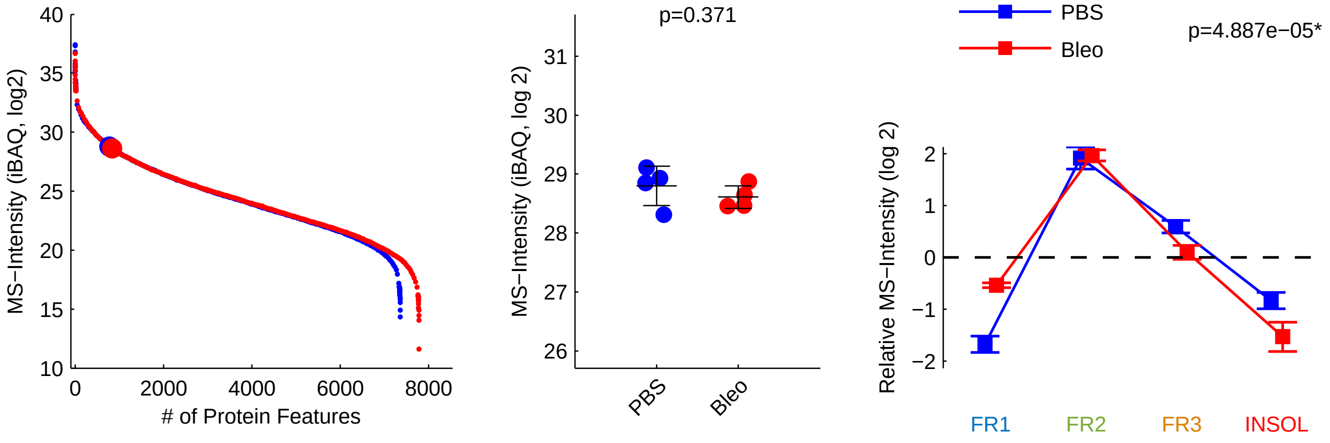

Q56A08 − Gpkow (id: 4281)

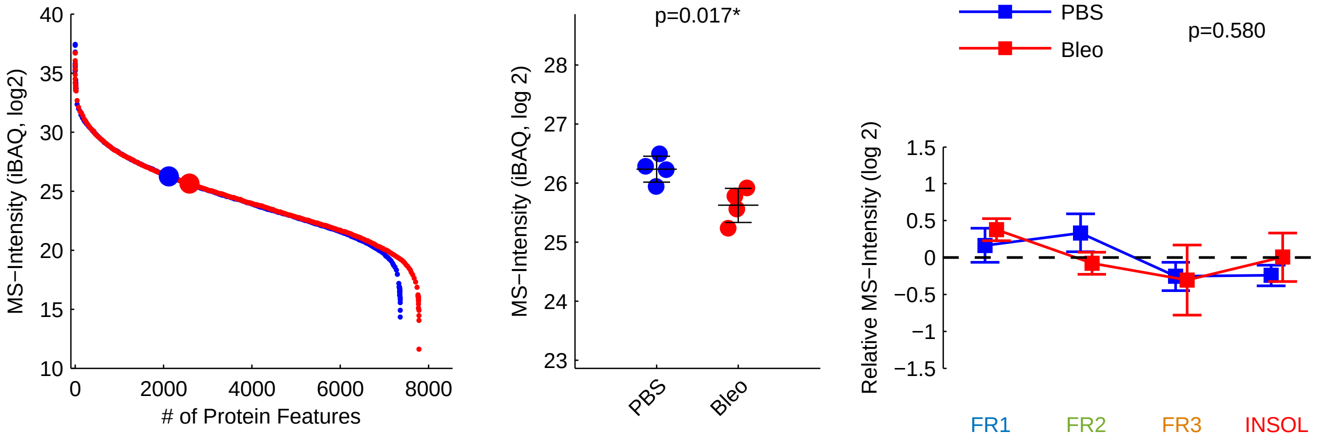

Q571E4 − Galns (id: 4283)

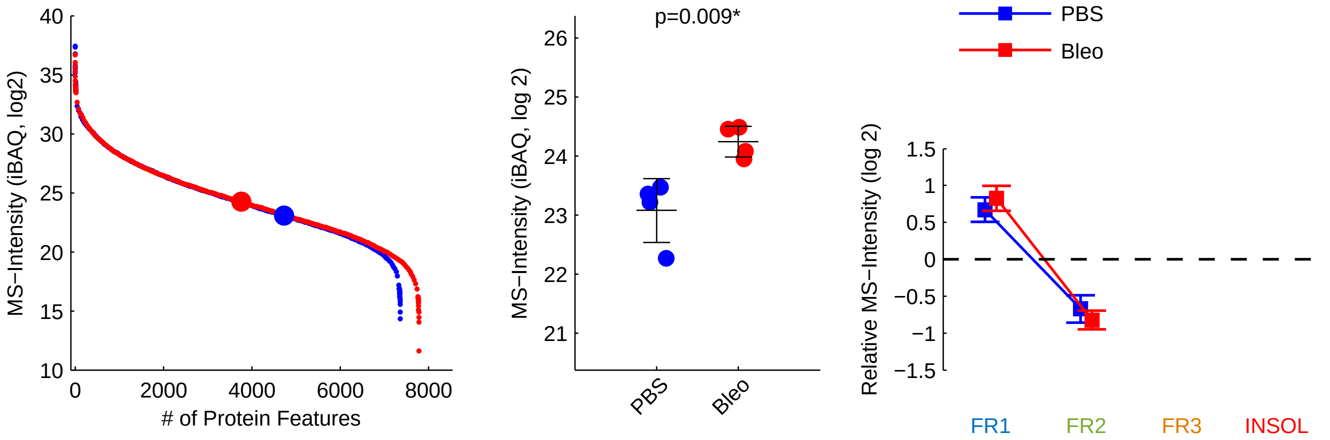

Q5EG47 − Prkaa1 (id: 4302)

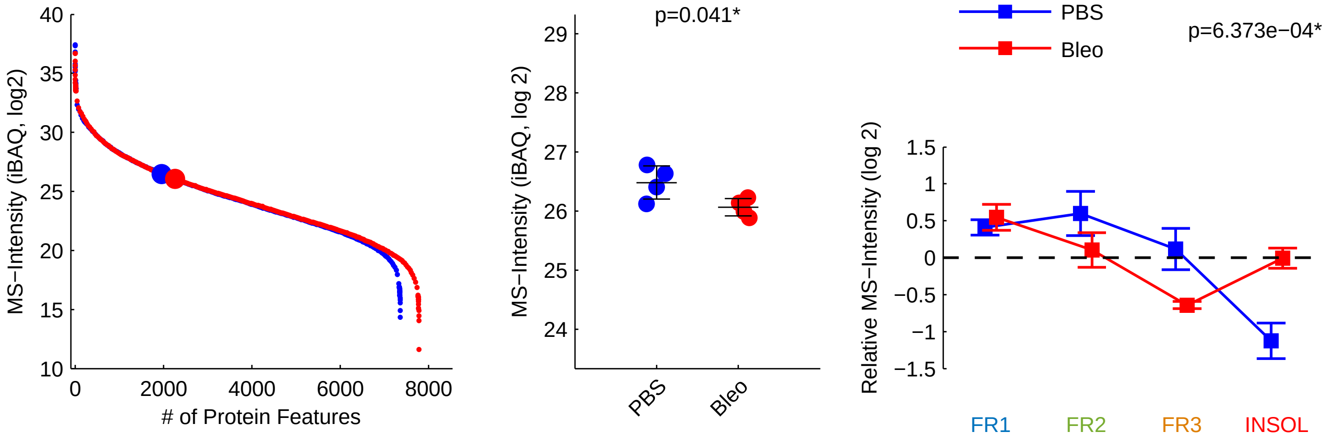

Q5F2E7 − Nufip2 (id: 4309)

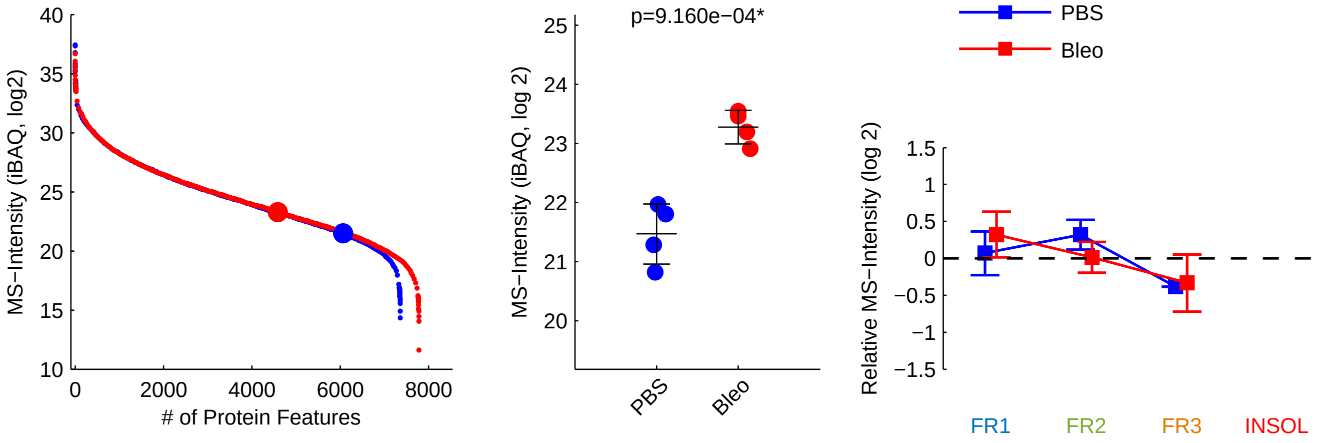

Q5F2E8 − Taok1 (id: 4310)

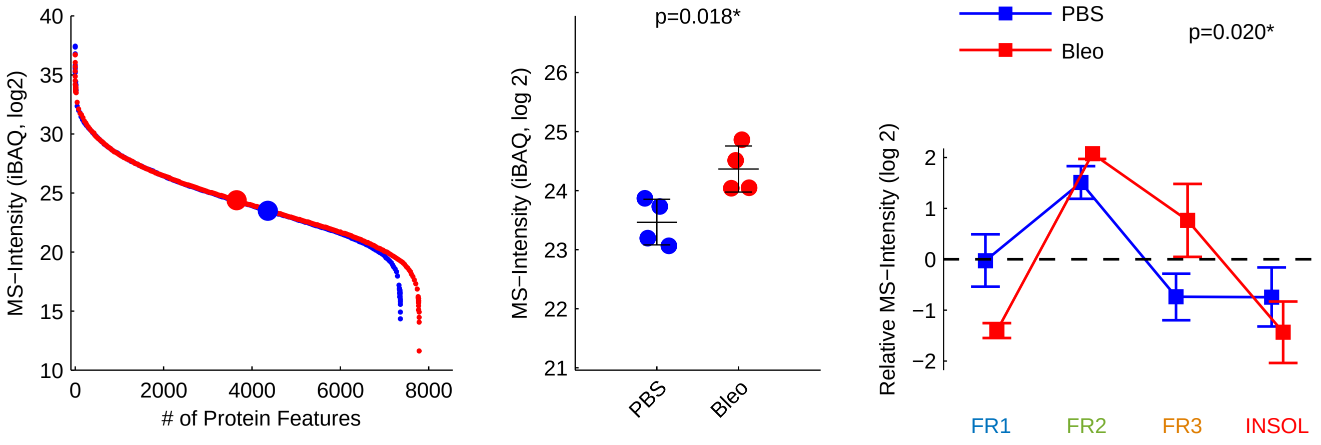

Q5FWI3 − Tmem2 (id: 4315)

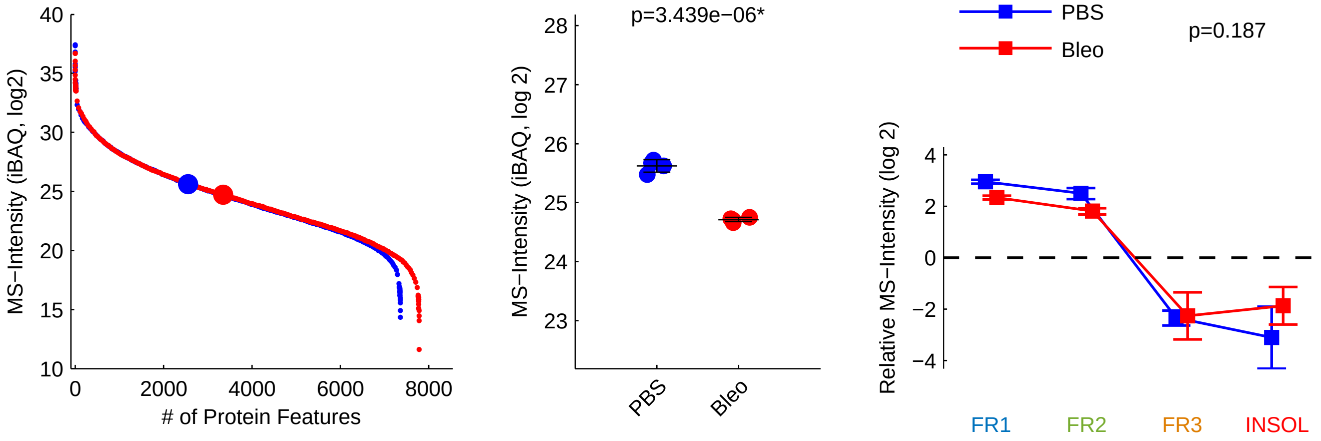

Q5H8C4 − Vps13a (id: 4318)

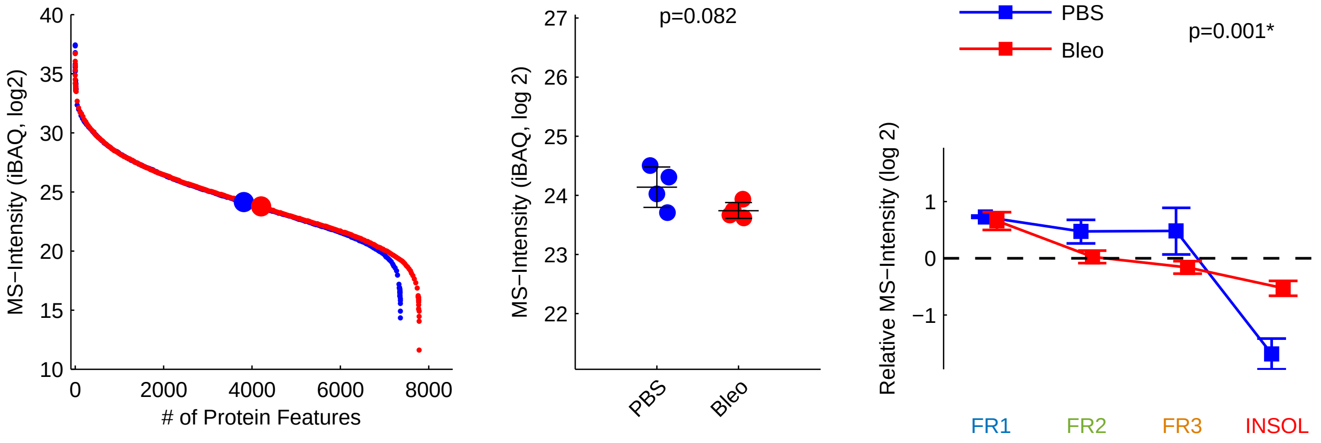

Q5HZI9 − Slc25a51 (id: 4321)

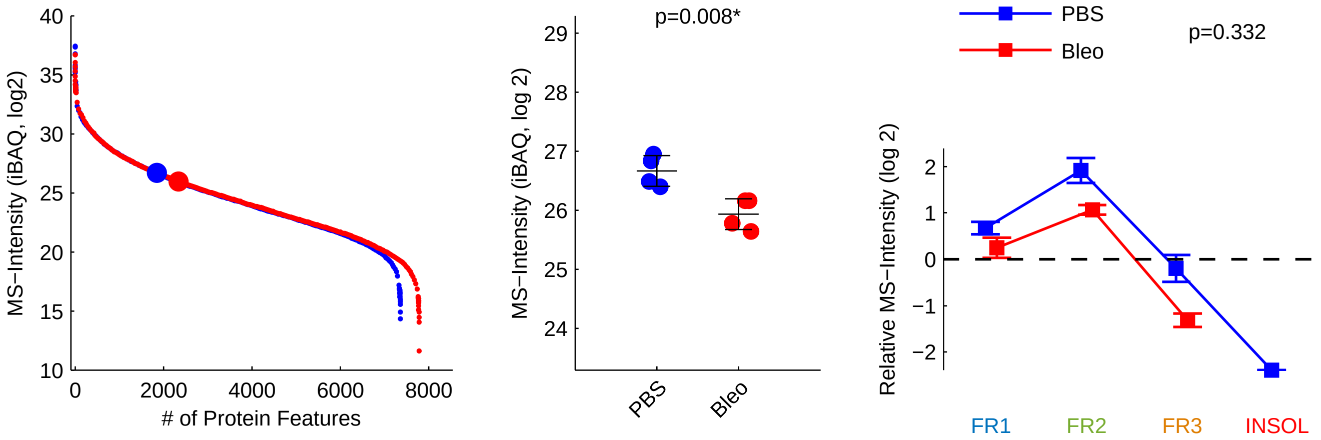

Q5PRF0 − Heatr5a (id: 4345)

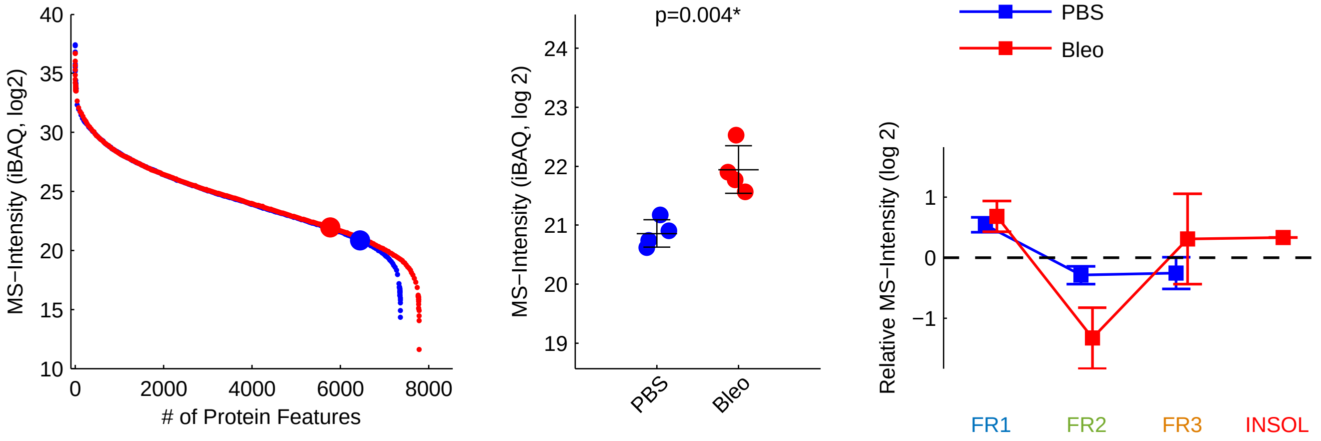

Q5QGU6 − Rtp3 (id: 4346)

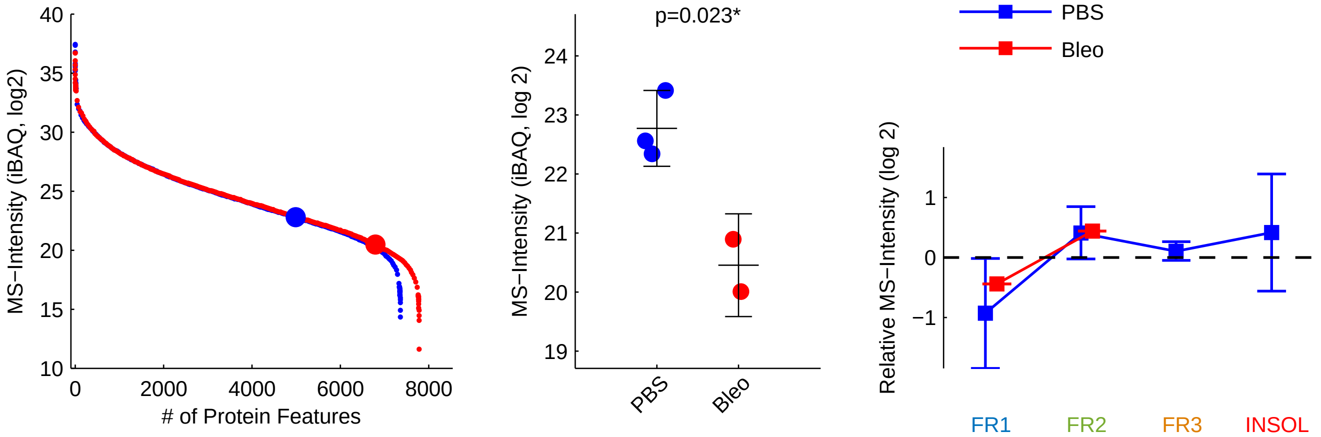

Q5SGK3 − Aox3l1 (id: 4358)

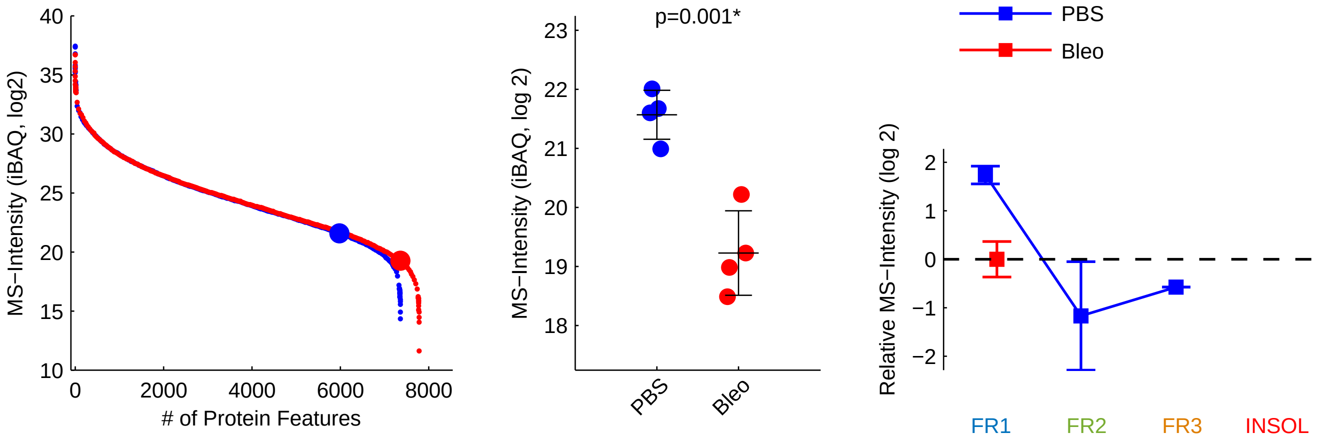

Q9EQ61 − Pes1 (id: 4361)

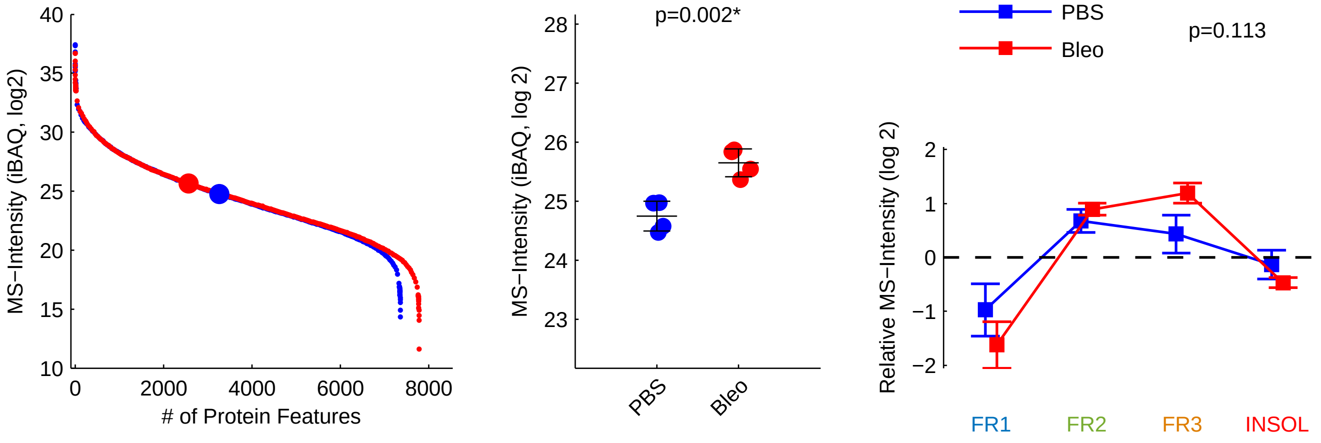

Q5SQ27 − Sec14l3 (id: 4362)

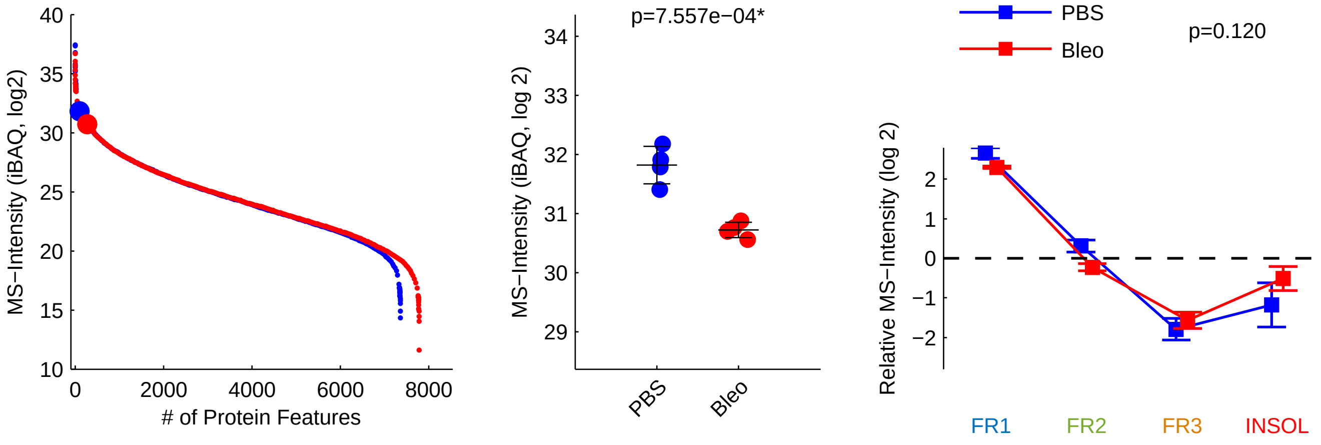

Q5SQY2 − Bod1 (id: 4365)

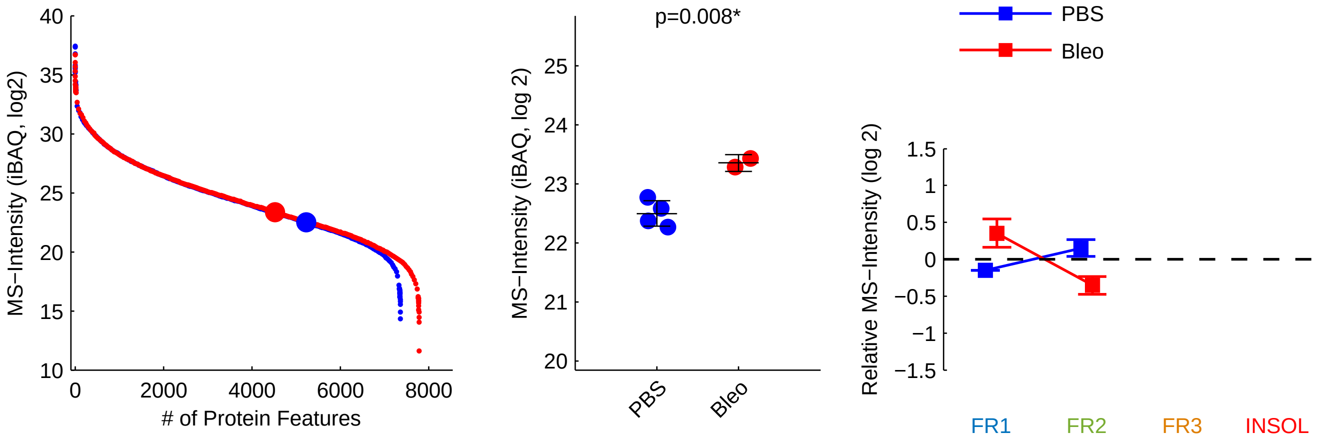

Q5SRX1 − Tom1l2 (id: 4366)

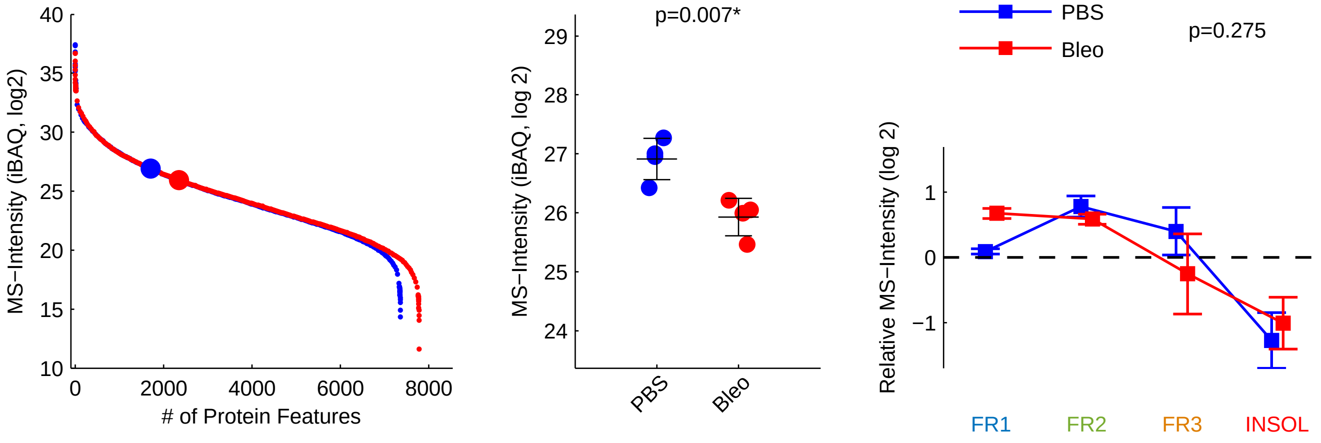

Q5SSW2 − Psme4 (id: 4372)

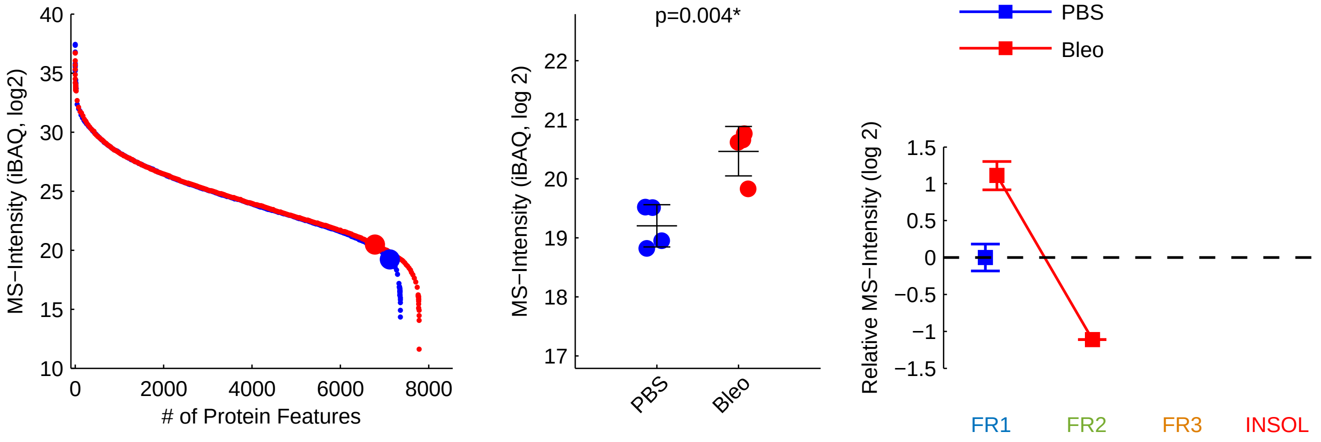

Q61545 − Ewsr1 (id: 4383)

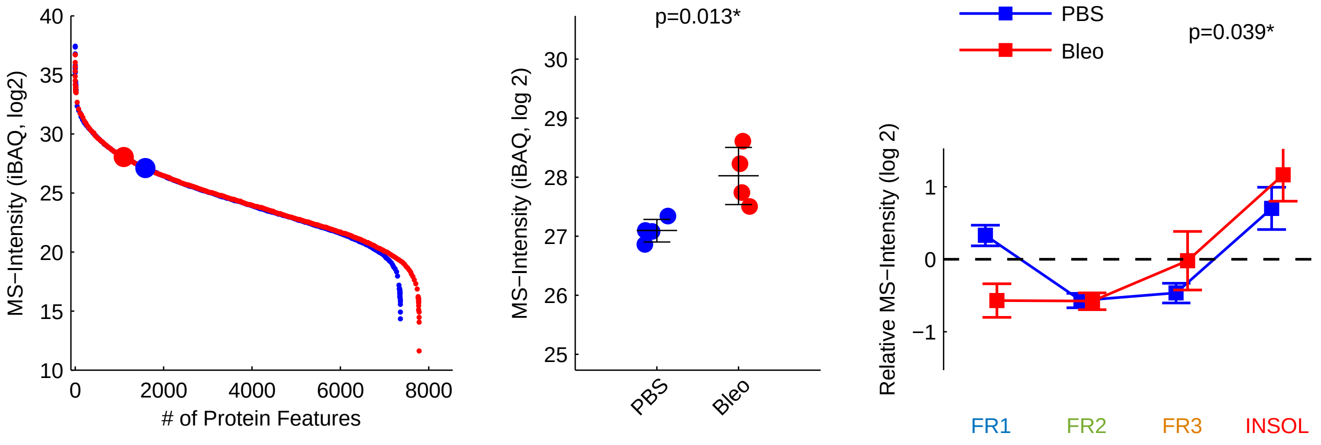

Q5SWU9 − Acaca (id: 4401)

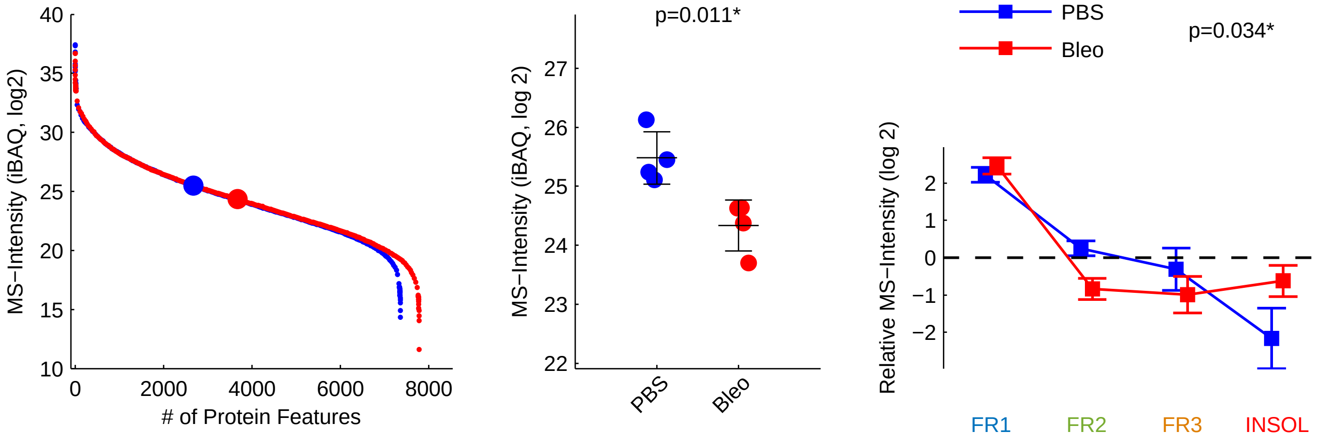

Q60716−2 − P4ha2 (id: 4406)

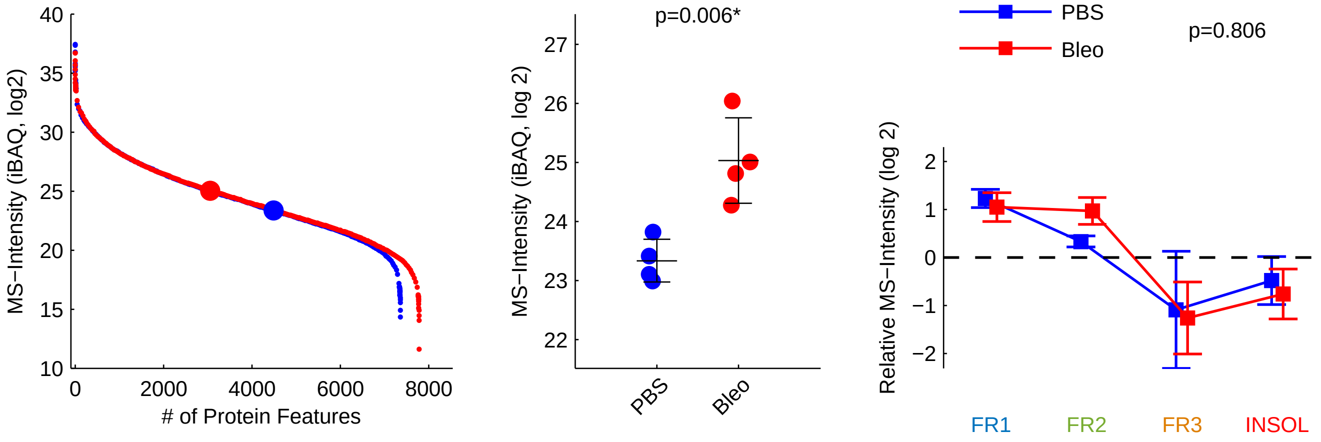

Q5SXC4 − Vezf1 (id: 4408)

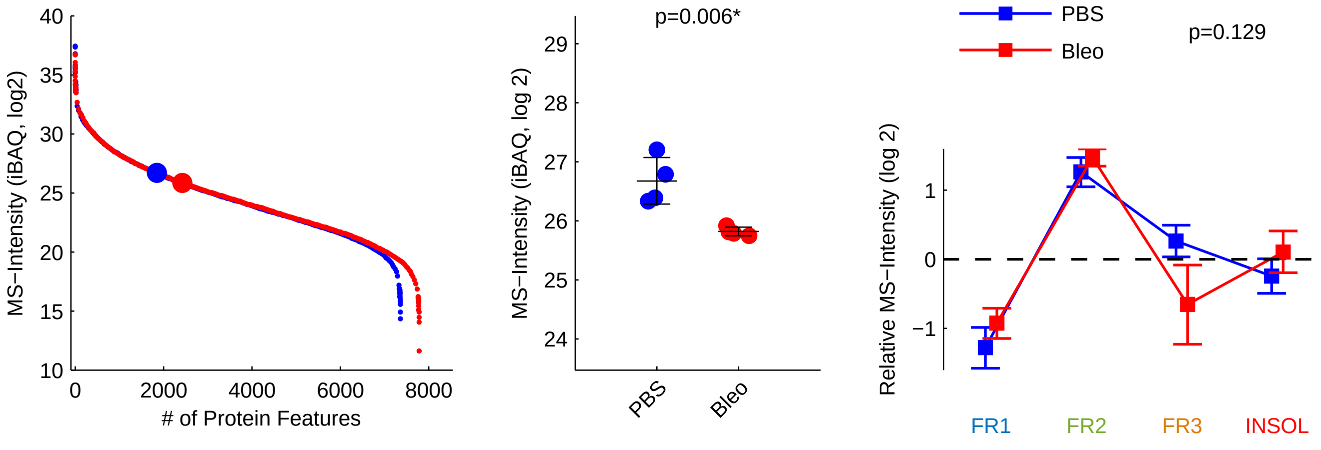

Q5SYD0 − Myo1d (id: 4413)

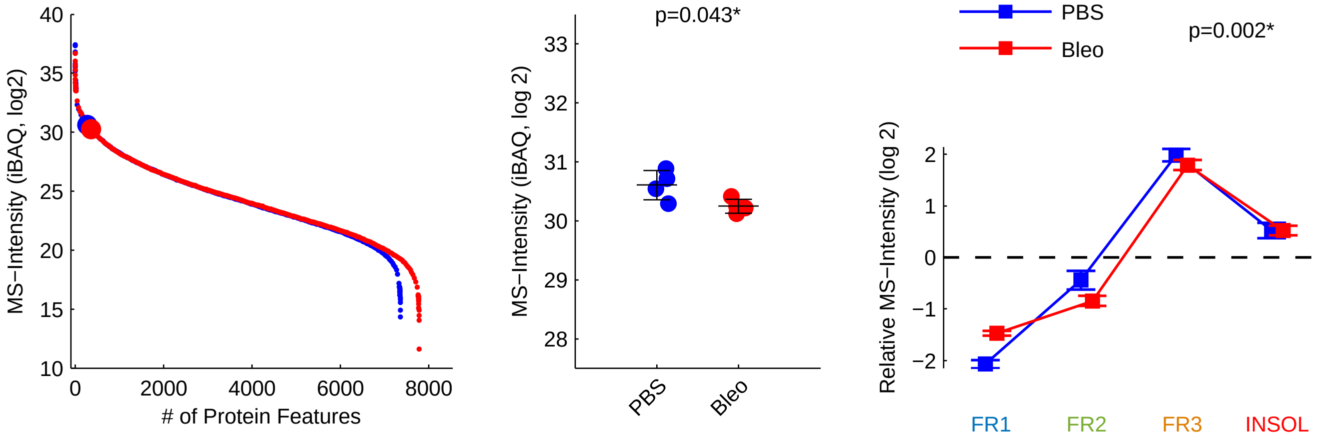

Q5U452 − 2810408M09Rik (id: 4415)

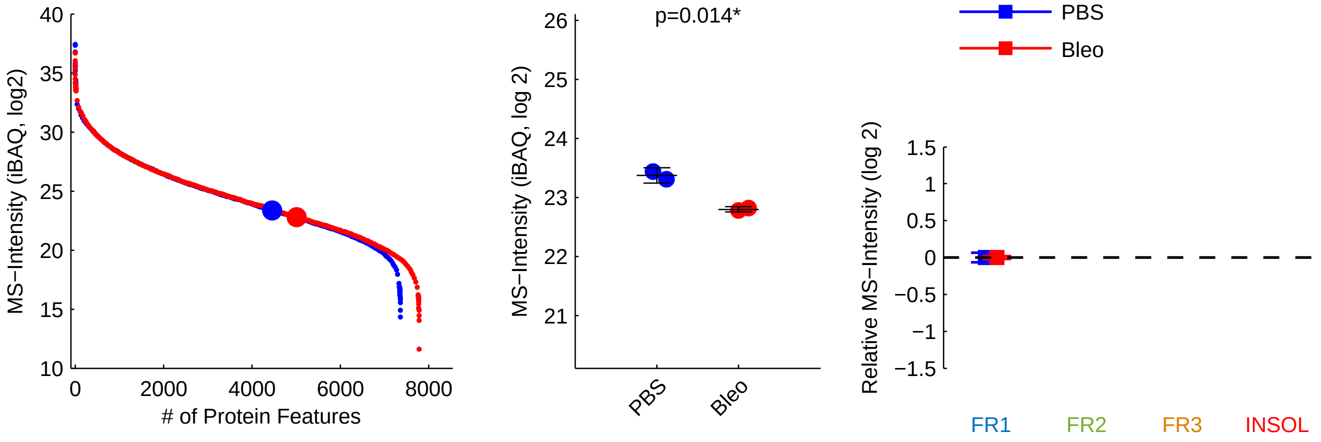

Q5XJY5 − Arcn1 (id: 4430)

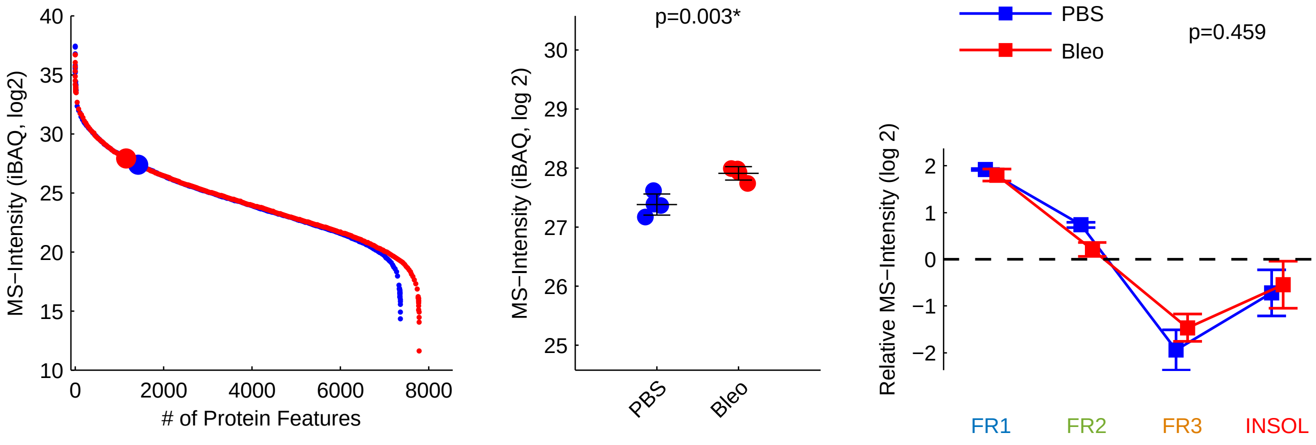

Q60520 − Sin3a (id: 4433)

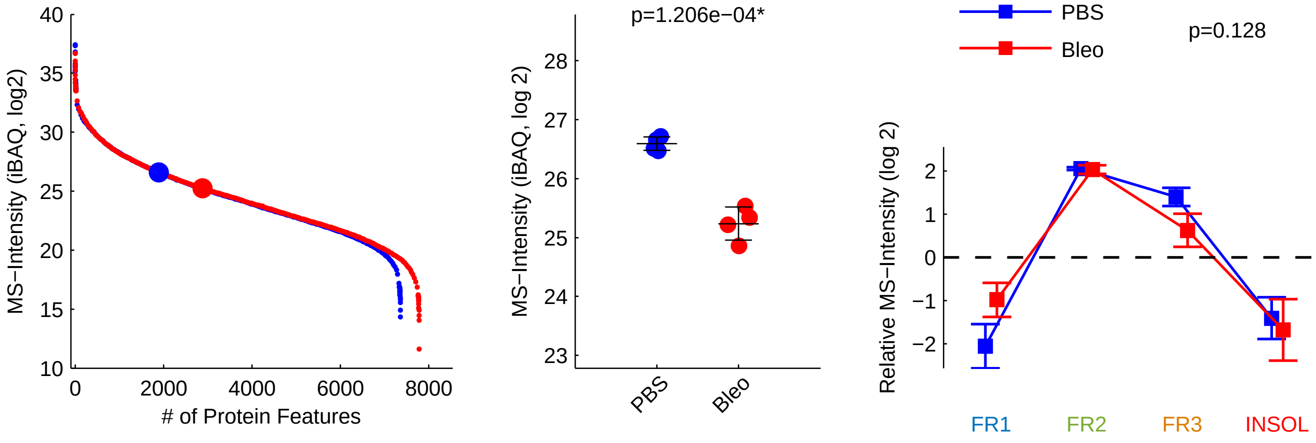

Q60590 − Orm1 (id: 4435)

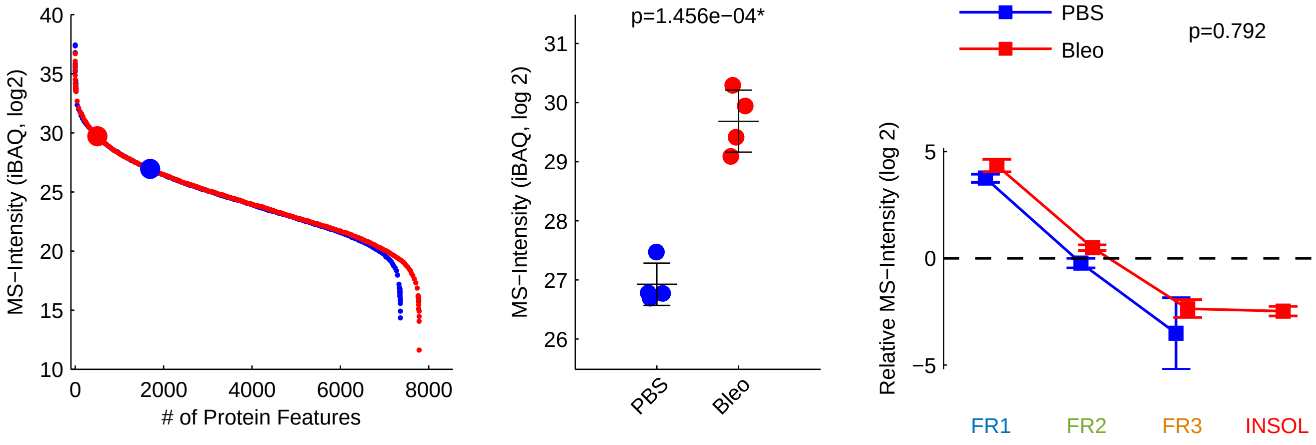

Q60598 − Cttn (id: 4438)

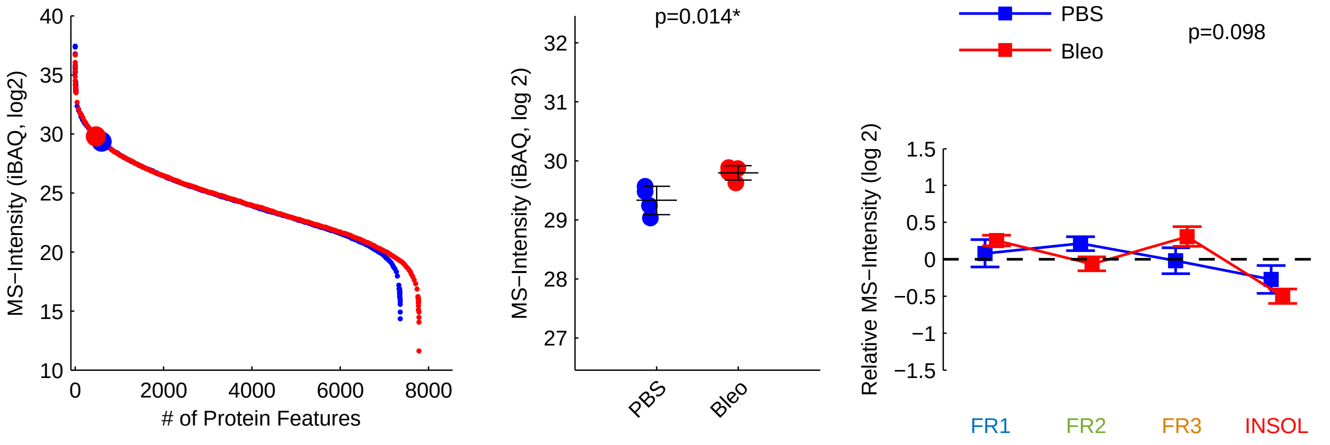

Q60605−2 − Myl6 (id: 4441)

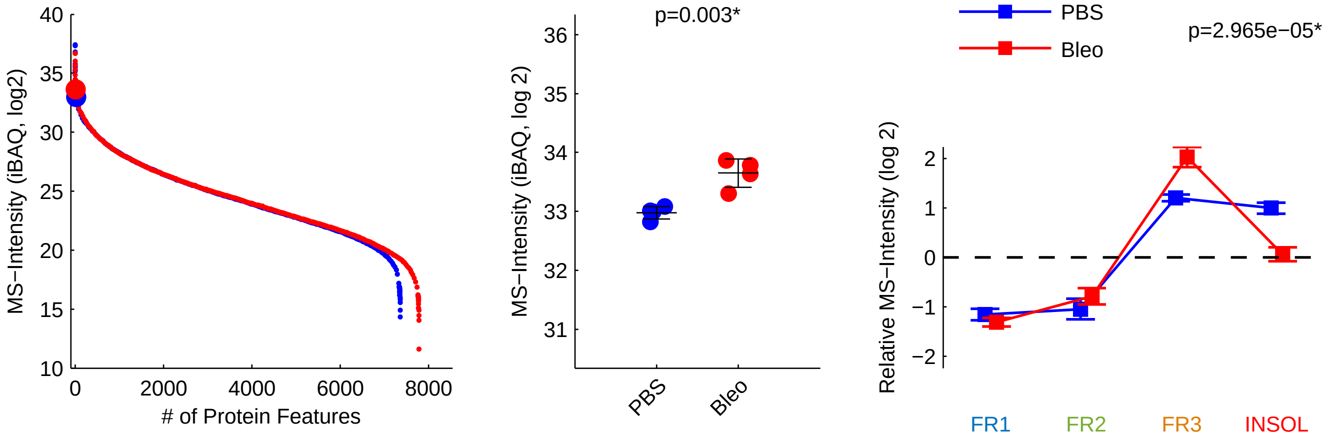

Q60634 − Flot2 (id: 4443)

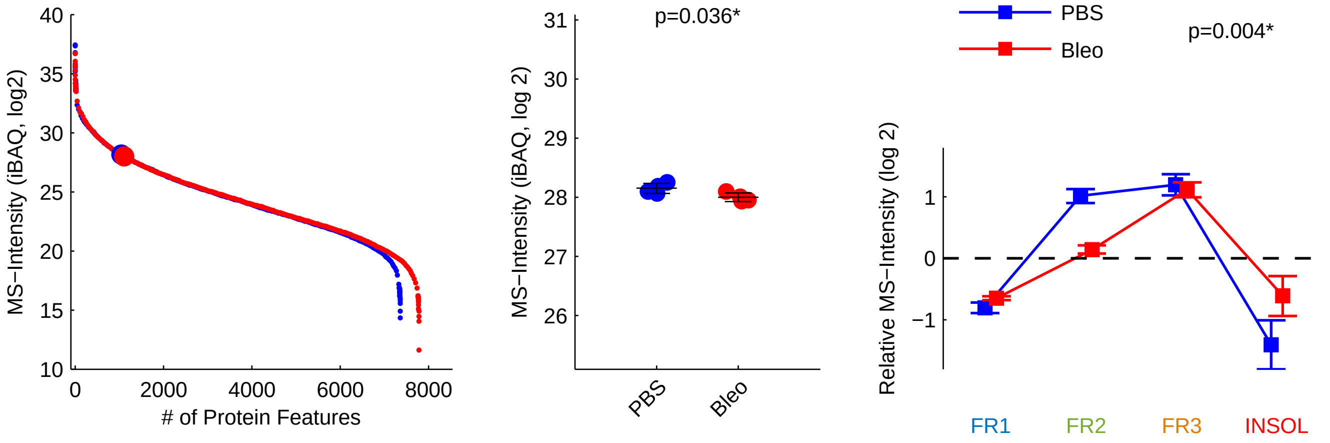

Q60668 − Hnrnpd (id: 4445)

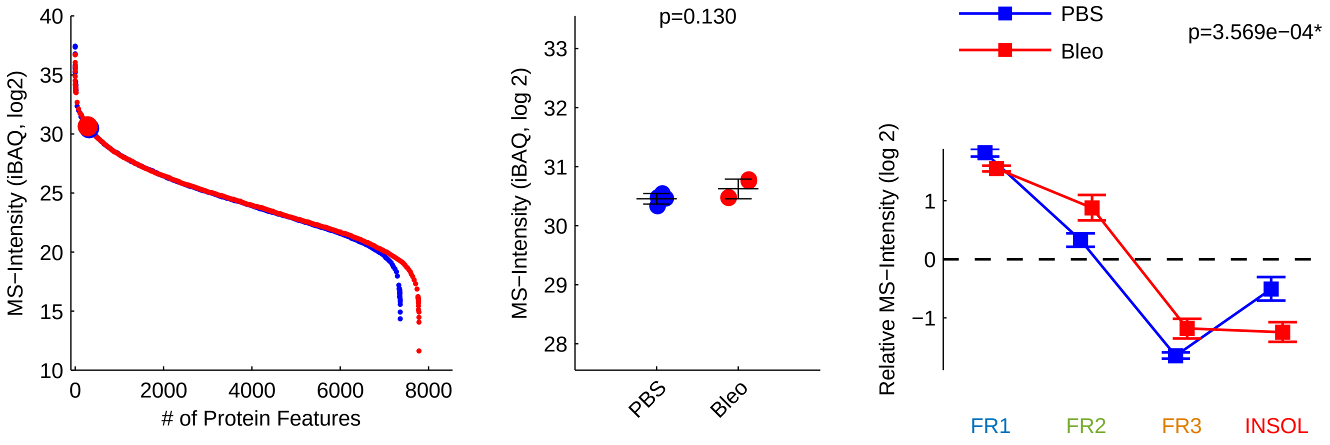

Q60692 − Psmb6 (id: 4447)

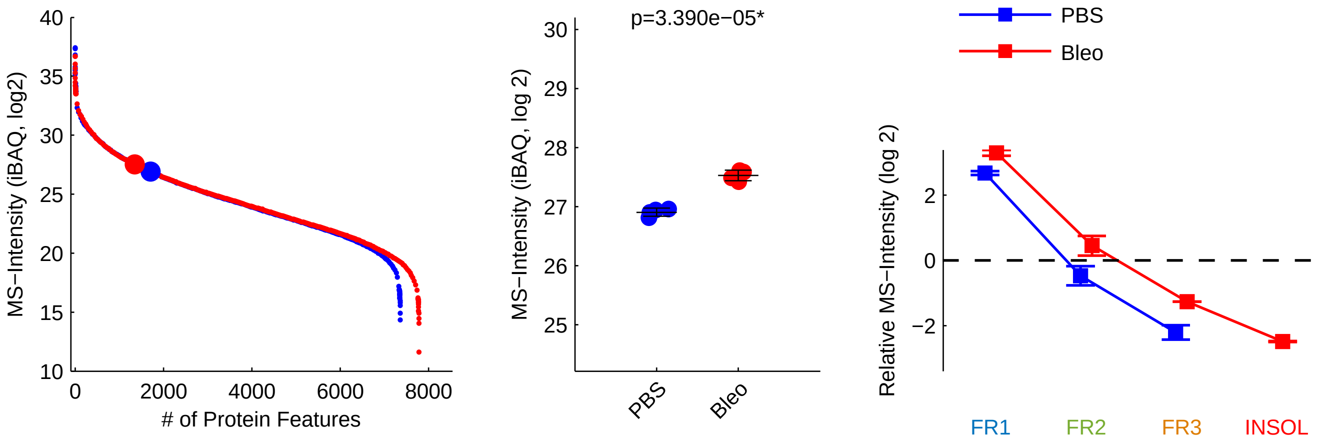

Q60715 − P4ha1 (id: 4452)

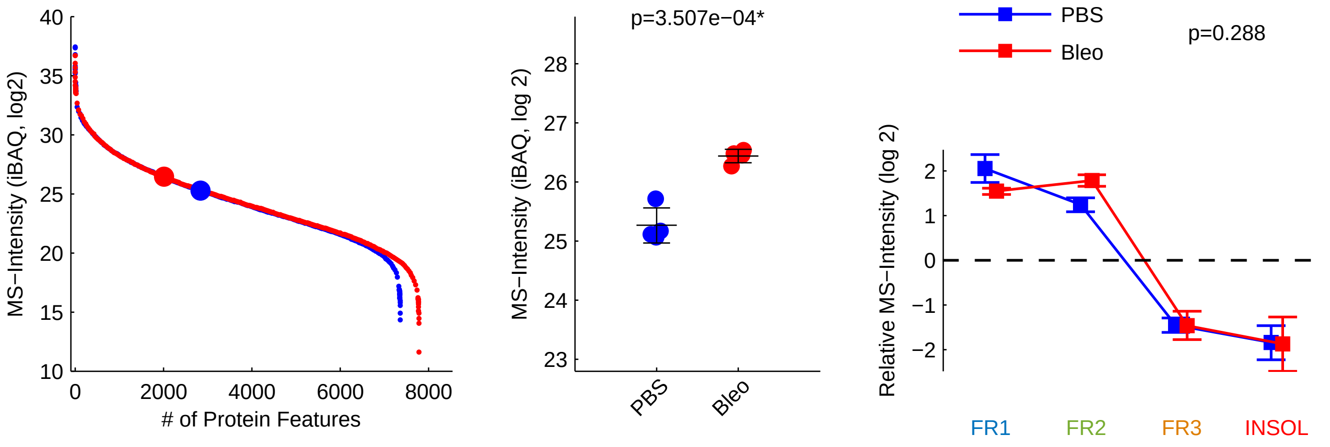

Q60738 − Slc30a1 (id: 4455)

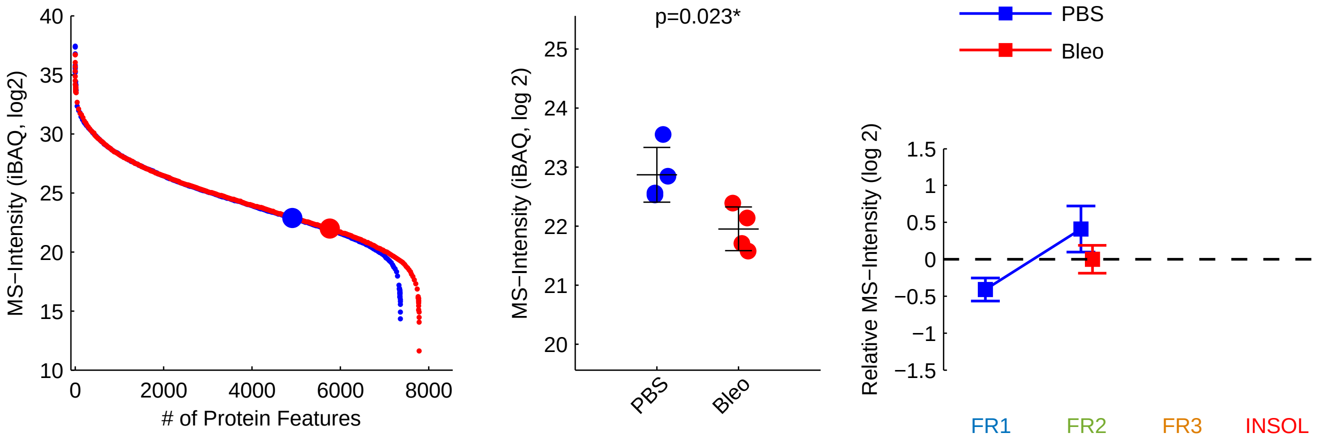

Q60760 − Grb10 (id: 4458)

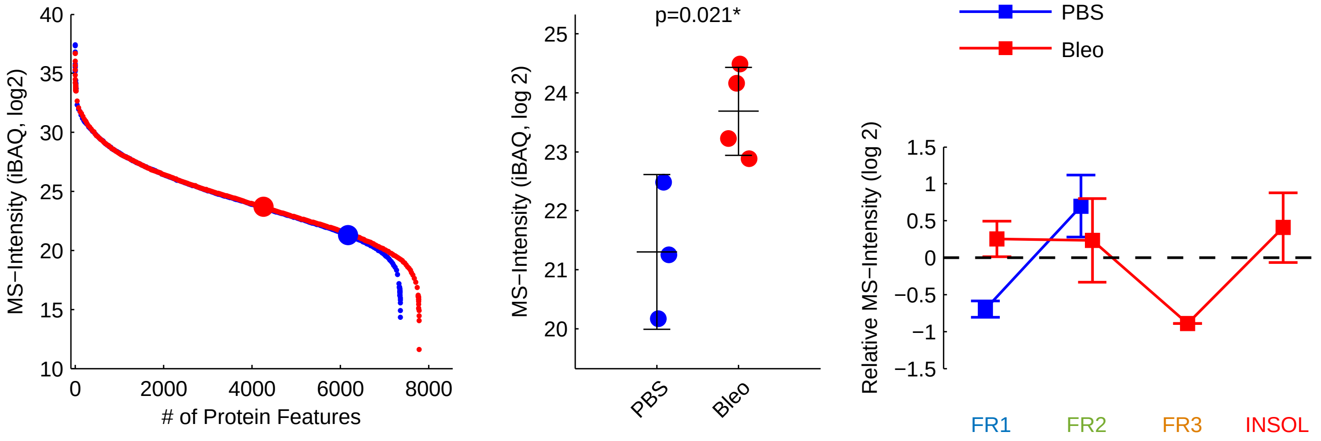

Q60766−2 − Irgm1 (id: 4459)

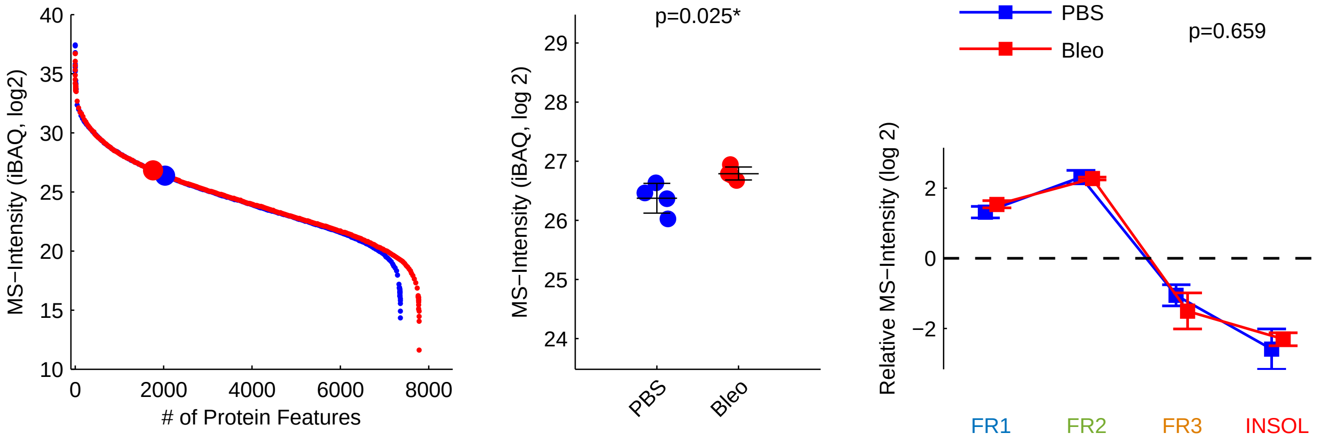

Q60770 − Stxbp3 (id: 4460)

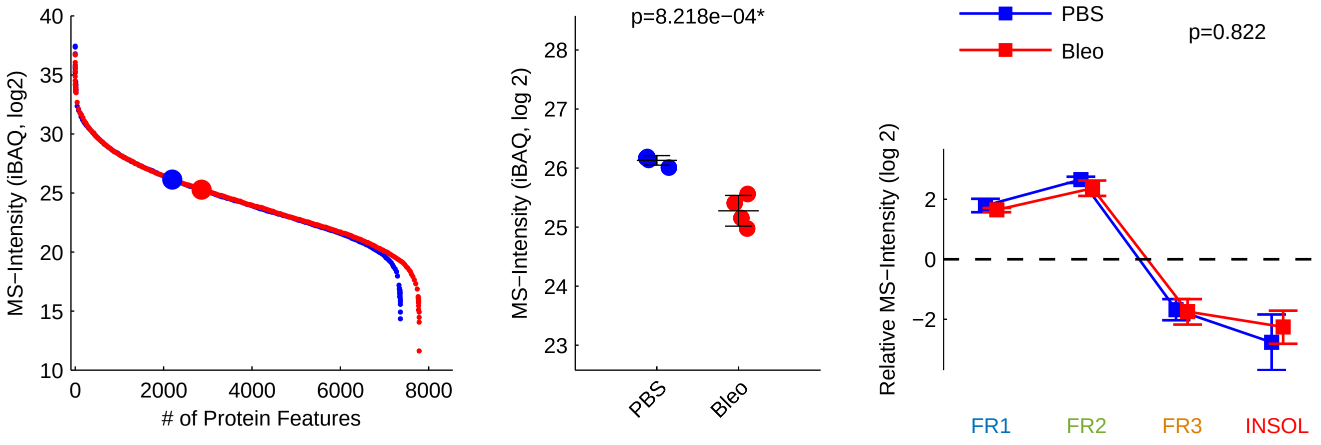

Q60790 − Rasa3 (id: 4465)

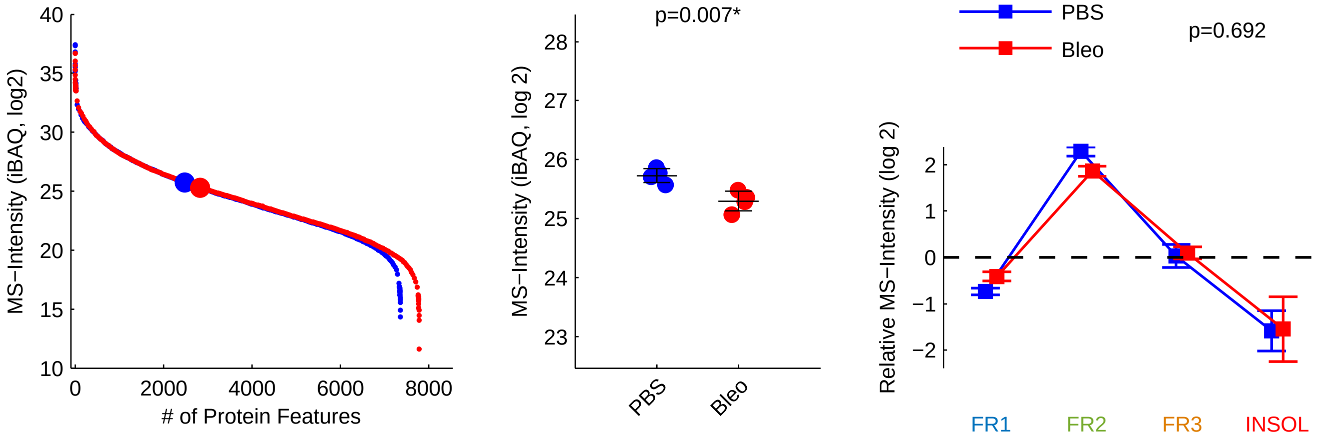

Q60823 − Akt2 (id: 4467)

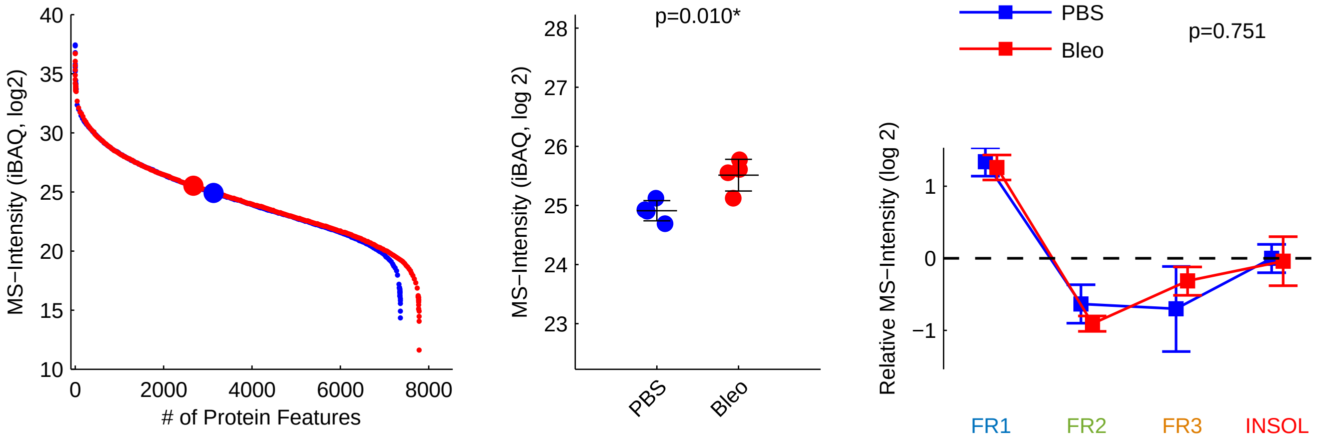

Q60864 − Stip1 (id: 4476)

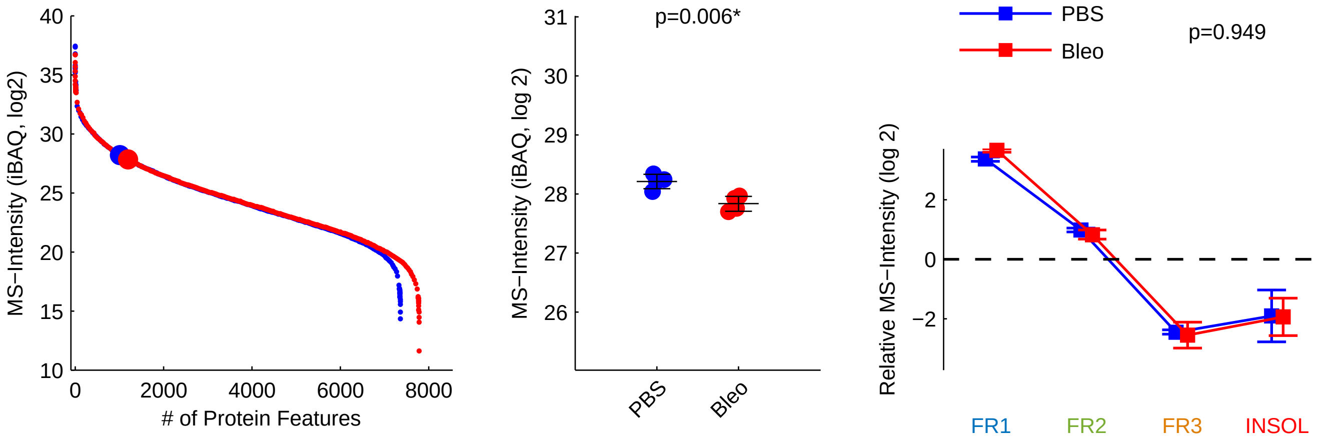

Q60865 − Caprin1 (id: 4477)

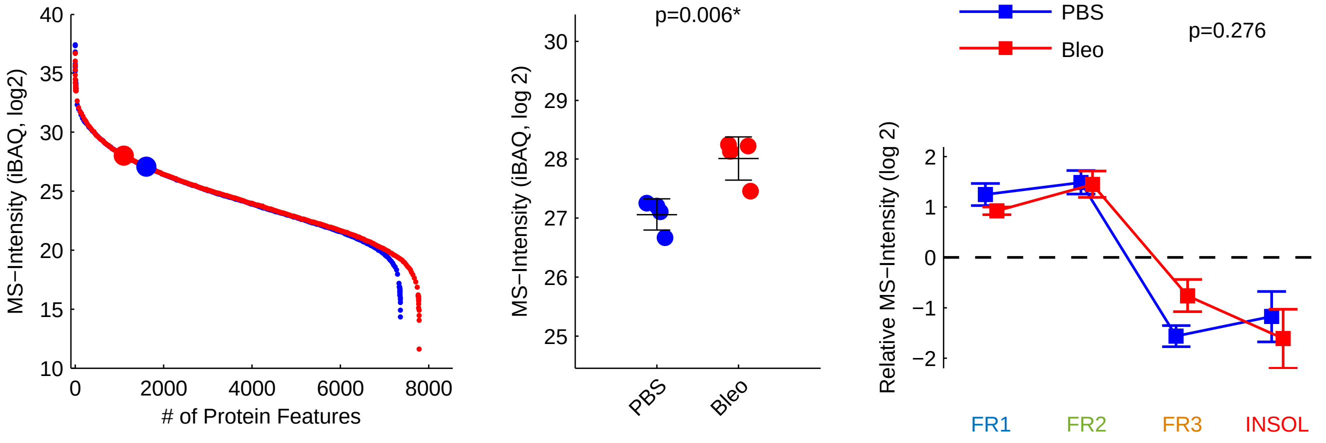

Q60872 − Eif1a (id: 4479)

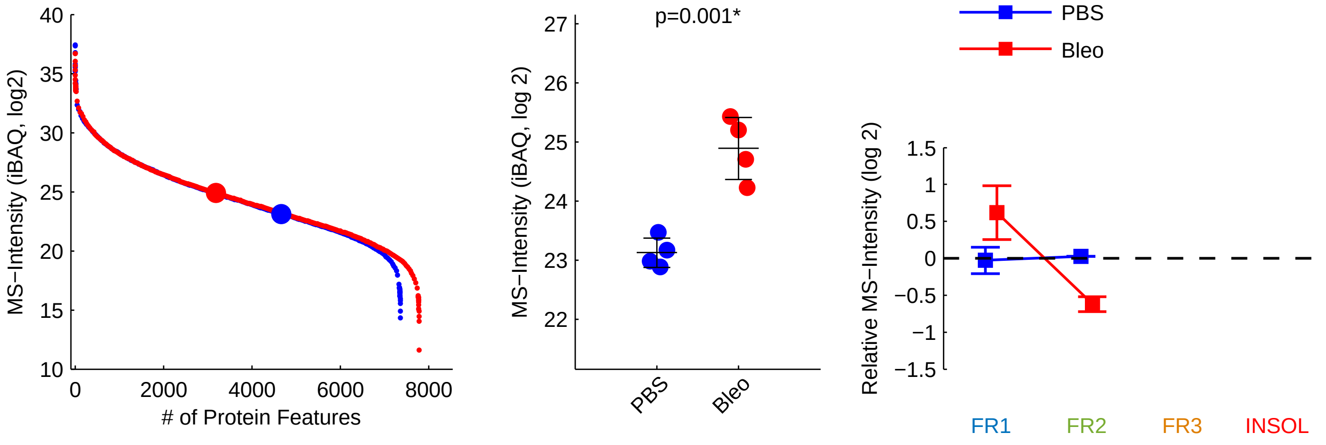

Q60902 − Eps15l1 (id: 4483)

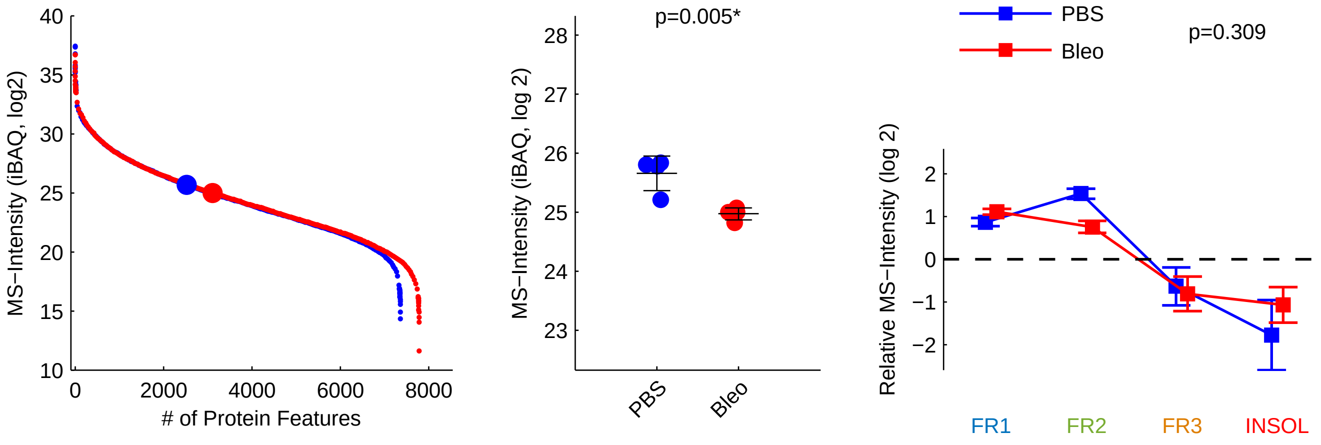

Q60930 − Vdac2 (id: 4485)

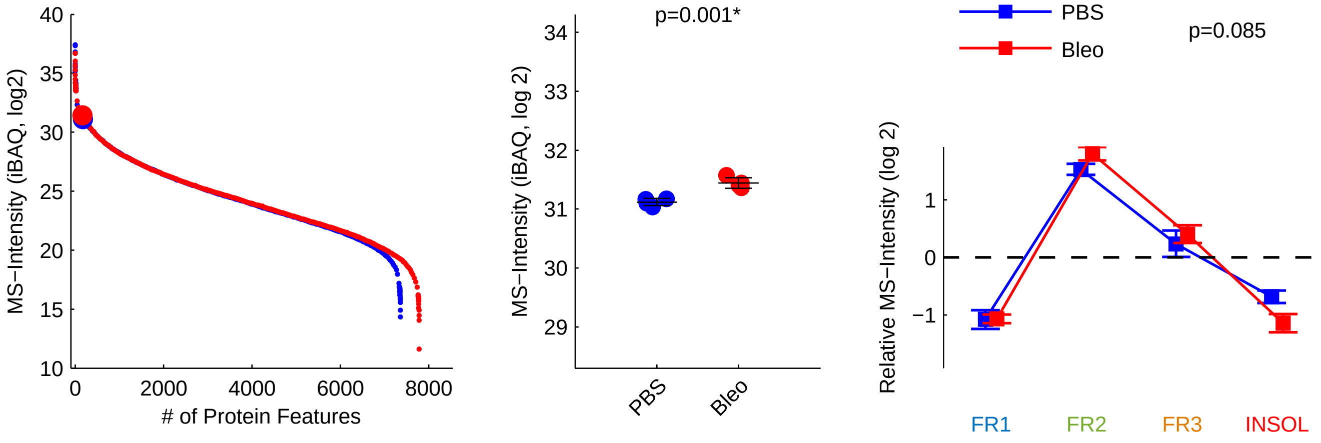

Q60953 − Pml (id: 4488)

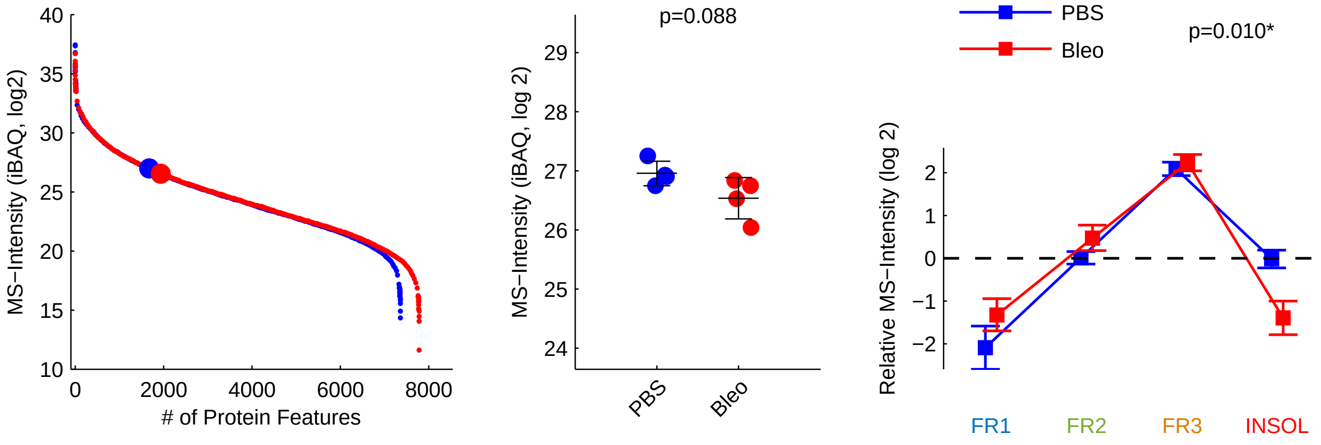

Q60963 − Pla2g7 (id: 4491)

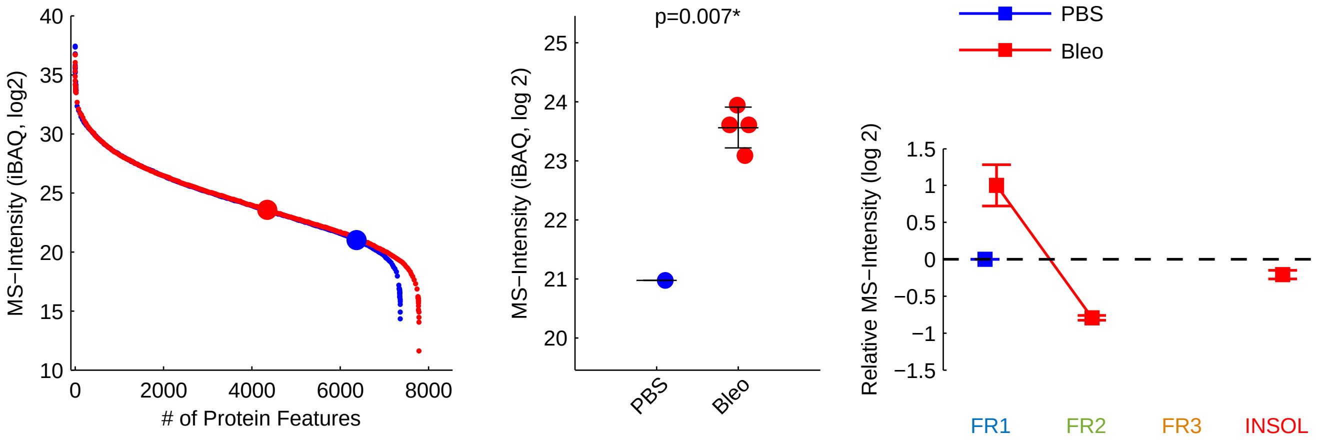

Q60994 − Adipoq (id: 4498)

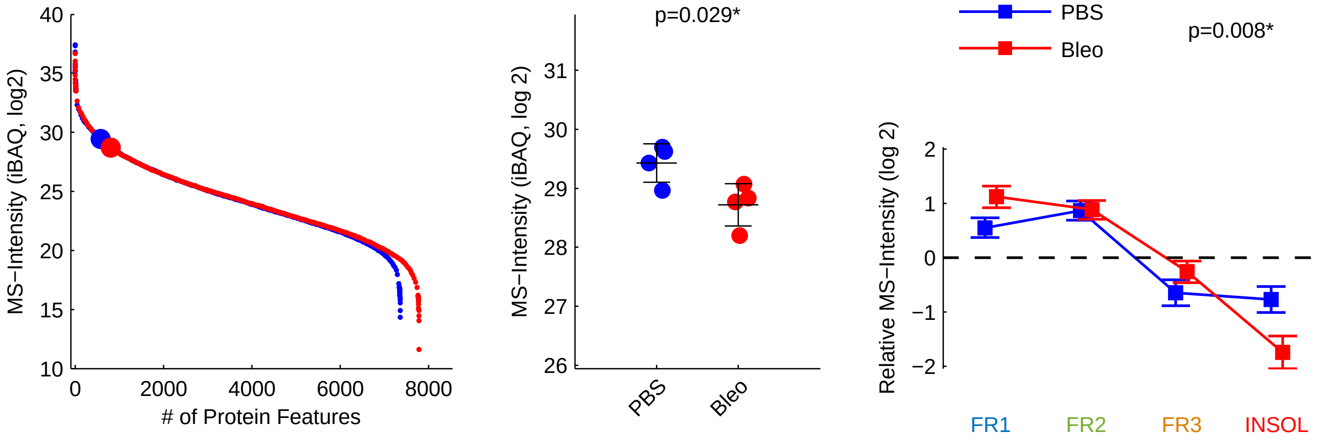

Q61001 − Lama5 (id: 4501)

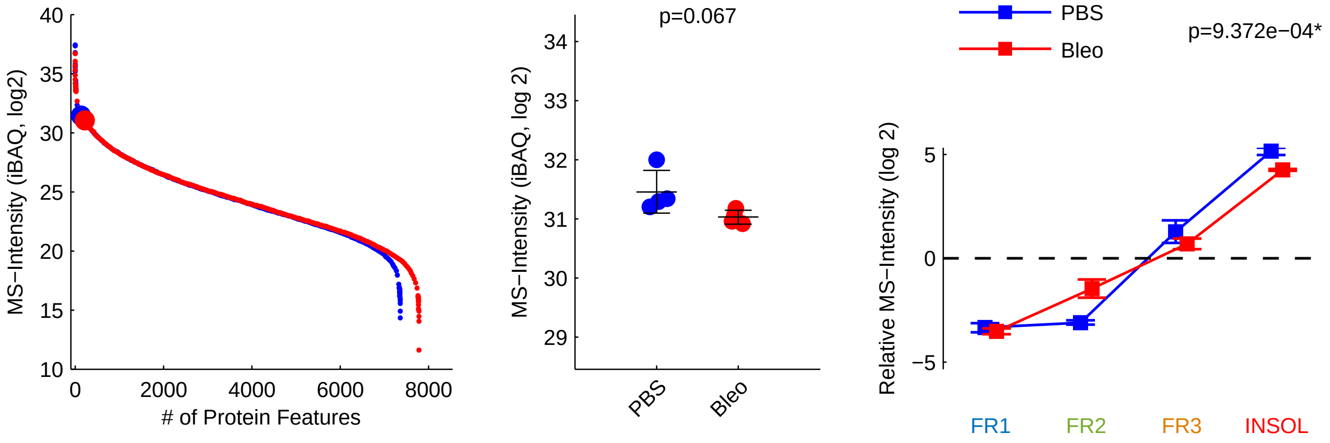

Q61009 − Scarb1 (id: 4502)

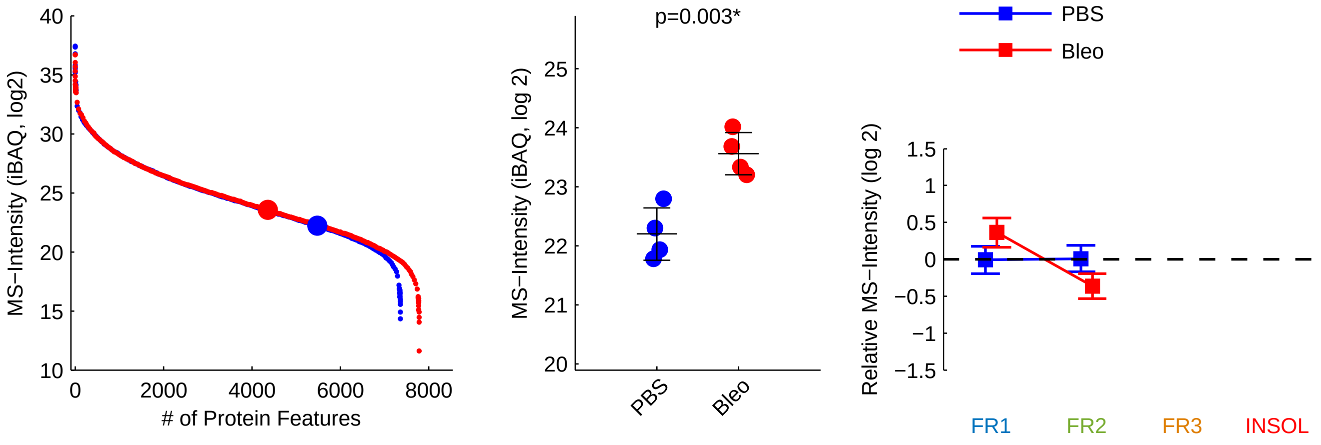

Q61017 − Gngt2 (id: 4503)

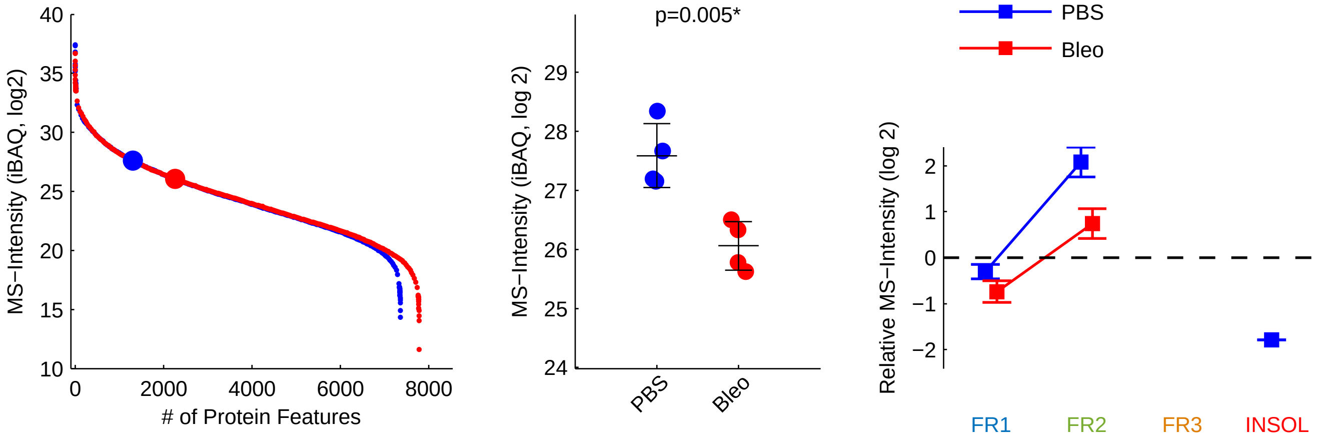

Q61029 − Tmpo (id: 4506)

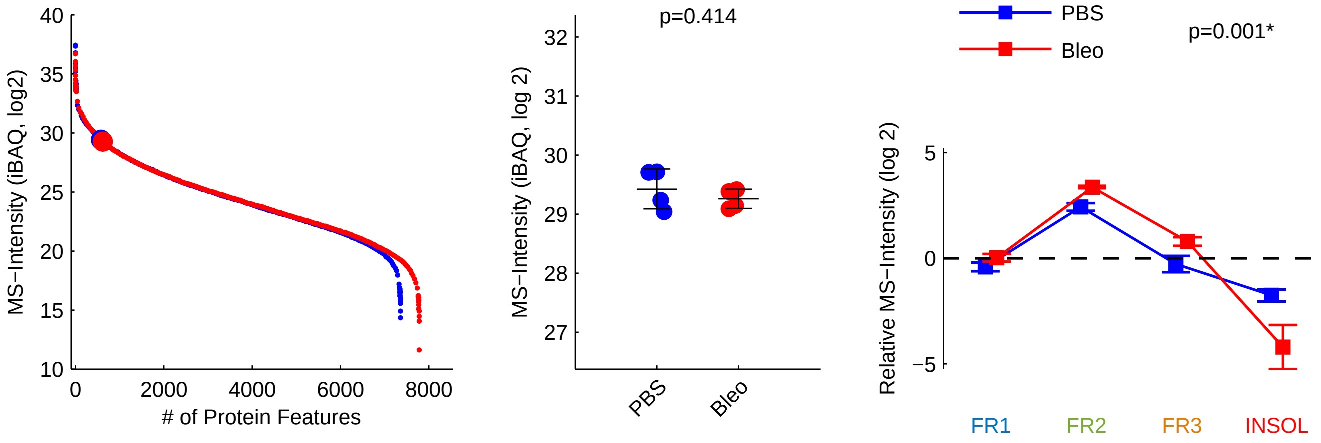

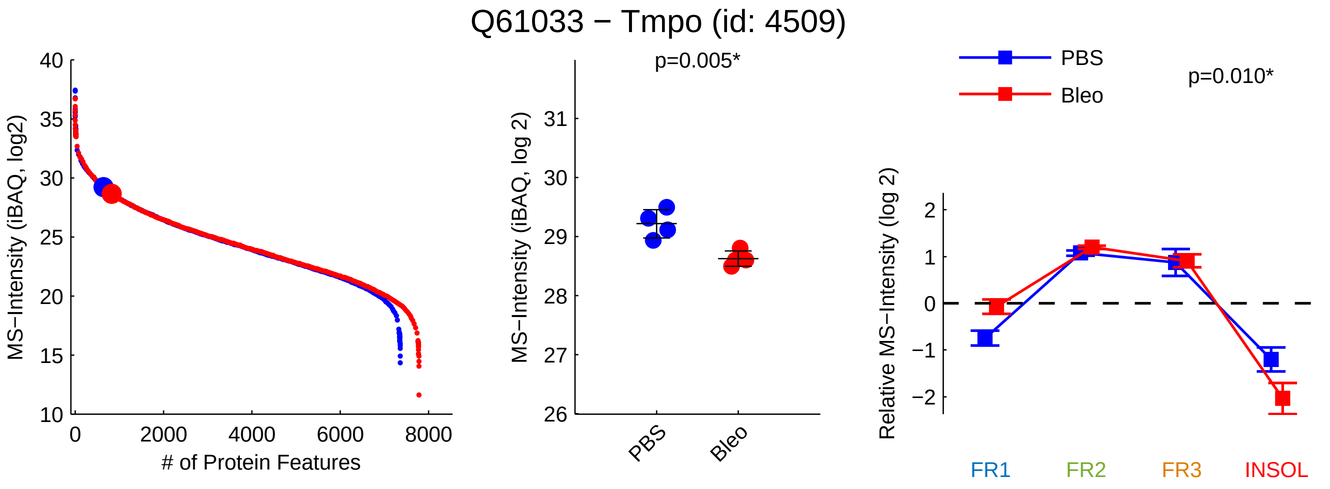
Q61033 − Tmpo (id: 4509)
MS−Intensity (iBAQ, log2)
# of Protein Features
p=0.005*
MS−Intensity (iBAQ, log 2)
PBS
Bleo
PBS
Bleo
p=0.010*
Relative MS−Intensity (log 2)
FR1
FR2
FR3
INSOL

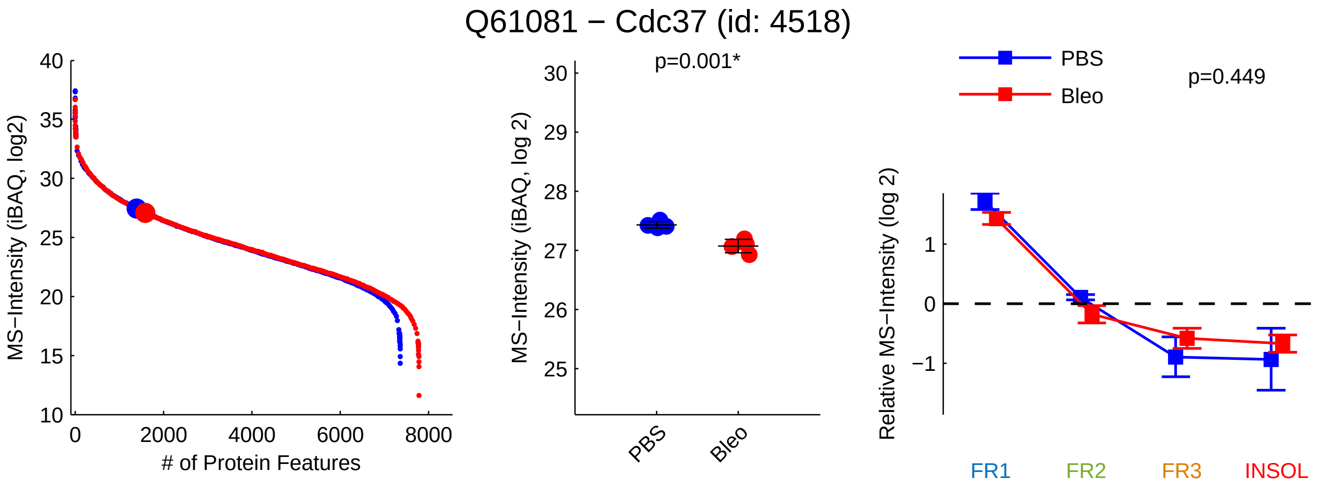
Q61081 − Cdc37 (id: 4518)
MS−Intensity (iBAQ, log2)
# of Protein Features
p=0.001*
MS−Intensity (iBAQ, log 2)
PBS
Bleo
PBS
Bleo
p=0.449
Relative MS−Intensity (log 2)
FR1
FR2
FR3
INSOL

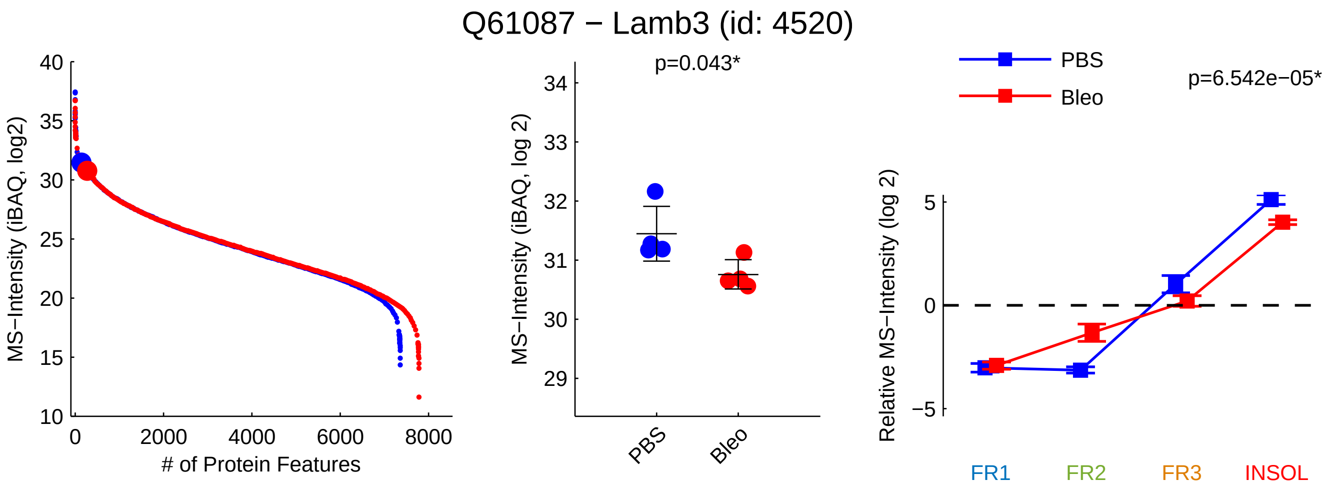
Q61087 − Lamb3 (id: 4520)
MS−Intensity (iBAQ, log2)
# of Protein Features
p=0.043*
MS−Intensity (iBAQ, log 2)
PBS
Bleo
PBS
Bleo
p=6.542e−05*
Relative MS−Intensity (log 2)
FR1
FR2
FR3
INSOL

Q61093 − Cybb (id: 4522)

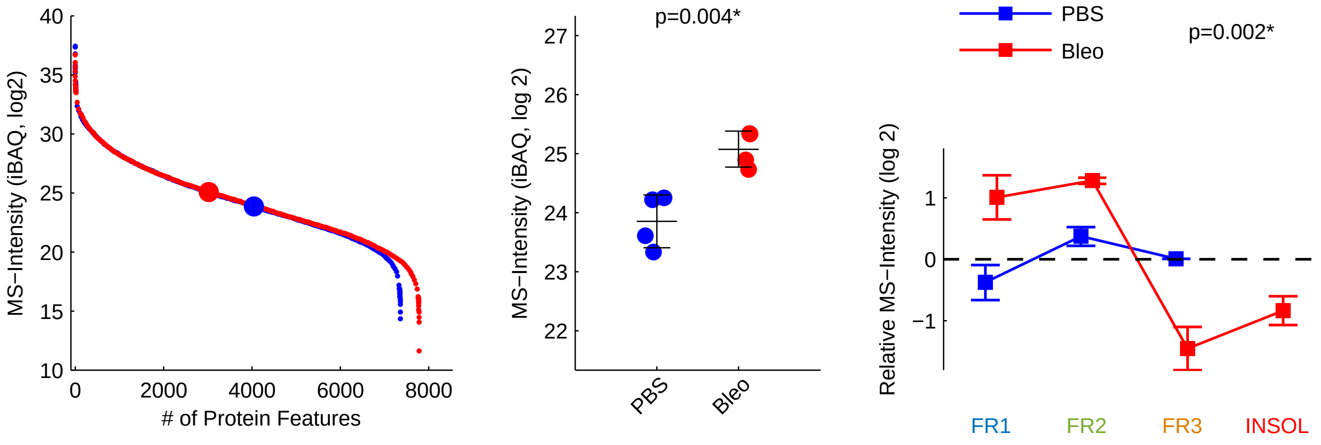

Q61129 − Cfi (id: 4528)

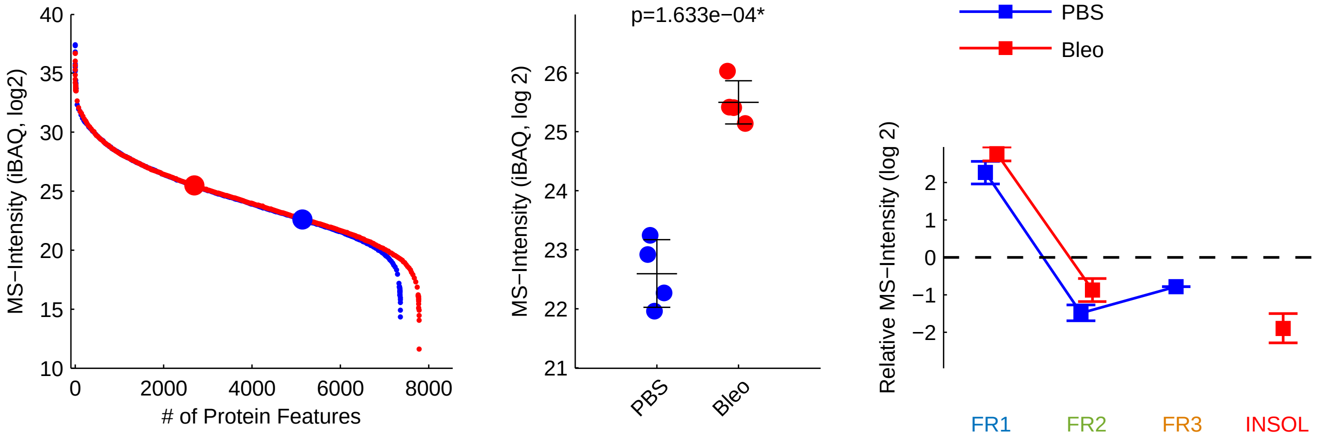

Q61136 − Prpf4b (id: 4530)

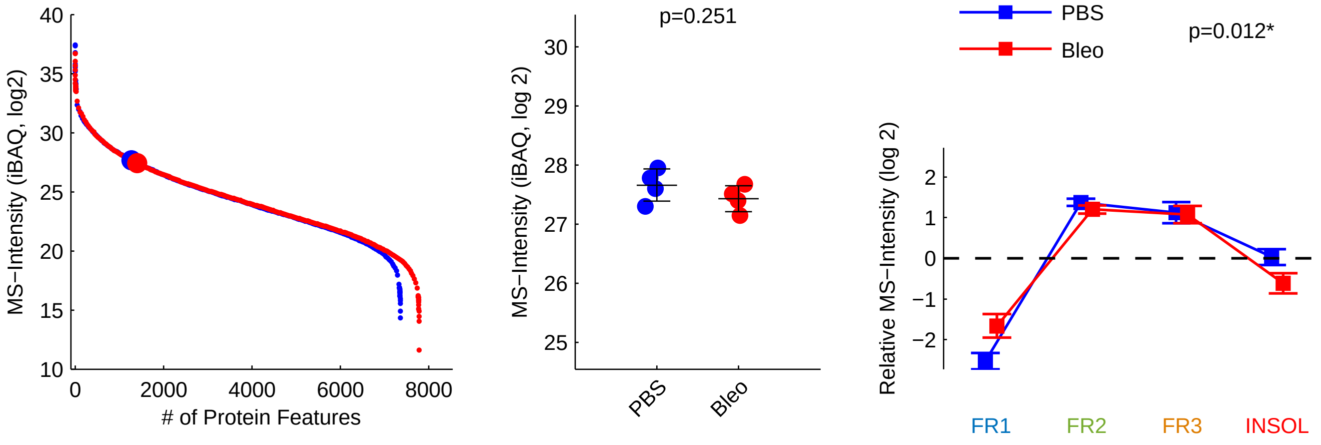

Q61140 − Bcar1 (id: 4531)

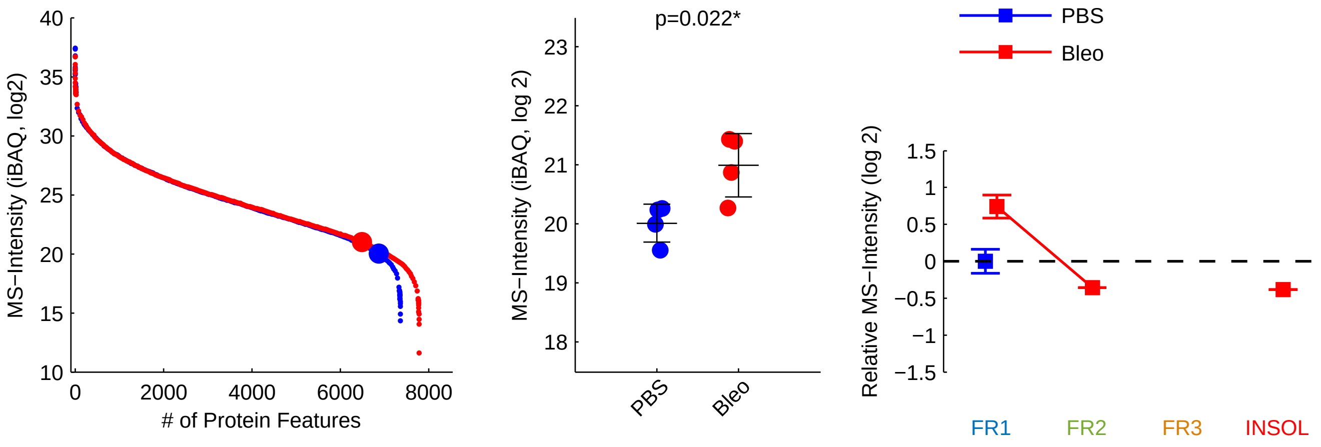

Q61142 − Spin1 (id: 4532)

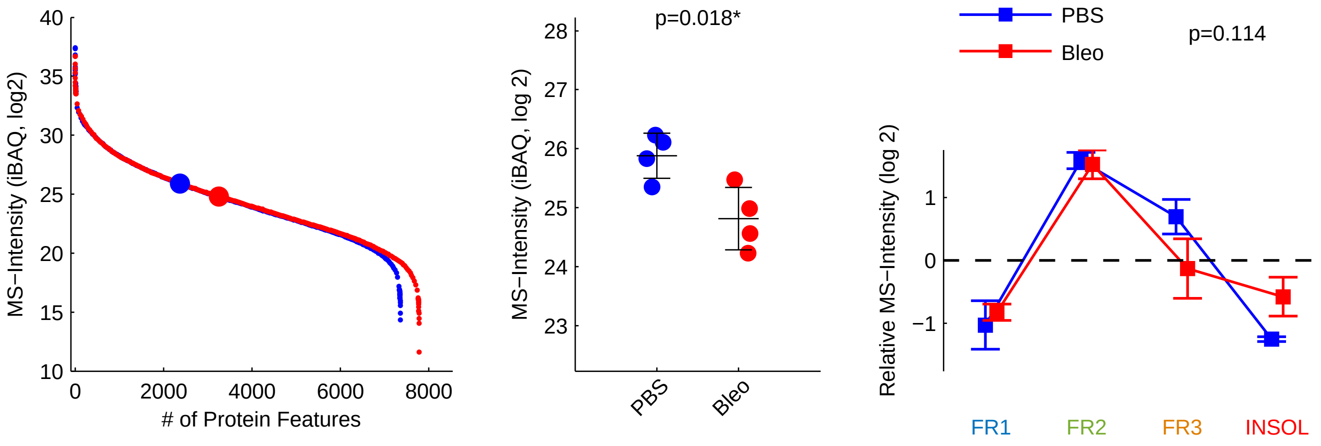

Q61161 − Map4k2 (id: 4537)

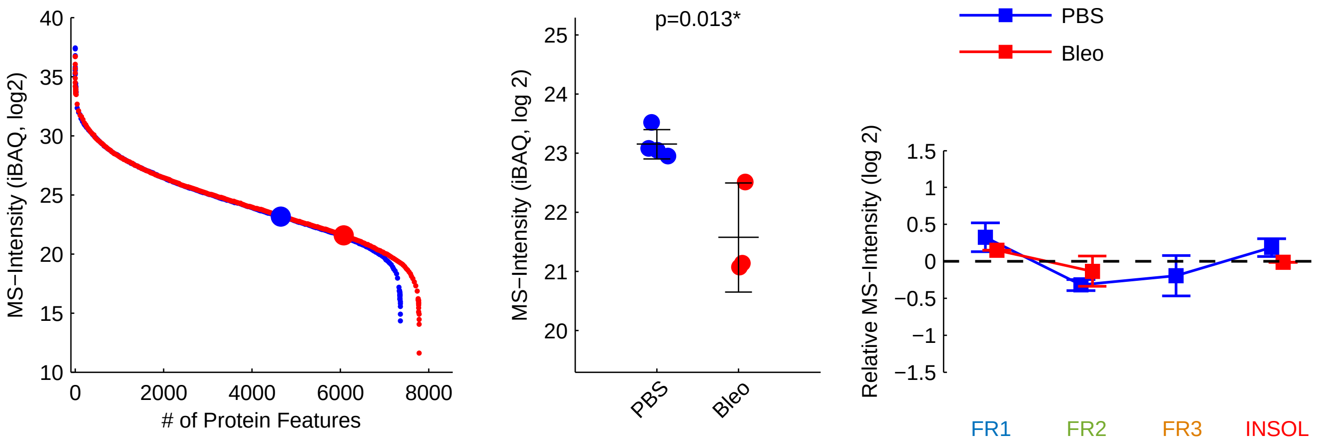

Q61164 − Ctcf (id: 4538)

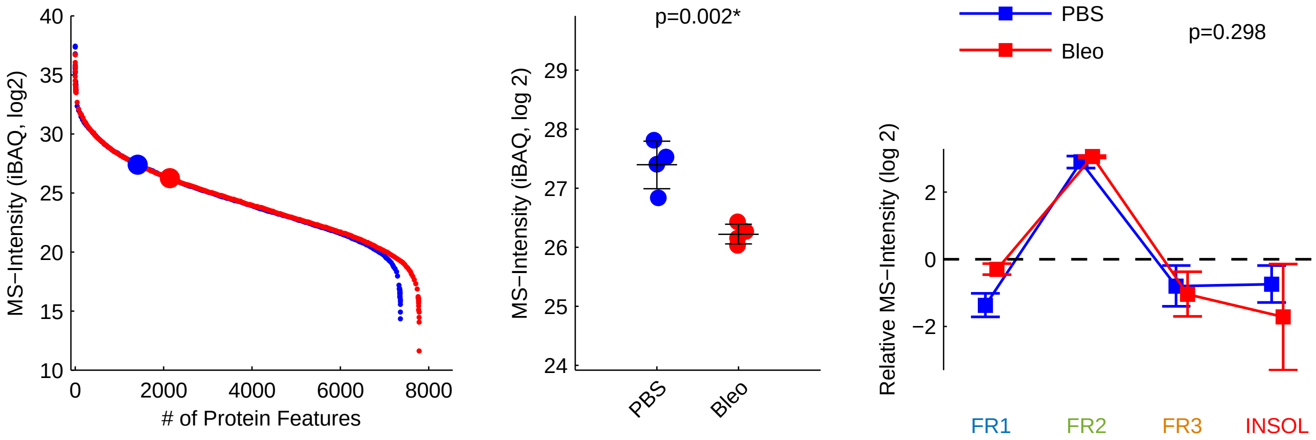

Q61176 − Arg1 (id: 4542)

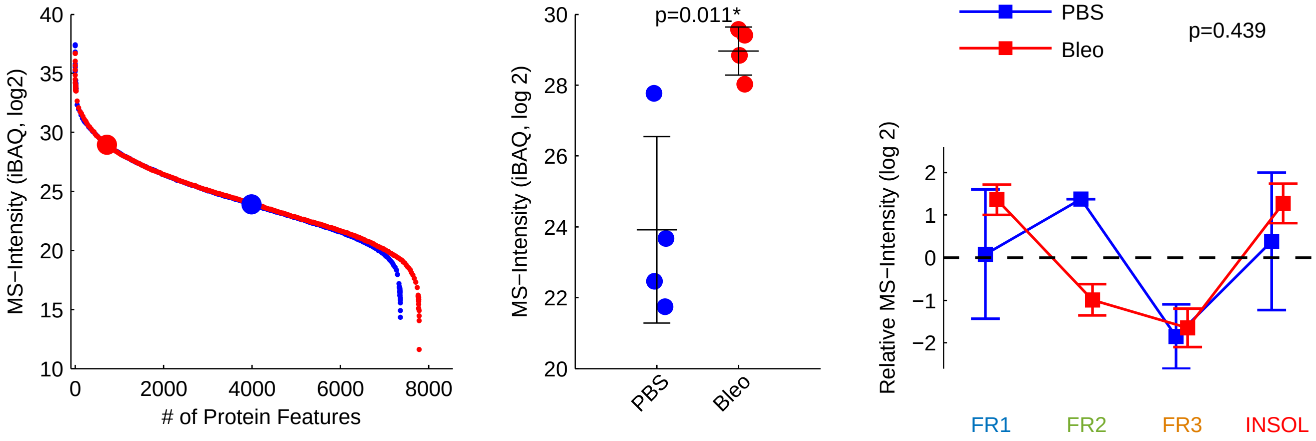

Q61191 − Hcfc1 (id: 4544)

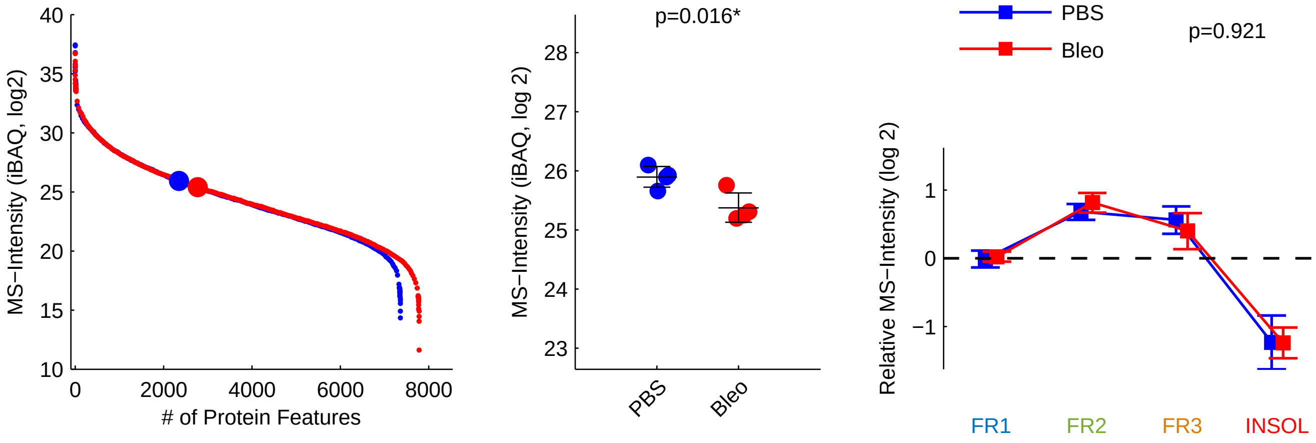

Q61235 − Sntb2 (id: 4551)

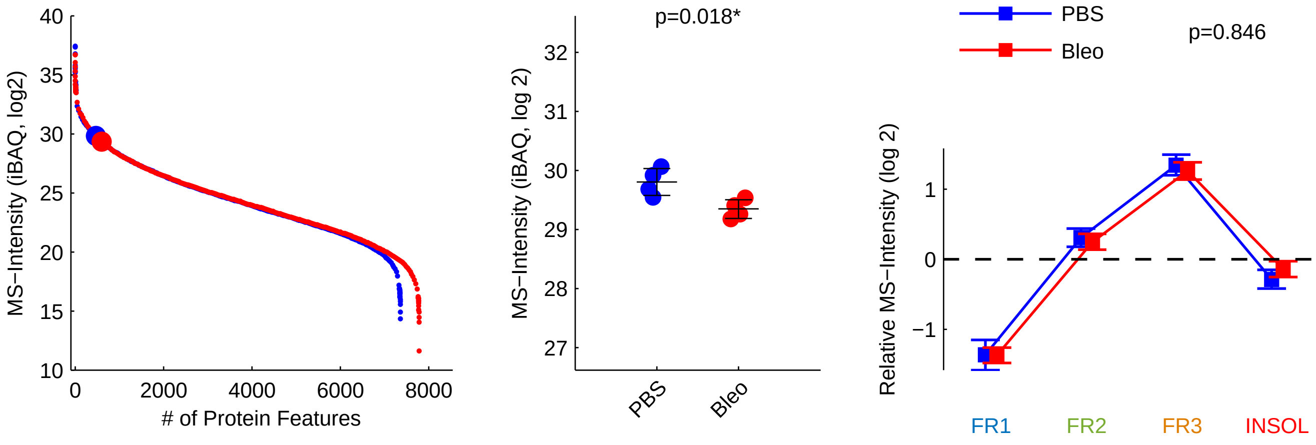

Q61247 − Serpinf2 (id: 4554)

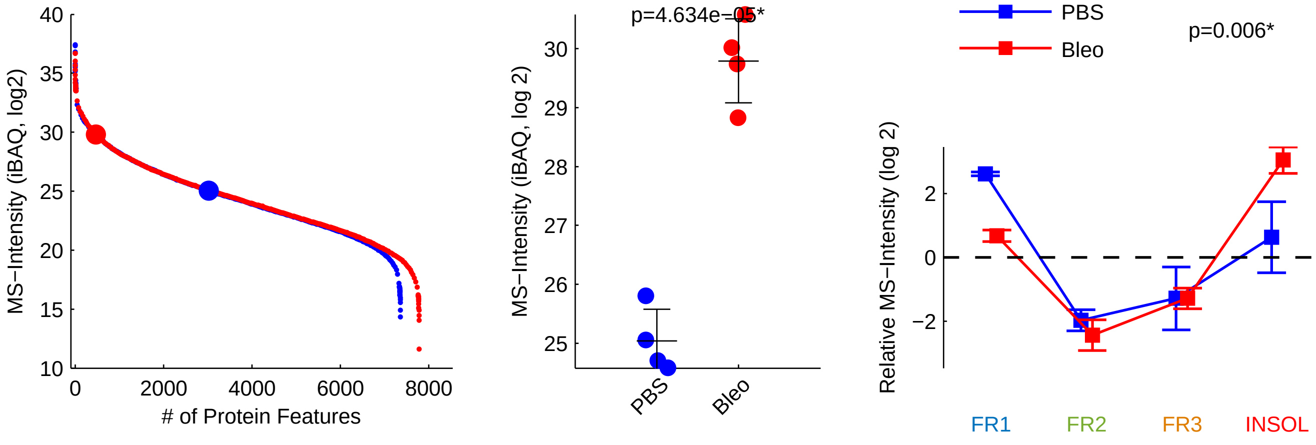

Q61288 − Acvrl1 (id: 4561)

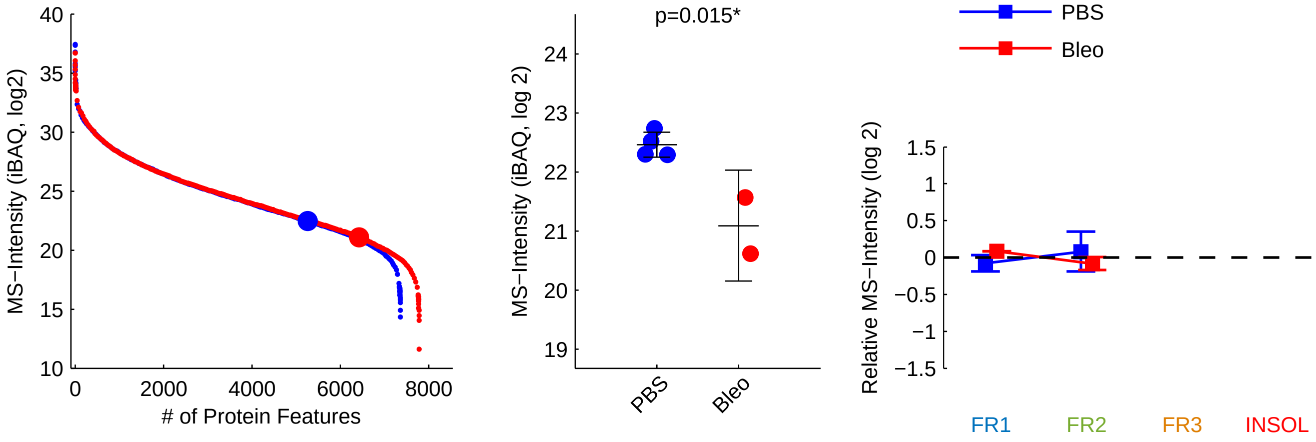

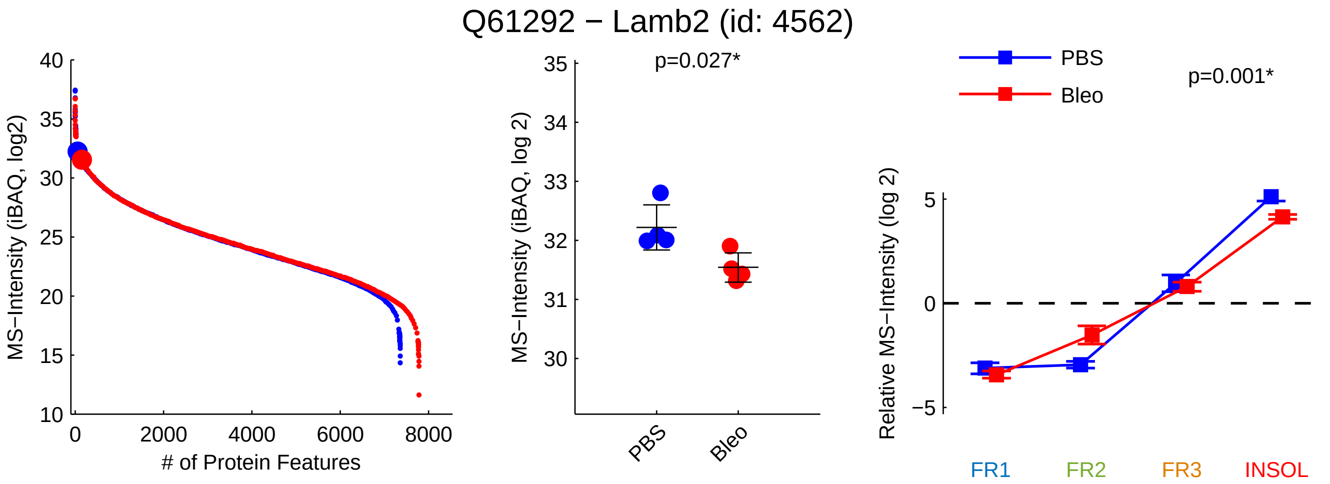
Q61292 − Lamb2 (id: 4562)
MS−Intensity (iBAQ, log2)
# of Protein Features
p=0.027*
MS−Intensity (iBAQ, log 2)
PBS
Bleo
p=0.001*
Relative MS−Intensity (log 2)
FR1
FR2
FR3
INSOL

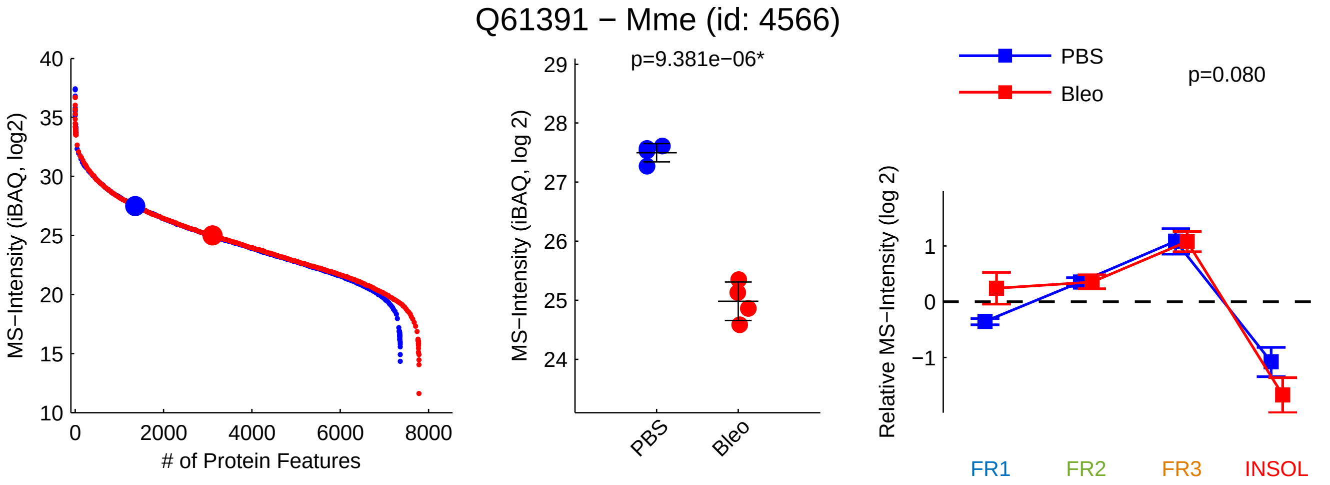
Q61391 − Mme (id: 4566)
MS−Intensity (iBAQ, log2)
# of Protein Features
p=9.381e−06*
MS−Intensity (iBAQ, log 2)
PBS
Bleo
p=0.080
Relative MS−Intensity (log 2)
FR1
FR2
FR3
INSOL

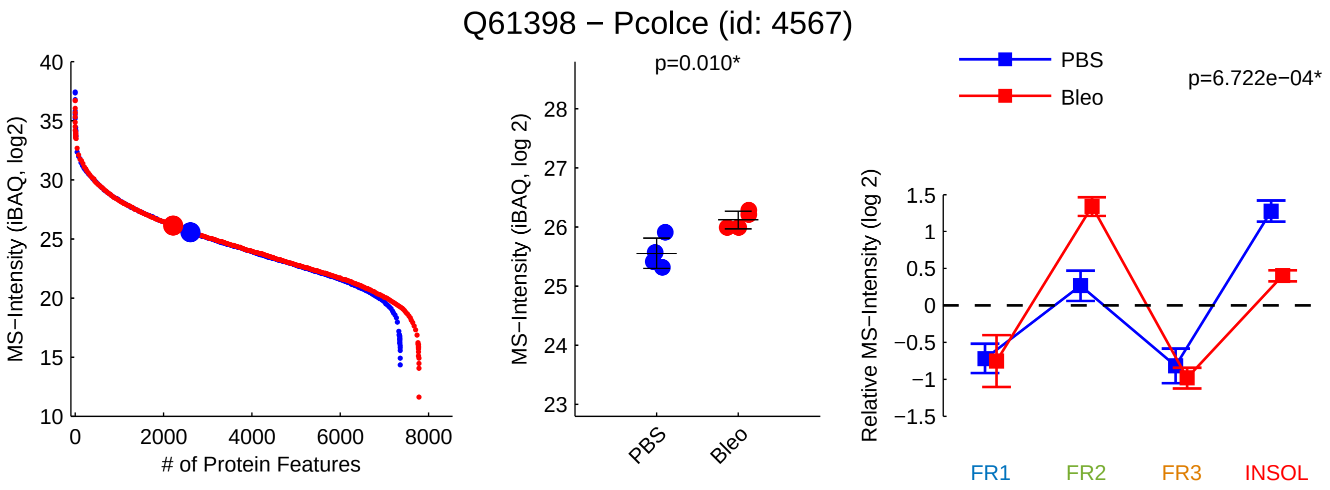
Q61398 − Pcolce (id: 4567)
MS−Intensity (iBAQ, log2)
# of Protein Features
p=0.010*
MS−Intensity (iBAQ, log 2)
PBS
Bleo
p=6.722e−04*
Relative MS−Intensity (log 2)
FR1
FR2
FR3
INSOL

Q61425 − Hadh (id: 4571)

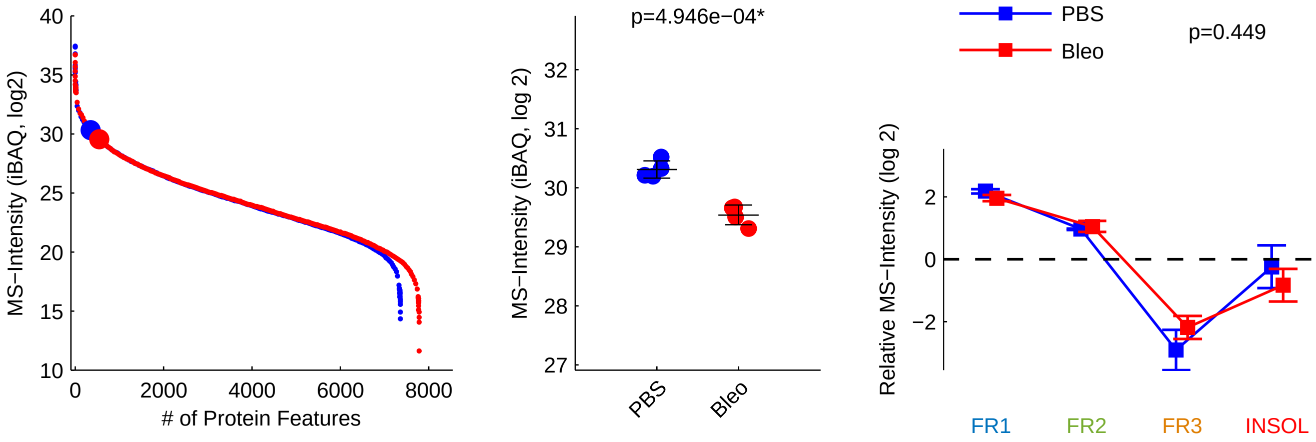

Q61508 − Ecm1 (id: 4579)

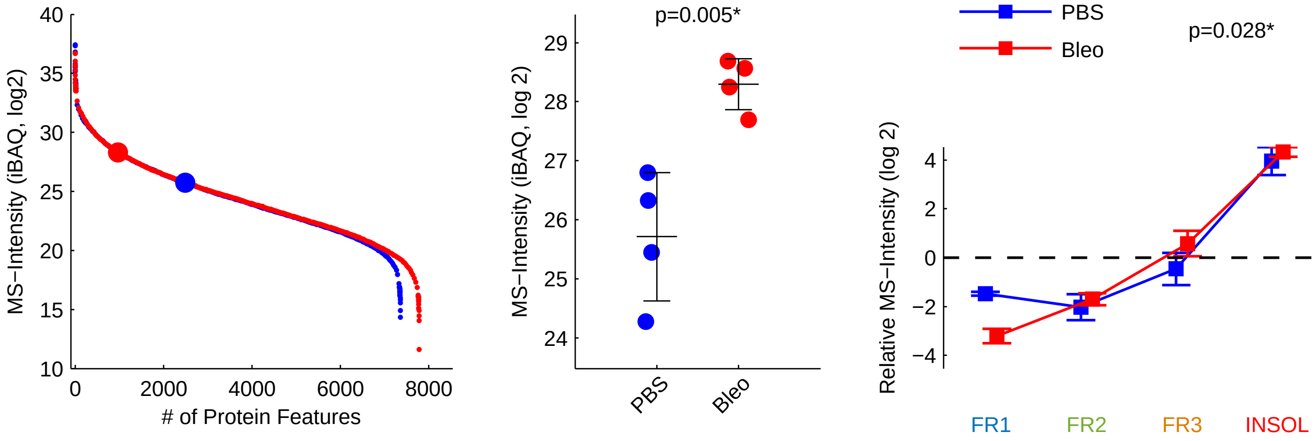

Q61553 − Fscn1 (id: 4584)

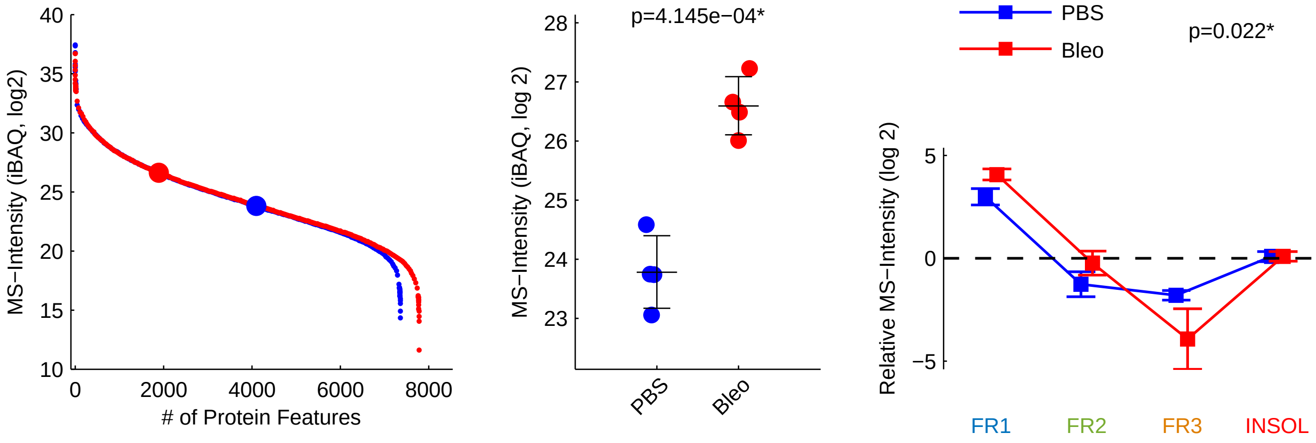

Q61576 − Fkbp10 (id: 4587)

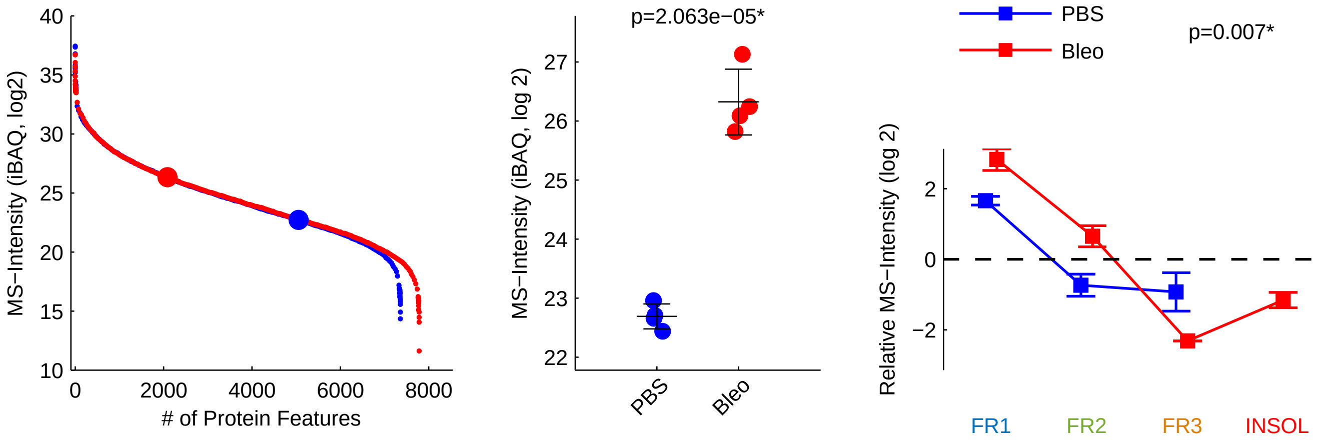

Q61635 − Ifi47 (id: 4597)

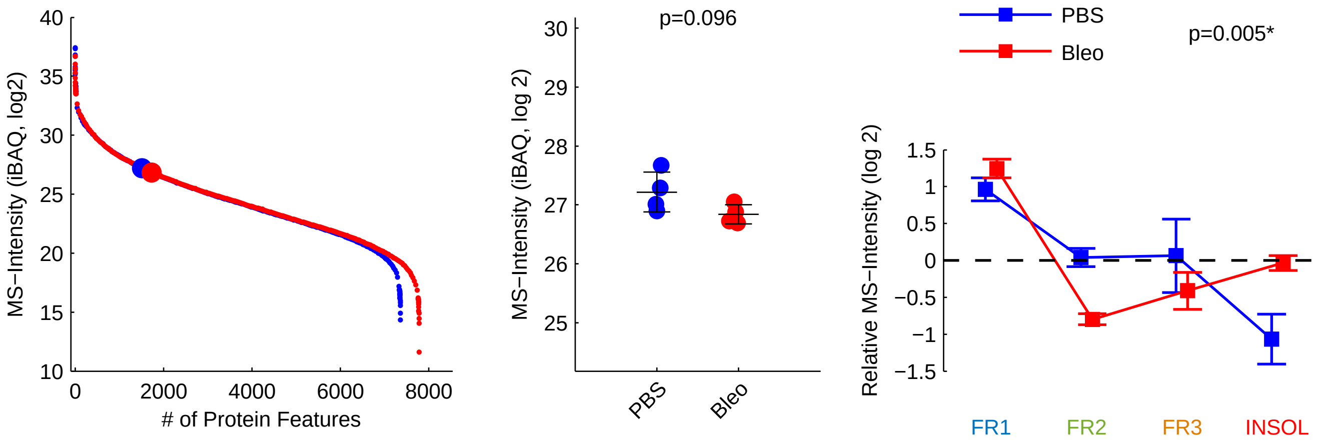

Q61646 − Hp (id: 4598)

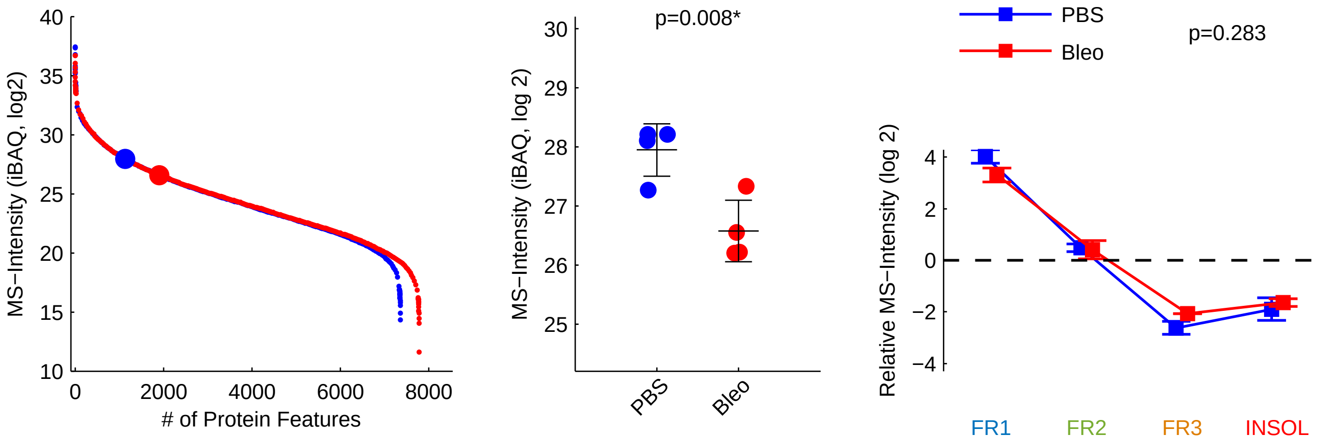

Q61656 − Ddx5 (id: 4600)

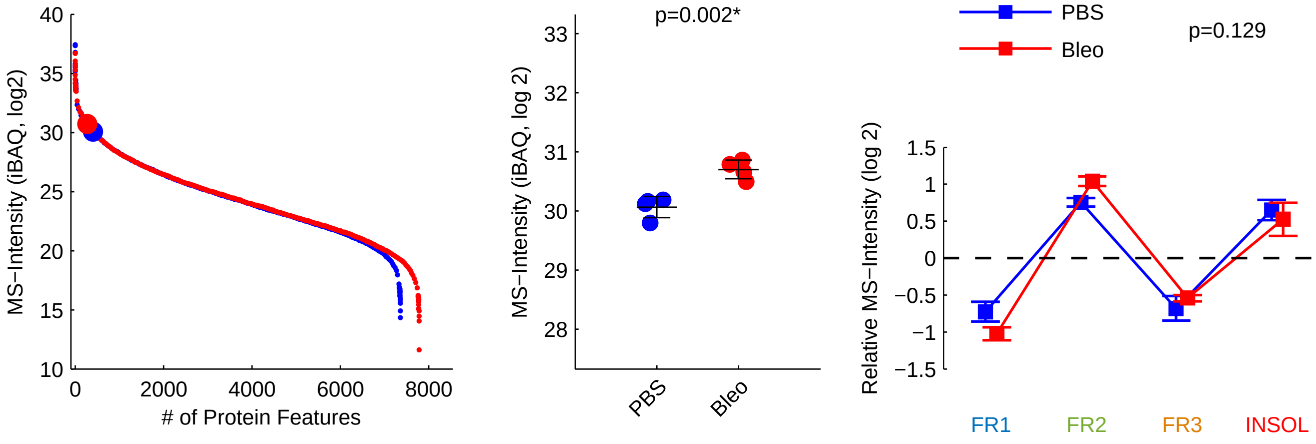

Q61696 − Hspa1a (id: 4604)

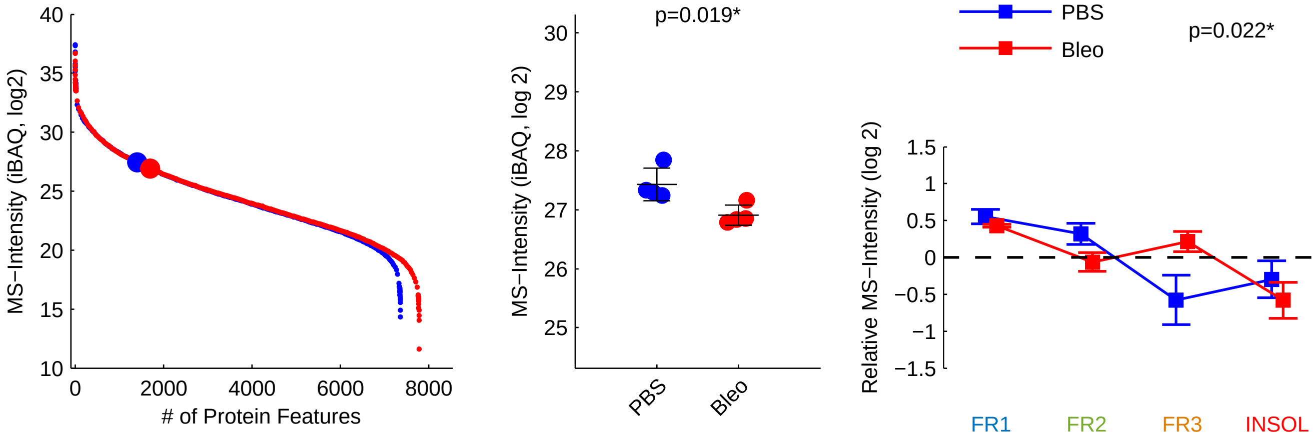

Q61699 − Hsph1 (id: 4605)

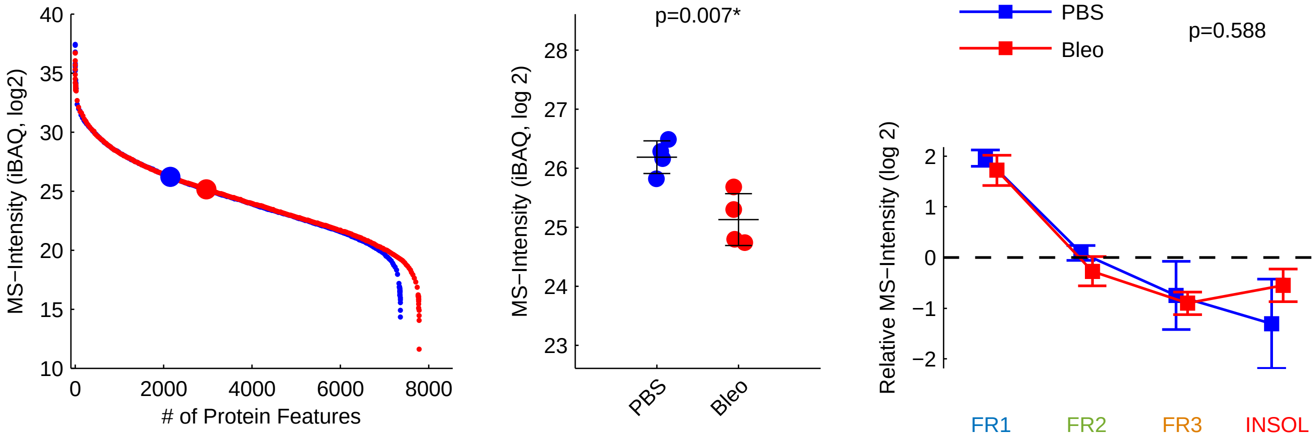

Q61702 − Itih1 (id: 4606)

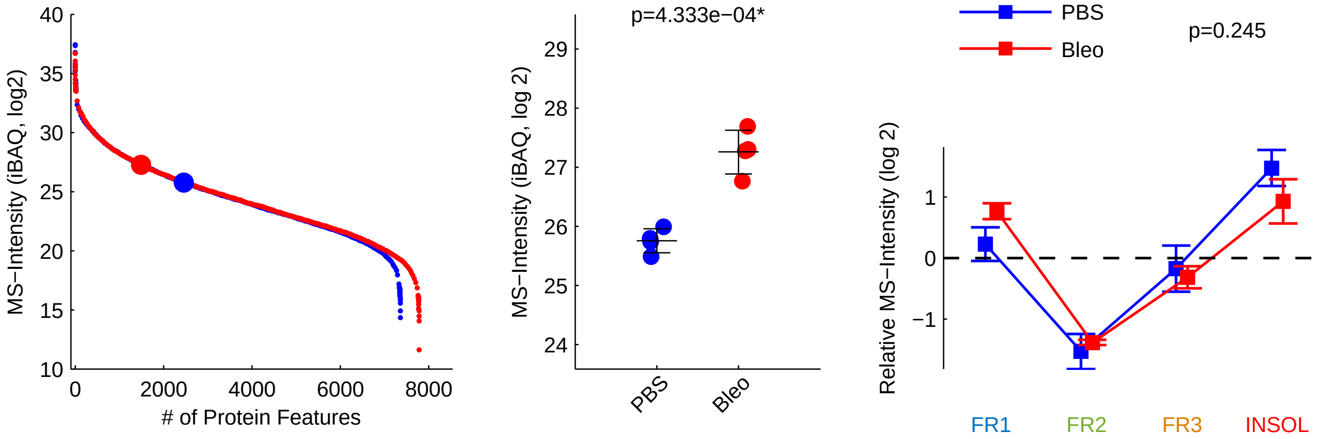

Q61704 − Itih3 (id: 4607)

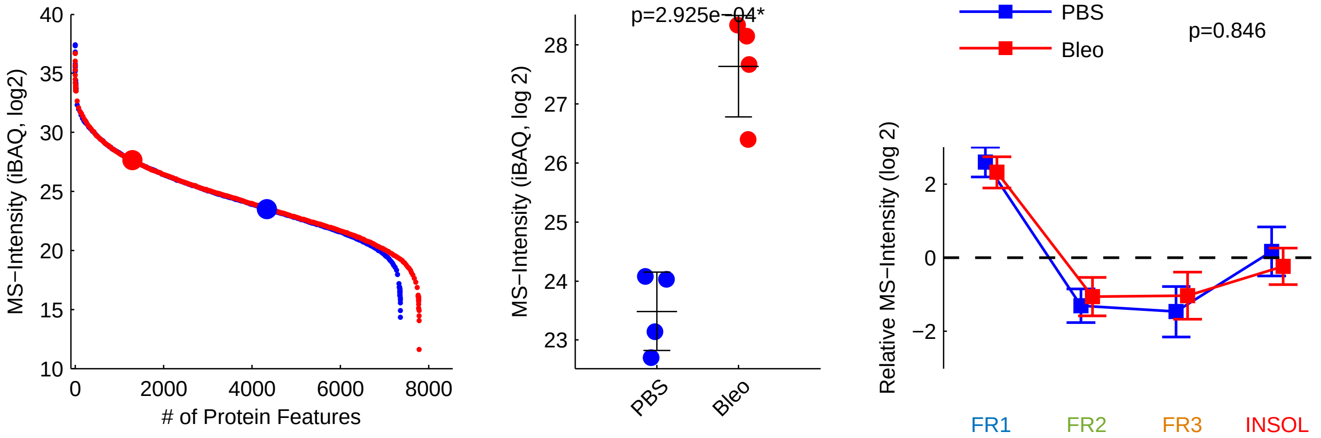

Q61730 − Il1rap (id: 4609)

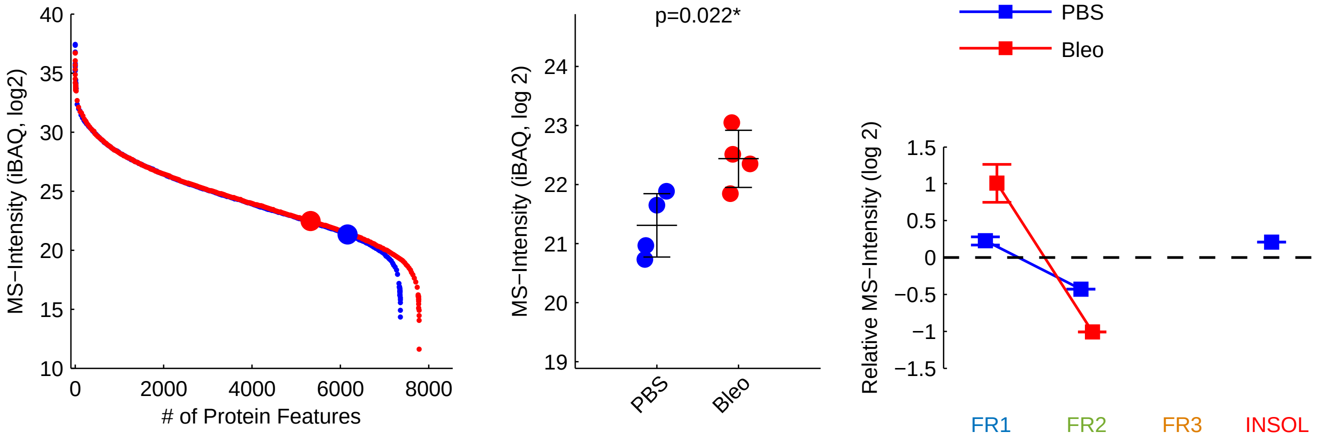

Q61735 − Cd47 (id: 4611)

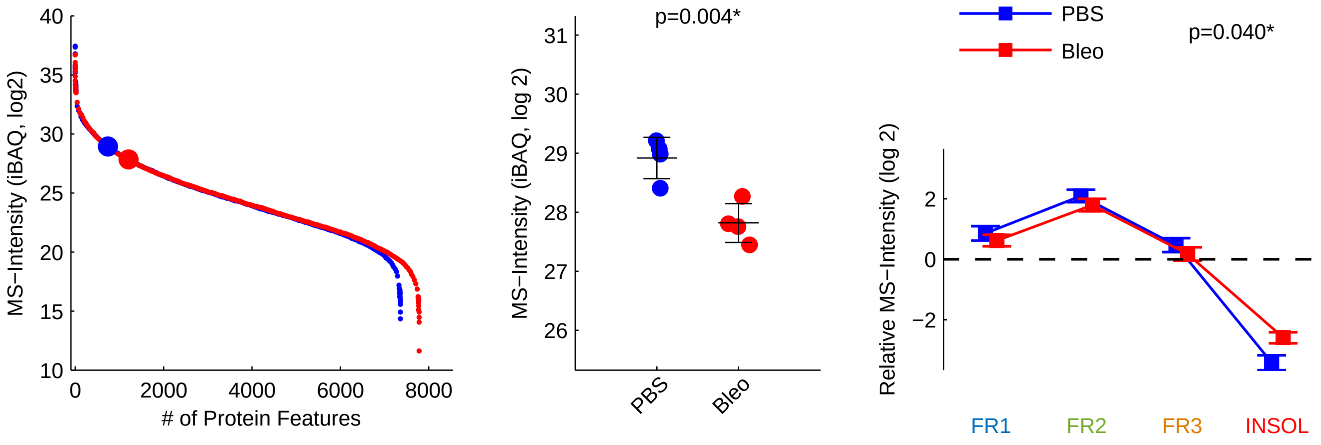

Q61771 − Kif3b (id: 4615)

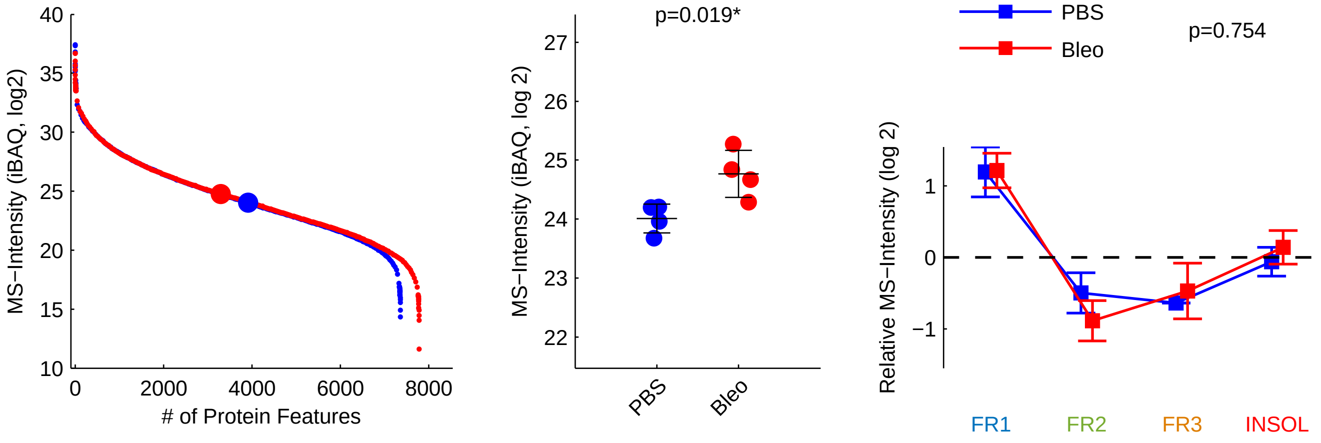

Q61792 − Lasp1 (id: 4617)

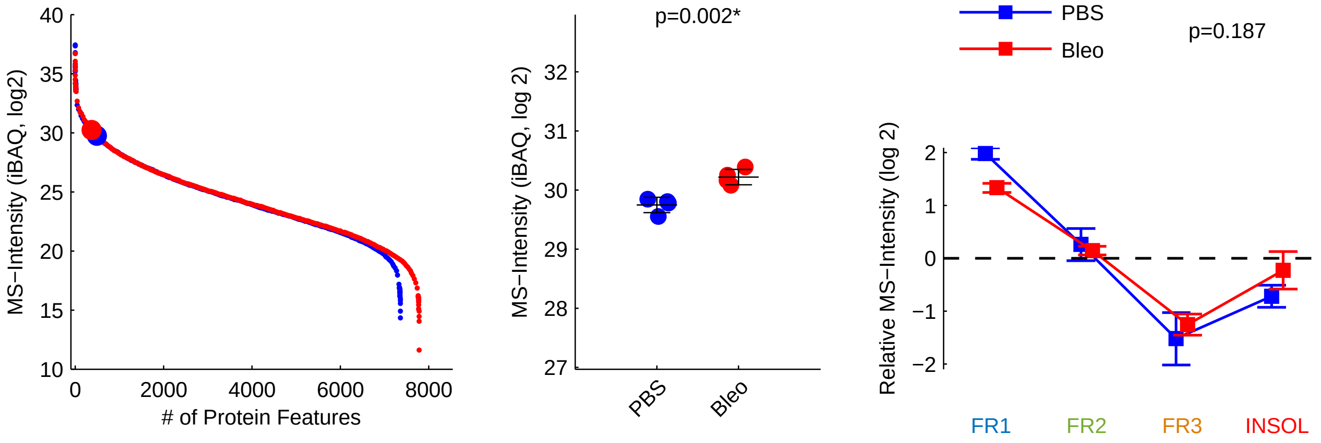

Q61830 − Mrc1 (id: 4622)

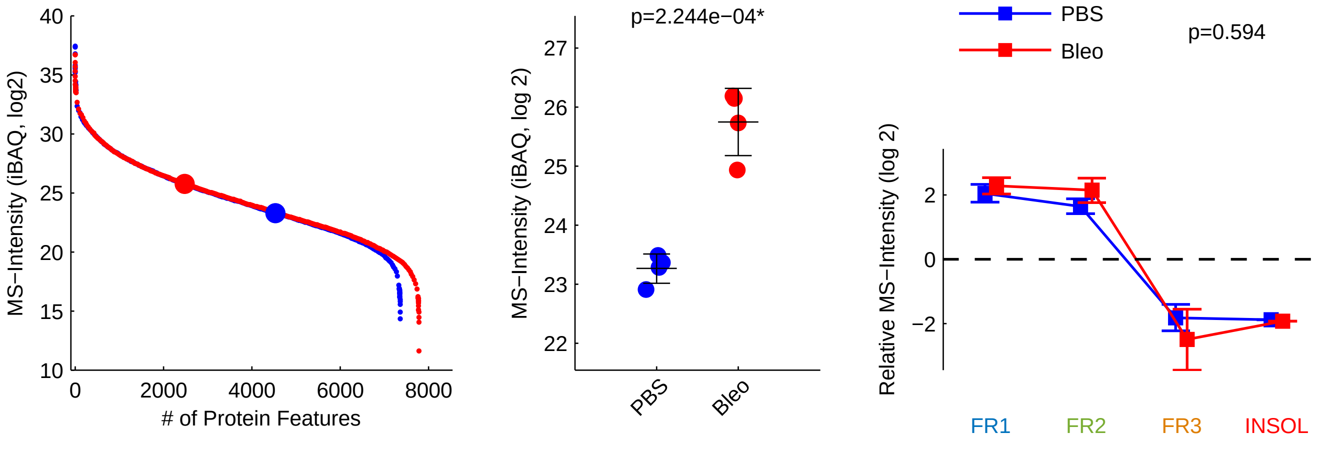

Q62000 − Ogn (id: 4633)

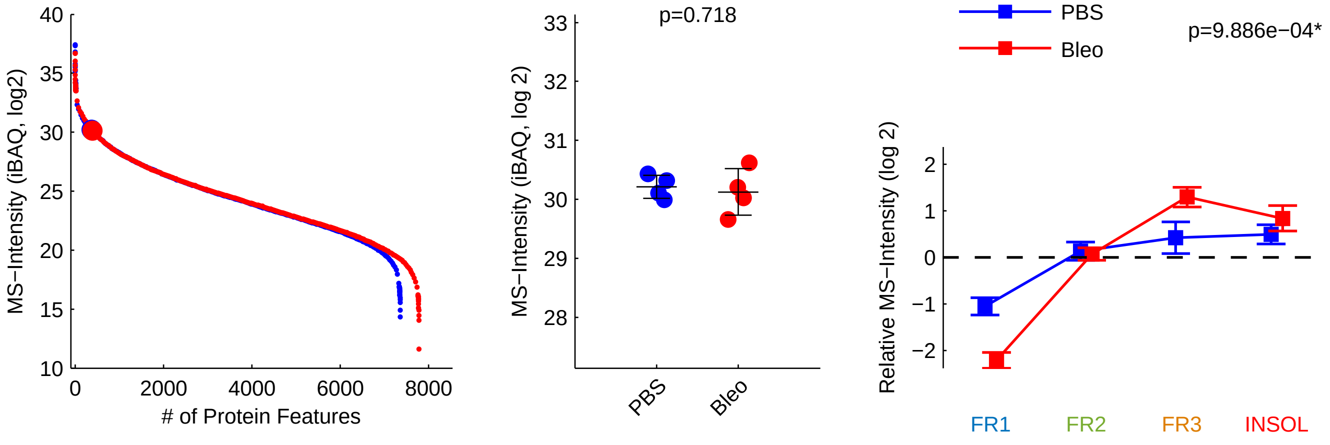

Q62074 − Prkci (id: 4641)

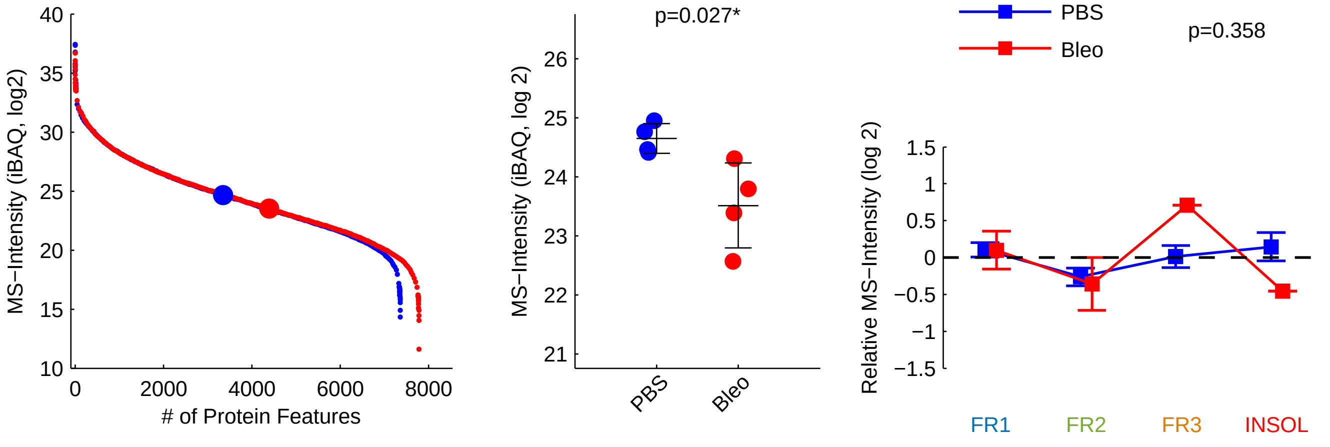

Q62084 − Ppp1r14b (id: 4642)

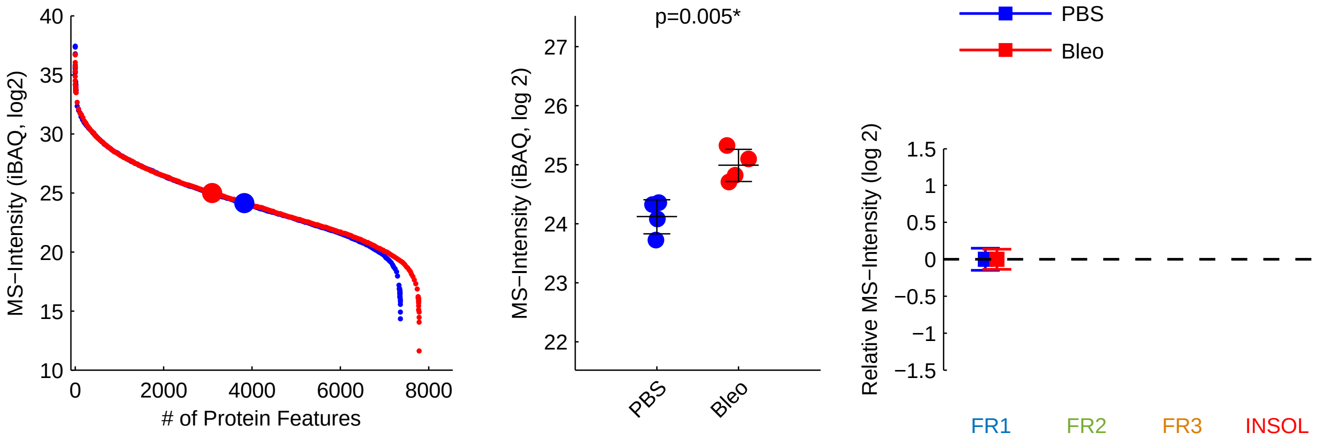

Q62087 − Pon3 (id: 4644)

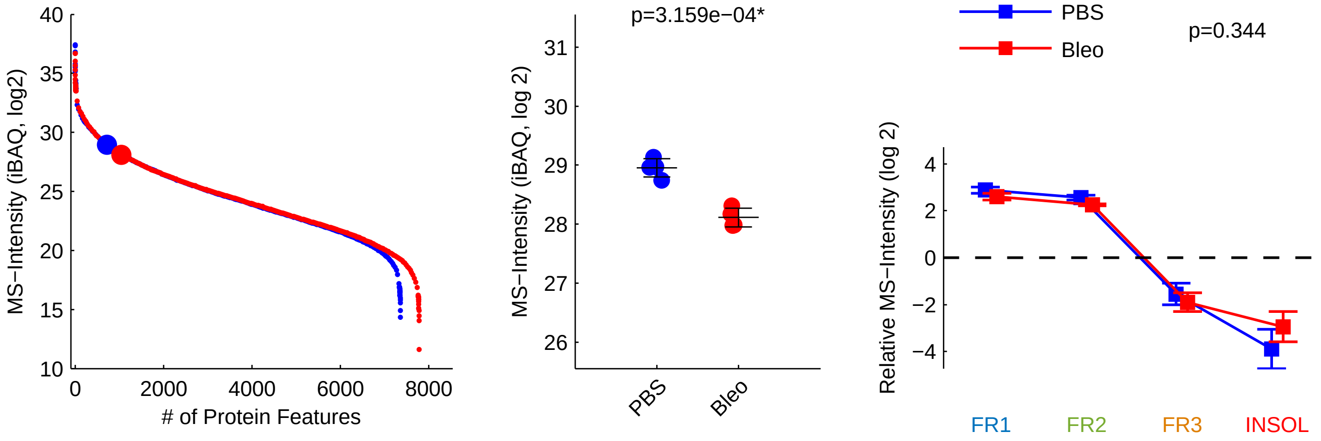

Q62095 − Ddx3y (id: 4646)

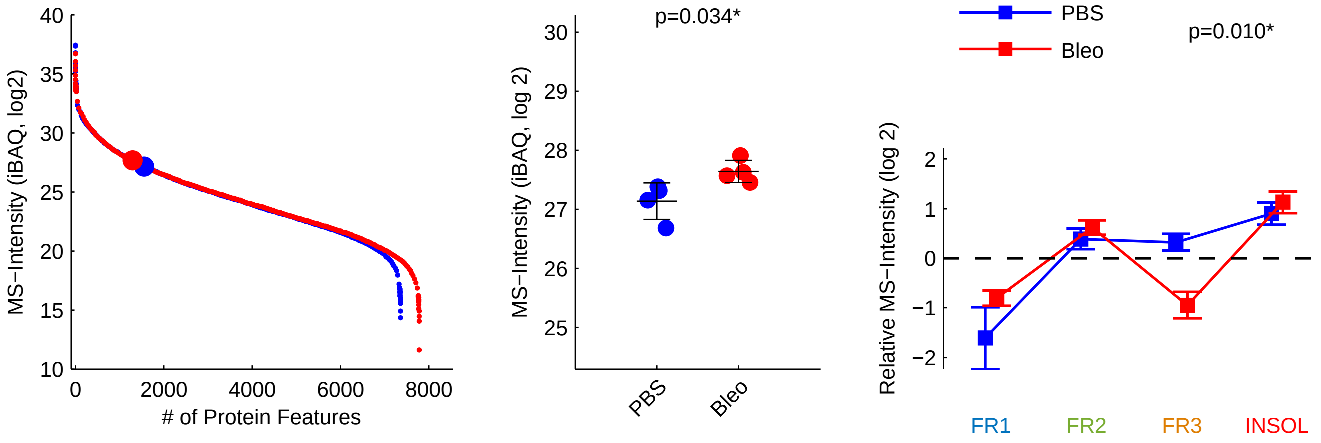

Q62165 − Dag1 (id: 4653)

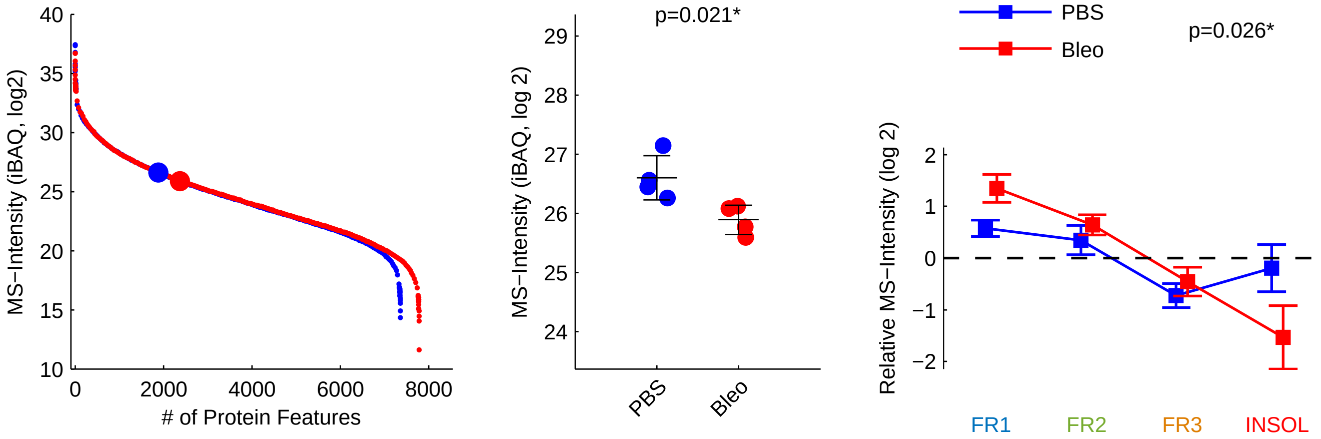

Q62167 − Ddx3x (id: 4654)

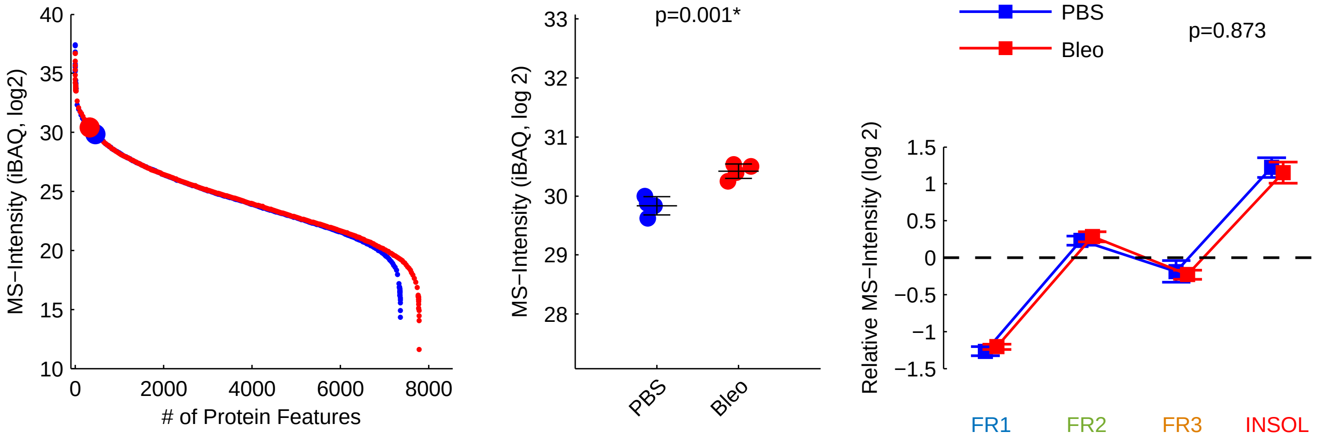

Q62181 − Sema3c (id: 4659)

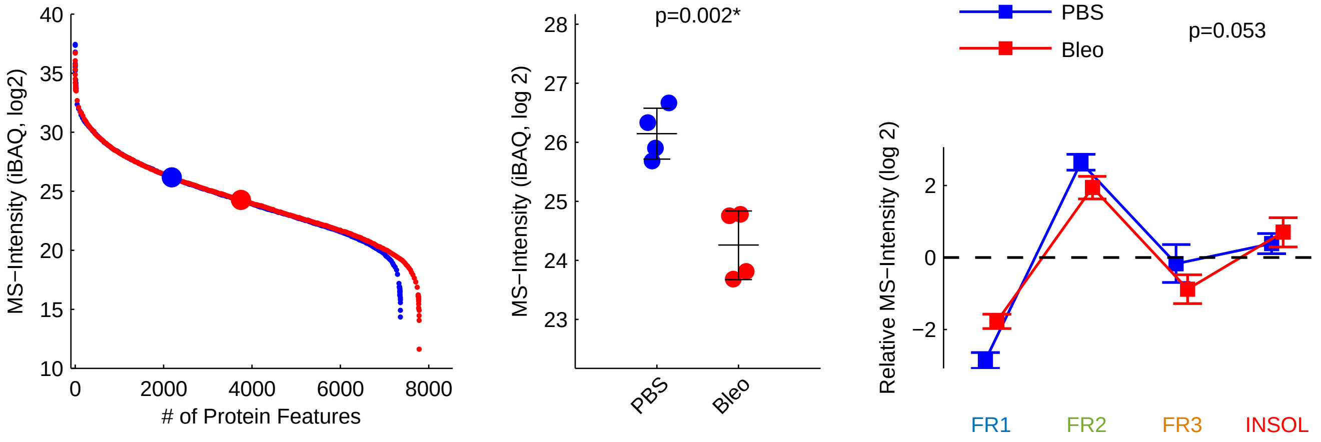

Q62186 − Ssr4 (id: 4660)

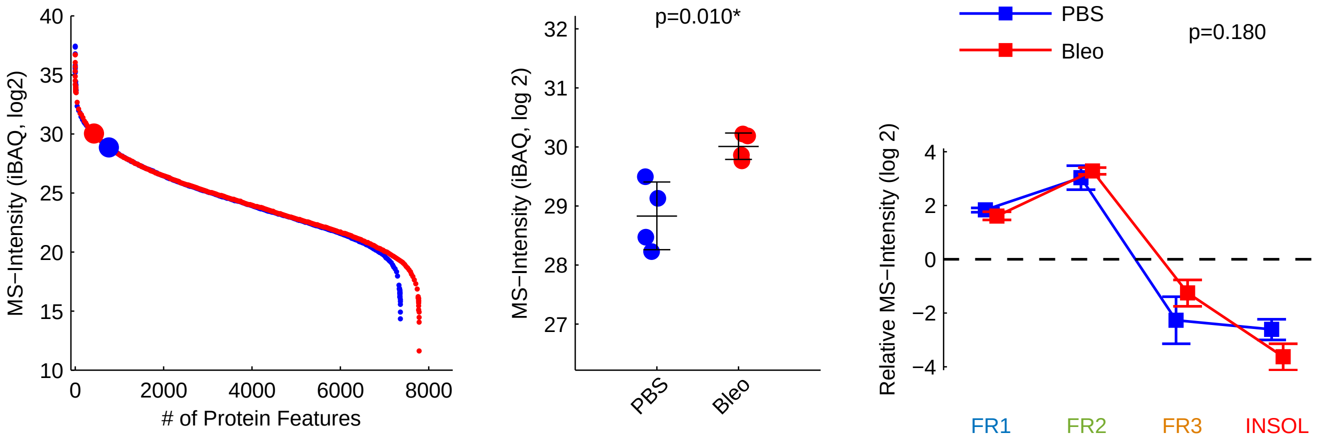

Q62219 − Tgfb1i1 (id: 4665)

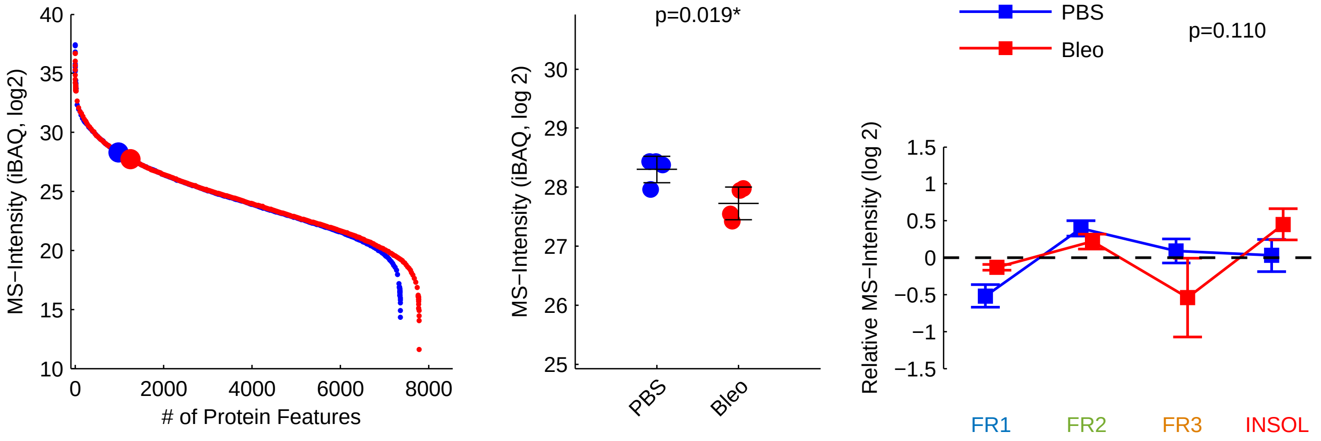

Q62356 − Fstl1 (id: 4680)

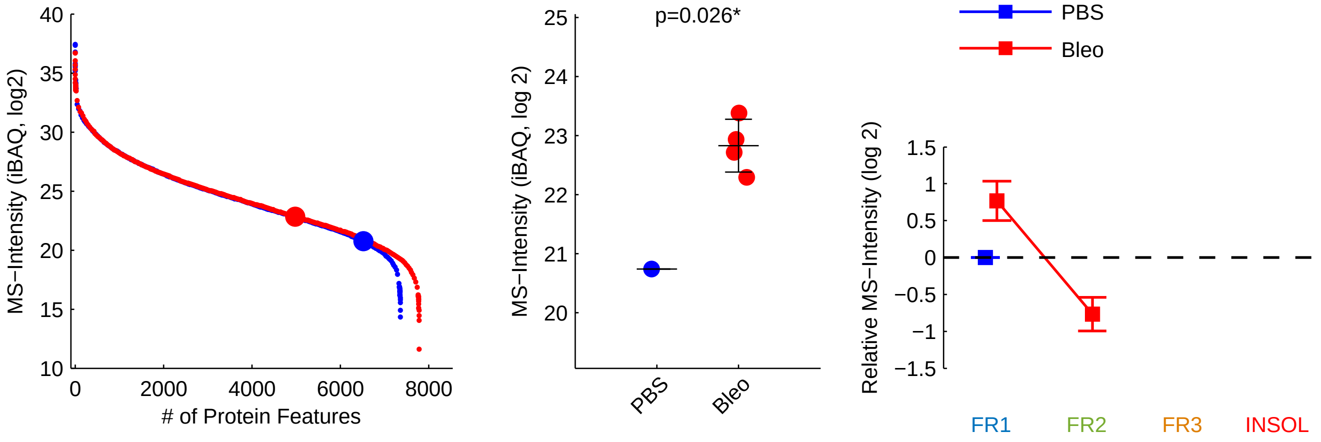

Q62426 − Cstb (id: 4691)

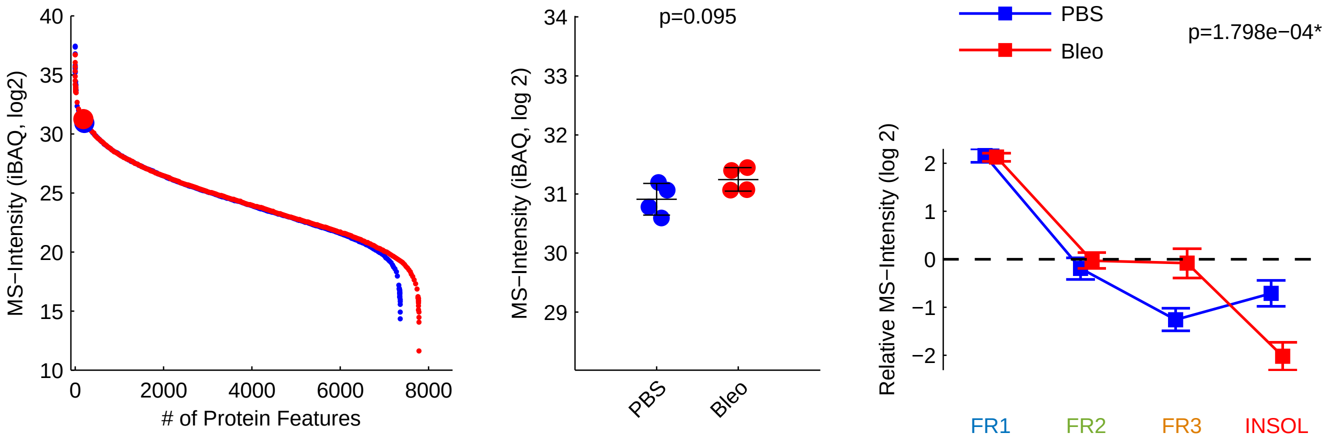

Q62465 − Vat1 (id: 4696)

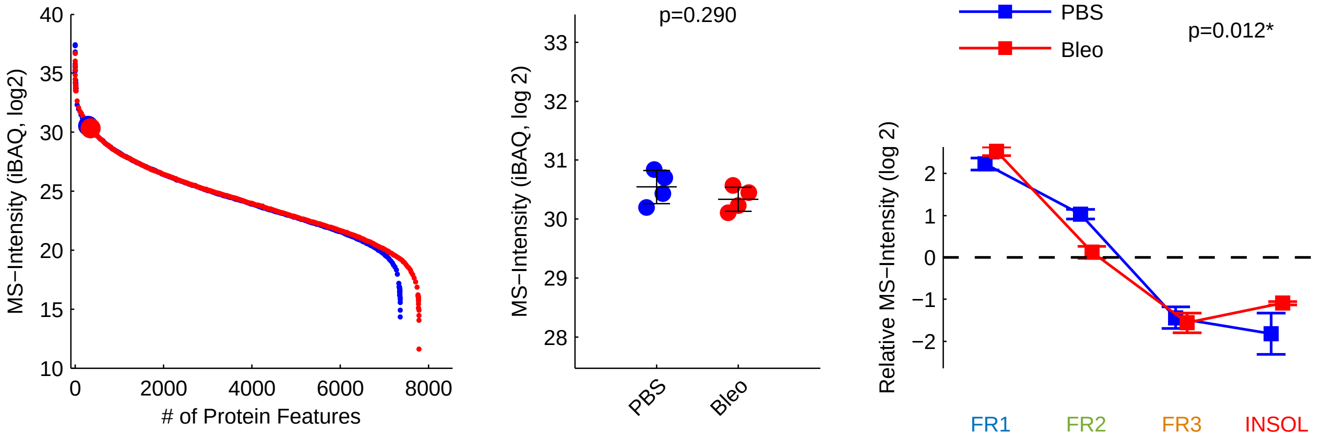

Q62470 − Itga3 (id: 4698)

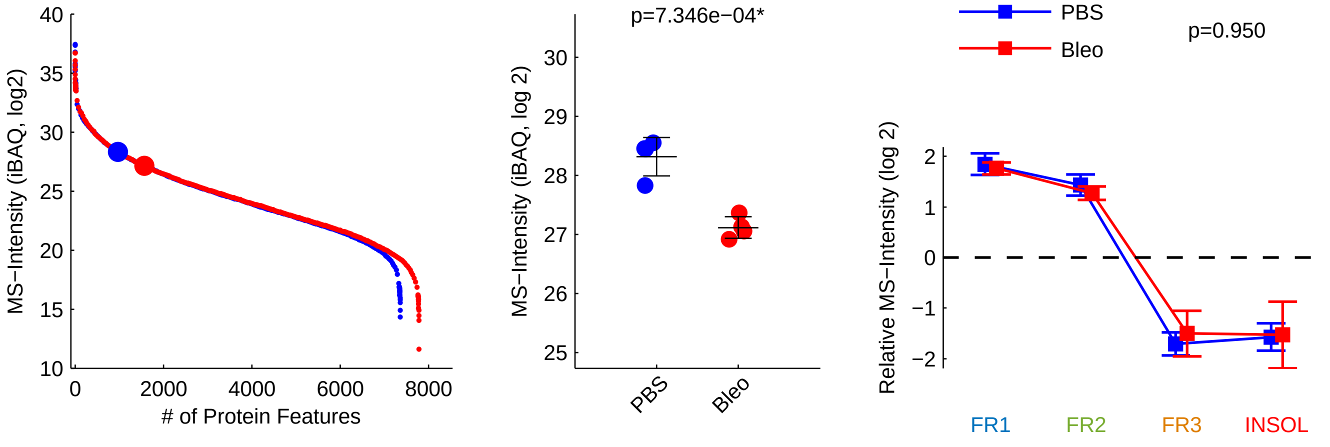

Q62523 − Zyx (id: 4701)

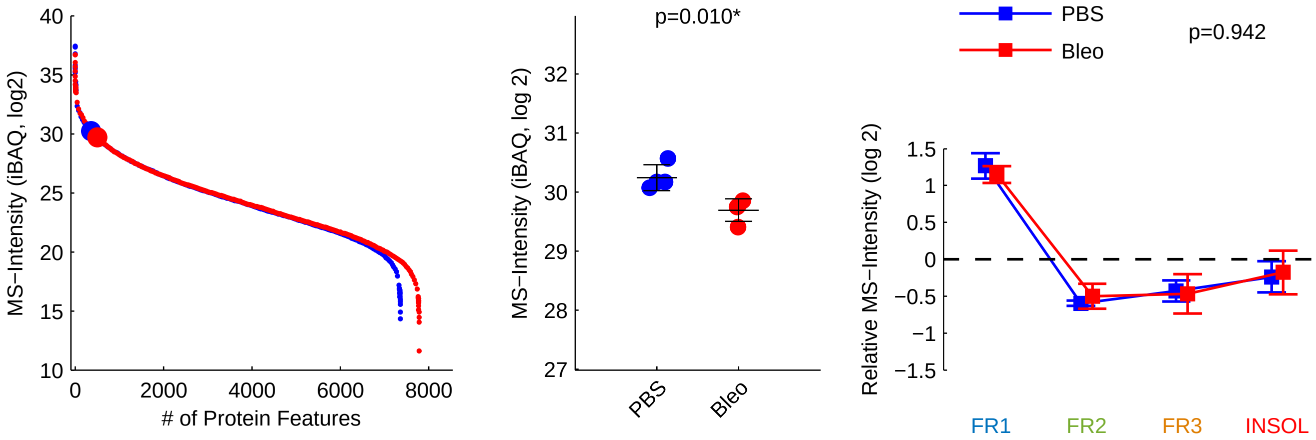

Q63810 − Ppp3r1 (id: 4703)

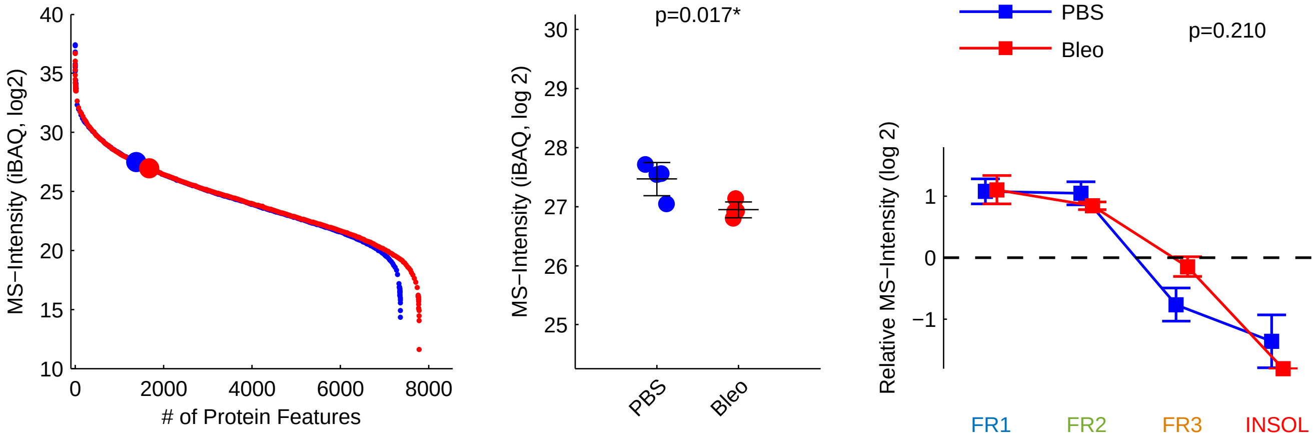

Q63870 − Col7a1 (id: 4708)

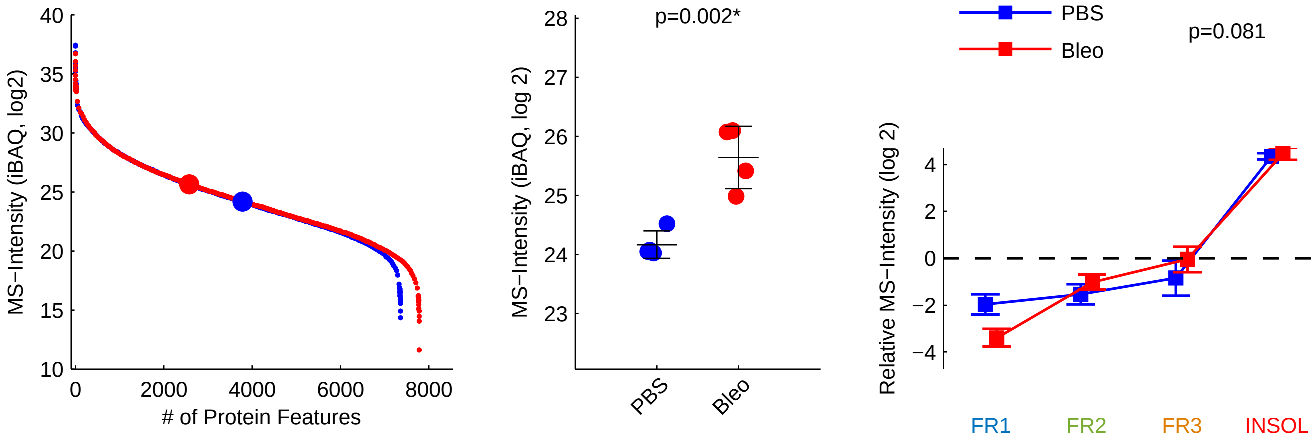

Q63918 − Sdpr (id: 4711)

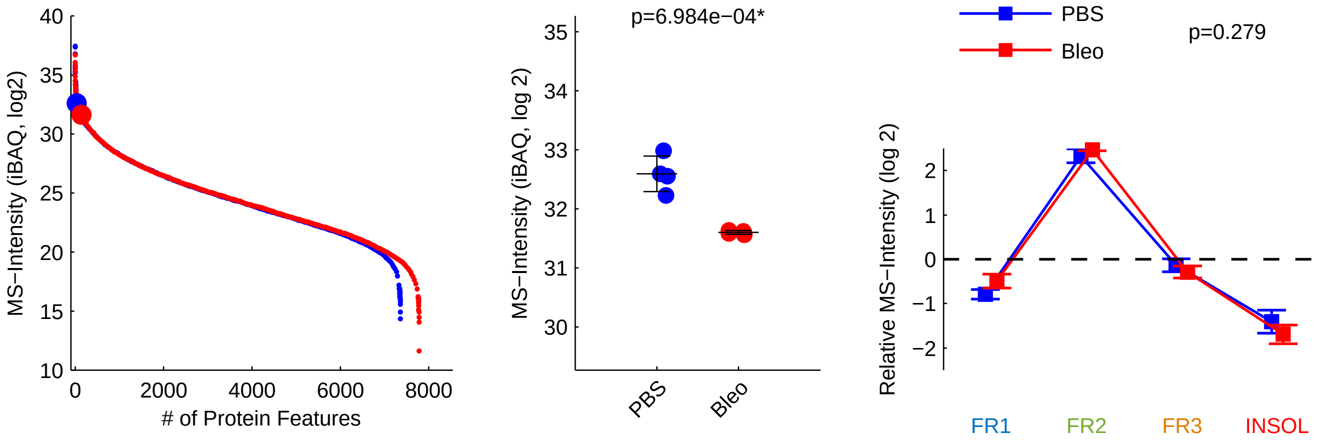

Q63ZW6 − Col4a5 (id: 4714)

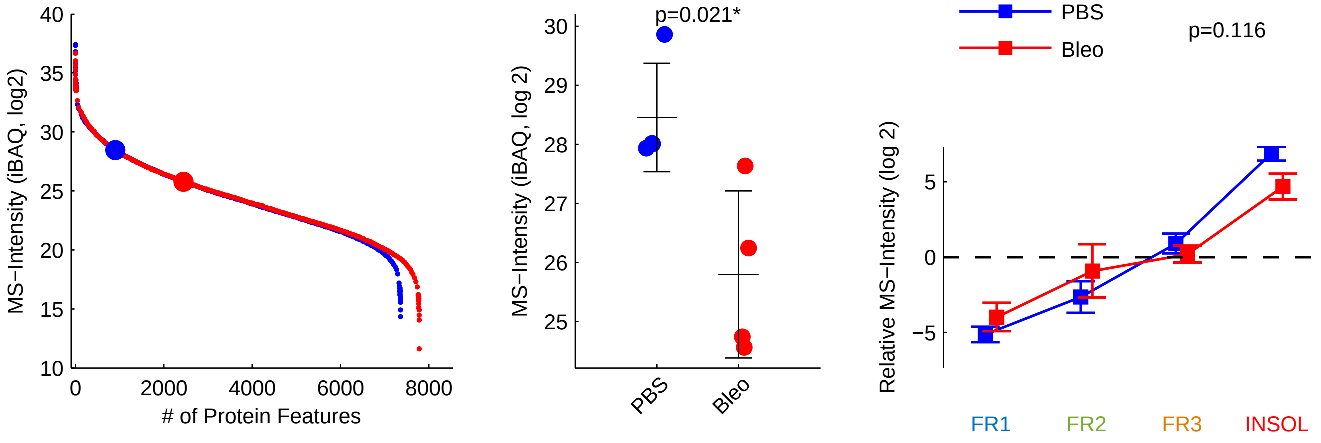

Q63ZW7 − Inadl (id: 4715)

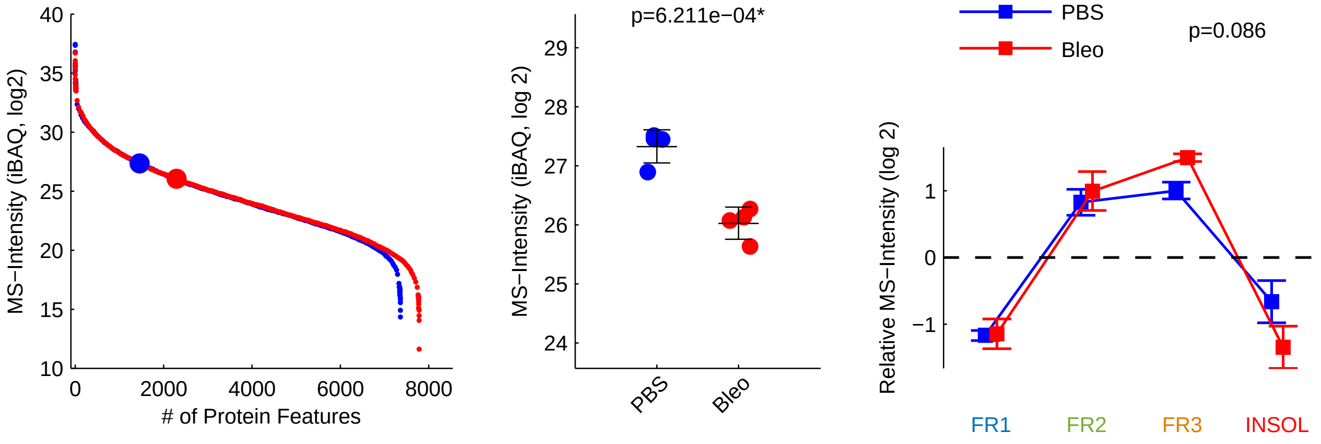

Q640N1 − Aebp1 (id: 4718)

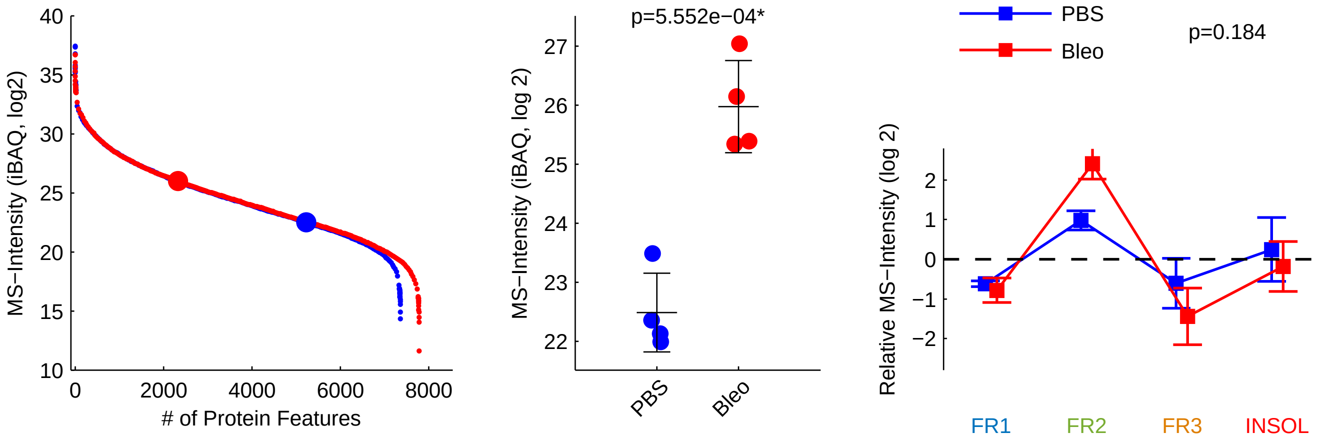

Q64152−2 − Btf3 (id: 4723)

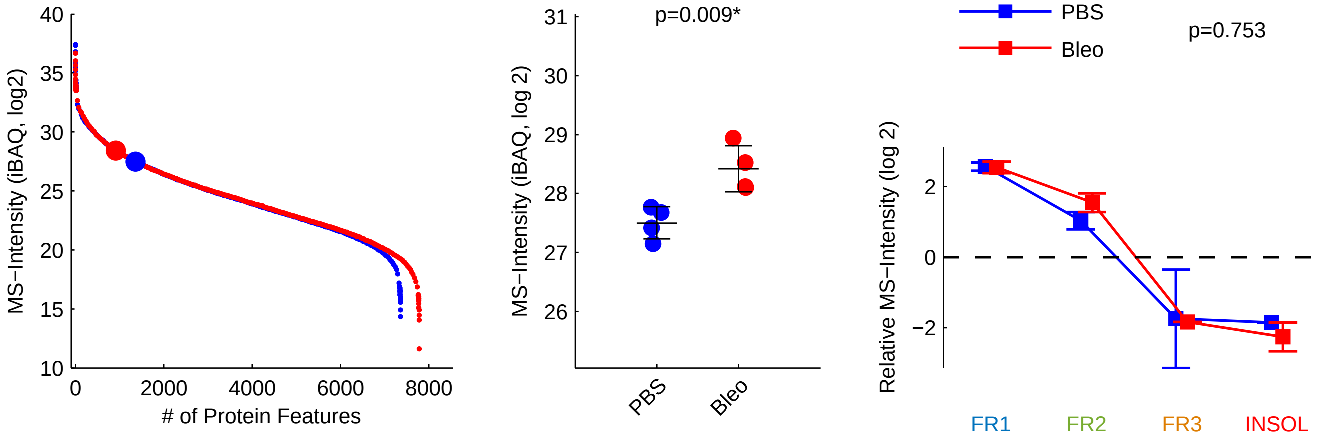

Q64191 − Aga (id: 4724)

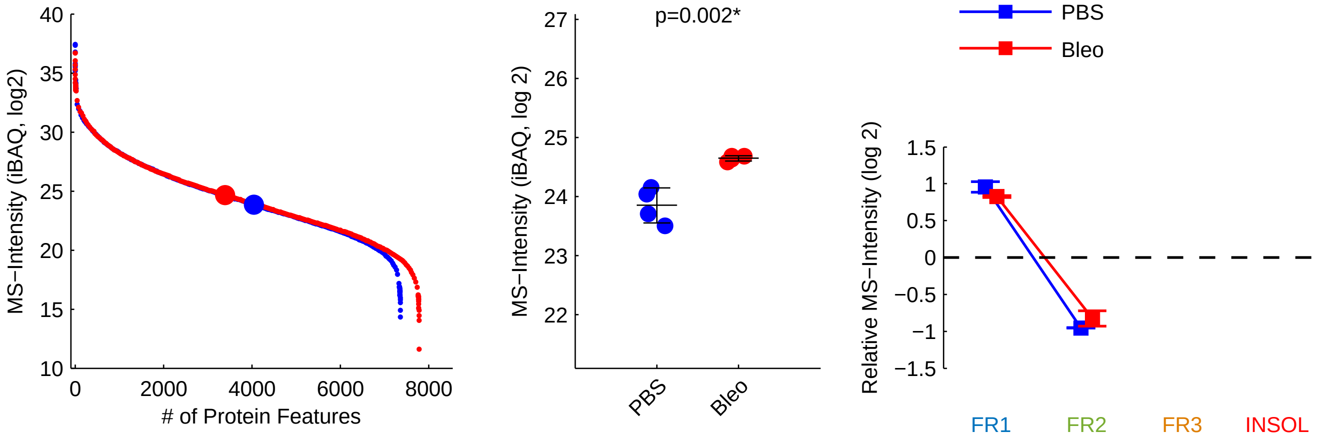

Q64282 − Ifit1 (id: 4731)

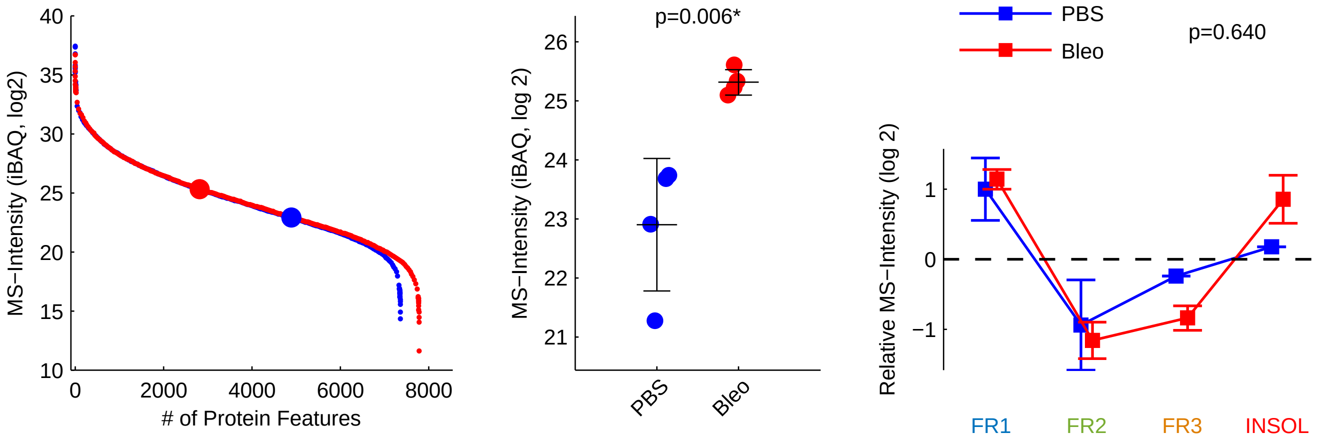

Q64310 − Surf4 (id: 4732)

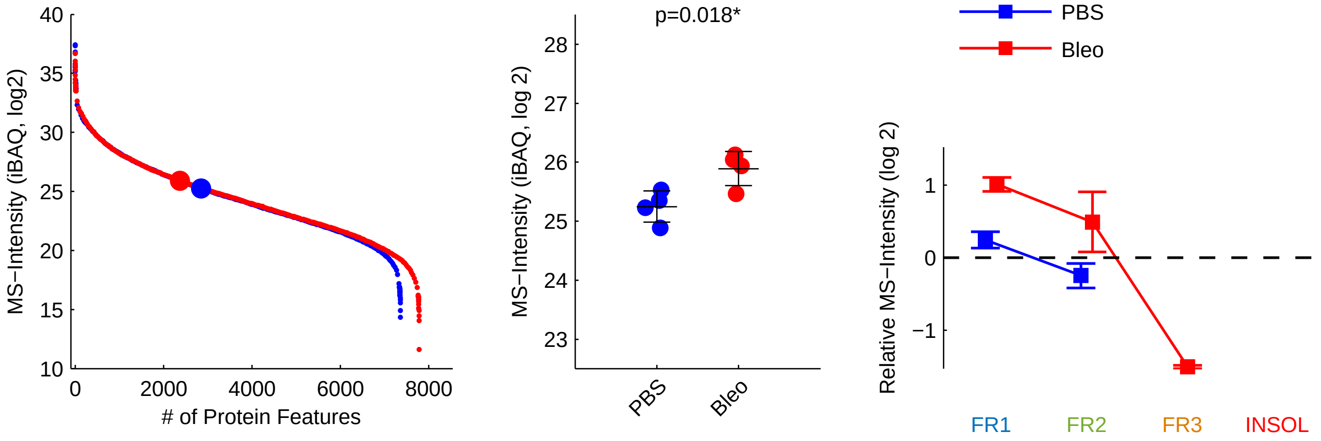

Q64314 − Cd34 (id: 4734)

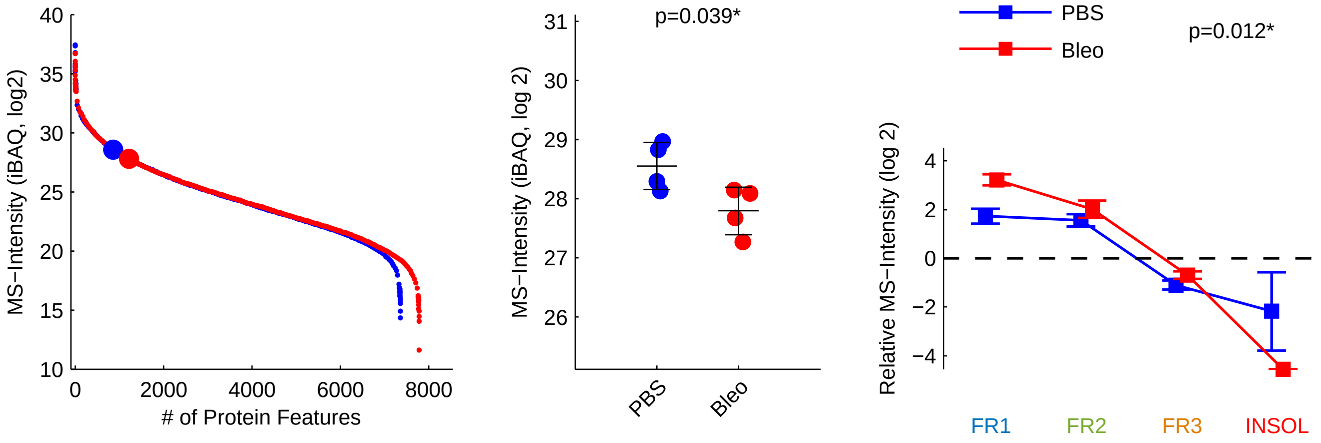

Q64324 − Stxbp2 (id: 4737)

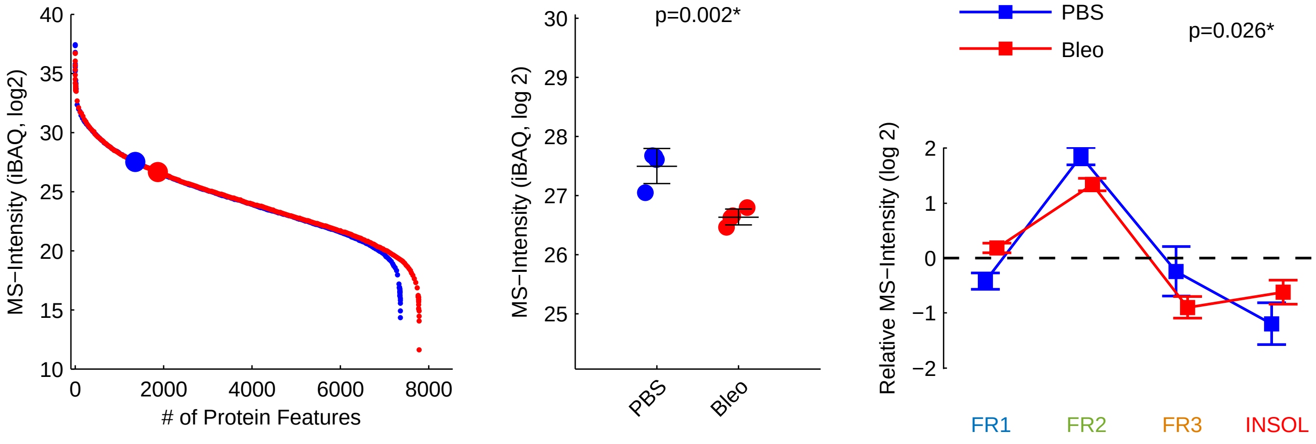

Q64339 − Isg15 (id: 4740)

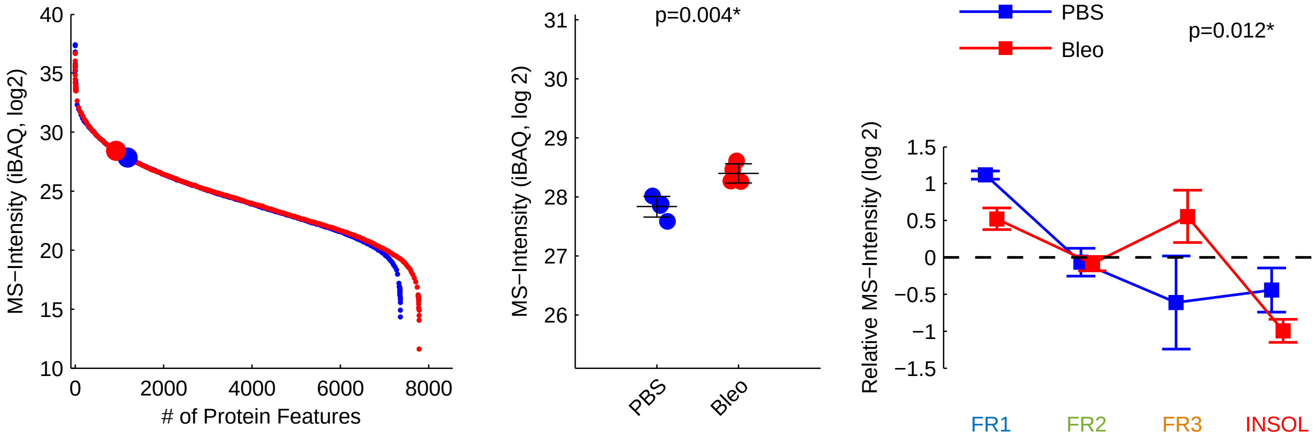

Q64345 − Ifit3 (id: 4742)

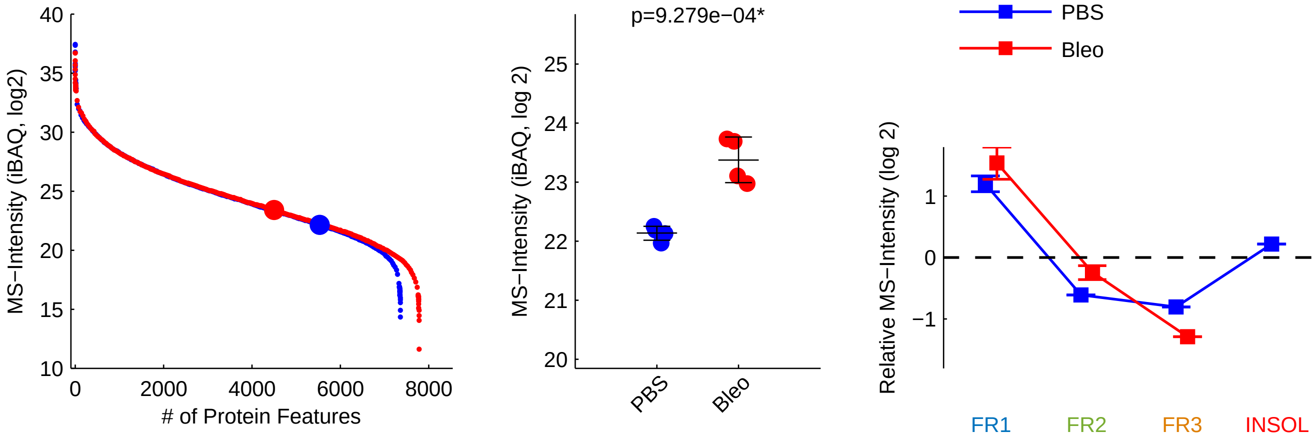

Q64378 − Fkbp5 (id: 4745)

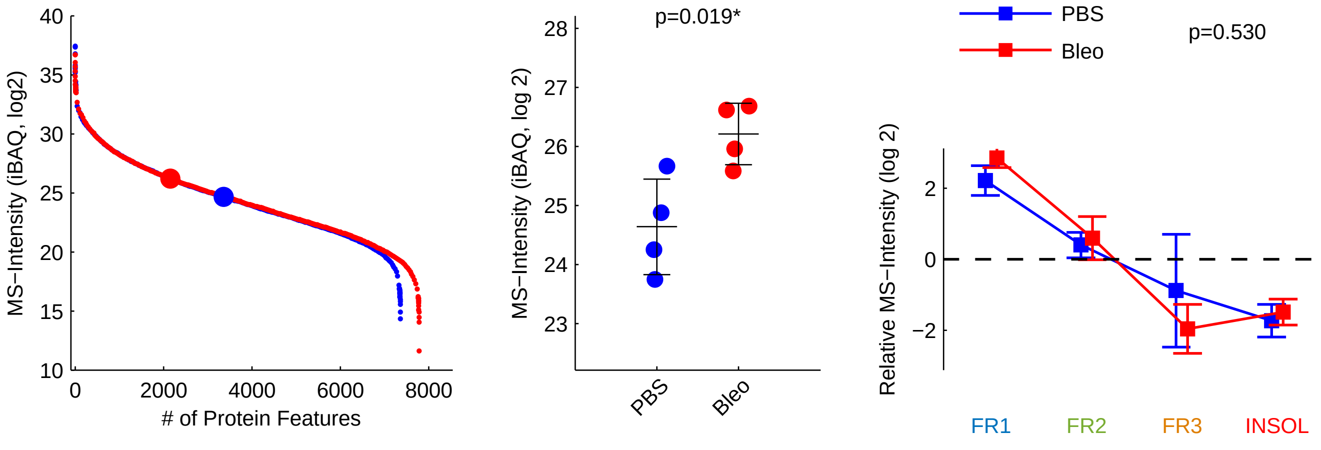

Q64435 − Ugt1a6 (id: 4747)

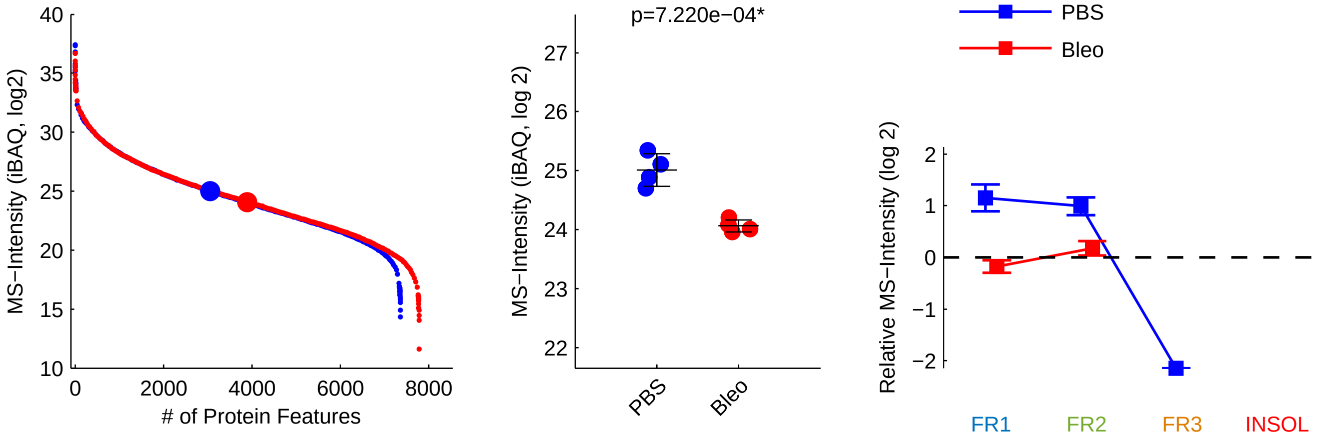

Q64442 − Sord (id: 4749)

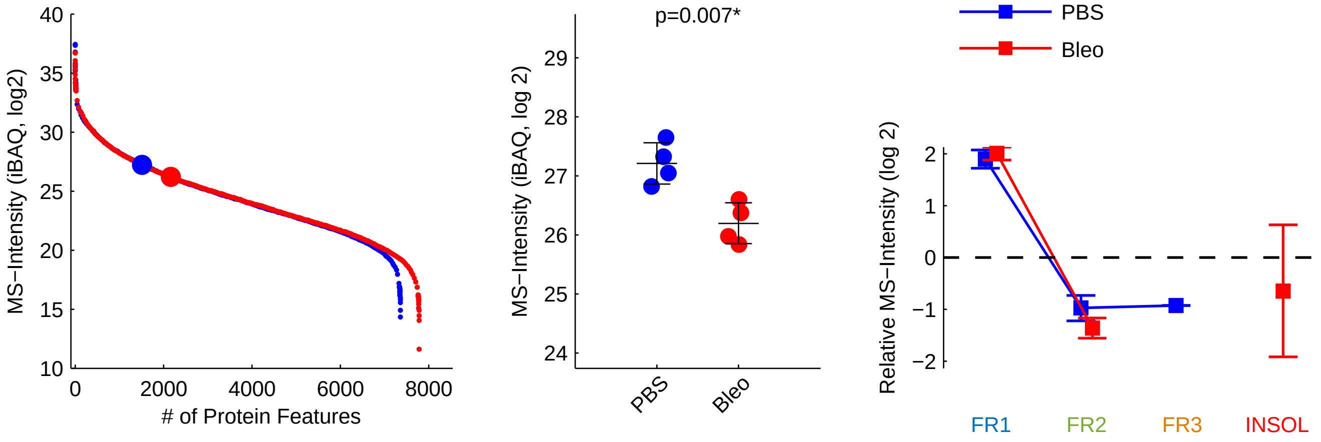

Q64444 − Ca4 (id: 4750)

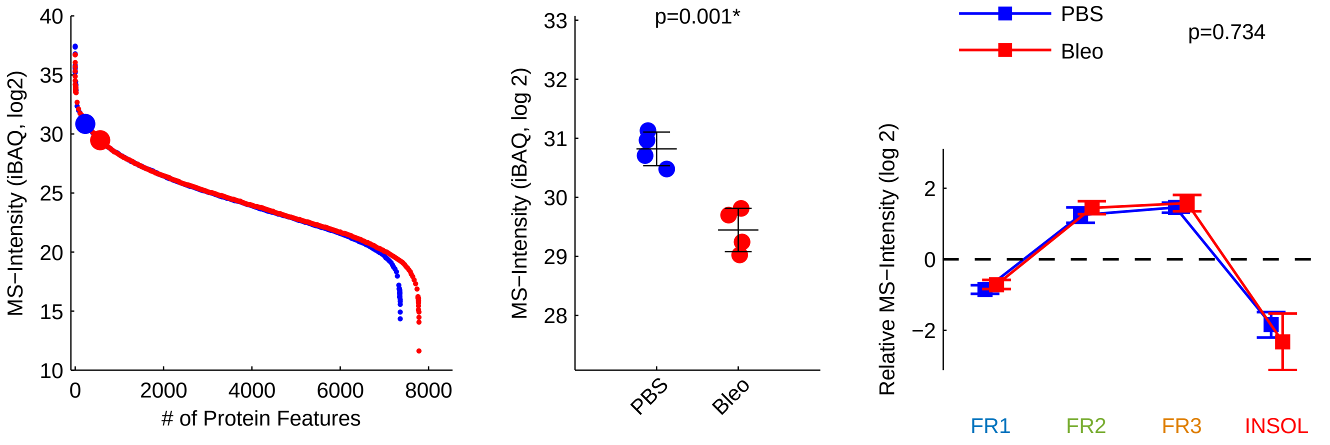

Q64449 − Mrc2 (id: 4752)

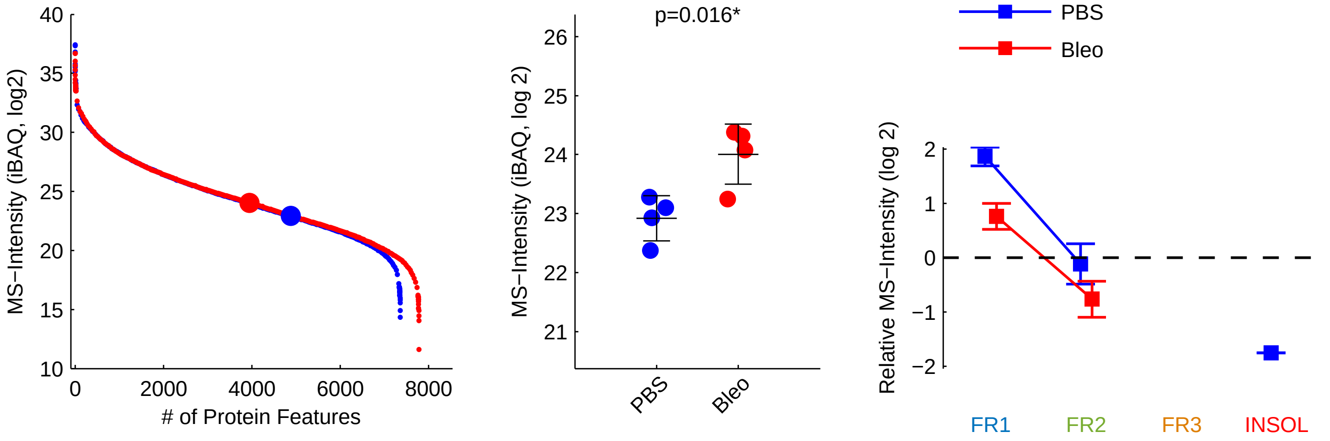

Q64462 − Cyp4b1 (id: 4753)

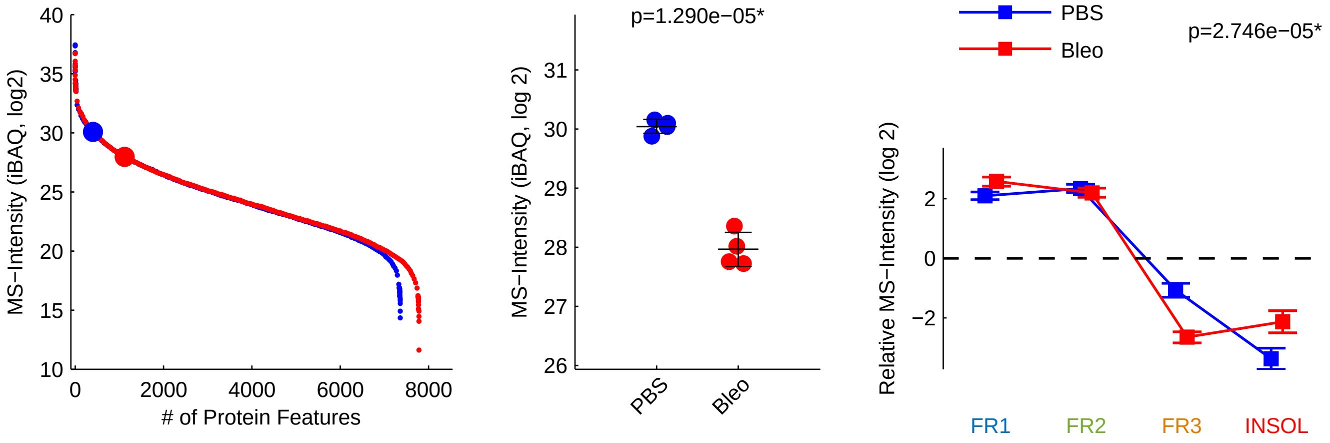

Q64471 − Gstt1 (id: 4755)

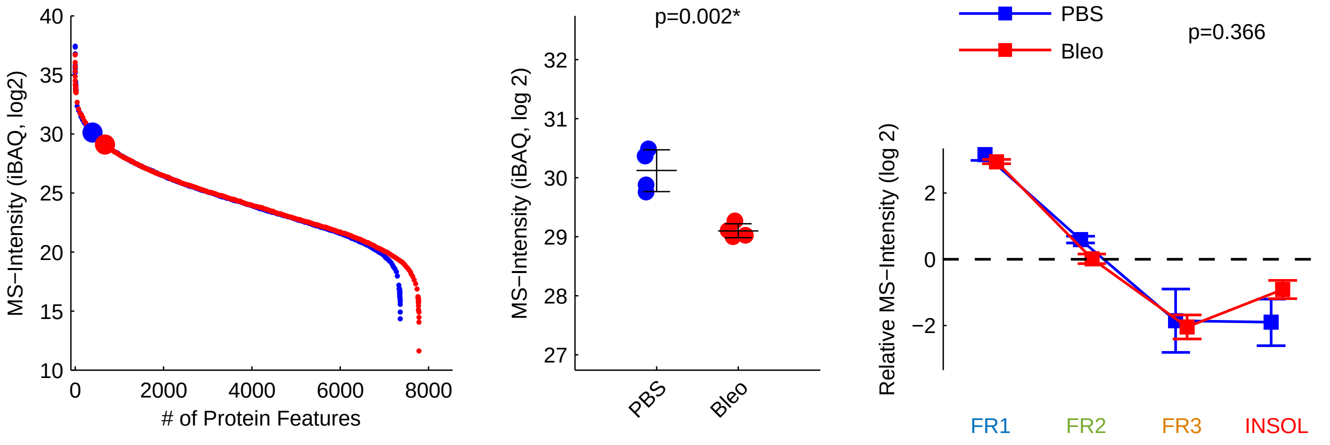

Q6ZWY9 − Hist1h2bc (id: 4757)

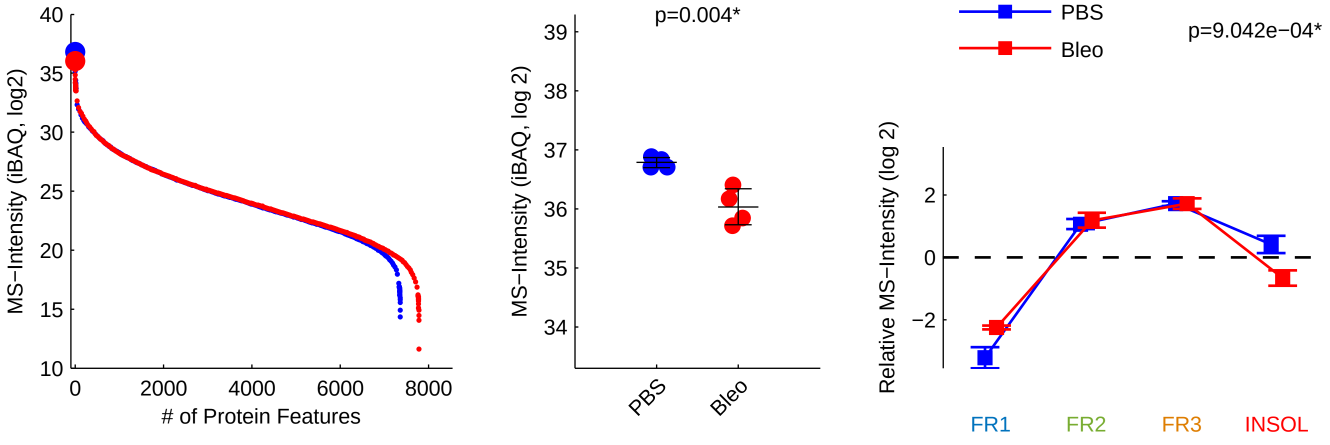

Q64514 − Tpp2 (id: 4759)

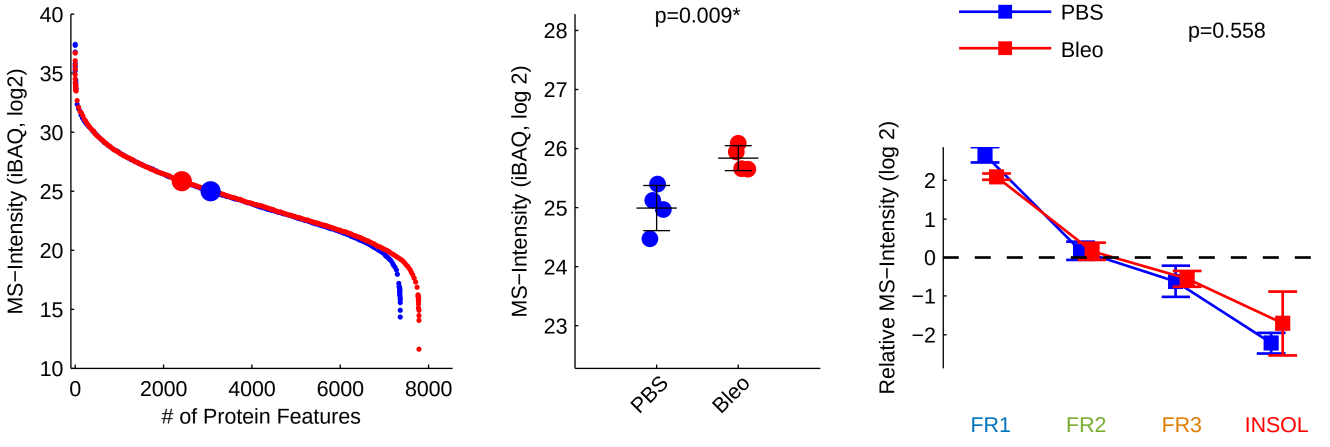

Q64516 − Gk (id: 4761)

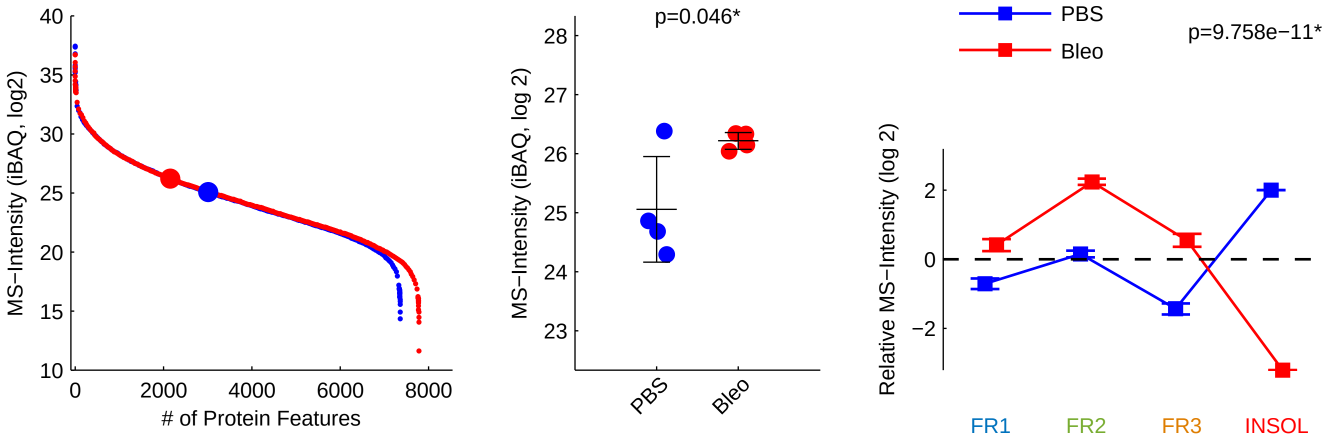

Q64521 − Gpd2 (id: 4764)

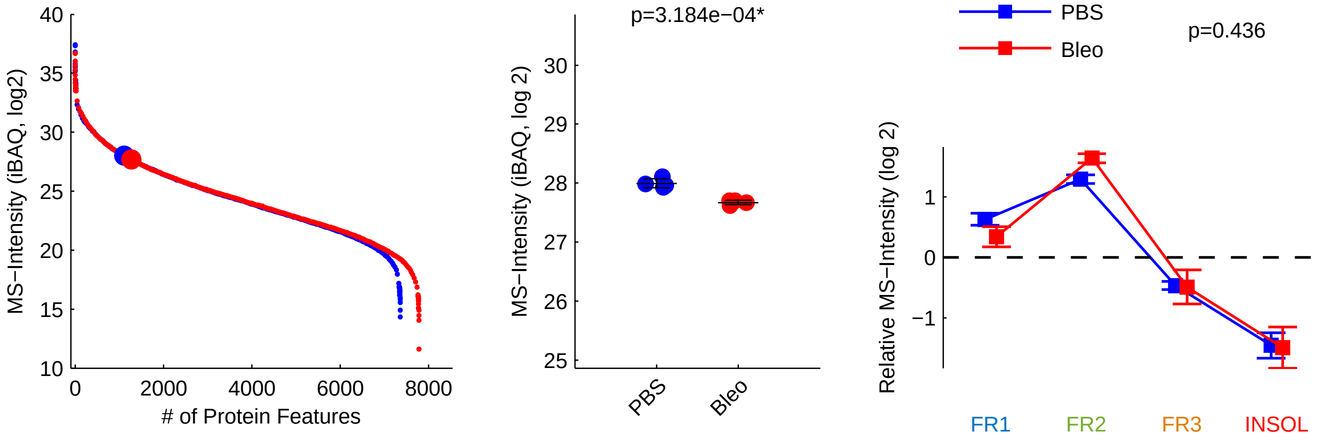

Q64524 − Hist2h2be (id: 4766)

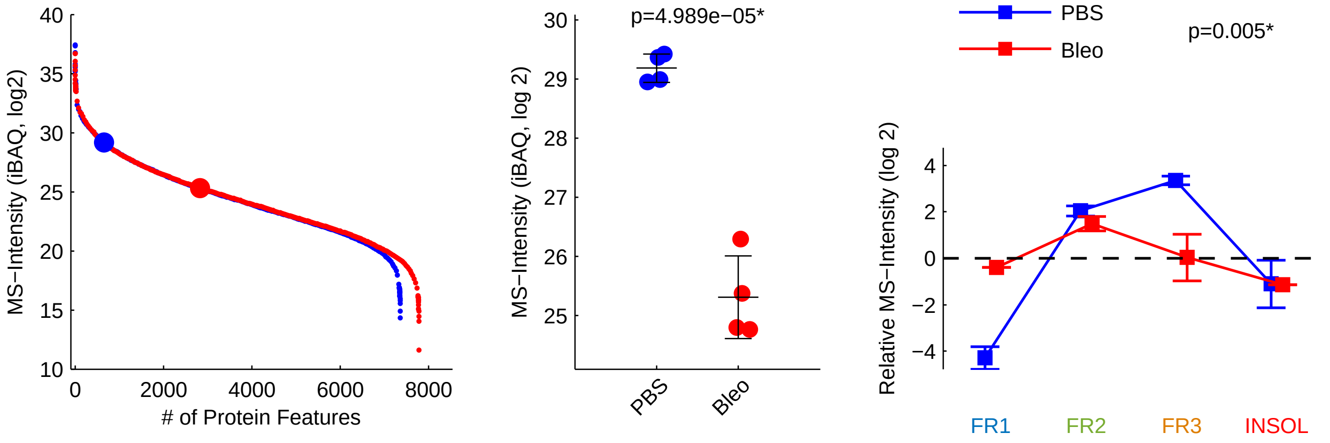

Q64704 − Stx3 (id: 4771)

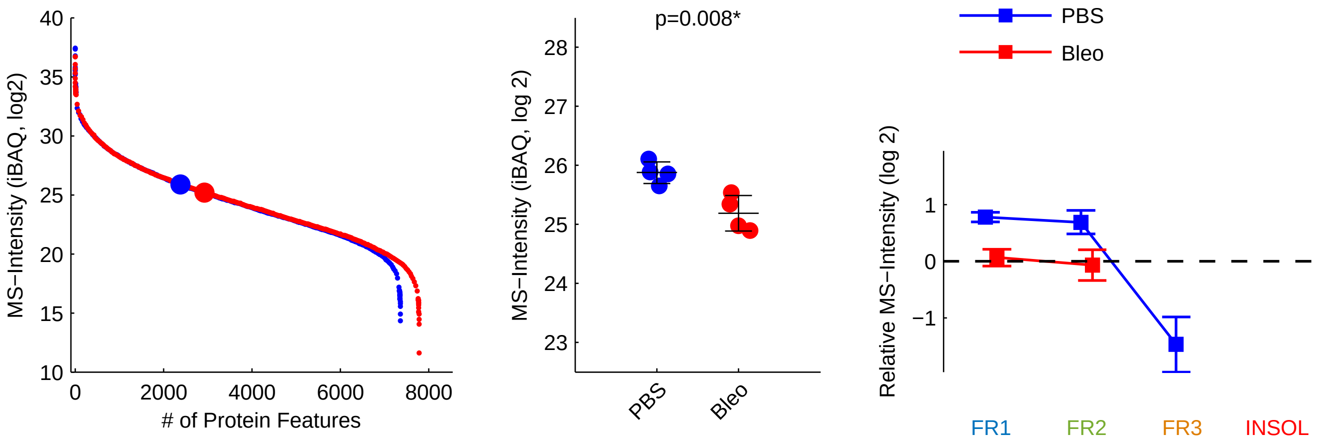

Q64726 − Azgp1 (id: 4772)

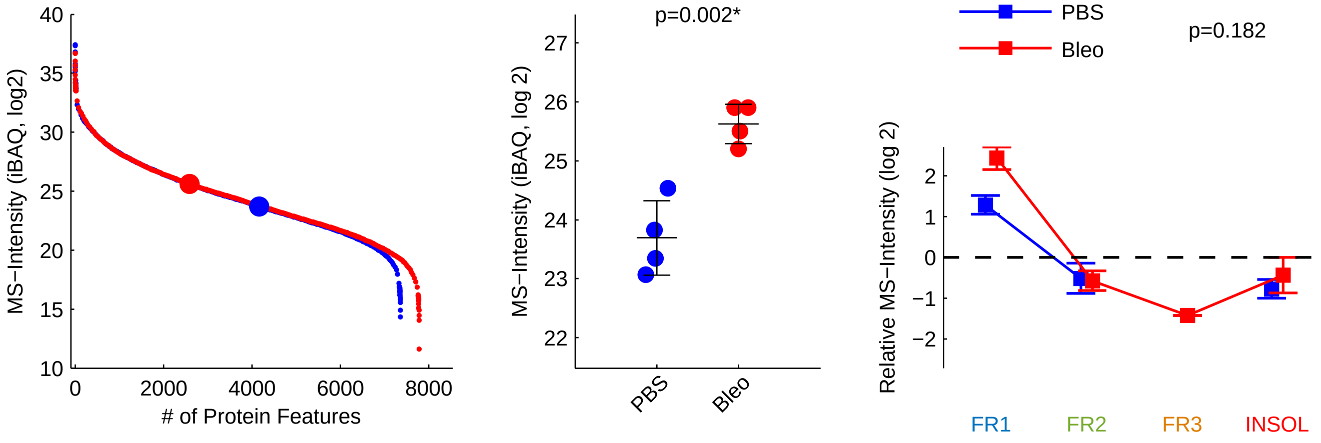

Q64727 − Vcl (id: 4773)

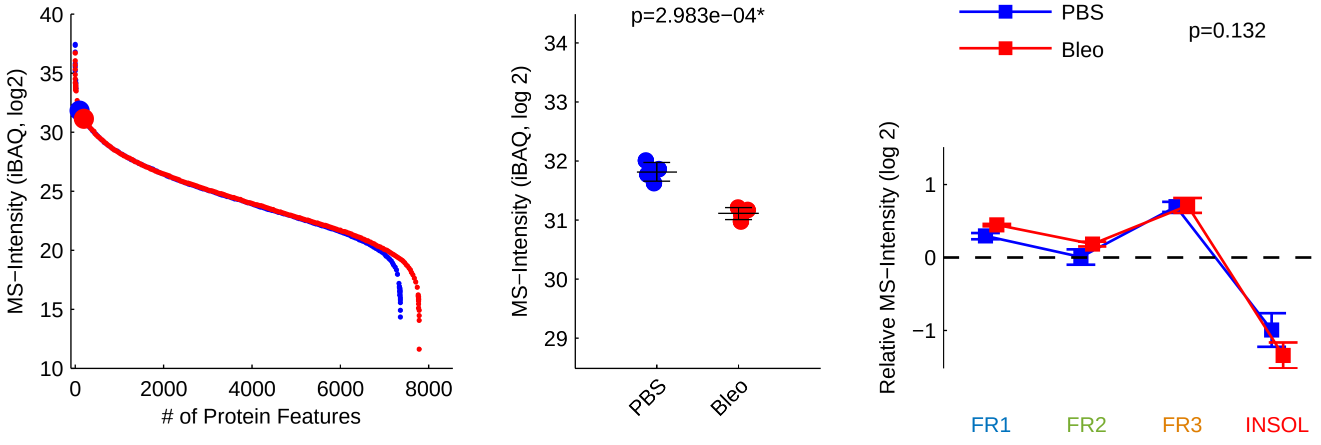

Q66K08 − Cilp (id: 4780)

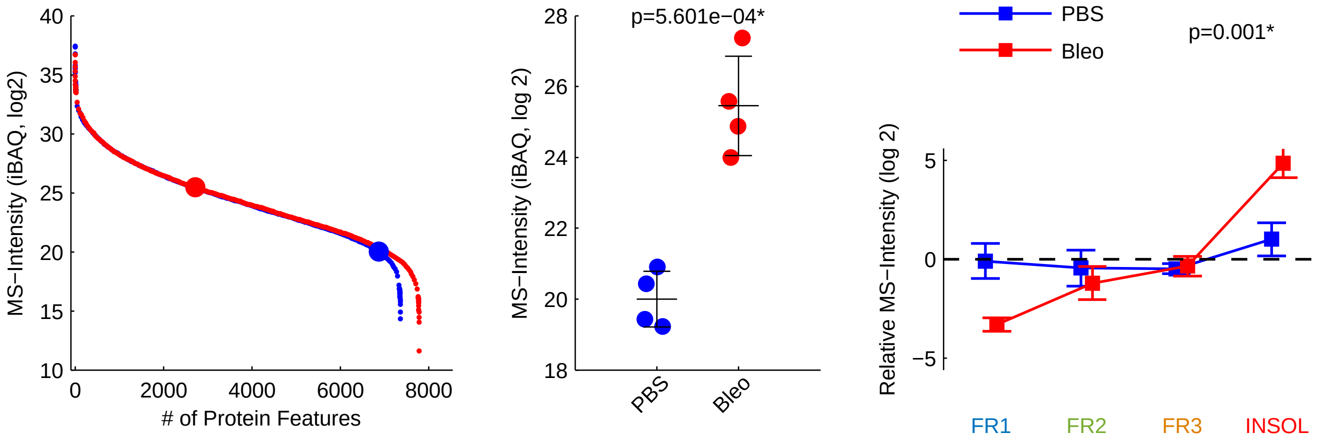

Q68FH0 − Pkp4 (id: 4787)

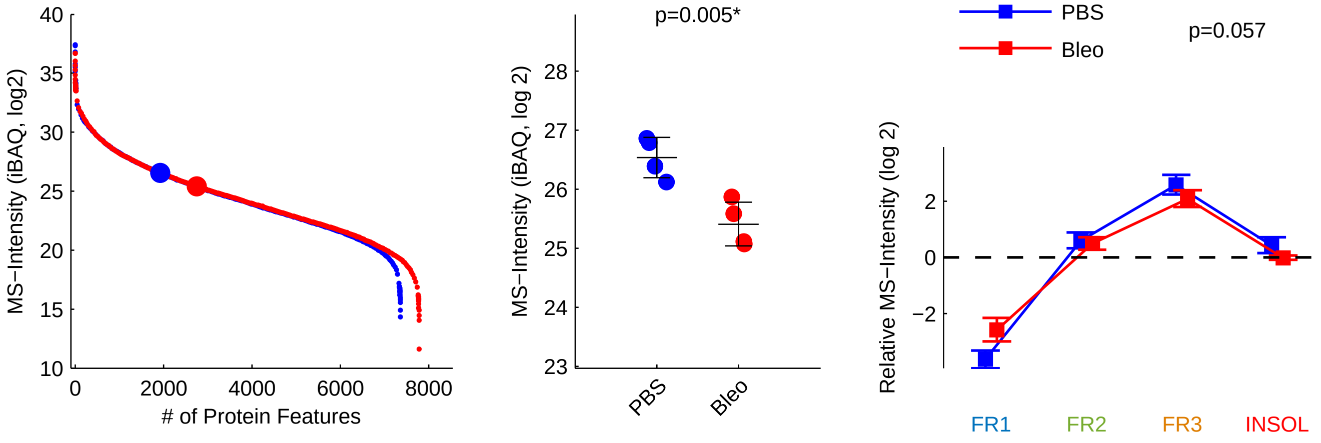

Q68FH4 − Galk2 (id: 4788)

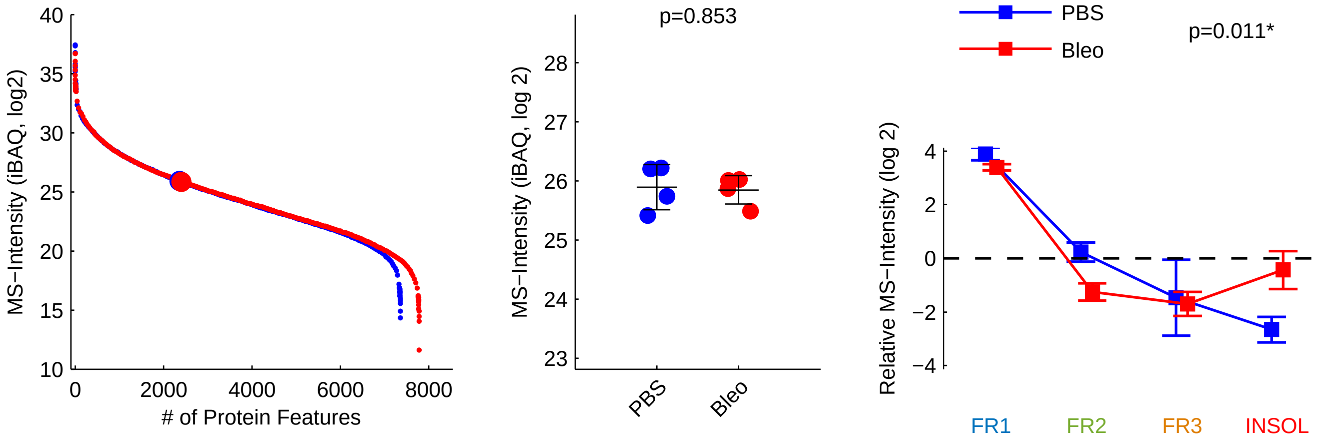

Q69Z99 − Znf512 (id: 4793)

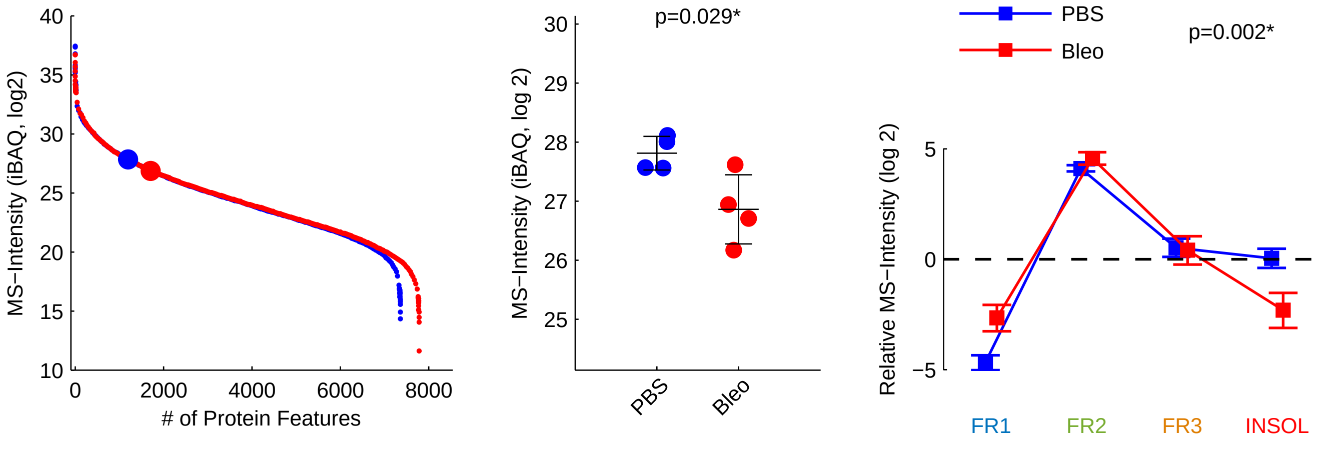

Q69ZR9−2 − Fam208a (id: 4802)

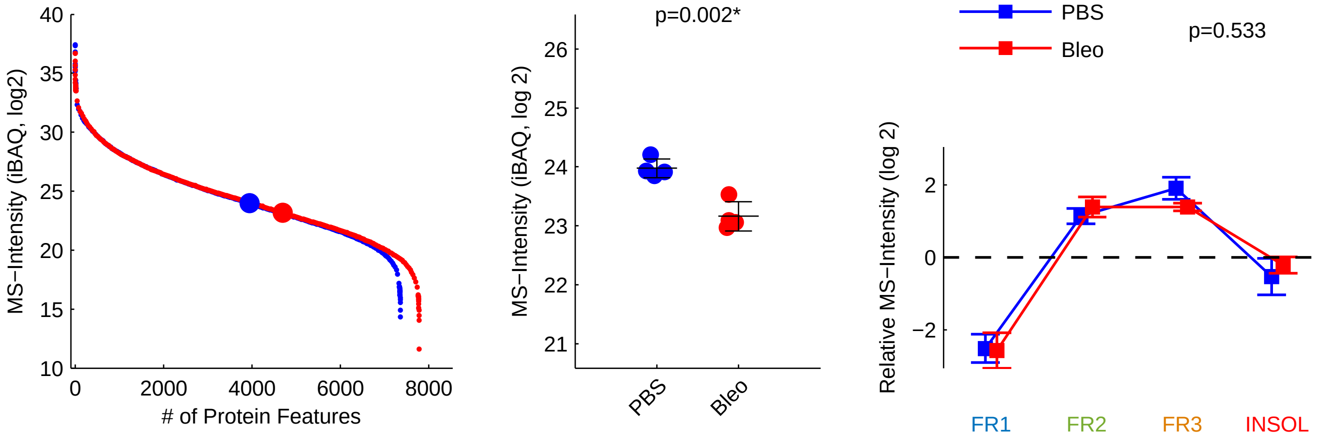

Q69ZX8 − Ablim3 (id: 4806)

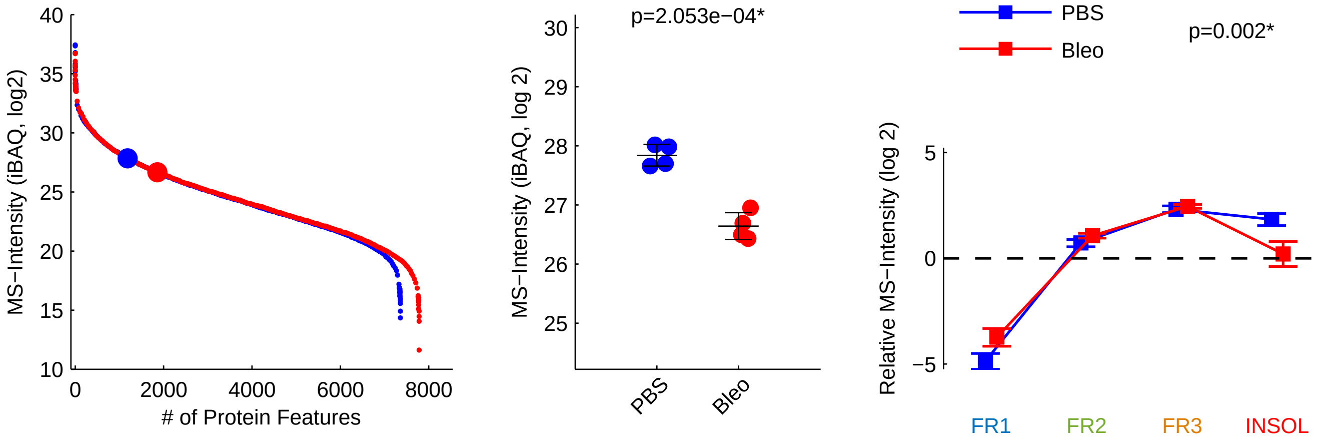

Q6A058 − Armcx2 (id: 4810)

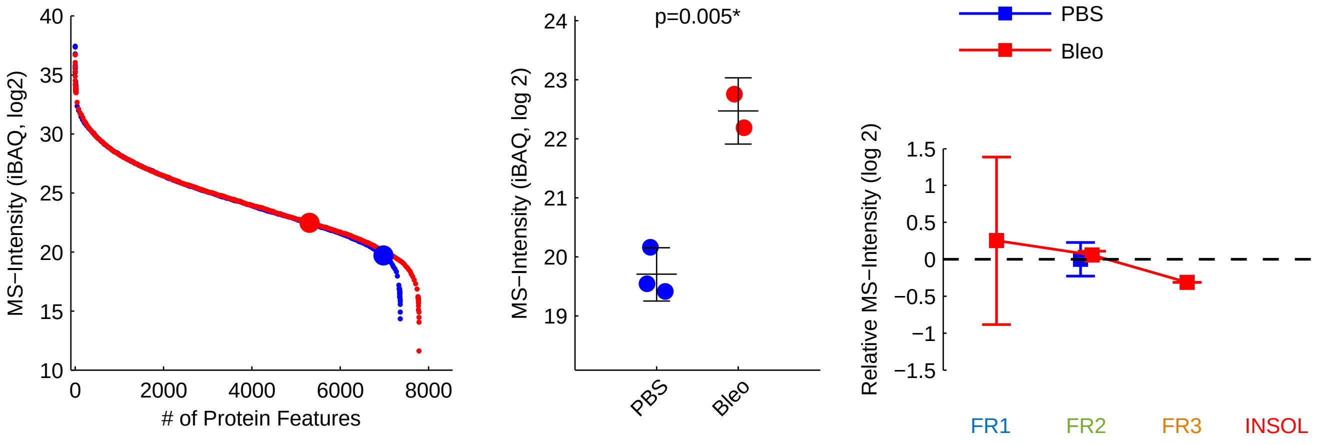

Q6A068 − Cdc5l (id: 4811)

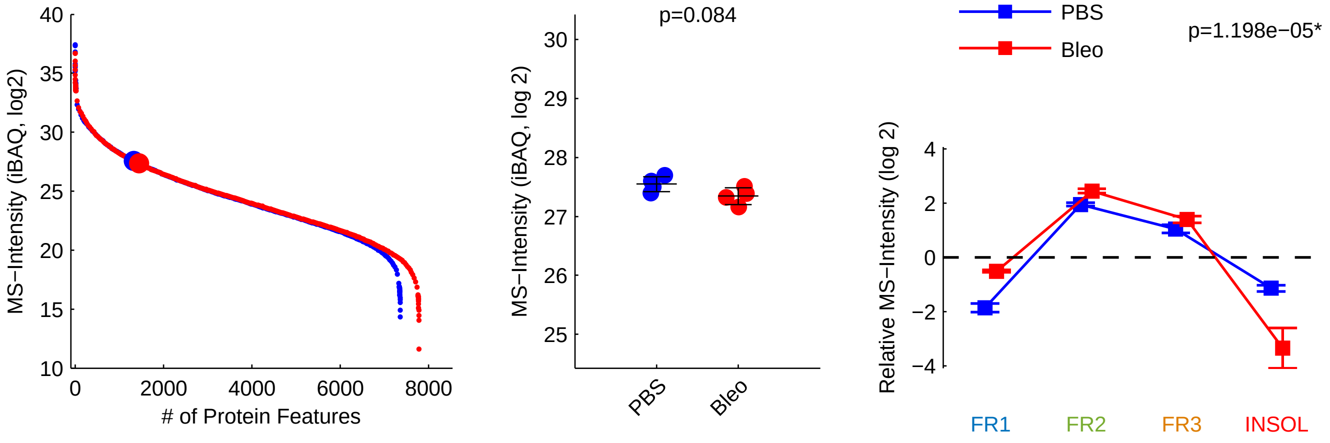

Q6A0D4 − Rftn1 (id: 4814)

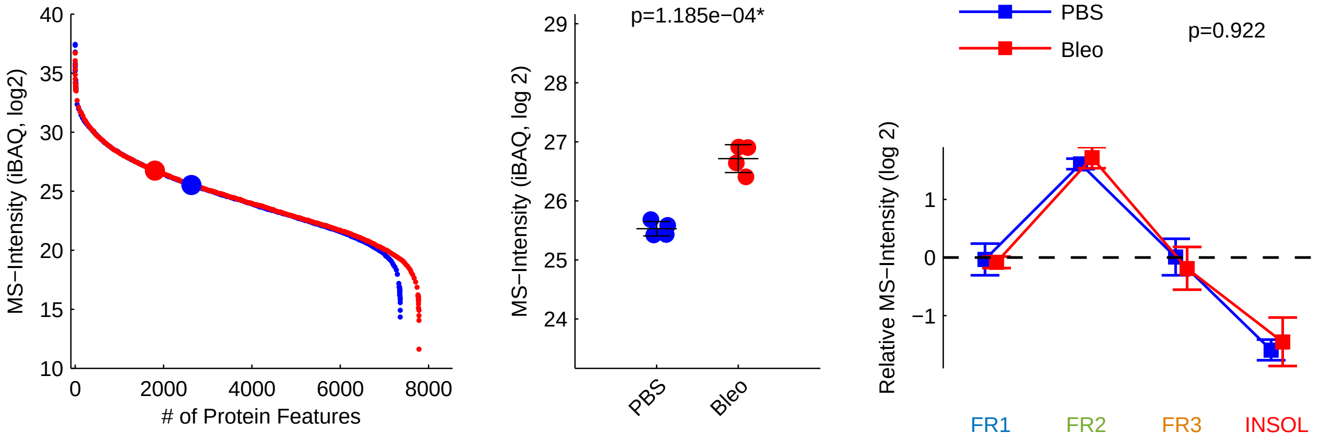

Q6DFV3 − Arhgap21 (id: 4816)

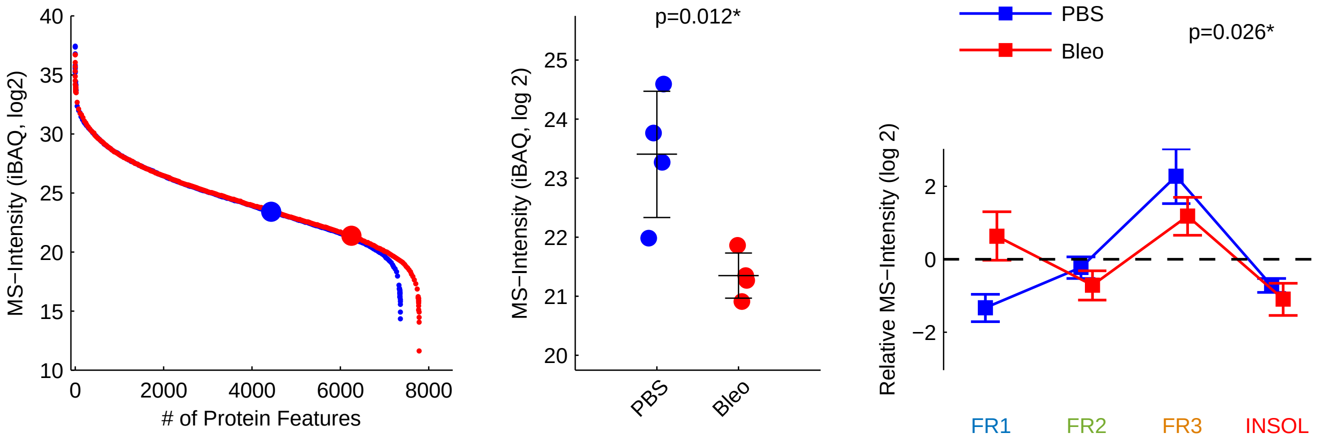

Q6DFX2 − Antxr2 (id: 4818)

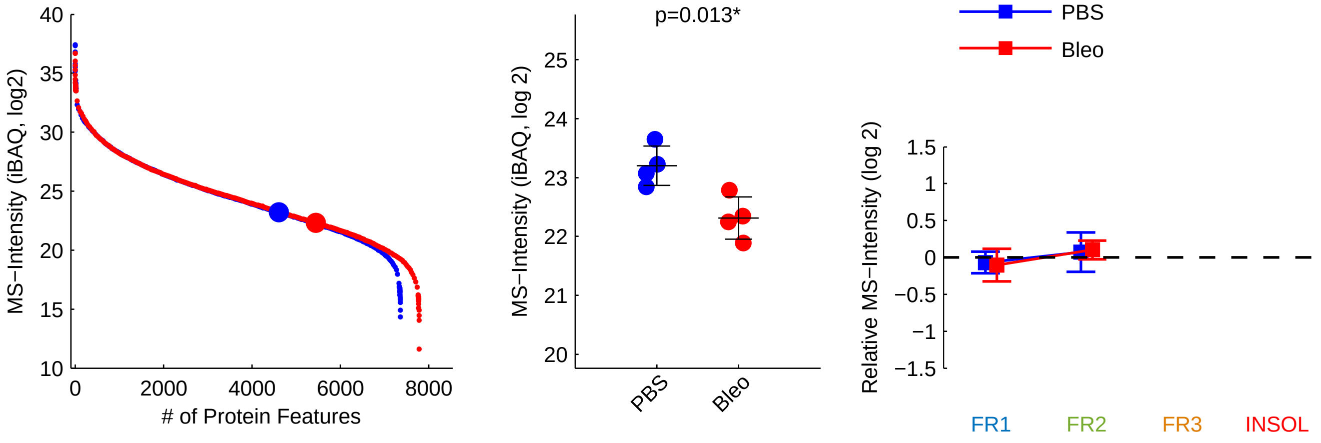

Q6DFZ1 − Gbf1 (id: 4819)

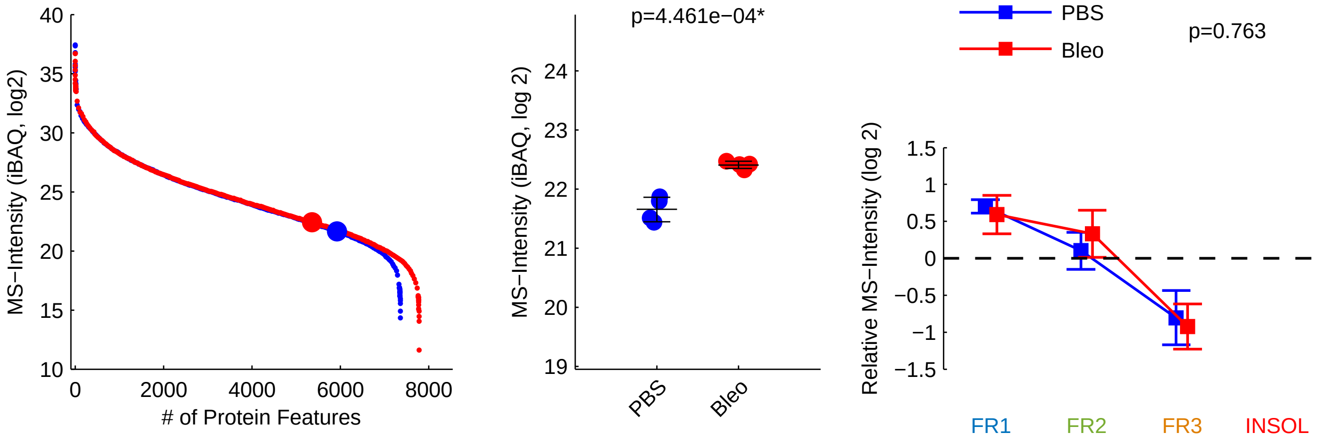

Q6GQS1 − Slc25a23 (id: 4830)

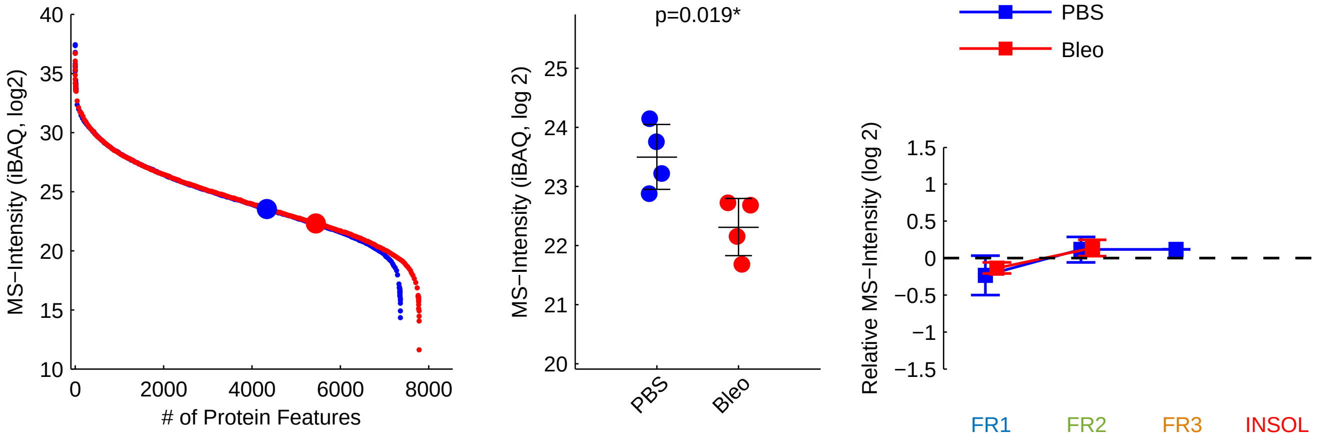

Q6GQT9 − Nomo1 (id: 4833)

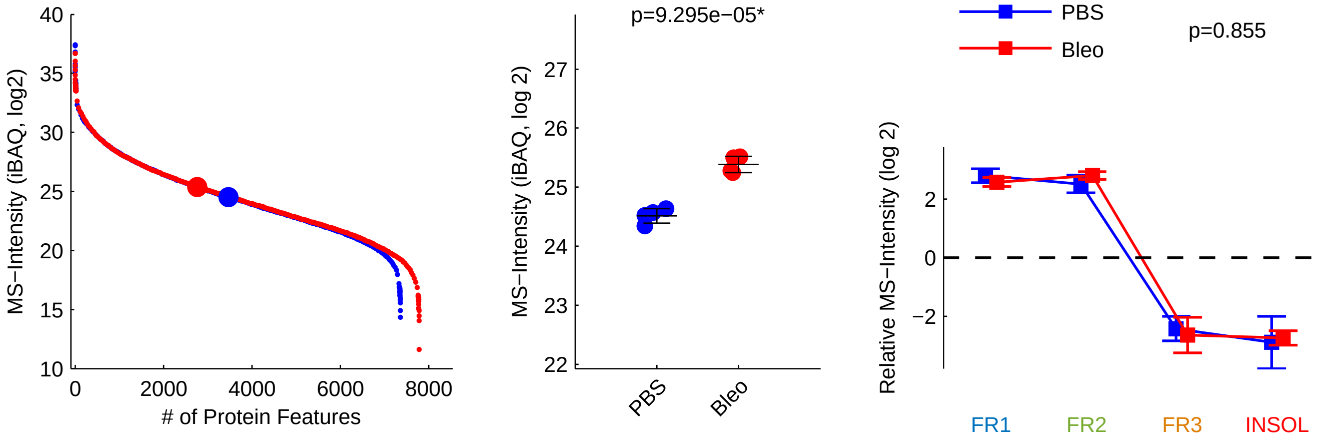

Q6GT24 − Prdx6 (id: 4834)

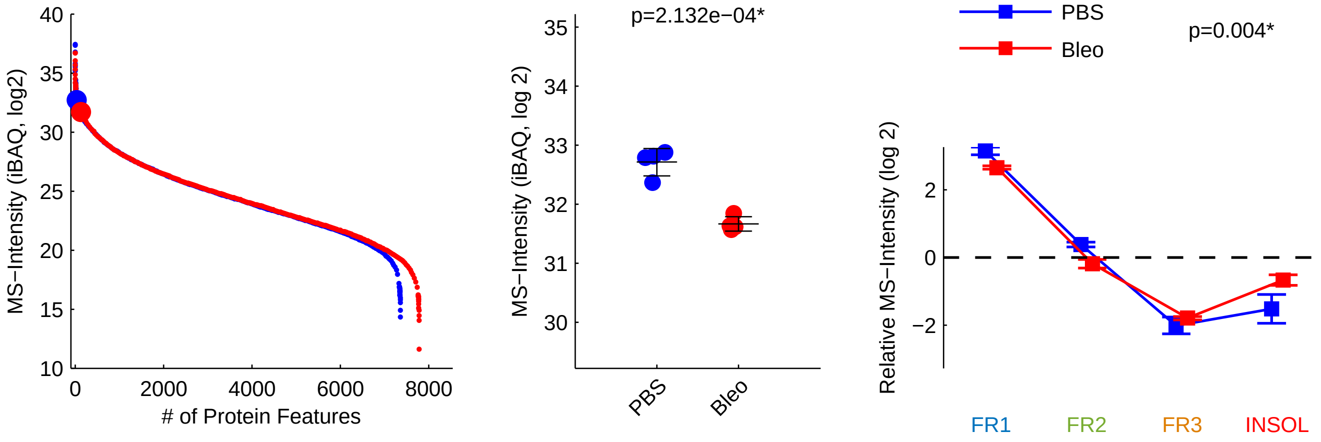

Q6GV12 − Kdsr (id: 4836)

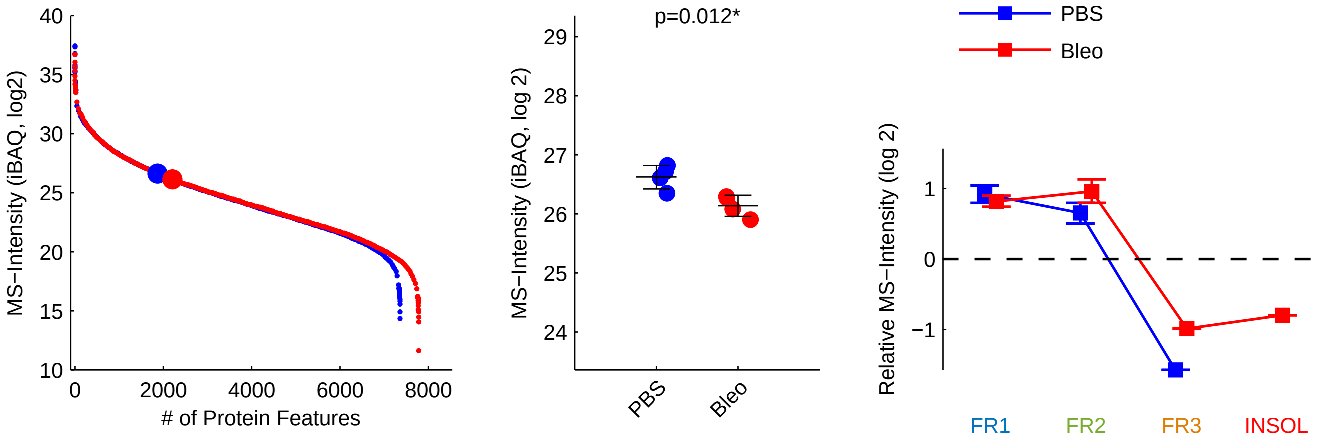

Q6IRU2 − Tpm4 (id: 4842)

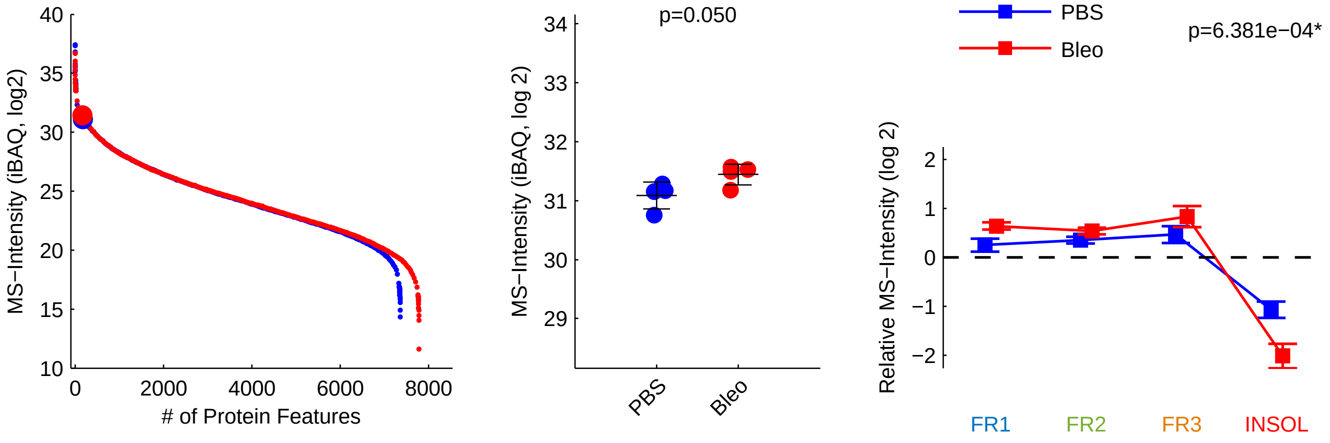

Q6IRU7 − Cep78 (id: 4844)

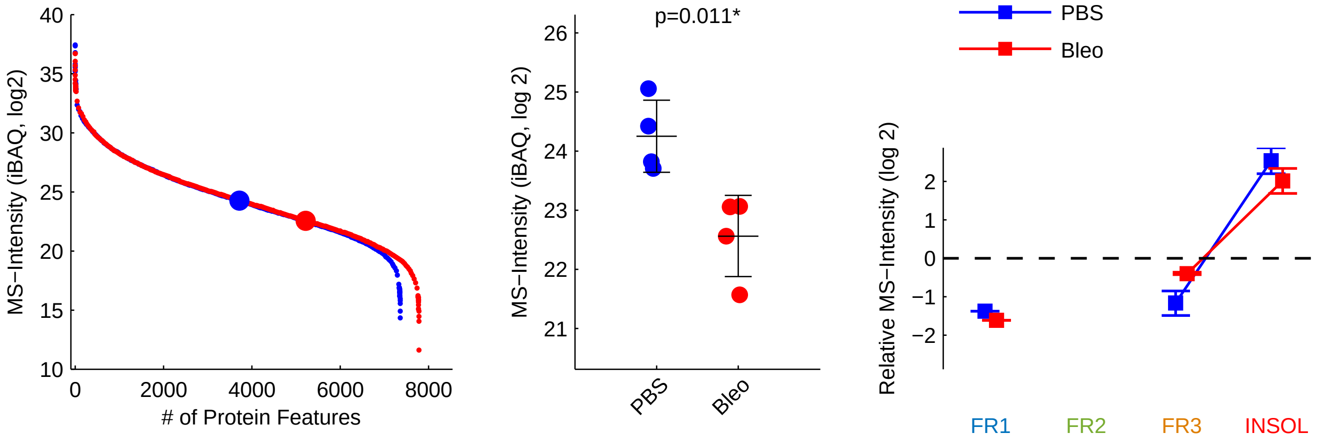

Q6KCD5 − Nipbl (id: 4847)

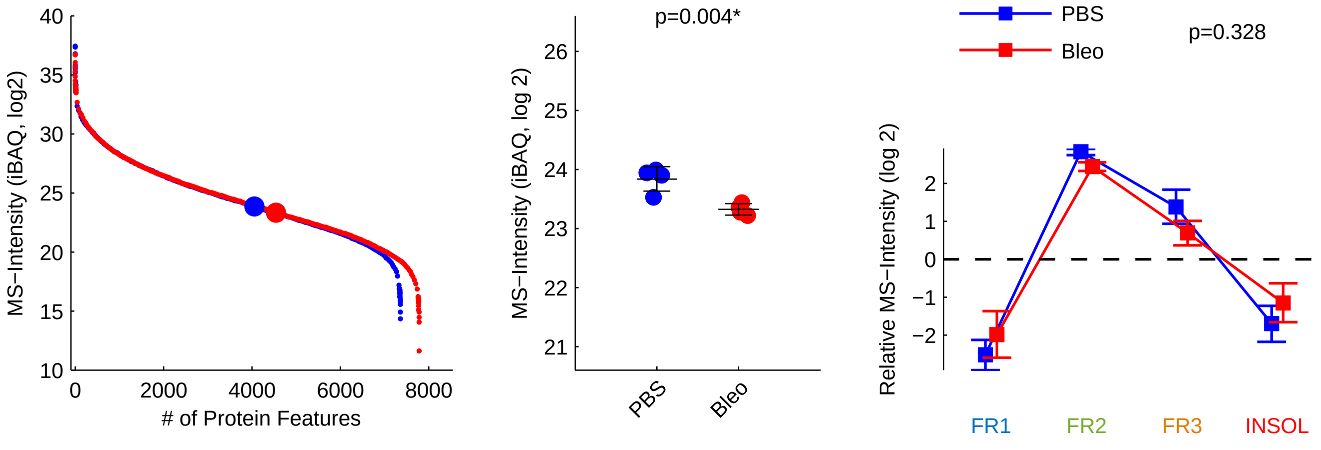

Q6NS46 − Pdcd11 (id: 4848)

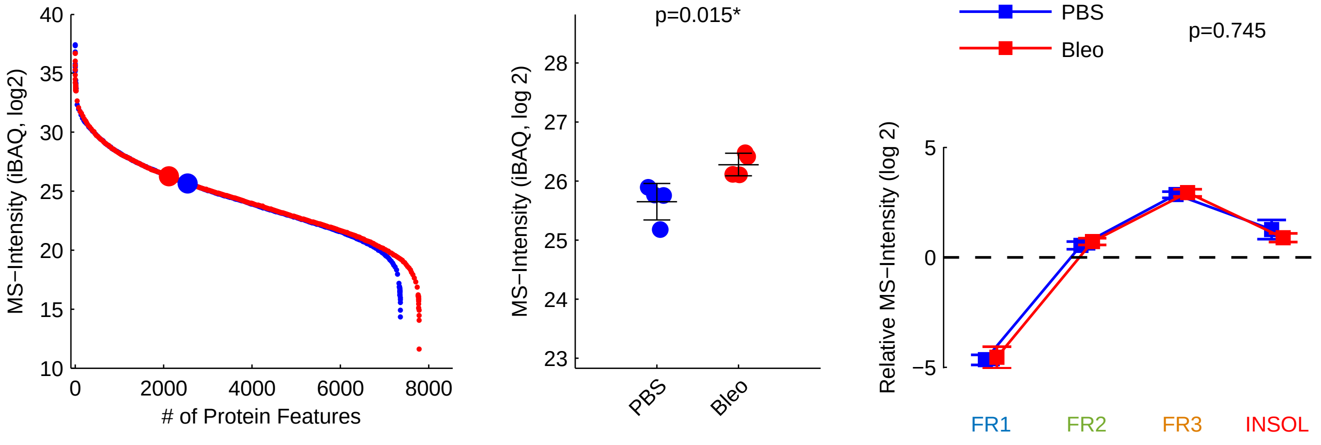

Q6NSR8 − Npepl1 (id: 4852)

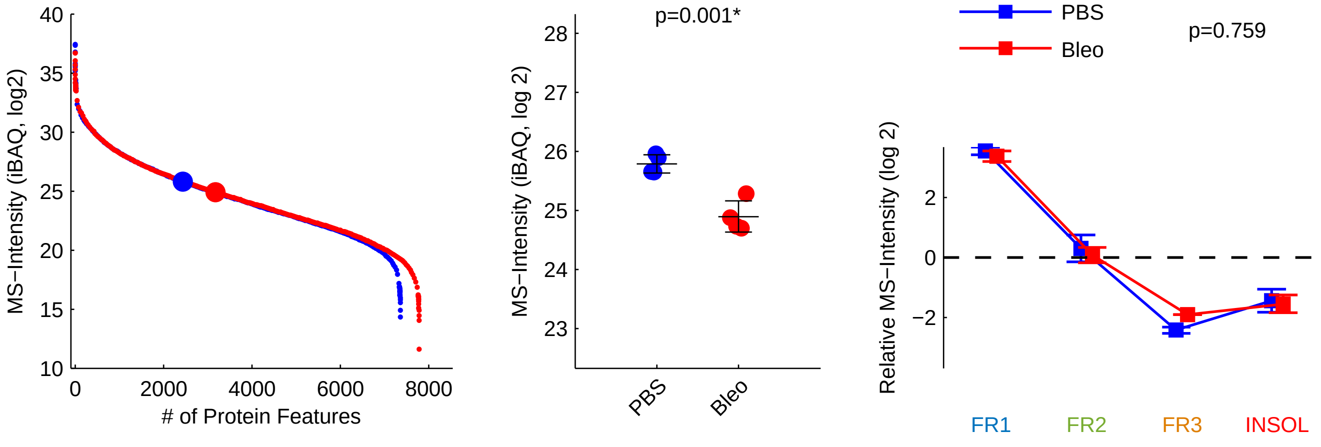

Q6NSU3 − Glt8d1 (id: 4853)

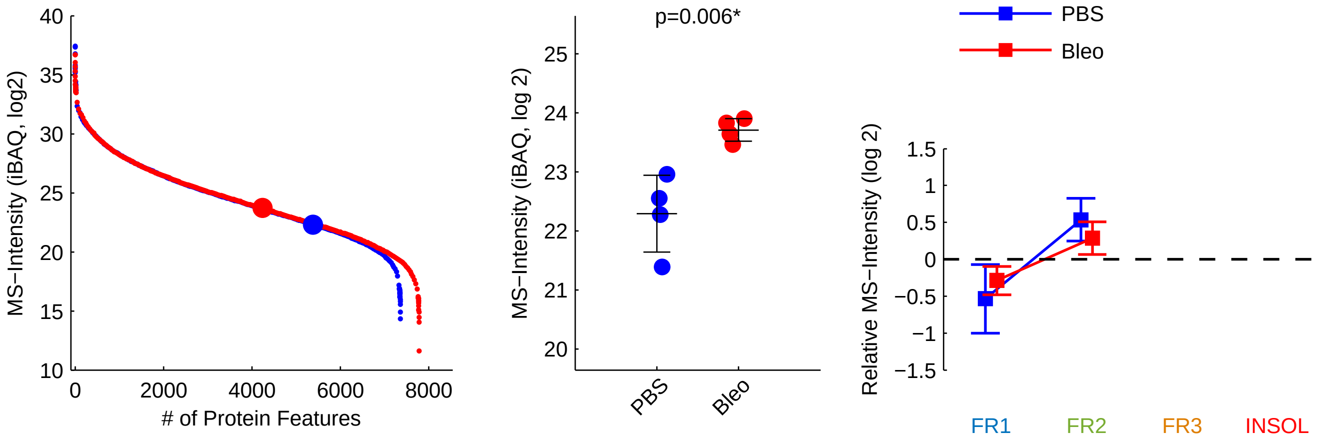

Q6NV99 − Haus6 (id: 4857)

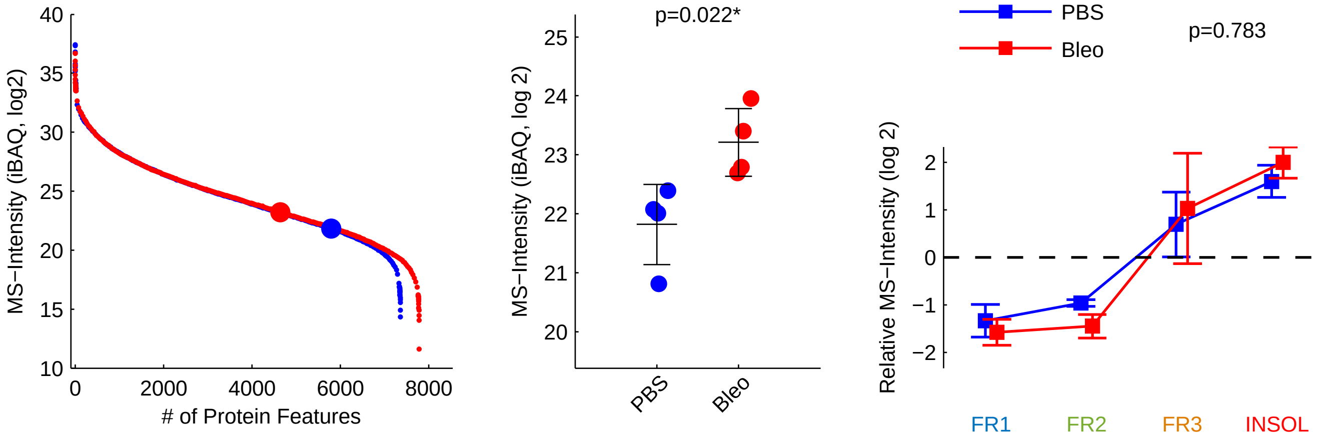

Q6NWW9 − Fndc3b (id: 4865)

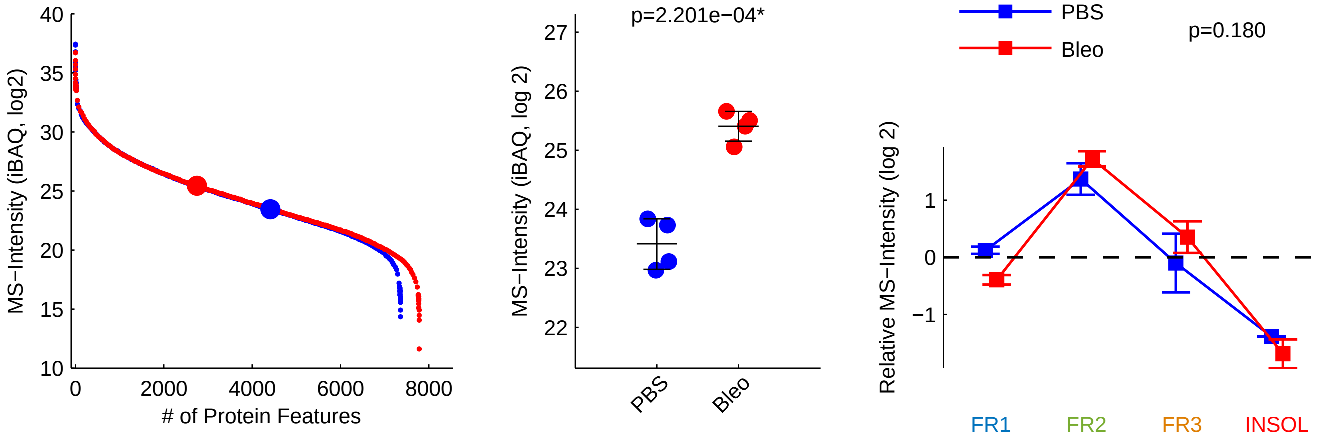

Q6NXL1 − Sec24d (id: 4870)

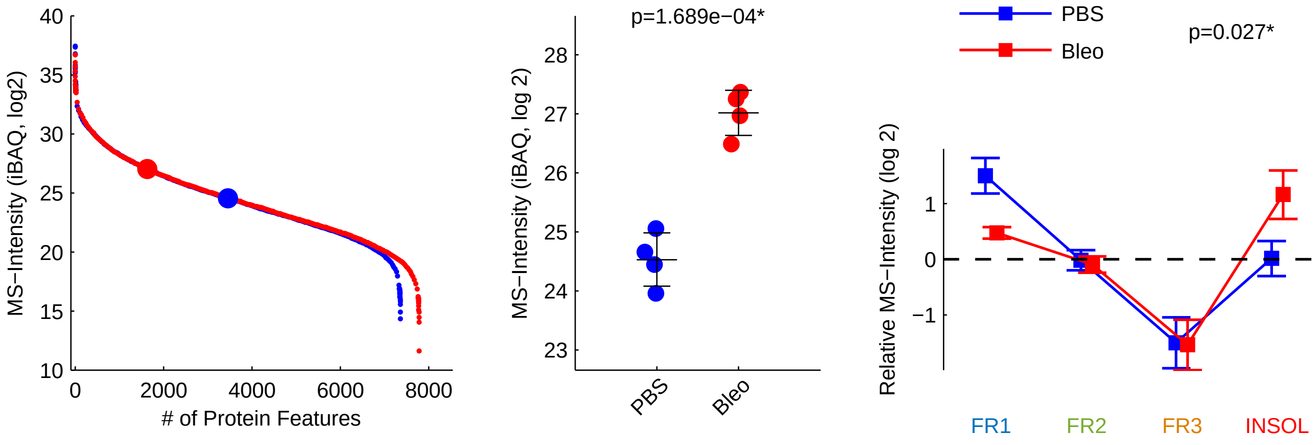

Q6P069 − Sri (id: 4883)

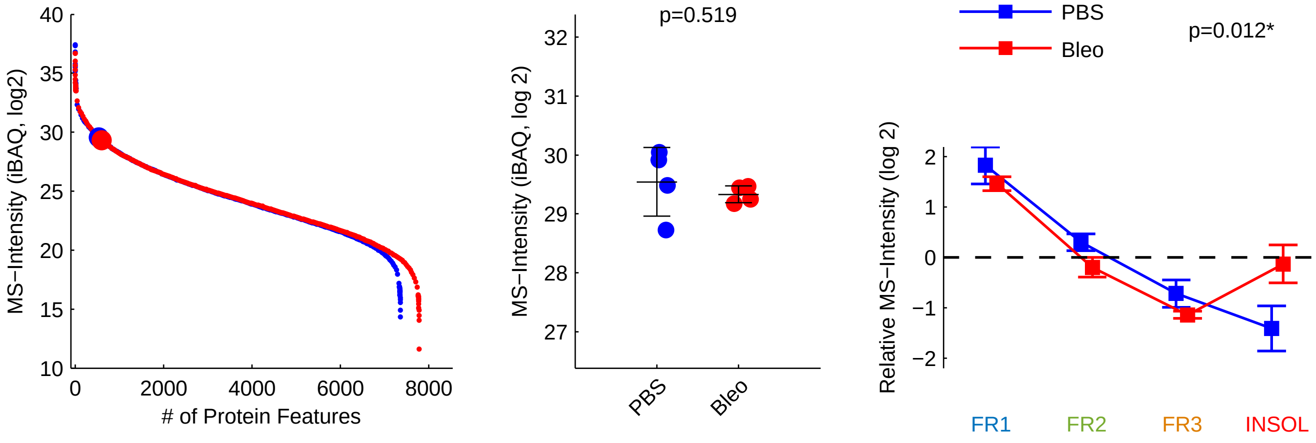

Q9WVL6 − Extl3 (id: 4890)

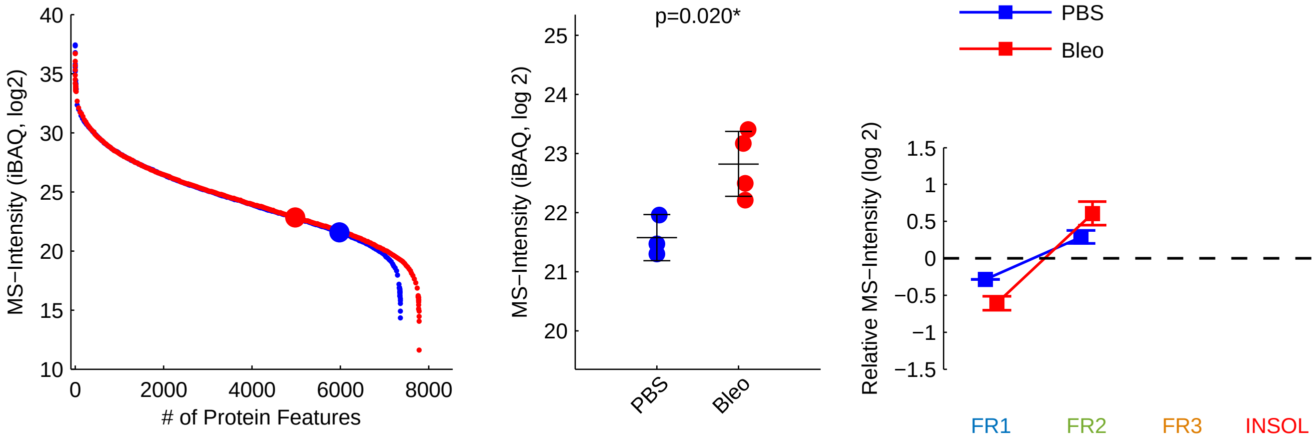

Q6P4T2 − Snrnp200 (id: 4905)

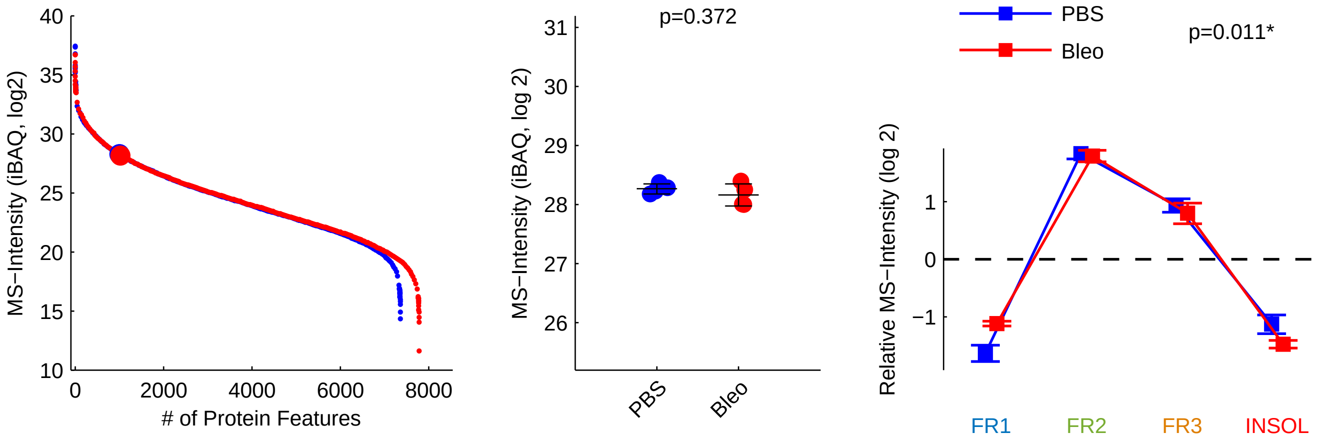

Q6P5B0 − Rrp12 (id: 4909)

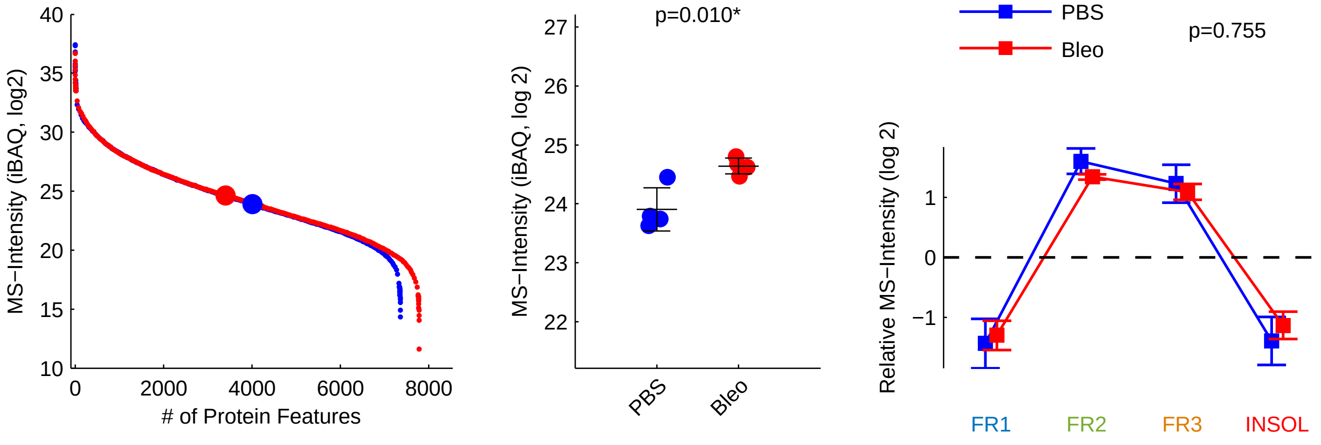

Q6P5E4 − Uggt1 (id: 4915)

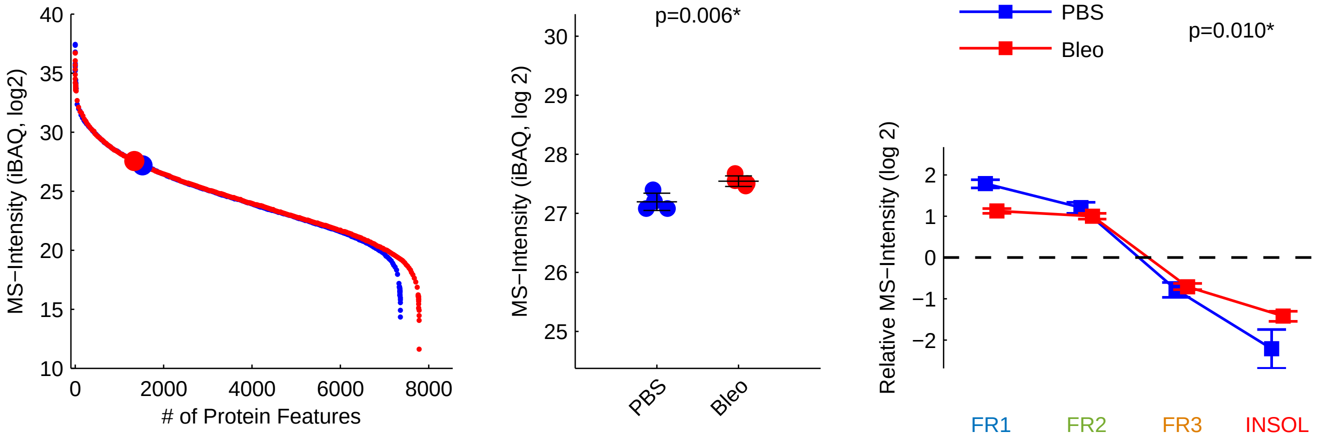

Q6P5F9 − Xpo1 (id: 4919)

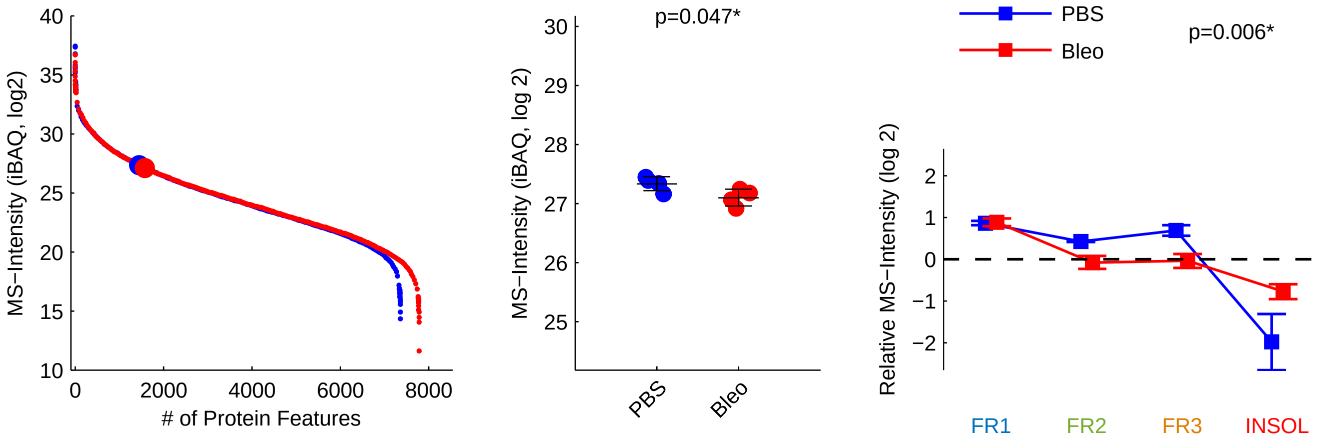

Q6P5H2 − Nes (id: 4921)

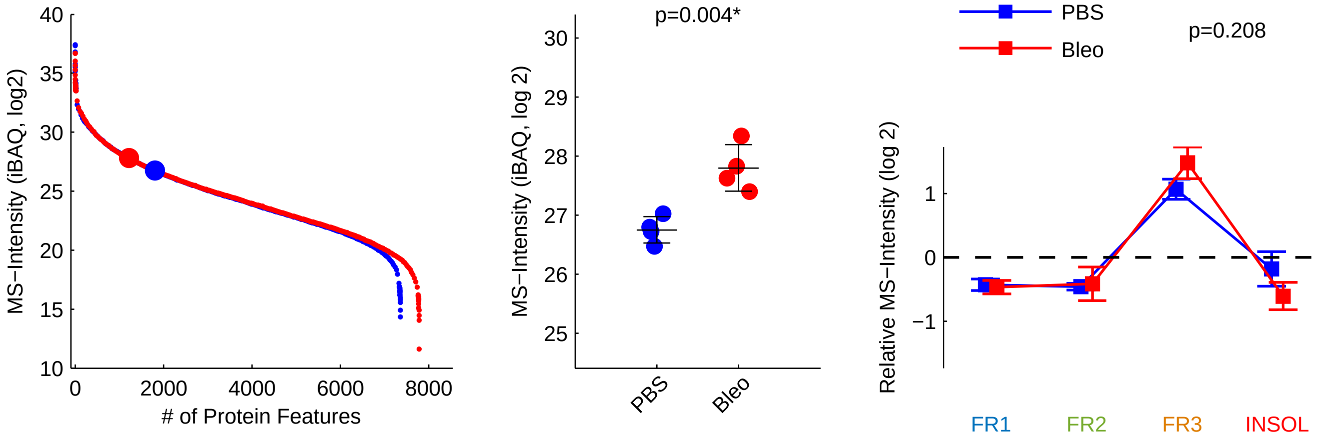

Q9JHS4 − Clpx (id: 4931)

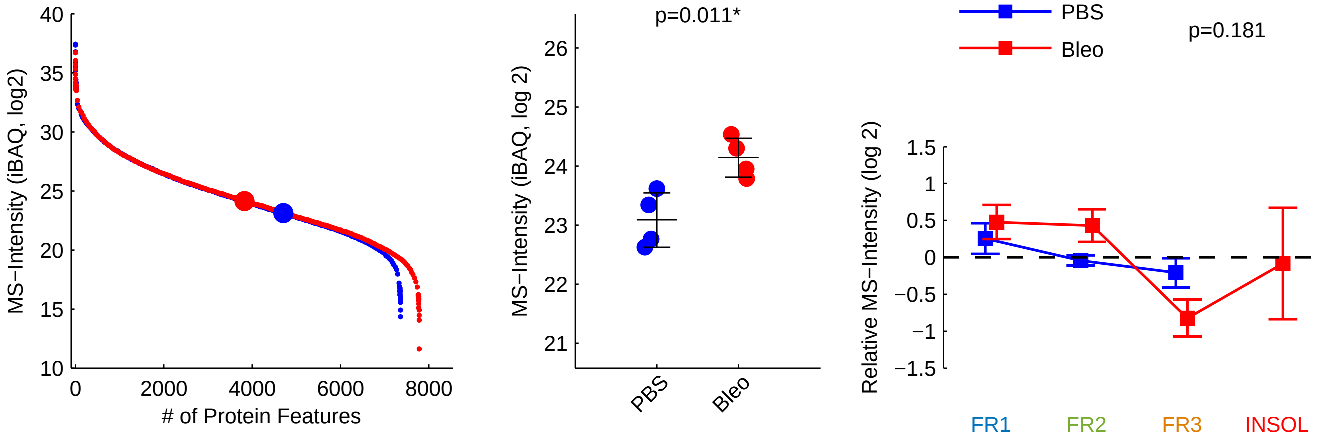

Q6P8P8 − Sftpc (id: 4932)

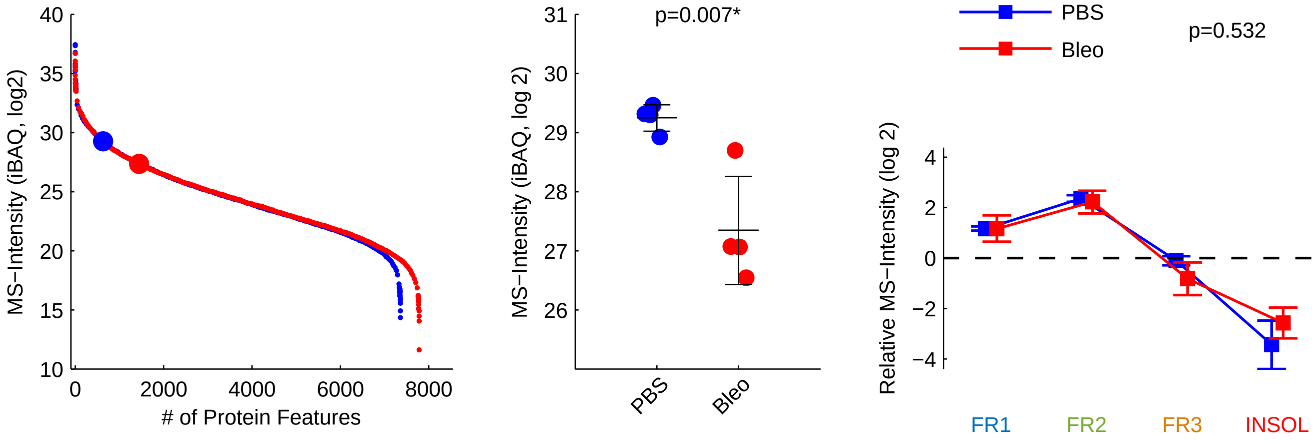

Q6P9J5 − Kank4 (id: 4935)

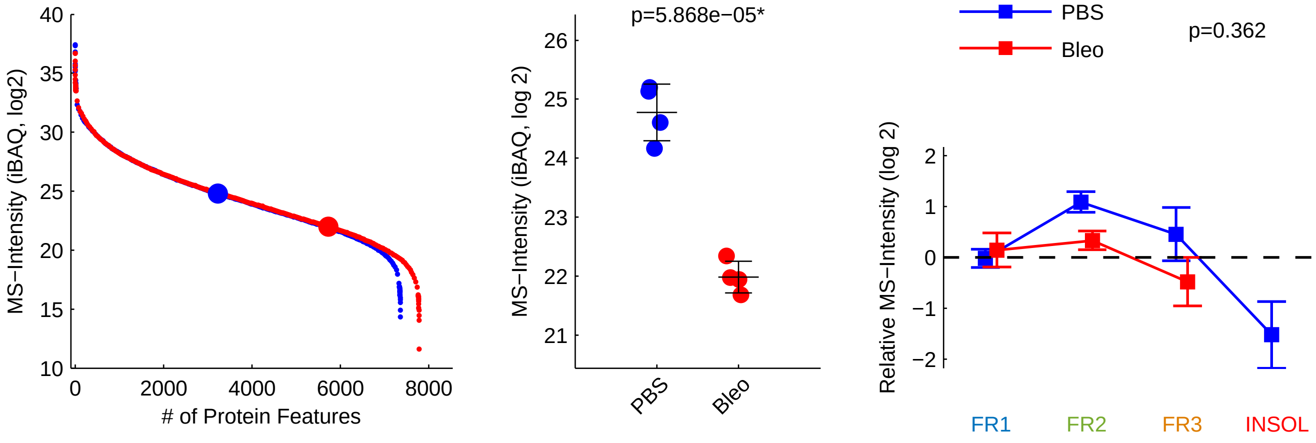

Q6P9Q4 − Fhod1 (id: 4938)

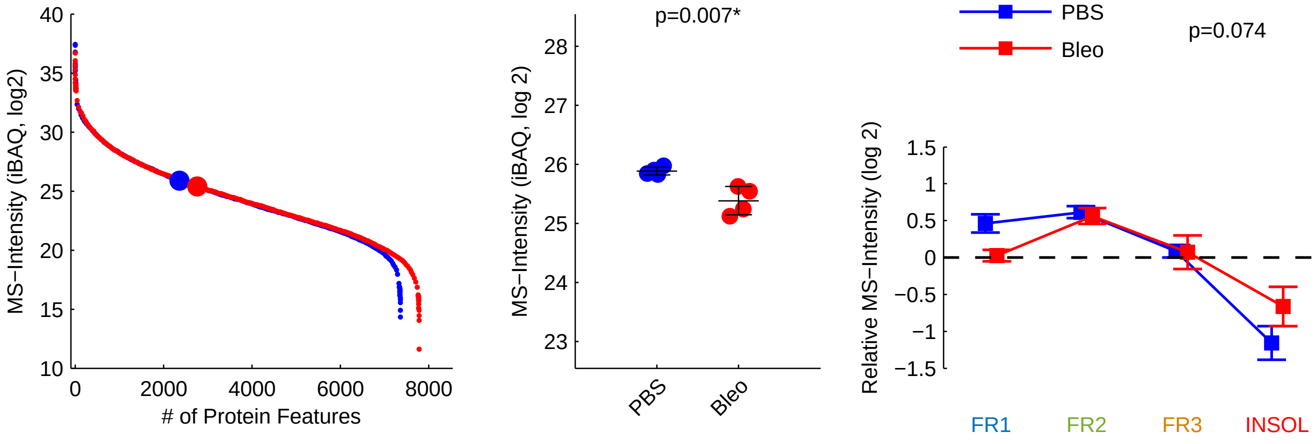

Q6P9R4 − Arhgef18 (id: 4942)

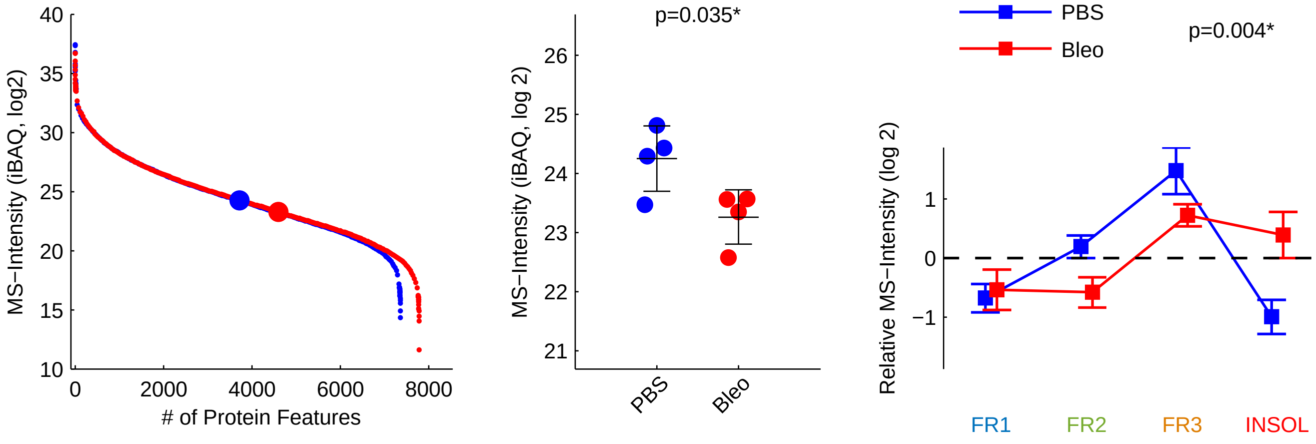

Q6PA06 − Atl2 (id: 4944)

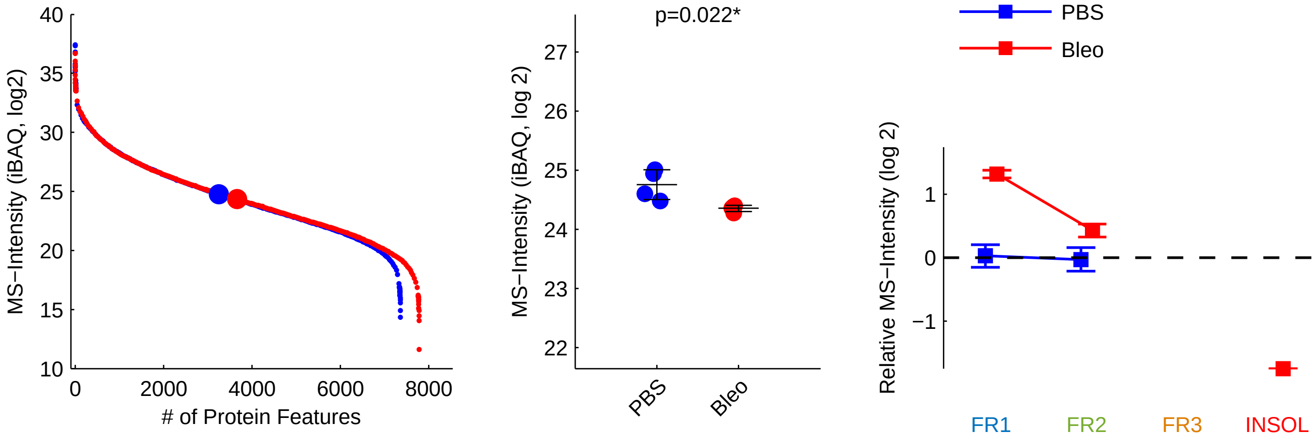

Q6PAV2 − Herc4 (id: 4954)

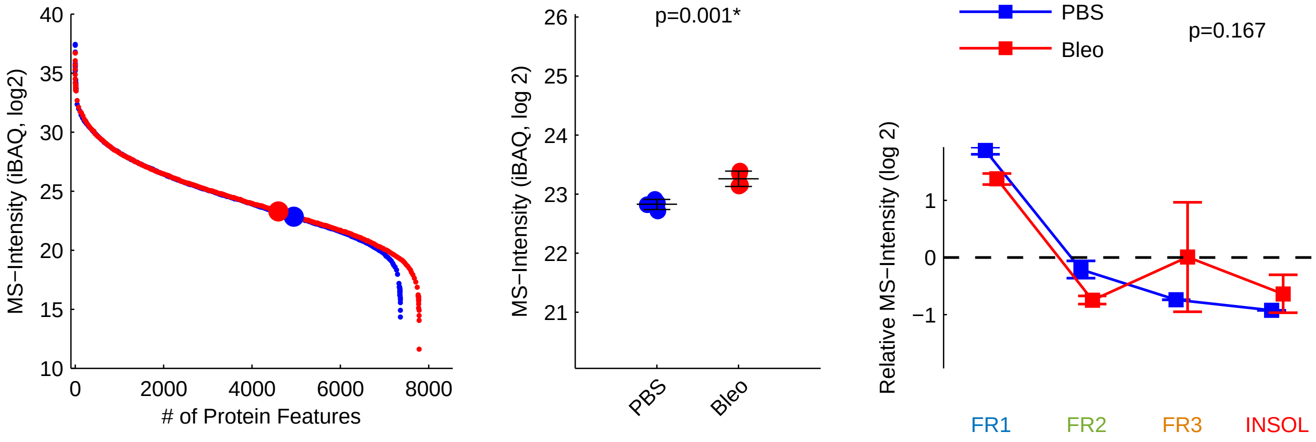

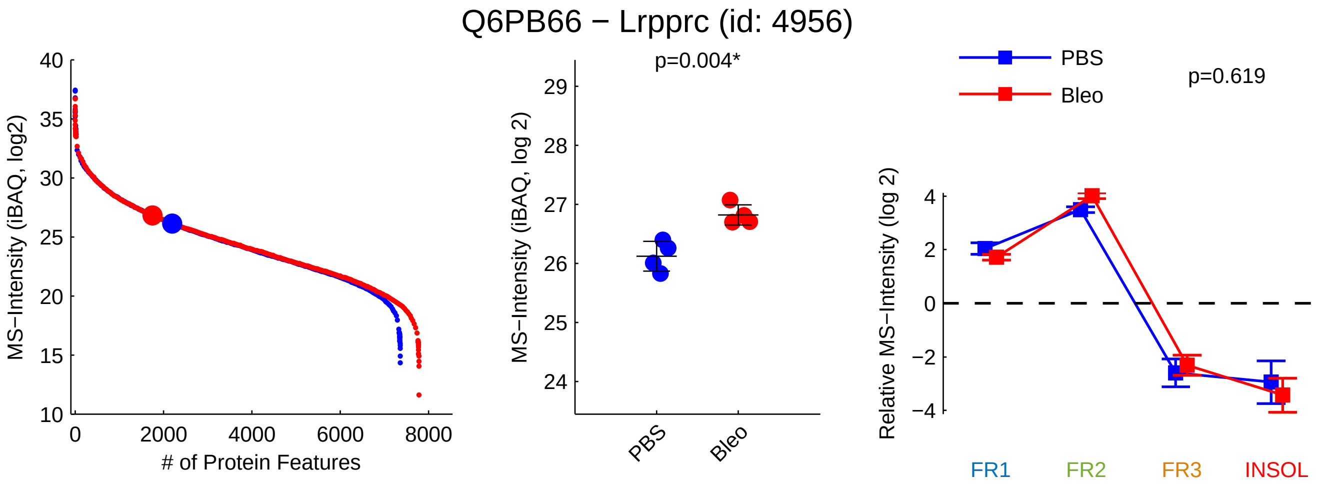

Q6PB66 − Lrpprc (id: 4956)
MS−Intensity (iBAQ, log2)
# of Protein Features
p=0.004*
MS−Intensity (iBAQ, log 2)
PBS
Bleo
p=0.619
Relative MS−Intensity (log 2)
FR1
FR2
FR3
INSOL

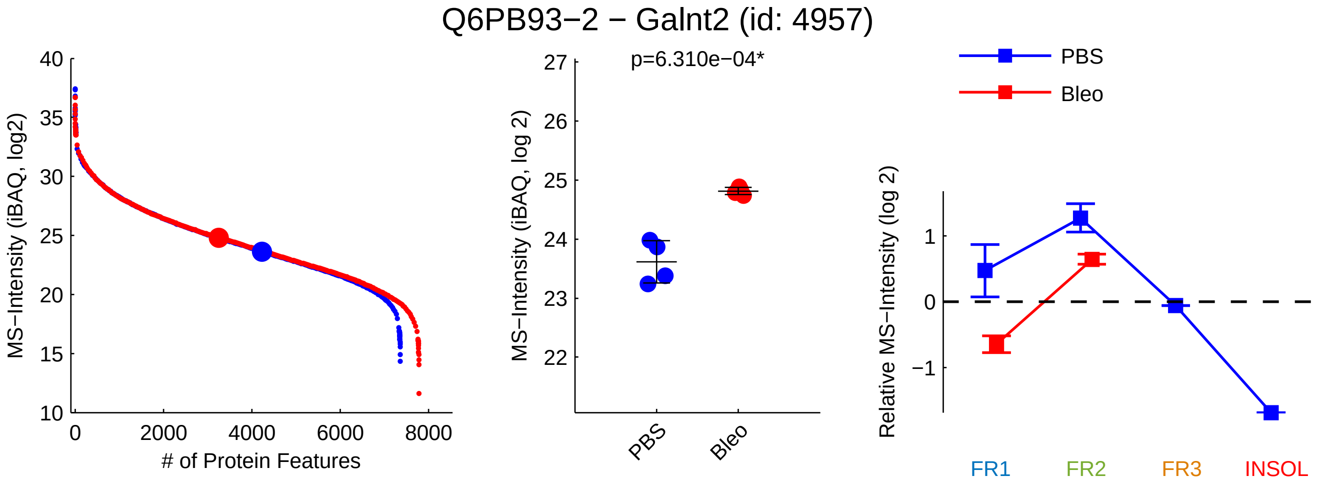

Q6PB93−2 − Galnt2 (id: 4957)
MS−Intensity (iBAQ, log2)
# of Protein Features
p=6.310e−04*
MS−Intensity (iBAQ, log 2)
PBS
Bleo
Relative MS−Intensity (log 2)
FR1
FR2
FR3
INSOL

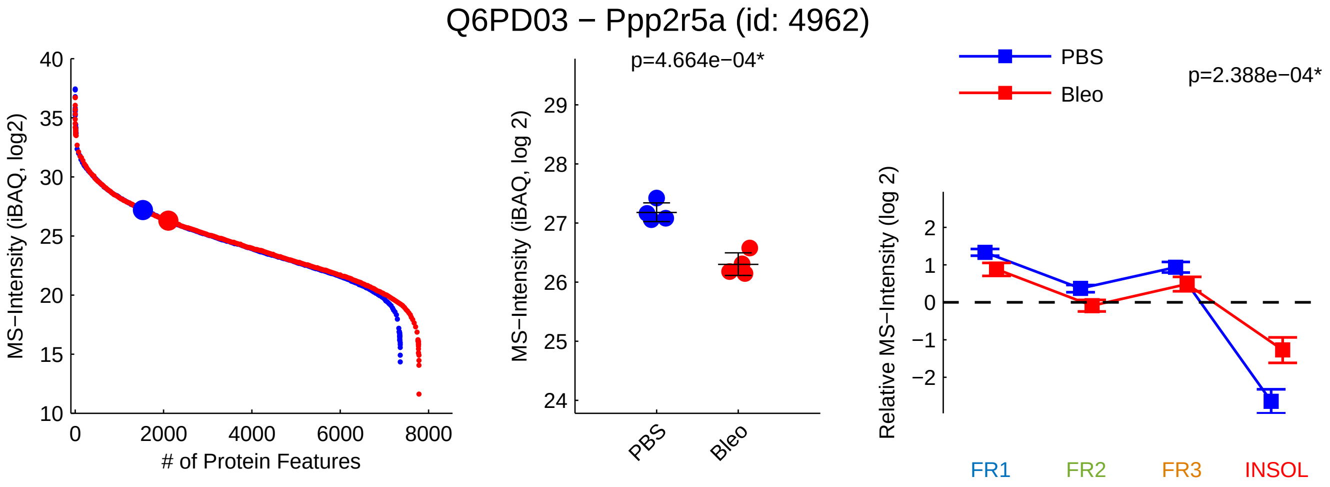

Q6PD03 − Ppp2r5a (id: 4962)
MS−Intensity (iBAQ, log2)
# of Protein Features
p=4.664e−04*
MS−Intensity (iBAQ, log 2)
PBS
Bleo
p=2.388e−04*
Relative MS−Intensity (log 2)
FR1
FR2
FR3
INSOL

Q6PDI5 − Ecm29 (id: 4968)

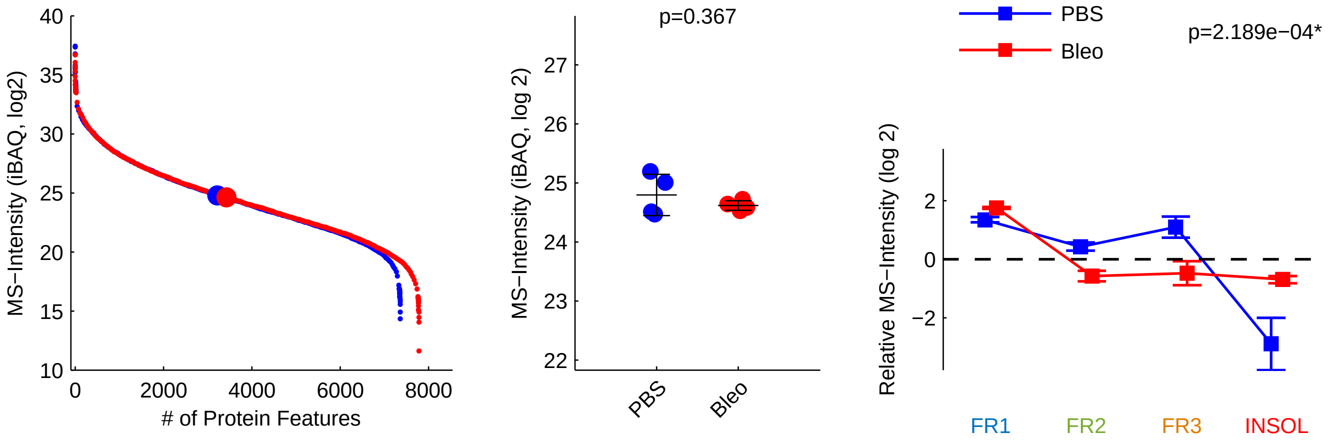

Q6PDL0 − Dync1li2 (id: 4970)

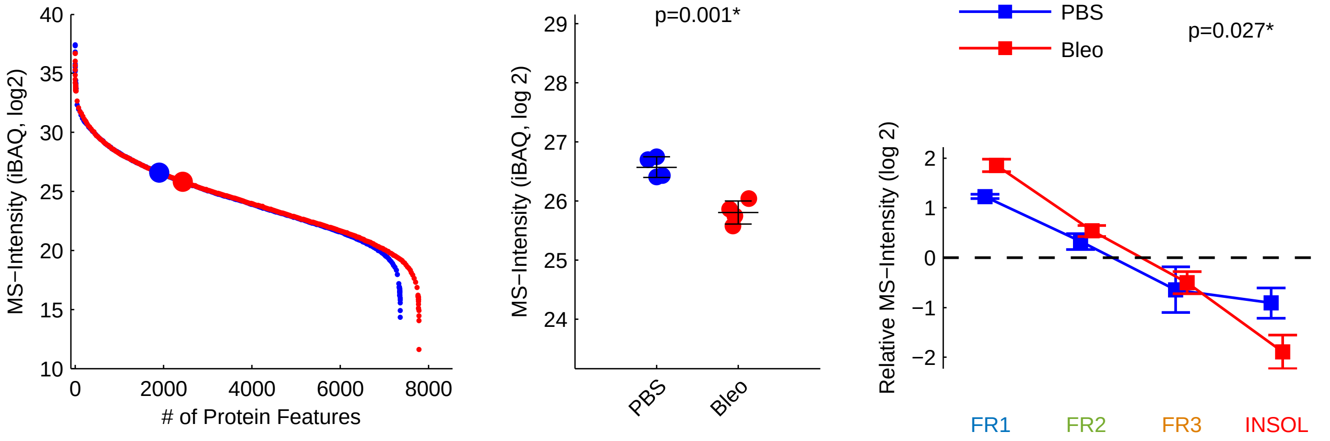

Q6PE01 − Snrnp40 (id: 4972)

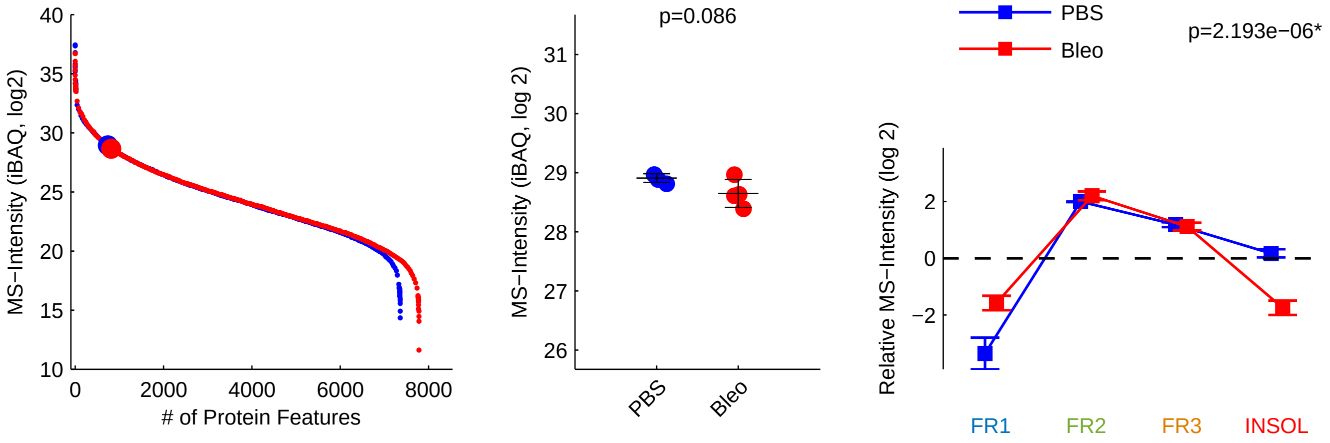

Q6PE54 − Dhx40 (id: 4974)

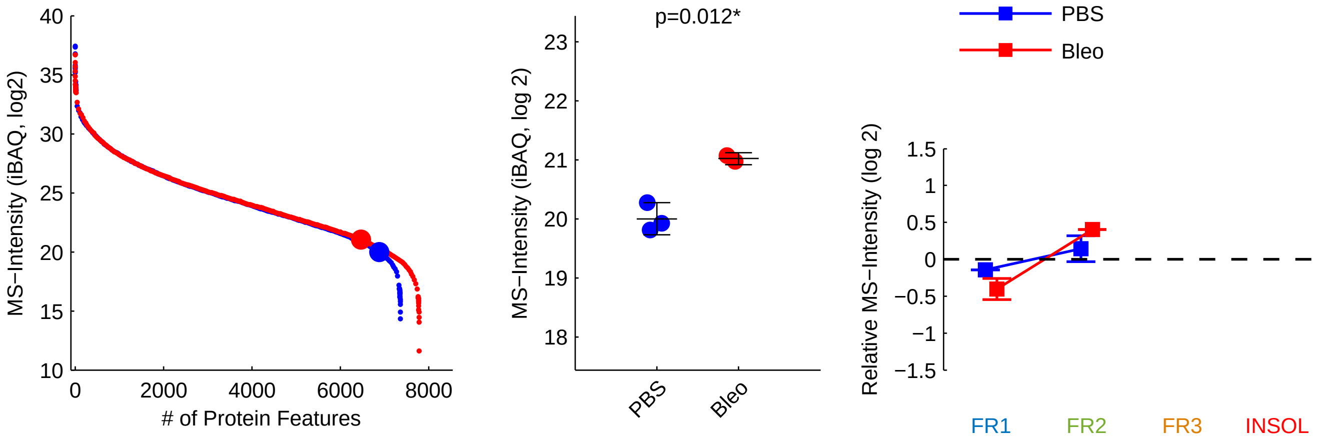

Q6PER3 − Mapre3 (id: 4978)

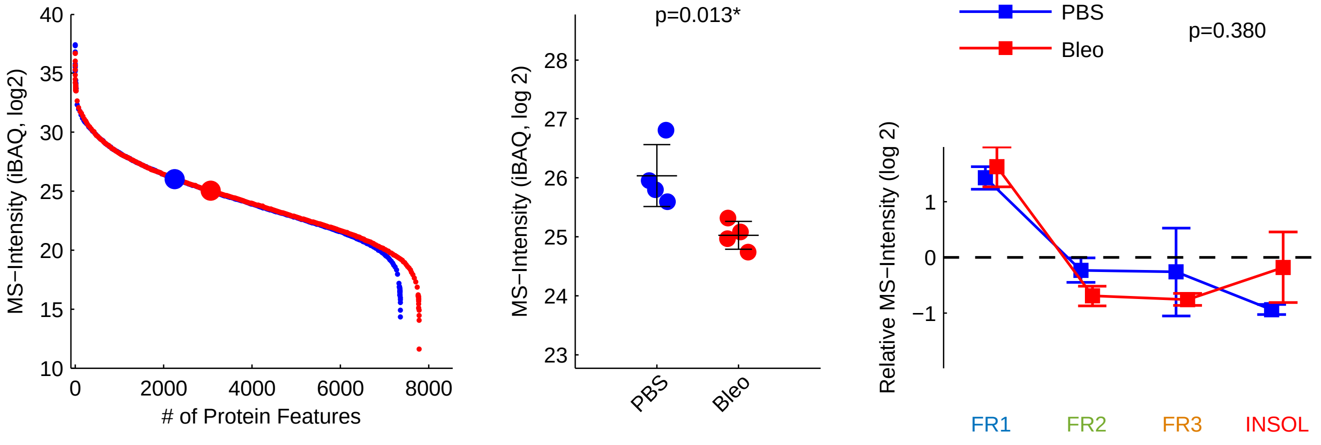

Q6PGC1 − Dhx29 (id: 4987)

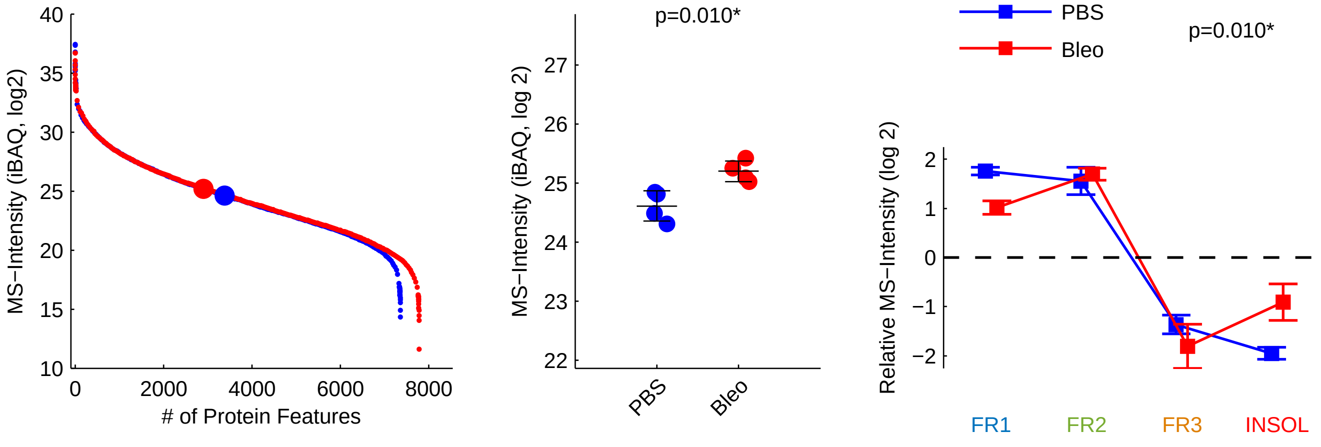

Q6PGF7 − Exoc8 (id: 4989)

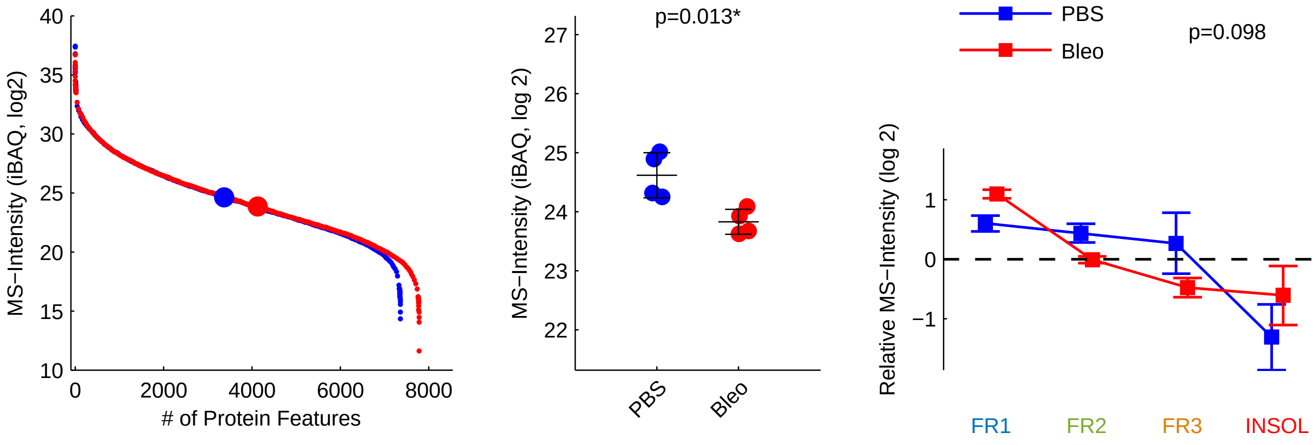

Q6PHN9 − Rab35 (id: 4997)

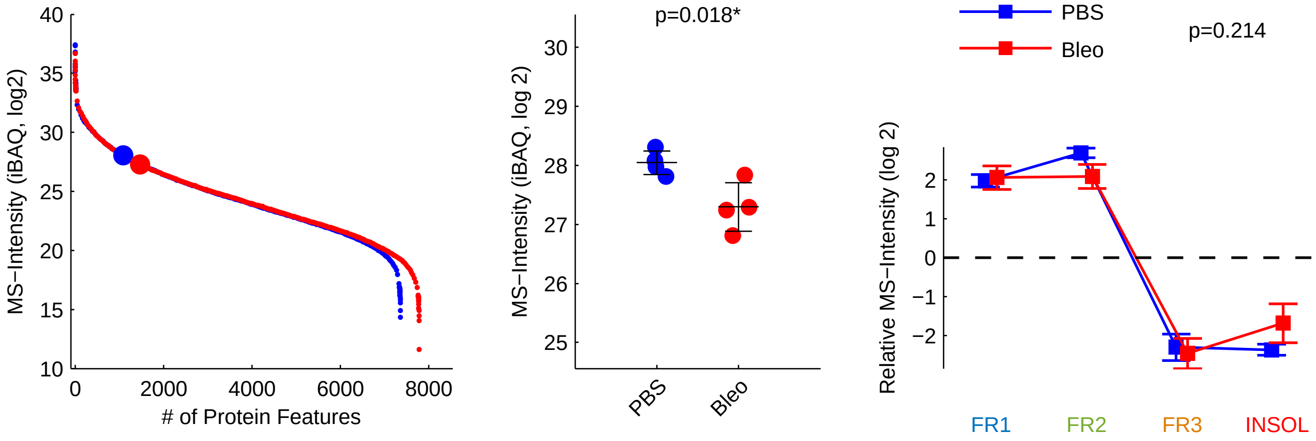

Q6PHU5 − Sort1 (id: 4999)

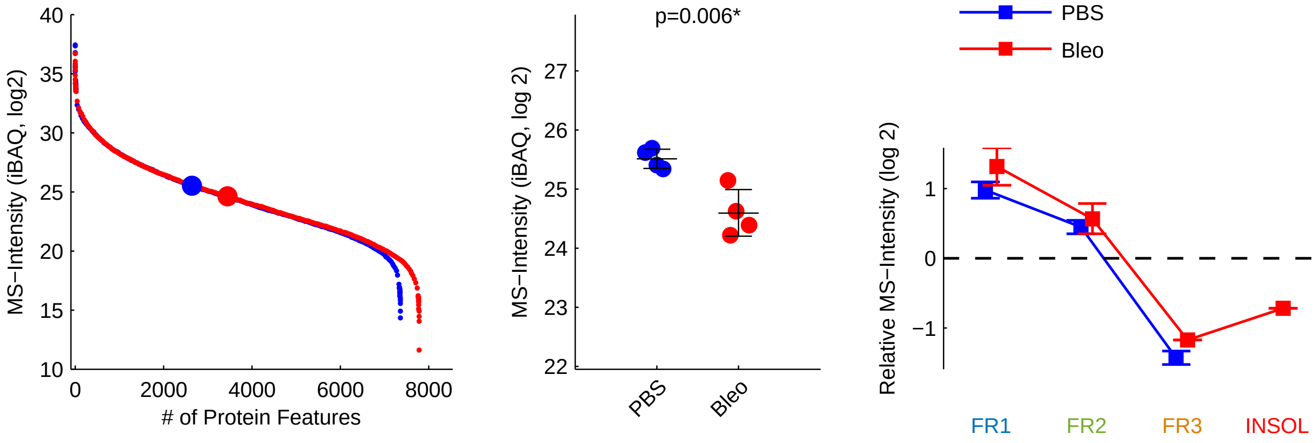

Q6PIU9 − (id: 5003)

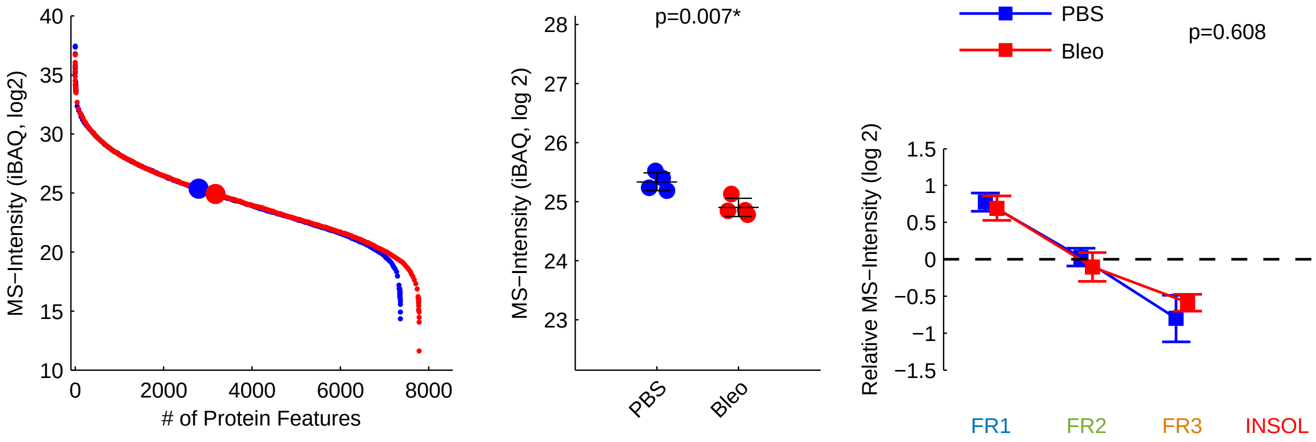

Q6Q477 − Atp2b4 (id: 5008)

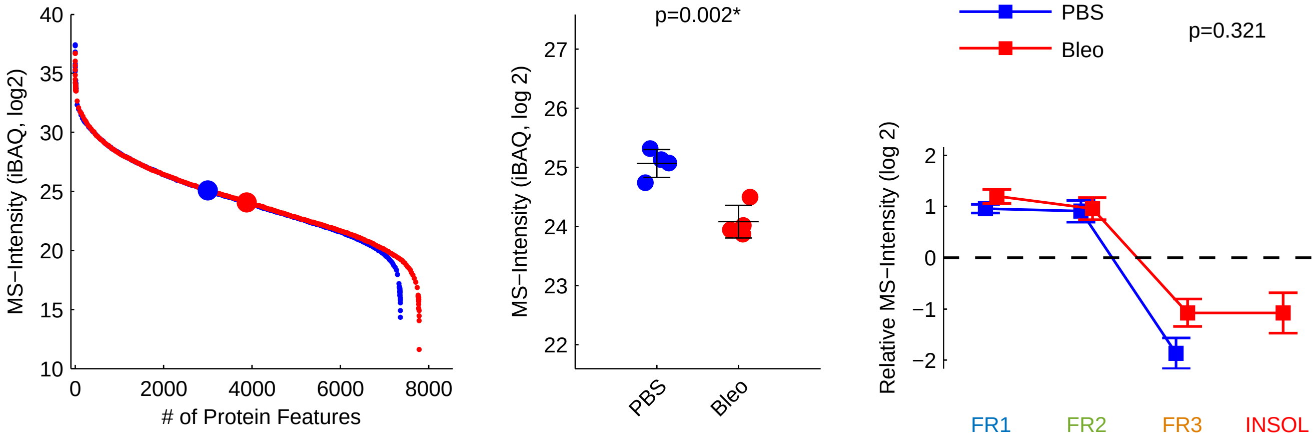

Q6R891 − Ppp1r9b (id: 5013)

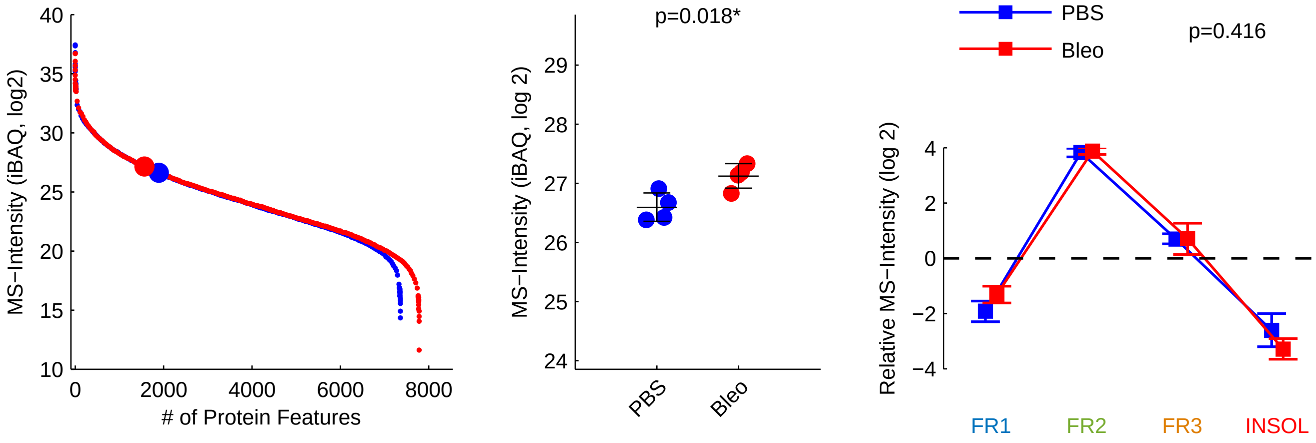

Q6RHR9 − Magi1 (id: 5014)

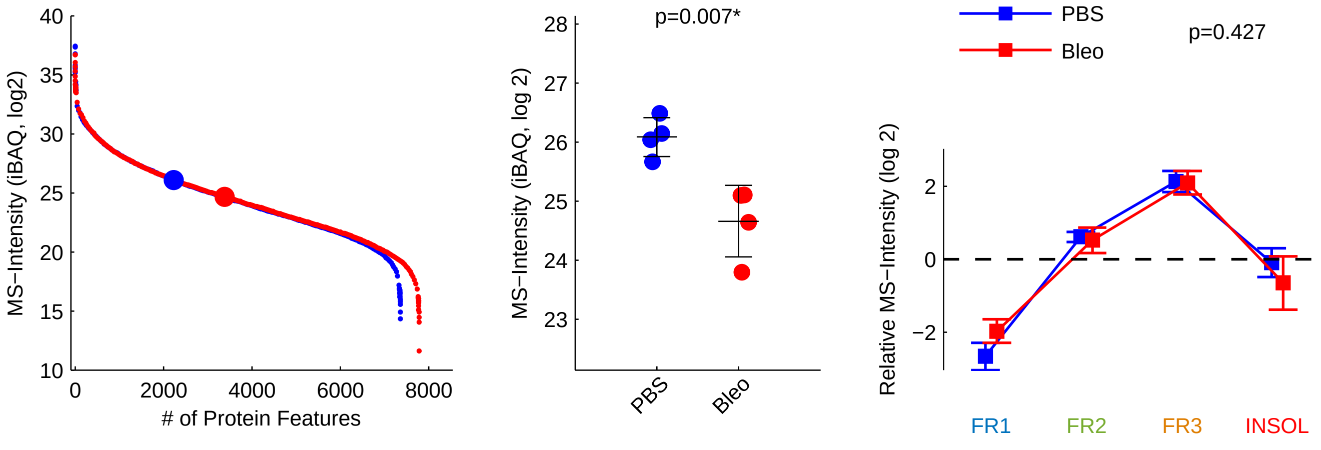

Q6S9I0 − Kng2 (id: 5016)

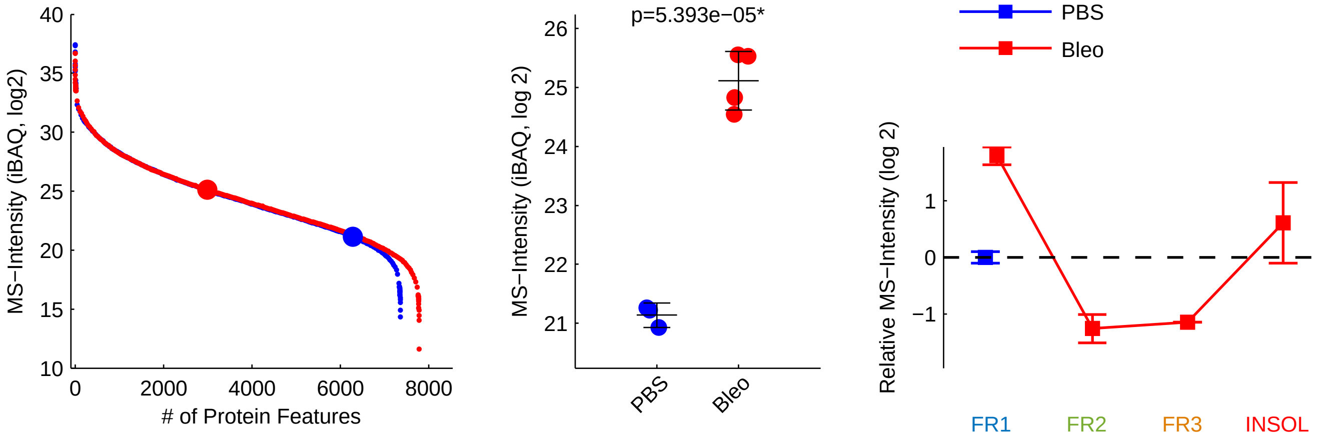

Q6VH22 − Ift172 (id: 5025)

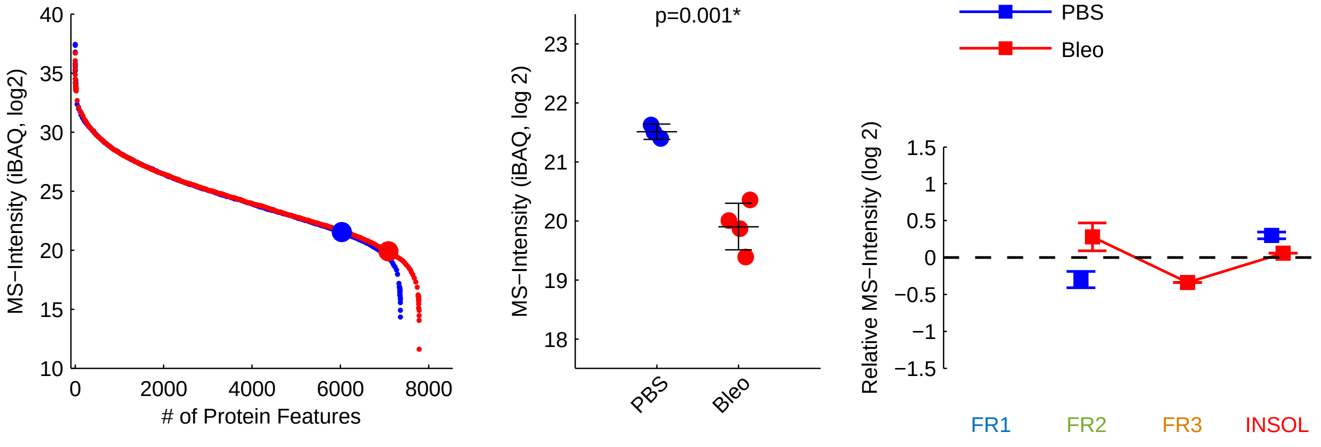

Q6WVG3 − Kctd12 (id: 5030)

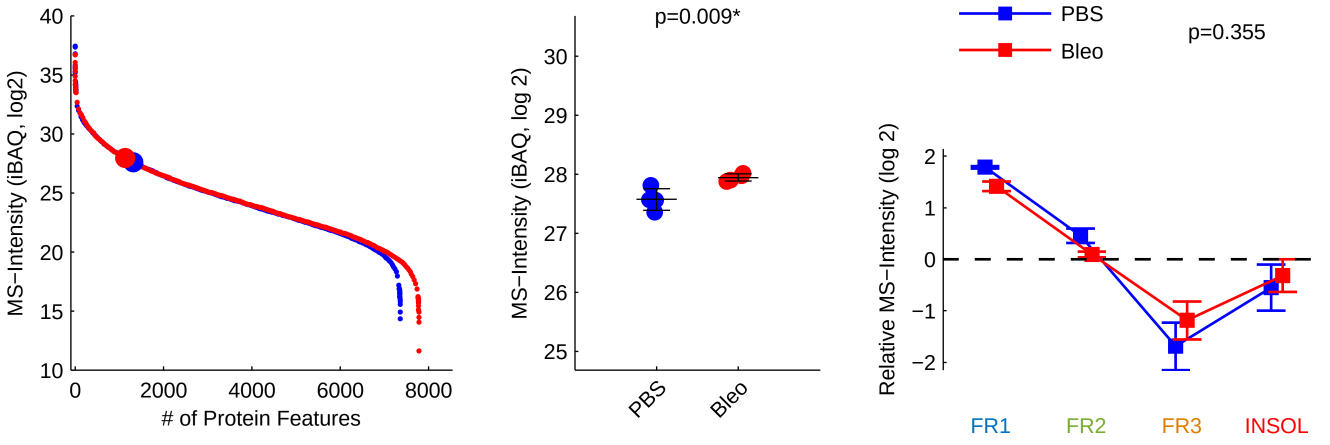

Q6ZQ03 − Fnbp4 (id: 5045)

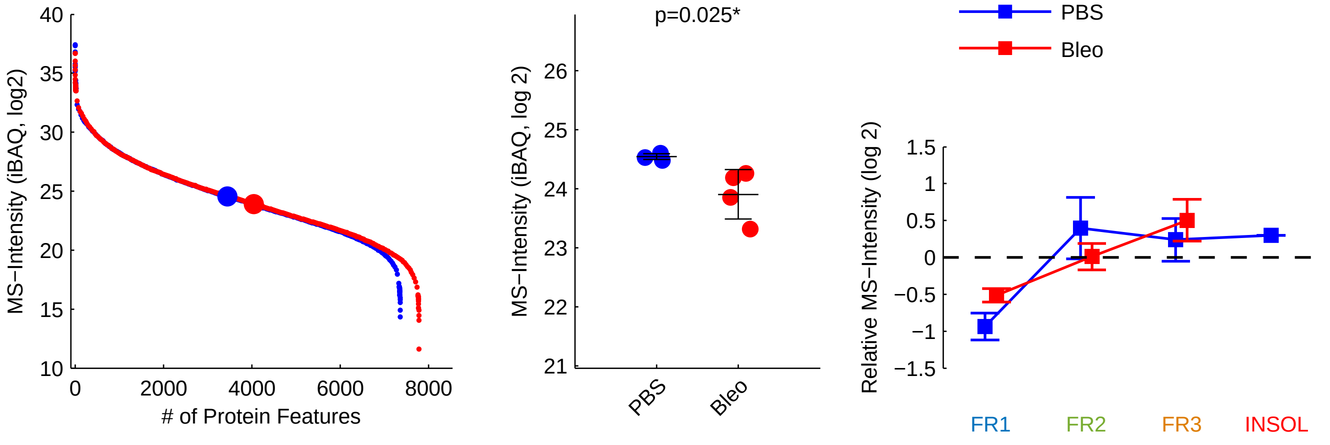

Q6ZQ08 − Cnot1 (id: 5048)

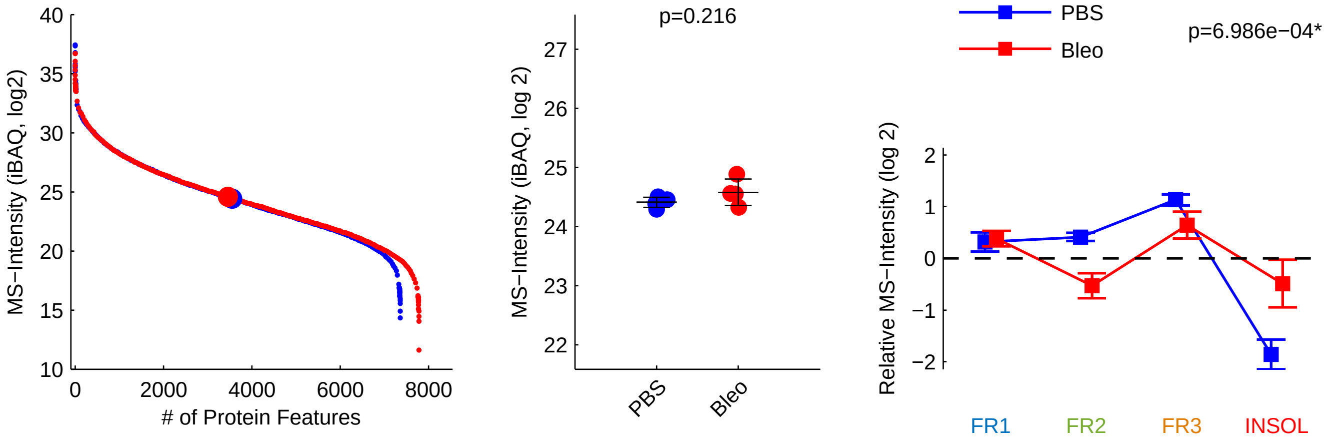

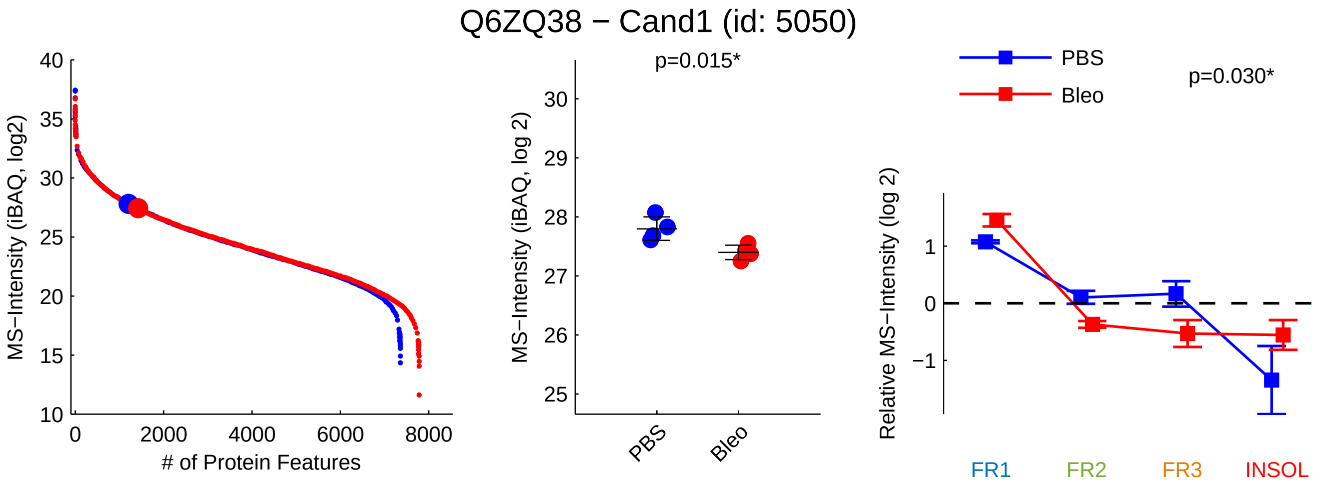
Q6ZQ38 − Cand1 (id: 5050)
MS−Intensity (iBAQ, log2)
# of Protein Features
p=0.015*
MS−Intensity (iBAQ, log 2)
PBS
Bleo
p=0.030*
Relative MS−Intensity (log 2)
FR1
FR2
FR3
INSOL

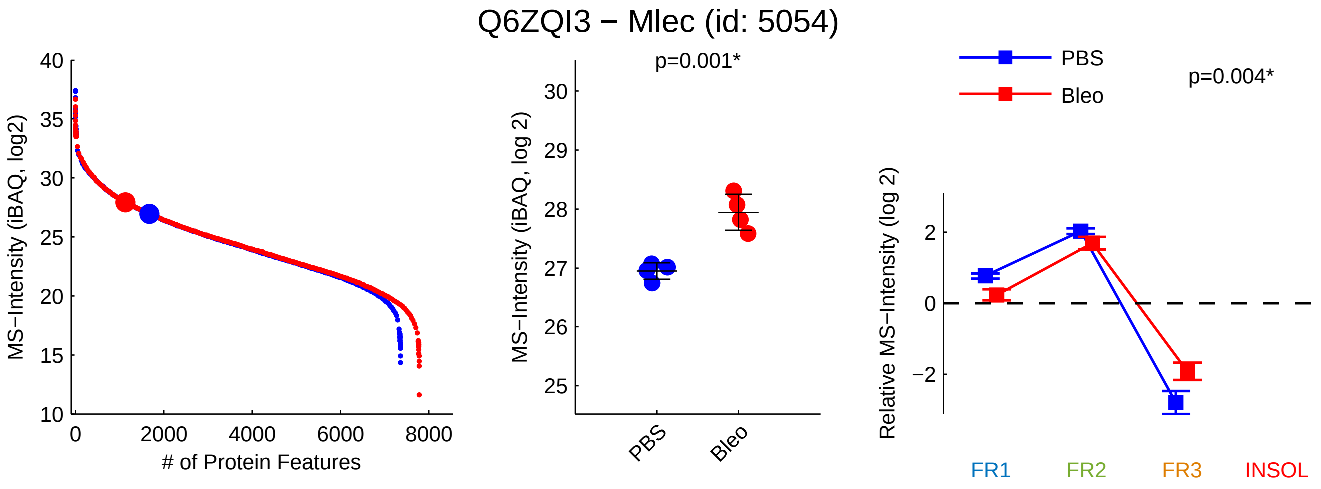
Q6ZQI3 − Mlec (id: 5054)
MS−Intensity (iBAQ, log2)
# of Protein Features
p=0.001*
MS−Intensity (iBAQ, log 2)
PBS
Bleo
p=0.004*
Relative MS−Intensity (log 2)
FR1
FR2
FR3
INSOL

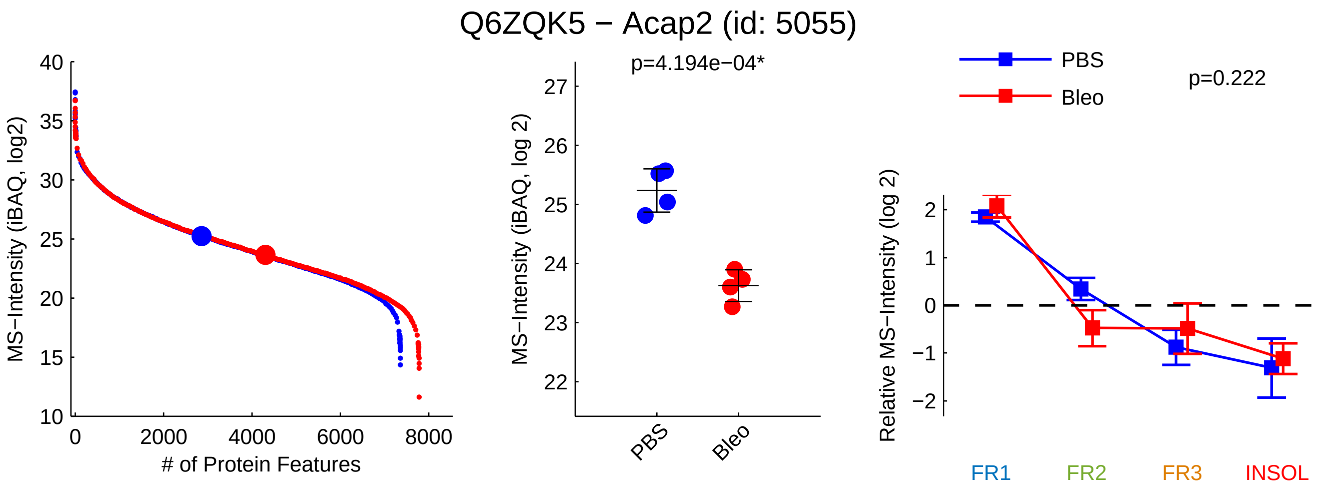
Q6ZQK5 − Acap2 (id: 5055)
MS−Intensity (iBAQ, log2)
# of Protein Features
p=4.194e−04*
MS−Intensity (iBAQ, log 2)
PBS
Bleo
p=0.222
Relative MS−Intensity (log 2)
FR1
FR2
FR3
INSOL

Q6ZQM8 − Ugt1a7c (id: 5057)

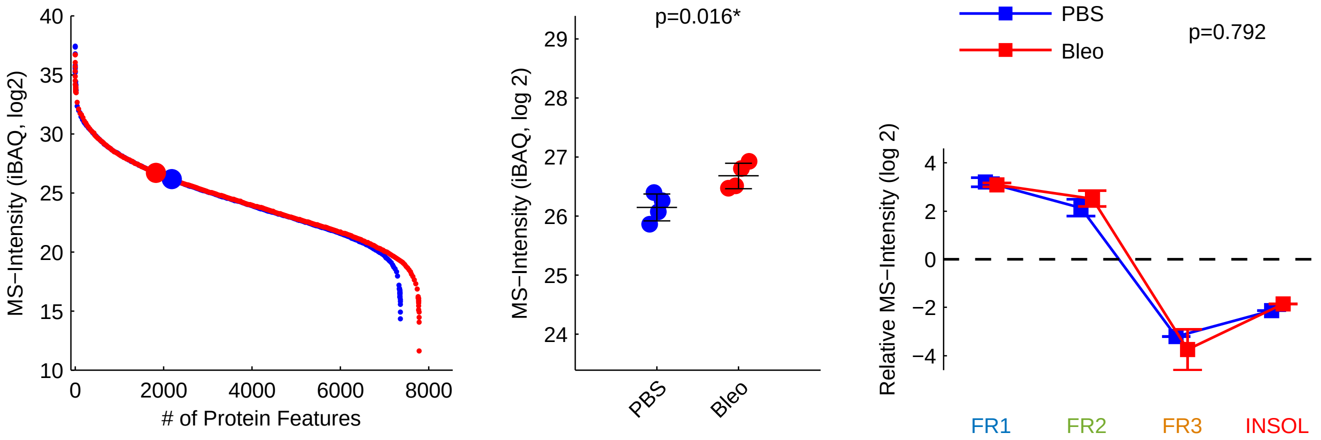

Q6ZWN5 − Rps9 (id: 5059)

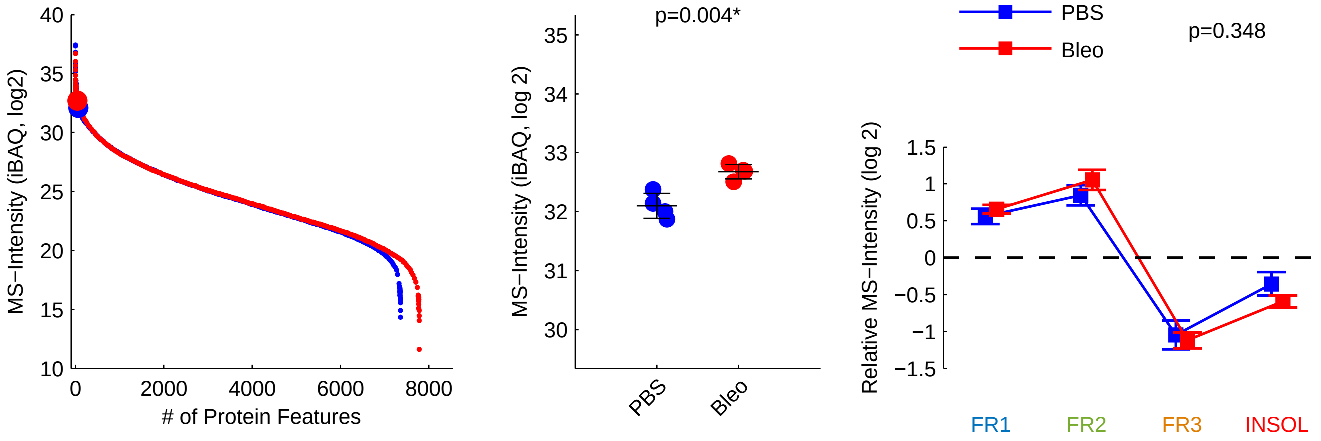

Q6ZWQ7 − Spcs3 (id: 5060)

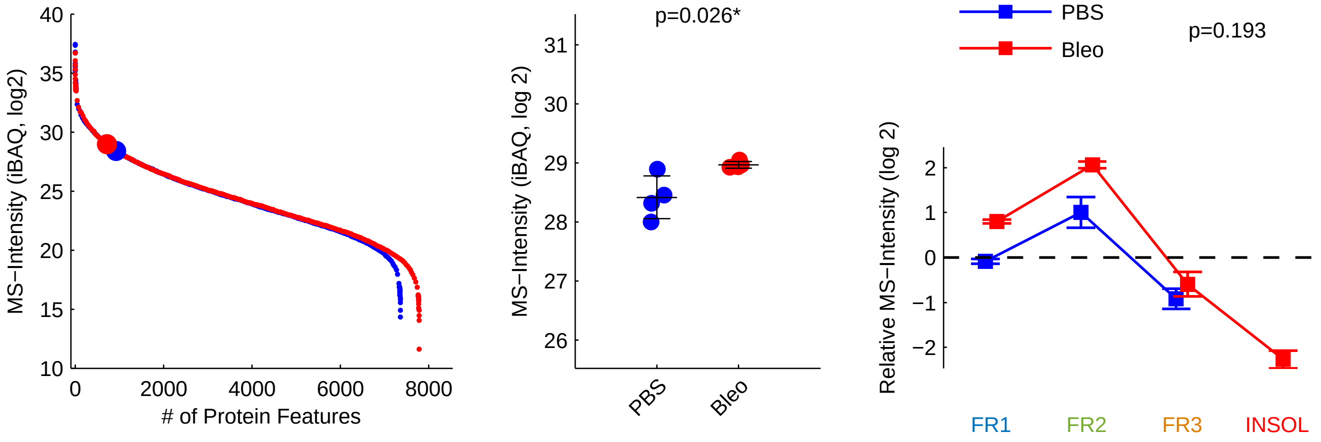

Q6ZWQ9 − Myl12a (id: 5061)

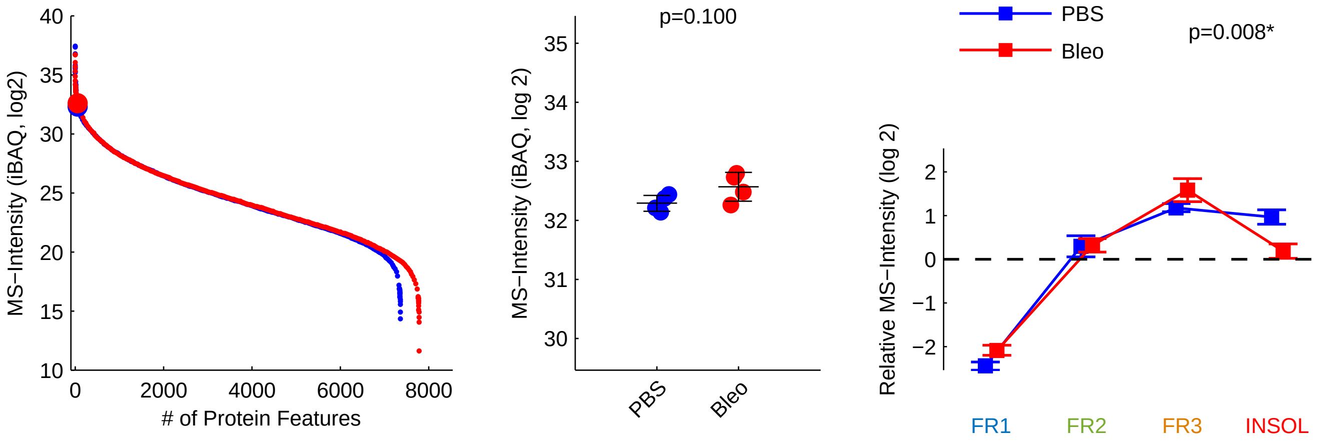

Q6ZWR6 − Syne1 (id: 5062)

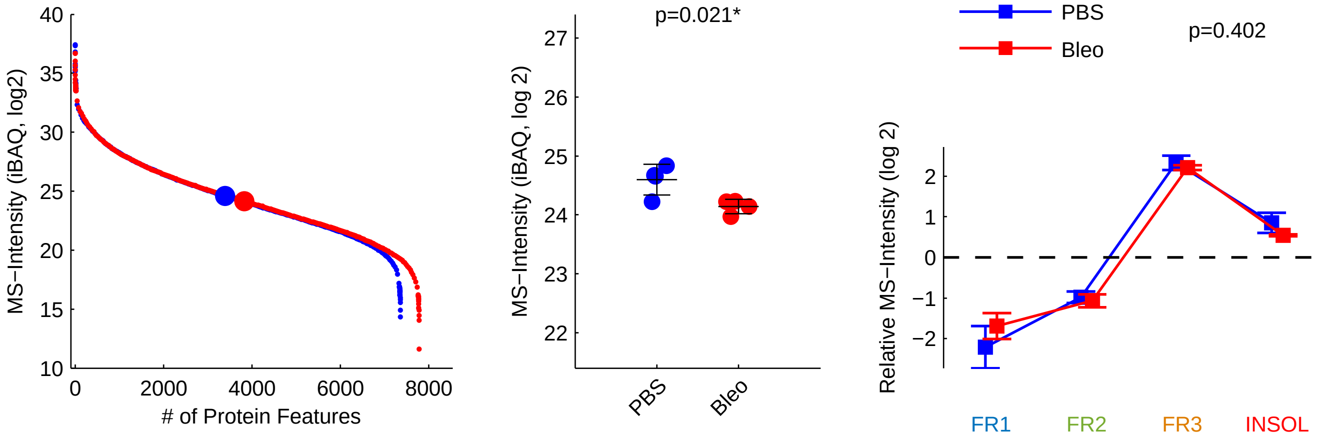

Q6ZWU9 − Rps27 (id: 5064)

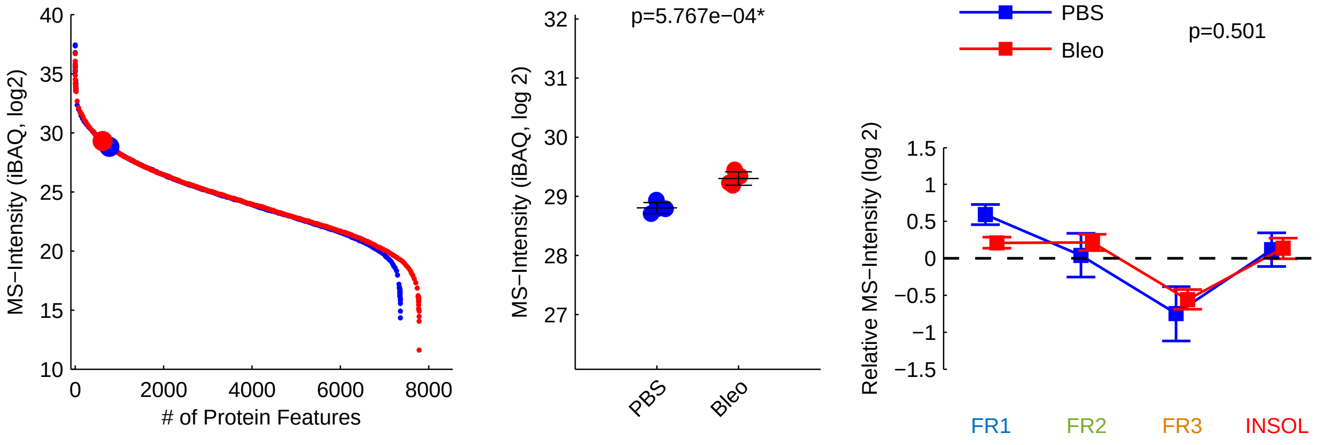

Q6ZWV3 − Rpl10 (id: 5065)

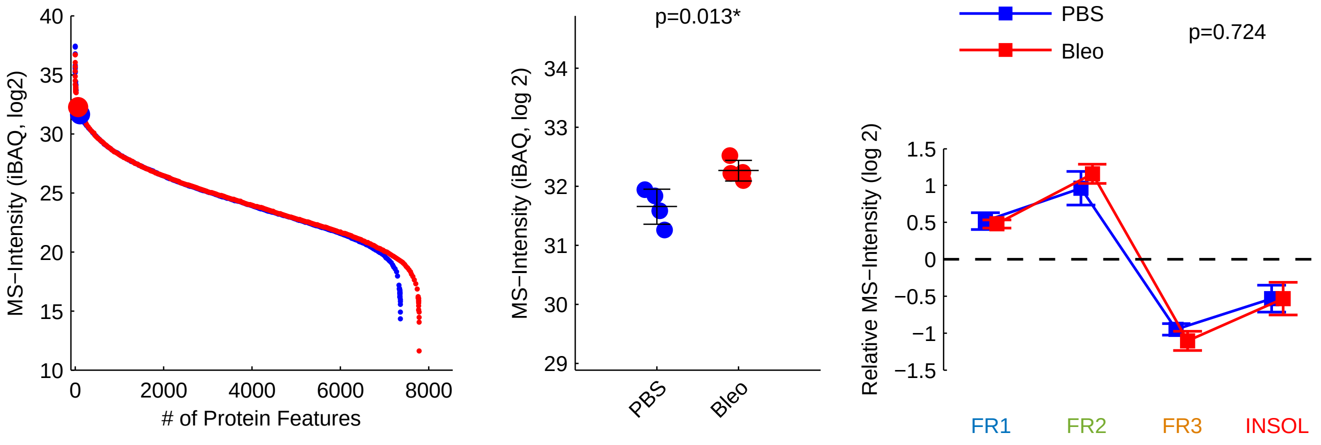

Q6ZWV7 − Rpl35 (id: 5066)

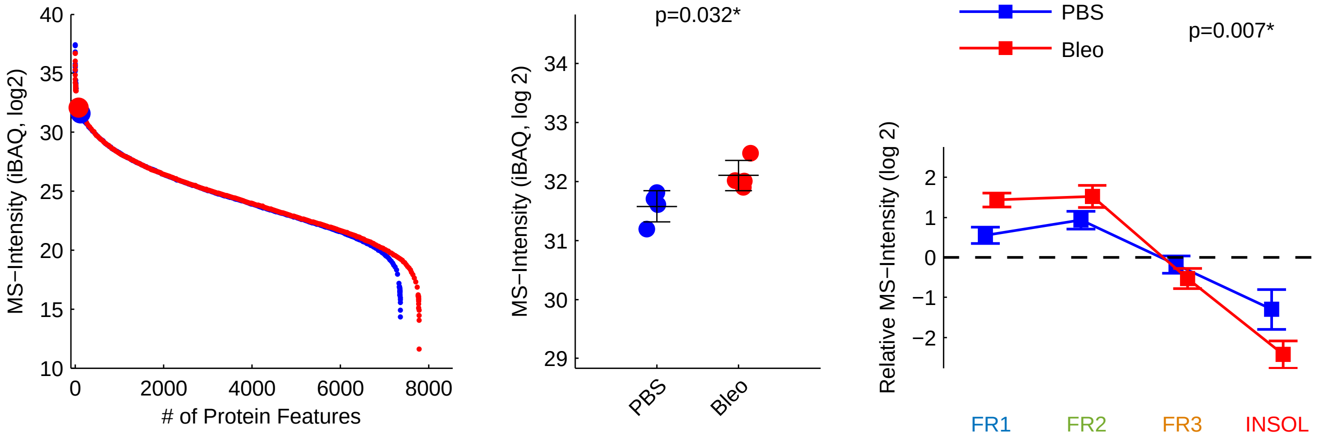

Q6ZWX6 − Eif2s1 (id: 5067)

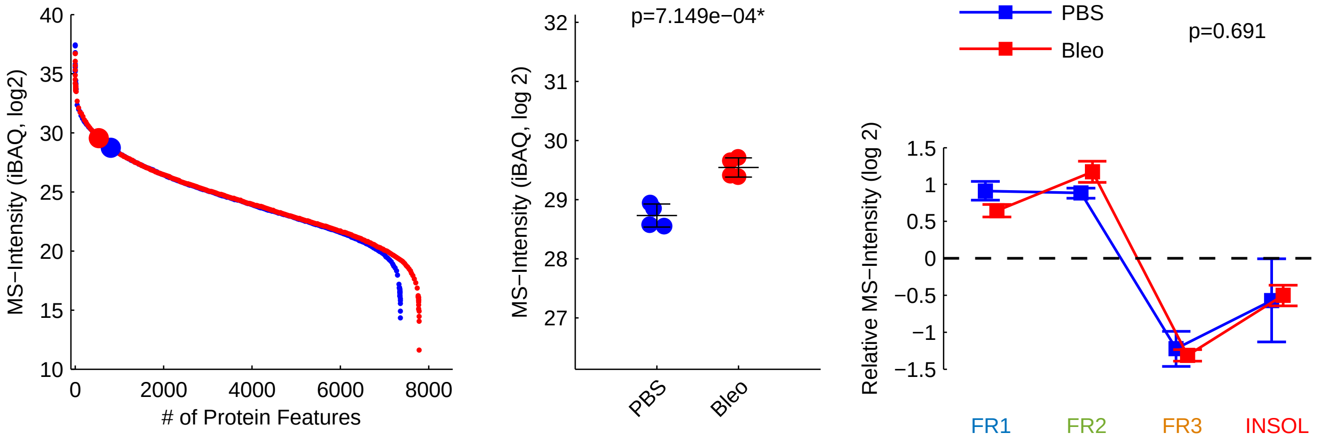

Q6ZWY3 − Rps27l (id: 5068)

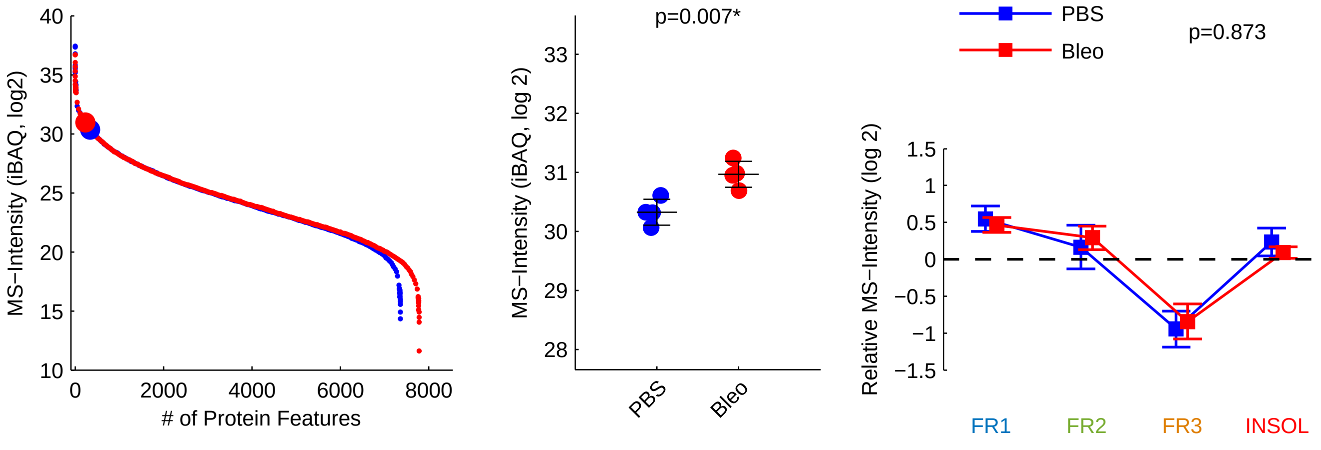

Q6ZWY8 − Tmsb10 (id: 5069)

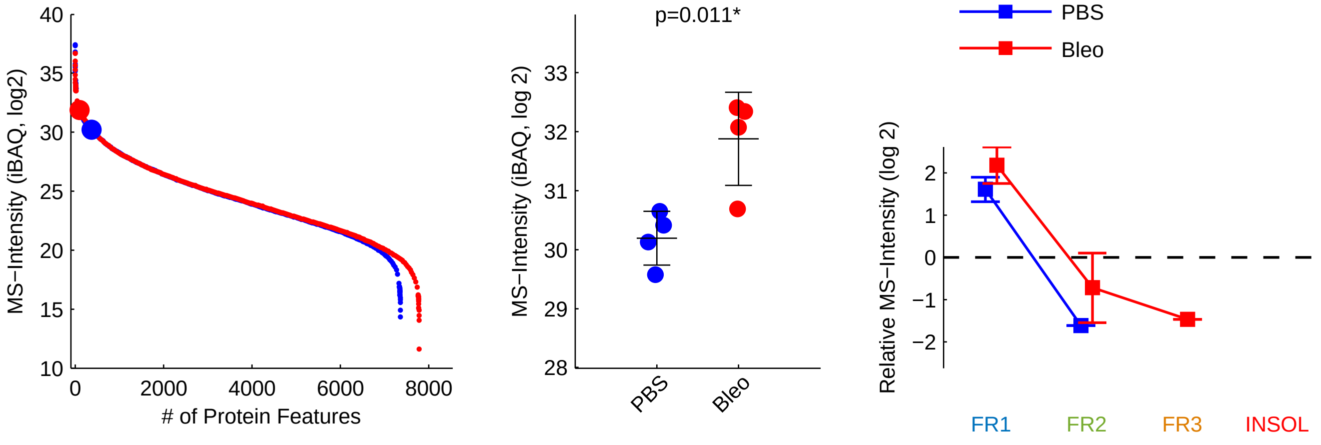

Q6ZWZ6 − Rps12 (id: 5071)

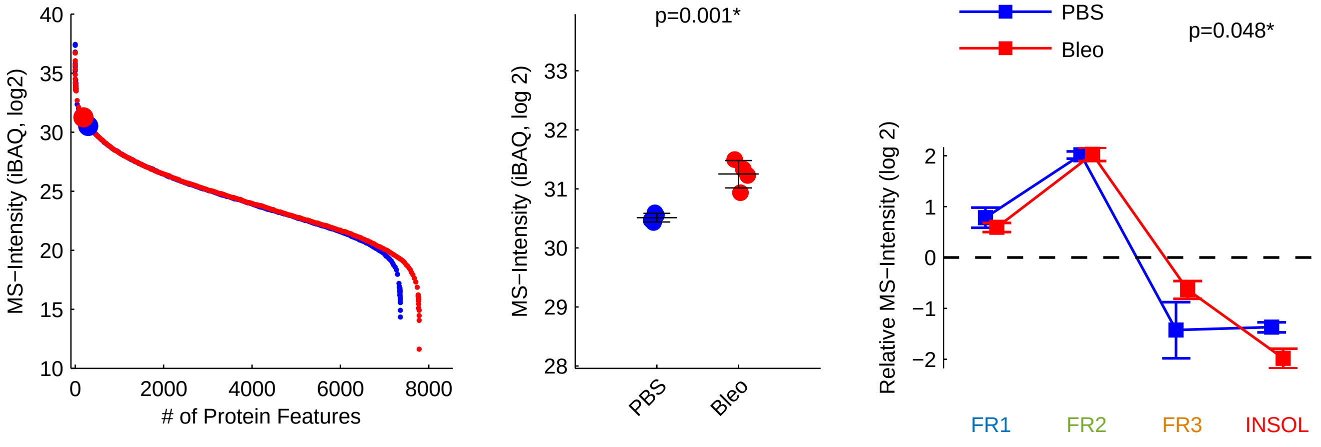

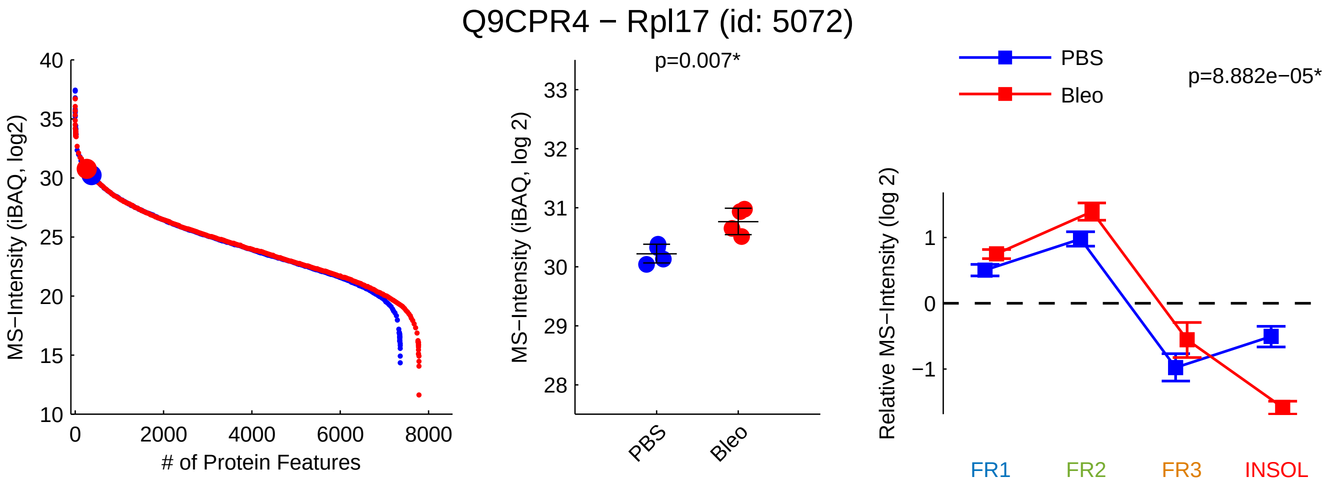

Q9CPR4 − Rpl17 (id: 5072)
MS−Intensity (iBAQ, log2)
# of Protein Features
p=0.007*
MS−Intensity (iBAQ, log 2)
PBS
Bleo
p=8.882e−05*
Relative MS−Intensity (log 2)
FR1
FR2
FR3
INSOL

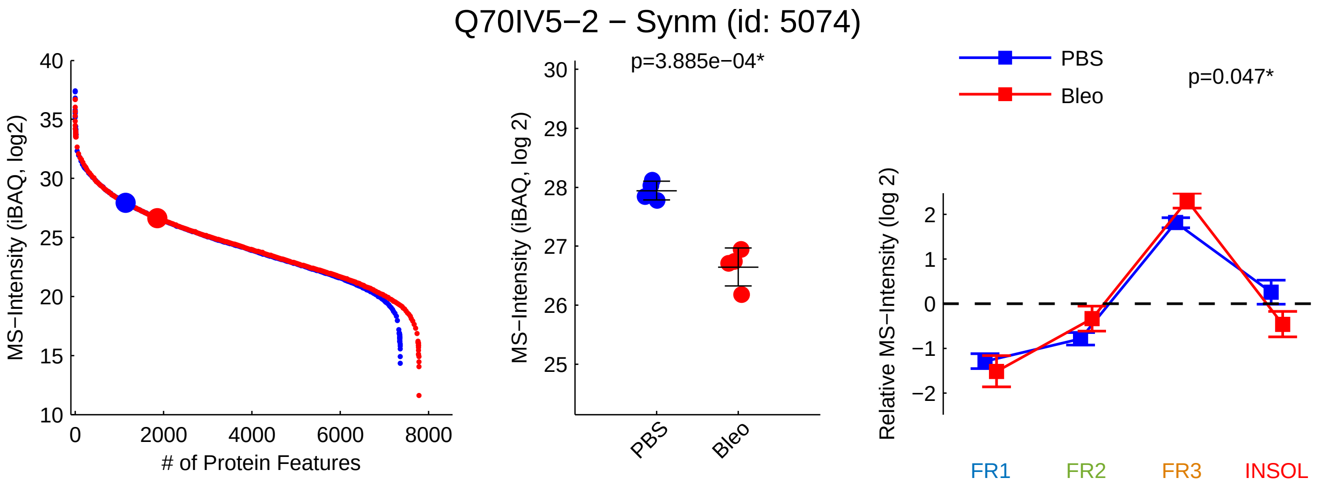

Q70IV5−2 − Synm (id: 5074)
MS−Intensity (iBAQ, log2)
# of Protein Features
p=3.885e−04*
MS−Intensity (iBAQ, log 2)
PBS
Bleo
p=0.047*
Relative MS−Intensity (log 2)
FR1
FR2
FR3
INSOL

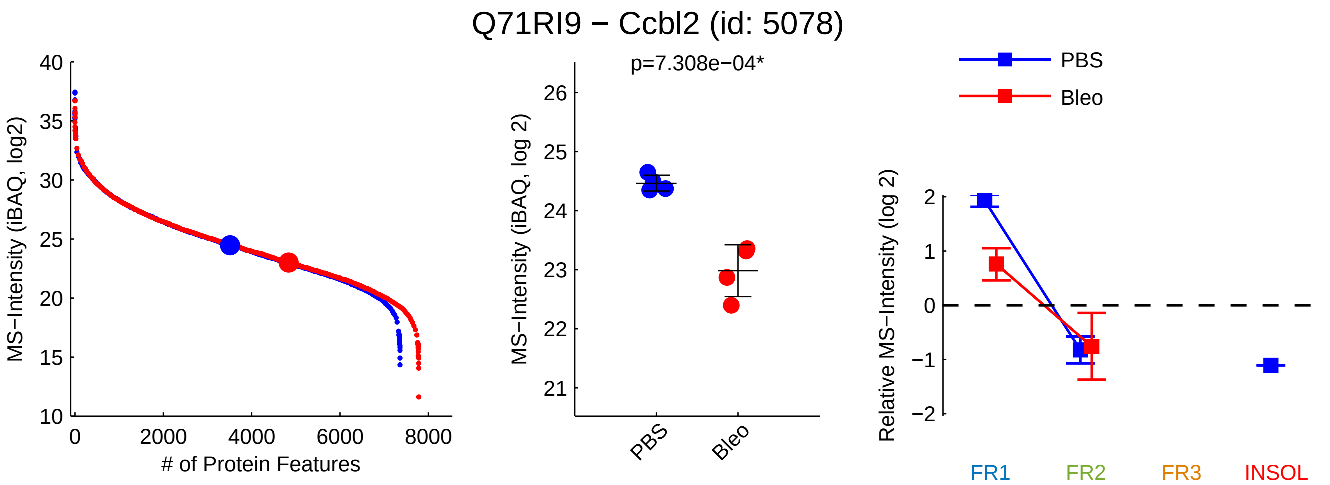

Q71RI9 − Ccbl2 (id: 5078)
MS−Intensity (iBAQ, log2)
# of Protein Features
p=7.308e−04*
MS−Intensity (iBAQ, log 2)
PBS
Bleo
Relative MS−Intensity (log 2)
FR1
FR2
FR3
INSOL

Q75N62 − Gimap8 (id: 5079)

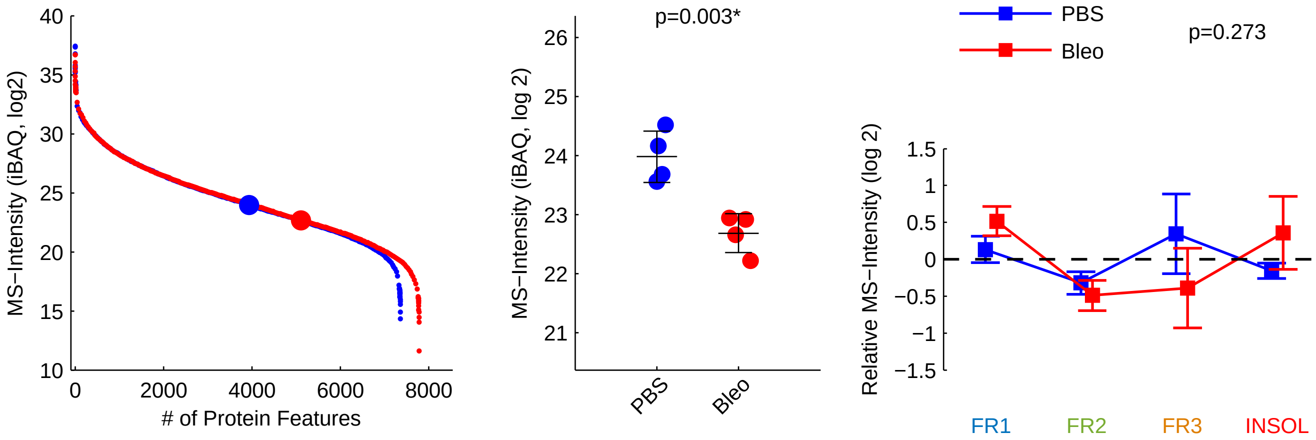

Q76MZ3 − Ppp2r1a (id: 5084)

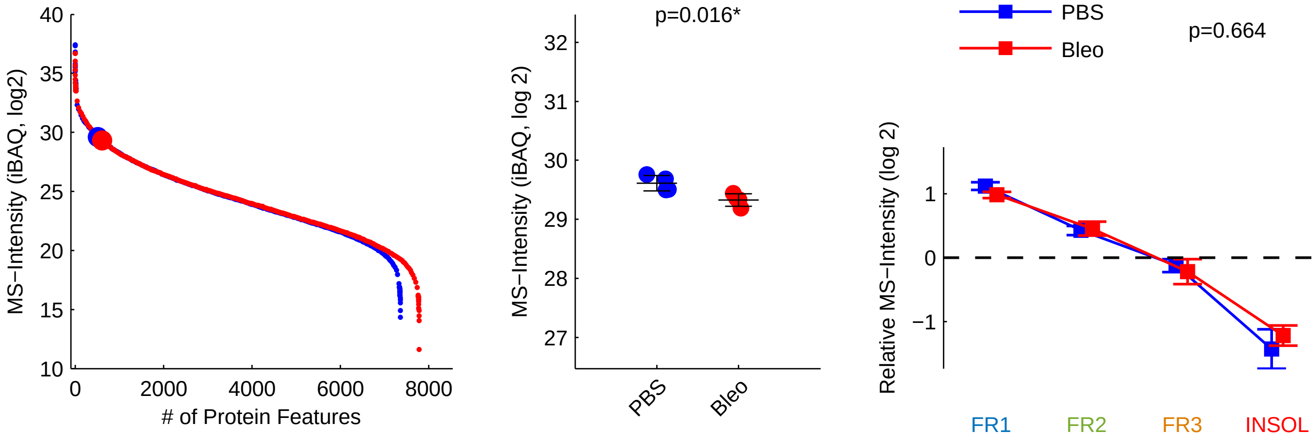

Q78IQ7 − Slc39a4 (id: 5089)

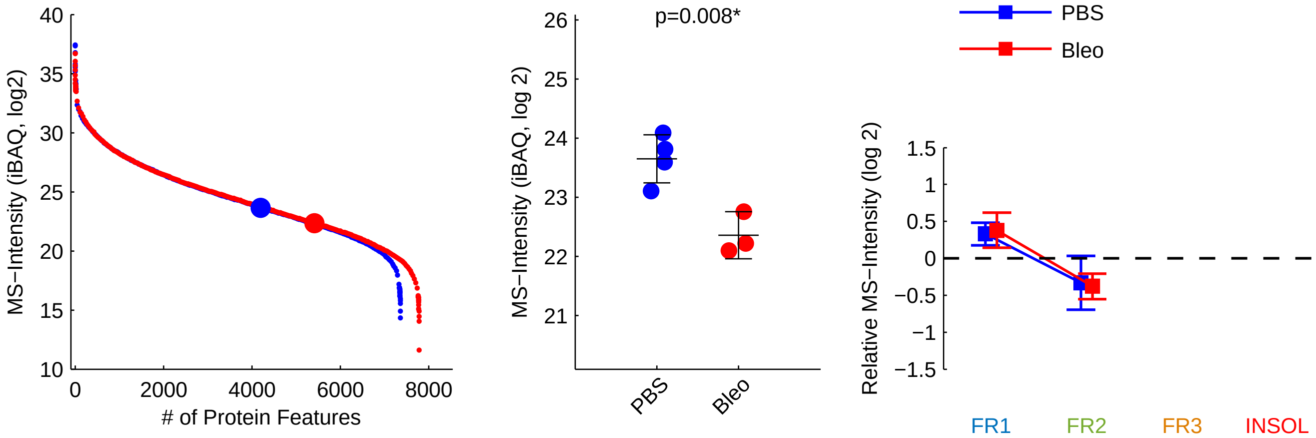

Q78IS1 − Tmed3 (id: 5090)

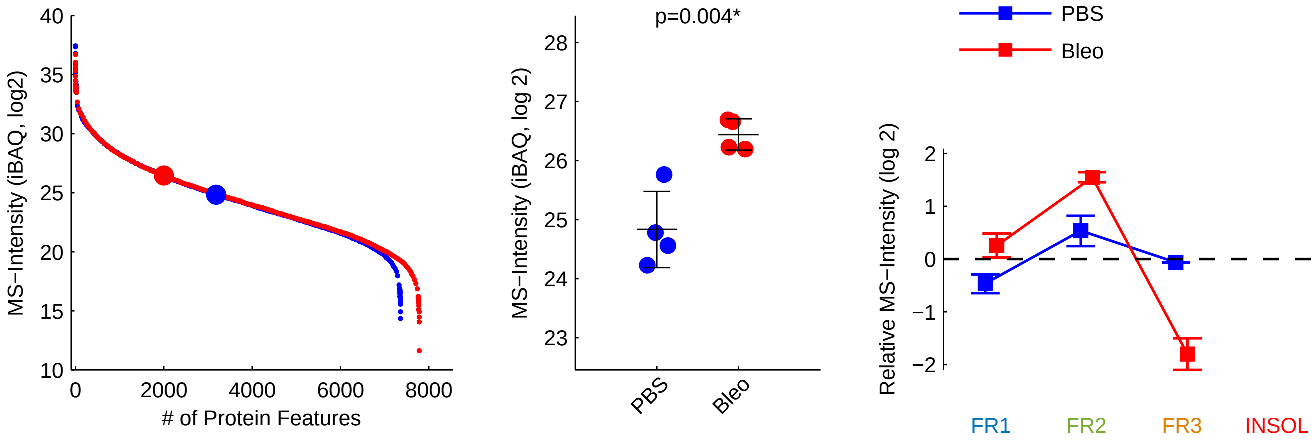

Q78PG9 − Ccdc25 (id: 5095)

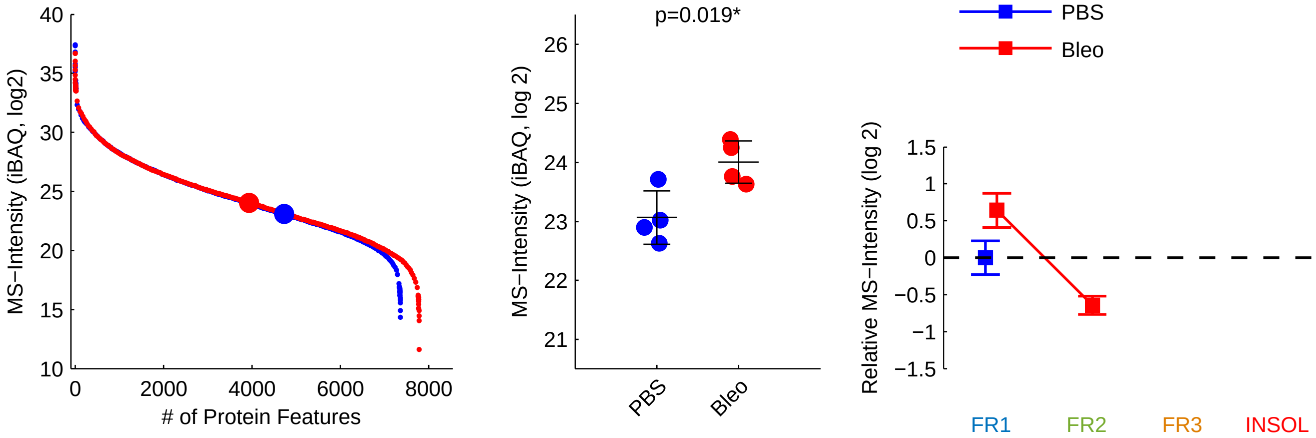

Q78PY7 − Snd1 (id: 5096)

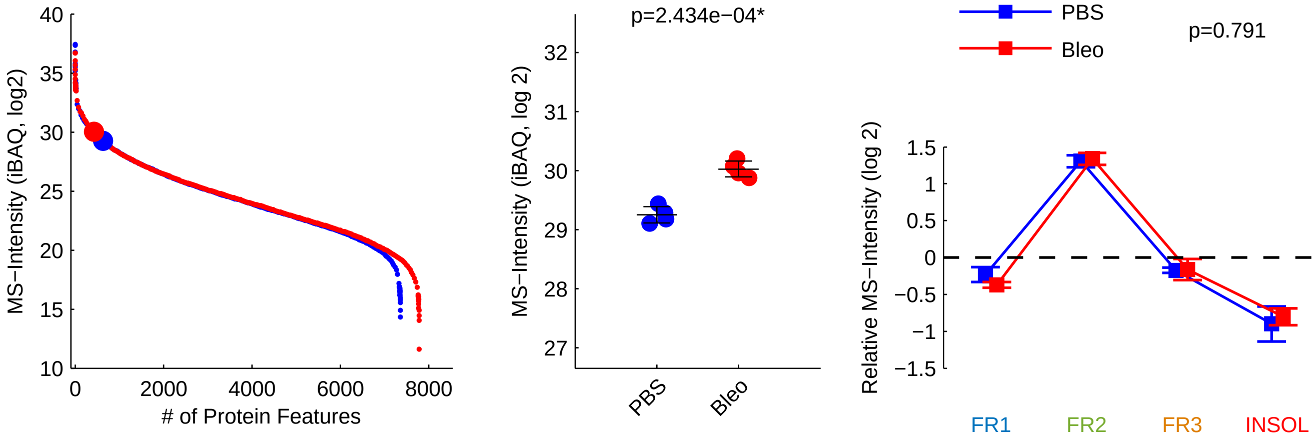

Q78XF5 − Ostc (id: 5097)

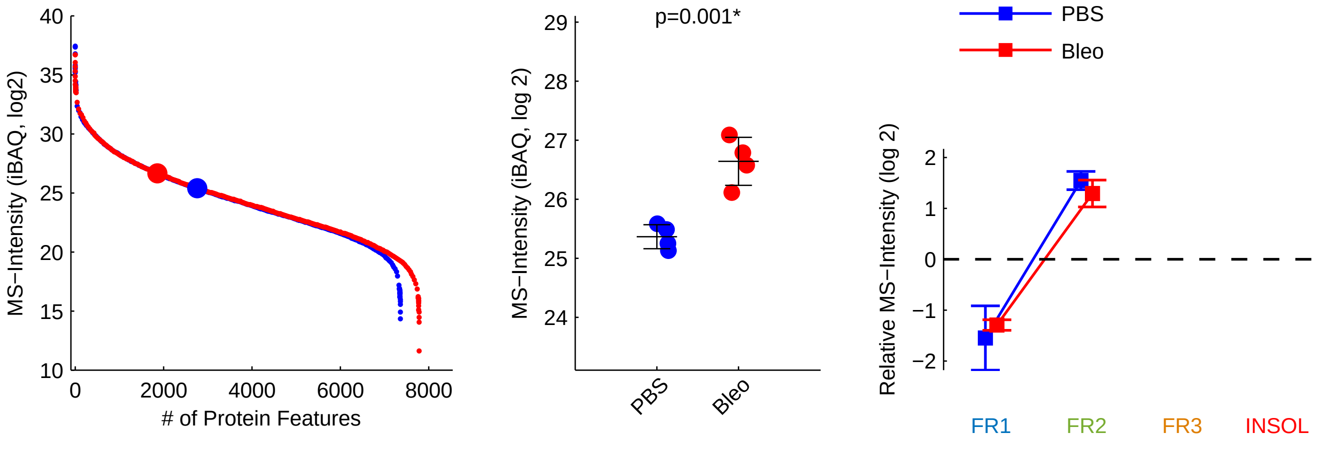

Q791T5 − Mtch1 (id: 5103)

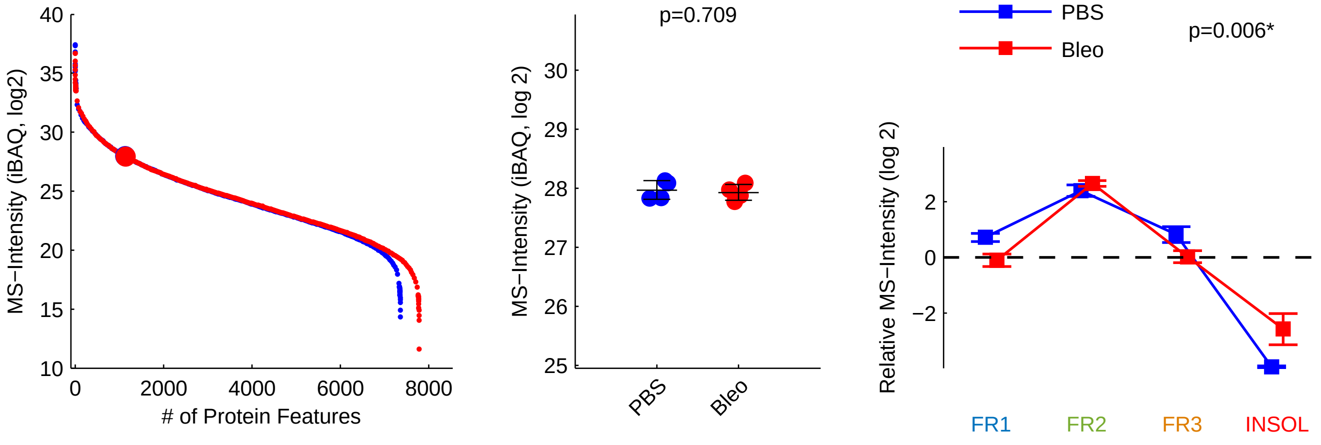

Q7JJ13 − Brd2 (id: 5105)

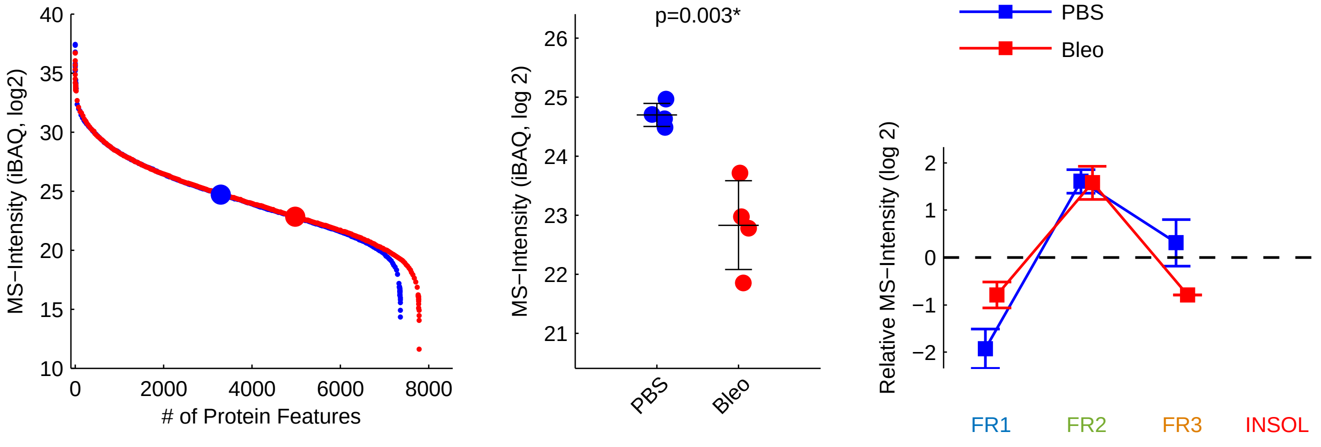

Q7TMB8 − Cyfip1 (id: 5111)

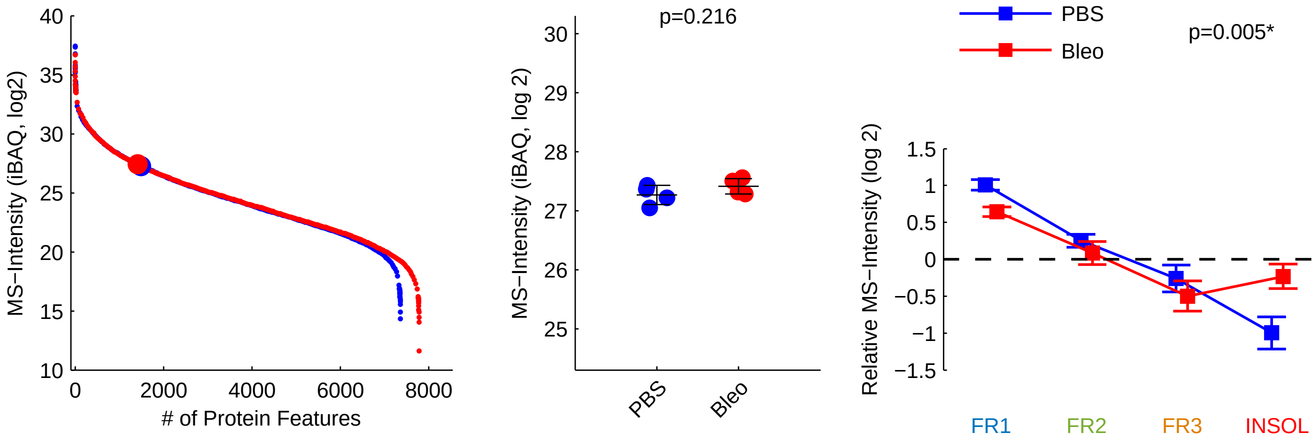

Q7TMK9−2 − Syncrip (id: 5118)

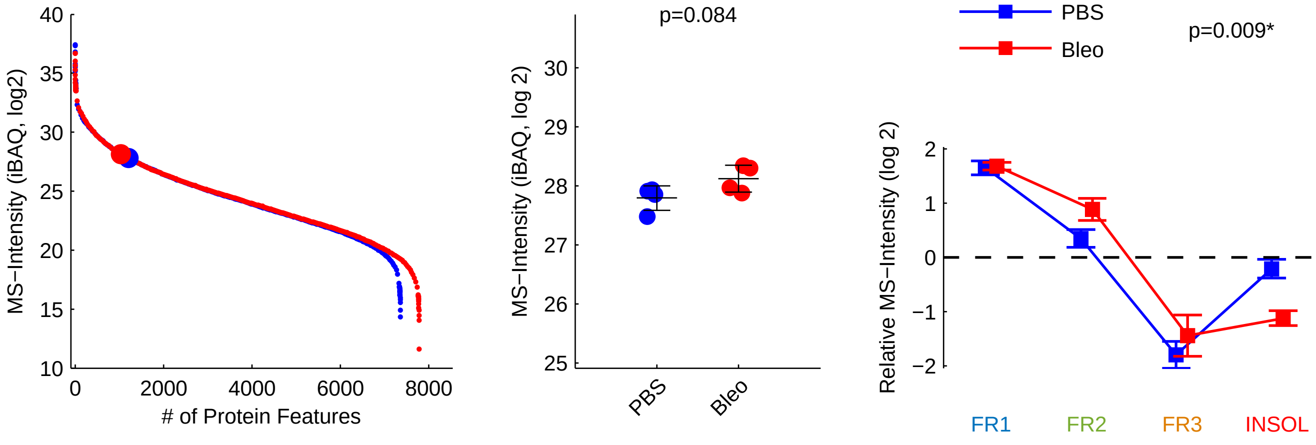

Q7TMR0 − Prcp (id: 5121)

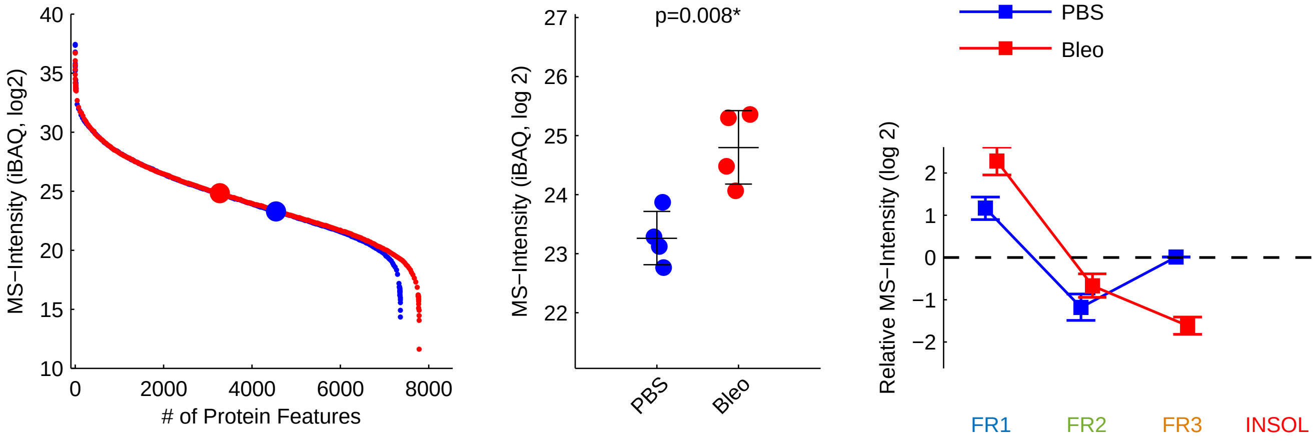

Q7TN29 − Smap2 (id: 5126)

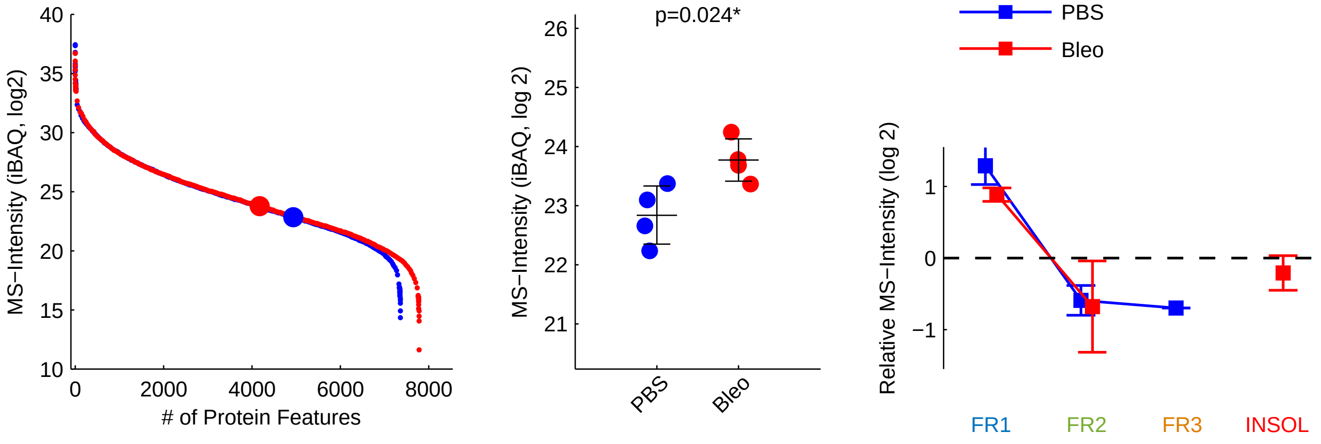

Q7TNC9 − Inpp5a (id: 5129)

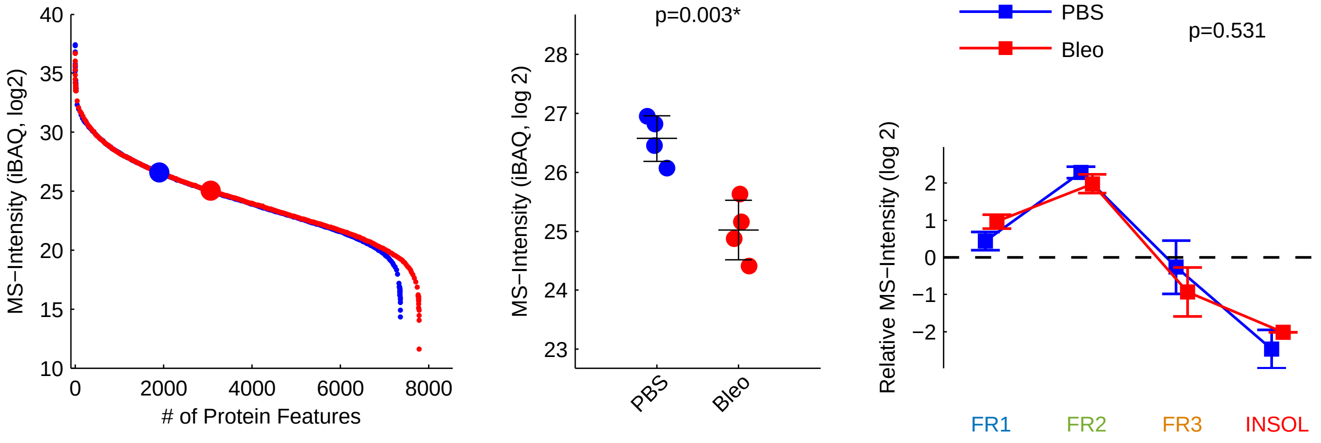

Q7TPD0 − Ints3 (id: 5137)

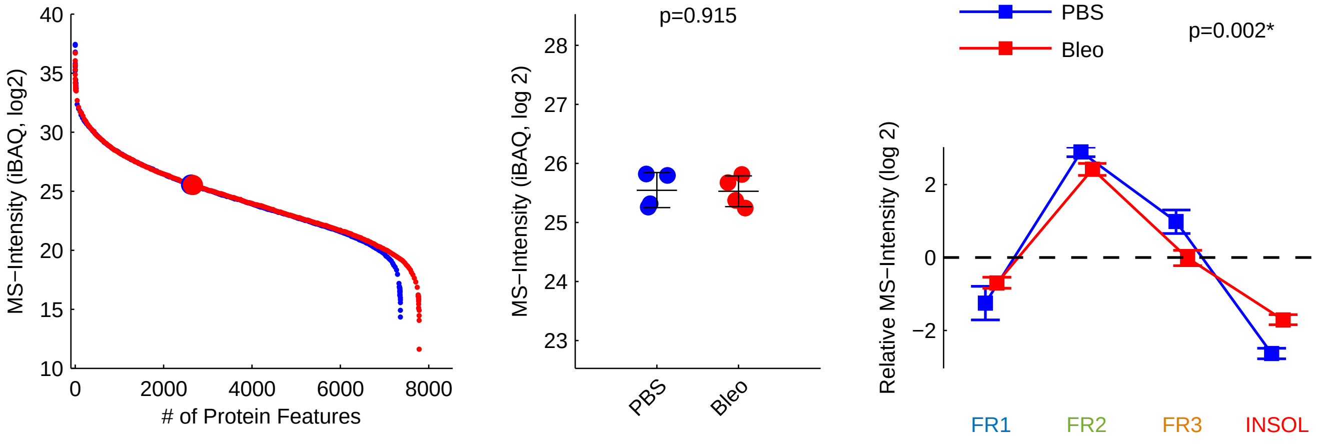

Q7TPM9 − Mtmr10 (id: 5140)

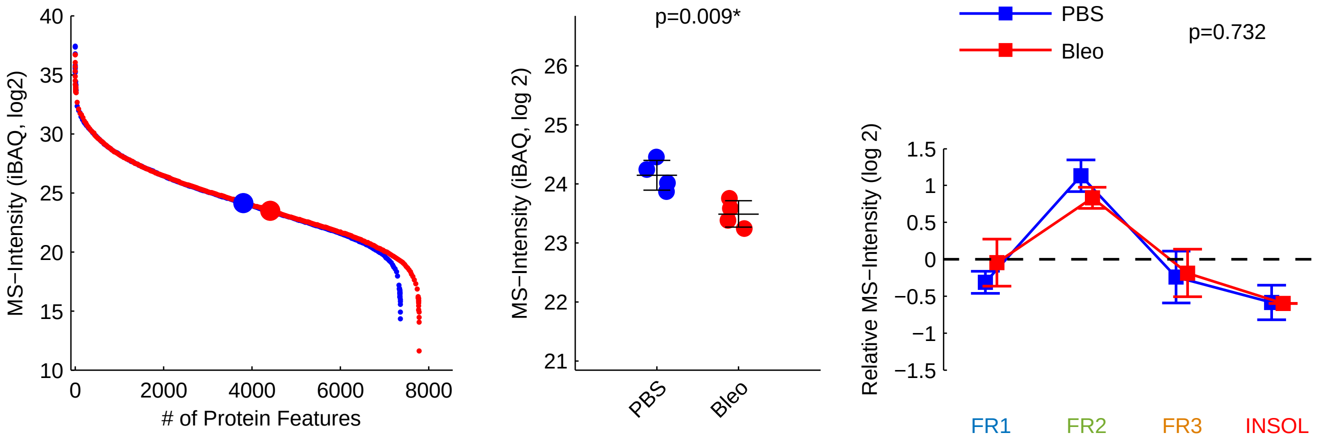

Q7TPN9 − Prr14 (id: 5141)

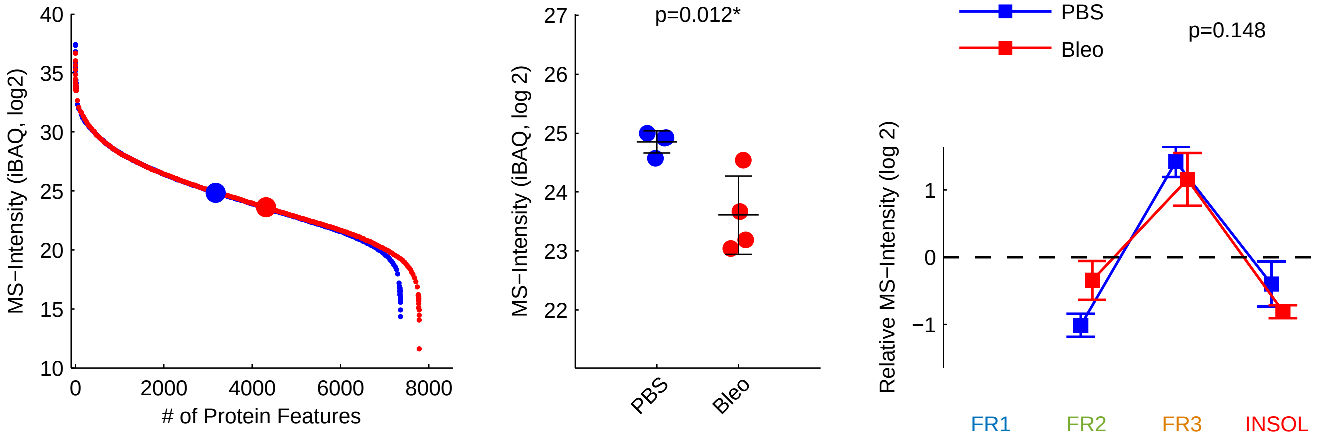

Q7TPQ3 − Shprh (id: 5142)

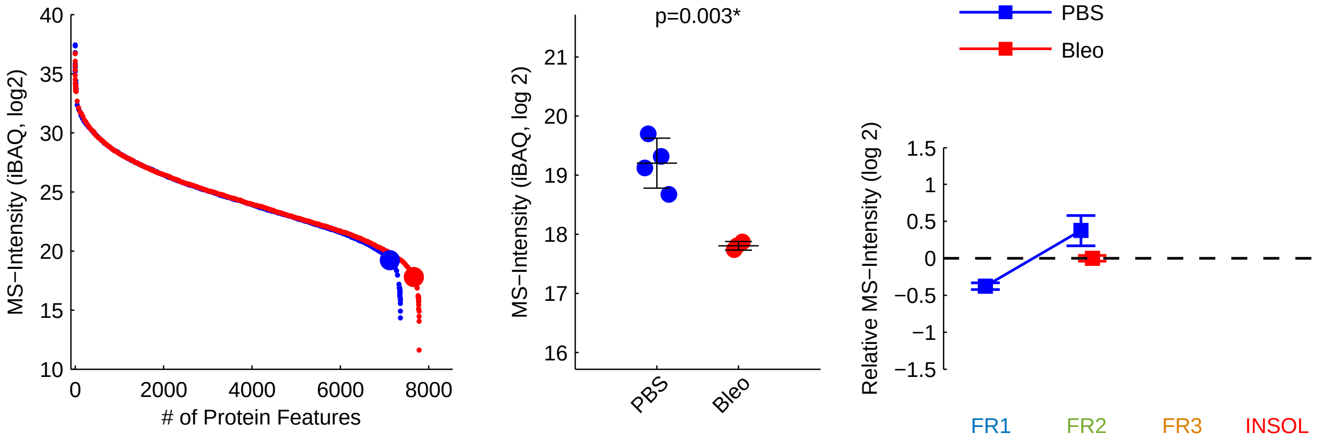

Q7TPR4 − Actn1 (id: 5143)

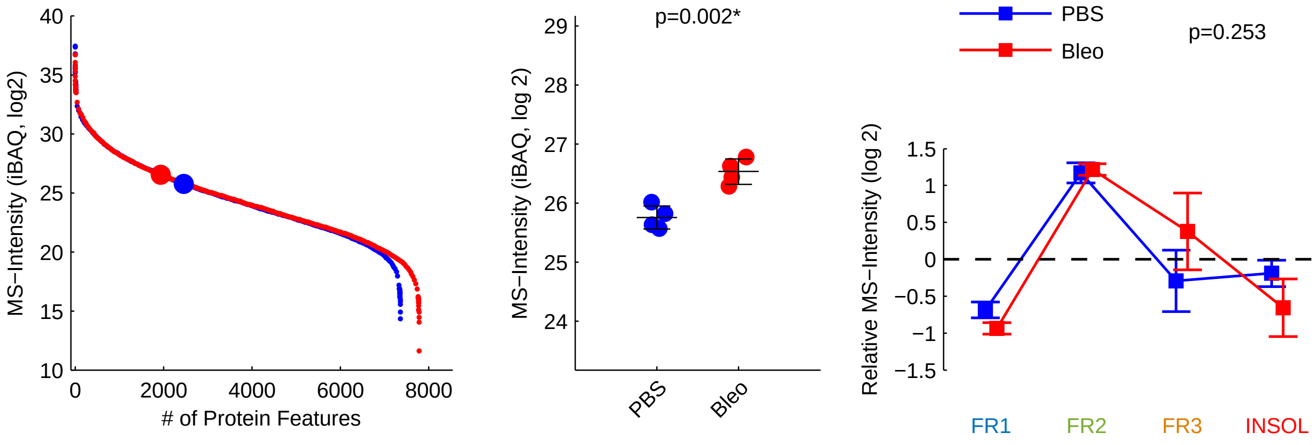

Q7TPV4 − Mybbp1a (id: 5145)

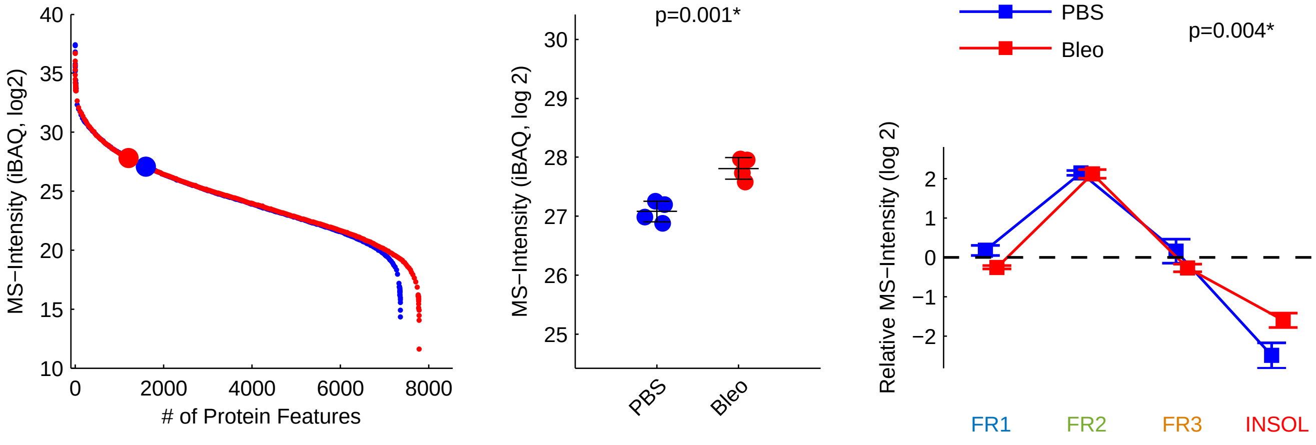

Q7TPW1 − Nexn (id: 5146)

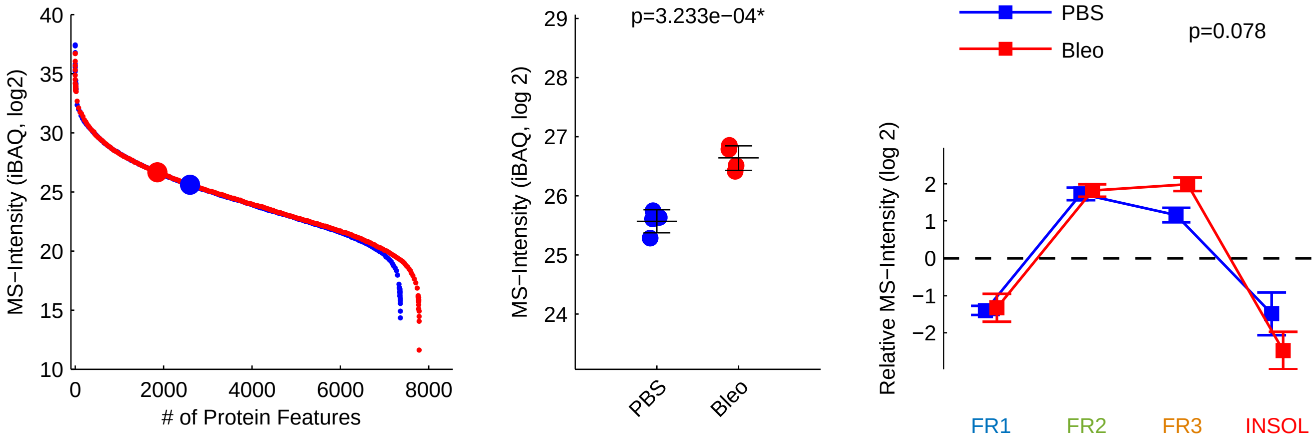

Q7TQ62 − Podn (id: 5148)

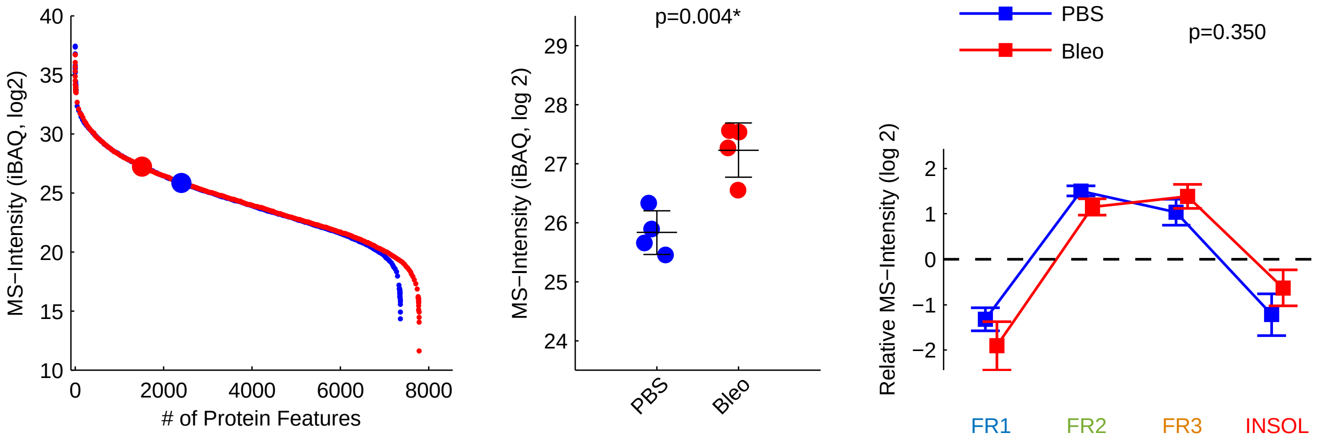

Q7TQD2 − Tppp (id: 5150)

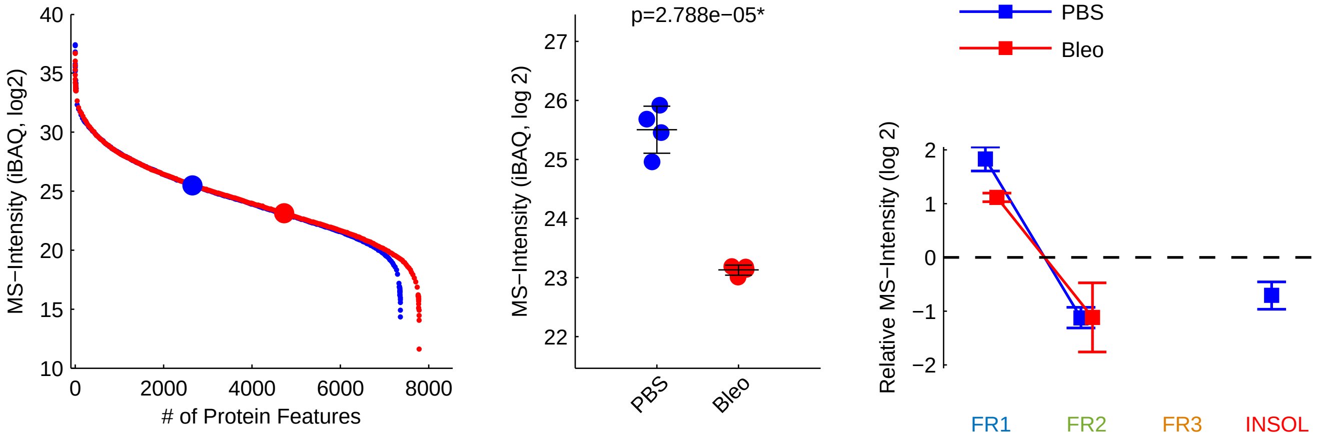

Q7TQF7 − Amph (id: 5152)

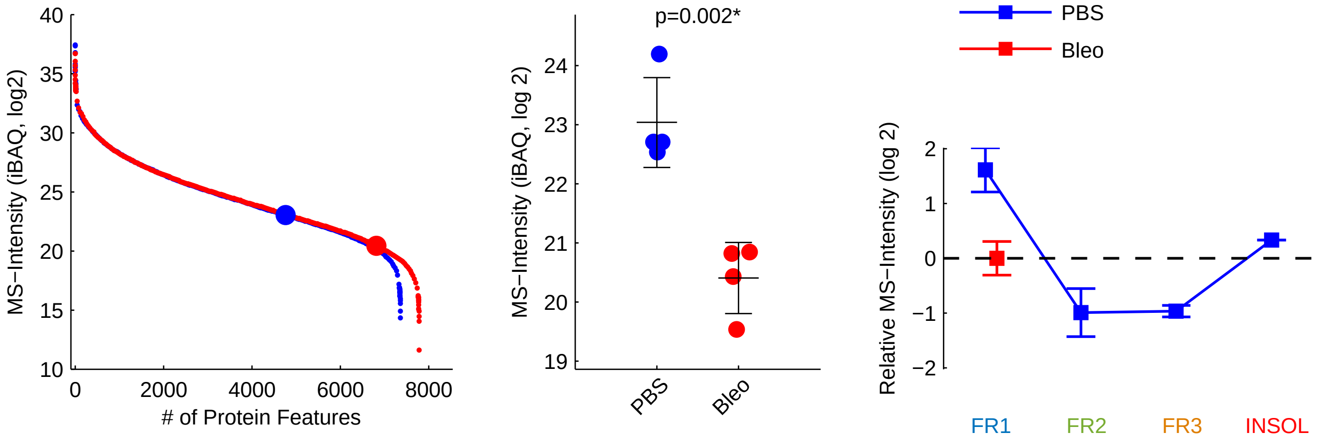

Q7TQH0 − Atxn2l (id: 5153)

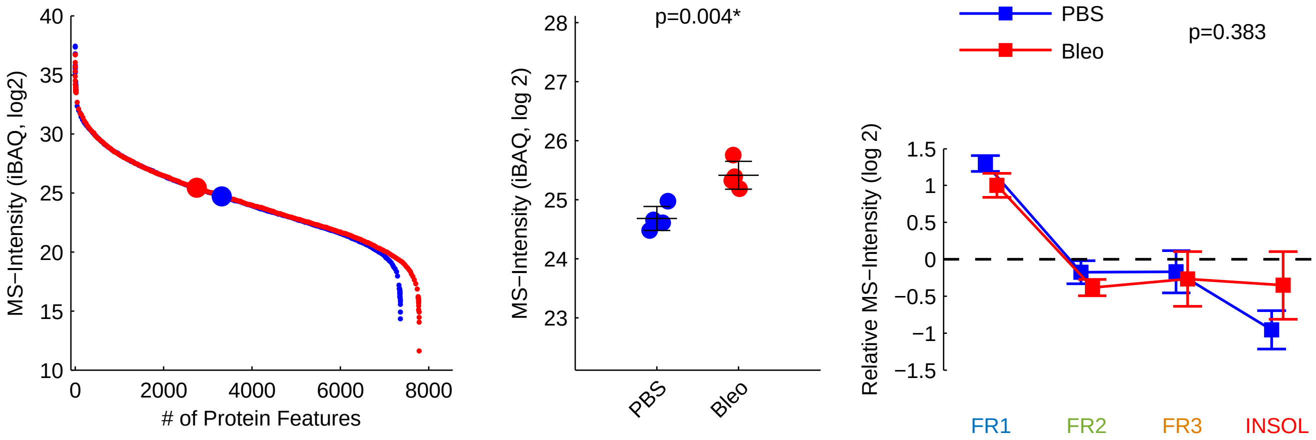

Q7TQK1 − Ints7 (id: 5156)

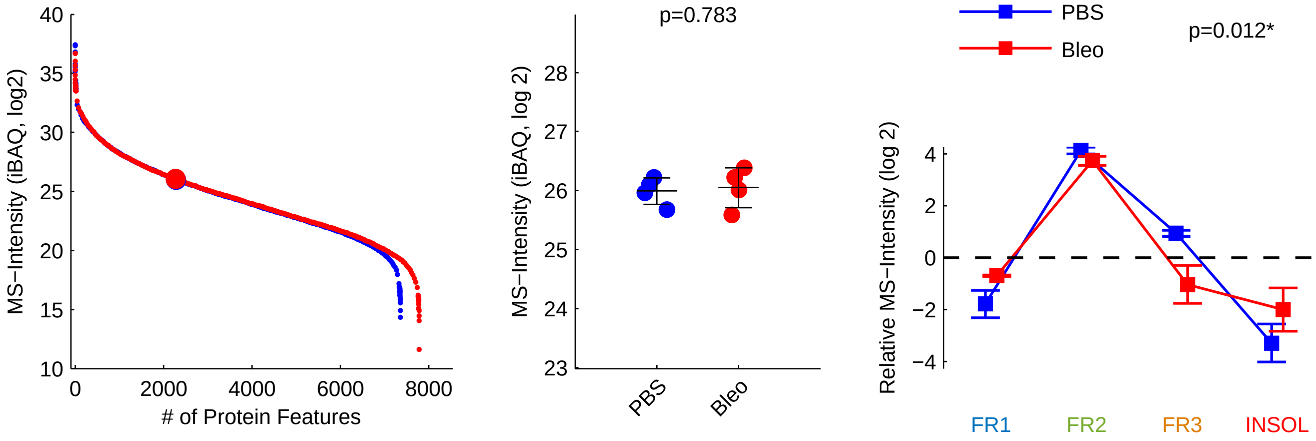

Q7TSG1 − Cep120 (id: 5165)

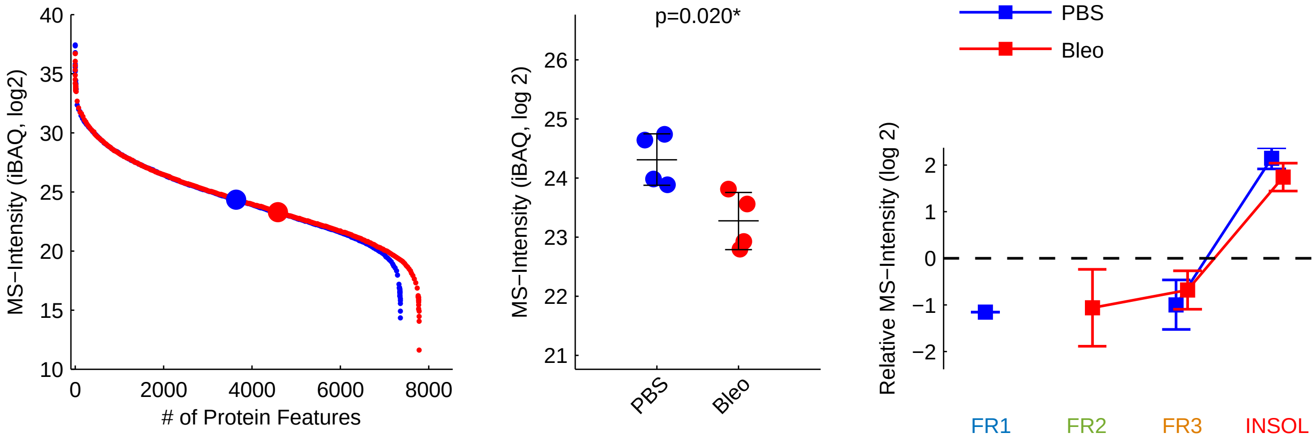

Q7TSV4 − Pgm2 (id: 5176)

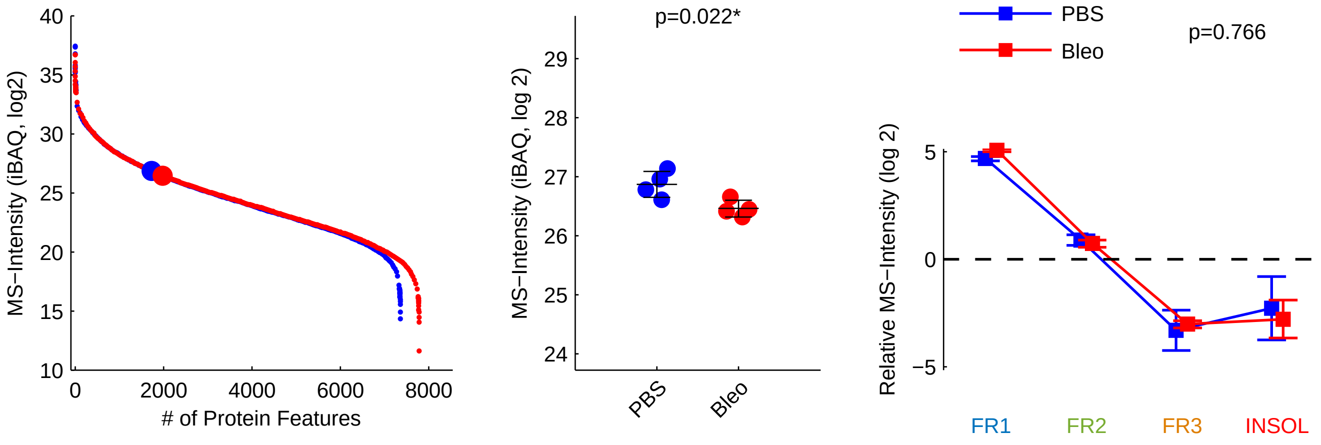

Q7TSZ8 − Nacc1 (id: 5178)

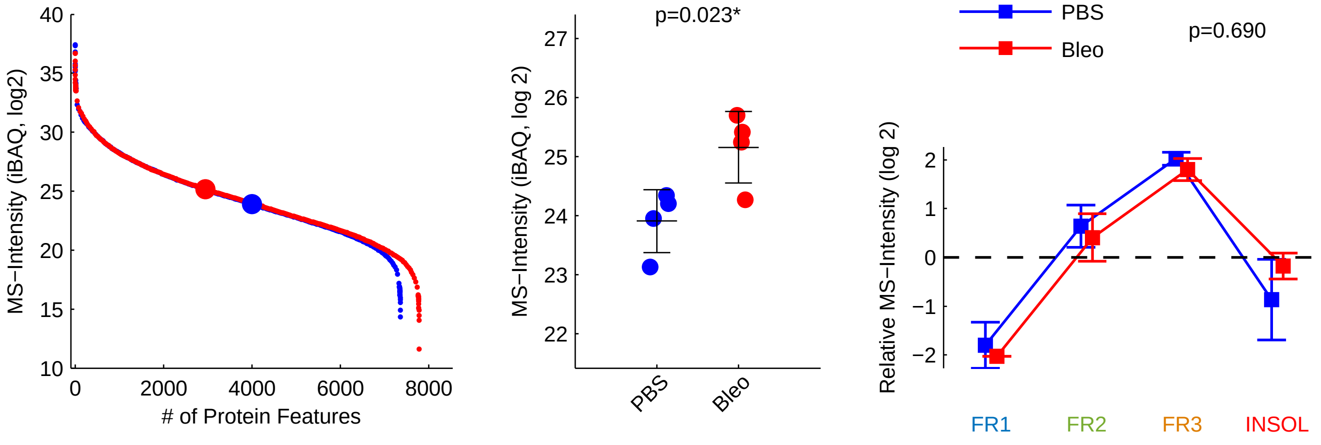

Q7TT37 − Ikbkap (id: 5182)

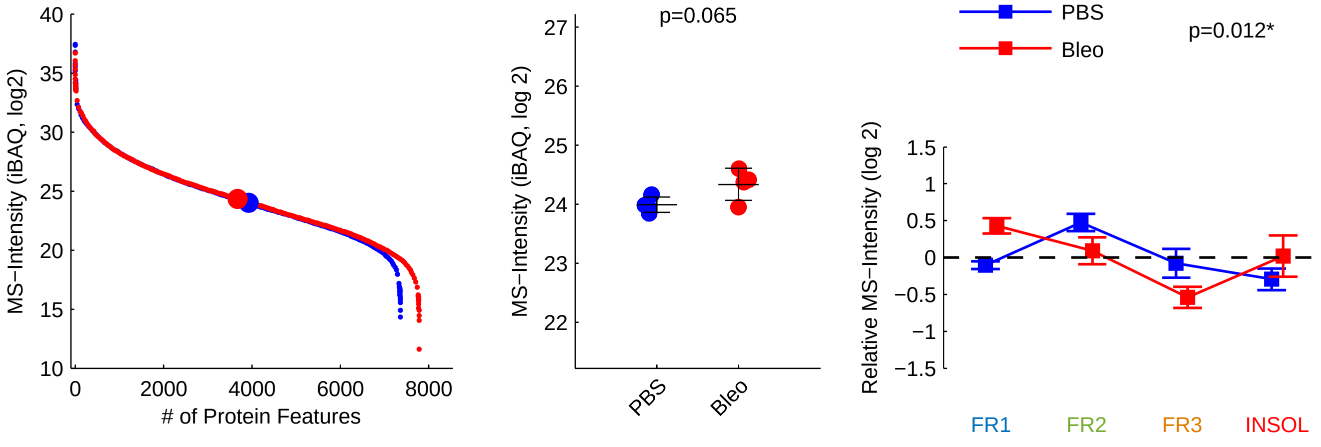

Q80SZ7 − Gng5 (id: 5187)

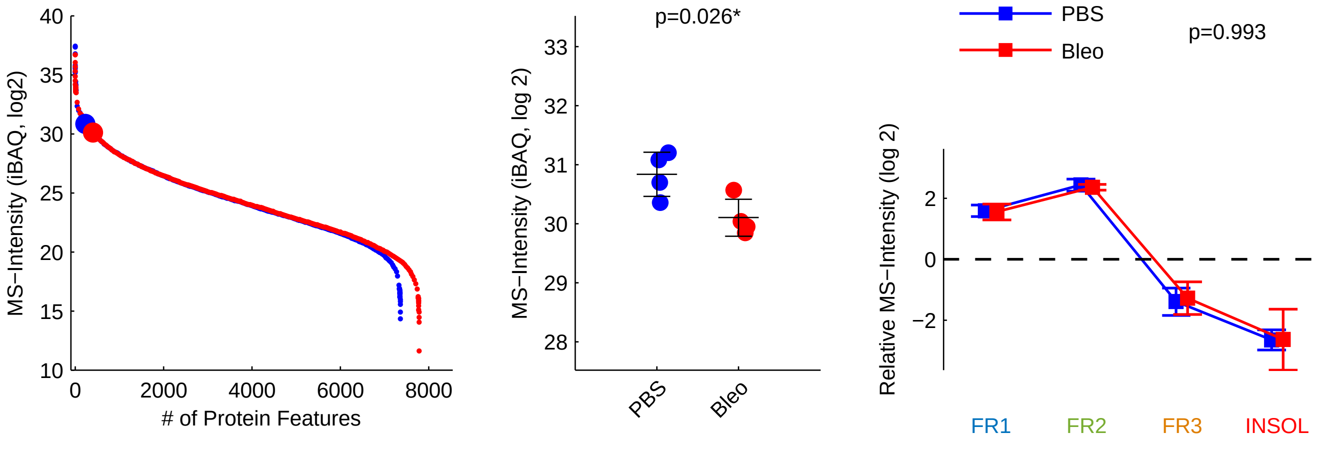

Q80TM9 − Nisch (id: 5200)

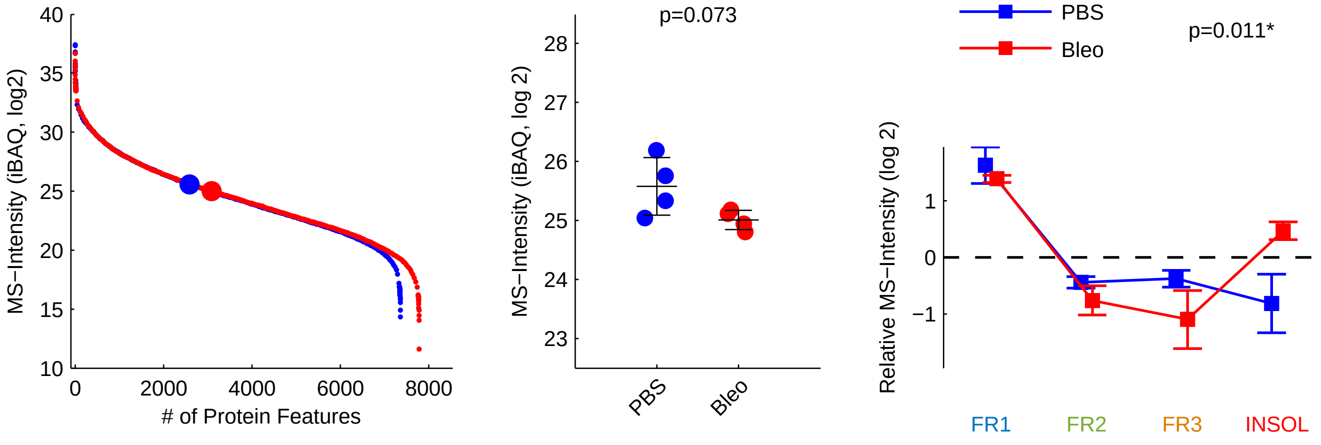

Q80TN4 − Dnajc16 (id: 5201)

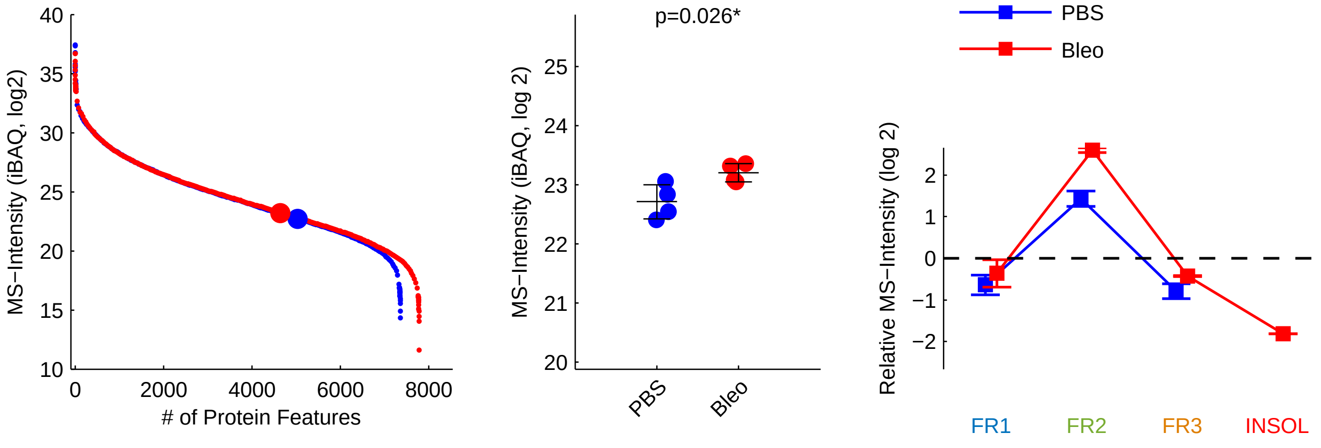

Q80U72−4 − Scrib (id: 5212)

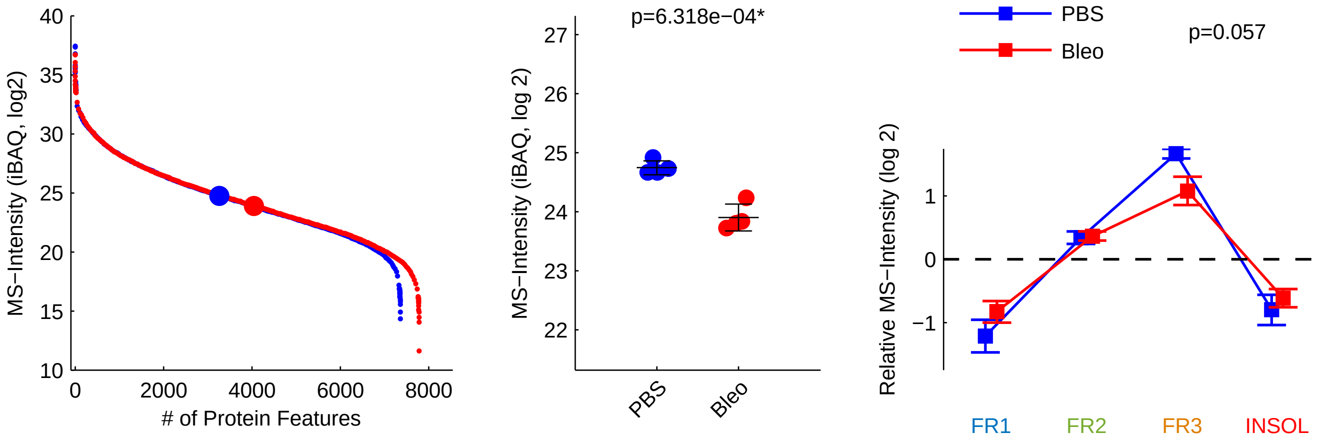

Q80UP5 − Ankrd13a (id: 5224)

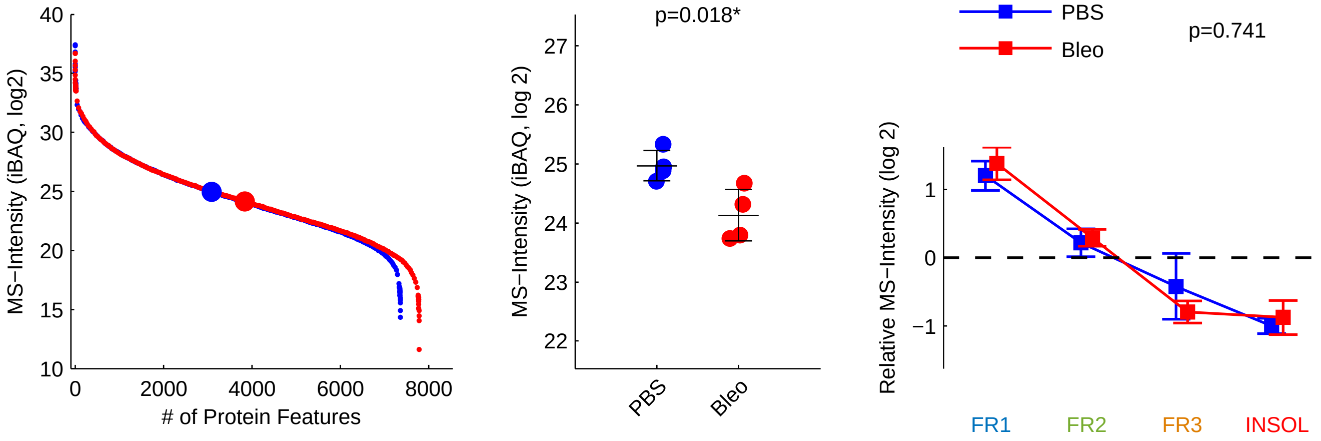

Q80UR4 − Prss34 (id: 5225)

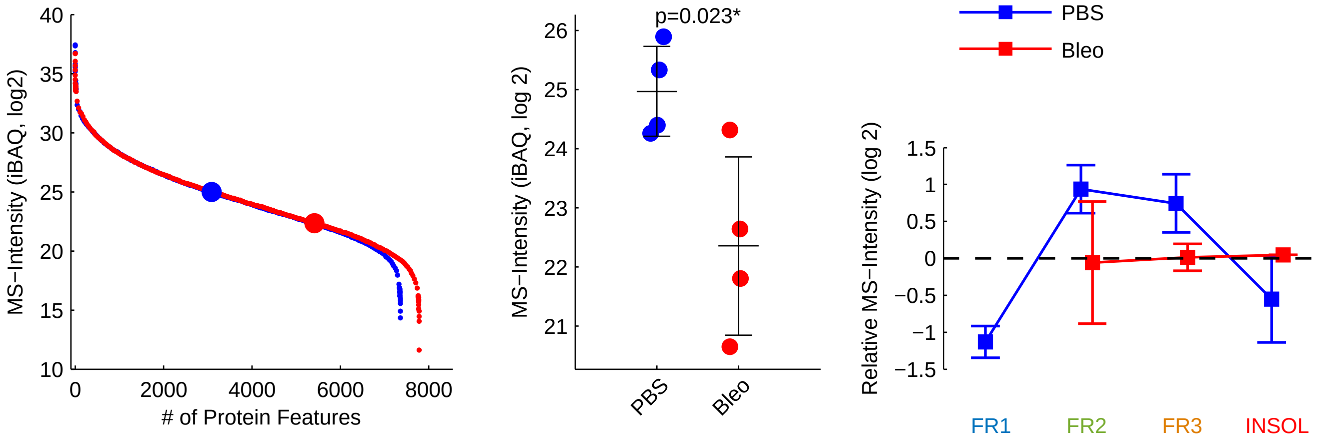

Q80UW8 − Polr2e (id: 5229)

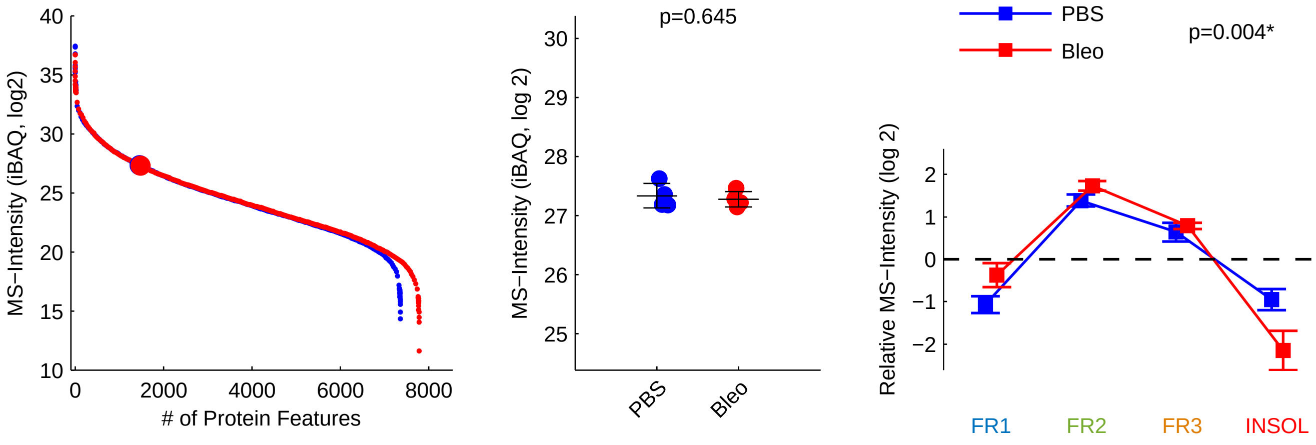

Q80V70 − Megf6 (id: 5237)

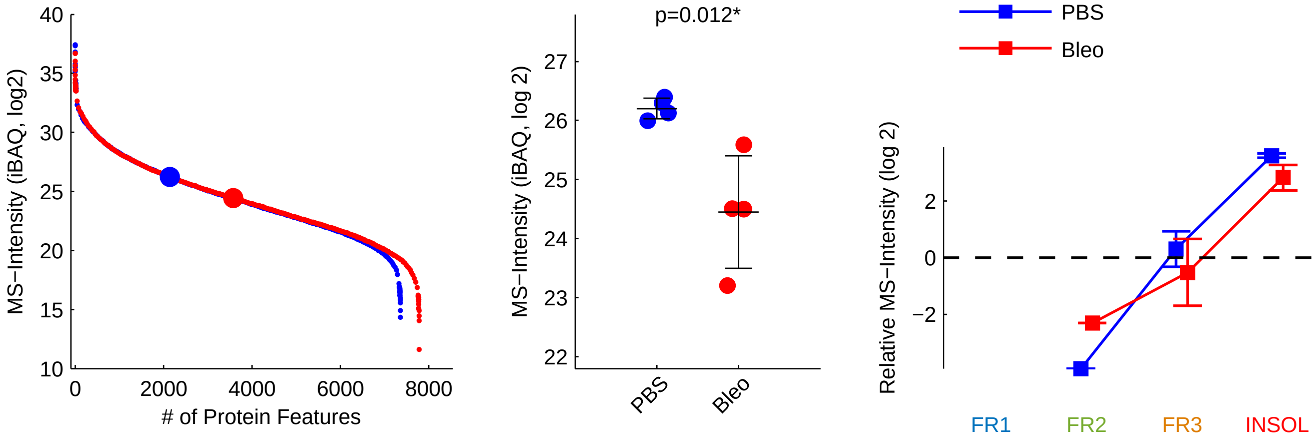

Q9JLM8 − Dclk1 (id: 5240)

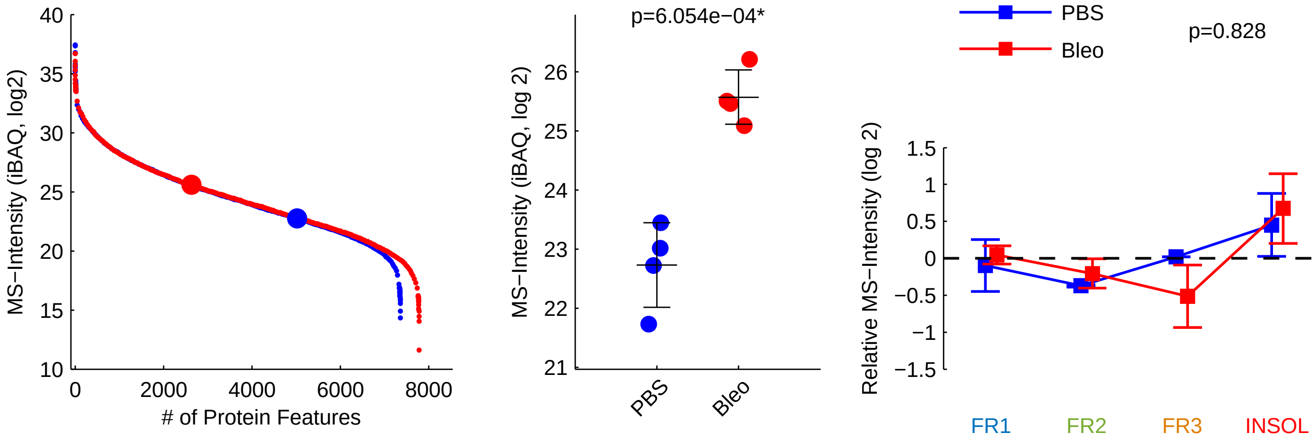

Q80VP0 − Tecpr1 (id: 5246)

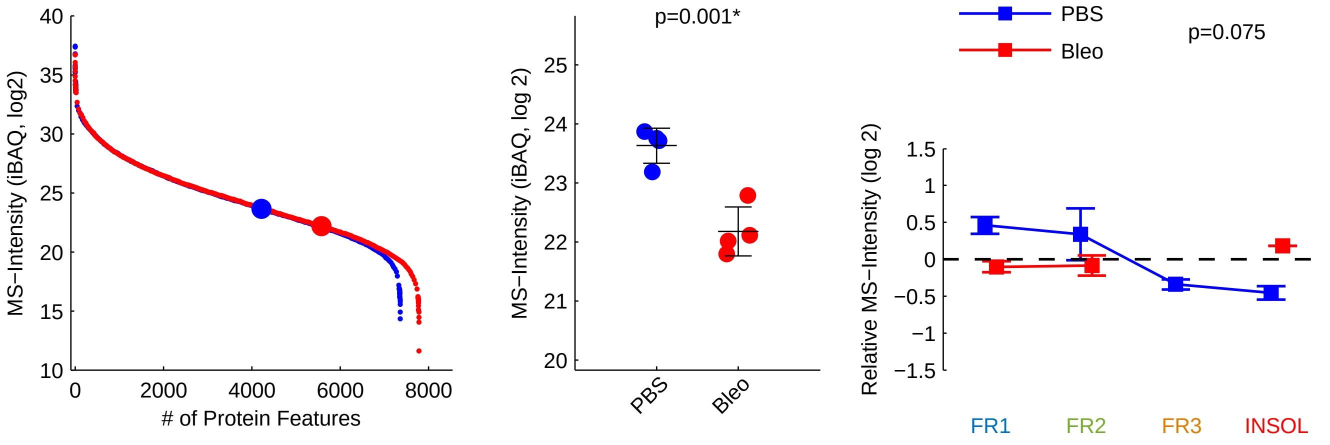

Q80VQ0 − Aldh3b1 (id: 5249)

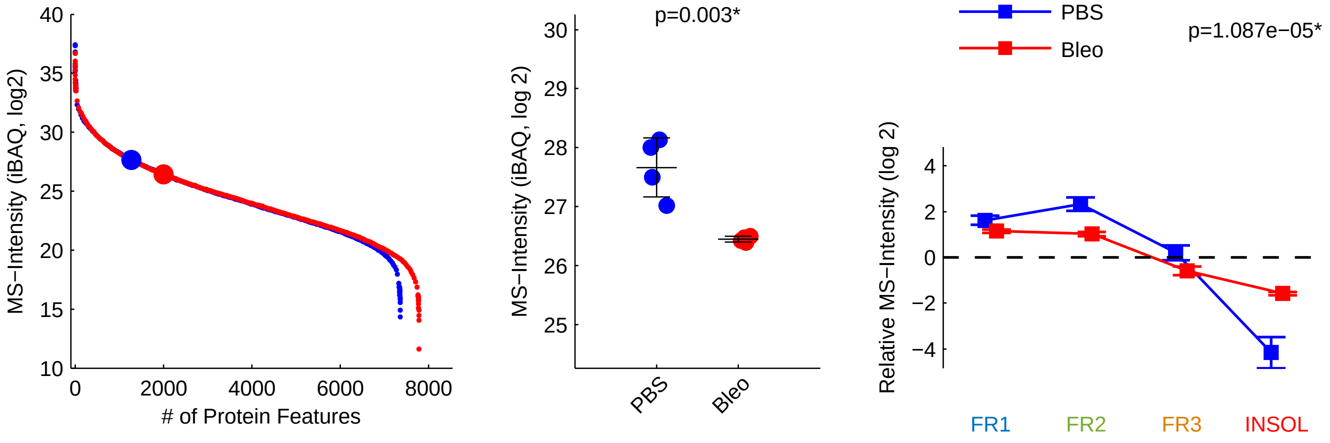

Q80VQ1 − Lrrc1 (id: 5250)

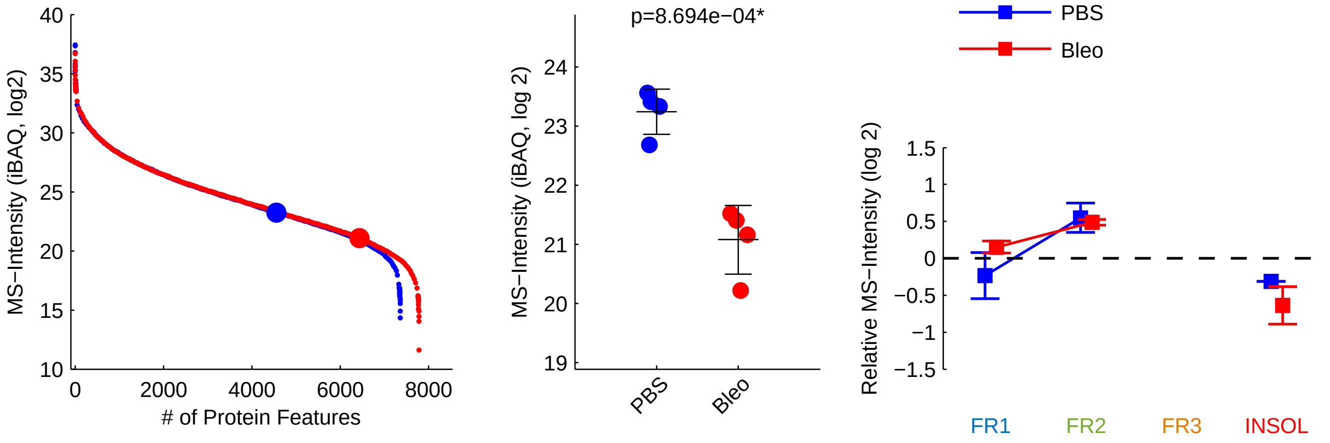

Q80W22 − Thnsl2 (id: 5255)

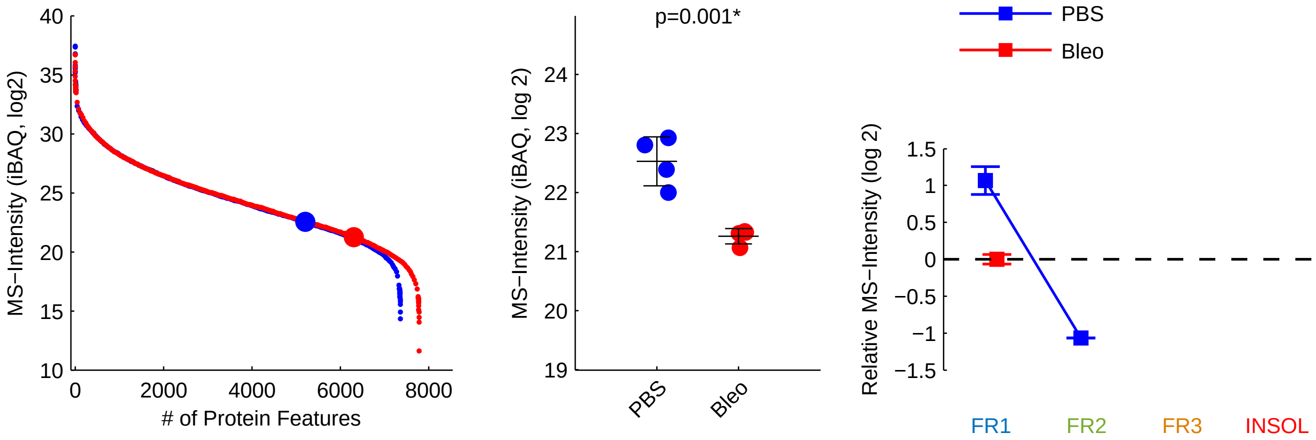

Q80WB5 − Wdyhv1 (id: 5261)

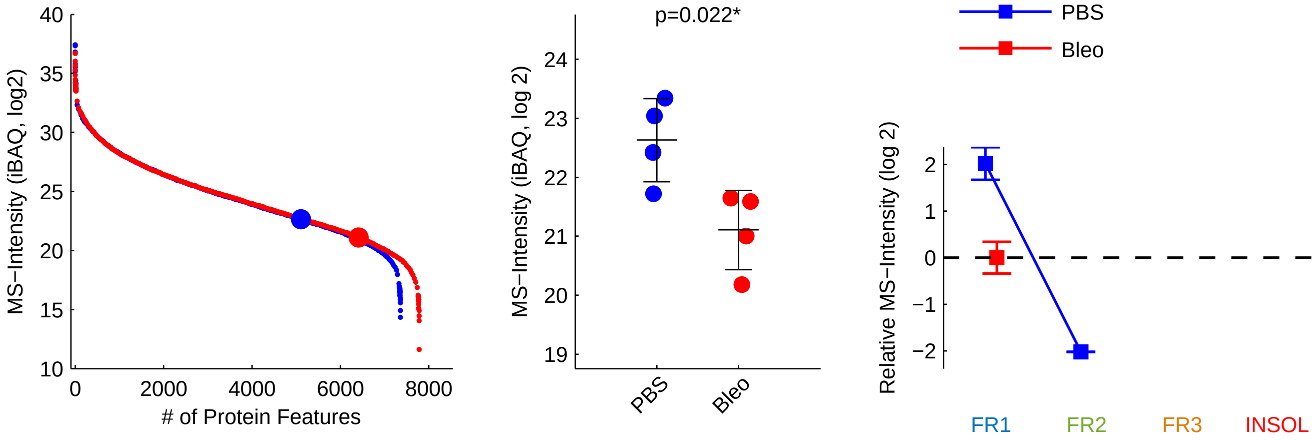

Q80WW9 − Ddrgk1 (id: 5269)

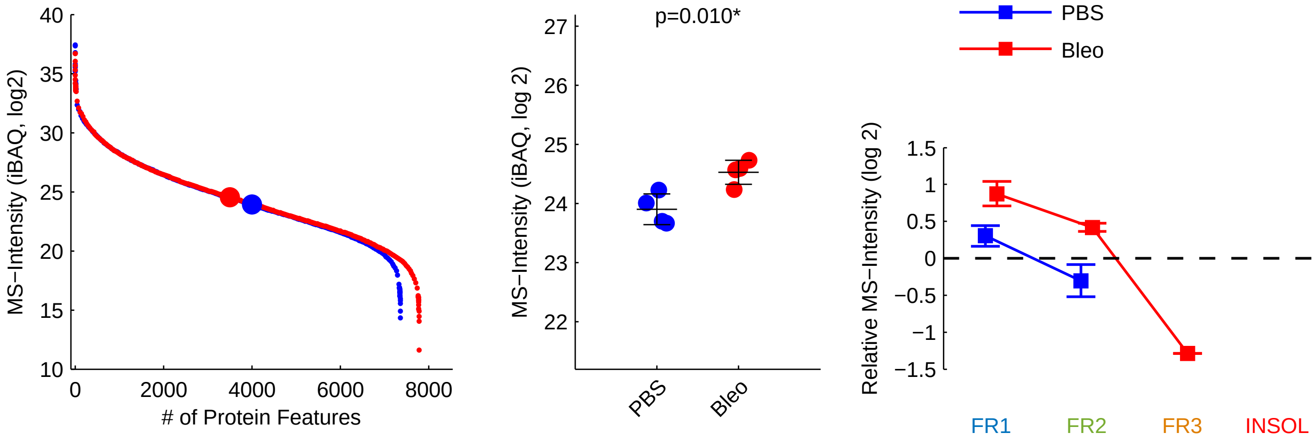

Q80X19 − Col14a1 (id: 5270)

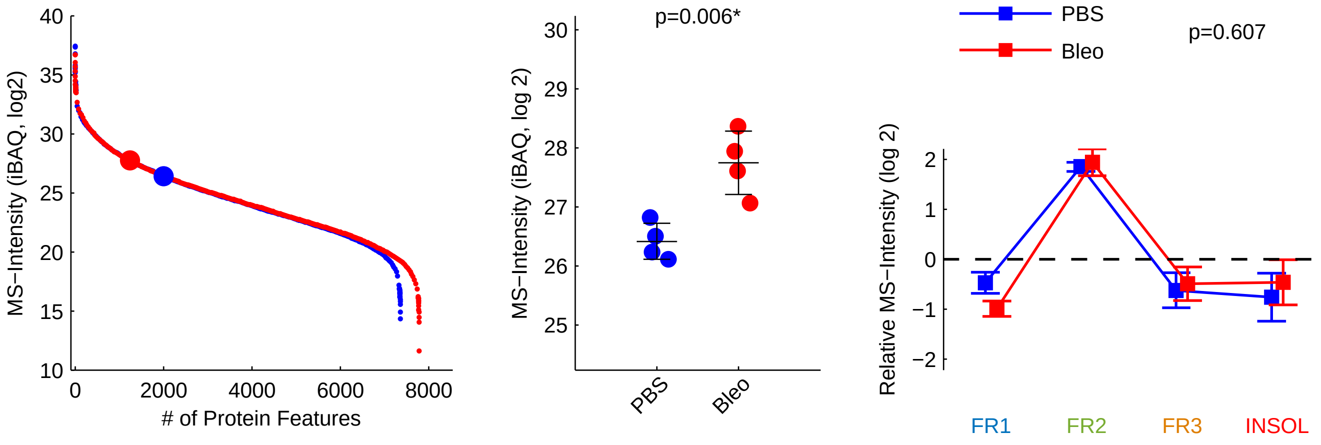

Q80X50 − Ubap2l (id: 5273)

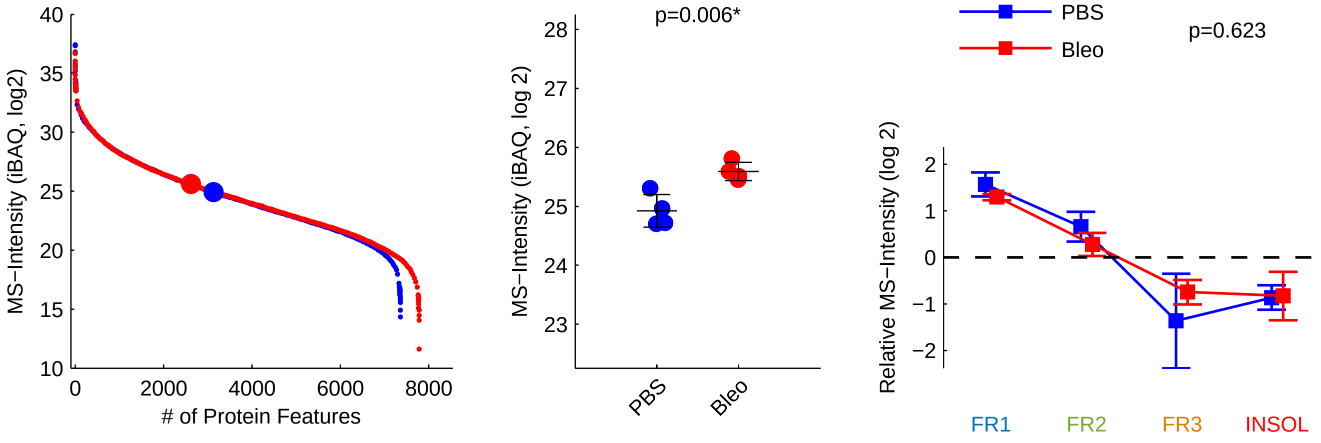

Q80X73 − Pelo (id: 5276)

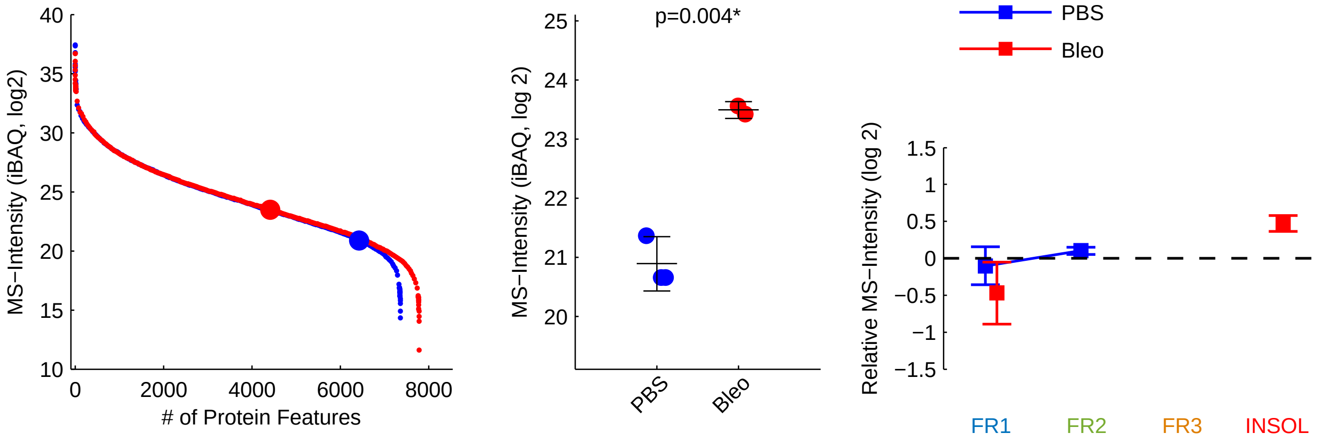

Q80X85 − Mrps7 (id: 5280)

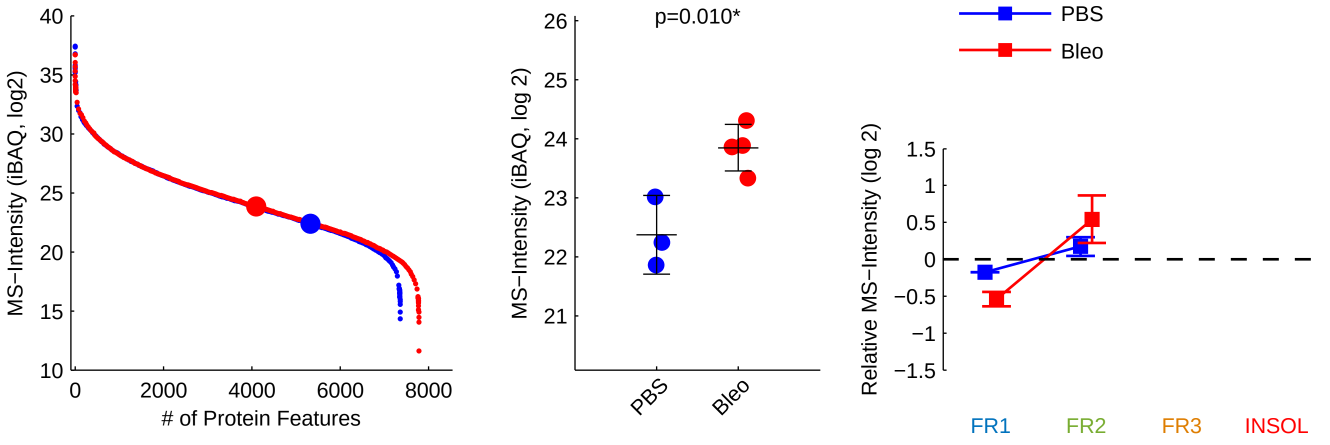

Q80X90 − Flnb (id: 5281)

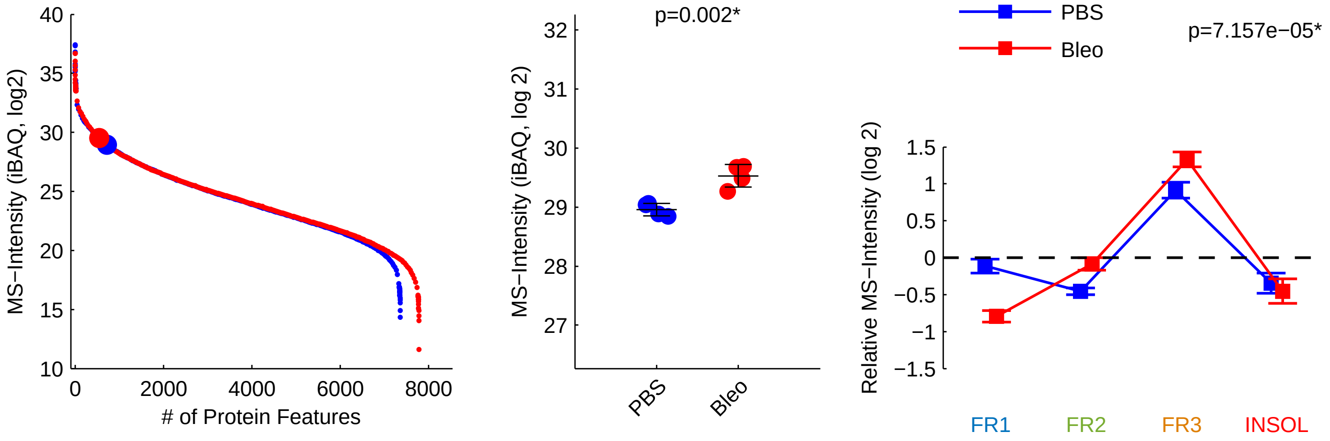

Q80X95 − Rraga (id: 5282)

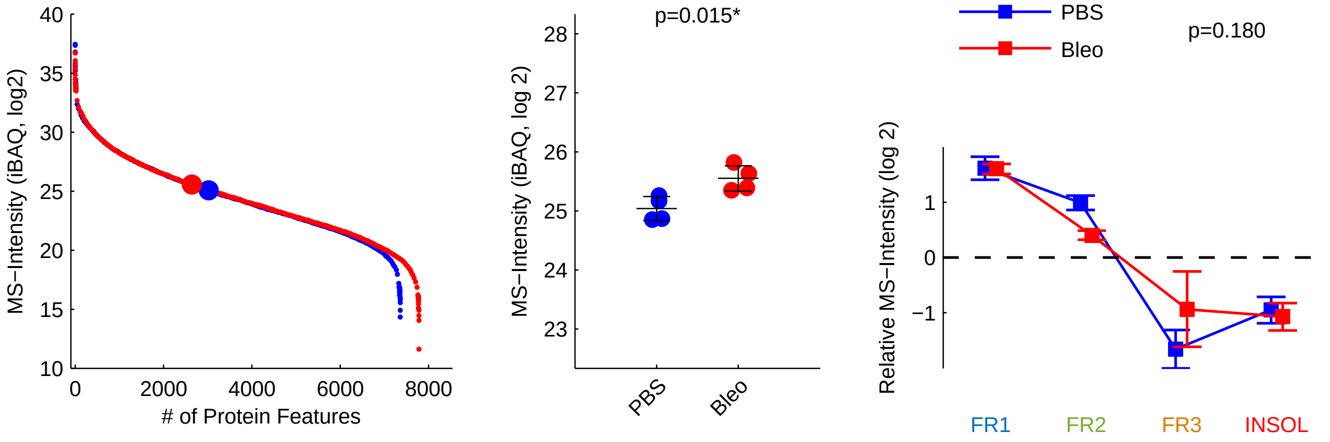

Q80XC3 − Usp6nl (id: 5285)

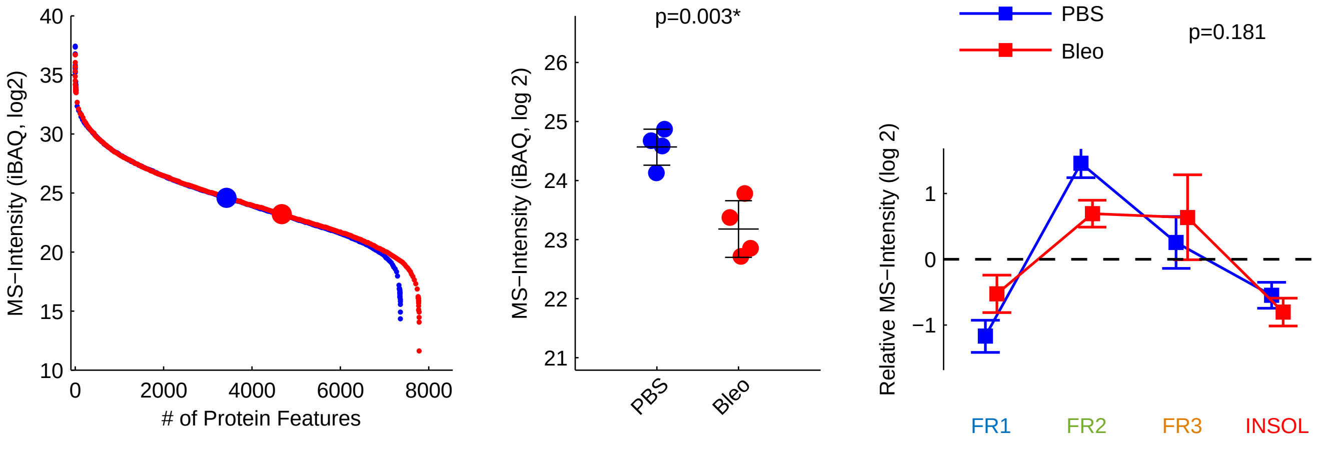

Q80XH4 − Wscd1 (id: 5287)

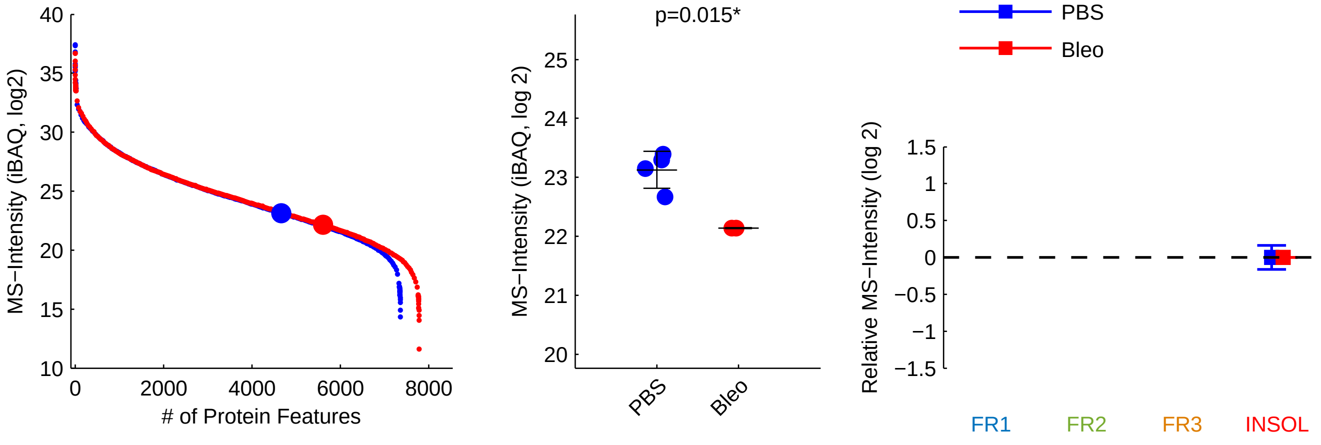

Q80XL6 − Acad11 (id: 5292)

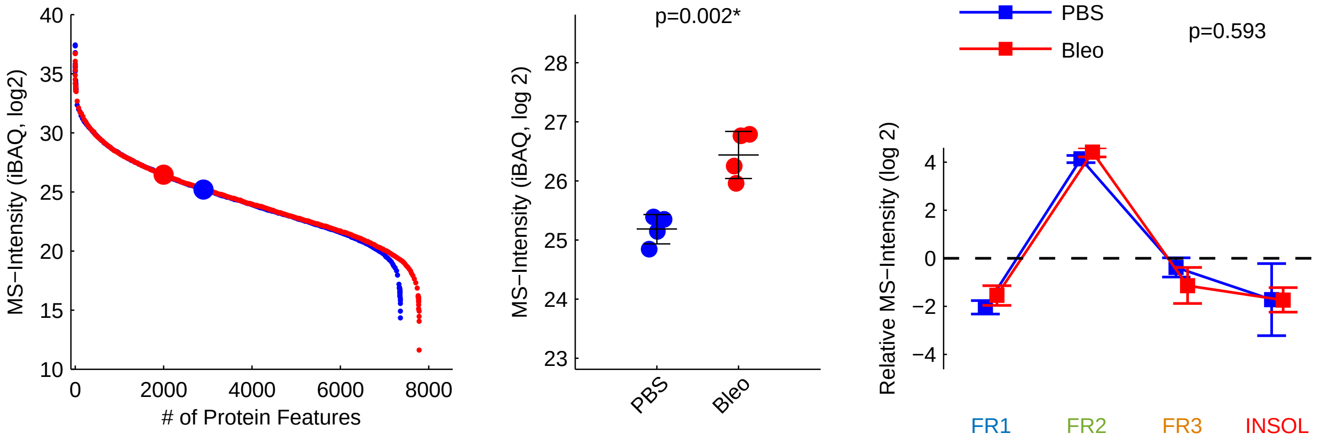

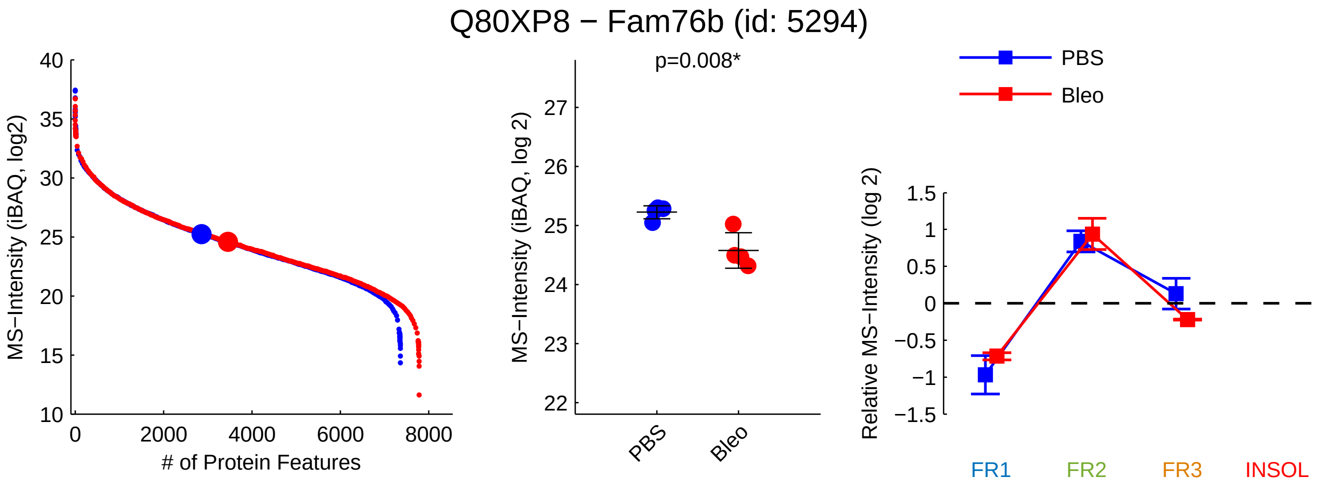
Q80XP8 − Fam76b (id: 5294)
MS−Intensity (iBAQ, log2)
# of Protein Features
p=0.008*
MS−Intensity (iBAQ, log 2)
PBS
Bleo
Relative MS−Intensity (log 2)
FR1
FR2
FR3
INSOL

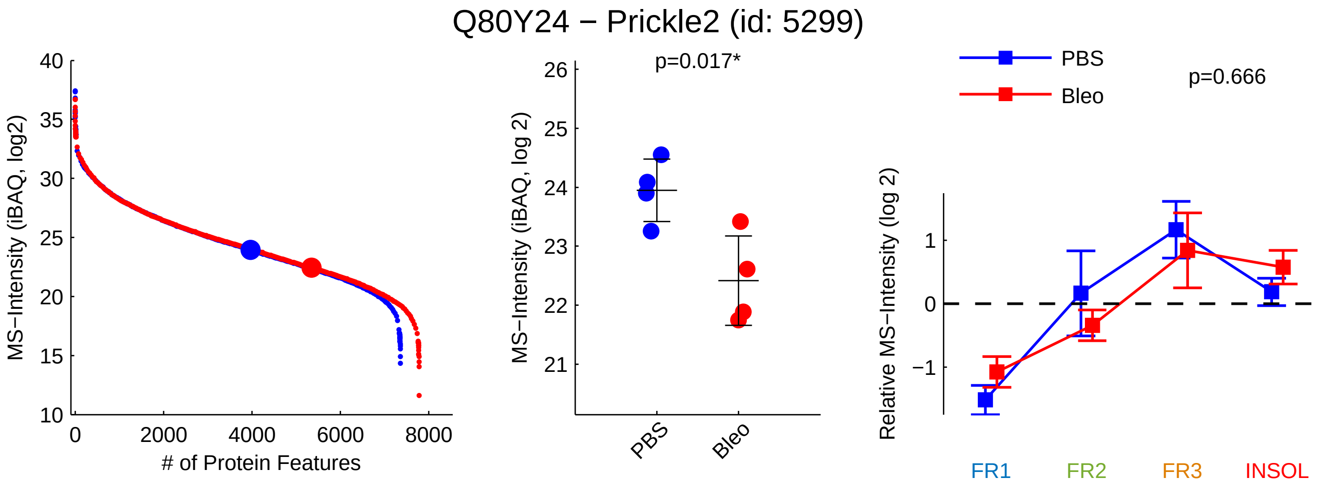
Q80Y24 − Prickle2 (id: 5299)
MS−Intensity (iBAQ, log2)
# of Protein Features
p=0.017*
MS−Intensity (iBAQ, log 2)
PBS
Bleo
p=0.666
Relative MS−Intensity (log 2)
FR1
FR2
FR3
INSOL

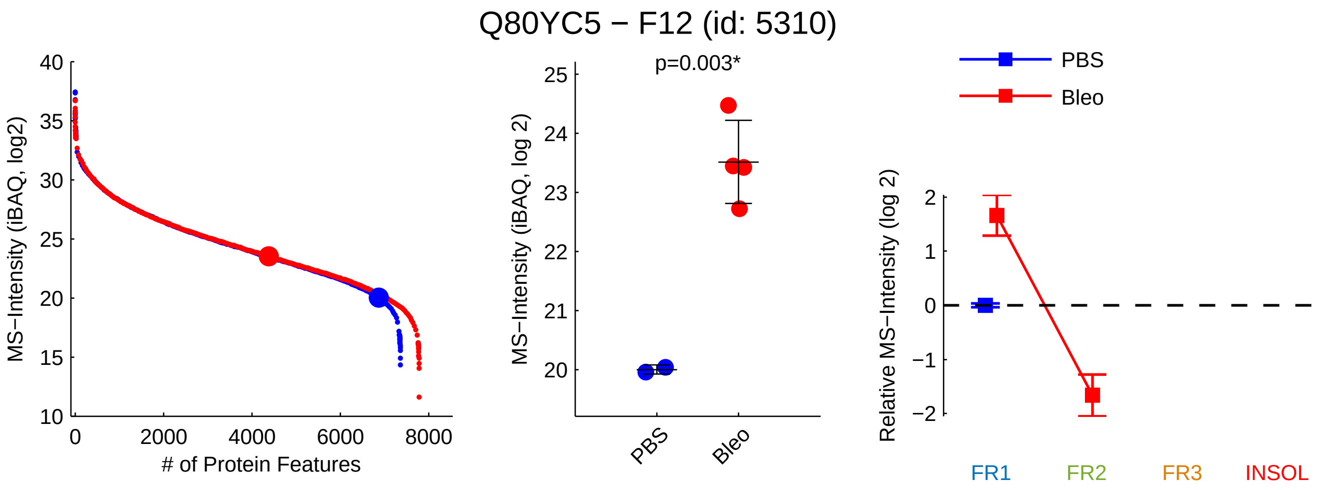
Q80YC5 − F12 (id: 5310)
MS−Intensity (iBAQ, log2)
# of Protein Features
p=0.003*
MS−Intensity (iBAQ, log 2)
PBS
Bleo
Relative MS−Intensity (log 2)
FR1
FR2
FR3
INSOL

Q80YQ1 − Thbs1 (id: 5314)

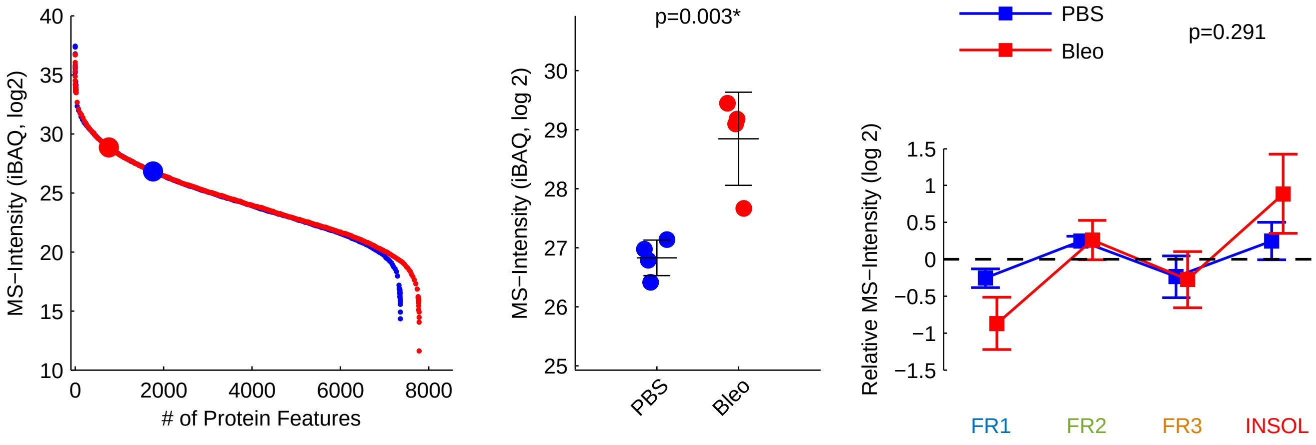

Q80YX1 − Tnc (id: 5322)

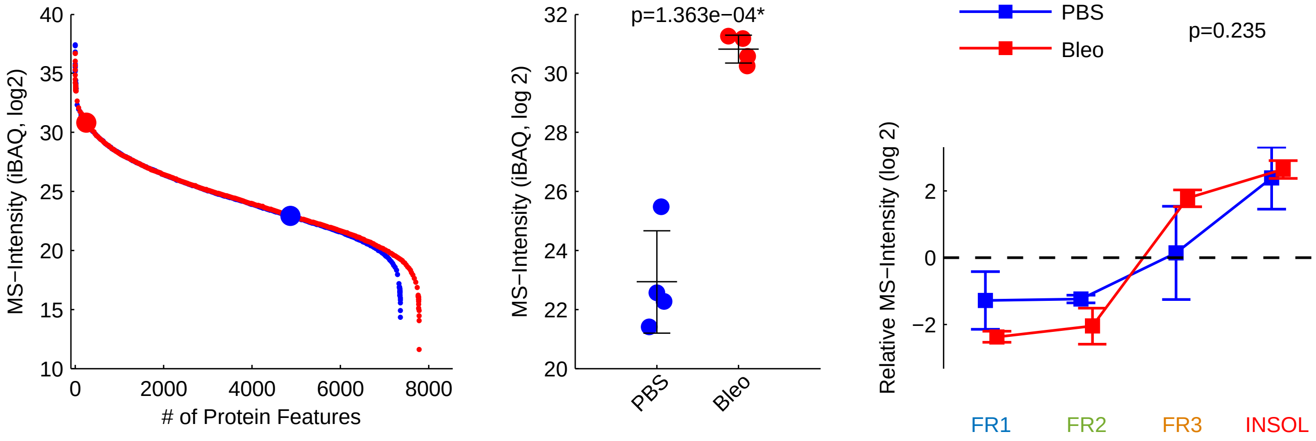

Q80Z96 − Vangl1 (id: 5325)

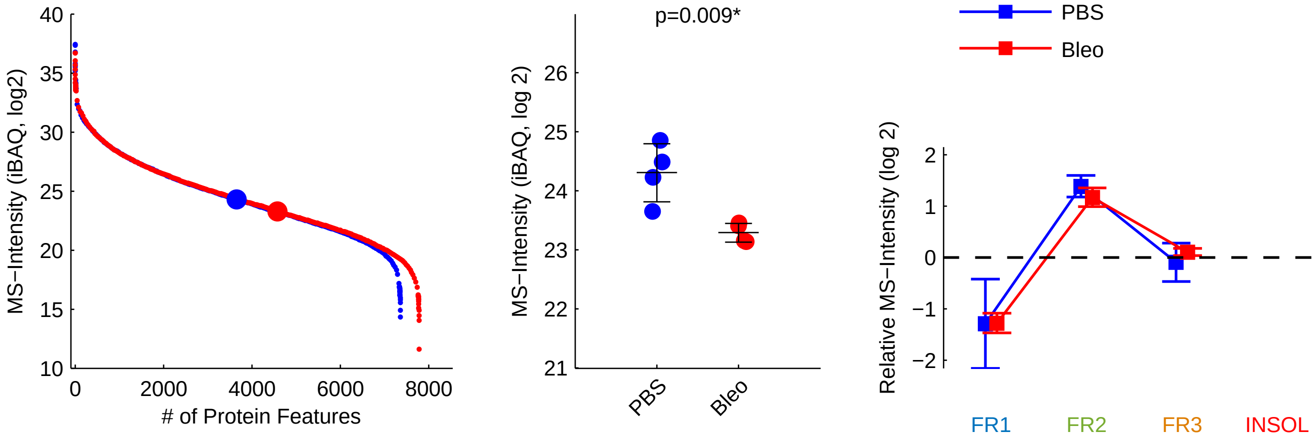

Q80ZJ1 − Rap2a (id: 5328)

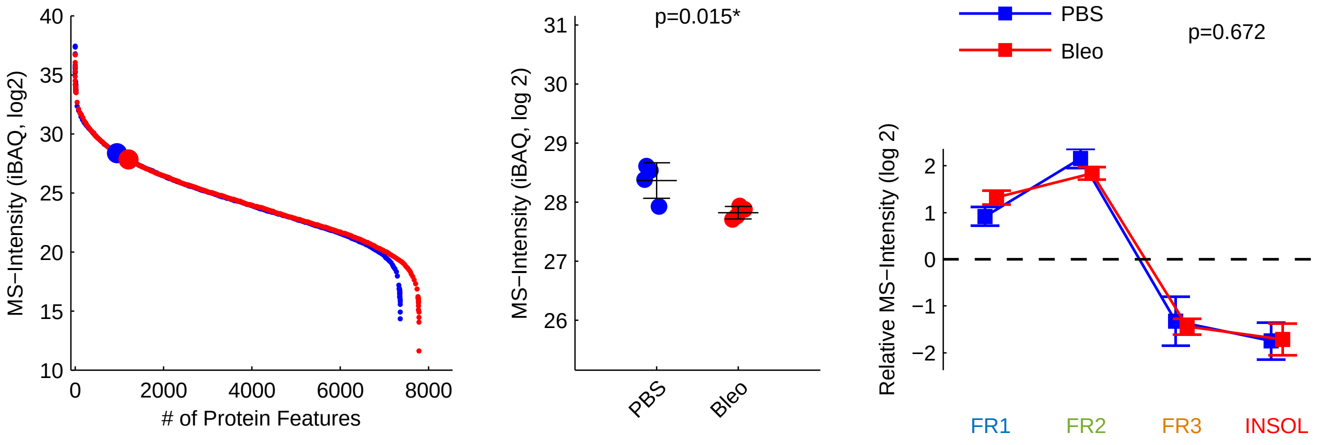

Q80ZX0 − Sec24b (id: 5335)

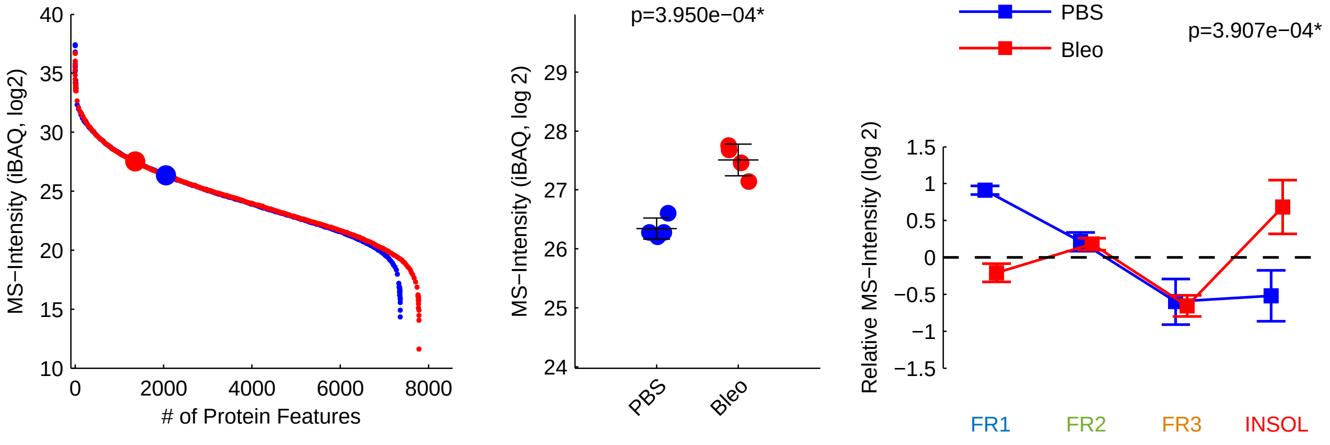

Q810D6 − Grwd1 (id: 5339)

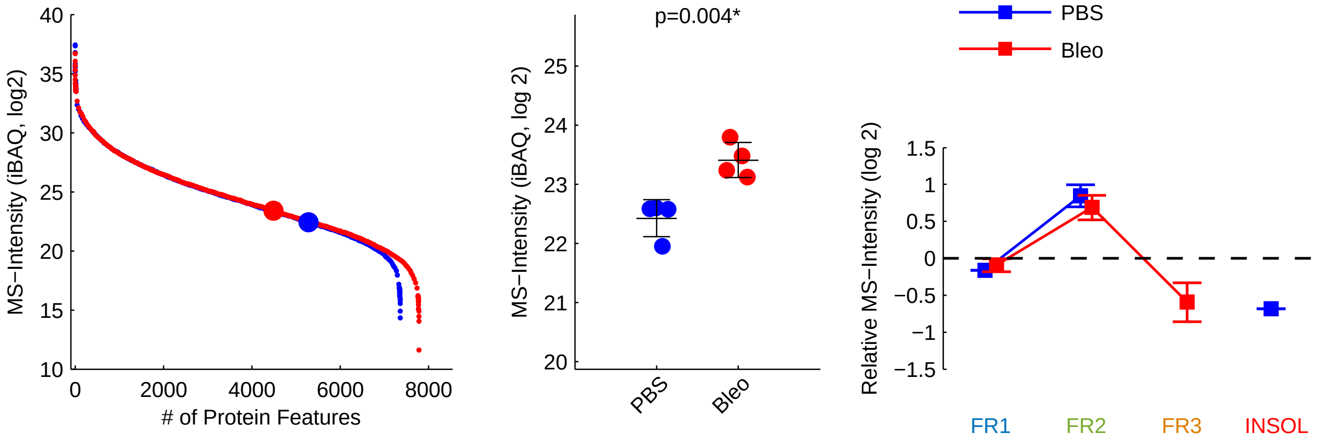

Q810Q5 − Nmes1 (id: 5342)

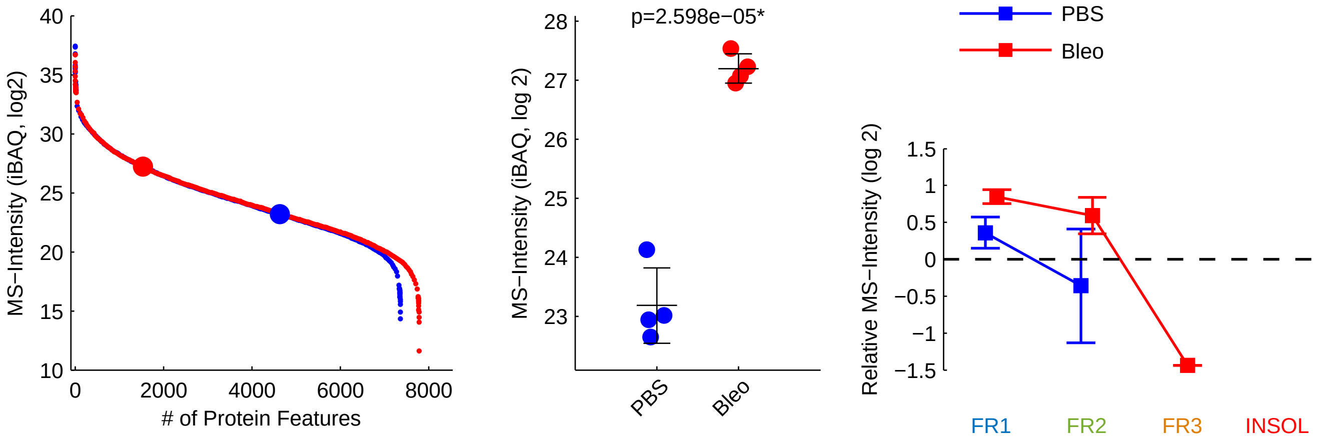

Q810S1 − Ccdc109b (id: 5343)

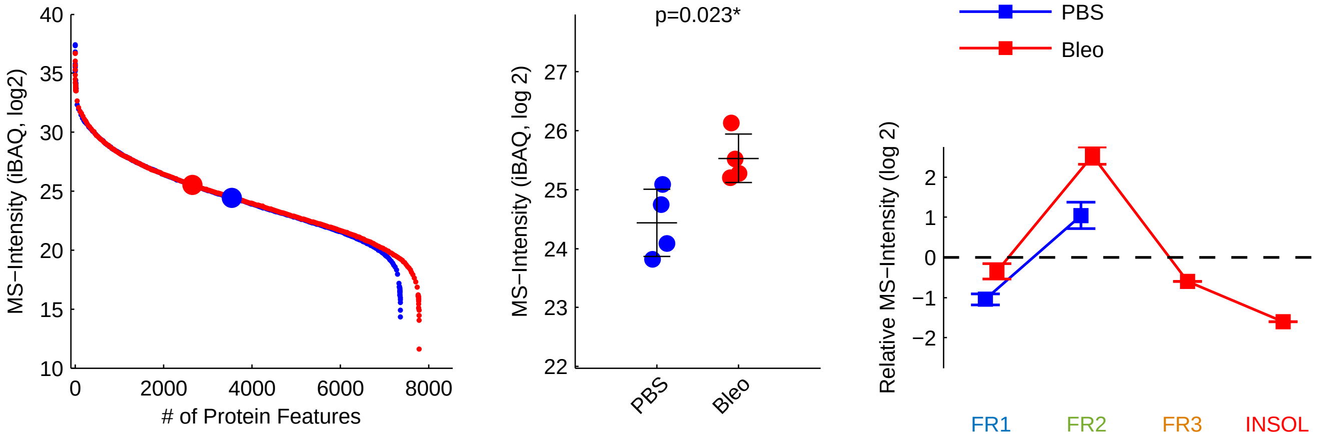

Q810V0 − Mphosph10 (id: 5346)

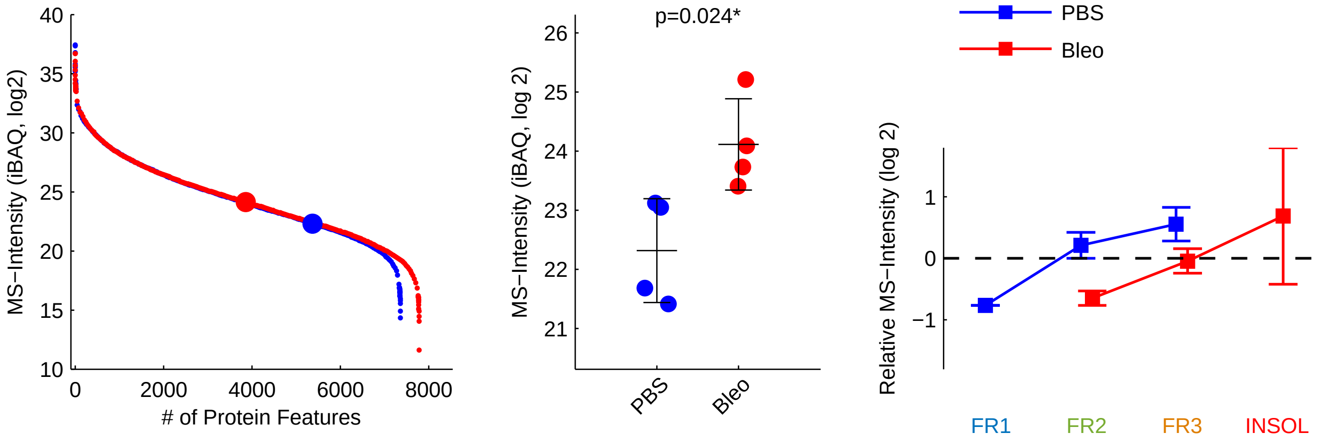

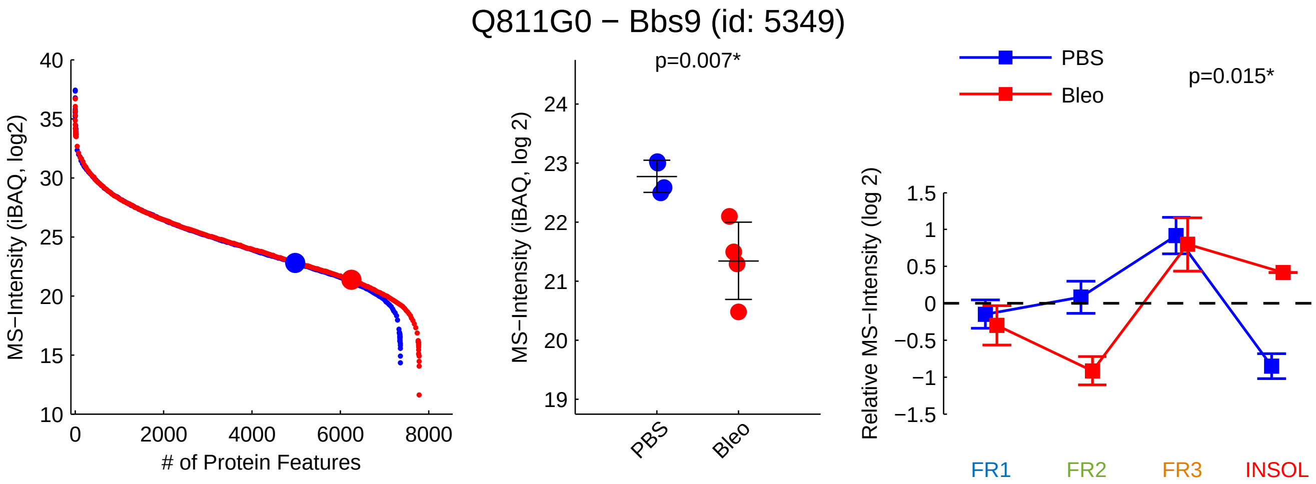
Q811G0 − Bbs9 (id: 5349)
MS−Intensity (iBAQ, log2)
# of Protein Features
p=0.007*
MS−Intensity (iBAQ, log 2)
PBS
Bleo
p=0.015*
Relative MS−Intensity (log 2)
FR1
FR2
FR3
INSOL

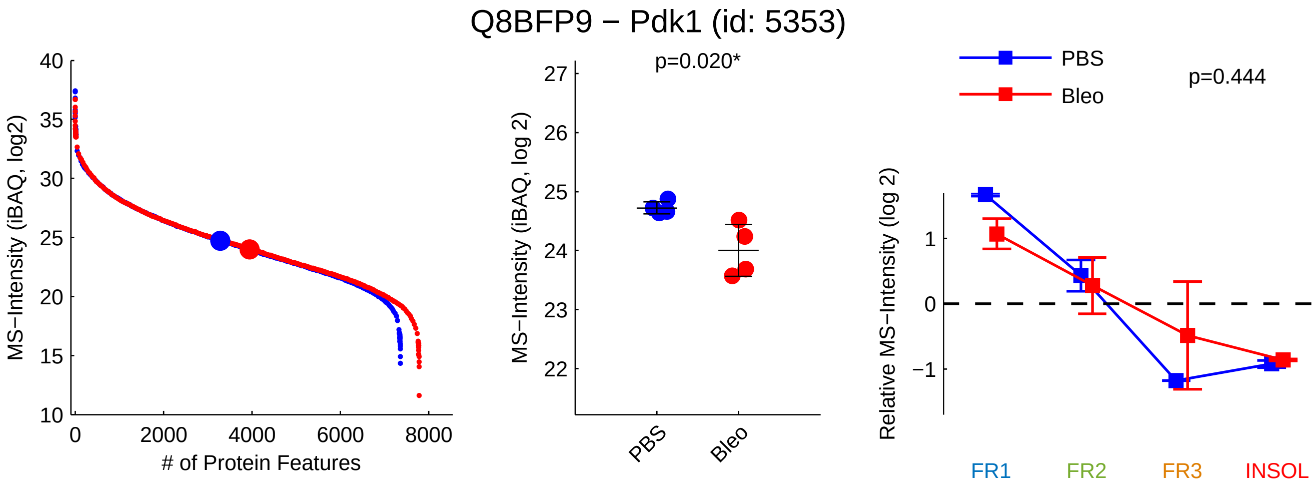
Q8BFP9 − Pdk1 (id: 5353)
MS−Intensity (iBAQ, log2)
# of Protein Features
p=0.020*
MS−Intensity (iBAQ, log 2)
PBS
Bleo
p=0.444
Relative MS−Intensity (log 2)
FR1
FR2
FR3
INSOL

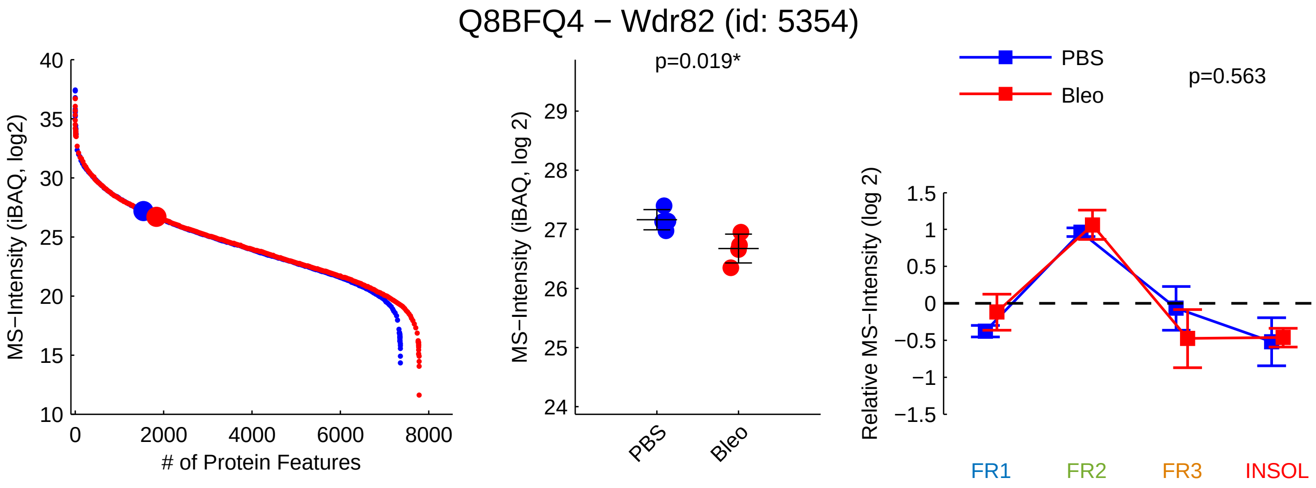
Q8BFQ4 − Wdr82 (id: 5354)
MS−Intensity (iBAQ, log2)
# of Protein Features
p=0.019*
MS−Intensity (iBAQ, log 2)
PBS
Bleo
p=0.563
Relative MS−Intensity (log 2)
FR1
FR2
FR3
INSOL

Q8BFR4 − Gns (id: 5357)

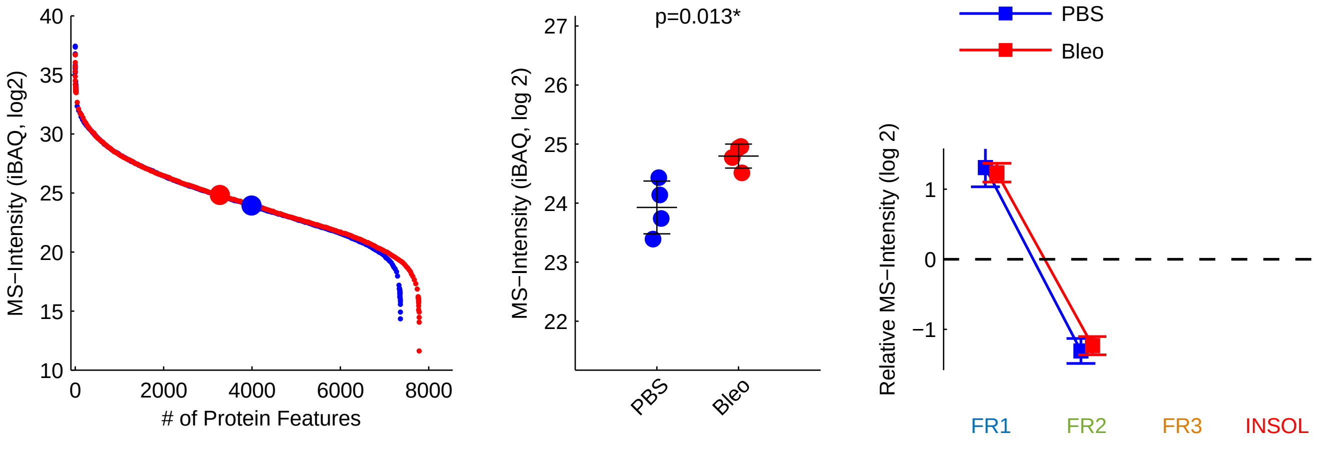

Q8BFS6 − Cpped1 (id: 5359)

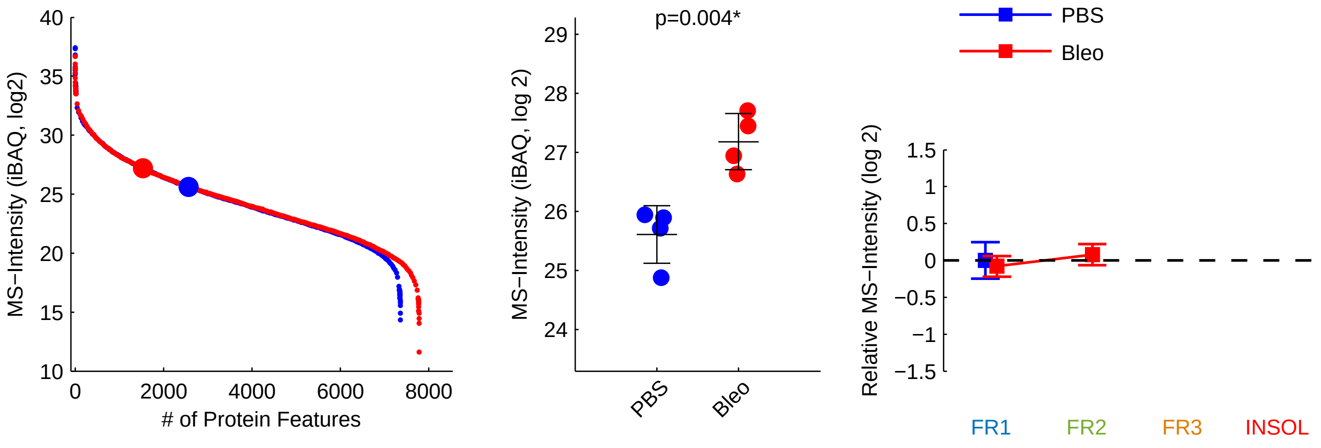

Q8BFY9 − Tnpo1 (id: 5367)

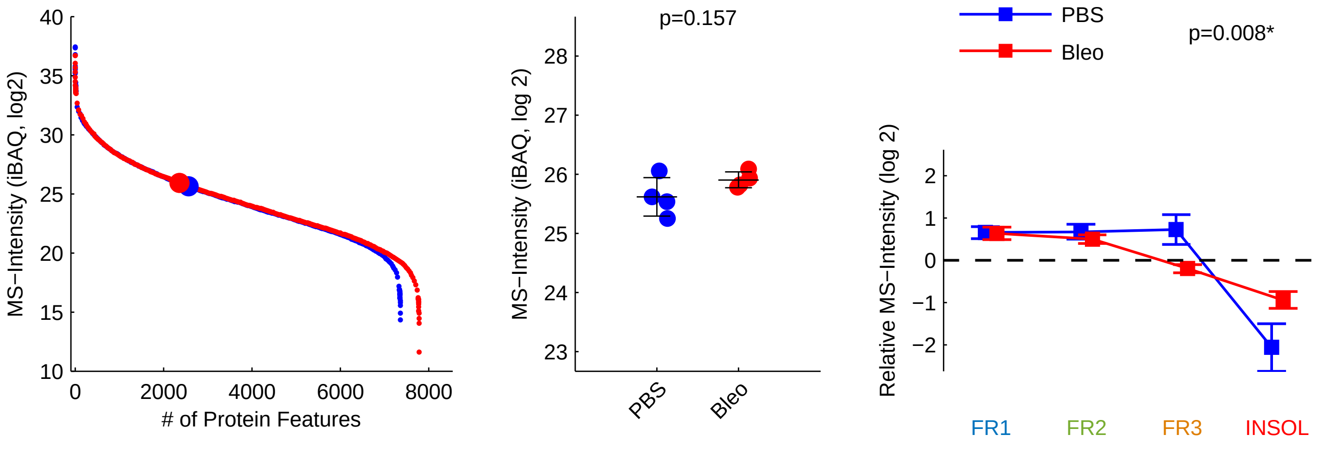

Q8BG05 − Hnrnpa3 (id: 5370)

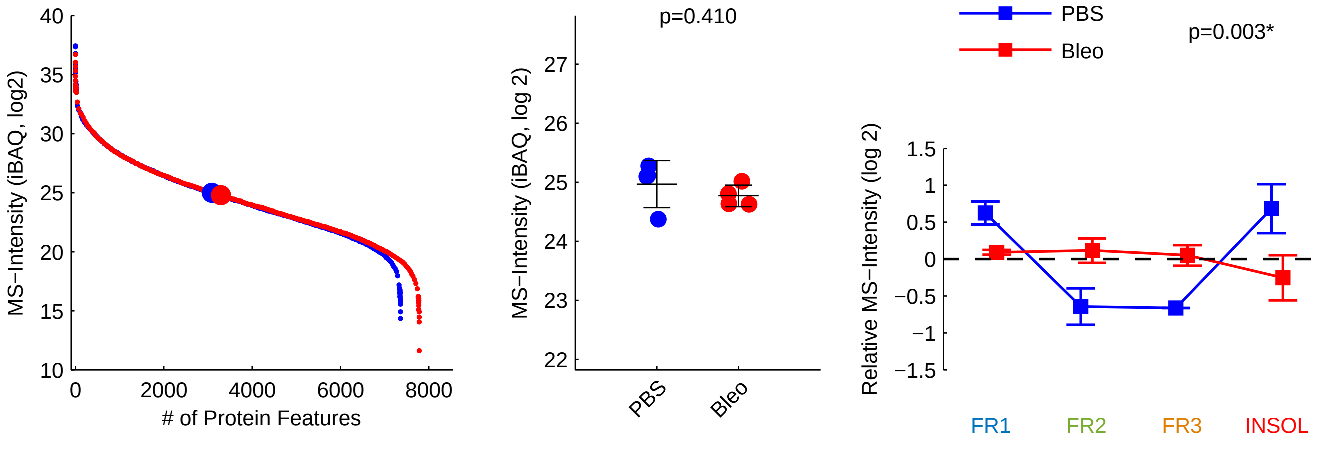

Q8BG05−2 − Hnrnpa3 (id: 5371)

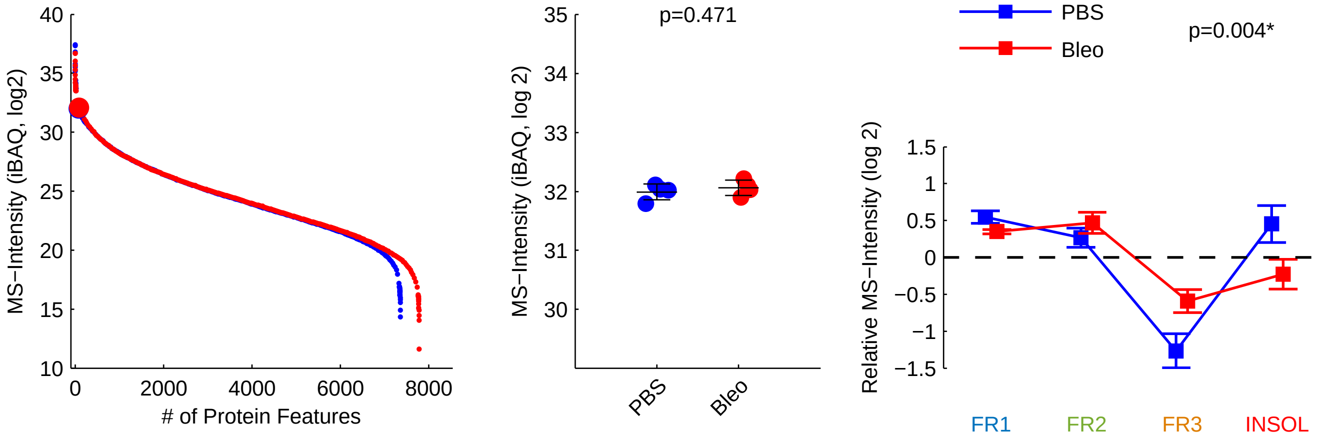

Q8BG13 − Rbm3 (id: 5373)

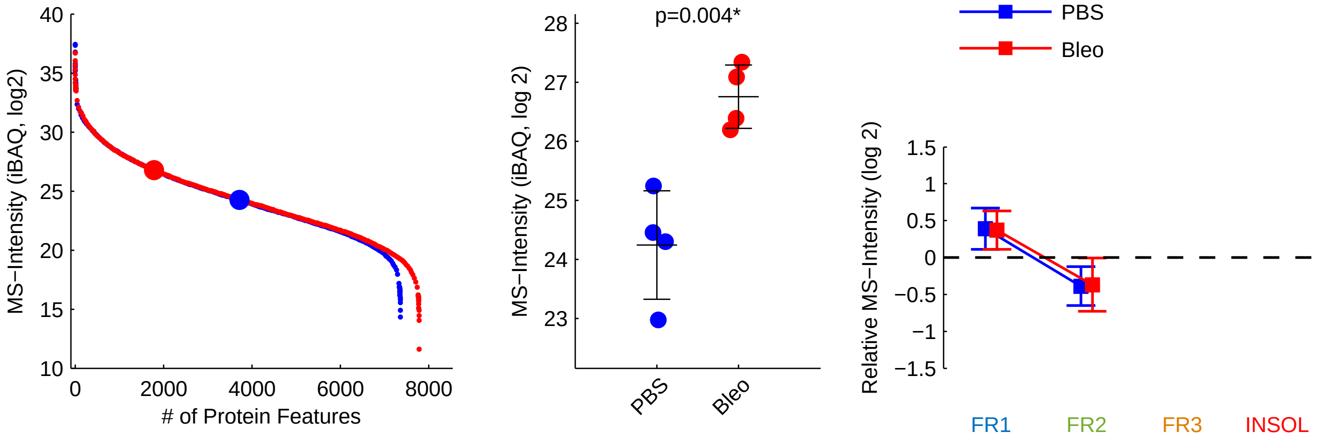

Q8BG32 − Psmd11 (id: 5377)

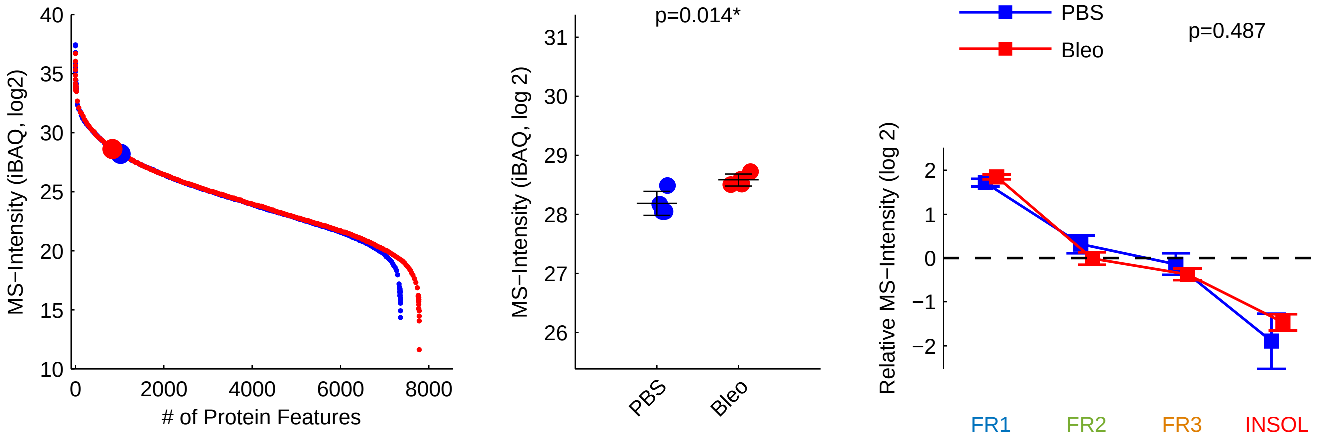

Q8BG51 − Rhot1 (id: 5380)

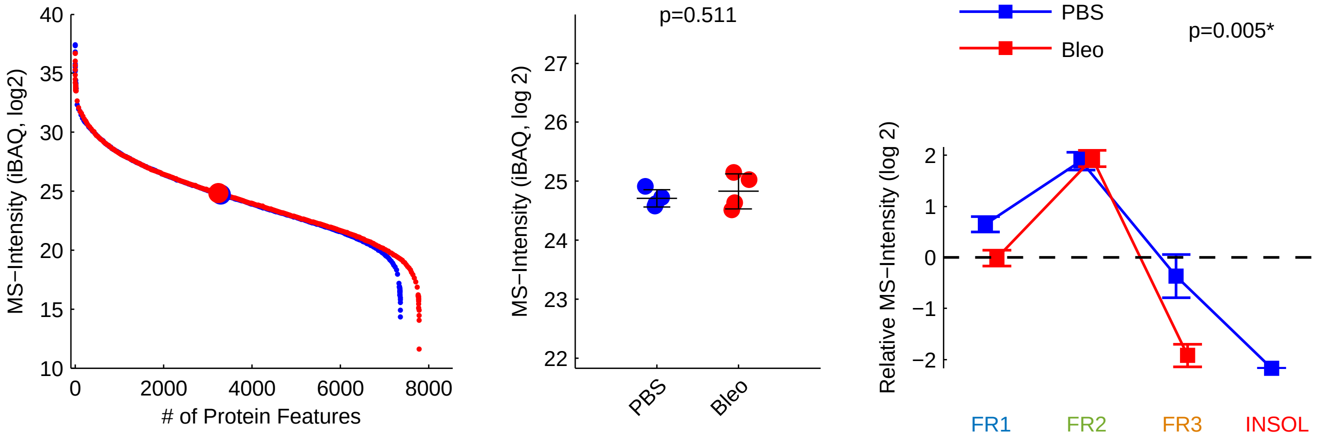

Q8BGC0 − Htatsf1 (id: 5394)

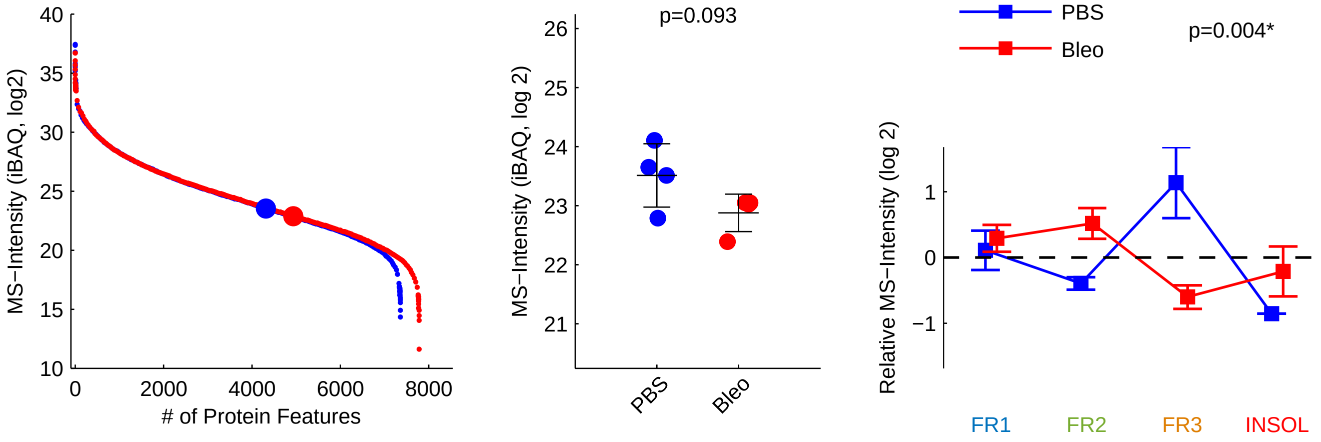

Q8BGQ7 − Aars (id: 5414)

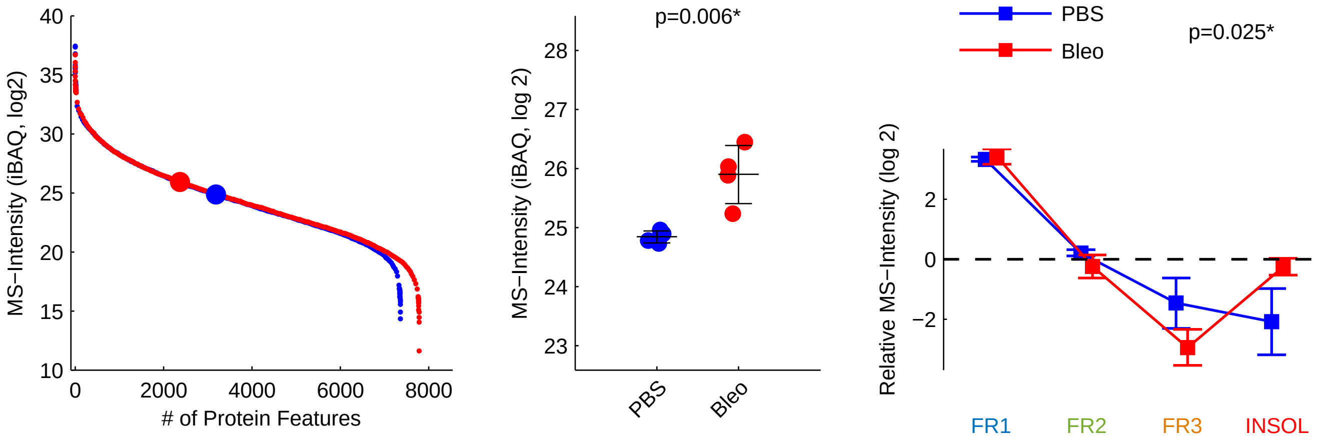

Q8BGR2 − Lrrc8d (id: 5415)

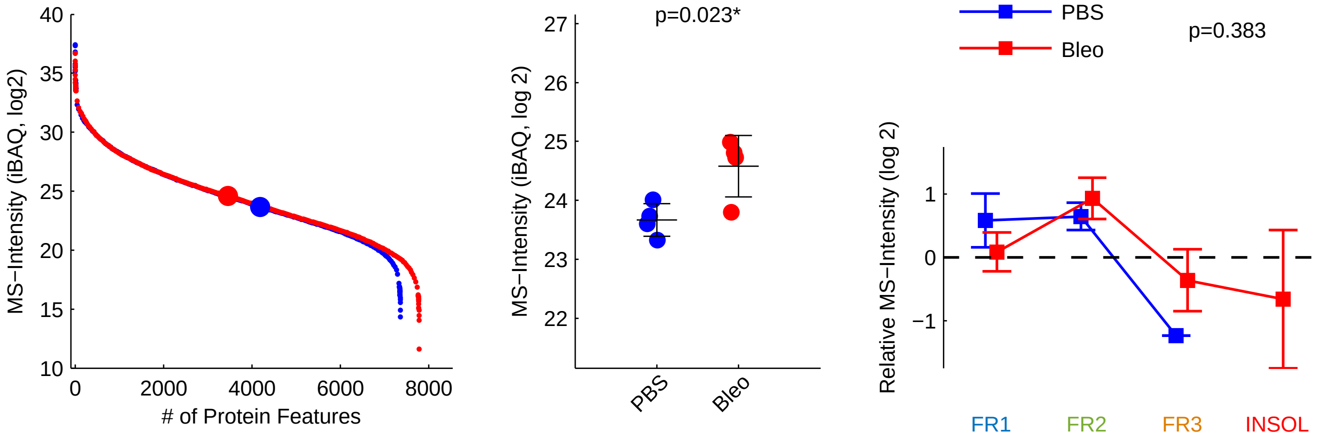

Q8BGS1 − Epb41l5 (id: 5420)

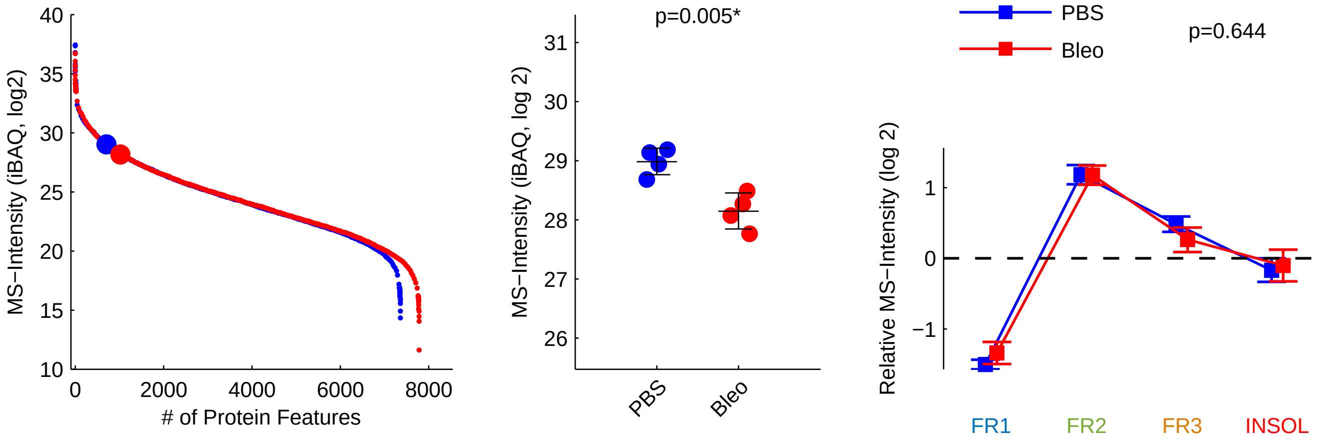

Q8BGU5 − Ccny (id: 5429)

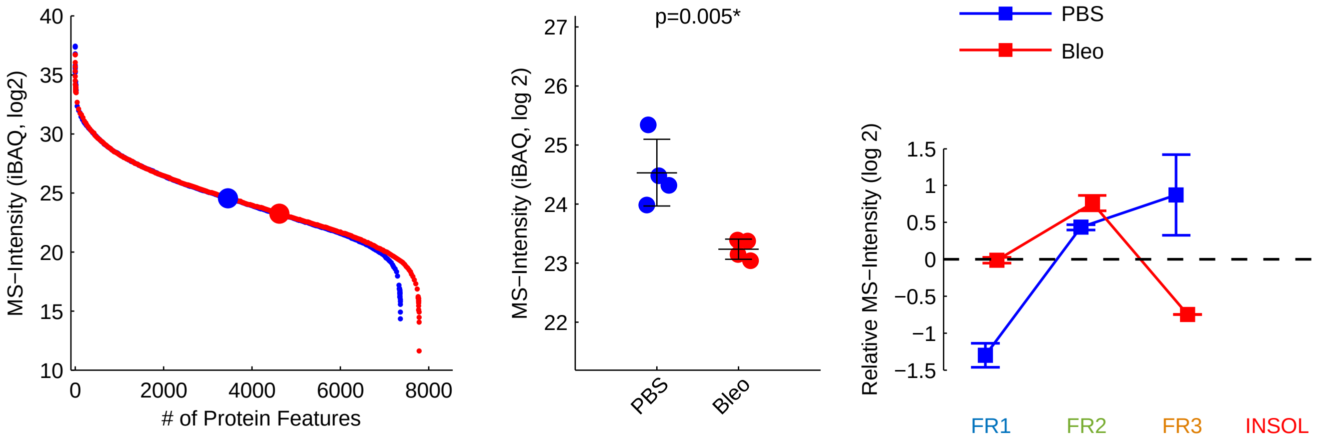

Q8BH40 − Stx7 (id: 5448)

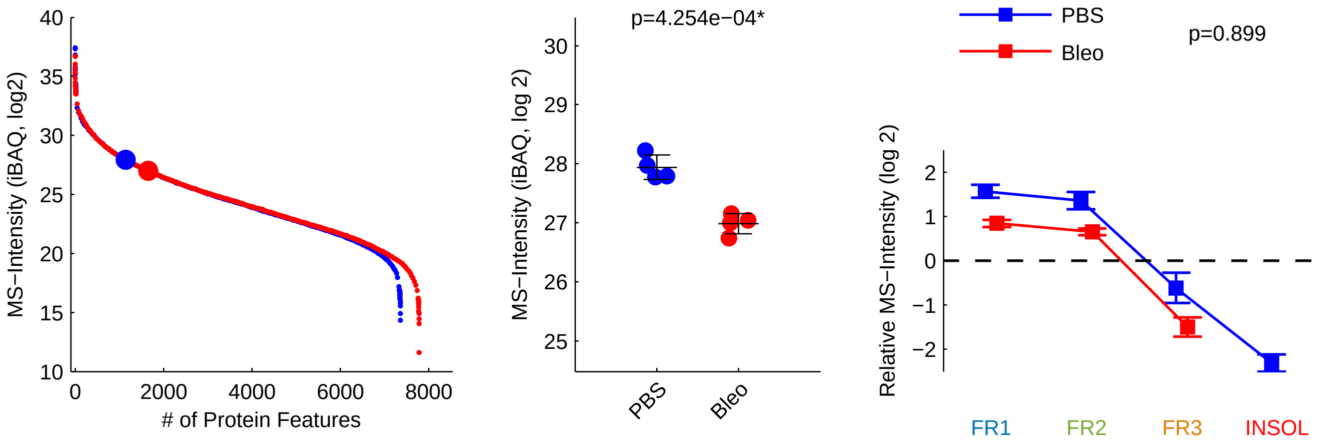

Q8BH57 − Wdr48 (id: 5454)

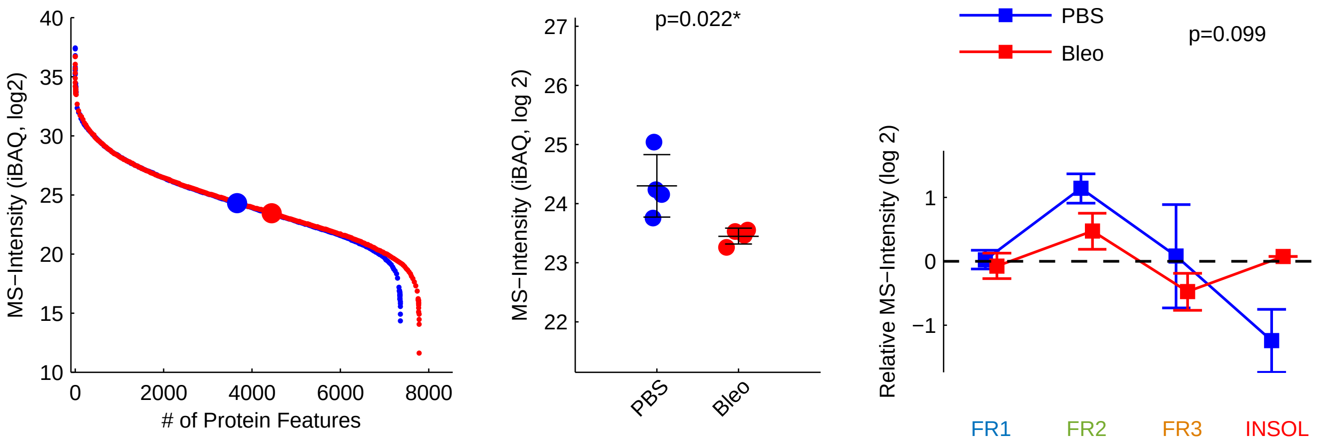

Q8BH61 − F13a1 (id: 5457)

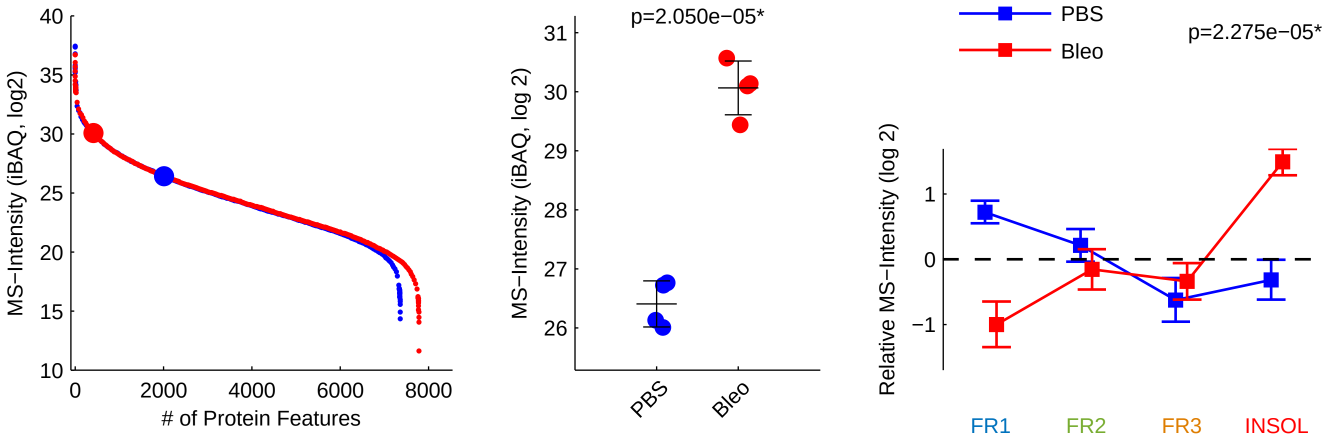

Q8BH64 − Ehd2 (id: 5458)

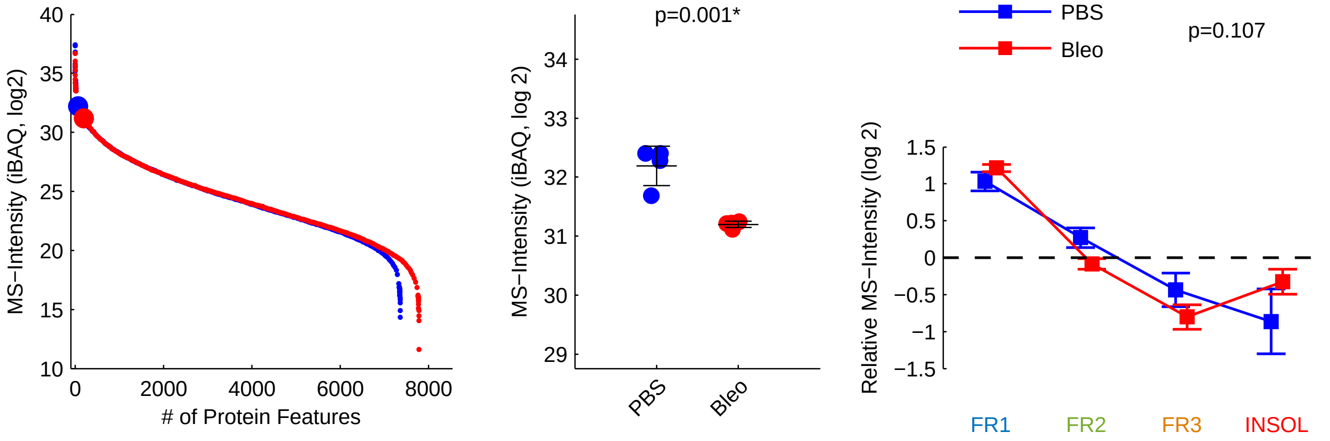

Q8BH69 − Sephs1 (id: 5460)

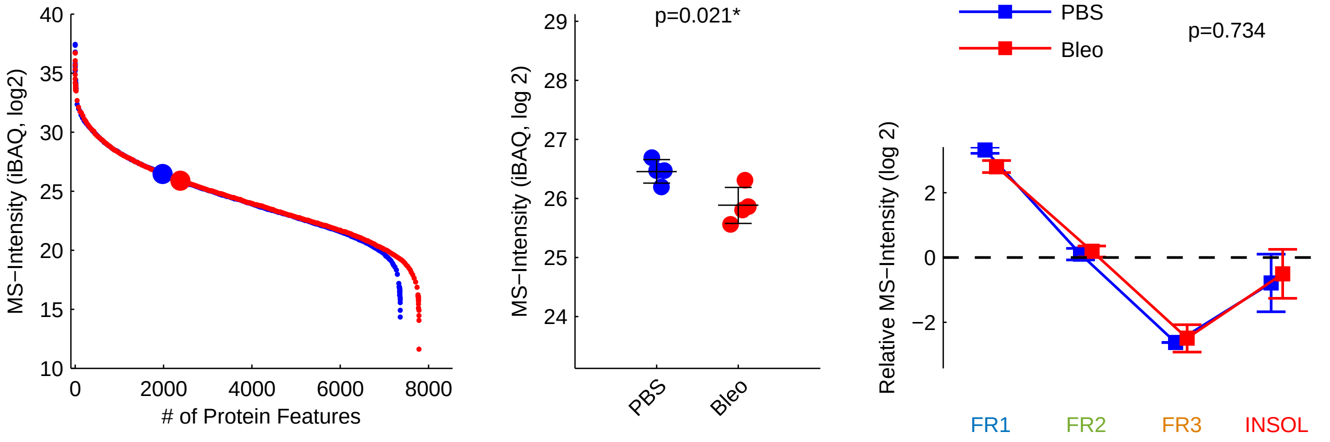

Q9QY76 − Vapb (id: 5464)

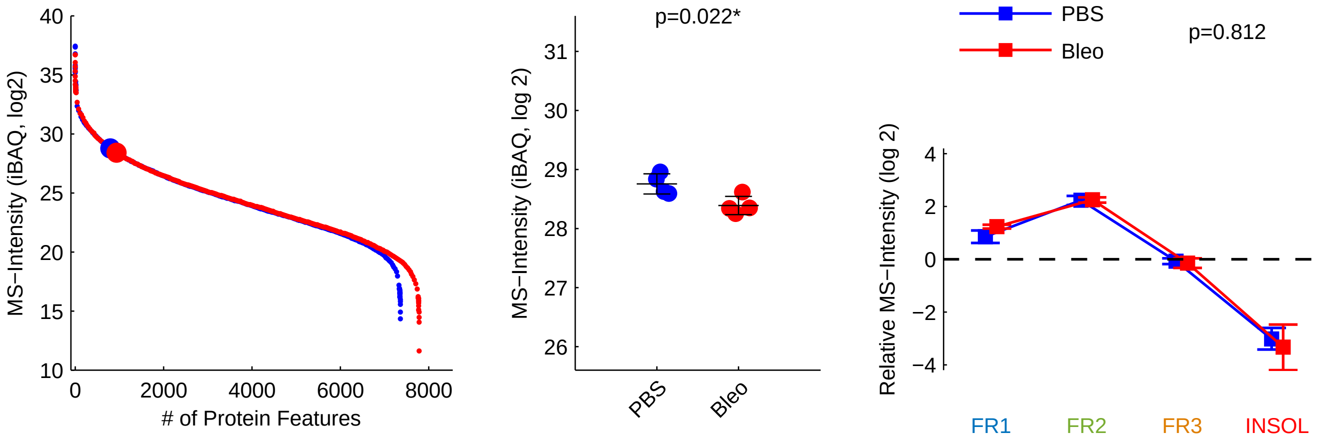

Q8BH95 − Echs1 (id: 5466)

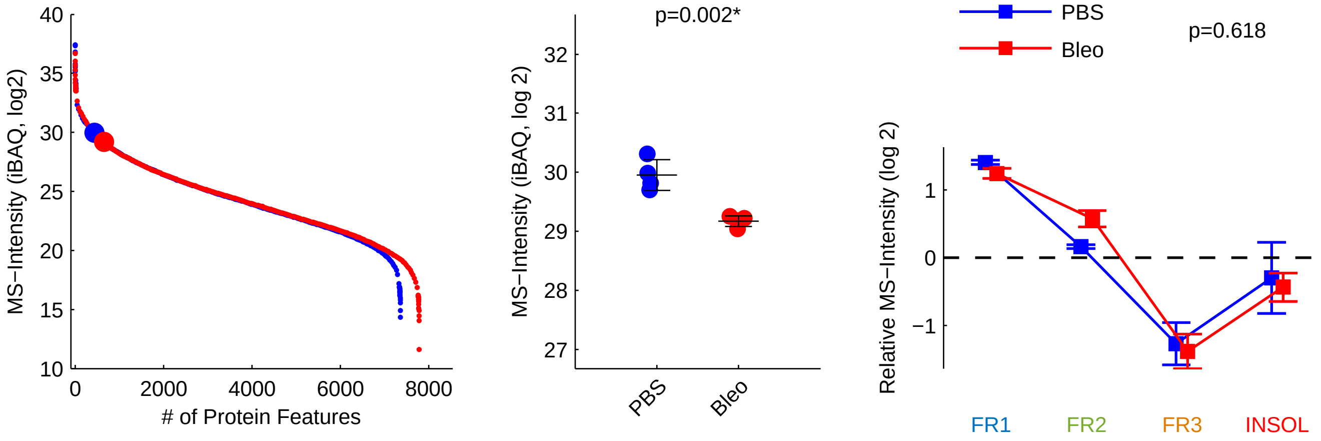

Q8BH97 − Rcn3 (id: 5467)

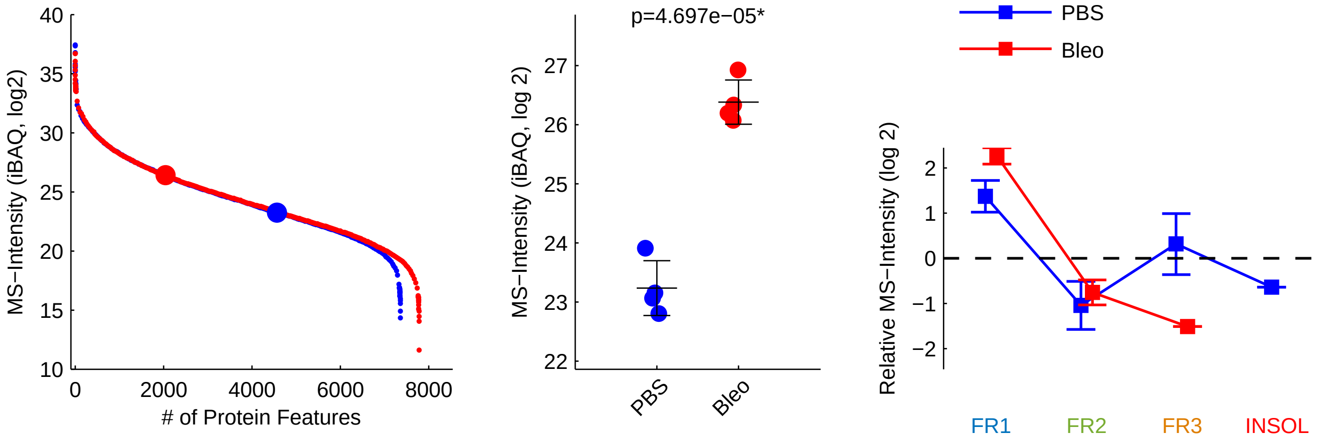

Q8BHC0 − Lyve1 (id: 5473)

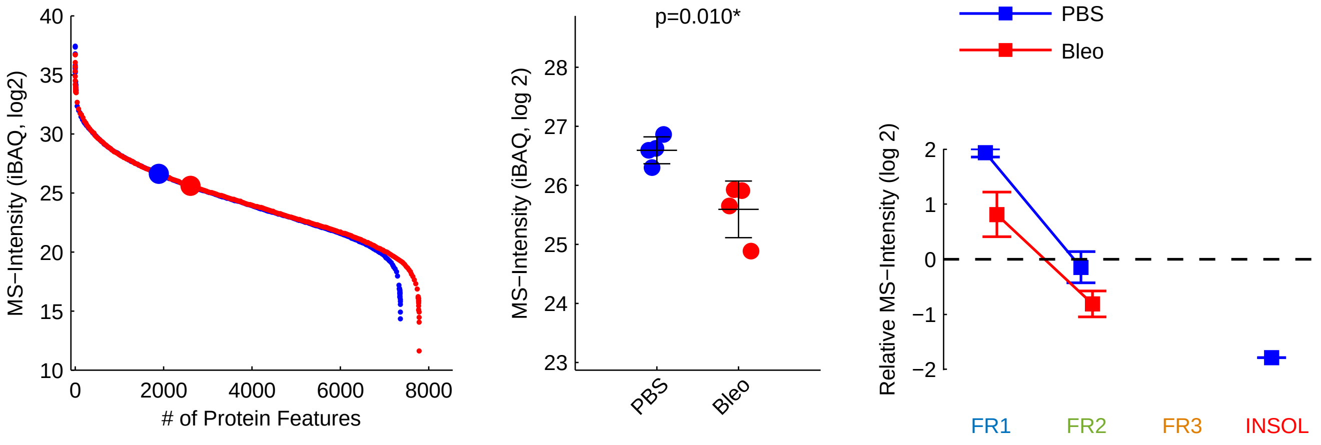

Q8BHC4 − Dcakd (id: 5474)

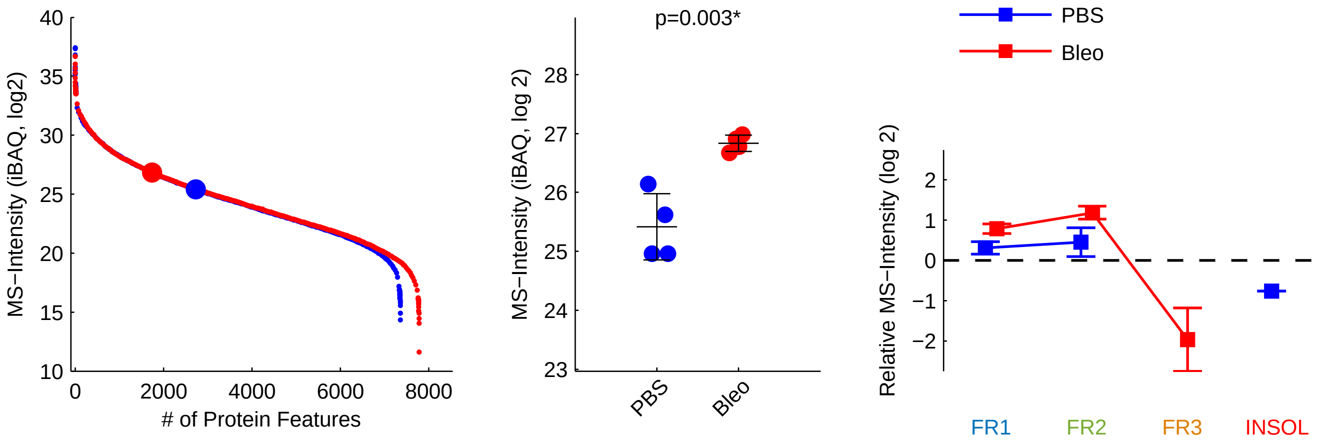

Q8BHD1 − Poc1b (id: 5477)

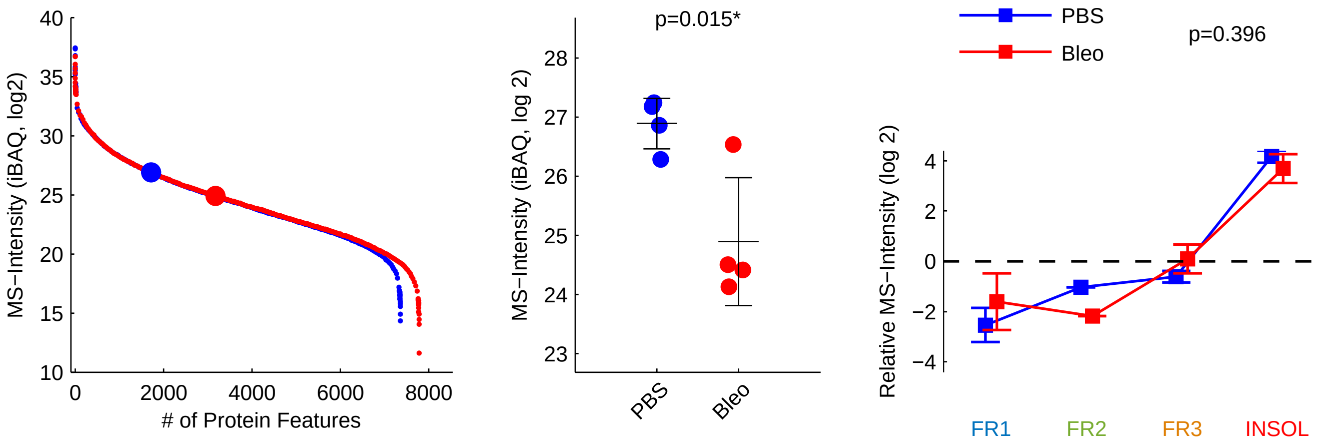

Q8BHG9 − Cggbp1 (id: 5483)

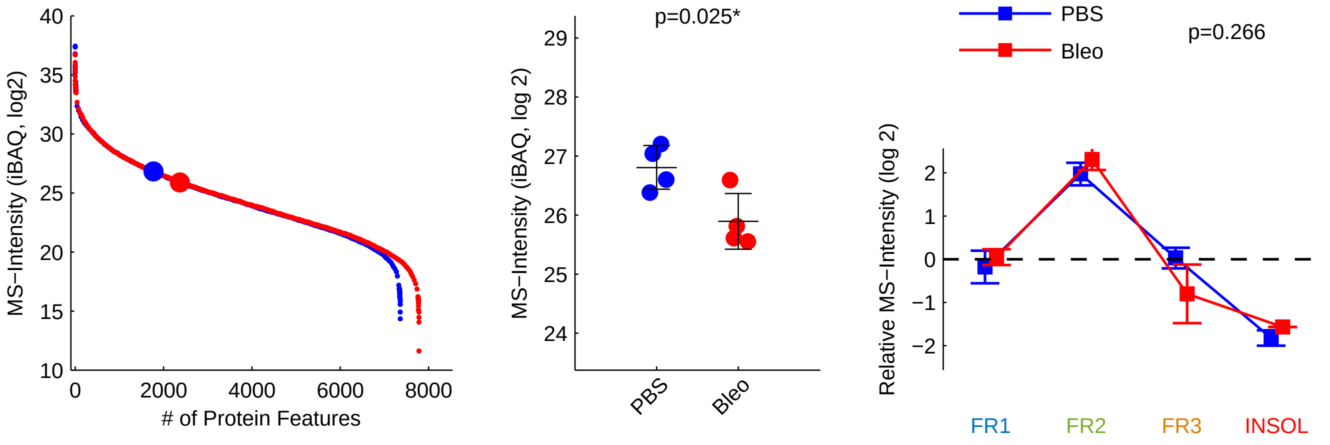

Q8BHL8 − Psmf1 (id: 5489)

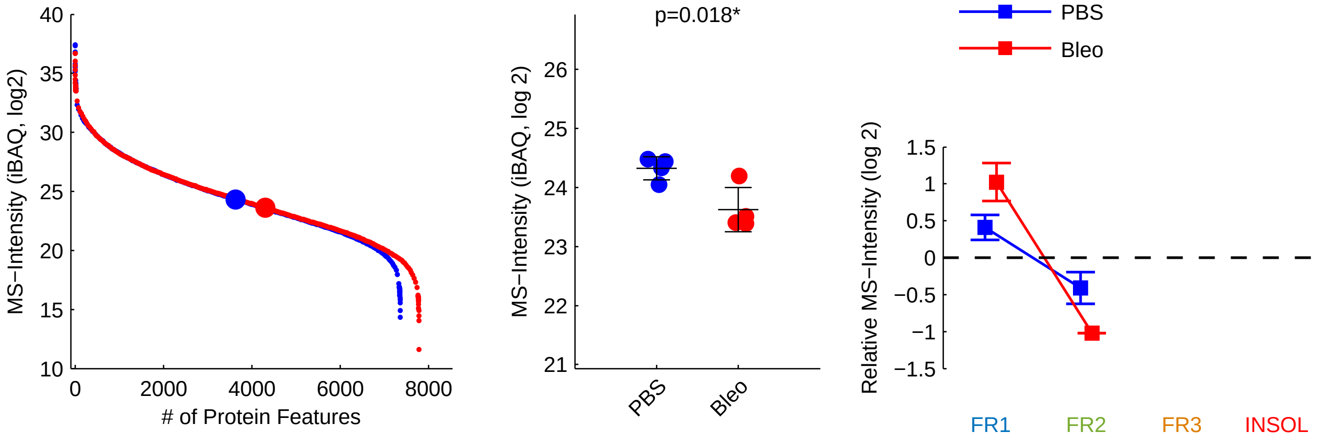

Q8BHZ0 − Fam49a (id: 5500)

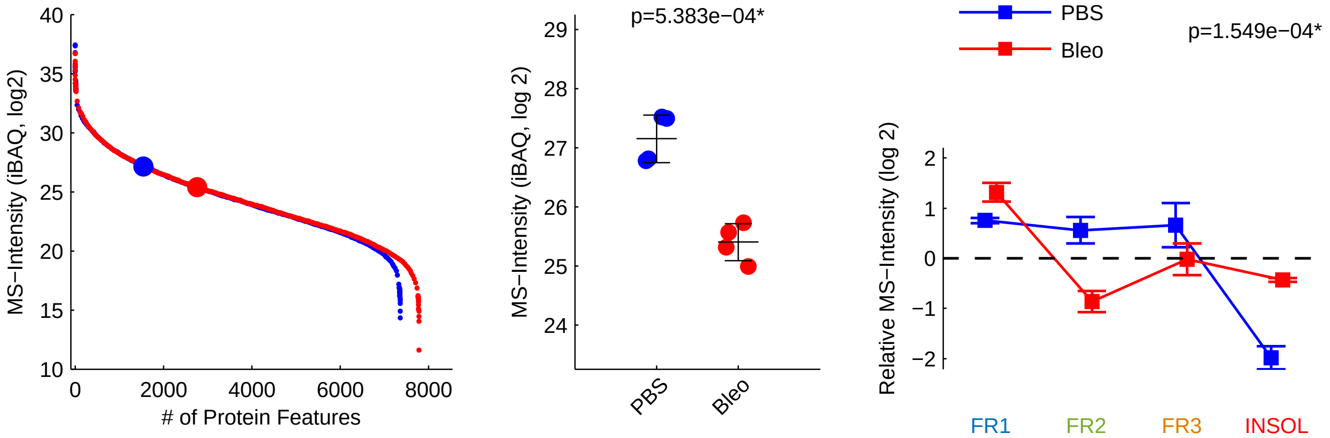

Q8BI08 − Mal2 (id: 5502)

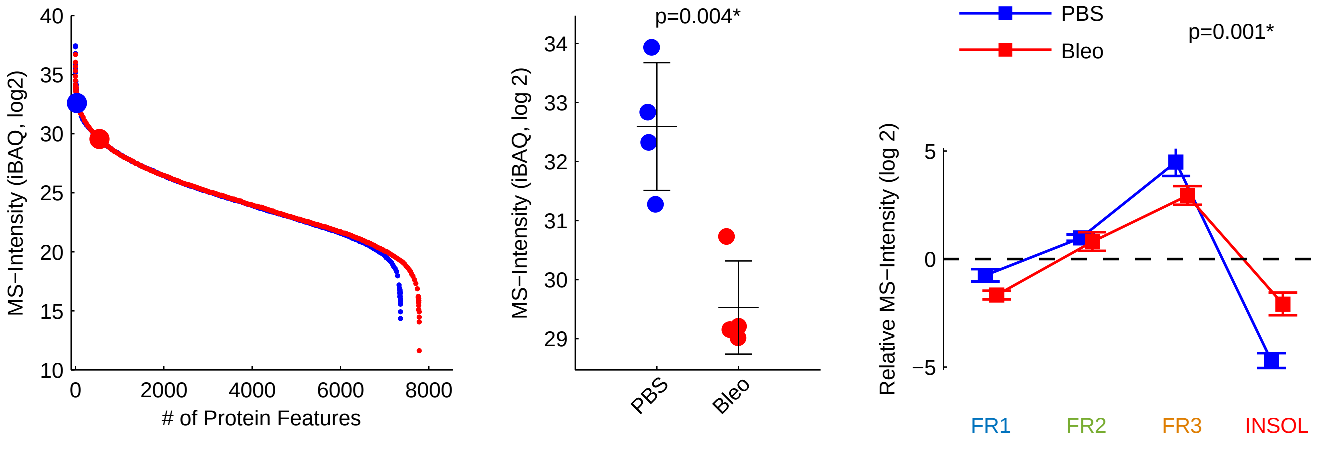

Q8BI22 − Cep128 (id: 5503)

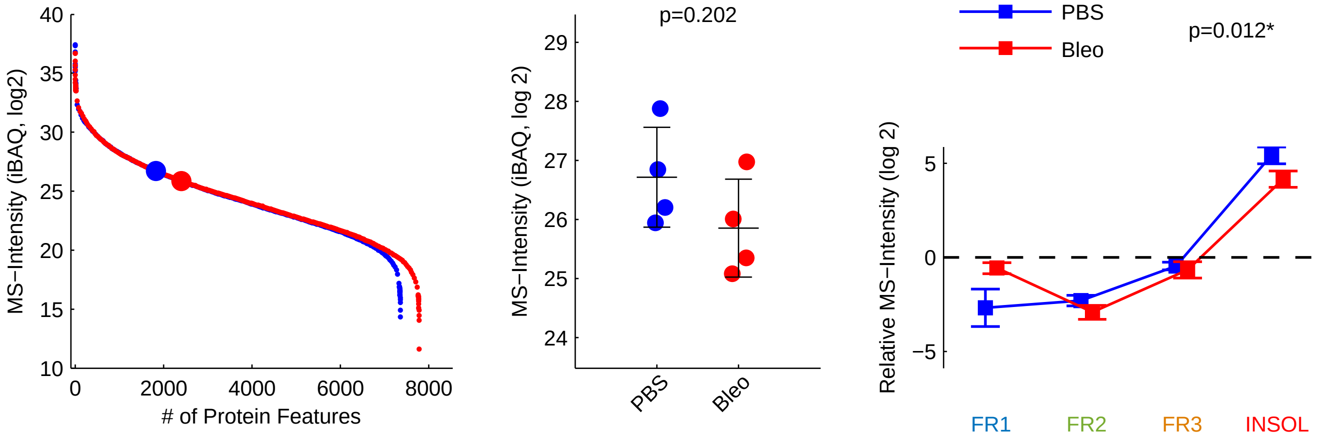

Q8BIG4 − Fbxo28 (id: 5509)

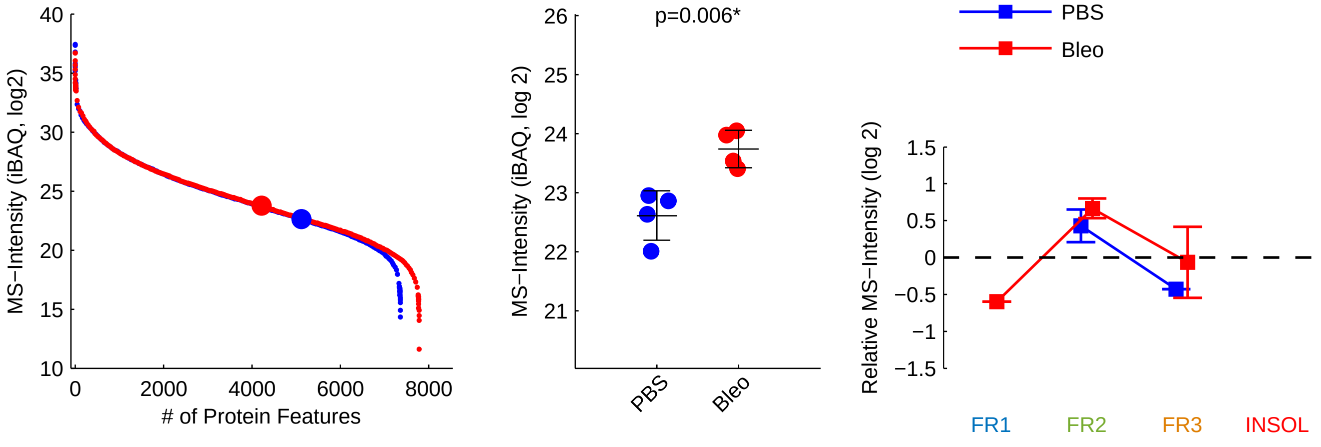

Q8BIJ6 − Iars2 (id: 5511)

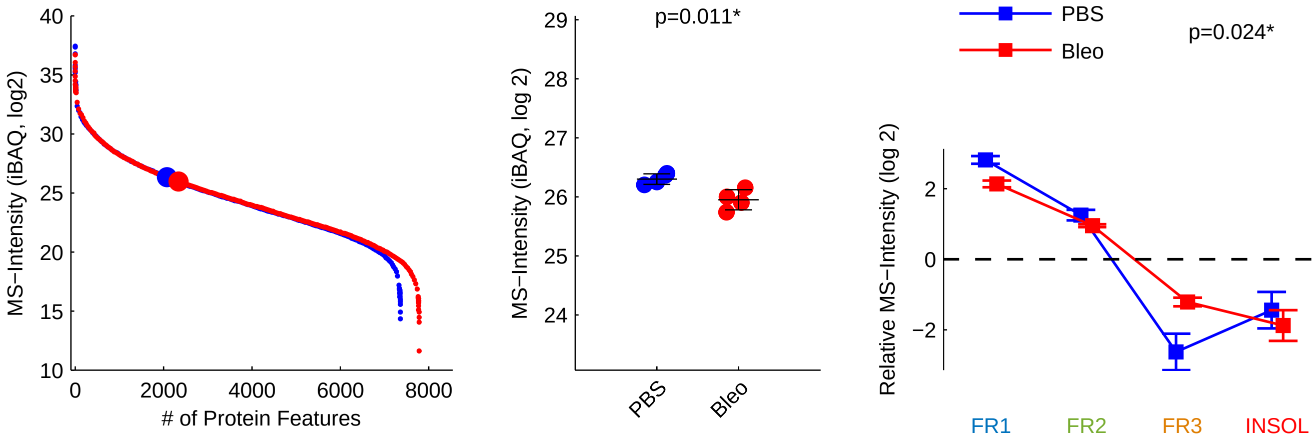

Q8BJQ9 − Csgalnact1 (id: 5530)

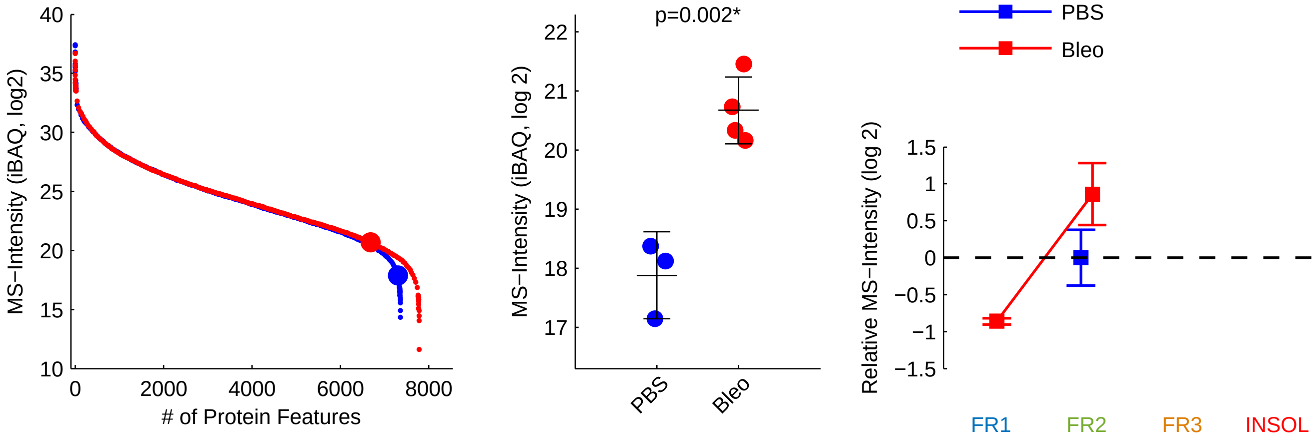

Q8BJW6 − Eif2a (id: 5534)

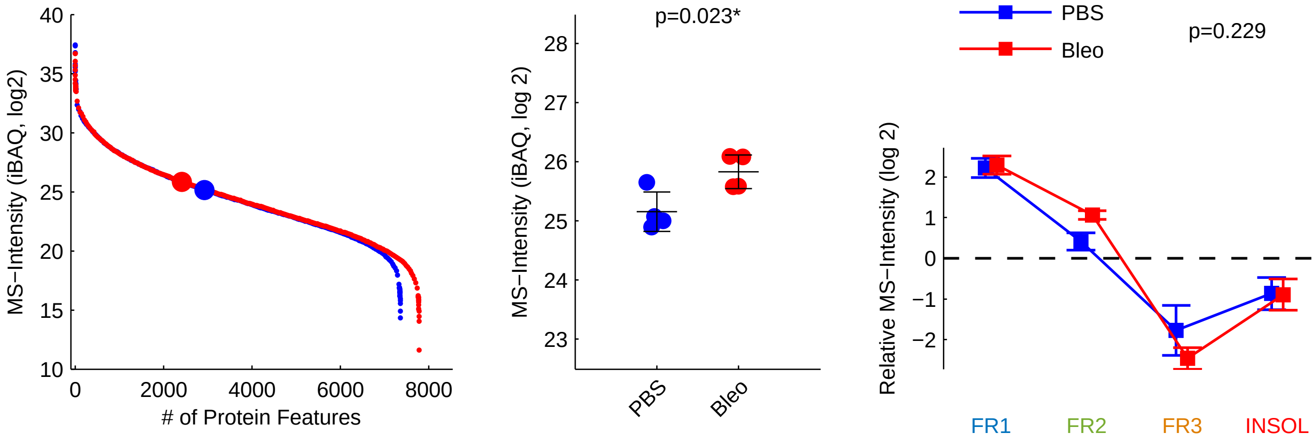

Q8BJY1 − Psmd5 (id: 5535)

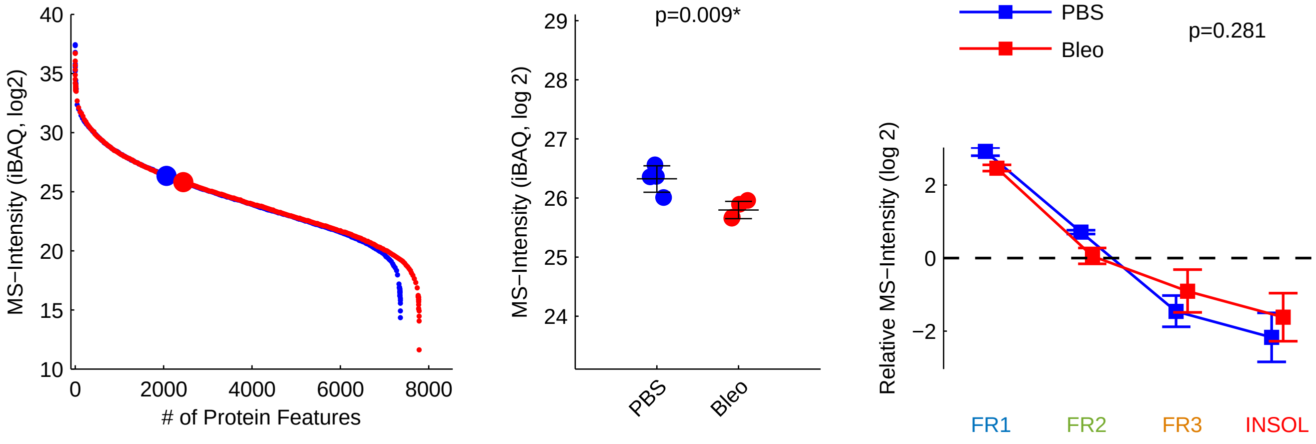

Q8BK62 − Olfml3 (id: 5542)

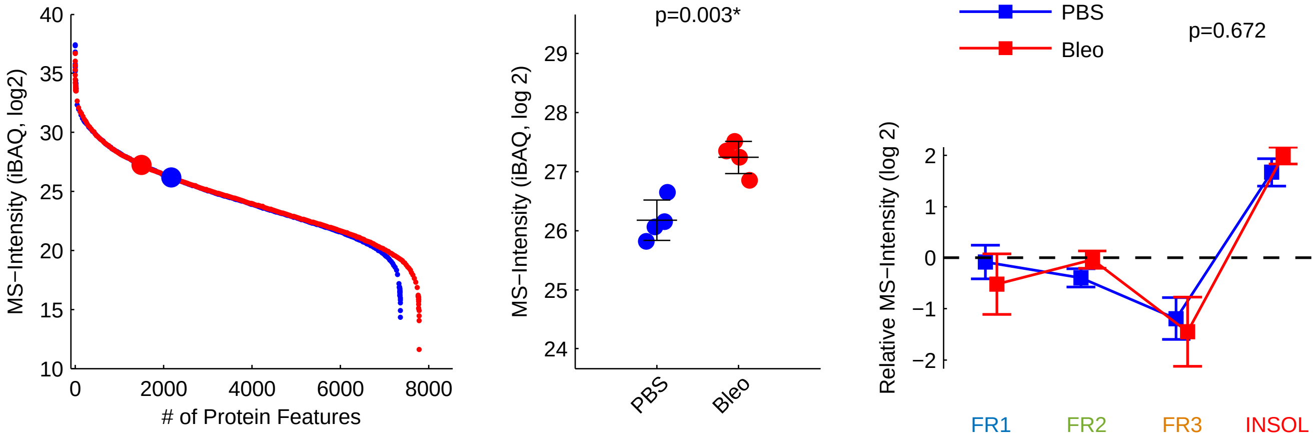

Q8BK64 − Ahsa1 (id: 5543)

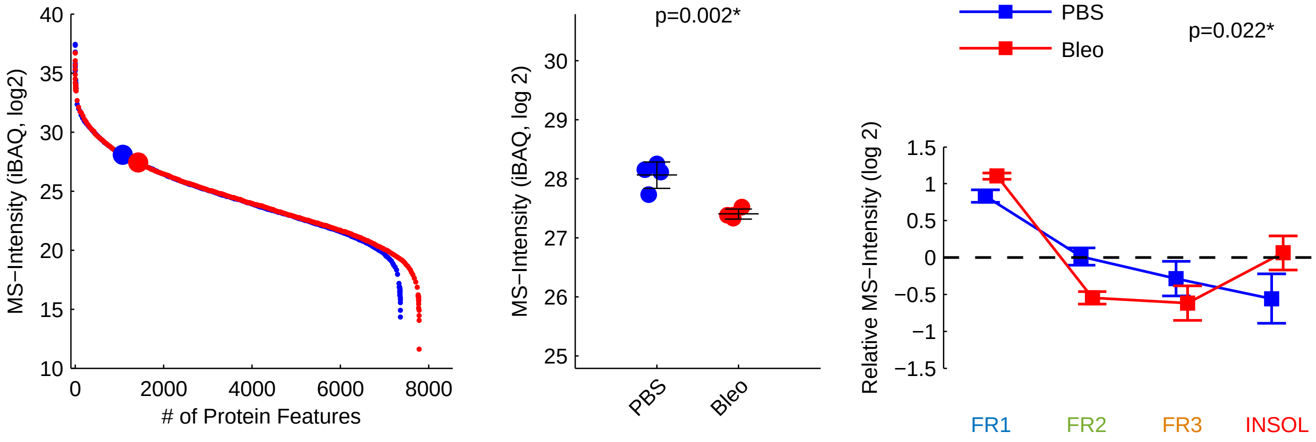

Q8BKC5 − Ipo5 (id: 5547)

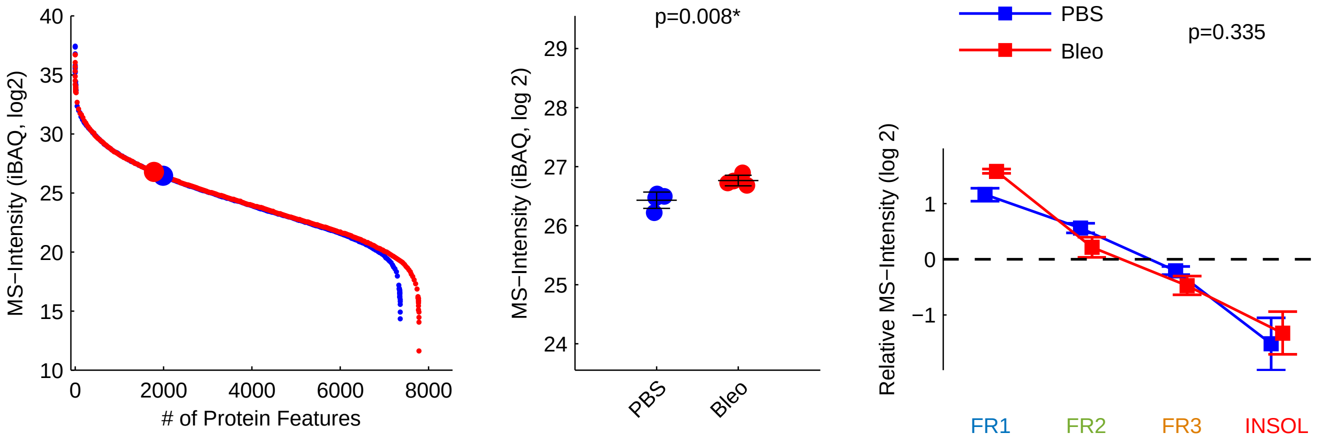

Q8BKX1 − Baiap2 (id: 5557)

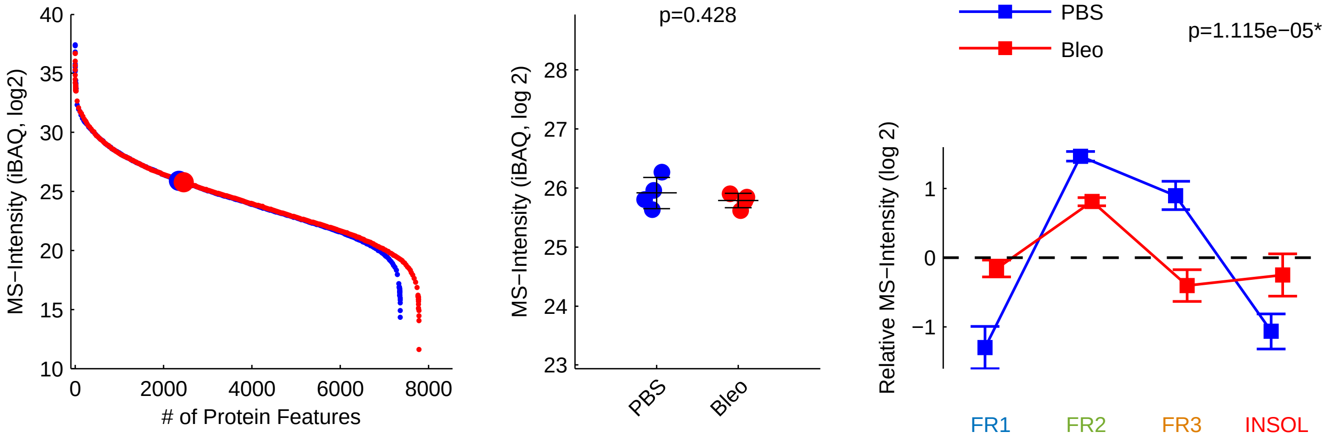

Q8BL97−3 − Srsf7 (id: 5568)

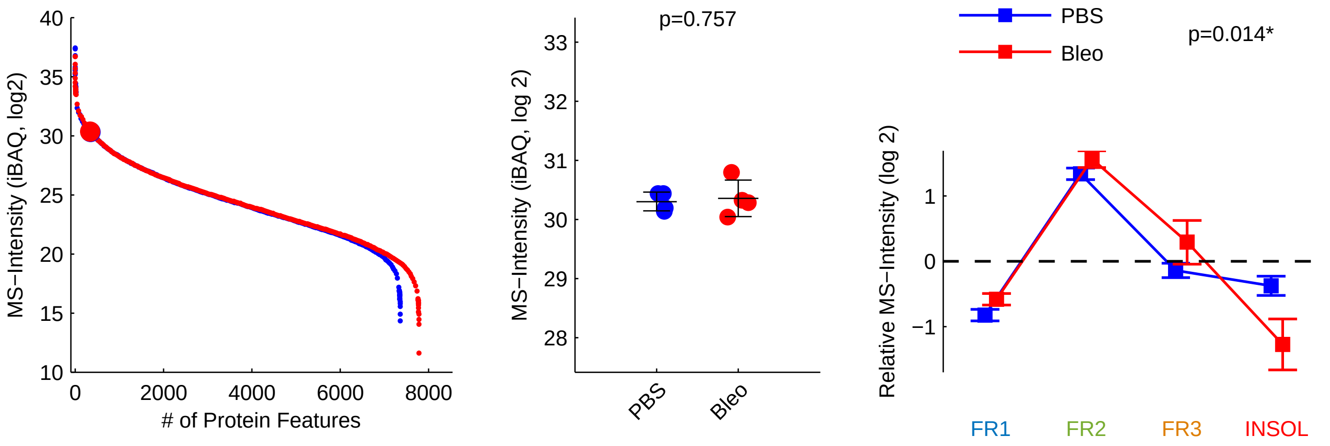

Q8BLN5 − Lss (id: 5574)

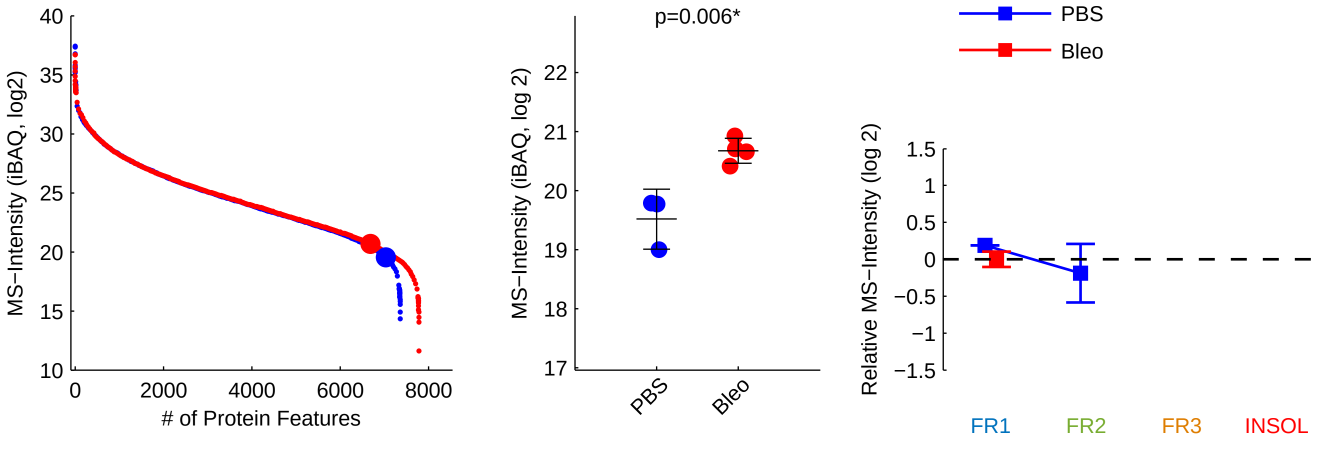

Q8BM55 − Tmem214 (id: 5581)

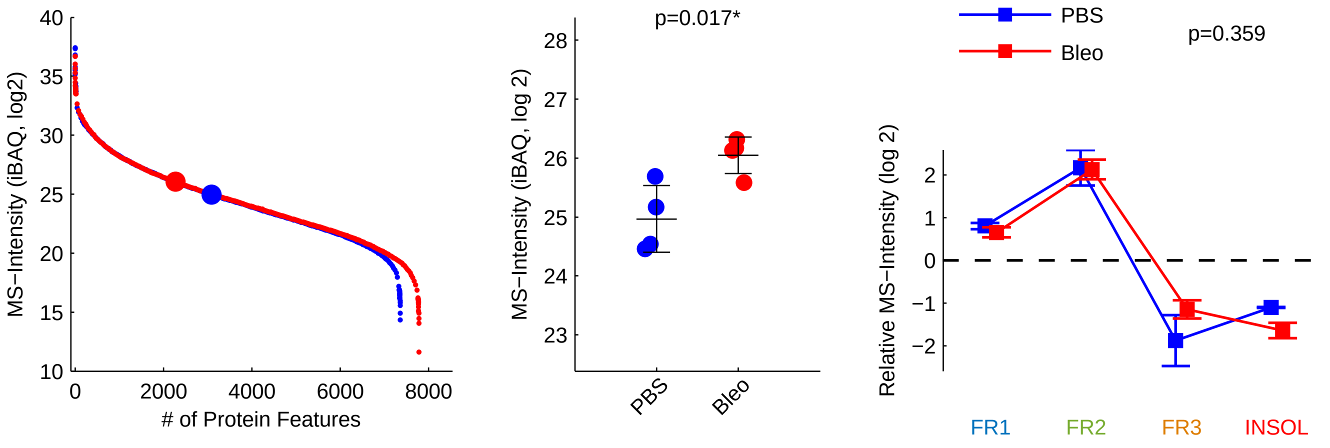

Q8BMA6 − Srp68 (id: 5586)

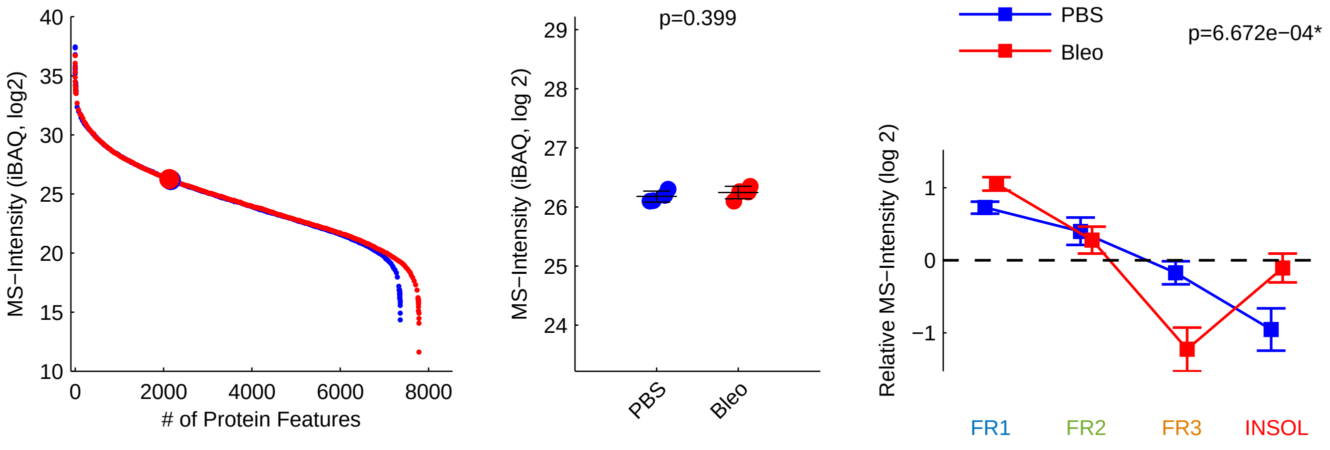

Q8BMJ2 − Lars (id: 5594)

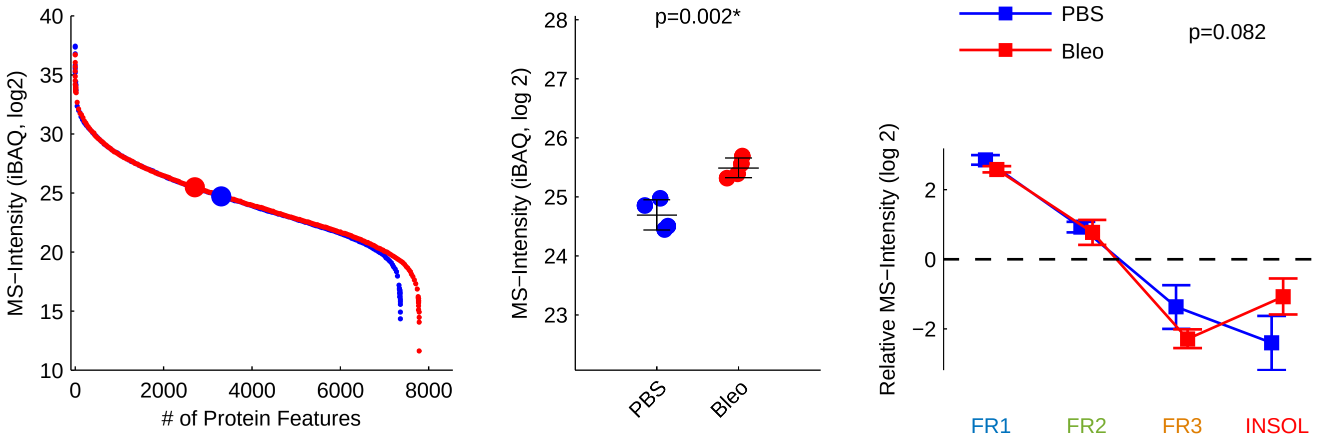

Q8BMK4 − Ckap4 (id: 5597)

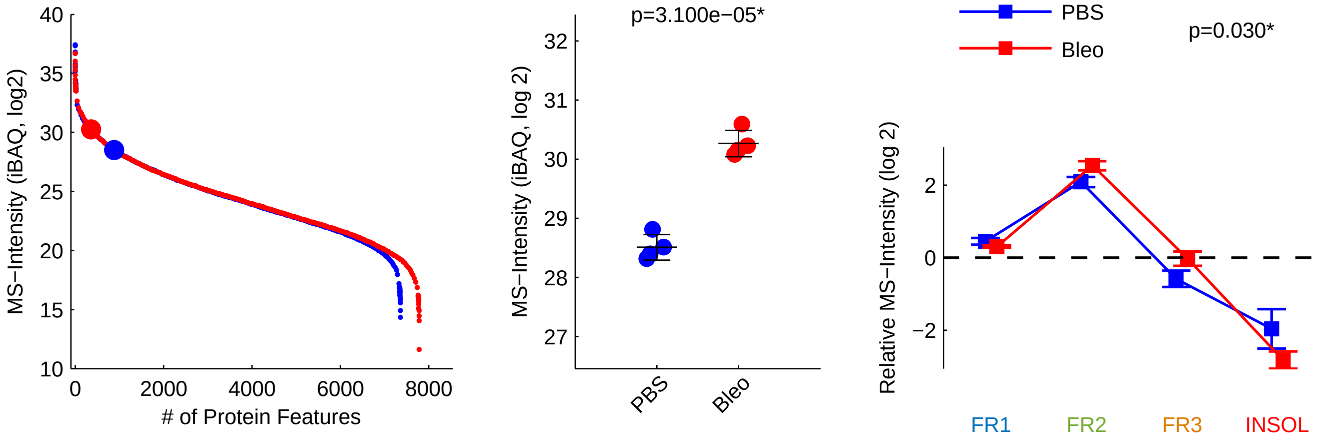

Q8BML9 − Qars (id: 5600)

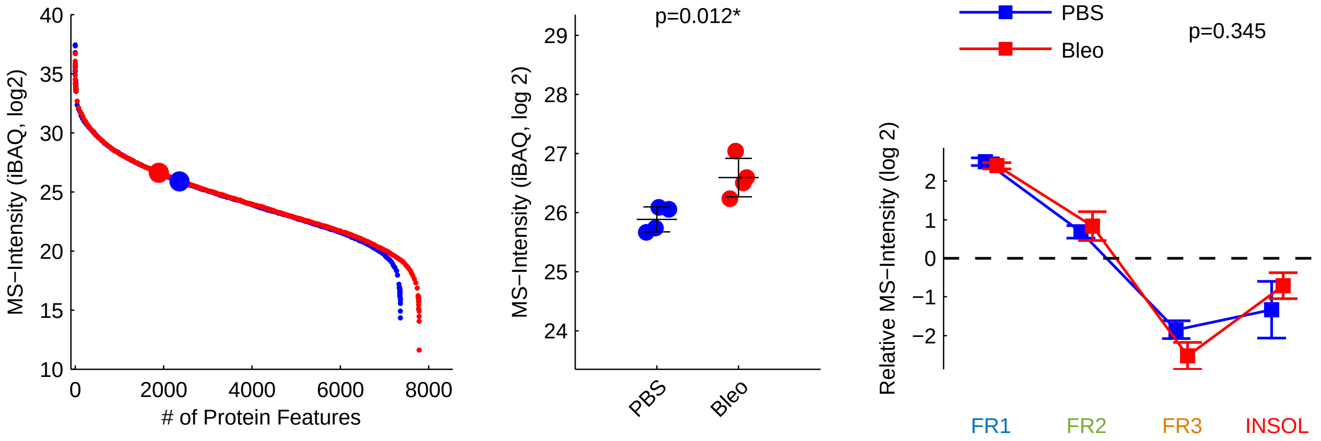

Q8BMP6 − Acbd3 (id: 5601)

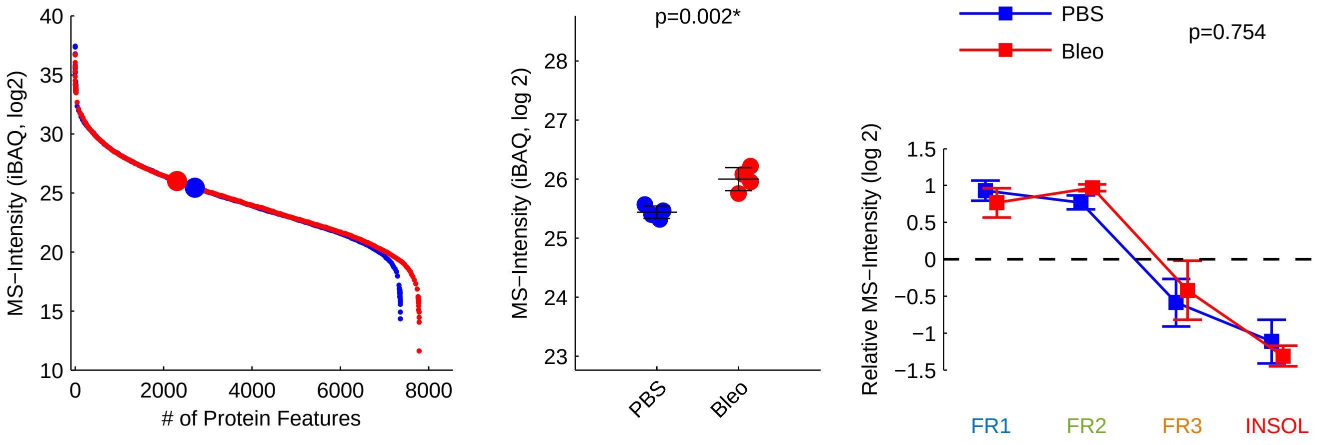

Q8BMQ2 − Gtf3c4 (id: 5602)

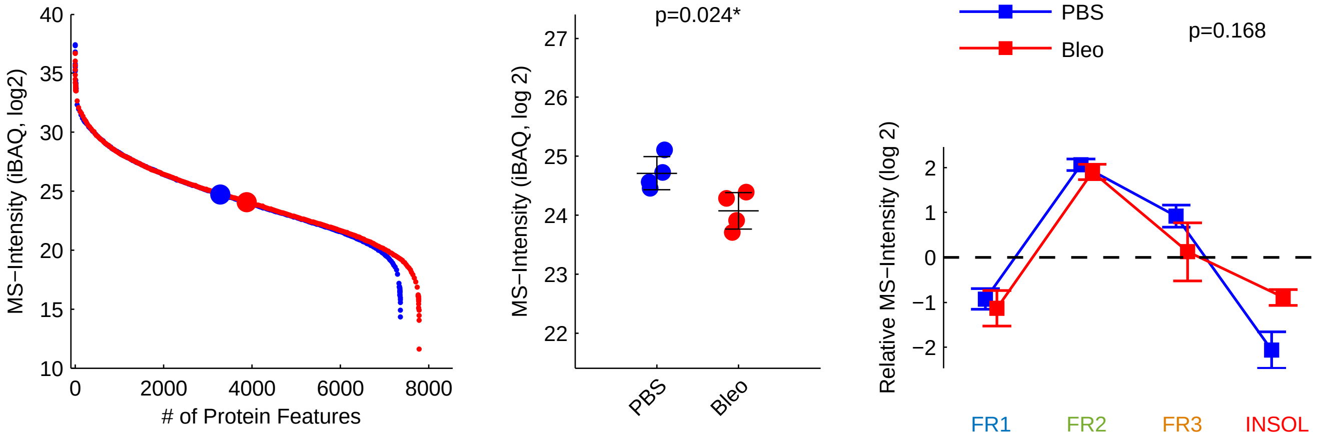

Q8BN21 − Vrk2 (id: 5607)

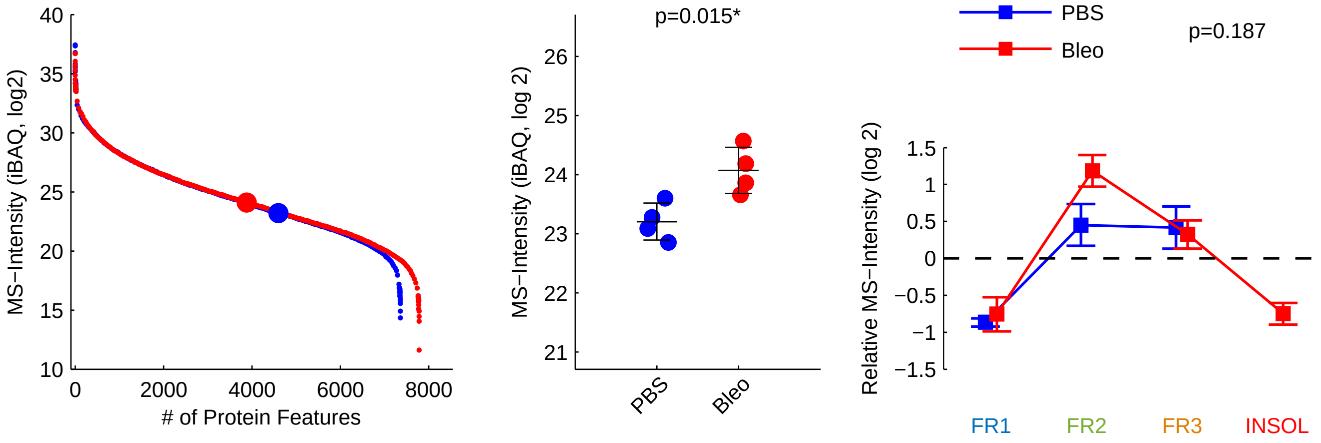

Q8BND5−3 − Qsox1 (id: 5612)

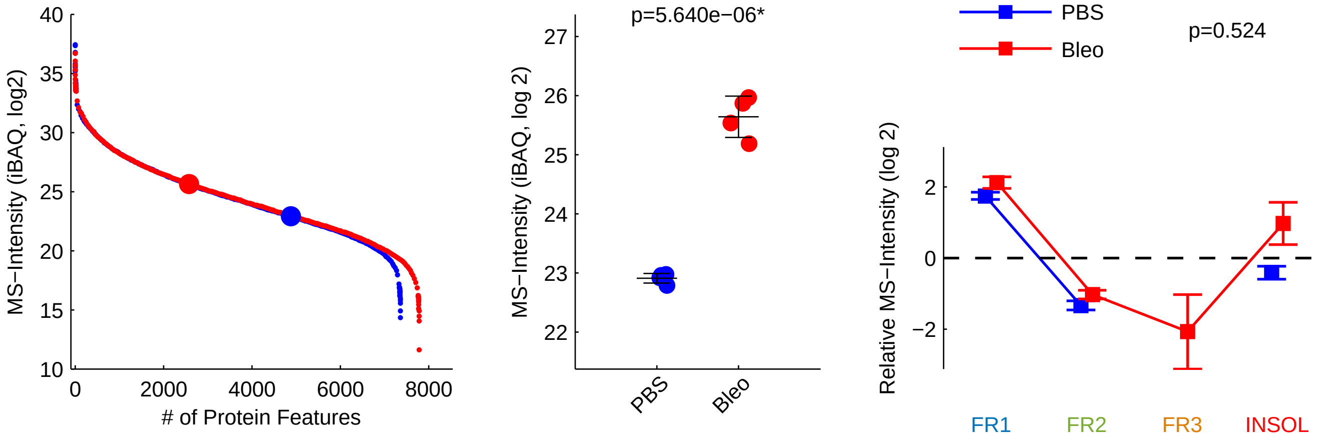

Q8BP47 − Nars (id: 5622)

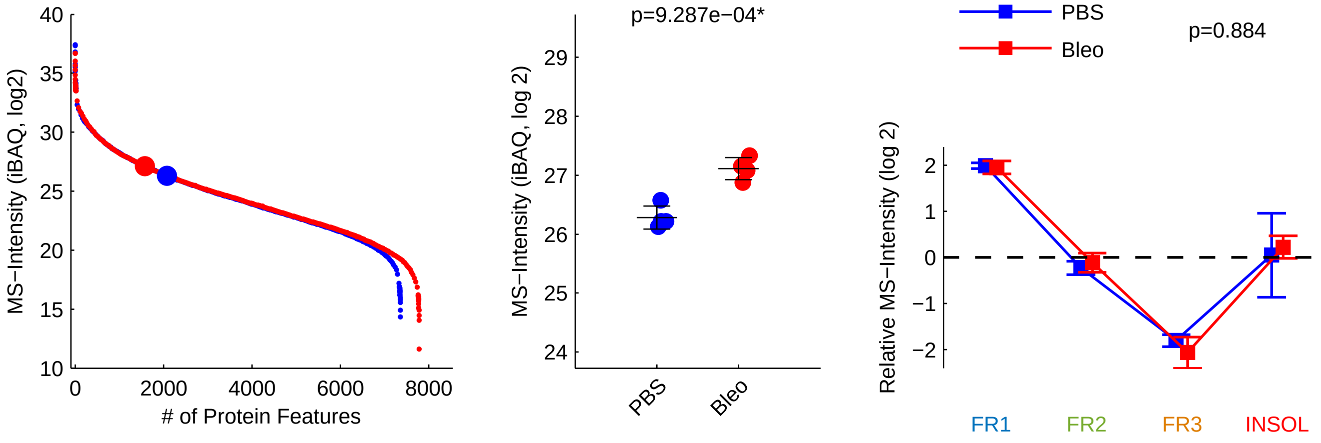

Q8BP48 − Metap1 (id: 5623)

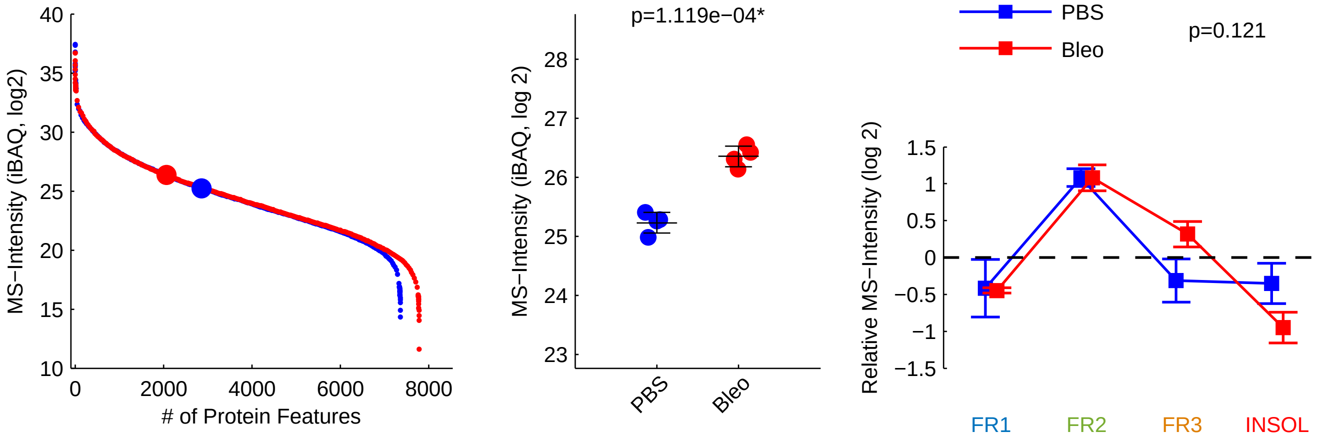

Q8BPB5 − Efemp1 (id: 5628)

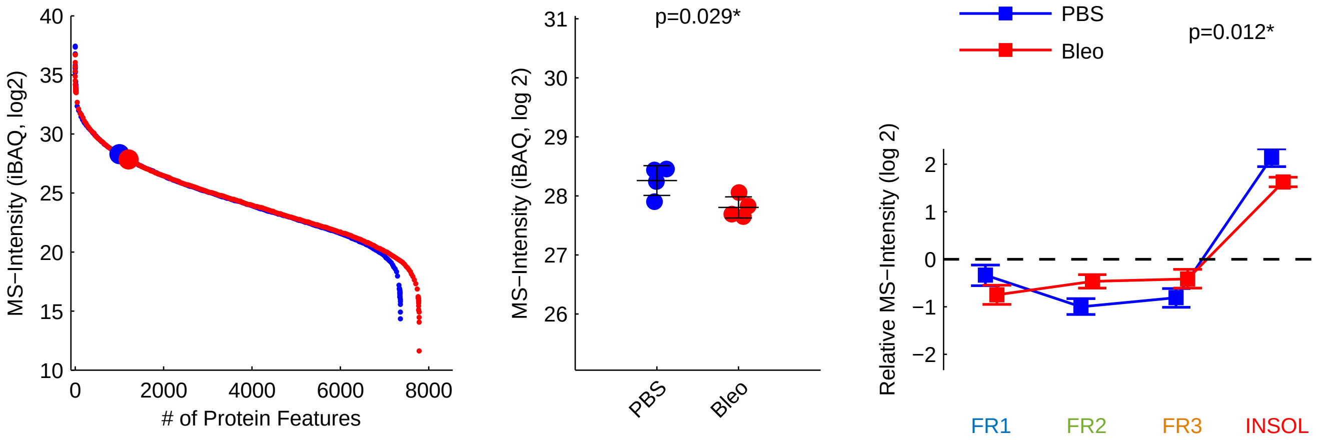

Q8BPU7 − Elmo1 (id: 5631)

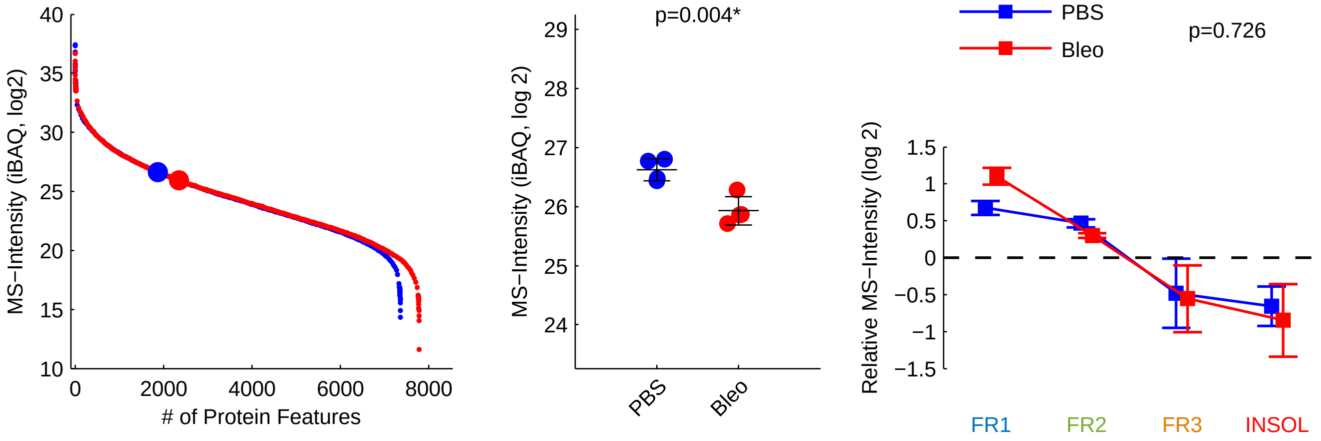

Q8BQN6 − Znf146 (id: 5638)

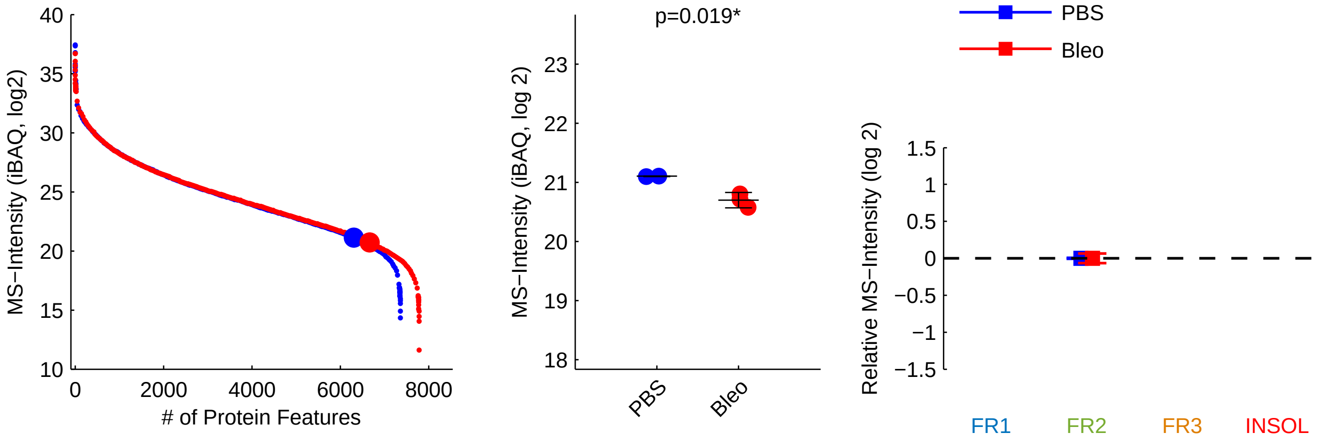

Q8BR65 − Suds3 (id: 5640)

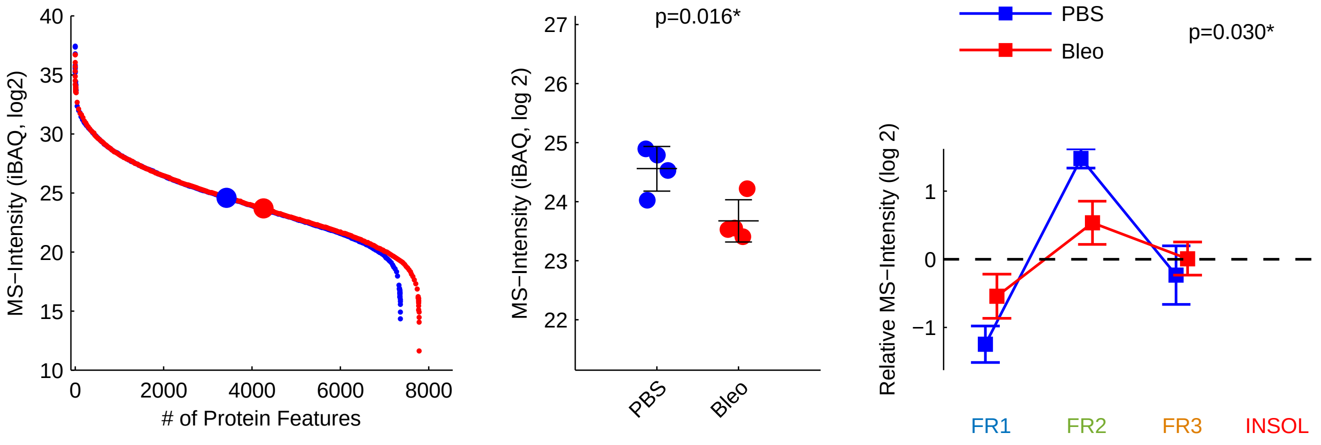

Q8BR90 − (id: 5641)

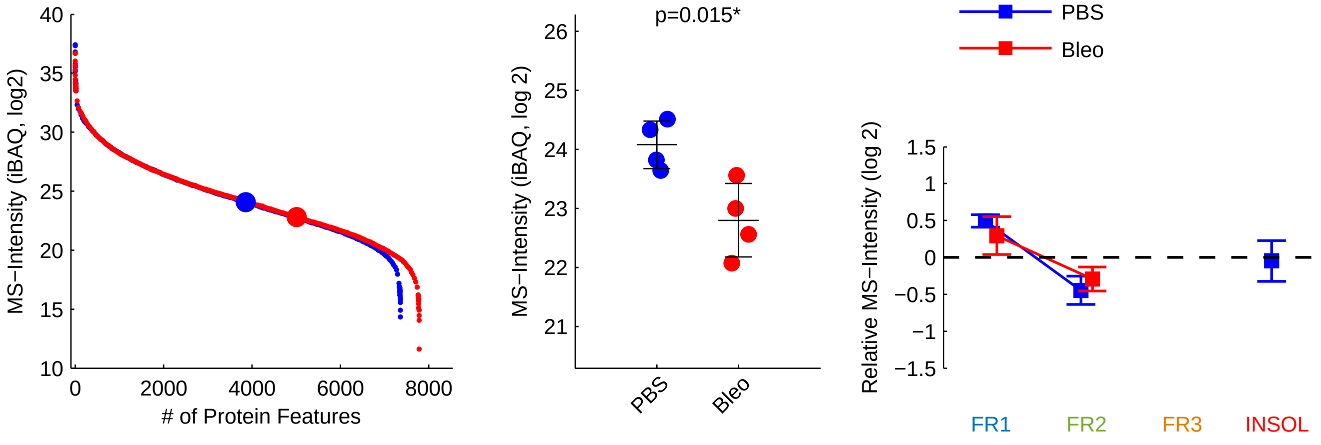

Q8BSE0 − Rmdn2 (id: 5657)

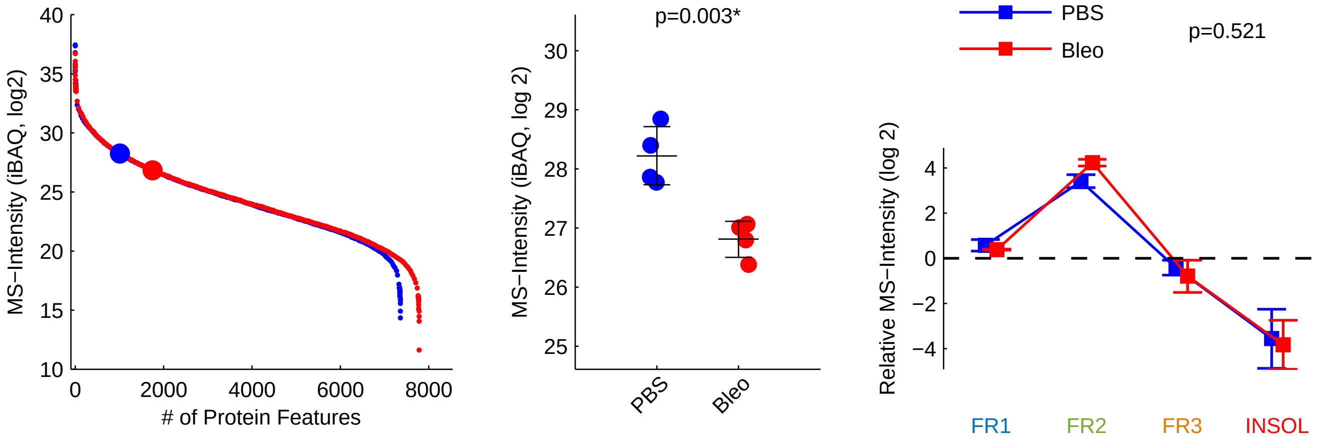

Q8BTW9 − Pak4 (id: 5675)

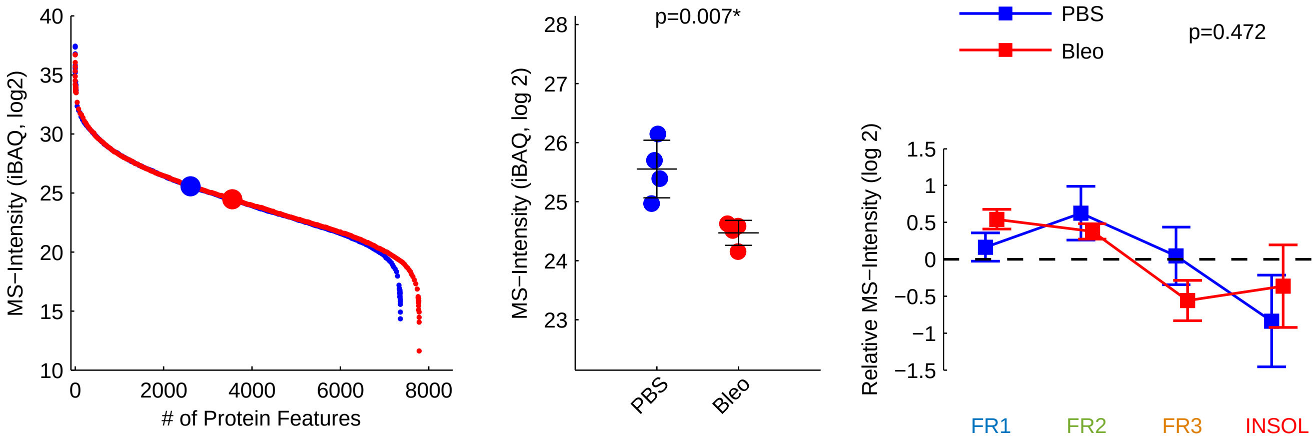

Q8BU03 − Pwp2 (id: 5683)

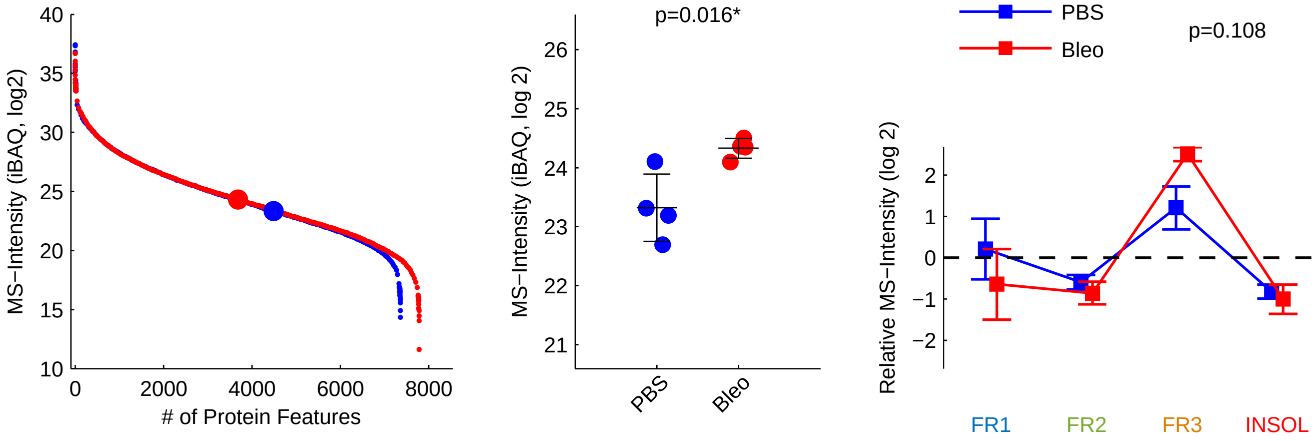

Q8BU30 − Iars (id: 5687)

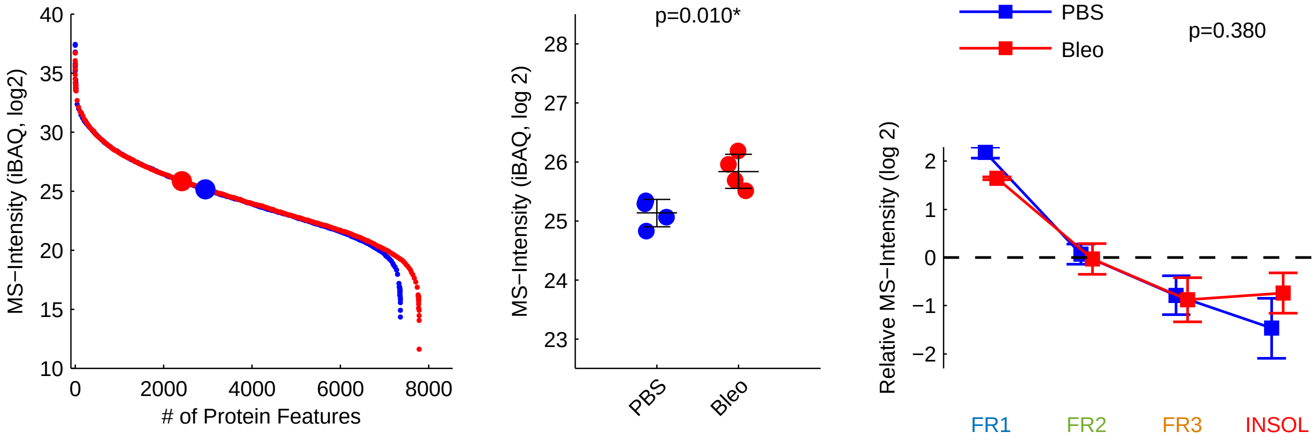

Q8BUK6 − Hook3 (id: 5696)

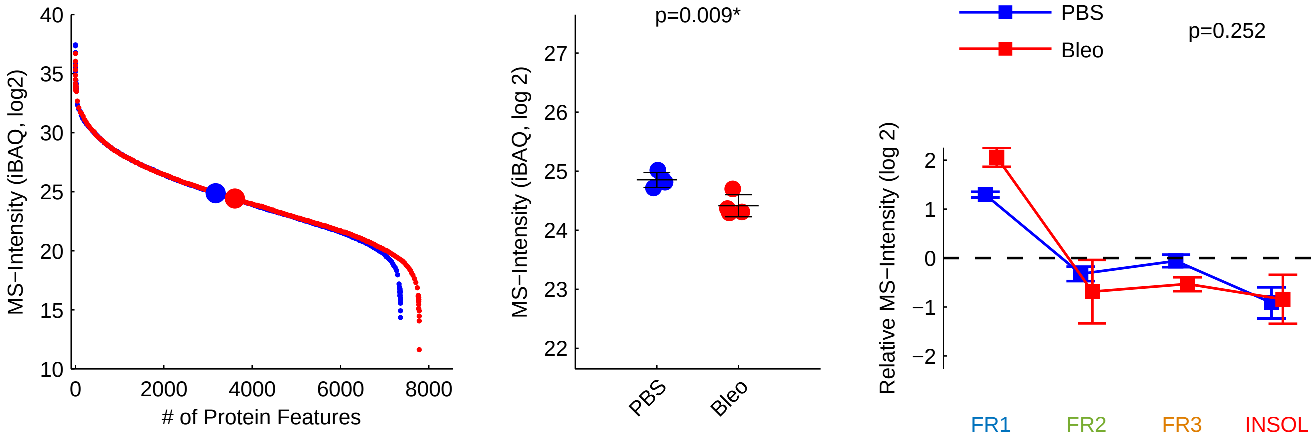

Q8BUR4 − Dock1 (id: 5698)

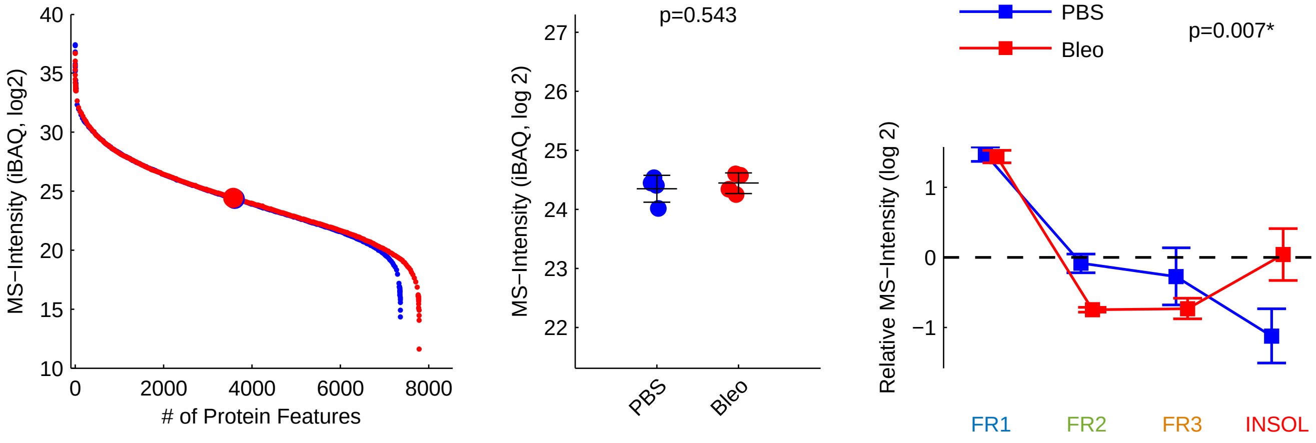

Q8BV49 − Pyhin1 (id: 5704)

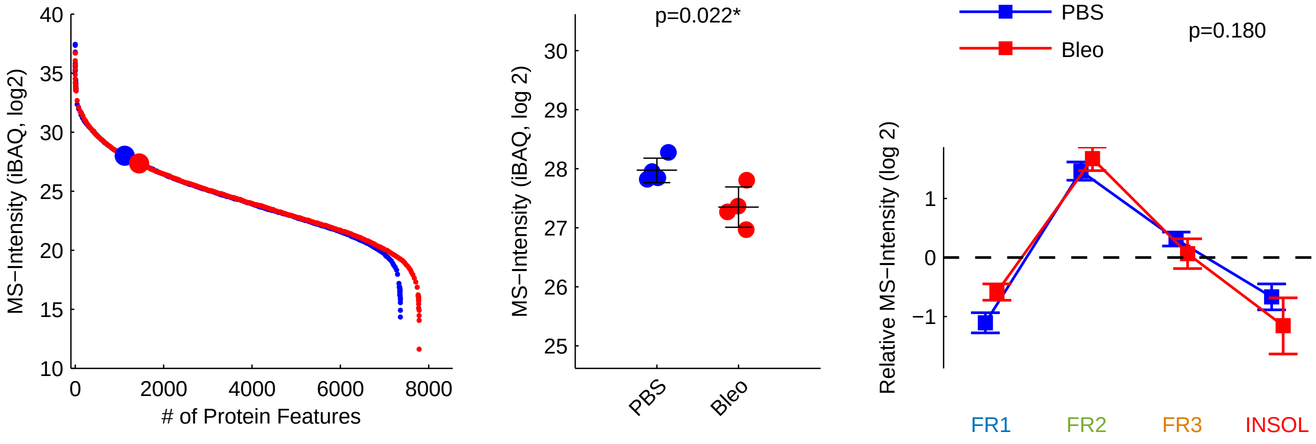

Q8BVI4 − Qdpr (id: 5711)

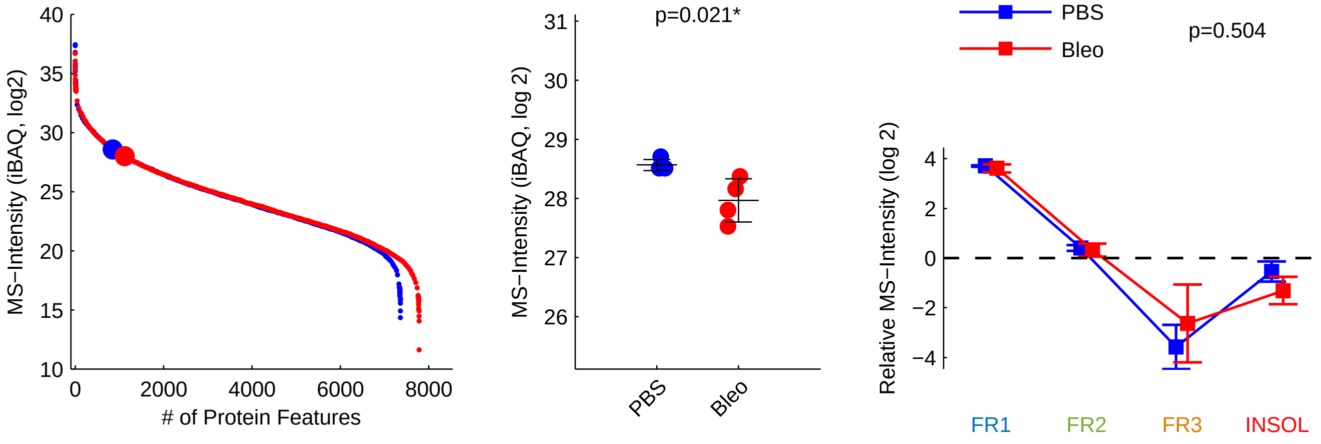

Q8BW75 − Maob (id: 5728)

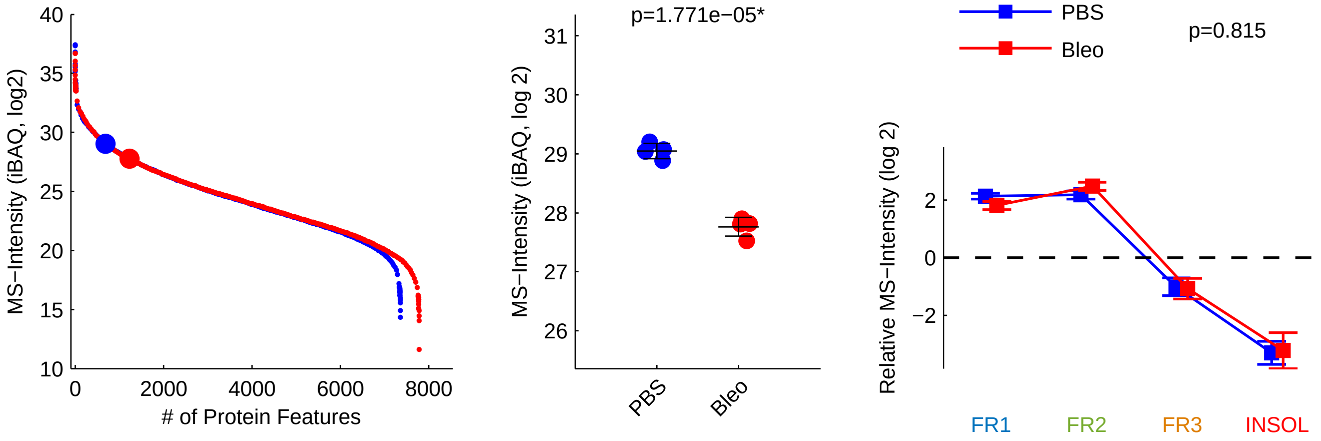

Q8BWF0 − Aldh5a1 (id: 5730)

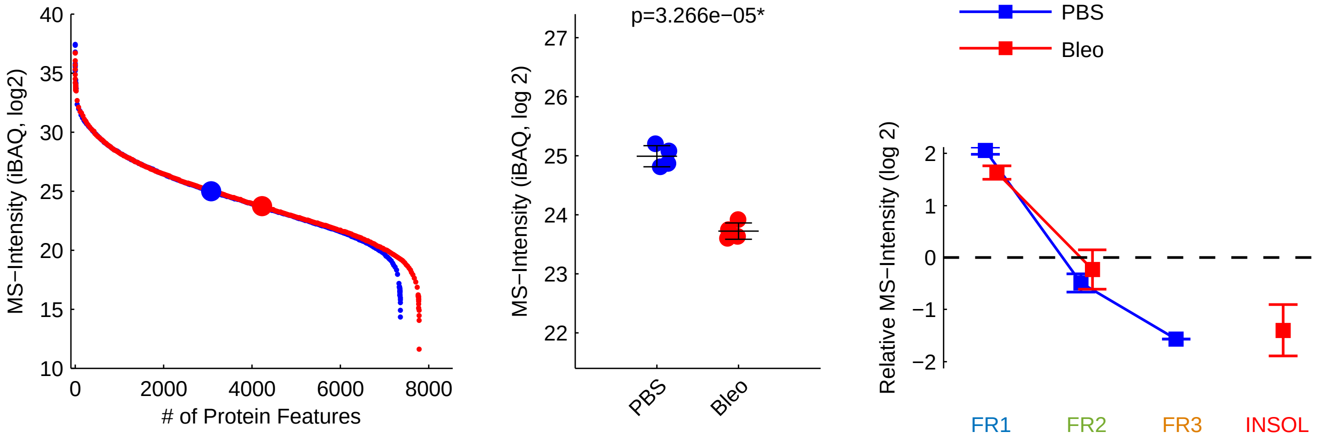

Q8BWG8 − Arrb1 (id: 5732)

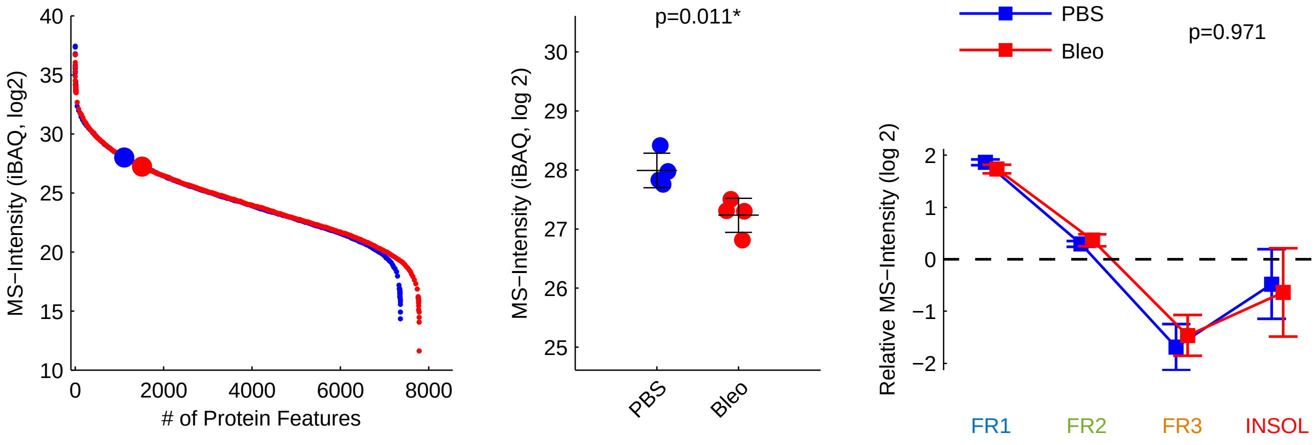

Q8BWT1 − Acaa2 (id: 5741)

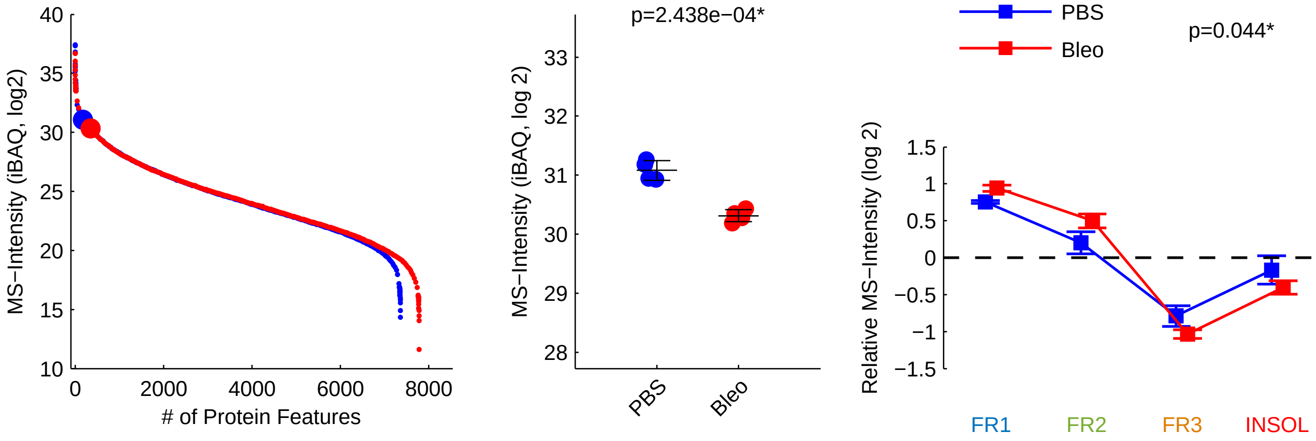

Q8BWY2 − Clec1a (id: 5743)

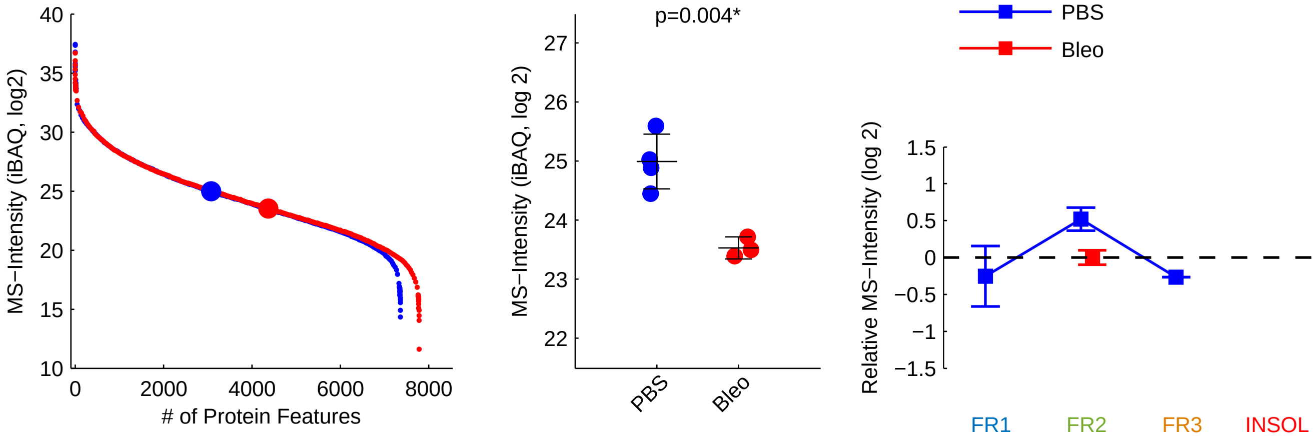

Q8BWY3 − Etf1 (id: 5744)

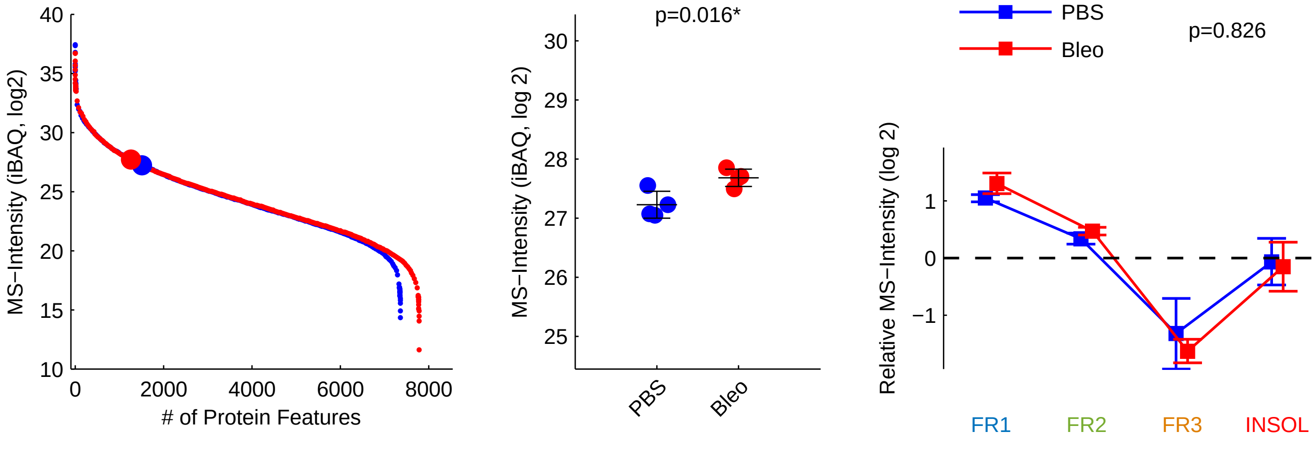

Q8BX09 − Rbbp5 (id: 5748)

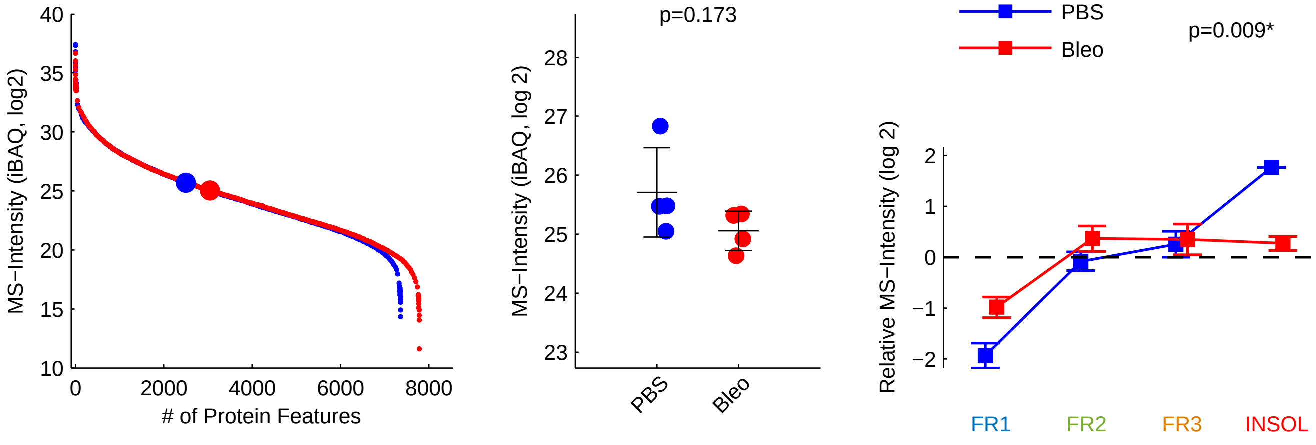

Q8BX10 − Pgam5 (id: 5749)

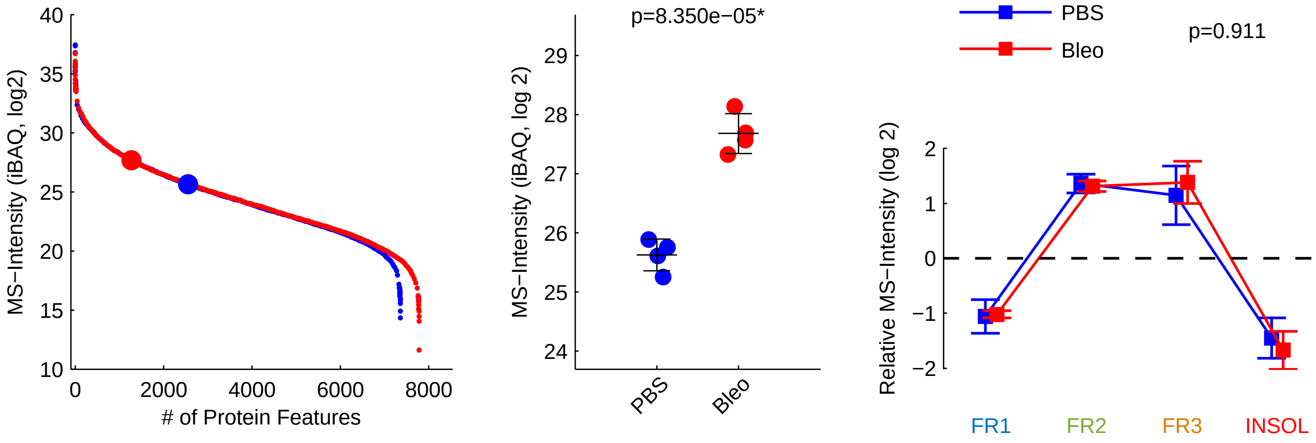

Q8BX70 − Vps13c (id: 5751)

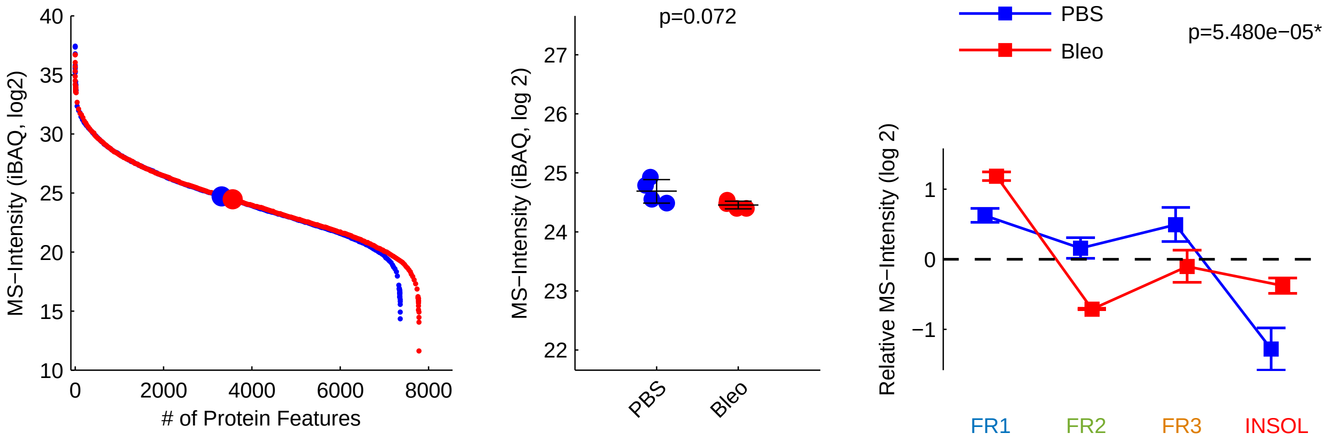

Q8BXK9 − Clic5 (id: 5756)

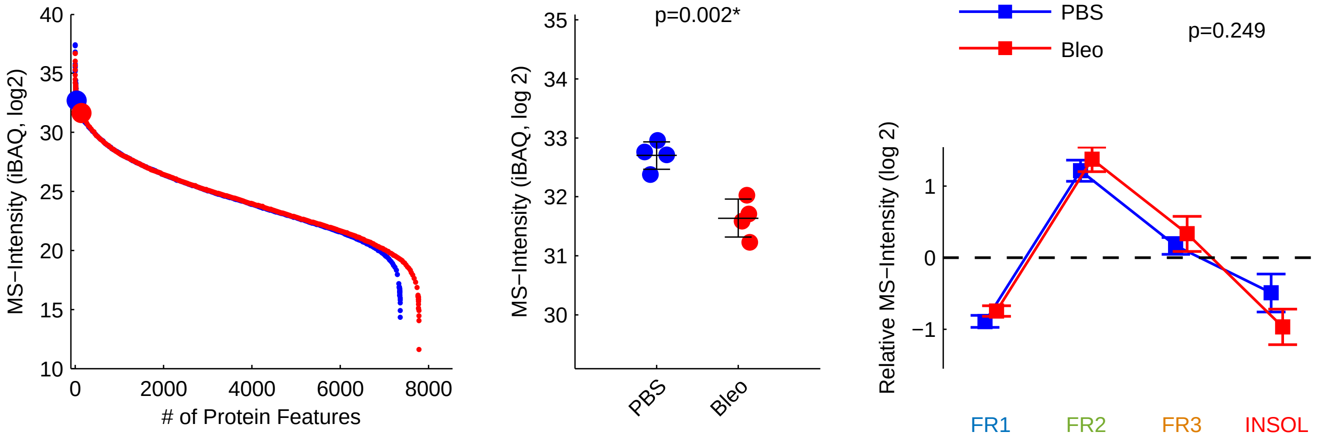

Q8BXR9 − Osbpl6 (id: 5760)

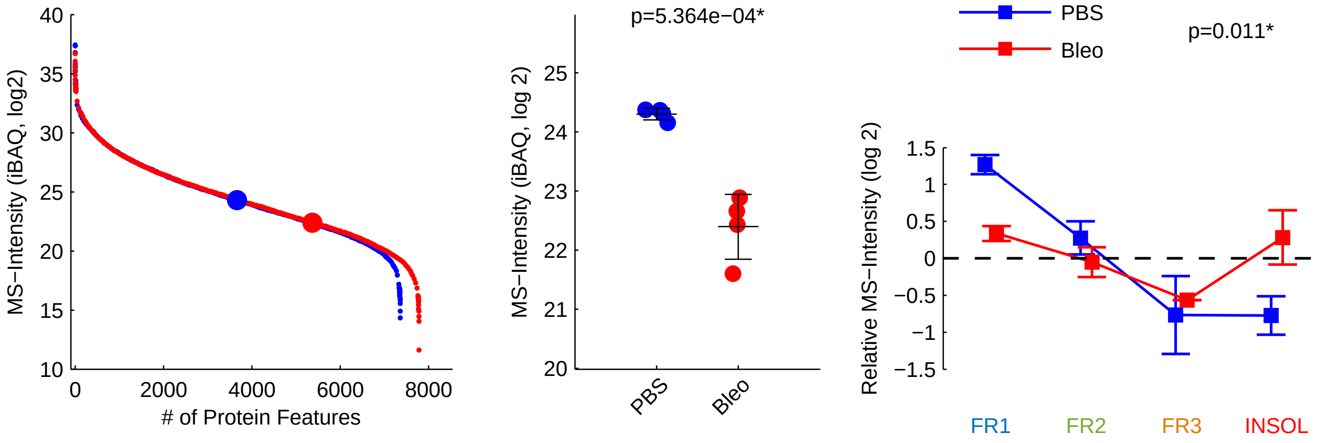

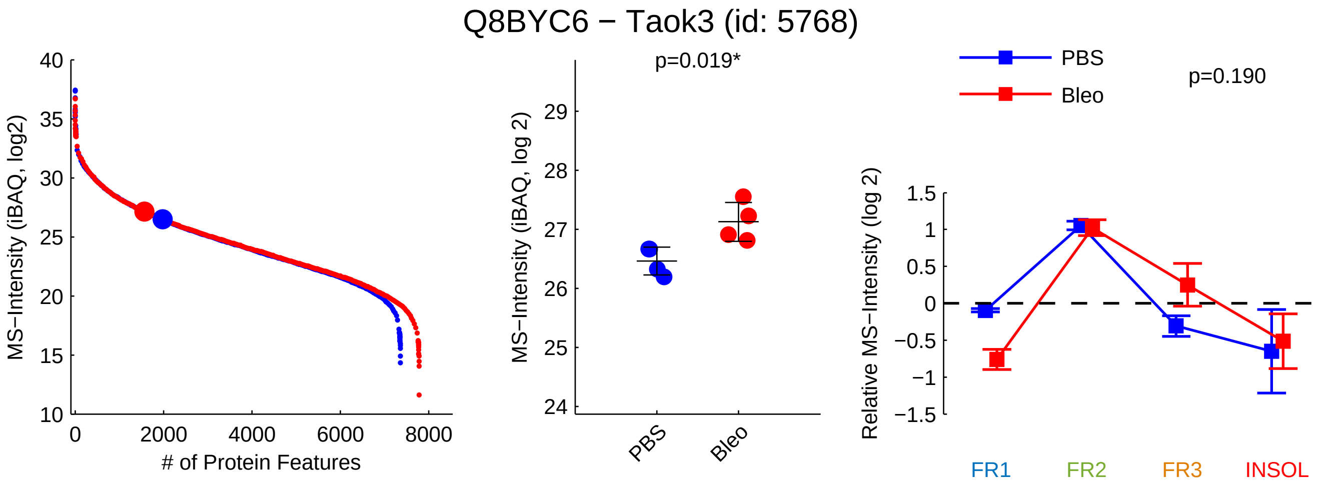
Q8BYC6 − Taok3 (id: 5768)
MS−Intensity (iBAQ, log2)
# of Protein Features
p=0.019*
MS−Intensity (iBAQ, log 2)
PBS
Bleo
p=0.190
Relative MS−Intensity (log 2)
FR1
FR2
FR3
INSOL

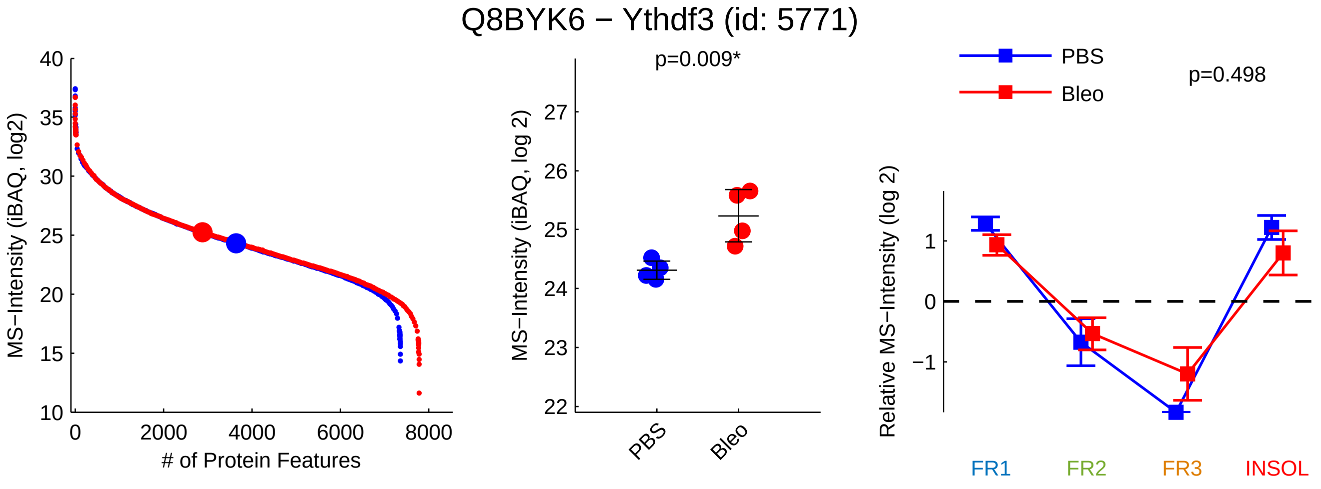
Q8BYK6 − Ythdf3 (id: 5771)
MS−Intensity (iBAQ, log2)
# of Protein Features
p=0.009*
MS−Intensity (iBAQ, log 2)
PBS
Bleo
p=0.498
Relative MS−Intensity (log 2)
FR1
FR2
FR3
INSOL

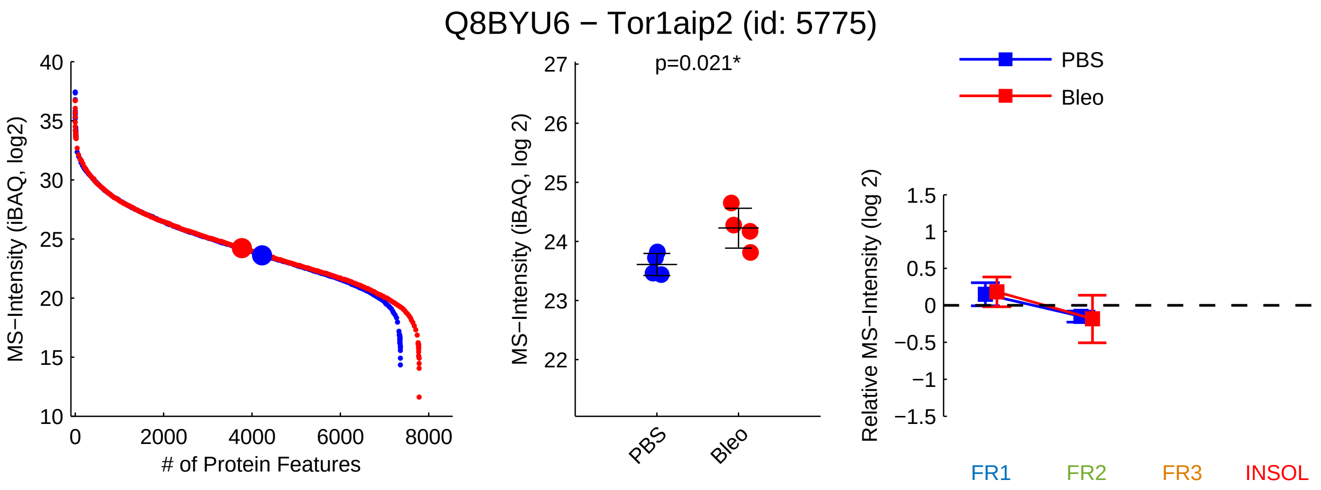
Q8BYU6 − Tor1aip2 (id: 5775)
MS−Intensity (iBAQ, log2)
# of Protein Features
p=0.021*
MS−Intensity (iBAQ, log 2)
PBS
Bleo
Relative MS−Intensity (log 2)
FR1
FR2
FR3
INSOL

Q8BYW1 − Arhgap25 (id: 5776)

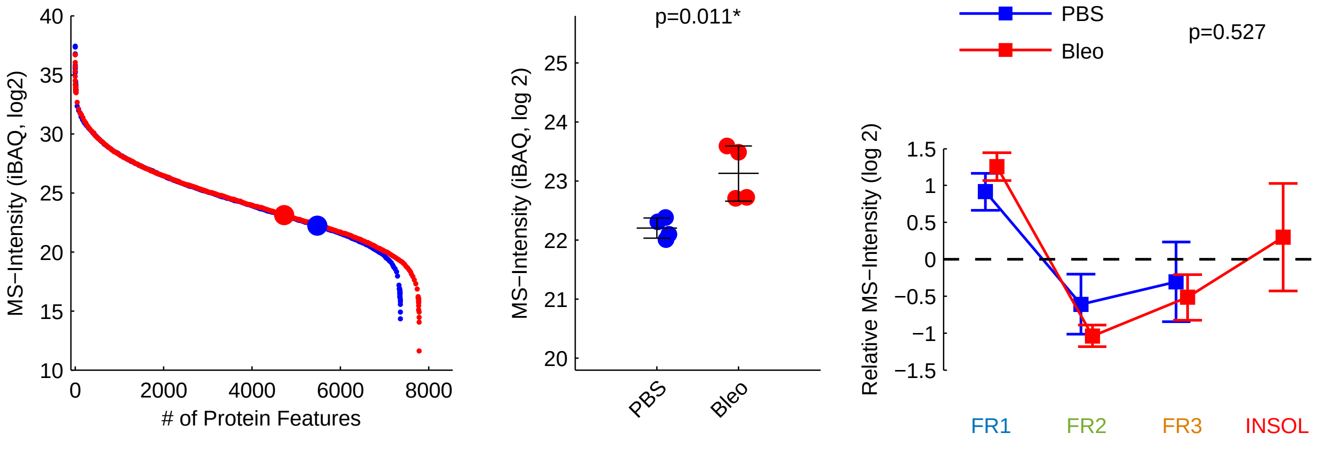

Q8BZ03 − Prkd2 (id: 5781)

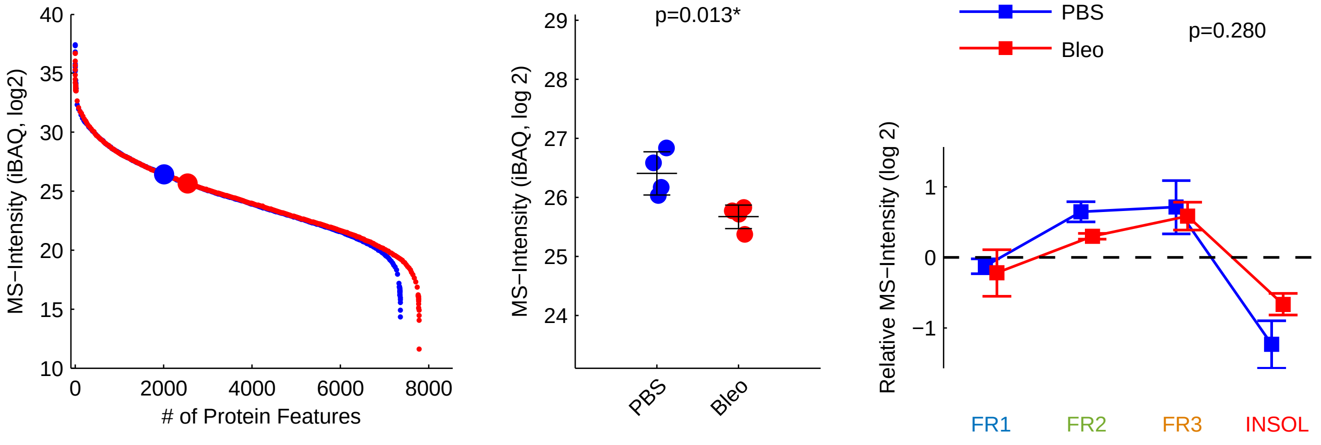

Q8BZF8 − Pgm5 (id: 5788)

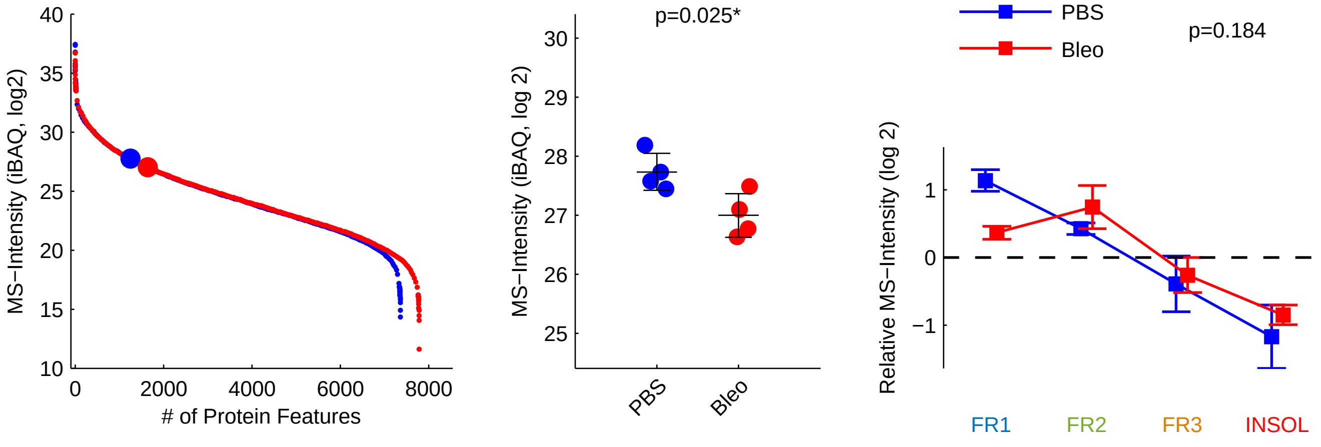

Q8BZI0 − Afap1l1 (id: 5789)

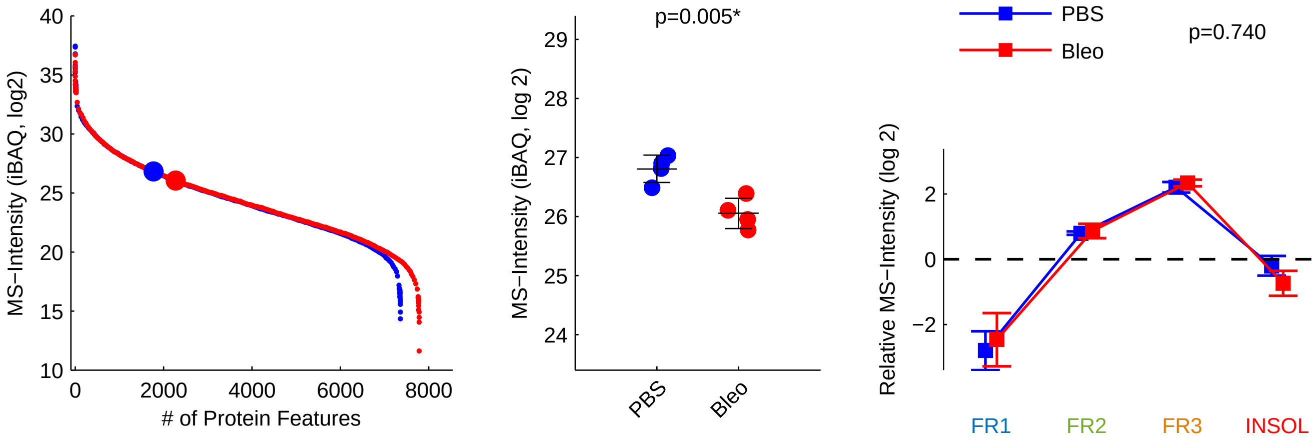

Q8BZW8 − Nhlrc2 (id: 5796)

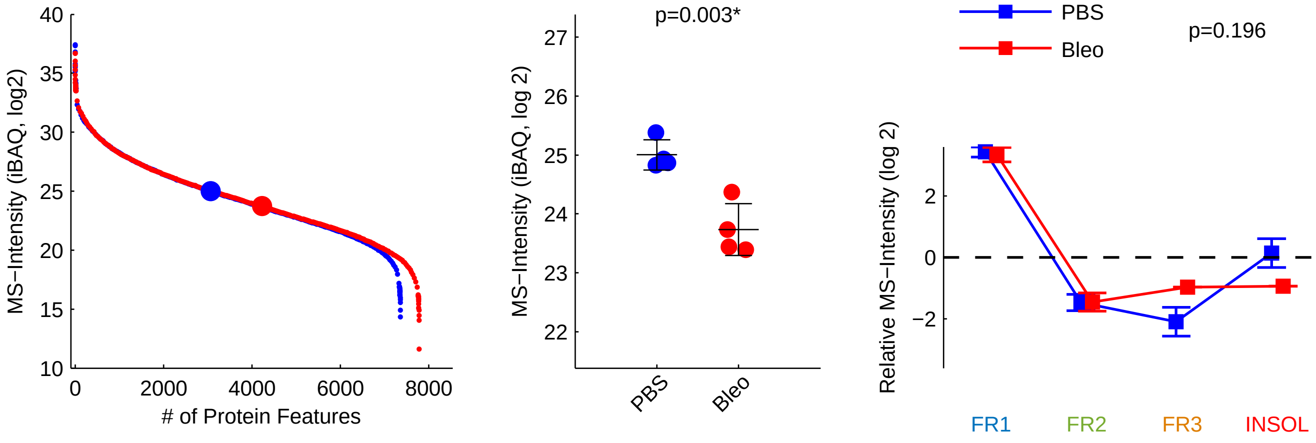

Q8C0M9 − Asrgl1 (id: 5816)

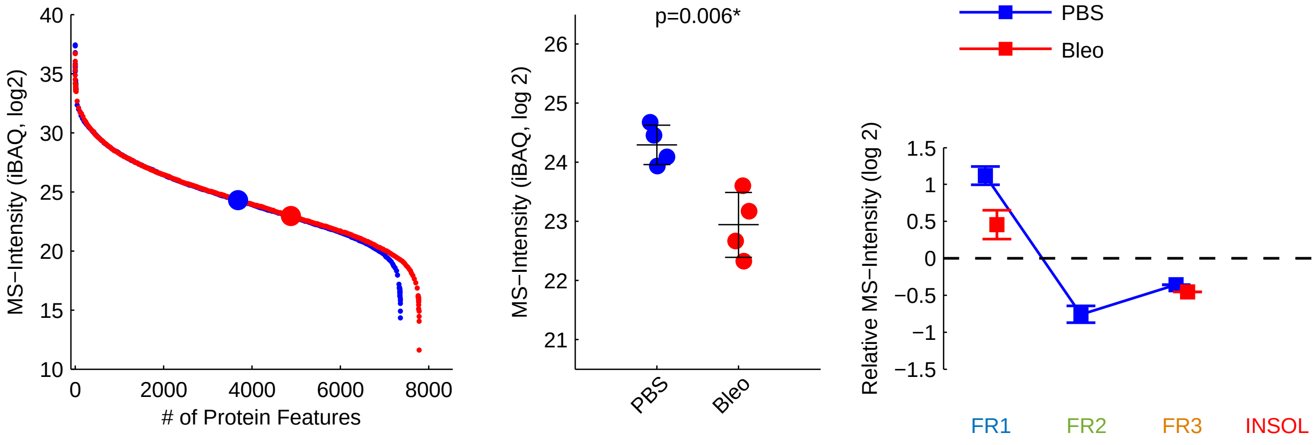

Q8C166 − Cpne1 (id: 5834)

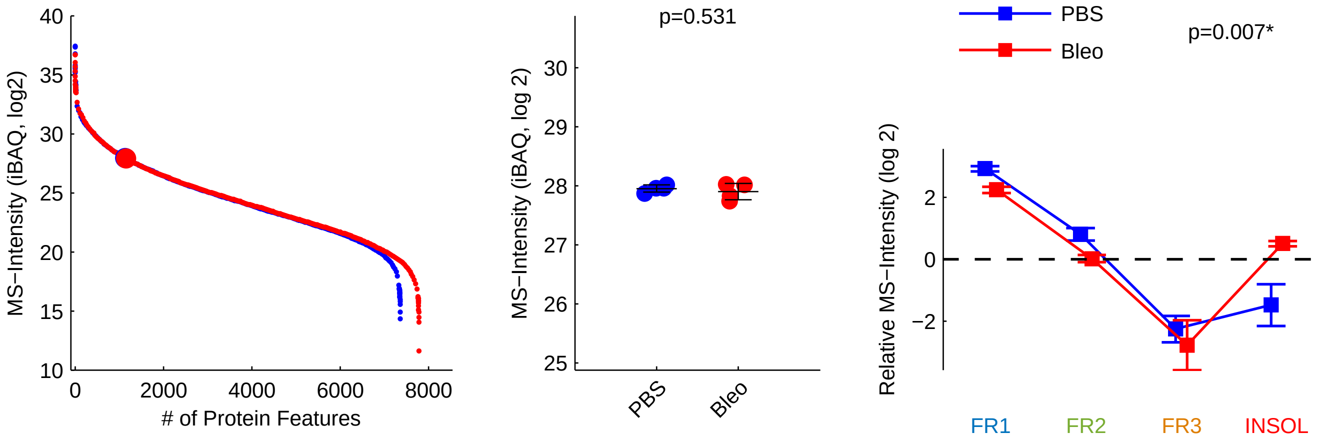

Q8C1D8 − Iws1 (id: 5840)

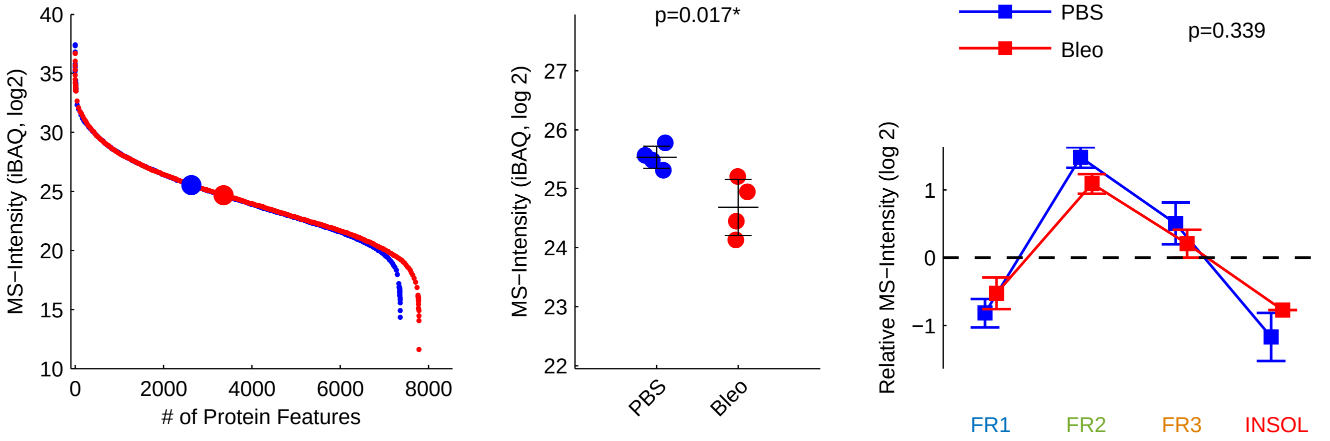

Q8C3J5 − Dock2 (id: 5854)

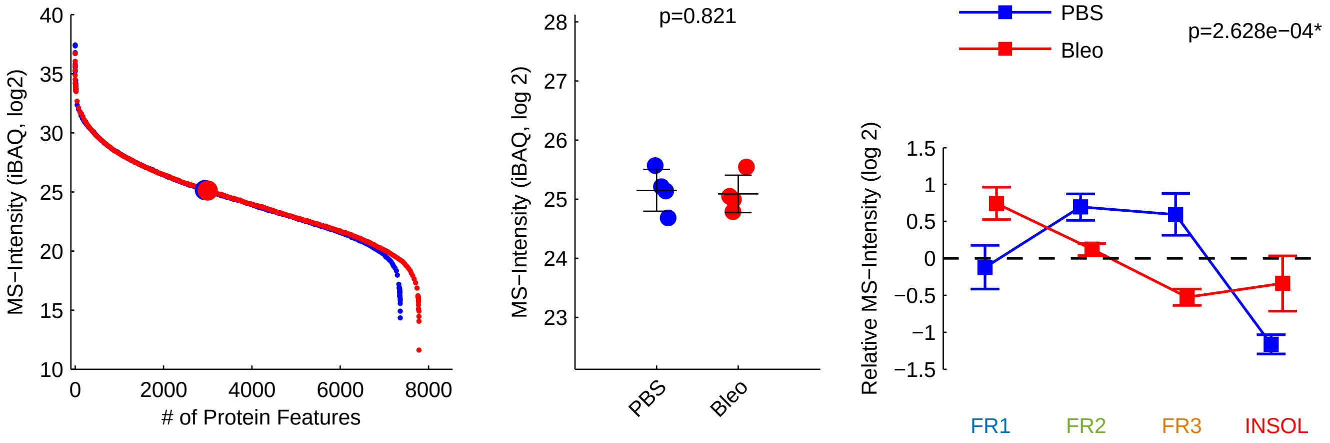

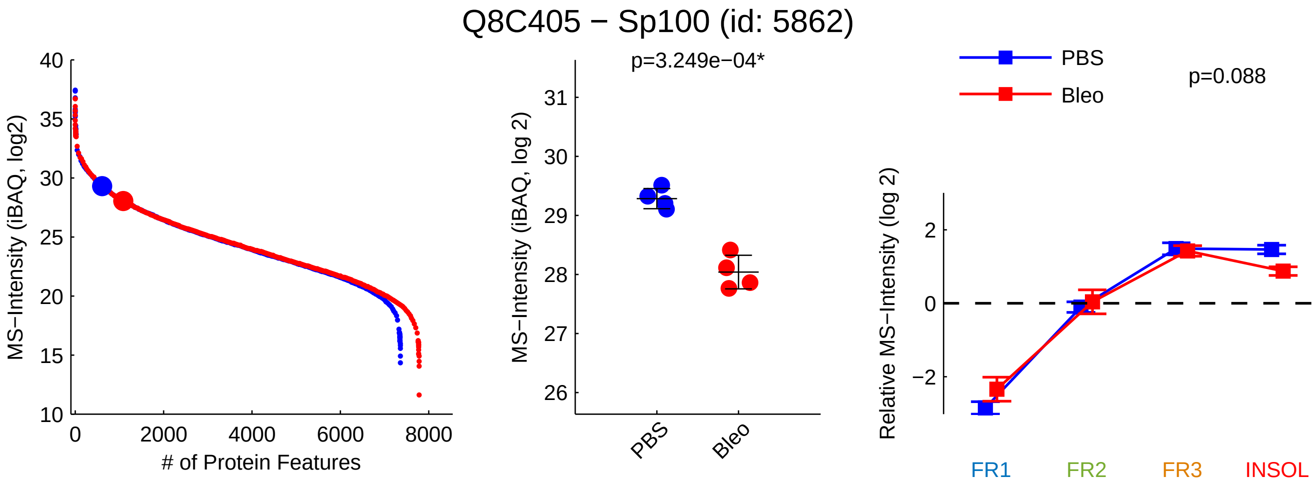
Q8C405 − Sp100 (id: 5862)
MS−Intensity (iBAQ, log2)
# of Protein Features
p=3.249e−04*
MS−Intensity (iBAQ, log 2)
PBS
Bleo
PBS
Bleo
p=0.088
Relative MS−Intensity (log 2)
FR1
FR2
FR3
INSOL

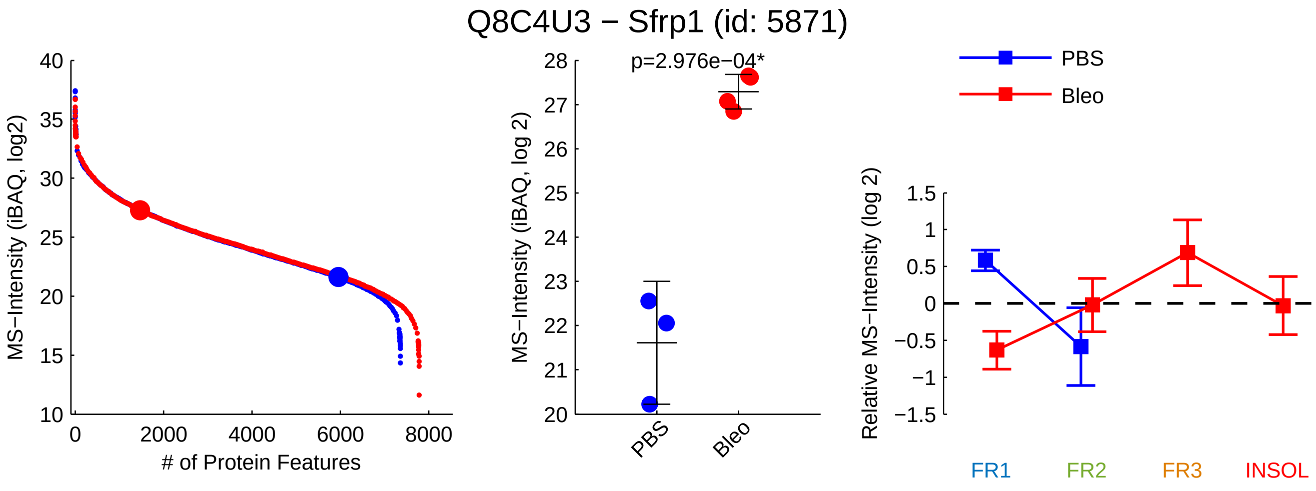
Q8C4U3 − Sfrp1 (id: 5871)
MS−Intensity (iBAQ, log2)
# of Protein Features
p=2.976e−04*
MS−Intensity (iBAQ, log 2)
PBS
Bleo
PBS
Bleo
Relative MS−Intensity (log 2)
FR1
FR2
FR3
INSOL

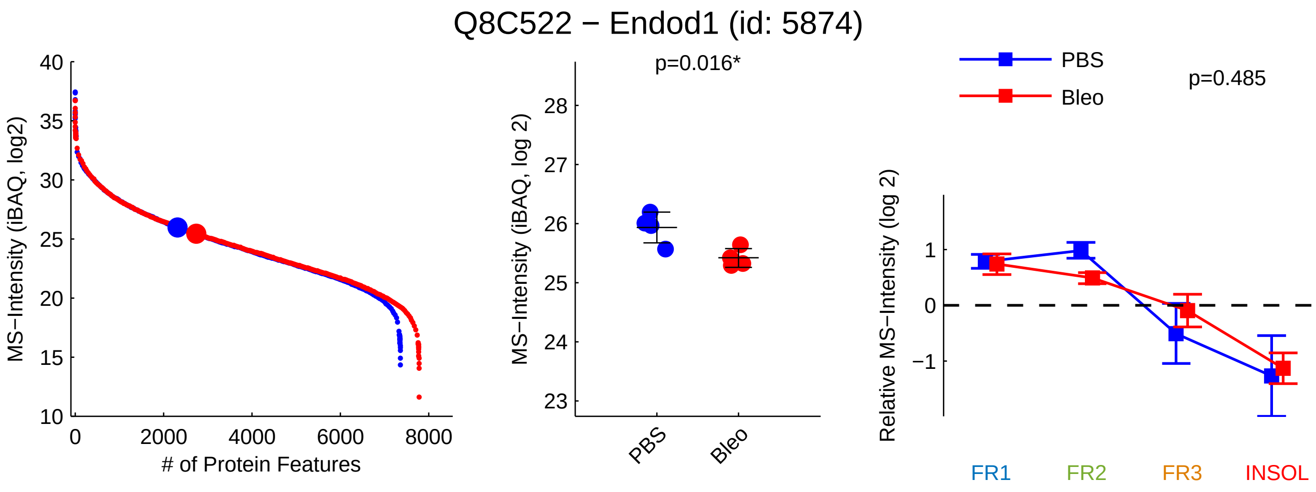
Q8C522 − Endod1 (id: 5874)
MS−Intensity (iBAQ, log2)
# of Protein Features
p=0.016*
MS−Intensity (iBAQ, log 2)
PBS
Bleo
PBS
Bleo
p=0.485
Relative MS−Intensity (log 2)
FR1
FR2
FR3
INSOL

Q8C5H8 − Nadk2 (id: 5877)

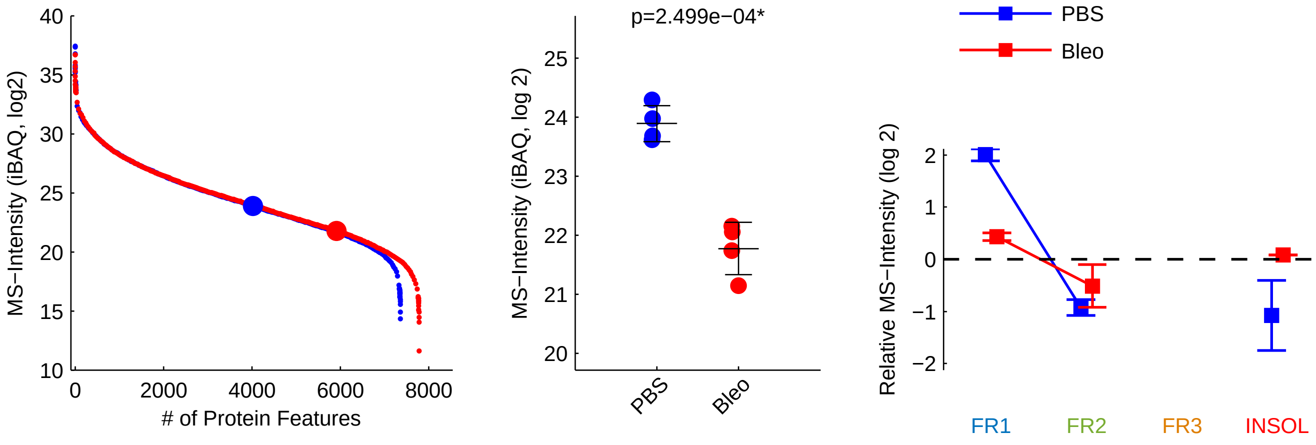

Q8C5L6 − Inpp5k (id: 5879)

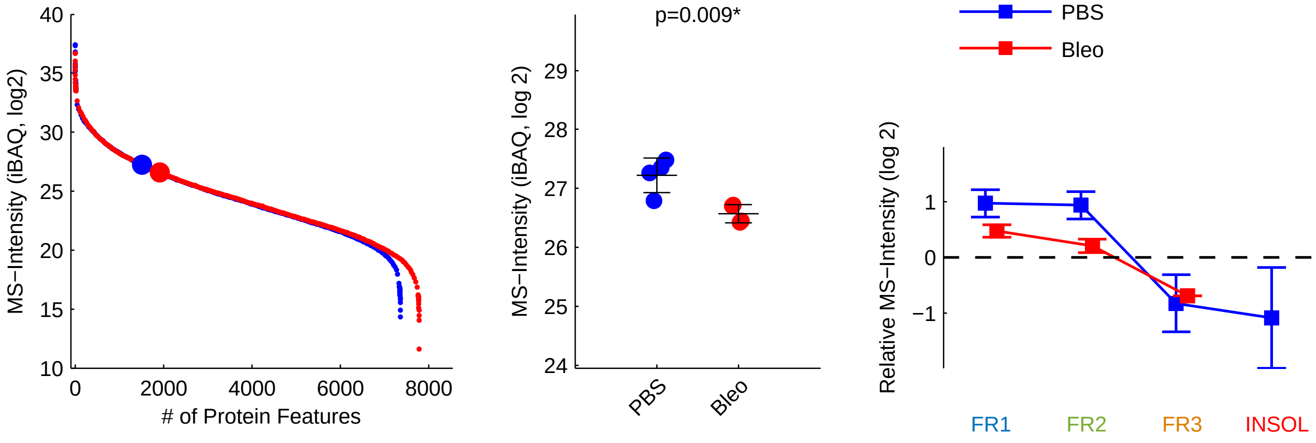

Q8C650 − Sept10 (id: 5890)

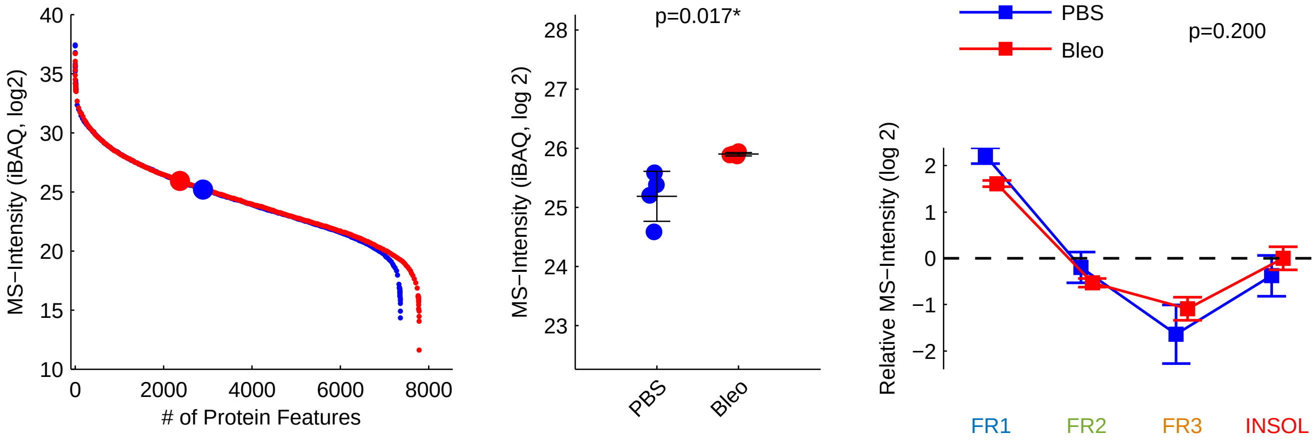

Q8C6B0 − Mettl7a1 (id: 5891)

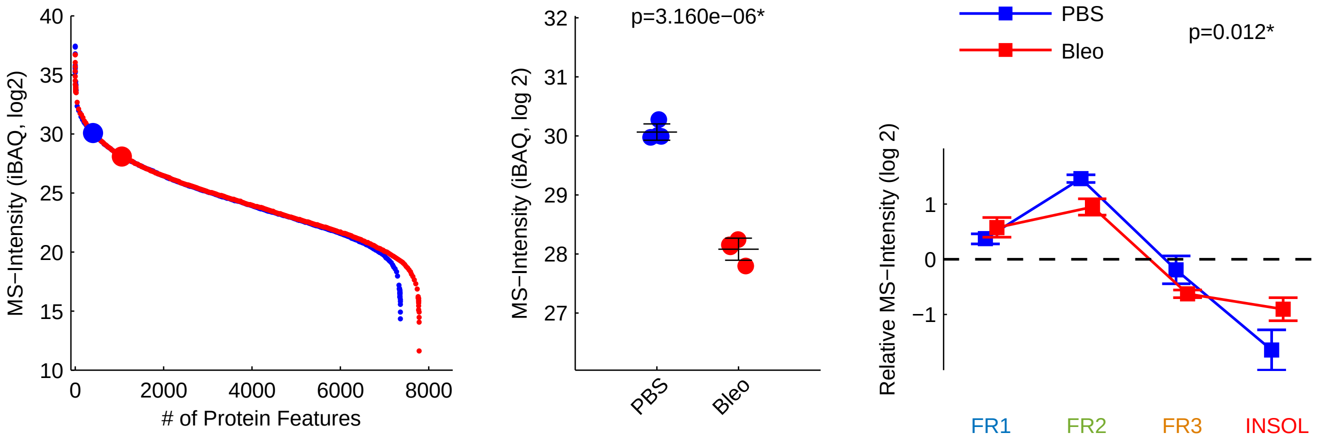

Q8C754 − Vps52 (id: 5901)

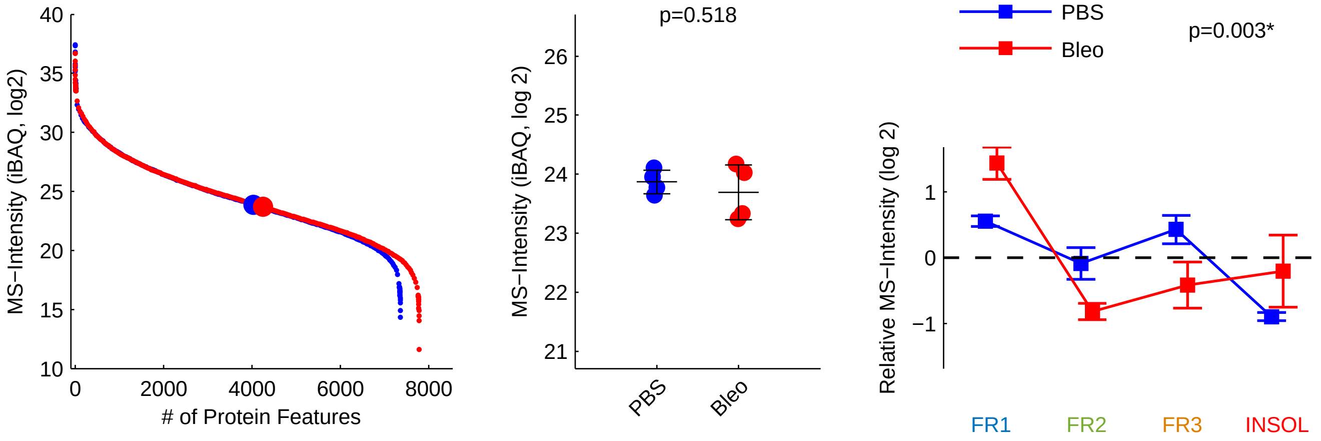

Q8C7H1 − Mmaa (id: 5909)

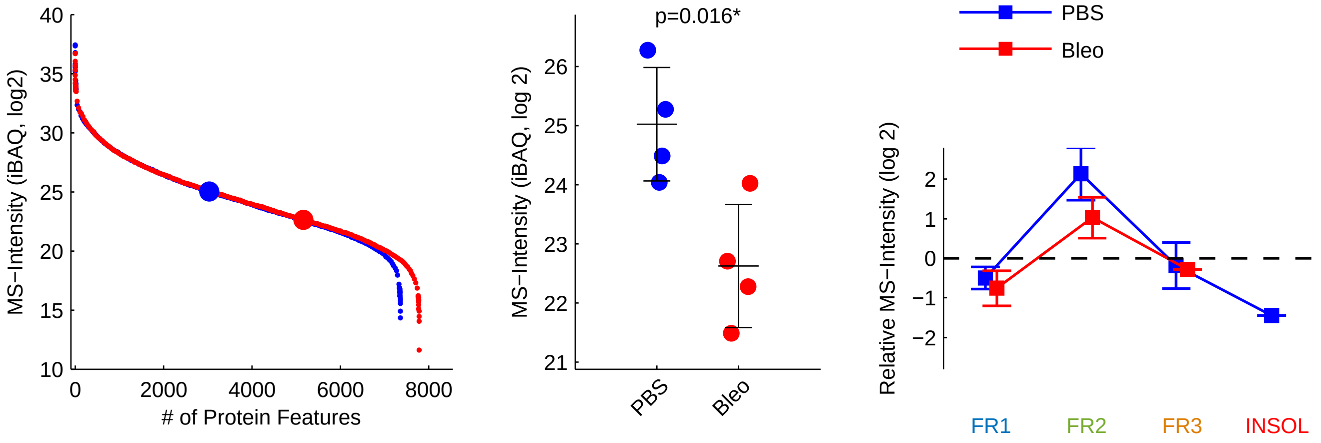

Q8C7X2−2 − Emc1 (id: 5915)

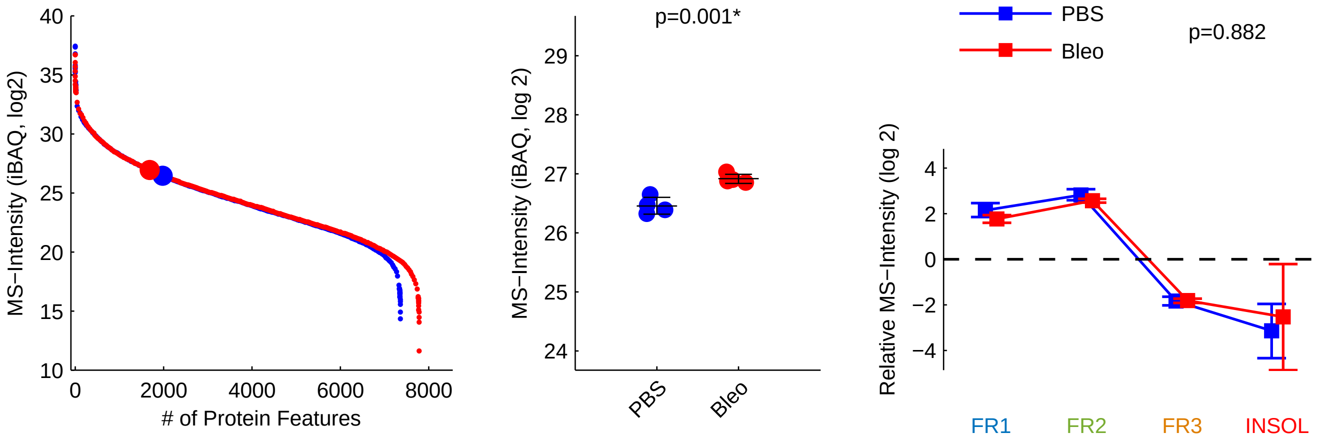

Q8C804 − Spice1 (id: 5916)

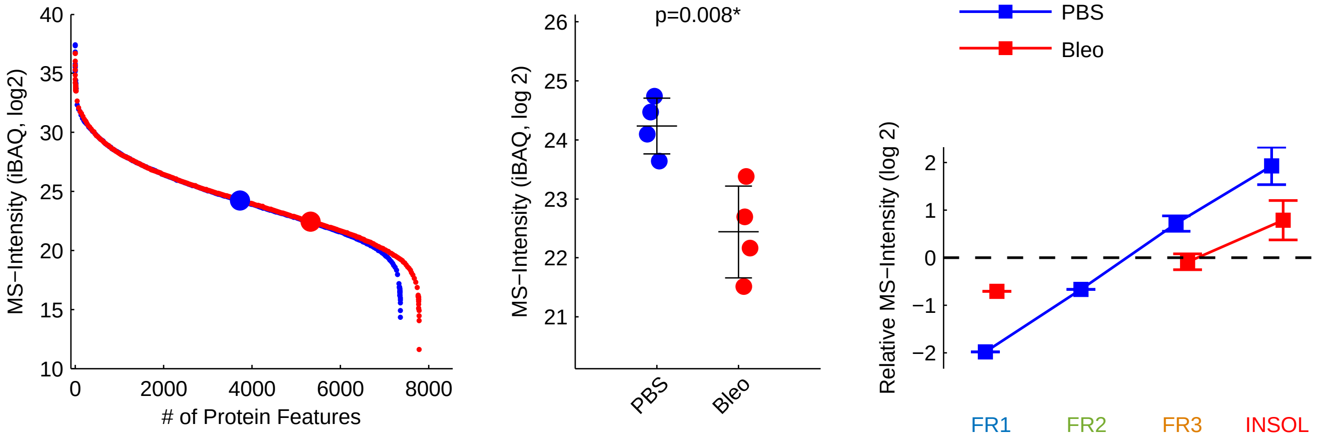

Q8C845 − Efhd2 (id: 5917)

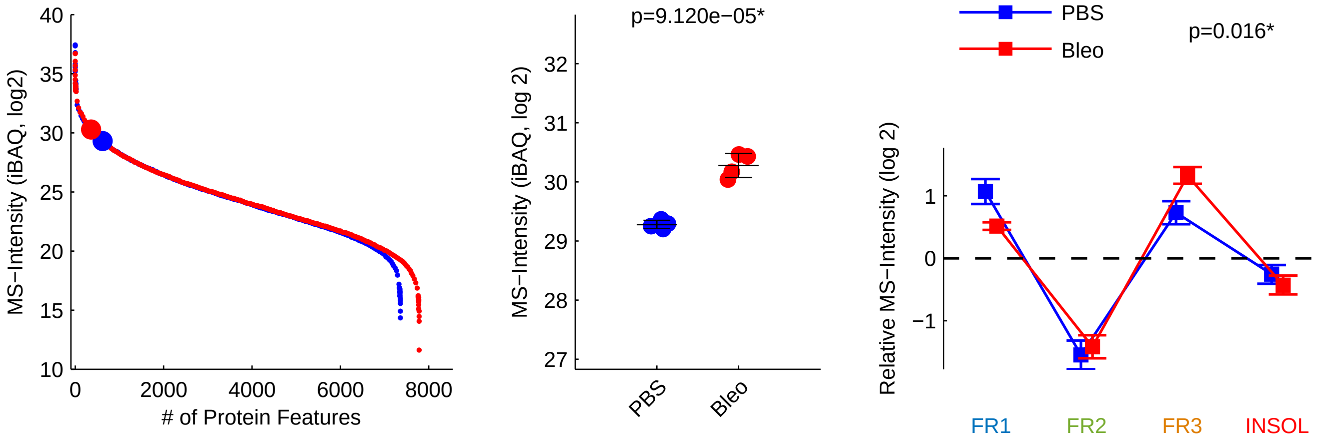

Q8C863 − Itch (id: 5919)

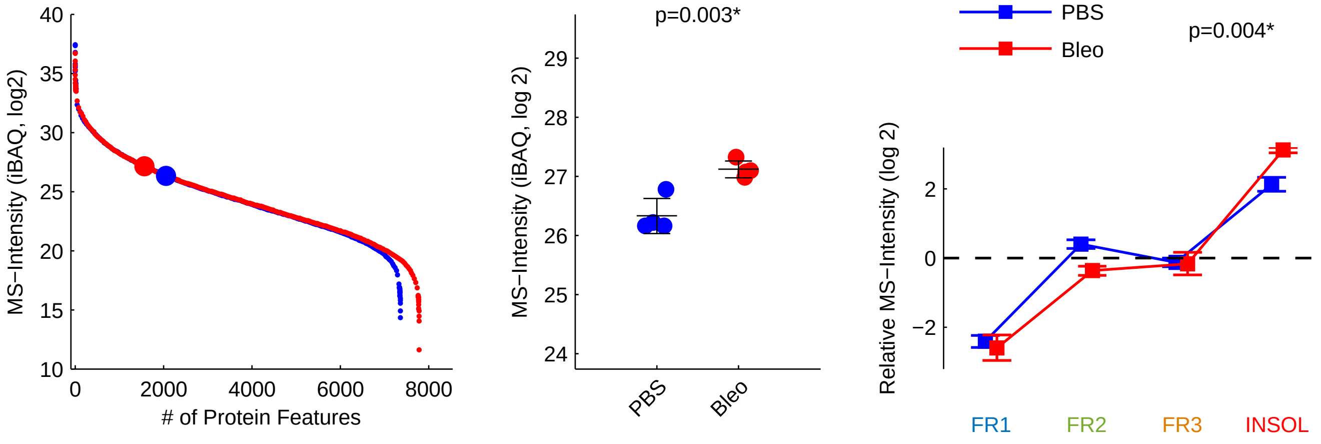

Q8C878 − Uba3 (id: 5920)

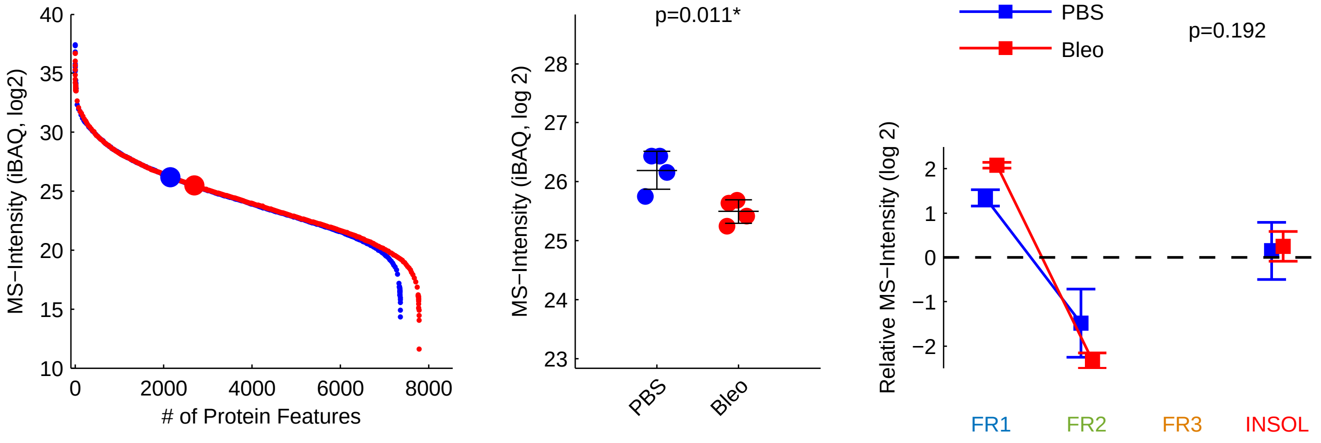

Q8C8U0−3 − Ppfibp1 (id: 5925)

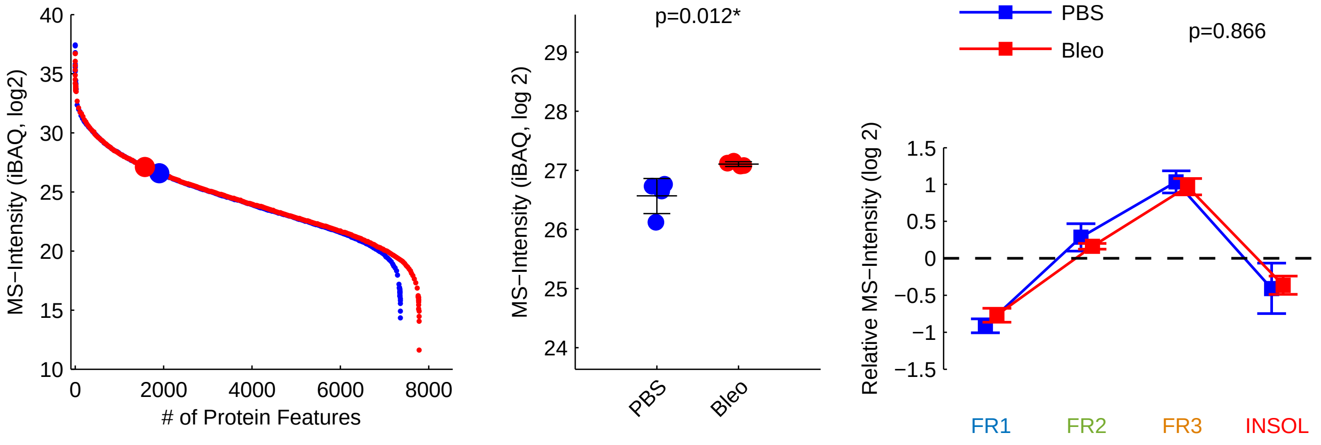

Q8CAA7 − Pgm2l1 (id: 5933)

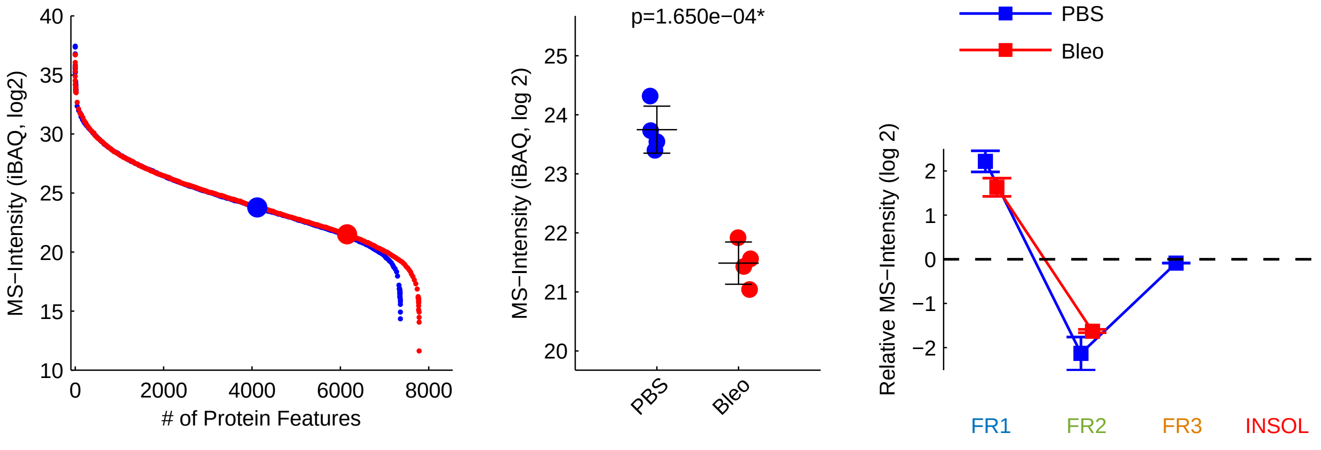

Q8CAH8 − Prkg2 (id: 5934)

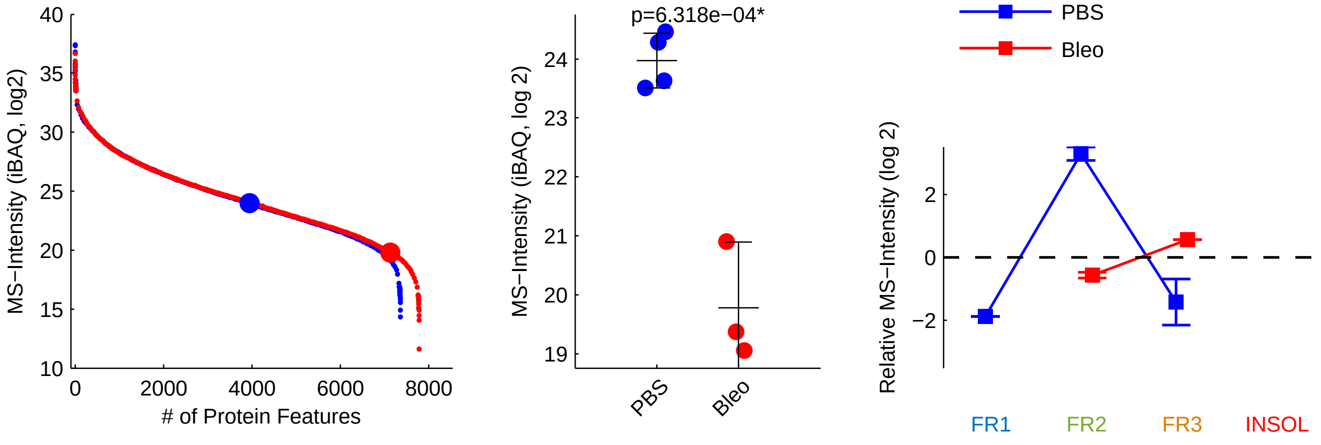

Q8CAQ8−2 − Immt (id: 5938)

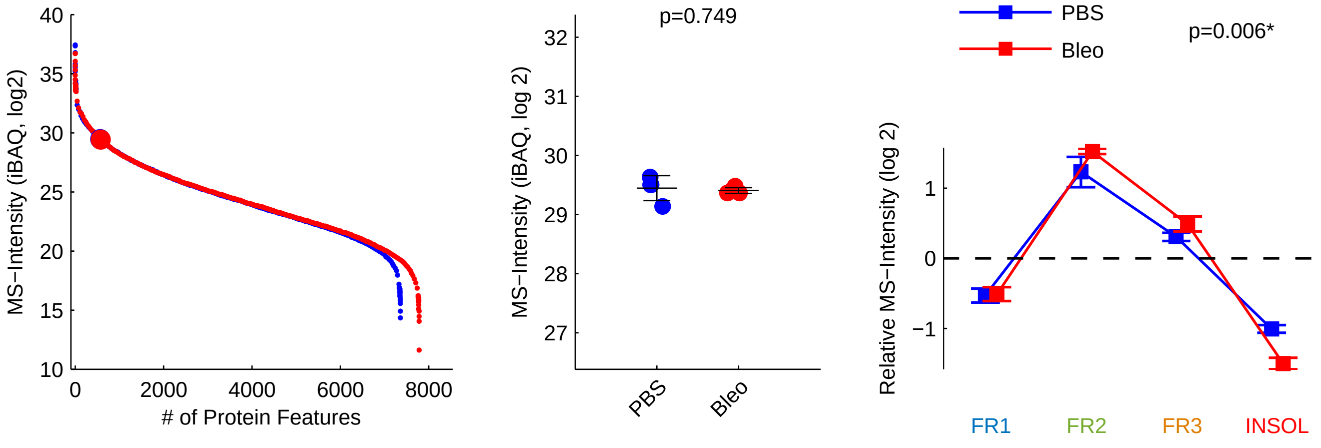

Q8CAY6 − Acat2 (id: 5941)

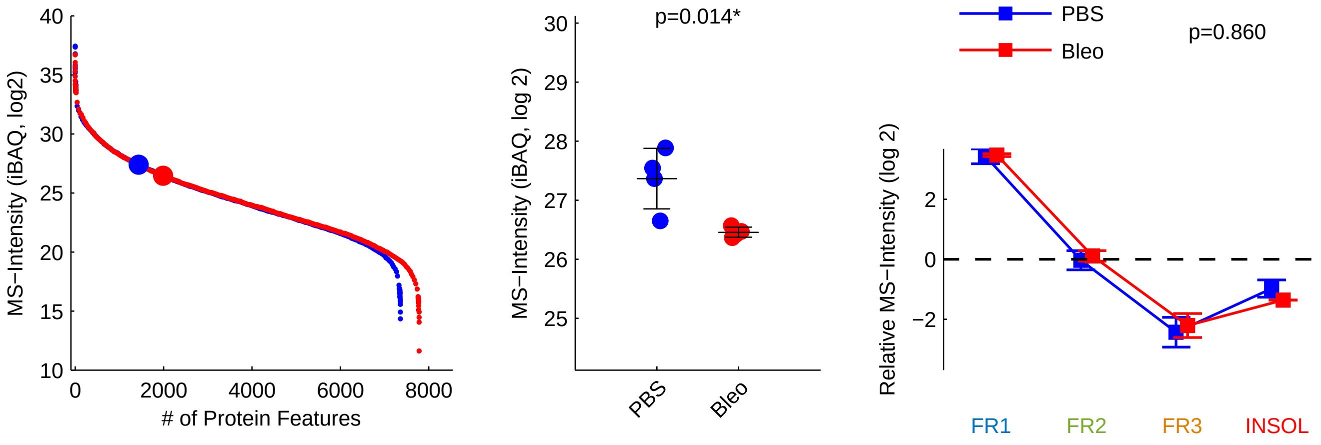

Q8CCH2 − Nhlrc3 (id: 5960)

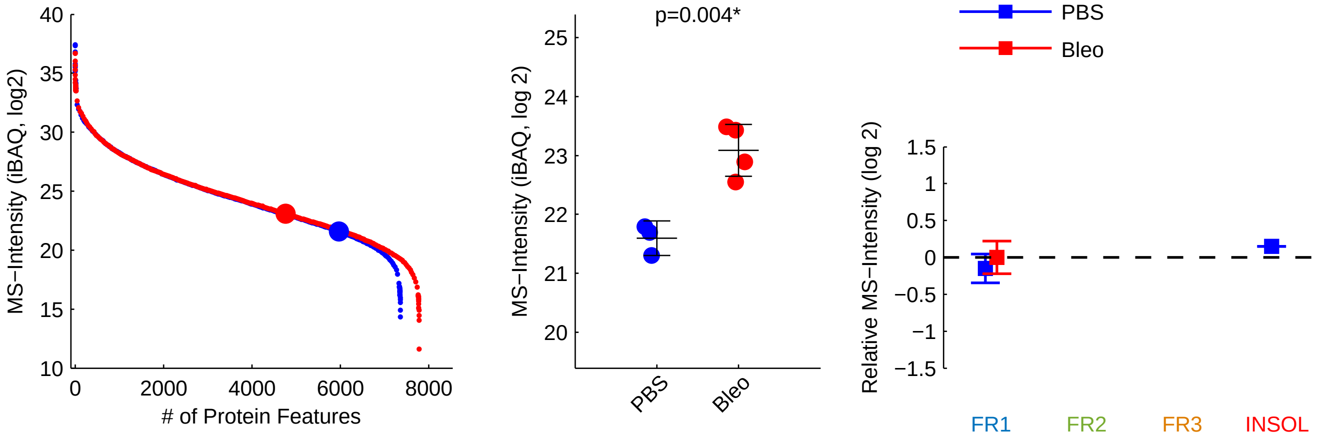

Q8CCK0 − H2afy2 (id: 5964)

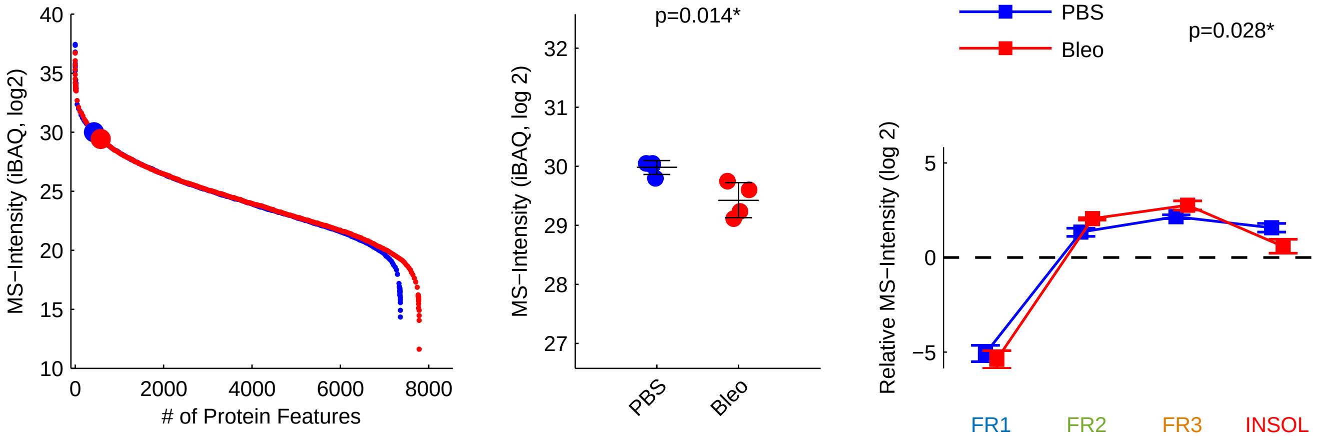

Q8CCP0 − Nemf (id: 5966)

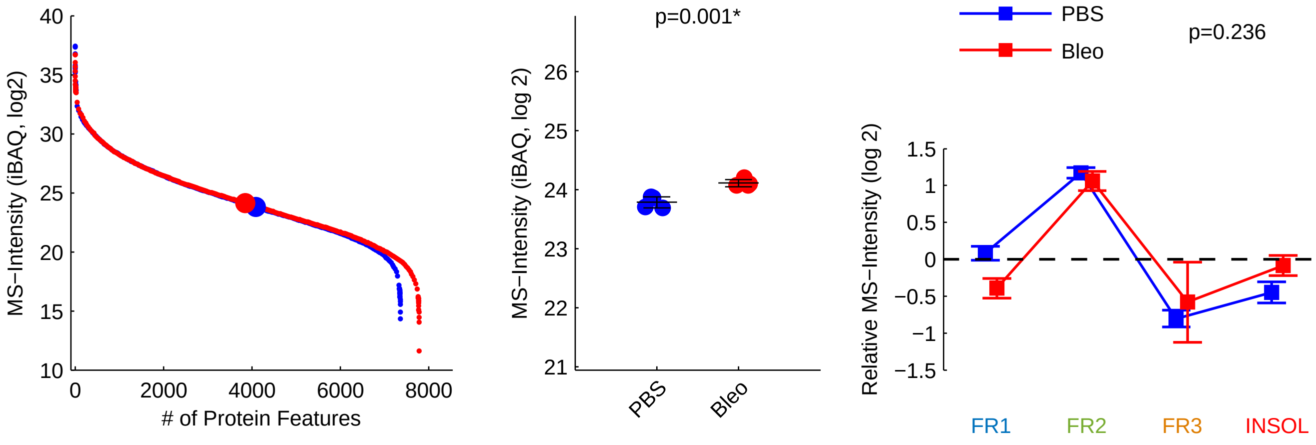

Q8CDN6 − Txnl1 (id: 5977)

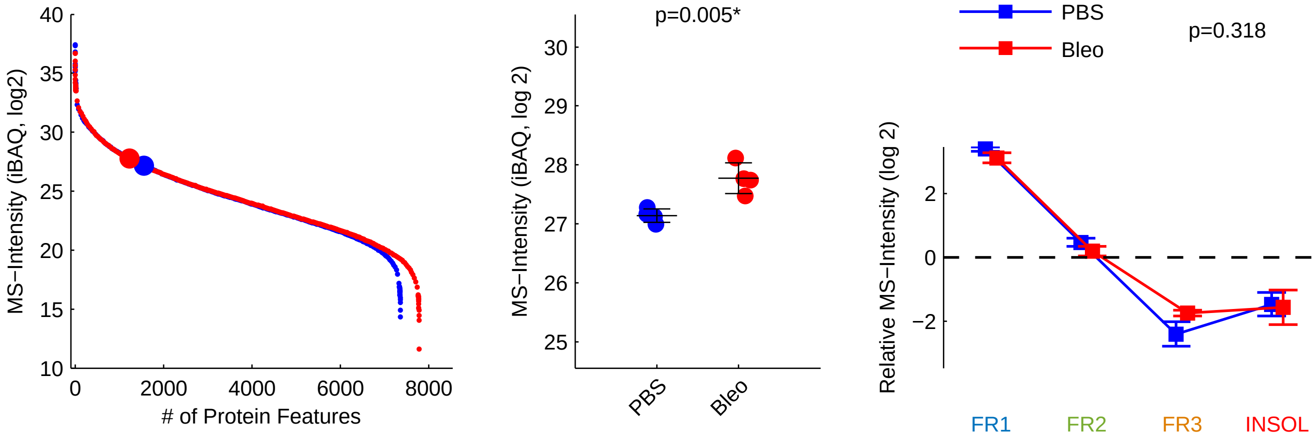

Q8CFB8 − Parp3 (id: 5997)

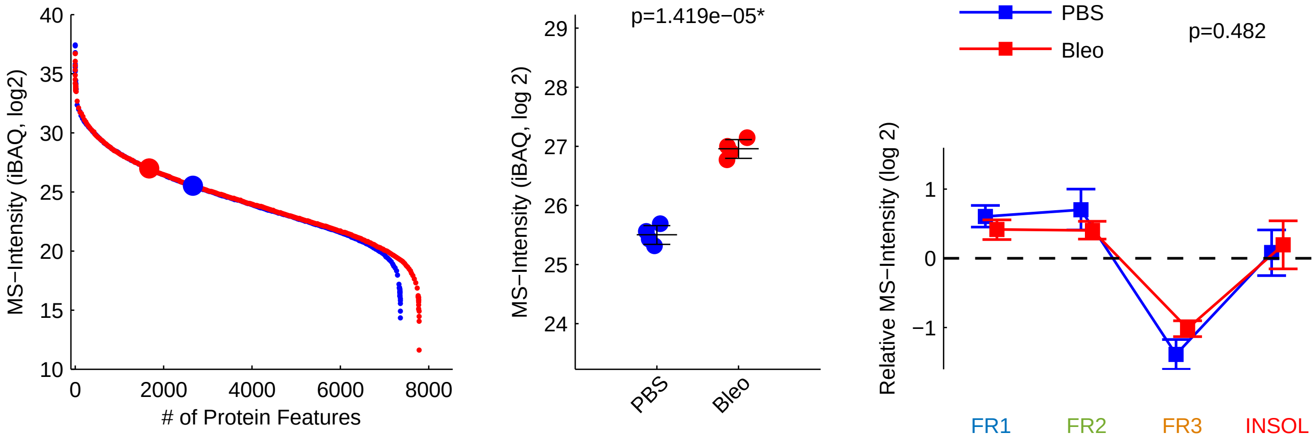

Q8CFD4 − Snx8 (id: 5999)

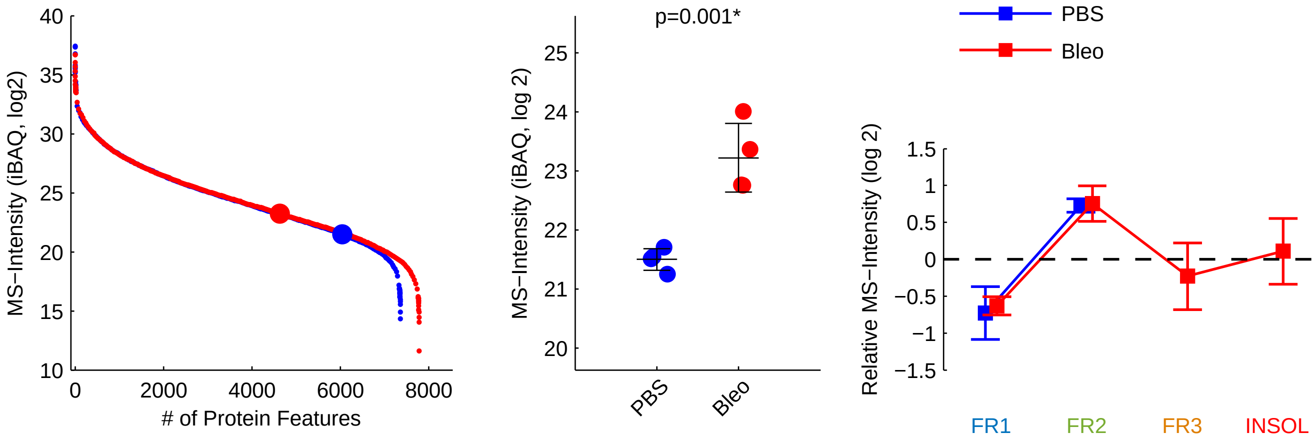

Q8CFI7 − Polr2b (id: 6003)

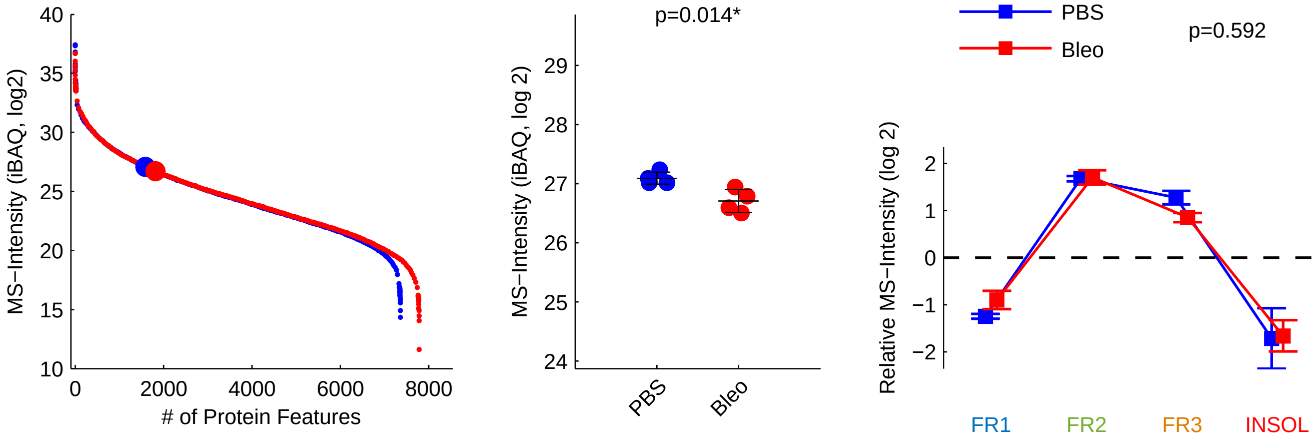

Q8CFR5 − Dtna (id: 6007)

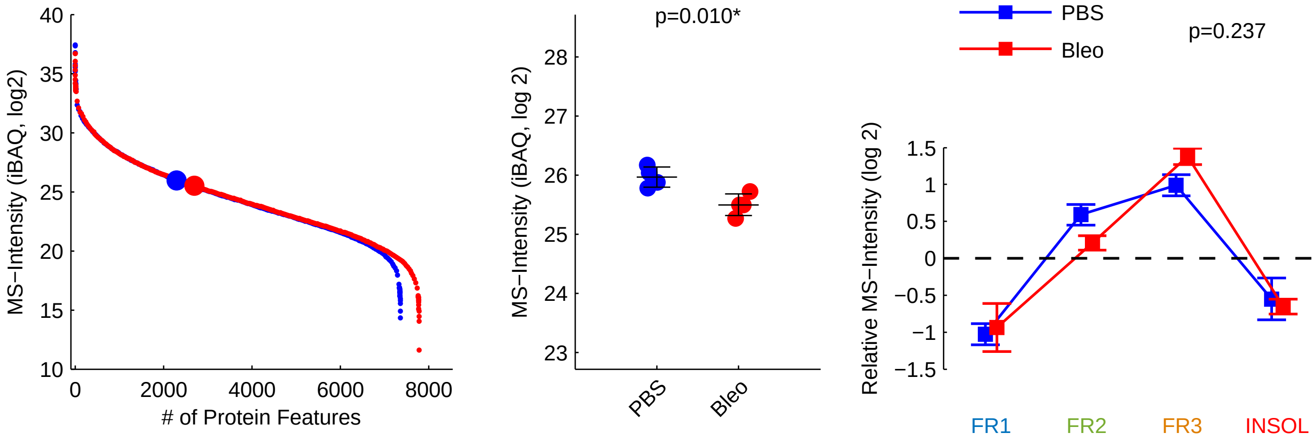

Q8CG03 − Pde5a (id: 6012)

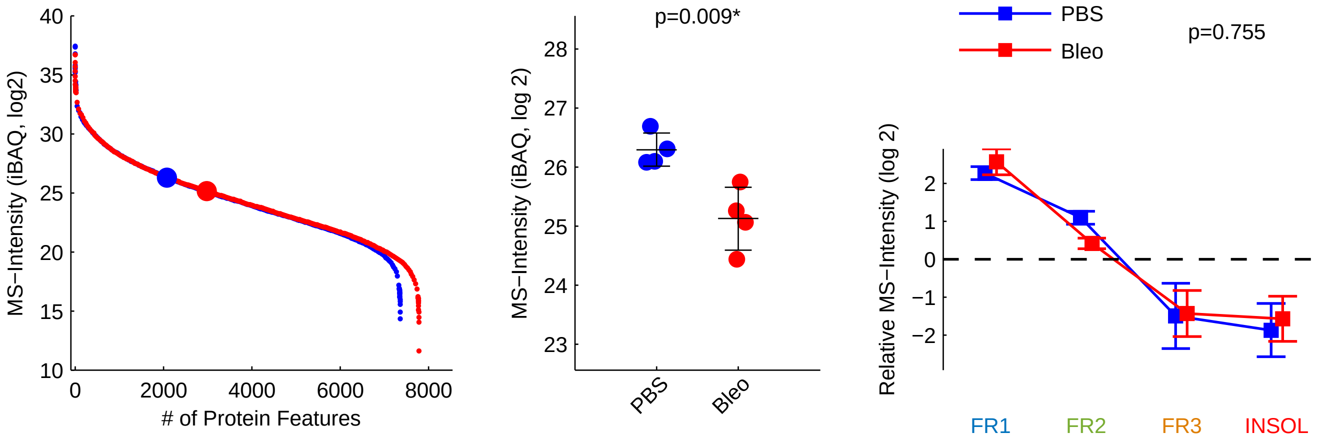

Q8CG29 − Myo1f (id: 6015)

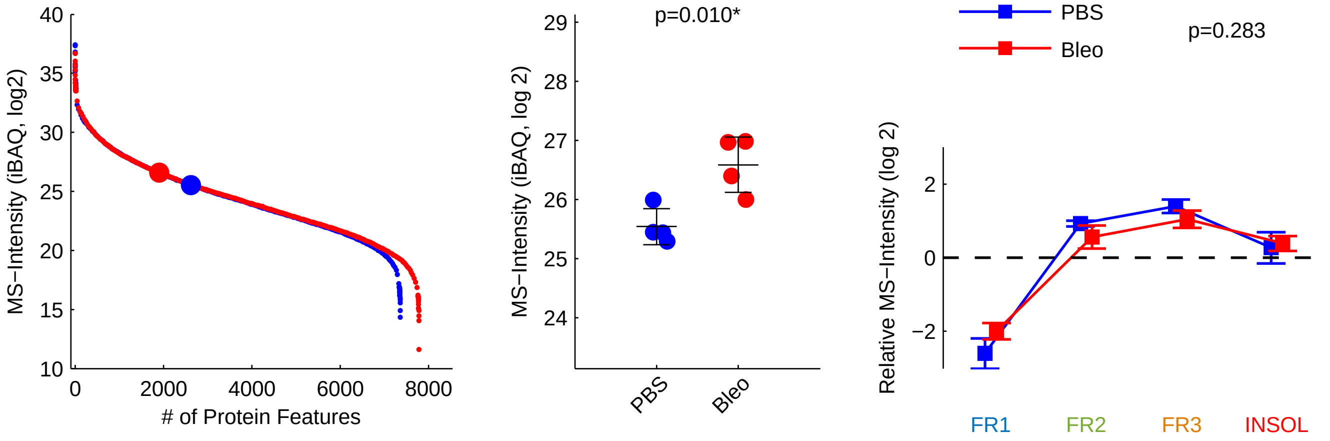

Q8CG70 − Leprel2 (id: 6019)

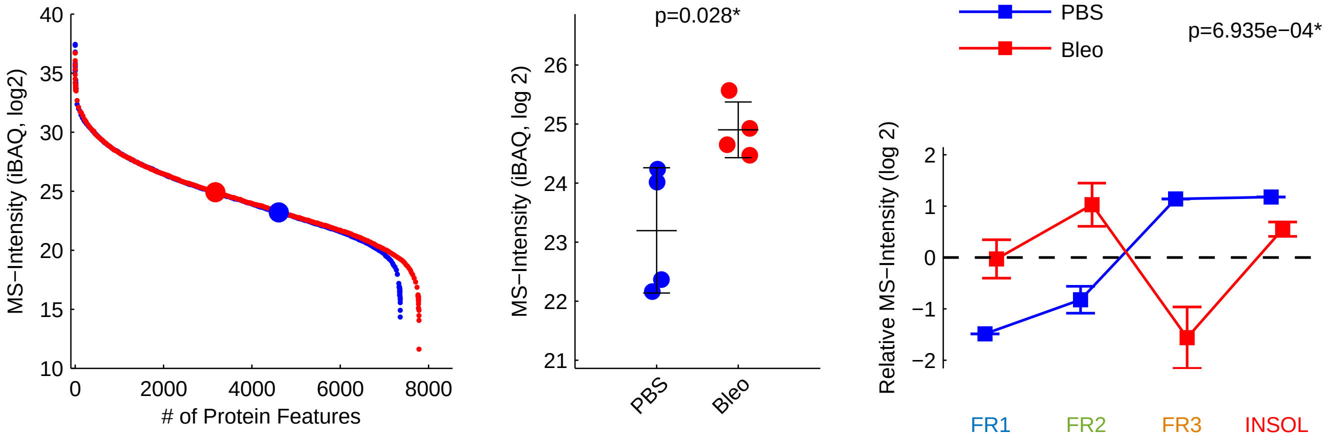

Q8CG72 − Adprhl2 (id: 6021)

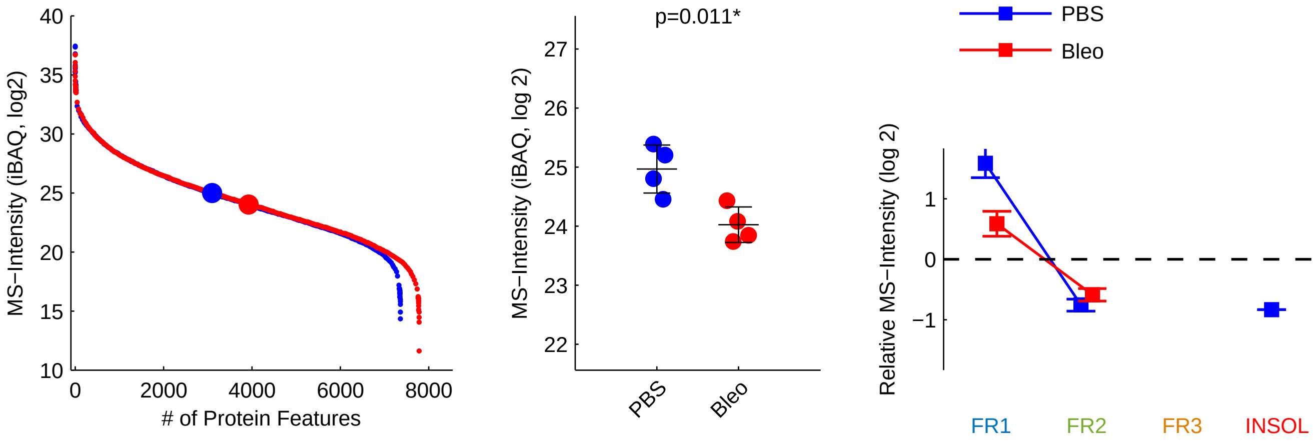

Q8CG85 − Mamdc2 (id: 6024)

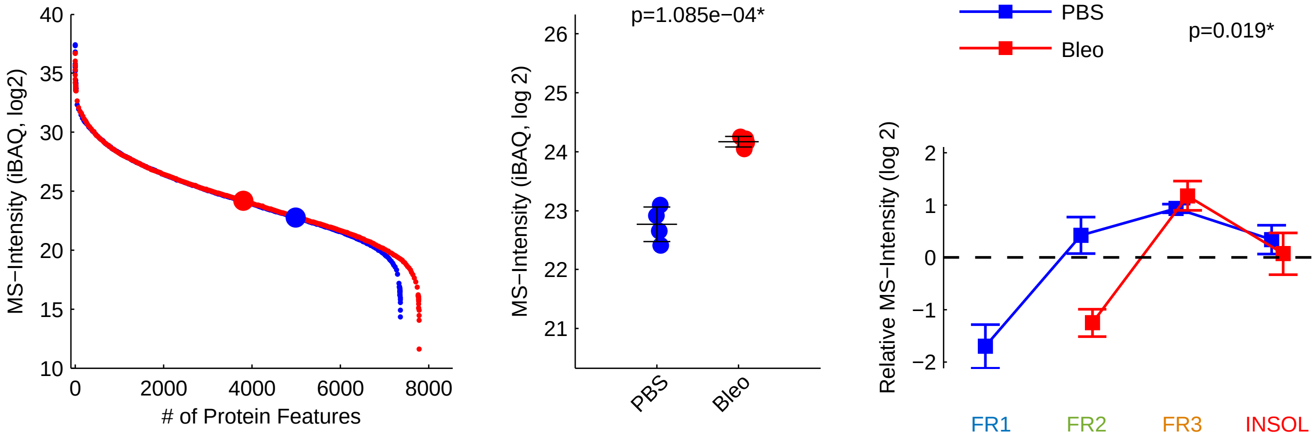

Q8CGA0 − Ppm1f (id: 6025)

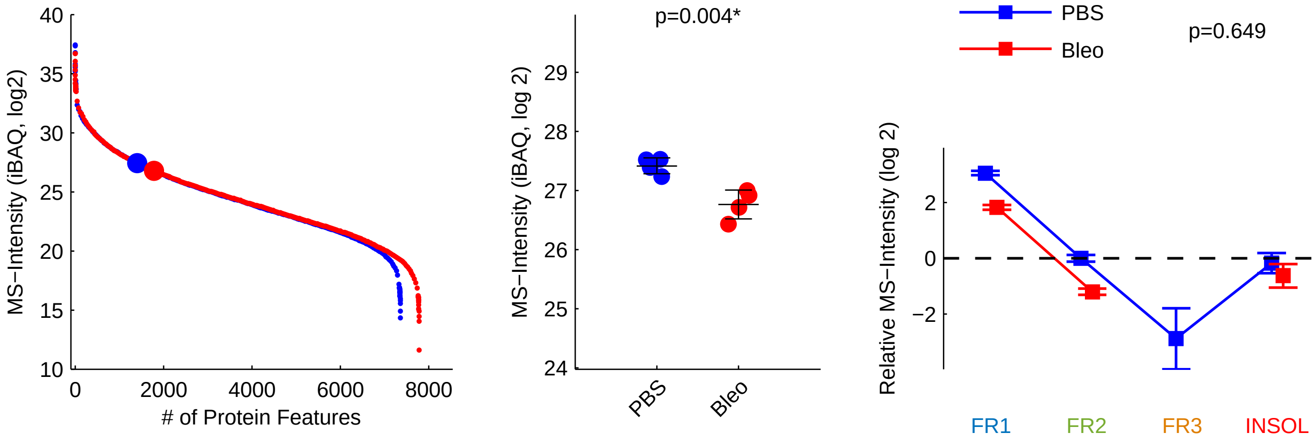

Q8CGB3−3 − Uaca (id: 6028)

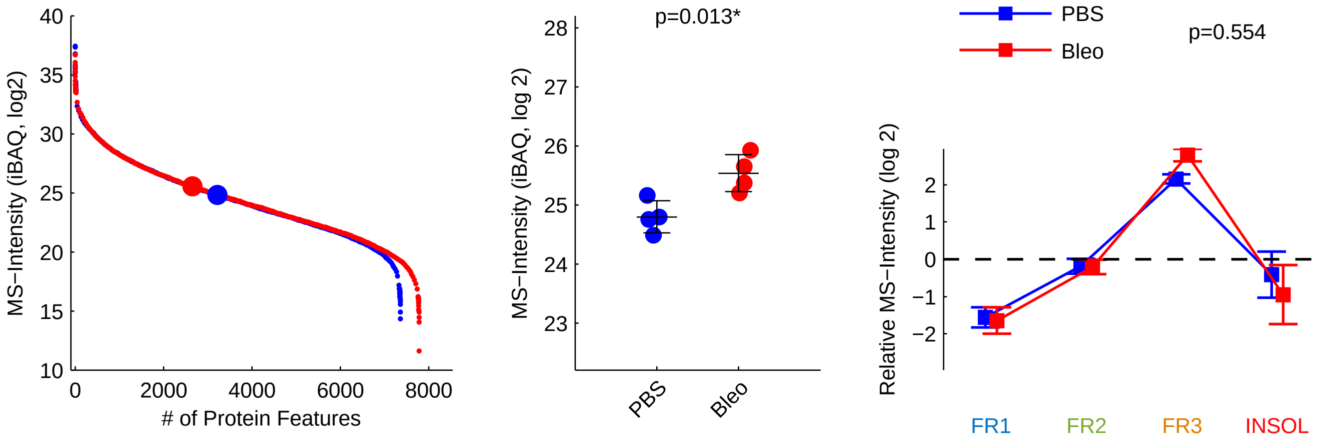

Q8CGB6 − Tenc1 (id: 6029)

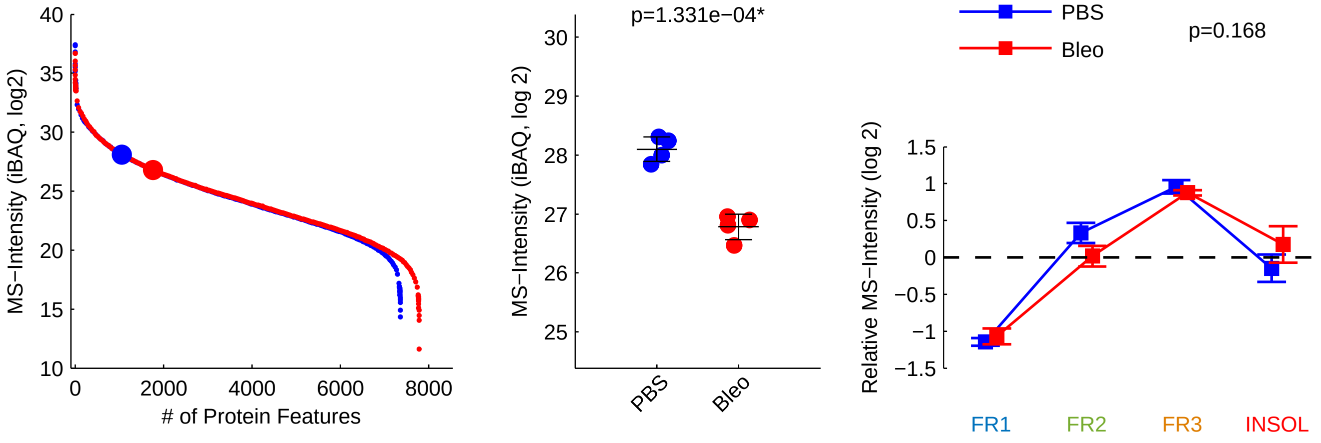

Q8CGC6 − Rbm28 (id: 6030)

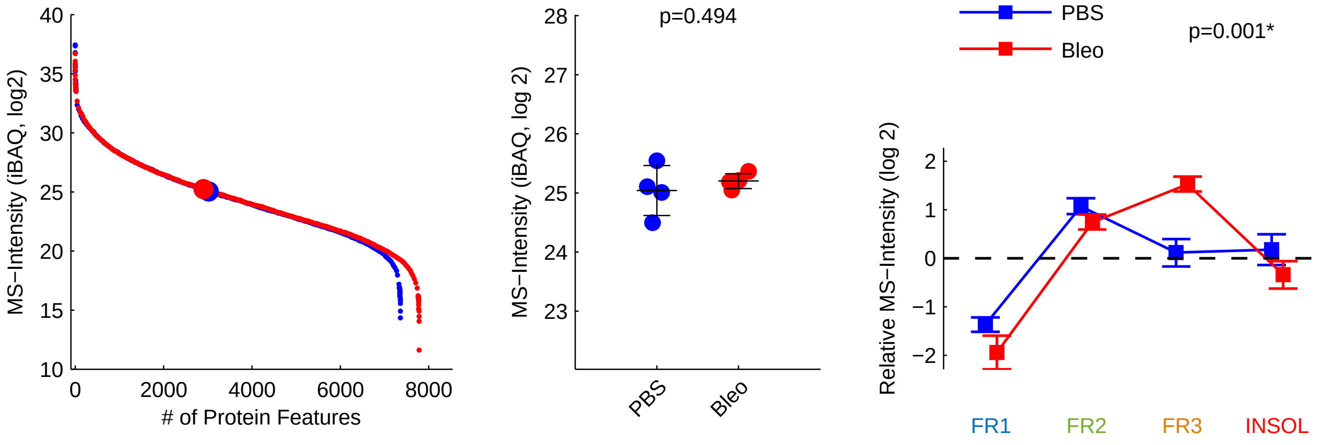

Q8CGF1 − Arhgap29 (id: 6033)

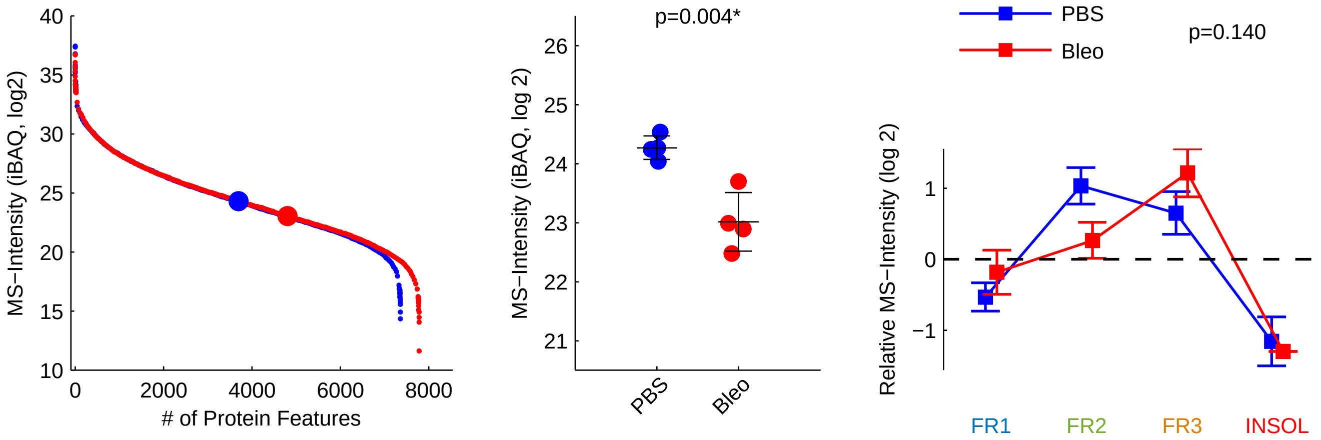

Q8CGK3 − Lonp1 (id: 6036)

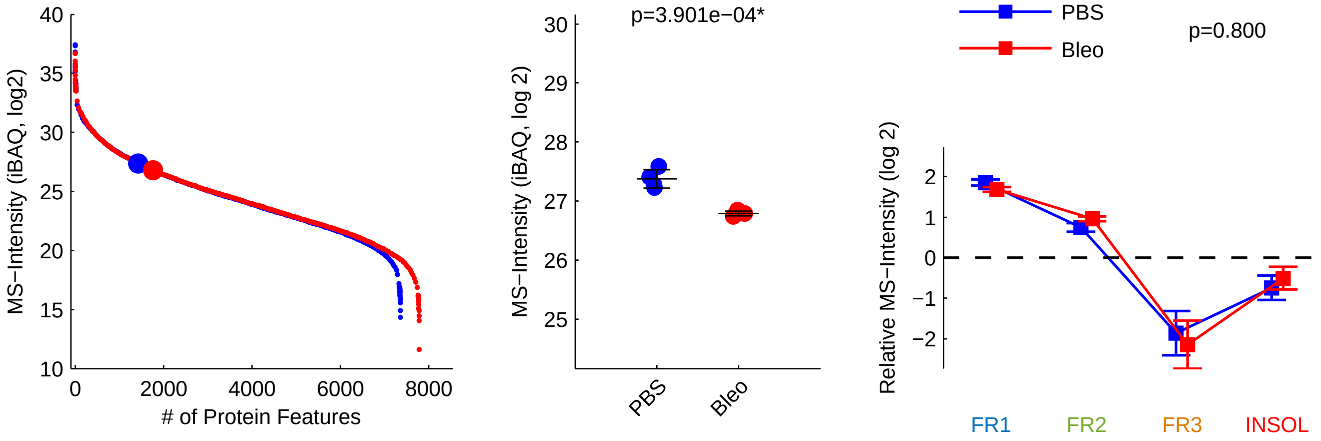

Q8CGN5 − Plin1 (id: 6037)

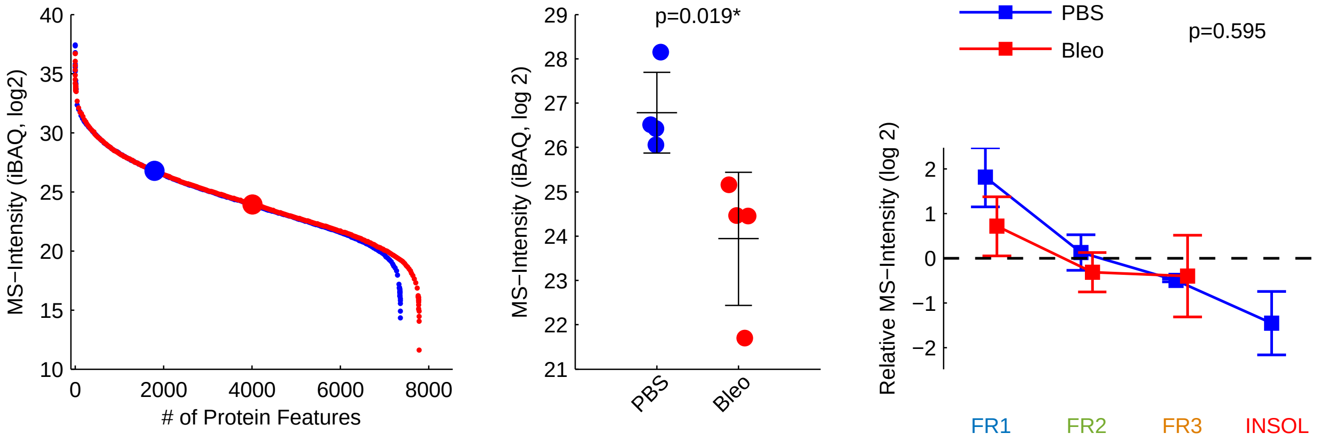

Q8CHI8 − Ep400 (id: 6051)

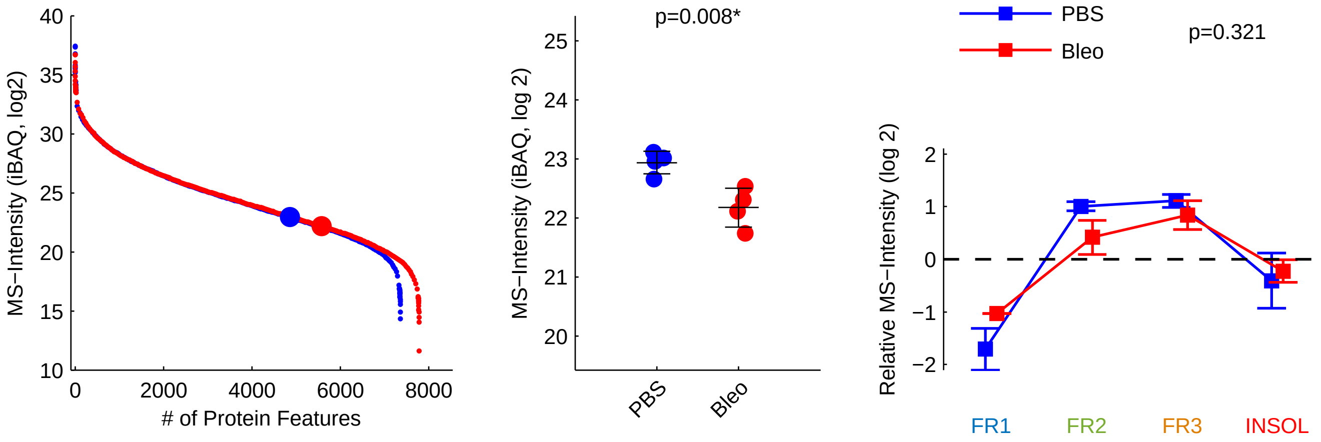

Q8CHT0 − Aldh4a1 (id: 6060)

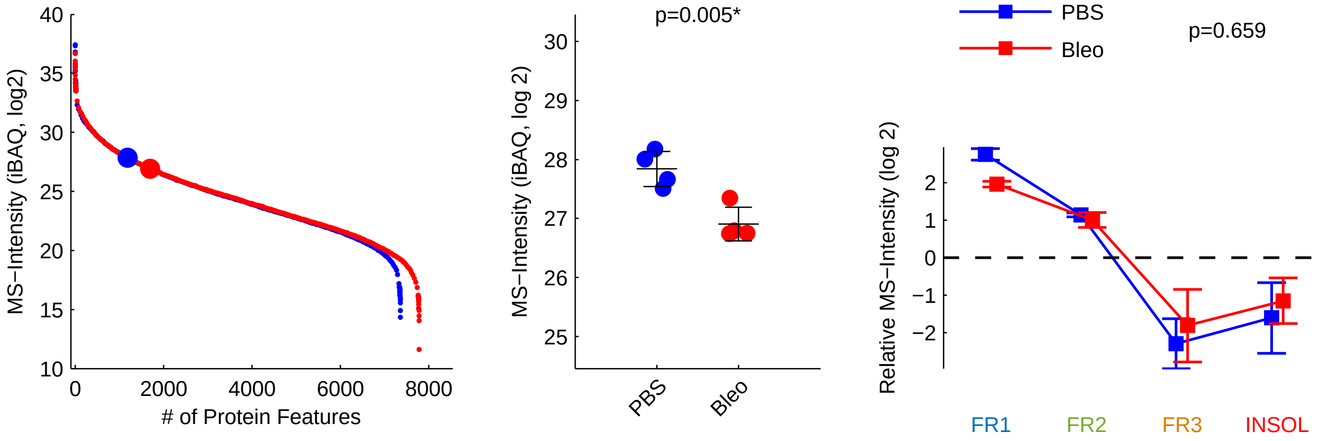

Q8CHU3−2 − Epn2 (id: 6062)

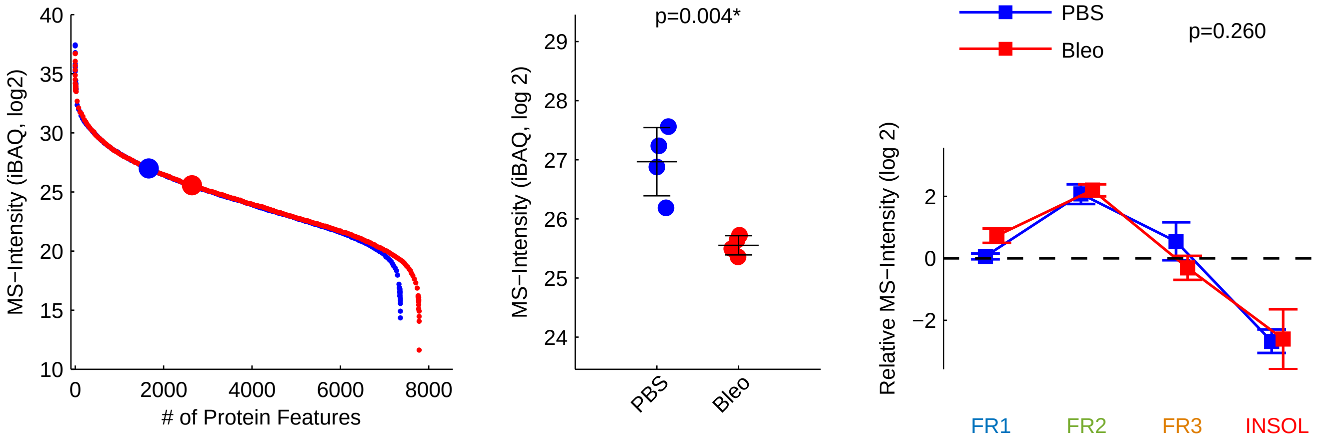

Q8CI94 − Pygb (id: 6082)

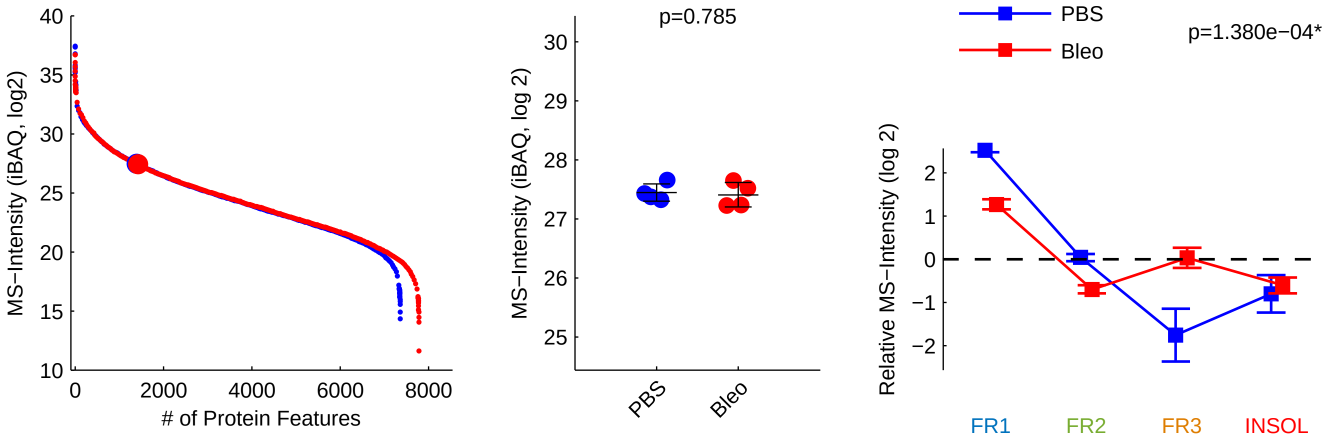

Q8CIB5 − Fermt2 (id: 6083)

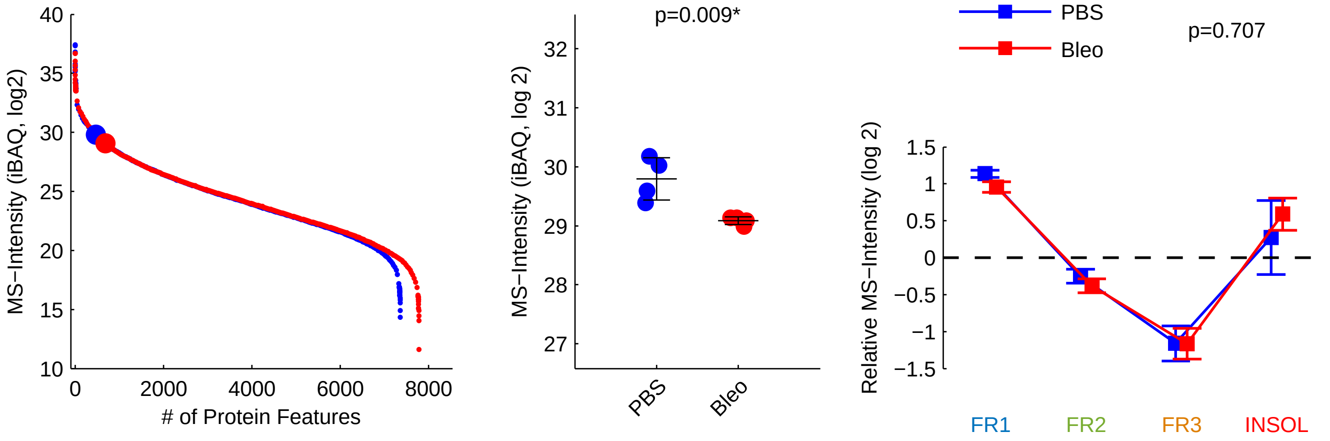

Q8CIE6 − Copa (id: 6085)

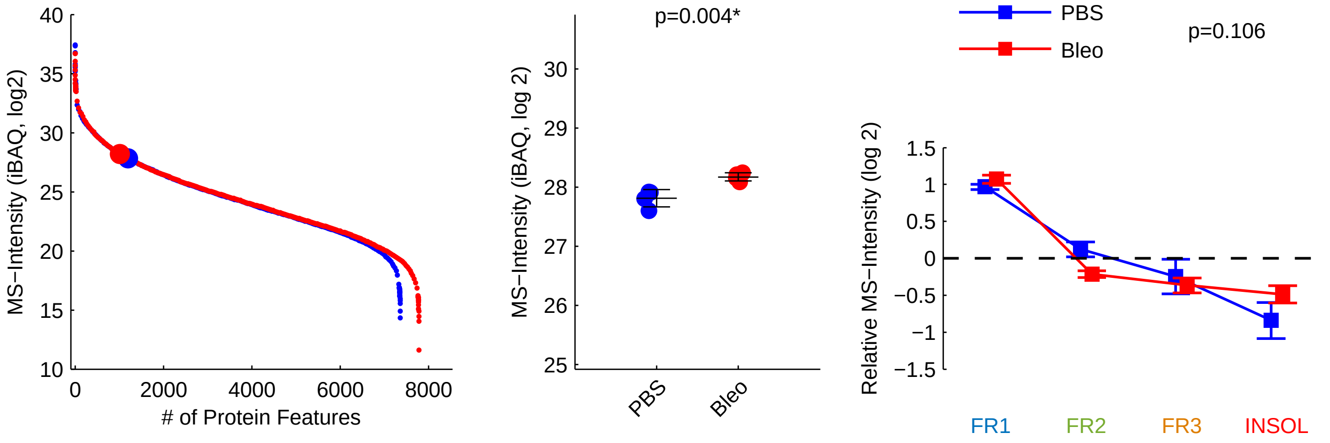

Q8CIG3 − Kdm1b (id: 6088)

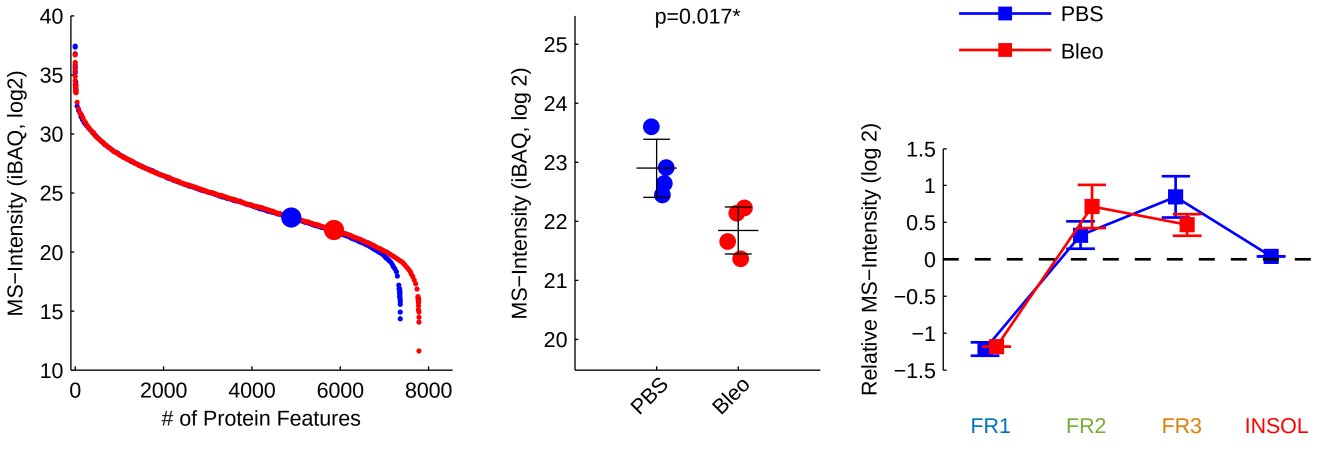

Q8CJ40 − Crocc (id: 6100)

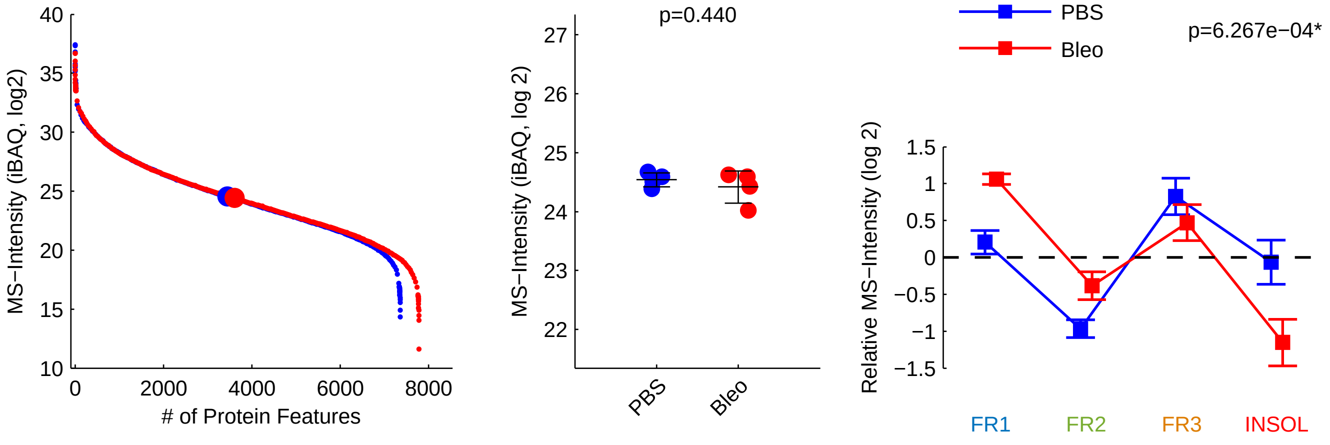

Q8CJ53 − Trip10 (id: 6102)

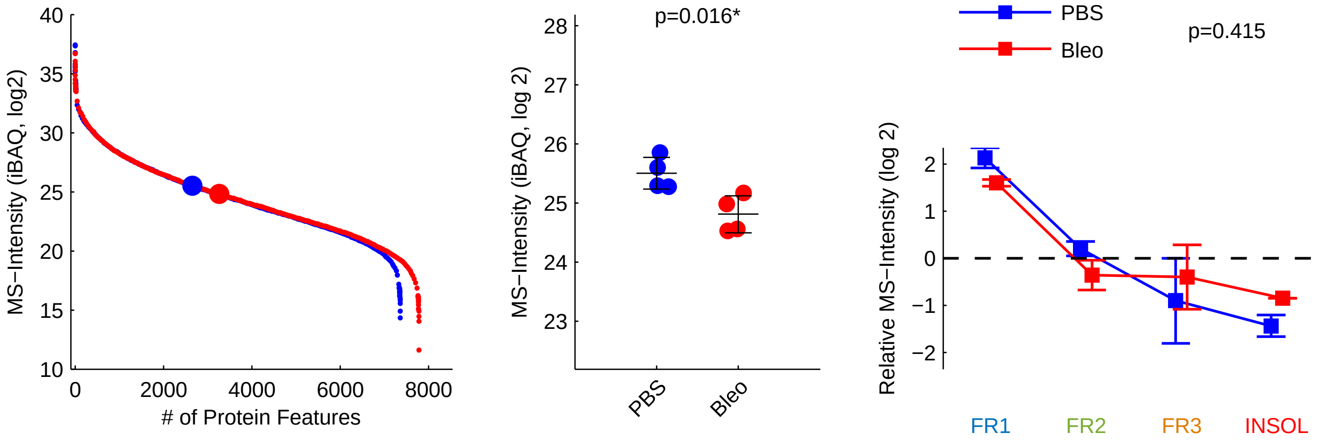

Q8CJ96 − Rassf8 (id: 6104)

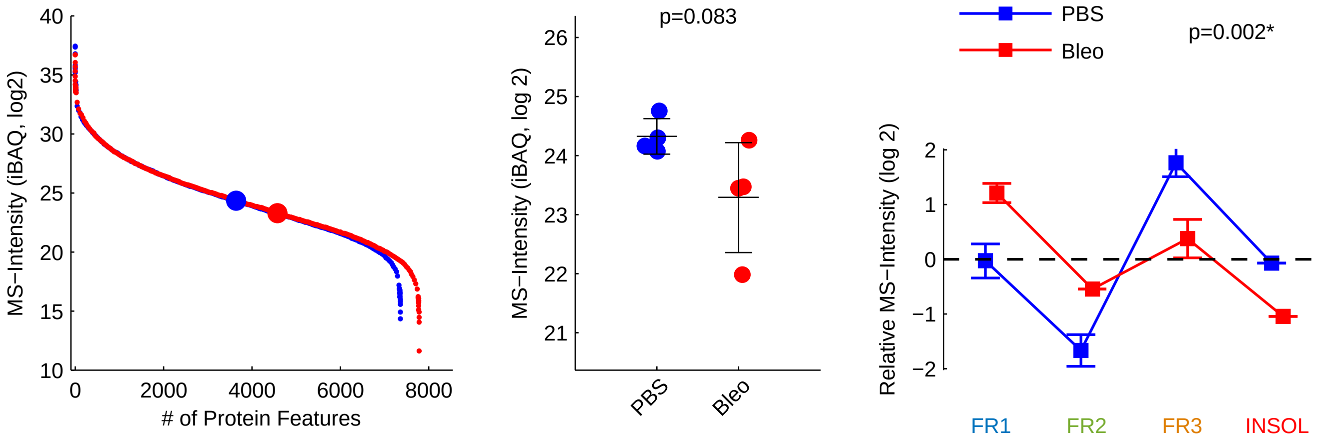

Q8CJG1 − Ago1 (id: 6109)

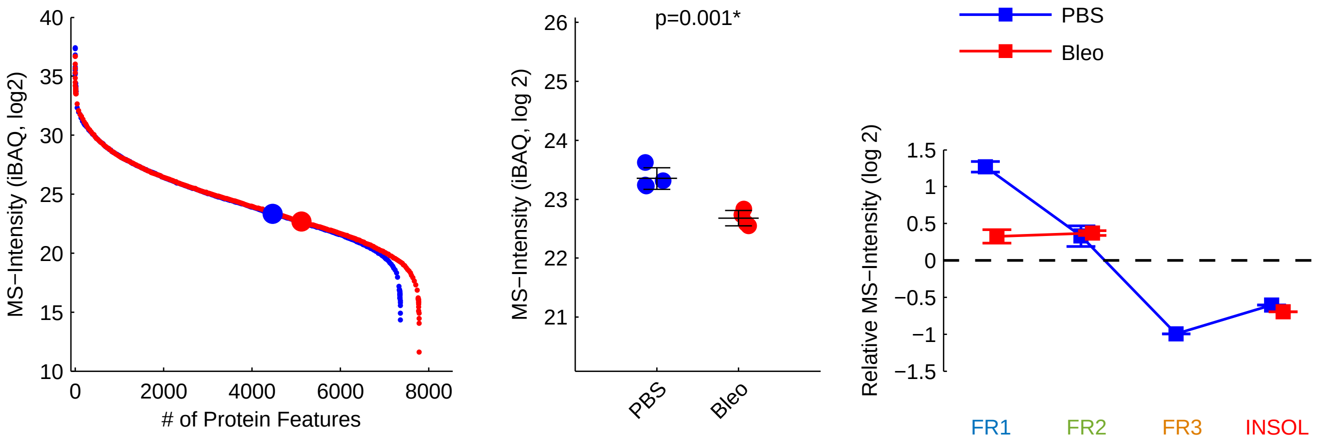

Q8JZM0 − Tfb1m (id: 6116)

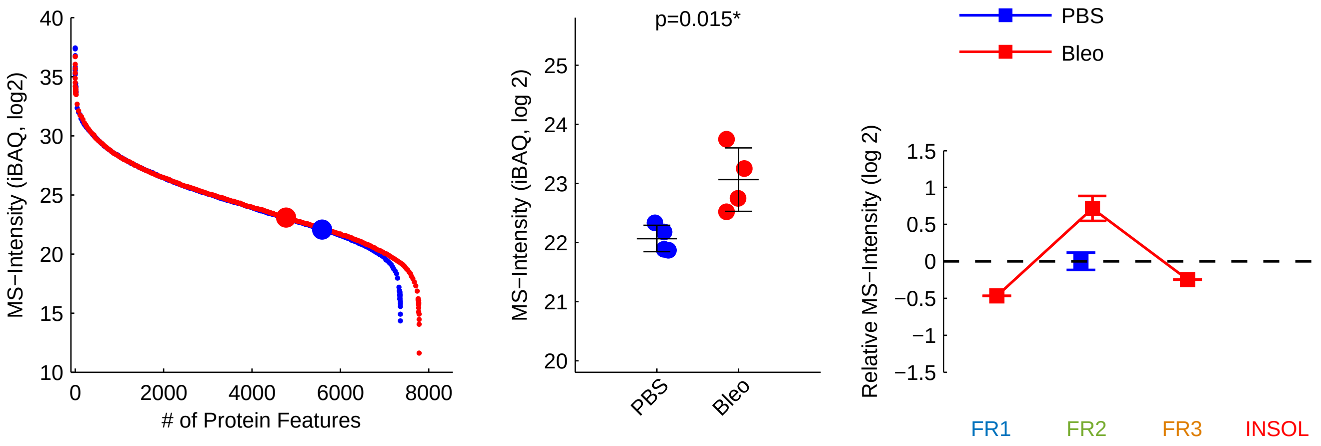

Q8JZN5 − Acad9 (id: 6120)

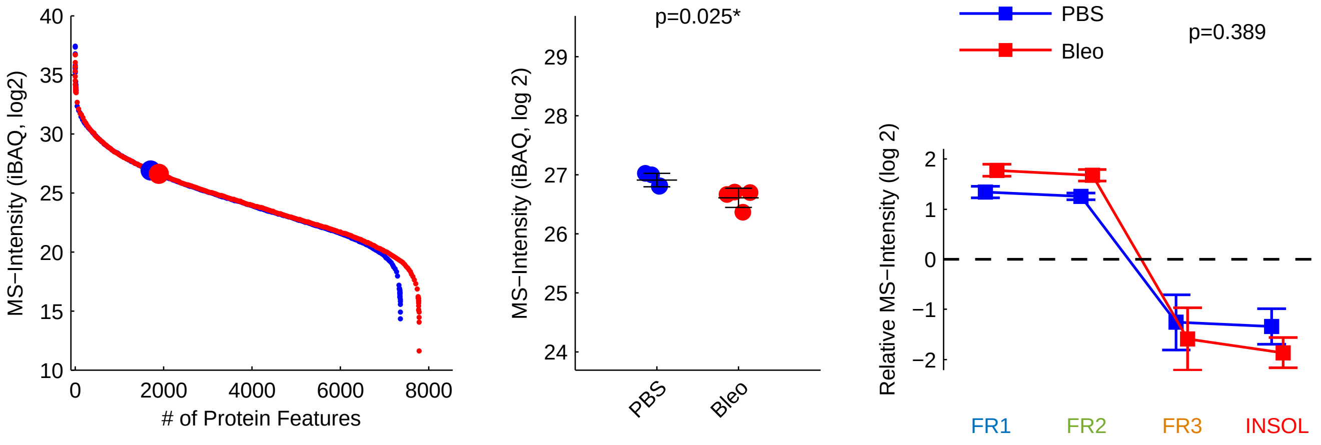

Q8JZQ9 − Eif3b (id: 6124)

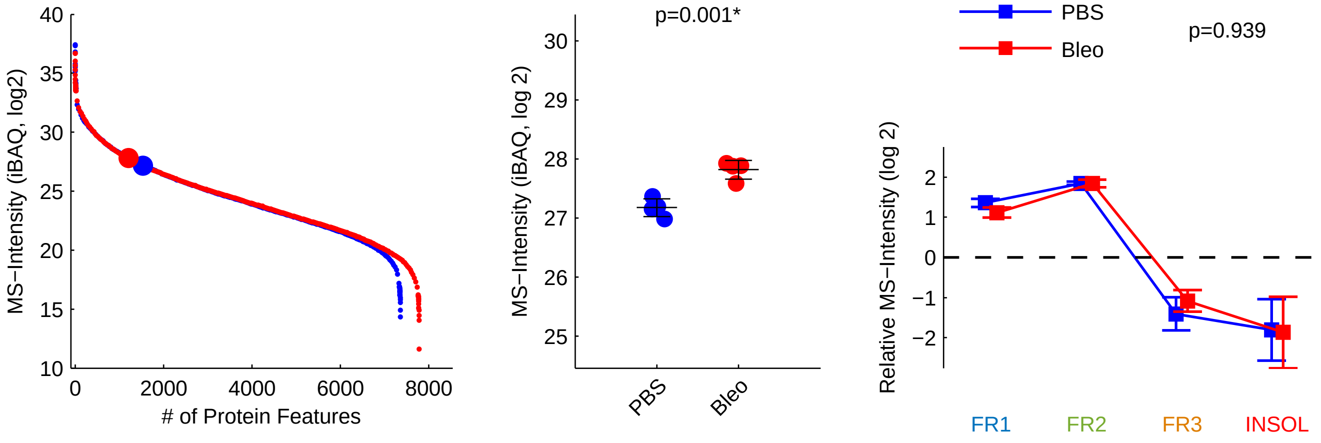

Q8JZR0 − Acsl5 (id: 6125)

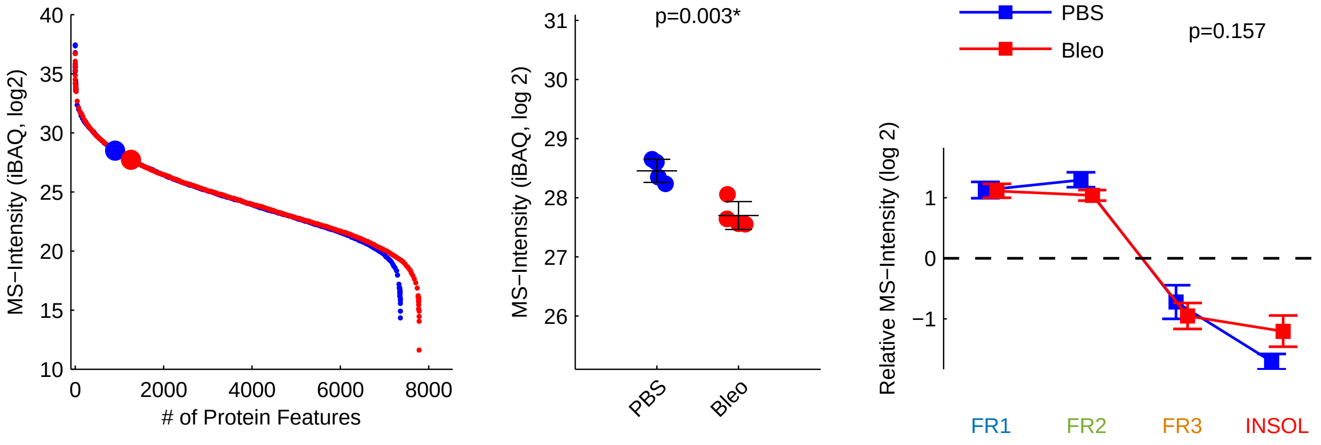

Q8JZV7 − Amdhd2 (id: 6130)

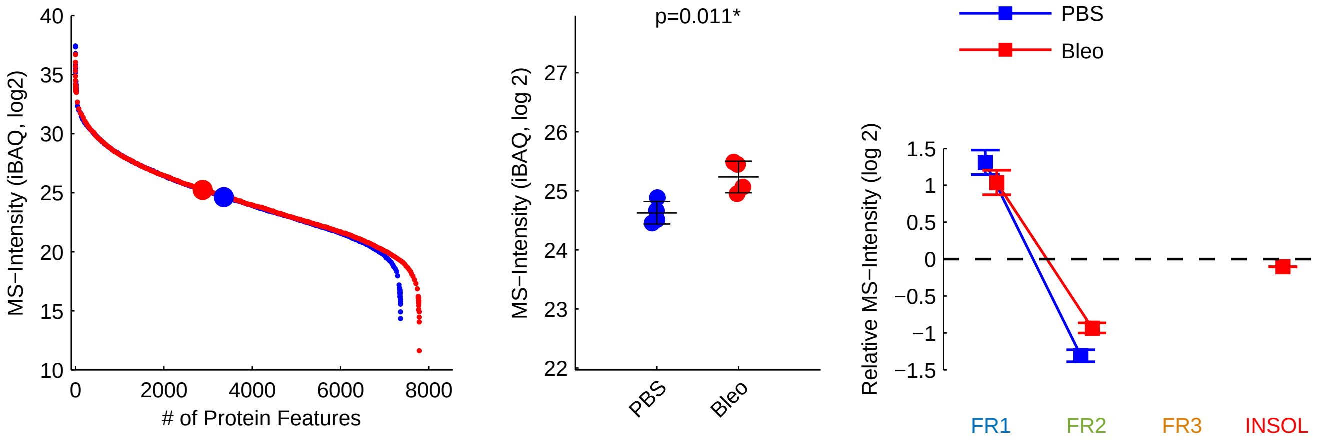

Q8JZX3 − Poc1a (id: 6131)

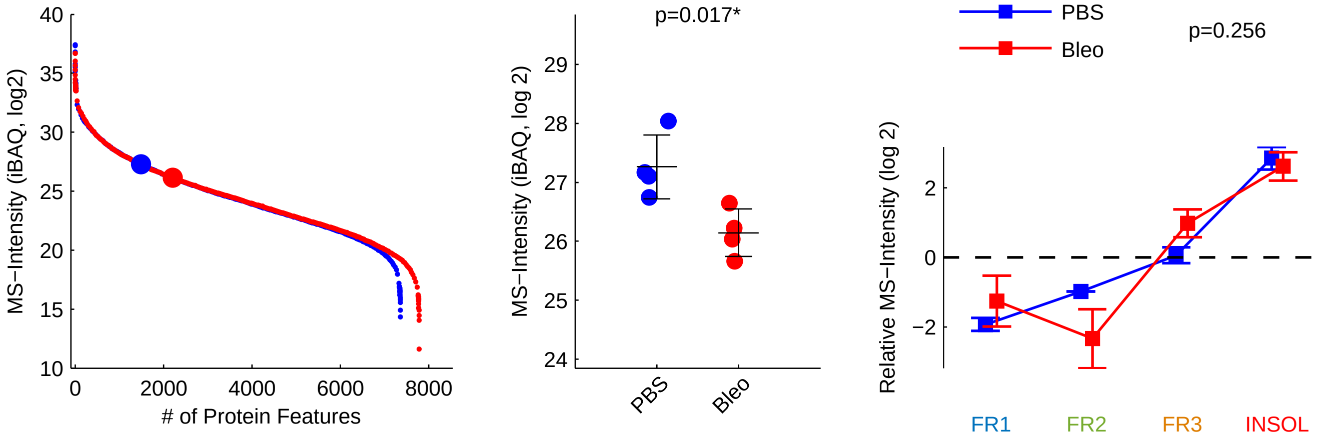

Q8JZX5 − Lrrc57 (id: 6133)

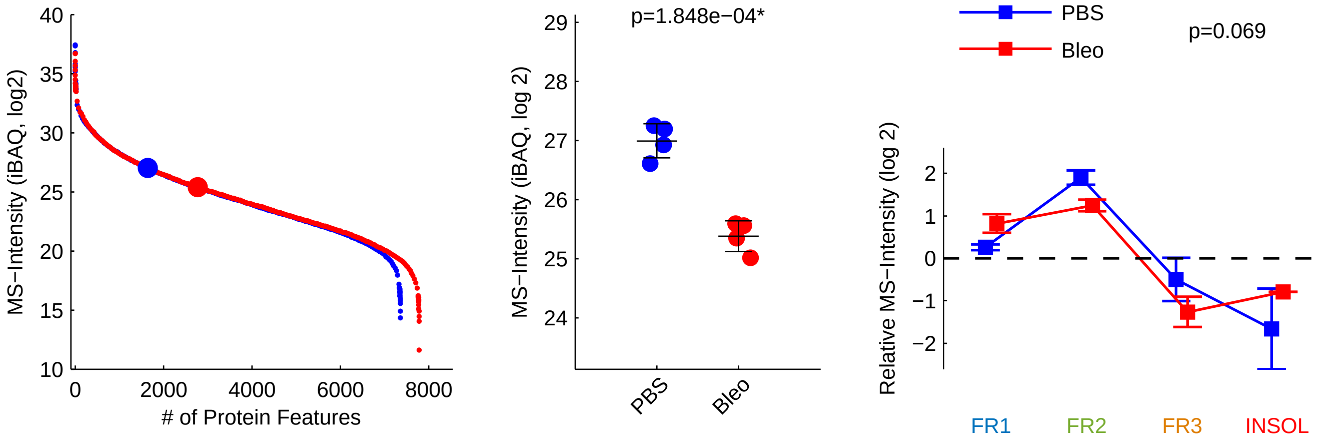

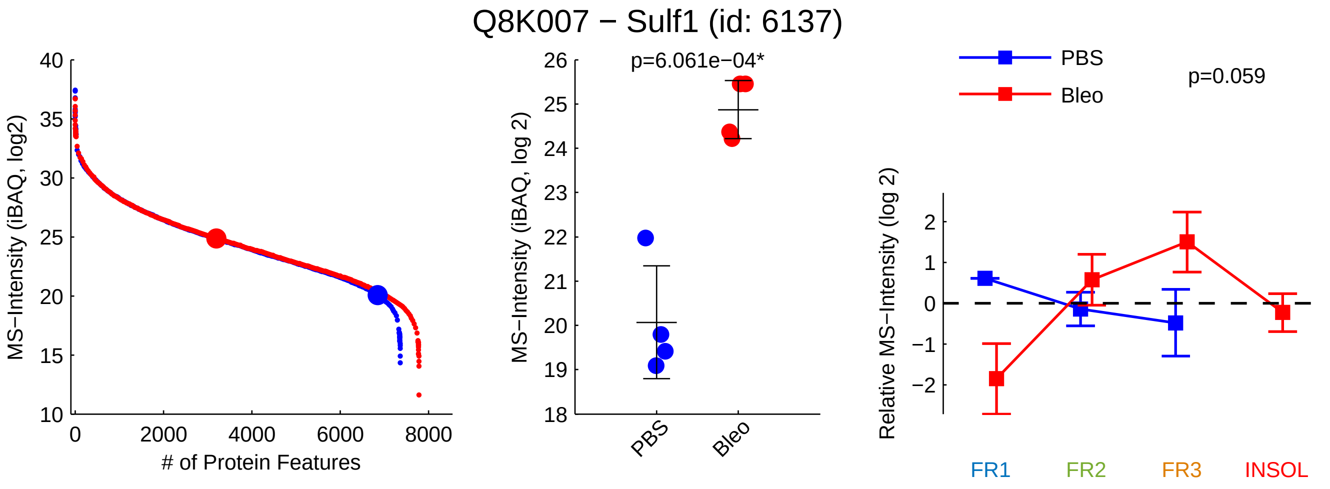
Q8K007 − Sulf1 (id: 6137)
MS−Intensity (iBAQ, log2)
# of Protein Features
p=6.061e−04*
MS−Intensity (iBAQ, log 2)
PBS
Bleo
p=0.059
Relative MS−Intensity (log 2)
FR1
FR2
FR3
INSOL

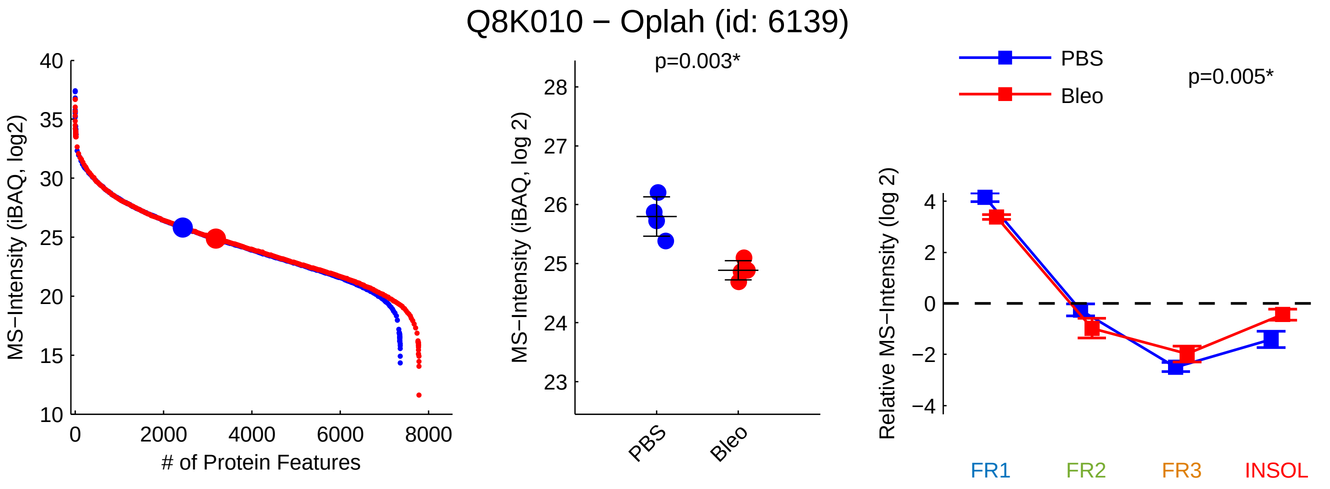
Q8K010 − Oplah (id: 6139)
MS−Intensity (iBAQ, log2)
# of Protein Features
p=0.003*
MS−Intensity (iBAQ, log 2)
PBS
Bleo
p=0.005*
Relative MS−Intensity (log 2)
FR1
FR2
FR3
INSOL

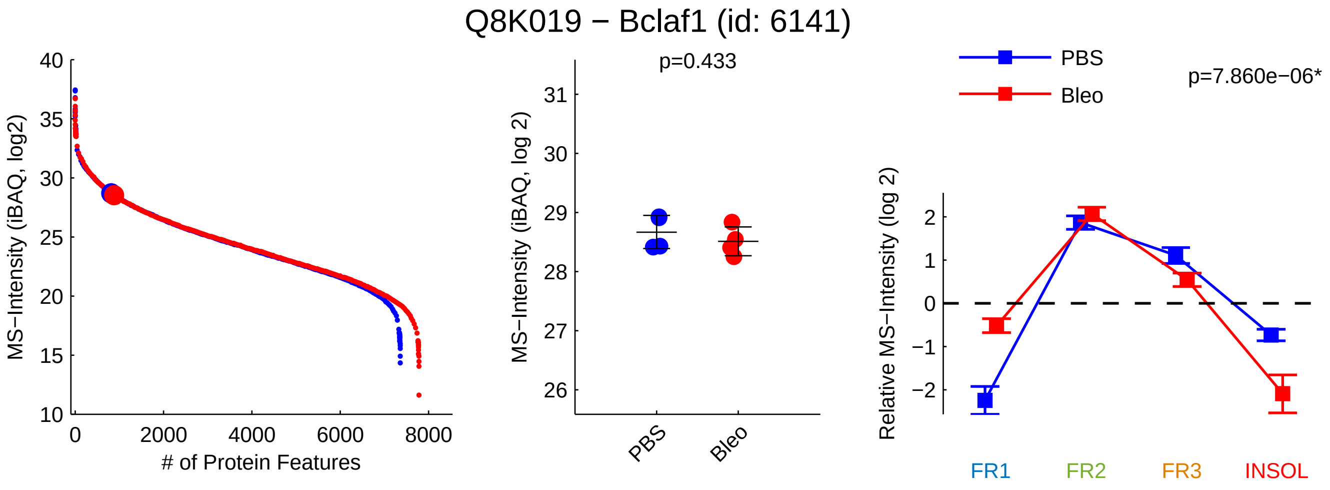
Q8K019 − Bclaf1 (id: 6141)
MS−Intensity (iBAQ, log2)
# of Protein Features
p=0.433
MS−Intensity (iBAQ, log 2)
PBS
Bleo
p=7.860e−06*
Relative MS−Intensity (log 2)
FR1
FR2
FR3
INSOL

Q8K0C4 − Cyp51a1 (id: 6151)

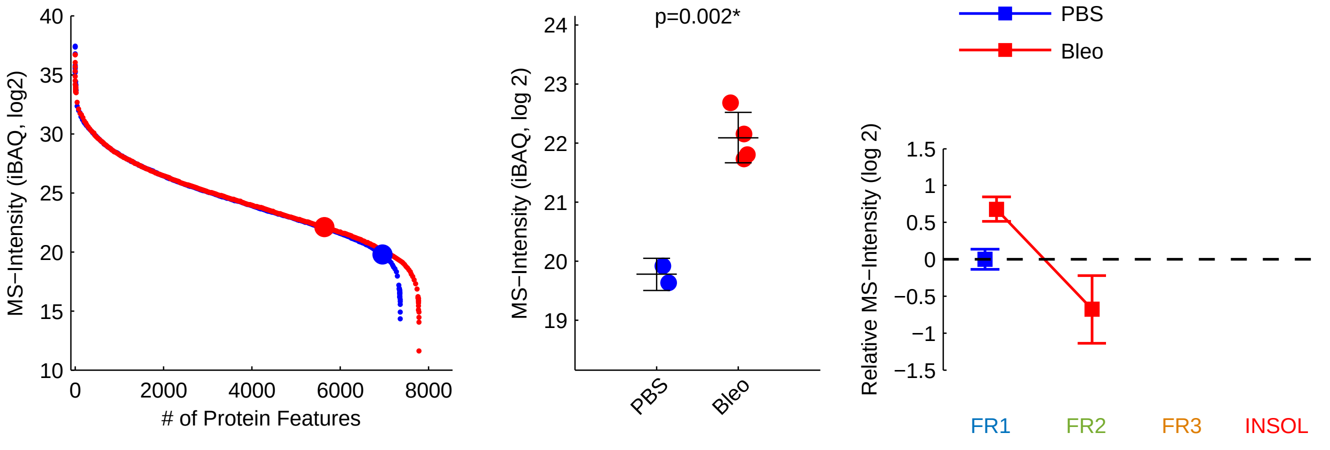

Q8K0E8 − Fgb (id: 6155)

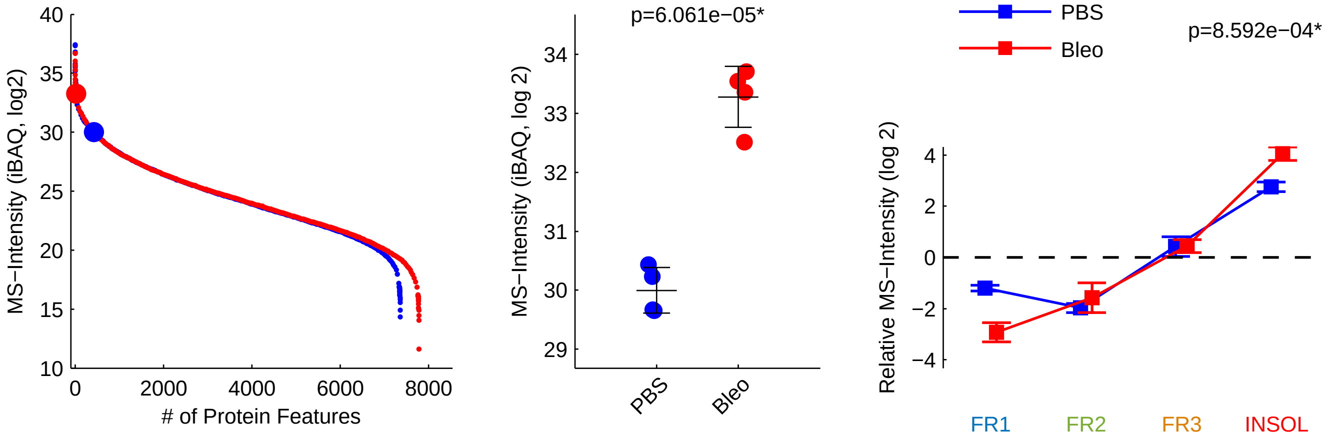

Q8K0L9 − Zbtb20 (id: 6158)

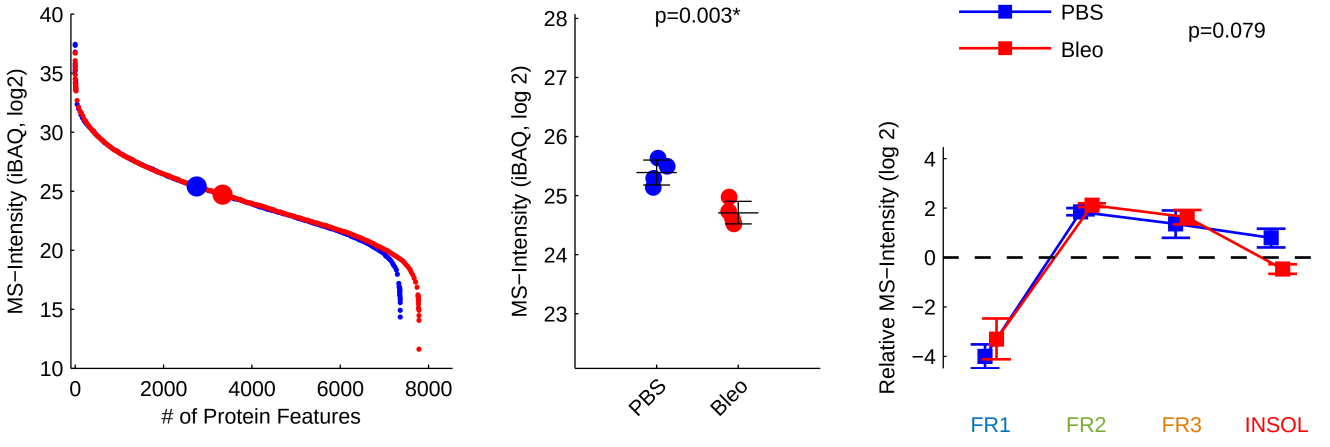

Q8K0Z7 − Taco1 (id: 6166)

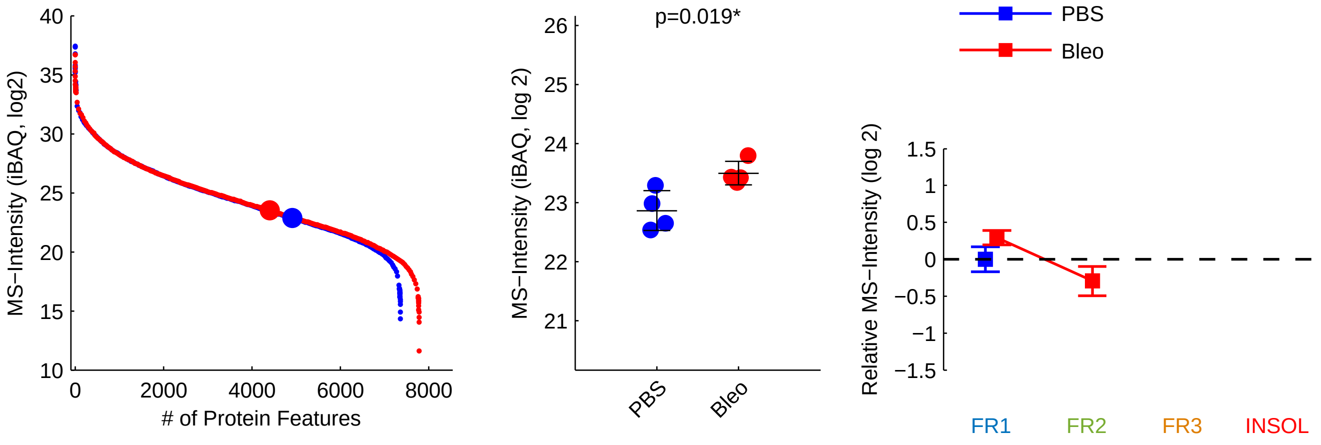

Q8K124 − Plekho2 (id: 6169)

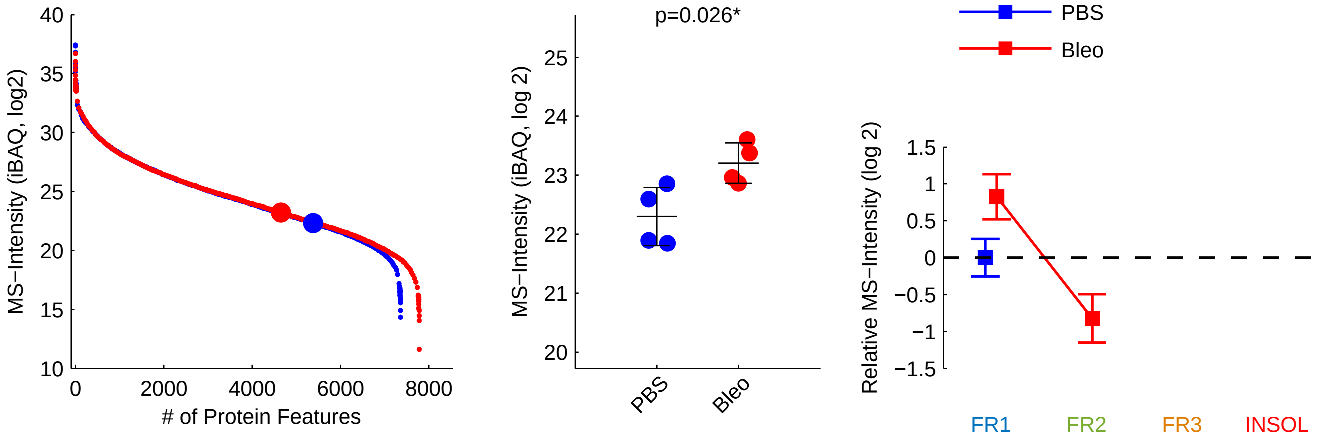

Q8K183 − Pdxk (id: 6171)

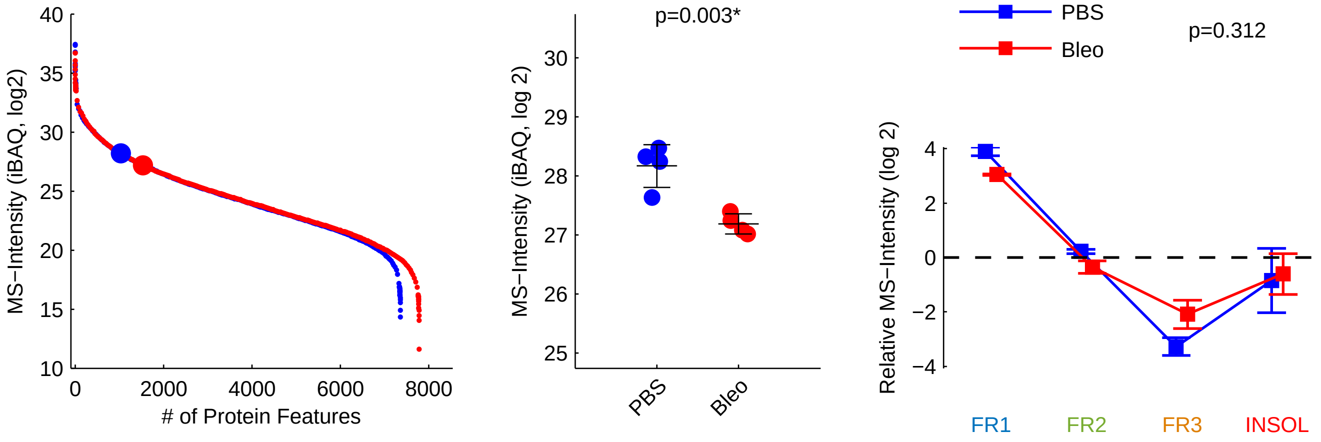

Q8K1B8 − Fermt3 (id: 6176)

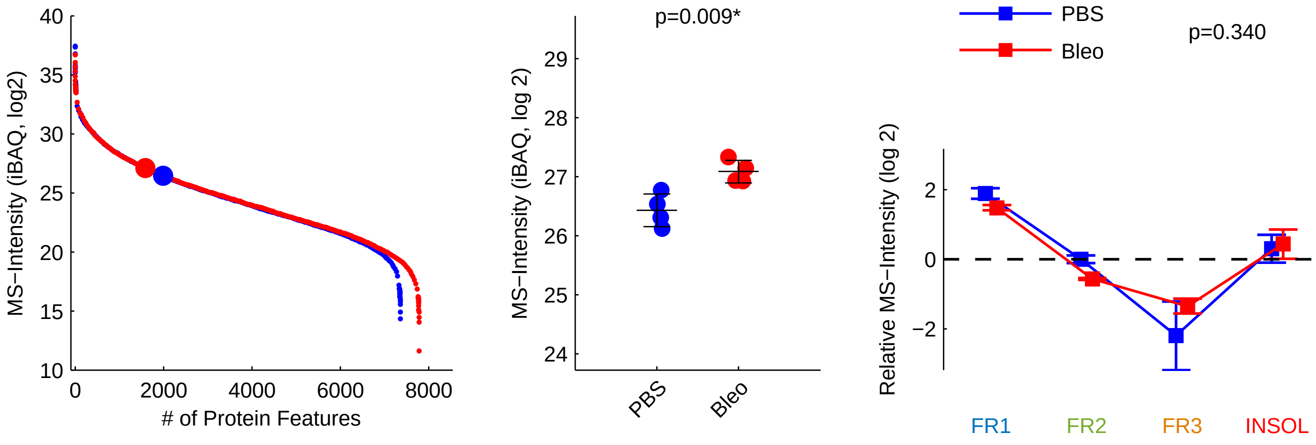

Q8K1M3 − Prkar2a (id: 6186)

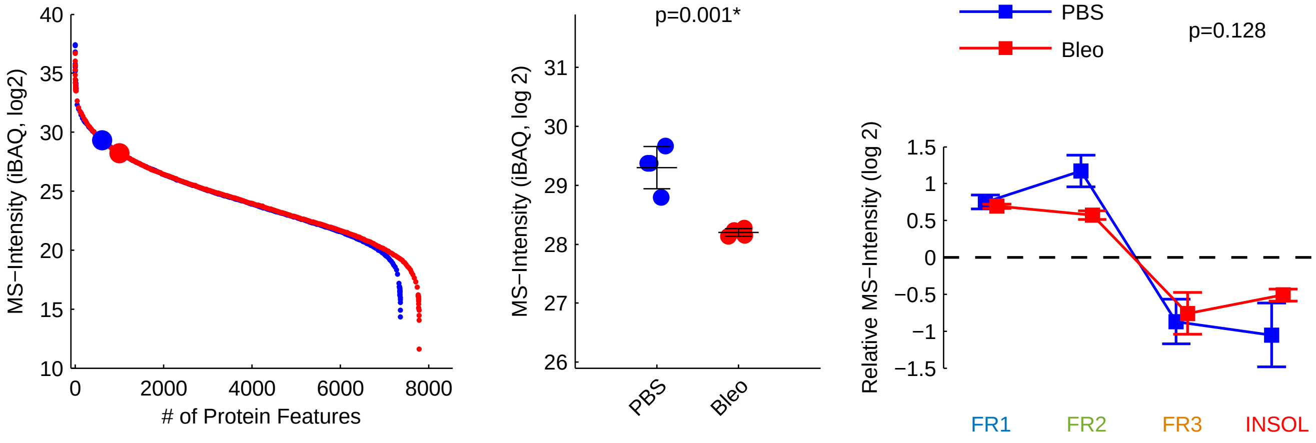

Q8K1M6−3 − Dnm1l (id: 6187)

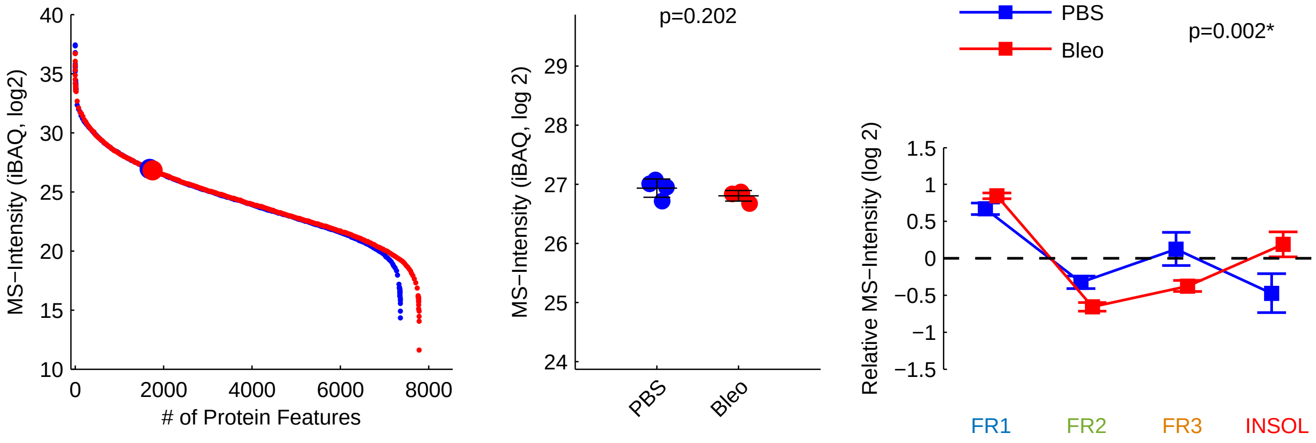

Q8K1X4 − Nckap1l (id: 6194)

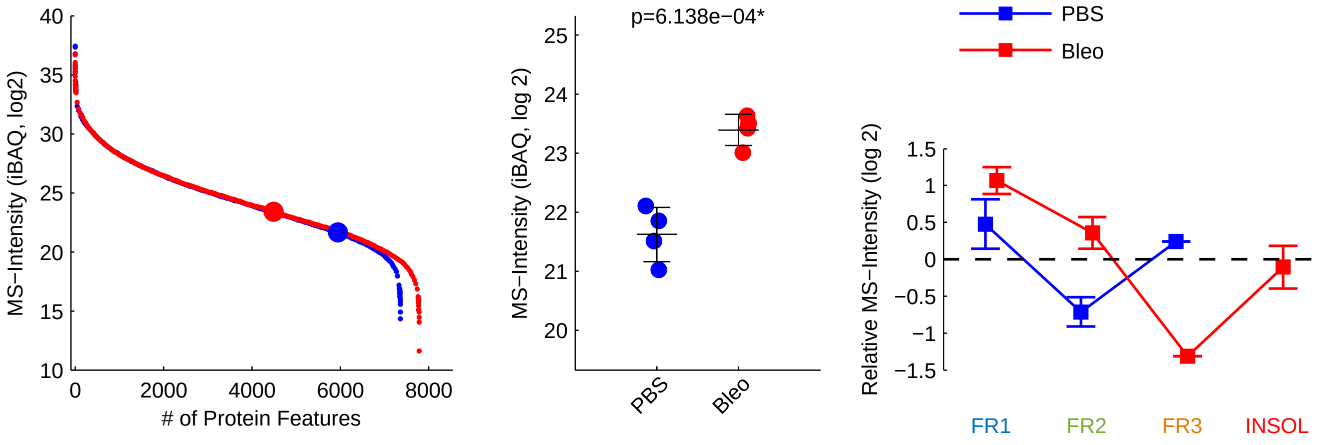

Q8K1Z0 − Coq9 (id: 6195)

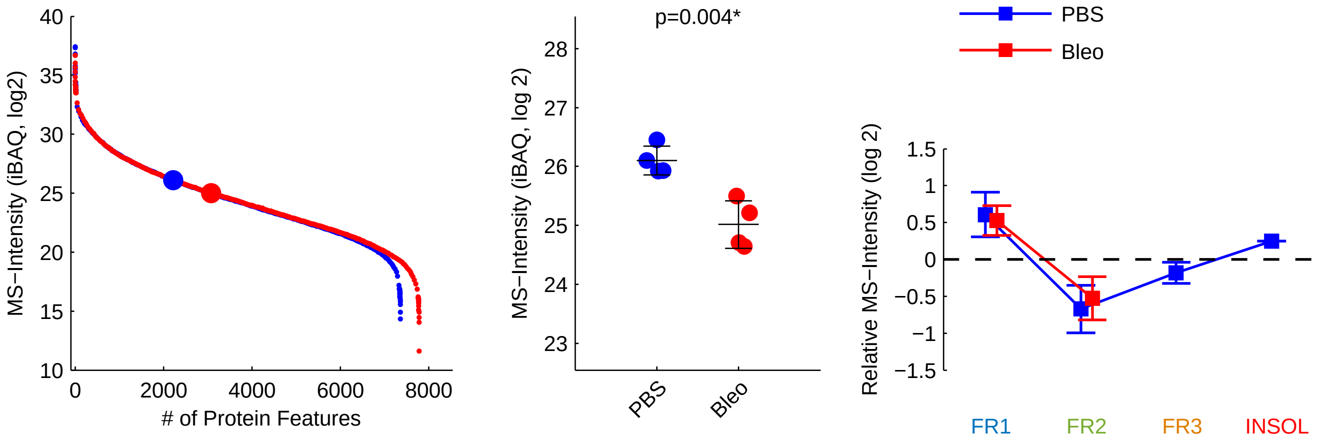

Q8K262 − Plac9 (id: 6204)

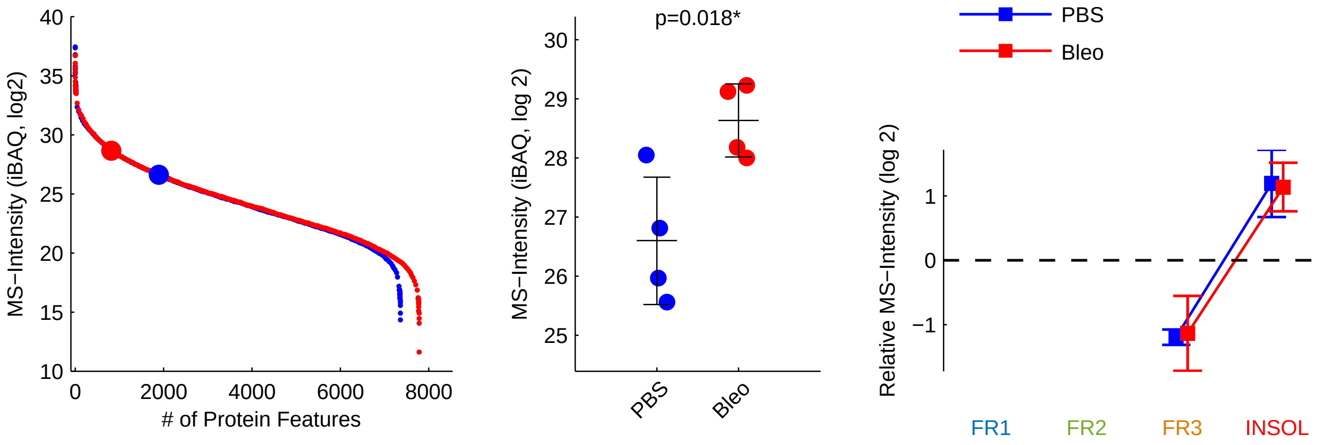

Q8K274 − Fn3krp (id: 6207)

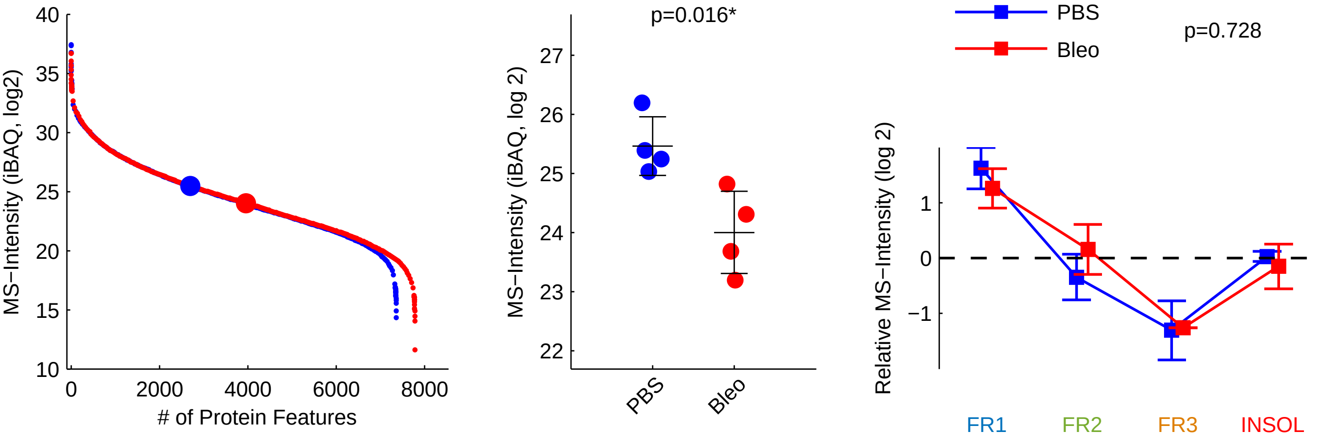

Q8K284 − Gtf3c1 (id: 6208)

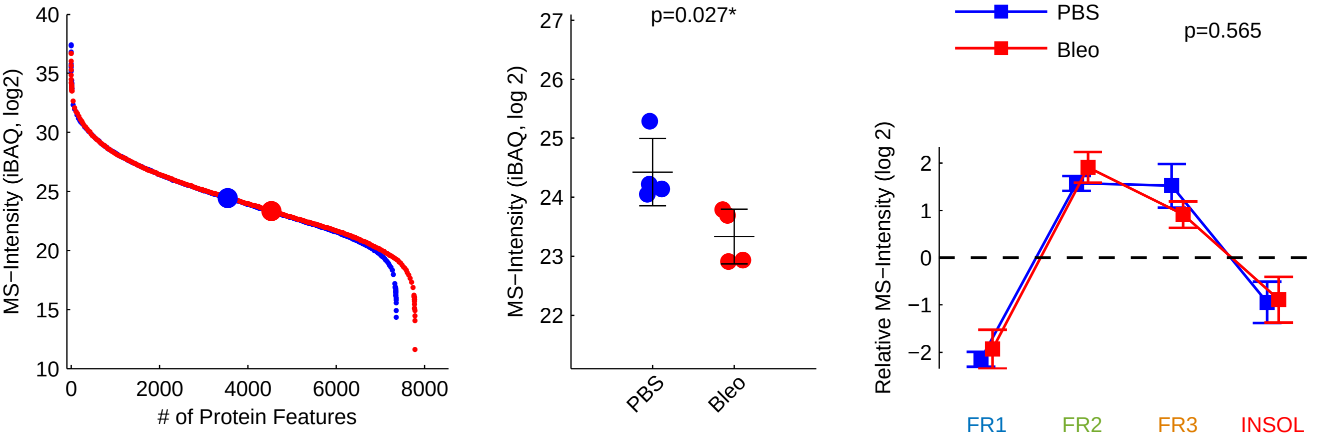

Q8K297 − Colgalt1 (id: 6209)

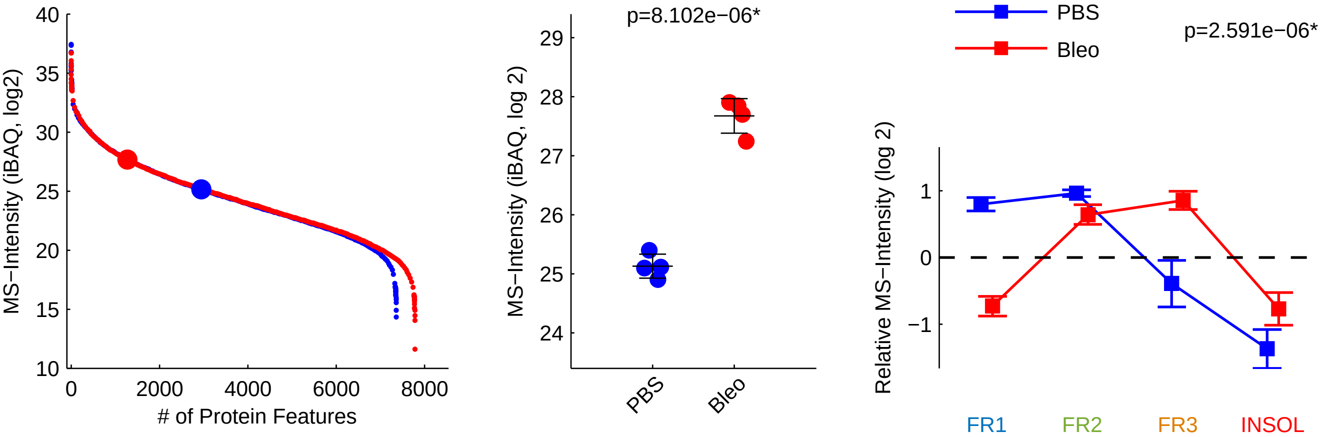

Q8K2A1 − Gulp1 (id: 6210)

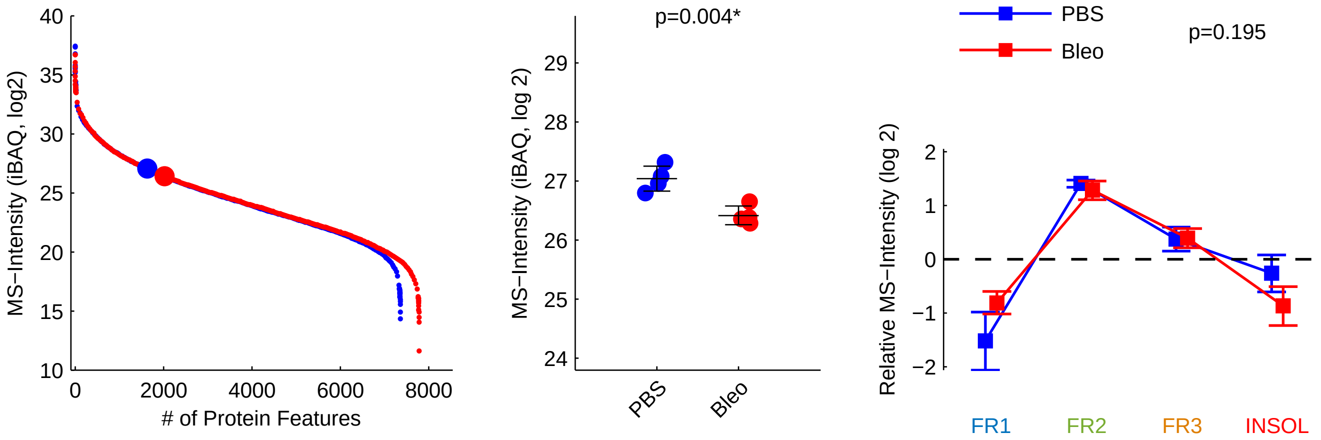

Q8K2B0 − Leprel4 (id: 6212)

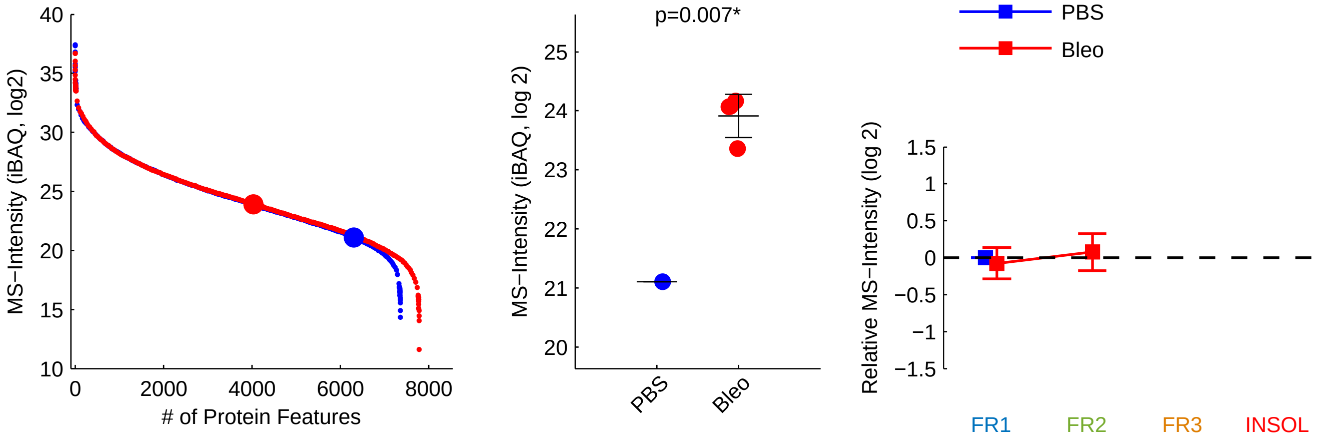

Q8K2C6 − Sirt5 (id: 6214)

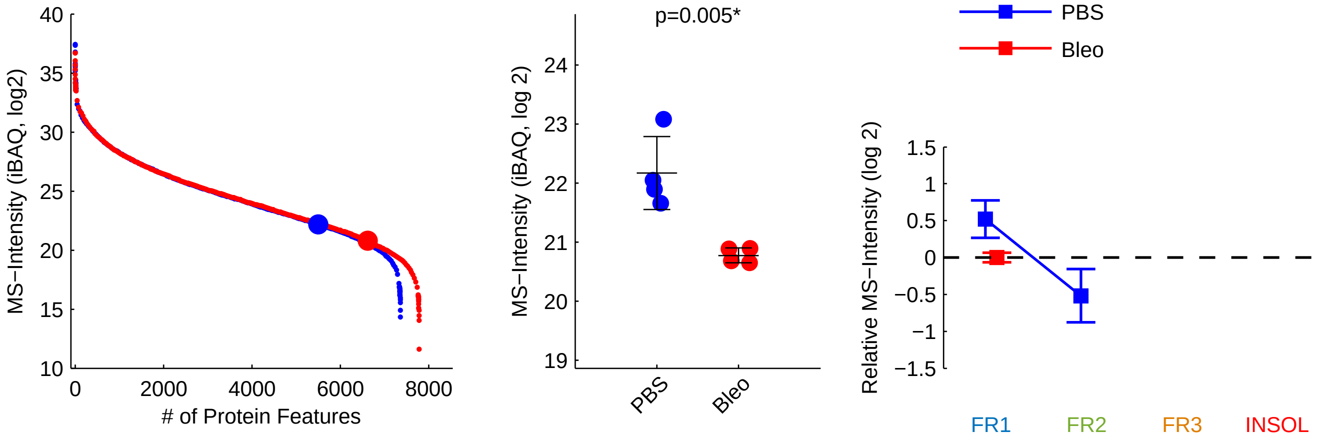

Q8K2C9 − ptplad1 (id: 6216)

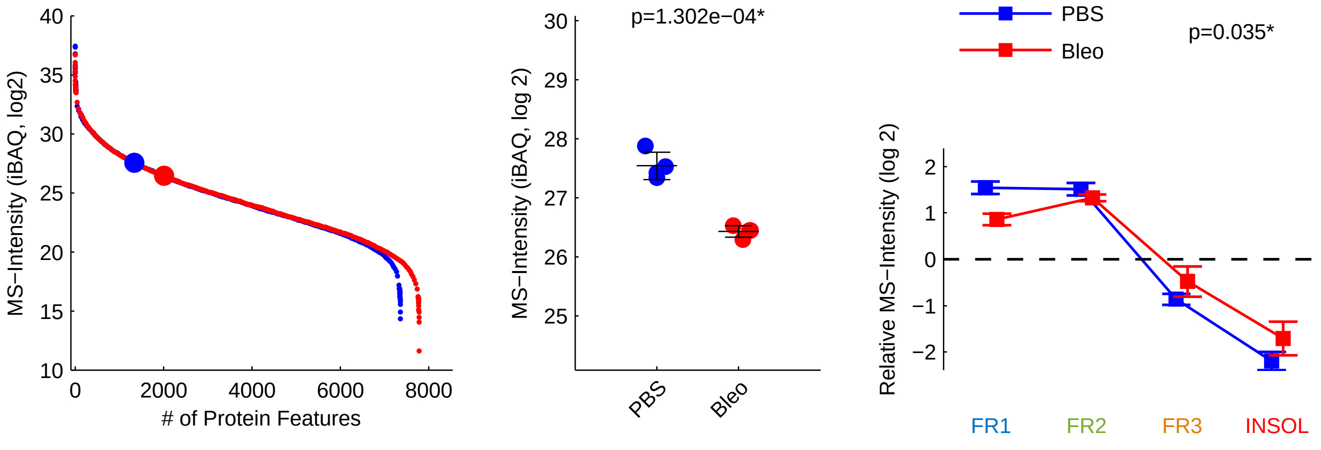

Q8K2F0 − Brd3 (id: 6219)

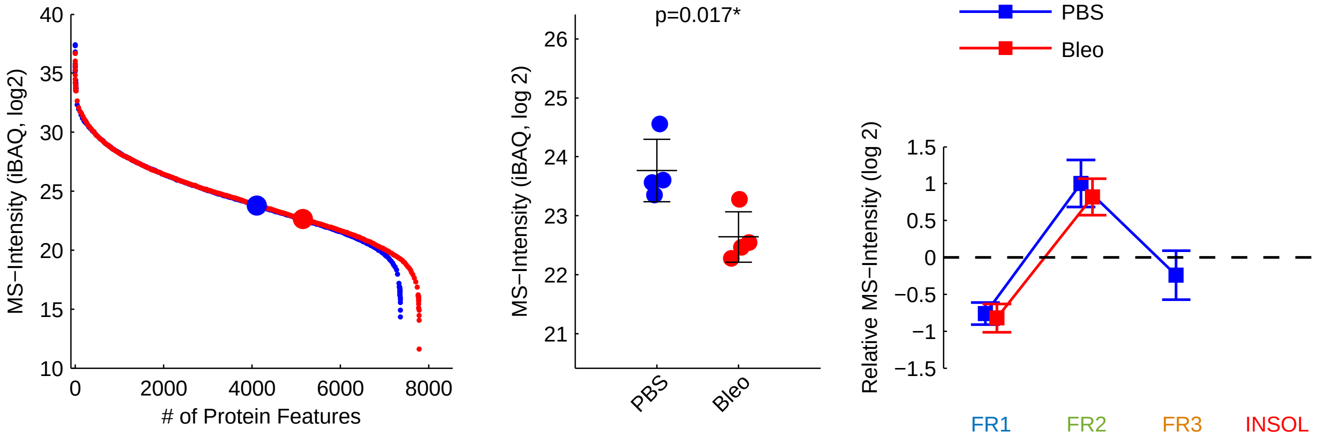

Q8K2I3 − Fmo2 (id: 6223)

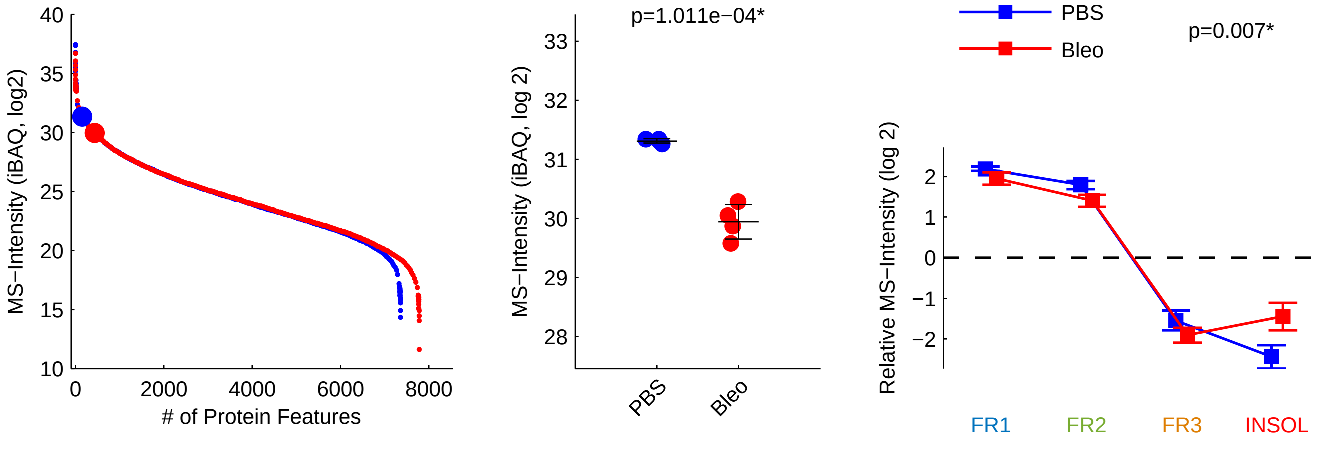

Q8K2Y3 − Eva1b (id: 6237)

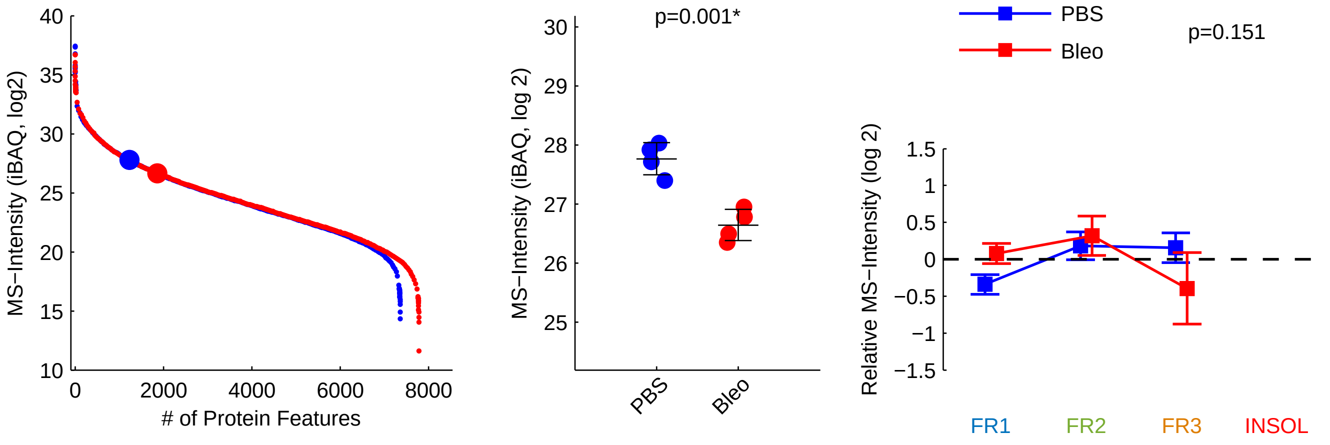

Q8K310 − Matr3 (id: 6242)

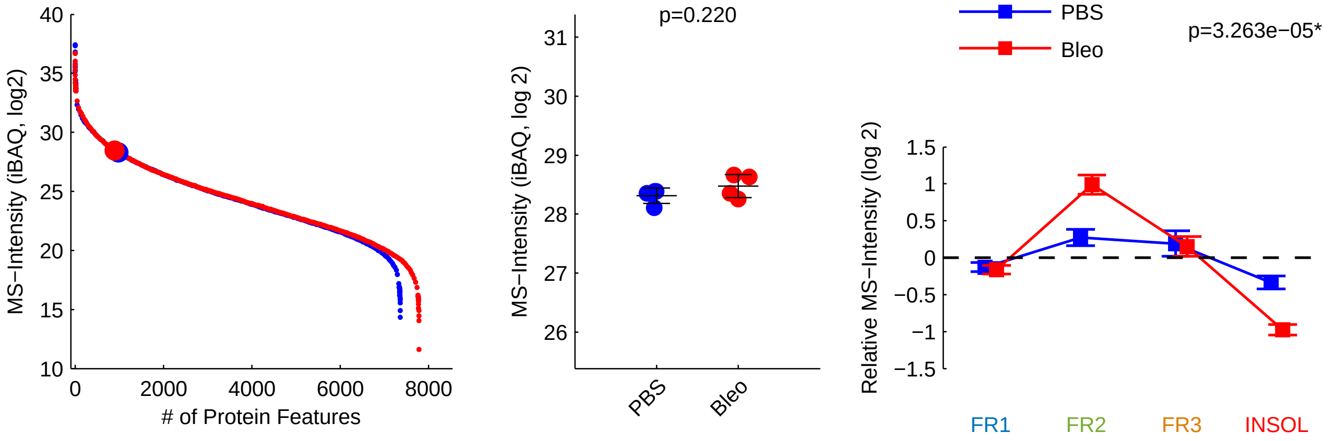

Q8K337 − Inpp5b (id: 6245)

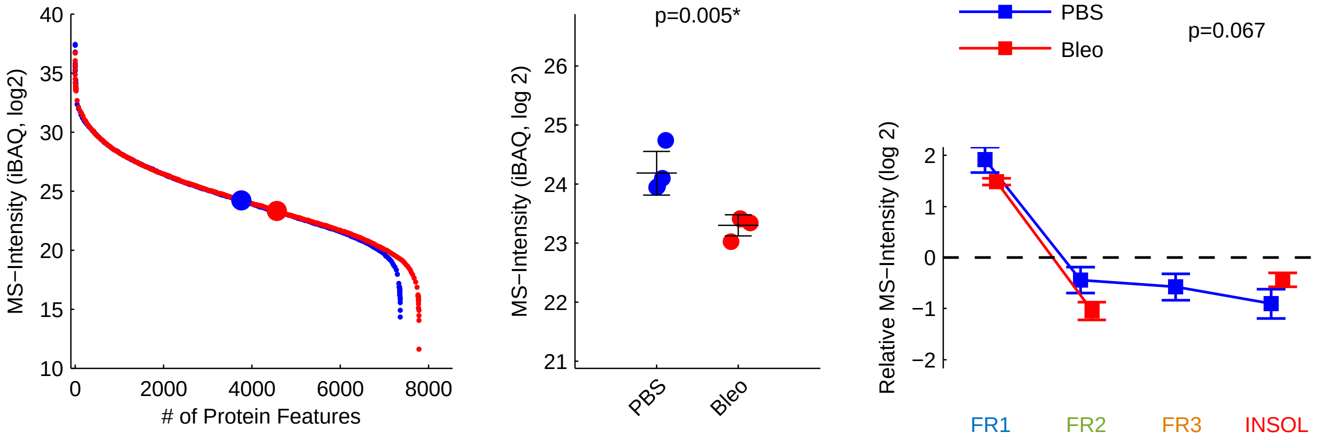

Q8K370 − Acad10 (id: 6249)

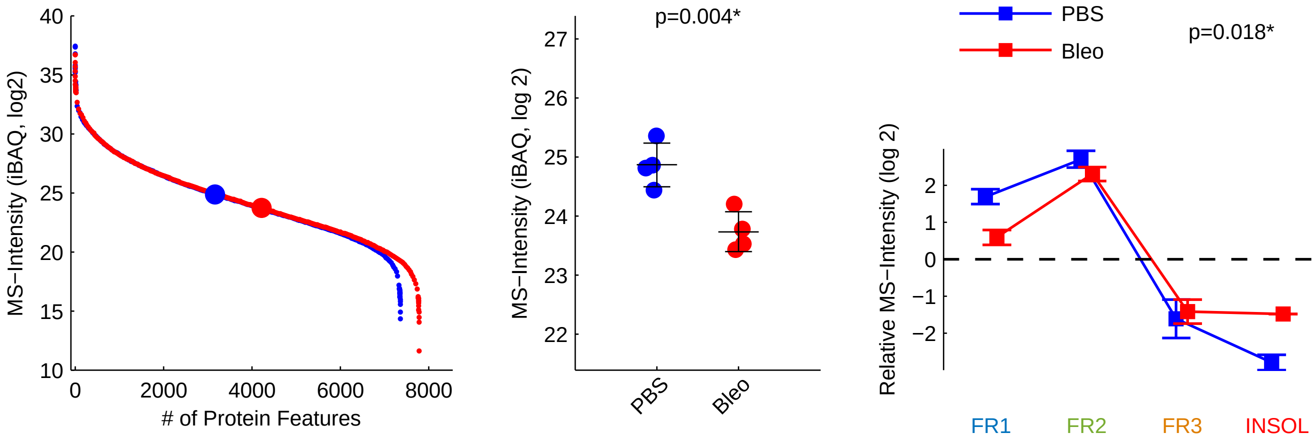

Q8K3A9 − Mepce (id: 6254)

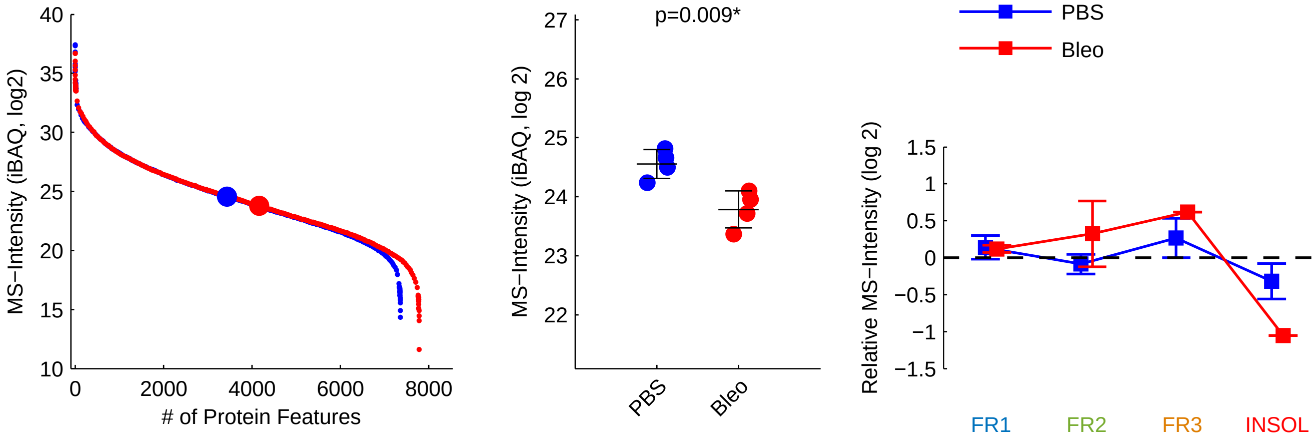

Q8K3G5 − Vrk3 (id: 6257)

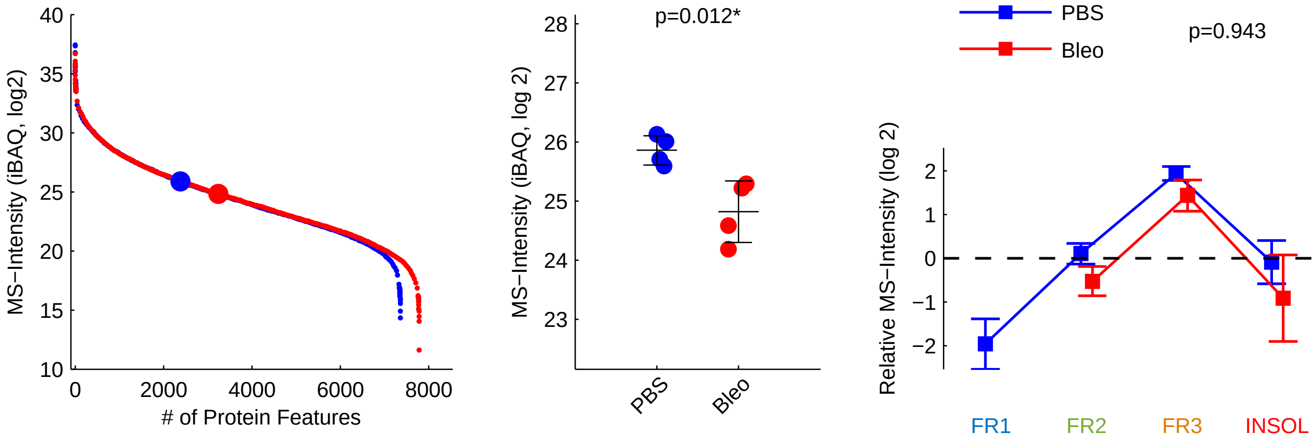

Q8K482 − Emilin2 (id: 6276)

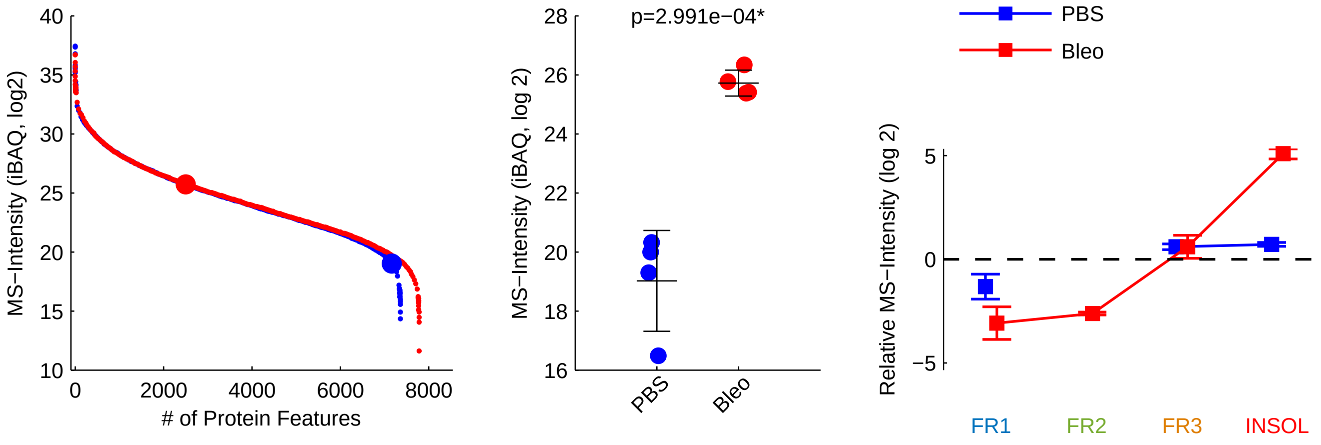

Q8K4F5 − Abhd11 (id: 6278)

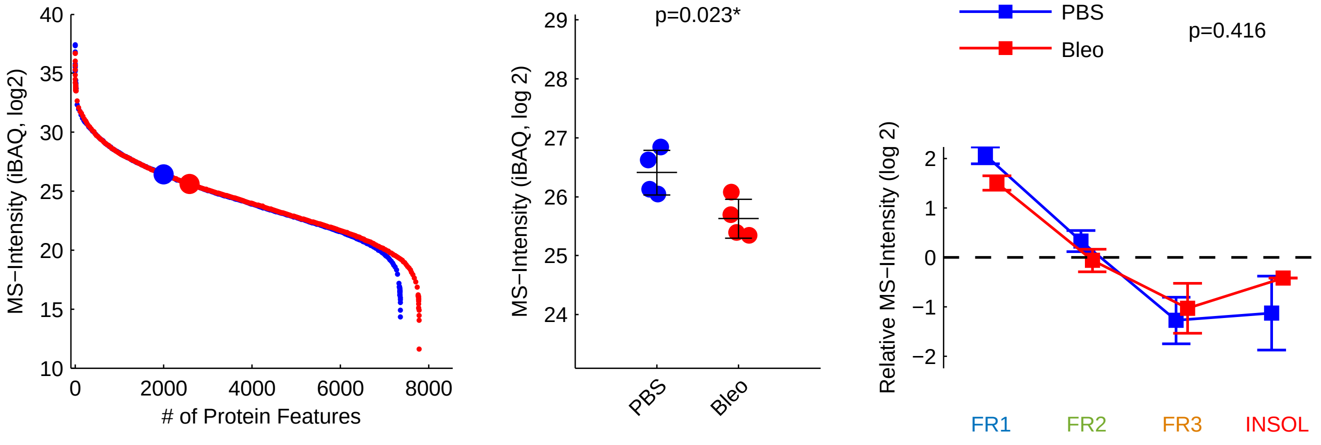

Q8K4L0 − Ddx54 (id: 6280)

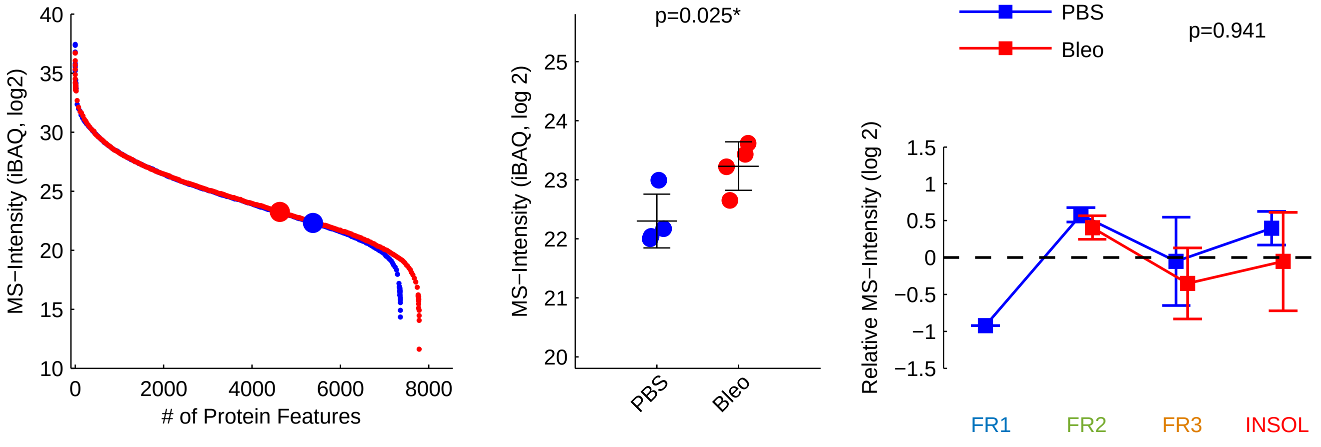

Q8K4P0 − Wdr33 (id: 6284)

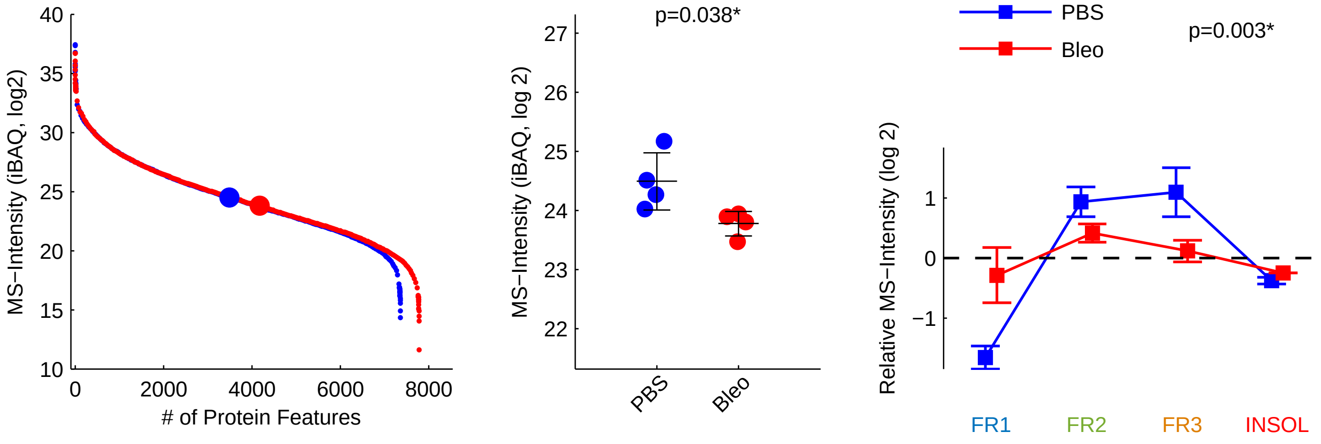

Q8K4R4 − Pitpnc1 (id: 6287)

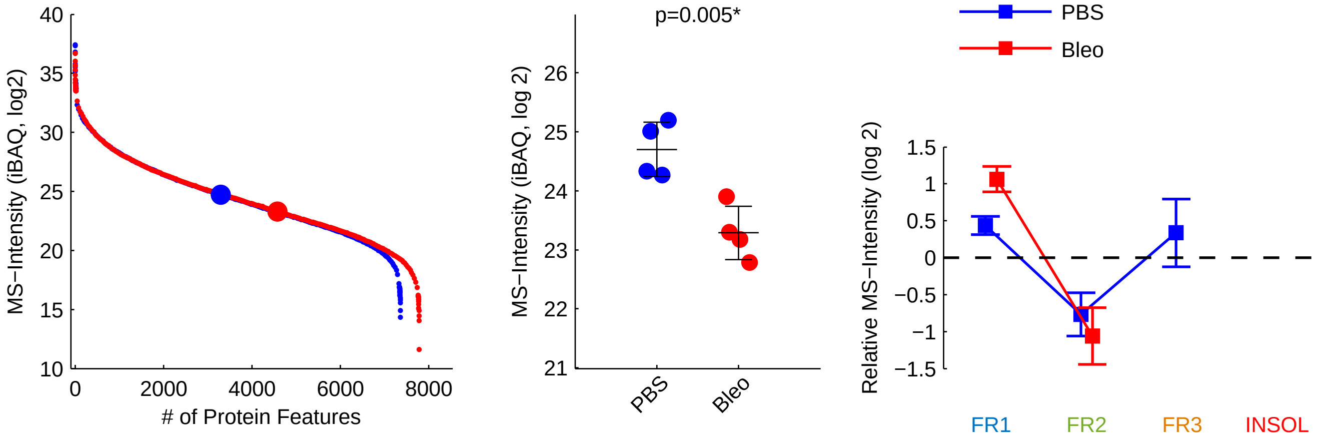

Q8K4Z3 − Apoa1bp (id: 6289)

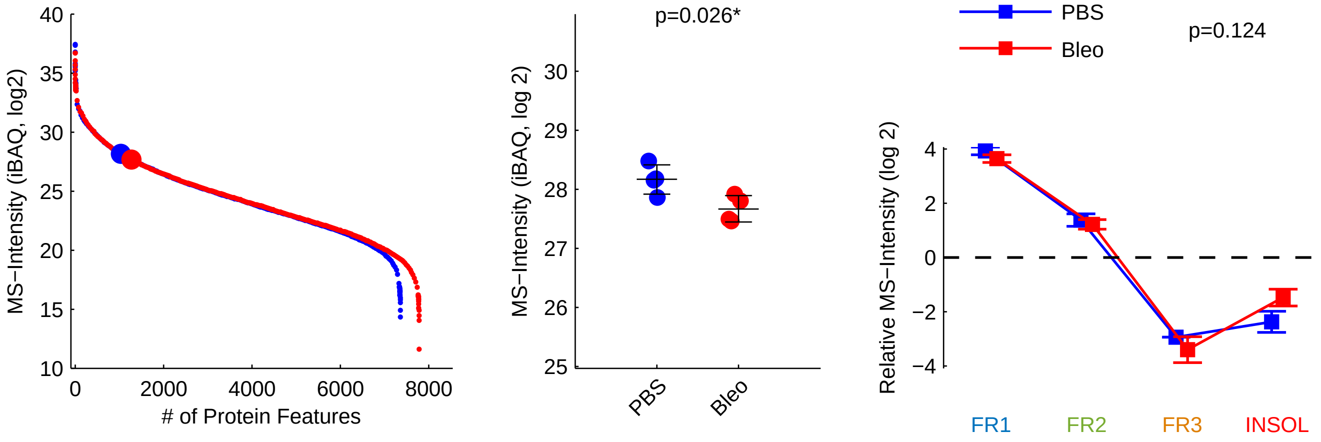

Q8K4Z5 − Sf3a1 (id: 6290)

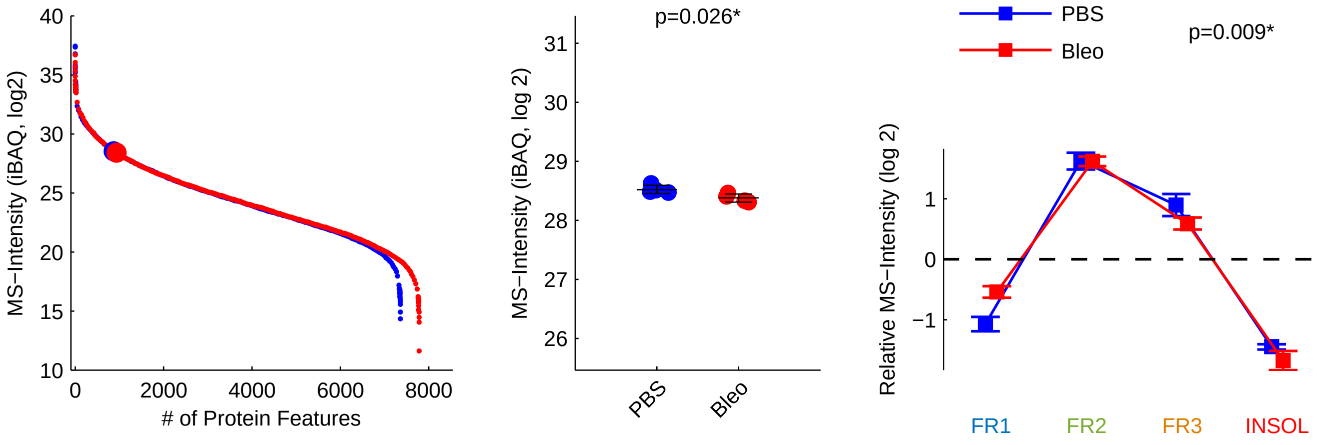

Q8QZR5 − Gpt (id: 6293)

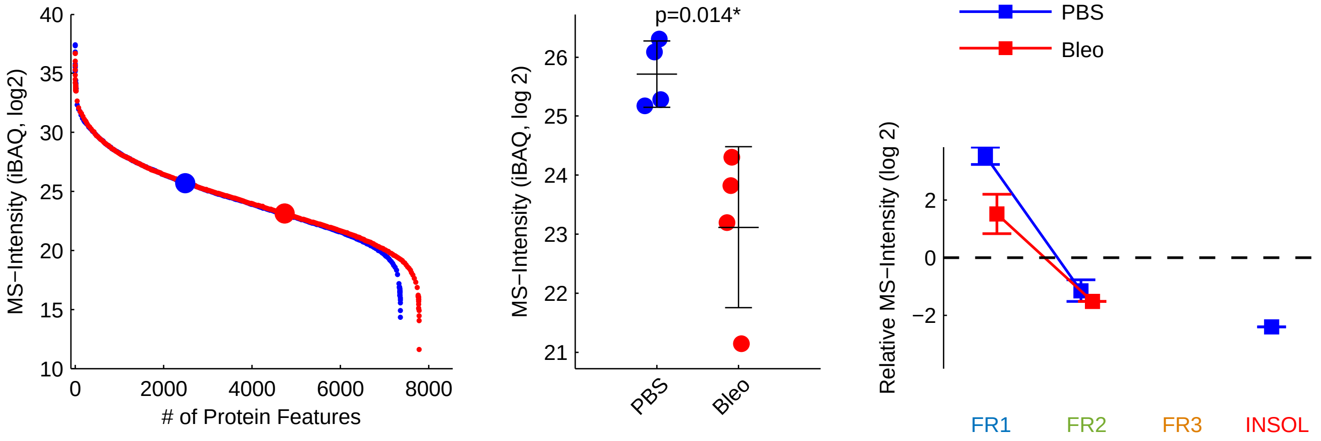

Q8QZS1 − Hibch (id: 6294)

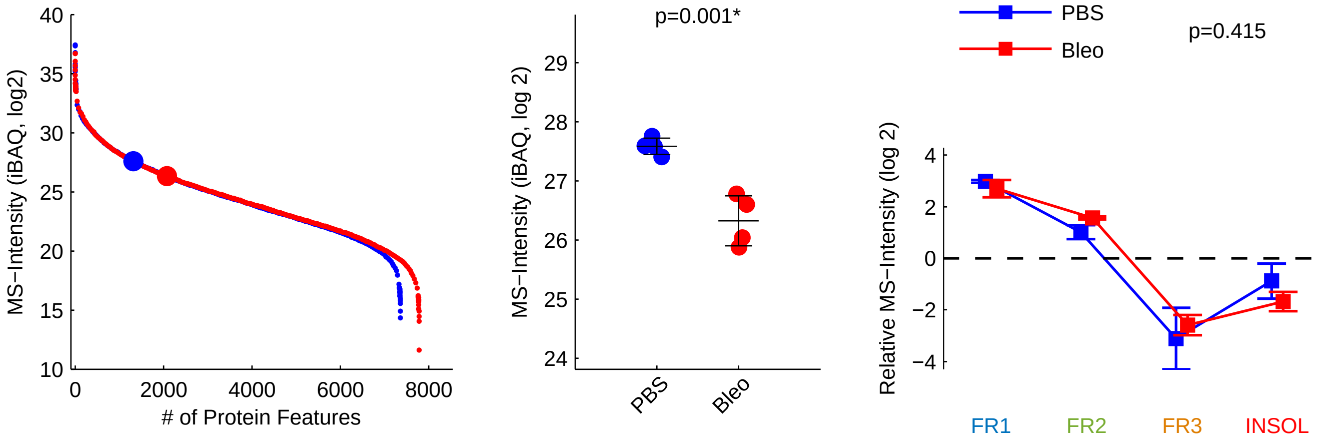

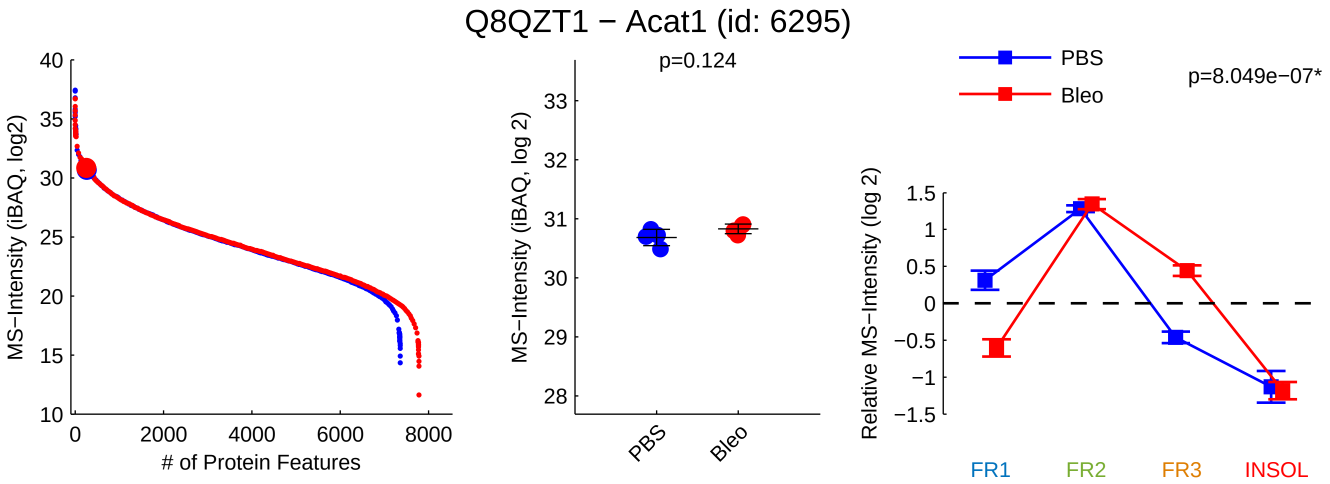
Q8QZT1 − Acat1 (id: 6295)
MS−Intensity (iBAQ, log2)
# of Protein Features
p=0.124
MS−Intensity (iBAQ, log 2)
PBS
Bleo
p=8.049e−07*
Relative MS−Intensity (log 2)
FR1
FR2
FR3
INSOL

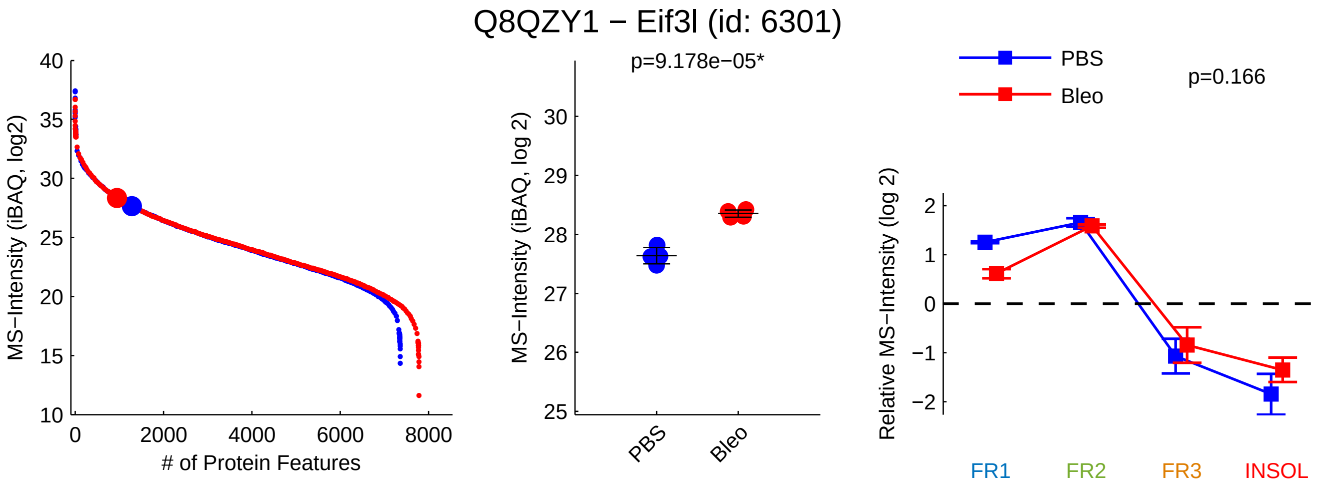
Q8QZY1 − Eif3l (id: 6301)
MS−Intensity (iBAQ, log2)
# of Protein Features
p=9.178e−05*
MS−Intensity (iBAQ, log 2)
PBS
Bleo
p=0.166
Relative MS−Intensity (log 2)
FR1
FR2
FR3
INSOL

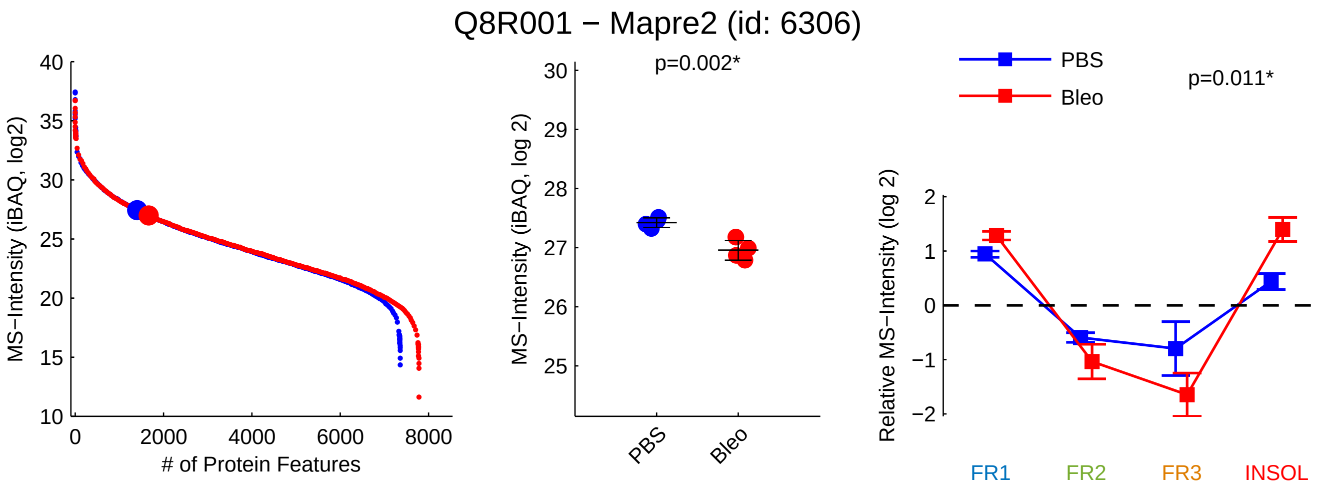
Q8R001 − Mapre2 (id: 6306)
MS−Intensity (iBAQ, log2)
# of Protein Features
p=0.002*
MS−Intensity (iBAQ, log 2)
PBS
Bleo
p=0.011*
Relative MS−Intensity (log 2)
FR1
FR2
FR3
INSOL

Q8R059 − Gale (id: 6311)

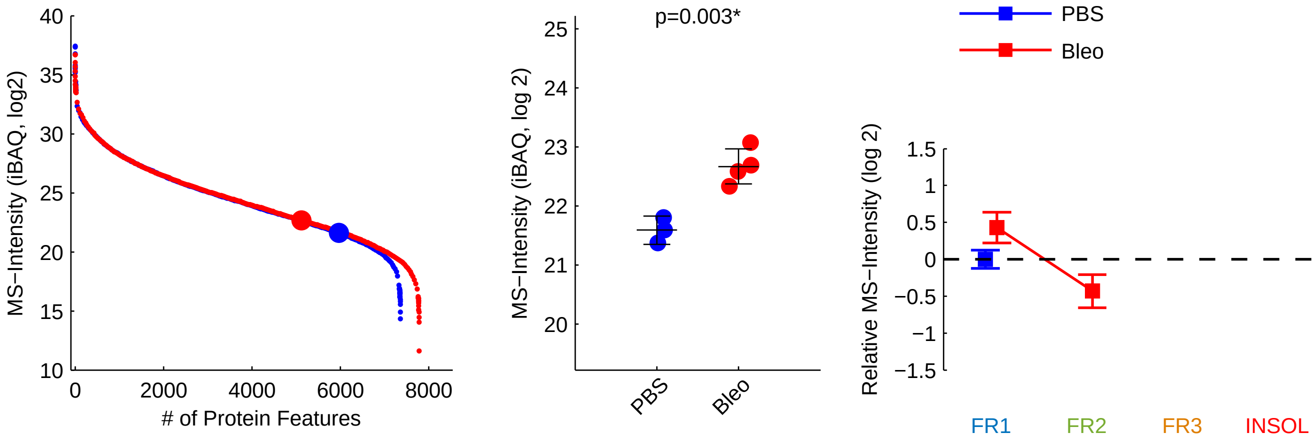

Q8R0F9 − Sec14l4 (id: 6319)

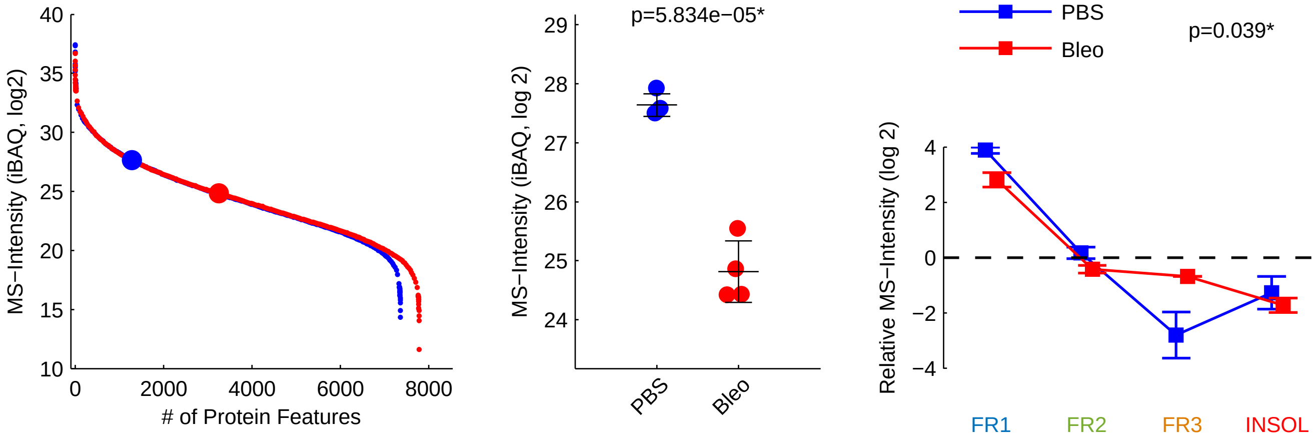

Q8R0X7 − Sgpl1 (id: 6332)

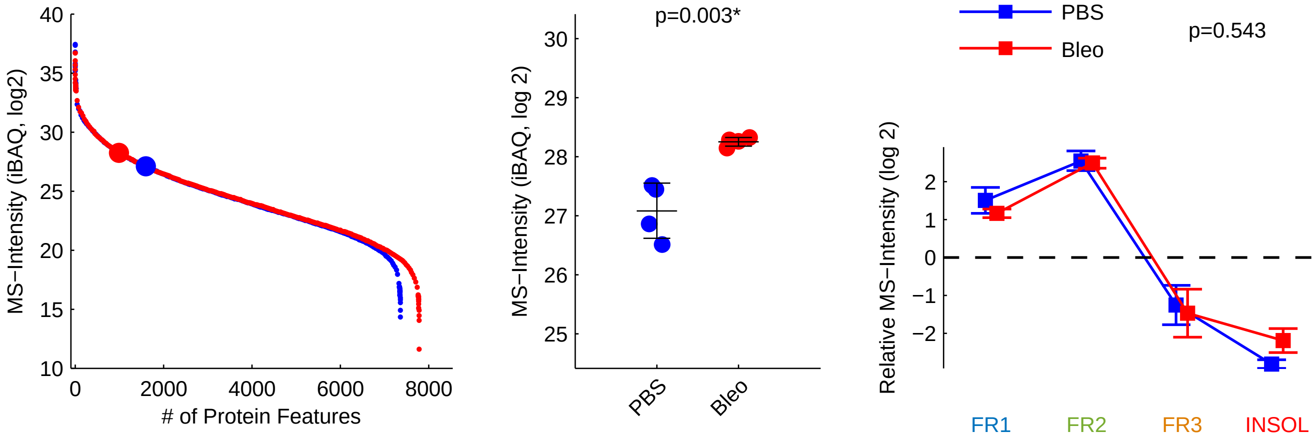

Q8R121 − Serpina10 (id: 6336)

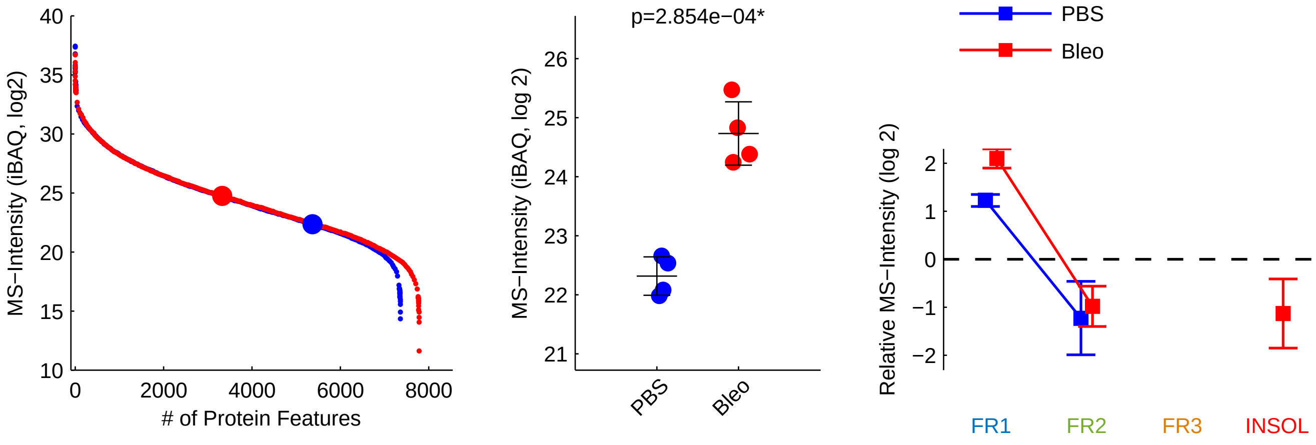

Q8R123 − Flad1 (id: 6337)

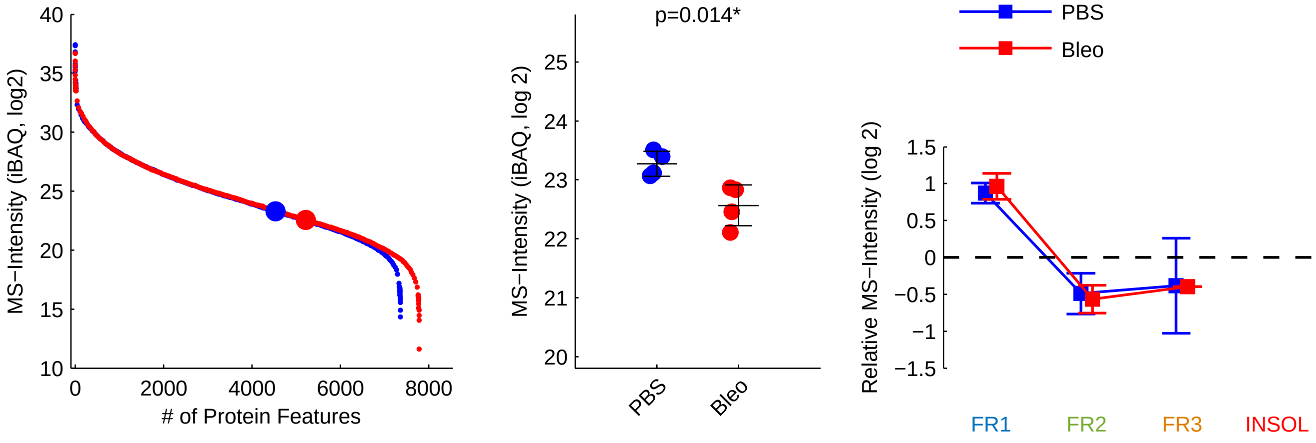

Q8R164 − Bphl (id: 6344)

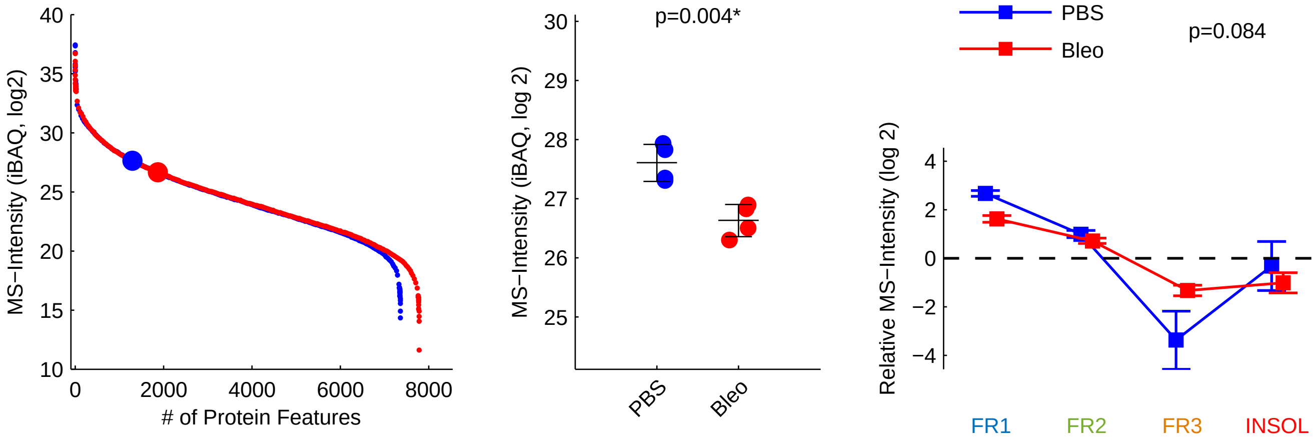

Q8R1B4 − Eif3c (id: 6348)

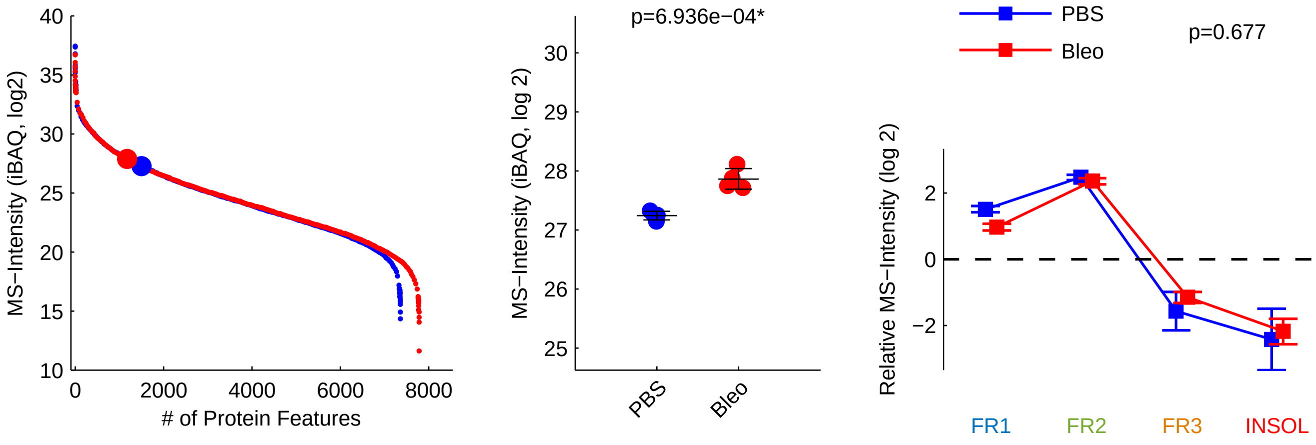

Q8R1F0 − D8Ertd738e (id: 6349)

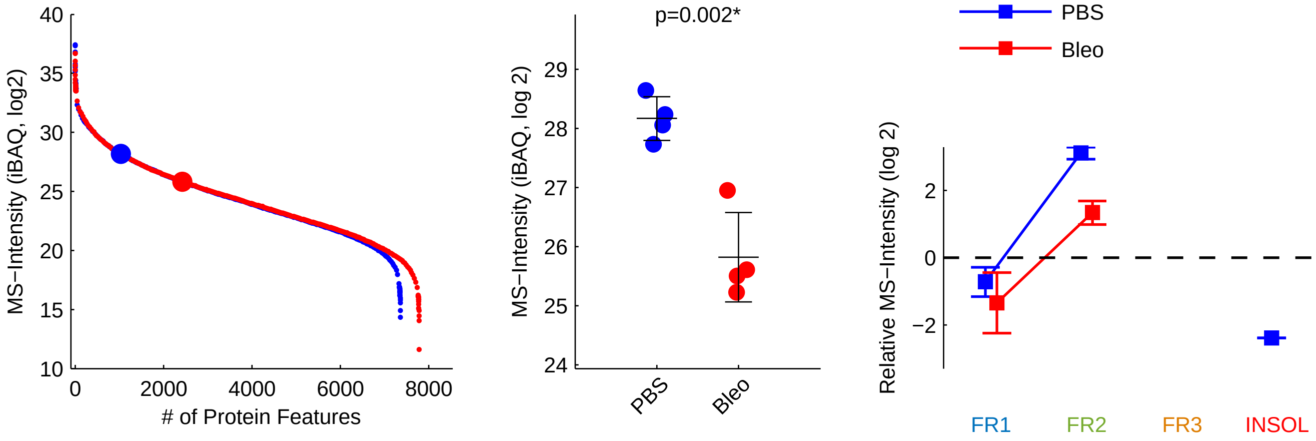

Q8R1H0 − Hopx (id: 6353)

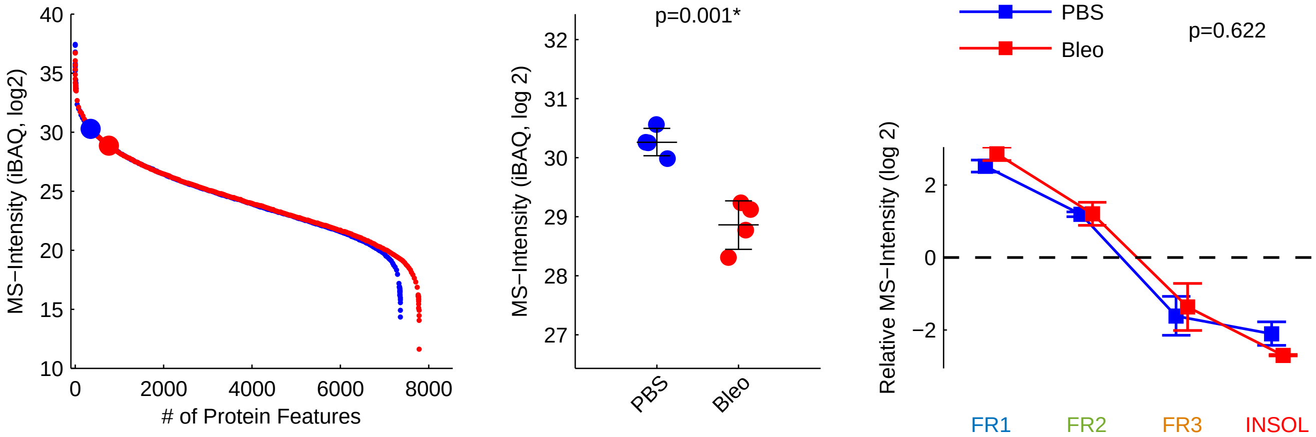

Q8R1I1 − Uqcr10 (id: 6354)

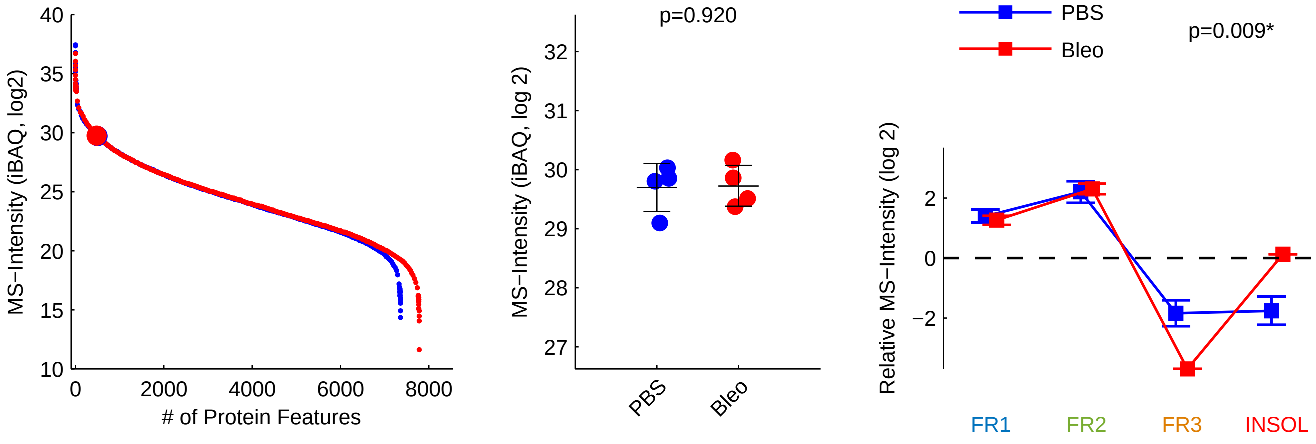

Q8R2N2 − Cirh1a (id: 6371)

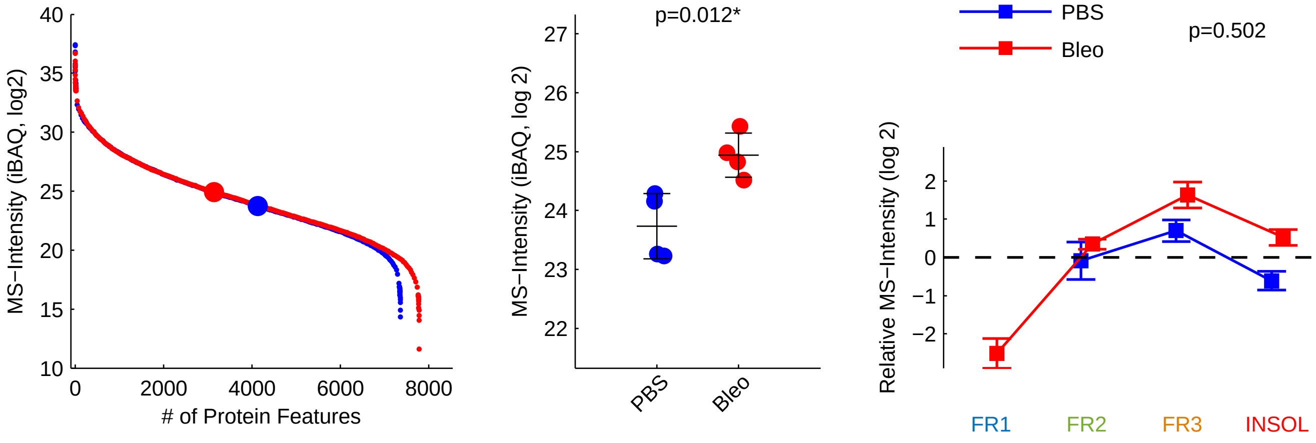

Q8R2Q8 − Bst2 (id: 6375)

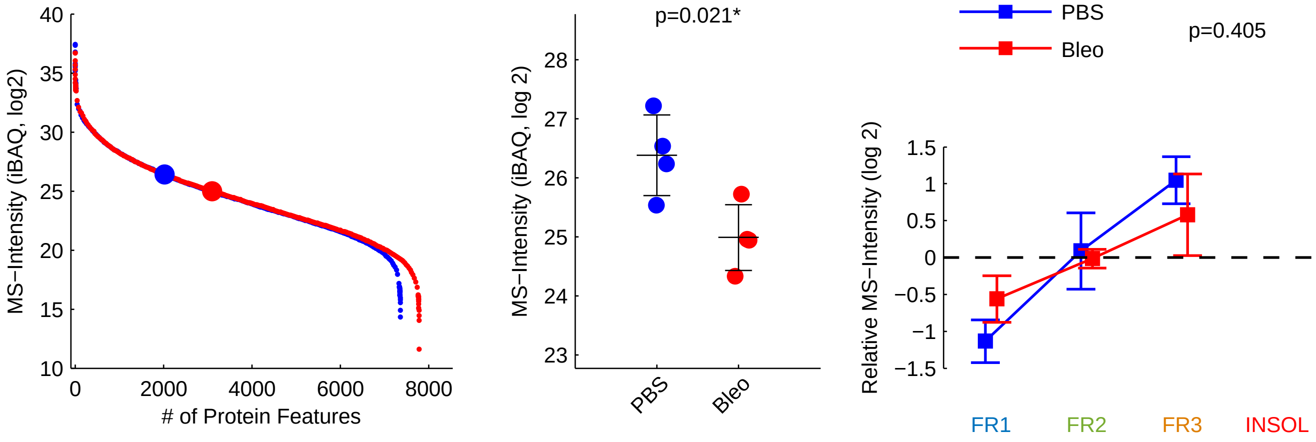

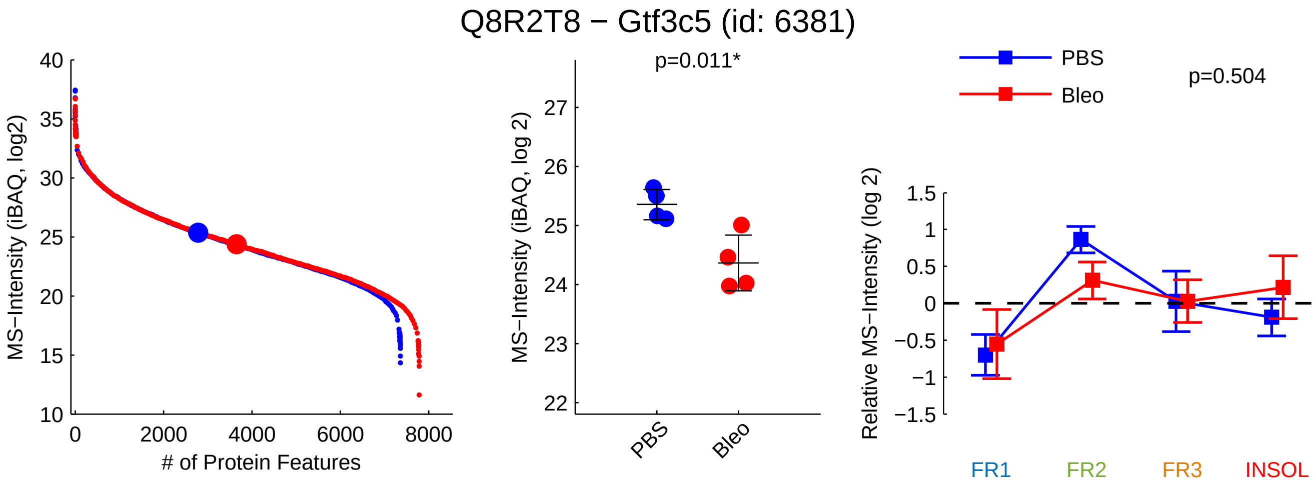
Q8R2T8 − Gtf3c5 (id: 6381)
MS−Intensity (iBAQ, log2)
# of Protein Features
p=0.011*
MS−Intensity (iBAQ, log 2)
PBS
Bleo
p=0.504
Relative MS−Intensity (log 2)
FR1
FR2
FR3
INSOL

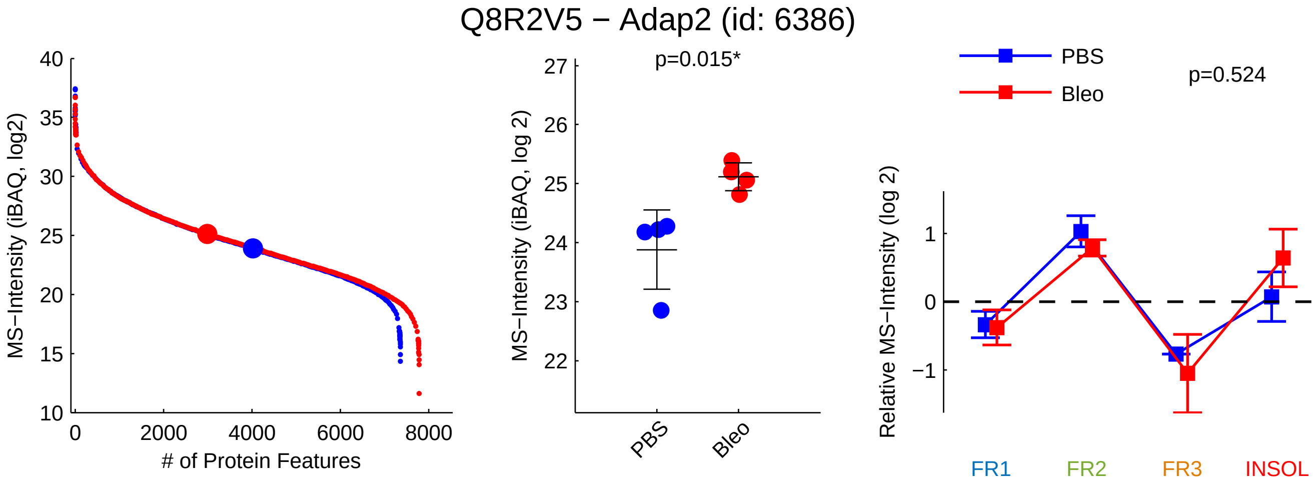
Q8R2V5 − Adap2 (id: 6386)
MS−Intensity (iBAQ, log2)
# of Protein Features
p=0.015*
MS−Intensity (iBAQ, log 2)
PBS
Bleo
p=0.524
Relative MS−Intensity (log 2)
FR1
FR2
FR3
INSOL

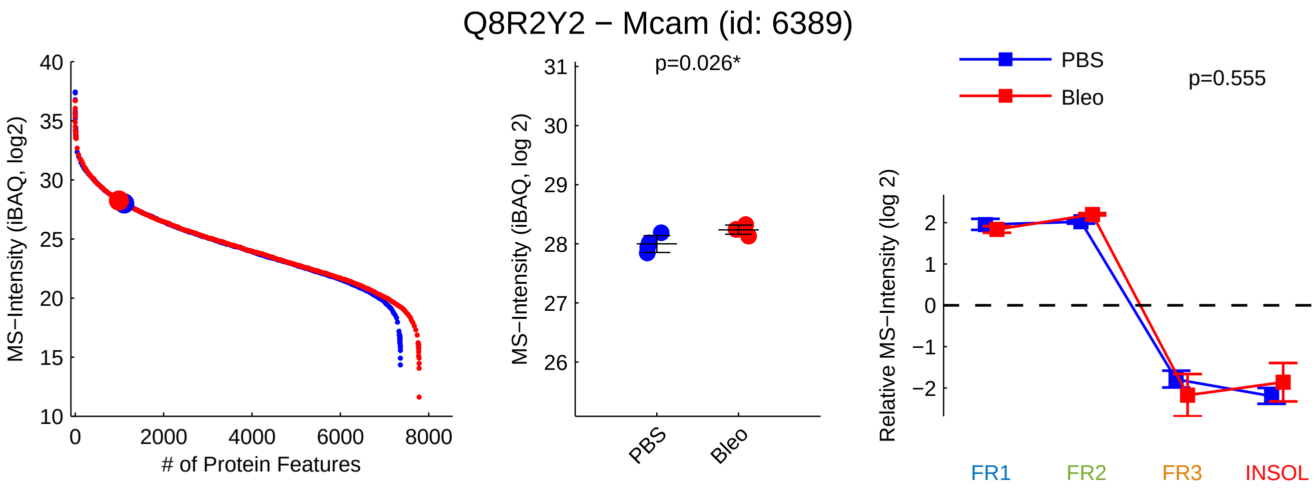
Q8R2Y2 − Mcam (id: 6389)
MS−Intensity (iBAQ, log2)
# of Protein Features
p=0.026*
MS−Intensity (iBAQ, log 2)
PBS
Bleo
p=0.555
Relative MS−Intensity (log 2)
FR1
FR2
FR3
INSOL

Q8R2Z5 − Vwa1 (id: 6393)

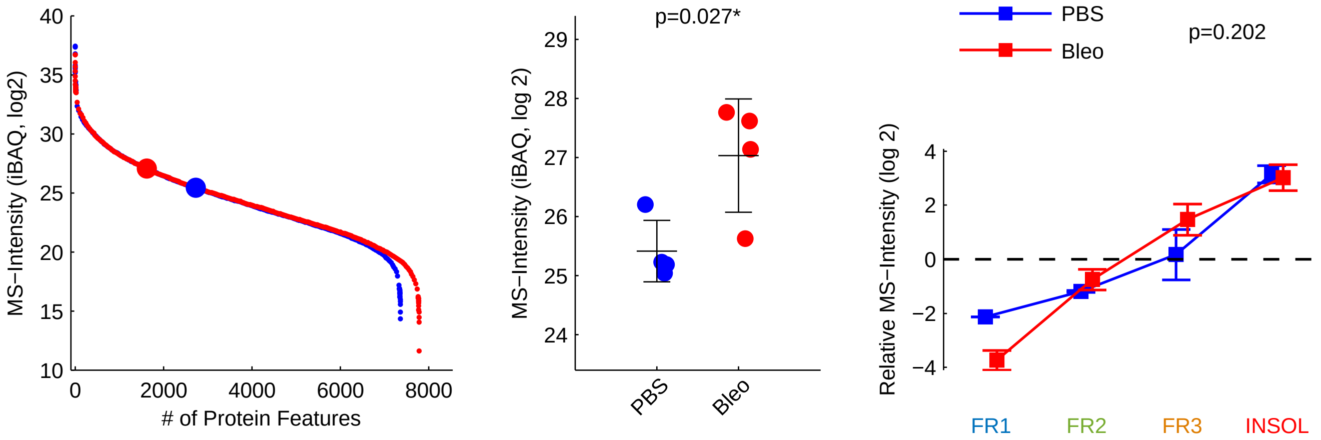

Q8R310 − Tmcc3 (id: 6395)

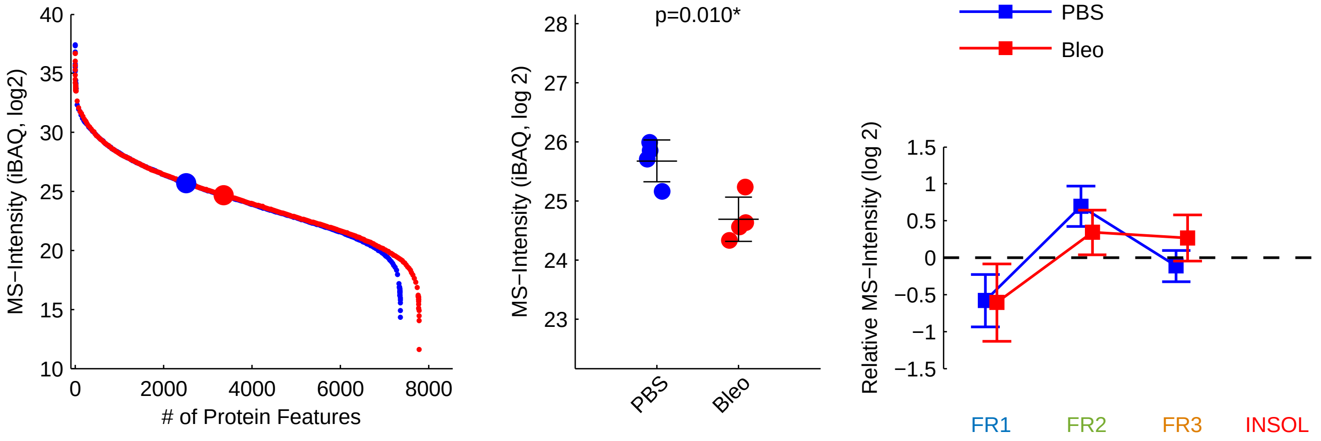

Q8R361 − Rab11fip5 (id: 6404)

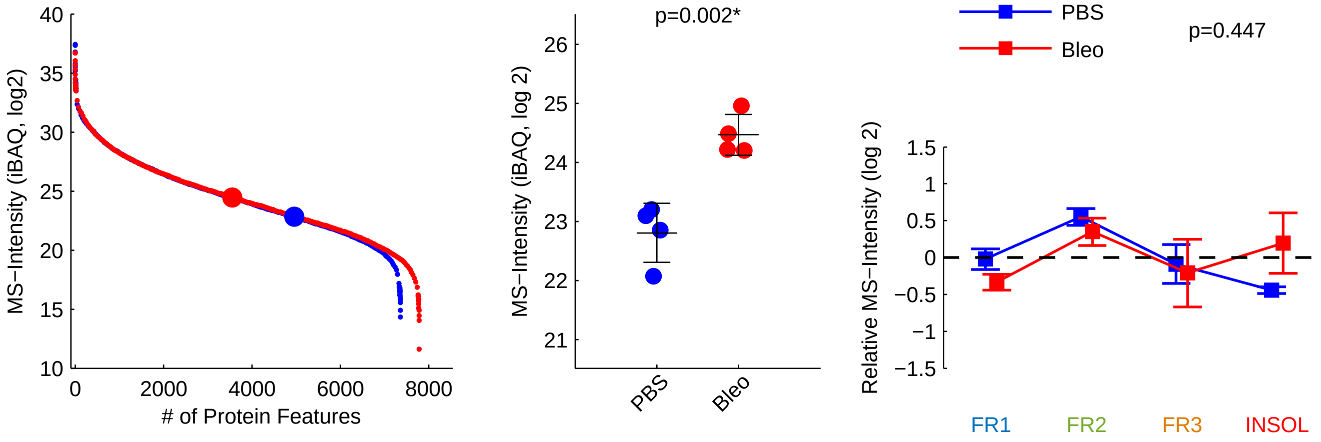

Q8R370 − Ushbp1 (id: 6405)

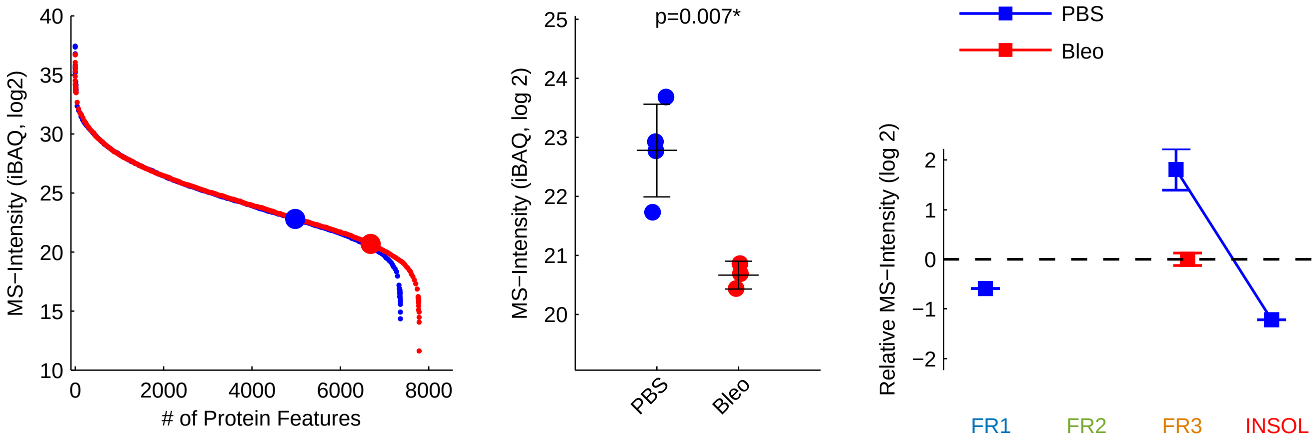

Q8R3D1 − Tbc1d13 (id: 6412)

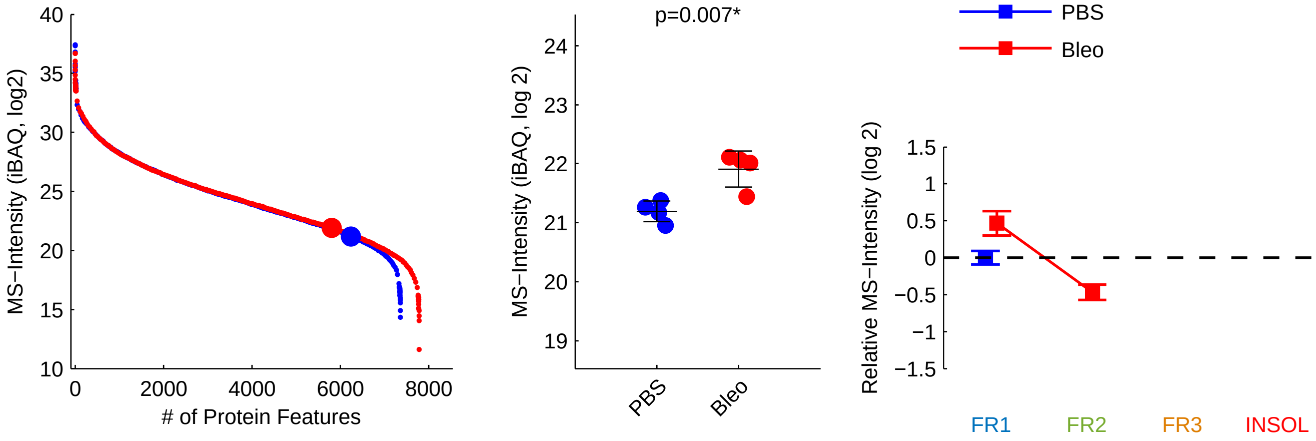

Q8R3G9 − Tspan8 (id: 6416)

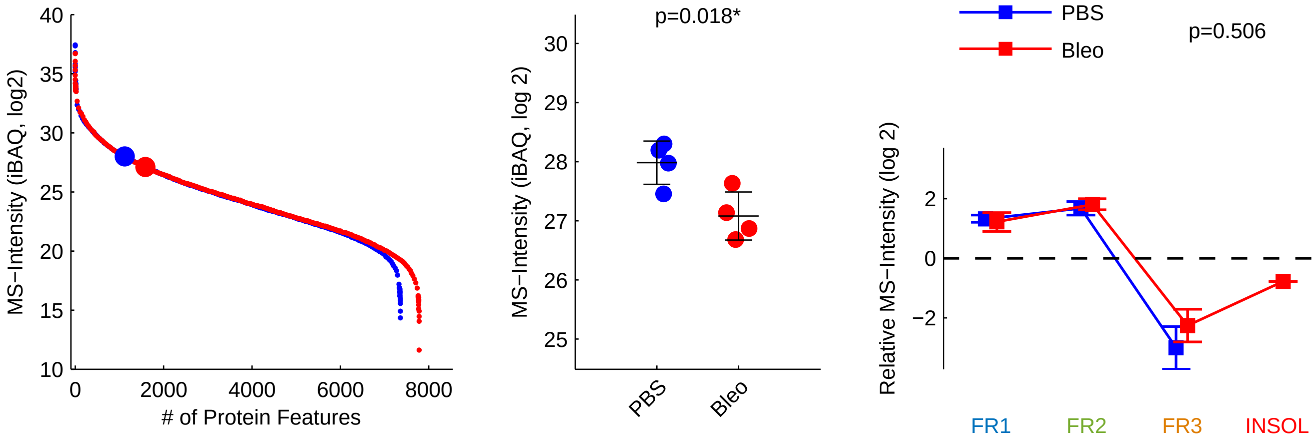

Q8R3H7 − Hs2st1 (id: 6417)

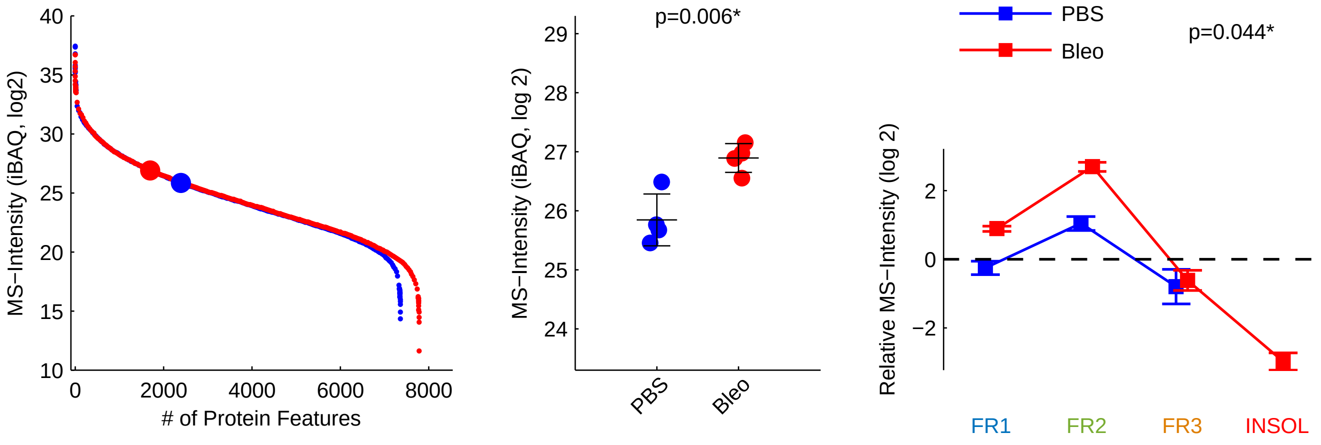

Q8R3V5 − Sh3glb2 (id: 6429)

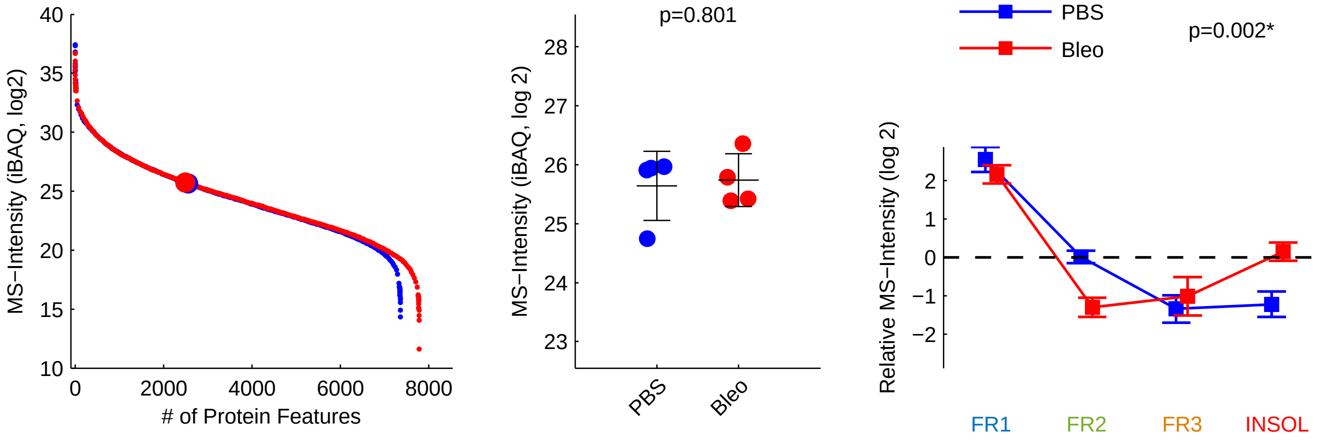

Q8R420 − Abca3 (id: 6436)

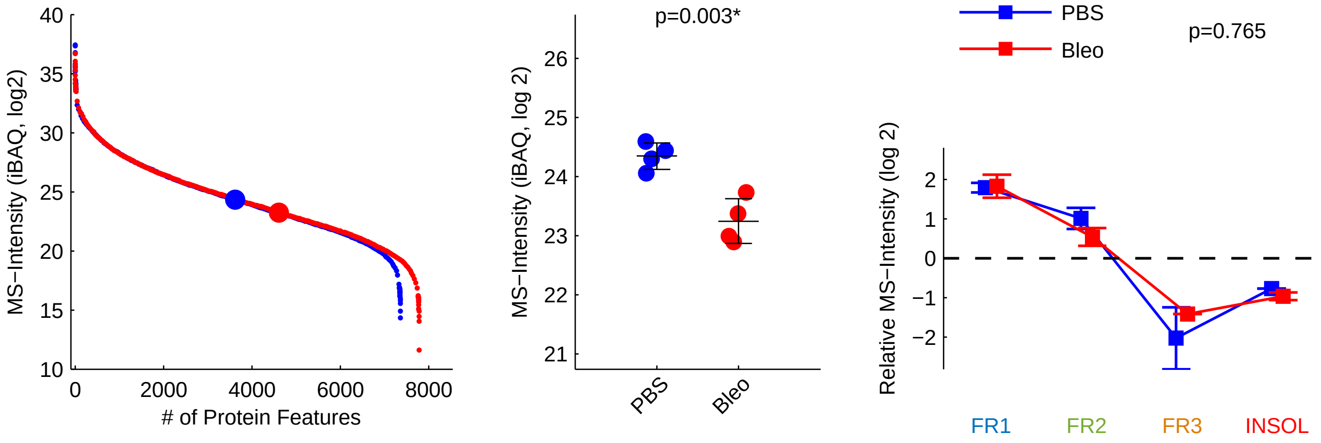

Q8R422 − Cd109 (id: 6437)

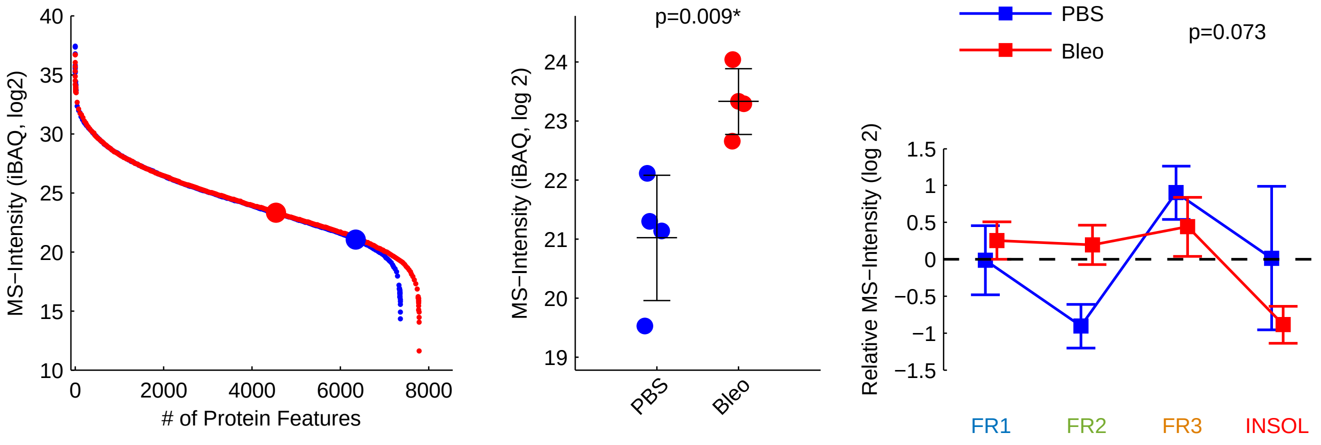

Q8R4W6 − Pcolce2 (id: 6447)

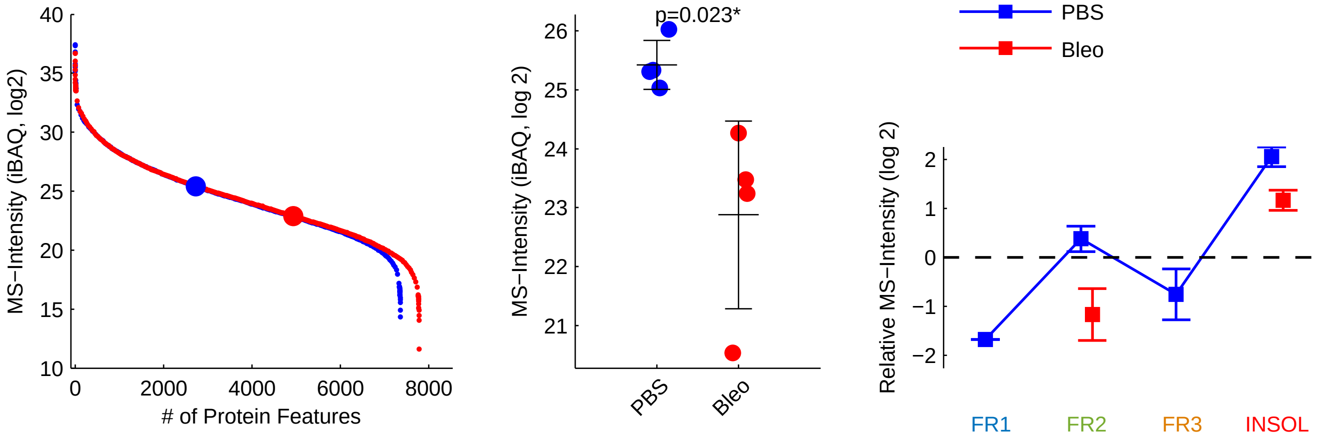

Q8R502 − Lrrc8c (id: 6450)

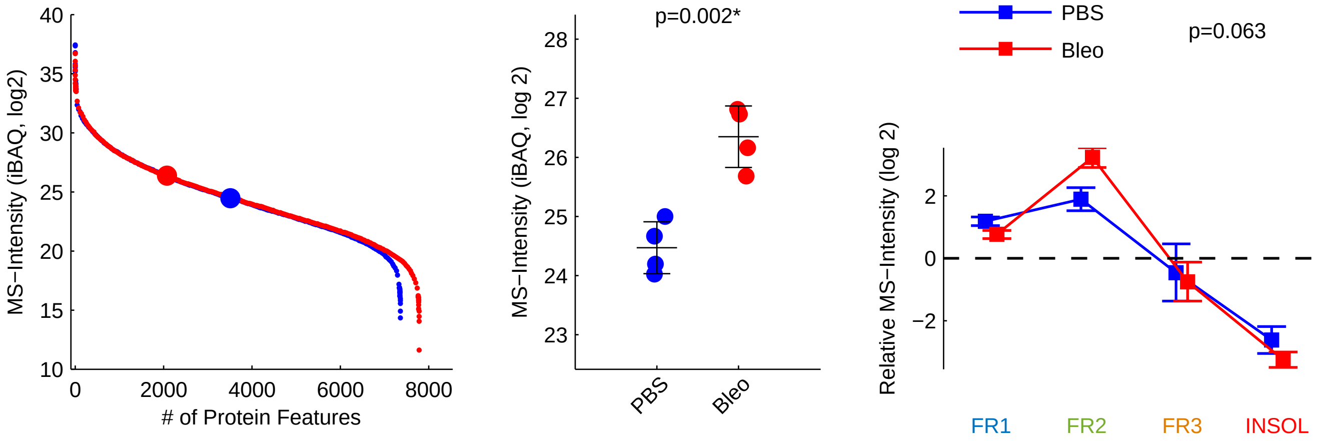

Q8R574 − Prpsap2 (id: 6454)

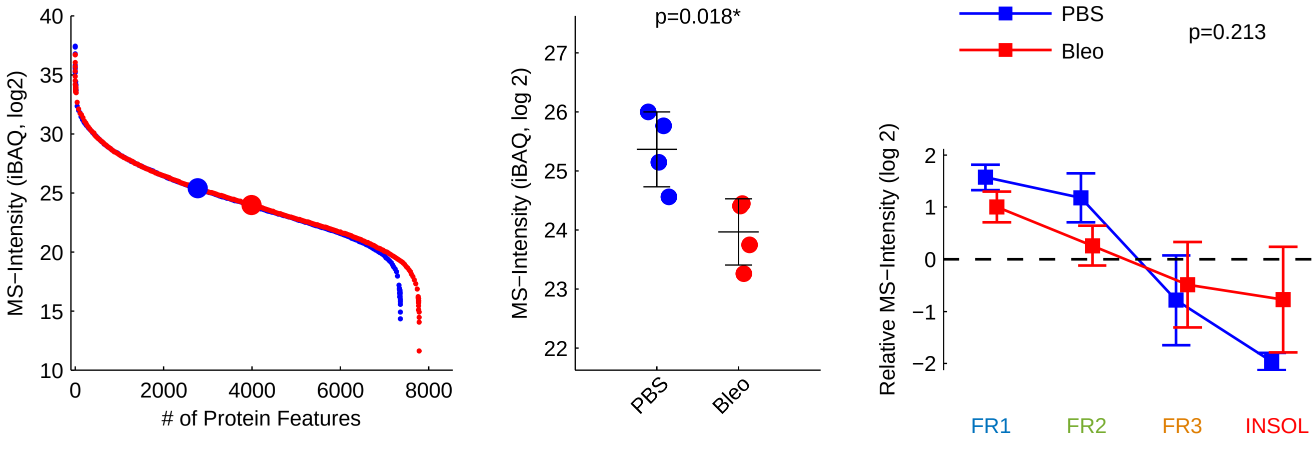

Q8R5A3 − Apbb1ip (id: 6456)

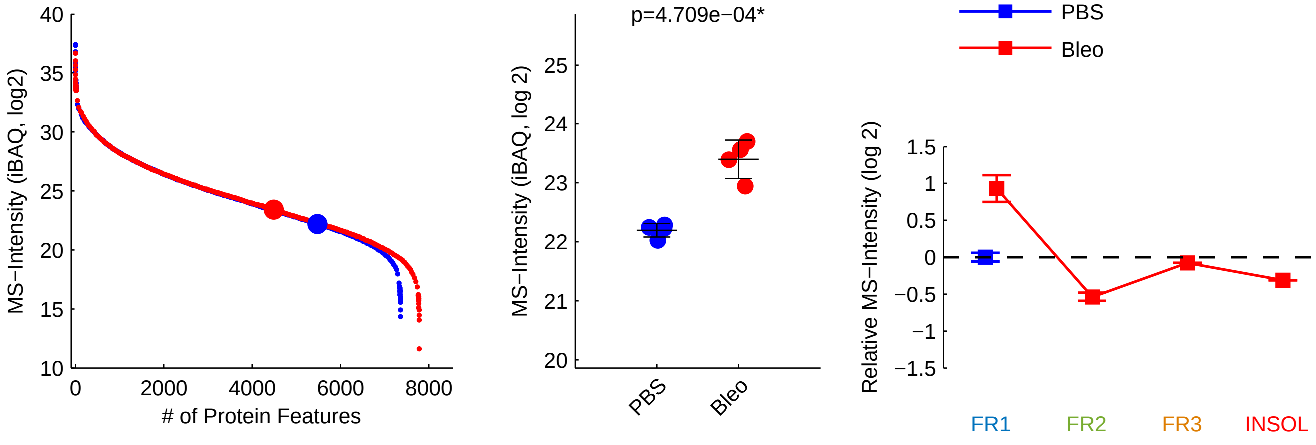

Q8R5C5 − Actr1b (id: 6457)

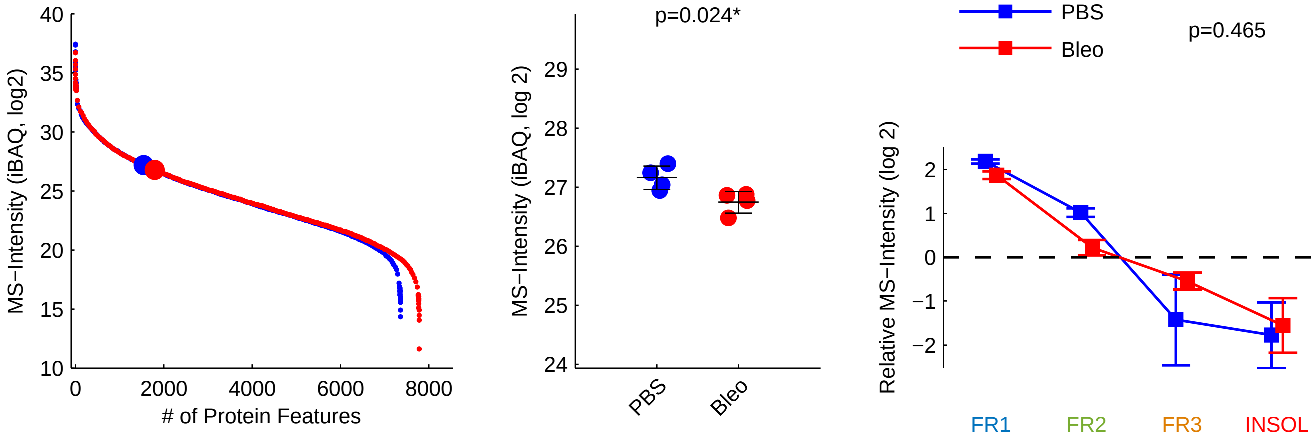

Q8R5F3 − Oard1 (id: 6458)

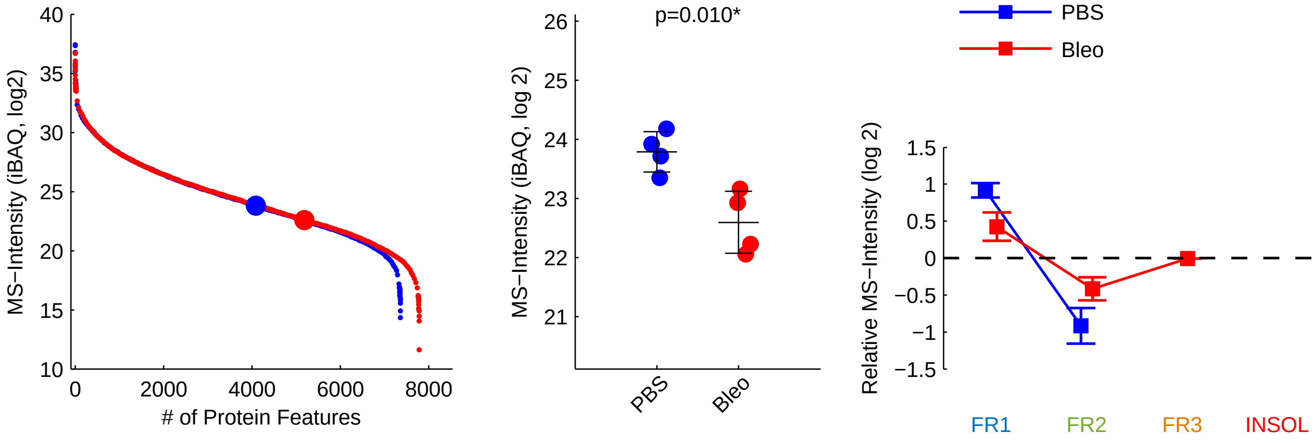

Q8R5G7 − Arap3 (id: 6460)

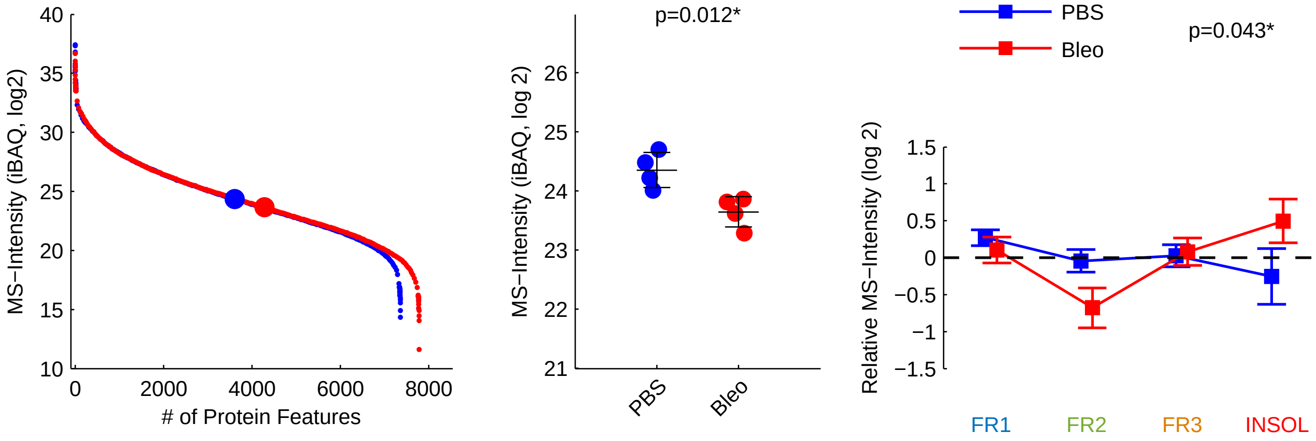

Q8R5L3 − Vps39 (id: 6467)

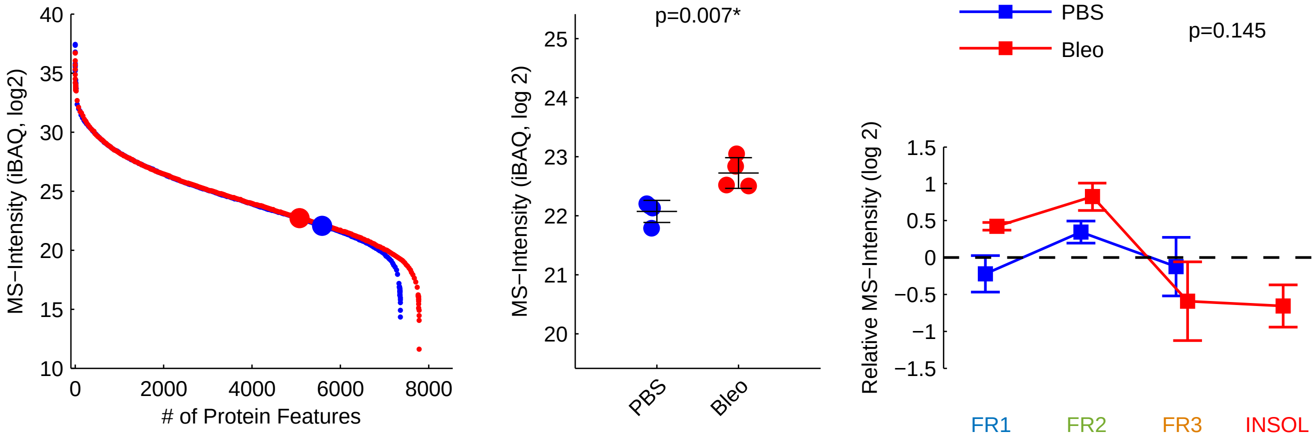

Q8VBZ3 − Clptm1 (id: 6476)

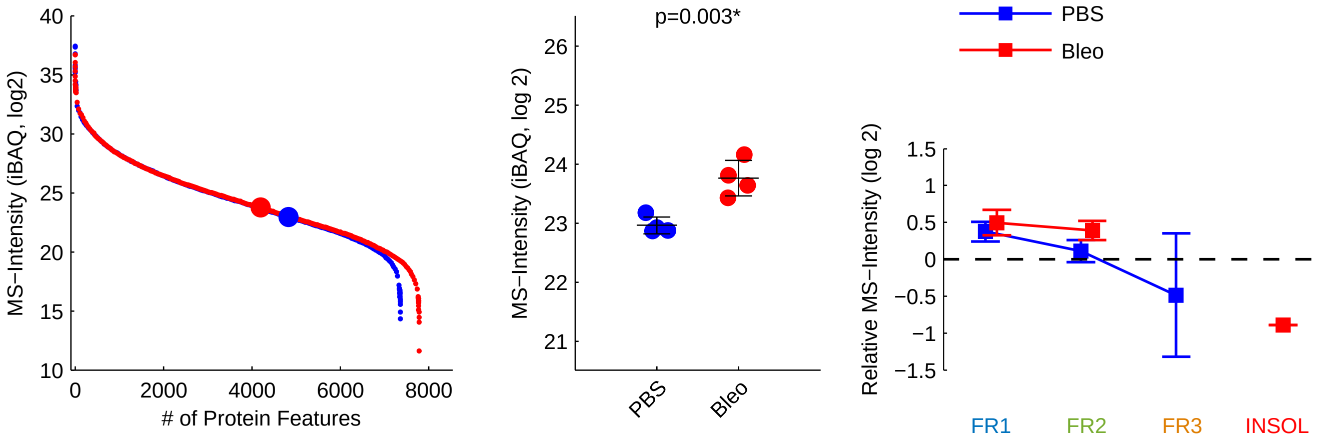

Q8VCA8 − Scrn2 (id: 6489)

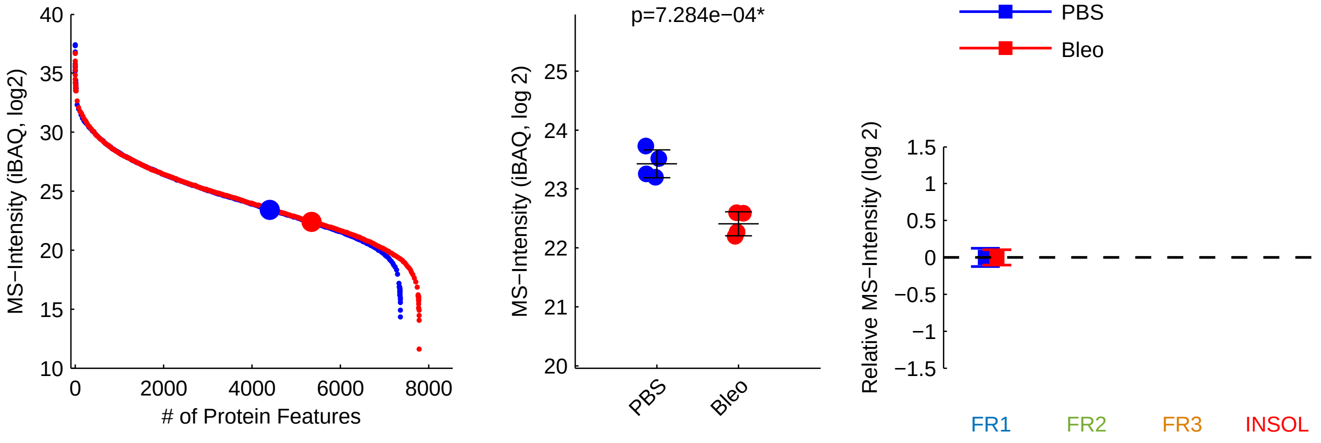

Q8VCC1 − Hpgd (id: 6492)

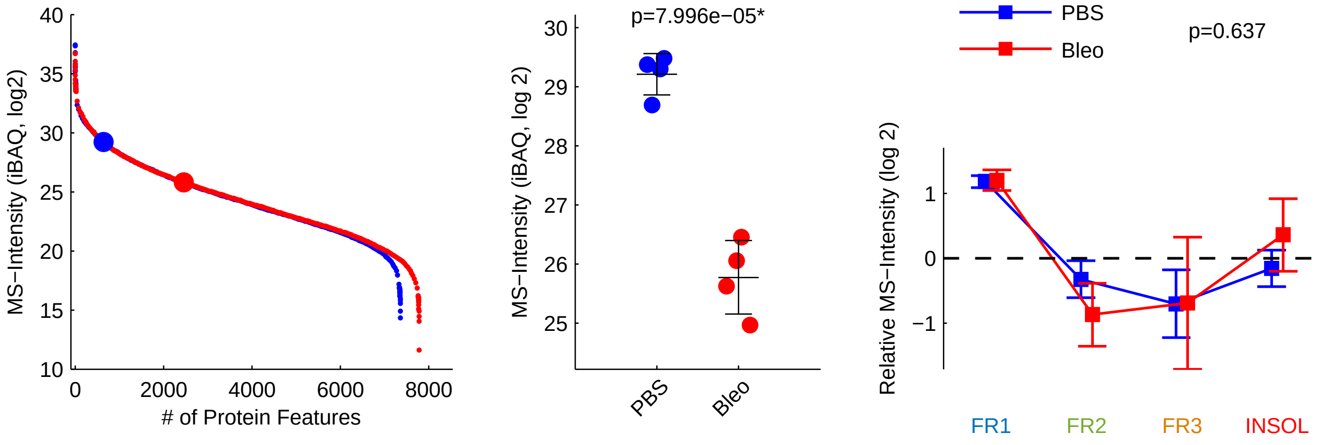

Q8VCC9 − Spon1 (id: 6494)

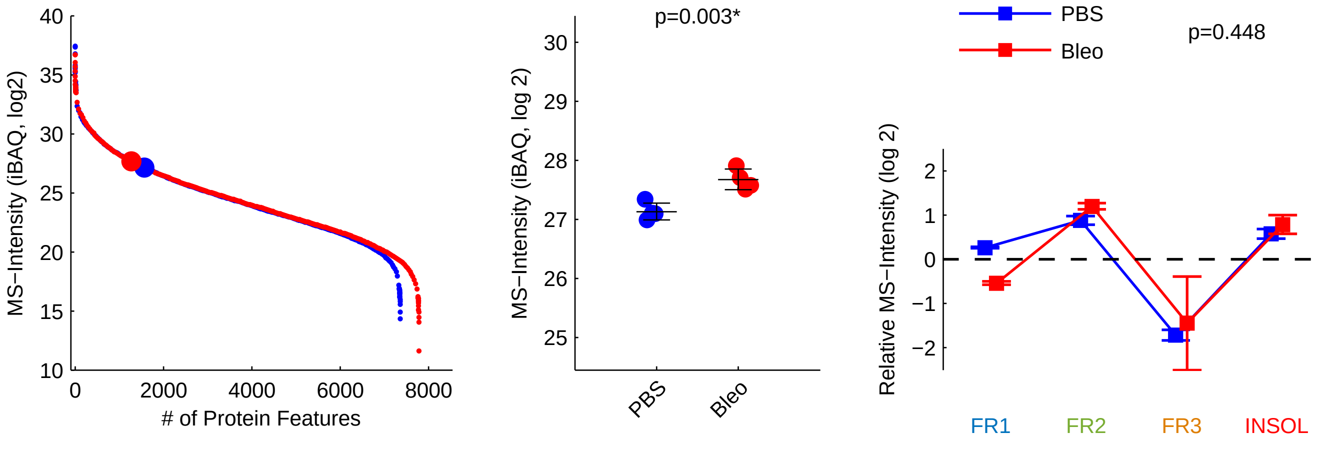

Q8VCM7 − Fgg (id: 6512)

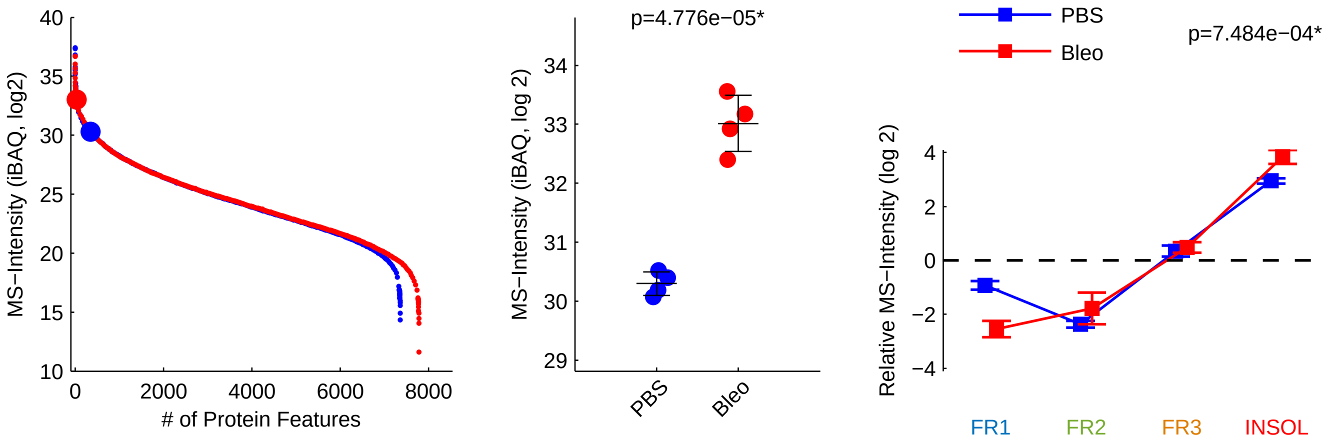

Q8VCP9 − Clec14a (id: 6518)

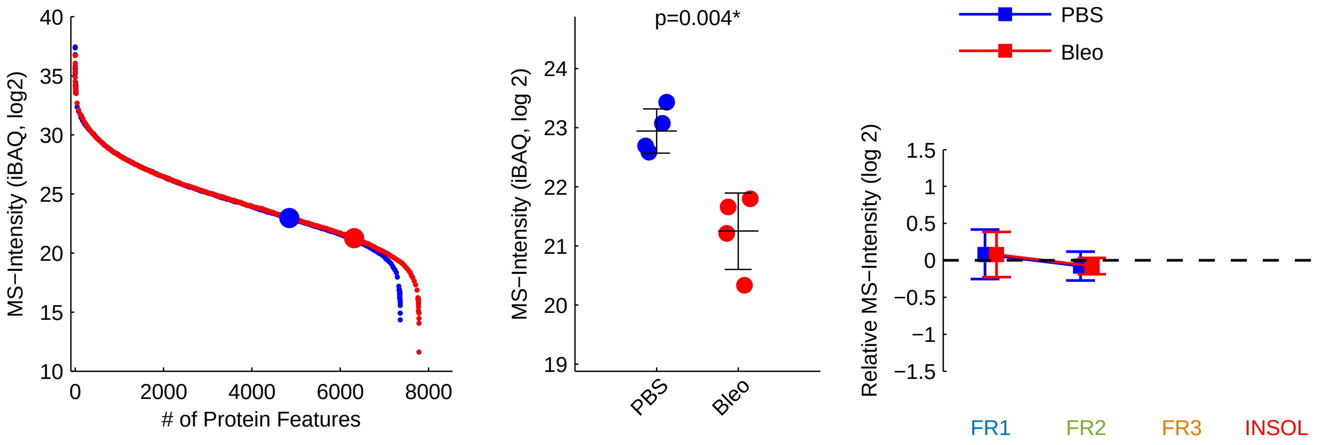

Q8VCT3 − Rnpep (id: 6523)

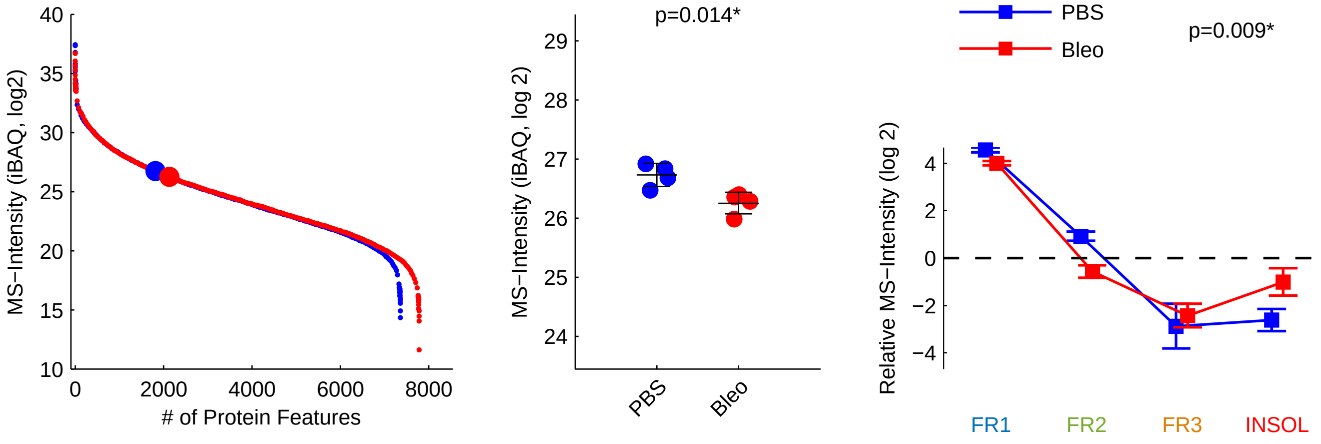

Q8VCT4 − Ces1d (id: 6524)

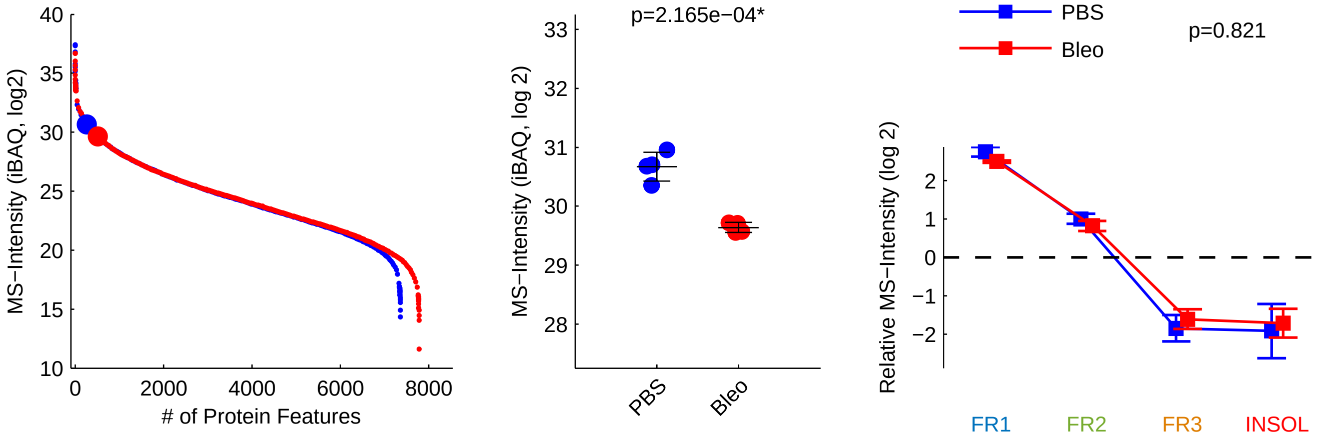

Q8VCU2 − Gpld1 (id: 6525)

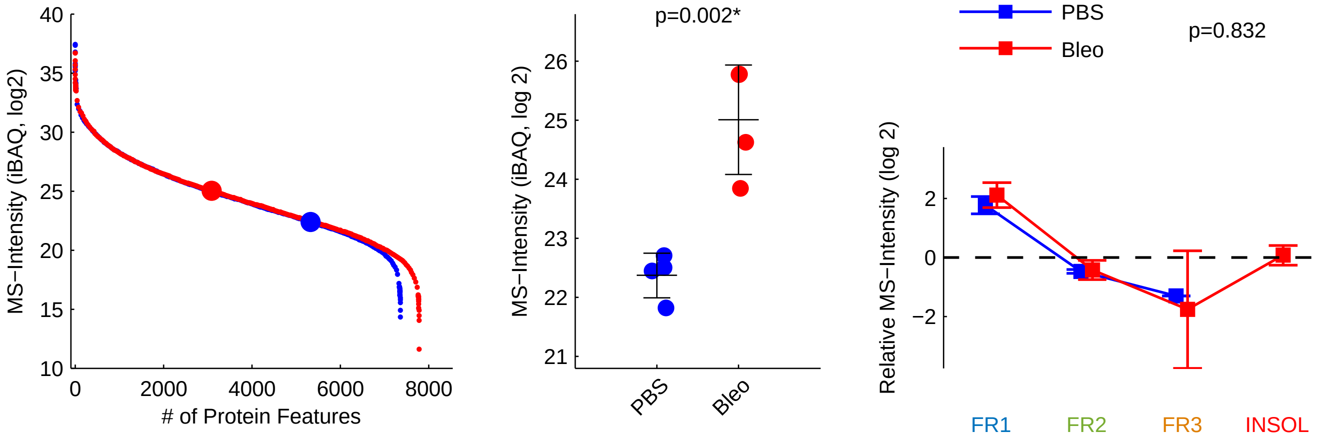

Q8VCW8 − Acsf2 (id: 6526)

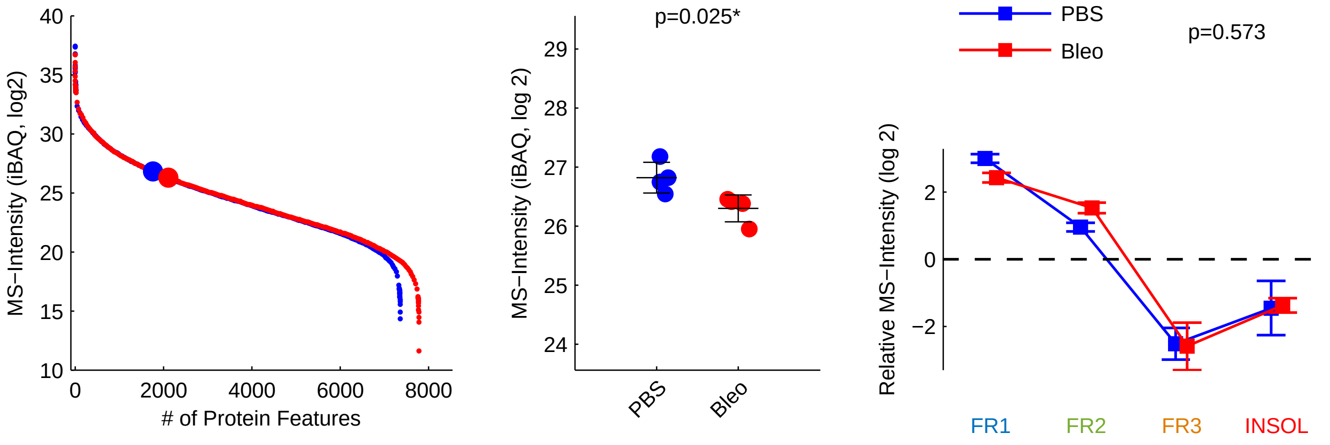

Q8VCX5 − Micu1 (id: 6528)

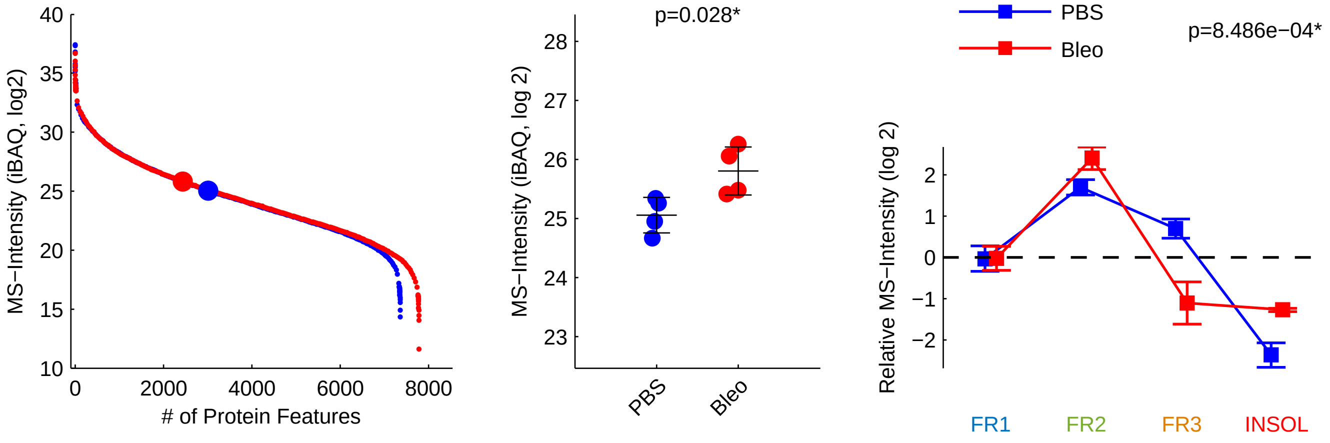

Q8VDD5 − Myh9 (id: 6538)

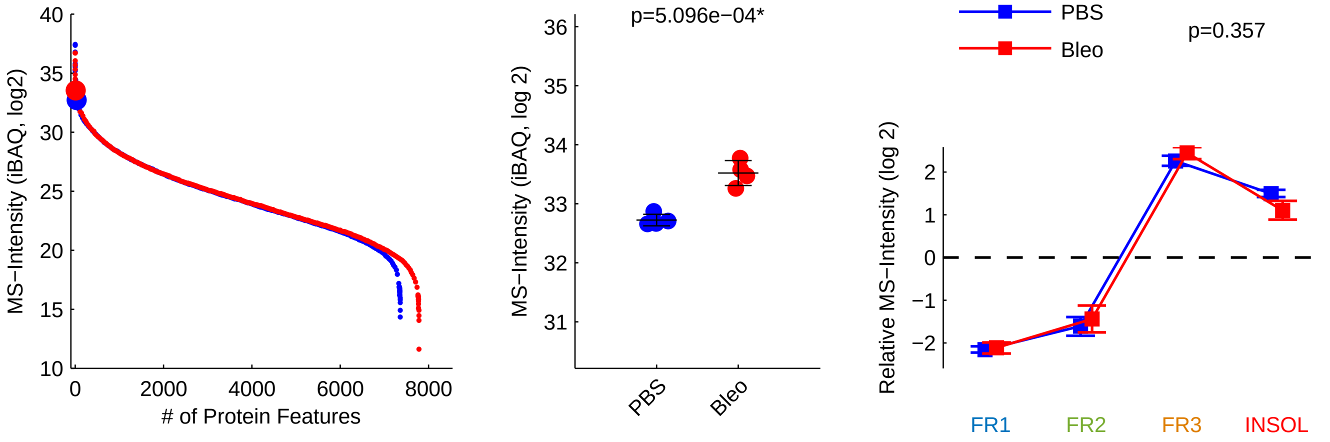

Q8VDF3 − Dapk2 (id: 6541)

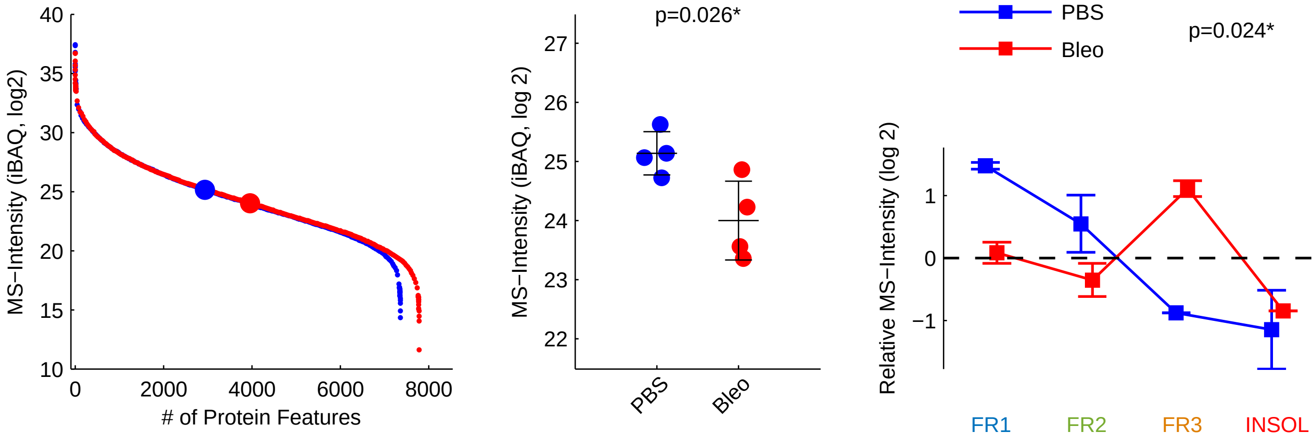

Q8VDJ3 − Hdlbp (id: 6546)

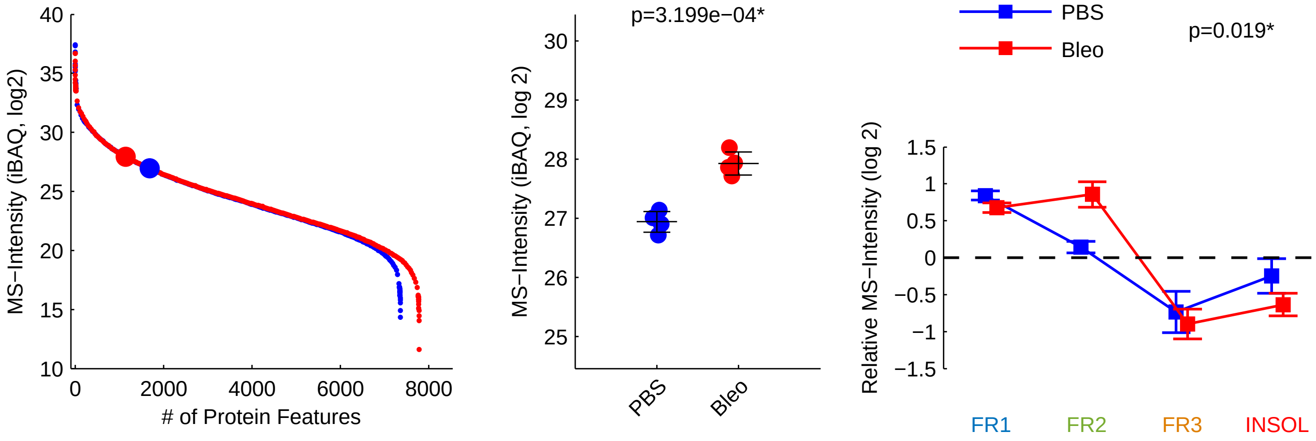

Q8VDM4 − Psmd2 (id: 6550)

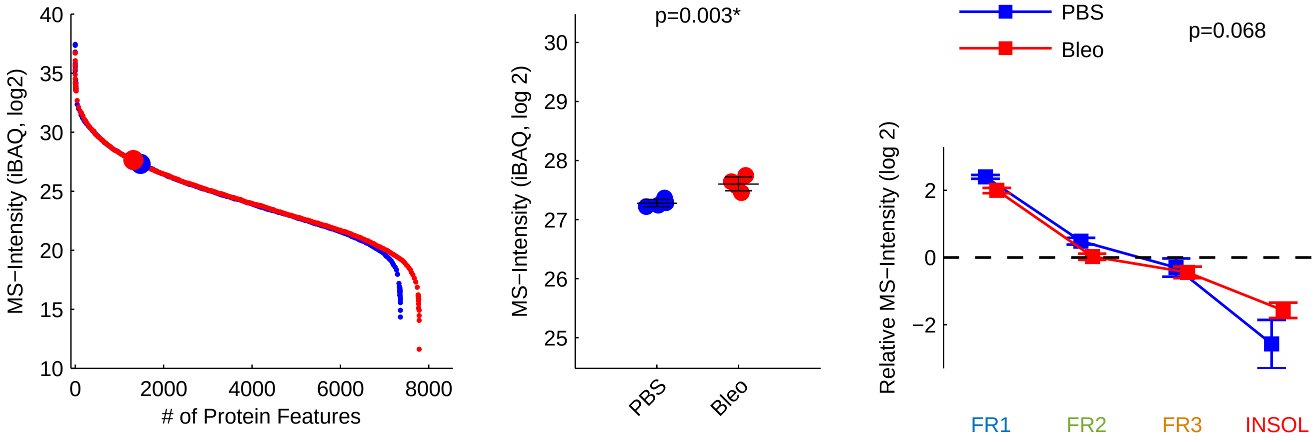

Q8VDP3 − Mical1 (id: 6554)

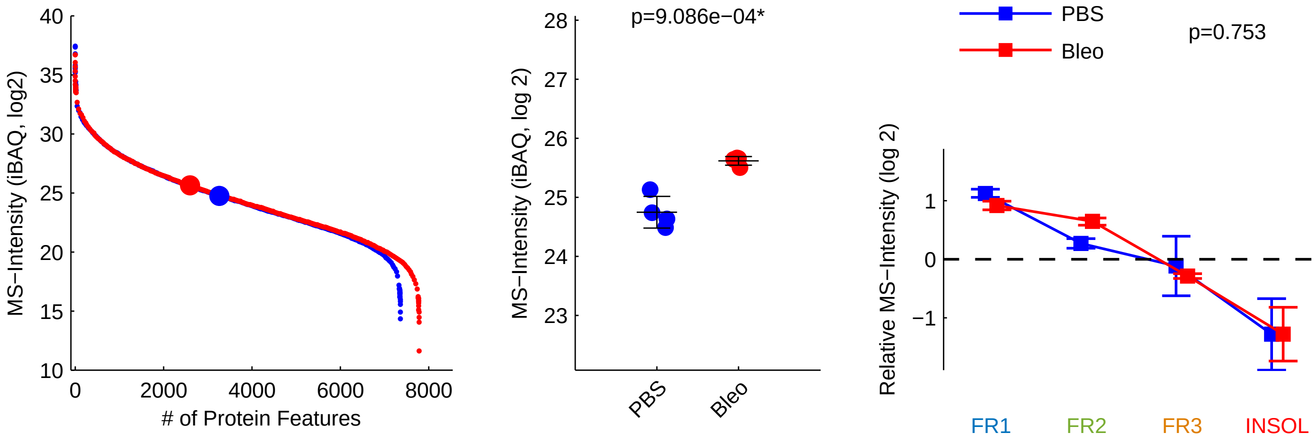

Q8VDQ1 − Ptgr2 (id: 6557)

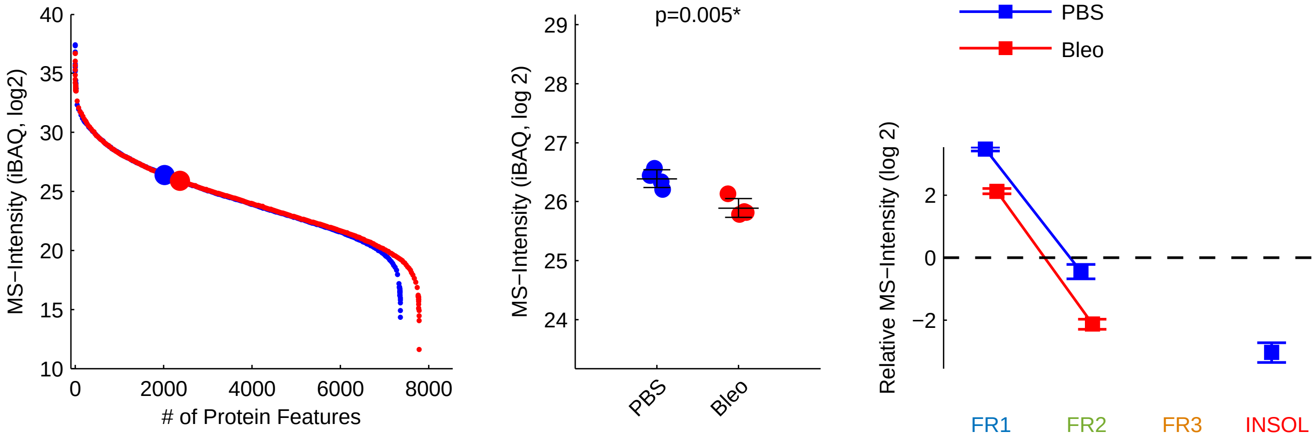

Q8VDW0 − Ddx39a (id: 6564)

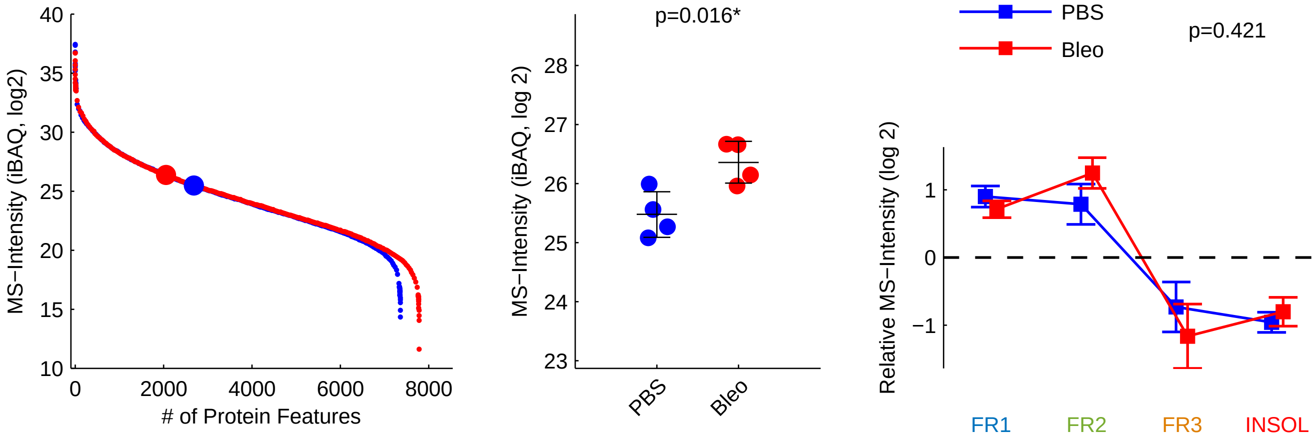

Q8VE22 − Mrps23 (id: 6572)

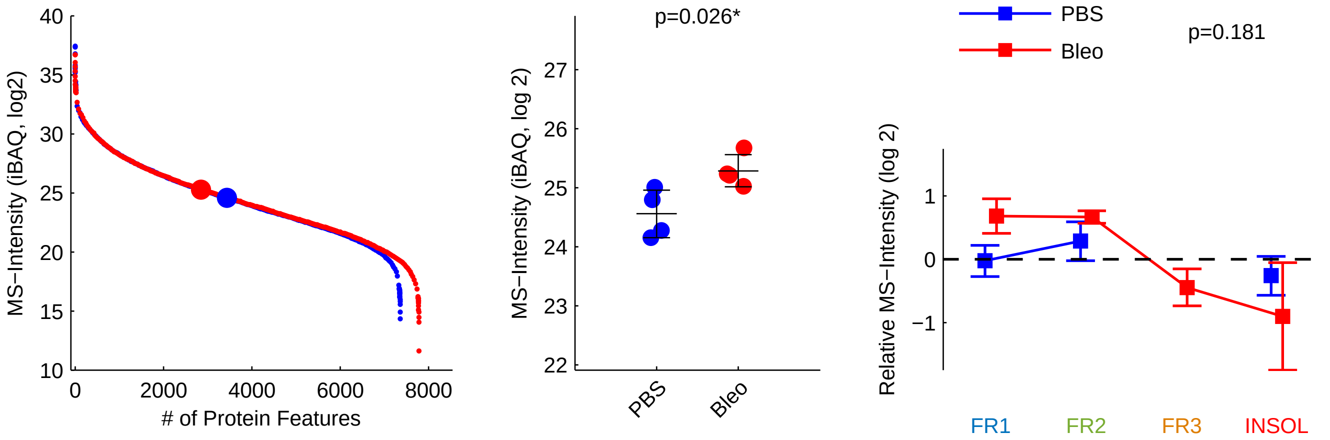

Q8VED9 − Lgalsl (id: 6586)

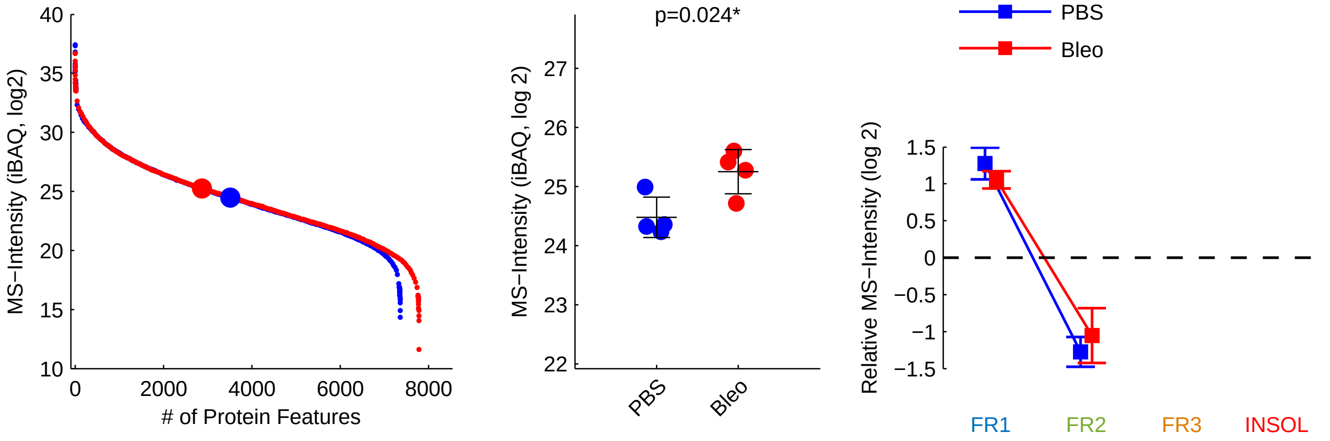

Q8VEE1 − Lmcd1 (id: 6588)

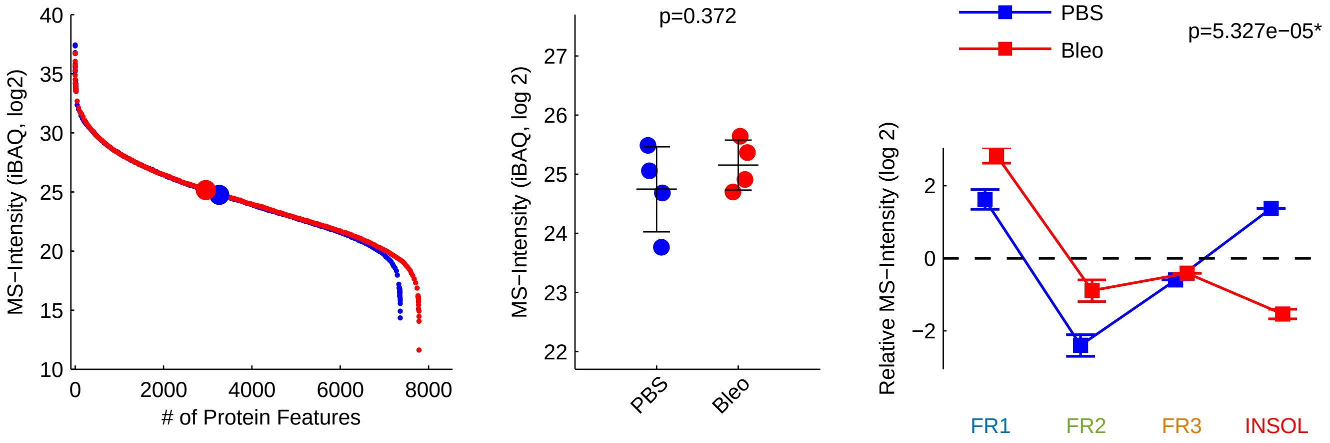

Q8VEK3 − Hnrnpu (id: 6597)

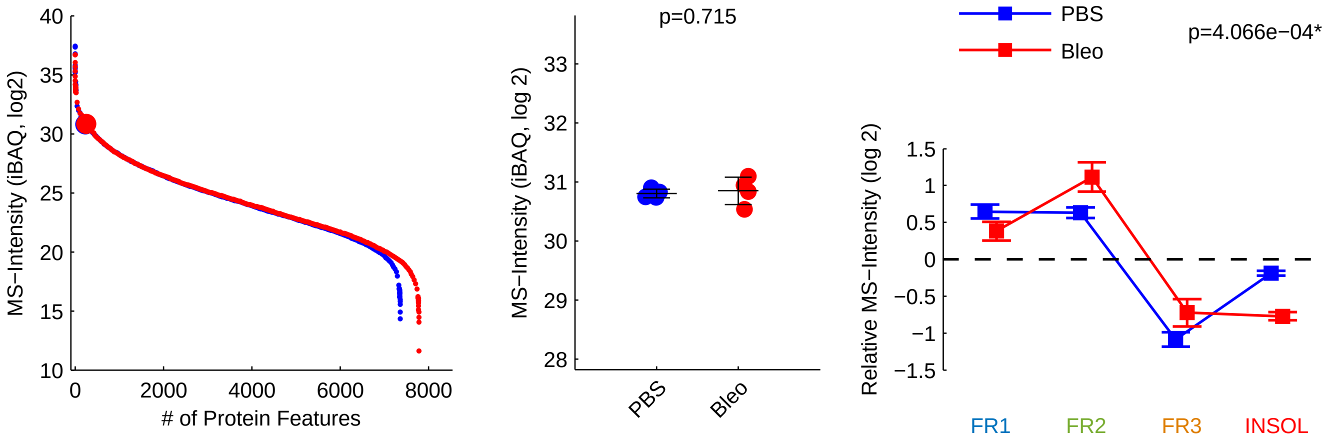

Q8VHE0 − Sec63 (id: 6605)

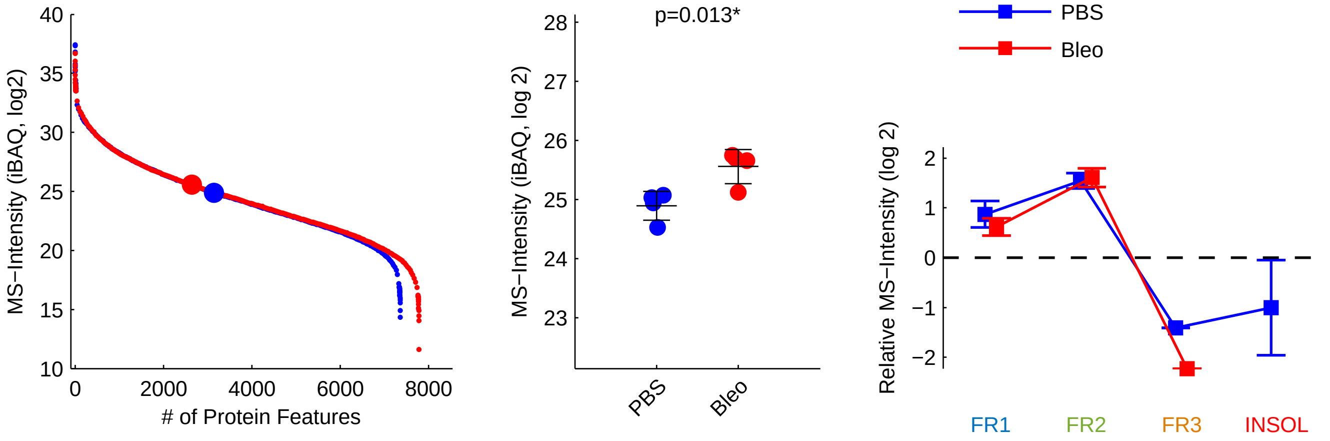

Q8VHI3 − Pofut2 (id: 6608)

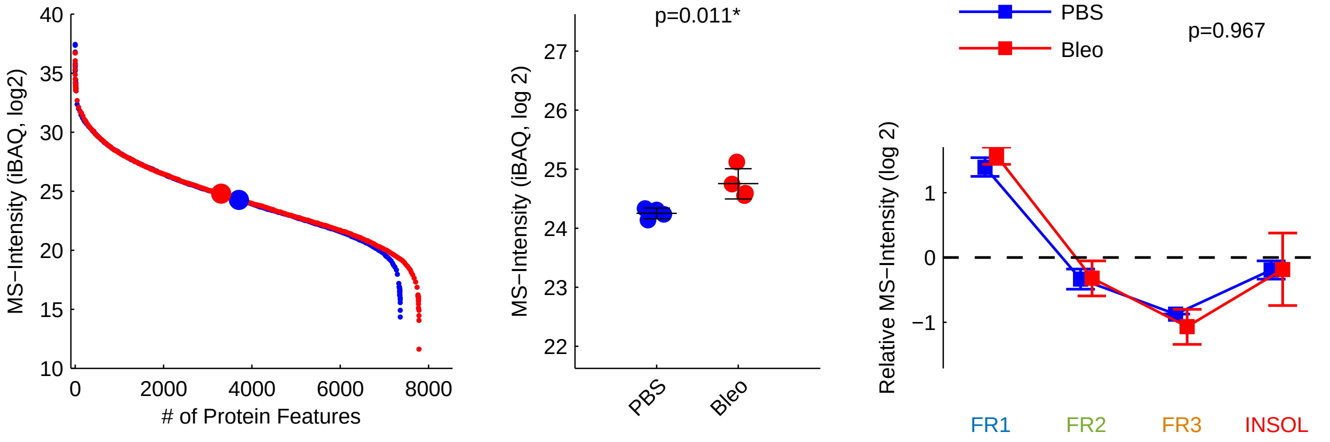

Q8VHM5 − Hnrnpr (id: 6615)

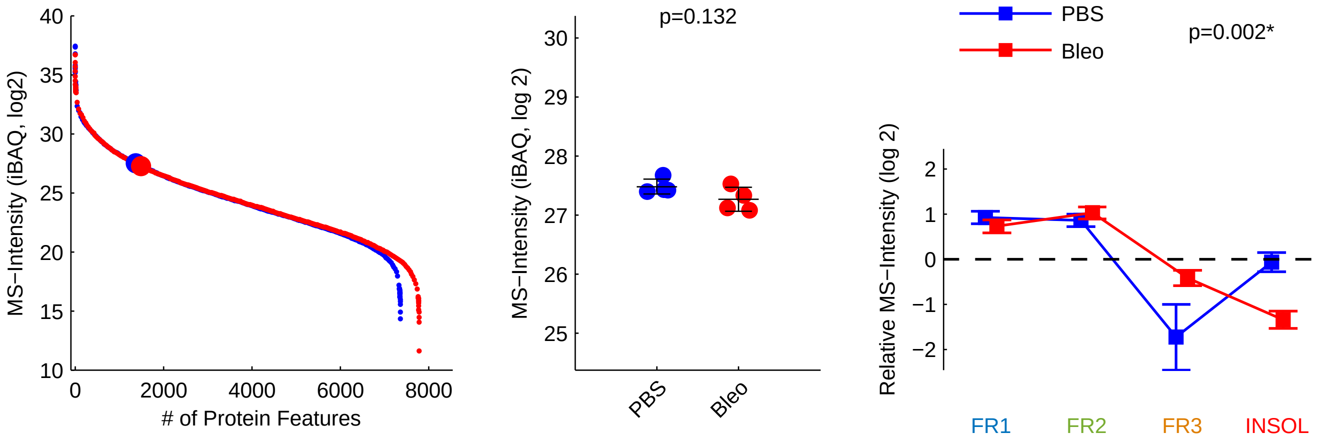

Q8VHQ3 − Ppp1r16b (id: 6617)

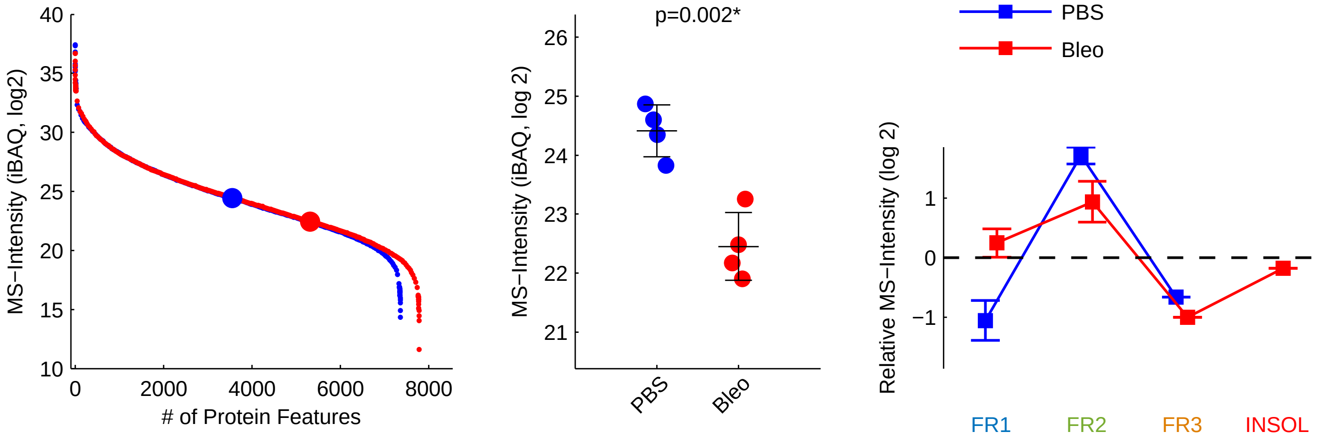

Q8VHY0 − Cspg4 (id: 6620)

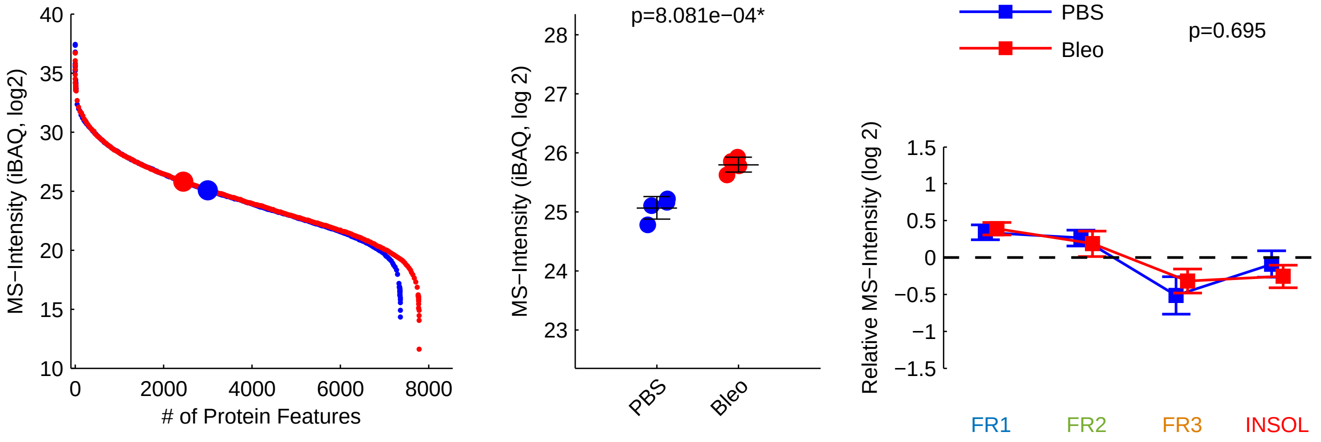

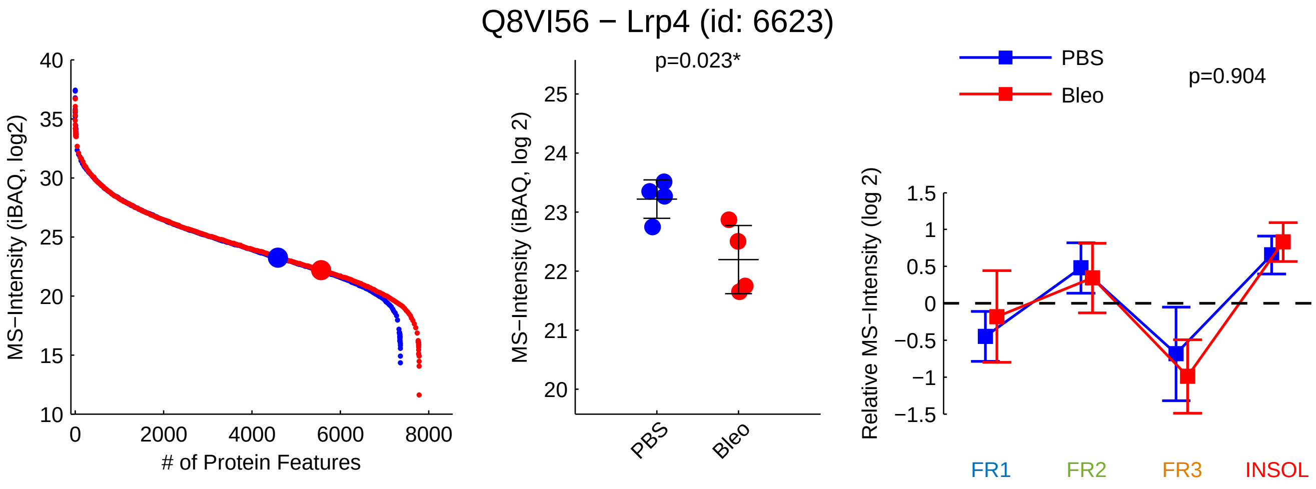
Q8VI56 − Lrp4 (id: 6623)
MS−Intensity (iBAQ, log2)
# of Protein Features
p=0.023*
MS−Intensity (iBAQ, log 2)
PBS
Bleo
p=0.904
Relative MS−Intensity (log 2)
FR1
FR2
FR3
INSOL

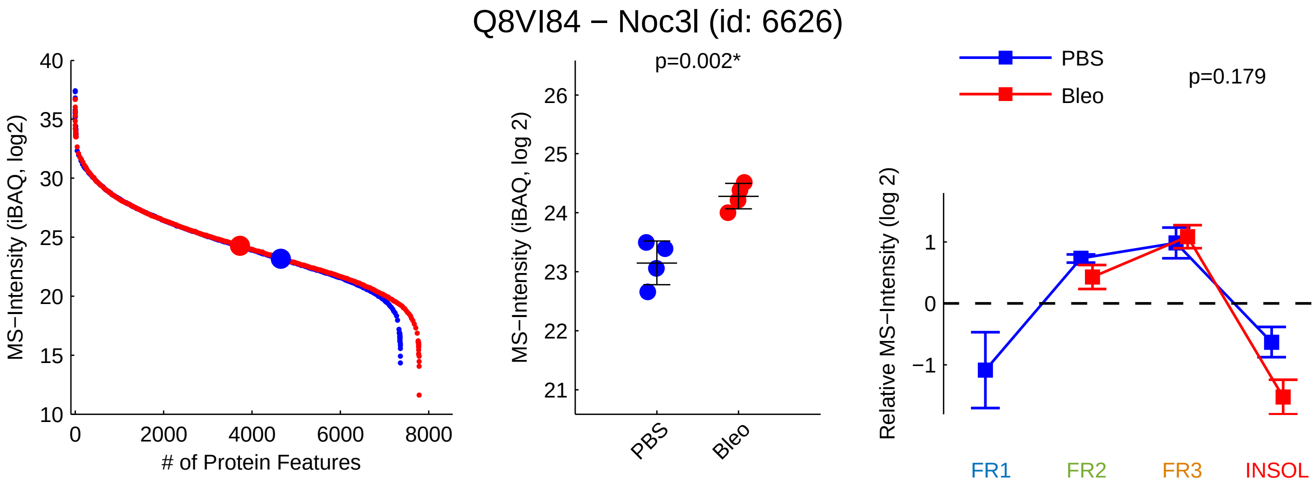
Q8VI84 − Noc3l (id: 6626)
MS−Intensity (iBAQ, log2)
# of Protein Features
p=0.002*
MS−Intensity (iBAQ, log 2)
PBS
Bleo
p=0.179
Relative MS−Intensity (log 2)
FR1
FR2
FR3
INSOL

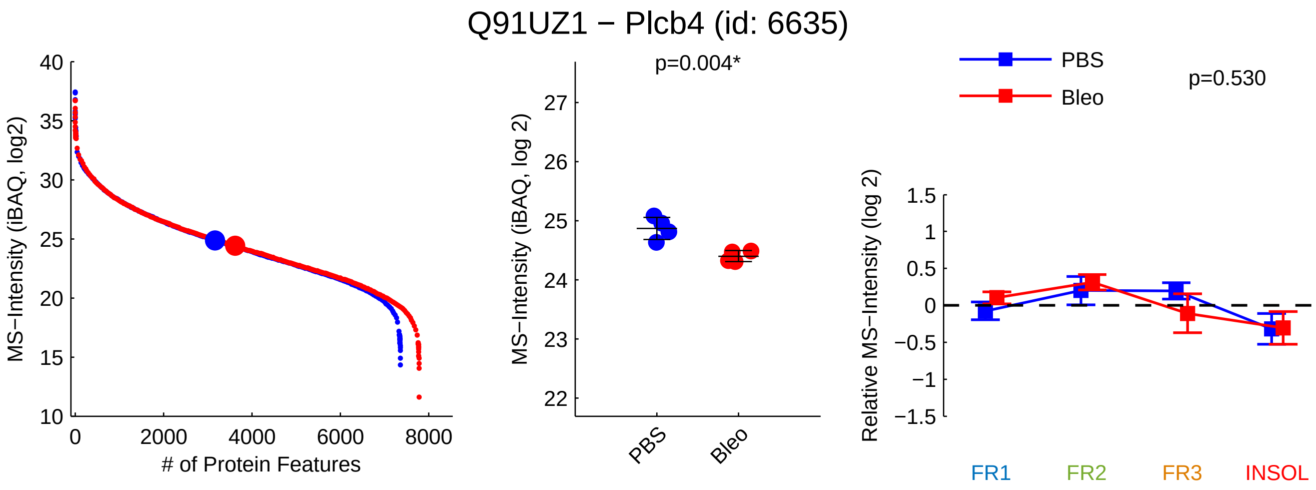
Q91UZ1 − Plcb4 (id: 6635)
MS−Intensity (iBAQ, log2)
# of Protein Features
p=0.004*
MS−Intensity (iBAQ, log 2)
PBS
Bleo
p=0.530
Relative MS−Intensity (log 2)
FR1
FR2
FR3
INSOL

Q91V09 − Wdr13 (id: 6639)

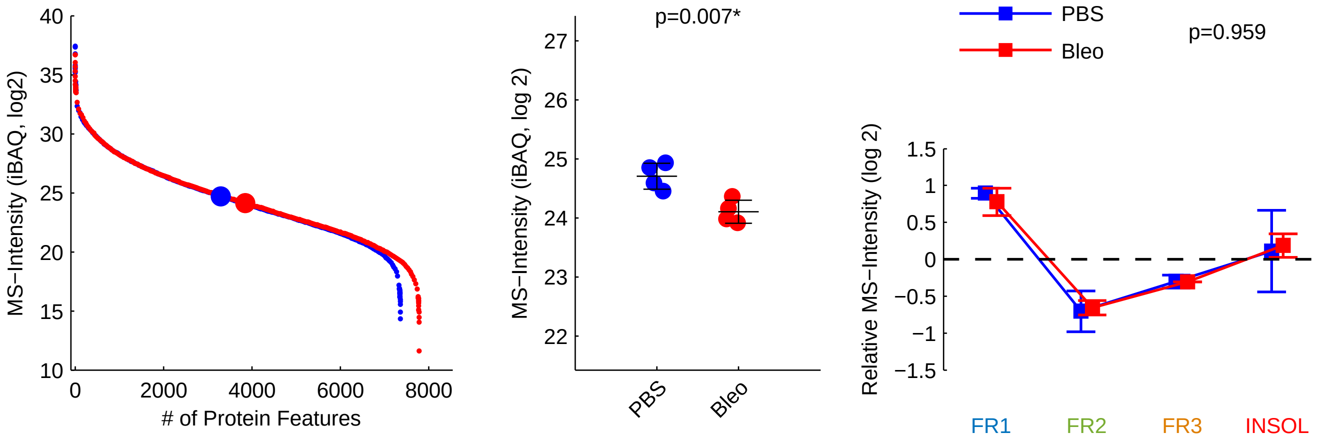

Q91V55 − Rps5 (id: 6645)

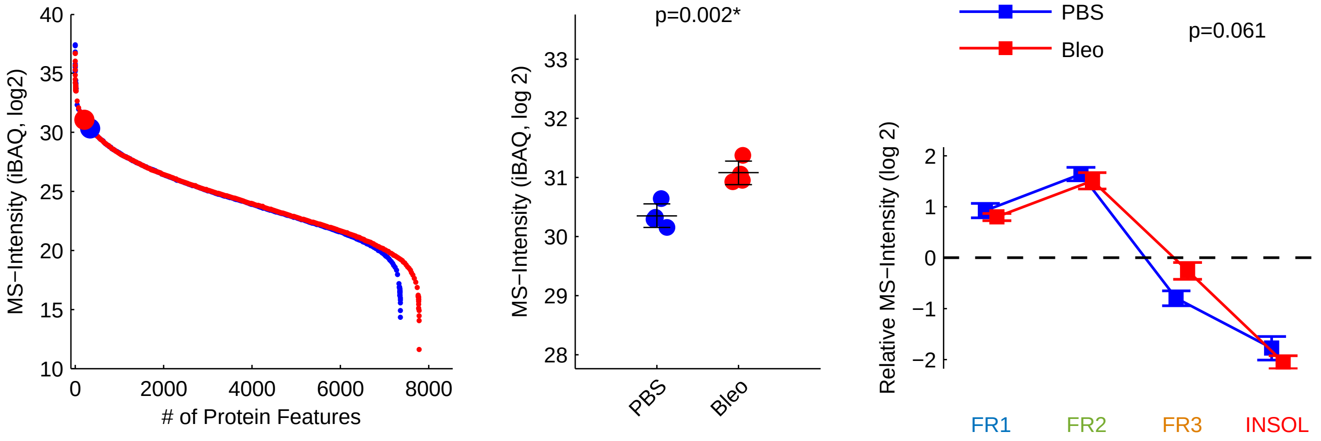

Q91V64 − Isoc1 (id: 6647)

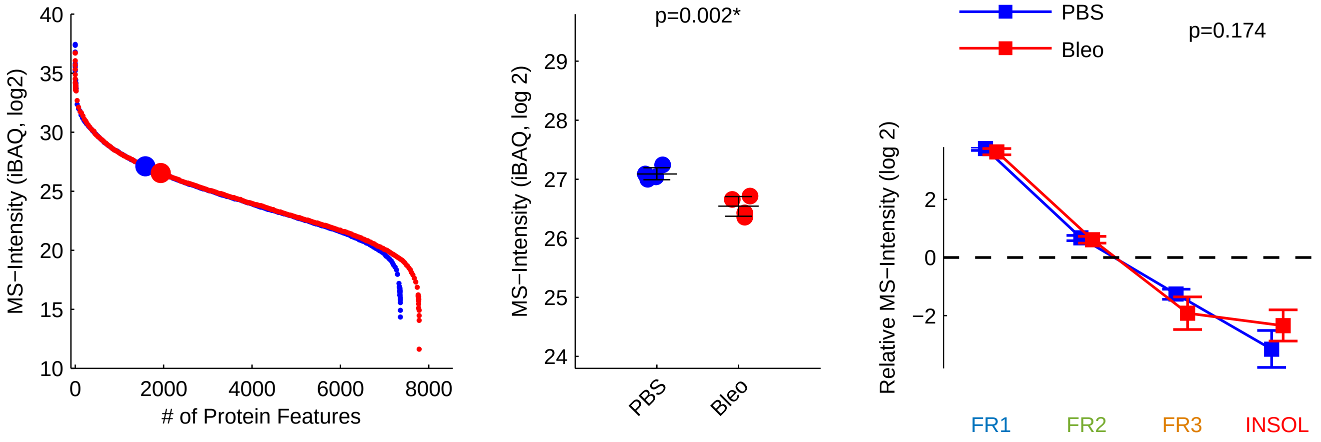

Q91V77 − S100a1 (id: 6649)

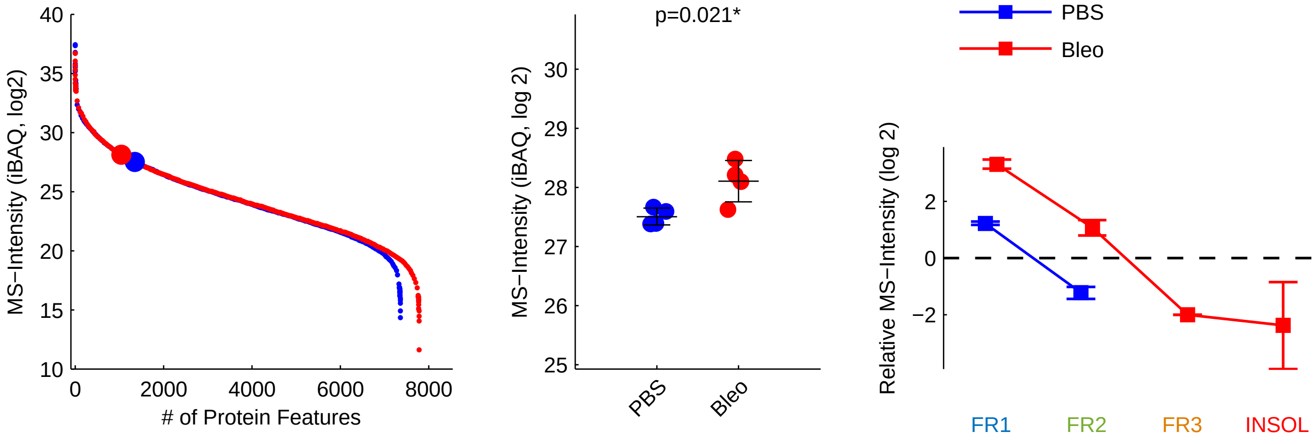

Q91V88−4 − Npnt (id: 6651)

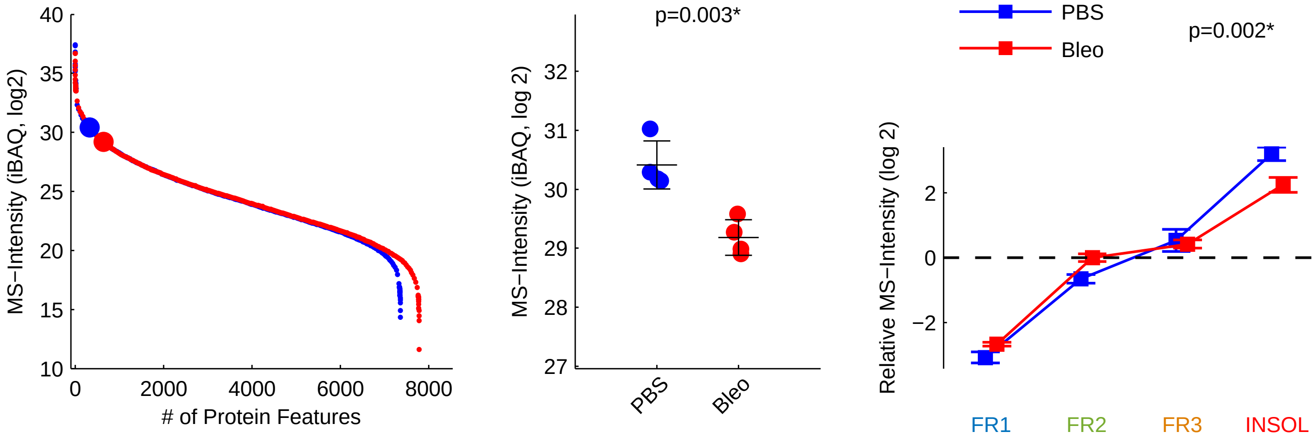

Q91VC7 − Ppp1r14a (id: 6659)

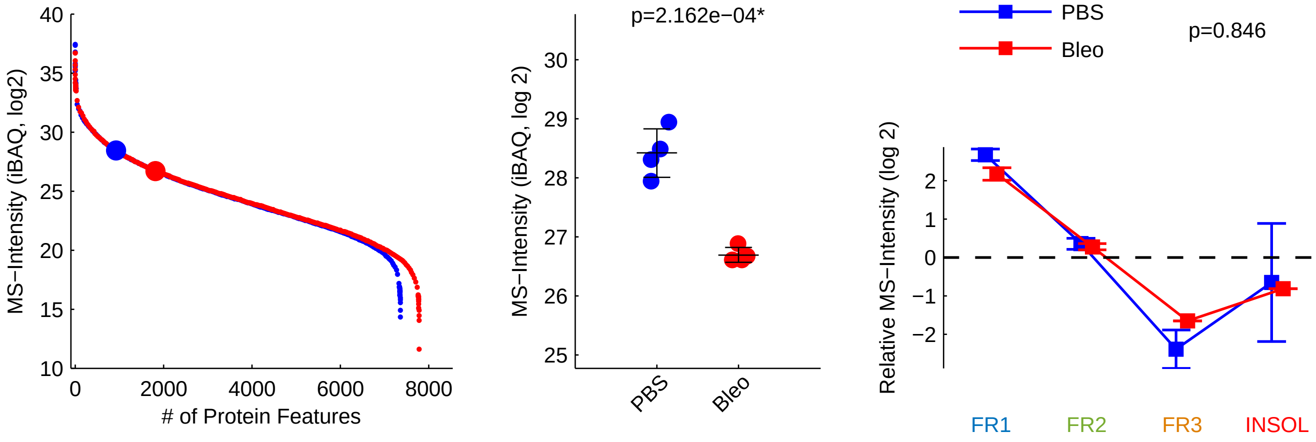

Q91VE6 − Mki67ip (id: 6664)

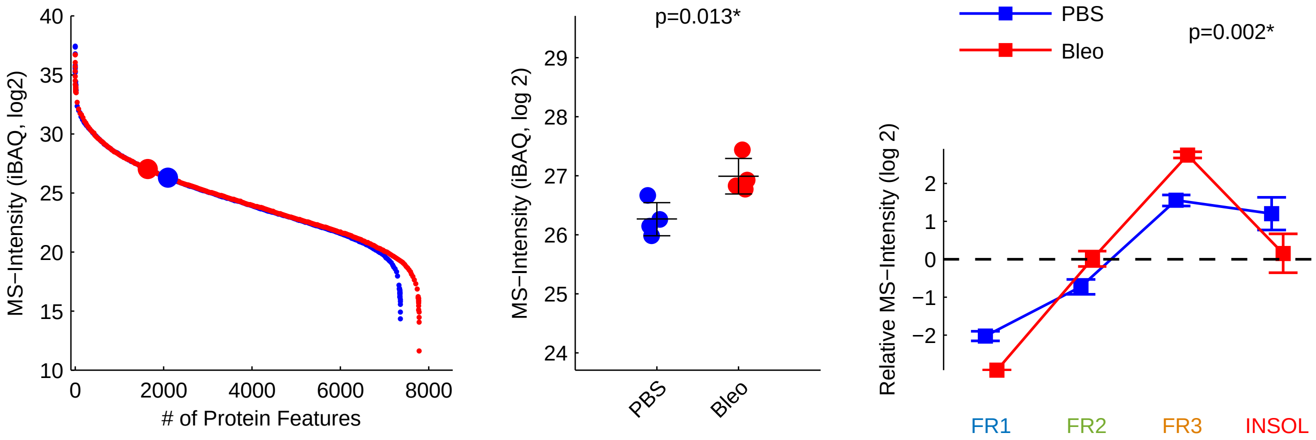

Q91VF2 − Hnmt (id: 6665)

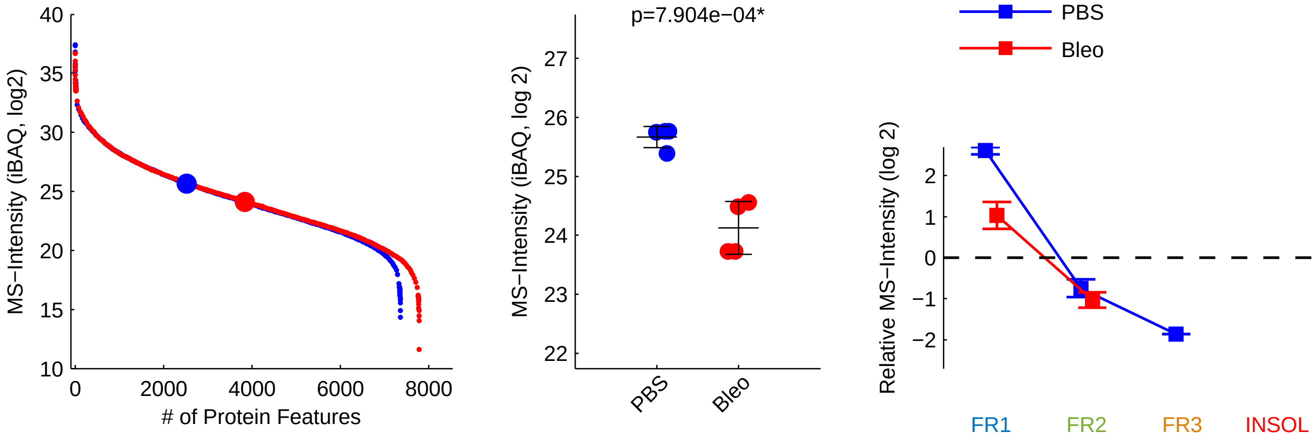

Q91VH2 − Snx9 (id: 6667)

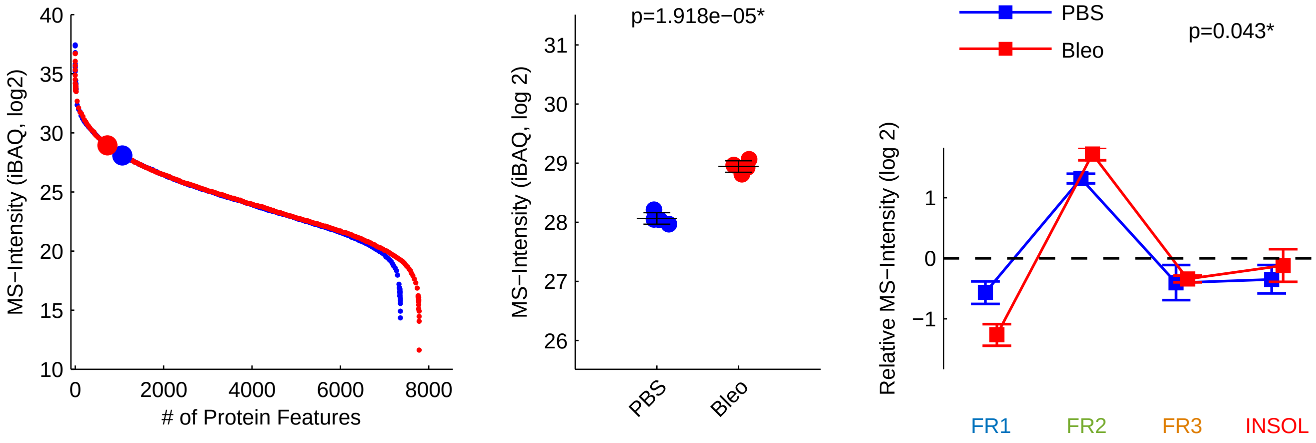

Q91VI7 − Rnh1 (id: 6669)

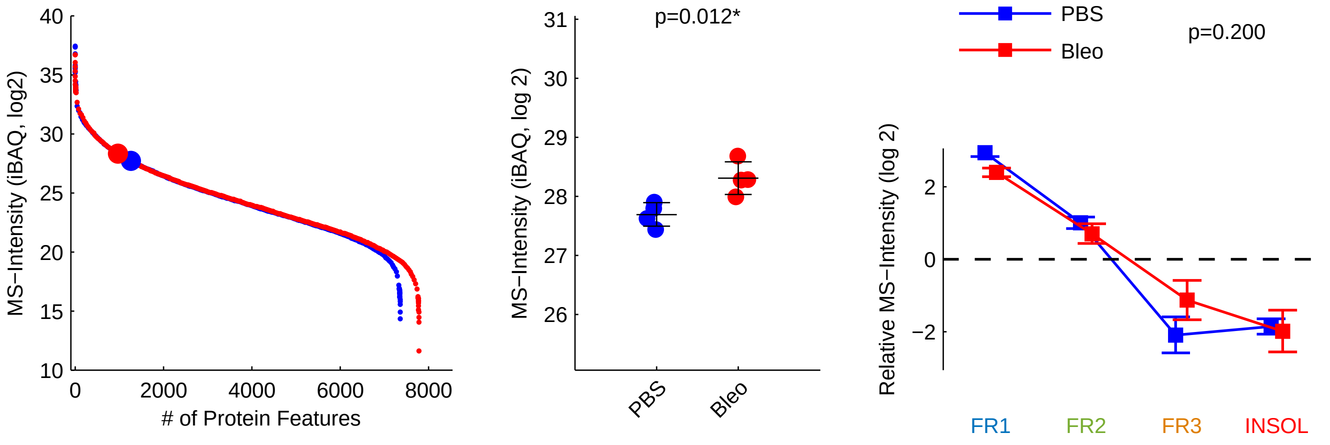

Q91VJ2 − Prkcdbp (id: 6671)

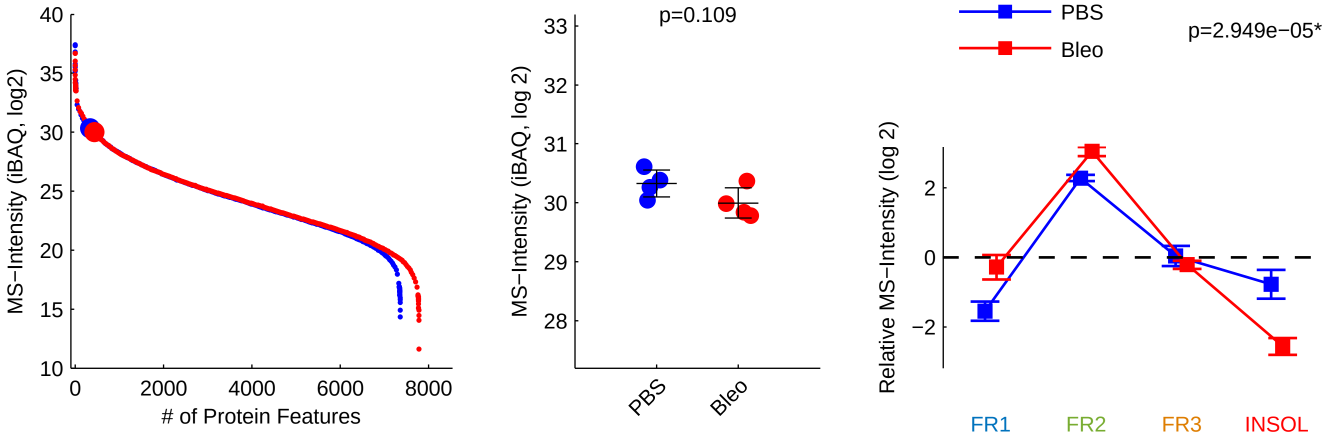

Q91VJ4 − Stk38 (id: 6672)

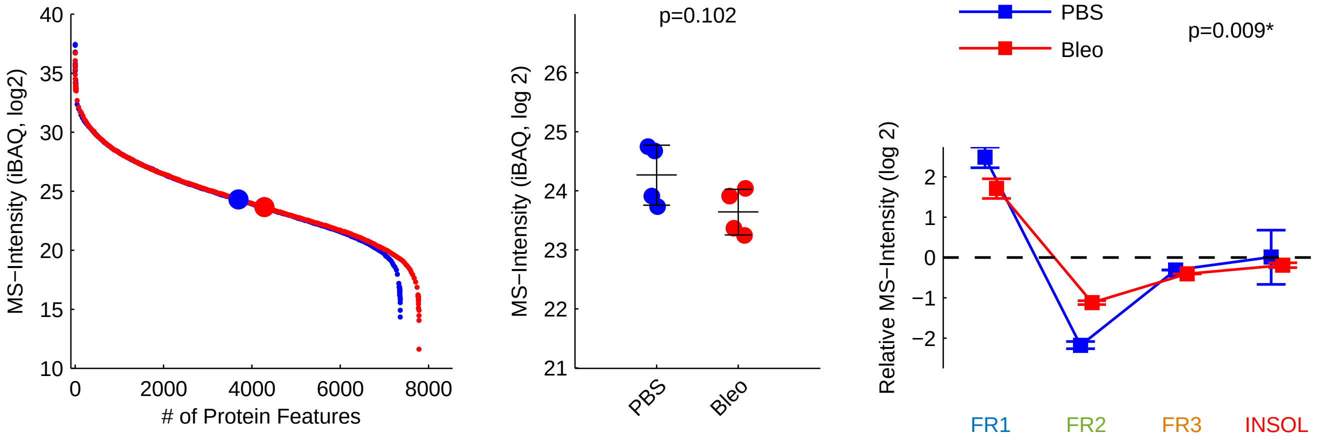

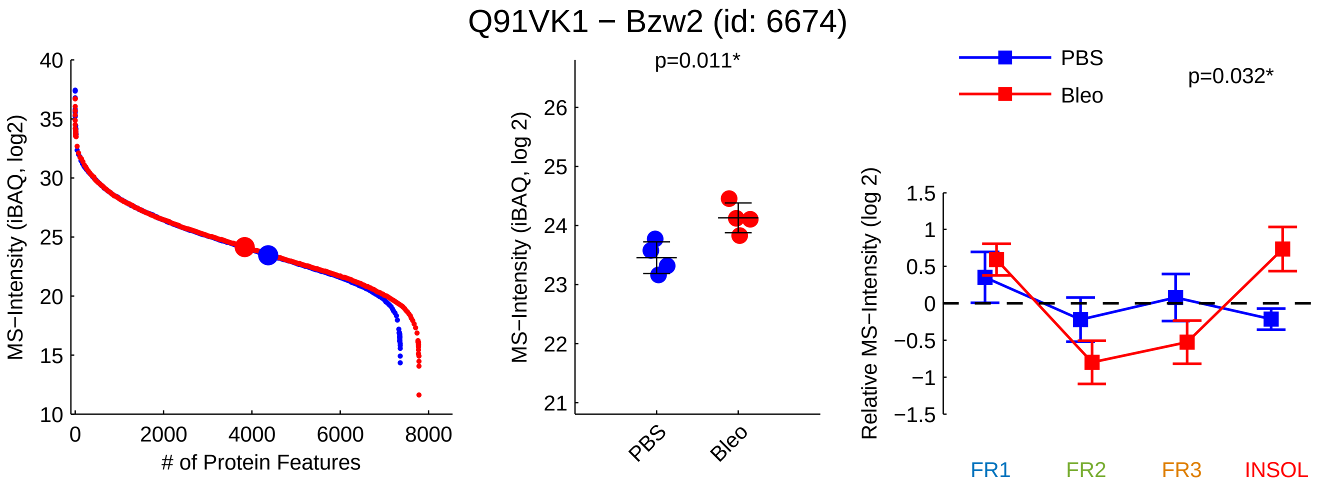
Q91VK1 − Bzw2 (id: 6674)
MS−Intensity (iBAQ, log2)
# of Protein Features
p=0.011*
MS−Intensity (iBAQ, log 2)
PBS
Bleo
PBS
Bleo
p=0.032*
Relative MS−Intensity (log 2)
FR1
FR2
FR3
INSOL

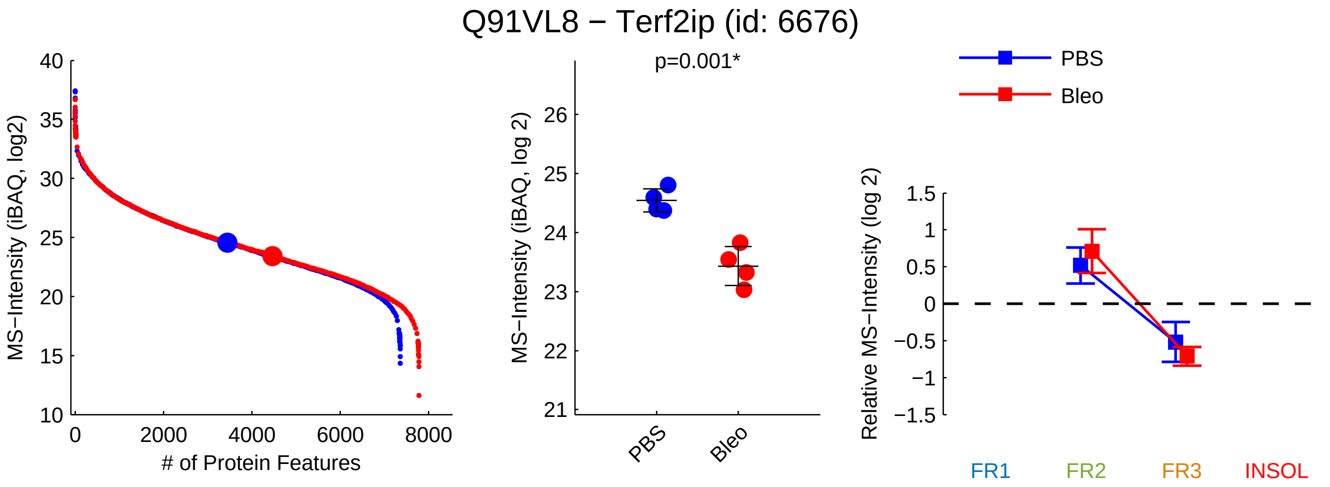
Q91VL8 − Terf2ip (id: 6676)
MS−Intensity (iBAQ, log2)
# of Protein Features
p=0.001*
MS−Intensity (iBAQ, log 2)
PBS
Bleo
PBS
Bleo
Relative MS−Intensity (log 2)
FR1
FR2
FR3
INSOL

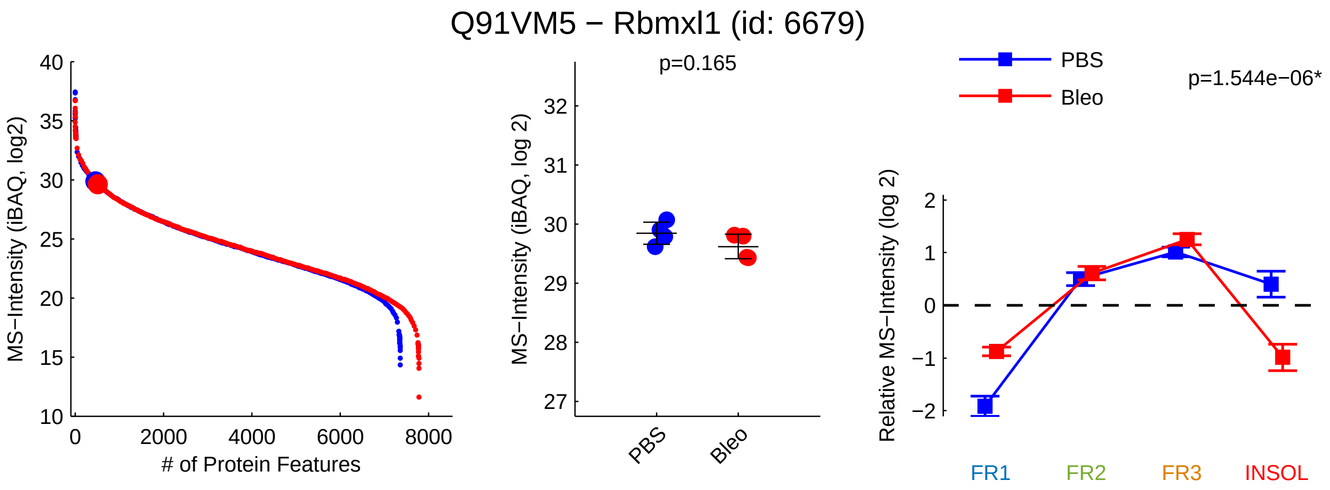
Q91VM5 − Rbmxl1 (id: 6679)
MS−Intensity (iBAQ, log2)
# of Protein Features
p=0.165
MS−Intensity (iBAQ, log 2)
PBS
Bleo
PBS
Bleo
p=1.544e−06*
Relative MS−Intensity (log 2)
FR1
FR2
FR3
INSOL

Q91VM9 − Ppa2 (id: 6680)

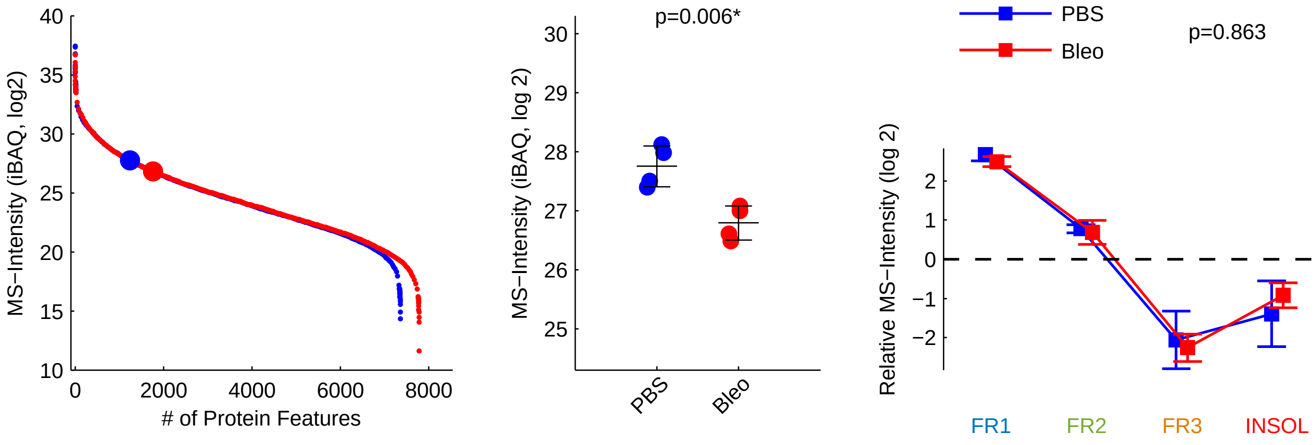

Q91VT4 − Cbr4 (id: 6689)

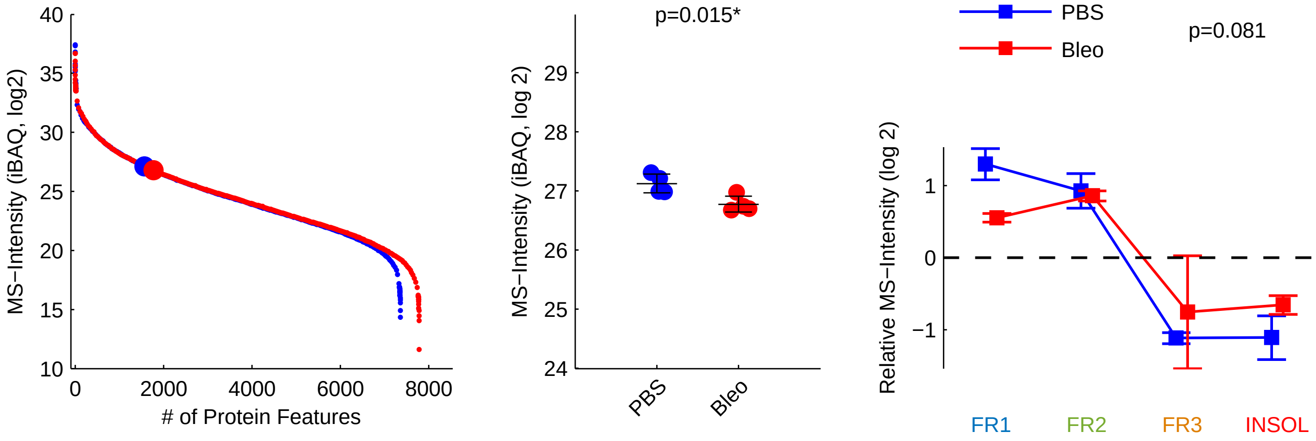

Q91VV4−2 − Dennd2d (id: 6691)

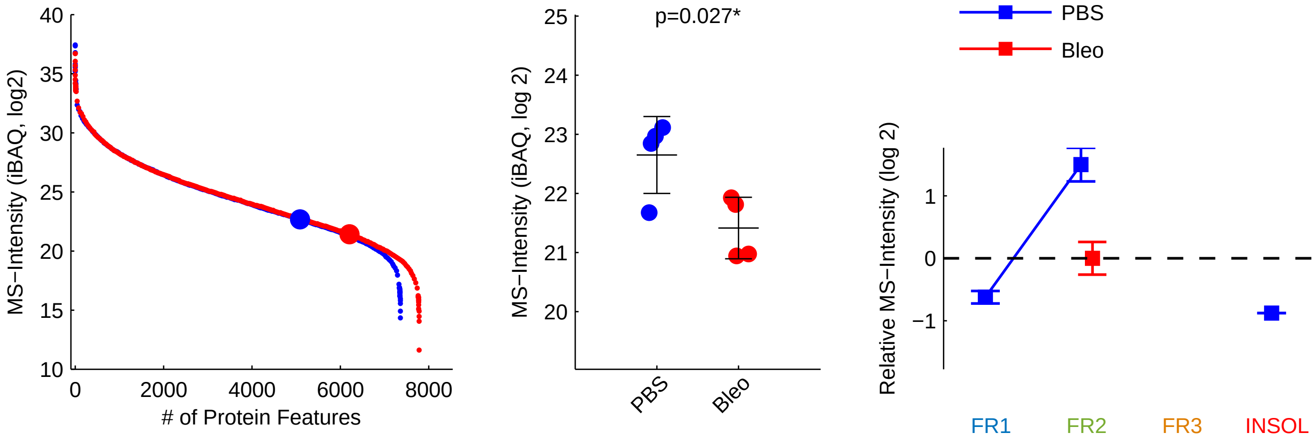

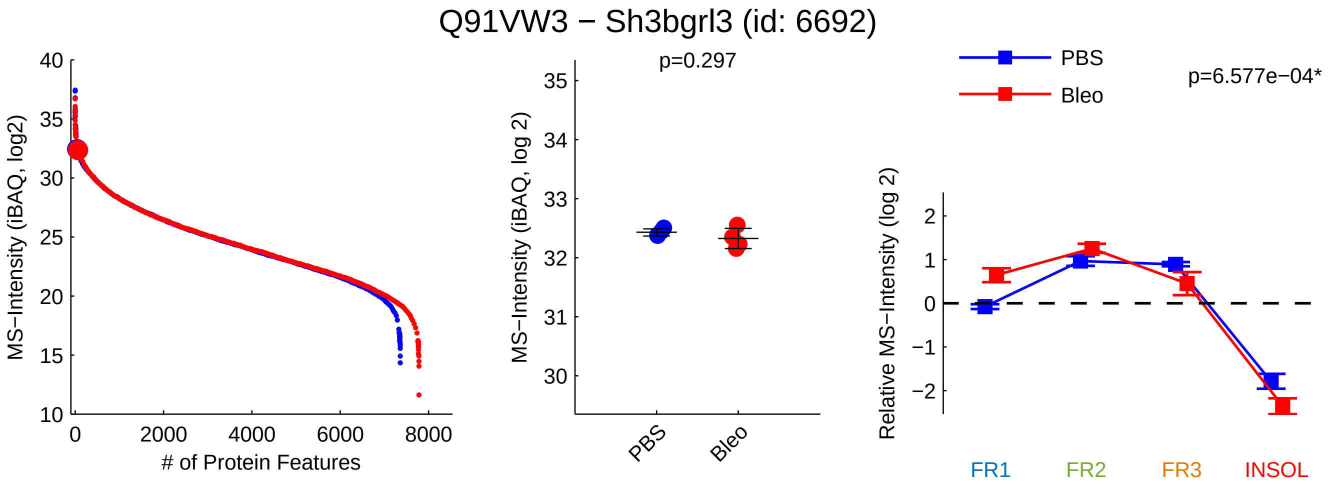
Q91VW3 − Sh3bgrl3 (id: 6692)
MS−Intensity (iBAQ, log2)
# of Protein Features
p=0.297
MS−Intensity (iBAQ, log 2)
PBS
Bleo
p=6.577e−04*
Relative MS−Intensity (log 2)
FR1
FR2
FR3
INSOL

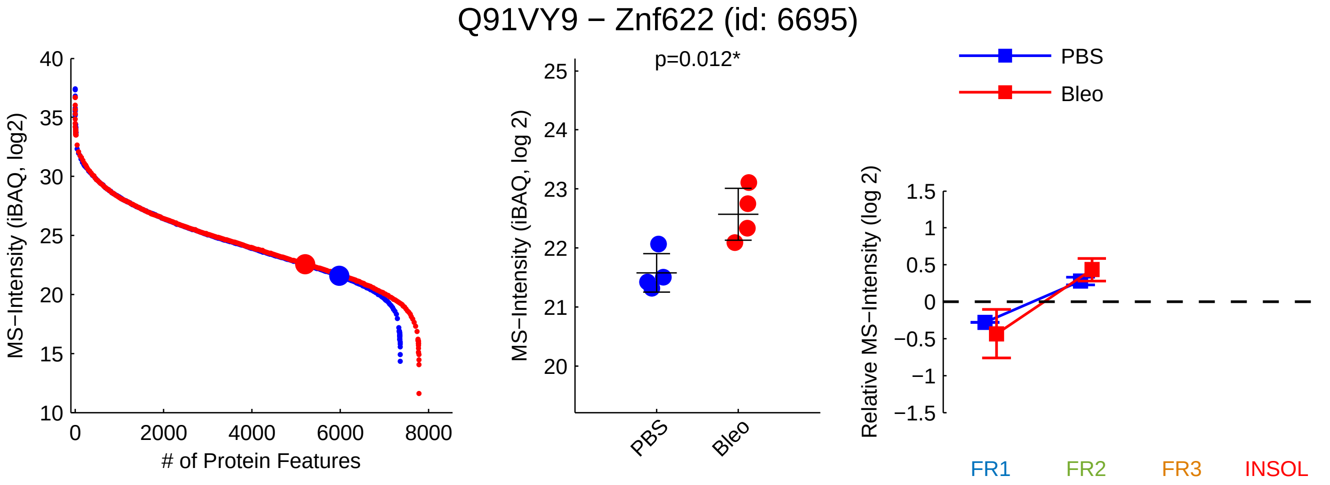
Q91VY9 − Znf622 (id: 6695)
MS−Intensity (iBAQ, log2)
# of Protein Features
p=0.012*
MS−Intensity (iBAQ, log 2)
PBS
Bleo
Relative MS−Intensity (log 2)
FR1
FR2
FR3
INSOL

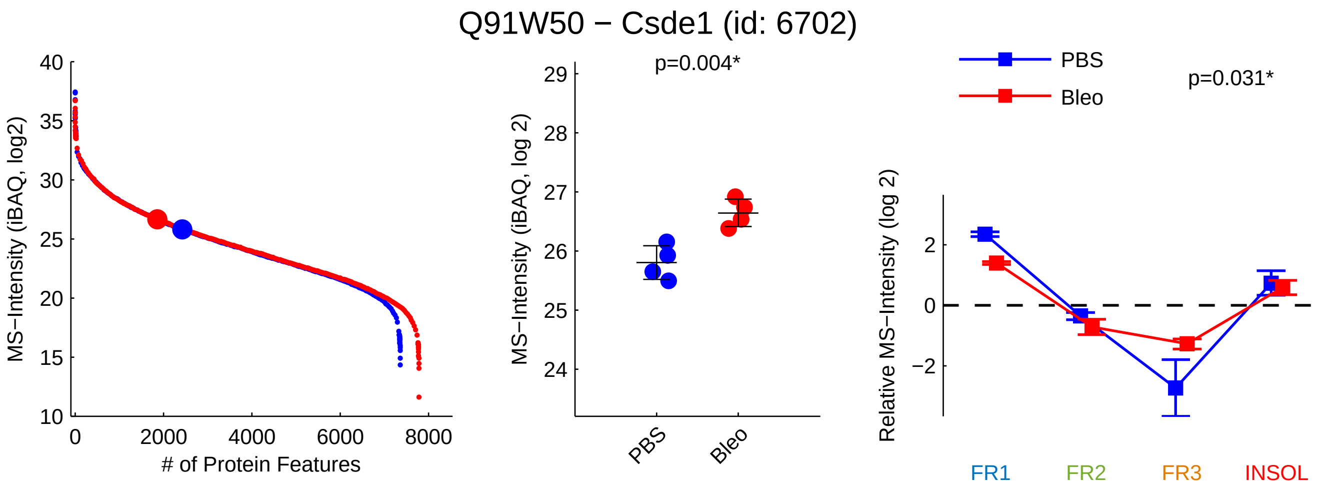
Q91W50 − Csde1 (id: 6702)
MS−Intensity (iBAQ, log2)
# of Protein Features
p=0.004*
MS−Intensity (iBAQ, log 2)
PBS
Bleo
p=0.031*
Relative MS−Intensity (log 2)
FR1
FR2
FR3
INSOL

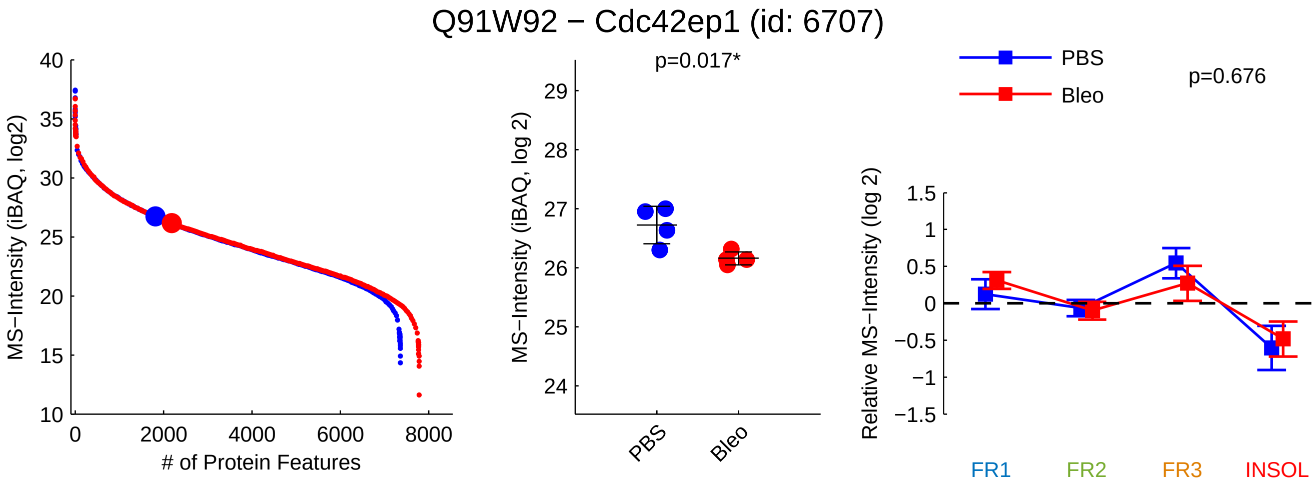
Q91W92 − Cdc42ep1 (id: 6707)
MS−Intensity (iBAQ, log2)
# of Protein Features
p=0.017*
MS−Intensity (iBAQ, log 2)
PBS
Bleo
p=0.676
Relative MS−Intensity (log 2)
FR1
FR2
FR3
INSOL

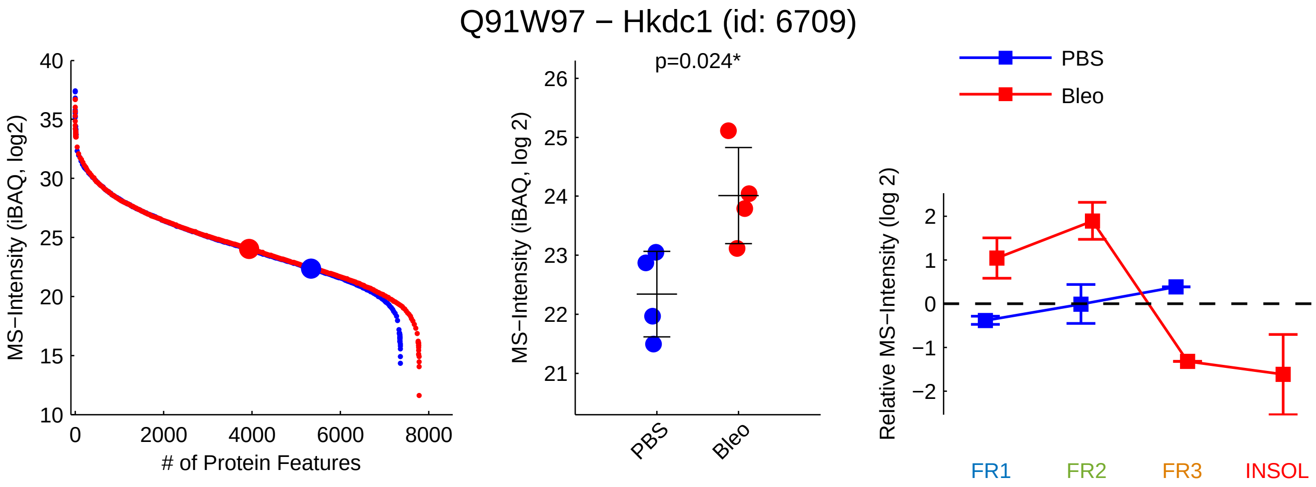
Q91W97 − Hkdc1 (id: 6709)
MS−Intensity (iBAQ, log2)
# of Protein Features
p=0.024*
MS−Intensity (iBAQ, log 2)
PBS
Bleo
Relative MS−Intensity (log 2)
FR1
FR2
FR3
INSOL

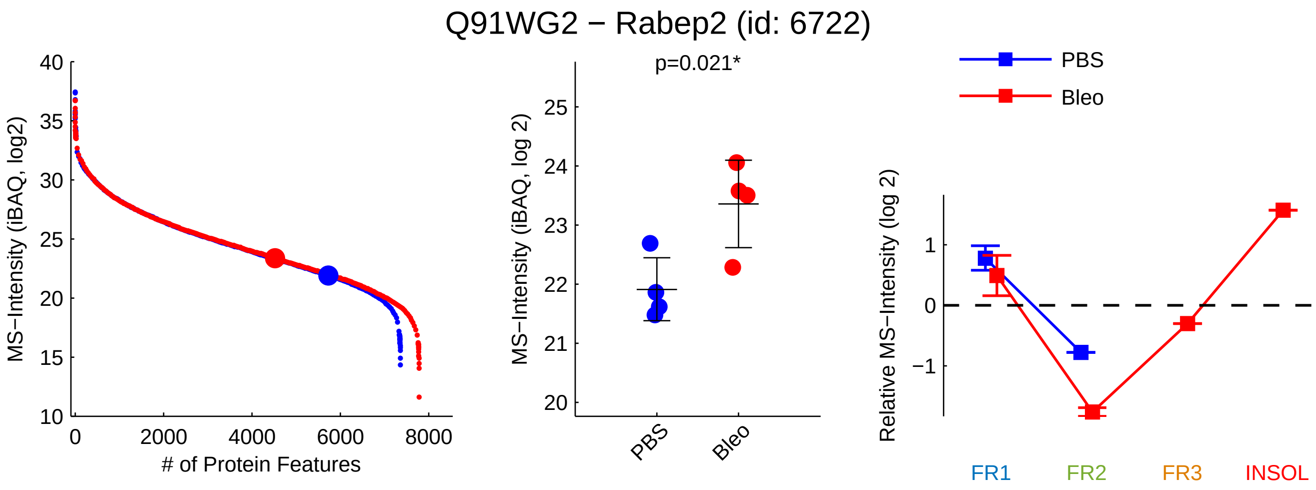
Q91WG2 − Rabep2 (id: 6722)
MS−Intensity (iBAQ, log2)
# of Protein Features
p=0.021*
MS−Intensity (iBAQ, log 2)
PBS
Bleo
Relative MS−Intensity (log 2)
FR1
FR2
FR3
INSOL

Q91WK2 − Eif3h (id: 6726)

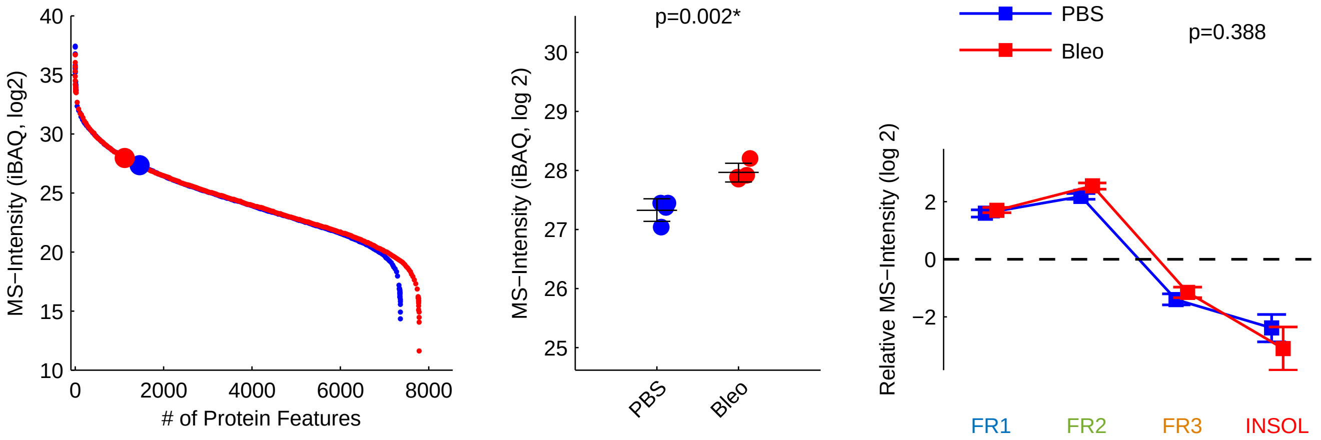

Q91WS0 − Cisd1 (id: 6736)

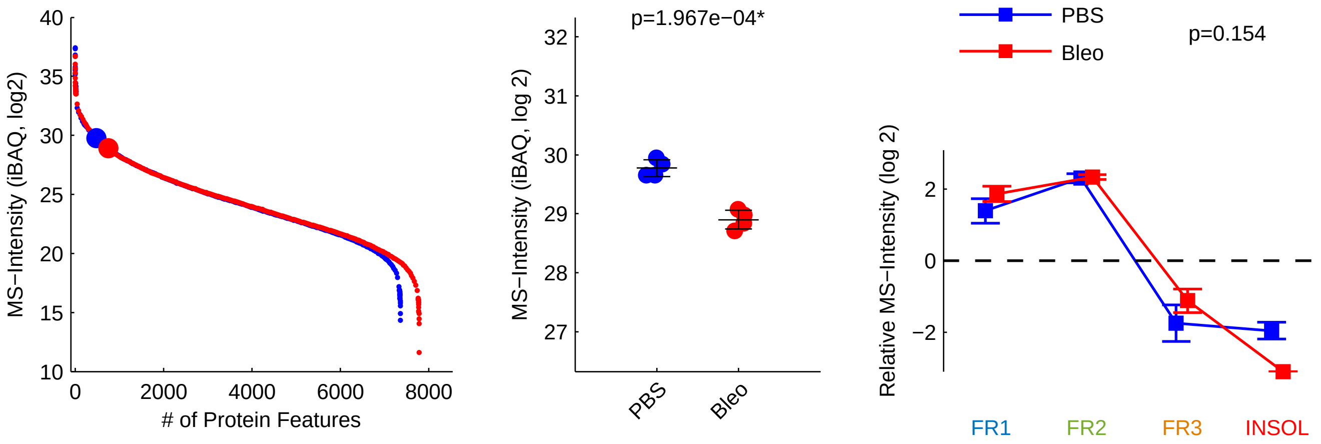

Q91WU0 − Ces1f (id: 6739)

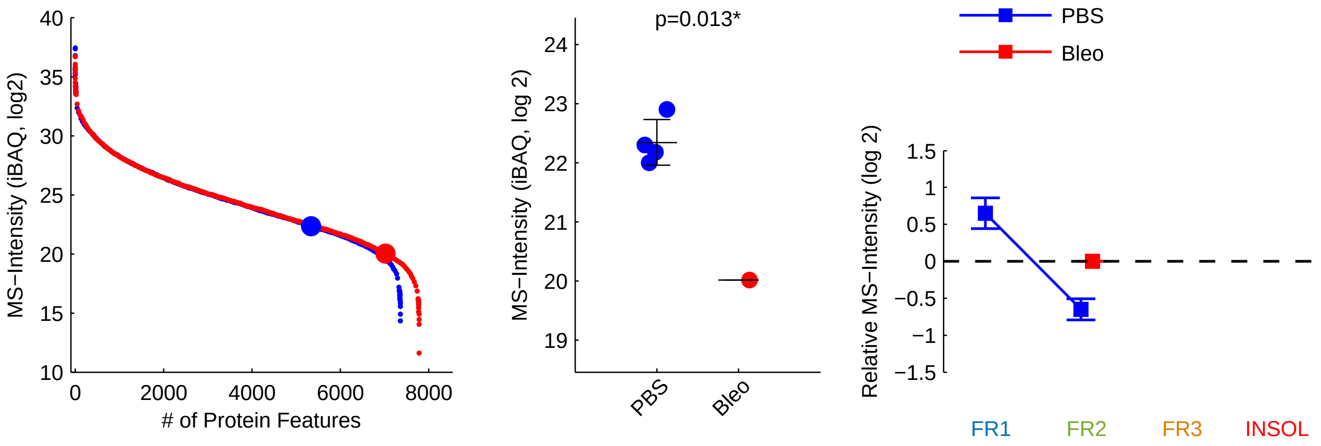

Q91WU5 − As3mt (id: 6740)

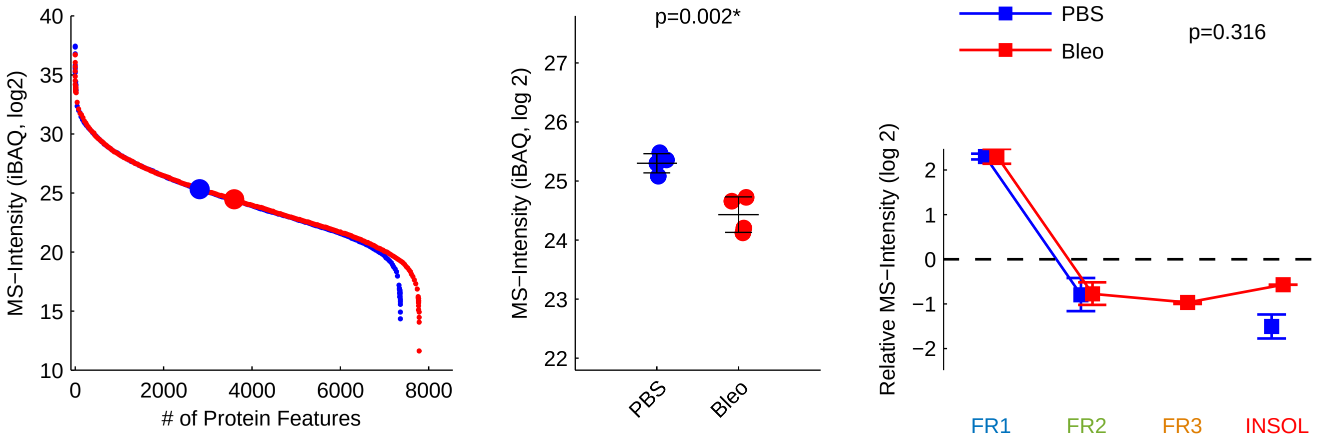

Q91X72 − Hpx (id: 6747)

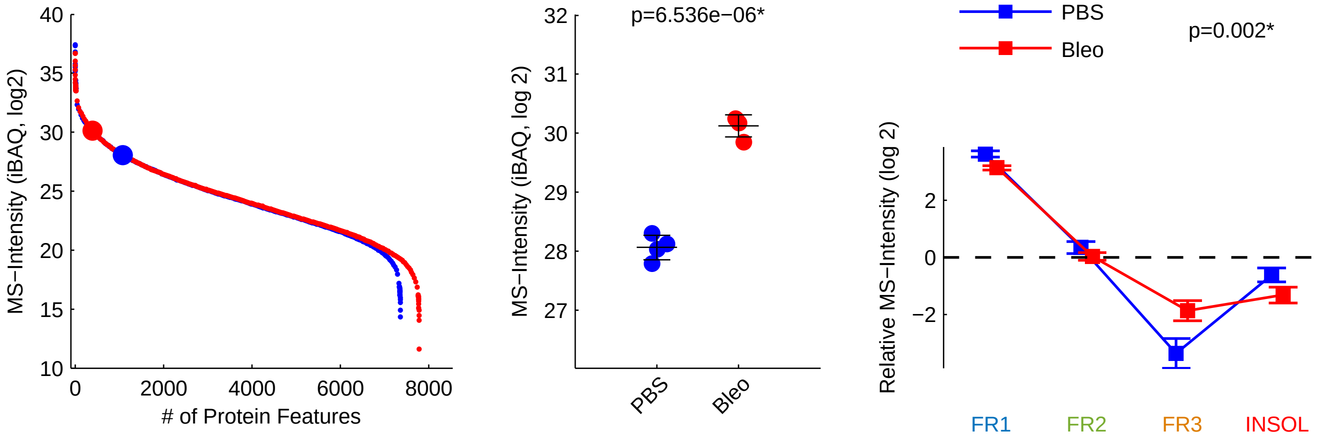

Q91X76 − Nt5dc2 (id: 6748)

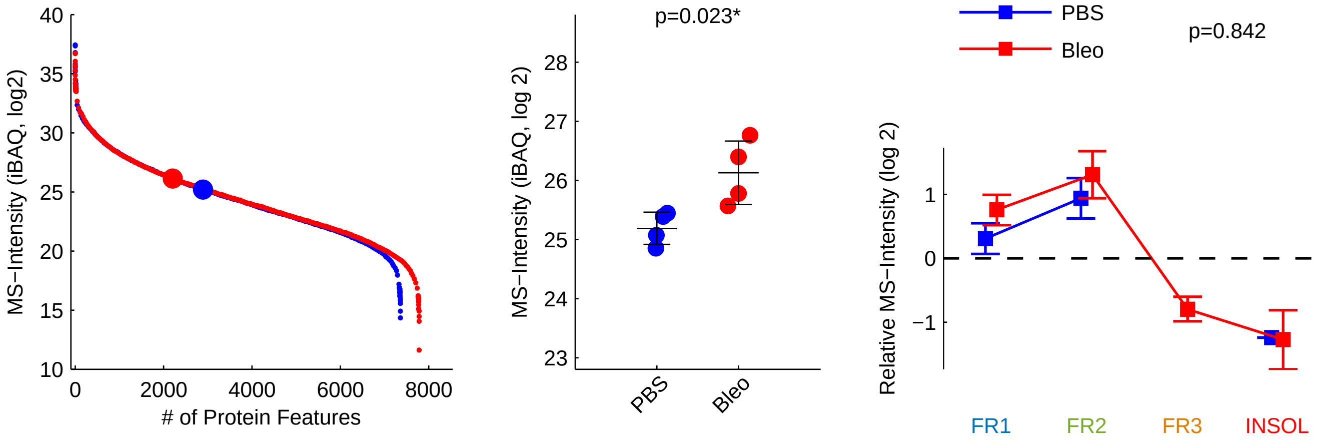

Q91X97 − Ncald (id: 6755)

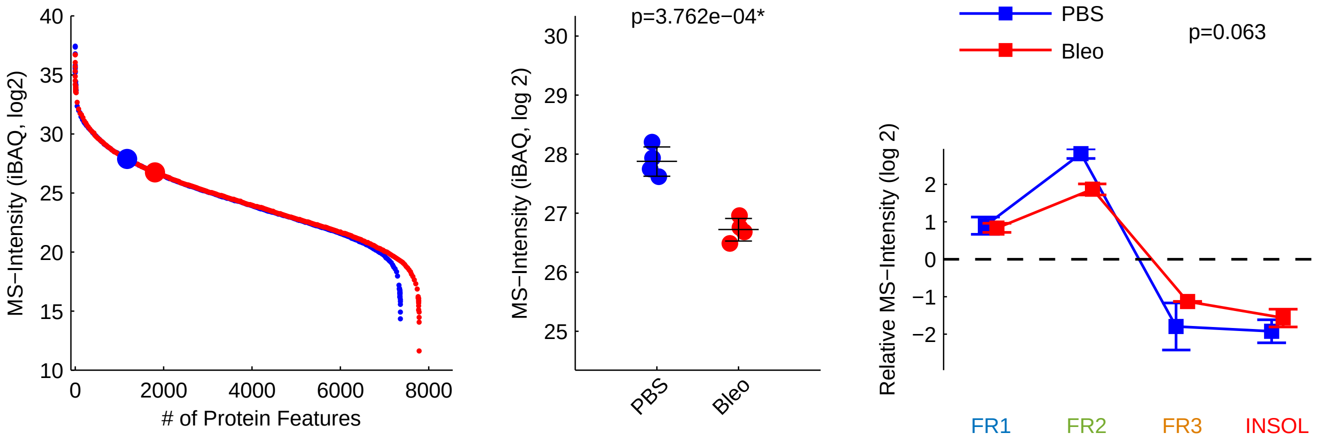

Q91XB7 − Yif1a (id: 6759)

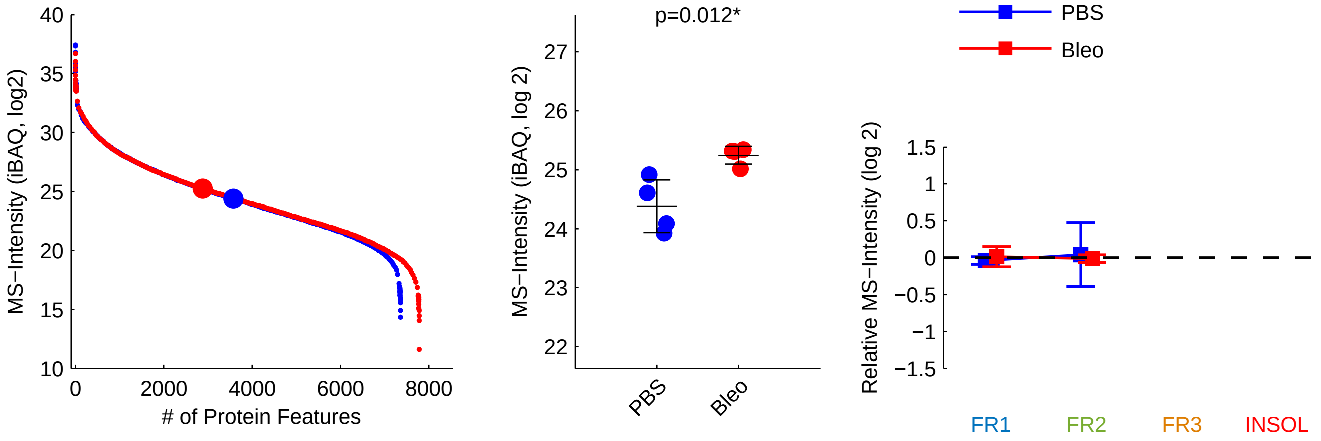

Q91XD2 − Lims2 (id: 6763)

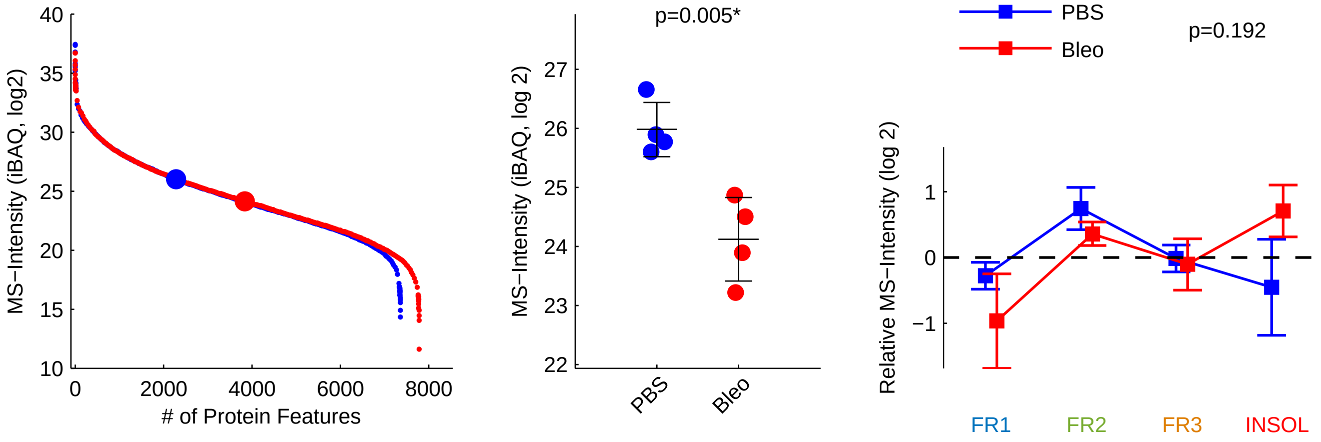

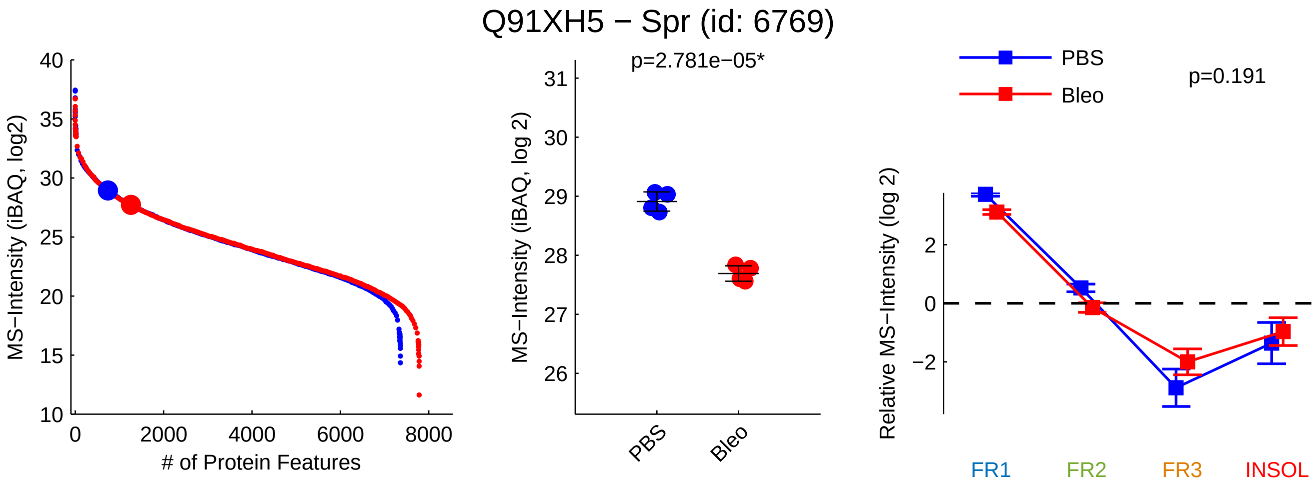
Q91XH5 − Spr (id: 6769)
MS−Intensity (iBAQ, log2)
# of Protein Features
0 2000 4000 6000 8000
10 15 20 25 30 35 40
p=2.781e−05*
MS−Intensity (iBAQ, log 2)
26 27 28 29 30 31
PBS
Bleo
PBS
Bleo
p=0.191
Relative MS−Intensity (log 2)
−2 0 2
FR1
FR2
FR3
INSOL

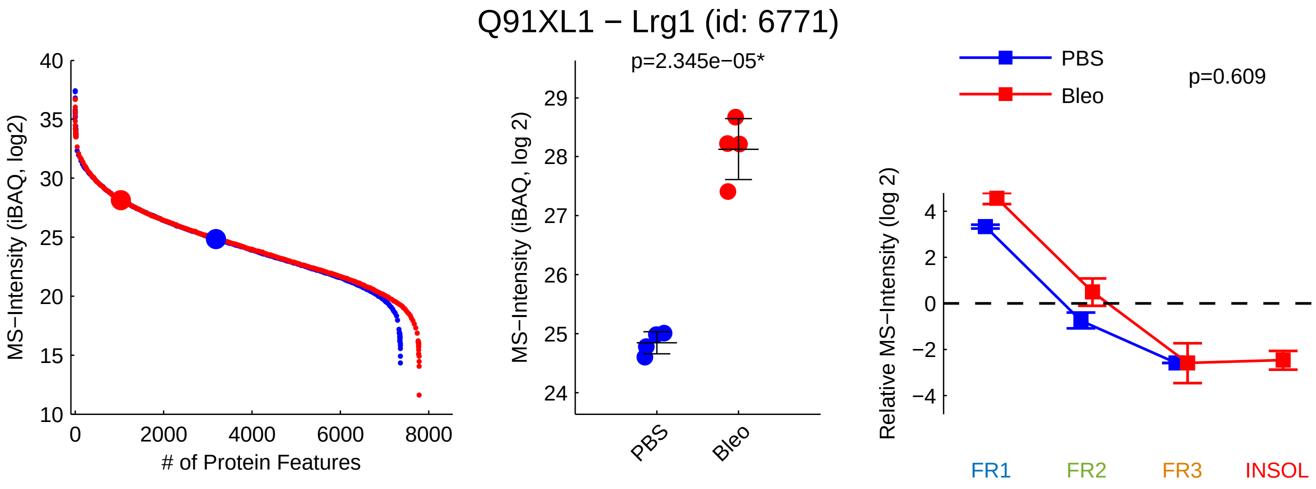
Q91XL1 − Lrg1 (id: 6771)
MS−Intensity (iBAQ, log2)
# of Protein Features
0 2000 4000 6000 8000
10 15 20 25 30 35 40
p=2.345e−05*
MS−Intensity (iBAQ, log 2)
24 25 26 27 28 29
PBS
Bleo
PBS
Bleo
p=0.609
Relative MS−Intensity (log 2)
−4 −2 0 2 4
FR1
FR2
FR3
INSOL

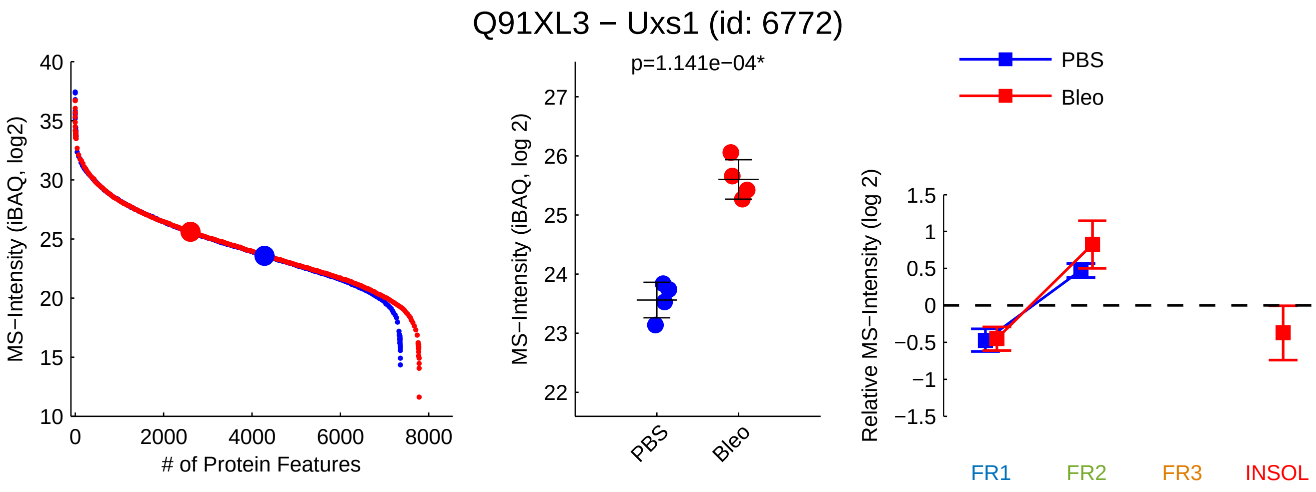
Q91XL3 − Uxs1 (id: 6772)
MS−Intensity (iBAQ, log2)
# of Protein Features
0 2000 4000 6000 8000
10 15 20 25 30 35 40
p=1.141e−04*
MS−Intensity (iBAQ, log 2)
22 23 24 25 26 27
PBS
Bleo
PBS
Bleo
Relative MS−Intensity (log 2)
−1.5 −1 −0.5 0 0.5 1 1.5
FR1
FR2
FR3
INSOL

Q91XV3 − Basp1 (id: 6776)

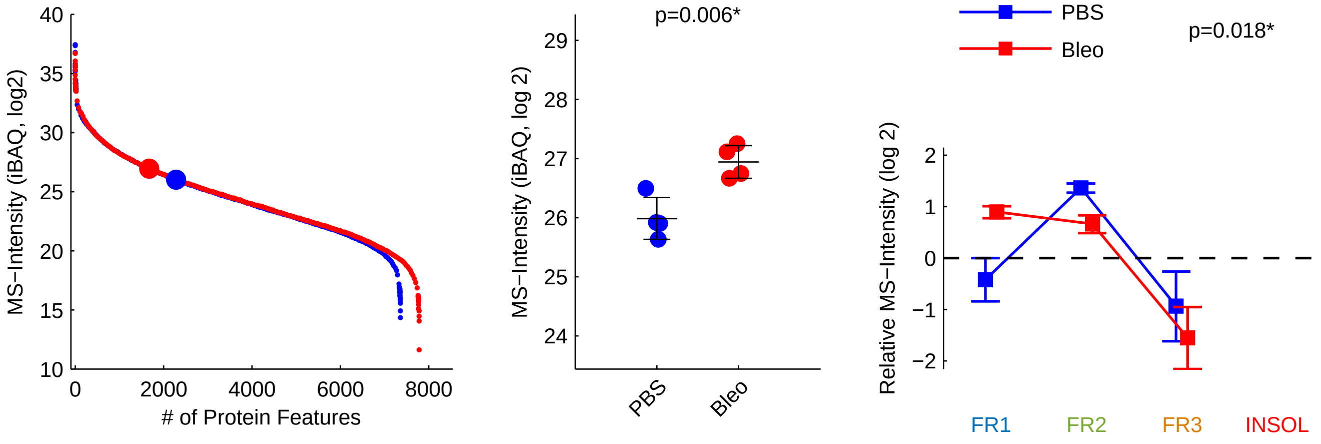

Q91YE7 − Rbm5 (id: 6783)

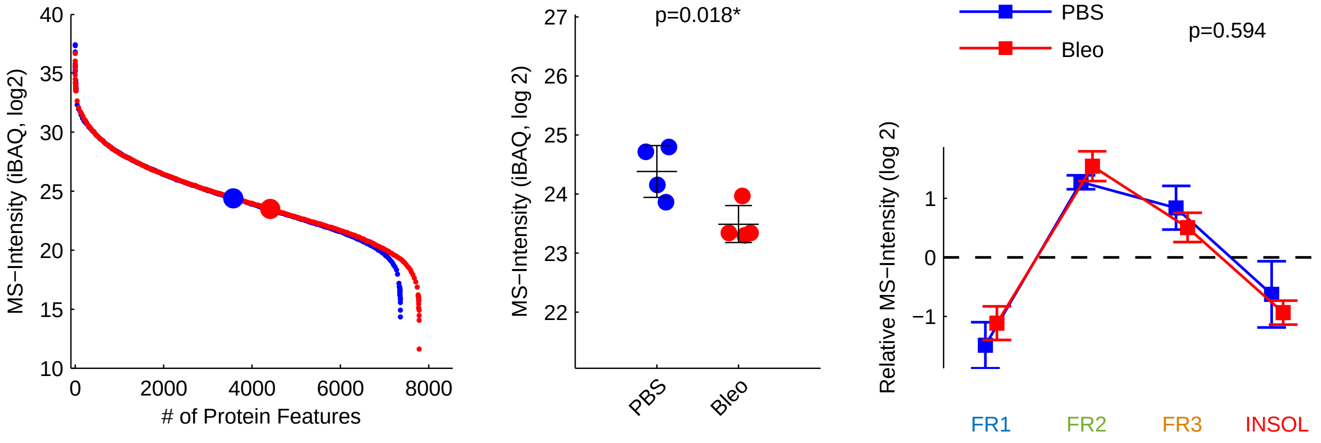

Q91YE8 − Synpo2 (id: 6784)

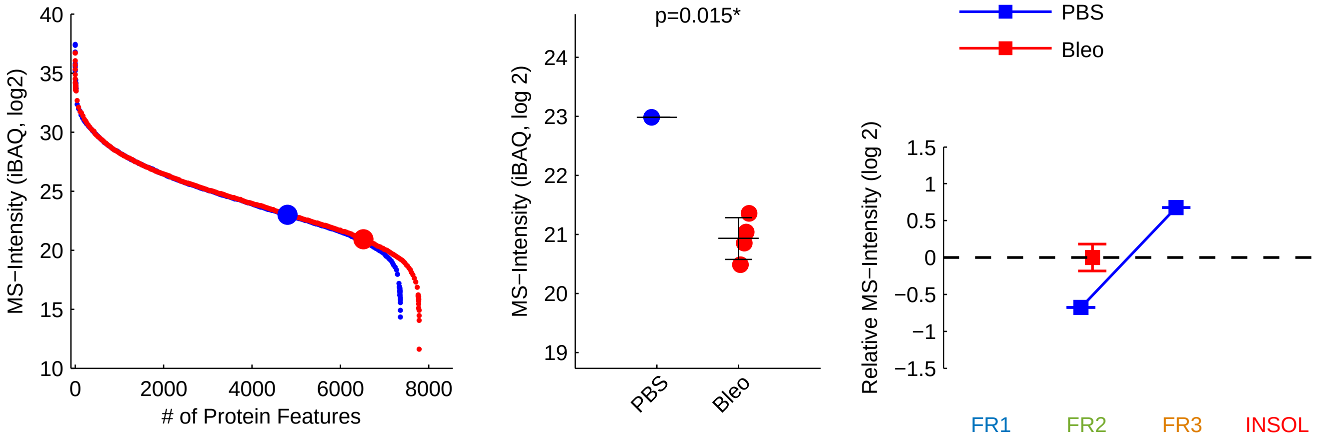

Q91YJ2 − Snx4 (id: 6789)

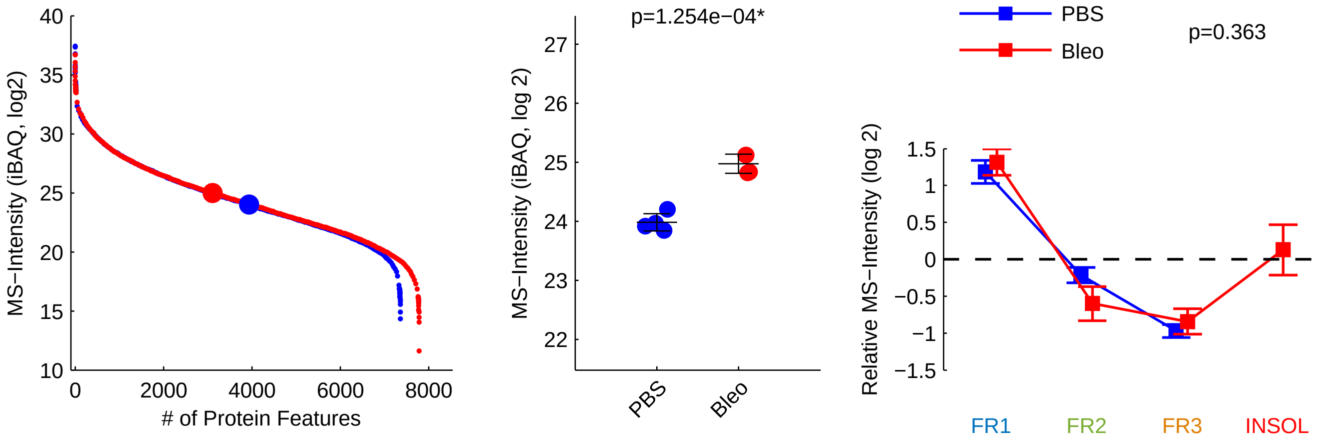

Q91YM4 − Tbrg4 (id: 6794)

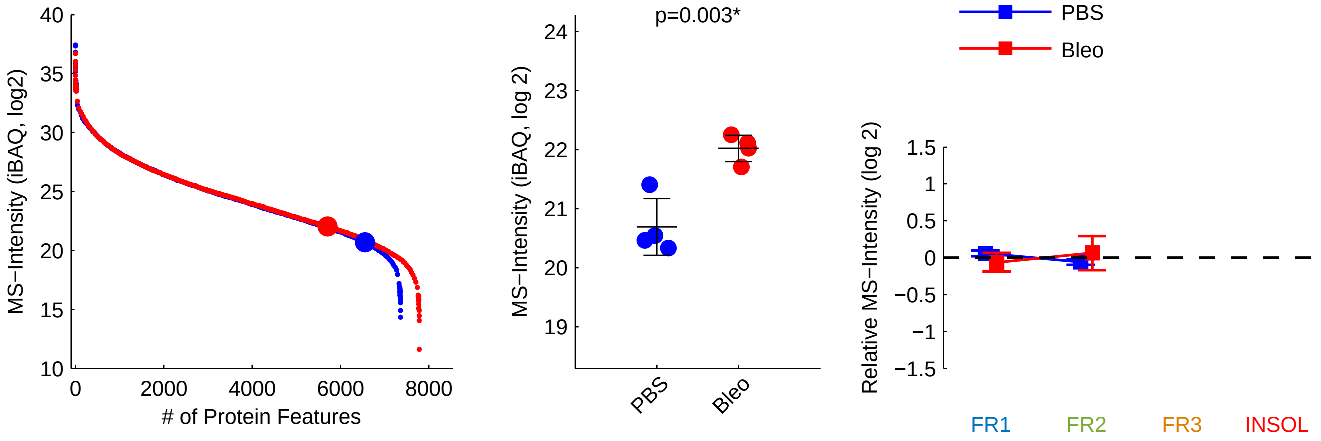

Q91YP0 − L2hgdh (id: 6797)

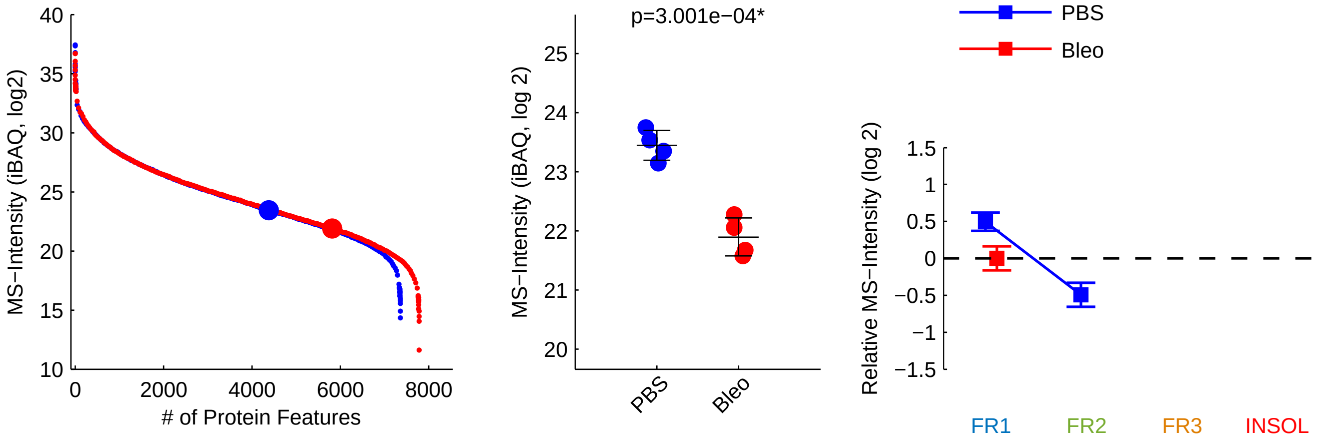

Q91YP3 − Dera (id: 6799)

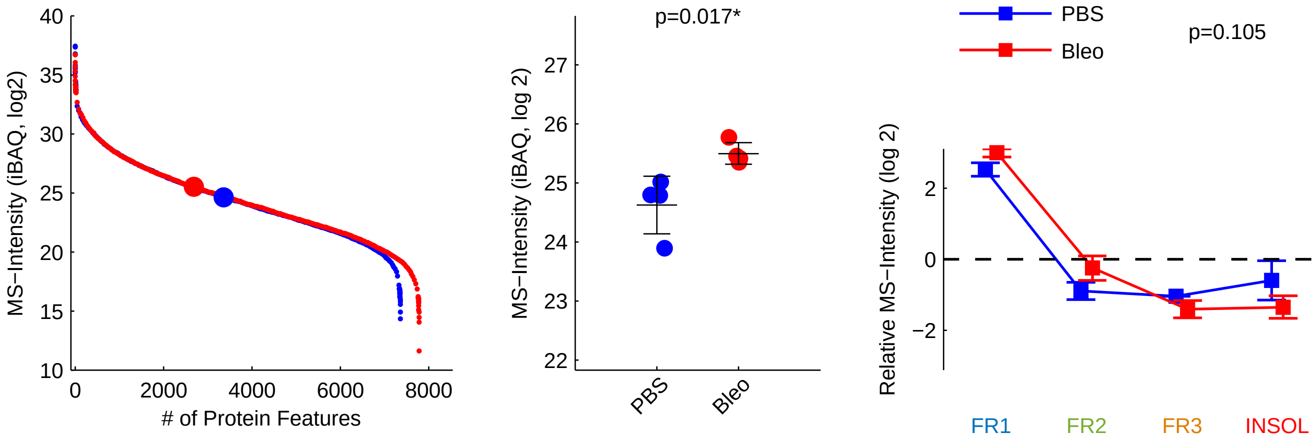

Q91YQ5 − Rpn1 (id: 6800)

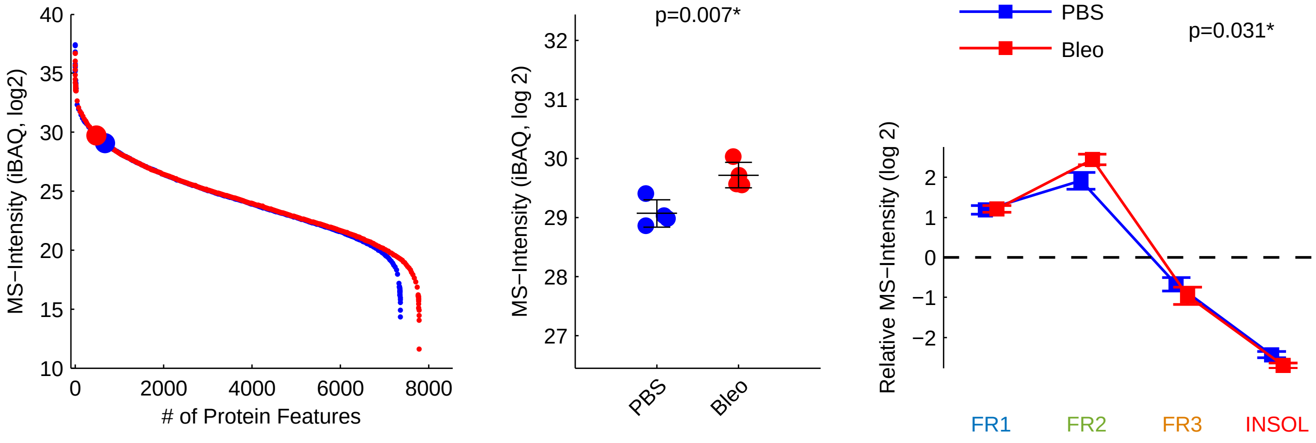

Q91YS8 − Camk1 (id: 6805)

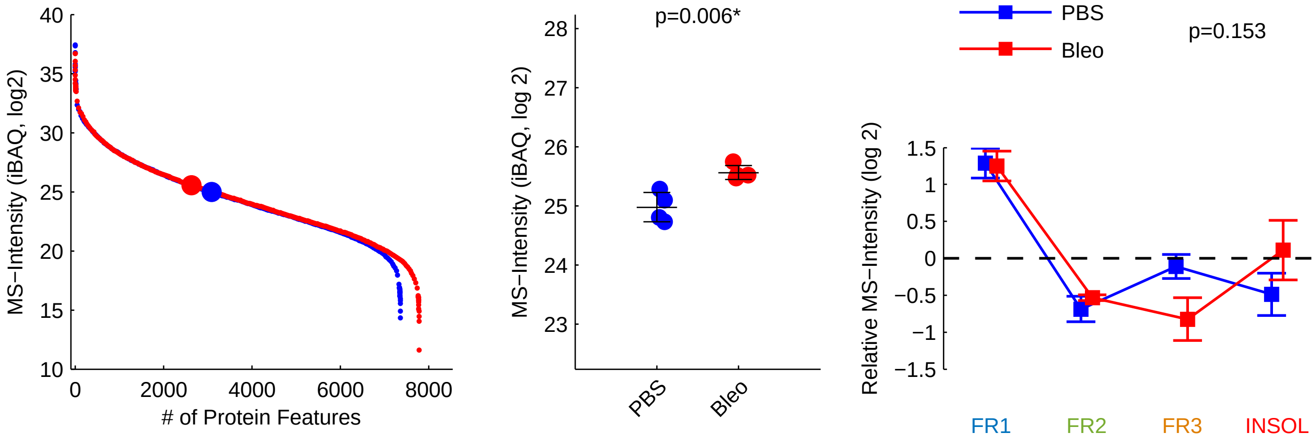

Q91Z40 − Gbp7 (id: 6817)

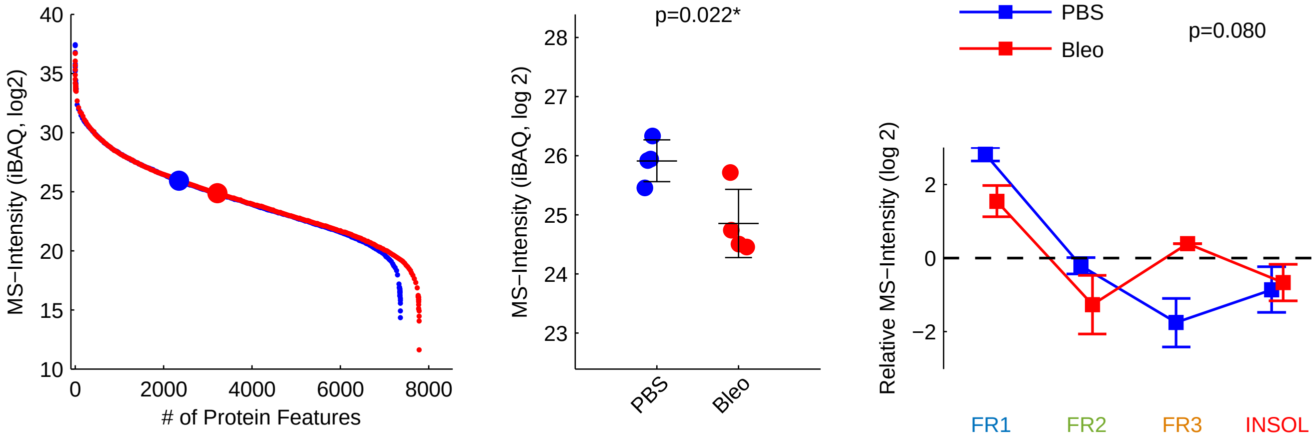

Q91Z53 − Grhpr (id: 6820)

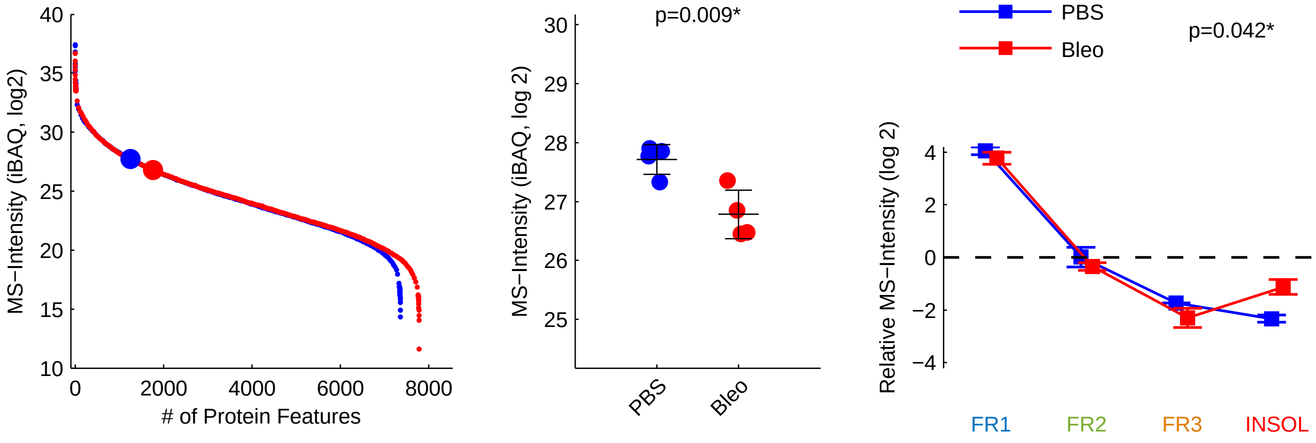

Q91Z58 − (id: 6821)

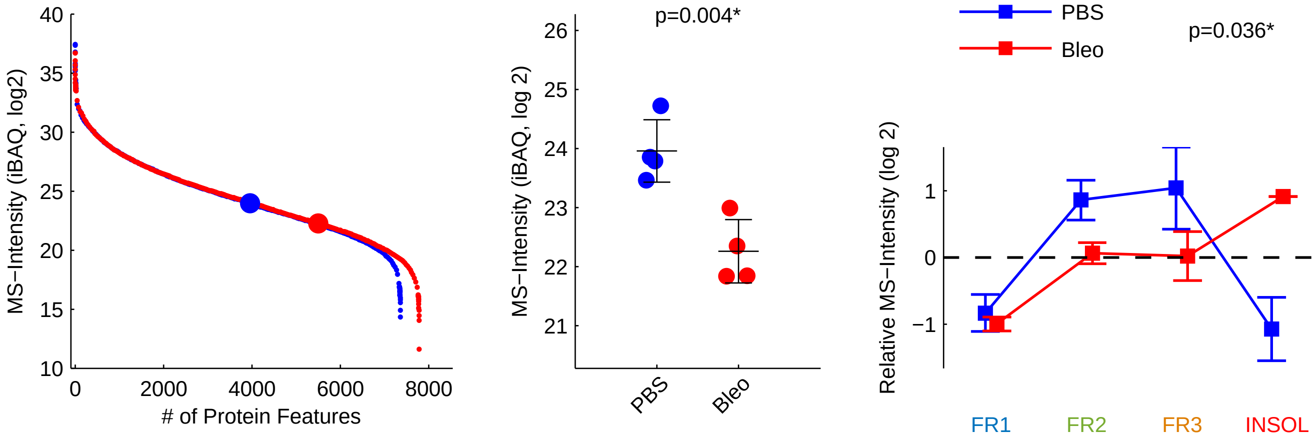

Q91Z96 − Bmp2k (id: 6825)

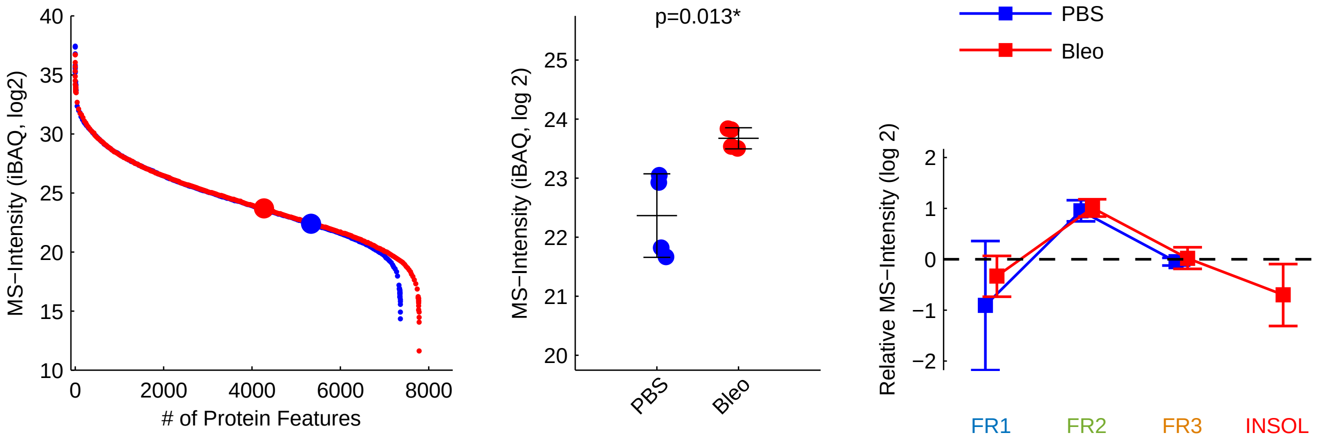

Q91ZA3 − Pcca (id: 6827)

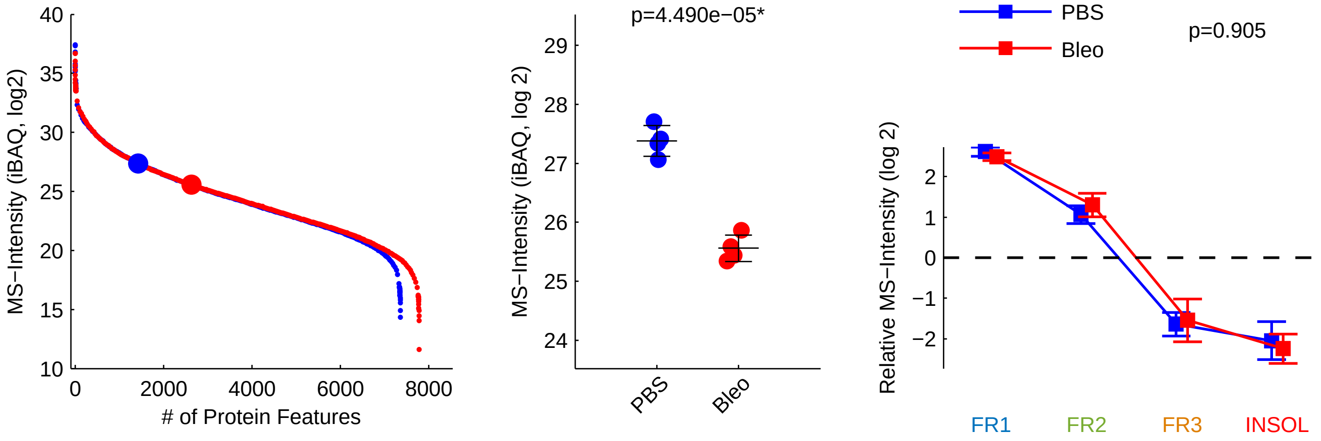

Q920A5 − Scpep1 (id: 6837)

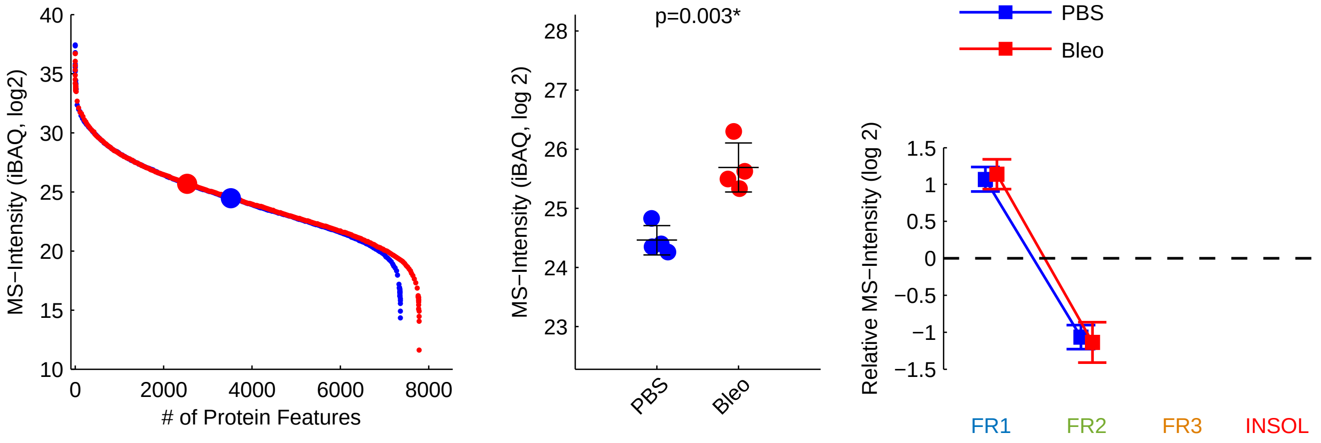

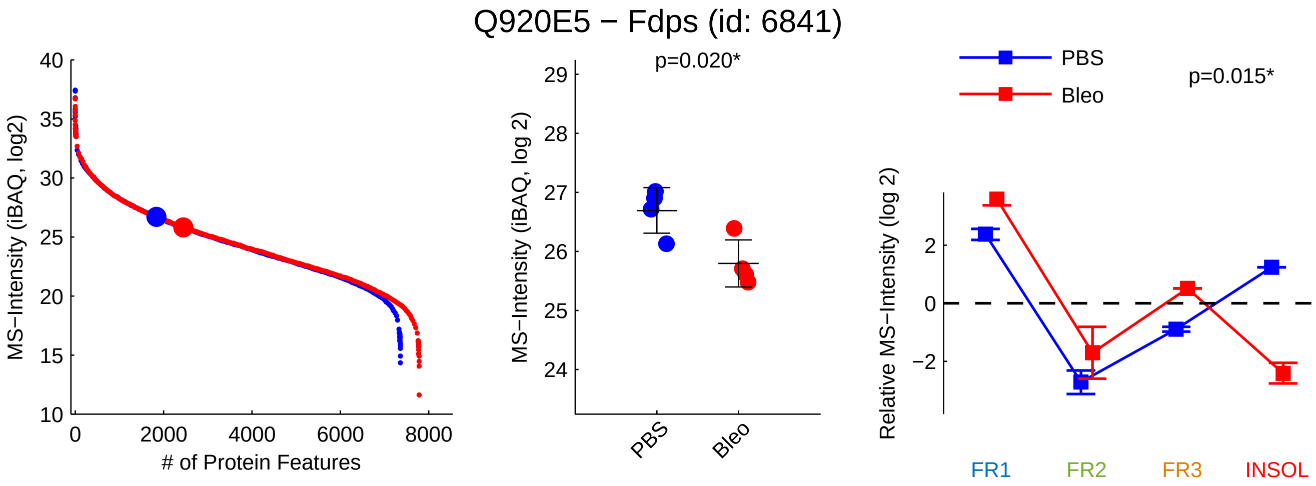
Q920E5 − Fdps (id: 6841)
MS−Intensity (iBAQ, log2)
# of Protein Features
0
2000
4000
6000
8000
10
15
20
25
30
35
40
p=0.020*
MS−Intensity (iBAQ, log 2)
24
25
26
27
28
29
PBS
Bleo
PBS
Bleo
p=0.015*
Relative MS−Intensity (log 2)
2
0
−2
FR1
FR2
FR3
INSOL

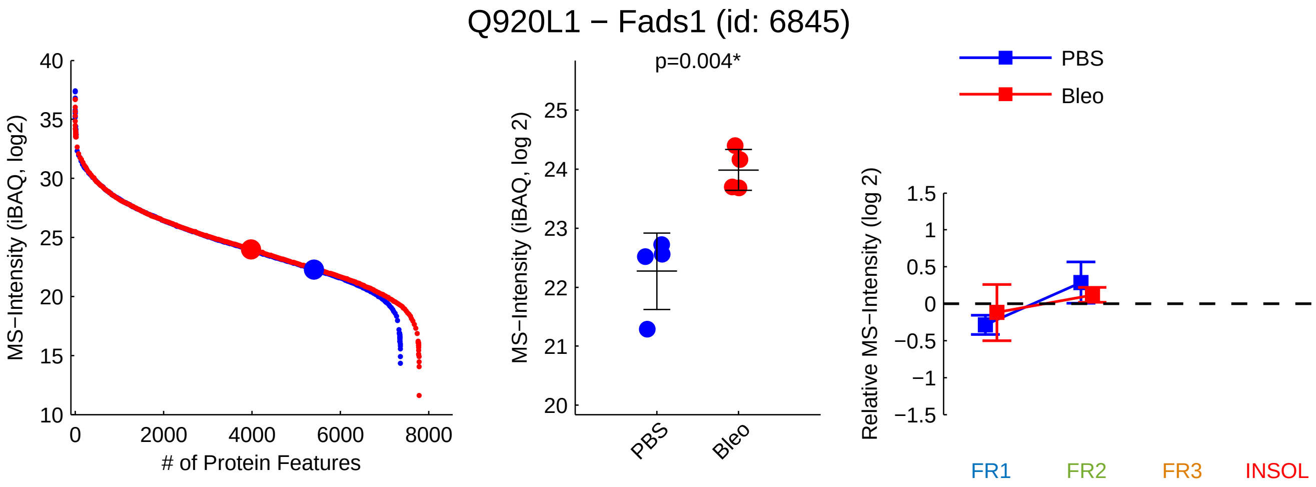
Q920L1 − Fads1 (id: 6845)
MS−Intensity (iBAQ, log2)
# of Protein Features
0
2000
4000
6000
8000
10
15
20
25
30
35
40
p=0.004*
MS−Intensity (iBAQ, log 2)
20
21
22
23
24
25
PBS
Bleo
PBS
Bleo
Relative MS−Intensity (log 2)
1.5
1
0.5
0
−0.5
−1
−1.5
FR1
FR2
FR3
INSOL

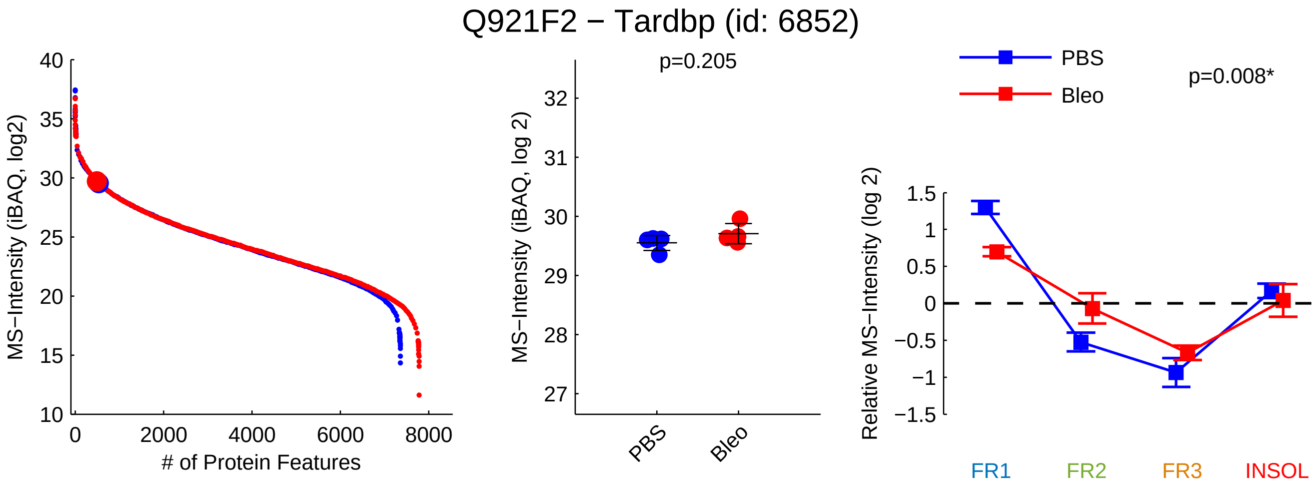
Q921F2 − Tardbp (id: 6852)
MS−Intensity (iBAQ, log2)
# of Protein Features
0
2000
4000
6000
8000
10
15
20
25
30
35
40
p=0.205
MS−Intensity (iBAQ, log 2)
27
28
29
30
31
32
PBS
Bleo
PBS
Bleo
p=0.008*
Relative MS−Intensity (log 2)
1.5
1
0.5
0
−0.5
−1
−1.5
FR1
FR2
FR3
INSOL

Q921I1 − Tf (id: 6859)

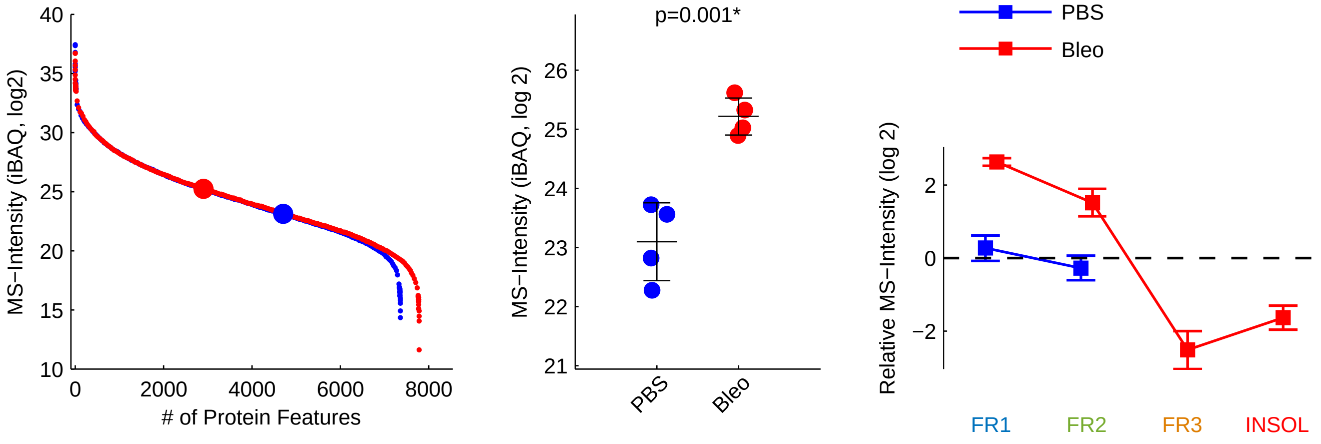

Q921I6 − Sh3bp4 (id: 6860)

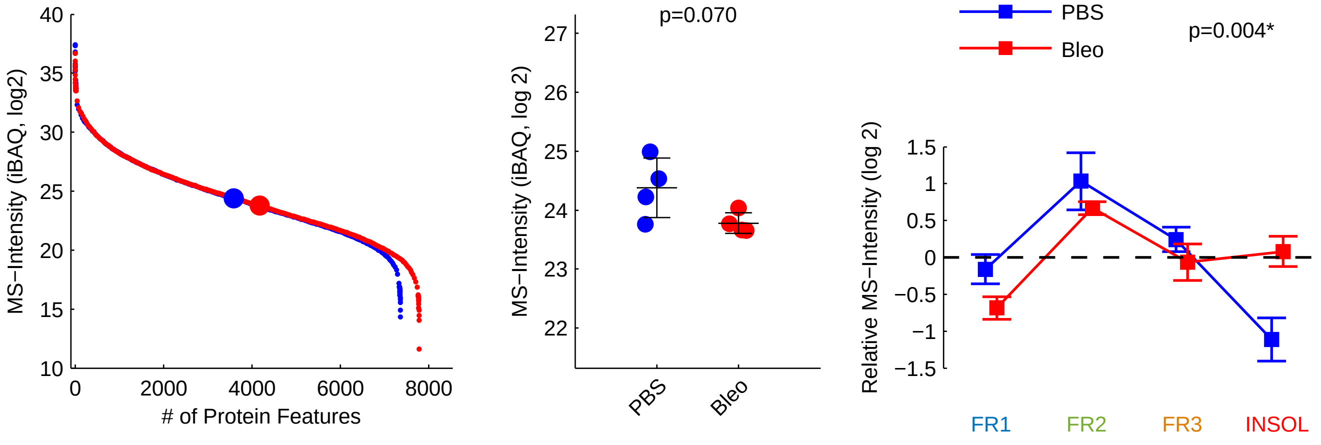

Q921I9 − Exosc4 (id: 6861)

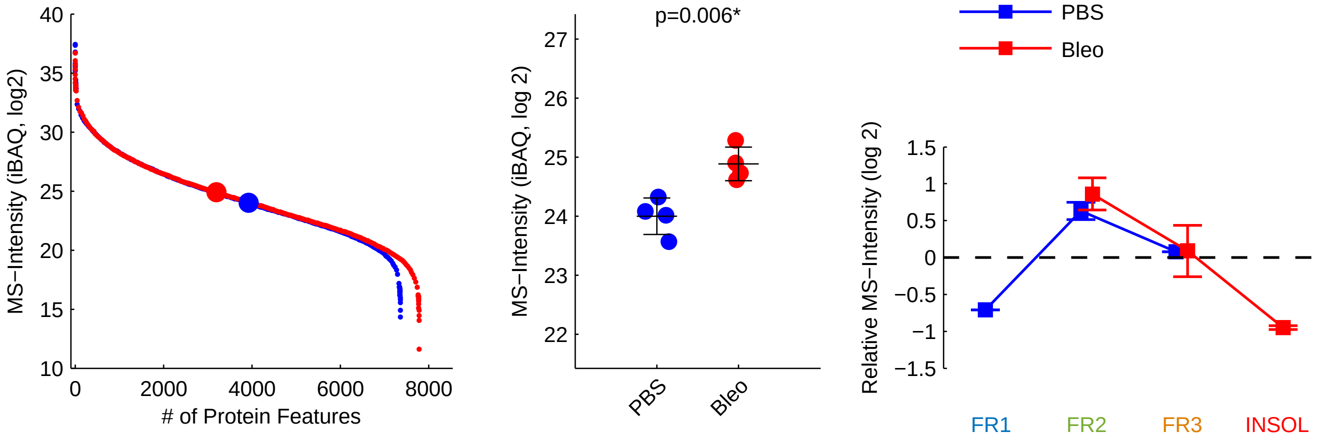

Q921J2 − Rheb (id: 6862)

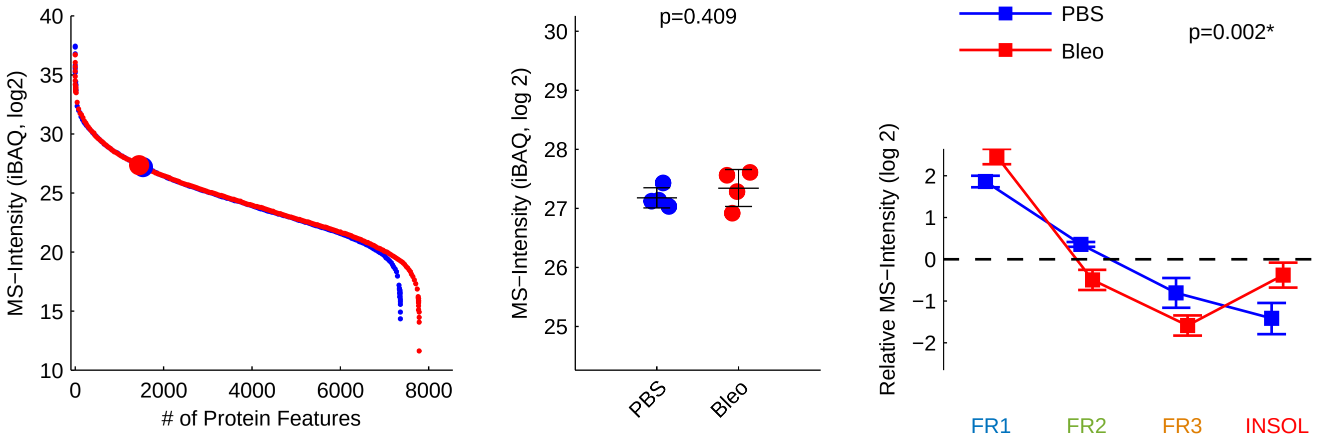

Q921K2 − Parp1 (id: 6863)

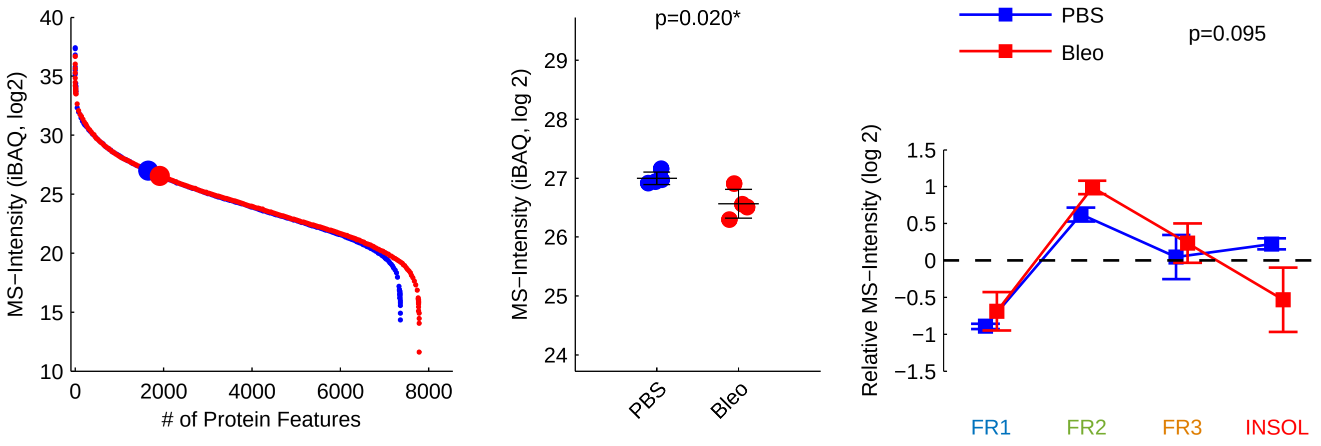

Q921K8 − Fam115c (id: 6864)

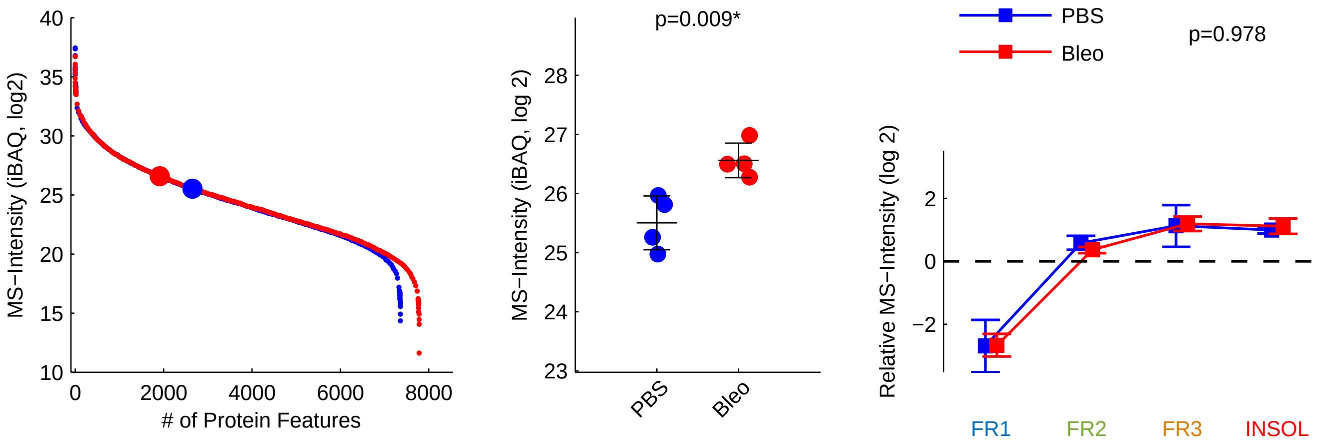

Q921L3 − Tmco1 (id: 6865)

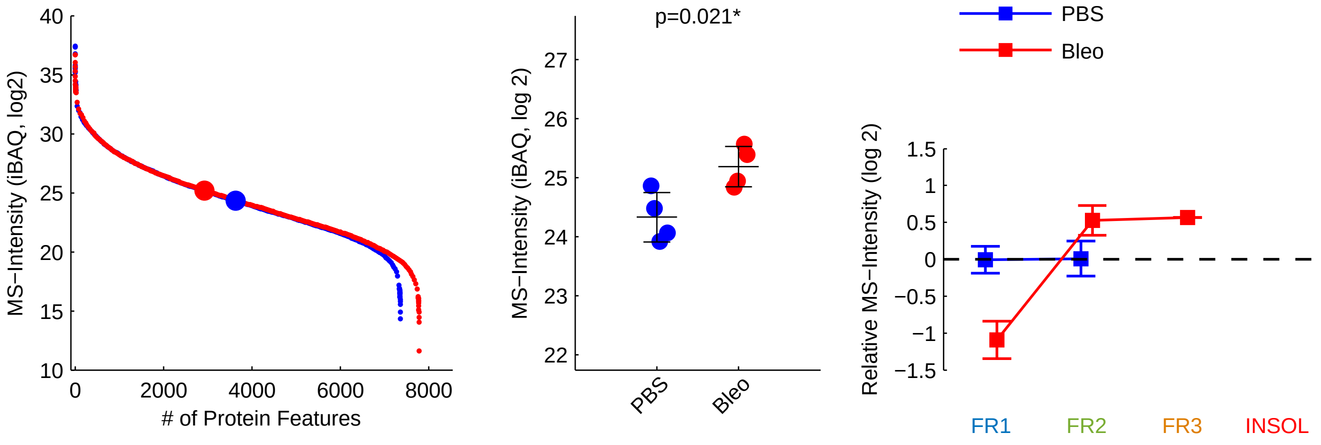

Q921T2−2 − Tor1aip1 (id: 6875)

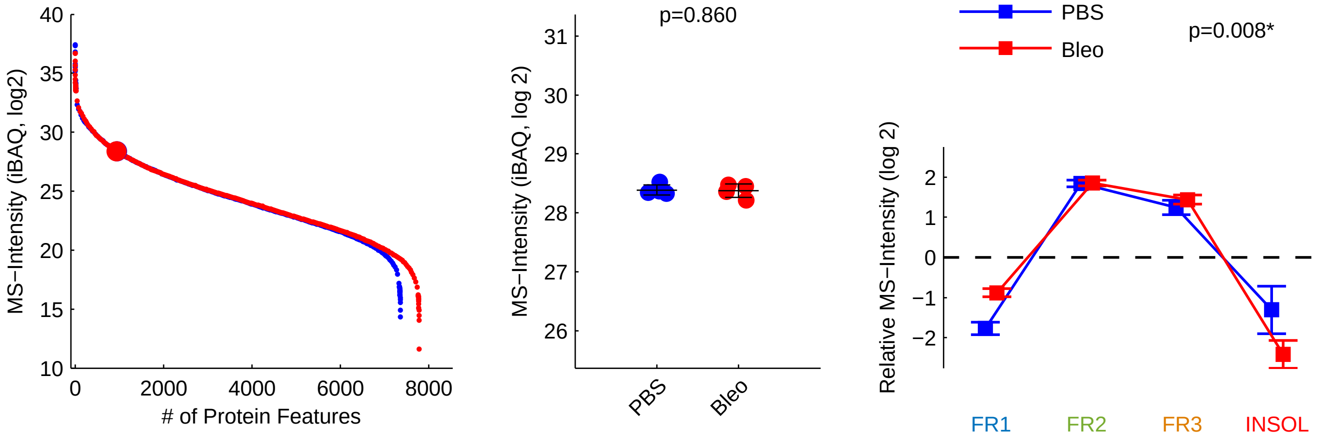

Q921X9 − Pdia5 (id: 6880)

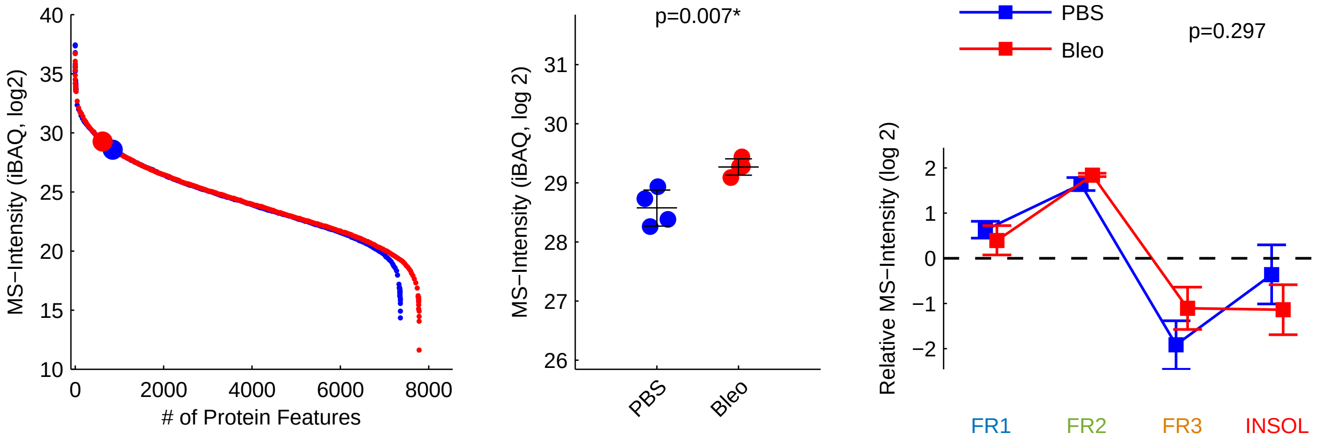

Q921Z5 − Tnfaip8 (id: 6883)

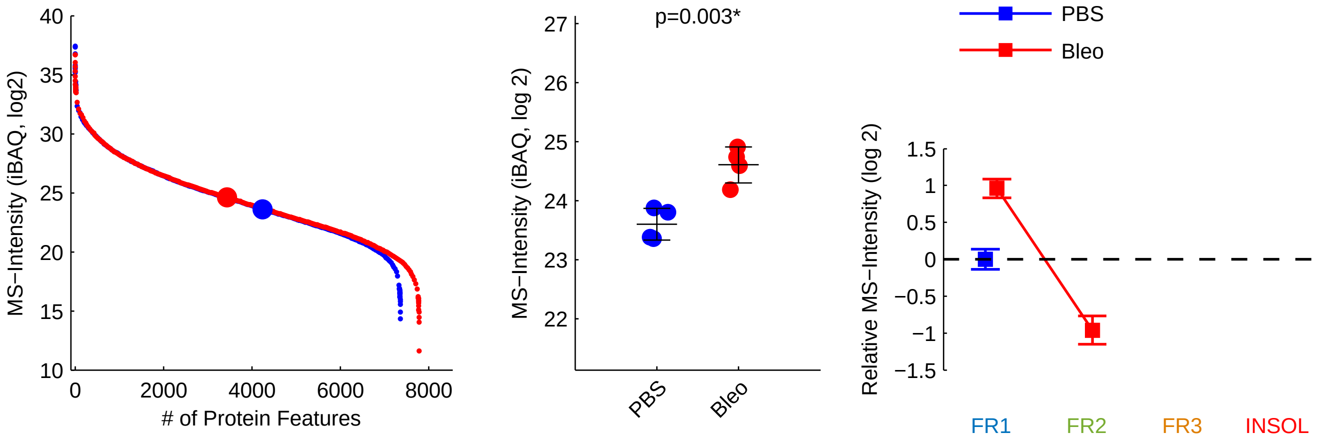

Q922F4 − Tubb6 (id: 6887)

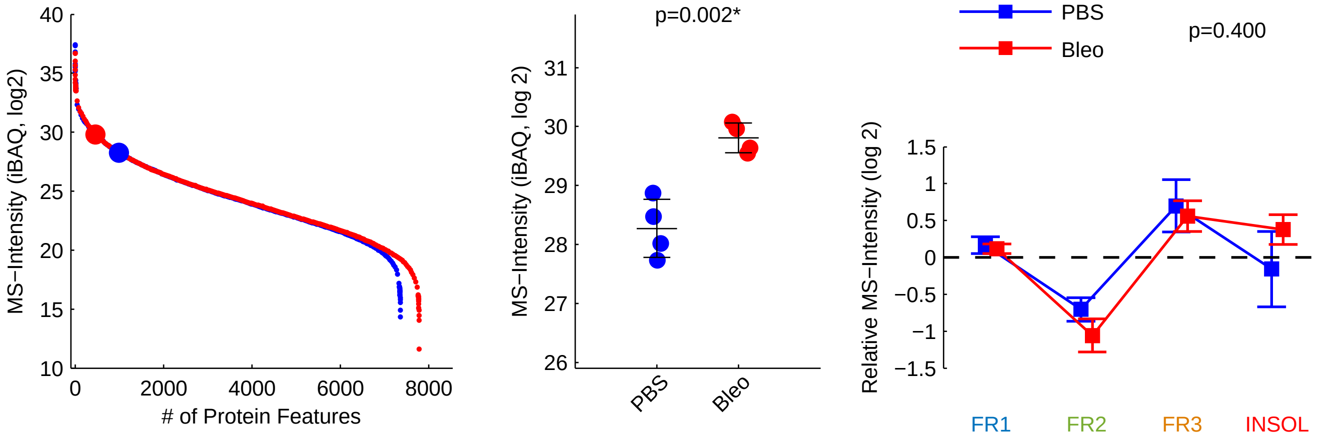

Q922H4 − Gmppa (id: 6891)

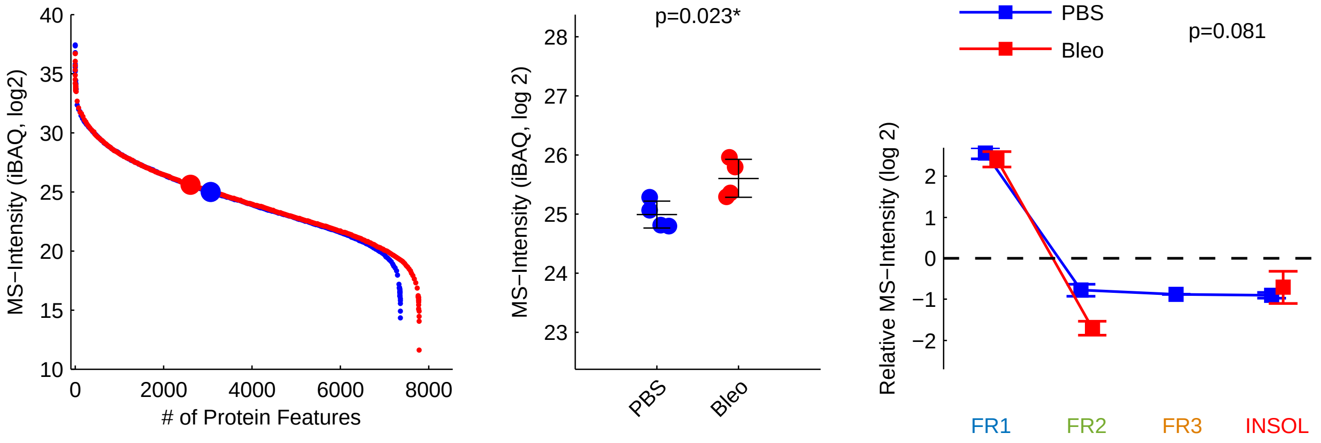

Q922J9 − Far1 (id: 6892)

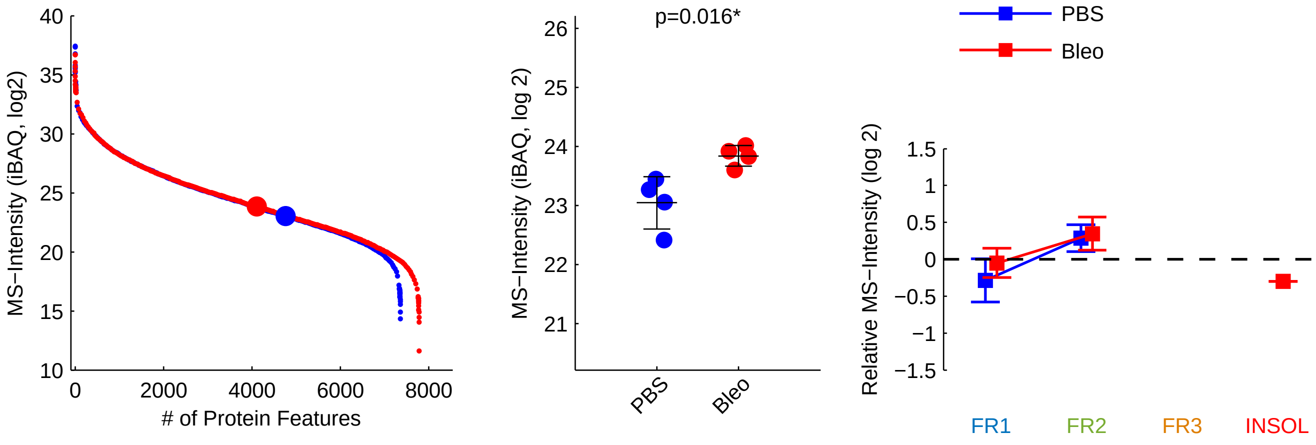

Q922M3 − Kctd10 (id: 6893)

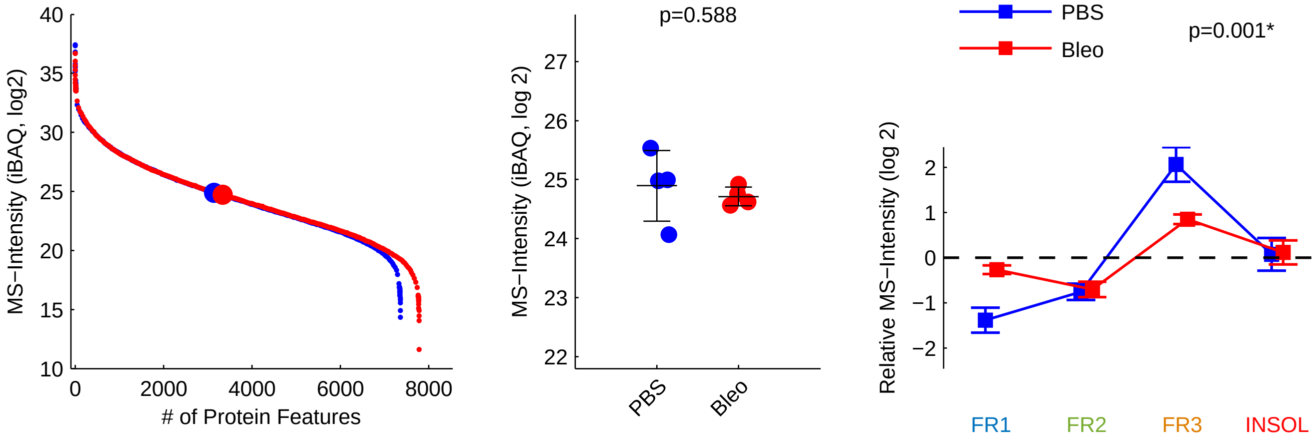

Q922Q8 − Lrrc59 (id: 6898)

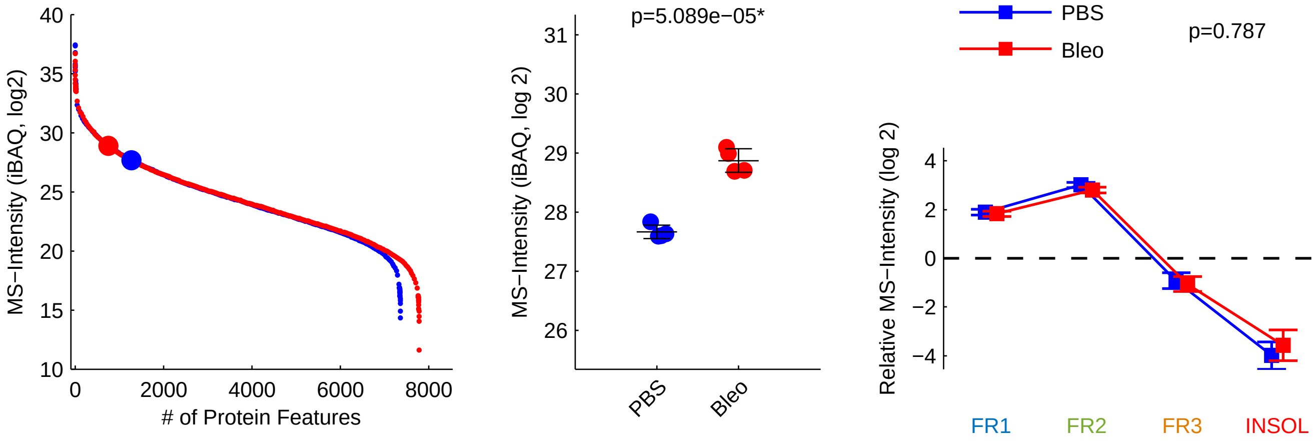

Q924T2 − Mrps2 (id: 6928)

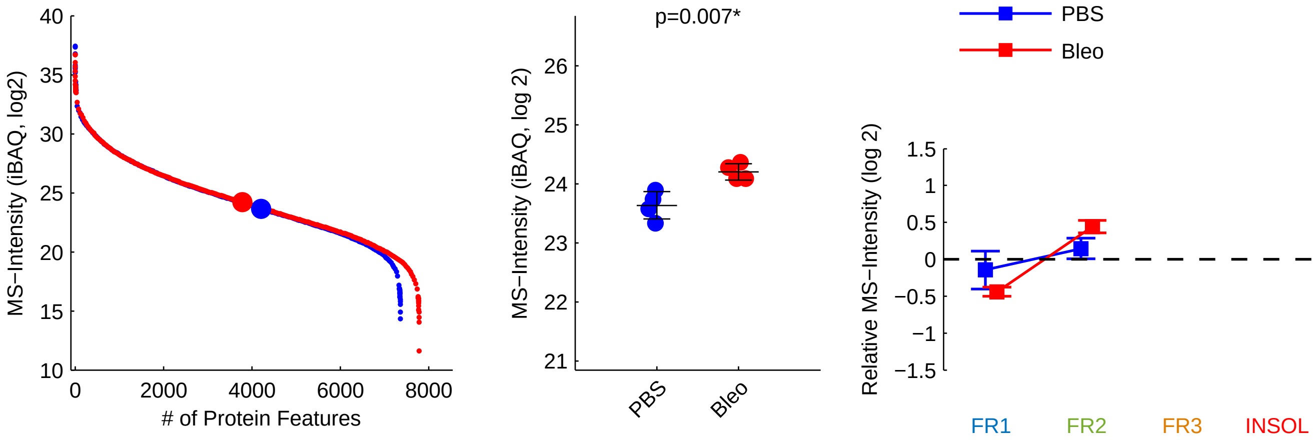

Q925F2 − Esam (id: 6936)

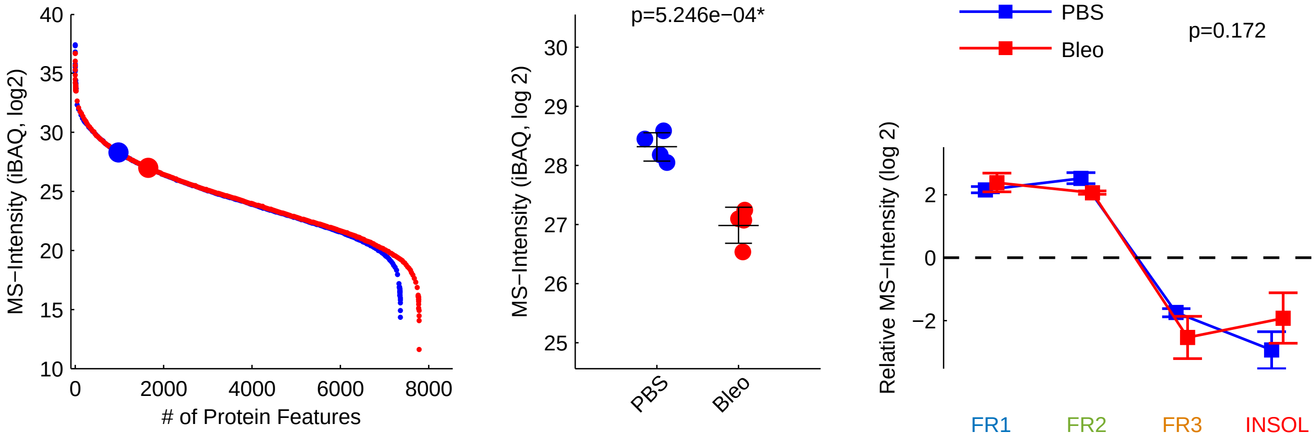

Q925N0 − Sfxn5 (id: 6941)

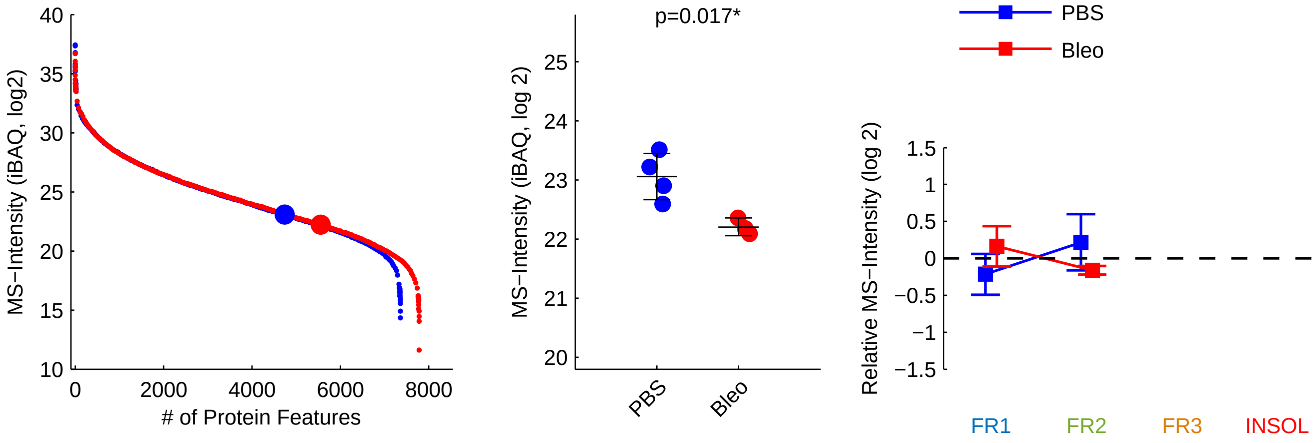

Q99020 − Hnrnpab (id: 6944)

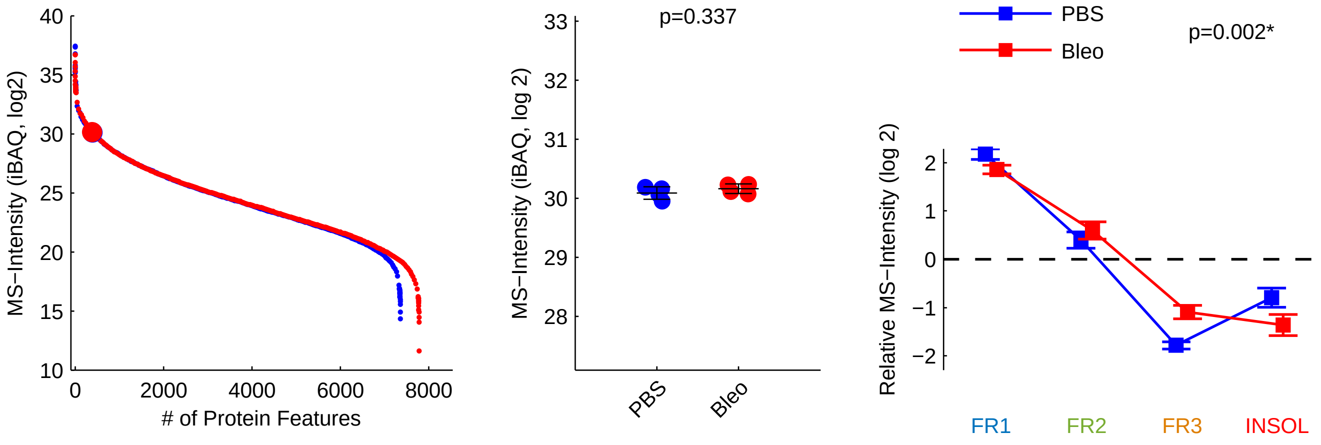

Q99104 − Myo5a (id: 6945)

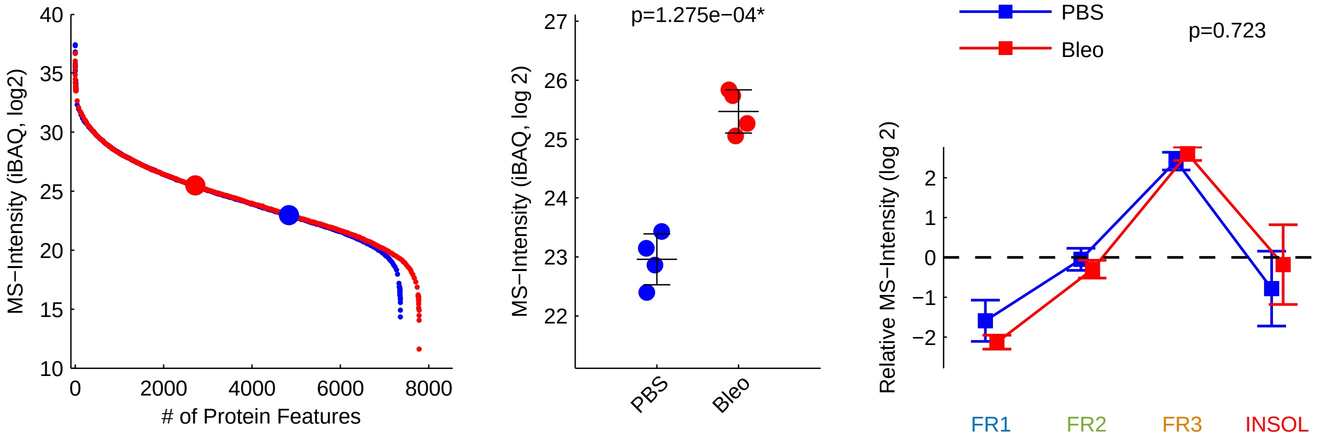

Q99J08 − Sec14l2 (id: 6948)

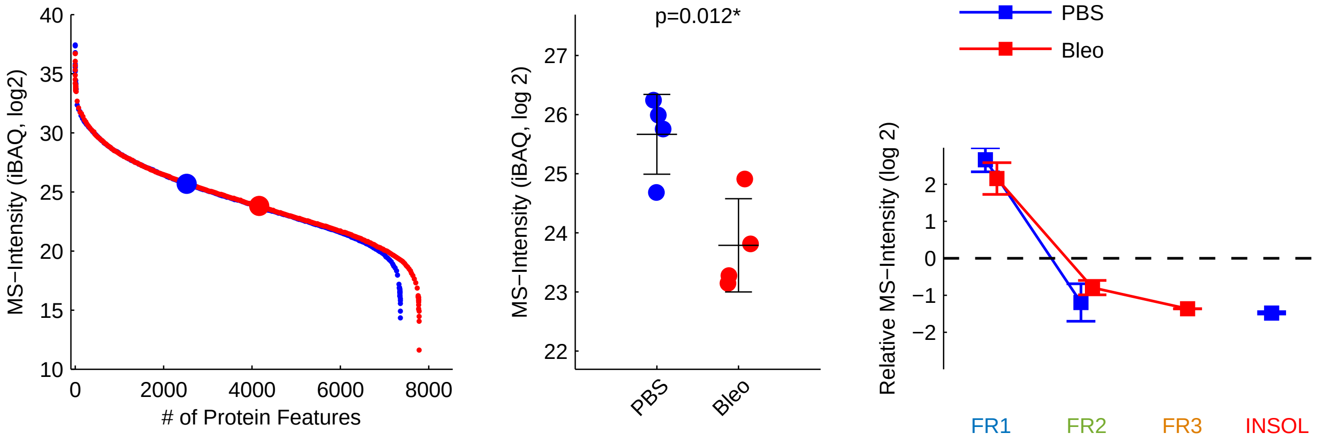

Q99J39 − Mlycd (id: 6955)

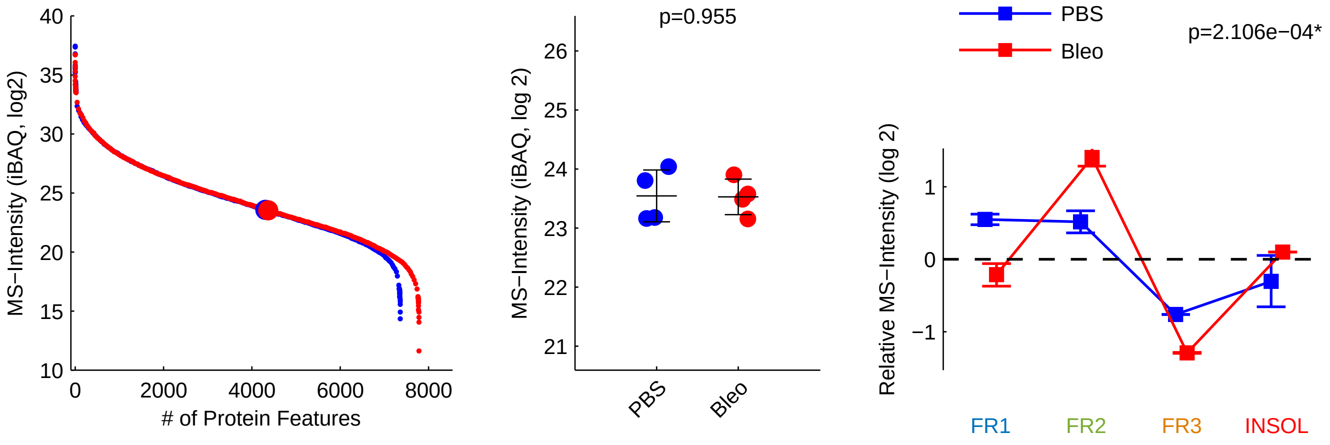

Q99JF8 − Psip1 (id: 6965)

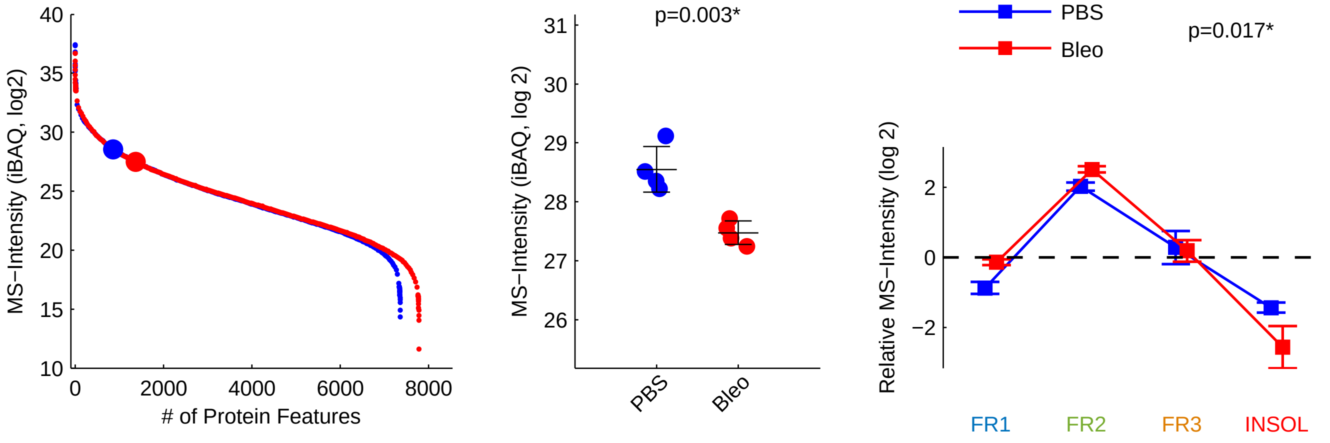

Q99JI4 − Psmd6 (id: 6968)

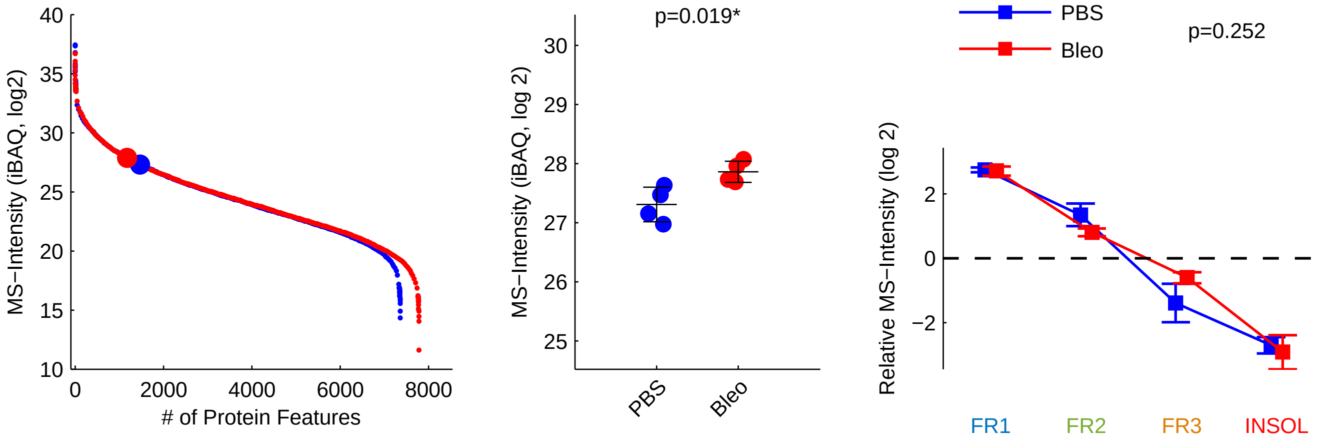

Q99JI6 − Rap1b (id: 6969)

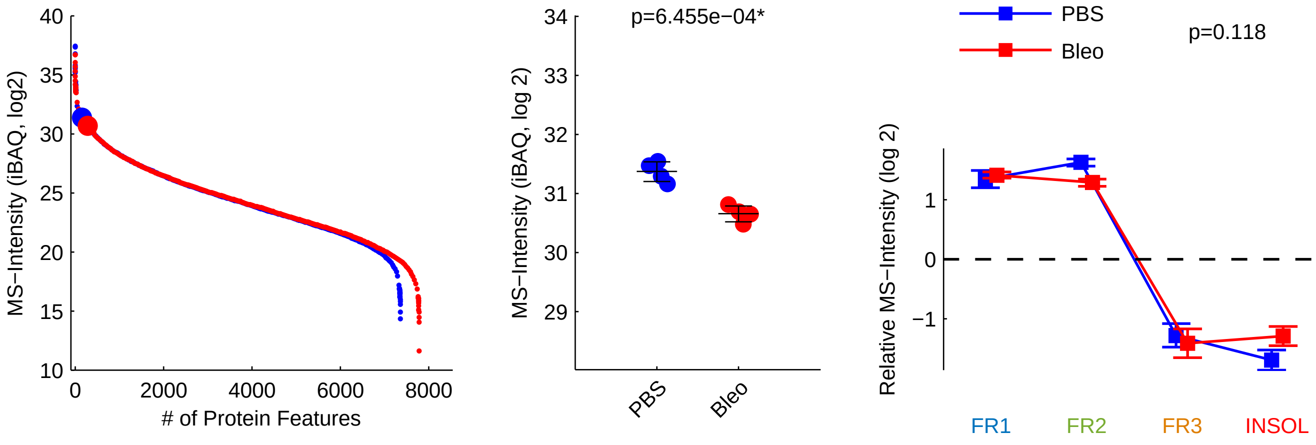

Q99JP6−2 − Homer3 (id: 6972)

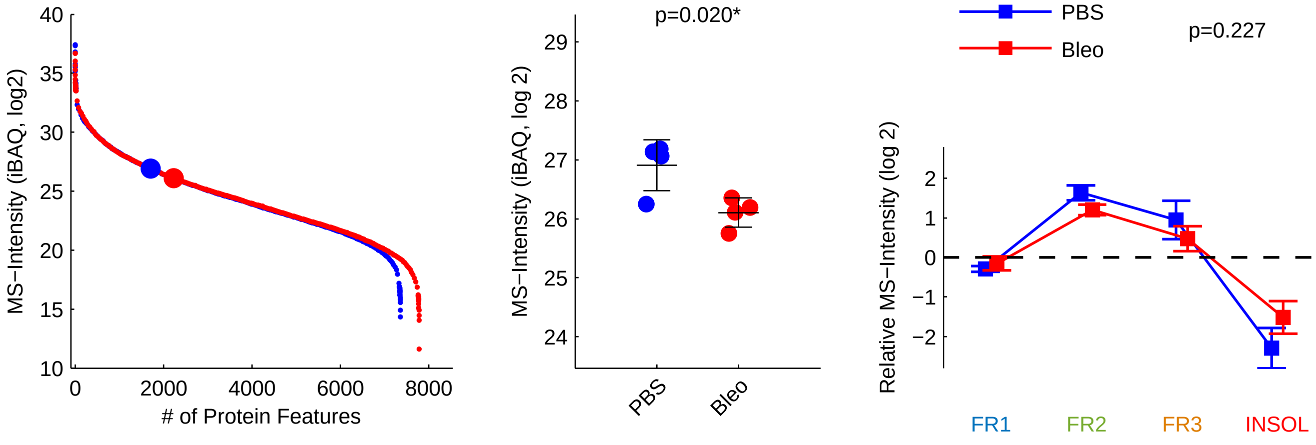

Q99JR8 − Smarcd2 (id: 6975)

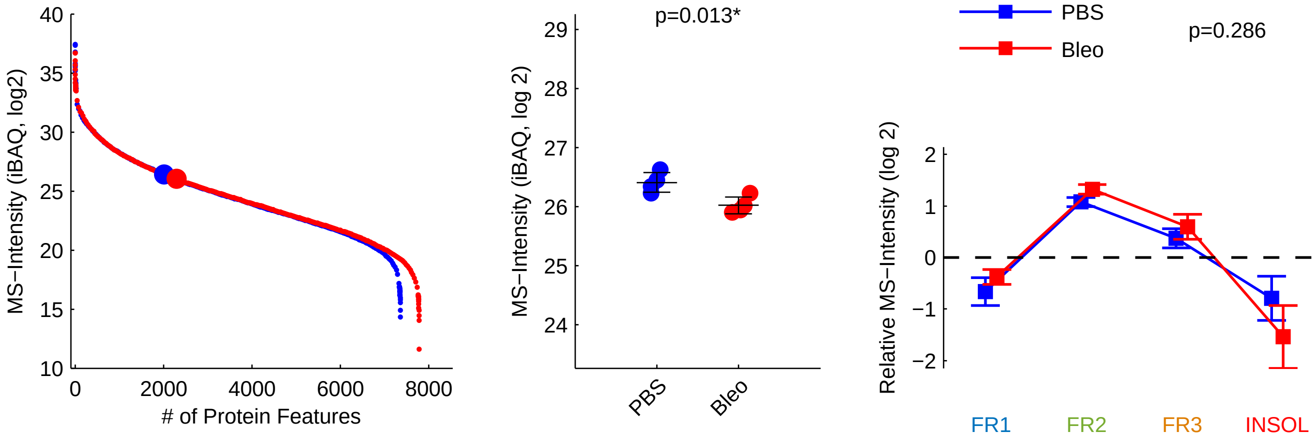

Q99JW2 − Acy1 (id: 6977)

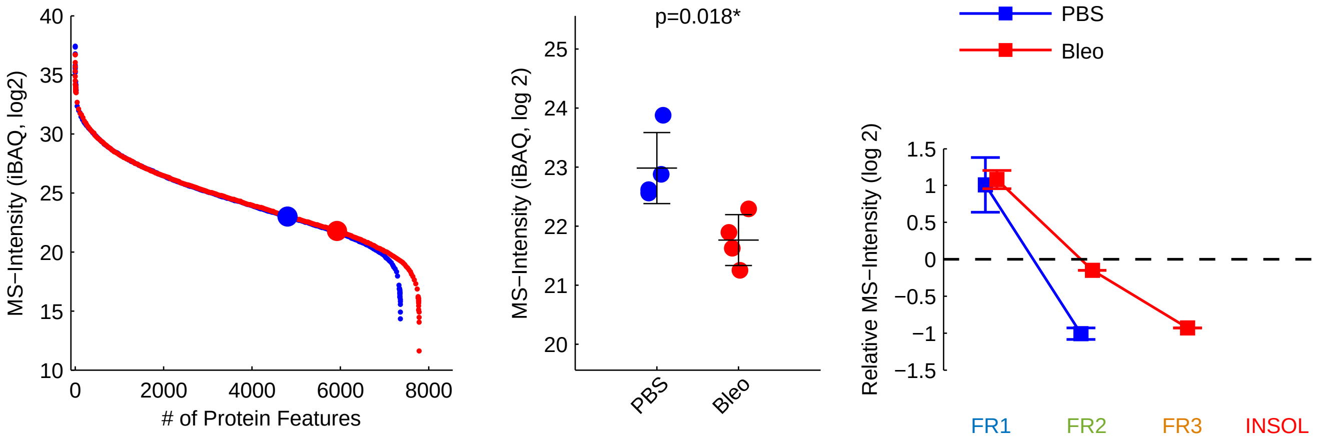

Q99JW5 − Epcam (id: 6979)

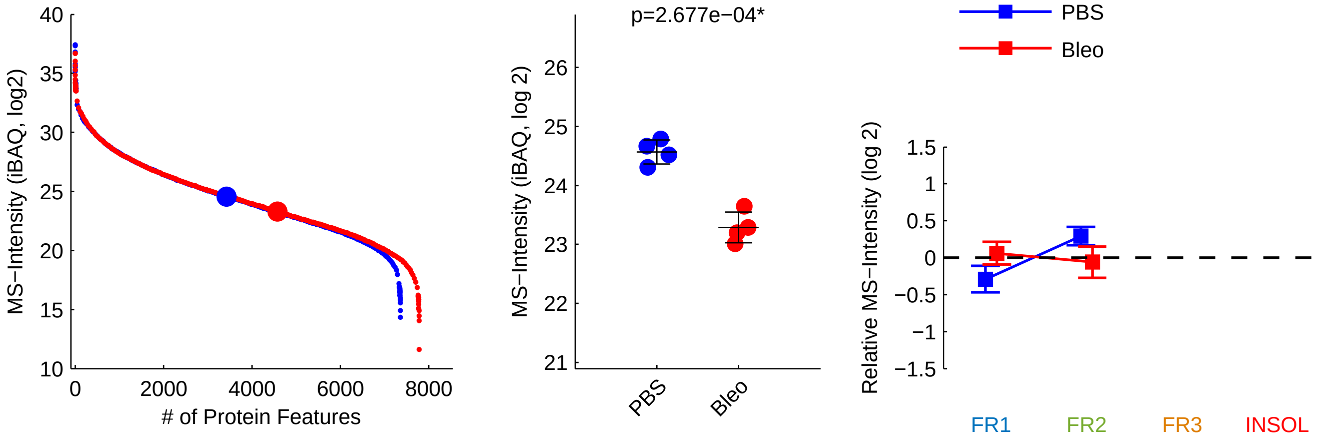

Q99JX4 − Eif3m (id: 6981)

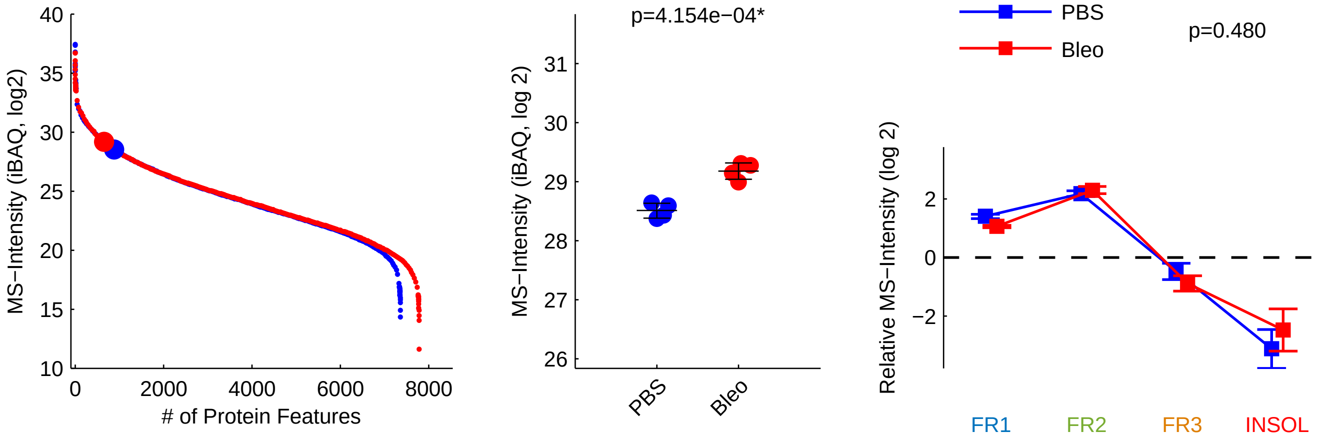

Q99JY0 − Hadhb (id: 6983)

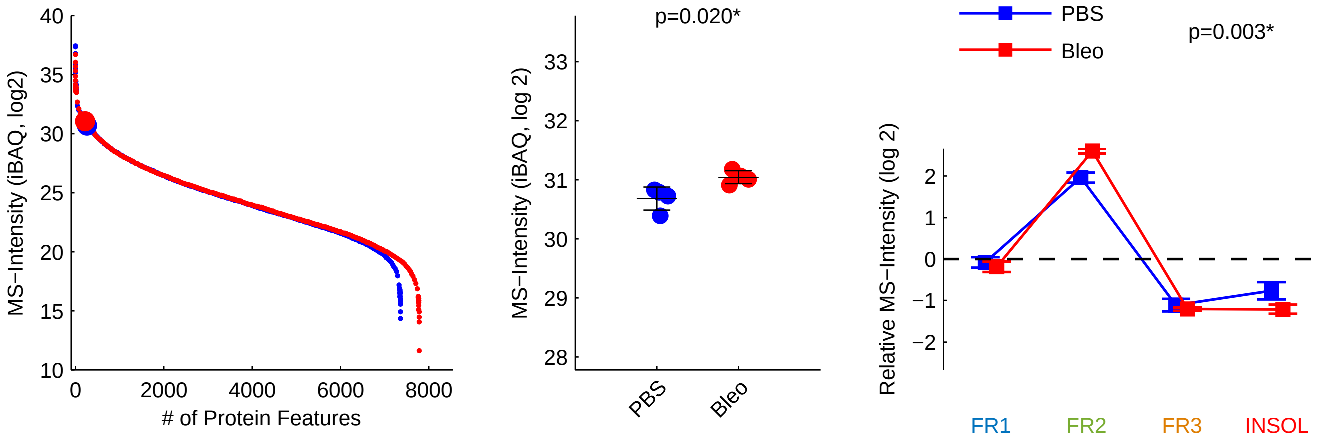

Q99JY6 − Higd1b (id: 6985)

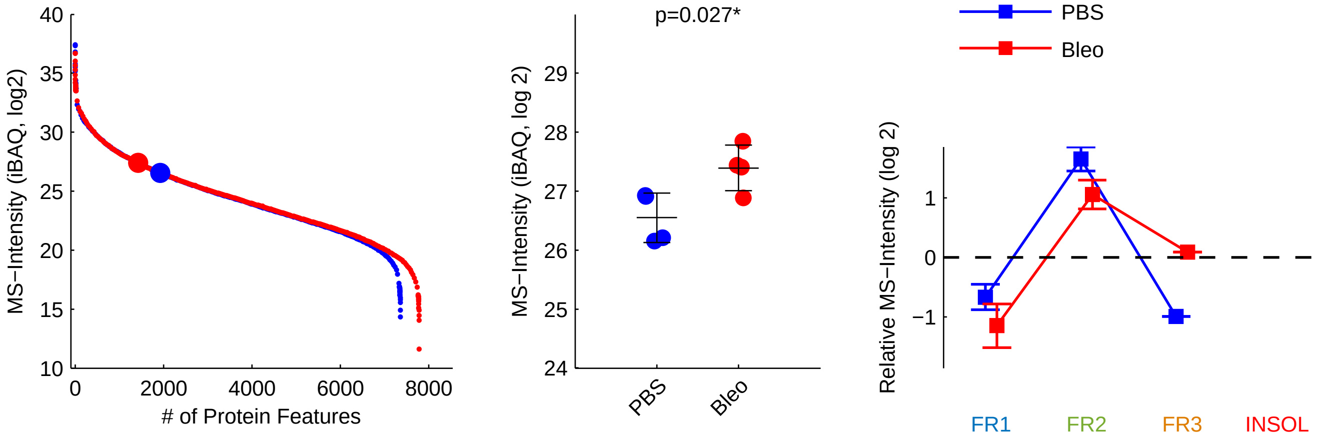

Q99JY8 − Ppap2b (id: 6986)

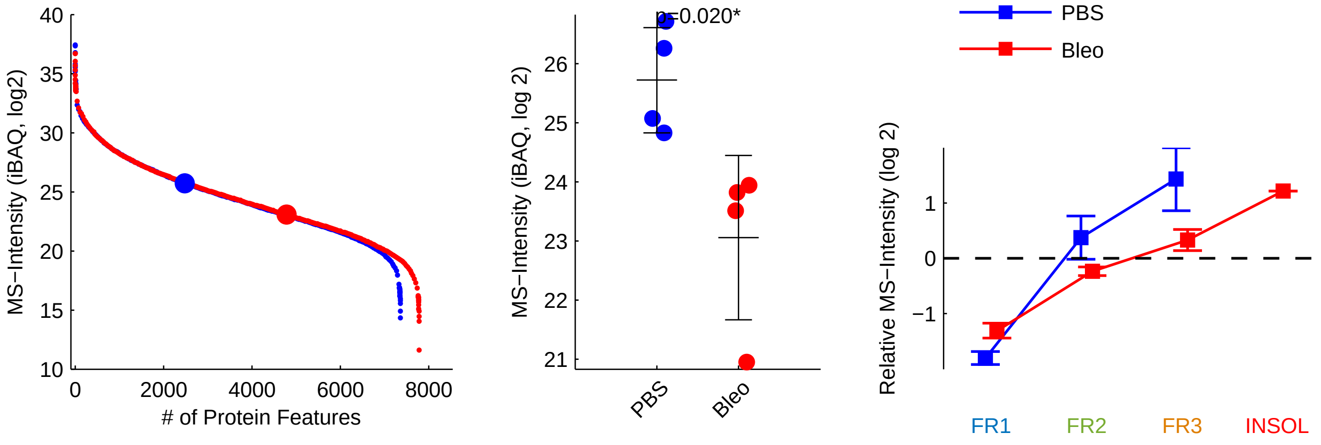

Q99JY9 − Actr3 (id: 6987)

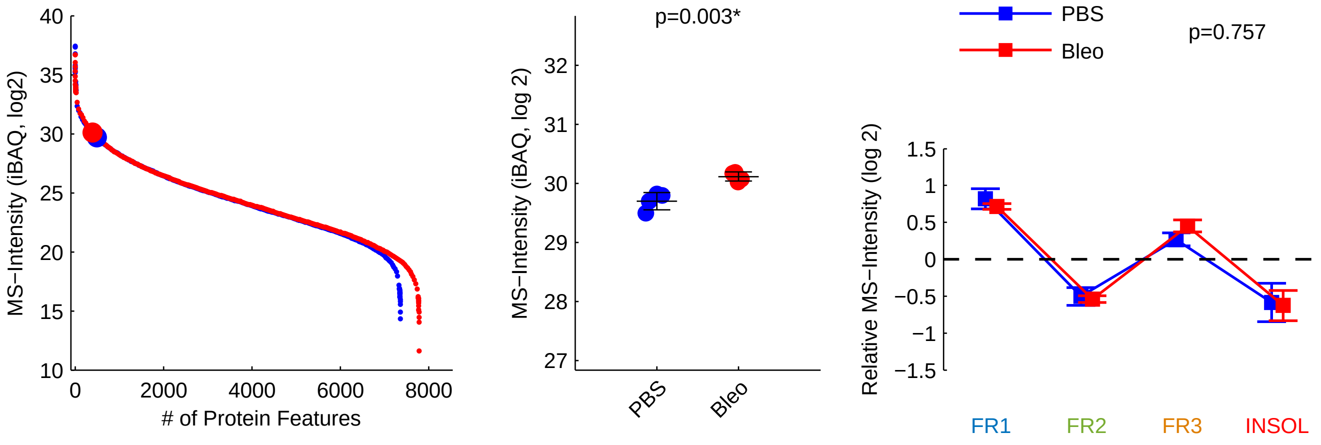

Q99K01−2 − Pdxdc1 (id: 6990)

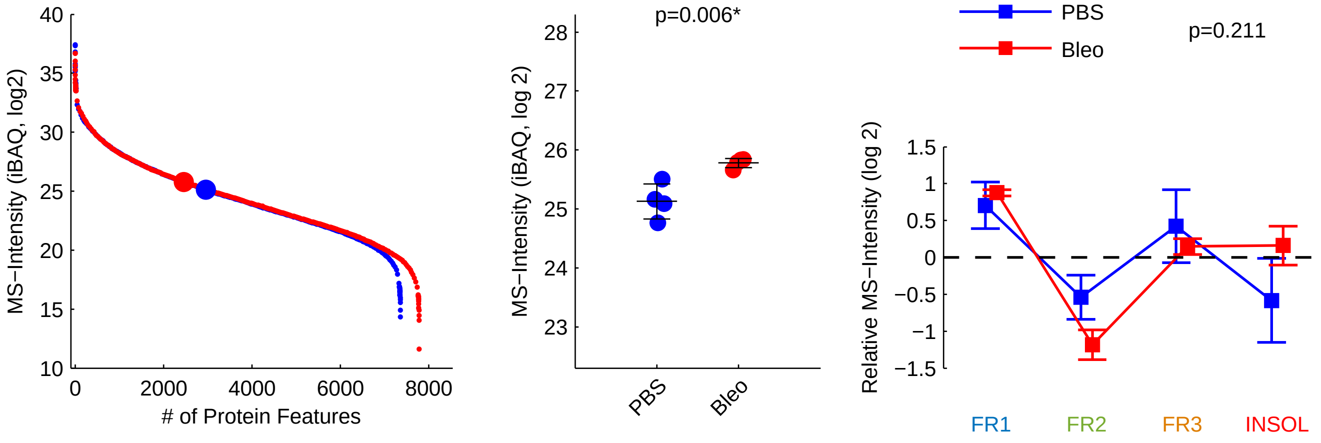

Q99K28 − Arfgap2 (id: 6992)

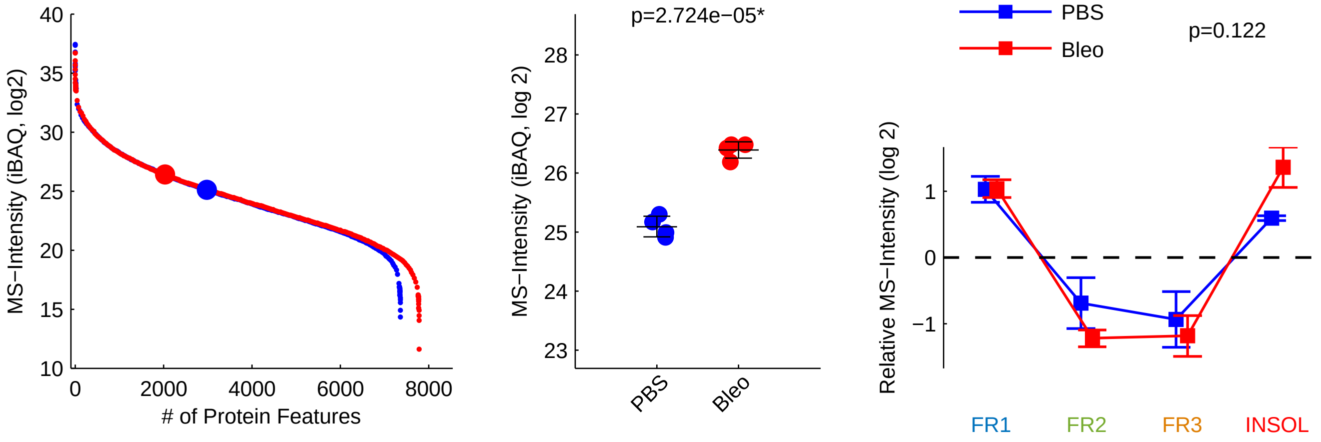

Q99K41 − Emilin1 (id: 6994)

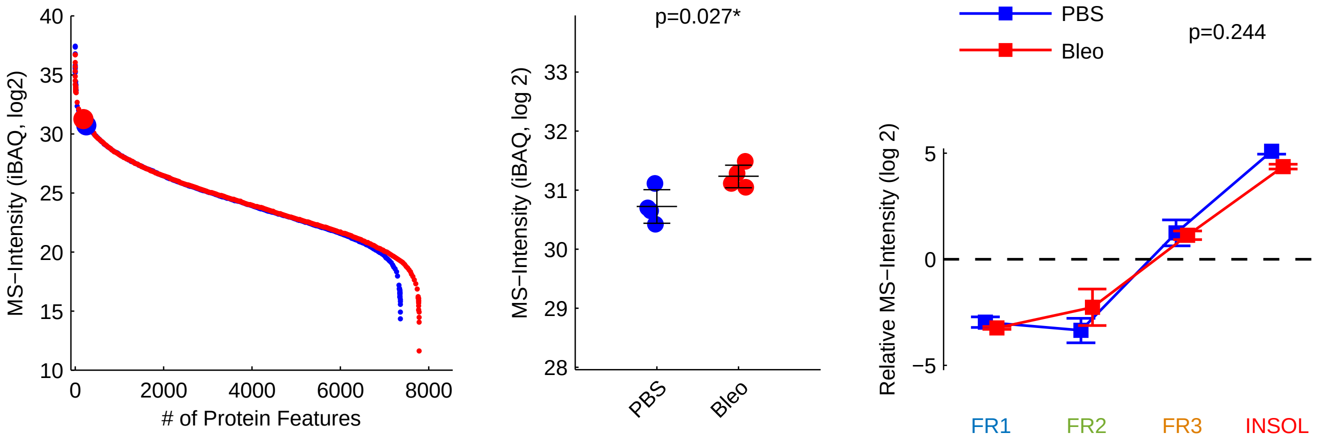

Q99K85 − Psat1 (id: 7003)

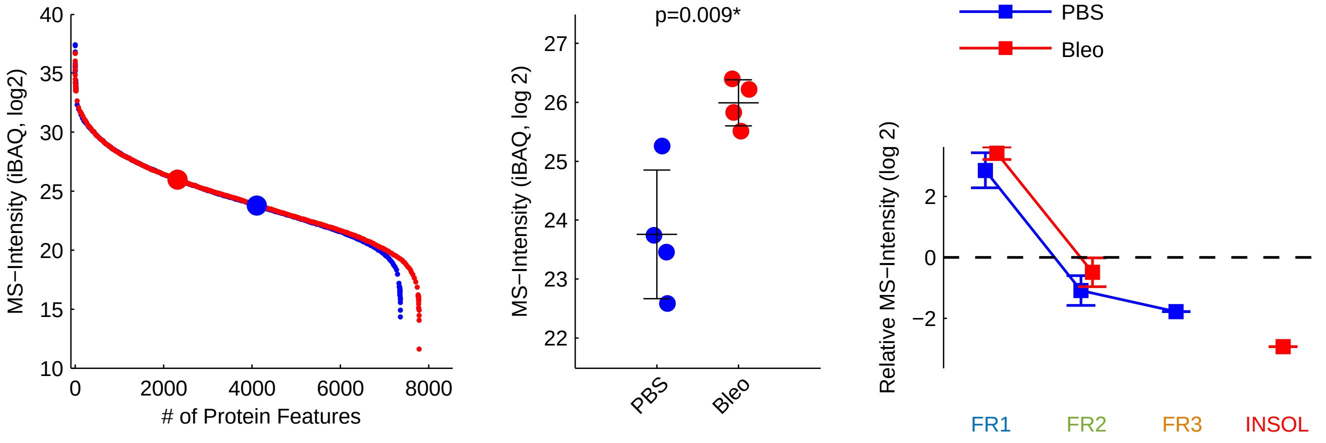

Q99KB8−2 − Hagh (id: 7005)

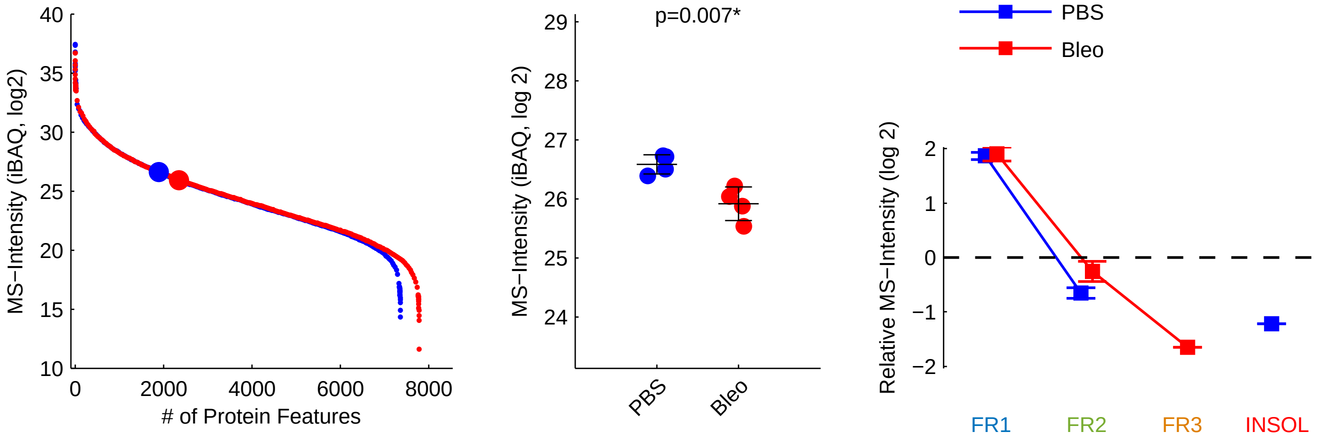

Q99KP3 − Cryl1 (id: 7022)

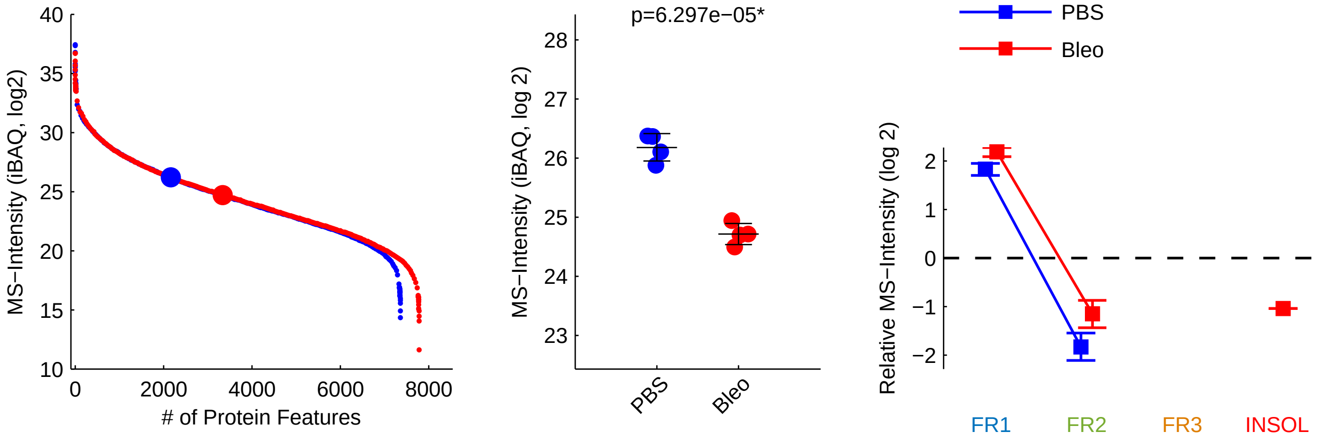

Q99KV1 − Dnajb11 (id: 7030)

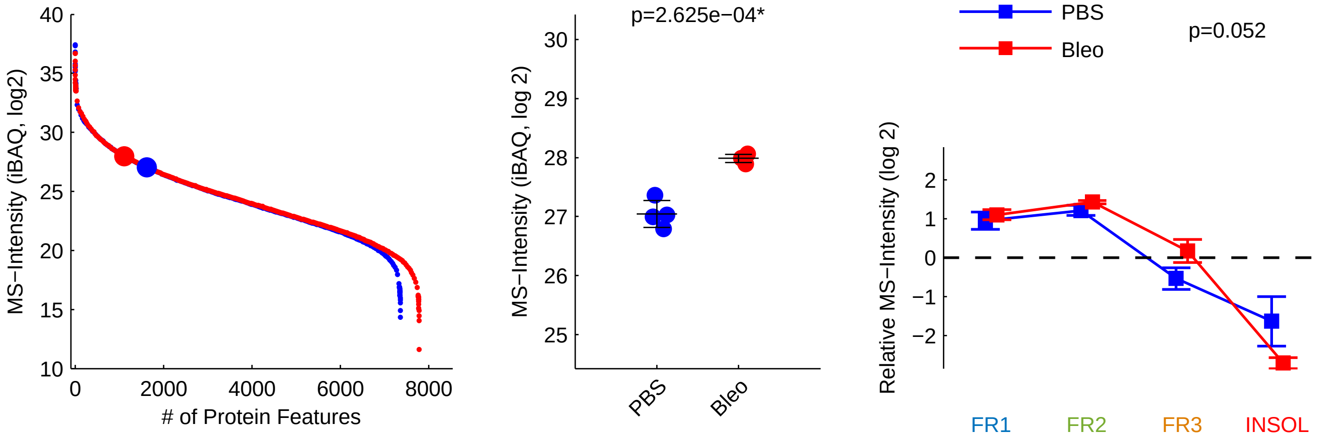

Q99L13 − Hibadh (id: 7038)

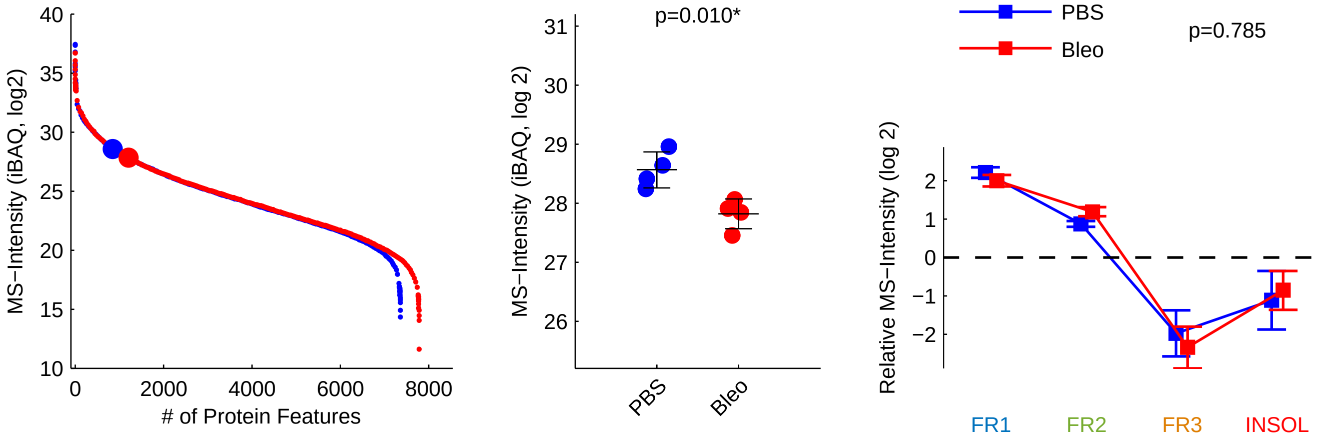

Q99L20 − Gstt3 (id: 7039)

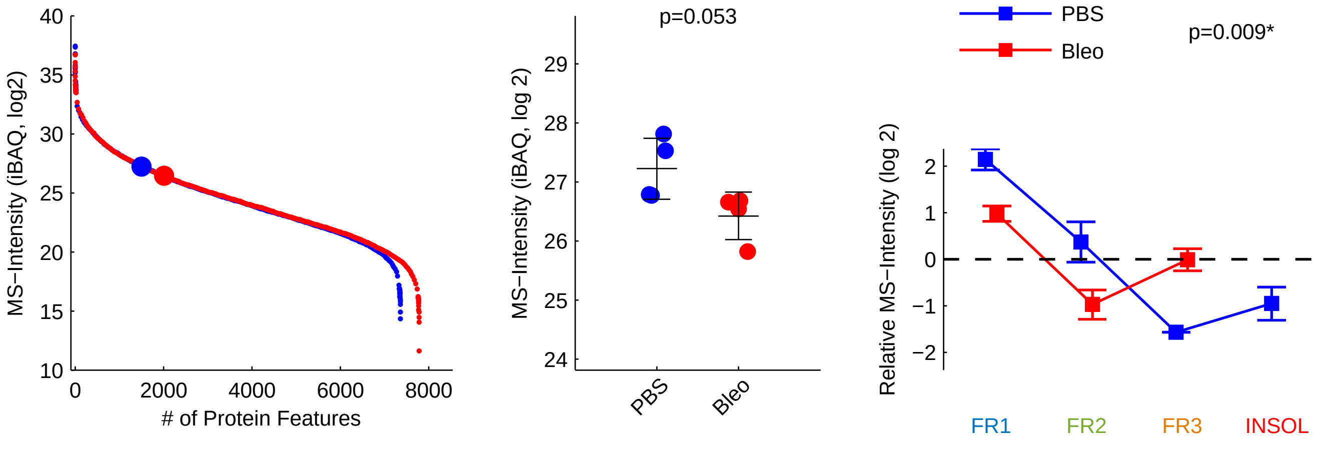

Q99L45 − Eif2s2 (id: 7042)

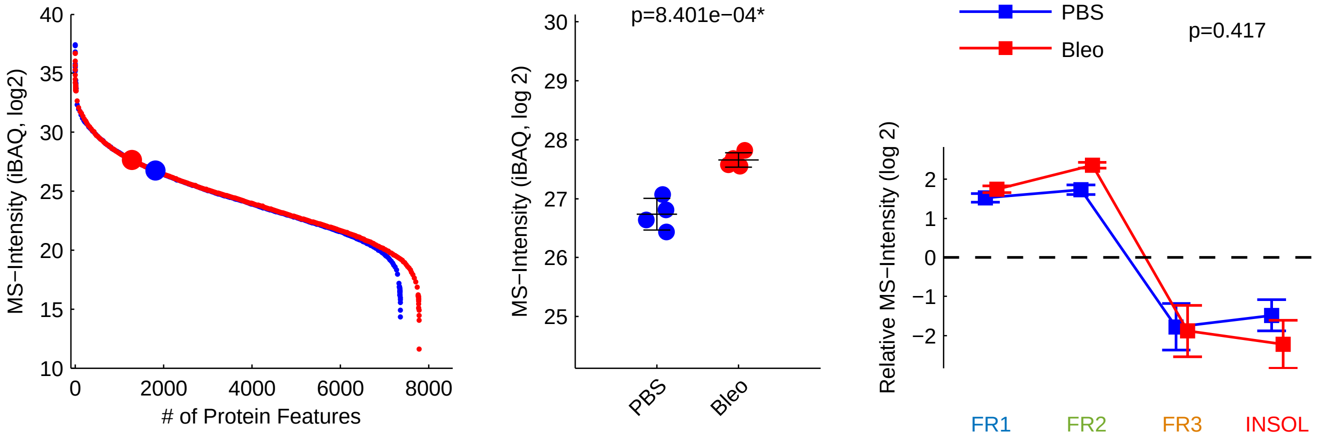

Q99L88 − Sntb1 (id: 7045)

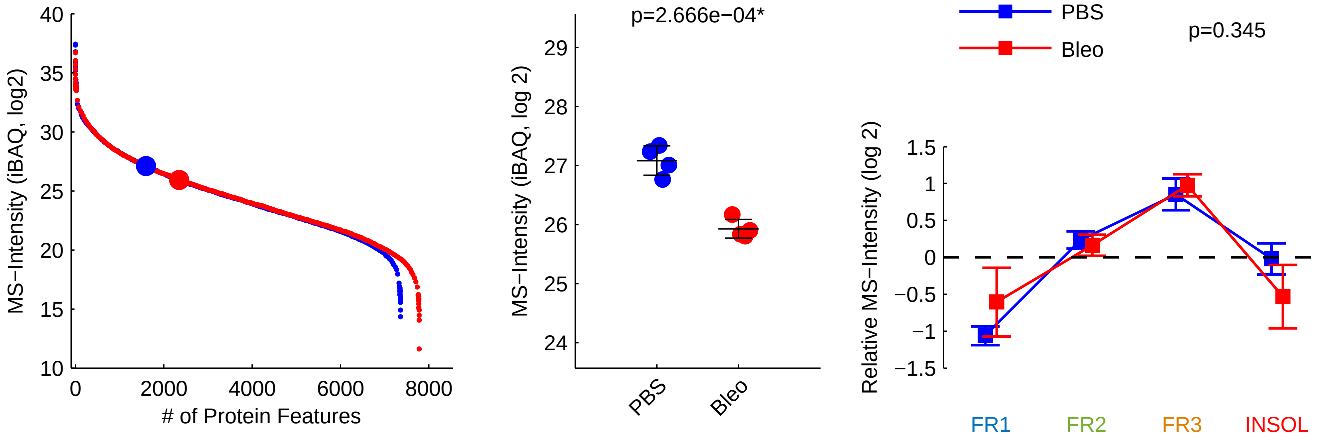

Q99LB2 − Dhrs4 (id: 7047)

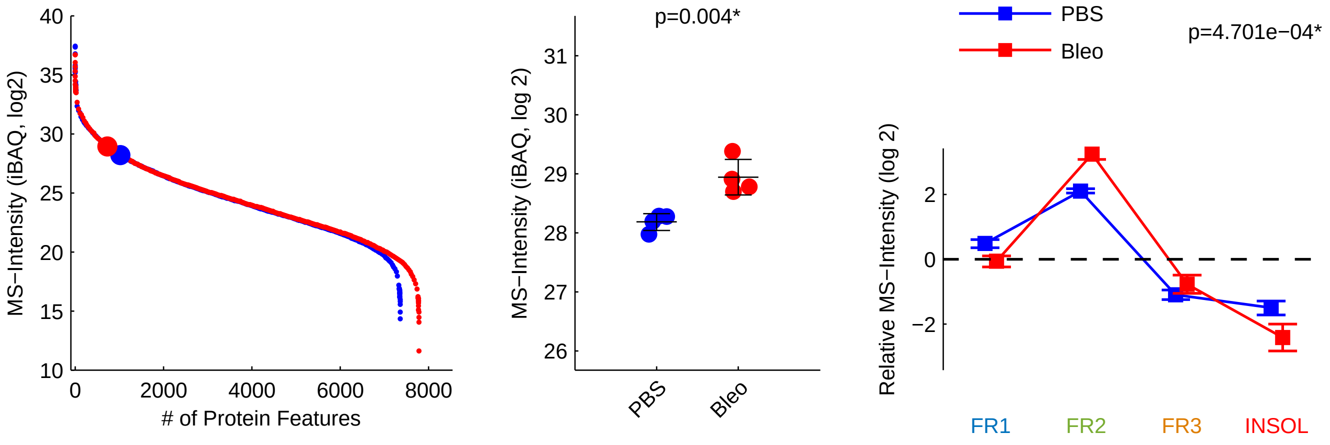

Q99LB4 − Capg (id: 7048)

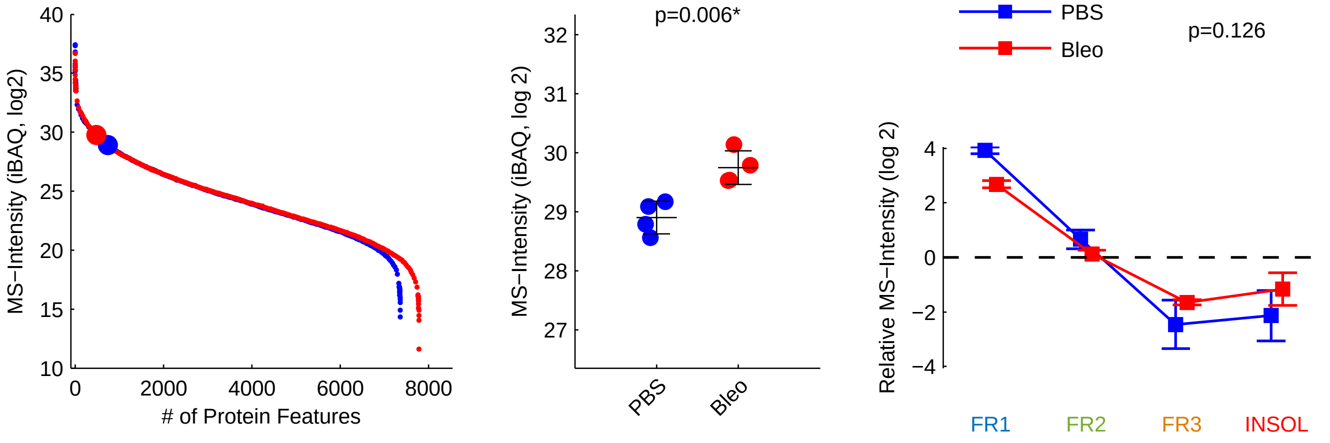

Q99LB7 − Sardh (id: 7050)

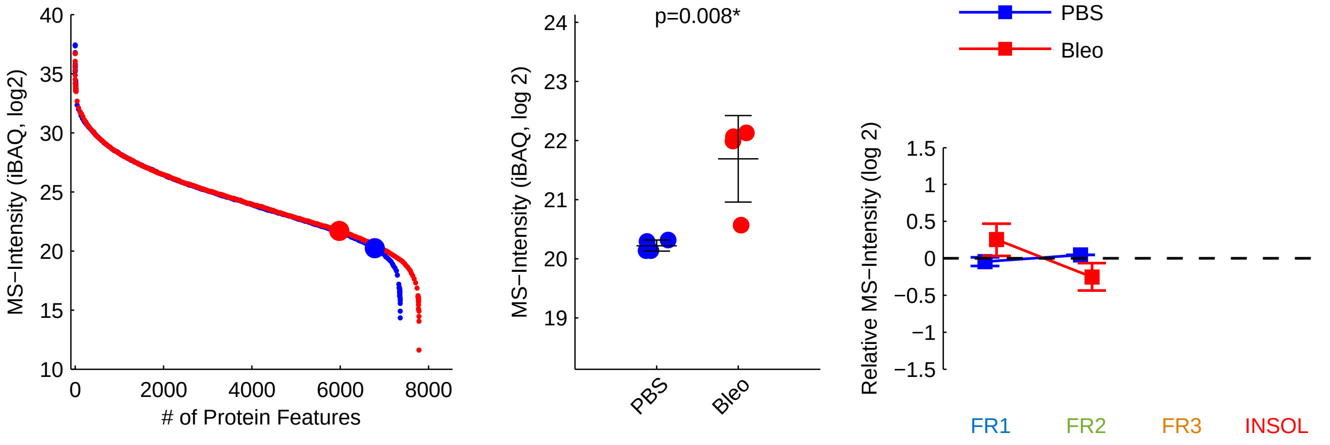

Q99LC5 − Etfa (id: 7053)

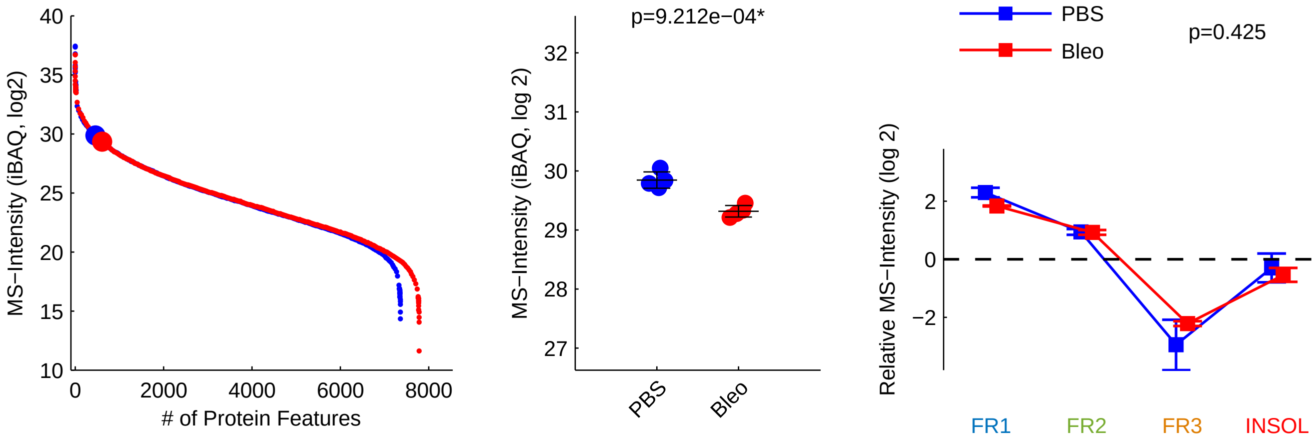

Q99LD8 − Ddah2 (id: 7056)

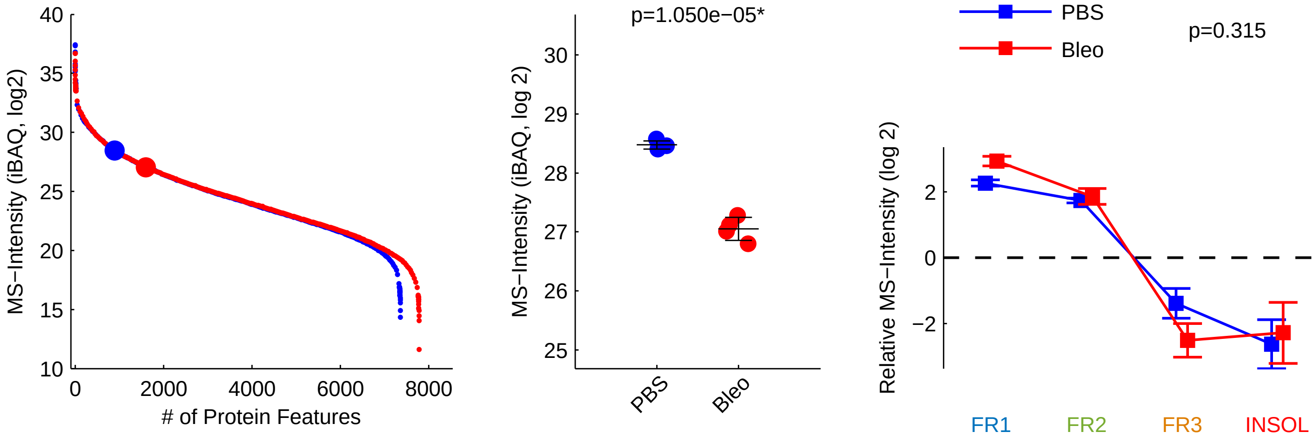

Q99LE6 − Abcf2 (id: 7058)

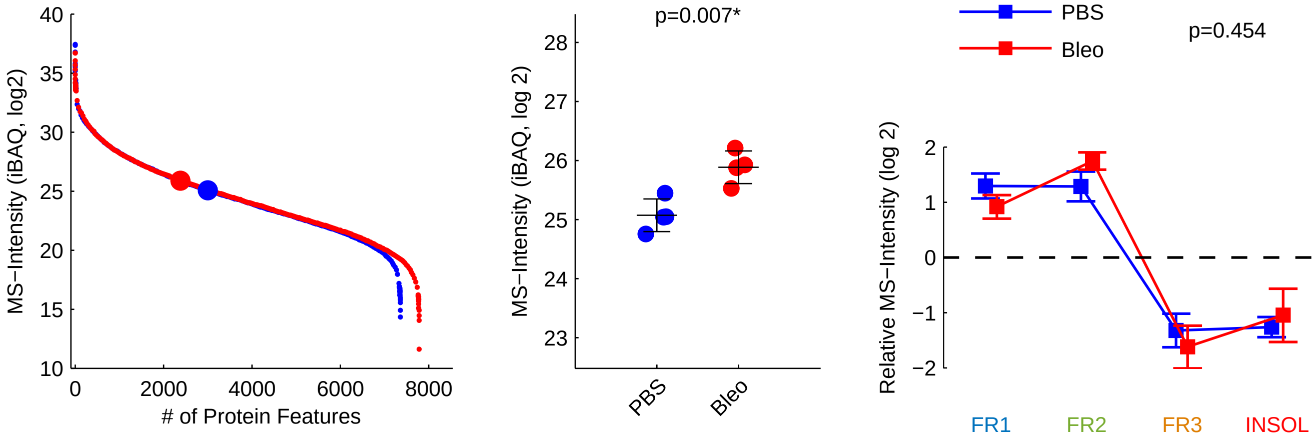

Q99LJ1 − Fuca1 (id: 7066)

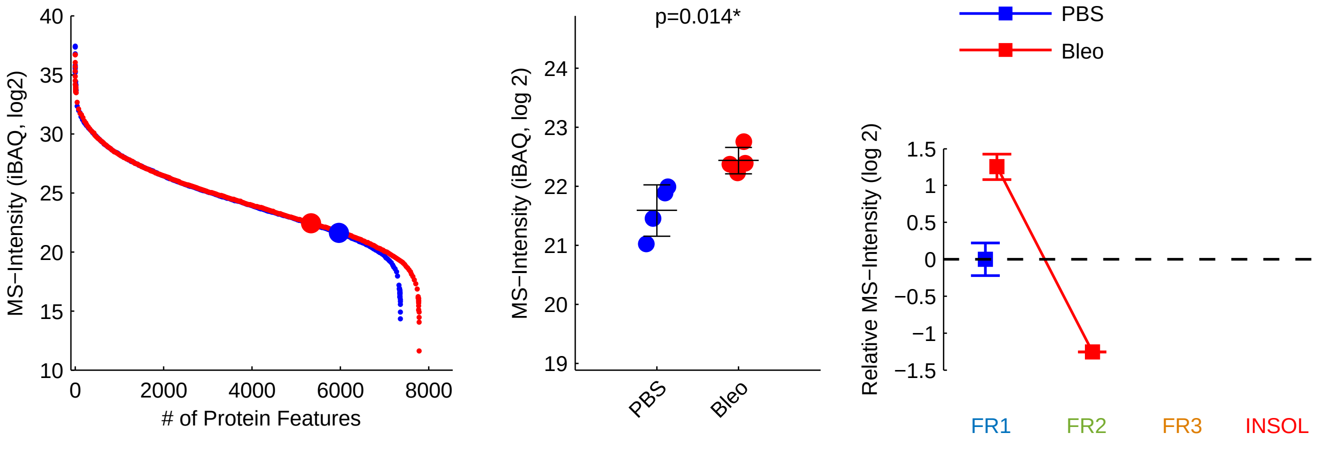

Q99LJ6 − Gpx7 (id: 7067)

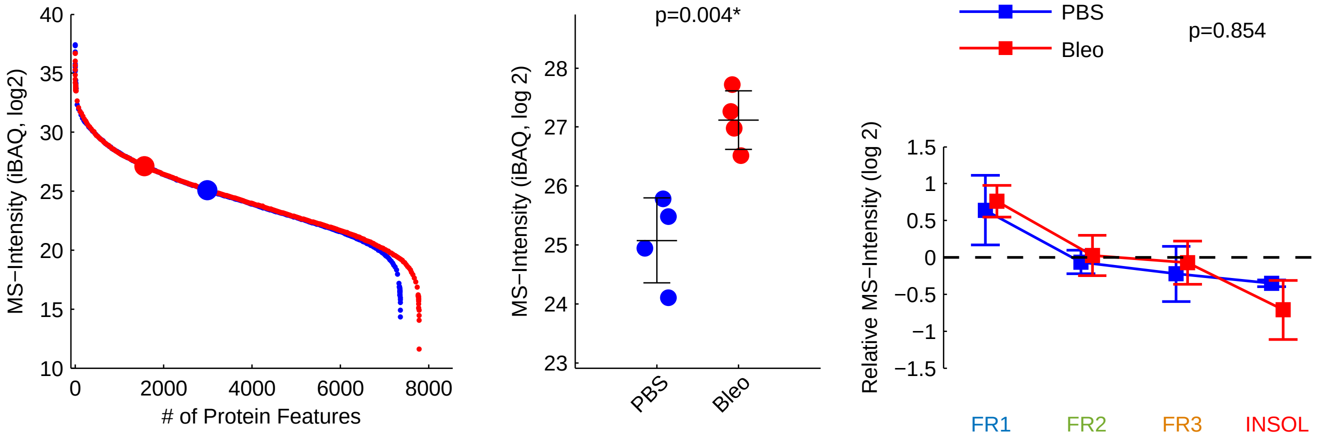

Q99LR1 − Abhd12 (id: 7074)

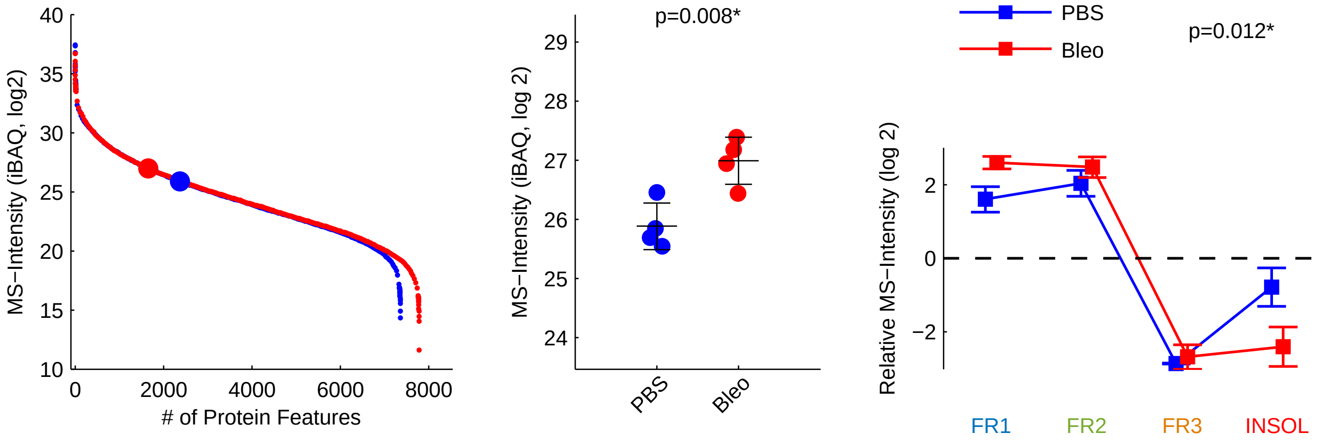

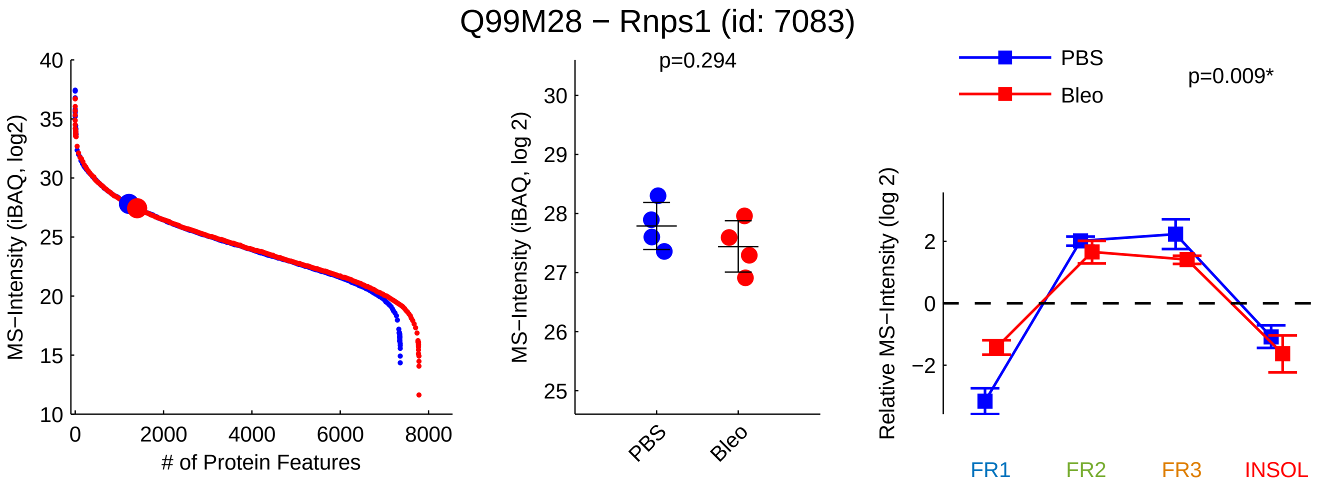

Q99M28 − Rnps1 (id: 7083)
MS−Intensity (iBAQ, log2)
# of Protein Features
p=0.294
MS−Intensity (iBAQ, log 2)
PBS
Bleo
PBS
Bleo
p=0.009*
Relative MS−Intensity (log 2)
FR1
FR2
FR3
INSOL

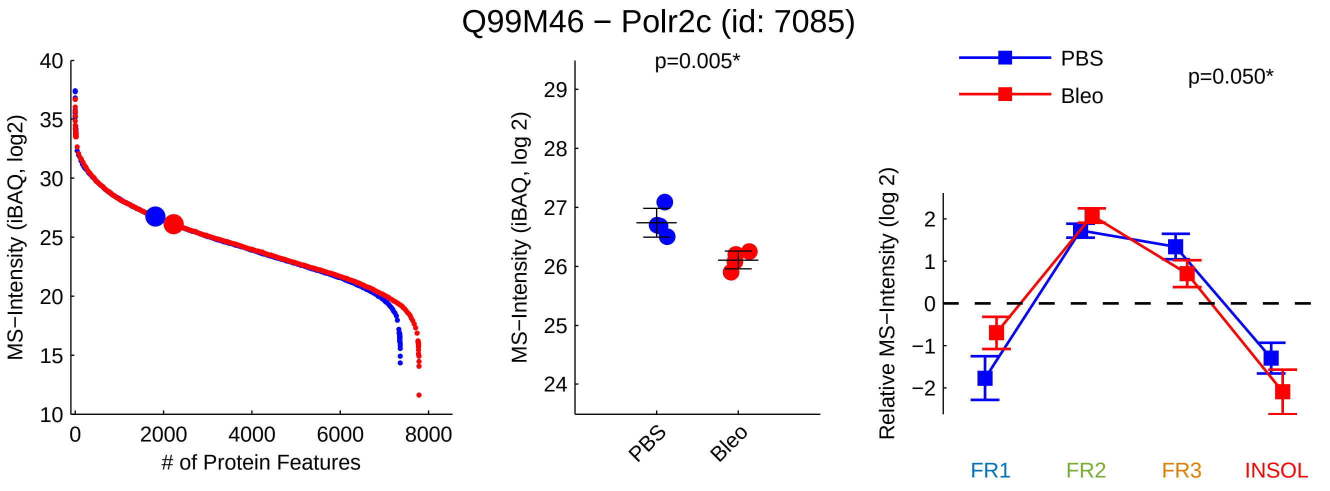

Q99M46 − Polr2c (id: 7085)
MS−Intensity (iBAQ, log2)
# of Protein Features
p=0.005*
MS−Intensity (iBAQ, log 2)
PBS
Bleo
PBS
Bleo
p=0.050*
Relative MS−Intensity (log 2)
FR1
FR2
FR3
INSOL

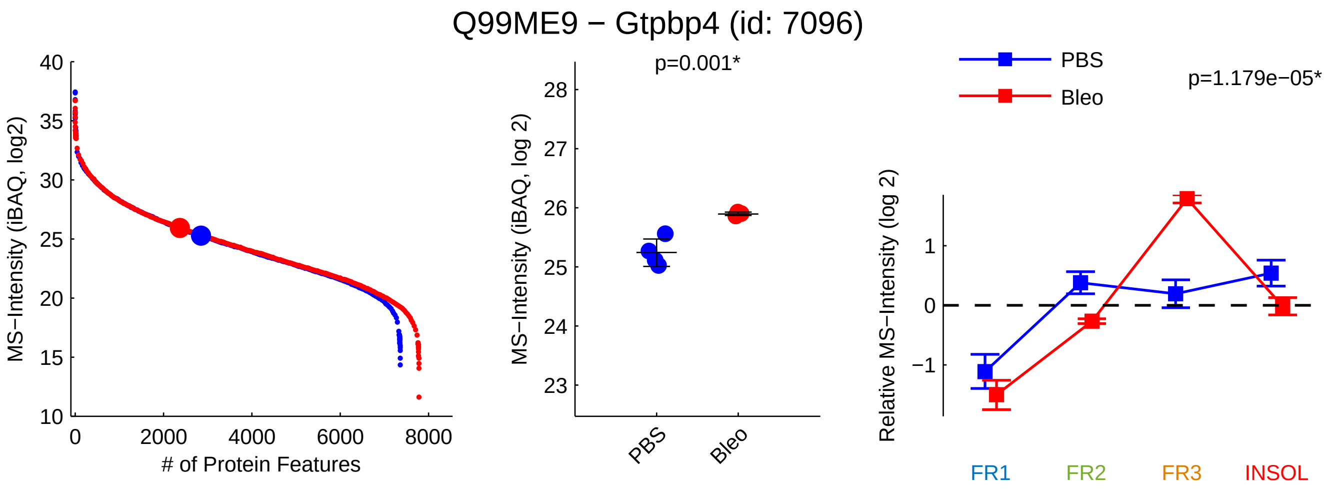

Q99ME9 − Gtpbp4 (id: 7096)
MS−Intensity (iBAQ, log2)
# of Protein Features
p=0.001*
MS−Intensity (iBAQ, log 2)
PBS
Bleo
PBS
Bleo
p=1.179e−05*
Relative MS−Intensity (log 2)
FR1
FR2
FR3
INSOL

Q99MJ9 − Ddx50 (id: 7098)

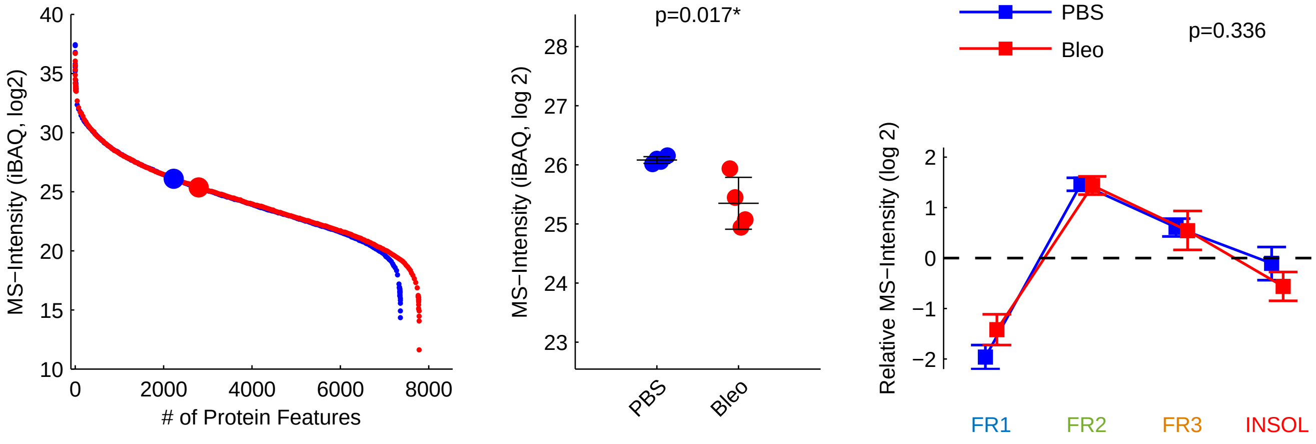

Q99MN9 − Pccb (id: 7101)

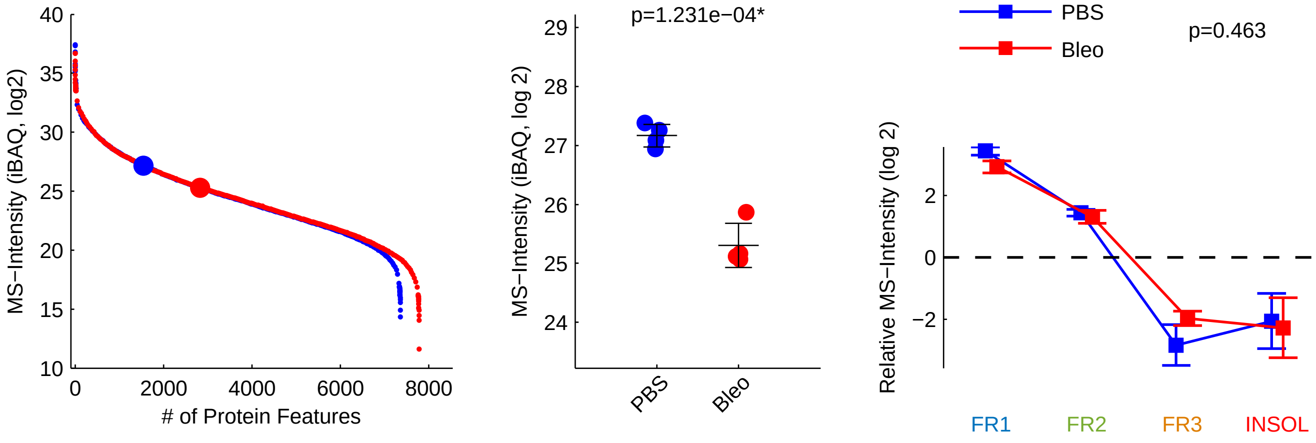

Q99MR8 − Mccc1 (id: 7107)

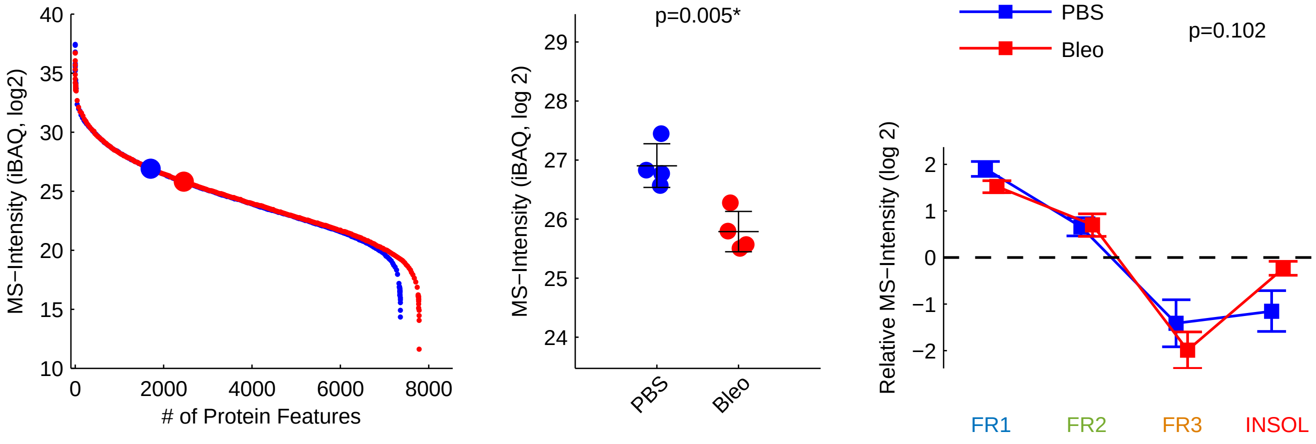

Q99N15 − Hsd17b10 (id: 7112)

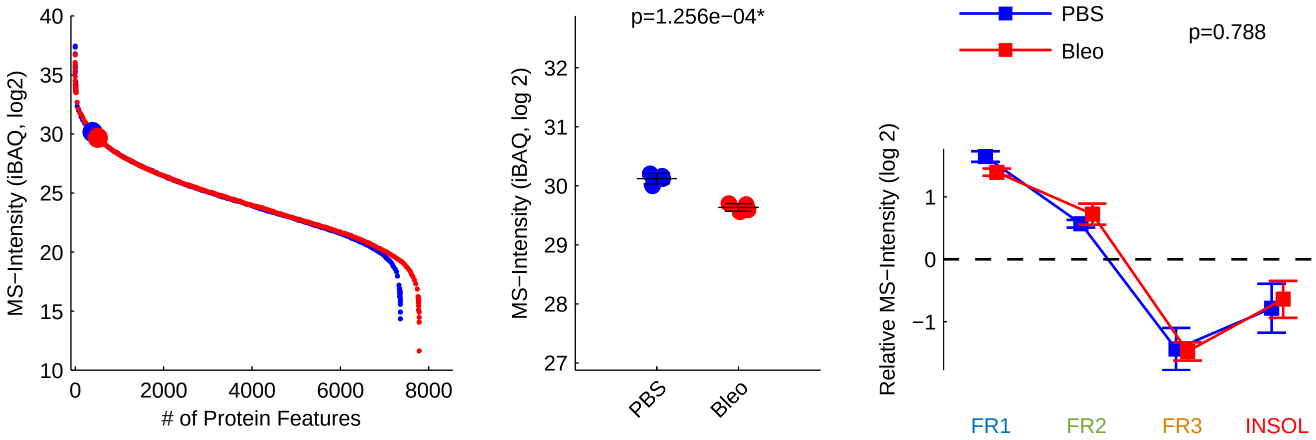

Q99N16 − Cyp4f3 (id: 7113)

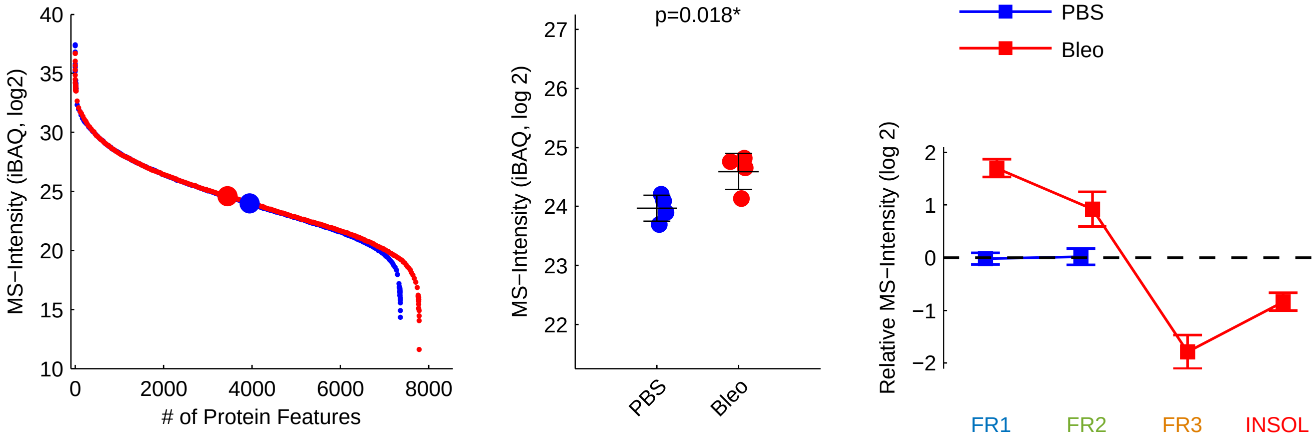

Q99N17 − Cyp4f16 (id: 7114)

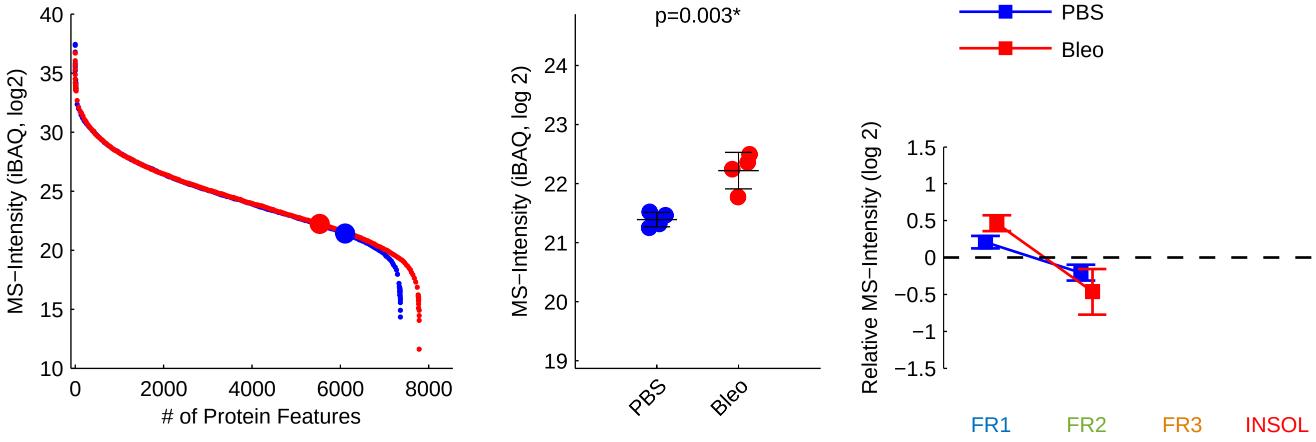

Q99N93 − Mrpl16 (id: 7123)

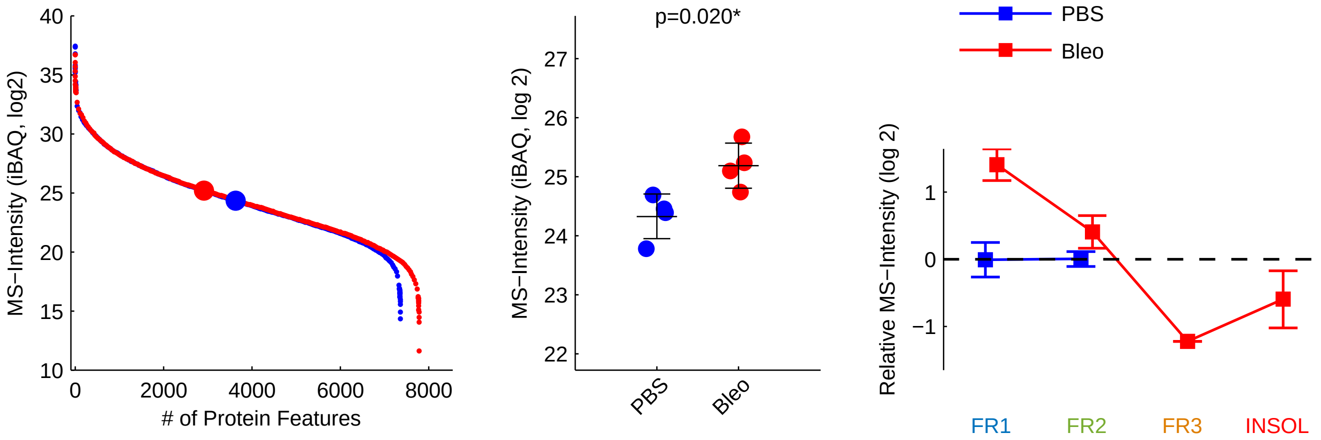

Q99NB1 − Acss1 (id: 7126)

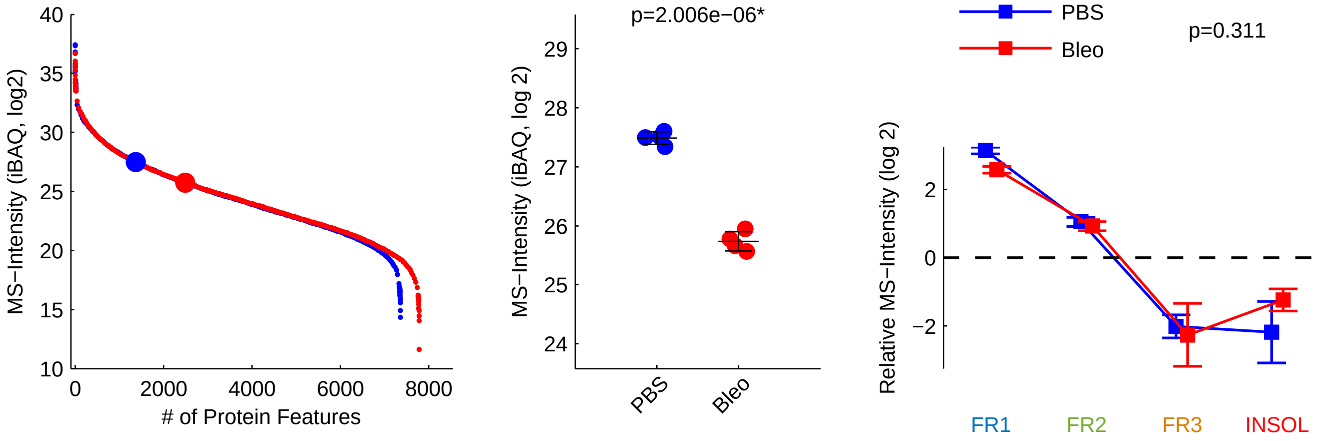

Q99P58 − Rab27b (id: 7133)

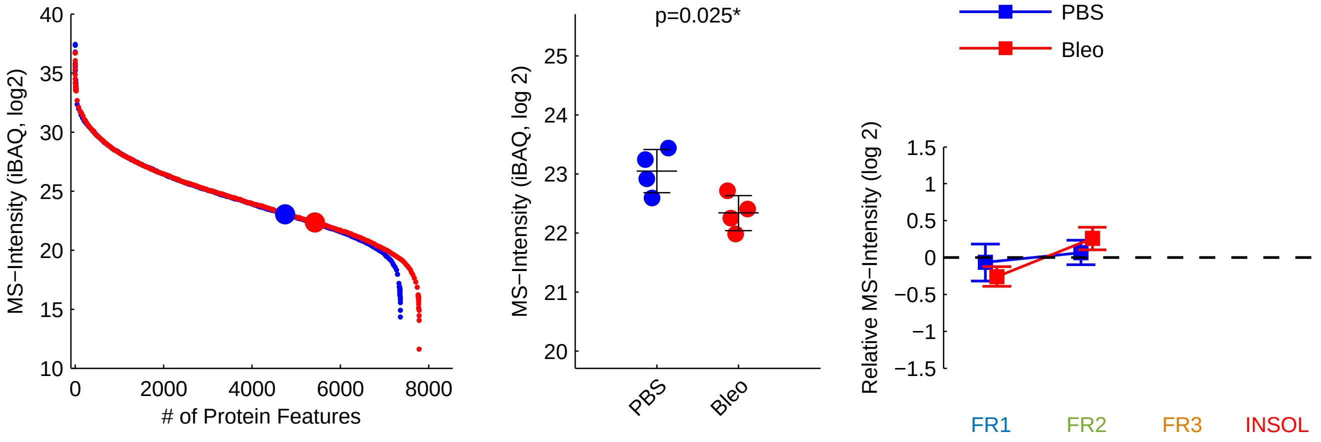

Q99P91 − Gpnmb (id: 7137)

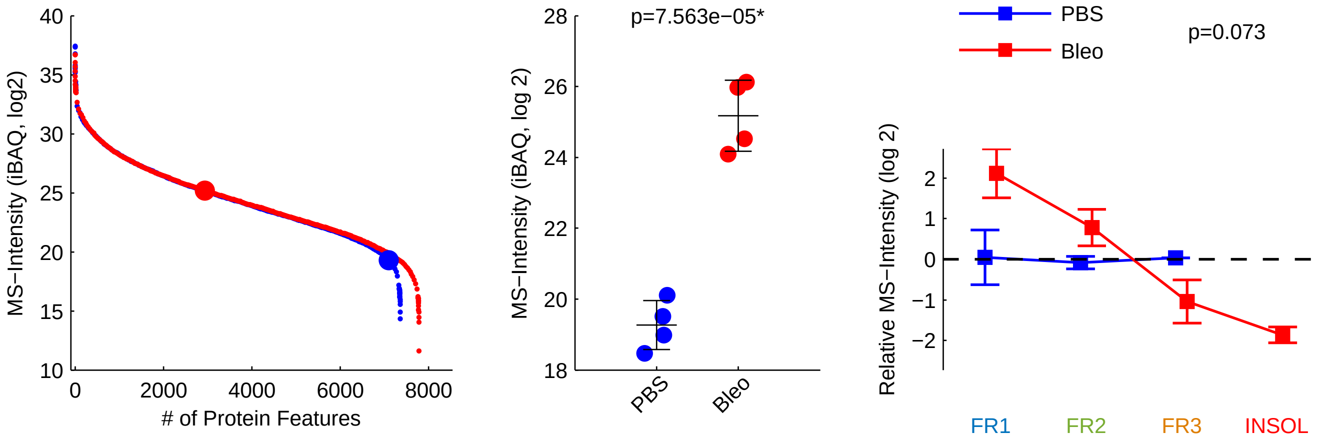

Q99PL6 − Ubxn6 (id: 7139)

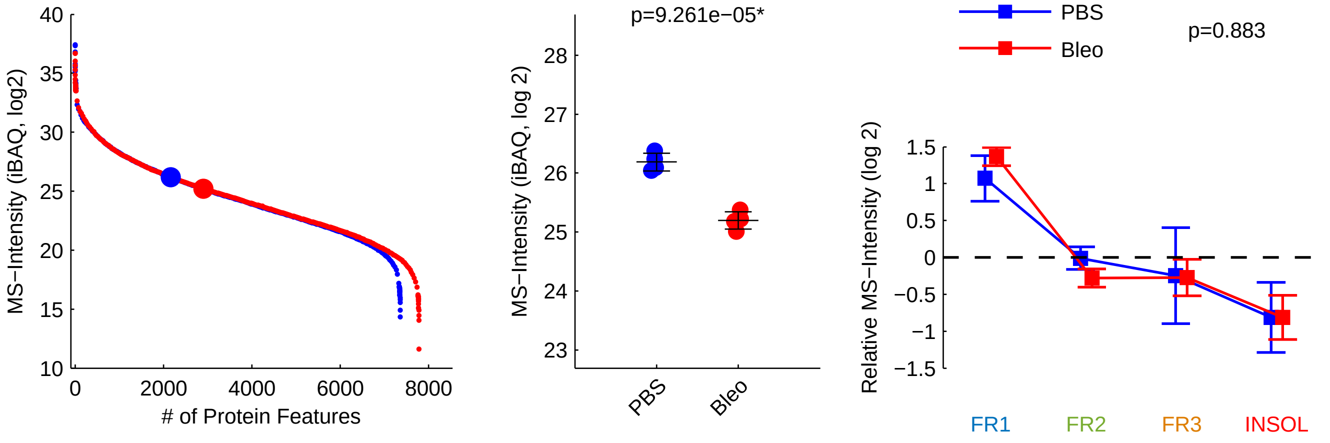

Q9CPQ1 − Cox6c (id: 7153)

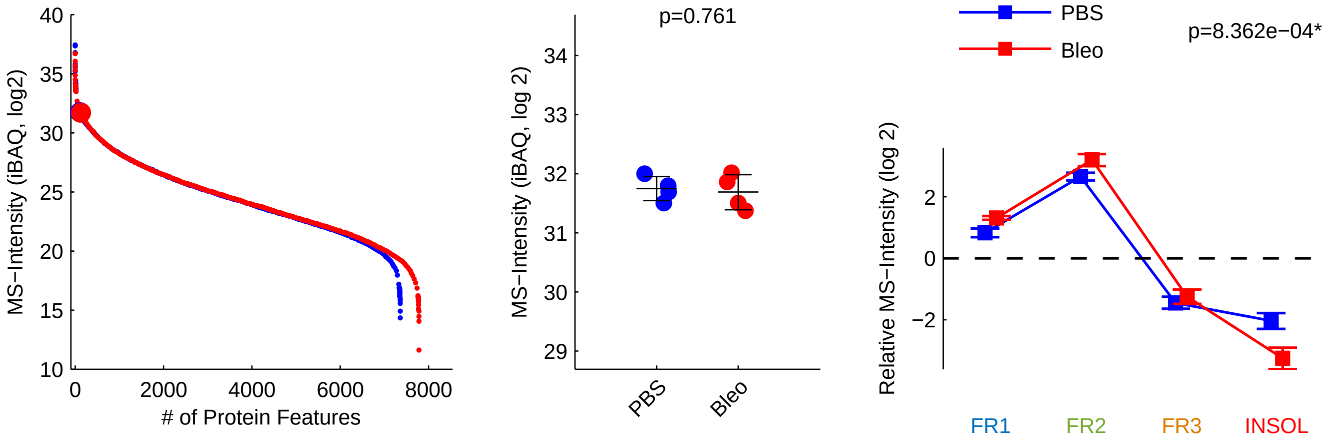

Q9CPQ3 − Tomm22 (id: 7154)

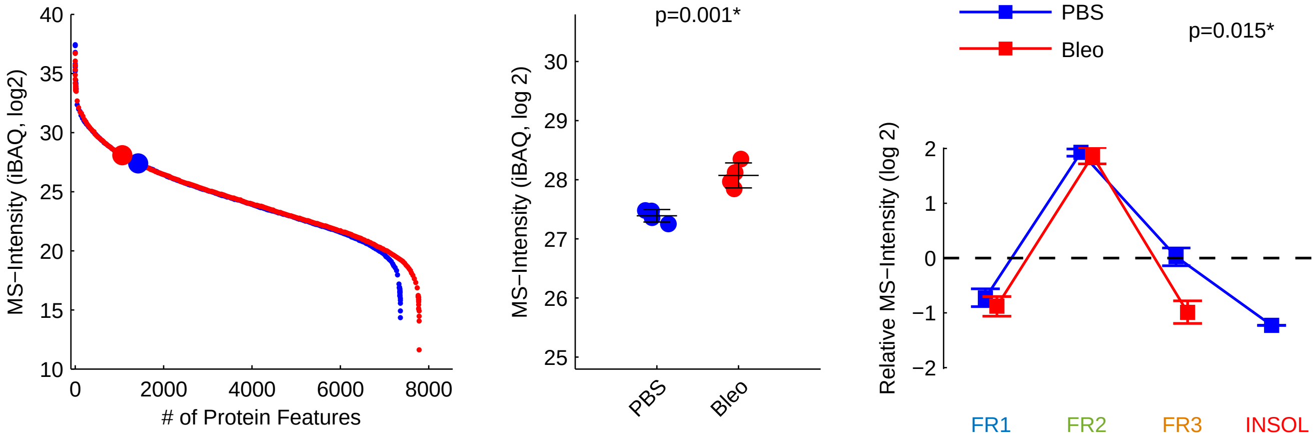

Q9CPU0 − Glo1 (id: 7166)

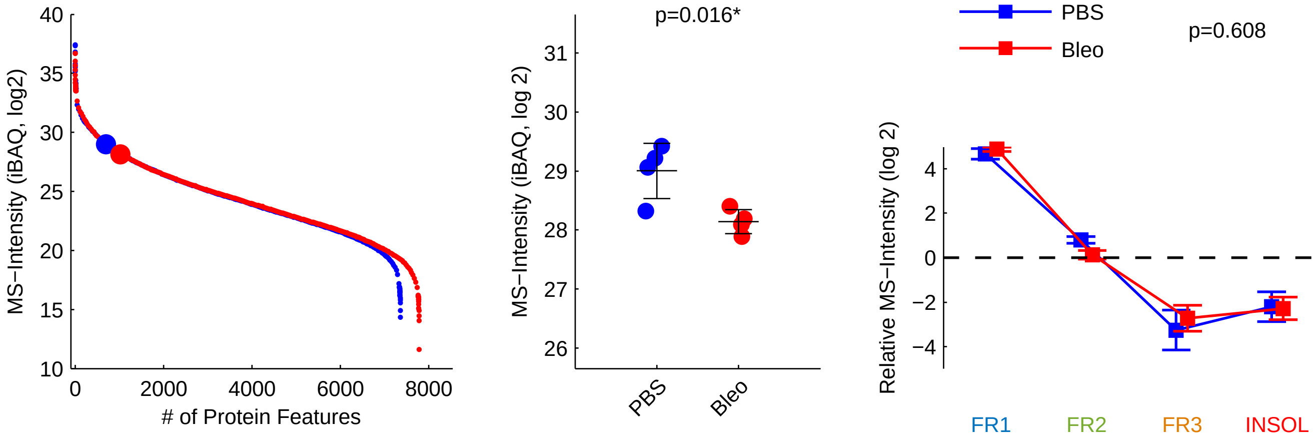

Q9CPV4 − Glod4 (id: 7169)

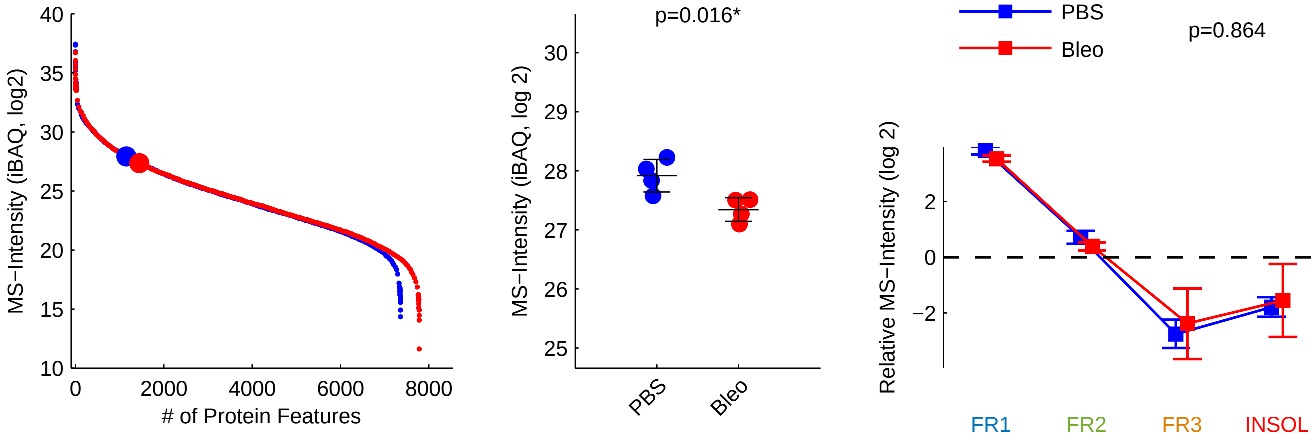

Q9CPW4 − Arpc5 (id: 7171)

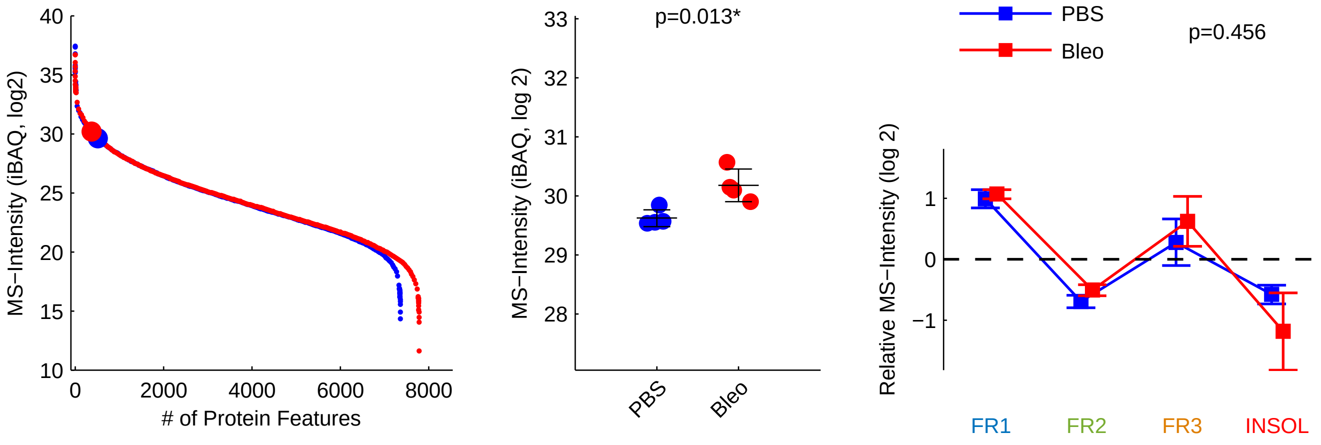

Q9CPY1 − Mrpl51 (id: 7178)

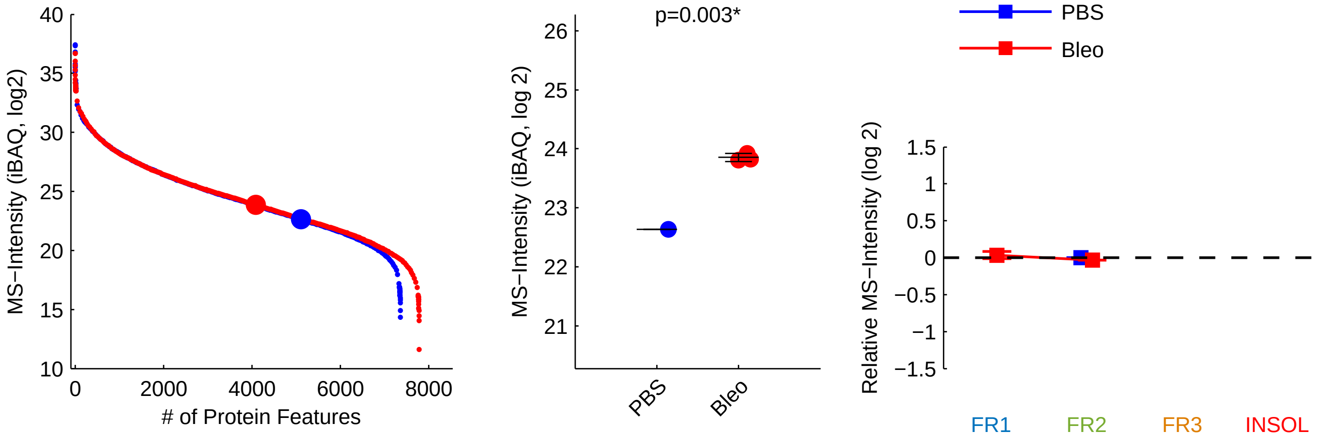

Q9CPY7 − Lap3 (id: 7180)

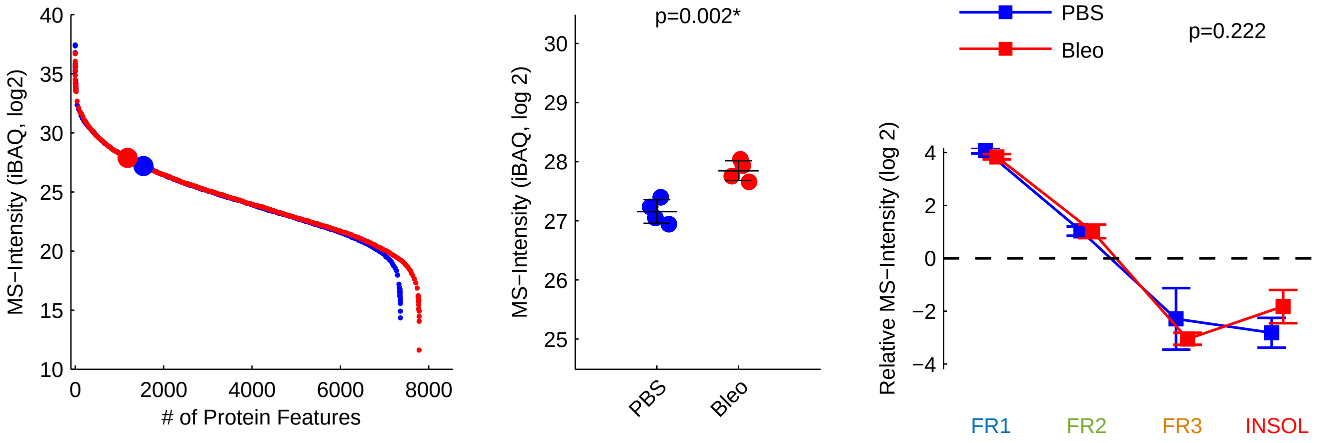

Q9CPZ8 − Cmc1 (id: 7181)

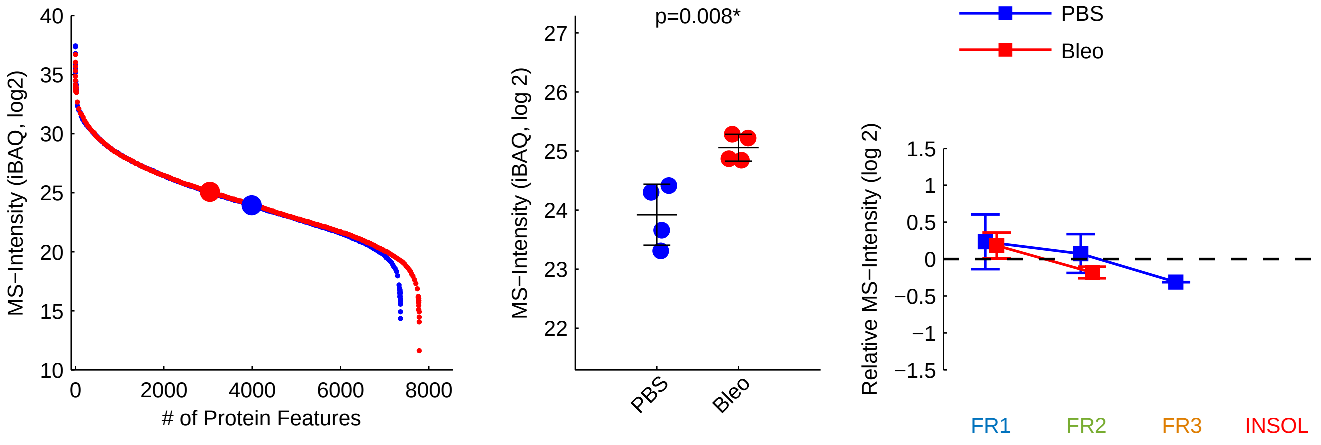

Q9CQ01 − Rnaset2 (id: 7183)

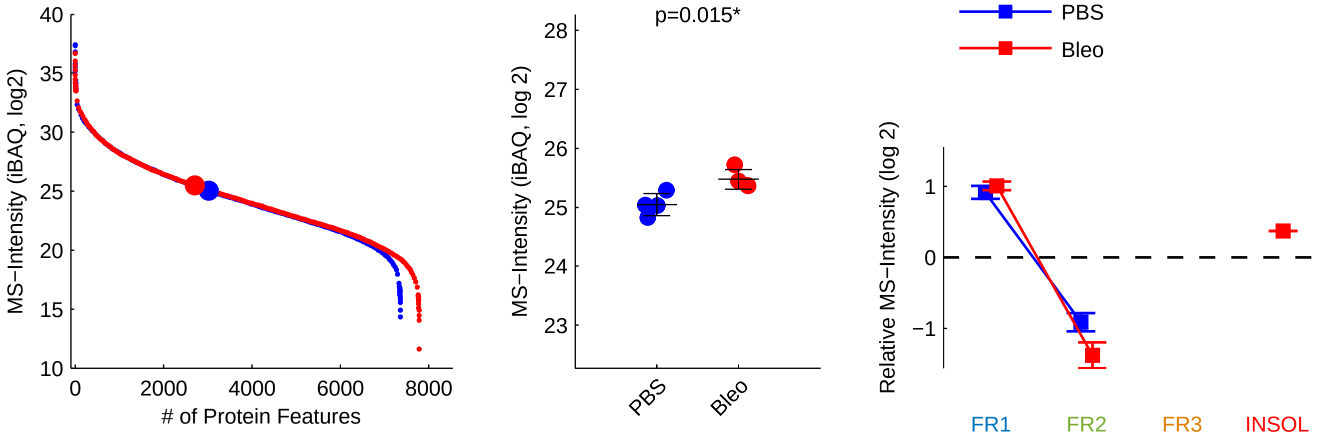

Q9CQ19 − Myl9 (id: 7187)

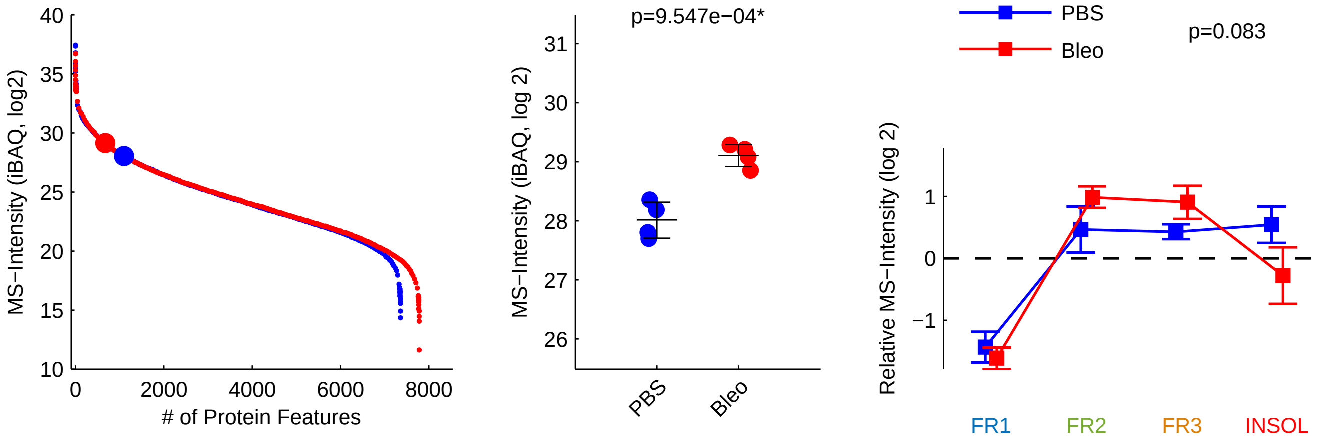

Q9CQ45 − Nenf (id: 7195)

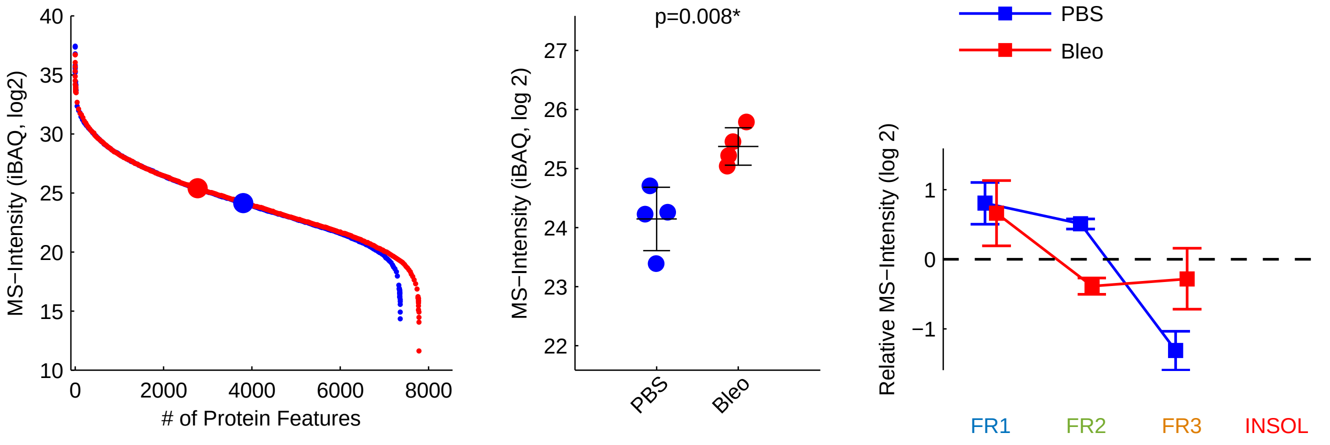

Q9CQ62 − Decr1 (id: 7201)

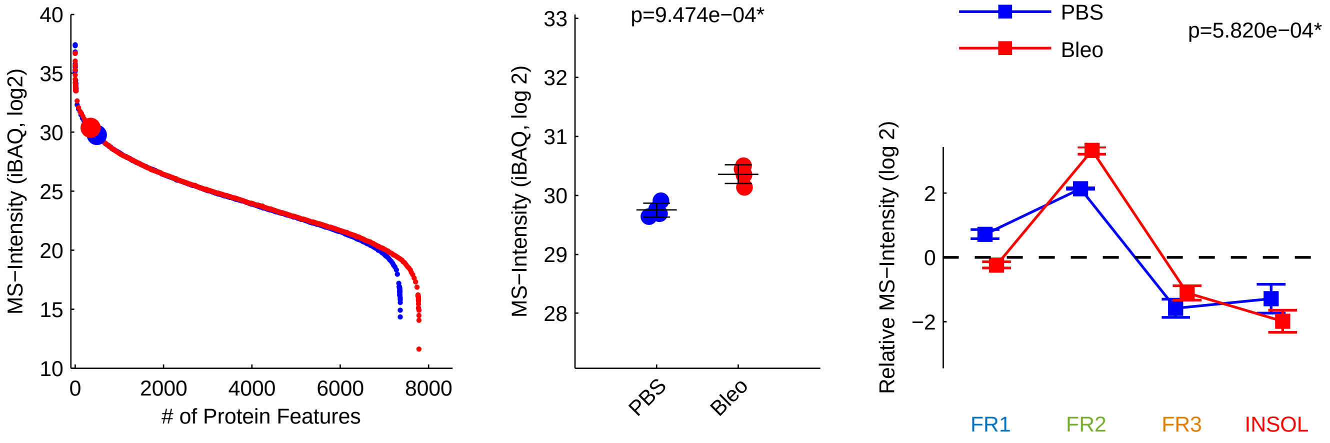

Q9CQ73 − Pkp2 (id: 7206)

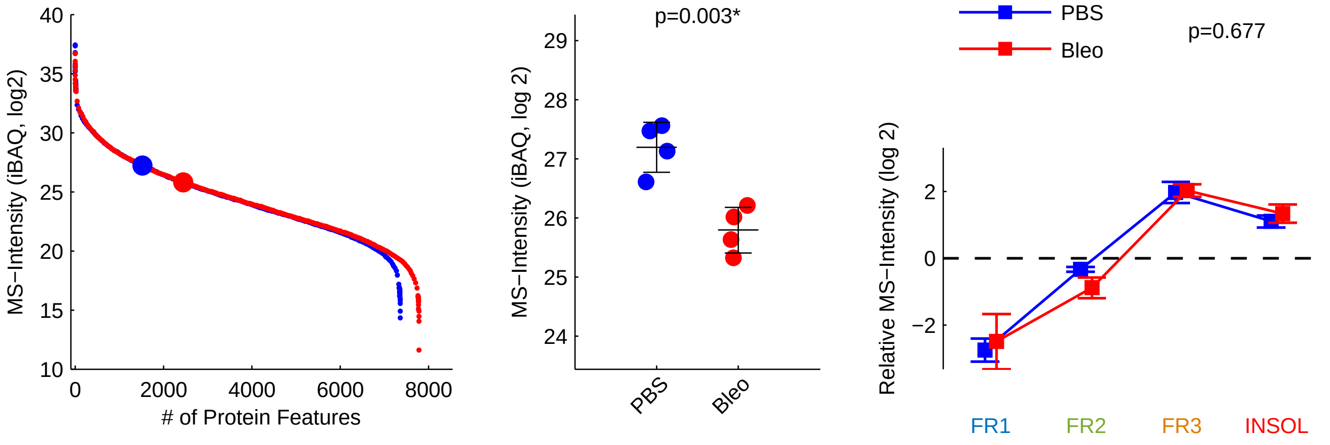

Q9CQ80 − Vps25 (id: 7209)

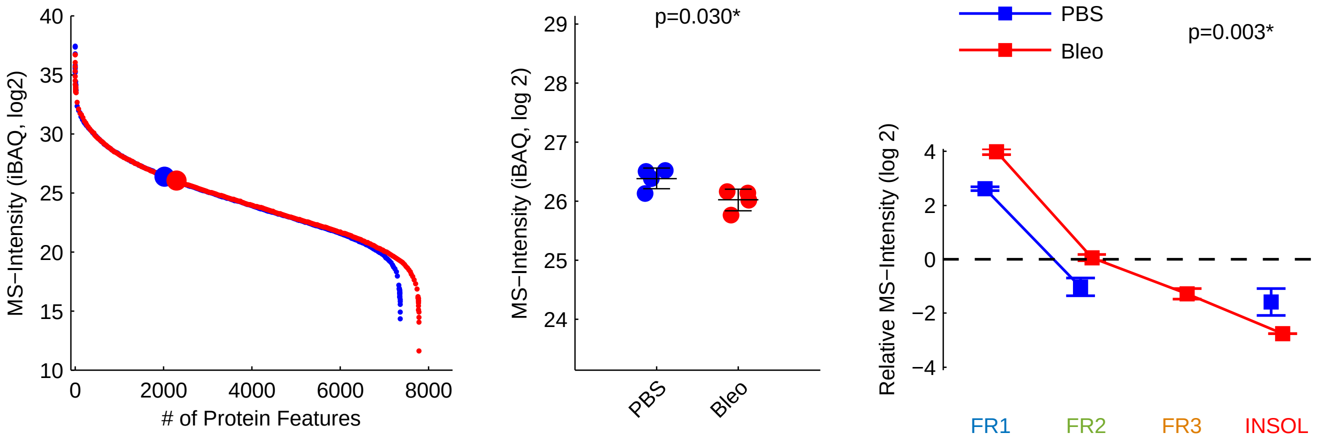

Q9CQ85 − Timm22 (id: 7210)

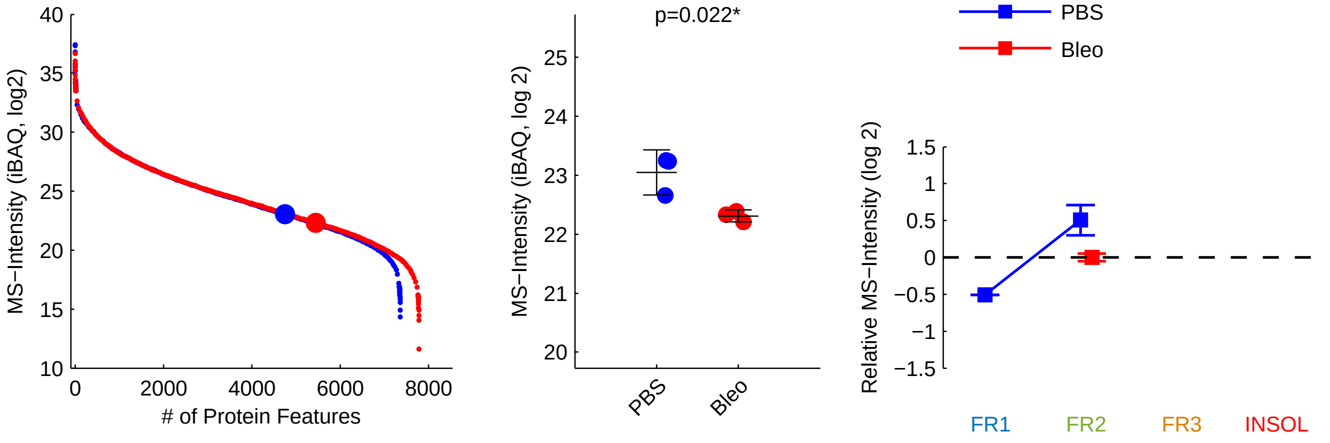

Q9CQC6 − Bzw1 (id: 7221)

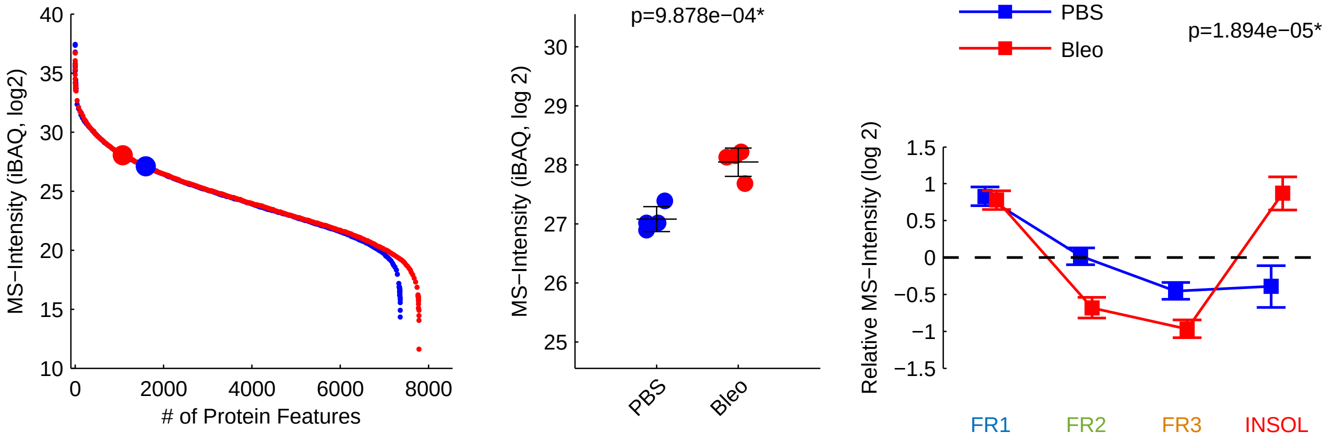

Q9CQC7 − Ndufb4 (id: 7222)

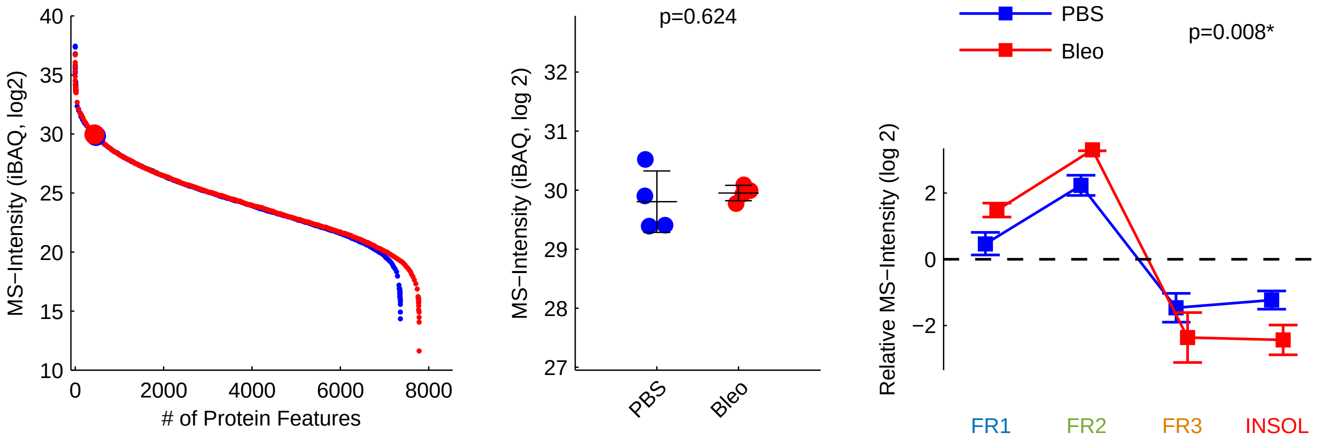

Q9CQH7 − Btf3l4 (id: 7238)

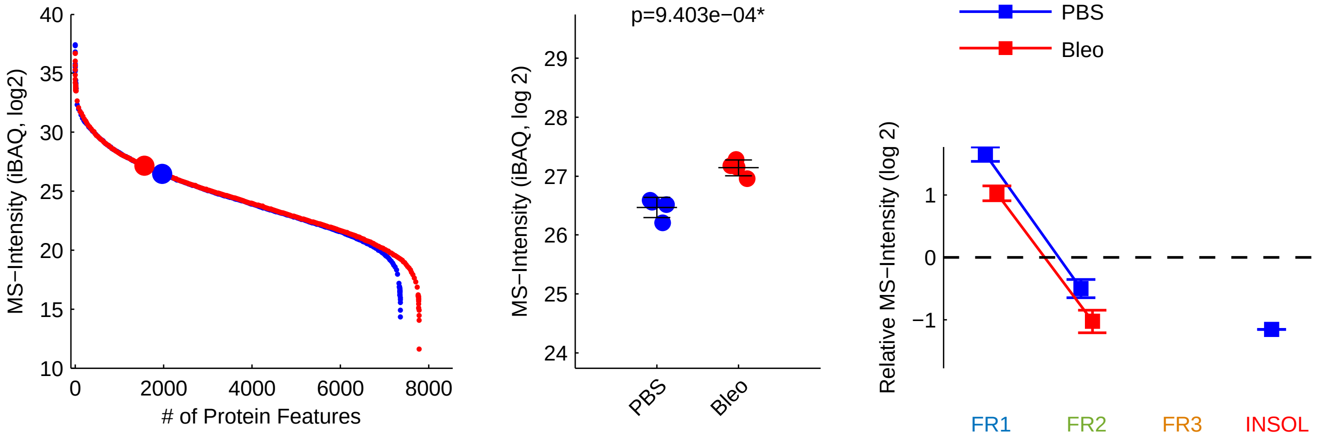

Q9CQI6 − Cotl1 (id: 7241)

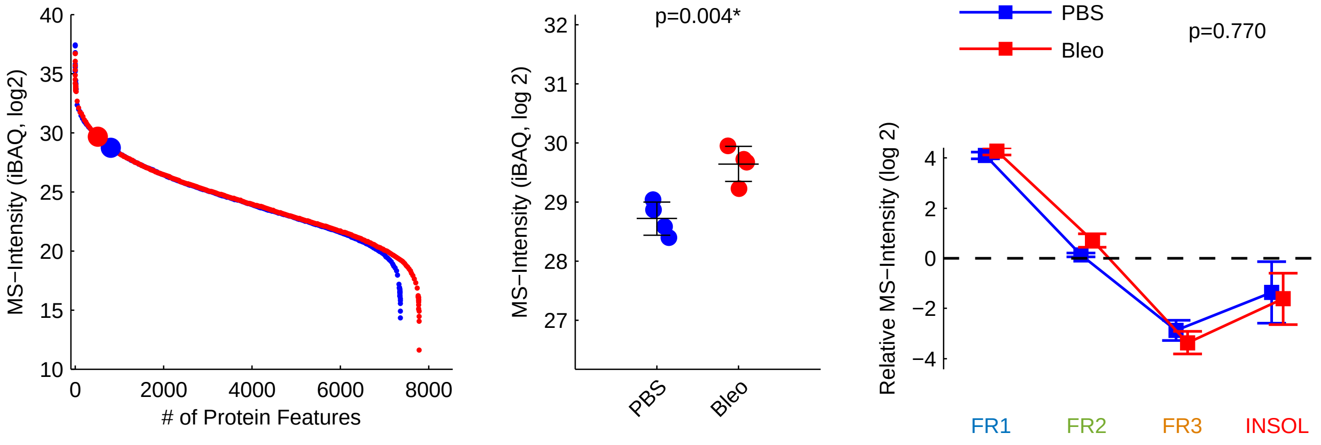

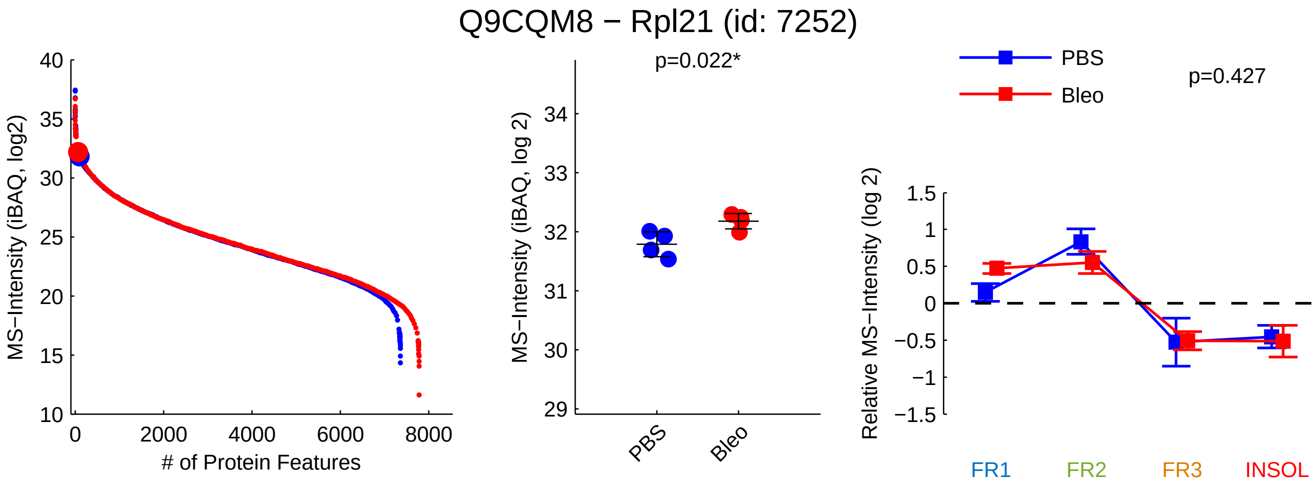
Q9CQM8 − Rpl21 (id: 7252)
MS−Intensity (iBAQ, log2)
# of Protein Features
p=0.022*
MS−Intensity (iBAQ, log 2)
PBS
Bleo
p=0.427
Relative MS−Intensity (log 2)
FR1
FR2
FR3
INSOL

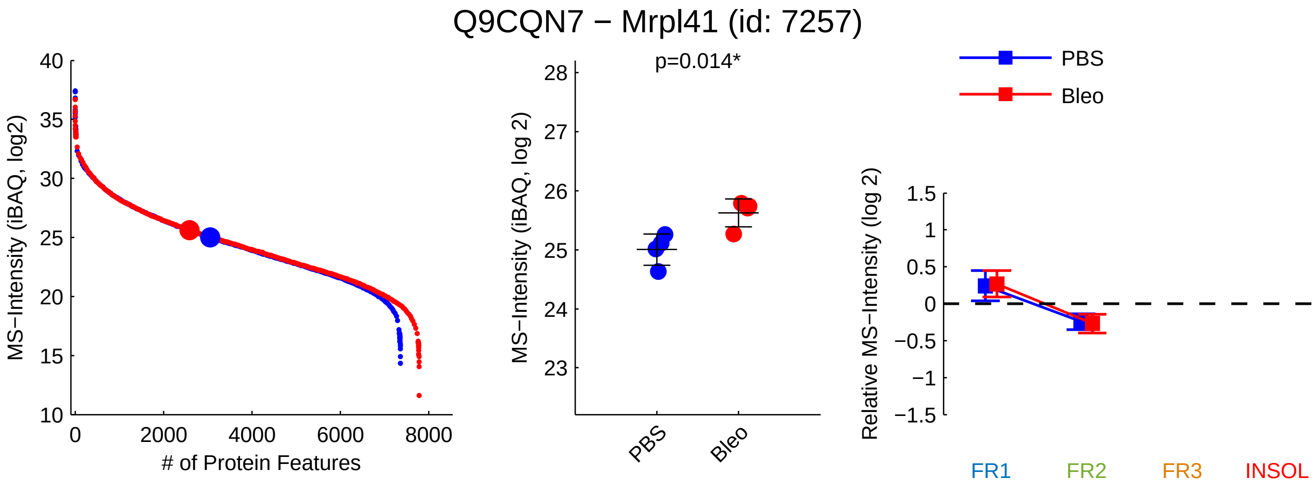
Q9CQN7 − Mrpl41 (id: 7257)
MS−Intensity (iBAQ, log2)
# of Protein Features
p=0.014*
MS−Intensity (iBAQ, log 2)
PBS
Bleo
Relative MS−Intensity (log 2)
FR1
FR2
FR3
INSOL

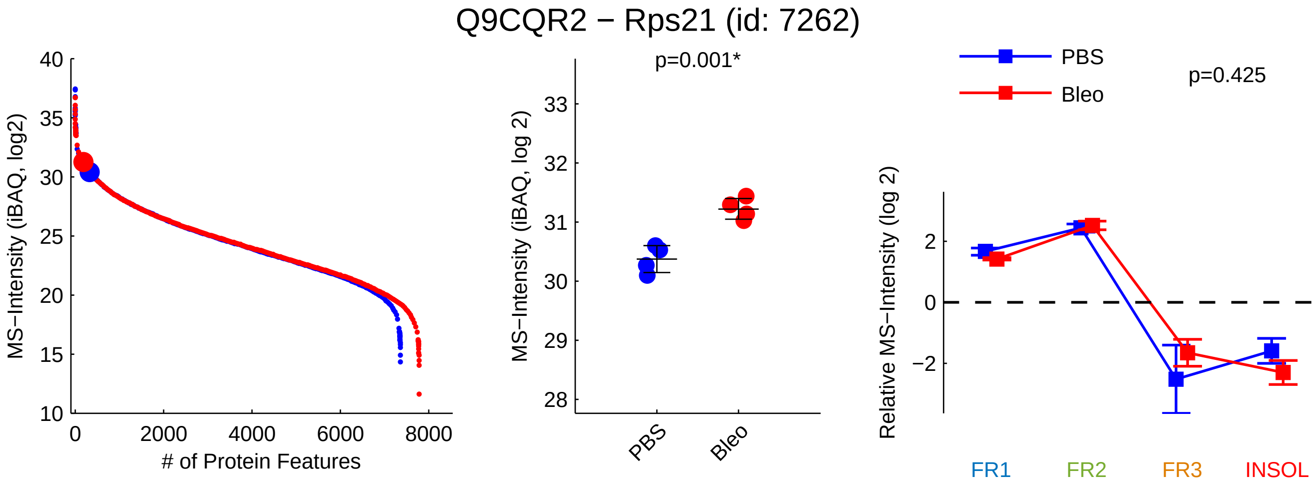
Q9CQR2 − Rps21 (id: 7262)
MS−Intensity (iBAQ, log2)
# of Protein Features
p=0.001*
MS−Intensity (iBAQ, log 2)
PBS
Bleo
p=0.425
Relative MS−Intensity (log 2)
FR1
FR2
FR3
INSOL

Q9CQR4 − Acot13 (id: 7263)

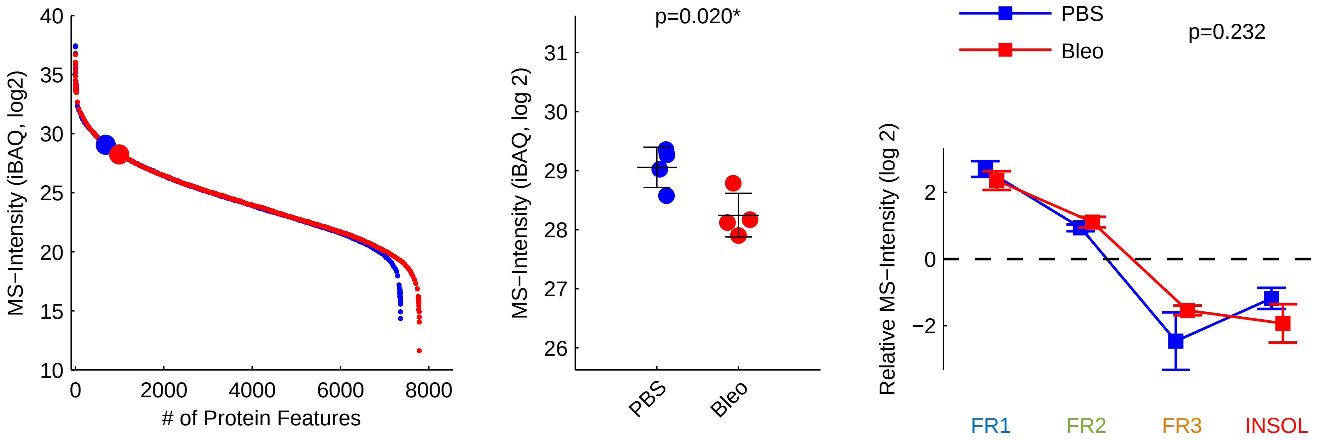

Q9CQU0 − Txndc12 (id: 7272)

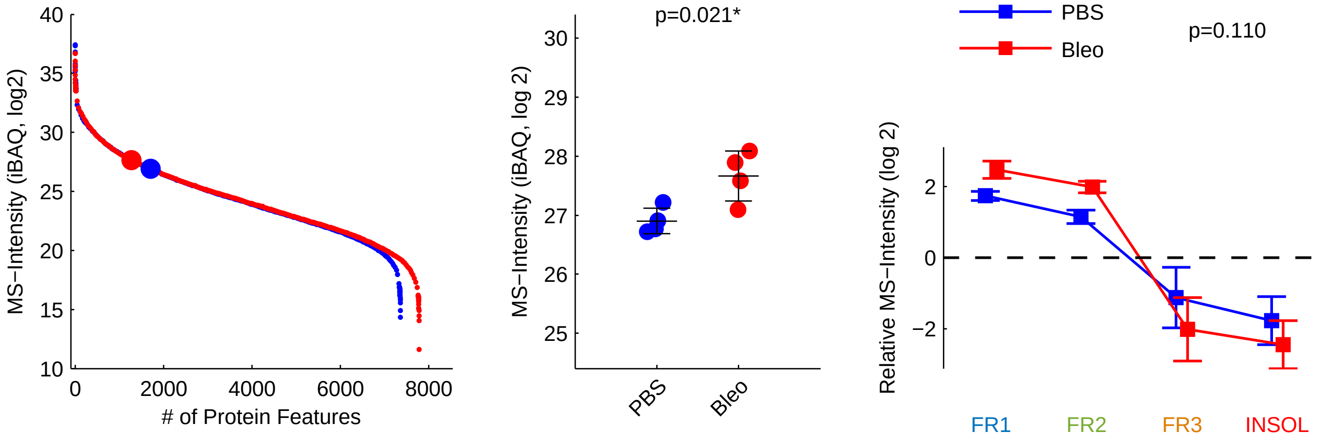

Q9CQX6 − Gm16286 (id: 7286)

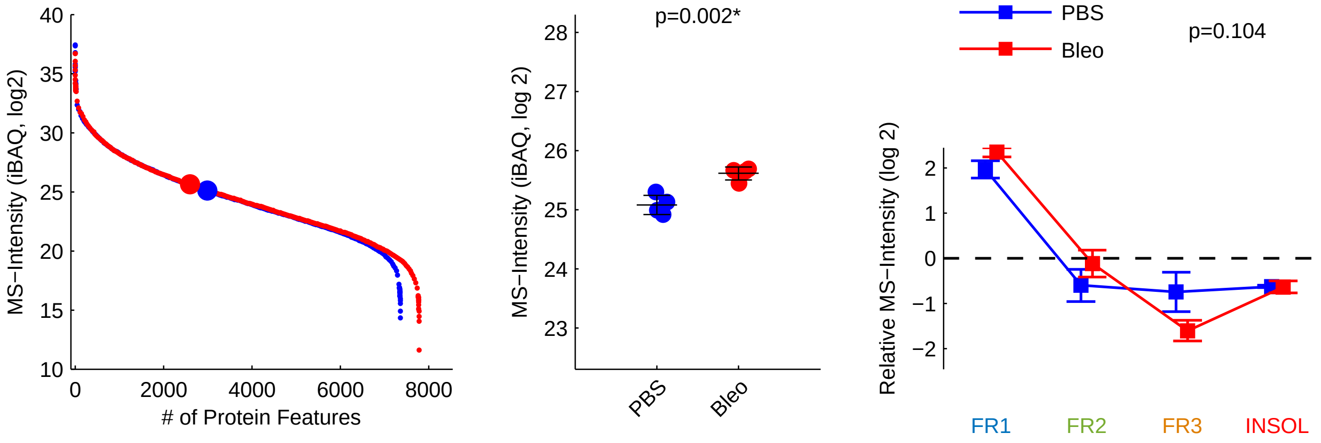

Q9CR09 − Ufc1 (id: 7295)

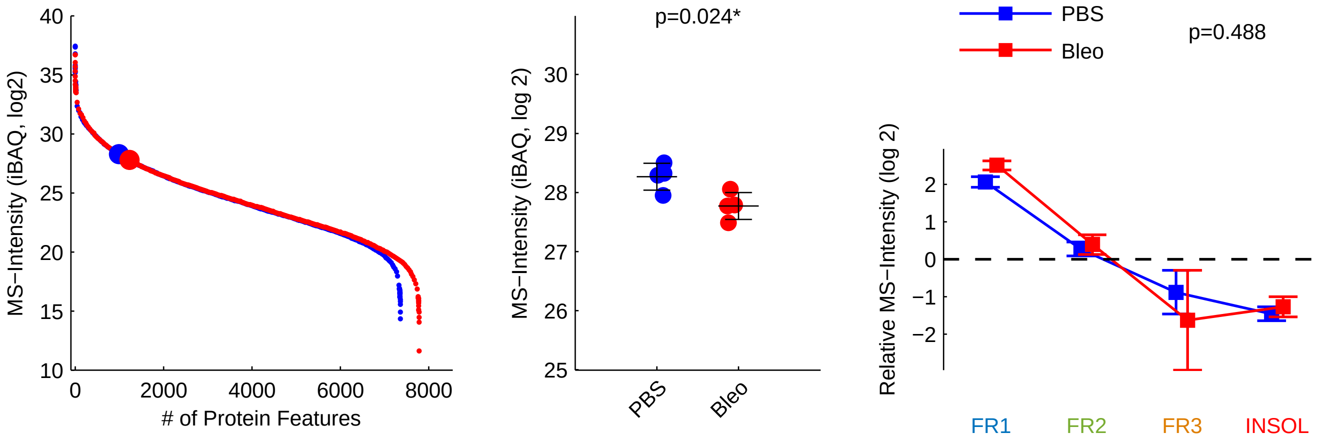

Q9CR11 − Yeats4 (id: 7296)

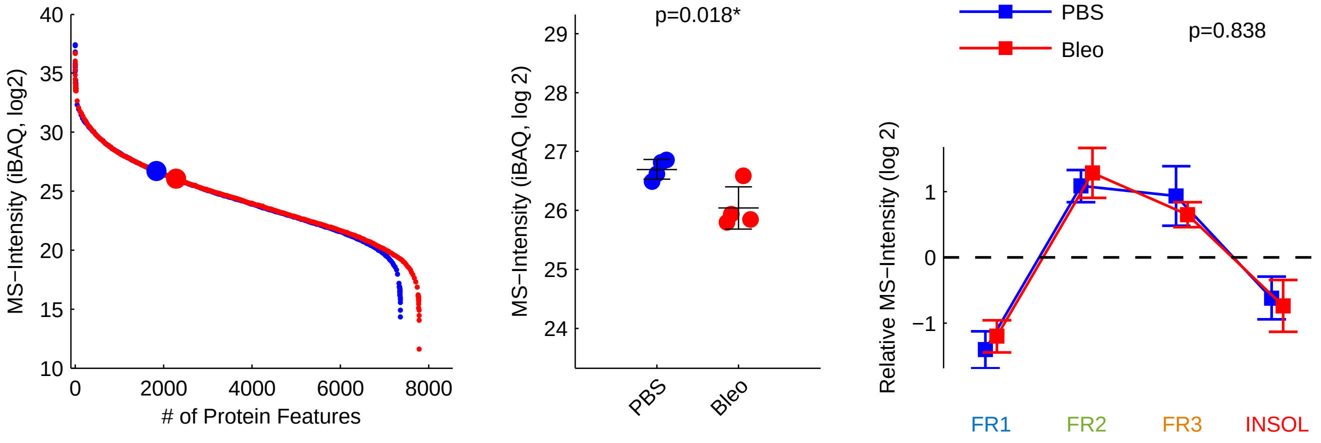

Q9CR13 − (id: 7297)

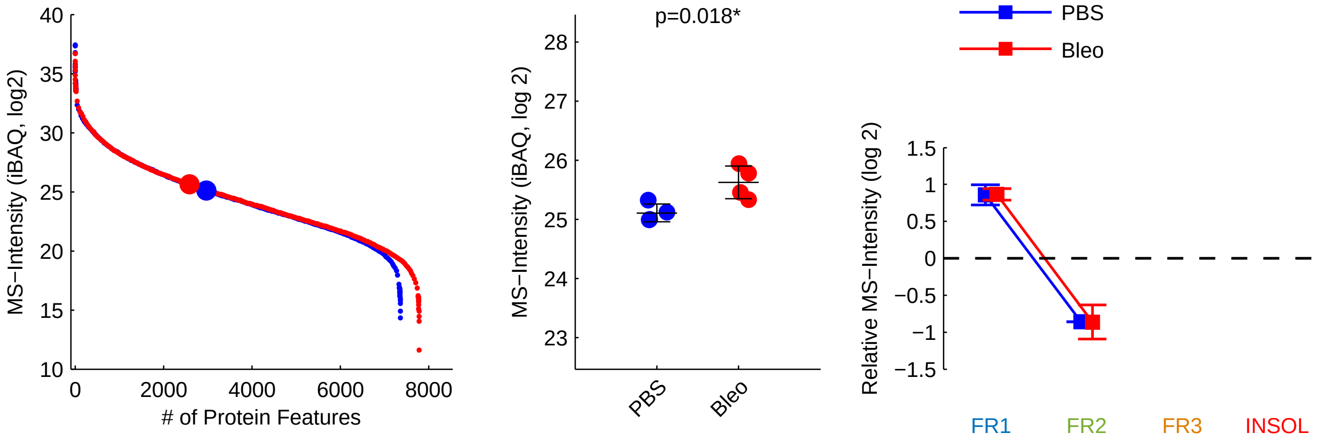

Q9CR16 − Ppid (id: 7298)

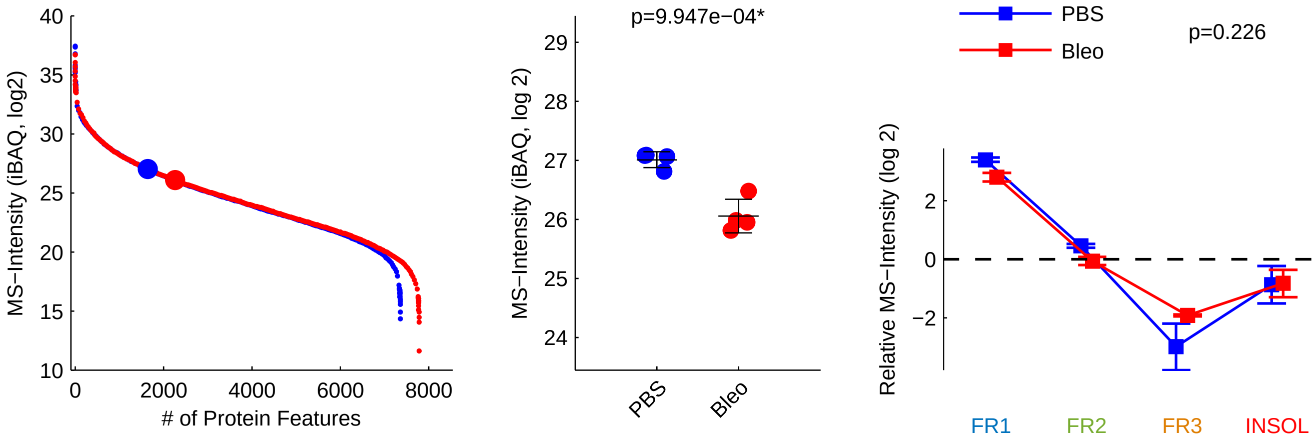

Q9CR26 − Vta1 (id: 7301)

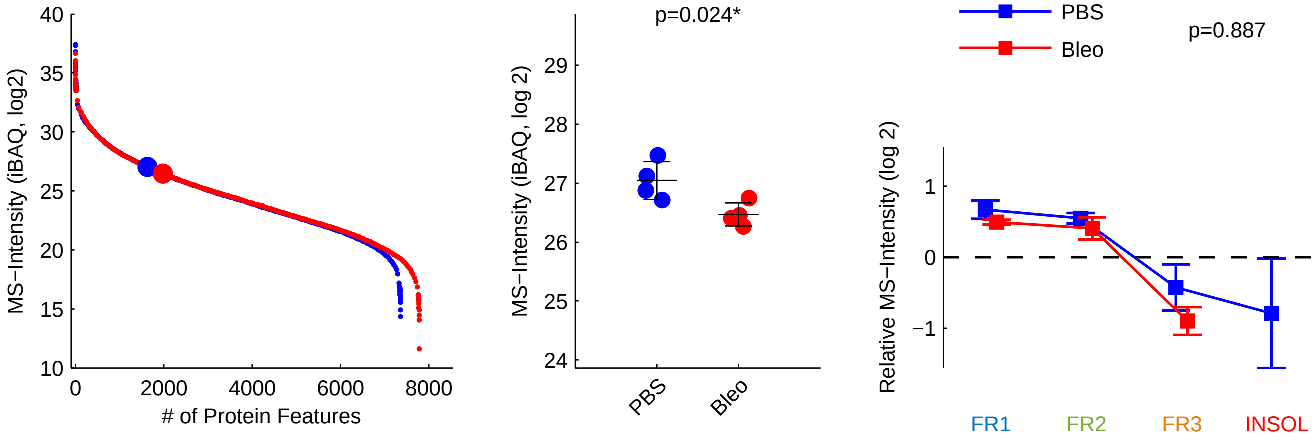

Q9CR47 − Nsa2 (id: 7306)

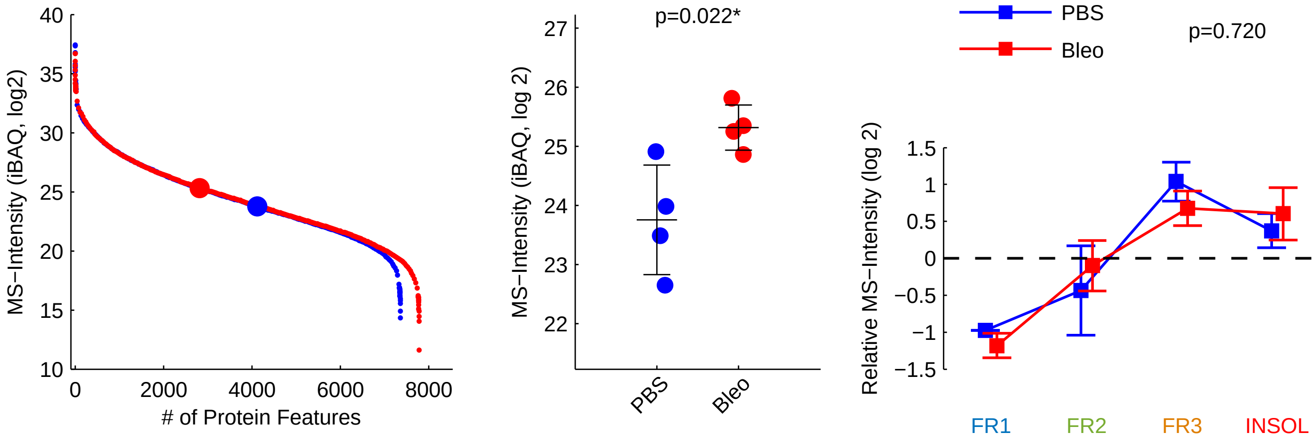

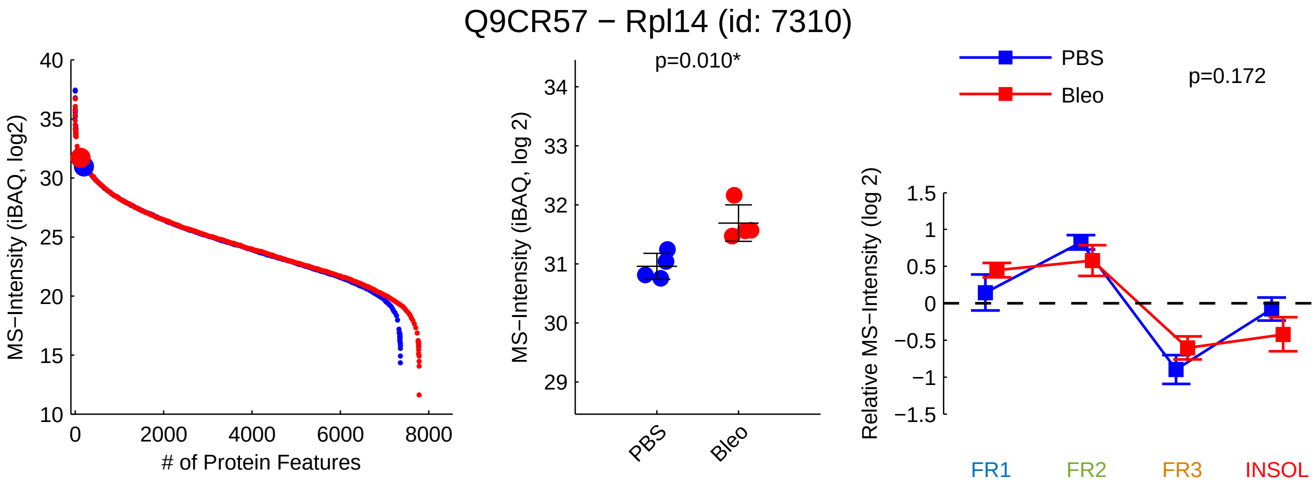
Q9CR57 − Rpl14 (id: 7310)
MS−Intensity (iBAQ, log2)
# of Protein Features
p=0.010*
MS−Intensity (iBAQ, log 2)
PBS
Bleo
p=0.172
Relative MS−Intensity (log 2)
FR1
FR2
FR3
INSOL

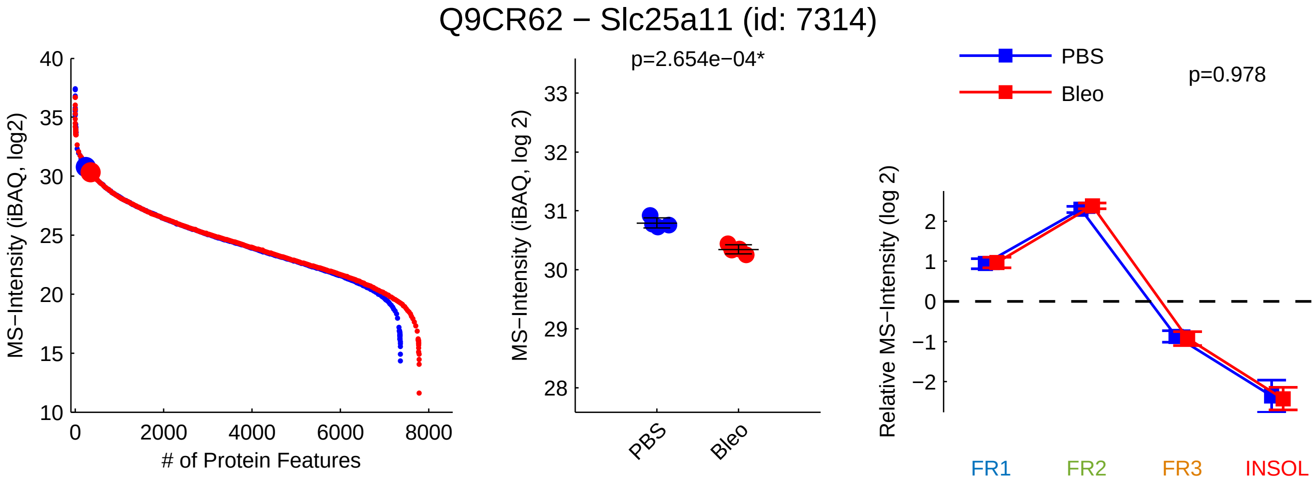
Q9CR62 − Slc25a11 (id: 7314)
MS−Intensity (iBAQ, log2)
# of Protein Features
p=2.654e−04*
MS−Intensity (iBAQ, log 2)
PBS
Bleo
p=0.978
Relative MS−Intensity (log 2)
FR1
FR2
FR3
INSOL

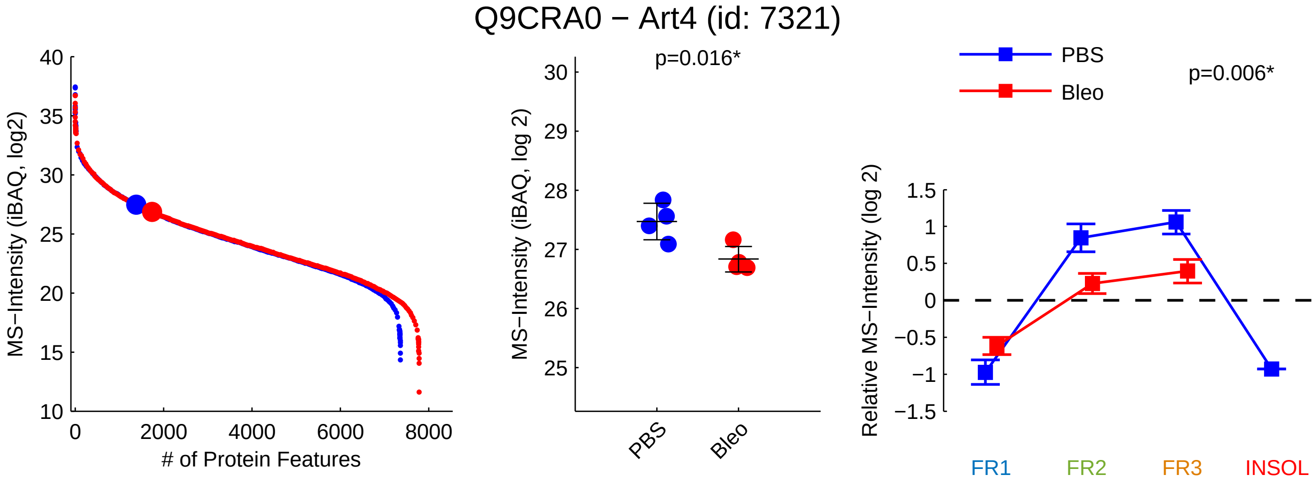
Q9CRA0 − Art4 (id: 7321)
MS−Intensity (iBAQ, log2)
# of Protein Features
p=0.016*
MS−Intensity (iBAQ, log 2)
PBS
Bleo
p=0.006*
Relative MS−Intensity (log 2)
FR1
FR2
FR3
INSOL

Q9CRB6 − Tppp3 (id: 7330)

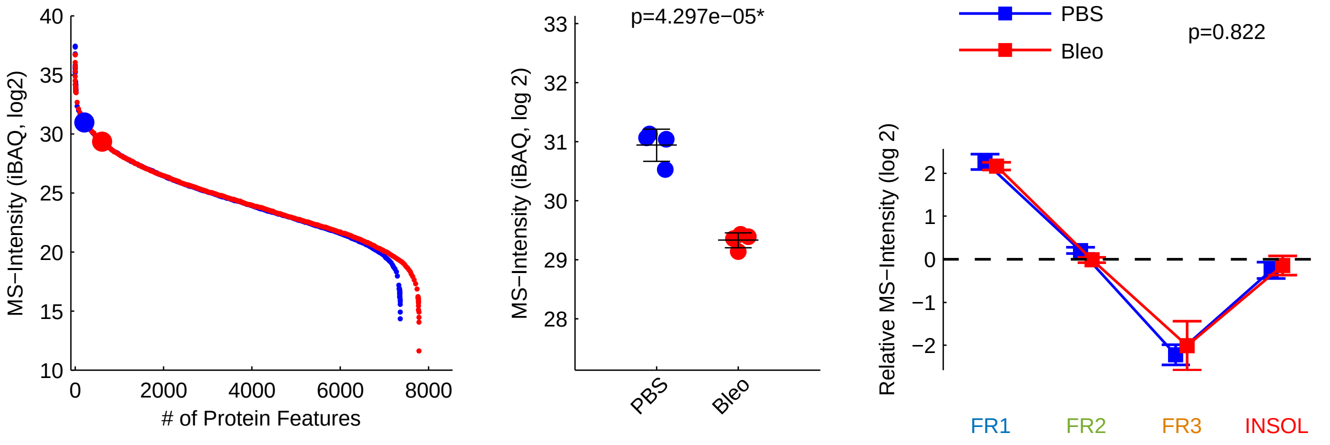

Q9CRD0 − Ociad1 (id: 7338)

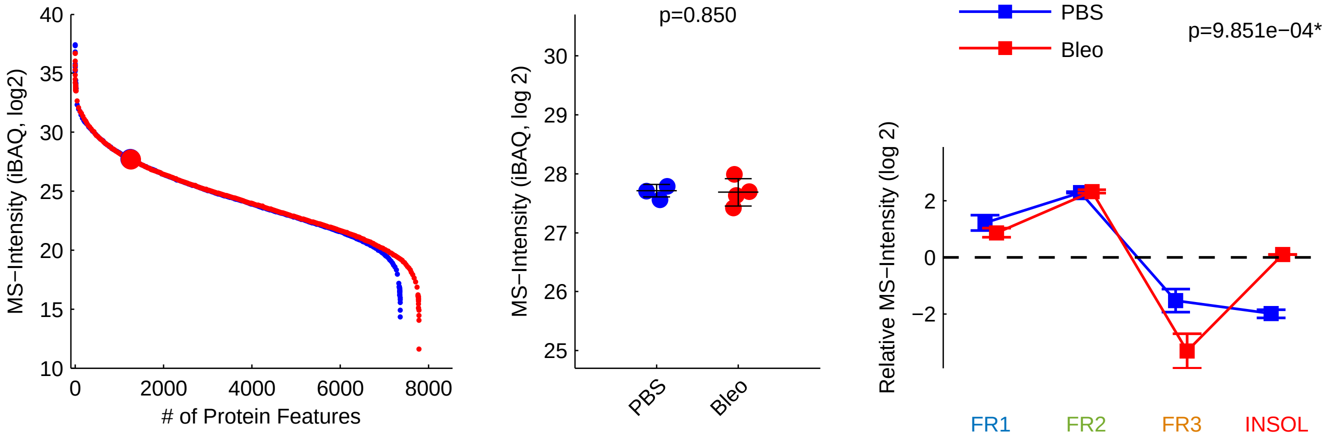

Q9CS00 − Cactin (id: 7342)

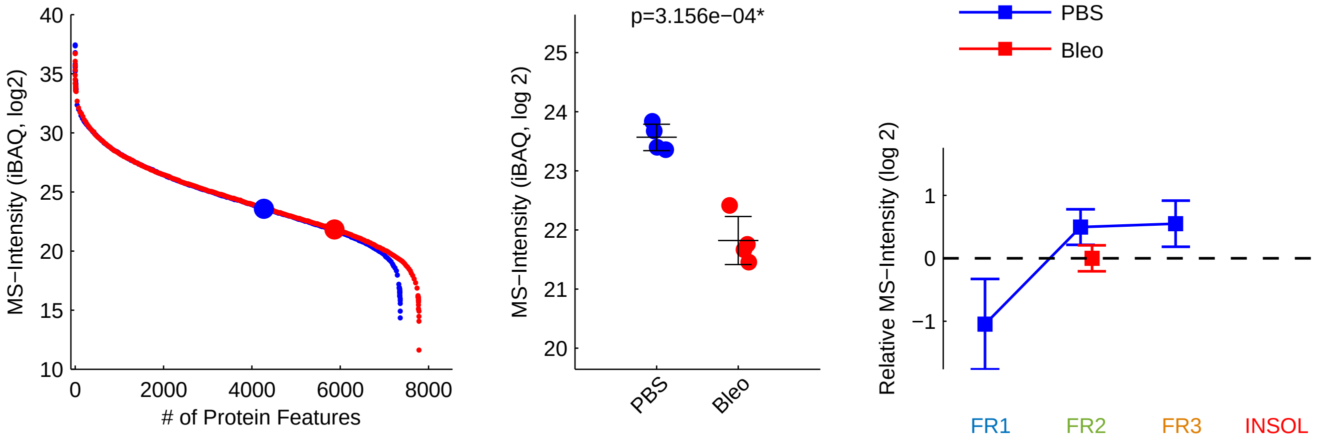

Q9CS42 − Prps2 (id: 7343)

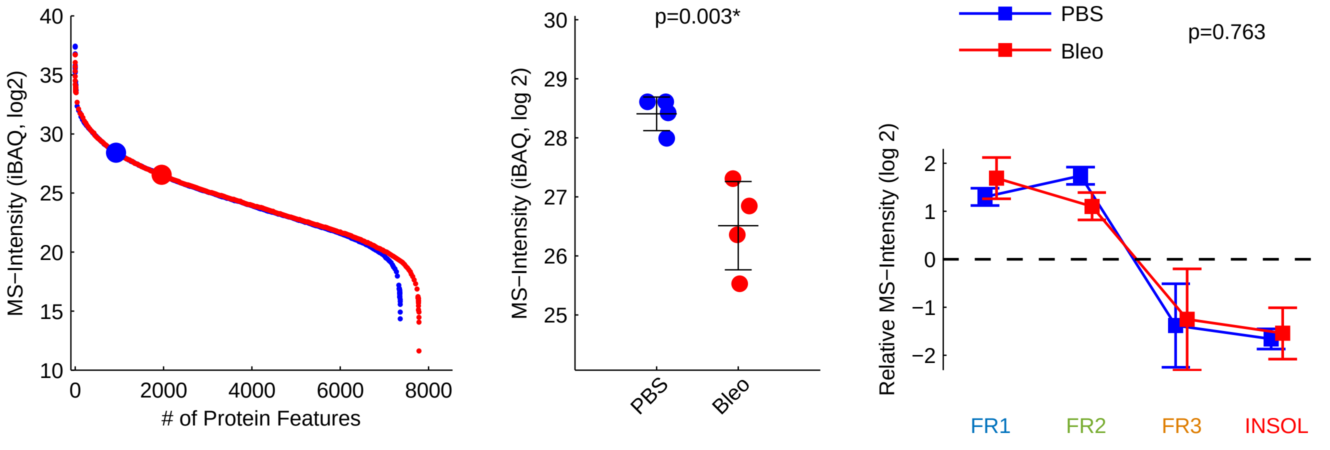

Q9CSB4 − Pard3b (id: 7345)

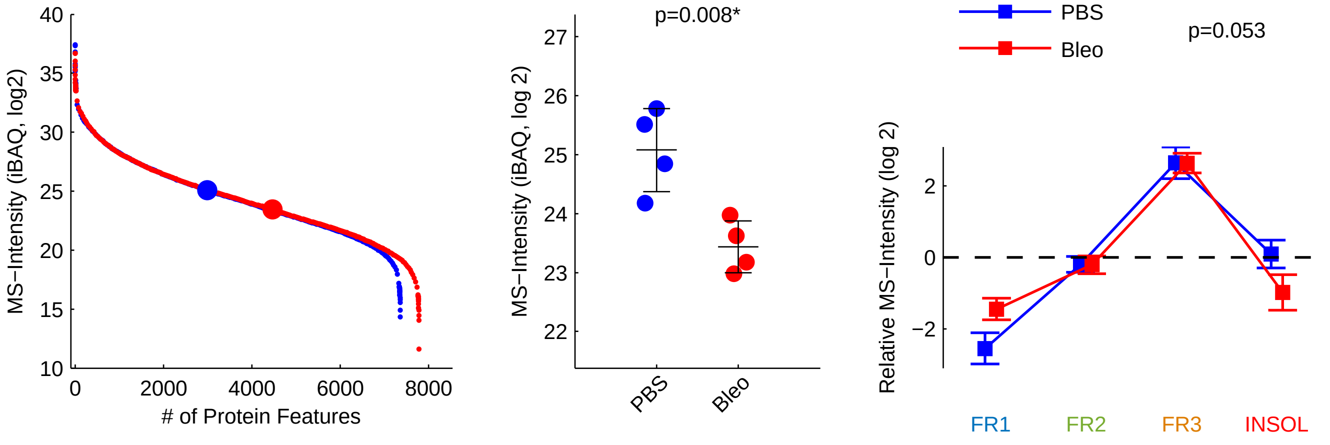

Q9CSN1 − Snw1 (id: 7347)

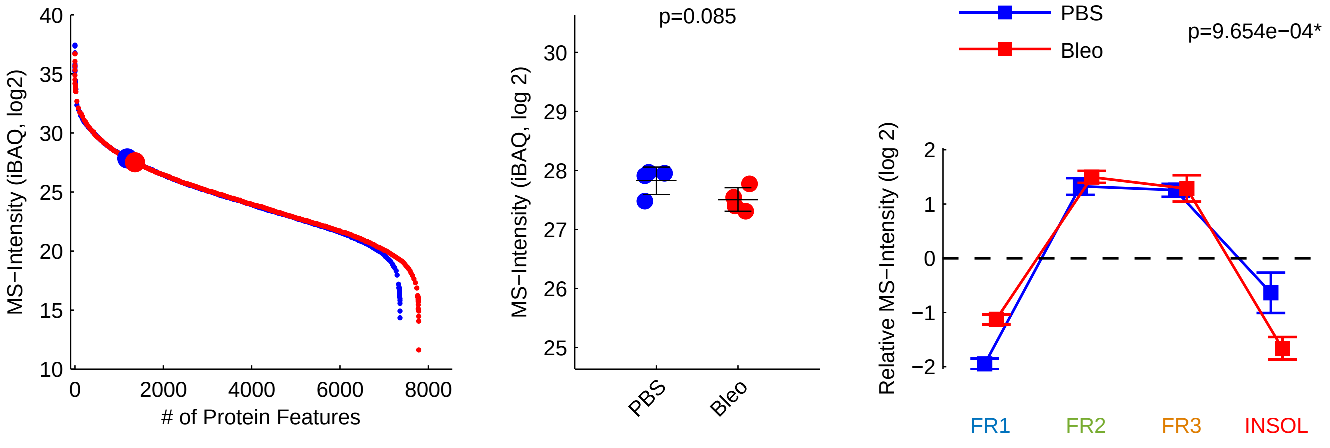

Q9CSU0 − Rprd1b (id: 7348)

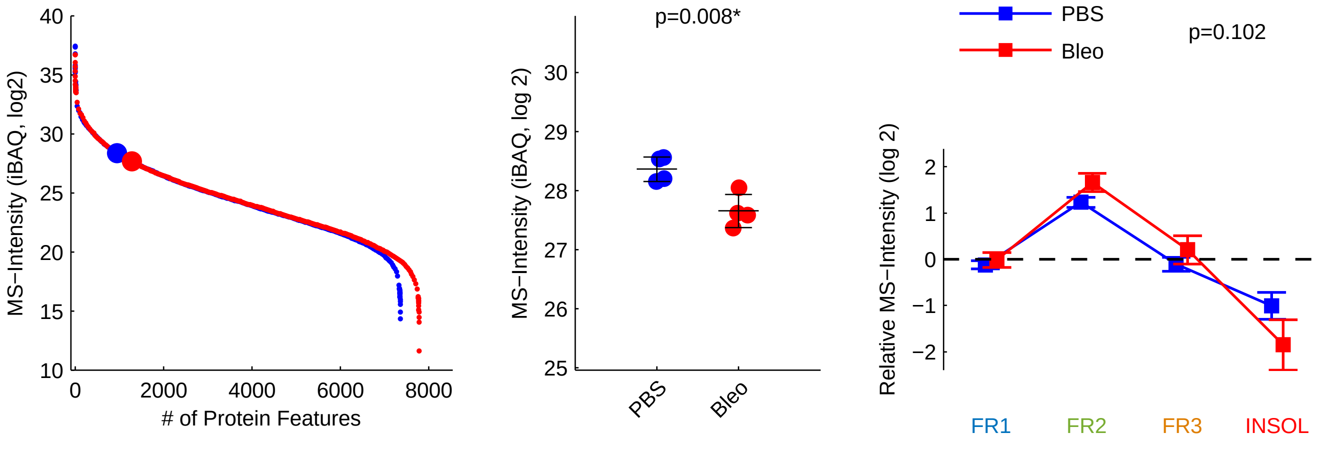

Q9CWF2 − Tubb2b (id: 7360)

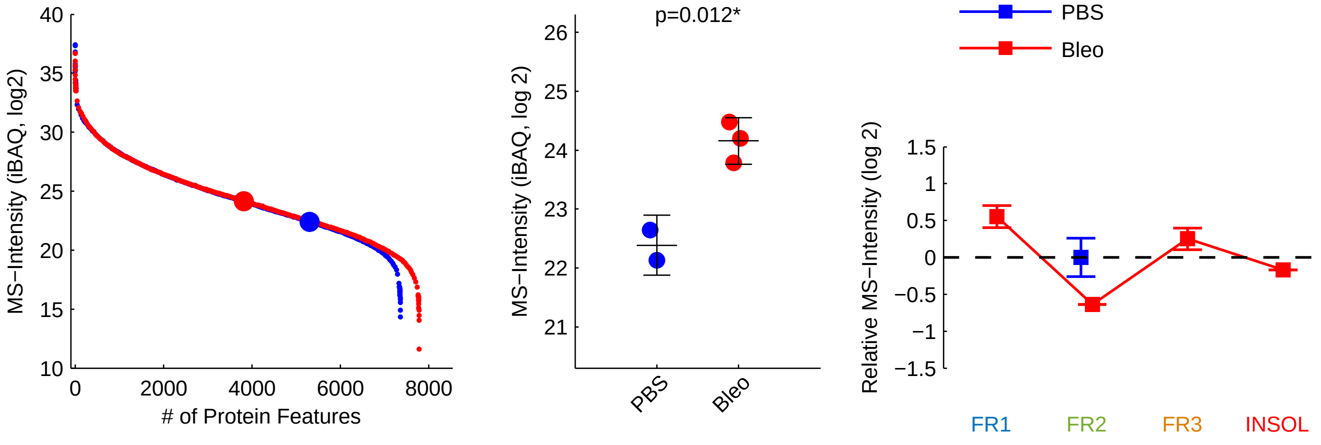

Q9CWS0 − Ddah1 (id: 7372)

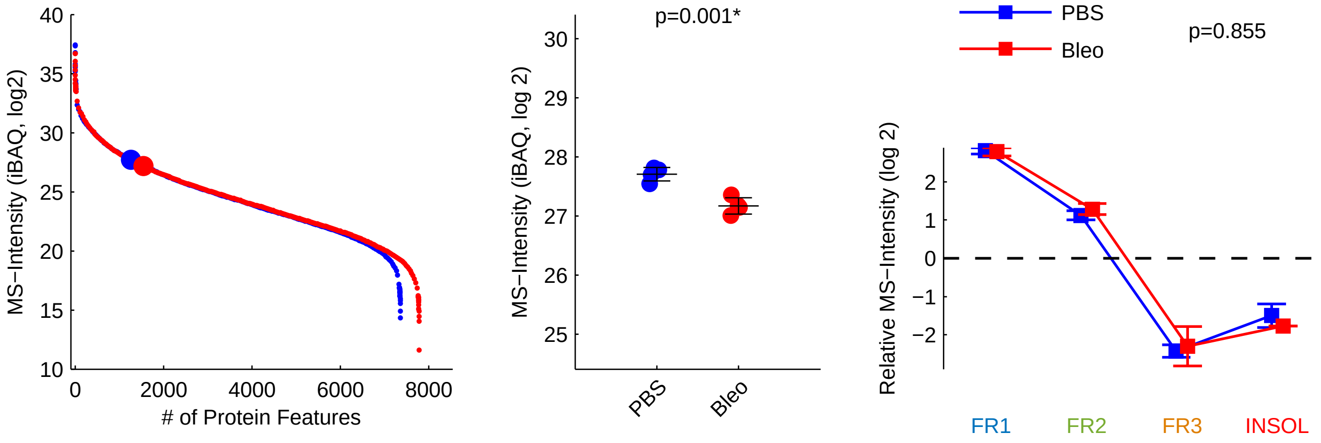

Q9CWW6 − Pin4 (id: 7376)

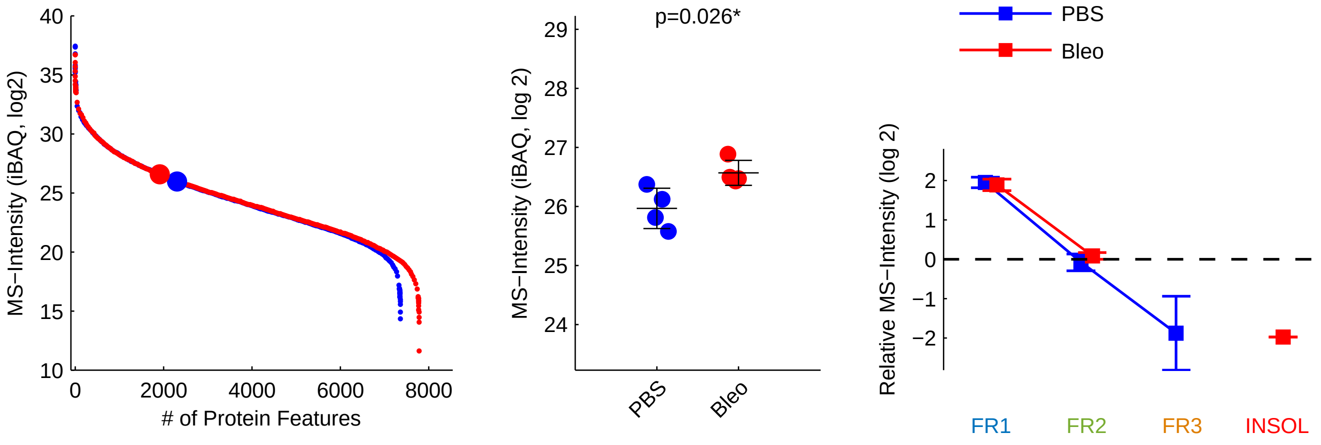

Q9CWZ7 − Napg (id: 7381)

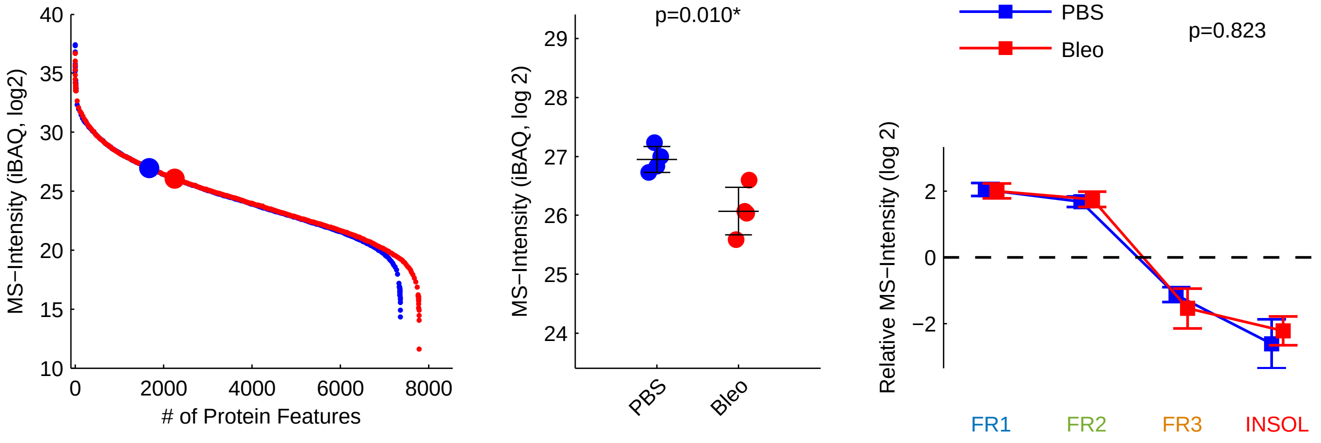

Q9CX00 − Ist1 (id: 7382)

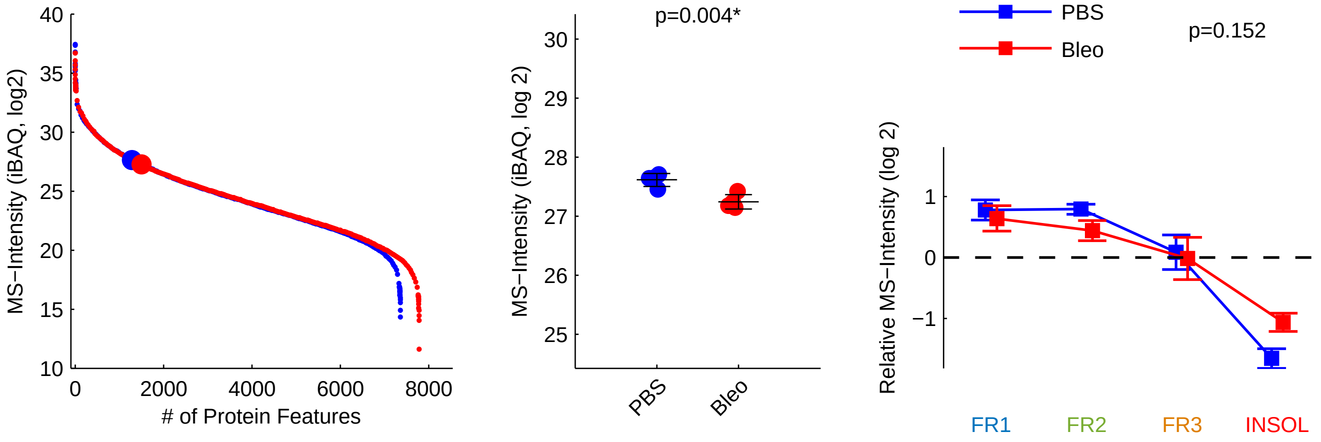

Q9CX30−2 − Yif1b (id: 7385)

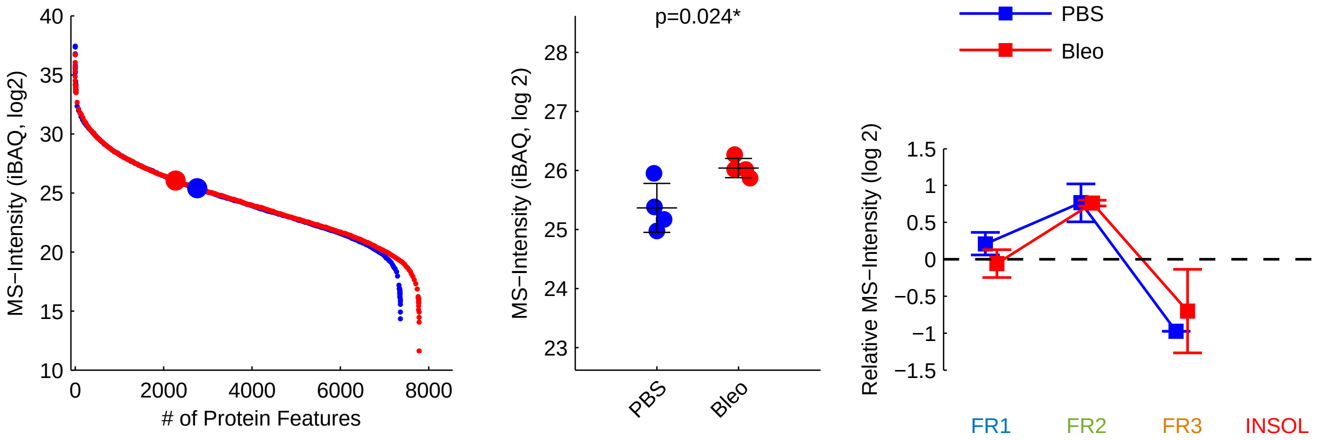

Q9CX34 − Sugt1 (id: 7386)

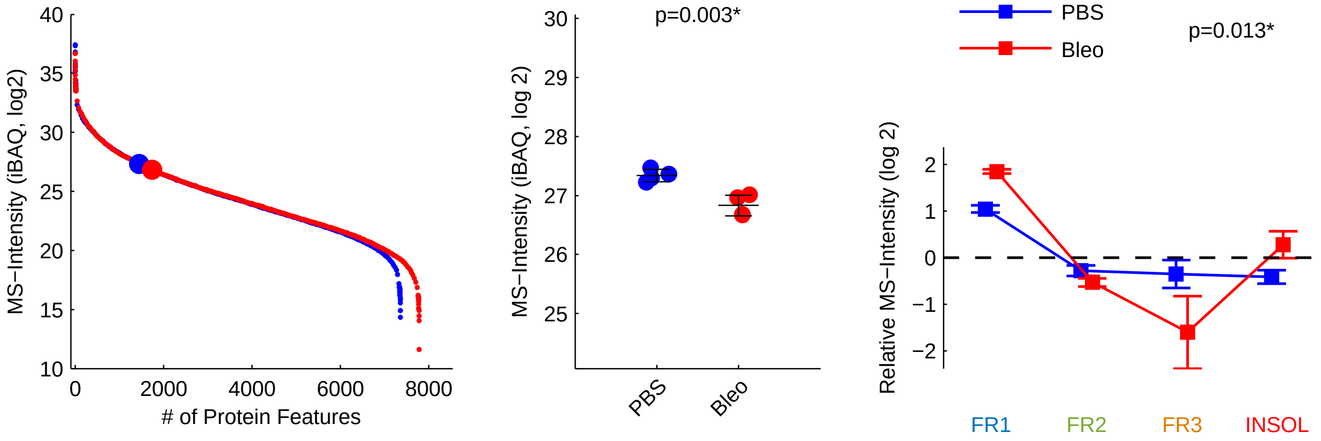

Q9CX56 − Psmd8 (id: 7388)

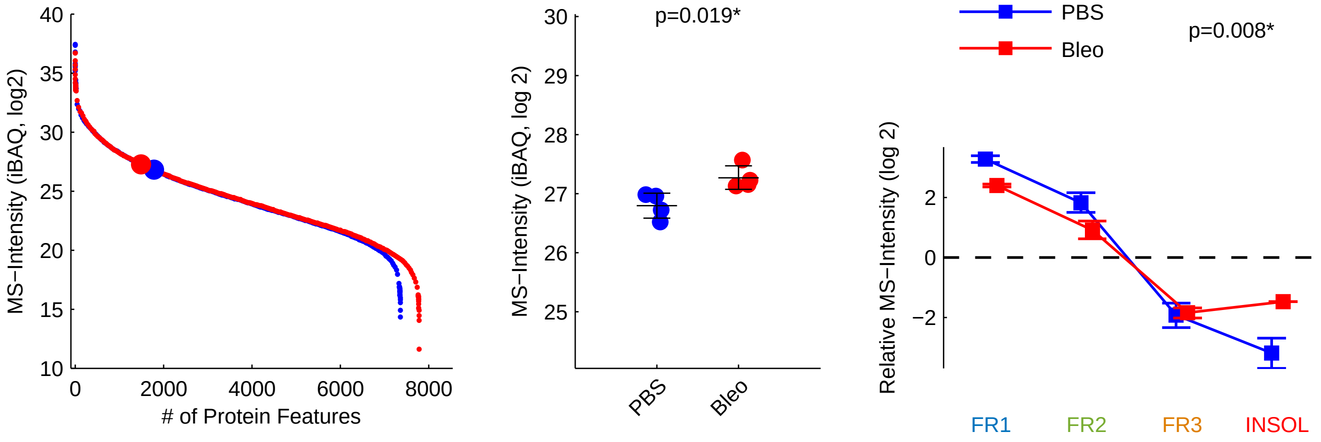

Q9CX83 − Armcx1 (id: 7390)

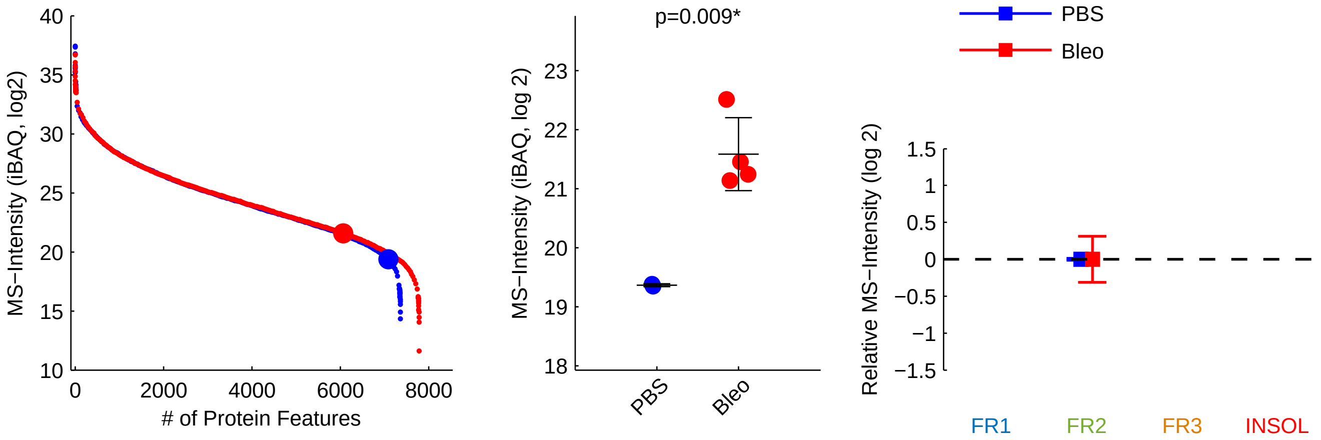

Q9CXF4 − Tbc1d15 (id: 7398)

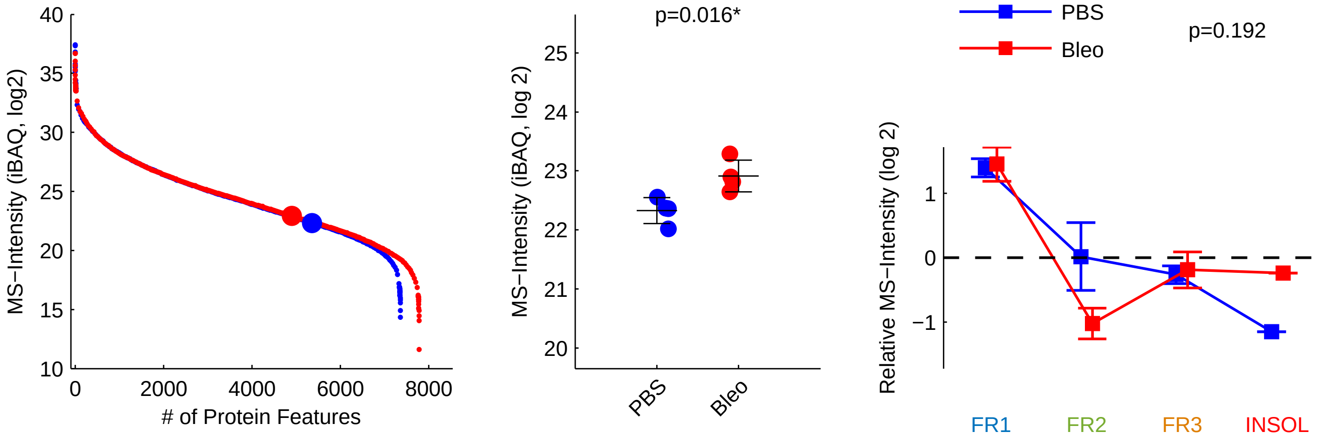

Q9CXG3 − Ppil4 (id: 7400)

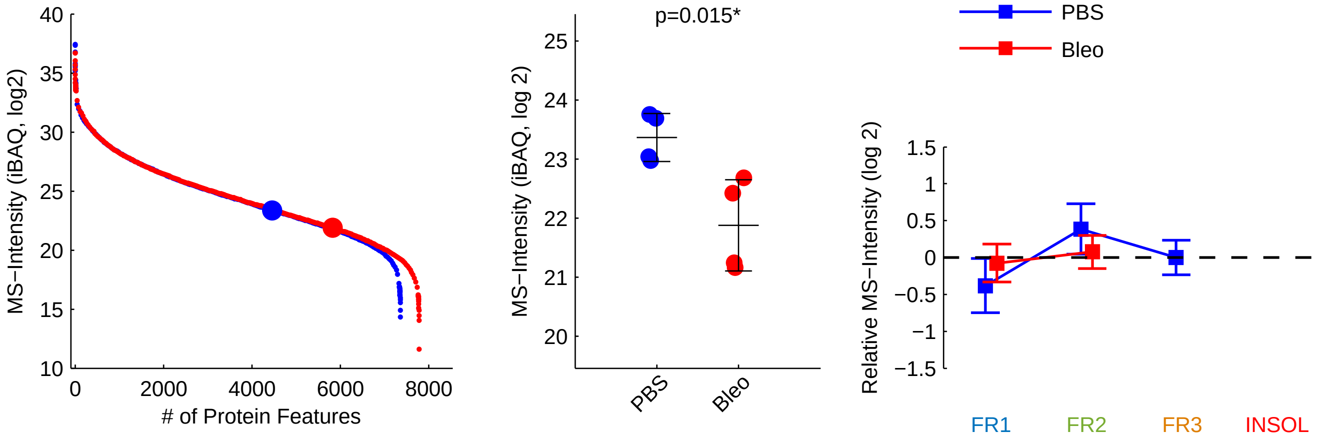

Q9CXK8 − Nip7 (id: 7404)

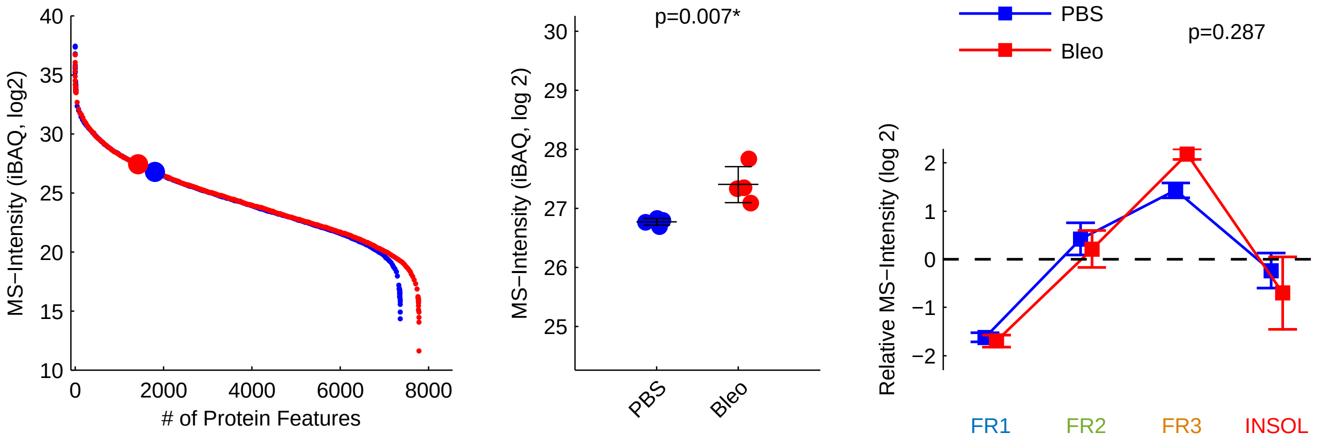

Q9CXR1 − Dhrs7 (id: 7407)

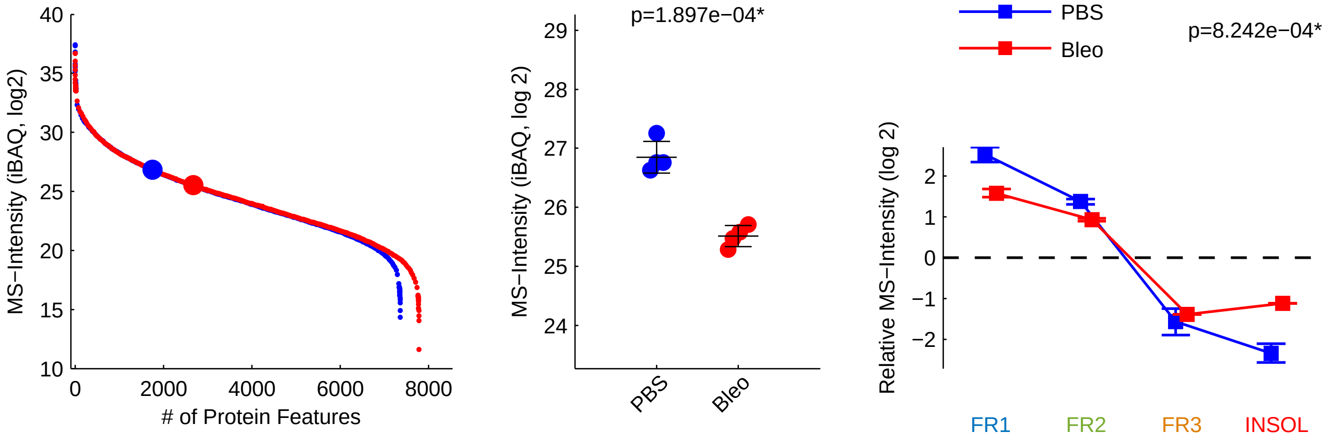

Q9CXS4 − Cenpv (id: 7408)

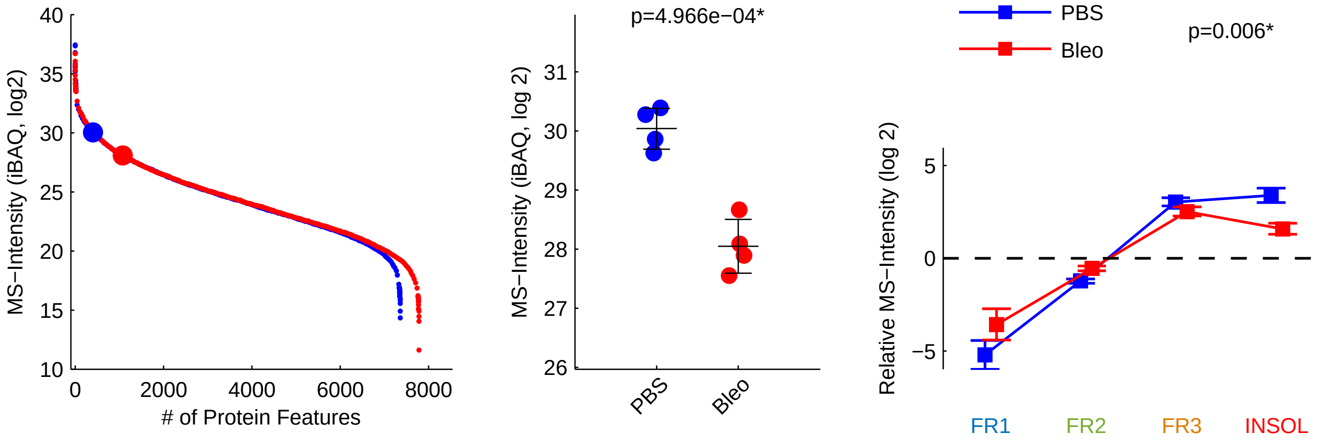

Q9CXW2 − Mrps22 (id: 7416)

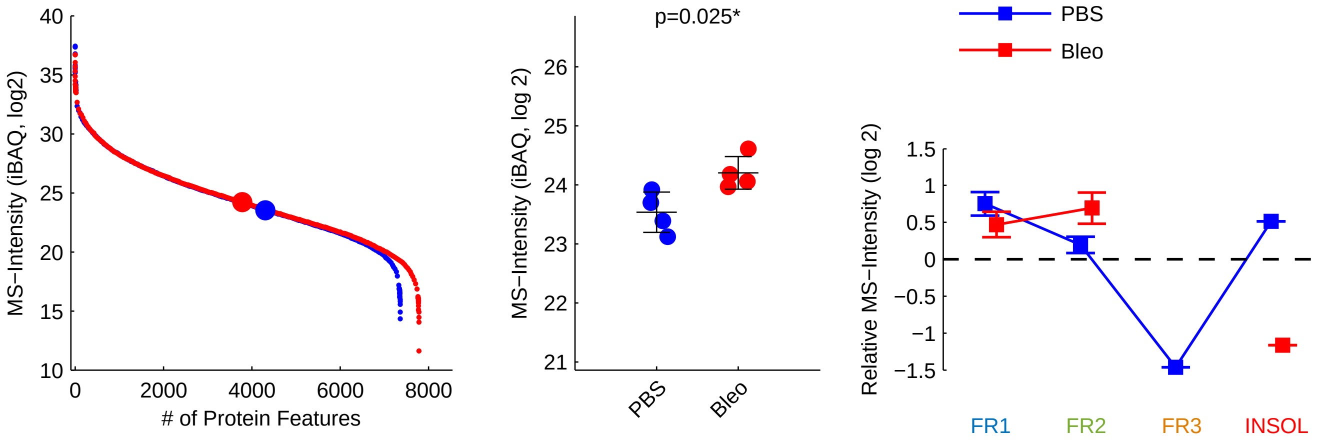

Q9CXW3 − Cacybp (id: 7417)

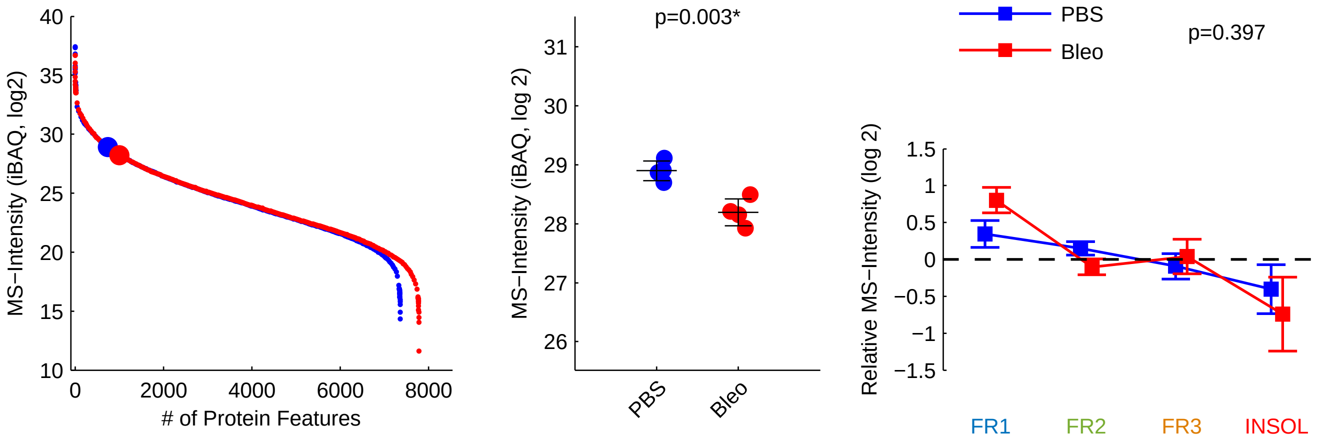

Q9CY27 − Tecr (id: 7423)

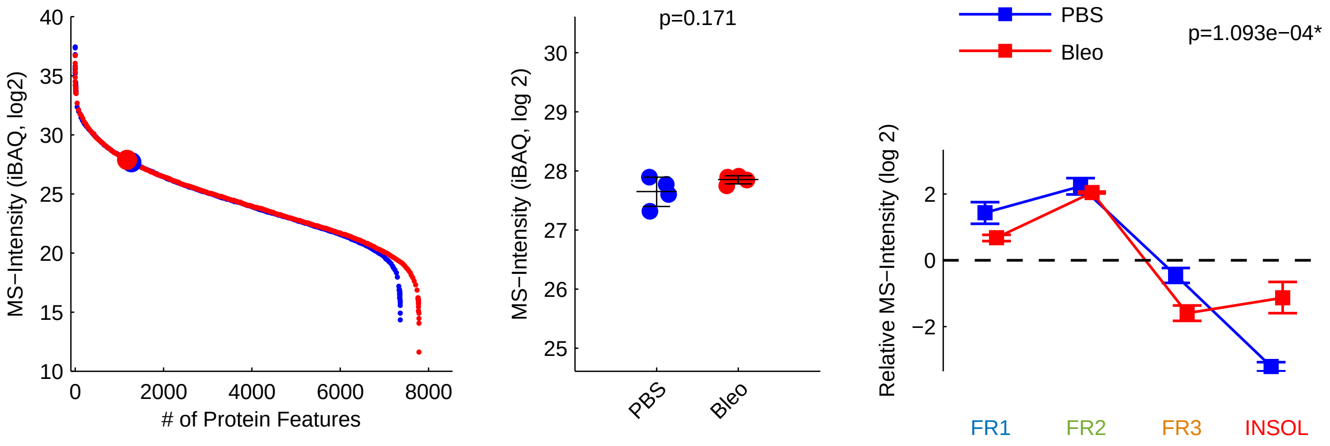

Q9CY58 − Serbp1 (id: 7428)

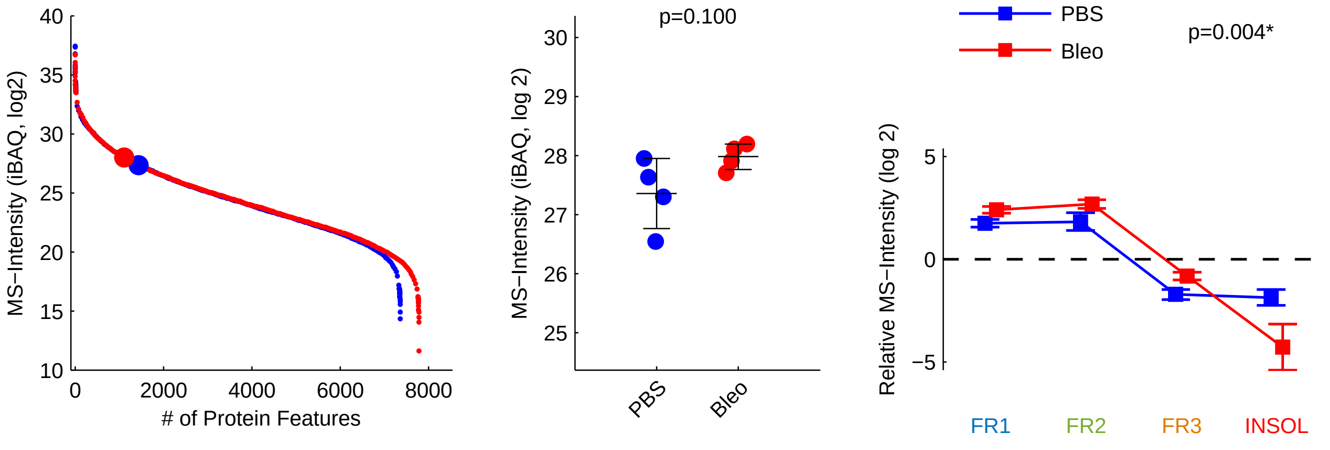

Q9CYA6 − Zcchc8 (id: 7436)

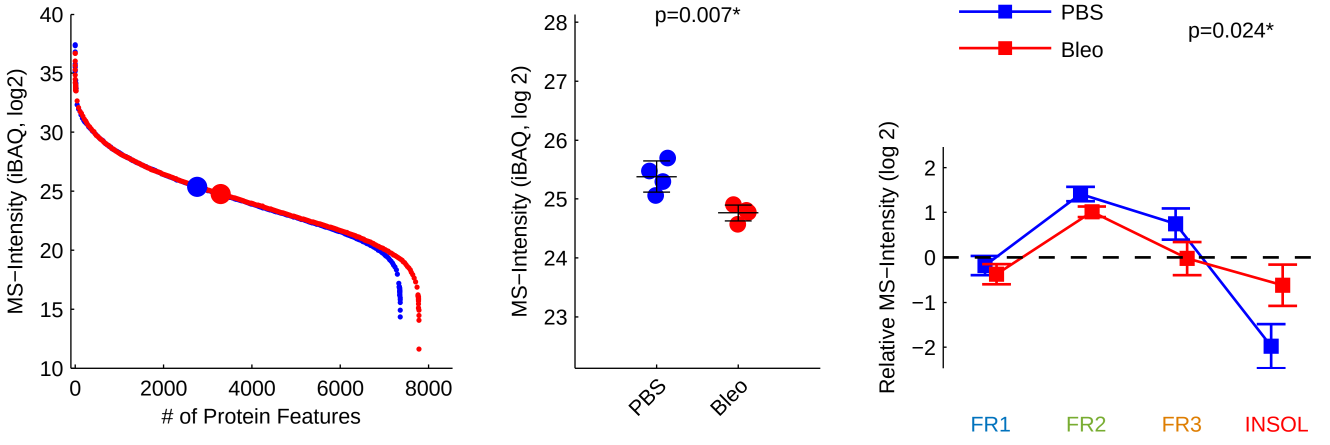

Q9CYD3 − Crtap (id: 7438)

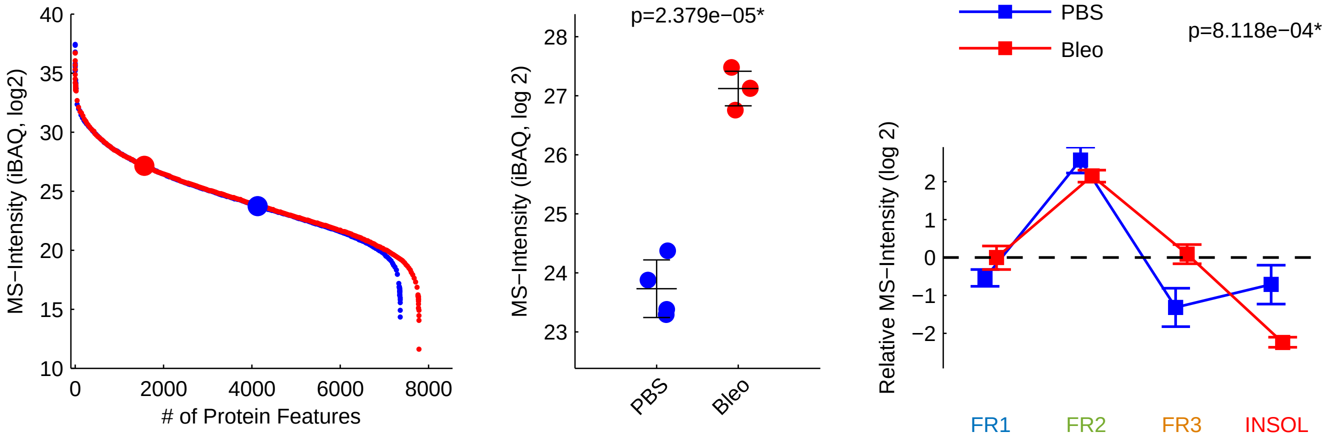

Q9CYI4 − Luc7l (id: 7442)

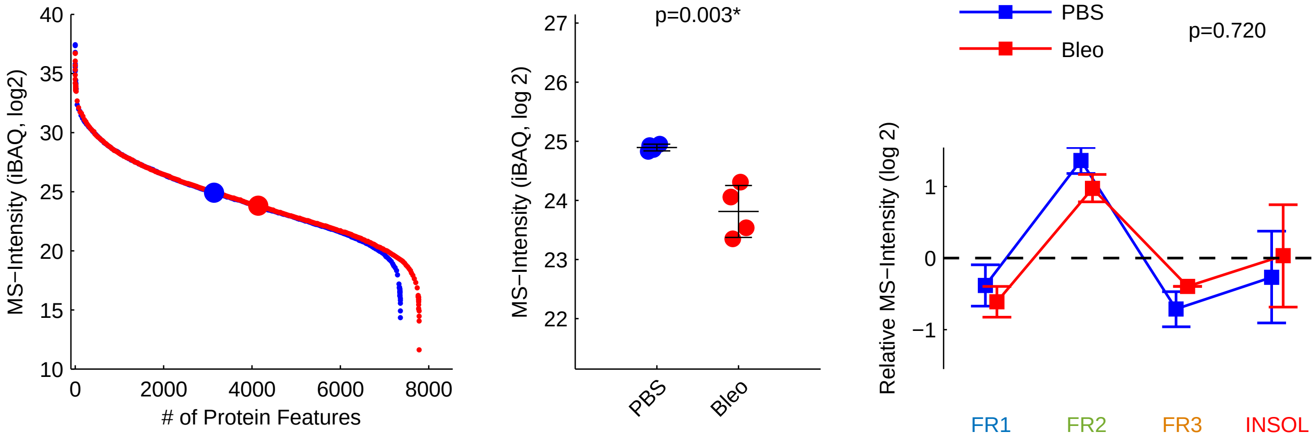

Q9CYL5 − Glipr2 (id: 7443)

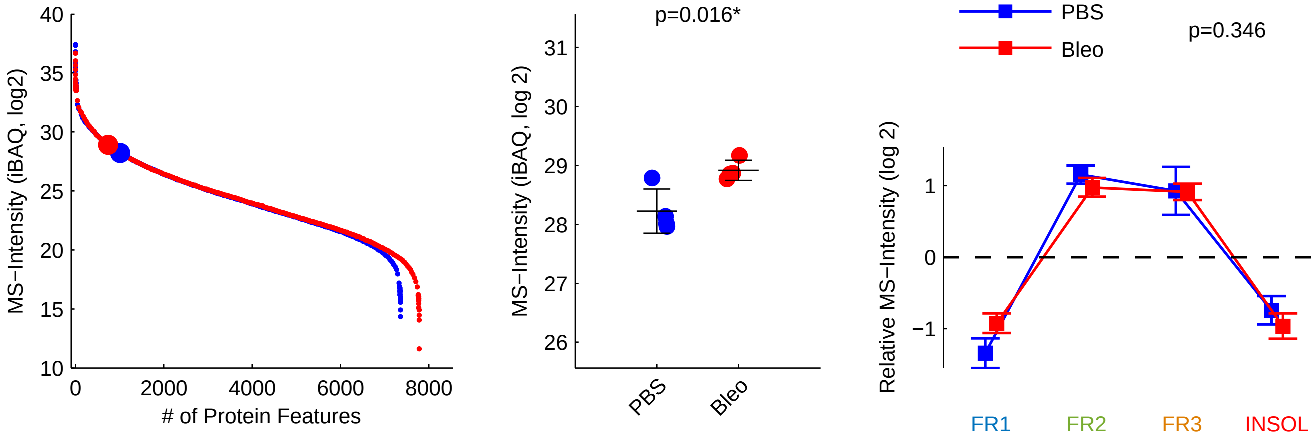

Q9CYT6 − Cap2 (id: 7447)

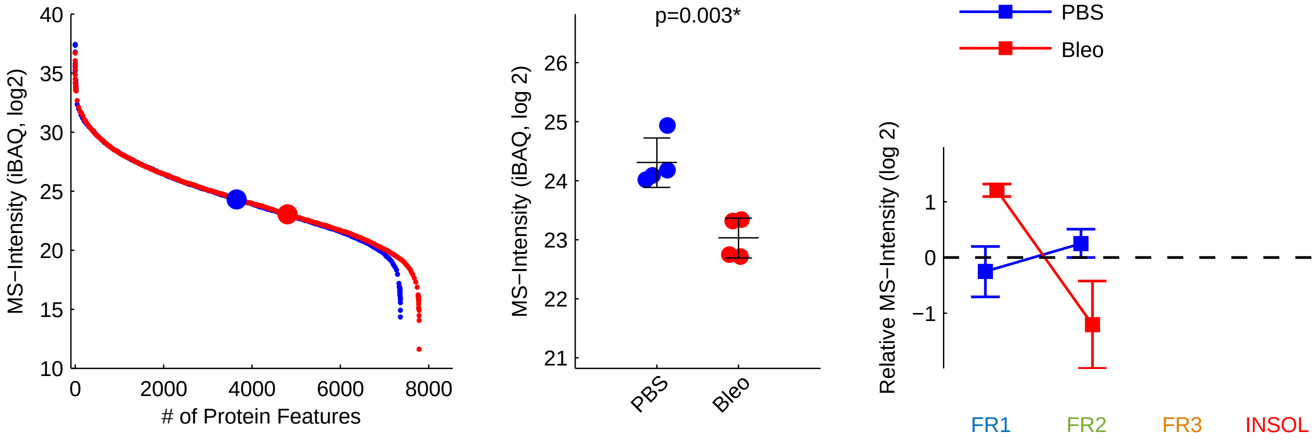

Q9CZD3 − Gars (id: 7464)

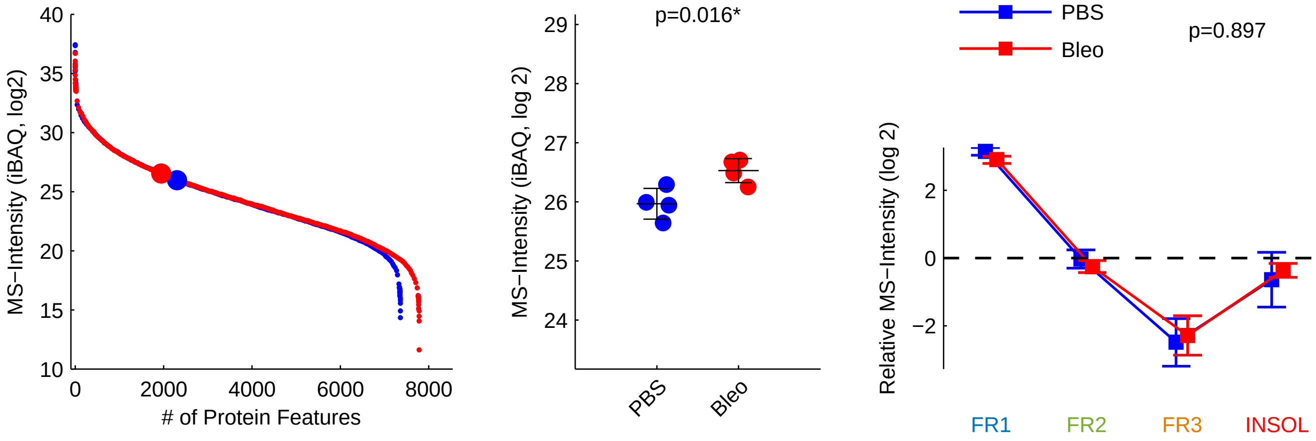

Q9CZJ2 − Hspa12b (id: 7473)

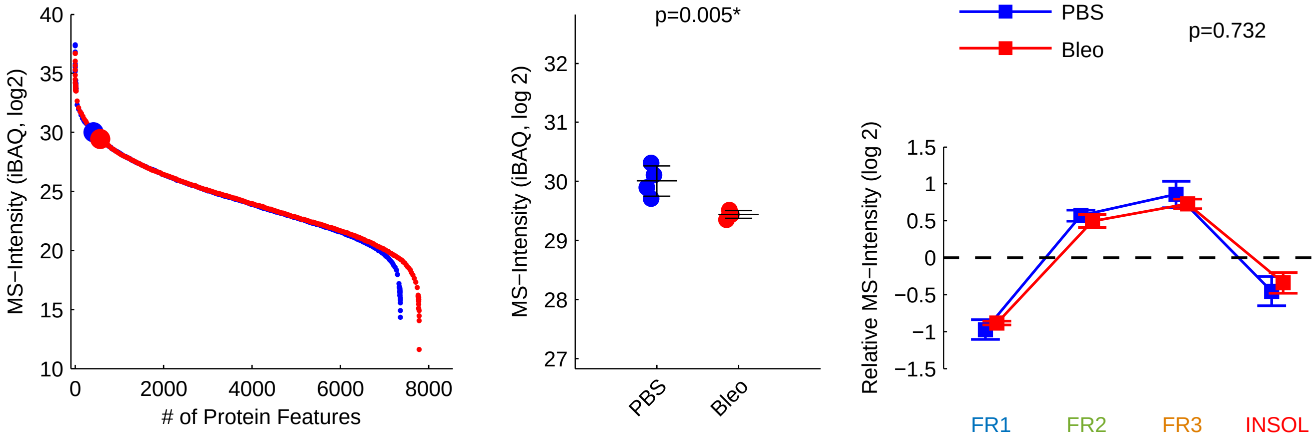

Q9CZN7 − Shmt2 (id: 7475)

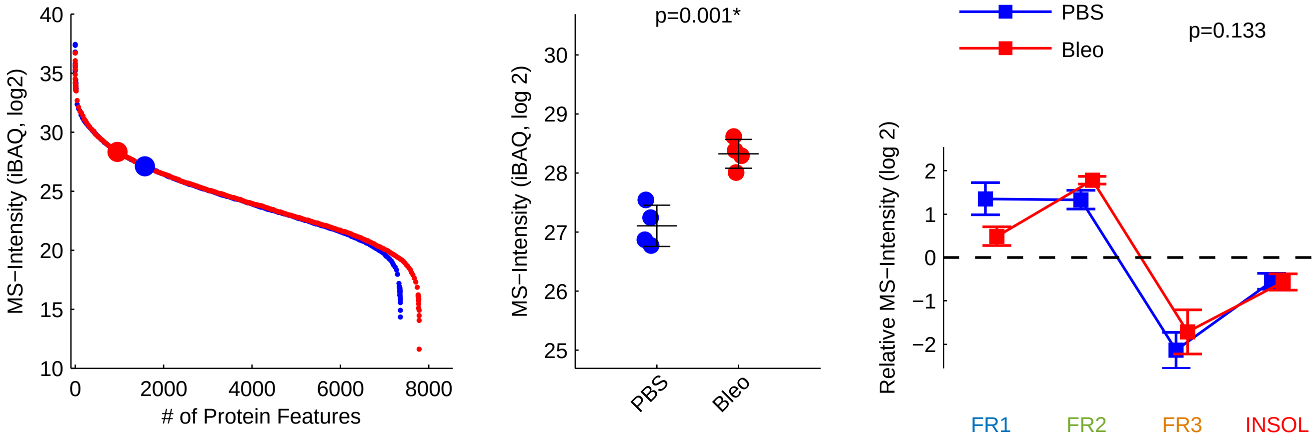

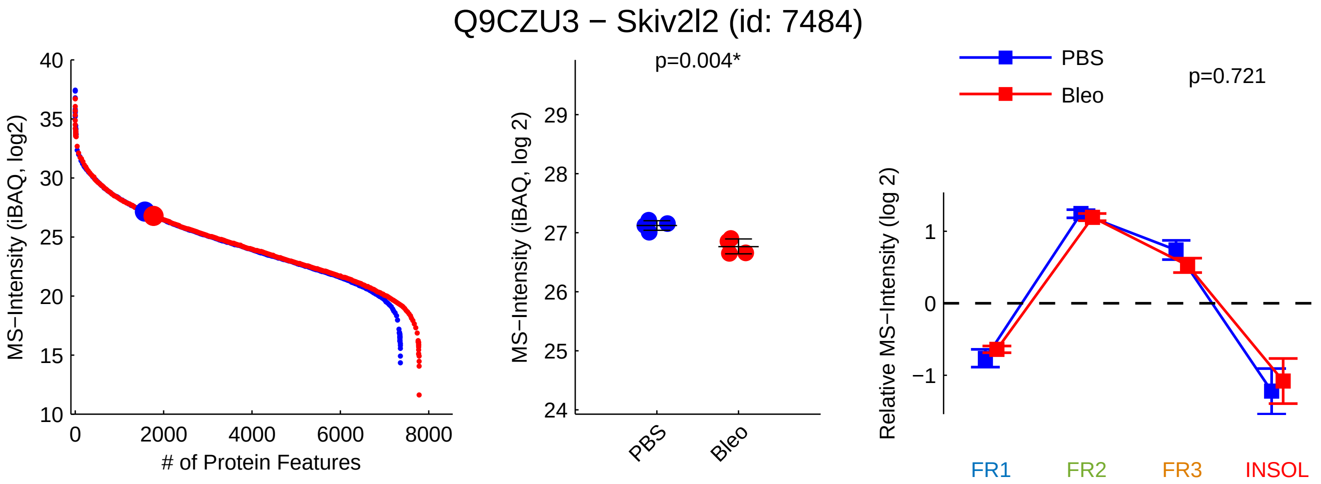
Q9CZU3 − Skiv2l2 (id: 7484)
MS−Intensity (iBAQ, log2)
# of Protein Features
p=0.004*
MS−Intensity (iBAQ, log 2)
PBS
Bleo
p=0.721
Relative MS−Intensity (log 2)
FR1
FR2
FR3
INSOL

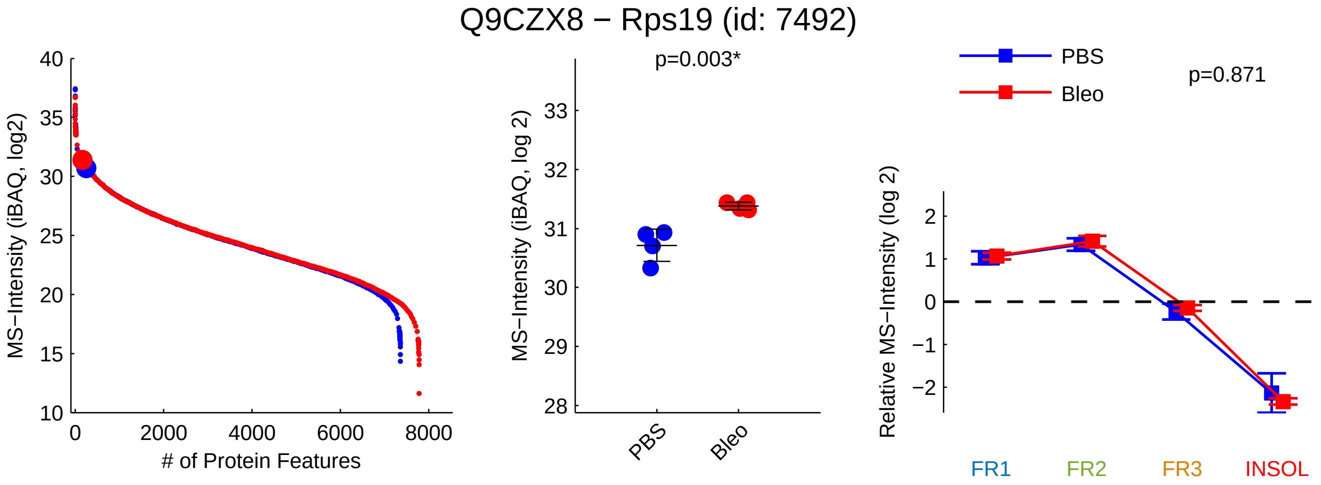
Q9CZX8 − Rps19 (id: 7492)
MS−Intensity (iBAQ, log2)
# of Protein Features
p=0.003*
MS−Intensity (iBAQ, log 2)
PBS
Bleo
p=0.871
Relative MS−Intensity (log 2)
FR1
FR2
FR3
INSOL

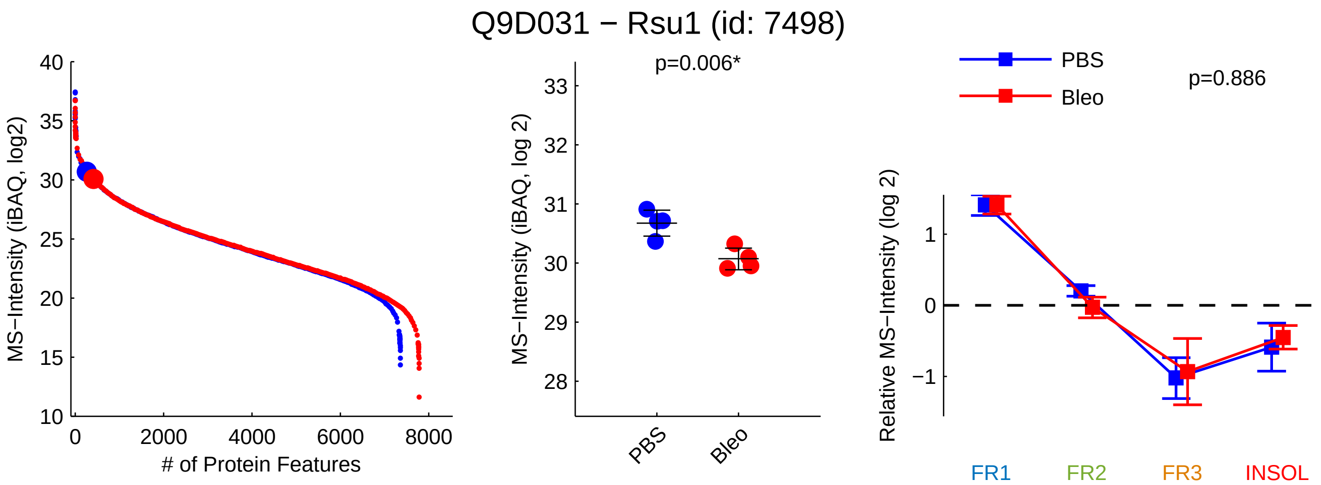
Q9D031 − Rsu1 (id: 7498)
MS−Intensity (iBAQ, log2)
# of Protein Features
p=0.006*
MS−Intensity (iBAQ, log 2)
PBS
Bleo
p=0.886
Relative MS−Intensity (log 2)
FR1
FR2
FR3
INSOL

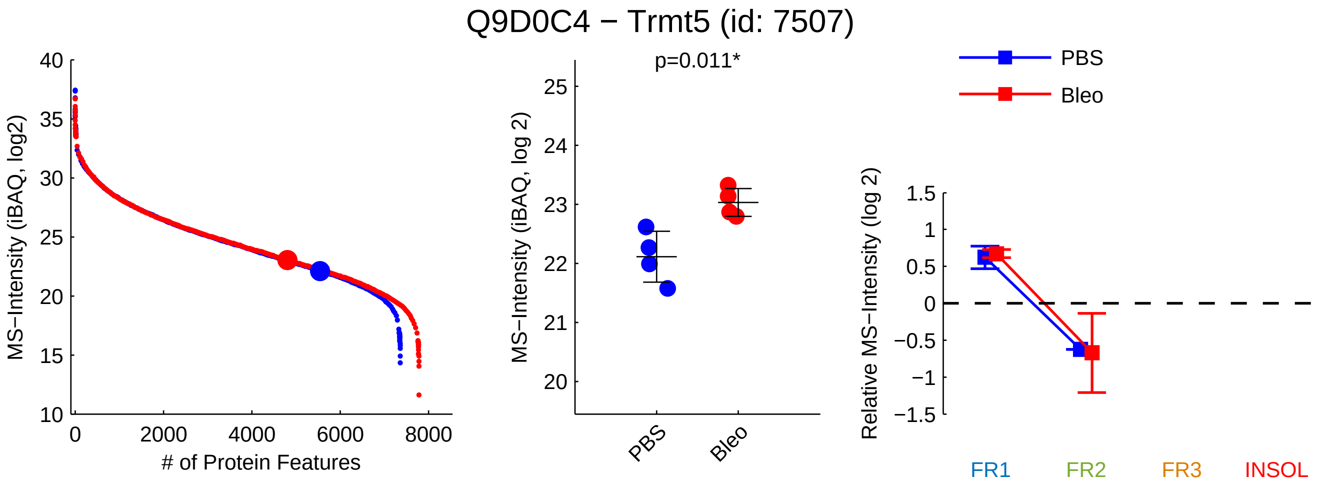
Q9D0C4 − Trmt5 (id: 7507)
MS−Intensity (iBAQ, log2)
# of Protein Features
p=0.011*
MS−Intensity (iBAQ, log 2)
PBS
Bleo
Relative MS−Intensity (log 2)
FR1
FR2
FR3
INSOL

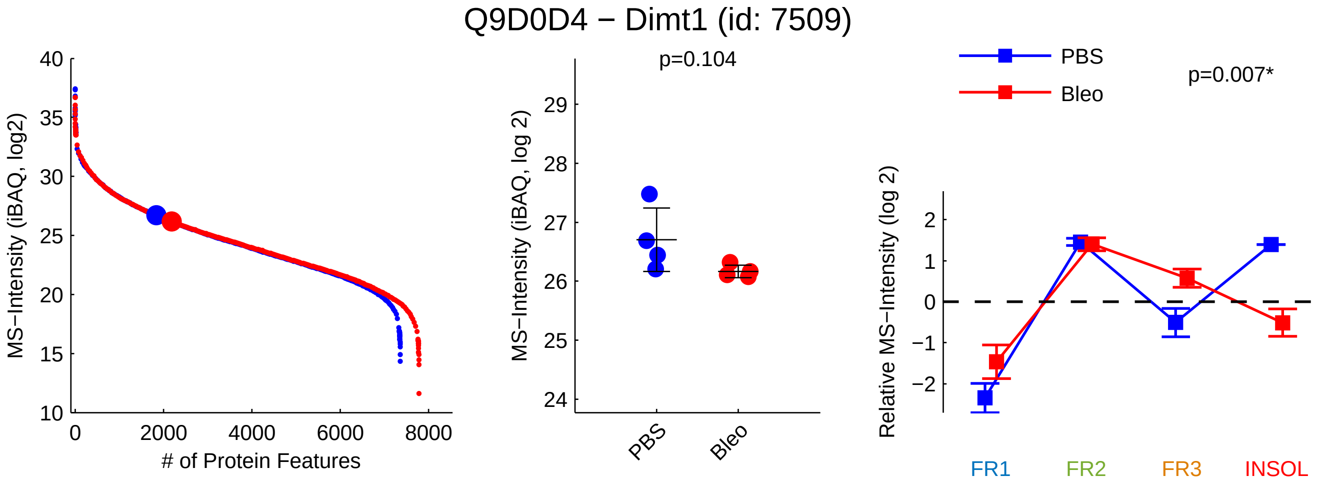
Q9D0D4 − Dimt1 (id: 7509)
MS−Intensity (iBAQ, log2)
# of Protein Features
p=0.104
MS−Intensity (iBAQ, log 2)
PBS
Bleo
p=0.007*
Relative MS−Intensity (log 2)
FR1
FR2
FR3
INSOL

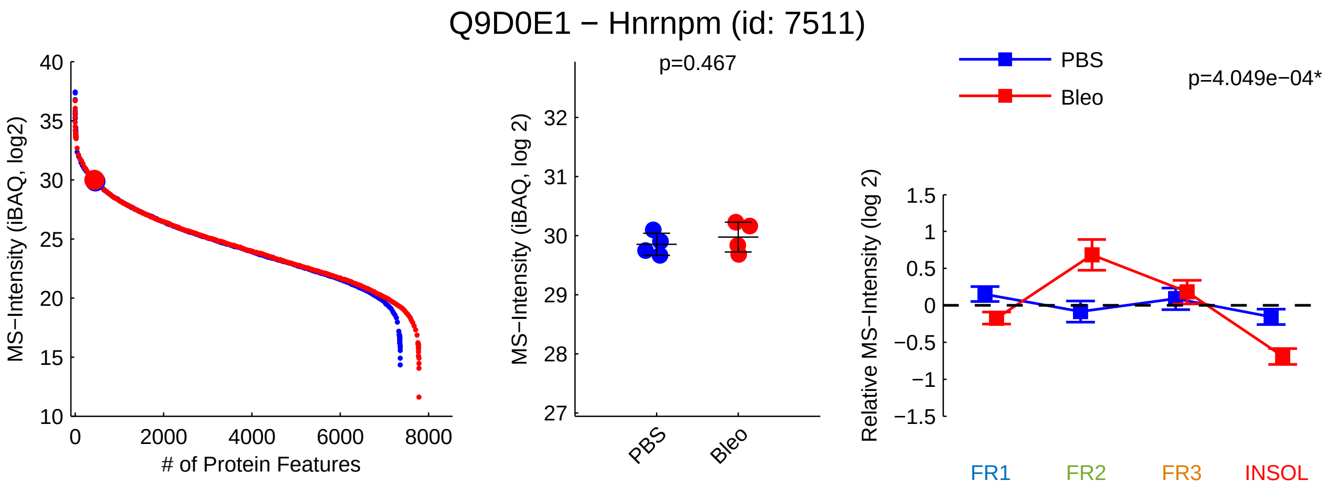
Q9D0E1 − Hnrnpm (id: 7511)
MS−Intensity (iBAQ, log2)
# of Protein Features
p=0.467
MS−Intensity (iBAQ, log 2)
PBS
Bleo
p=4.049e−04*
Relative MS−Intensity (log 2)
FR1
FR2
FR3
INSOL

Q9D0I8 − Mrto4 (id: 7519)

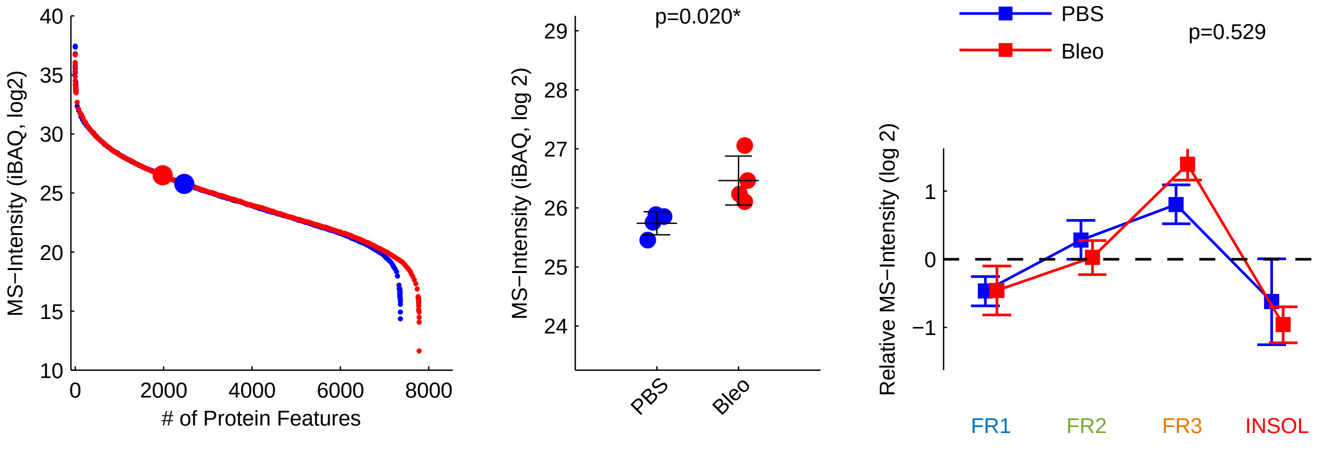

Q9D0I9 − Rars (id: 7520)

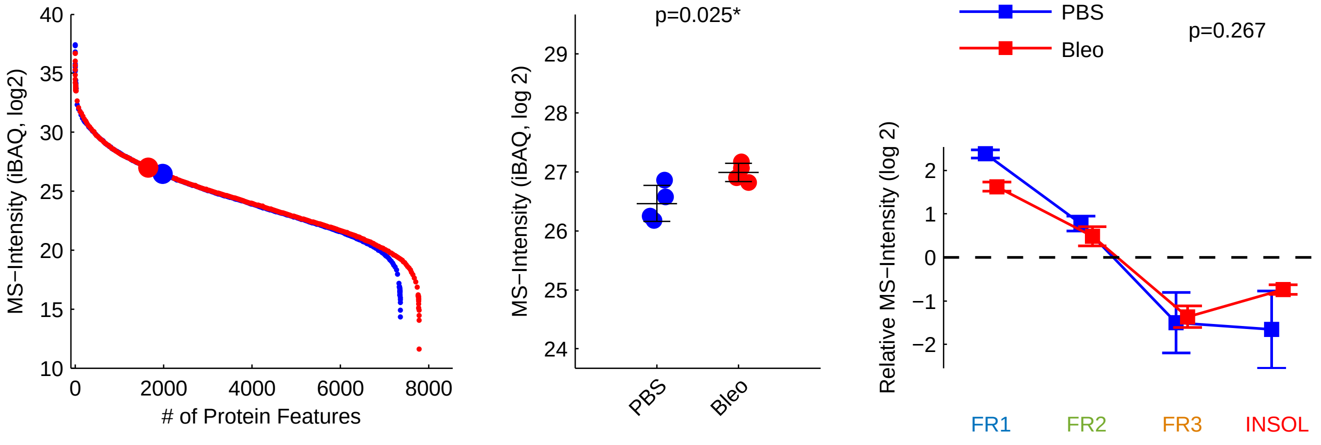

Q9D0M3 − Cyc1 (id: 7530)

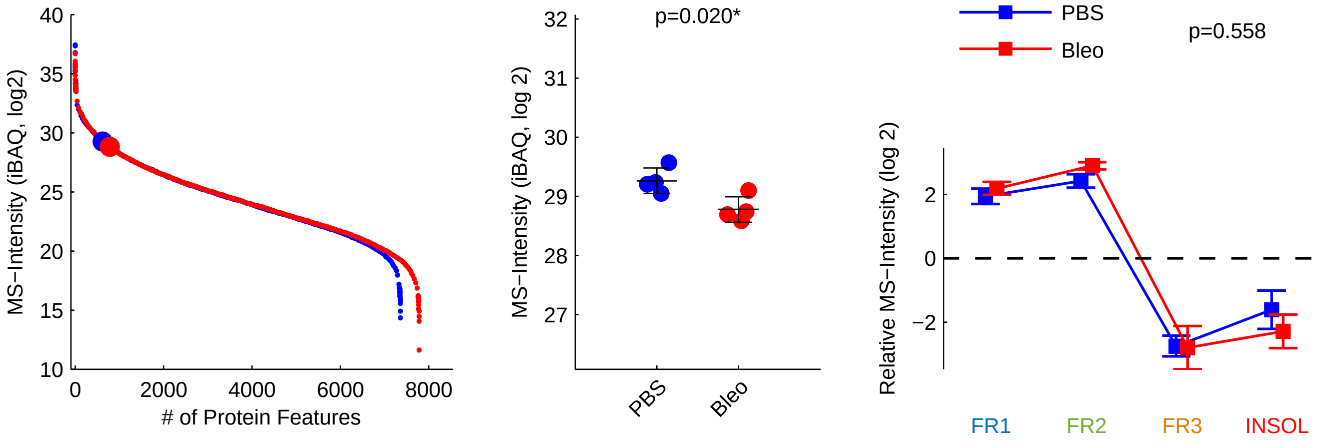

Q9D0R4 − Ddx56 (id: 7536)

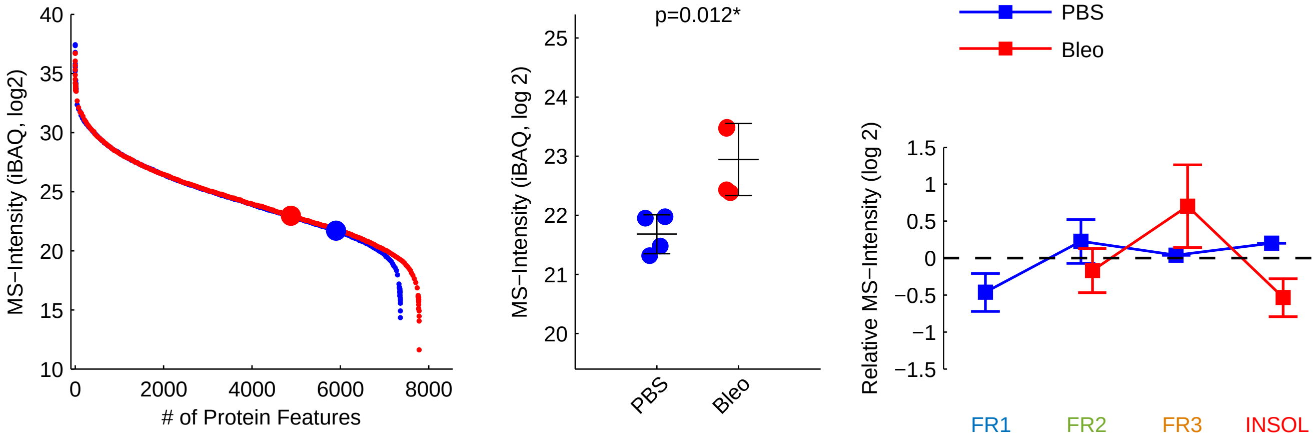

Q9D168 − Ints12 (id: 7548)

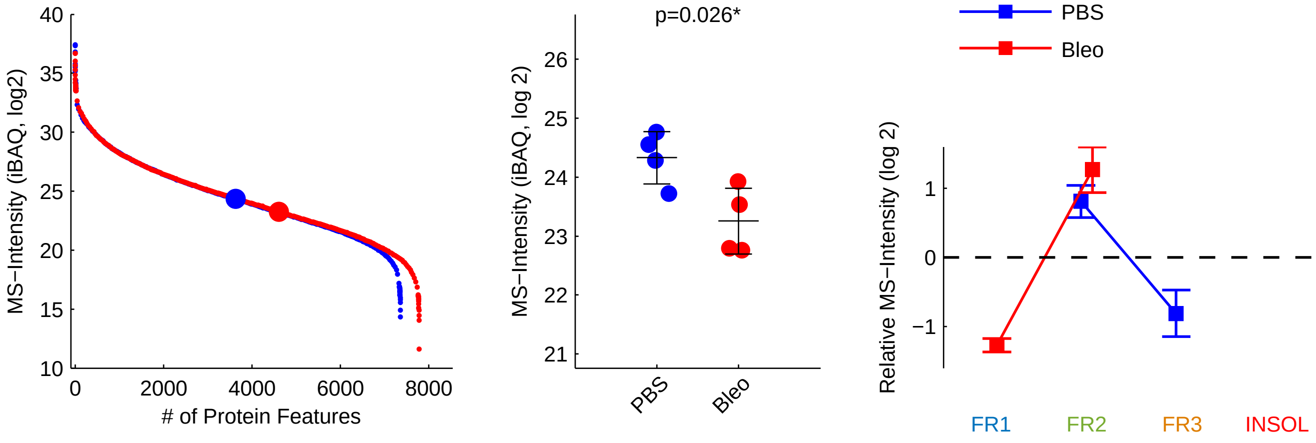

Q9D172 − D10Jhu81e (id: 7549)

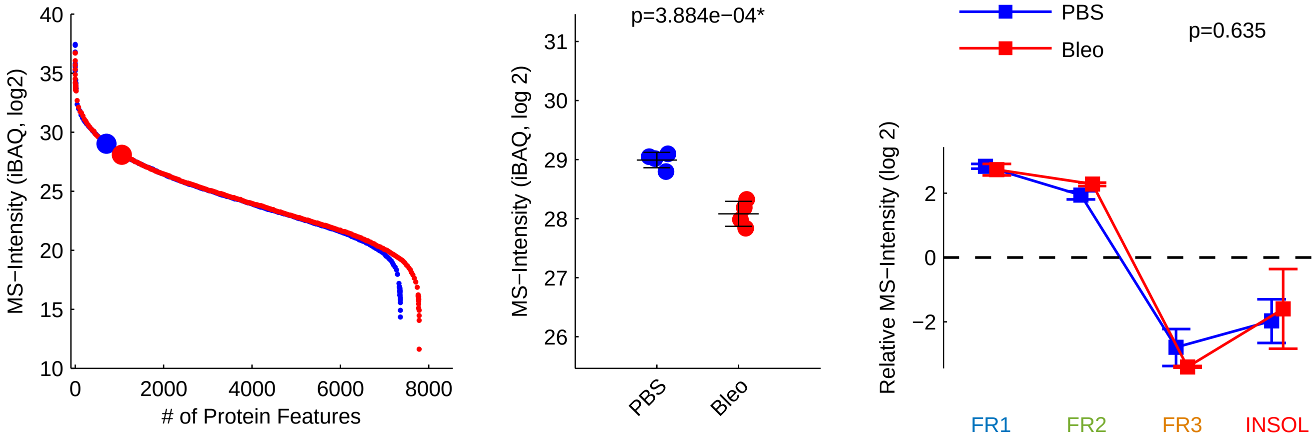

Q9D1D4 − Tmed10 (id: 7558)

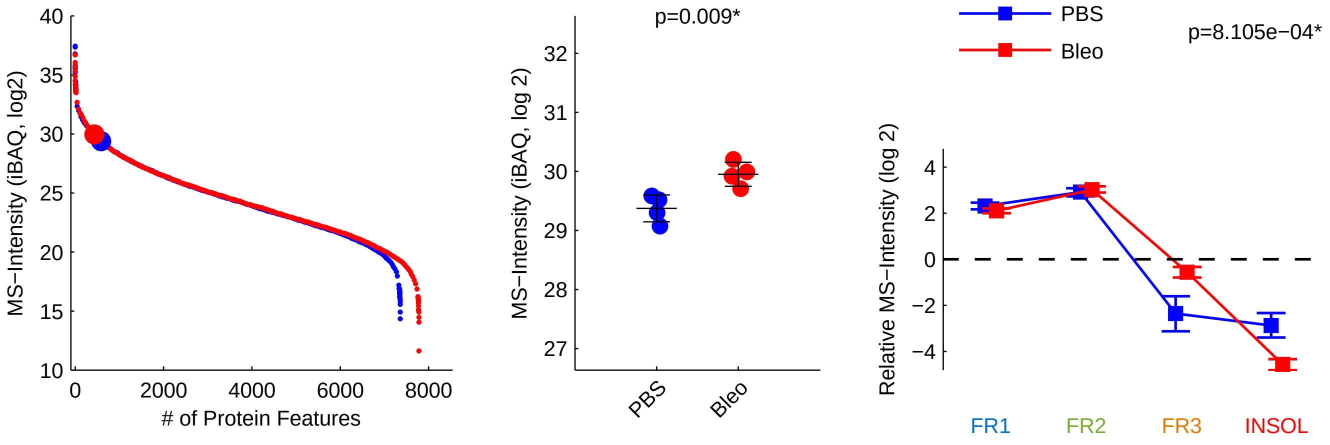

Q9D1I5 − Mcee (id: 7571)

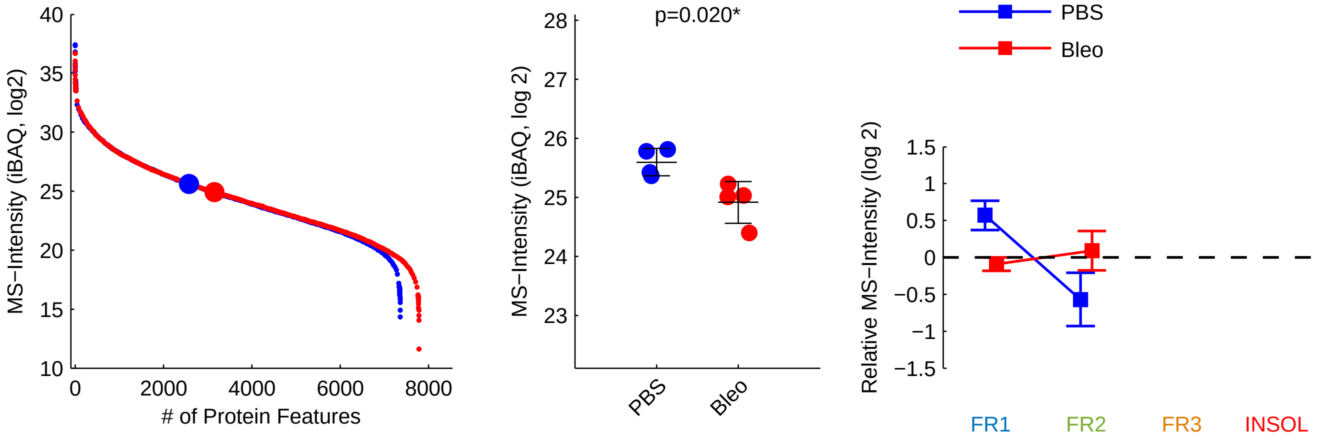

Q9D1I6 − Mrpl14 (id: 7572)

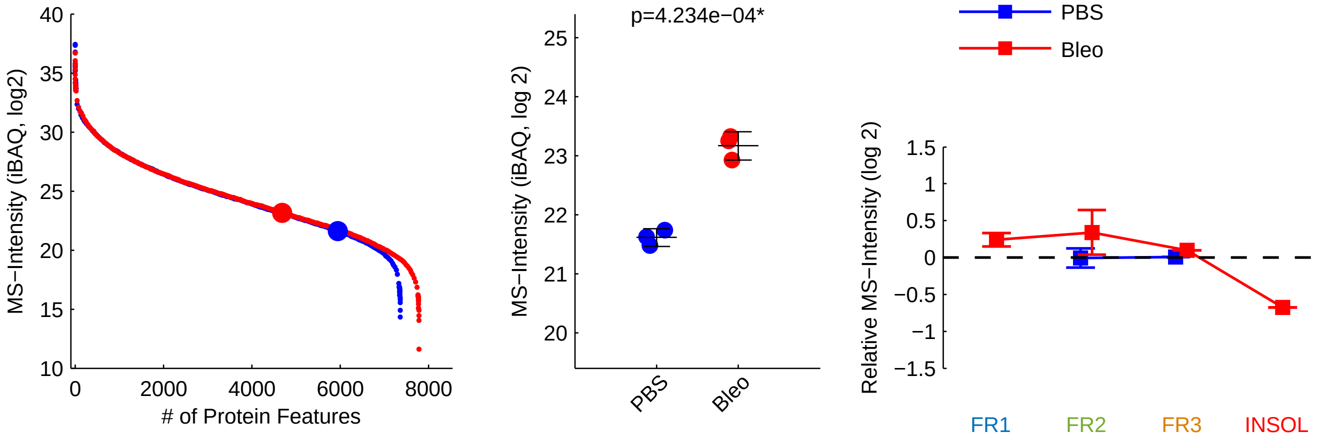

Q9D1L0 − Chchd2 (id: 7576)

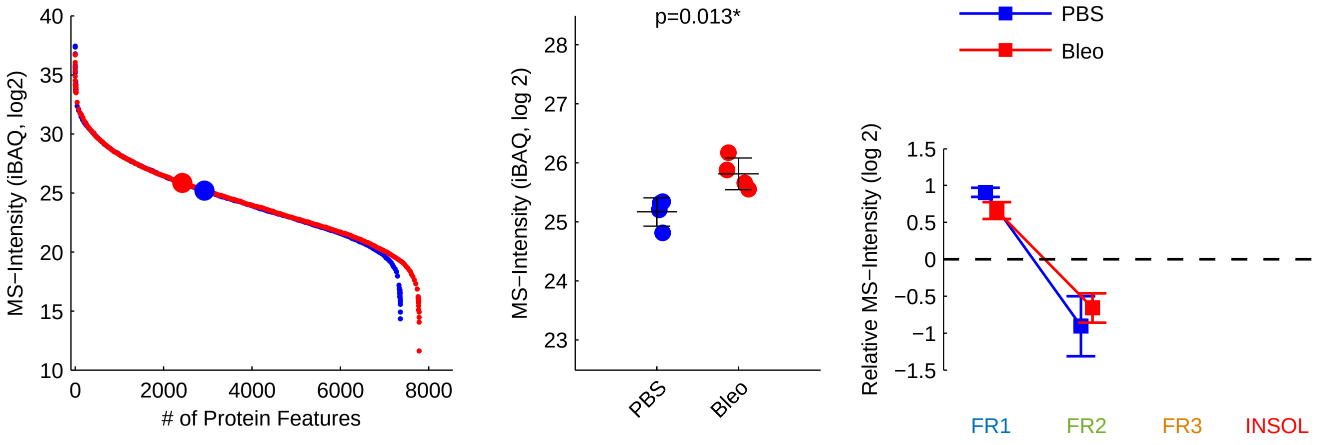

Q9D1M4 − Eef1e1 (id: 7580)

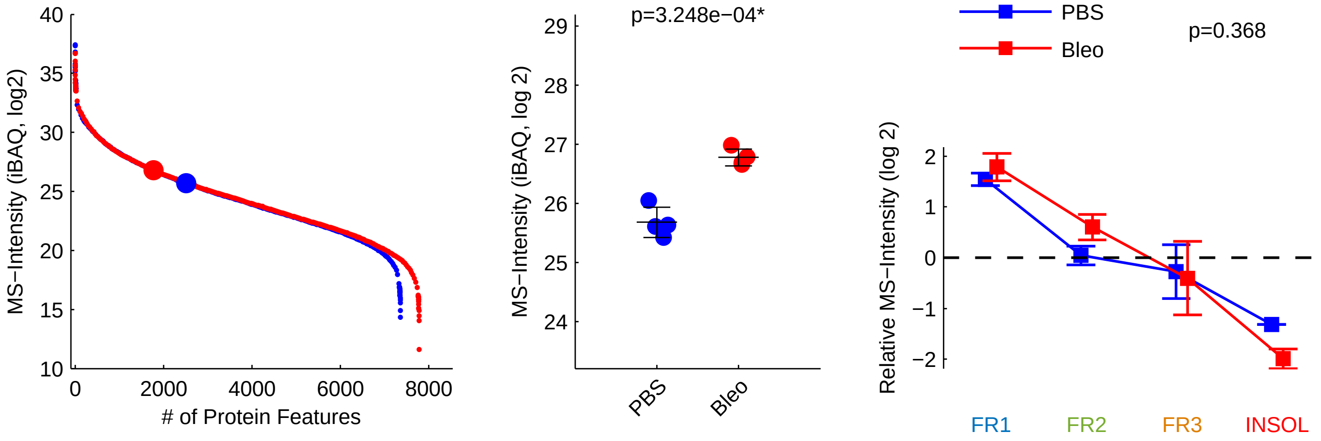

Q9D1P4 − Chordc1 (id: 7586)

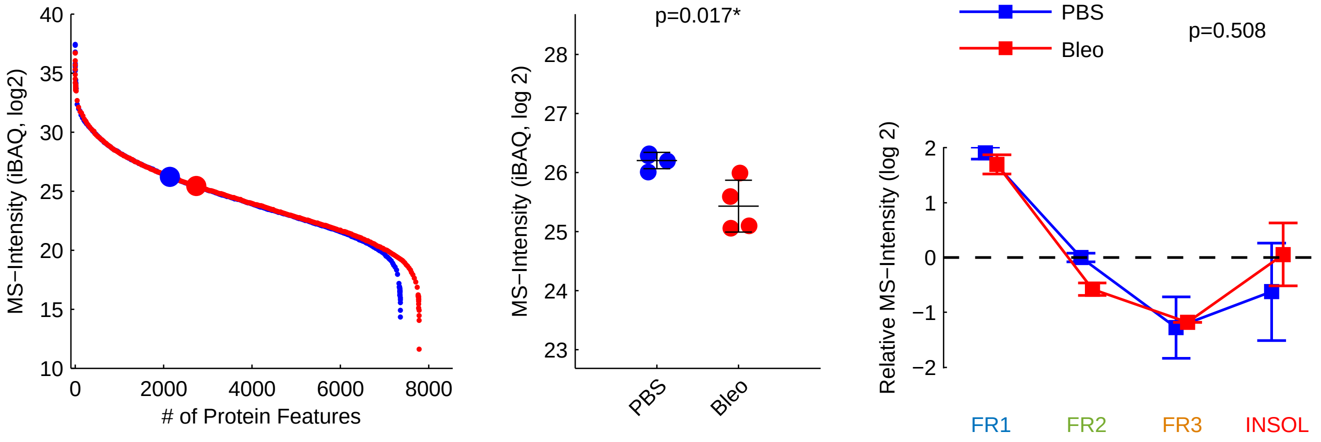

Q9D1Q6 − Erp44 (id: 7589)

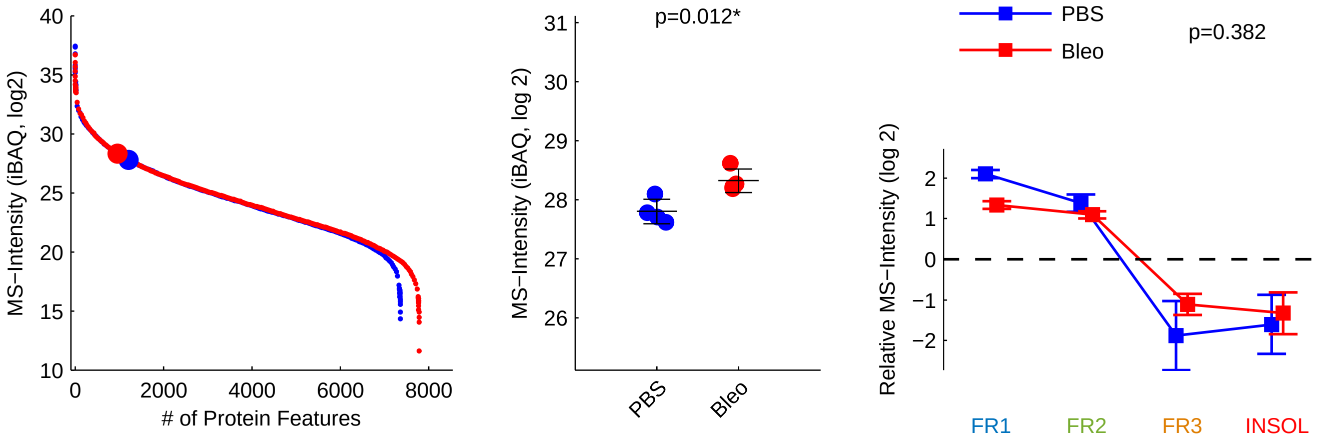

Q9D1R9 − Rpl34 (id: 7592)

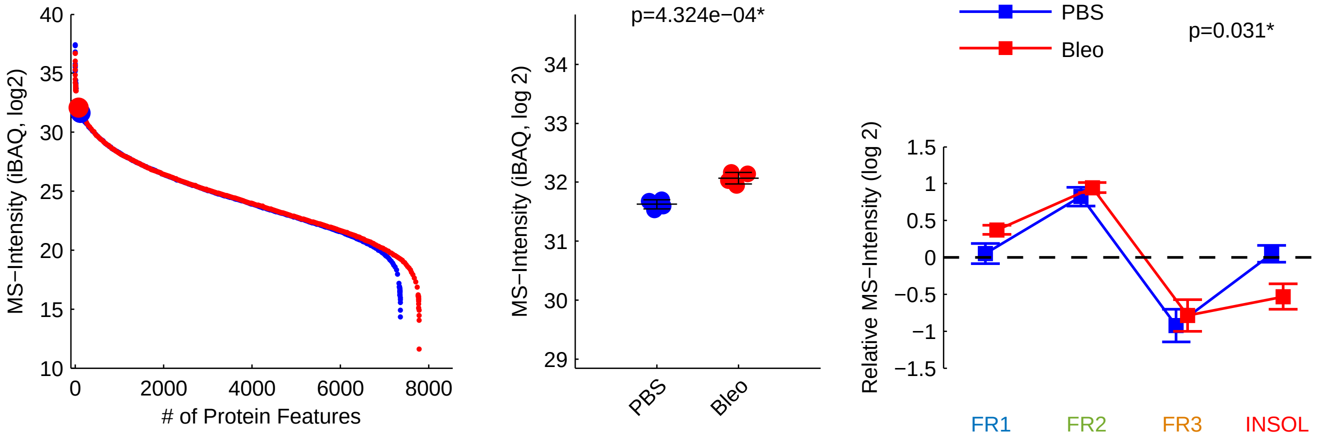

Q9D236 − Htra3 (id: 7595)

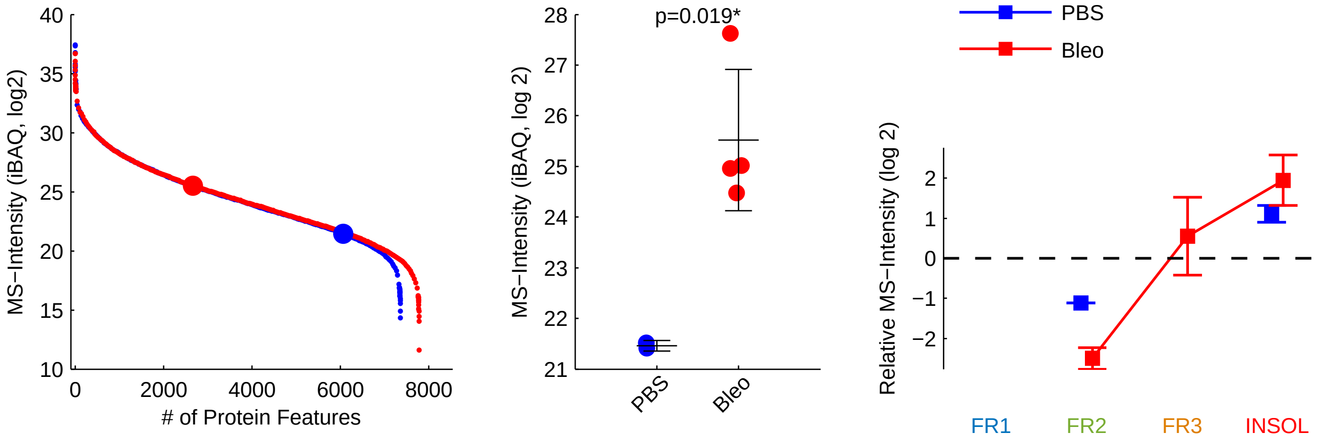

Q9D281 − Fam114a1 (id: 7598)

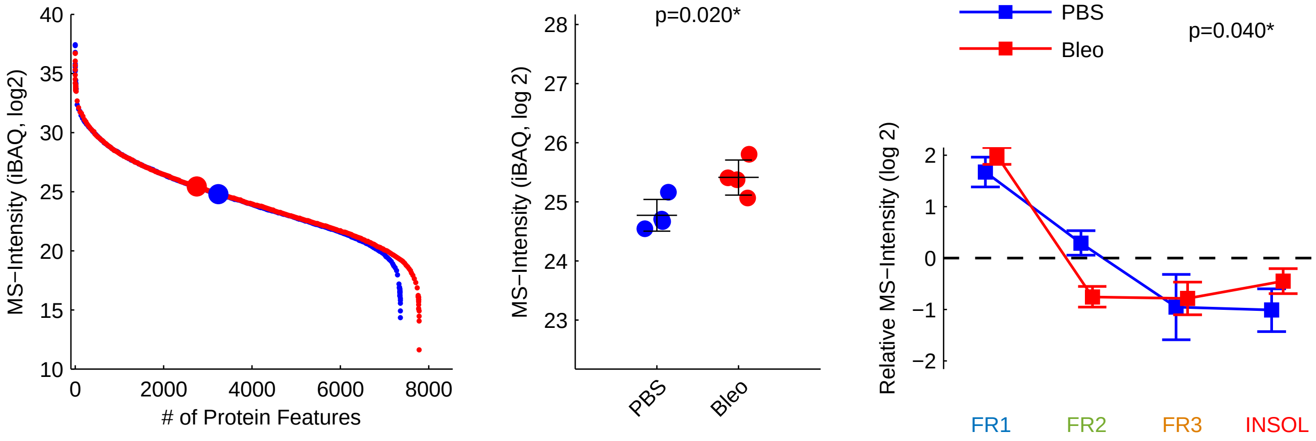

Q9D2Q8 − S100a14 (id: 7609)

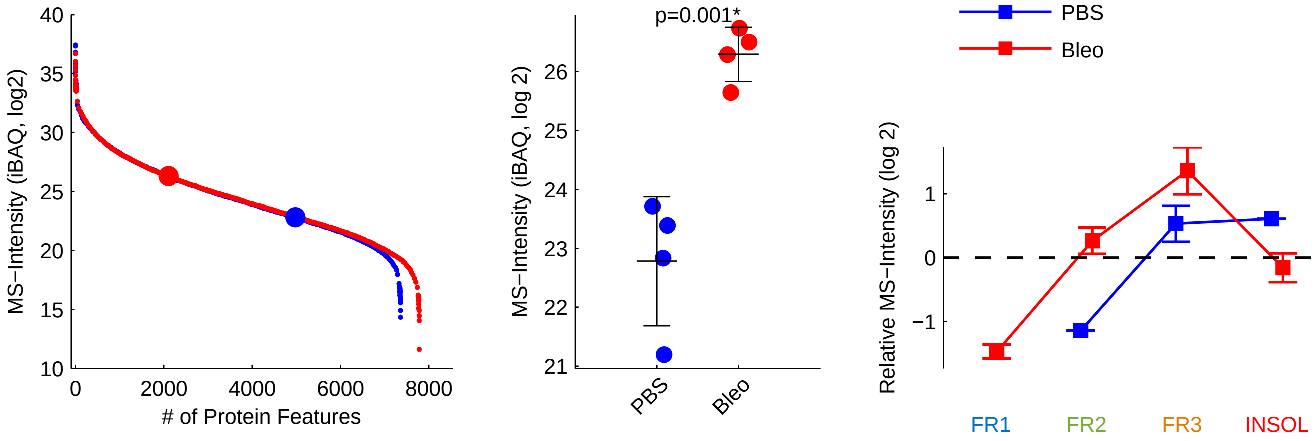

Q9D2Y4 − Mlkl (id: 7616)

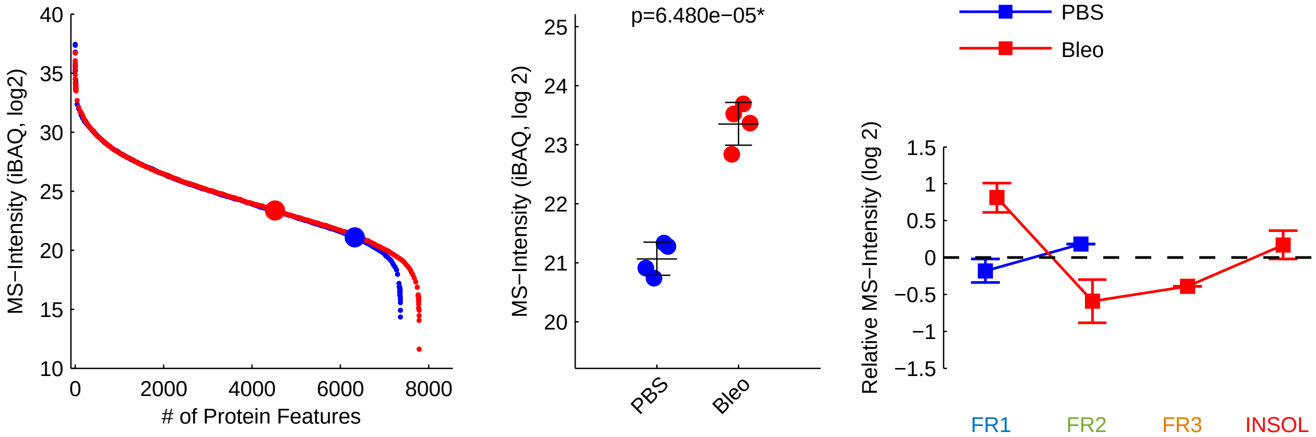

Q9D379 − Ephx1 (id: 7621)

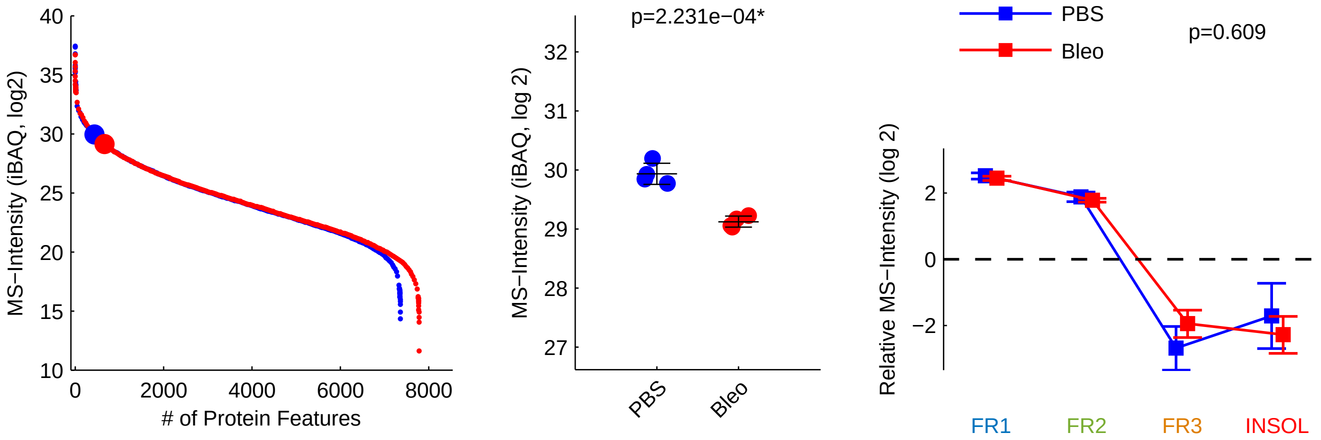

Q9D3D0 − Ttpal (id: 7626)

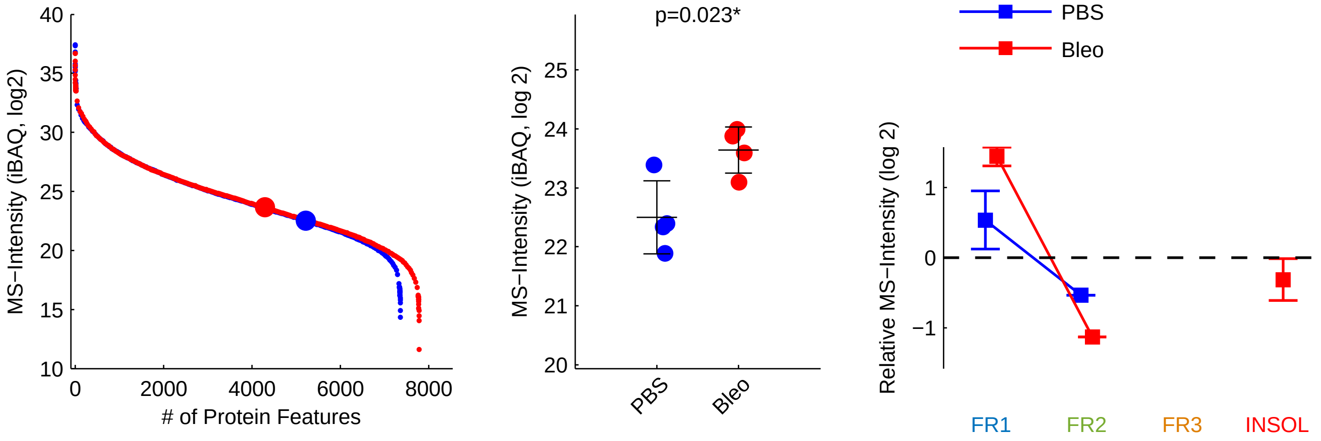

Q9D3P8 − Plgrkt (id: 7631)

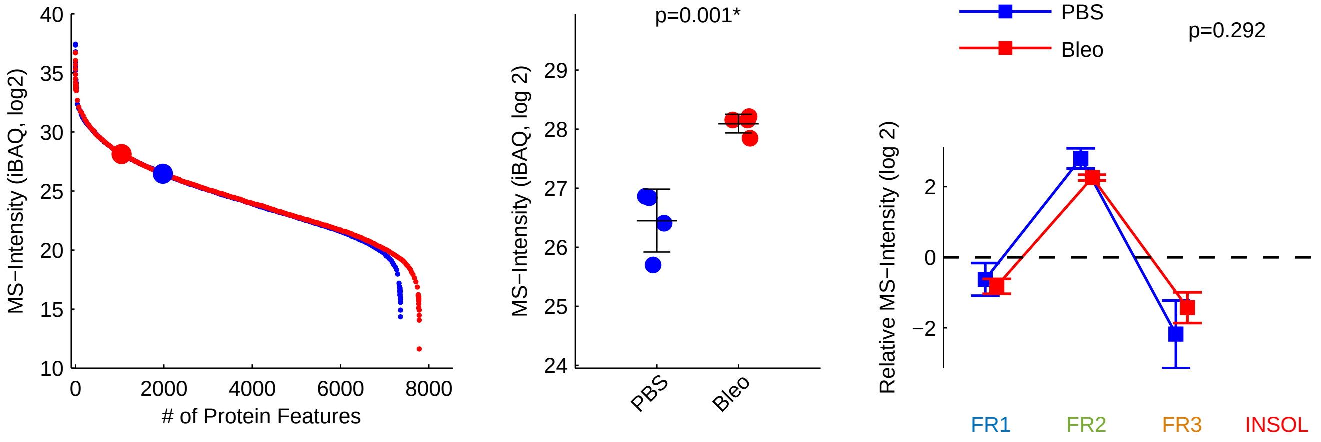

Q9D4H1 − Exoc2 (id: 7642)

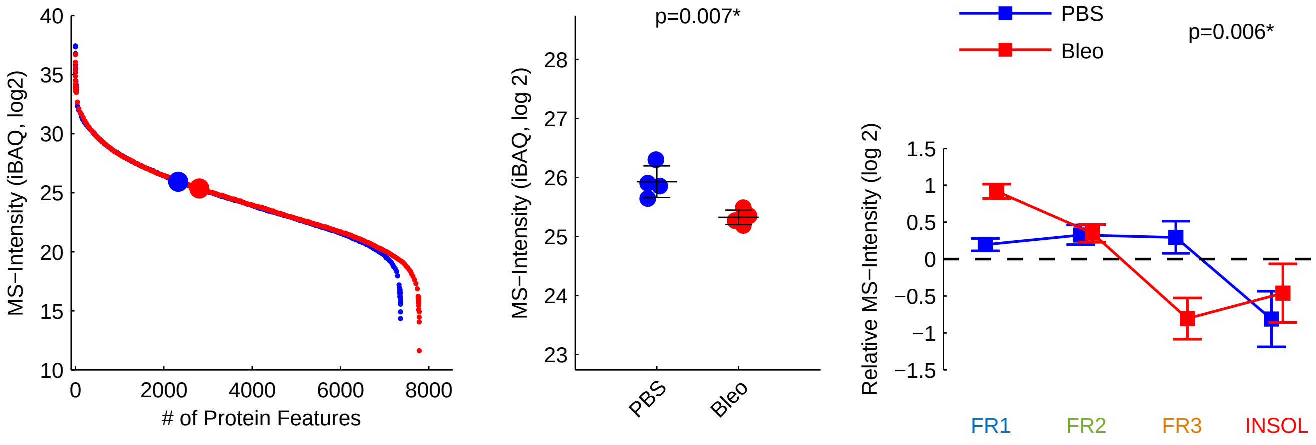

Q9D5T0 − Atad1 (id: 7656)

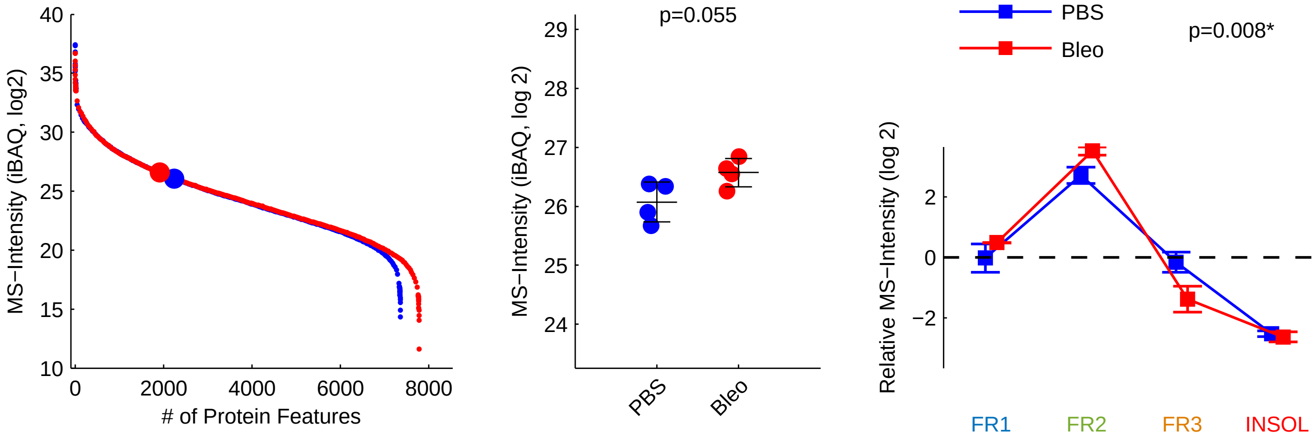

Q9D5V5 − Cul5 (id: 7657)

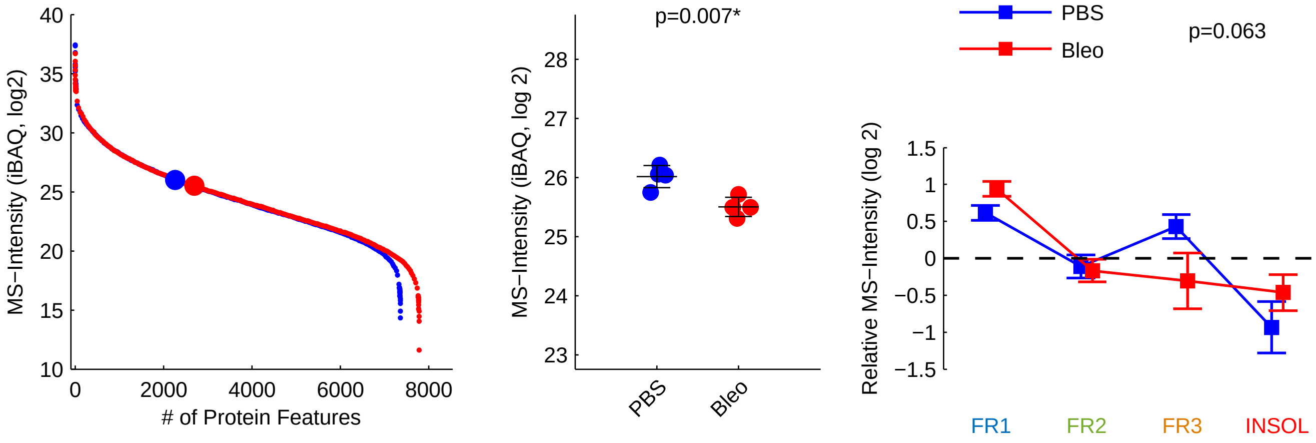

Q9D658 − Ptp4a3 (id: 7662)

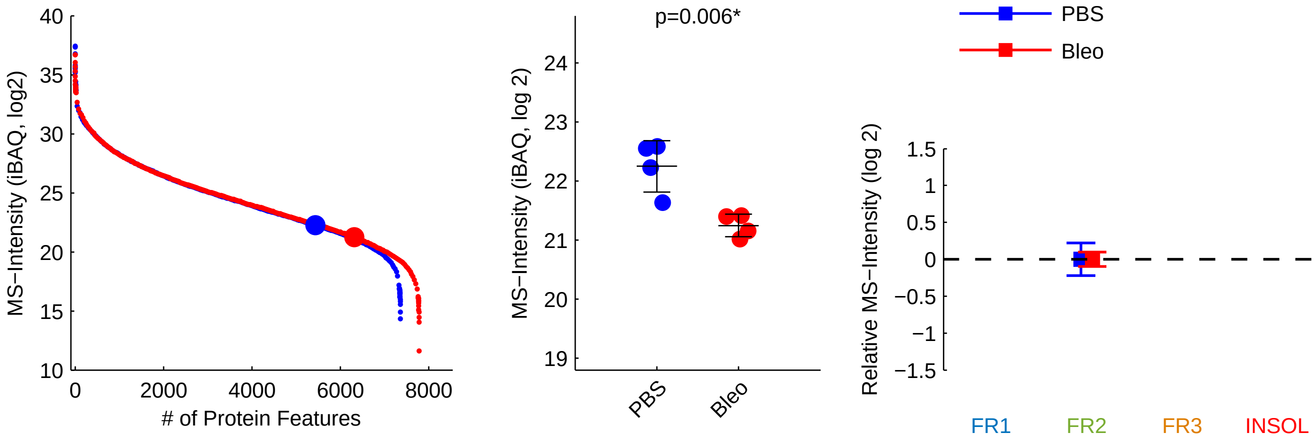

Q9D662 − Sec23b (id: 7663)

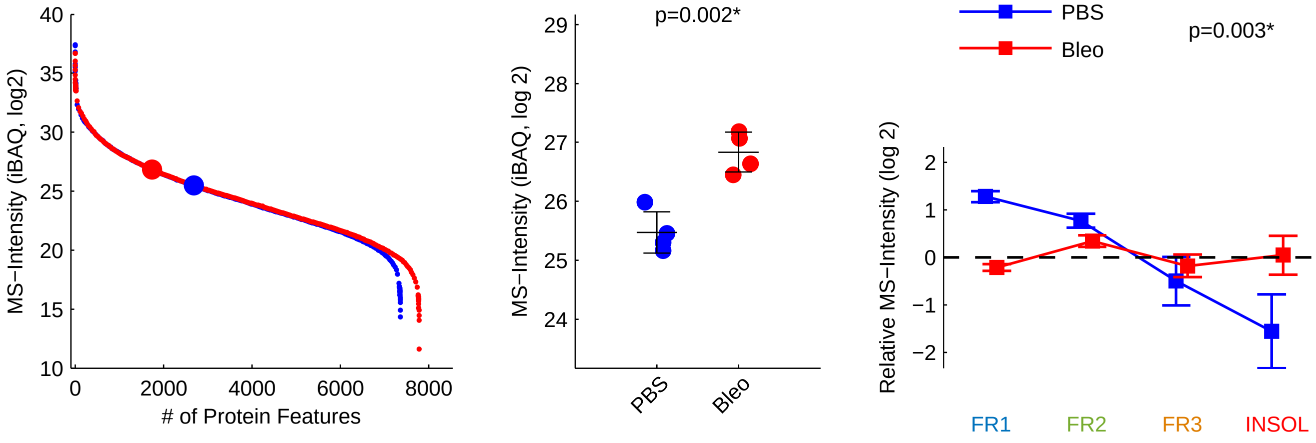

Q9D6Y7 − Msra (id: 7685)

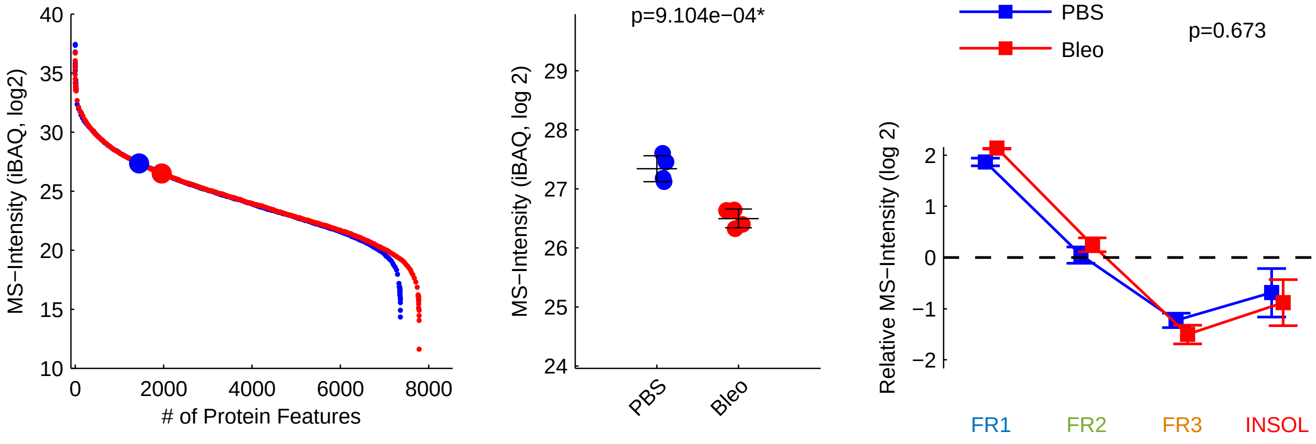

Q9D6Y9 − Gbe1 (id: 7686)

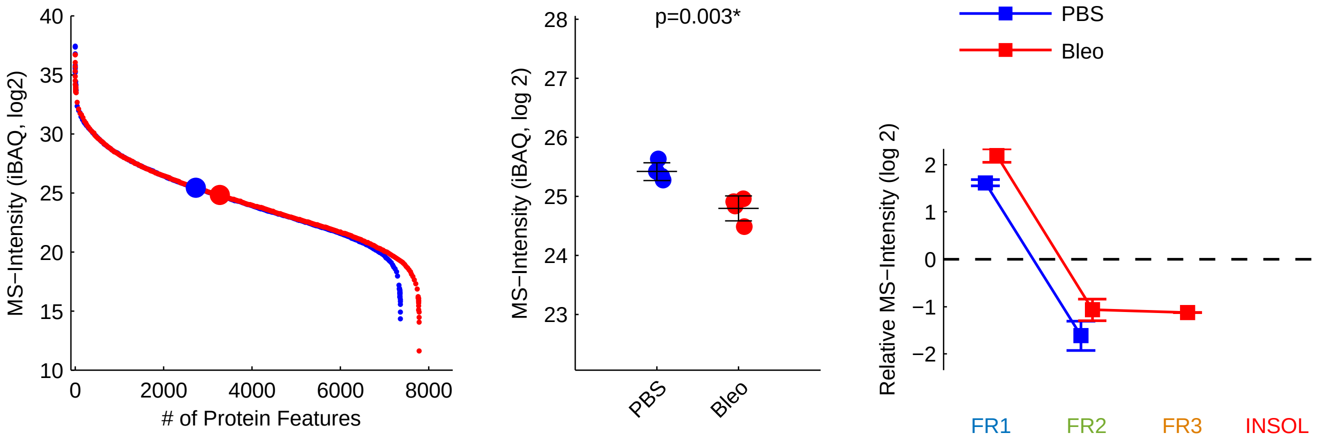

Q9D7B7 − Gpx8 (id: 7698)

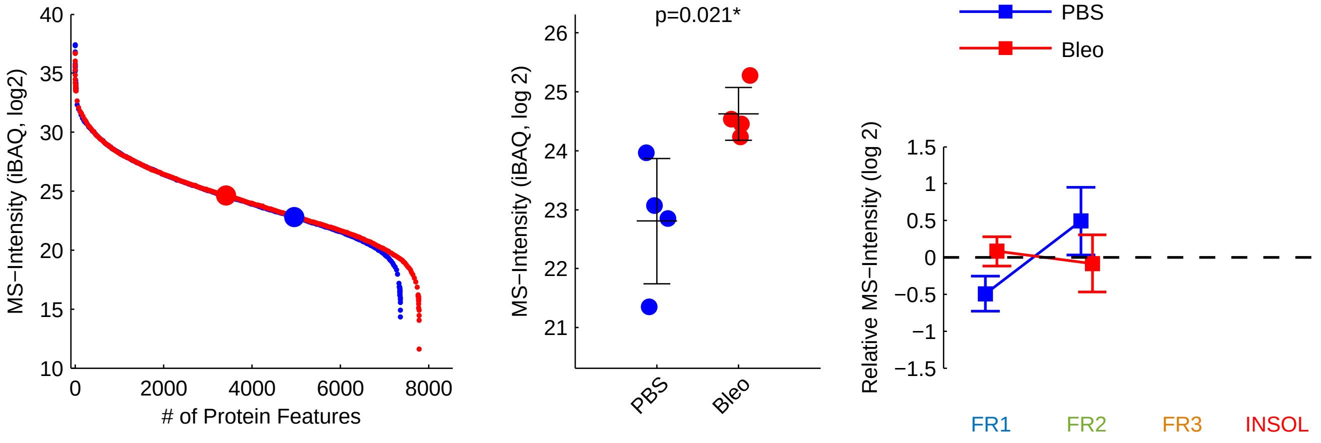

Q9D7E3 − Ovca2 (id: 7699)

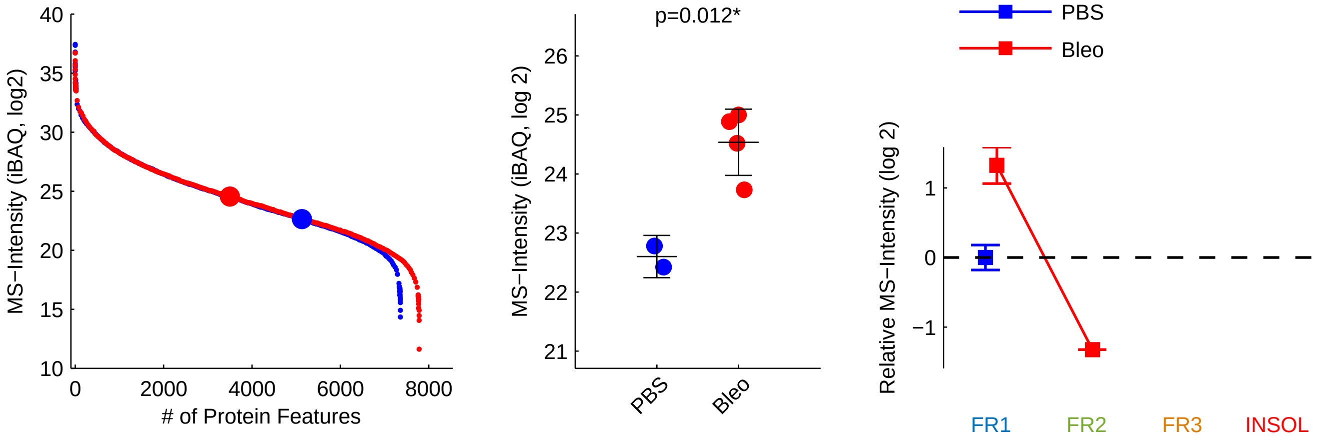

Q9D7H3 − RtcA (id: 7701)

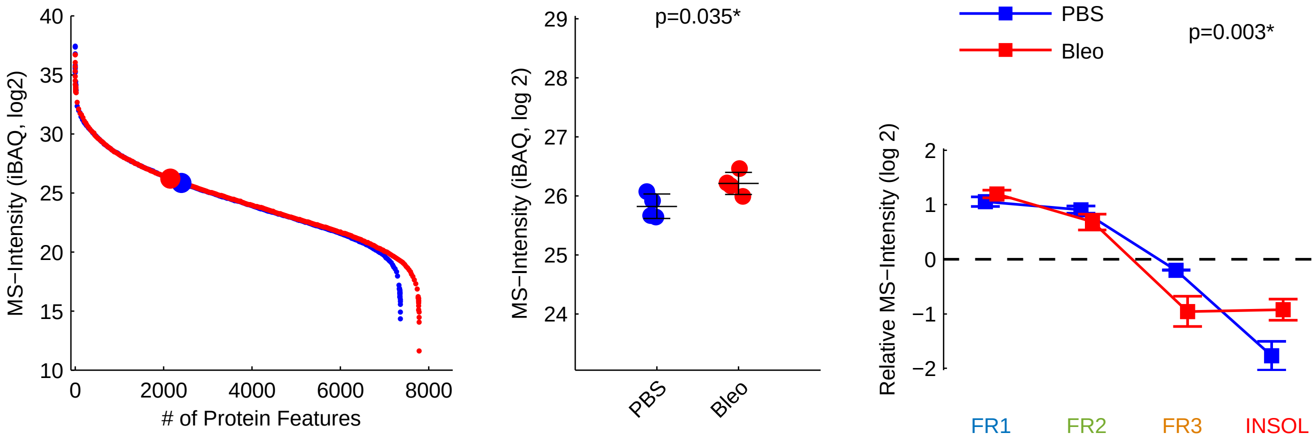

Q9D7J9 − Echdc3 (id: 7705)

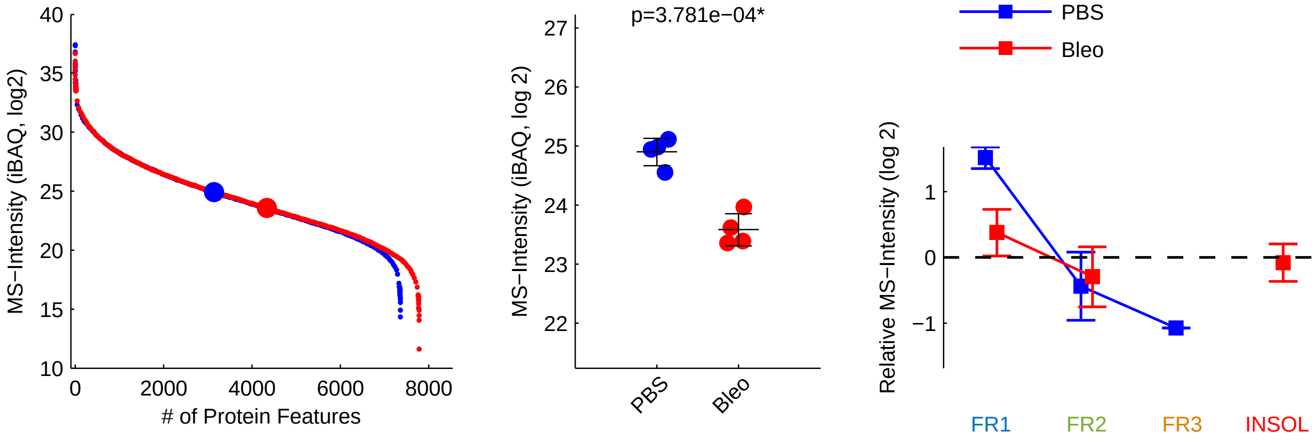

Q9D7N3 − Mrps9 (id: 7708)

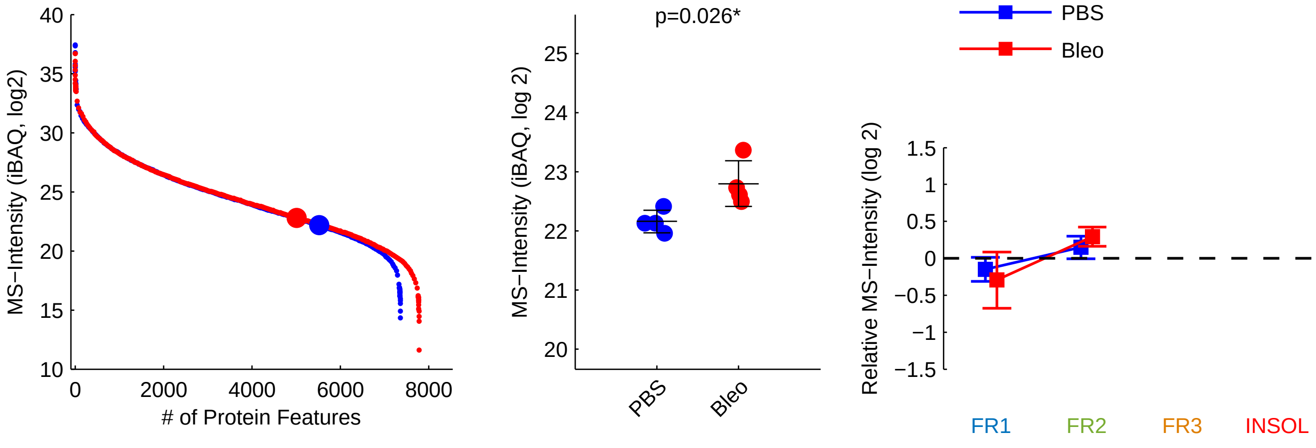

Q9D7N9 − Apmap (id: 7710)

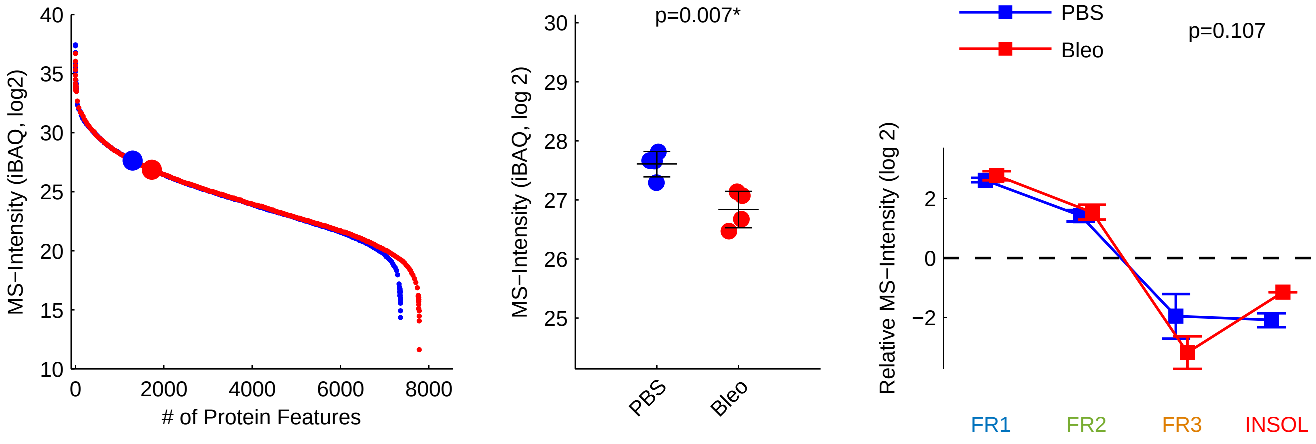

Q9D7P7 − Clic3 (id: 7712)

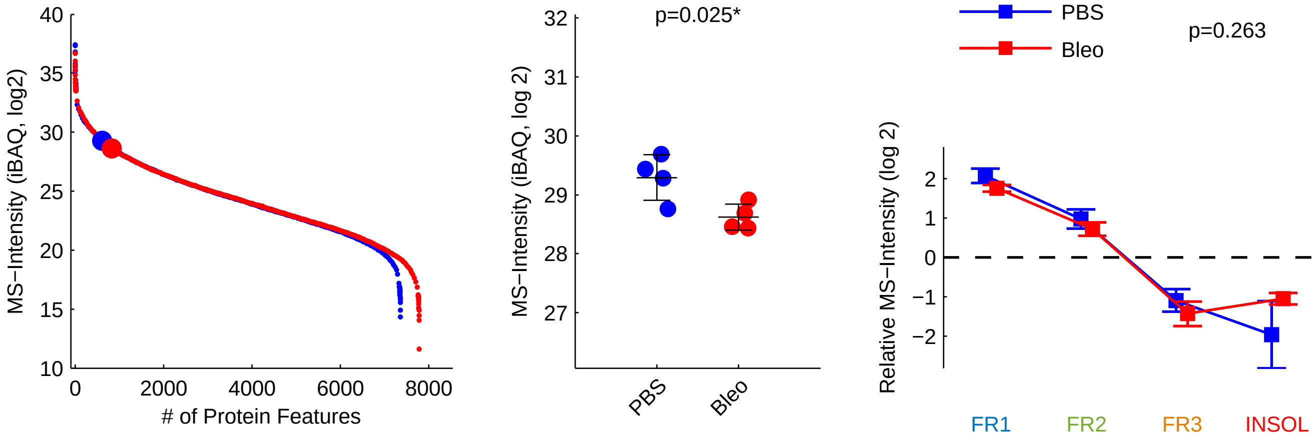

Q9D7S7 − Rpl22l1 (id: 7714)

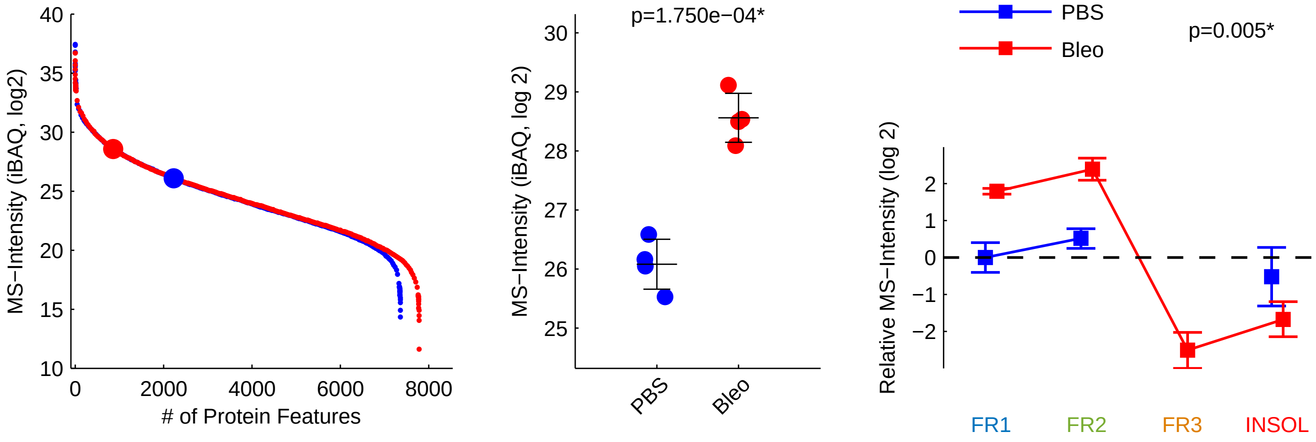

Q9D7V9 − Naaa (id: 7716)

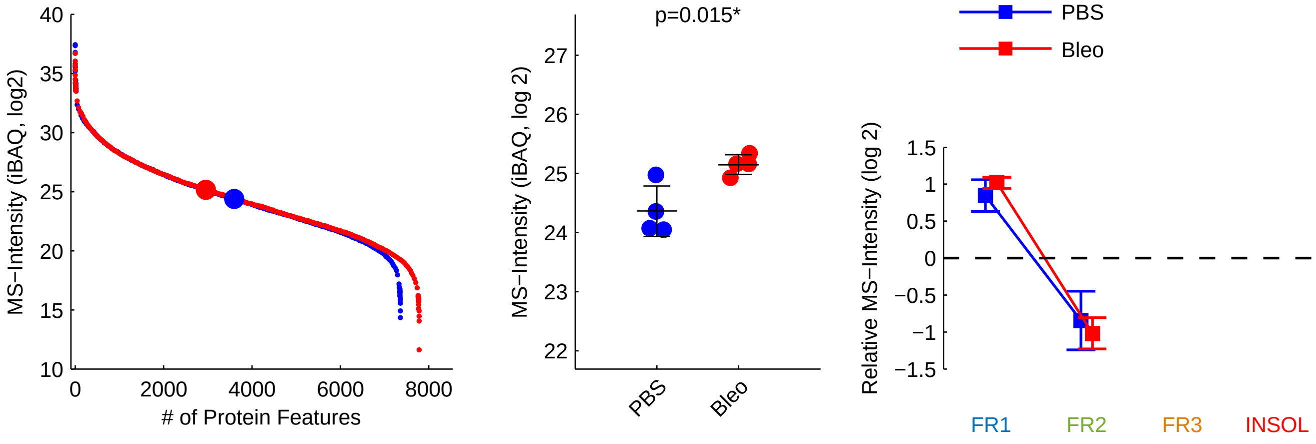

Q9D819 − Ppa1 (id: 7723)

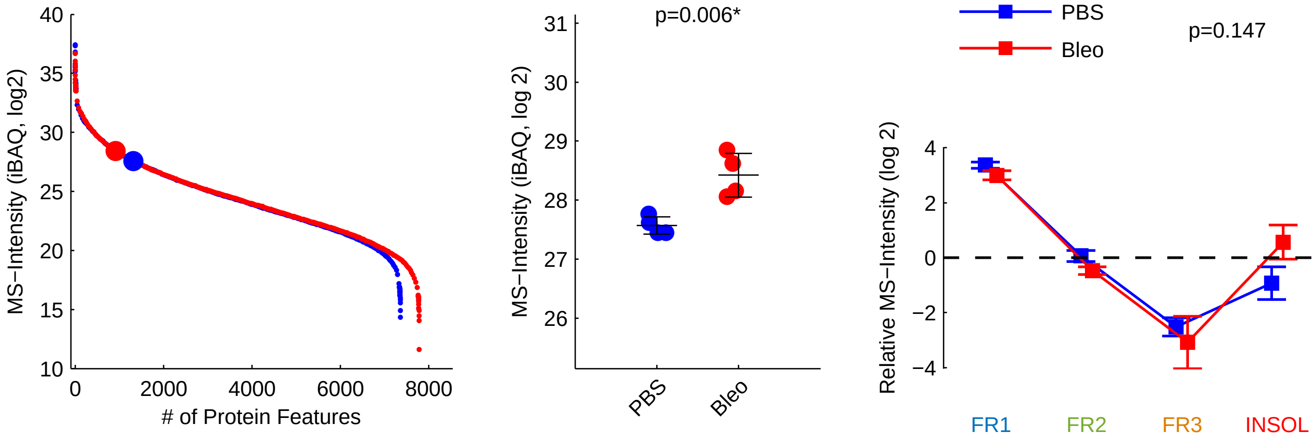

Q9D8E6 − Rpl4 (id: 7741)

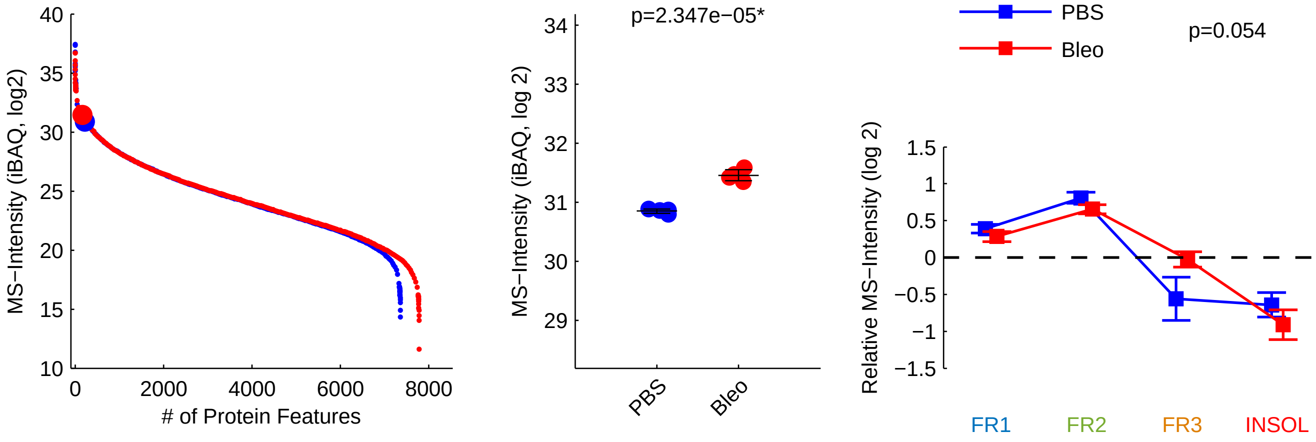

Q9D8M4 − Rpl7l1 (id: 7744)

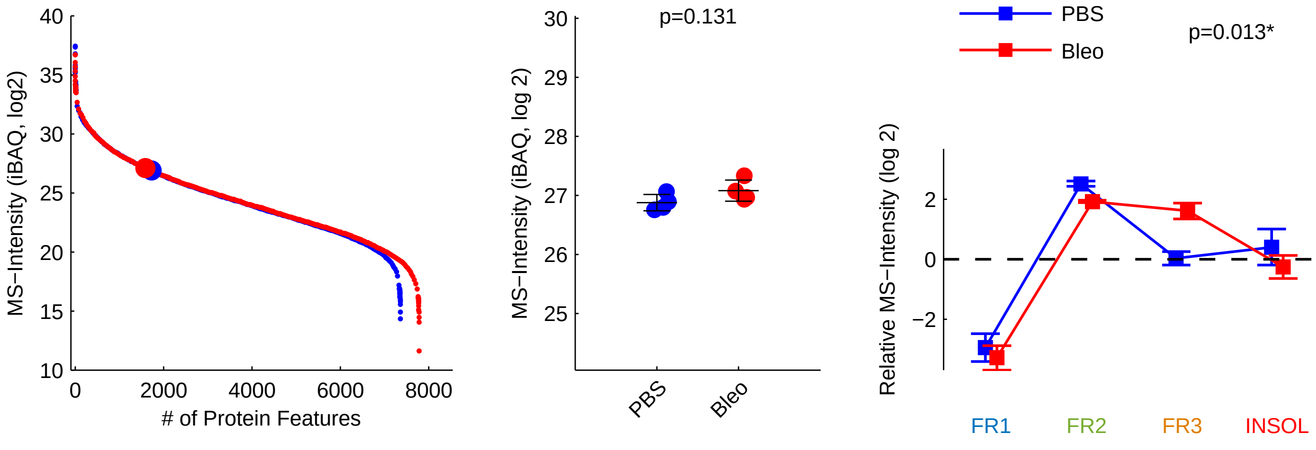

Q9D8S3 − Arfgap3 (id: 7747)

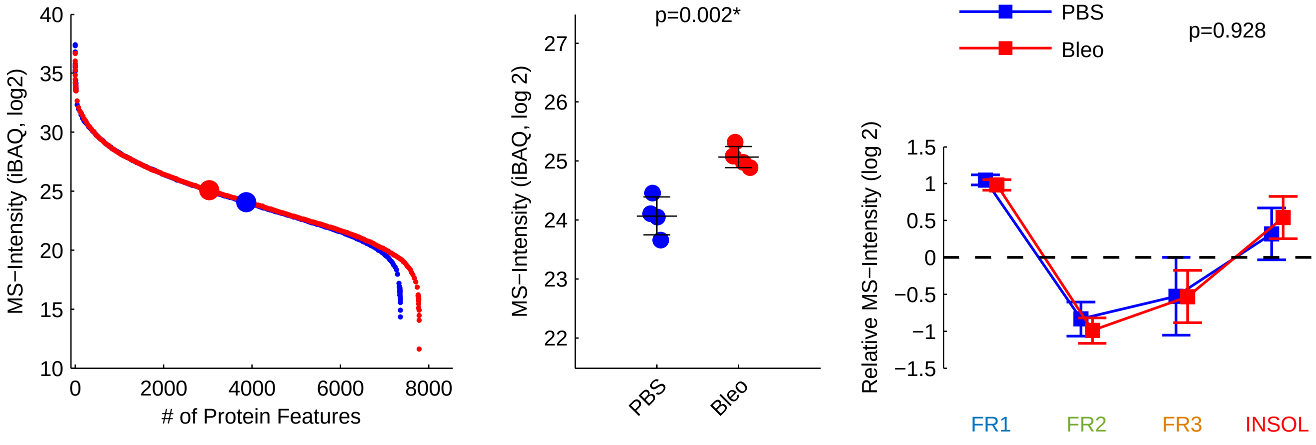

Q9D8W5 − Psmd12 (id: 7755)

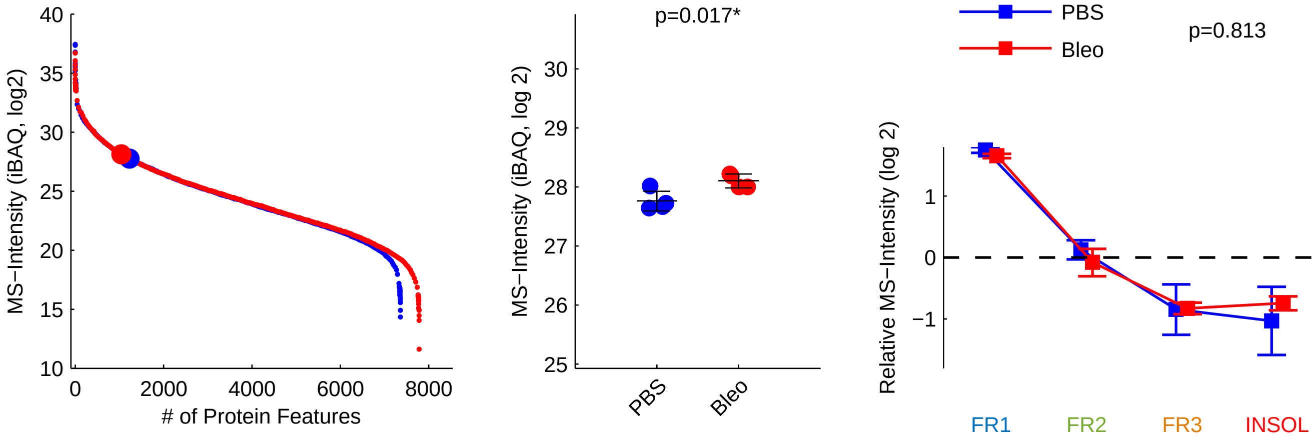

Q9D937 − 1810009A15Rik (id: 7767)

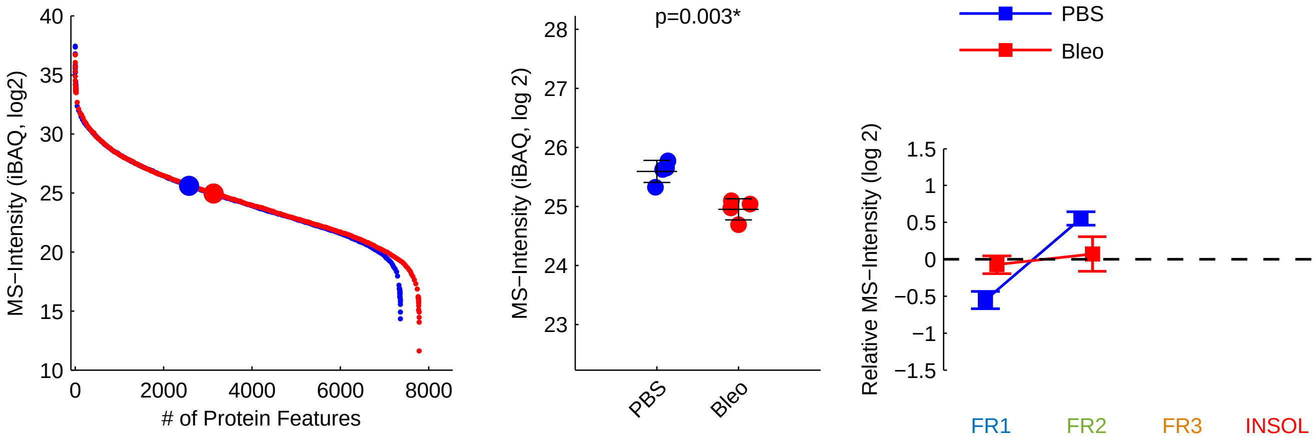

Q9D952 − Evpl (id: 7769)

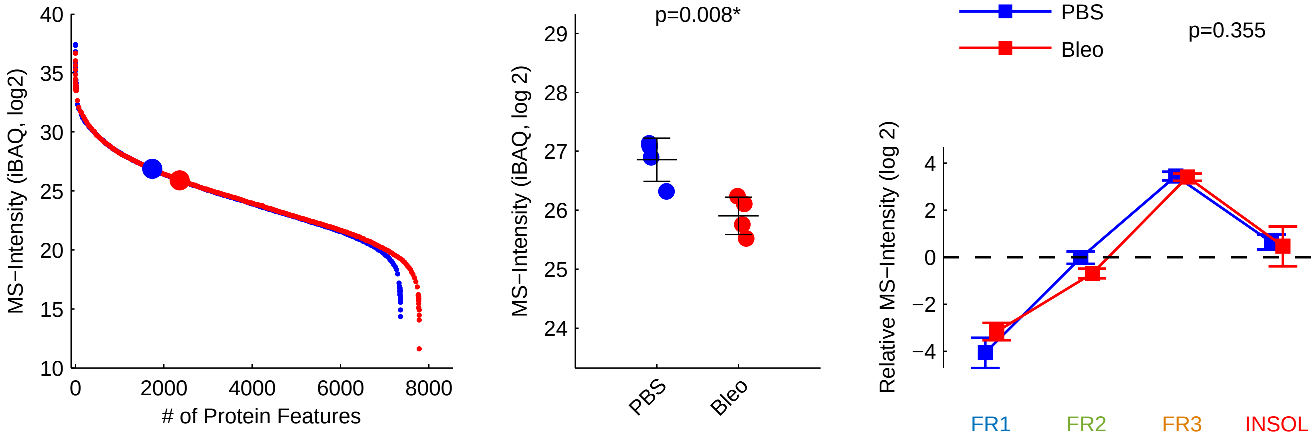

Q9D9M2 − Usp12 (id: 7775)

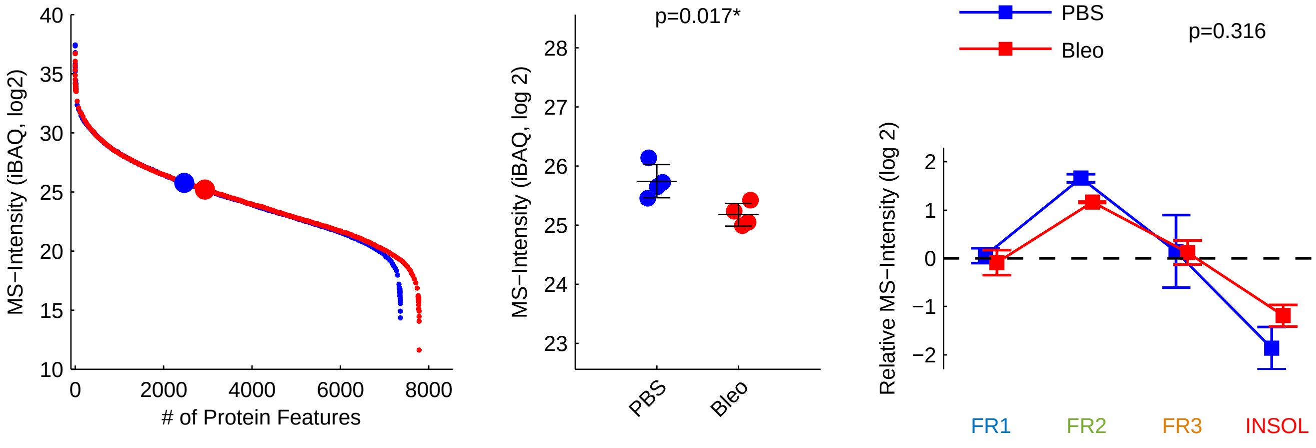

Q9D9V3 − Echdc1 (id: 7777)

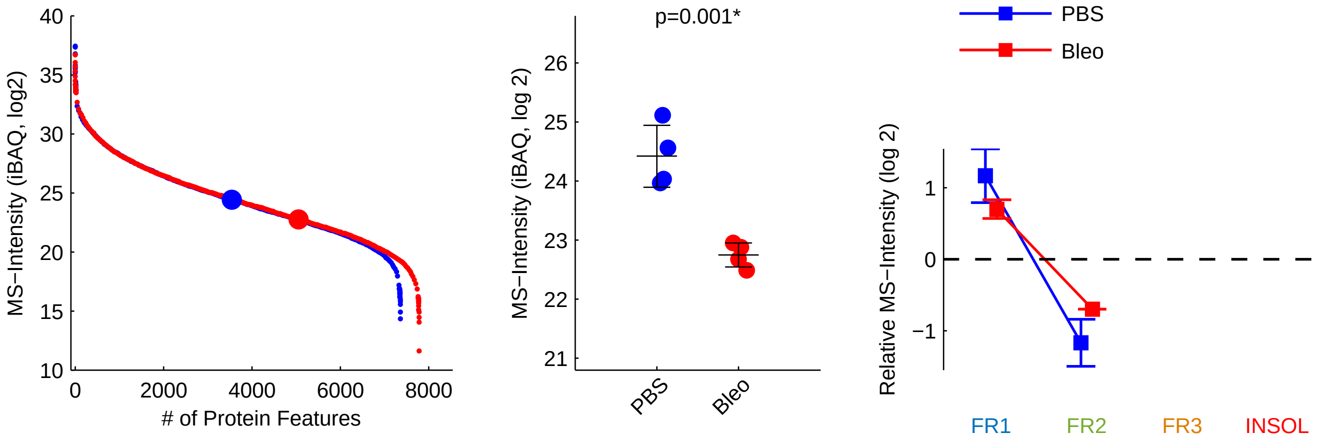

Q9DAM7 − (id: 7791)

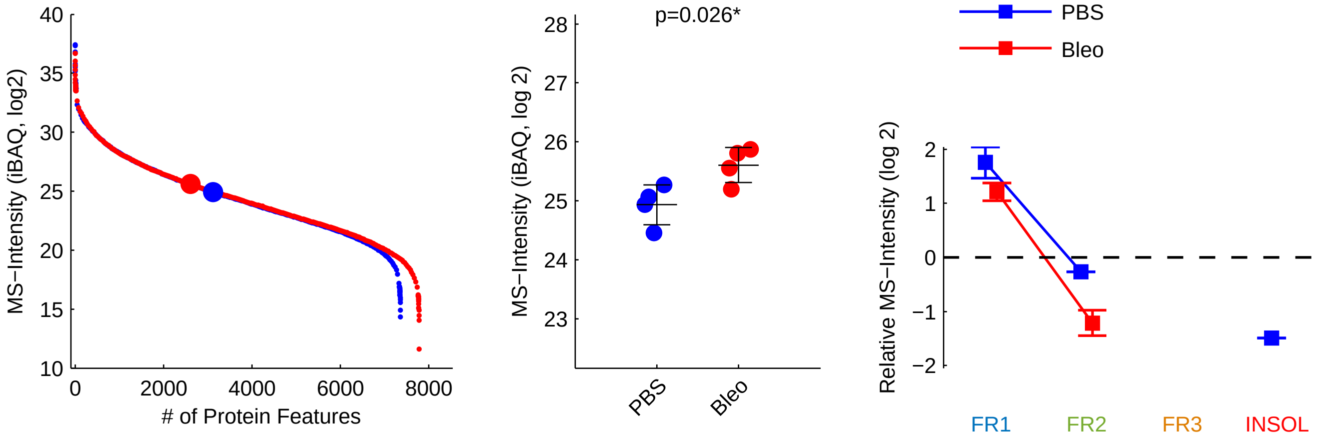

Q9DAR7 − Dcps (id: 7792)

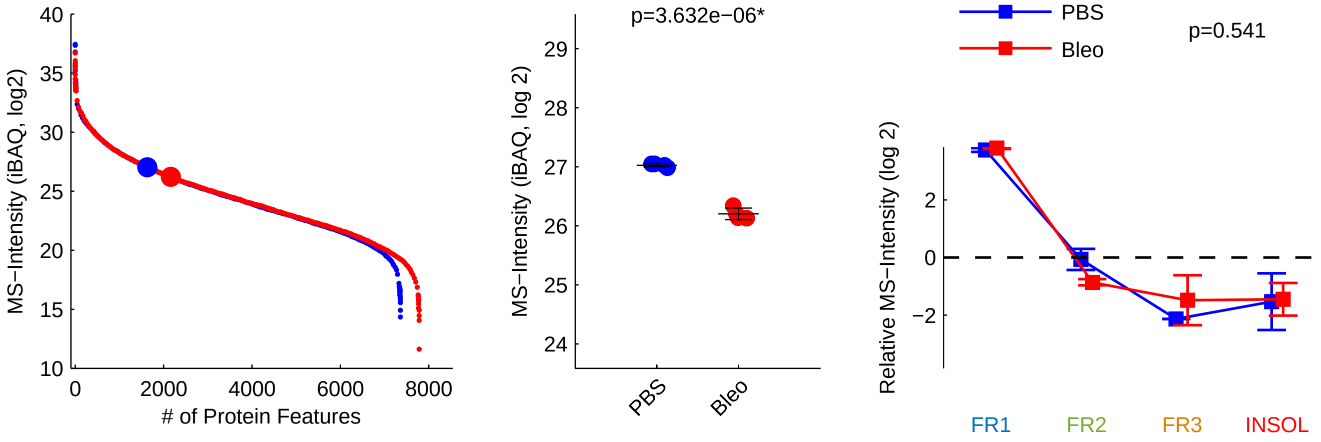

Q9DAS9 − Gng12 (id: 7794)

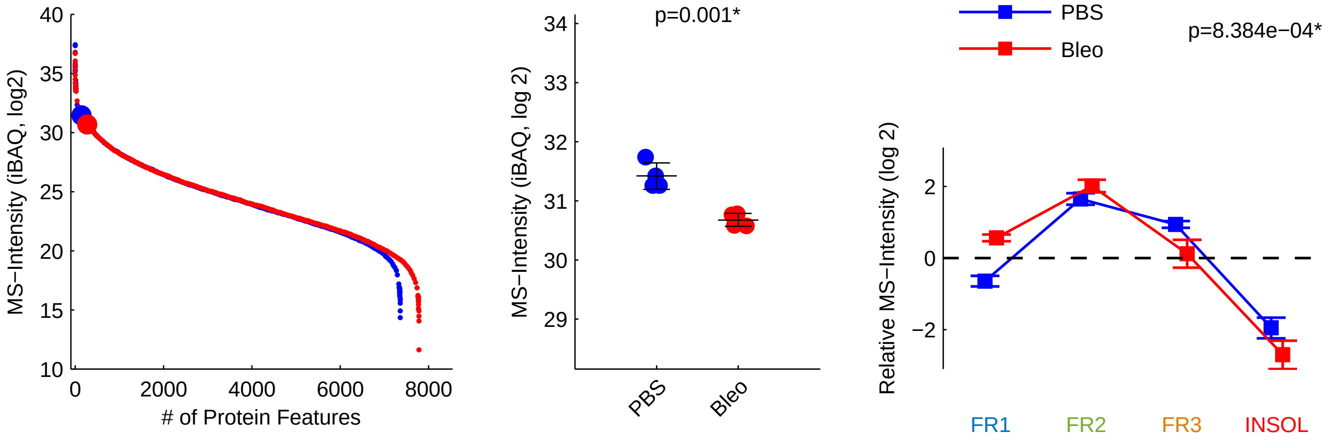

Q9DAV6 − Serpinb9b (id: 7797)

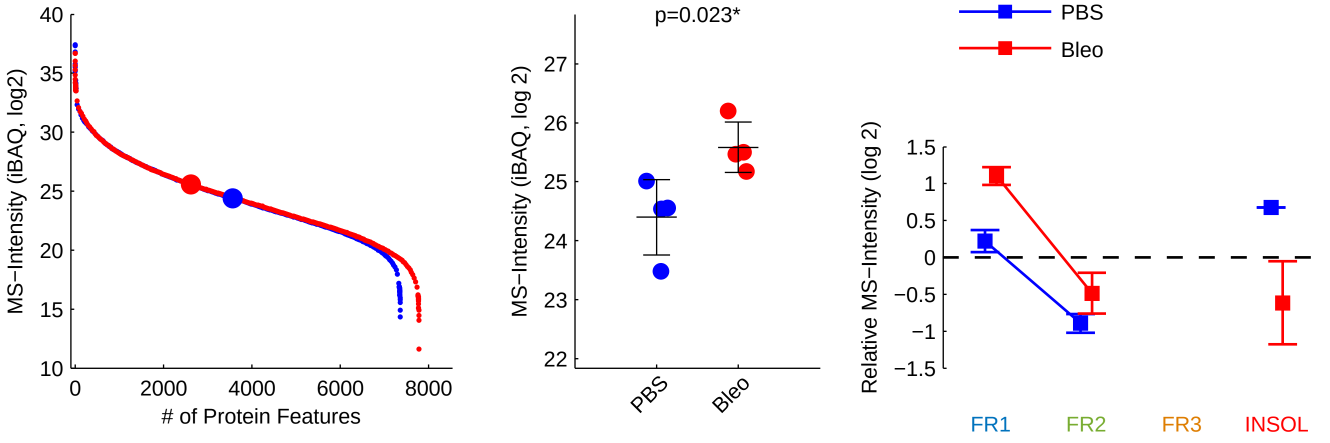

Q9DB16 − Cab39l (id: 7805)

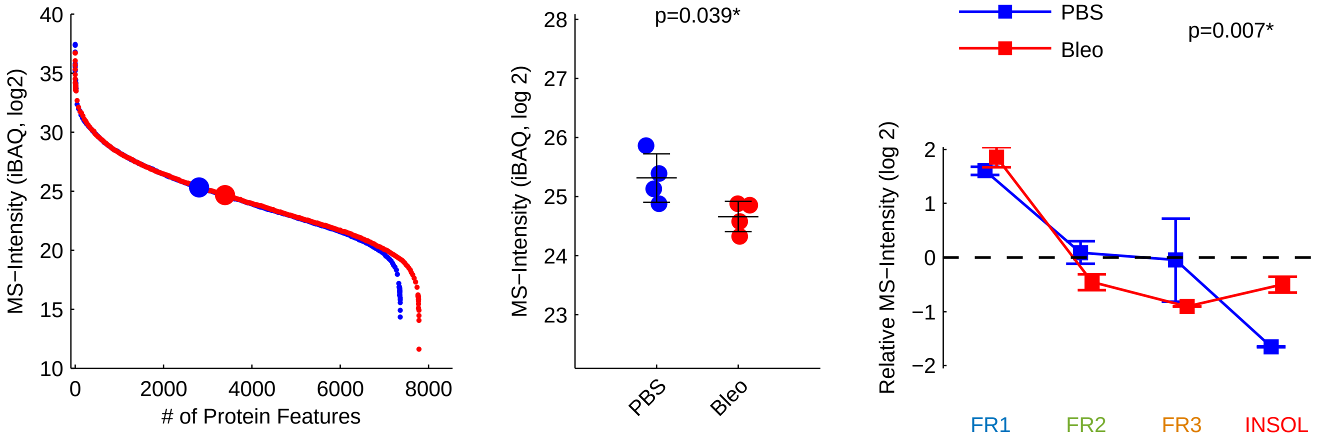

Q9DB29 − Iah1 (id: 7810)

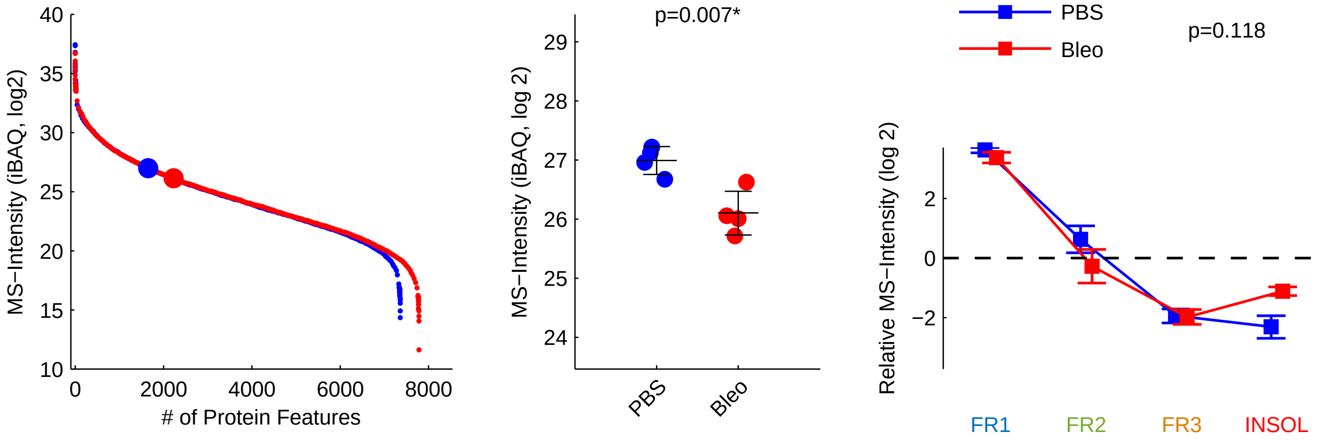

Q9DB73 − Cyb5r1 (id: 7815)

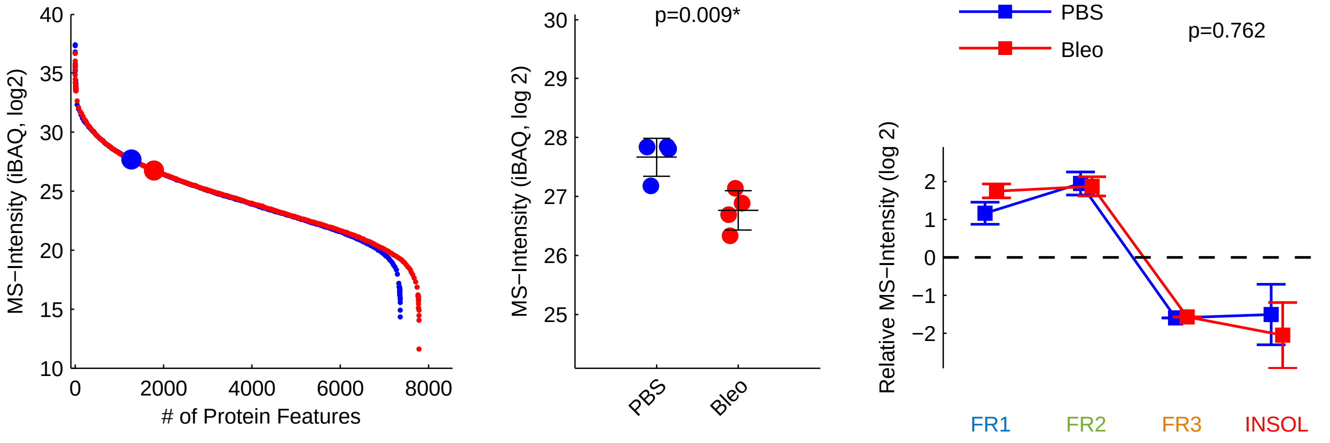

Q9DB77 − Uqcrc2 (id: 7816)

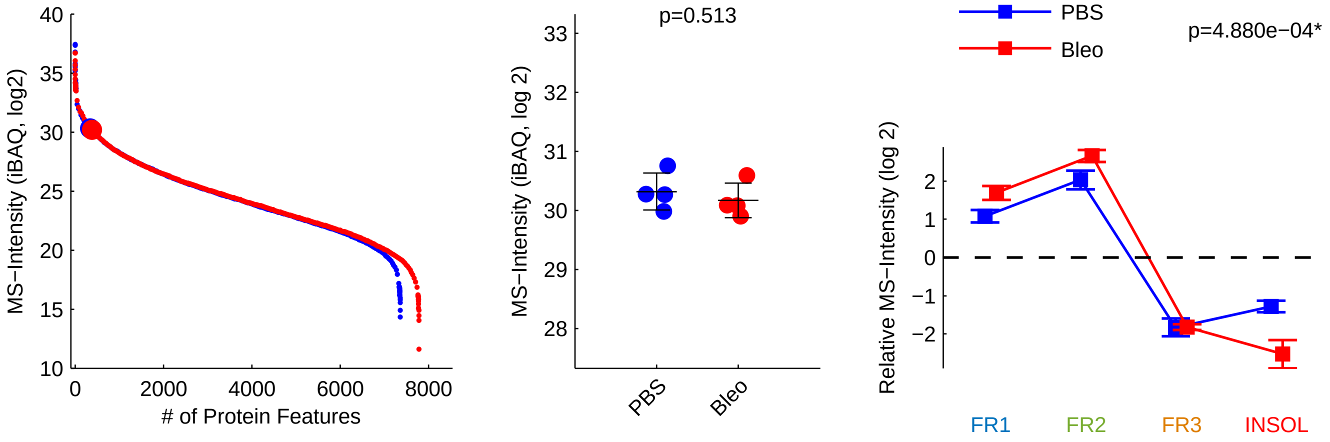

Q9DBB5 − Eif4e3 (id: 7822)

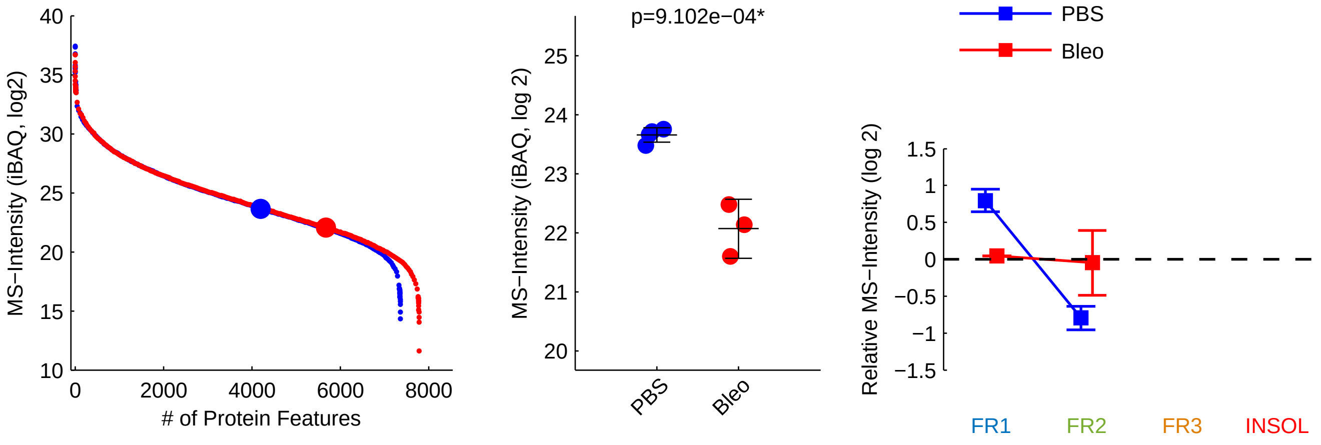

Q9DBB8 − Dhdh (id: 7823)

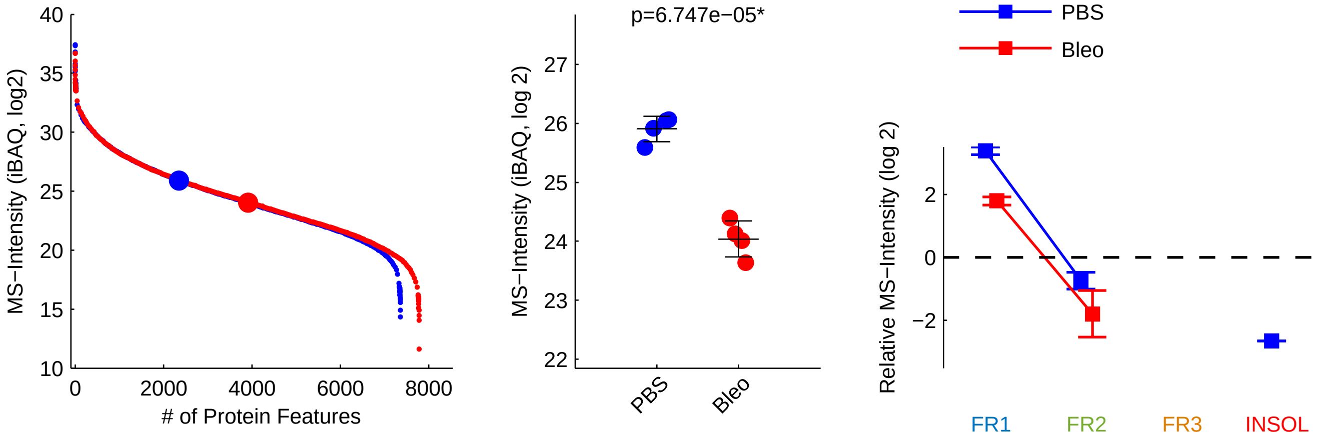

Q9DBD0 − Ica (id: 7828)

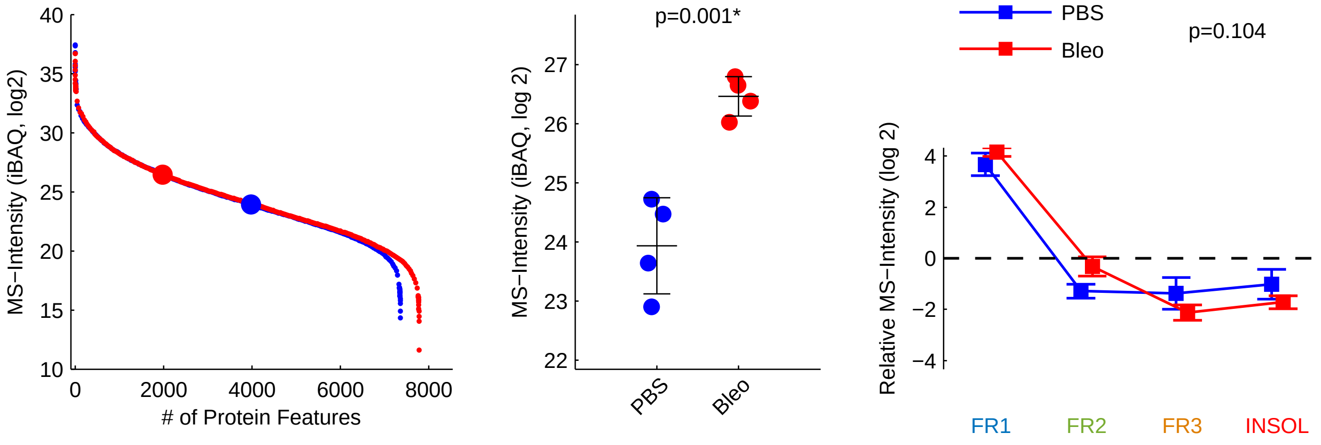

Q9DBE0 − Csad (id: 7830)

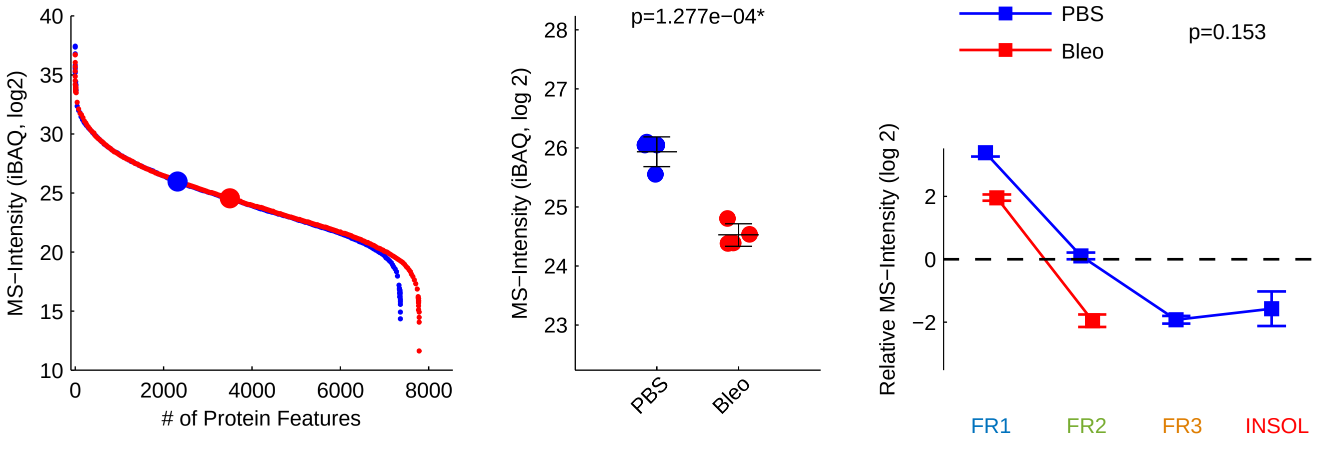

Q9DBE9 − Ftsj3 (id: 7832)

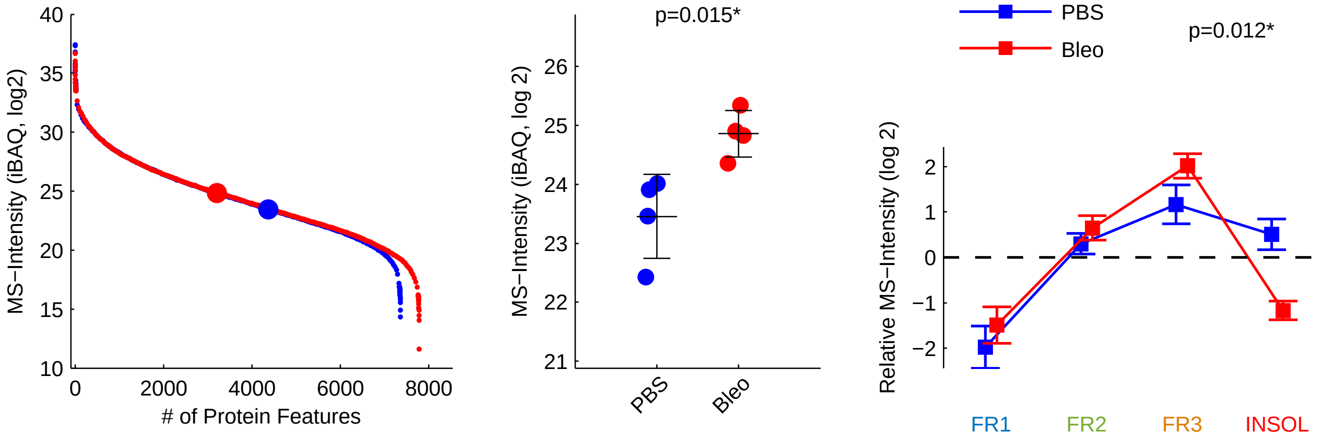

Q9DBF1−2 − Aldh7a1 (id: 7833)

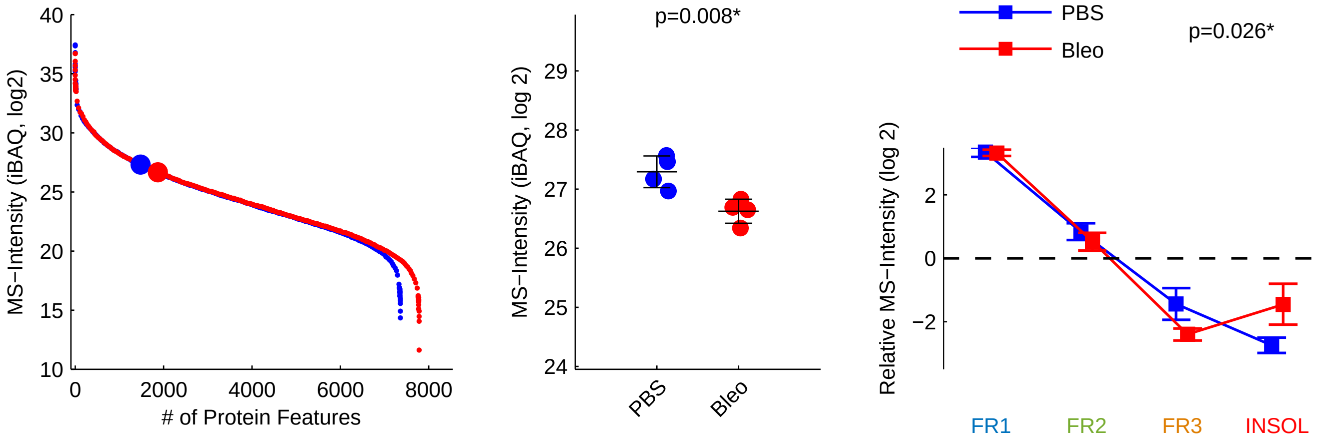

Q9DBG5 − Plin3 (id: 7837)

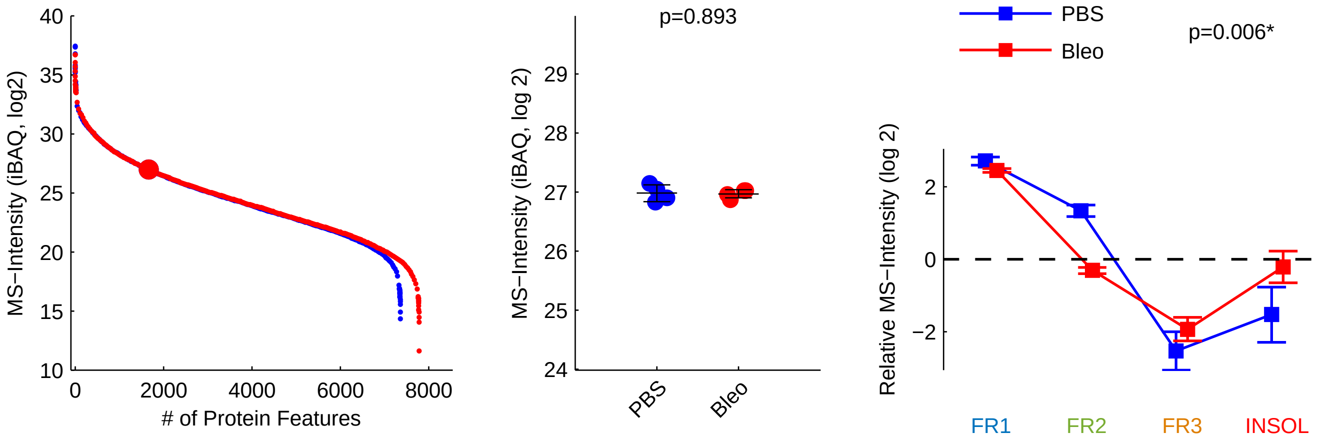

Q9DBG6 − Rpn2 (id: 7838)

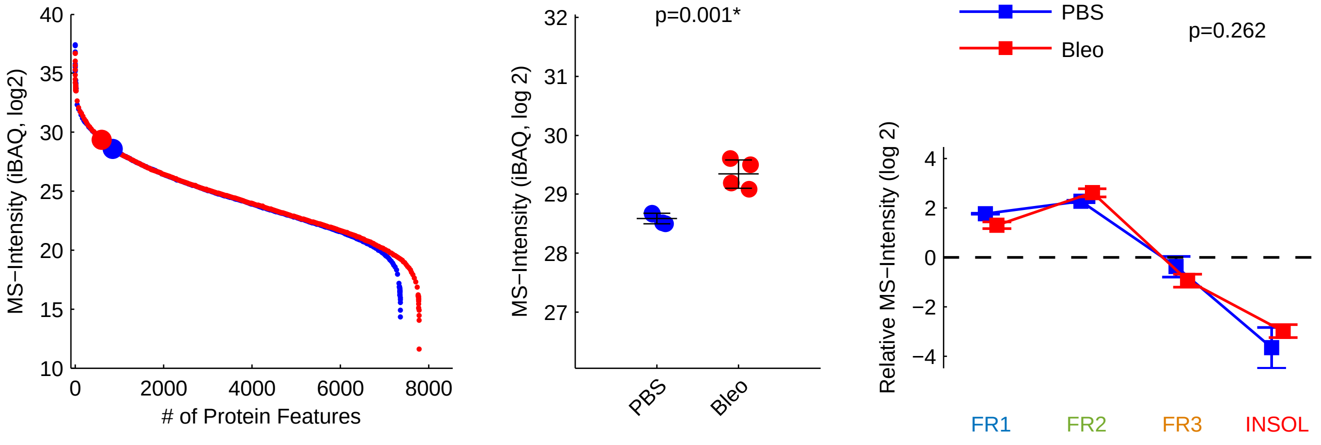

Q9DBG7 − Srpr (id: 7839)

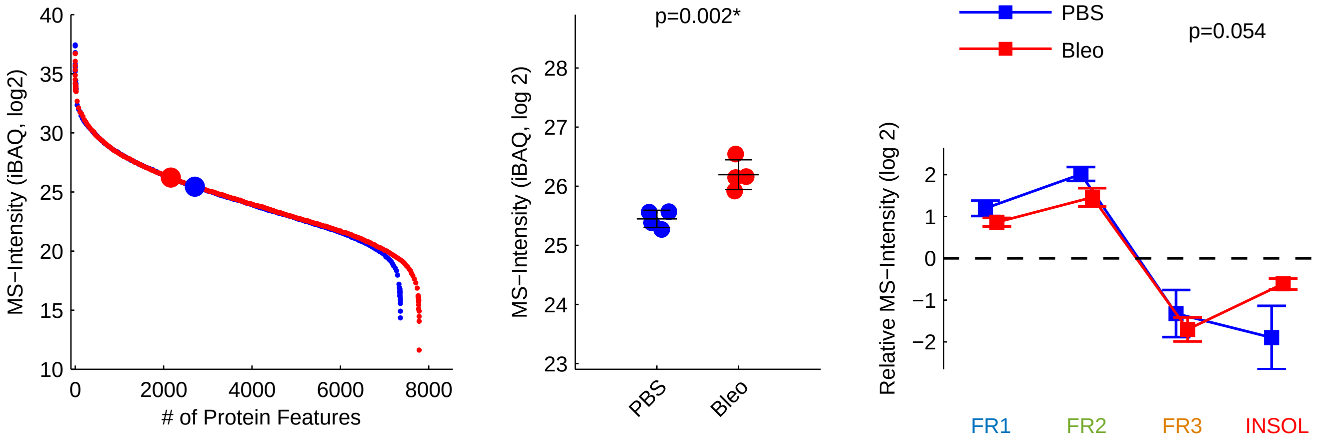

Q9DBJ3 − Baiap2l1 (id: 7844)

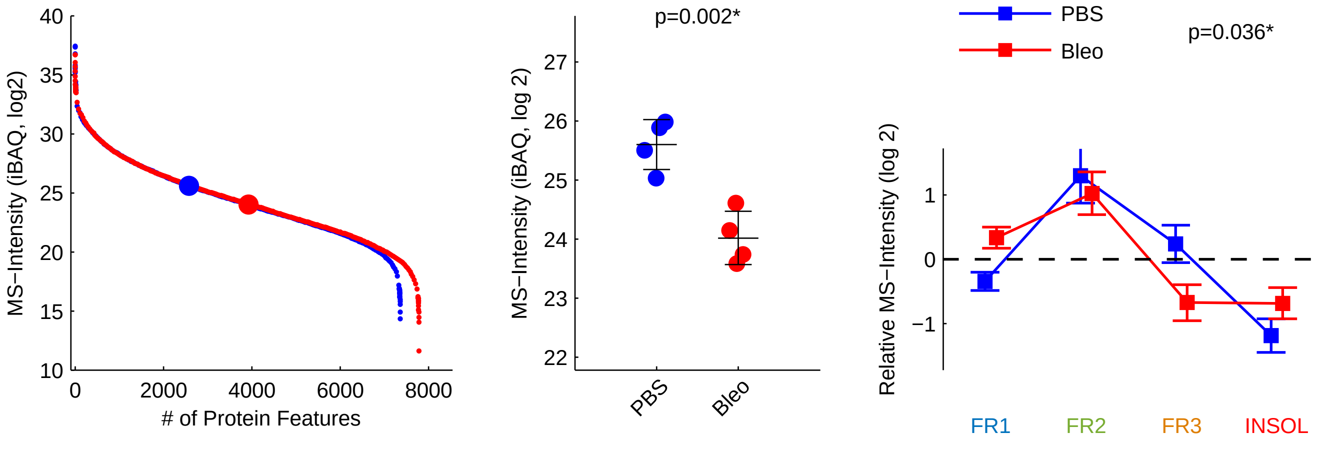

Q9DBK7 − Uba7 (id: 7845)

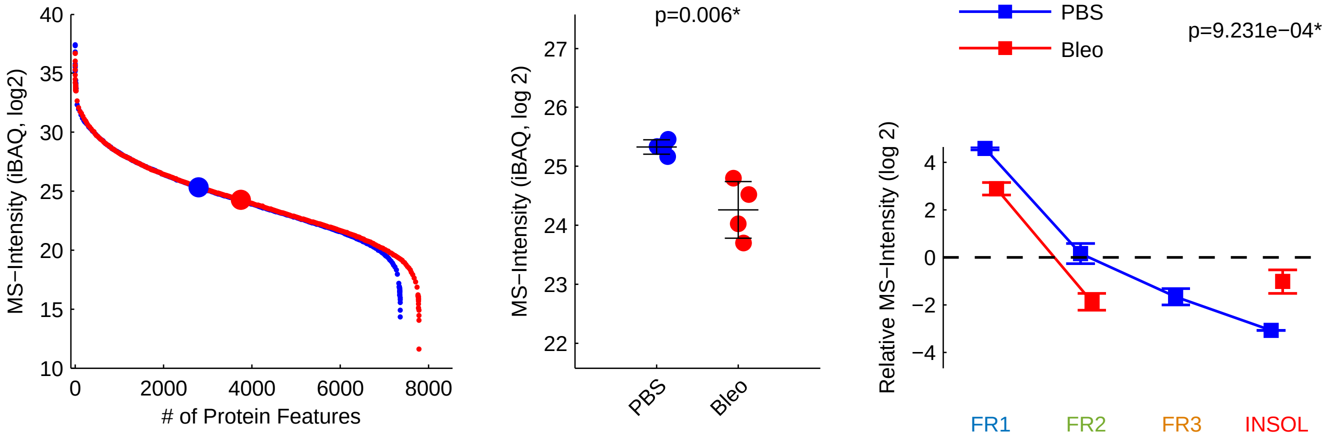

Q9DBM2 − Ehhadh (id: 7850)

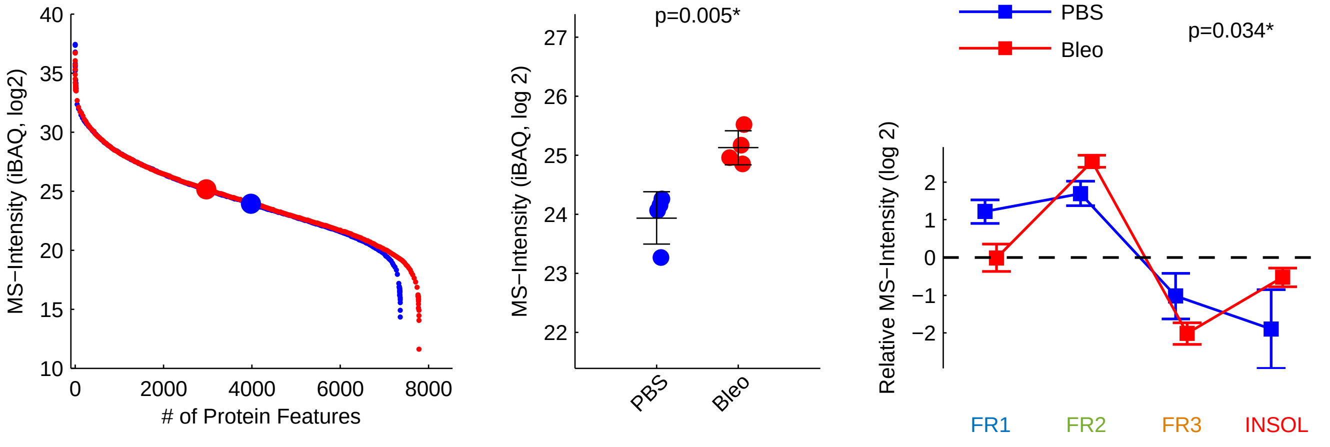

Q9DBP0 − Slc34a2 (id: 7852)

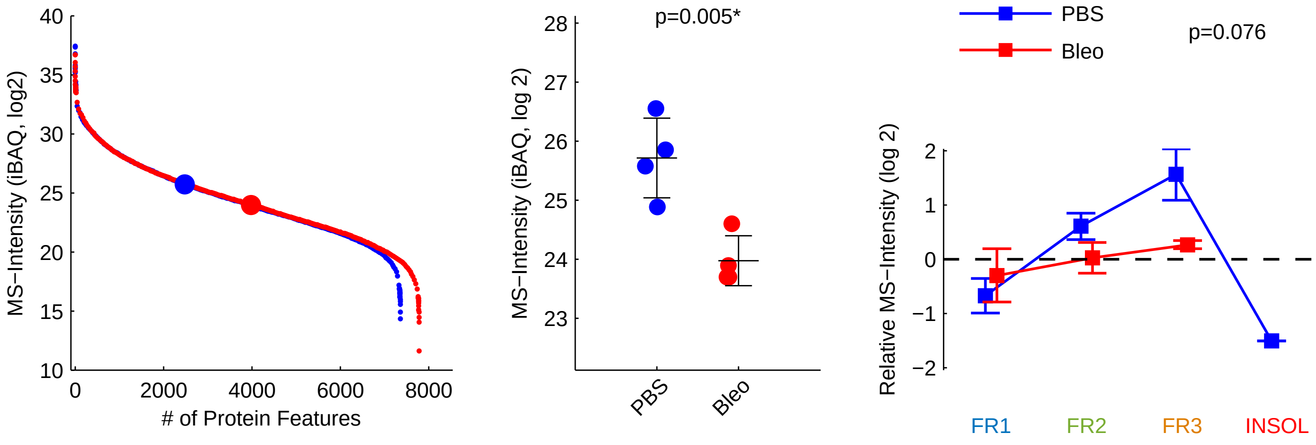

Q9DBP5 − Cmpk1 (id: 7853)

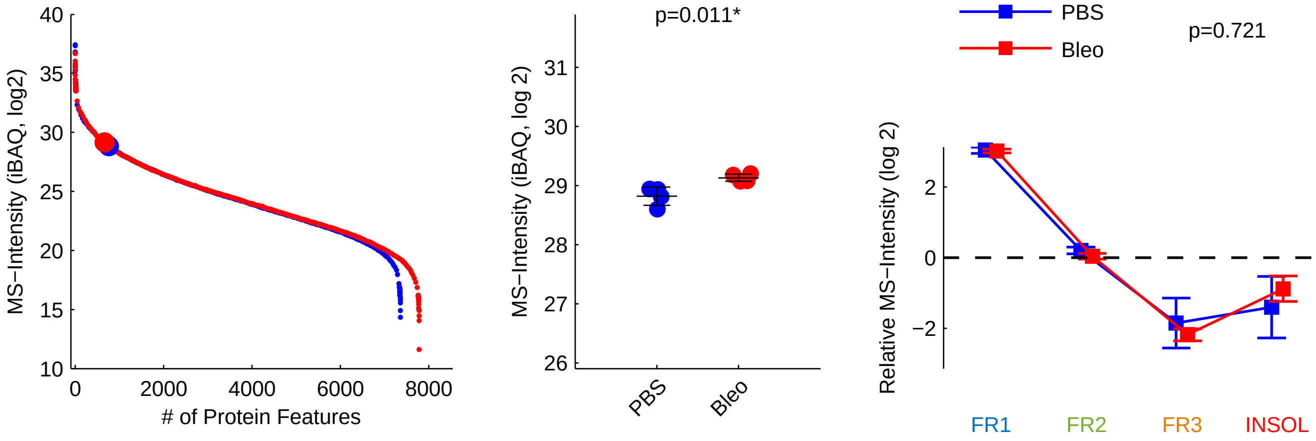

Q9DBR1 − Xrn2 (id: 7856)

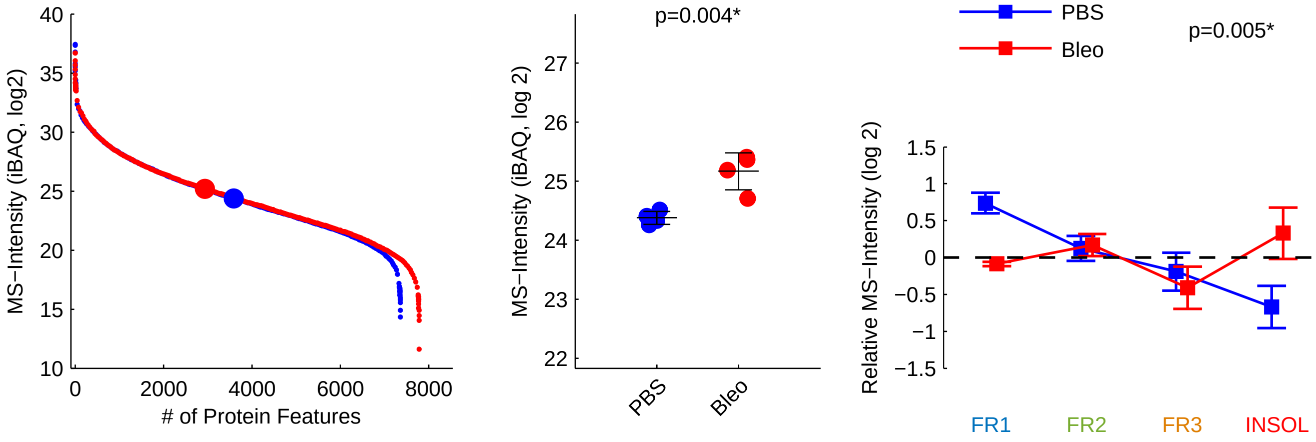

Q9DBR7 − Ppp1r12a (id: 7857)

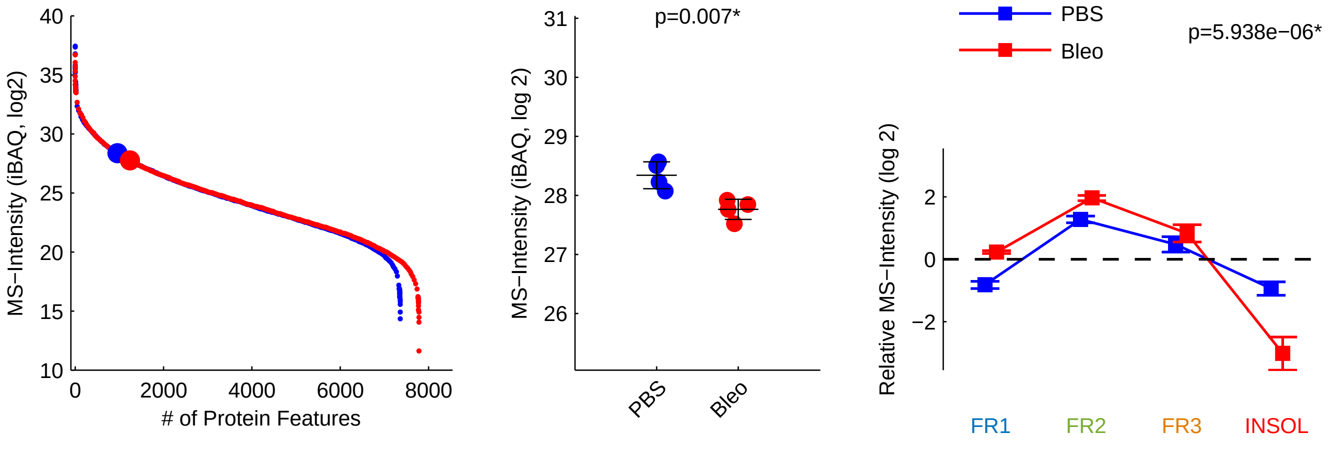

Q9DBS1 − Tmem43 (id: 7858)

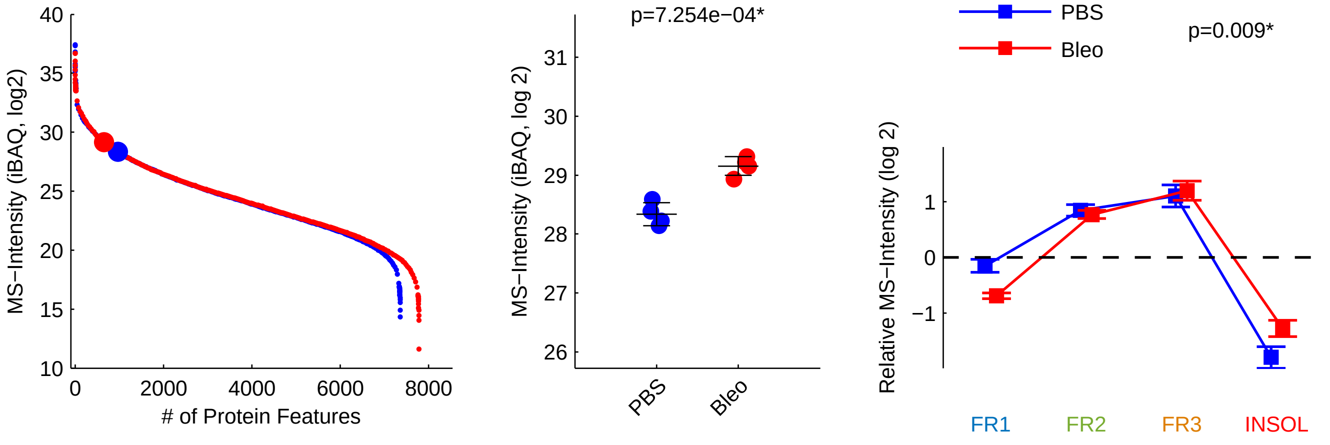

Q9DBS4 − Acoxl (id: 7860)

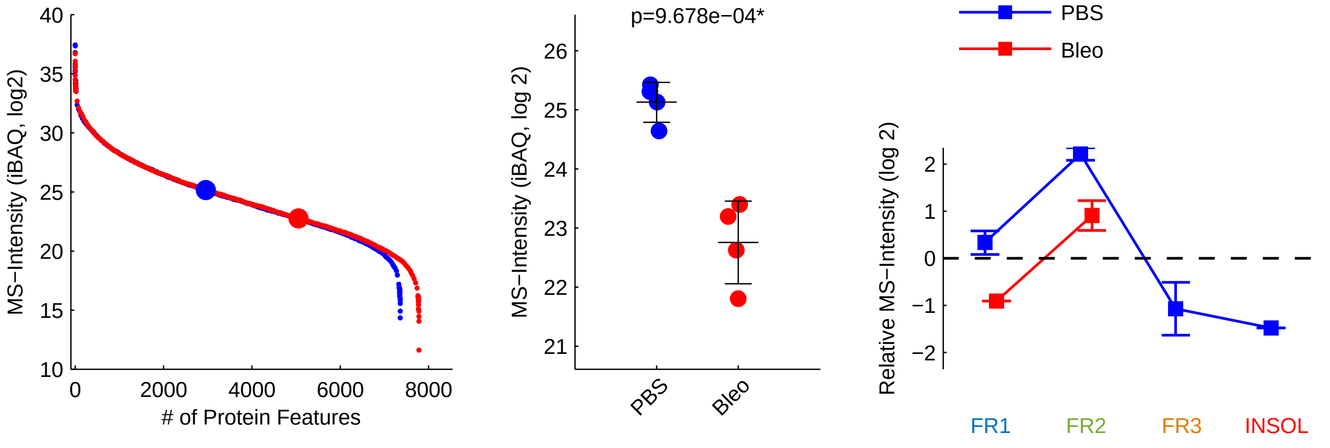

Q9DBS8 − Poc5 (id: 7862)

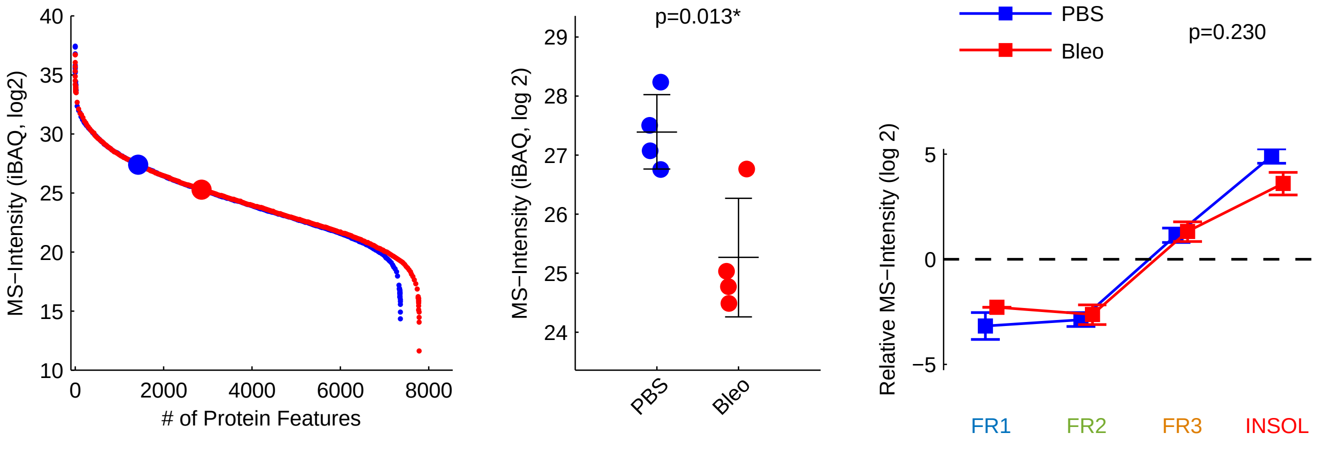

Q9DBW0 − Cyp4v2 (id: 7866)

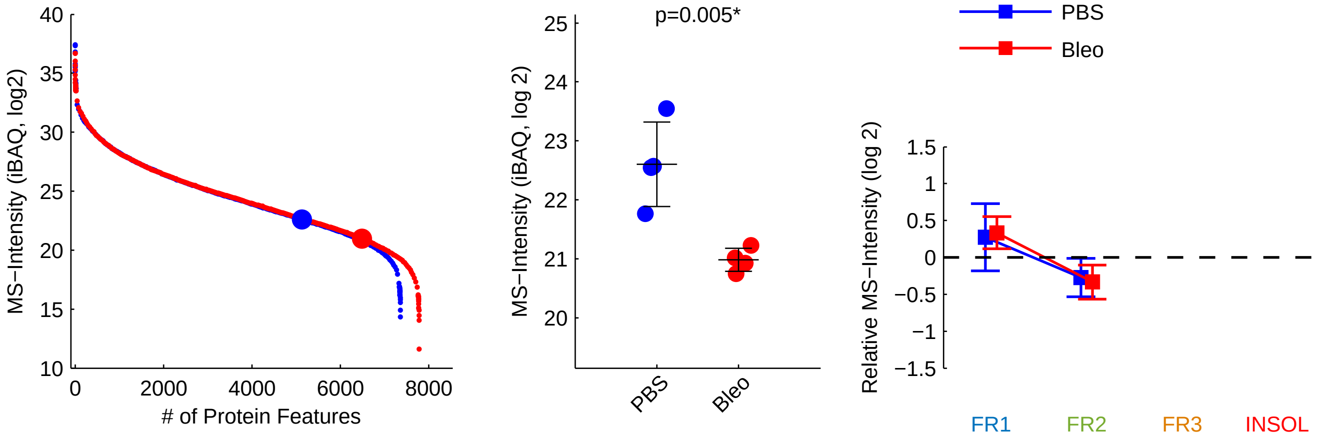

Q9DBX6 − Cyp2s1 (id: 7870)

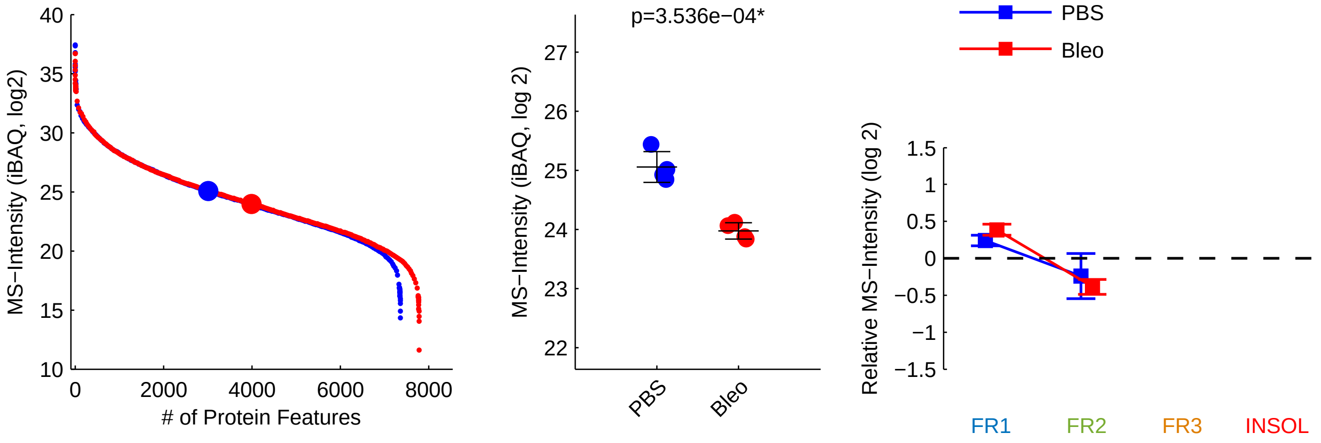

Q9DBZ1−2 − Ikbip (id: 7874)

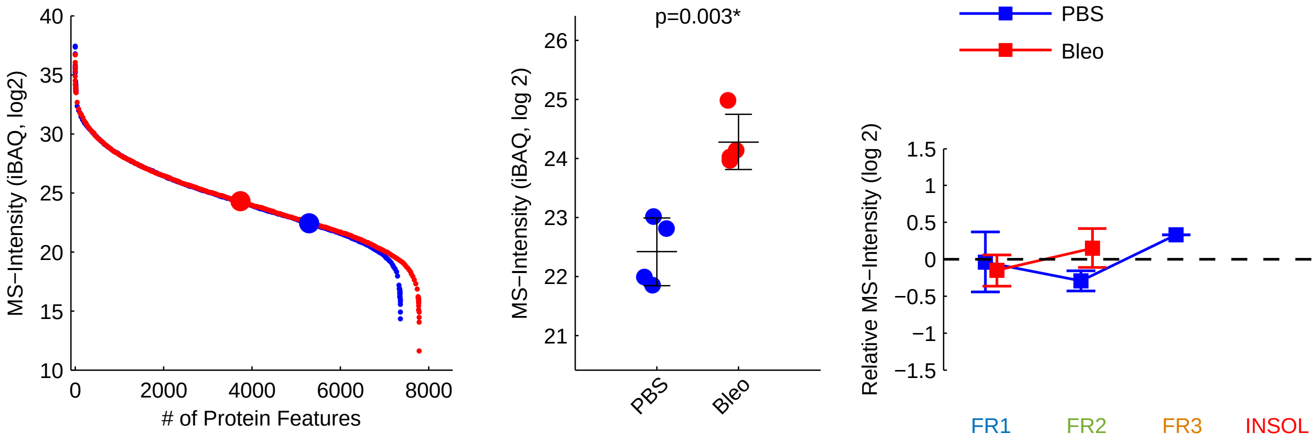

Q9DBZ5 − Eif3k (id: 7875)

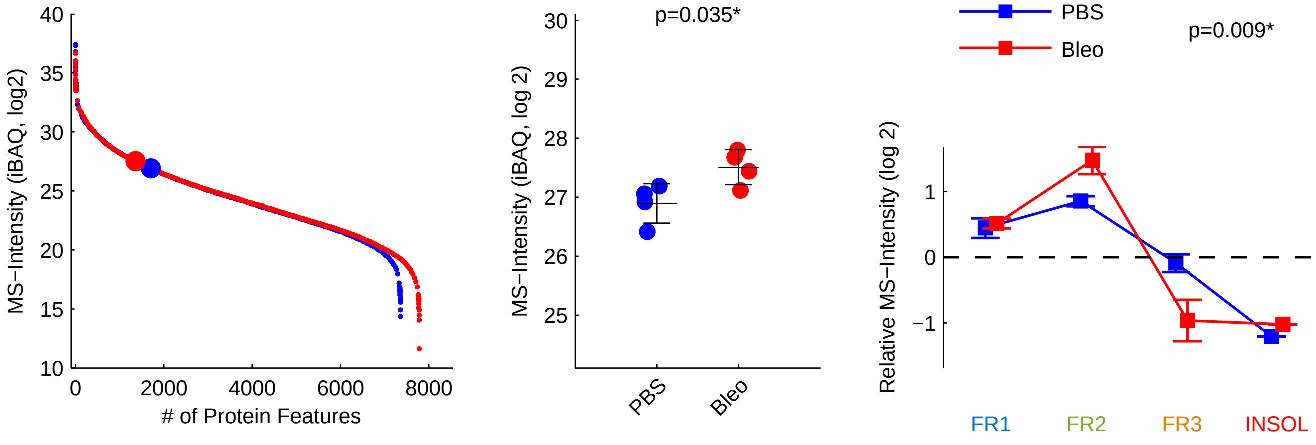

Q9DC07 − Nebl (id: 7877)

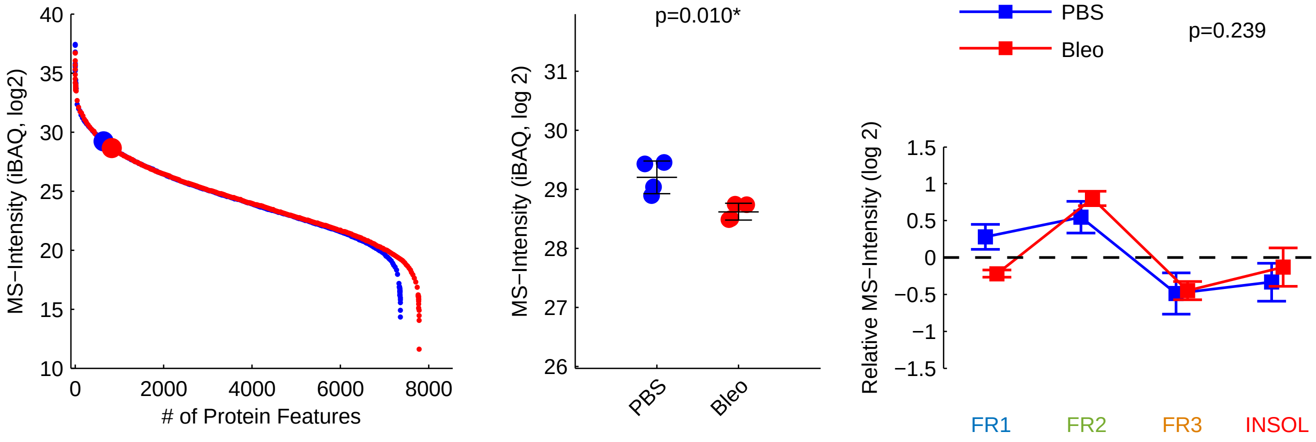

Q9DC23 − Dnajc10 (id: 7879)

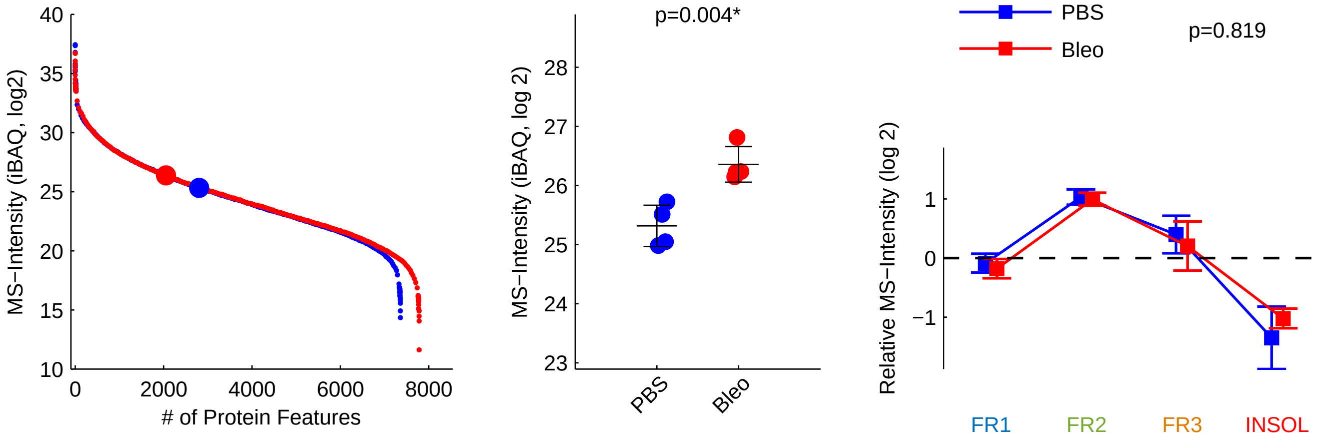

Q9DC51 − Gnai3 (id: 7888)

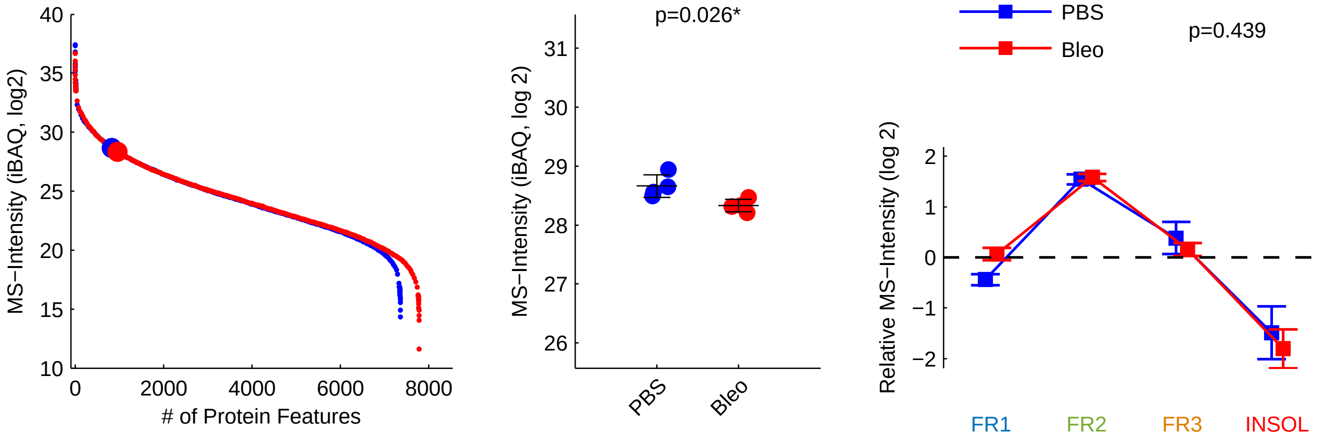

Q9DC61 − Pmpca (id: 7889)

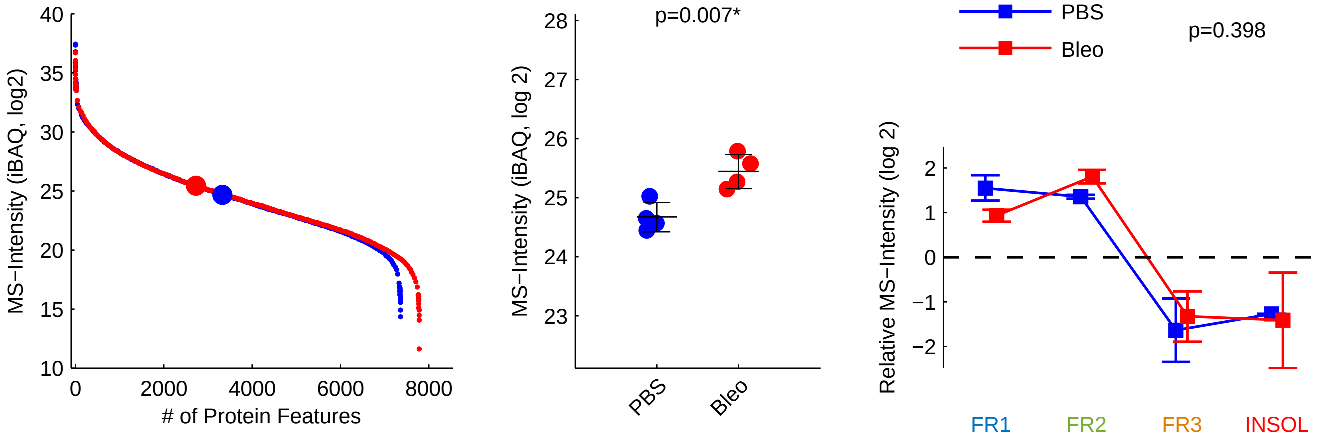

Q9DCA5 − Brix1 (id: 7895)

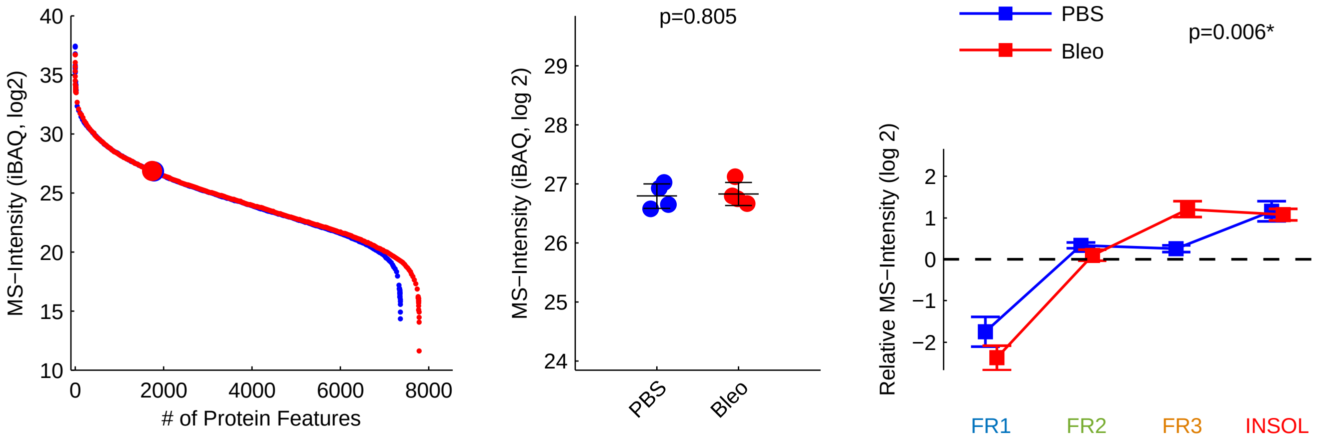

Q9DCB1 − Hmgn3 (id: 7896)

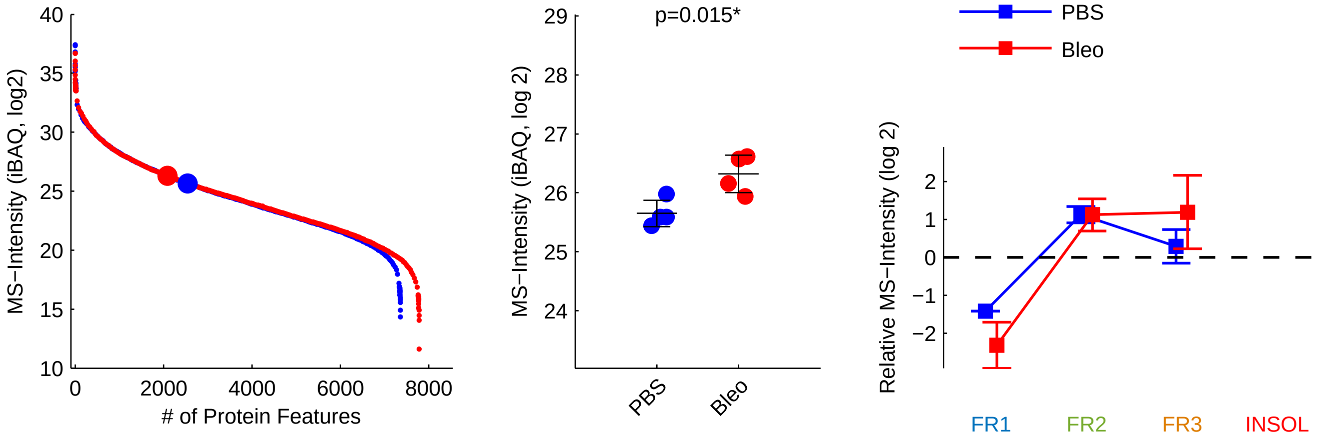

Q9DCC4 − Pycrl (id: 7898)

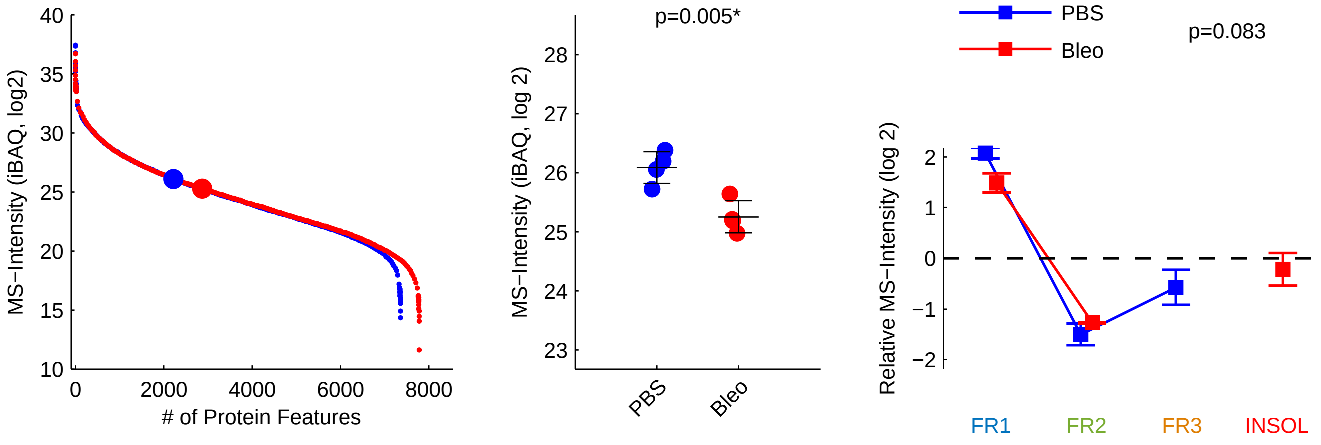

Q9DCC7 − Isoc2b (id: 7900)

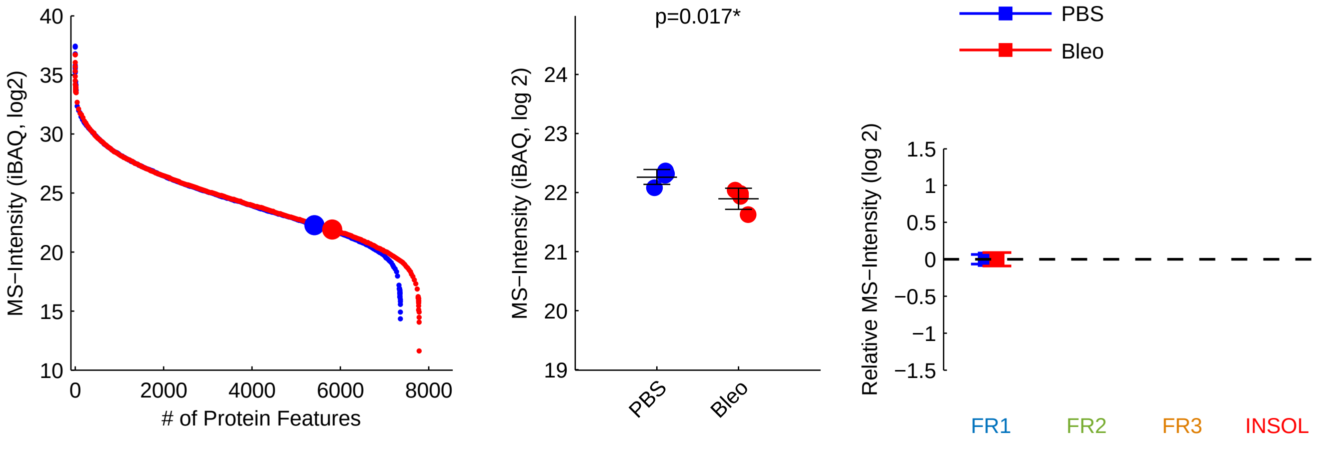

Q9DCC8 − Tomm20 (id: 7901)

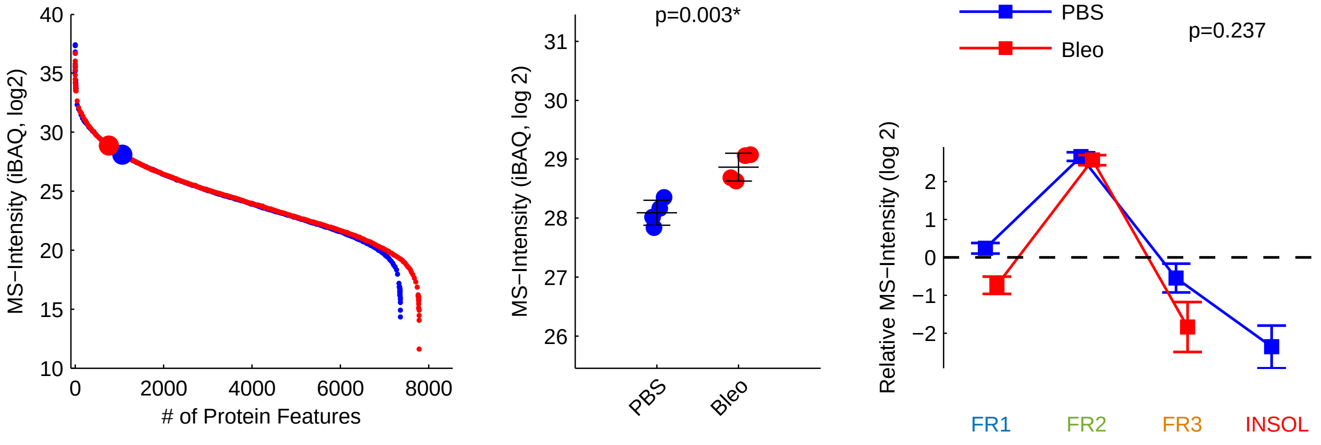

Q9DCF9 − Ssr3 (id: 7908)

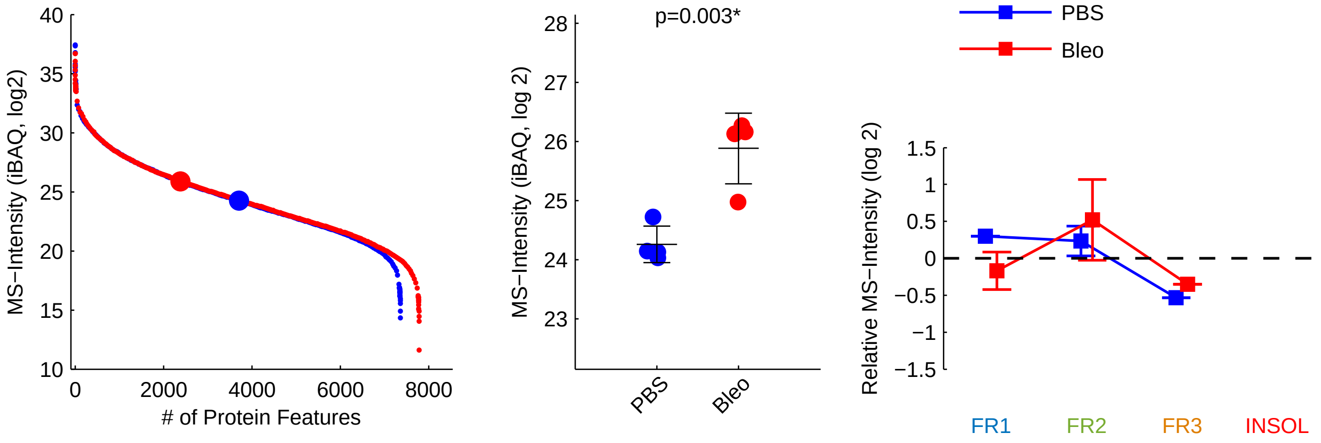

Q9DCH4 − Eif3f (id: 7910)

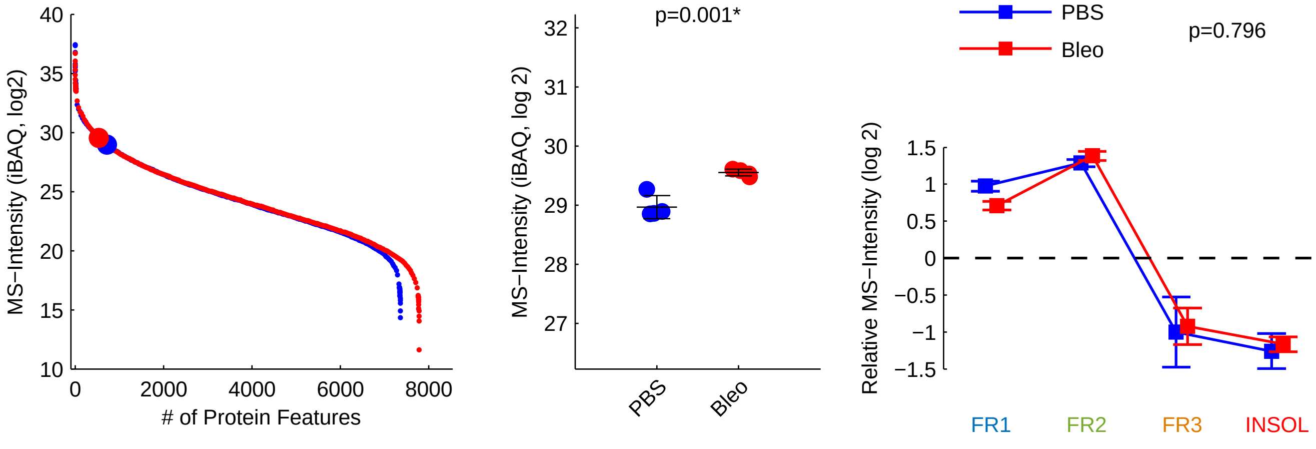

Q9DCJ5 − Ndufa8 (id: 7912)

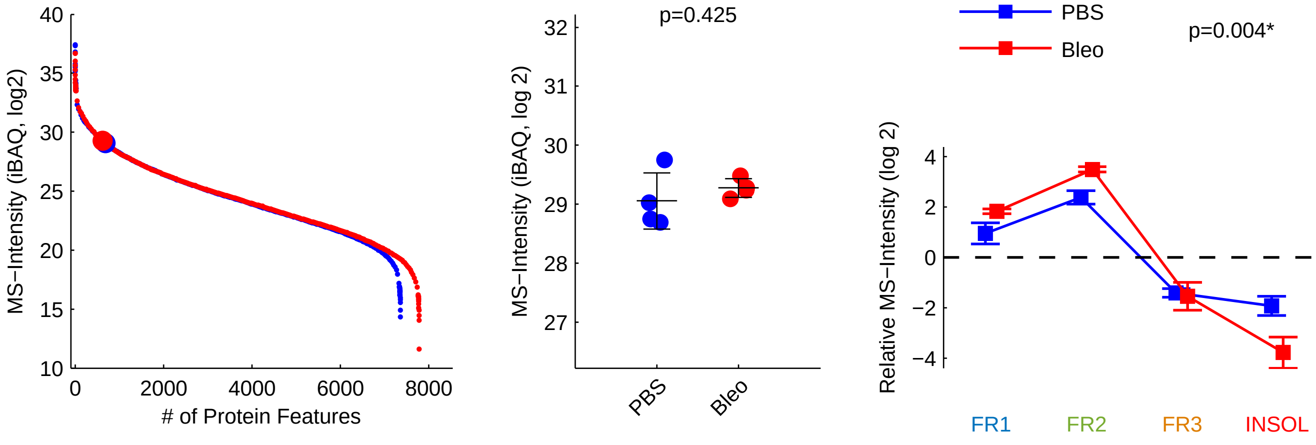

Q9DCJ9 − Npl (id: 7913)

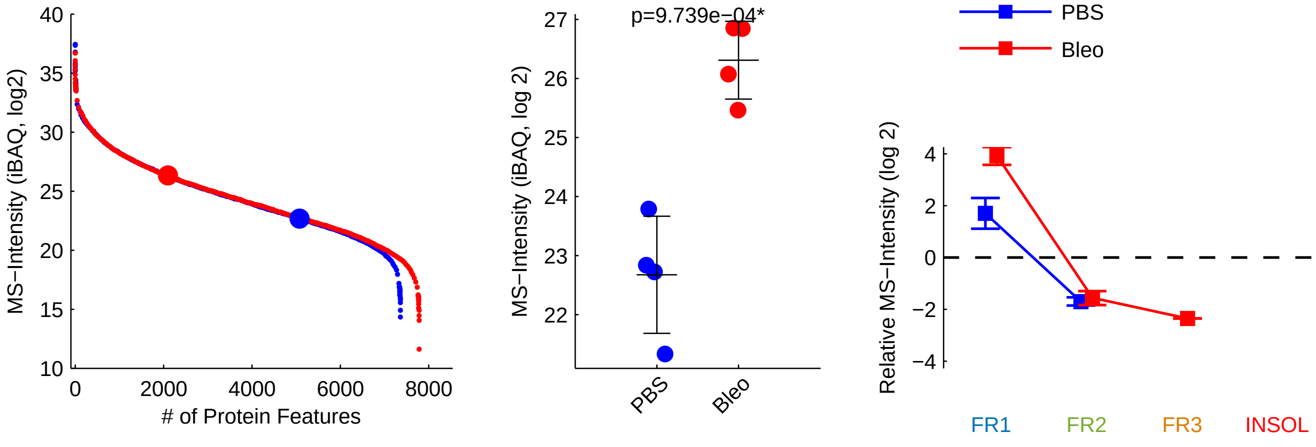

Q9DCL9 − Paics (id: 7915)

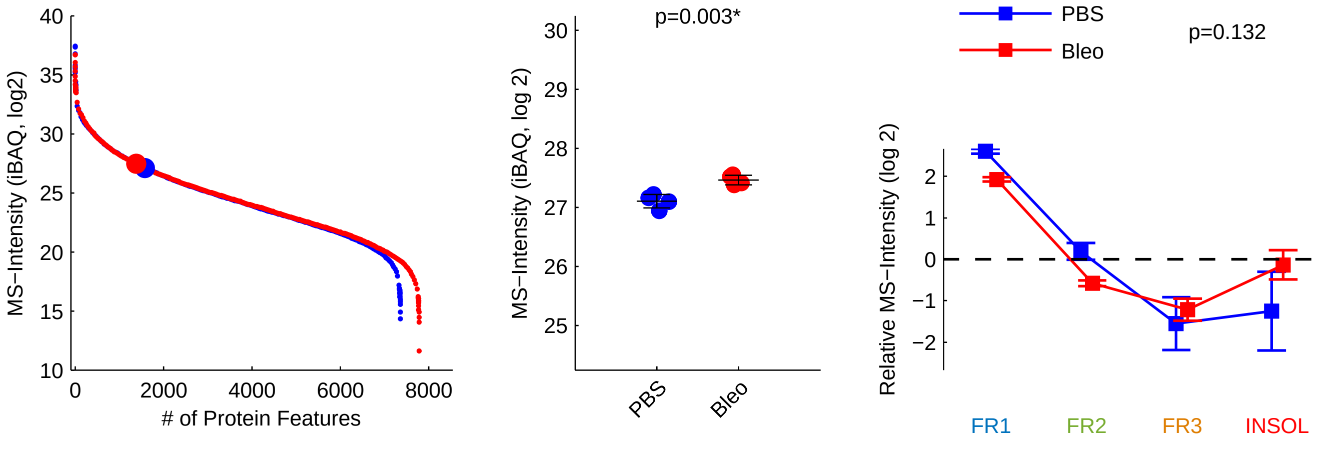

Q9DCM2 − Gstk1 (id: 7917)

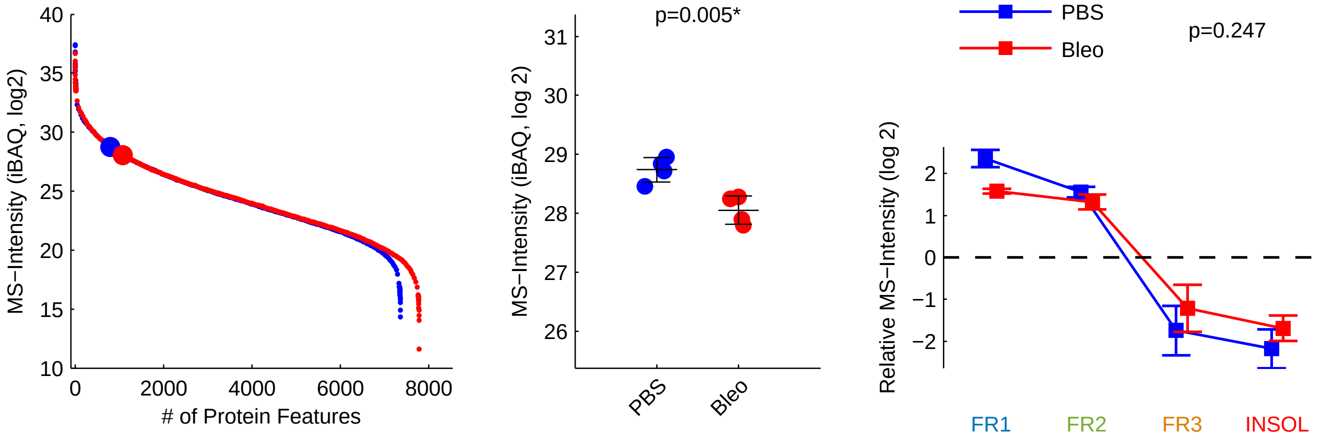

Q9DCR2 − Ap3s1 (id: 7921)

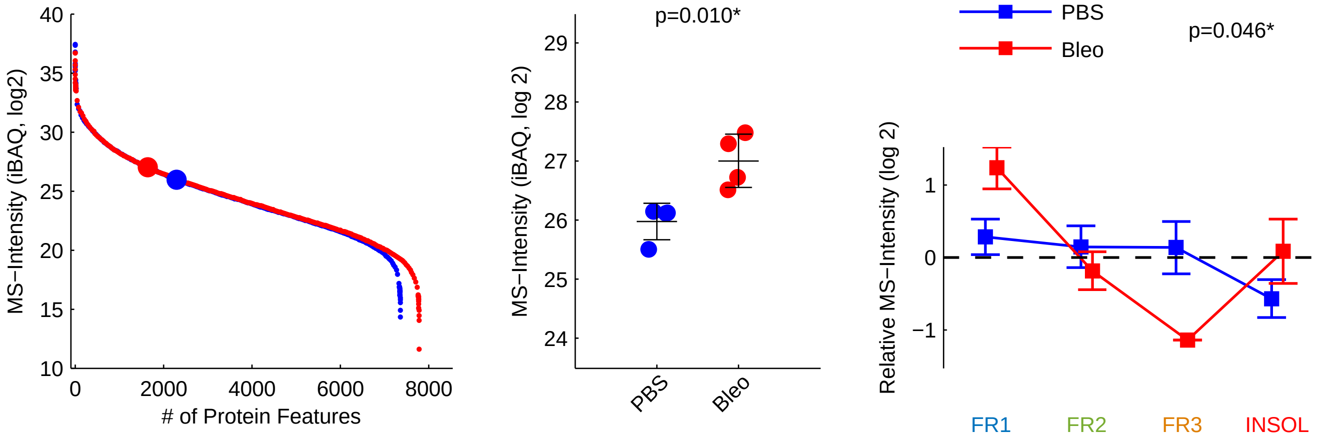

Q9DCS3 − Mecr (id: 7923)

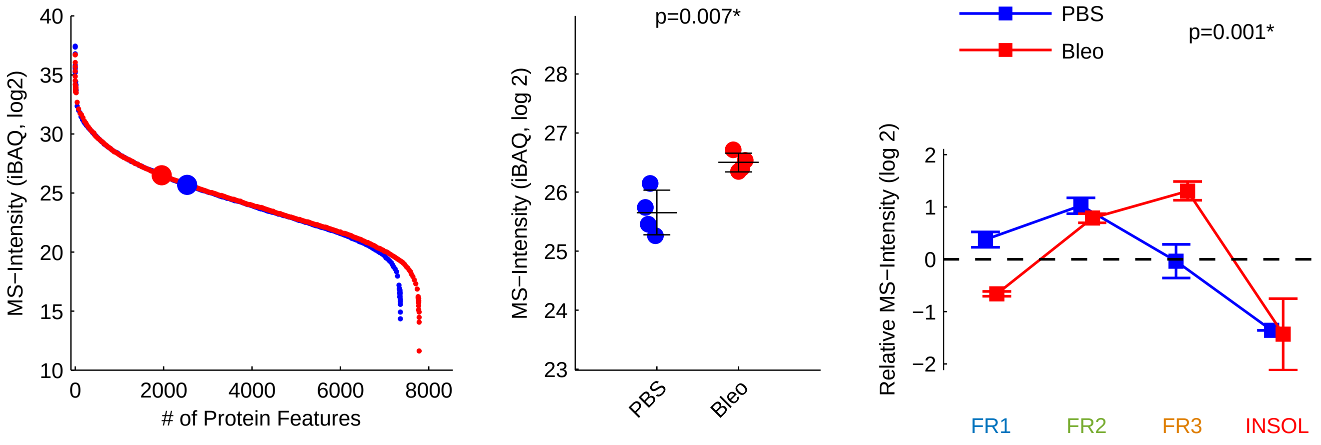

Q9DCT5 − Sdf2 (id: 7927)

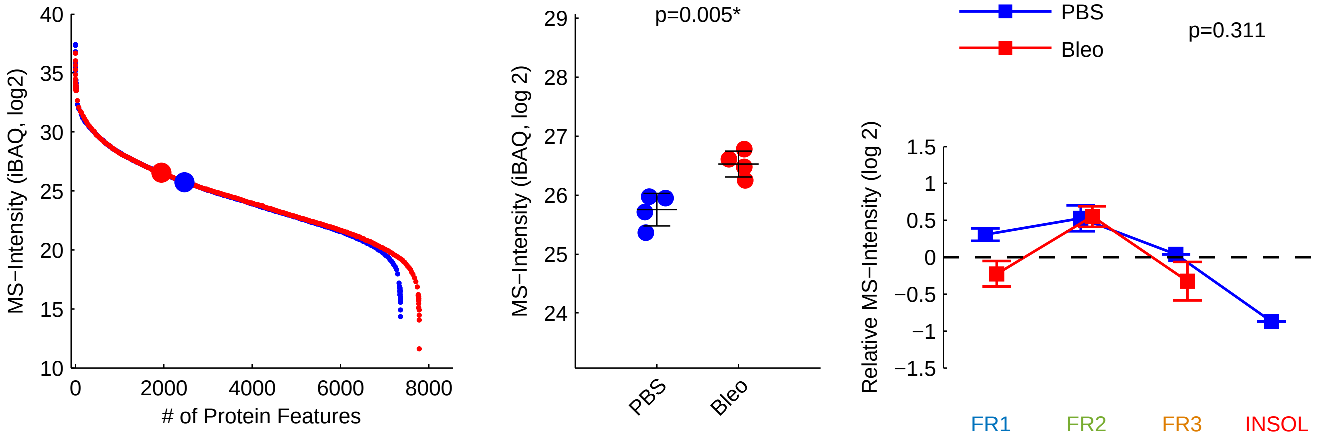

Q9DCT8 − Crip2 (id: 7928)

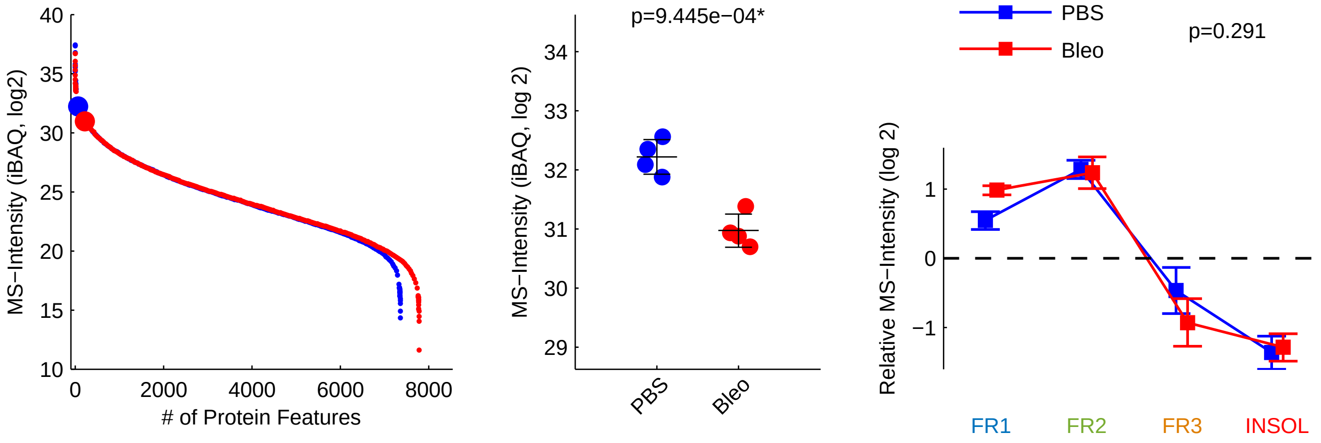

Q9DCW4 − Etfb (id: 7932)

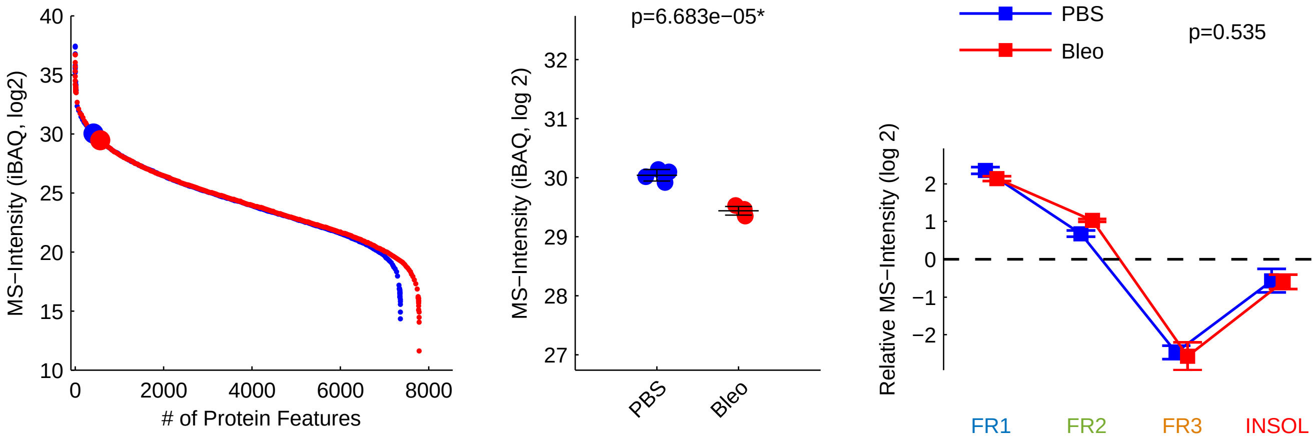

Q9DCX8 − Iyd (id: 7935)

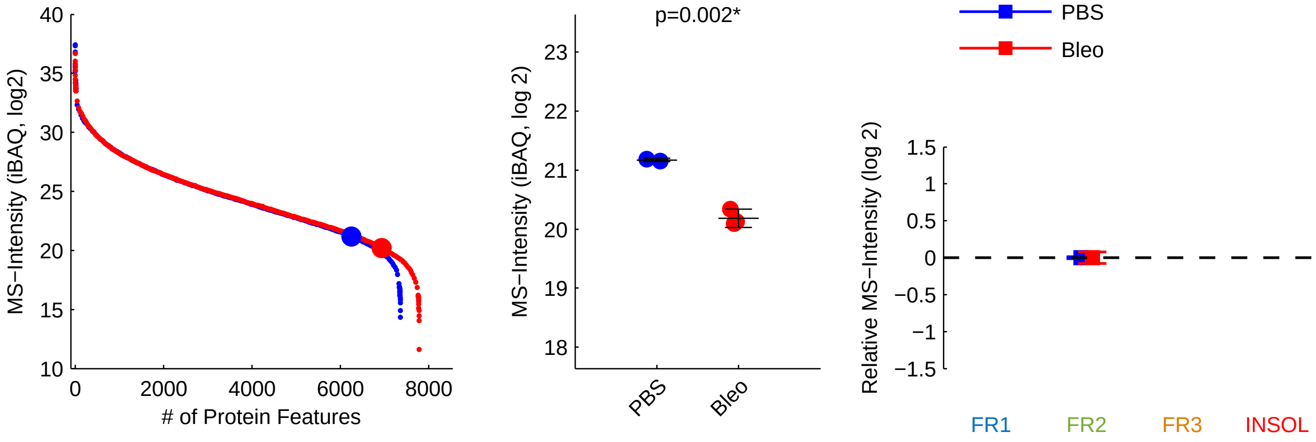

Q9DD18 − Dtd1 (id: 7940)

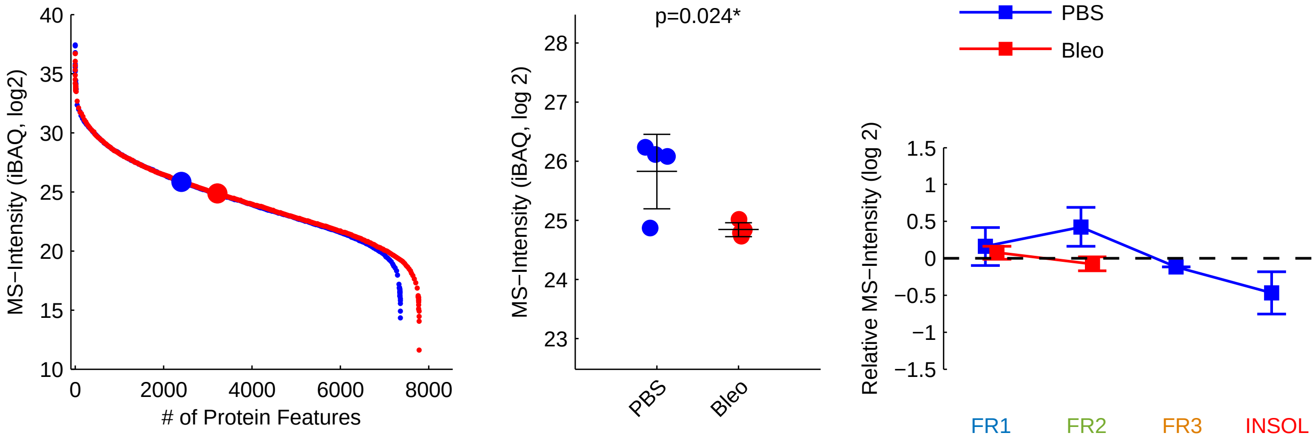

Q9EP89 − Lactb (id: 7949)

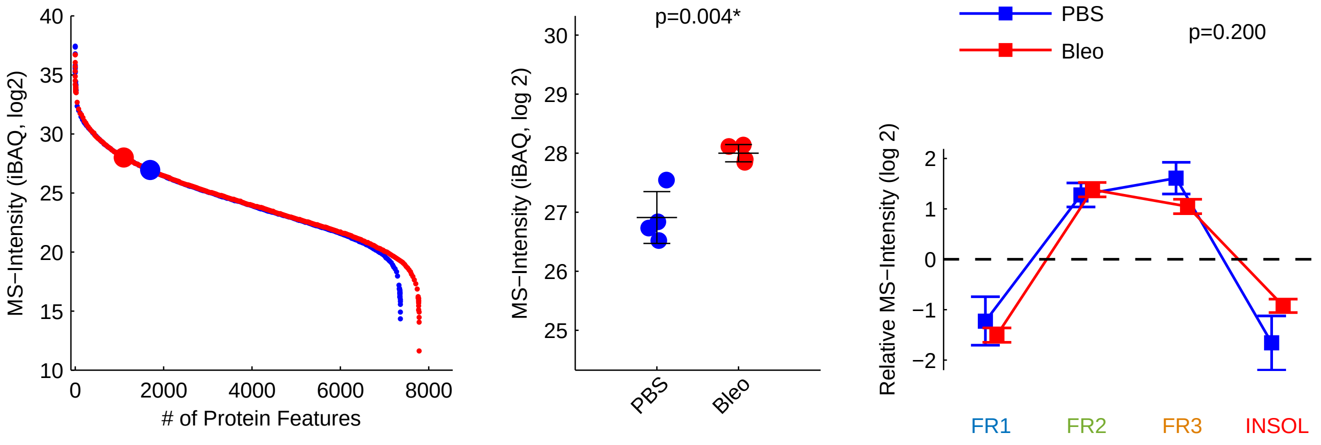

Q9EPB4 − Pycard (id: 7952)

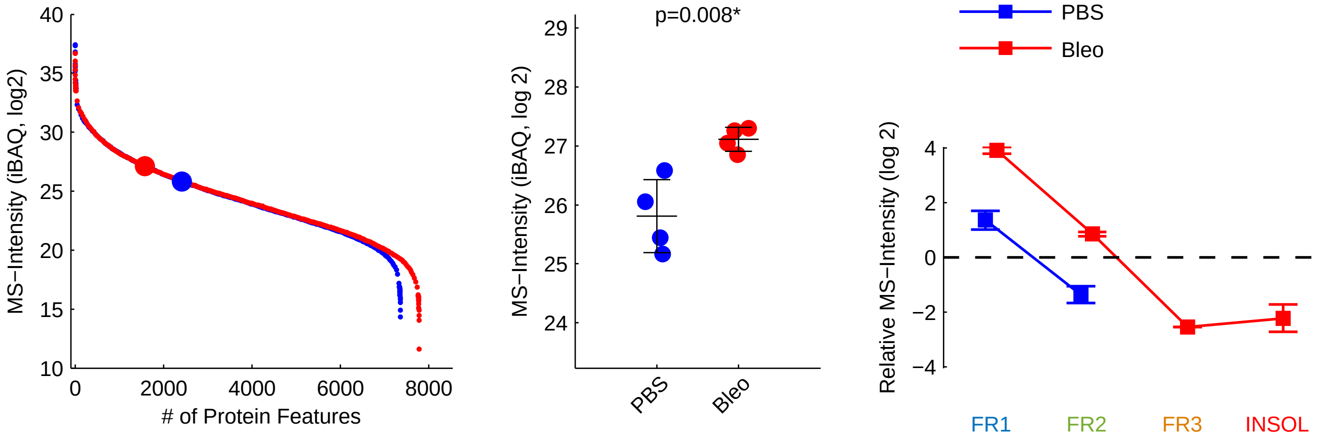

Q9EPC1 − Parva (id: 7953)

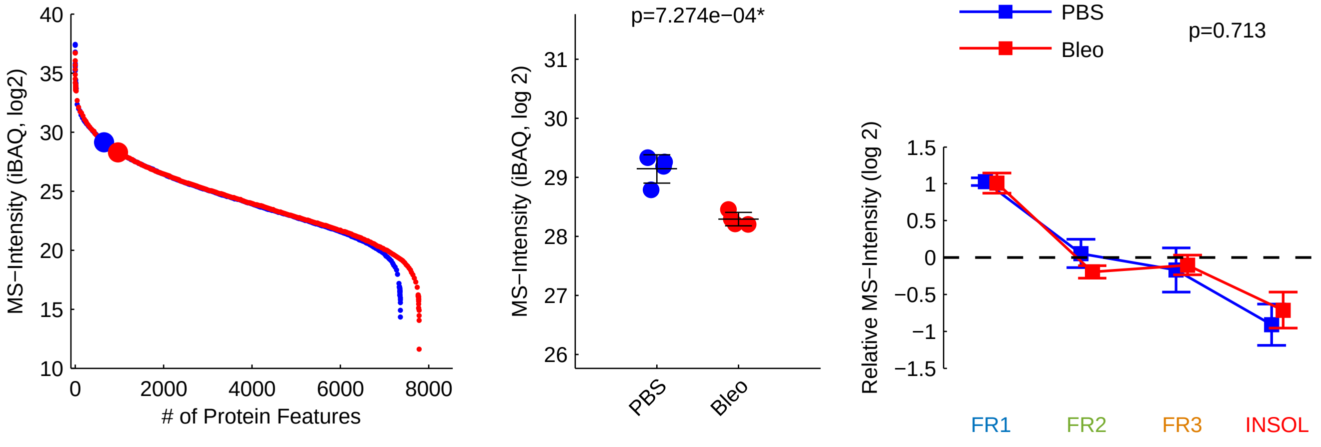

Q9EPJ9 − Arfgap1 (id: 7956)

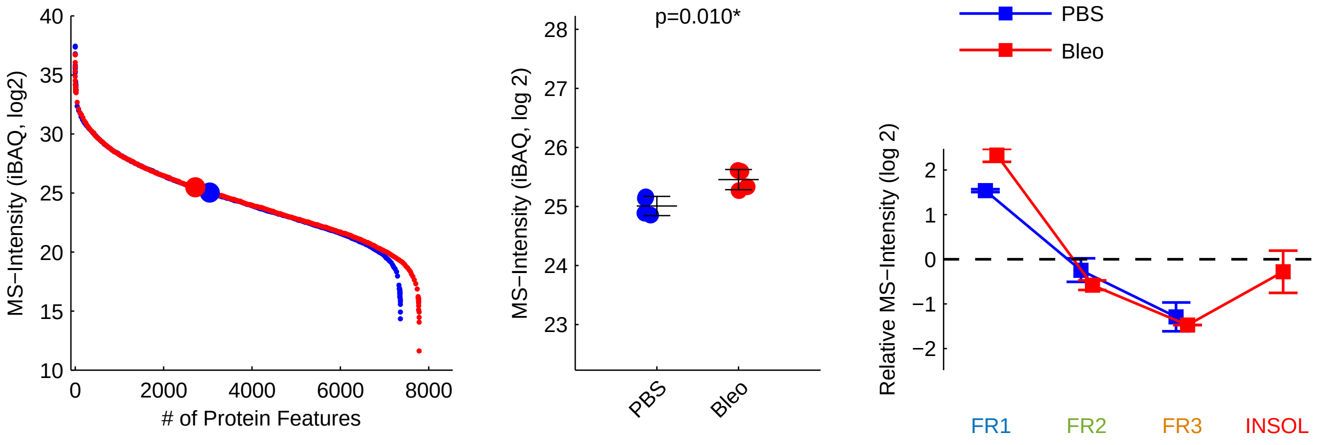

Q9EPK2 − Rp2 (id: 7957)

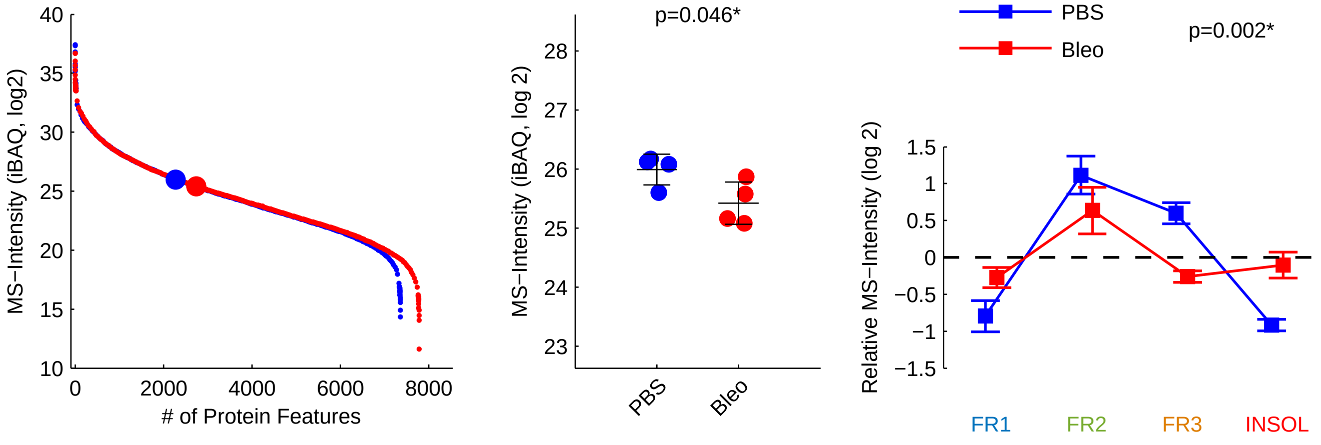

Q9EPK6 − Sil1 (id: 7959)

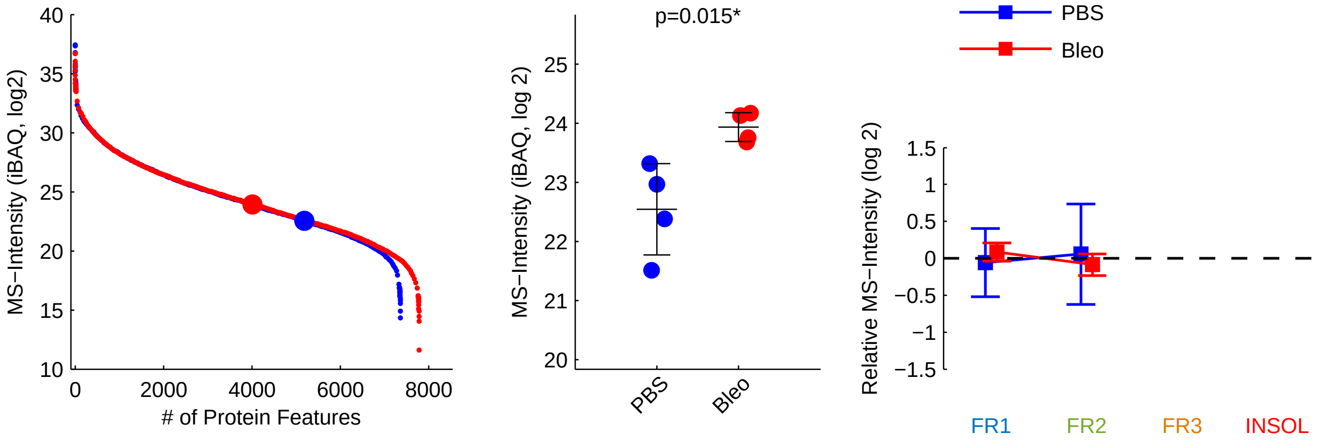

Q9EPL9 − Acox3 (id: 7963)

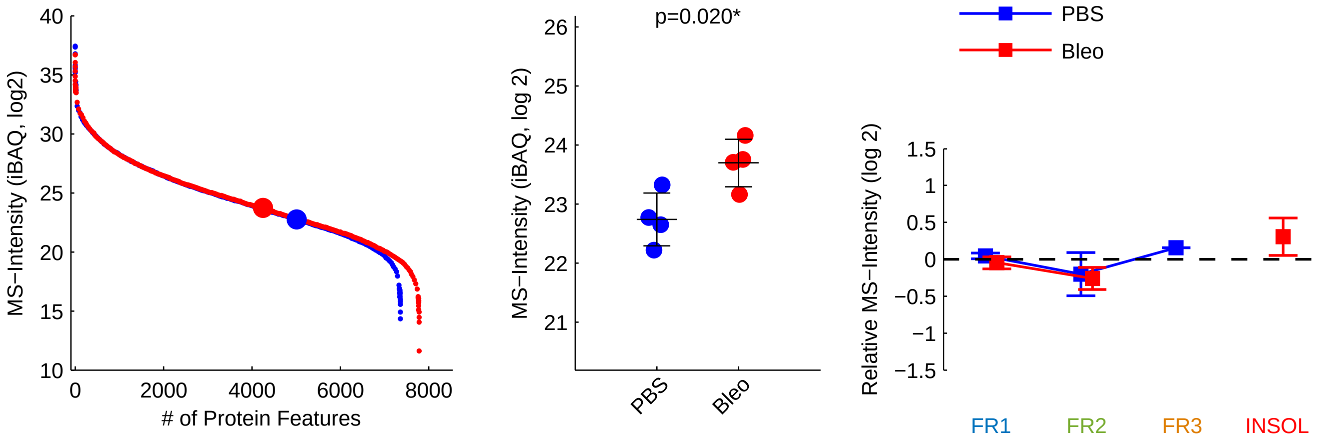

Q9EPR5 − Sorcs2 (id: 7967)

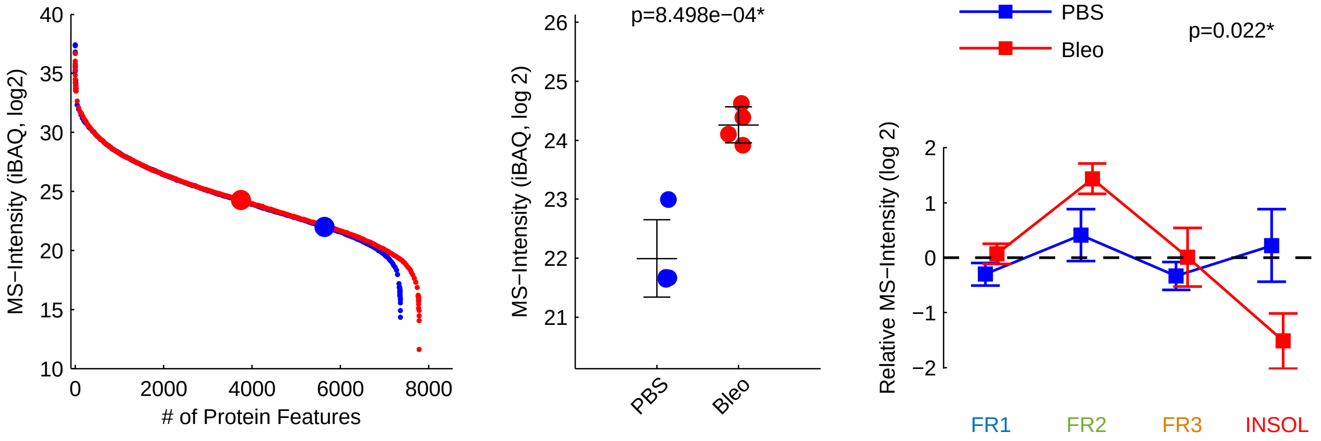

Q9EQ06 − Hsd17b11 (id: 7974)

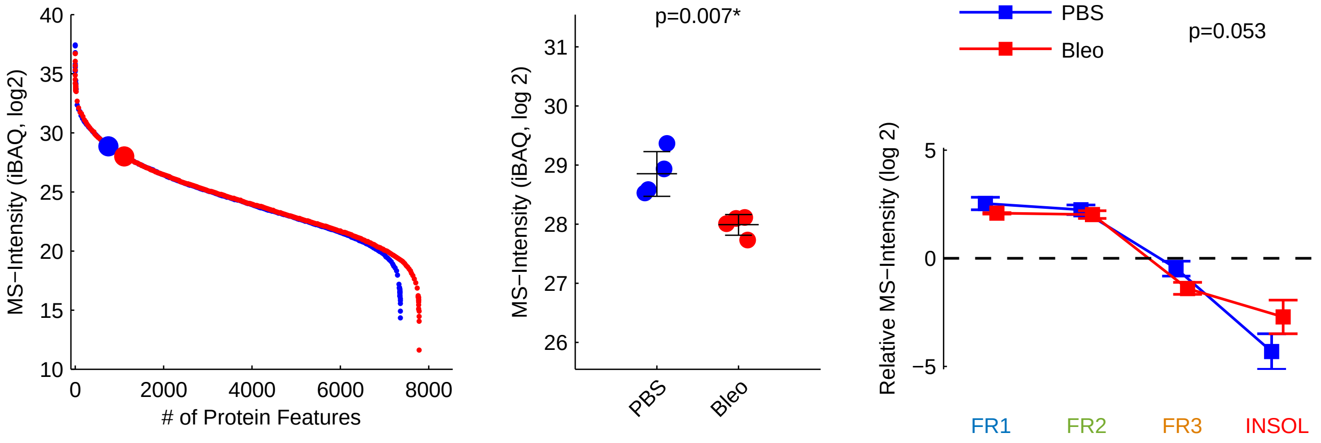

Q9EQ20 − Aldh6a1 (id: 7976)

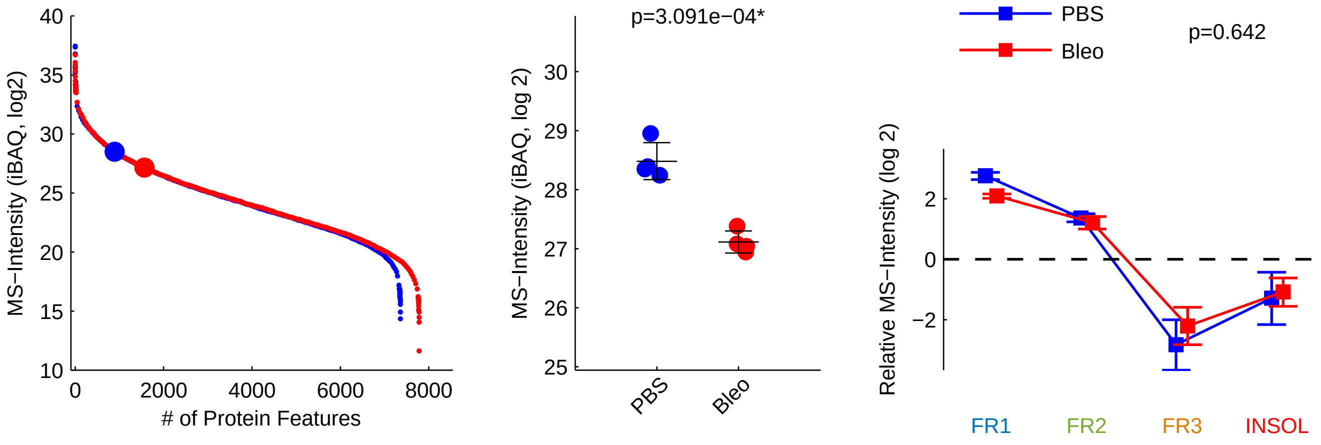

Q9EQC5 − Scyl1 (id: 7979)

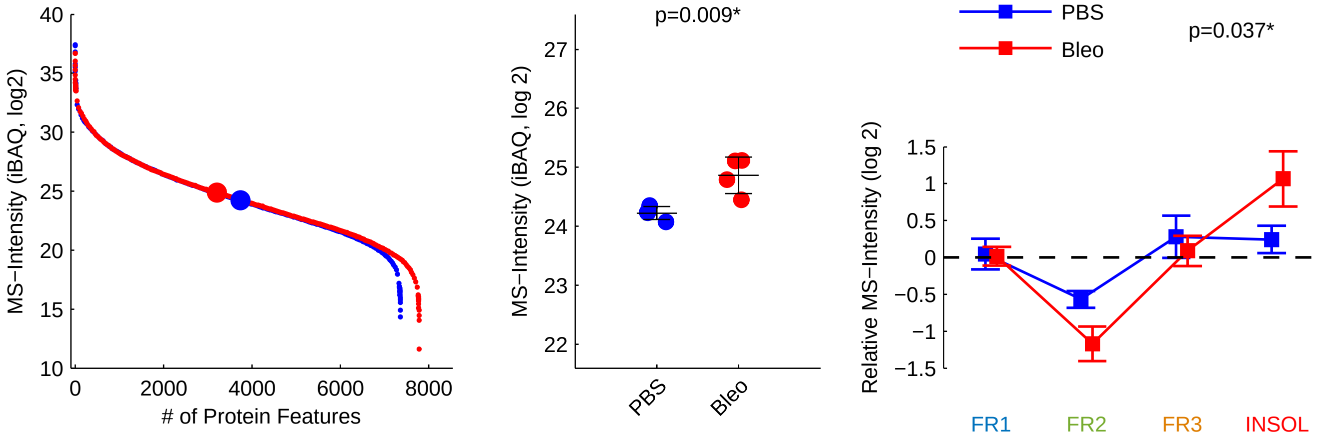

Q9EQF6 − Dpysl5 (id: 7982)

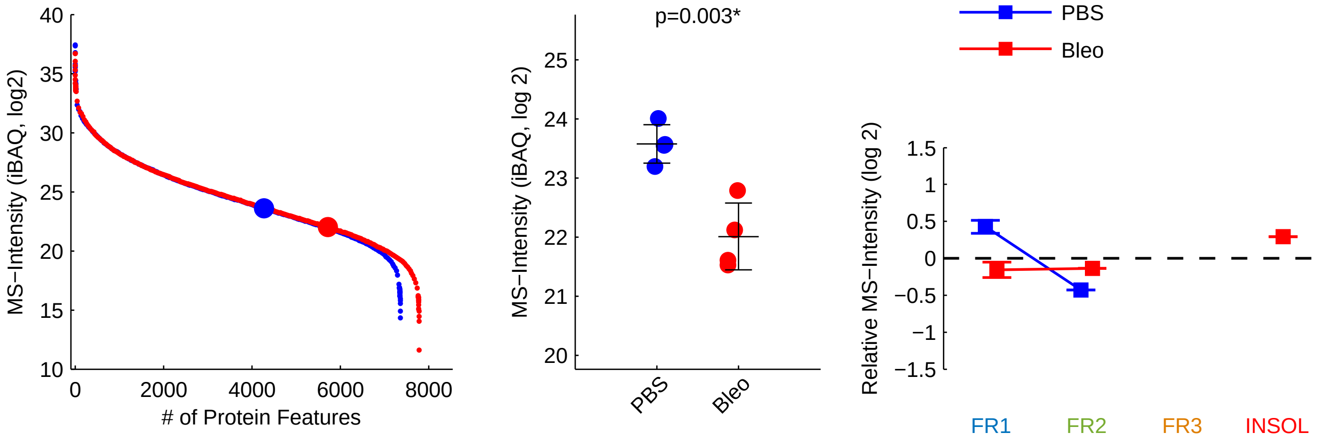

Q9EQG9 − Col4a3bp (id: 7985)

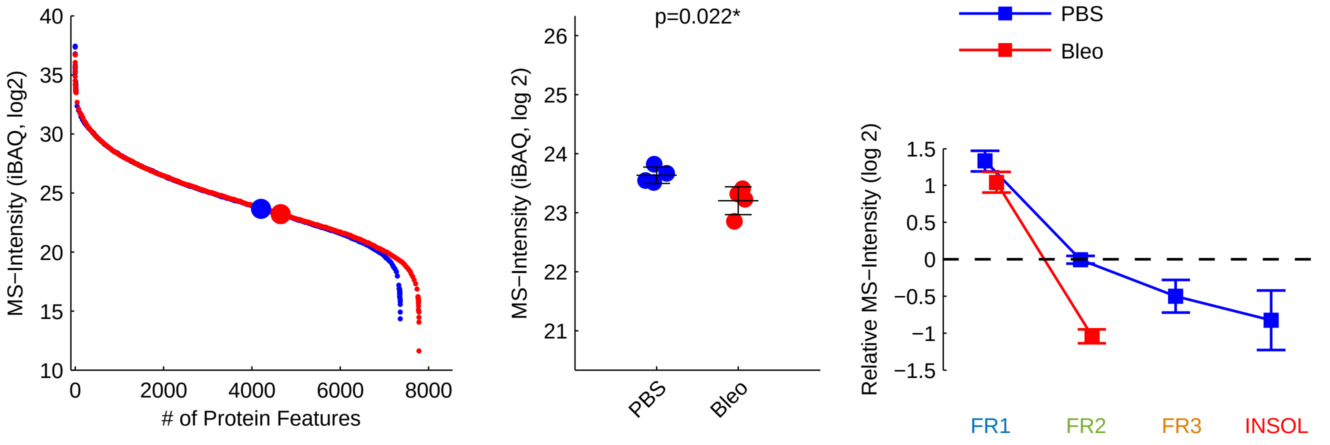

Q9EQI8 − Mrpl46 (id: 7990)

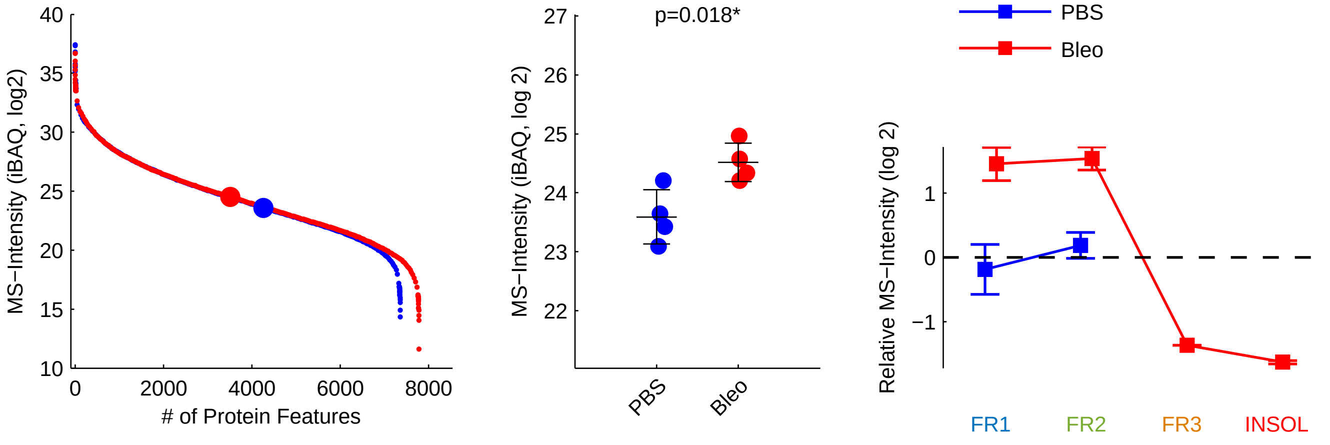

Q9EQK5 − Mvp (id: 7993)

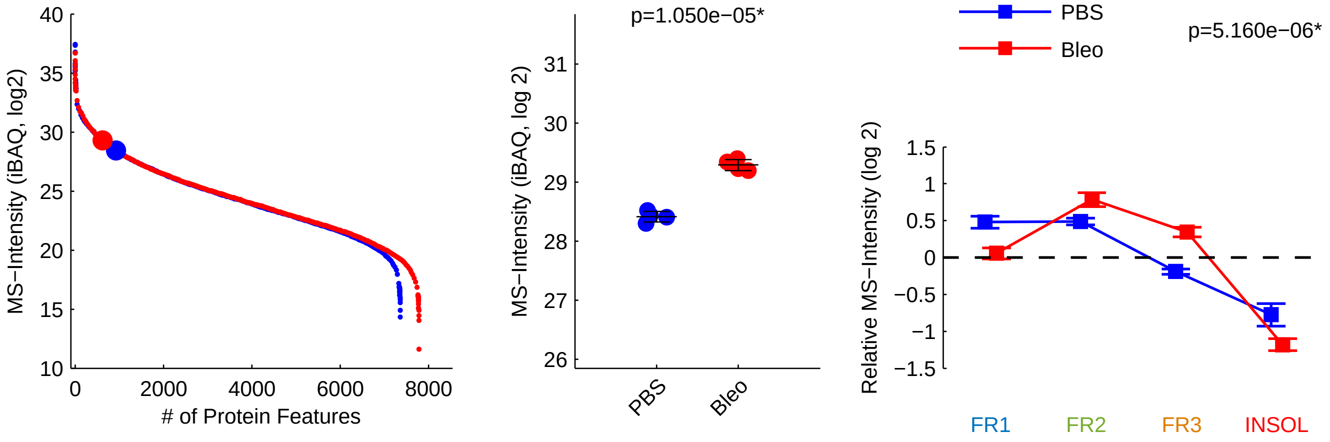

Q9EQP2 − Ehd4 (id: 7996)

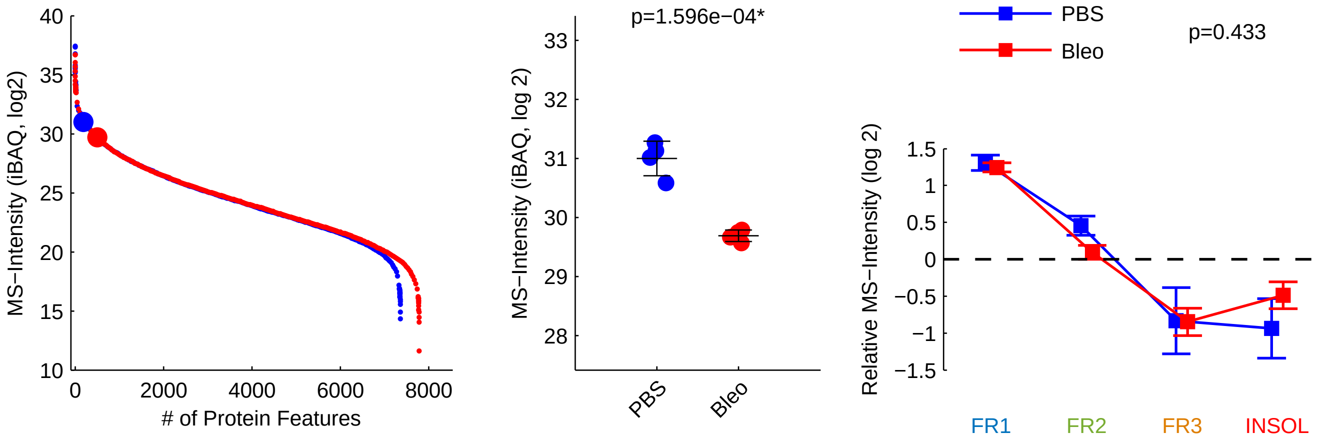

Q9EQQ9 − Mgea5 (id: 7998)

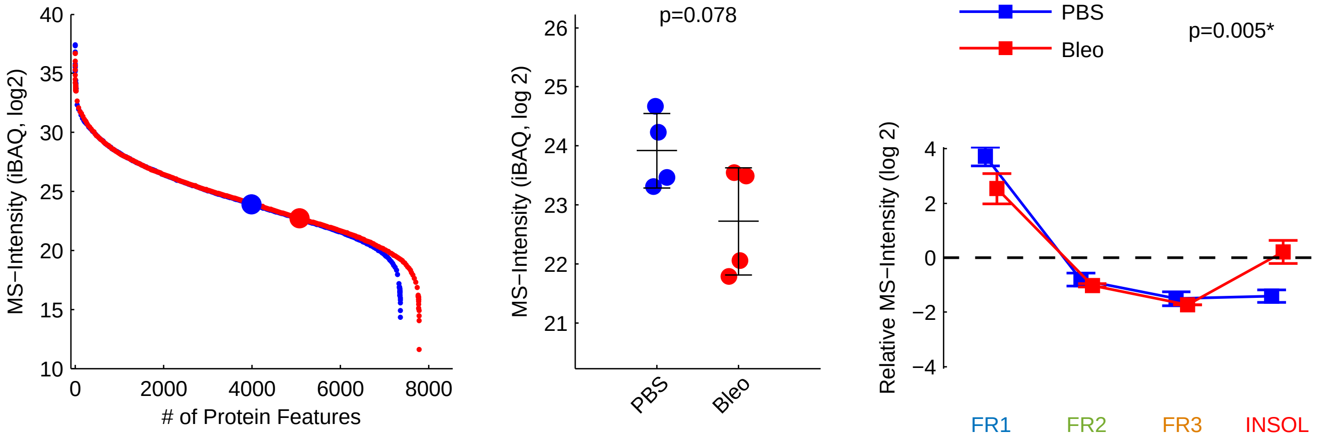

Q9EQX4 − Aif1l (id: 8001)

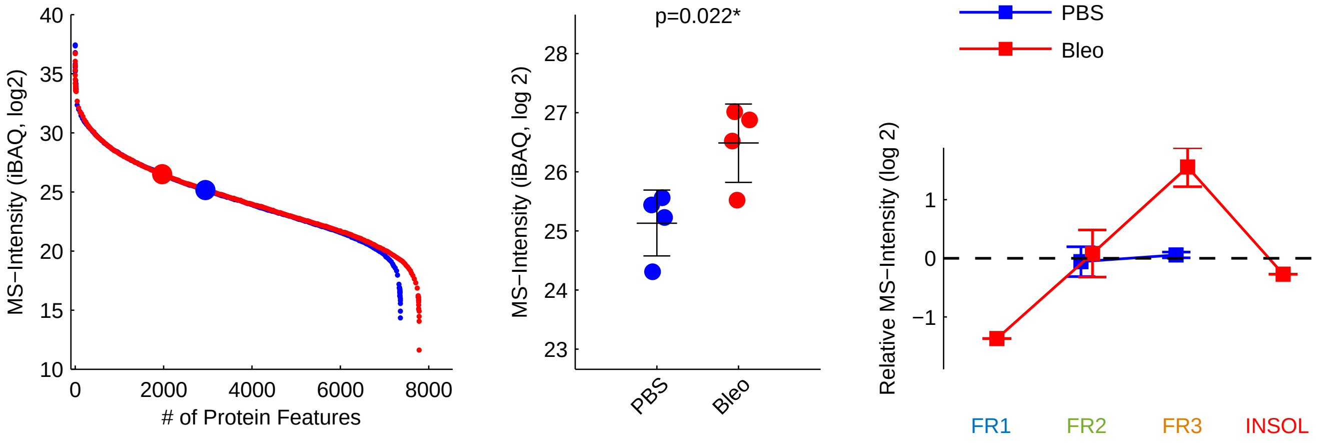

Q9ER00 − Stx12 (id: 8002)

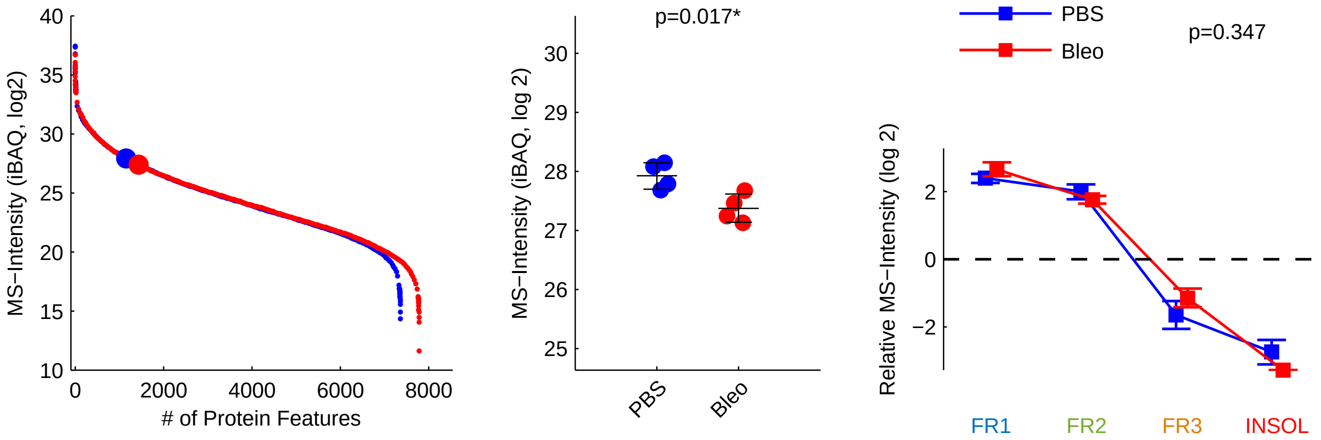

Q9ERD7 − Tubb3 (id: 8015)

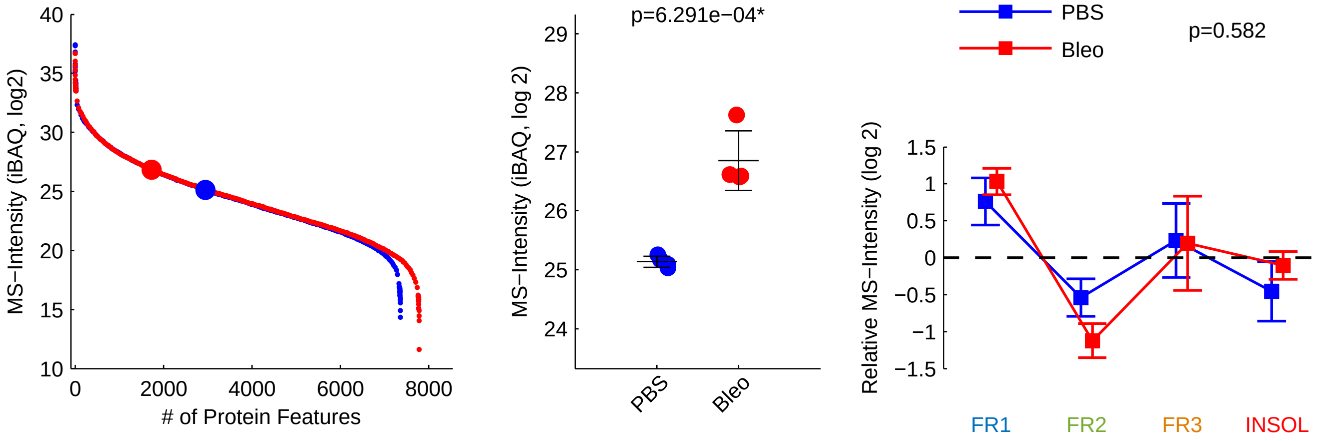

Q9ERE7 − Mesdc2 (id: 8016)

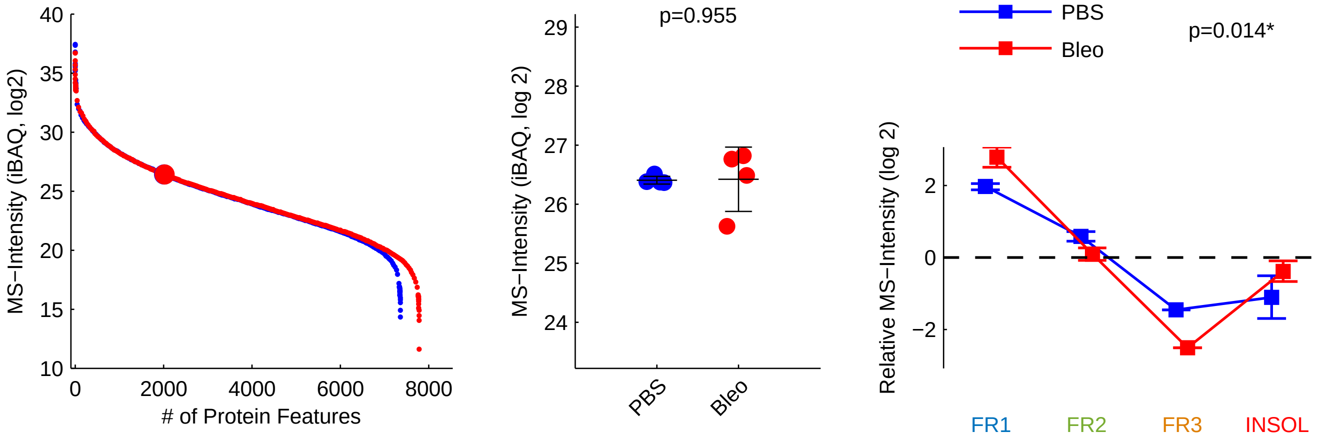

Q9ERL9 − Gucy1a3 (id: 8025)

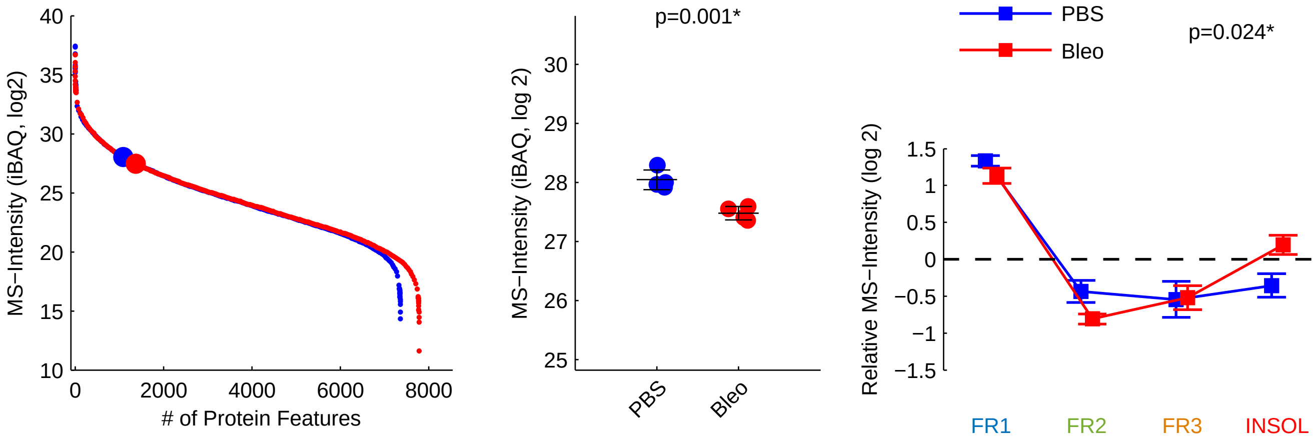

Q9ERN0 − Scamp2 (id: 8026)

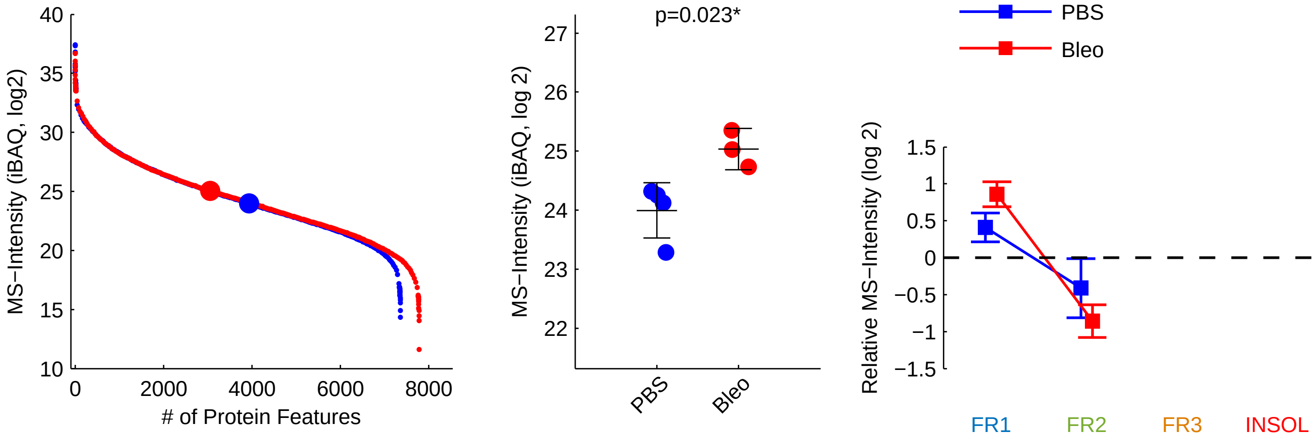

Q9ERR7 − Sep15 (id: 8027)

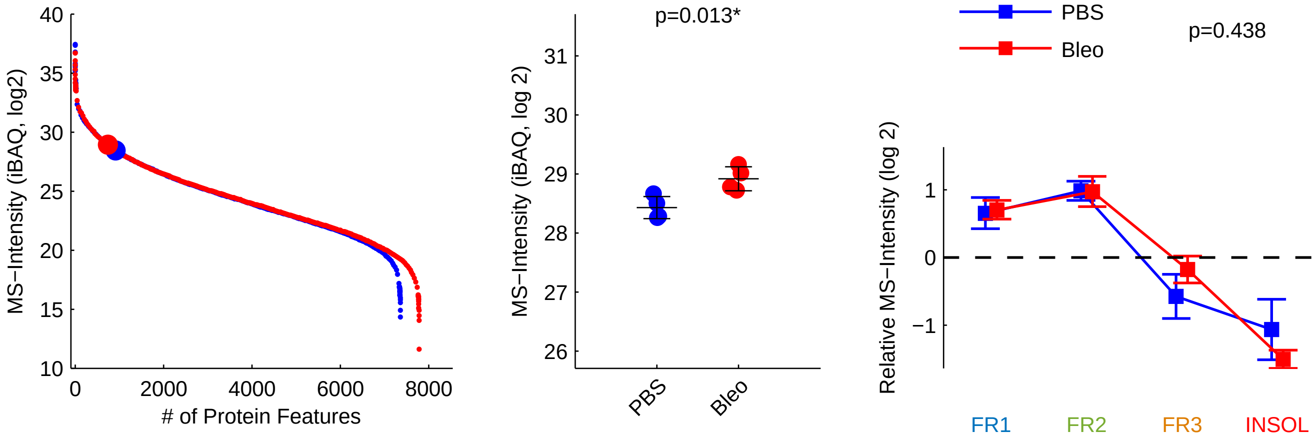

Q9ERS5 − Plekha2 (id: 8030)

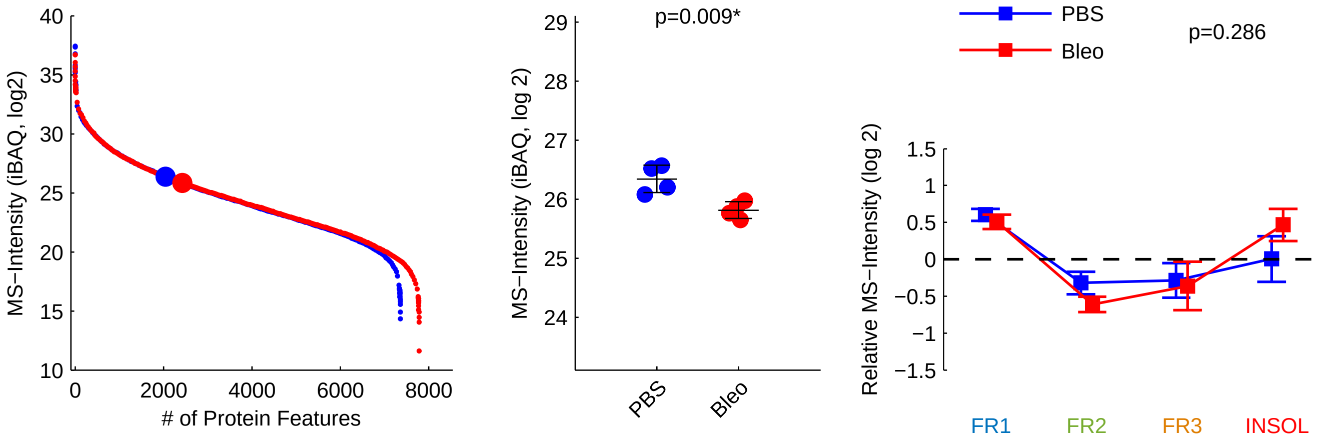

Q9ERU3 − Znf22 (id: 8031)

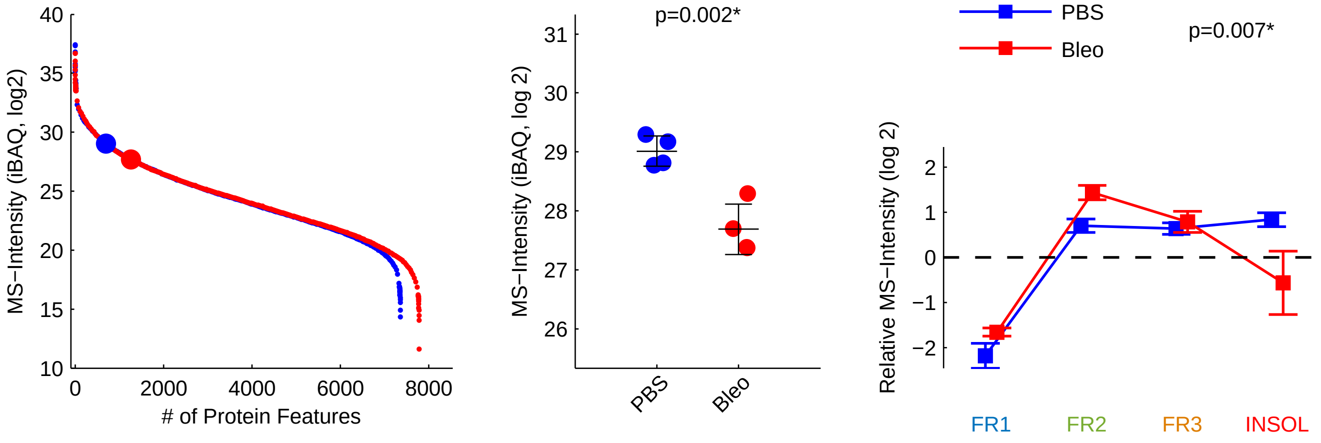

Q9ES46 − Parvb (id: 8039)

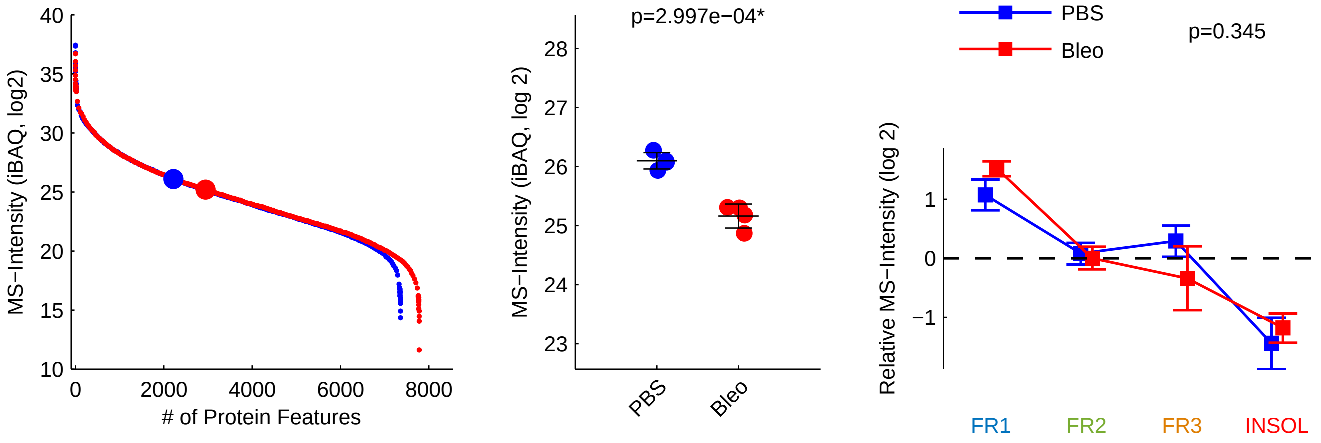

Q9ES52 − Inpp5d (id: 8040)

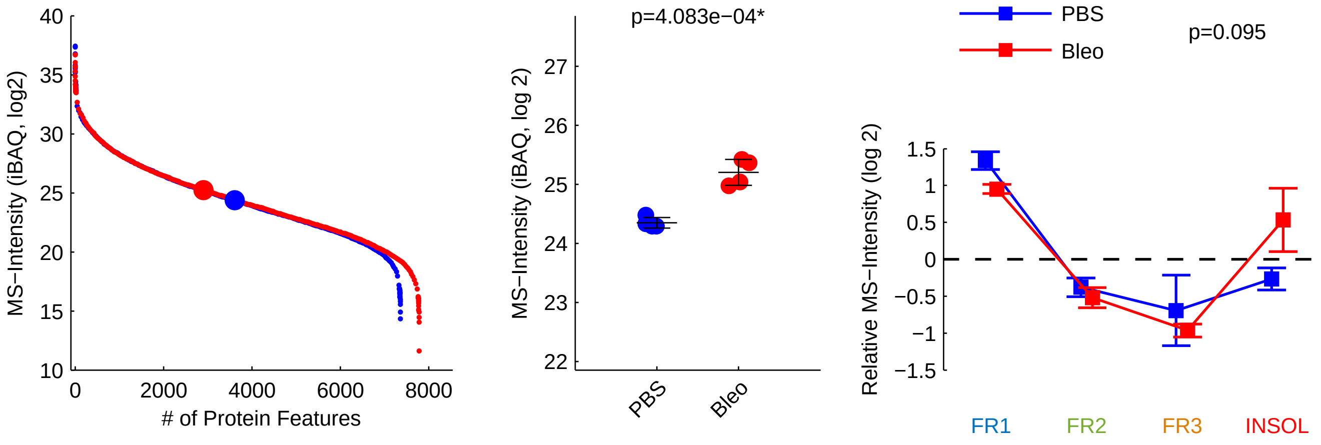

Q9ESB3 − Hrg (id: 8045)

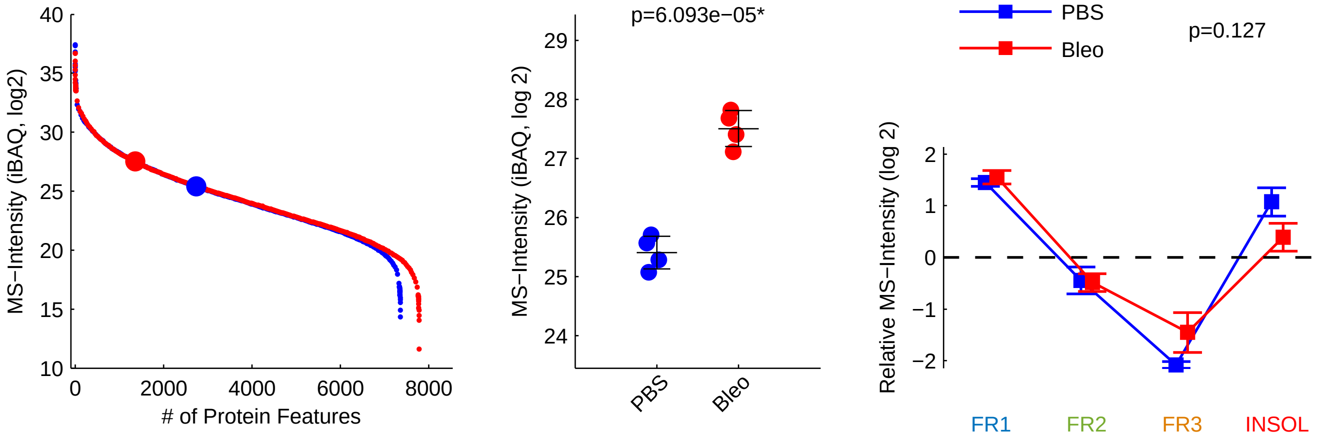

Q9ESD7−2 − Dysf (id: 8047)

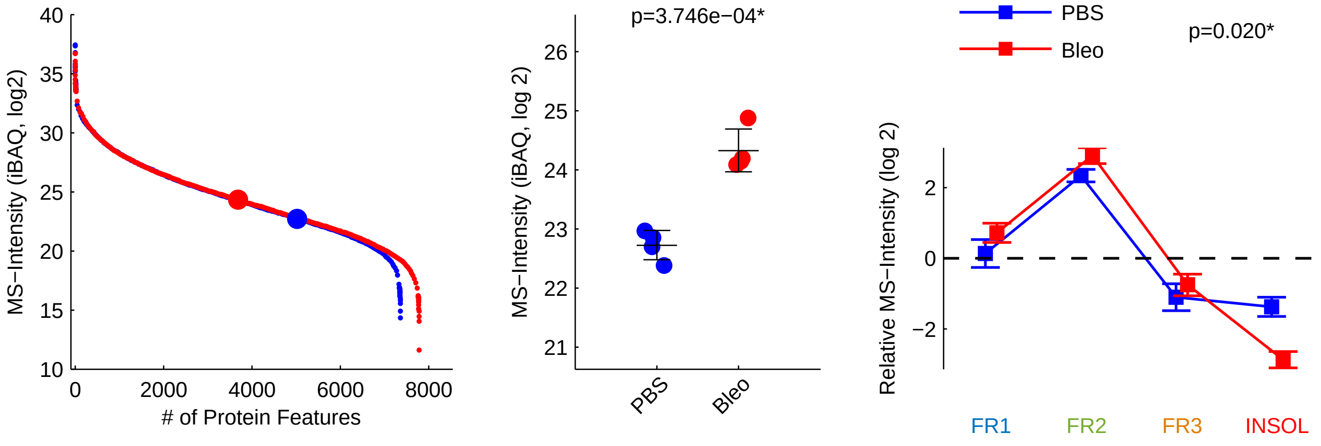

Q9ESP1 − Sdf2l1 (id: 8051)

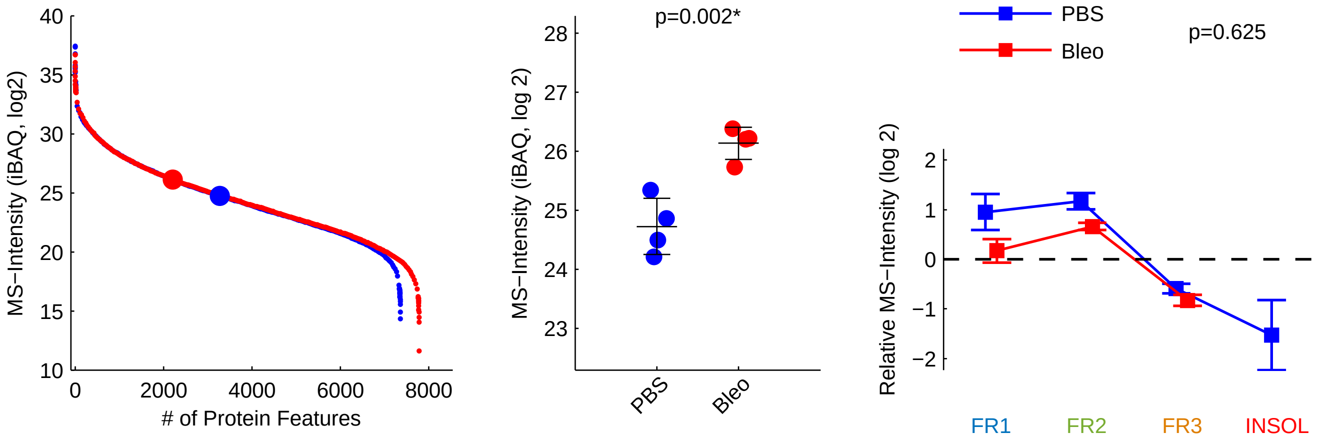

Q9ESX5 − Dkc1 (id: 8058)

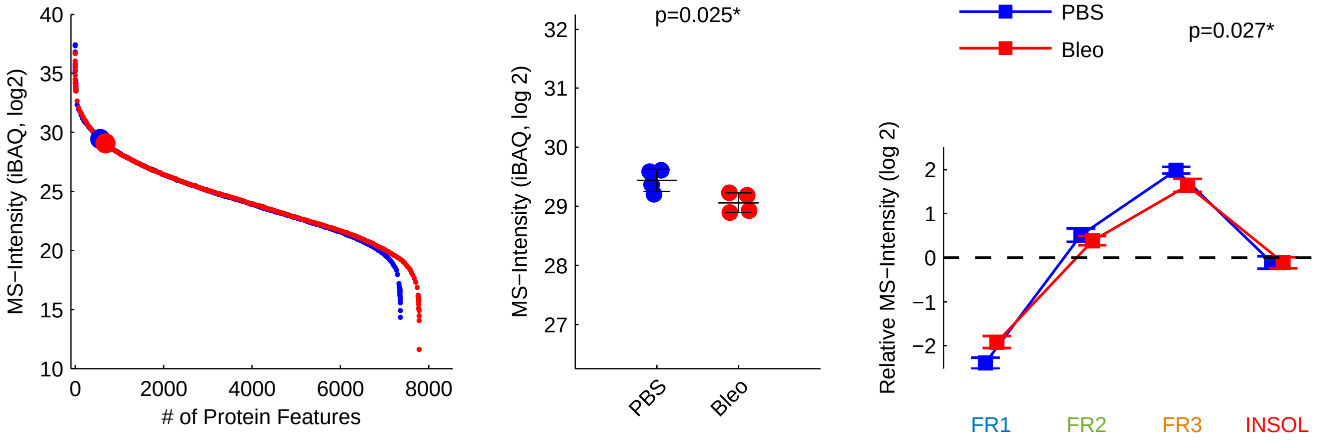

Q9ET30 − Tm9sf3 (id: 8063)

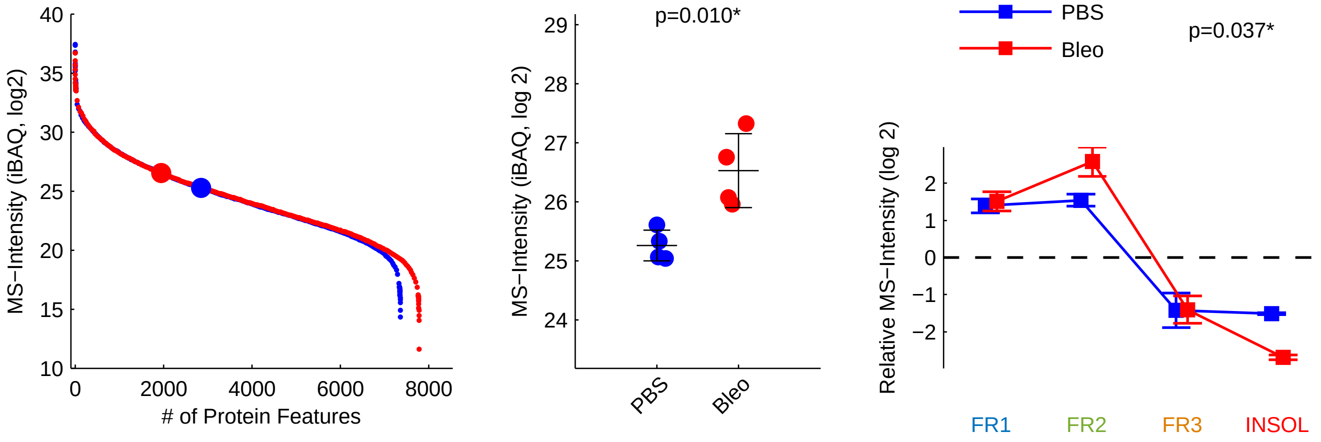

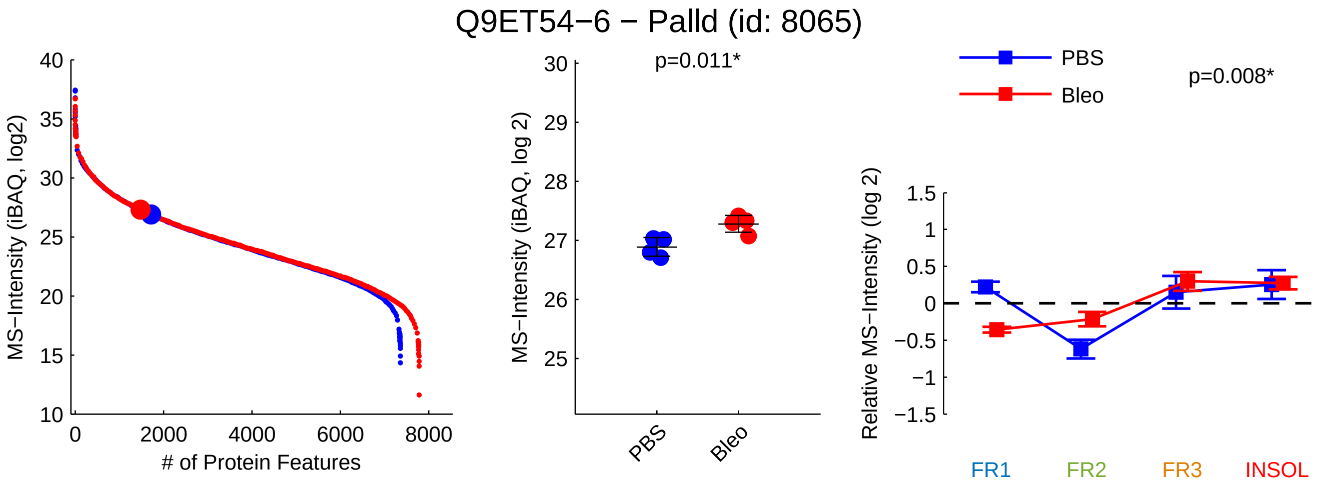
Q9ET54−6 − Palld (id: 8065)
MS−Intensity (iBAQ, log2)
# of Protein Features
MS−Intensity (iBAQ, log 2)
p=0.011*
PBS
Bleo
Relative MS−Intensity (log 2)
p=0.008*
FR1
FR2
FR3
INSOL

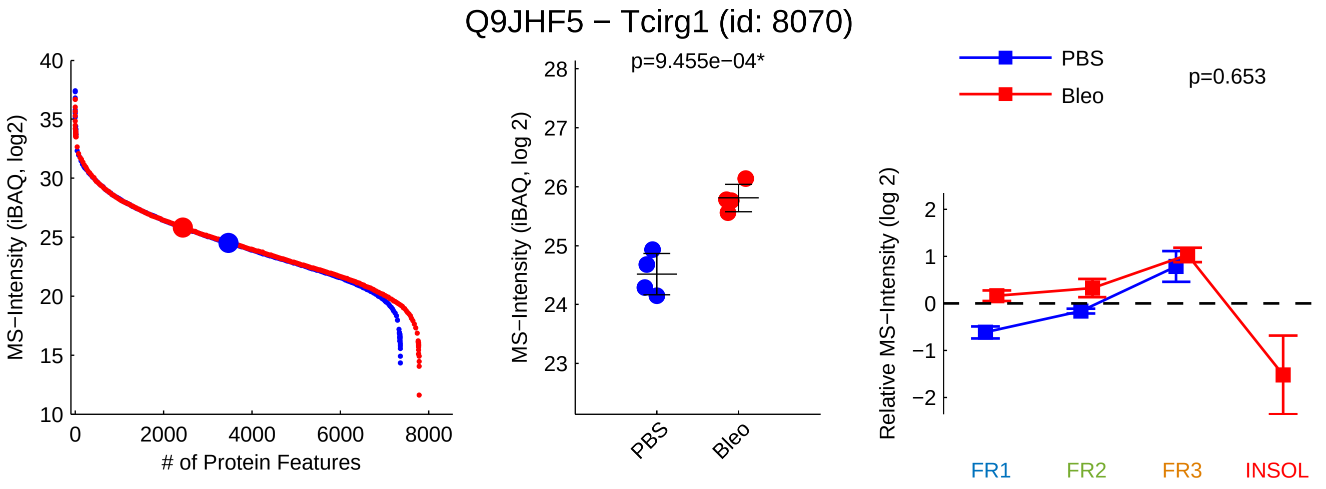
Q9JHF5 − Tcirg1 (id: 8070)
MS−Intensity (iBAQ, log2)
# of Protein Features
MS−Intensity (iBAQ, log 2)
p=9.455e−04*
PBS
Bleo
Relative MS−Intensity (log 2)
p=0.653
FR1
FR2
FR3
INSOL

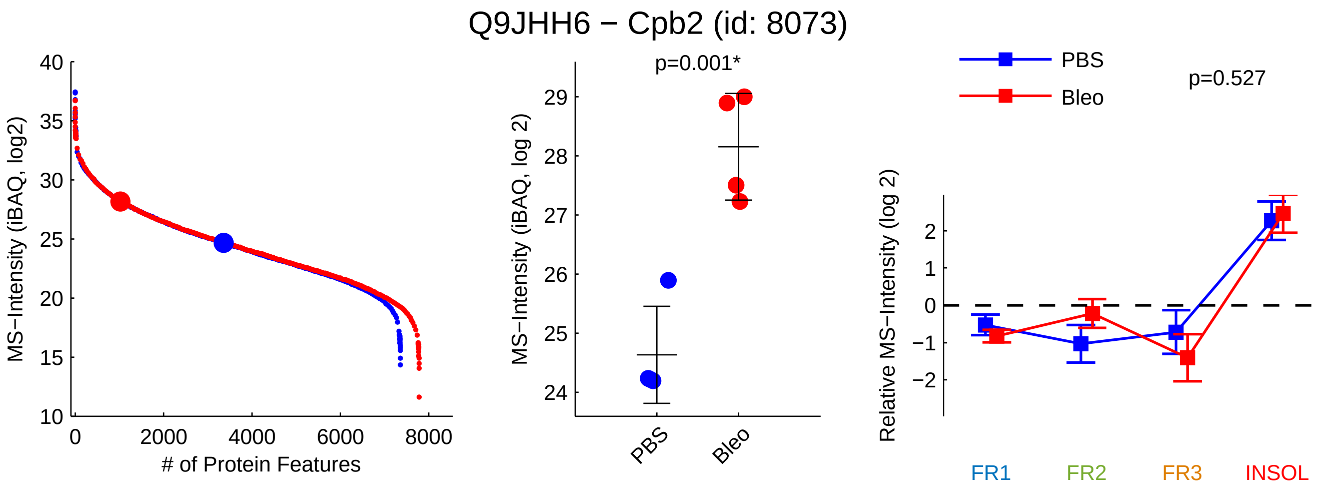
Q9JHH6 − Cpb2 (id: 8073)
MS−Intensity (iBAQ, log2)
# of Protein Features
MS−Intensity (iBAQ, log 2)
p=0.001*
PBS
Bleo
Relative MS−Intensity (log 2)
p=0.527
FR1
FR2
FR3
INSOL

Q9JHI0 − Mmp19 (id: 8075)

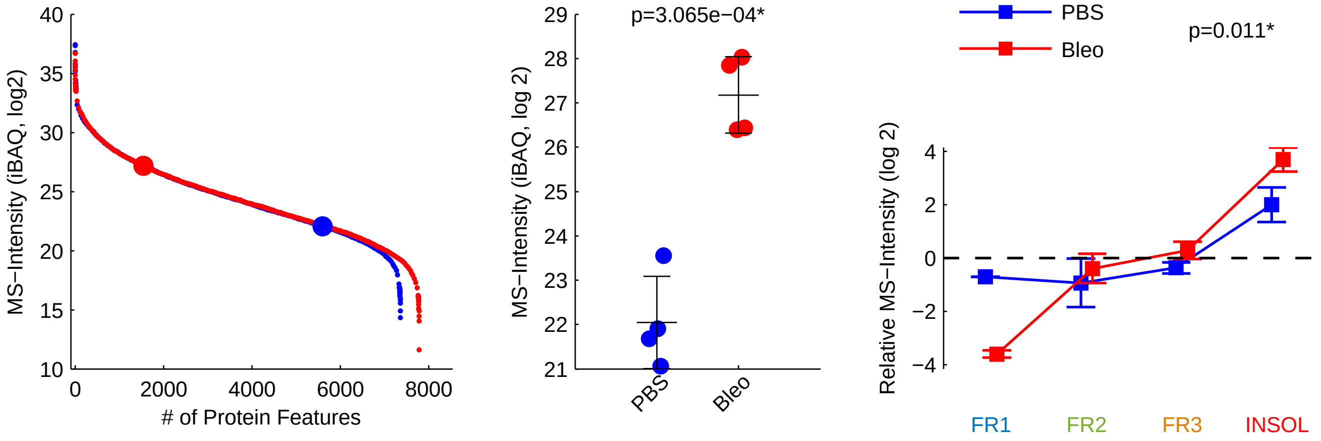

Q9JHI5 − Ivd (id: 8076)

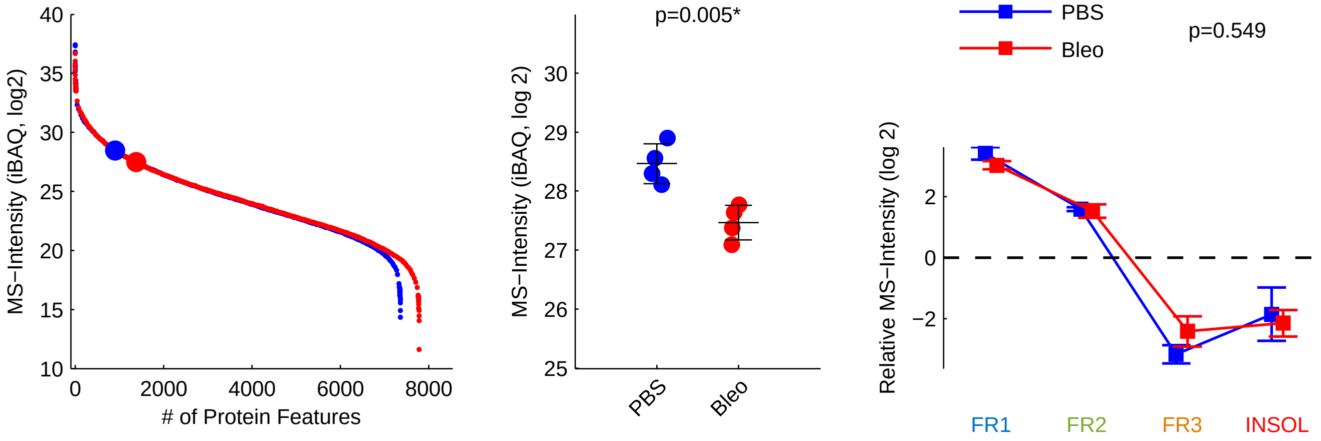

Q9JHI8 − Nox4 (id: 8078)

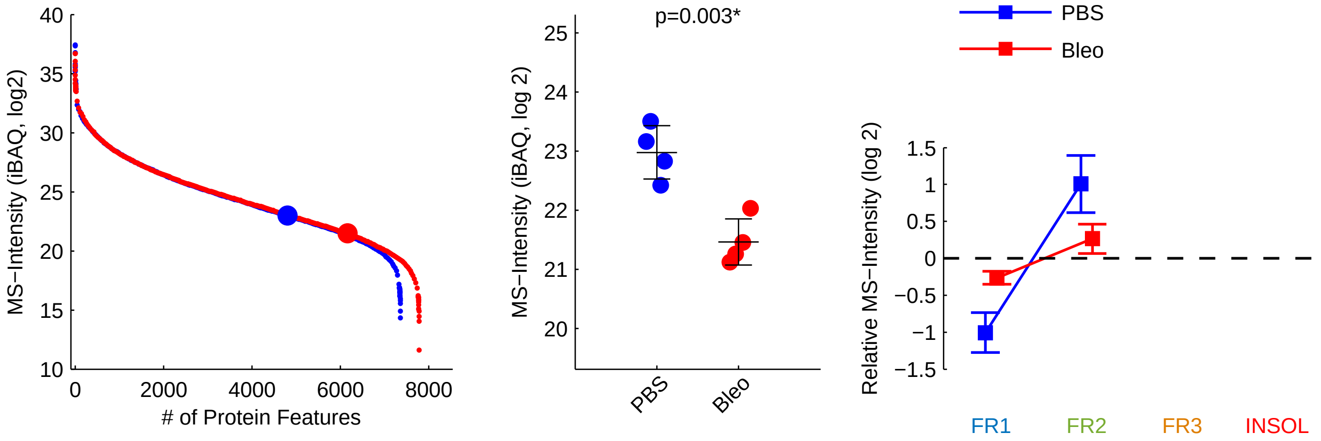

Q9JHJ0 − Tmod3 (id: 8080)

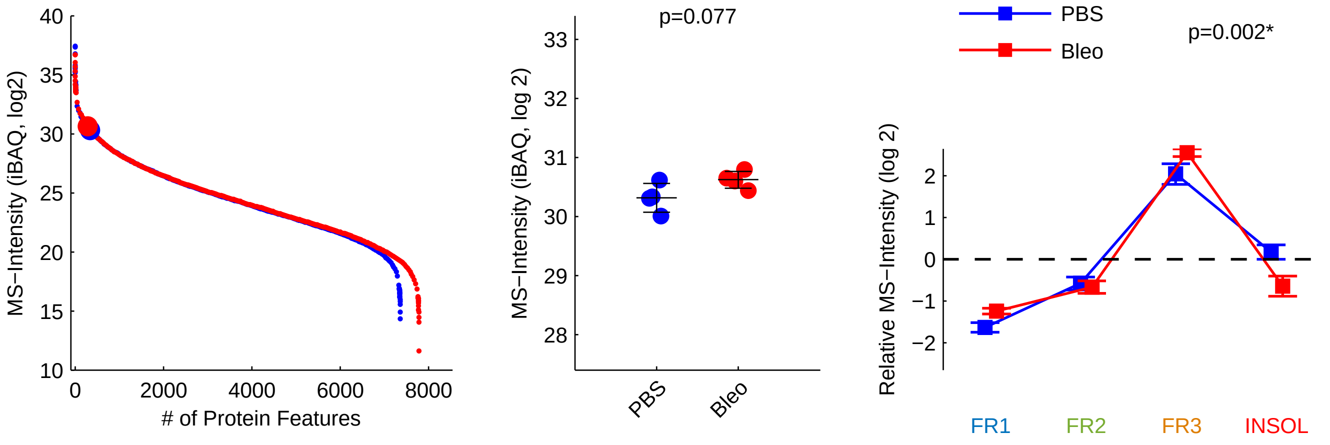

Q9JHL1 − Slc9a3r2 (id: 8083)

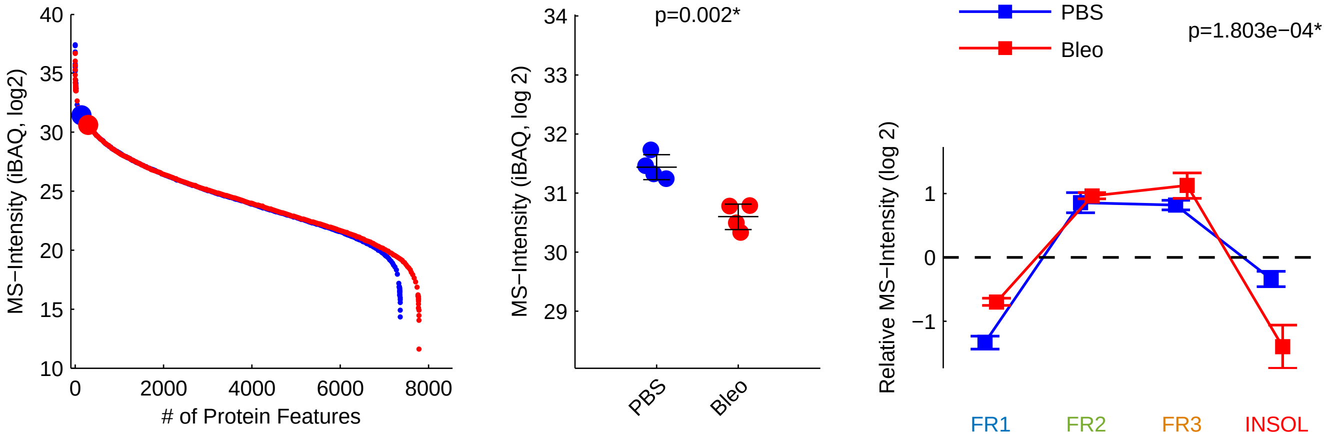

Q9JHP7 − Kdelc1 (id: 8086)

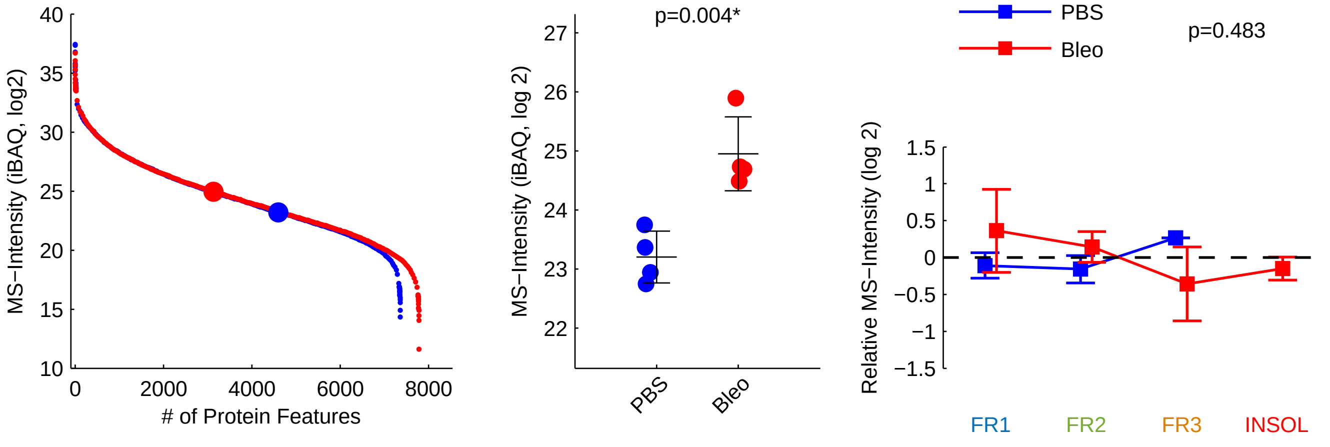

Q9JHQ0 − Anxa9 (id: 8087)

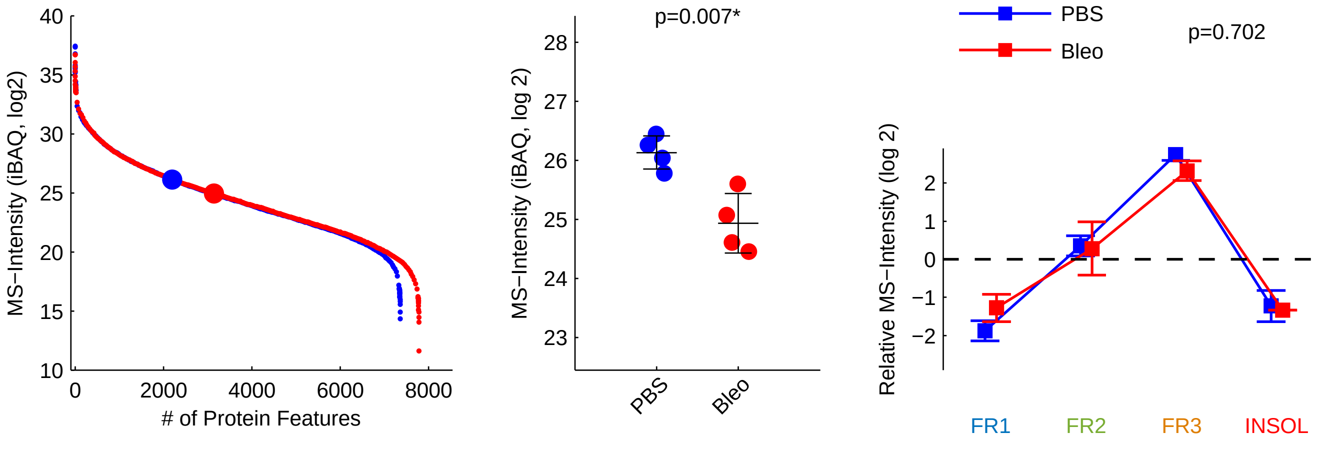

Q9JHU4 − Dync1h1 (id: 8092)

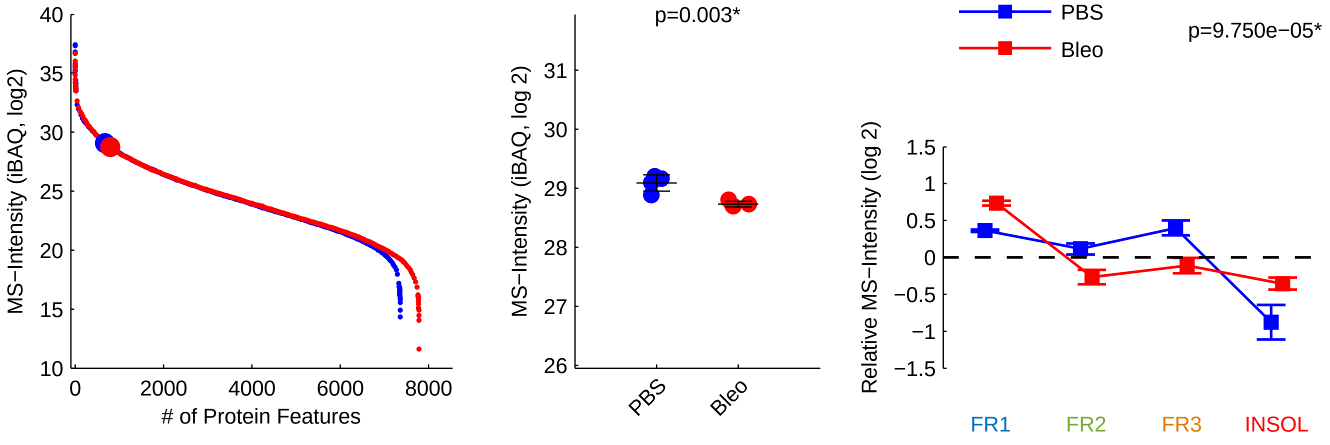

Q9JHU9 − Isyna1 (id: 8093)

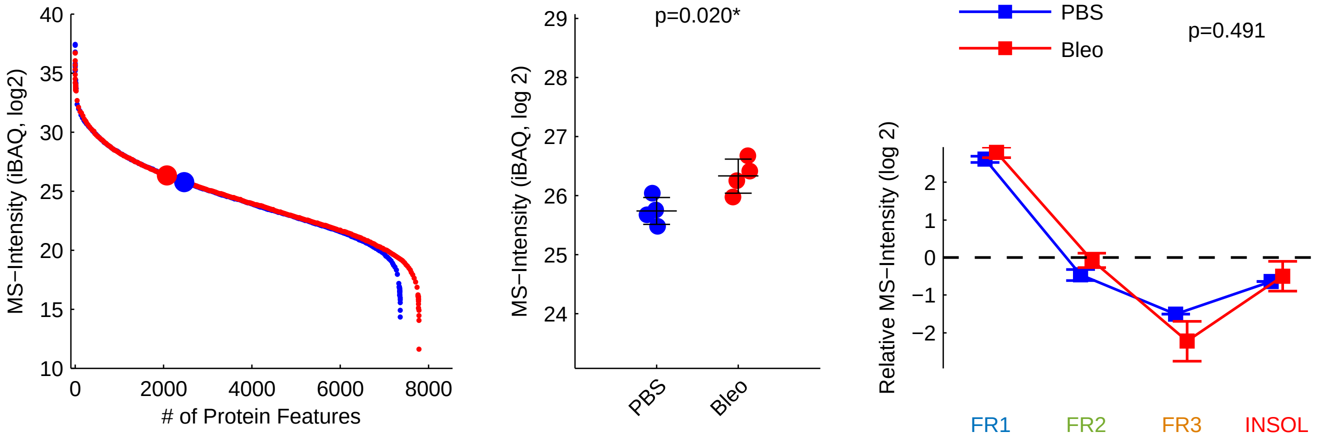

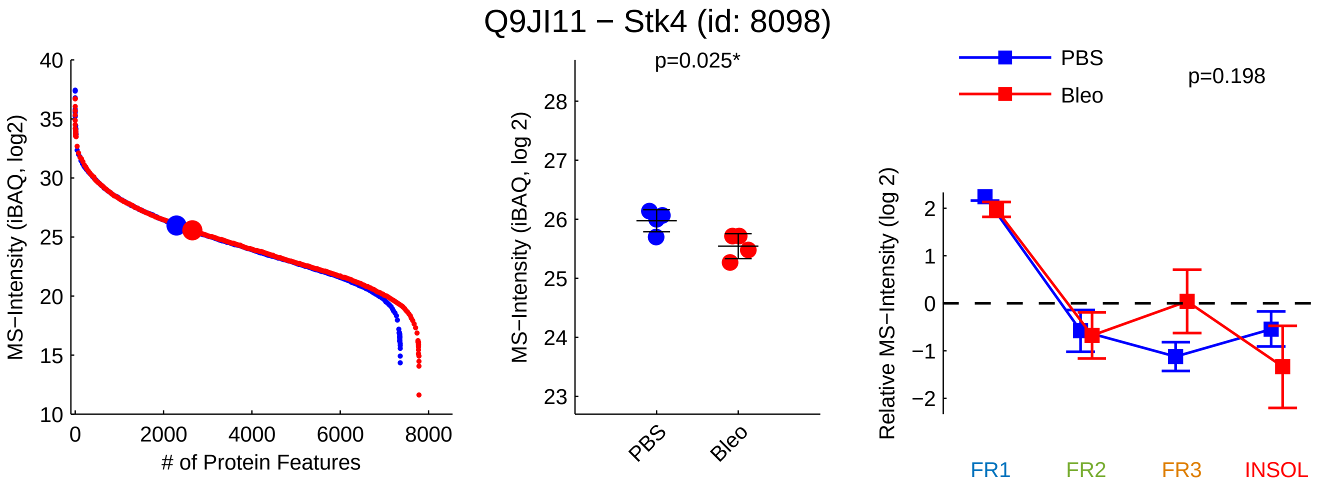
Q9JI11 − Stk4 (id: 8098)
MS−Intensity (iBAQ, log2)
# of Protein Features
p=0.025*
MS−Intensity (iBAQ, log 2)
PBS
Bleo
p=0.198
Relative MS−Intensity (log 2)
FR1
FR2
FR3
INSOL

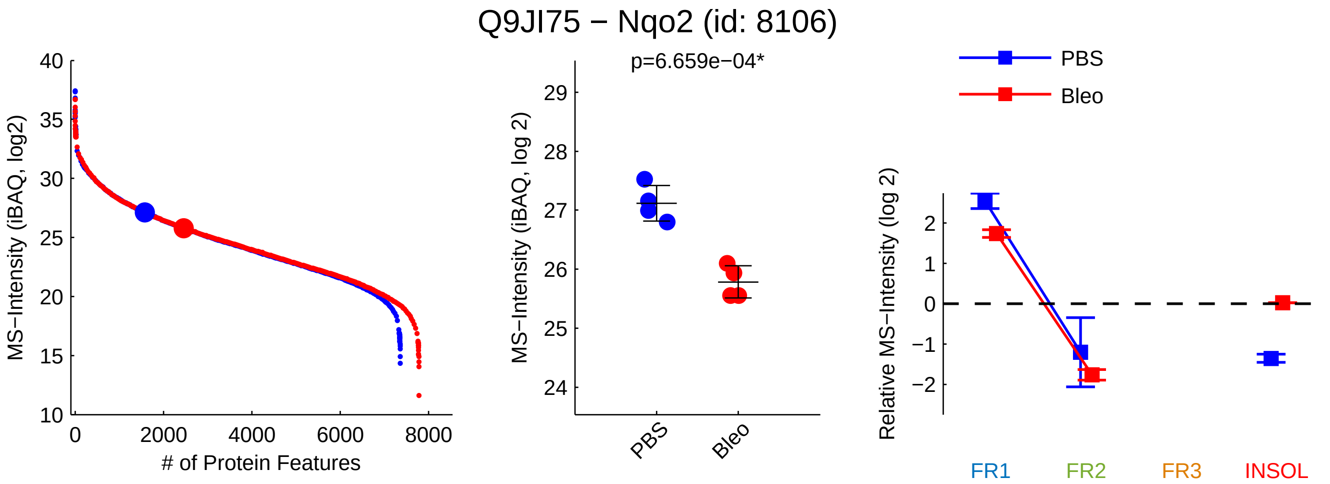
Q9JI75 − Nqo2 (id: 8106)
MS−Intensity (iBAQ, log2)
# of Protein Features
p=6.659e−04*
MS−Intensity (iBAQ, log 2)
PBS
Bleo
Relative MS−Intensity (log 2)
FR1
FR2
FR3
INSOL

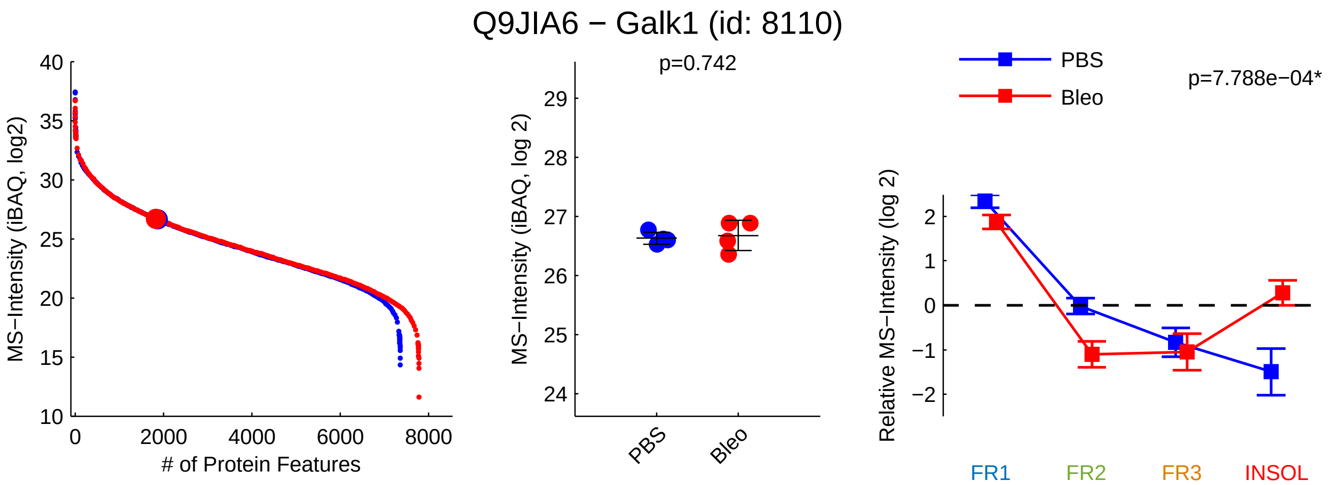
Q9JIA6 − Galk1 (id: 8110)
MS−Intensity (iBAQ, log2)
# of Protein Features
p=0.742
MS−Intensity (iBAQ, log 2)
PBS
Bleo
p=7.788e−04*
Relative MS−Intensity (log 2)
FR1
FR2
FR3
INSOL

Q9JIF7 − Copb1 (id: 8113)

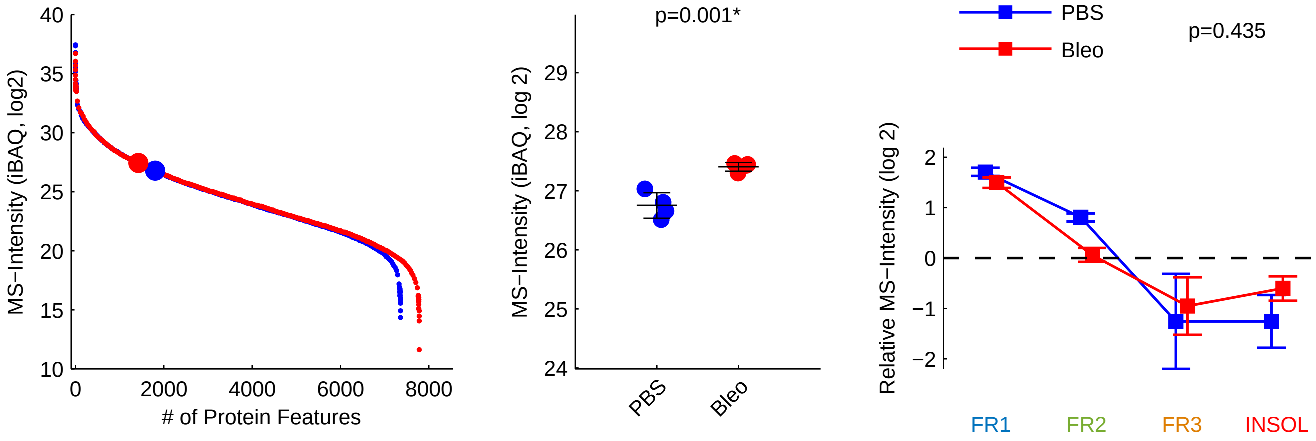

Q9JII6 − Akr1a1 (id: 8118)

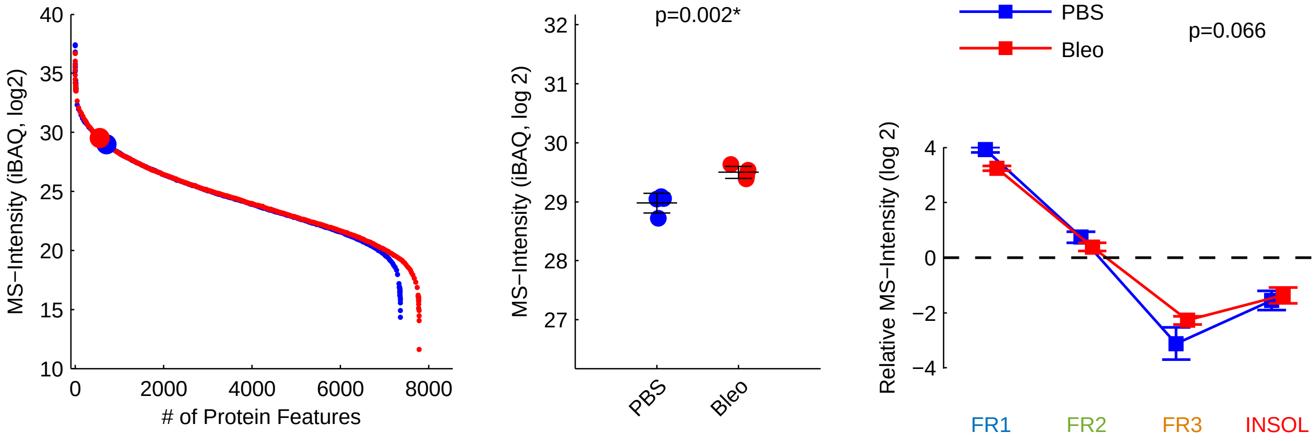

Q9JIK5 − Ddx21 (id: 8119)

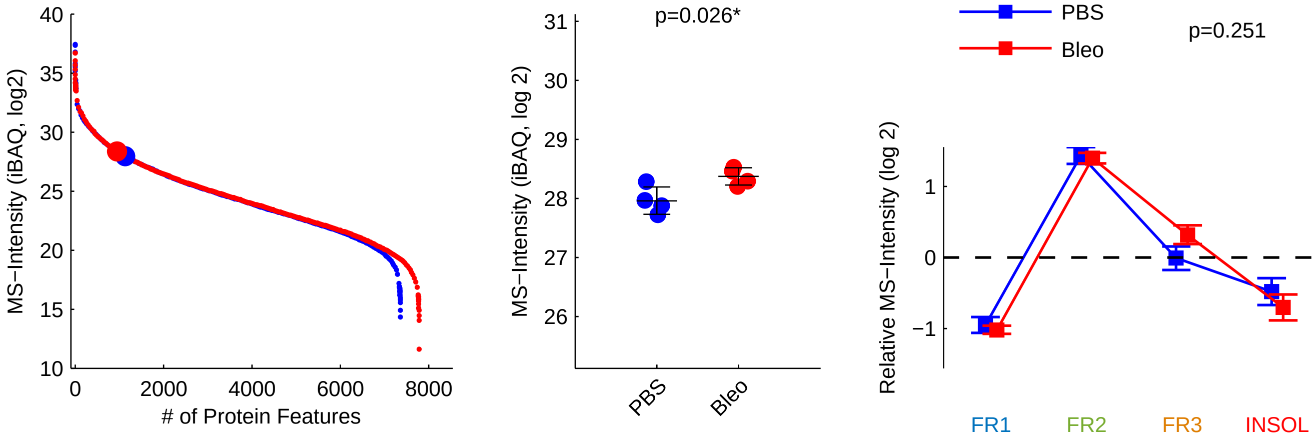

Q9JJA4 − Wdr12 (id: 8132)

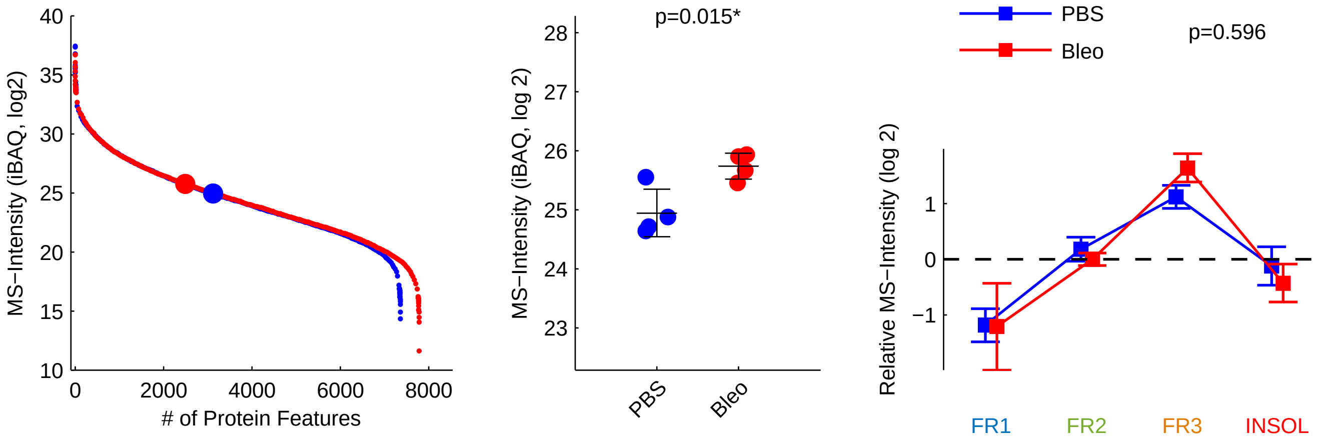

Q9JJI8 − Rpl38 (id: 8139)

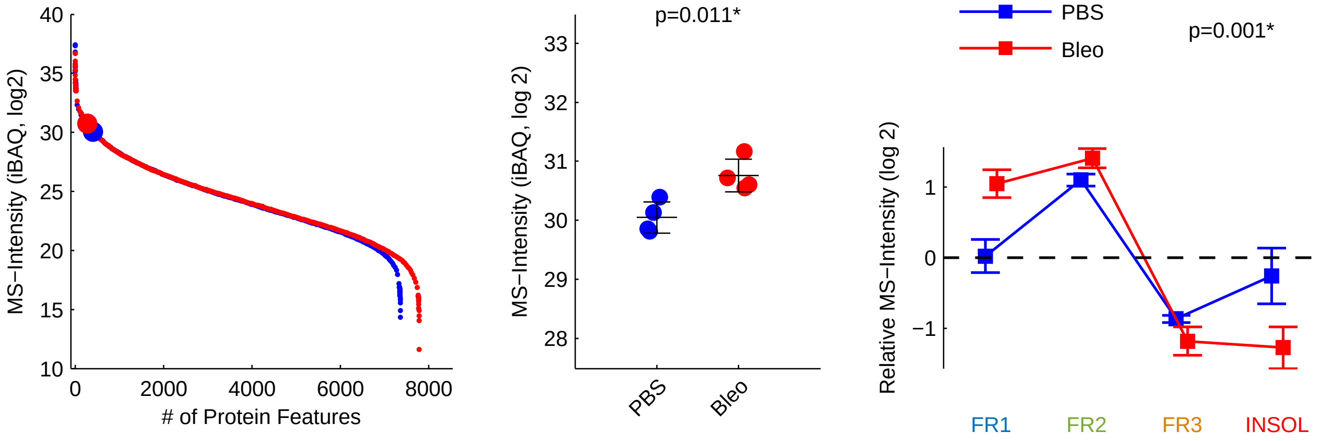

Q9JJN5 − Cpn1 (id: 8140)

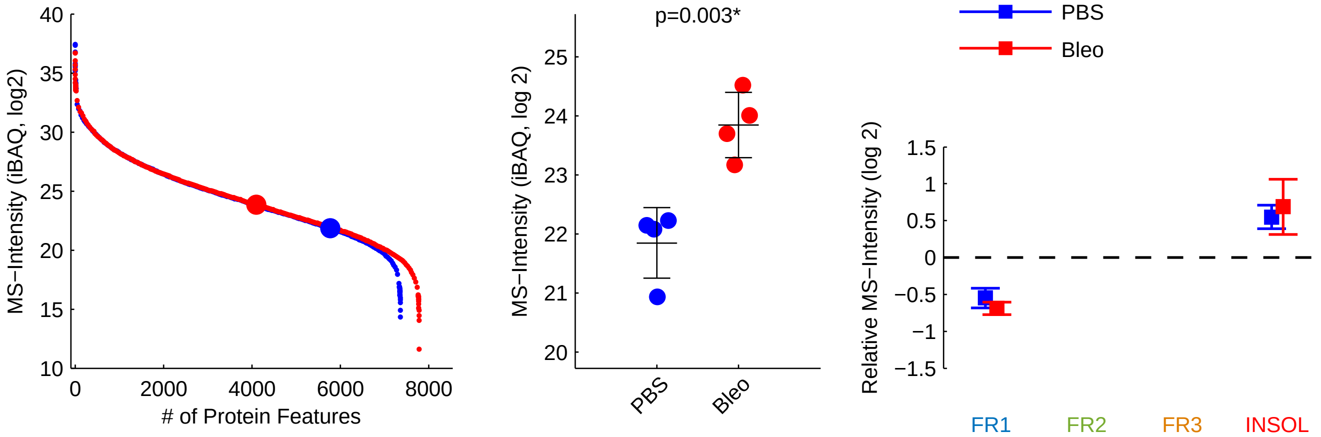

Q9JJR5 − Akip1 (id: 8143)

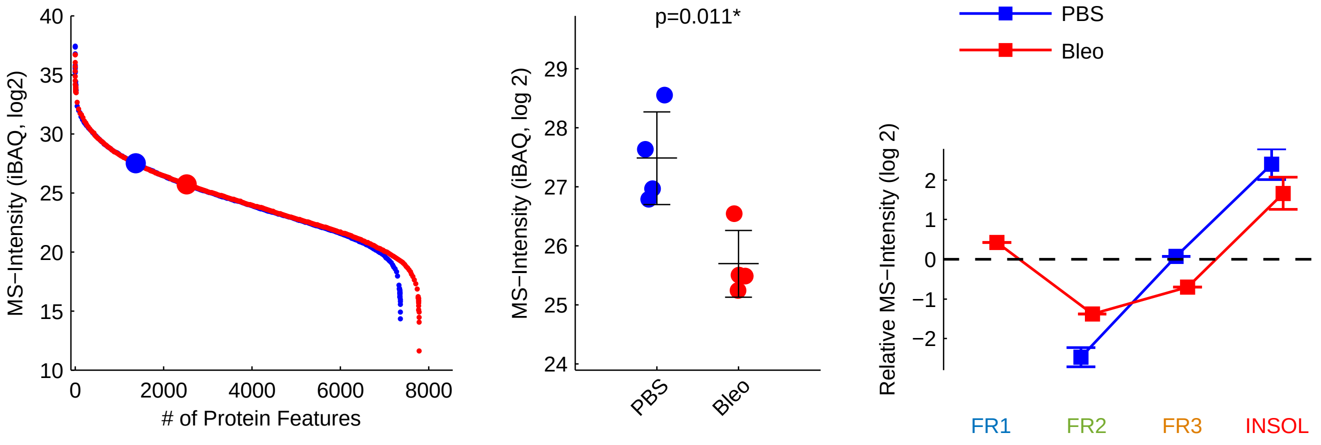

Q9JJZ2 − Tuba8 (id: 8150)

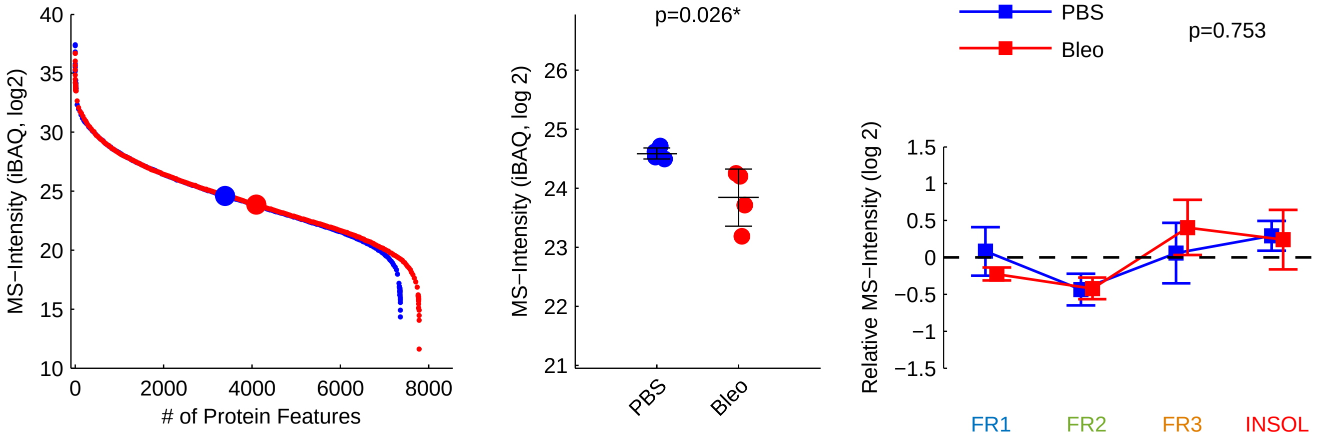

Q9JK38 − Gnpnat1 (id: 8157)

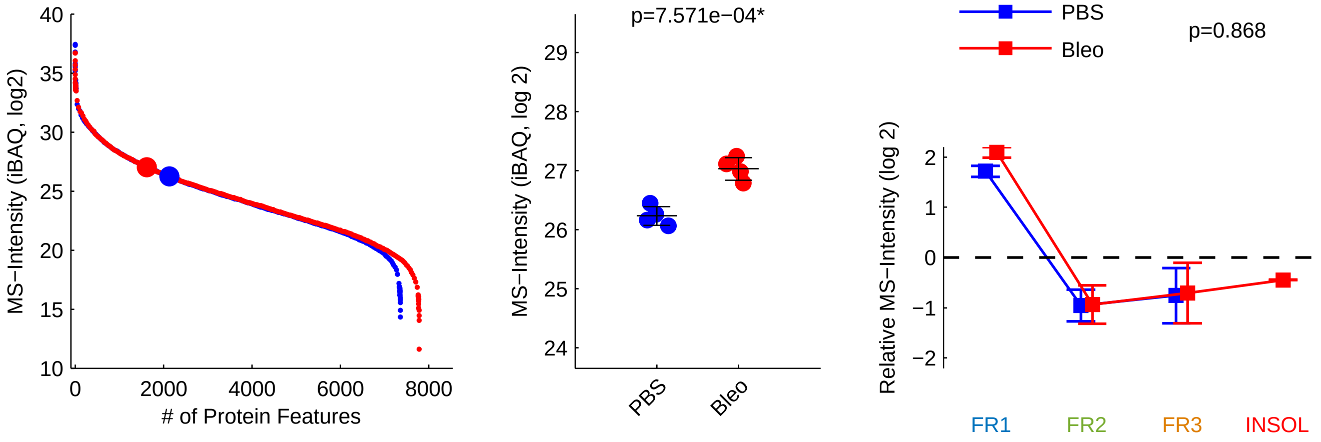

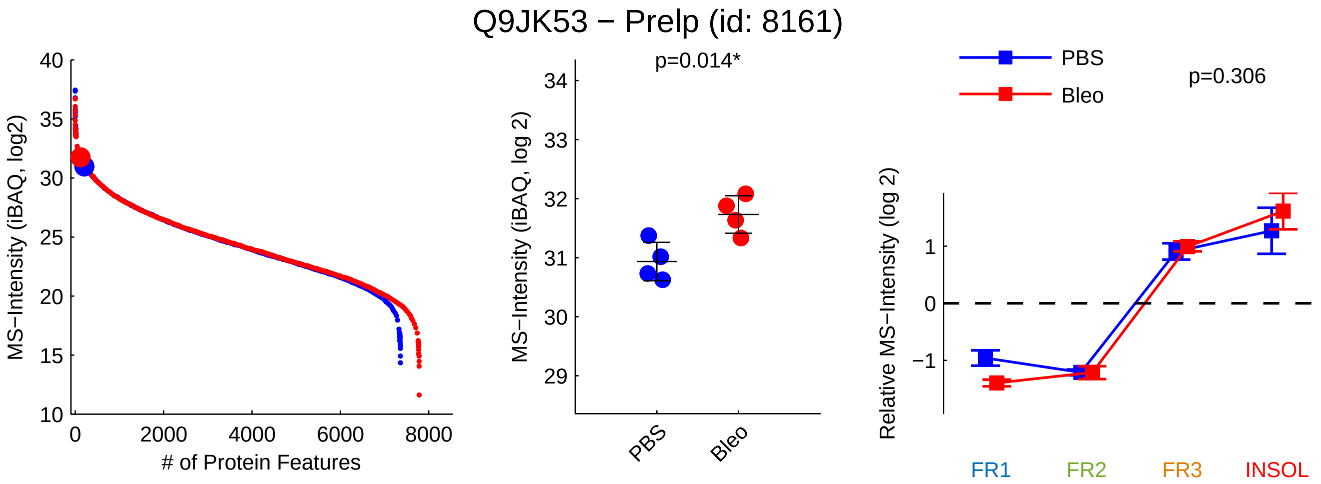

Q9JK53 − Prelp (id: 8161)
MS−Intensity (iBAQ, log2)
# of Protein Features
p=0.014*
MS−Intensity (iBAQ, log 2)
PBS
Bleo
p=0.306
Relative MS−Intensity (log 2)
FR1
FR2
FR3
INSOL

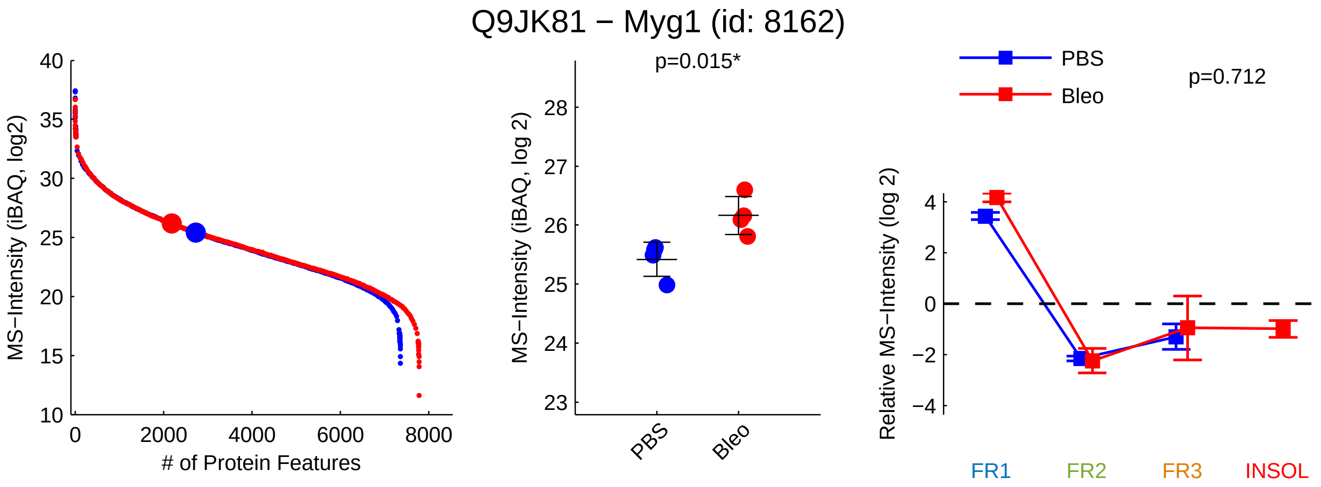

Q9JK81 − Myg1 (id: 8162)
MS−Intensity (iBAQ, log2)
# of Protein Features
p=0.015*
MS−Intensity (iBAQ, log 2)
PBS
Bleo
p=0.712
Relative MS−Intensity (log 2)
FR1
FR2
FR3
INSOL

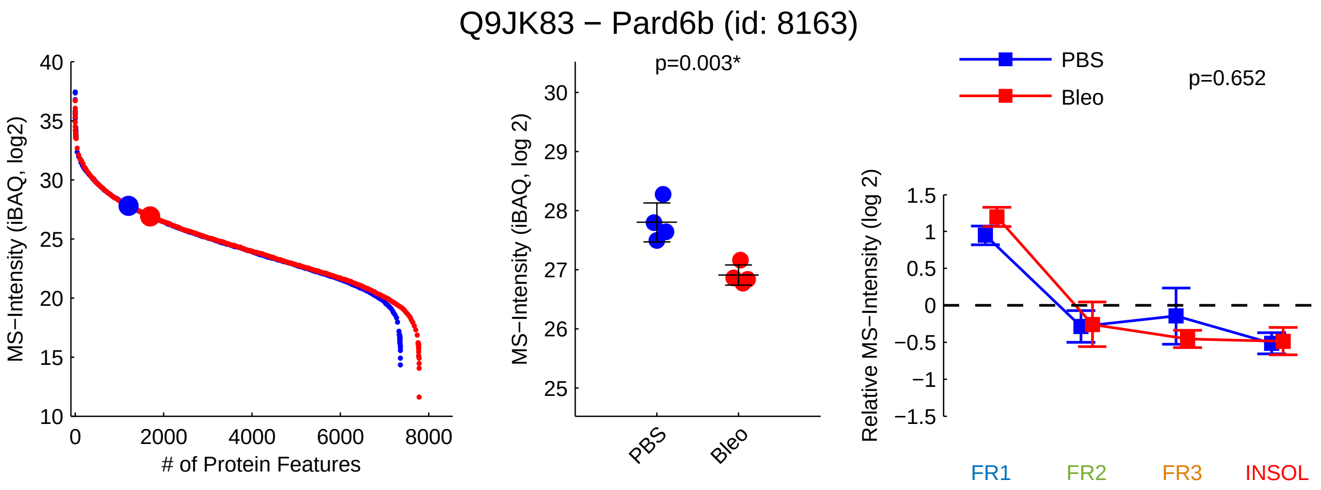

Q9JK83 − Pard6b (id: 8163)
MS−Intensity (iBAQ, log2)
# of Protein Features
p=0.003*
MS−Intensity (iBAQ, log 2)
PBS
Bleo
p=0.652
Relative MS−Intensity (log 2)
FR1
FR2
FR3
INSOL

Q9JK92 − Hspb8 (id: 8165)

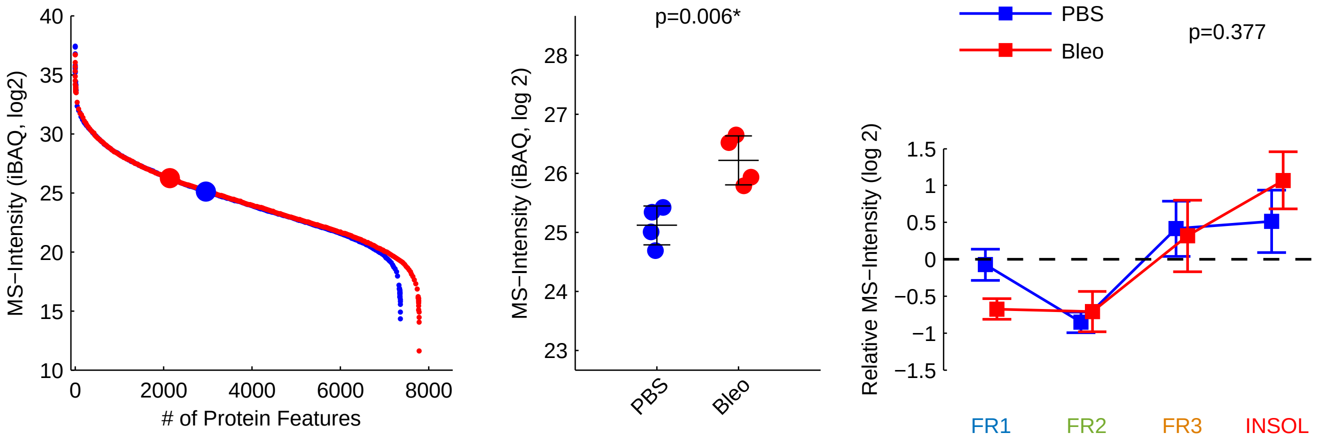

Q9JKB3−2 − Ybx3 (id: 8167)

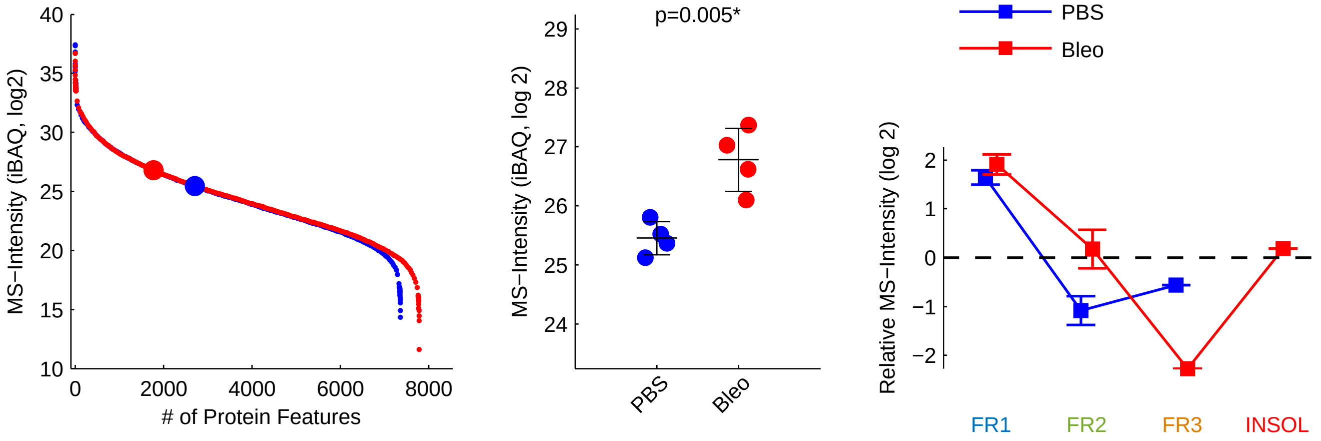

Q9JKF7 − Mrpl39 (id: 8171)

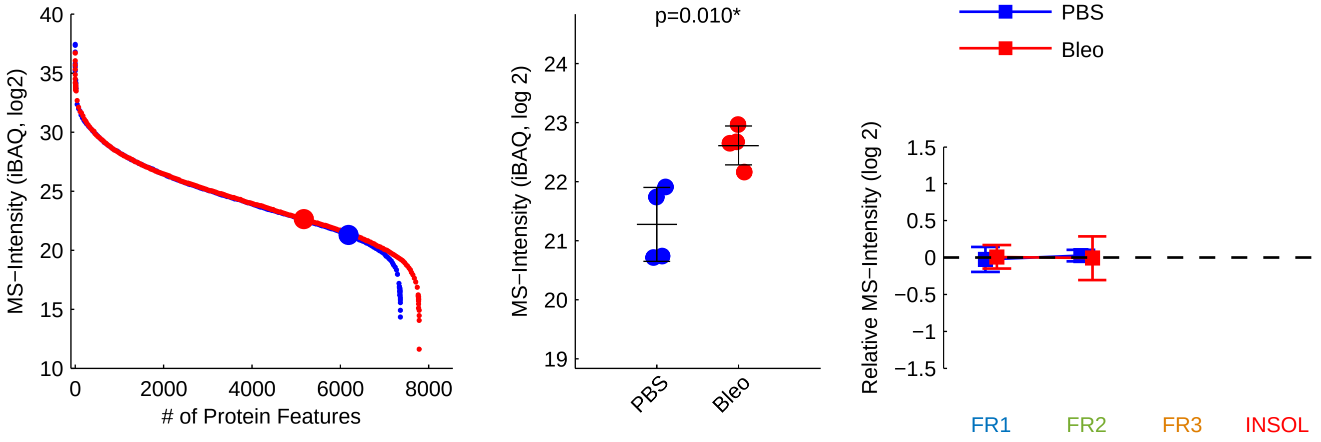

Q9JKJ9 − Cyp39a1 (id: 8172)

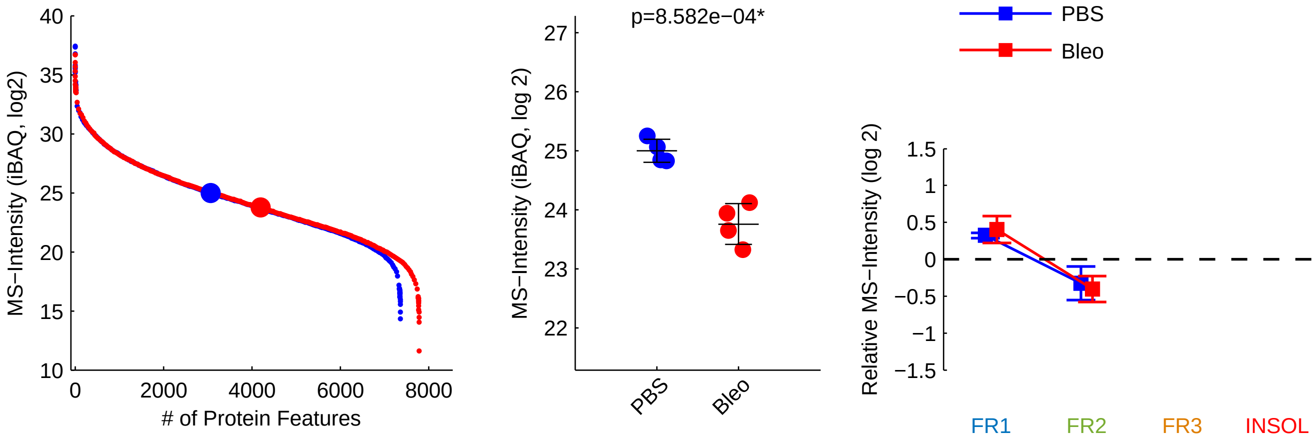

Q9JKX4 − Aatf (id: 8187)

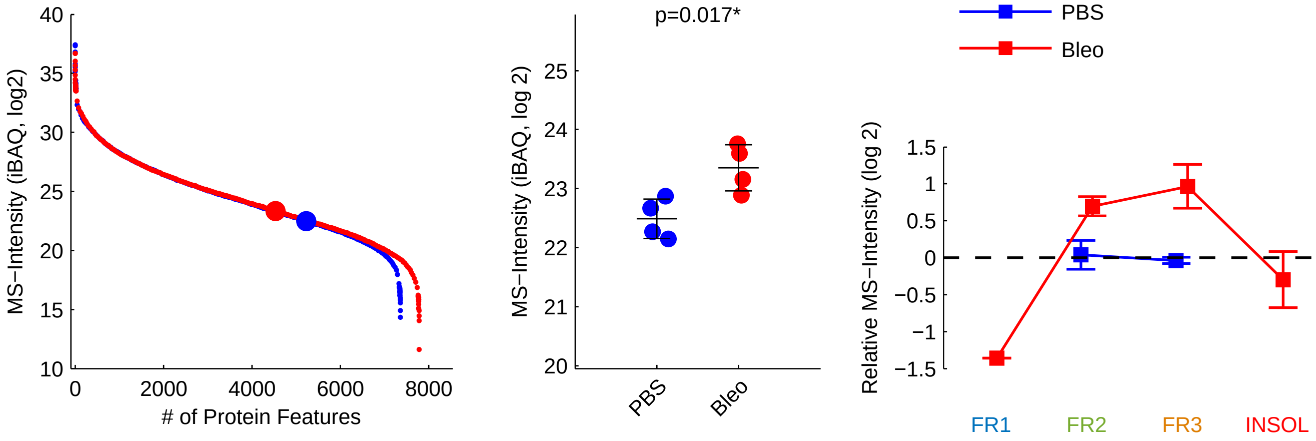

Q9JLB2 − Mpp5 (id: 8201)

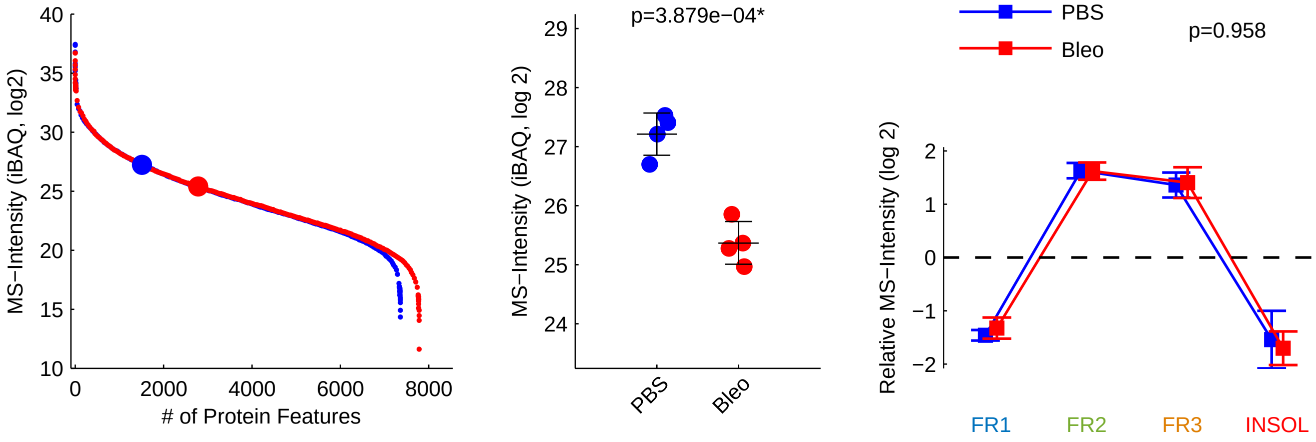

Q9JLF6 − Tgm1 (id: 8202)

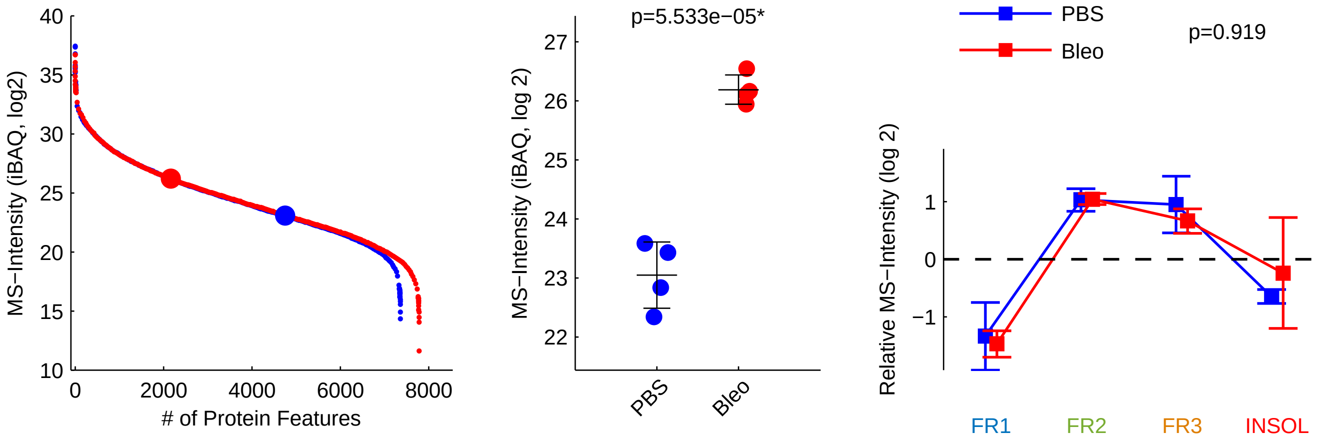

Q9JLI2 − Col5a3 (id: 8204)

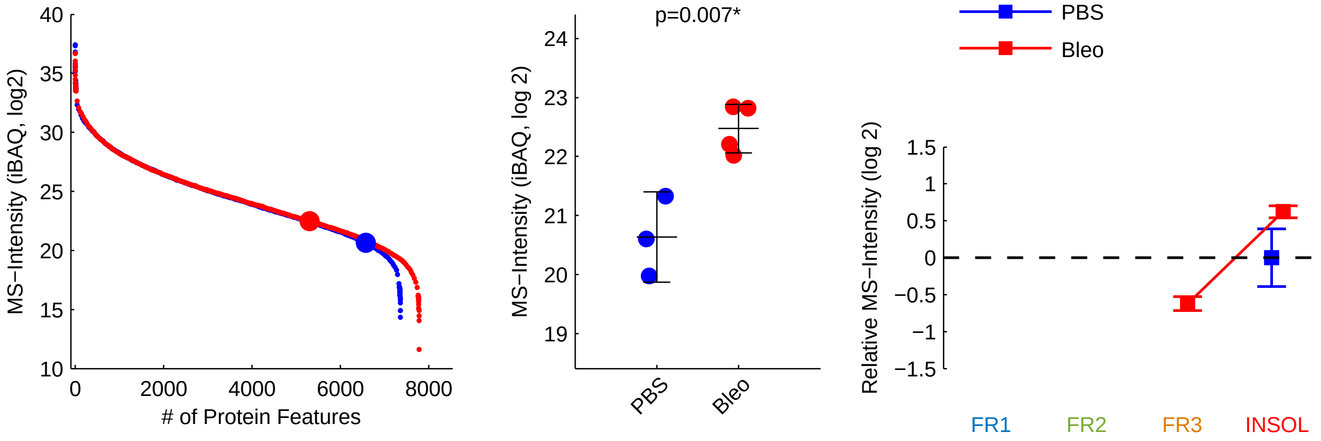

Q9JLJ2 − Aldh9a1 (id: 8208)

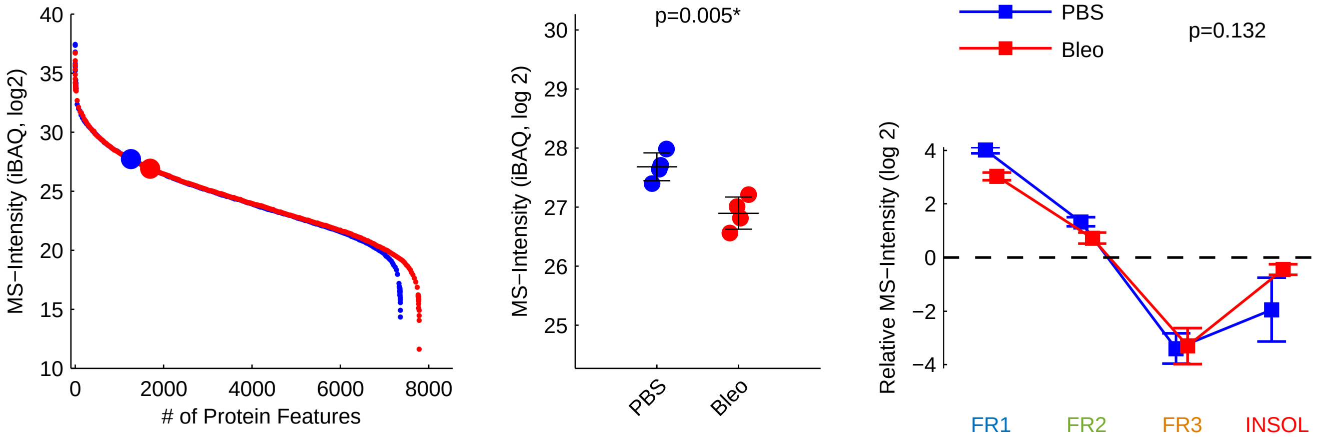

Q9JLN9 − Mtor (id: 8211)

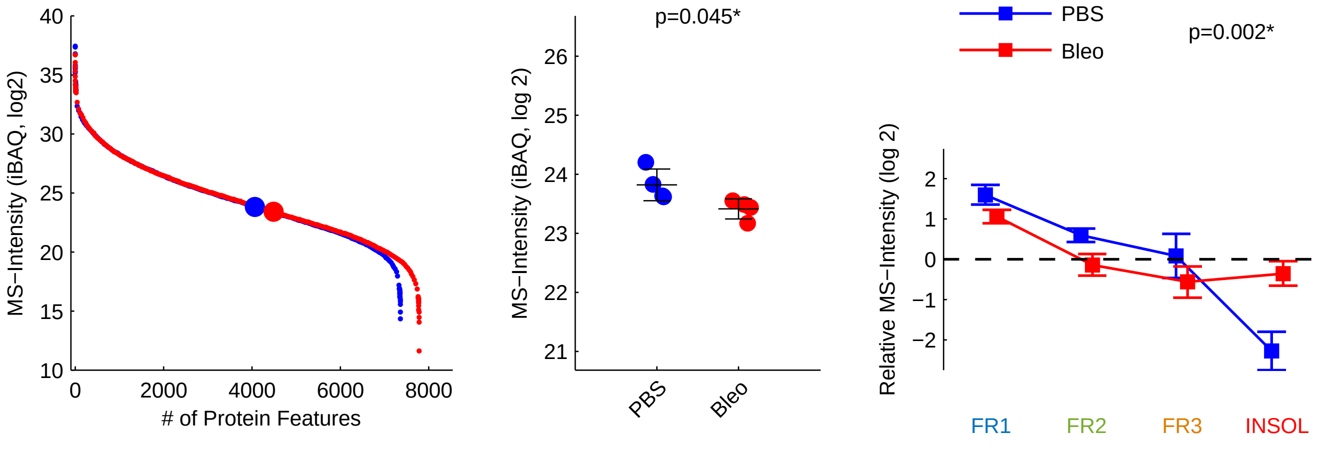

Q9JLV1 − Bag3 (id: 8215)

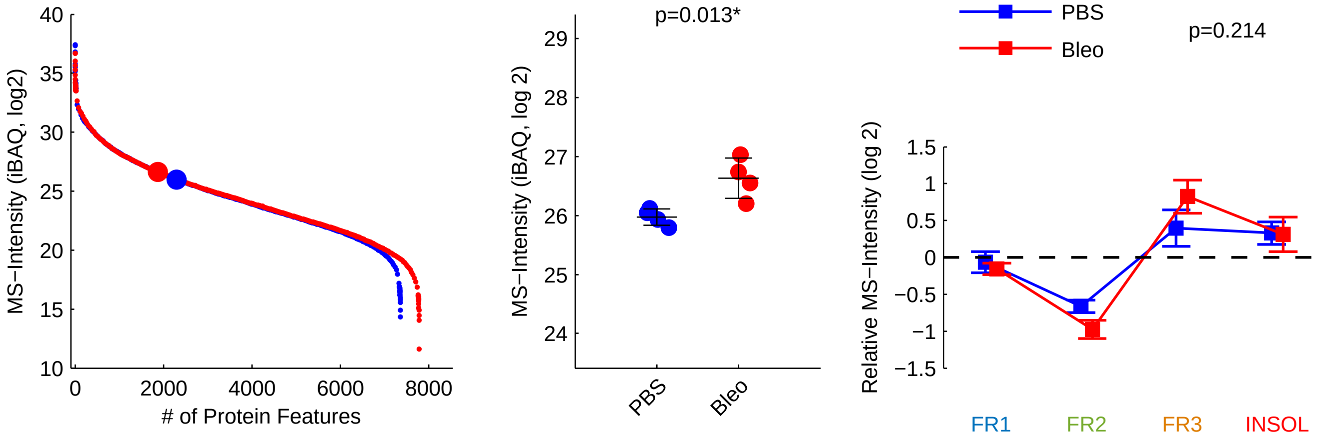

Q9JLZ3 − Auh (id: 8219)

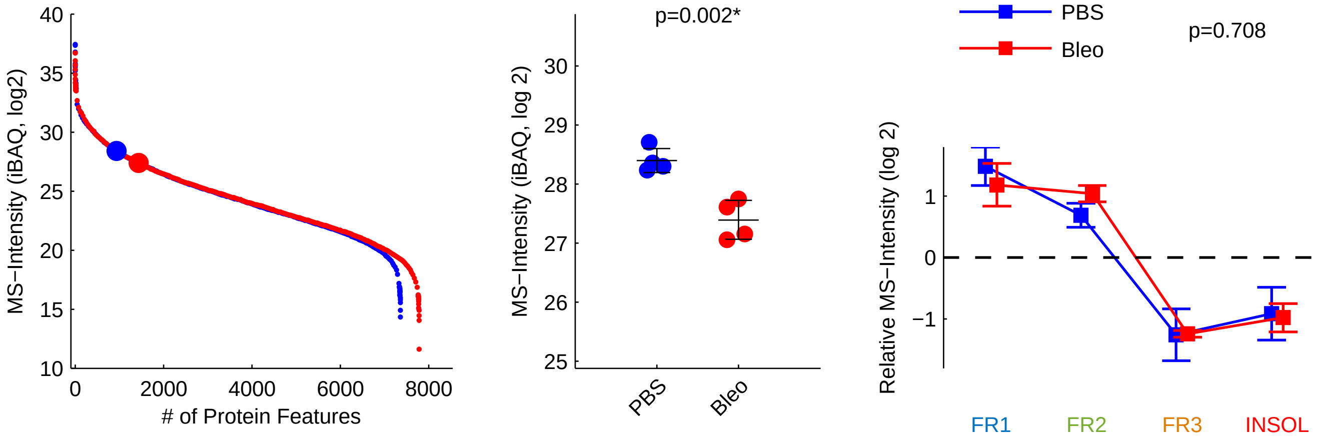

Q9JM51 − Ptges (id: 8224)

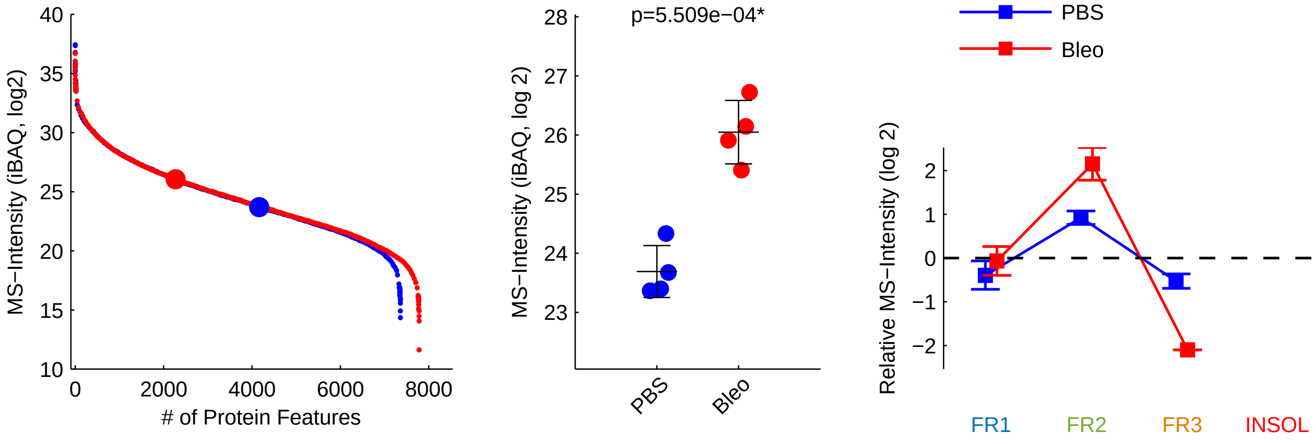

Q9JM58 − Crlf1 (id: 8225)

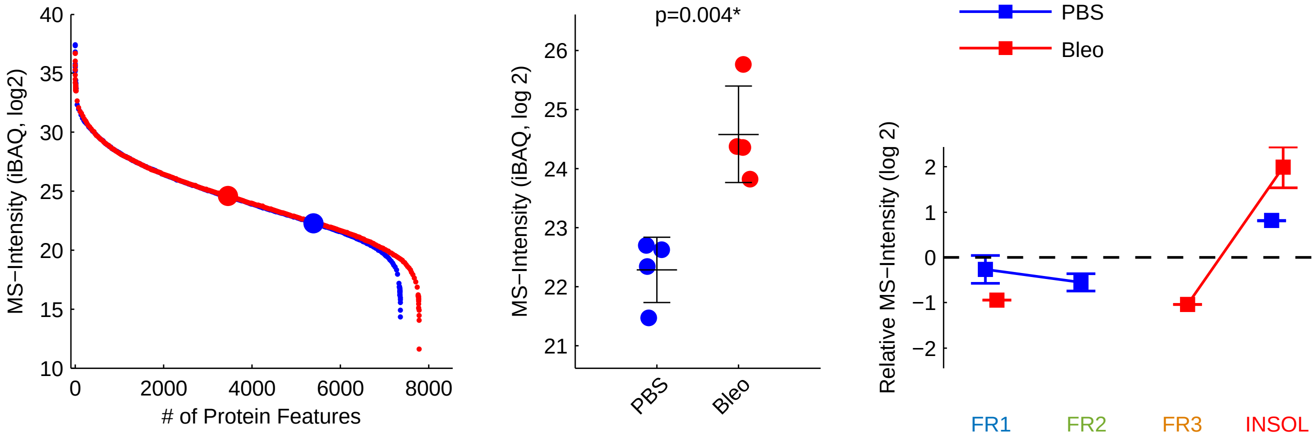

Q9JMC3 − Dnaja4 (id: 8237)

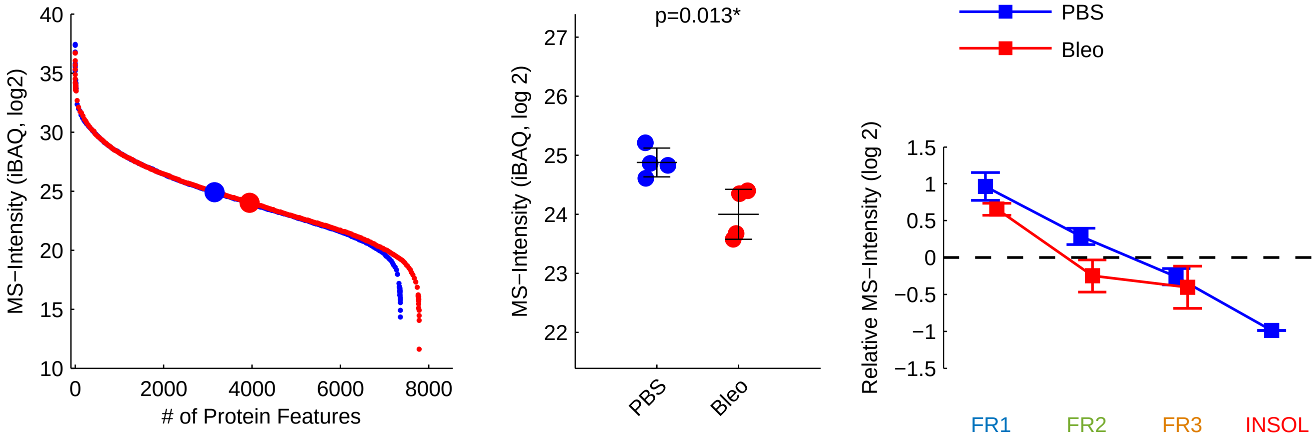

Q9JMC8 − Epb41l4b (id: 8238)

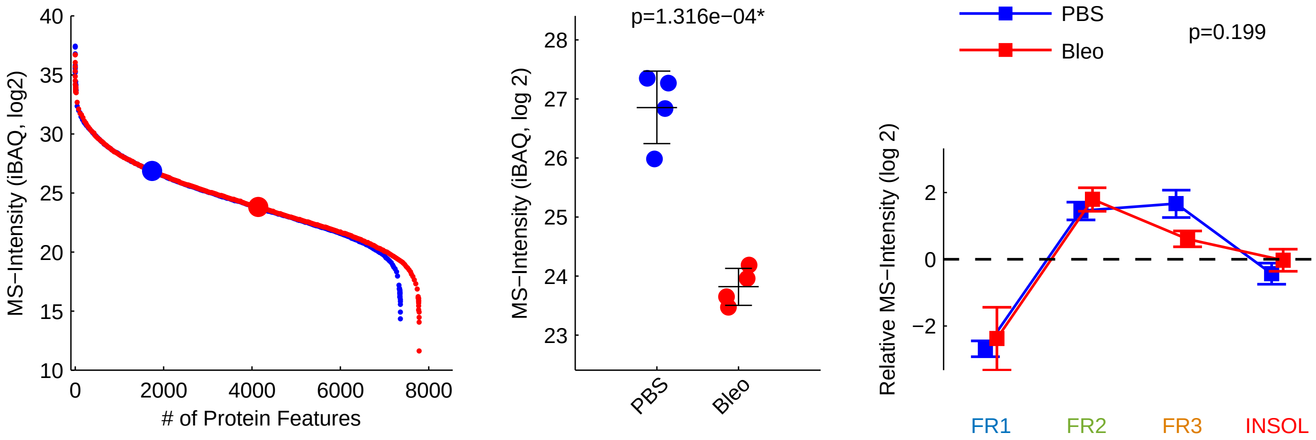

Q9JMG7 − Hdgfrp3 (id: 8243)

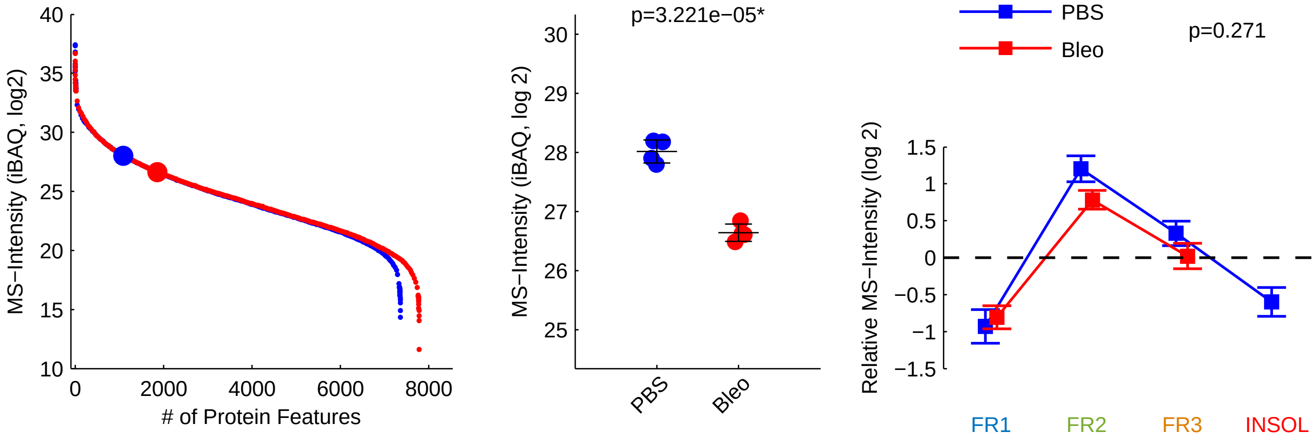

Q9JMK2 − Csnk1e (id: 8245)

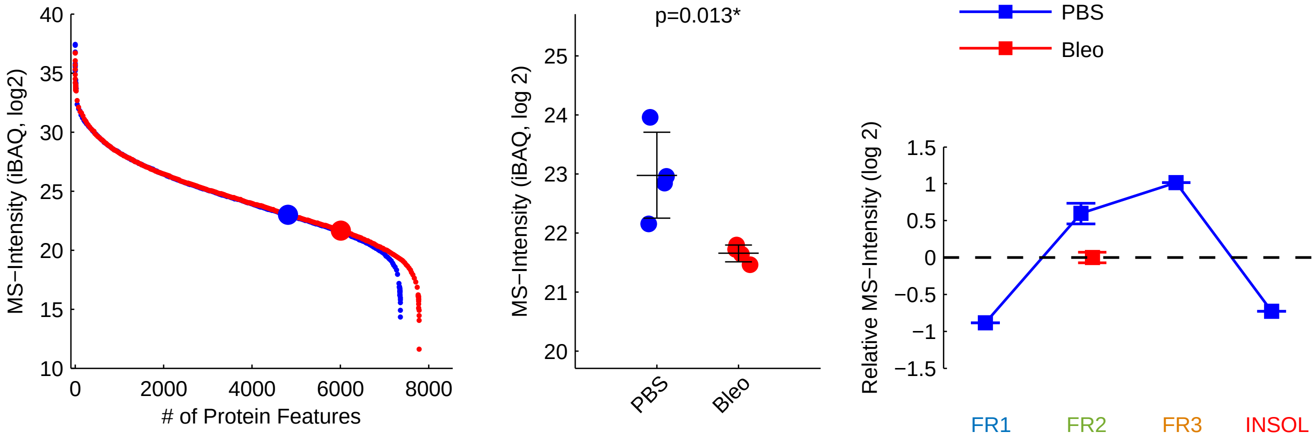

Q9QUG9 − Rasgrp2 (id: 8246)

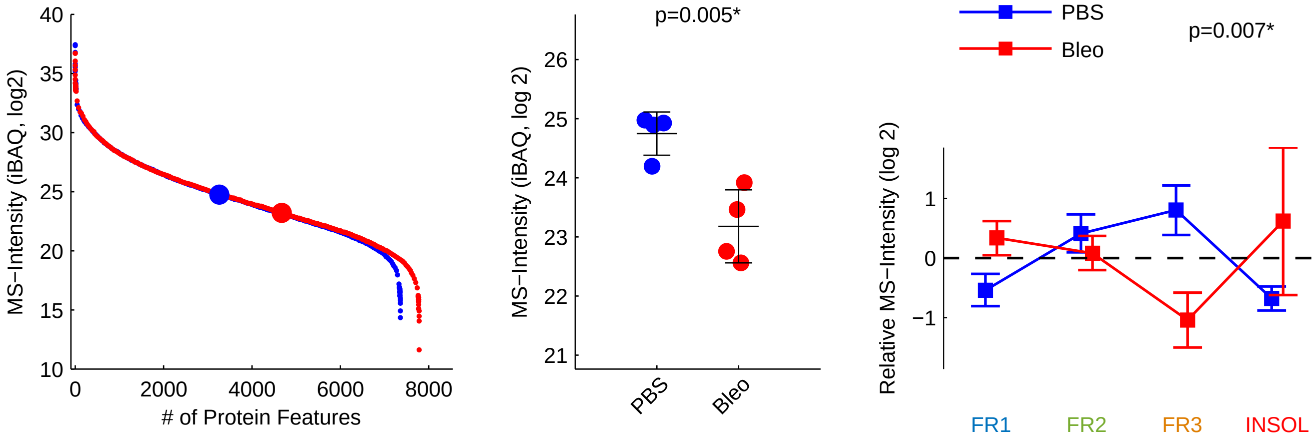

Q9QUH0 − Glrx (id: 8247)

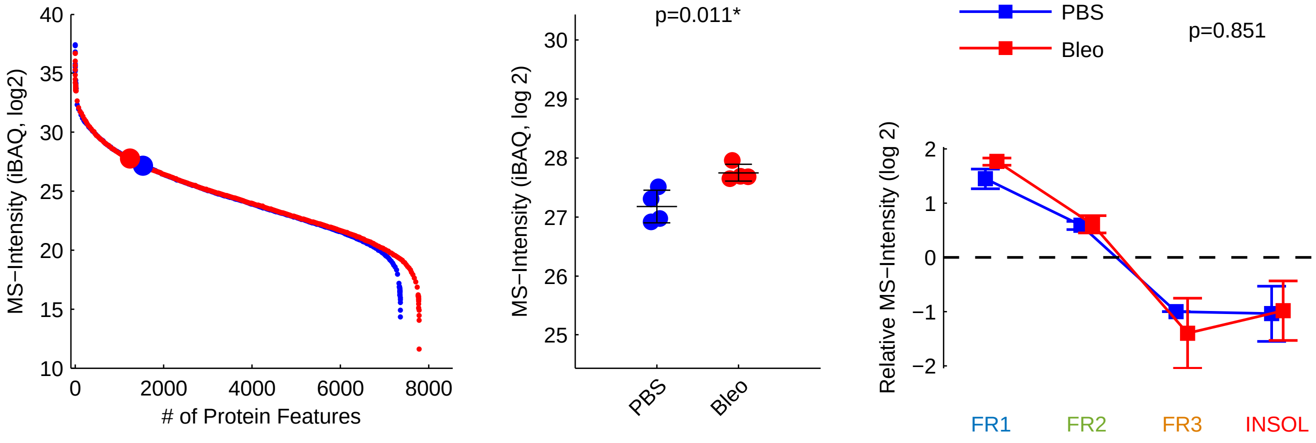

Q9QUP5 − Hapln1 (id: 8254)

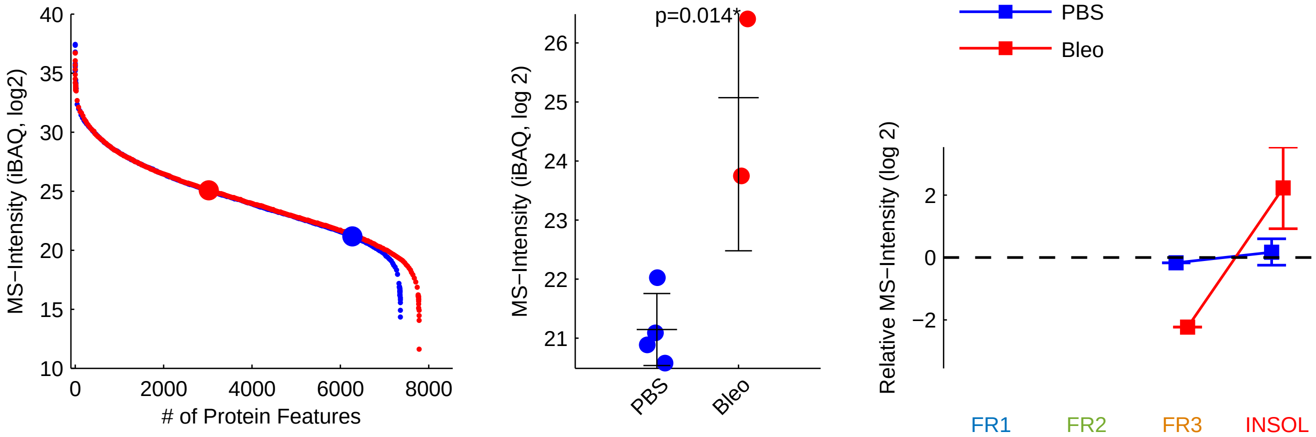

Q9QUR8 − Sema7a (id: 8257)

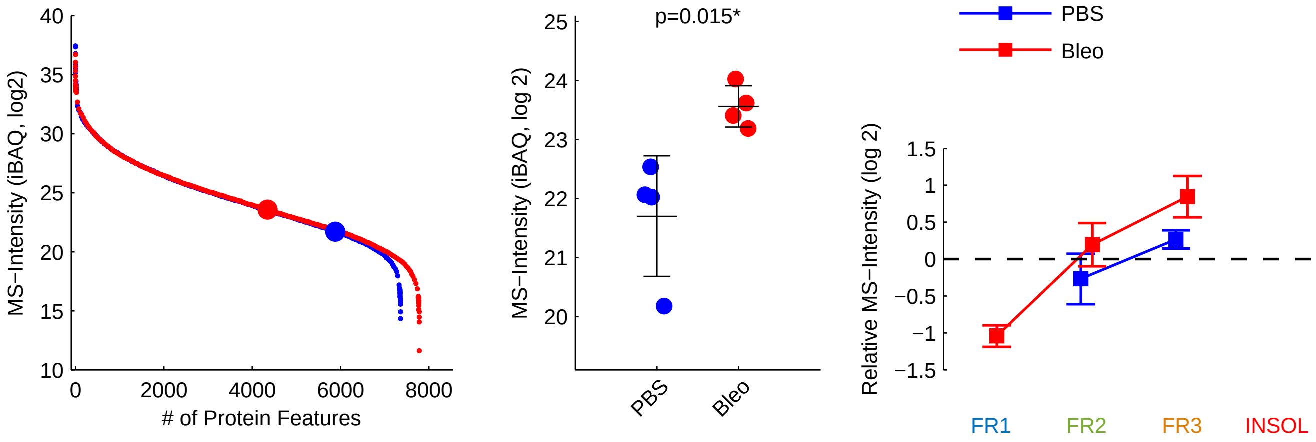

Q9QWH1 − Phc2 (id: 8260)

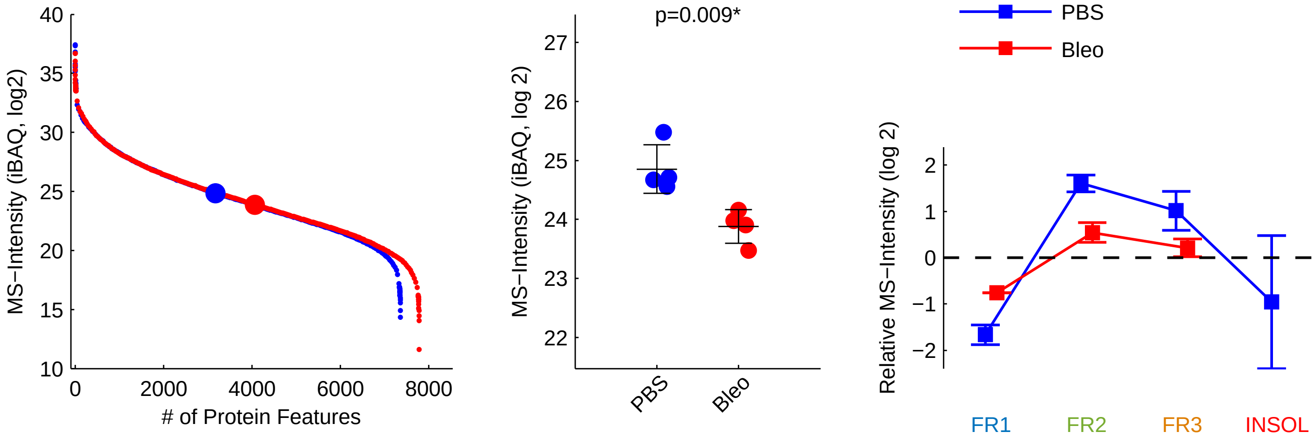

Q9QWR8 − Naga (id: 8262)

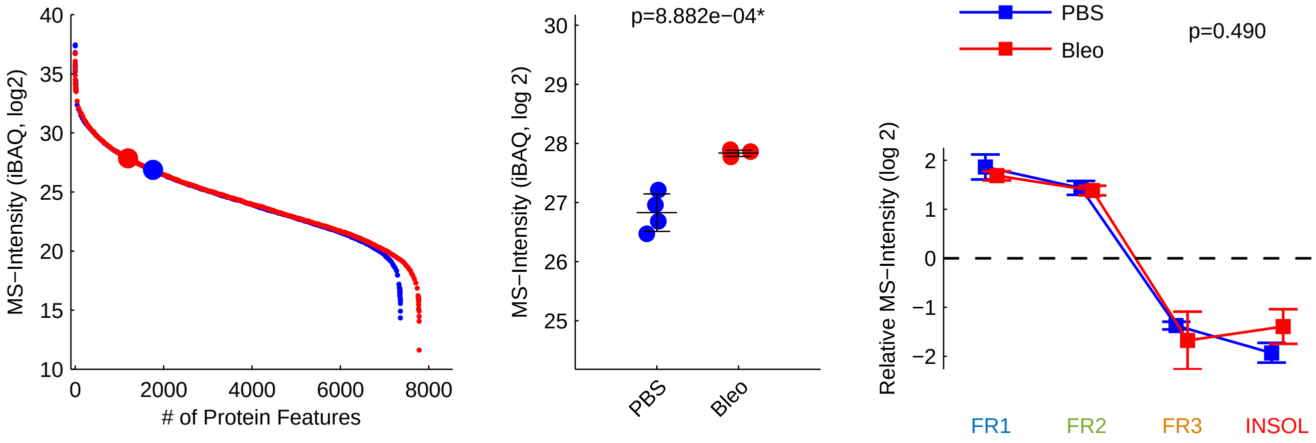

Q9QWV4 − Mlf1 (id: 8263)

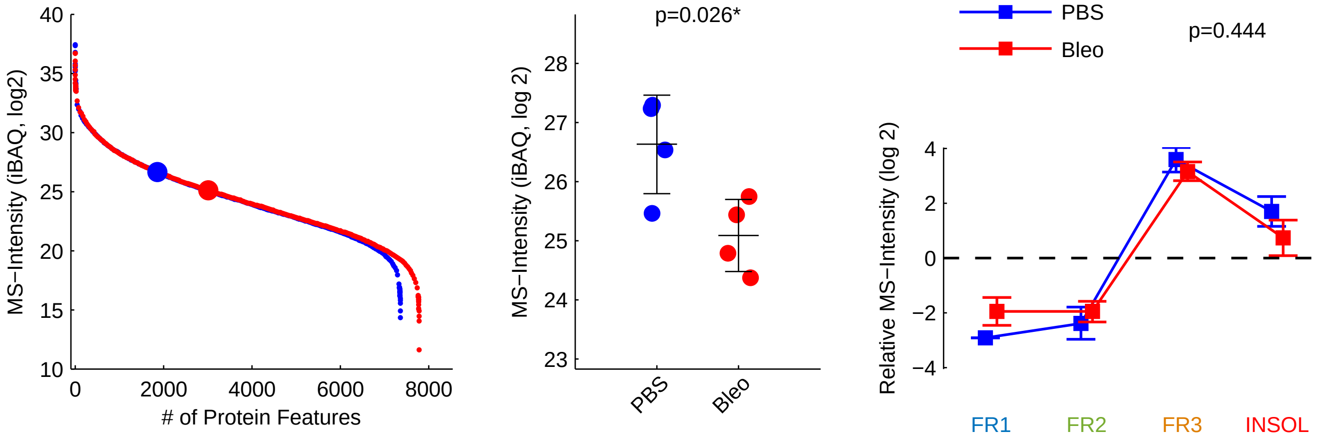

Q9QX60−2 − Dguok (id: 8267)

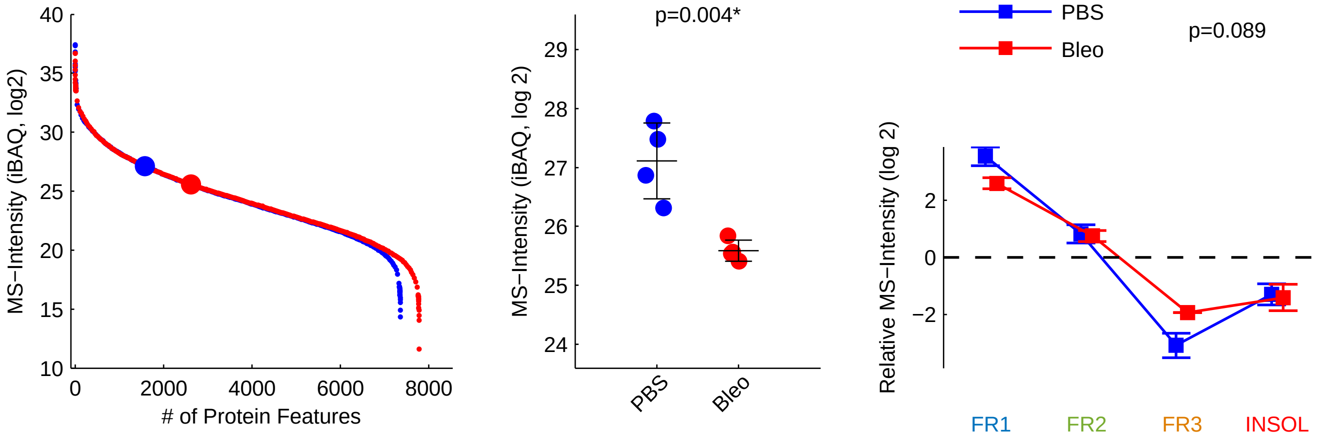

Q9QXB9 − Drg2 (id: 8269)

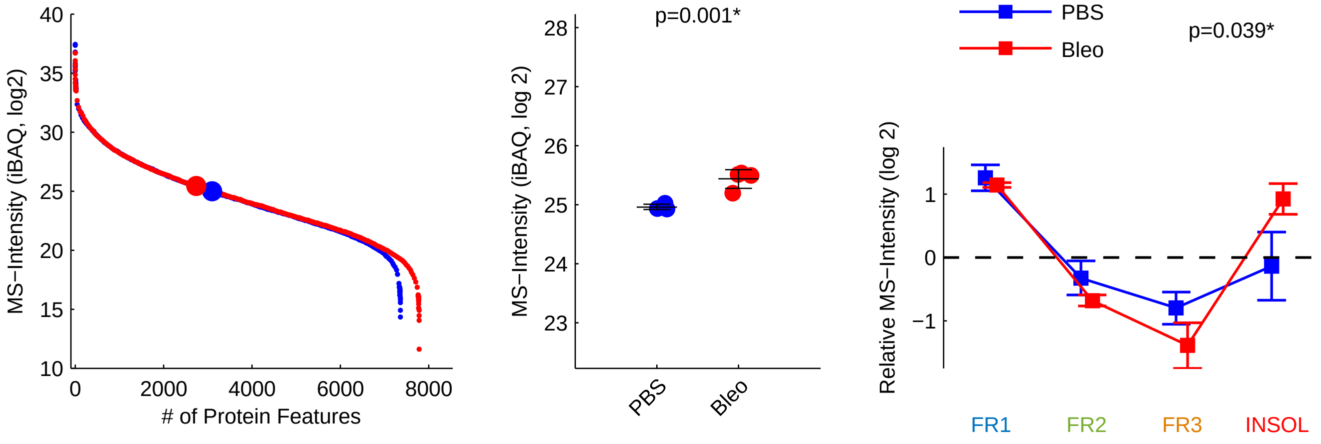

Q9QXC1 − Fetub (id: 8270)

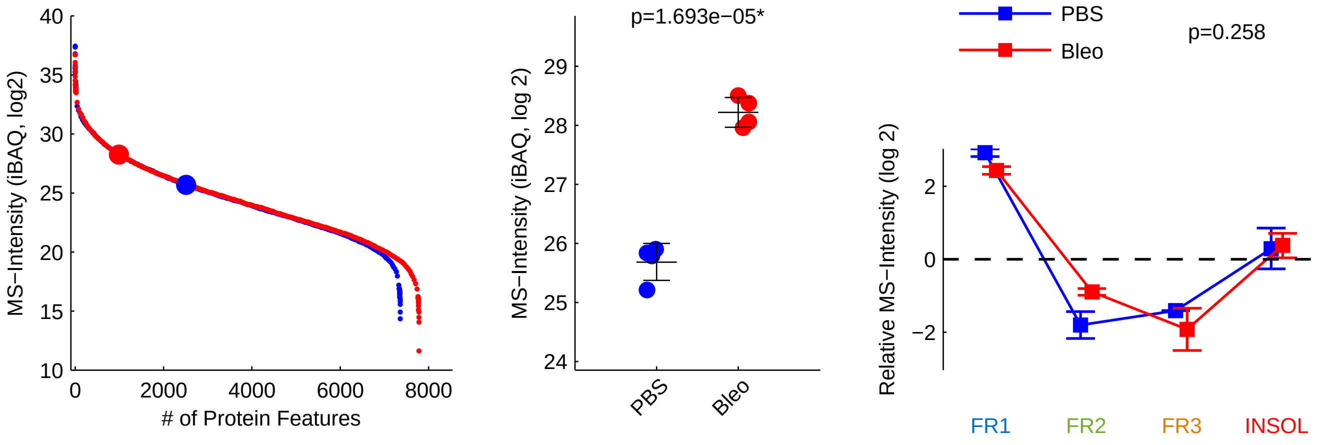

Q9QXK3 − Copg2 (id: 8280)

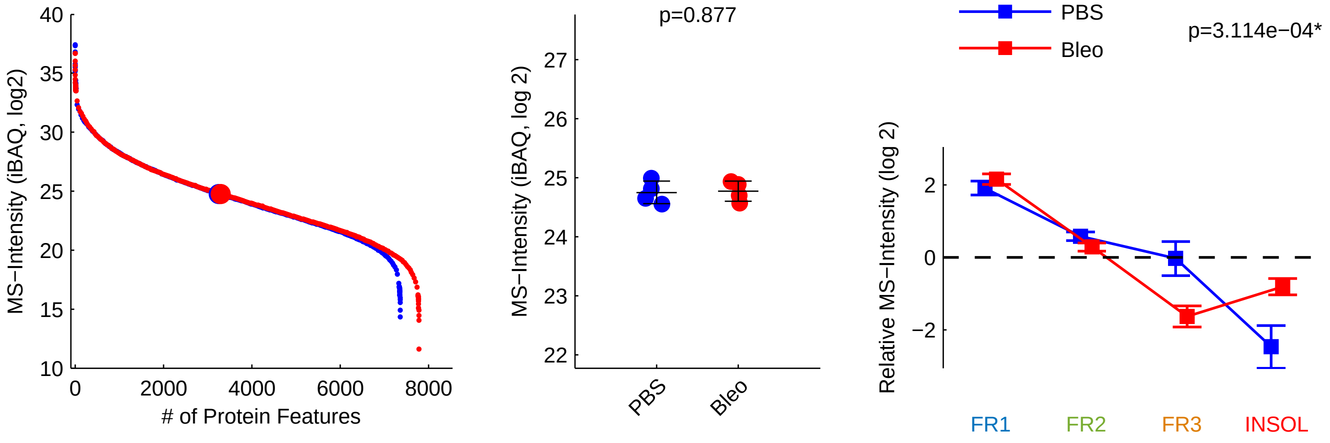

Q9QXS6−3 − Dbn1 (id: 8288)

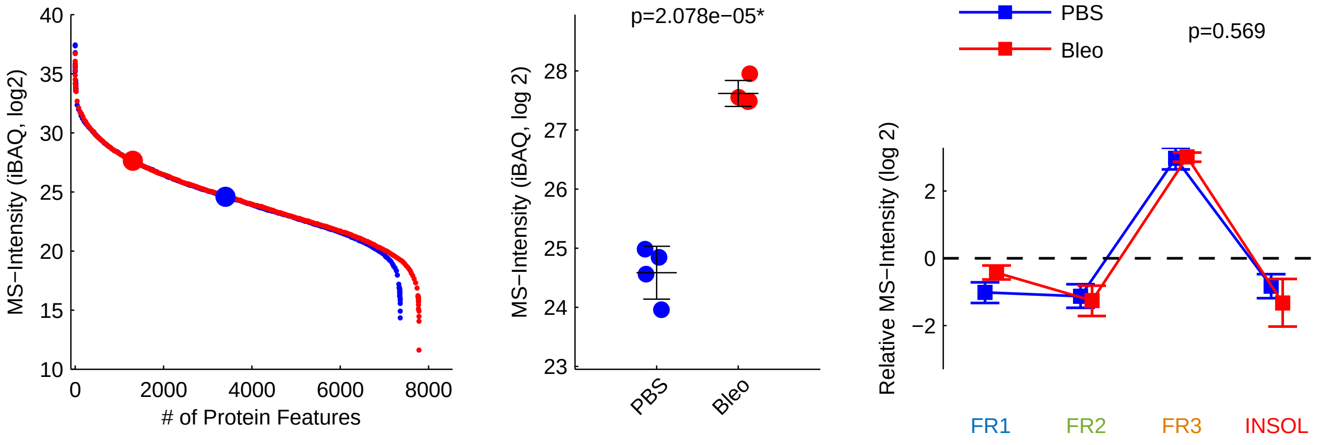

Q9QYA2 − Tomm40 (id: 8300)

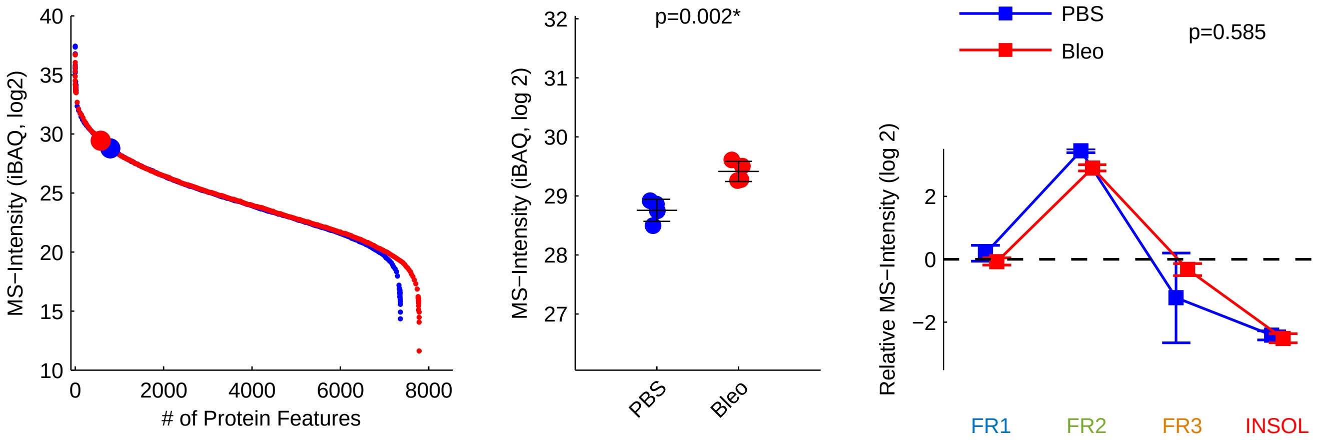

Q9QYB1 − Clic4 (id: 8301)

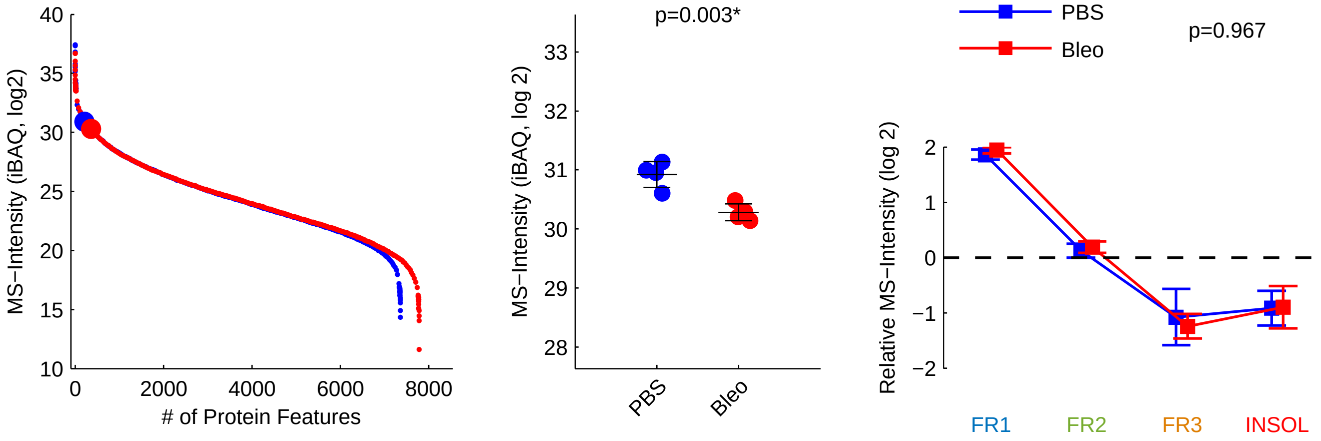

Q9QYB2 − Dach1 (id: 8302)

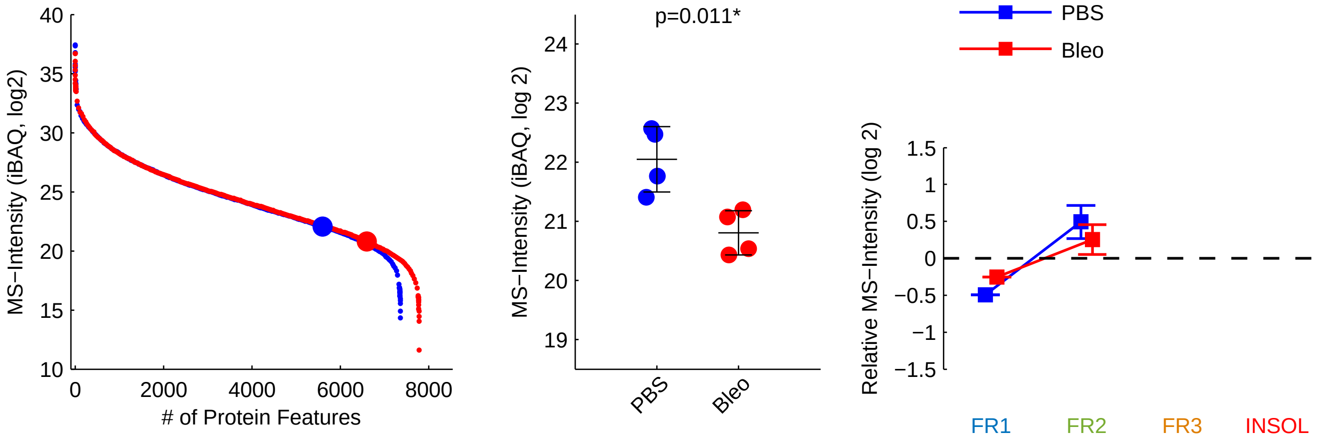

Q9QYB5−2 − Add3 (id: 8304)

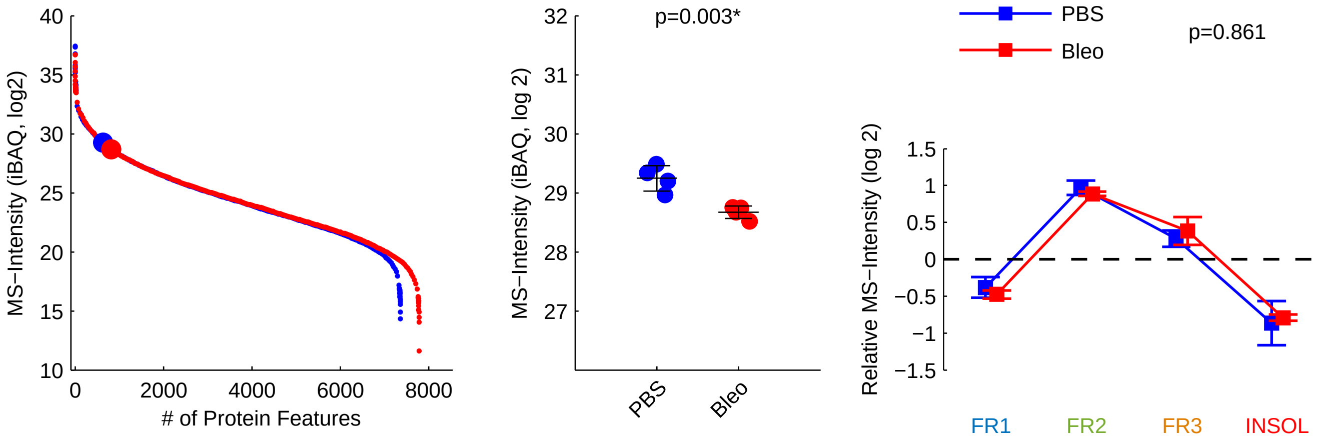

Q9QYC0 − Add1 (id: 8306)

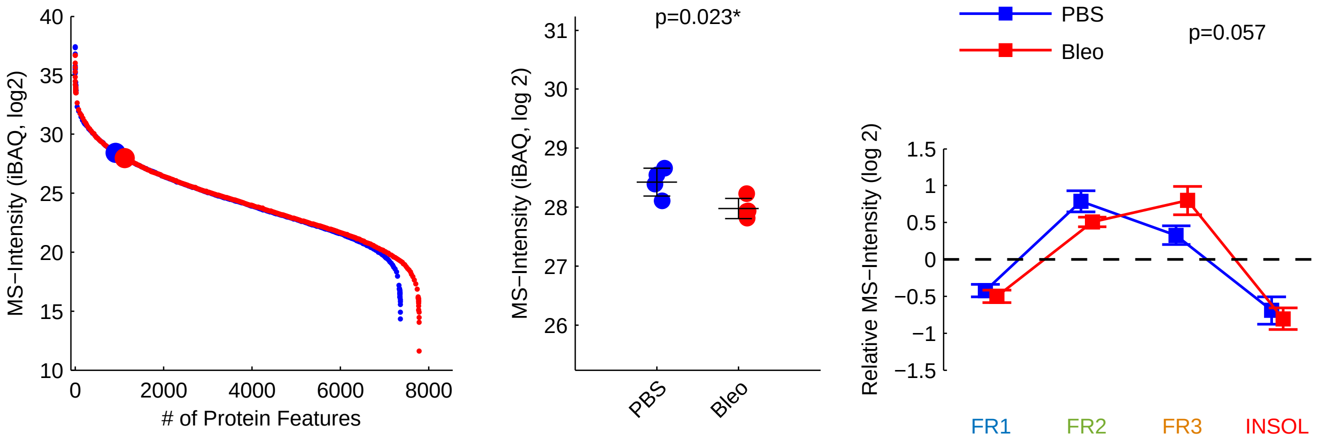

Q9QYC7 − Ggcx (id: 8307)

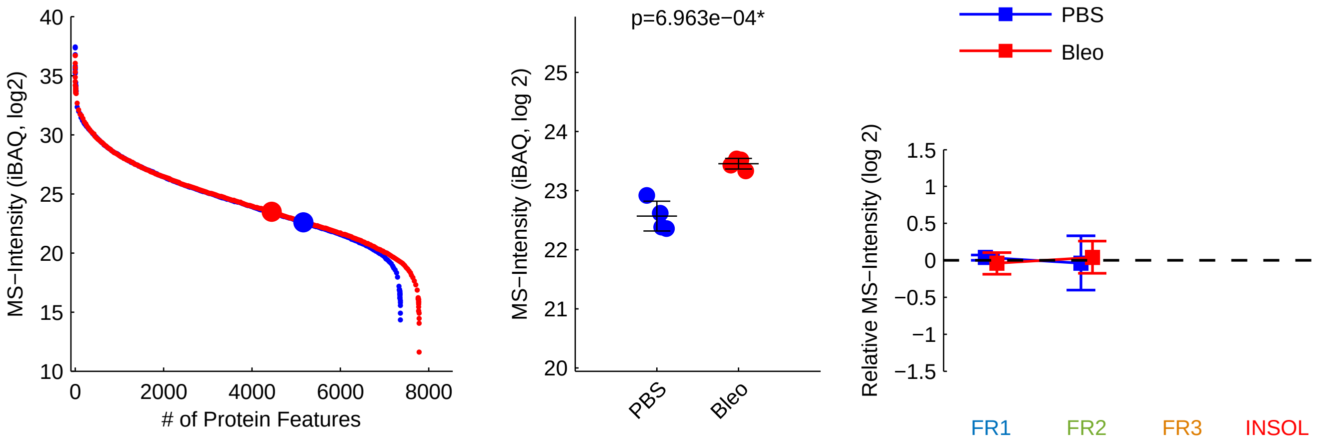

Q9QYI5 − Dnajb2 (id: 8315)

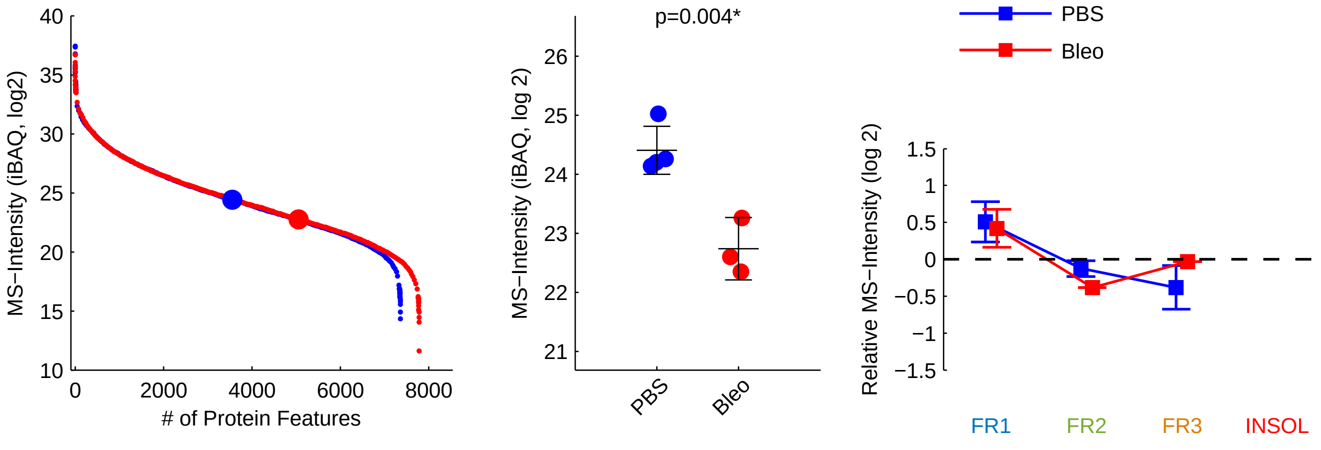

Q9QYR9 − Acot2 (id: 8321)

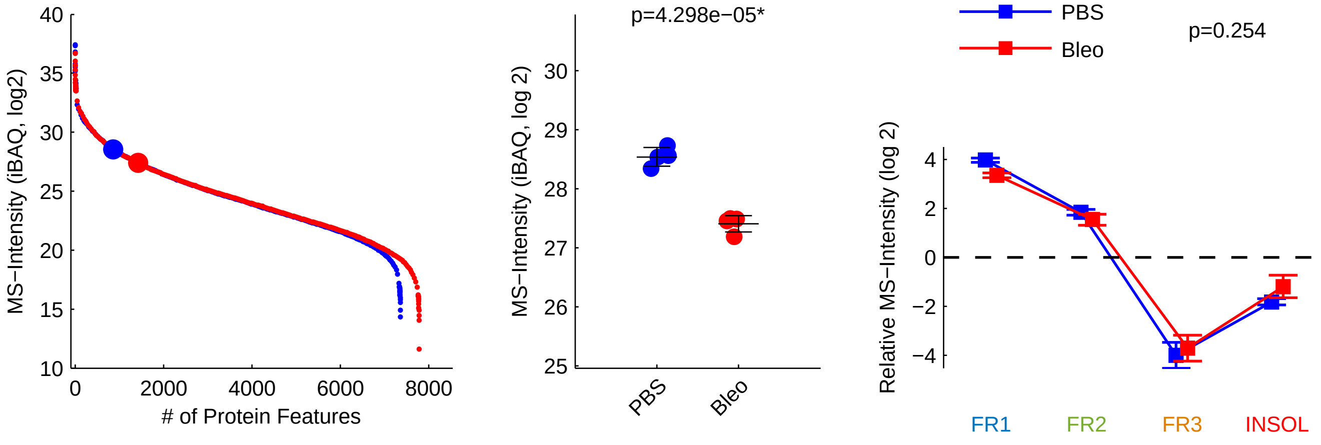

Q9QZB7 − Actr10 (id: 8332)

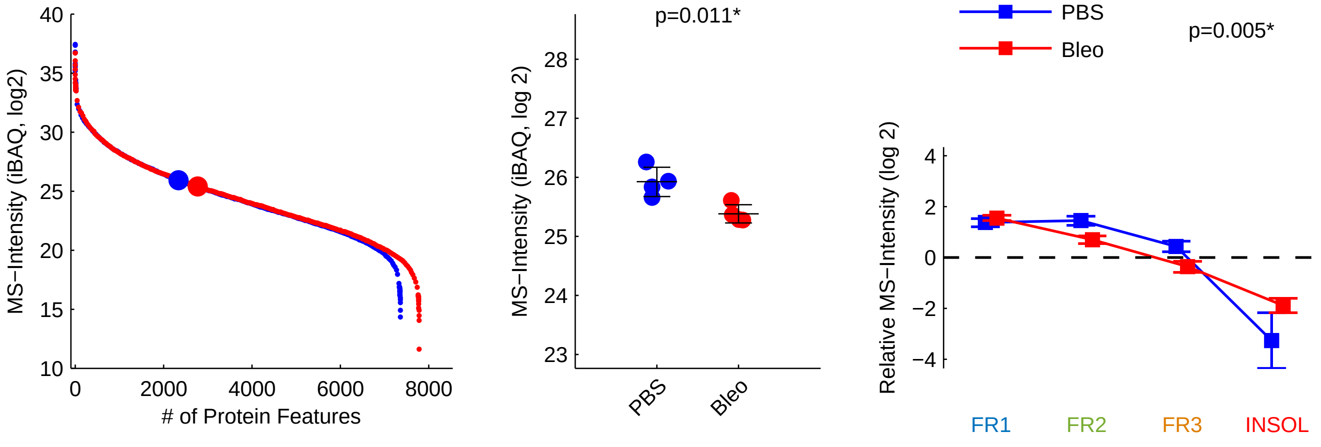

Q9QZD9 − Eif3i (id: 8337)

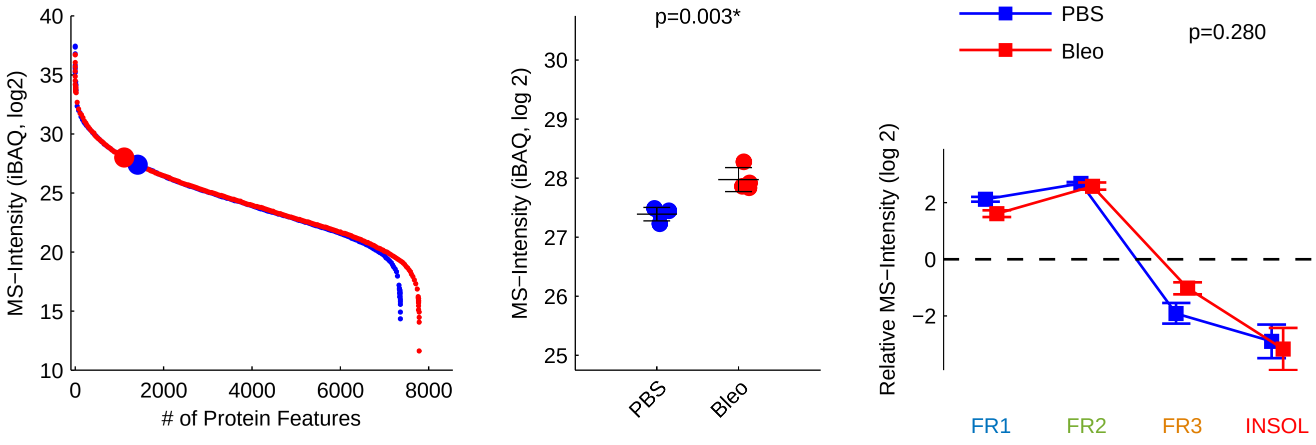

Q9QZE5 − Copg1 (id: 8338)

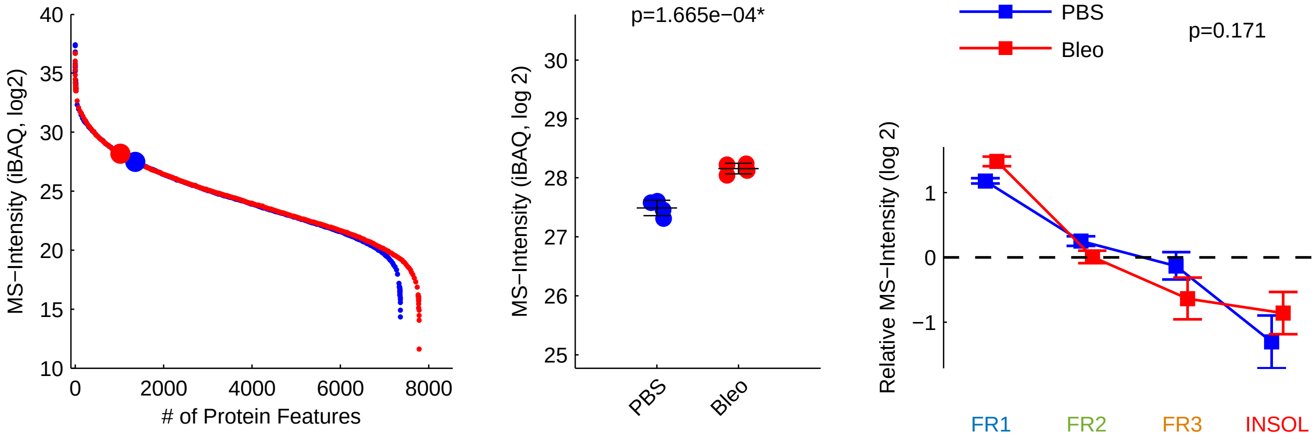

Q9QZK7 − Dok3 (id: 8345)

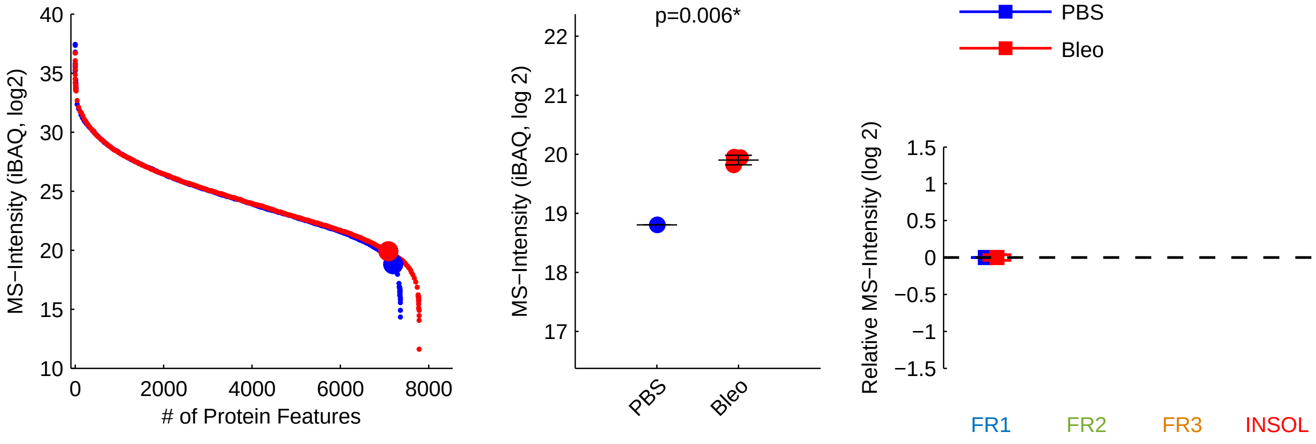

Q9QZS3 − Numb (id: 8352)

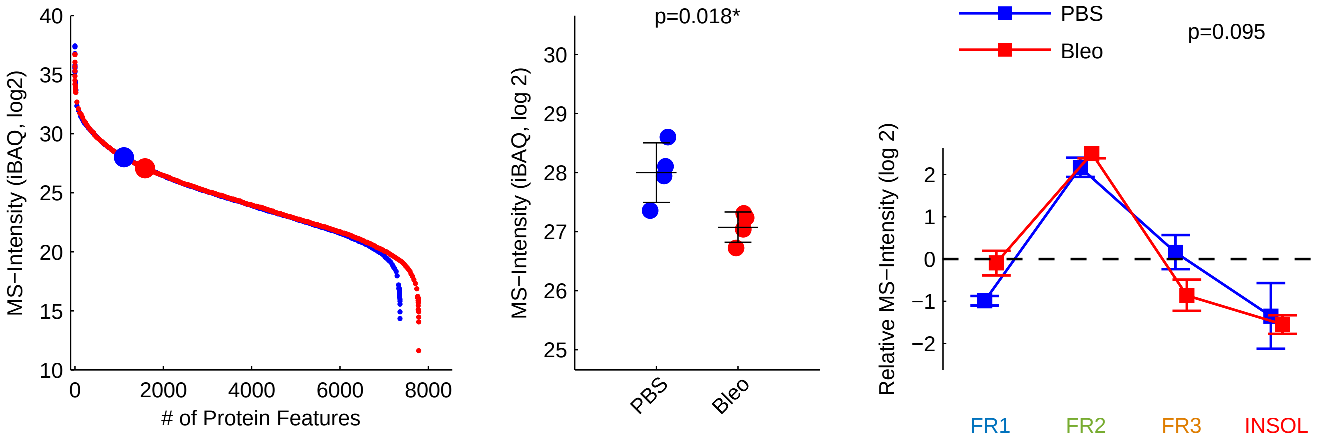

Q9R059 − Fhl3 (id: 8362)

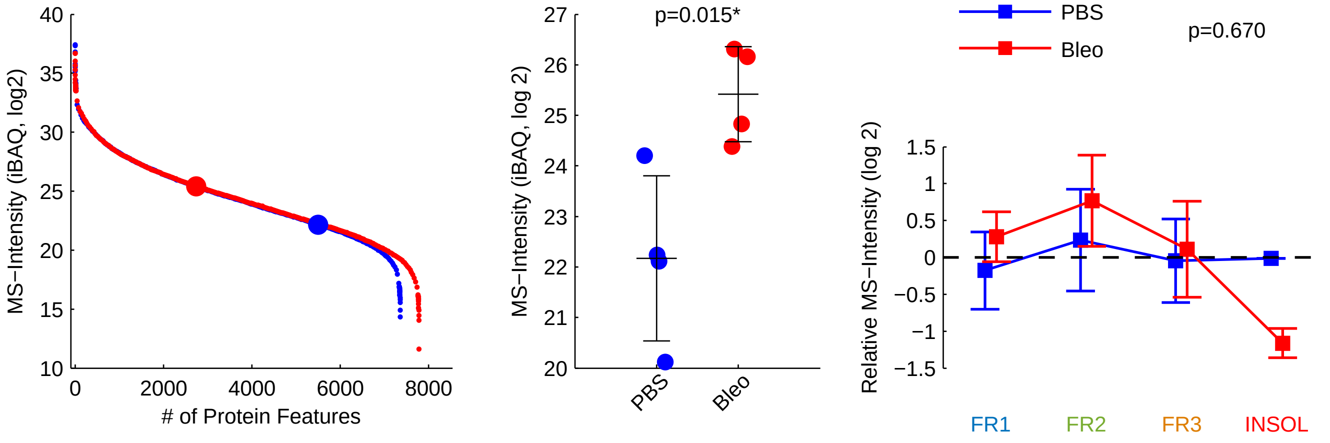

Q9R061 − Nubp2 (id: 8364)

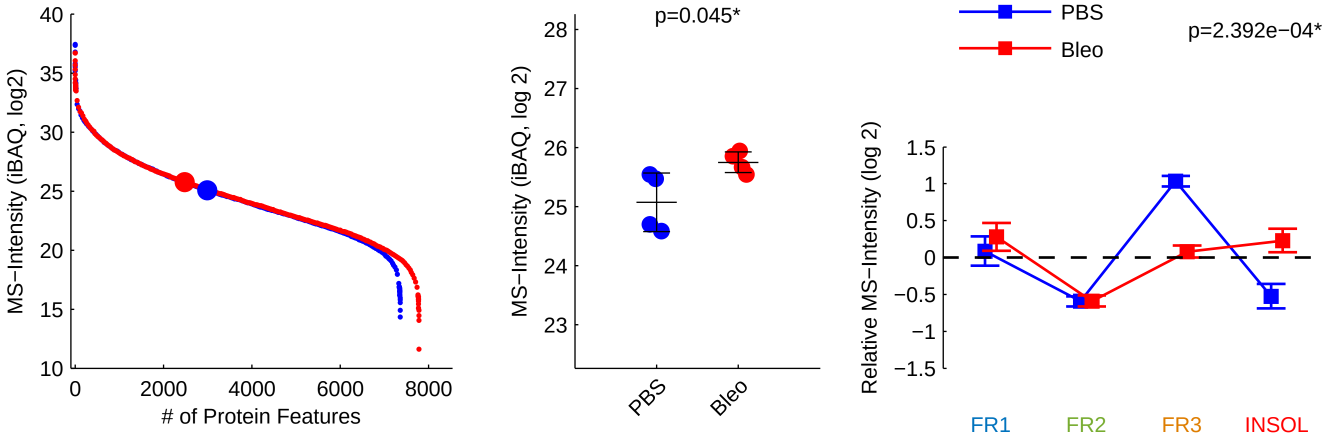

Q9R069 − Bcam (id: 8366)

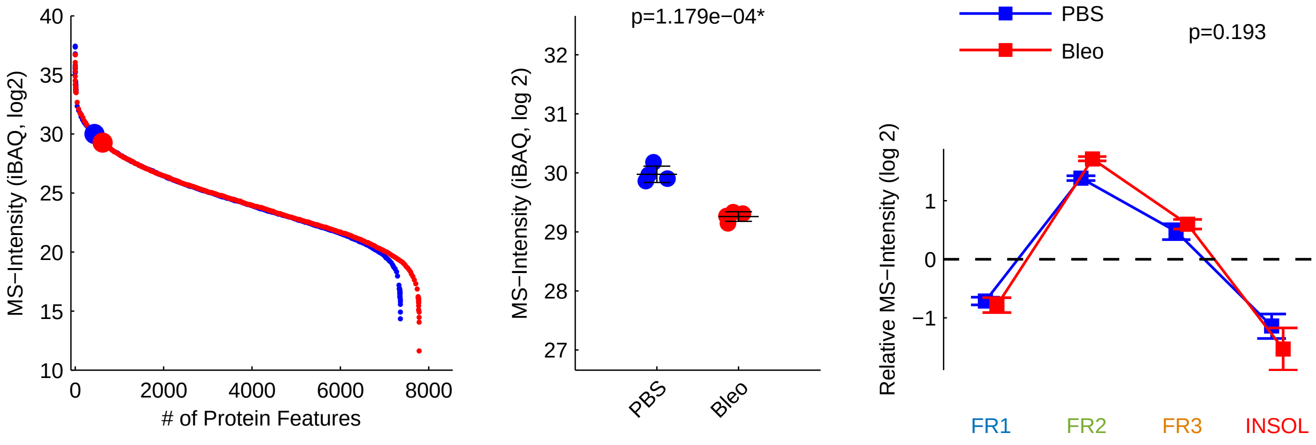

Q9R078 − Prkab1 (id: 8367)

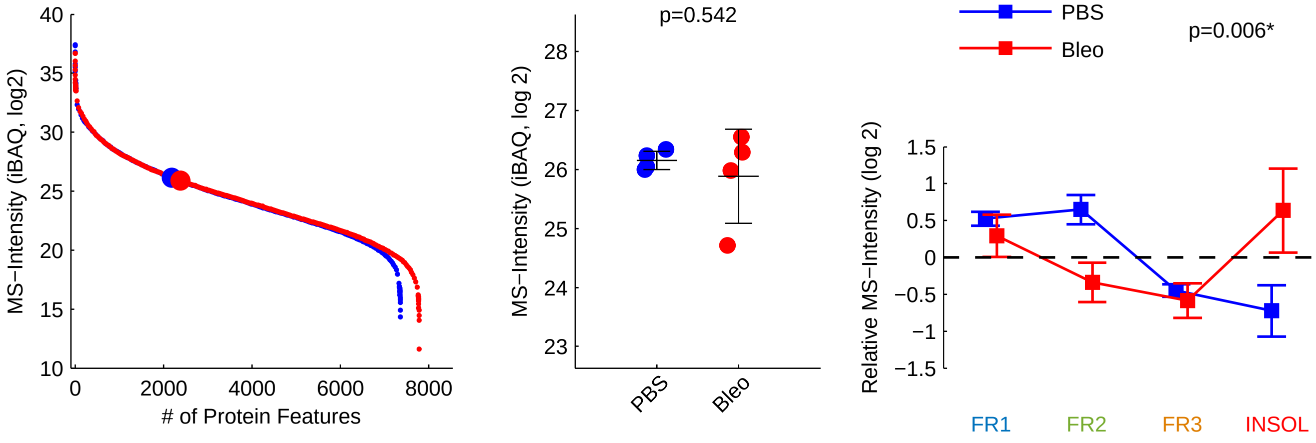

Q9R099 − Tbl2 (id: 8369)

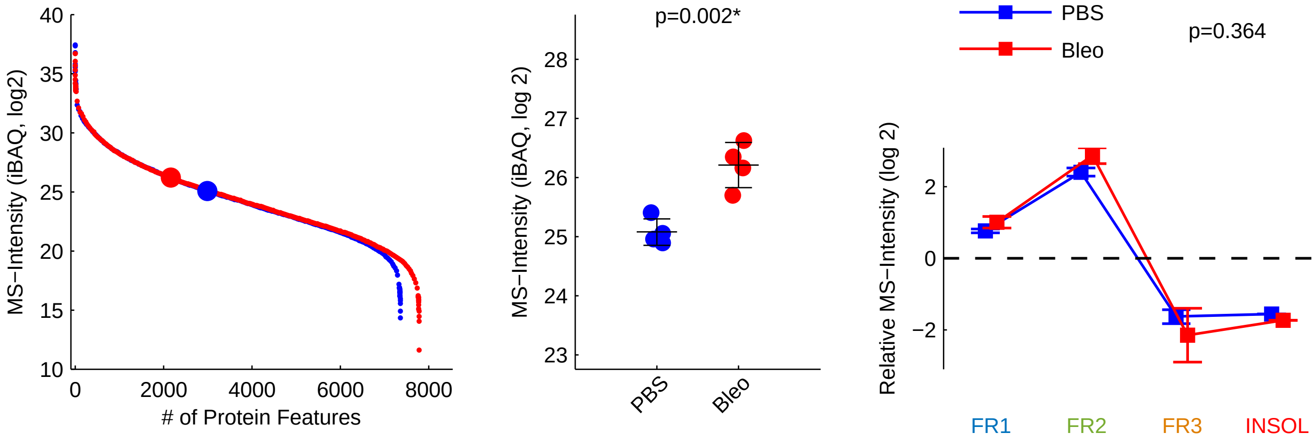

Q9R0B6 − Lamc3 (id: 8371)

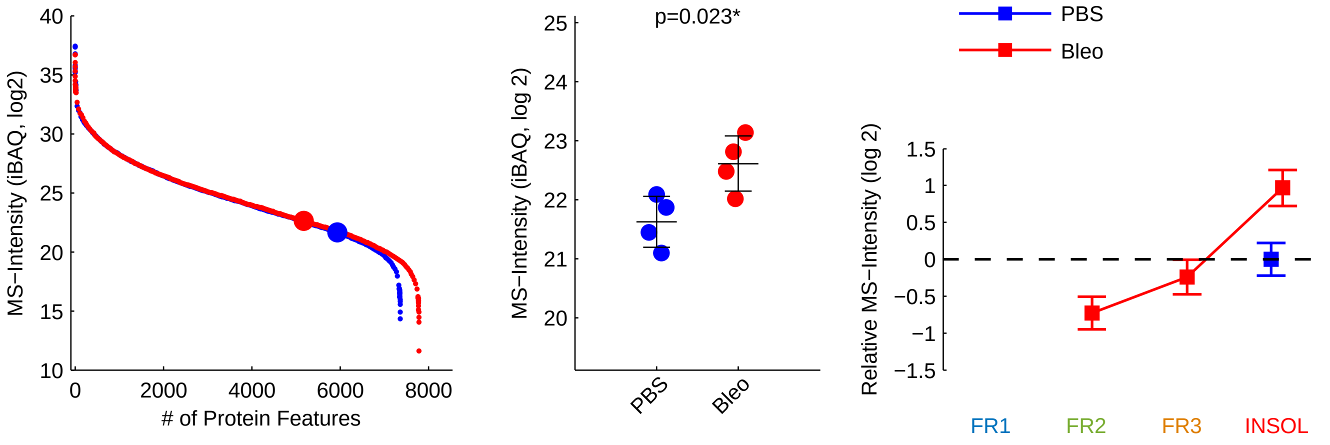

Q9R0C8 − Vav3 (id: 8373)

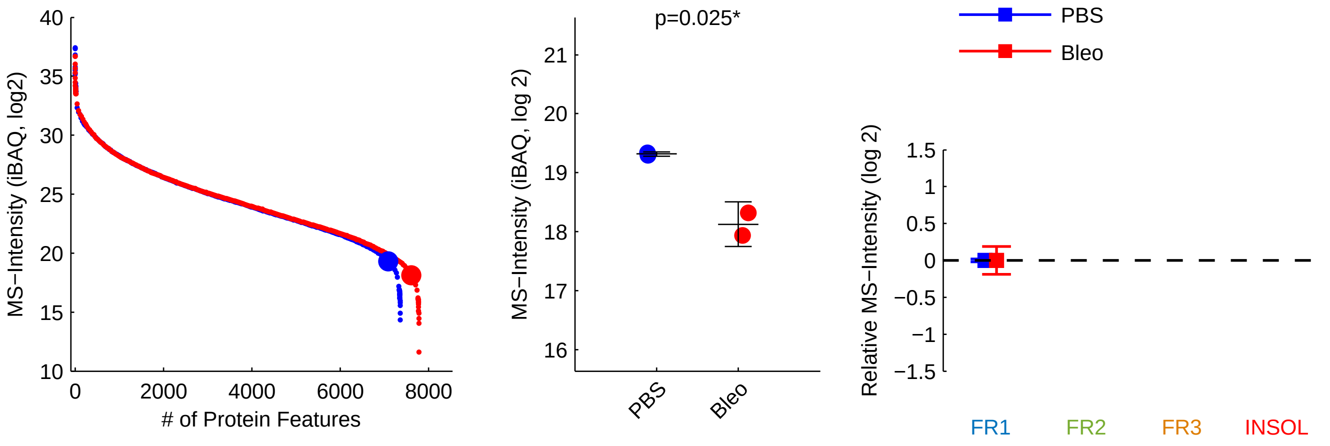

Q9R0E1 − Plod3 (id: 8374)

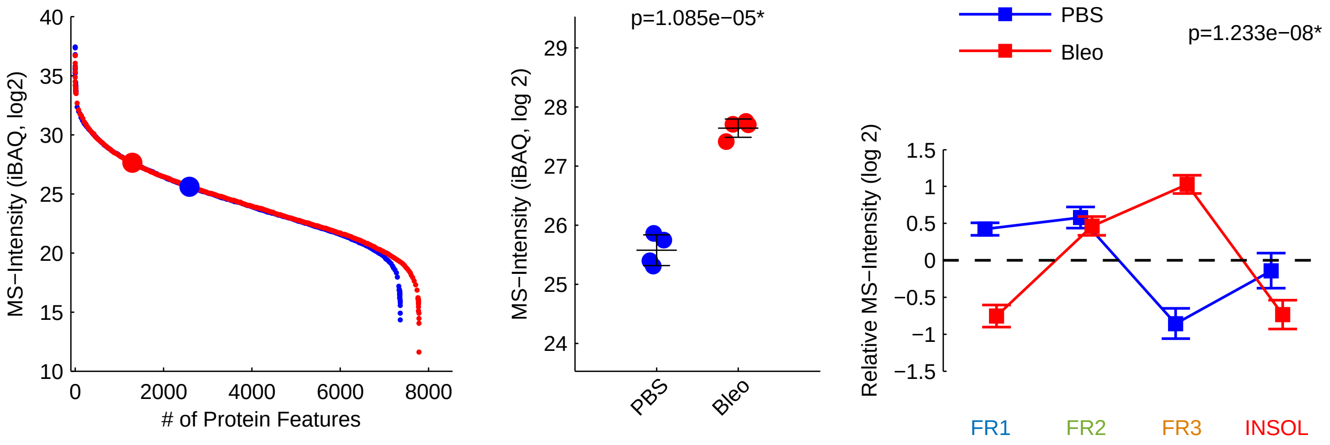

Q9R0E2 − Plod1 (id: 8375)

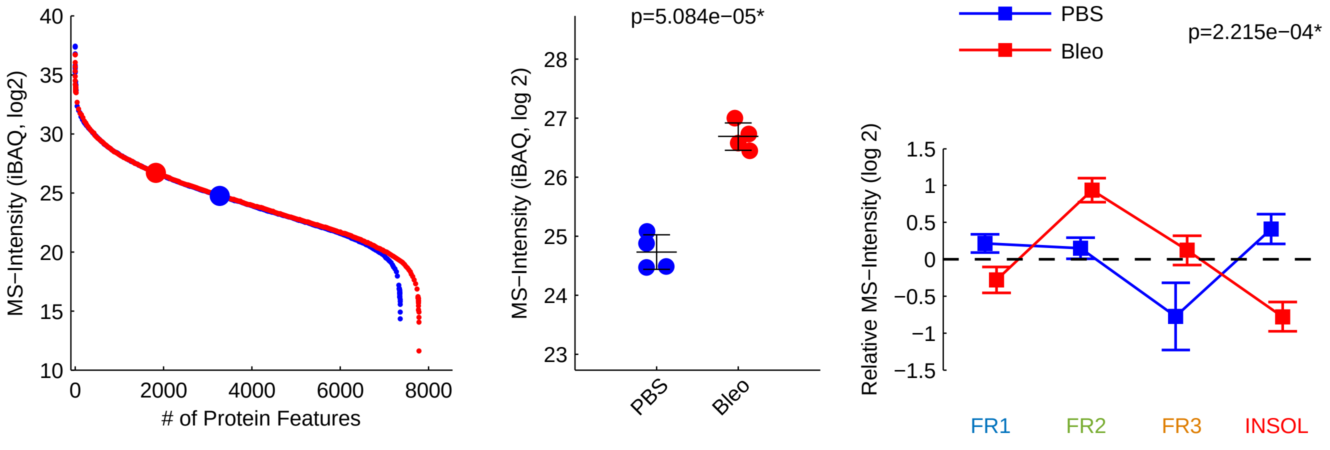

Q9R0H0−2 − Acox1 (id: 8377)

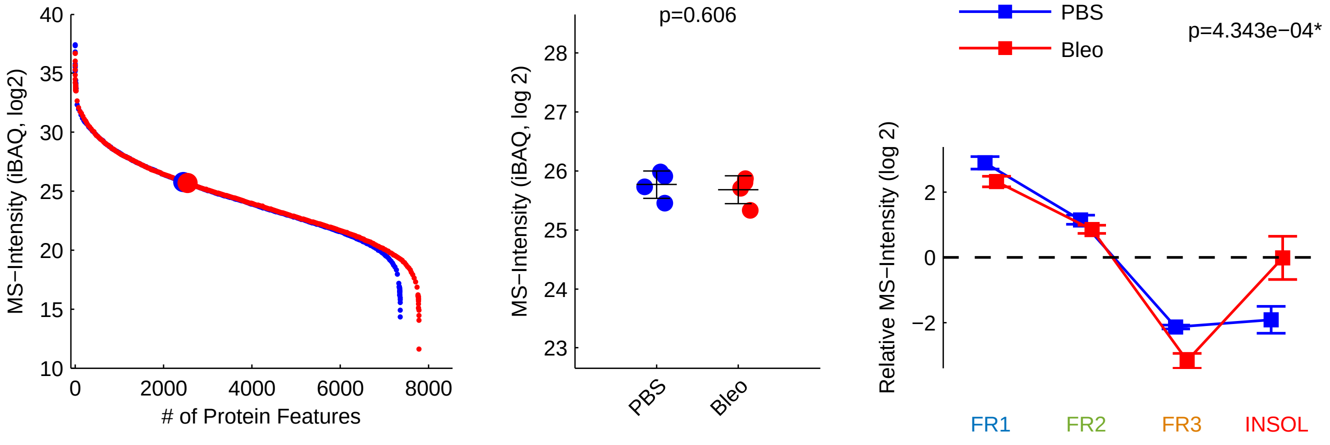

Q9R0M4 − Podxl (id: 8380)

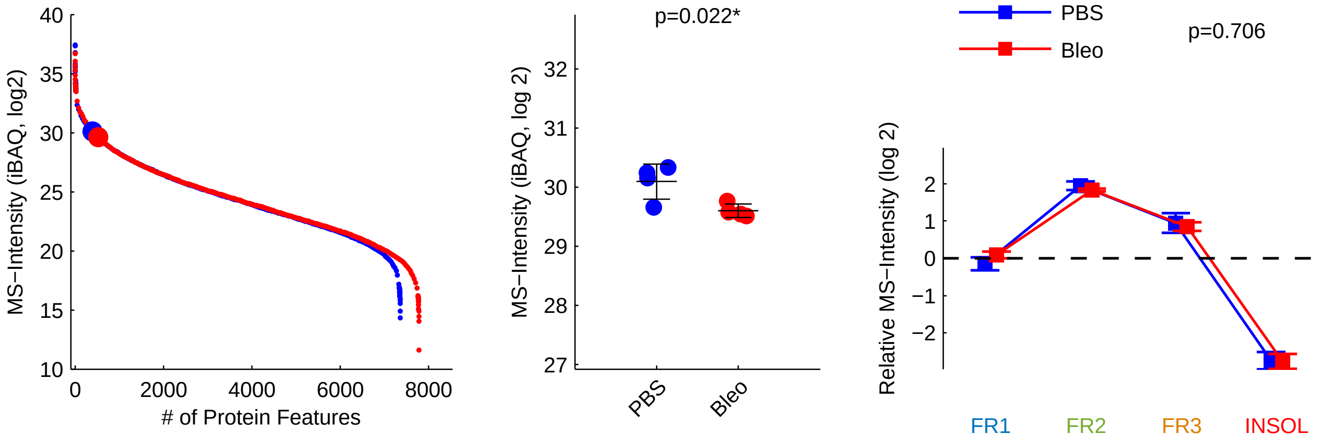

Q9R0Q6 − Arpc1a (id: 8387)

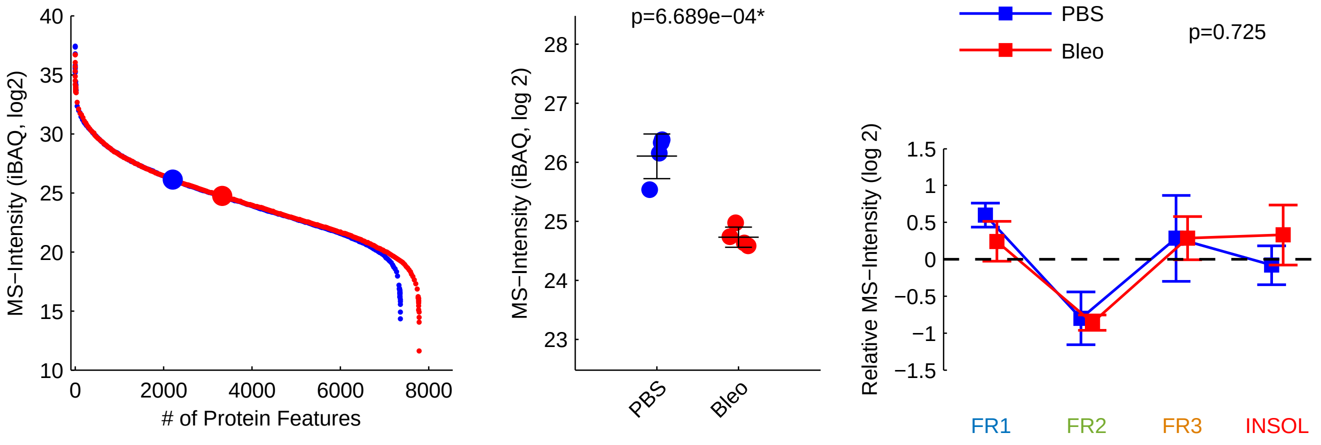

Q9R111 − Gda (id: 8391)

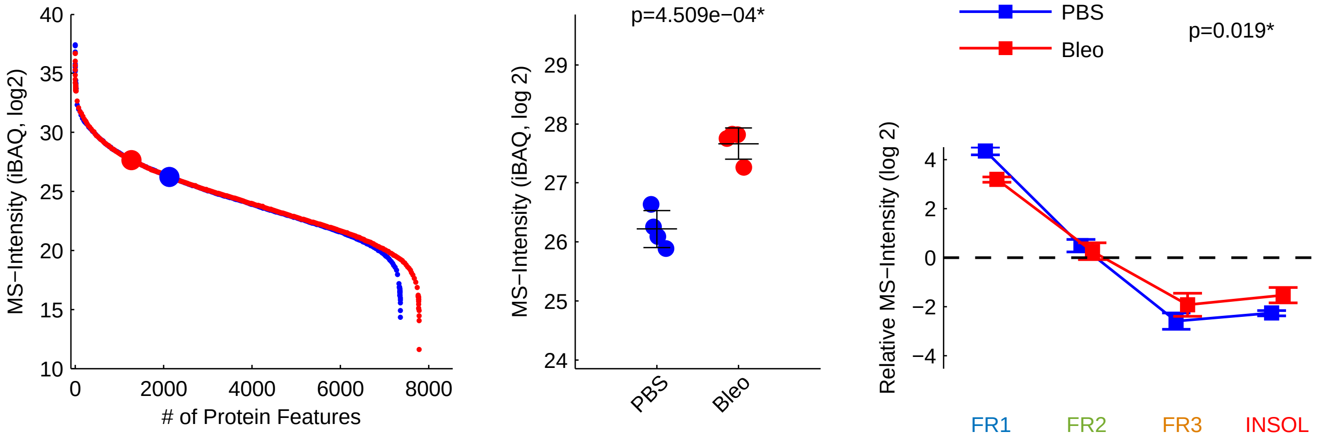

Q9R1S0 − B9d1 (id: 8411)

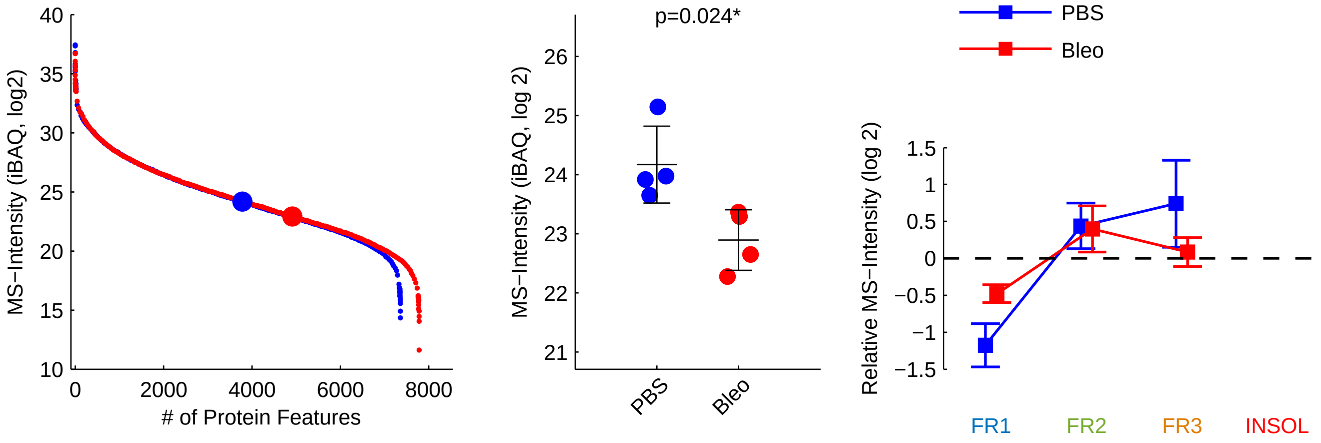

Q9R1W5 − Calcrl (id: 8415)

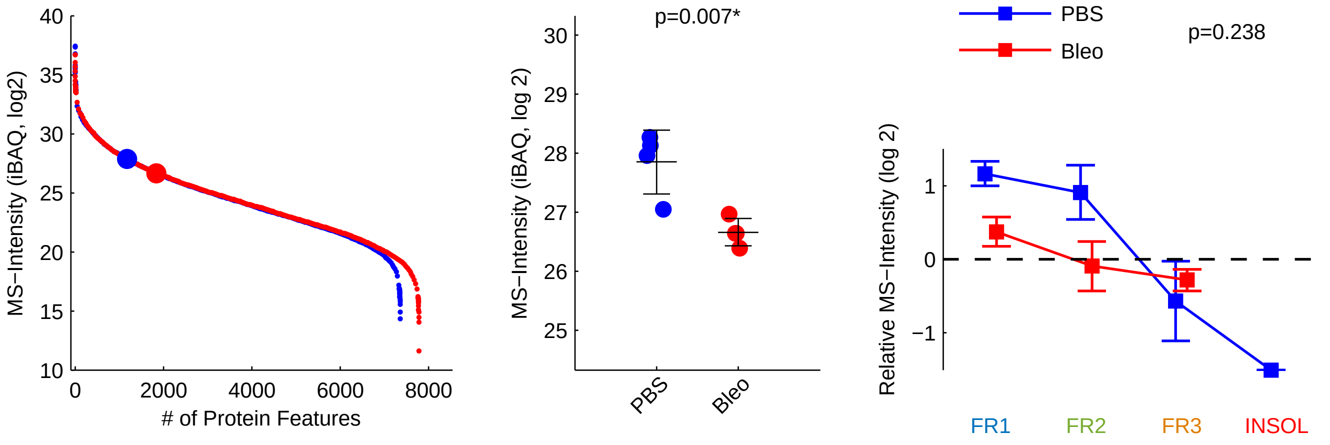

Q9R1Z8 − Sorbs3 (id: 8419)

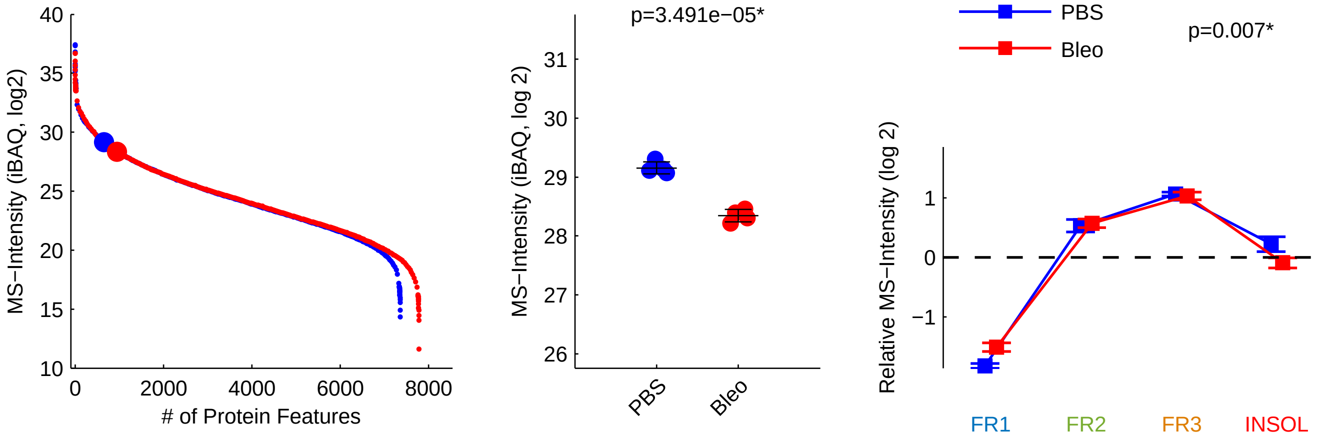

Q9R226 − Khdrbs3 (id: 8420)

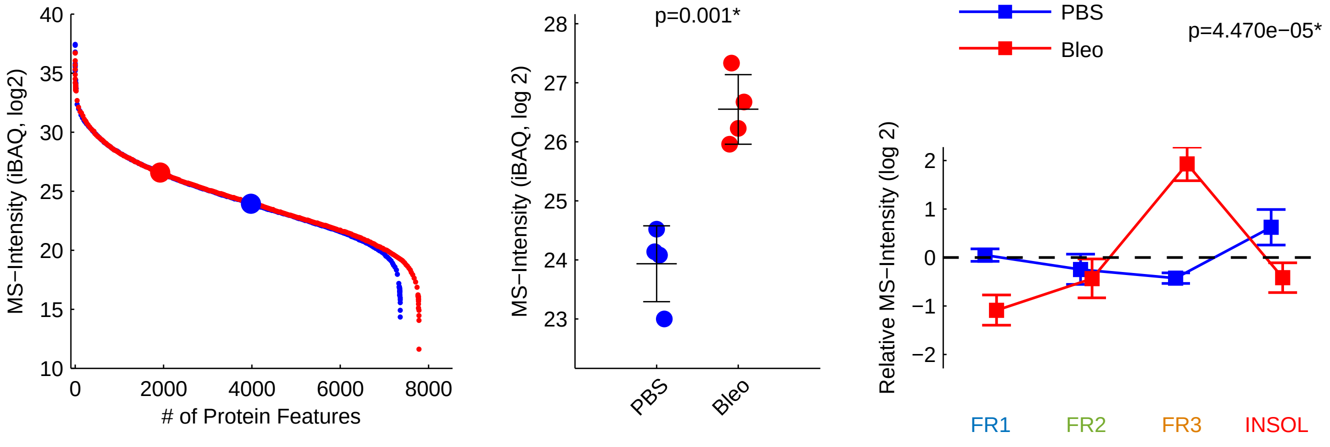

Q9R257 − Hebp1 (id: 8421)

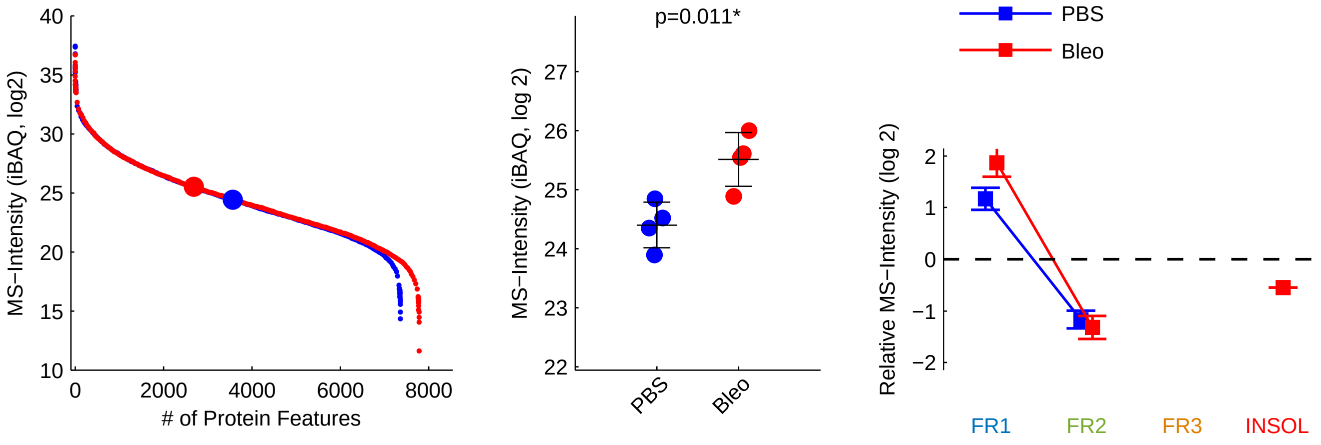

Q9WTI7−3 − Myo1c (id: 8424)

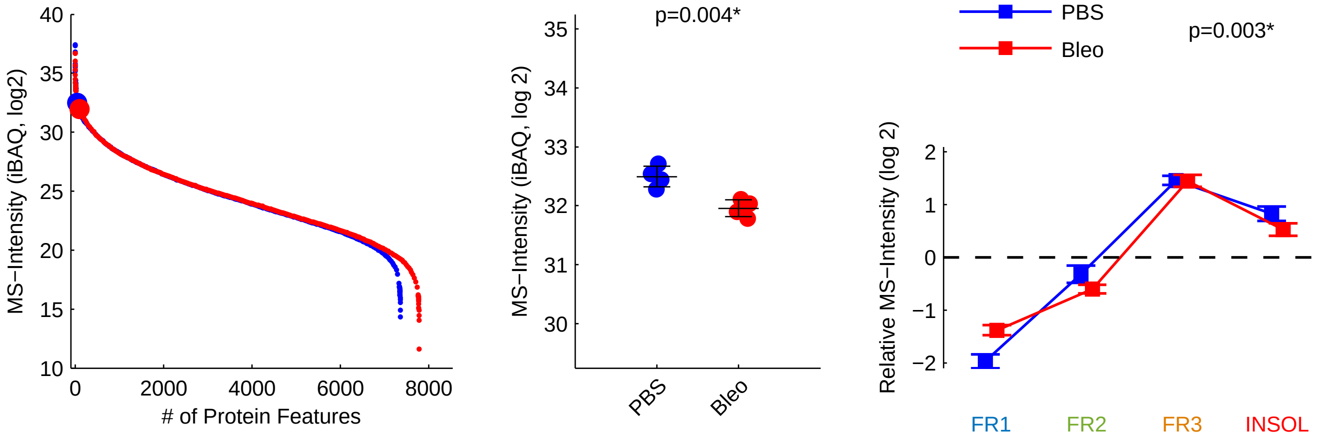

Q9WTK5 − Nfkb2 (id: 8427)

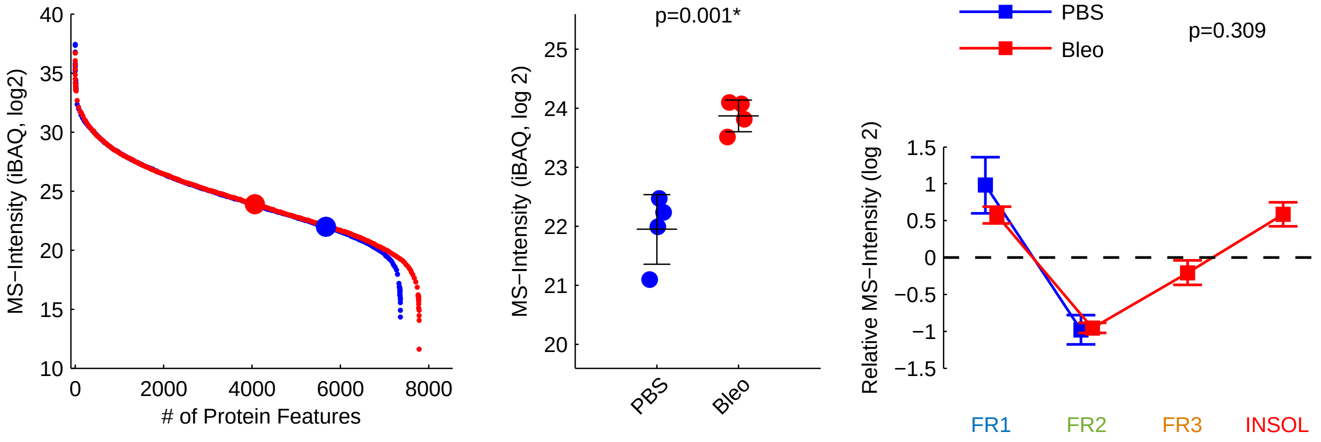

Q9WTL2 − Rab25 (id: 8428)

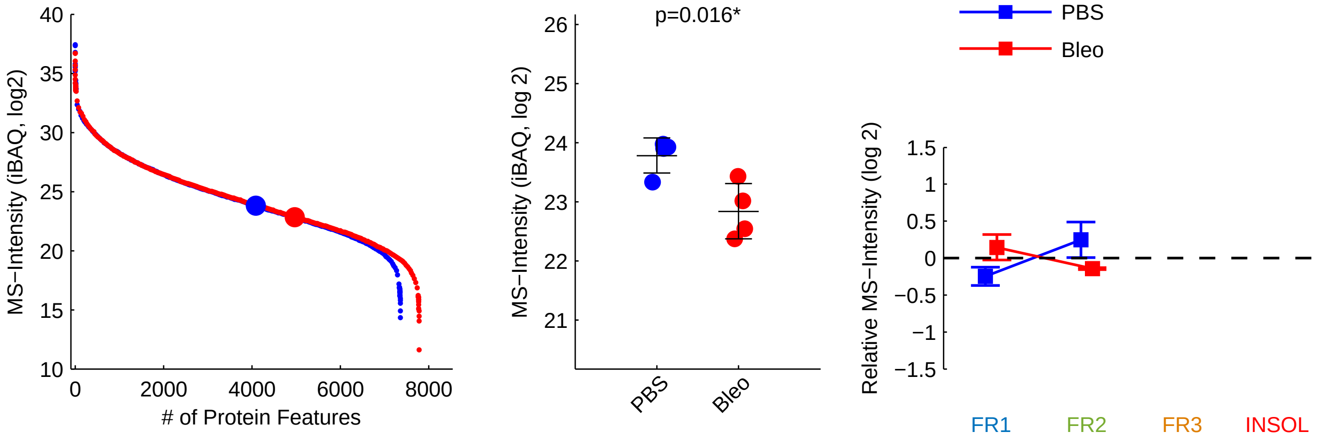

Q9WTN0 − Ggps1 (id: 8432)

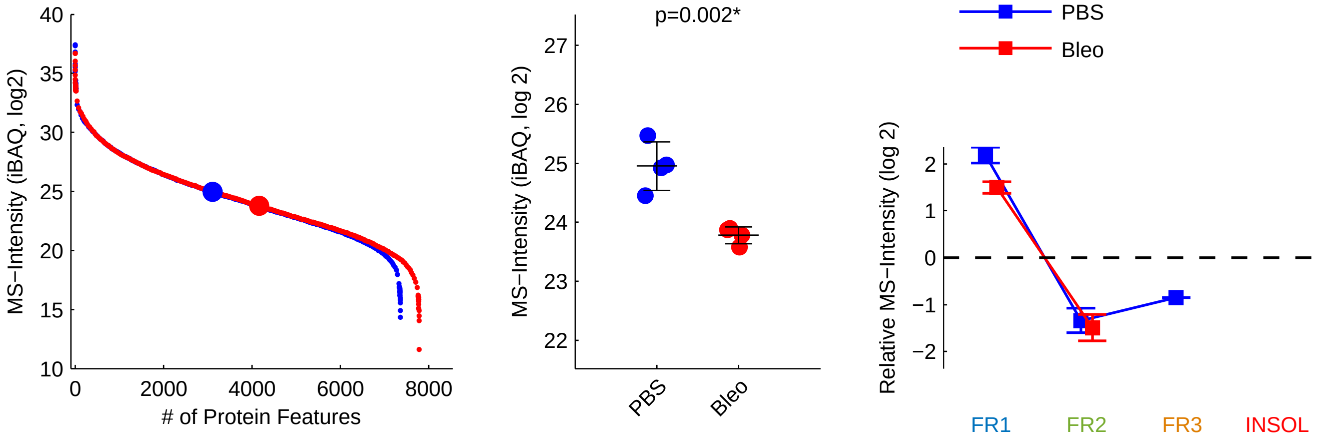

Q9WTP6 − Ak2 (id: 8433)

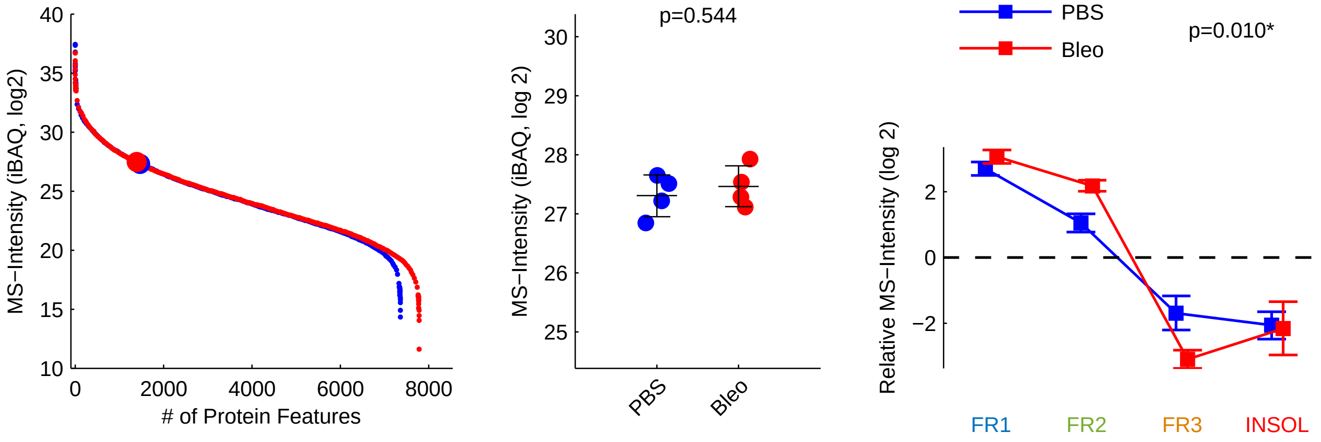

Q9WTP7 − Ak3 (id: 8434)

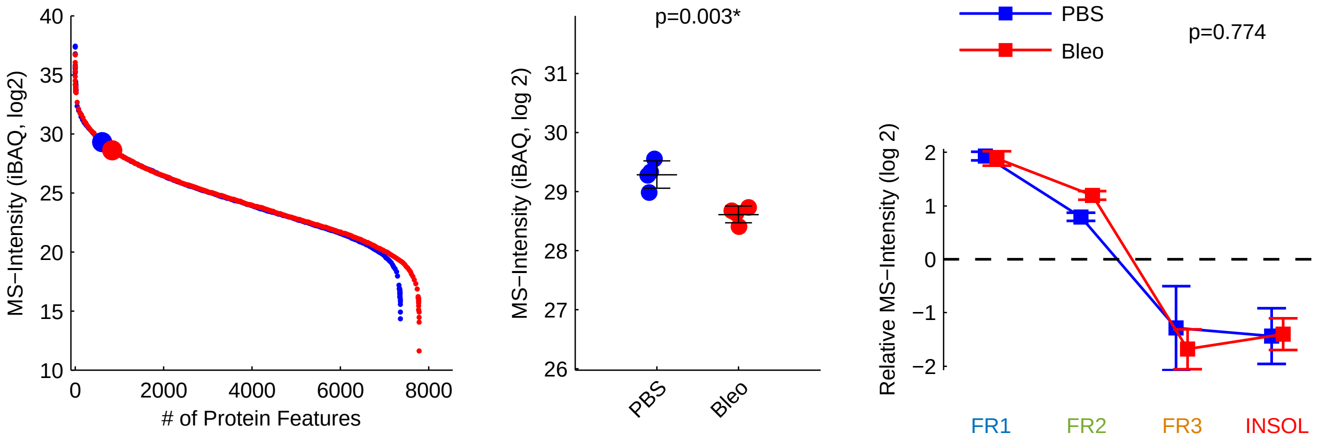

Q9WTQ5 − Akap12 (id: 8435)

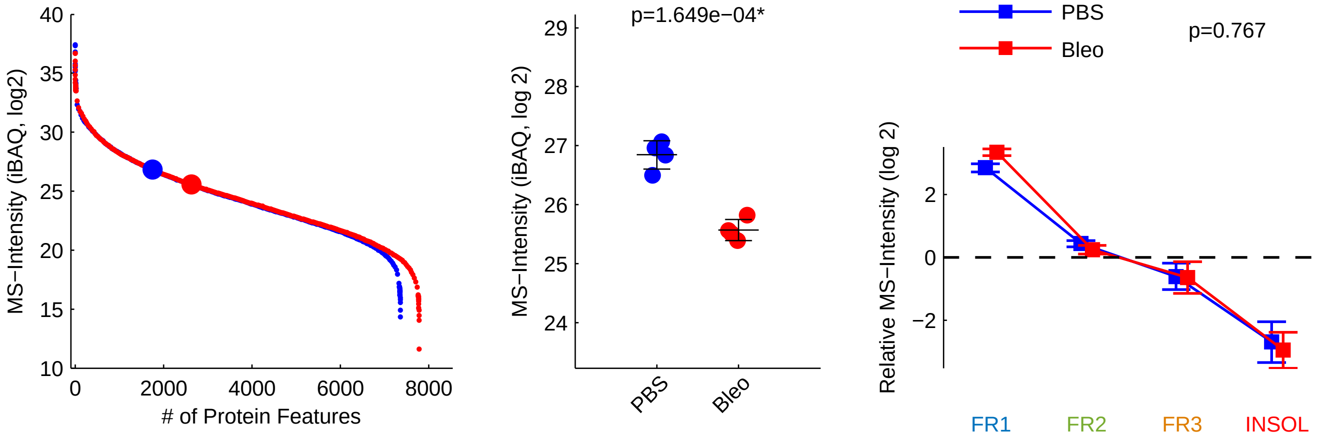

Q9WTR5 − Cdh13 (id: 8437)

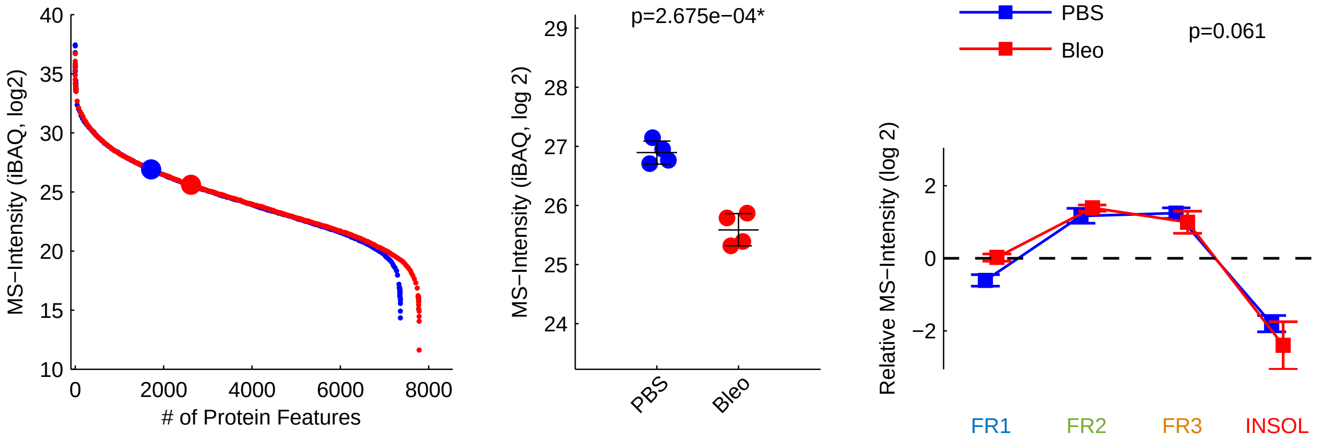

Q9WTU0 − Phf2 (id: 8439)

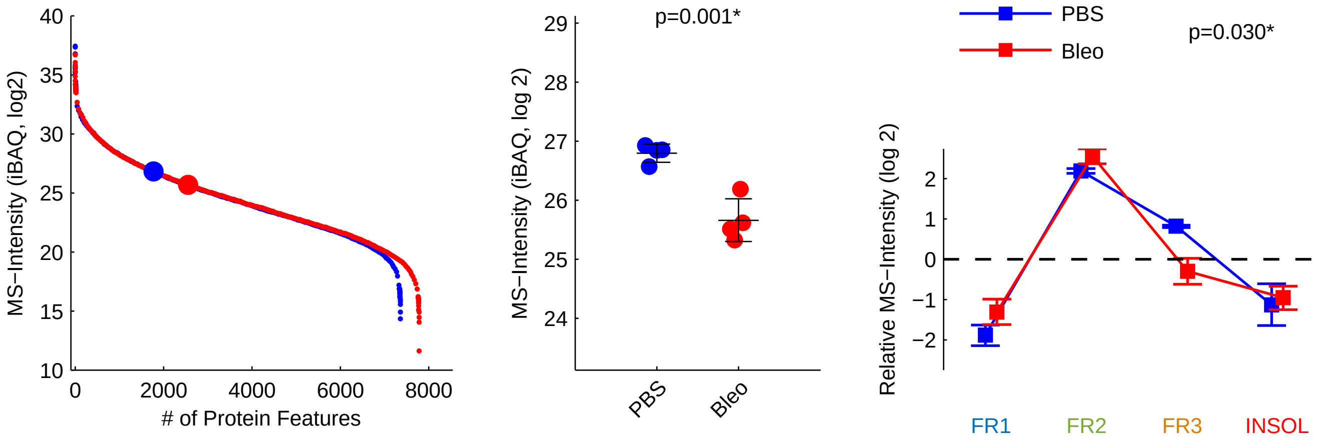

Q9WTV6 − Usp18 (id: 8441)

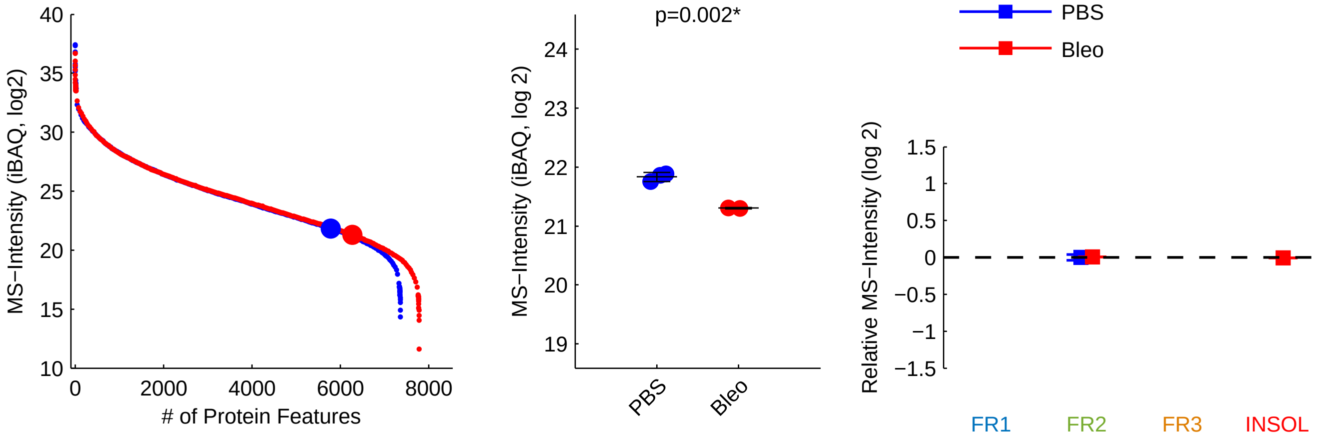

Q9WTX2 − Prkra (id: 8442)

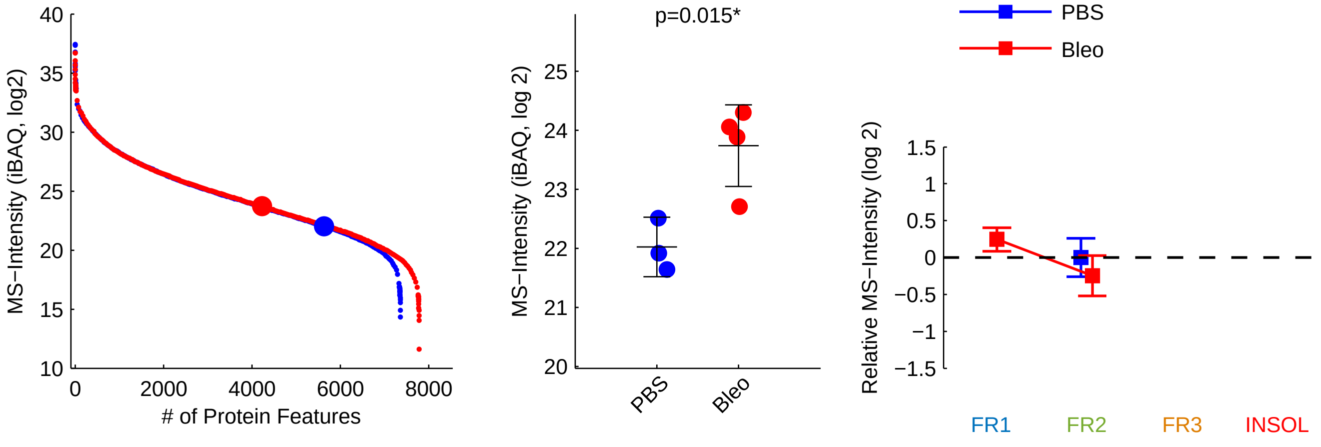

Q9WTY4 − Aqp5 (id: 8446)

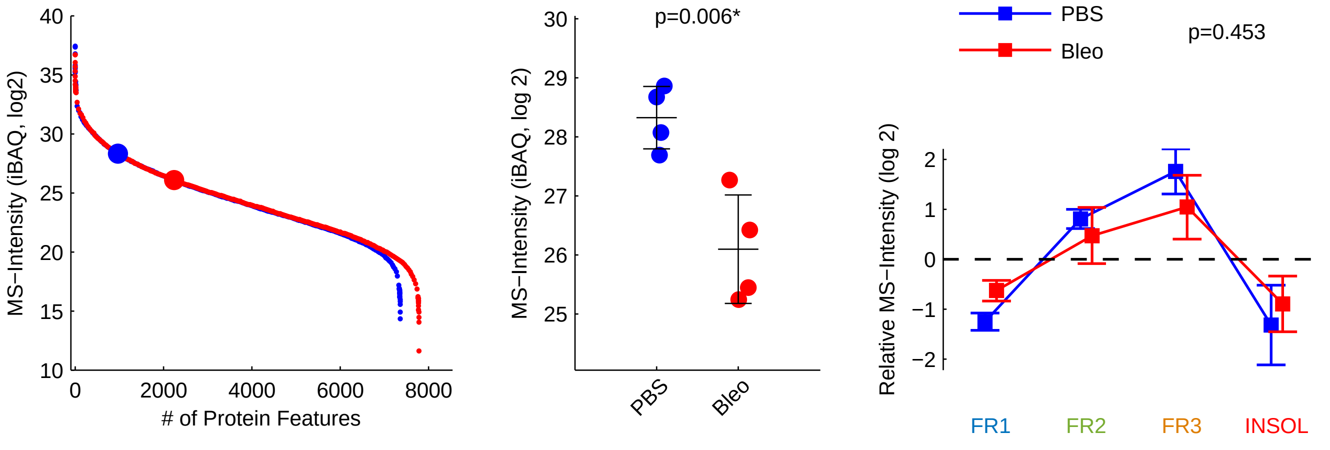

Q9WUA3 − Pfkp (id: 8456)

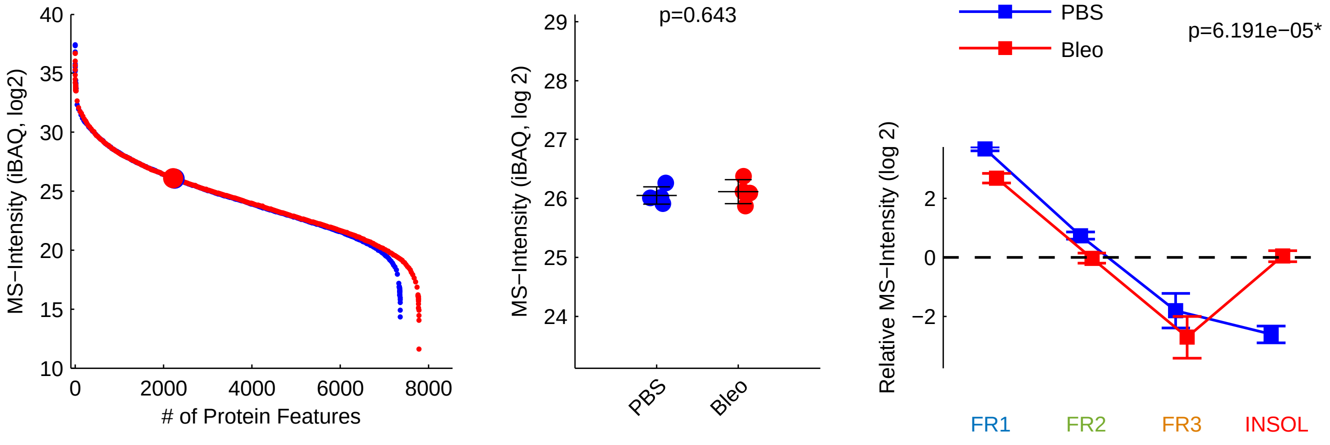

Q9WUD0 − Cyp2b10 (id: 8460)

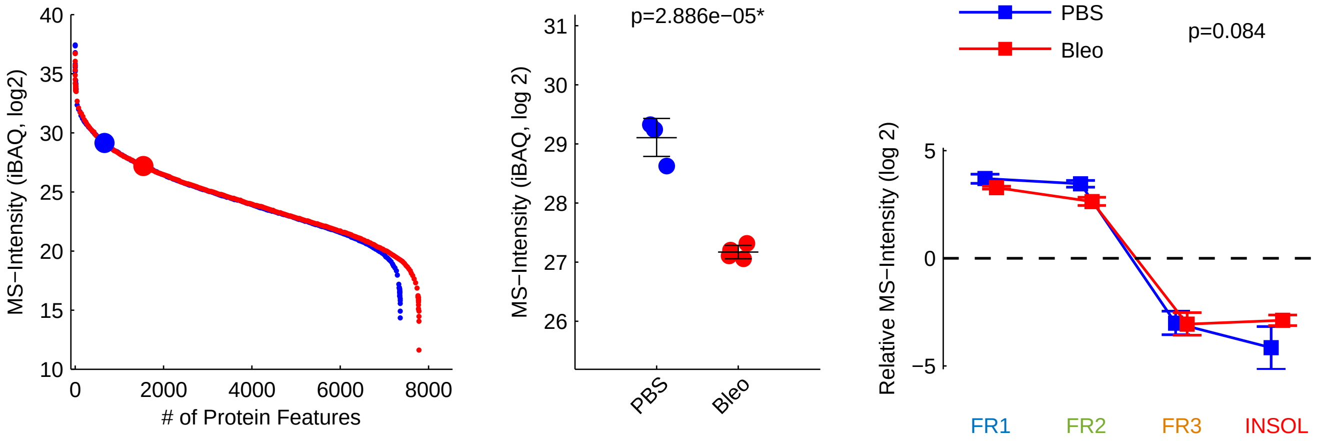

Q9WUM5 − Suclg1 (id: 8470)

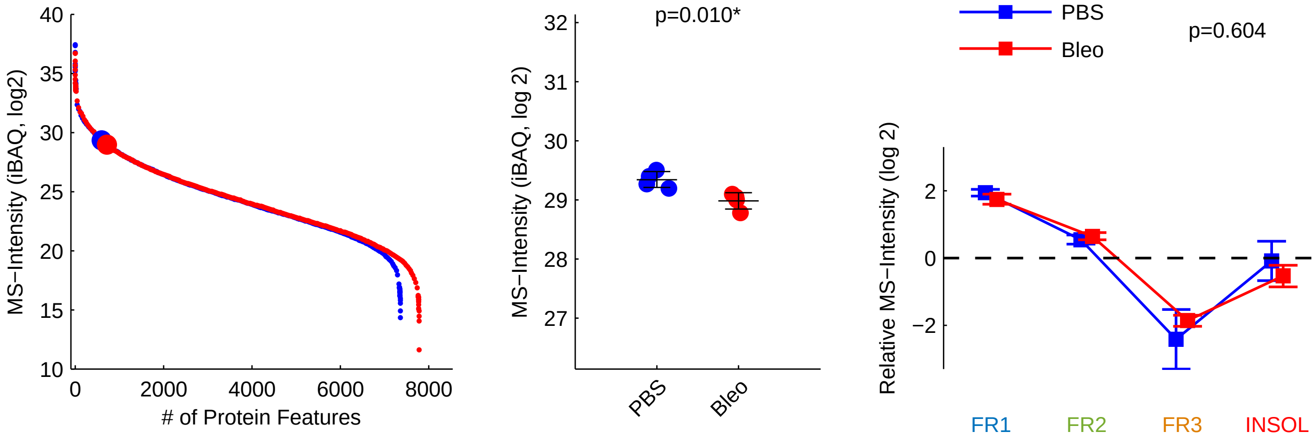

Q9WUP0 − Ramp2 (id: 8472)

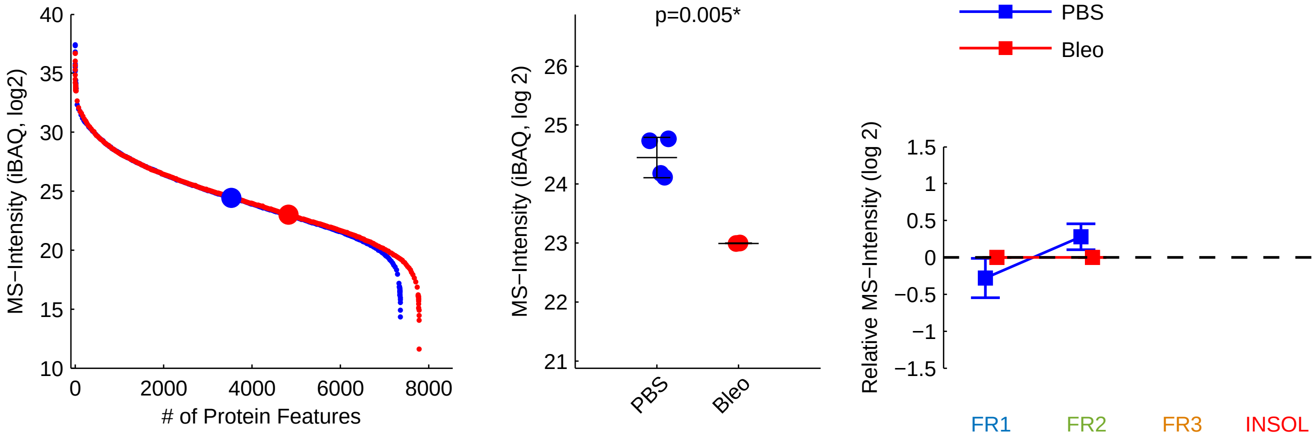

Q9WUP7 − Uchl5 (id: 8473)

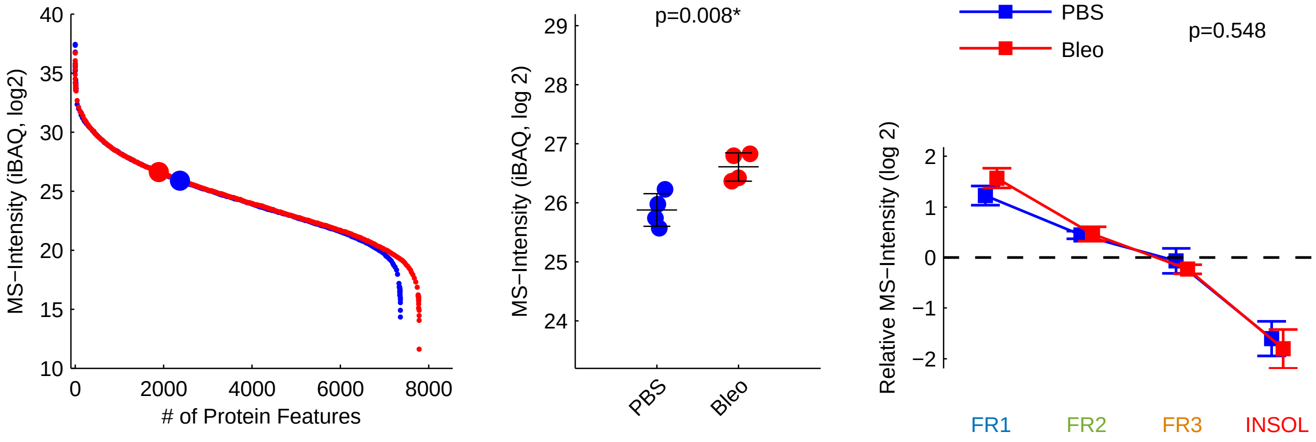

Q9WUU7 − Ctsz (id: 8479)

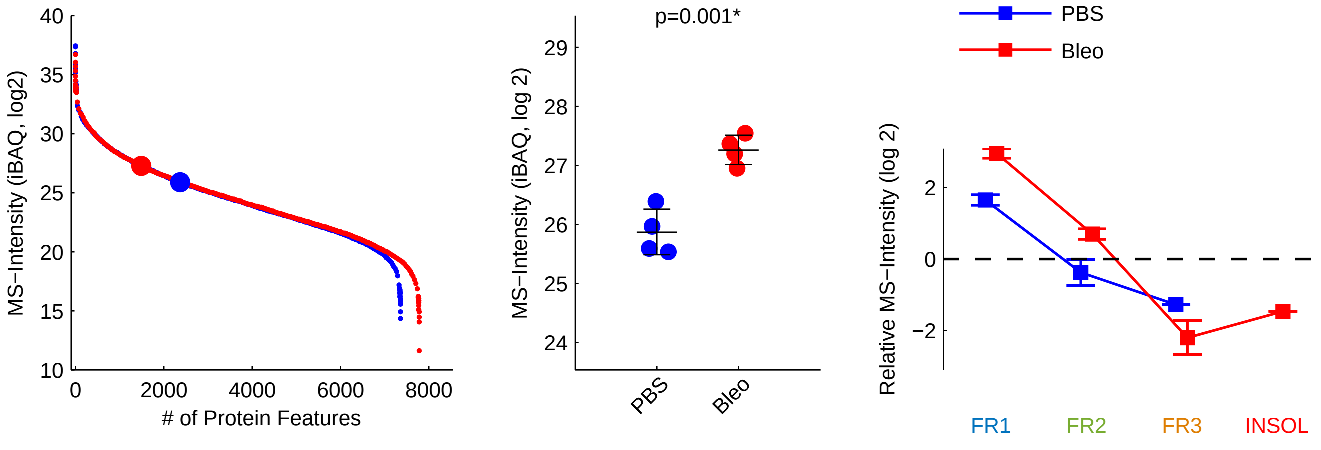

Q9WVE8 − Pacsin2 (id: 8504)

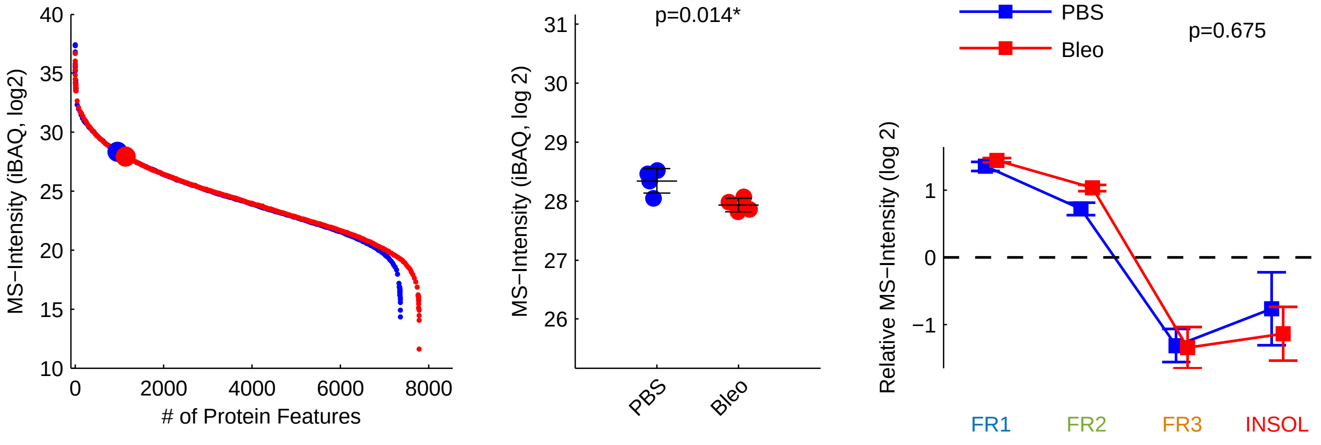

Q9WVJ2 − Psmd13 (id: 8511)

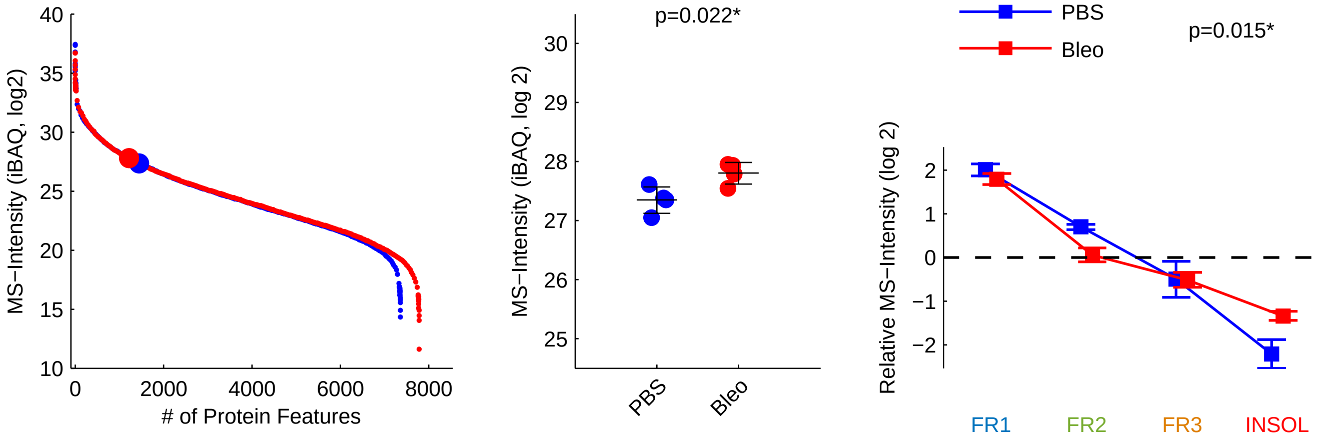

Q9WVK4 − Ehd1 (id: 8513)

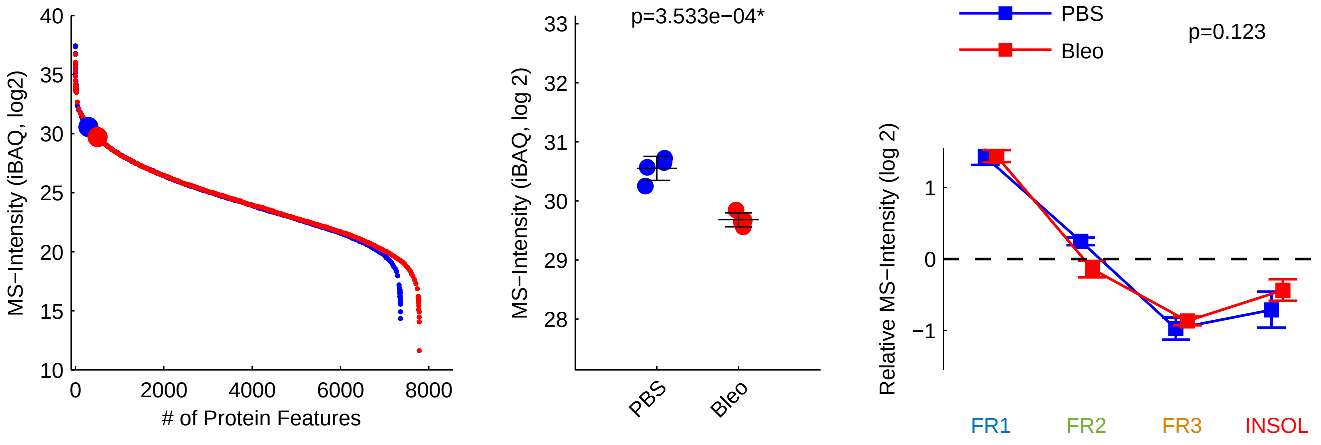

Q9WVL0 − Gstz1 (id: 8514)

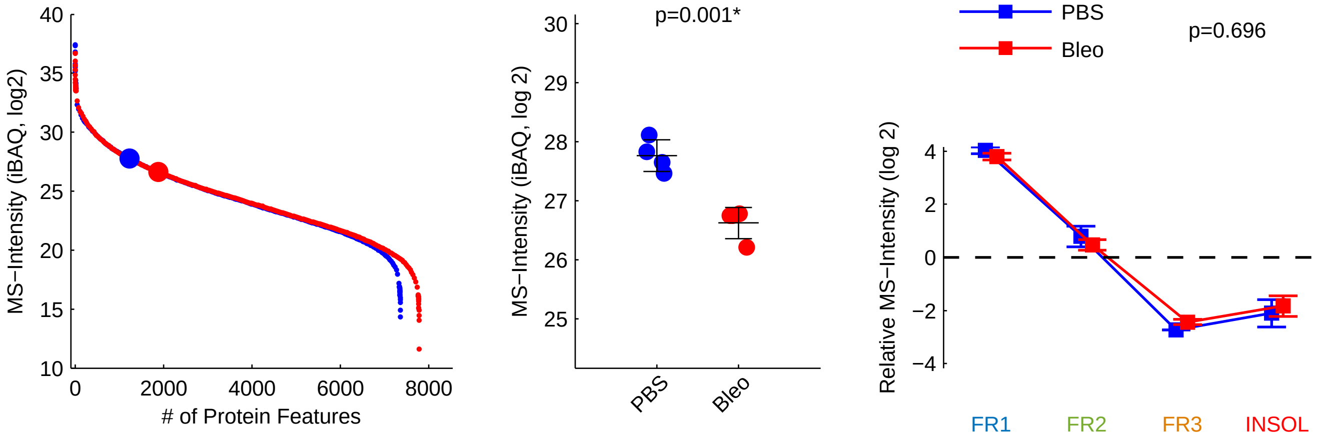

Q9WVP1 − Ap1m2 (id: 8519)

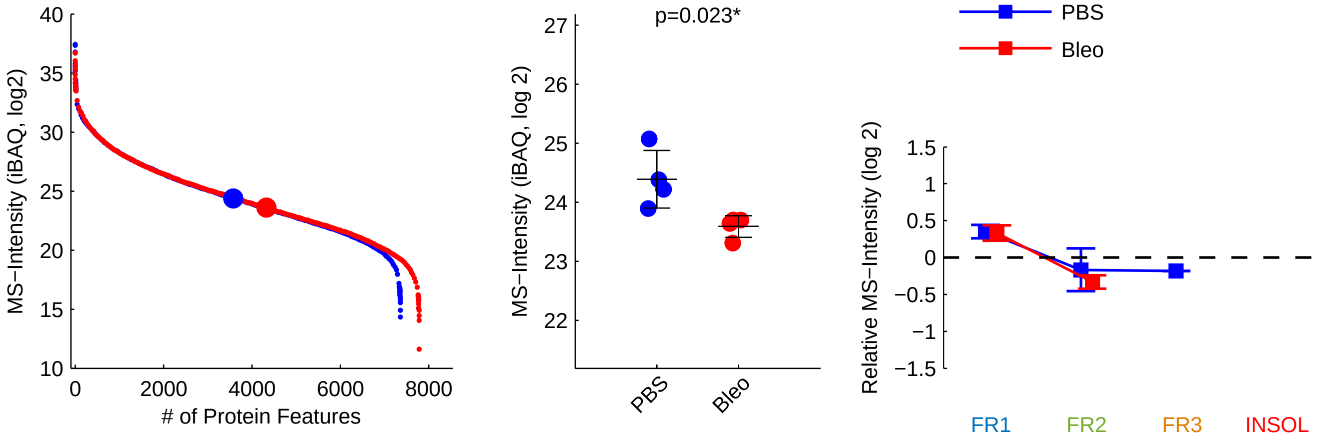

Q9Z0J1 − Reck (id: 8537)

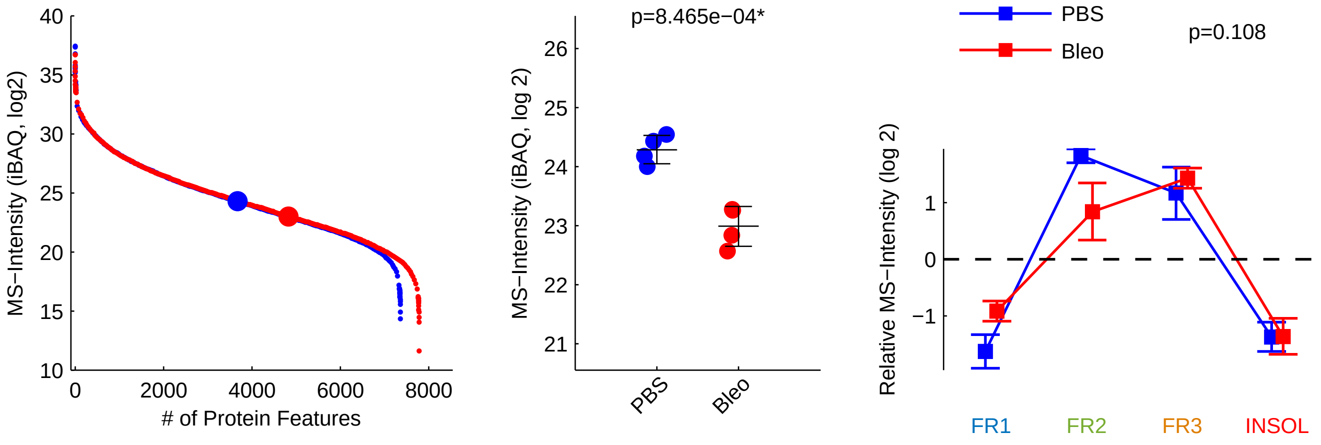

Q9Z0M5 − Lipa (id: 8541)

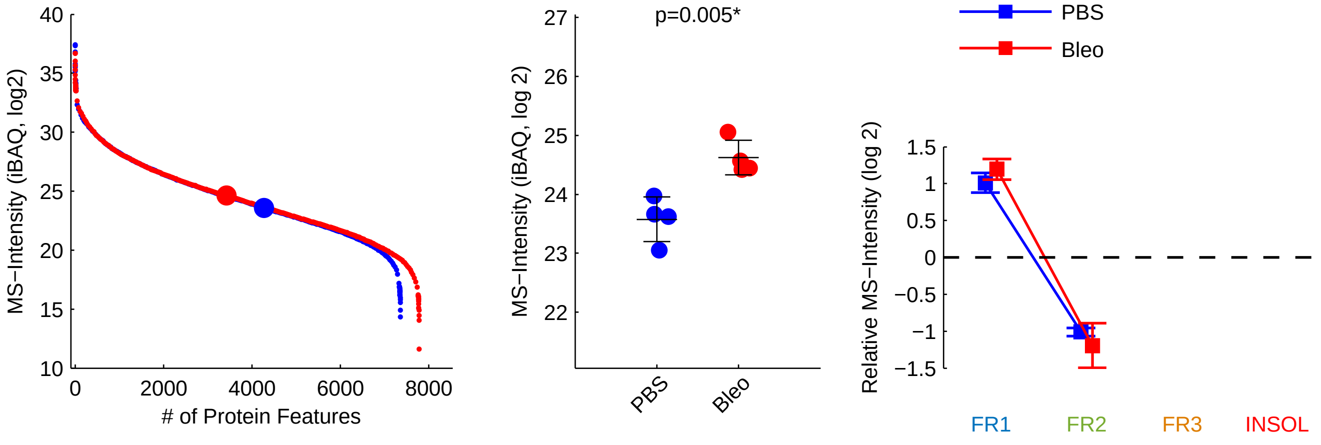

Q9Z0N1 − Eif2s3x (id: 8542)

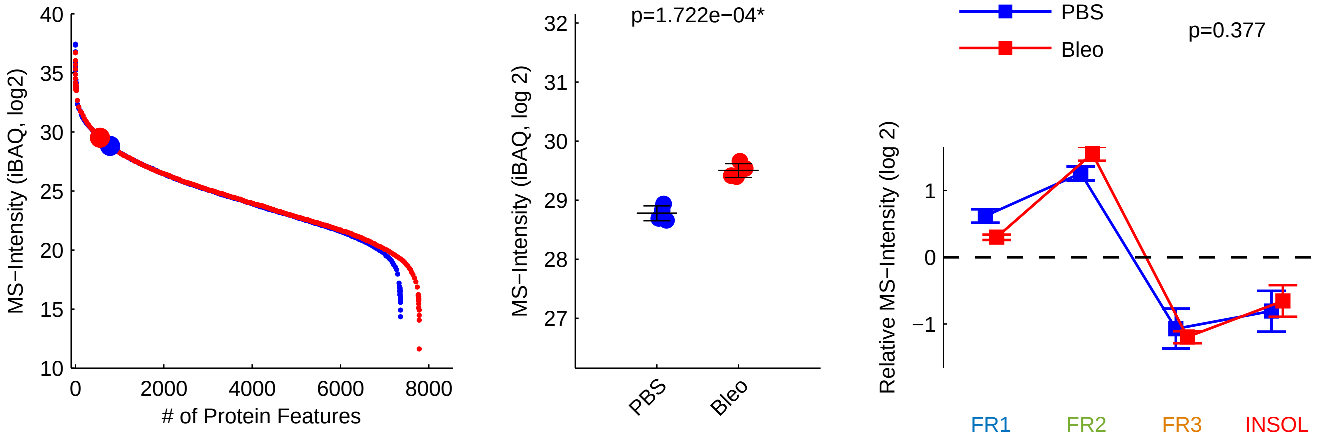

Q9Z0P4 − Palm (id: 8543)

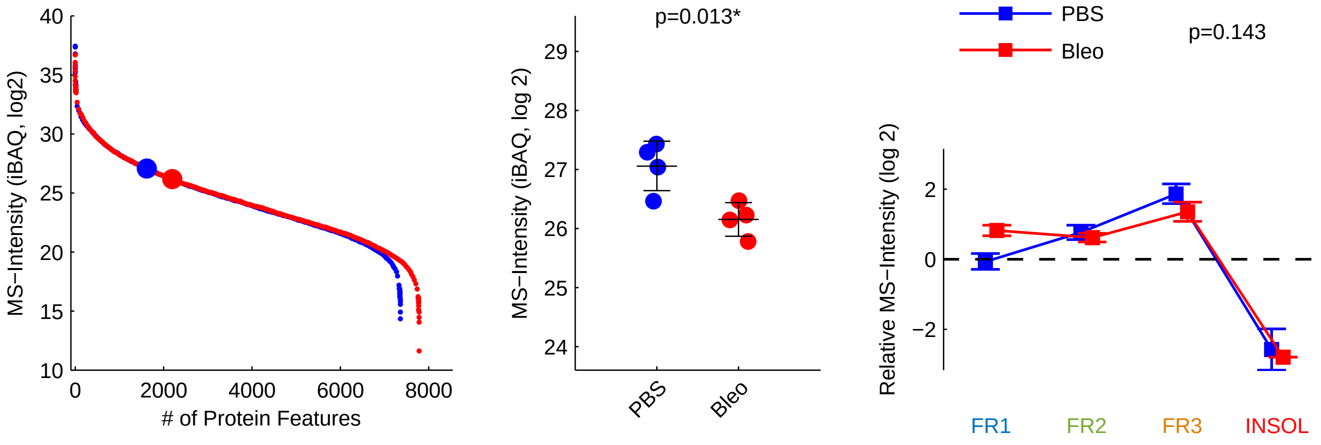

Q9Z0P5 − Twf2 (id: 8544)

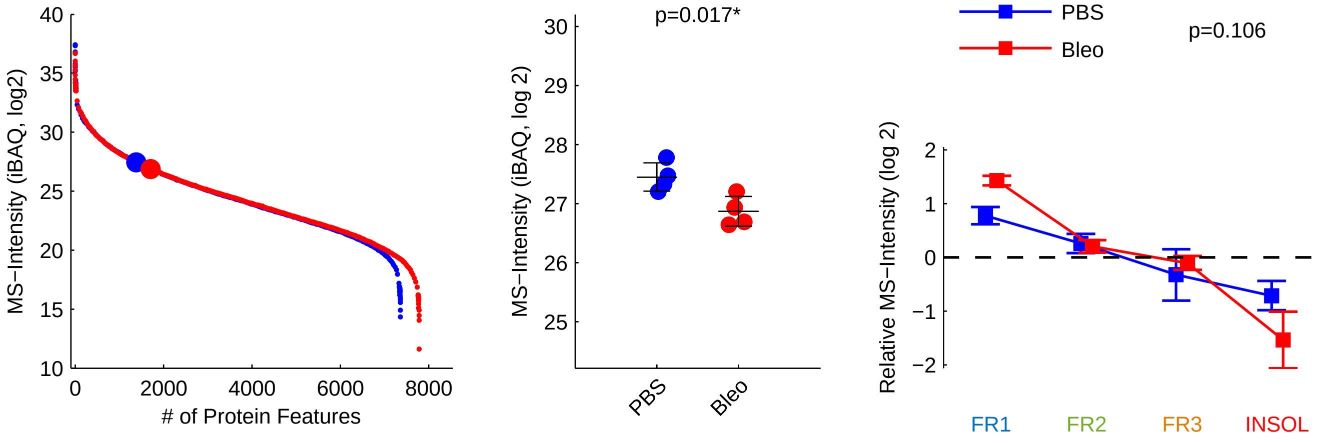

Q9Z0R9 − Fads2 (id: 8546)

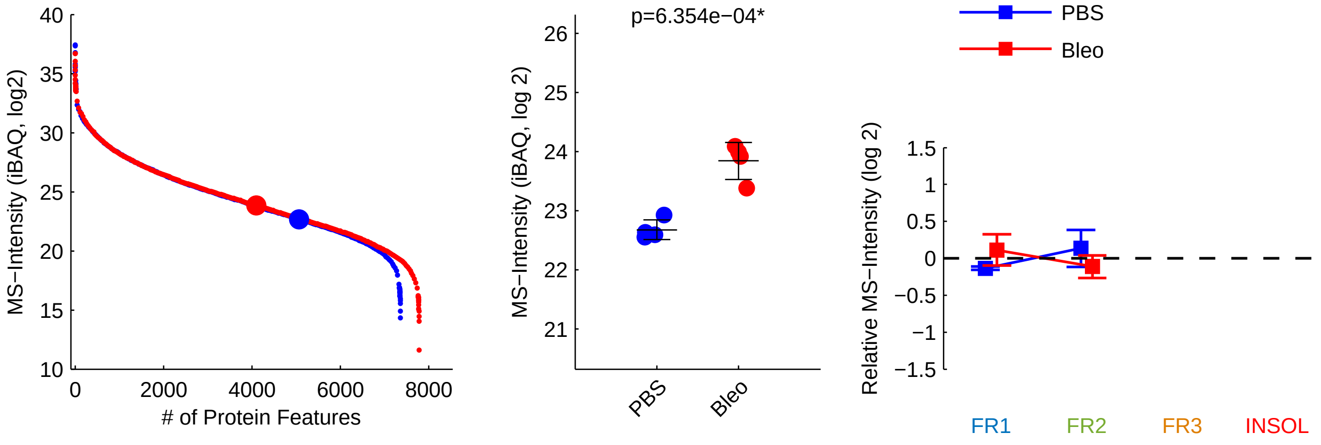

Q9Z0U1 − Tjp2 (id: 8549)

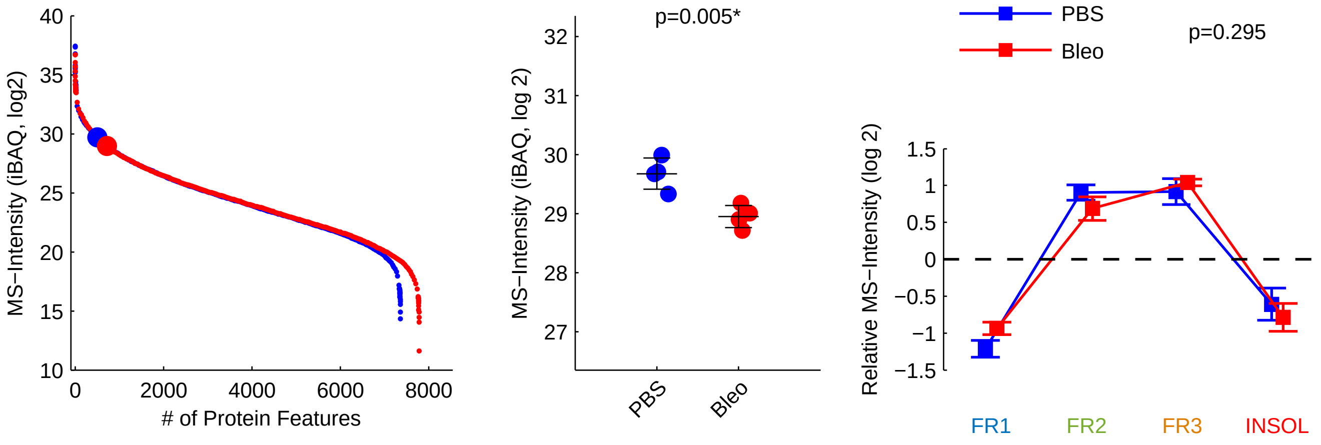

Q9Z0X4 − Pde3a (id: 8553)

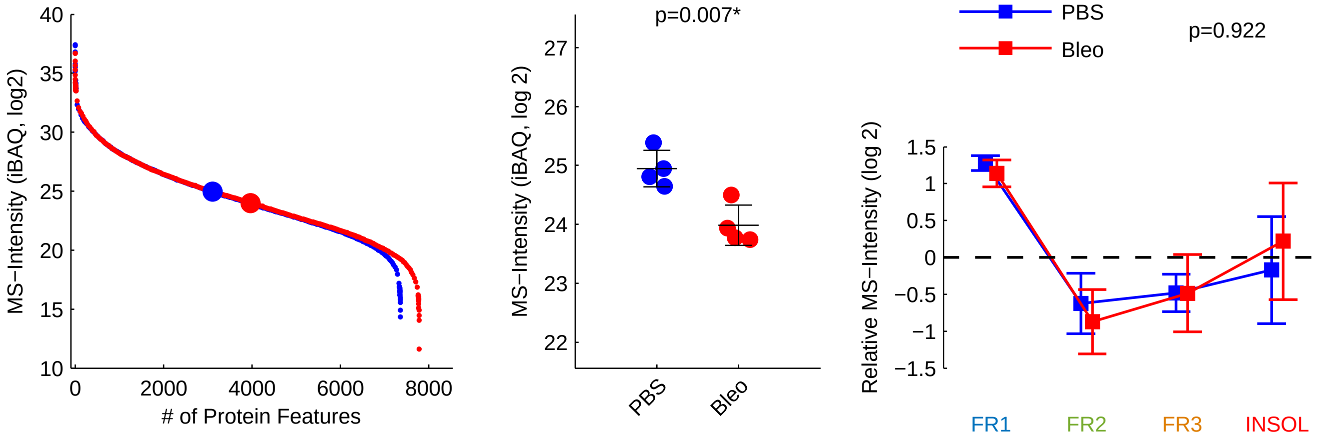

Q9Z100 − Cpxm1 (id: 8556)

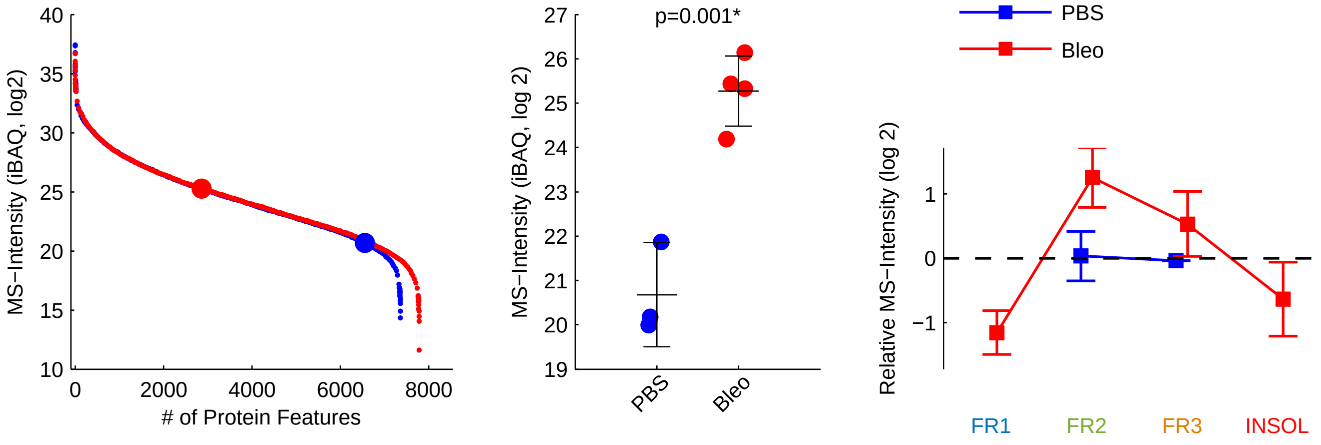

Q9Z110 − Aldh18a1 (id: 8557)

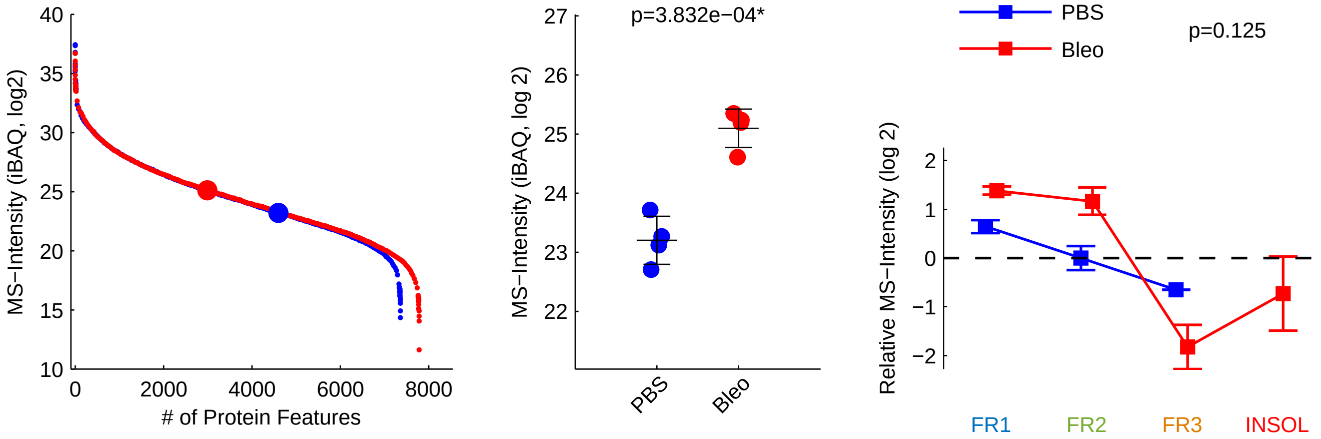

Q9Z1A1 − Tfg (id: 8566)

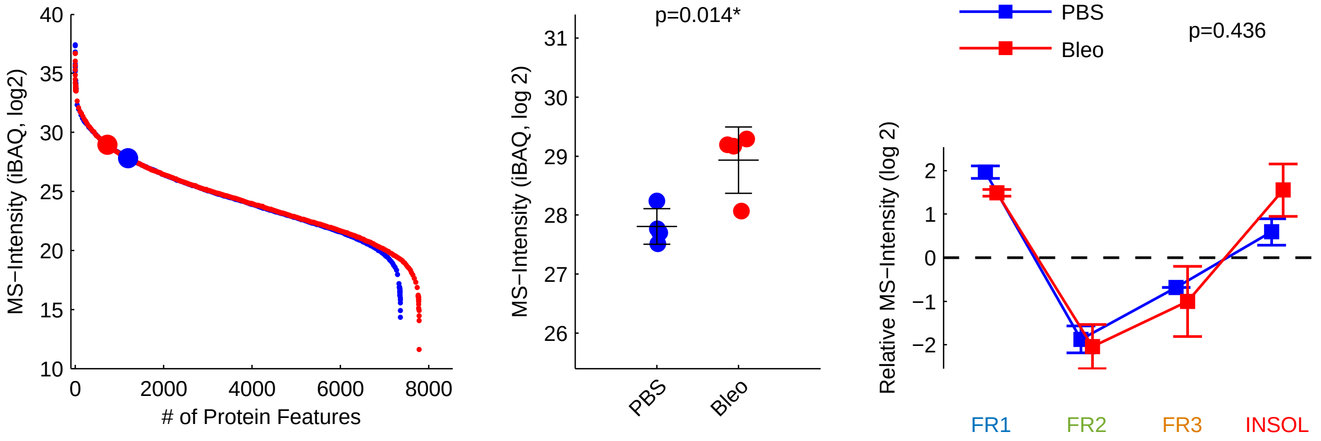

Q9Z1D1 − Eif3g (id: 8572)

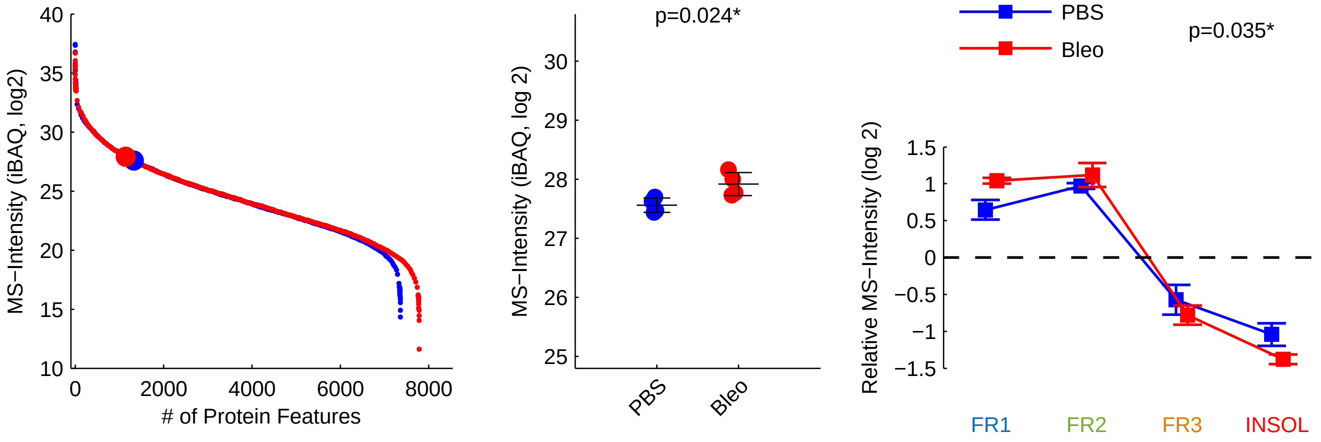

Q9Z1G3 − Atp6v1c1 (id: 8574)

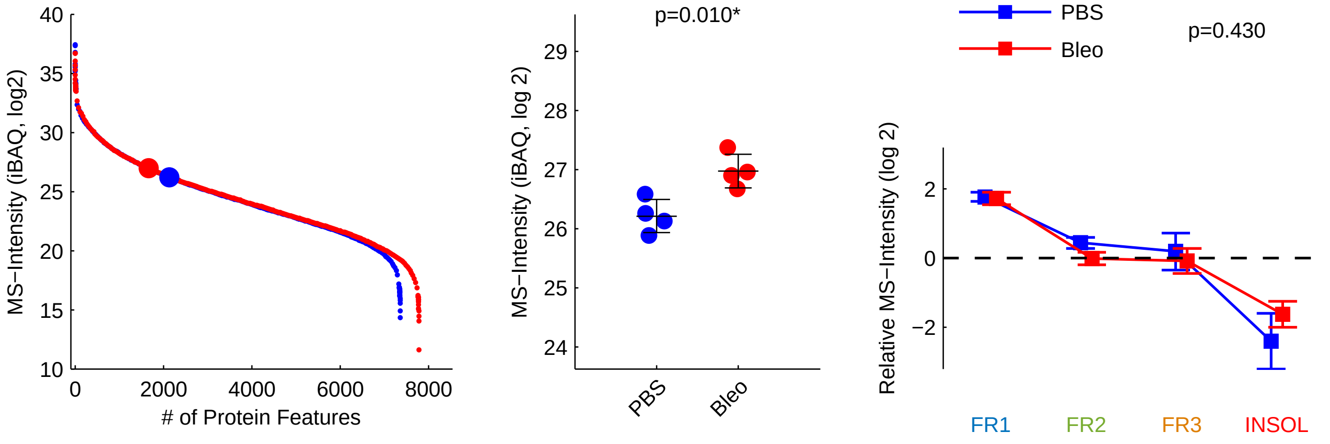

Q9Z1M8 − Ik (id: 8579)

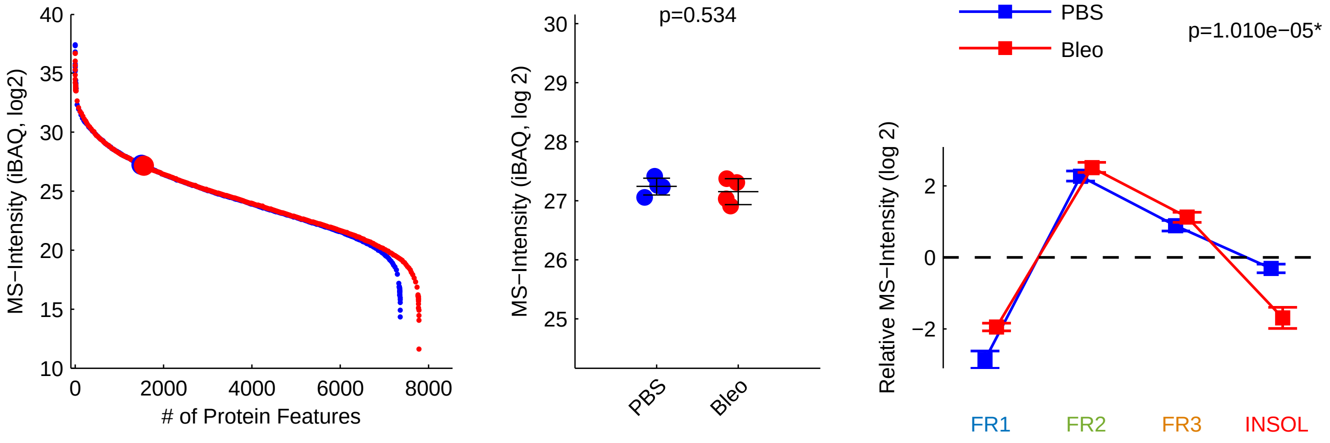

Q9Z1P7 − Kank3 (id: 8583)

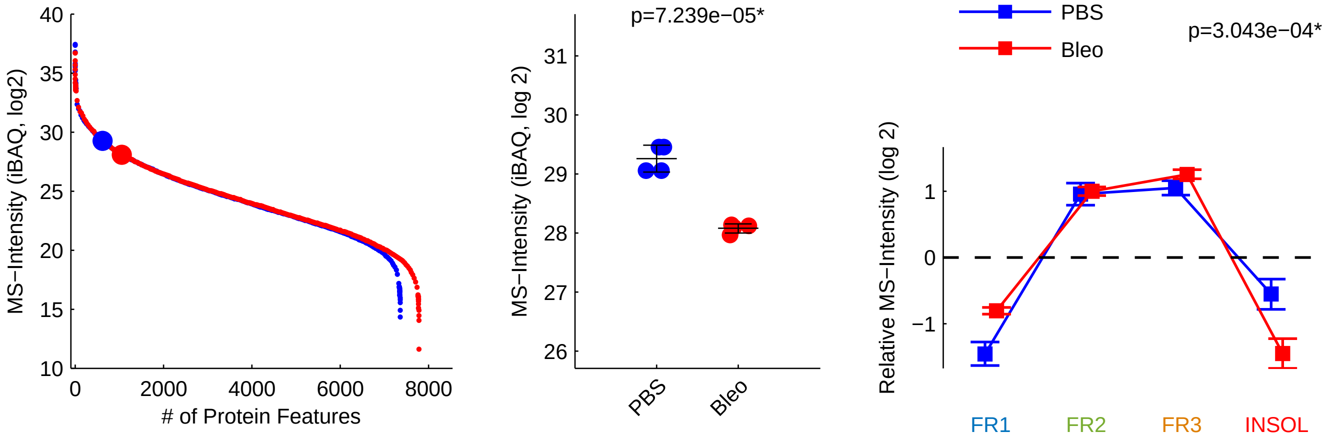

Q9Z1Q2 − Abhd16a (id: 8584)

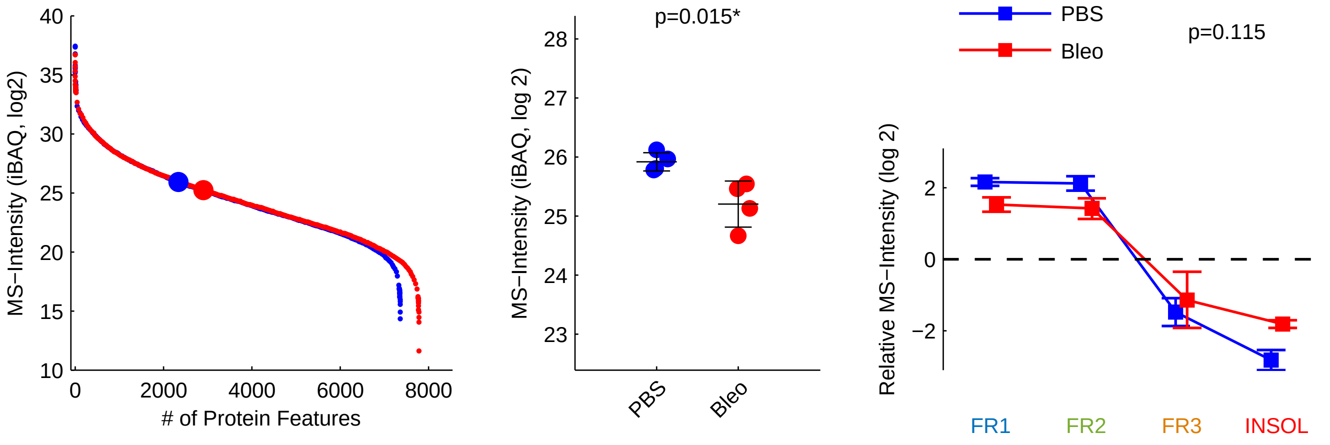

Q9Z1Q9 − Vars (id: 8586)

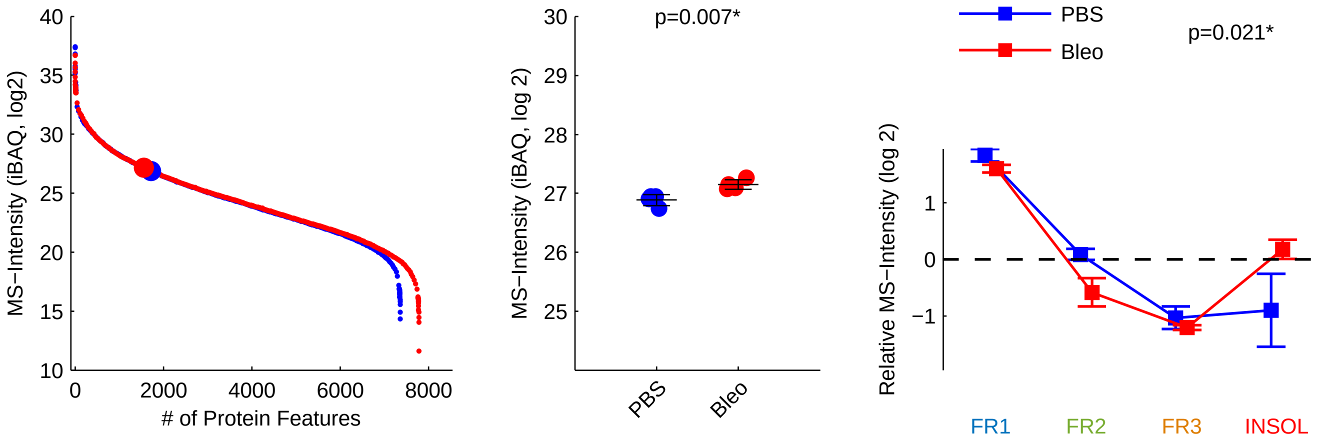

Q9Z1X4−3 − Ilf3 (id: 8591)

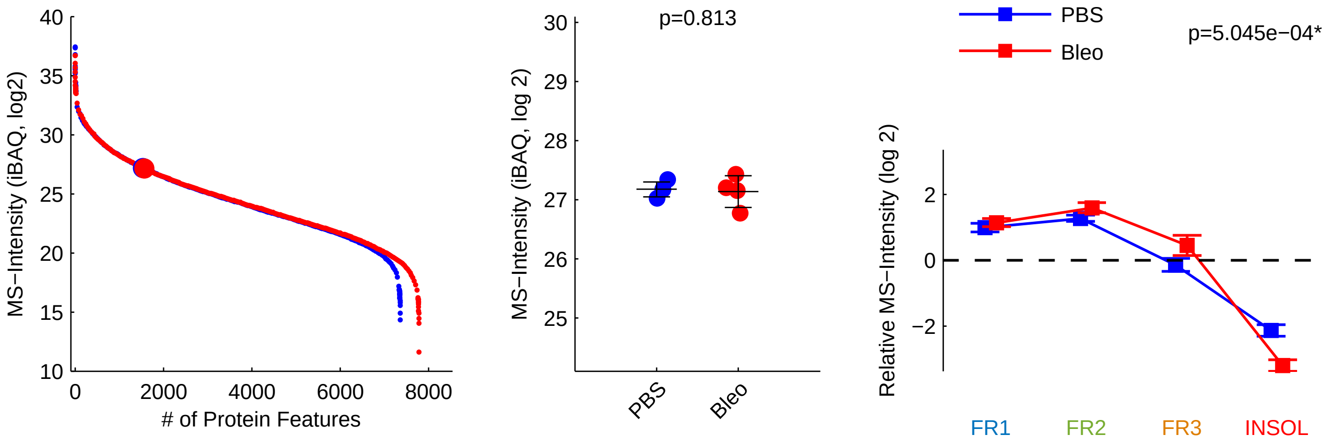

Q9Z247 − Fkbp9 (id: 8598)

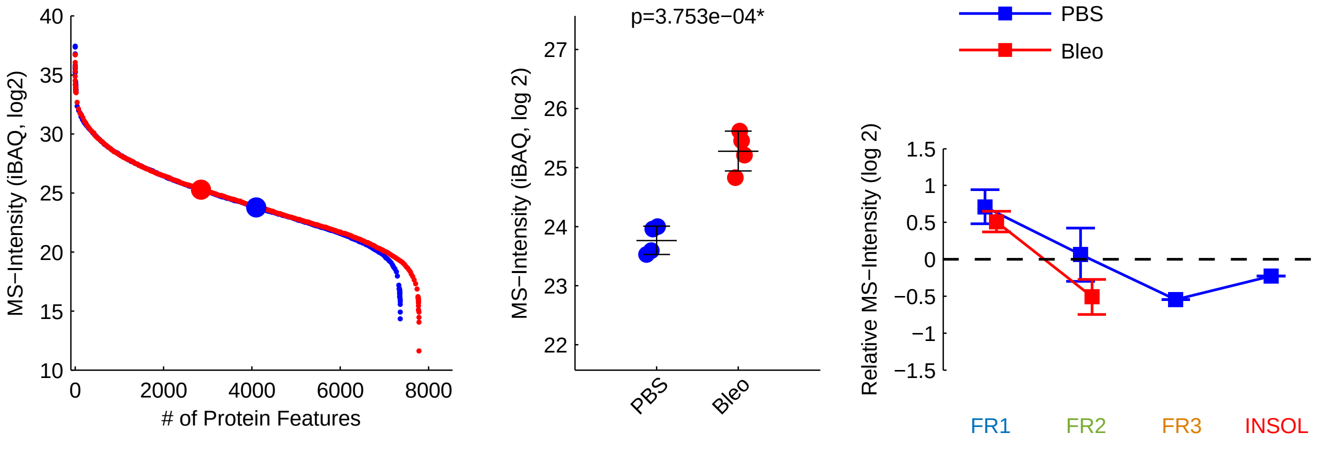

Q9Z277 − Baz1b (id: 8600)

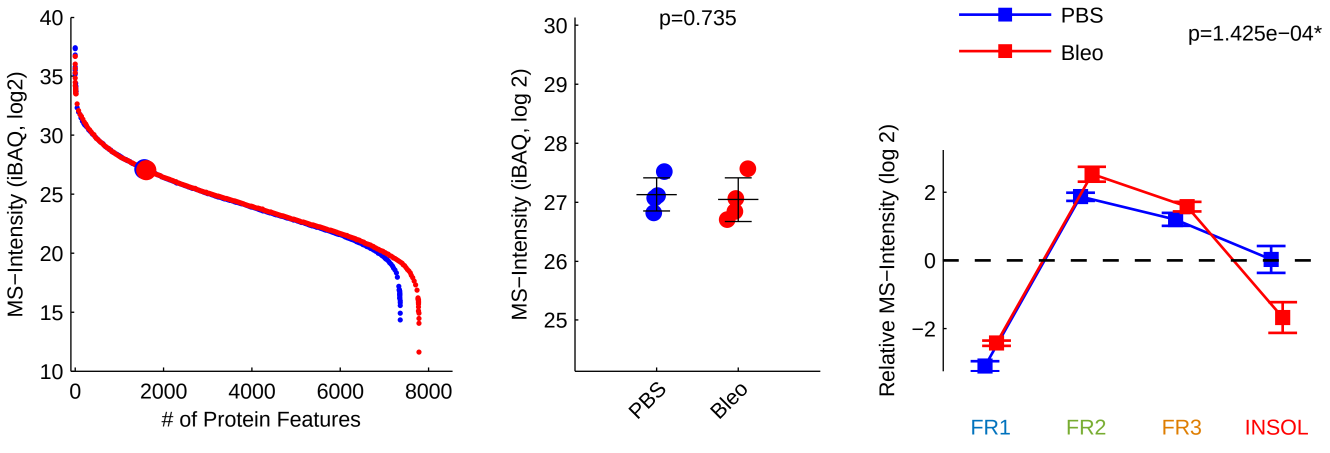

Q9Z2D0 − Mtmr9 (id: 8603)

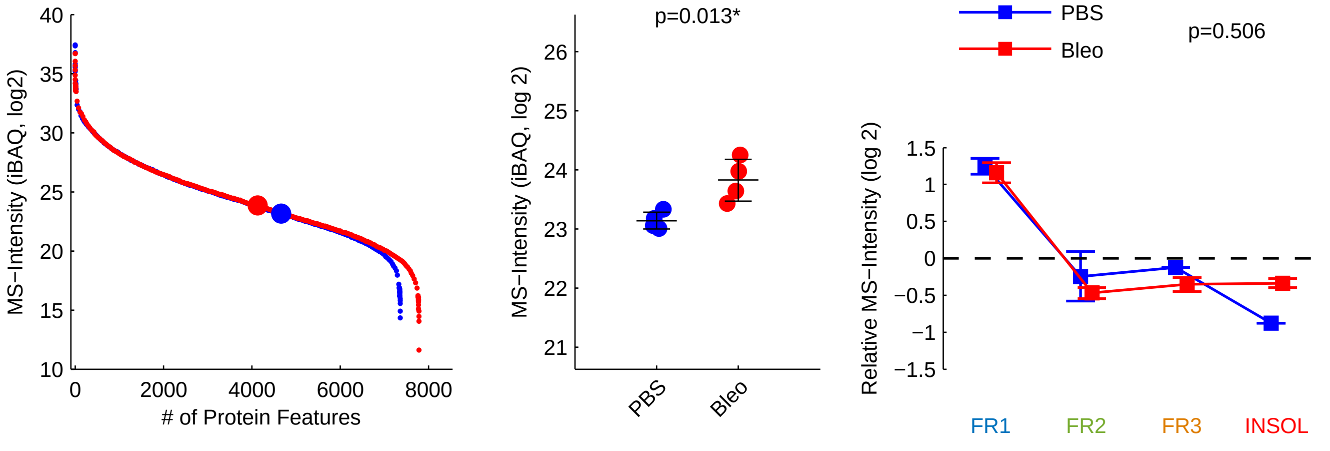

Q9Z2D6−2 − Mecp2 (id: 8605)

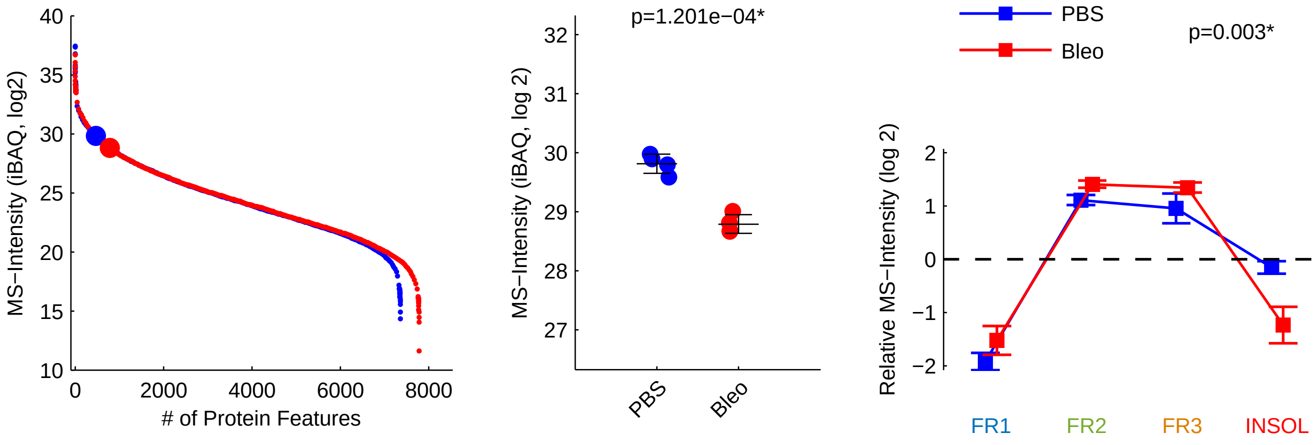

Q9Z2I8 − Suclg2 (id: 8614)

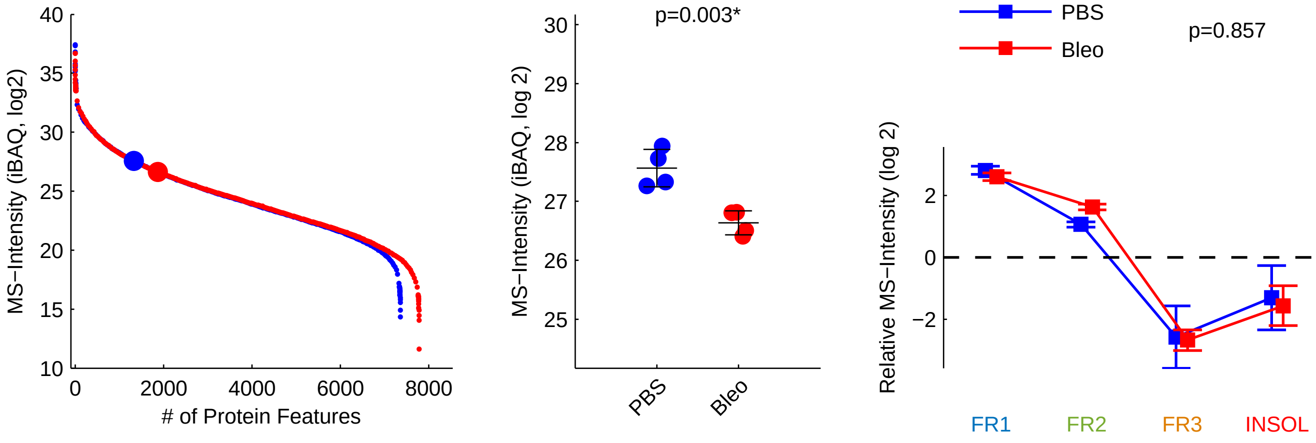

Q9Z2L7 − Crlf3 (id: 8617)

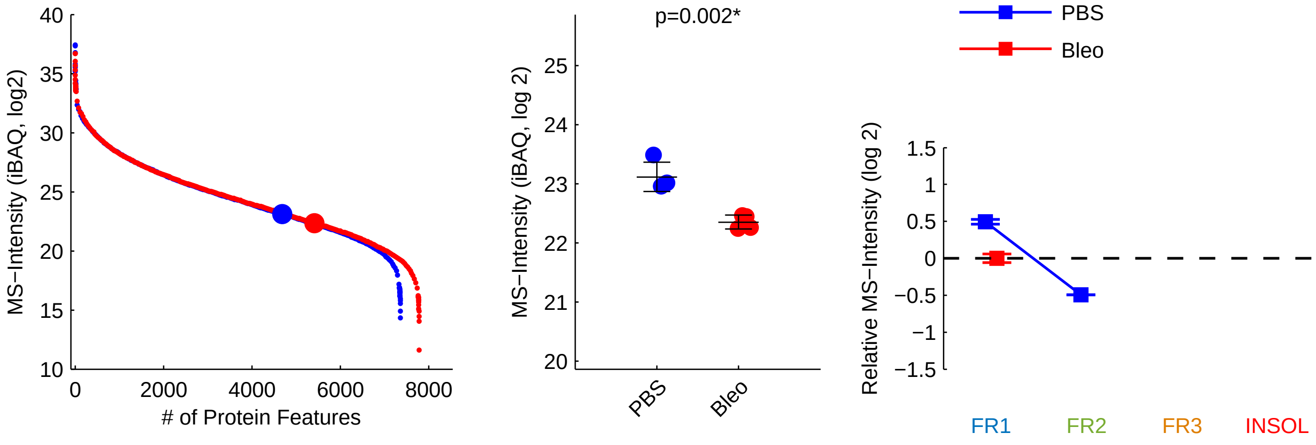

Q9Z2M7 − Pmm2 (id: 8619)

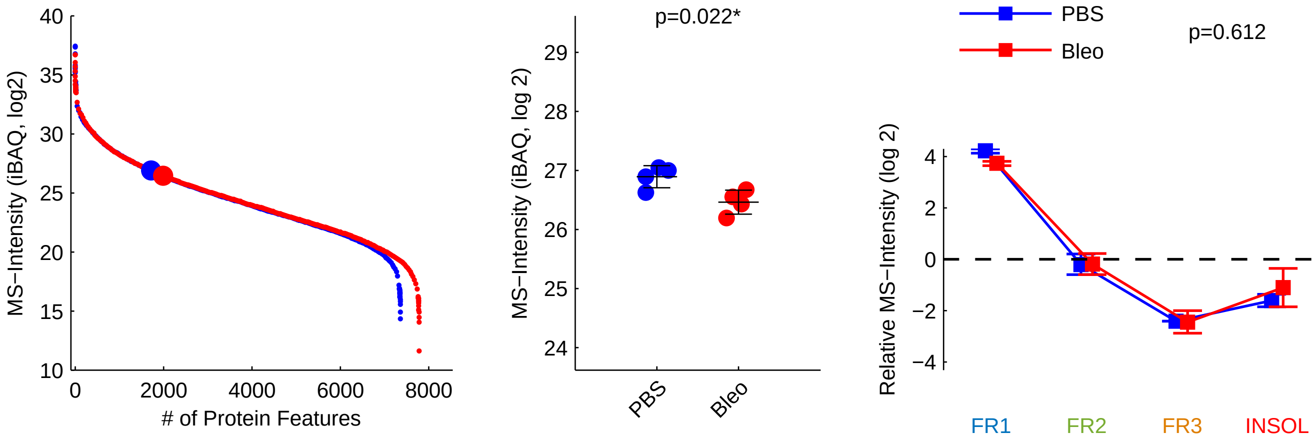

Q9Z2X1 − Hnrnpf (id: 8630)

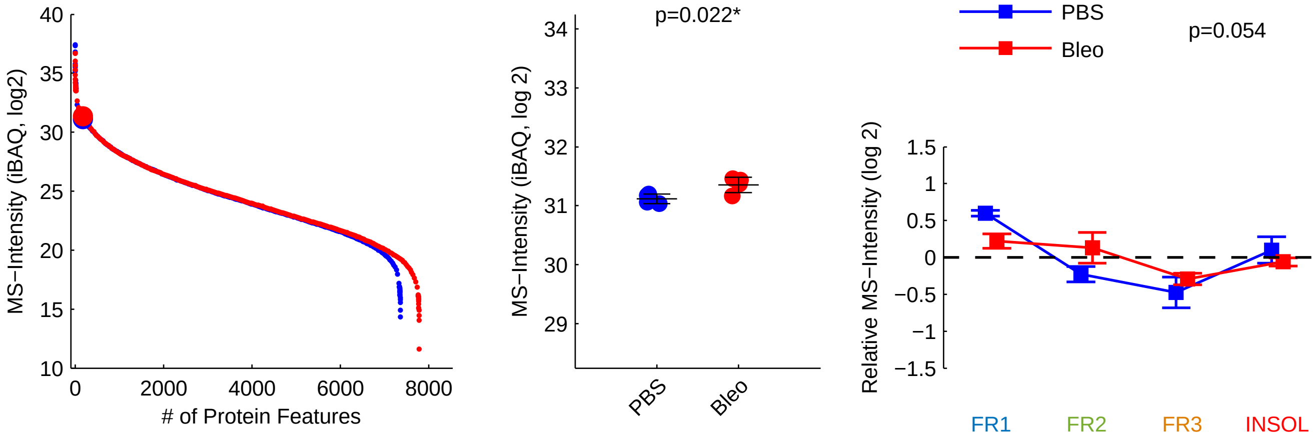

Q9Z2Z6 − Slc25a20 (id: 8636)

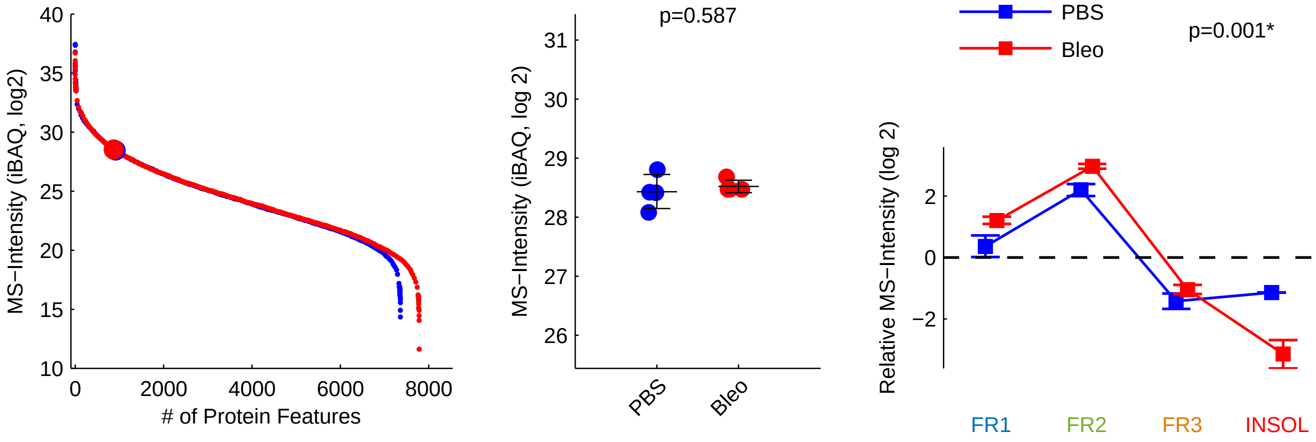

Supplement: Supplementary file 2 [file Solubility plots_TableEV1.pdf › Table EV1/Solubility plots_TableEV1.pdf]
