# Supplementary material for: Time- and compartment-resolved proteome profiling of the extracellular niche in lung injury and repair
Source: Mol Syst Biol. 2015 Jul 14;11(7):819. doi: 10.15252/msb.20156123 (PMC4547847; doi:10.15252/msb.20156123)

A2A4P0 − Dhx8 (id: 10)

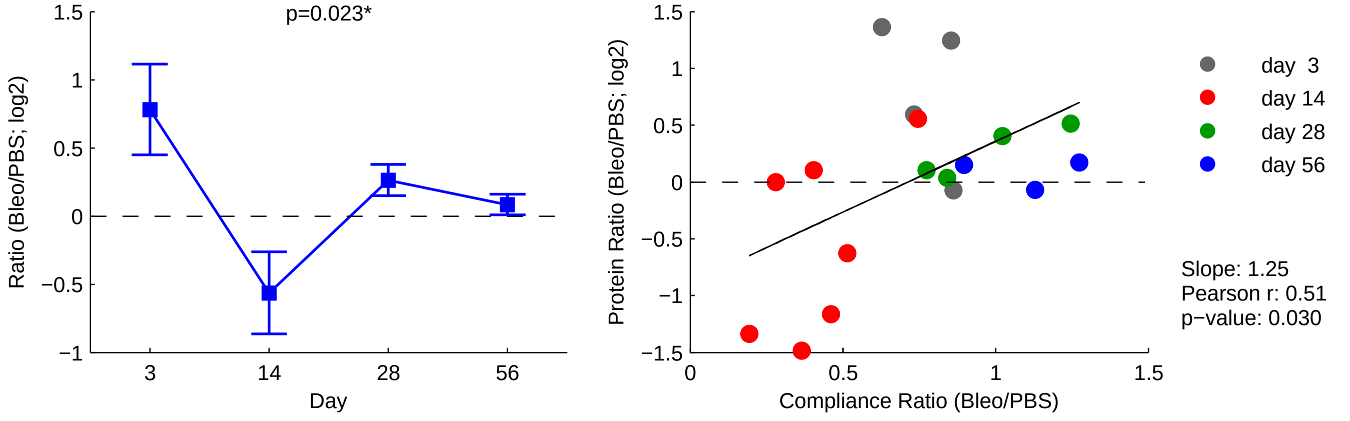

A2A547 − Rpl19 (id: 15)

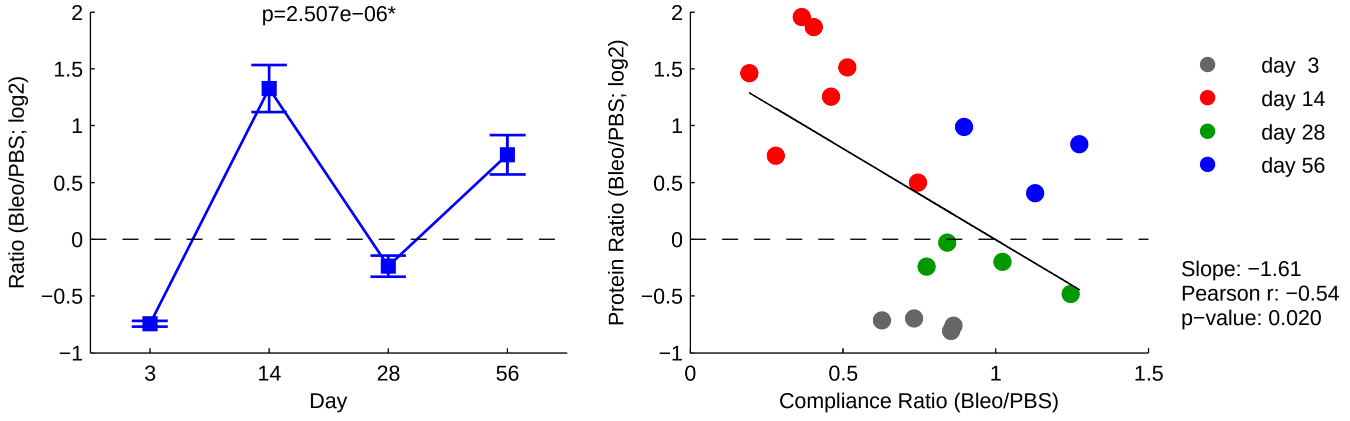

A2A5R8 − Stau1 (id: 17)

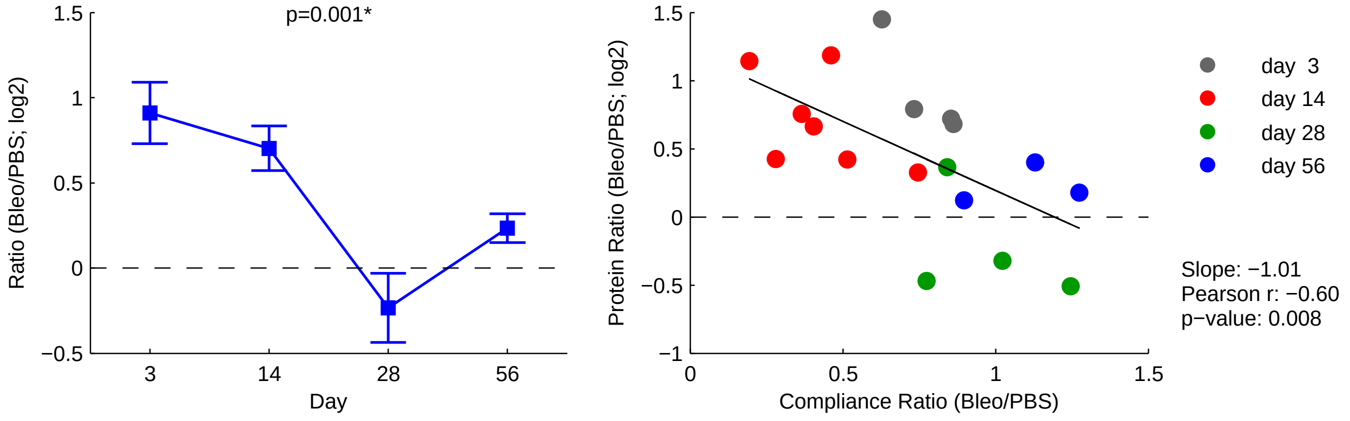

A2A5V2 − Sh3bp1 (id: 18)

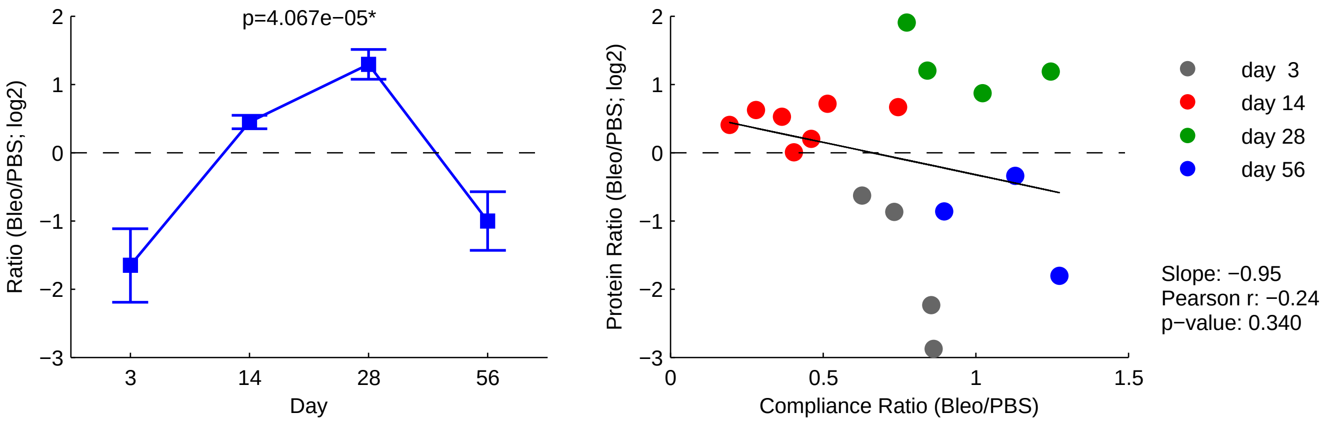

A2A654 − Bptf (id: 21)

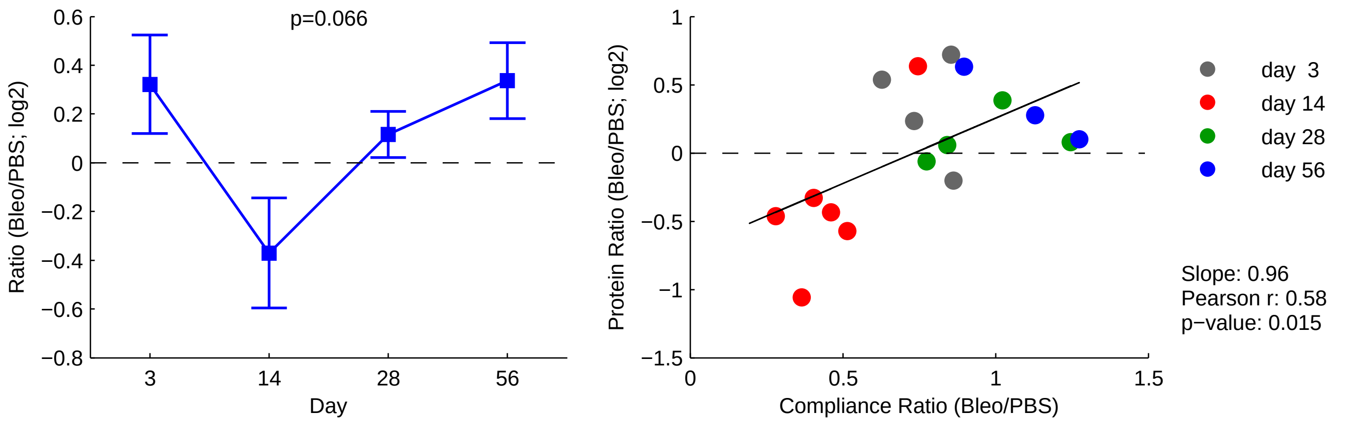

A2A6I3 − Tnnt3 (id: 25)

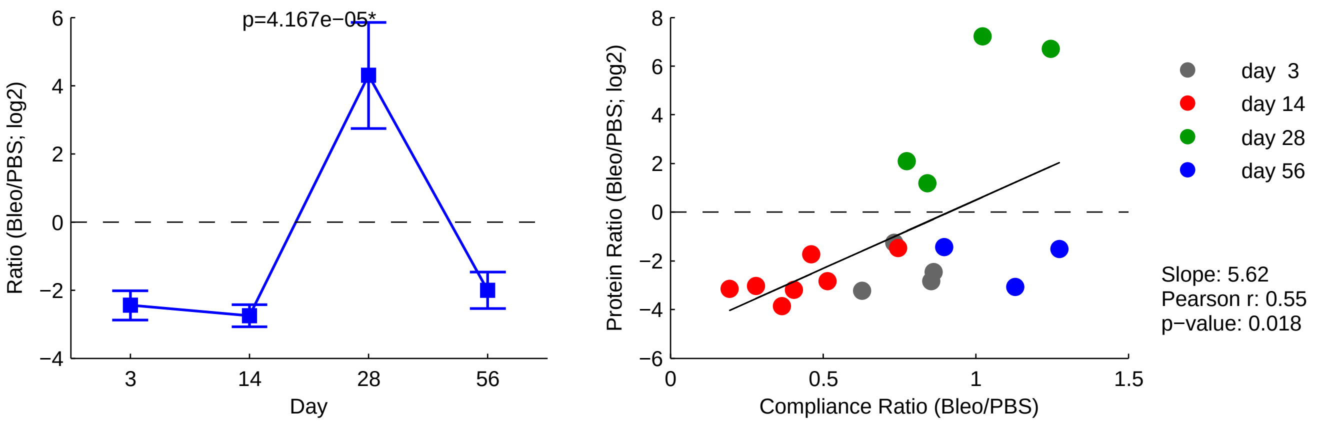

A2A6Q8 − Myl4 (id: 29)

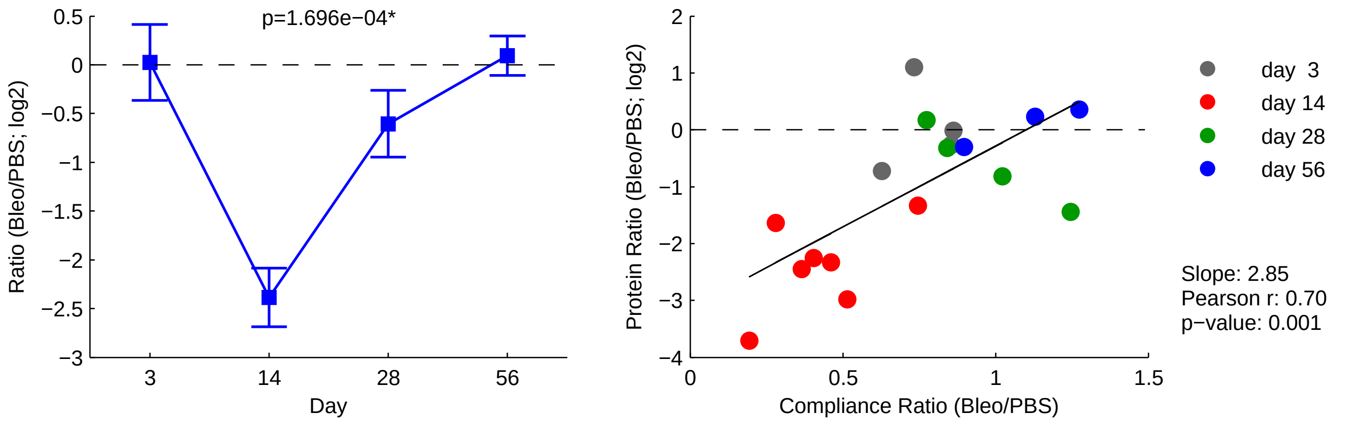

A2A7B5 − Prdm2 (id: 34)

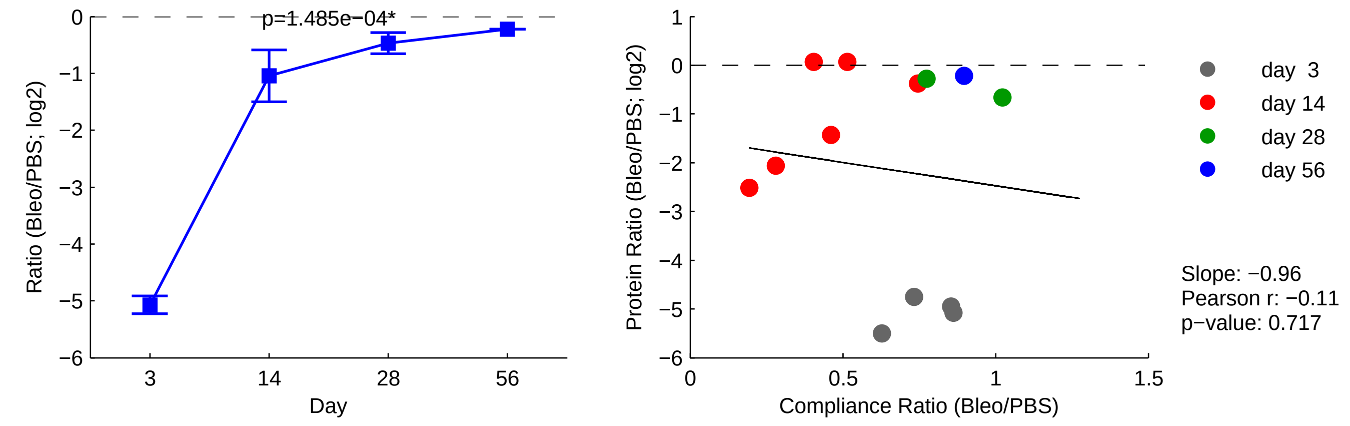

A2A7S8−2 − Kiaa1522 (id: 36)

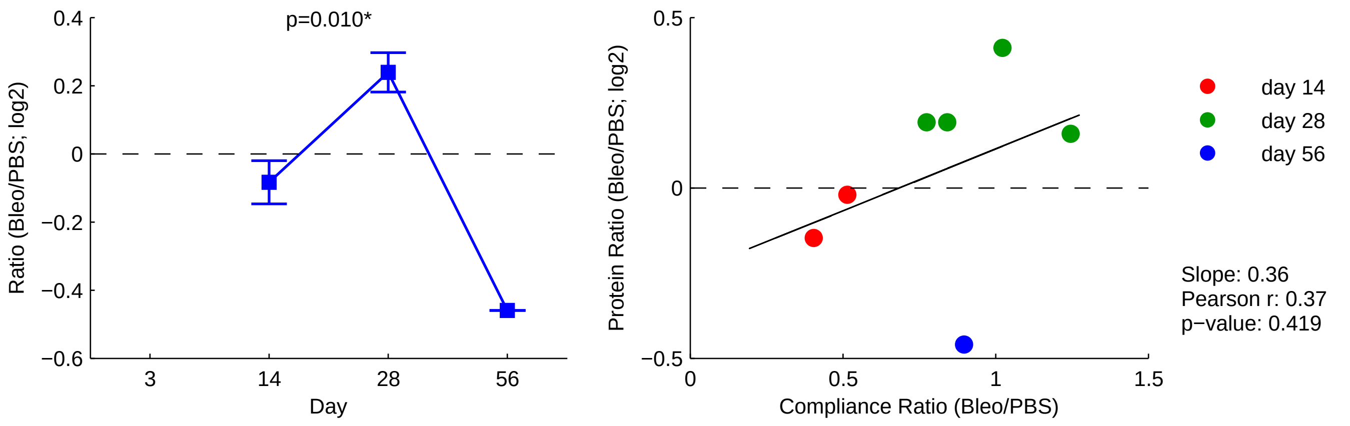

A2A838 − Epb4.1 (id: 37)

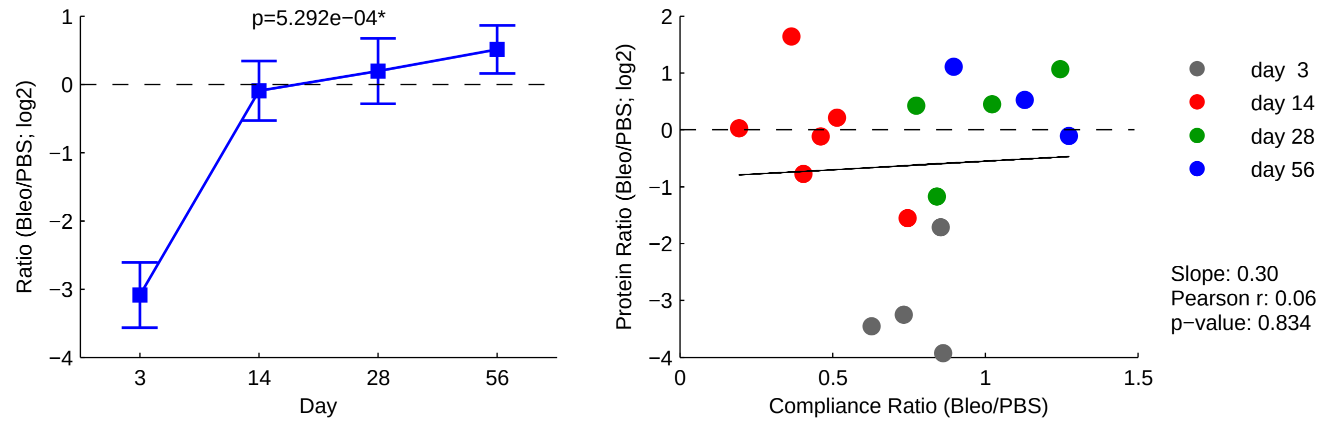

A2A863−2 − Itgb4 (id: 39)

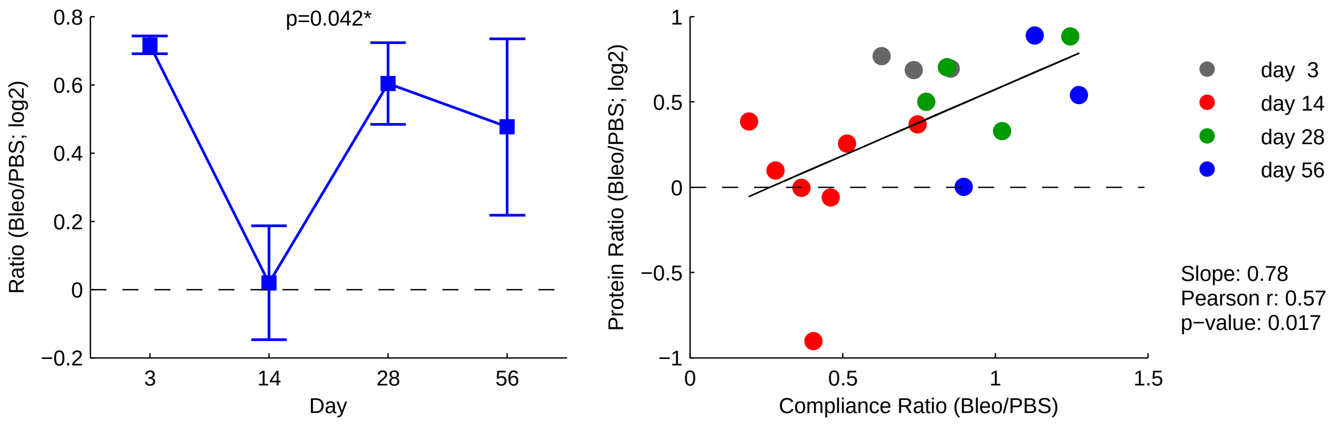

A2A8L5 − Ptprf (id: 42)

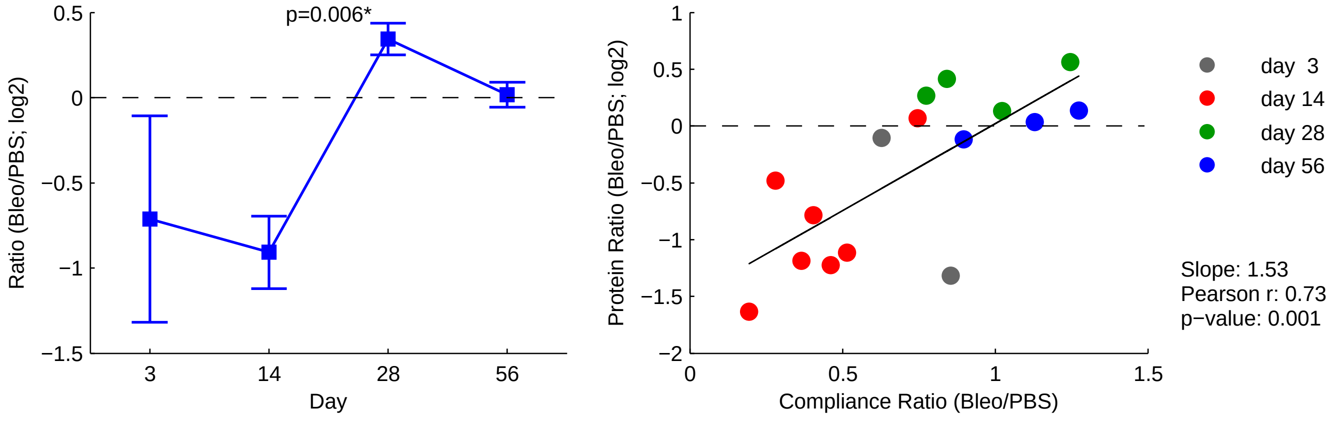

F6UHR6 − Osbpl9 (id: 47)

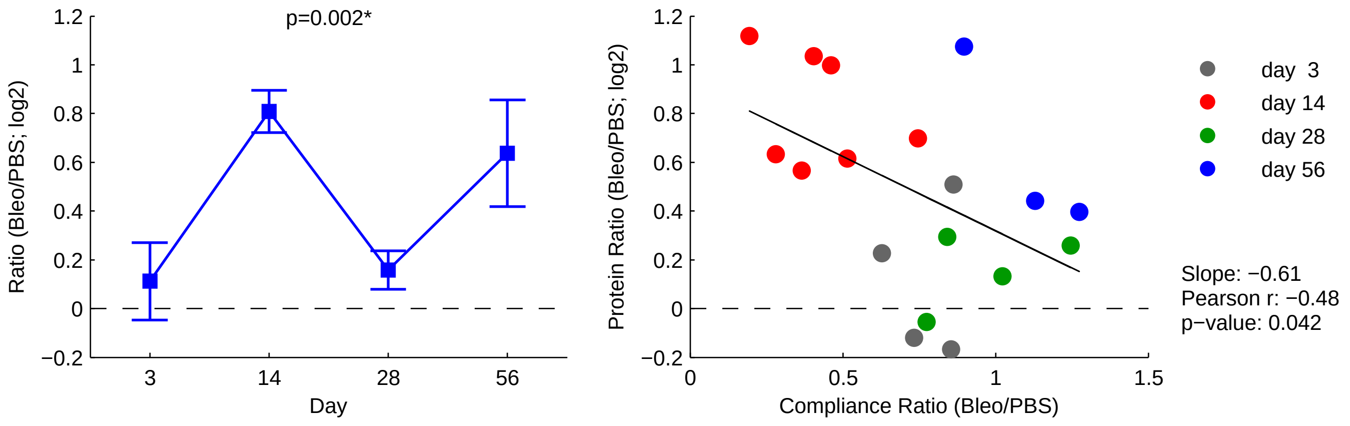

A2A9I0 − Gosr2 (id: 52)

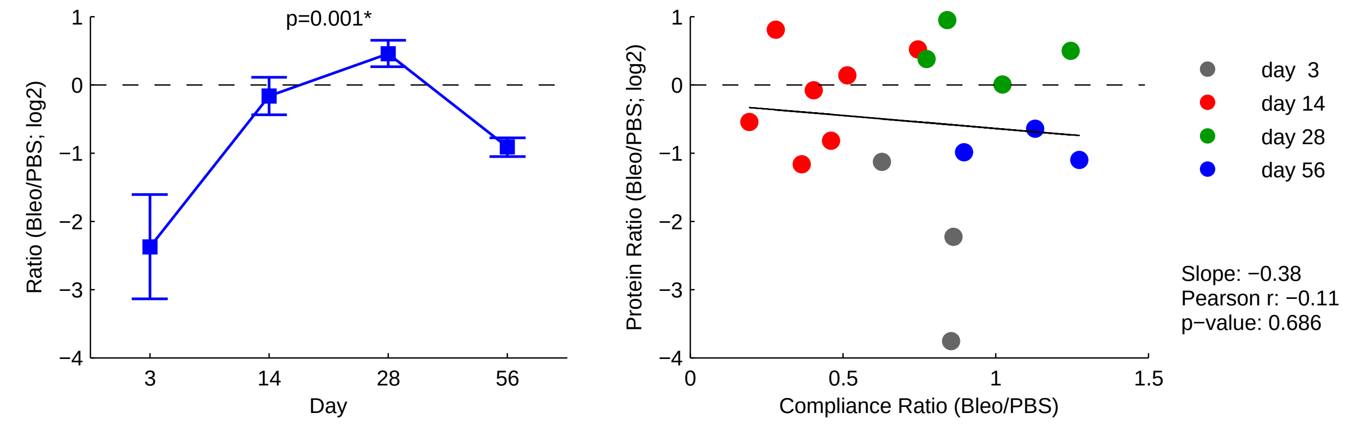

E9PX48 − Dock7 (id: 54)

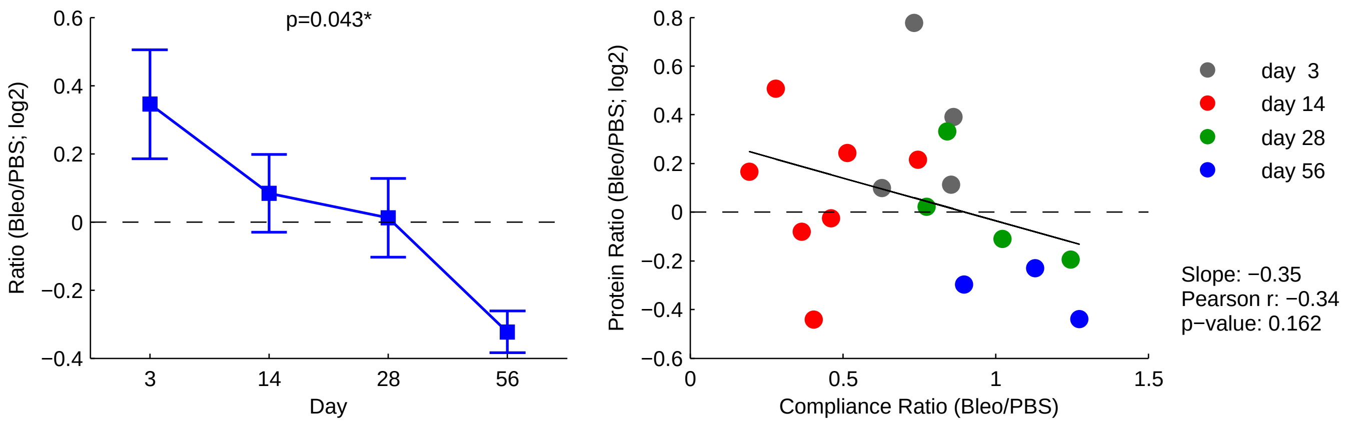

Q8BHG1 − Nrd1 (id: 56)

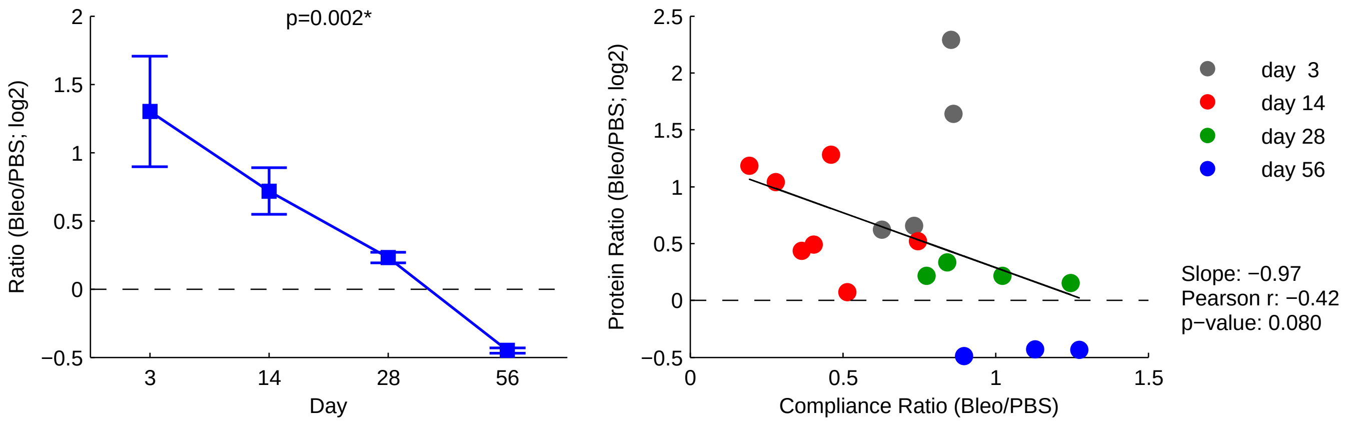

A2A9Z1 − Dmd (id: 58)

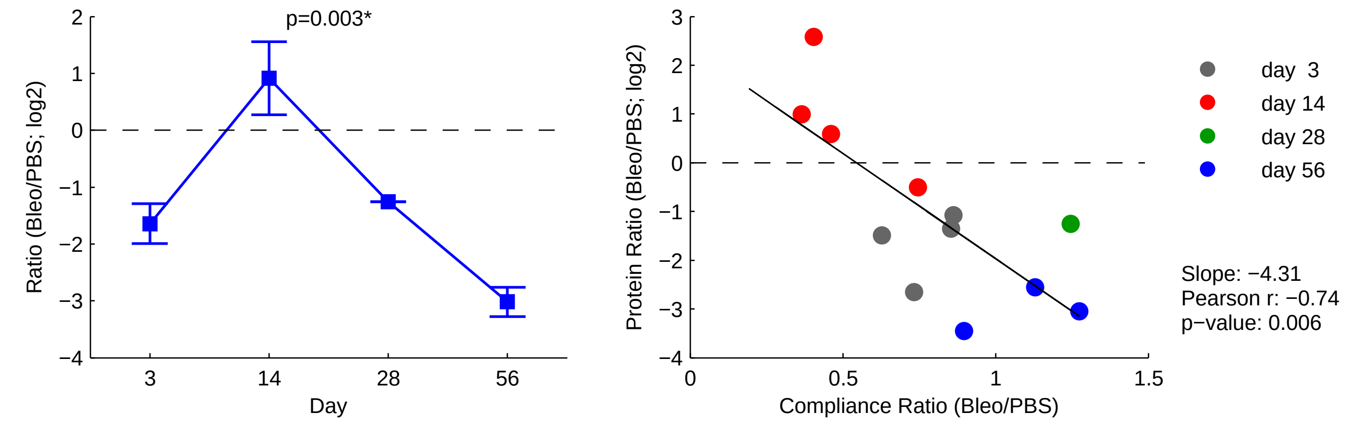

A2AAE1−6 − Kiaa1109 (id: 60)

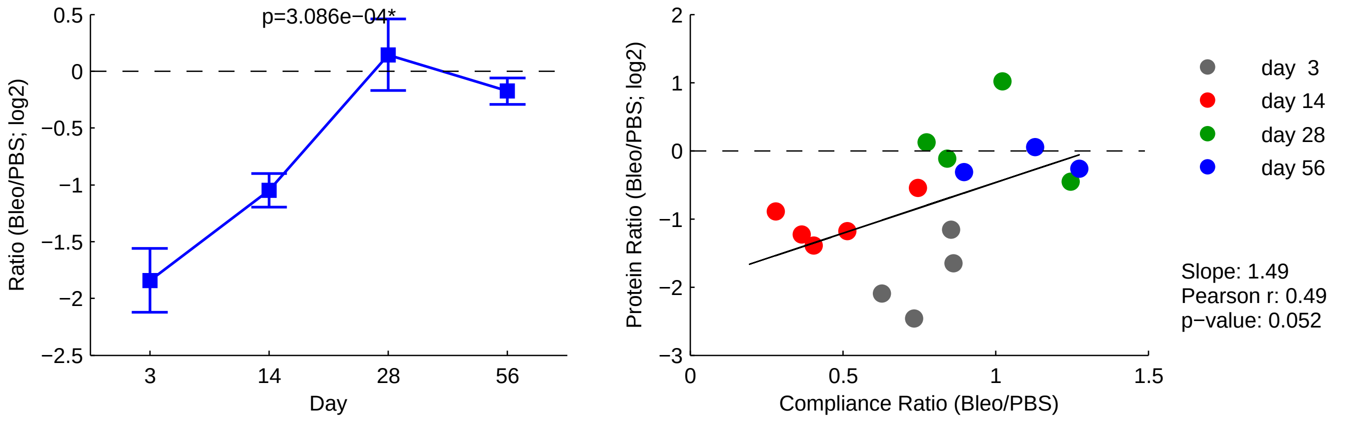

A2AAY5 − Sh3pxd2b (id: 63)

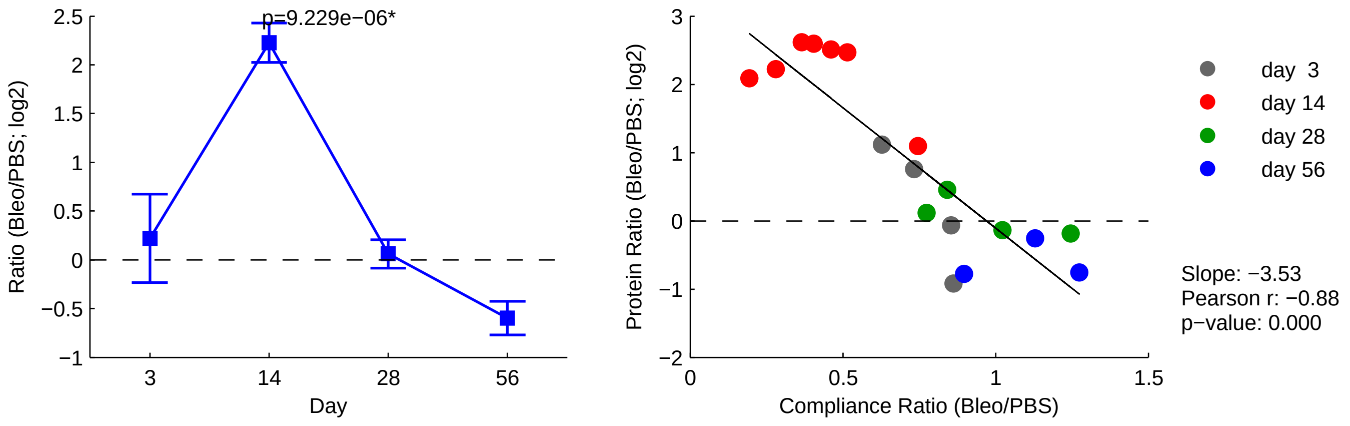

A2AB60 − Fmnl1 (id: 65)

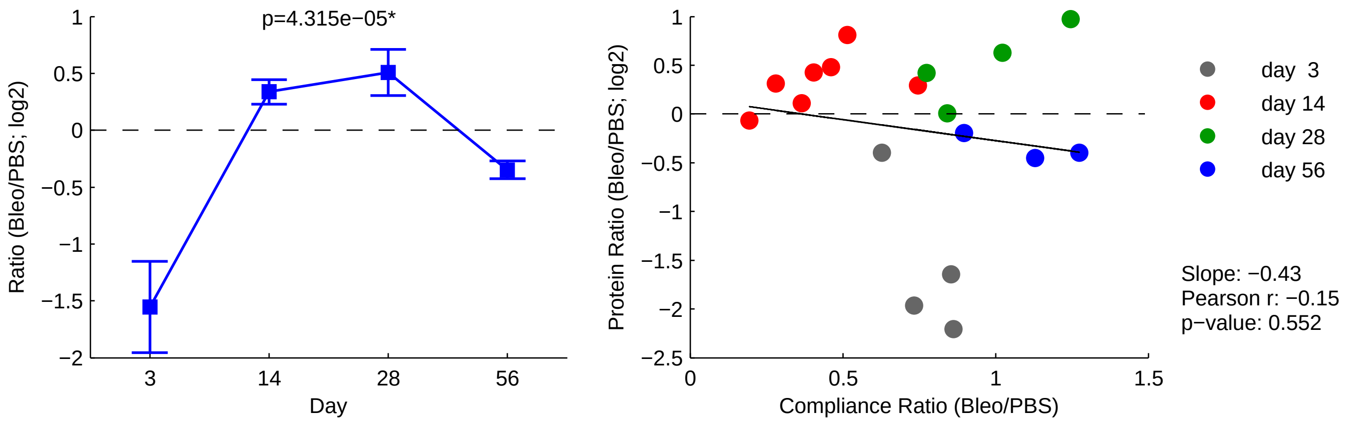

A2ABV5 − Med14 (id: 67)

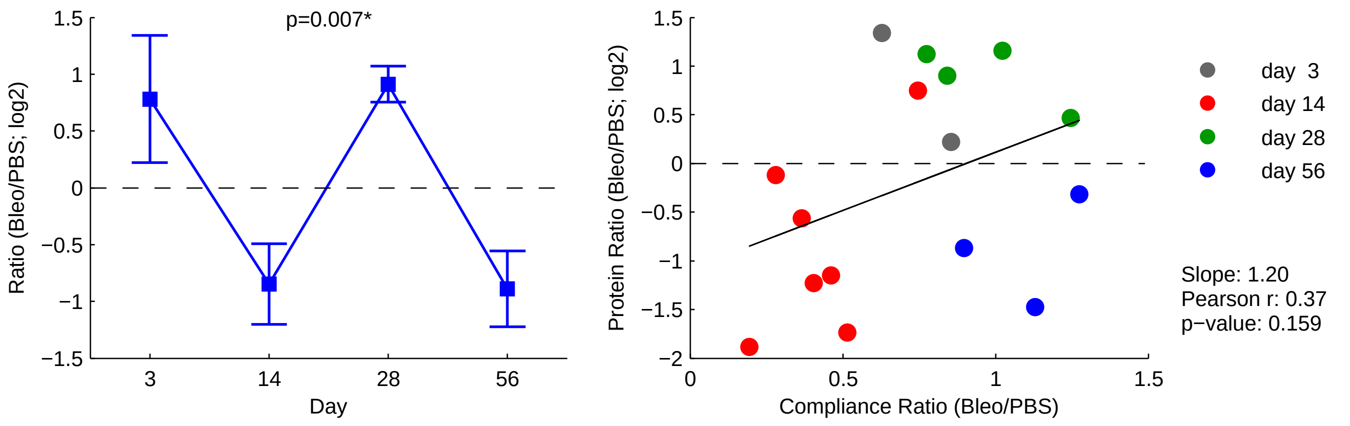

A2AC93−2 − Dnai2 (id: 70)

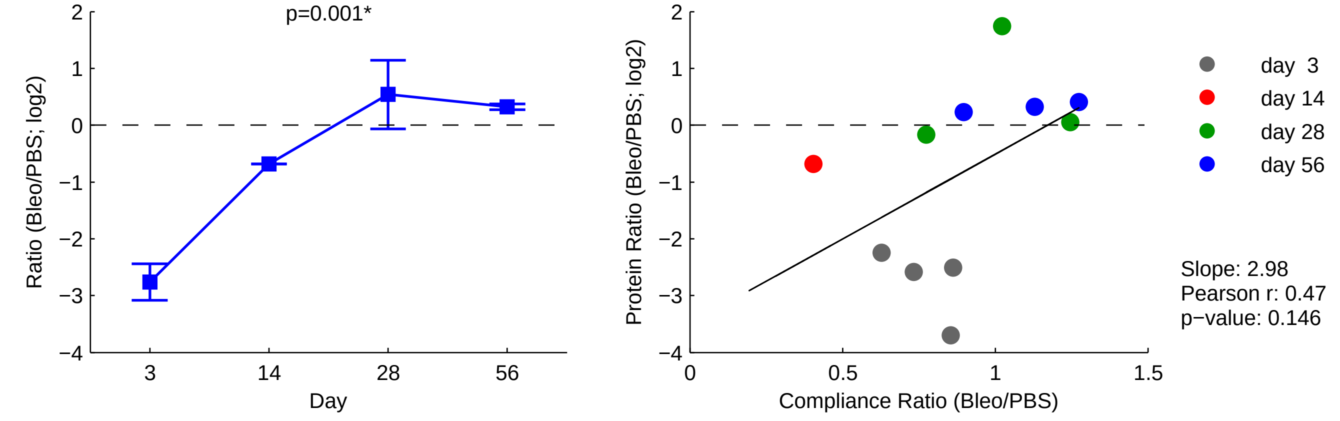

Q8BQZ4 − Ralgapb (id: 71)

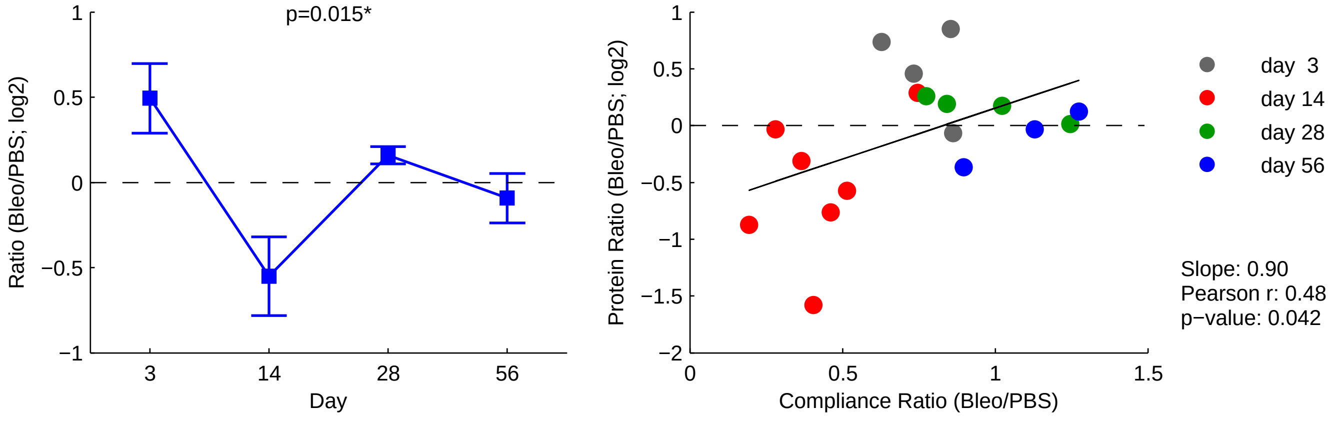

A2ACP1−2 − Ttc39a (id: 73)

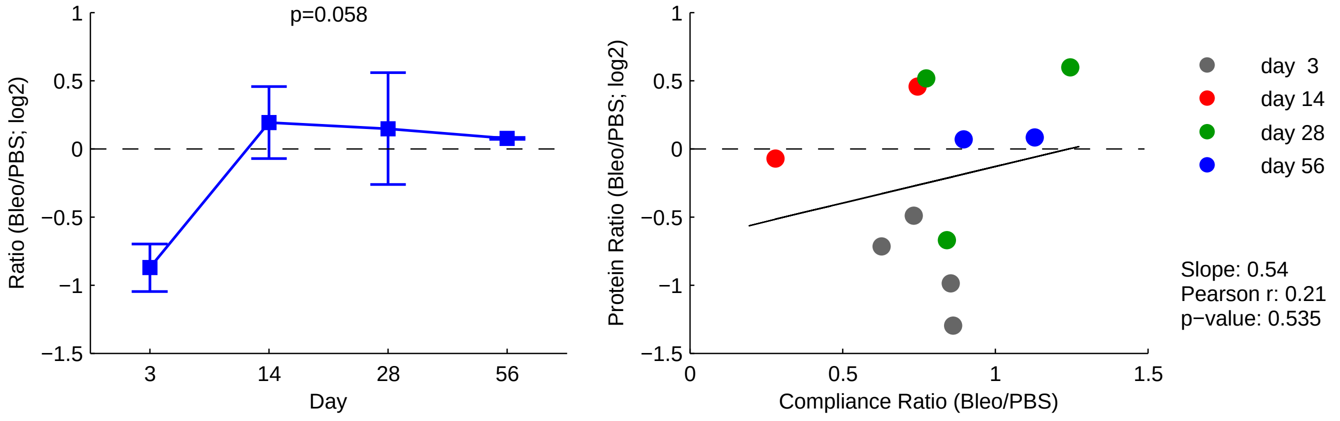

A2ADR8 − Ppp1r8 (id: 81)

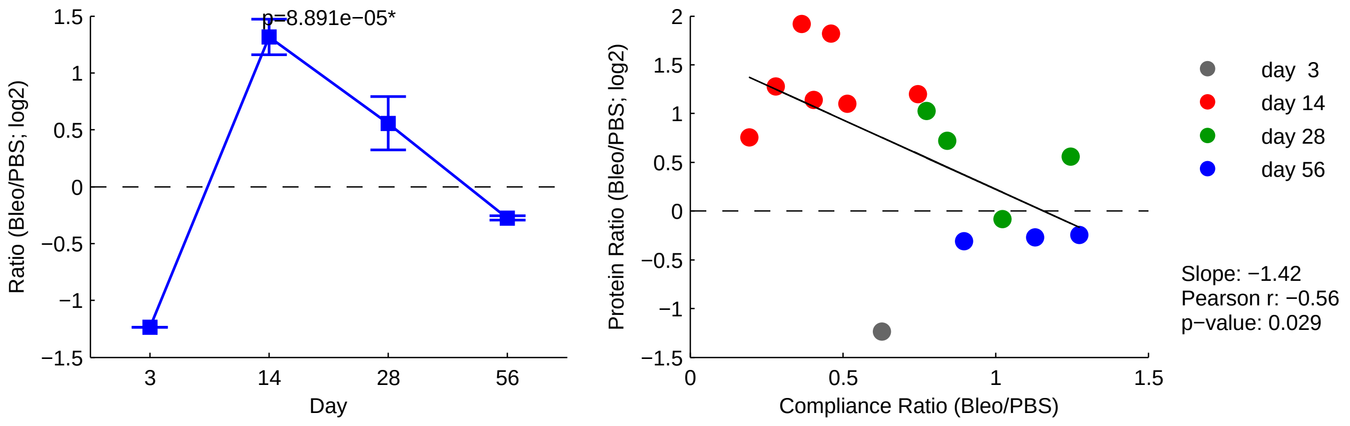

Q80Z25 − Ofd1 (id: 85)

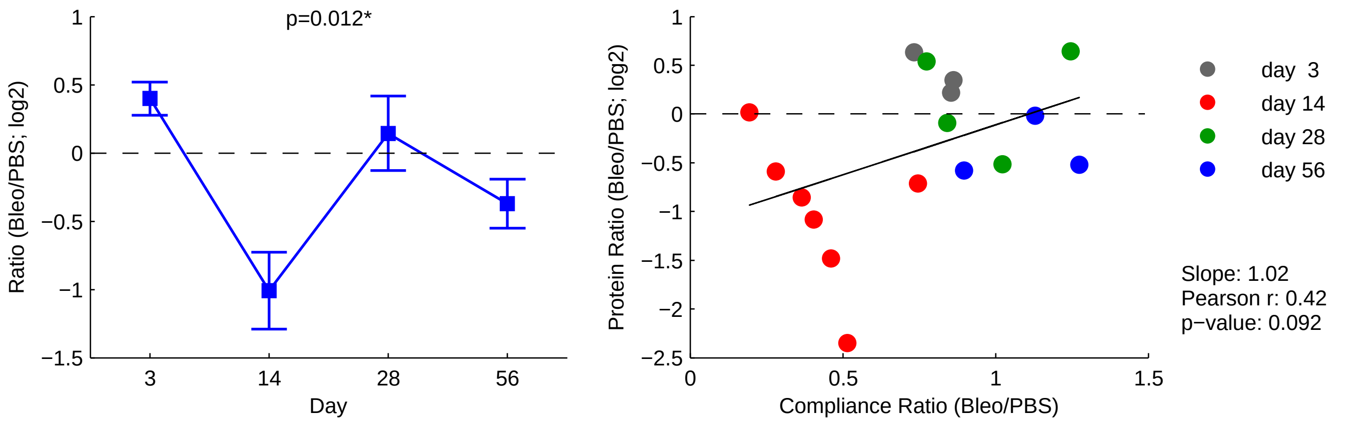

A2AEX8 − Fhl1 (id: 90)

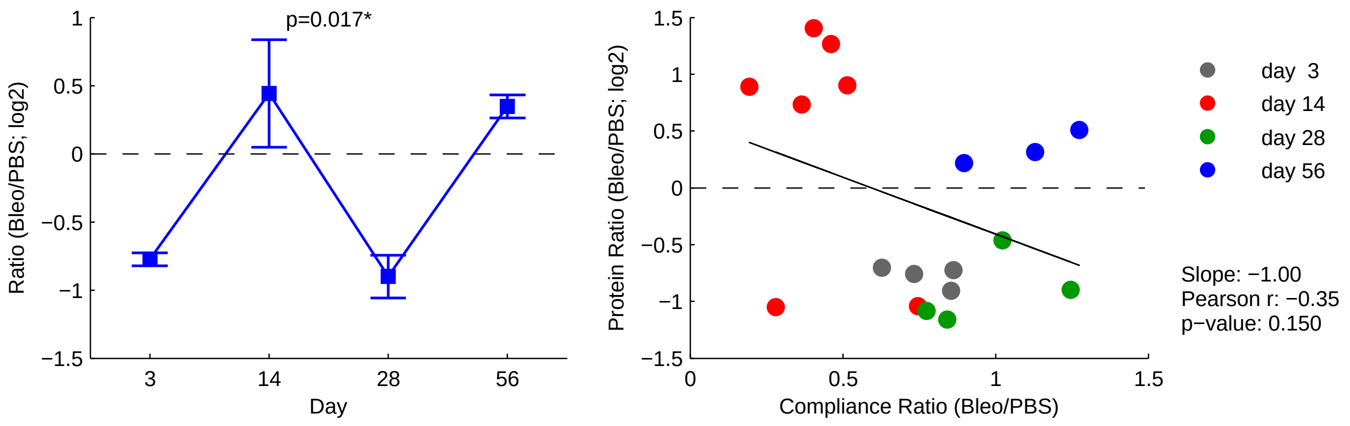

O35638 − Stag2 (id: 93)

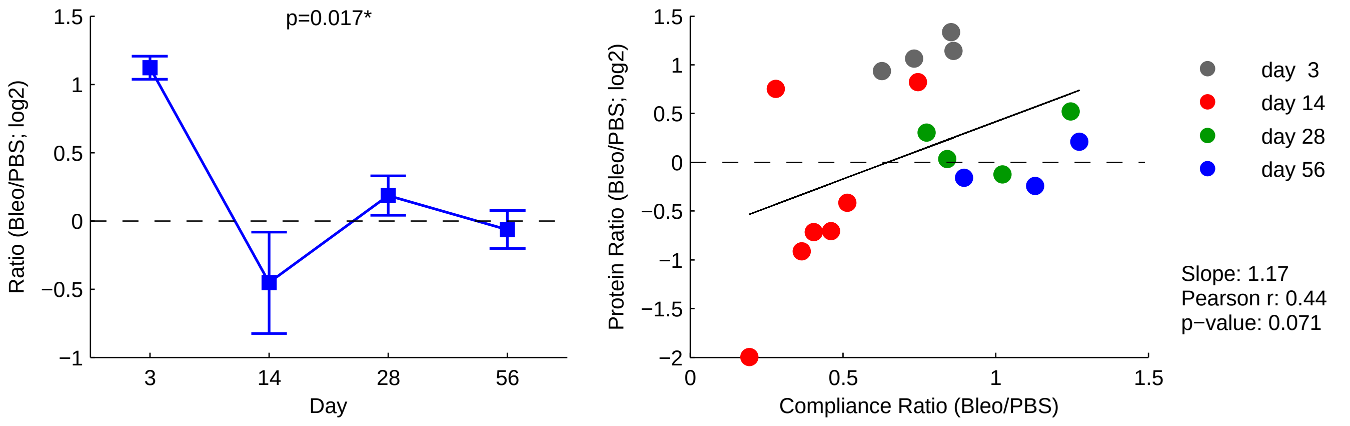

A2AFI8 − Reps2 (id: 94)

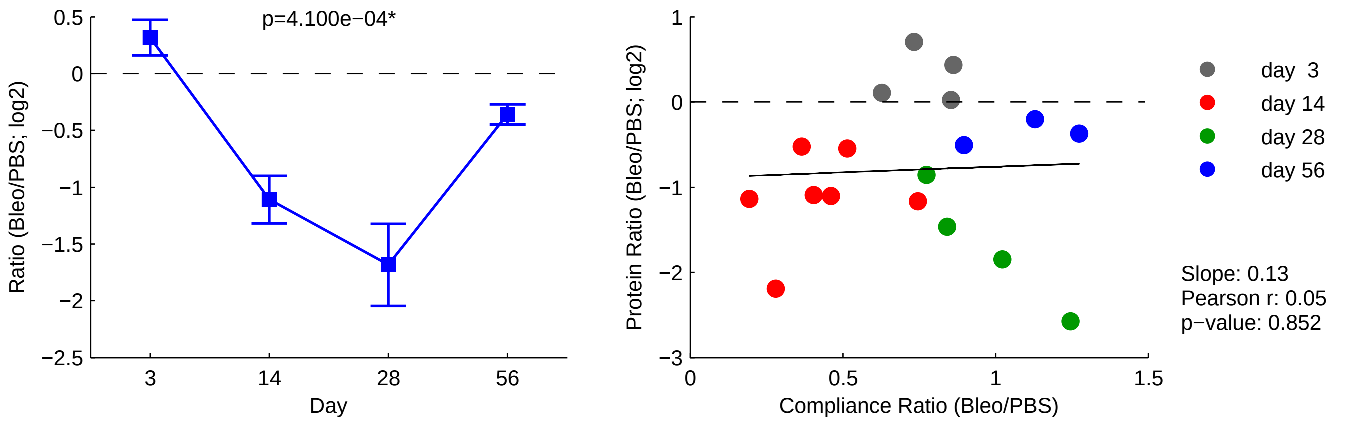

A2AFJ1 − Rbbp7 (id: 95)

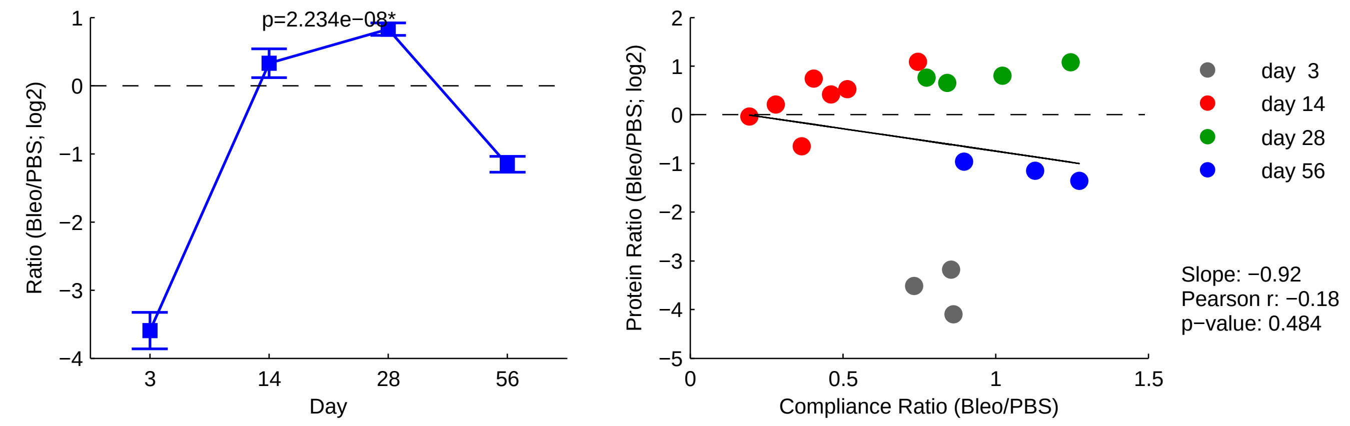

Q9R0M6 − Rab9a (id: 97)

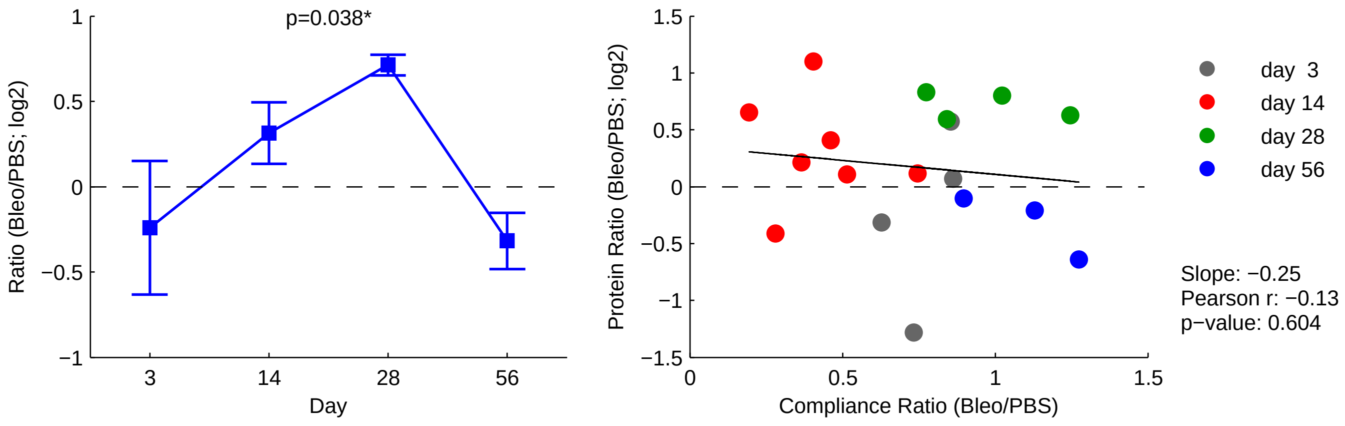

A2AFS3 − Kiaa1324 (id: 99)

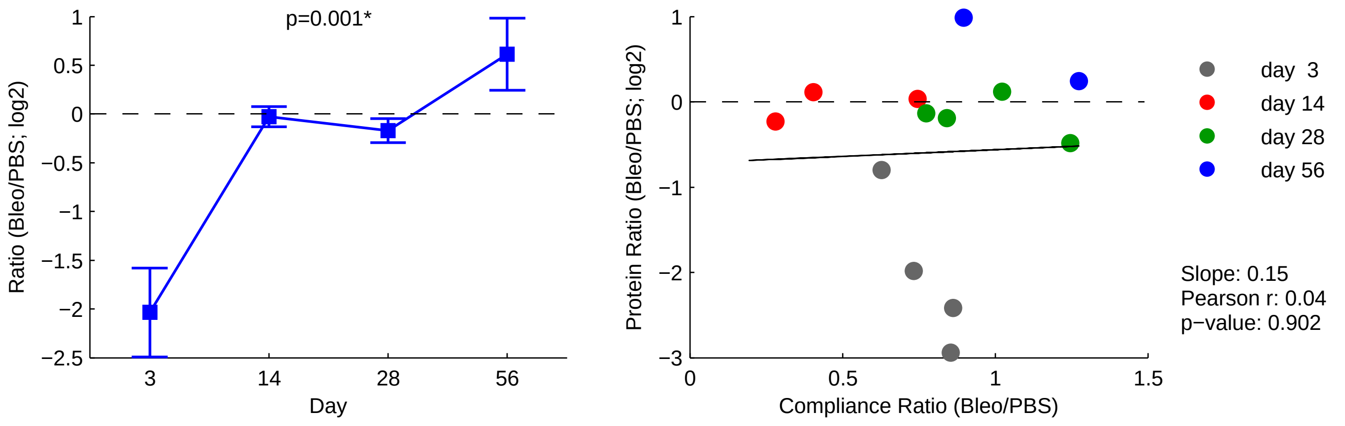

Q64430 − Atp7a (id: 103)

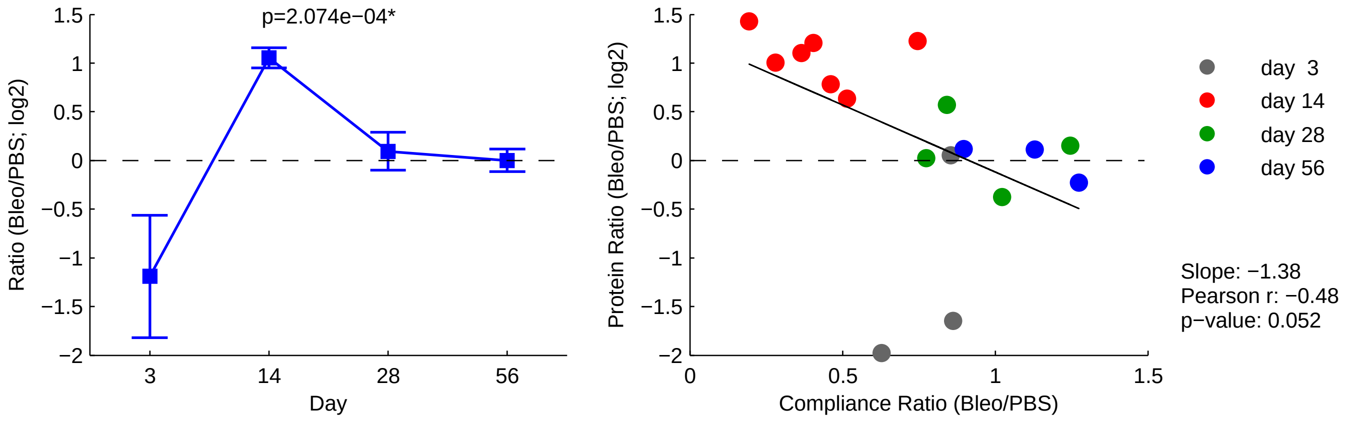

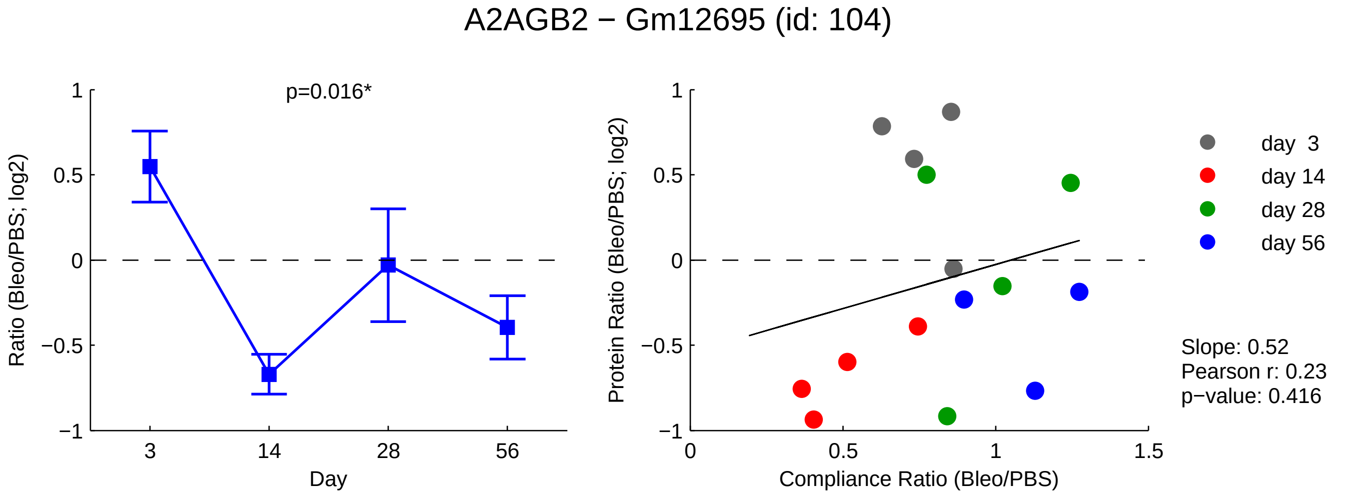
A2AGB2 − Gm12695 (id: 104)
p=0.016*
Ratio (Bleo/PBS; log2)
Day
Protein Ratio (Bleo/PBS; log2)
Compliance Ratio (Bleo/PBS)
day 3
day 14
day 28
day 56
Slope: 0.52
Pearson r: 0.23
p−value: 0.416

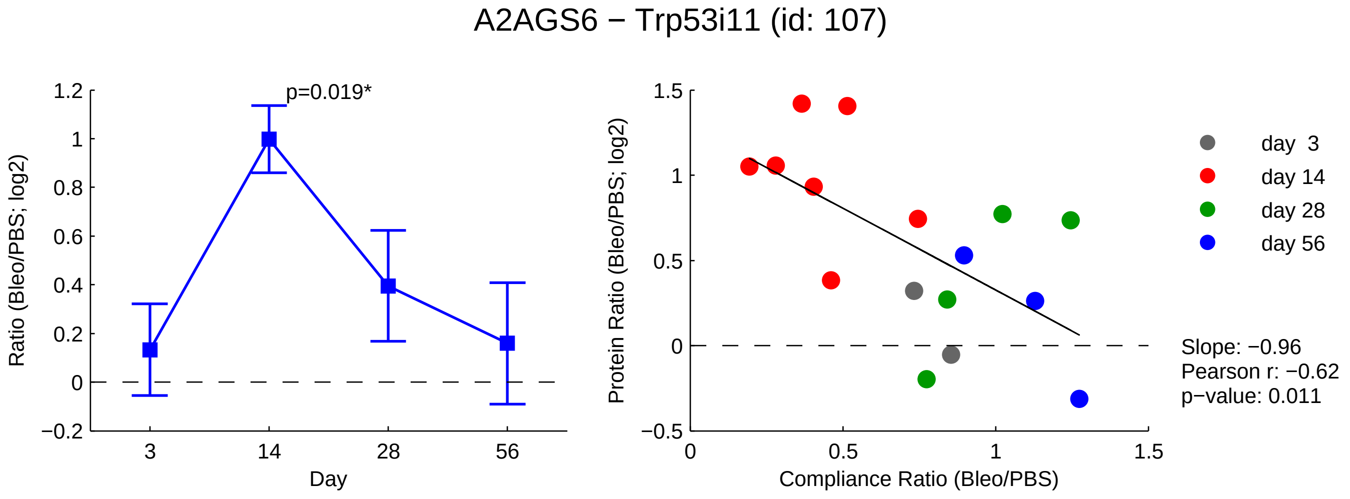
A2AGS6 − Trp53i11 (id: 107)
p=0.019*
Ratio (Bleo/PBS; log2)
Day
Protein Ratio (Bleo/PBS; log2)
Compliance Ratio (Bleo/PBS)
day 3
day 14
day 28
day 56
Slope: −0.96
Pearson r: −0.62
p−value: 0.011

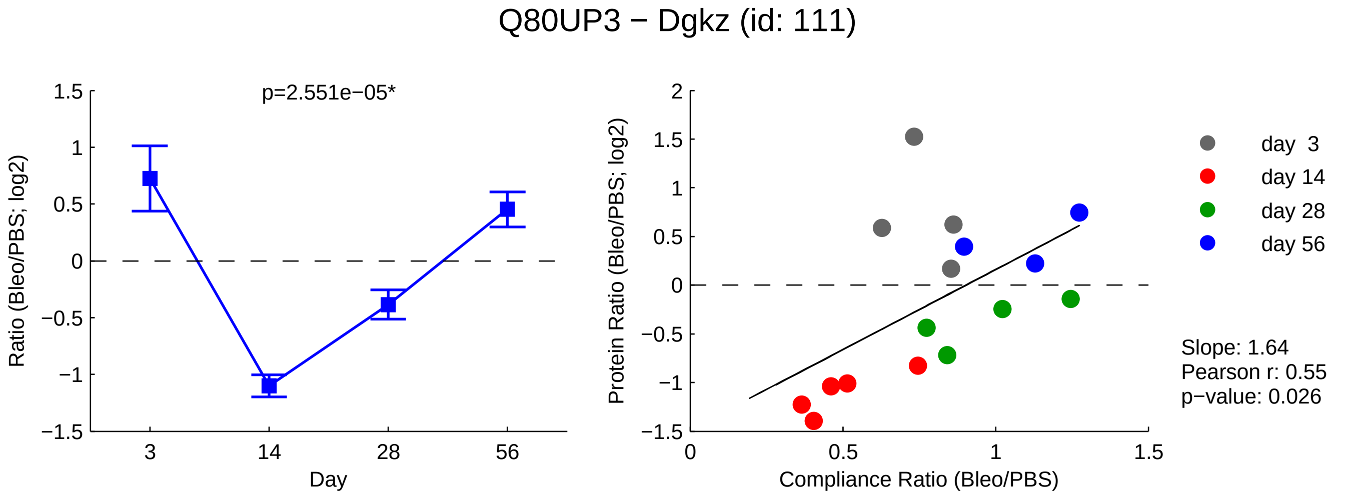
Q80UP3 − Dgkz (id: 111)
p=2.551e−05*
Ratio (Bleo/PBS; log2)
Day
Protein Ratio (Bleo/PBS; log2)
Compliance Ratio (Bleo/PBS)
day 3
day 14
day 28
day 56
Slope: 1.64
Pearson r: 0.55
p−value: 0.026

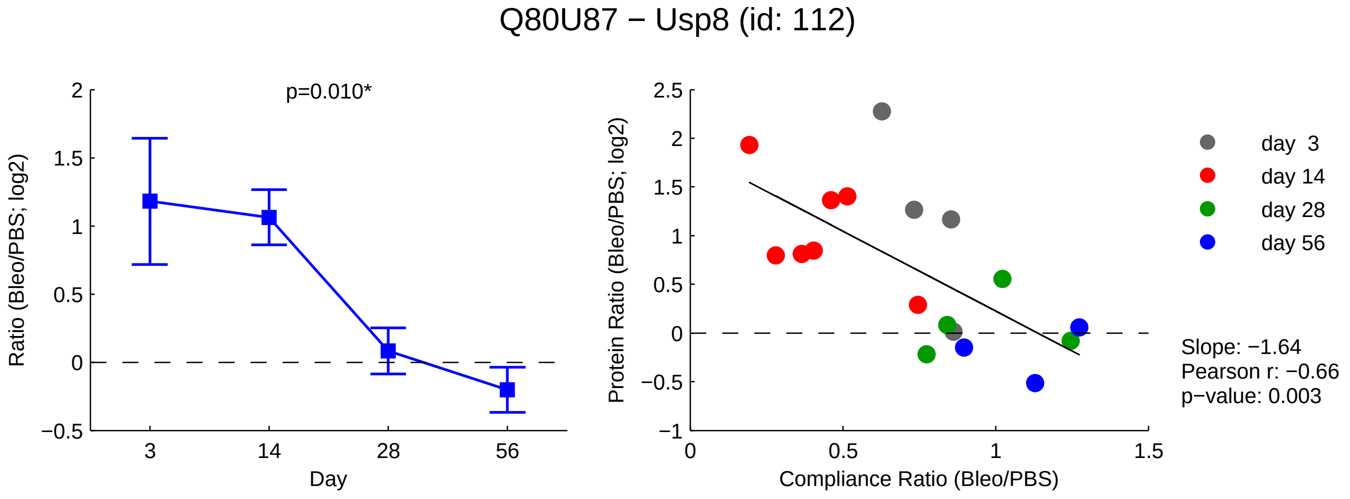

Q80U87 − Usp8 (id: 112)
p=0.010*
Ratio (Bleo/PBS; log2)
Day
Protein Ratio (Bleo/PBS; log2)
Compliance Ratio (Bleo/PBS)
day 3
day 14
day 28
day 56
Slope: −1.64
Pearson r: −0.66
p−value: 0.003

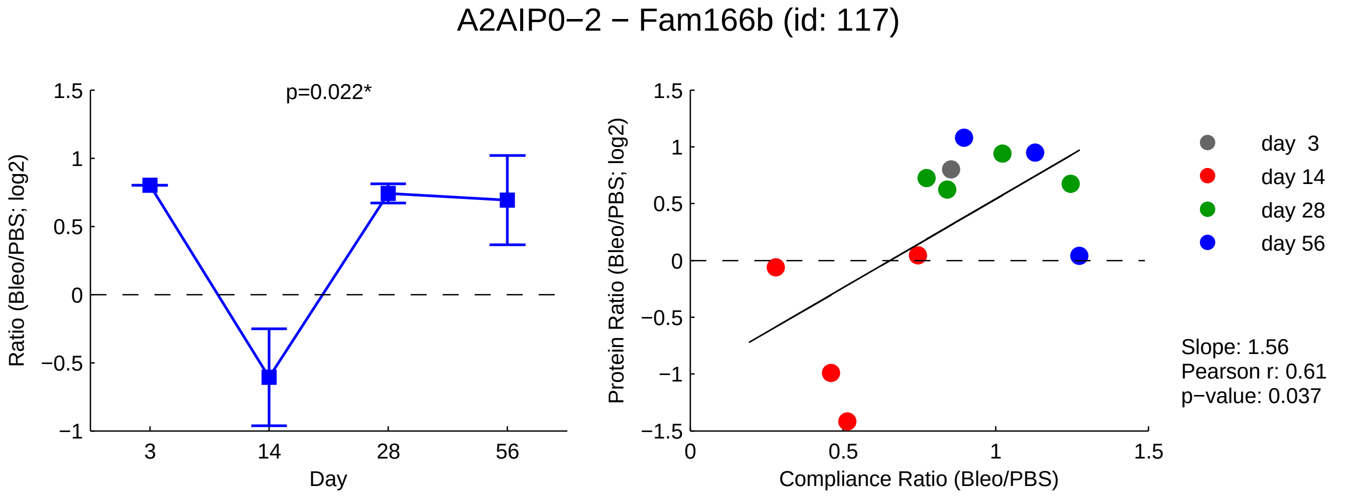

A2AIP0−2 − Fam166b (id: 117)
p=0.022*
Ratio (Bleo/PBS; log2)
Day
Protein Ratio (Bleo/PBS; log2)
Compliance Ratio (Bleo/PBS)
day 3
day 14
day 28
day 56
Slope: 1.56
Pearson r: 0.61
p−value: 0.037

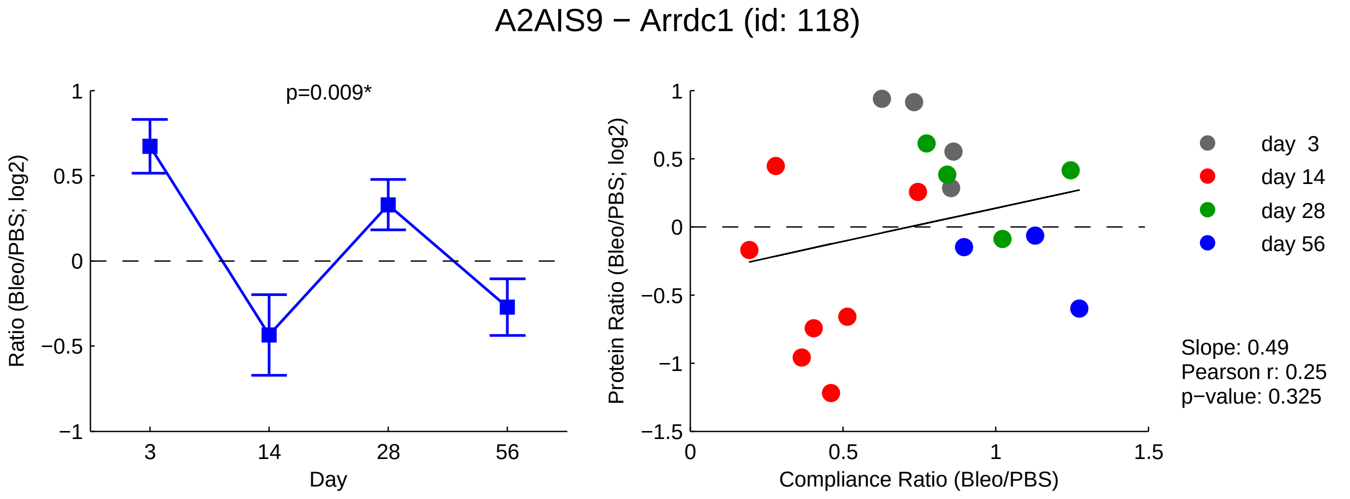

A2AIS9 − Arrdc1 (id: 118)
p=0.009*
Ratio (Bleo/PBS; log2)
Day
Protein Ratio (Bleo/PBS; log2)
Compliance Ratio (Bleo/PBS)
day 3
day 14
day 28
day 56
Slope: 0.49
Pearson r: 0.25
p−value: 0.325

A2AIV2 − Kiaa1429 (id: 120)

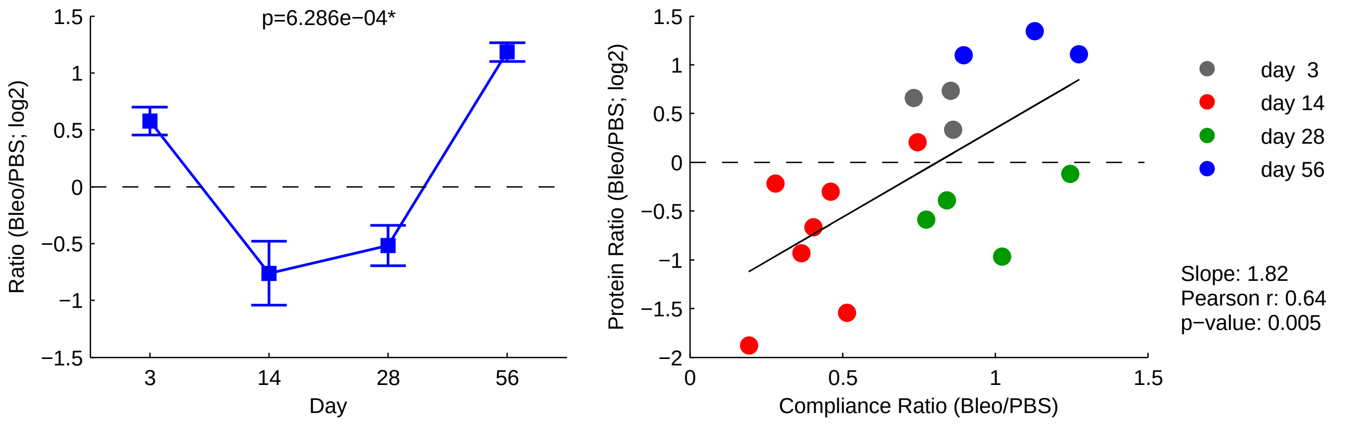

E9QAT4 − Sec16a (id: 122)

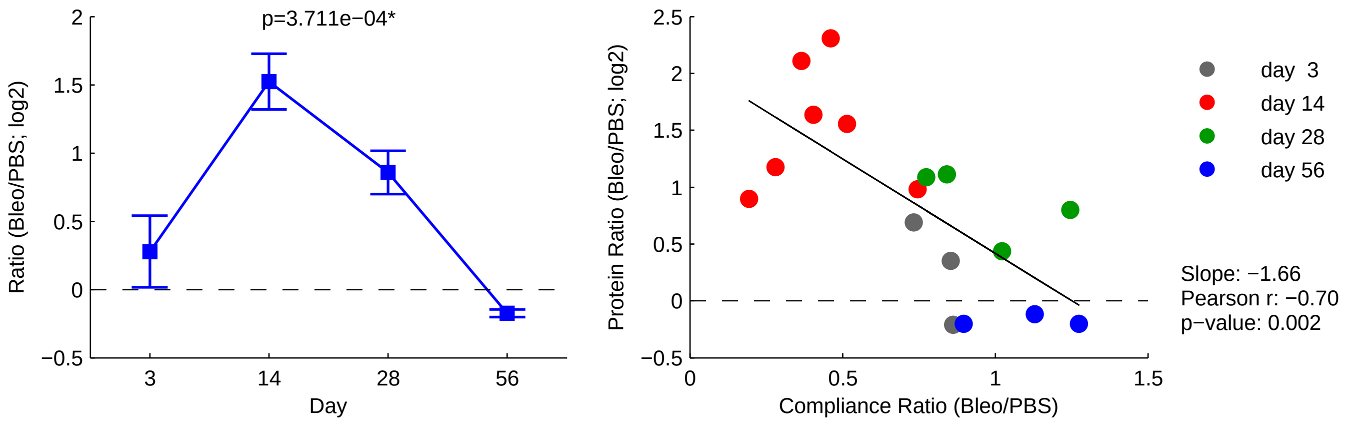

A2AJ15 − Man1b1 (id: 123)

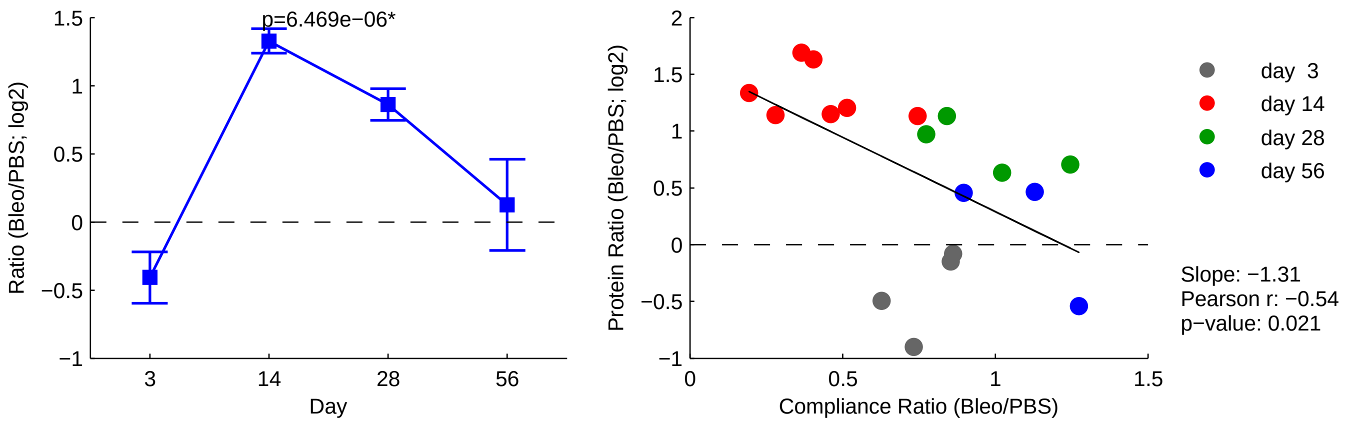

A2AJI0 − Map7d1 (id: 131)

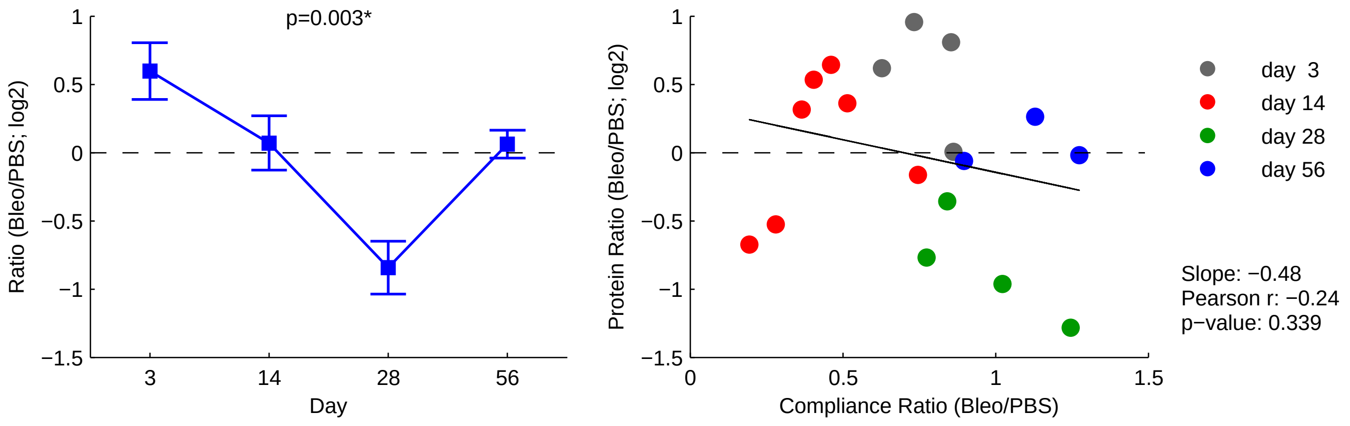

Q8BR76 − Tmem67 (id: 133)

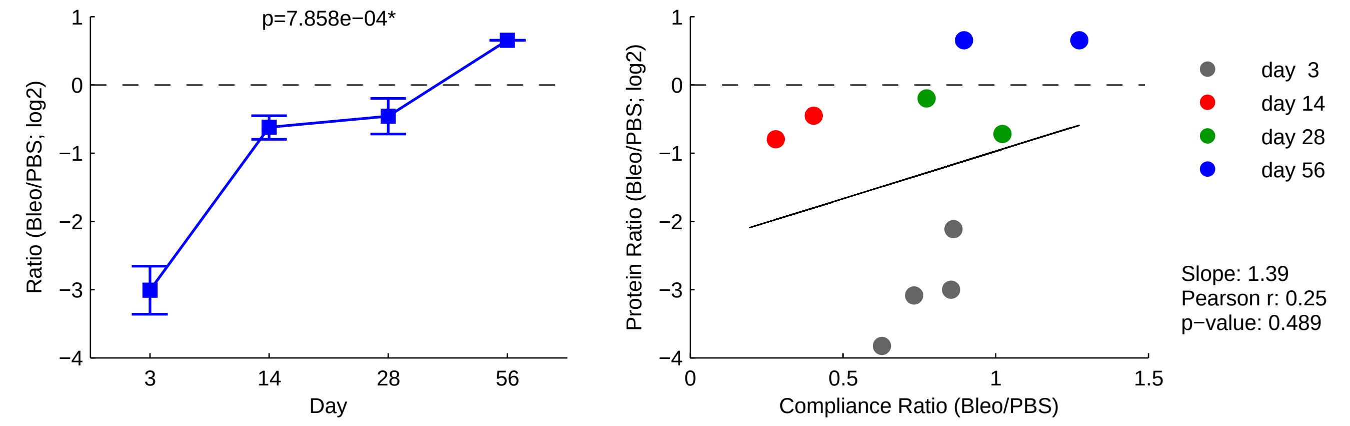

Q3UV70 − Pdp1 (id: 134)

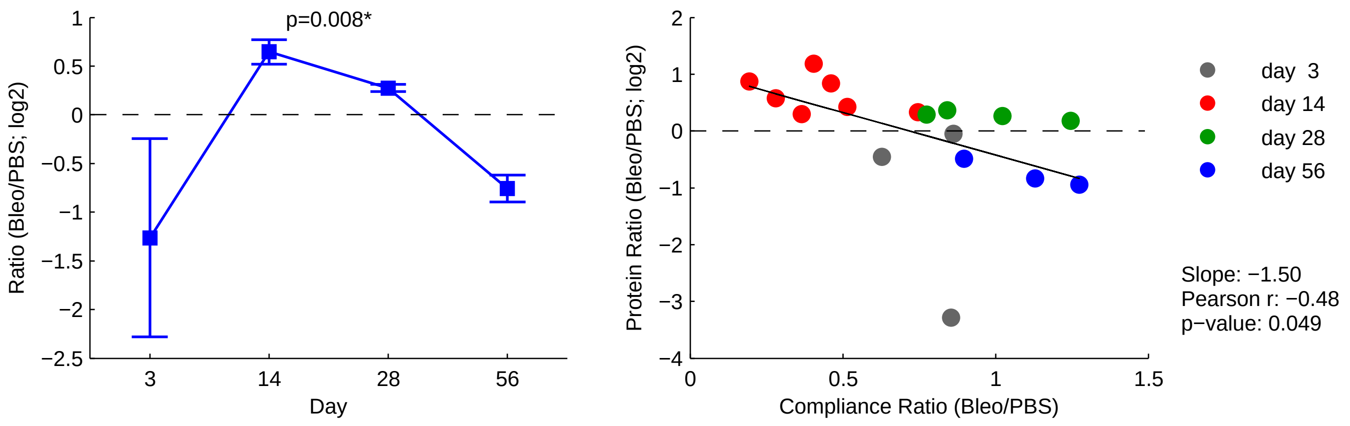

A2AJT4 − Pnisr (id: 135)

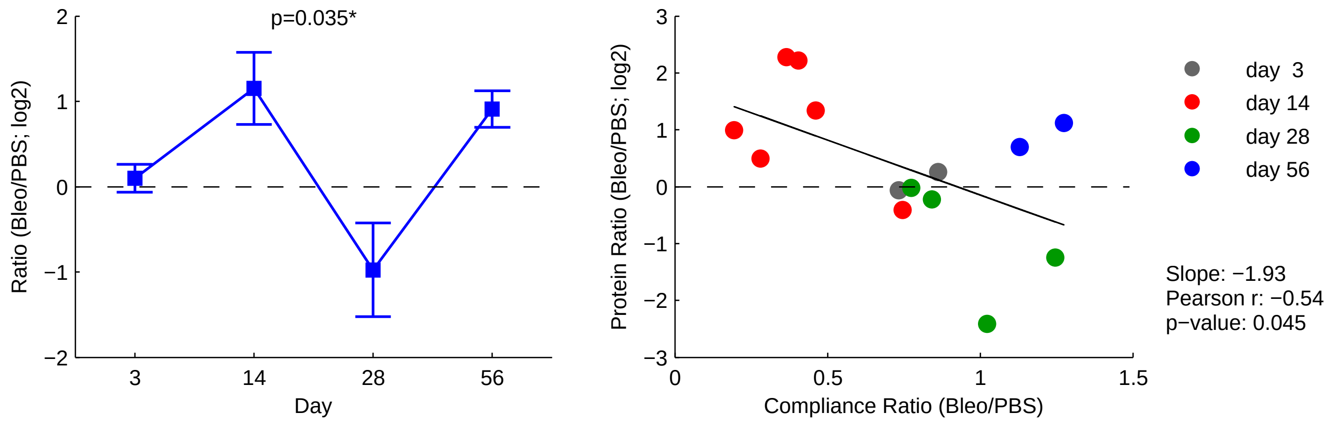

A2AJY2 − Col15a1 (id: 136)

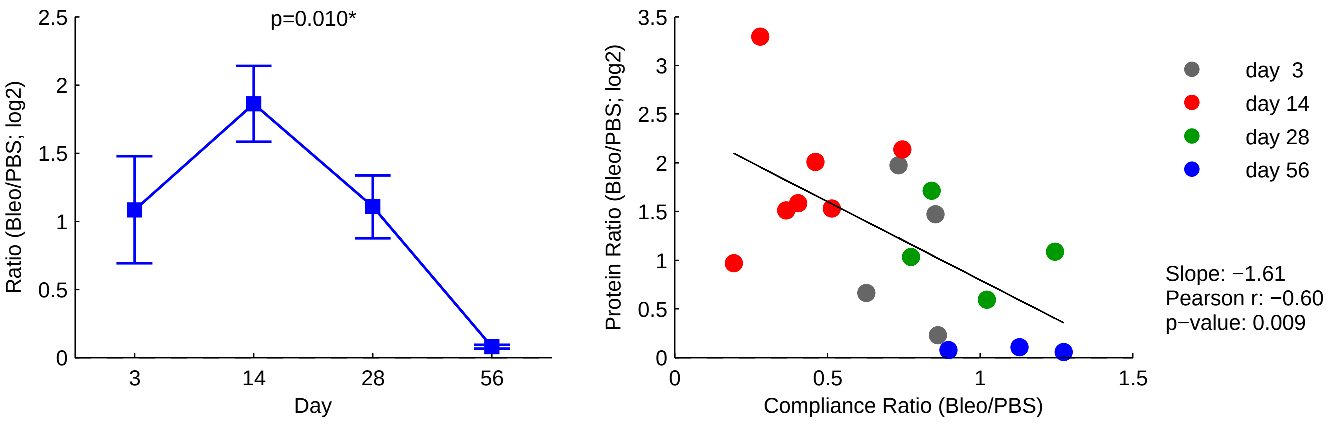

A2AKD7 − Snta1 (id: 137)

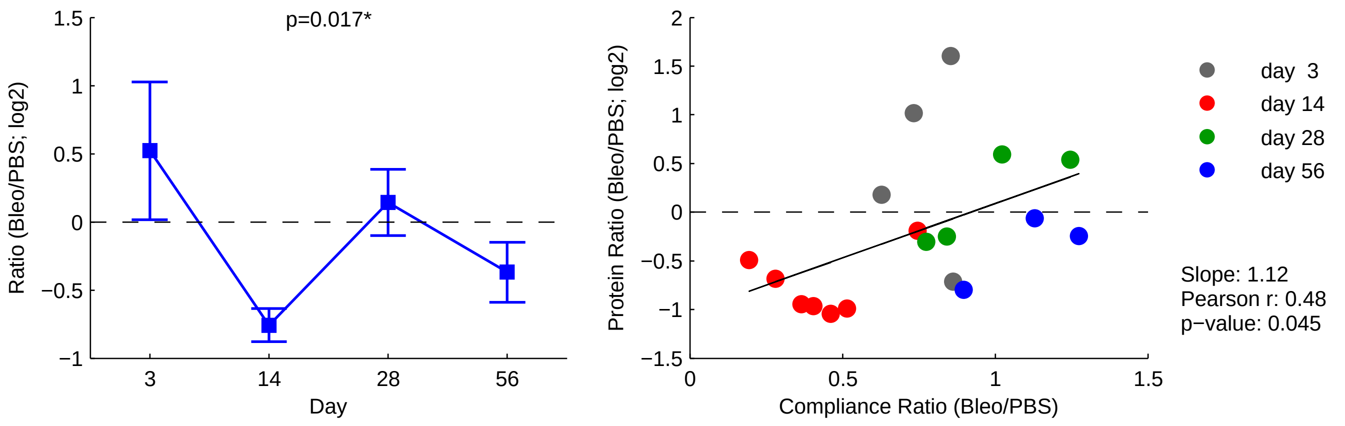

E9PYJ6 − Focad (id: 138)

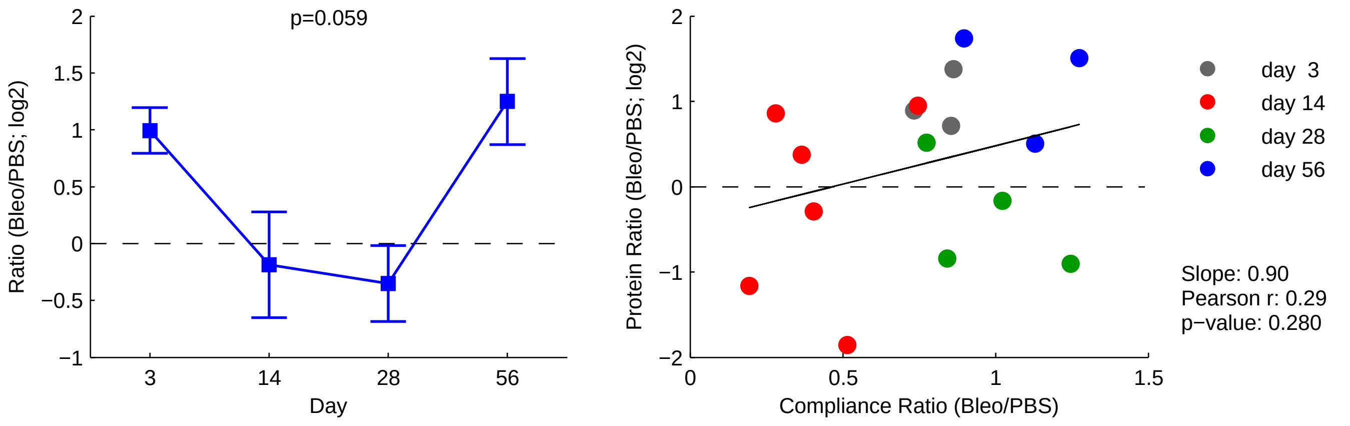

A2AKU9 − Atp5c1 (id: 140)

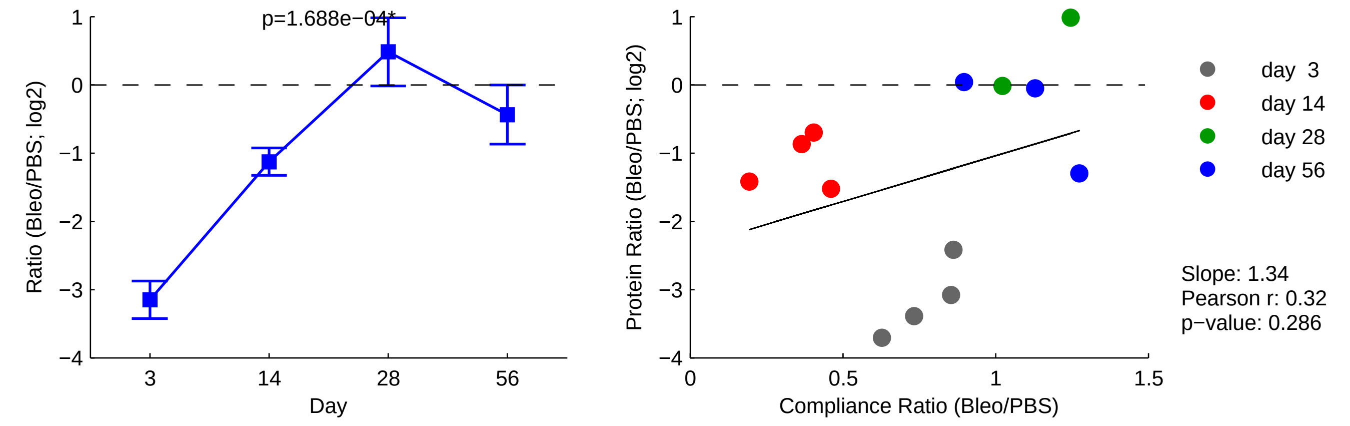

A2AL12 − Hnrnpa3 (id: 143)

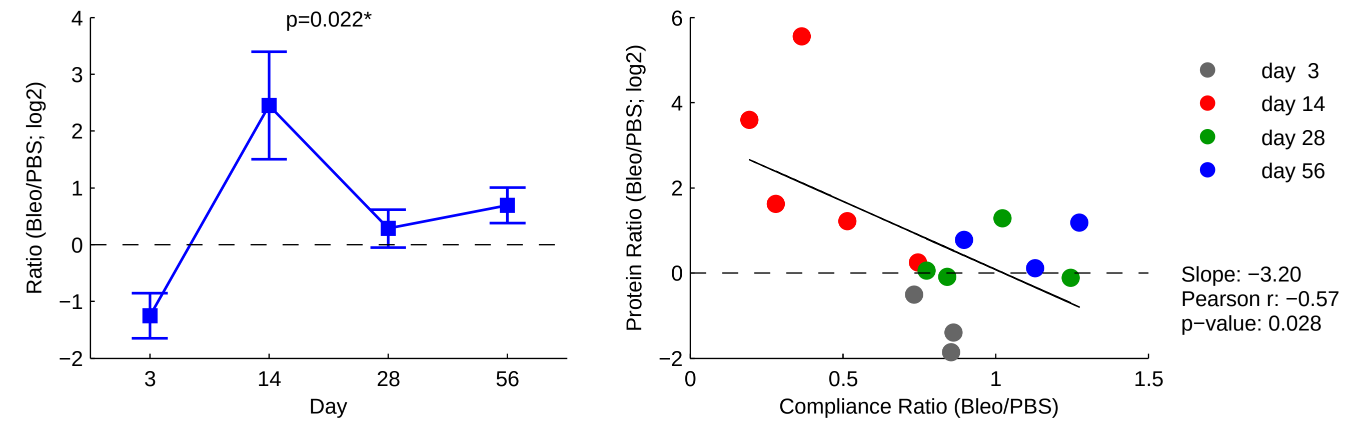

A2AL36 − Cntrl (id: 144)

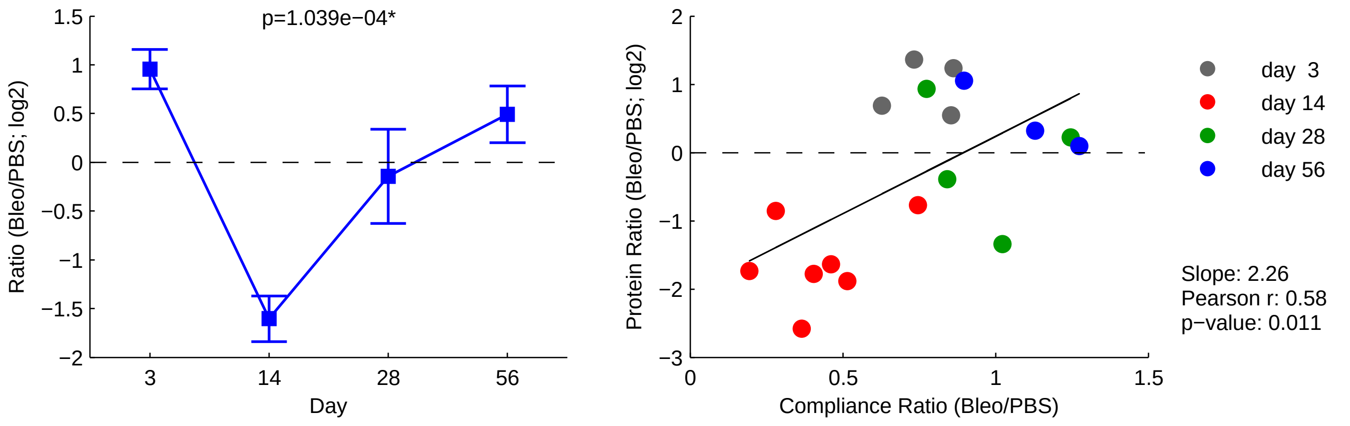

Q8C0I1 − Agps (id: 145)

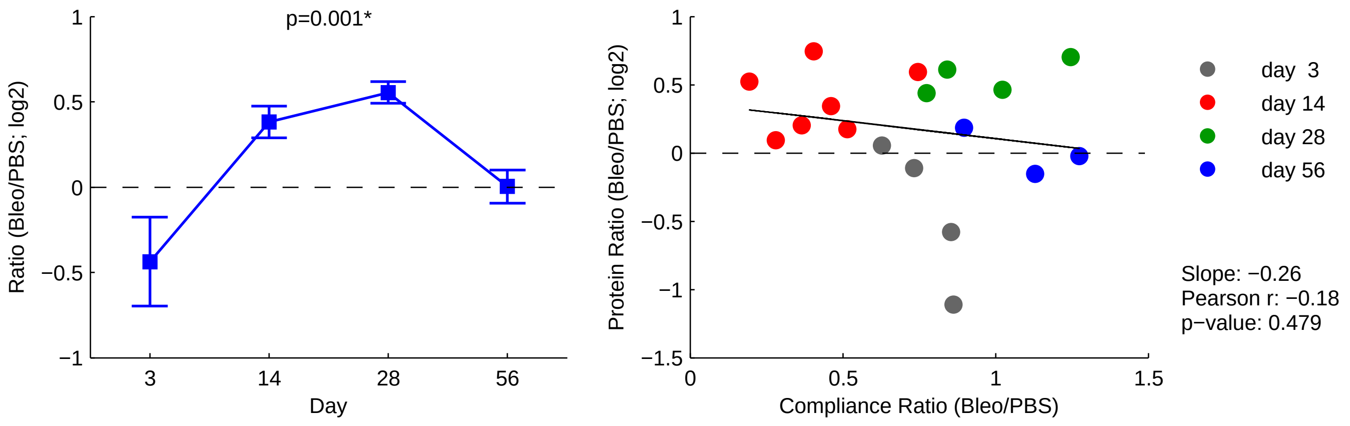

Q8CBM2 − Asph (id: 146)

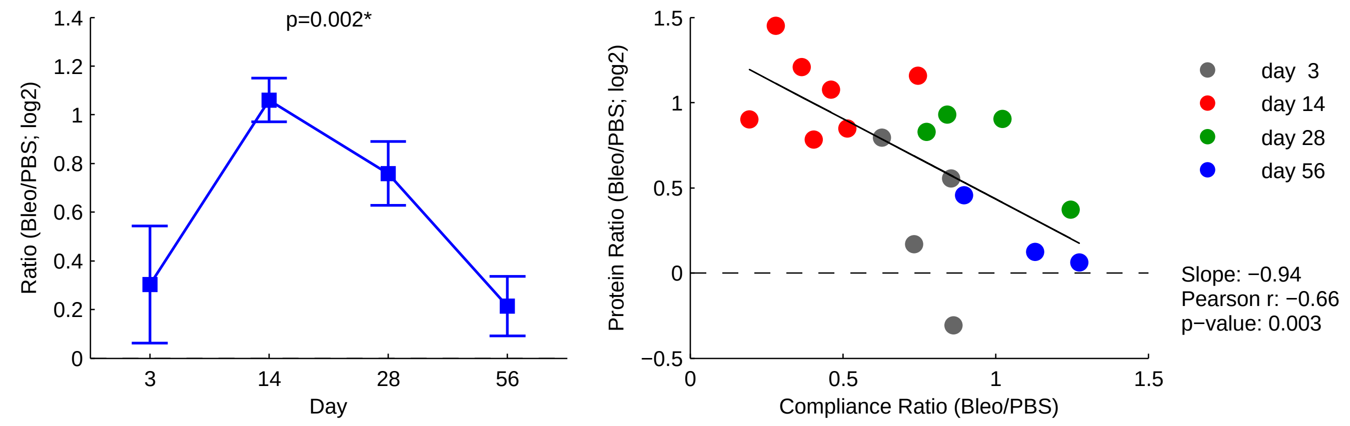

P09925 − Surf1 (id: 148)

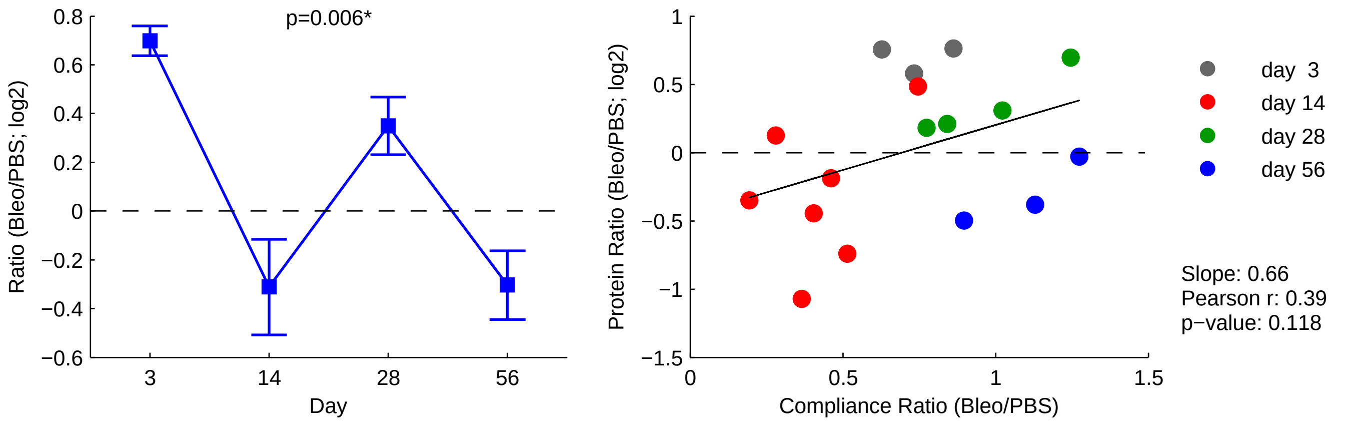

A2ALR9 − Usp48 (id: 149)

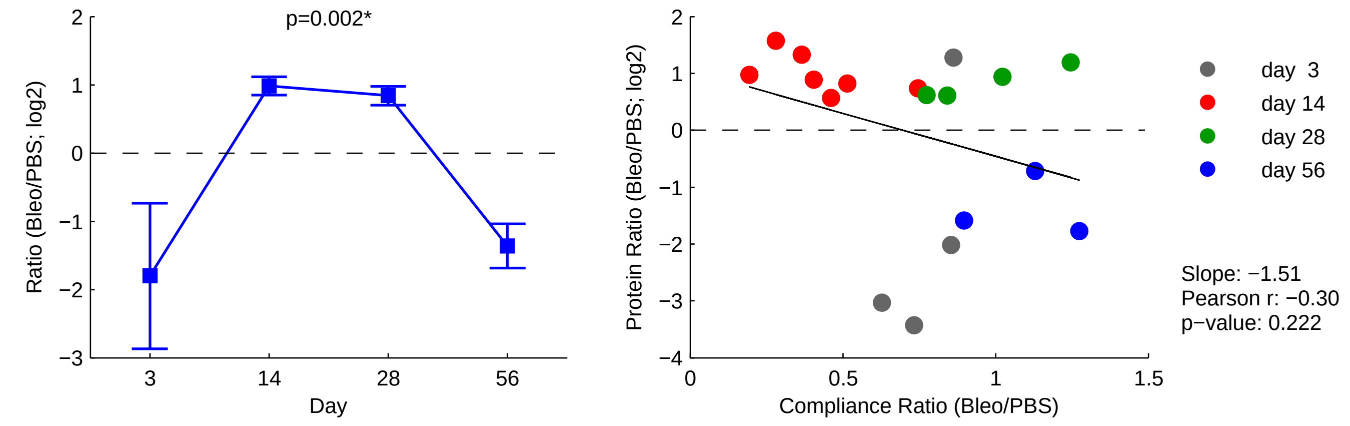

A2ALW5−2 − Dnajc25 (id: 151)

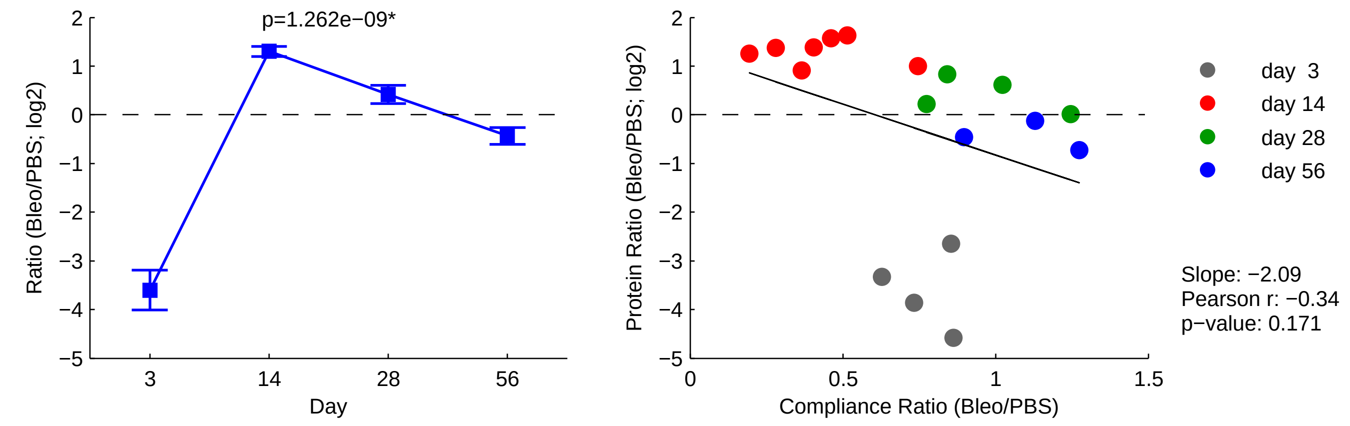

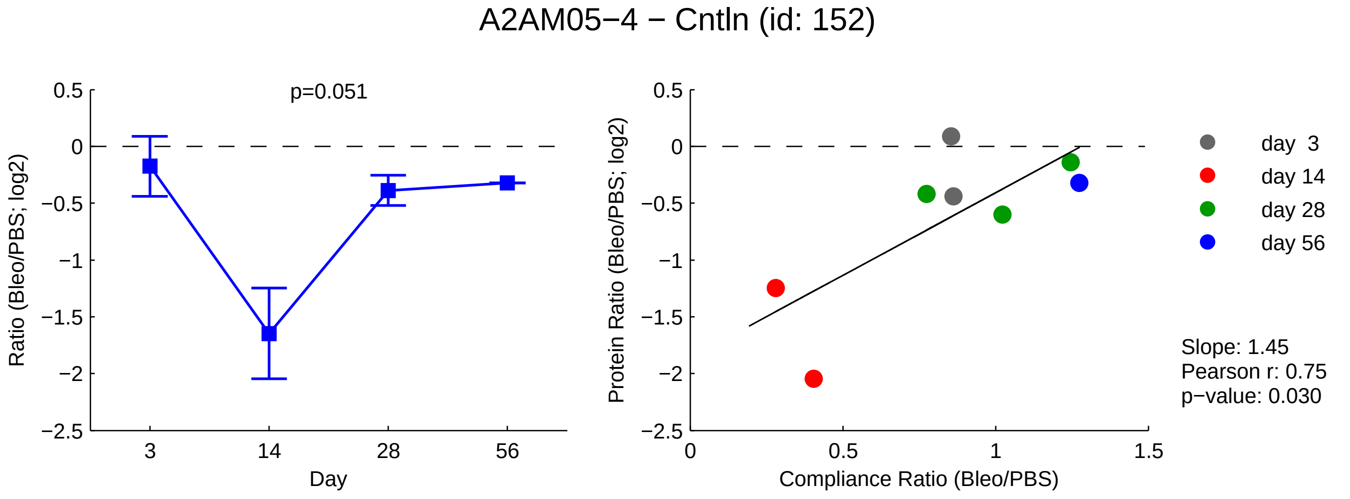
A2AM05−4 − Cntln (id: 152)
p=0.051
Ratio (Bleo/PBS; log2)
Day
Protein Ratio (Bleo/PBS; log2)
Compliance Ratio (Bleo/PBS)
day 3
day 14
day 28
day 56
Slope: 1.45
Pearson r: 0.75
p−value: 0.030

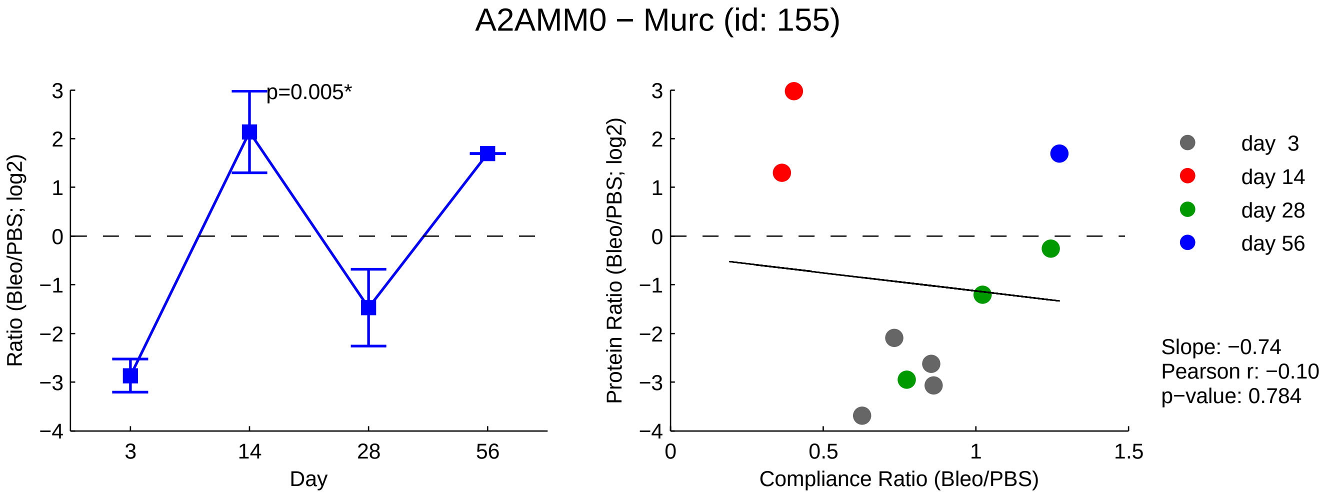
A2AMM0 − Murc (id: 155)
p=0.005*
Ratio (Bleo/PBS; log2)
Day
Protein Ratio (Bleo/PBS; log2)
Compliance Ratio (Bleo/PBS)
day 3
day 14
day 28
day 56
Slope: −0.74
Pearson r: −0.10
p−value: 0.784

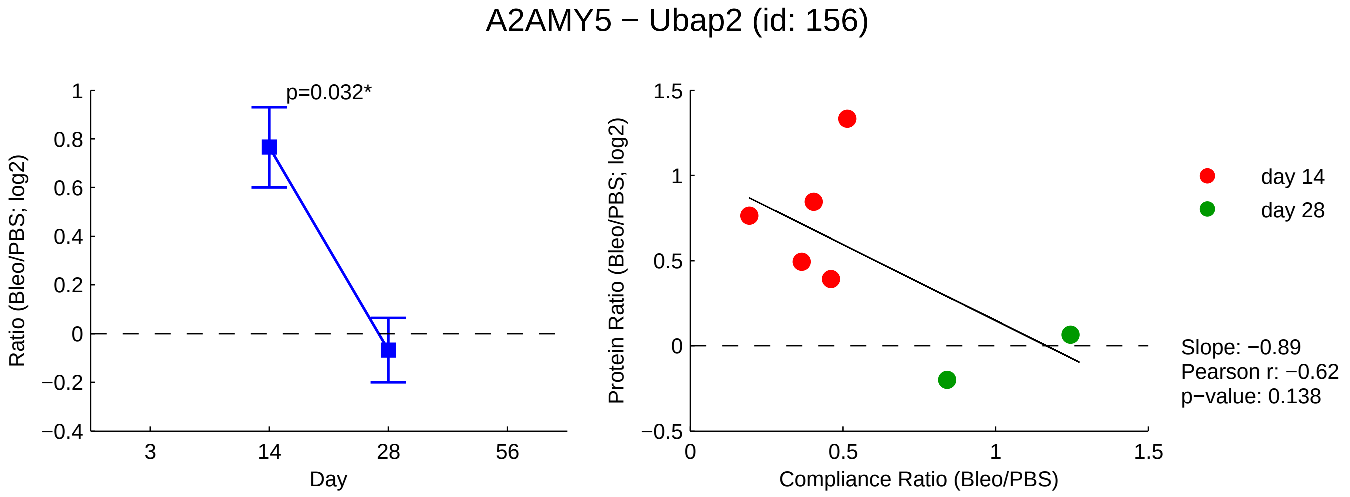
A2AMY5 − Ubap2 (id: 156)
p=0.032*
Ratio (Bleo/PBS; log2)
Day
Protein Ratio (Bleo/PBS; log2)
Compliance Ratio (Bleo/PBS)
day 14
day 28
Slope: −0.89
Pearson r: −0.62
p−value: 0.138

A2AN08−3 − Ubr4 (id: 157)

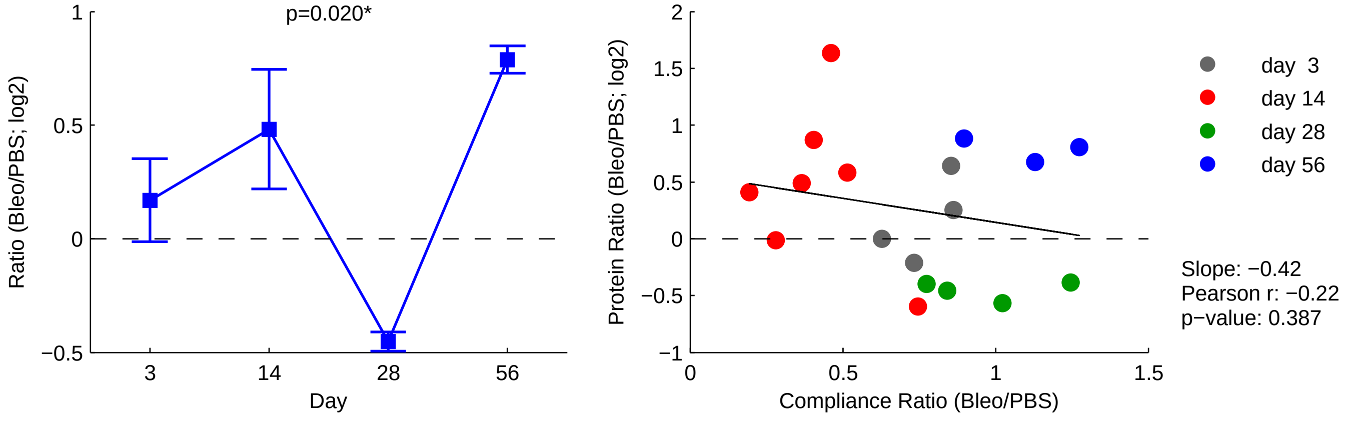

A2ANY6 − Mdn1 (id: 159)

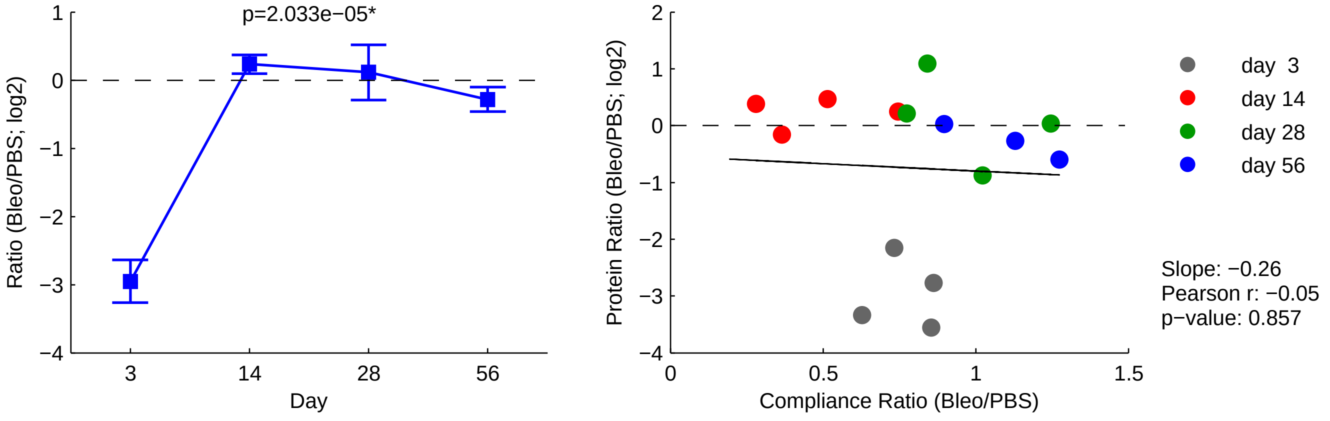

I7HJQ8 − Cd99l2 (id: 160)

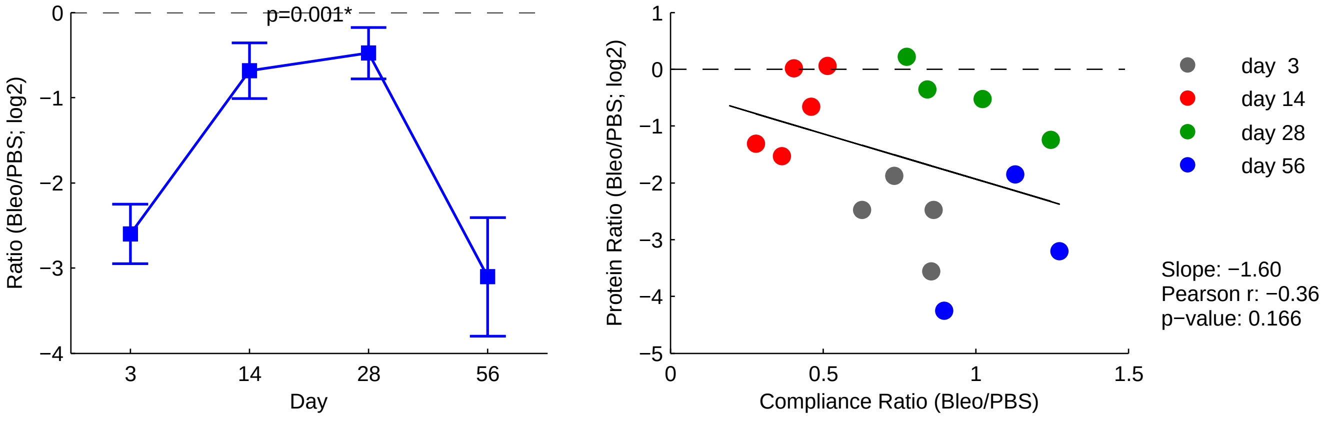

Q8BYK8 − Zc3h6 (id: 163)

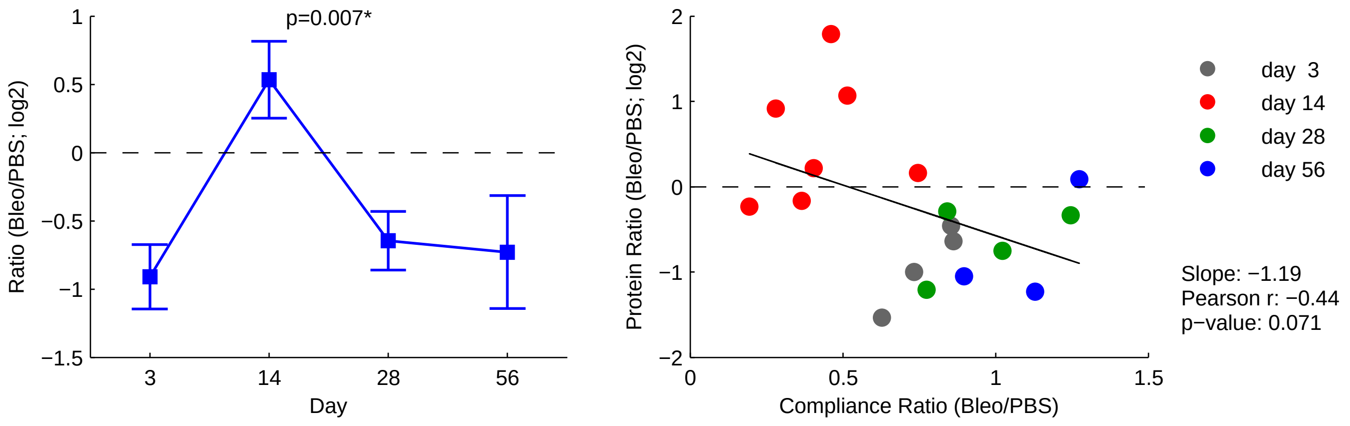

Q9QY24−2 − Zbp1 (id: 164)

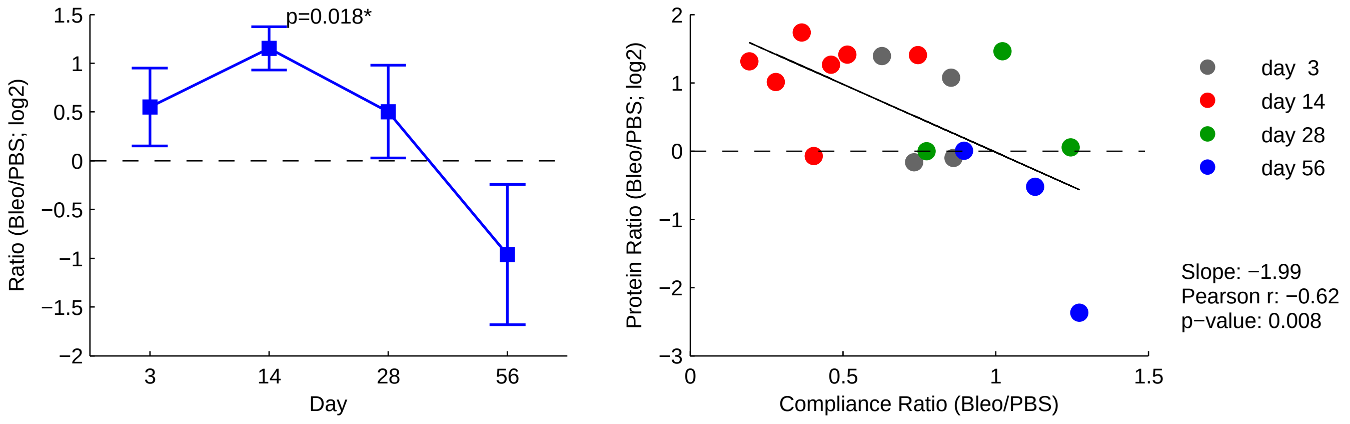

A2API8 − Akap2 (id: 166)

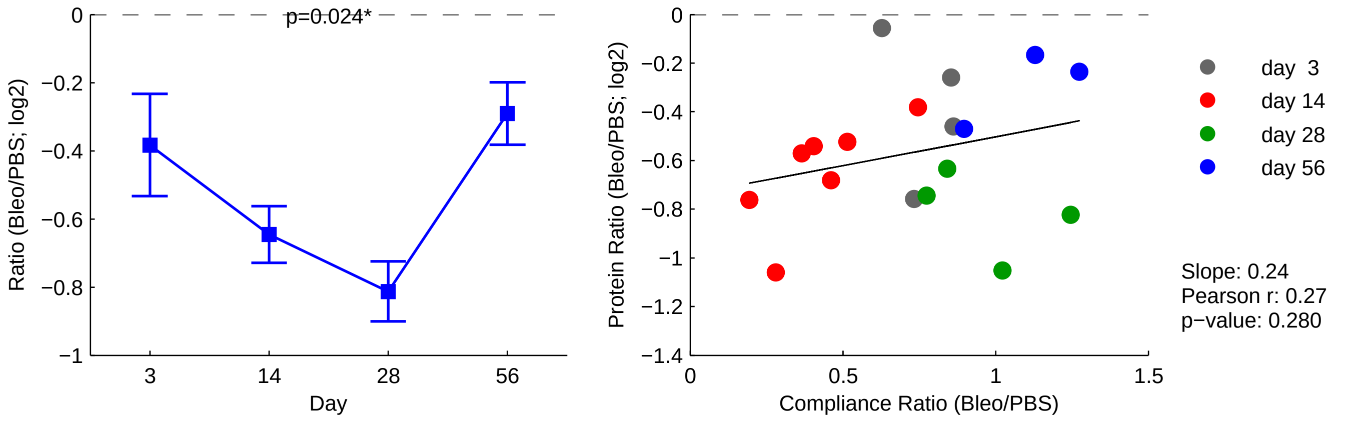

A2APY7 − Ndufaf5 (id: 169)

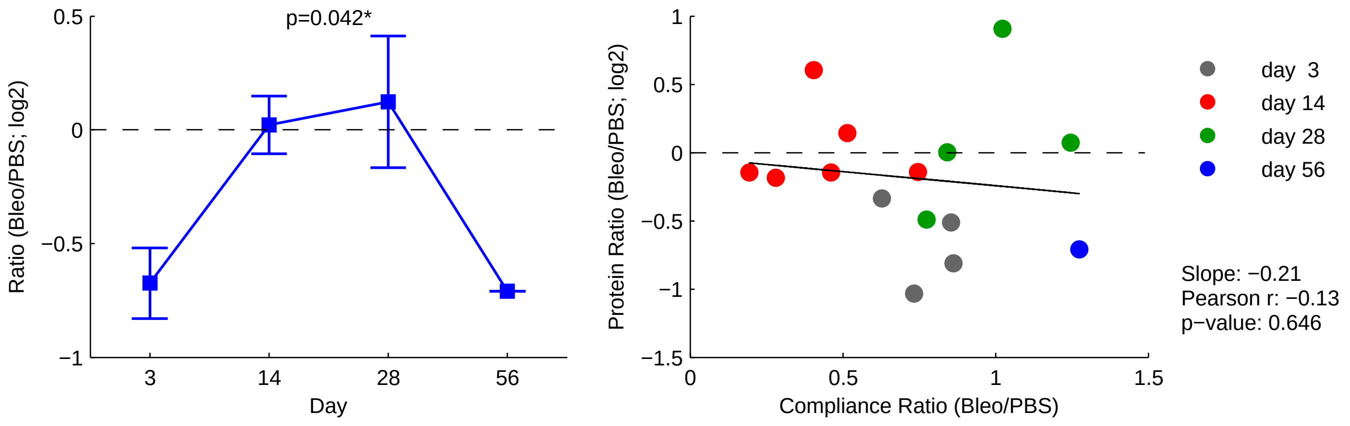

A2AQ25 − Skt (id: 172)

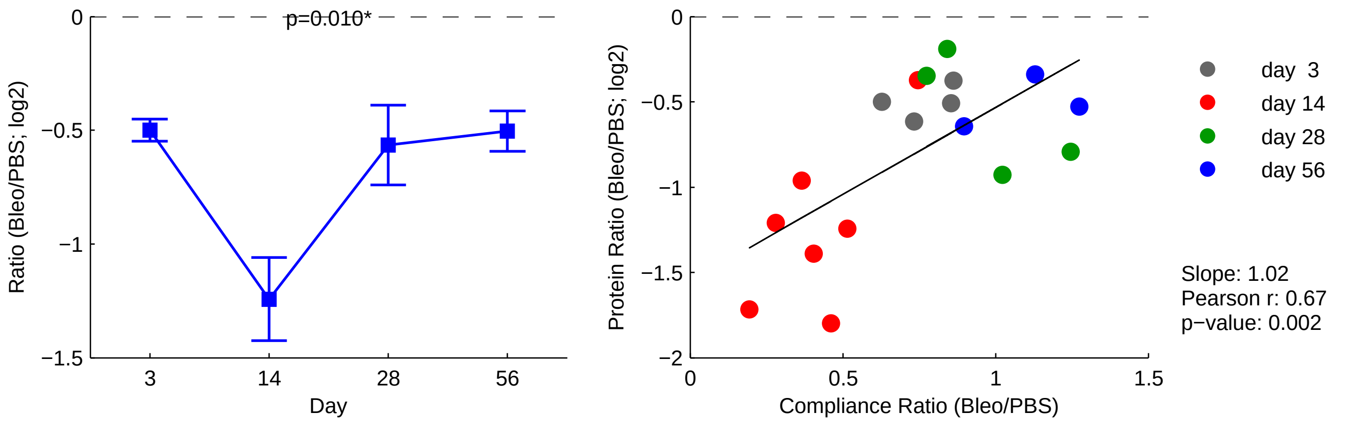

A2AQB2 − Neb (id: 176)

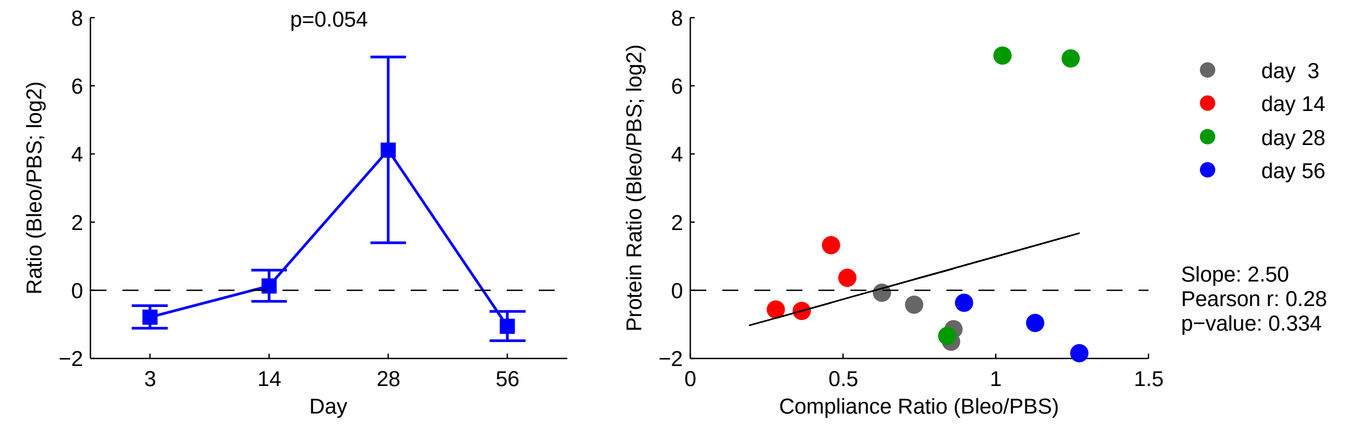

Q8BXN9−2 − Tmem87a (id: 179)

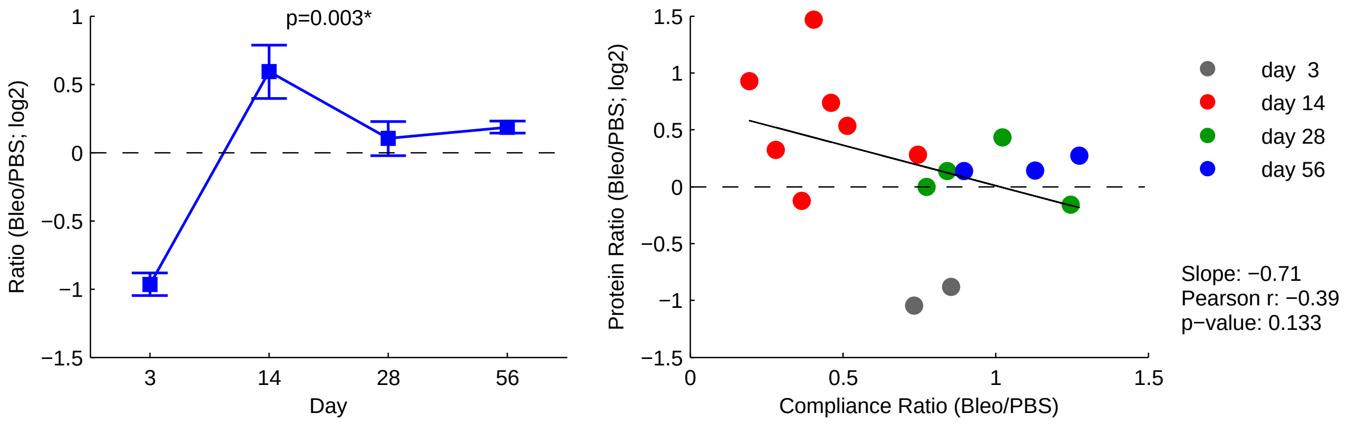

A2ARA8 − Itga8 (id: 184)

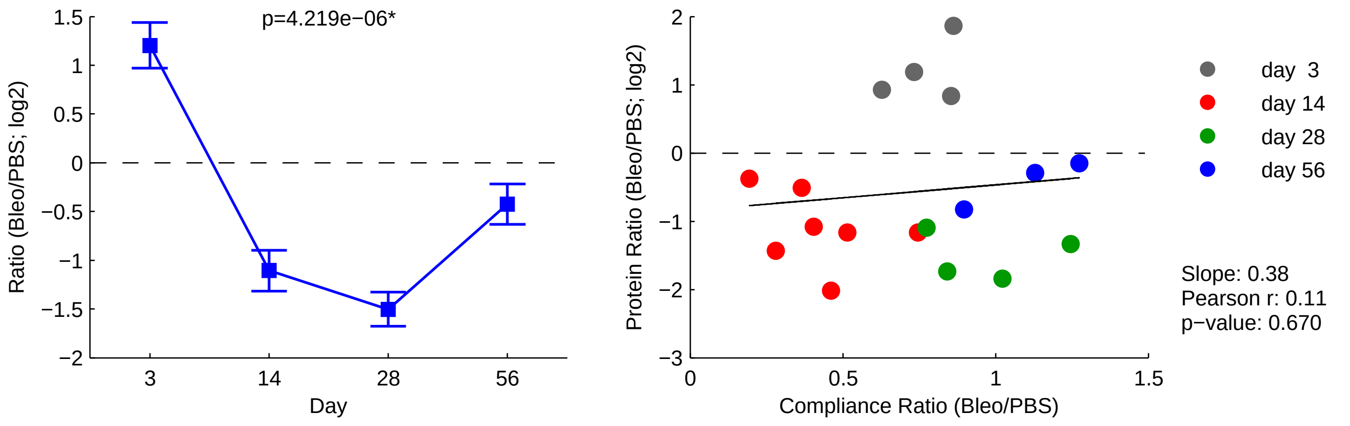

A2ARV4 − Lrp2 (id: 187)

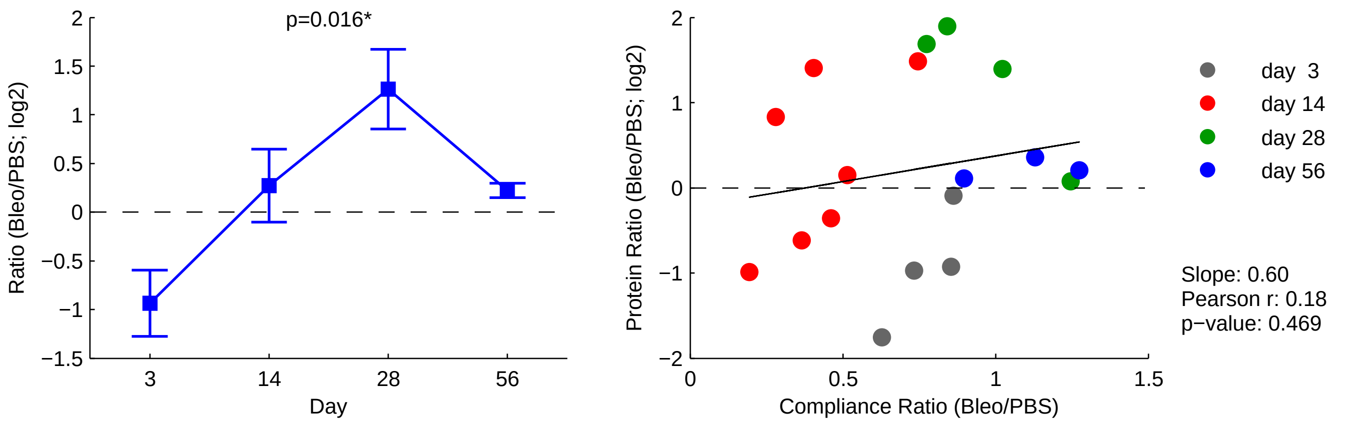

Q68FH0−2 − Pkp4 (id: 189)

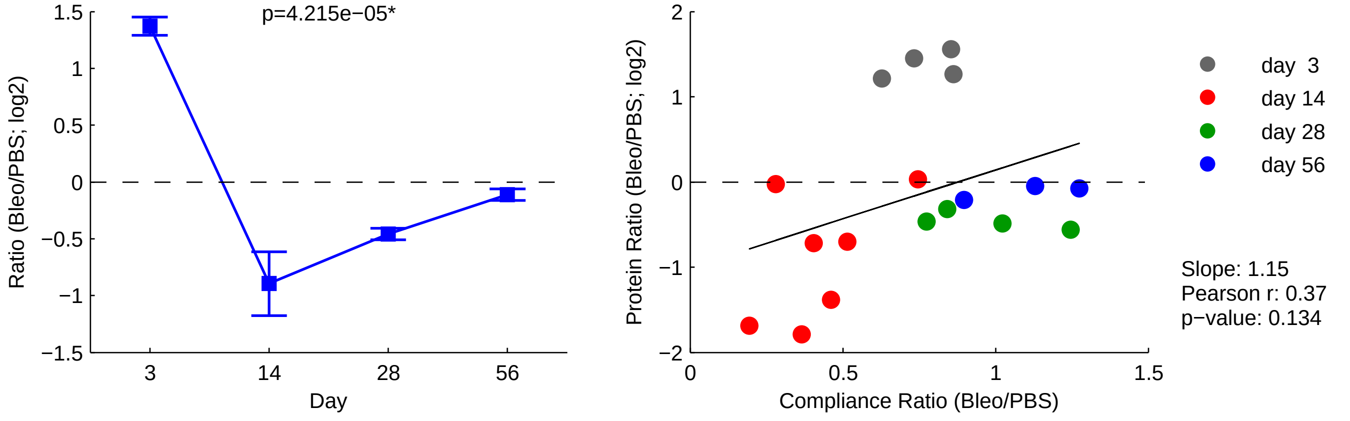

P28660 − Nckap1 (id: 191)

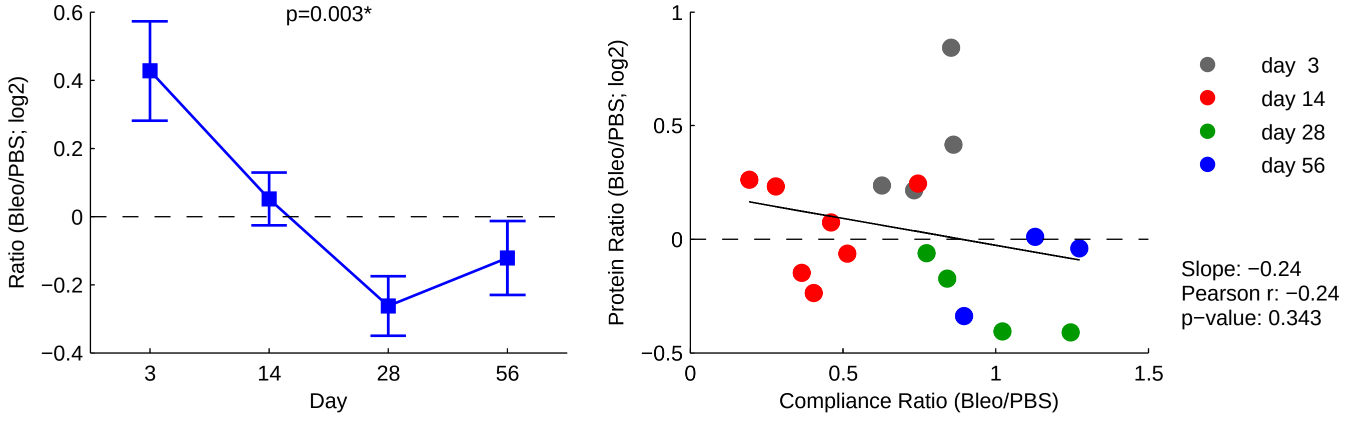

A2ASS6 − Ttn (id: 193)

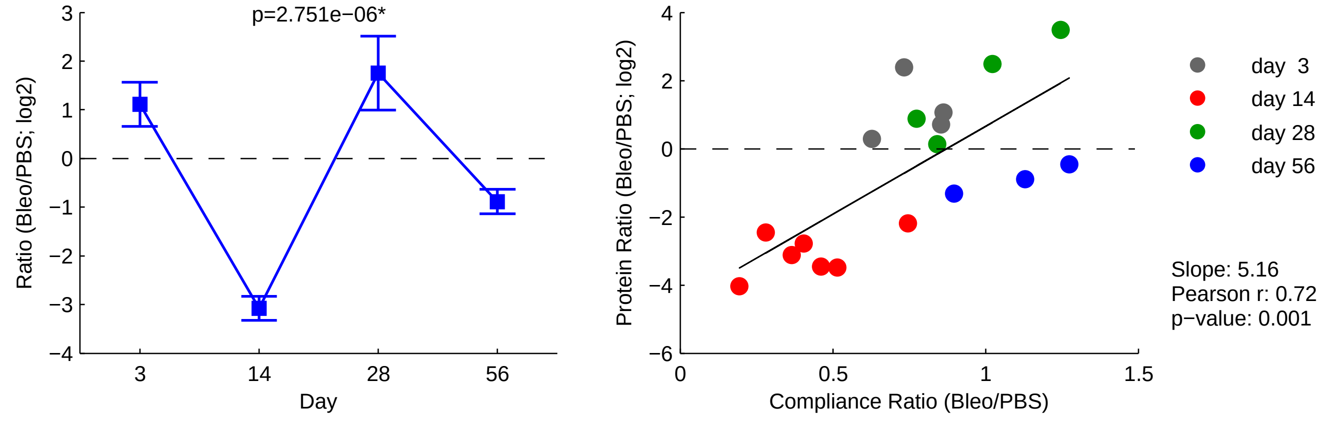

A2AT37 − Upf2 (id: 199)

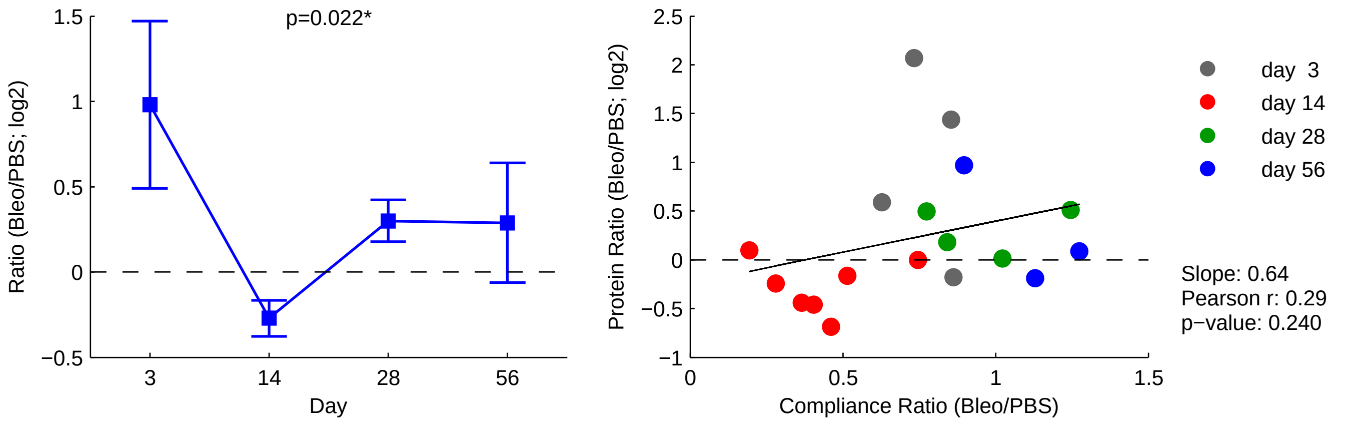

A2ATI9 − Gorasp2 (id: 201)

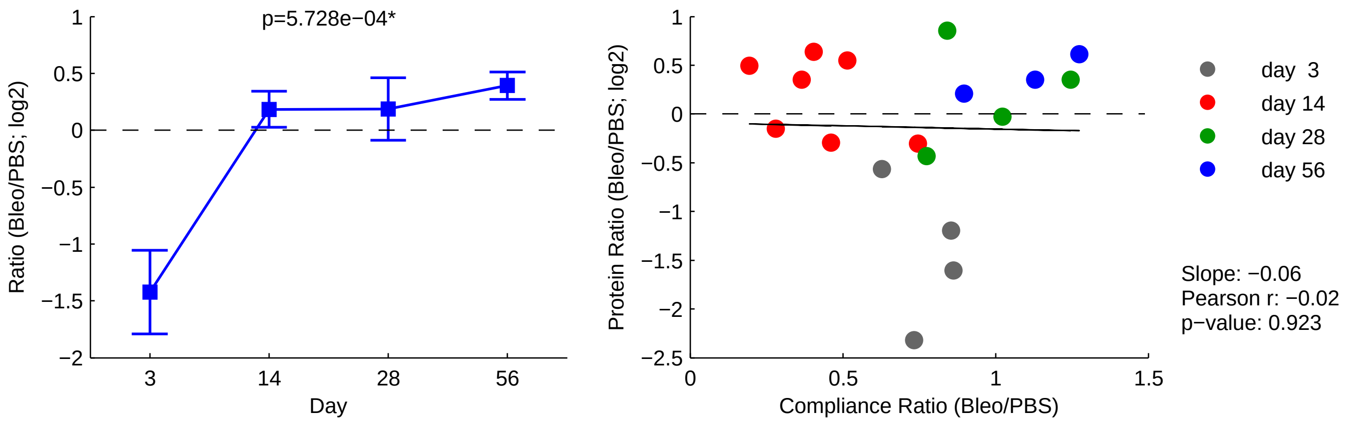

A2ATK9 − Fam171a1 (id: 202)

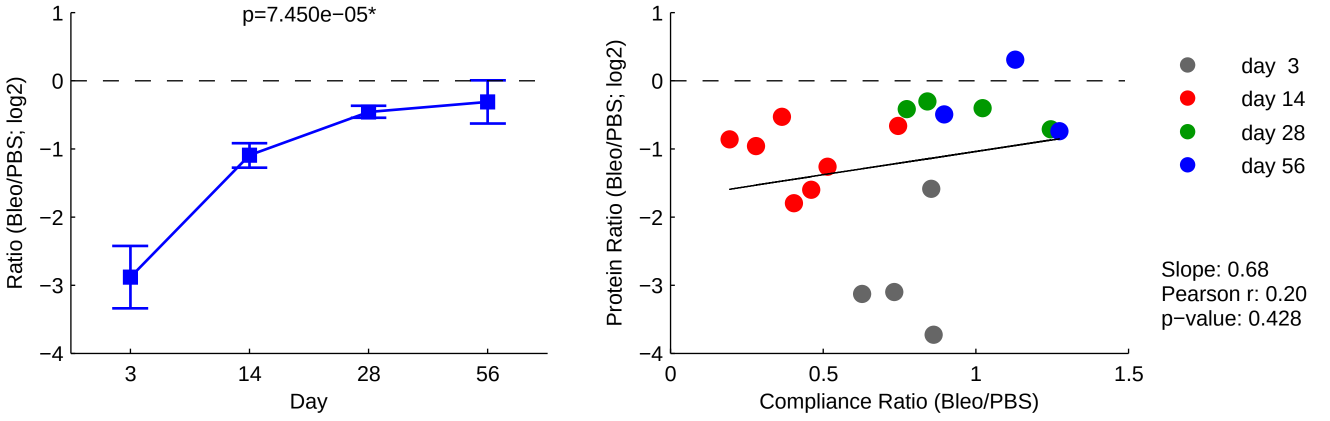

A2AU71 − Armc3 (id: 206)

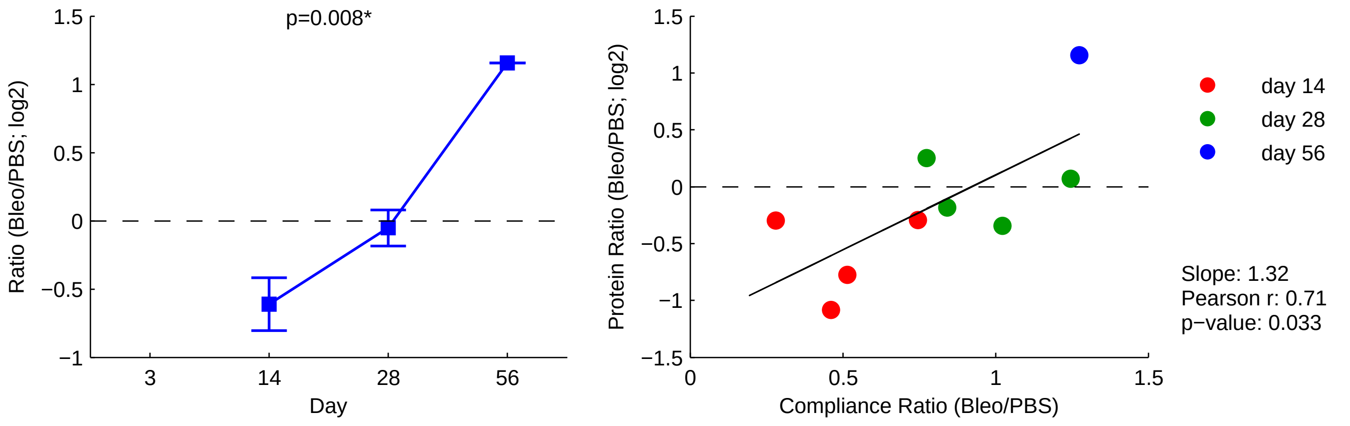

A2AU91 − Trp53bp1 (id: 207)

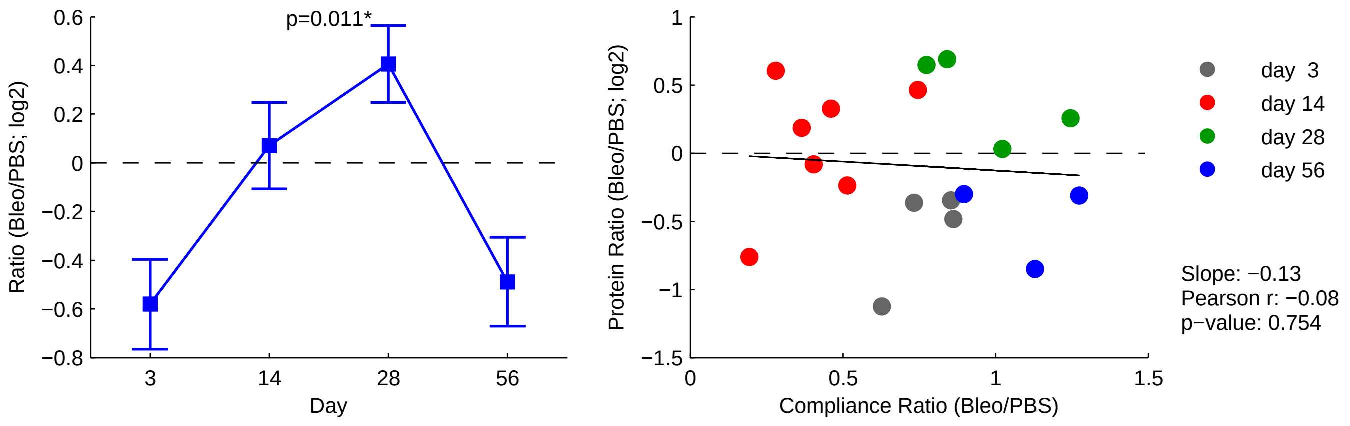

A2AUE1 − Dnajc5 (id: 209)

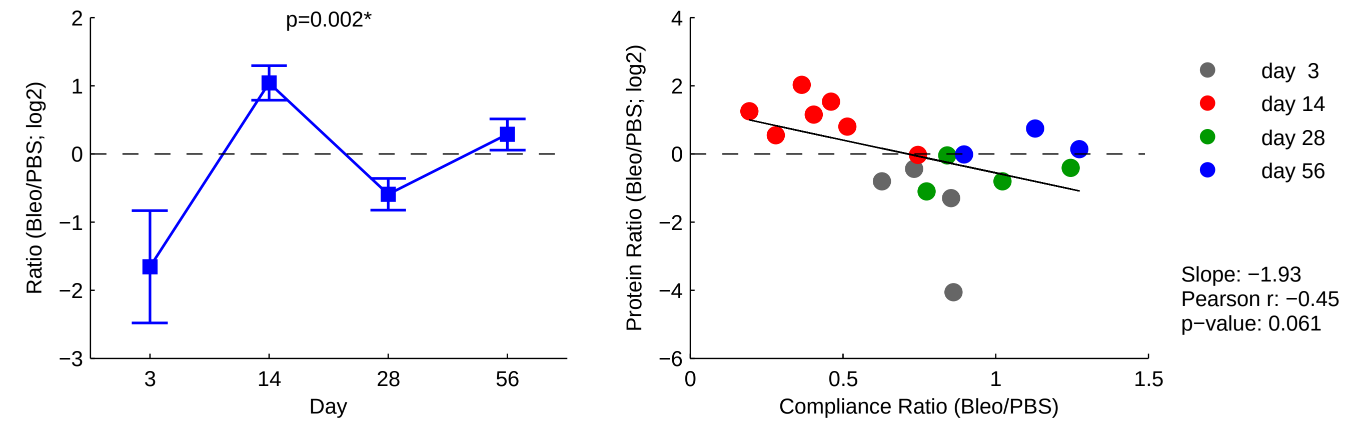

A2AUX5 − Dab2ip (id: 214)

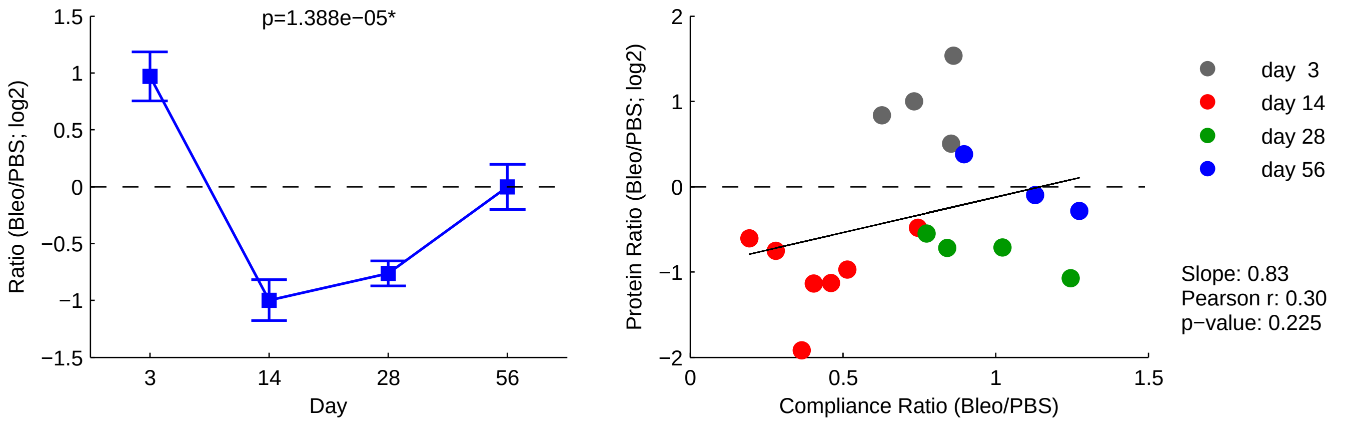

A2AVA0 − Svep1 (id: 216)

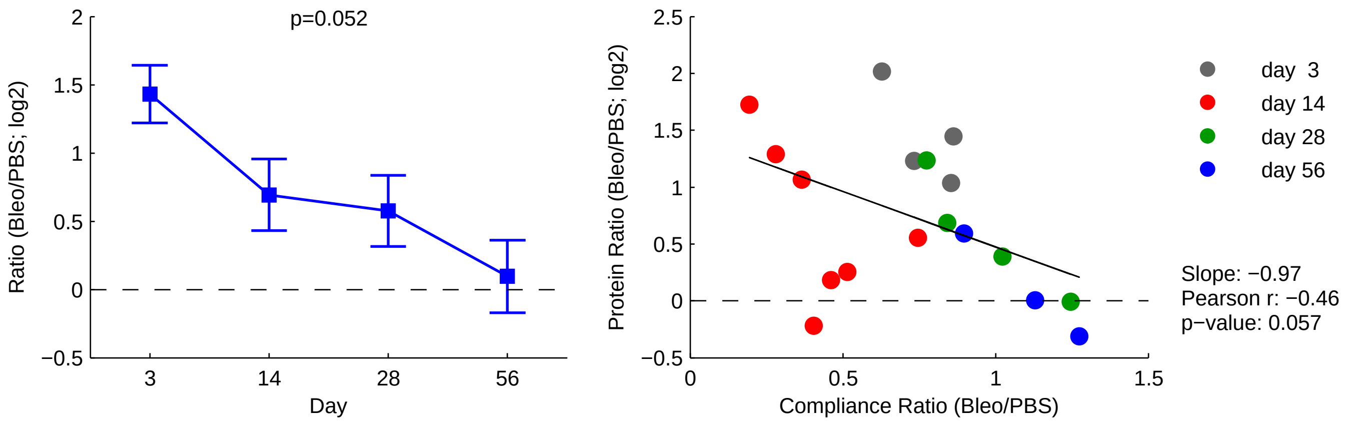

A2AVJ7 − Rrbp1 (id: 217)

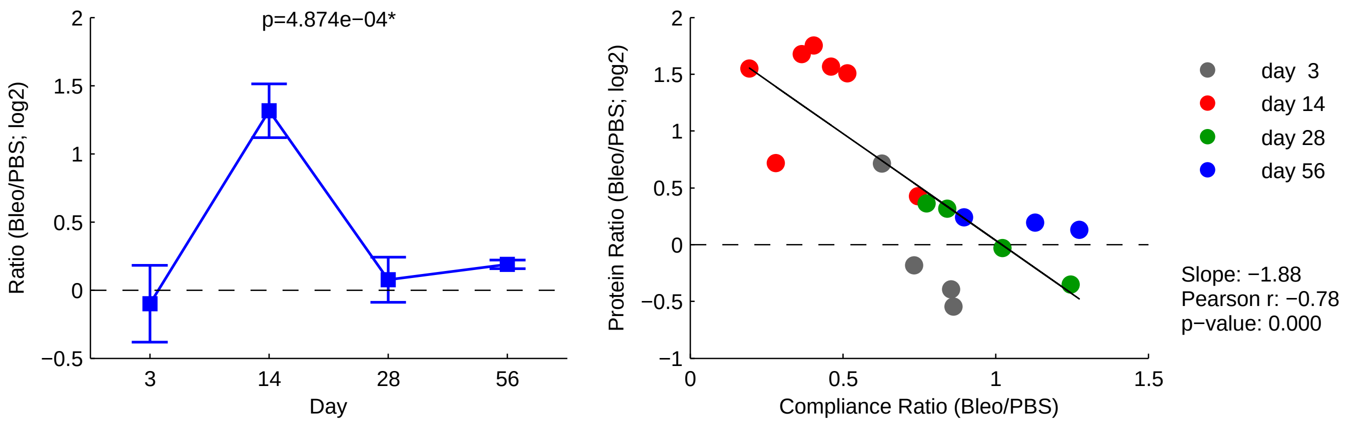

A2AWF8 − Ptprj (id: 221)

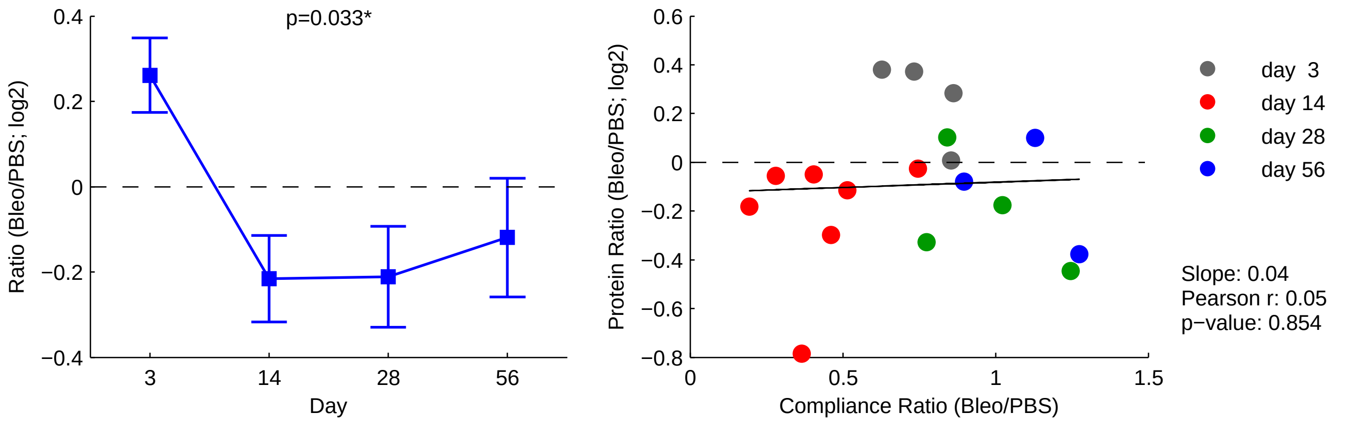

P59326 − Ythdf1 (id: 222)

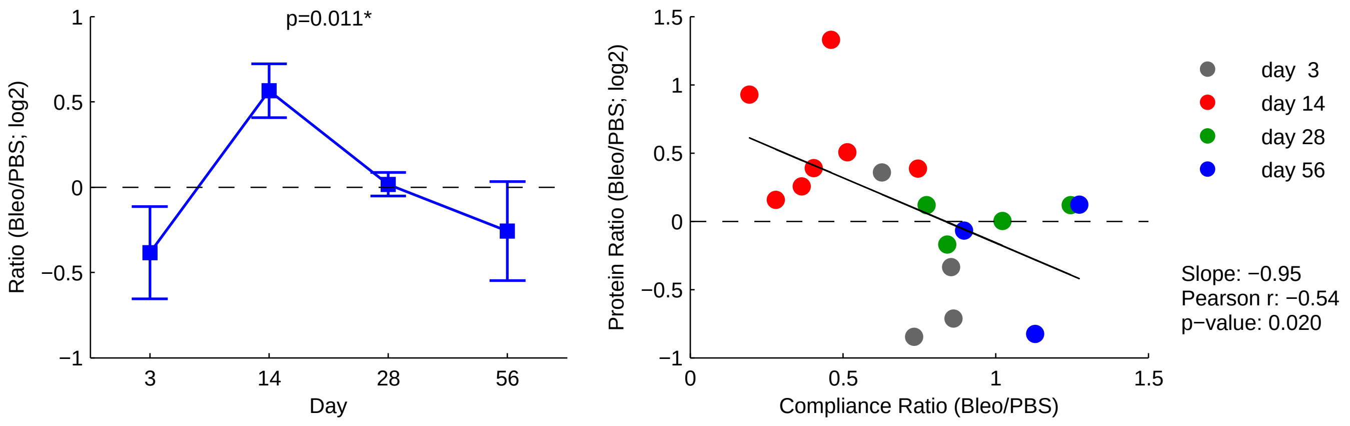

A2AWT5 − Ubtf (id: 224)

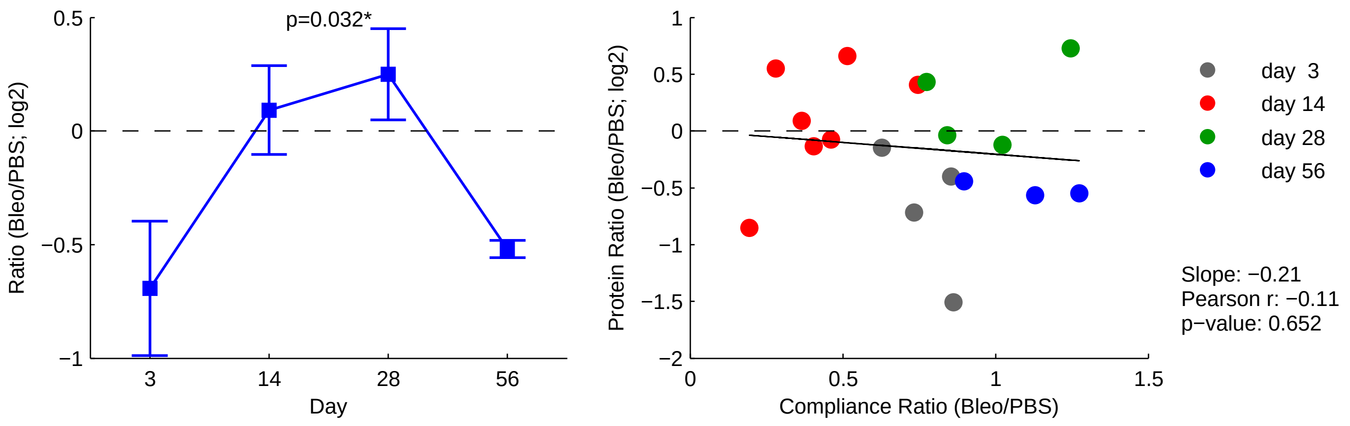

A2AX52 − Col6a4 (id: 226)

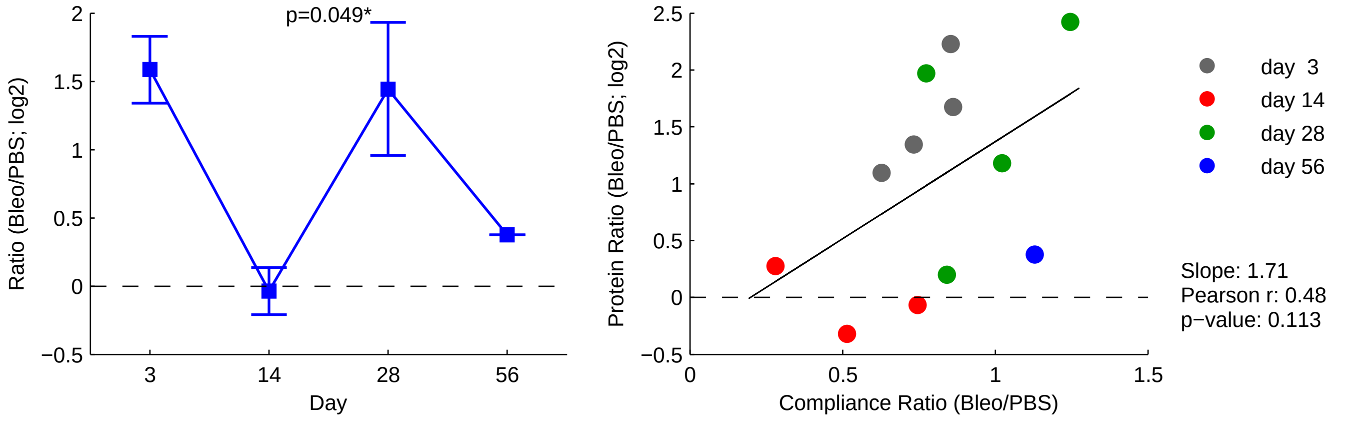

A2BEF2 − Dlg3 (id: 231)

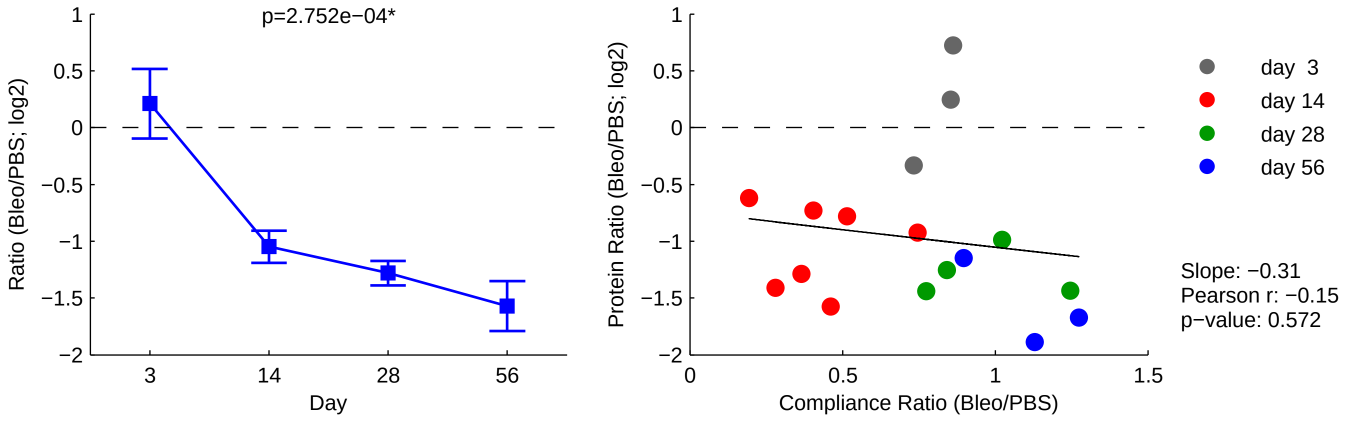

P97863−3 − Nfib (id: 232)

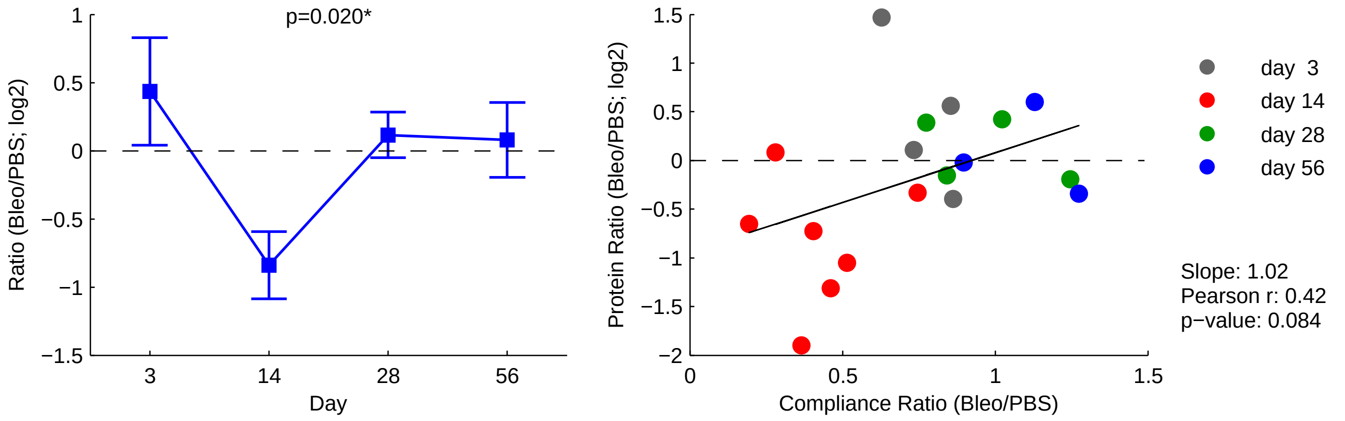

A2BH40−3 − Arid1a (id: 233)

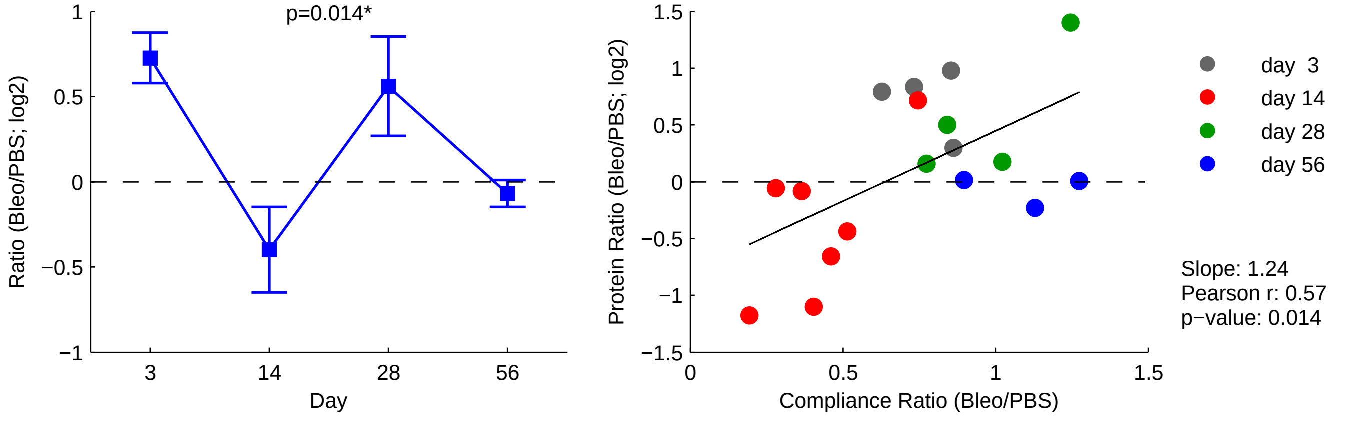

P35283 − Rab12 (id: 238)

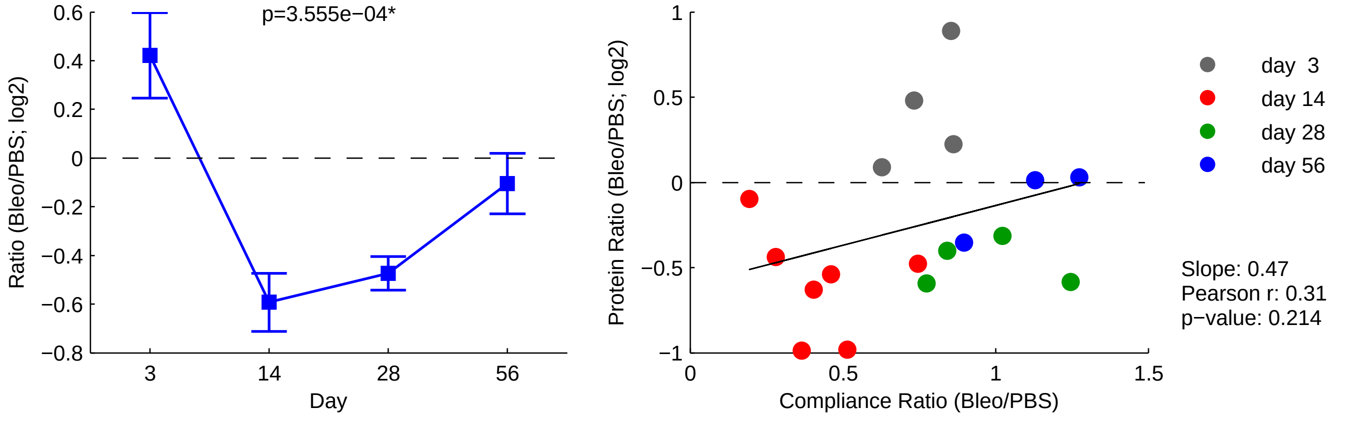

A2RSX9 − Arfip1 (id: 245)

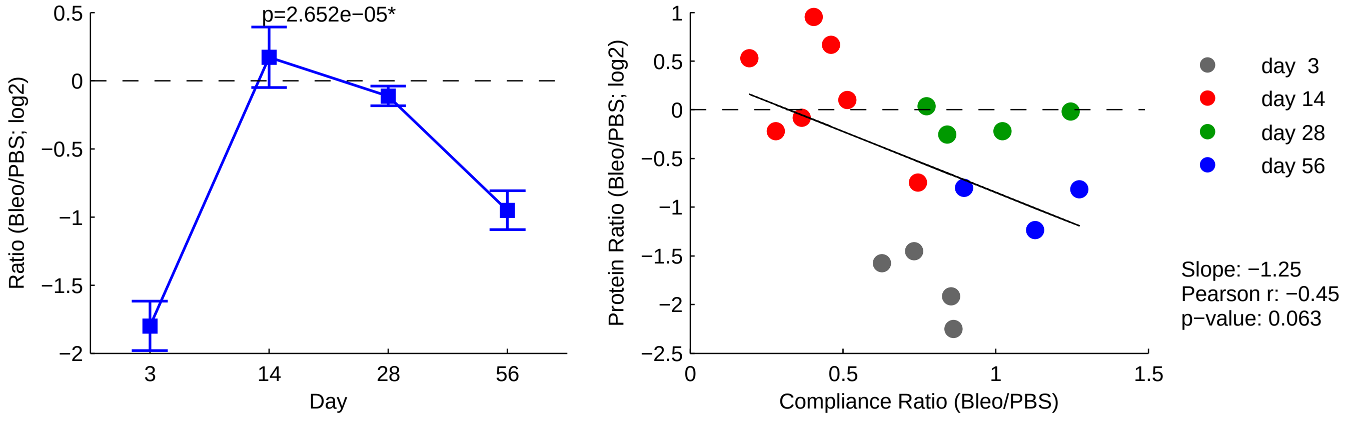

A2RT60 − Htra4 (id: 248)

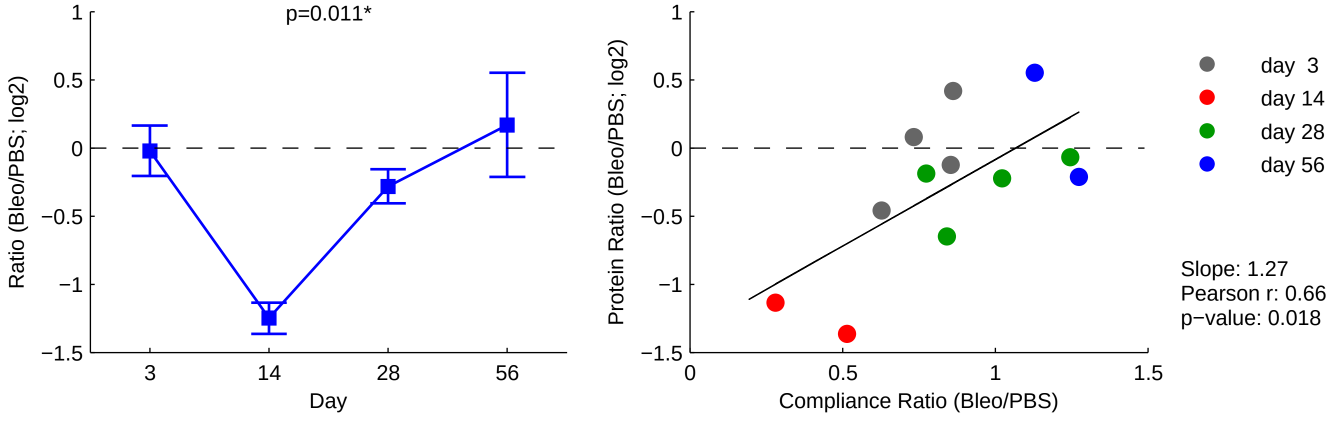

A2RT67 − Dennd3 (id: 249)

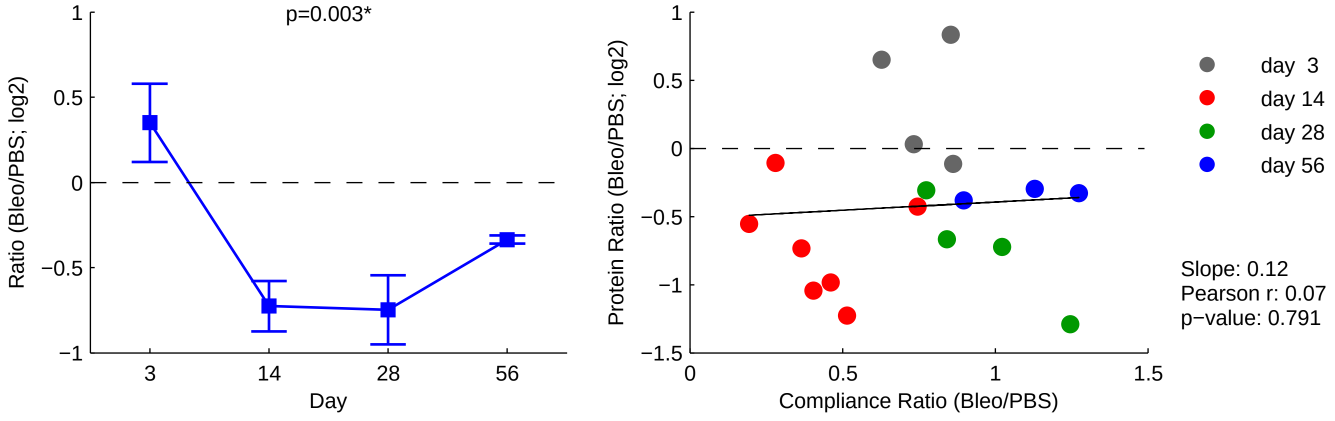

Q60952 − Cep250 (id: 257)

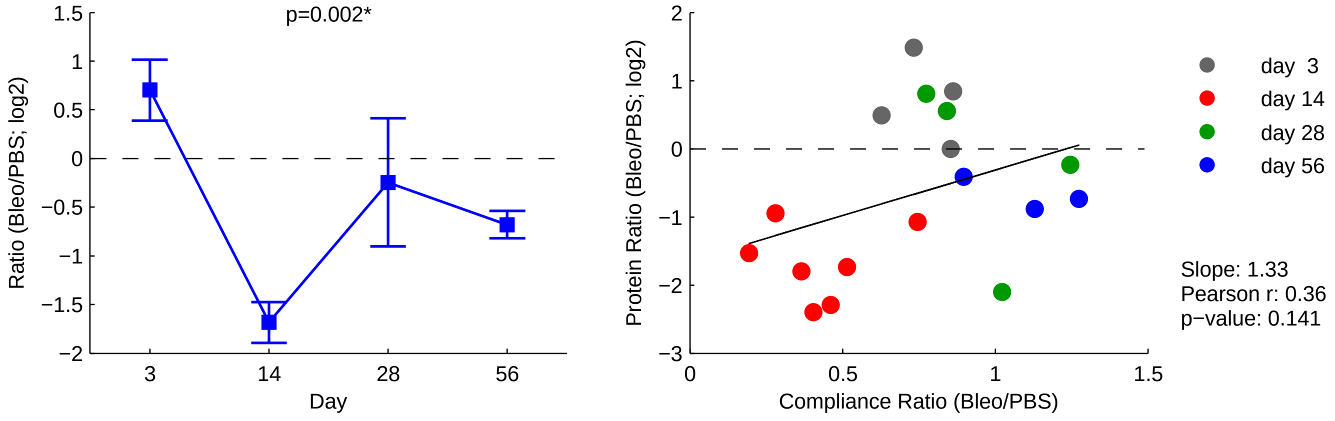

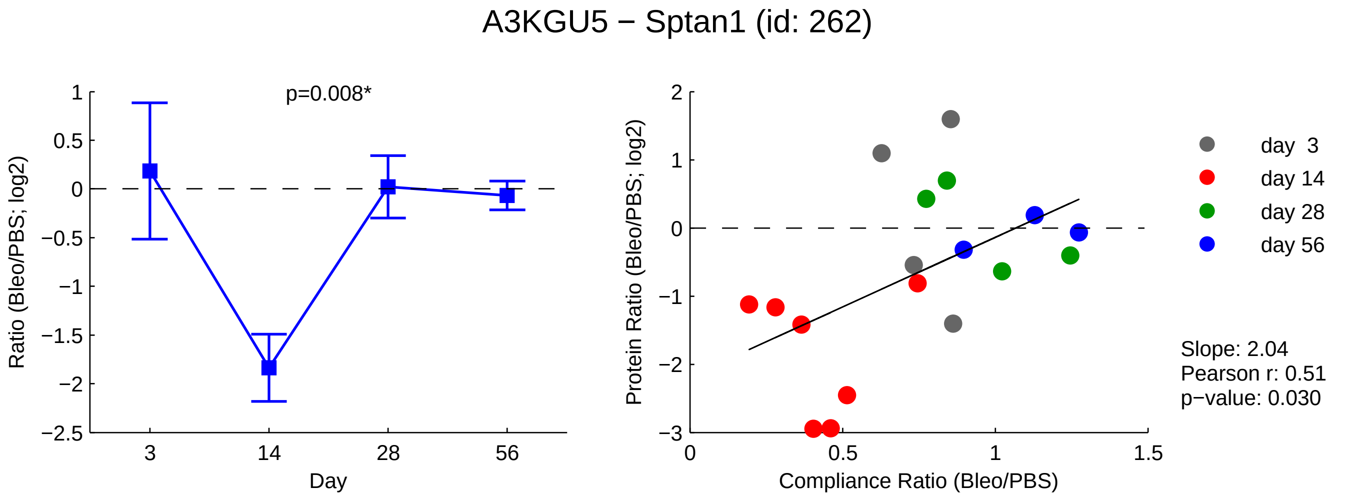
A3KGU5 − Sptan1 (id: 262)
p=0.008*
Ratio (Bleo/PBS; log2)
Day
3
14
28
56
Protein Ratio (Bleo/PBS; log2)
Compliance Ratio (Bleo/PBS)
day 3
day 14
day 28
day 56
Slope: 2.04
Pearson r: 0.51
p−value: 0.030

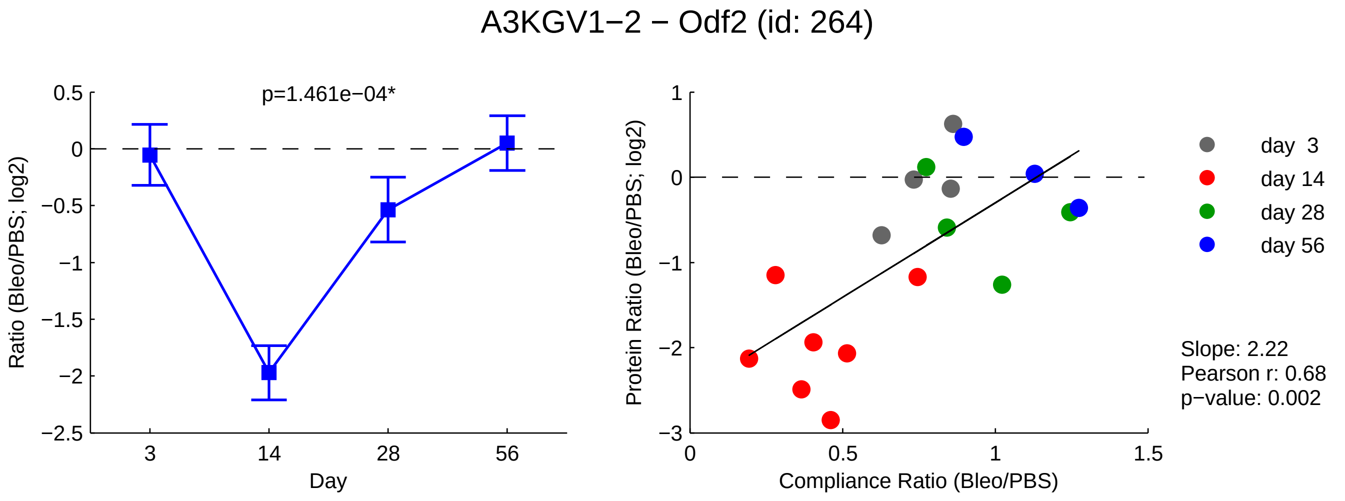
A3KGV1−2 − Odf2 (id: 264)
p=1.461e−04*
Ratio (Bleo/PBS; log2)
Day
3
14
28
56
Protein Ratio (Bleo/PBS; log2)
Compliance Ratio (Bleo/PBS)
day 3
day 14
day 28
day 56
Slope: 2.22
Pearson r: 0.68
p−value: 0.002

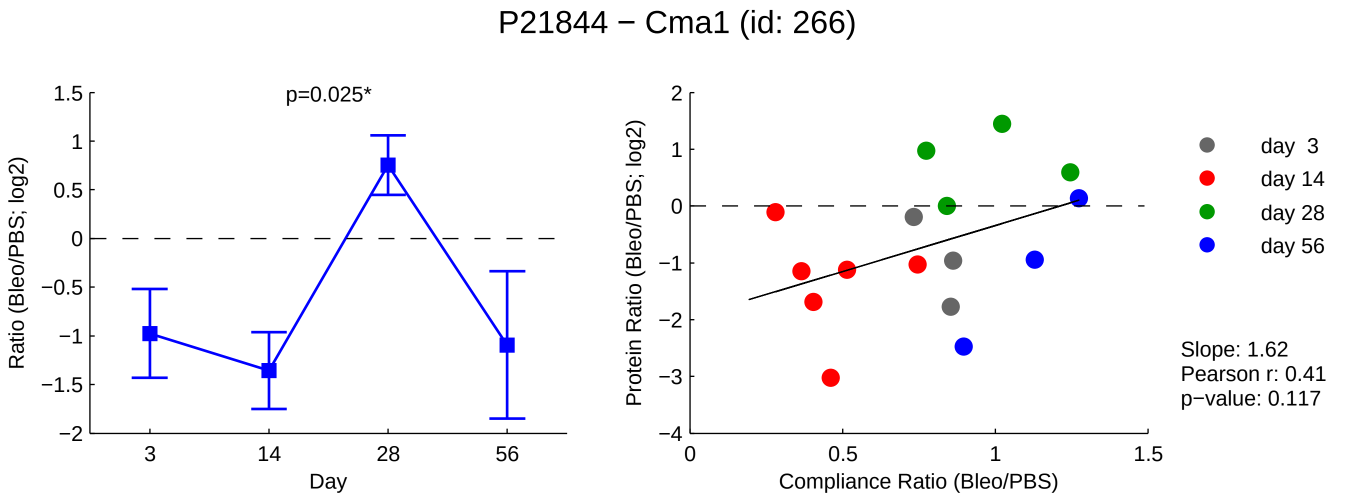
P21844 − Cma1 (id: 266)
p=0.025*
Ratio (Bleo/PBS; log2)
Day
3
14
28
56
Protein Ratio (Bleo/PBS; log2)
Compliance Ratio (Bleo/PBS)
day 3
day 14
day 28
day 56
Slope: 1.62
Pearson r: 0.41
p−value: 0.117

B7ZNY3 − Pard3 (id: 268)

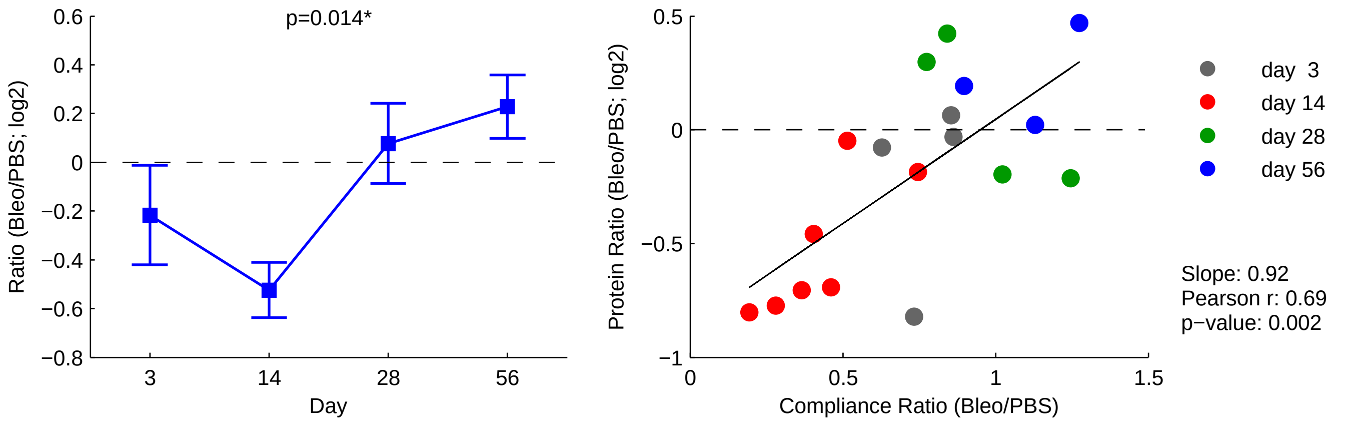

E9Q1B5 − Col6a5 (id: 269)

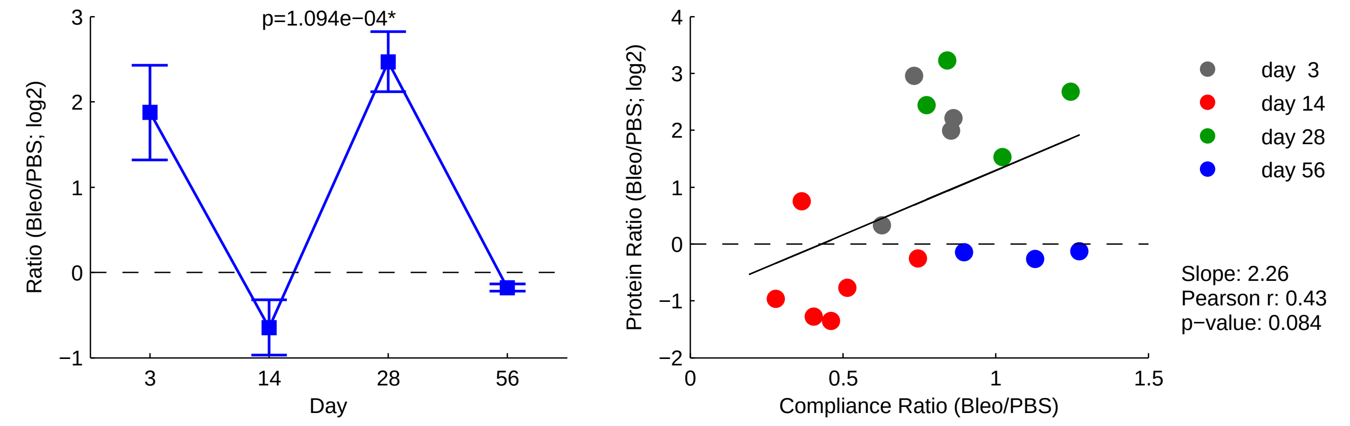

A6H5X4−3 − Phf11 (id: 270)

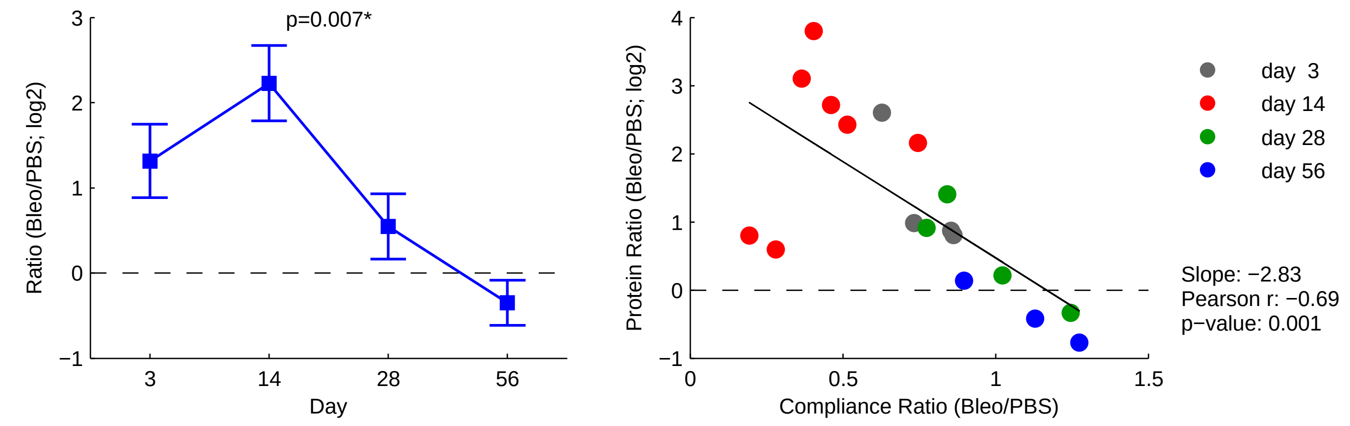

A6H699 − Poc1b (id: 277)

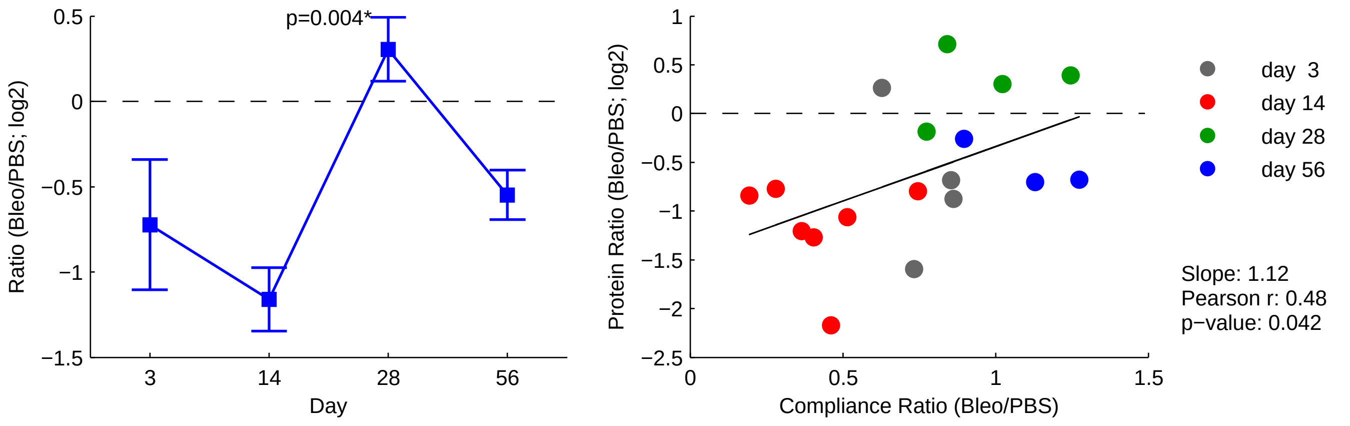

A6H6E2 − Mmrn2 (id: 279)

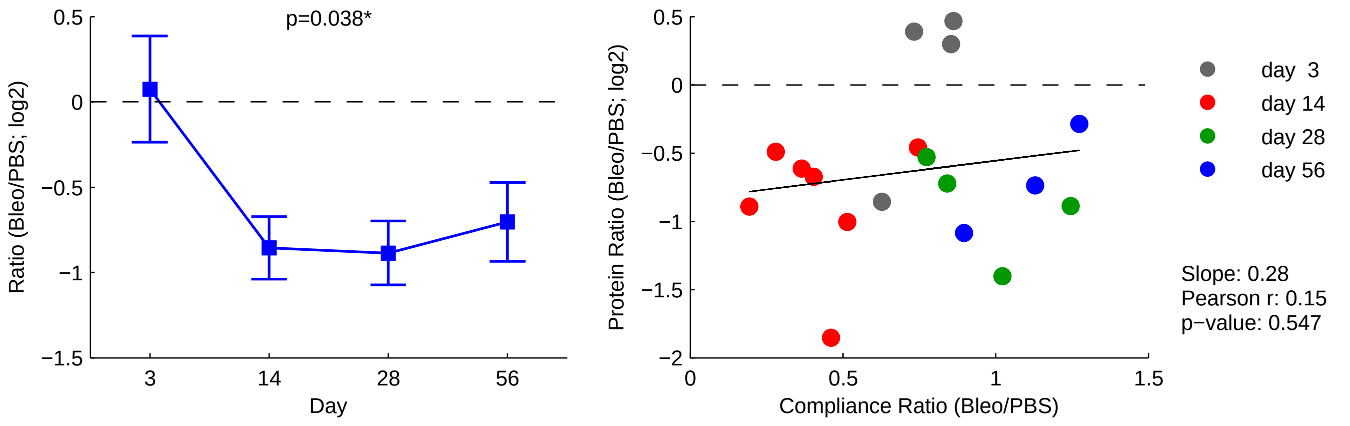

Q3V1T4 − Lepre1 (id: 282)

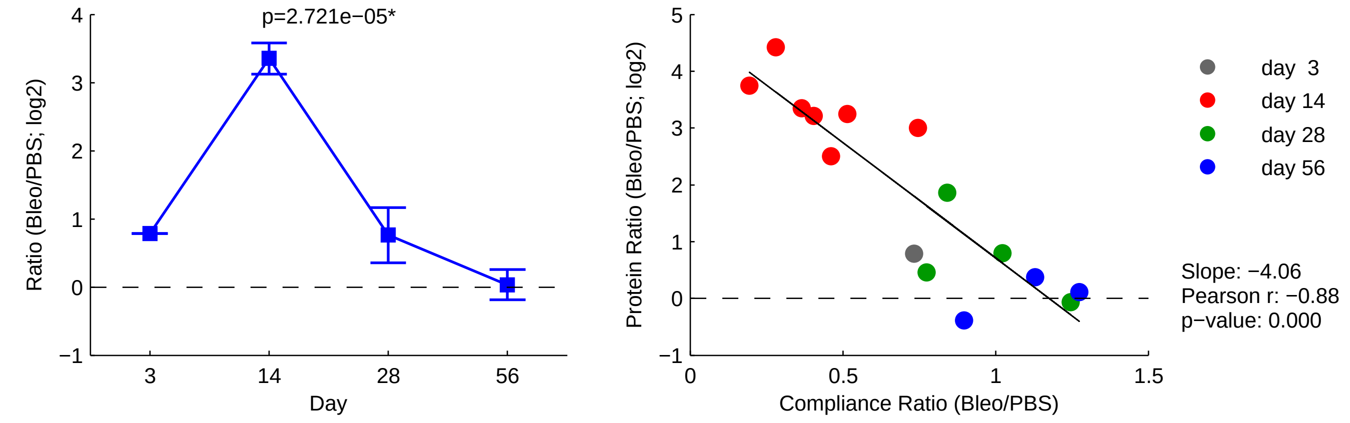

A6PWD2 − Fhad1 (id: 283)

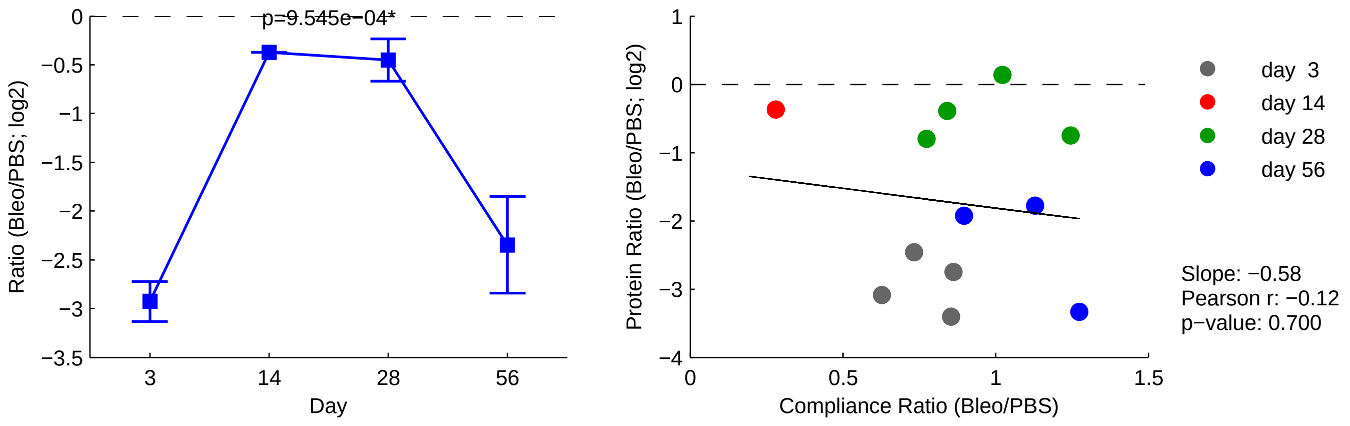

A6PWX9 − Uqcc (id: 286)

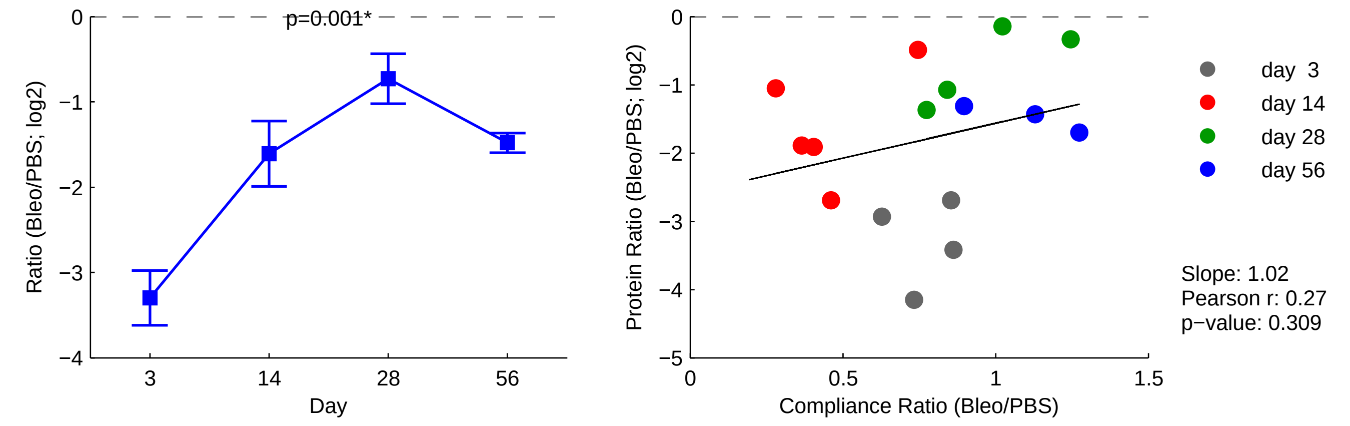

A6X8Z5 − Arhgap31 (id: 289)

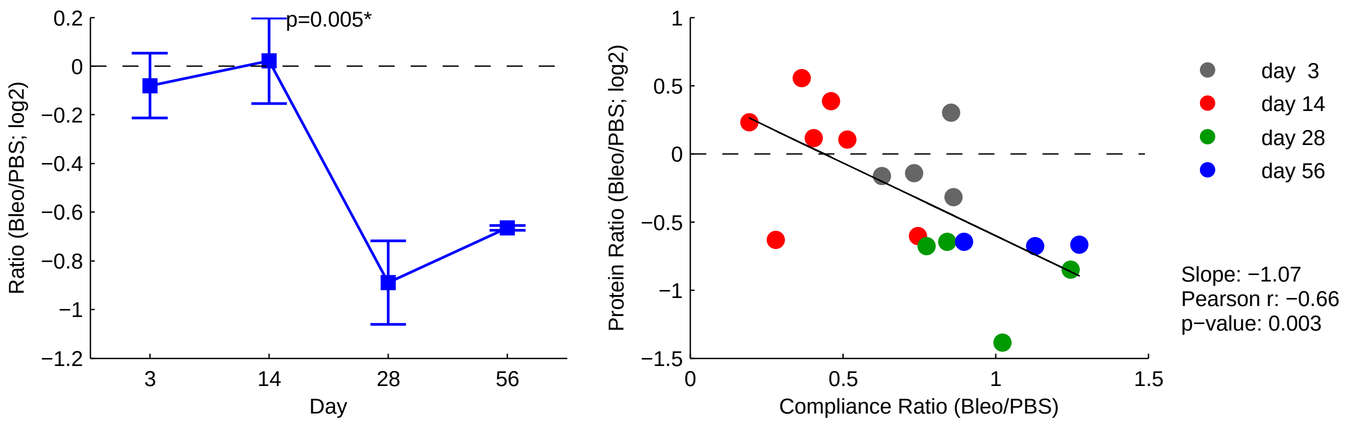

E9Q5L2 − Itih4 (id: 291)

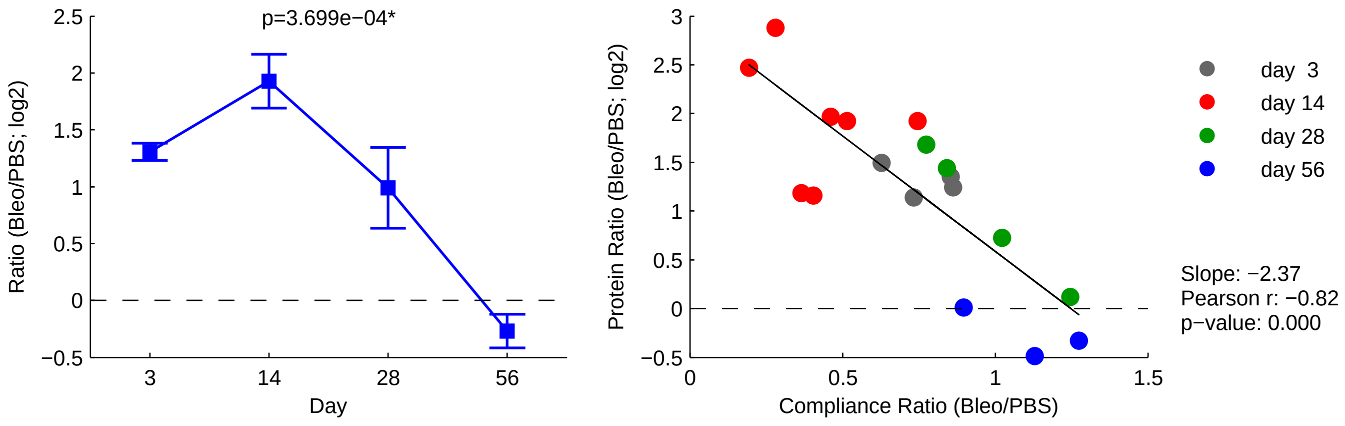

Q9JL15 − Lgals8 (id: 295)

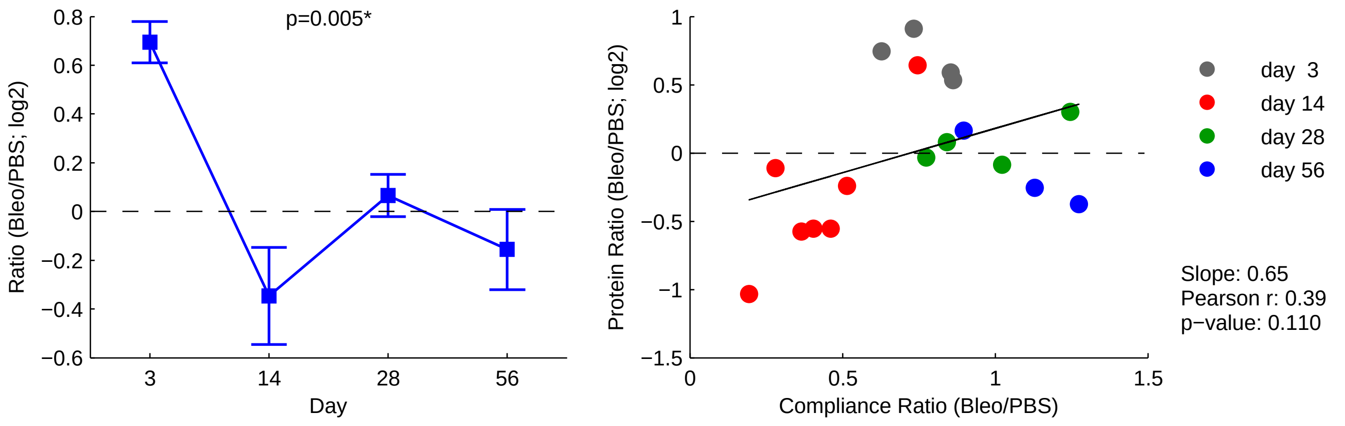

A8Y5N8 − Plekhf2 (id: 298)

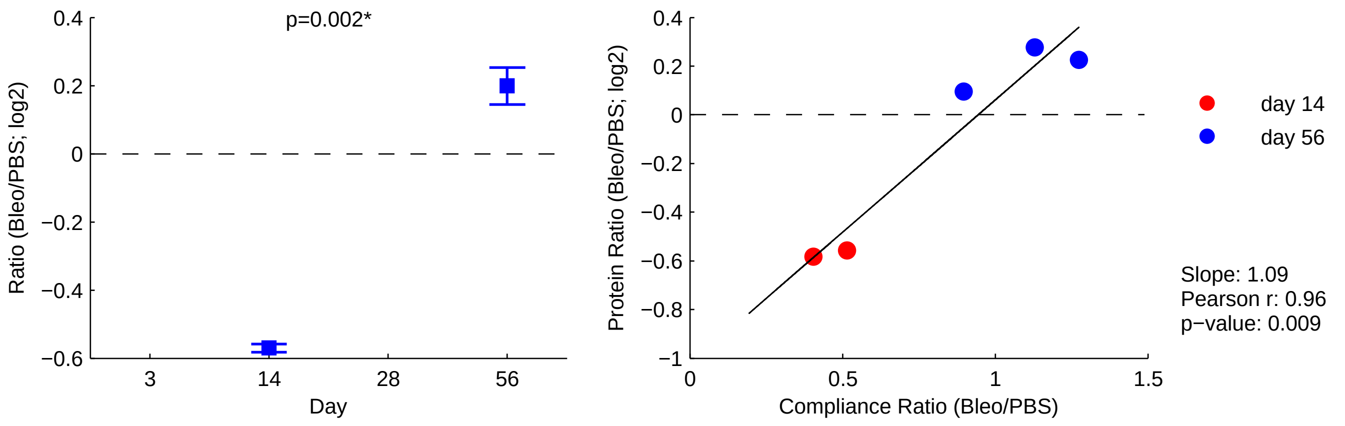

Q8CFI5 − Pars2 (id: 300)

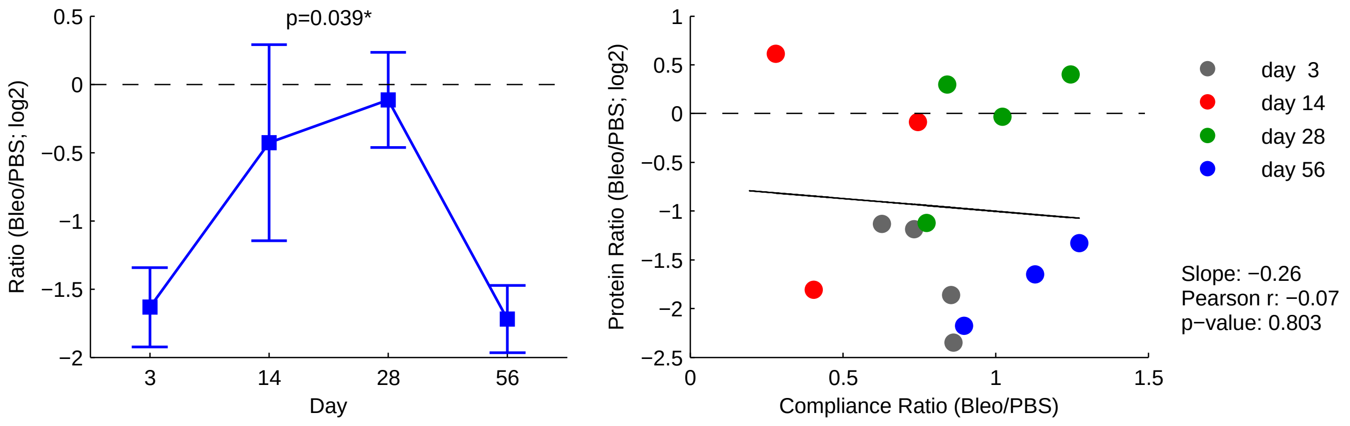

Q8JZN1 − Mgl2 (id: 301)

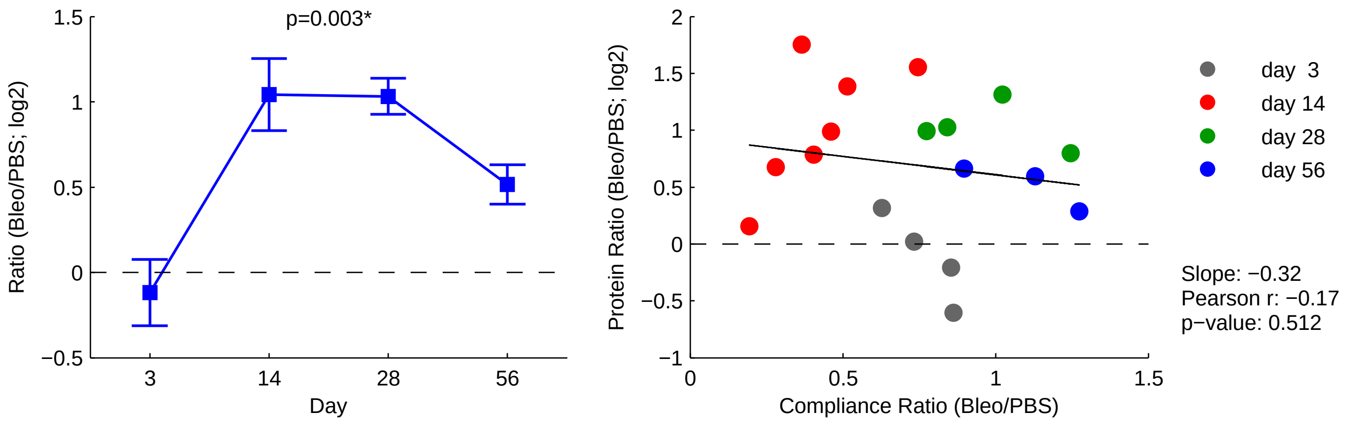

B0R091 − Chp1 (id: 308)

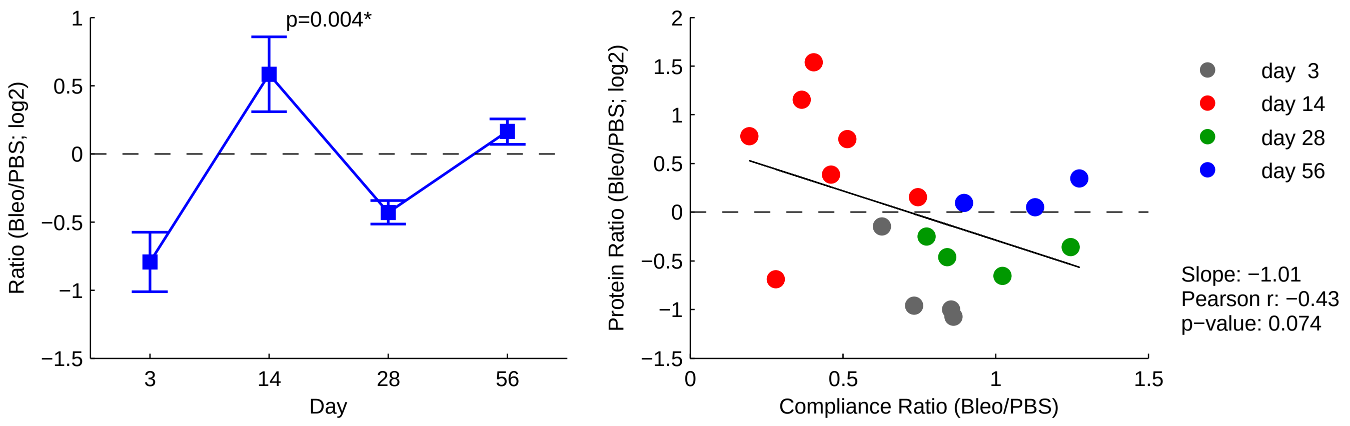

Q9ESU6 − Brd4 (id: 311)

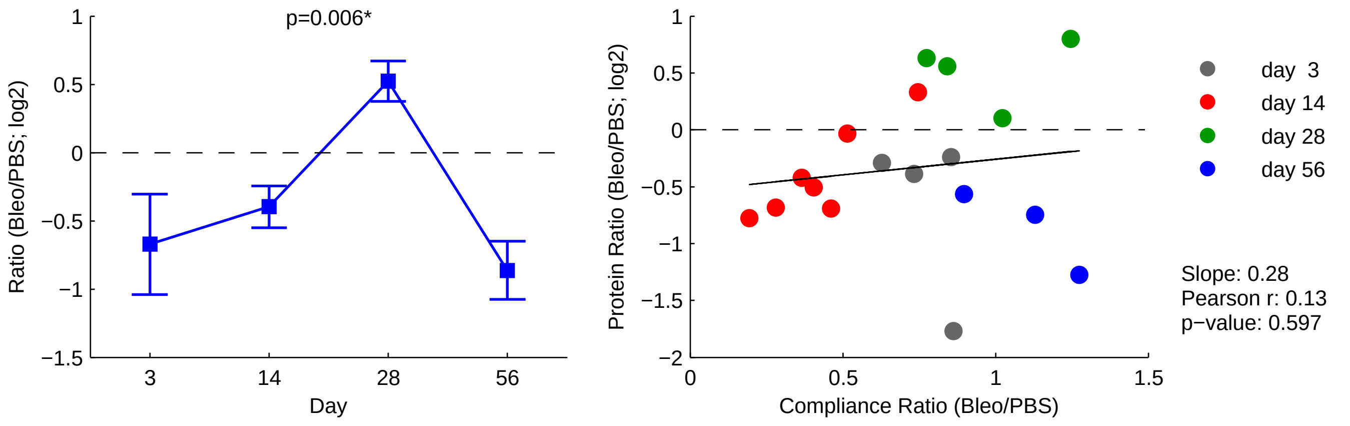

Q61414 − Krt15 (id: 315)

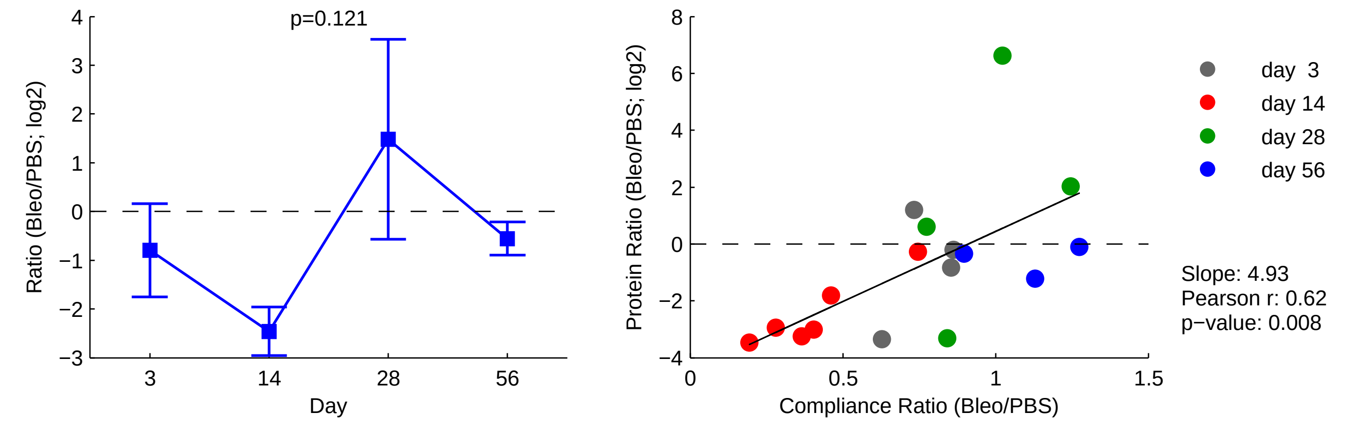

Q9D7X3 − Dusp3 (id: 316)

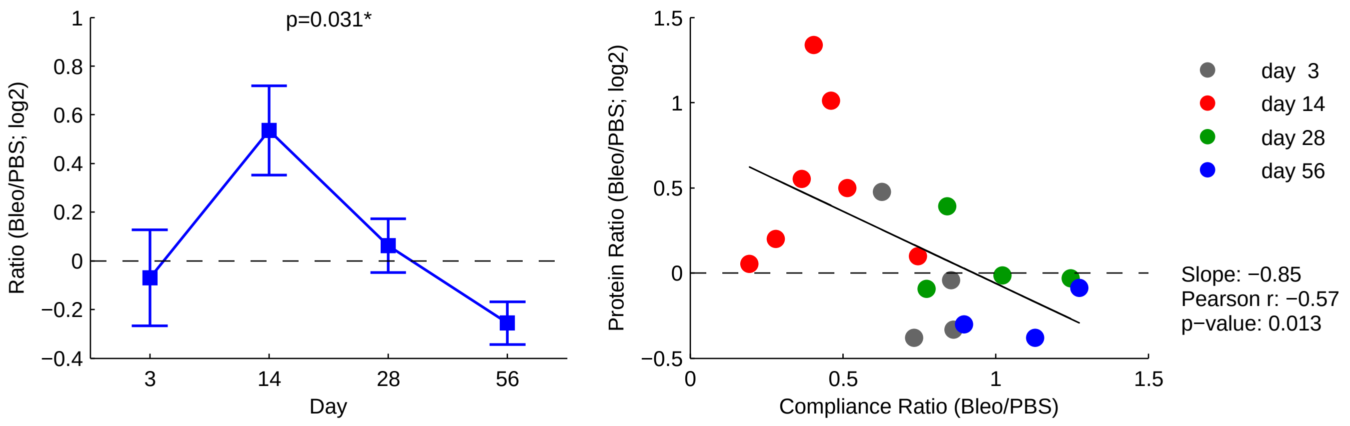

O08573−2 − Lgals9 (id: 317)

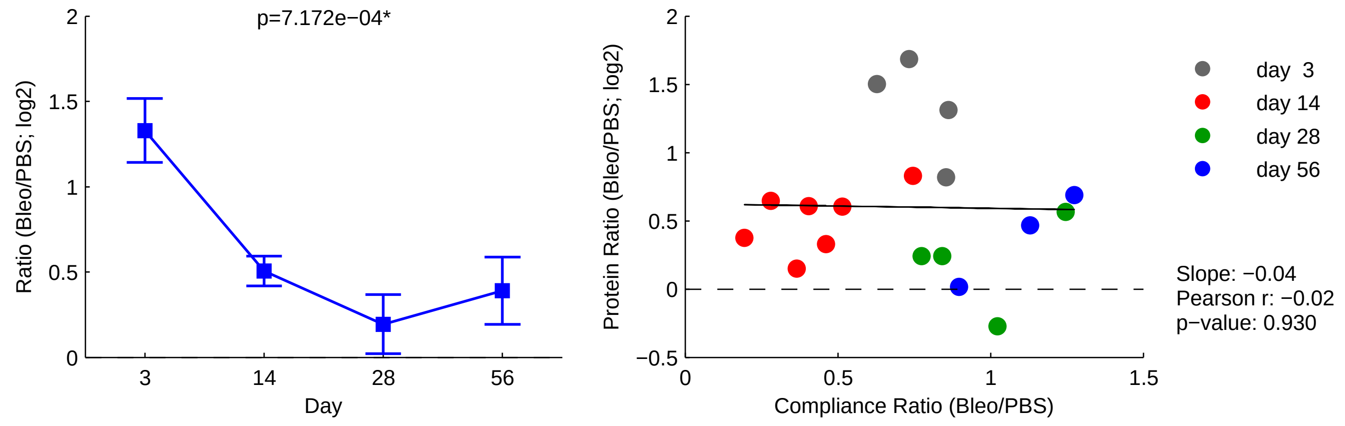

B2RWE3 − Cisd3 (id: 322)

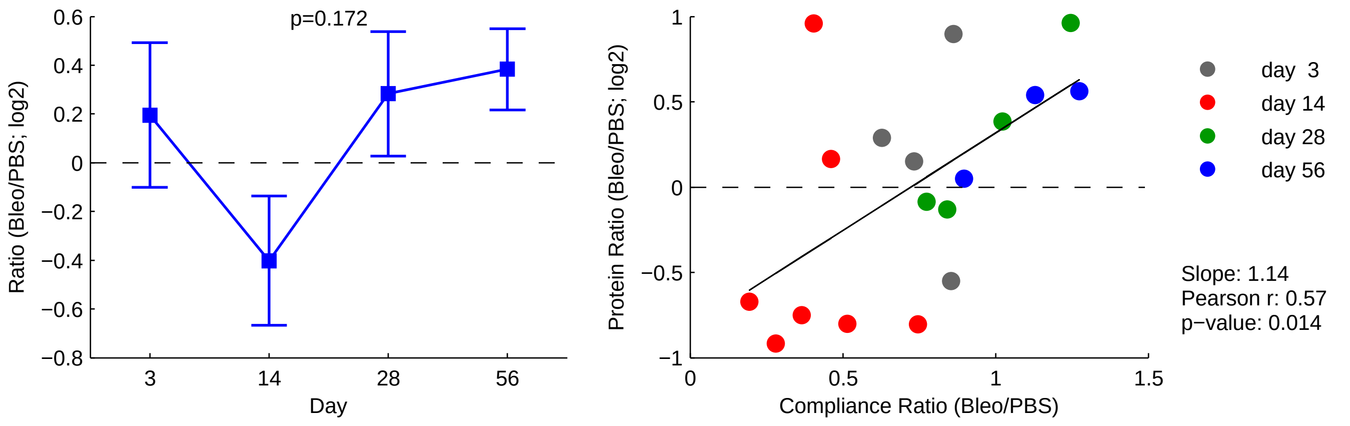

F7C528 − Chd3 (id: 323)

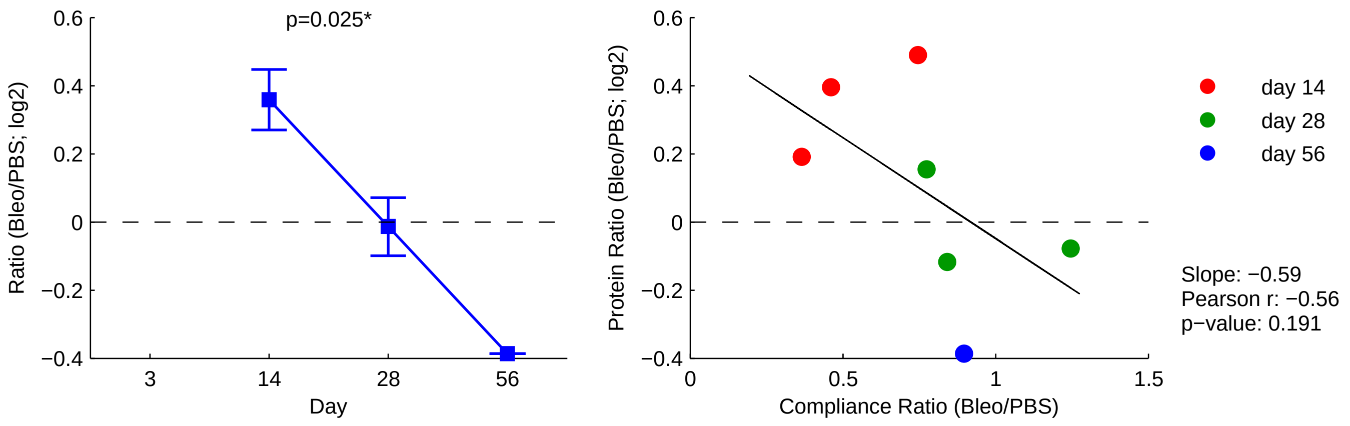

B1AR51 − Dnahc9 (id: 324)

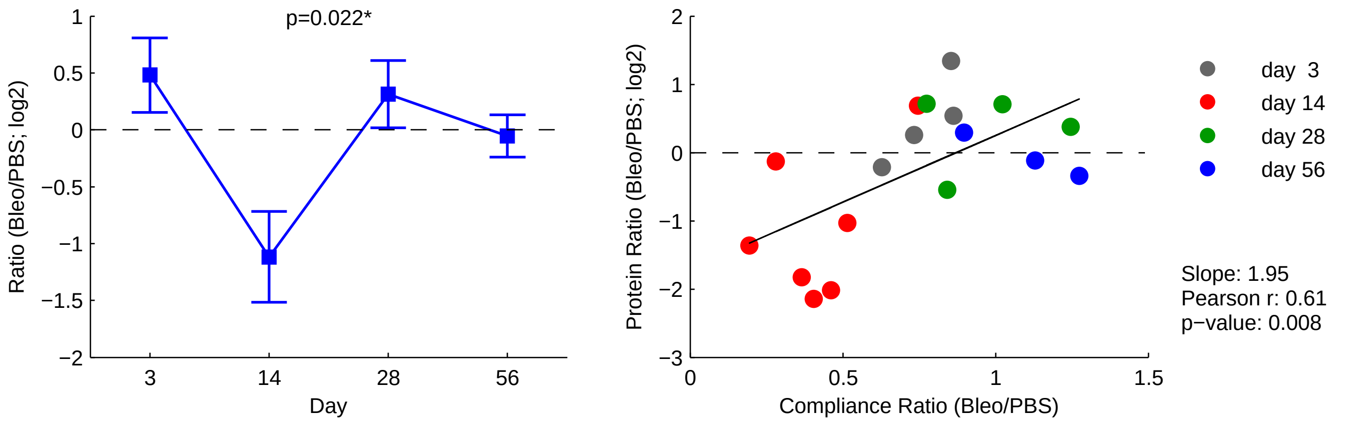

B1ART1 − Vps13d (id: 327)

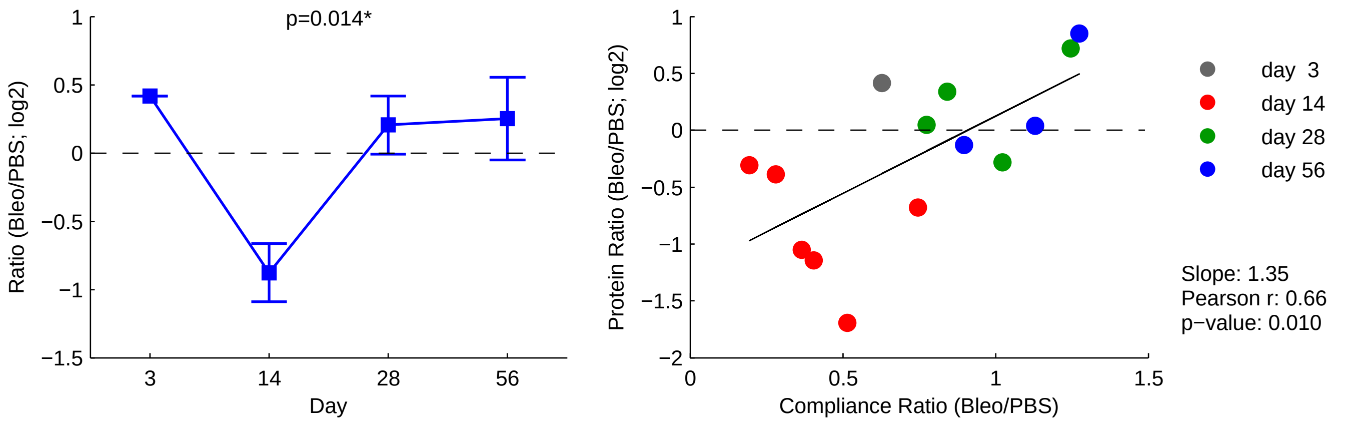

B1ASA5 − Zfp362 (id: 329)

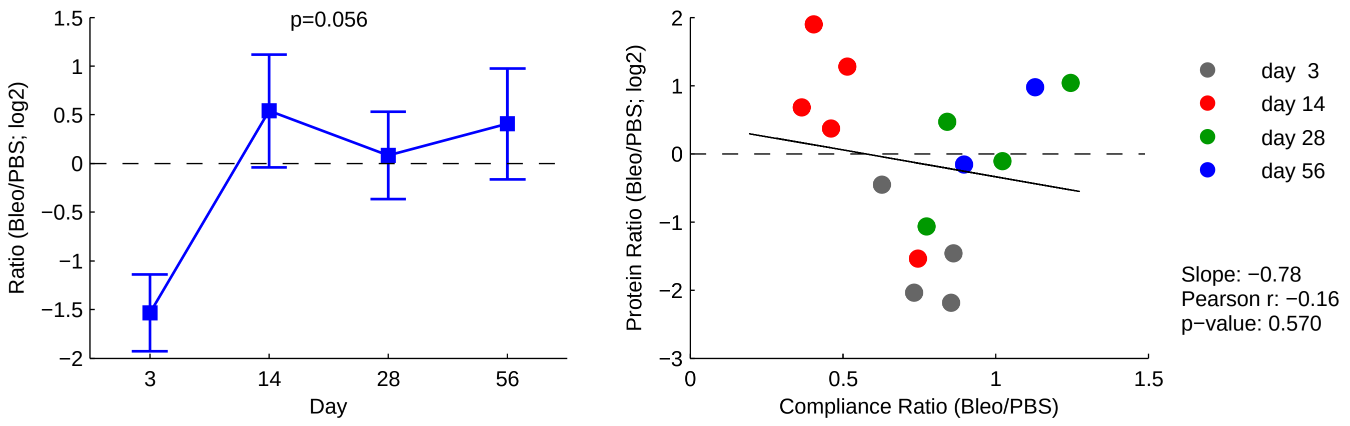

B1ASP2 − Jak1 (id: 332)

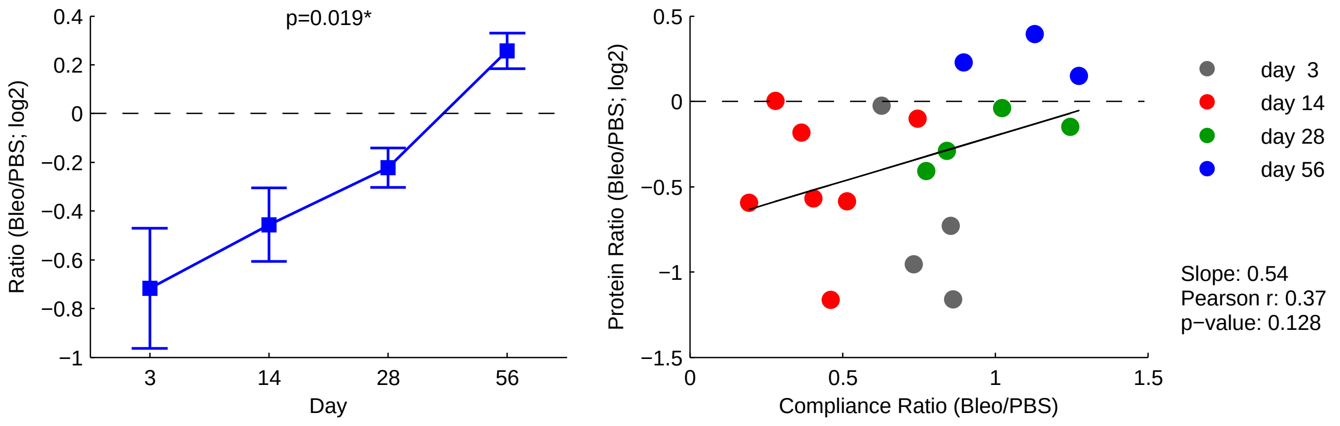

Q9D186 − Apoo (id: 333)

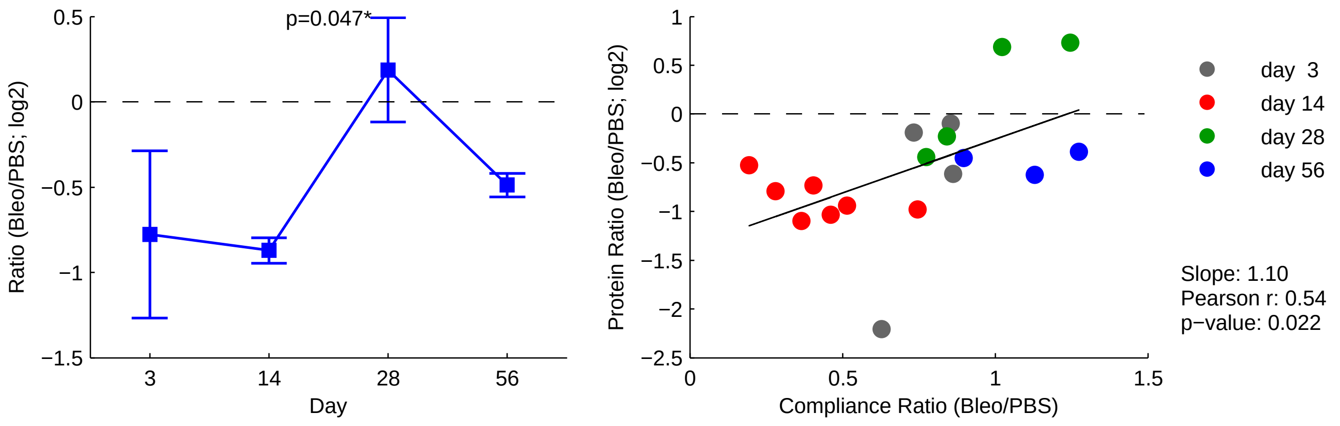

P47740 − Aldh3a2 (id: 340)

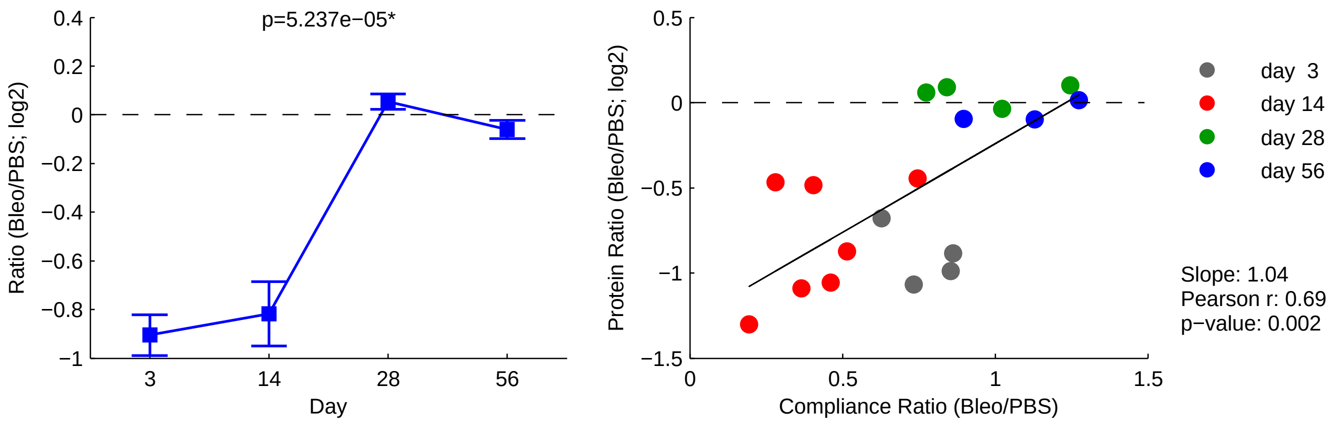

Q60780−2 − Gas7 (id: 341)

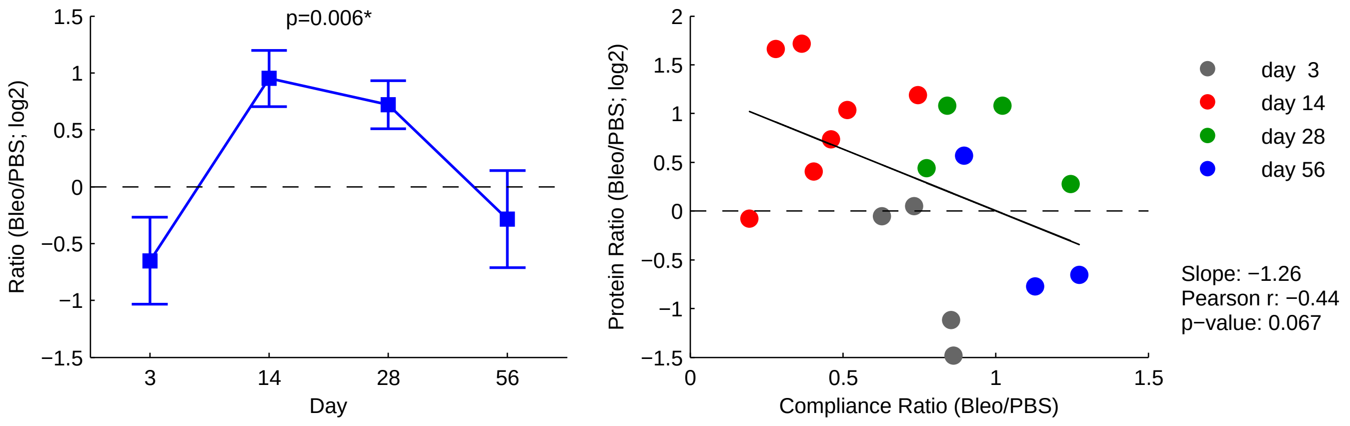

B1ATP7 − Elac2 (id: 342)

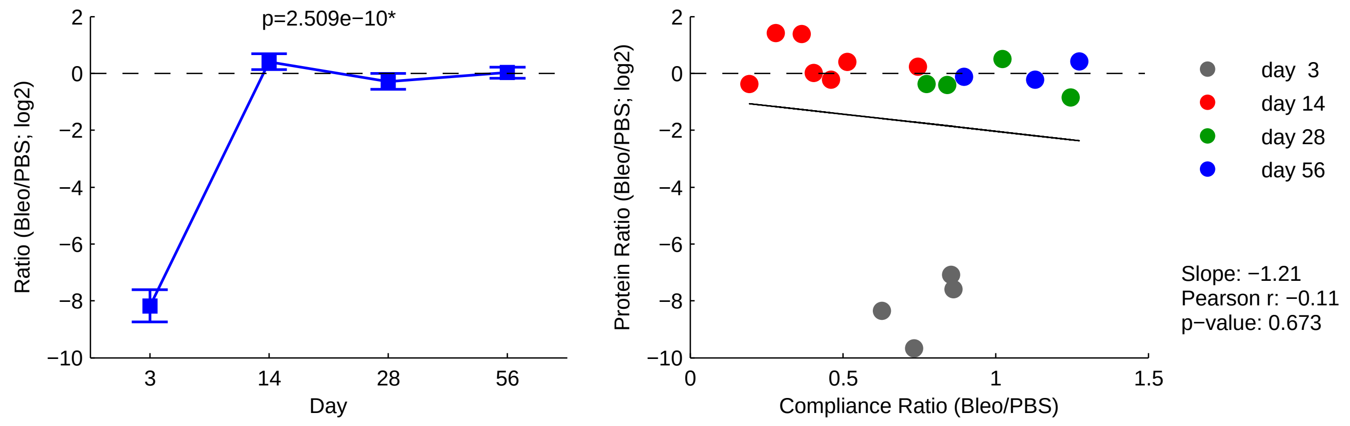

B1ATS5 − Atp2a3 (id: 343)

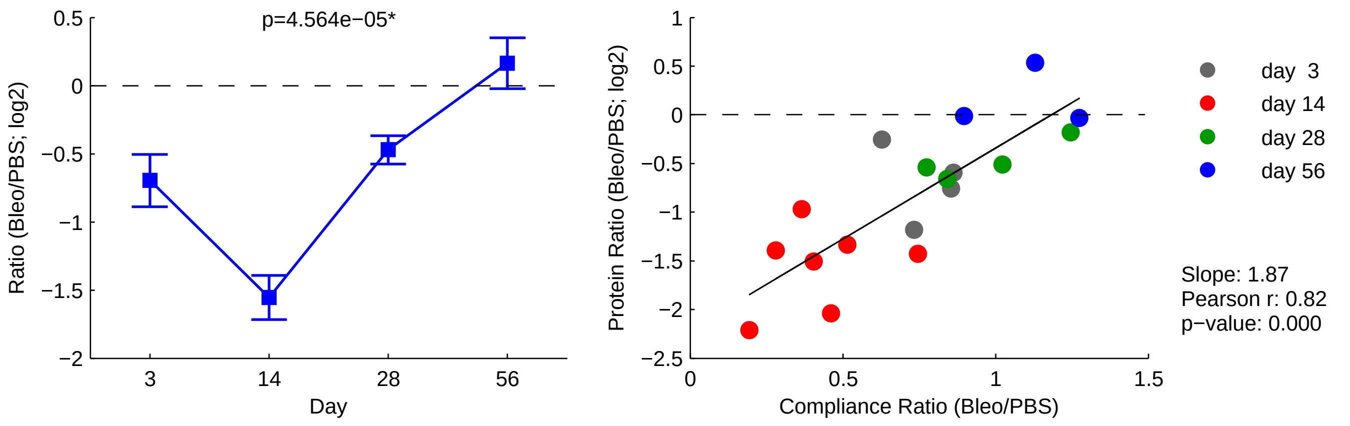

B1ATZ0 − Hgs (id: 345)

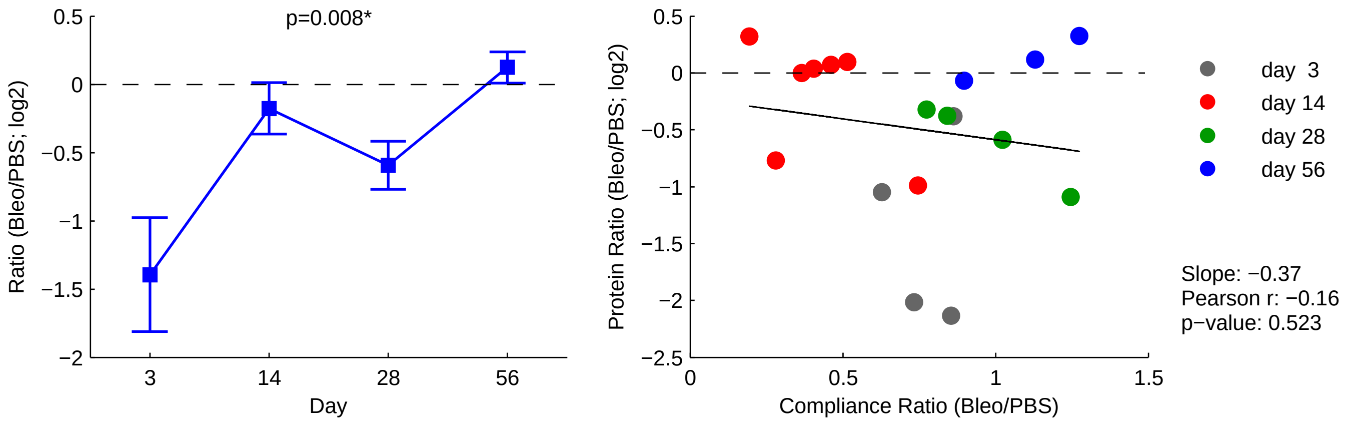

Q9CWP6−2 − Mospd2 (id: 346)

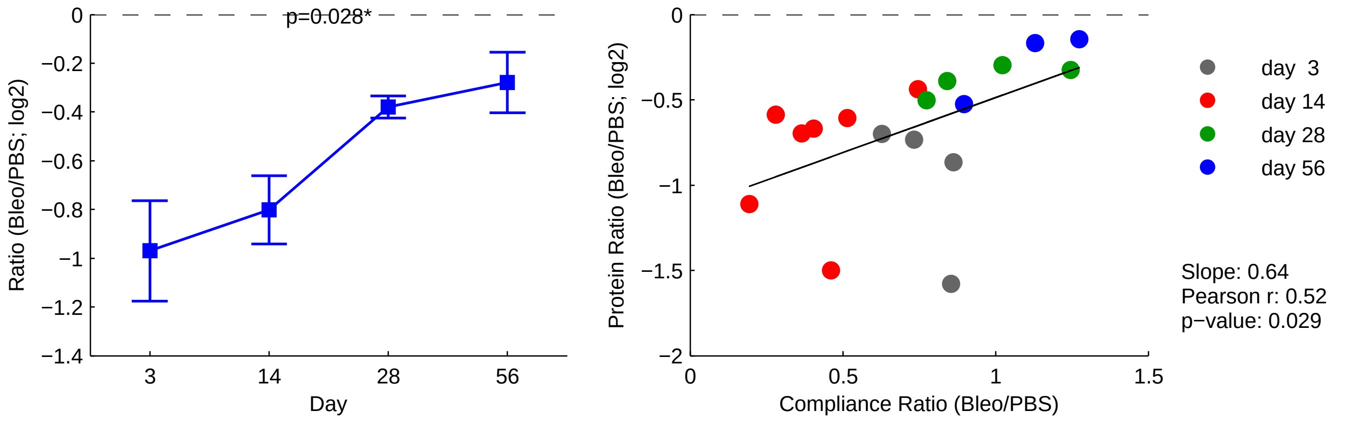

B1AUY7 − Naa10 (id: 352)

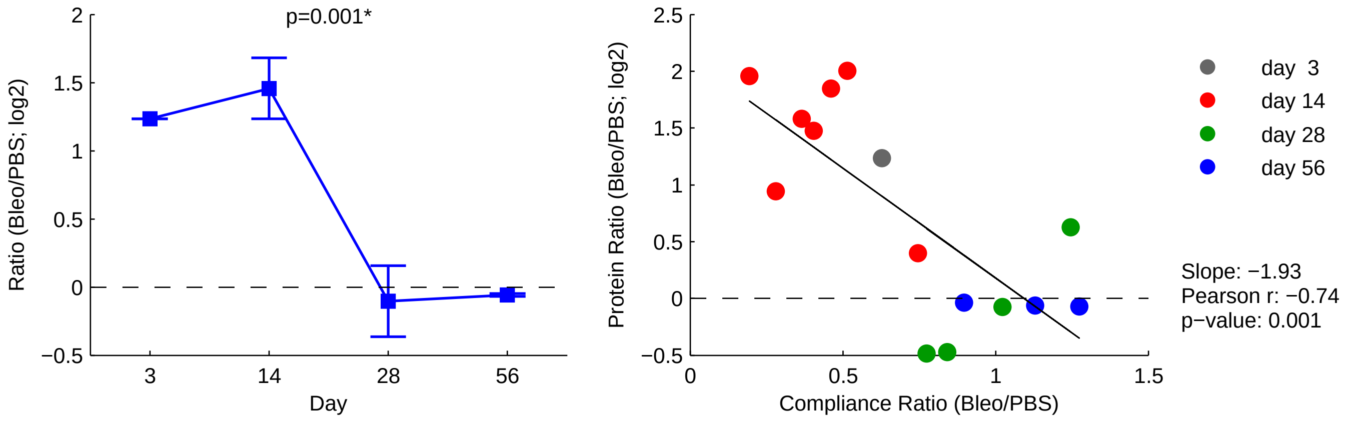

B1AV66 − Yipf6 (id: 354)

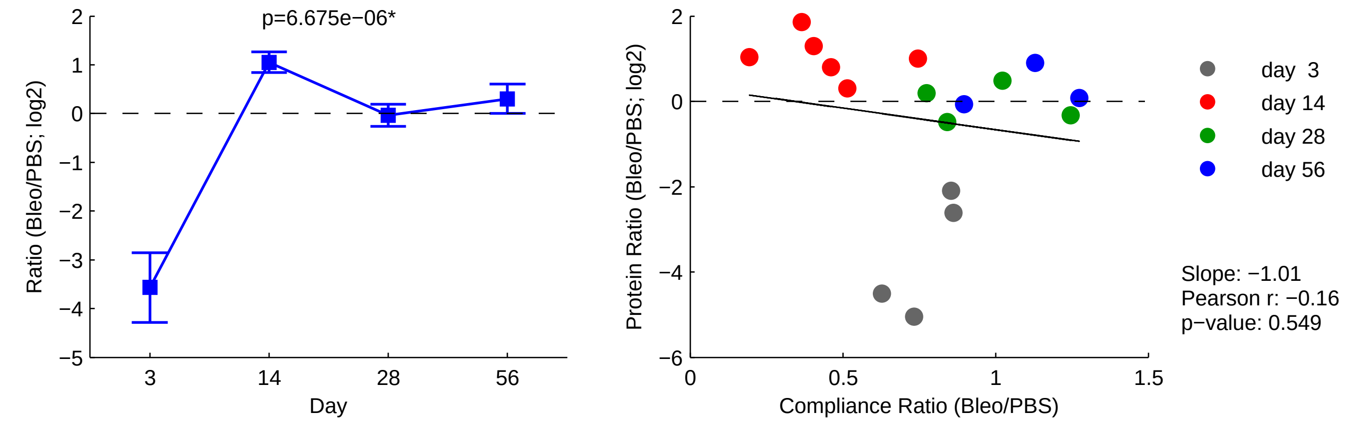

B1AVD1 − Xpnpep2 (id: 355)

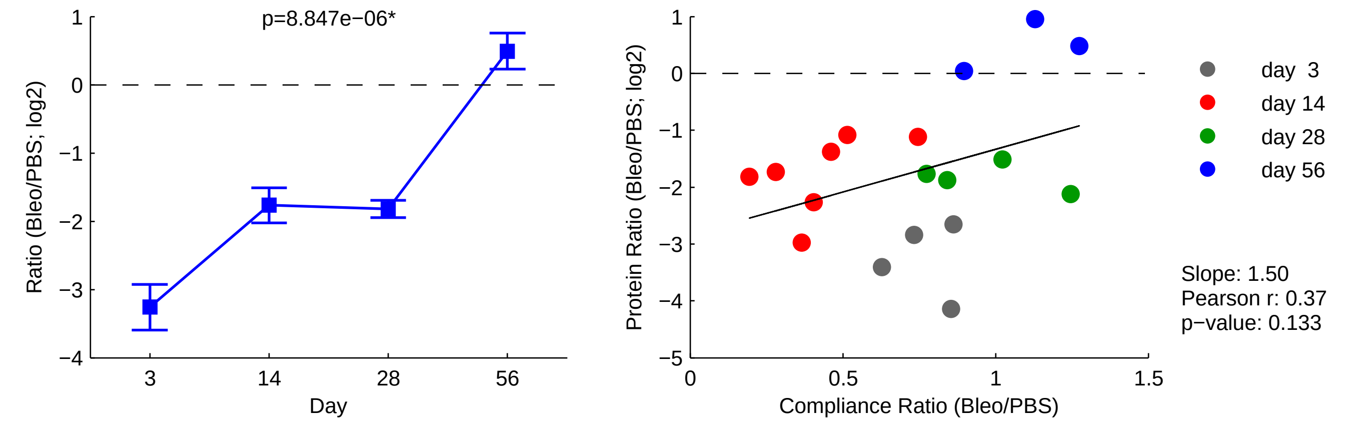

B1AVF4 − Mid2 (id: 356)

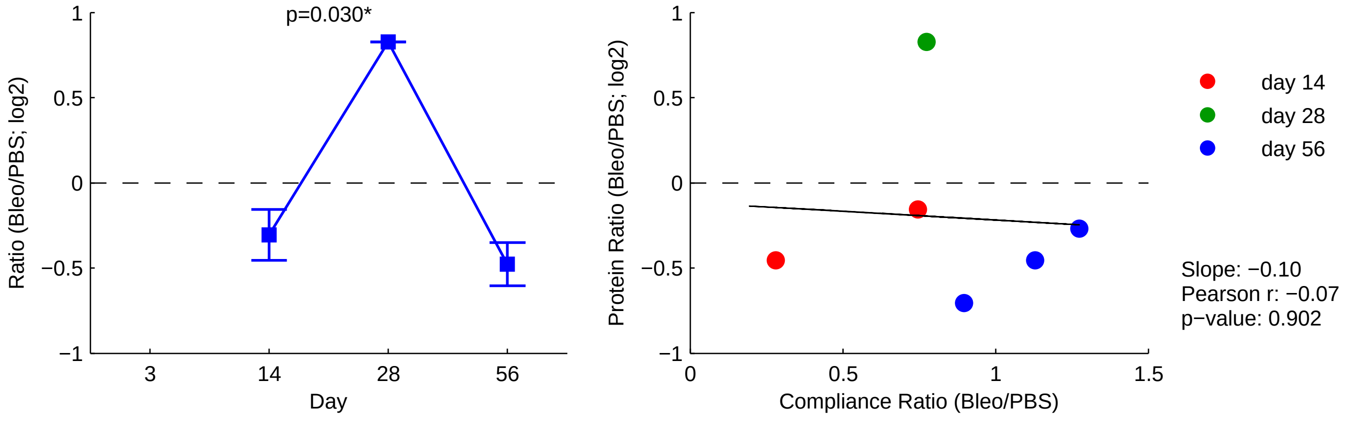

B1AVK5 − Col4a6 (id: 361)

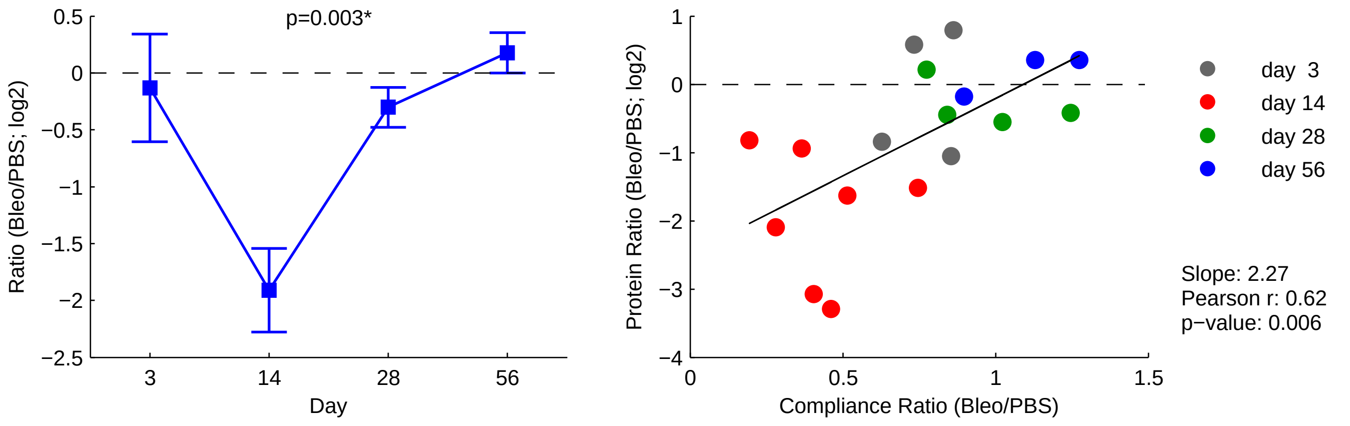

B1AW21 − Mtm1 (id: 364)

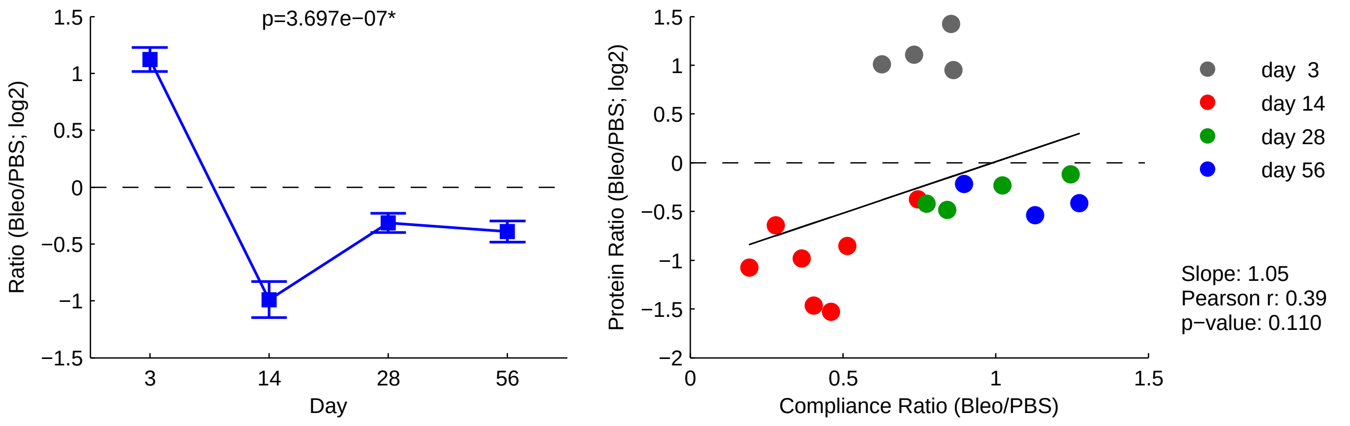

B1AWE0 − Clta (id: 367)

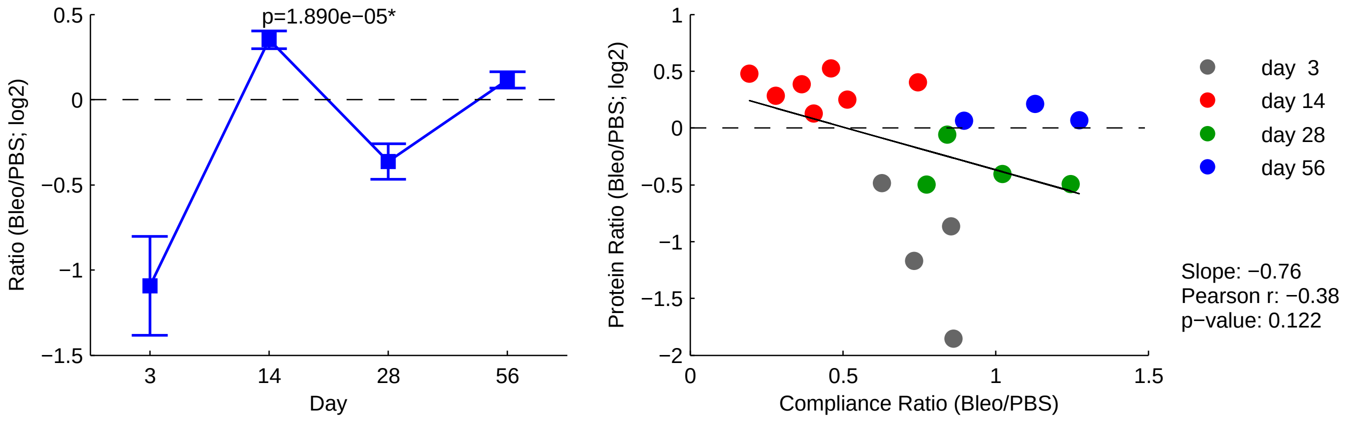

Q02858 − Tek (id: 368)

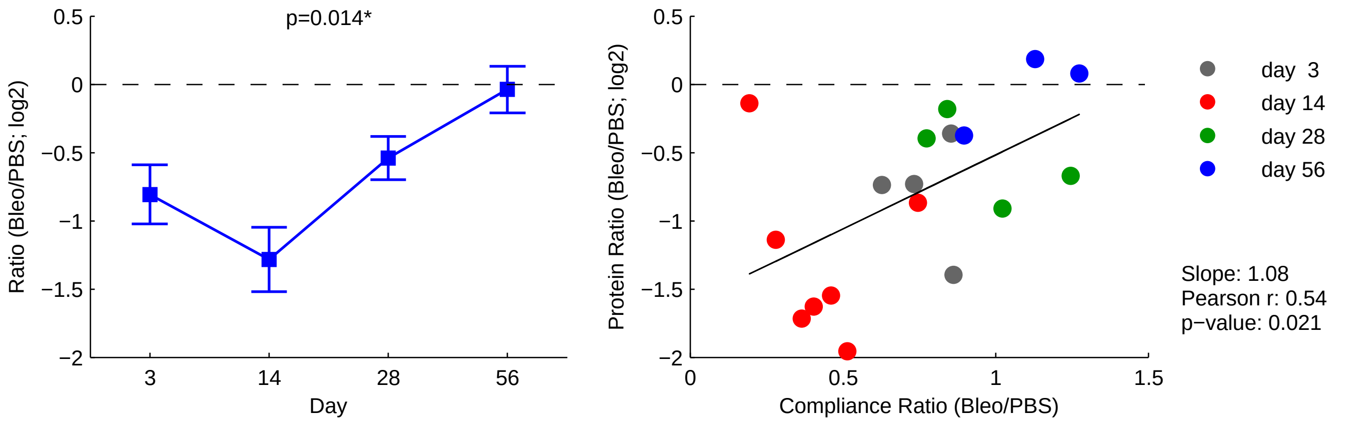

B1AXP6 − Tomm5 (id: 371)

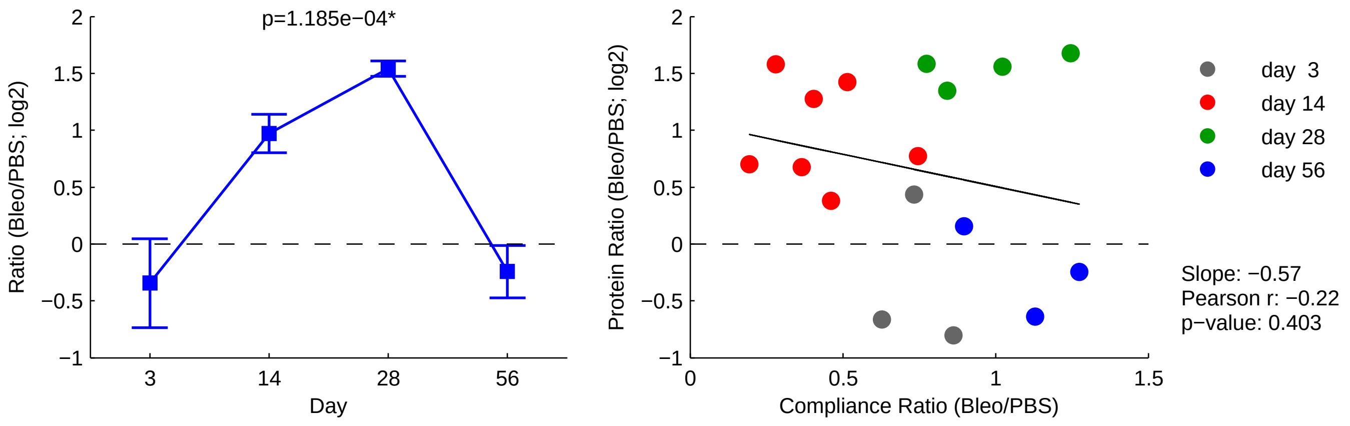

B1AY85 − Plxdc2 (id: 374)

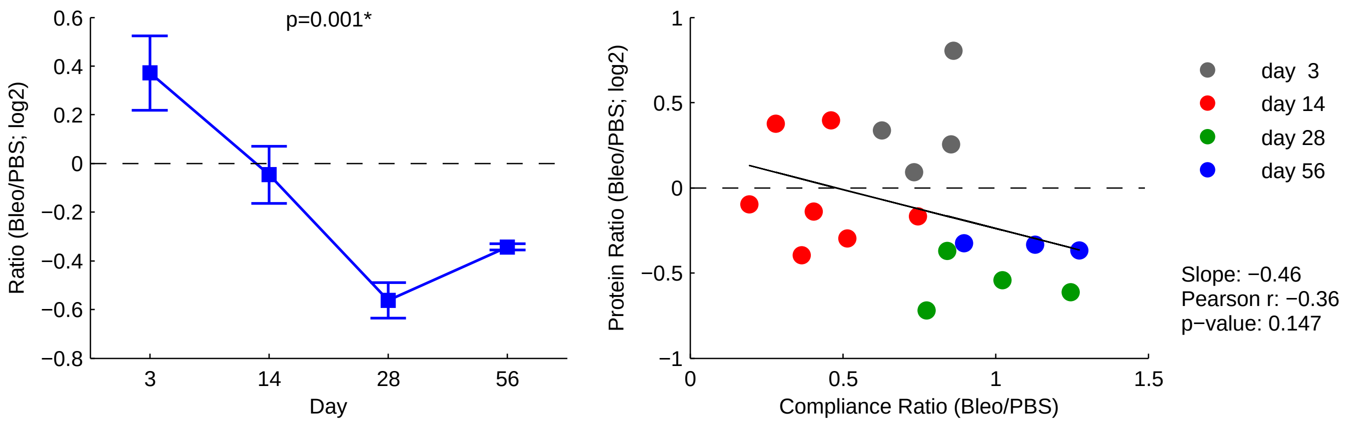

B1AZ46 − Baiap2 (id: 380)

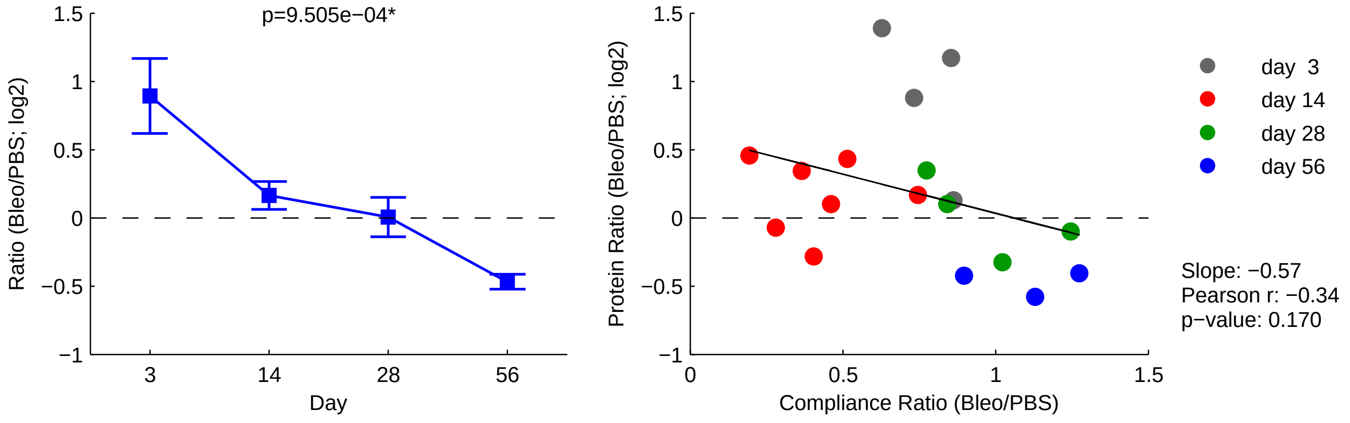

B1AZA5 − Tmem245 (id: 381)

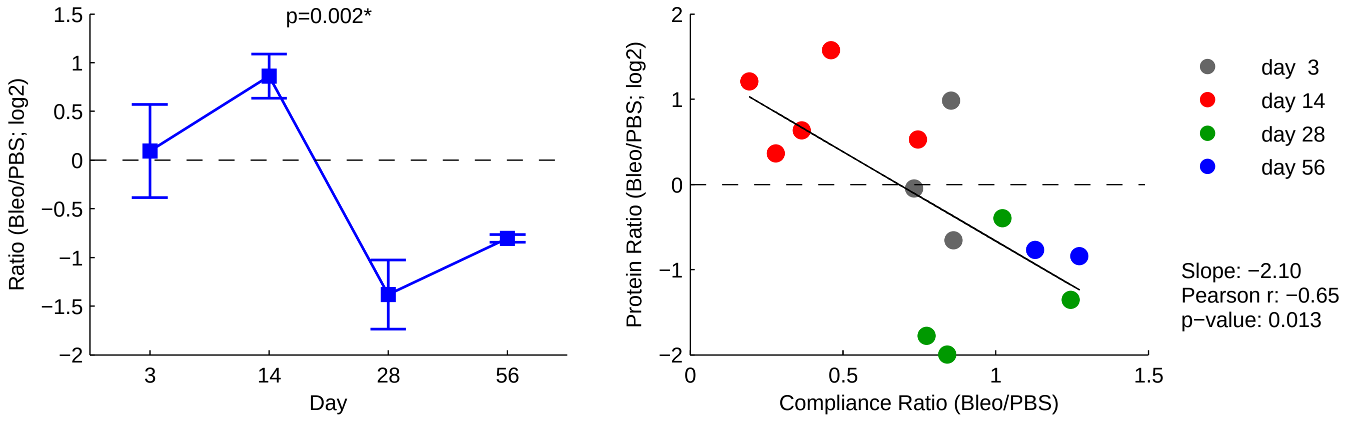

B1AZI6 − Thoc2 (id: 382)

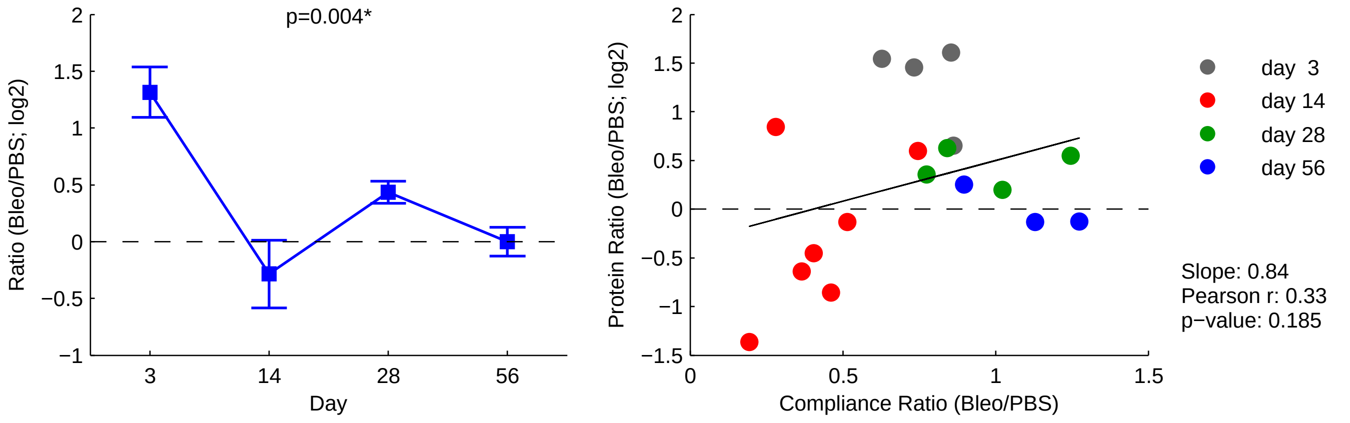

B1AZP2−3 − Dlgap4 (id: 383)

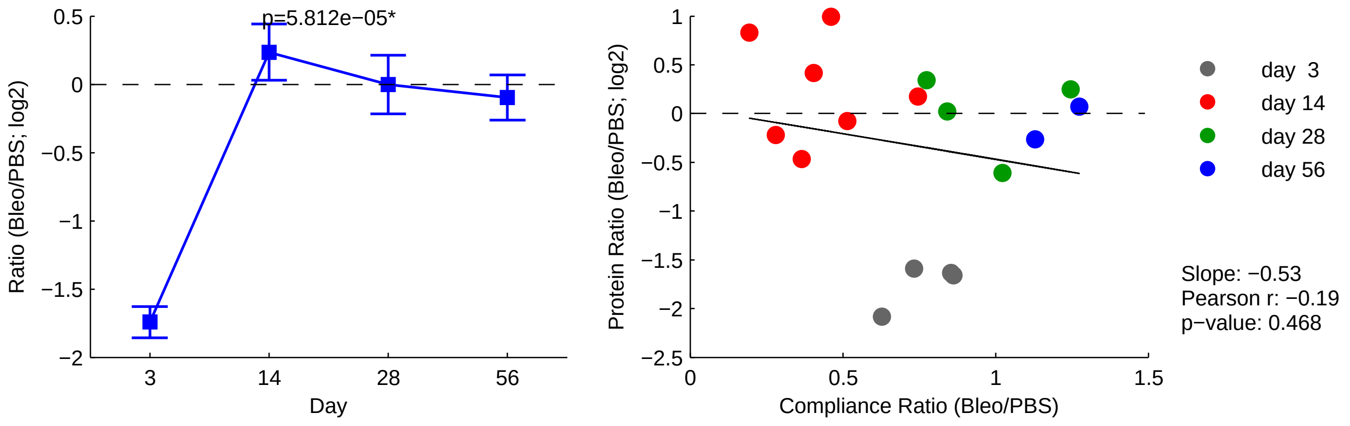

B1B1A8 − Mylk (id: 385)

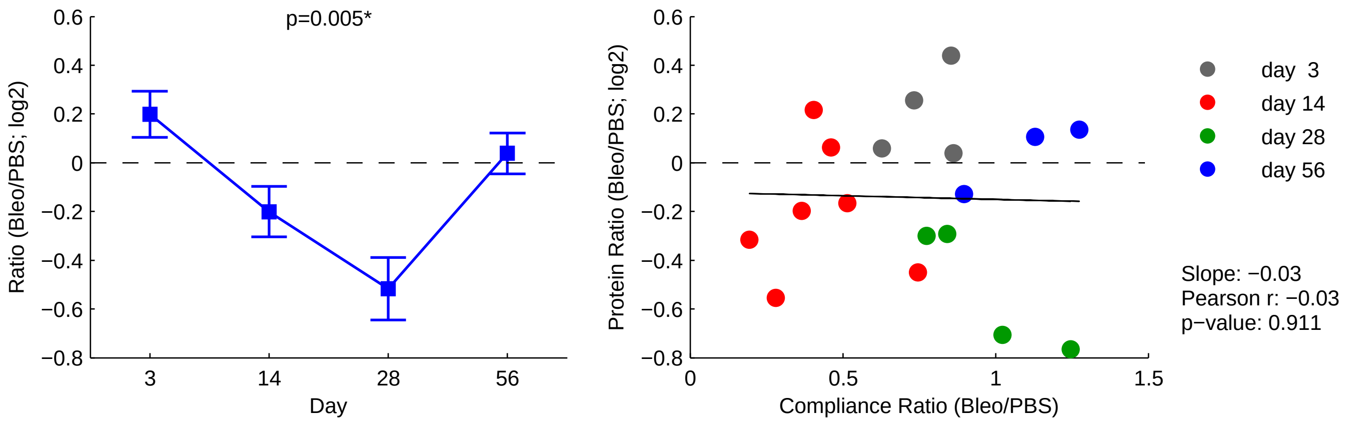

B1B1D8 − Mrpl2 (id: 386)

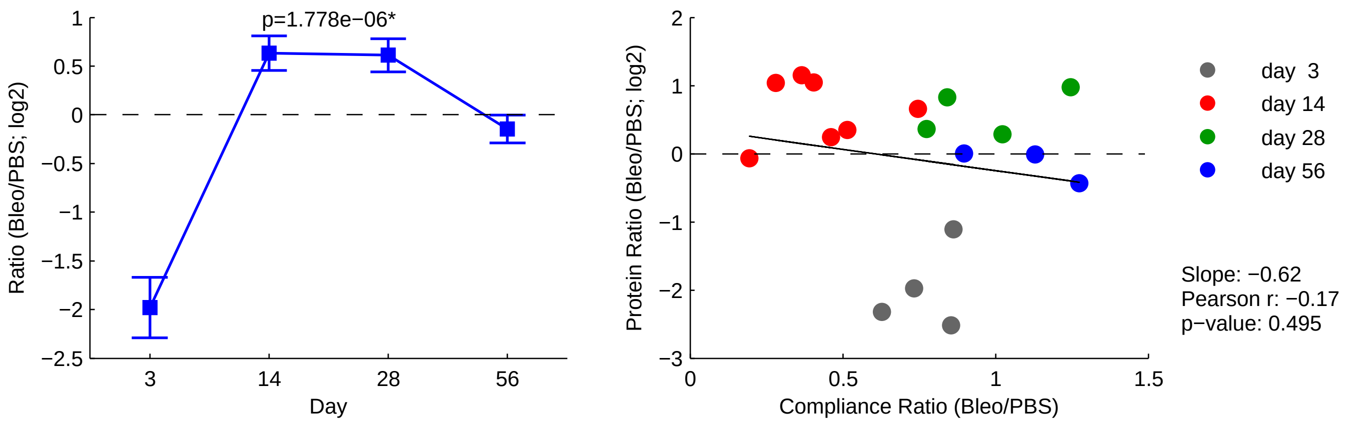

Q9D9T8 − Efhc1 (id: 389)

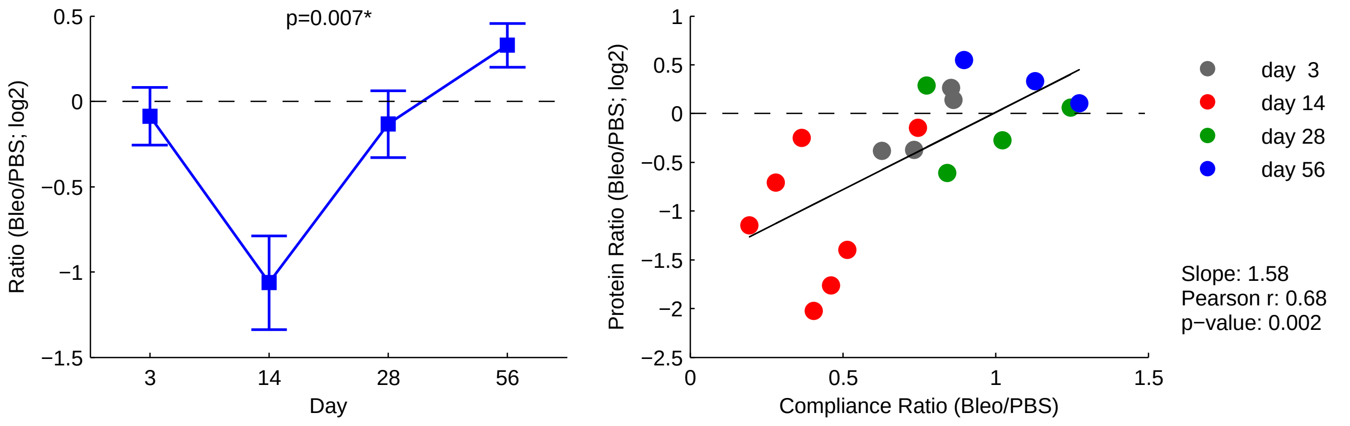

B2KF29 − Clps (id: 392)

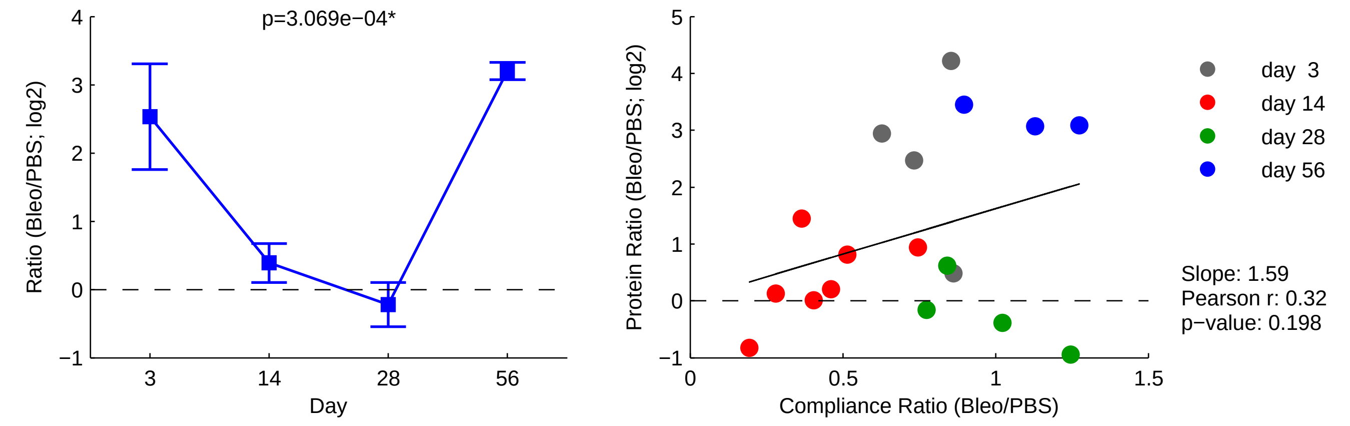

B2M1R6 − Hnrnpk (id: 396)

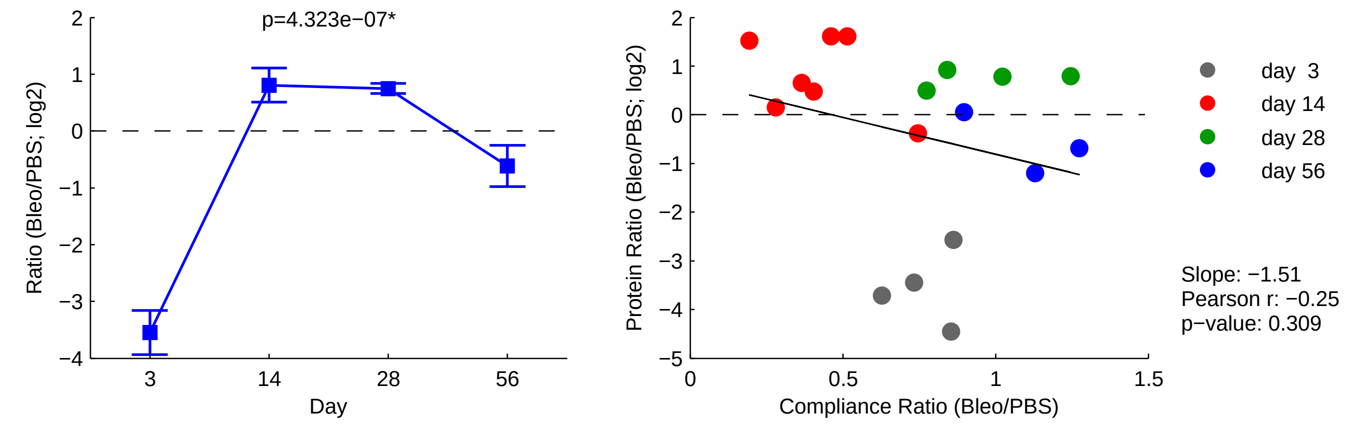

E9PW14 − Mdm1 (id: 399)

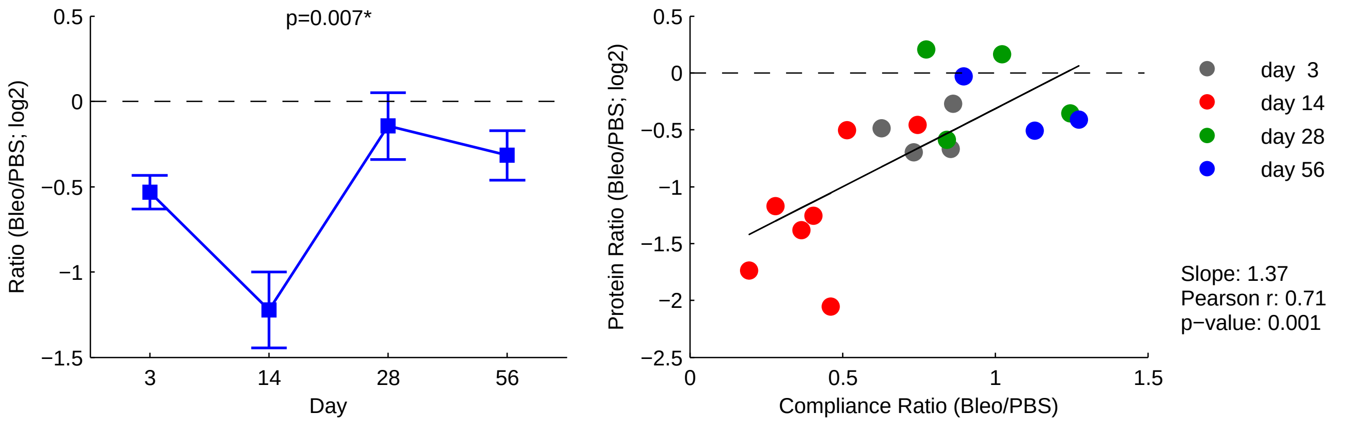

B2RQ71 − Dip2c (id: 402)

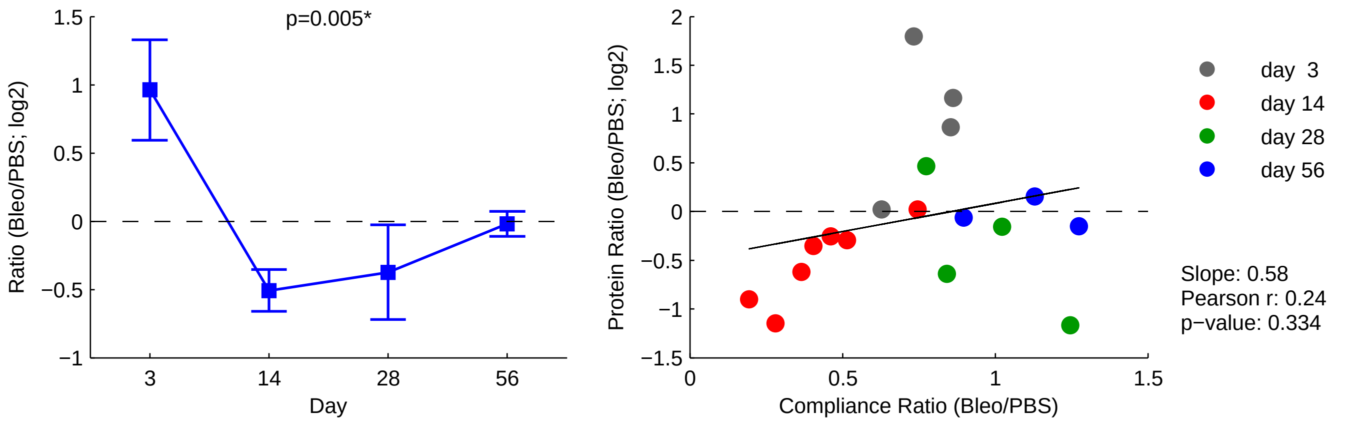

E9QAI5 − Cad (id: 404)

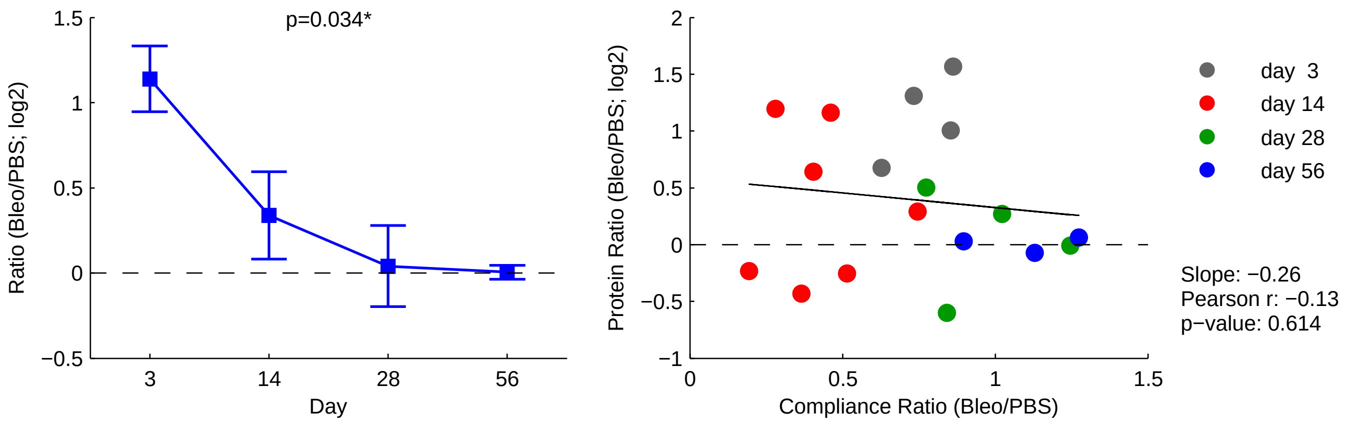

B2RQE8 − Arhgap42 (id: 406)

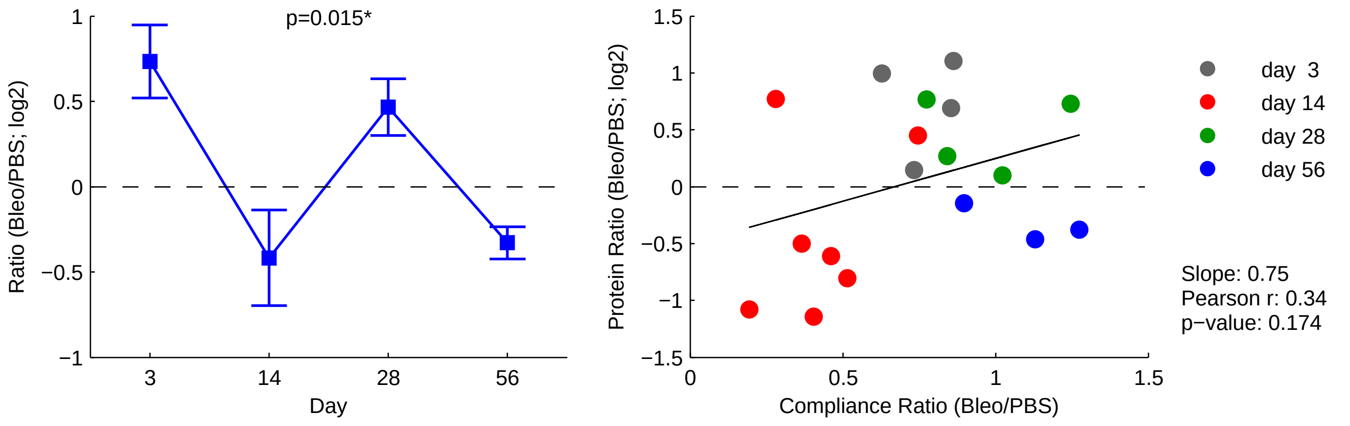

B2RQG2 − Phf3 (id: 407)

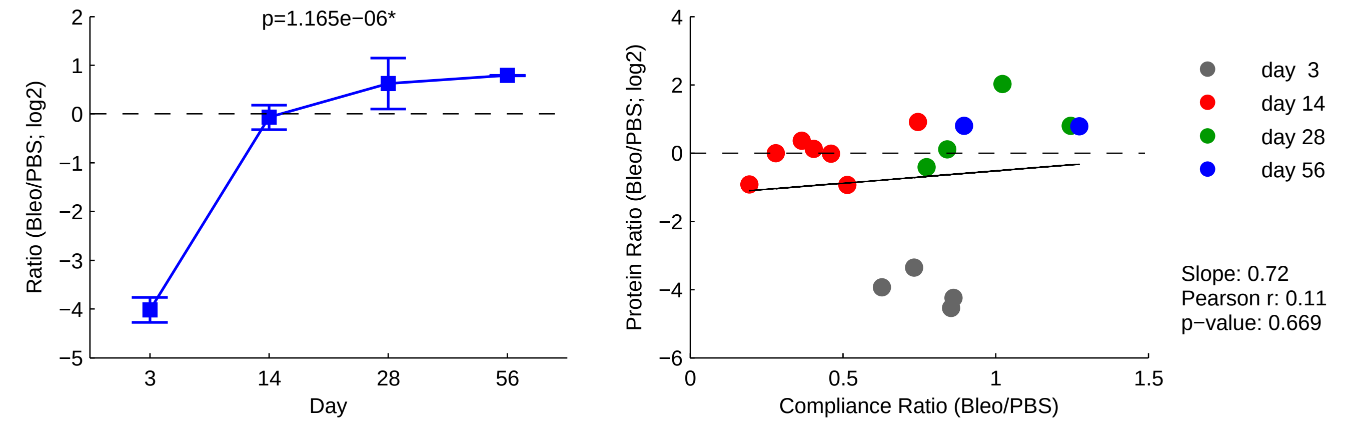

B2RQS1 − Strn3 (id: 409)

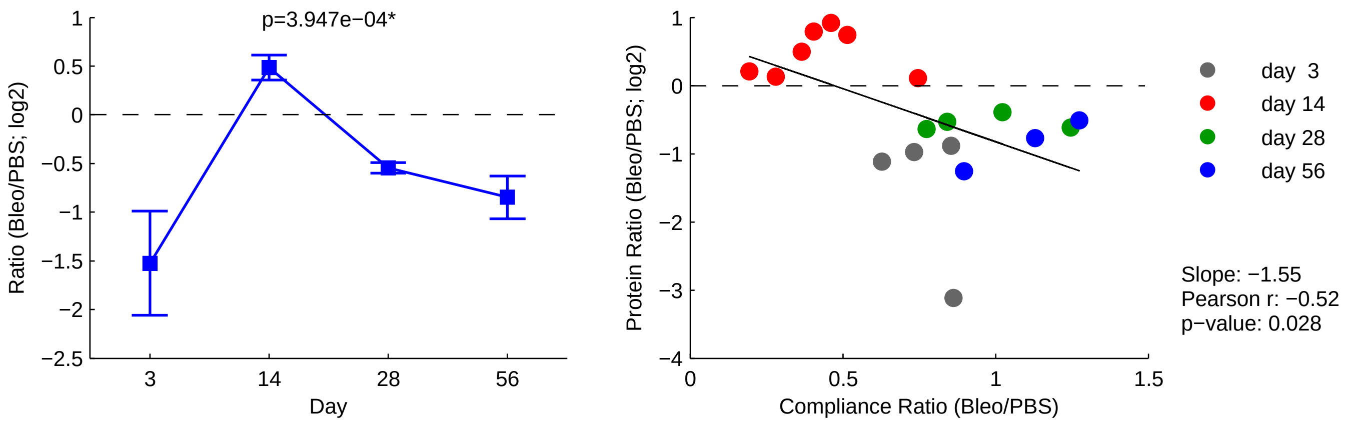

Q8C4Y1 − Max (id: 416)

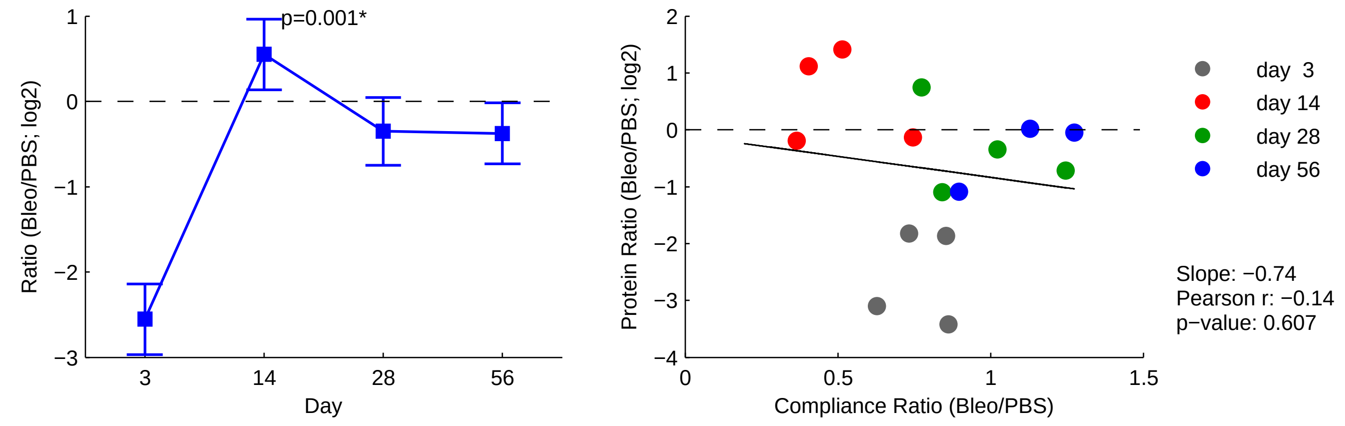

Q9DC53 − Cpne8 (id: 417)

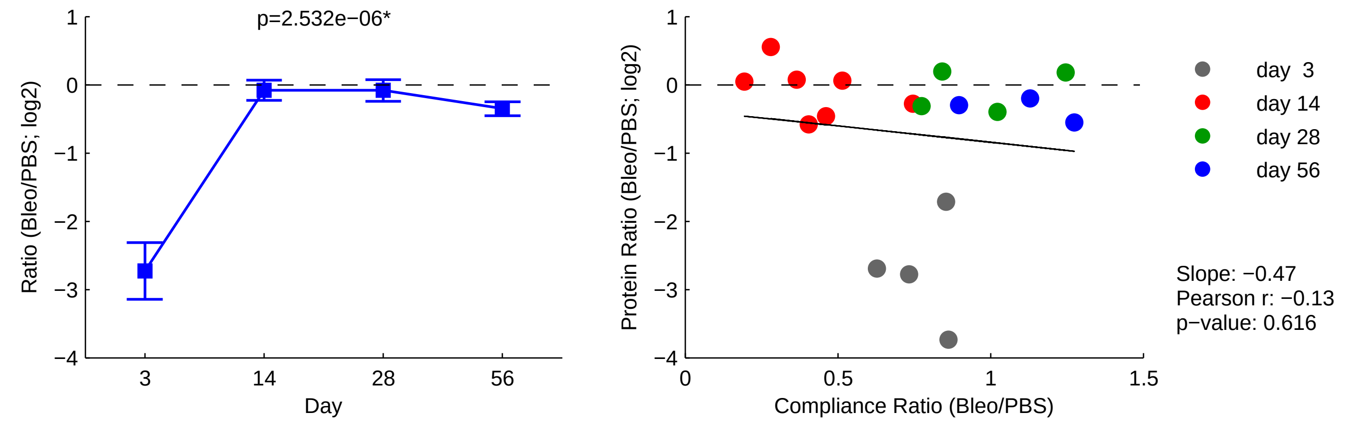

B2RSH2 − Gnai1 (id: 419)

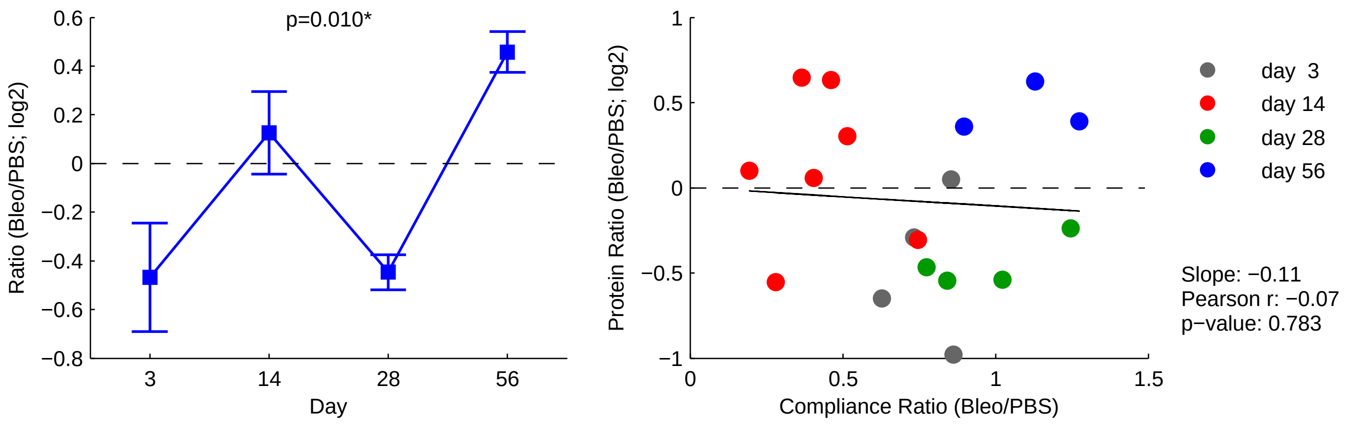

B2RSU6 − Cgnl1 (id: 420)

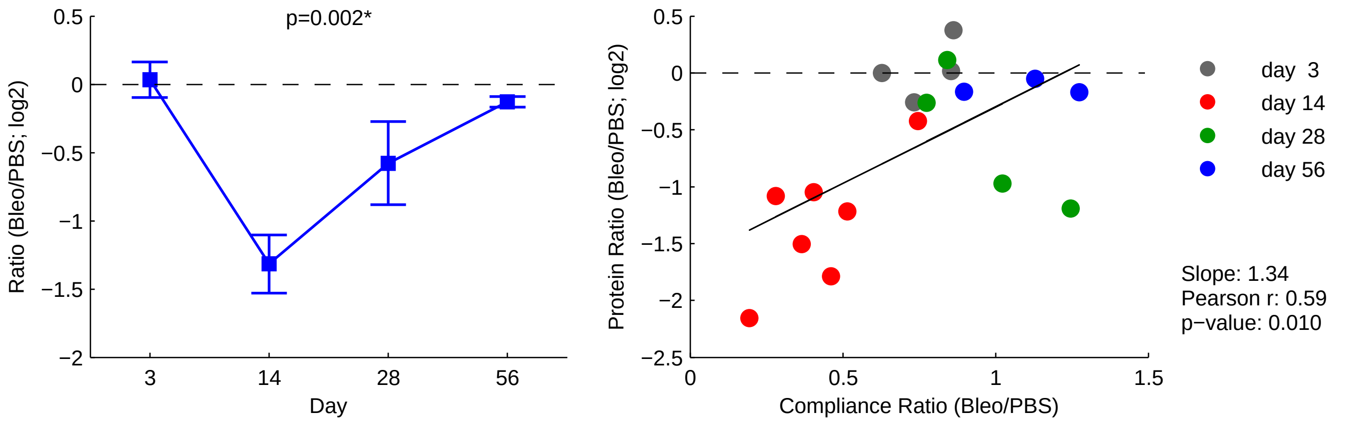

Q8CHG3 − Gcc2 (id: 421)

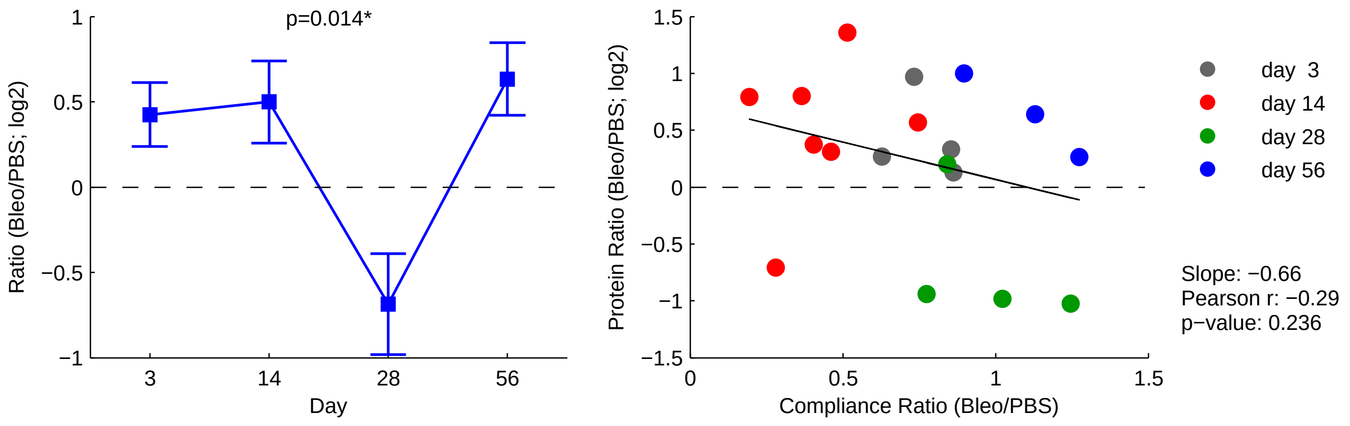

B2RU80 − Ptprb (id: 422)

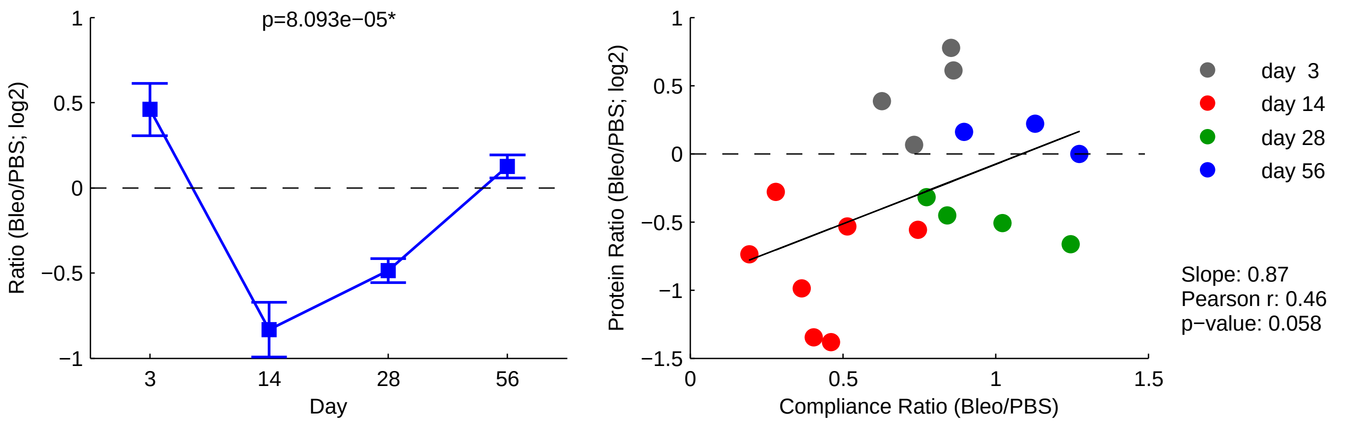

B2RUR8 − Otud7b (id: 427)

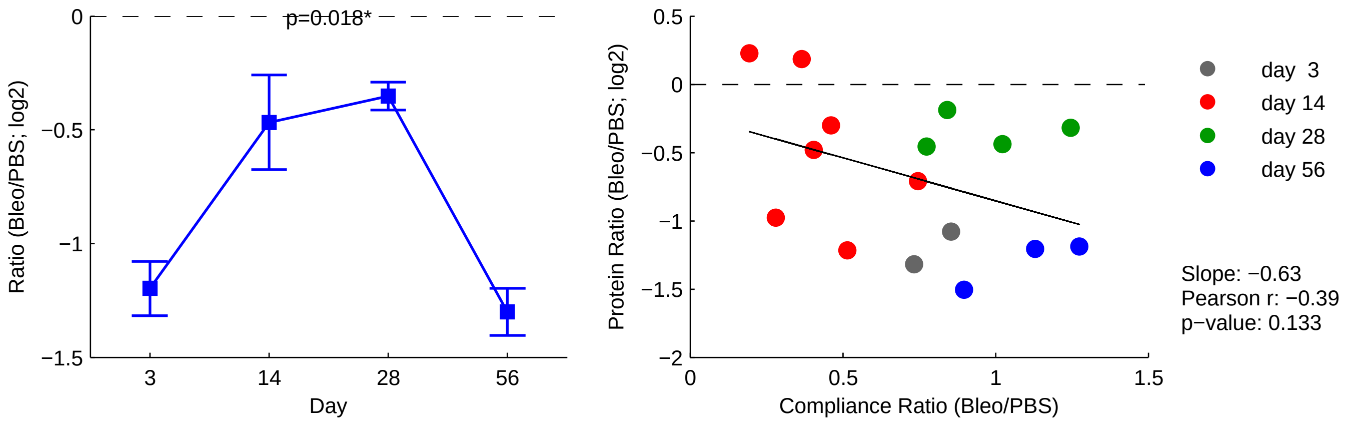

B2RXC2 − Itpkb (id: 432)

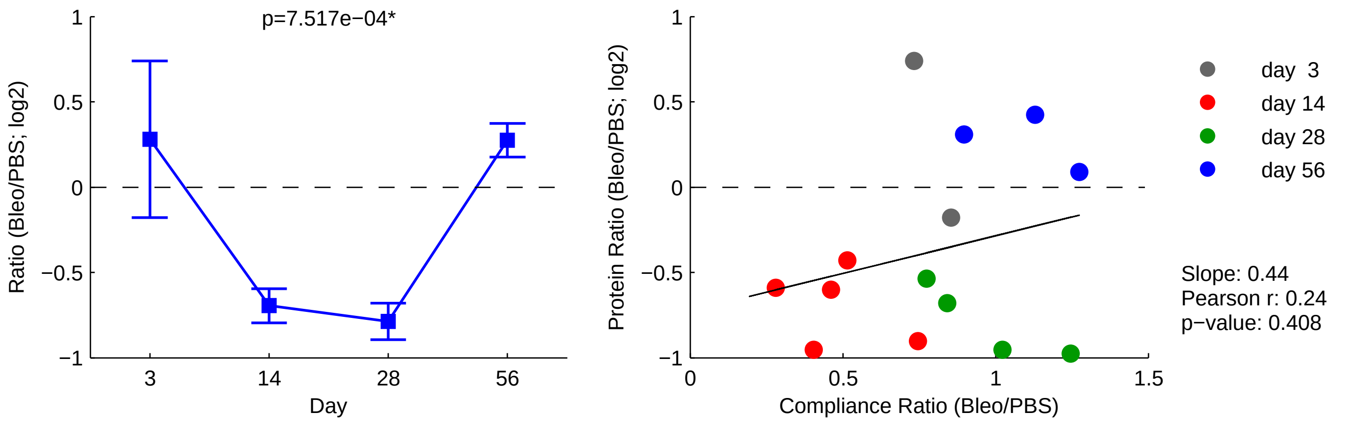

B2RXP1 − Lrch3 (id: 435)

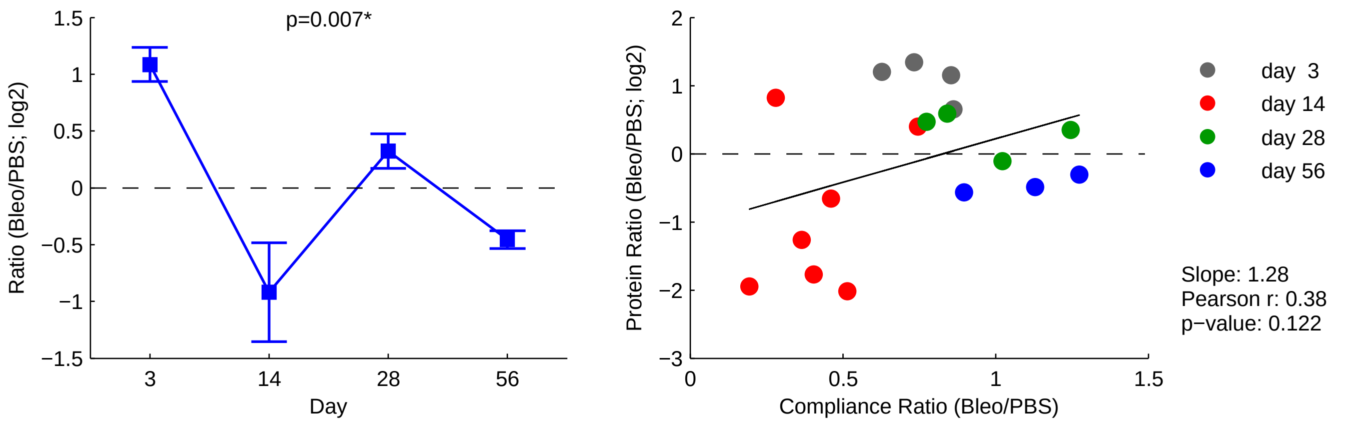

B2RXS4 − Plxnb2 (id: 437)

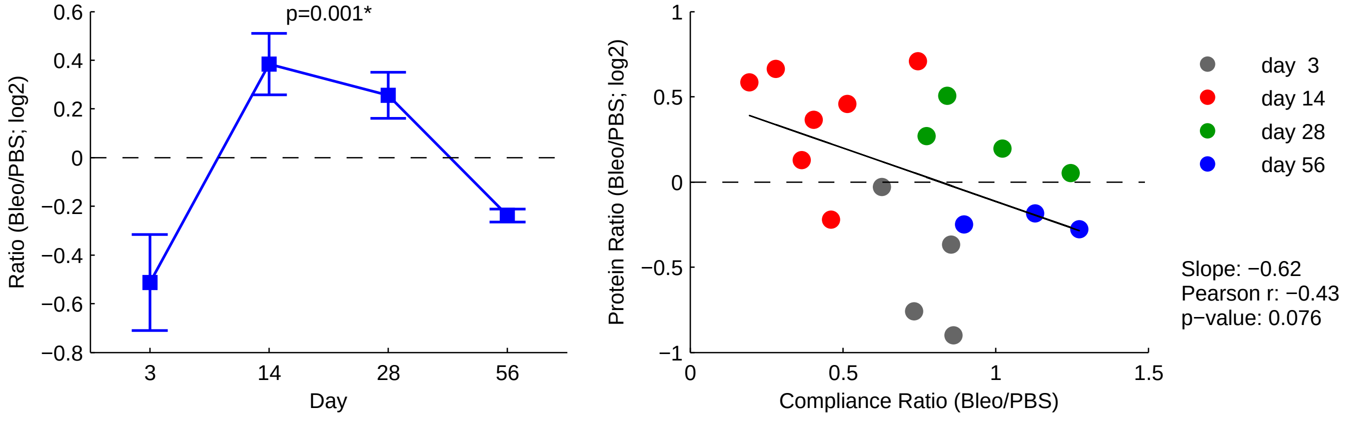

B2RXW8 − Ppfia1 (id: 439)

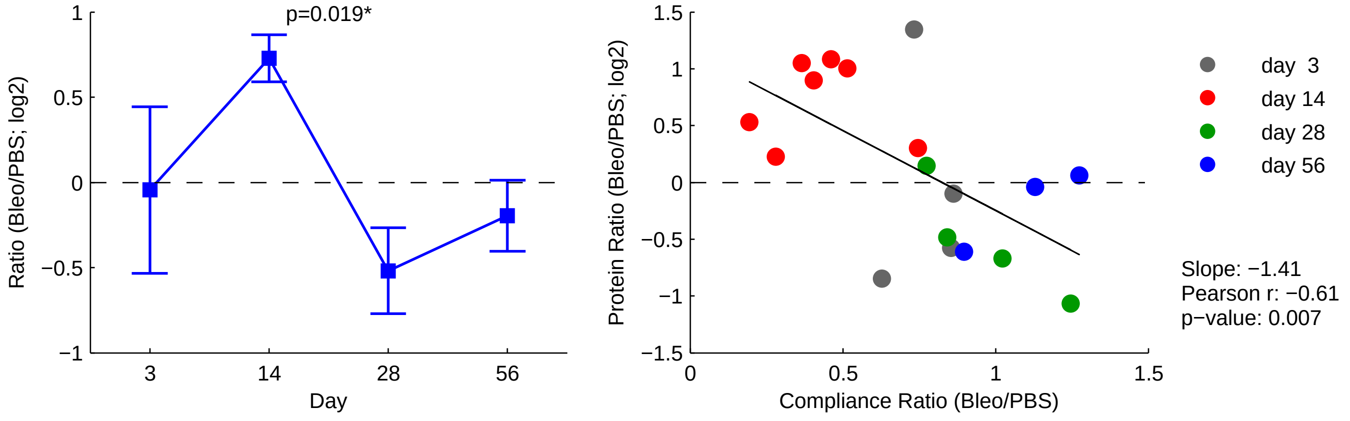

Q6ZPE2 − Sbf1 (id: 440)

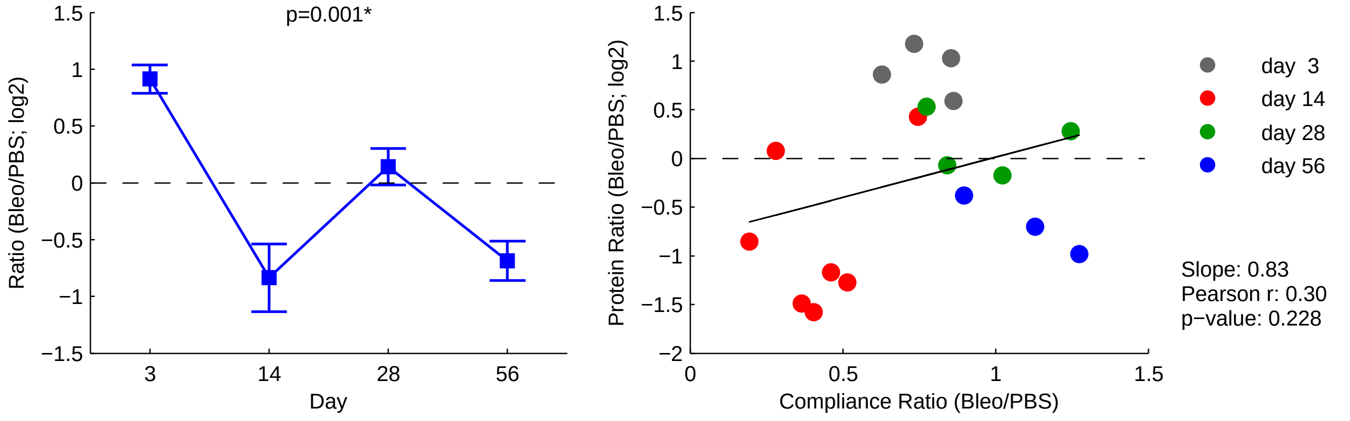

B2RY50 − Armc4 (id: 442)

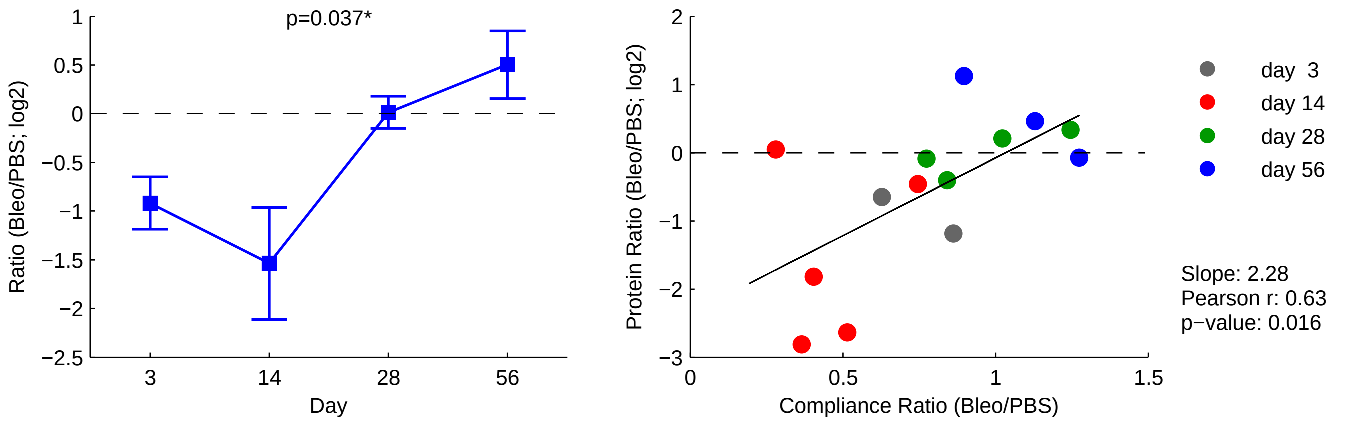

B7FAU9 − Flna (id: 448)

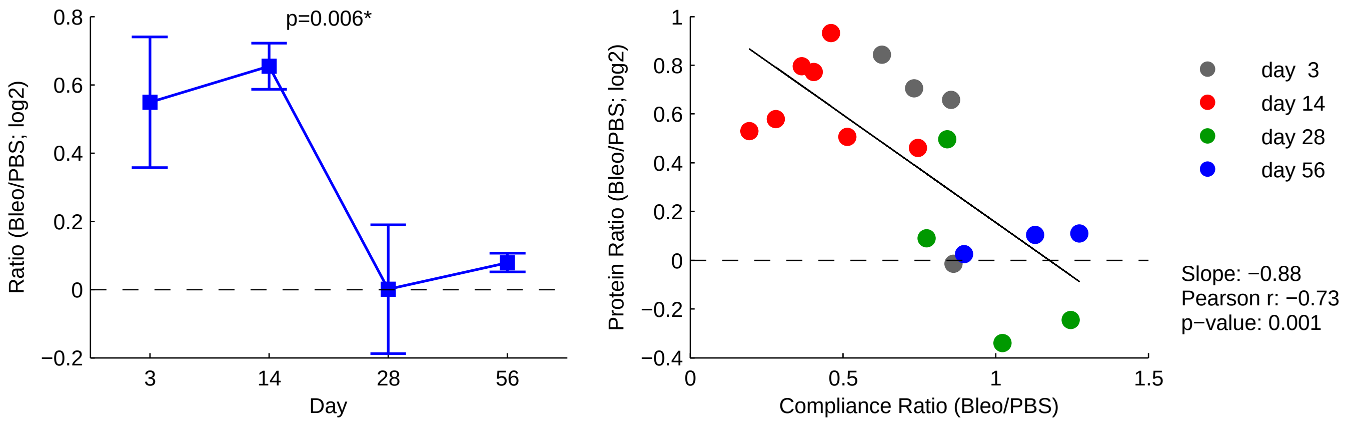

B7ZCI2 − Nebl (id: 453)

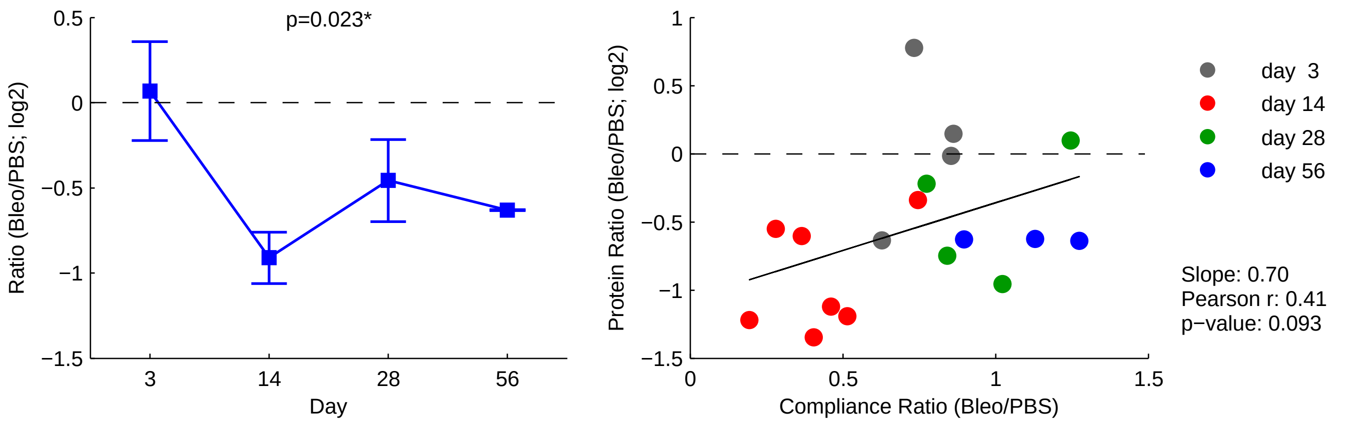

Q80TH2−1 − Erbb2ip (id: 459)

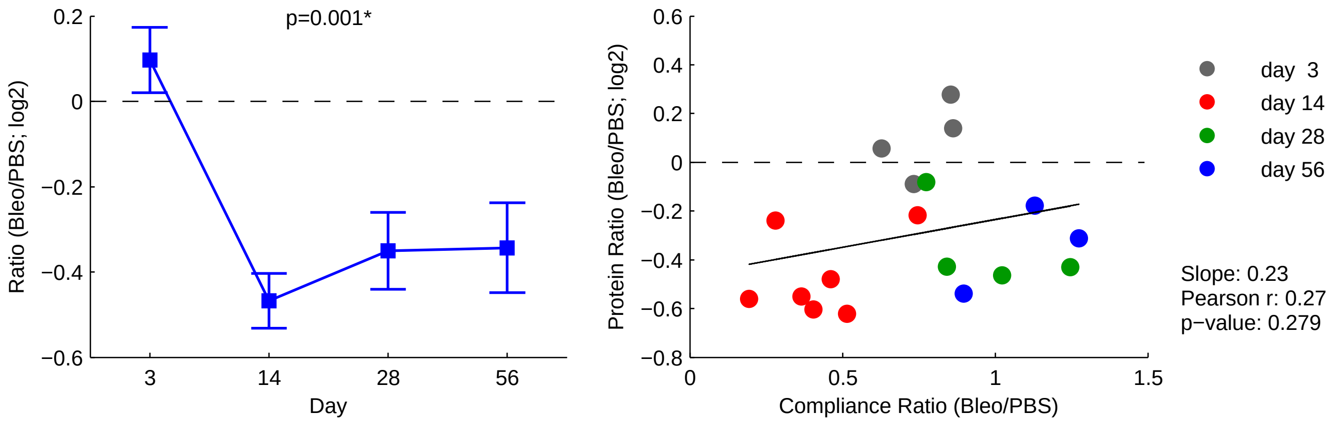

B7ZP47 − Wapal (id: 460)

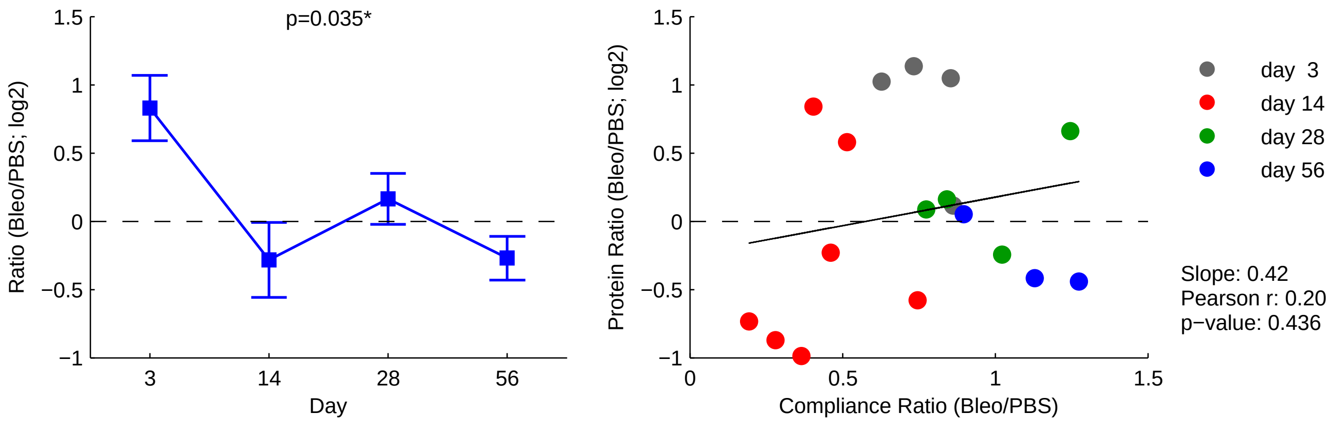

Q8CEE0−2 − Cep57 (id: 463)

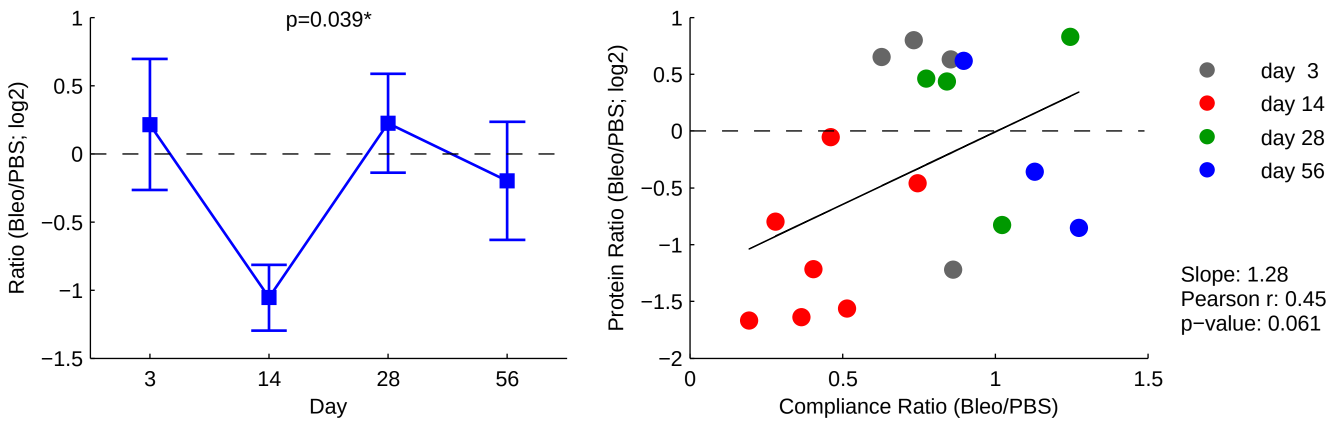

B8JJN0 − Gm20547 (id: 465)

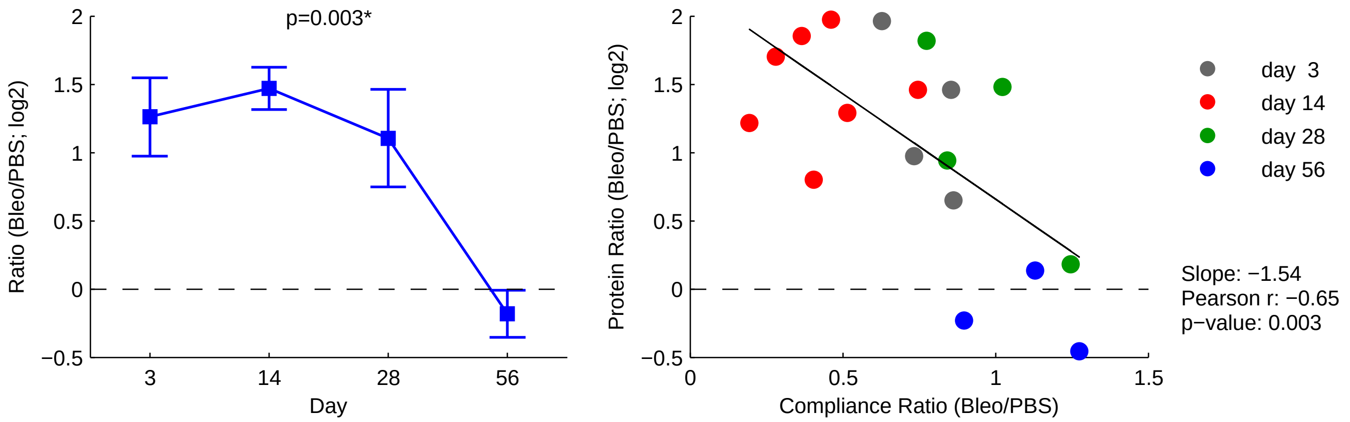

B8JK39 − Itga9 (id: 466)

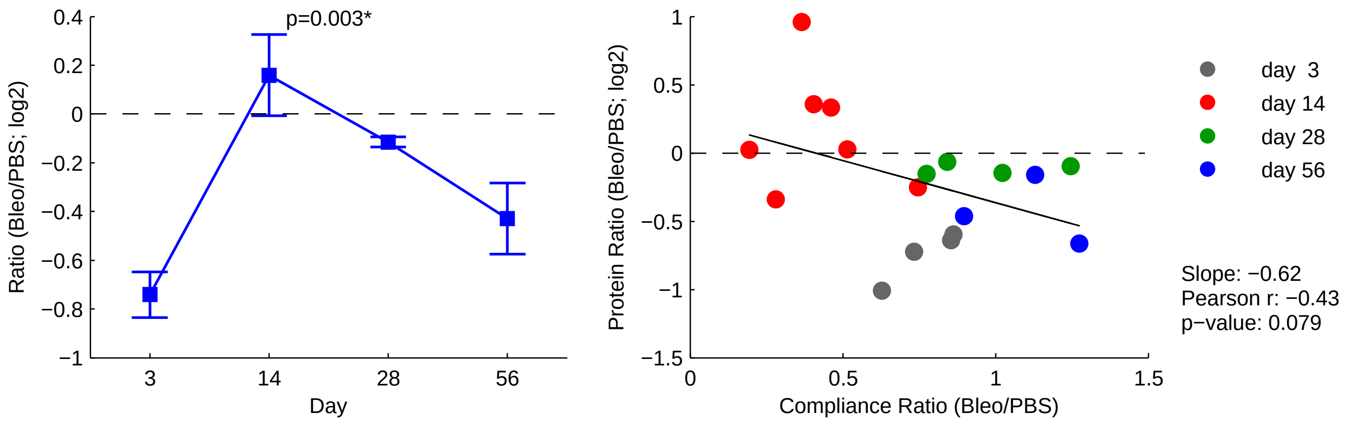

G3XA57 − Rab11fip2 (id: 474)

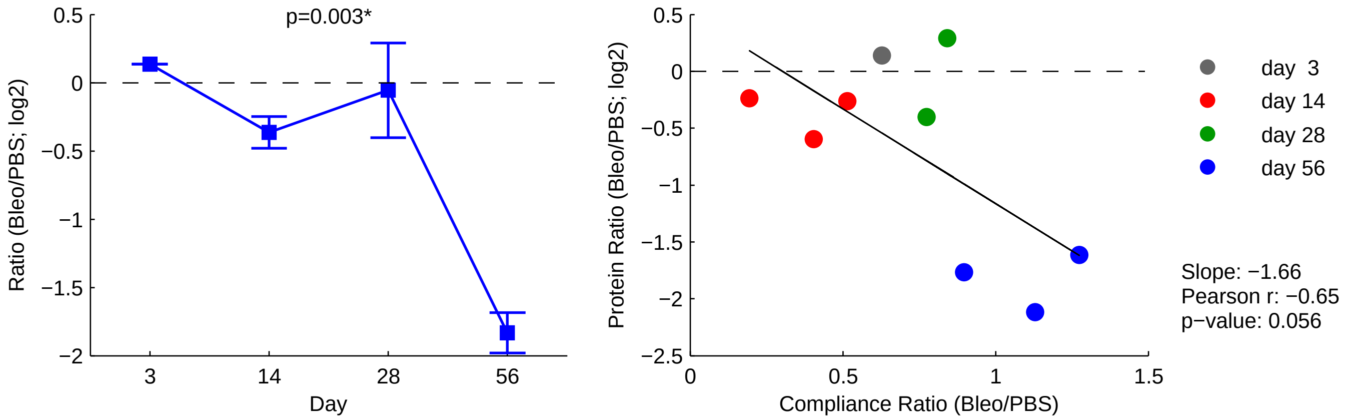

Q8C176 − Taf2 (id: 480)

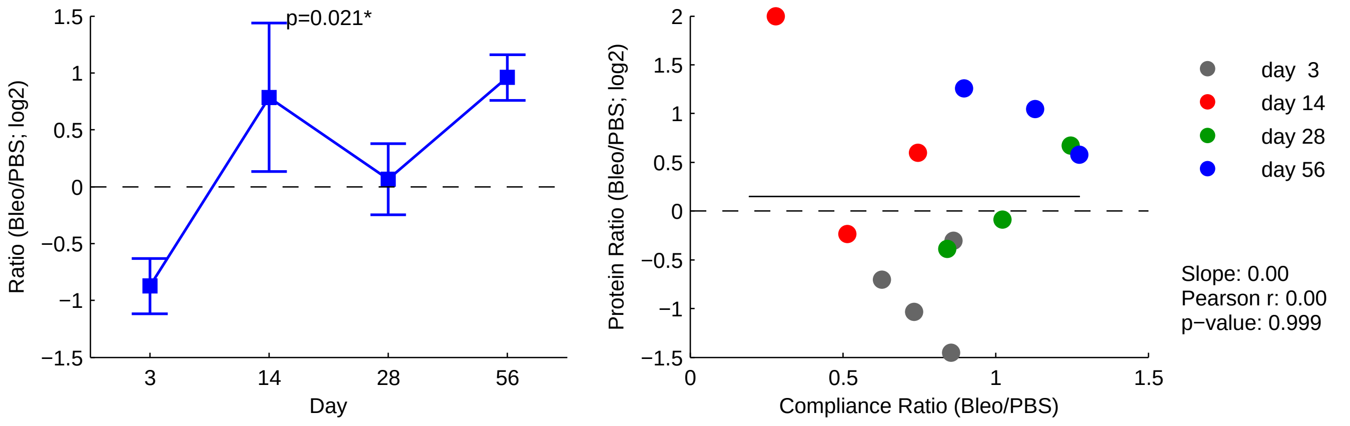

Q80TL7−2 − Mon2 (id: 483)

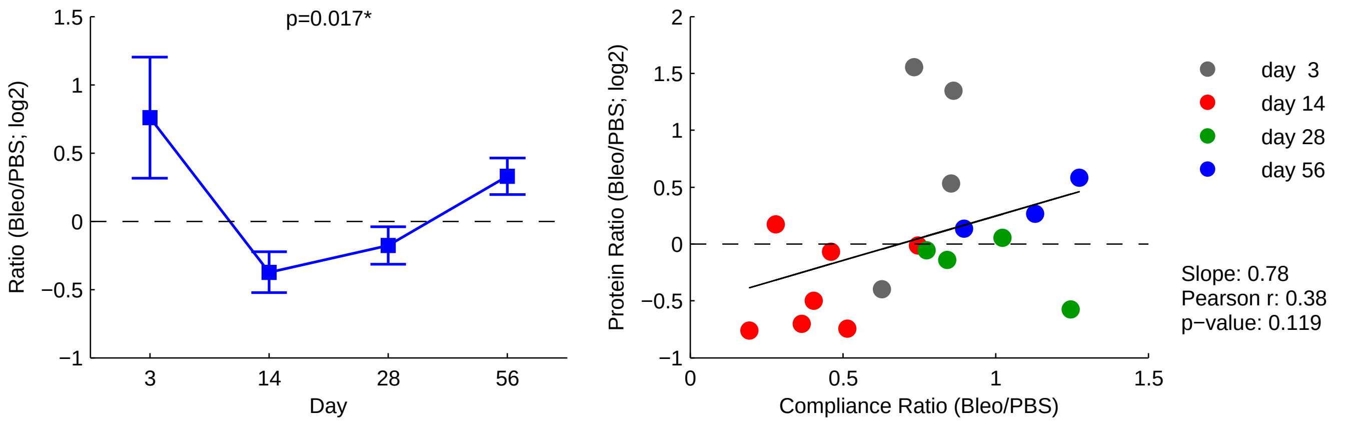

B9EKR1 − Ptprz1 (id: 484)

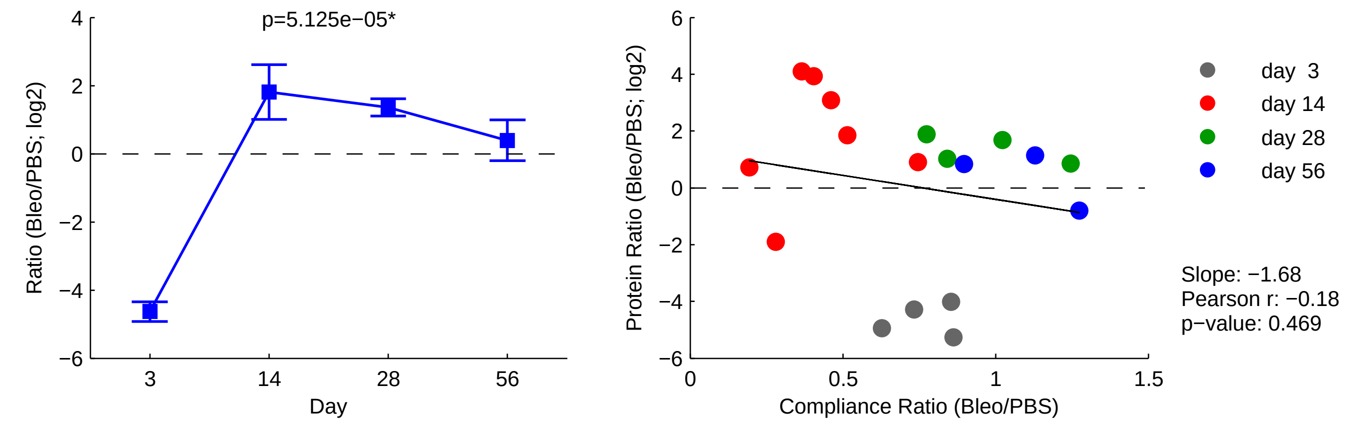

C5H3H7 − Ager (id: 486)

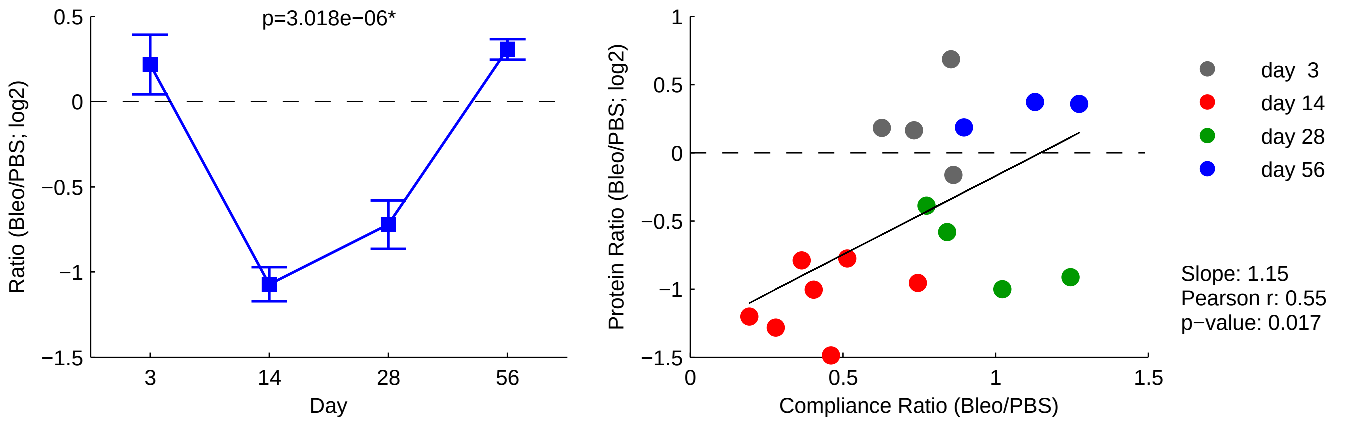

CON__P00761 − (id: 494)

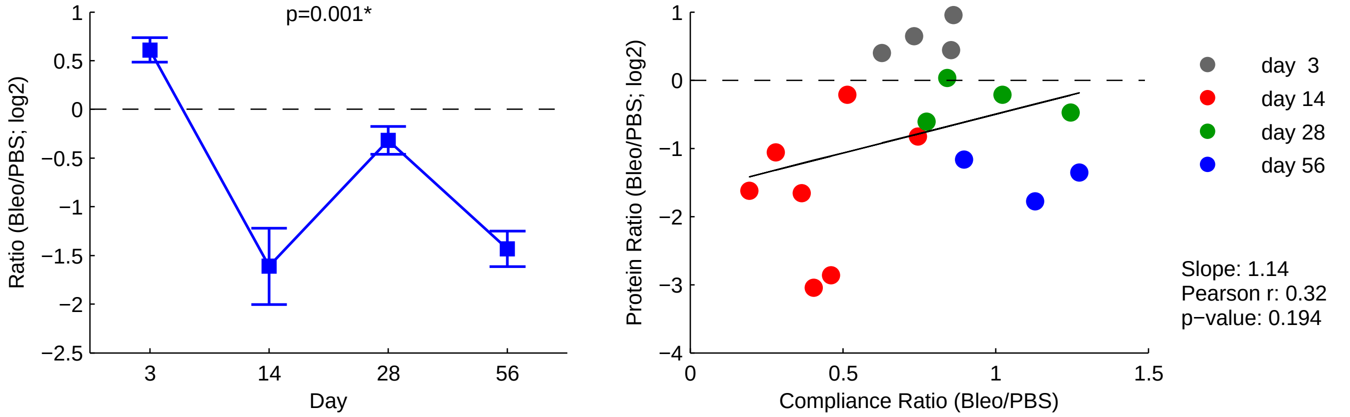

CON__P01966 − (id: 495)

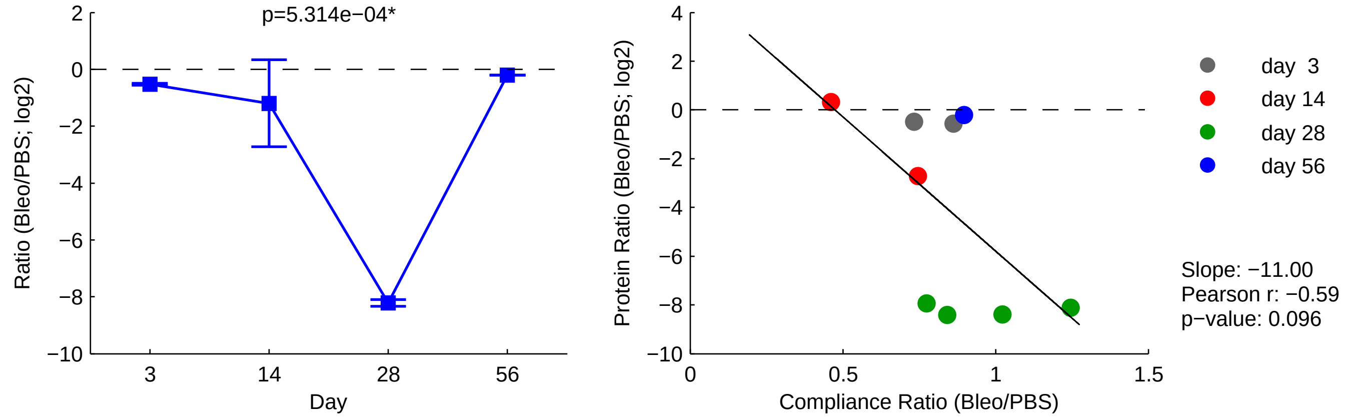

CON__P02533 − (id: 496)

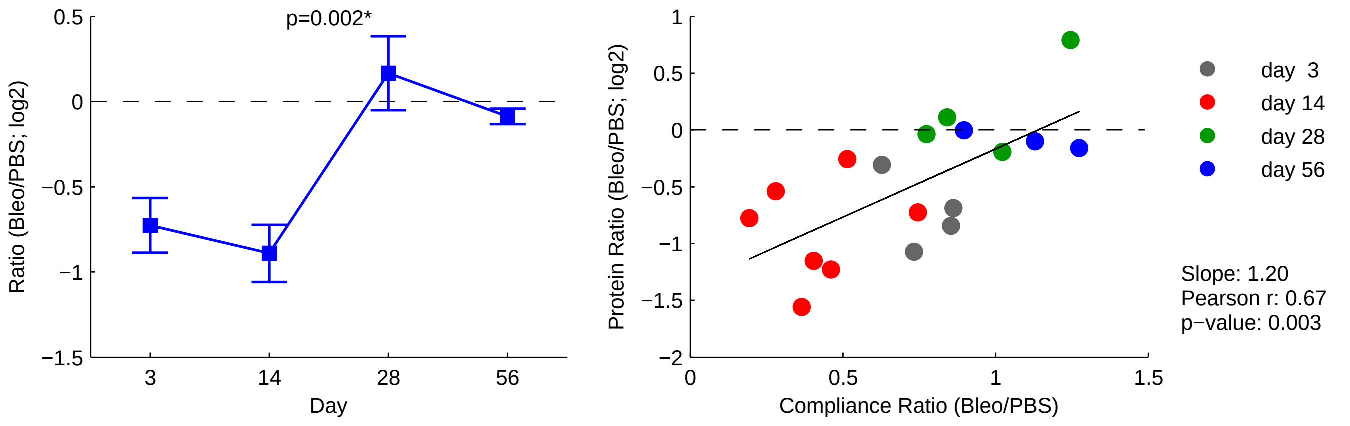

CON__P02538 − (id: 497)

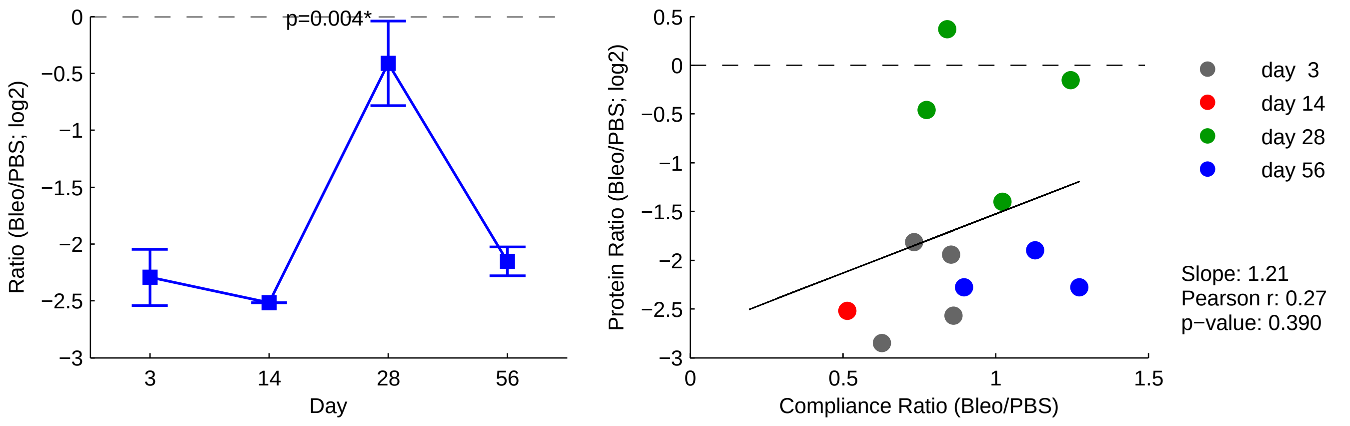

CON__P02662 − (id: 499)

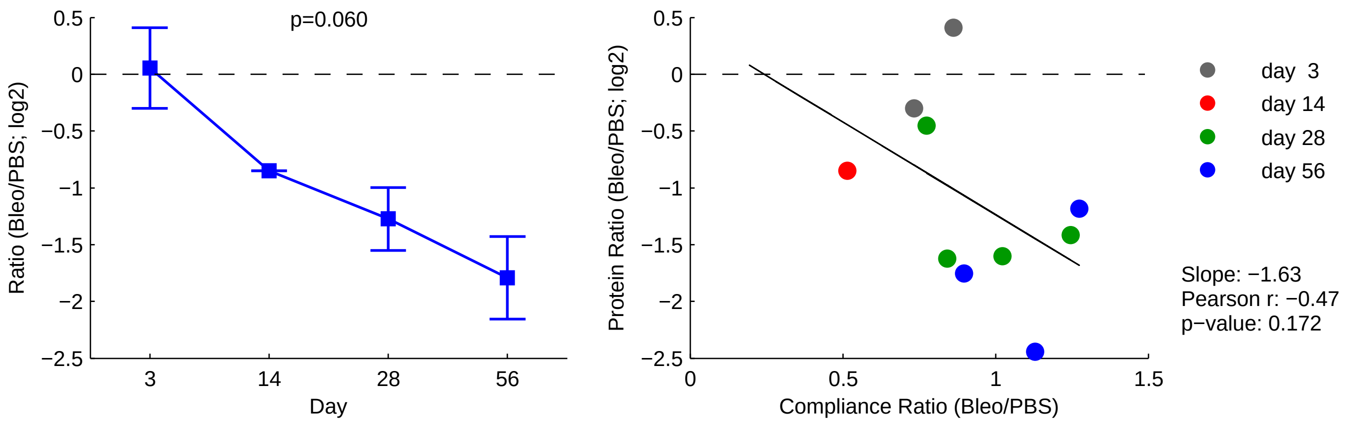

CON__P02769 − (id: 505)

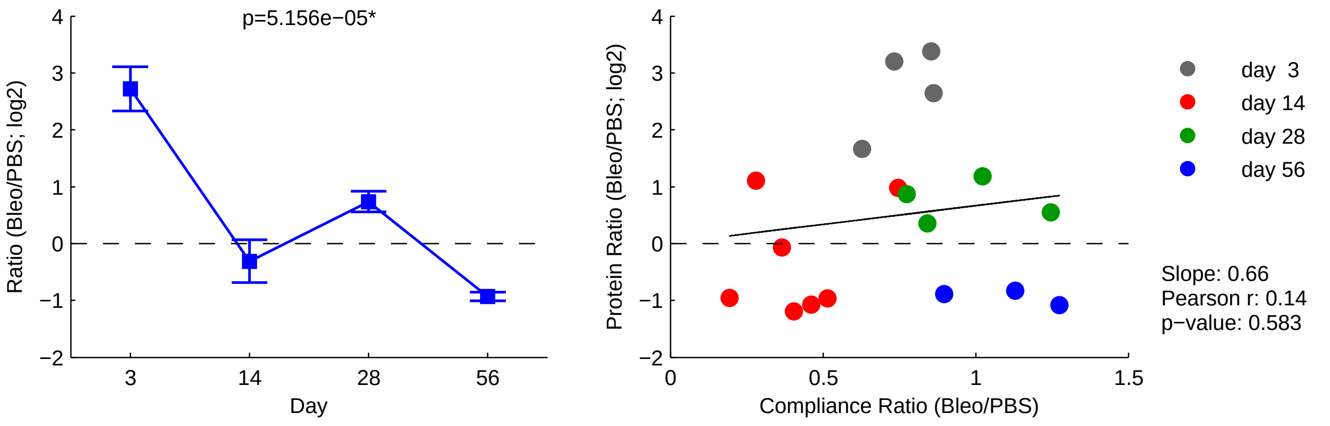

CON__P04259 − (id: 507)

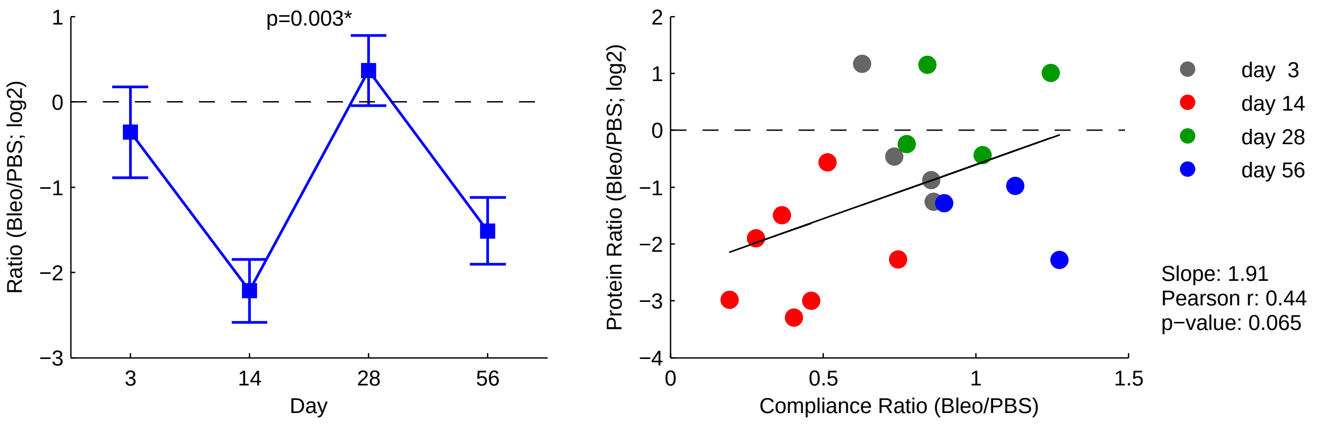

P05784 − Krt18 (id: 509)

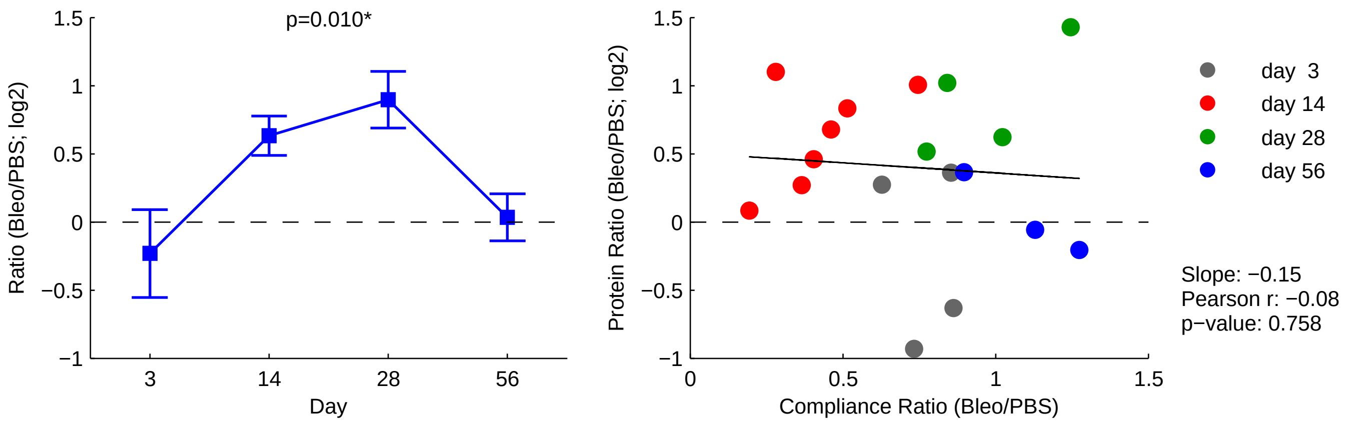

CON__P08727 − (id: 513)

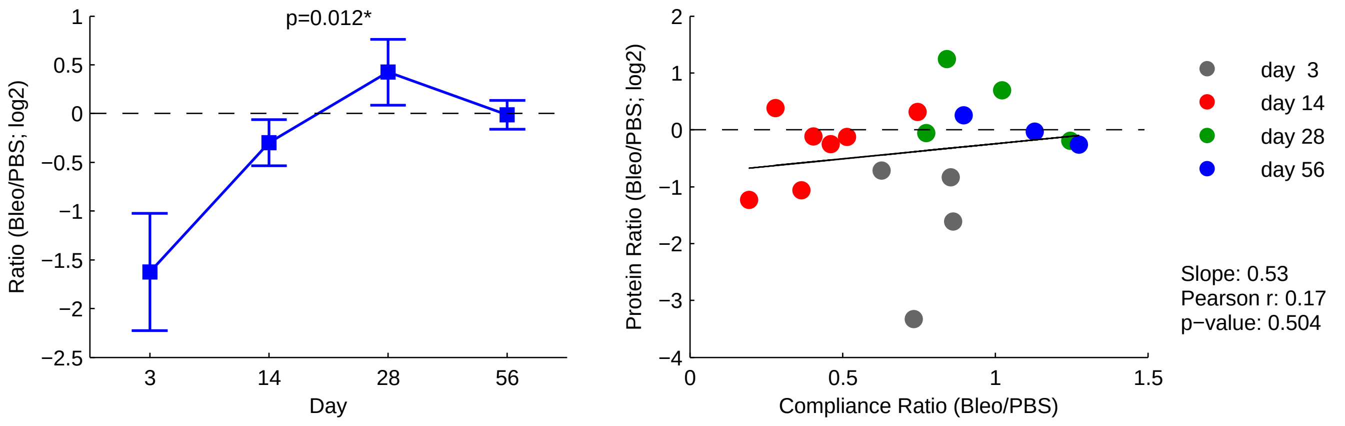

CON__P08779 − (id: 515)

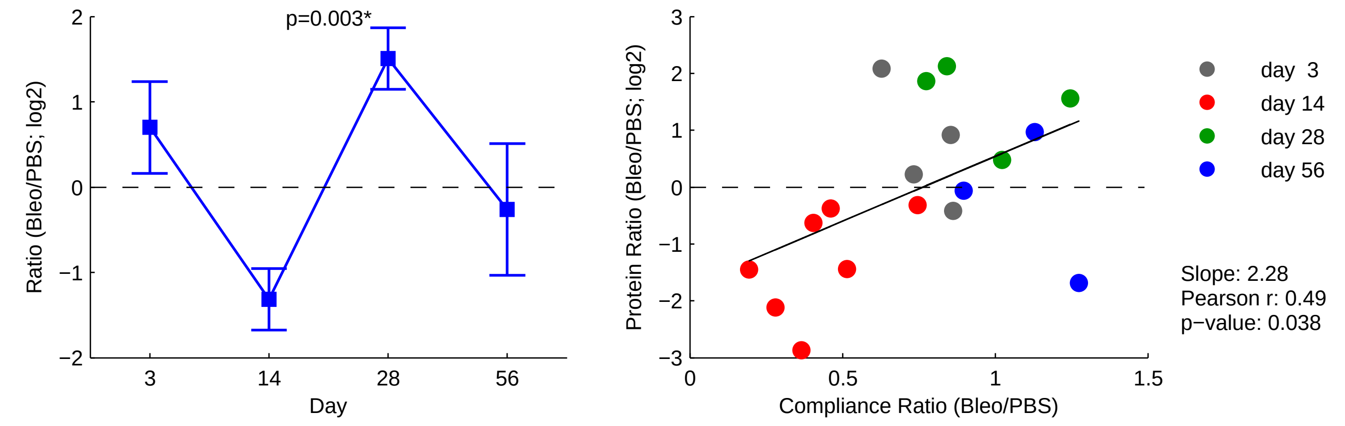

CON__P15636 − (id: 518)

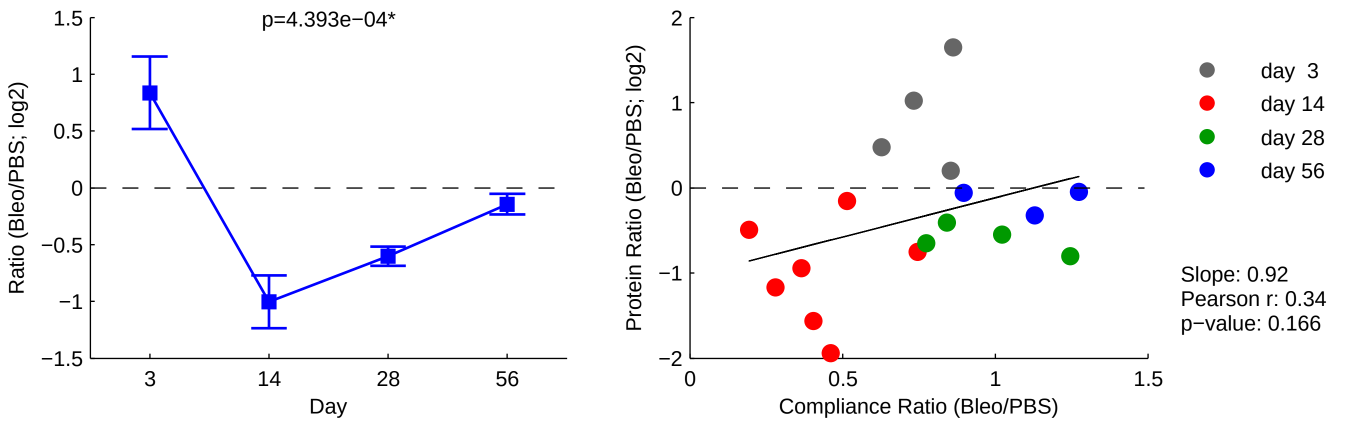

P19001 − Krt19 (id: 519)

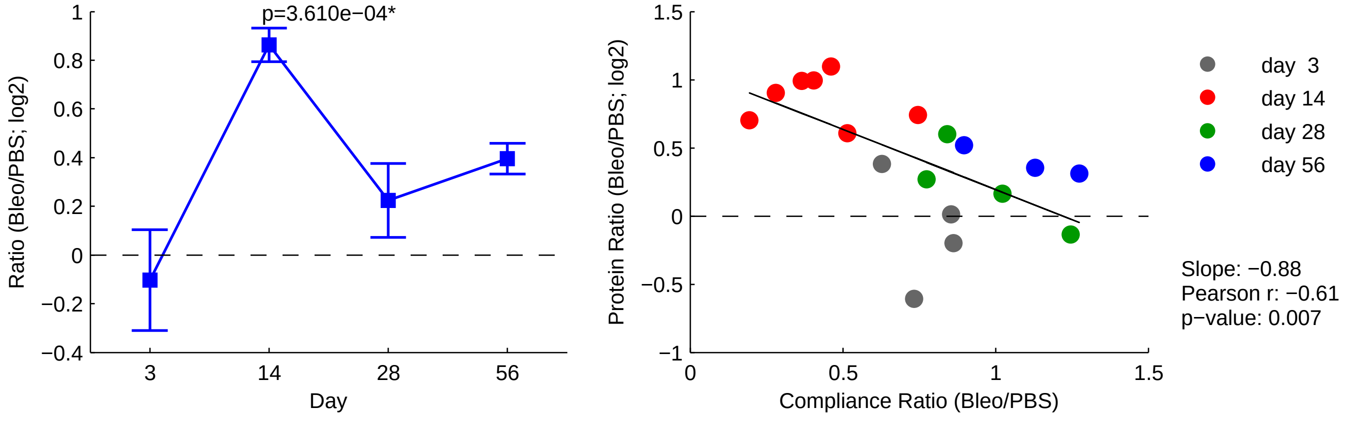

Q922U2 − Krt5 (id: 549)

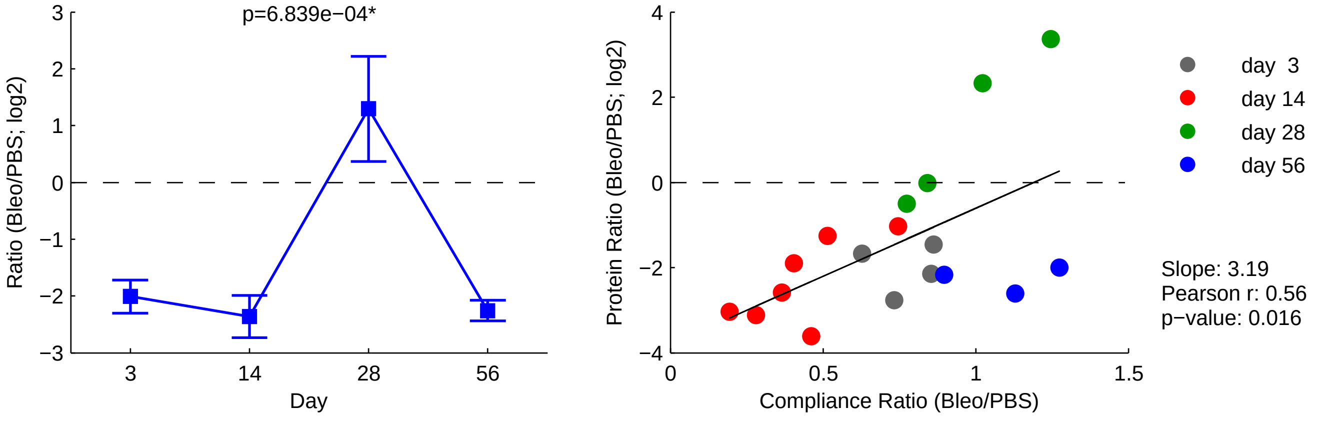

Q9DCV7 − Krt7 (id: 552)

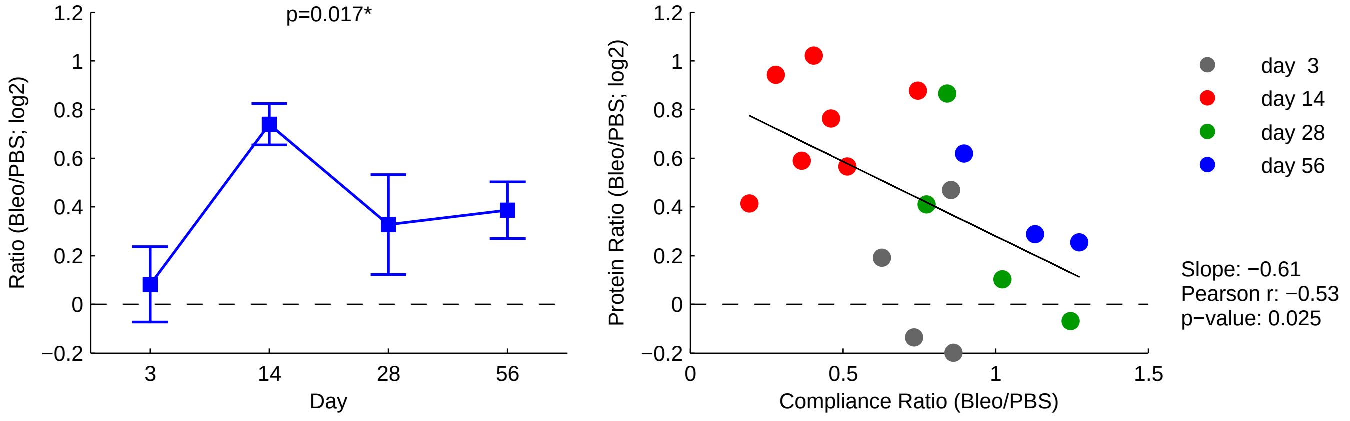

Q9QWL7 − Krt17 (id: 555)

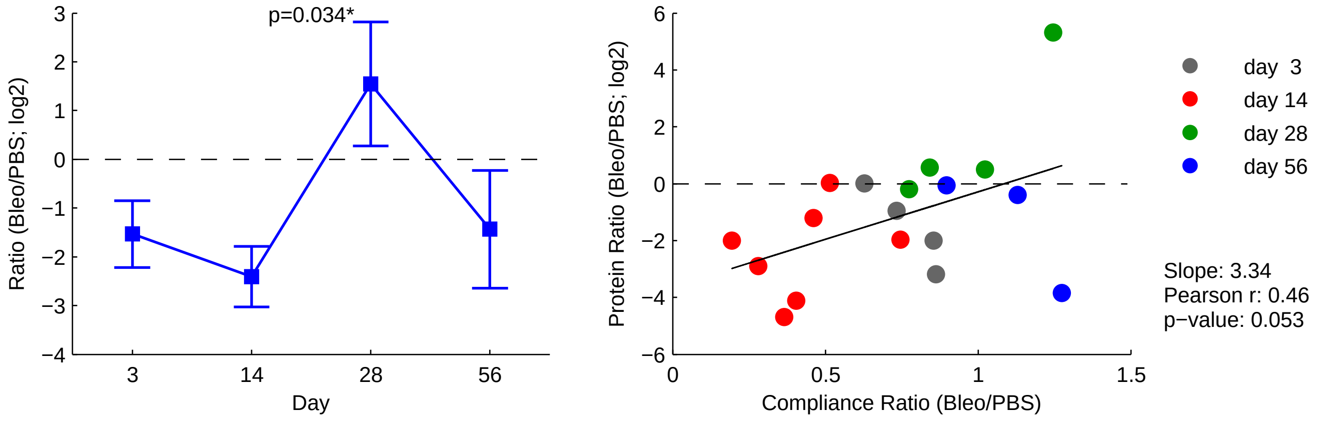

D0QMC3 − Mndal (id: 557)

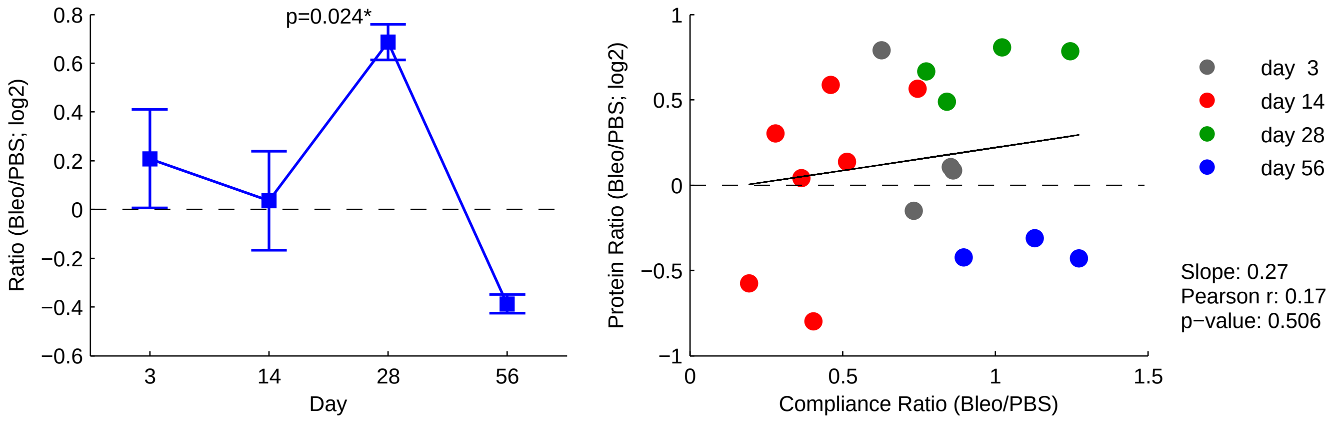

D3YTN4 − Gimap4 (id: 559)

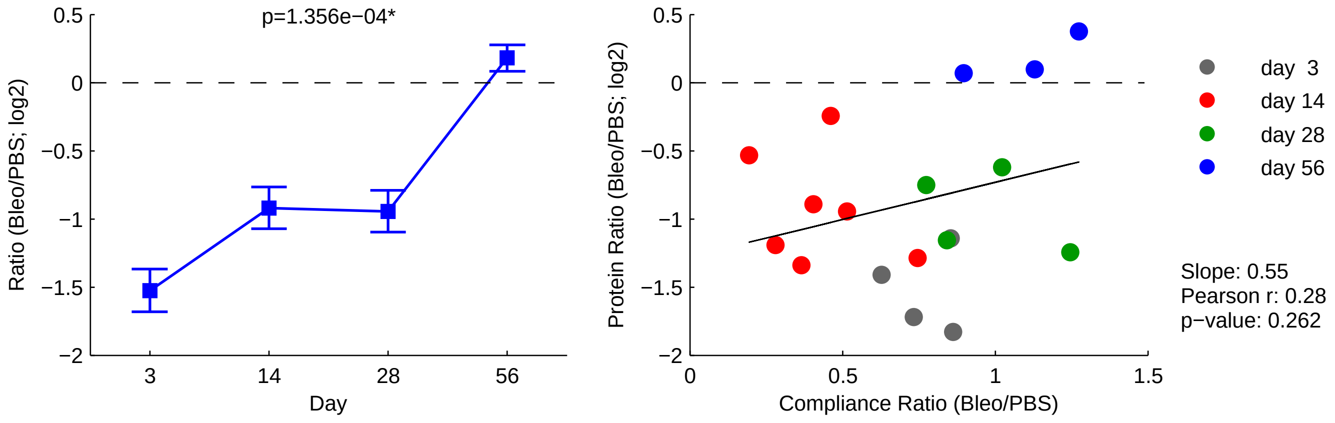

D3YWT0 − Sec11a (id: 564)

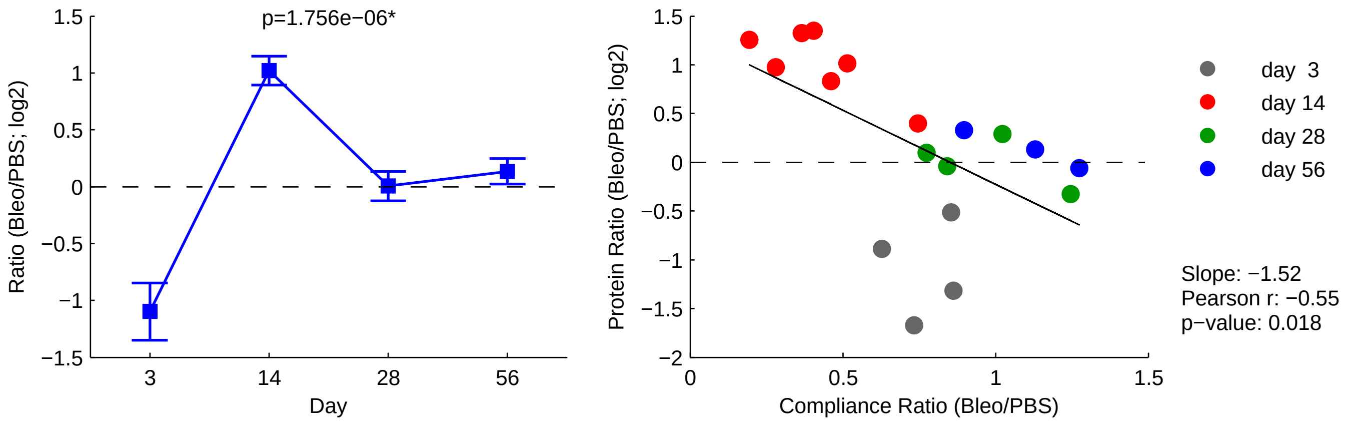

D3Z634 − Lphn3 (id: 569)

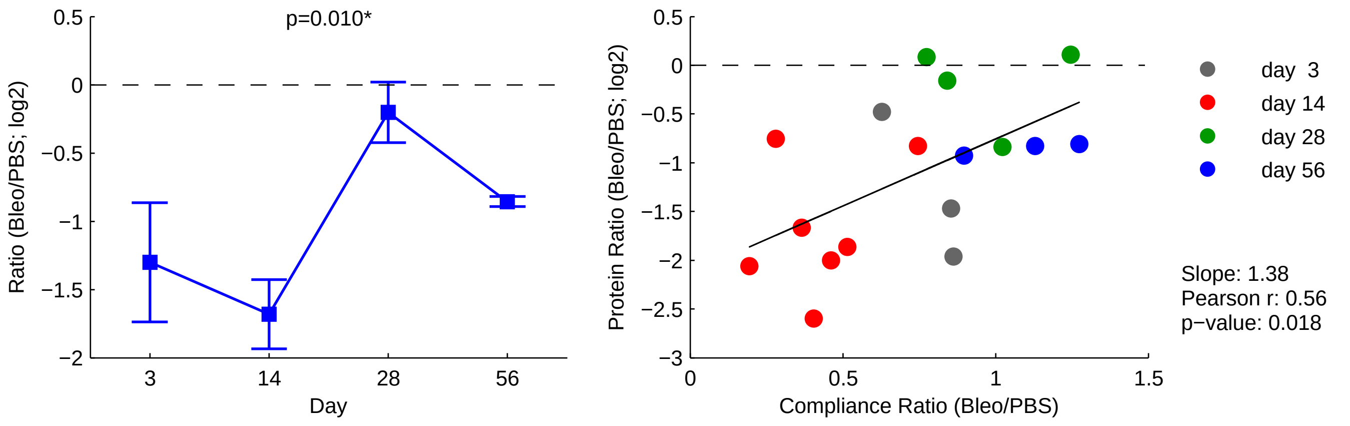

D3YU58 − Anapc7 (id: 576)

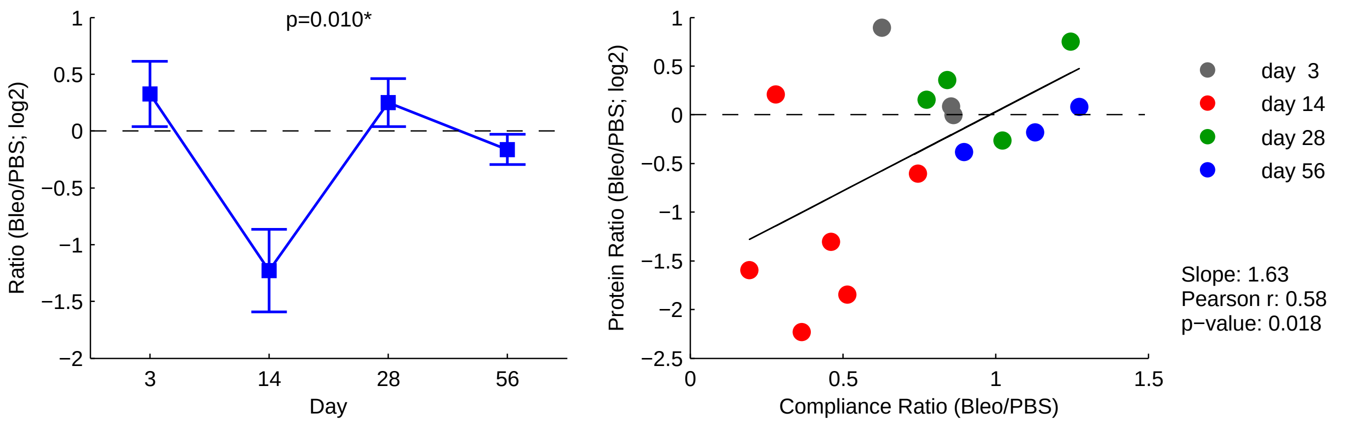

D3YU60 − Mgst1 (id: 578)

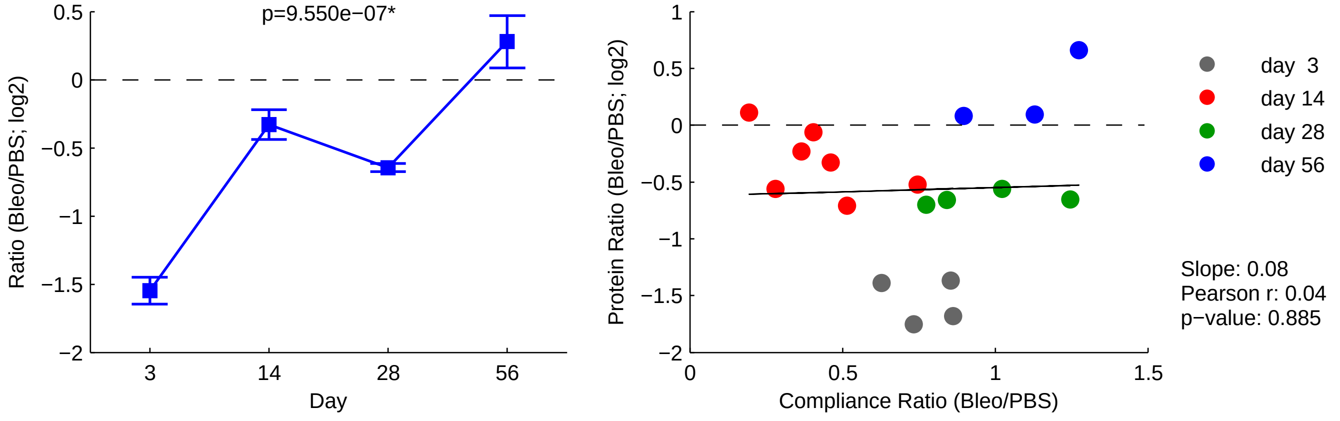

D3YUC0 − Lig1 (id: 581)

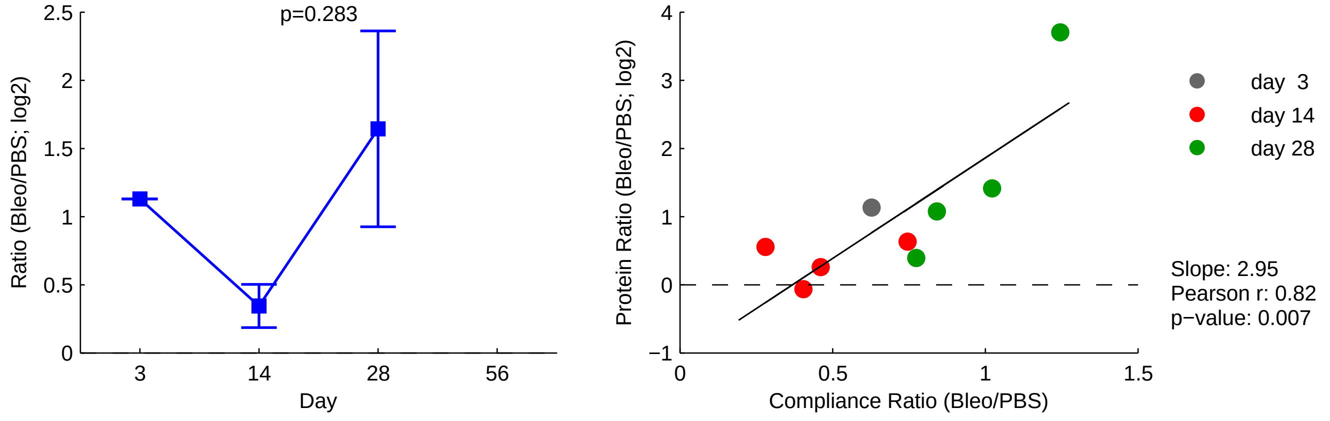

D3YUM1 − Ndufv1 (id: 590)

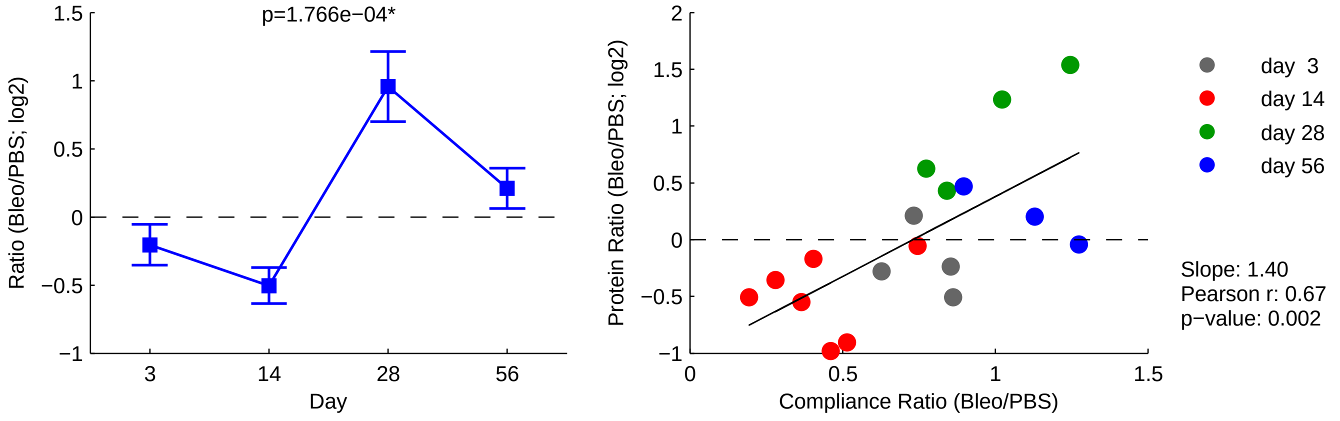

Q0P5W1 − Vps8 (id: 592)

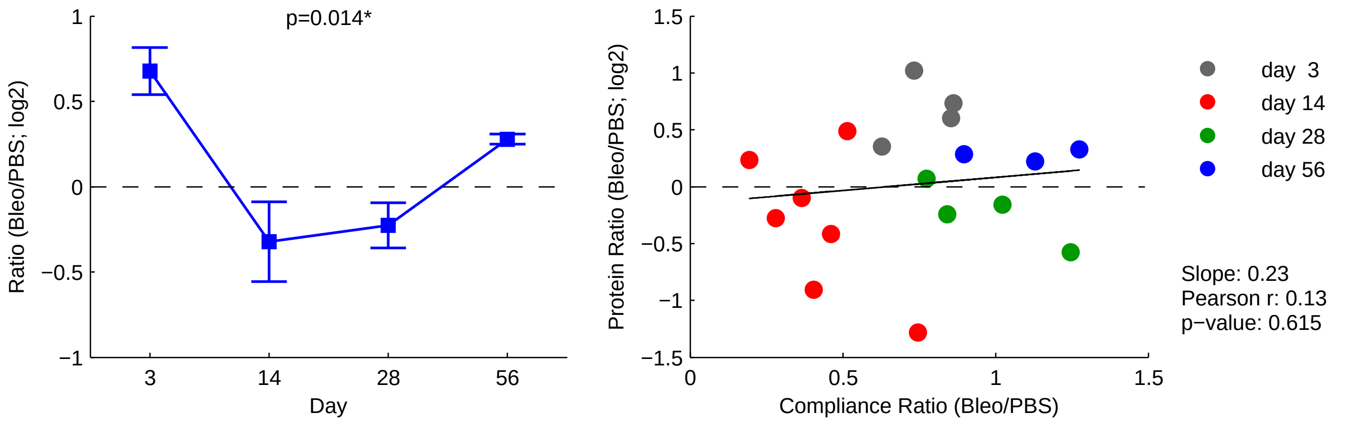

D3YUS5 − Rasal2 (id: 596)

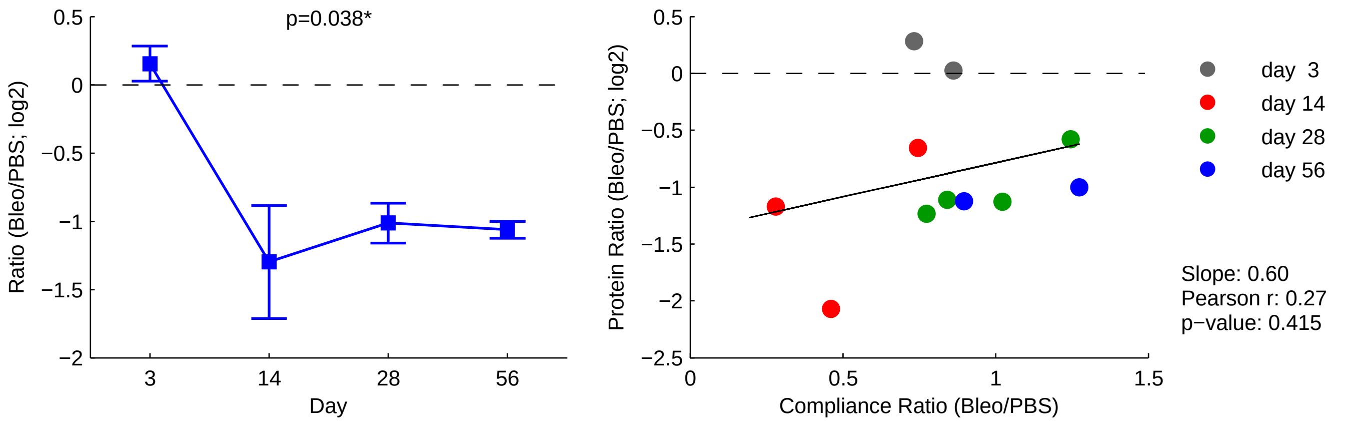

D3YUY3 − Pip5k1a (id: 600)

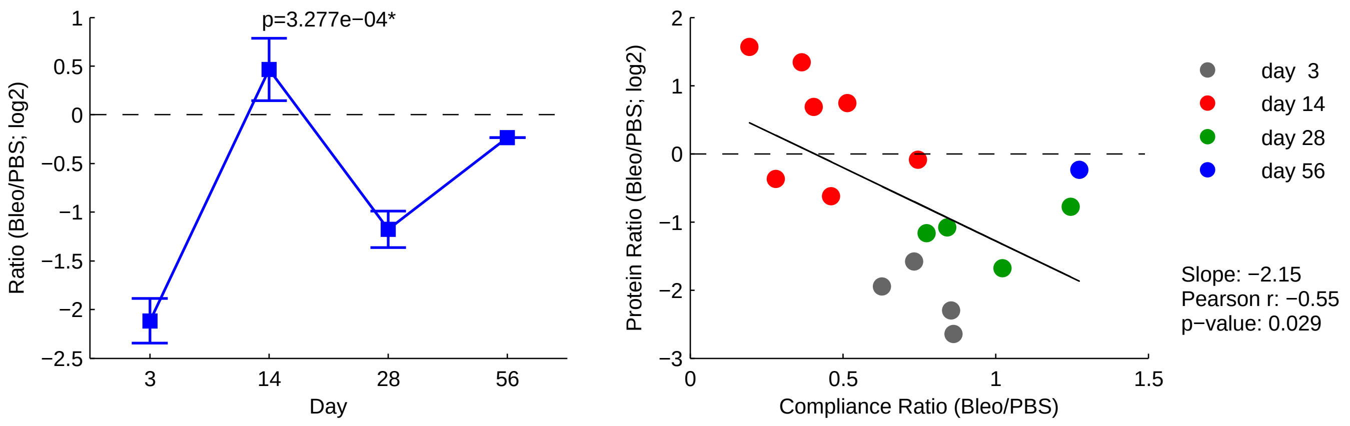

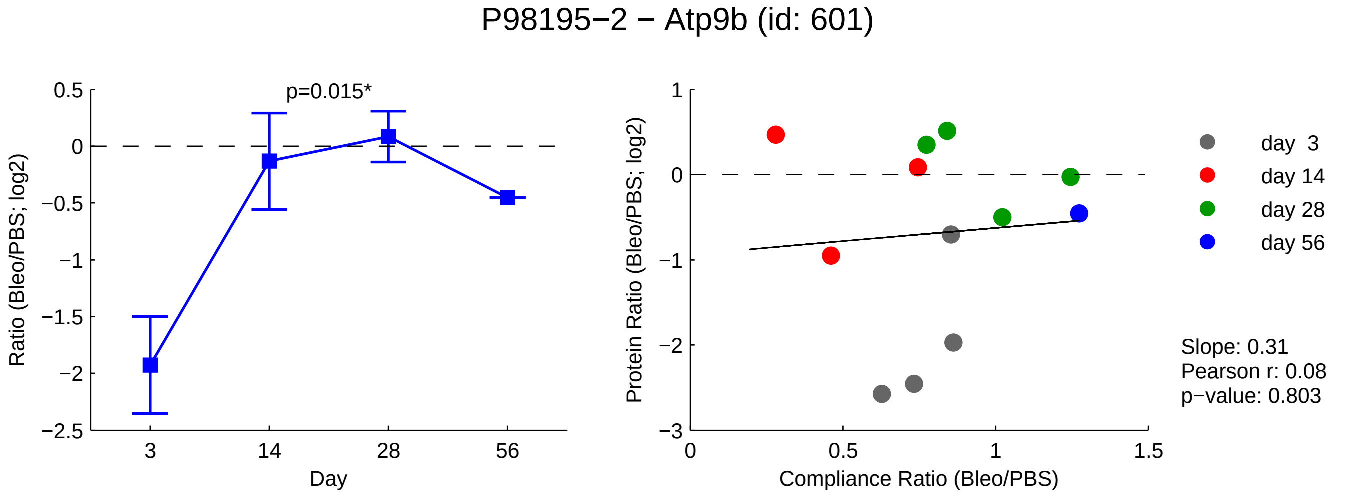
P98195−2 − Atp9b (id: 601)
p=0.015*
Ratio (Bleo/PBS; log2)
Day
Protein Ratio (Bleo/PBS; log2)
Compliance Ratio (Bleo/PBS)
day 3
day 14
day 28
day 56
Slope: 0.31
Pearson r: 0.08
p−value: 0.803

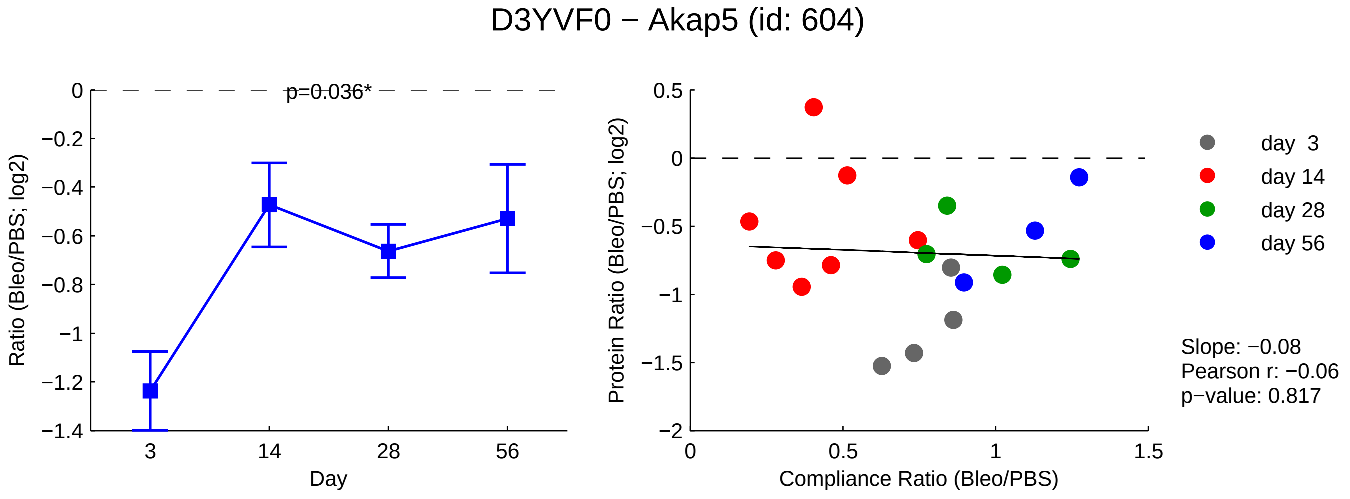
D3YVF0 − Akap5 (id: 604)
p=0.036*
Ratio (Bleo/PBS; log2)
Day
Protein Ratio (Bleo/PBS; log2)
Compliance Ratio (Bleo/PBS)
day 3
day 14
day 28
day 56
Slope: −0.08
Pearson r: −0.06
p−value: 0.817

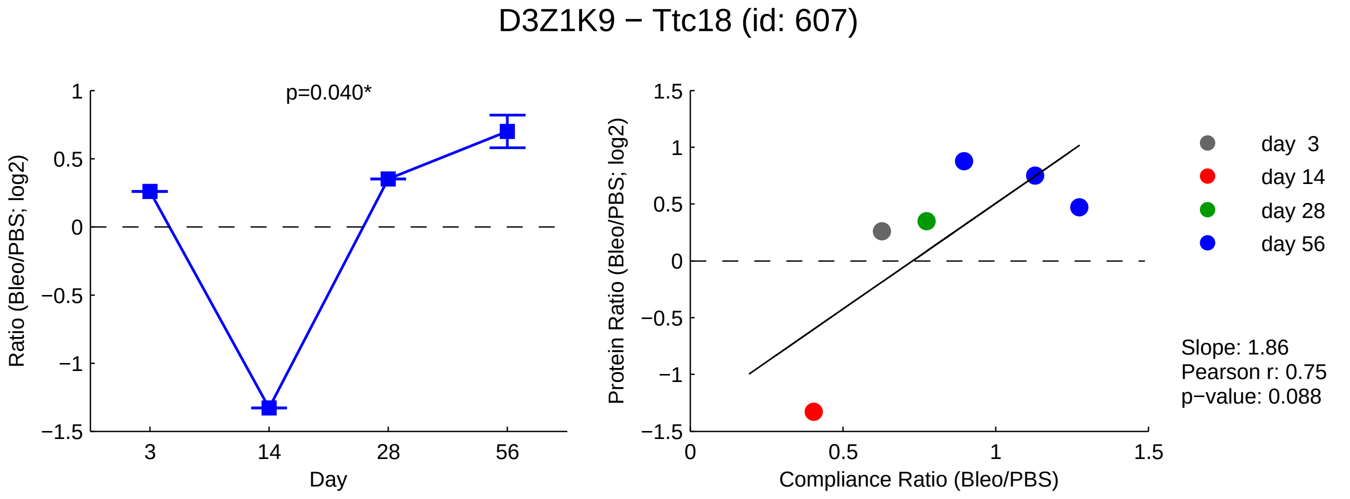
D3Z1K9 − Ttc18 (id: 607)
p=0.040*
Ratio (Bleo/PBS; log2)
Day
Protein Ratio (Bleo/PBS; log2)
Compliance Ratio (Bleo/PBS)
day 3
day 14
day 28
day 56
Slope: 1.86
Pearson r: 0.75
p−value: 0.088

D3YVV9 − Synpo2 (id: 617)

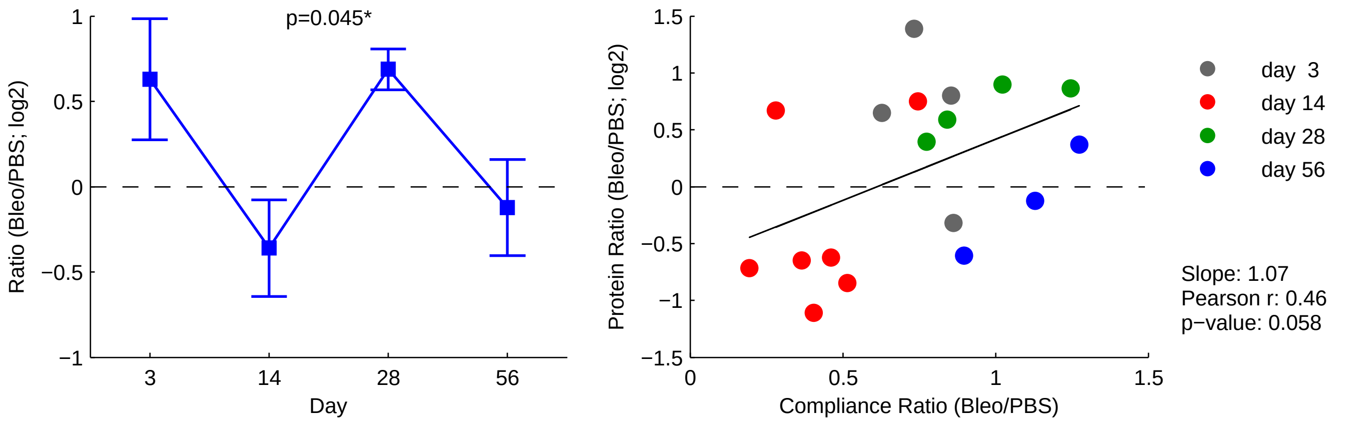

D3YVW2 − Golim4 (id: 618)

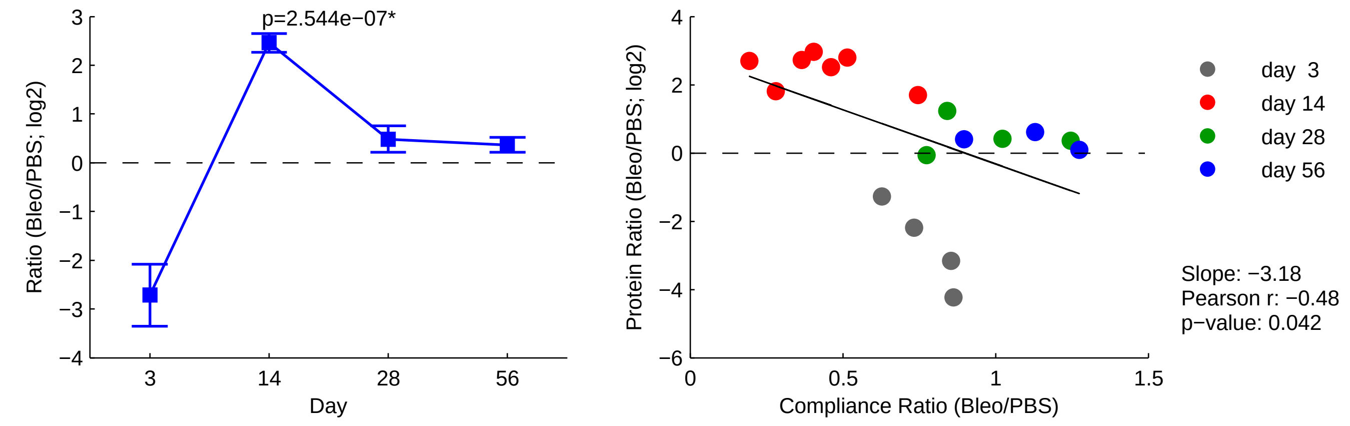

D6RFN5 − Ninj1 (id: 621)

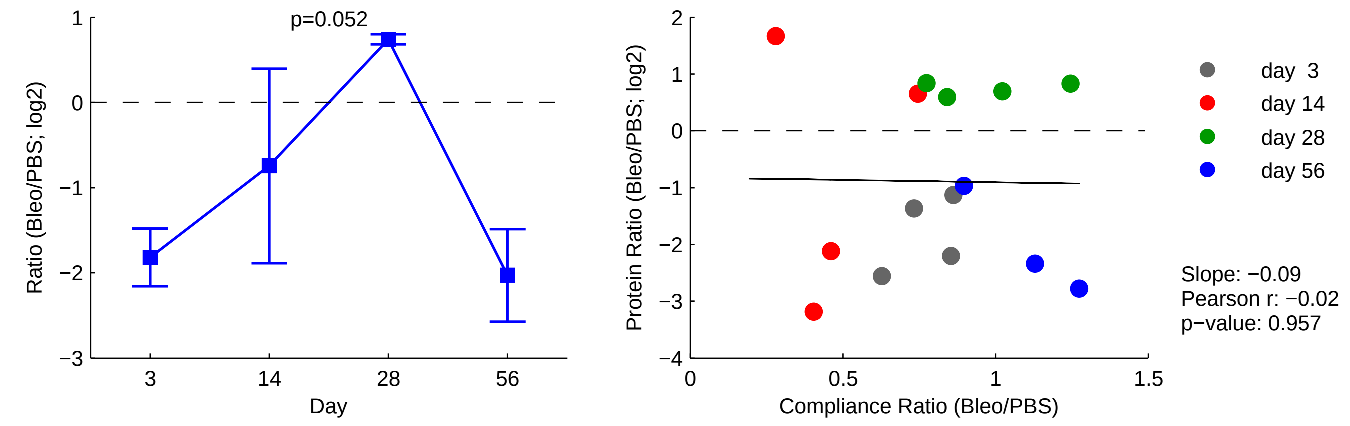

D3YW48 − Capns1 (id: 622)

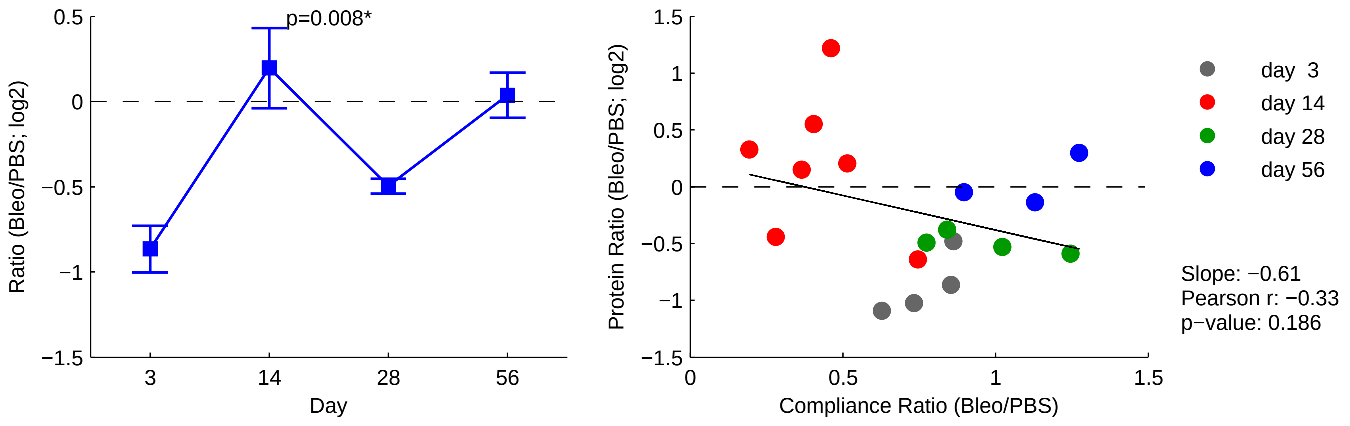

Q61838 − A2m (id: 623)

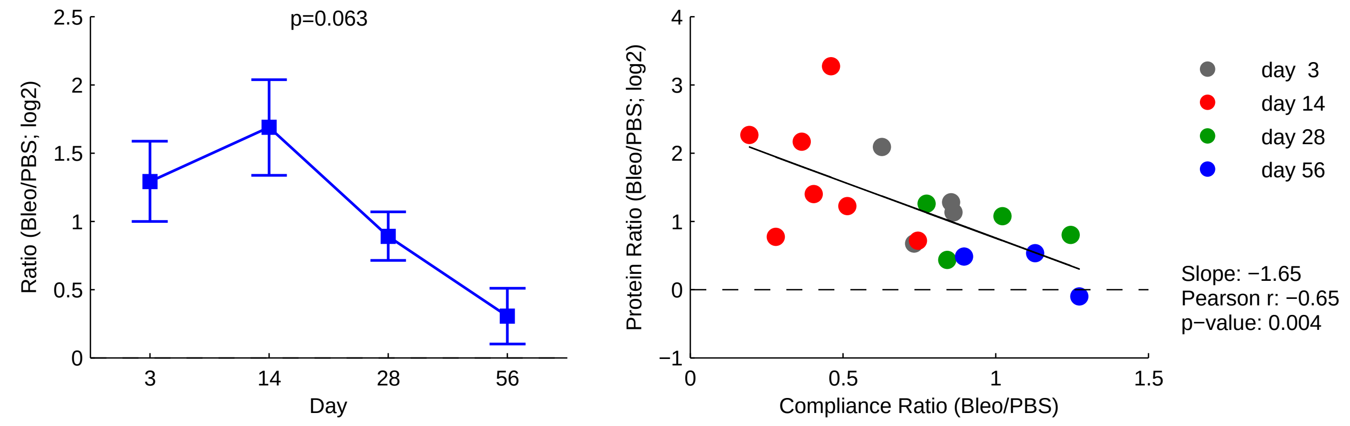

D3YWB8 − Rps6kb2 (id: 624)

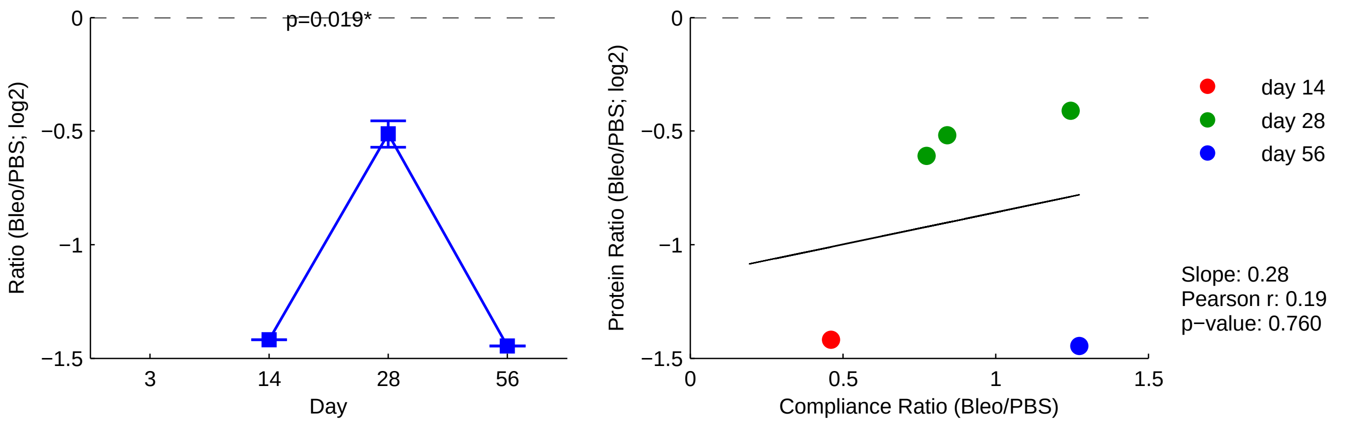

Q69Z89−2 − Radil (id: 628)

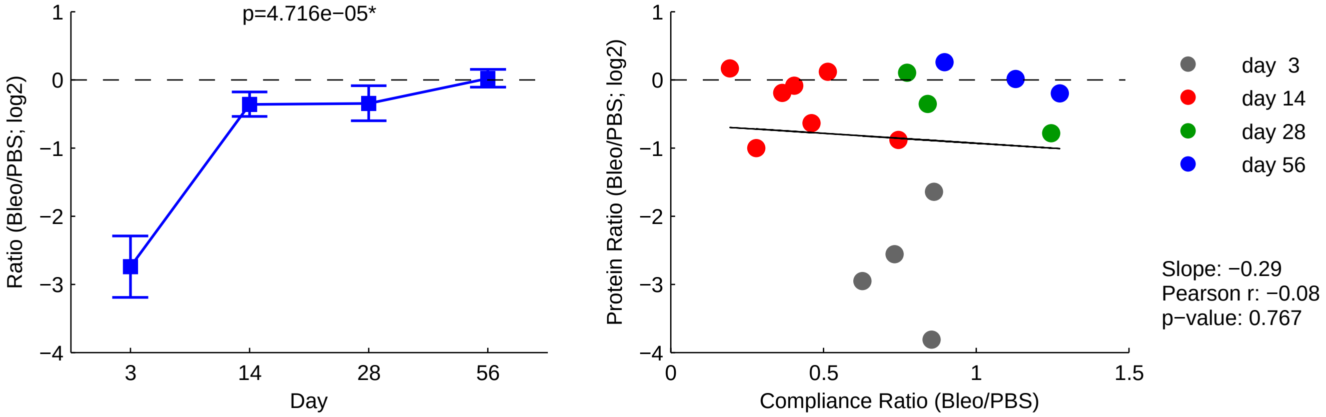

P62751 − Rpl23a (id: 630)

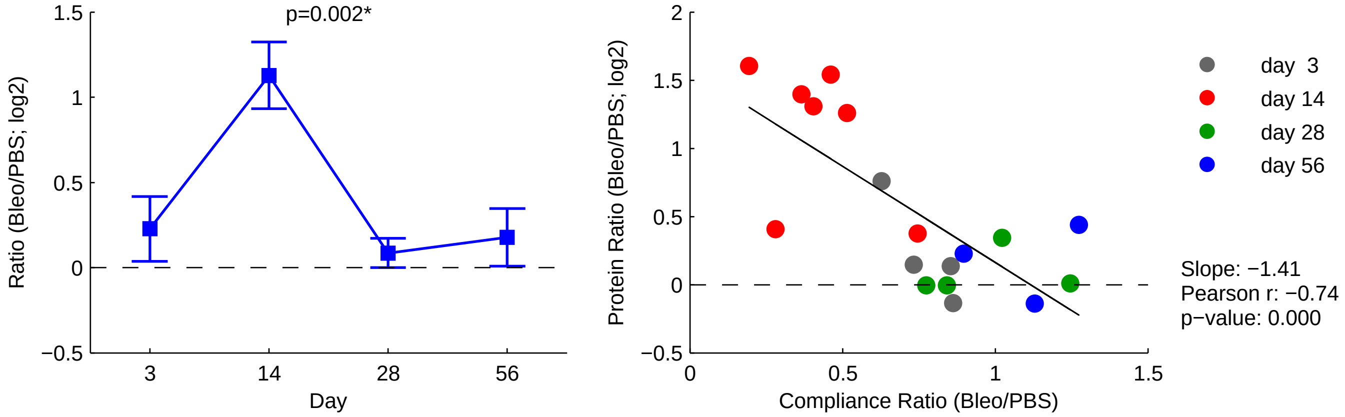

D3YWS8 − Leng8 (id: 632)

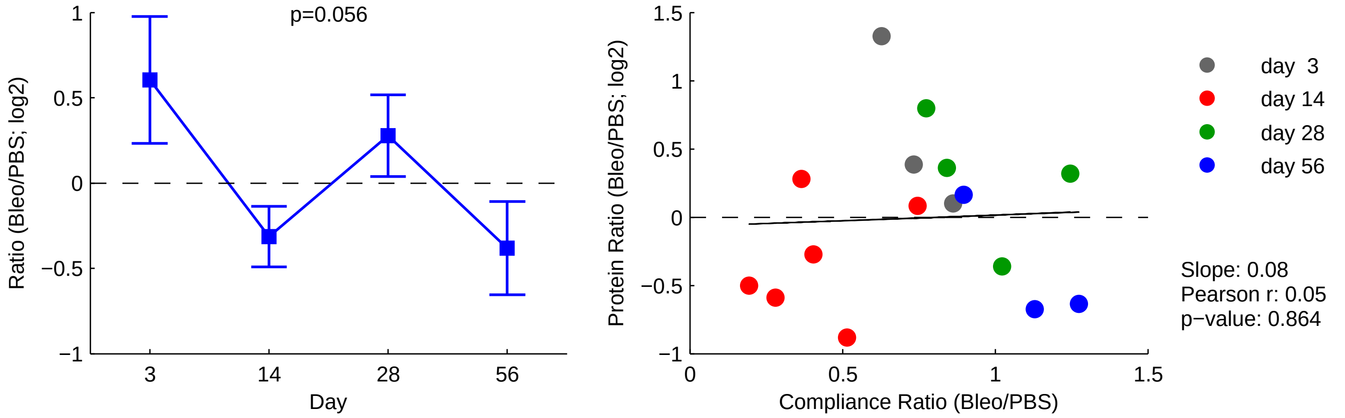

D3YWT1 − Hnrnph3 (id: 634)

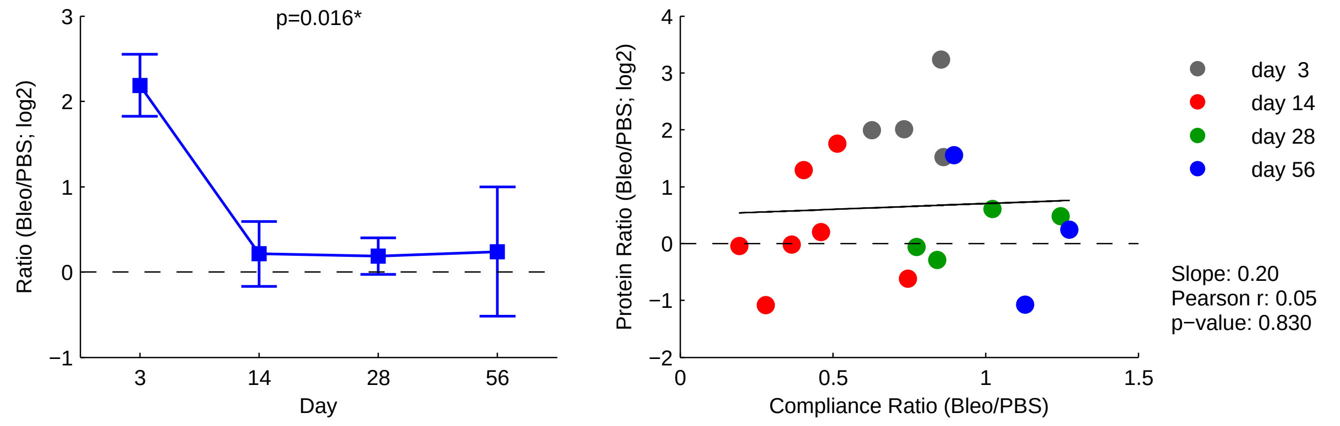

D3YWX2 − Ylpm1 (id: 635)

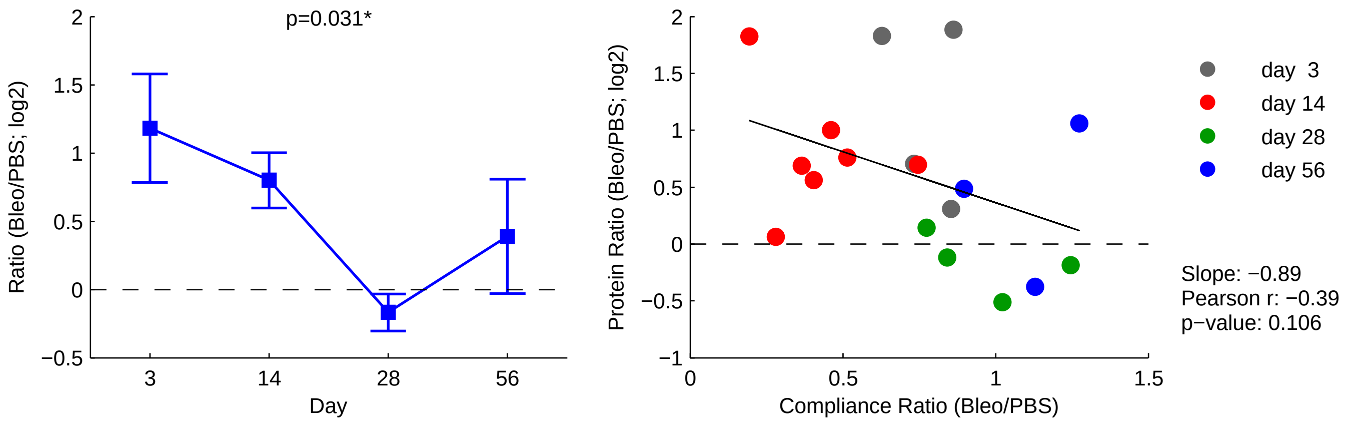

Q7SIG6−2 − Asap2 (id: 638)

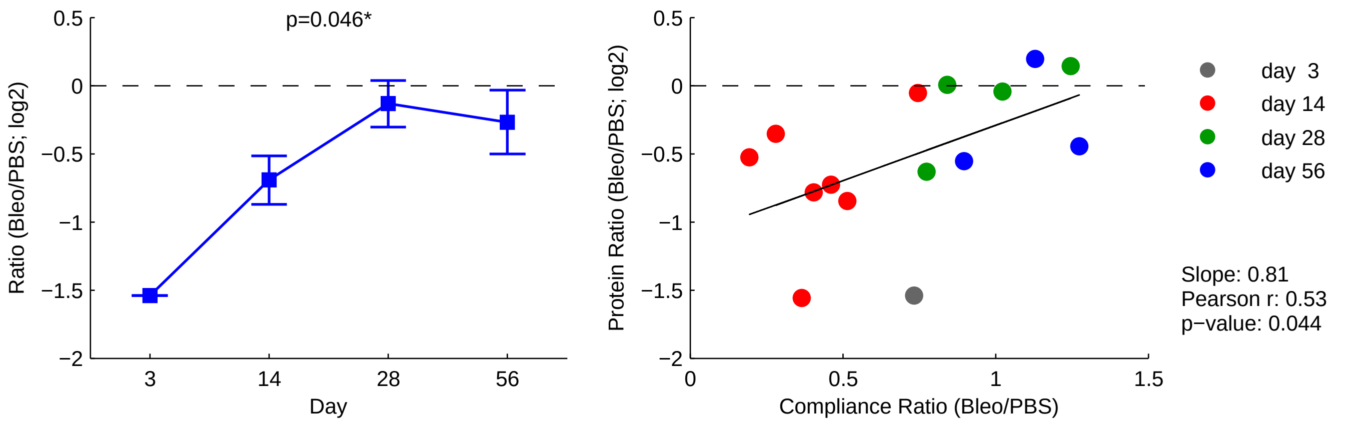

D3YX90 − Adamts17 (id: 639)

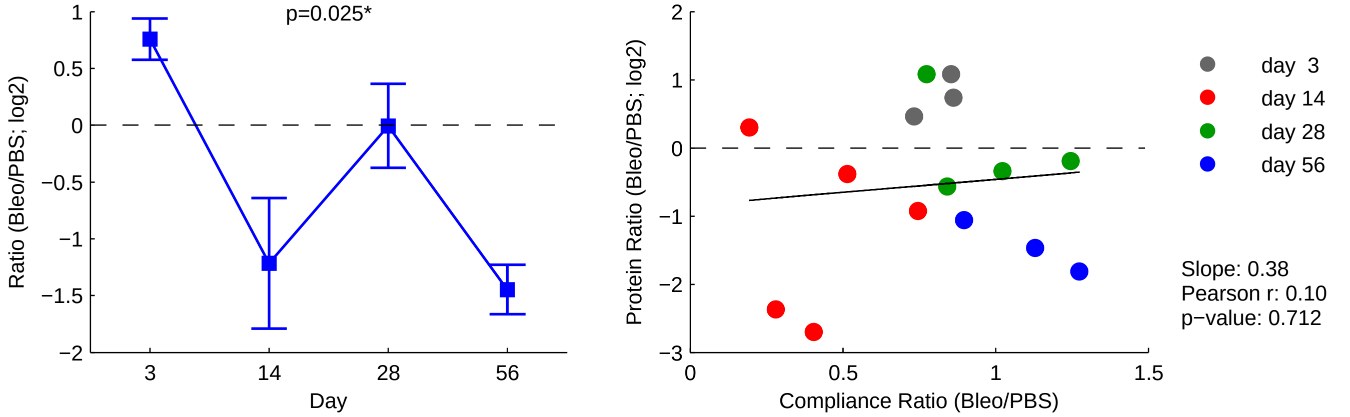

H3BL23 − Ube2e2 (id: 640)

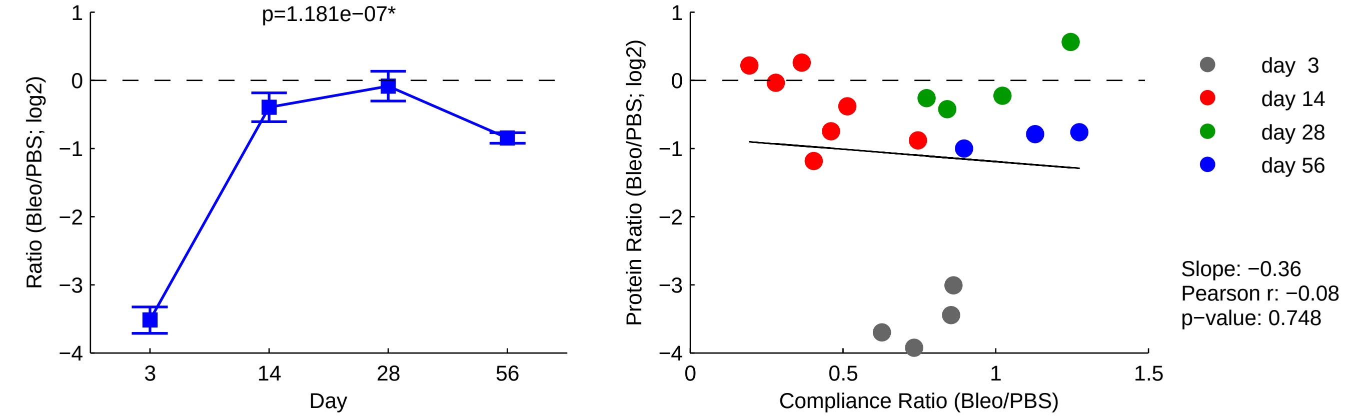

D3YXF5 − C7 (id: 641)

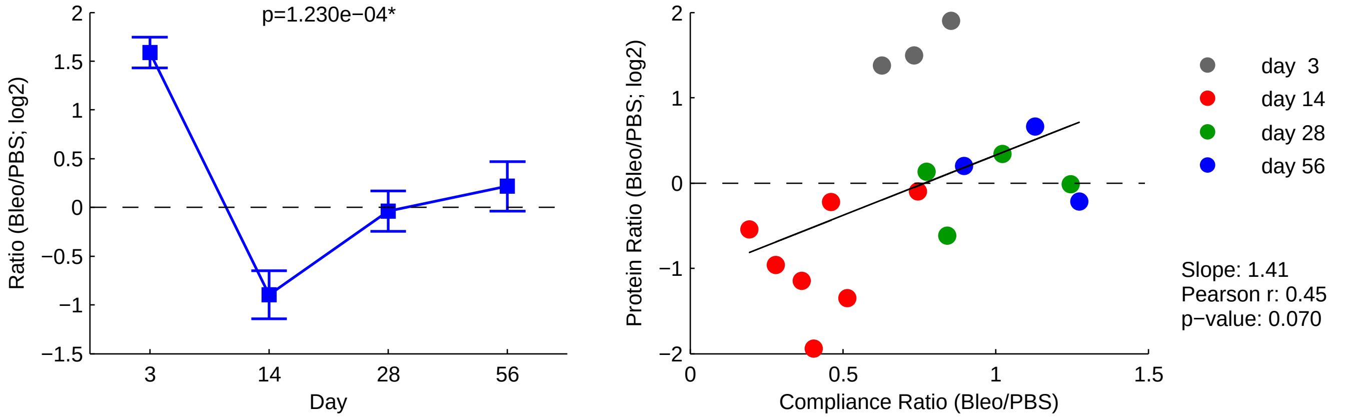

D3YXG0 − Hmcn1 (id: 642)

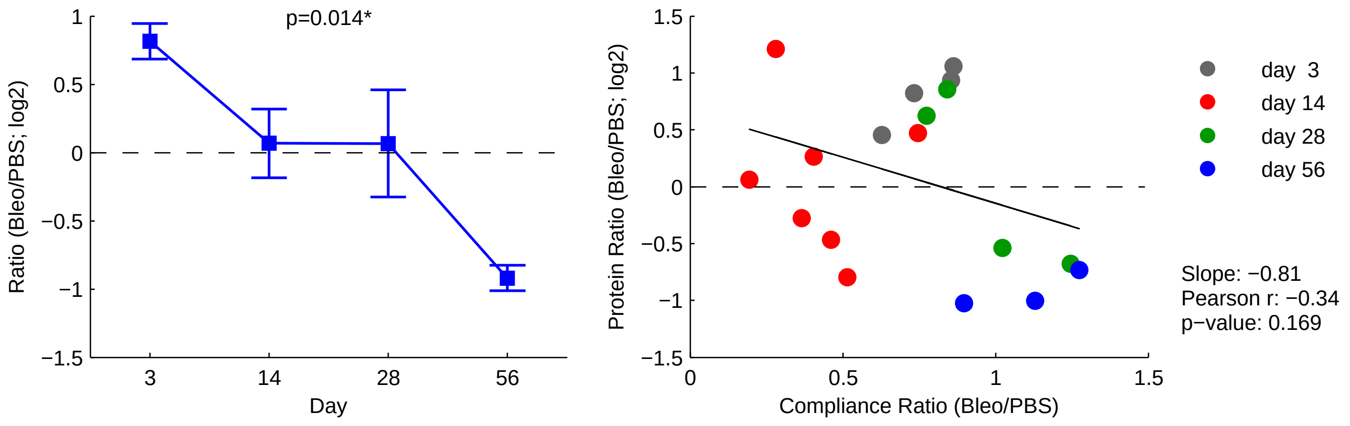

Q3UV74 − Itgb2l (id: 643)

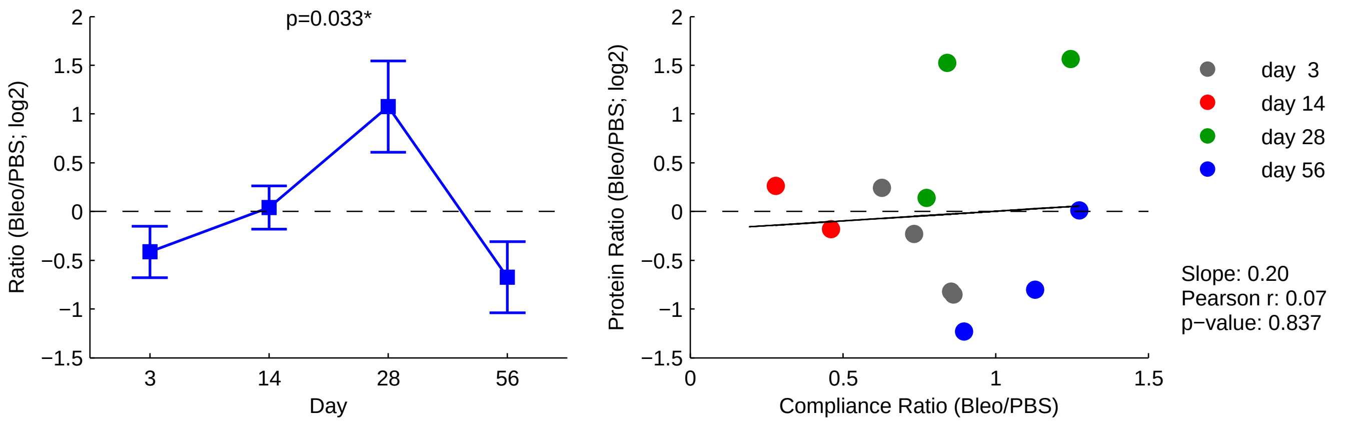

F6T9K1 − Drap1 (id: 656)

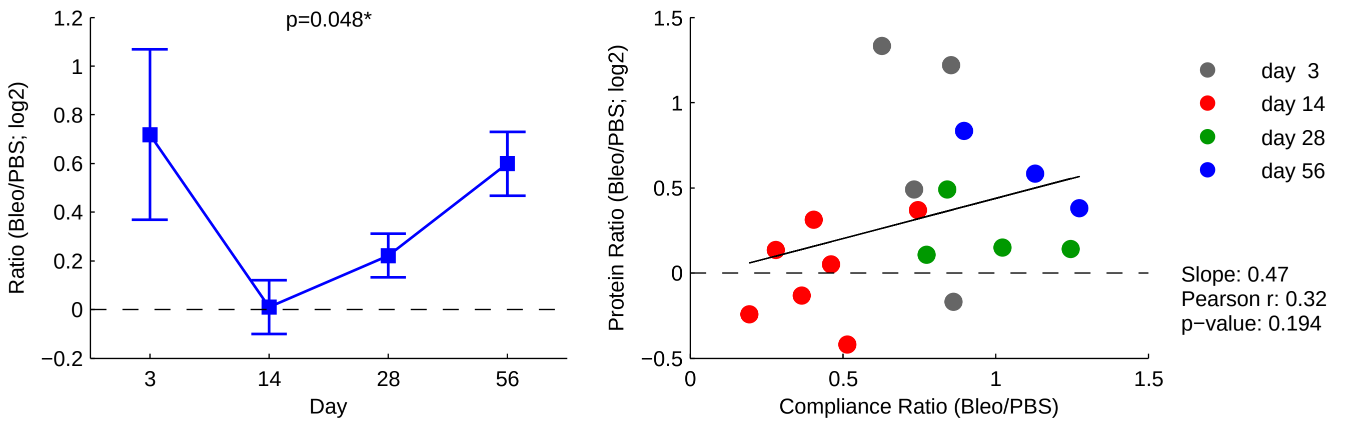

Q91ZD4 − Vangl2 (id: 660)

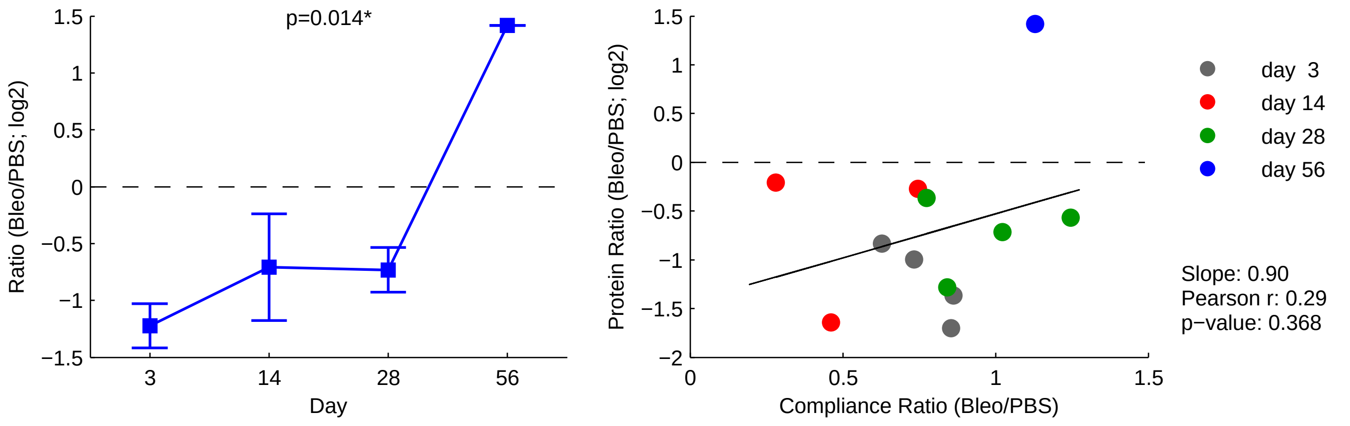

D3YYK8 − Mapre2 (id: 664)

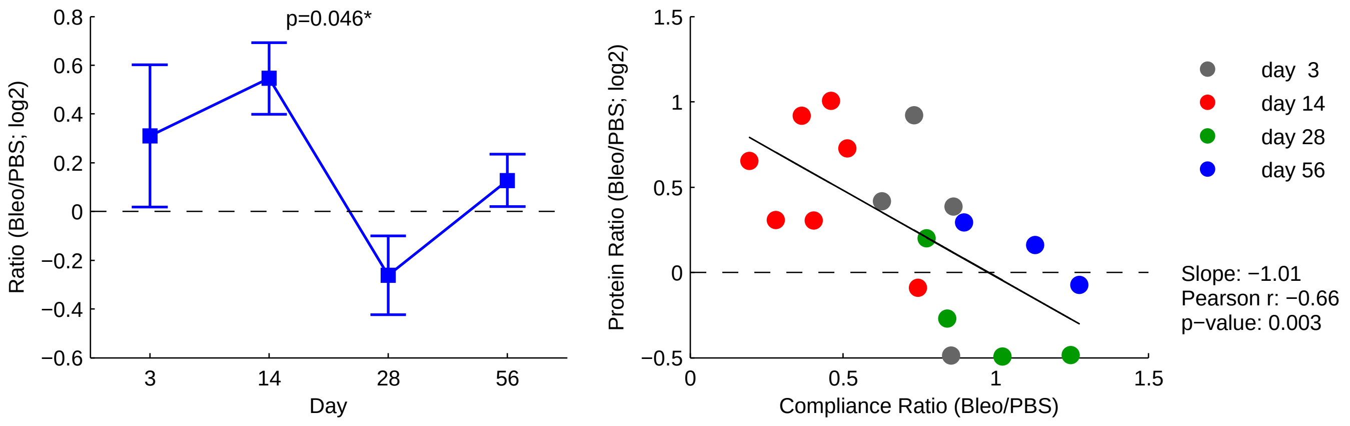

D3YYN7 − Atp1a2 (id: 666)

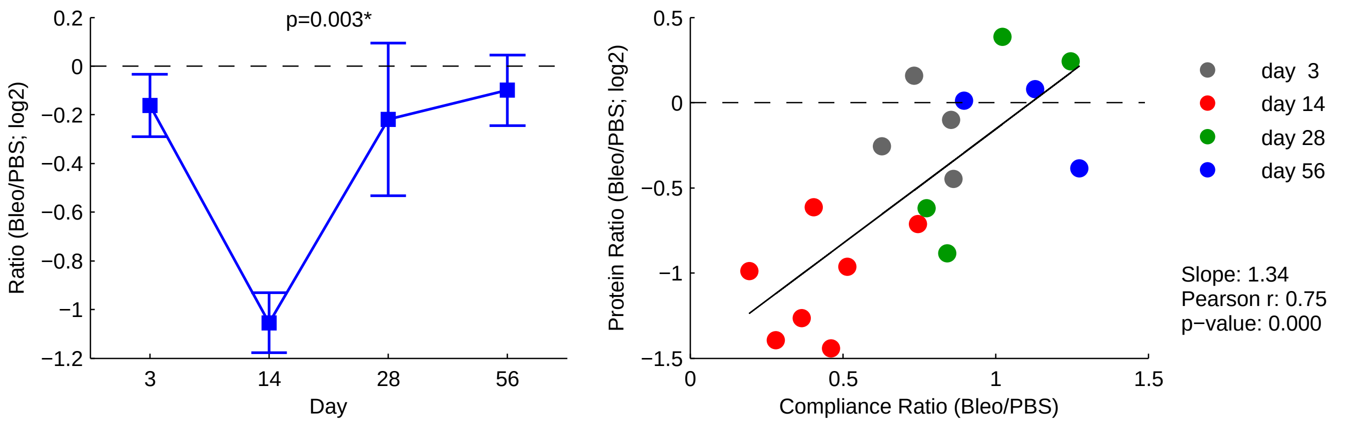

D3YYN8 − Cdc42bpa (id: 667)

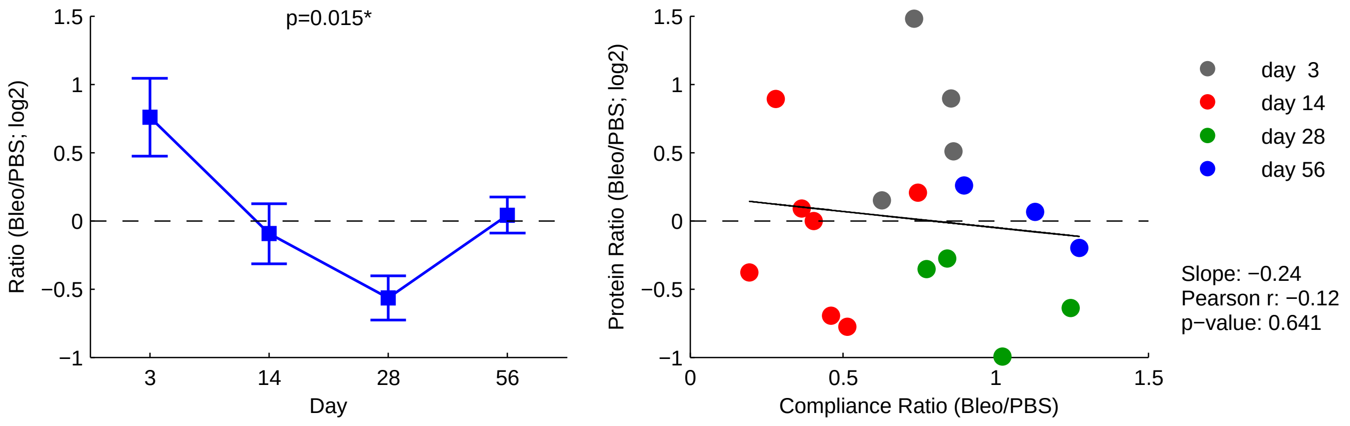

D3YYP4 − Nr2f2 (id: 668)

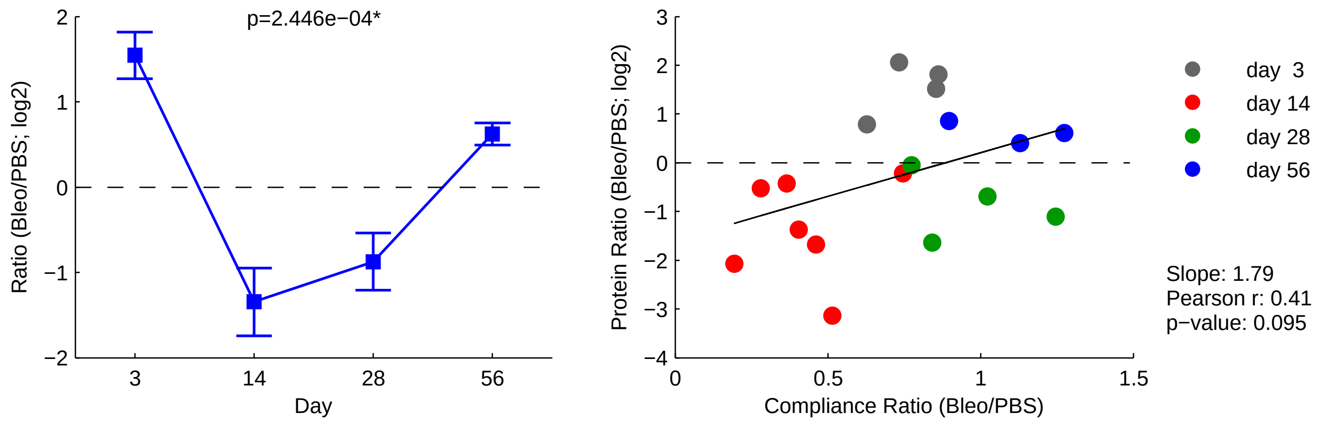

D3YYQ8 − Dnahc10 (id: 669)

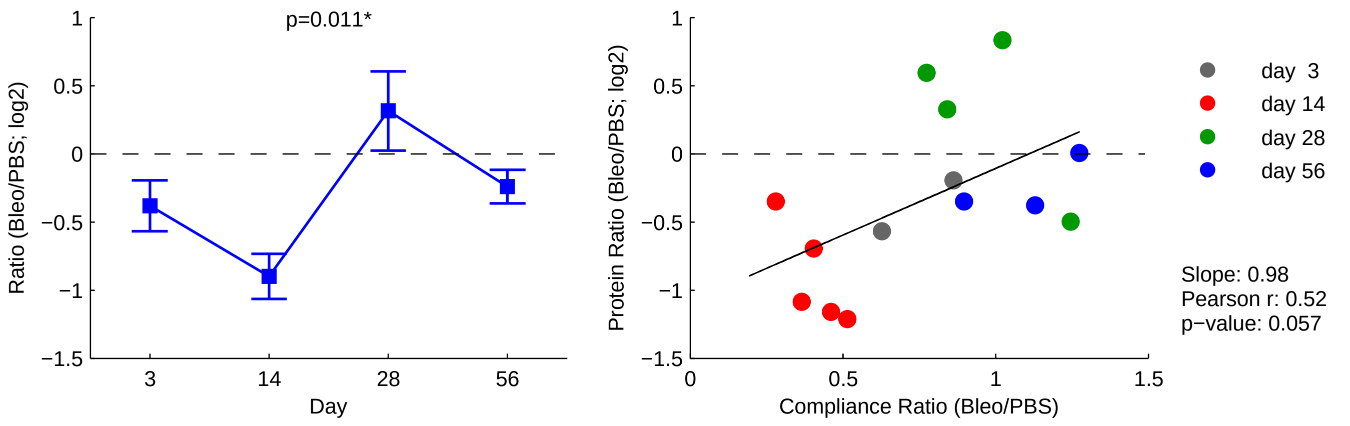

O35678 − Mgll (id: 670)

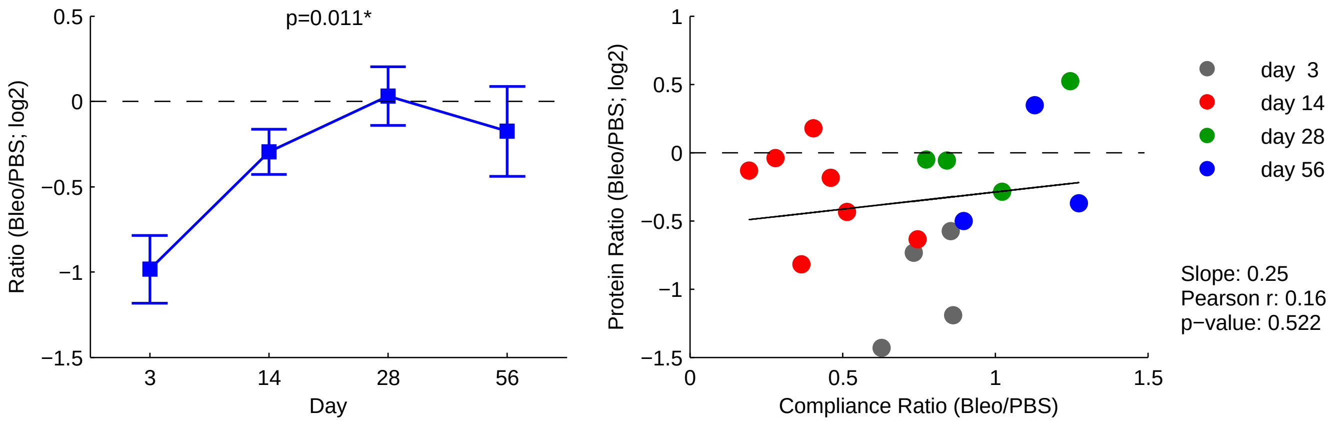

D3YYT0 − Cdh2 (id: 671)

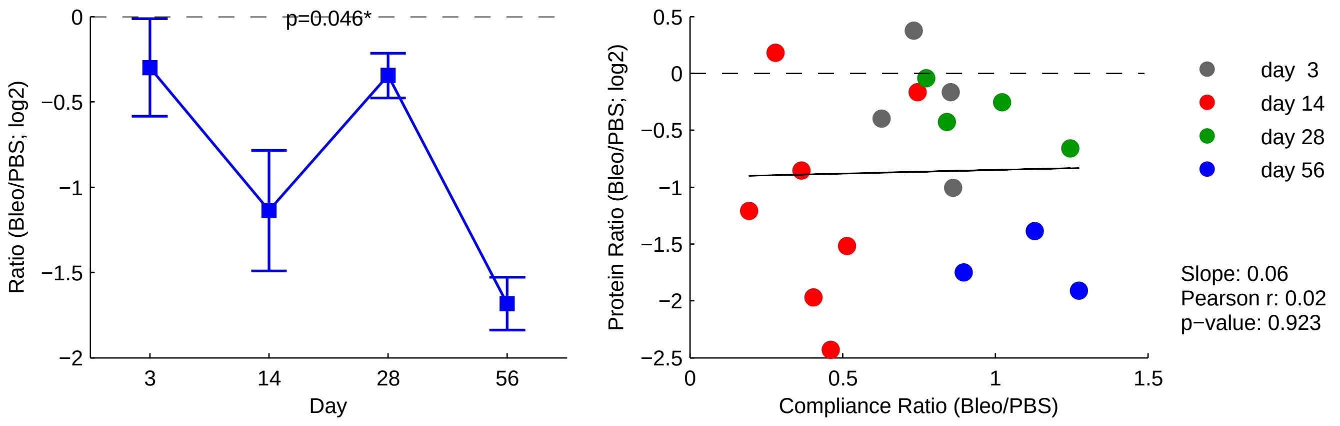

D3YYT1 − Glyr1 (id: 672)

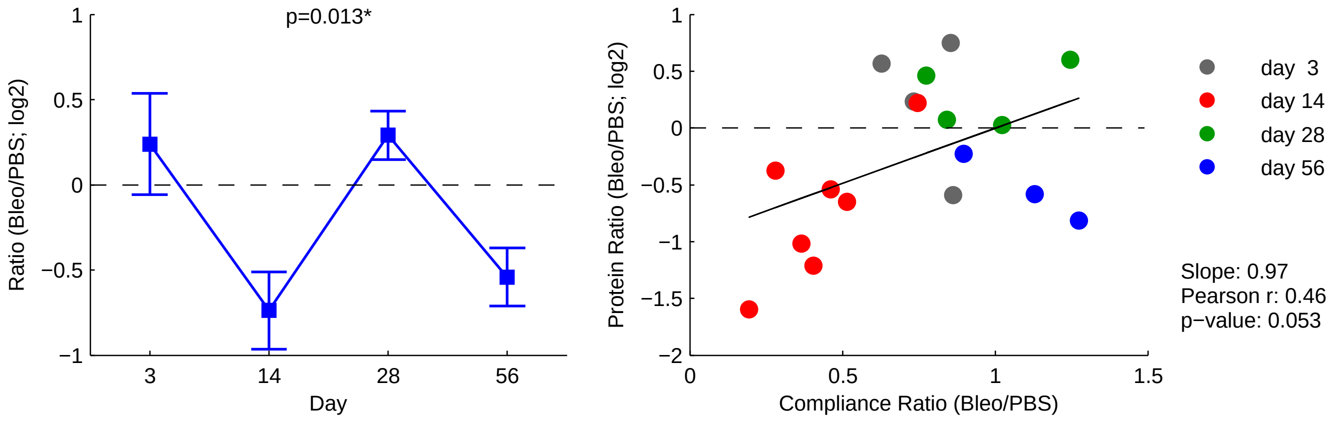

D3YYU9 − Lsr (id: 674)

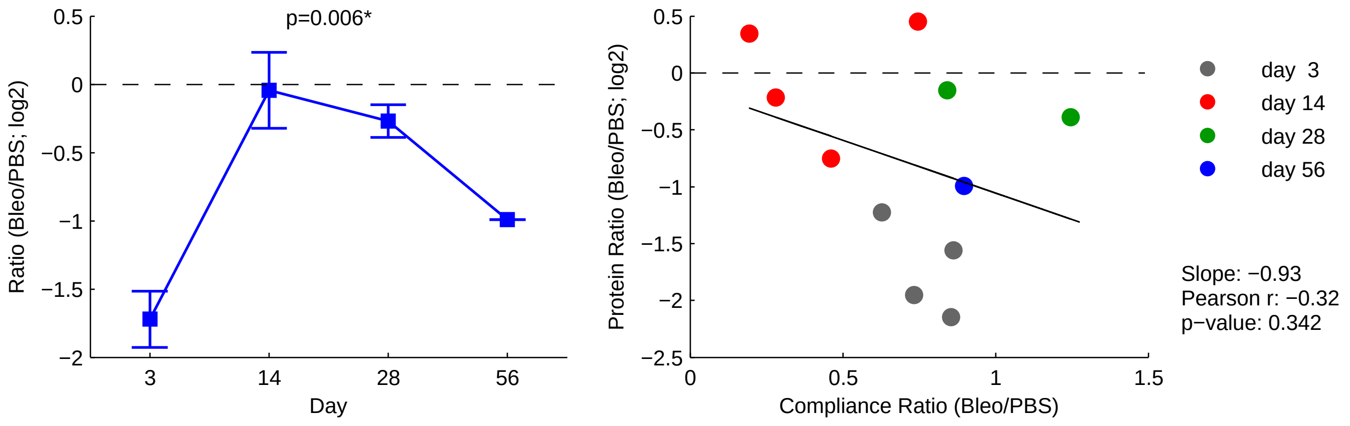

D3YYW8 − Rbm42 (id: 675)

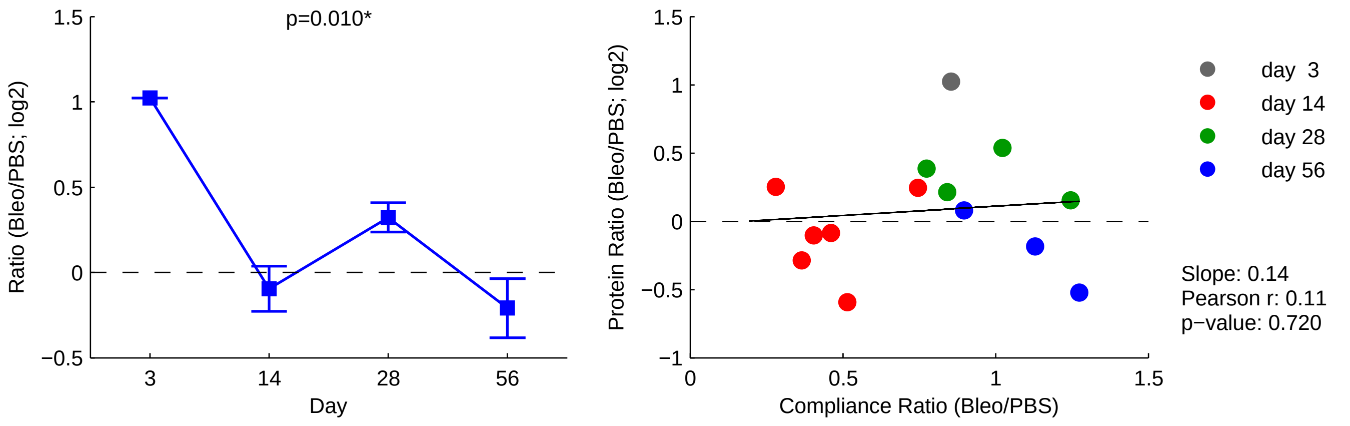

D3YYY8 − Arhgef26 (id: 676)

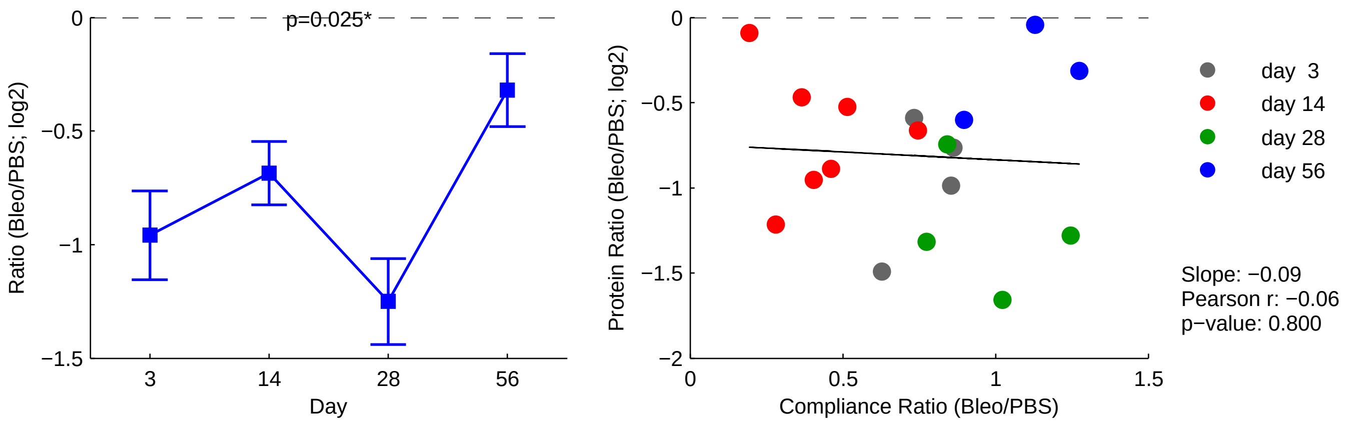

D3YZ08 − Terf2 (id: 678)

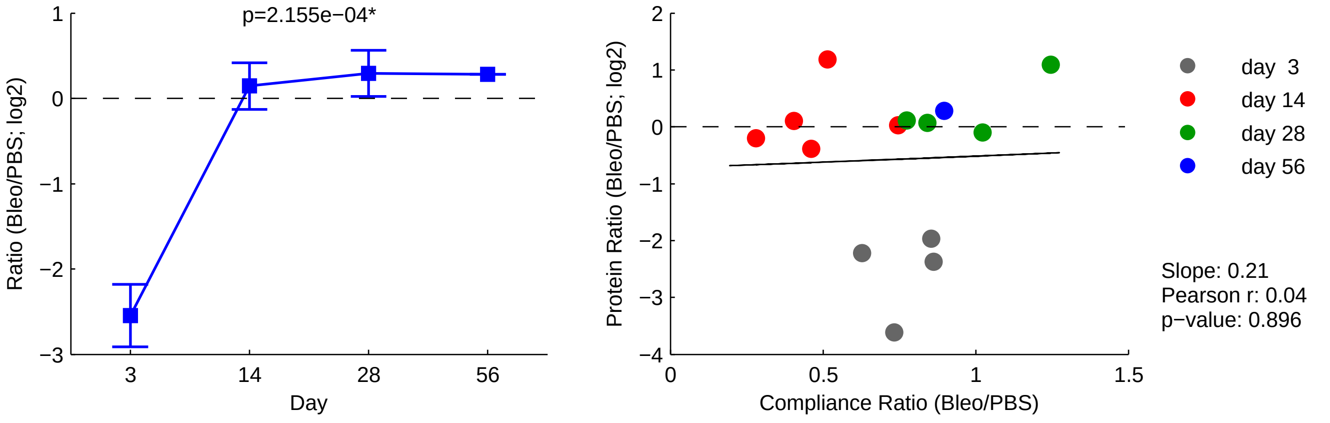

D3YZ54 − Hacl1 (id: 681)

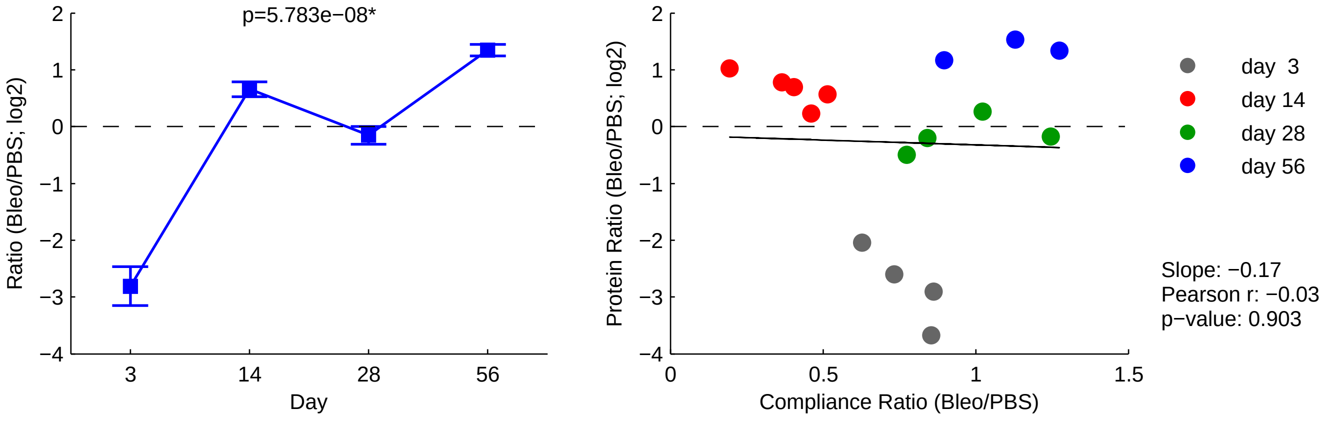

D3YZ62 − Myo5a (id: 683)

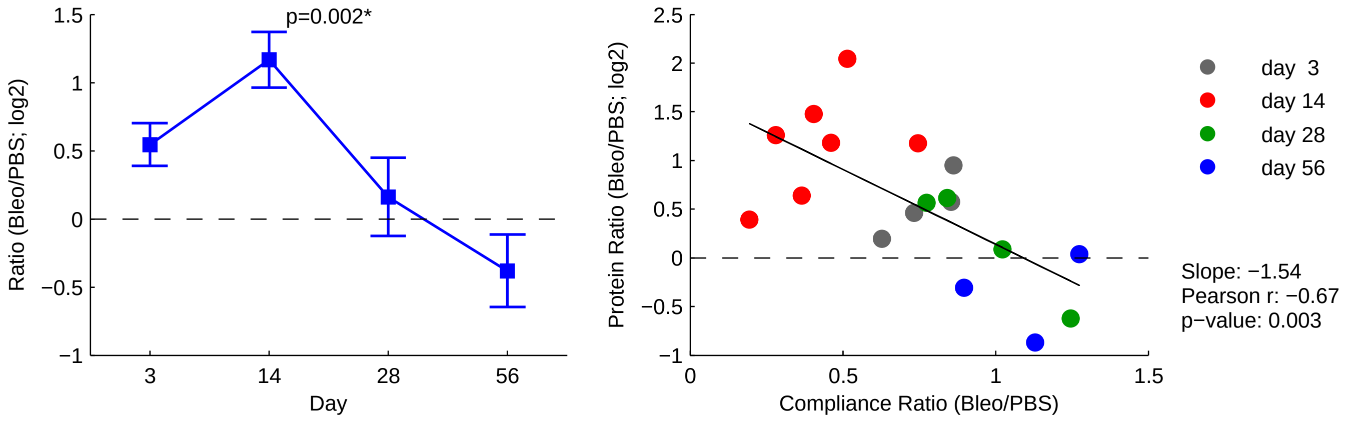

D3YZC2 − Men1 (id: 685)

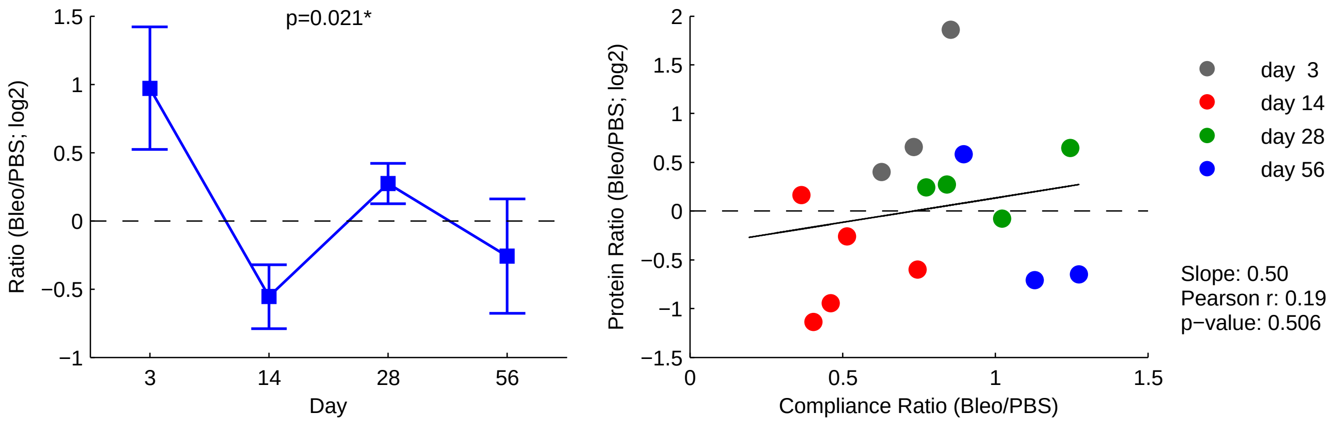

Q80UV9−3 − Taf1 (id: 687)

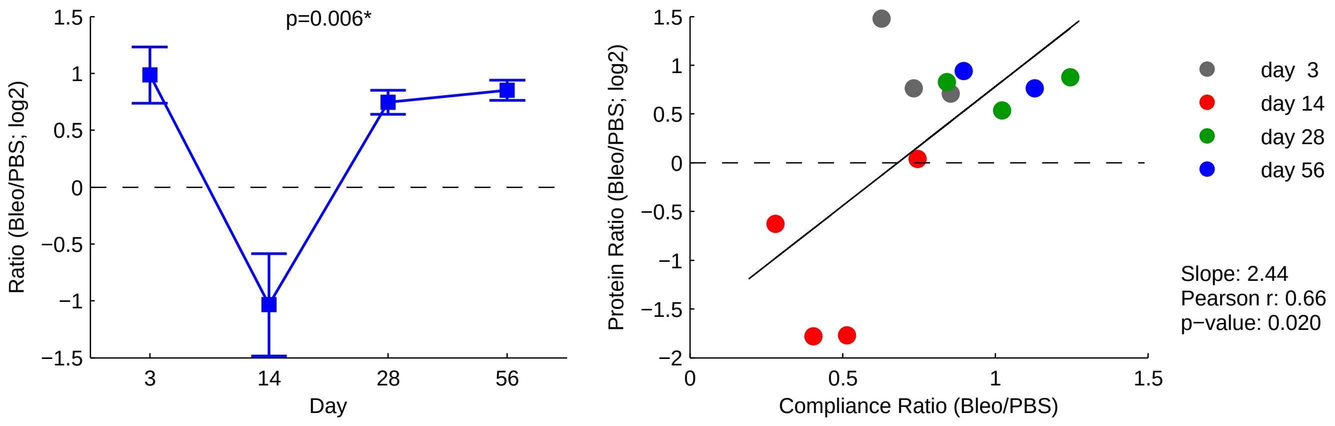

D3YZN4 − Spg7 (id: 688)

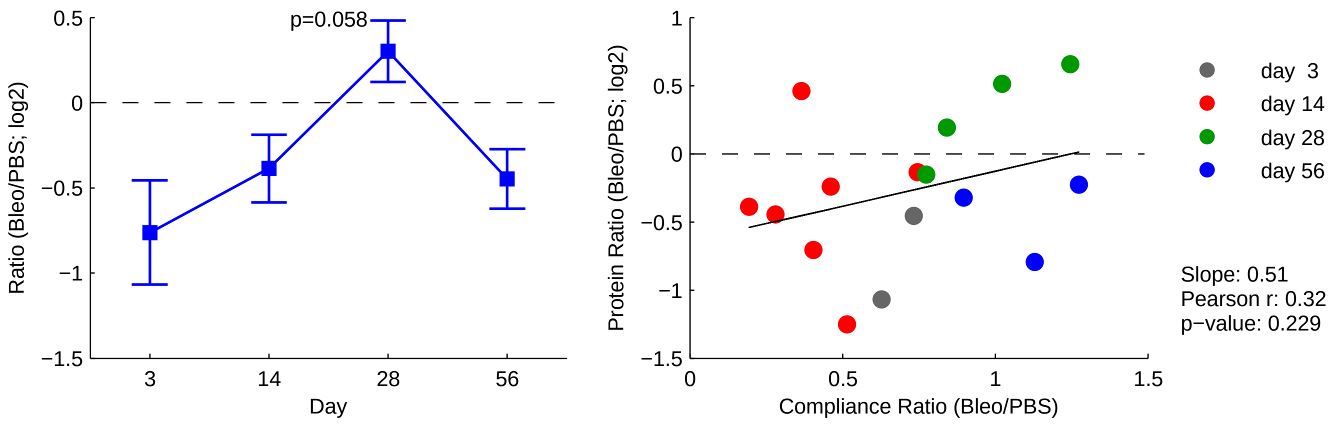

F7ABX5 − Bad (id: 692)

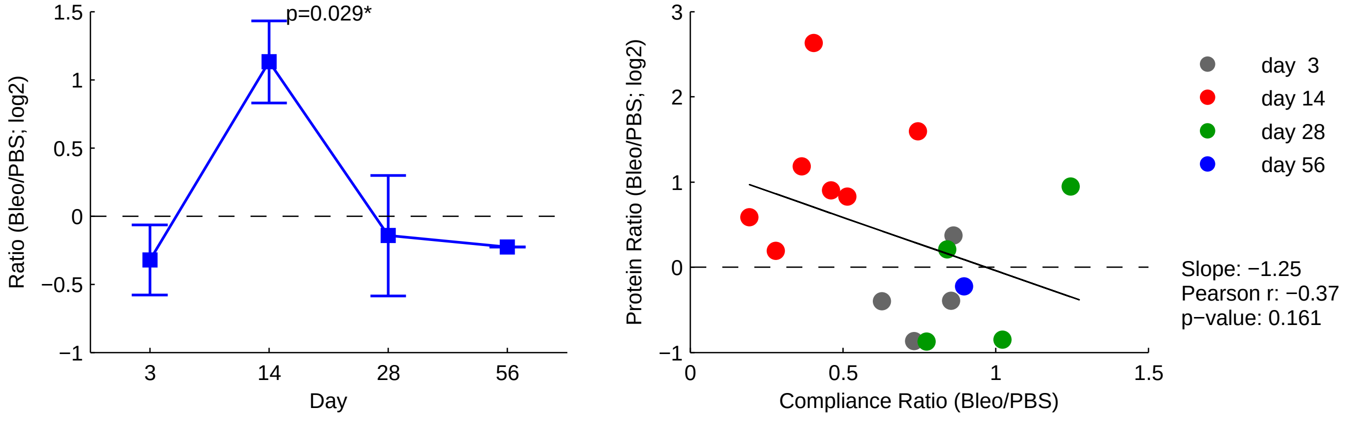

E9Q7G1 − Tmed7 (id: 693)

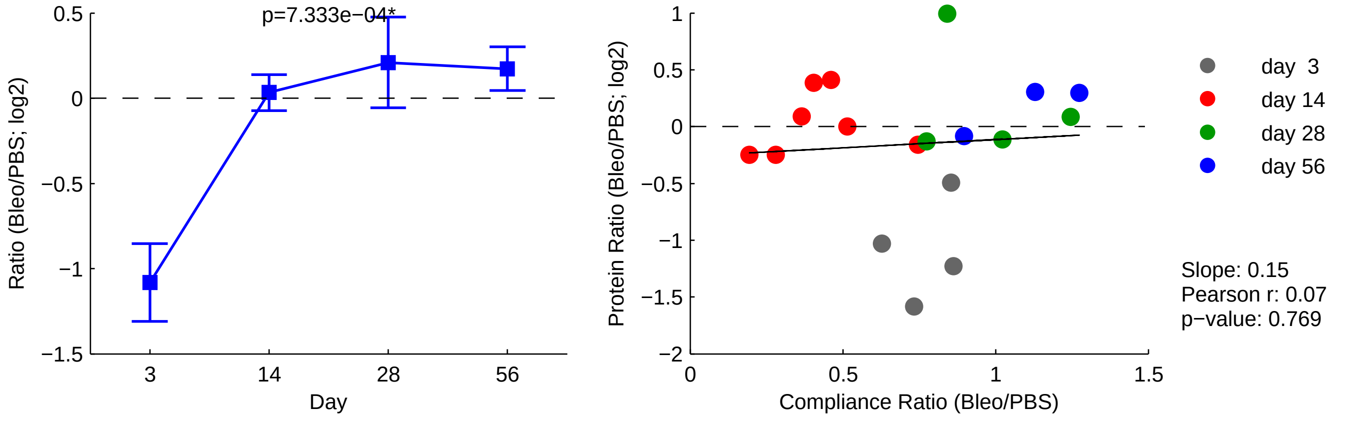

D3Z030 − Lrrc16a (id: 694)

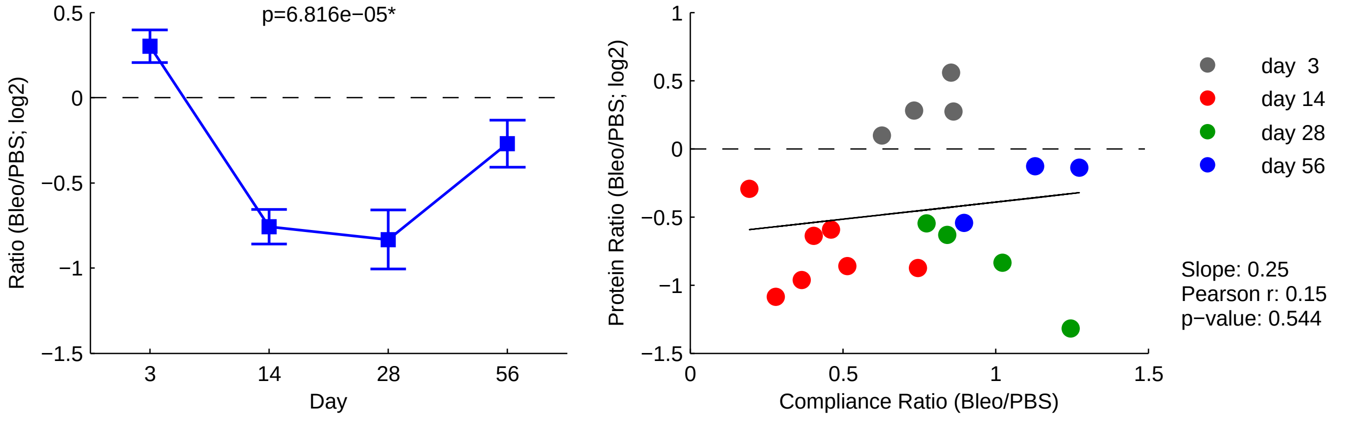

D3Z079 − Stxbp5 (id: 696)

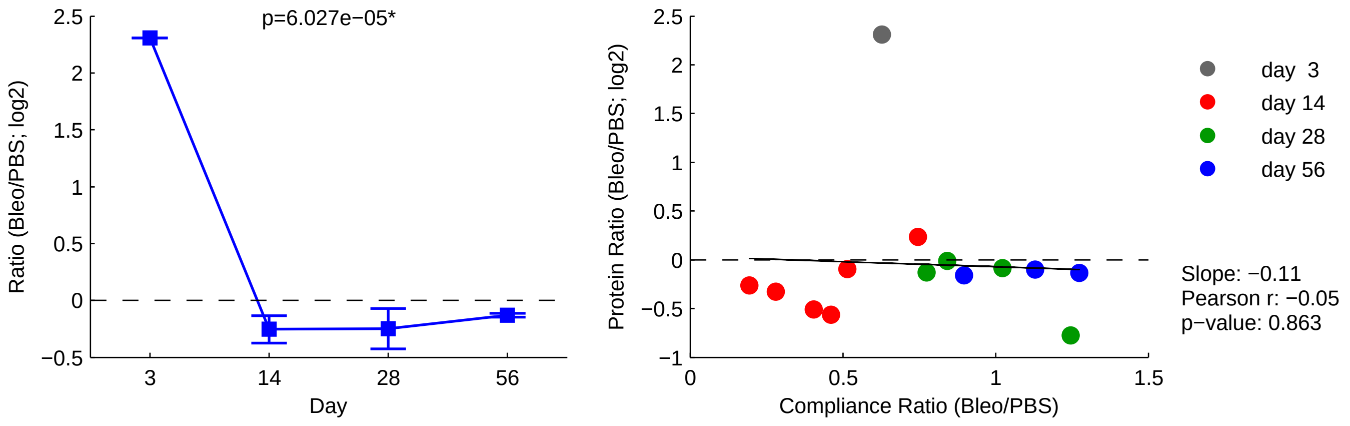

D3Z0A2 − Prmt1 (id: 697)

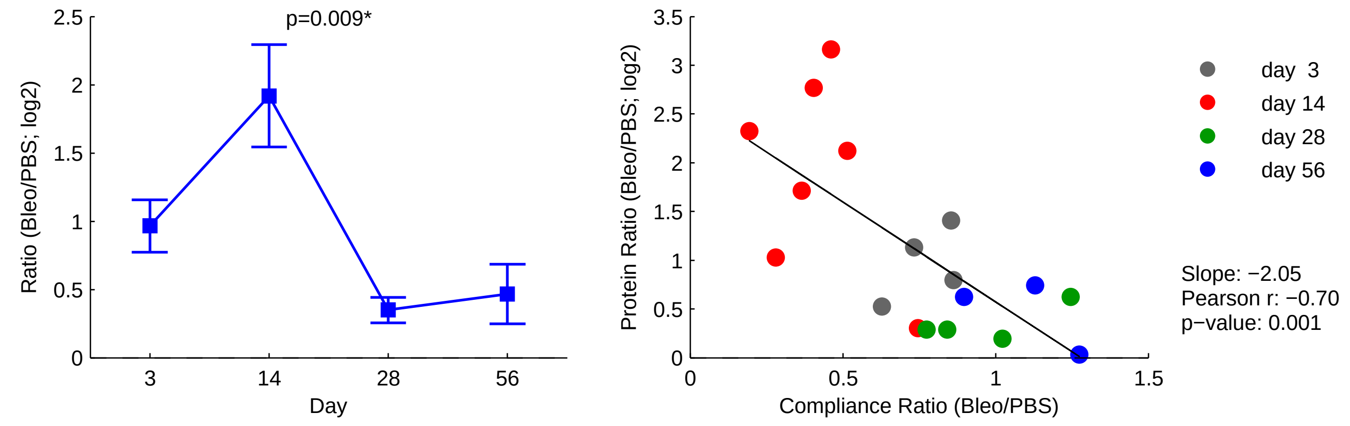

D3Z0F5 − Cops6 (id: 699)

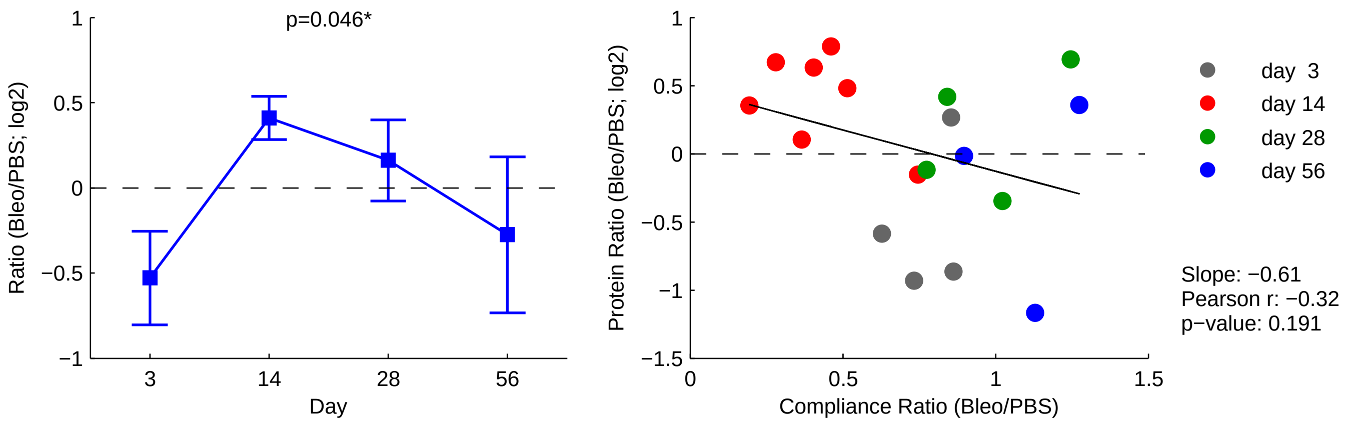

D3Z0M9 − Ddx23 (id: 706)

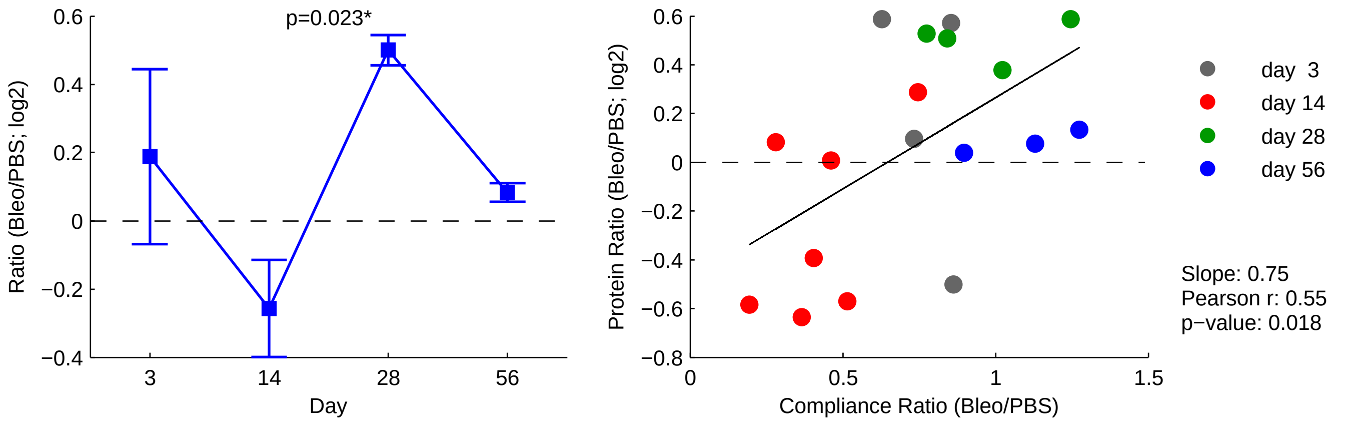

D3Z0R8 − Ttc7 (id: 708)

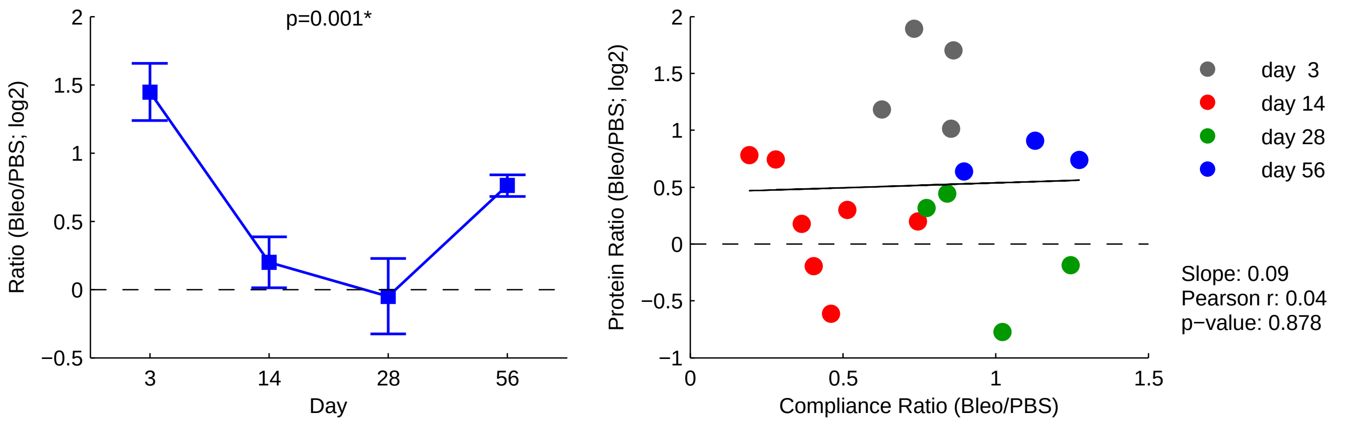

D3Z0T0 − Taf6 (id: 710)

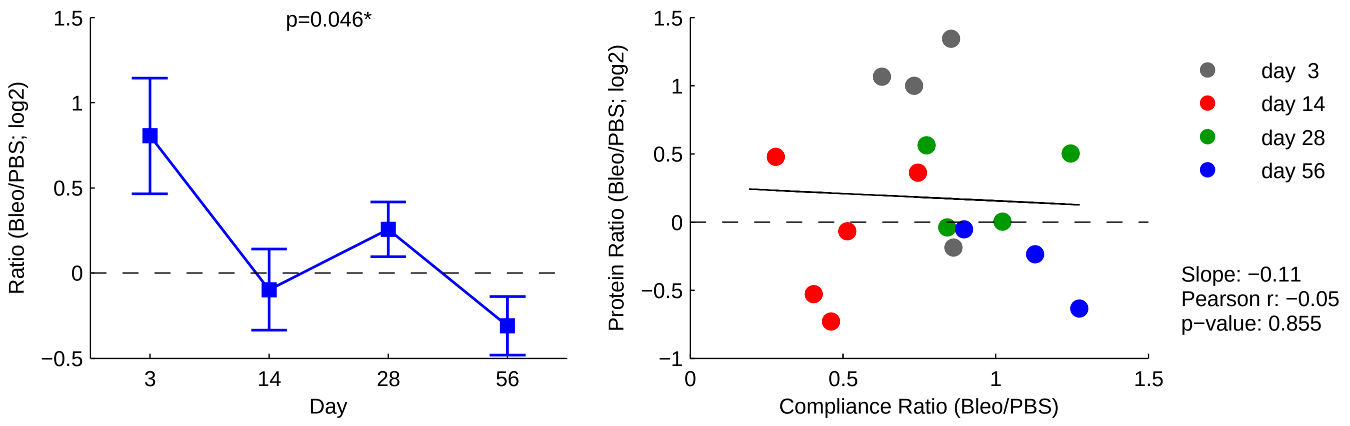

E9PWB1 − Phldb1 (id: 713)

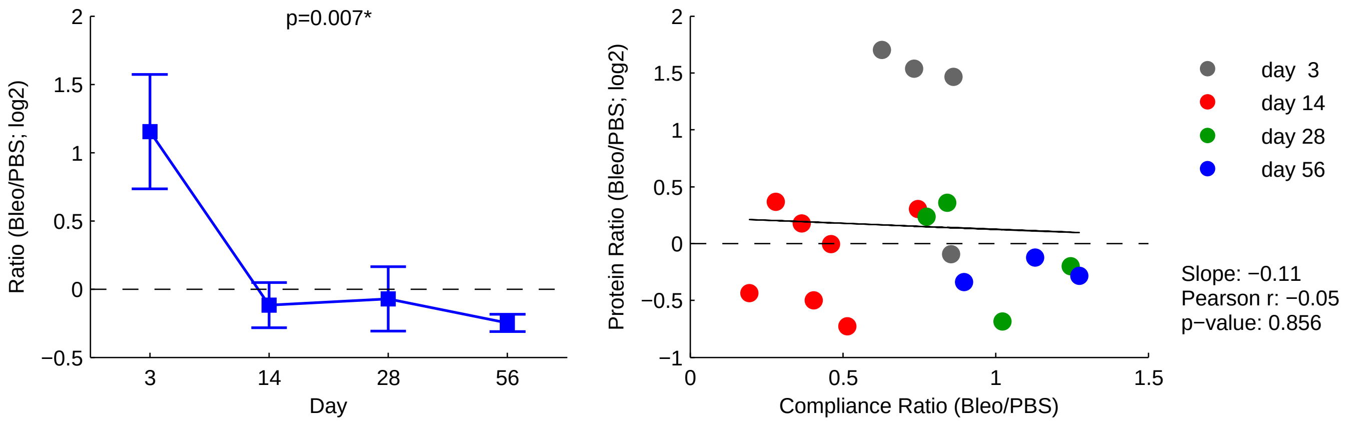

D3Z183 − Mfsd6 (id: 717)

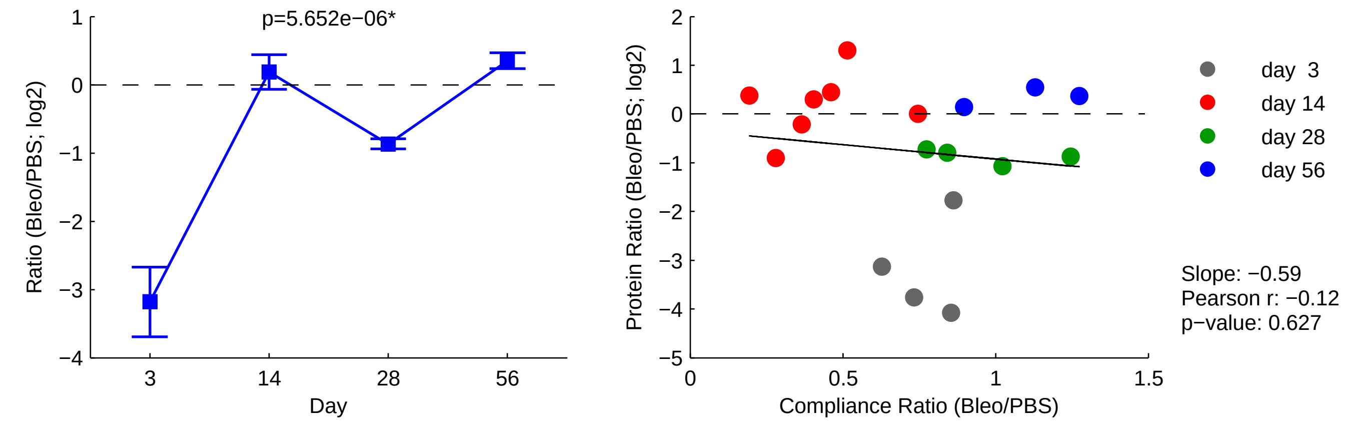

D3Z191 − Tmem106b (id: 718)

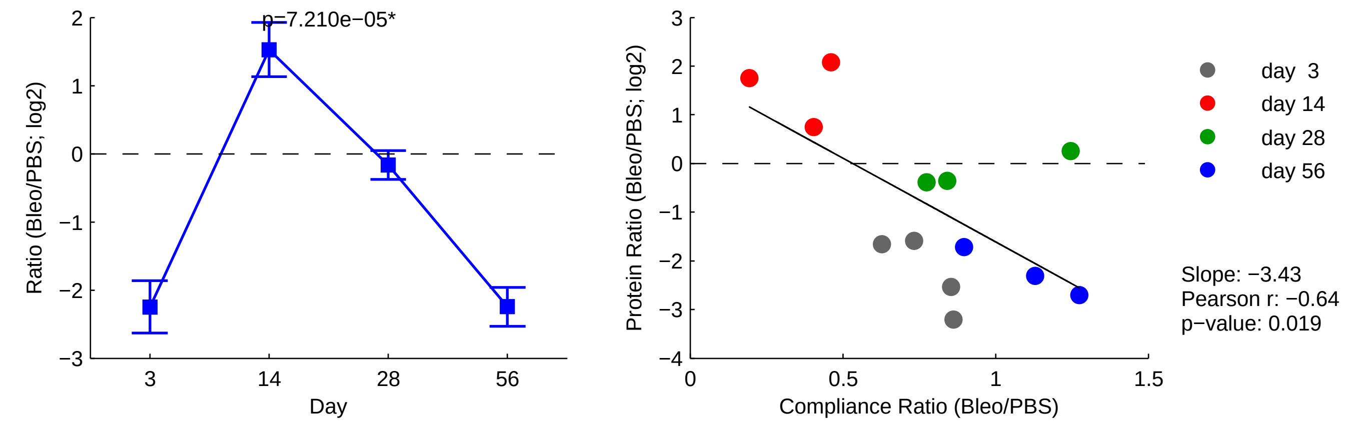

Q6P2L6−4 − Whsc1l1 (id: 719)

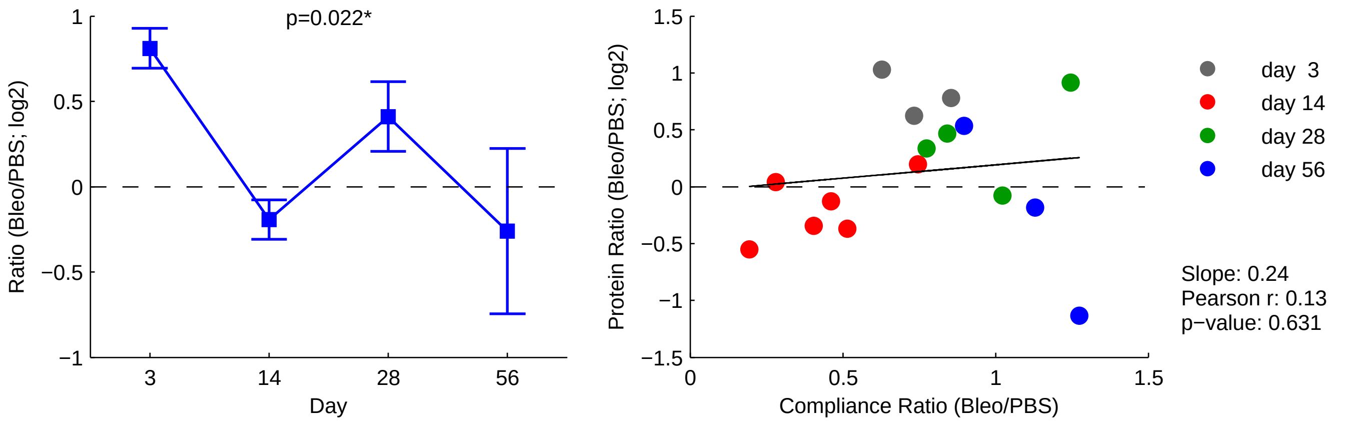

D3Z2E7 − AI607873 (id: 729)

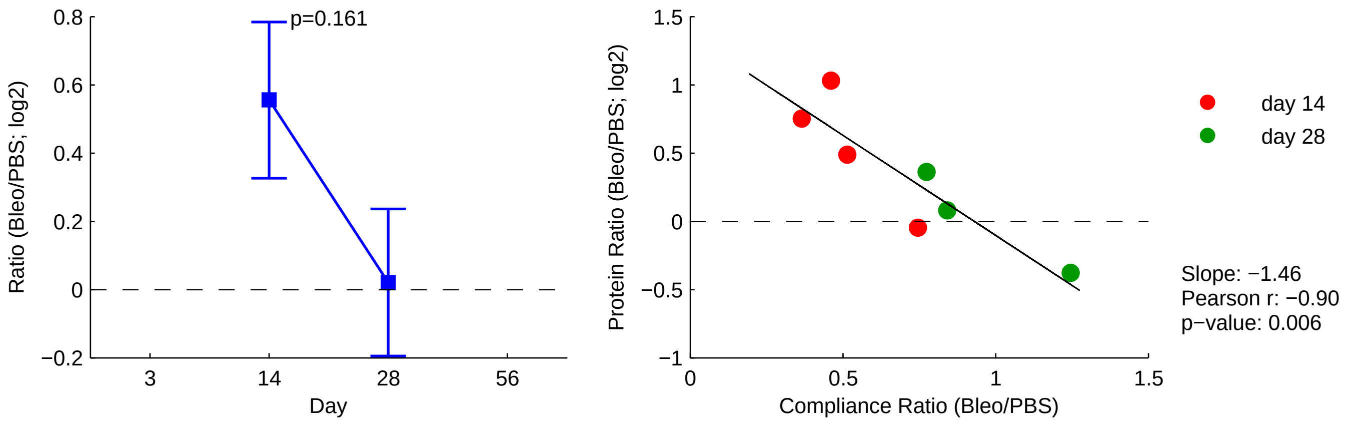

D3Z2J6 − Tmx2 (id: 731)

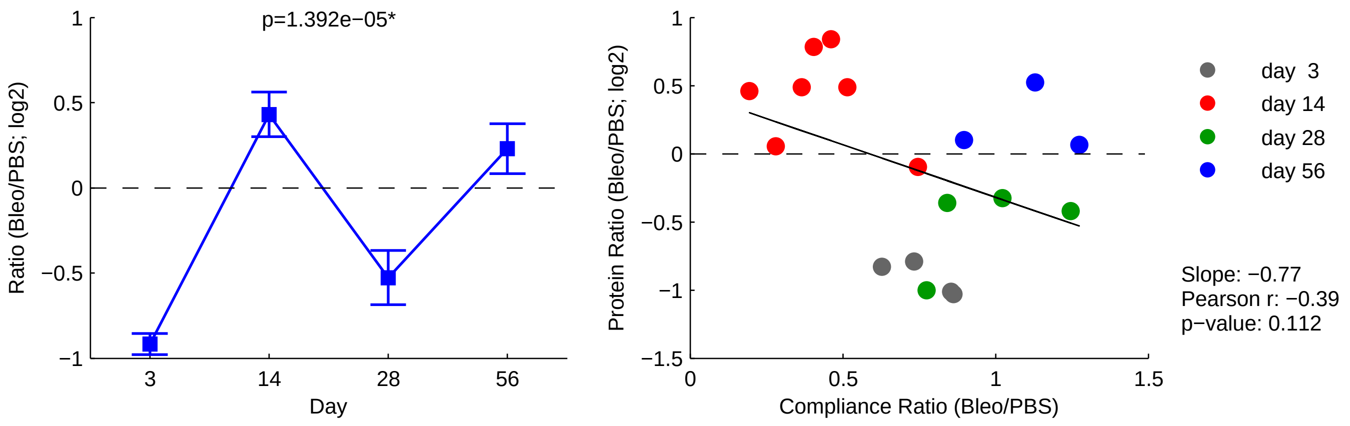

D3Z2P2 − Chi3l1 (id: 733)

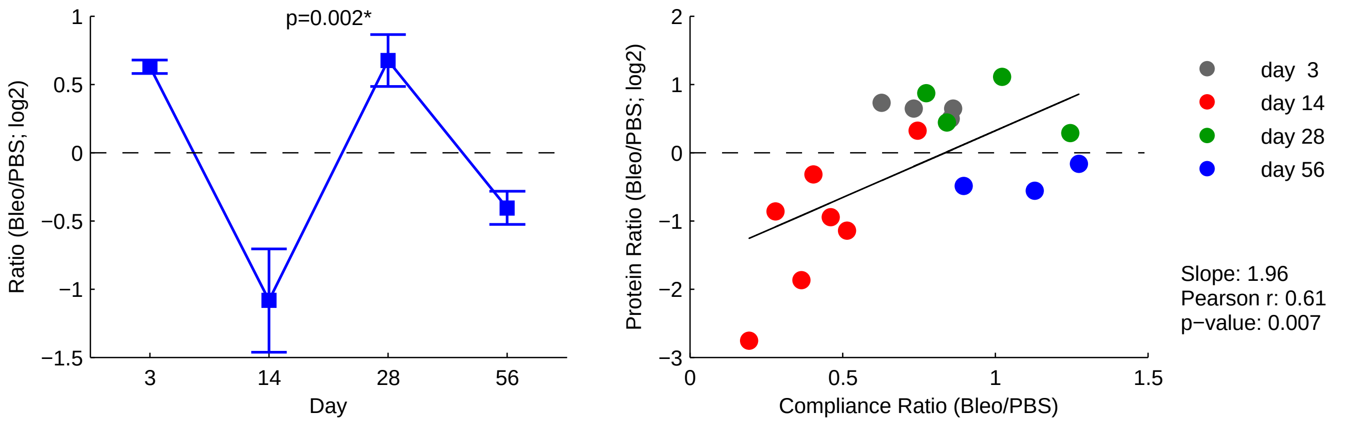

Q922J3 − Clip1 (id: 736)

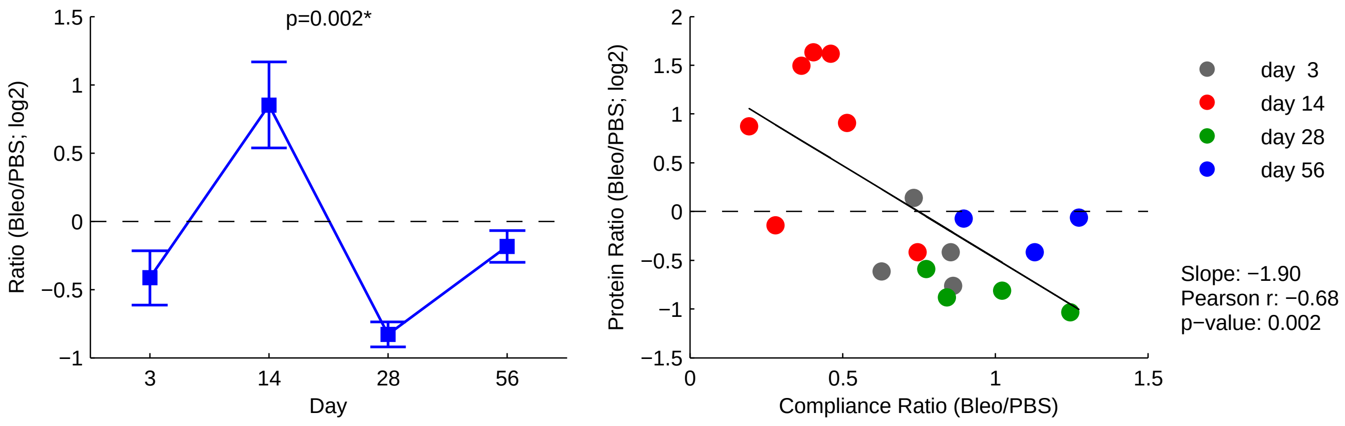

D3Z3A0 − Ppp1r2 (id: 737)

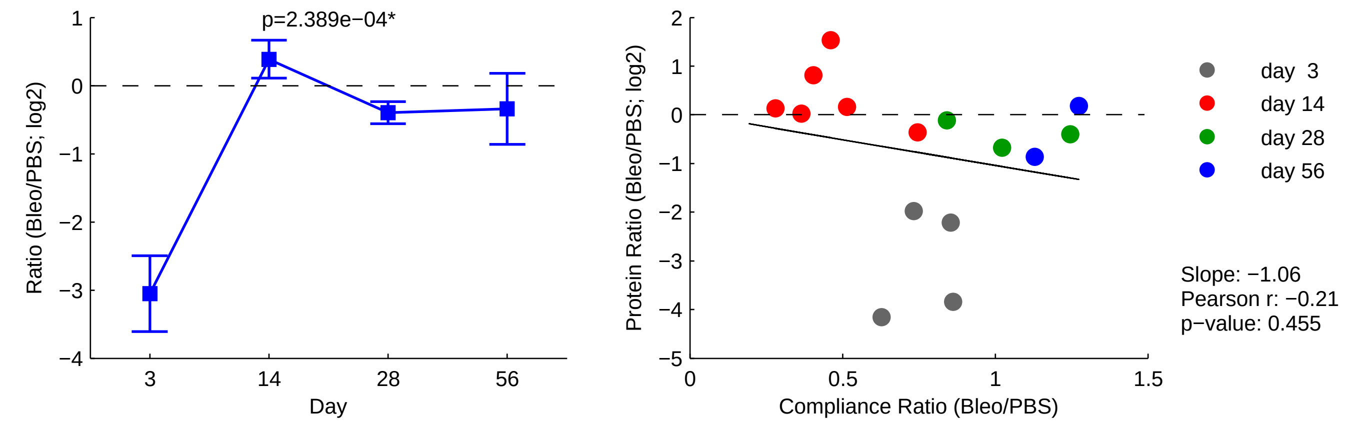

D3Z3J6 − Paip1 (id: 741)

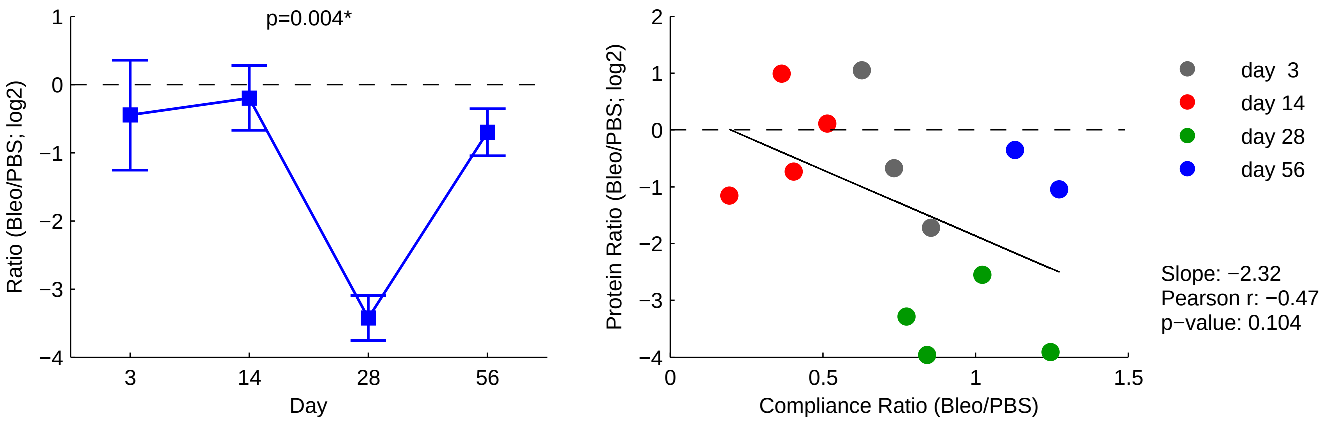

Q921U8−2 − Smtn (id: 743)

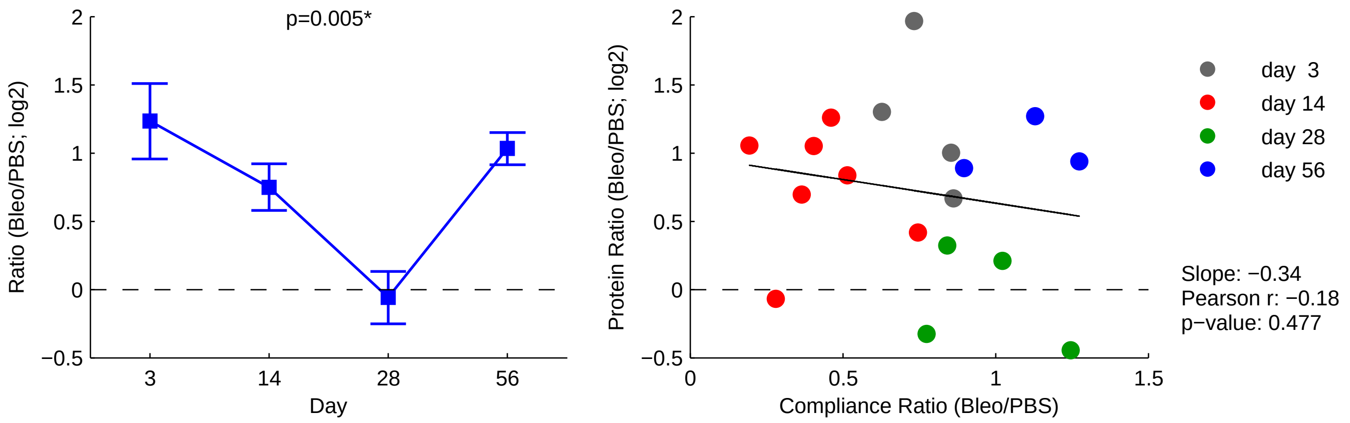

D3Z3R1 − Gm5745 (id: 744)

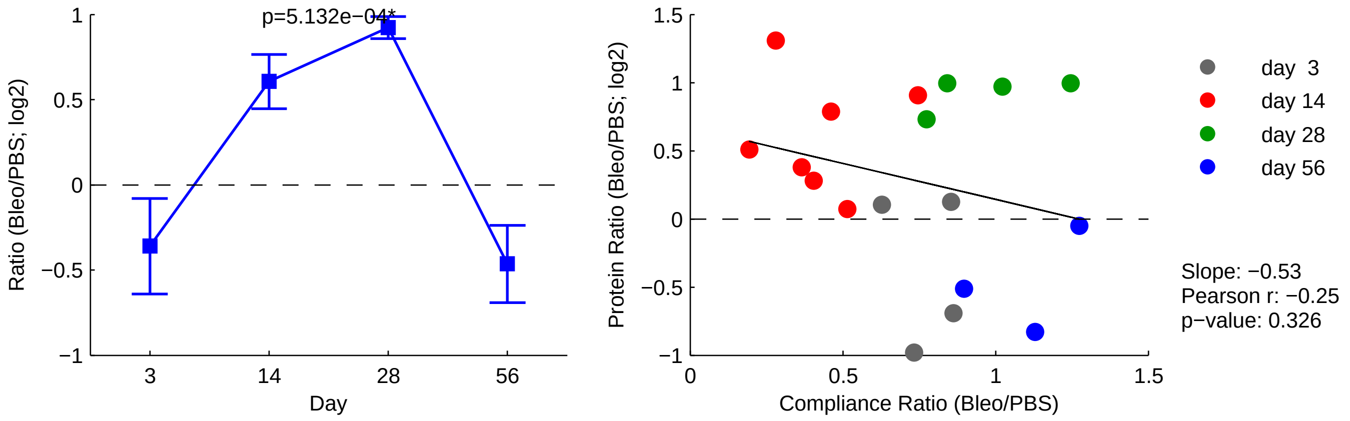

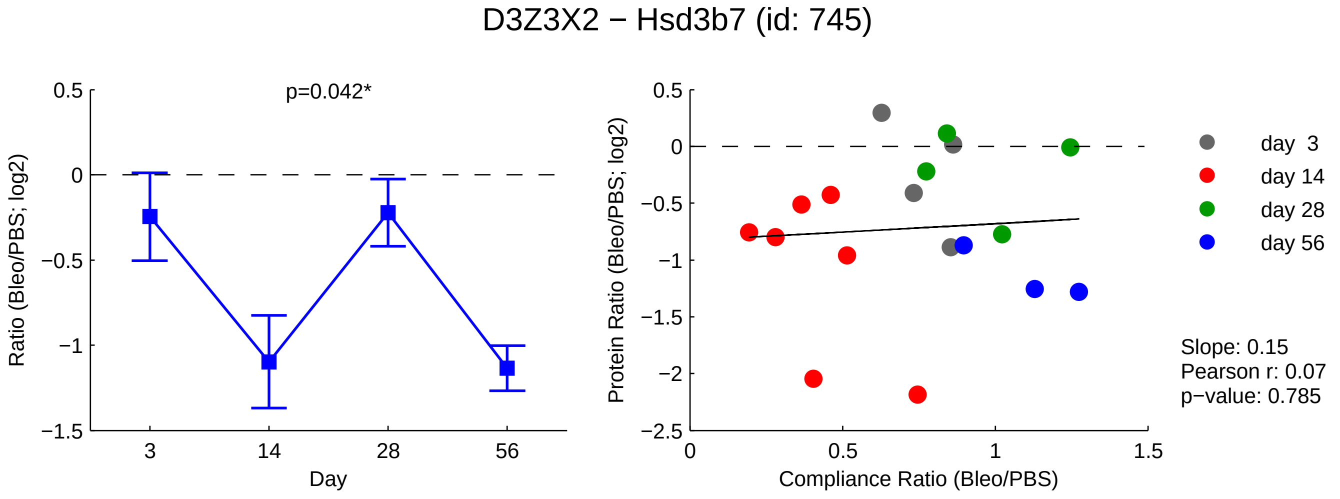
D3Z3X2 − Hsd3b7 (id: 745)
p=0.042*
Ratio (Bleo/PBS; log2)
Day
Protein Ratio (Bleo/PBS; log2)
Compliance Ratio (Bleo/PBS)
day 3
day 14
day 28
day 56
Slope: 0.15
Pearson r: 0.07
p−value: 0.785

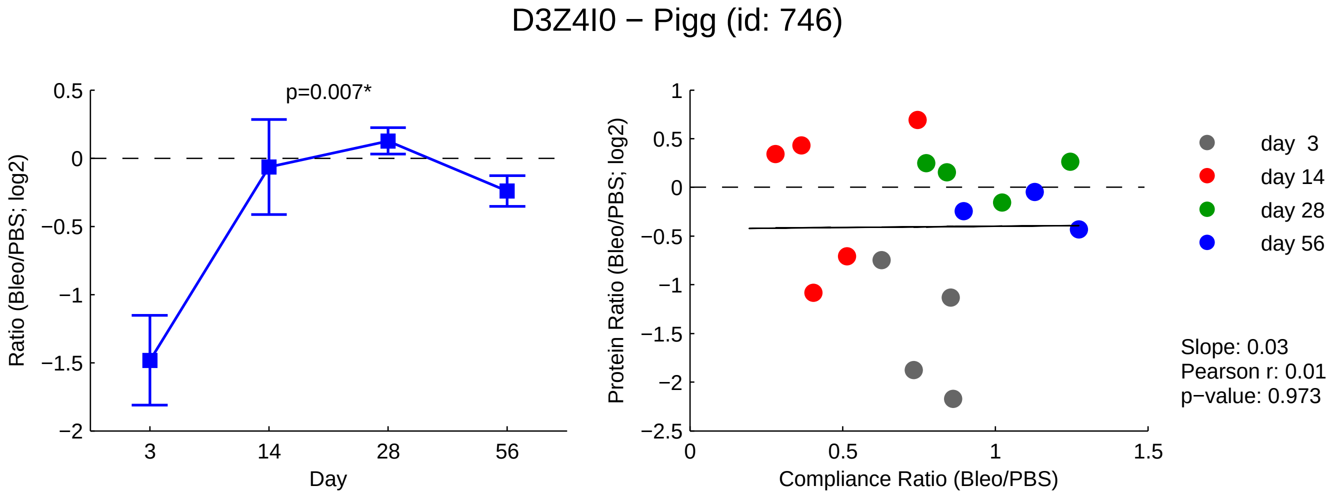
D3Z4I0 − Pigg (id: 746)
p=0.007*
Ratio (Bleo/PBS; log2)
Day
Protein Ratio (Bleo/PBS; log2)
Compliance Ratio (Bleo/PBS)
day 3
day 14
day 28
day 56
Slope: 0.03
Pearson r: 0.01
p−value: 0.973

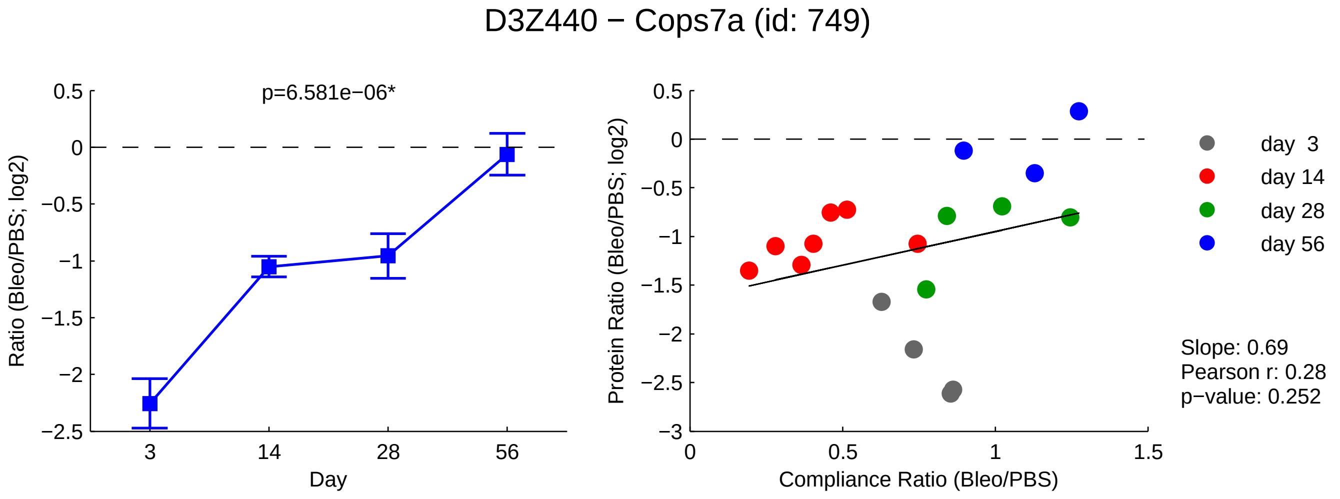
D3Z440 − Cops7a (id: 749)
p=6.581e−06*
Ratio (Bleo/PBS; log2)
Day
Protein Ratio (Bleo/PBS; log2)
Compliance Ratio (Bleo/PBS)
day 3
day 14
day 28
day 56
Slope: 0.69
Pearson r: 0.28
p−value: 0.252

D3Z481 − Fam126a (id: 751)

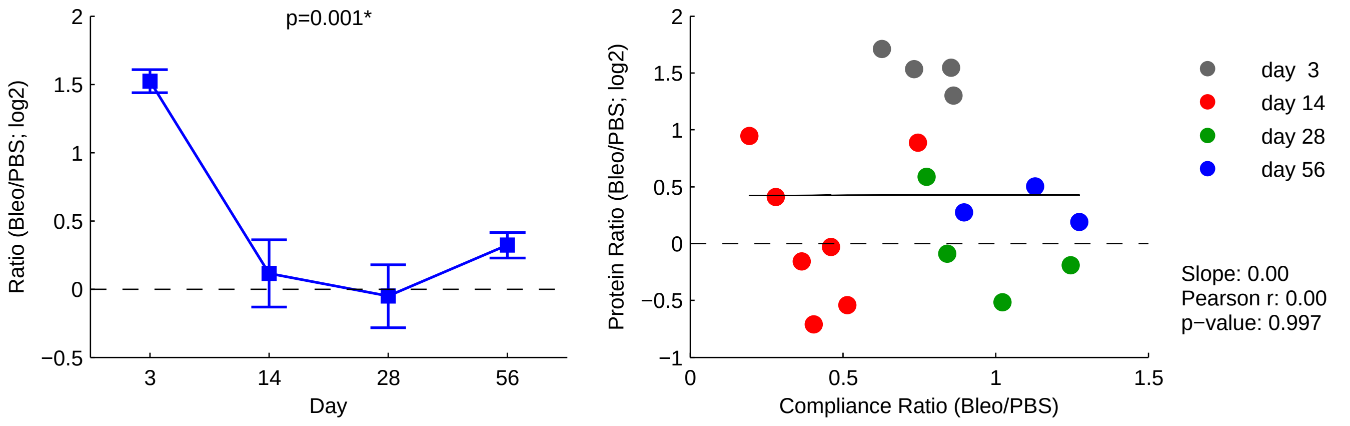

D3Z4C3 − Zbtb7a (id: 753)

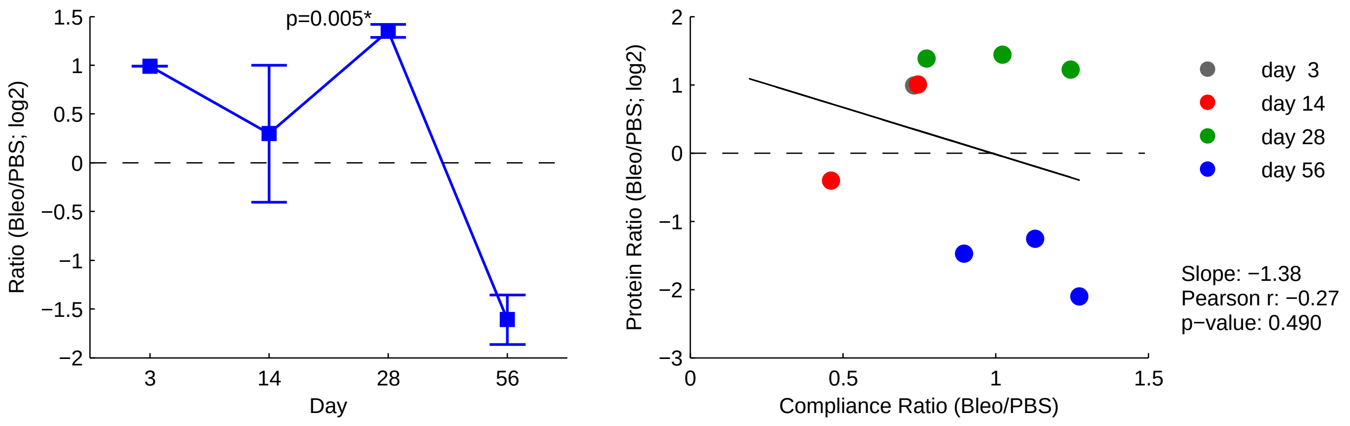

D3Z4C9 − Mnf1 (id: 754)

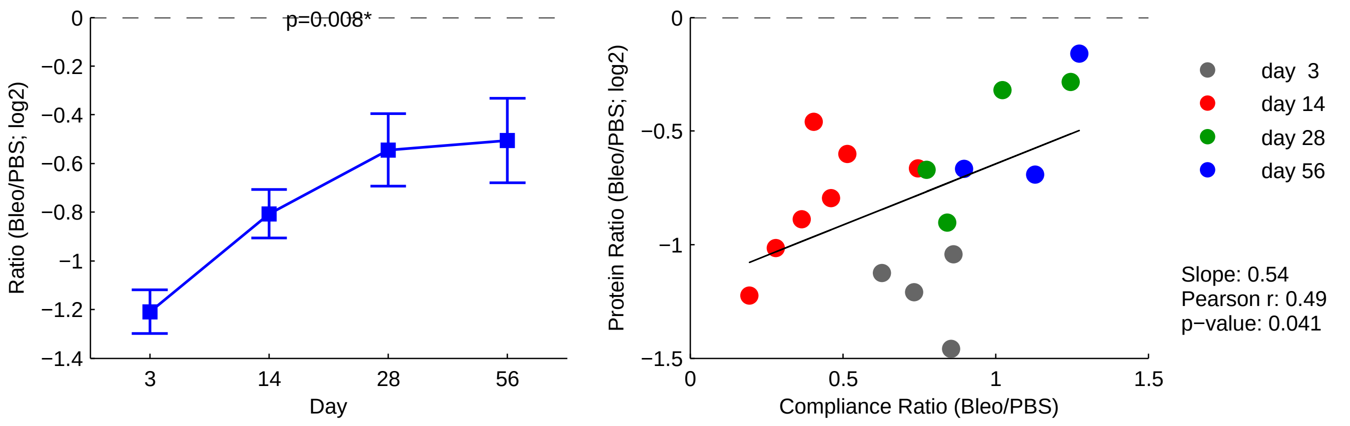

F8WJ99 − Mfap5 (id: 756)

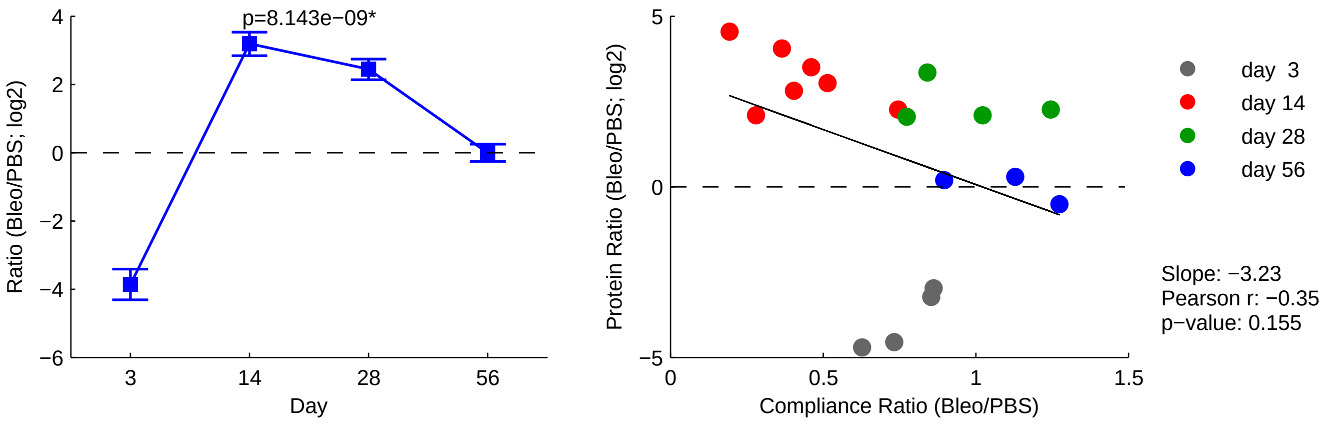

D3Z4E3 − Dhx32 (id: 758)

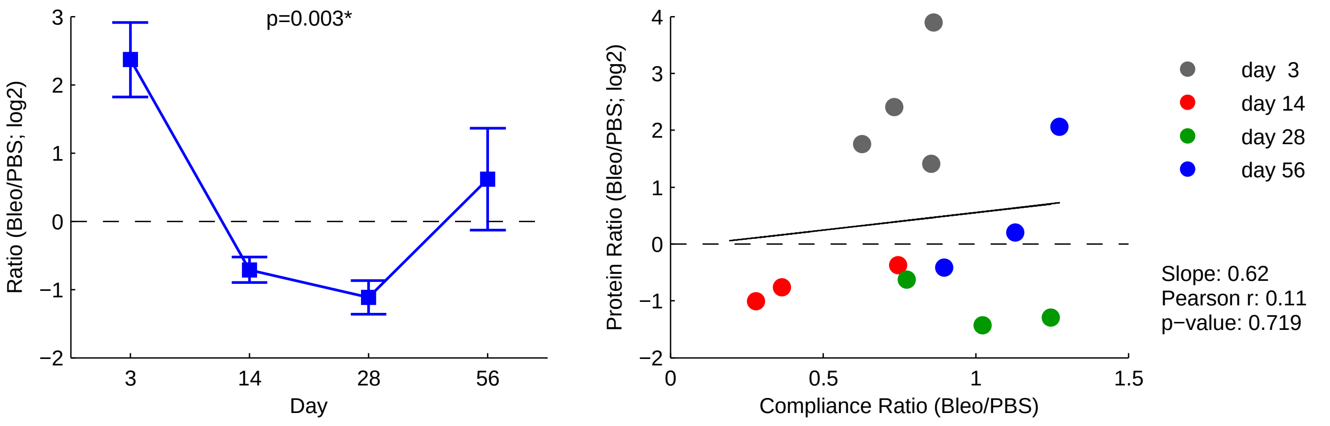

D3Z6H3 − Dctn6 (id: 759)

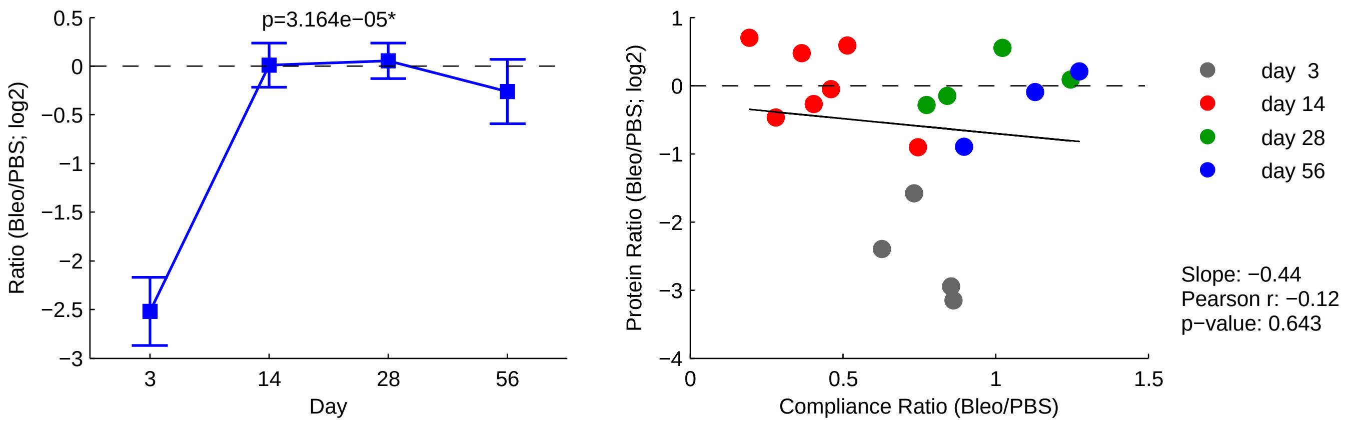

P59481 − Lman2l (id: 765)

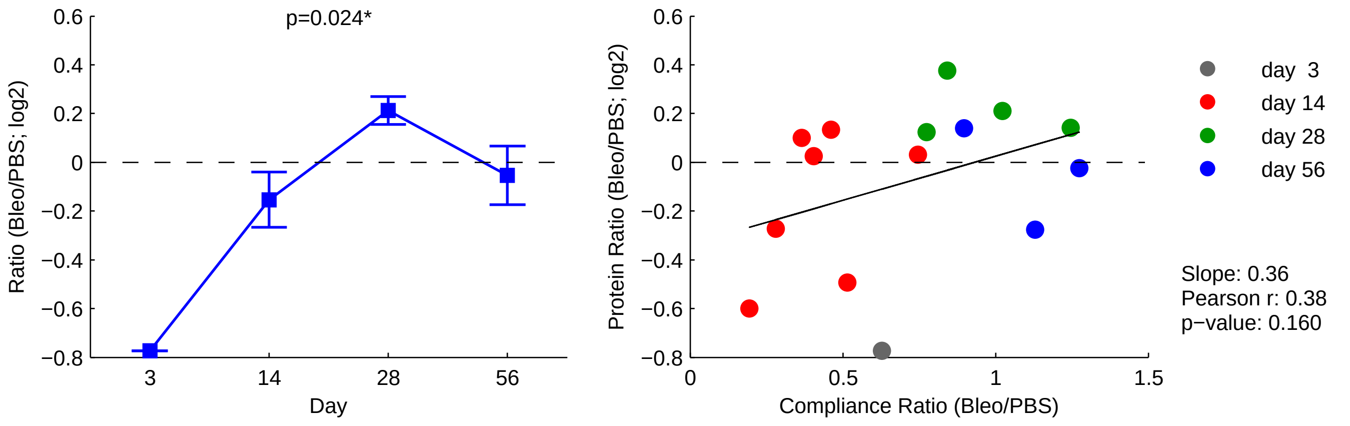

D3Z598 − Ltbp4 (id: 771)

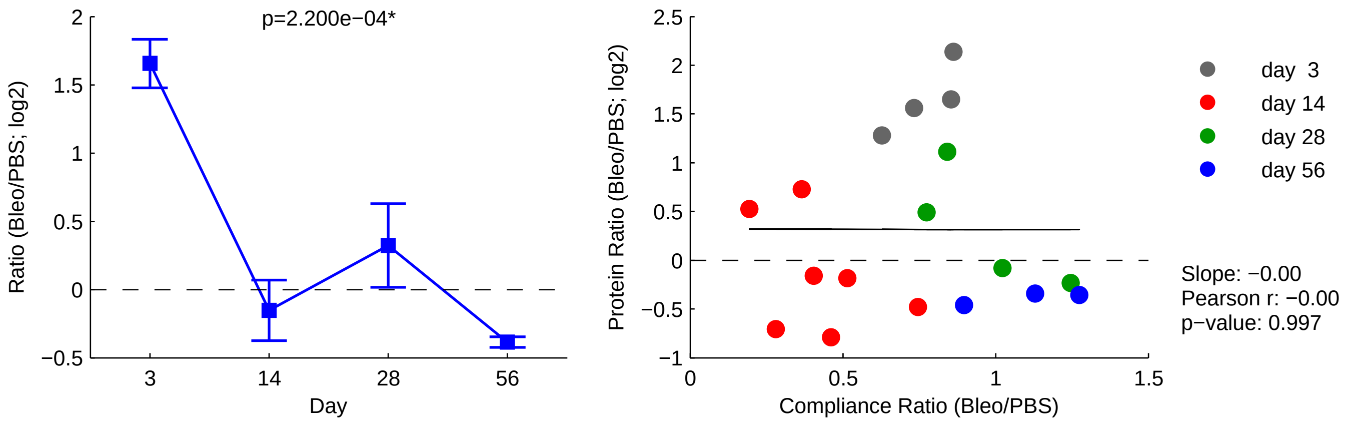

D3Z5F7 − Gm20521 (id: 773)

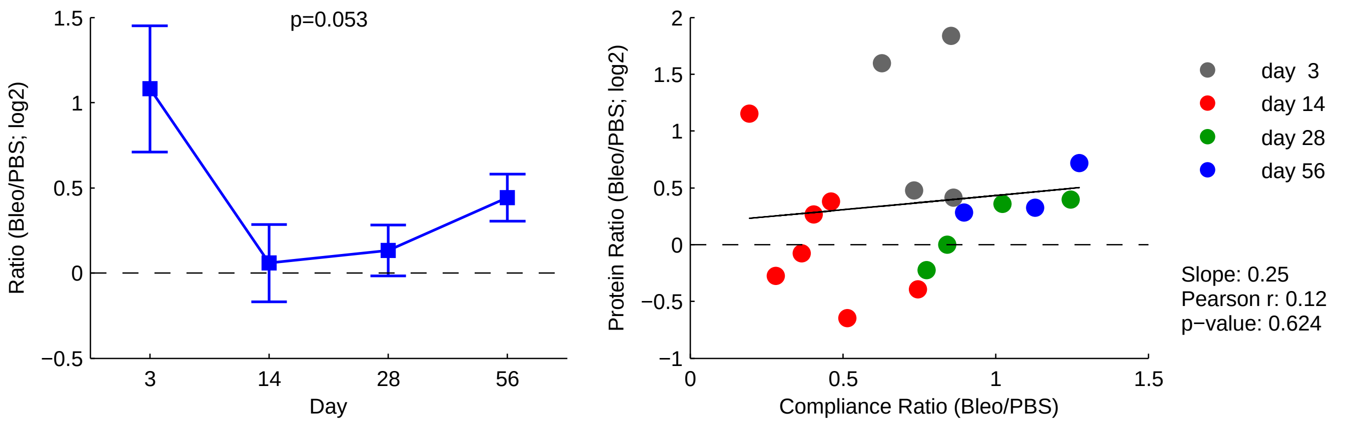

D3Z5I1 − Zc3hav1 (id: 774)

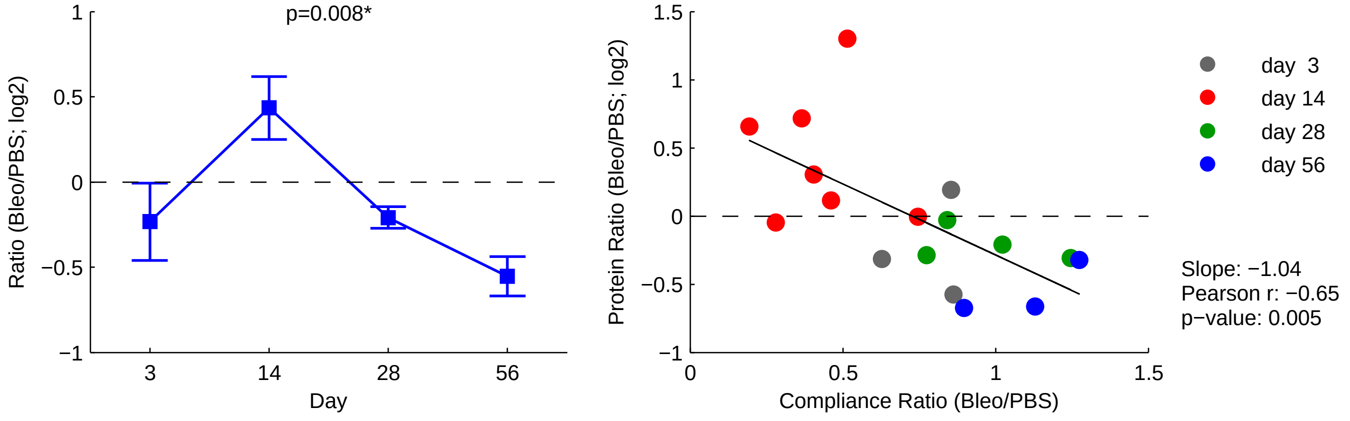

Q02357 − Ank1 (id: 776)

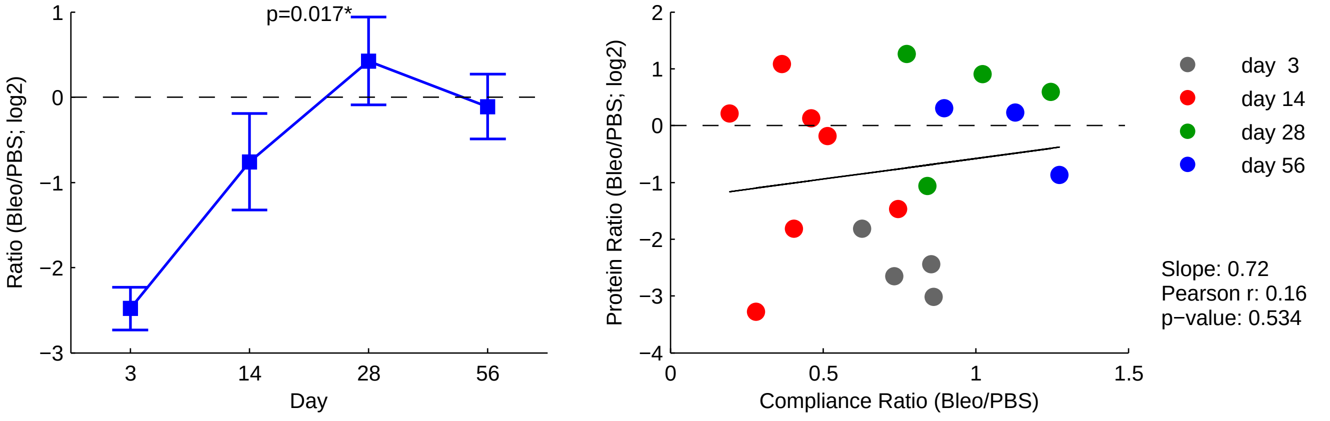

Q61103 − Dpf2 (id: 778)

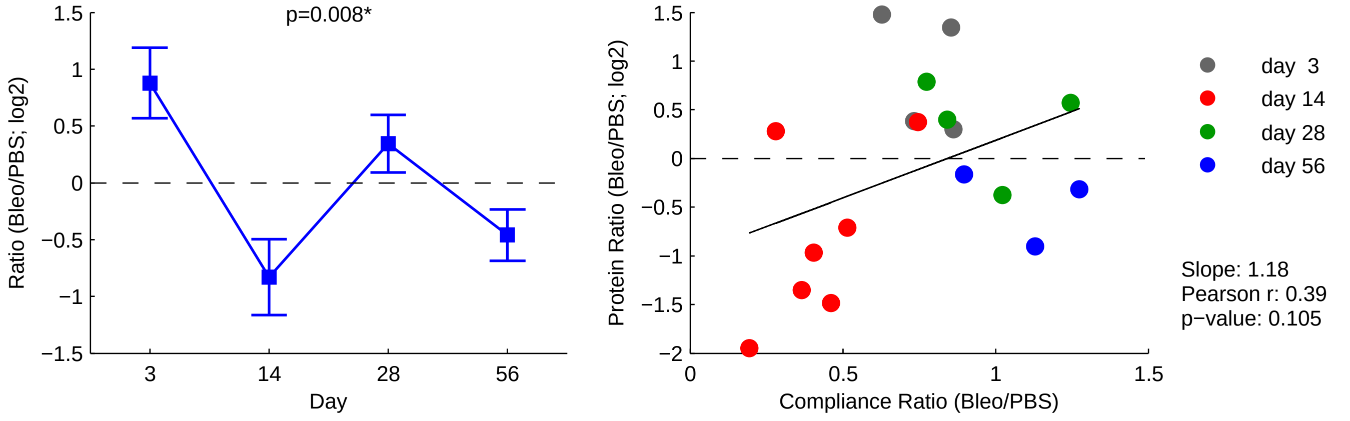

D3Z656 − Synj1 (id: 783)

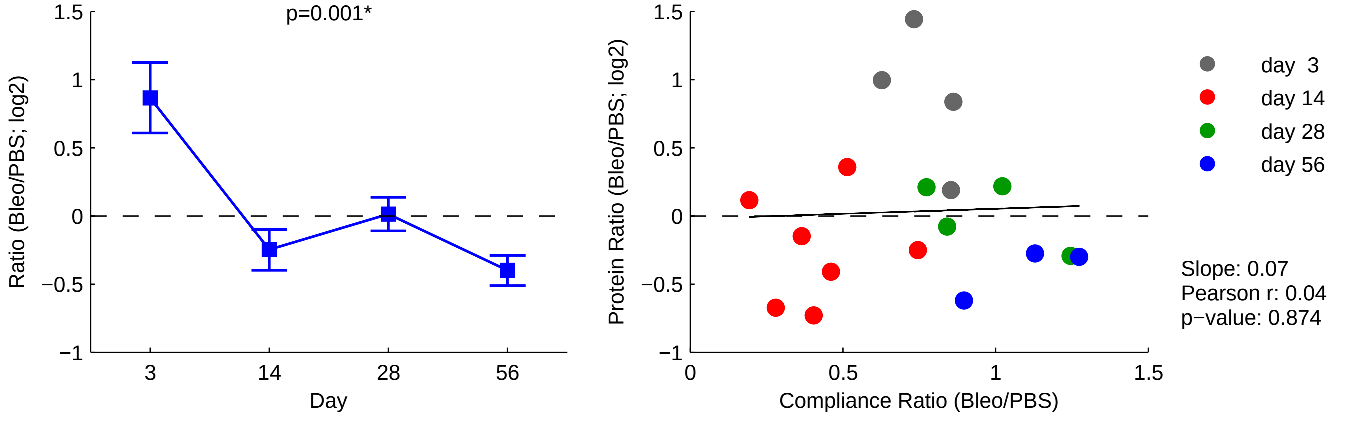

Q9DCT6−2 − Bap18 (id: 785)

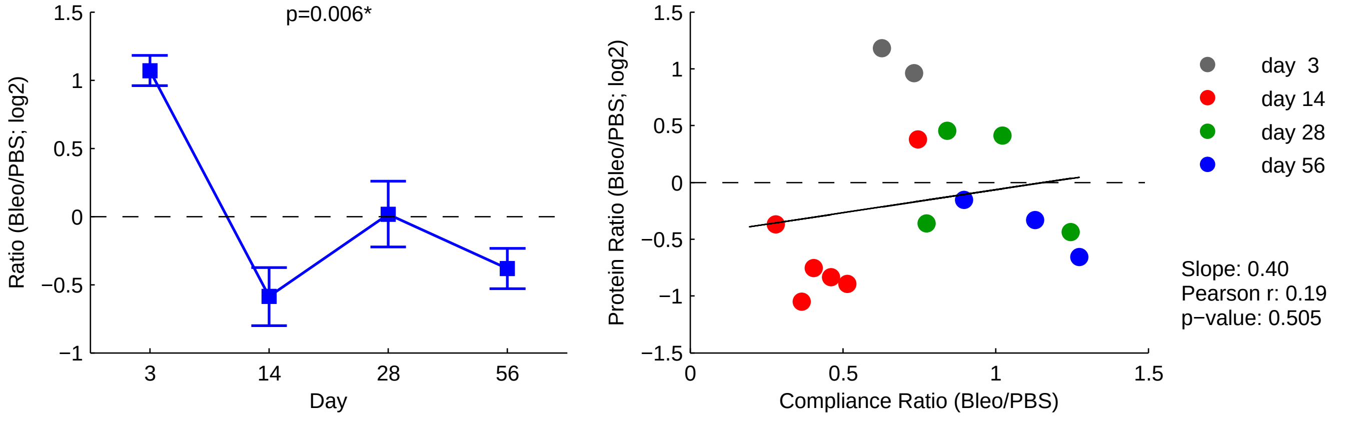

H7BWX9 − Sumo2 (id: 797)

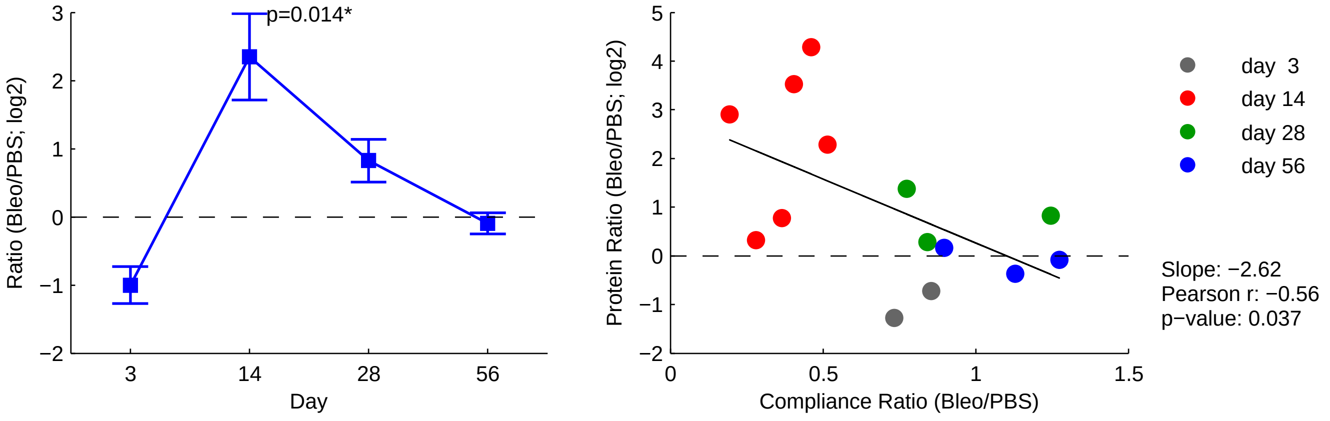

D3Z7C8 − Bcat2 (id: 800)

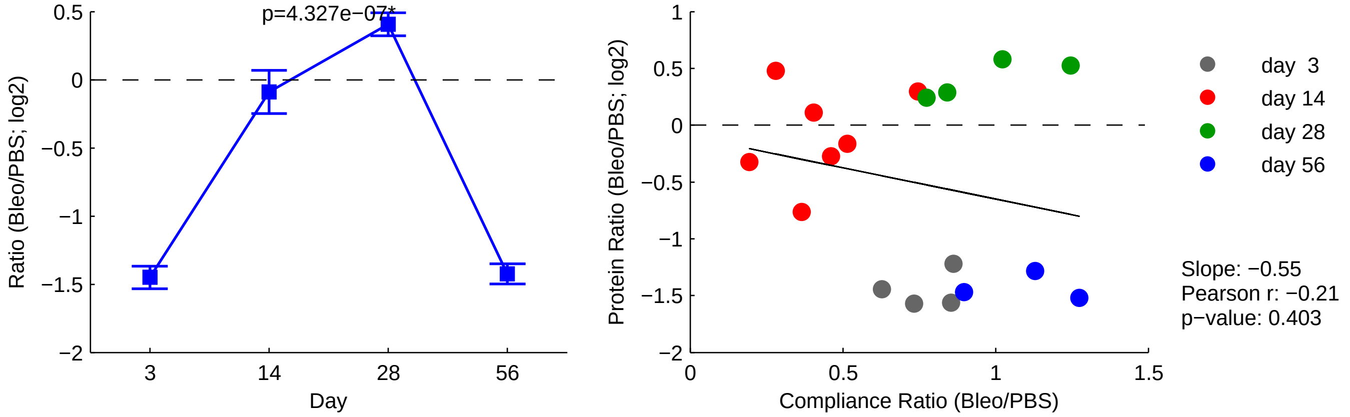

D3Z7E5 − Gsk3a (id: 802)

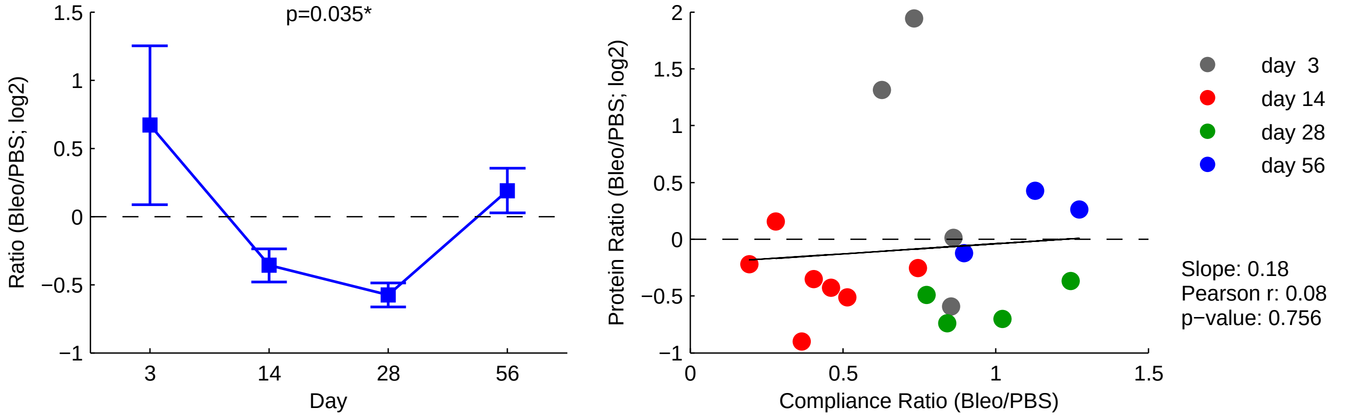

Q9CPT5 − Nop16 (id: 805)

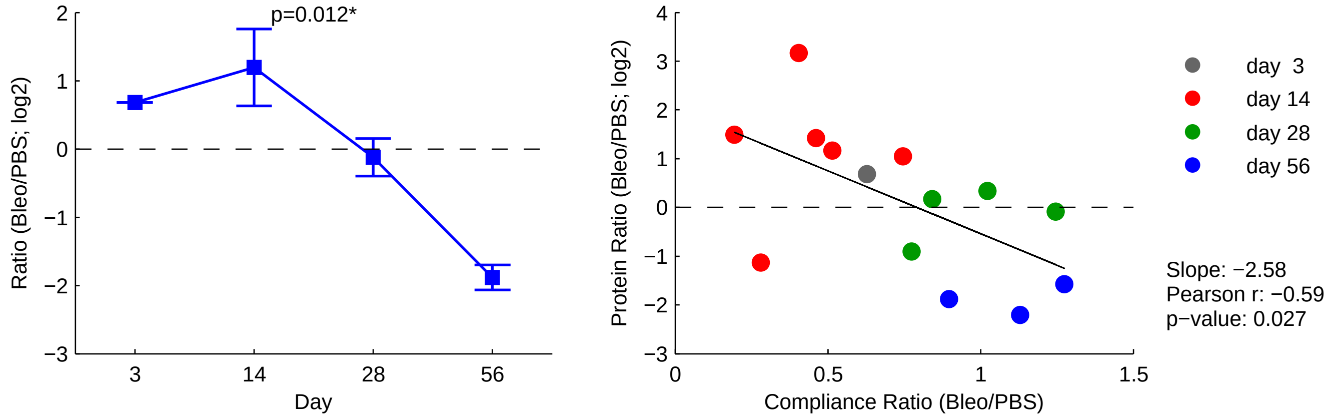

D3Z7P3 − Gls (id: 806)

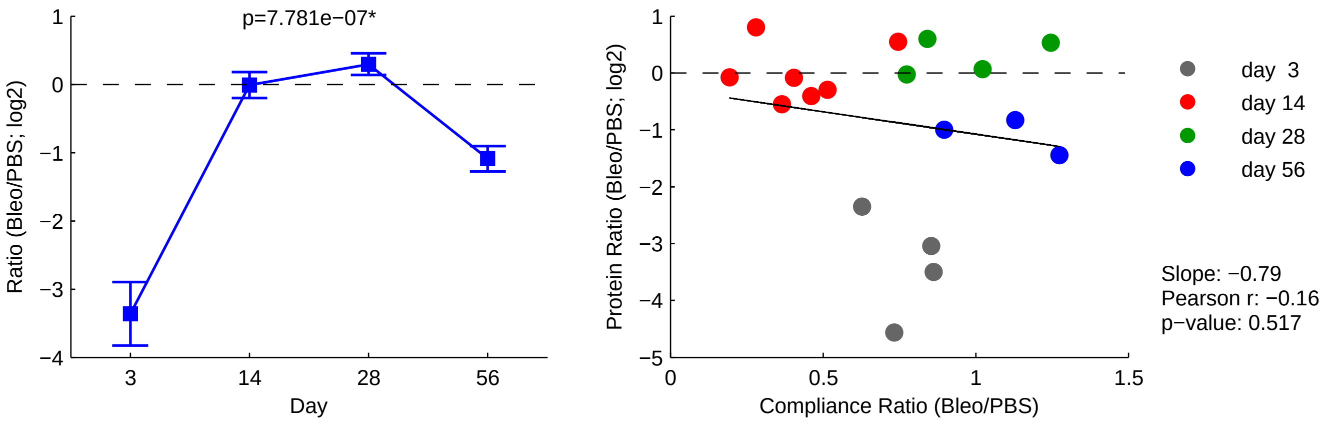

D3Z7P3−2 − (id: 807)

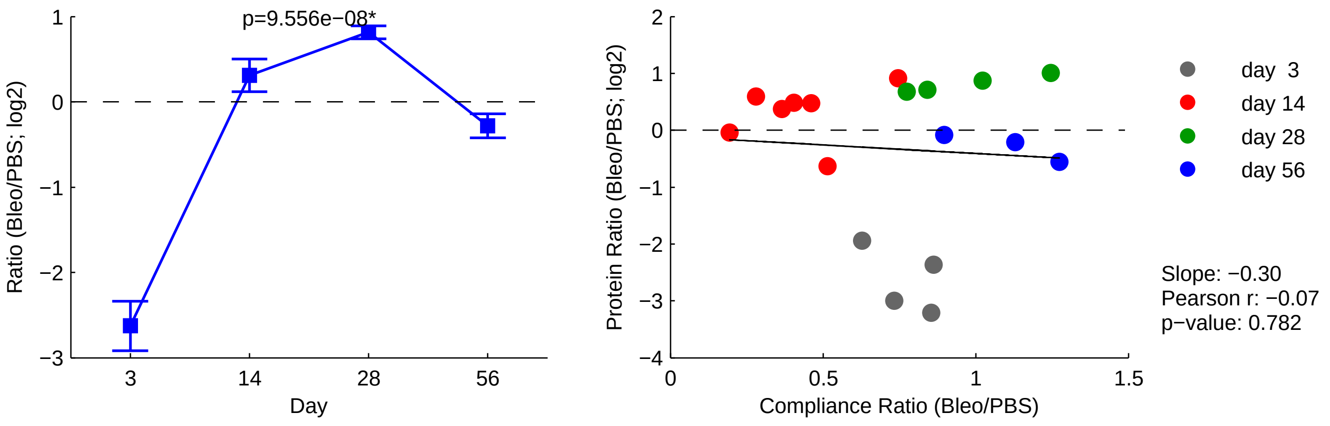

D3Z7Q2 − Smim20 (id: 808)

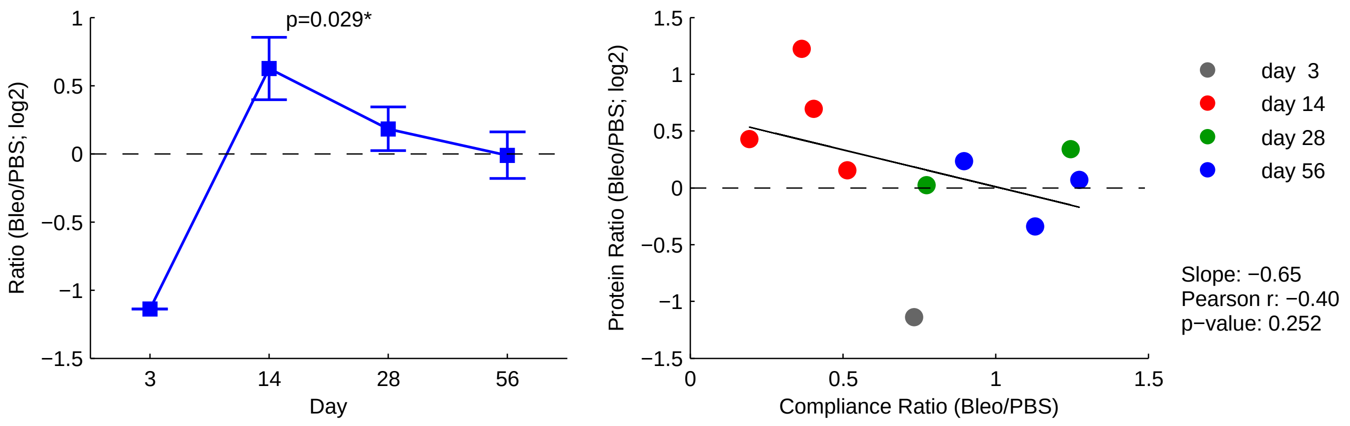

Q3TBD2 − Hmha1 (id: 809)

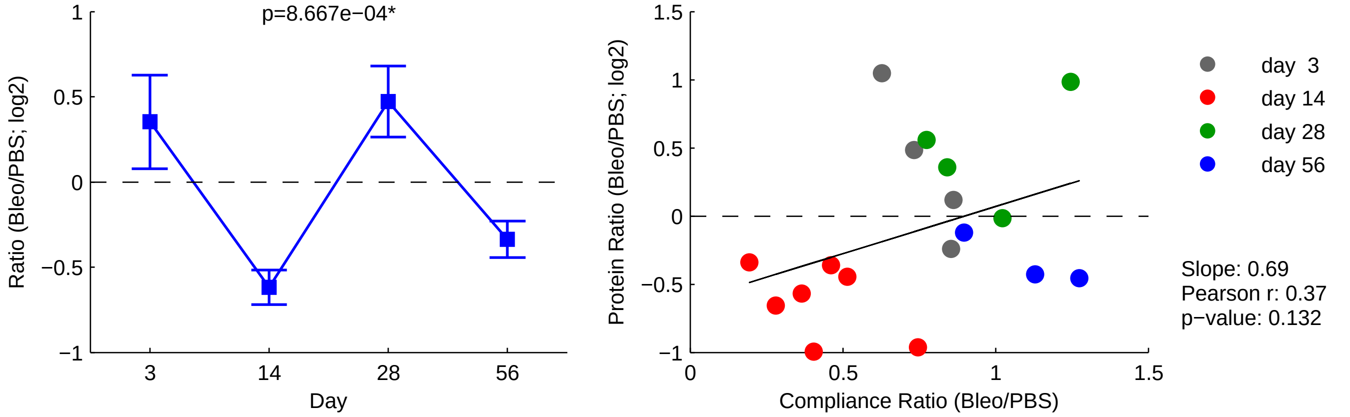

D3Z7V3 − Slmap (id: 811)

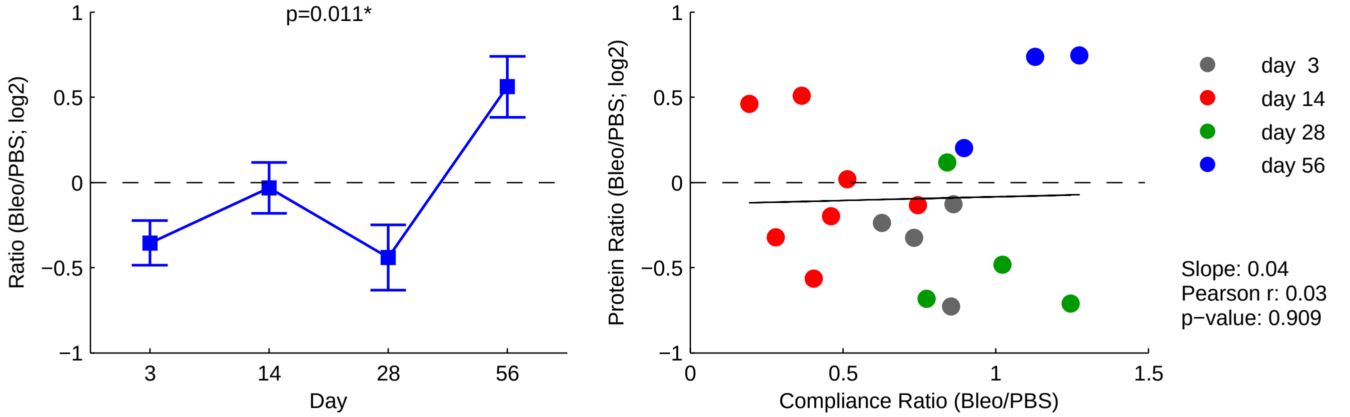

D5MCW4 − Cuta (id: 813)

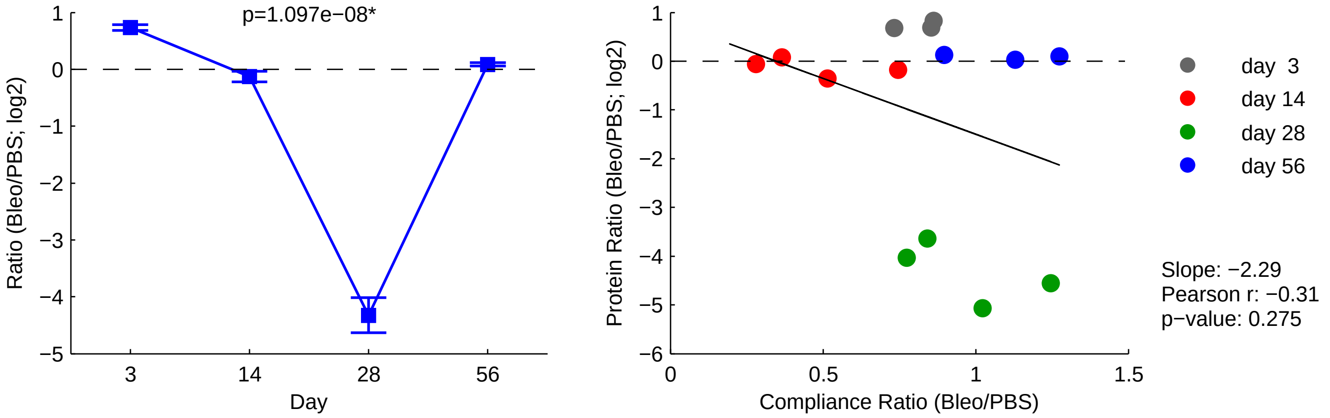

D6REH1 − Pxmp2 (id: 817)

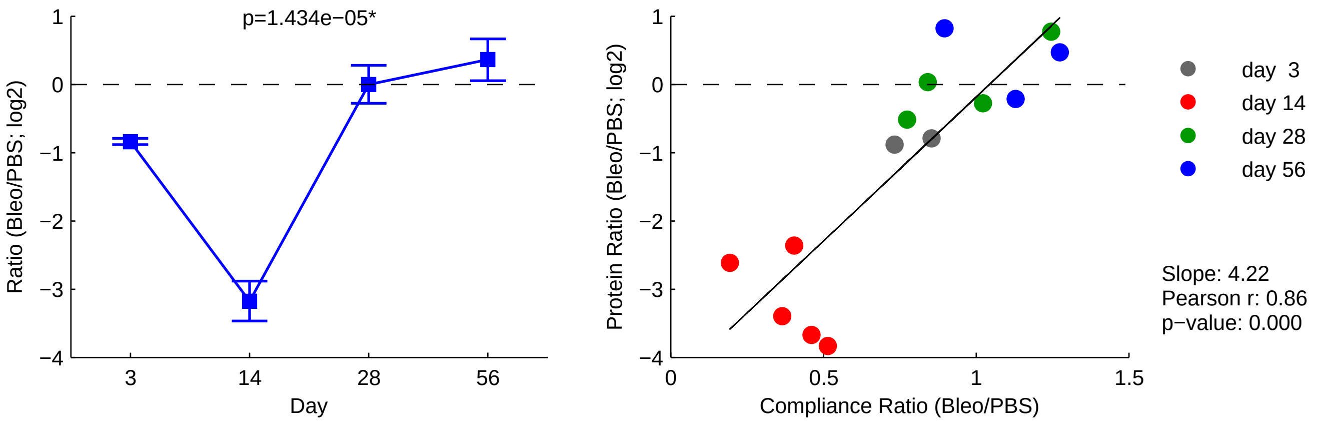

Q9Z210 − Pex11b (id: 823)

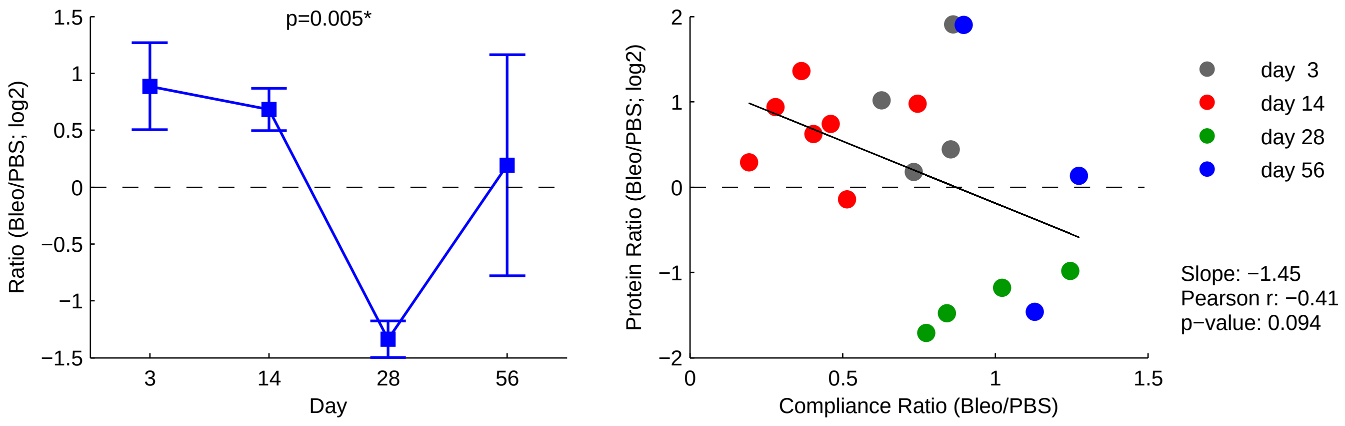

E0CX78 − Fcrla (id: 833)

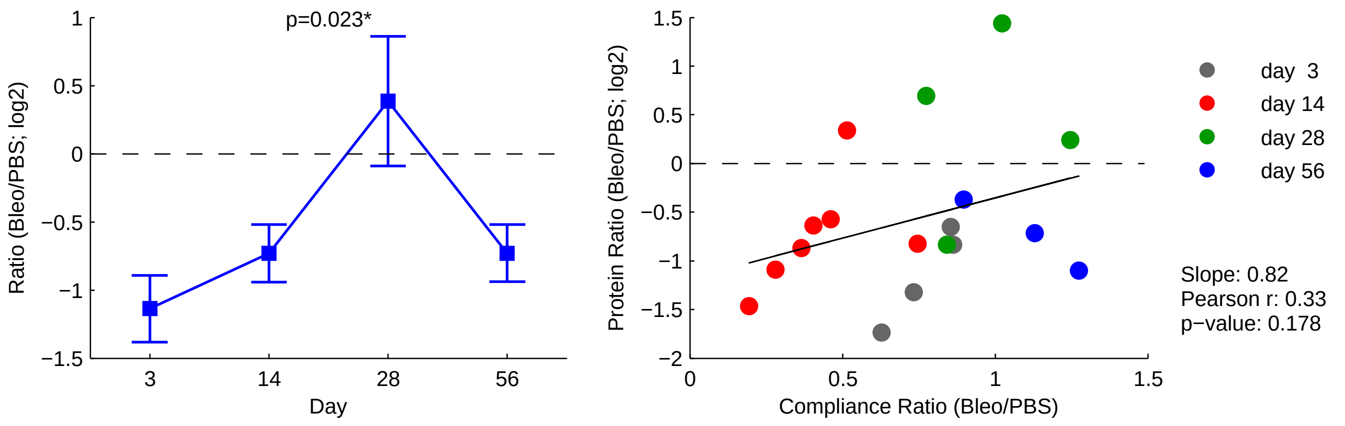

E0CZE8 − Tmbim6 (id: 834)

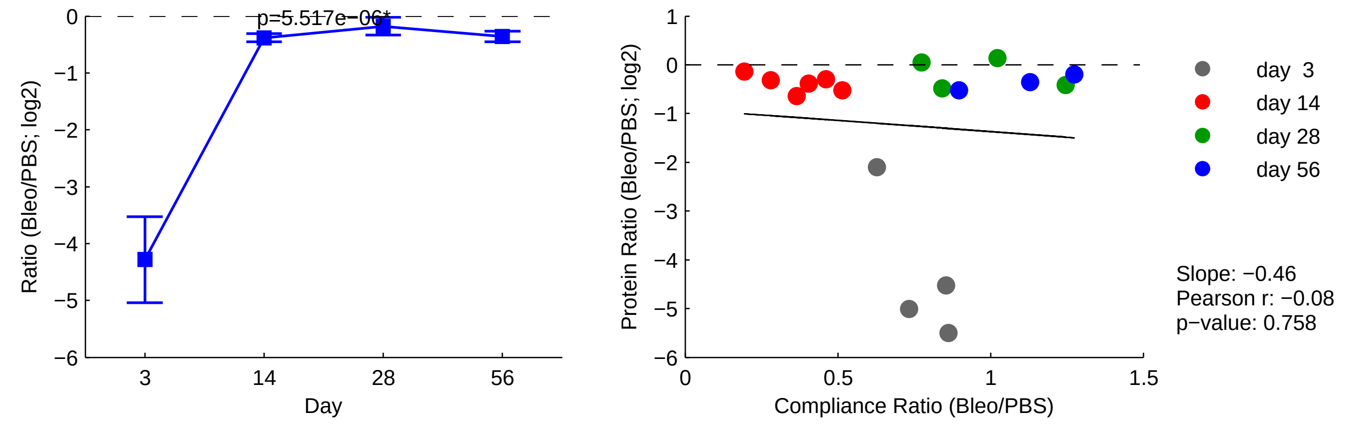

Q61301−2 − Ctnna2 (id: 835)

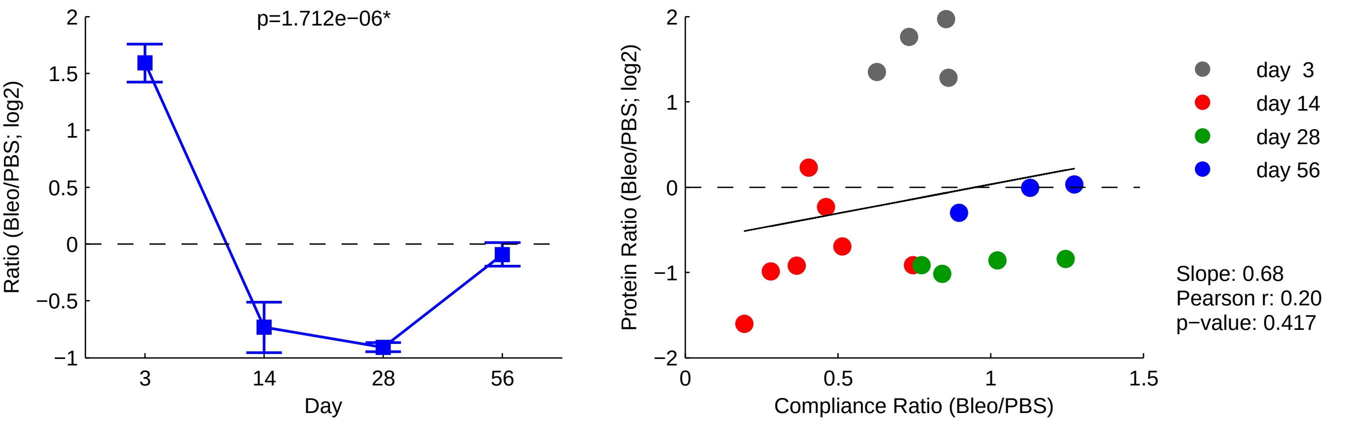

E0CZD9 − Zfpl1 (id: 838)

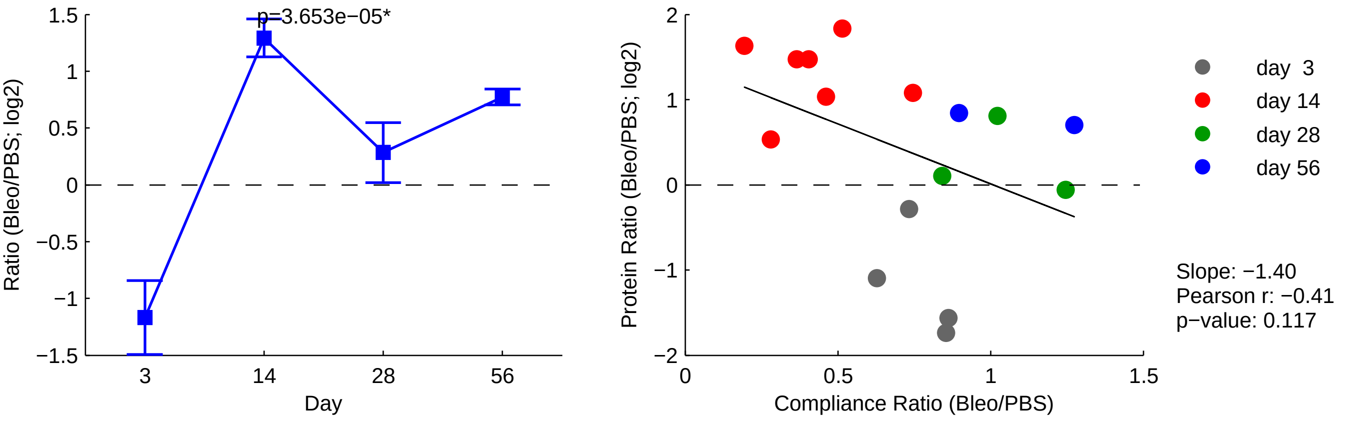

G3XA21 − Mroh1 (id: 855)

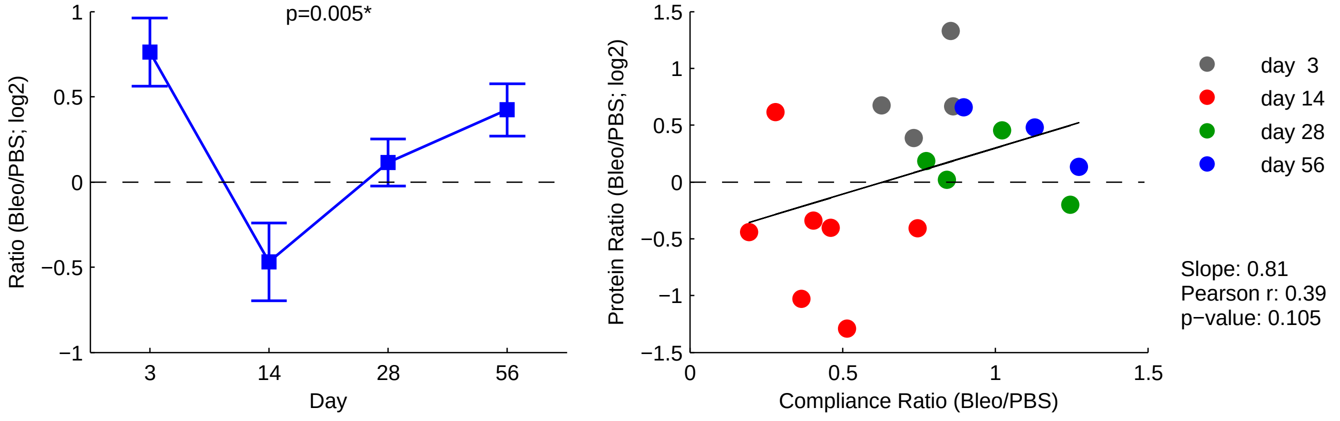

P28740−2 − Kif2a (id: 856)

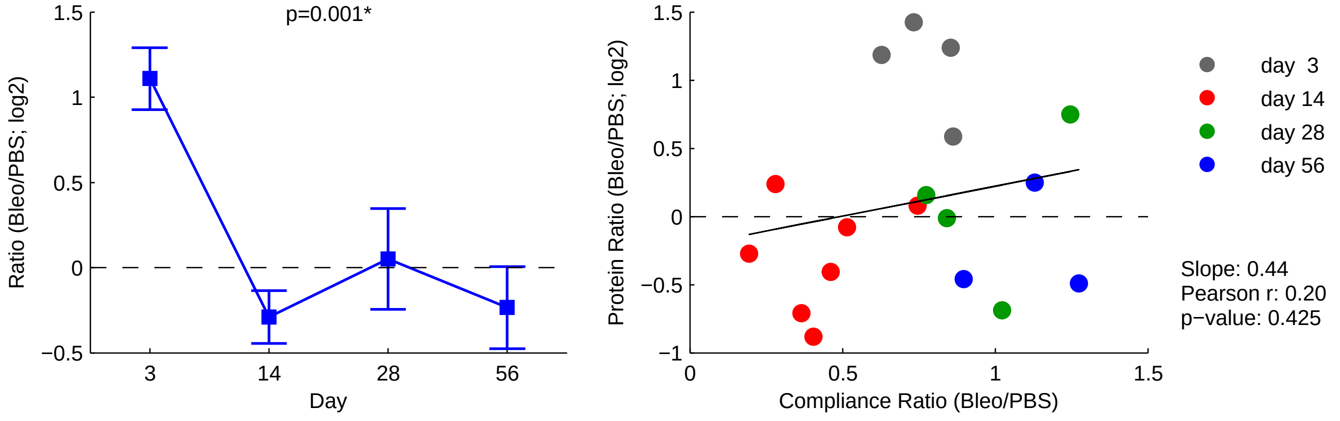

E0CZ73 − Ocln (id: 857)

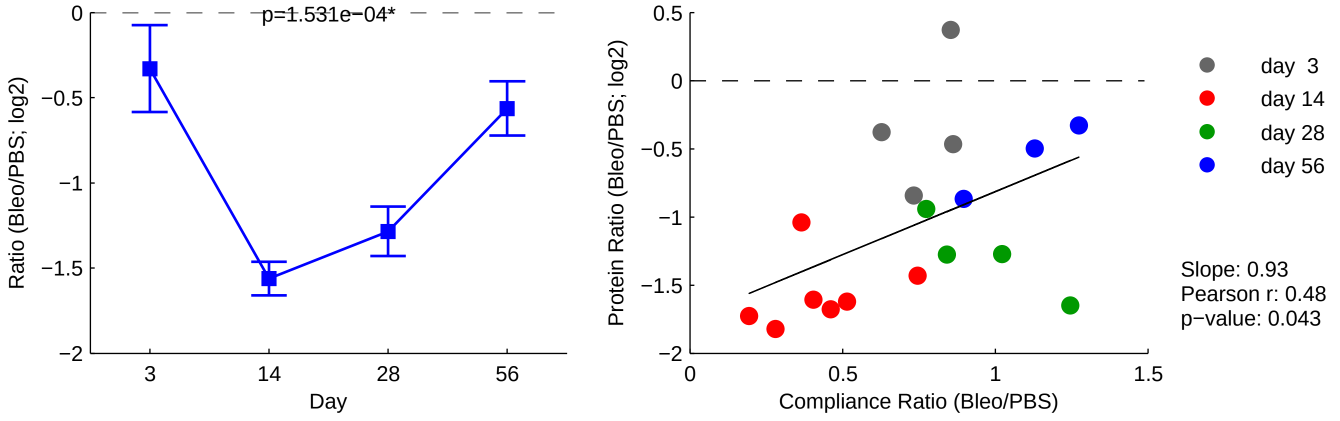

E0CZ81 − Tbce (id: 858)

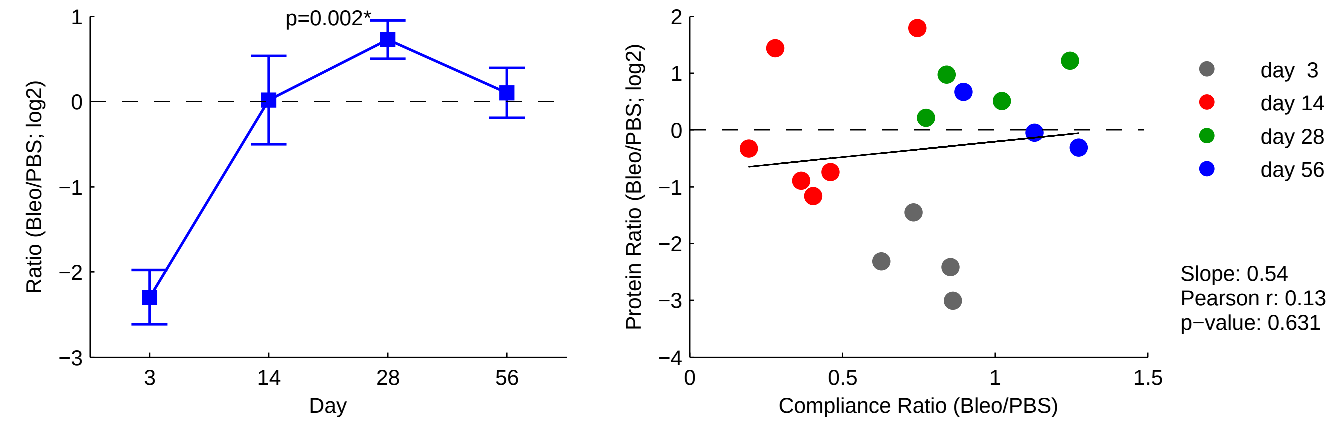

E2QRQ2 − Arhgef38 (id: 862)

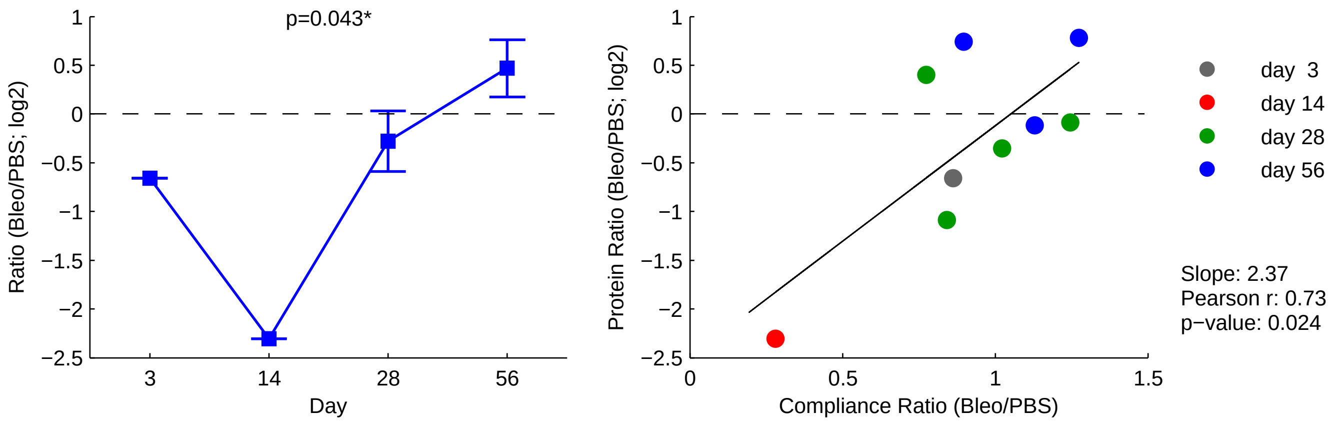

P70170−3 − Abcc9 (id: 871)

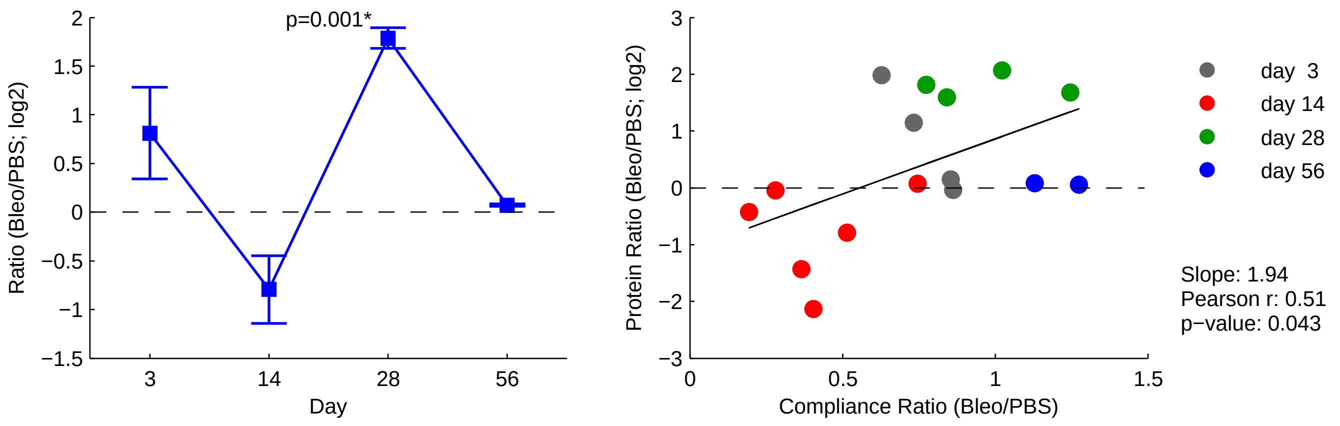

E9PUK3 − Fnbp1l (id: 874)

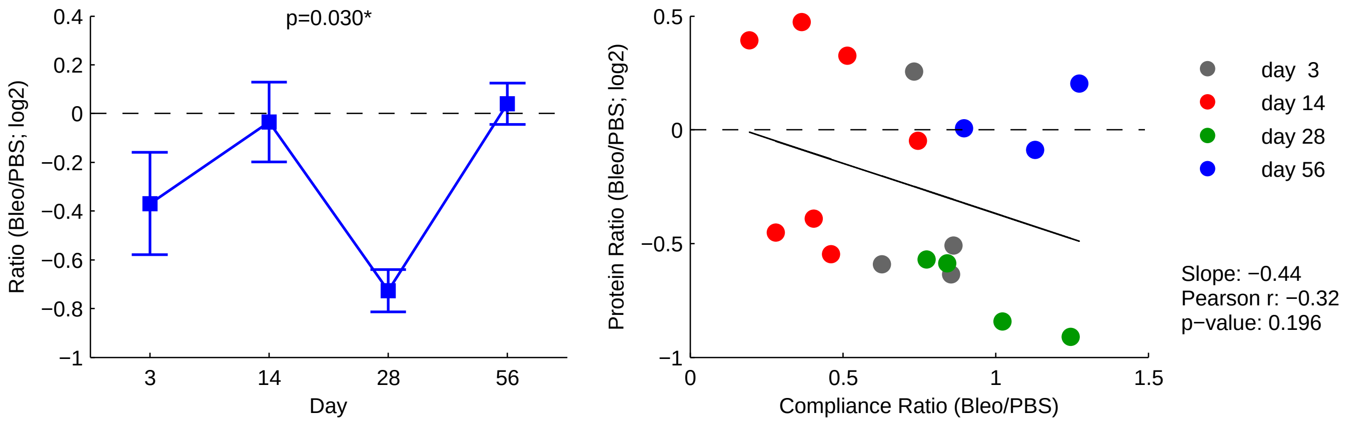

E9PUM4 − Tln2 (id: 875)

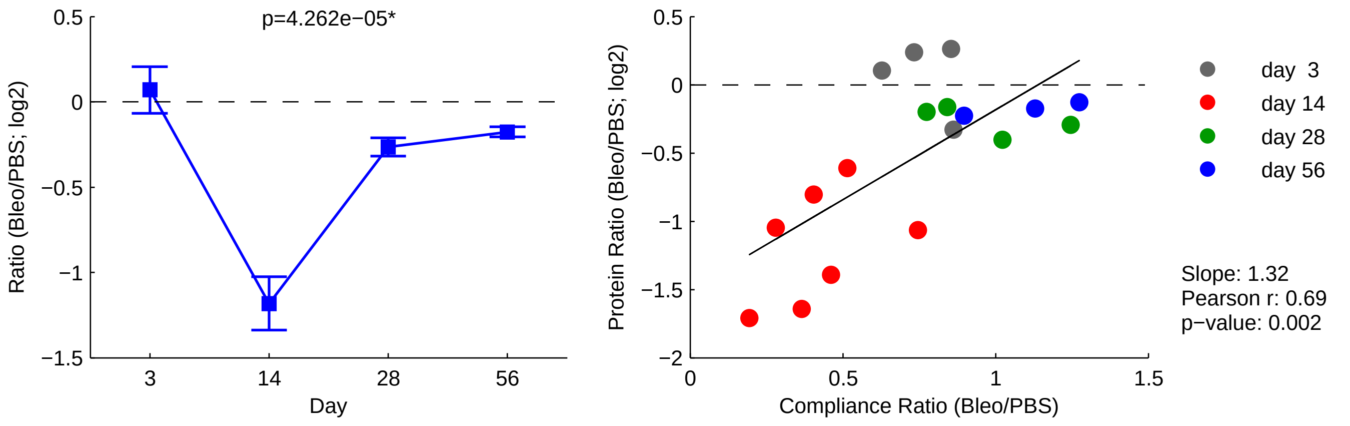

E9PUM5 − Gm4788 (id: 876)

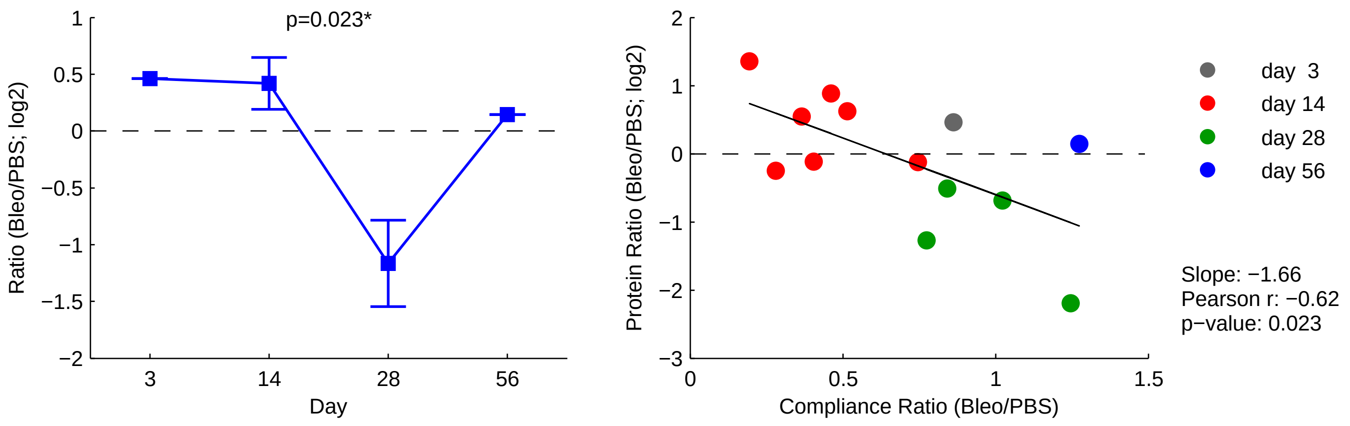

E9PUN5 − Ror2 (id: 877)

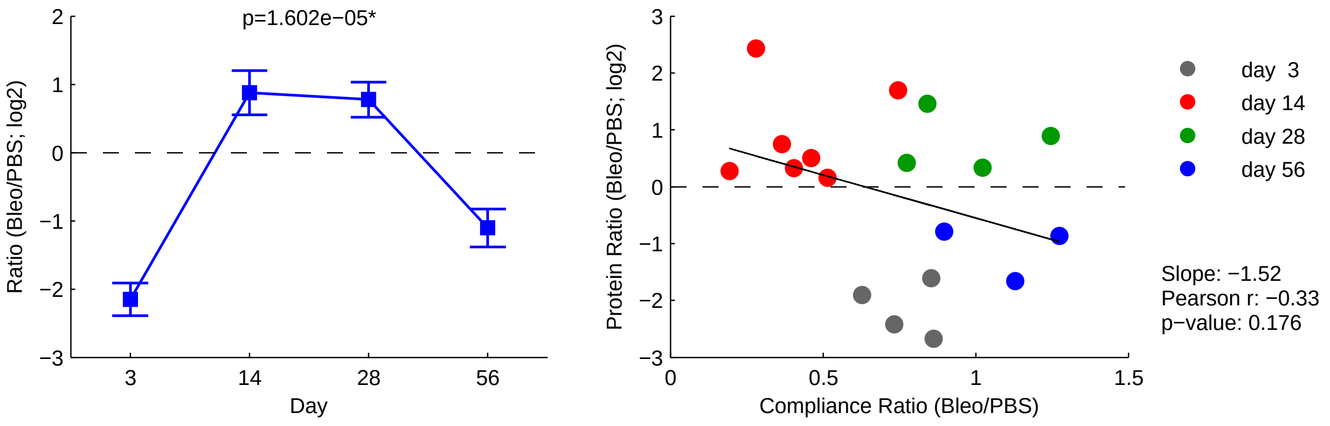

Q921M4−2 − Golga2 (id: 880)

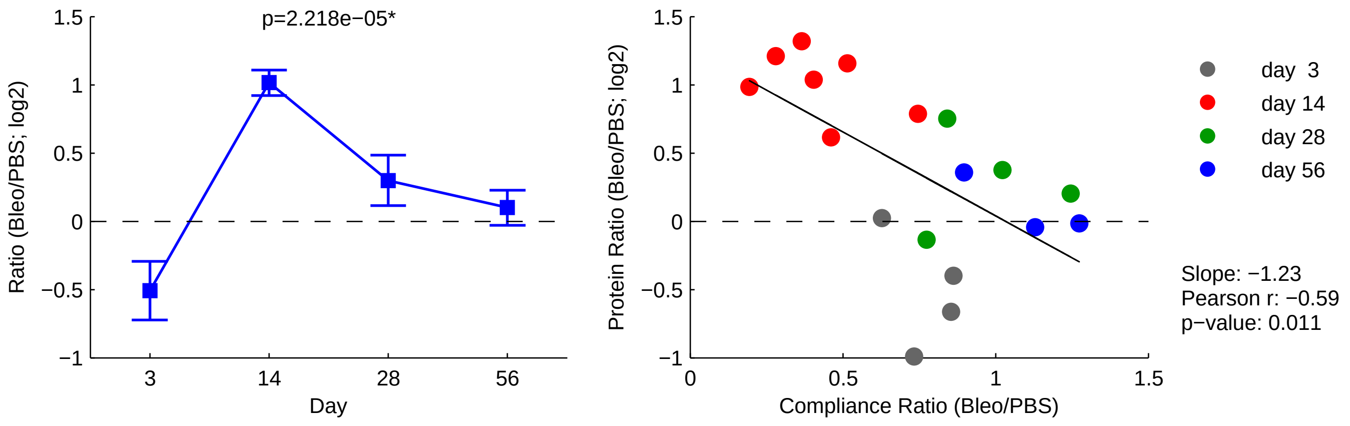

E9PUQ9 − Piezo1 (id: 883)

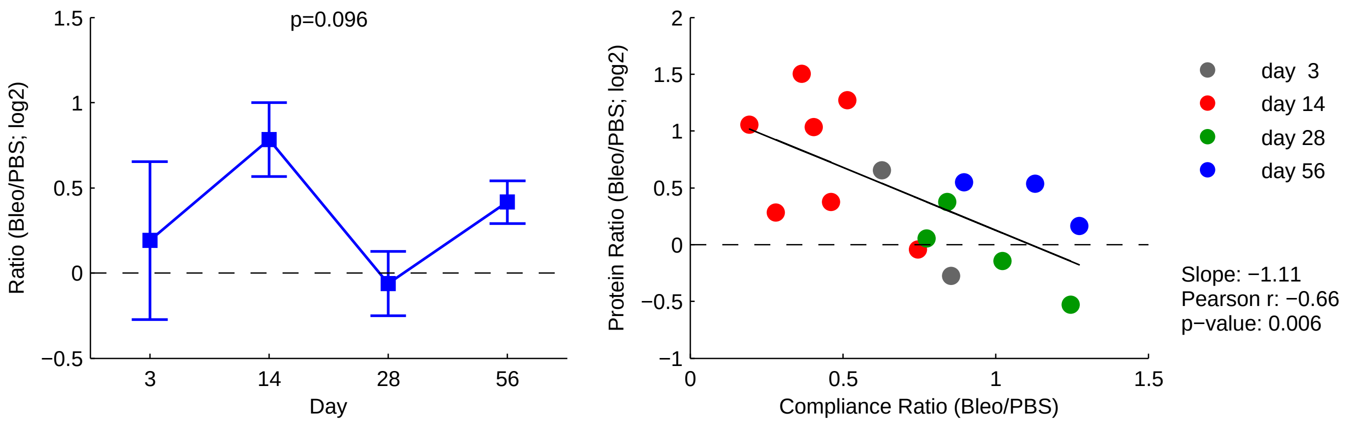

E9Q1M6 − Ankhd1 (id: 884)

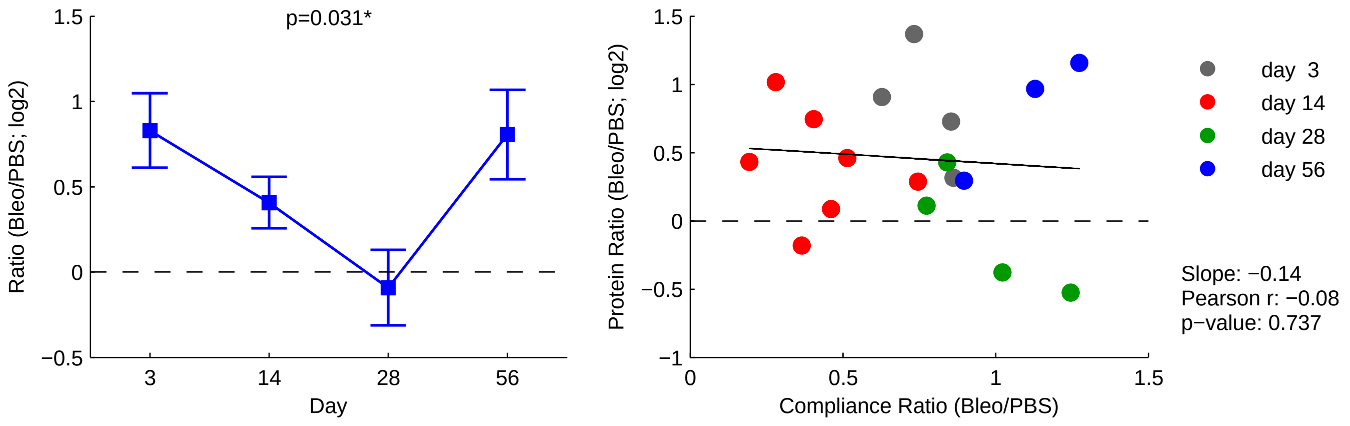

E9PUR4 − Lama3 (id: 885)

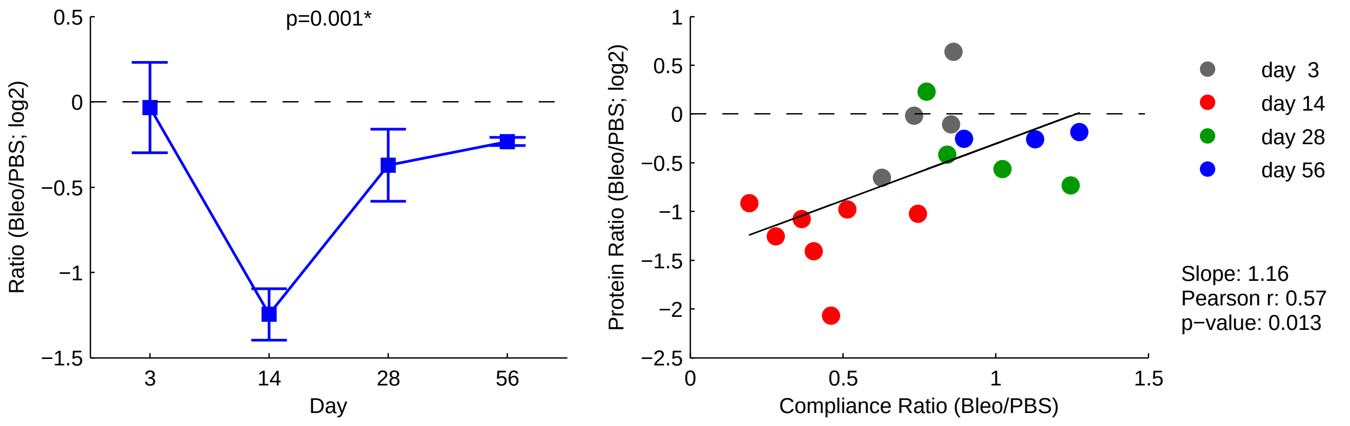

Q9EPK7−2 − Xpo7 (id: 889)

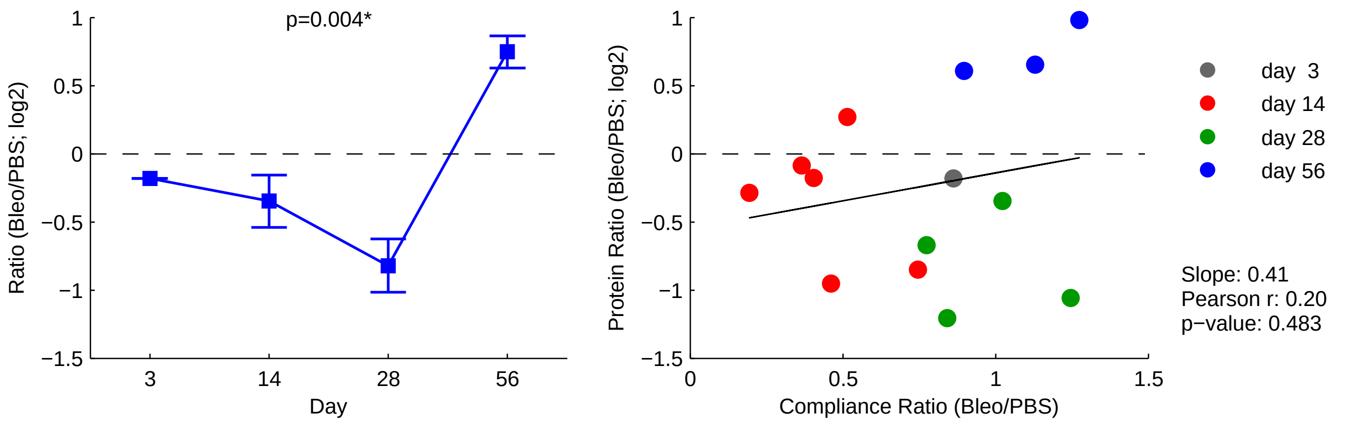

E9PV03 − Nbeal1 (id: 891)

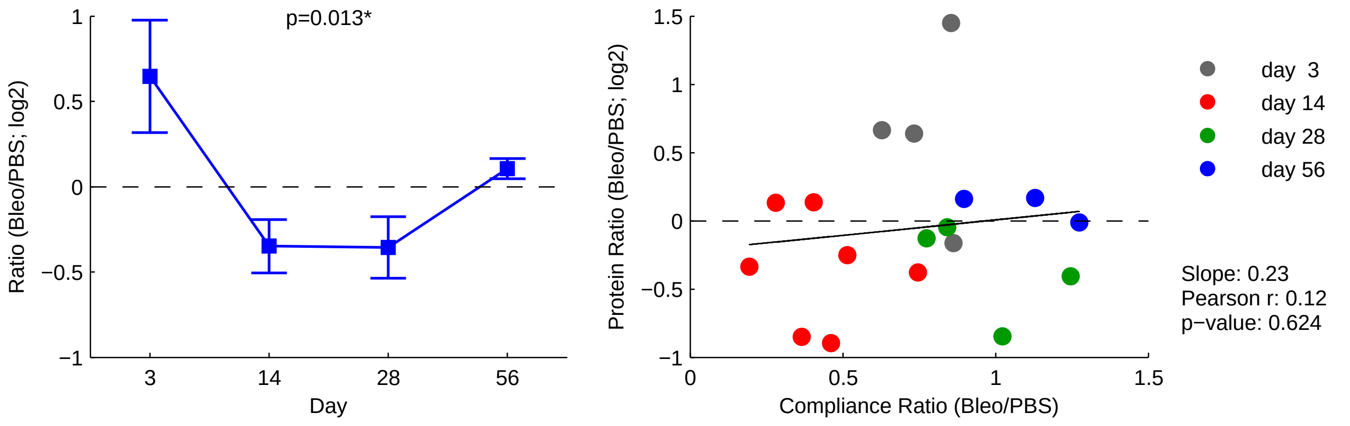

E9PV24 − Fga (id: 892)

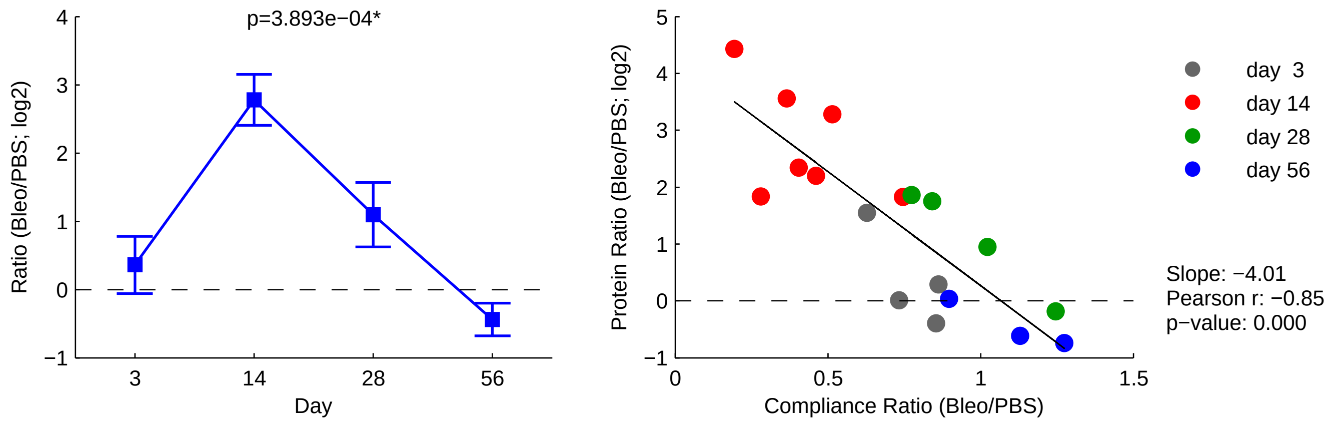

E9PV38 − Ces2g (id: 893)

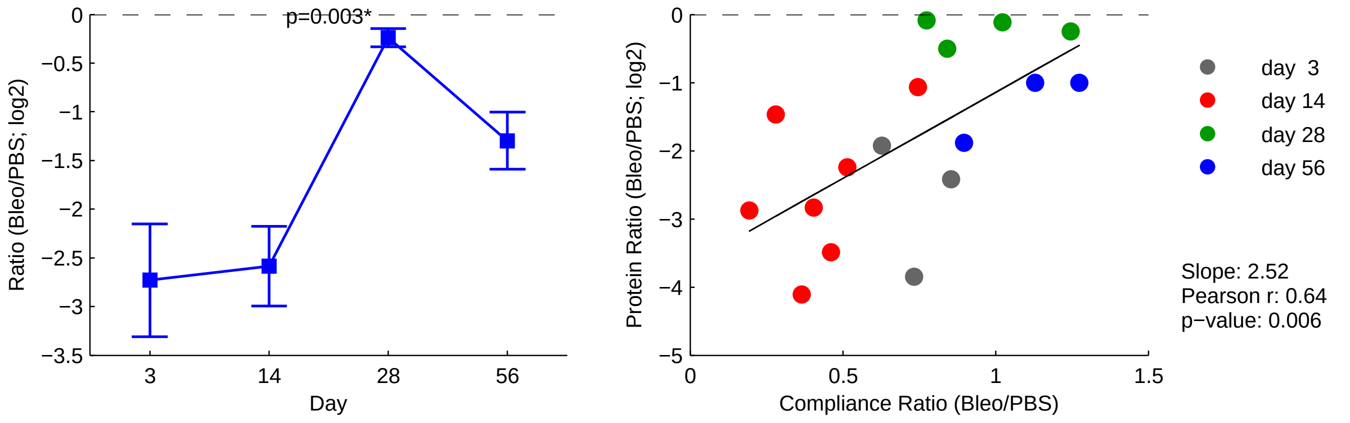

O08808 − Diaph1 (id: 894)

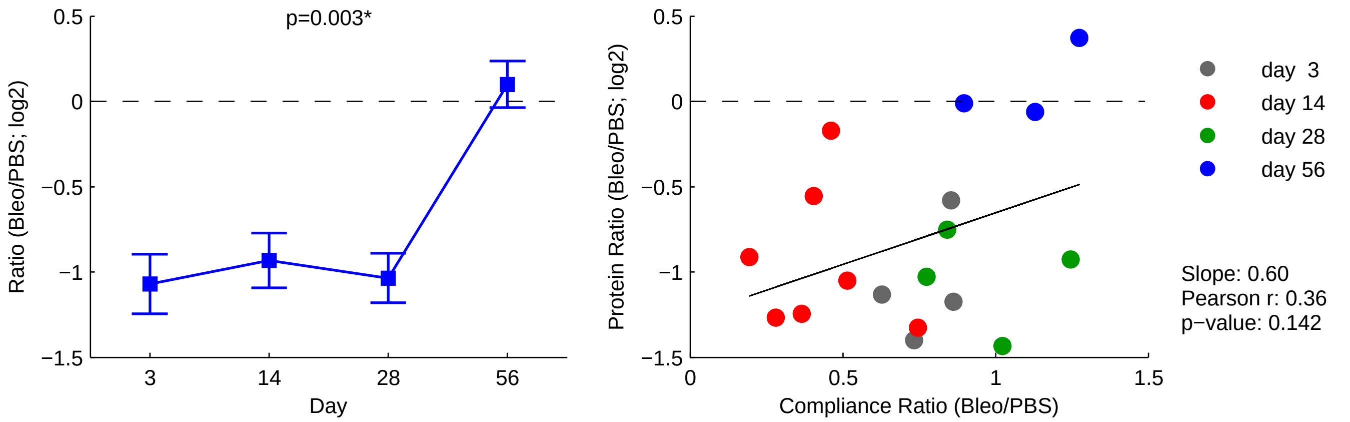

E9PV44 − Atpif1 (id: 895)

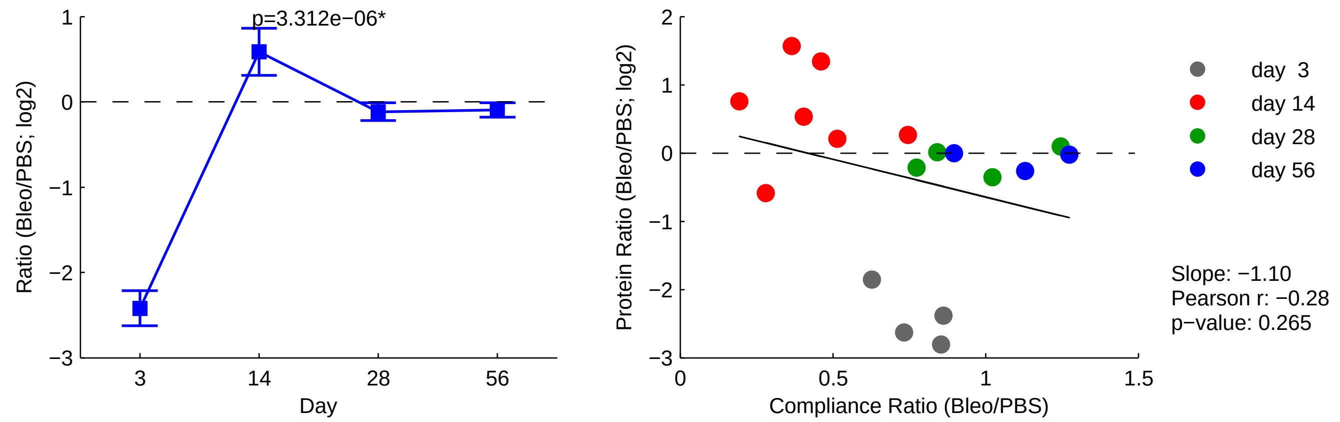

E9PVA6 − Git2 (id: 902)

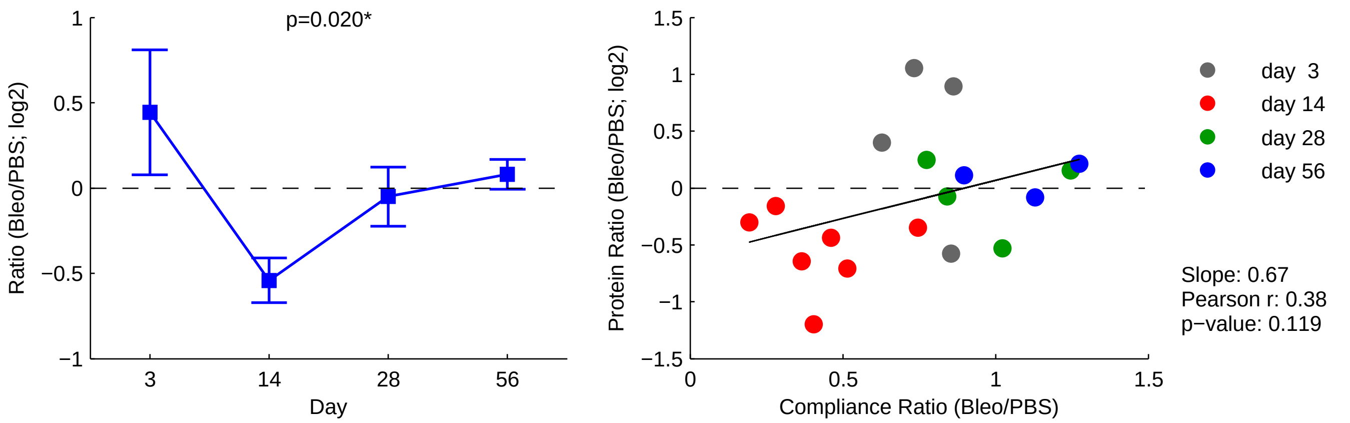

E9PVA8 − Gcn1l1 (id: 903)

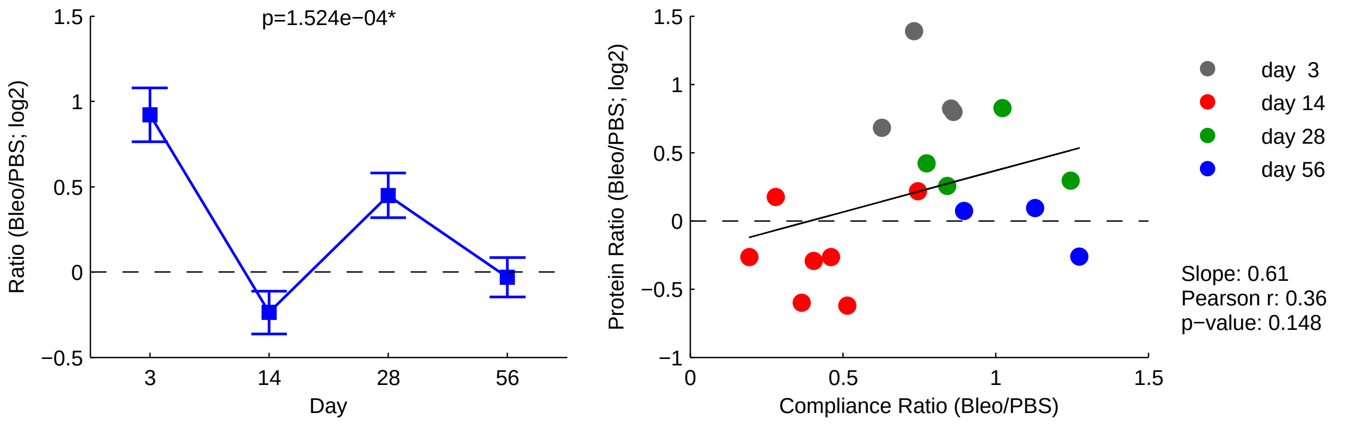

E9PVC6 − Eif4g1 (id: 905)

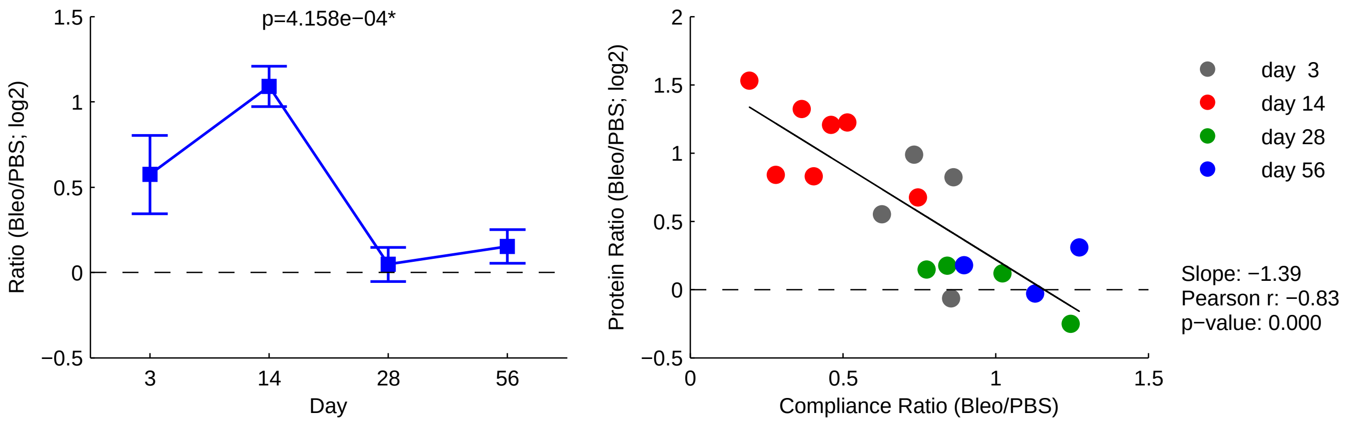

O35495−2 − Cdk14 (id: 906)

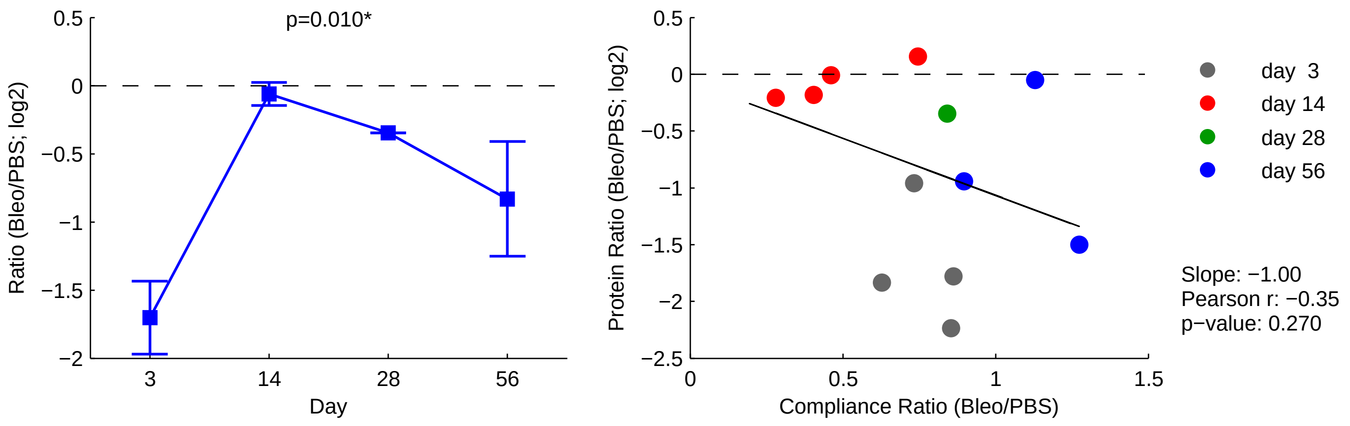

F8VPL5 − Map4k4 (id: 908)

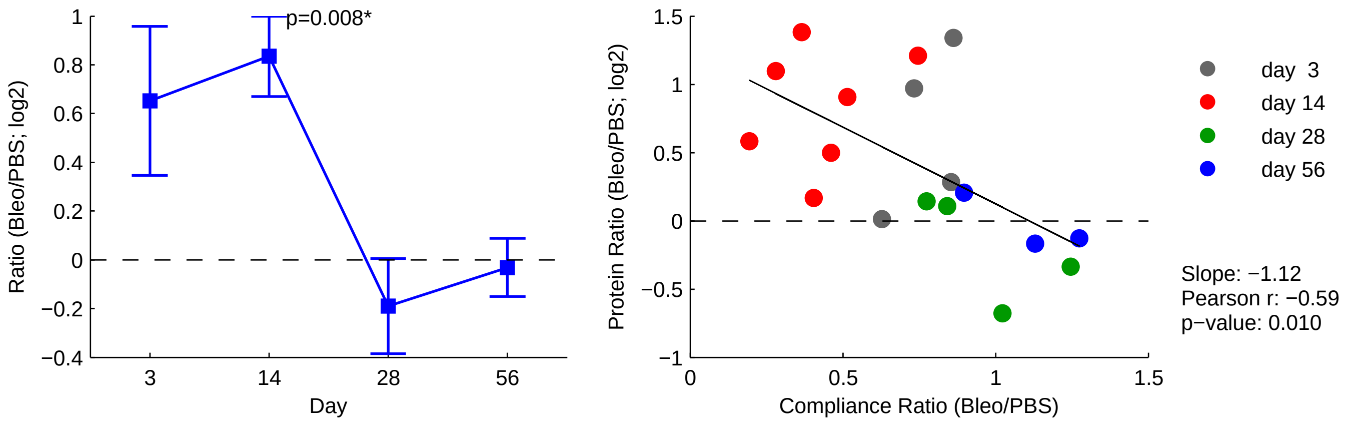

E9PVM7 − Gstm5 (id: 911)

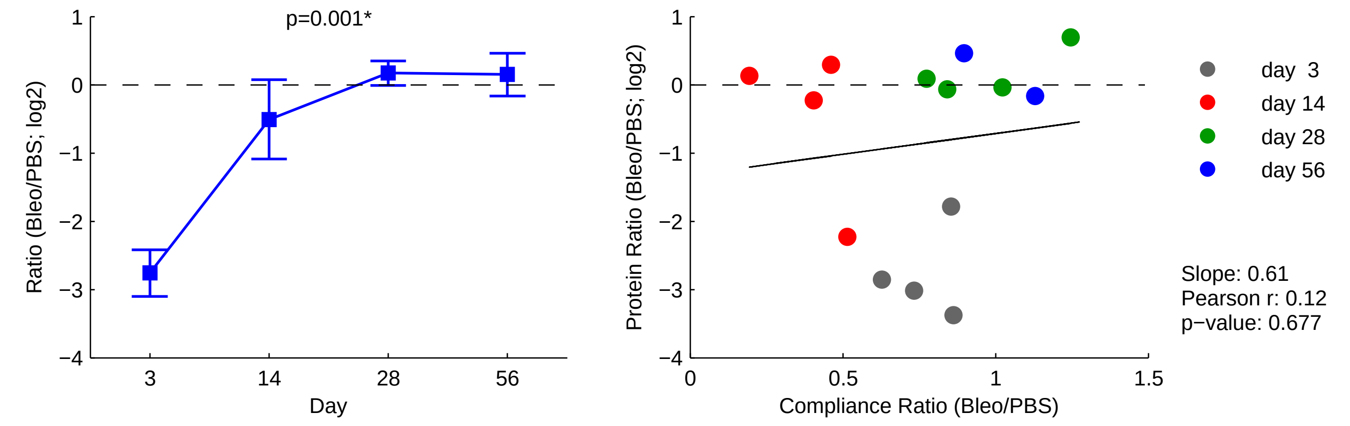

E9PVN6 − Gm20498 (id: 912)

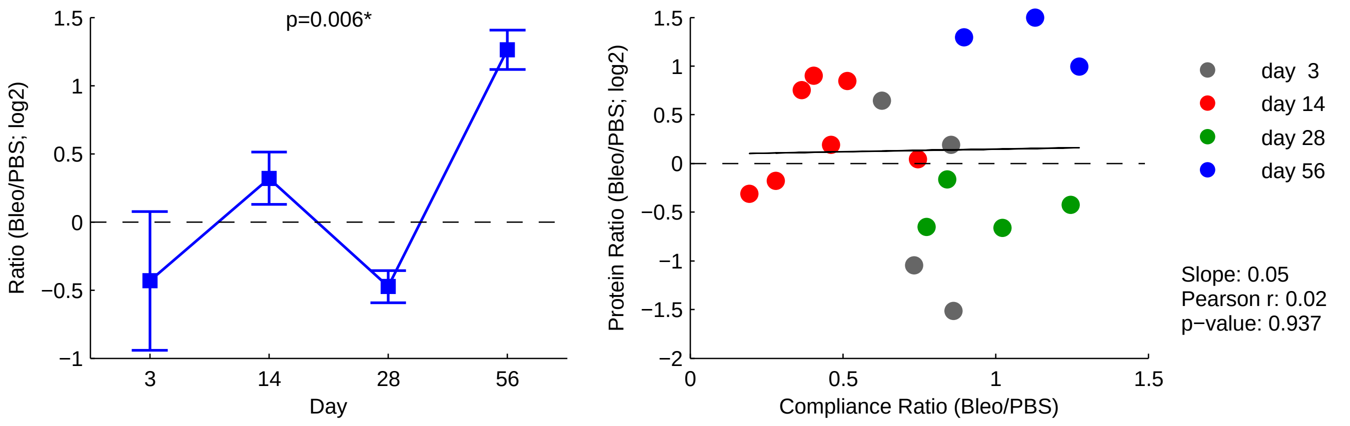

E9QAH1 − Golgb1 (id: 918)

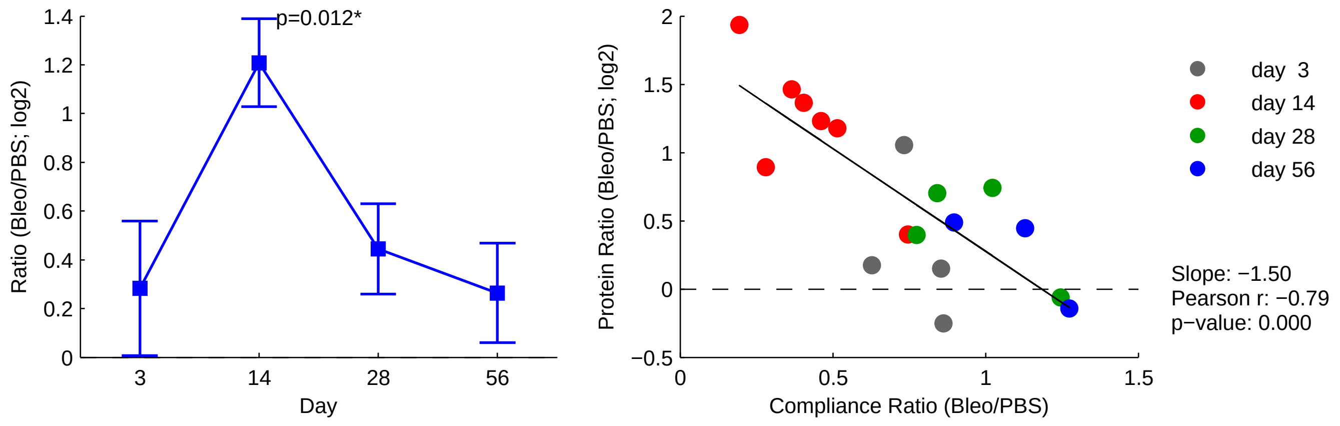

E9PW03 − Gpaa1 (id: 919)

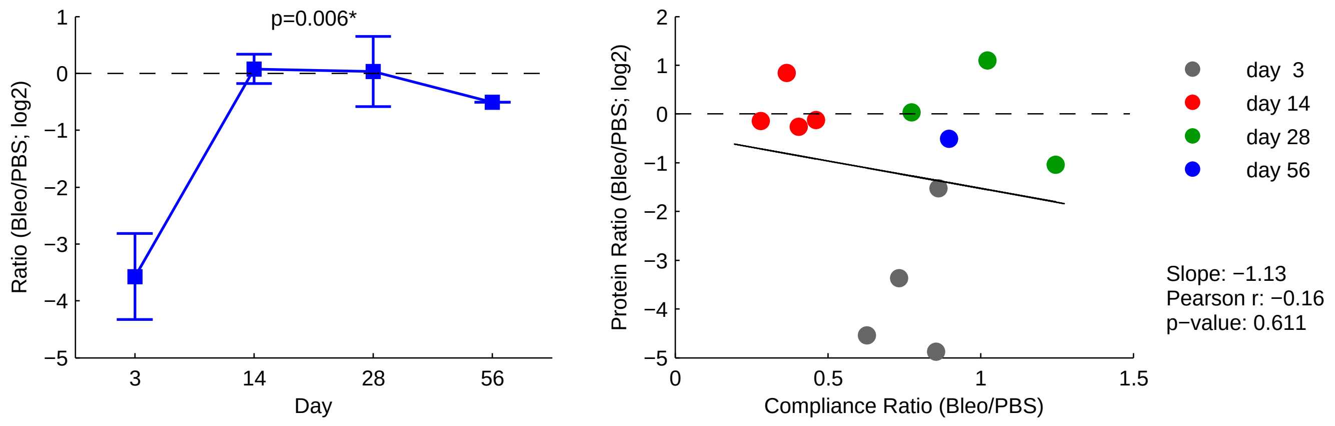

E9PW09 − Depdc1b (id: 920)

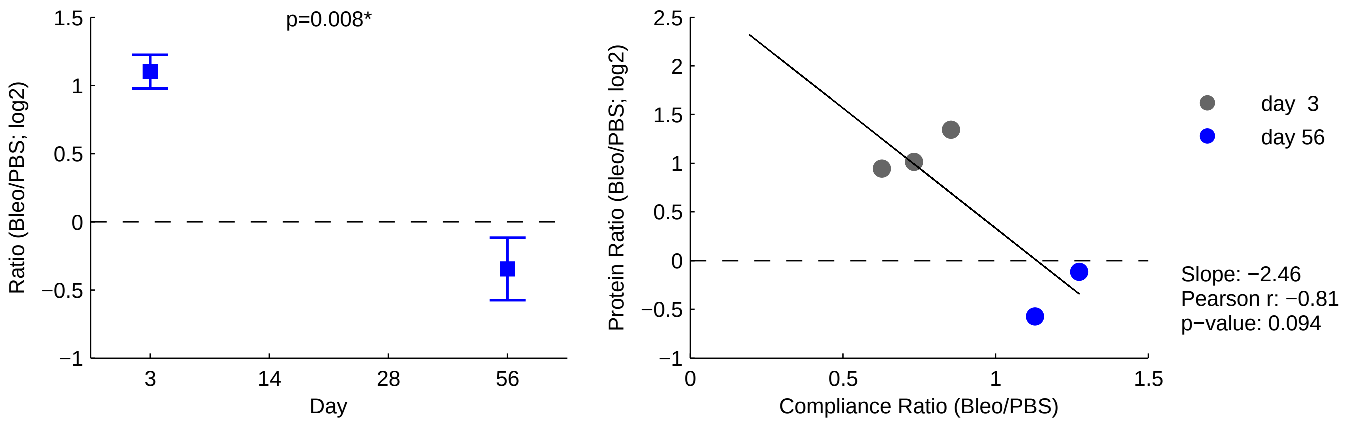

Q9CY57−5 − Chtop (id: 923)

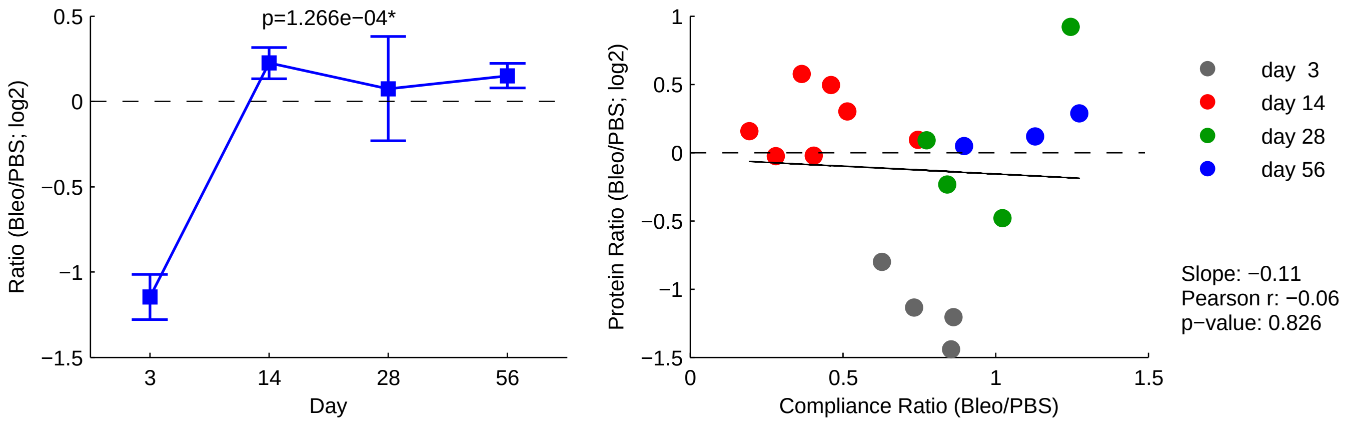

E9PWE8 − Dpysl3 (id: 928)

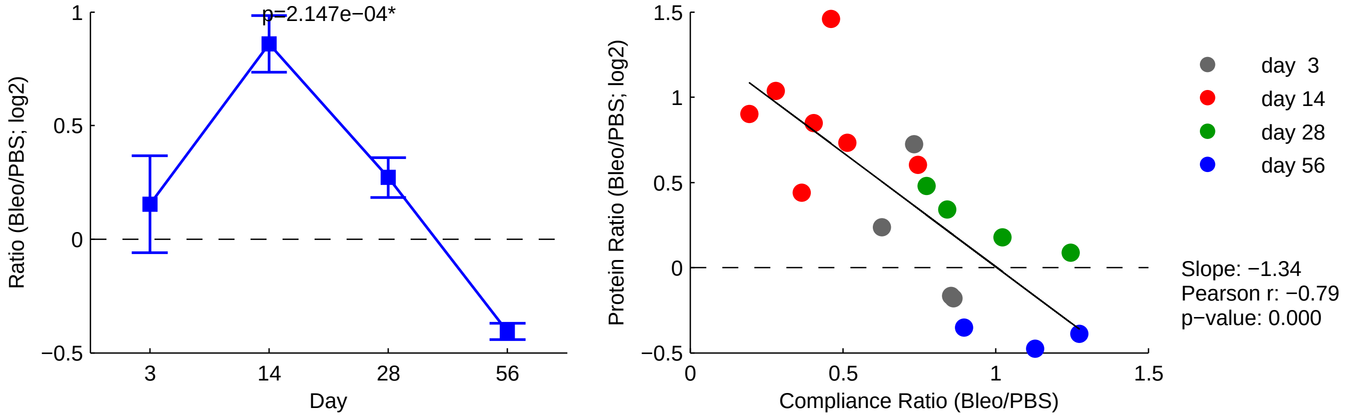

E9PWF0 − Thbs3 (id: 929)

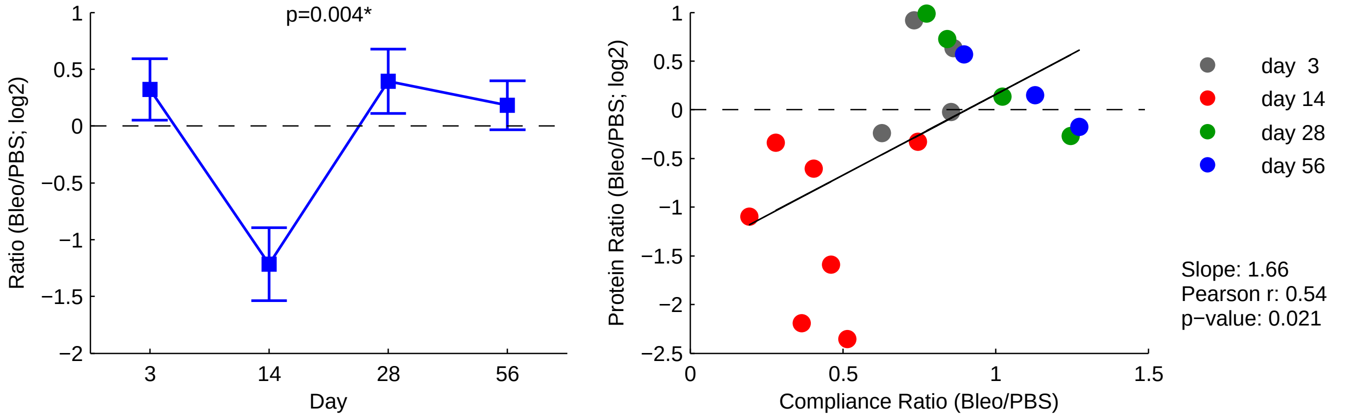

Q9DBR4−3 − Apbb2 (id: 934)

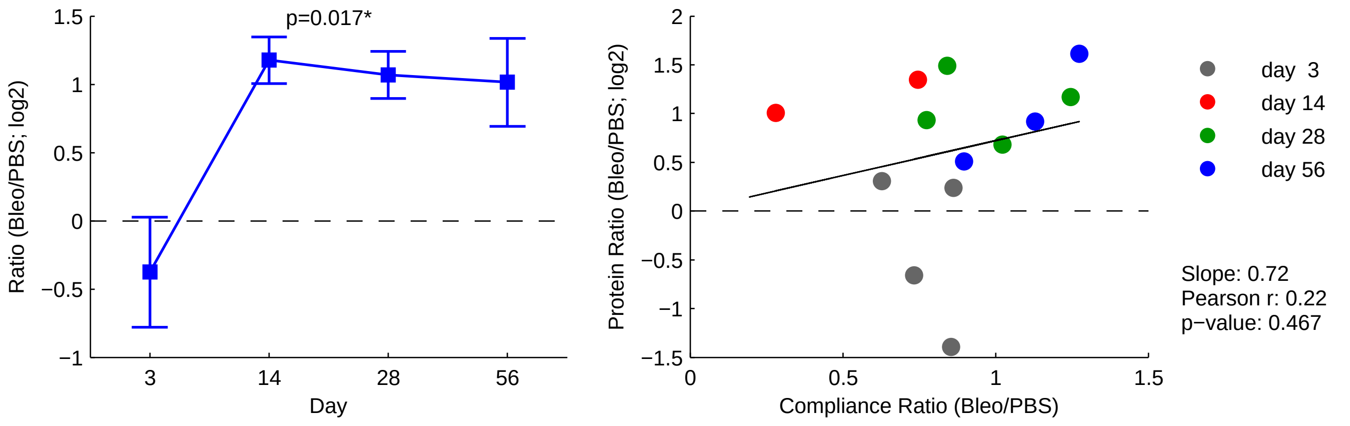

G3UW40 − Mcc (id: 935)

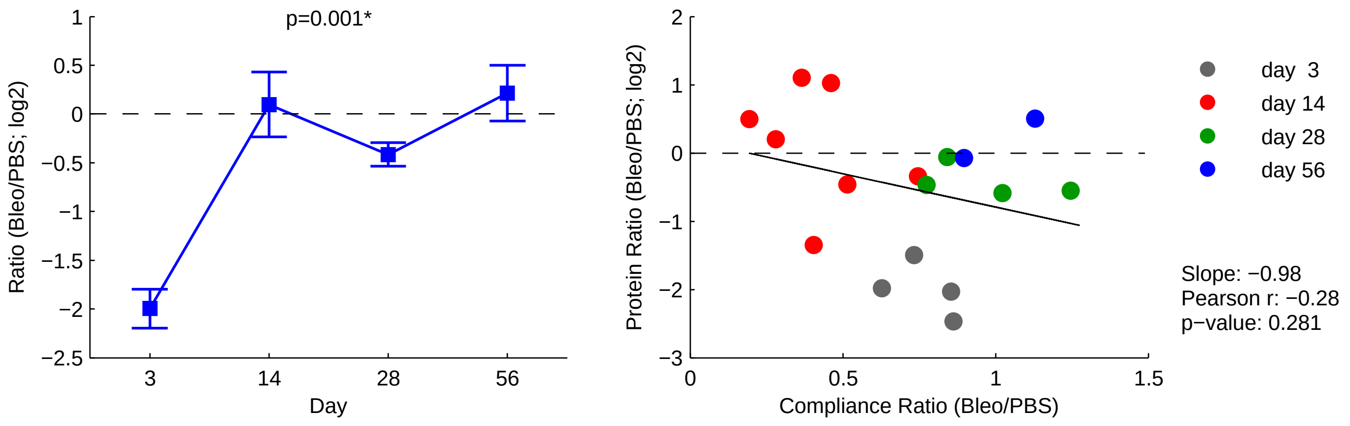

E9PWK7 − Ephb4 (id: 938)

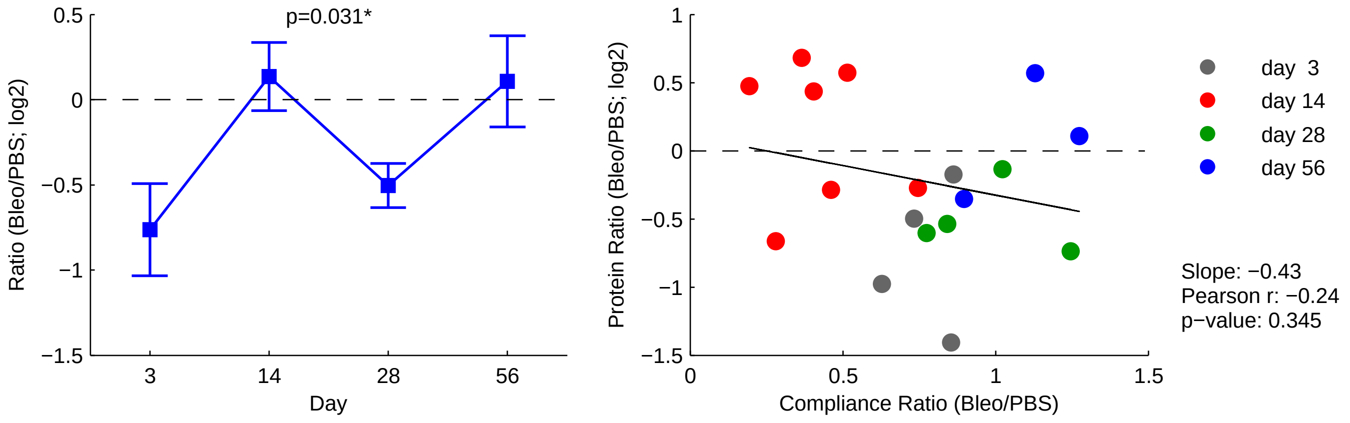

E9PZA7 − Trrap (id: 941)

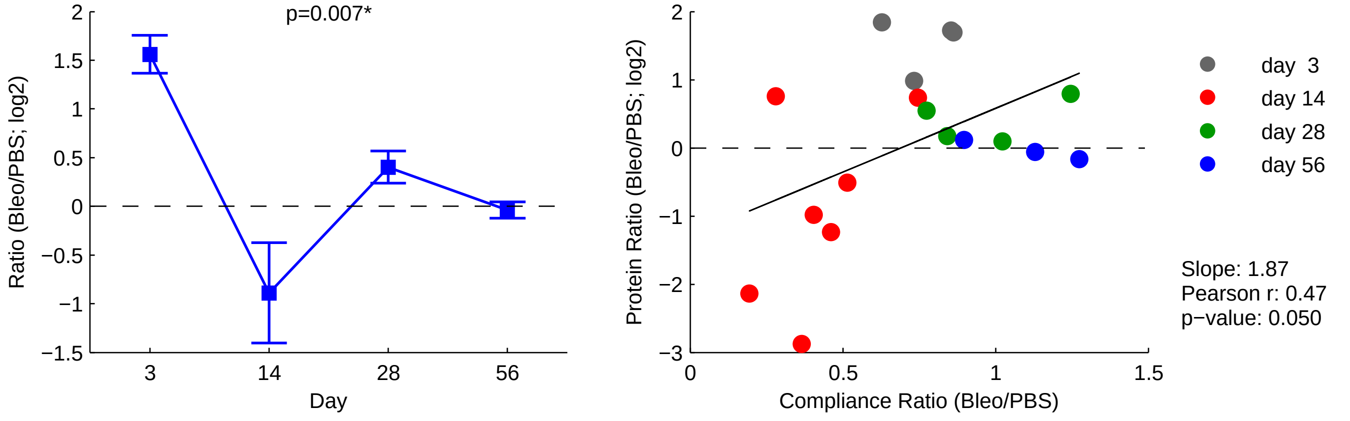

E9PWV3 − Rps6ka1 (id: 943)

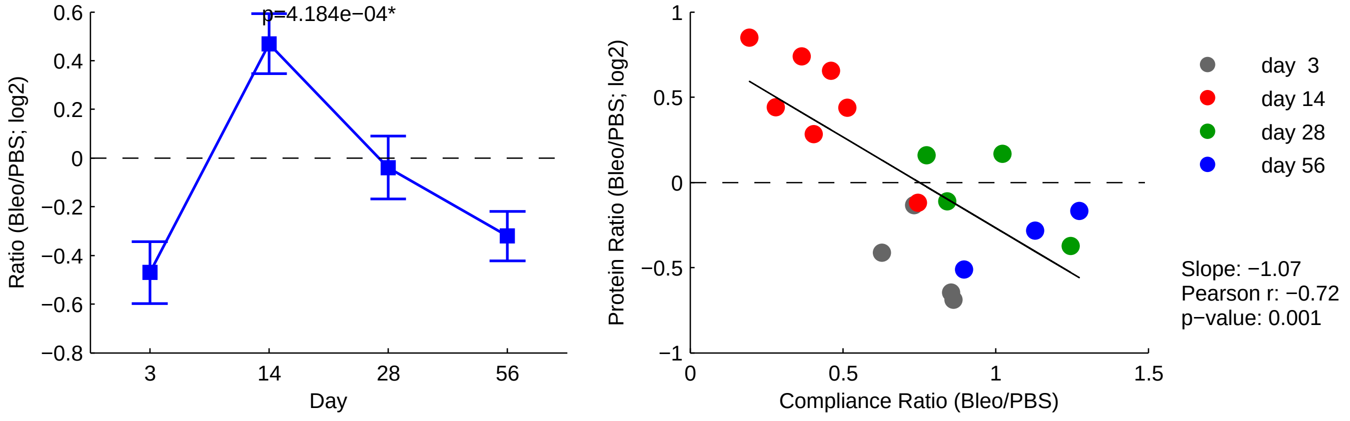

Q8BLI0 − Adamtsl1 (id: 945)

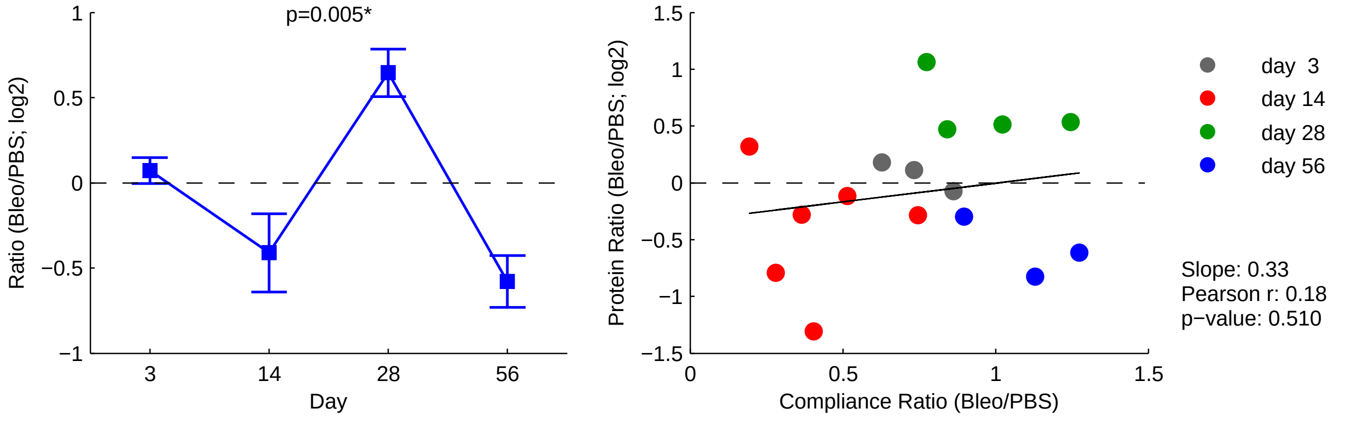

E9PWW9 − Rsf1 (id: 946)

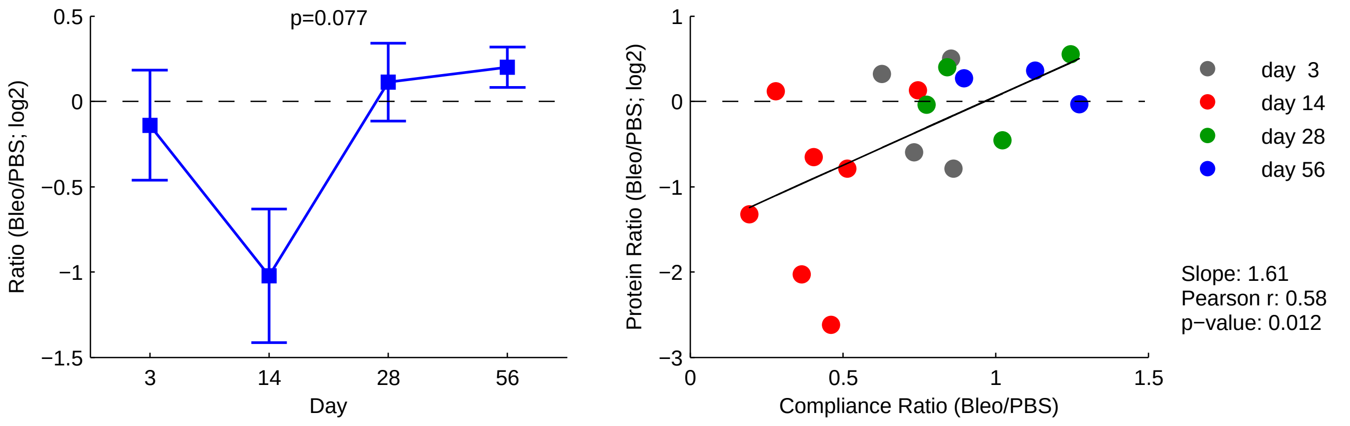

Q9CQ09 − Rpl3l (id: 947)

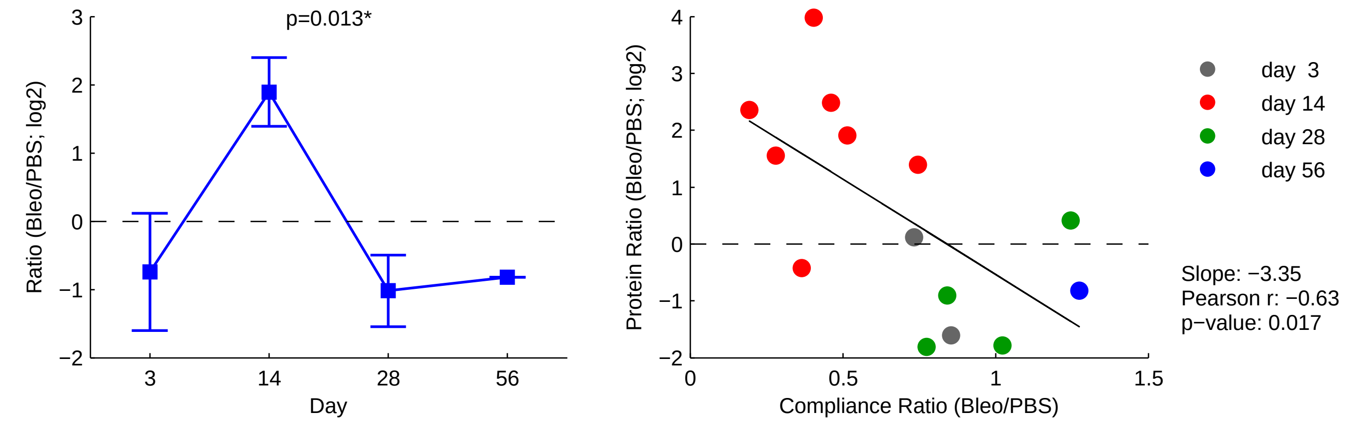

Q9D3W1 − Rtdr1 (id: 948)

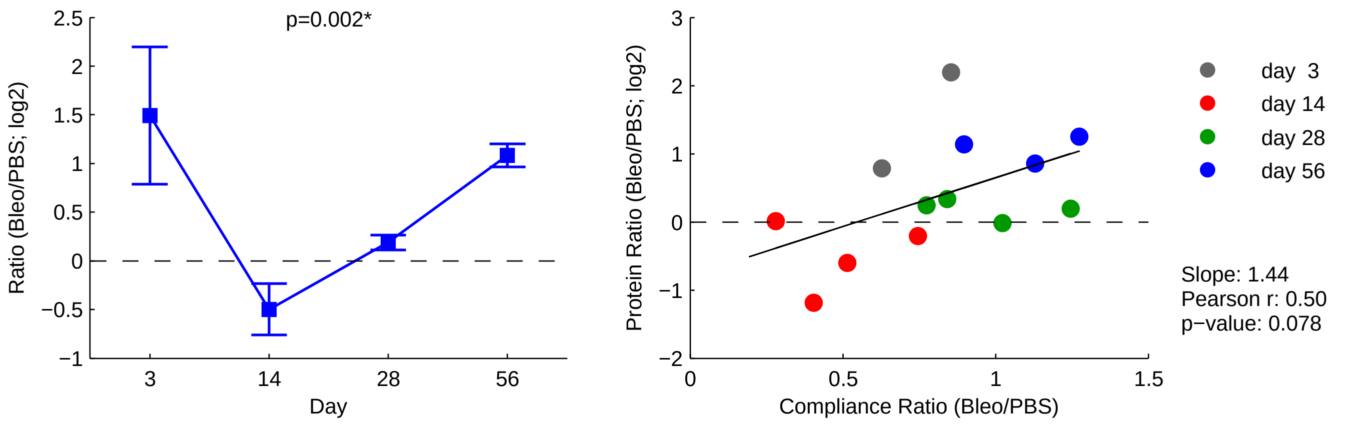

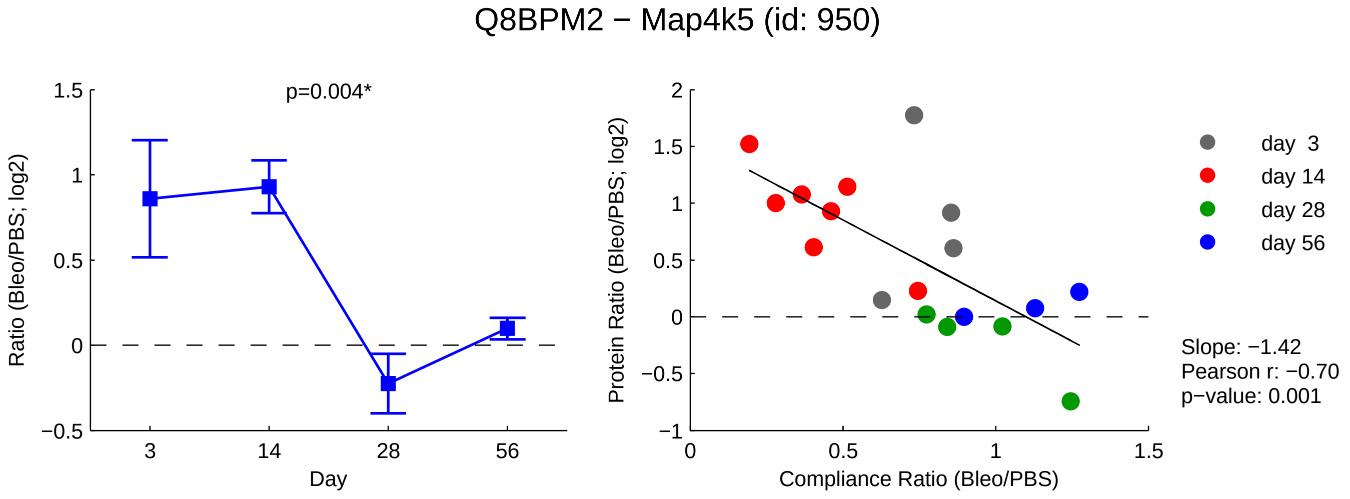

Q8BPM2 − Map4k5 (id: 950)
p=0.004*
Ratio (Bleo/PBS; log2)
Day
Protein Ratio (Bleo/PBS; log2)
Compliance Ratio (Bleo/PBS)
day 3
day 14
day 28
day 56
Slope: −1.42
Pearson r: −0.70
p−value: 0.001

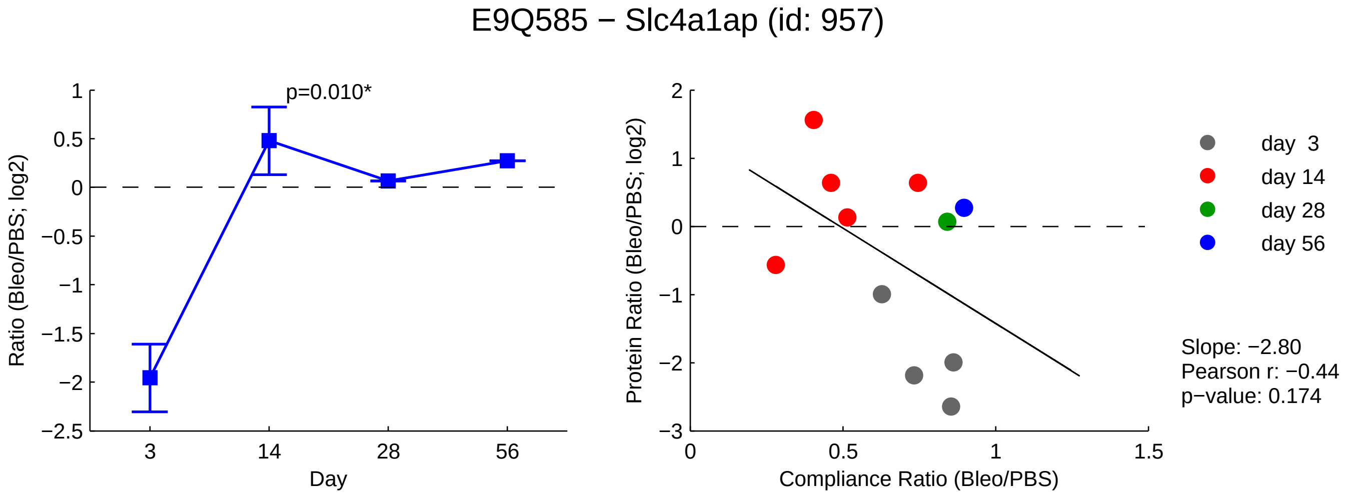

E9Q585 − Slc4a1ap (id: 957)
p=0.010*
Ratio (Bleo/PBS; log2)
Day
Protein Ratio (Bleo/PBS; log2)
Compliance Ratio (Bleo/PBS)
day 3
day 14
day 28
day 56
Slope: −2.80
Pearson r: −0.44
p−value: 0.174

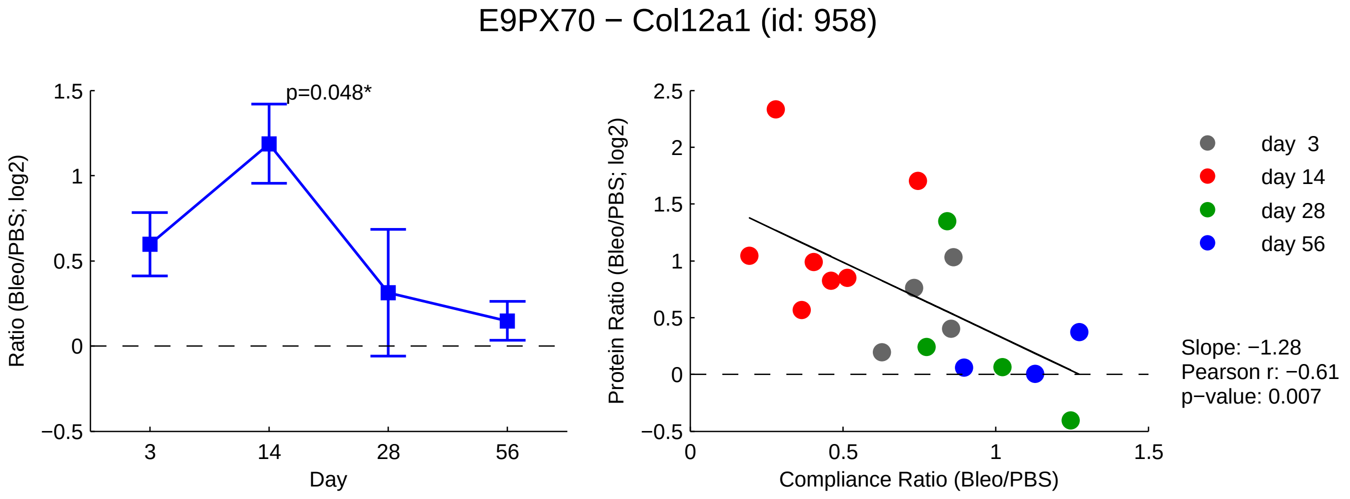

E9PX70 − Col12a1 (id: 958)
p=0.048*
Ratio (Bleo/PBS; log2)
Day
Protein Ratio (Bleo/PBS; log2)
Compliance Ratio (Bleo/PBS)
day 3
day 14
day 28
day 56
Slope: −1.28
Pearson r: −0.61
p−value: 0.007

E9Q705 − Bola3 (id: 959)

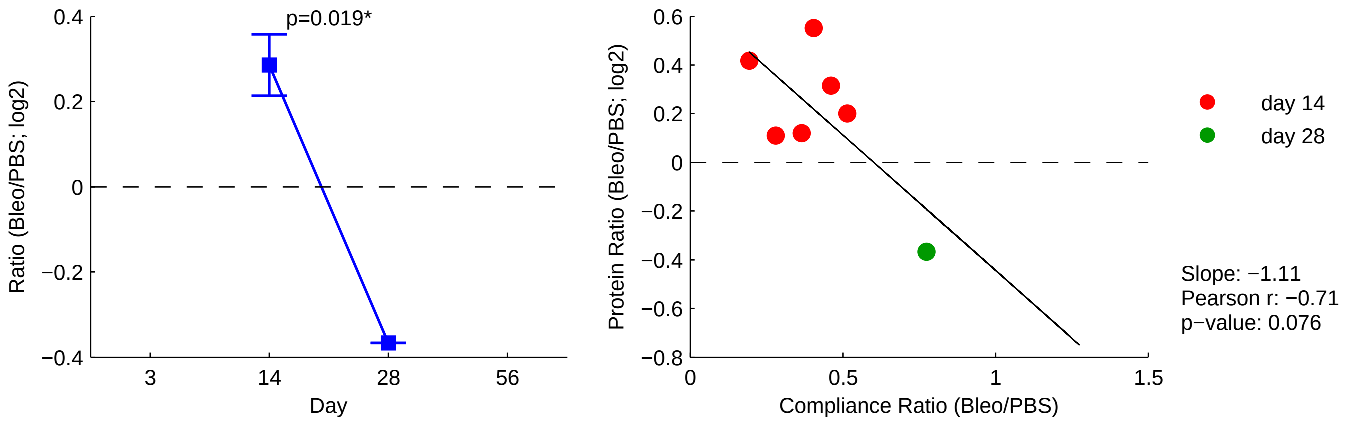

E9Q0D4 − Sbf2 (id: 965)

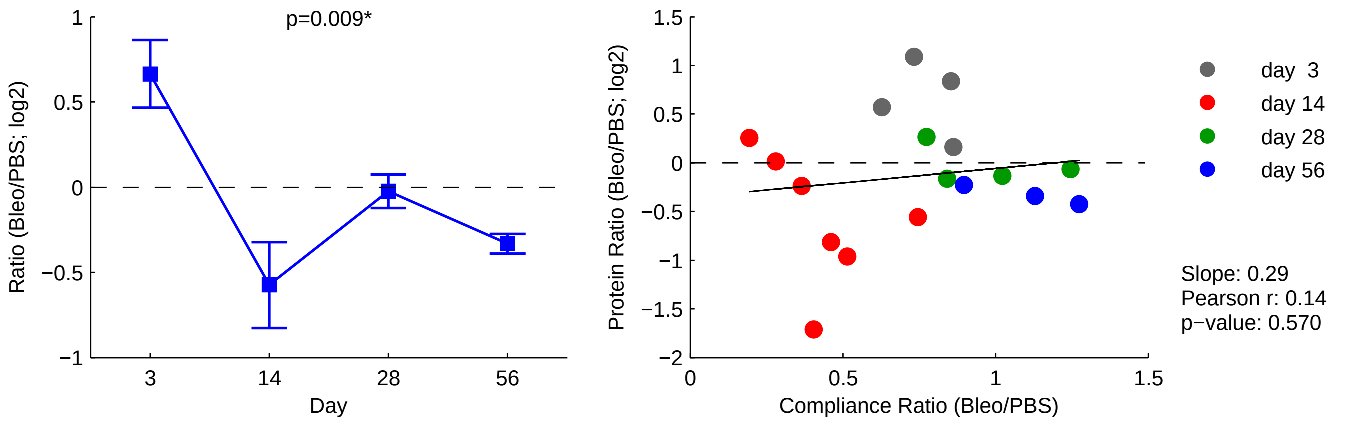

Q9Z0F8 − Adam17 (id: 968)

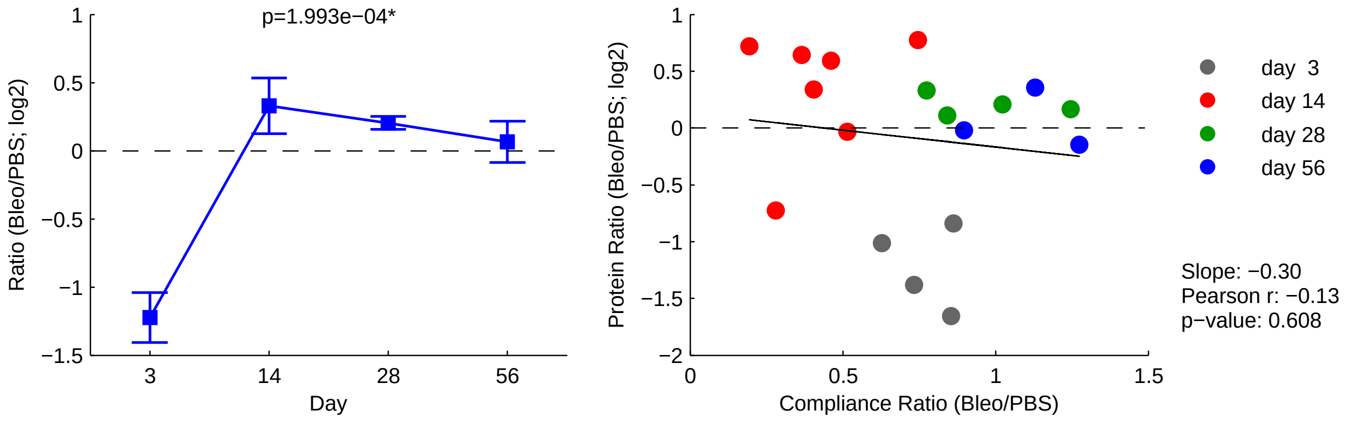

E9PXX7 − Txndc5 (id: 970)

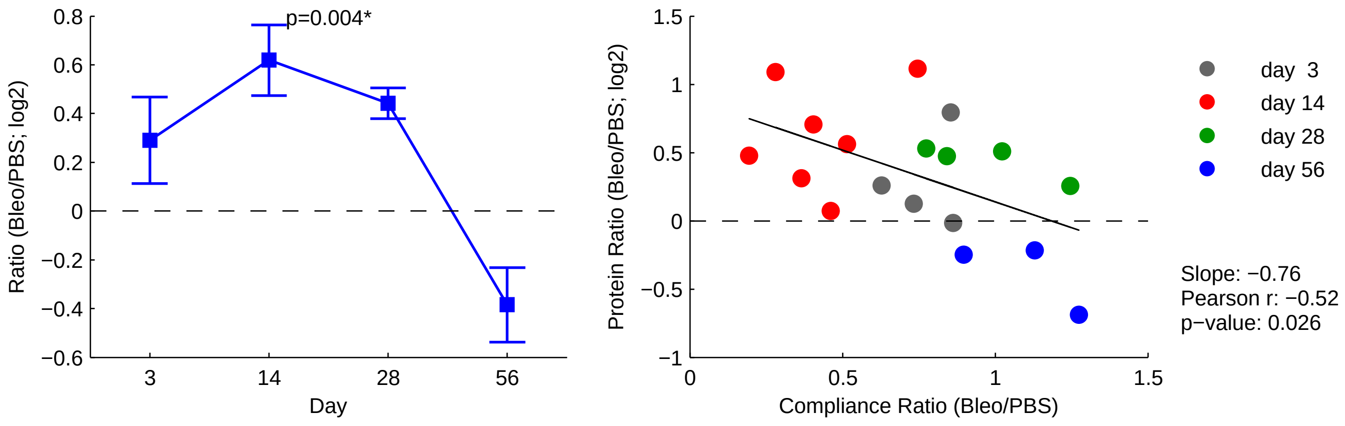

Q9WU42−2 − Ncor2 (id: 977)

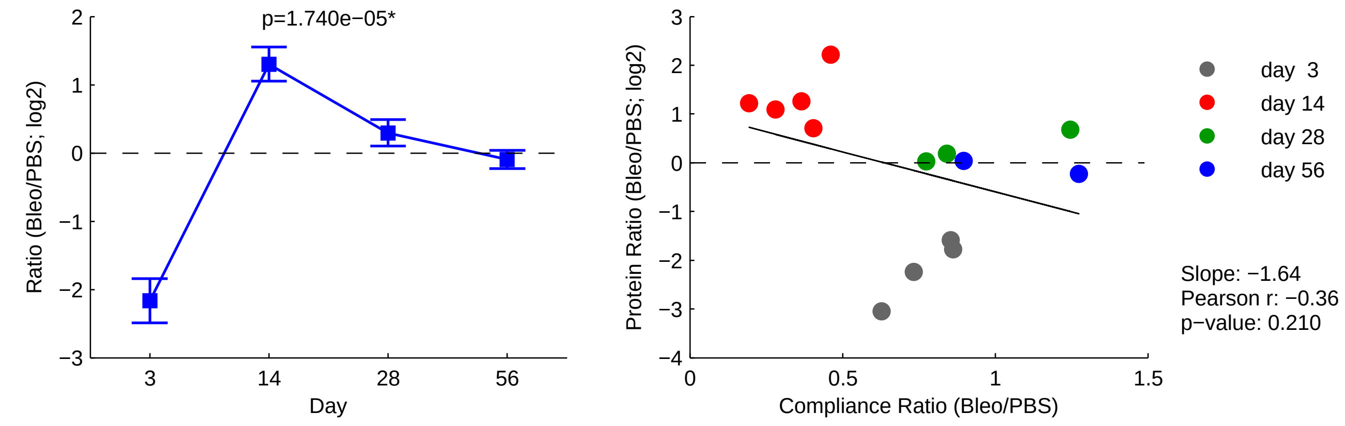

Q60649 − Clpb (id: 978)

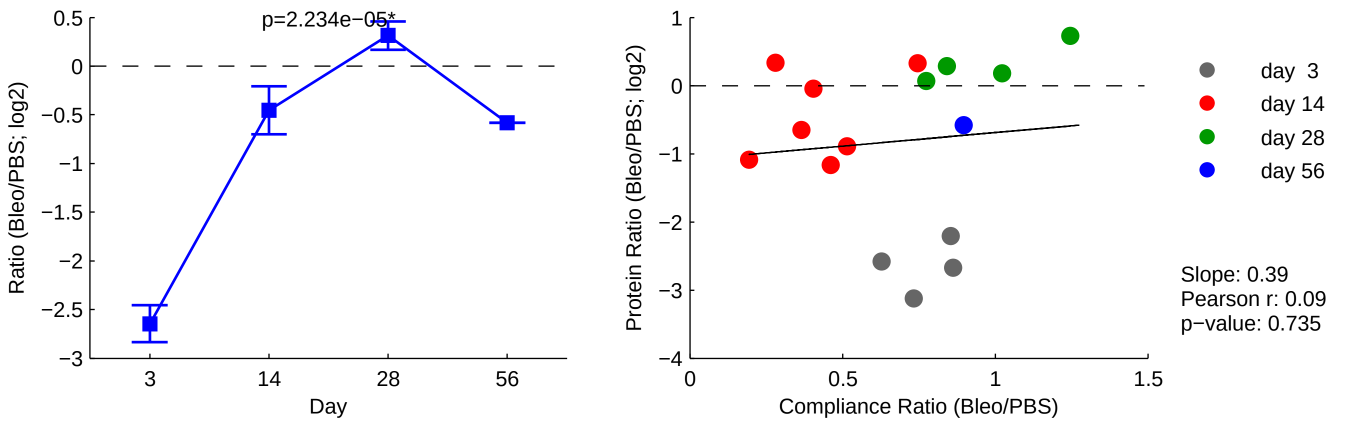

E9PYB0 − Ahnak2 (id: 980)

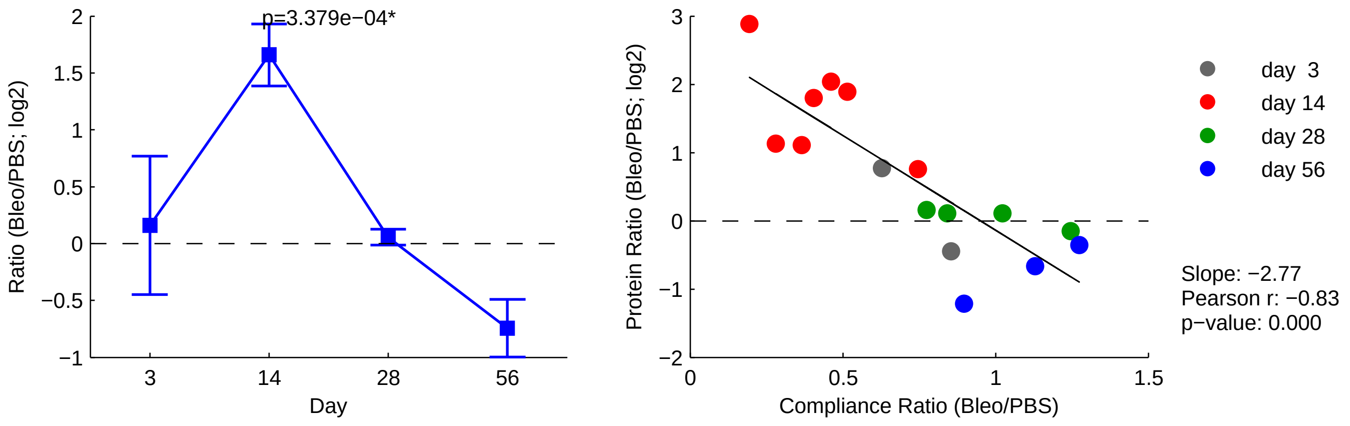

E9PYG6 − Rasa1 (id: 985)

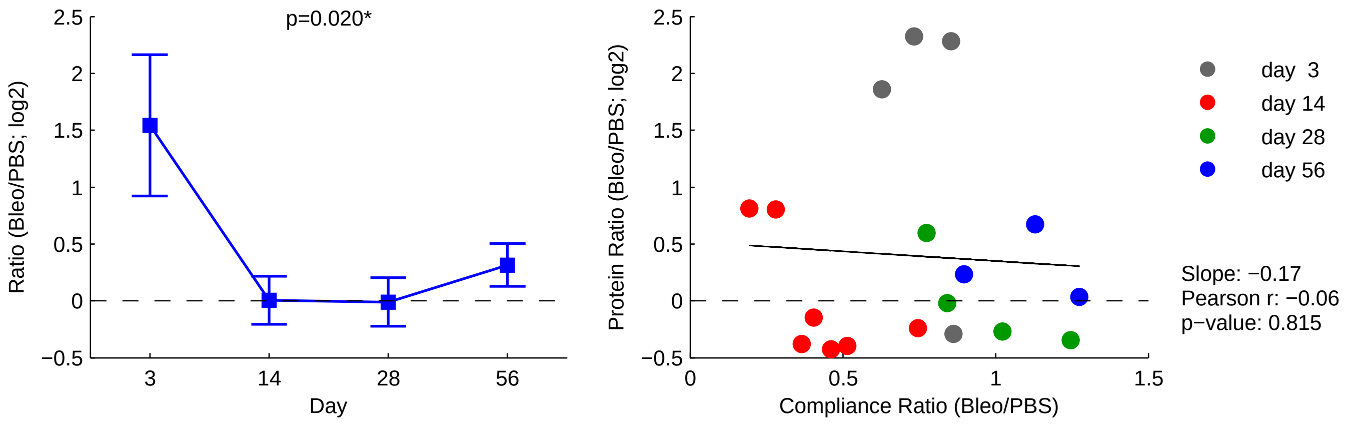

G3XA35 − Vcan (id: 986)

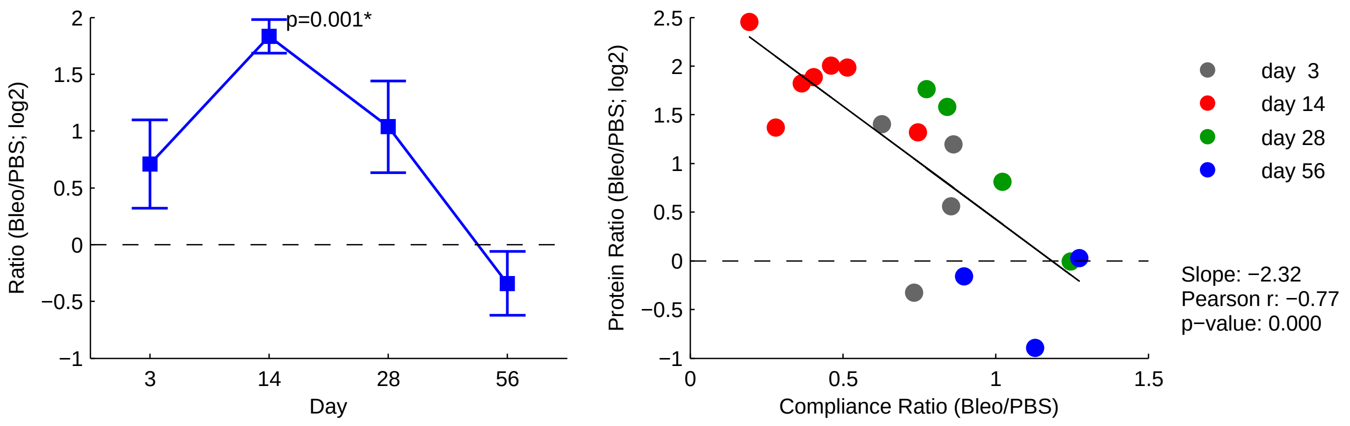

Q91V12 − Acot7 (id: 987)

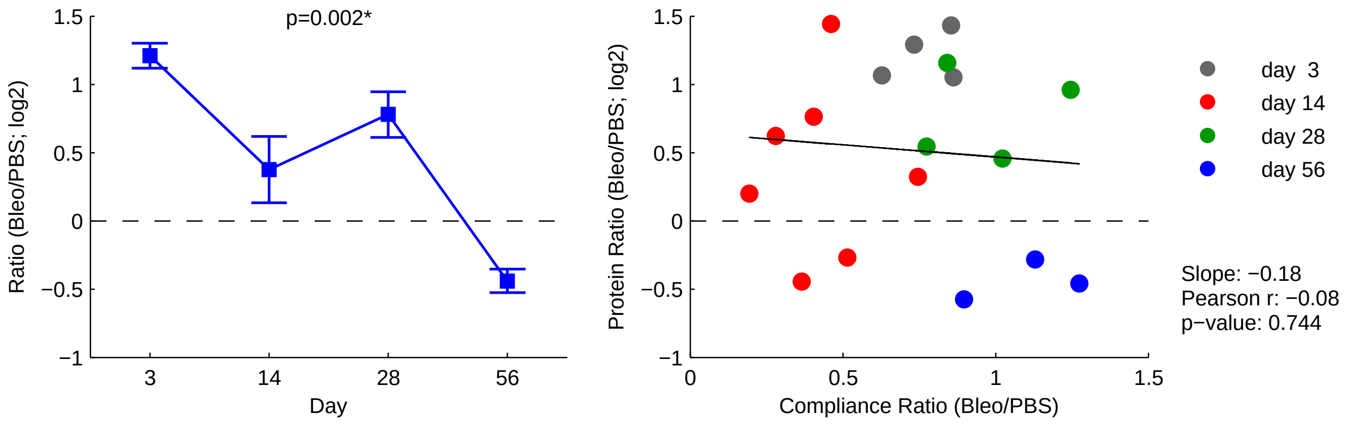

E9PYI8 − Usp14 (id: 989)

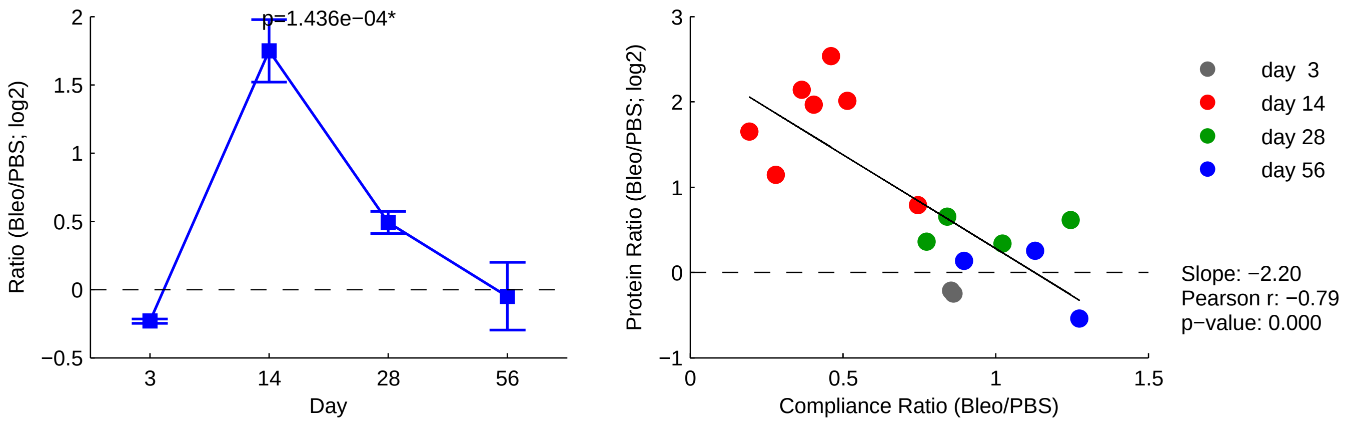

Q7TPD3−2 − Robo2 (id: 991)

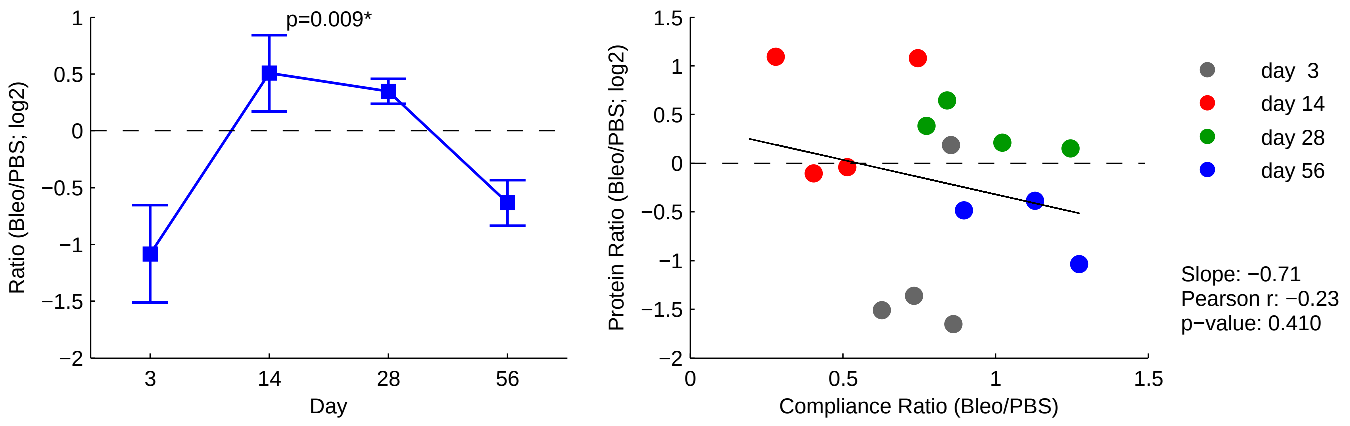

Q9CXW4 − Rpl11 (id: 993)

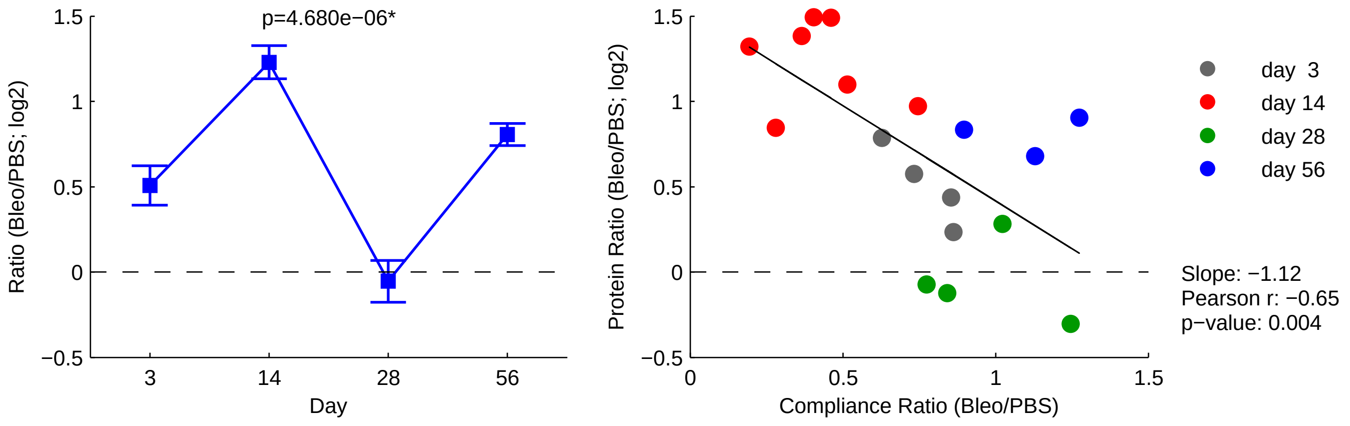

Q8R5M8−4 − Cadm1 (id: 994)

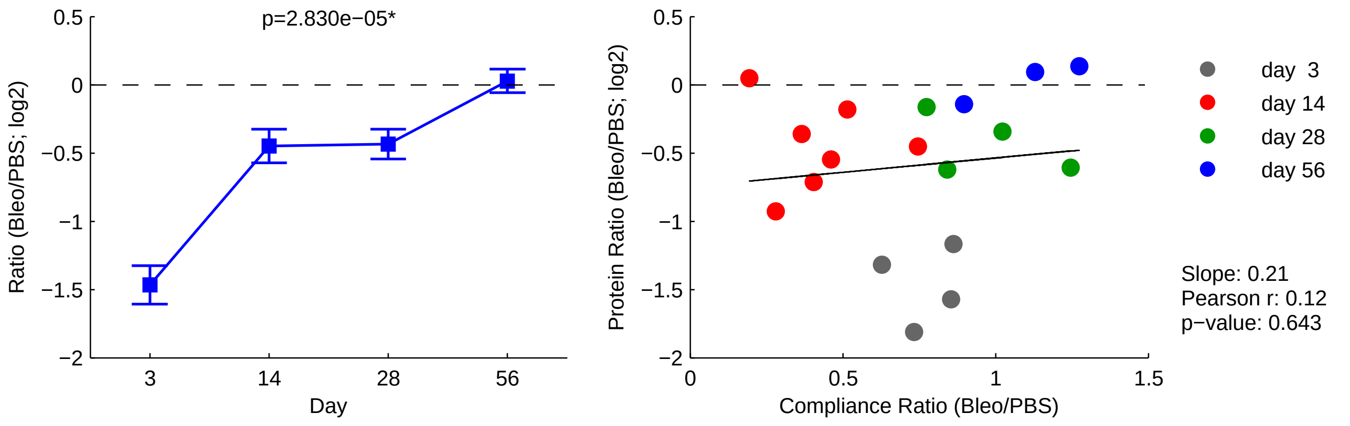

E9PYS6 − Arhgap4 (id: 995)

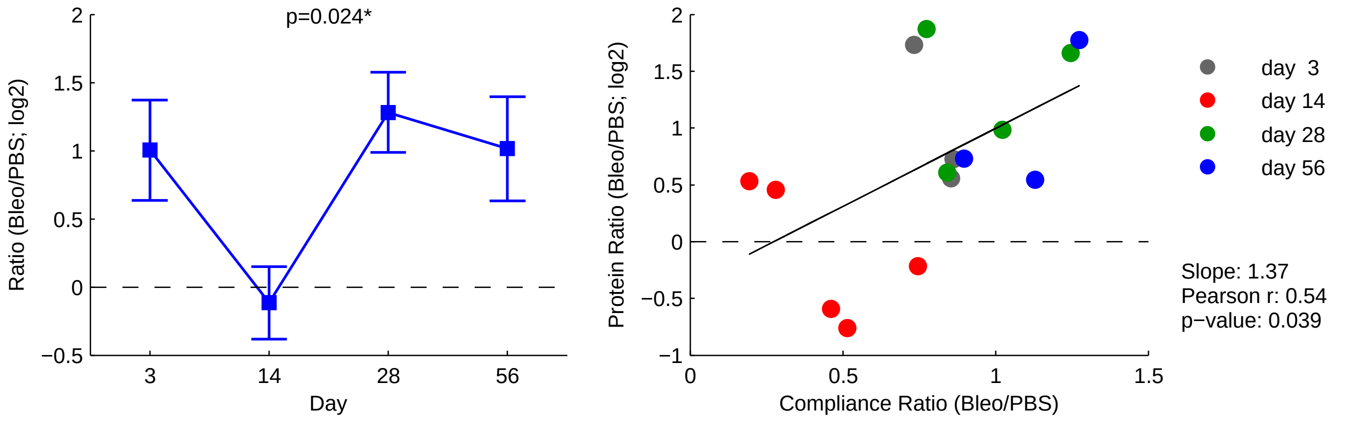

P97393 − Arhgap5 (id: 996)

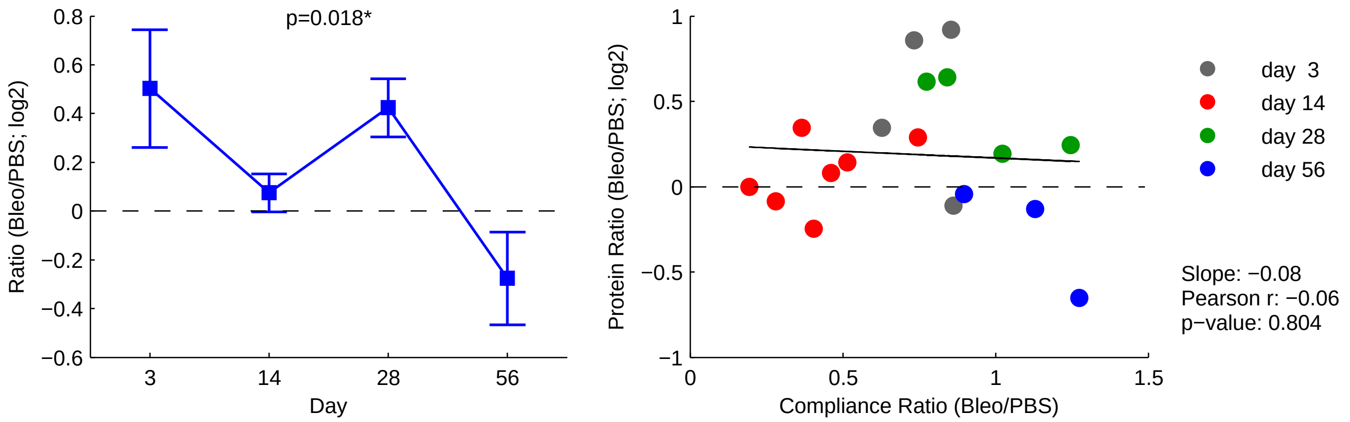

E9PZ00 − Psap (id: 998)

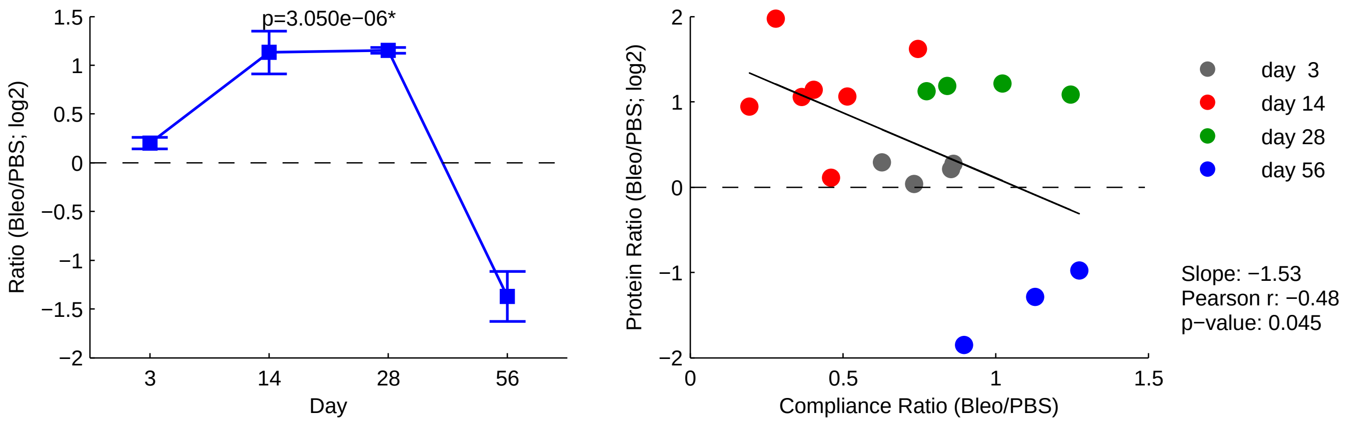

E9PZ16 − Hspg2 (id: 999)

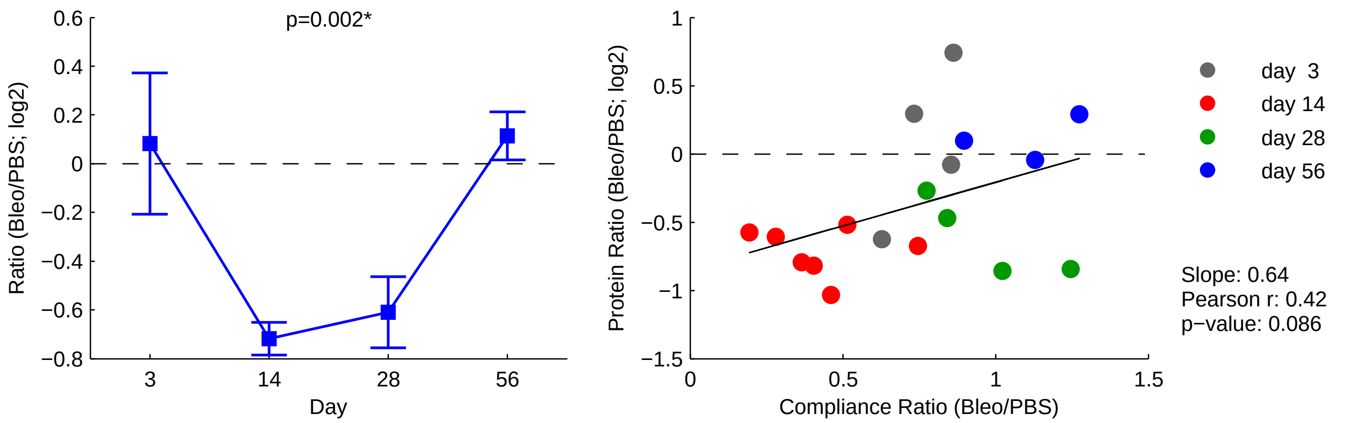

Q91W59−2 − Rbms1 (id: 1000)

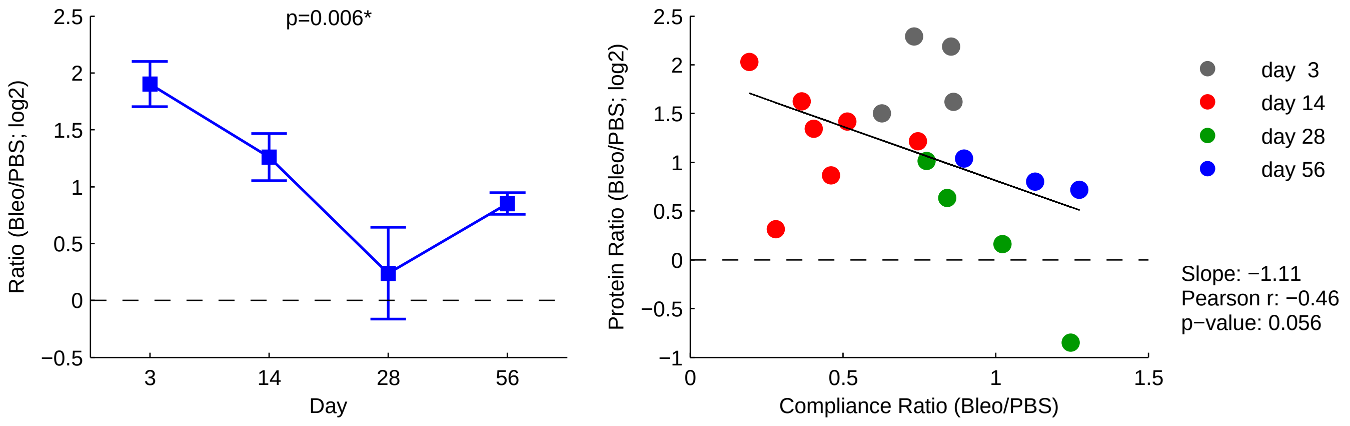

E9PZE9 − Spats2l (id: 1005)

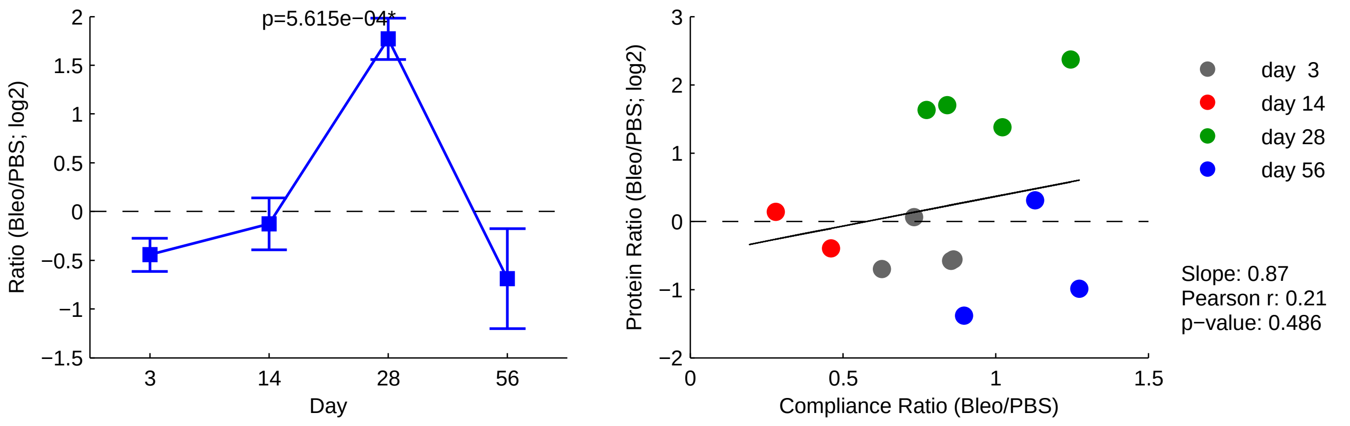

E9PZF0 − Gm20390 (id: 1006)

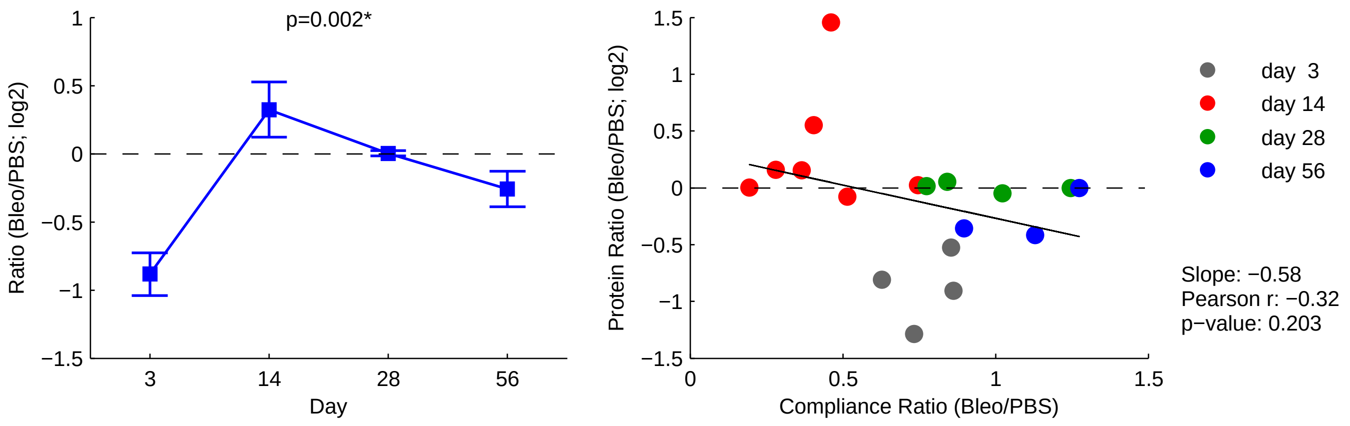

E9PZM4 − Chd2 (id: 1008)

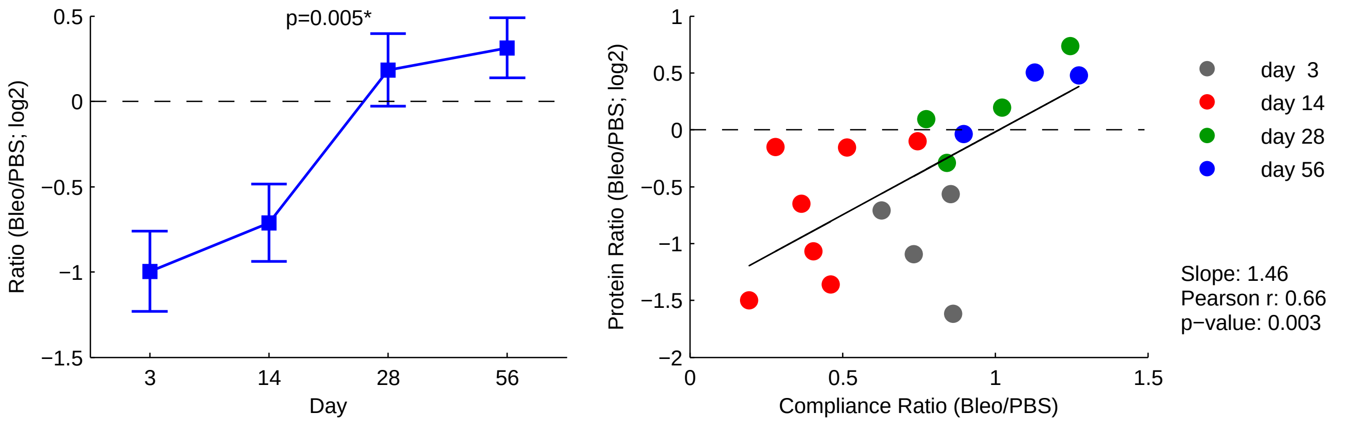

E9PZM7 − Scaf11 (id: 1009)

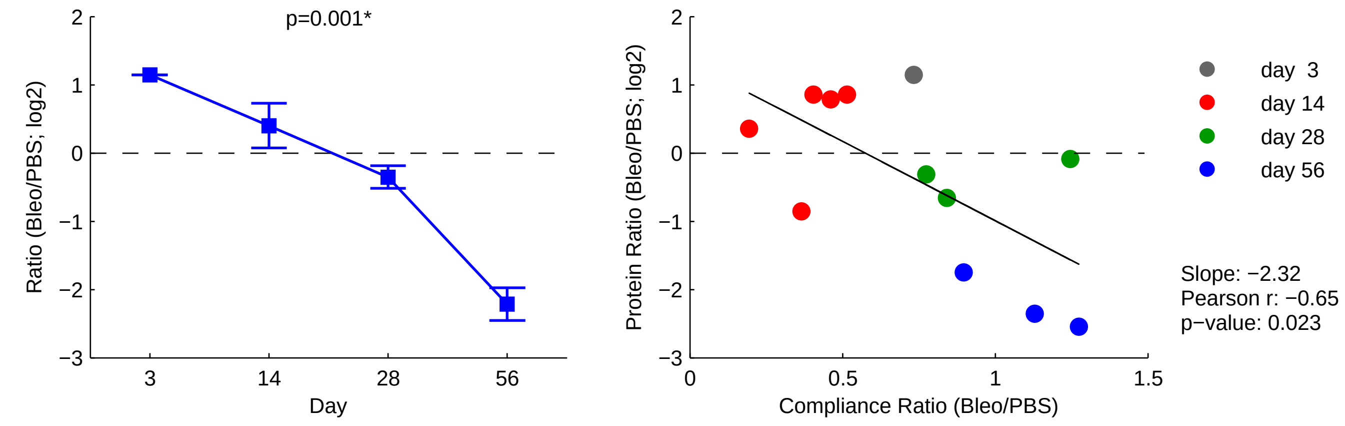

Q61387 − Cox7a2l (id: 1014)

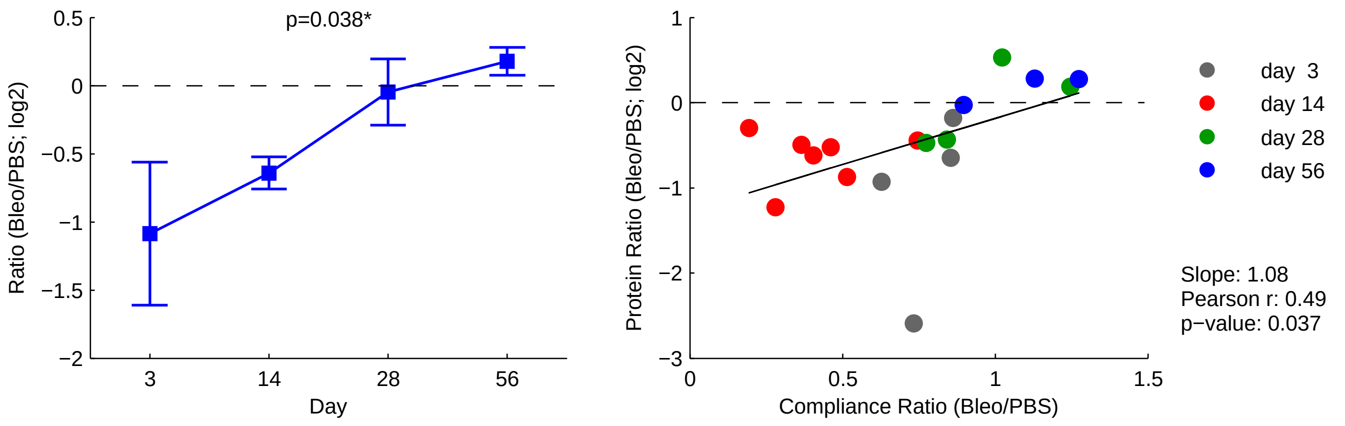

E9Q035 − Gm20425 (id: 1020)

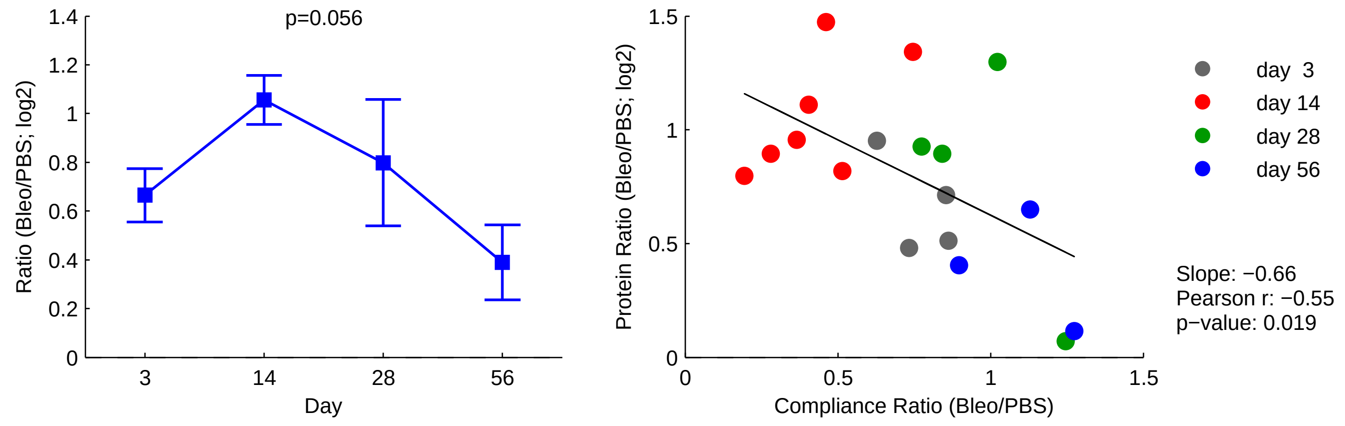

E9Q0M7 − Atp11b (id: 1029)

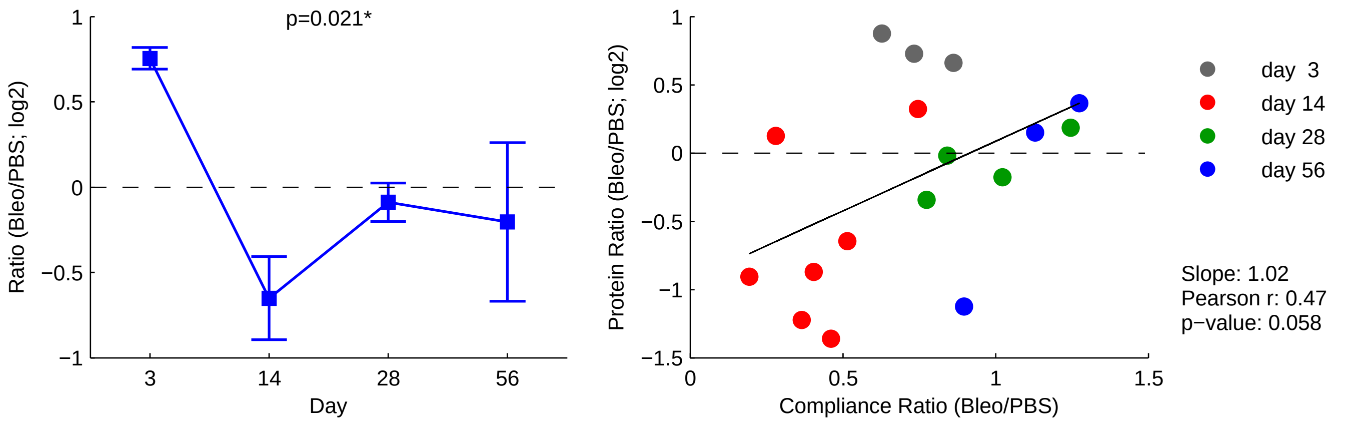

E9Q0S6 − Tns1 (id: 1031)

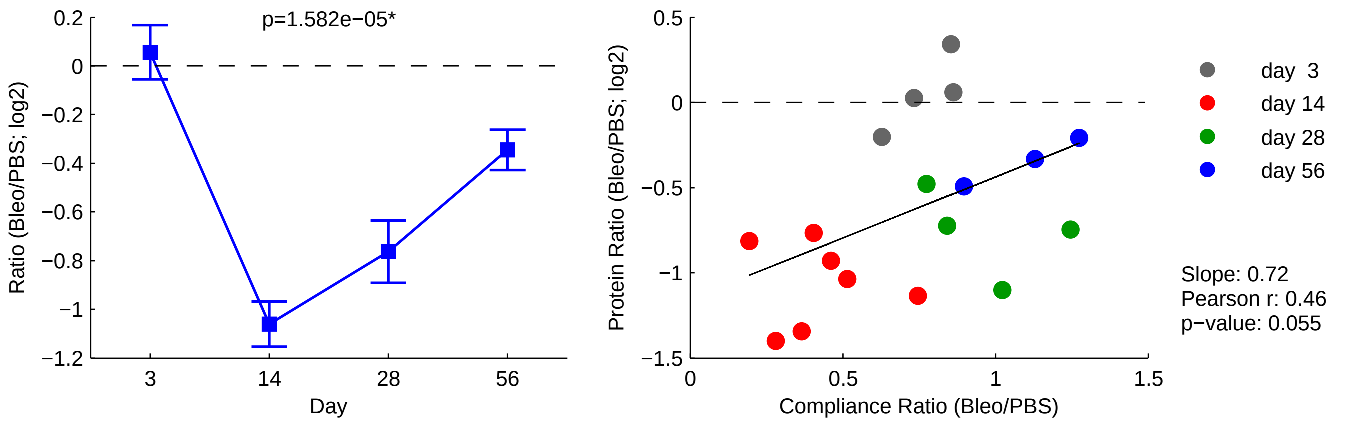

E9Q0W5 − Fam3c (id: 1032)

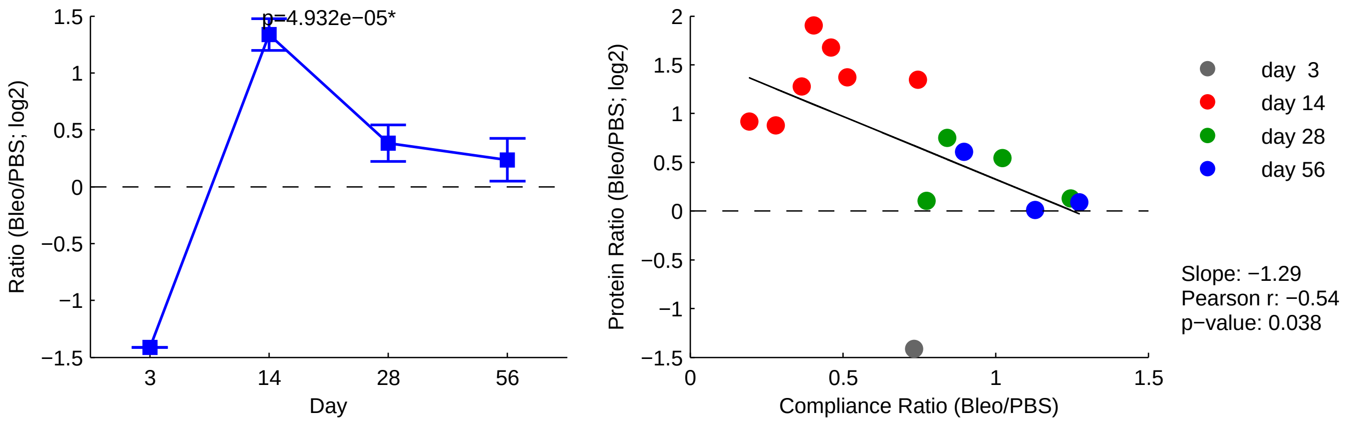

Q8BL65−2 − Ablim2 (id: 1033)

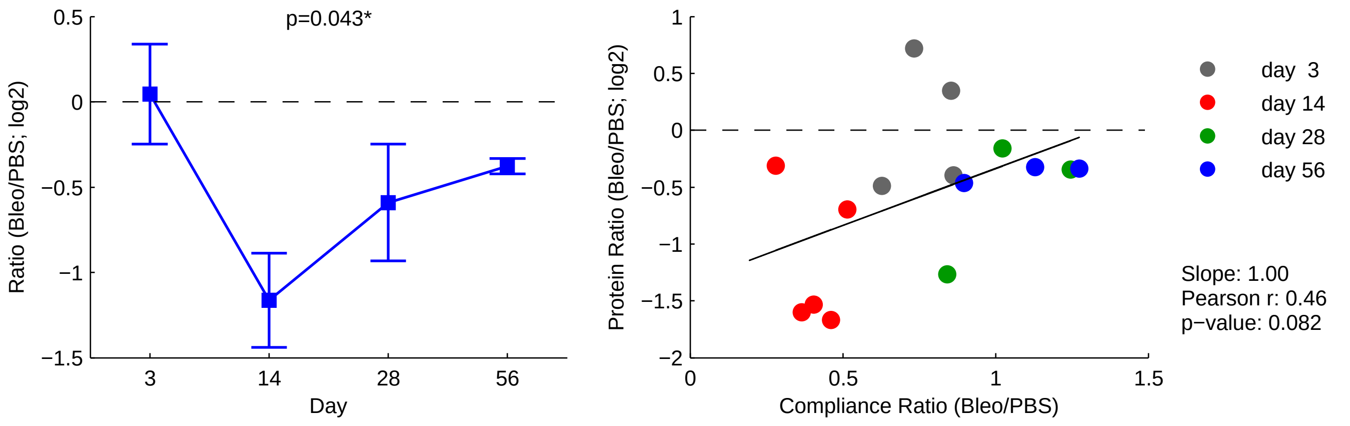

E9Q132 − Rpl24 (id: 1036)

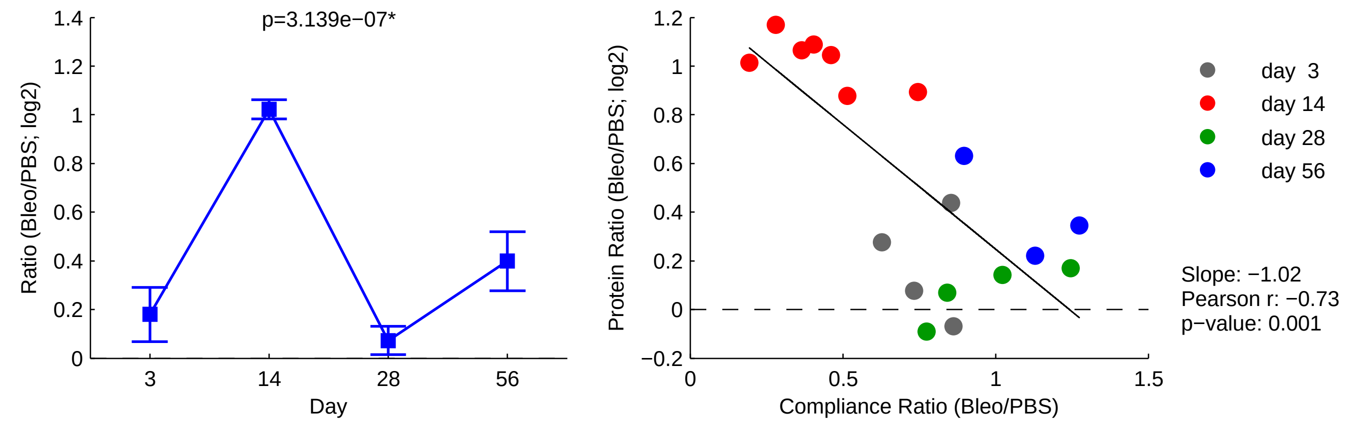

E9Q137 − Tex264 (id: 1037)

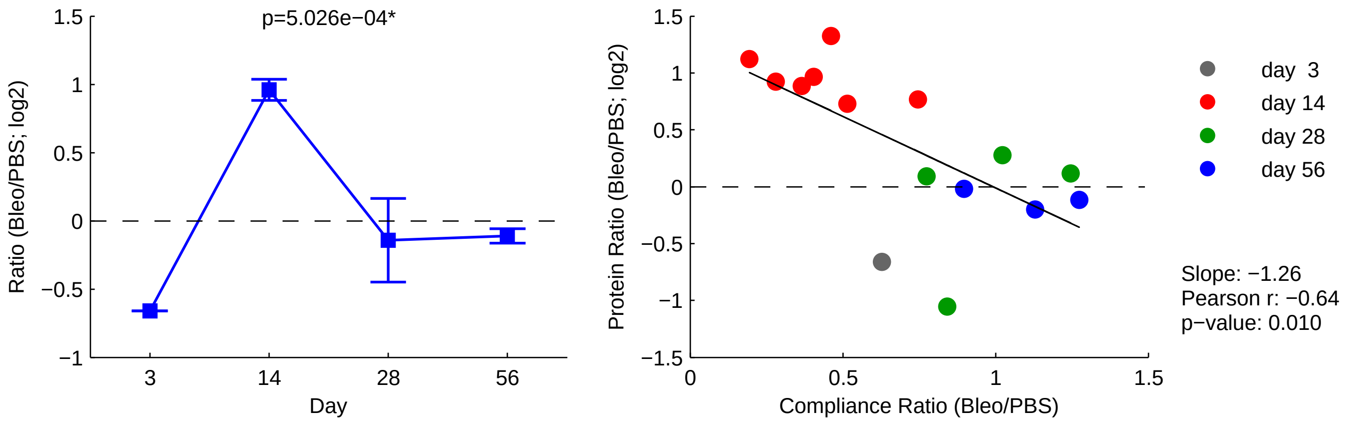

E9Q175 − Myo6 (id: 1041)

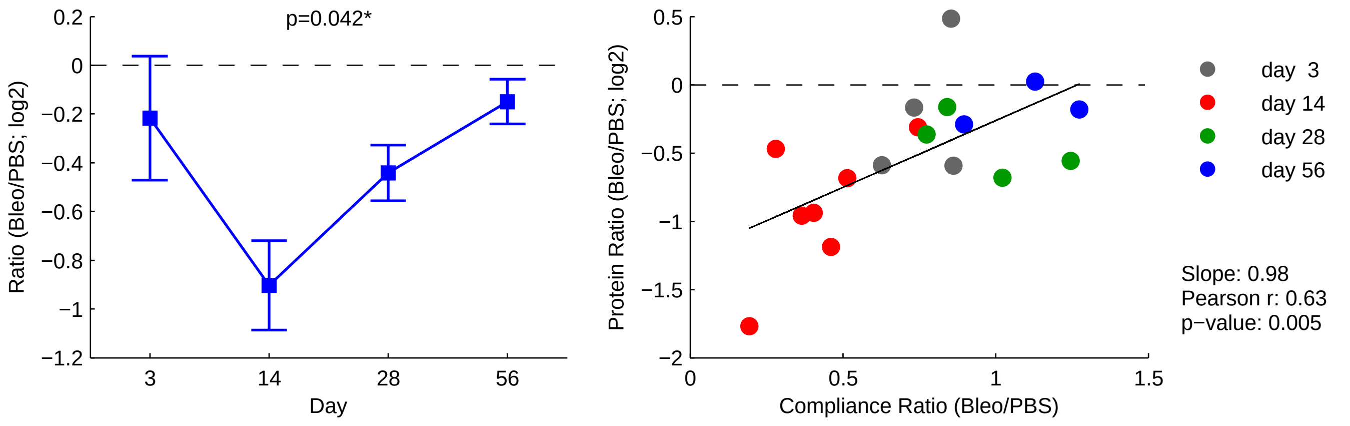

E9Q1D6 − Ltbp2 (id: 1045)

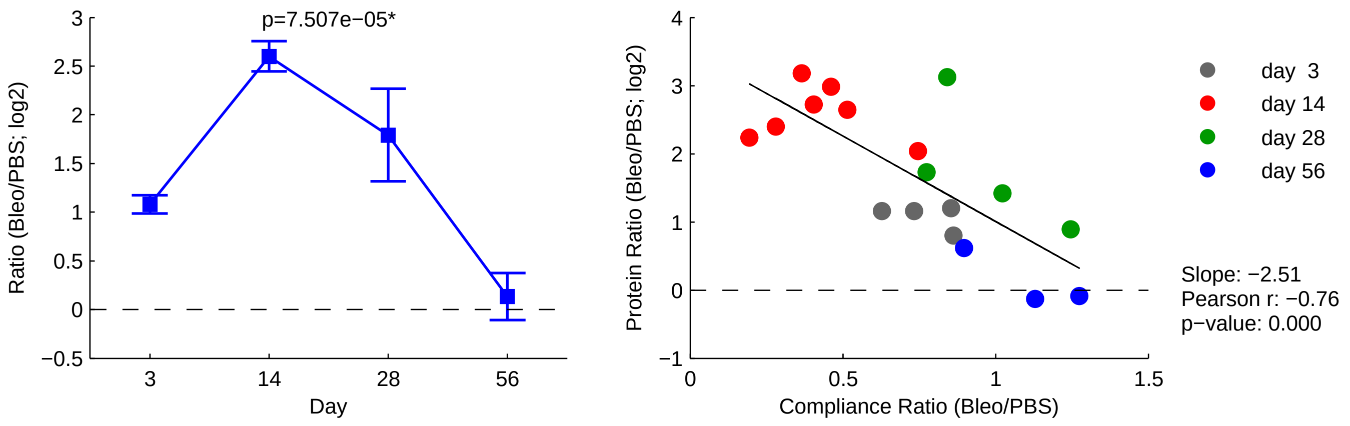

E9Q1F5 − Myo5c (id: 1047)

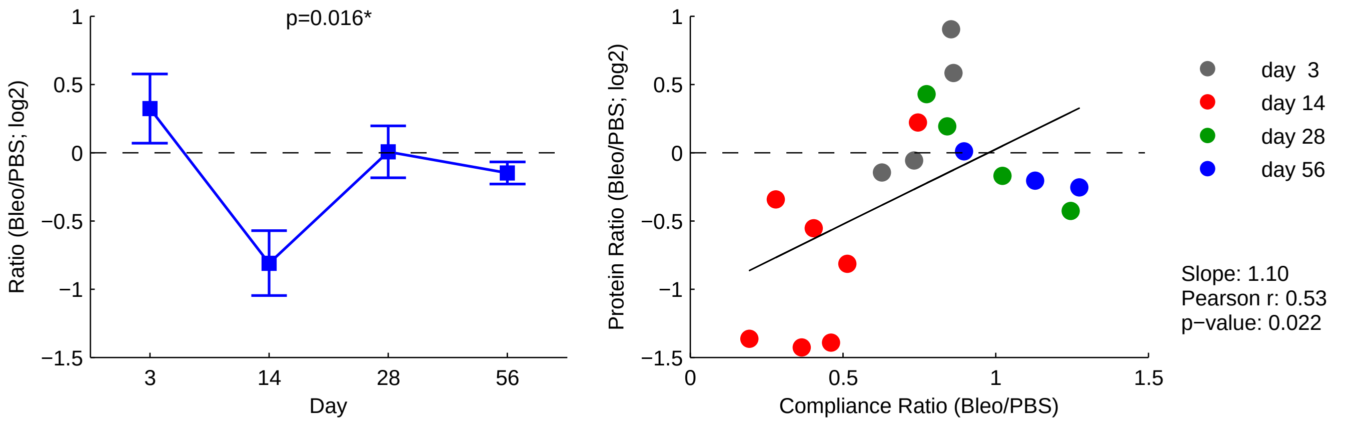

E9Q1P8 − Irf2bp2 (id: 1054)

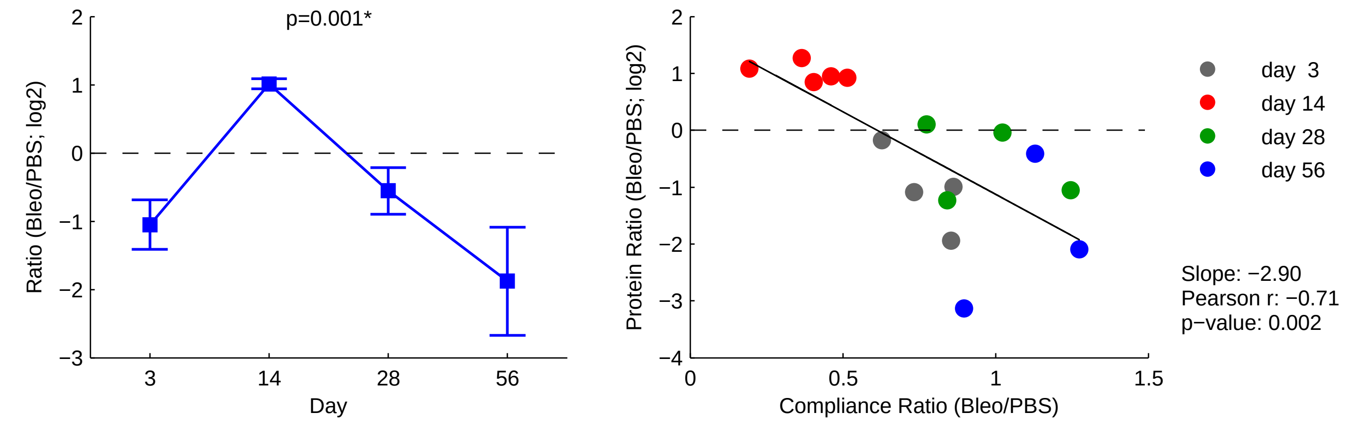

E9Q1S3 − Sec23a (id: 1055)

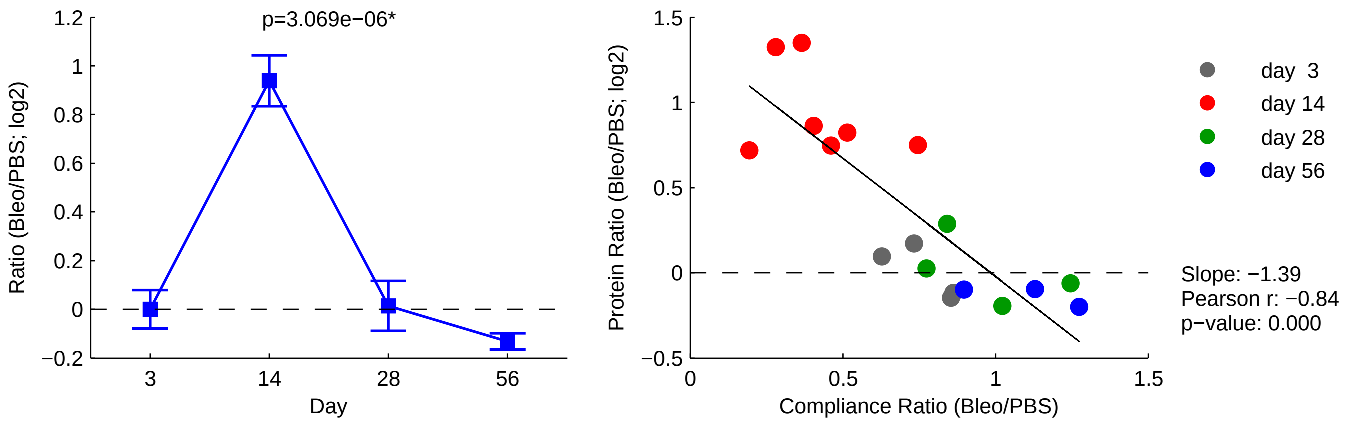

E9Q1W3 − Neb (id: 1057)

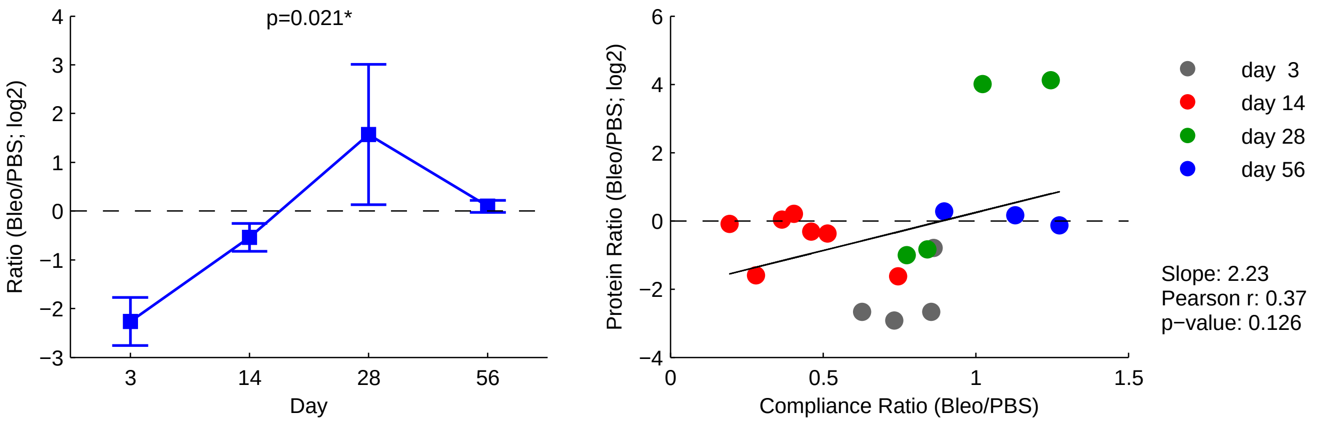

E9Q1Y3 − Apob (id: 1058)

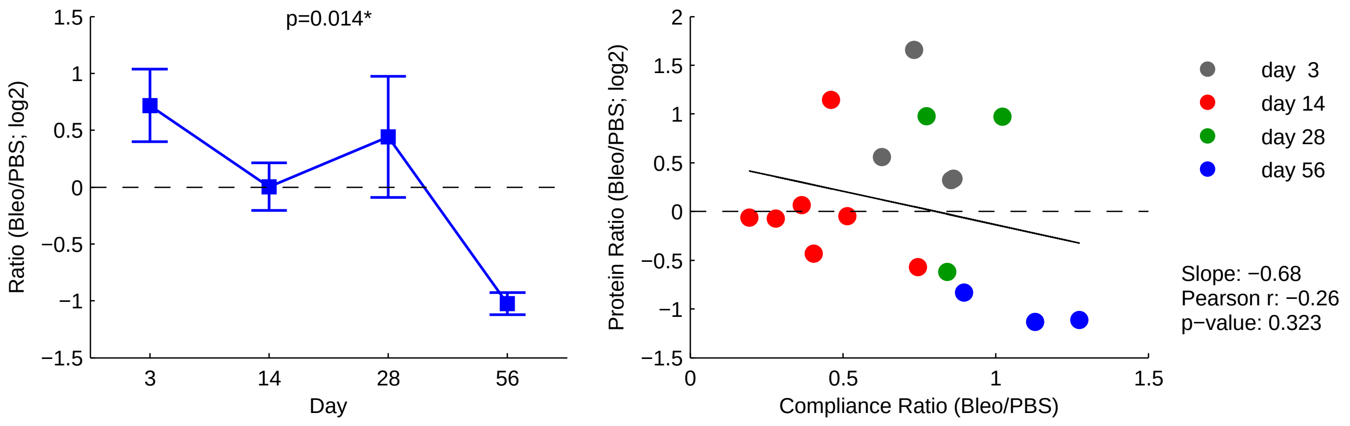

E9Q467 − Abcc4 (id: 1060)

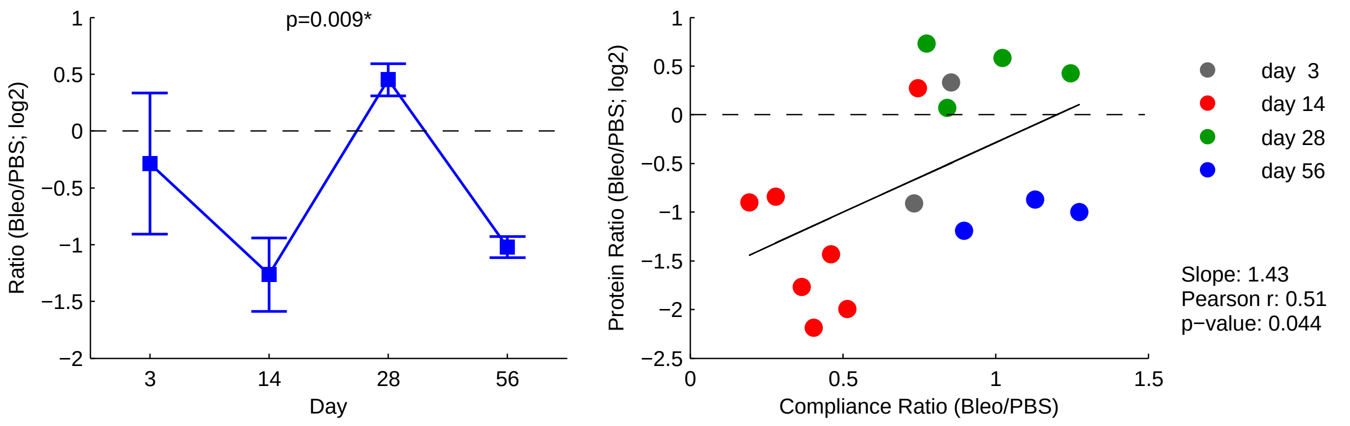

E9Q2E4 − Gm15800 (id: 1066)

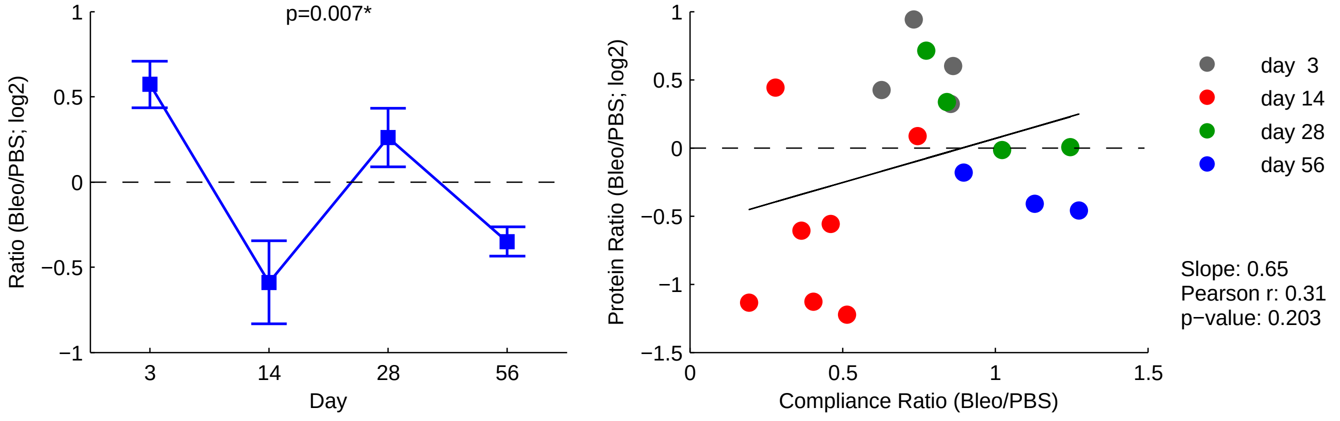

E9Q2M9 − Wdfy4 (id: 1069)

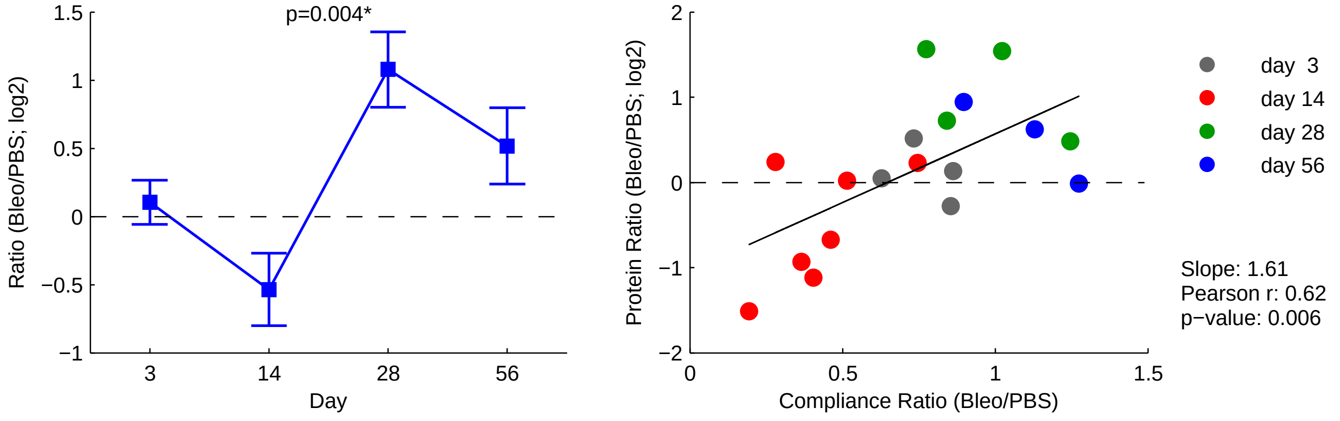

E9Q2X6 − Smc4 (id: 1074)

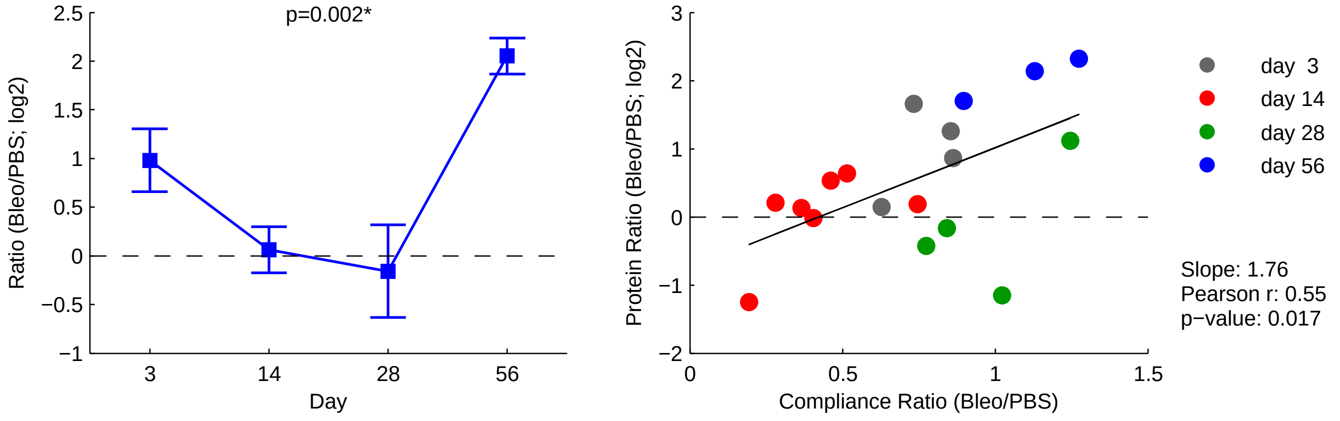

Q8VC91 − Ikbkg (id: 1075)

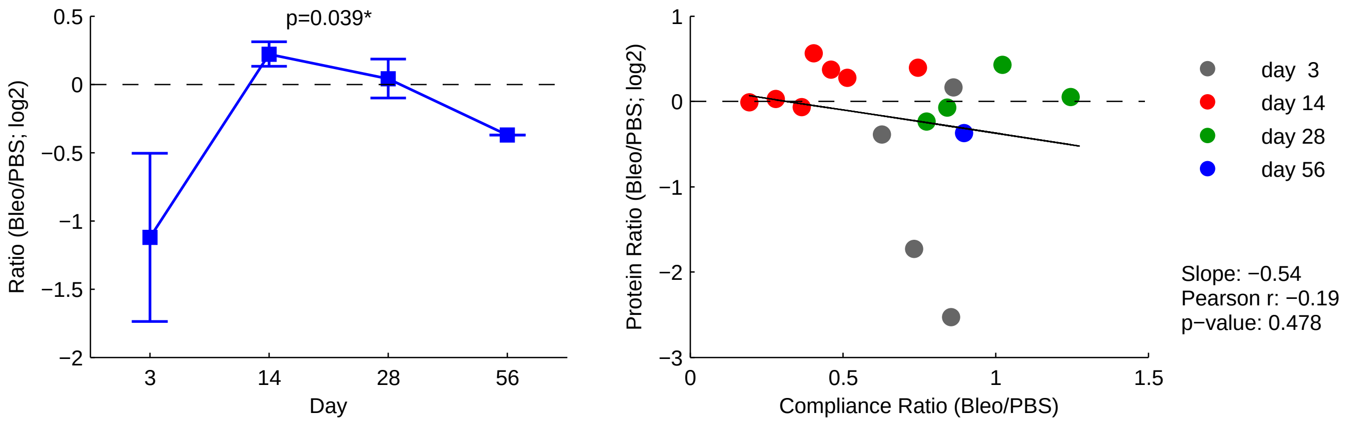

E9Q309 − Cep350 (id: 1076)

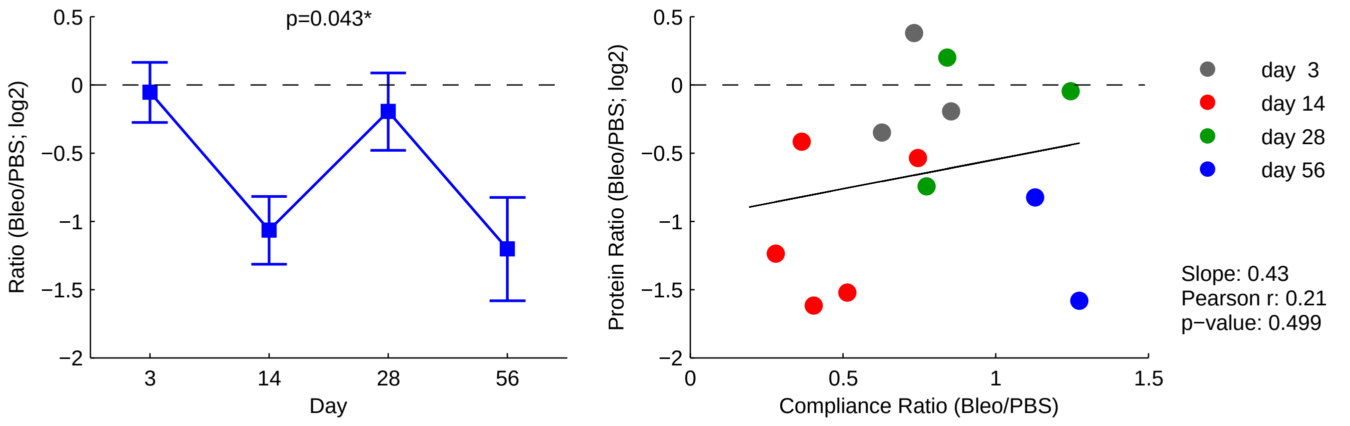

E9Q3E2 − Synpo (id: 1083)

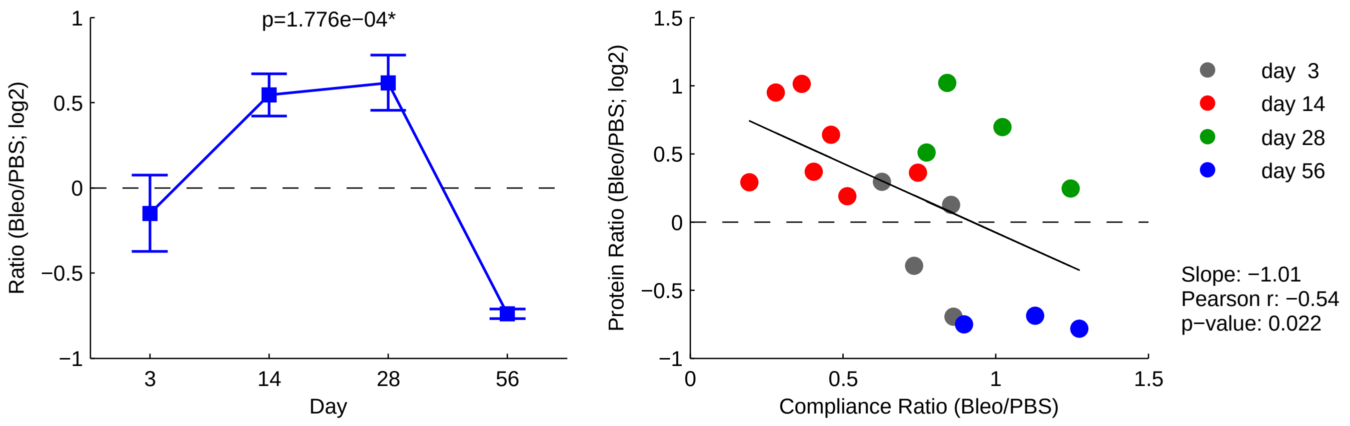

E9Q3G7 − Atp11a (id: 1084)

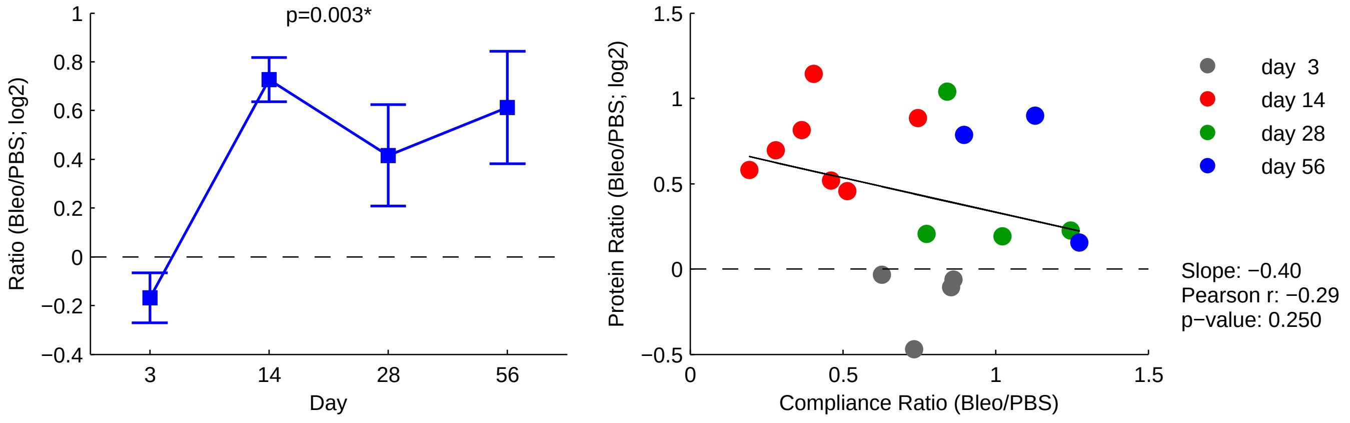

E9Q3H6 − Coq10a (id: 1086)

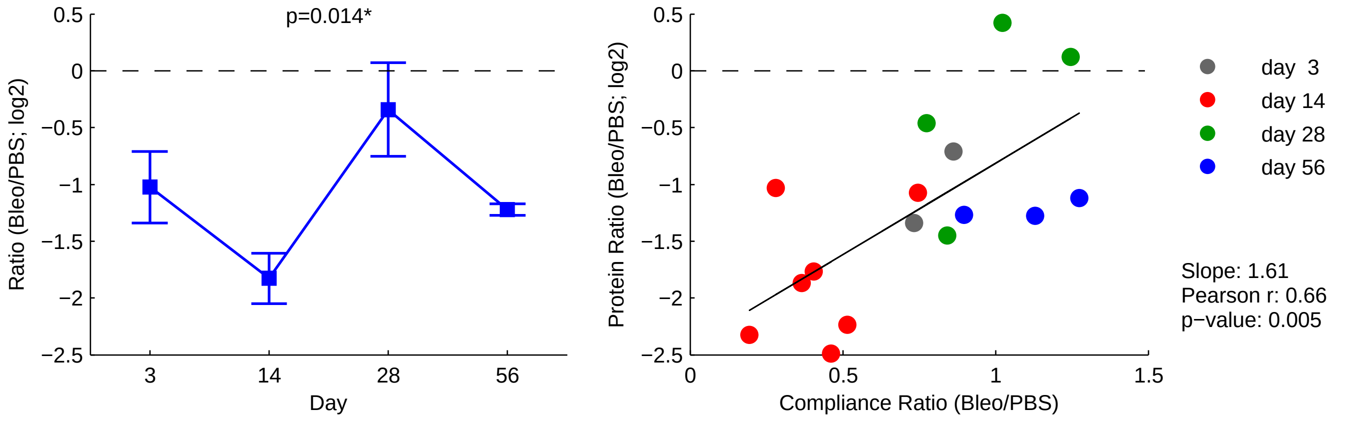

E9Q3K3 − Agap1 (id: 1087)

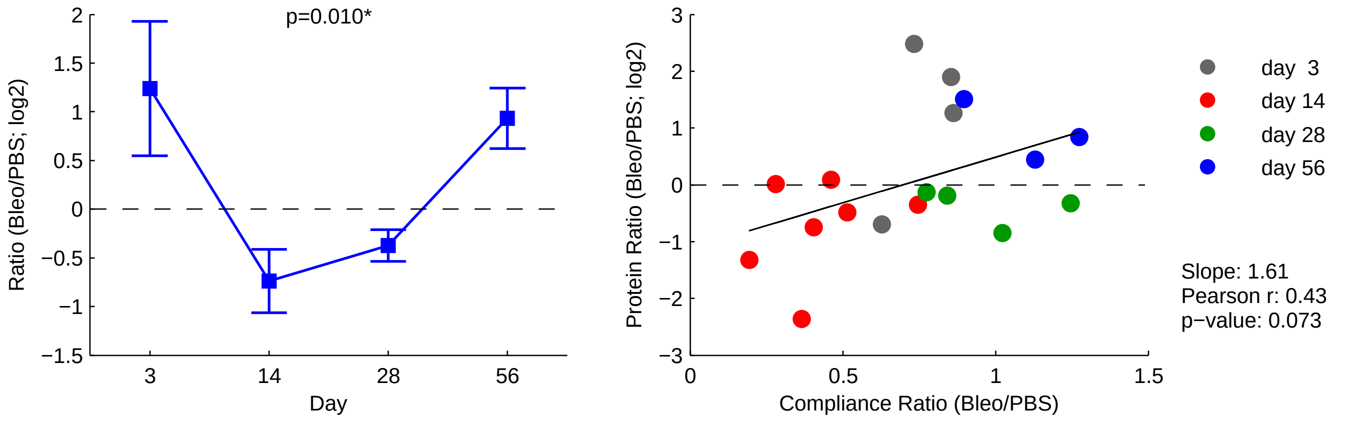

E9Q3L2 − Pi4ka (id: 1088)

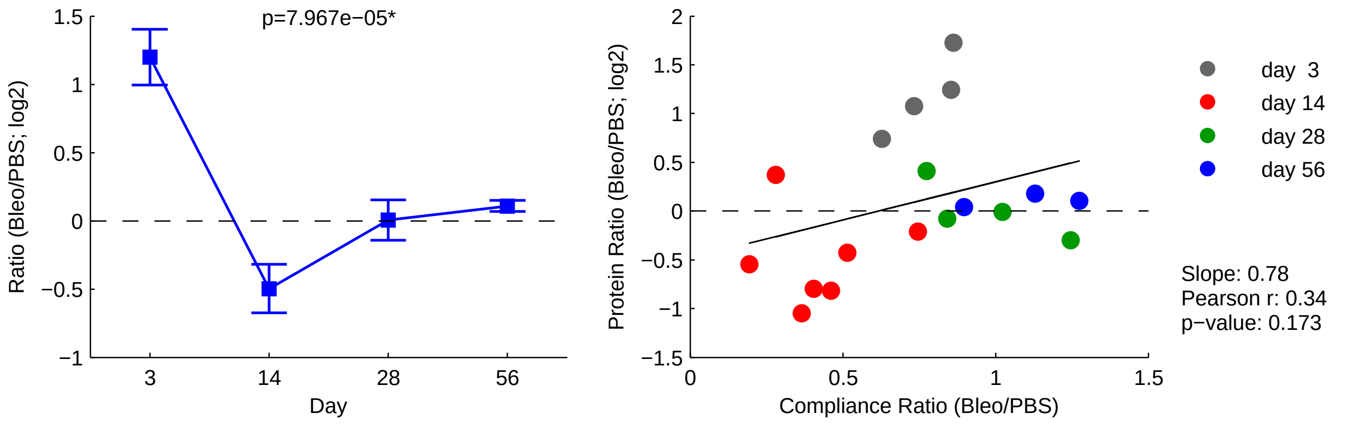

E9Q3M0 − Smad2 (id: 1089)

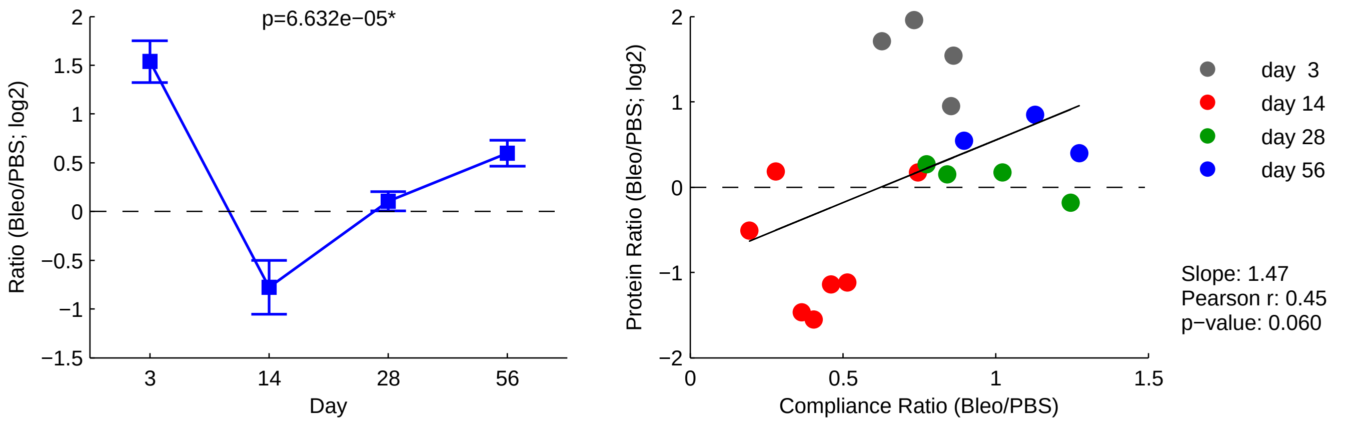

E9Q586 − Dctn1 (id: 1090)

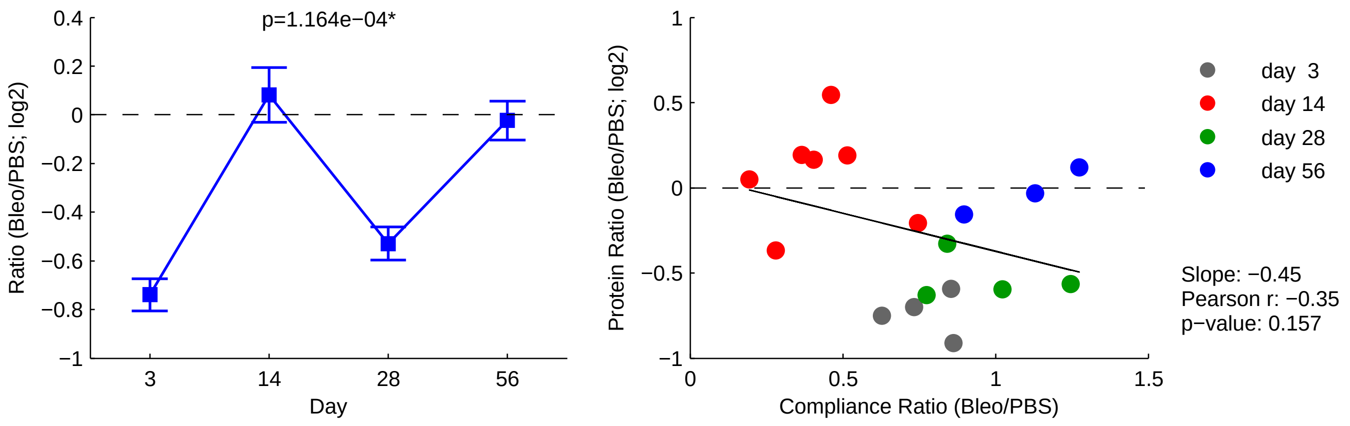

E9Q3N9 − Efemp2 (id: 1093)

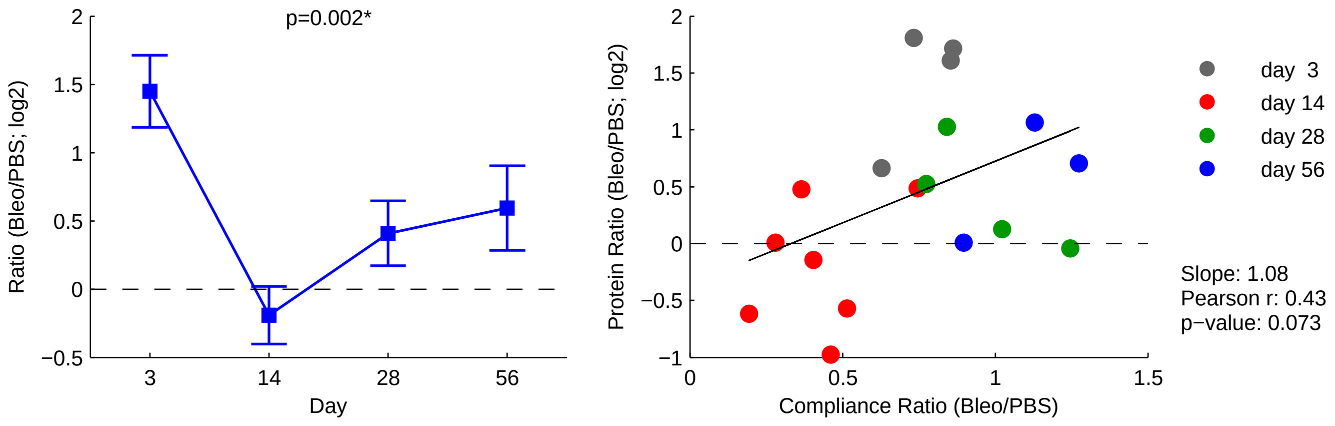

E9Q3Q6 − Alcam (id: 1094)

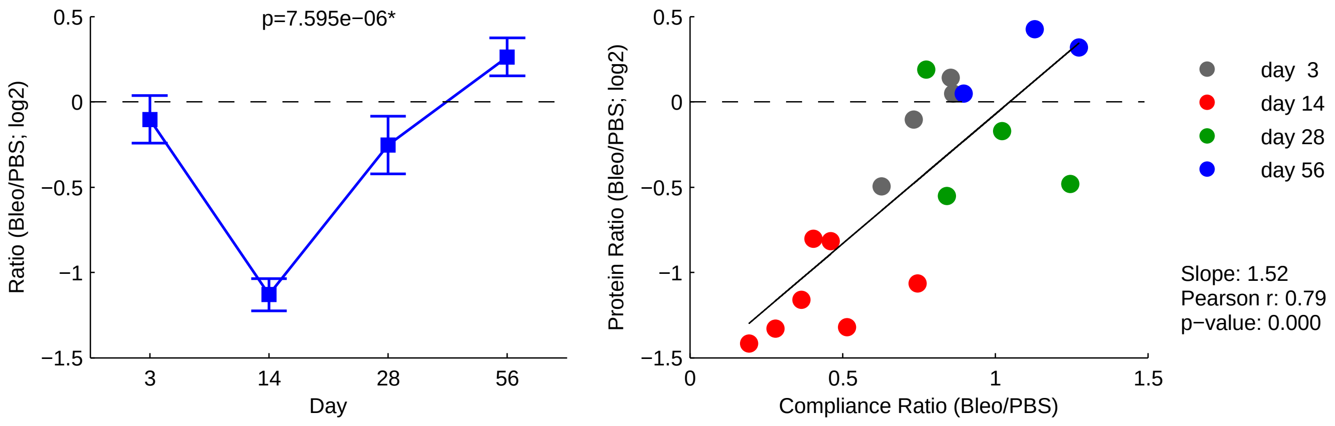

E9Q3Y4 − Lrba (id: 1102)

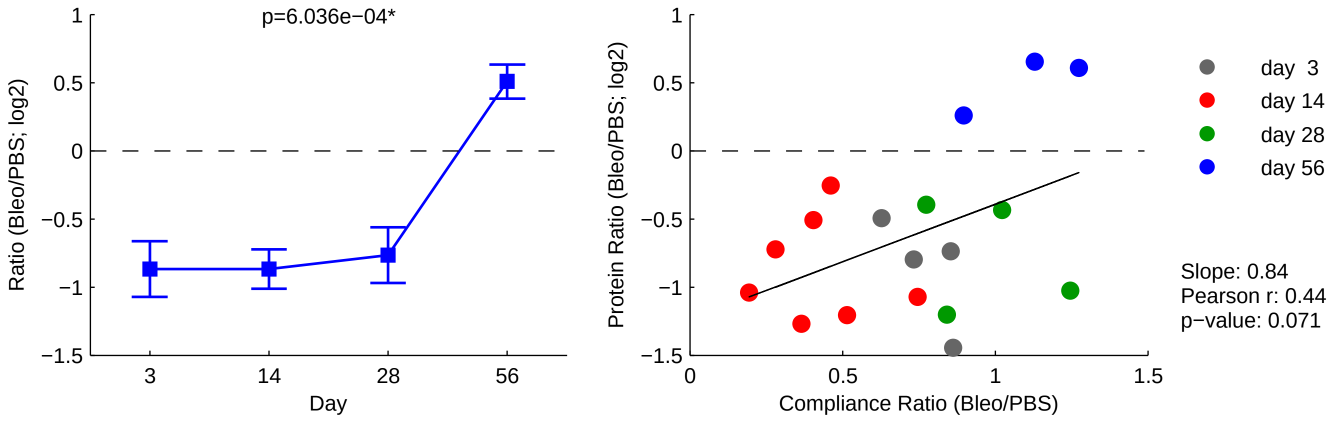

E9Q3Z5 − Svil (id: 1103)

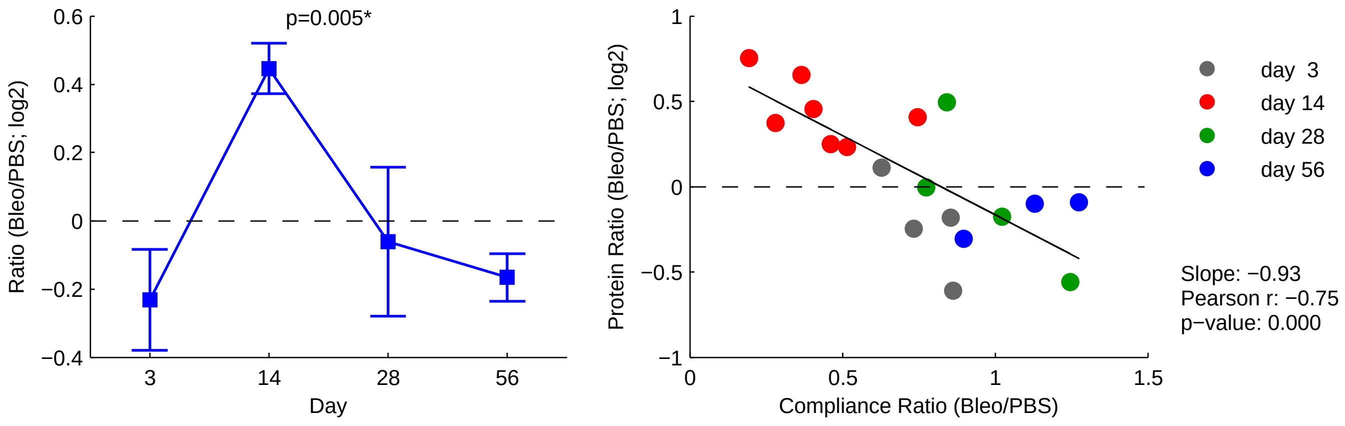

F6U7V1 − Ryr2 (id: 1104)

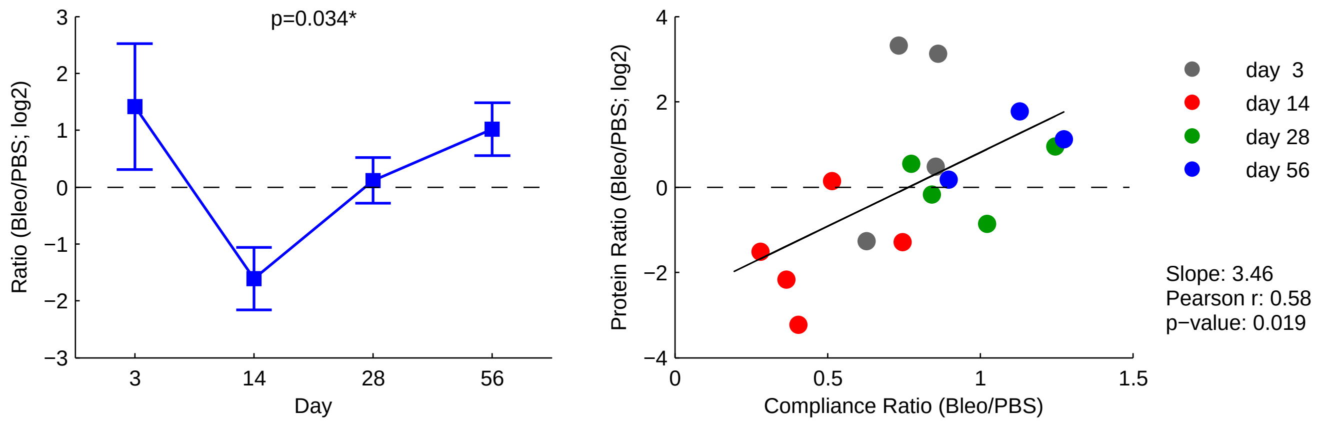

E9Q411 − Nbas (id: 1105)

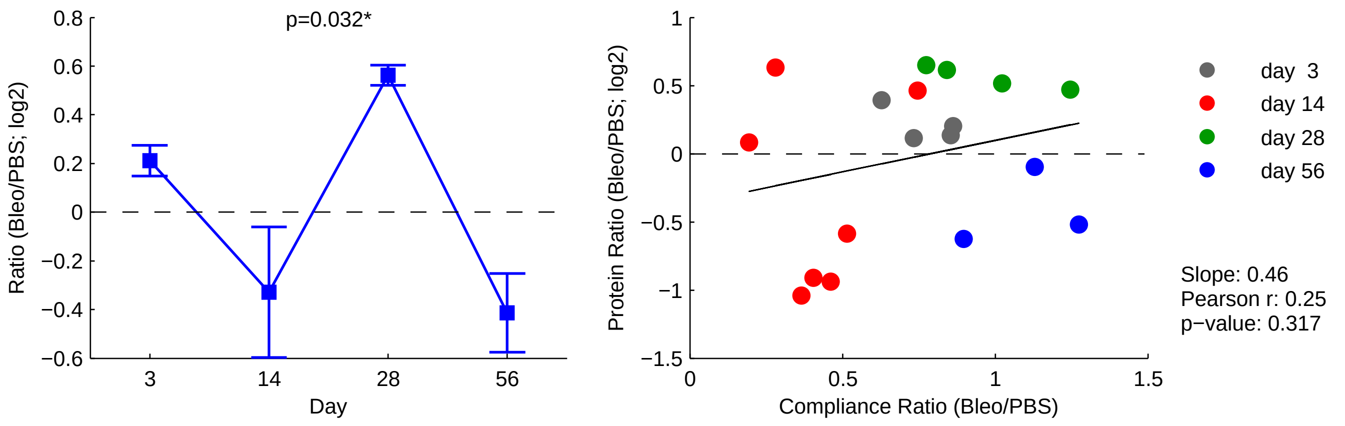

P08101−2 − Fcgr2b (id: 1107)

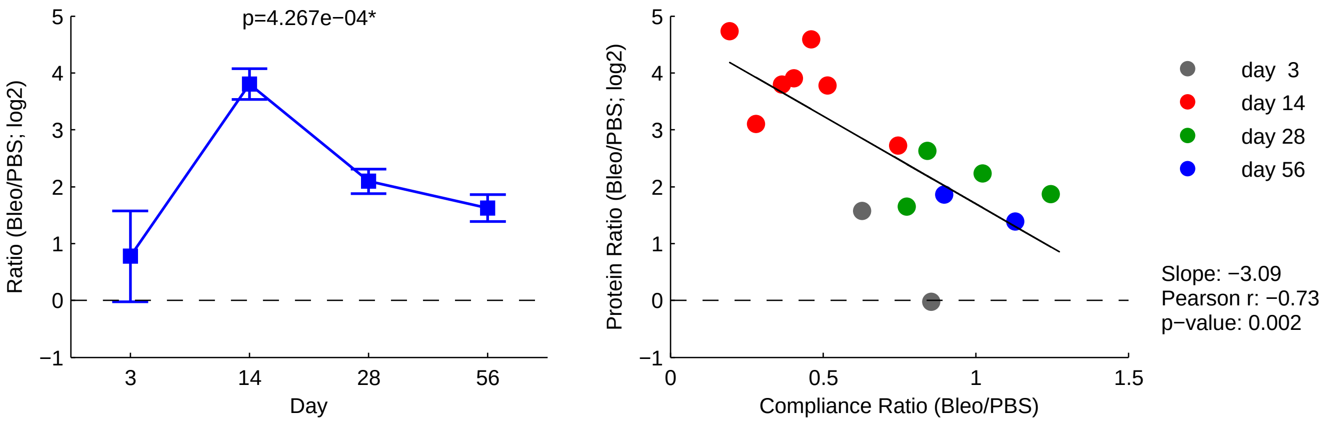

E9Q448 − Tpm1 (id: 1110)

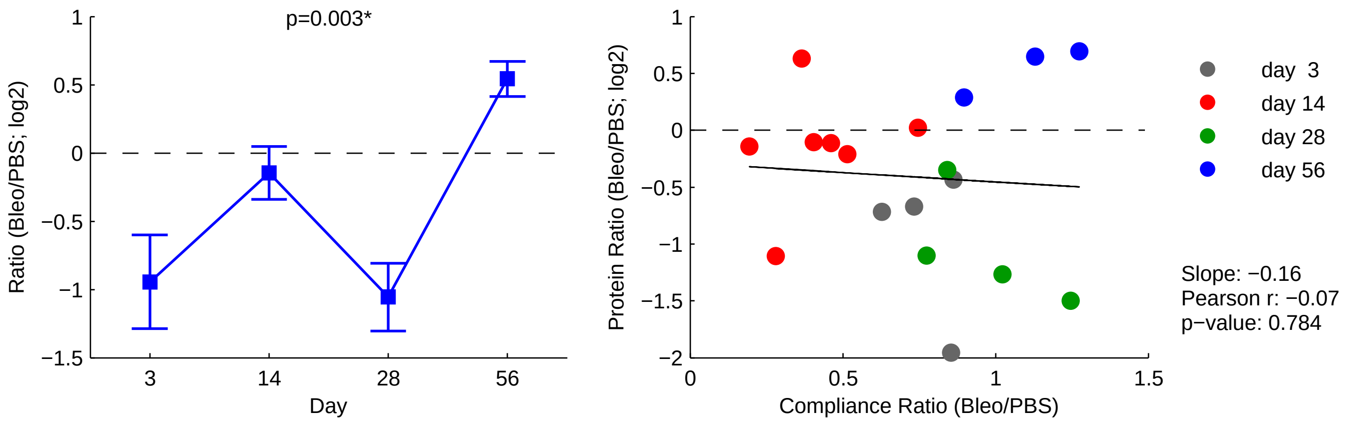

Q9CQ56 − Use1 (id: 1114)

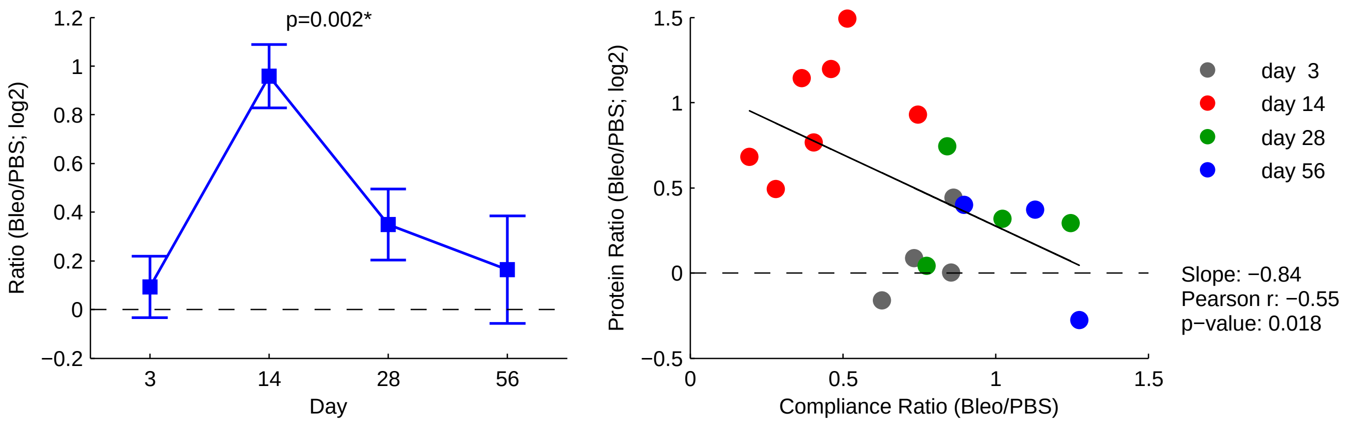

E9Q4P1 − Wdfy1 (id: 1120)

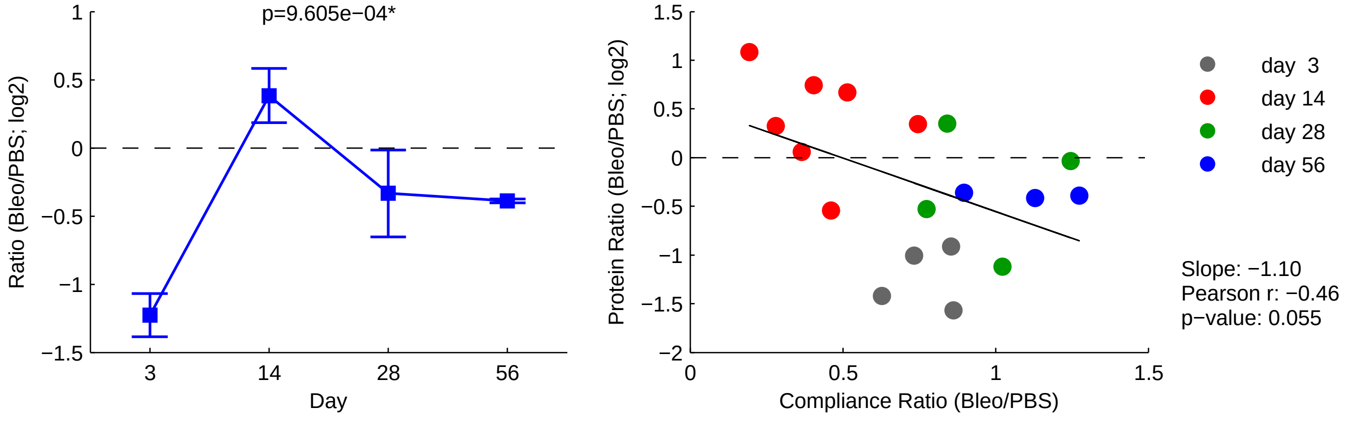

E9Q4S3 − Nin (id: 1121)

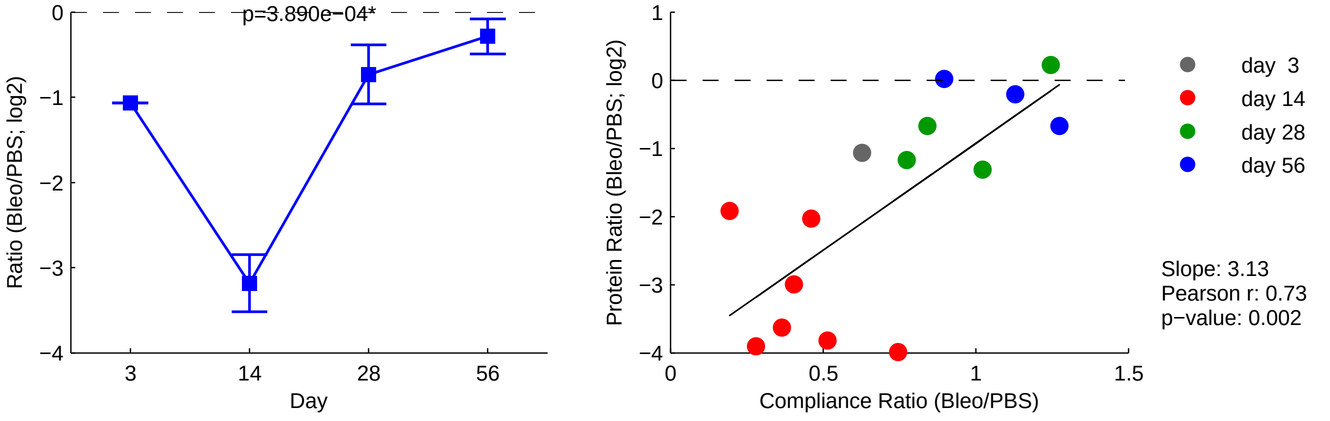

O70566 − Diaph2 (id: 1122)

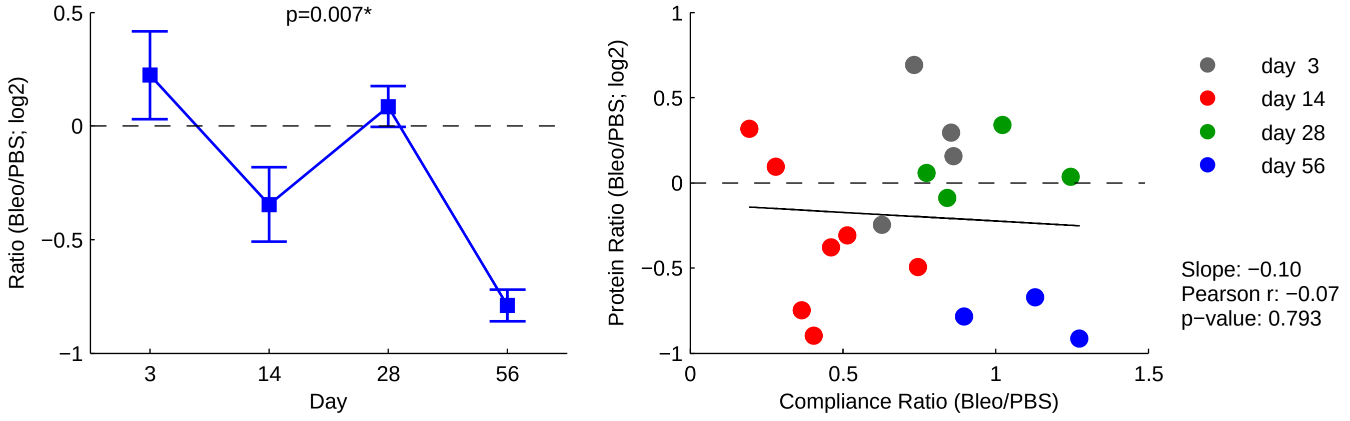

E9Q4X2 − Uggt2 (id: 1124)

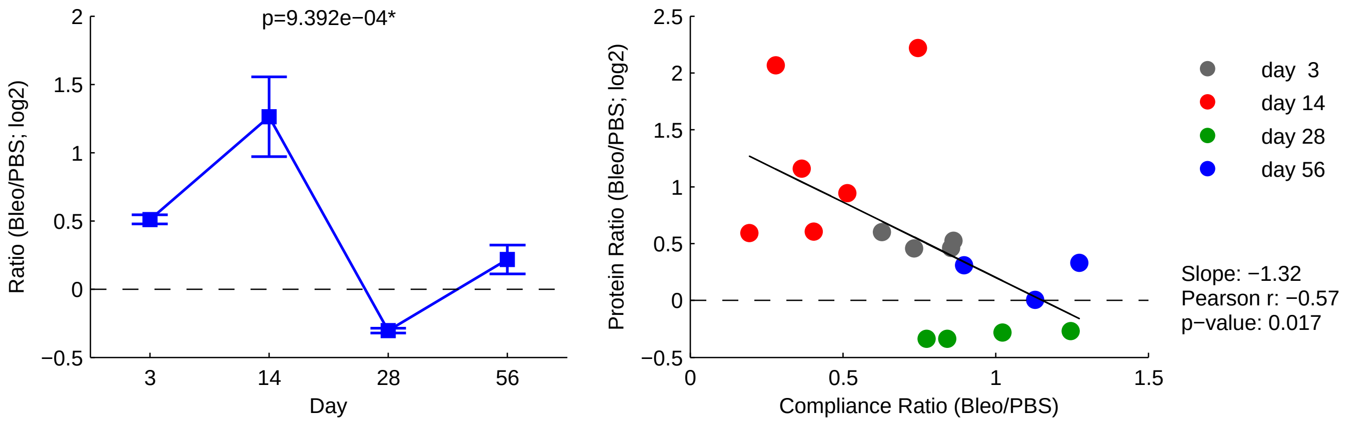

E9Q4Y4 − Cep192 (id: 1126)

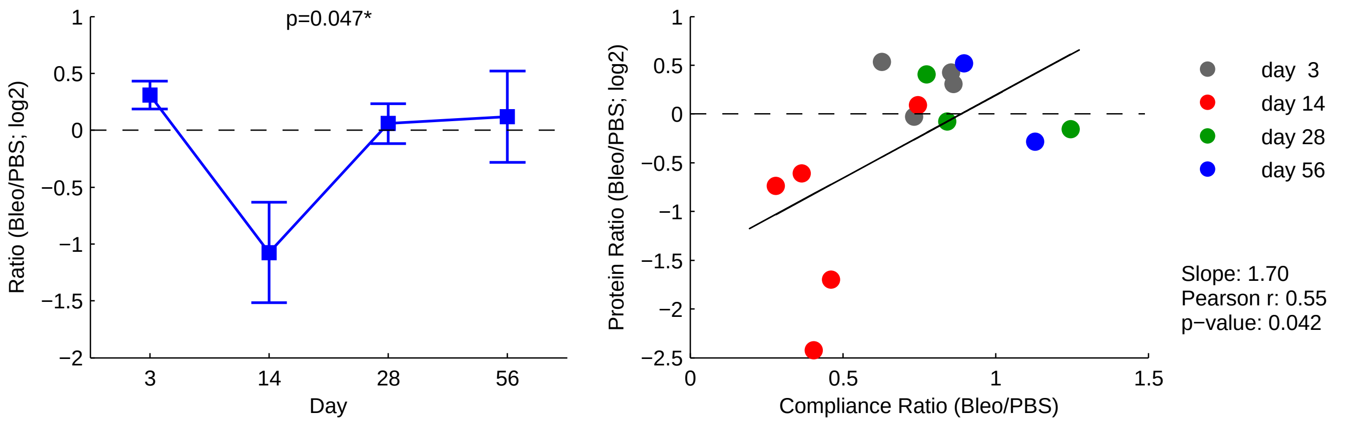

E9Q512 − Trip11 (id: 1129)

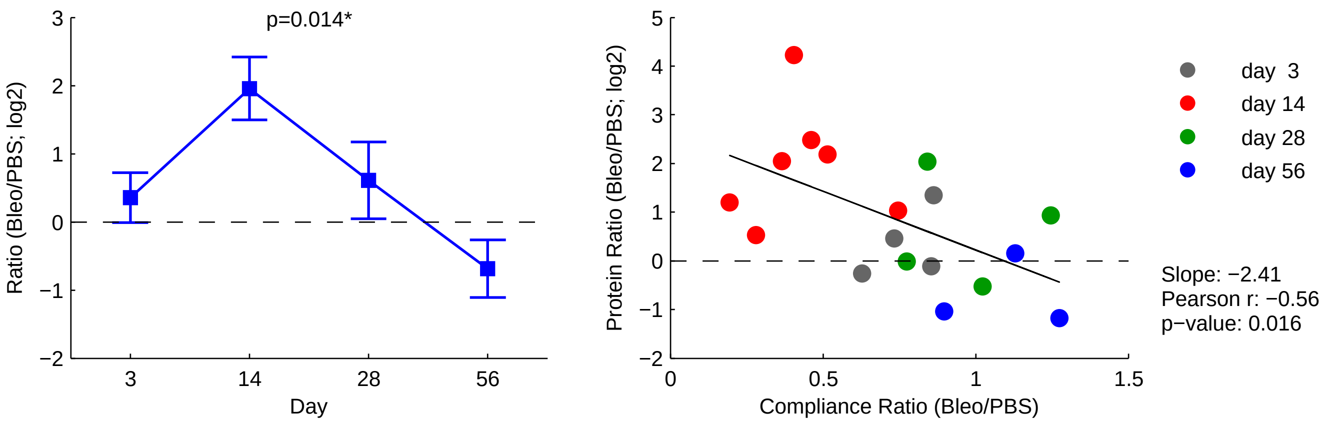

E9Q557 − Dsp (id: 1130)

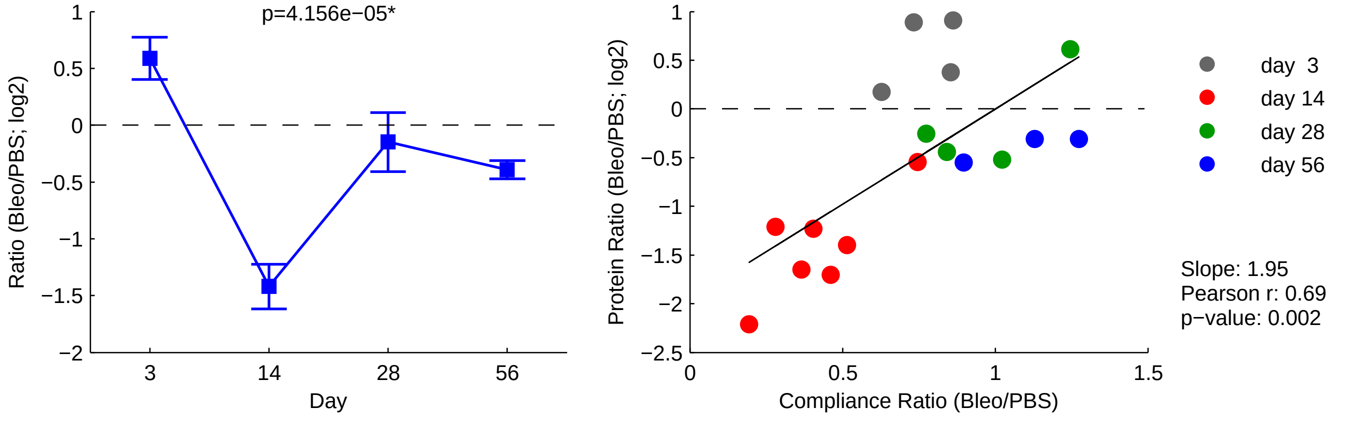

E9QNH6 − Myo1b (id: 1131)

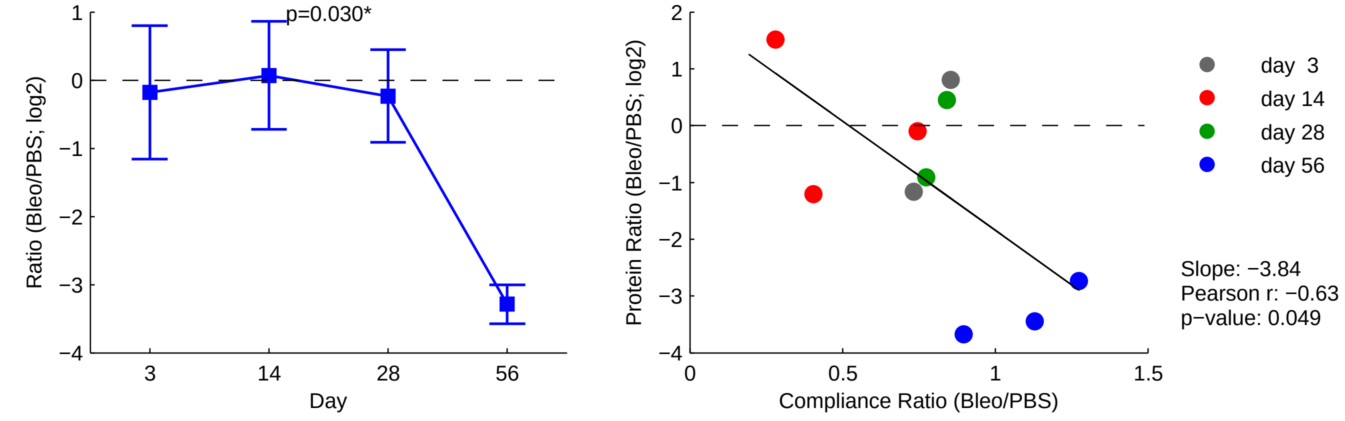

E9Q5C9 − Nolc1 (id: 1136)

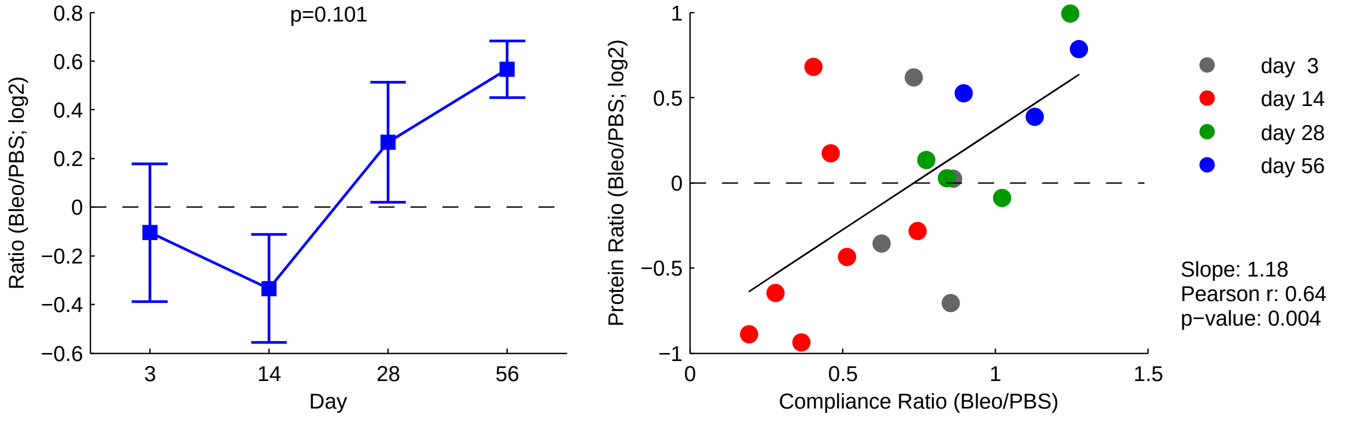

P69566 − Ranbp9 (id: 1137)

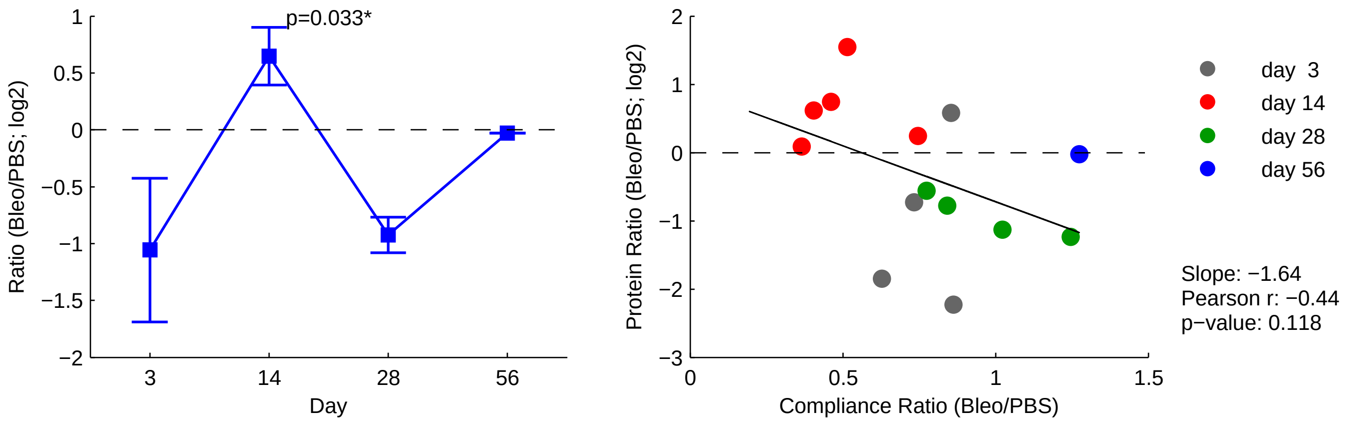

E9Q5I7 − Eng (id: 1144)

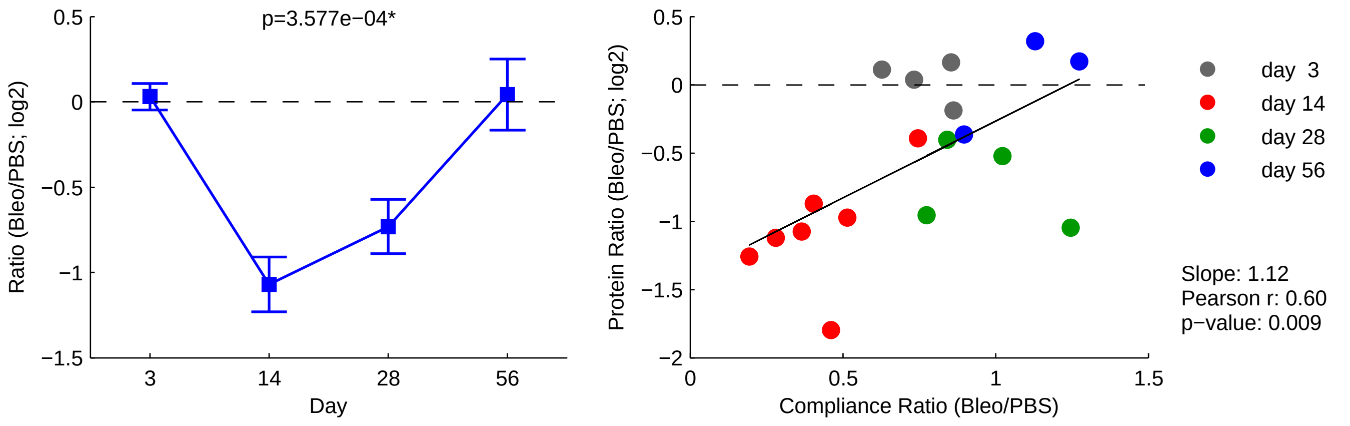

Q8K0Z5 − Tpm3 (id: 1145)

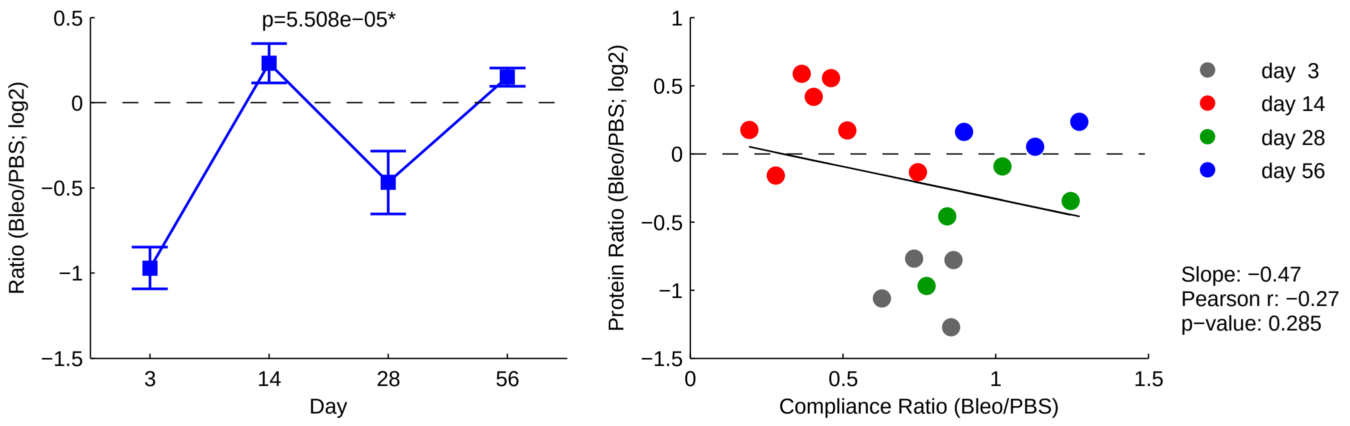

Q5SSH7−2 − Zzef1 (id: 1151)

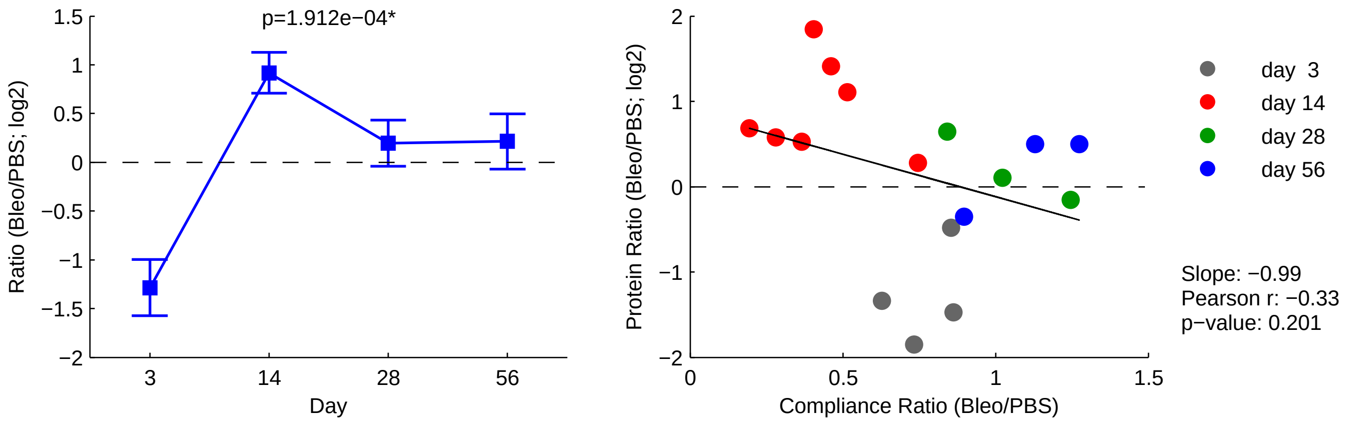

Q684R7−2 − Frem1 (id: 1152)

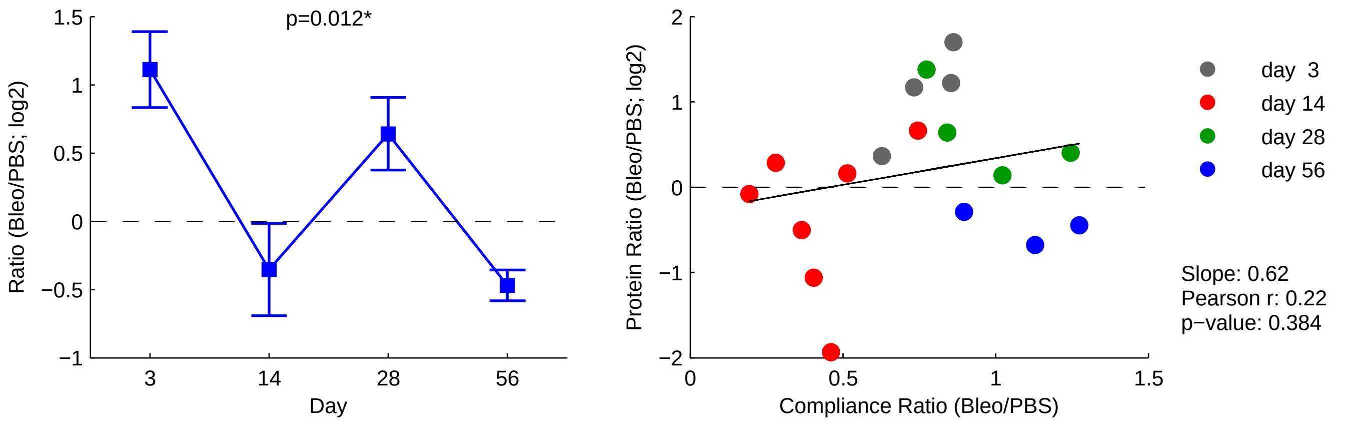

E9Q616 − Ahnak (id: 1154)

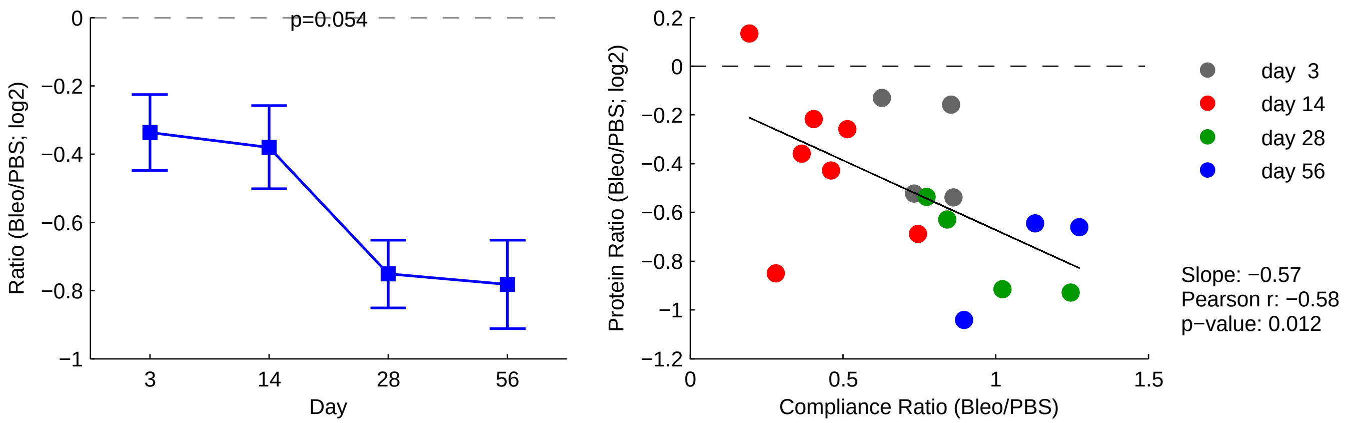

E9Q638 − Adam9 (id: 1156)

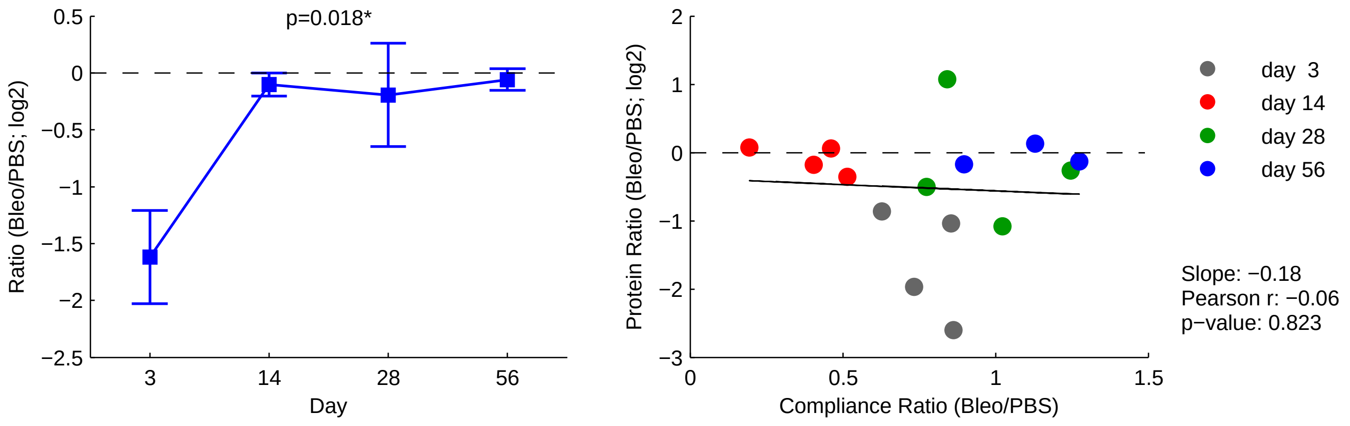

Q6NZN0−5 − Rbm26 (id: 1157)

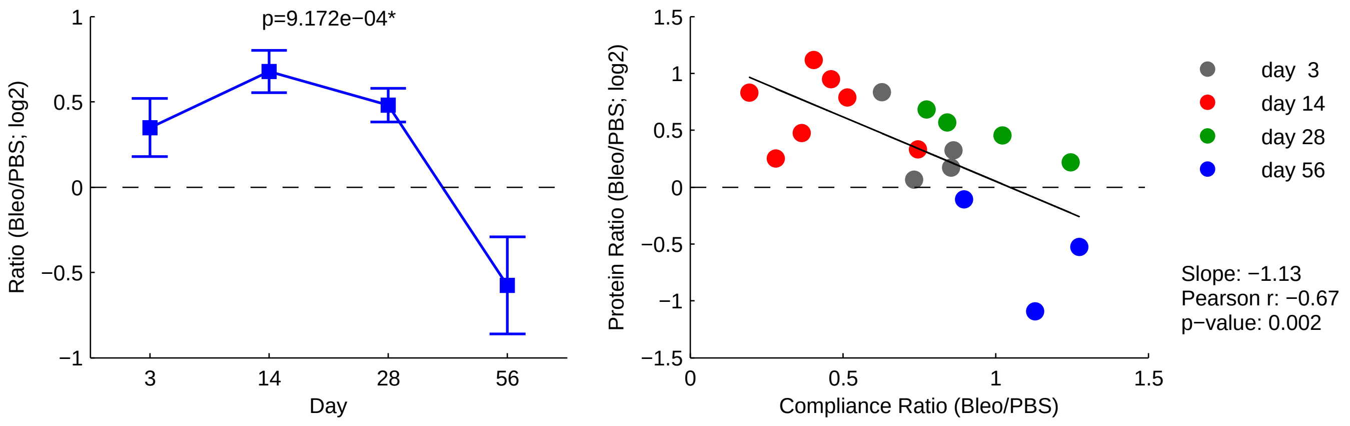

Q8CGE9 − Rgs12 (id: 1158)

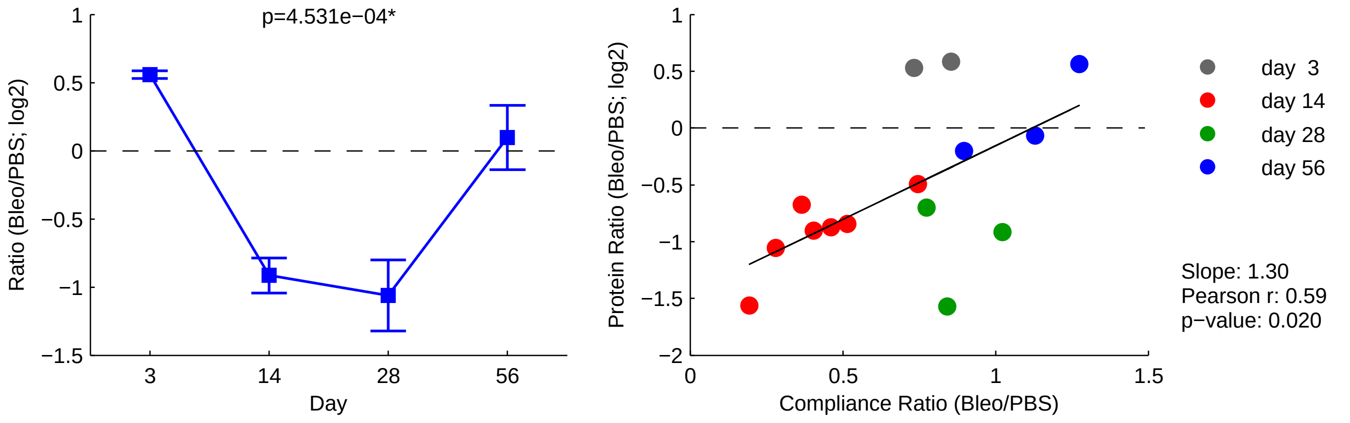

Q8C6K9 − Col6a6 (id: 1163)

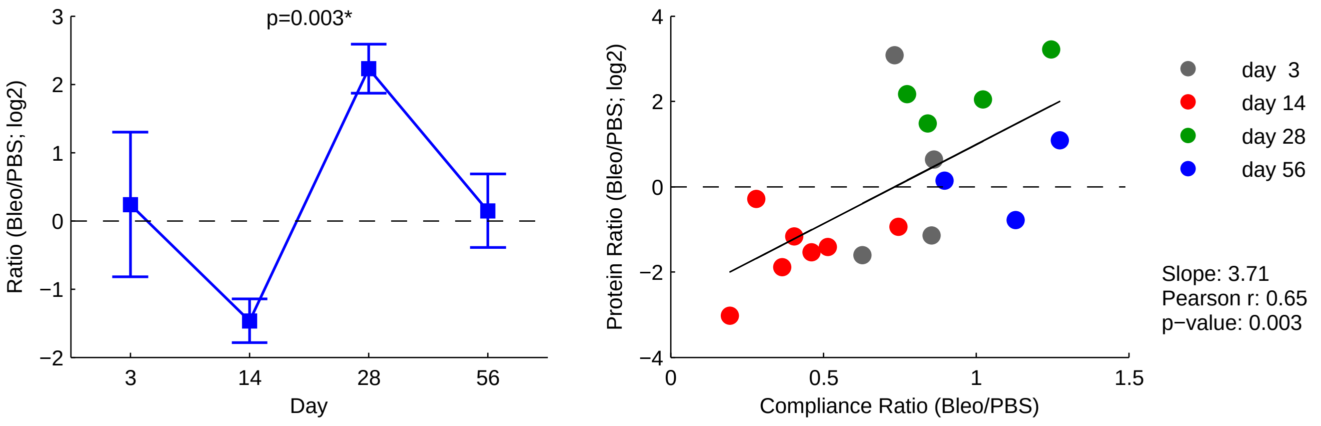

Q8CG14 − C1sa (id: 1164)

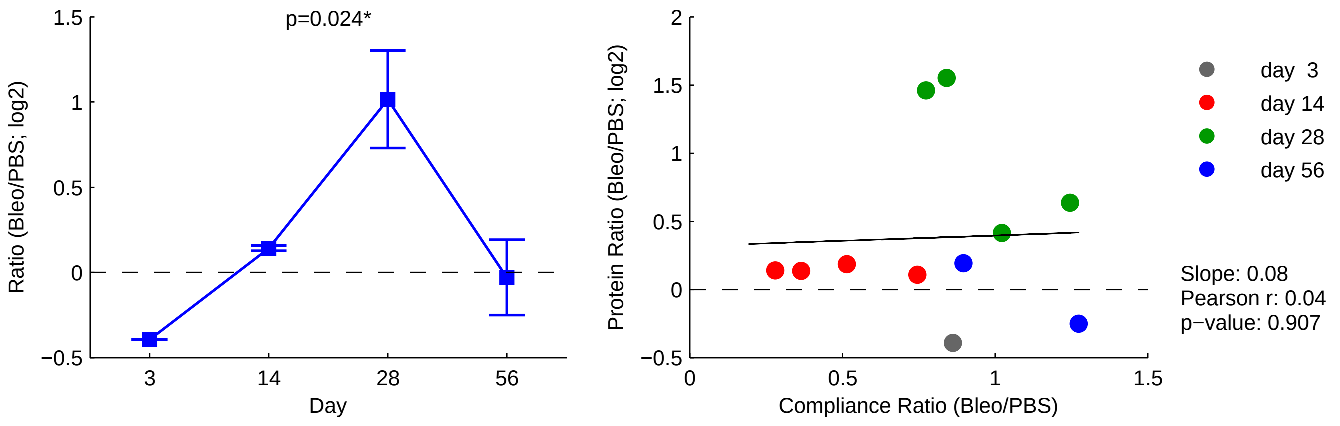

E9Q6C7 − Lphn2 (id: 1165)

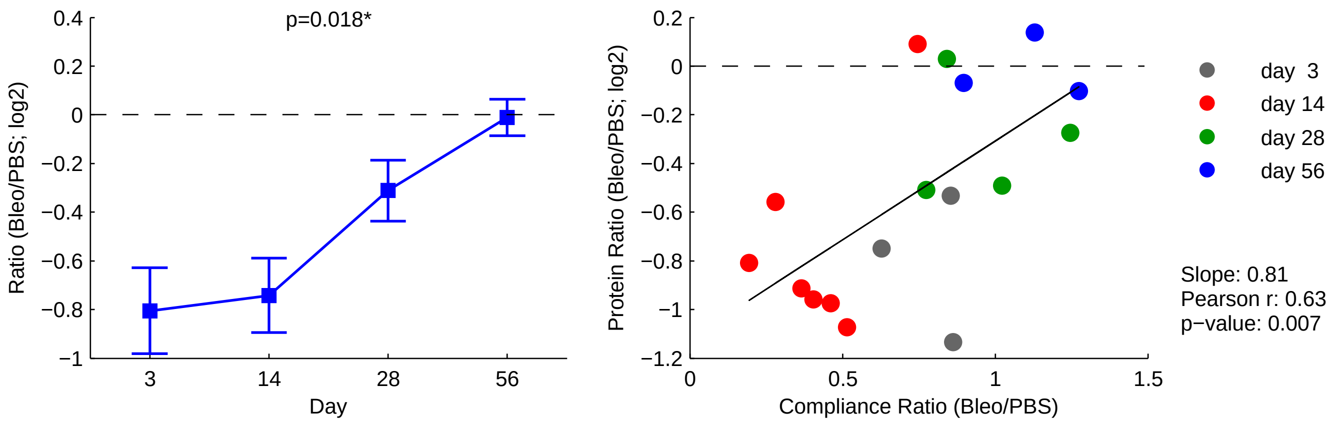

Q91X70 − C6 (id: 1166)

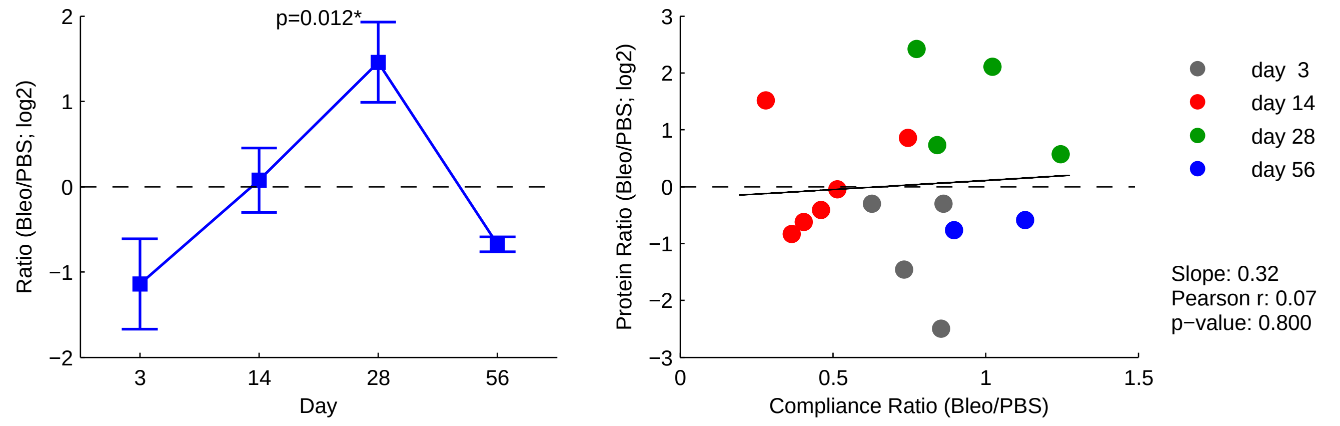

E9Q6H8 − Plekha5 (id: 1169)

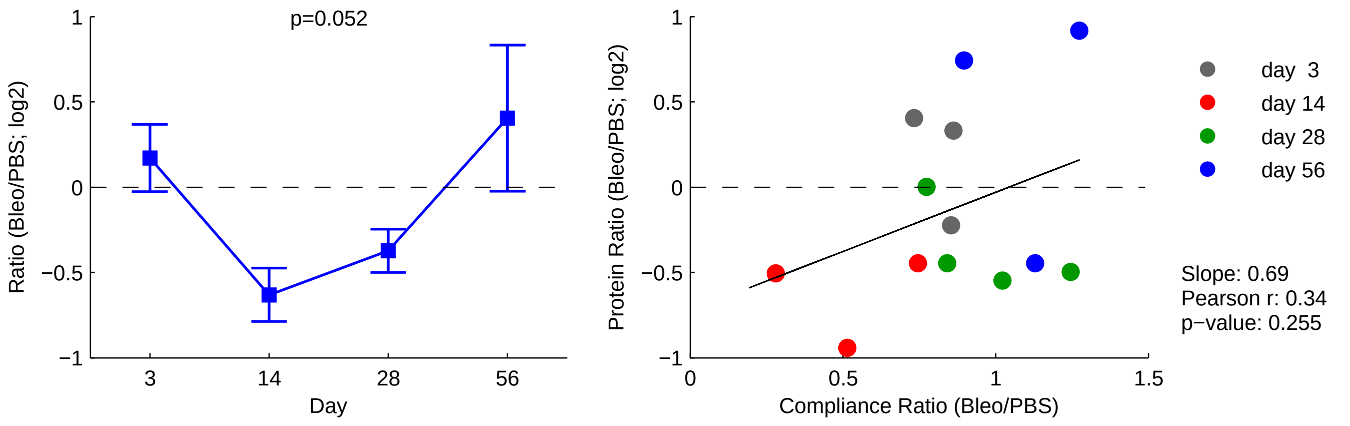

Q80TE7 − Lrrc7 (id: 1173)

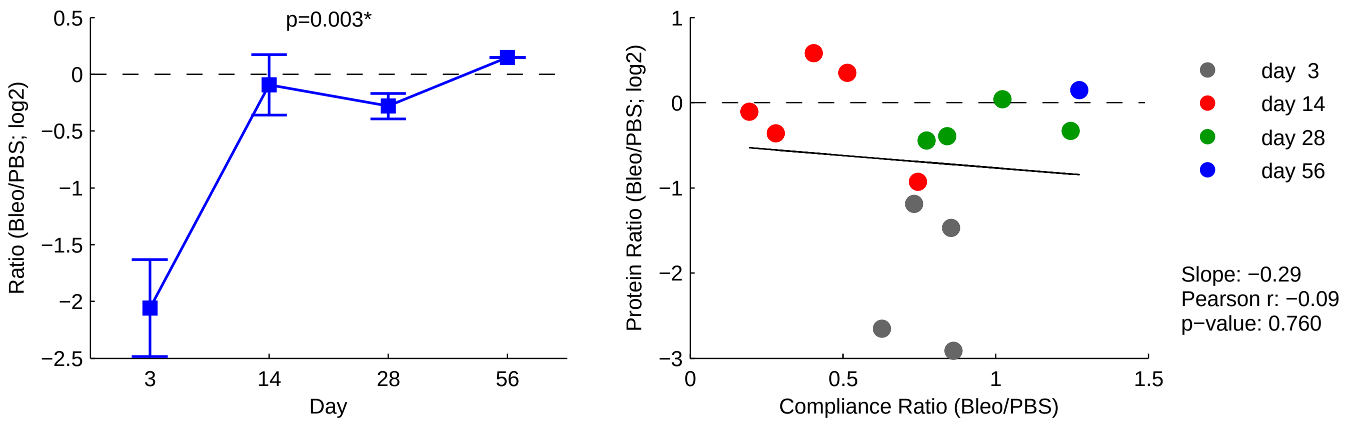

E9Q6P5 − Ttc7b (id: 1176)

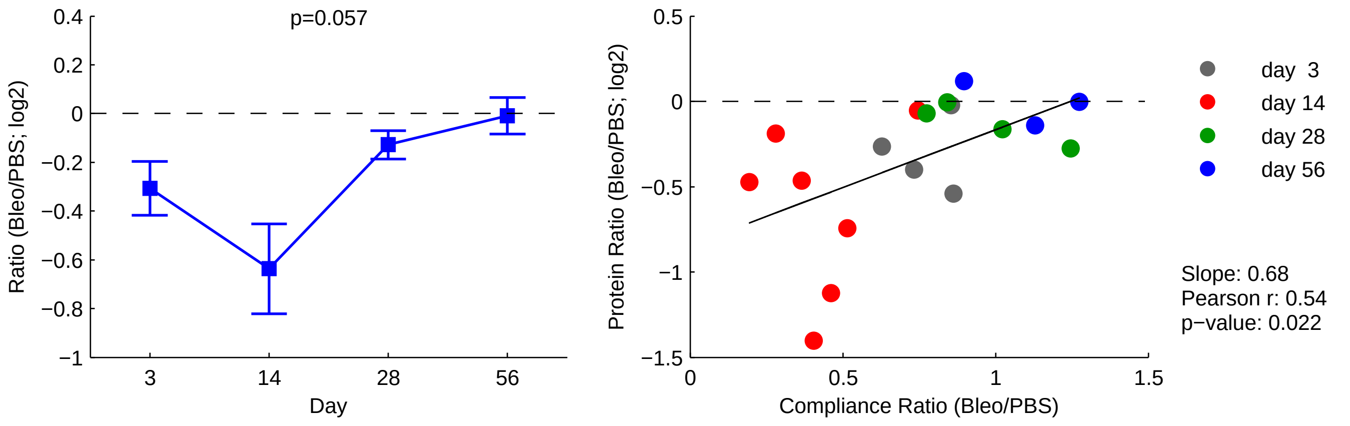

E9Q6R7 − Utrn (id: 1177)

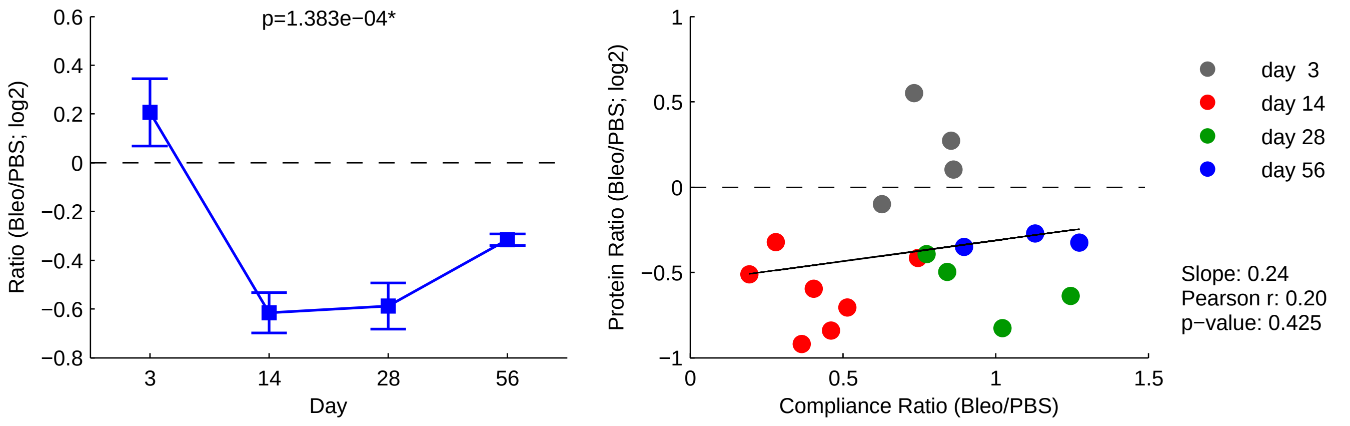

E9Q6U4 − Pfdn4 (id: 1178)

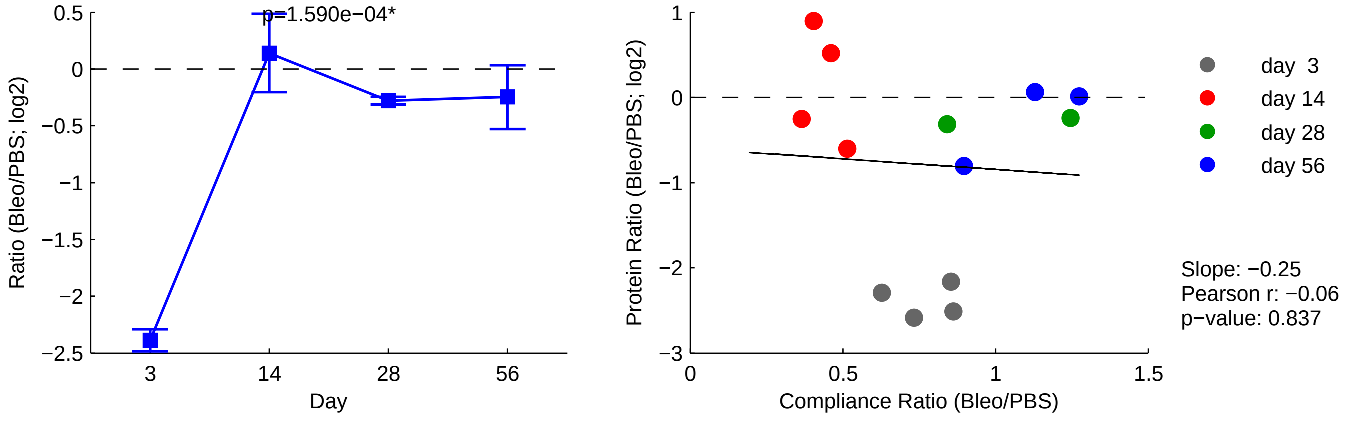

P25425−10 − Pou2f1 (id: 1180)

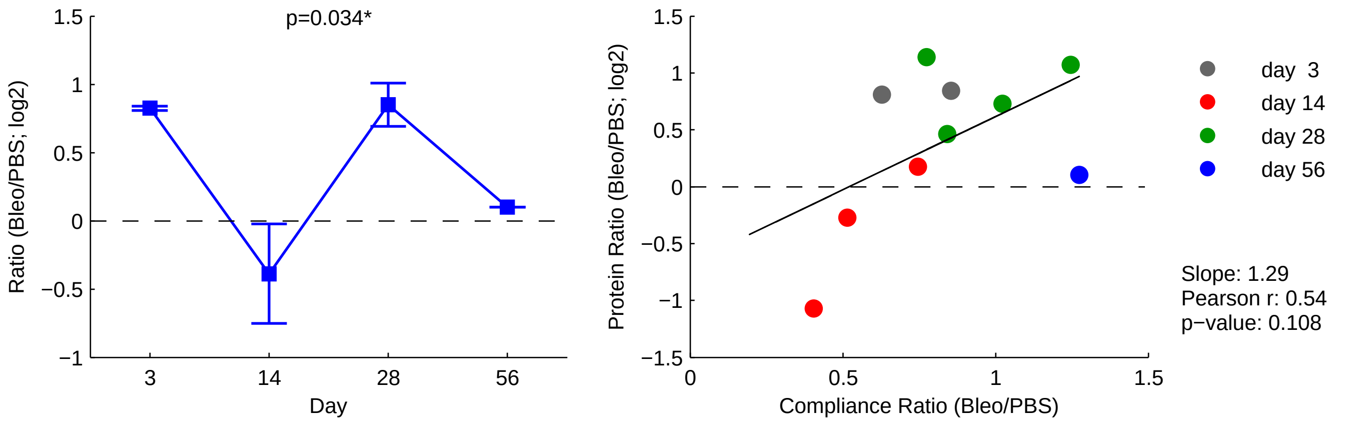

E9Q718 − Plod2 (id: 1181)

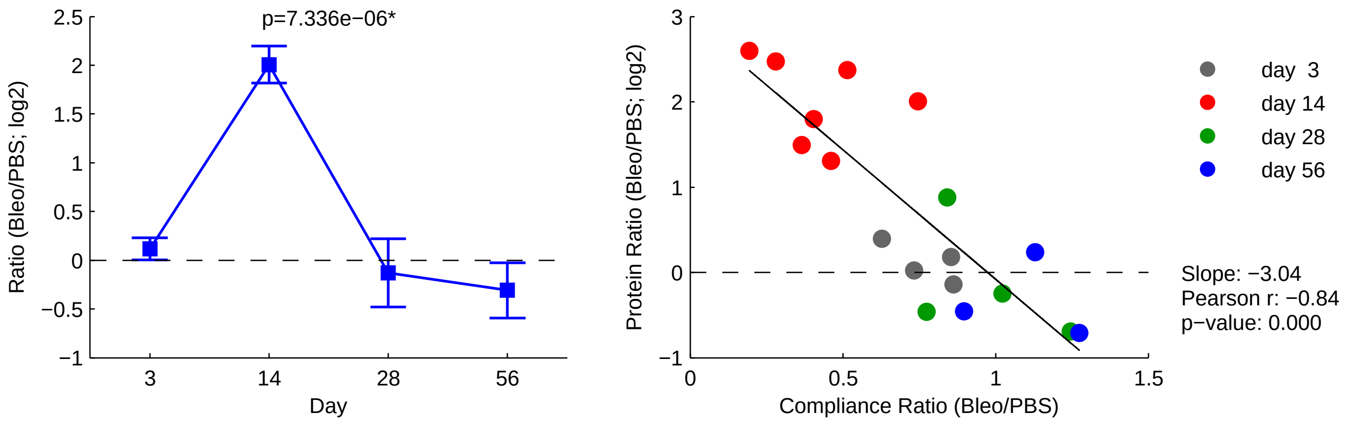

E9Q784 − Zc3h13 (id: 1182)

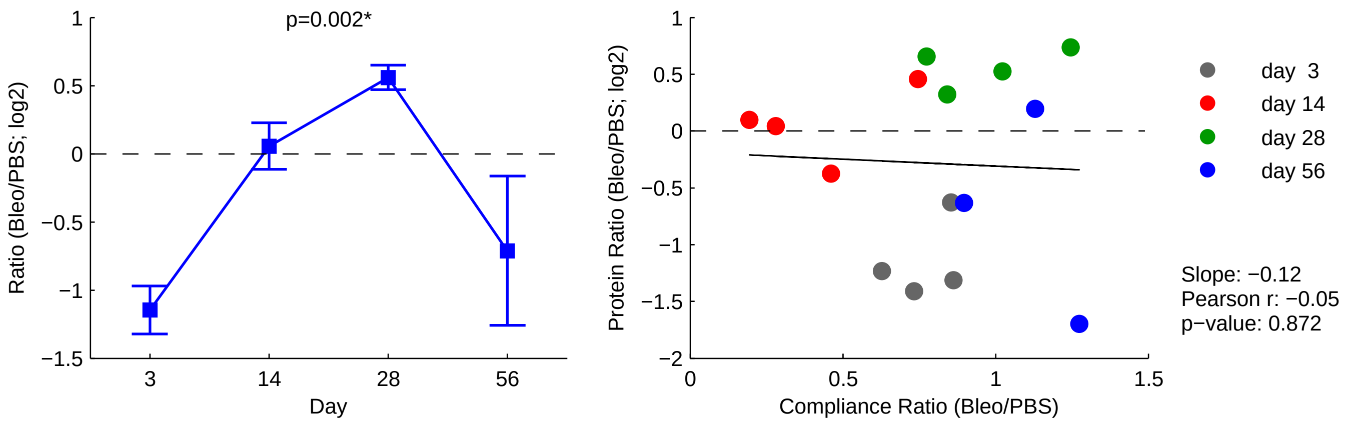

E9Q7G0 − Numa1 (id: 1188)

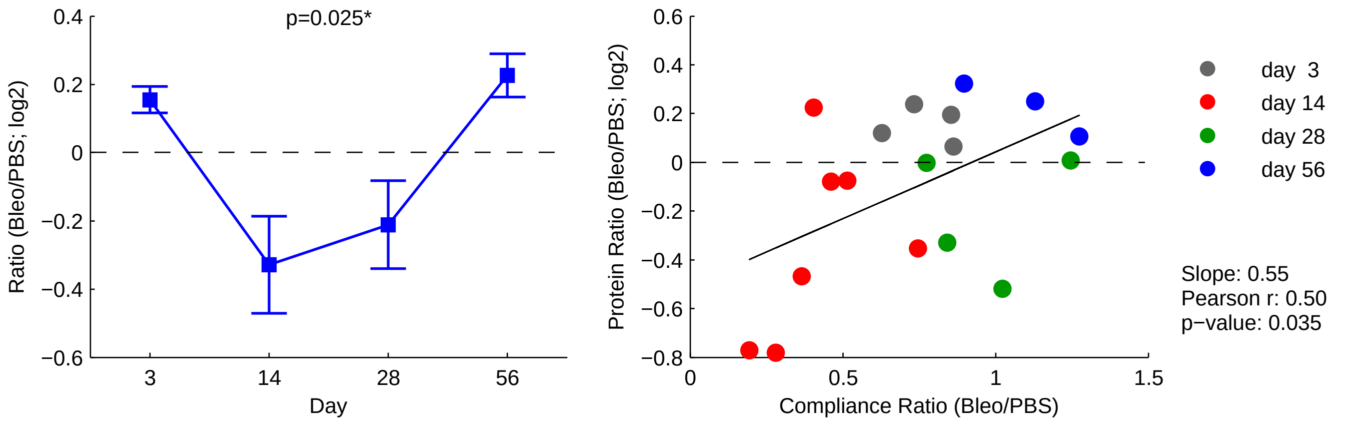

E9Q7H6 − Tars2 (id: 1189)

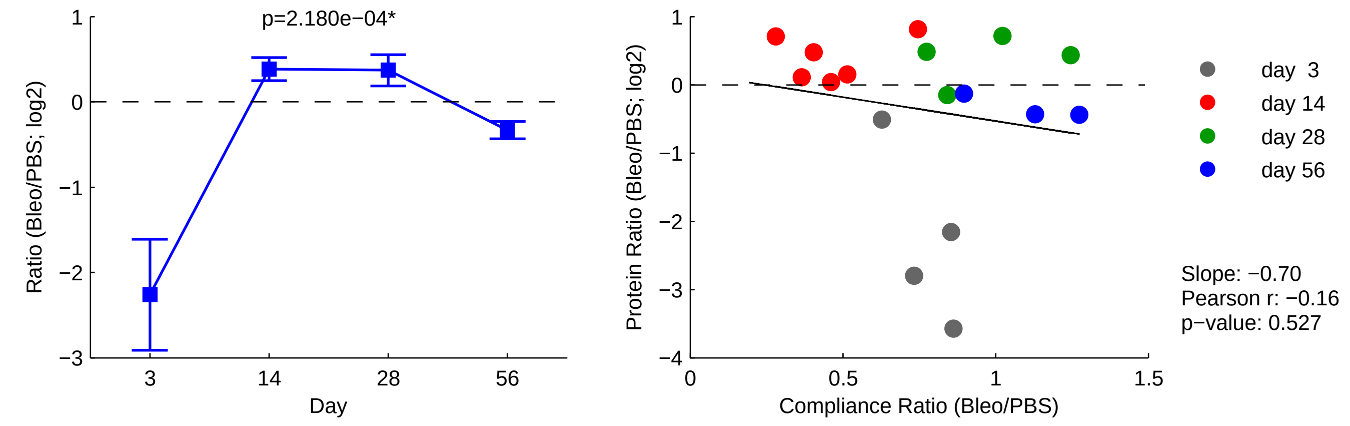

E9Q7X6−2 − Heg1 (id: 1199)

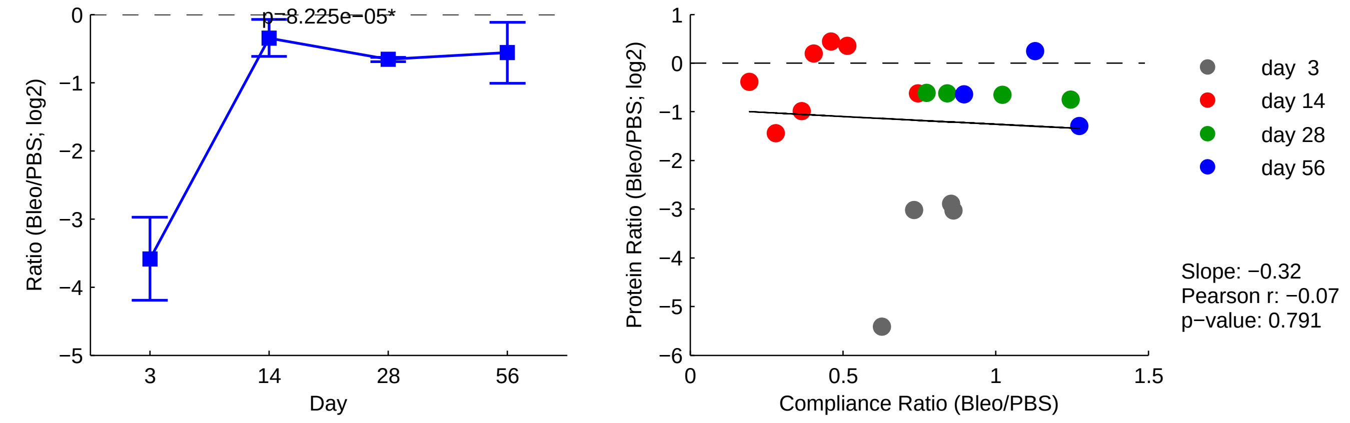

Q9QZQ1−2 − Mllt4 (id: 1203)

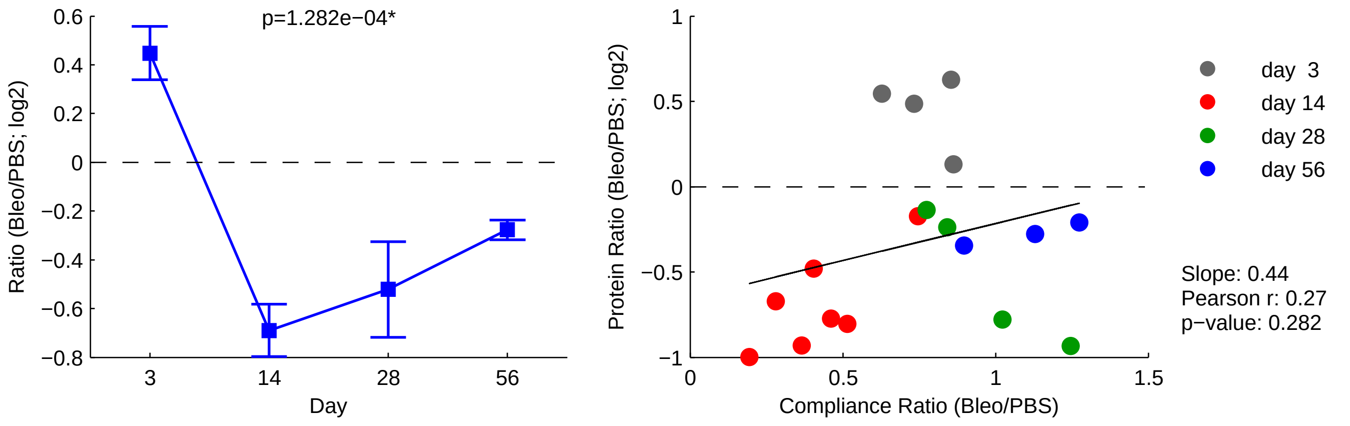

E9Q855 − Scamp3 (id: 1204)

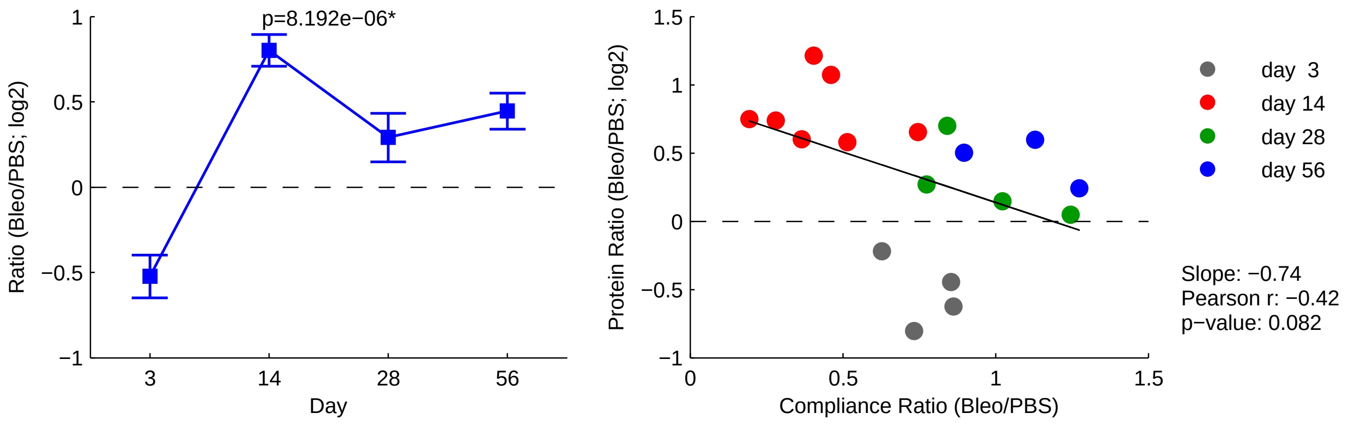

E9Q8I9 − Fry (id: 1211)

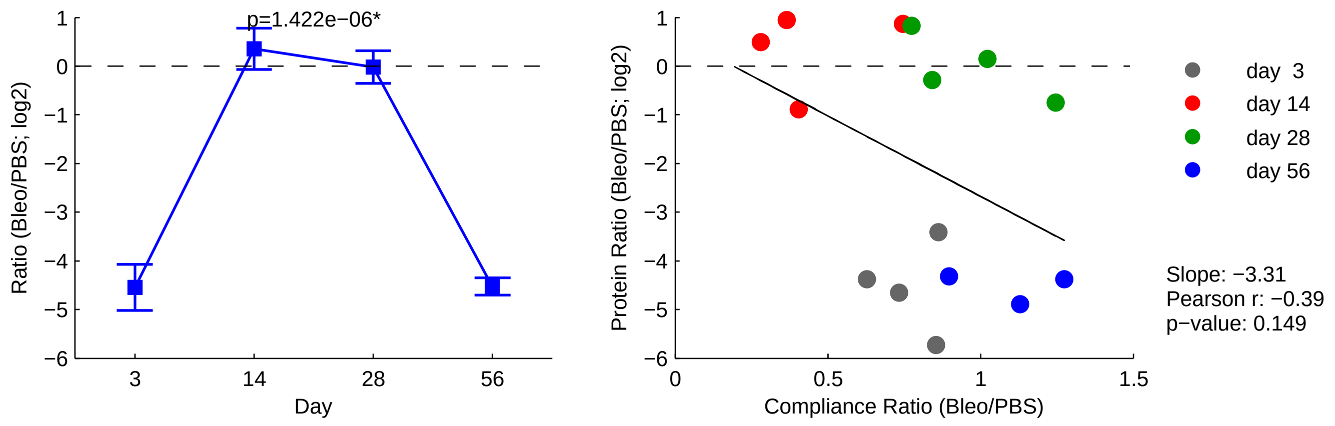

Q6ZPZ3−2 − Zc3h4 (id: 1213)

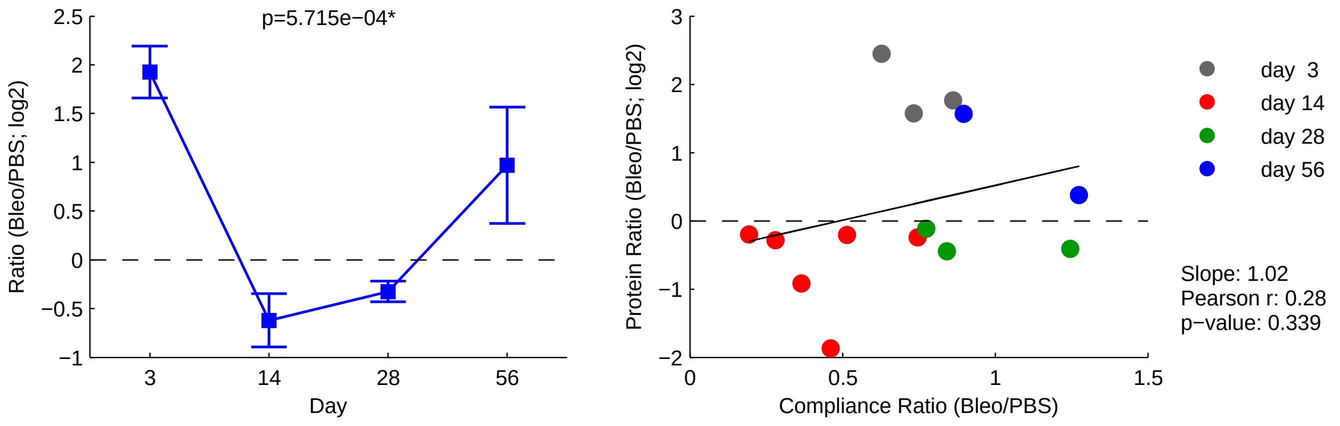

Q8BRT1 − Clasp2 (id: 1216)

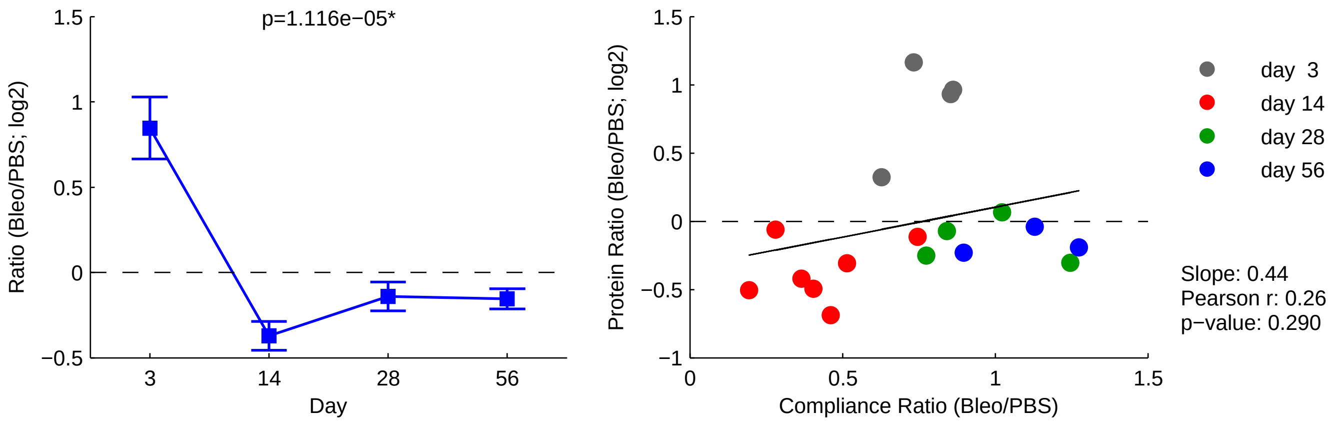

E9Q8Q6 − Ccdc141 (id: 1218)

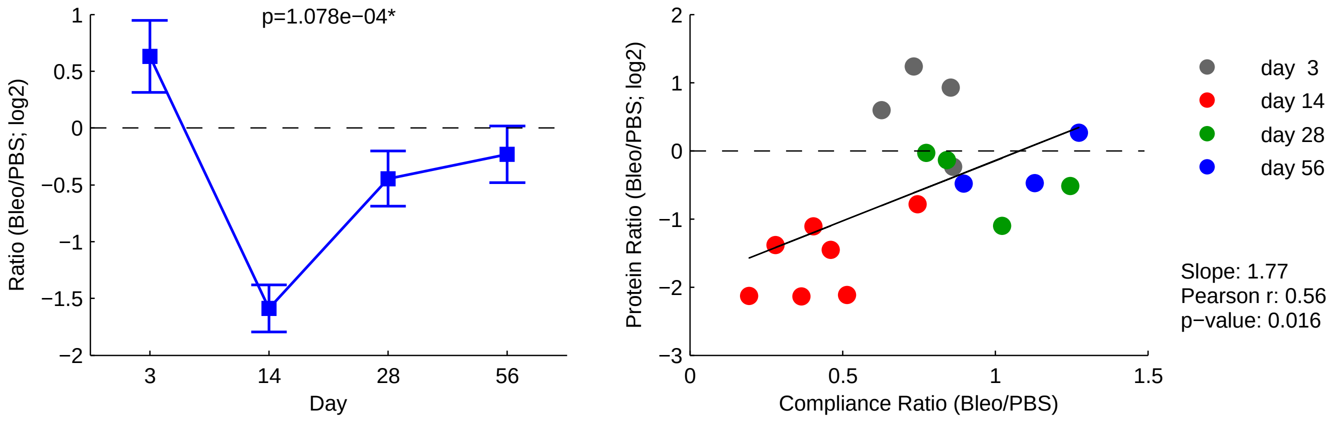

E9Q933 − Tmem11 (id: 1224)

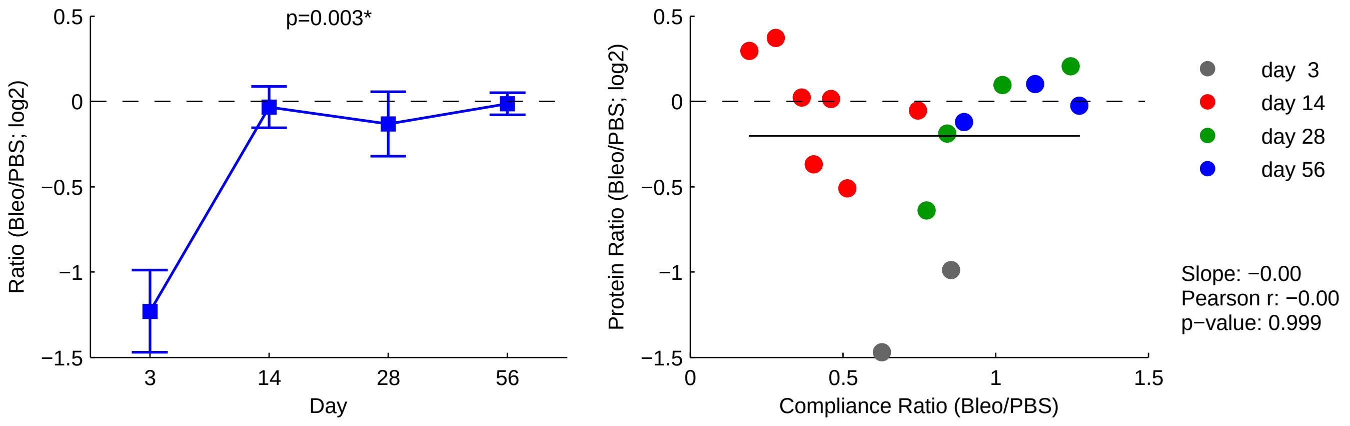

E9Q983 − Svil (id: 1225)

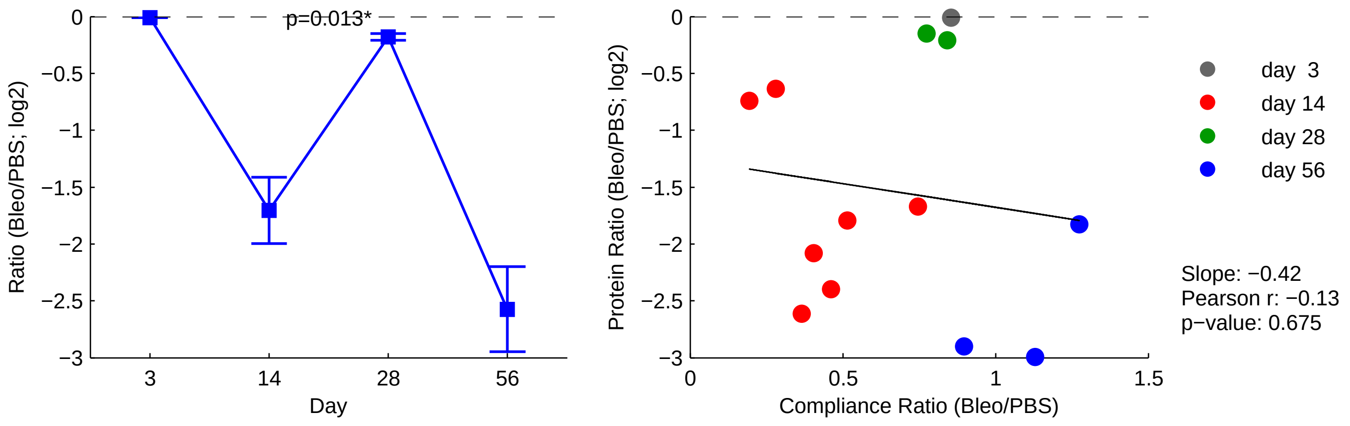

E9Q9C1 − Ablim1 (id: 1231)

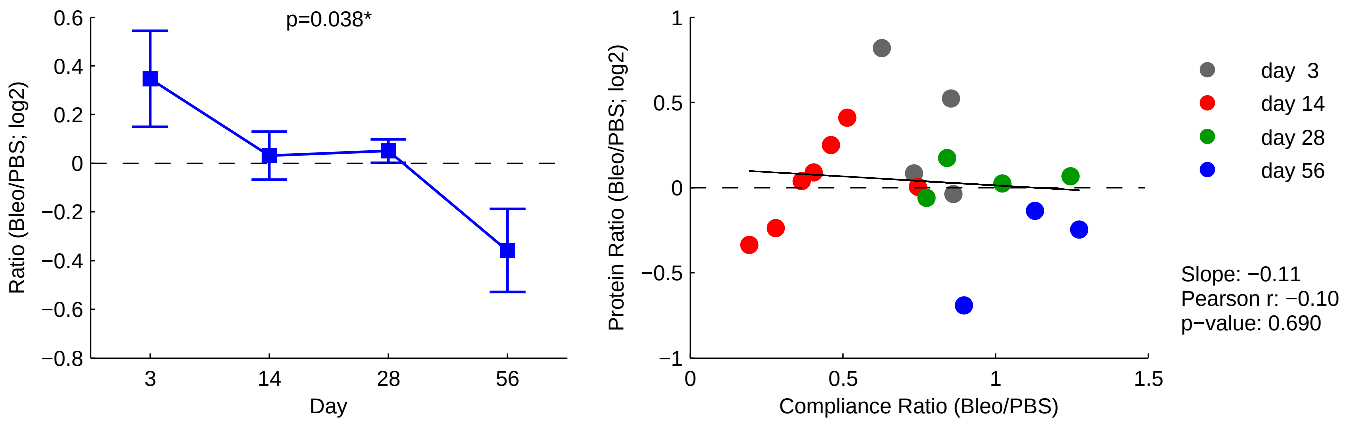

E9Q9C5 − Atp6v0c (id: 1233)

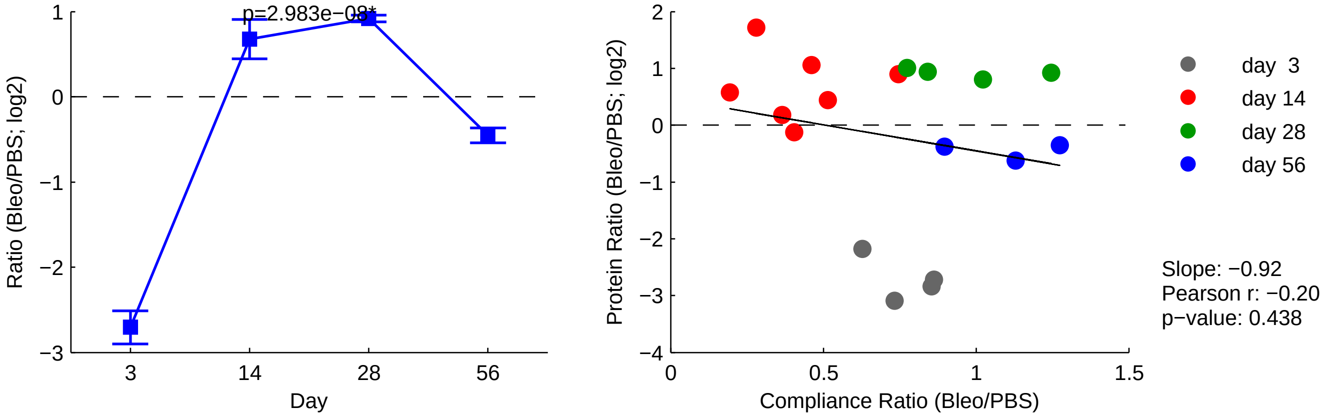

E9Q9E9 − 1190002N15Rik (id: 1237)

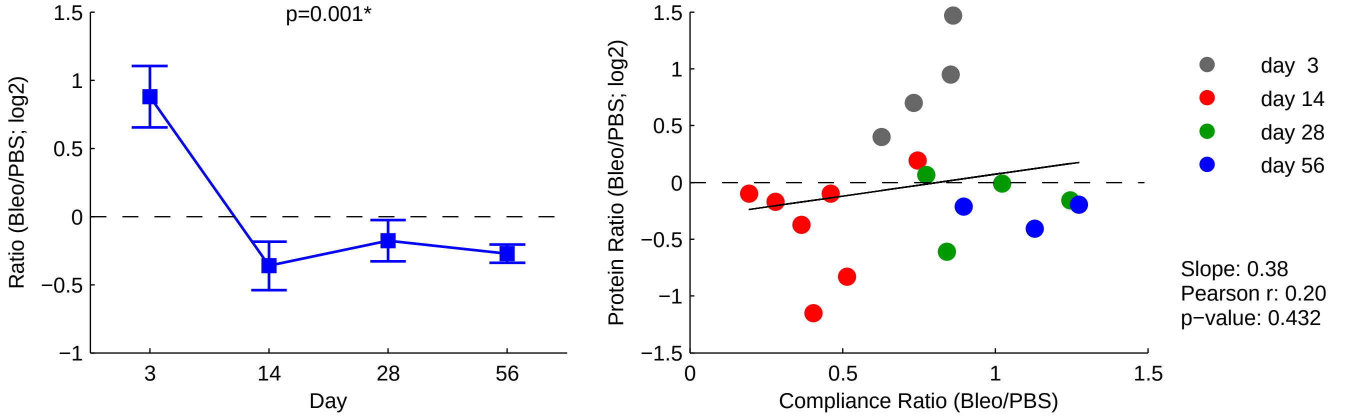

E9Q9M5 − Usp19 (id: 1244)

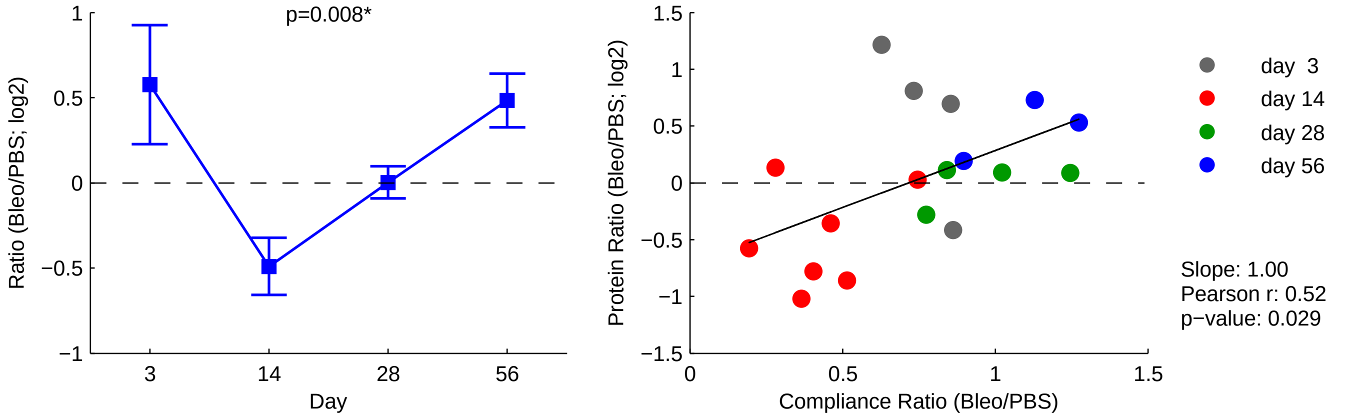

E9Q9T8 − Mybpc3 (id: 1248)

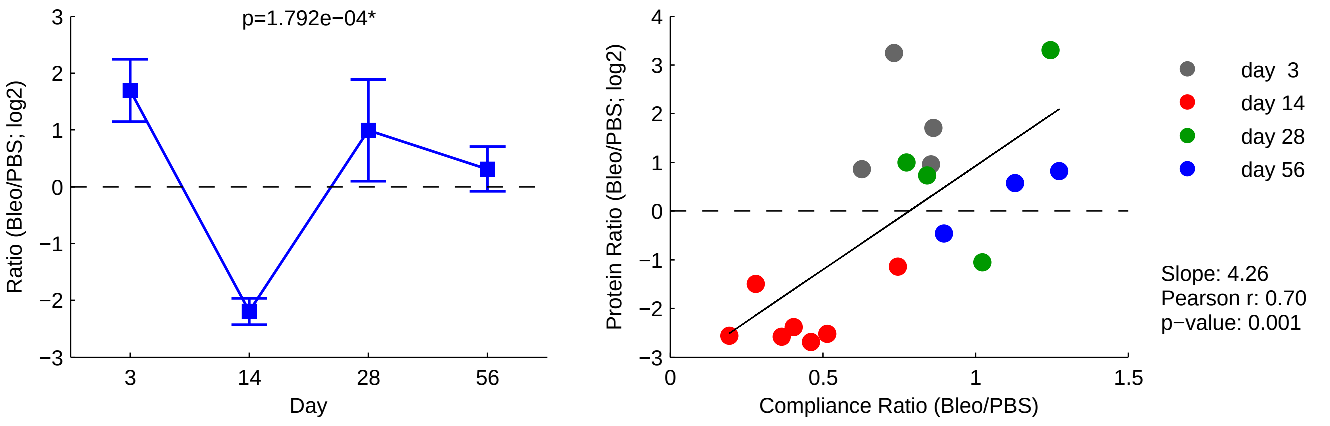

E9Q9W4 − Slc27a6 (id: 1250)

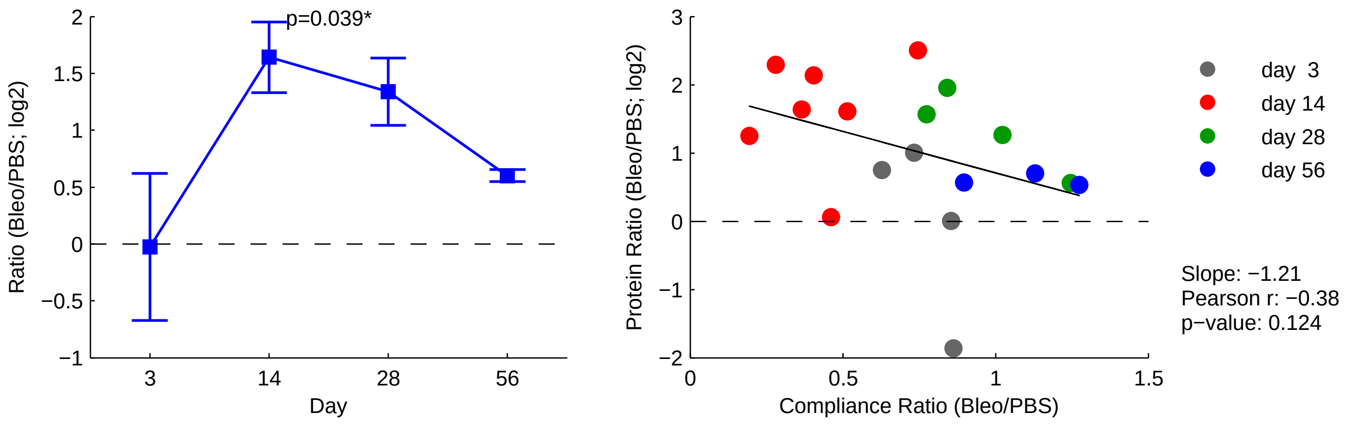

E9QA15 − Cald1 (id: 1251)

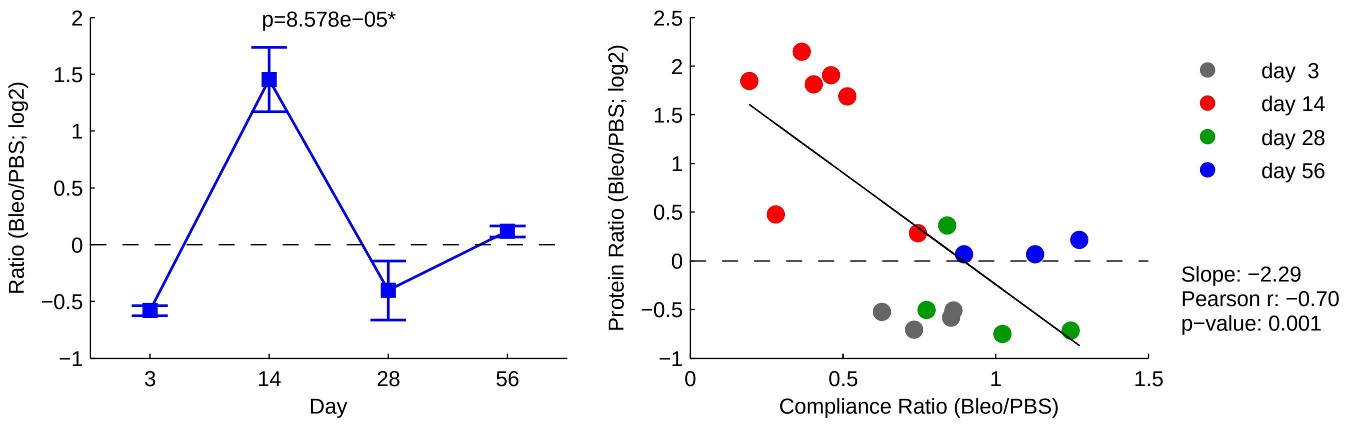

E9QA16 − Cald1 (id: 1252)

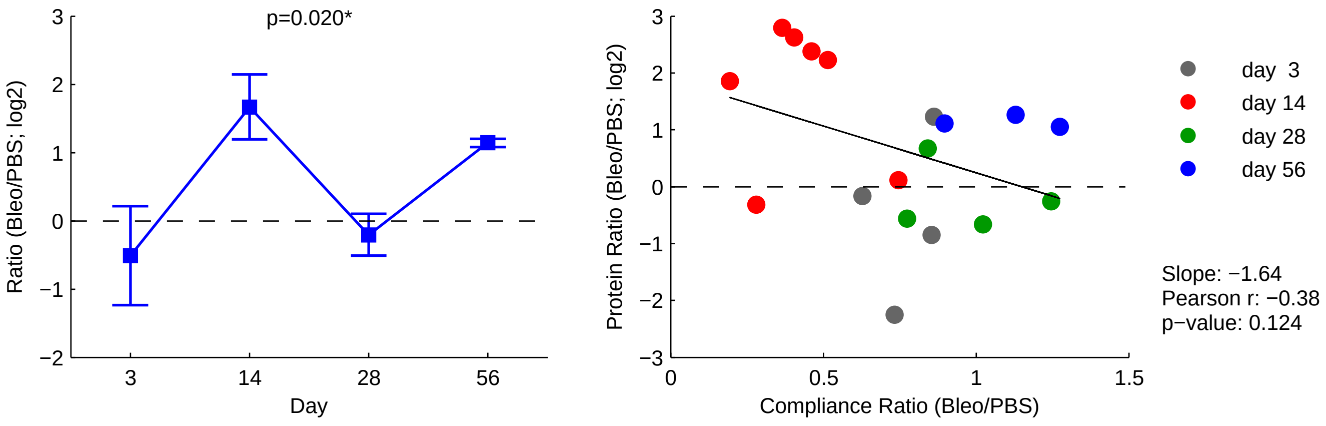

E9QAF9 − Tanc1 (id: 1256)

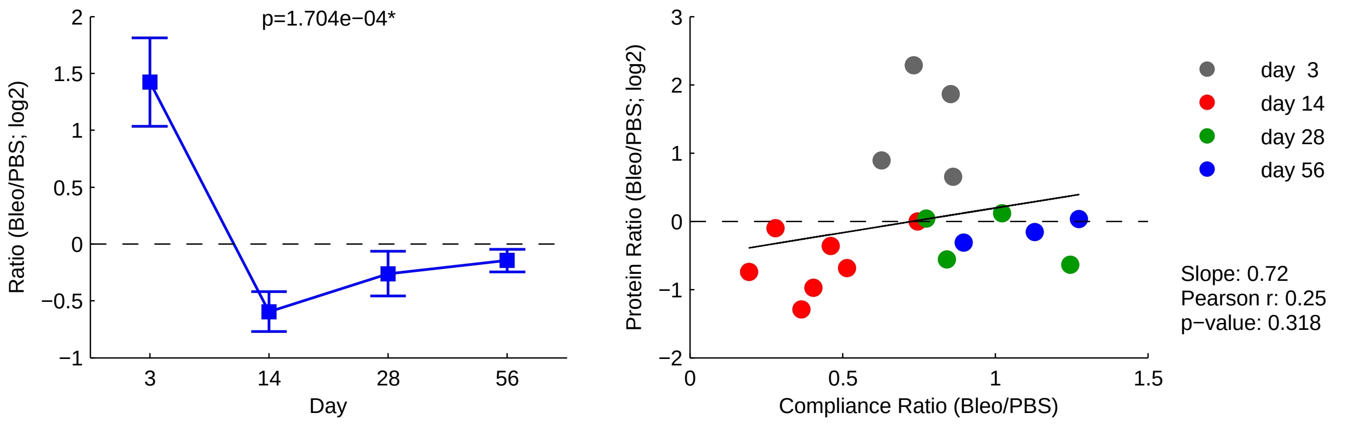

E9QAP7 − Taf4a (id: 1258)

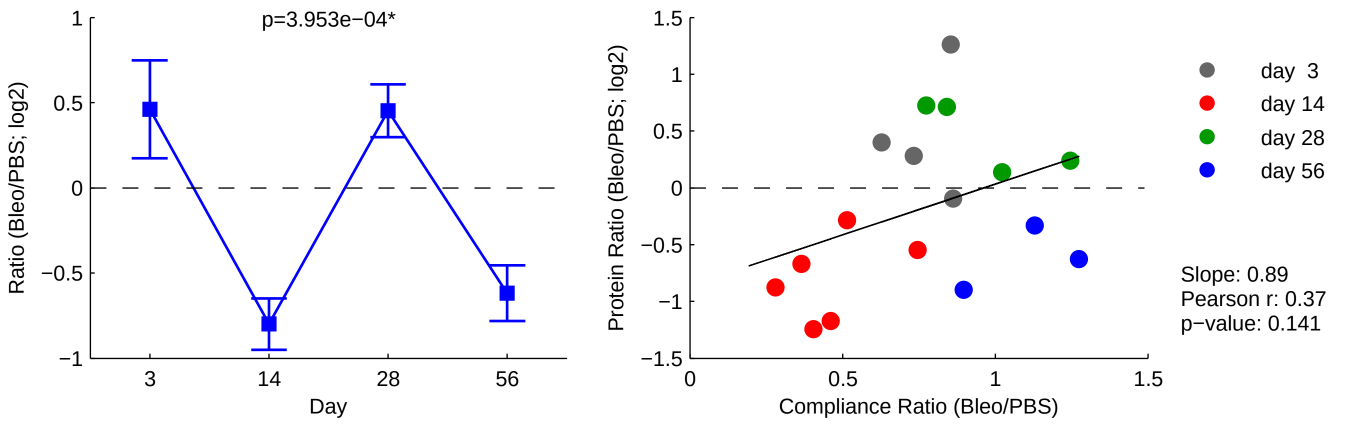

E9QAQ3 − Arhgap26 (id: 1259)

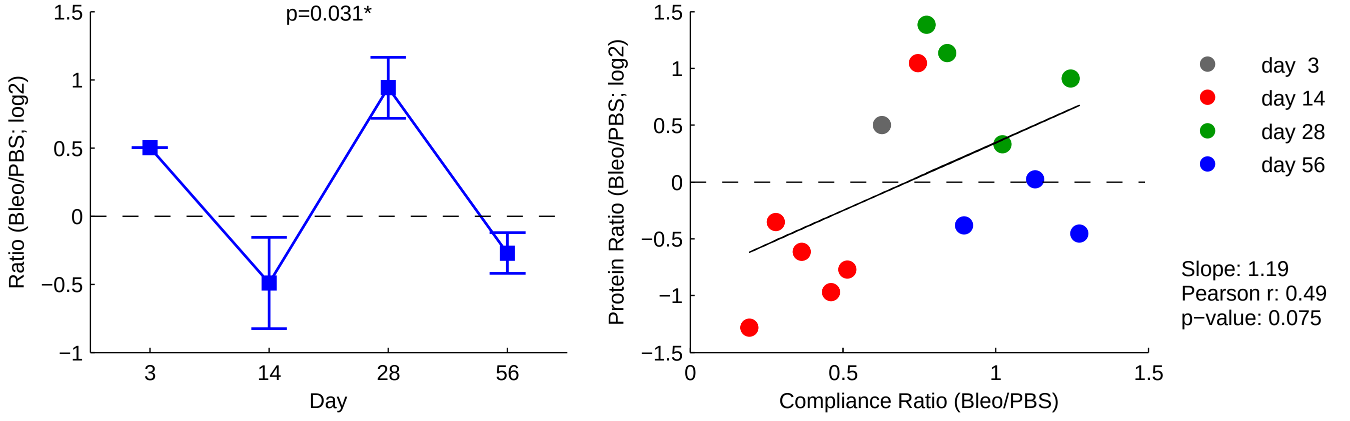

Q9WV60 − Gsk3b (id: 1260)

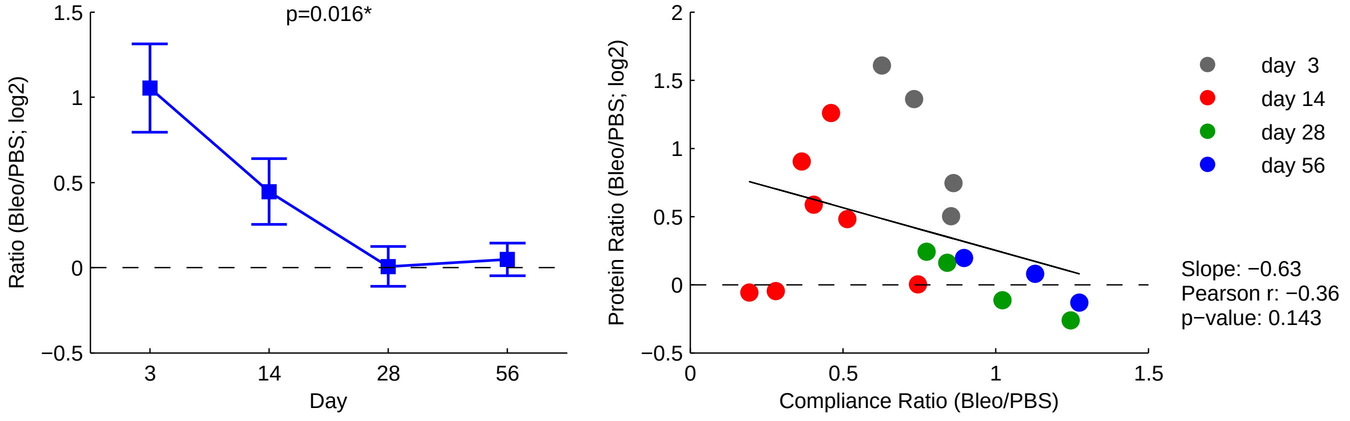

E9QAT0 − Fmr1 (id: 1262)

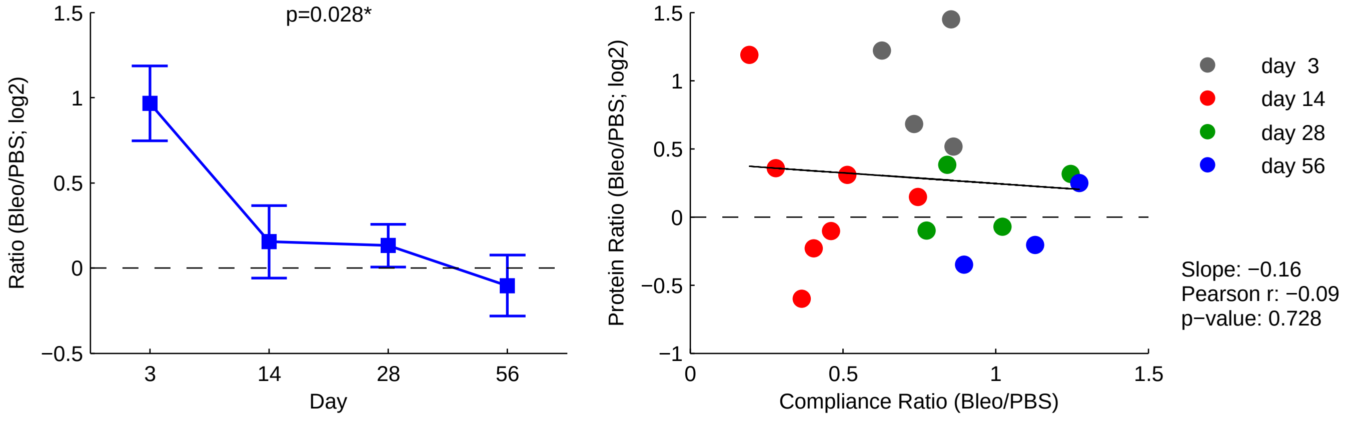

E9QAZ2 − Gm10020 (id: 1263)

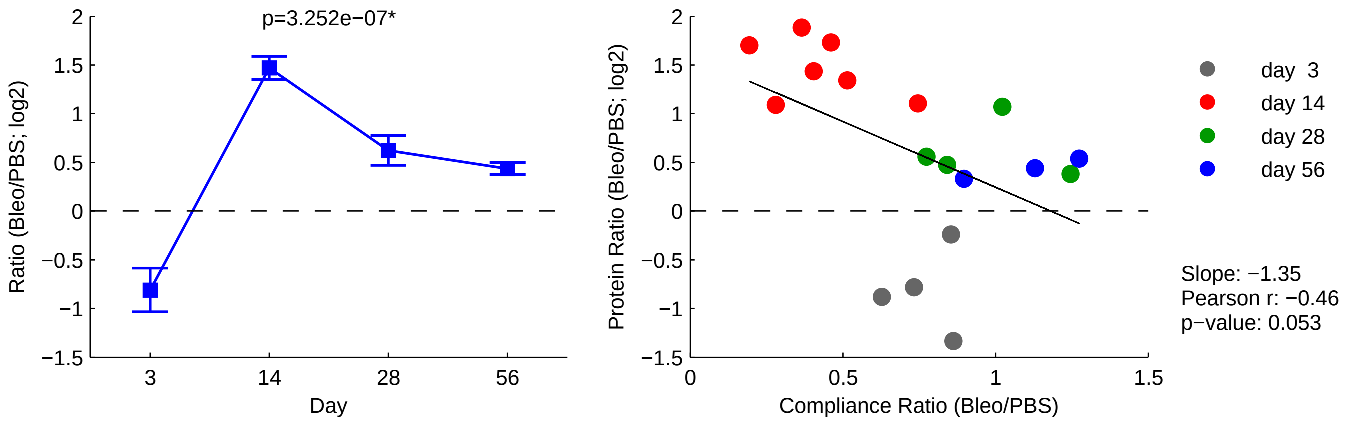

P13595−2 − Ncam1 (id: 1264)

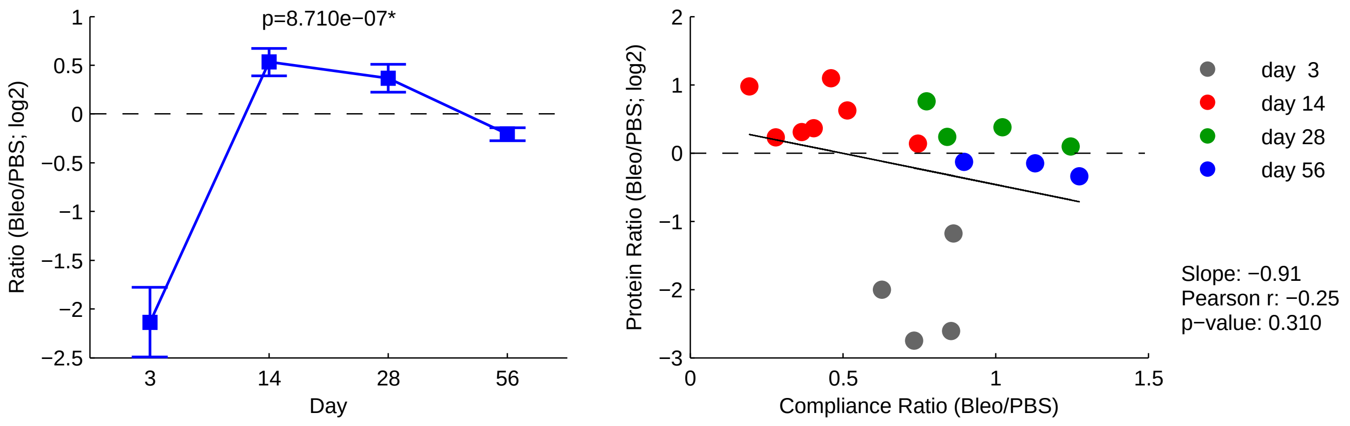

Q68FL6 − Mars (id: 1265)

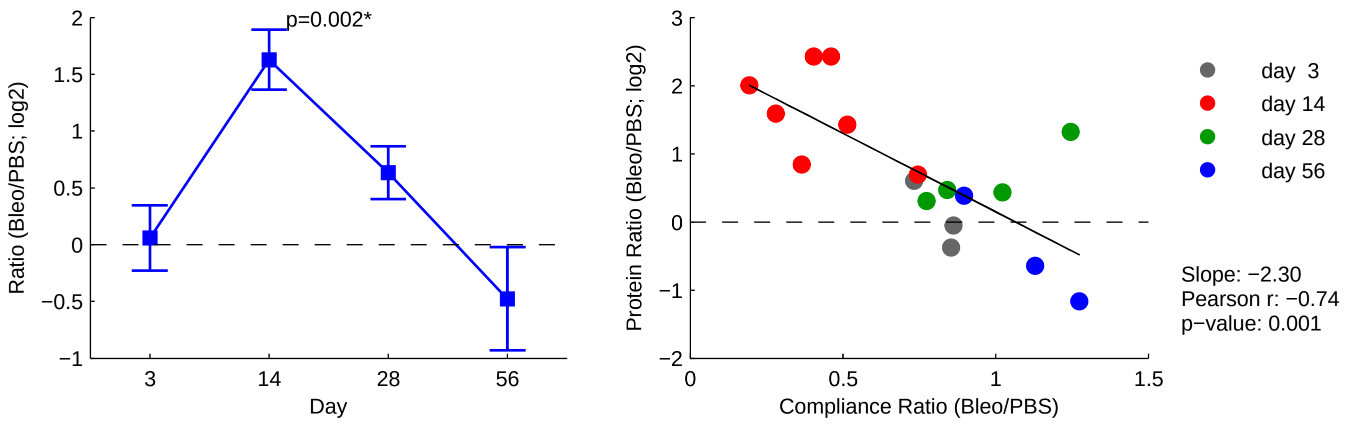

Q80ZK0 − Mrps10 (id: 1266)

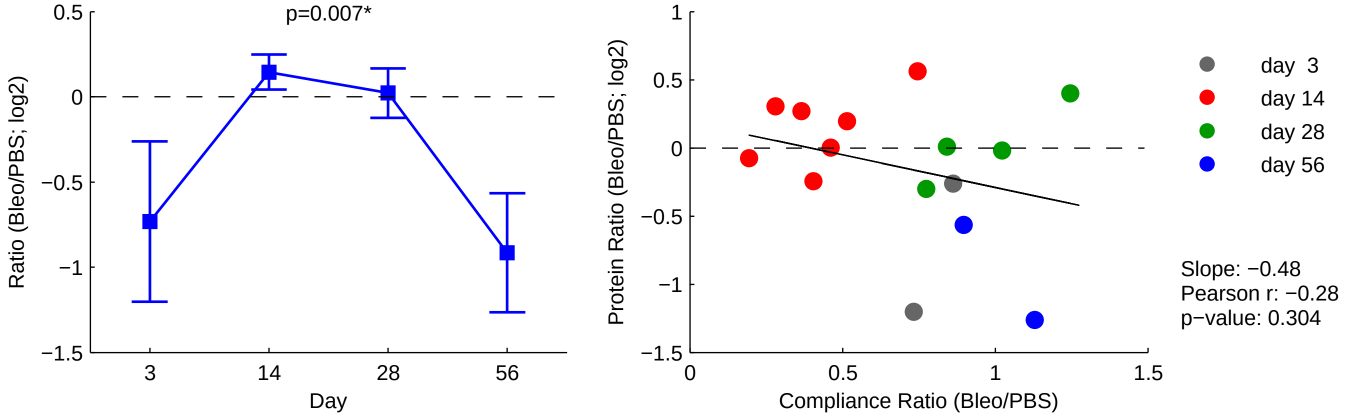

Q9R117 − Tyk2 (id: 1267)

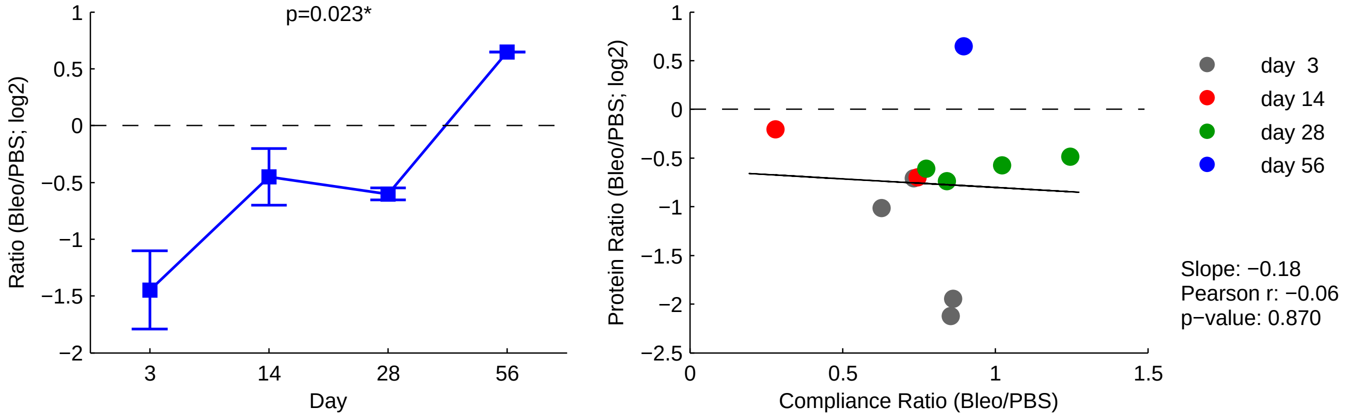

E9QJS7 − Cd97 (id: 1268)

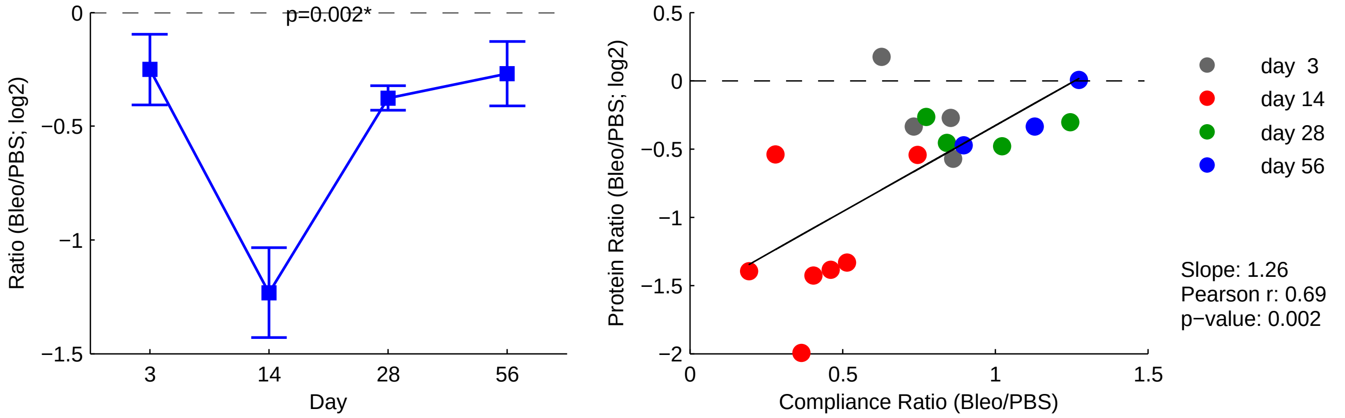

Q8R4E4 − Myoz3 (id: 1271)

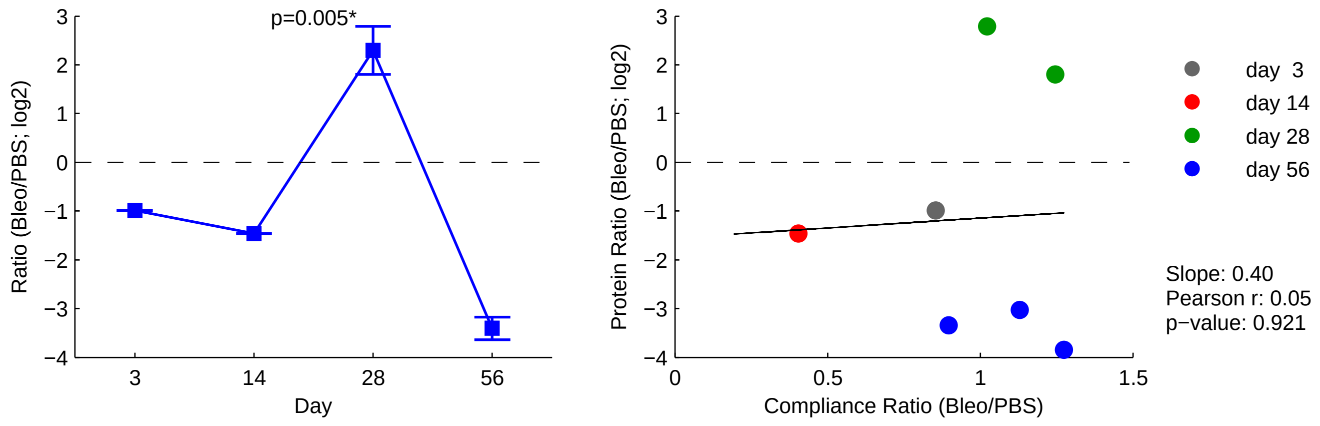

Q9QXJ2 − Stat2 (id: 1275)

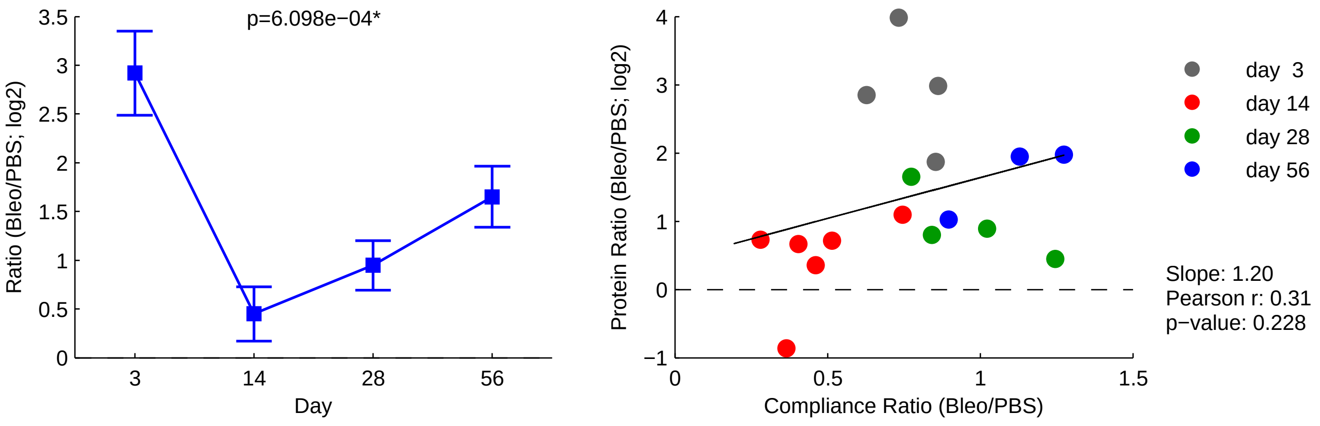

E9QK82 − Mpz (id: 1279)

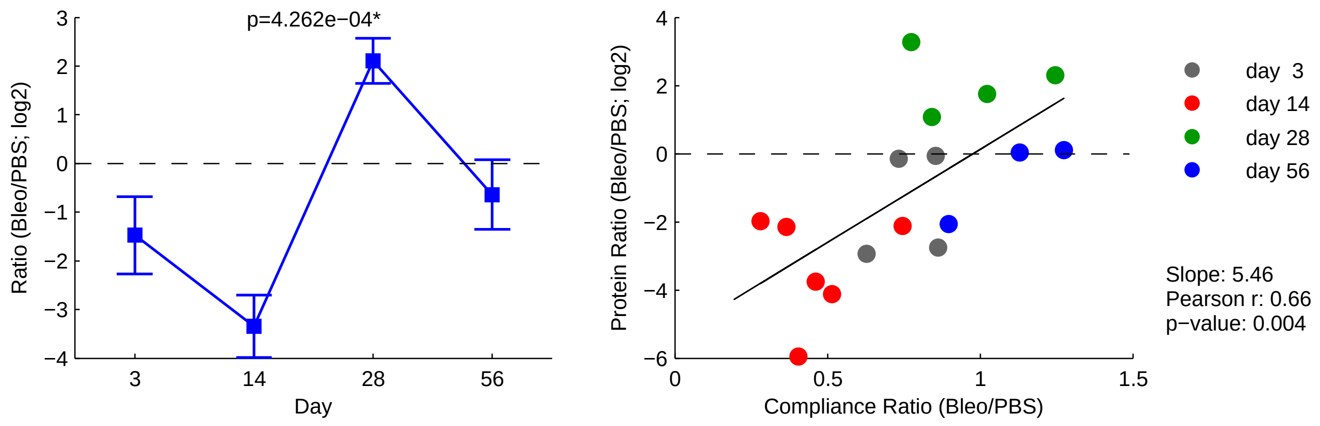

E9QKE4 − Rab3gap2 (id: 1286)

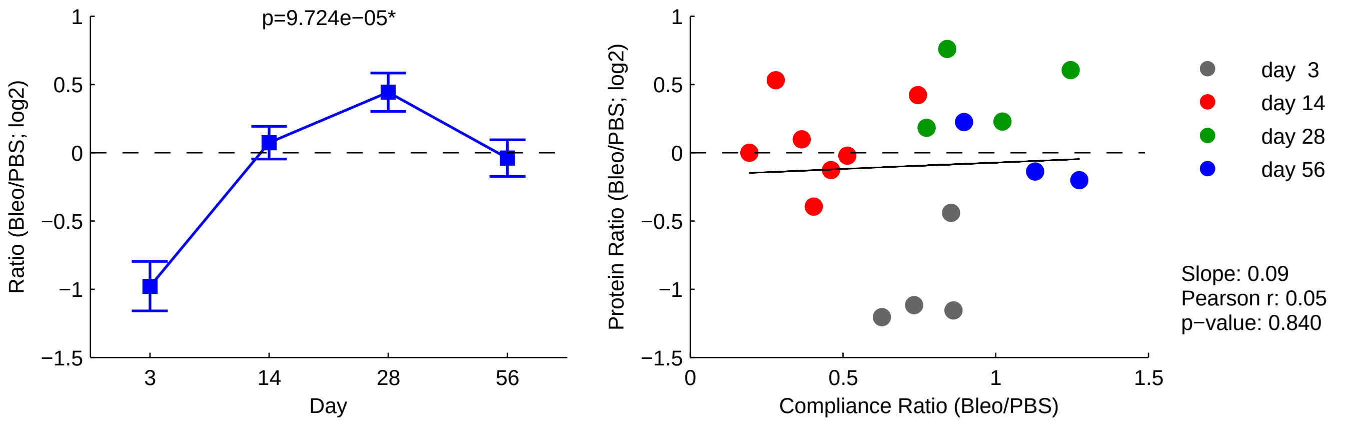

Q9CWH5 − Trmt11 (id: 1288)

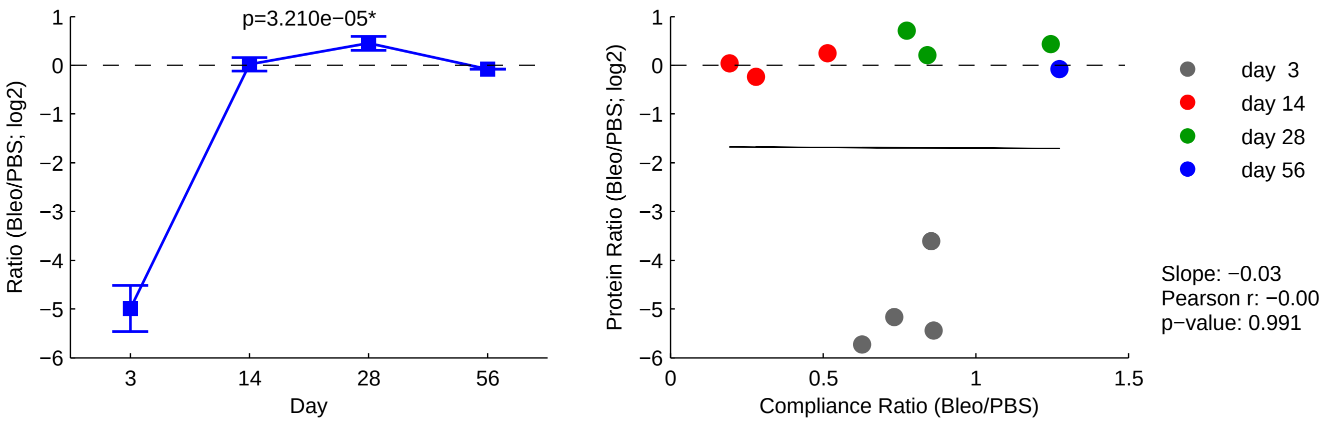

Q80TV8 − Clasp1 (id: 1290)

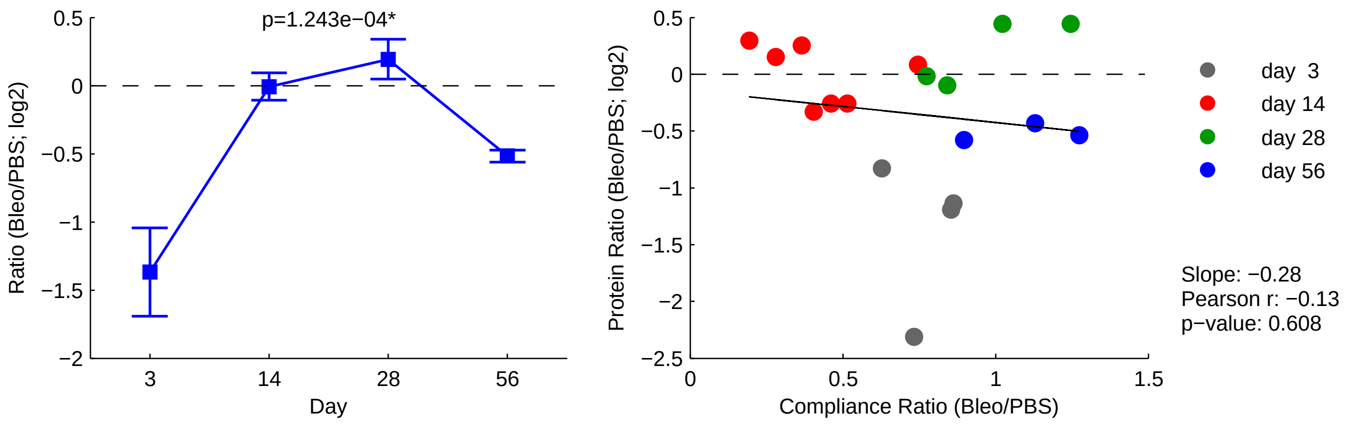

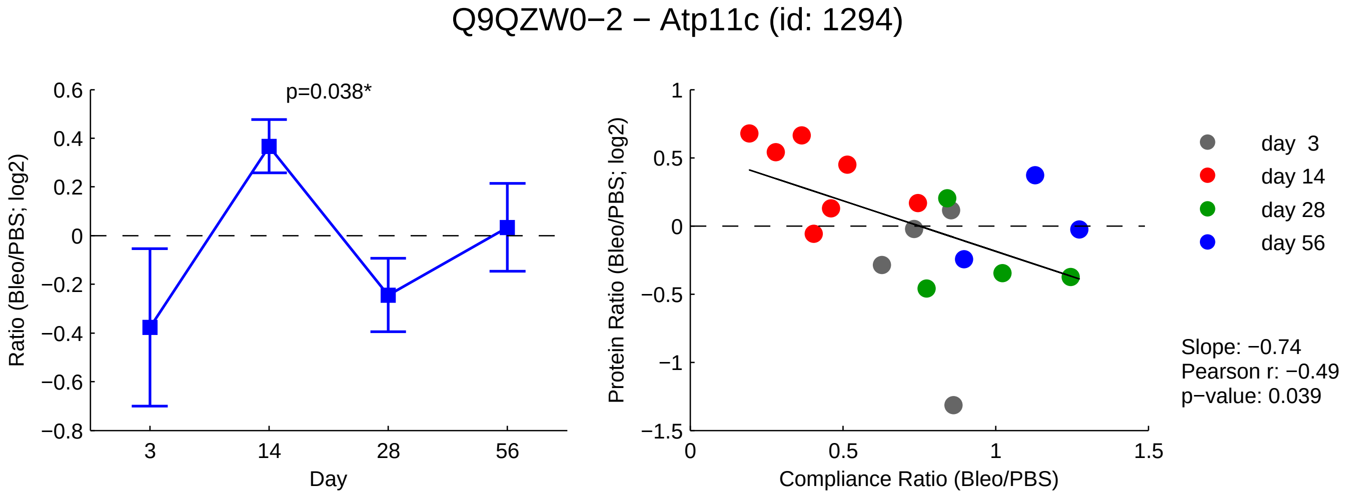
Q9QZW0−2 − Atp11c (id: 1294)
p=0.038*
Ratio (Bleo/PBS; log2)
Day
Protein Ratio (Bleo/PBS; log2)
Compliance Ratio (Bleo/PBS)
day 3
day 14
day 28
day 56
Slope: −0.74
Pearson r: −0.49
p−value: 0.039

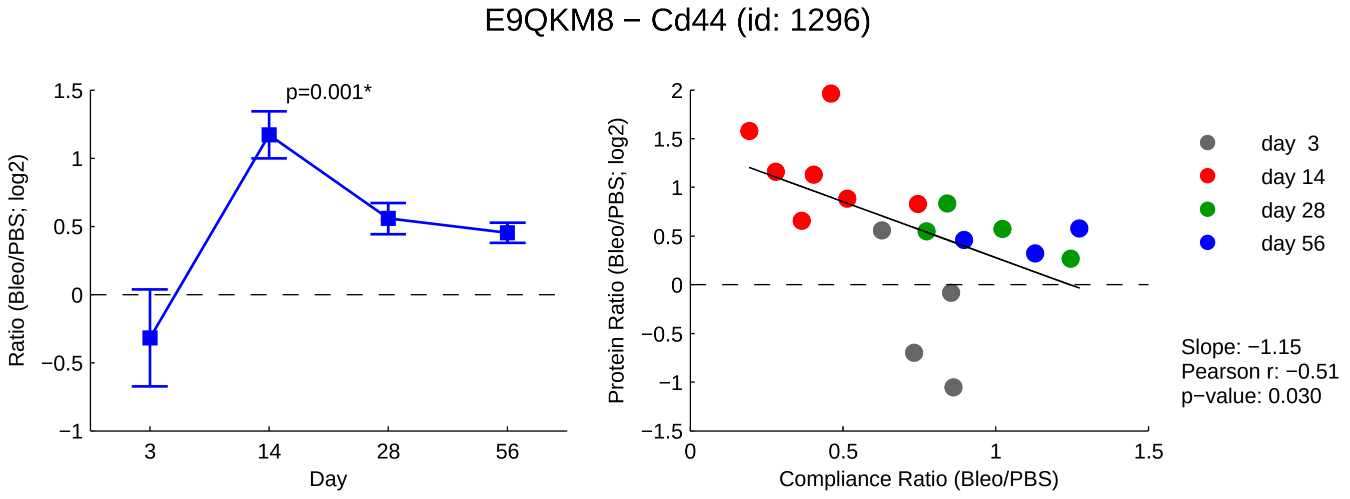
E9QKM8 − Cd44 (id: 1296)
p=0.001*
Ratio (Bleo/PBS; log2)
Day
Protein Ratio (Bleo/PBS; log2)
Compliance Ratio (Bleo/PBS)
day 3
day 14
day 28
day 56
Slope: −1.15
Pearson r: −0.51
p−value: 0.030

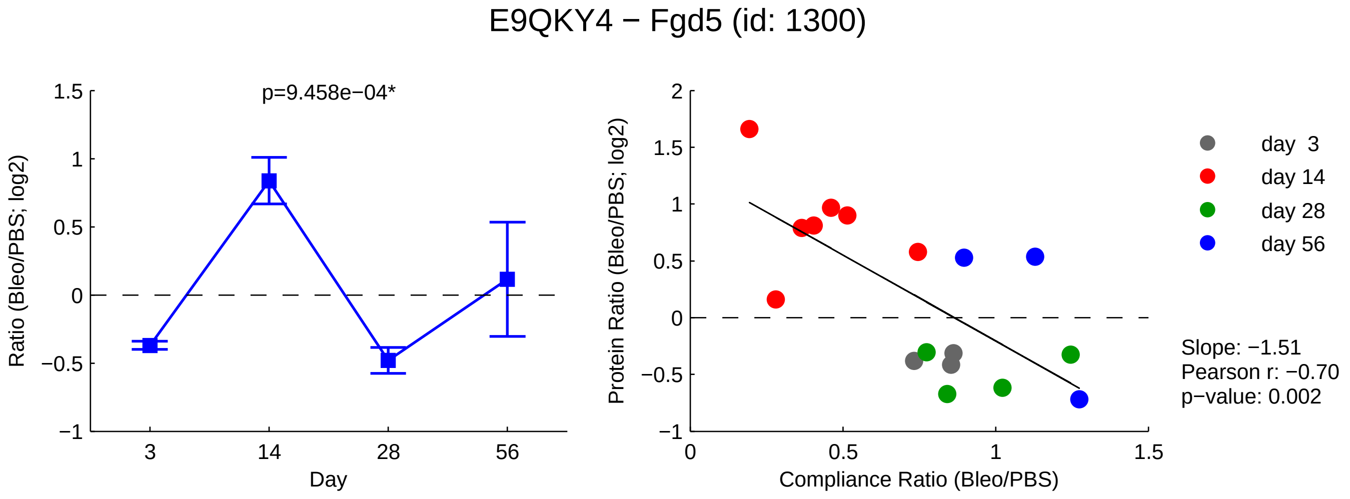
E9QKY4 − Fgd5 (id: 1300)
p=9.458e−04*
Ratio (Bleo/PBS; log2)
Day
Protein Ratio (Bleo/PBS; log2)
Compliance Ratio (Bleo/PBS)
day 3
day 14
day 28
day 56
Slope: −1.51
Pearson r: −0.70
p−value: 0.002

E9QKZ2 − Ipo9 (id: 1301)

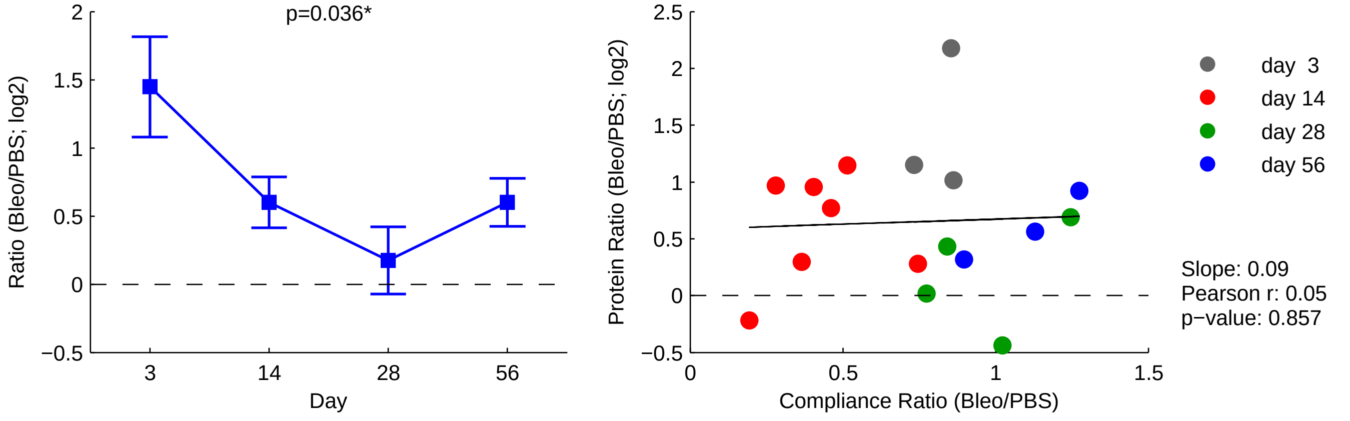

P98078−2 − Dab2 (id: 1302)

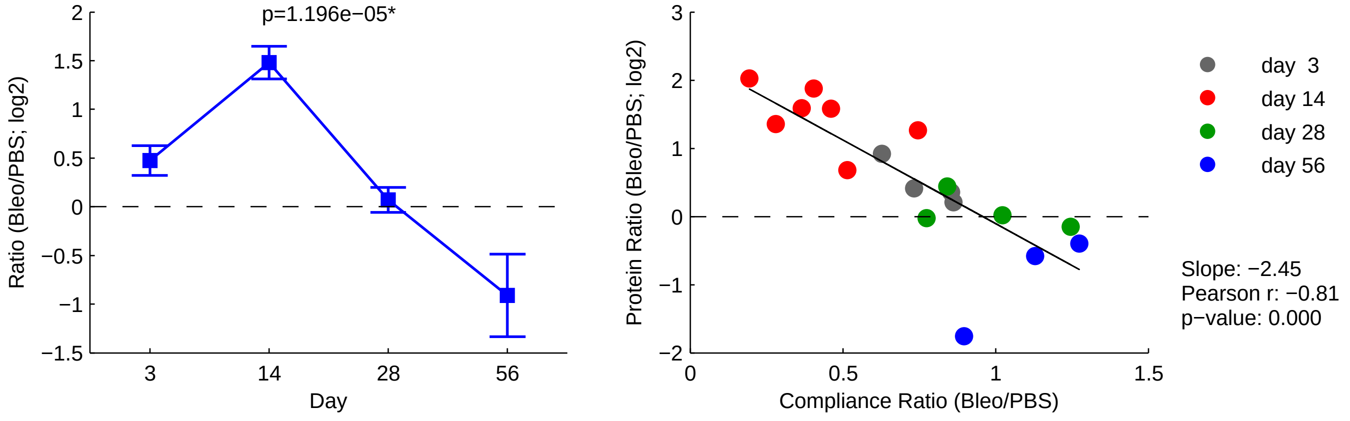

Q8CIC2 − Nupl2 (id: 1303)

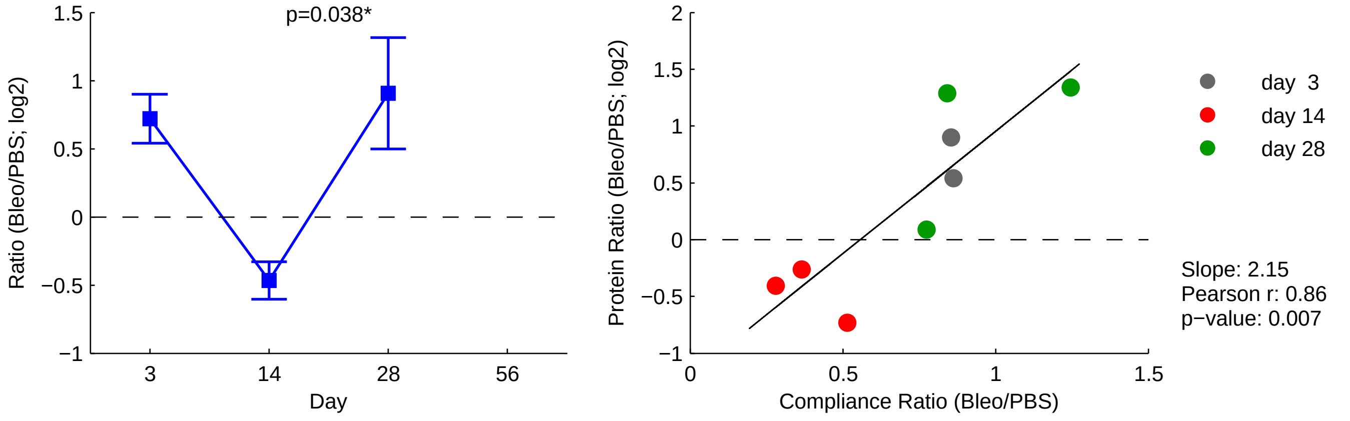

E9QL65 − Cog3 (id: 1304)

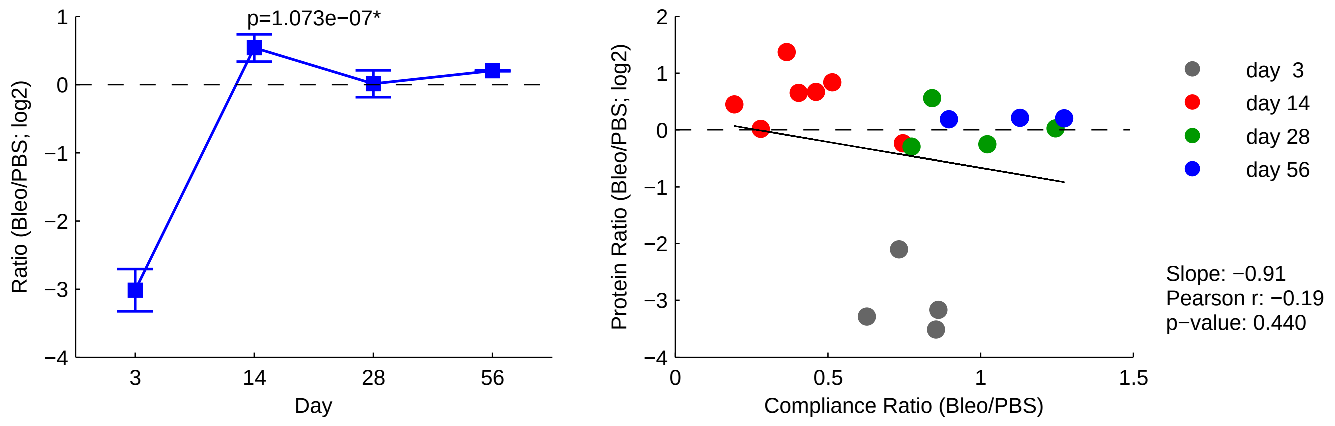

Q91YX5 − Lpgat1 (id: 1305)

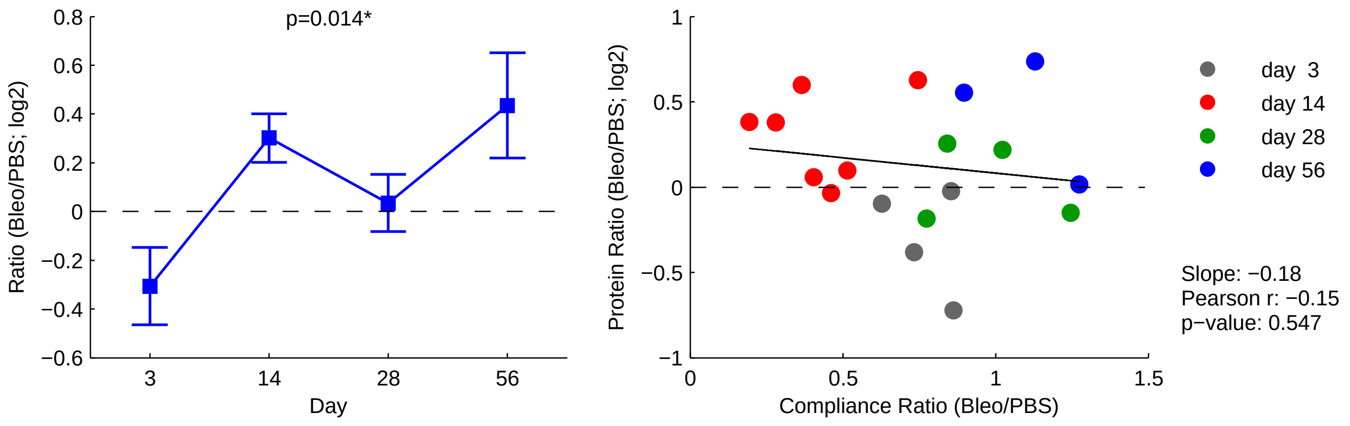

Q0GNC1−3 − Inf2 (id: 1307)

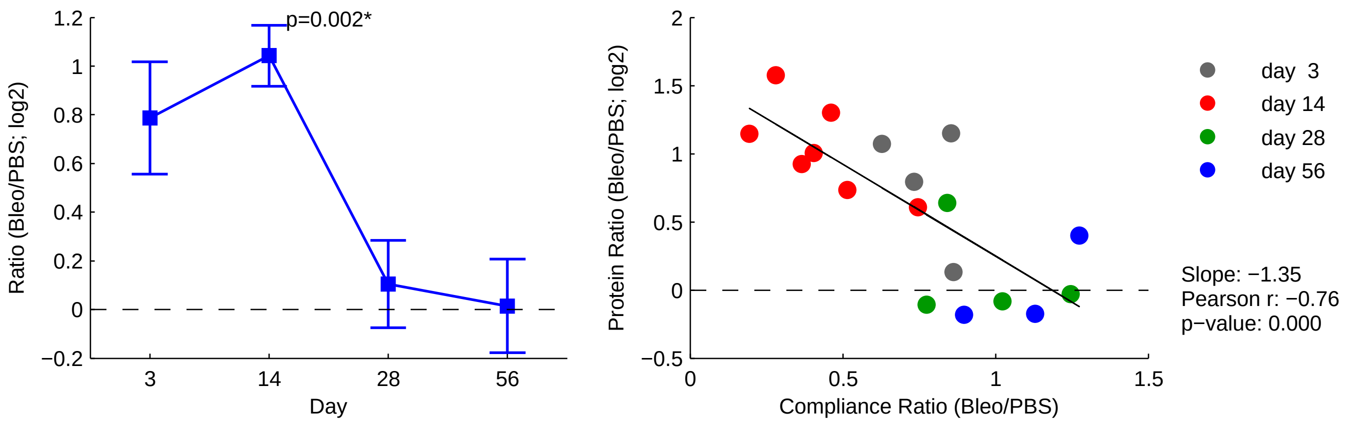

E9QLJ5 − Plec (id: 1311)

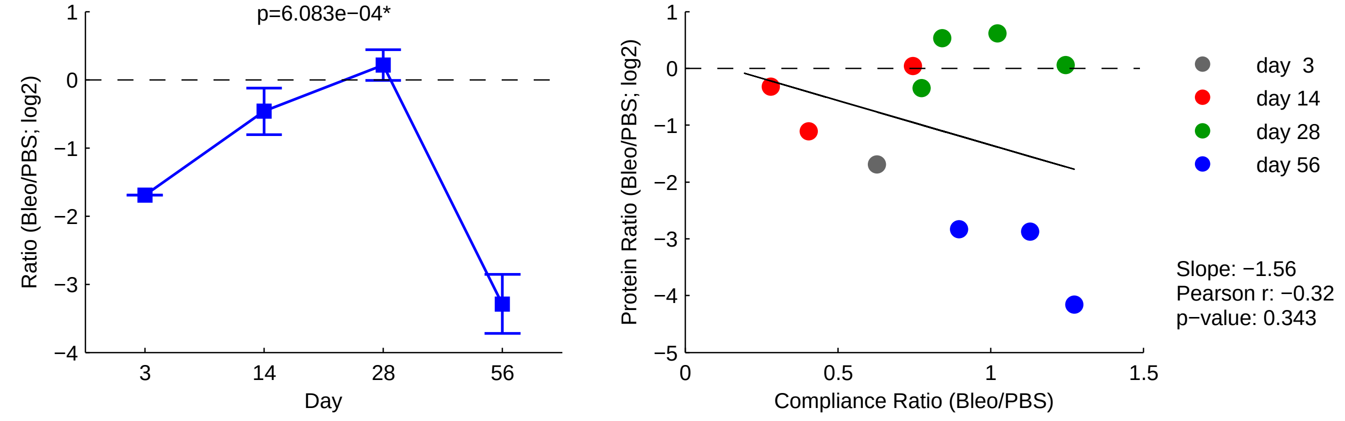

E9QLQ7 − Rab7l1 (id: 1313)

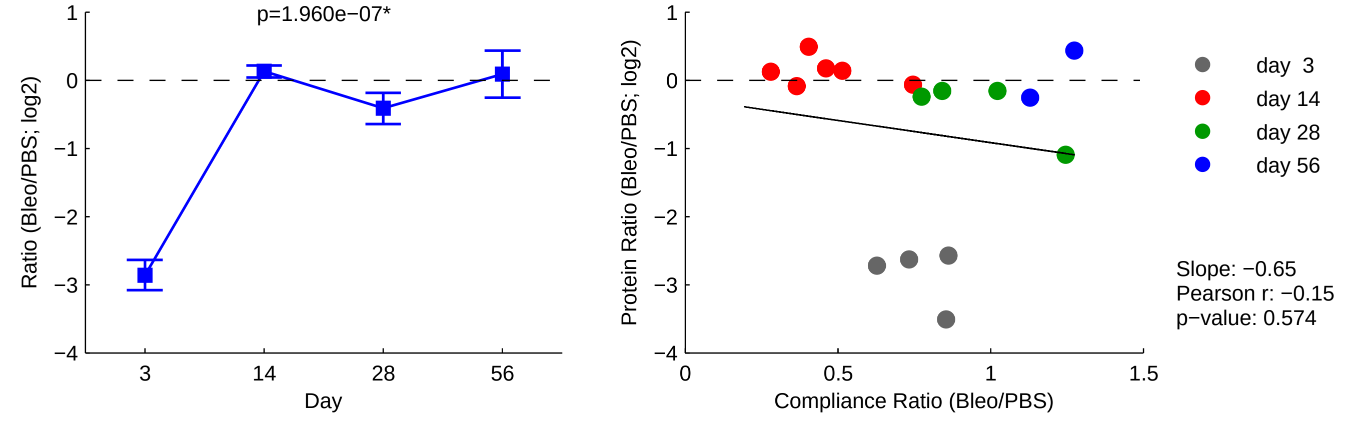

E9QM21 − Bak1 (id: 1316)

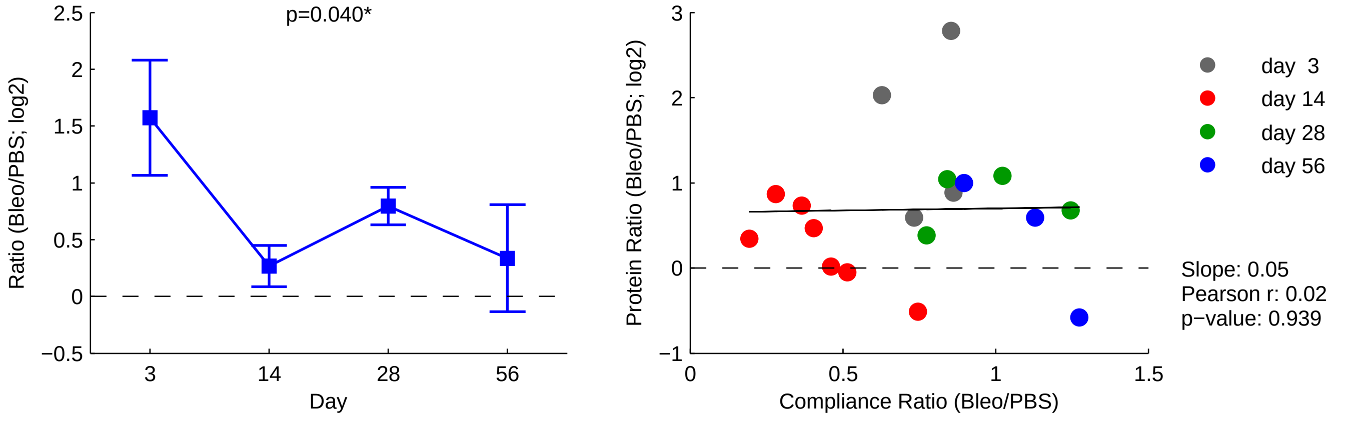

P55012 − Slc12a2 (id: 1317)

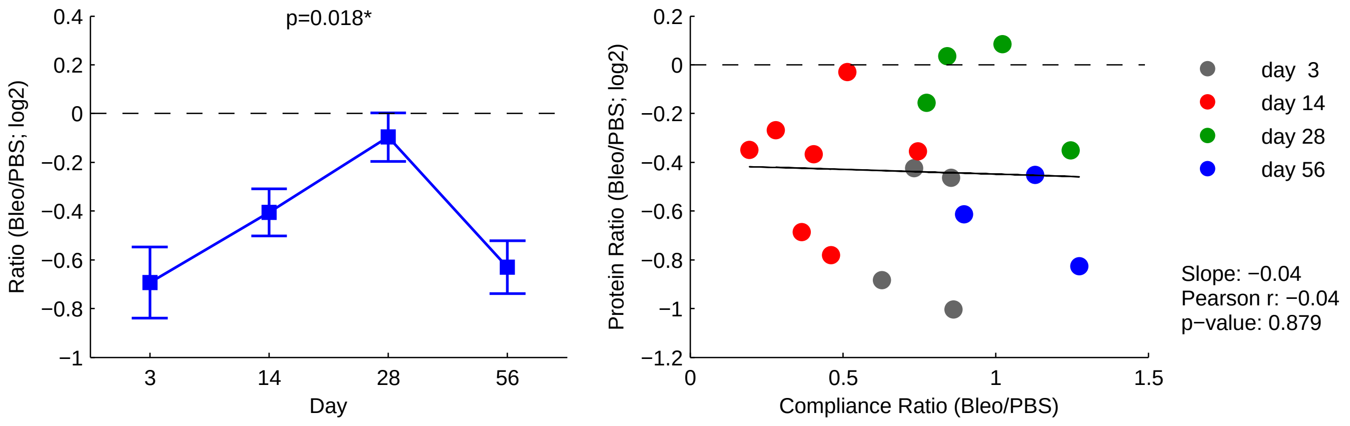

O70305−3 − Atxn2 (id: 1318)

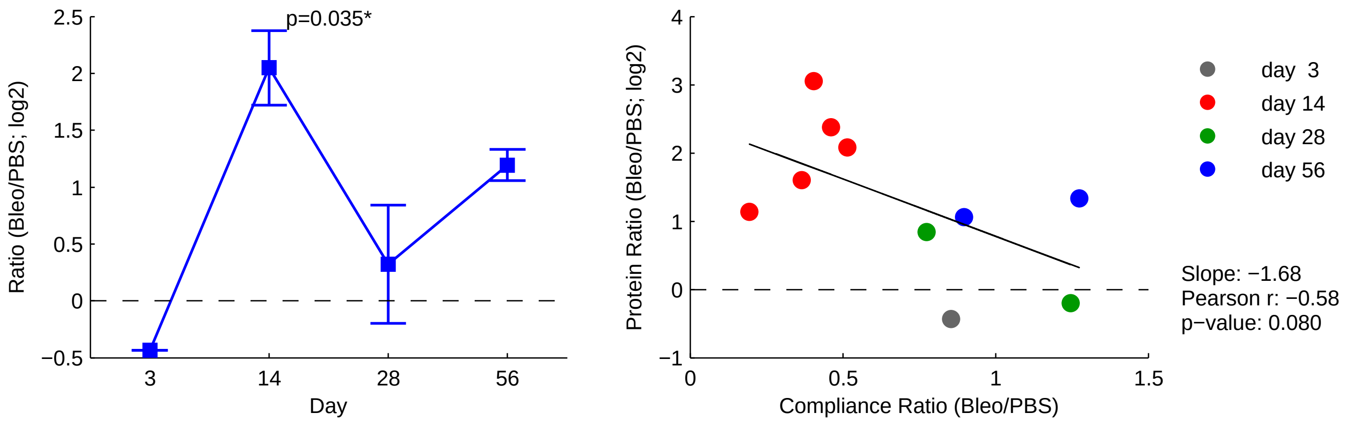

E9QM99 − Dock10 (id: 1319)

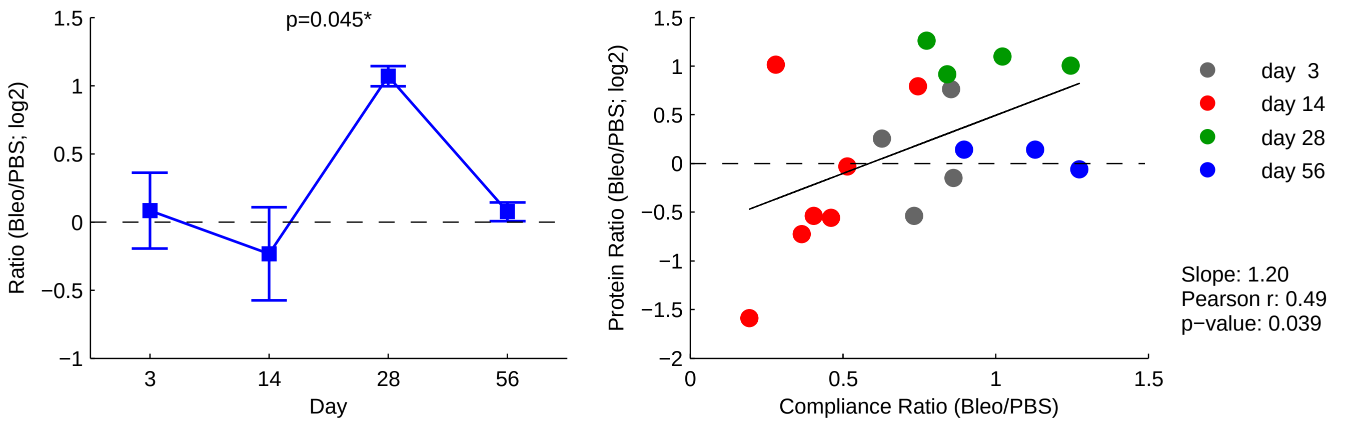

E9QMC1 − Cgn (id: 1322)

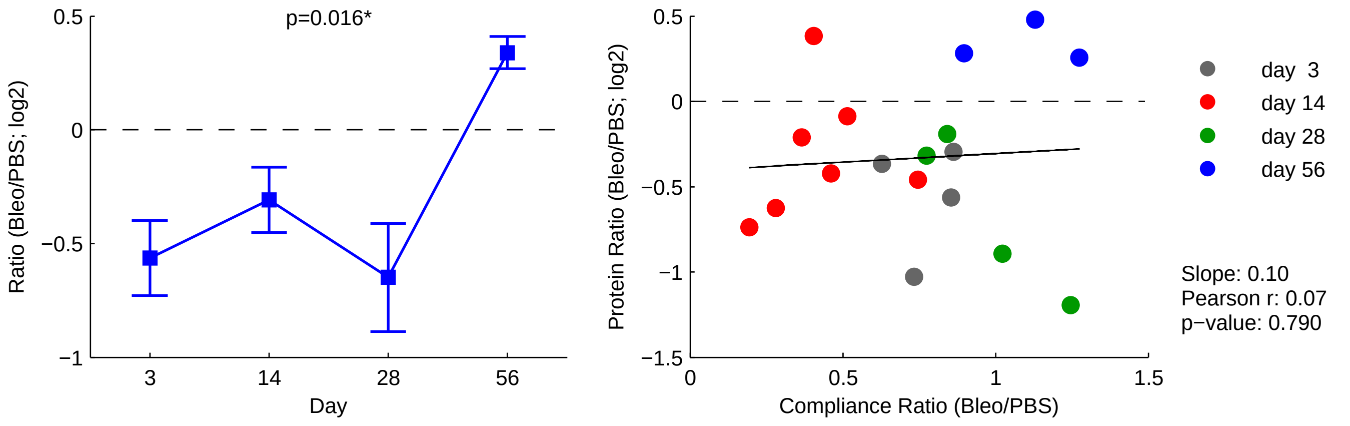

E9QME5 − Trim33 (id: 1324)

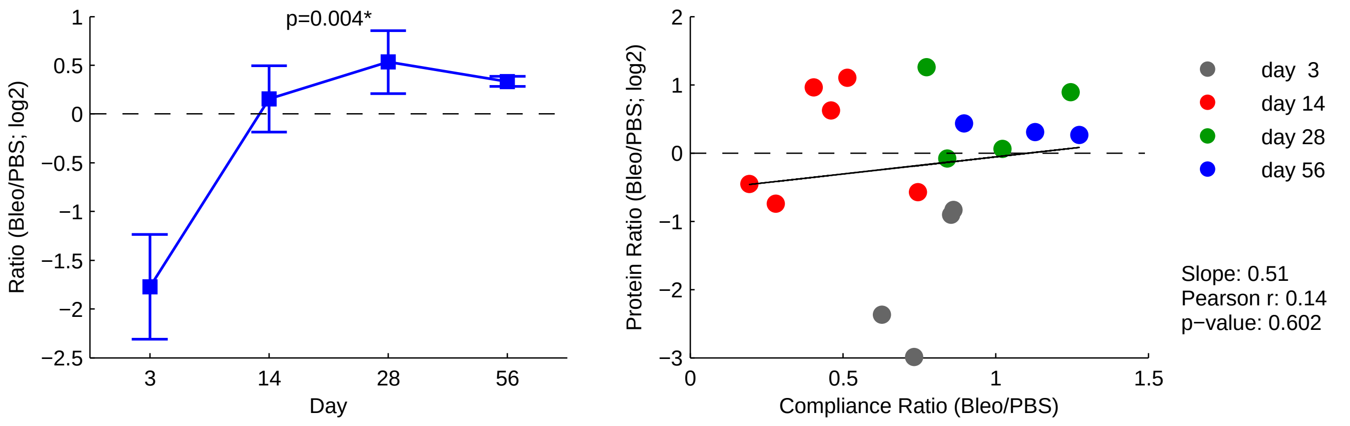

E9QMH7 − Ikbip (id: 1327)

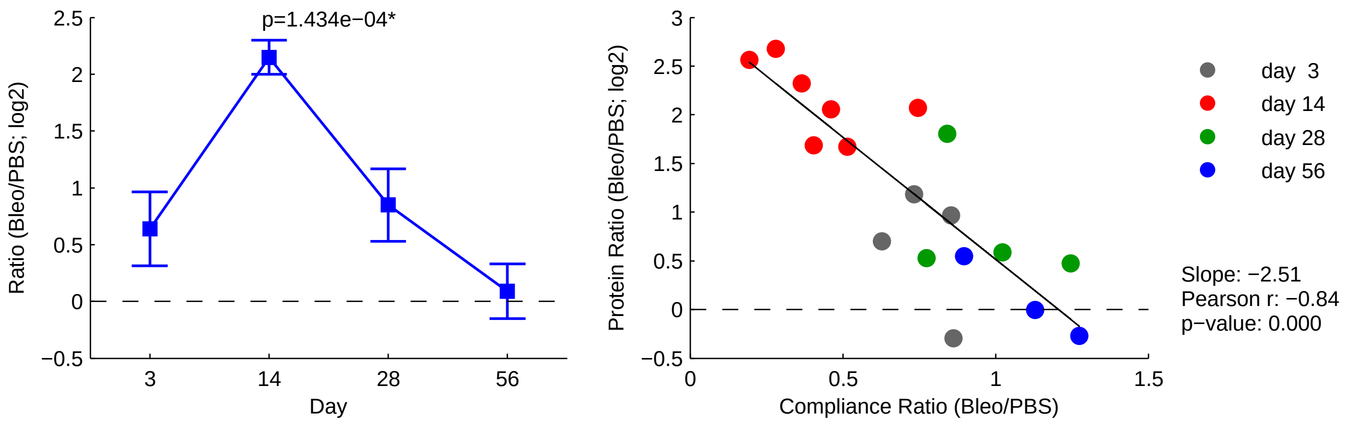

H3BKE6 − Asap1 (id: 1328)

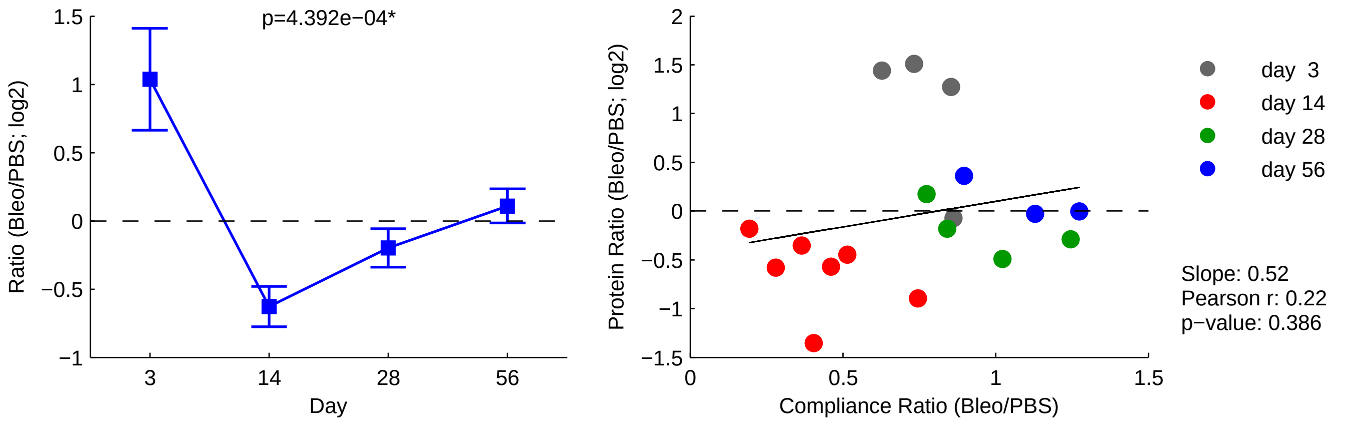

Q8BH86 − 9030617O03Rik (id: 1329)

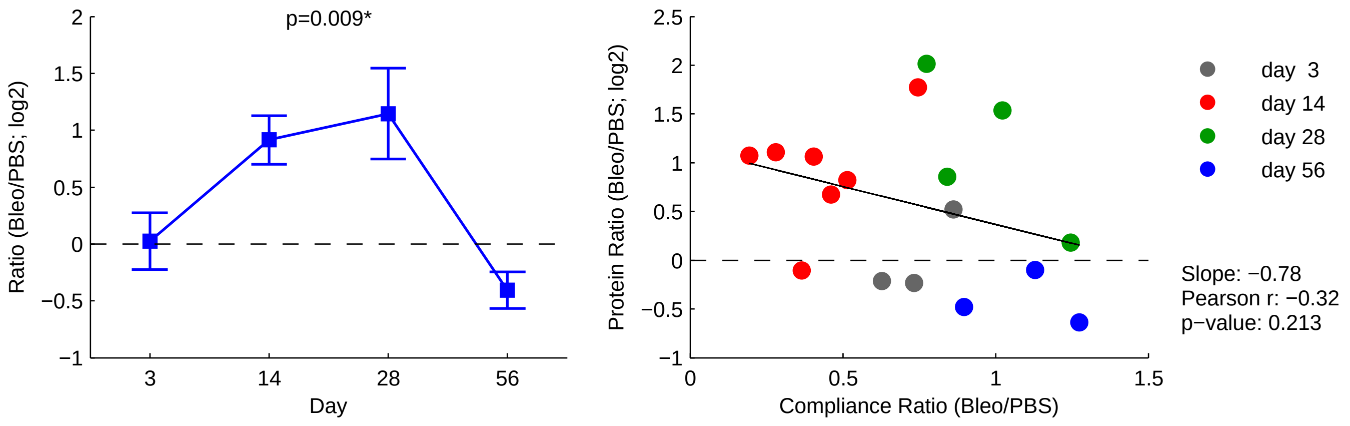

Q61464−4 − Zfml (id: 1330)

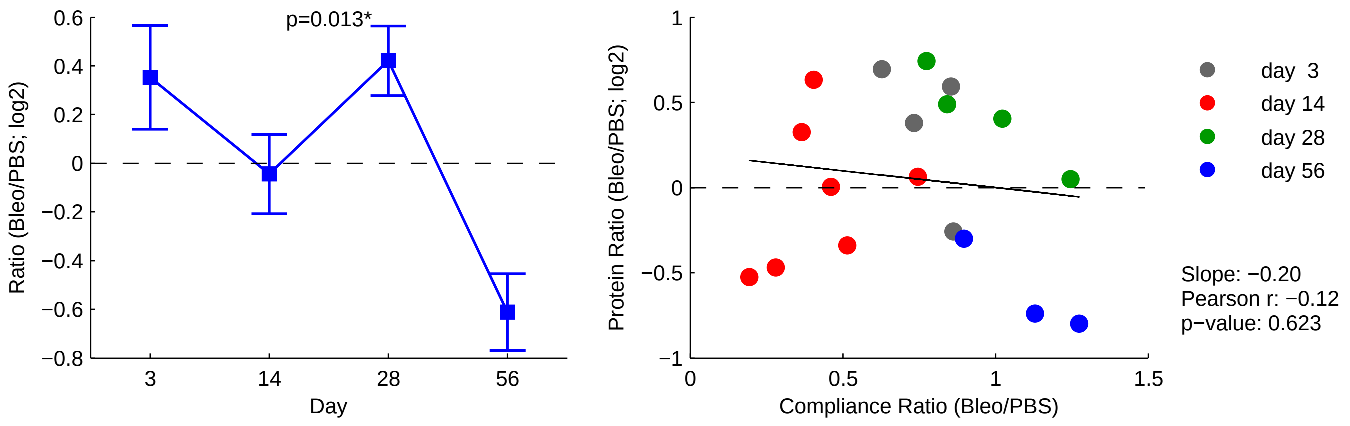

Q05512−3 − Mark2 (id: 1332)

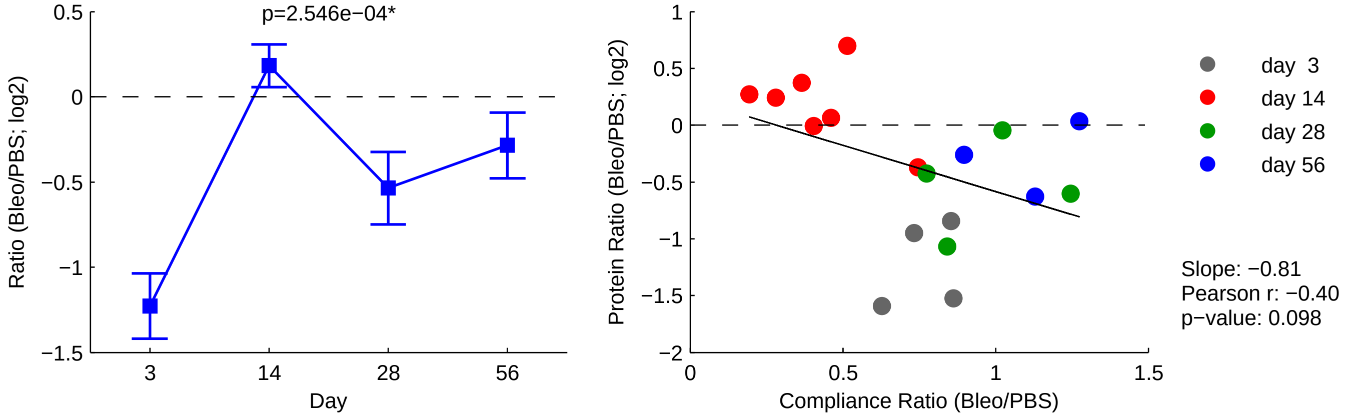

Q4KML4 − Abracl (id: 1334)

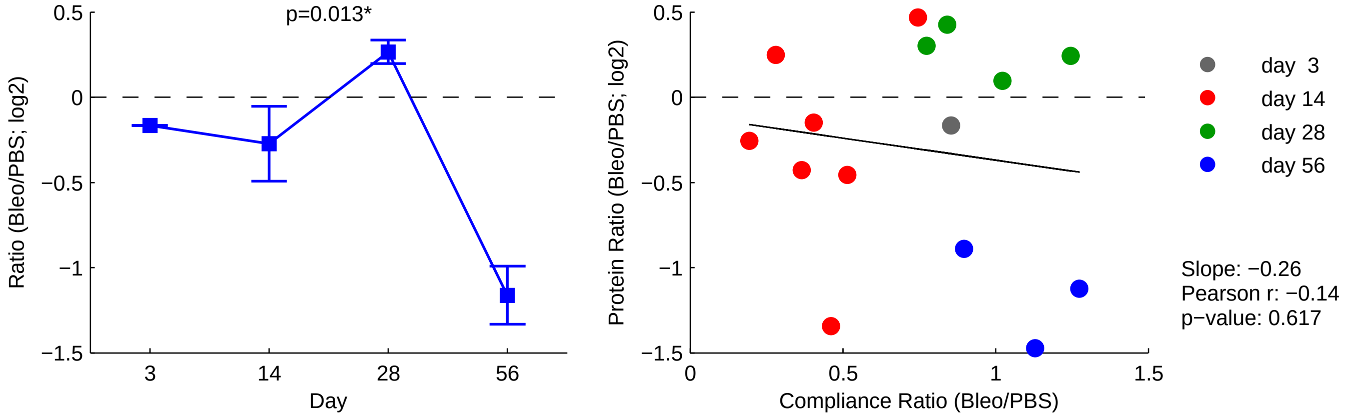

E9QMY1 − Cd200 (id: 1336)

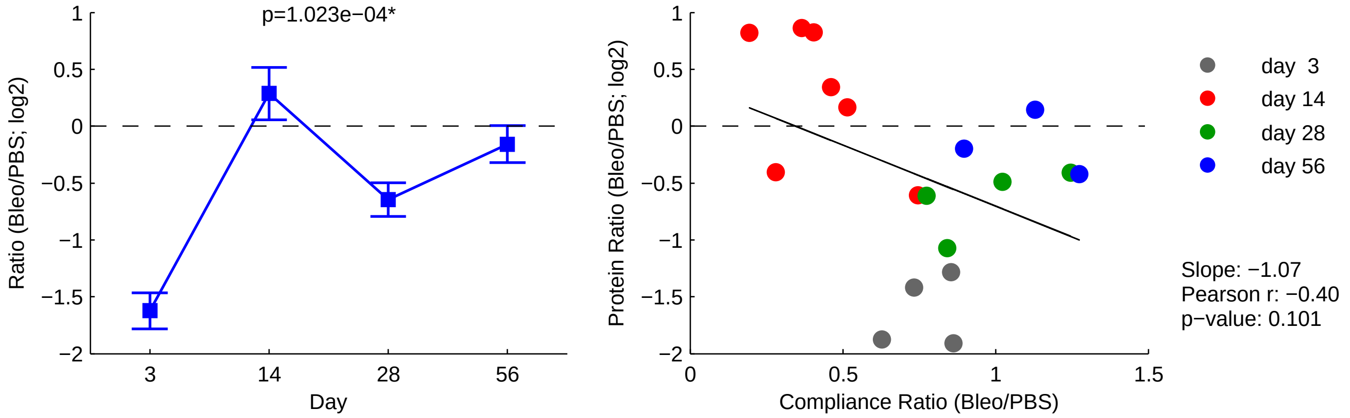

P05622 − Pdgfrb (id: 1339)

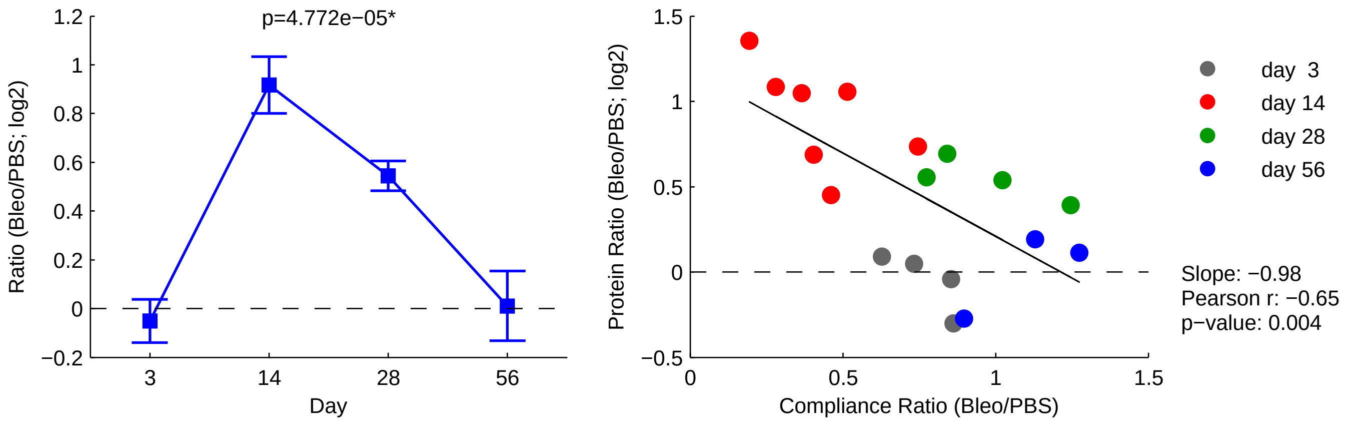

E9QN37 − Mpeg1 (id: 1341)

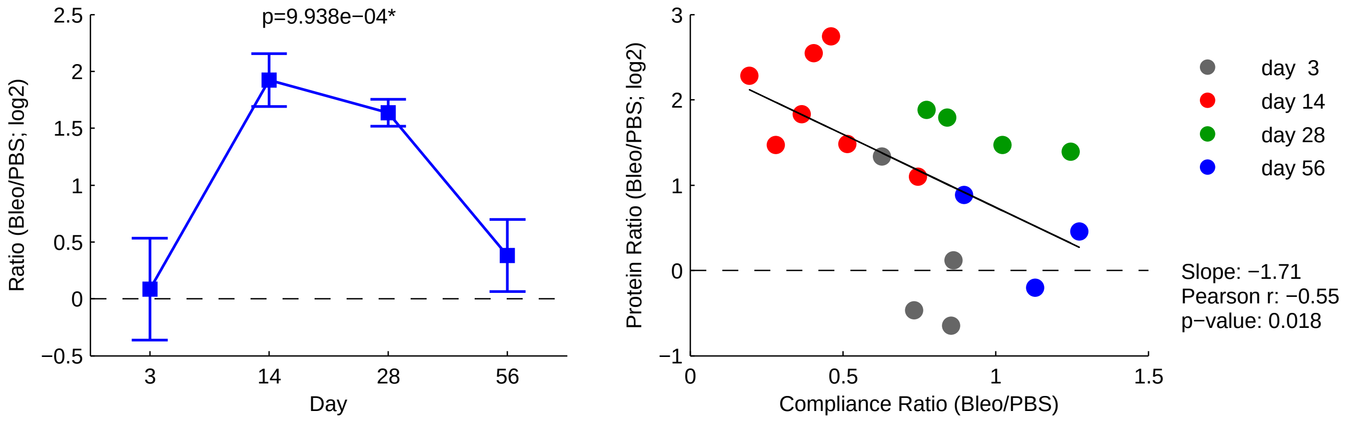

P02469 − Lamb1 (id: 1343)

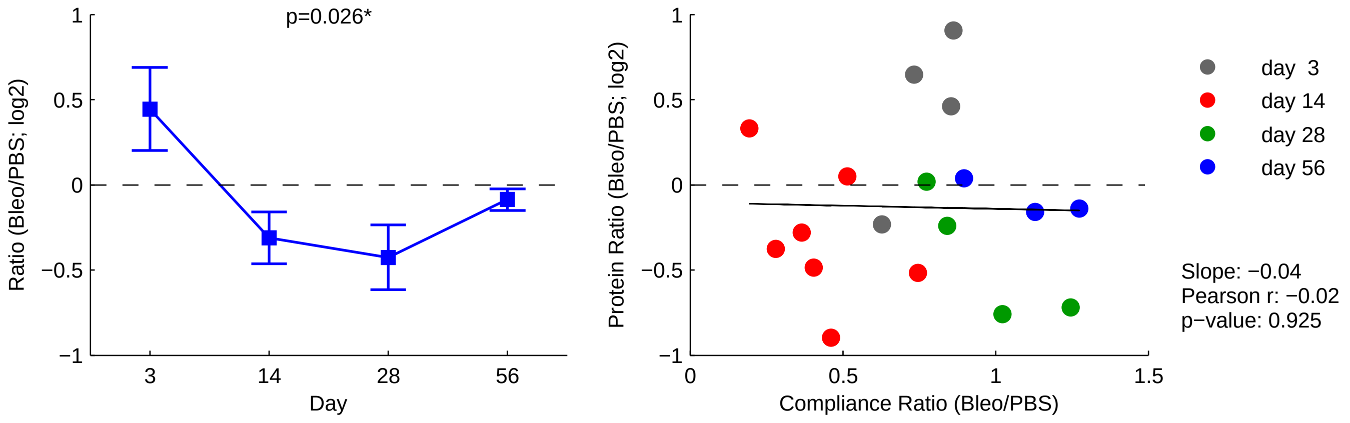

E9QNA7 − Sorbs1 (id: 1345)

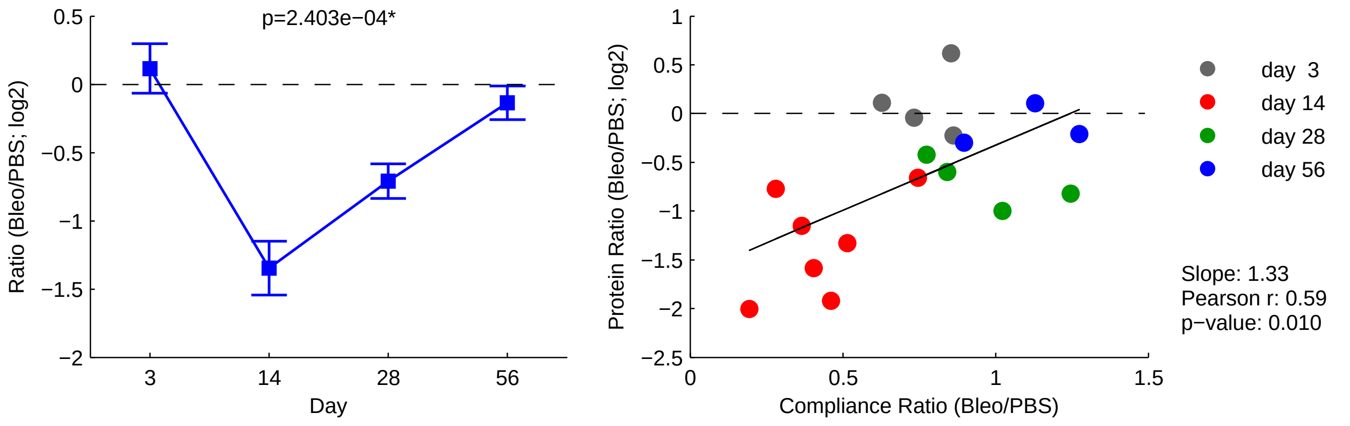

Q6PHS6 − Snx13 (id: 1348)

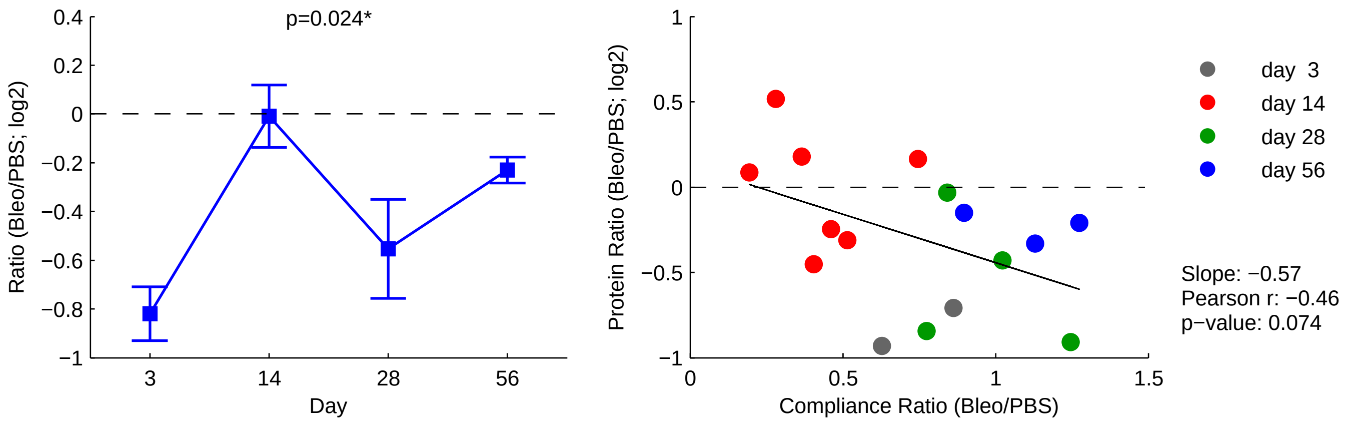

E9QNN1 − Dhx9 (id: 1350)

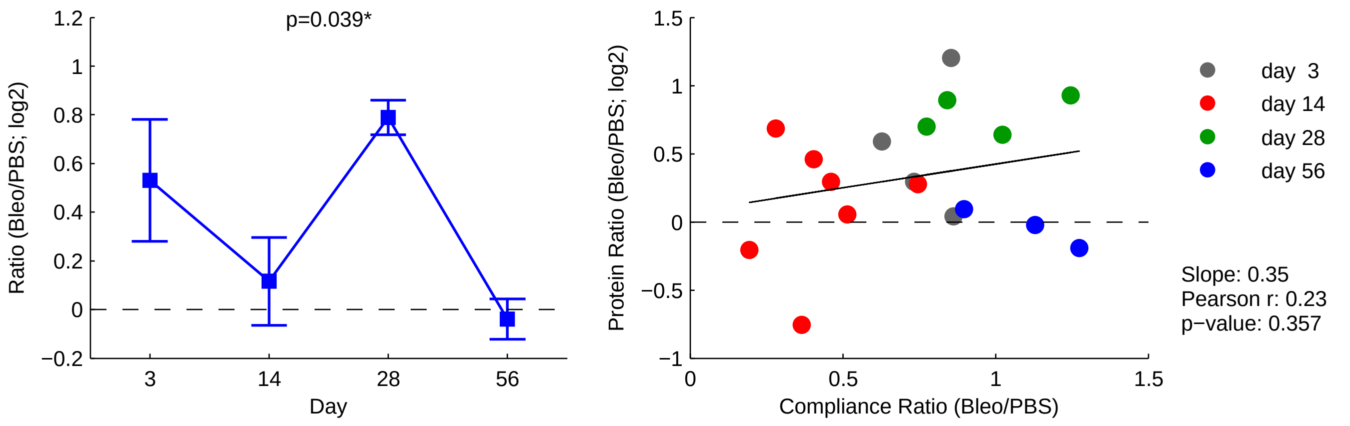

E9QNP0 − Kxd1 (id: 1351)

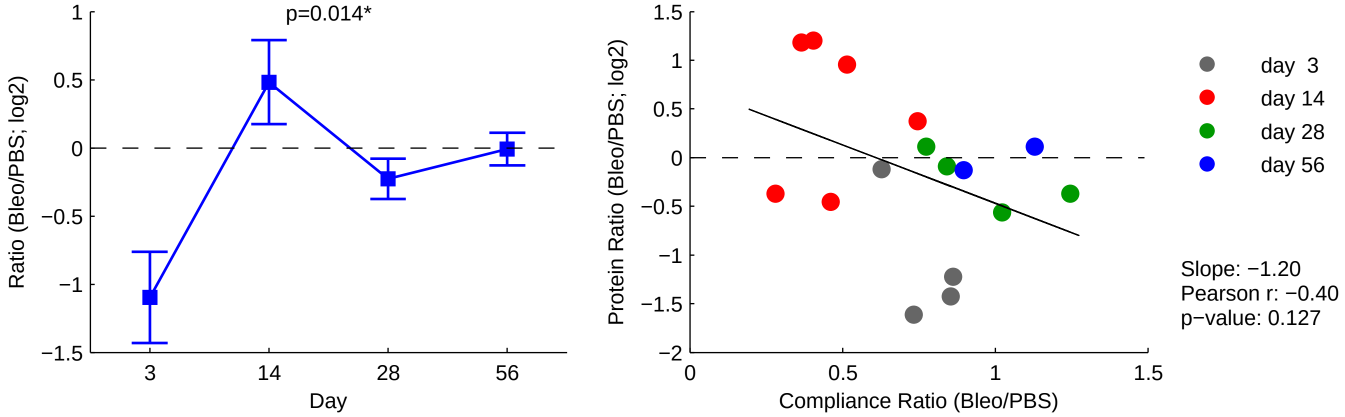

E9QNQ4 − Rapgef2 (id: 1353)

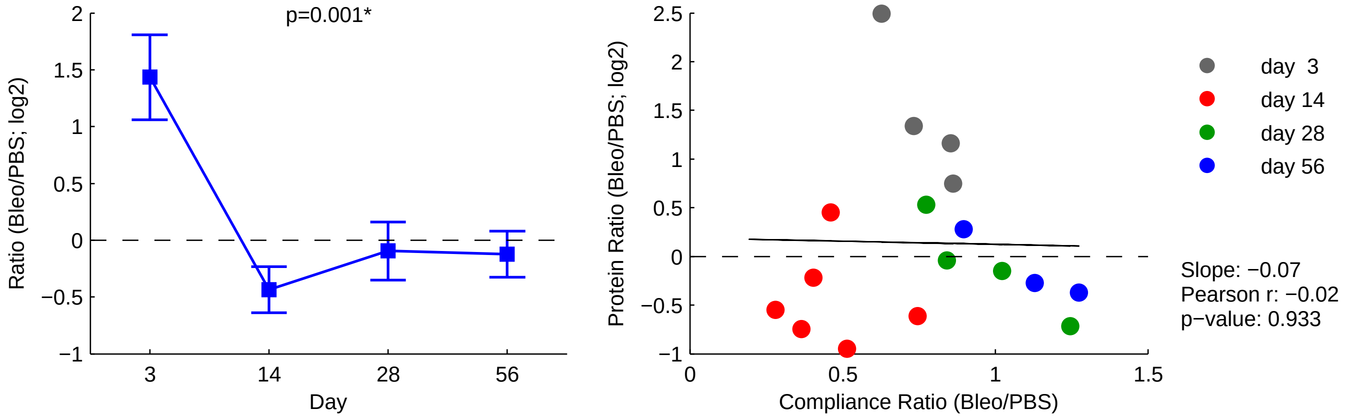

Q8R2G4−8 − Art3 (id: 1354)

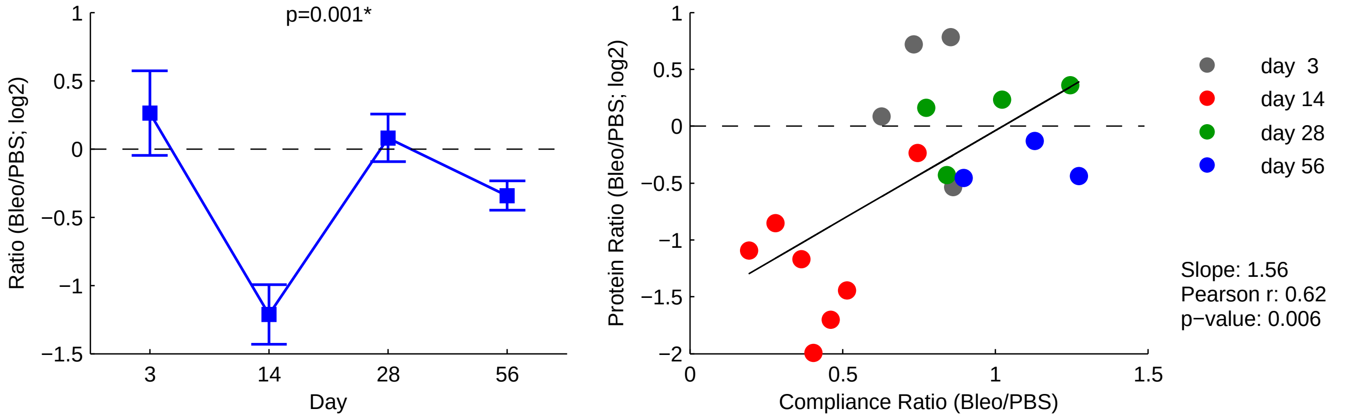

Q80YQ2 − Med23 (id: 1355)

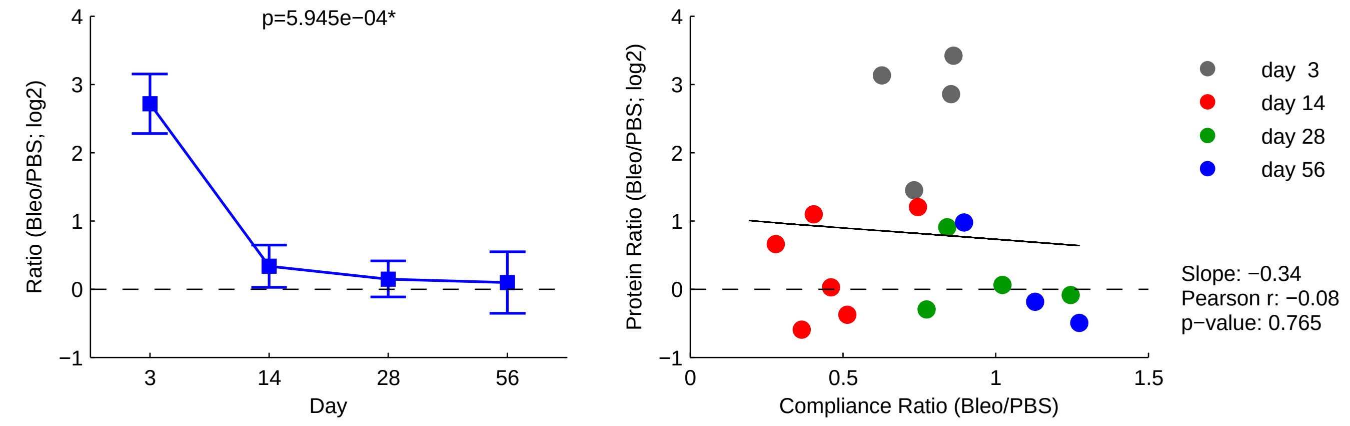

E9QNX9 − Igf1r (id: 1358)

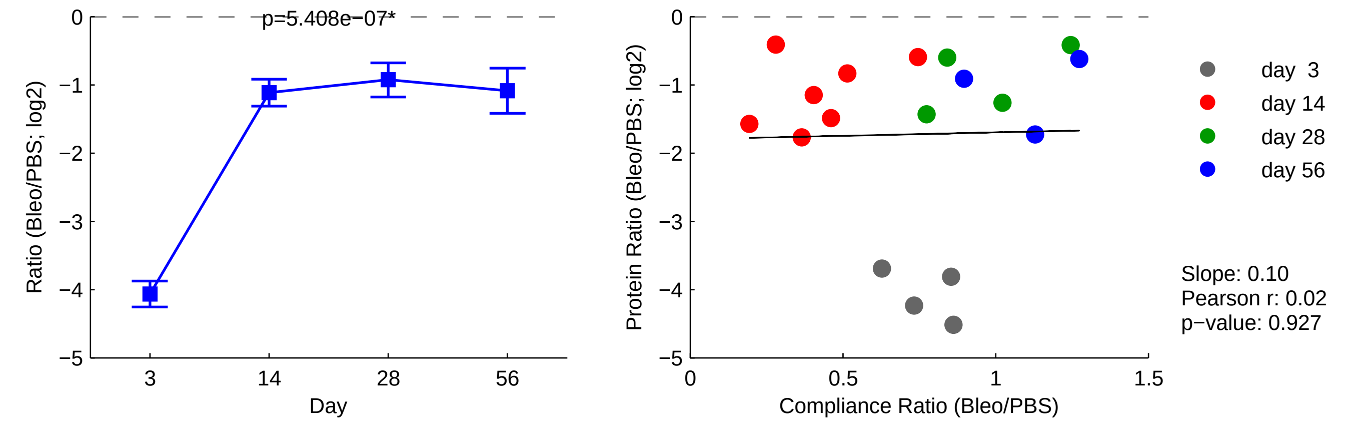

E9QP46 − Syne2 (id: 1363)

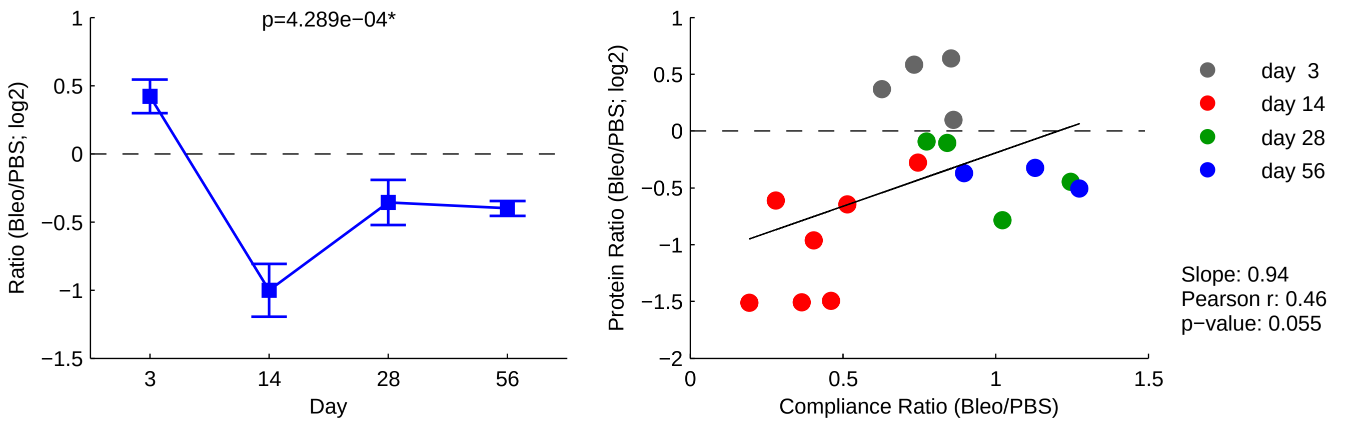

Q99MS7 − Ehbp1l1 (id: 1364)

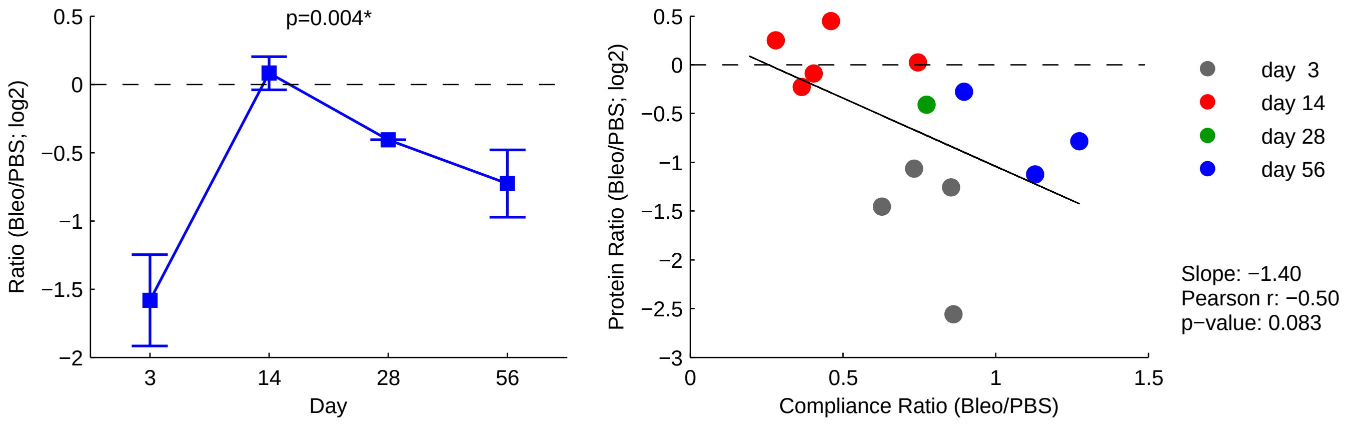

P55937−1 − Golga3 (id: 1368)

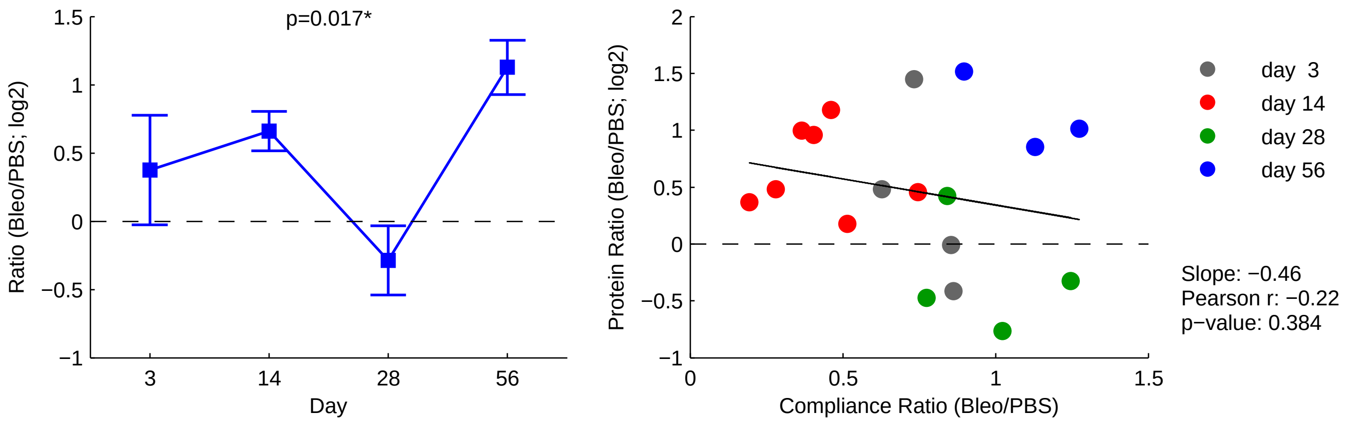

G5E8R3 − Pcx (id: 1371)

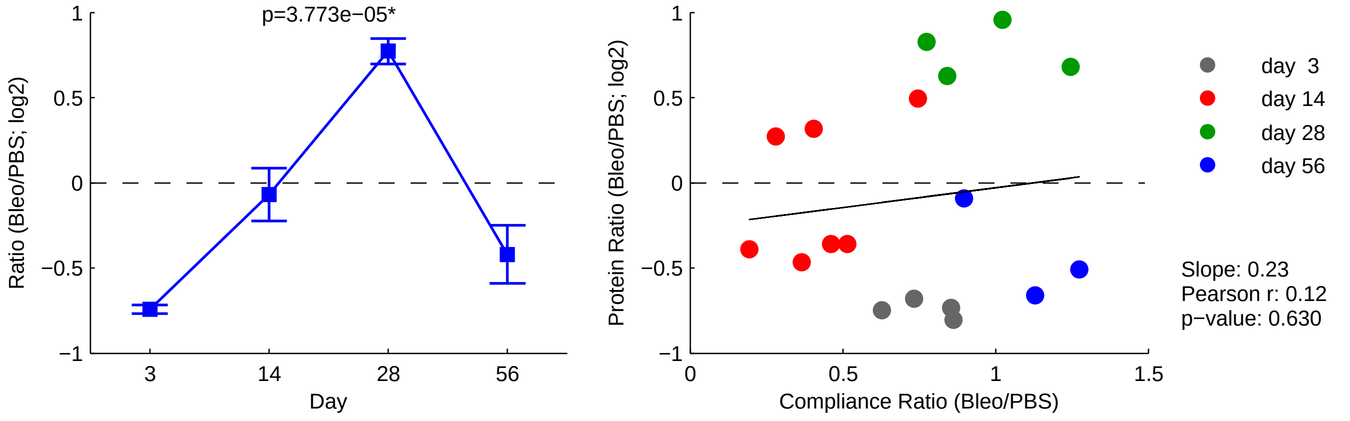

E9QPH0 − Prkg2 (id: 1374)

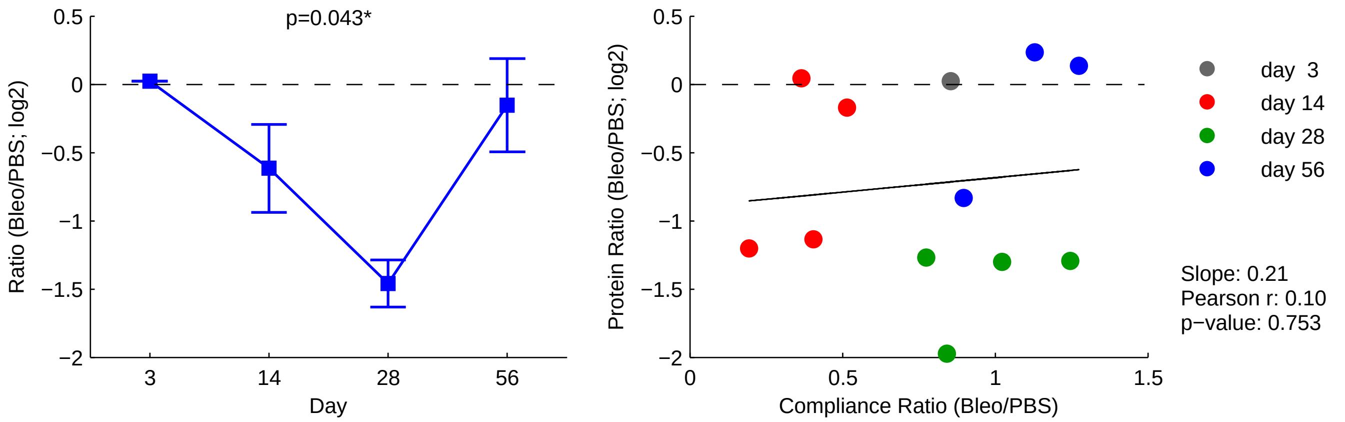

Q6A026 − Pds5a (id: 1375)

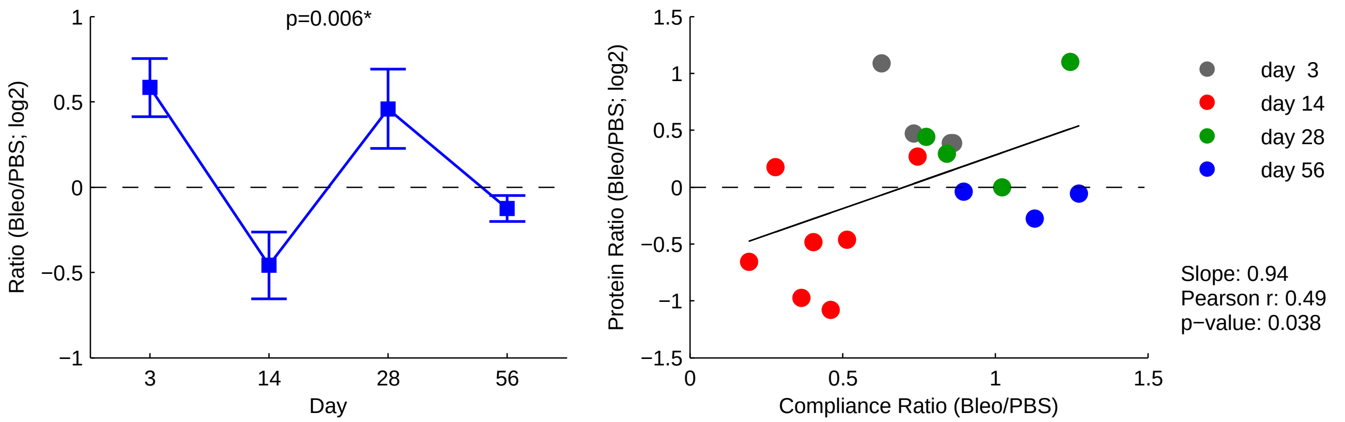

Q8VDR9 − Dock6 (id: 1377)

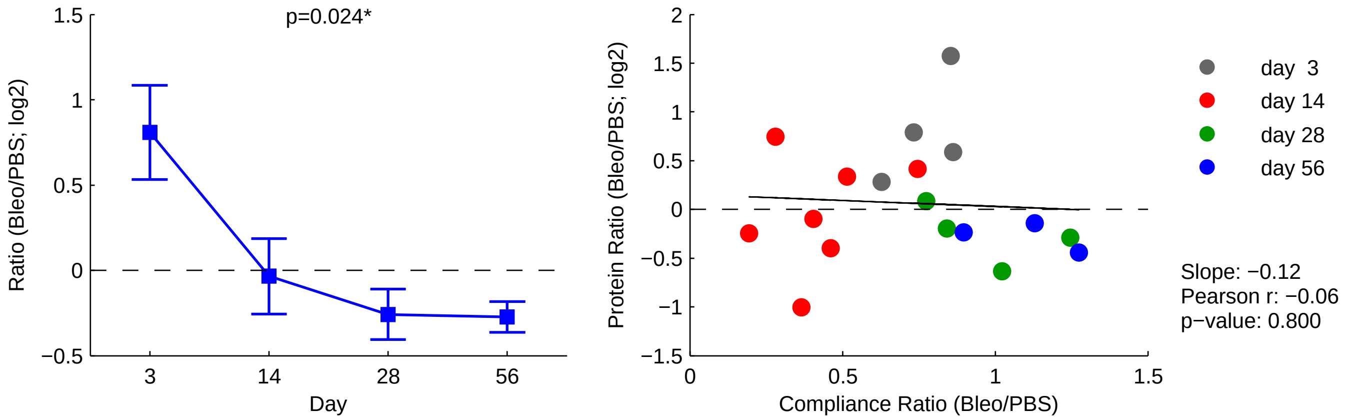

E9QPQ8 − Mrpl48 (id: 1378)

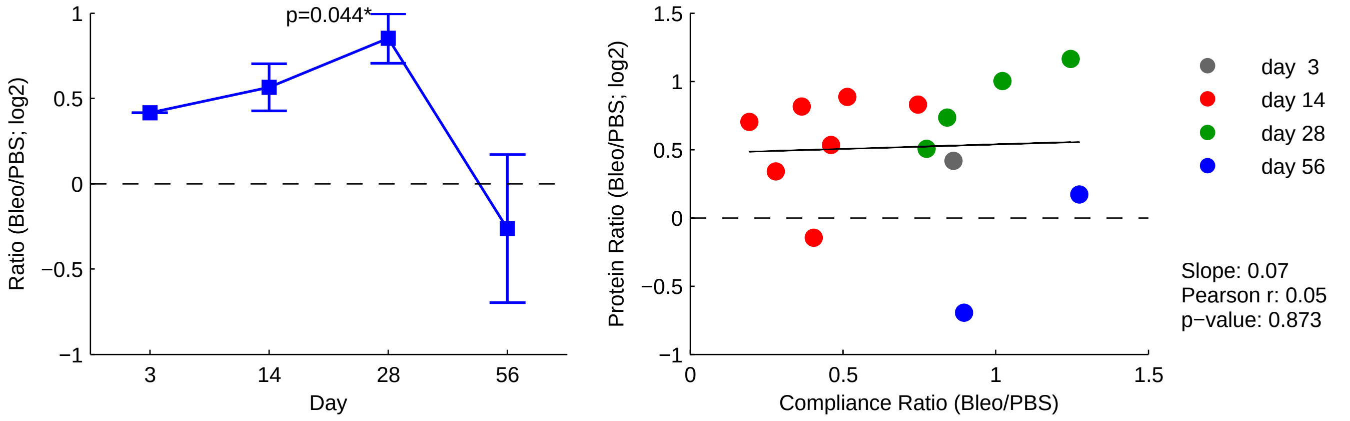

Q8CIZ8 − Vwf (id: 1379)

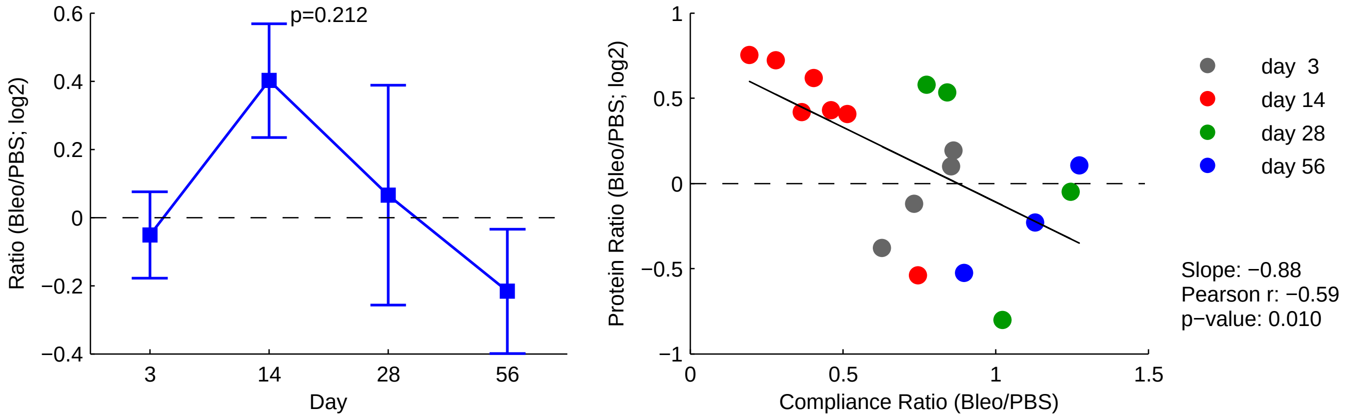

Q9CXZ1 − Ndufs4 (id: 1381)

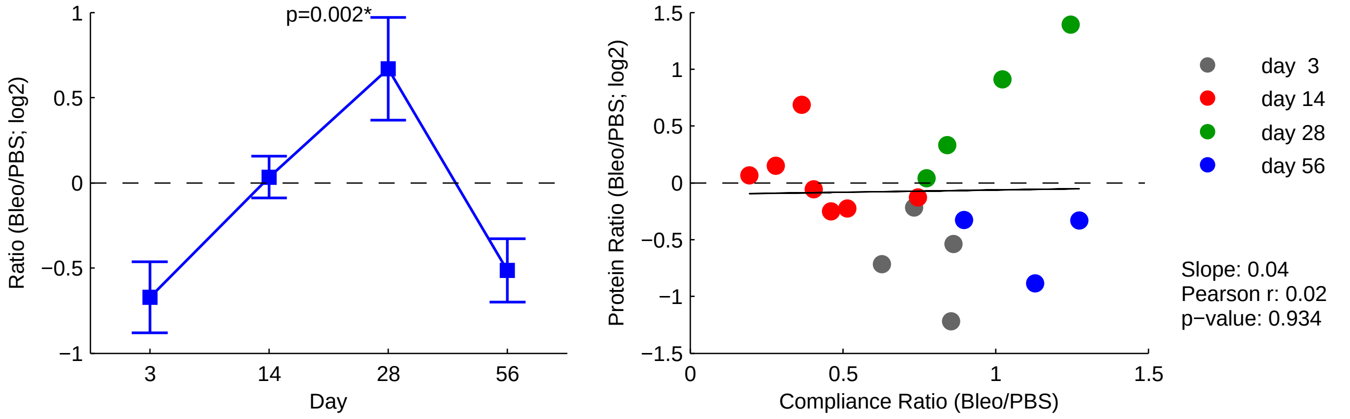

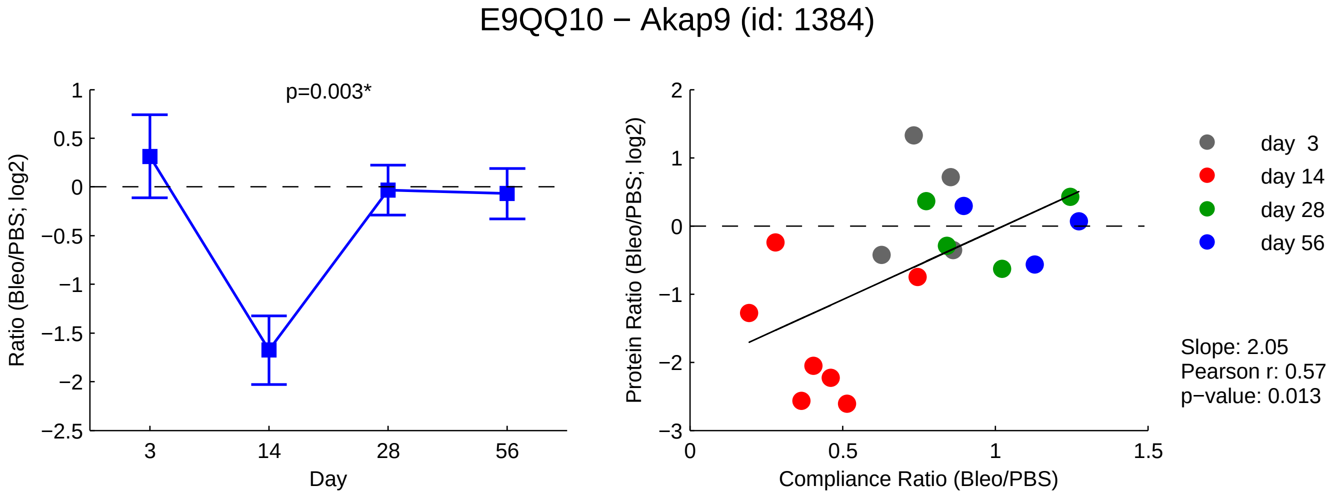
E9QQ10 − Akap9 (id: 1384)
p=0.003*
Ratio (Bleo/PBS; log2)
Day
Protein Ratio (Bleo/PBS; log2)
Compliance Ratio (Bleo/PBS)
day 3
day 14
day 28
day 56
Slope: 2.05
Pearson r: 0.57
p−value: 0.013

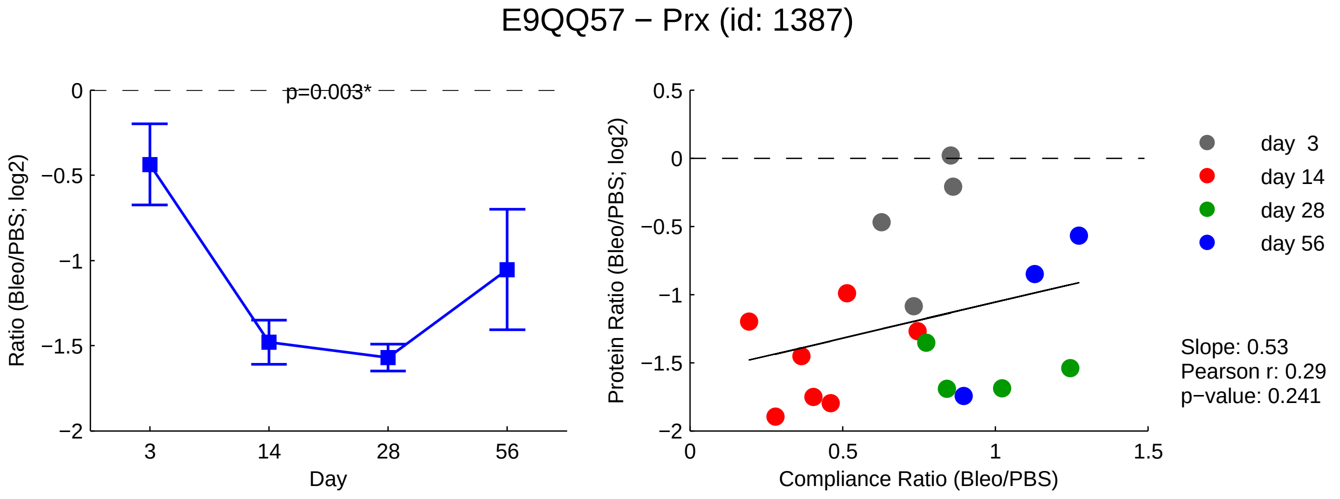
E9QQ57 − Prx (id: 1387)
p=0.003*
Ratio (Bleo/PBS; log2)
Day
Protein Ratio (Bleo/PBS; log2)
Compliance Ratio (Bleo/PBS)
day 3
day 14
day 28
day 56
Slope: 0.53
Pearson r: 0.29
p−value: 0.241

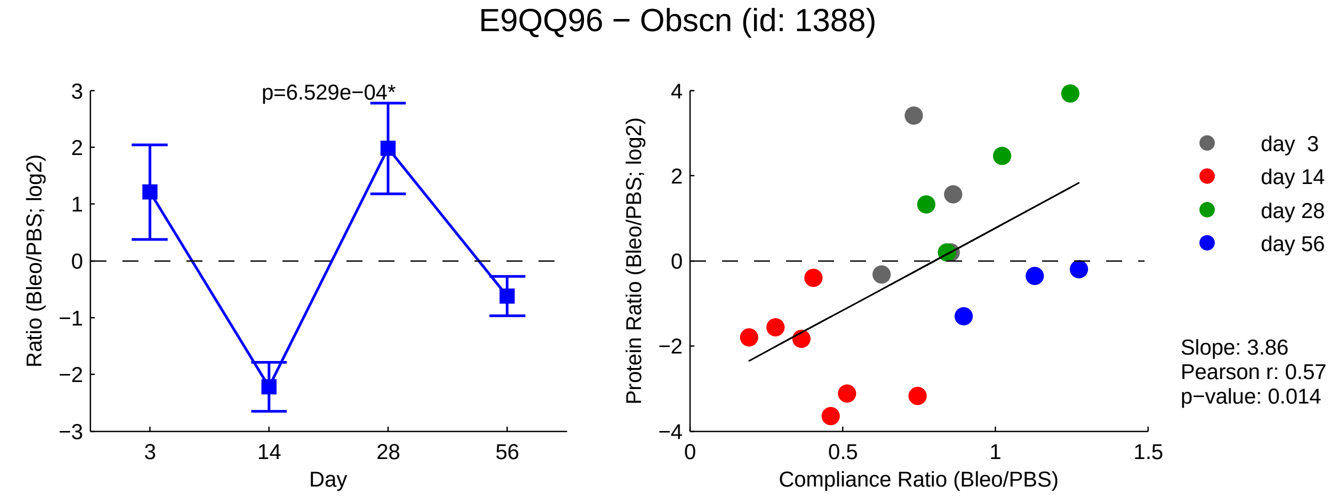
E9QQ96 − Obscn (id: 1388)
p=6.529e−04*
Ratio (Bleo/PBS; log2)
Day
Protein Ratio (Bleo/PBS; log2)
Compliance Ratio (Bleo/PBS)
day 3
day 14
day 28
day 56
Slope: 3.86
Pearson r: 0.57
p−value: 0.014

F2Z3U3 − Raph1 (id: 1389)

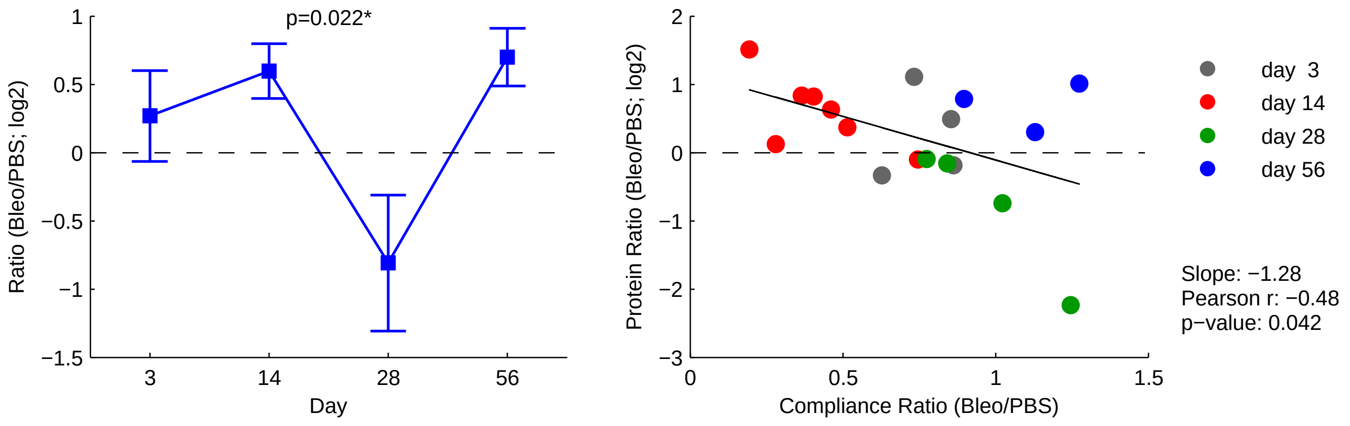

Q3TRL2 − Pdpk1 (id: 1393)

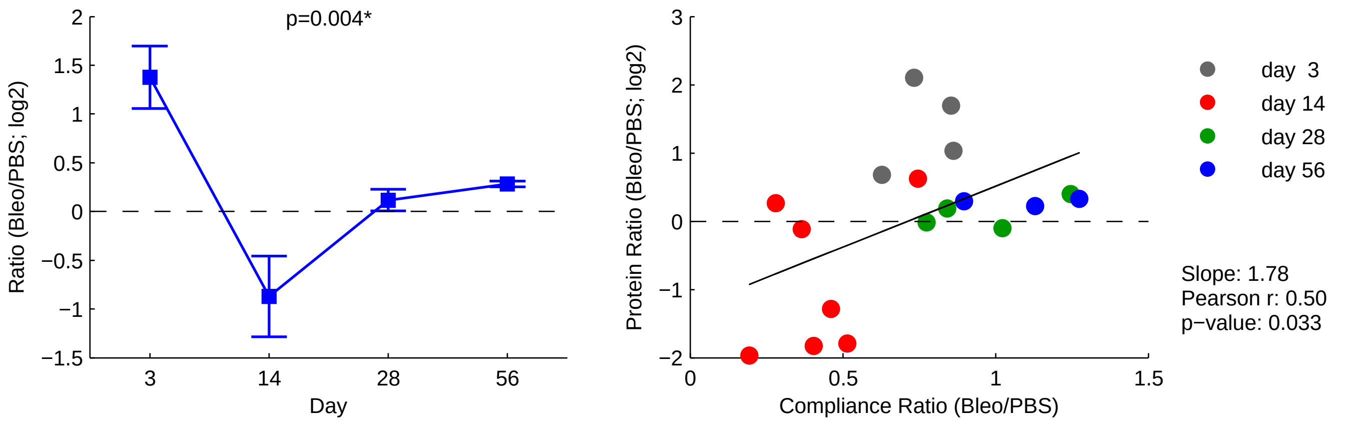

F2Z4B3 − Cdk2ap1 (id: 1400)

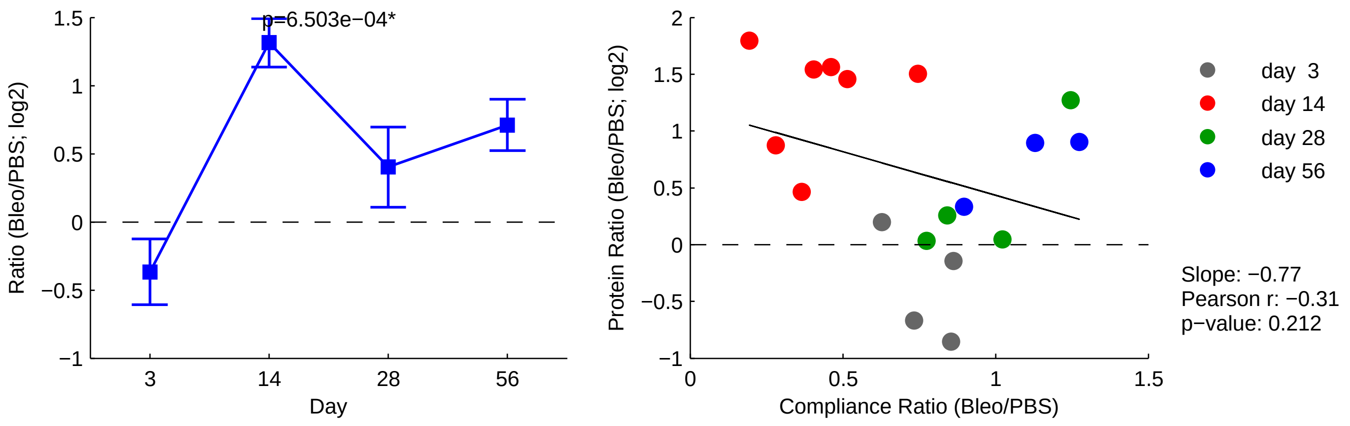

F6RN19 − Tmem259 (id: 1418)

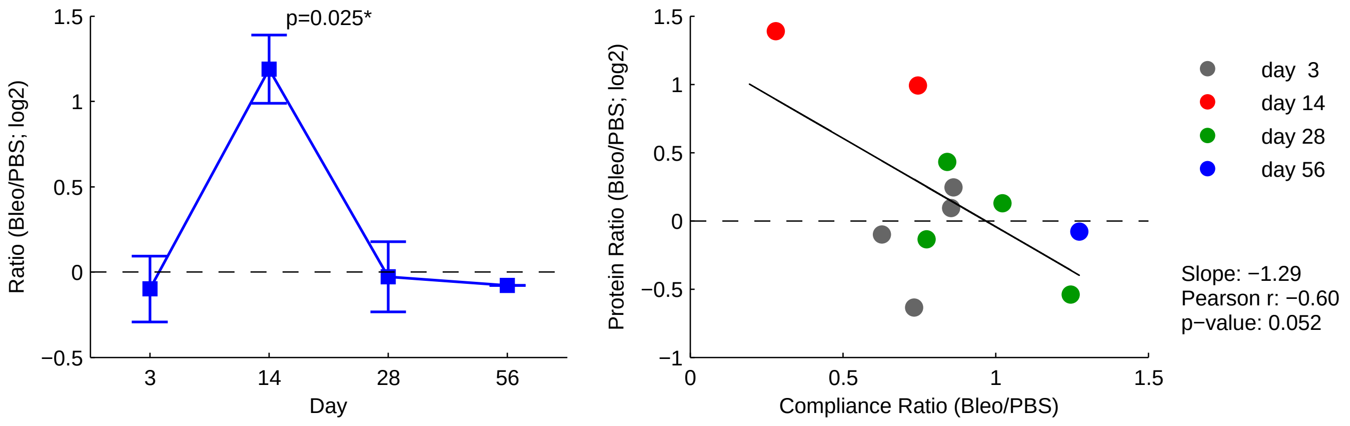

Q9ER58 − Spock2 (id: 1424)

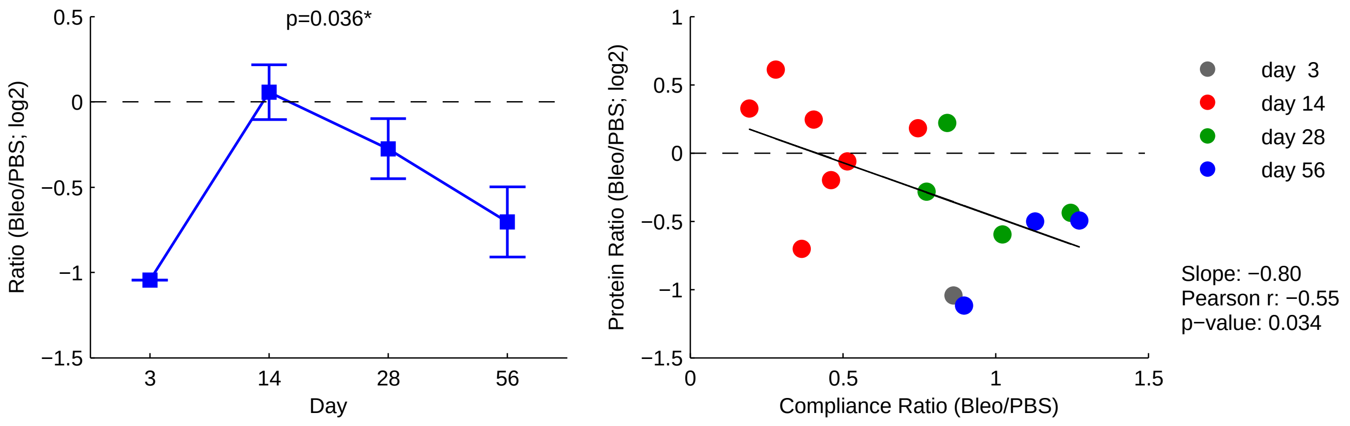

F6SQH7 − Poldip2 (id: 1425)

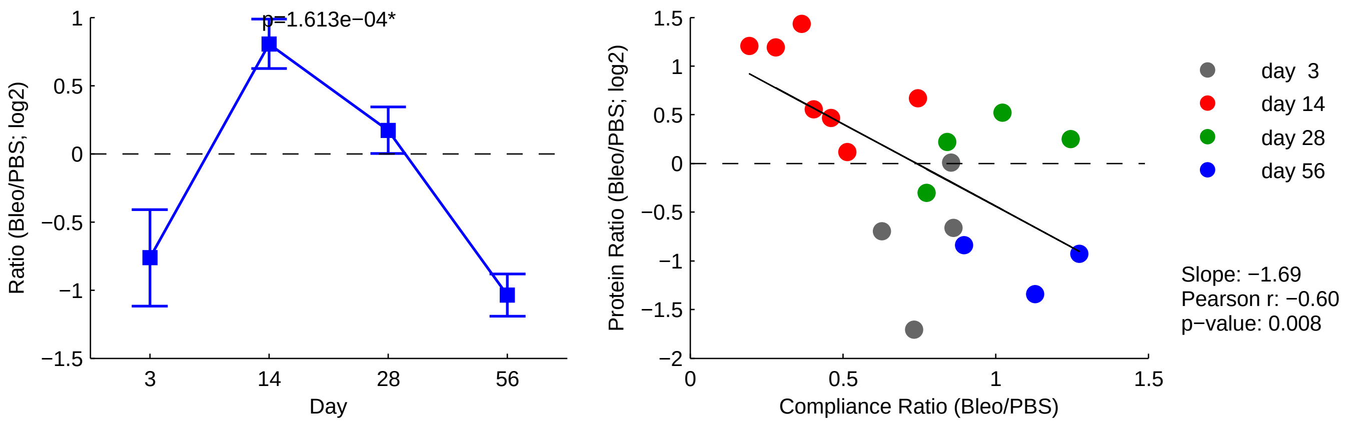

Q60739−2 − Bag1 (id: 1428)

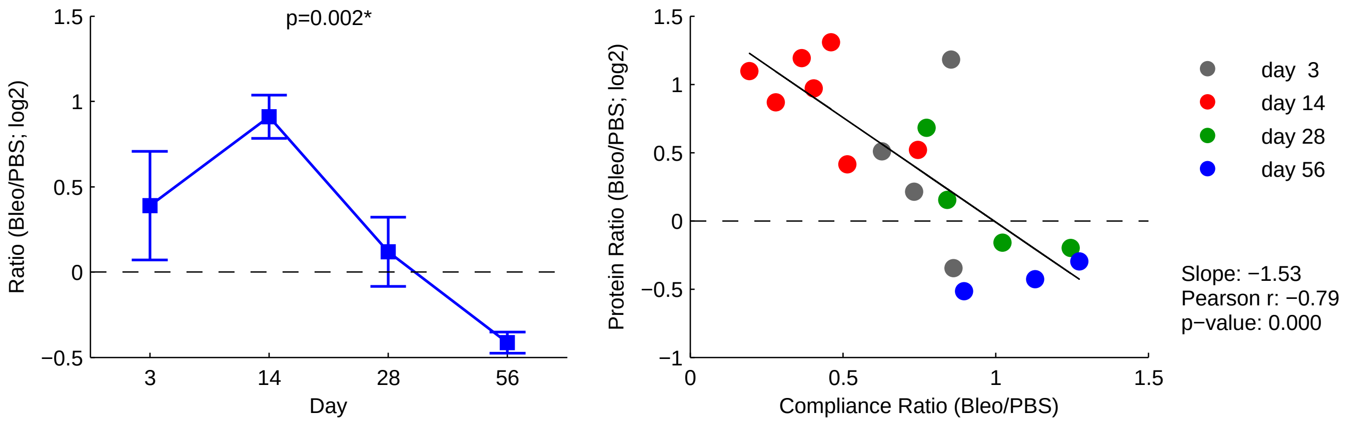

F6TQW2 − Ighg2c (id: 1433)

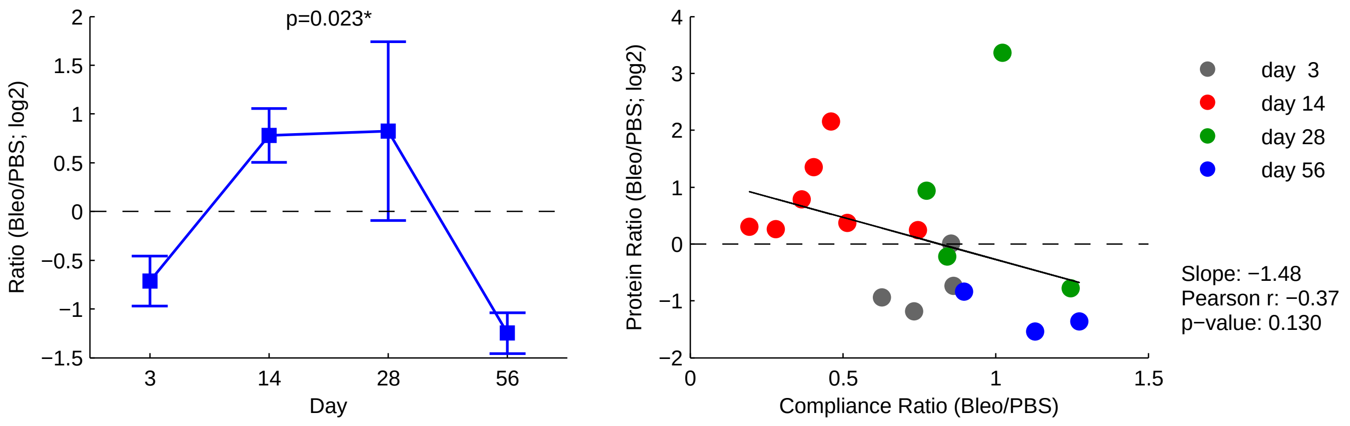

F6V6T4 − Tmed2 (id: 1441)

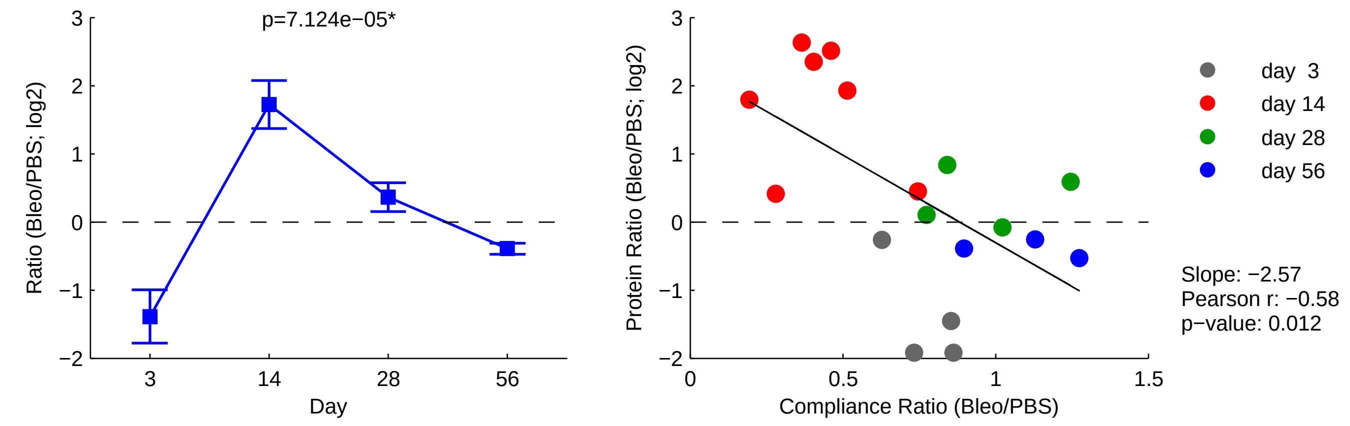

F6VG99 − Lmo7 (id: 1442)

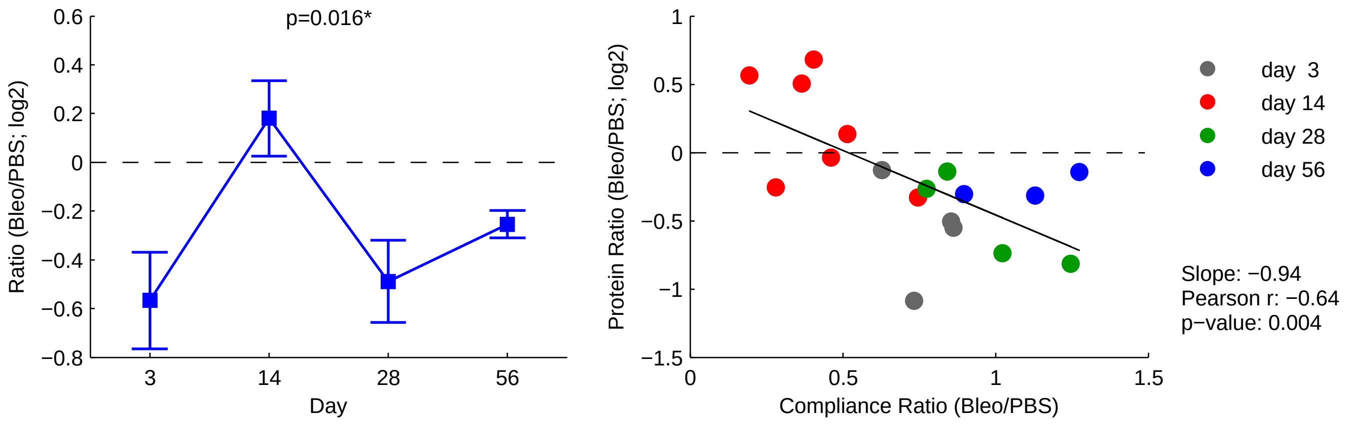

F6WBA1 − 2210011C24Rik (id: 1447)

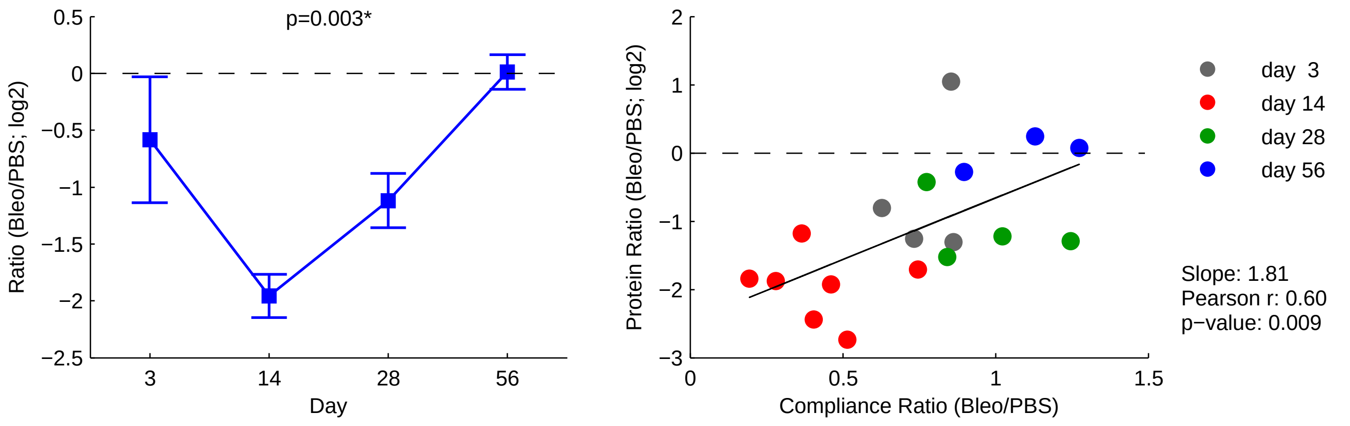

Q8K4I3 − Arhgef6 (id: 1452)

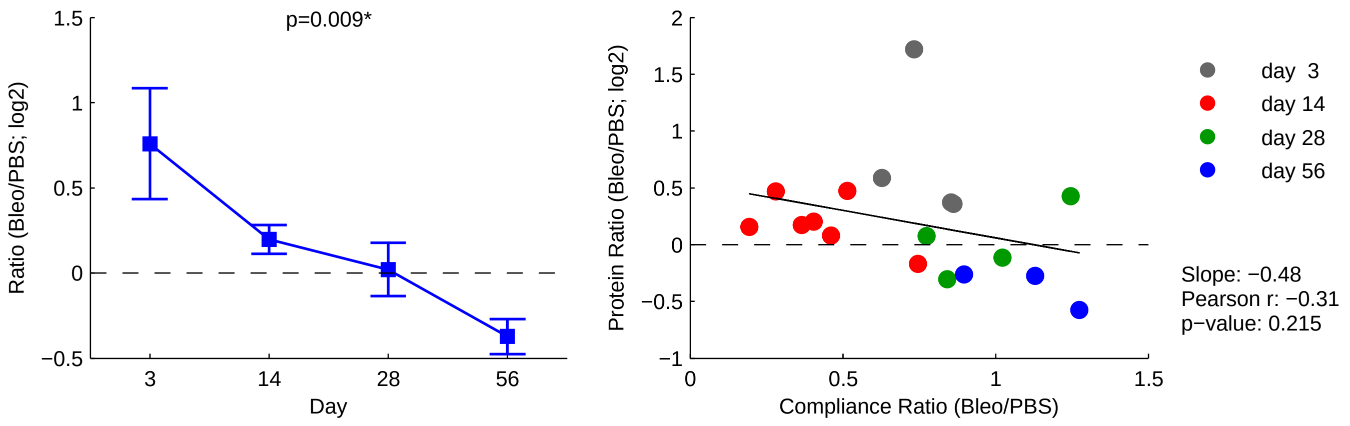

F7CJY7 − Stx16 (id: 1453)

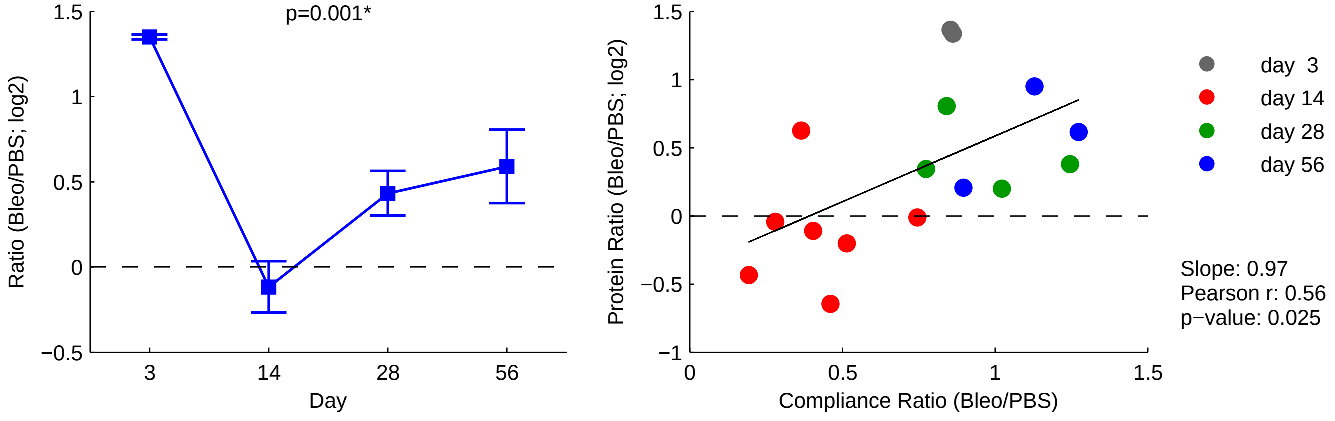

F6XLV1 − E030010N08Rik (id: 1456)

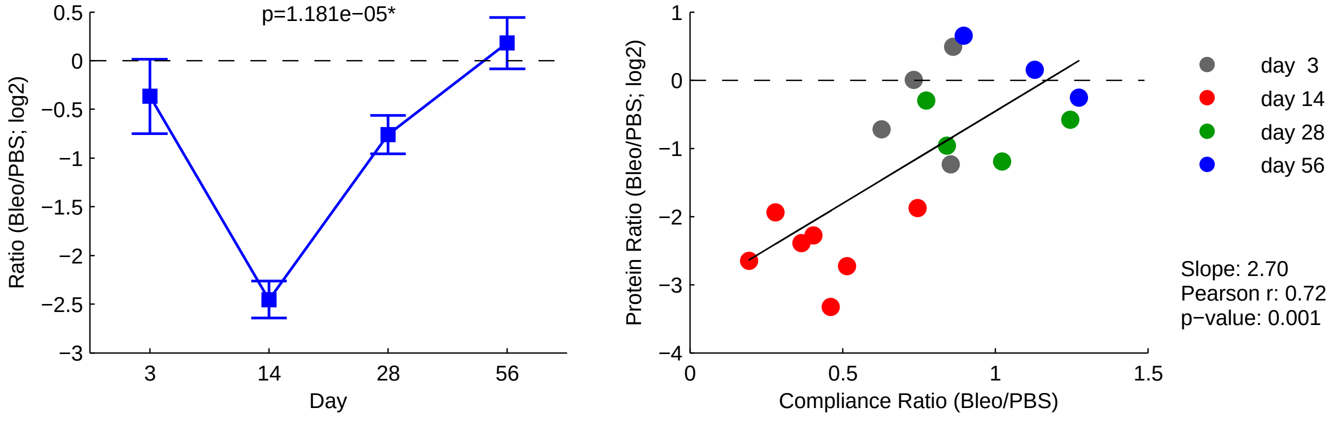

P61961 − Ufm1 (id: 1458)

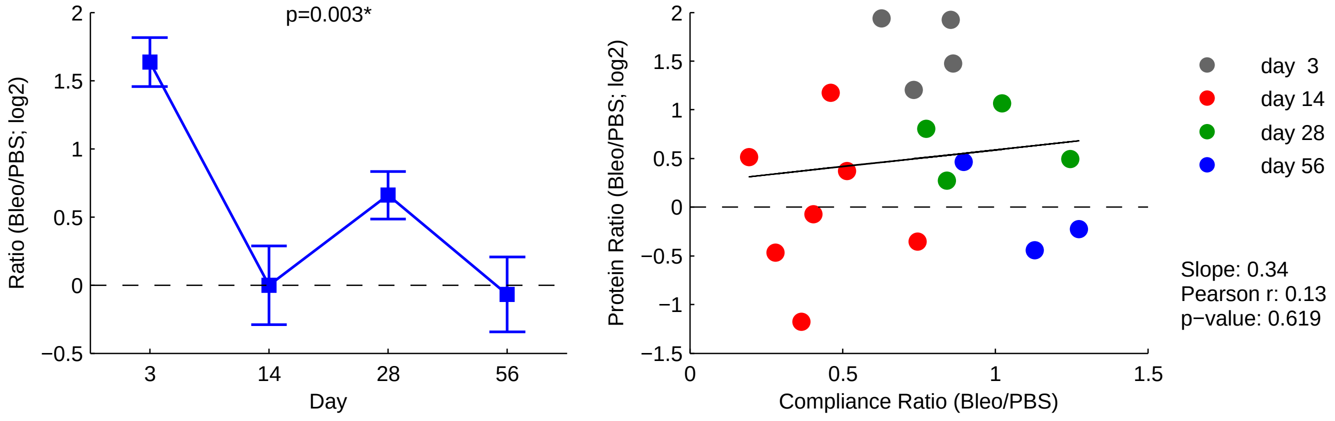

F6XYI9 − Tbp (id: 1460)

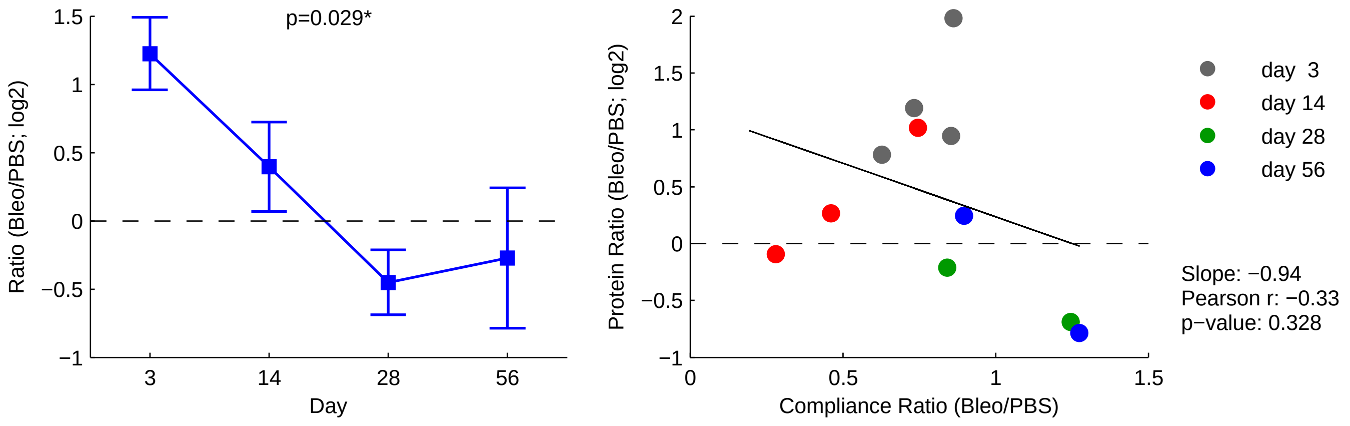

P62270 − Rps18 (id: 1467)

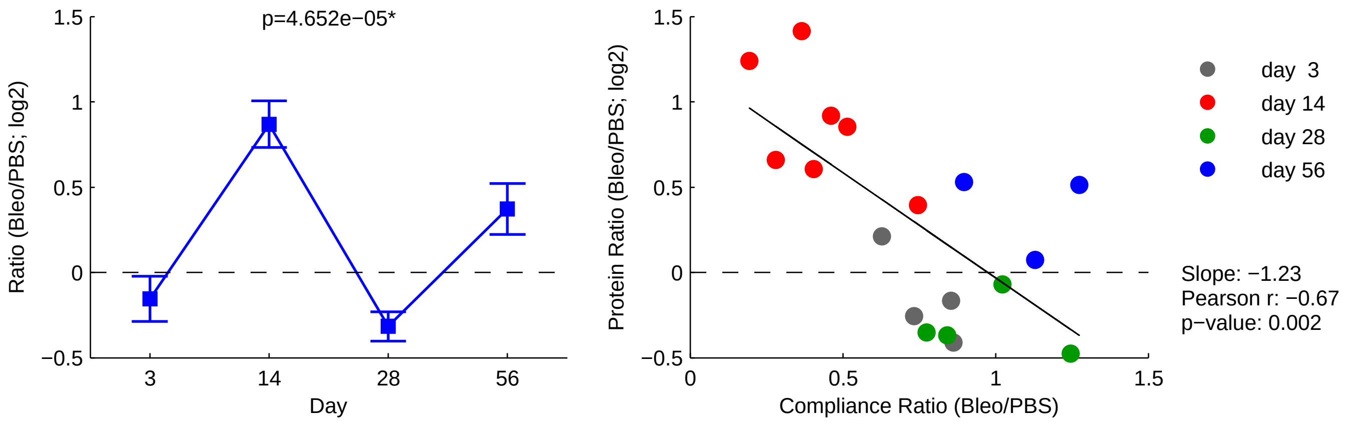

F6ZFT1 − Ndufab1 (id: 1473)

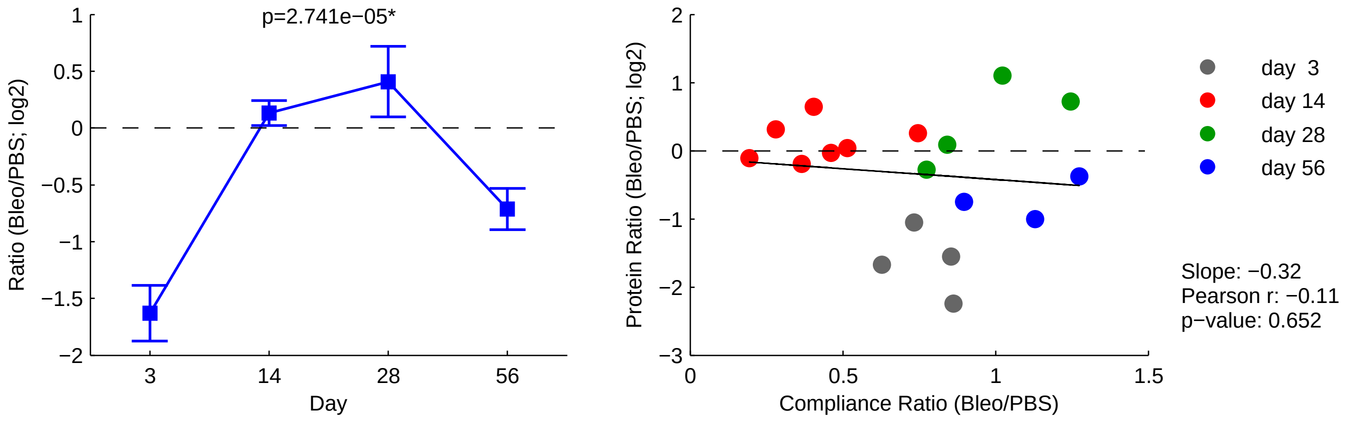

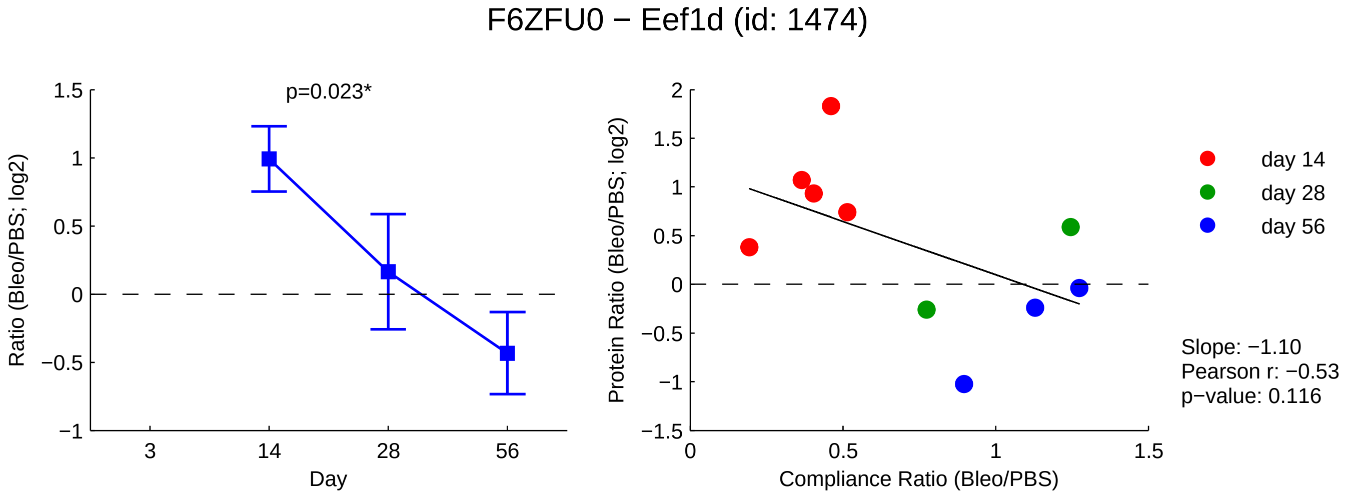
F6ZFU0 − Eef1d (id: 1474)
p=0.023*
Ratio (Bleo/PBS; log2)
Day
Protein Ratio (Bleo/PBS; log2)
Compliance Ratio (Bleo/PBS)
day 14
day 28
day 56
Slope: −1.10
Pearson r: −0.53
p−value: 0.116

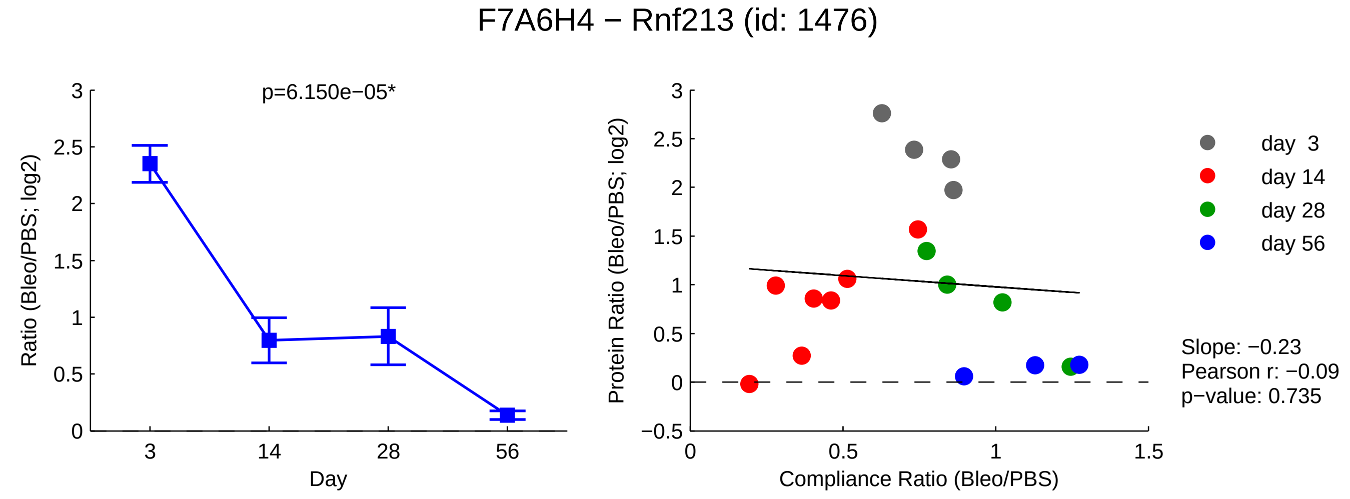
F7A6H4 − Rnf213 (id: 1476)
p=6.150e−05*
Ratio (Bleo/PBS; log2)
Day
Protein Ratio (Bleo/PBS; log2)
Compliance Ratio (Bleo/PBS)
day 3
day 14
day 28
day 56
Slope: −0.23
Pearson r: −0.09
p−value: 0.735

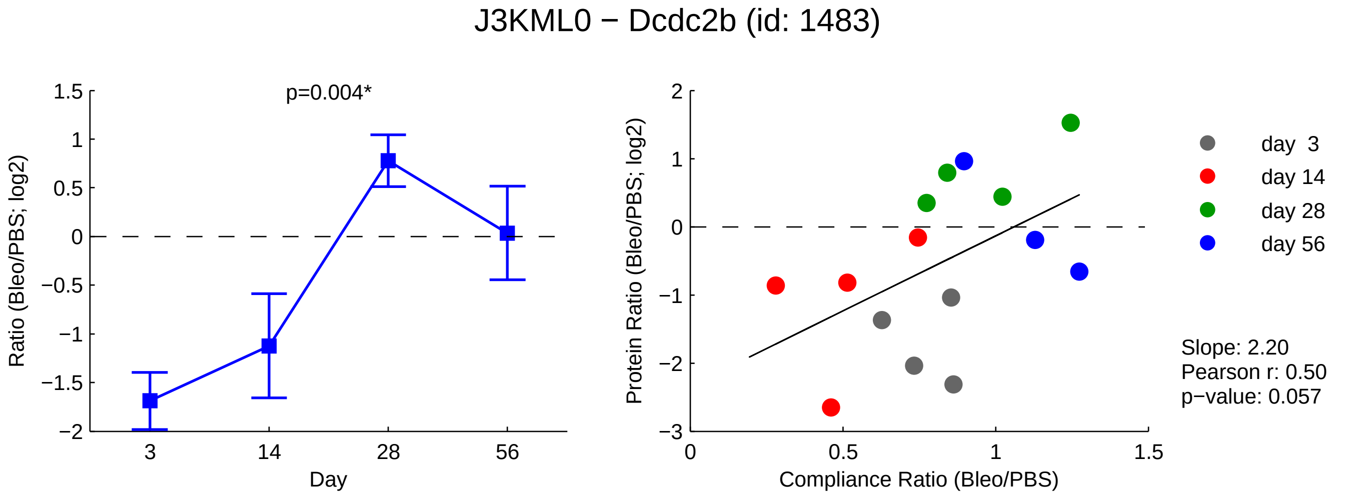
J3KML0 − Dcdc2b (id: 1483)
p=0.004*
Ratio (Bleo/PBS; log2)
Day
Protein Ratio (Bleo/PBS; log2)
Compliance Ratio (Bleo/PBS)
day 3
day 14
day 28
day 56
Slope: 2.20
Pearson r: 0.50
p−value: 0.057

F7BE84 − Ankrd50 (id: 1484)

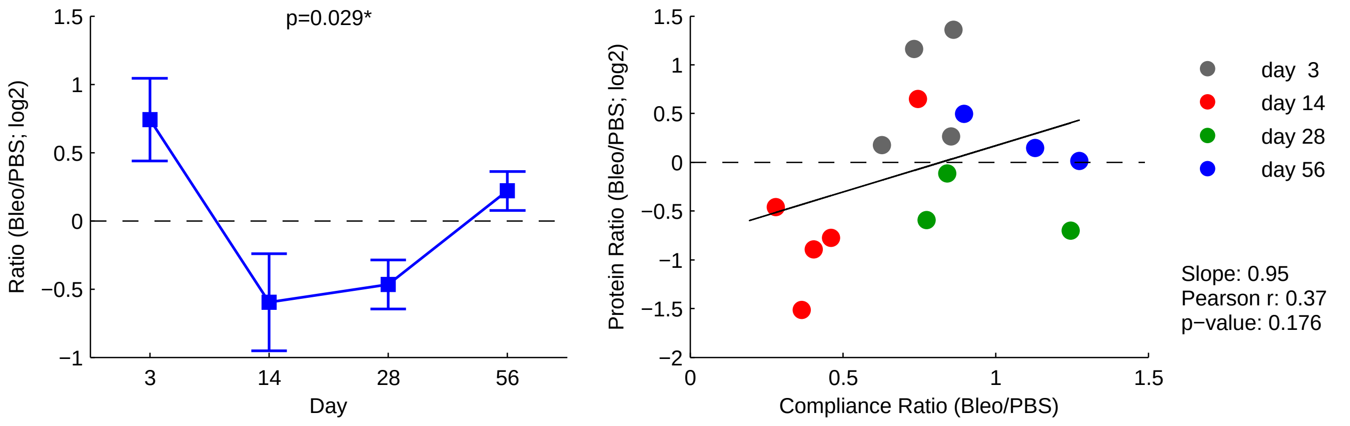

F7BQE4 − Arhgef10 (id: 1489)

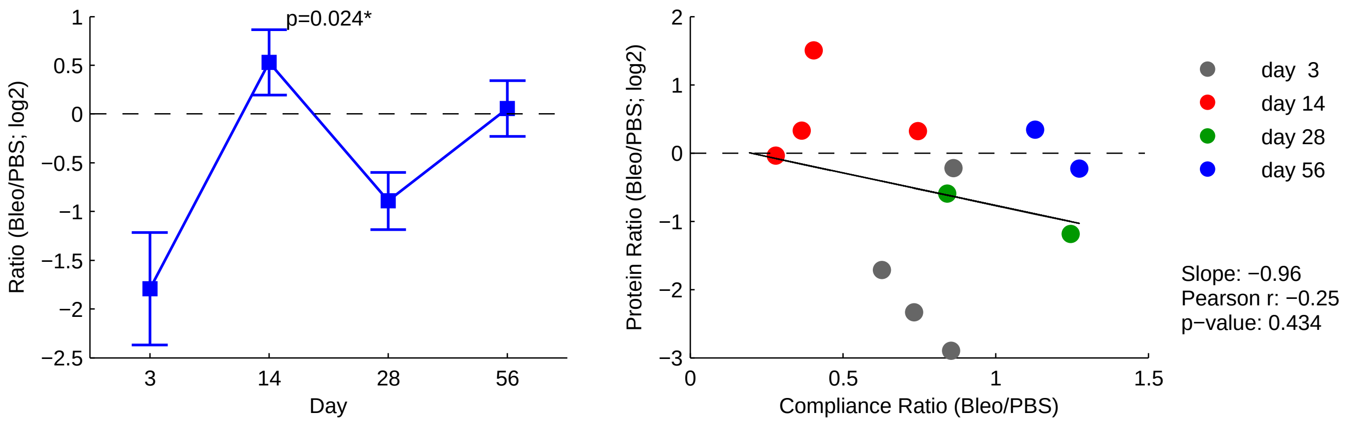

F7DBB3 − Ahnak2 (id: 1496)

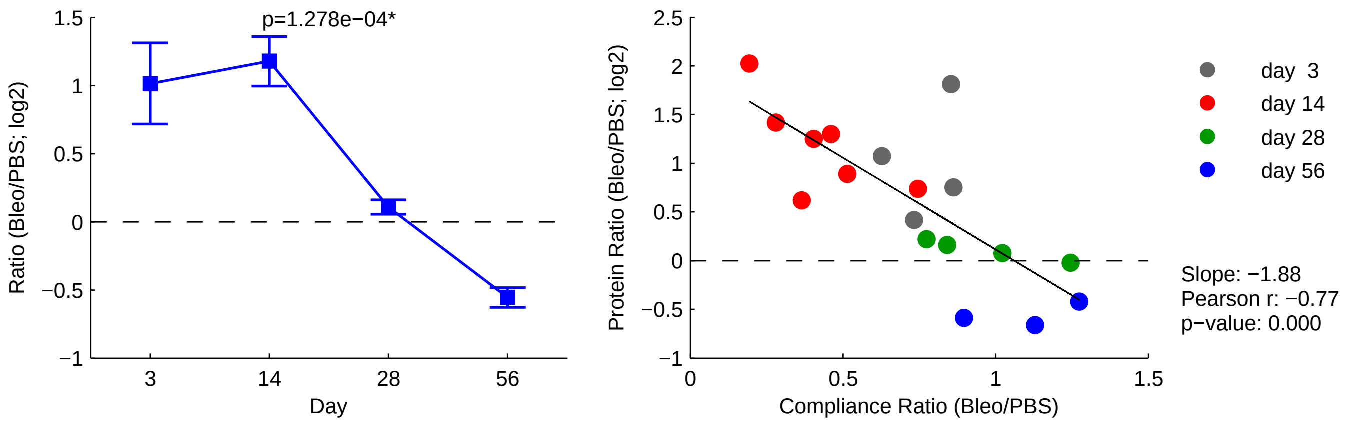

P05532−2 − Kit (id: 1498)

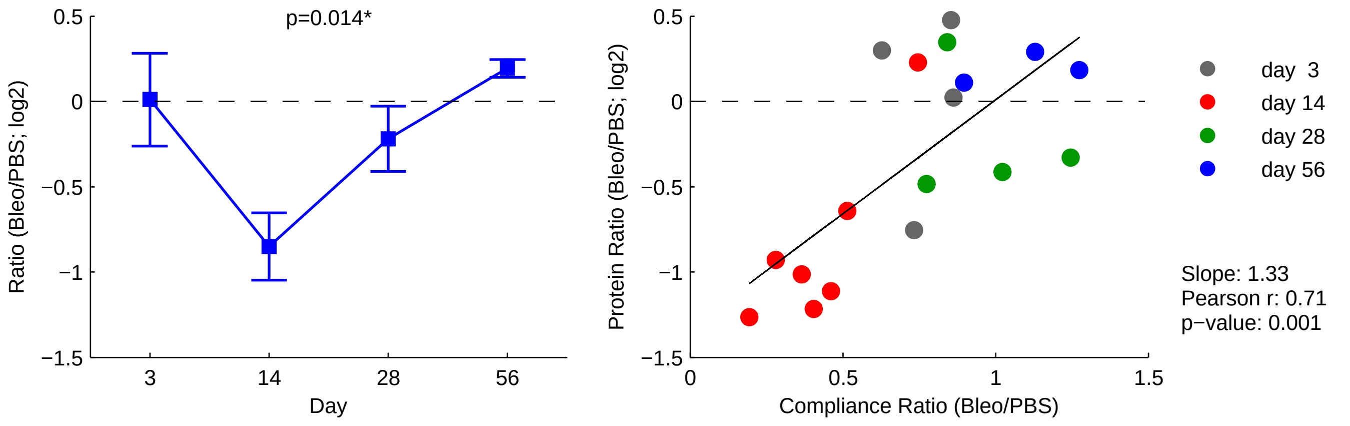

Q8C437−3 − Pex5l (id: 1501)

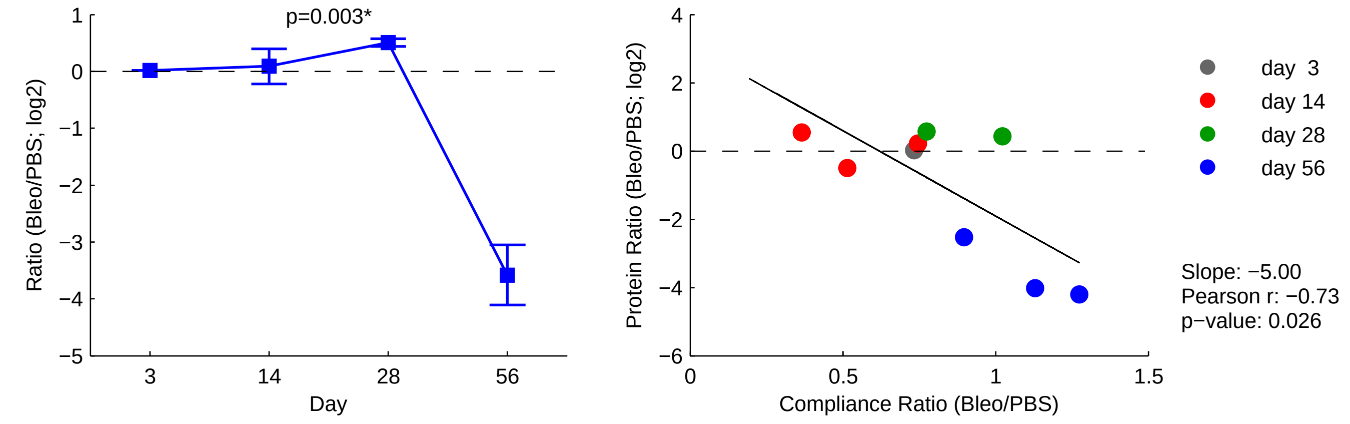

P56671 − Maz (id: 1504)

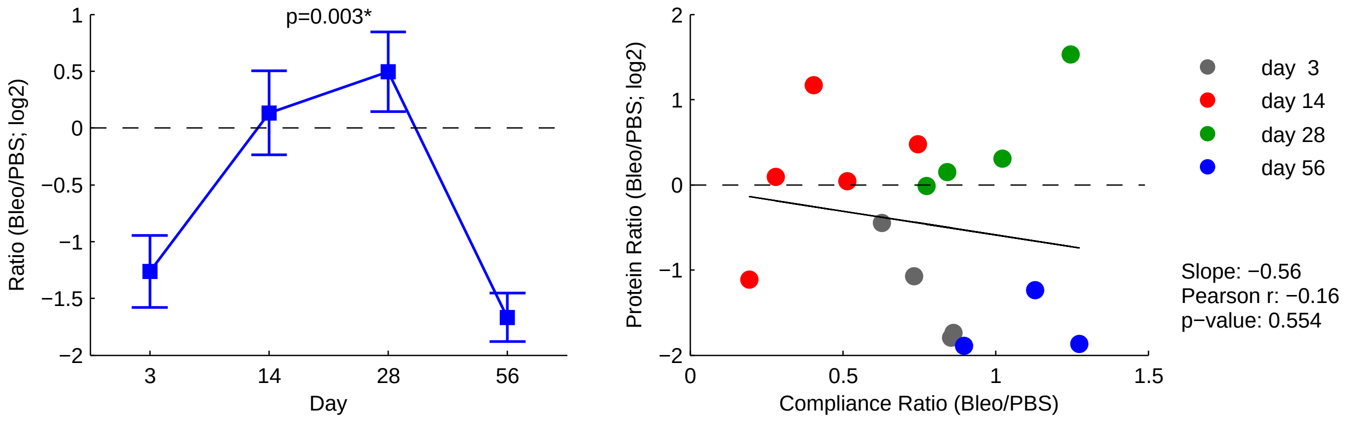

F8VPK5 − Rock2 (id: 1505)

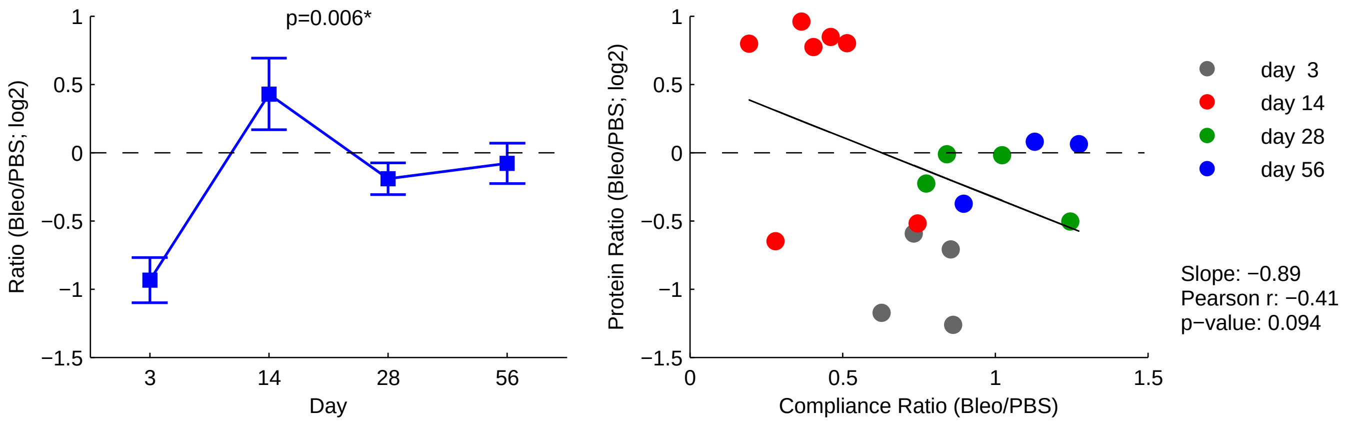

Q61194 − Pik3c2a (id: 1506)

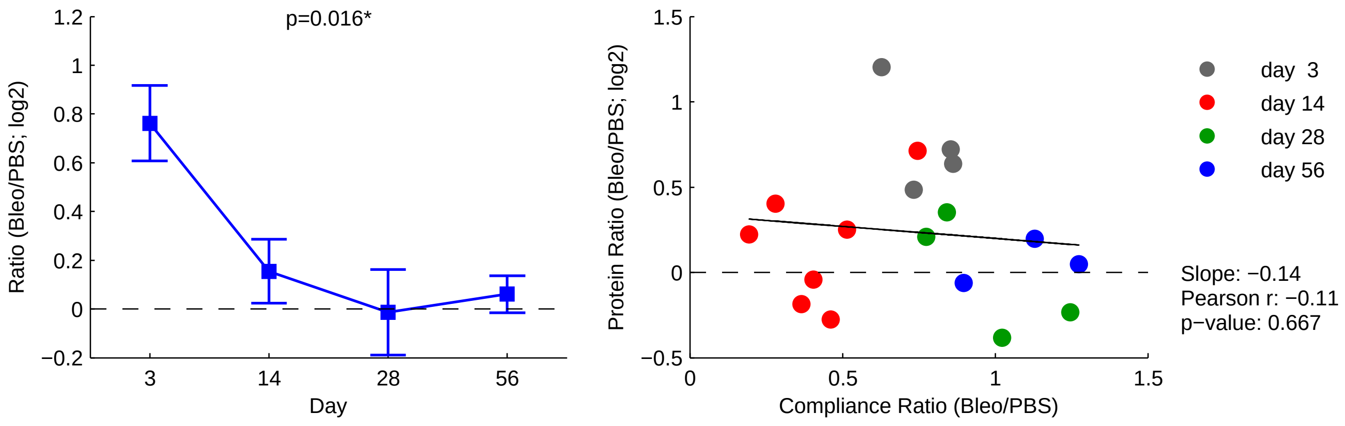

F8VPN7 − Dock9 (id: 1509)

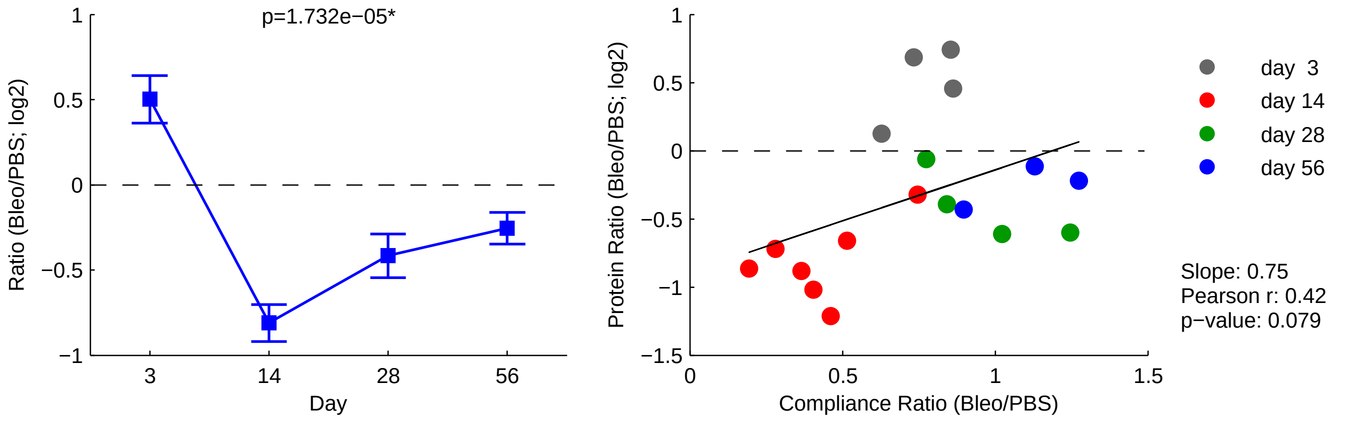

P45481 − Crebbp (id: 1513)

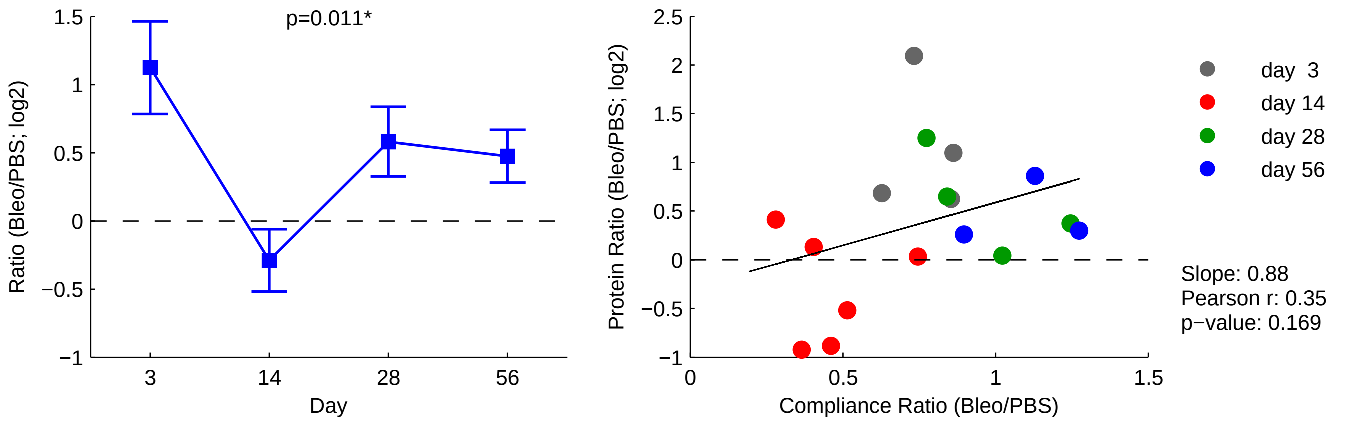

P97366 − Evi5 (id: 1514)

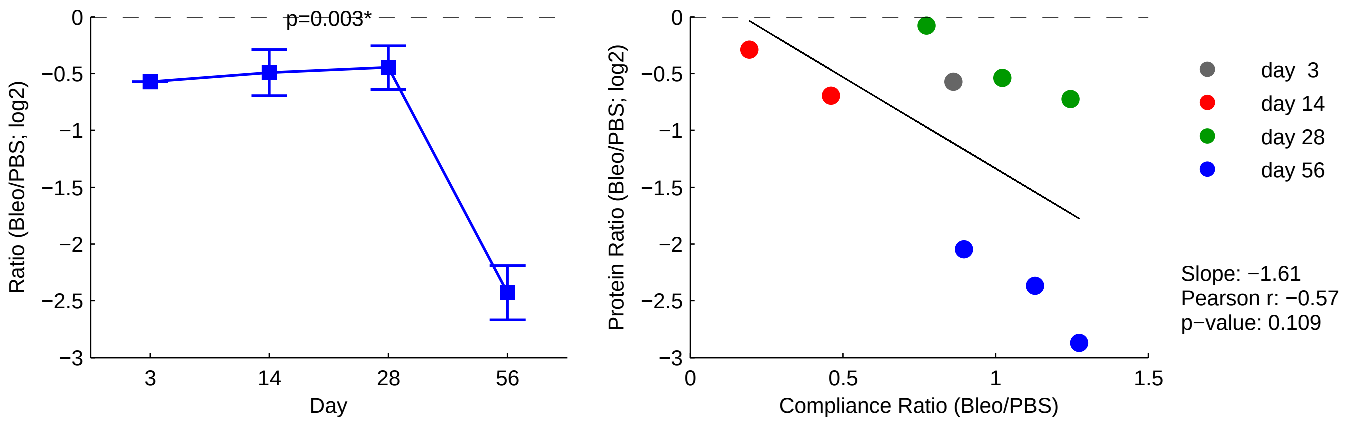

F8VPV0 − Pcnt (id: 1516)

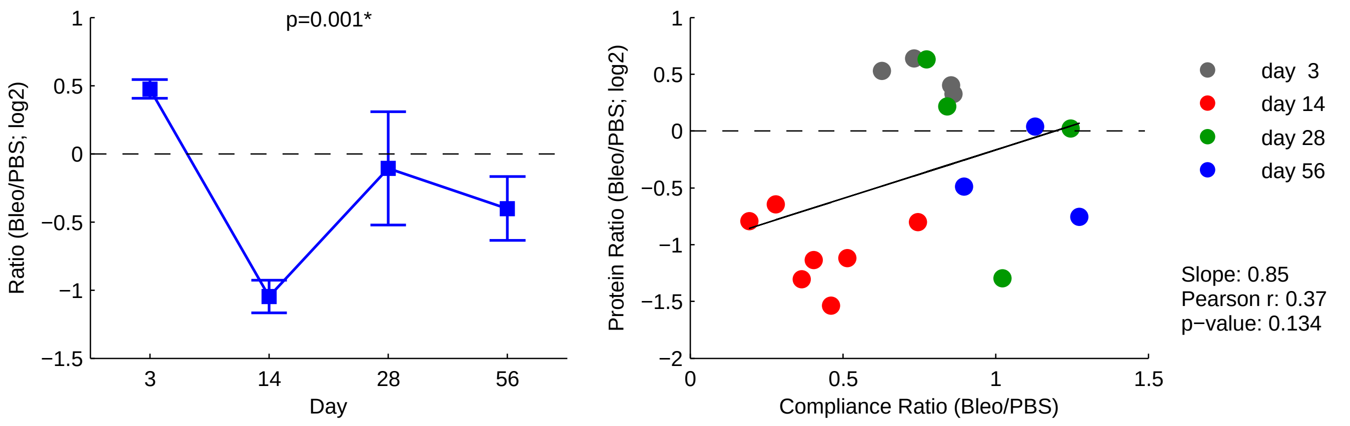

Q8C092 − Taf5 (id: 1517)

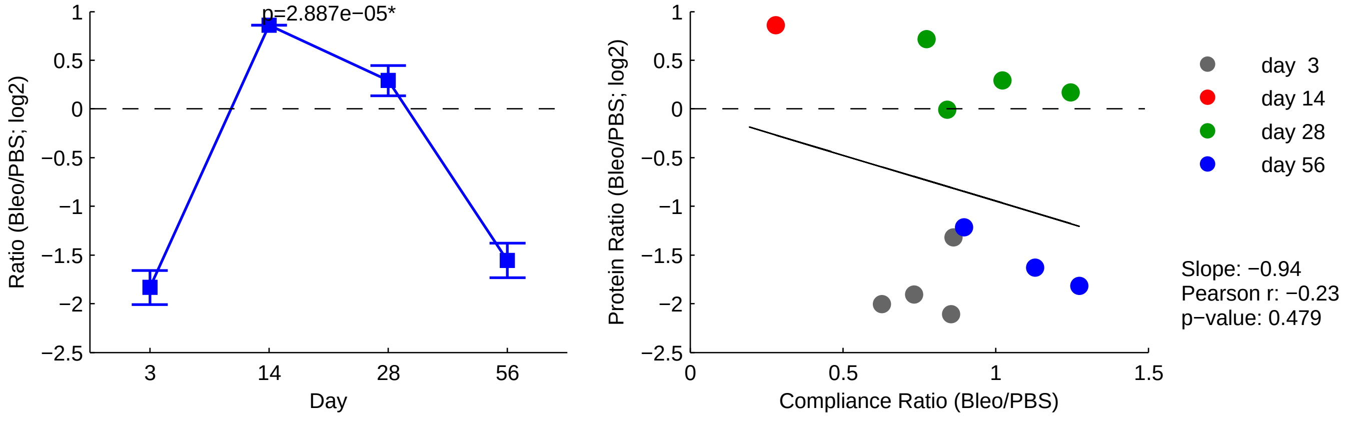

Q8VI36 − Pxn (id: 1522)

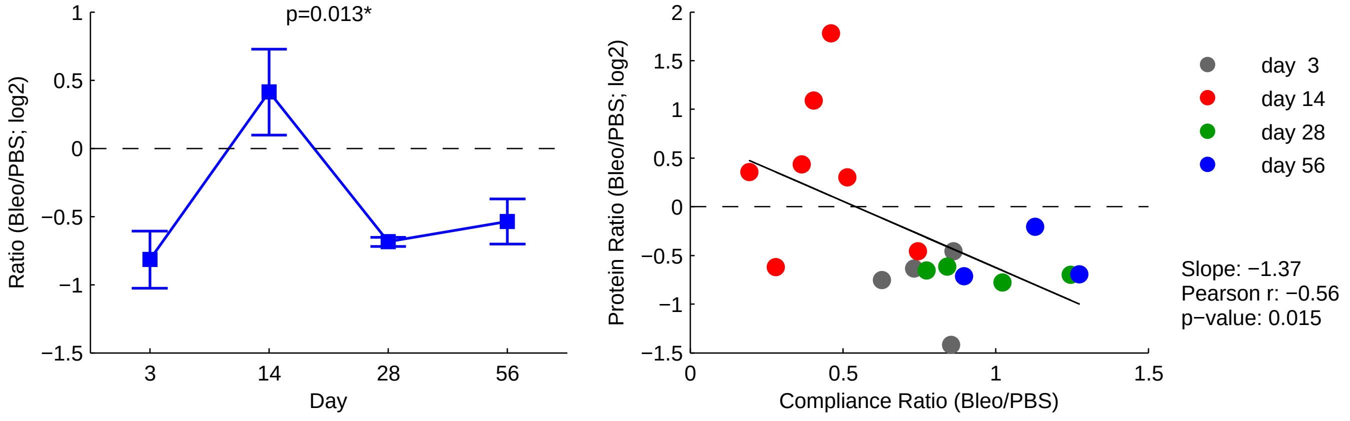

F8VQ40 − Lama1 (id: 1524)

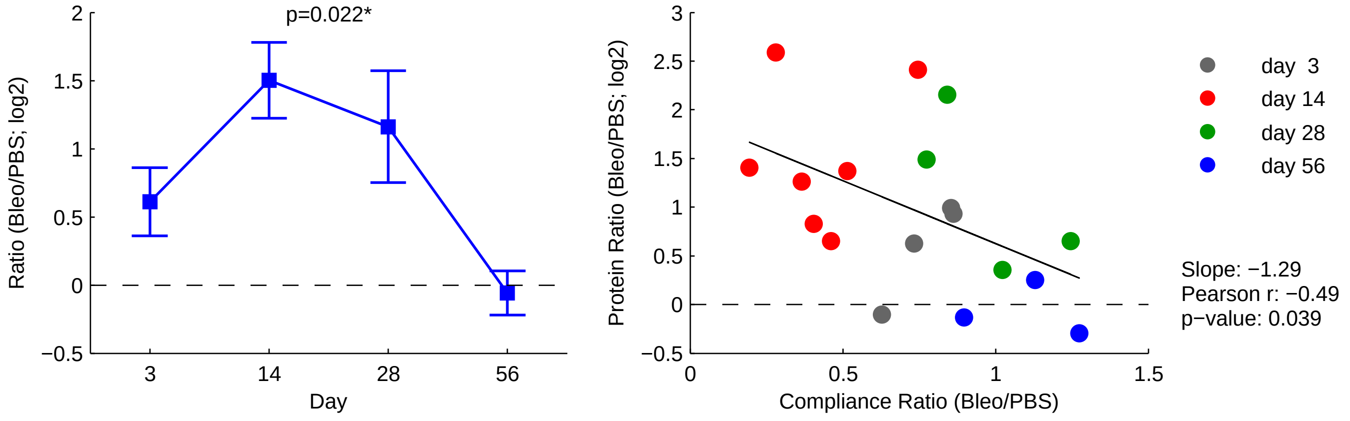

F8VQ43 − Lama2 (id: 1525)

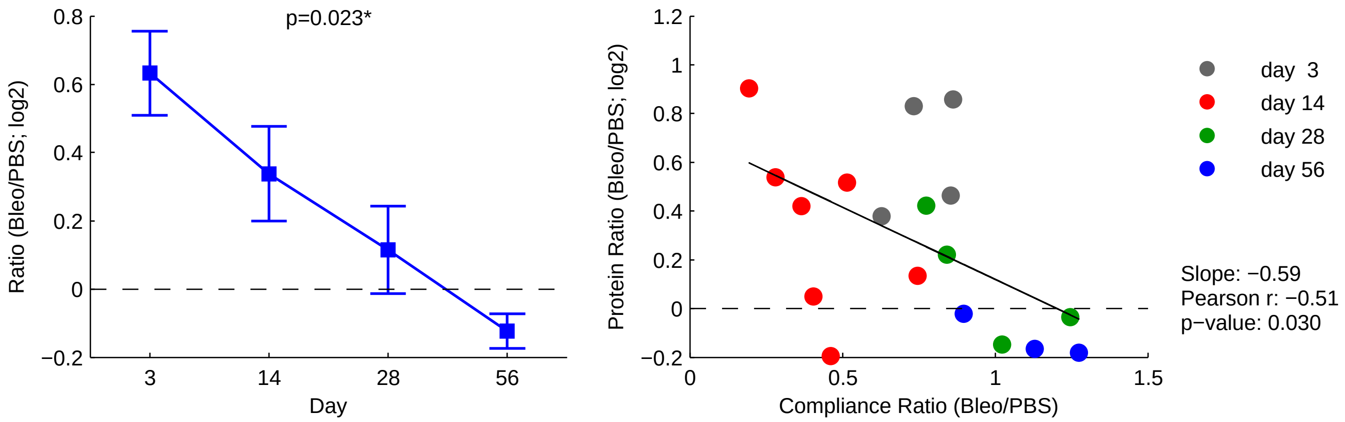

Q6Y685−2 − Tacc1 (id: 1530)

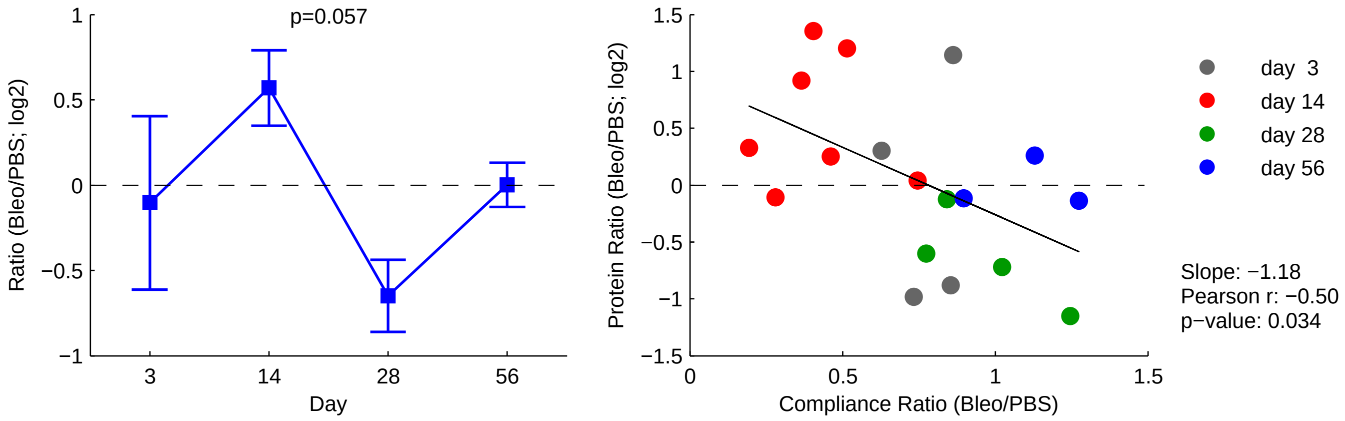

F8VQA4 − Pam (id: 1531)

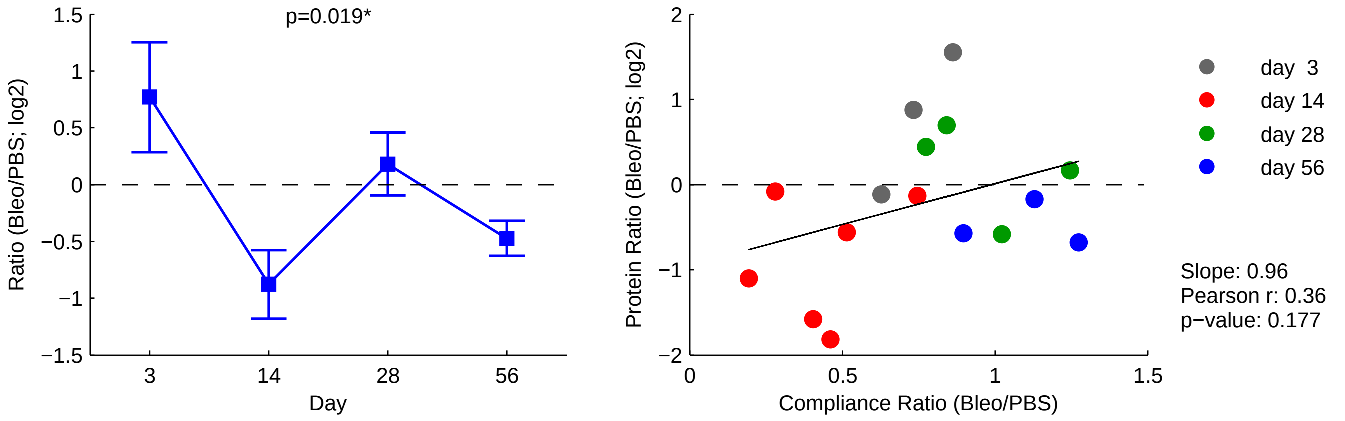

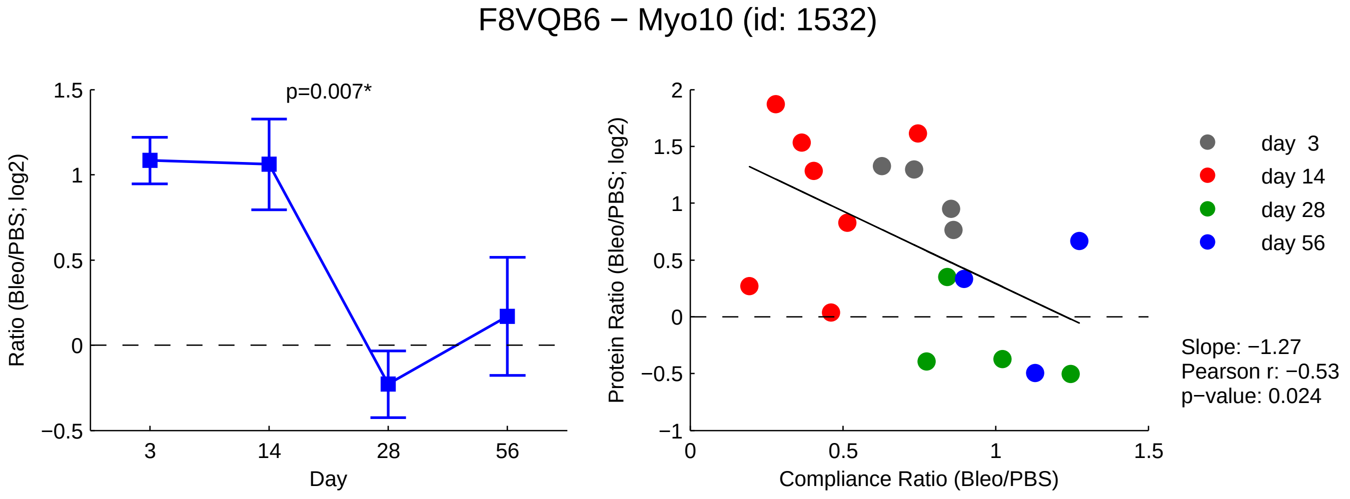
F8VQB6 − Myo10 (id: 1532)
p=0.007*
Ratio (Bleo/PBS; log2)
Day
Protein Ratio (Bleo/PBS; log2)
Compliance Ratio (Bleo/PBS)
day 3
day 14
day 28
day 56
Slope: −1.27
Pearson r: −0.53
p−value: 0.024

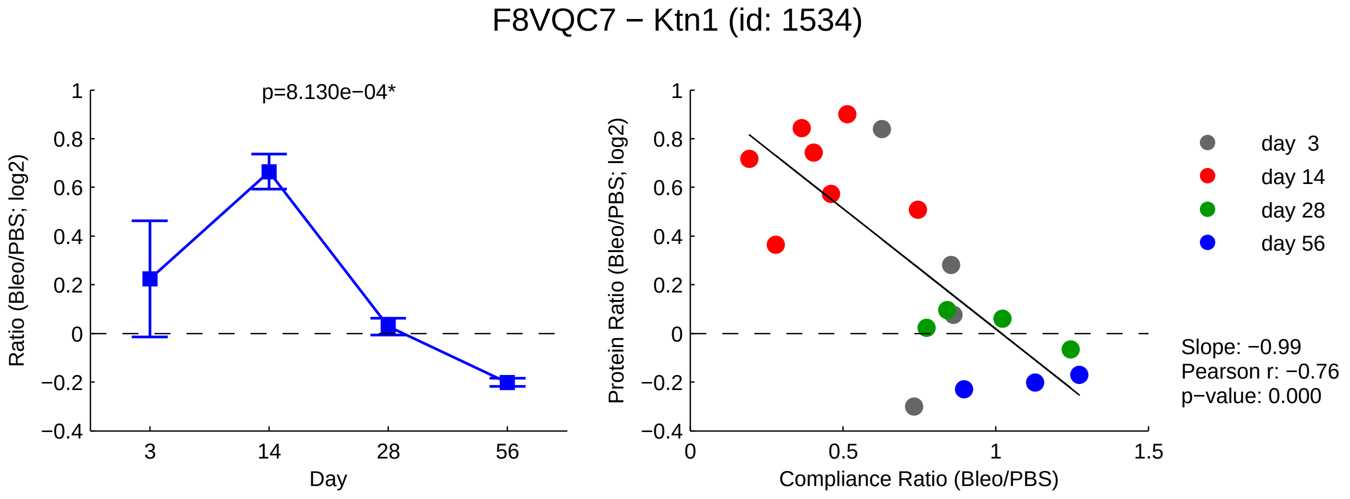
F8VQC7 − Ktn1 (id: 1534)
p=8.130e−04*
Ratio (Bleo/PBS; log2)
Day
Protein Ratio (Bleo/PBS; log2)
Compliance Ratio (Bleo/PBS)
day 3
day 14
day 28
day 56
Slope: −0.99
Pearson r: −0.76
p−value: 0.000

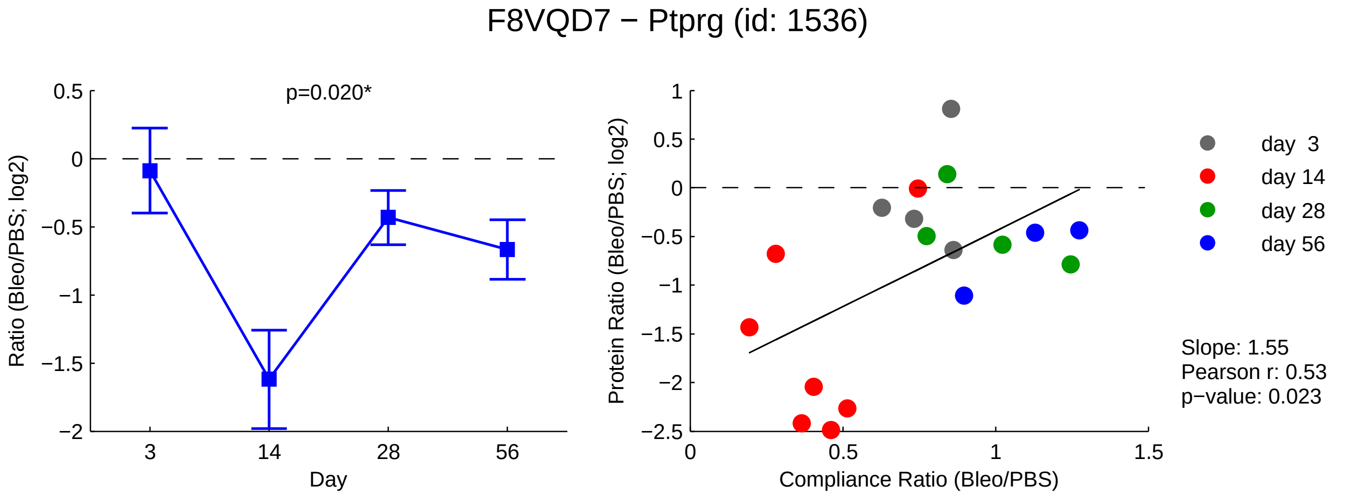
F8VQD7 − Ptprg (id: 1536)
p=0.020*
Ratio (Bleo/PBS; log2)
Day
Protein Ratio (Bleo/PBS; log2)
Compliance Ratio (Bleo/PBS)
day 3
day 14
day 28
day 56
Slope: 1.55
Pearson r: 0.53
p−value: 0.023

Q8VHH5 − Agap3 (id: 1537)

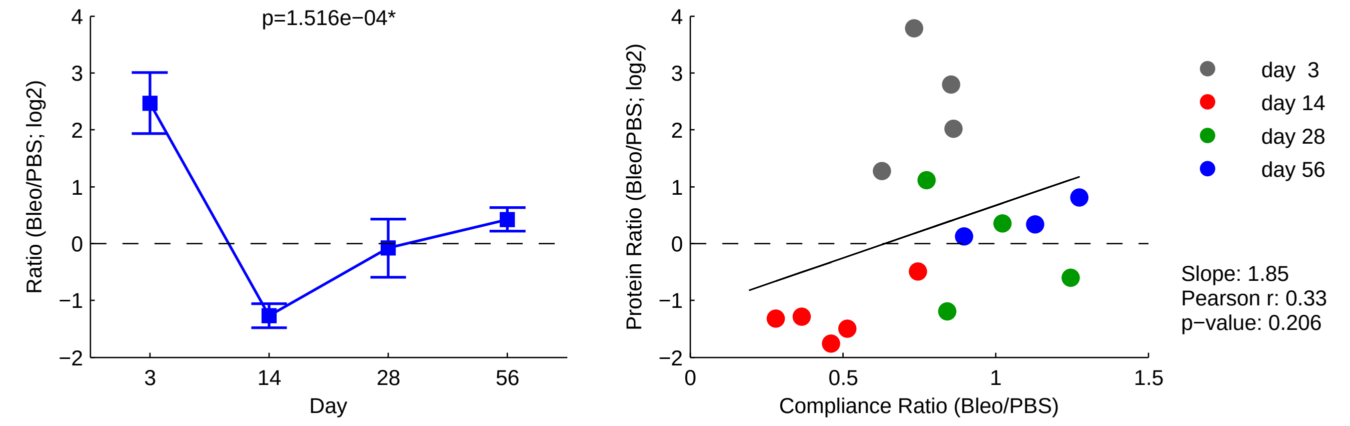

F8VQF9 − Trappc10 (id: 1538)

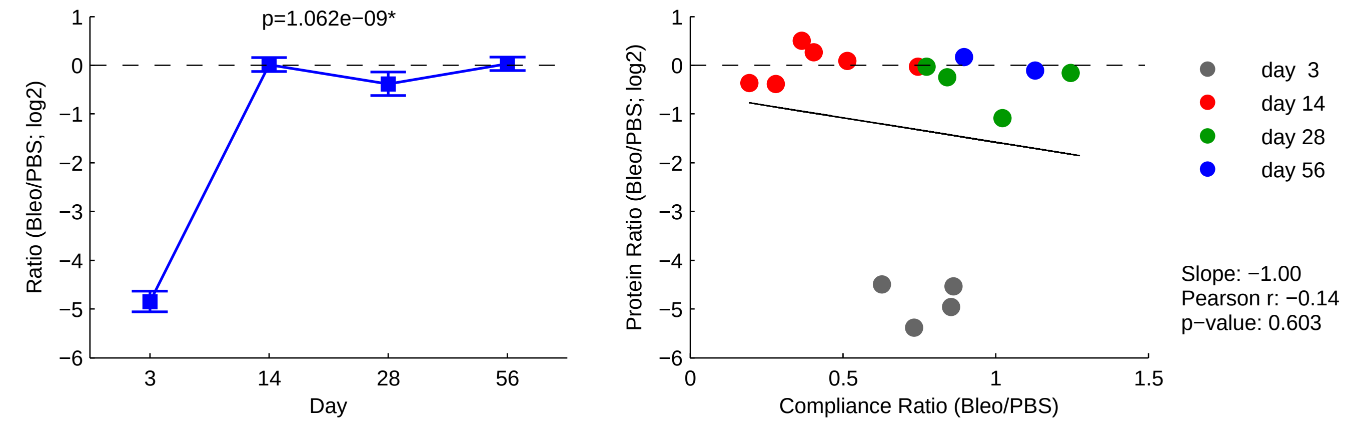

F8VQJ3 − Lamc1 (id: 1539)

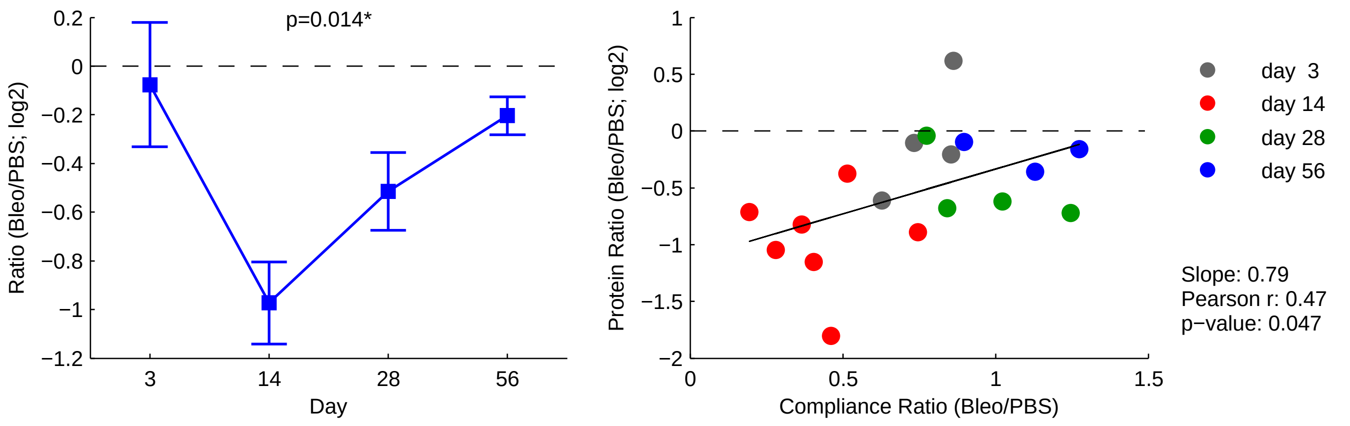

F8VQK3 − Gucy1a2 (id: 1540)

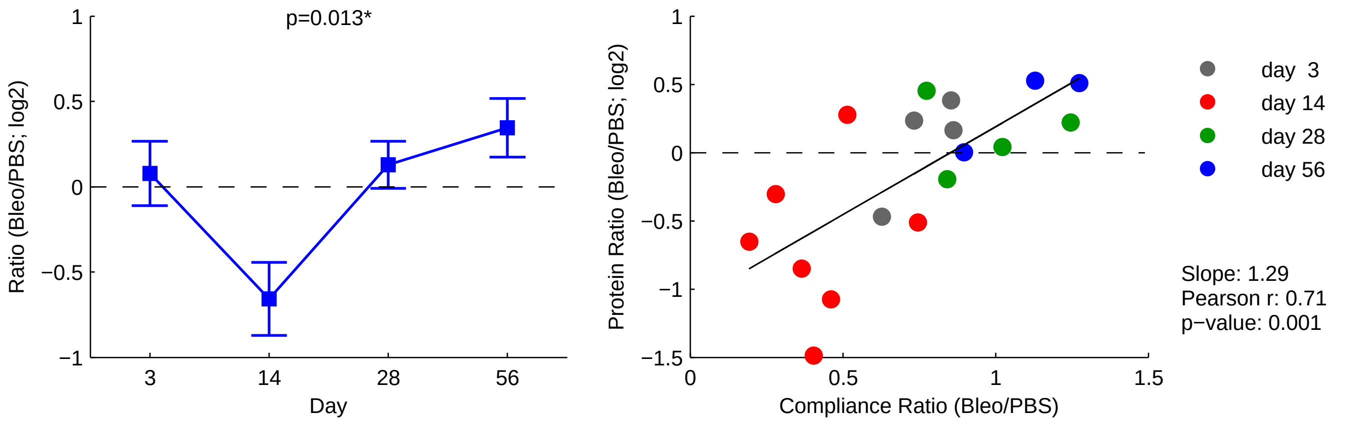

Q9D684−2 − Rin2 (id: 1543)

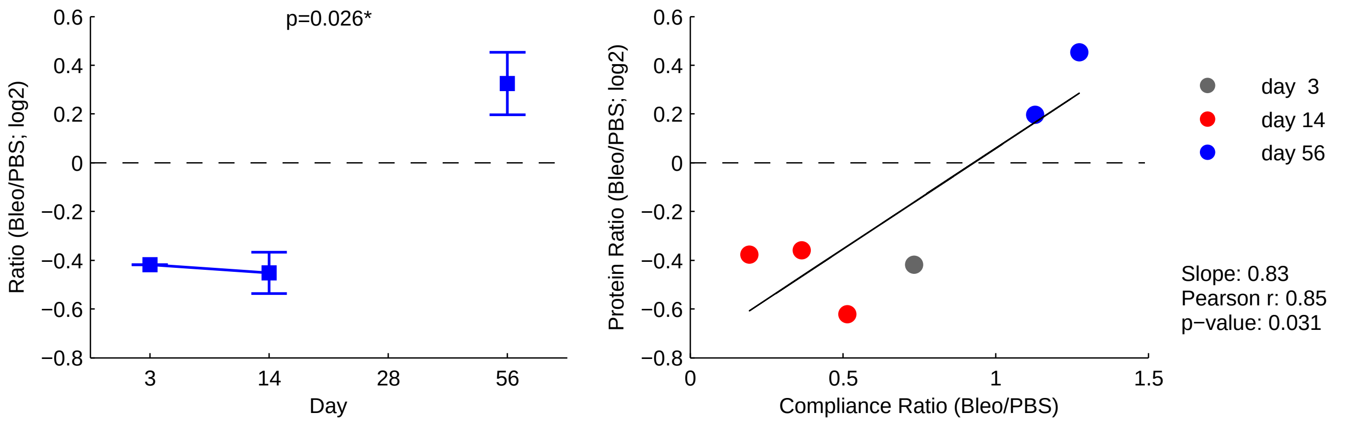

F8WGK6 − Sftpb (id: 1546)

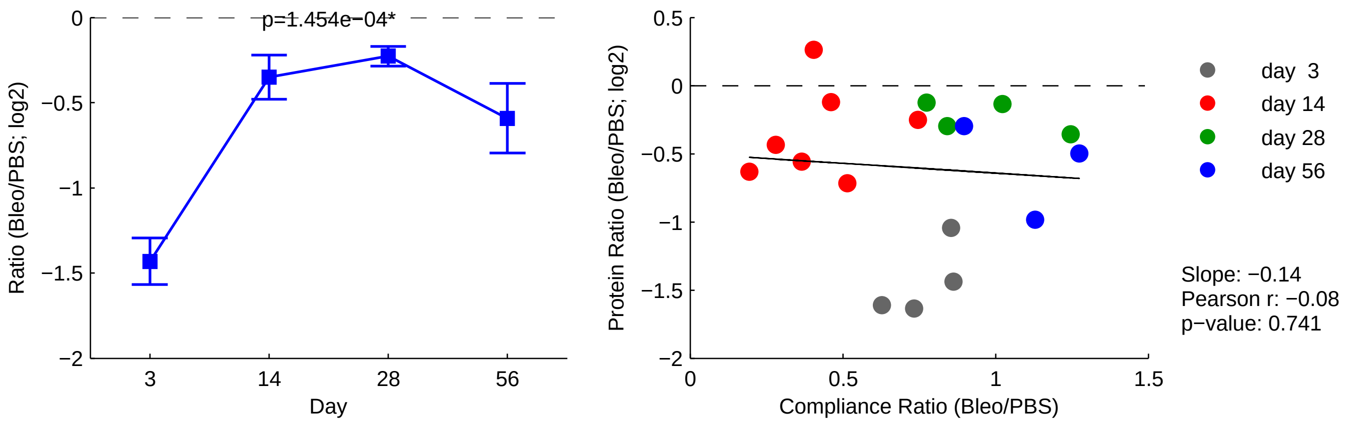

Q09014 − Ncf1 (id: 1550)

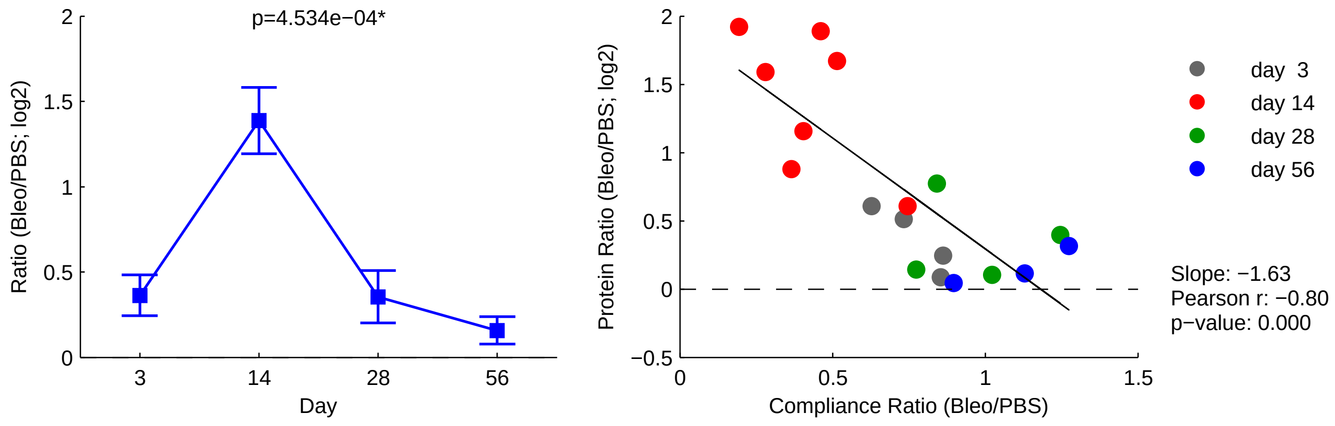

F8WHW3 − Tmem55b (id: 1558)

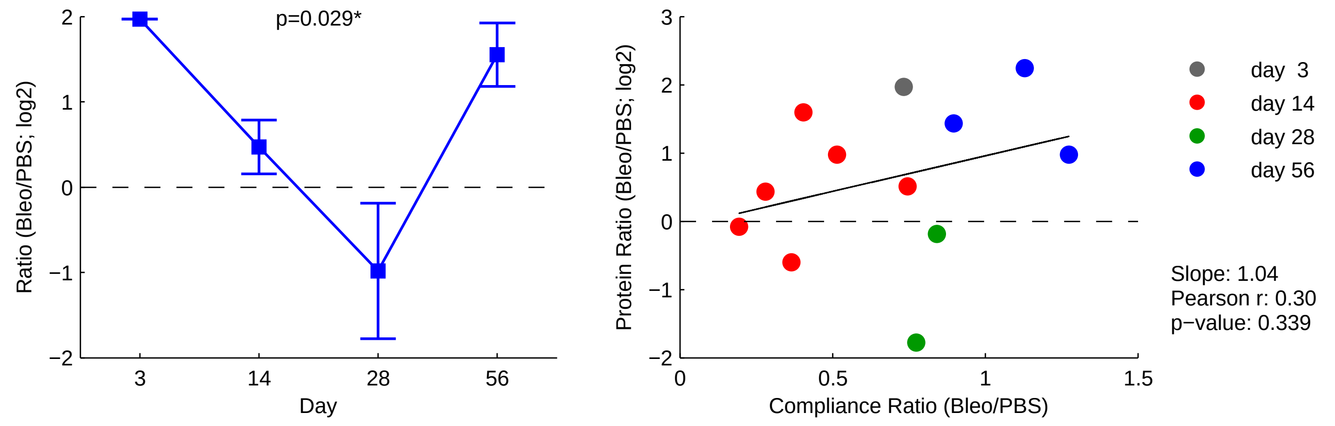

O70161−3 − Pip5k1c (id: 1559)

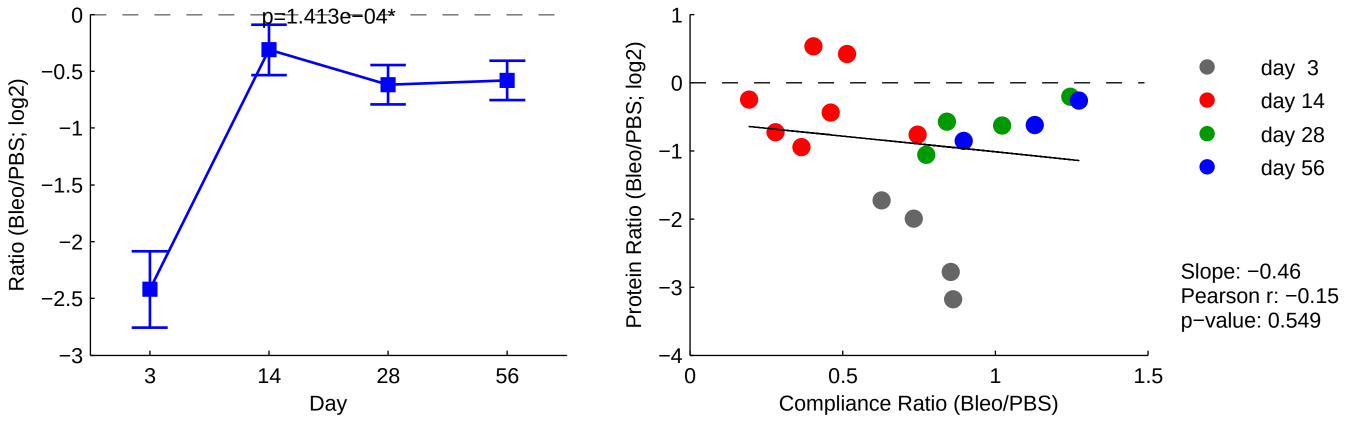

F8WI30 − Snx7 (id: 1563)

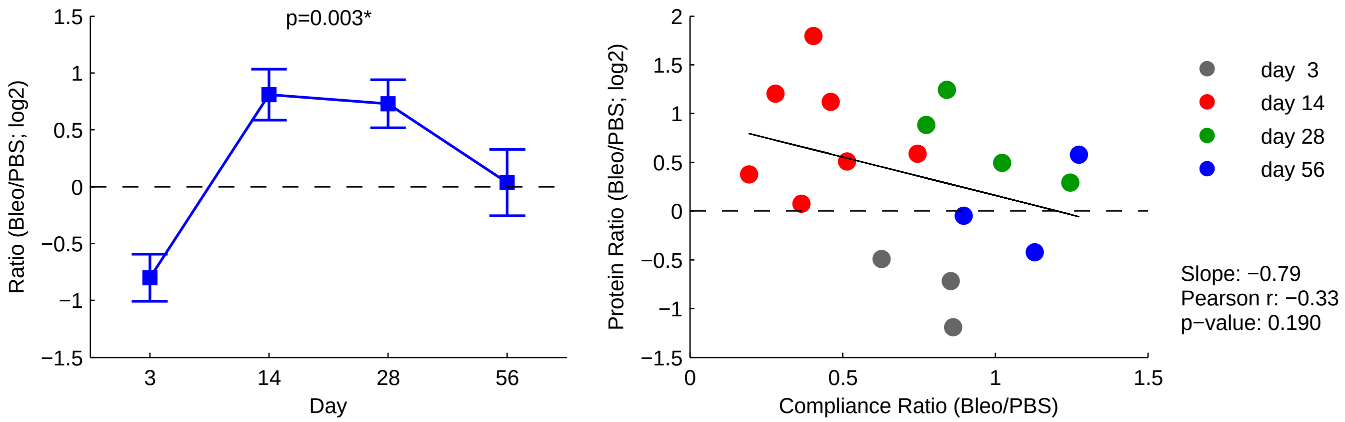

F8WID5 − Tpm1 (id: 1569)

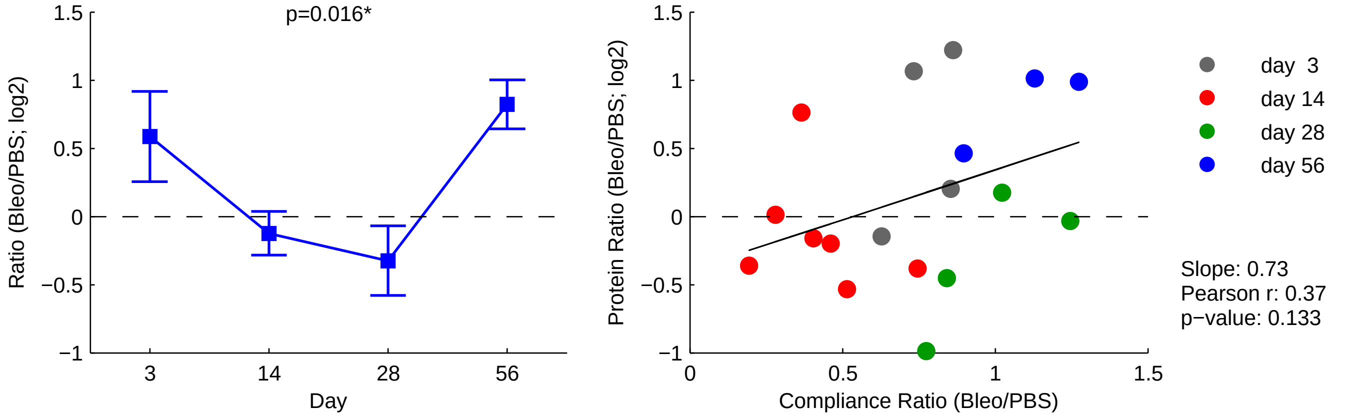

Q9JIS8 − Slc12a4 (id: 1571)

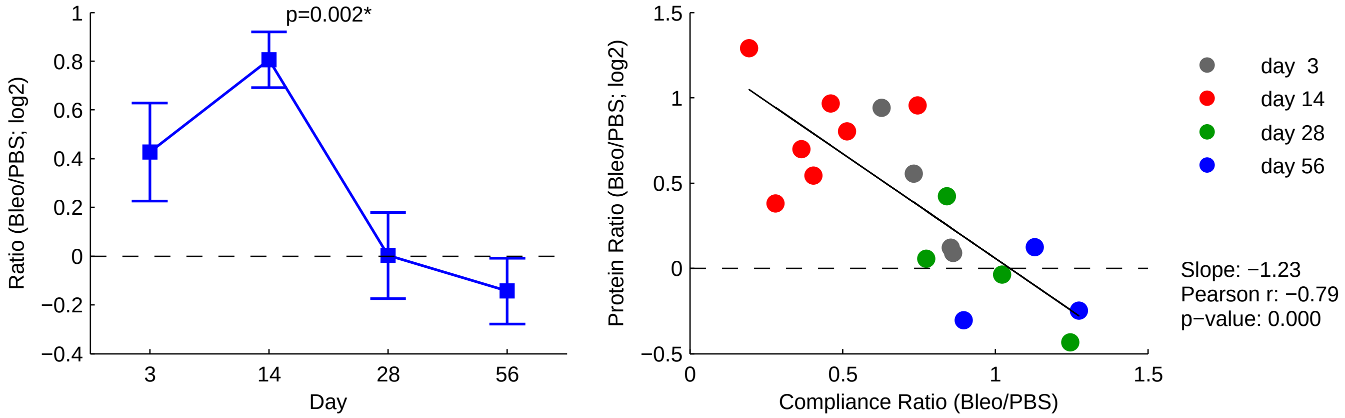

F8WIK5 − Plekha2 (id: 1572)

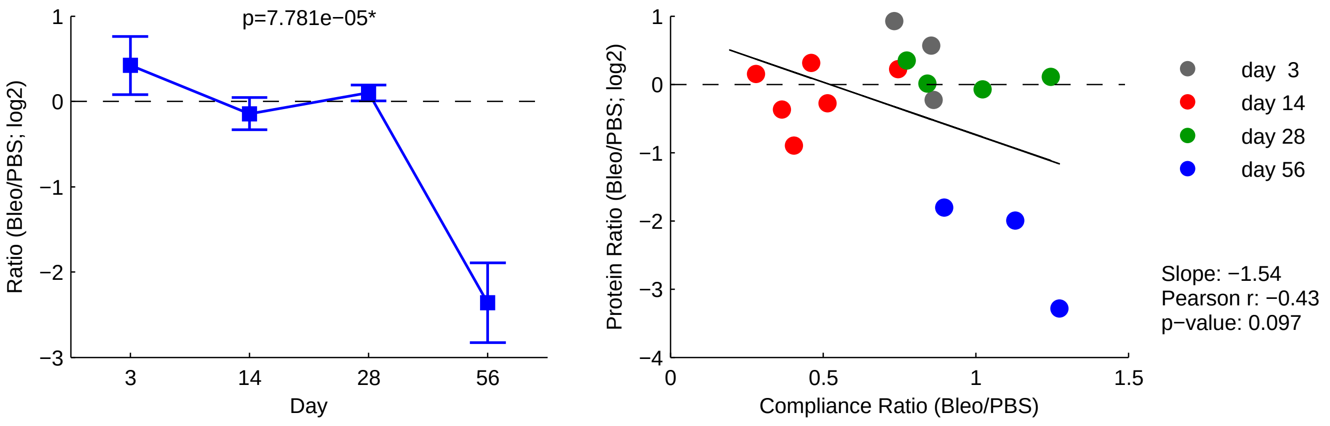

P10923 − Spp1 (id: 1573)

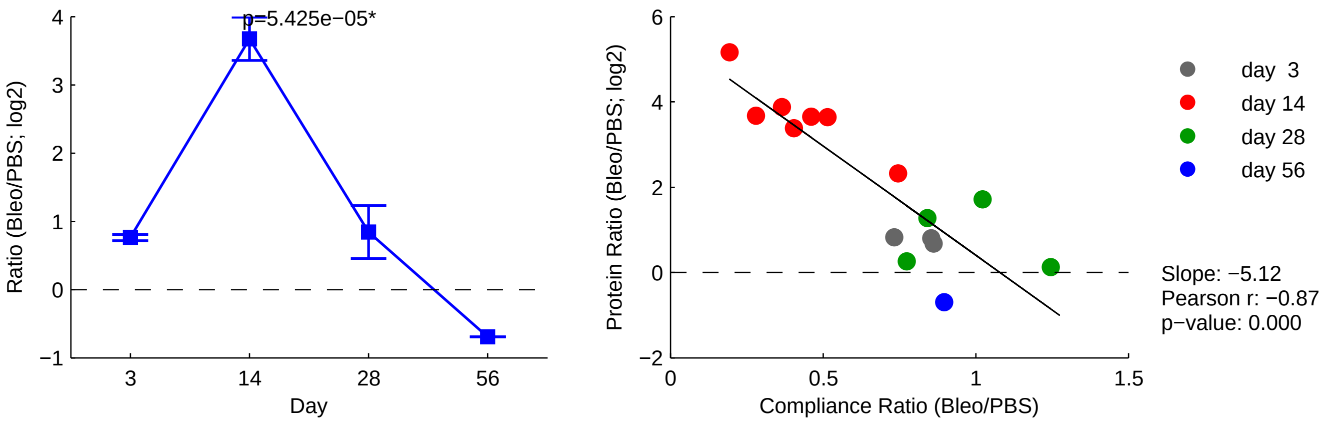

F8WIT2 − Anxa6 (id: 1575)

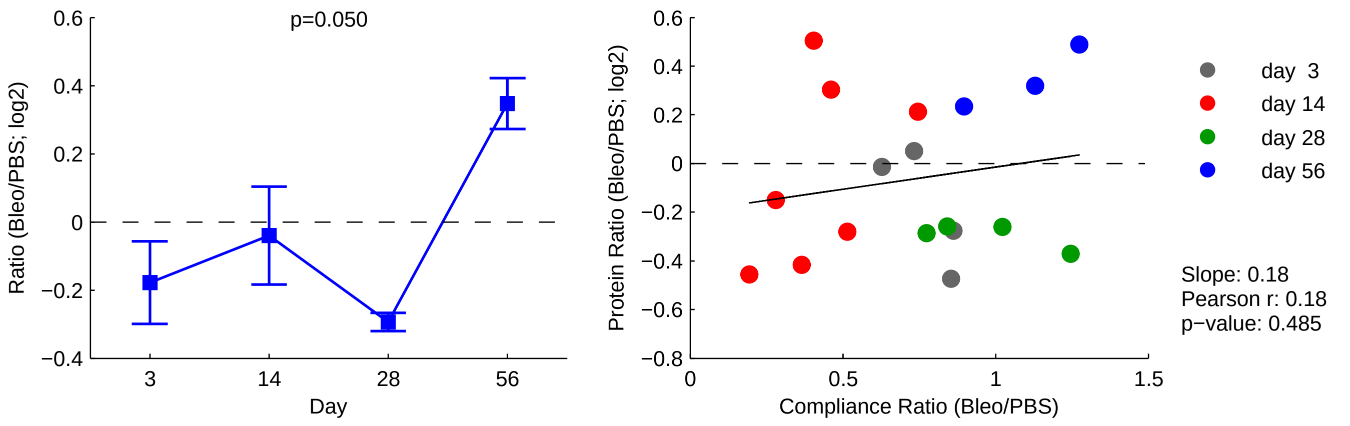

H7BXB9 − 1700037H04Rik (id: 1576)

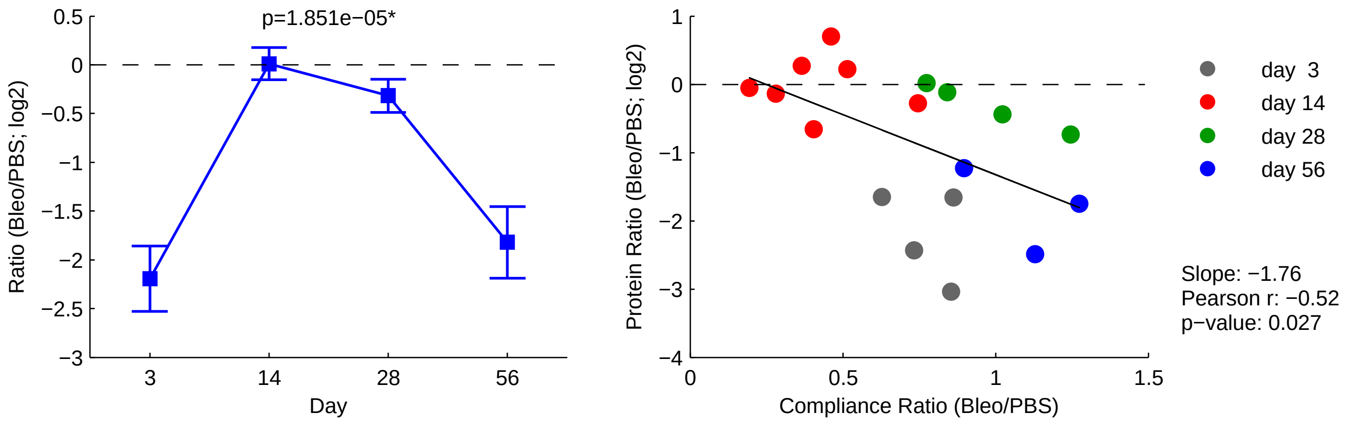

F8WIX8 − Hist1h2al (id: 1577)

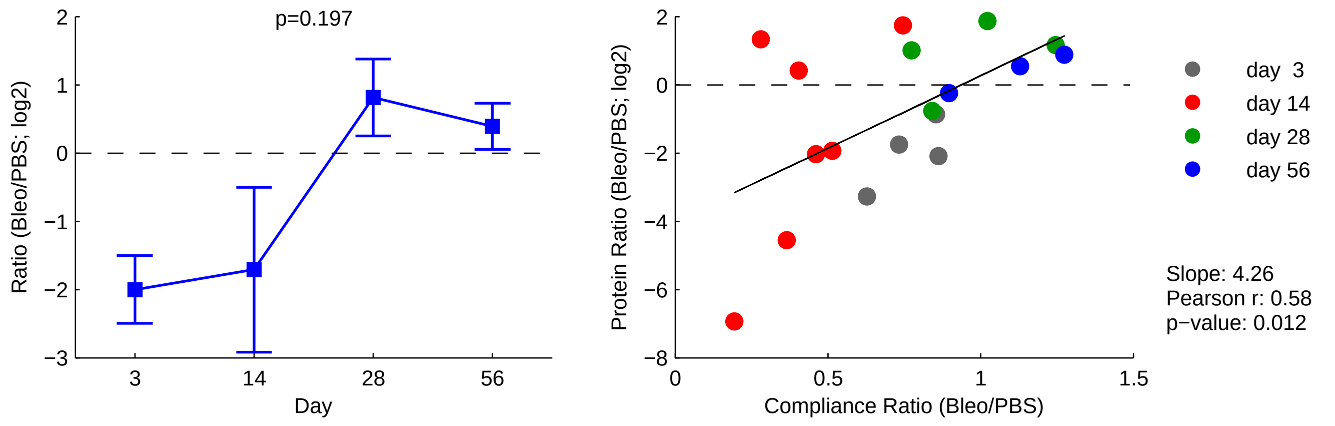

Q8BXR9−2 − Osbpl6 (id: 1578)

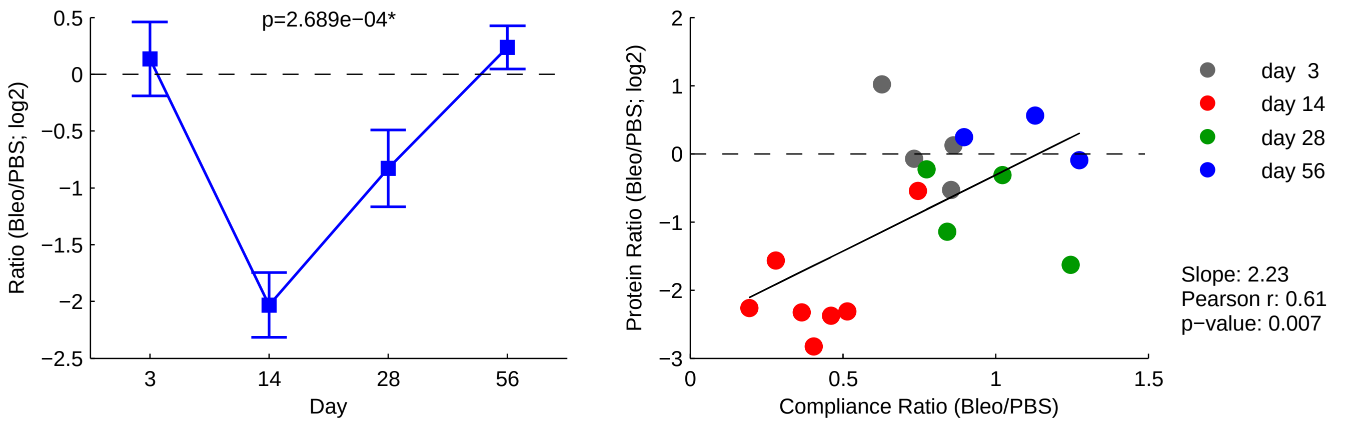

Q69ZZ6−2 − Tmcc1 (id: 1581)

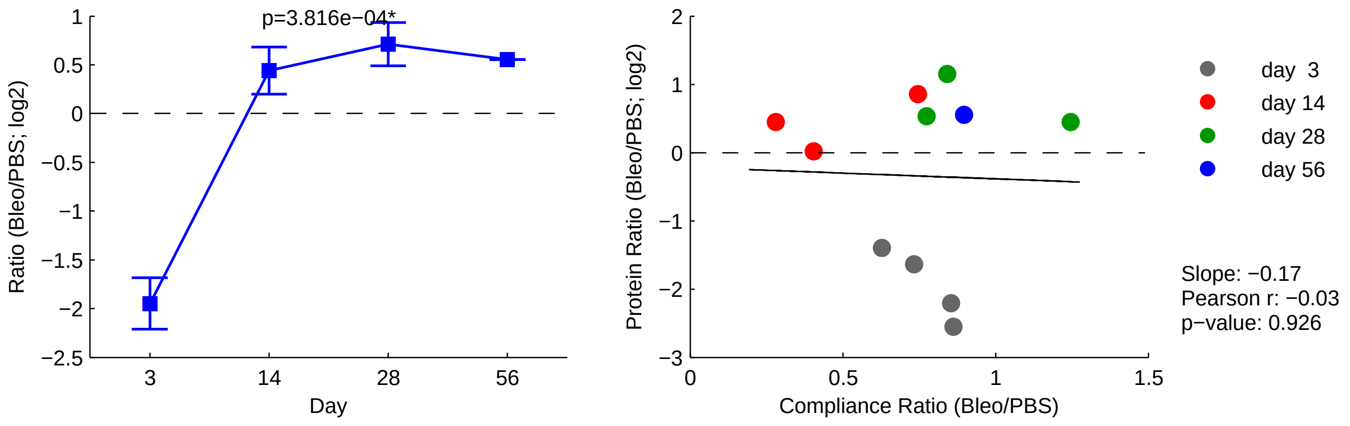

Q9ESV0 − Ddx24 (id: 1582)

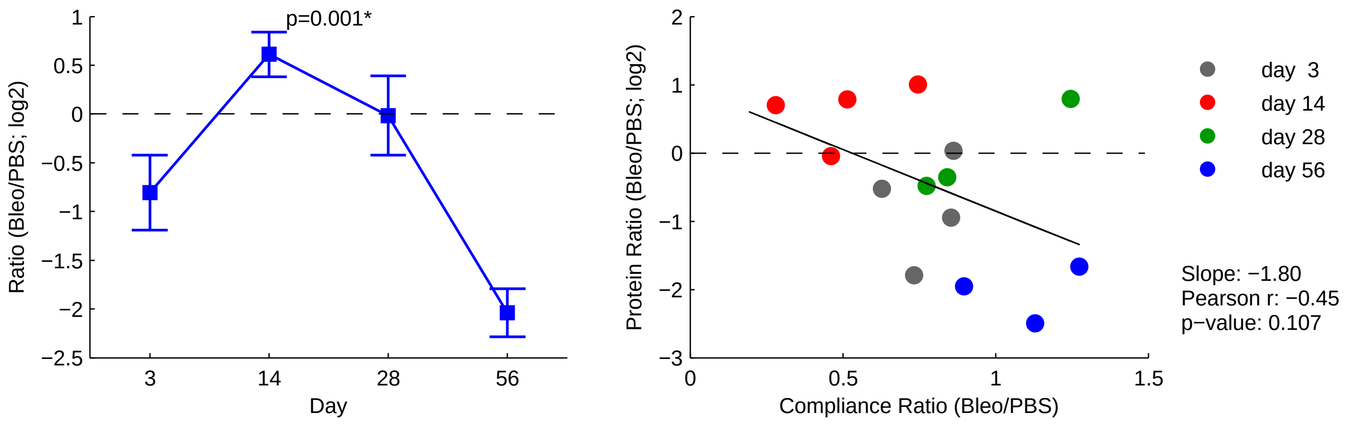

Q80X82 − Sympk (id: 1583)

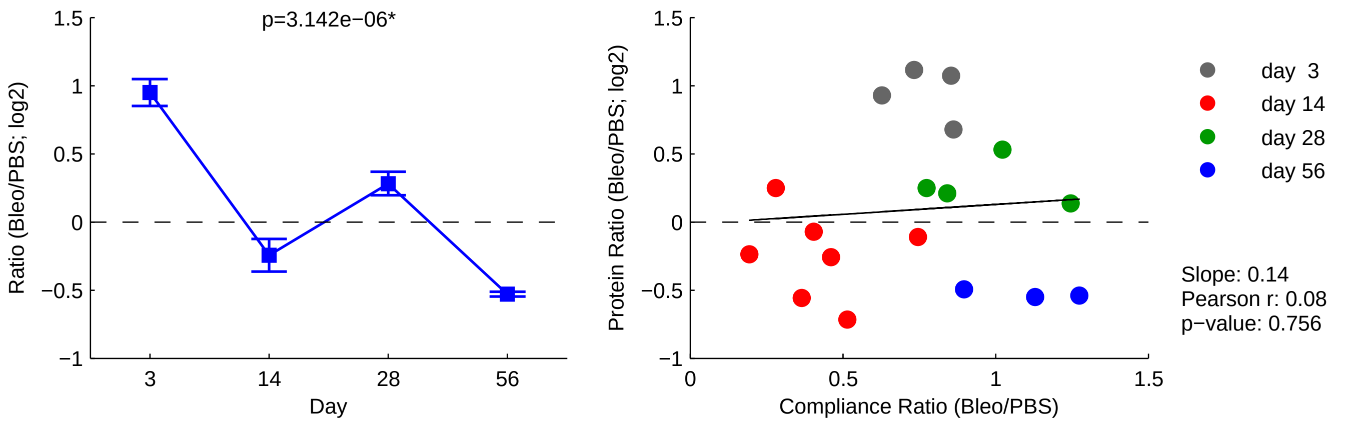

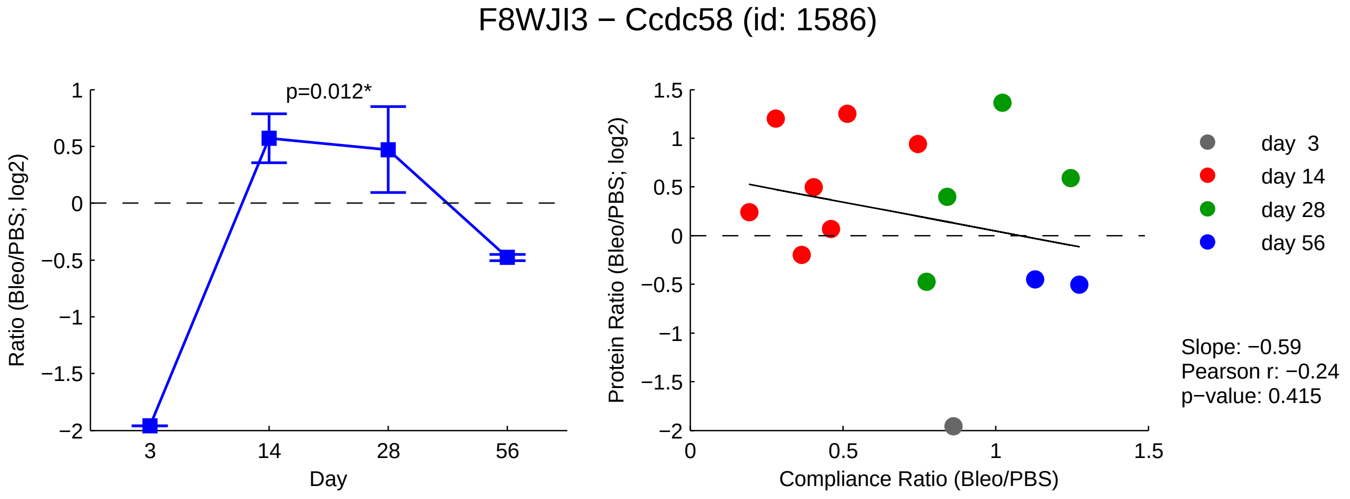
F8WJI3 − Ccdc58 (id: 1586)
p=0.012*
Ratio (Bleo/PBS; log2)
Day
Protein Ratio (Bleo/PBS; log2)
Compliance Ratio (Bleo/PBS)
day 3
day 14
day 28
day 56
Slope: −0.59
Pearson r: −0.24
p−value: 0.415

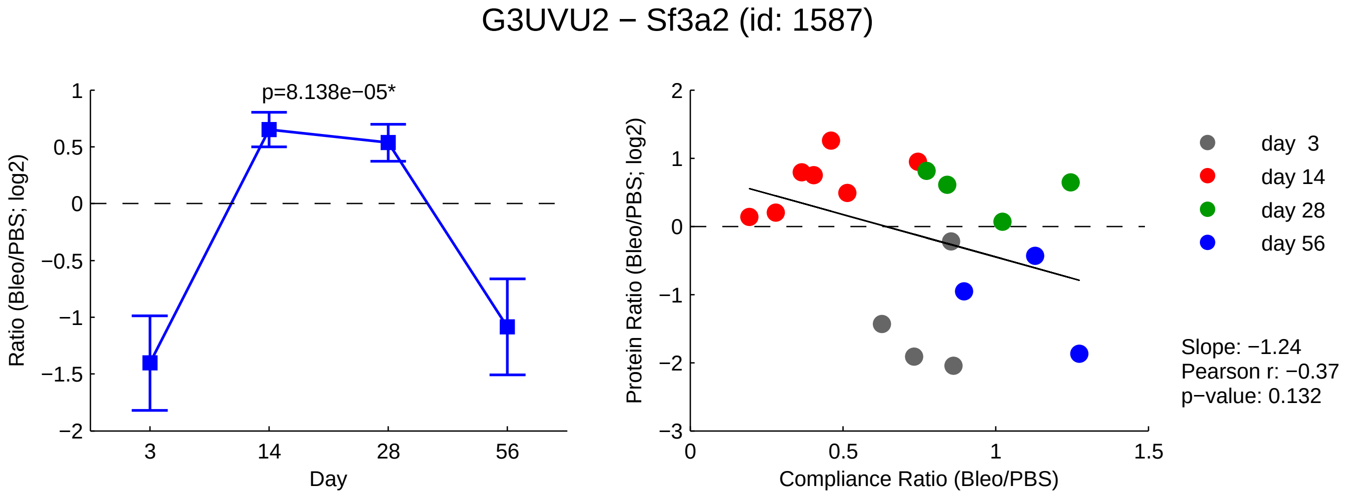
G3UVU2 − Sf3a2 (id: 1587)
p=8.138e−05*
Ratio (Bleo/PBS; log2)
Day
Protein Ratio (Bleo/PBS; log2)
Compliance Ratio (Bleo/PBS)
day 3
day 14
day 28
day 56
Slope: −1.24
Pearson r: −0.37
p−value: 0.132

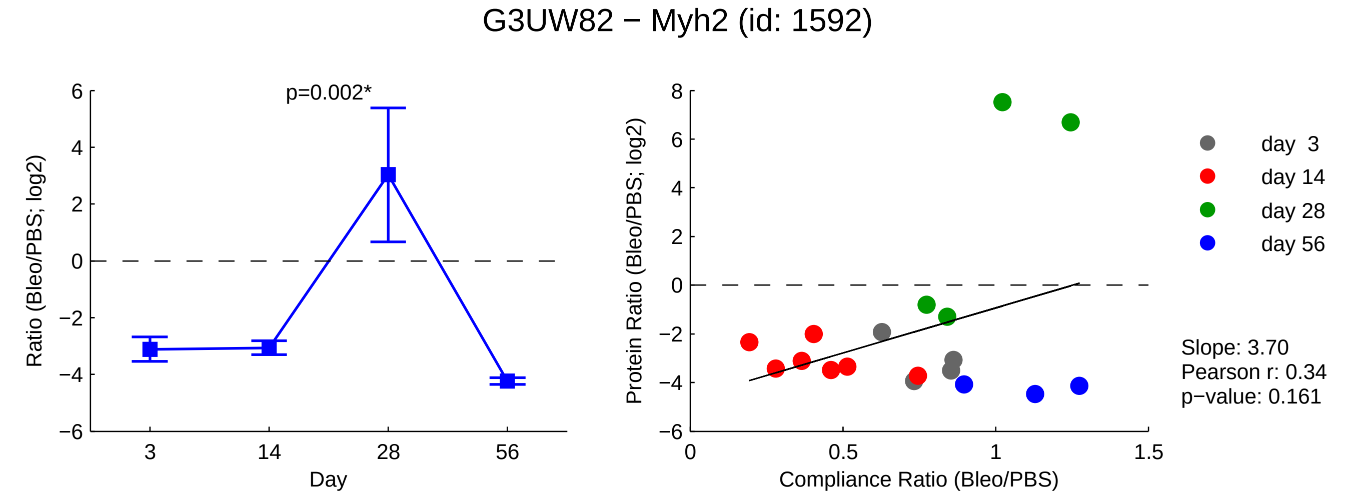
G3UW82 − Myh2 (id: 1592)
p=0.002*
Ratio (Bleo/PBS; log2)
Day
Protein Ratio (Bleo/PBS; log2)
Compliance Ratio (Bleo/PBS)
day 3
day 14
day 28
day 56
Slope: 3.70
Pearson r: 0.34
p−value: 0.161

Q9CZR2 − Naalad2 (id: 1594)

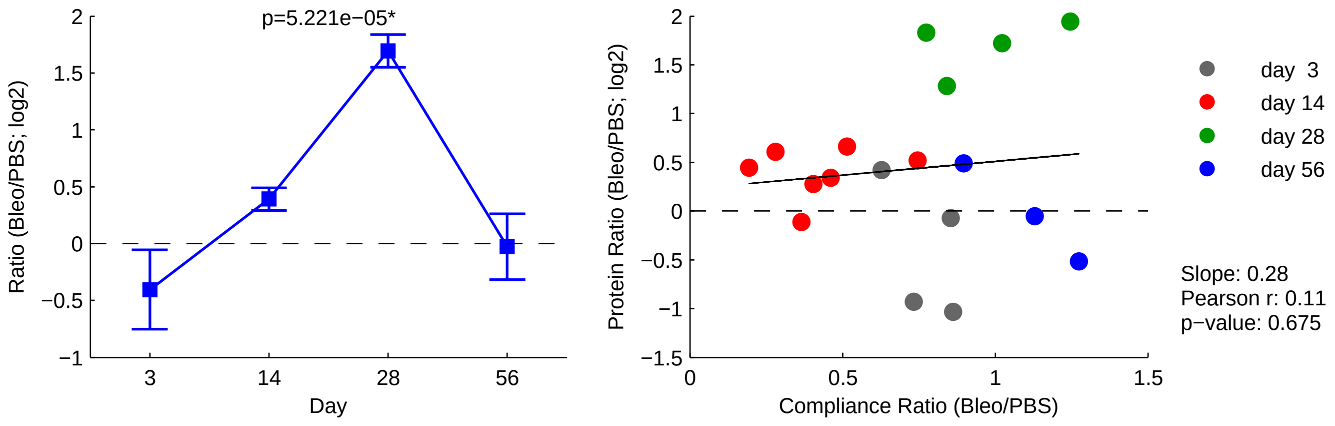

P62897 − Cycs (id: 1596)

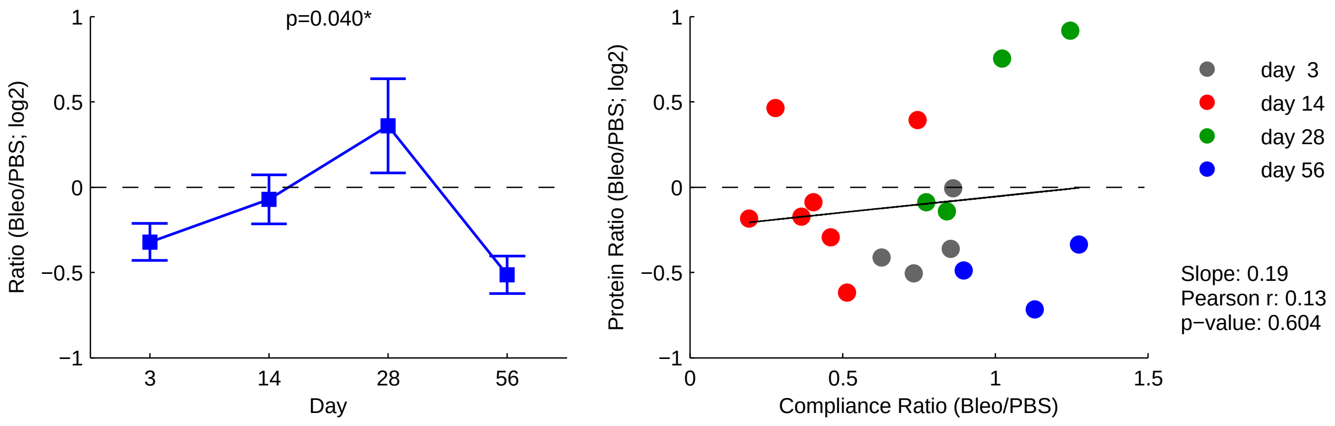

G3UWG7 − Ccdc53 (id: 1597)

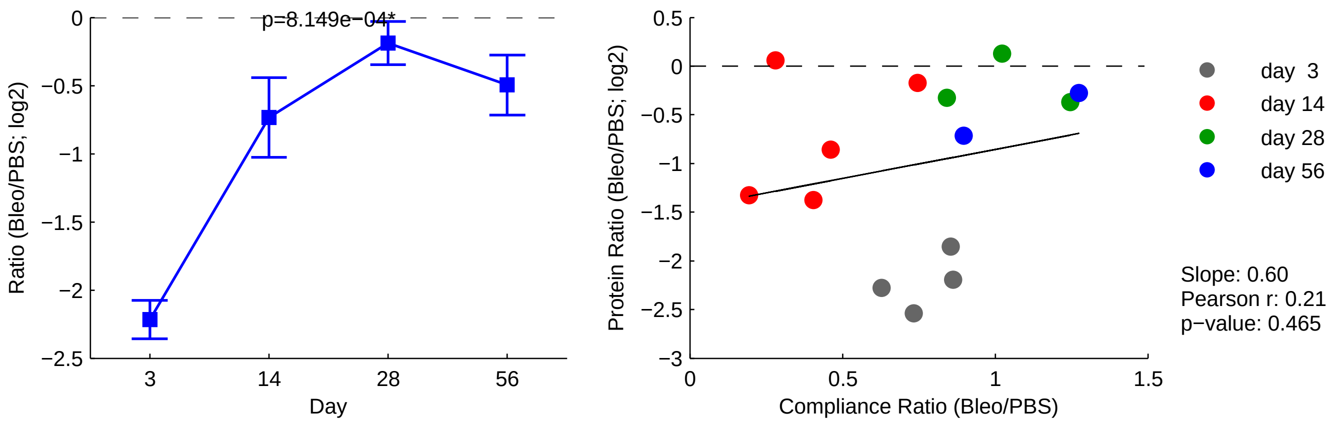

Q7TNP2 − Ppp2r1b (id: 1601)

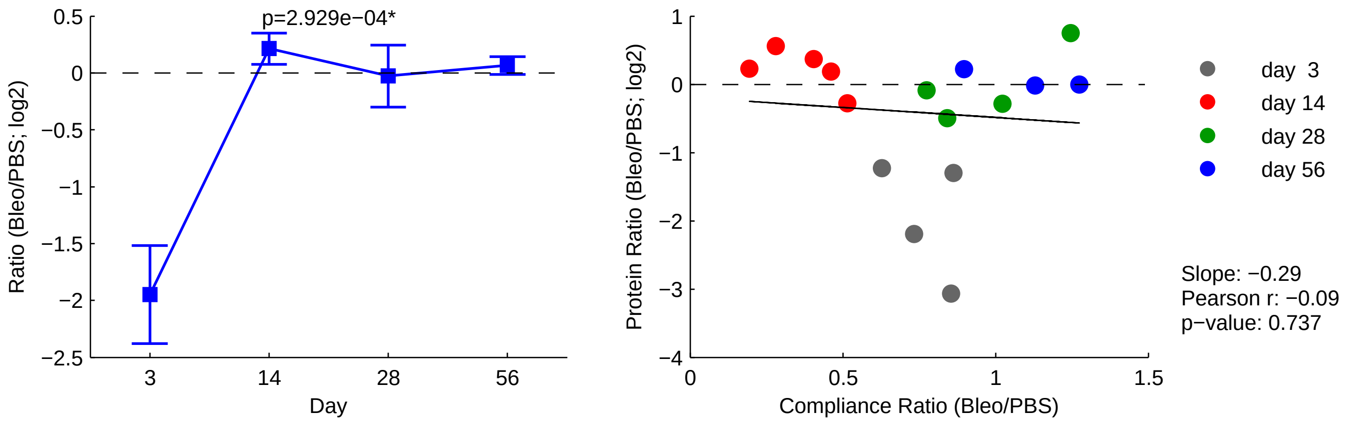

P35601−2 − Rfc1 (id: 1604)

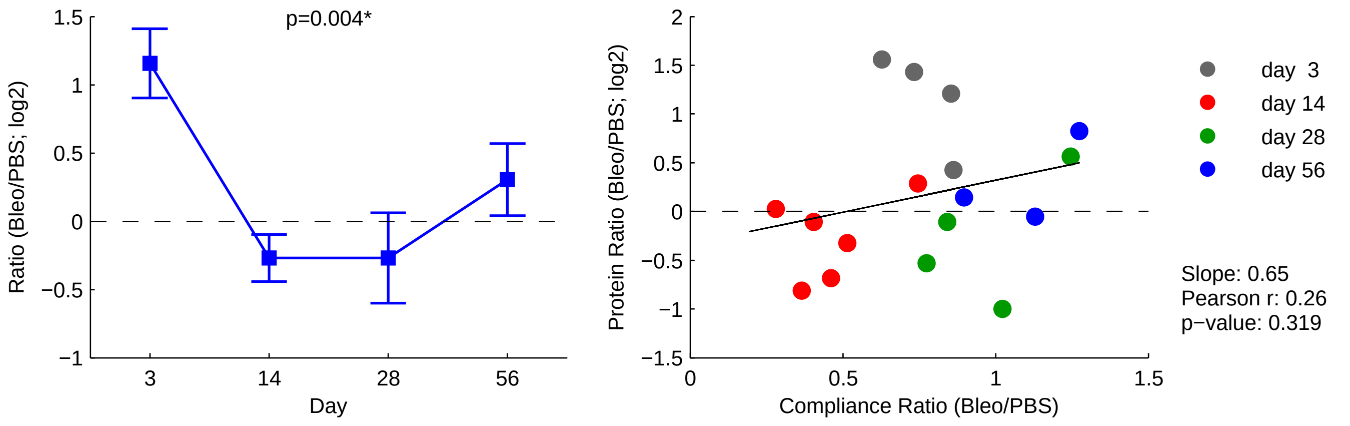

Q99LD4 − Gps1 (id: 1615)

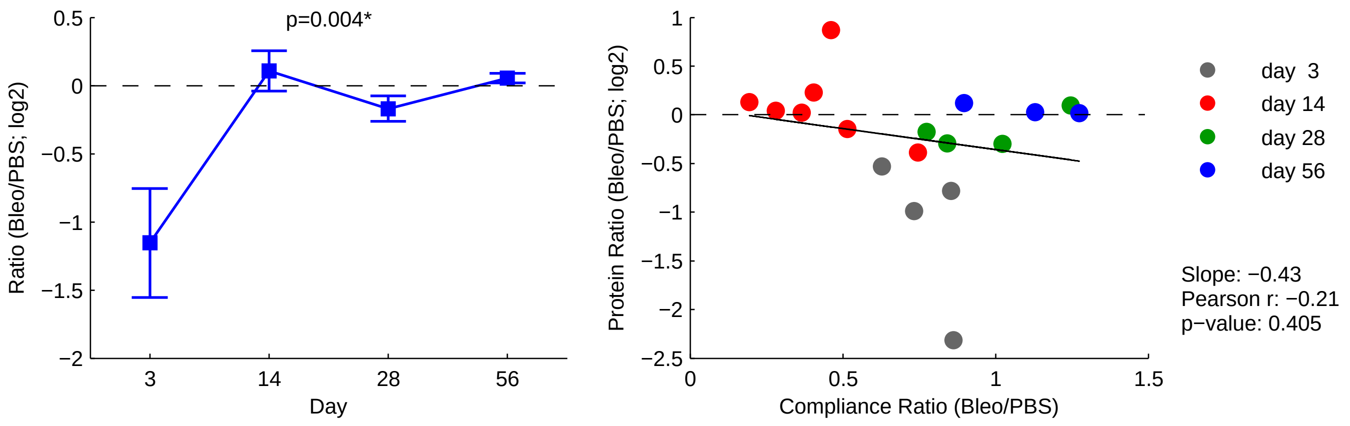

G3UXZ5 − Psme1 (id: 1616)

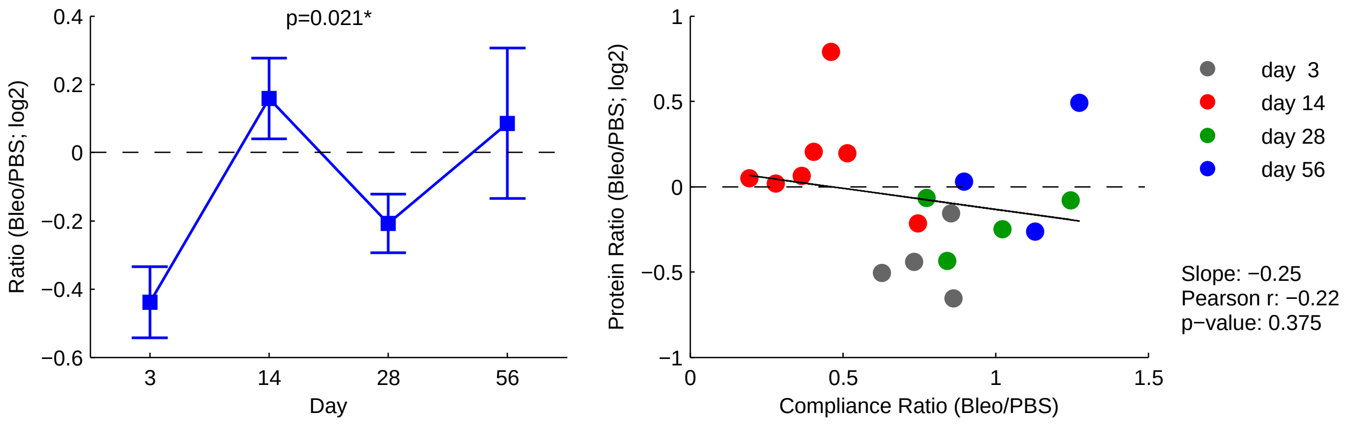

Q64487−3 − Ptprd (id: 1618)

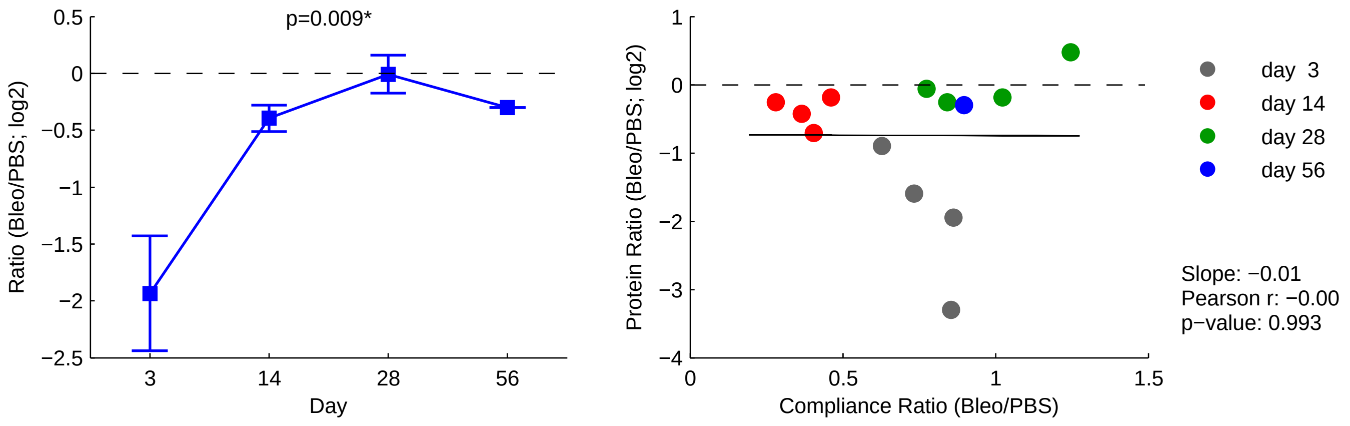

G3UYG6 − Gigyf2 (id: 1620)

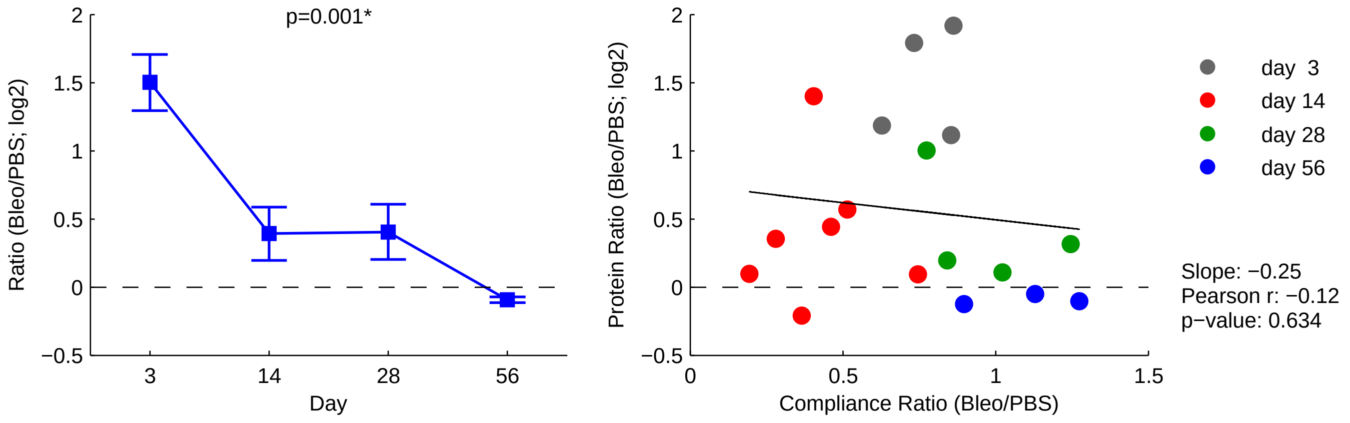

G3UYJ7 − Gm20441 (id: 1621)

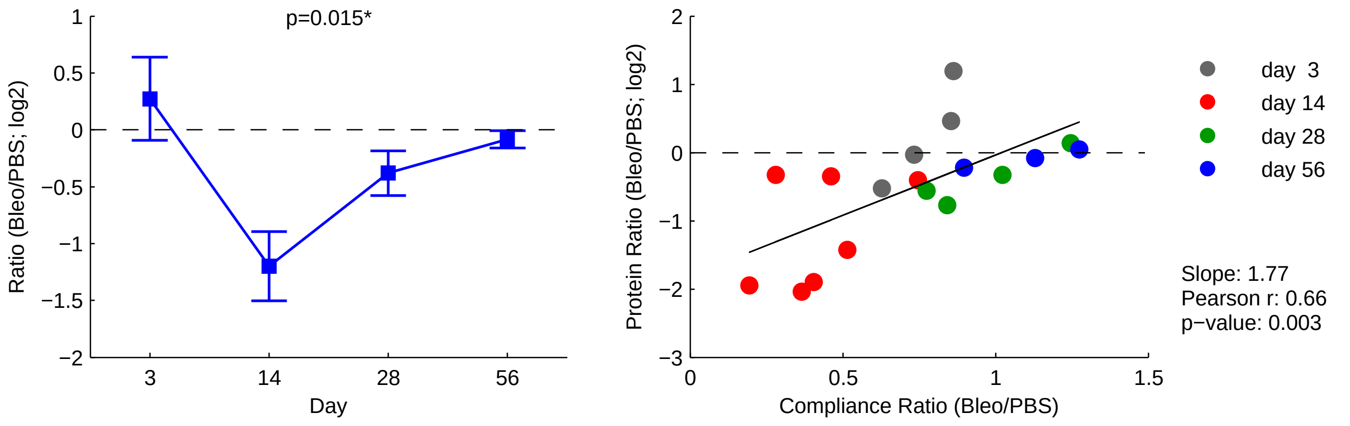

Q6VNB8 − Wdfy3 (id: 1624)

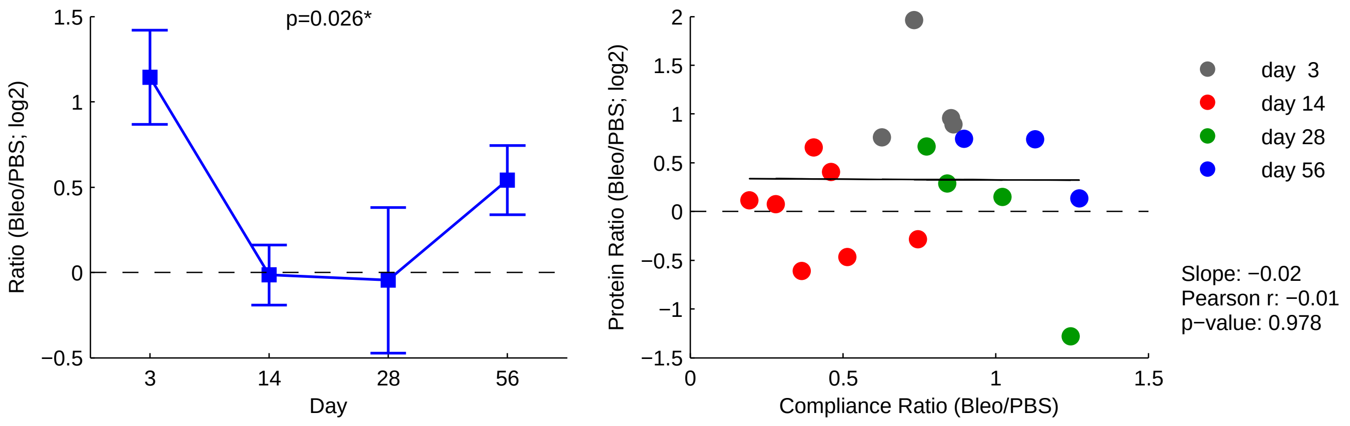

G3UZY2 − Txn2 (id: 1632)

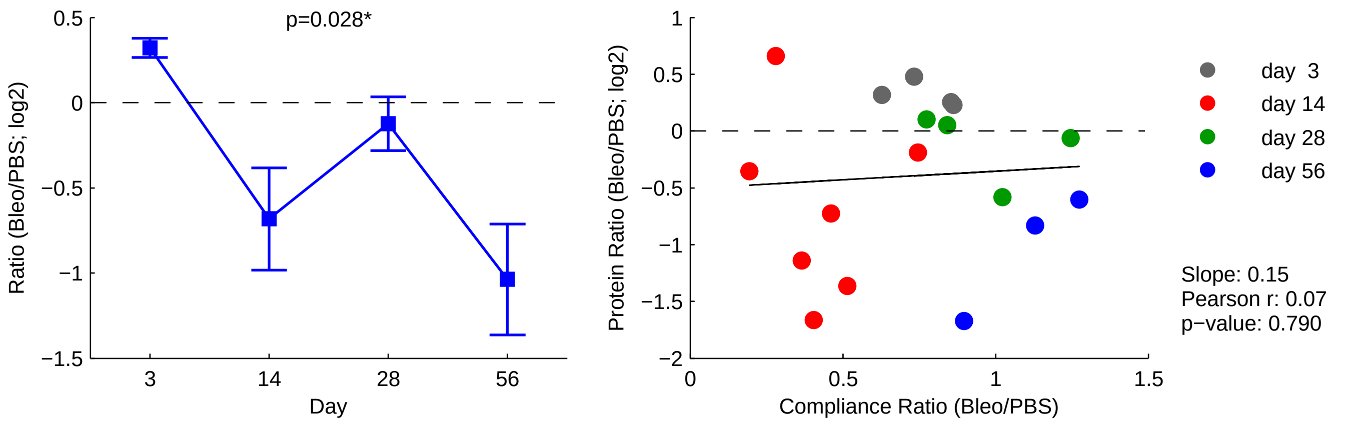

G3X8Q1 − Cabin1 (id: 1638)

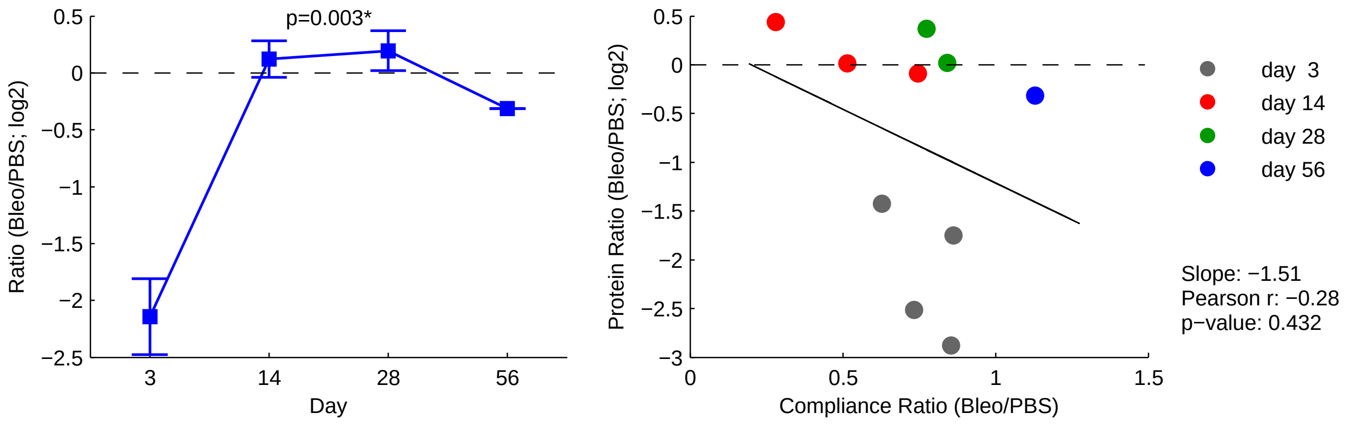

G3X9T8 − Cp (id: 1639)

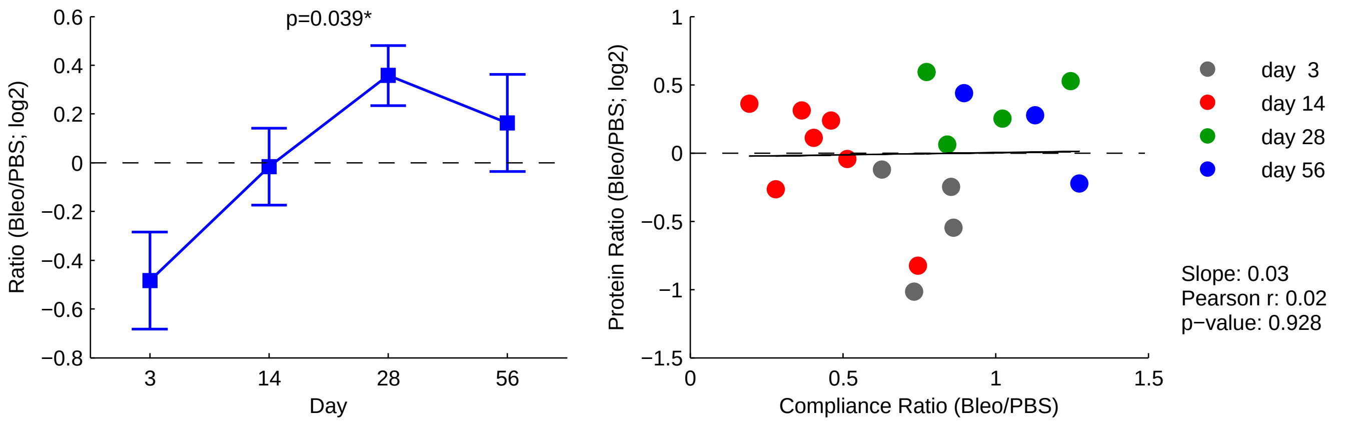

G3X8R0 − Reep5 (id: 1640)

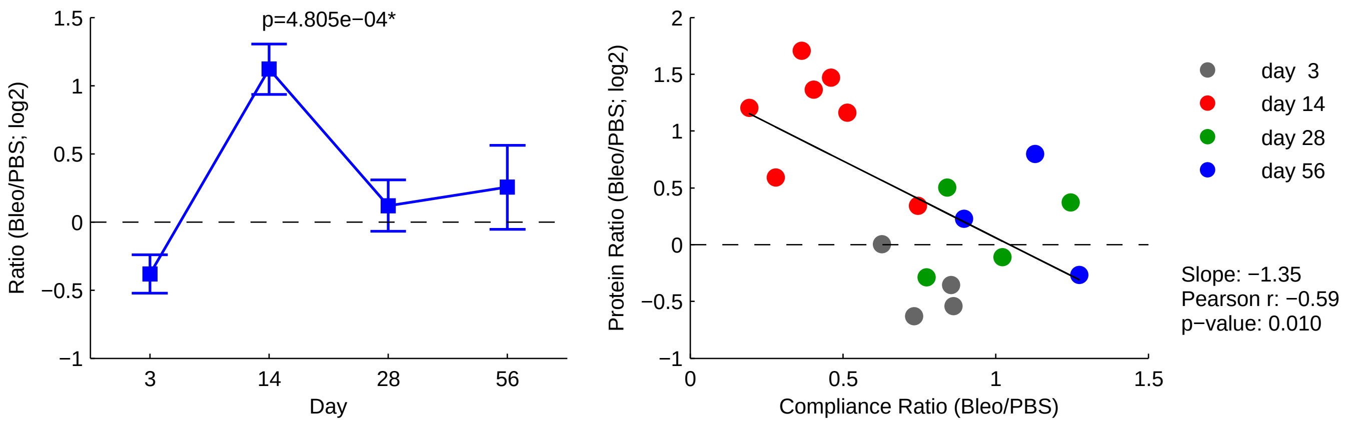

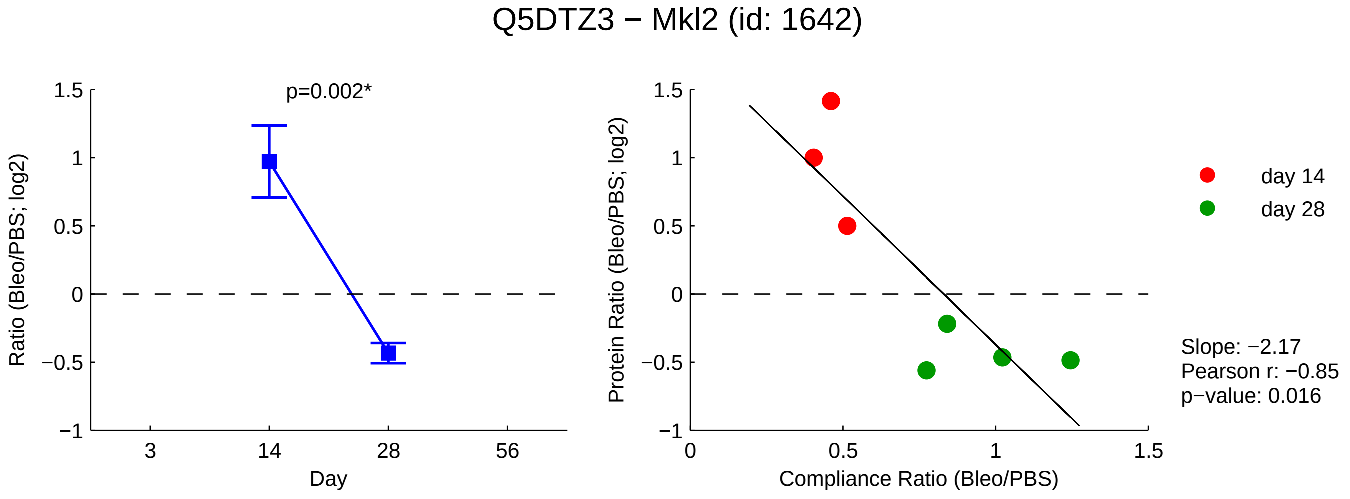
Q5DTZ3 − Mkl2 (id: 1642)
p=0.002*
Ratio (Bleo/PBS; log2)
Day
Protein Ratio (Bleo/PBS; log2)
Compliance Ratio (Bleo/PBS)
day 14
day 28
Slope: −2.17
Pearson r: −0.85
p−value: 0.016

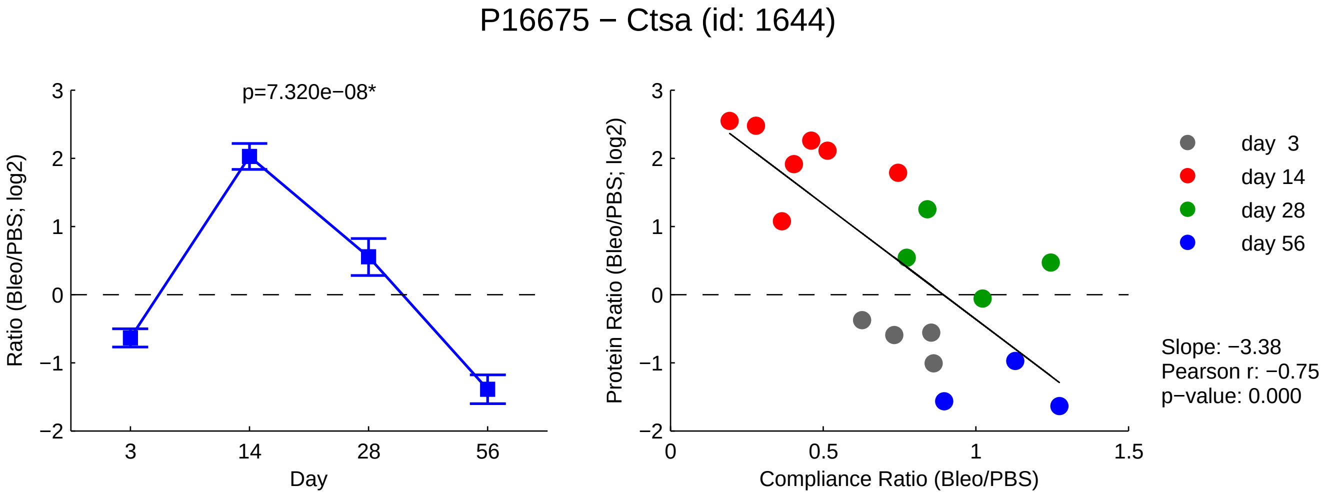
P16675 − Ctsa (id: 1644)
p=7.320e−08*
Ratio (Bleo/PBS; log2)
Day
Protein Ratio (Bleo/PBS; log2)
Compliance Ratio (Bleo/PBS)
day 3
day 14
day 28
day 56
Slope: −3.38
Pearson r: −0.75
p−value: 0.000

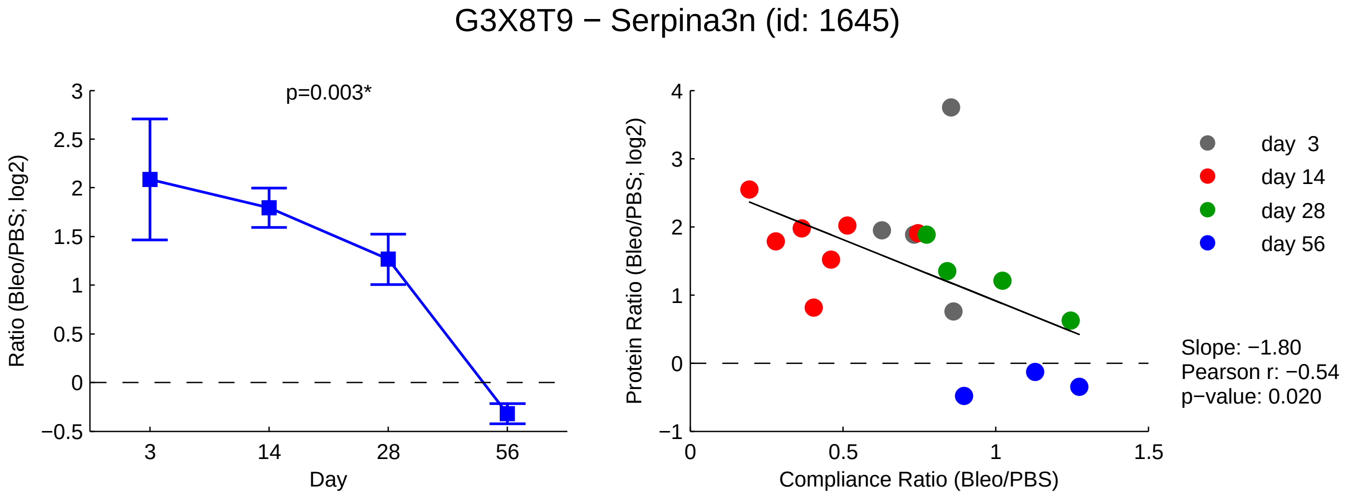
G3X8T9 − Serpina3n (id: 1645)
p=0.003*
Ratio (Bleo/PBS; log2)
Day
Protein Ratio (Bleo/PBS; log2)
Compliance Ratio (Bleo/PBS)
day 3
day 14
day 28
day 56
Slope: −1.80
Pearson r: −0.54
p−value: 0.020

O70458−2 − Osmr (id: 1647)

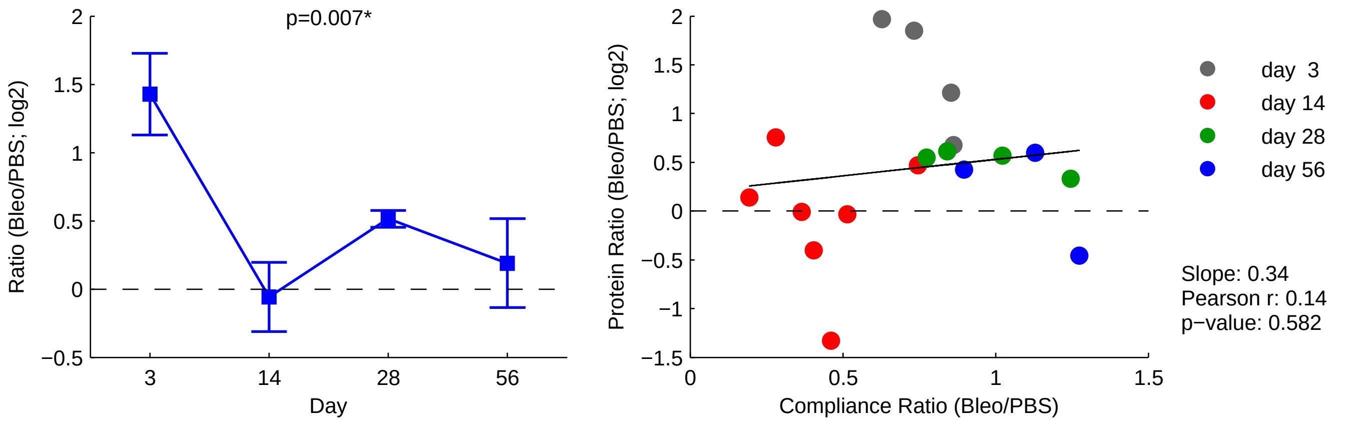

G3X8X0 − Dhx16 (id: 1651)

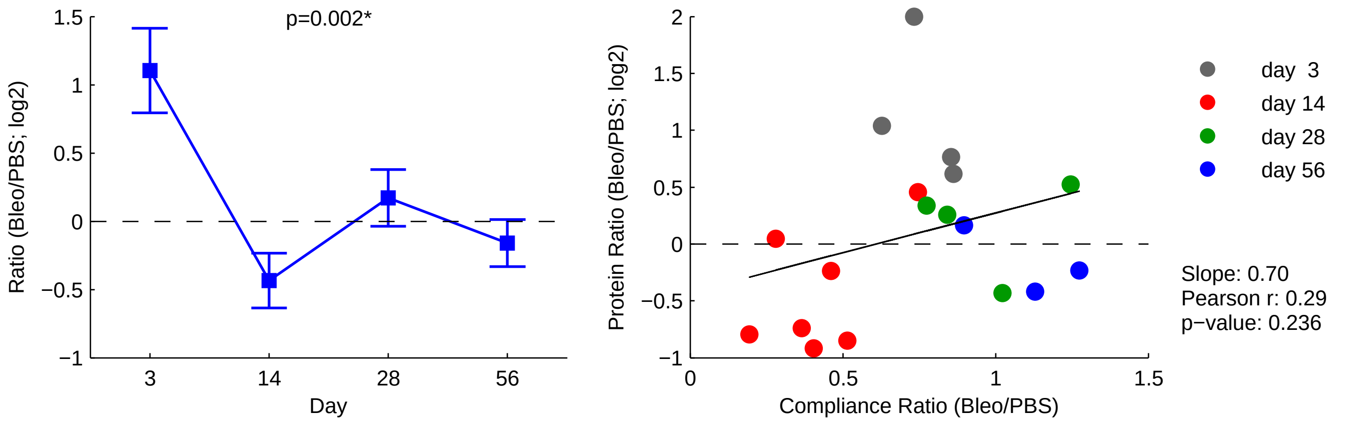

Q62230 − Siglec1 (id: 1652)

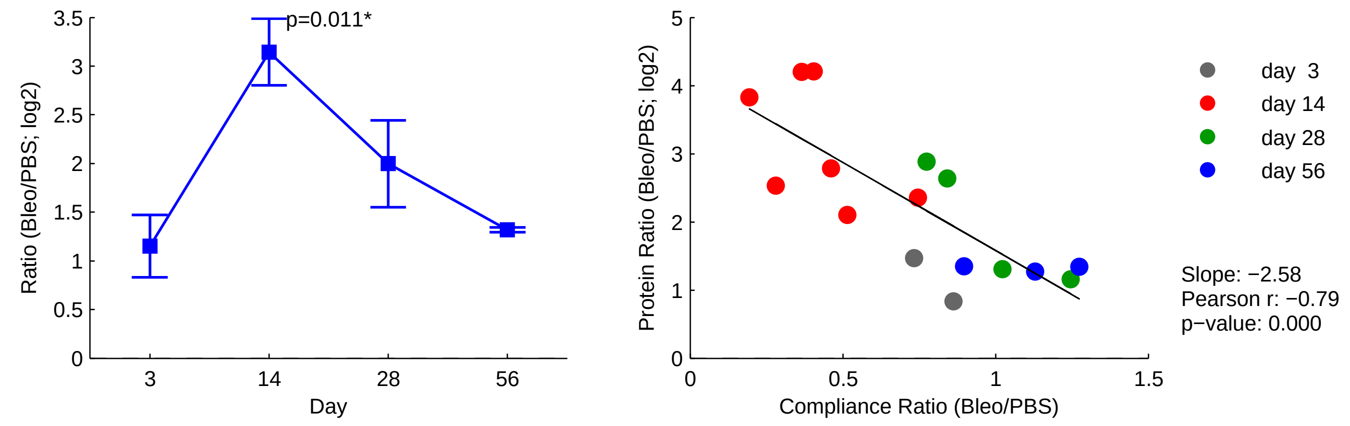

G3X8Y3 − Naa15 (id: 1654)

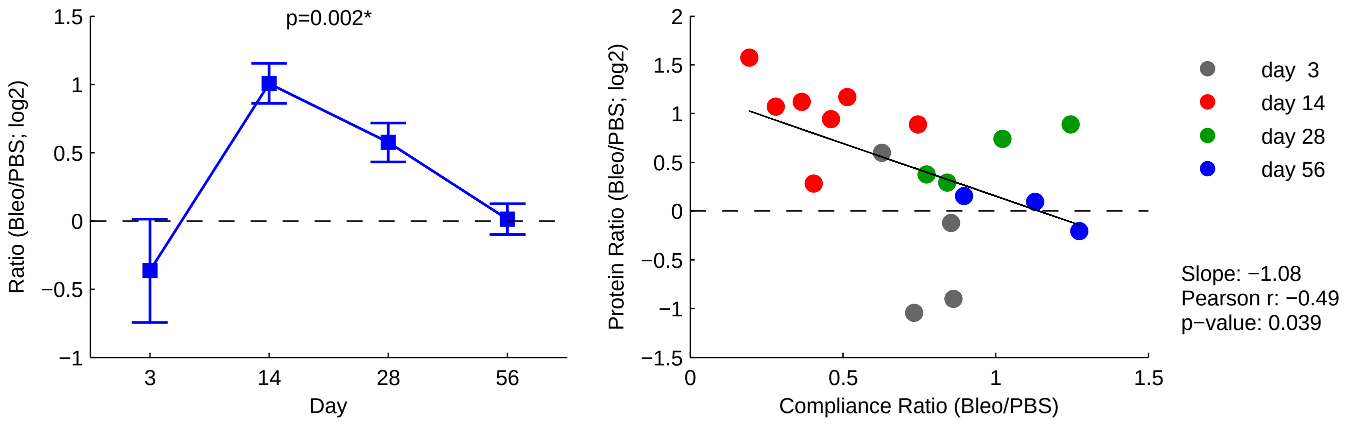

Q9QUN7 − Tlr2 (id: 1656)

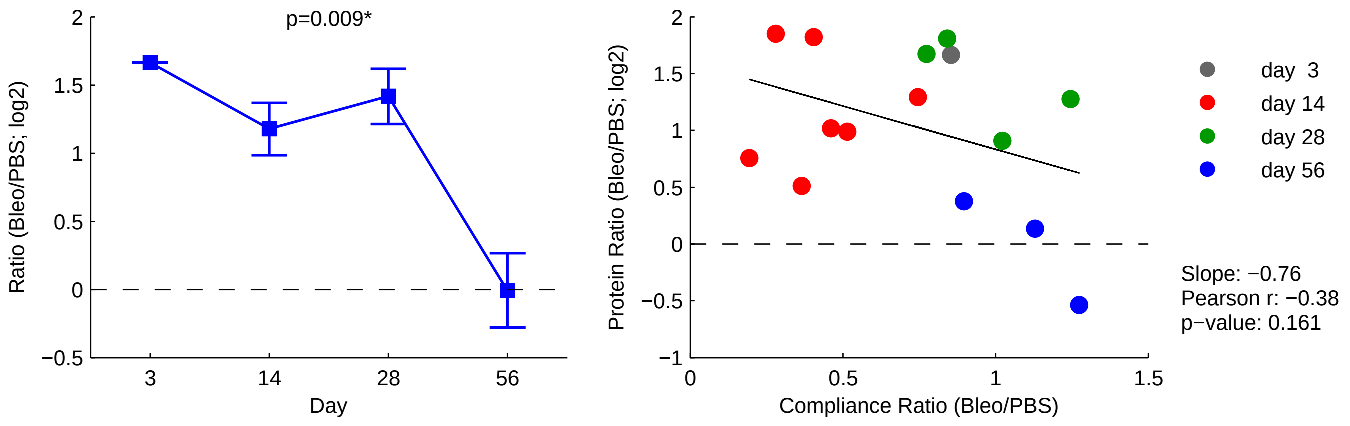

Q9DBR3 − Armc8 (id: 1658)

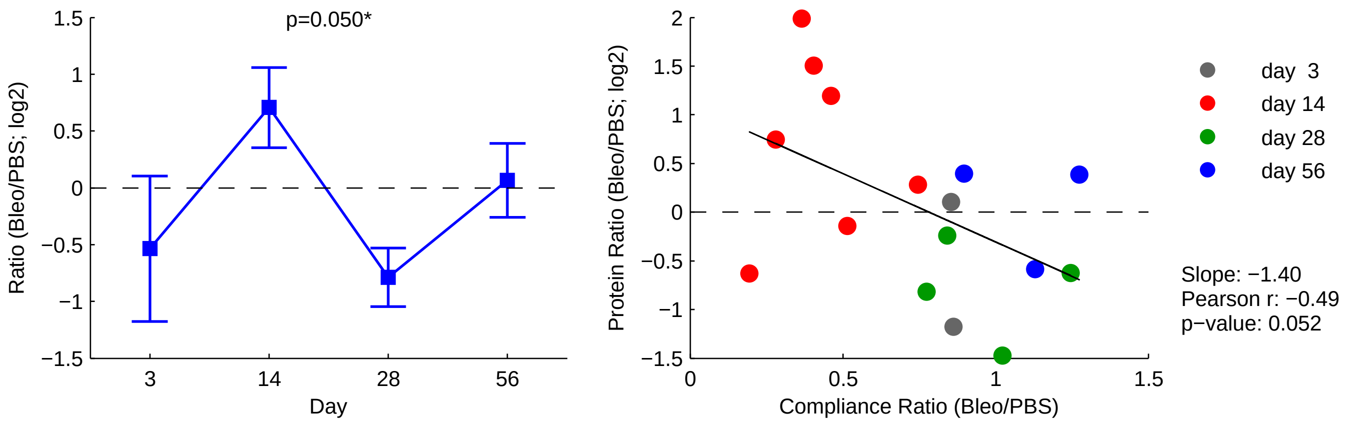

G3X922 − Dnajc13 (id: 1659)

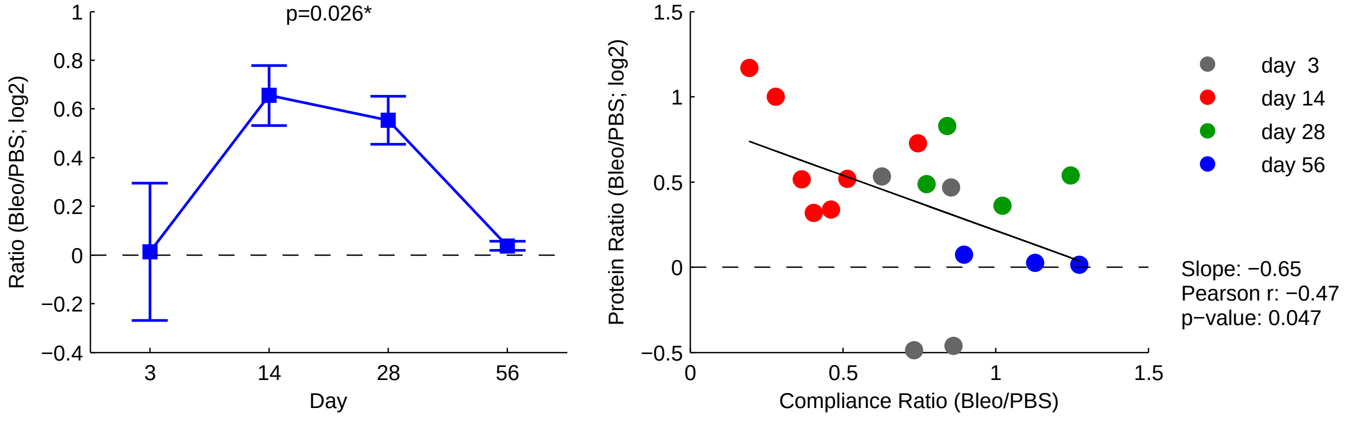

G3X924 − Plvap (id: 1660)

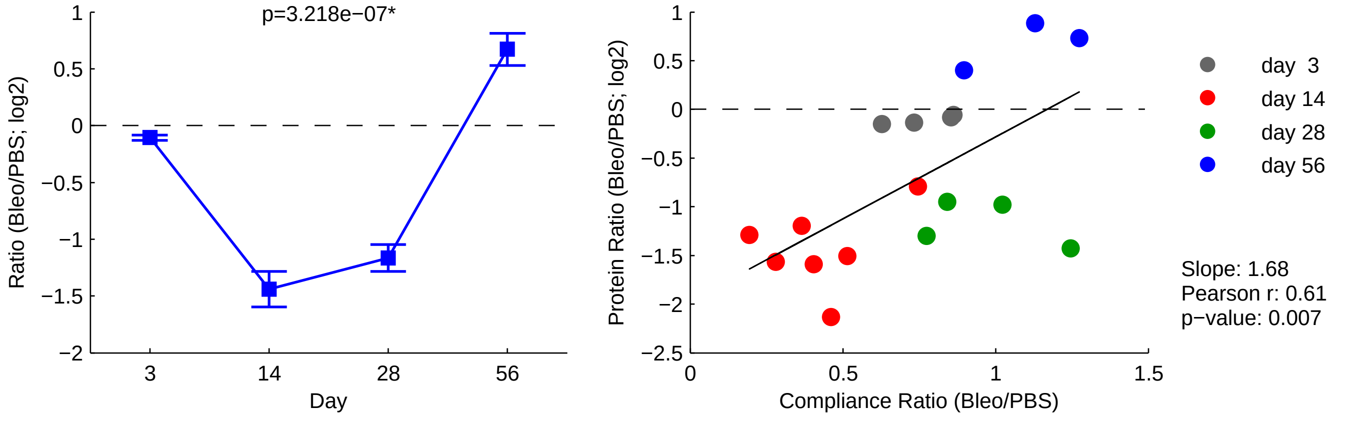

G3X928 − Sec23ip (id: 1662)

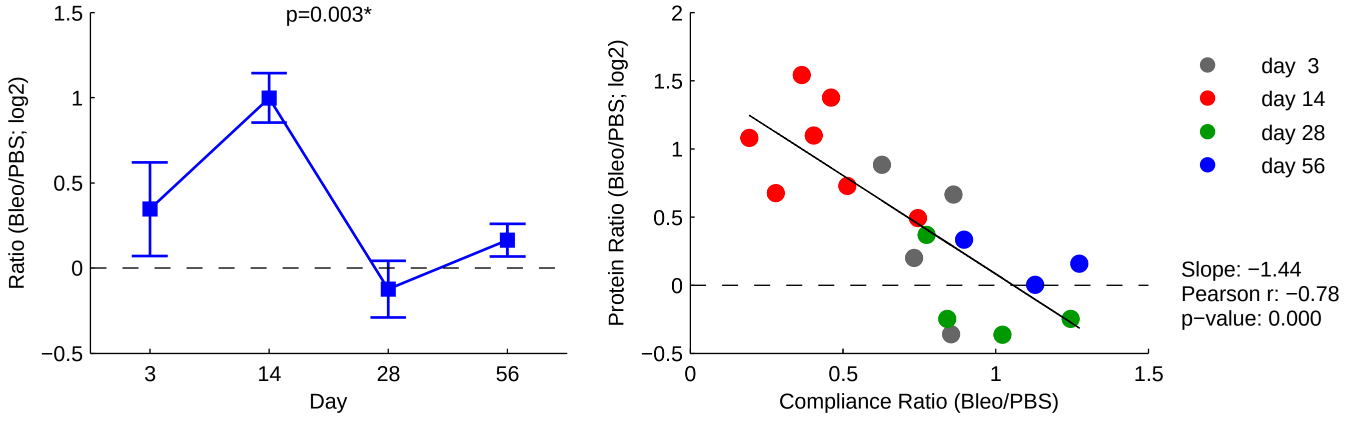

G3X951 − Ccdc151 (id: 1667)

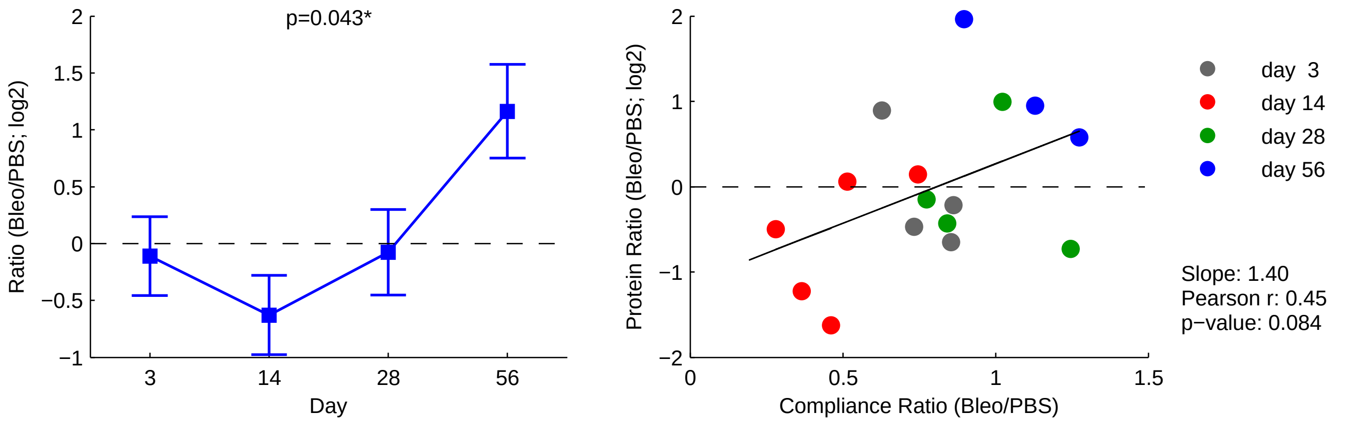

G3X972 − Sec24c (id: 1672)

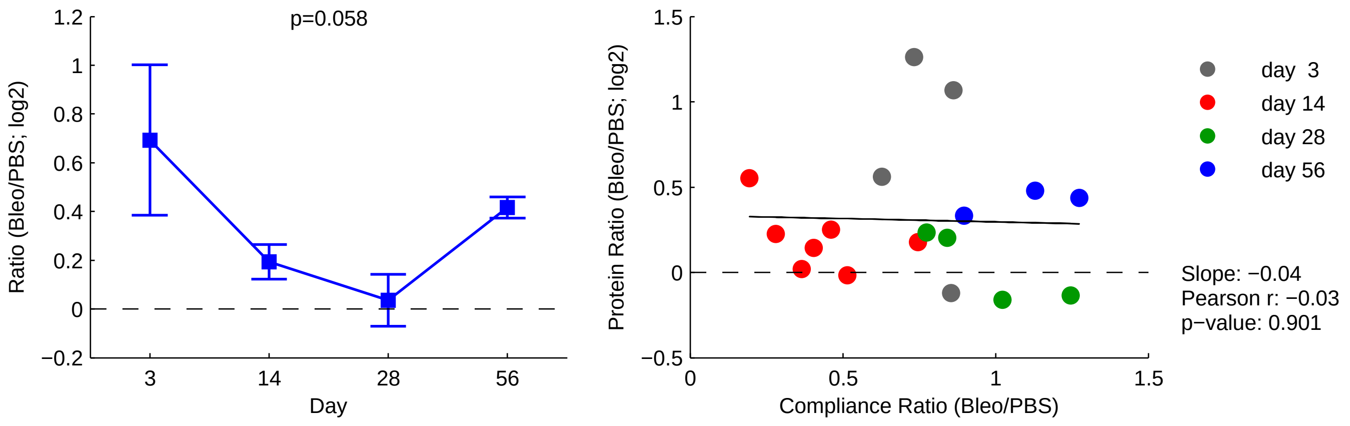

G3X973 − Stab1 (id: 1673)

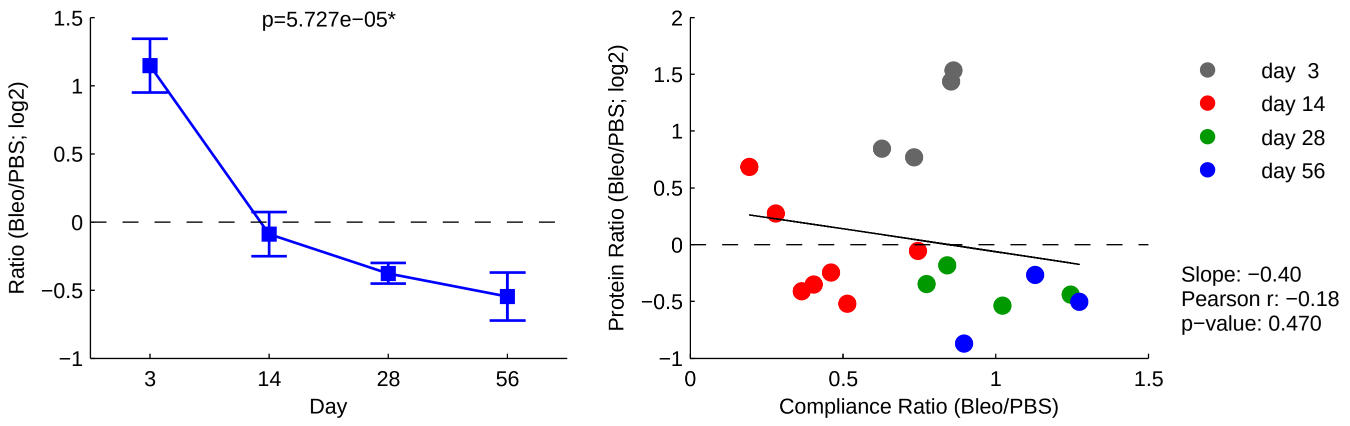

Q61703 − Itih2 (id: 1674)

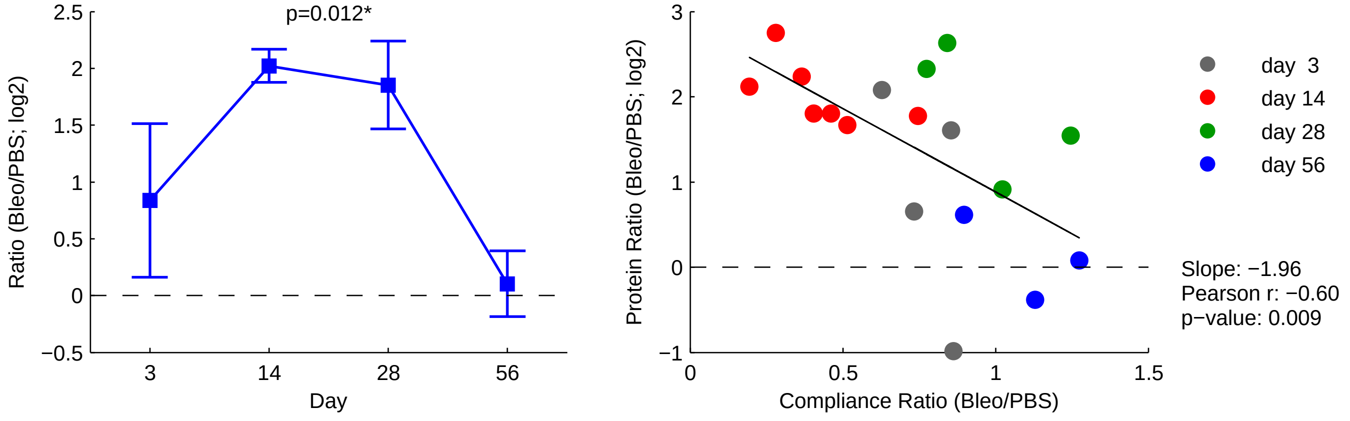

G3X982 − Aox3 (id: 1675)

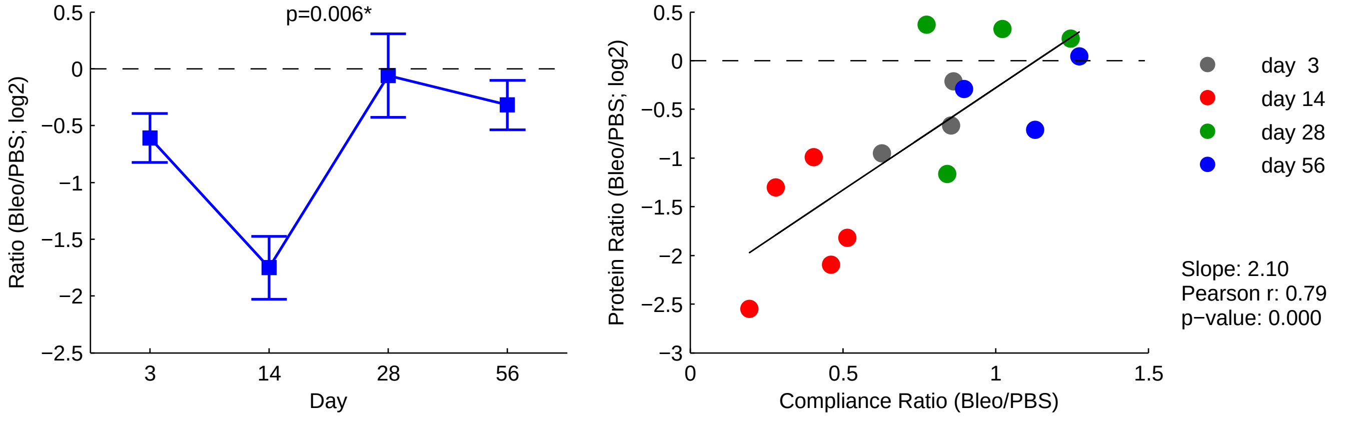

G3X9F4 − Tmem143 (id: 1684)

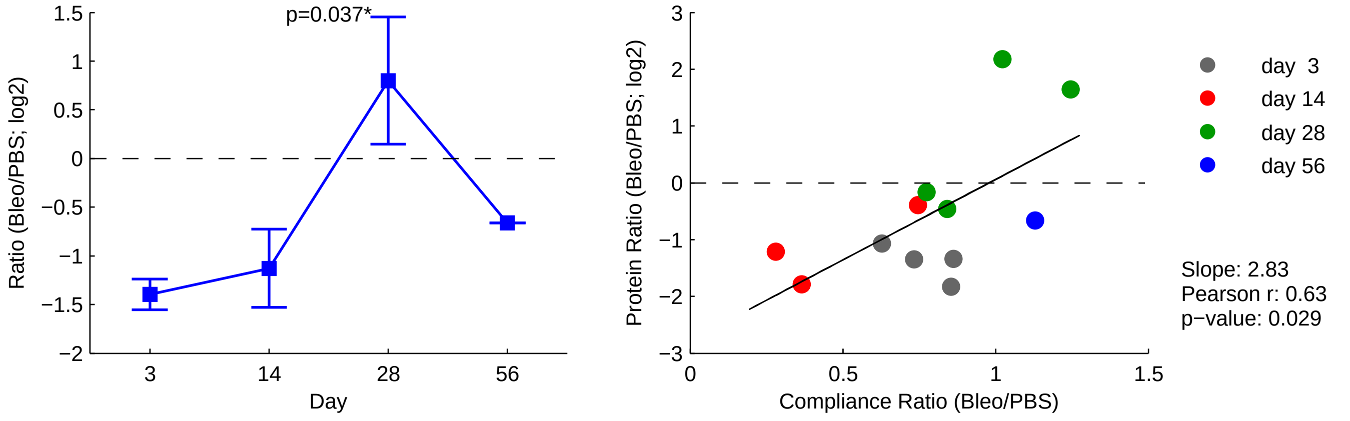

G3X9F6 − Sh3tc1 (id: 1685)

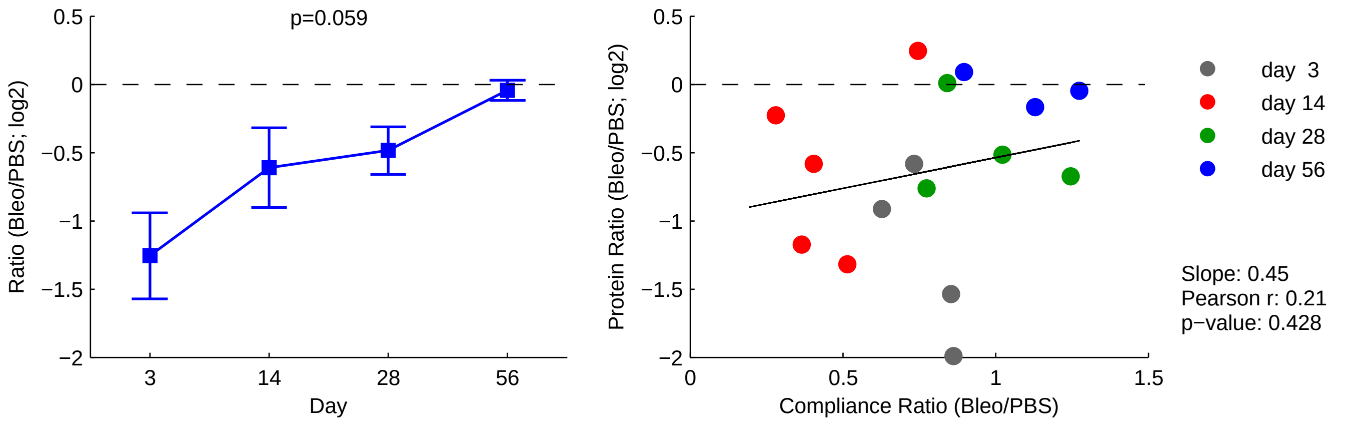

Q9JM52−2 − Mink1 (id: 1686)

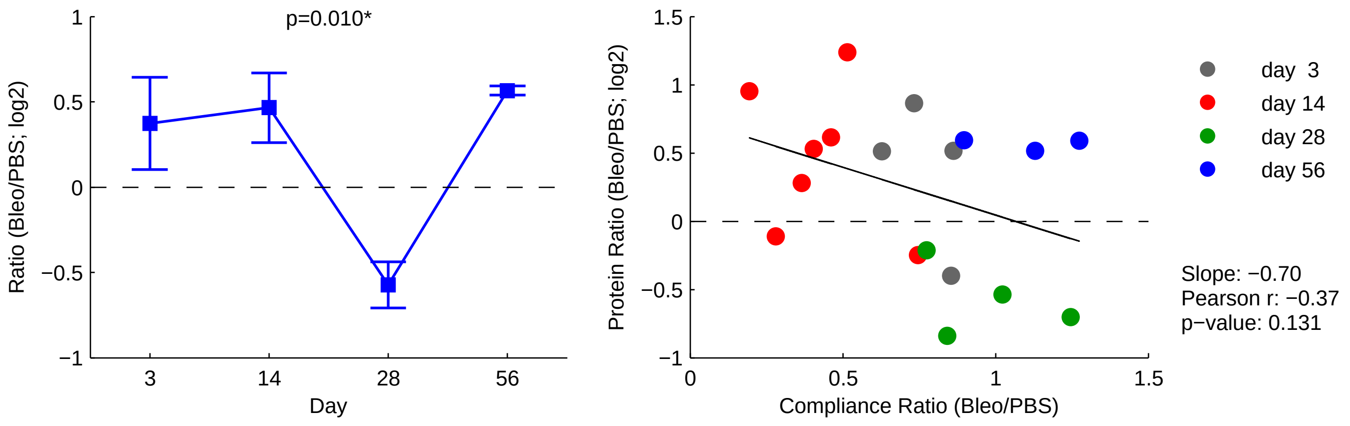

Q3TCR7 − Dnm2 (id: 1687)

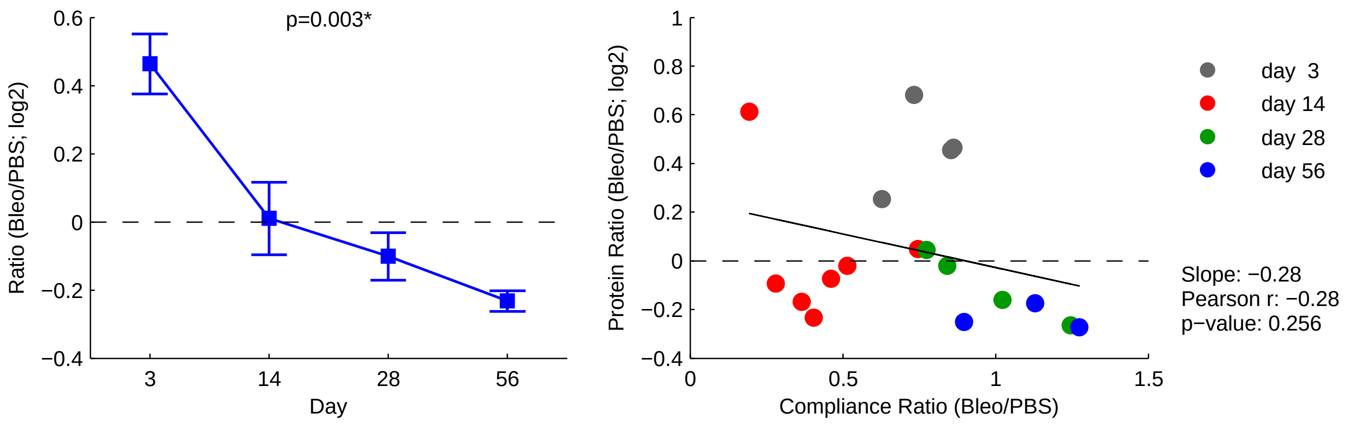

G3X9J0 − Sipa1l3 (id: 1690)

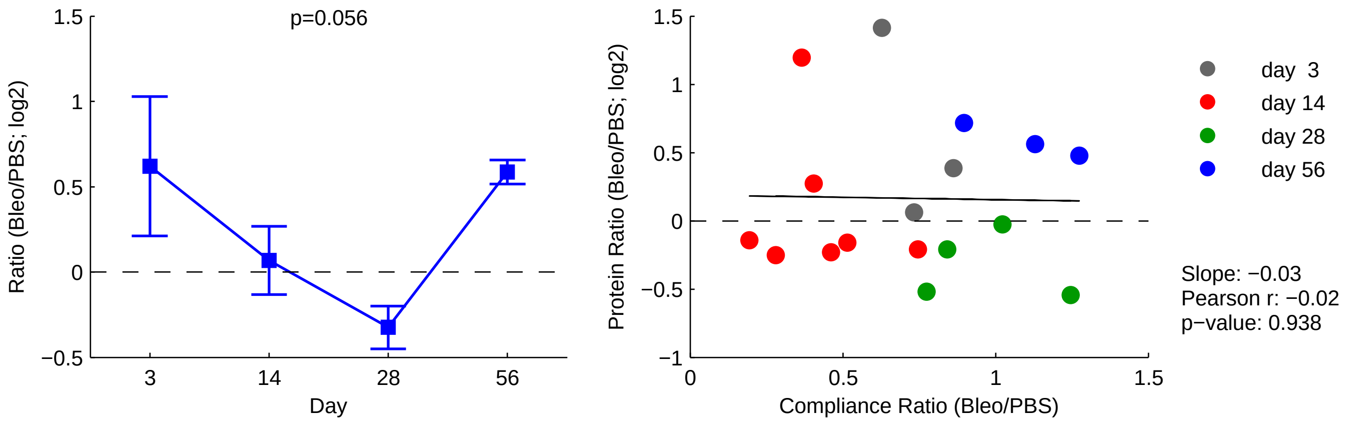

G3X9K3 − Arfgef1 (id: 1694)

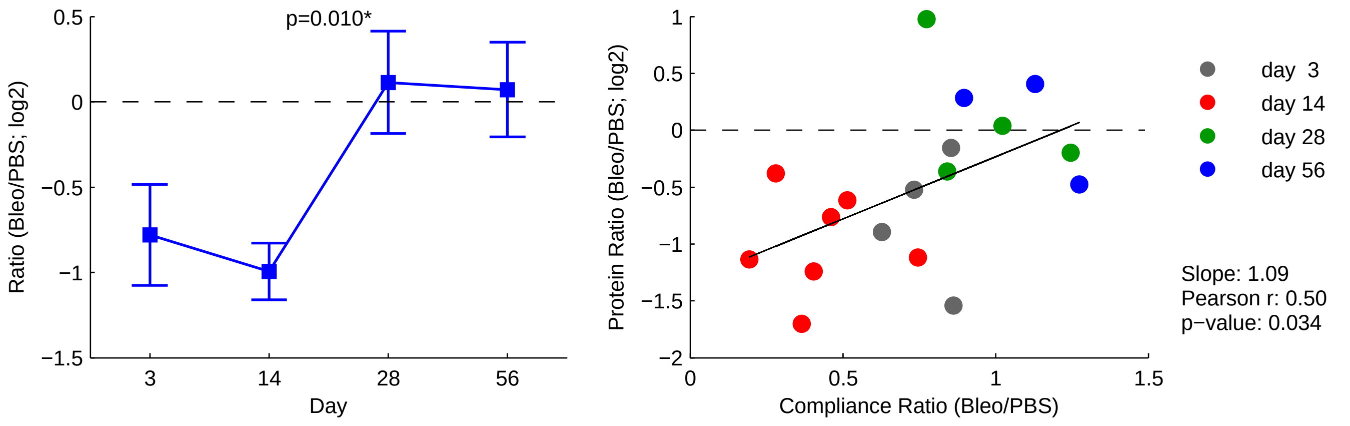

Q61738−5 − Itga7 (id: 1699)

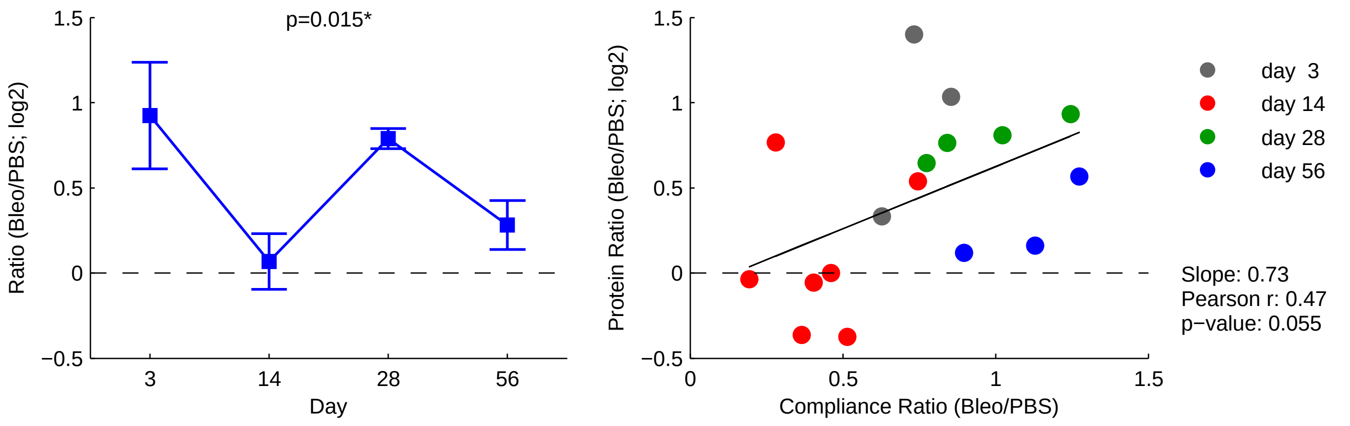

P06802−2 − Enpp1 (id: 1701)

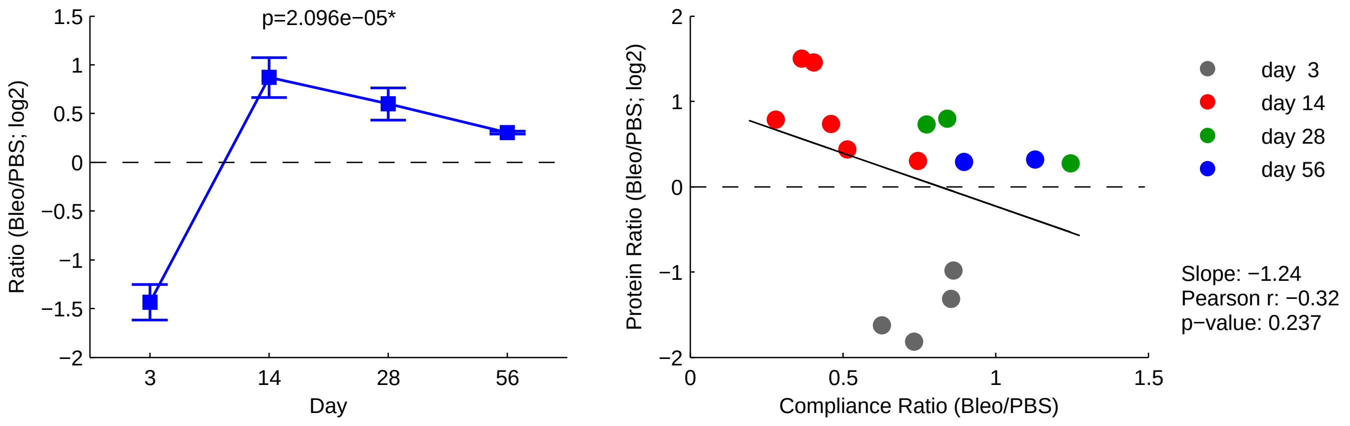

G3X9V2 − Ctnnd1 (id: 1703)

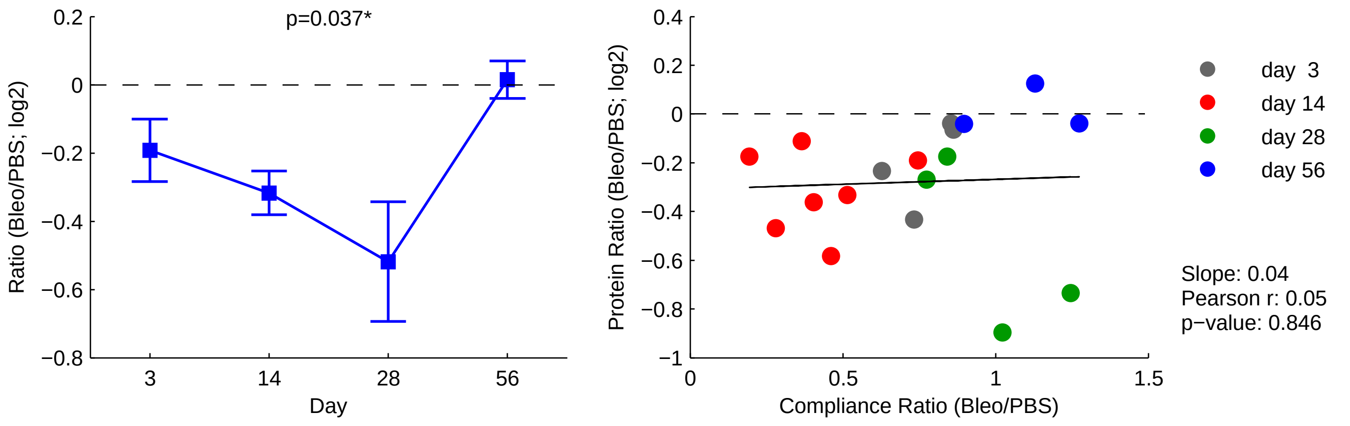

G5E8G0 − Hnrnpd (id: 1705)

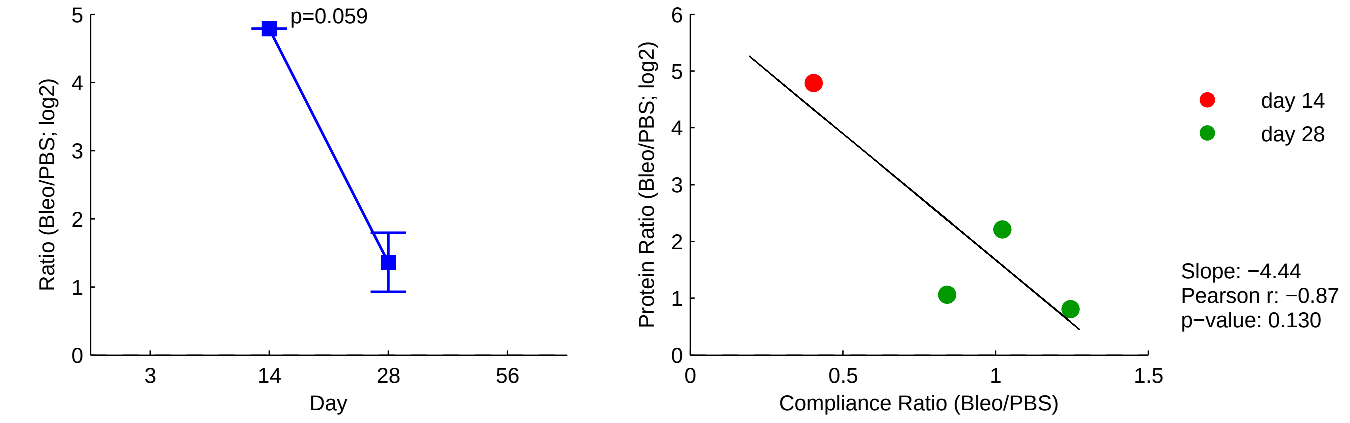

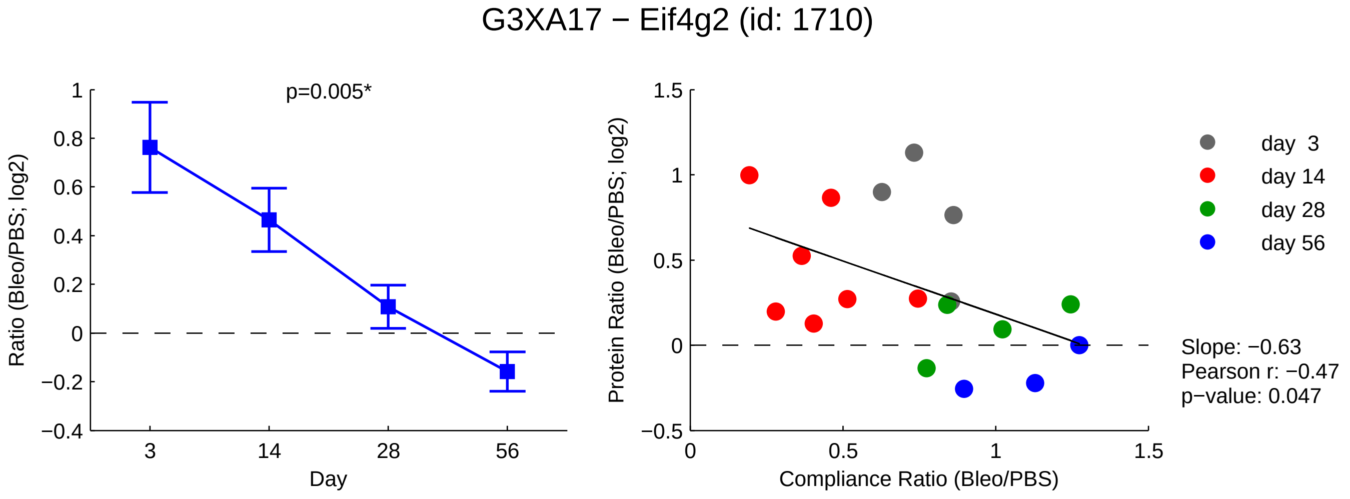
G3XA17 − Eif4g2 (id: 1710)
p=0.005*
Ratio (Bleo/PBS; log2)
Day
3
14
28
56
Protein Ratio (Bleo/PBS; log2)
Compliance Ratio (Bleo/PBS)
day 3
day 14
day 28
day 56
Slope: −0.63
Pearson r: −0.47
p−value: 0.047

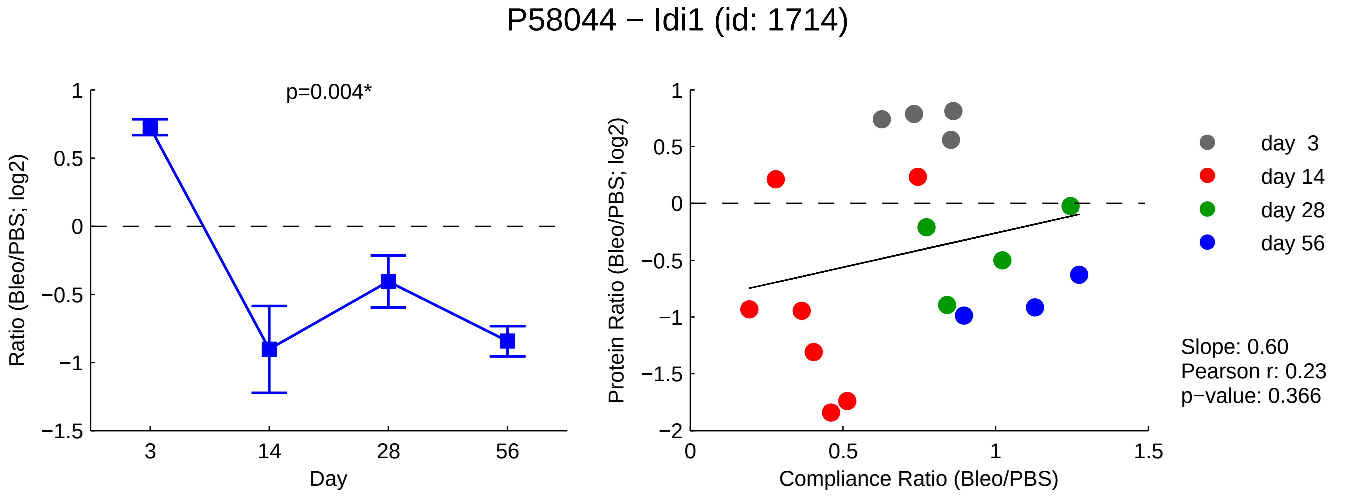
P58044 − Idi1 (id: 1714)
p=0.004*
Ratio (Bleo/PBS; log2)
Day
3
14
28
56
Protein Ratio (Bleo/PBS; log2)
Compliance Ratio (Bleo/PBS)
day 3
day 14
day 28
day 56
Slope: 0.60
Pearson r: 0.23
p−value: 0.366

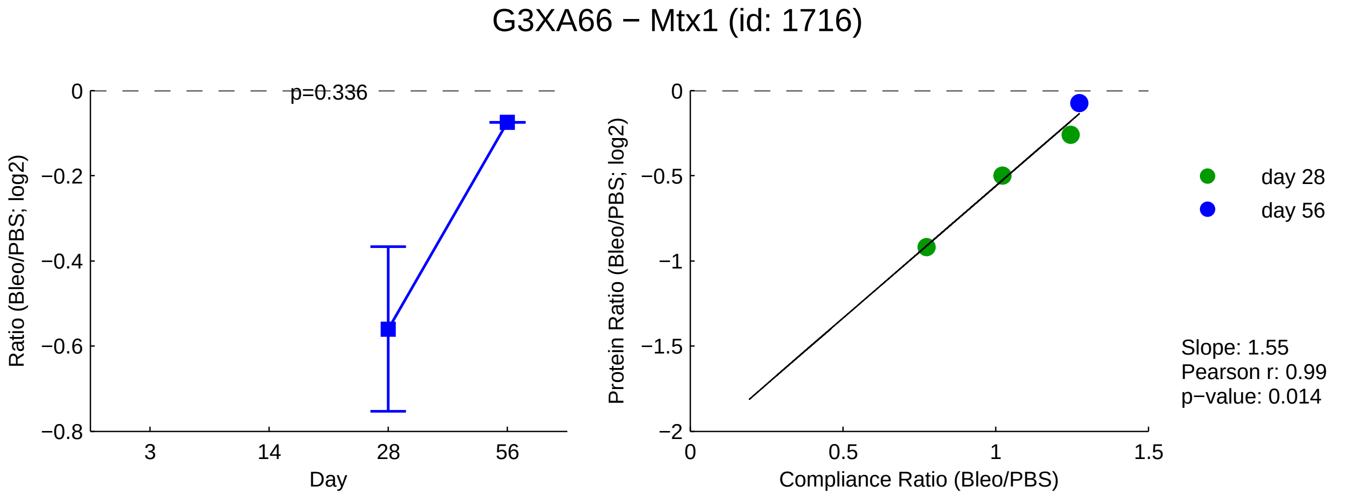
G3XA66 − Mtx1 (id: 1716)
p=0.336
Ratio (Bleo/PBS; log2)
Day
3
14
28
56
Protein Ratio (Bleo/PBS; log2)
Compliance Ratio (Bleo/PBS)
day 28
day 56
Slope: 1.55
Pearson r: 0.99
p−value: 0.014

G5E814 − Ndufa11 (id: 1717)

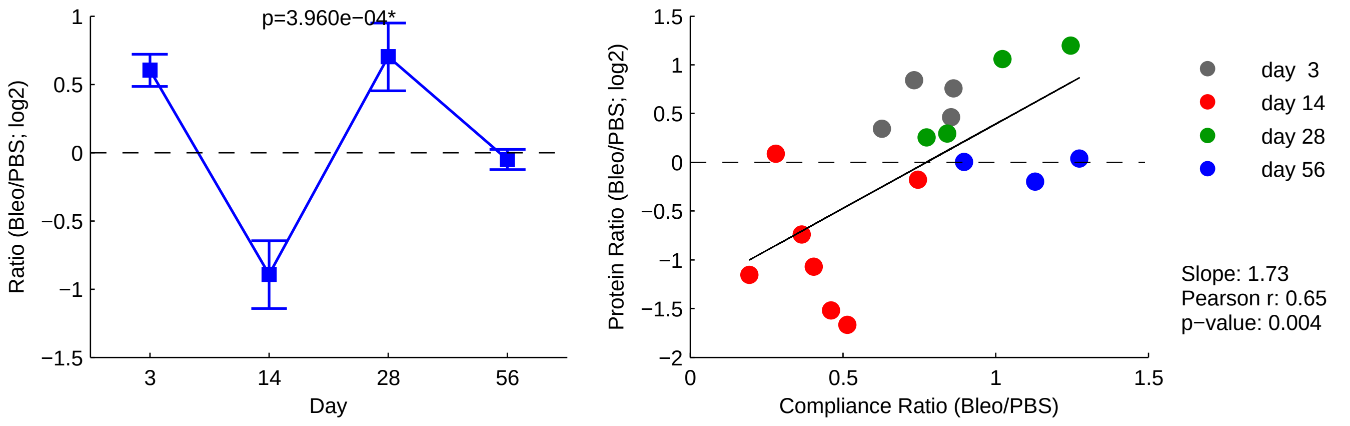

Q60676 − Ppp5c (id: 1718)

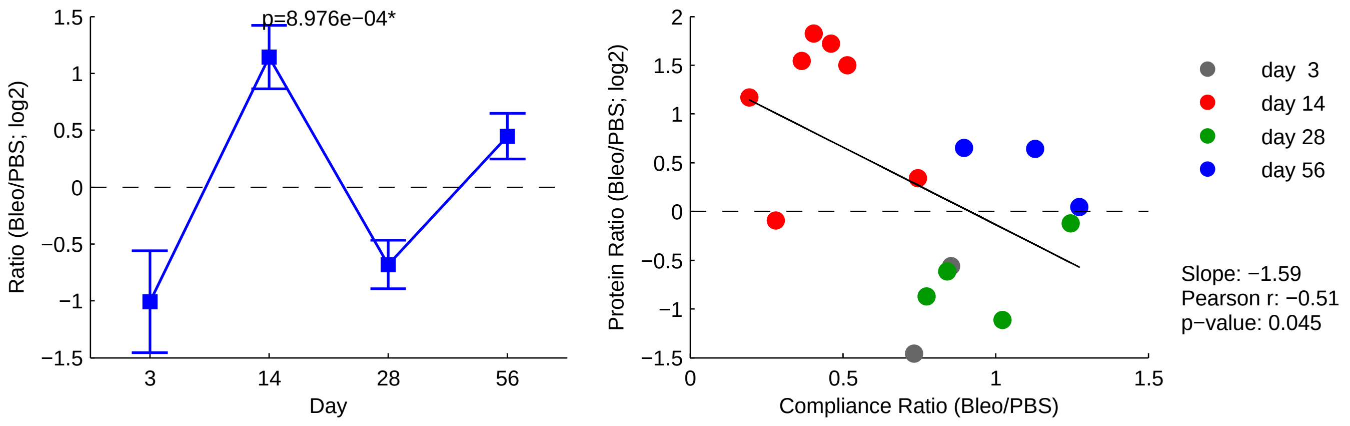

G5E829 − Atp2b1 (id: 1720)

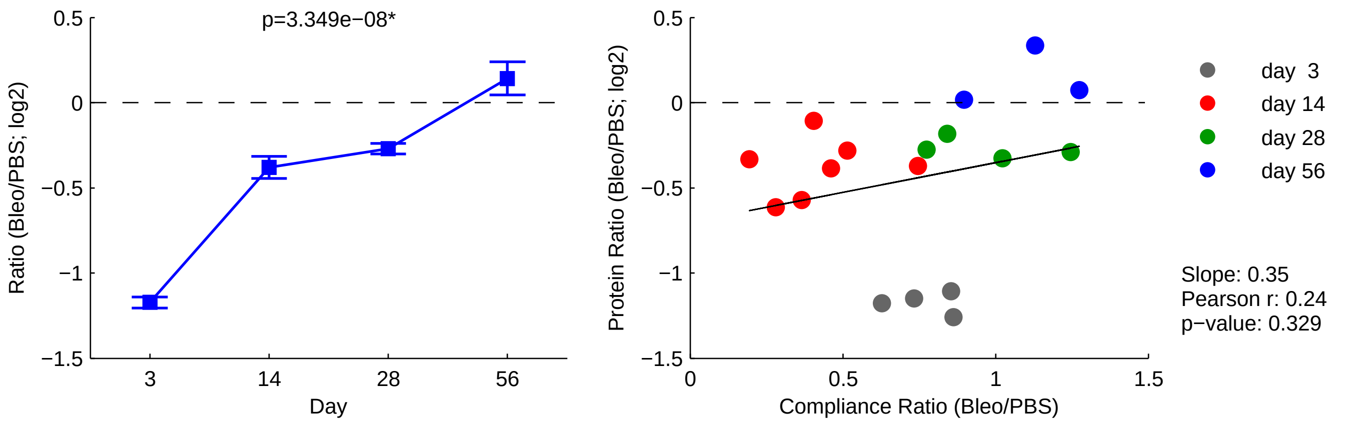

P15331 − Prph (id: 1722)

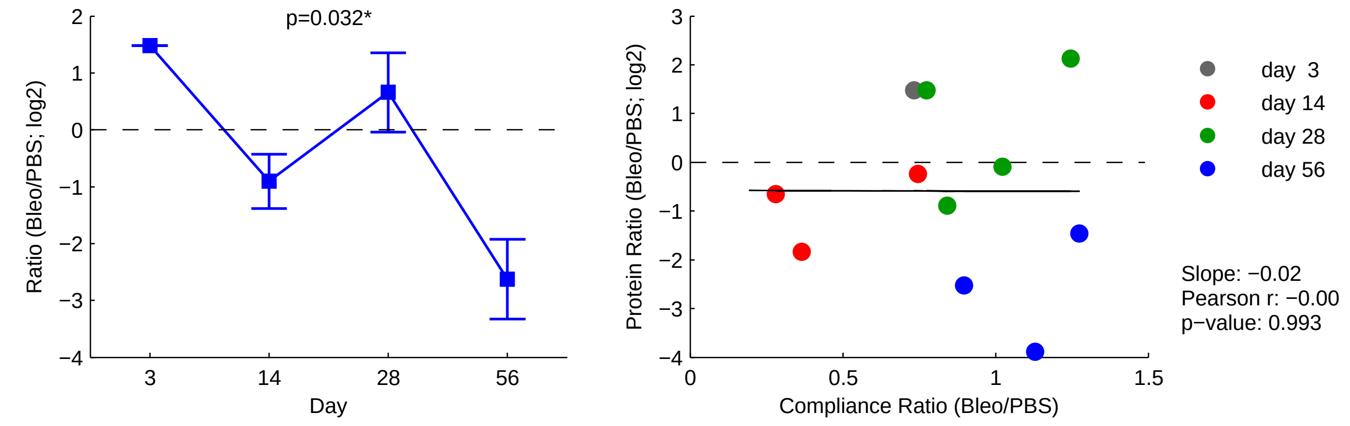

O55134 − Pcdh12 (id: 1723)

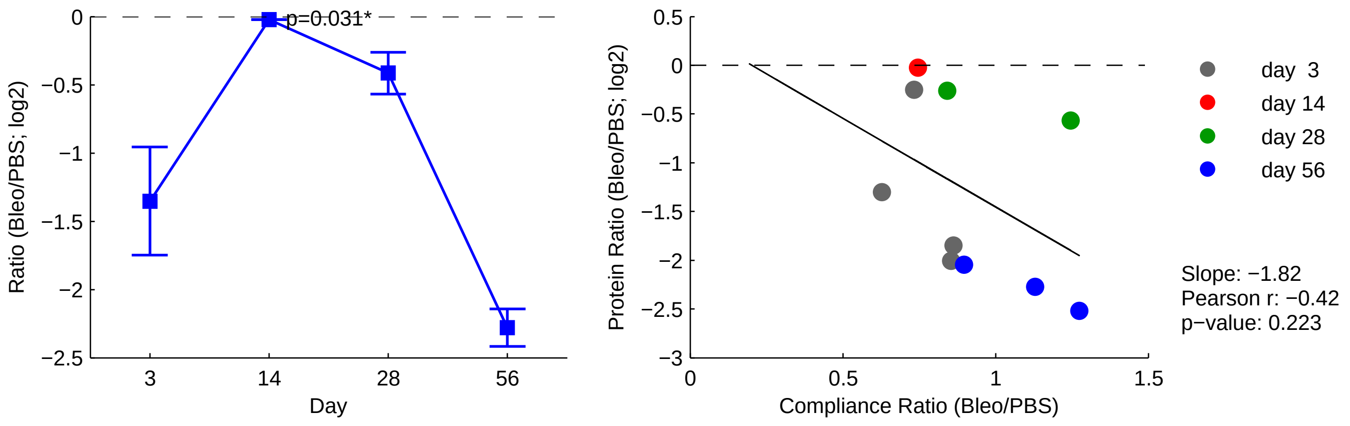

G5E850 − Cyb5 (id: 1724)

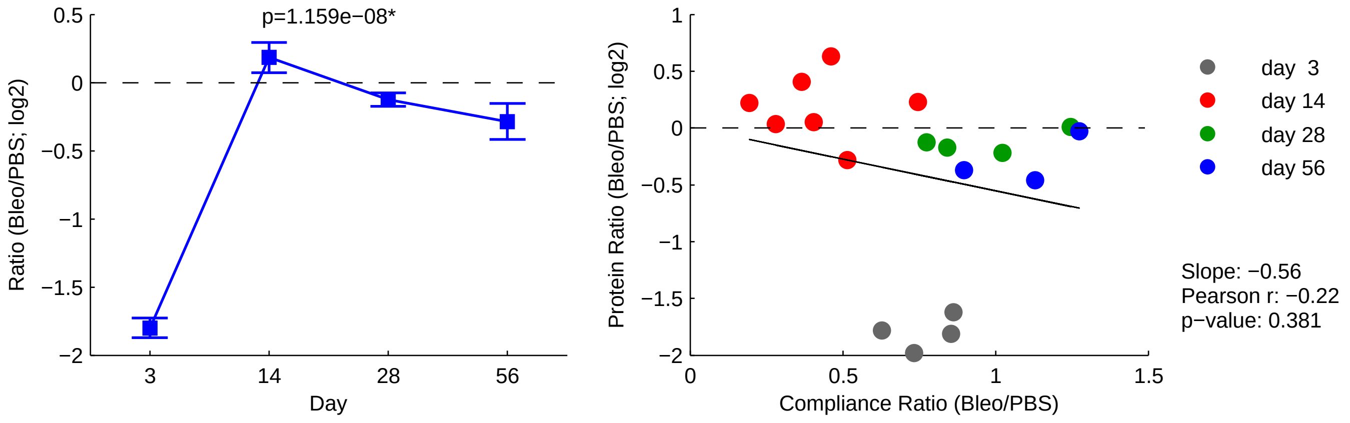

G5E861 − Sclt1 (id: 1727)

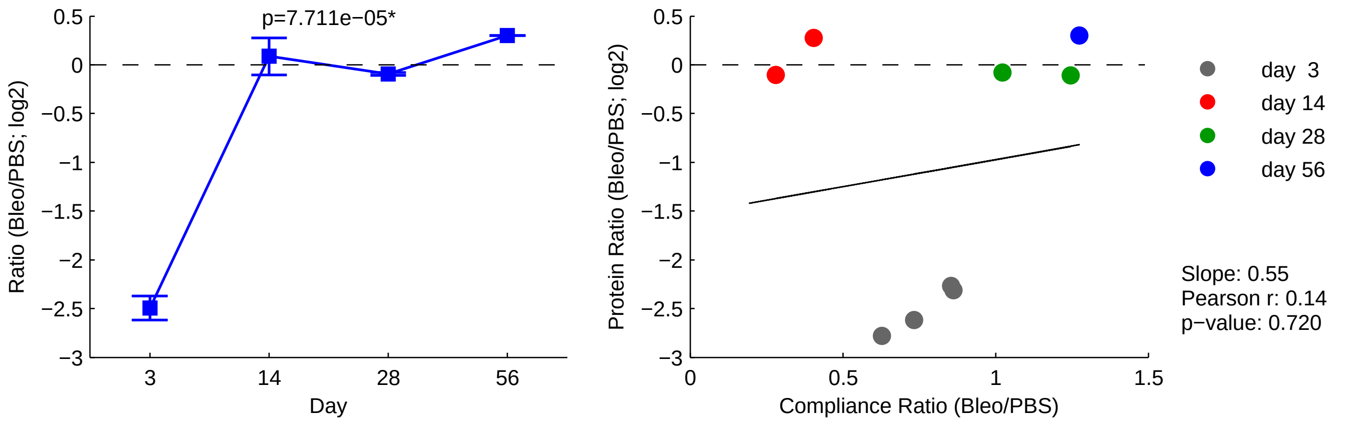

G5E870 − Trip12 (id: 1729)

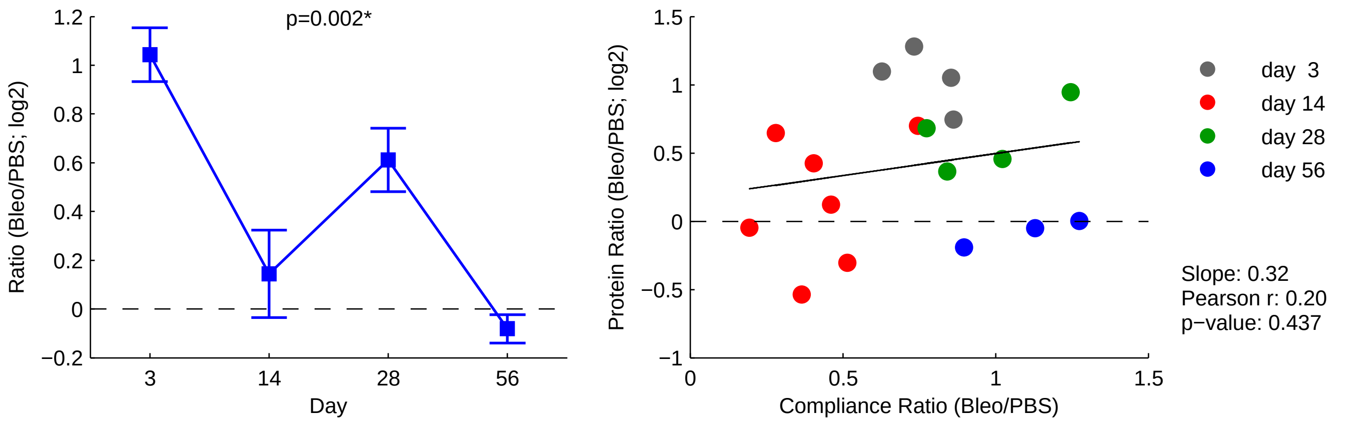

Q3TUH1 − Tamm41 (id: 1731)

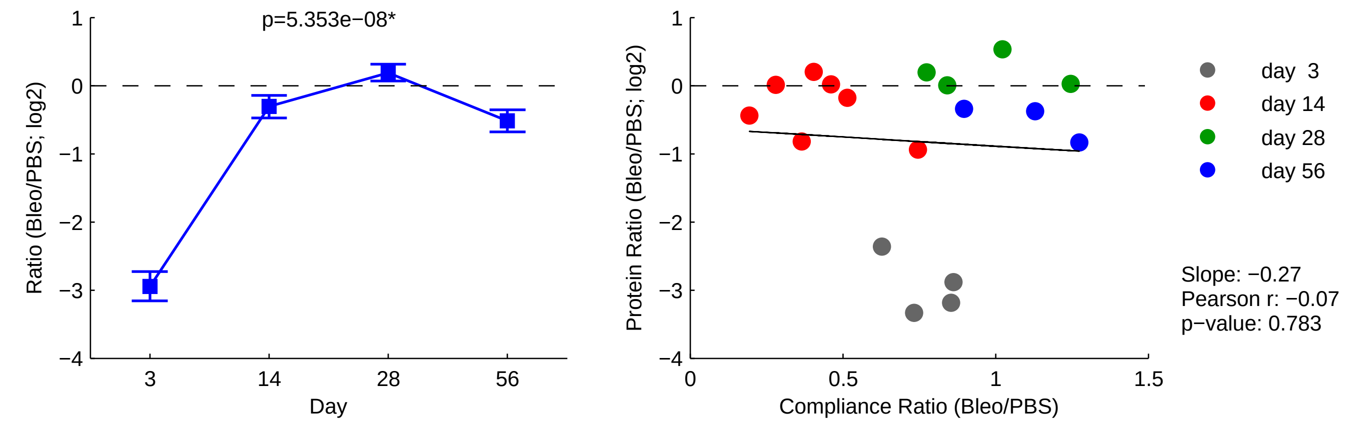

G5E895 − Akr1b10 (id: 1734)

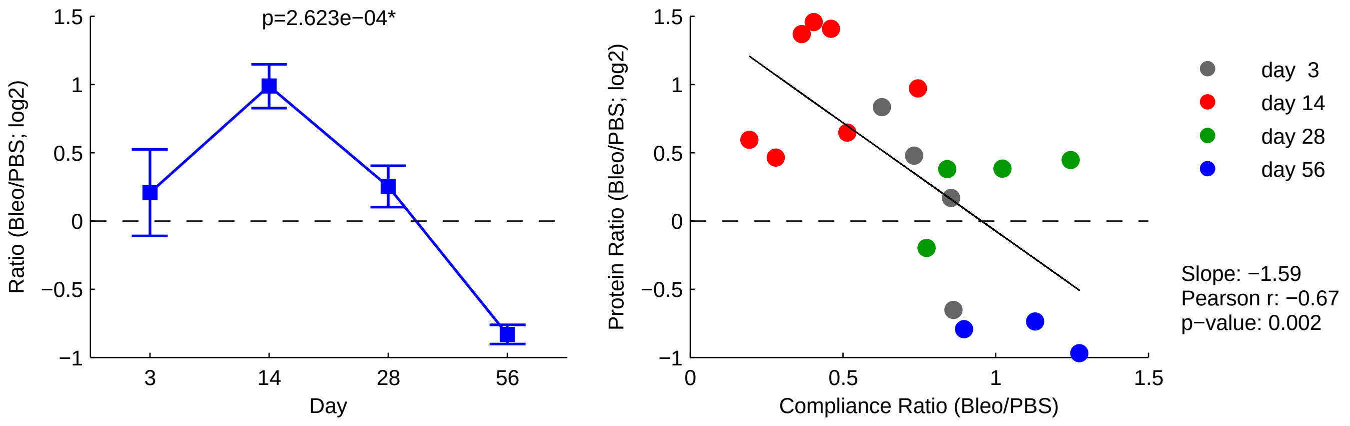

G5E896 − Edc4 (id: 1735)

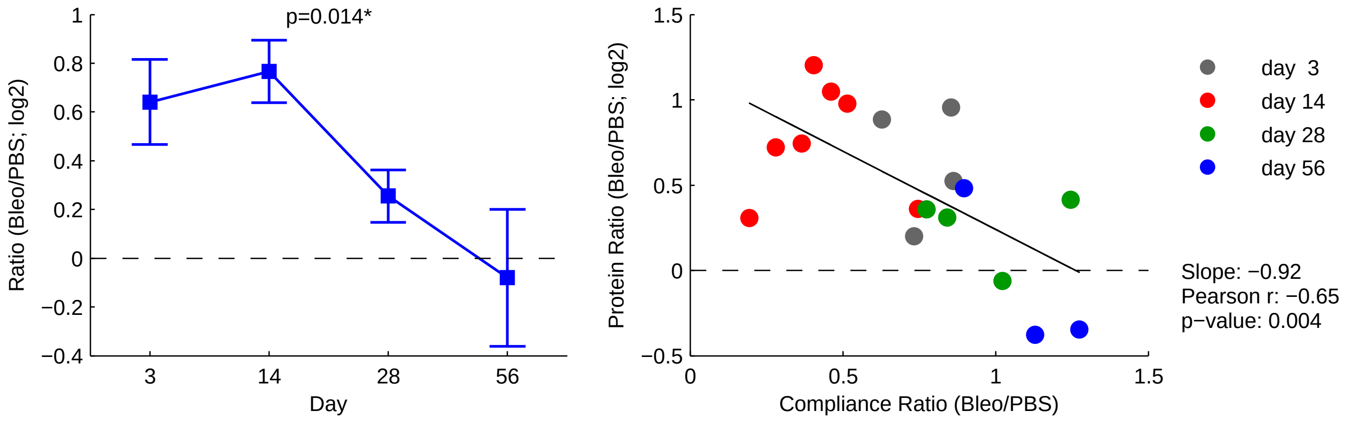

G5E898 − Ppl (id: 1737)

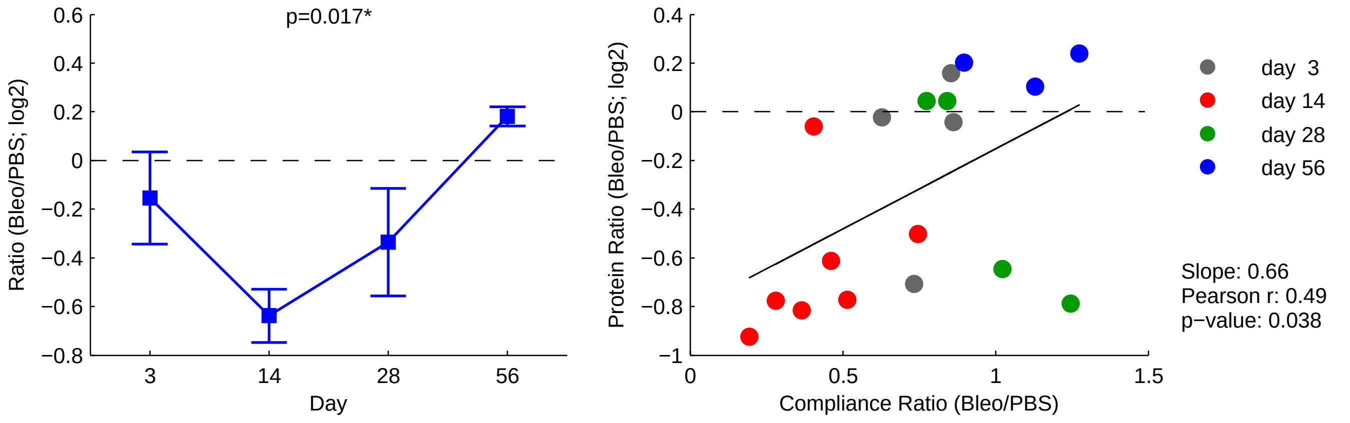

G5E8A3 − Slc44a3 (id: 1740)

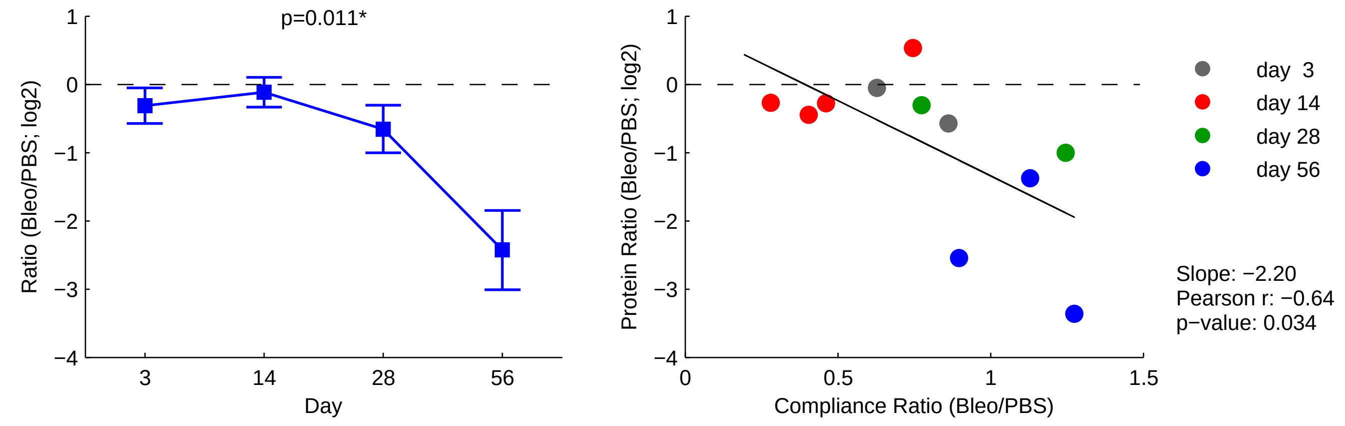

Q8BRH0 − Tmtc3 (id: 1744)

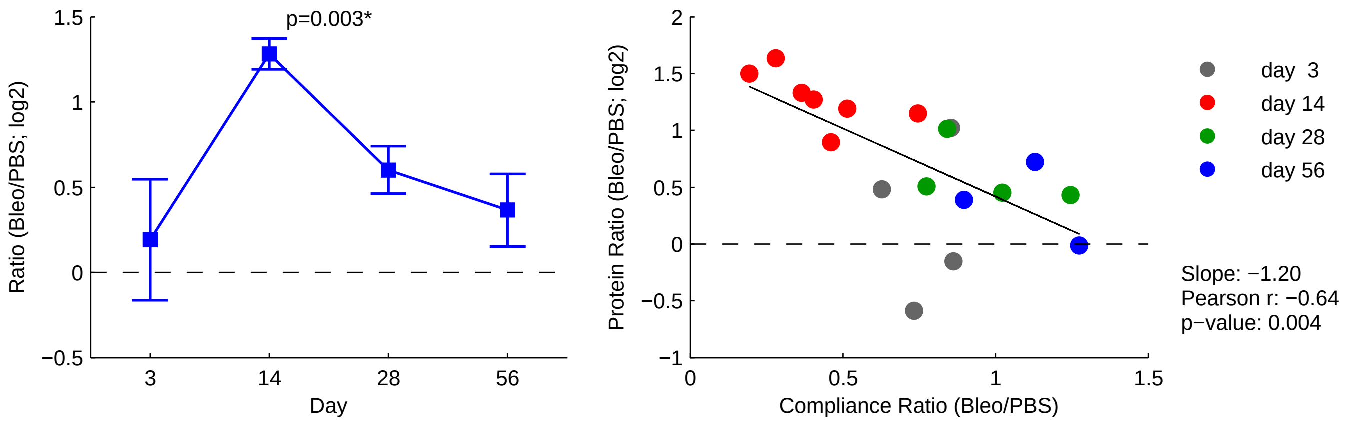

G5E8C6 − Vill (id: 1745)

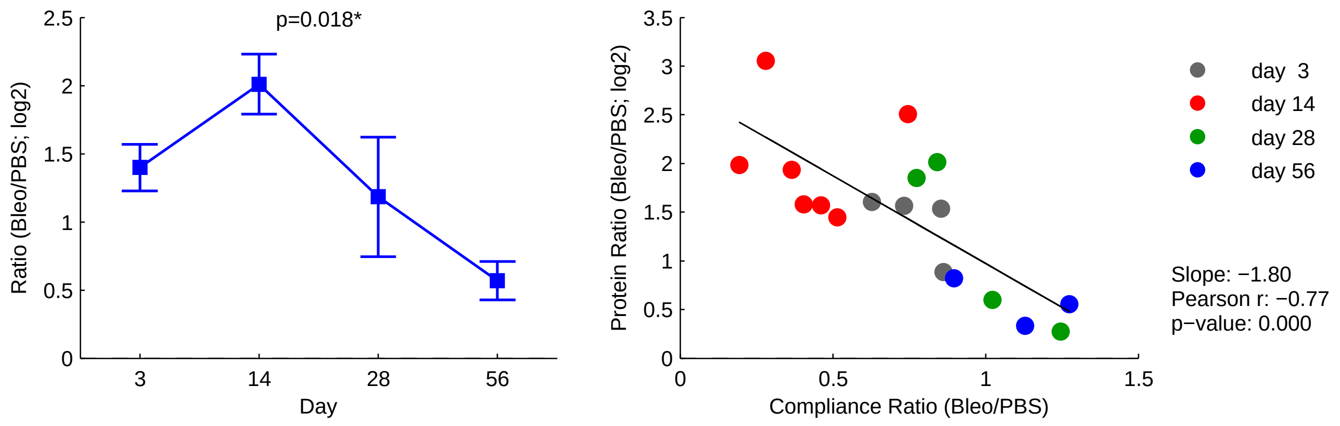

Q8CGZ0 − Cherp (id: 1750)

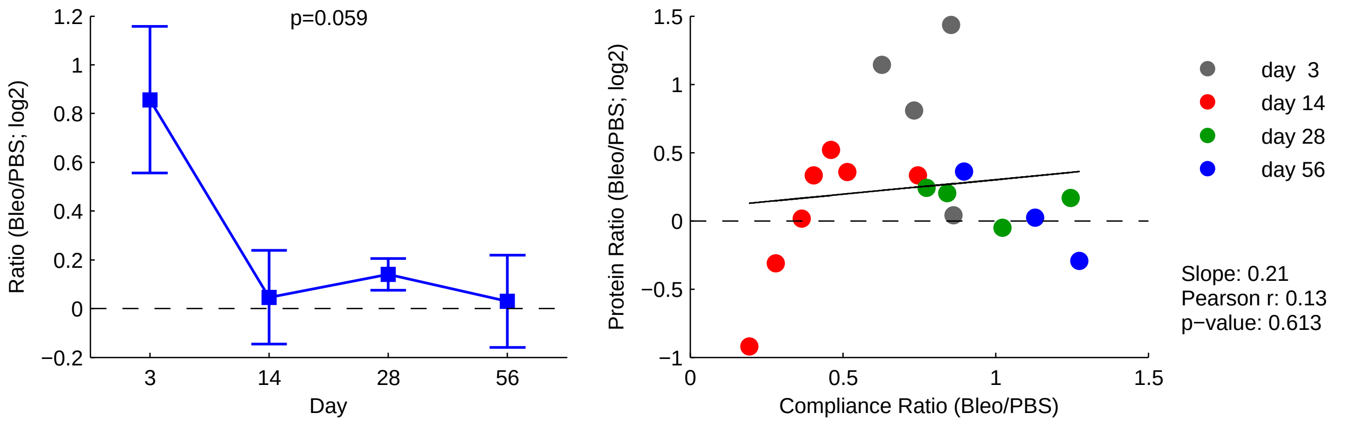

G5E8J3 − Wdr11 (id: 1752)

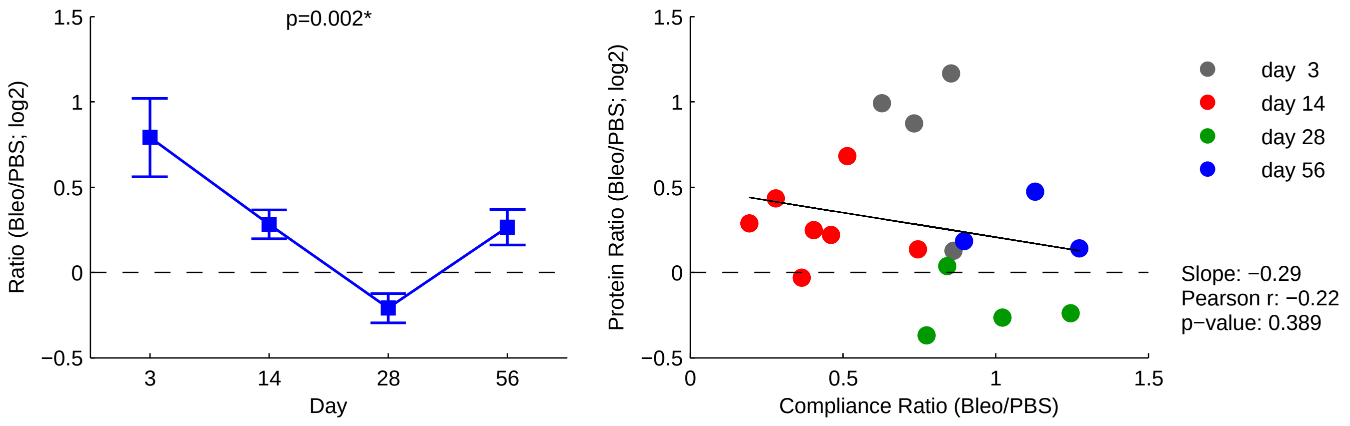

Q62136 − Ptpn21 (id: 1753)

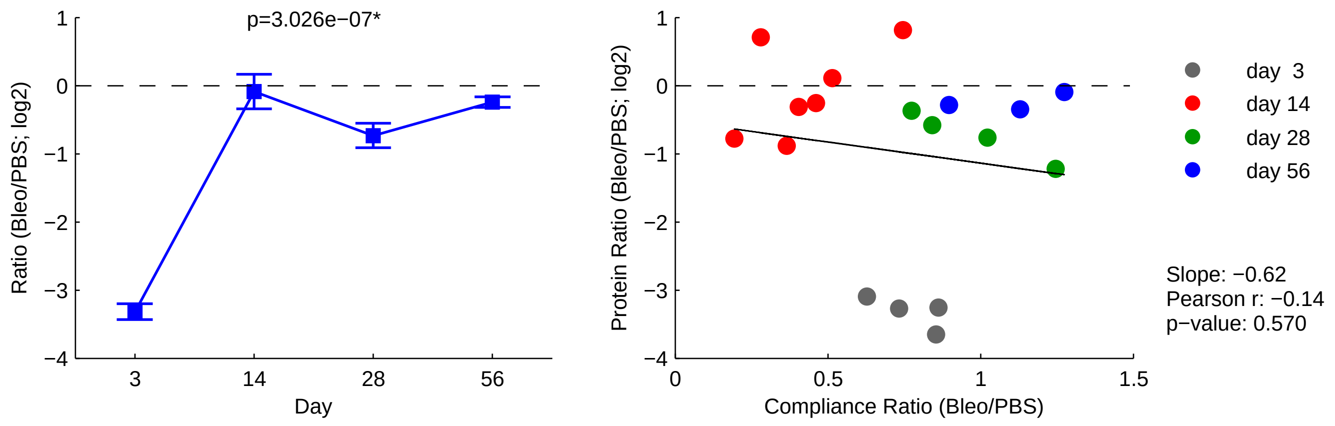

G5E8J6 − Hrc (id: 1754)

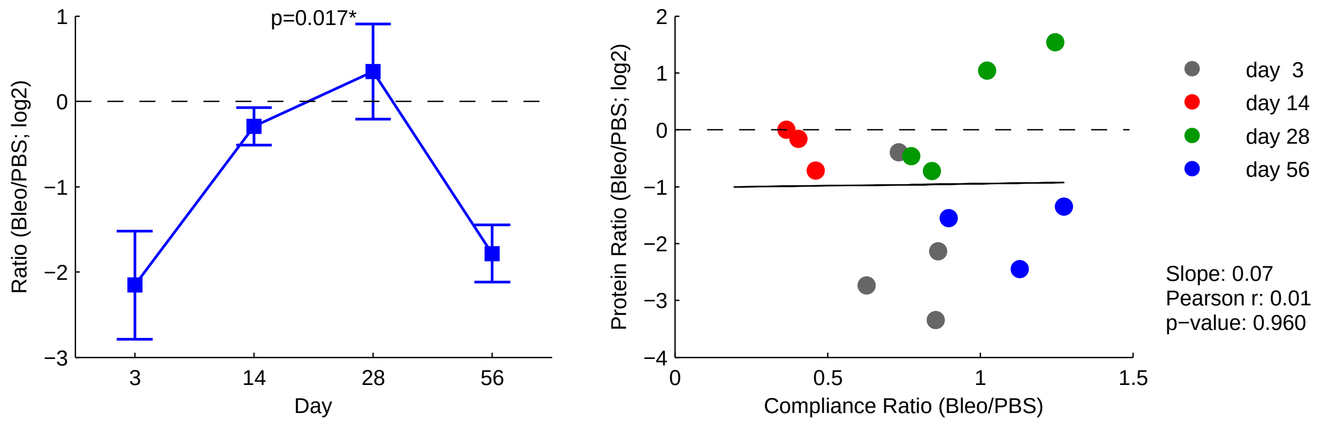

G5E8K5−2 − Ank3 (id: 1757)

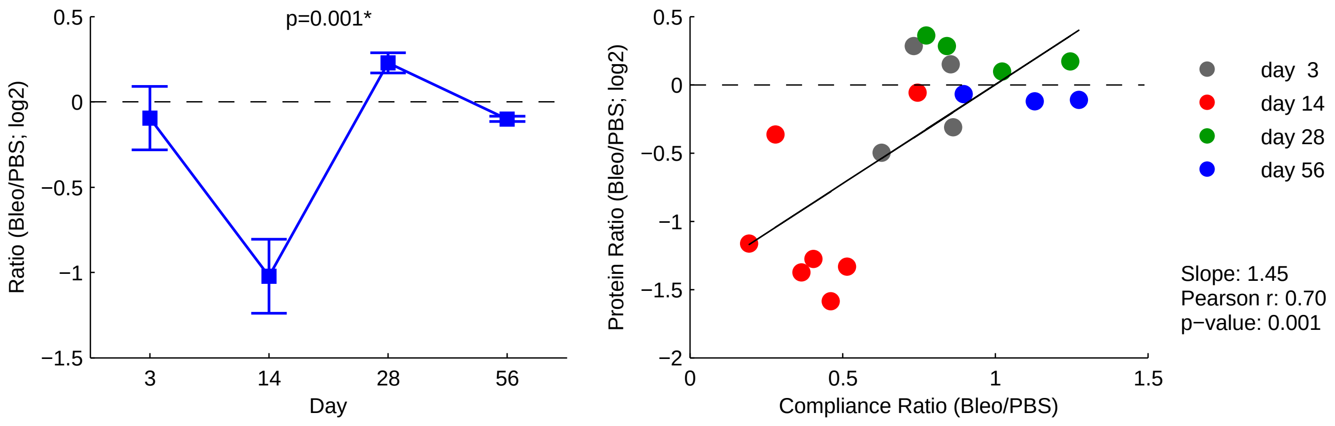

G5E8M9 − Icam2 (id: 1762)

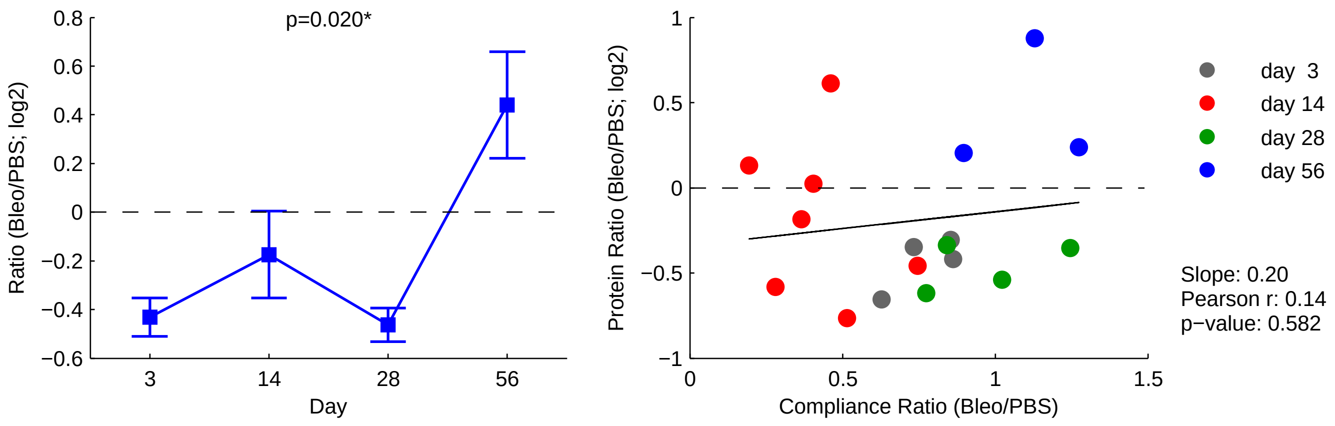

G5E8N7 − Pnkp (id: 1763)

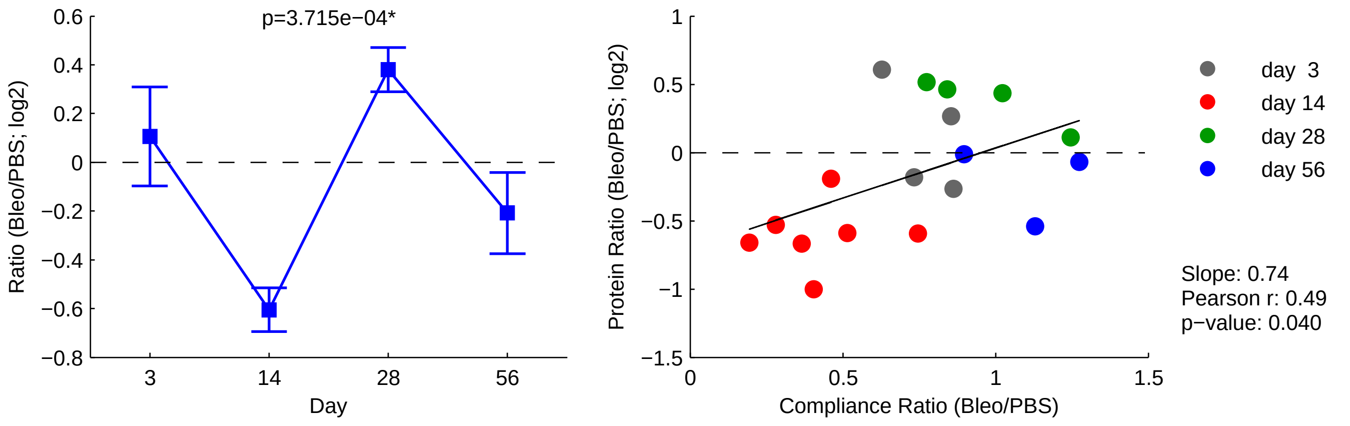

G5E8Q8 − Gpr116 (id: 1765)

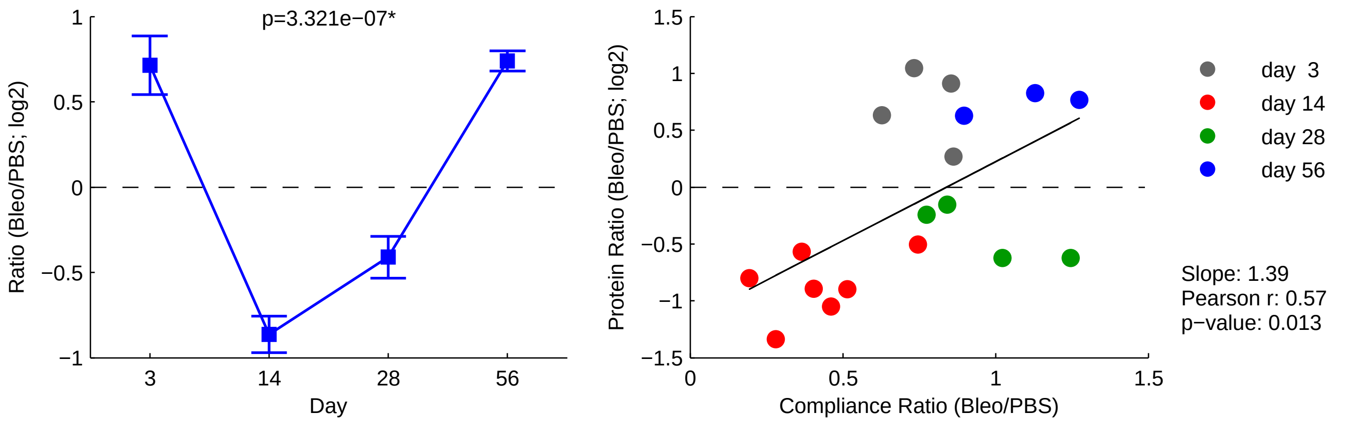

G5E8R2 − Tpm1 (id: 1766)

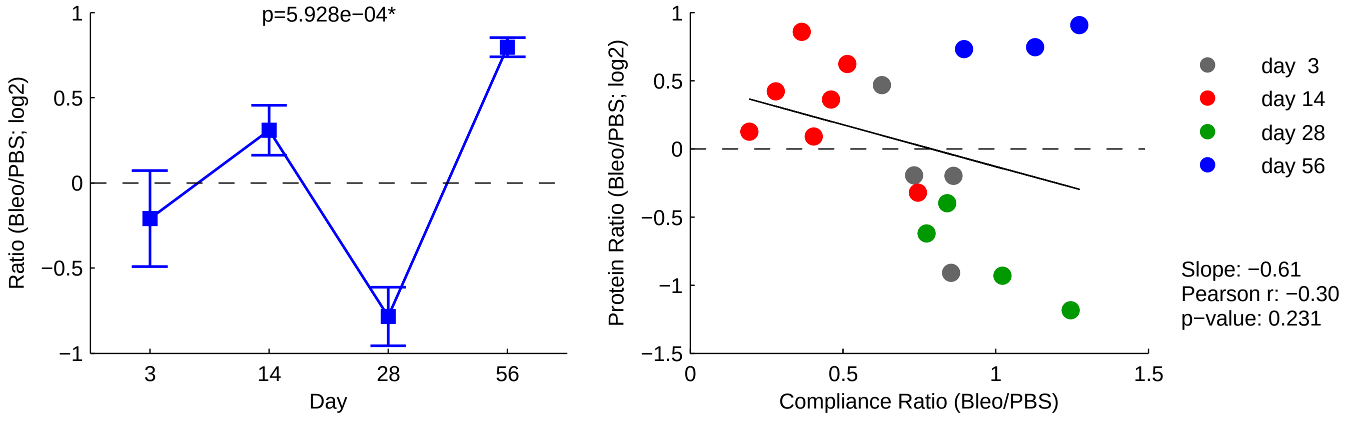

Q9D4H9−3 − Phf14 (id: 1768)

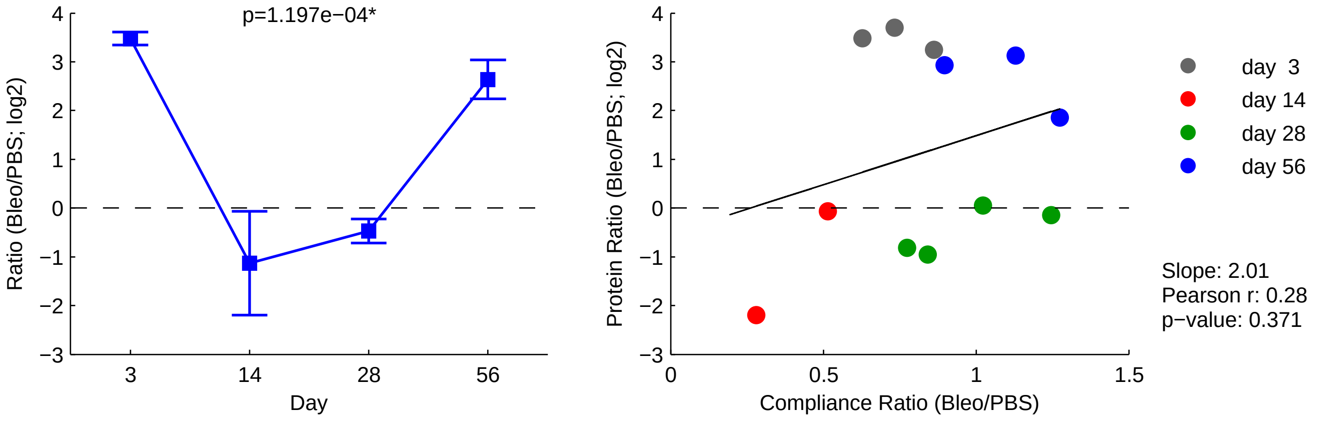

Q9EQJ9 − Magi3 (id: 1771)

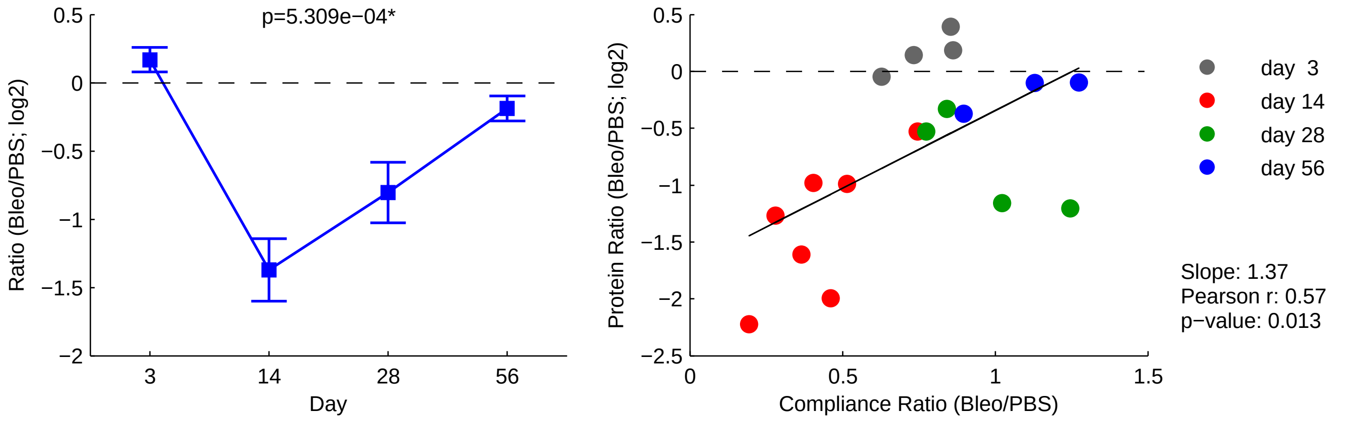

G5E8W8 − Zfp185 (id: 1775)

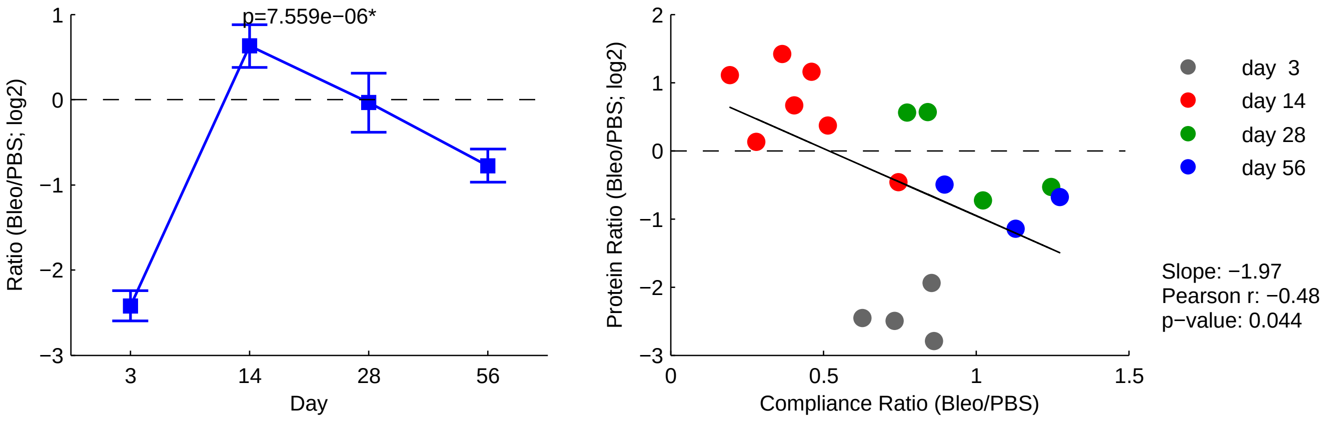

G5E924 − Hnrnpl (id: 1778)

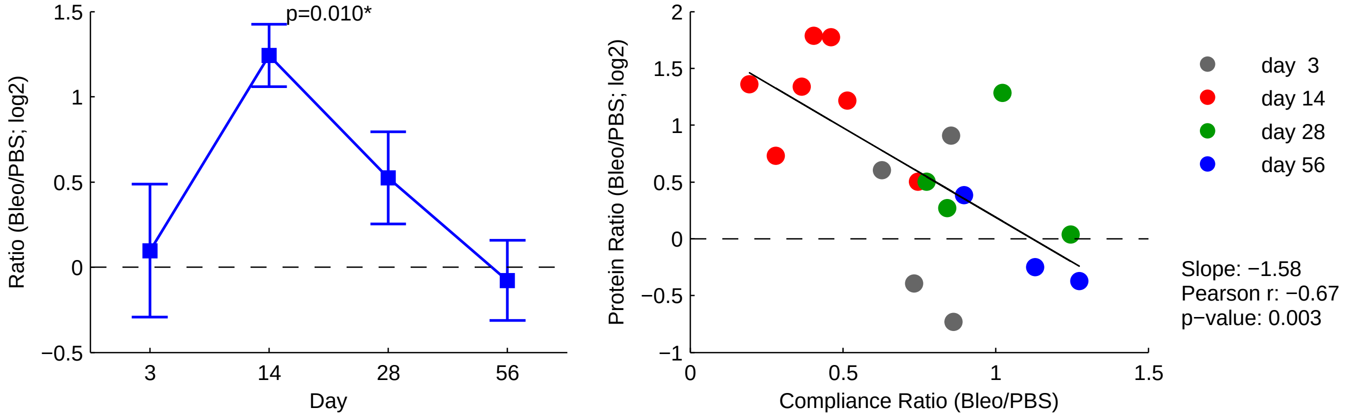

H3BIX4 − Nptn (id: 1780)

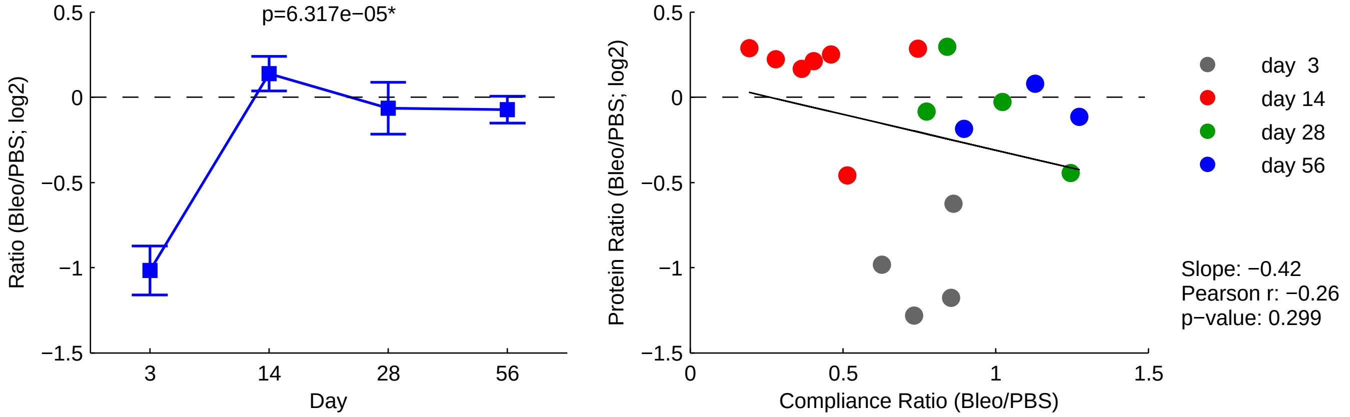

Q8BIE6 − Frmd4a (id: 1781)

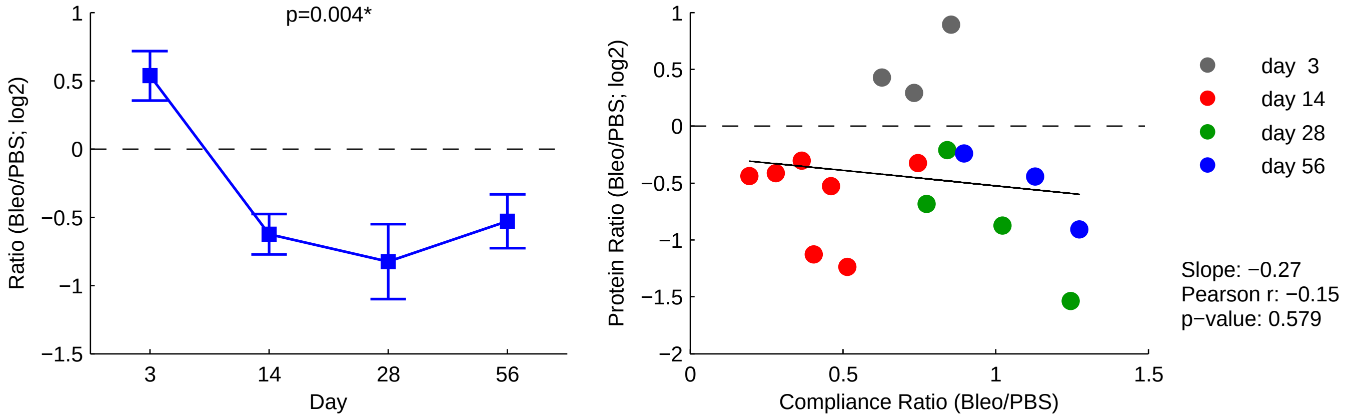

Q8R088 − Golph3l (id: 1782)

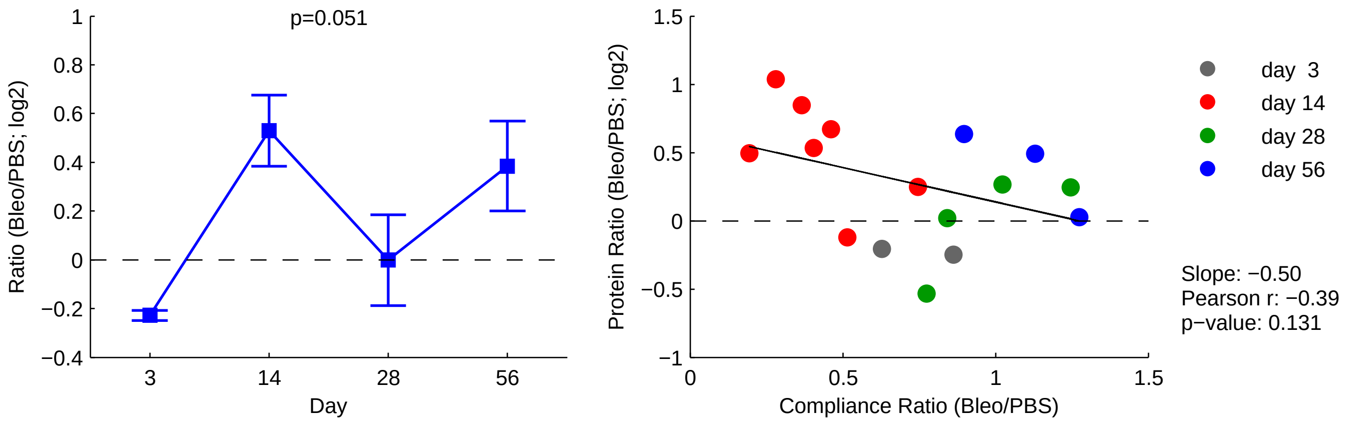

H3BJ97 − Tinagl1 (id: 1786)

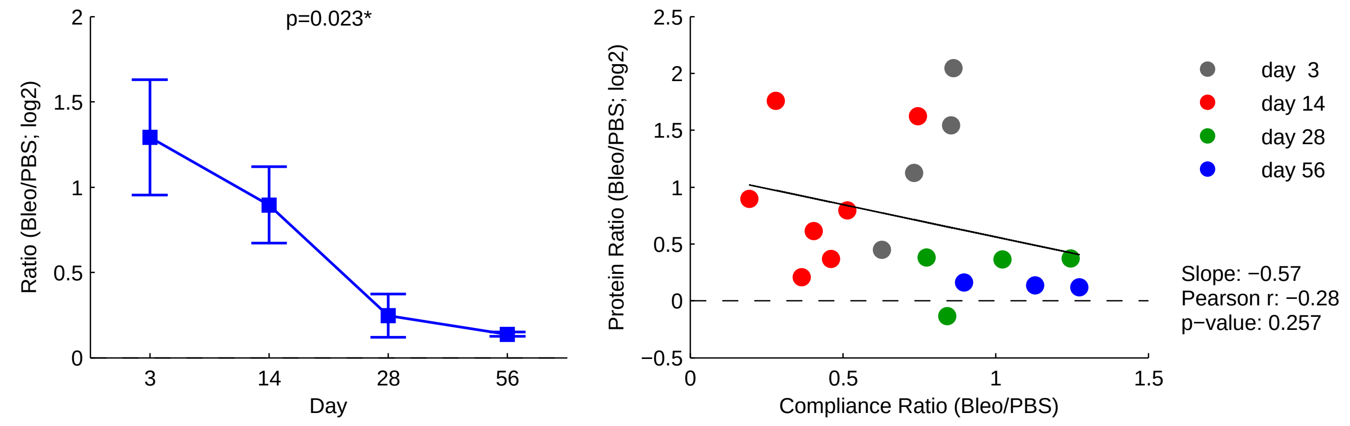

H3BJD6 − Ppp1r9a (id: 1788)

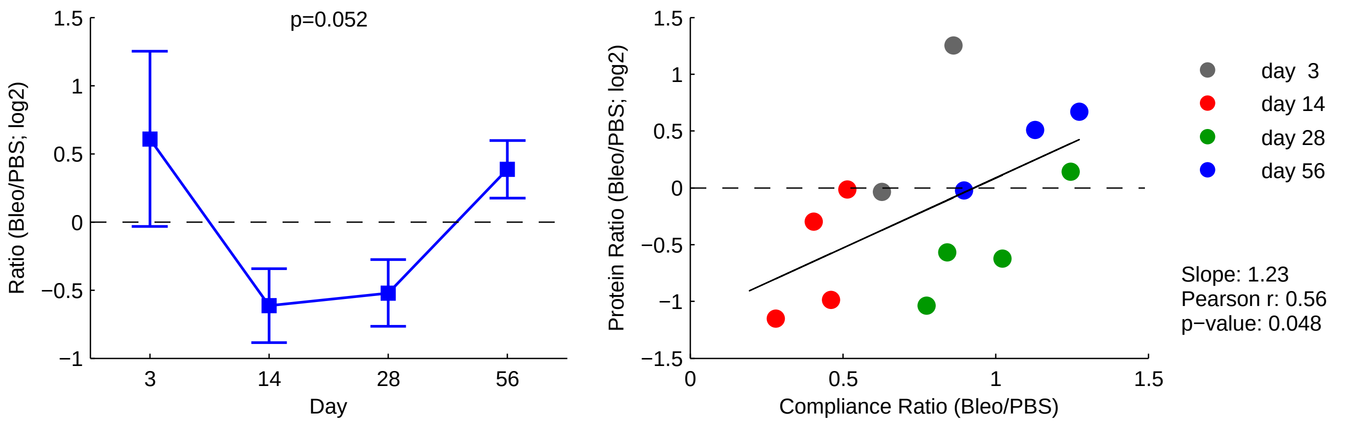

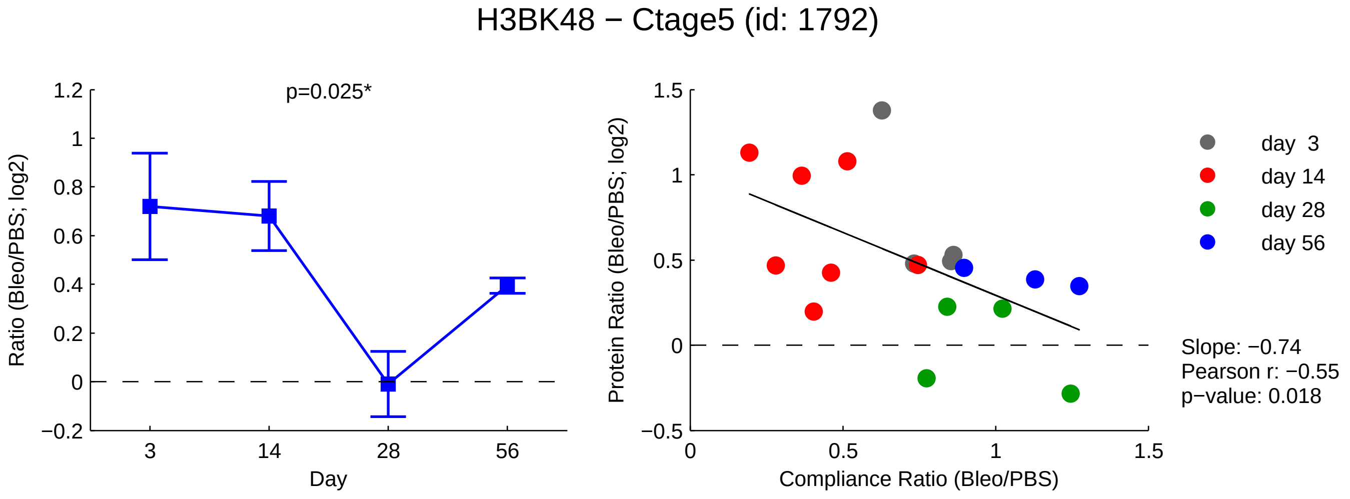
H3BK48 − Ctage5 (id: 1792)
p=0.025*
Ratio (Bleo/PBS; log2)
Day
3
14
28
56
Protein Ratio (Bleo/PBS; log2)
Compliance Ratio (Bleo/PBS)
day 3
day 14
day 28
day 56
Slope: −0.74
Pearson r: −0.55
p−value: 0.018

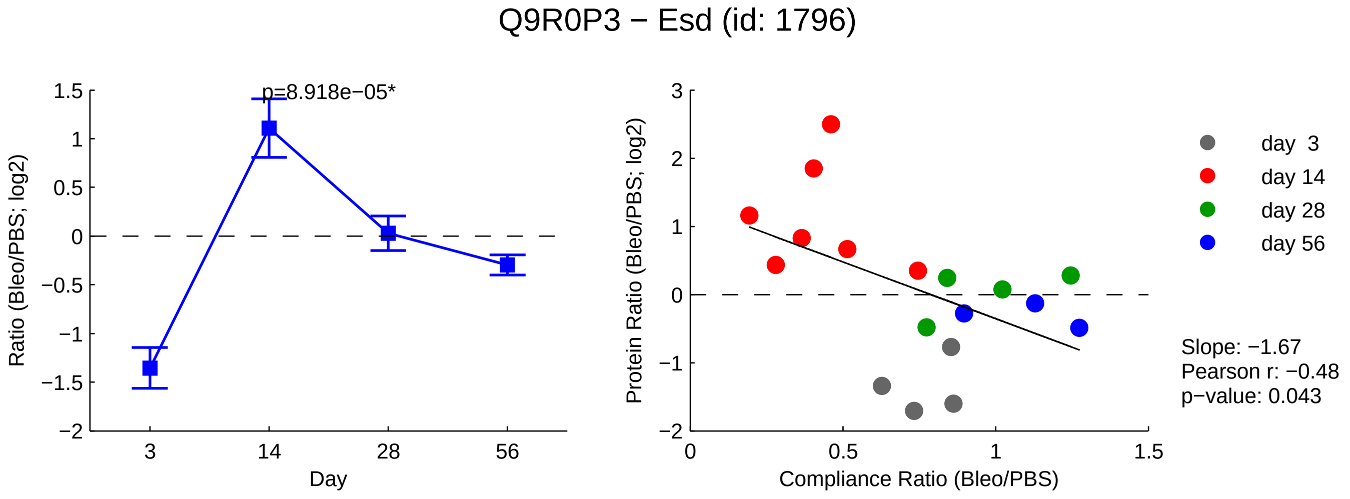
Q9R0P3 − Esd (id: 1796)
p=8.918e−05*
Ratio (Bleo/PBS; log2)
Day
3
14
28
56
Protein Ratio (Bleo/PBS; log2)
Compliance Ratio (Bleo/PBS)
day 3
day 14
day 28
day 56
Slope: −1.67
Pearson r: −0.48
p−value: 0.043

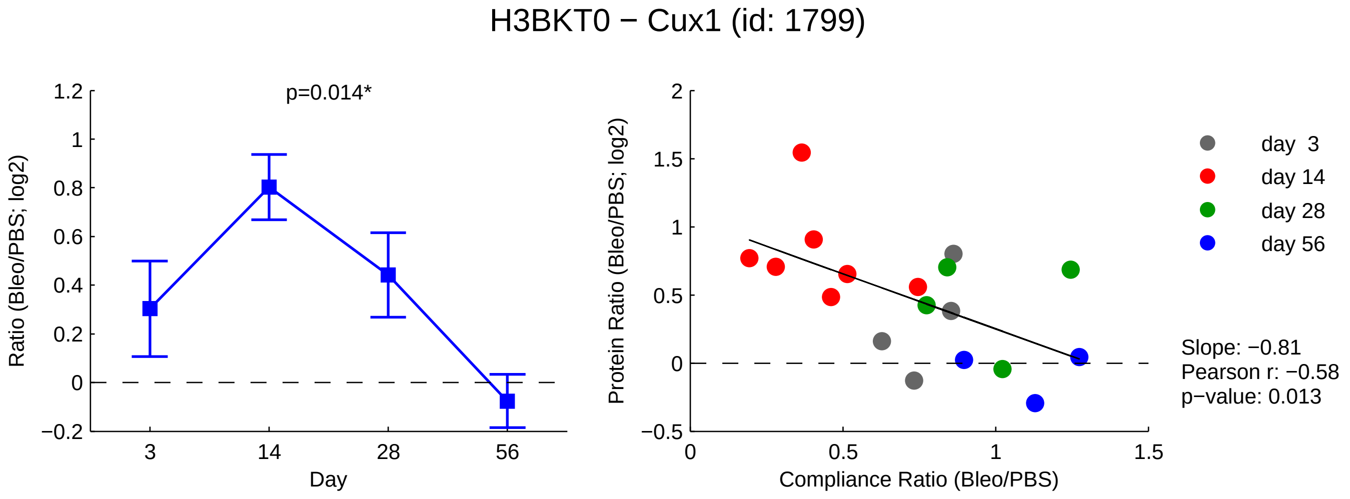
H3BKT0 − Cux1 (id: 1799)
p=0.014*
Ratio (Bleo/PBS; log2)
Day
3
14
28
56
Protein Ratio (Bleo/PBS; log2)
Compliance Ratio (Bleo/PBS)
day 3
day 14
day 28
day 56
Slope: −0.81
Pearson r: −0.58
p−value: 0.013

Q8C172 − Cers6 (id: 1801)

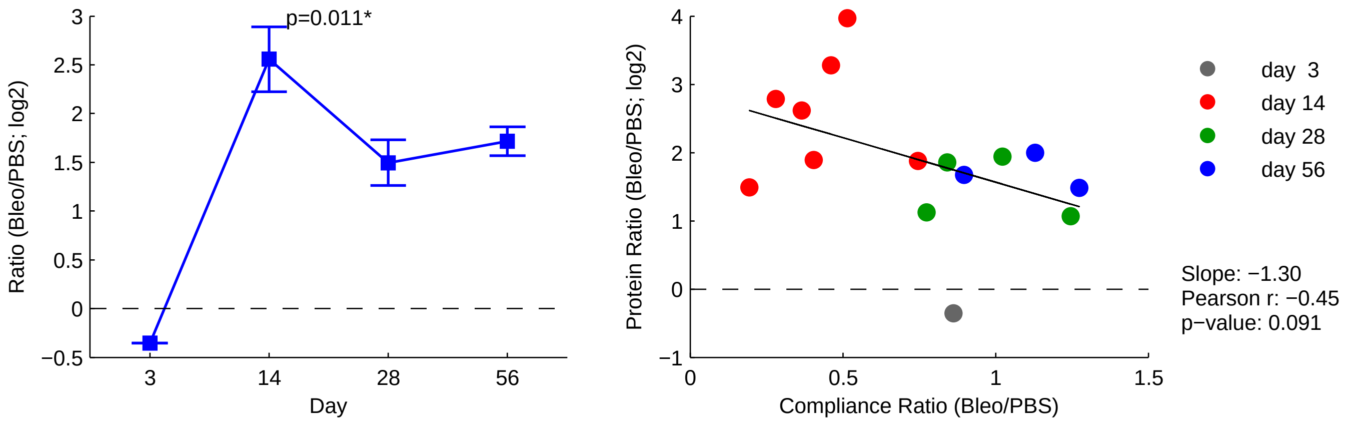

H3BL30 − Atpaf1 (id: 1802)

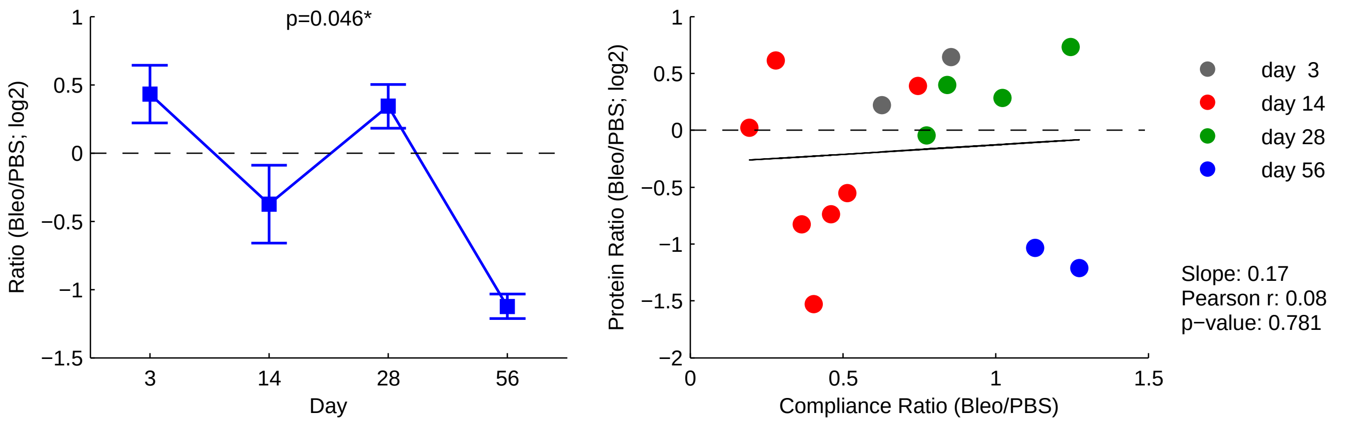

P40224−1 − Cxcl12 (id: 1815)

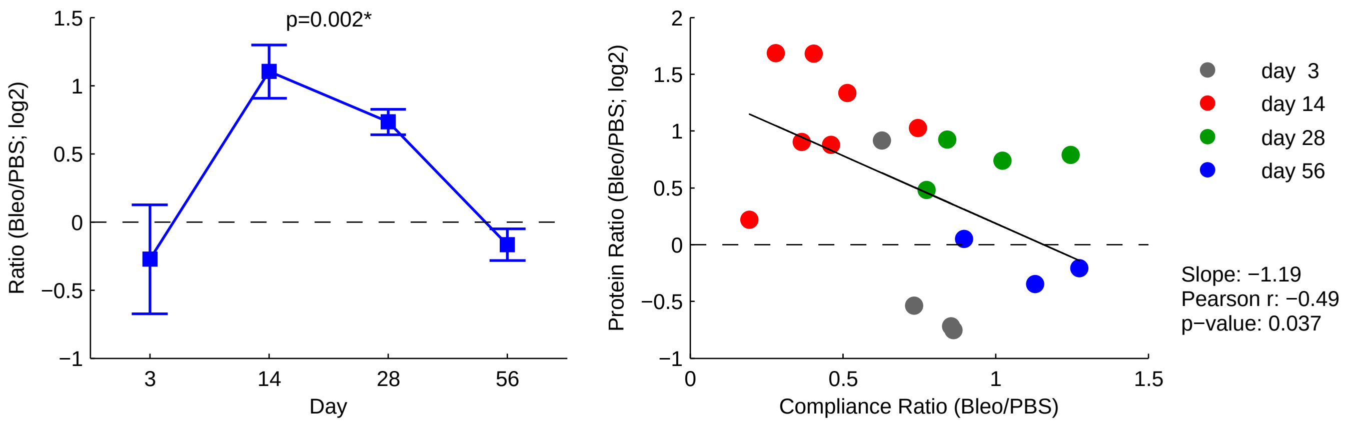

Q6ZPG2 − Wdr90 (id: 1816)

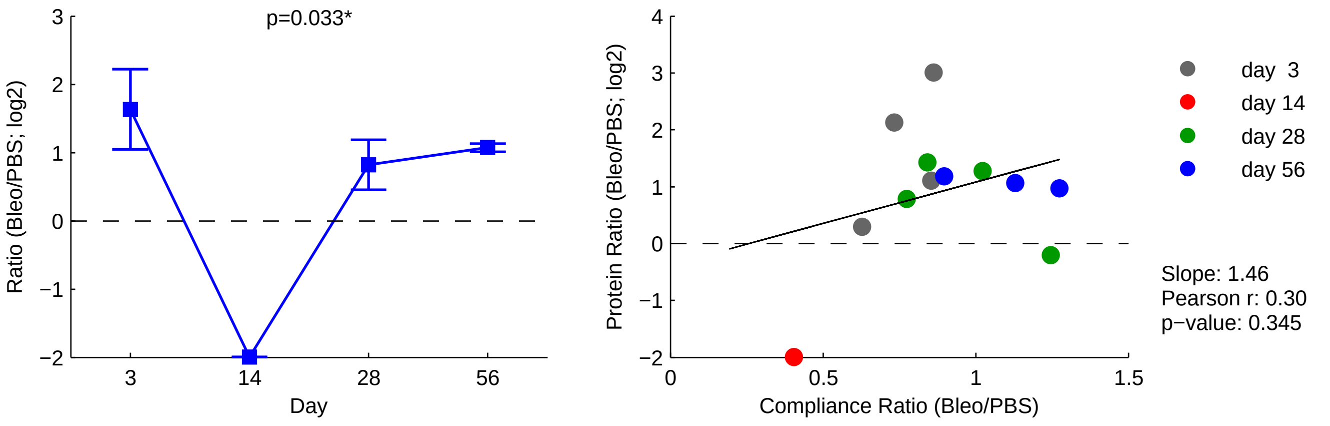

H7BX88 − Crat (id: 1819)

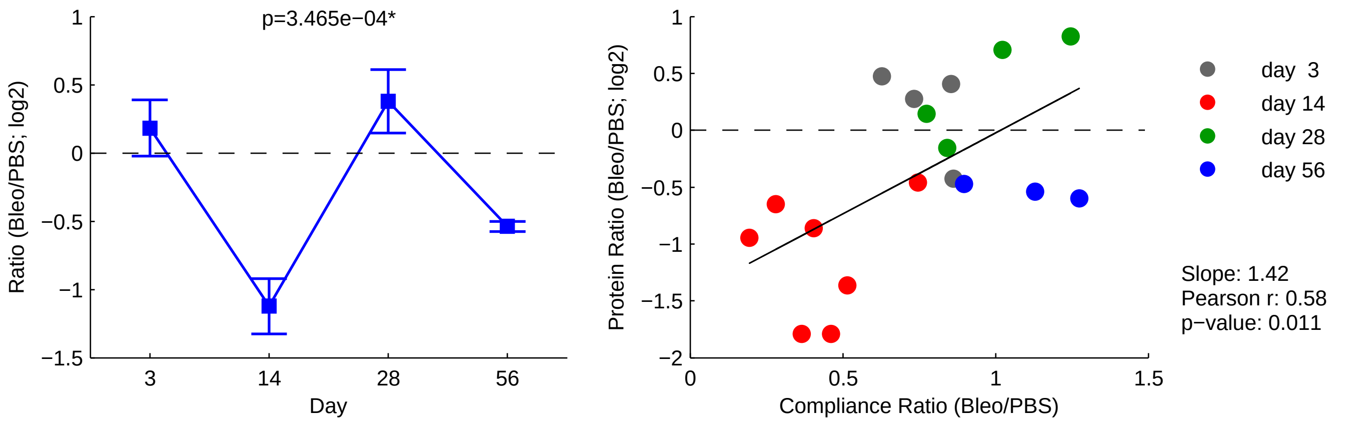

H9H9S3 − Zeb2 (id: 1823)

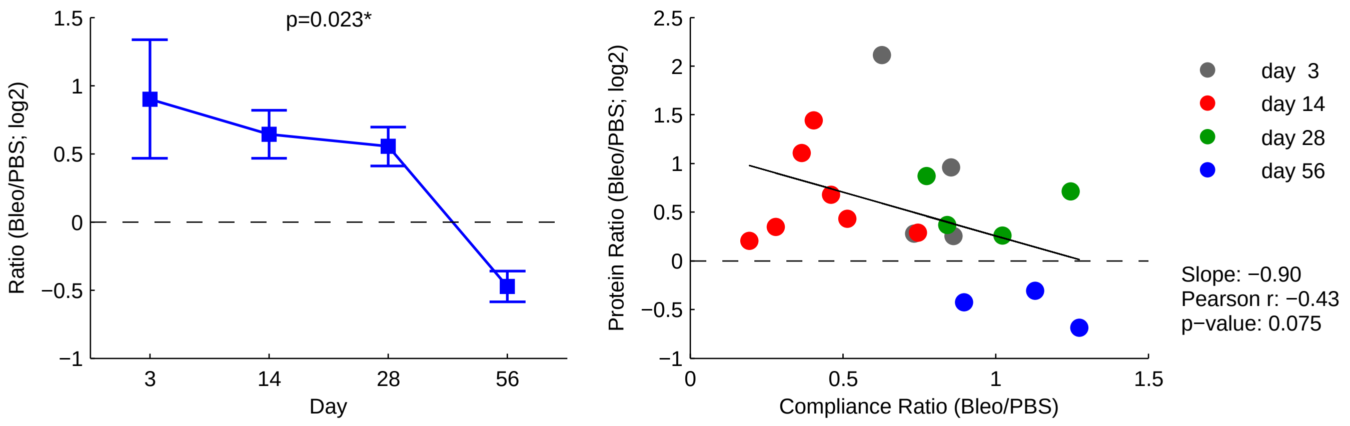

H9KUX8 − Birc6 (id: 1824)

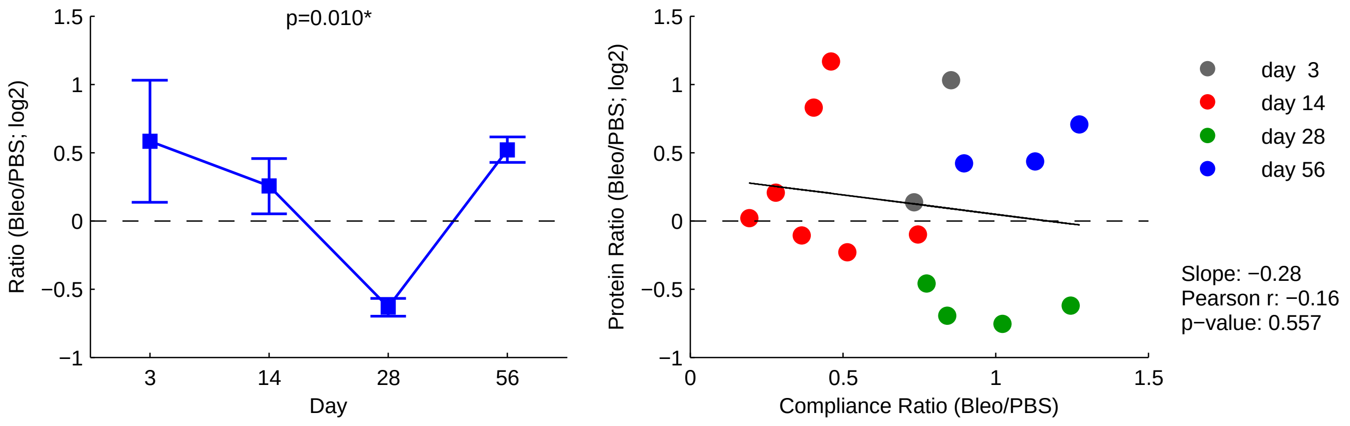

H9KV21 − Arih1 (id: 1827)

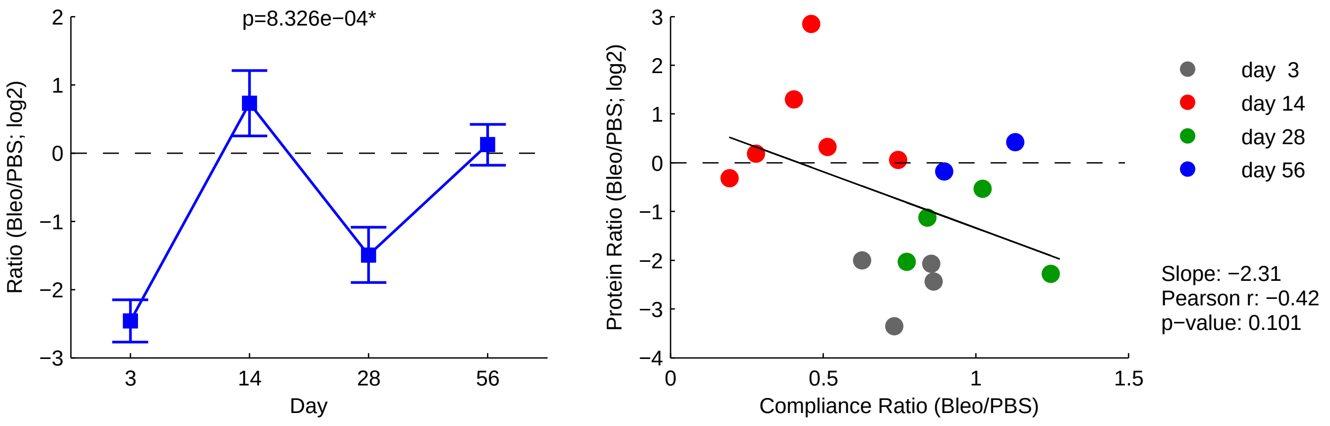

Q8K1E0 − Stx5 (id: 1828)

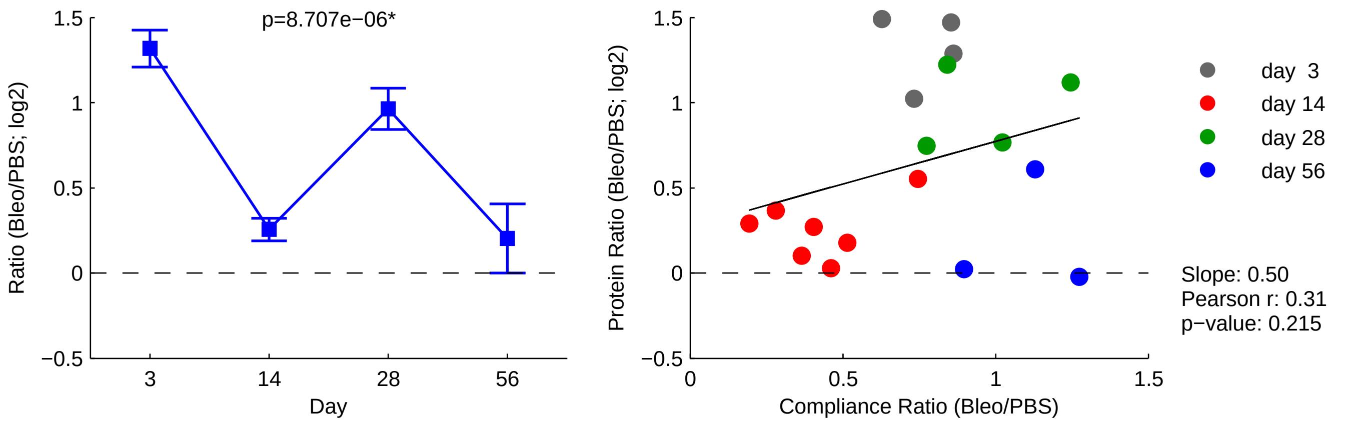

Q9D924 − Isca1 (id: 1829)

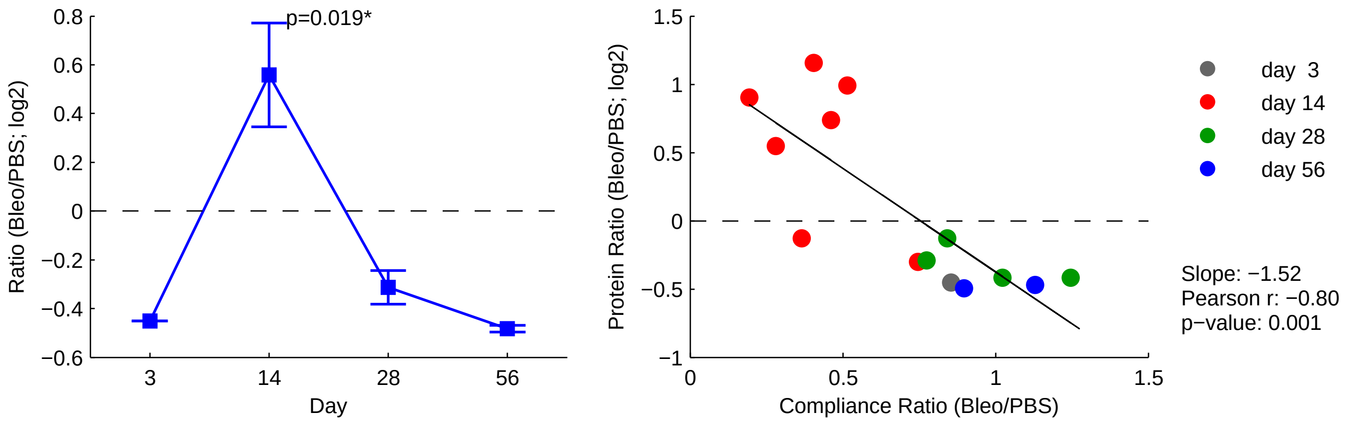

J3QML2 − Abcc3 (id: 1842)

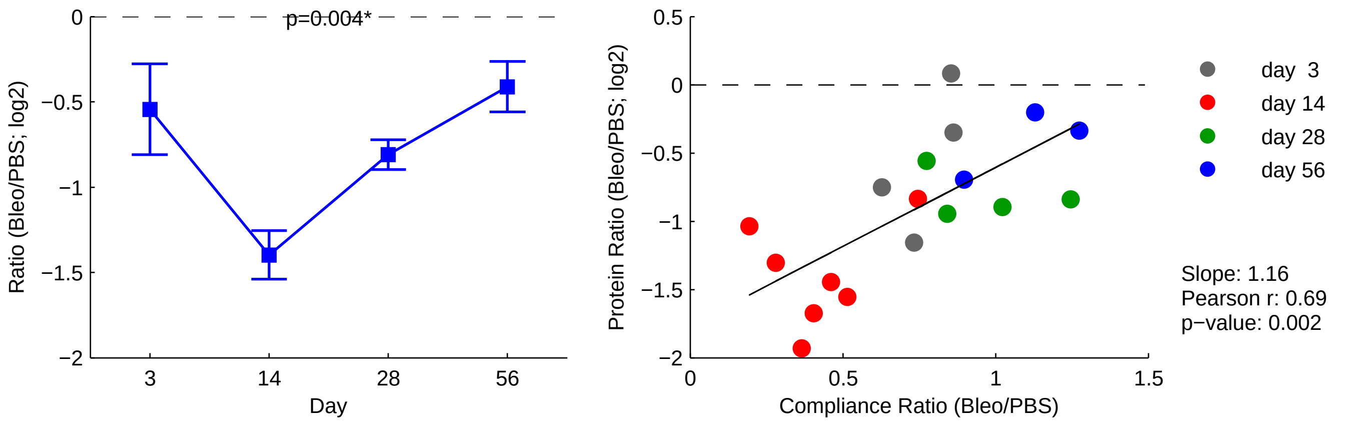

J3QMX0 − Rpp14 (id: 1844)

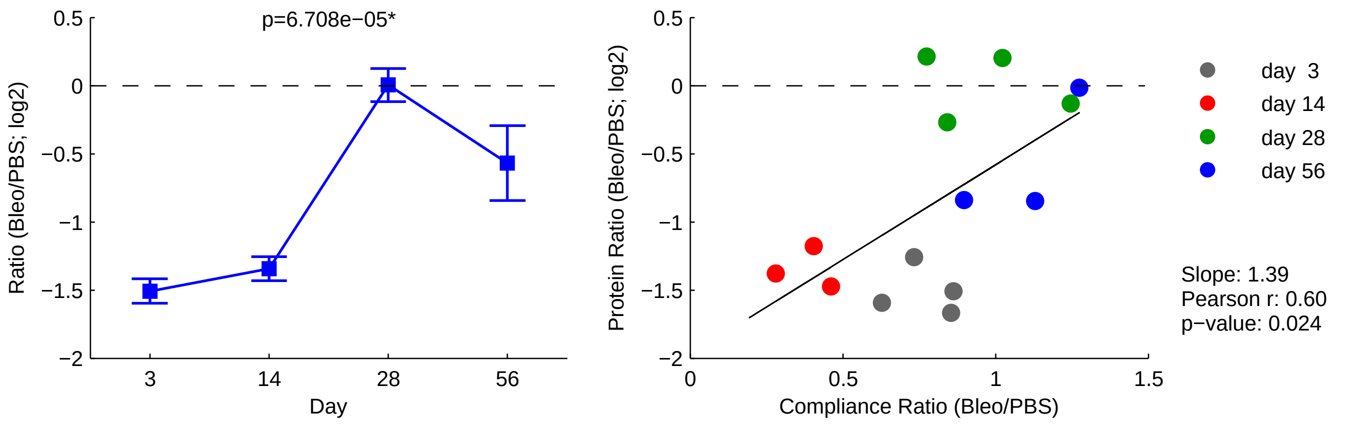

Q99PN3 − Trim26 (id: 1848)

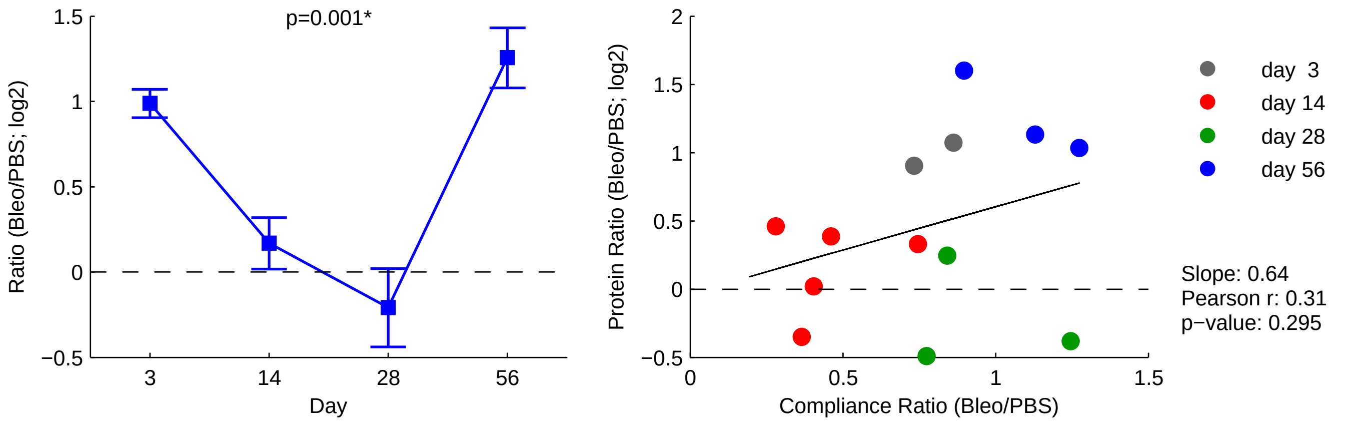

Q5I012−4 − Slc38a10 (id: 1850)

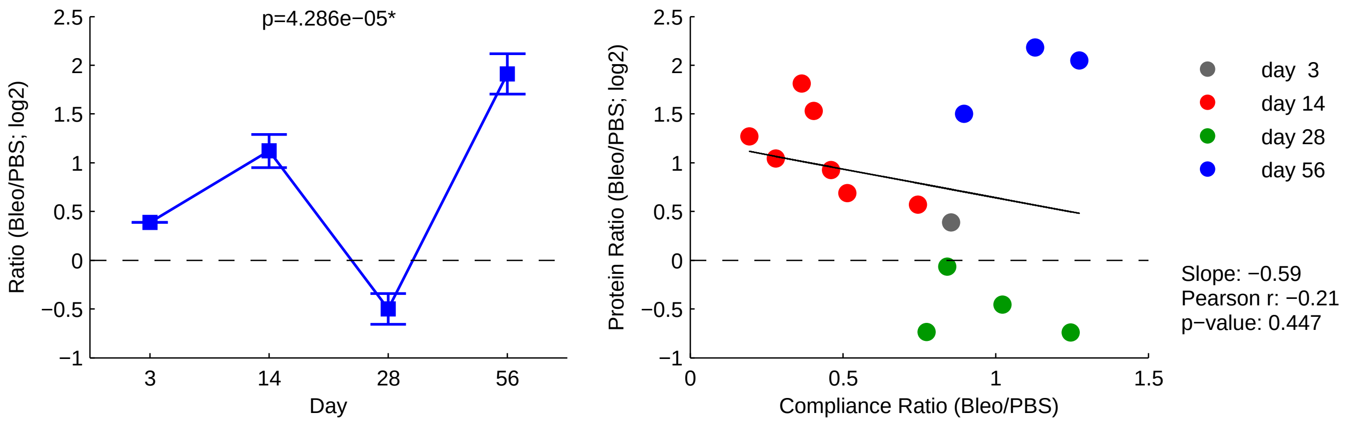

Q8BIH0−2 − Sap130 (id: 1851)

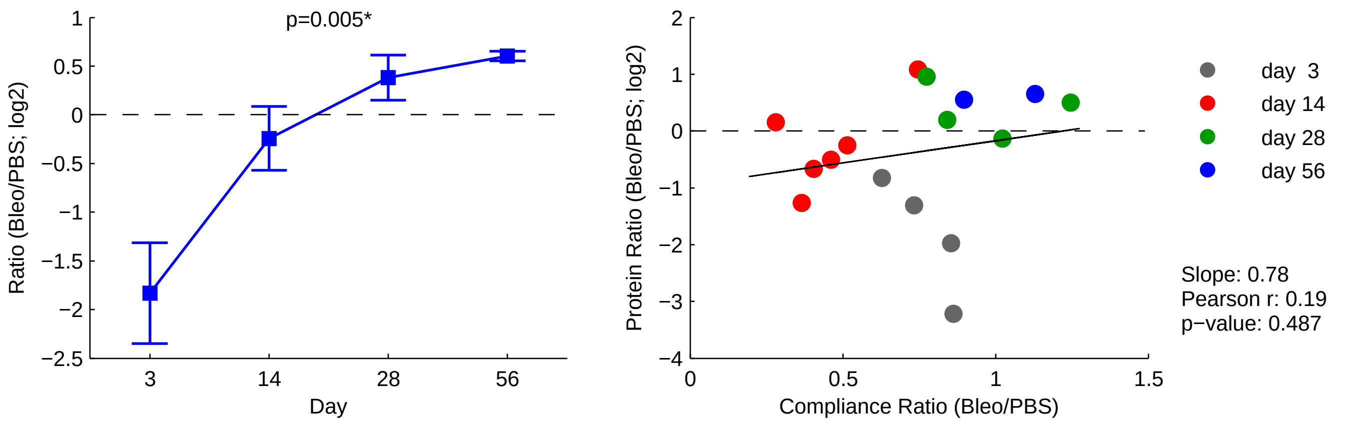

Q8BG05−2 − Gm9242 (id: 1856)

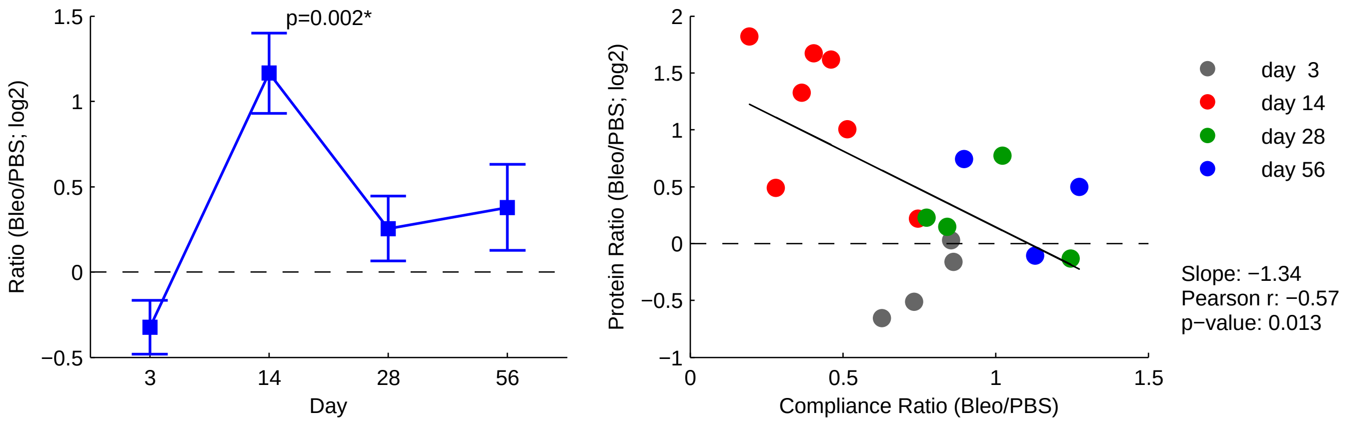

Q9CR86 − Carhsp1 (id: 1860)

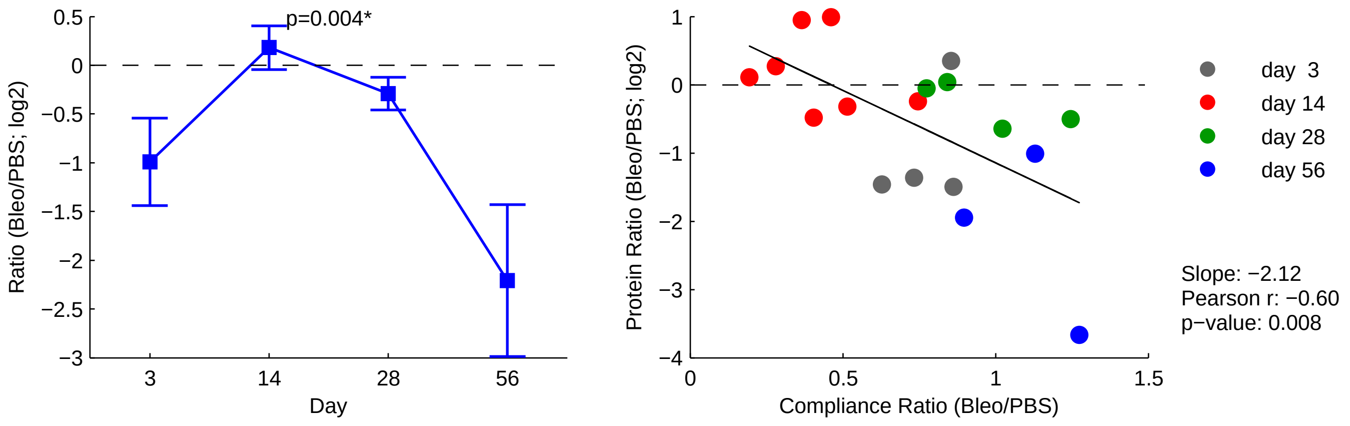

P50752−2 − Tnnt2 (id: 1862)

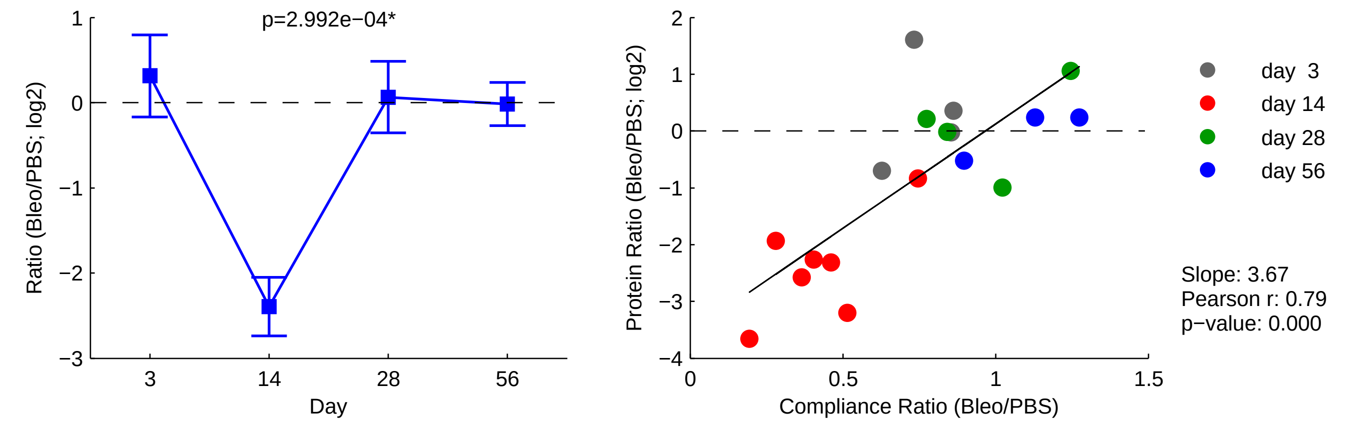

Q6P4S8 − Ints1 (id: 1865)

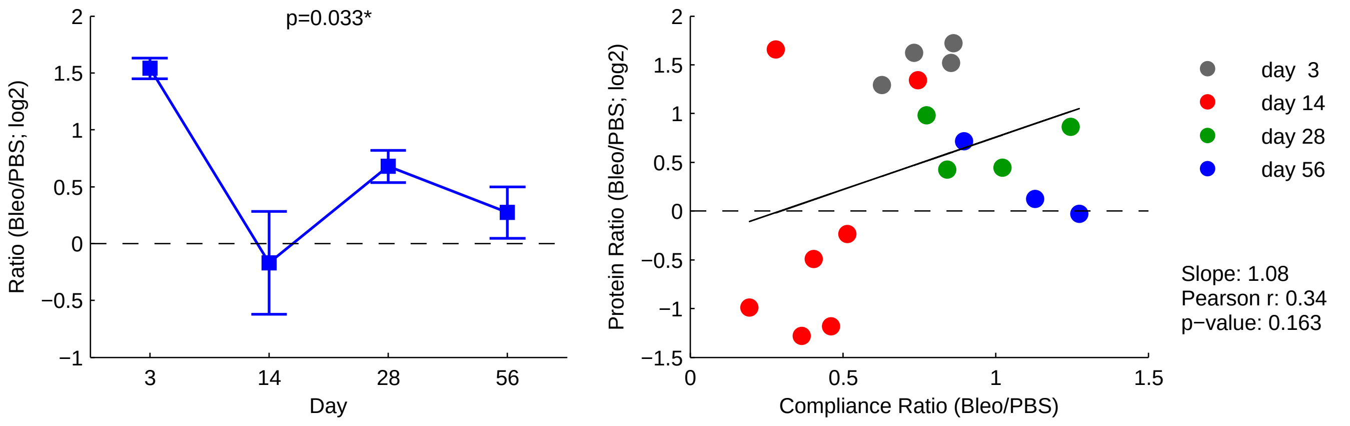

Q8CFE3 − Rcor1 (id: 1866)

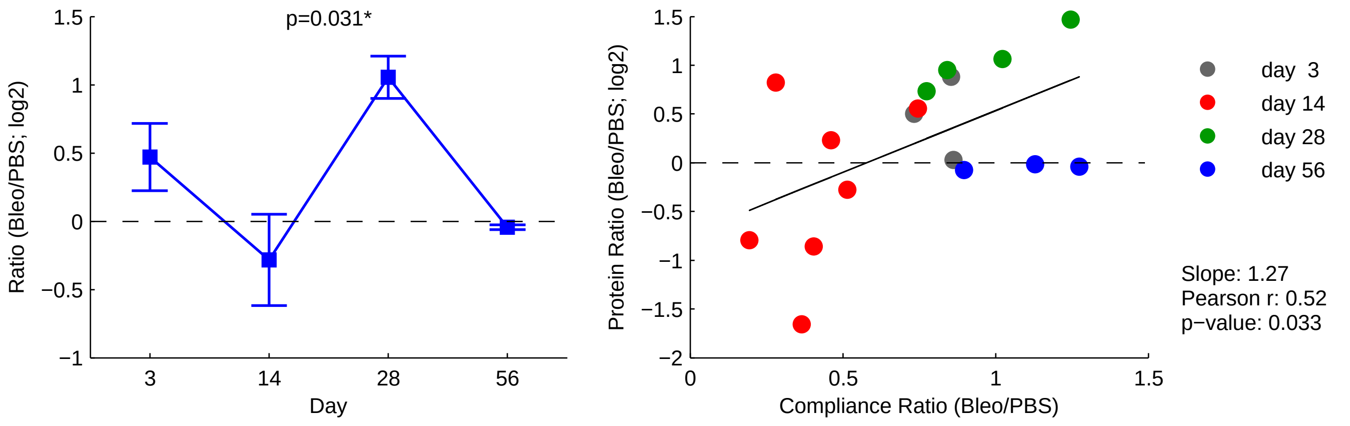

K3W4Q8 − Bsg (id: 1867)

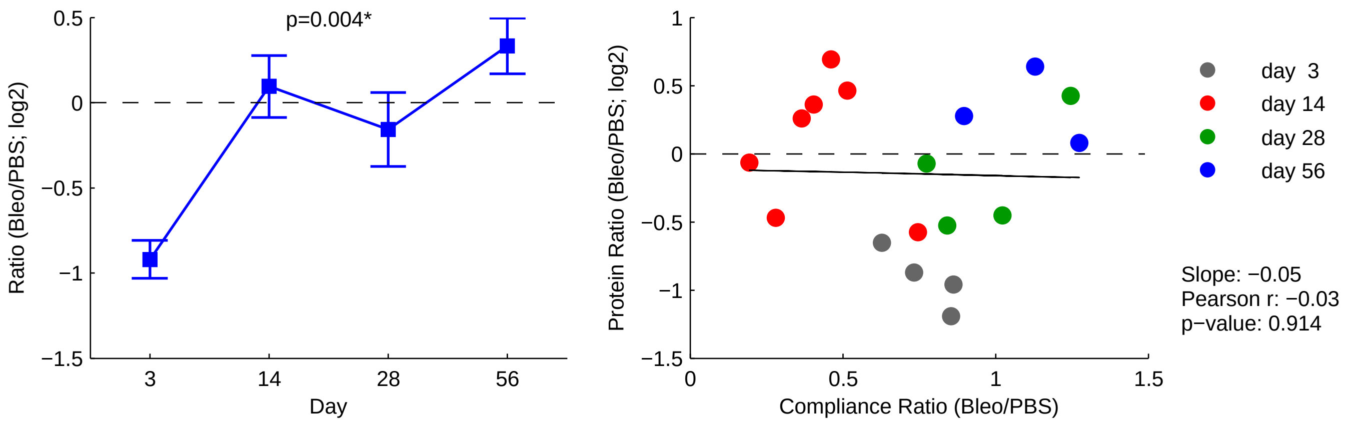

Q91WZ8 − Dtnbp1 (id: 1869)

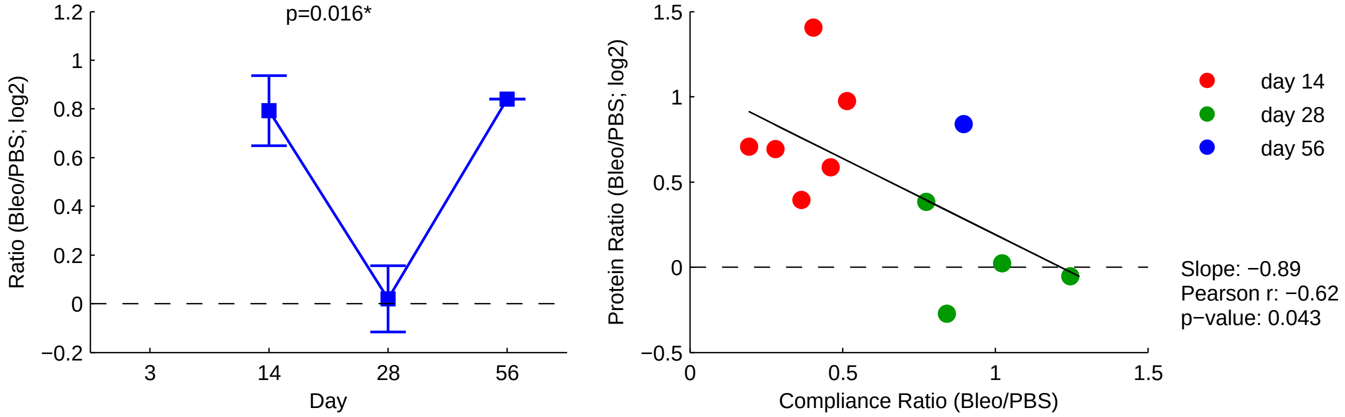

L7N1X8 − Gbp6 (id: 1878)

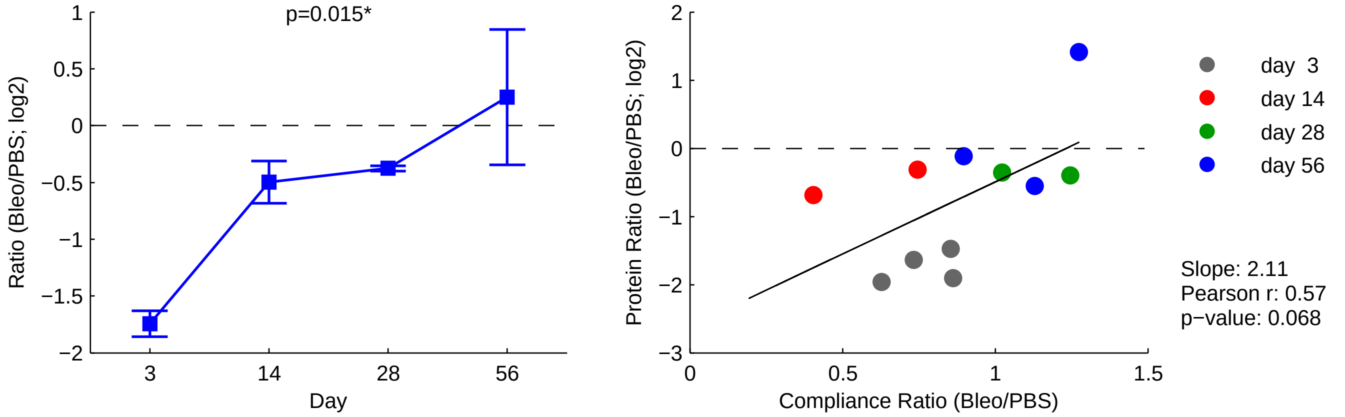

O08529 − Capn2 (id: 1889)

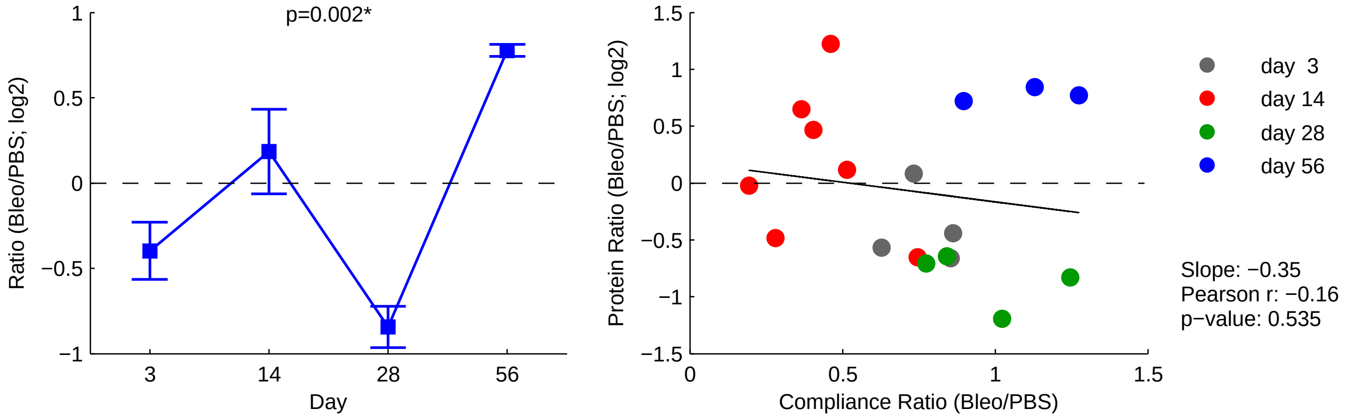

O08532−3 − Cacna2d1 (id: 1891)

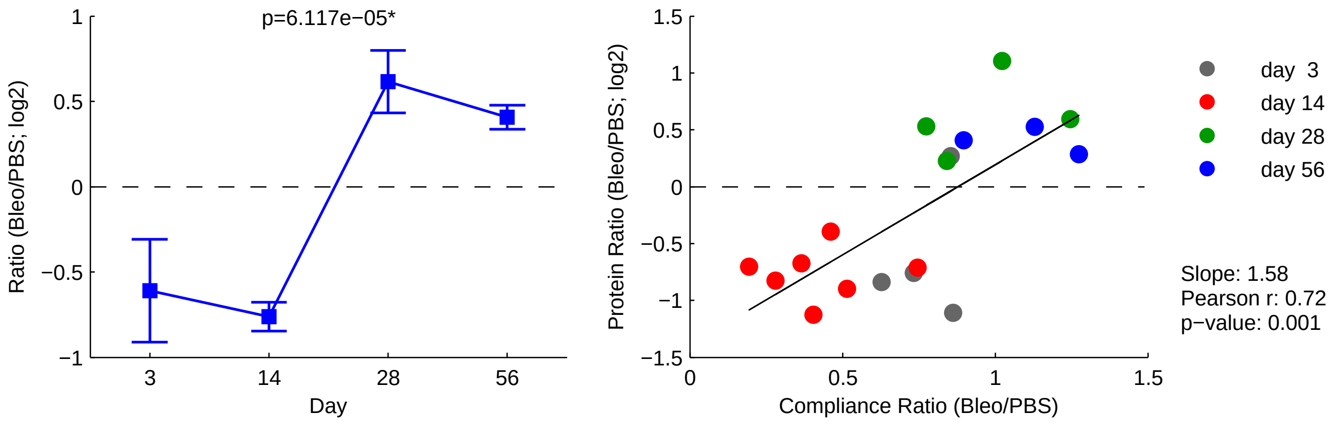

O08538 − Angpt1 (id: 1892)

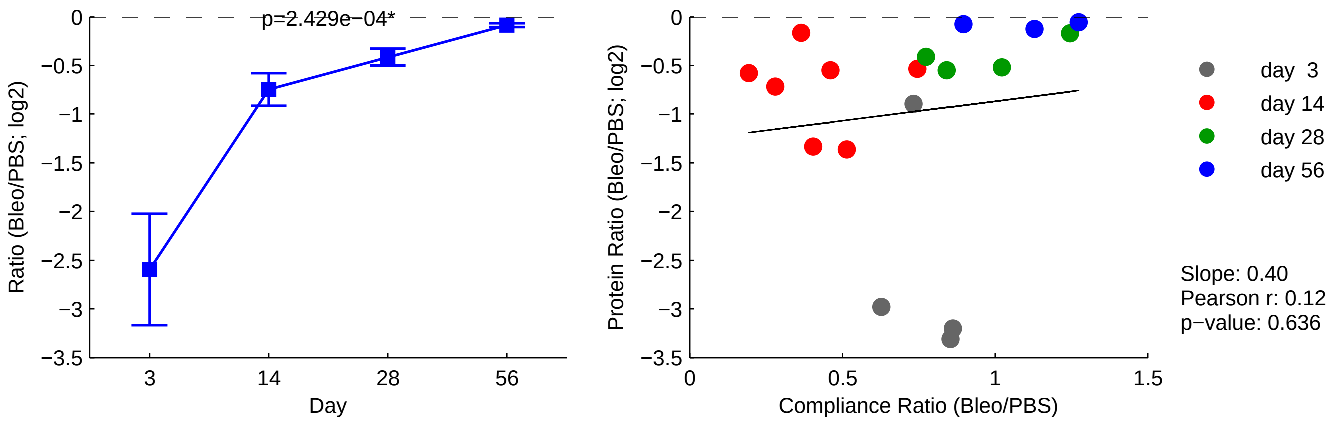

O08539−2 − Bin1 (id: 1893)

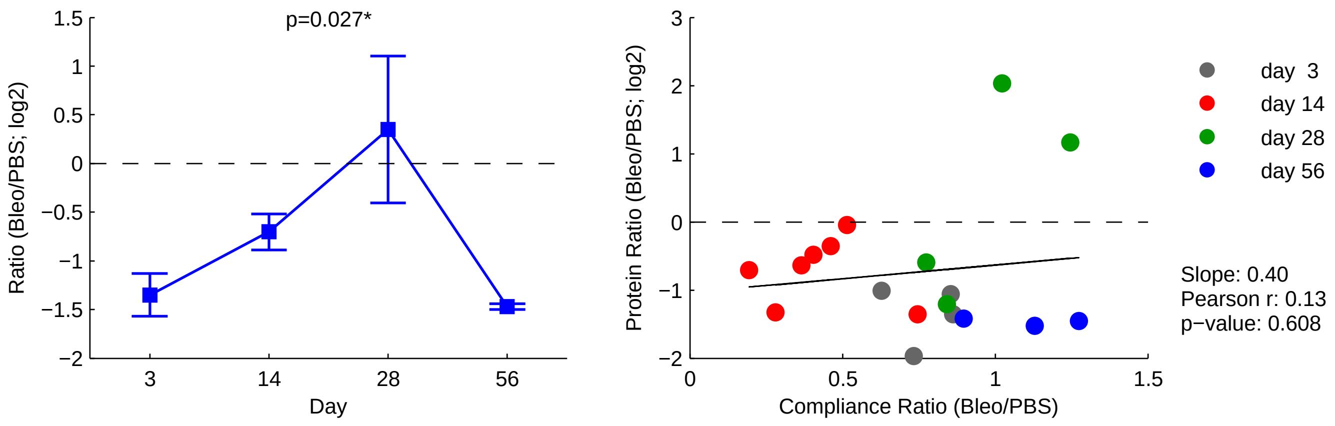

O08547 − Sec22b (id: 1894)

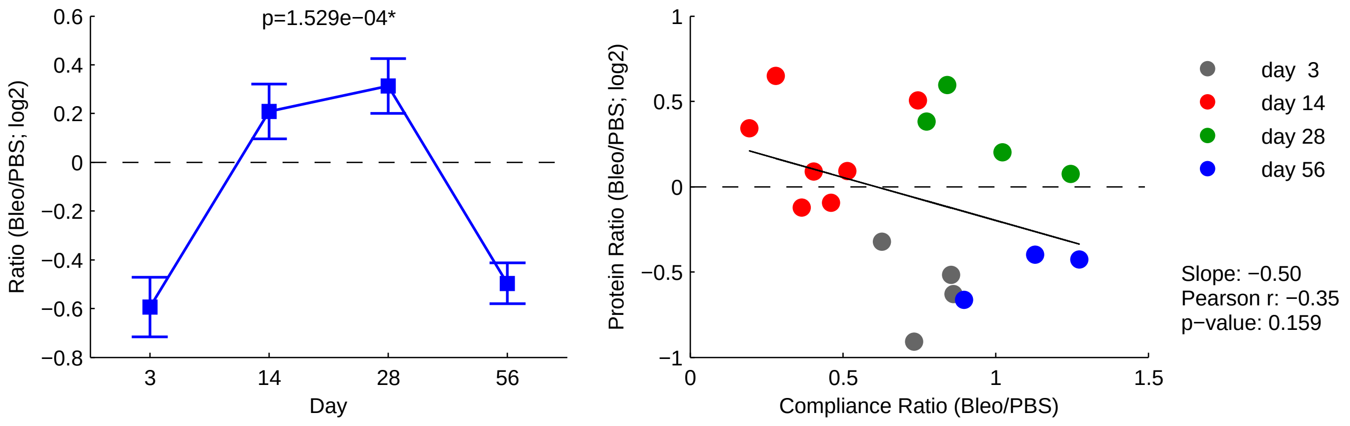

Q3THM8 − Emd (id: 1896)

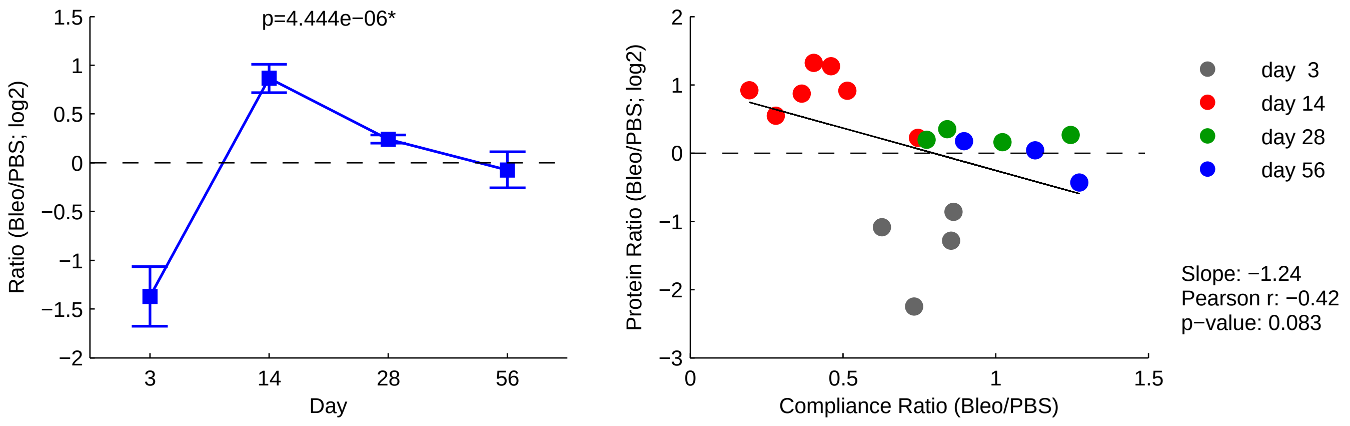

O08582 − Gtpbp1 (id: 1898)

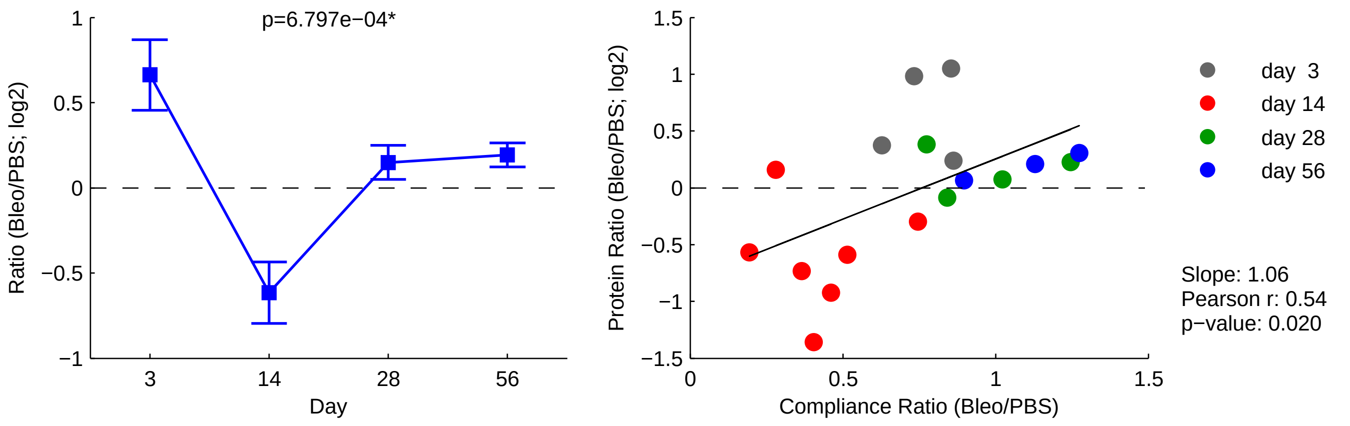

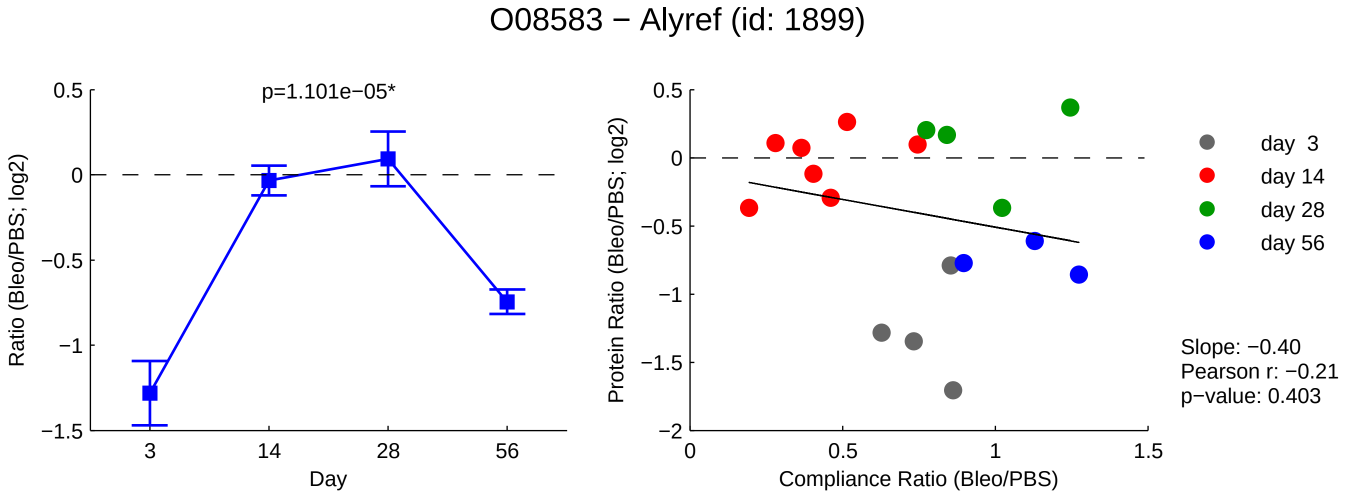
O08583 − Alyref (id: 1899)
p=1.101e−05*
Ratio (Bleo/PBS; log2)
0.5
0
−0.5
−1
−1.5
3
14
28
56
Day
Protein Ratio (Bleo/PBS; log2)
0.5
0
−0.5
−1
−1.5
−2
0
0.5
1
1.5
Compliance Ratio (Bleo/PBS)
day 3
day 14
day 28
day 56
Slope: −0.40
Pearson r: −0.21
p−value: 0.403

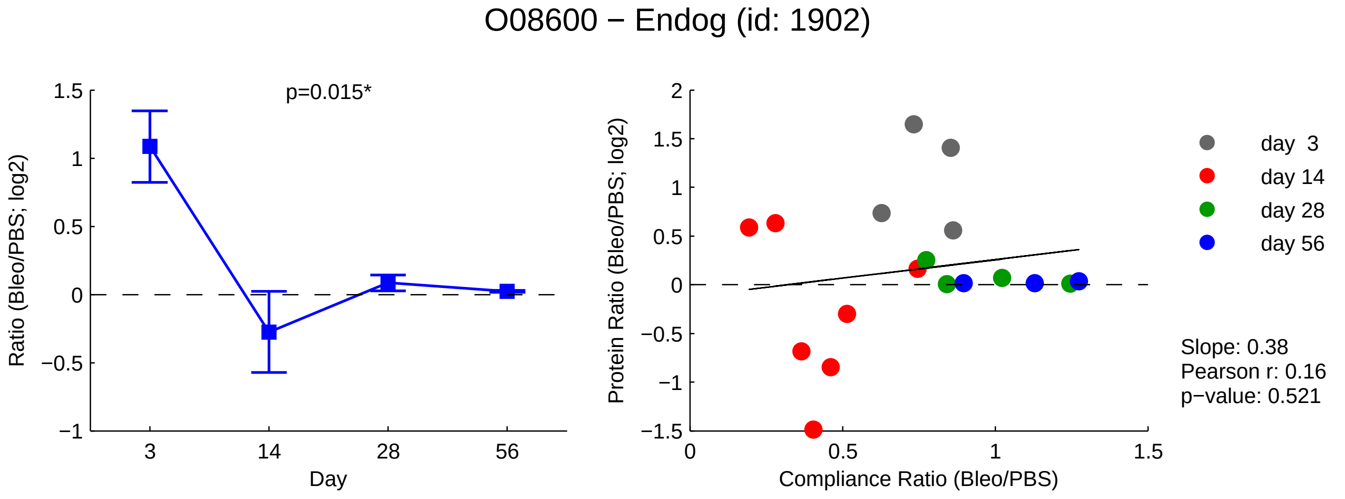
O08600 − Endog (id: 1902)
p=0.015*
Ratio (Bleo/PBS; log2)
1.5
1
0.5
0
−0.5
−1
3
14
28
56
Day
Protein Ratio (Bleo/PBS; log2)
2
1.5
1
0.5
0
−0.5
−1
−1.5
0
0.5
1
1.5
Compliance Ratio (Bleo/PBS)
day 3
day 14
day 28
day 56
Slope: 0.38
Pearson r: 0.16
p−value: 0.521

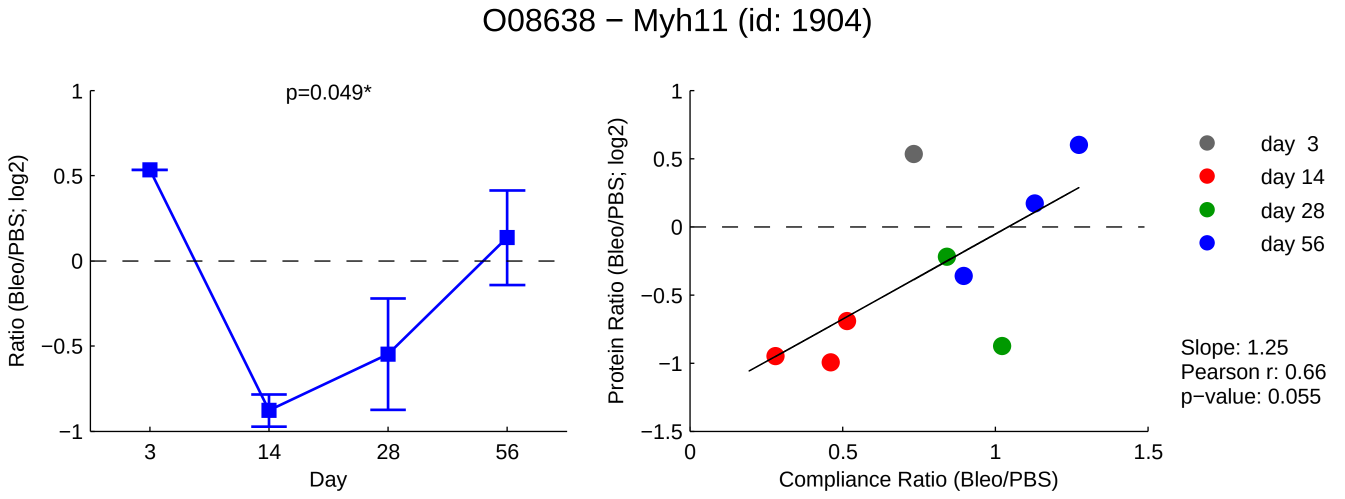
O08638 − Myh11 (id: 1904)
p=0.049*
Ratio (Bleo/PBS; log2)
1
0.5
0
−0.5
−1
3
14
28
56
Day
Protein Ratio (Bleo/PBS; log2)
1
0.5
0
−0.5
−1
−1.5
0
0.5
1
1.5
Compliance Ratio (Bleo/PBS)
day 3
day 14
day 28
day 56
Slope: 1.25
Pearson r: 0.66
p−value: 0.055

O08663 − Metap2 (id: 1905)

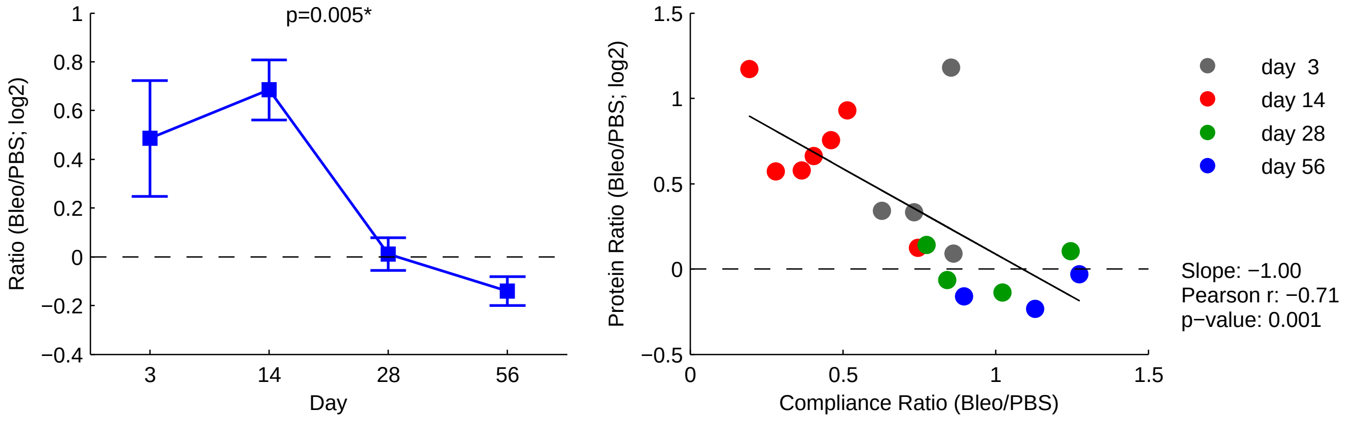

O08677−2 − (id: 1907)

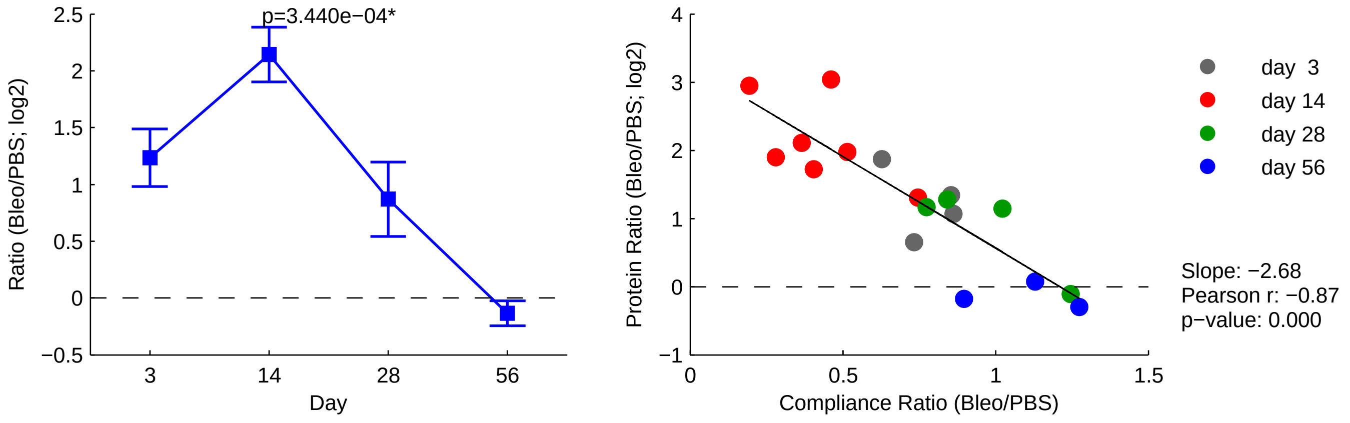

O08691 − Arg2 (id: 1909)

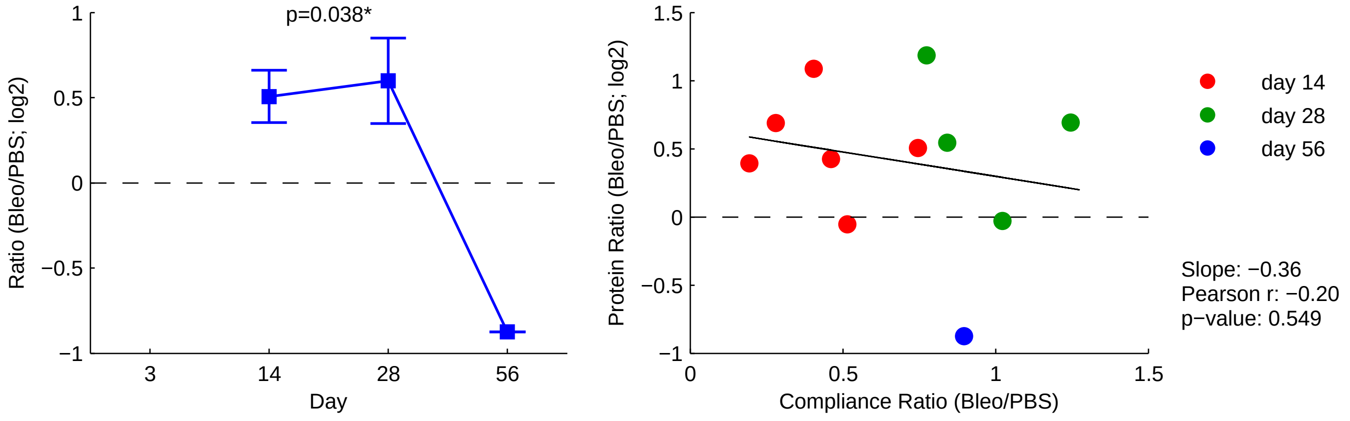

O08692 − Ngp (id: 1910)

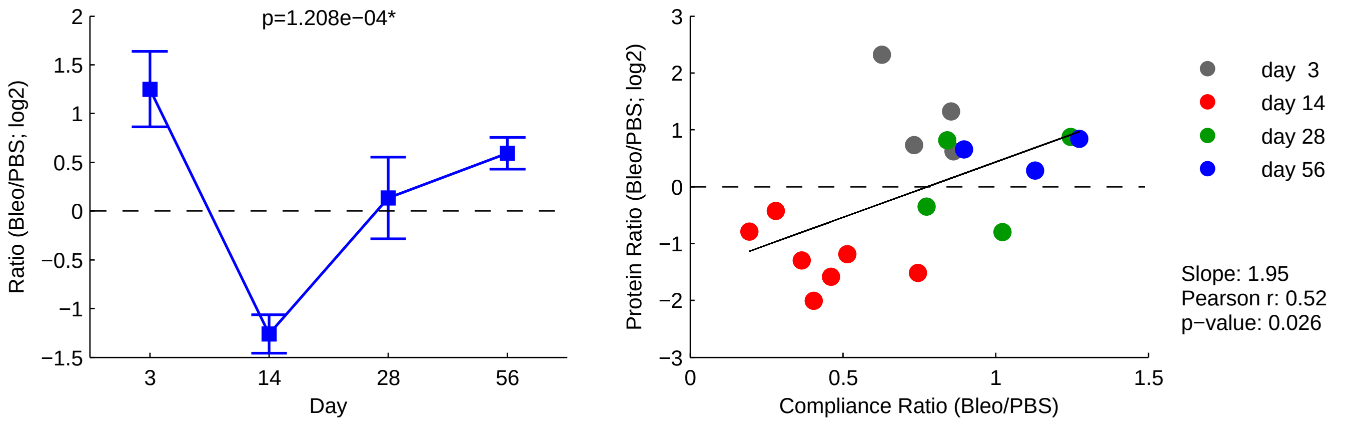

O08739 − Ampd3 (id: 1913)

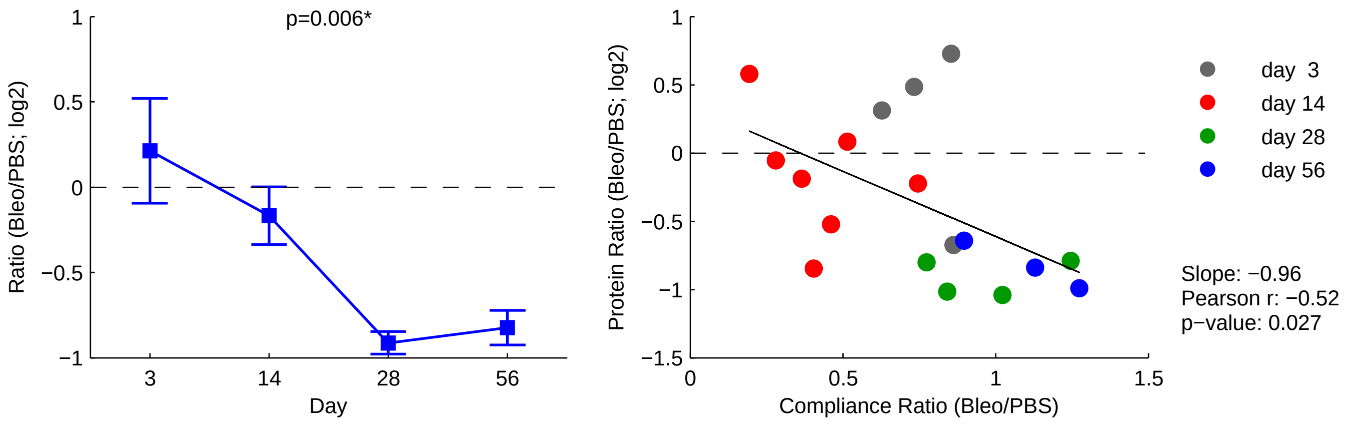

O08749 − Dld (id: 1915)

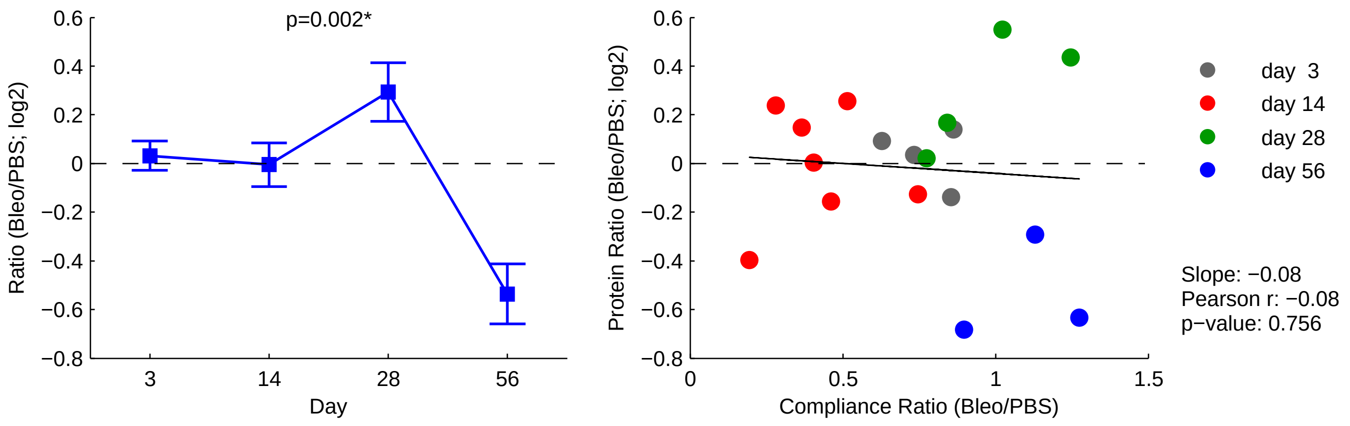

O08795−2 − Prkcsh (id: 1919)

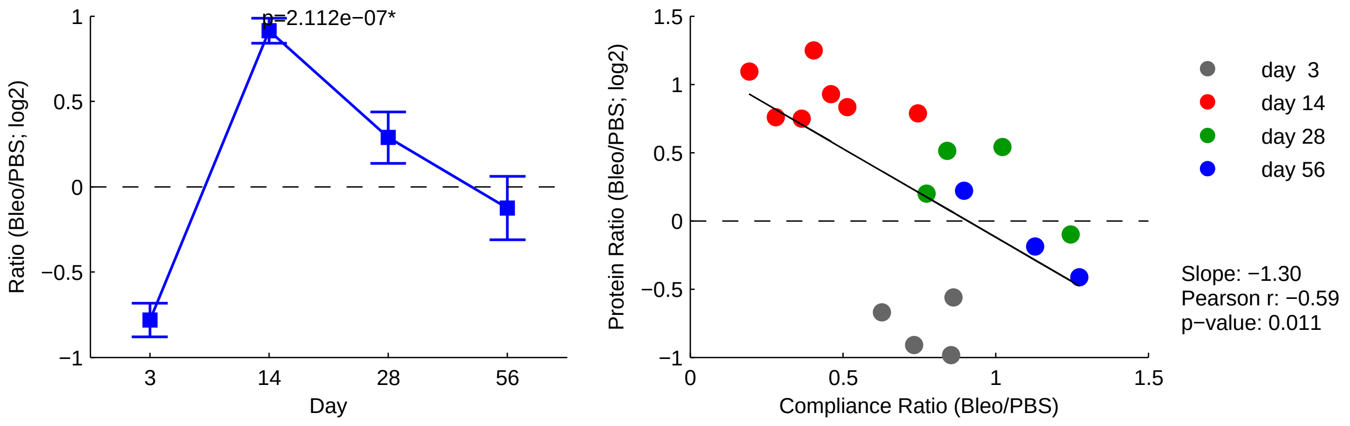

O08804 − Serpinb6b (id: 1922)

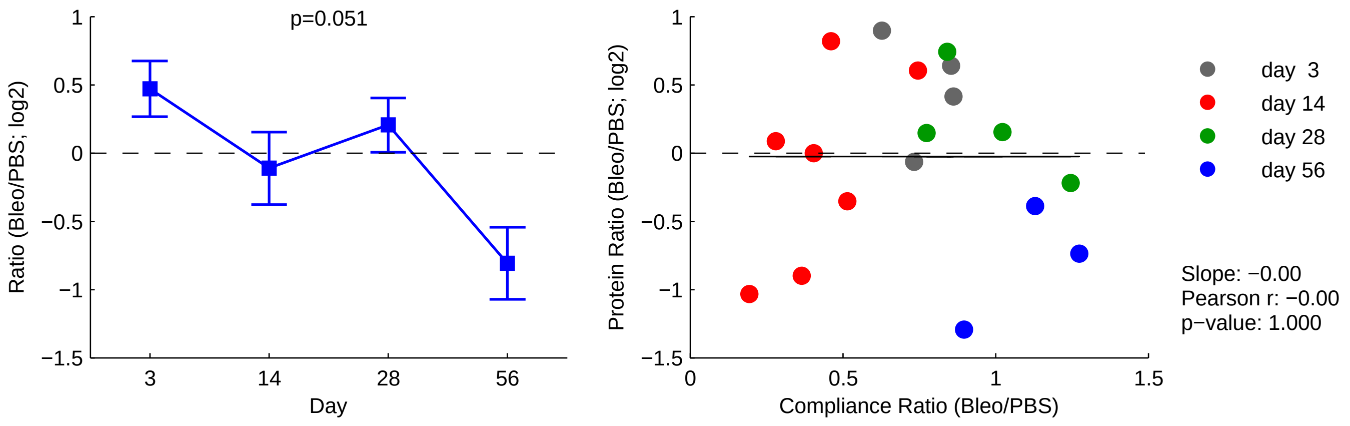

O08807 − Prdx4 (id: 1923)

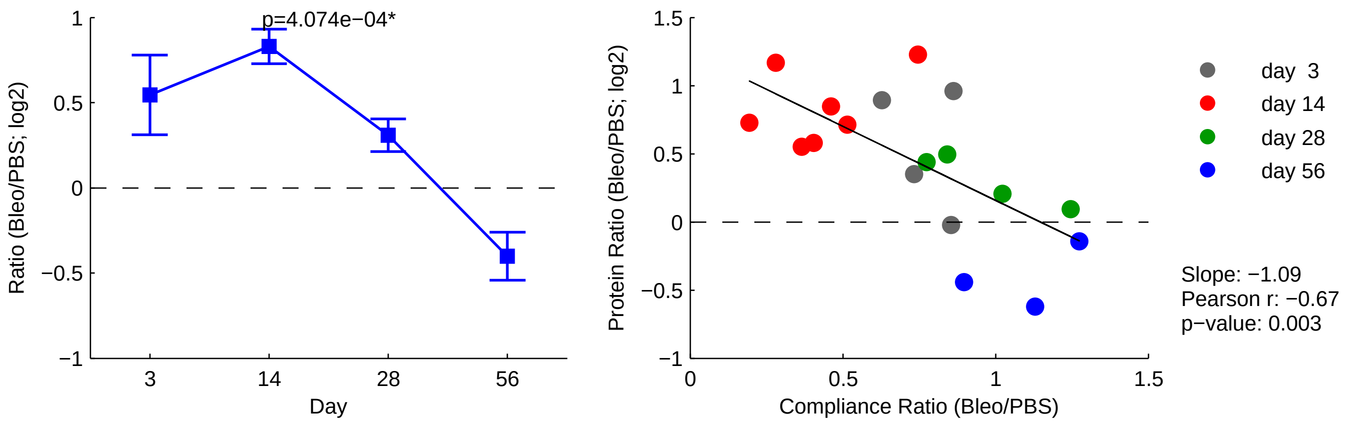

O08914 − Faah (id: 1929)

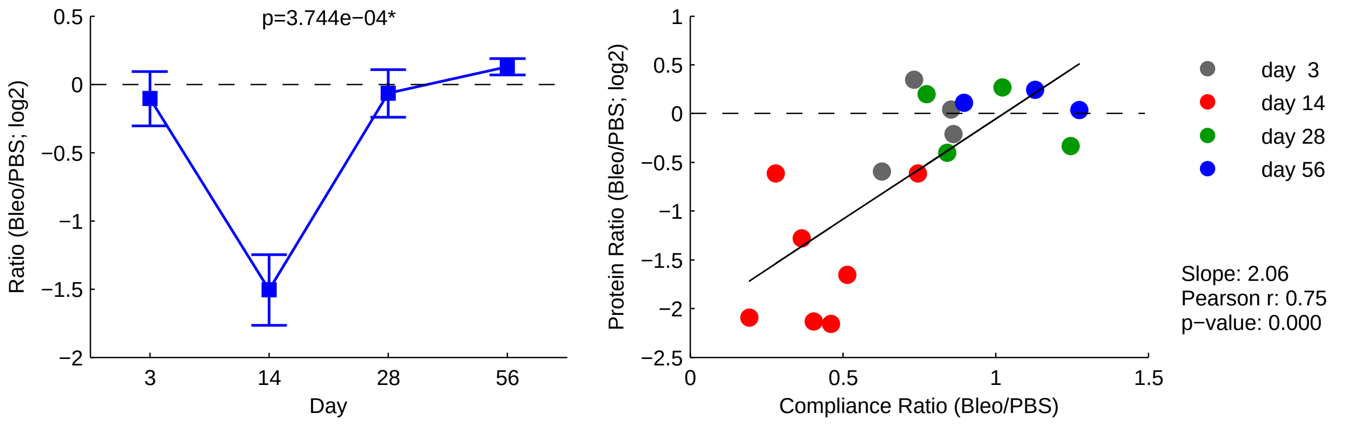

O08915 − Aip (id: 1930)

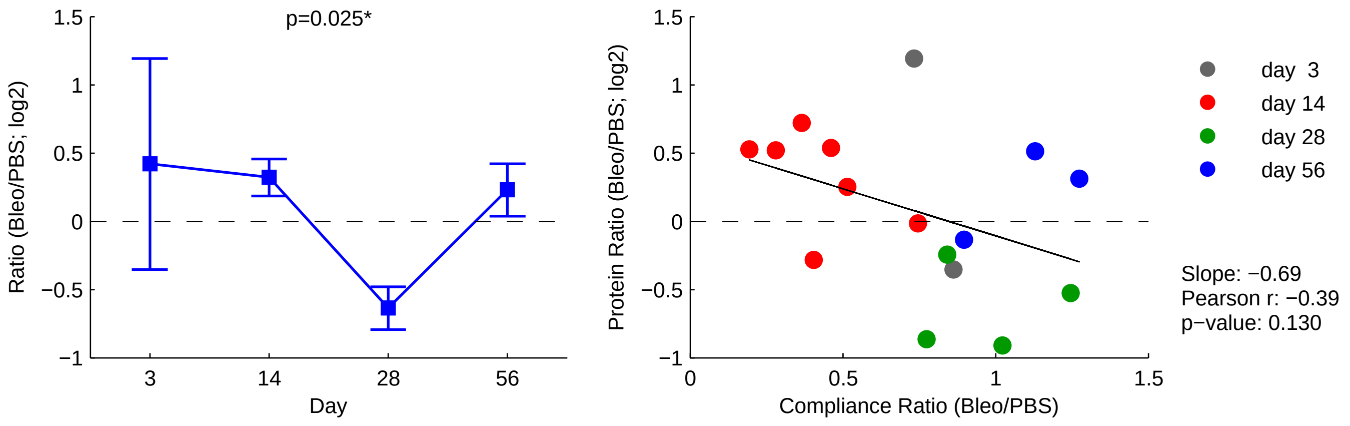

O08917 − Flot1 (id: 1931)

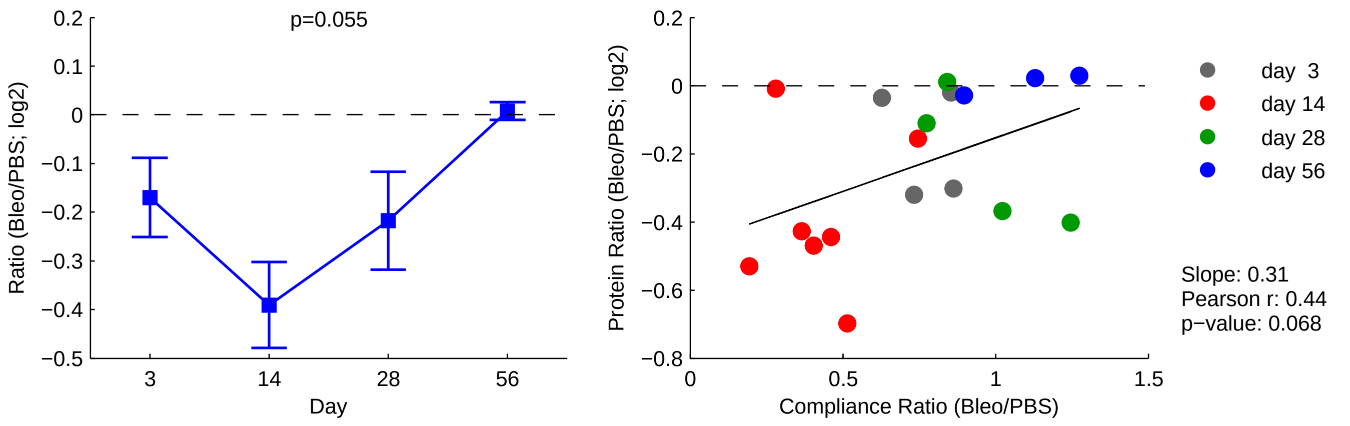

O08967 − Cyth3 (id: 1933)

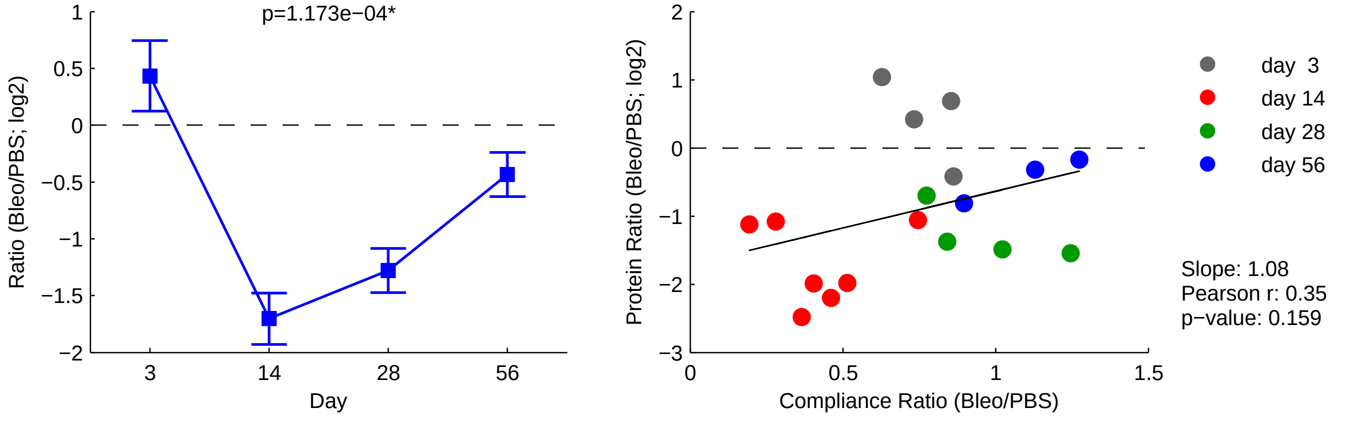

Q3TMX0 − Sdcbp (id: 1935)

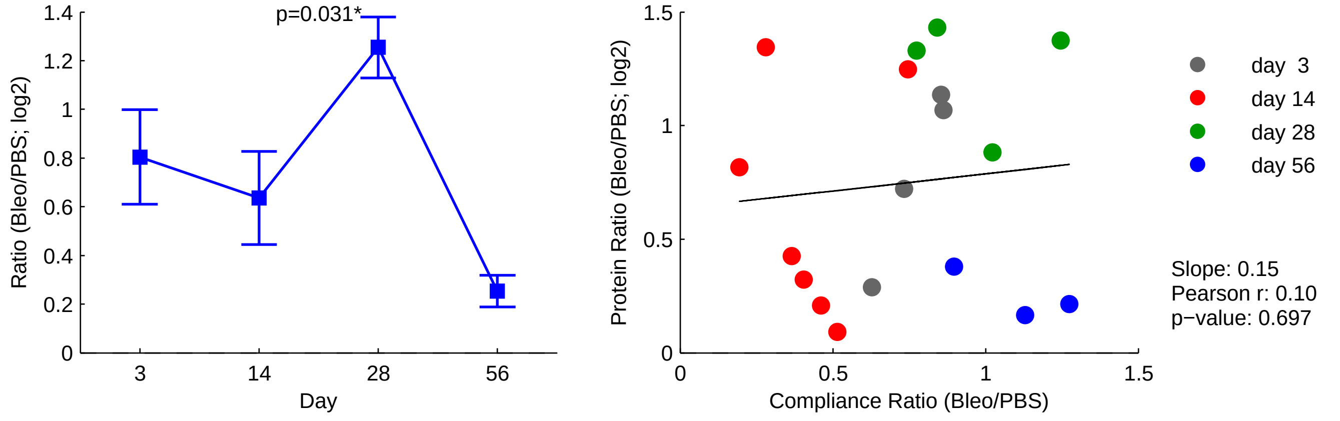

O08997 − Atox1 (id: 1936)

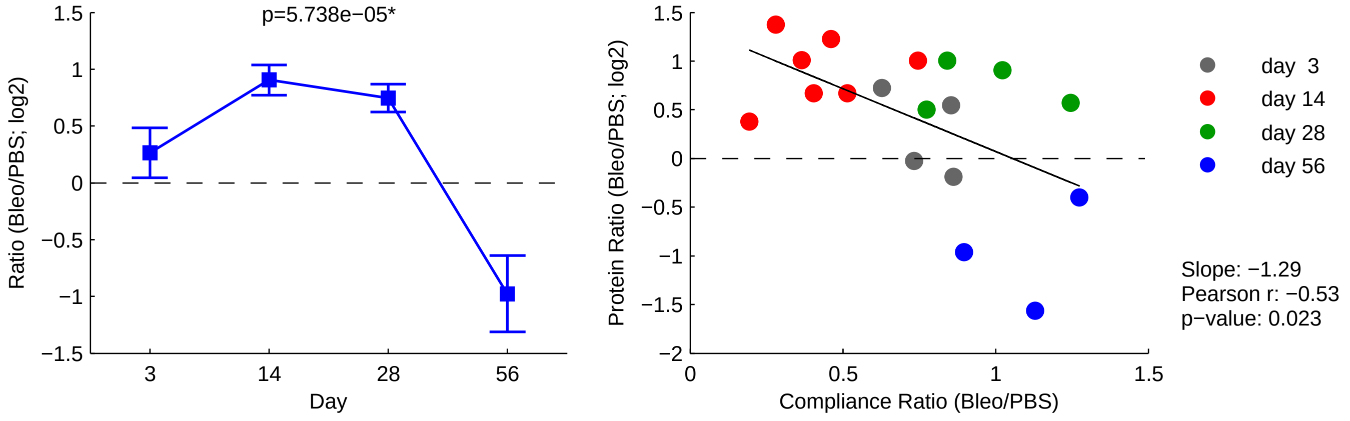

O09005 − Degs1 (id: 1937)

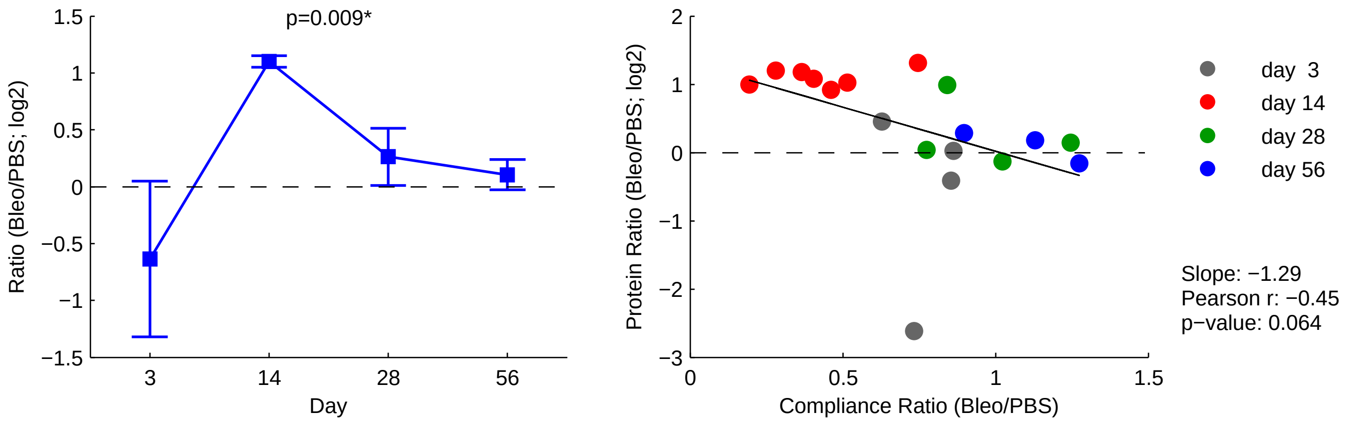

O09043 − Napsa (id: 1940)

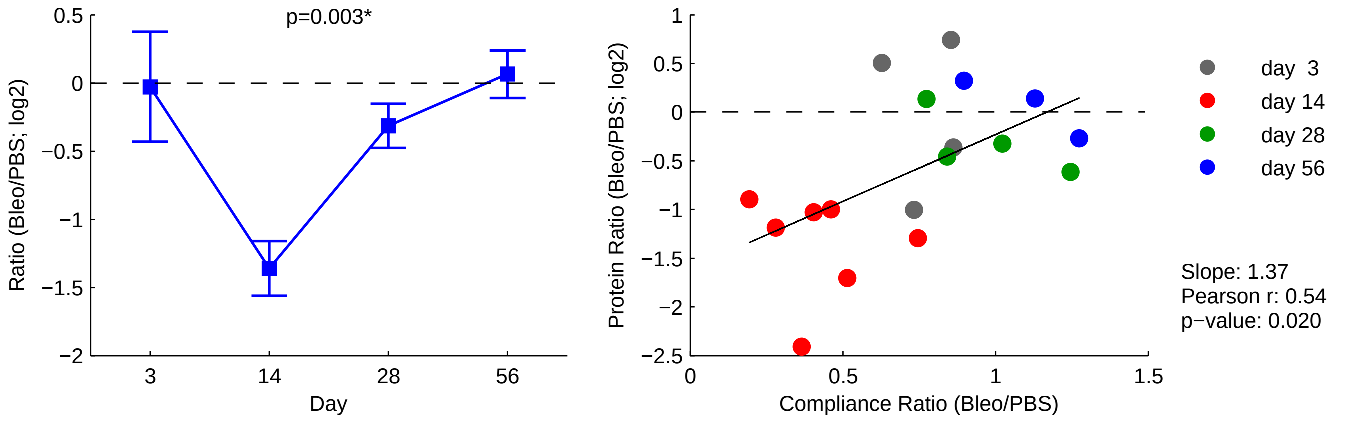

O09044 − Snap23 (id: 1941)

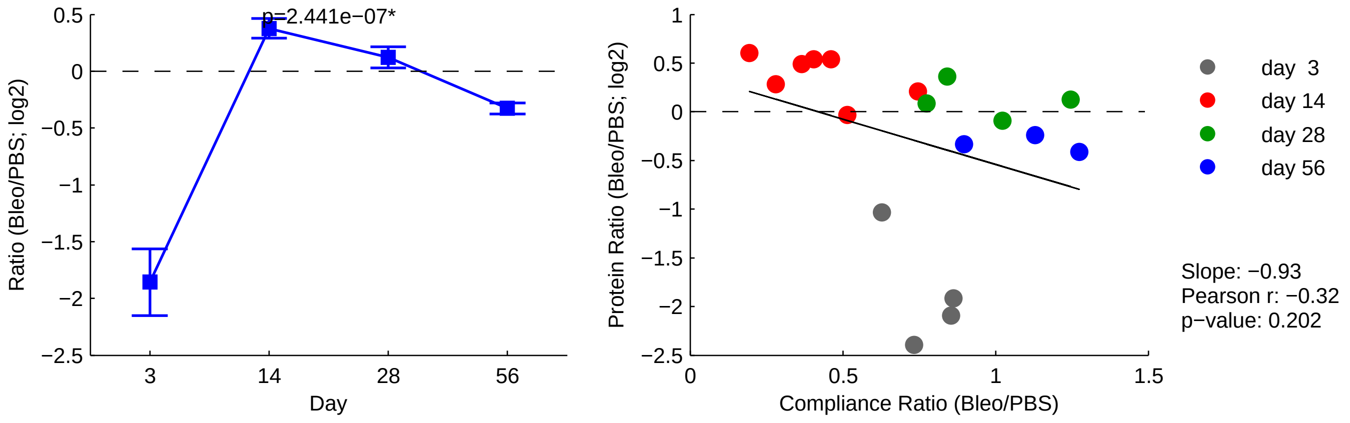

O09061 − Psmb1 (id: 1943)

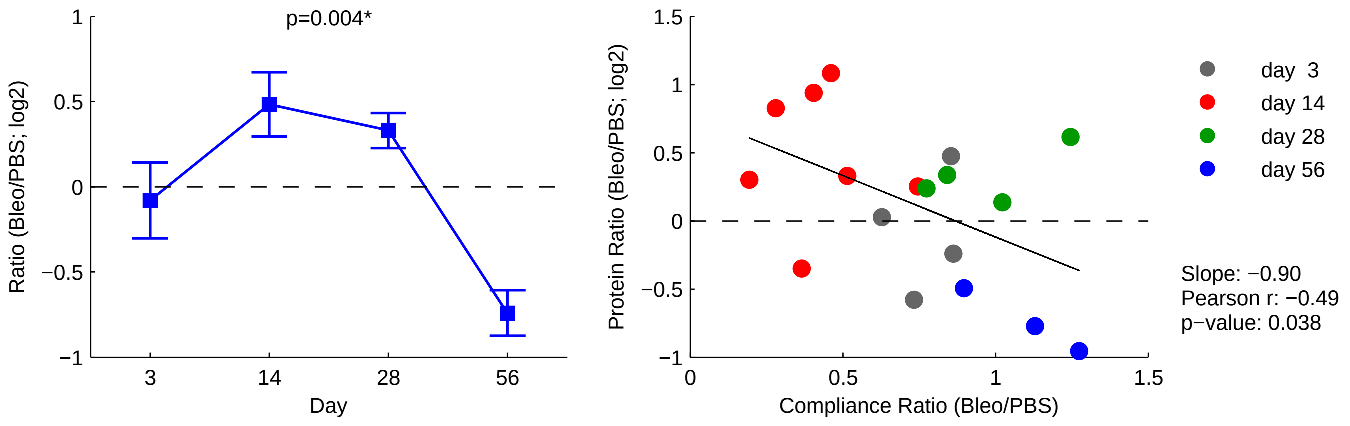

O09106 − Hdac1 (id: 1944)

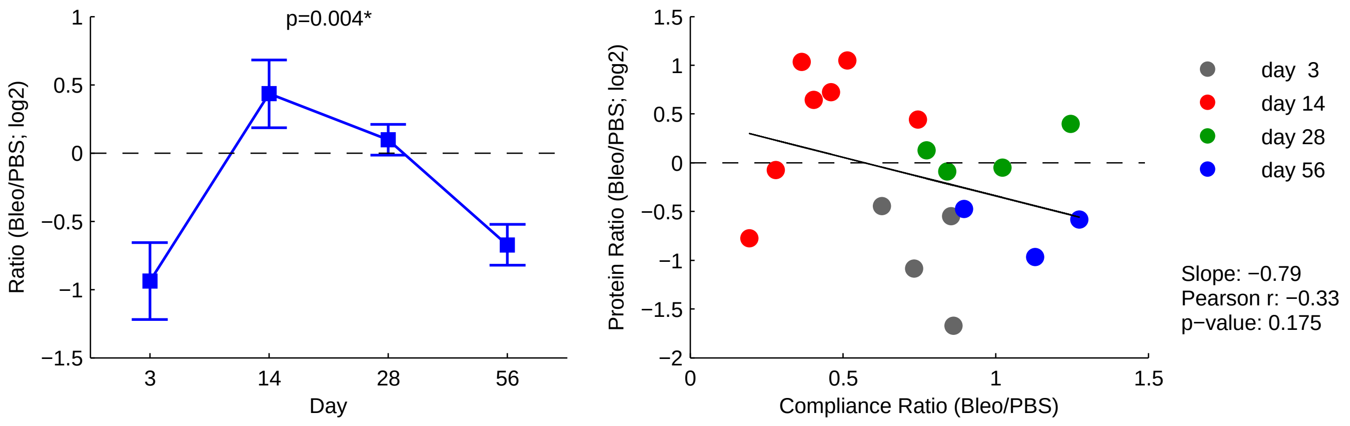

O09110 − Map2k3 (id: 1945)

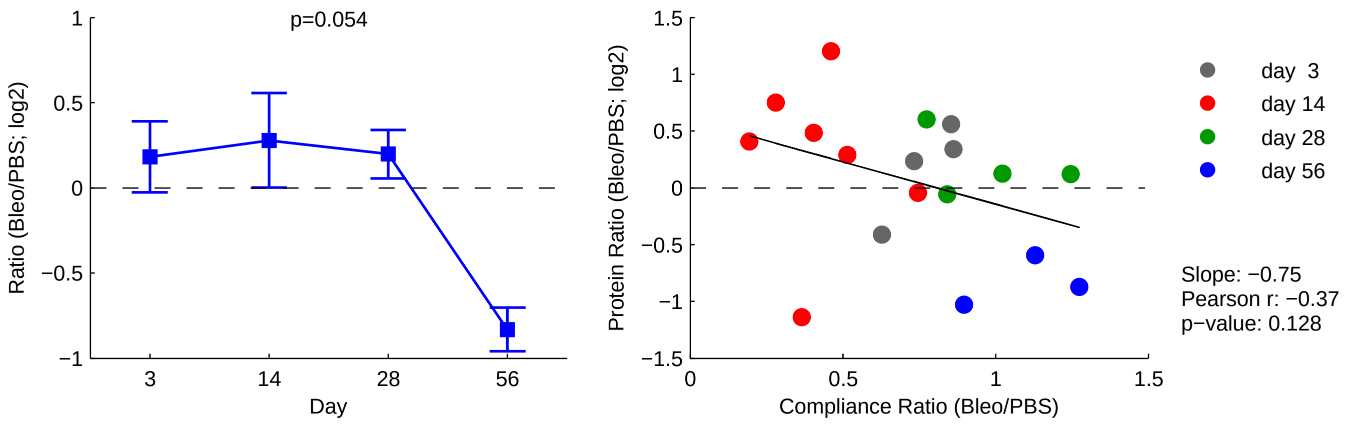

O09111 − Ndufb11 (id: 1946)

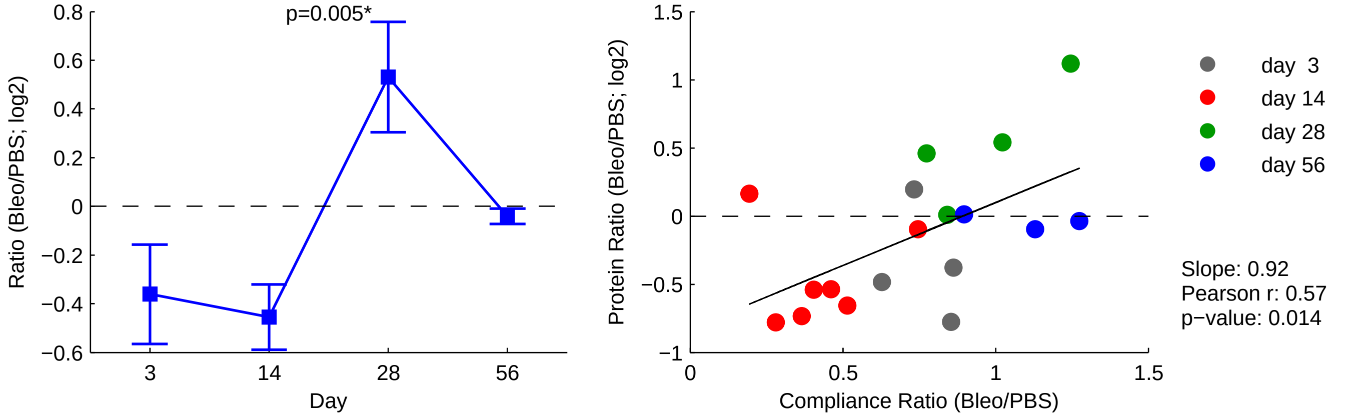

O09117−2 − Sypl (id: 1947)

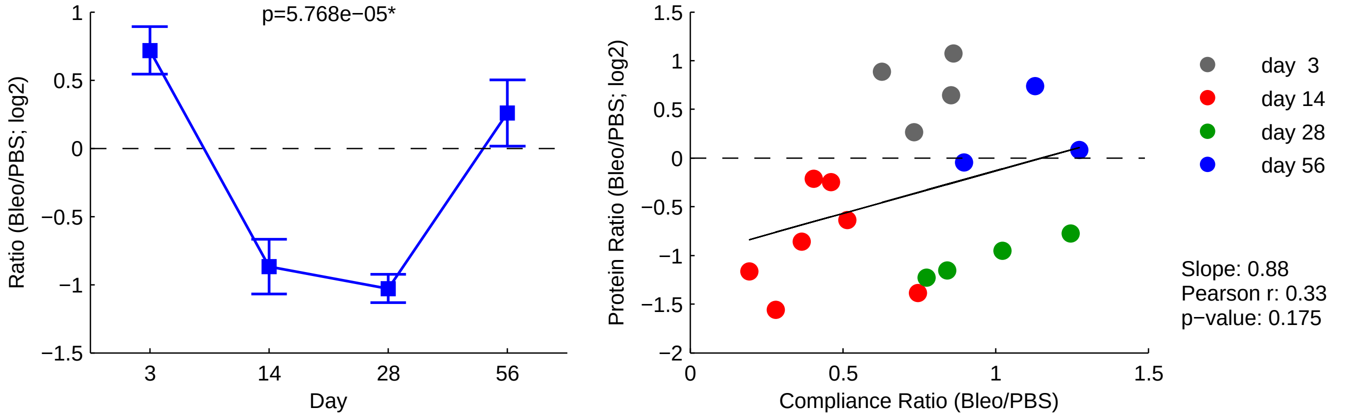

O09131 − Gsto1 (id: 1951)

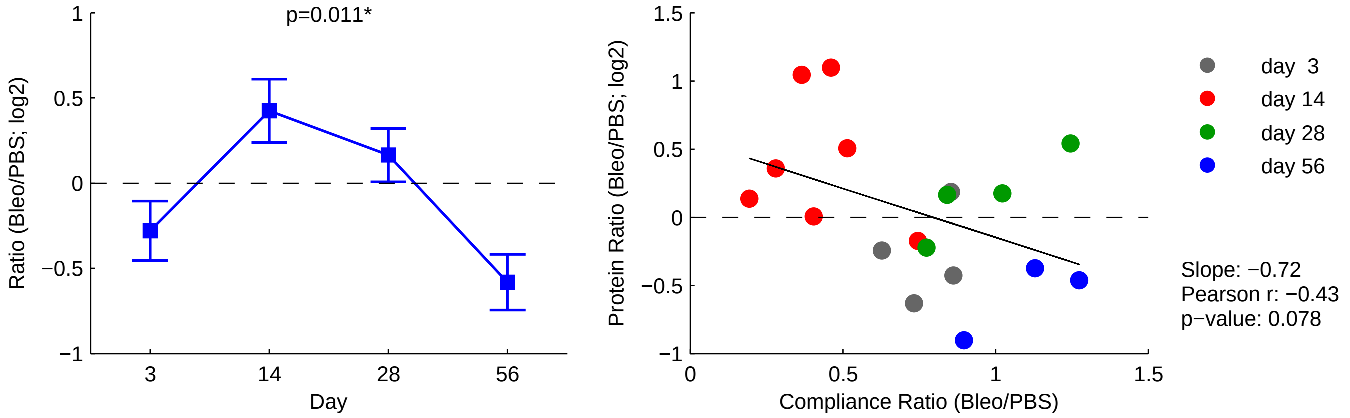

O09159 − Man2b1 (id: 1952)

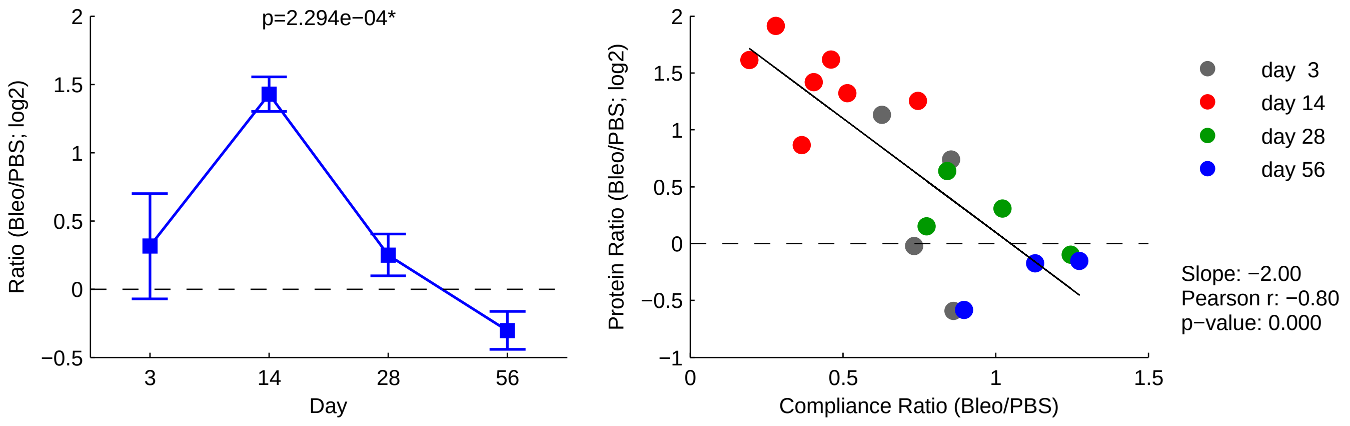

O09161 − Casq2 (id: 1953)

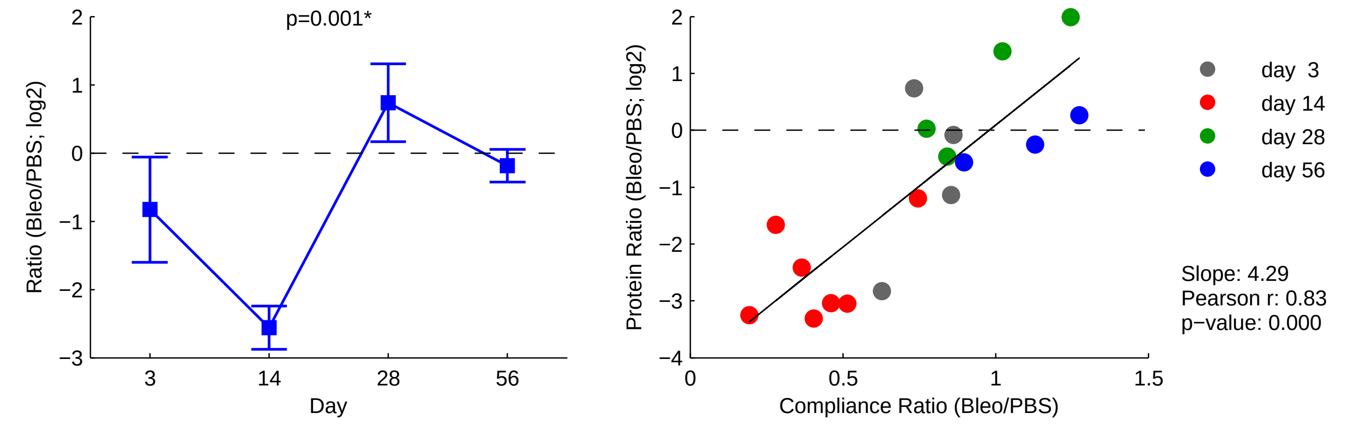

O09164 − Sod3 (id: 1954)

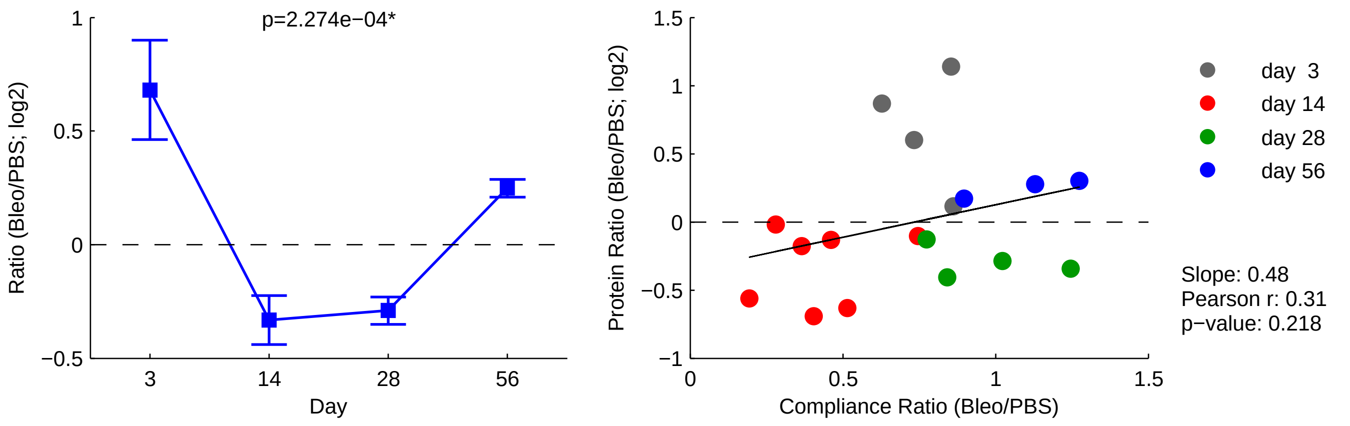

O09165 − Casq1 (id: 1955)

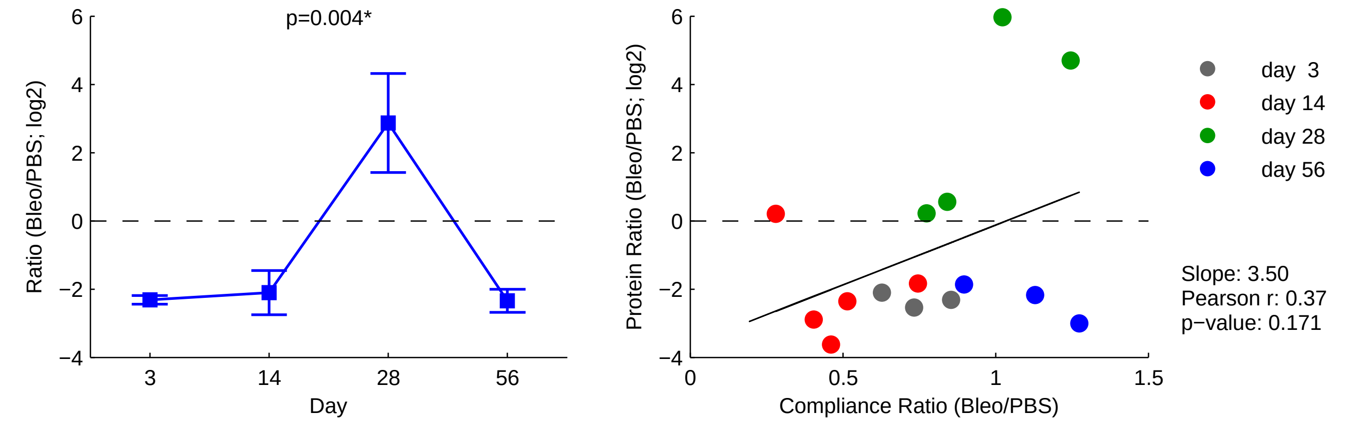

Q9CQM8 − Rpl21 (id: 1956)

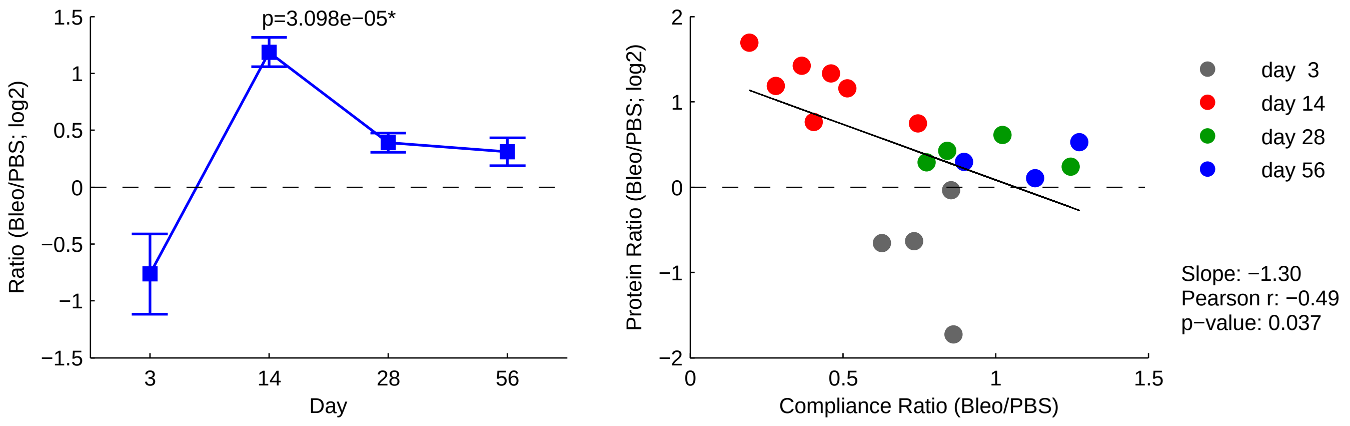

O09174 − Amacr (id: 1958)

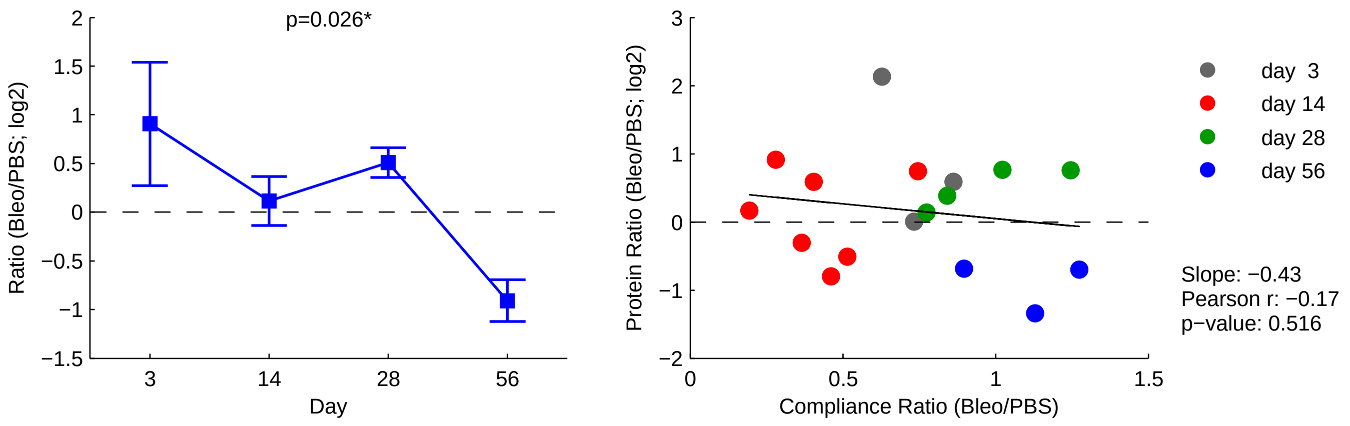

O35075 − Dscr3 (id: 1960)

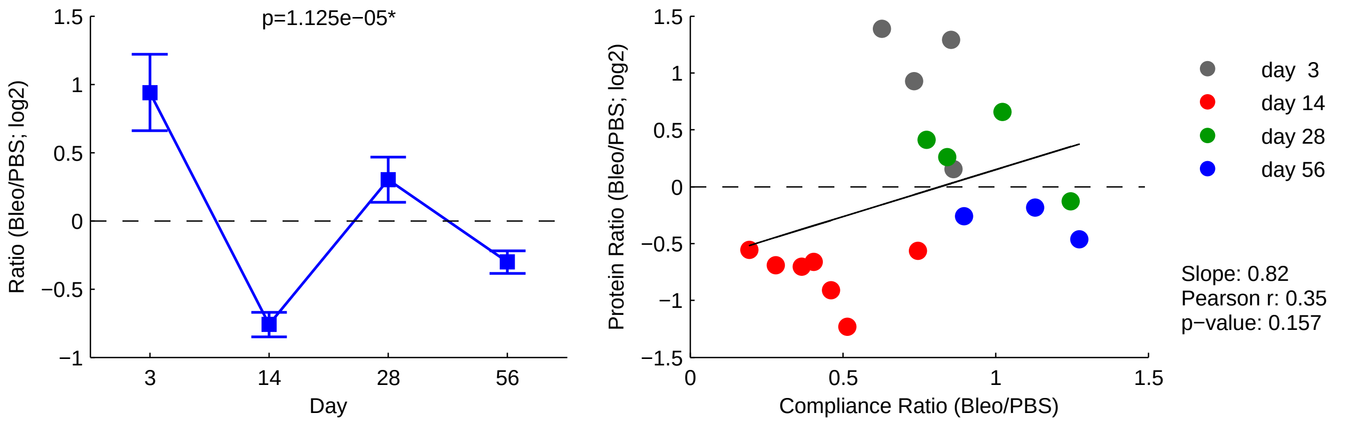

O35083 − Agpat1 (id: 1961)

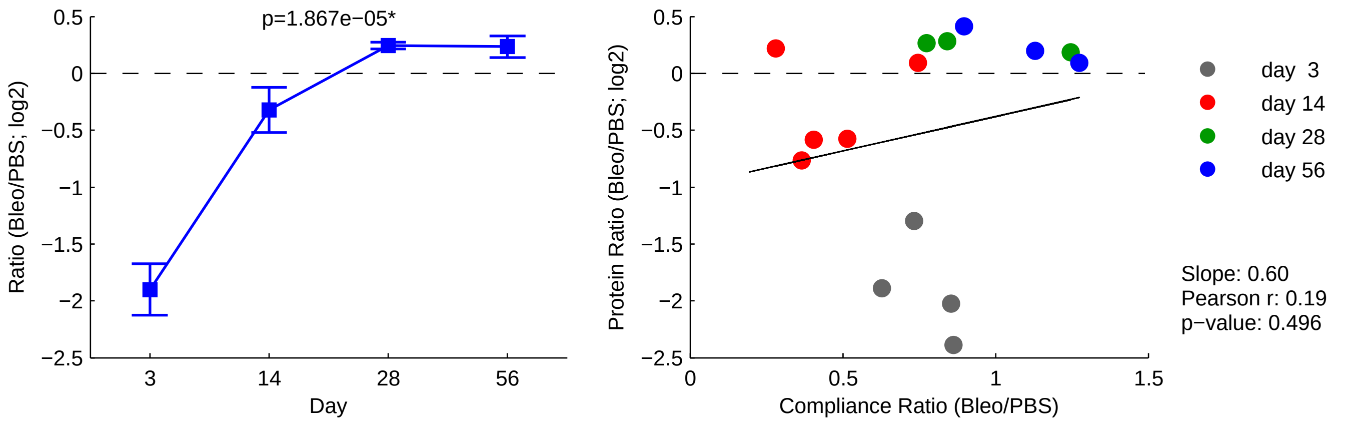

O35114 − Scarb2 (id: 1963)

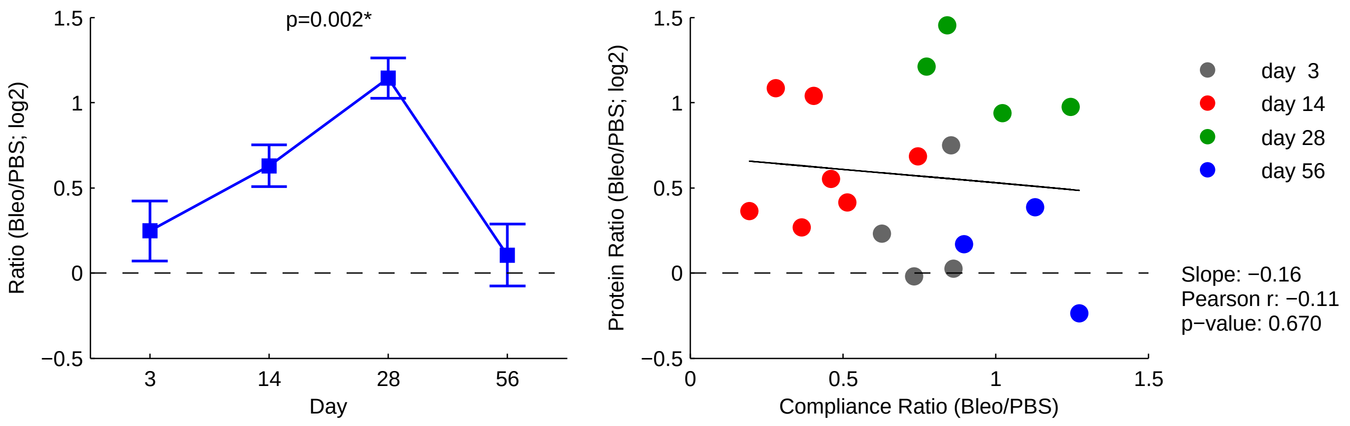

O35127 − Grcc10 (id: 1965)

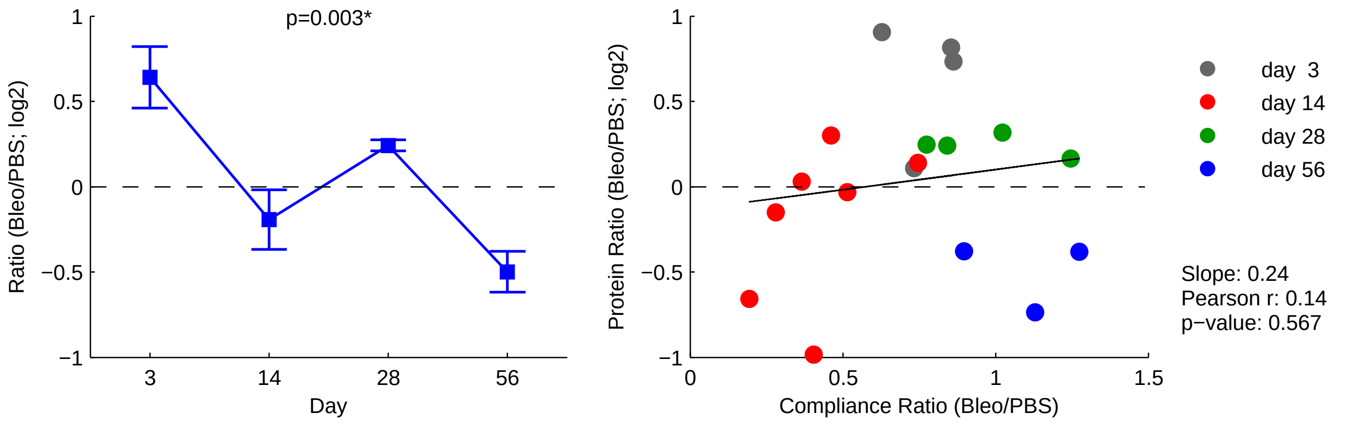

O35129 − Phb2 (id: 1966)

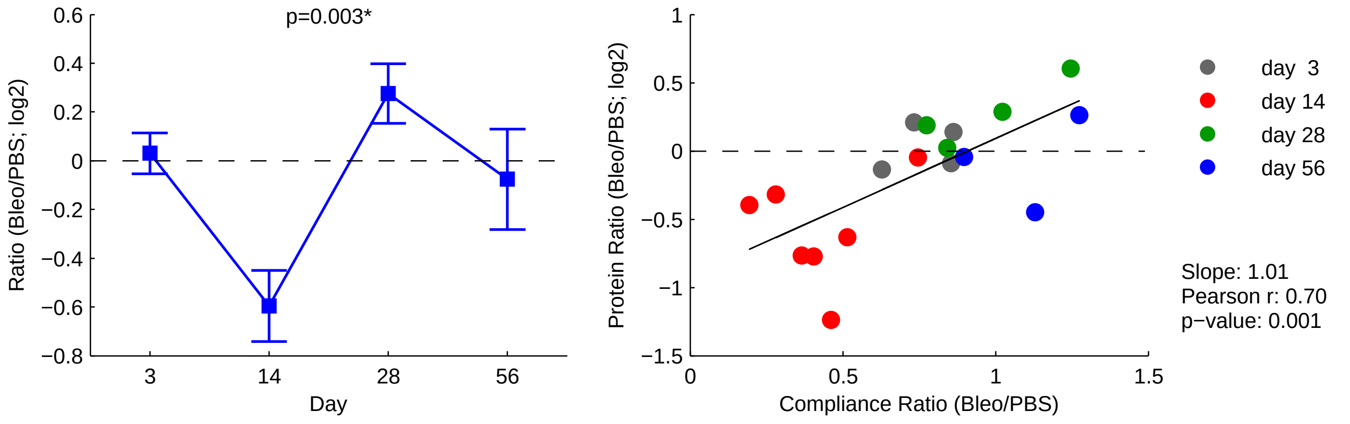

O35153 − Bet1l (id: 1969)

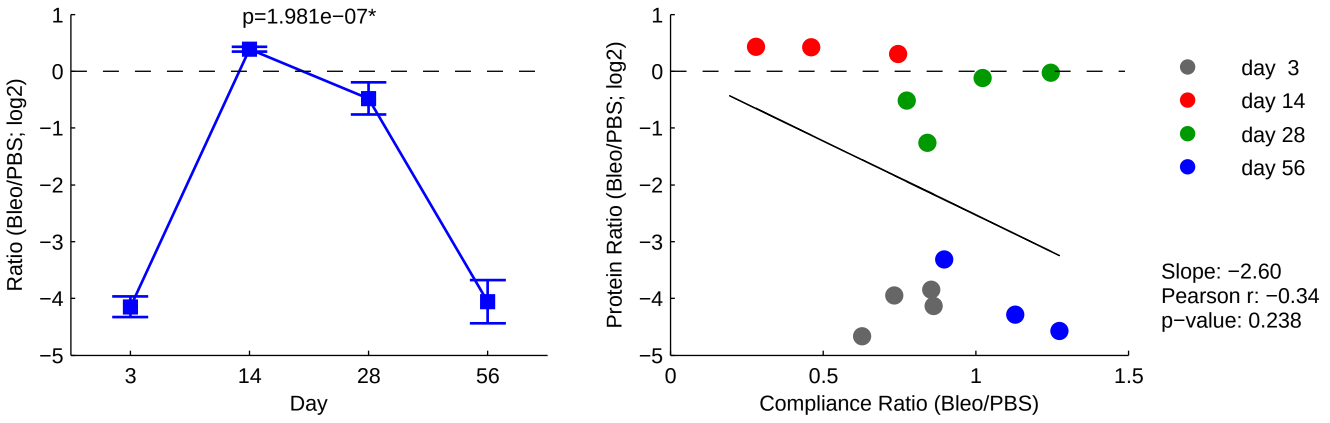

O35231 − Kifc3 (id: 1976)

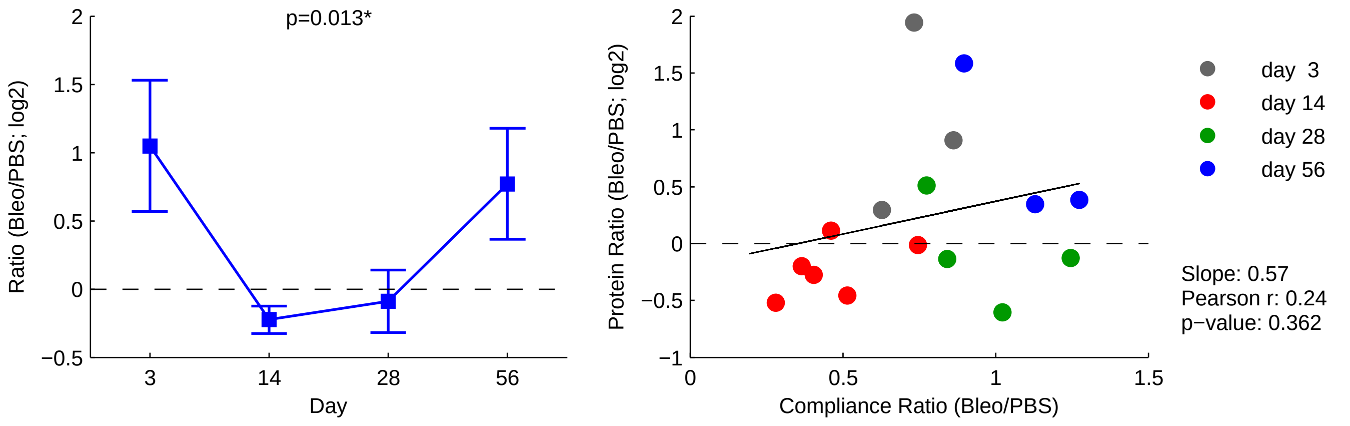

O35239 − Ptpn9 (id: 1977)

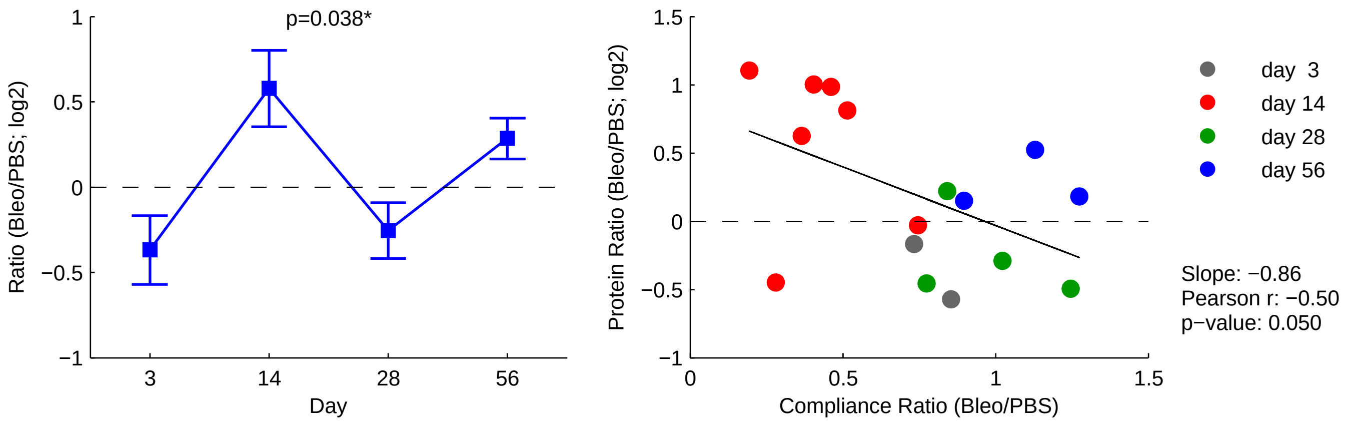

O35286 − Dhx15 (id: 1979)

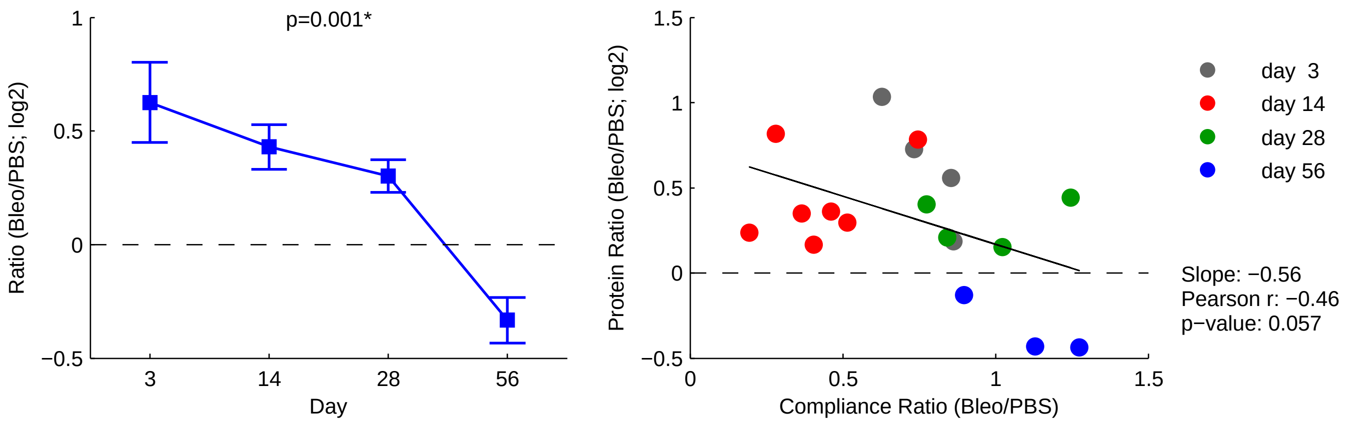

O35309 − Nmi (id: 1981)

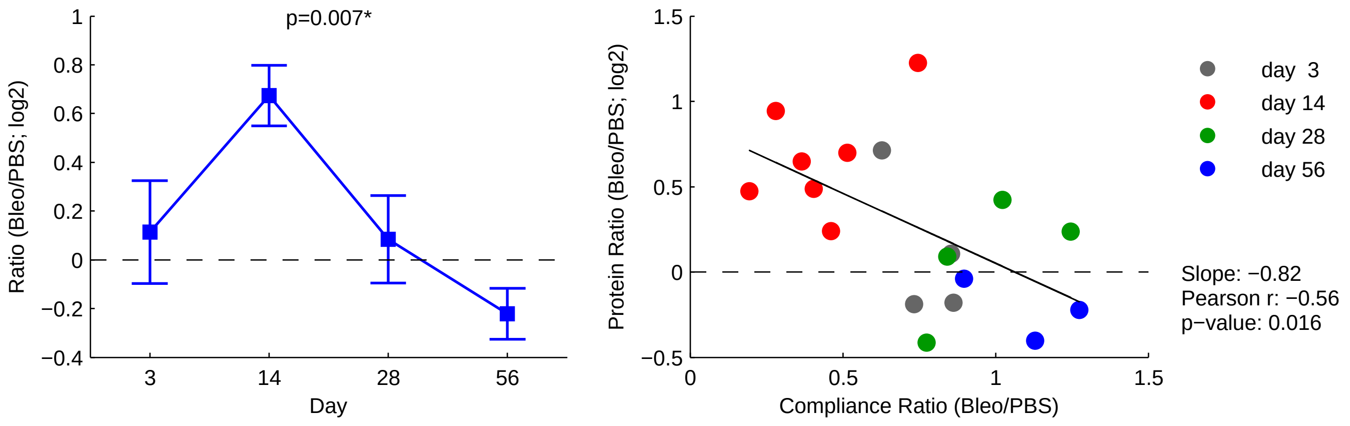

O35326 − Srsf5 (id: 1982)

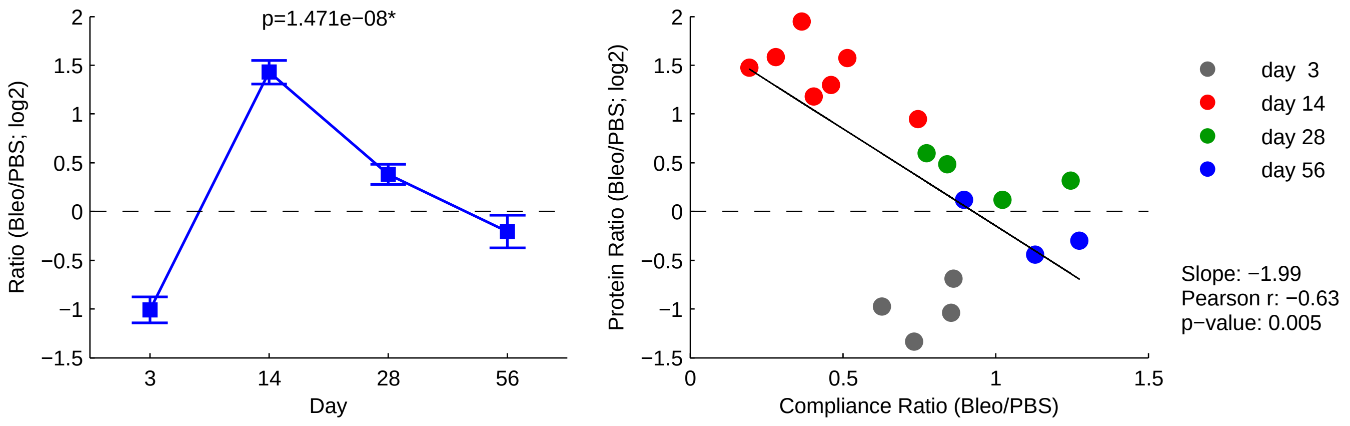

O35344 − Kpna3 (id: 1984)

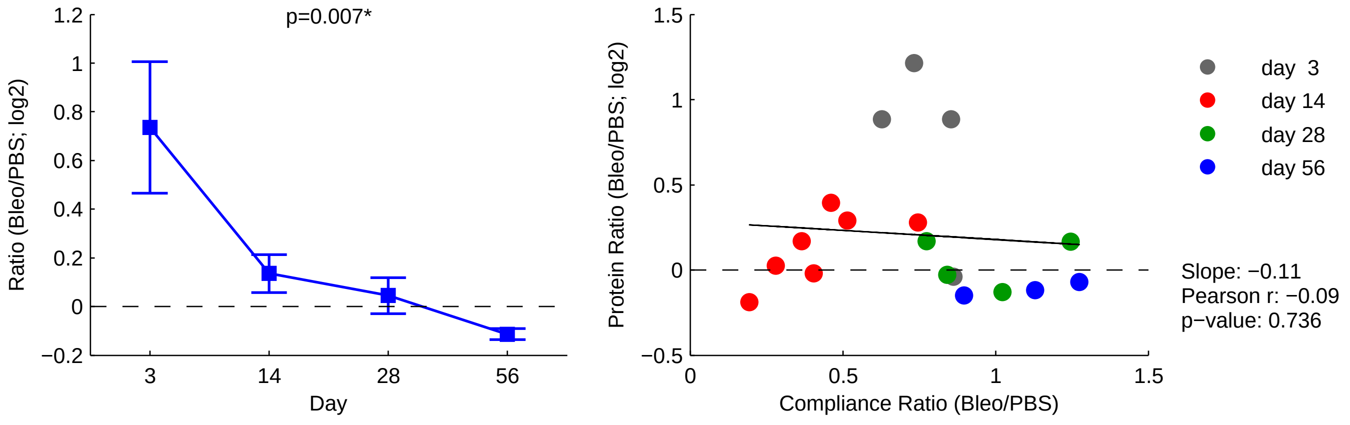

O35345 − Kpna6 (id: 1985)

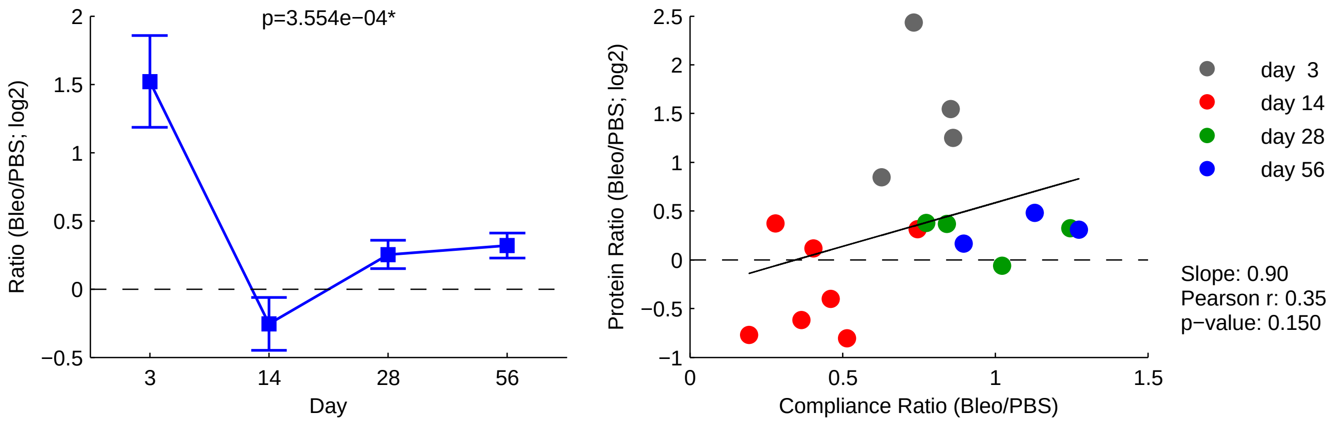

O35368 − Ifi203 (id: 1987)

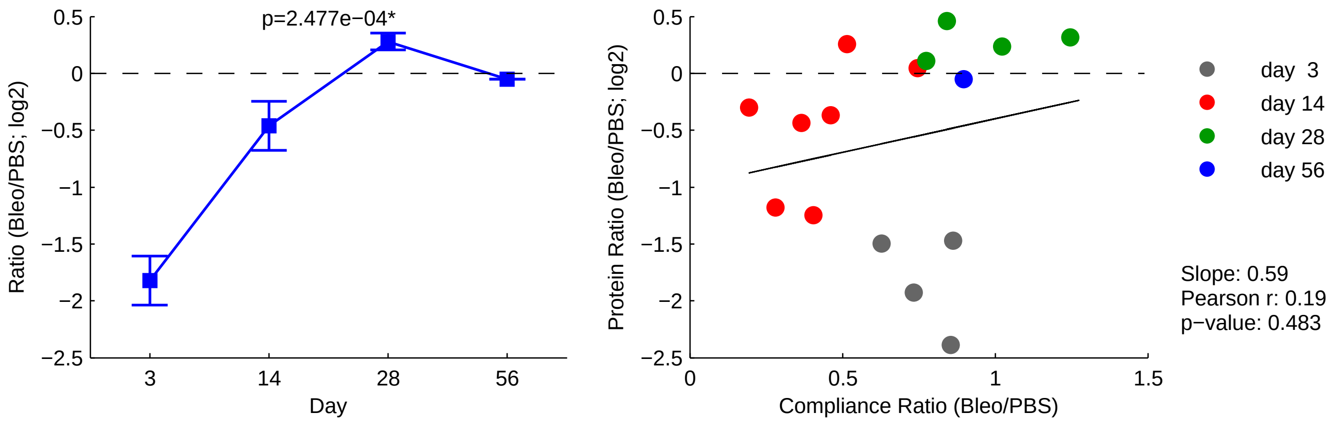

O35381 − Anp32a (id: 1990)

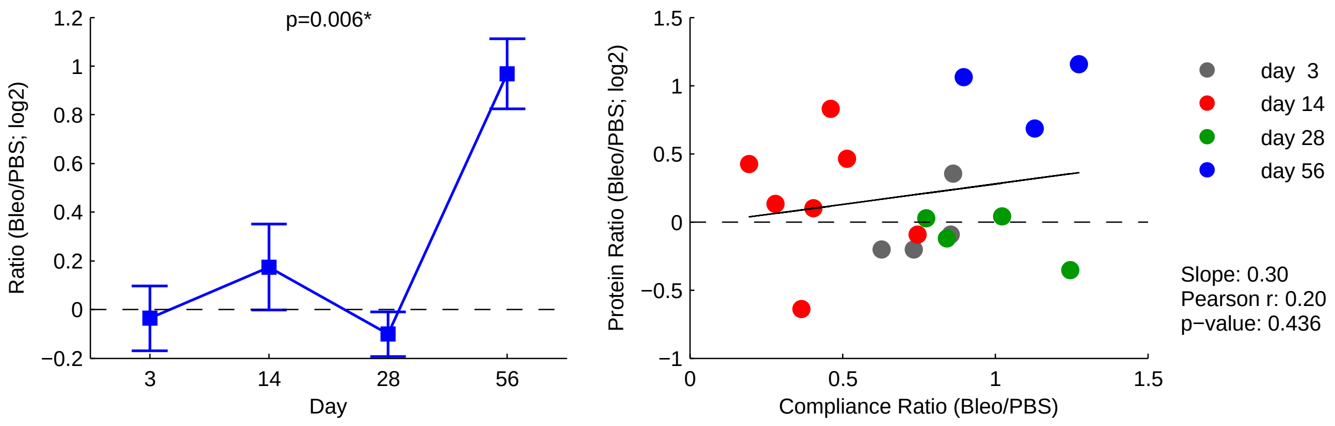

O35405 − Pld3 (id: 1993)

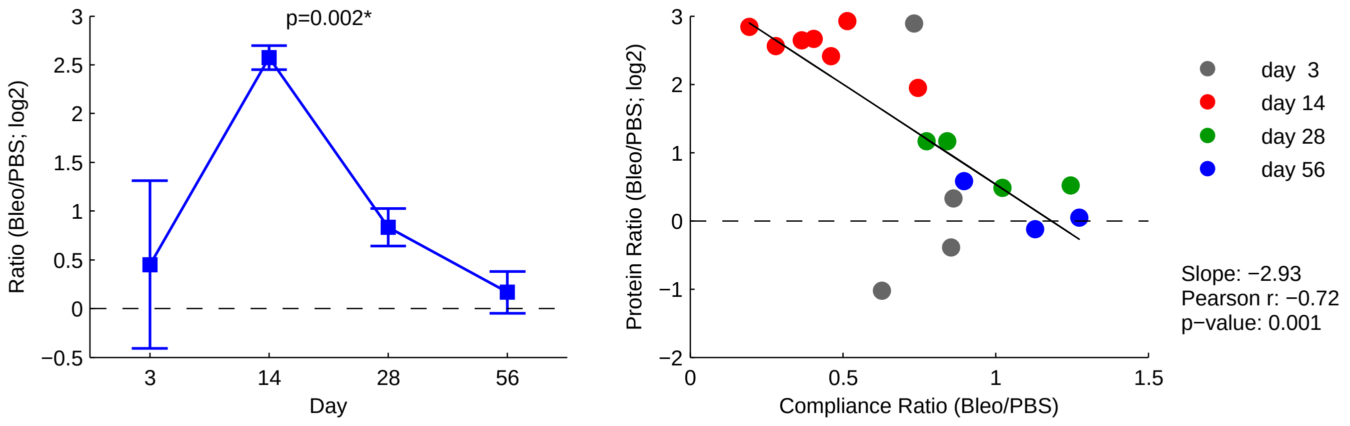

O35435 − Dhodh (id: 1995)

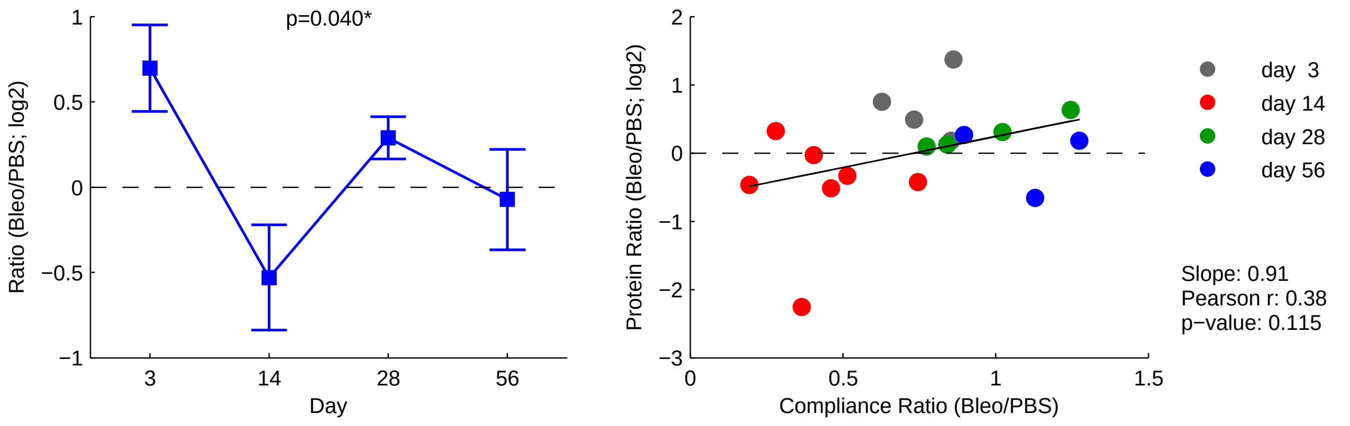

O35452 − Tnxb (id: 1996)

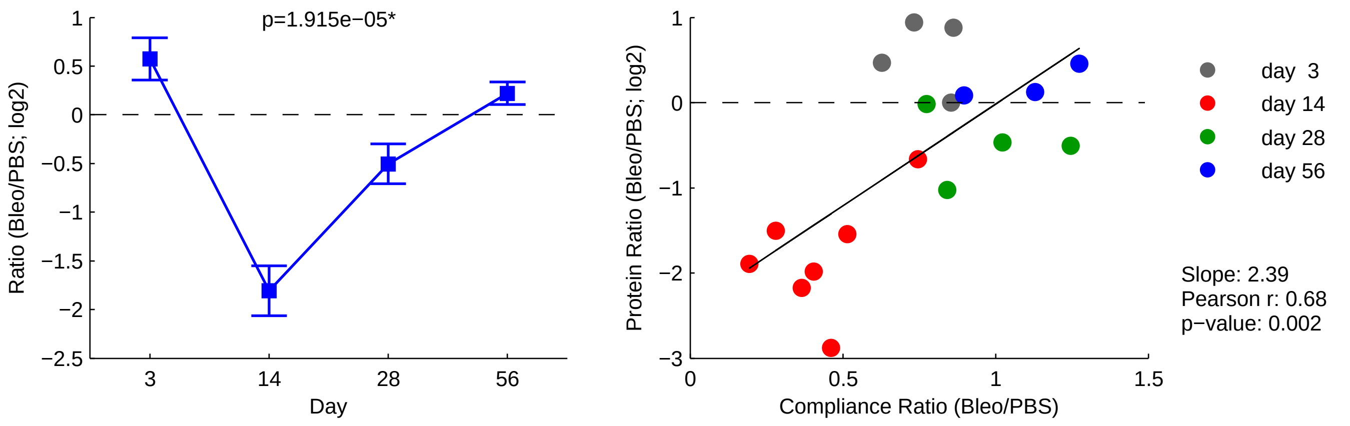

O35459 − Ech1 (id: 1997)

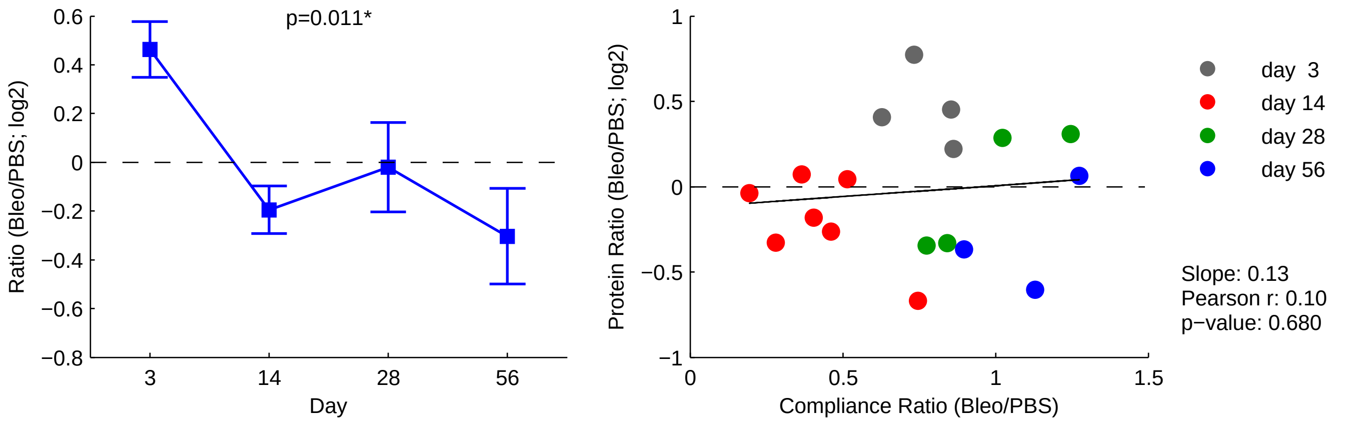

O35465−2 − Fkbp8 (id: 1998)

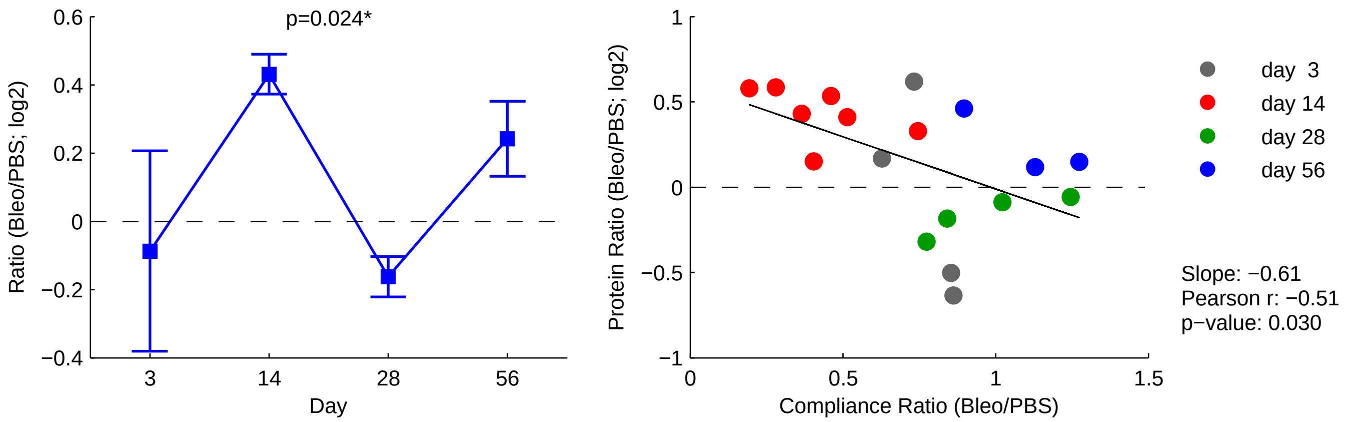

O35488 − Slc27a2 (id: 1999)

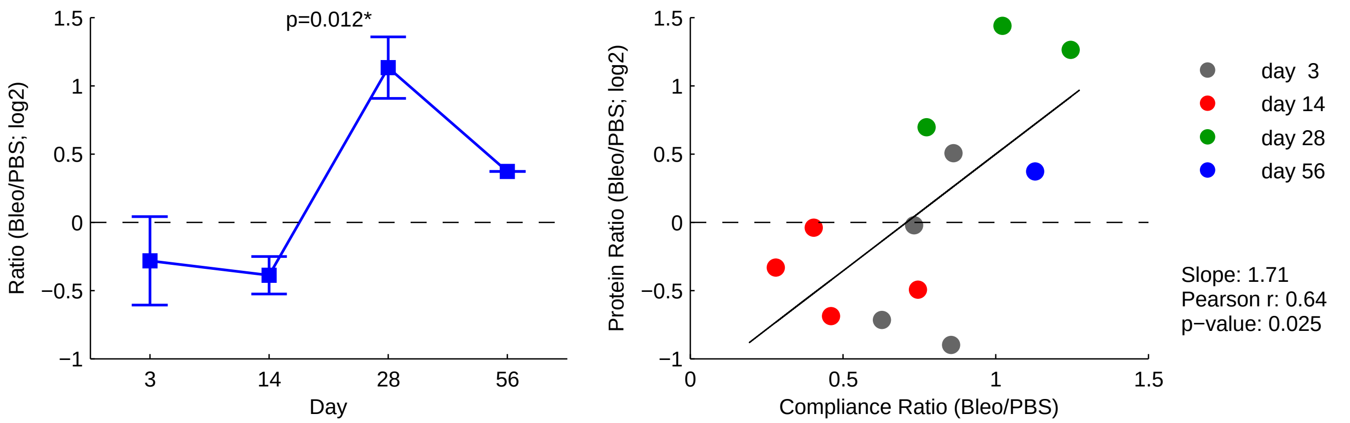

O35566 − Cd151 (id: 2002)

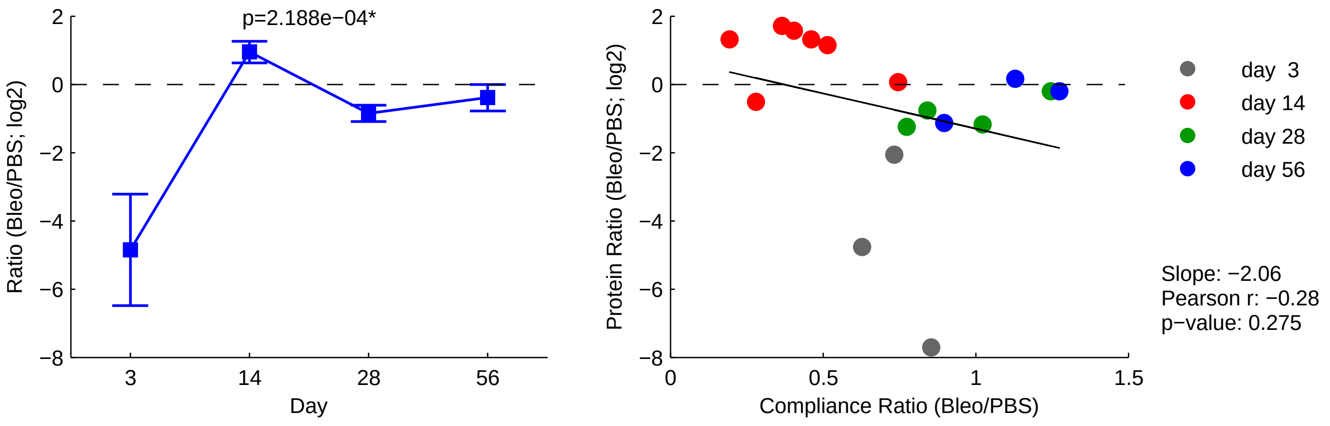

O35593 − Psmd14 (id: 2003)

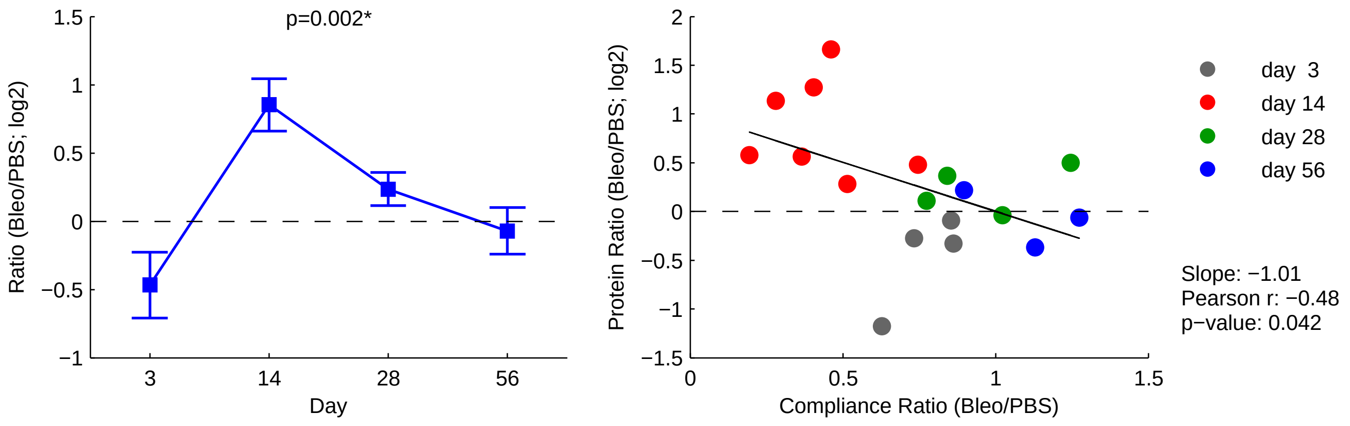

O35598 − Adam10 (id: 2005)

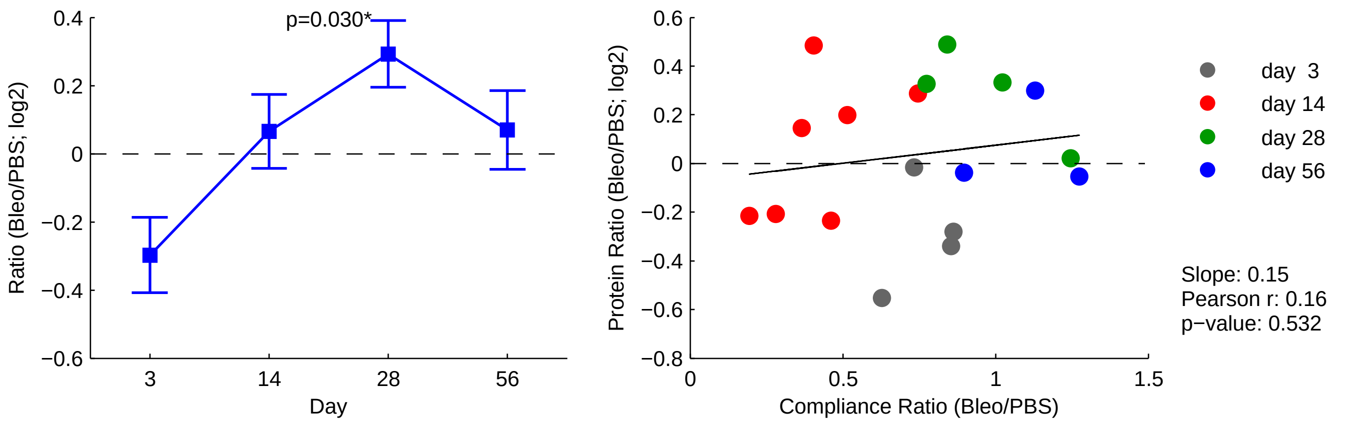

O35601−2 − Fyb (id: 2007)

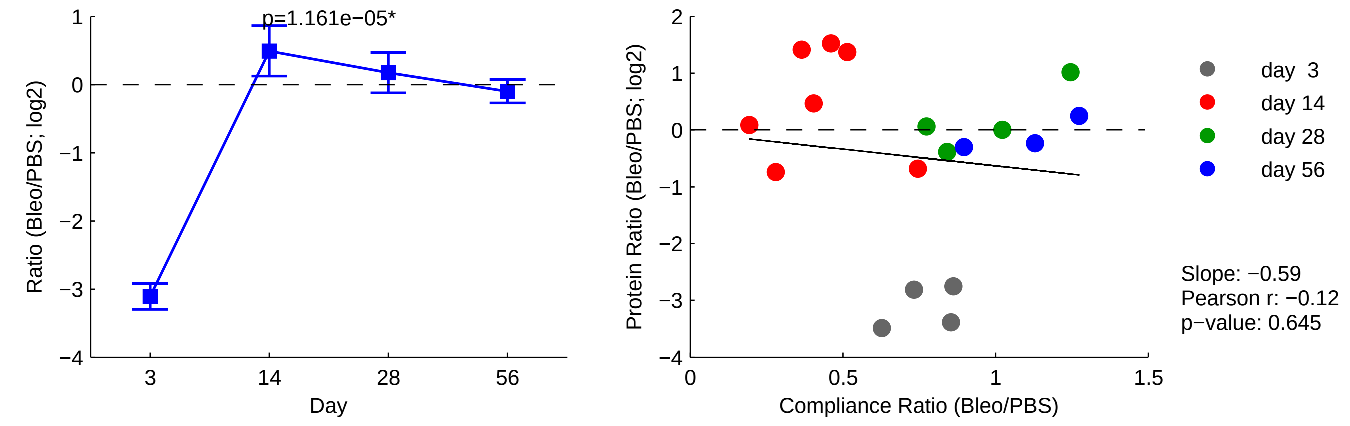

O35604 − Npc1 (id: 2008)

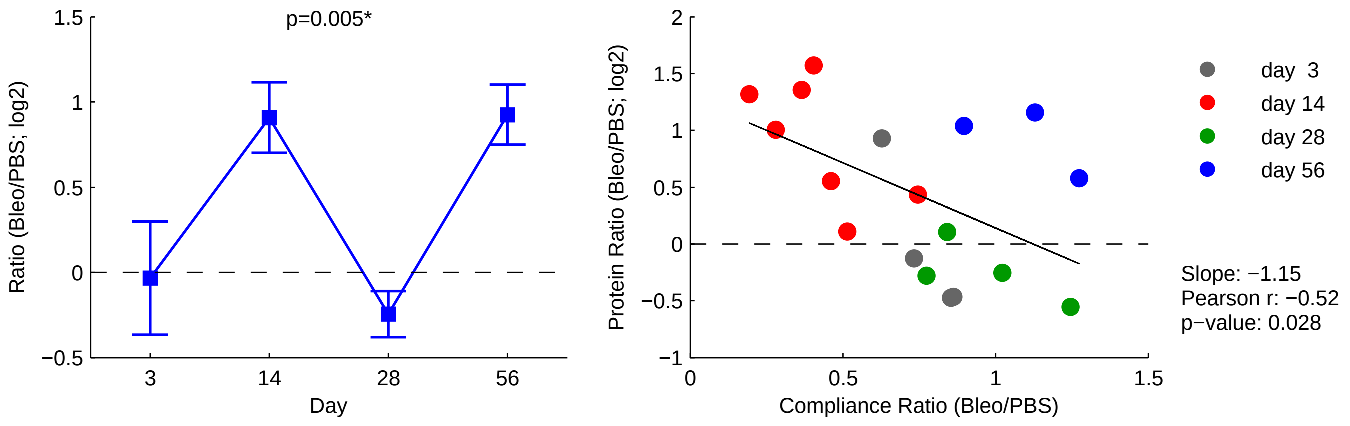

O35608 − Angpt2 (id: 2010)

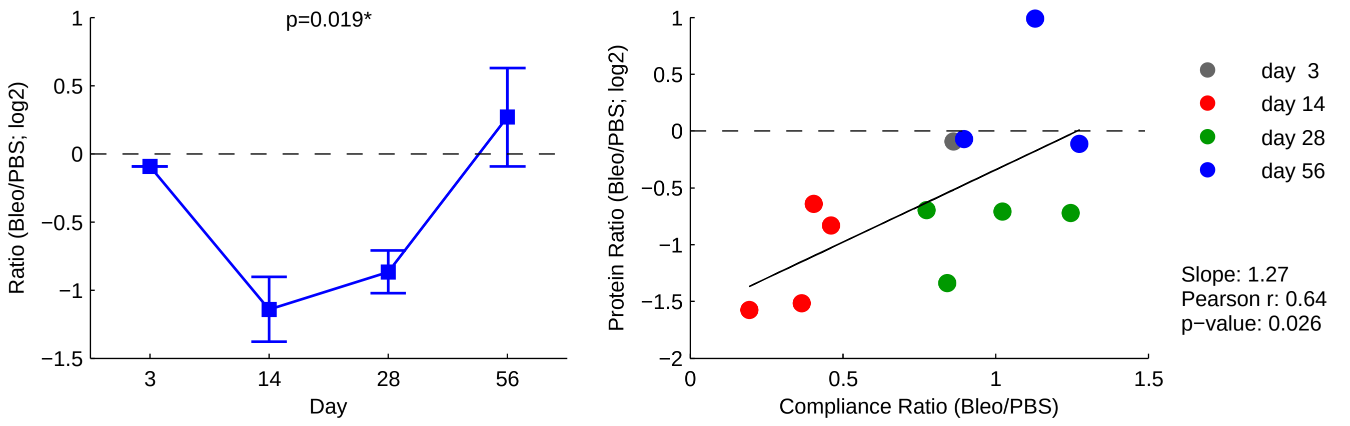

O35623 − Bet1 (id: 2011)

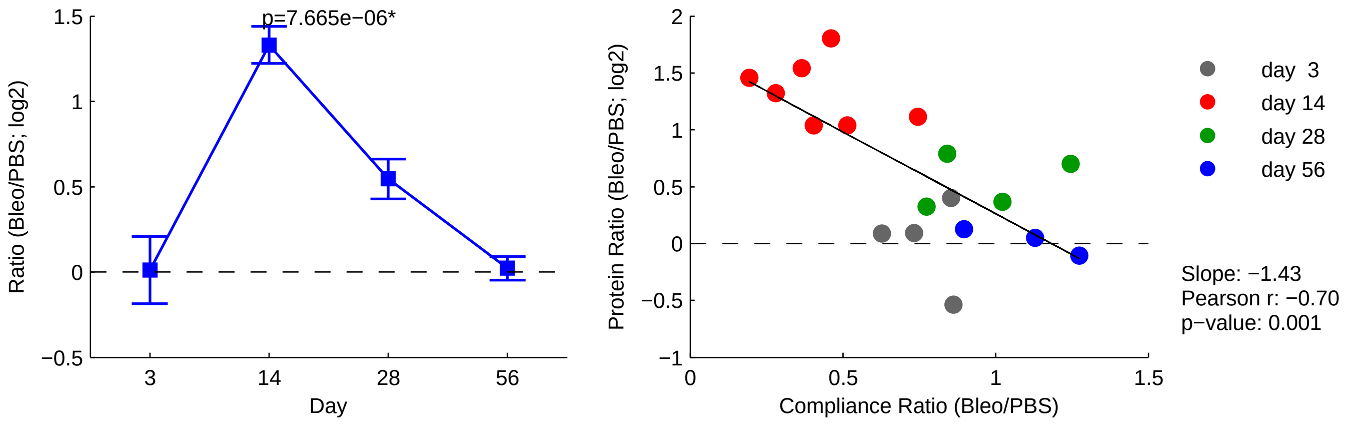

O35632 − Hyal2 (id: 2012)

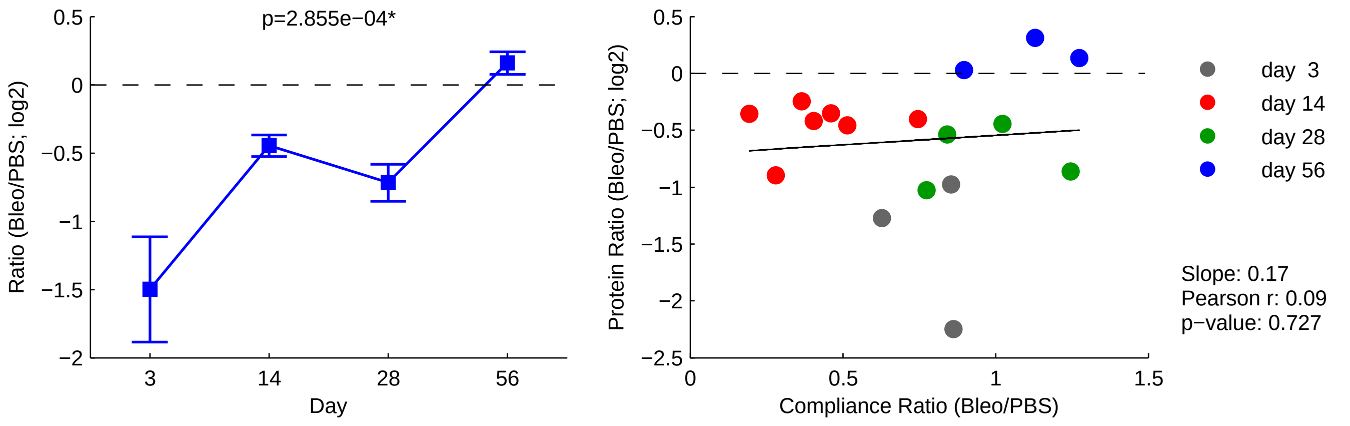

O35643 − Ap1b1 (id: 2015)

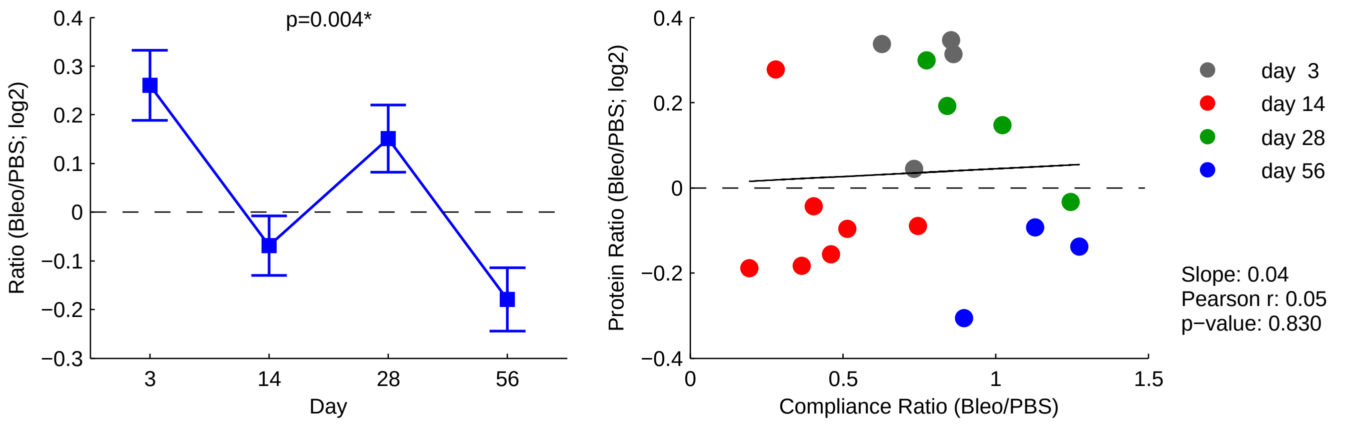

O35682 − Myadm (id: 2020)

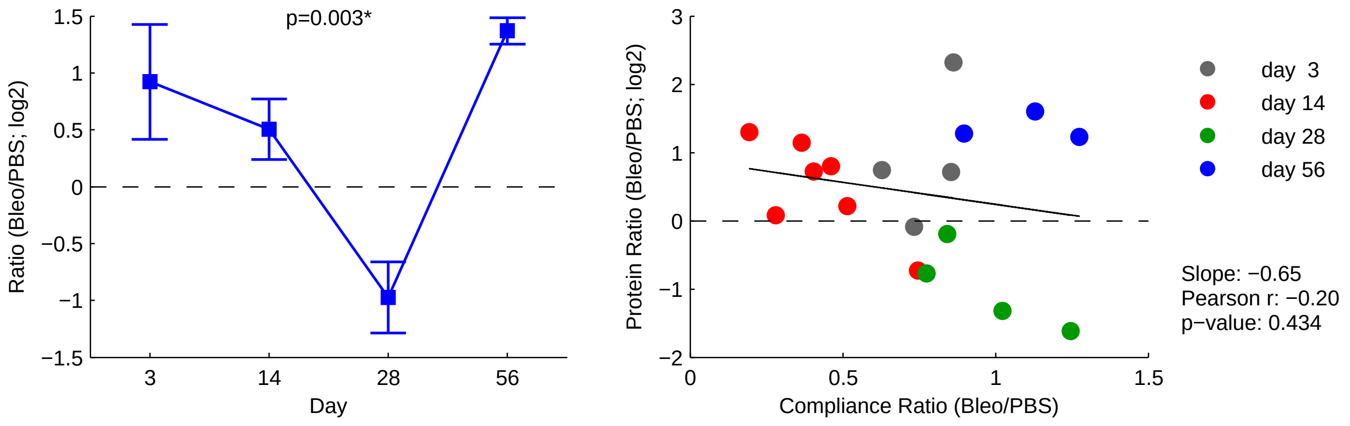

O35691 − Pnn (id: 2023)

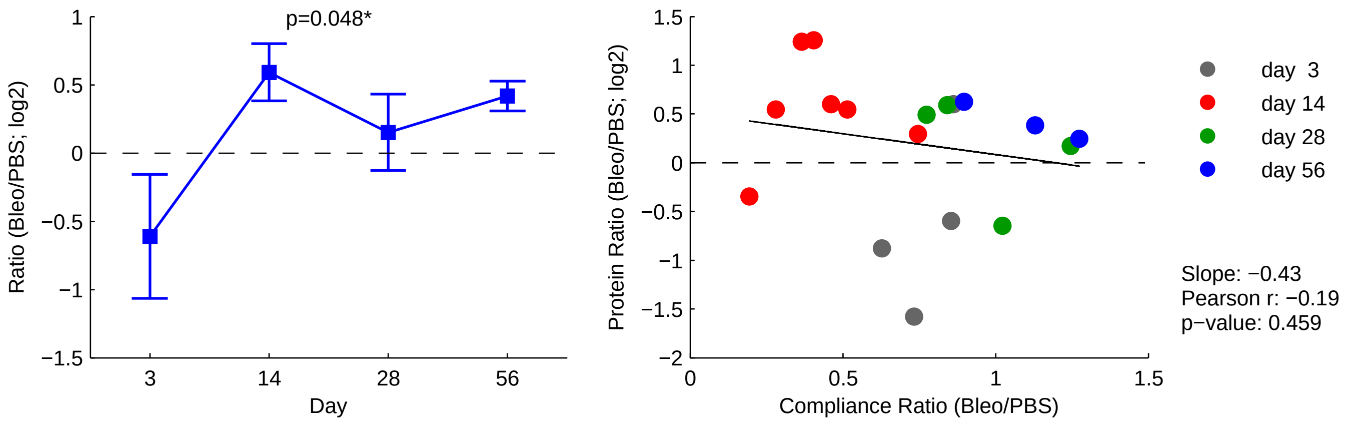

O35704 − Sptlc1 (id: 2025)

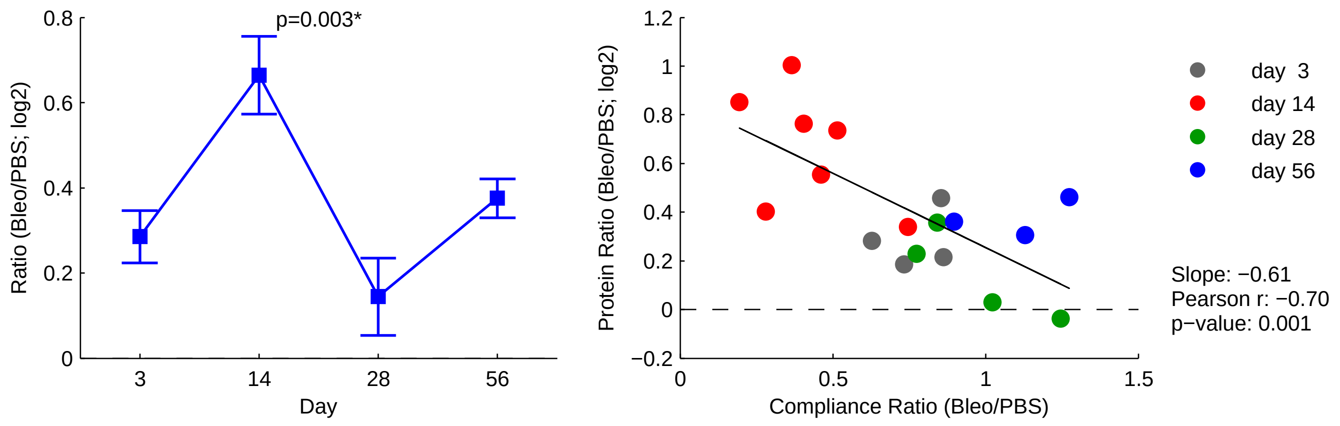

O35737 − Hnrnph1 (id: 2027)

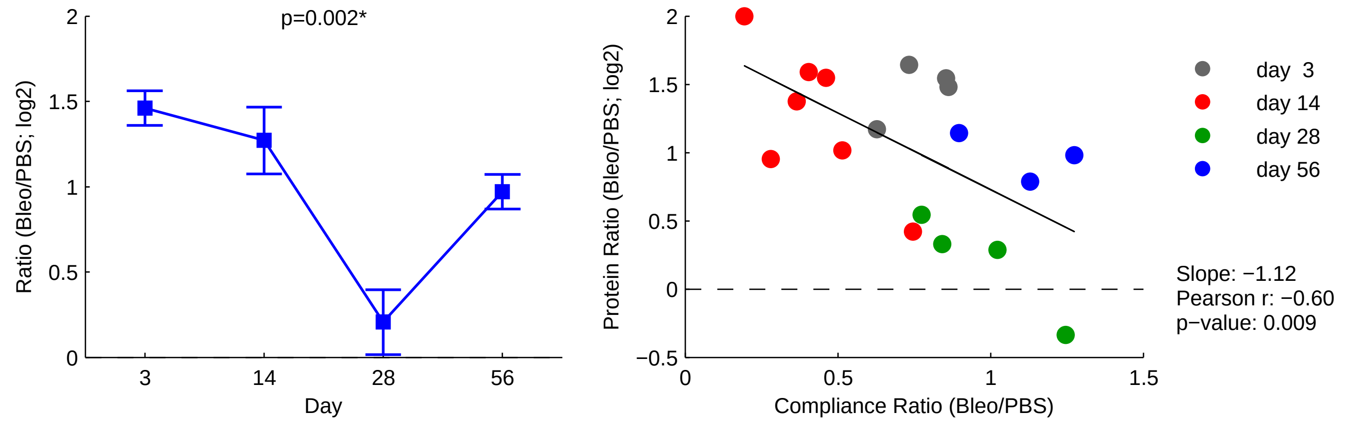

O35841 − Api5 (id: 2029)

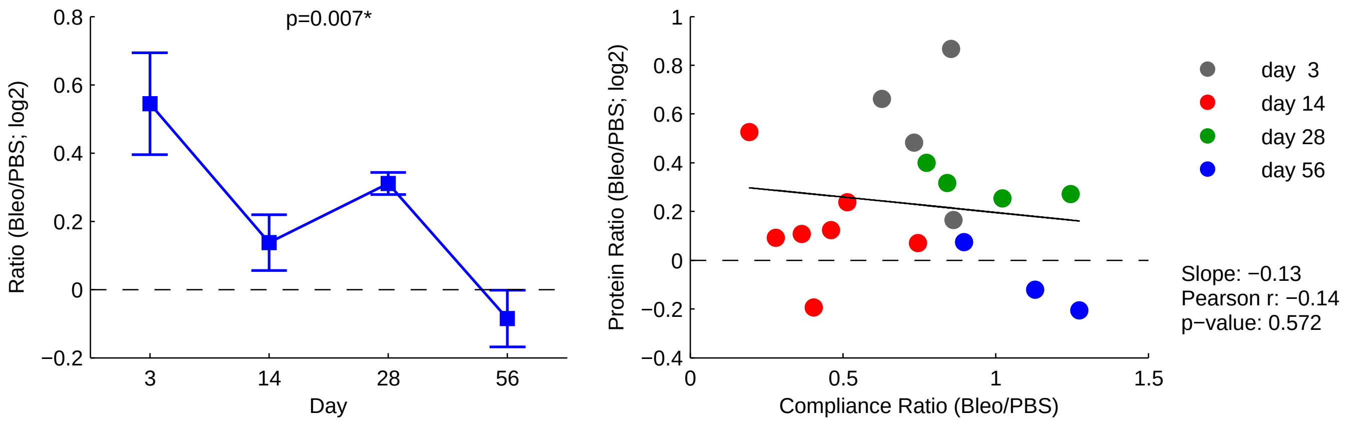

O35857 − Timm44 (id: 2030)

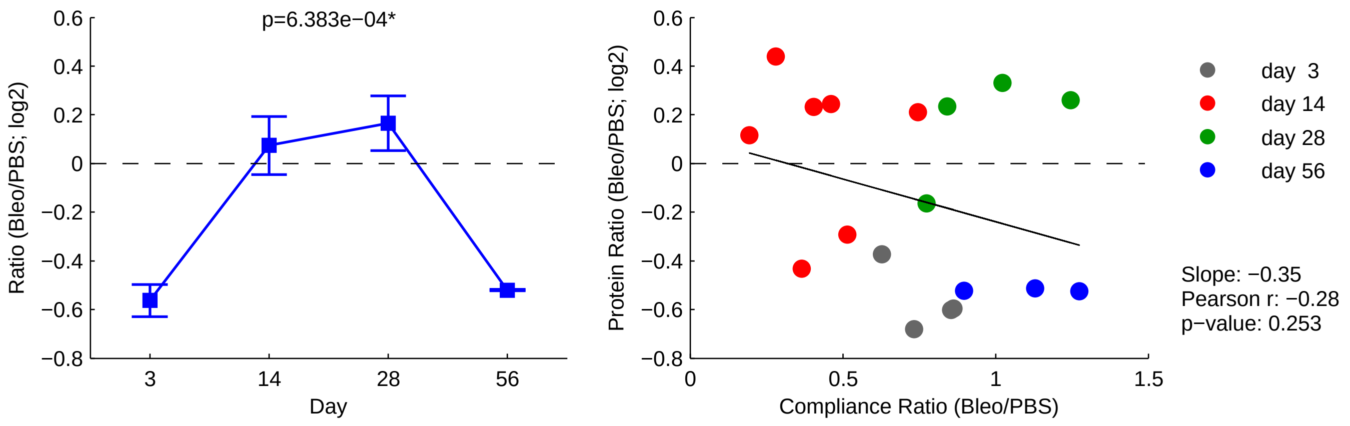

Q5SSP3 − Slc1a4 (id: 2032)

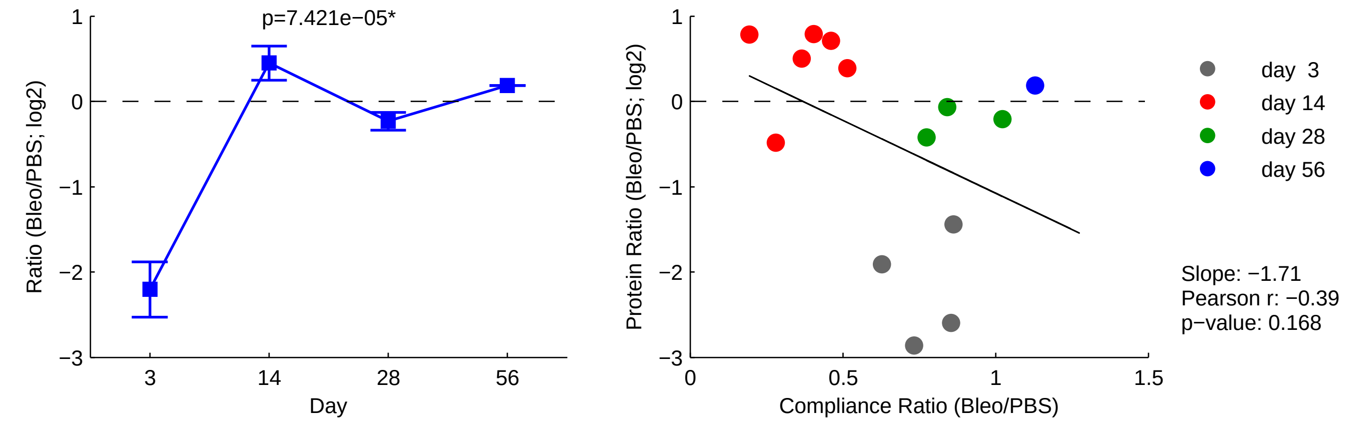

O35887 − Calu (id: 2033)

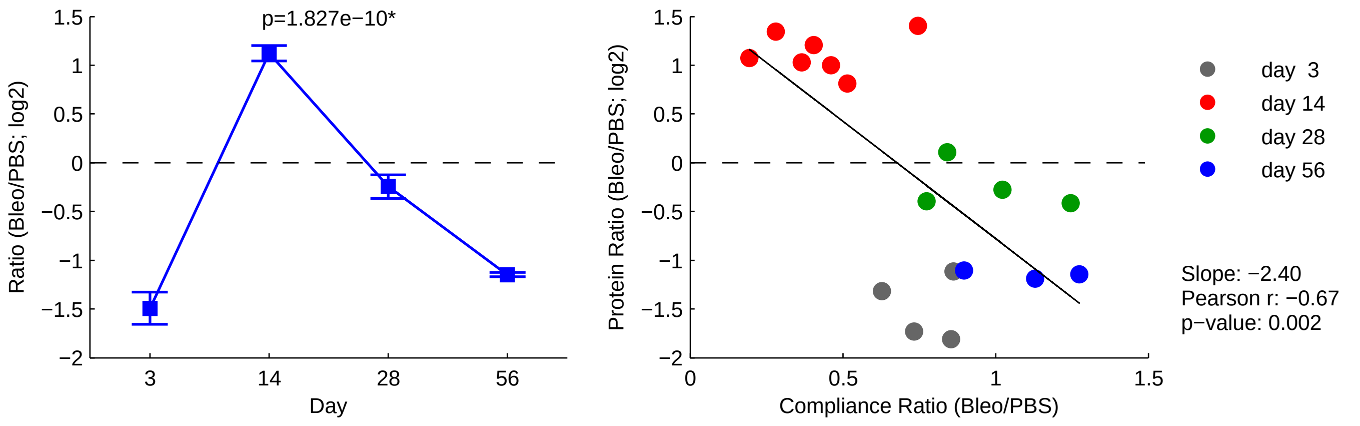

O35900 − Lsm2 (id: 2034)

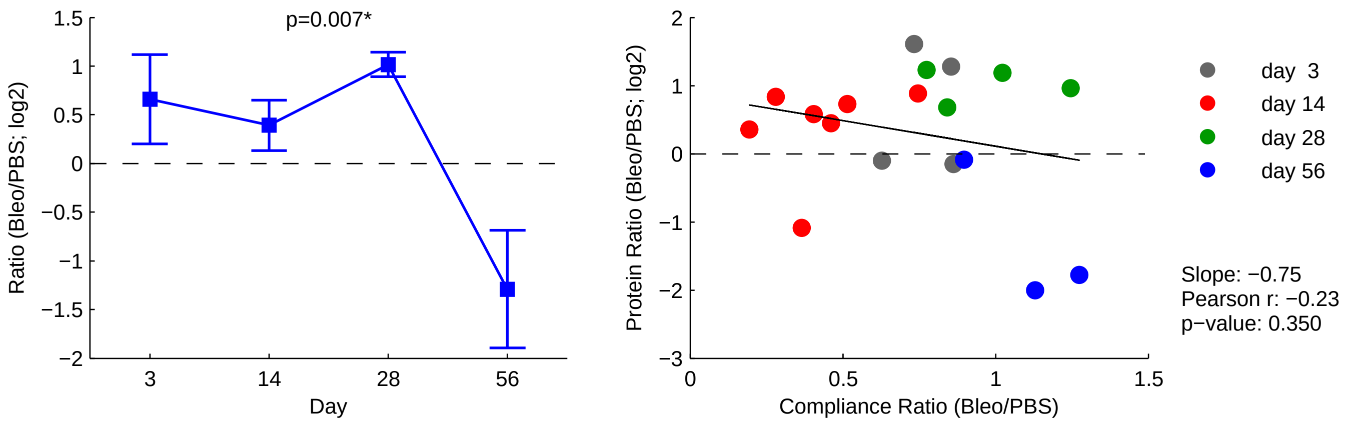

O35930 − Gp1ba (id: 2036)

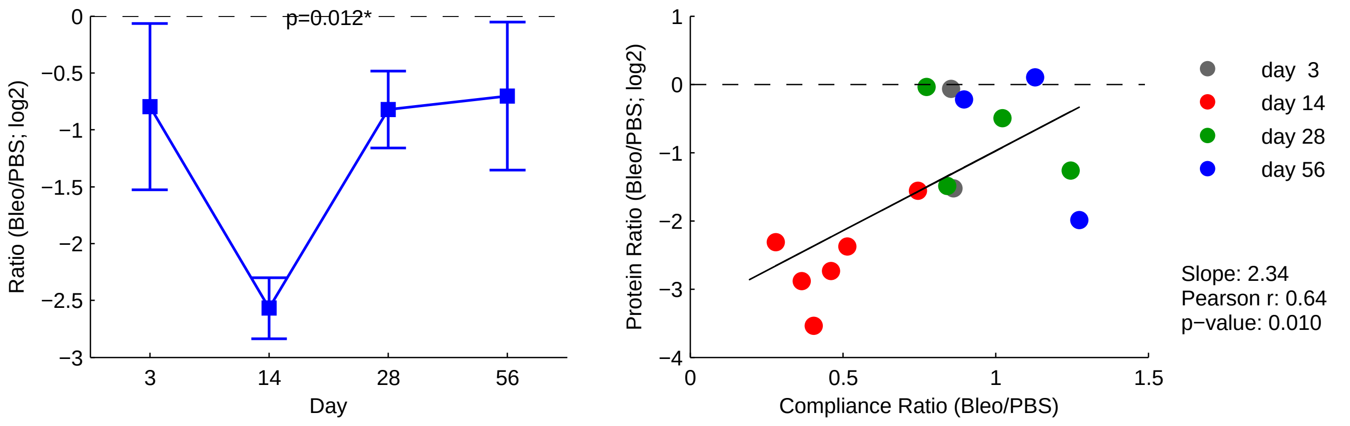

O35943 − Fxn (id: 2037)

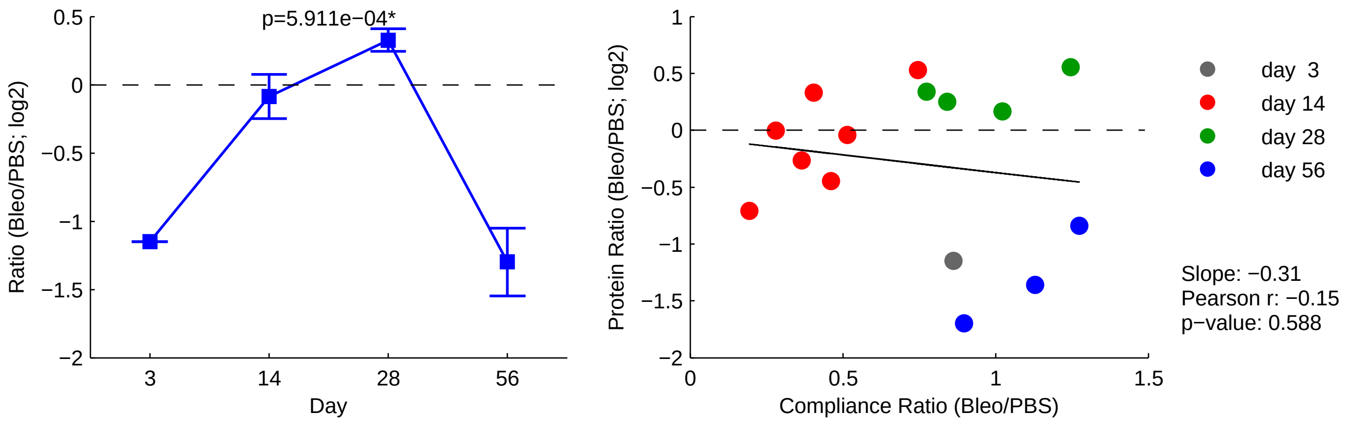

O35963 − Rab33b (id: 2041)

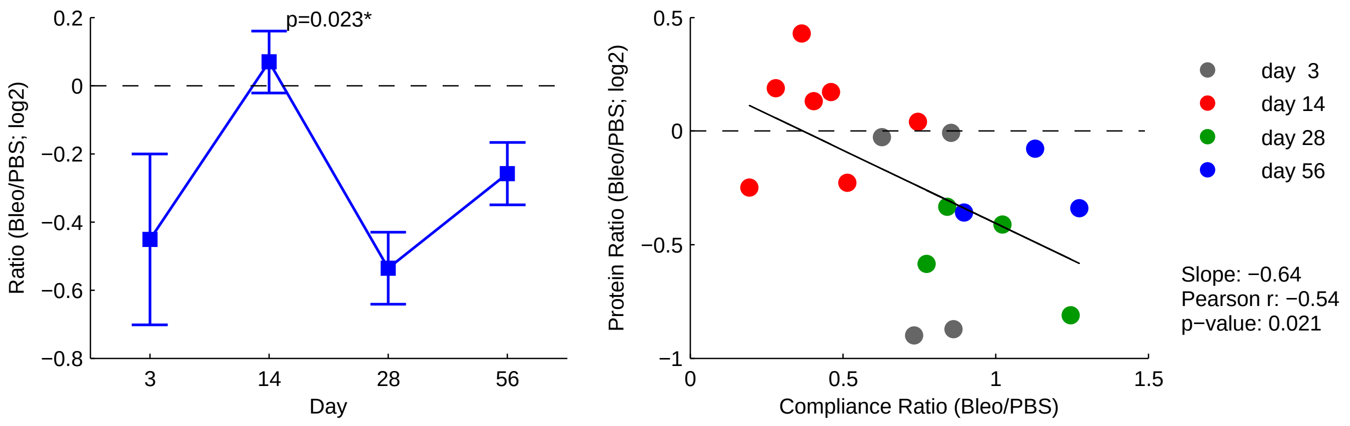

O35972 − Mrpl23 (id: 2042)

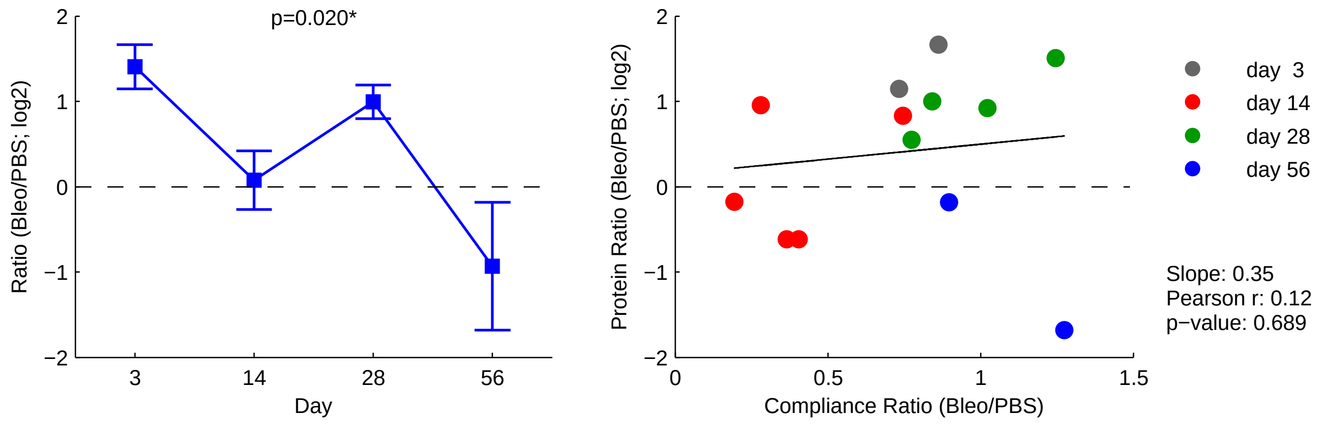

O54774 − Ap3d1 (id: 2048)

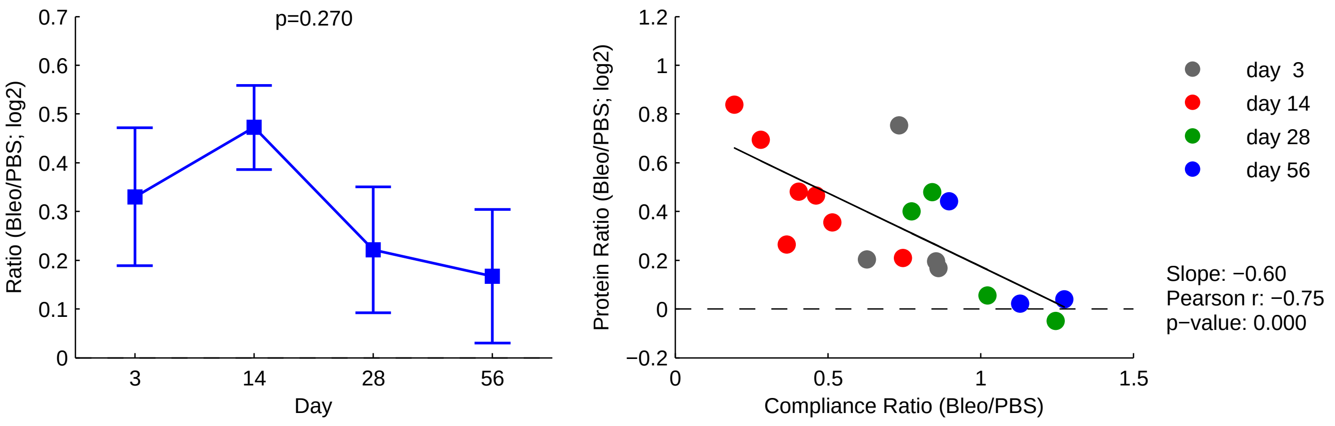

O54782 − Man2b2 (id: 2050)

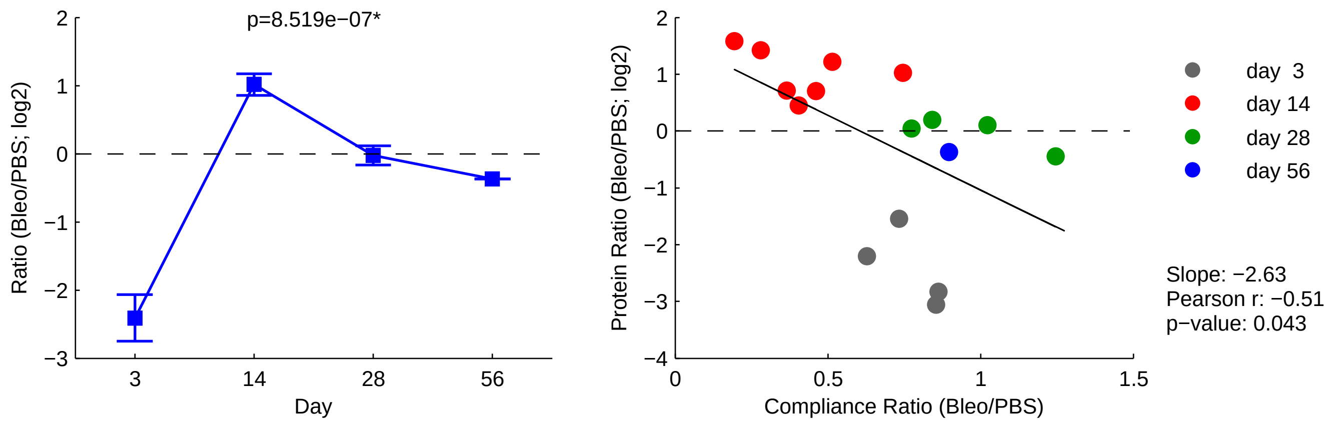

O54784 − Dapk3 (id: 2051)

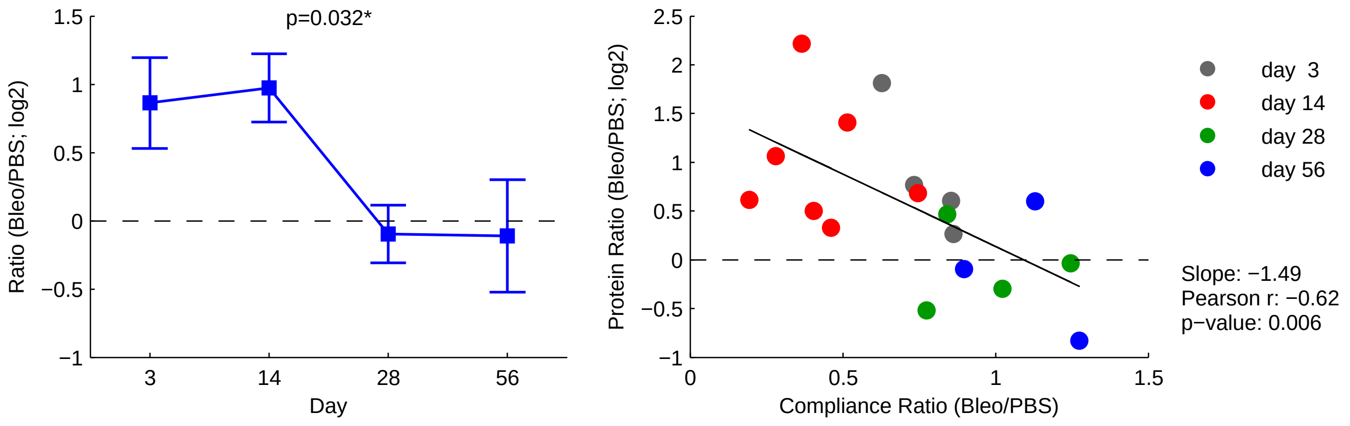

O54785 − Limk2 (id: 2052)

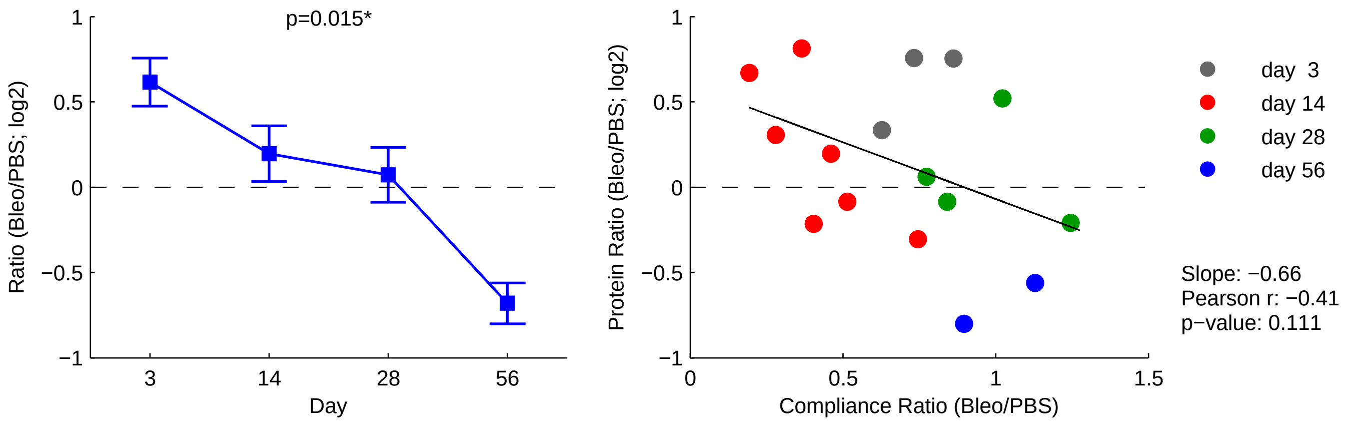

O54791 − Maff (id: 2056)

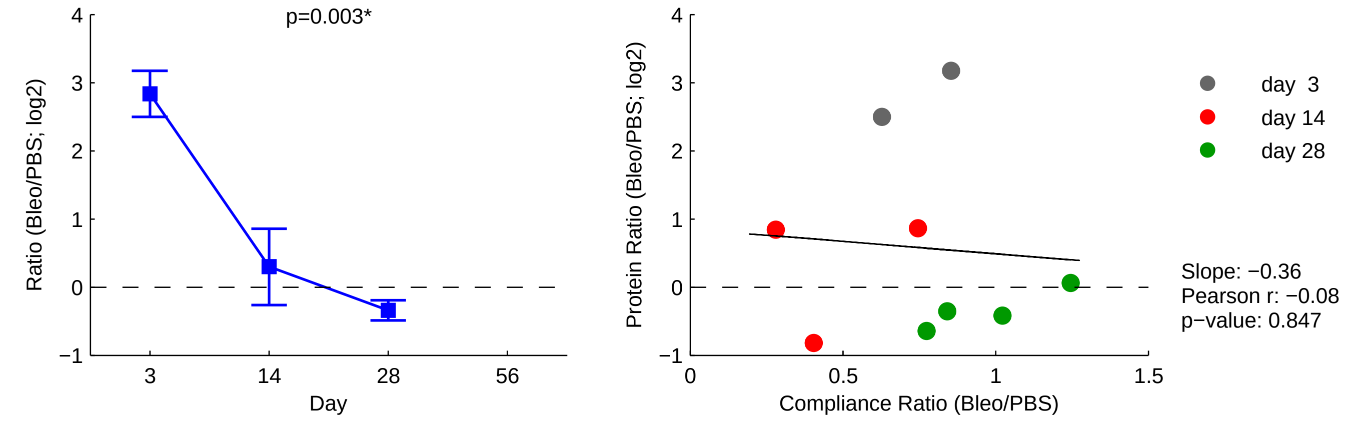

O54825 − Bysl (id: 2058)

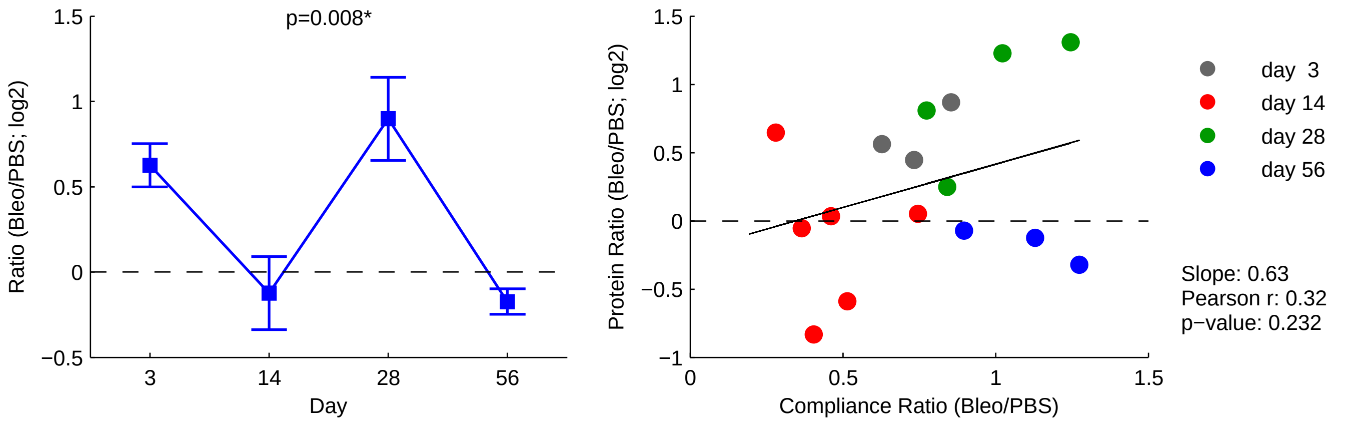

O54834−3 − Arhgap6 (id: 2060)

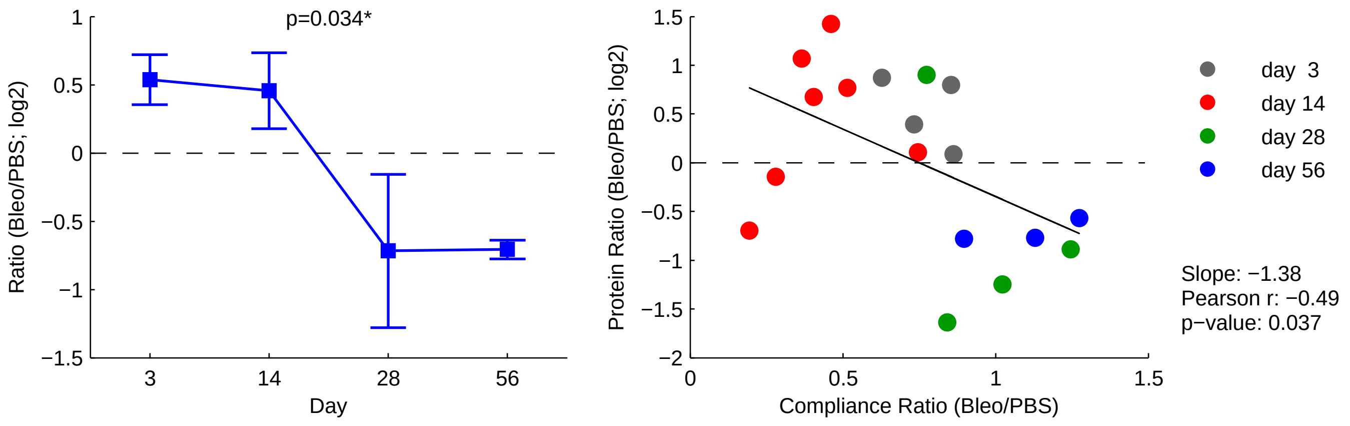

O54907 − Tnfsf12 (id: 2063)

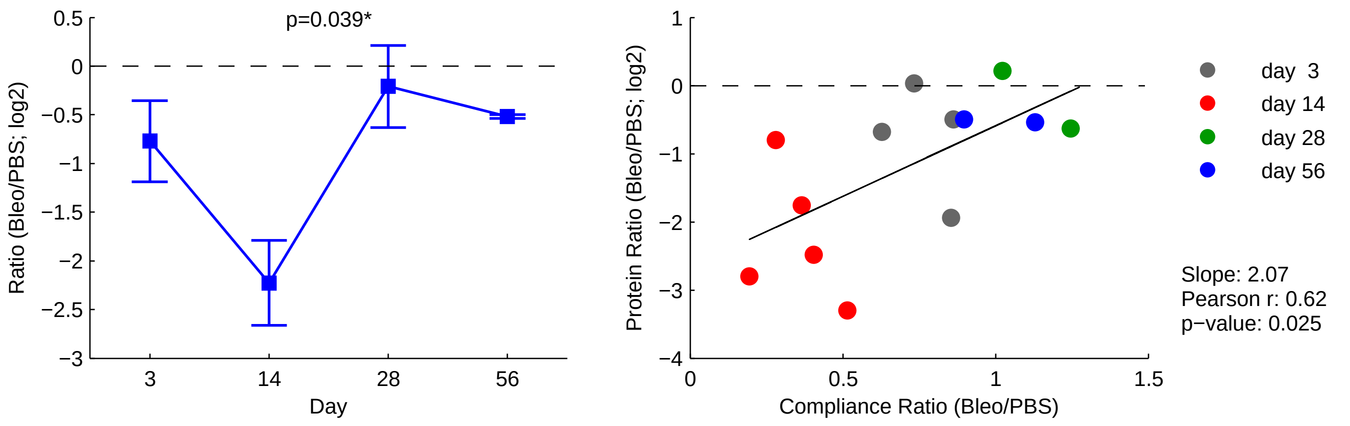

O54940 − Bnip2 (id: 2066)

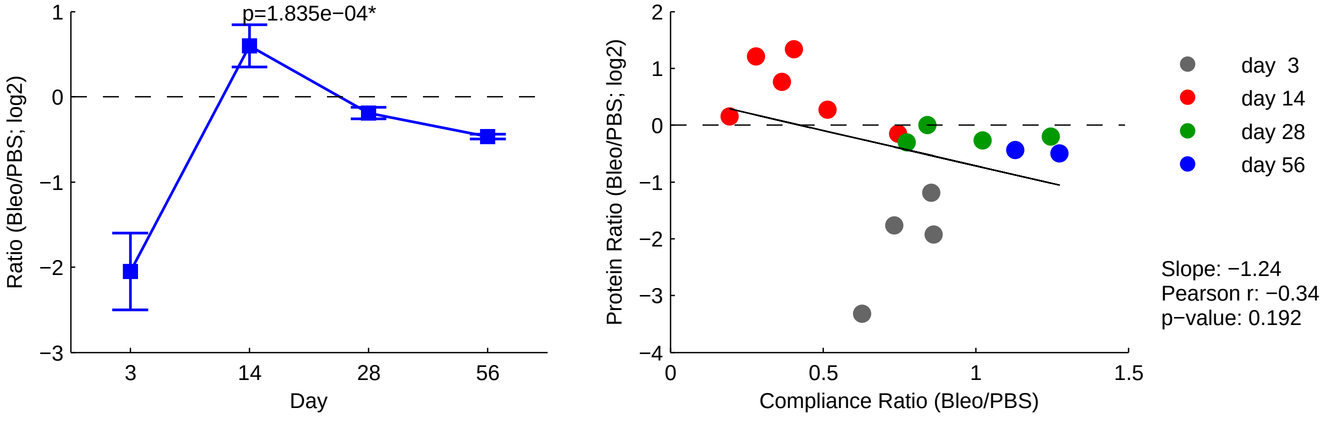

O54946−2 − Dnajb6 (id: 2069)

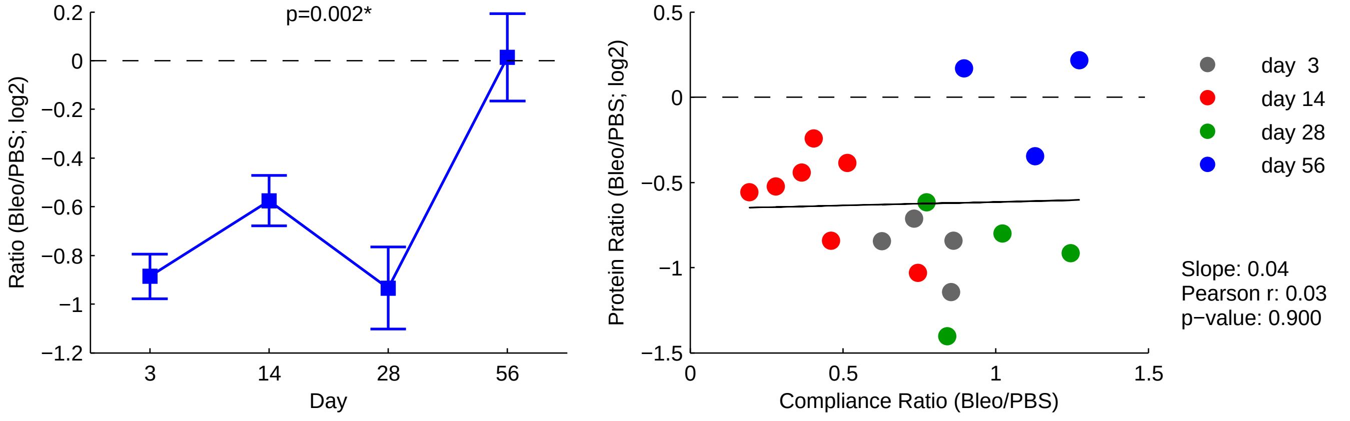

O54950 − Prkag1 (id: 2070)

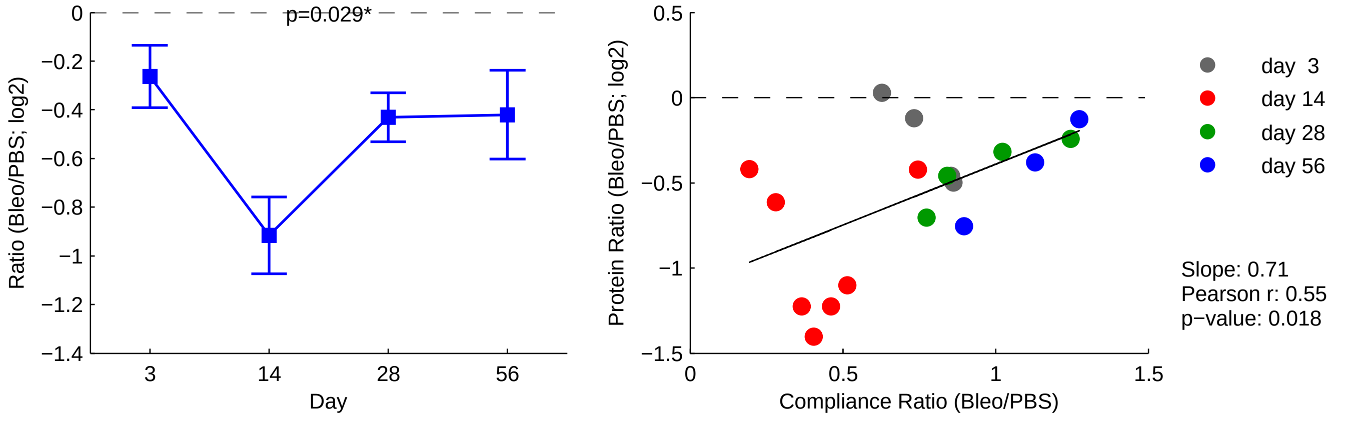

O54962 − Banf1 (id: 2071)

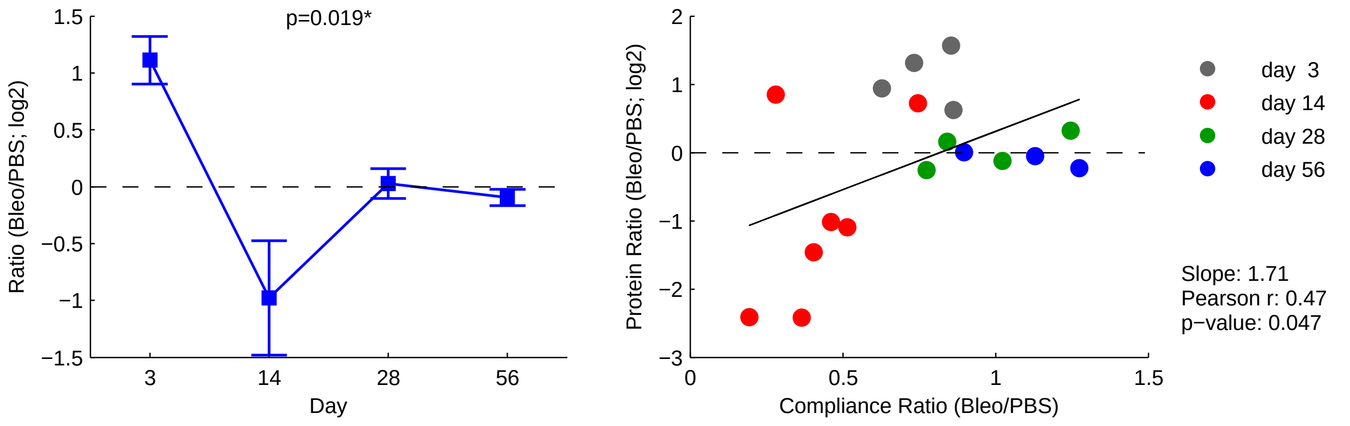

O54984 − Asna1 (id: 2074)

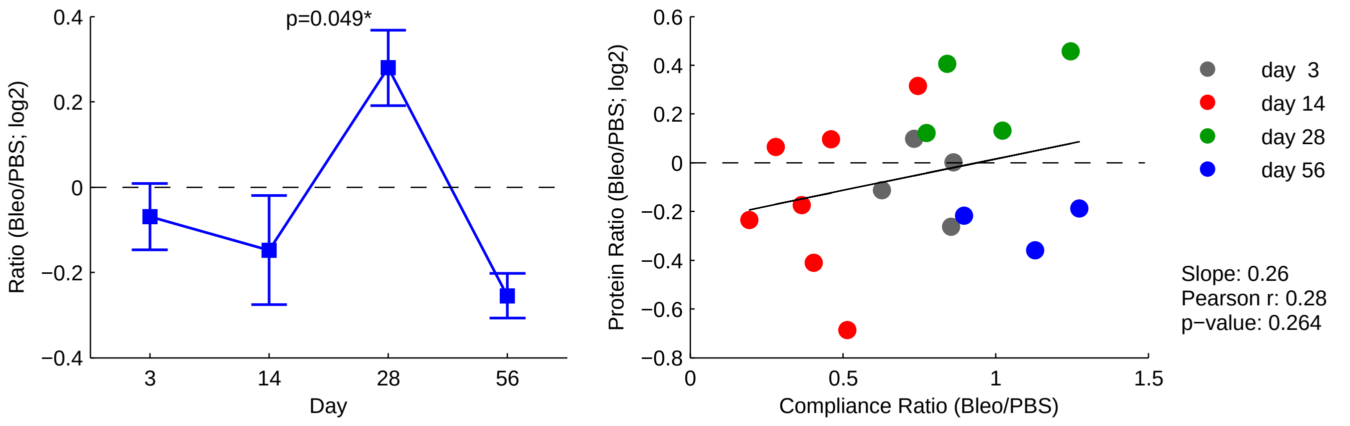

O54988 − Slk (id: 2075)

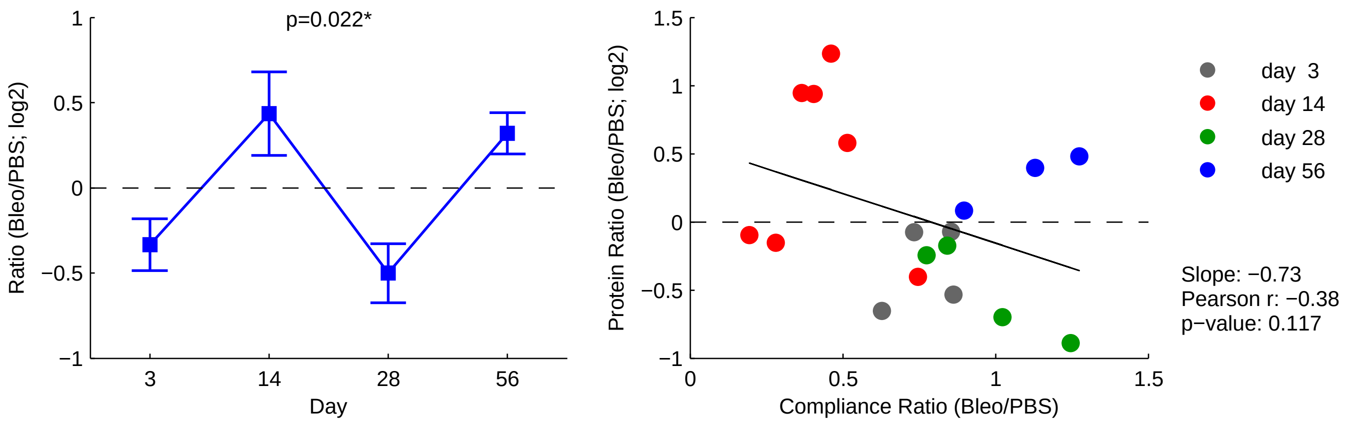

O54998 − Fkbp7 (id: 2076)

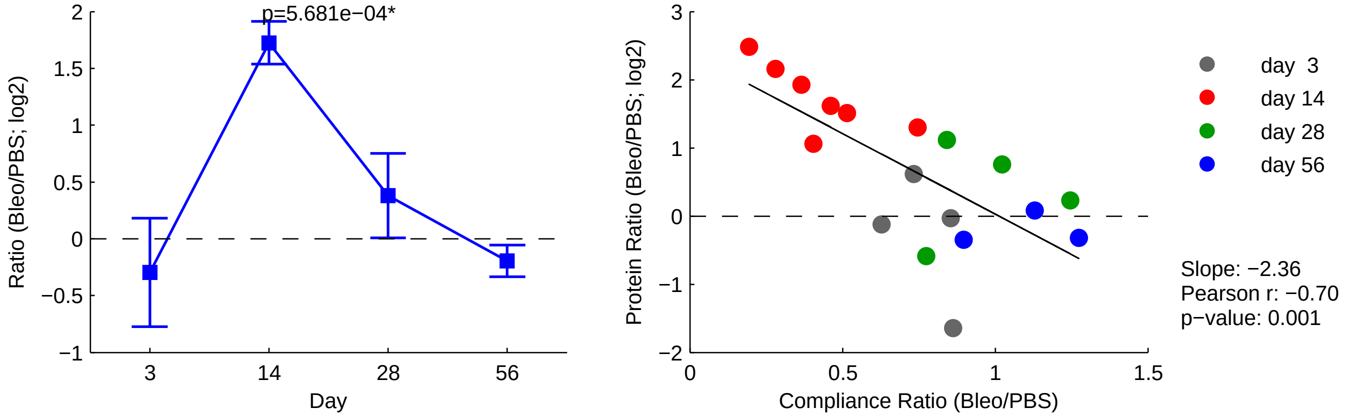

O55013 − Trappc3 (id: 2078)

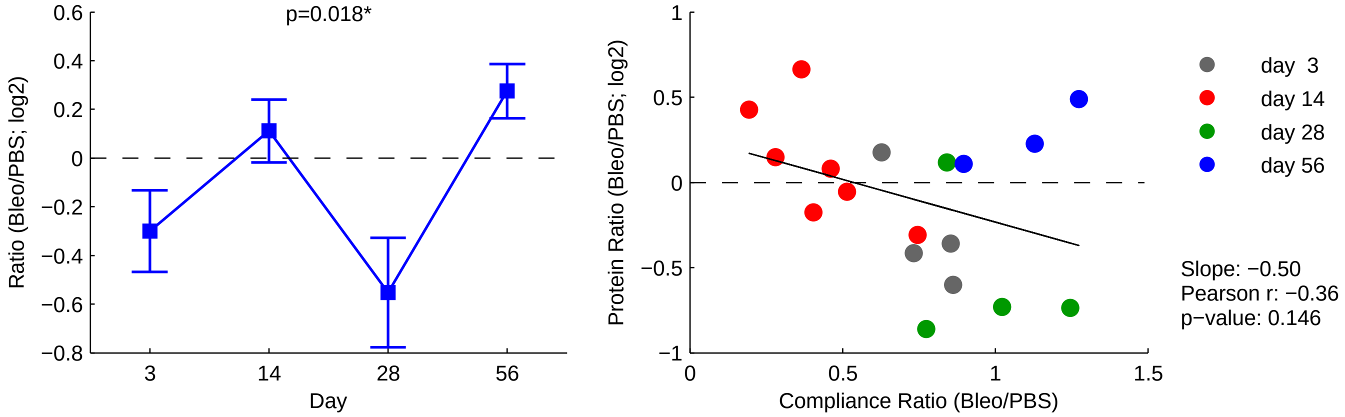

O55022 − Pgrmc1 (id: 2079)

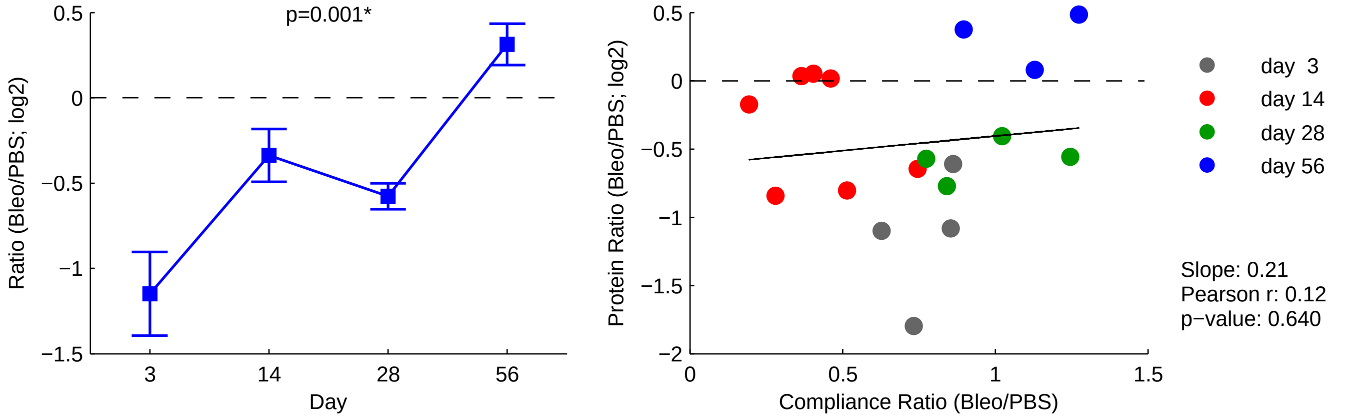

O55026 − Entpd2 (id: 2081)

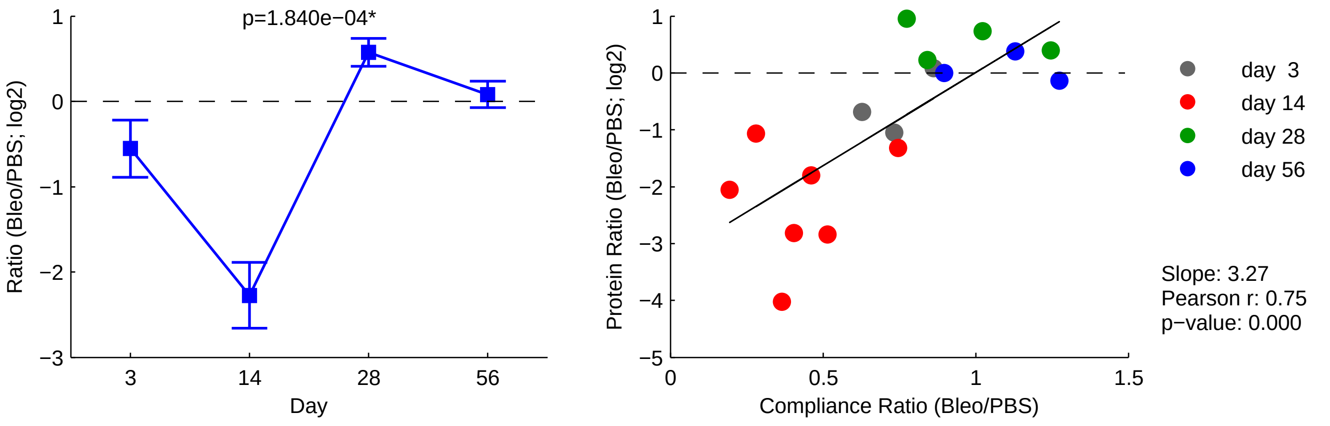

O55028 − Bckdk (id: 2082)

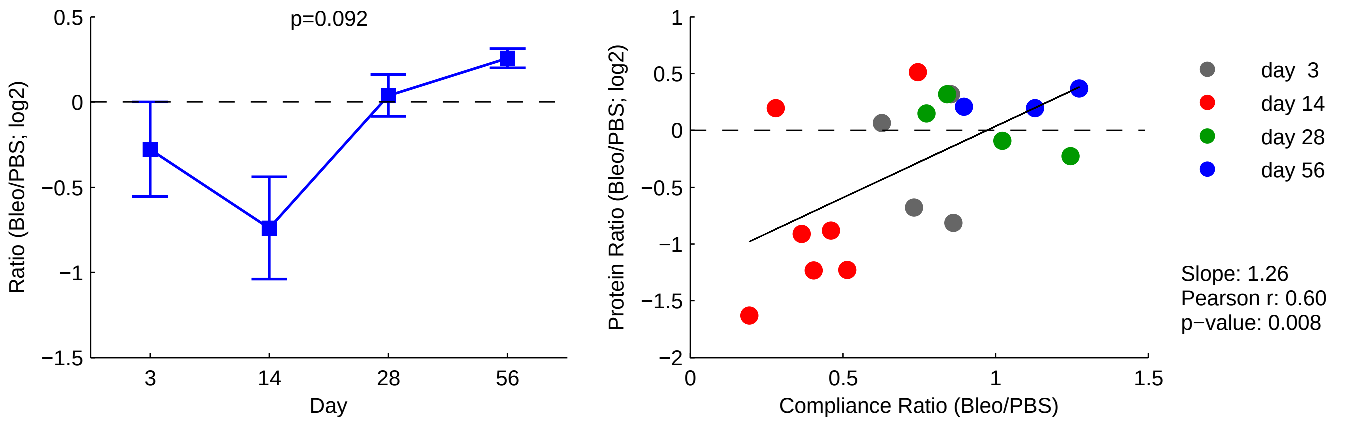

O55029 − Copb2 (id: 2083)

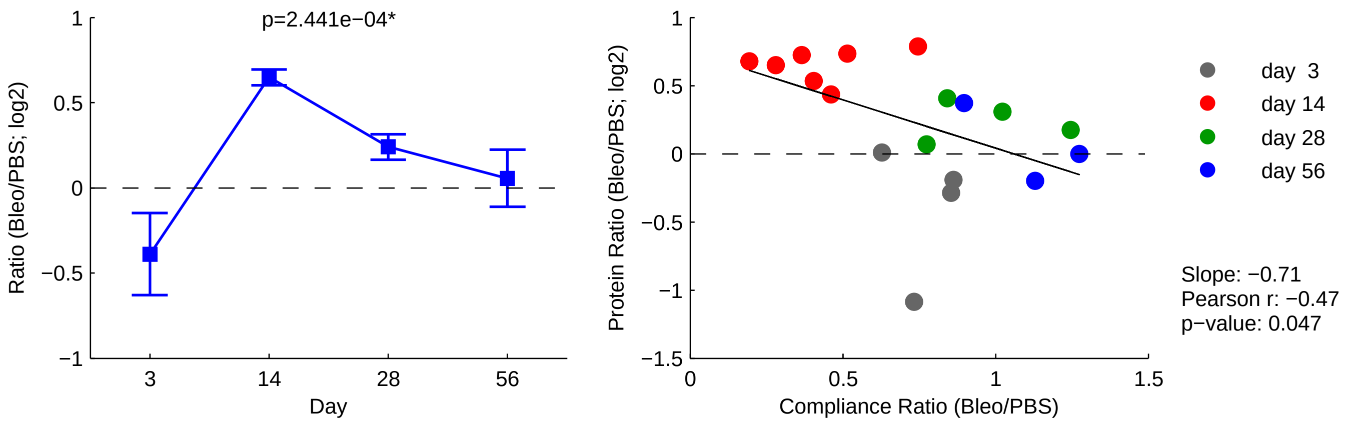

O55033 − Nck2 (id: 2084)

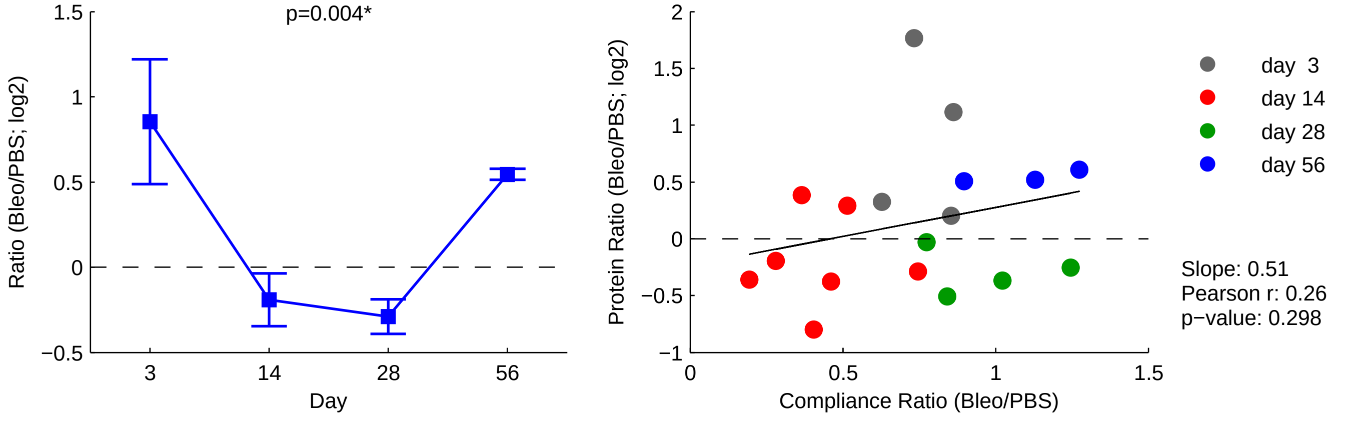

O55042−2 − Snca (id: 2085)

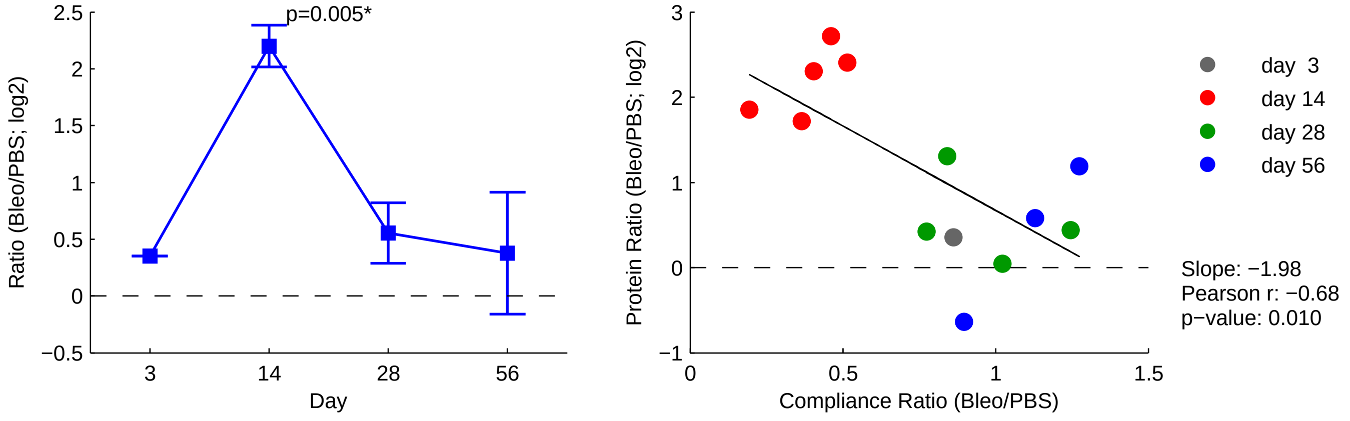

O55074 − Akap7 (id: 2087)

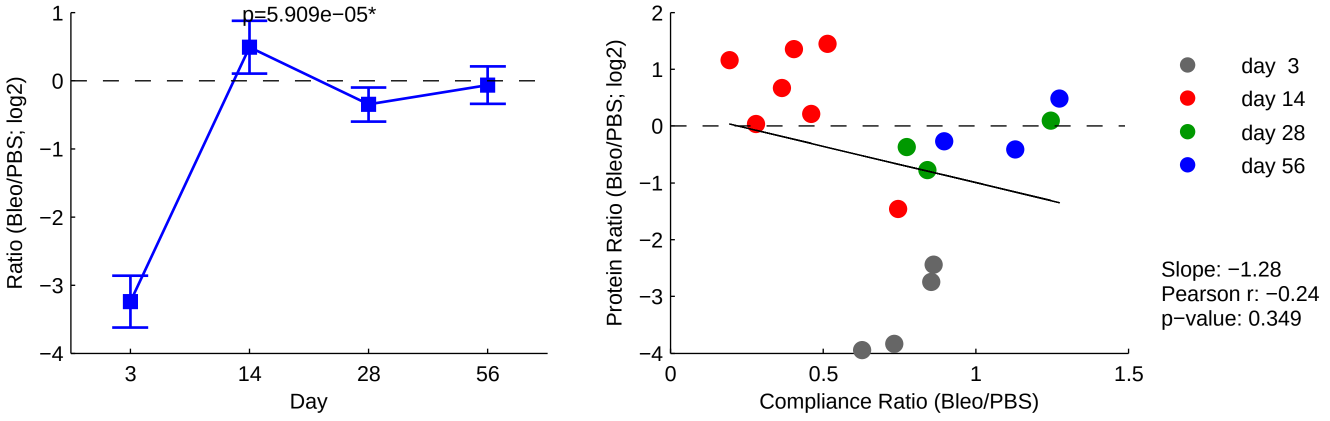

O55103−2 − (id: 2089)

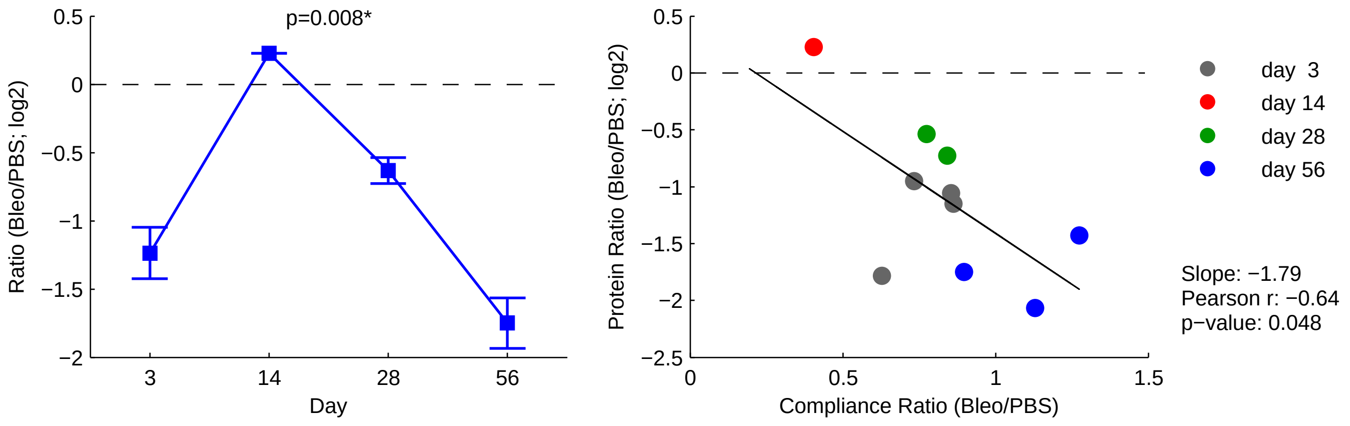

O55106 − Strn (id: 2090)

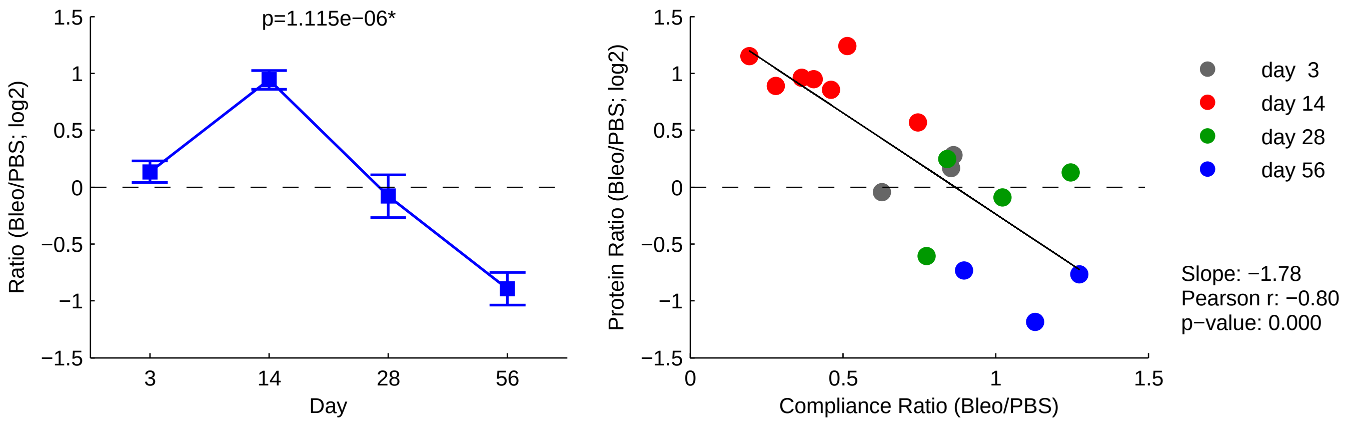

O55111 − Dsg2 (id: 2091)

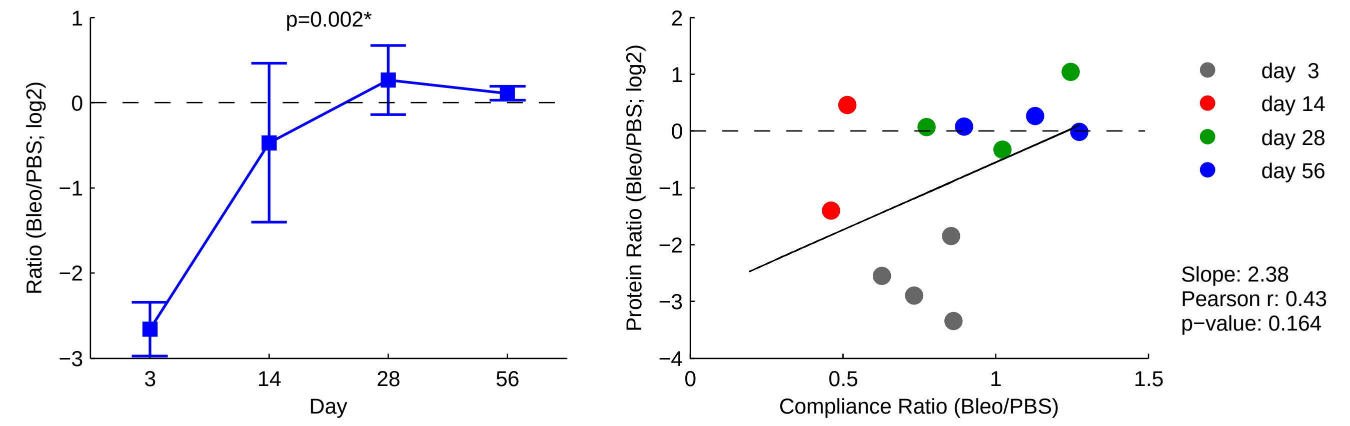

O55125 − Nipsnap1 (id: 2092)

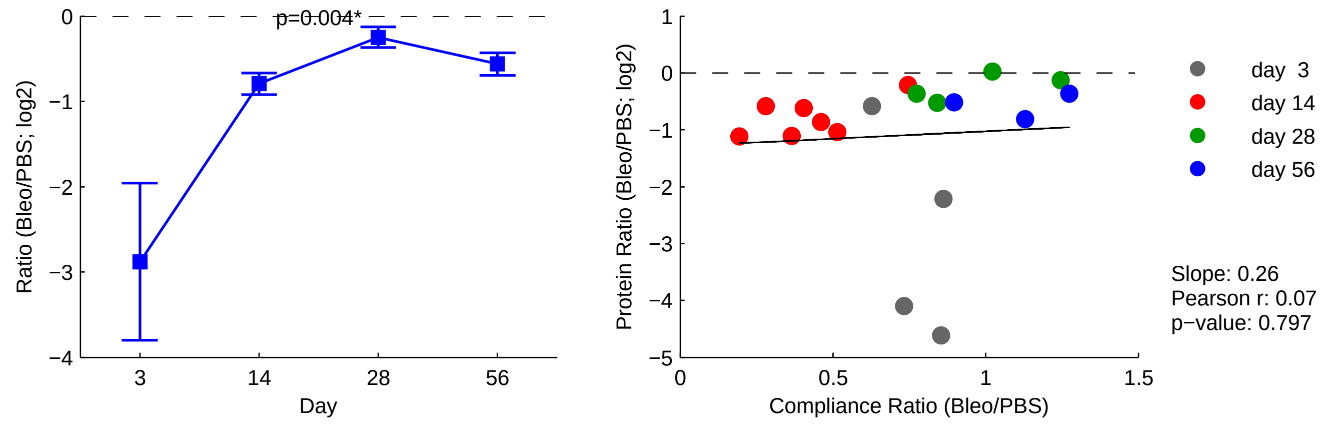

O55142 − Rpl35a (id: 2095)

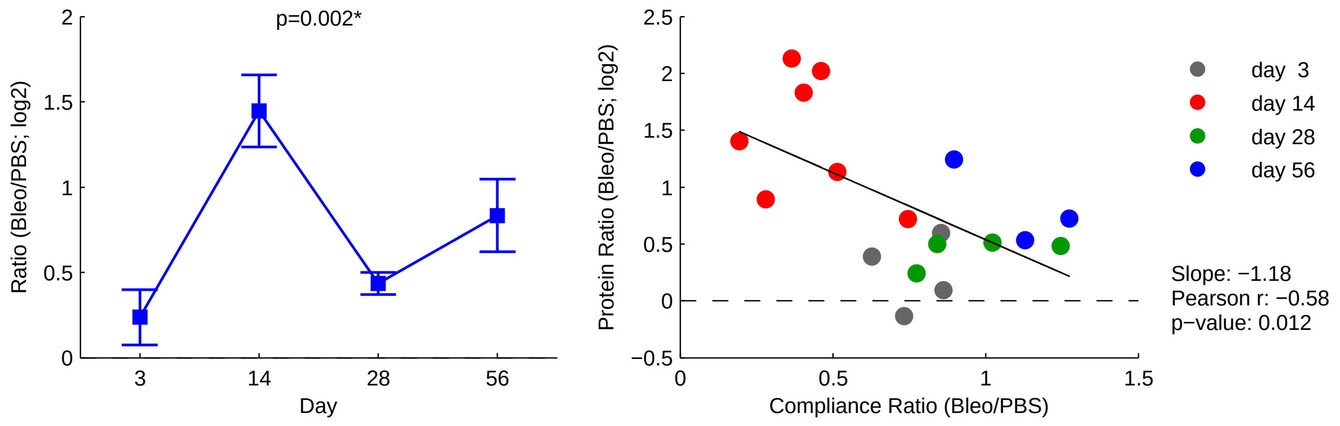

O55143 − Atp2a2 (id: 2096)

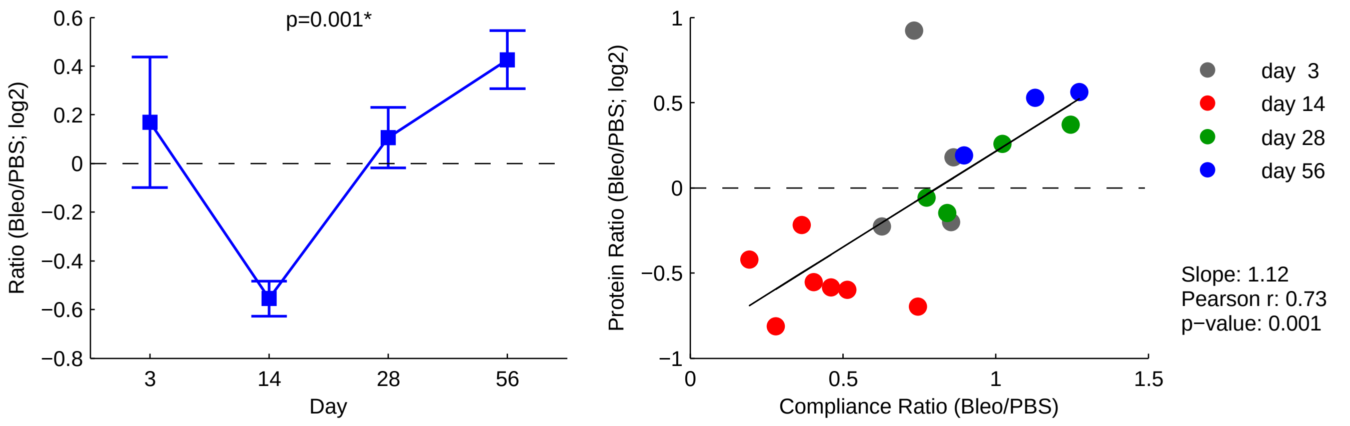

O55143−2 − Atp2a2 (id: 2097)

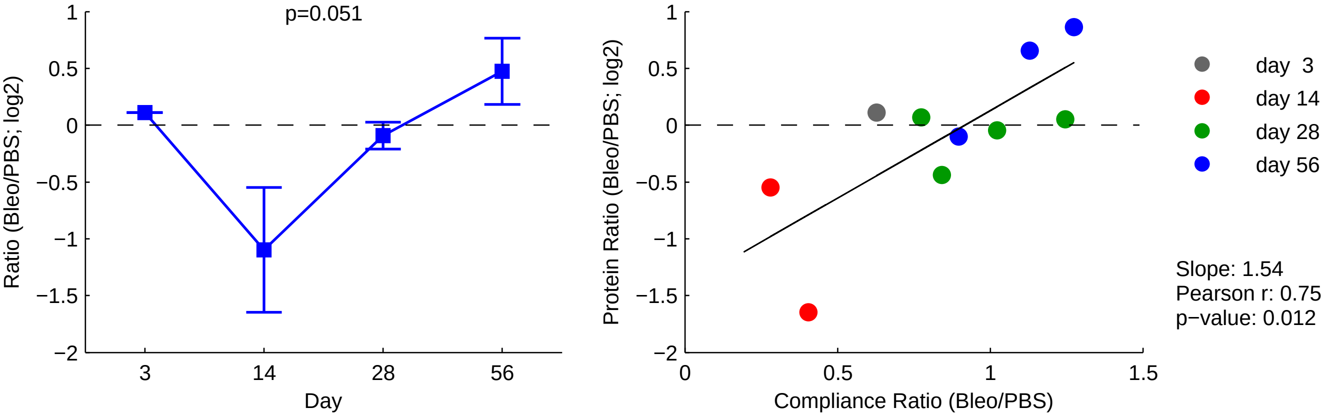

O55226 − Chad (id: 2103)

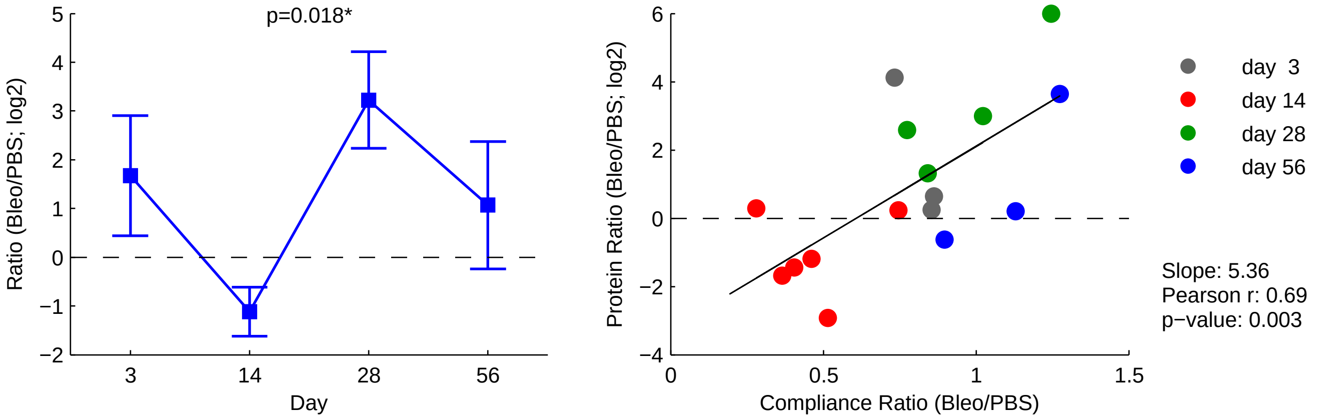

O55234 − Psmb5 (id: 2104)

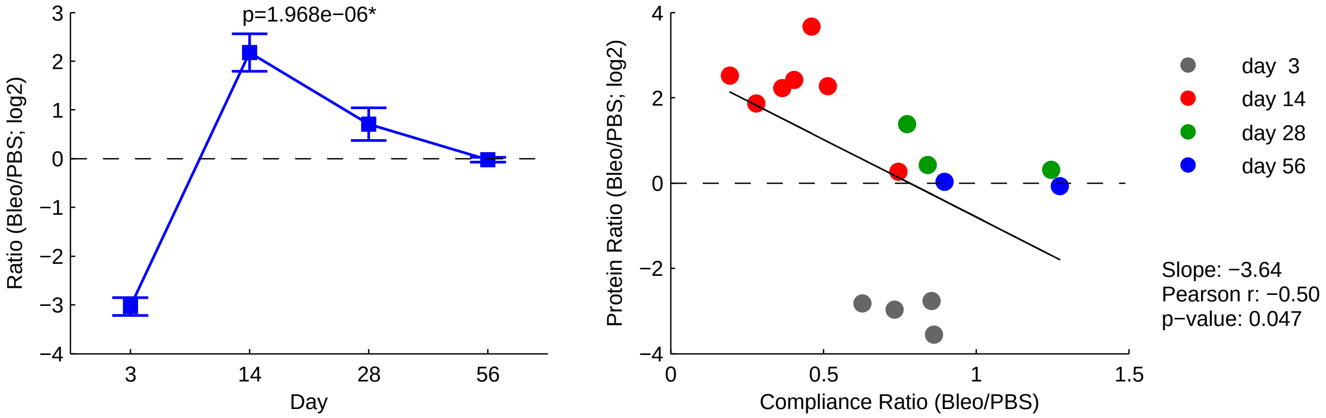

O70138 − Mmp8 (id: 2106)

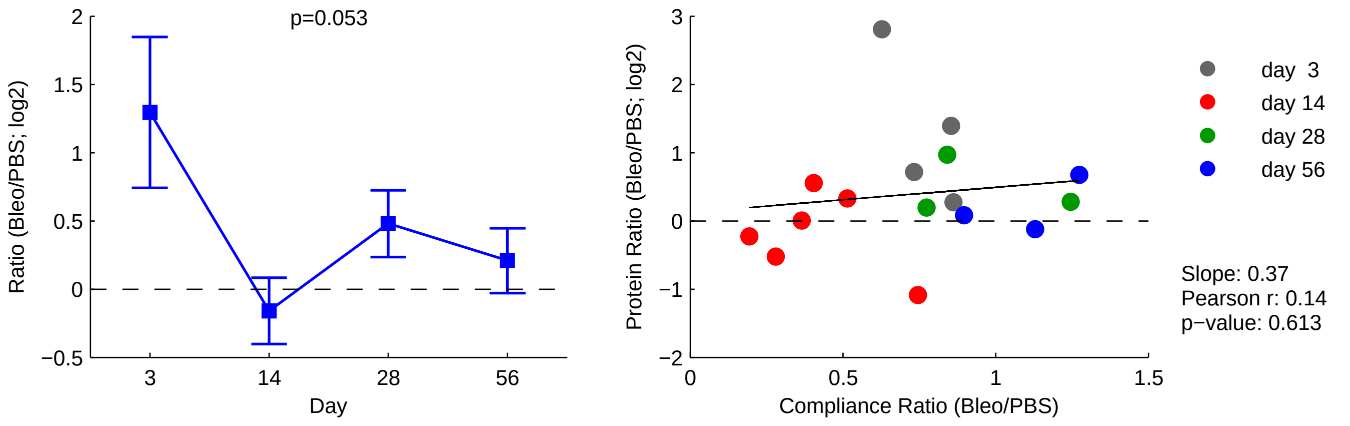

O70145 − Ncf2 (id: 2107)

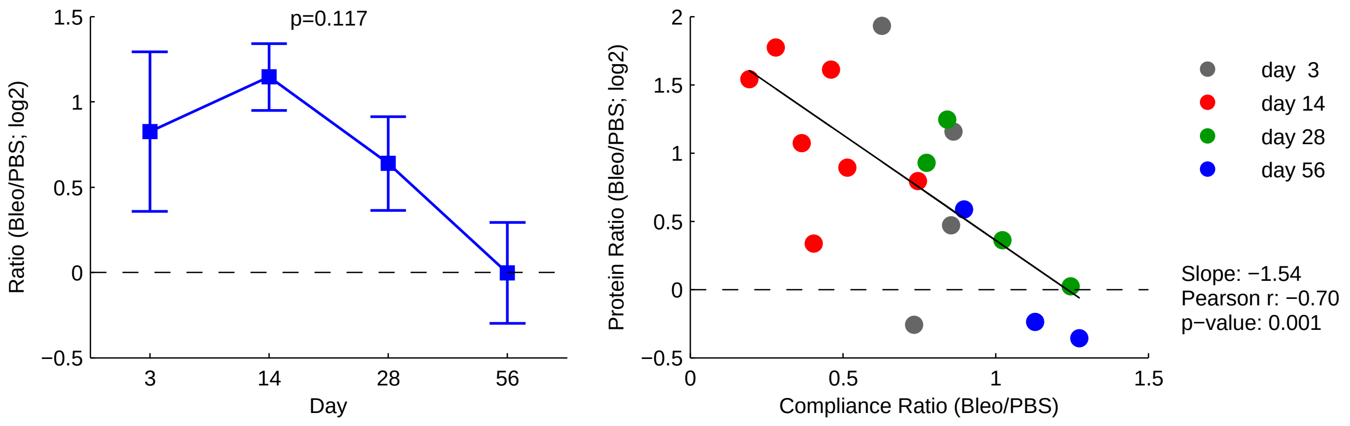

O70194 − Eif3d (id: 2110)

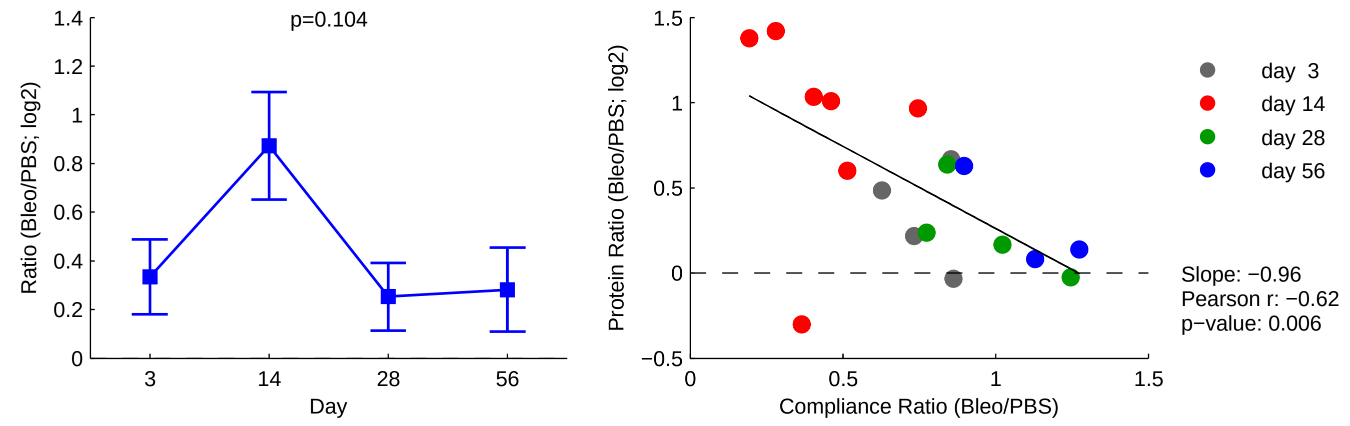

O70200 − Aif1 (id: 2111)

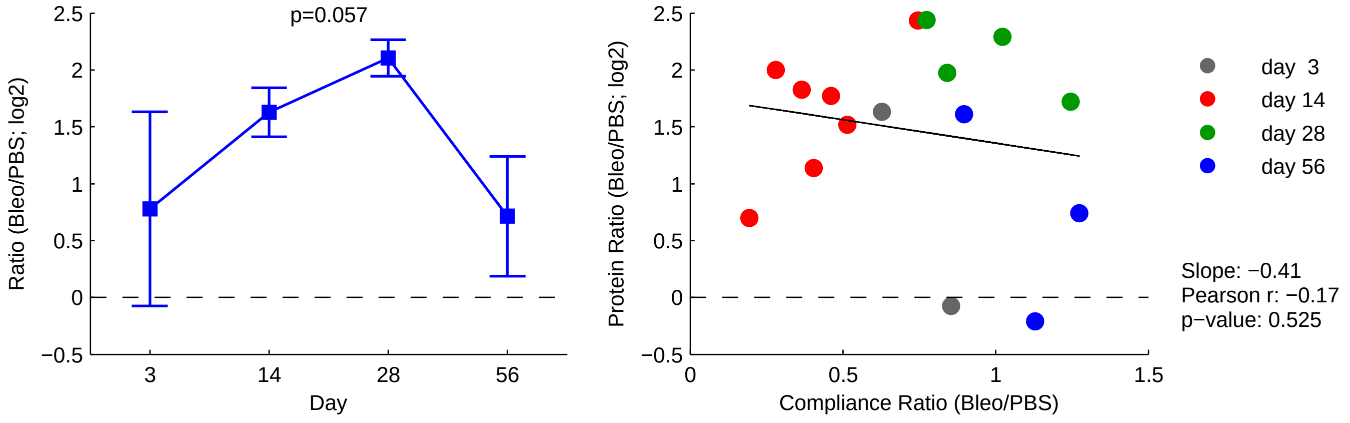

O70251 − Eef1b (id: 2114)

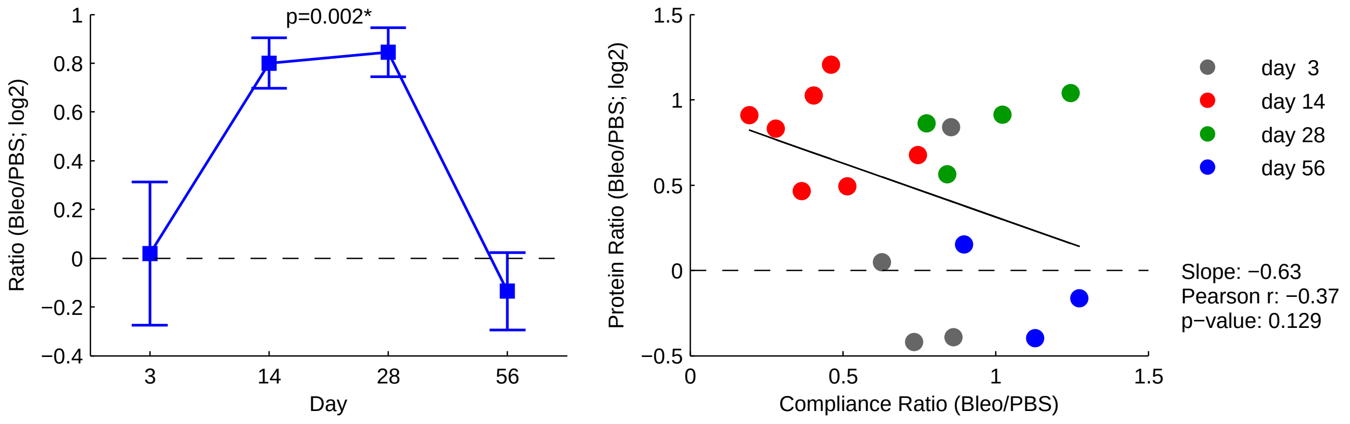

O70252 − Hmox2 (id: 2115)

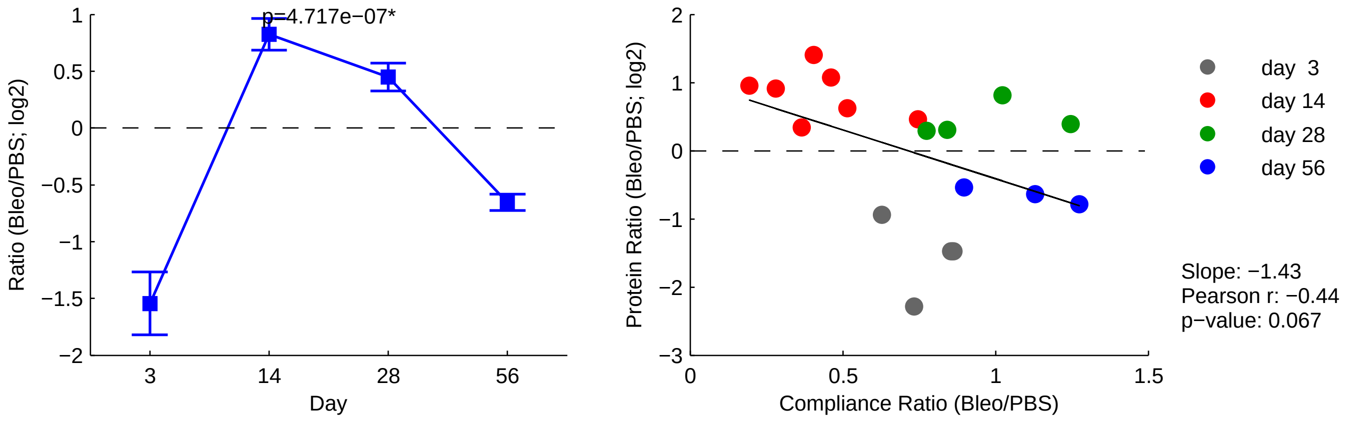

O70274 − Ptp4a2 (id: 2116)

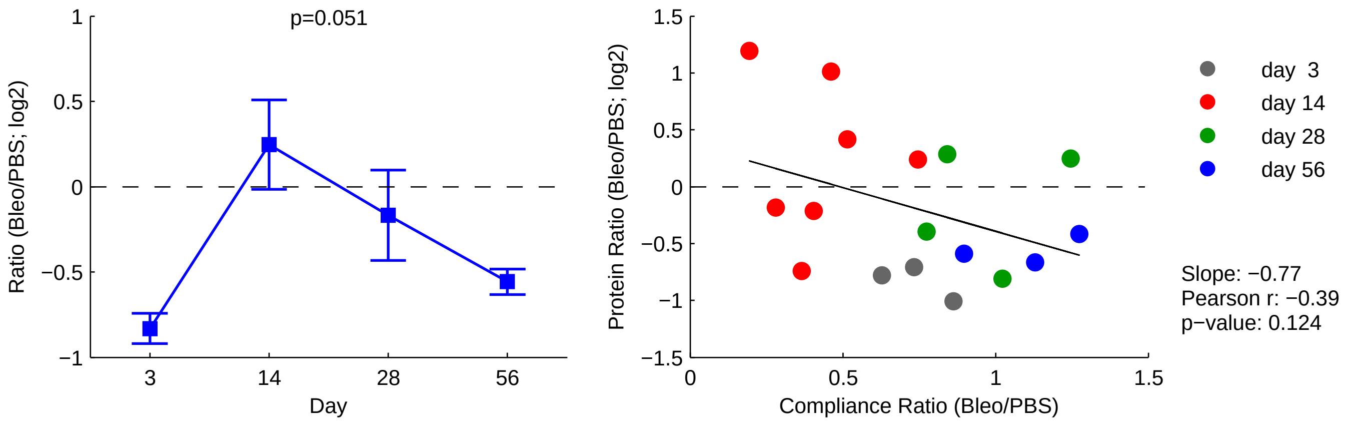

O70281 − Tpst1 (id: 2117)

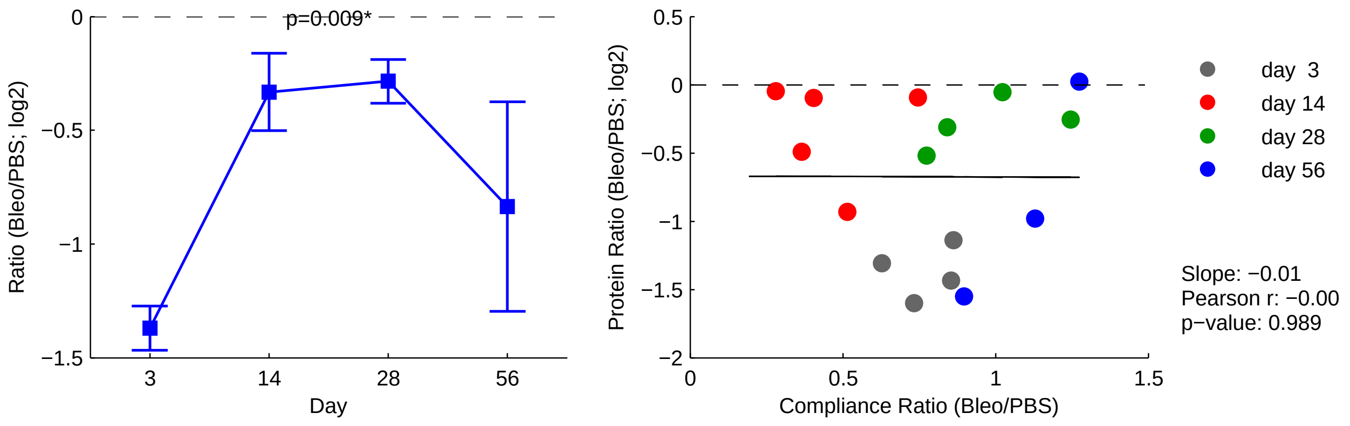

O70310 − Nmt1 (id: 2121)

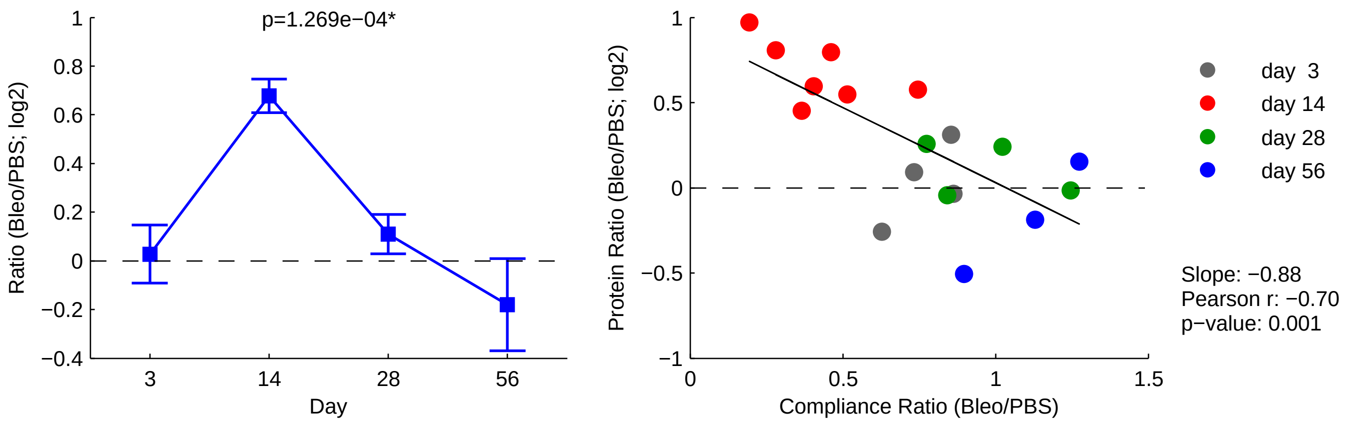

O70318 − Epb41l2 (id: 2122)

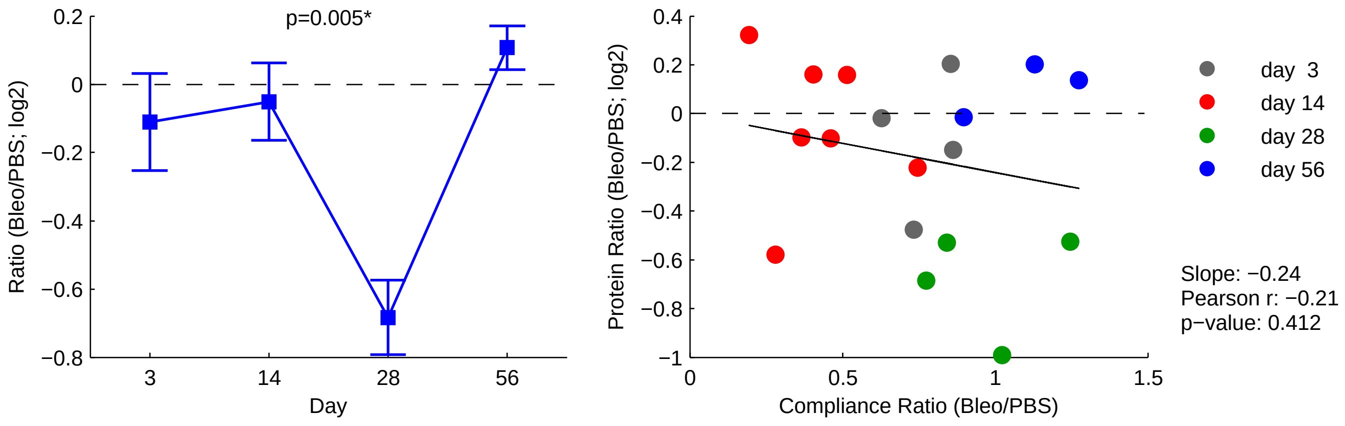

O70325−2 − Gpx4 (id: 2124)

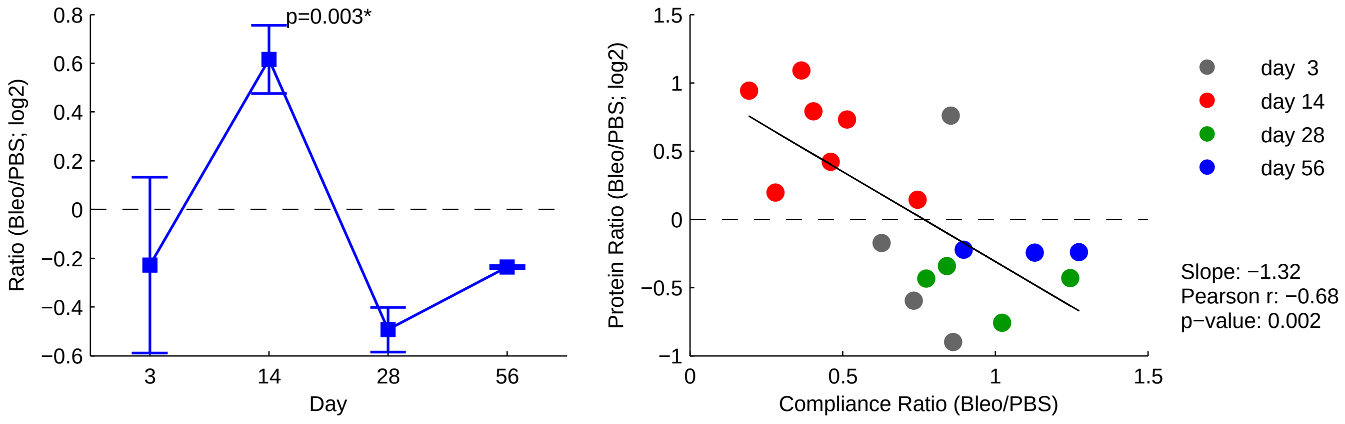

O70370 − Ctss (id: 2125)

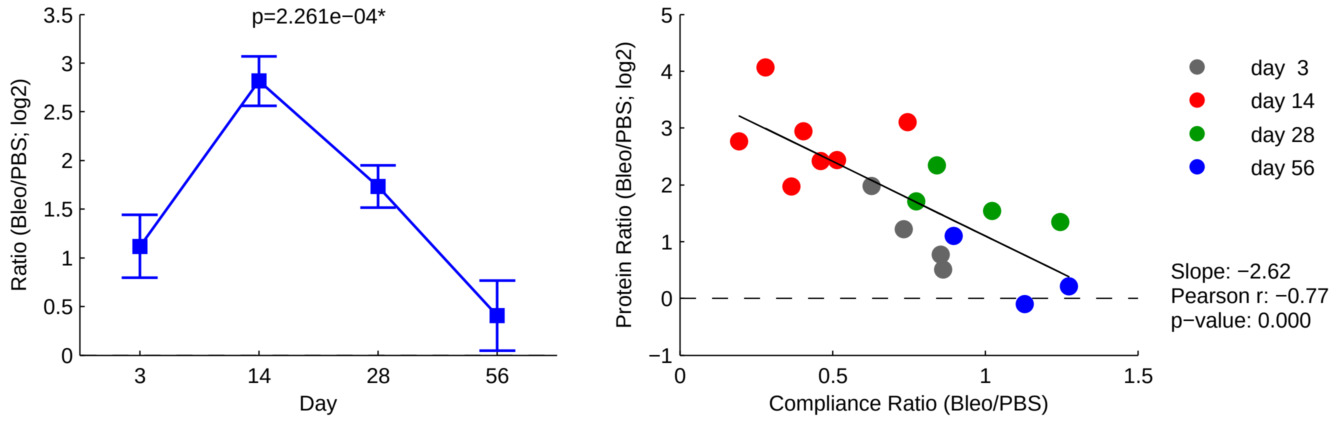

O70378 − Emc8 (id: 2127)

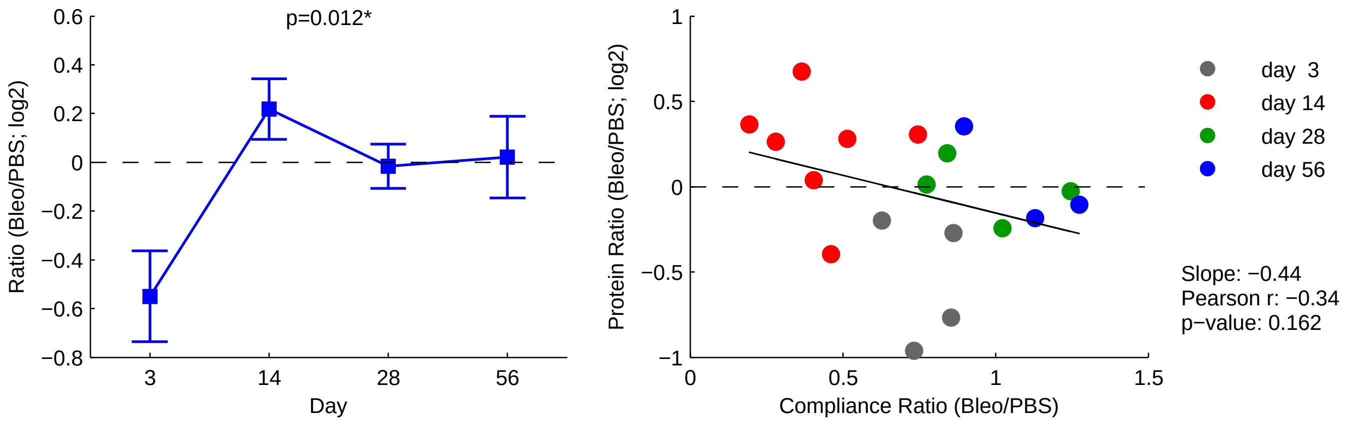

O70400 − Pdlim1 (id: 2128)

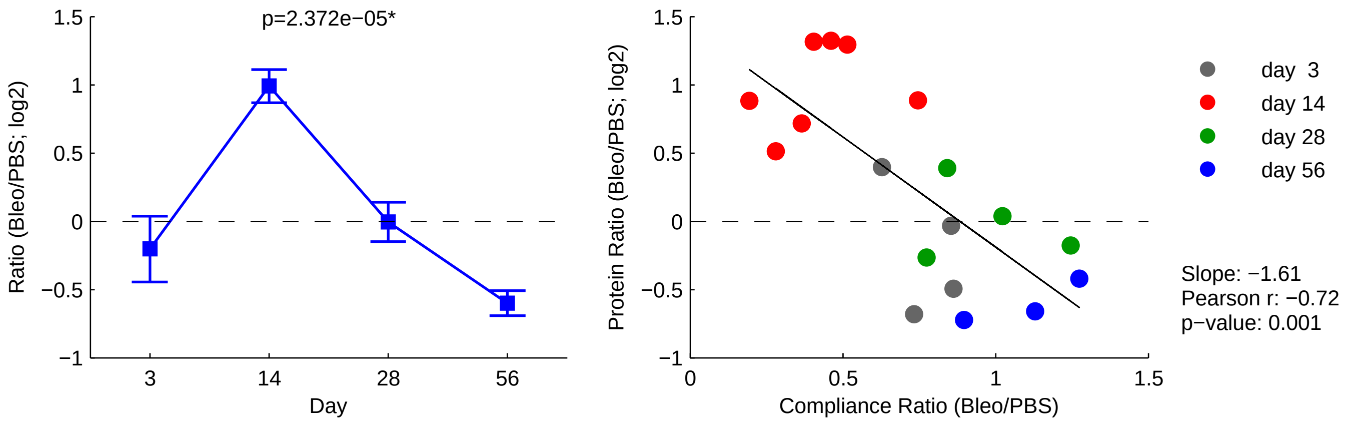

O70404 − Vamp8 (id: 2129)

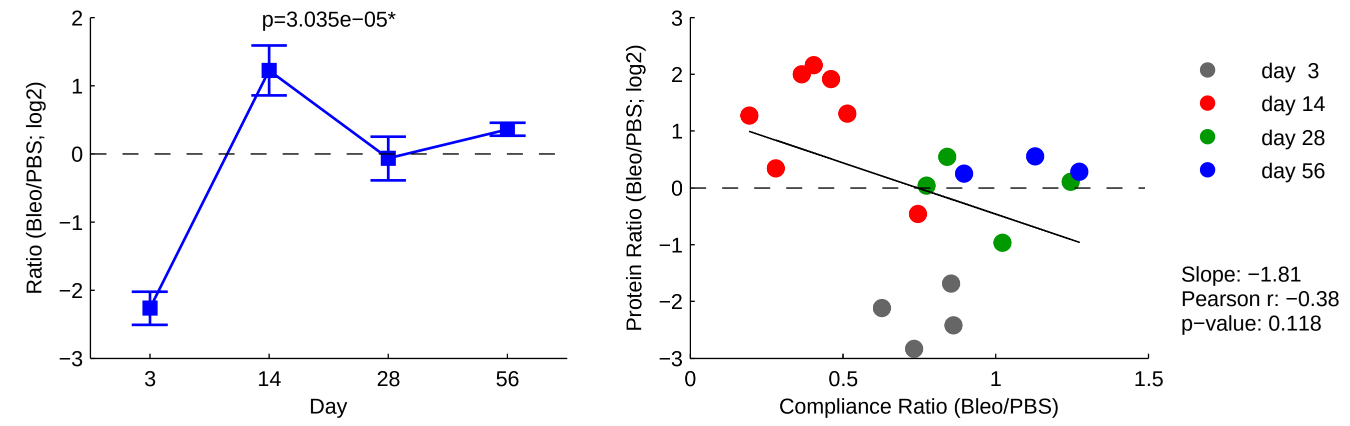

O70423 − Aoc3 (id: 2131)

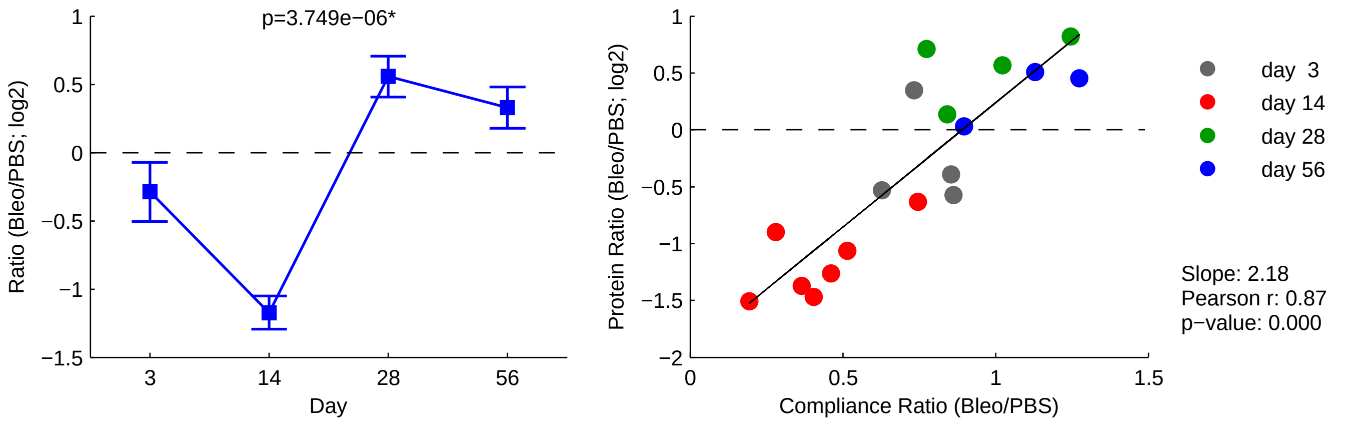

O70435 − Psma3 (id: 2133)

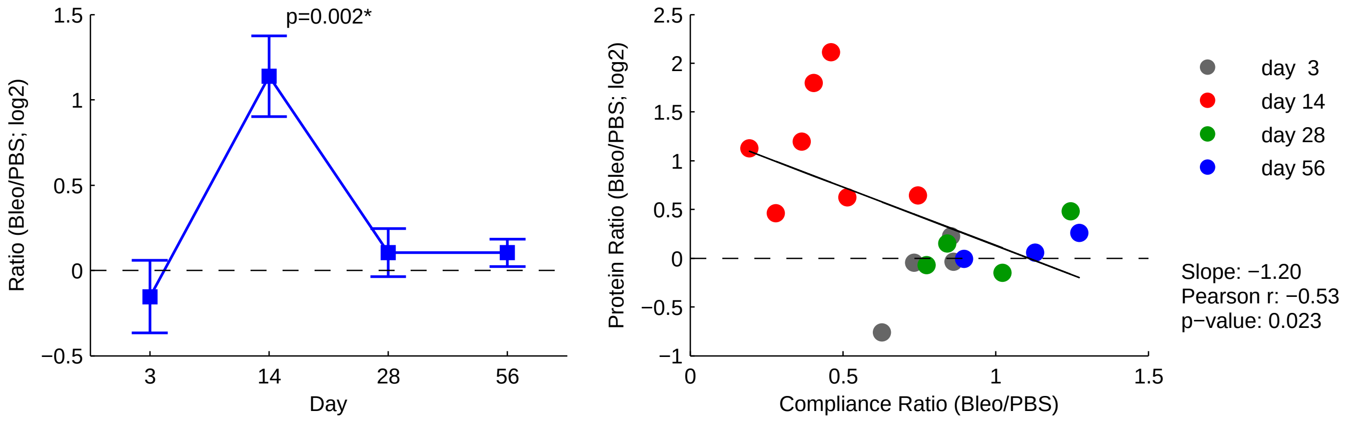

O70456 − Sfn (id: 2136)

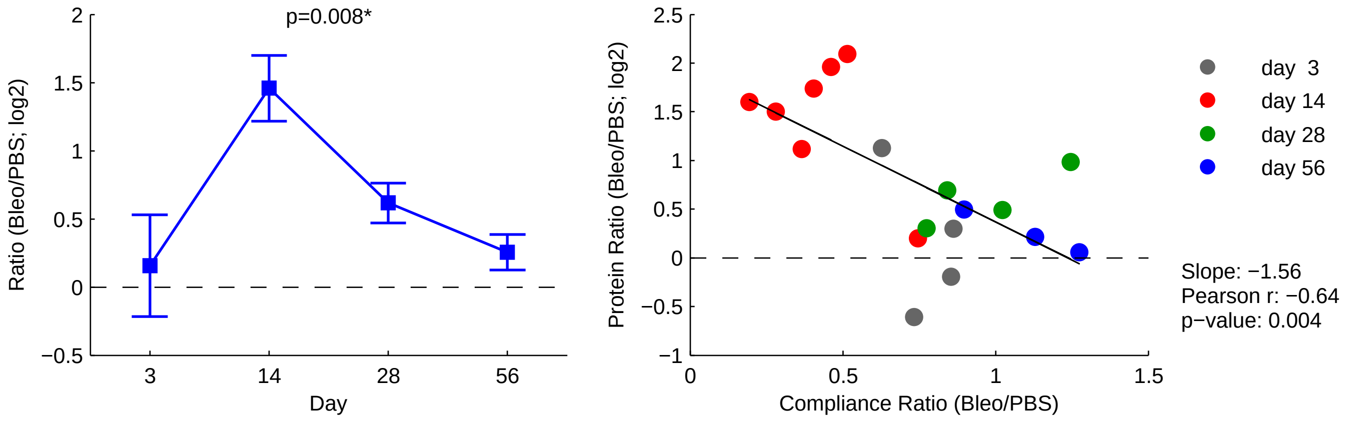

O70475 − Ugdh (id: 2138)

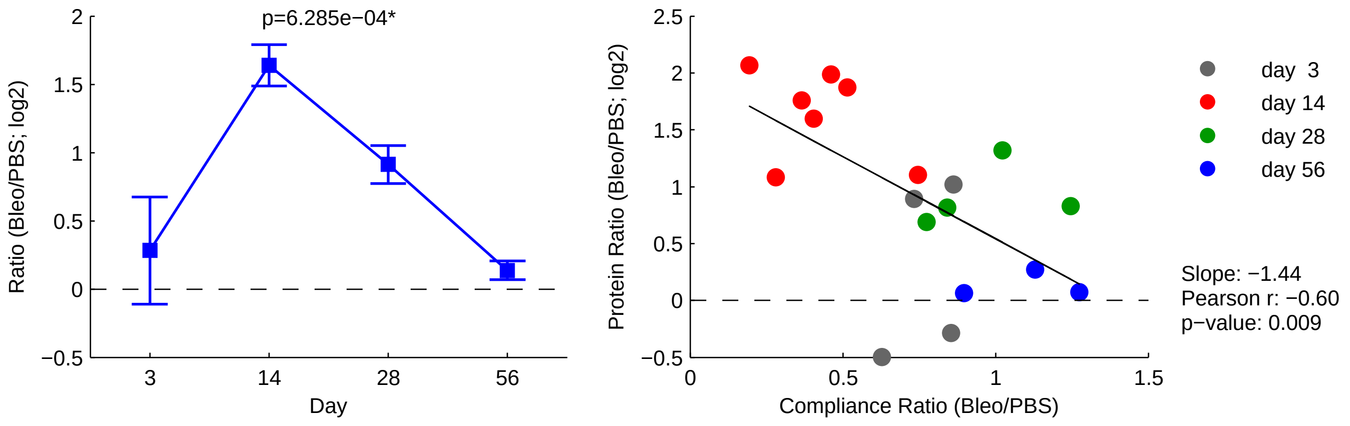

Q8BSN6 − Vamp4 (id: 2141)

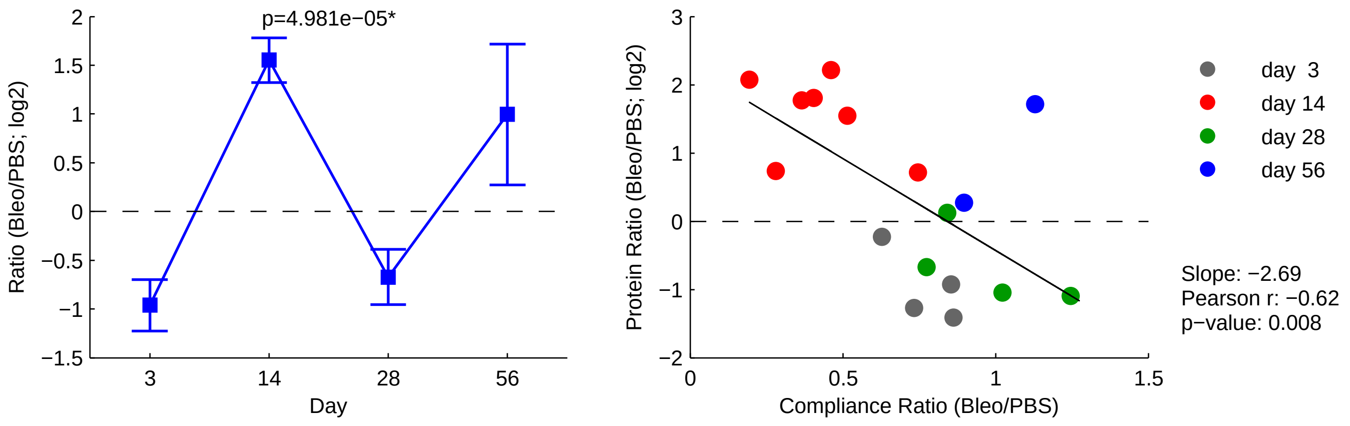

O70496 − Clcn7 (id: 2144)

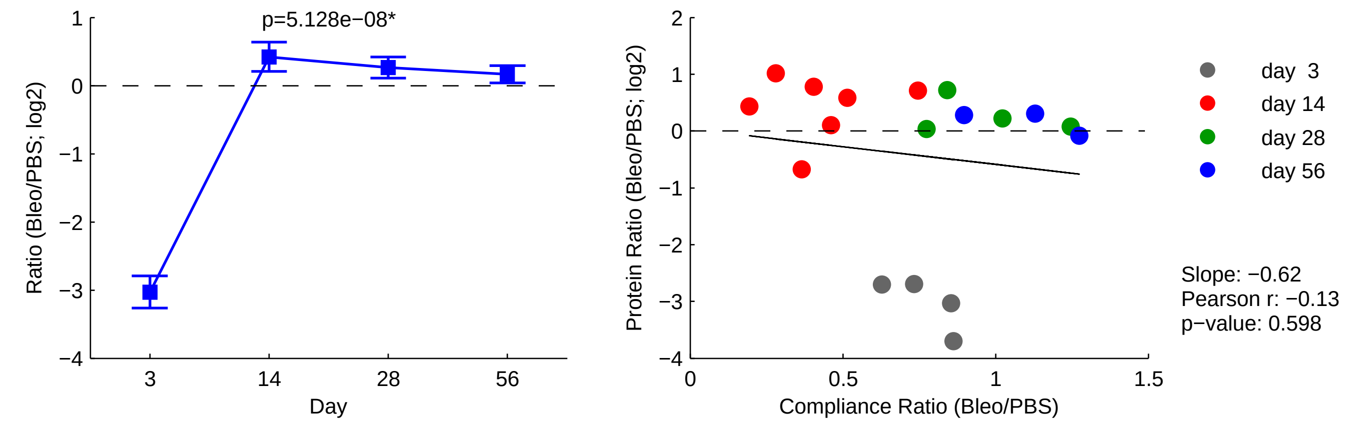

O70503 − Hsd17b12 (id: 2145)

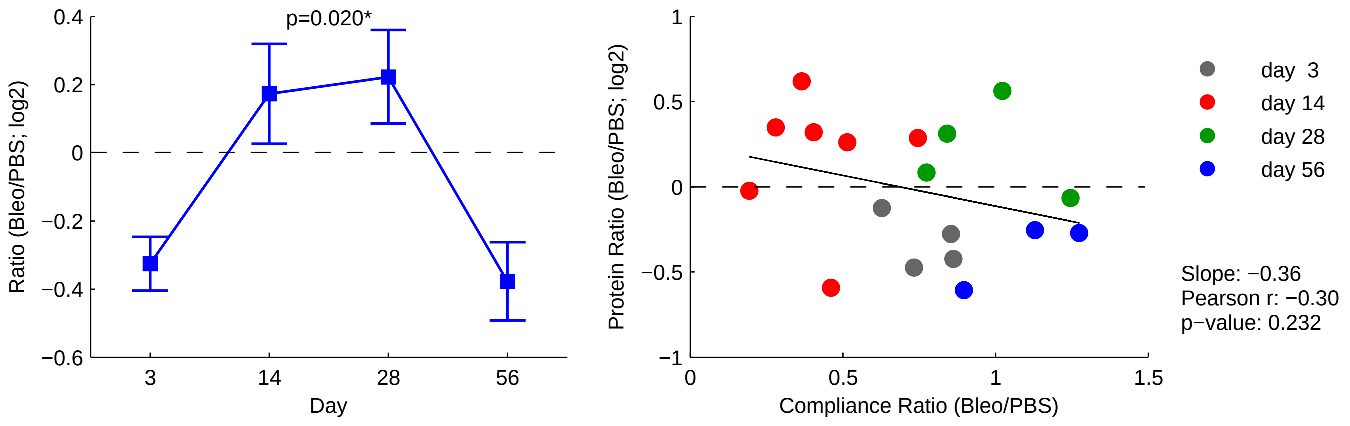

O70548 − Tcap (id: 2148)

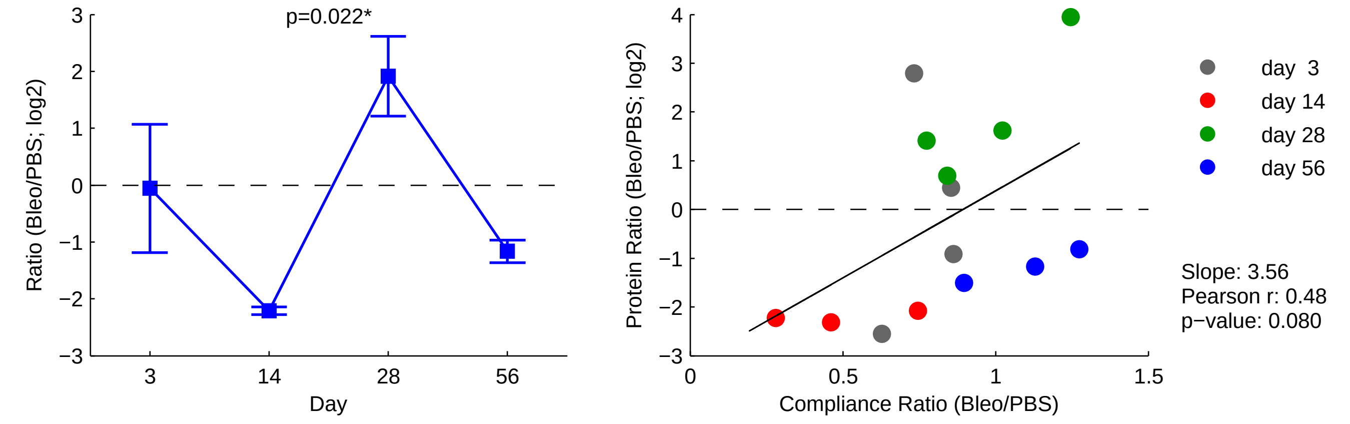

O70551 − Srpk1 (id: 2149)

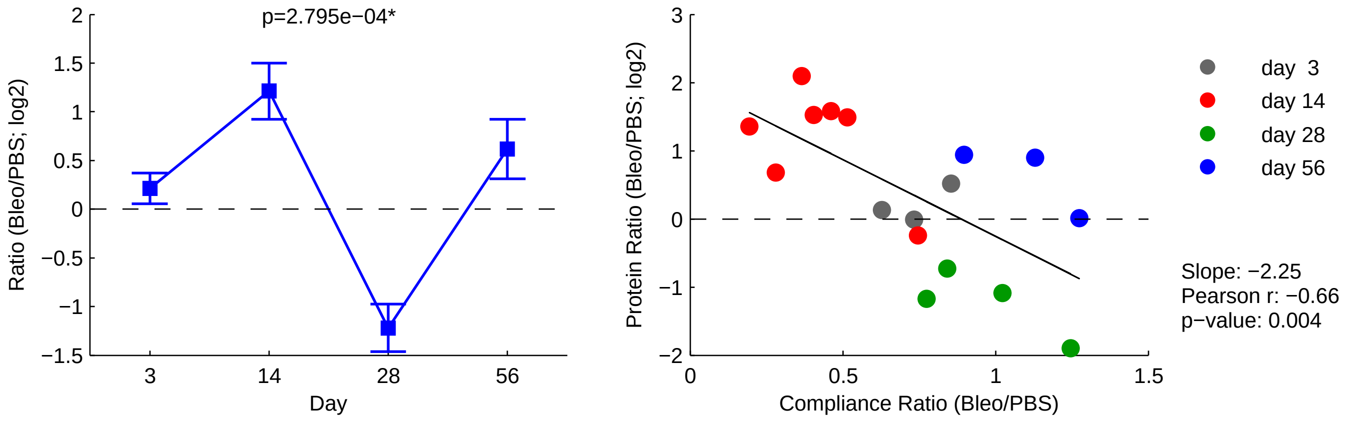

O70579 − Slc25a17 (id: 2153)

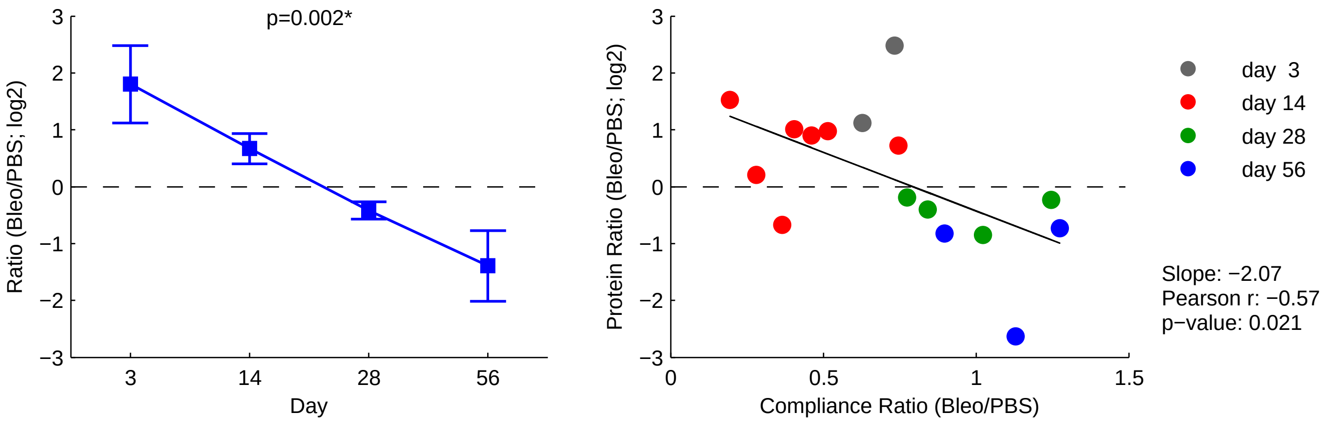

O70589−3 − Cask (id: 2155)

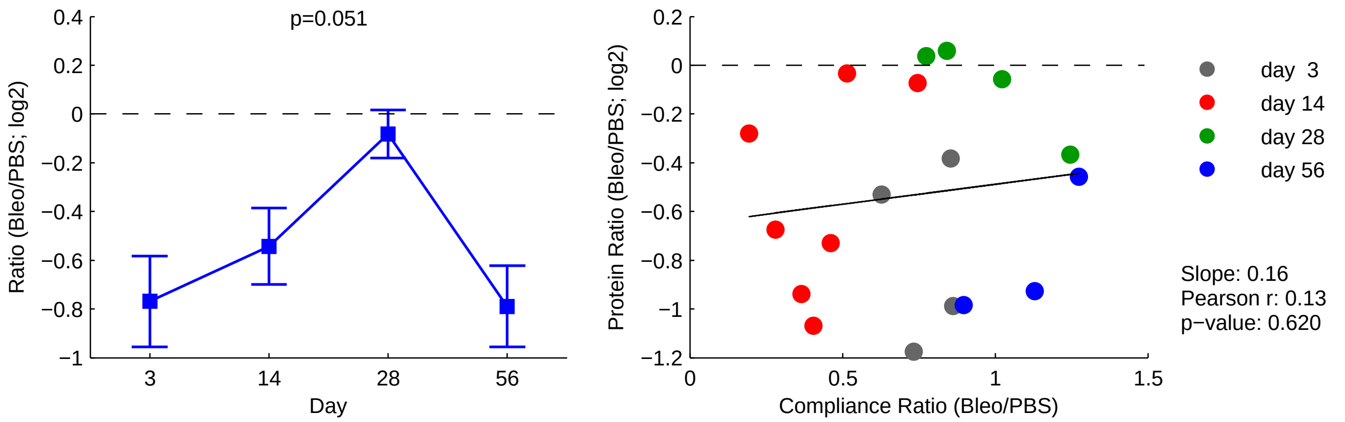

O70591 − Pfdn2 (id: 2156)

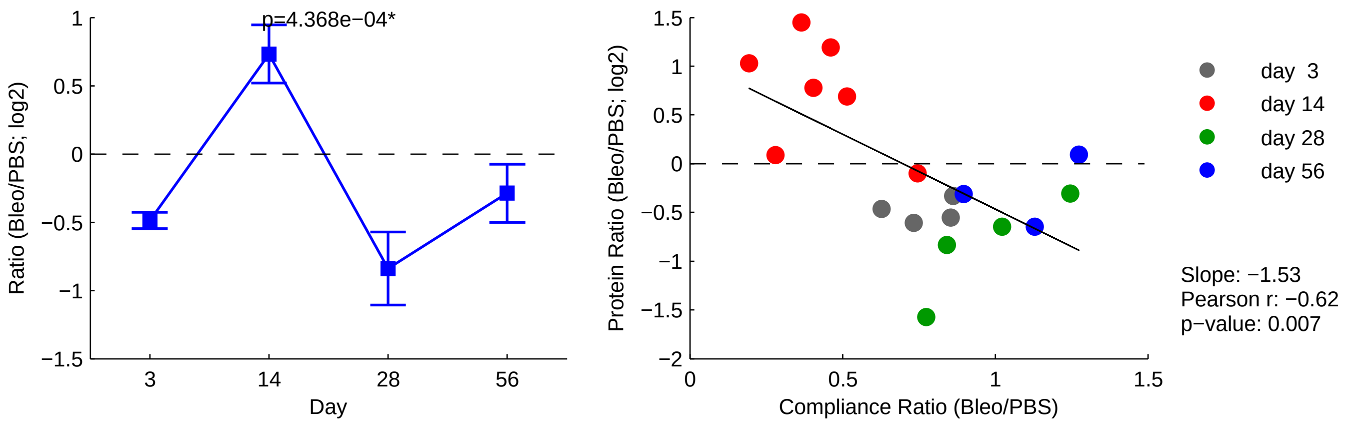

O78196 − H2−Eb1 (id: 2158)

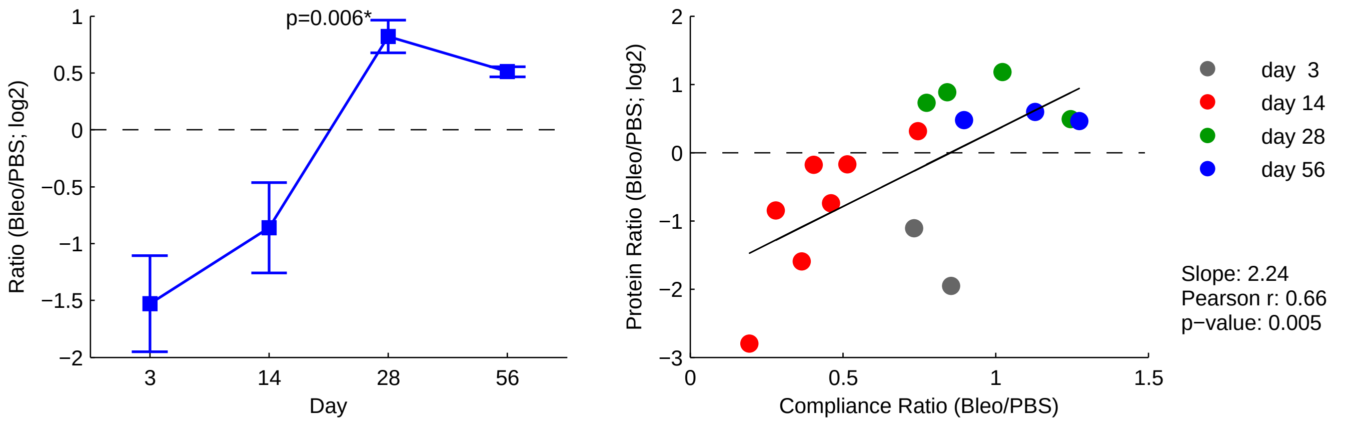

O88200 − Clec11a (id: 2161)

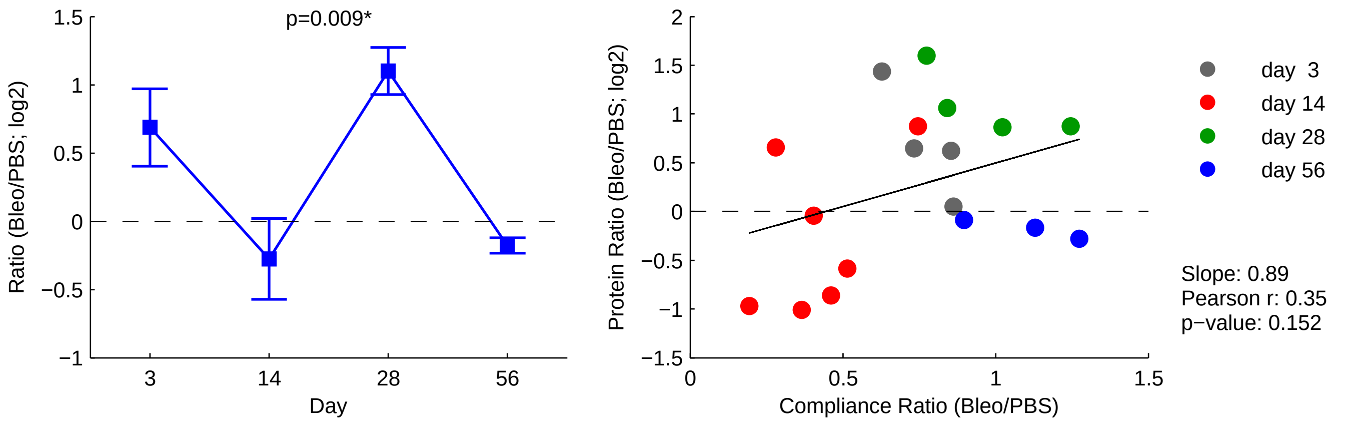

O88207 − Col5a1 (id: 2162)

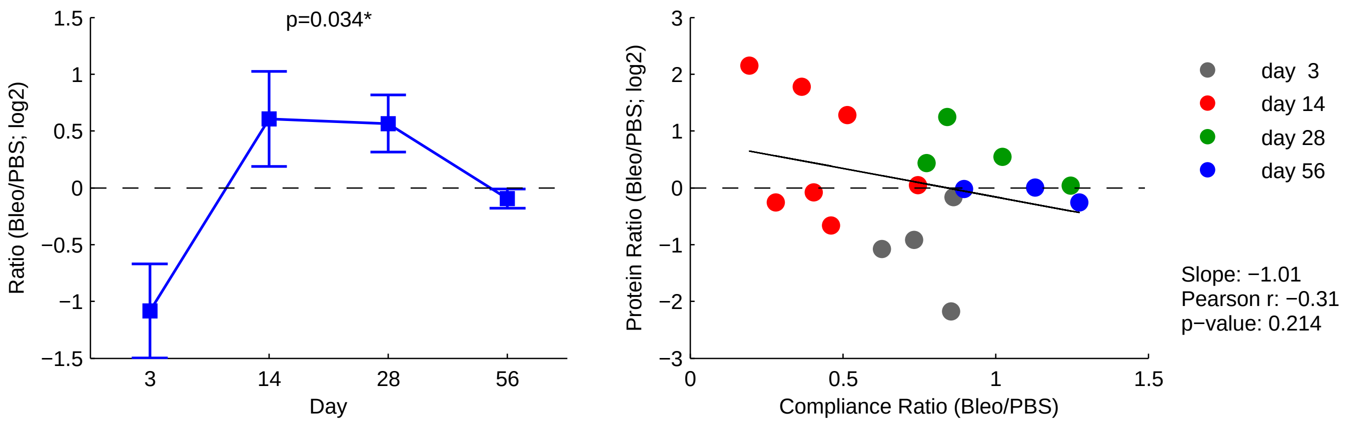

O88286 − Wiz (id: 2165)

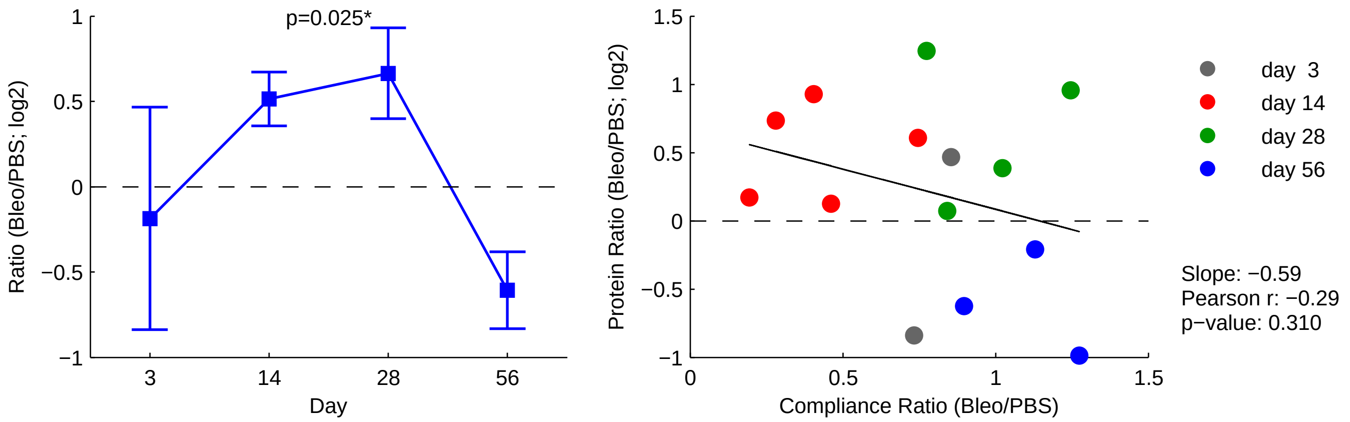

O88322 − Nid2 (id: 2169)

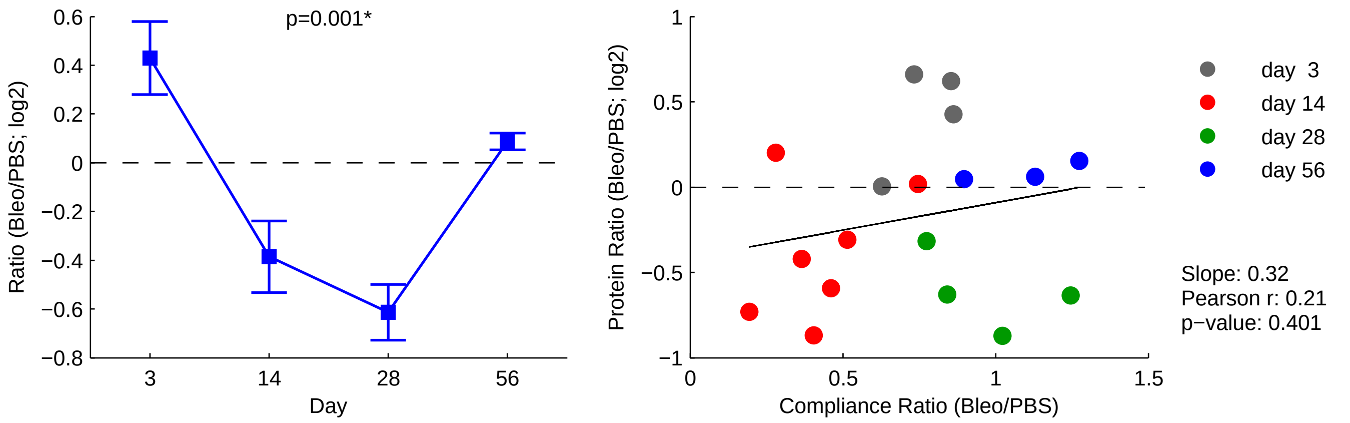

O88325 − Naglu (id: 2170)

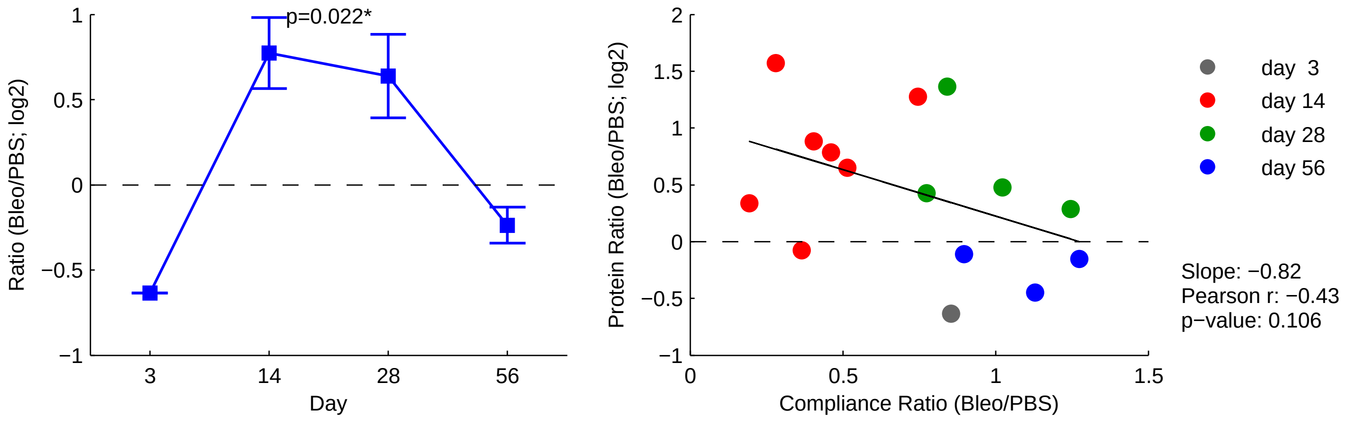

O88342 − Wdr1 (id: 2173)

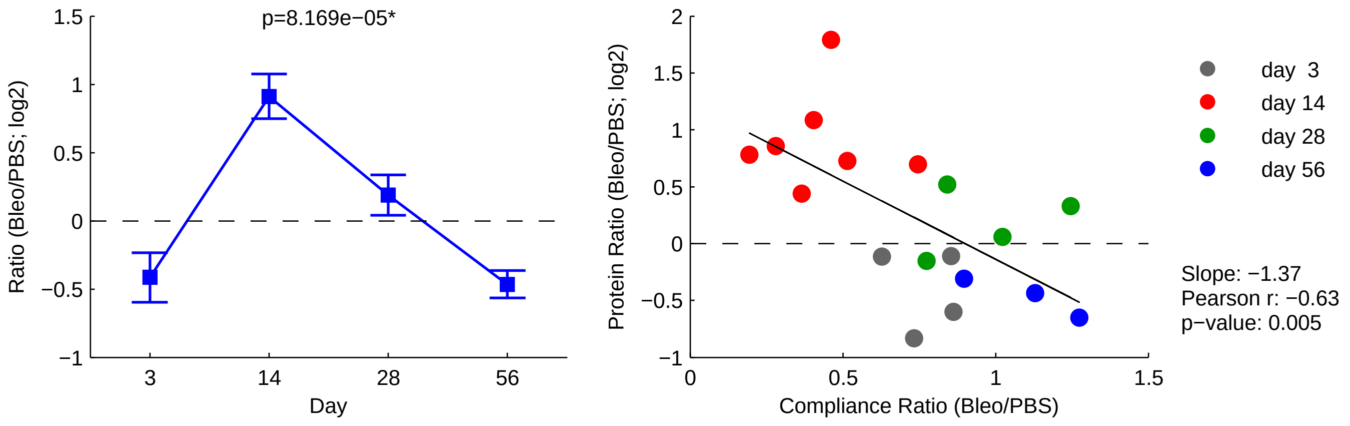

Q5D0E0 − Ikbkb (id: 2174)

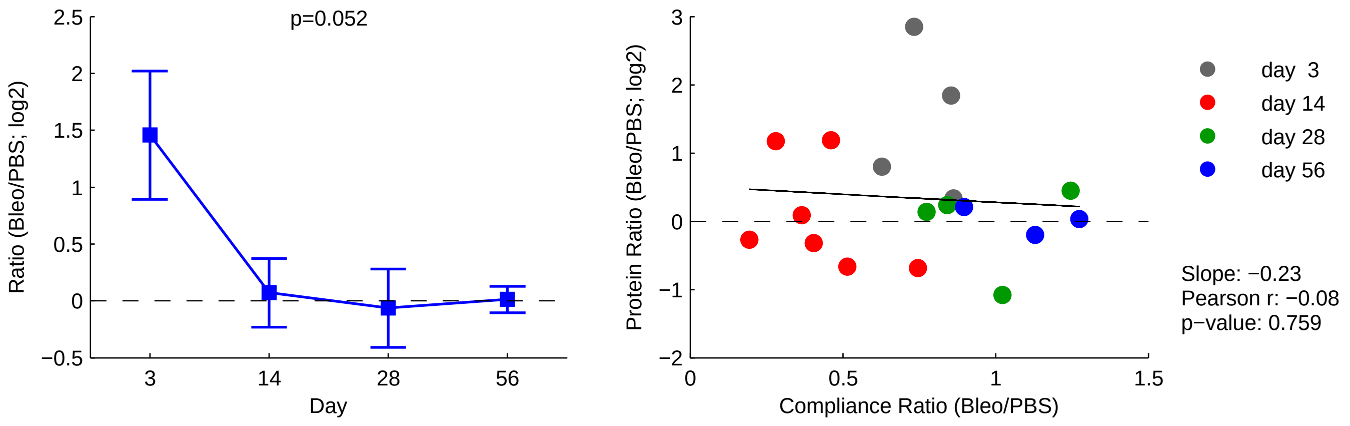

O88396 − Grpel2 (id: 2176)

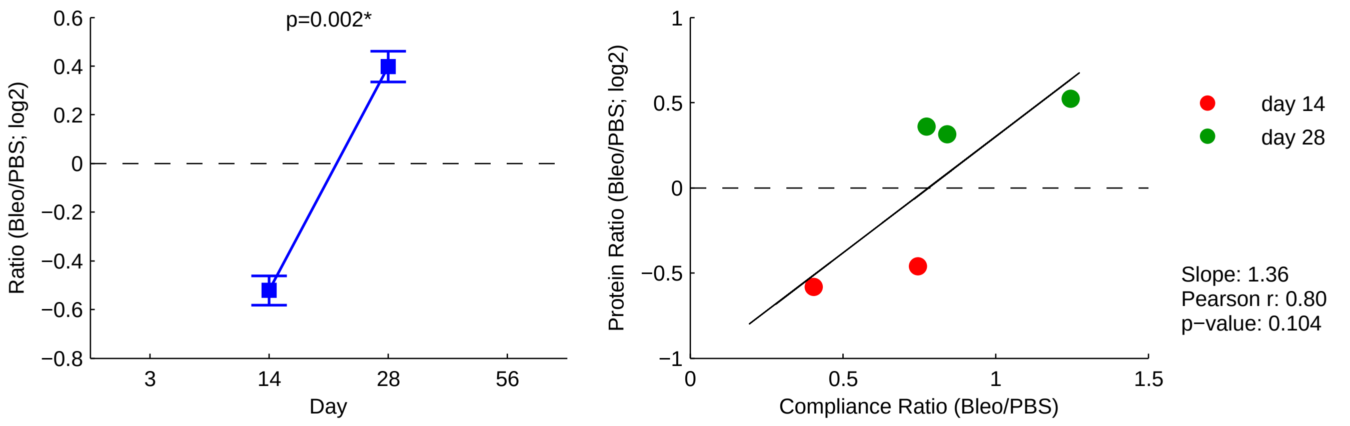

O88413 − Tulp3 (id: 2177)

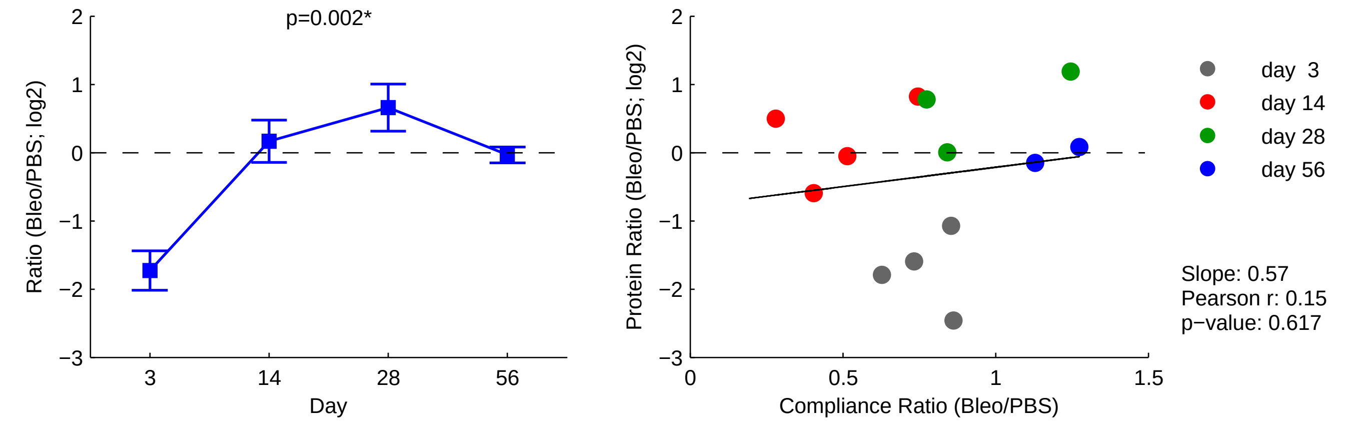

O88428 − Papss2 (id: 2178)

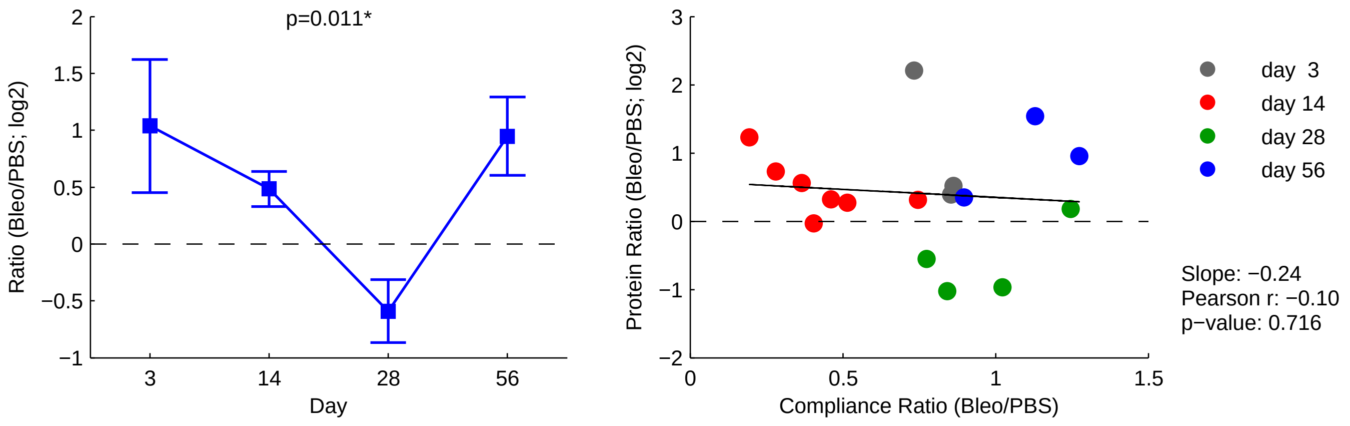

O88441 − Mtx2 (id: 2179)

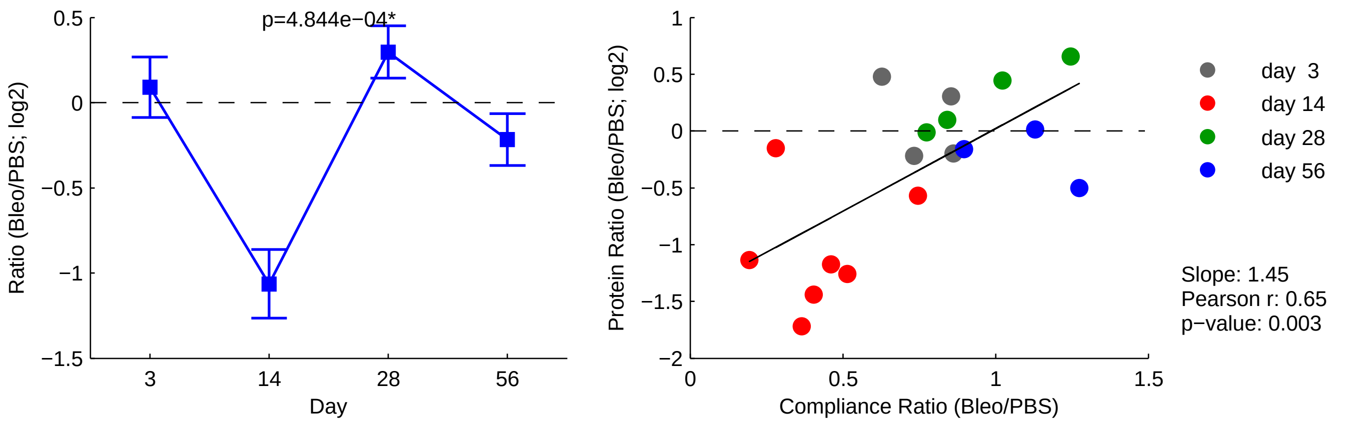

O88448 − Klc2 (id: 2180)

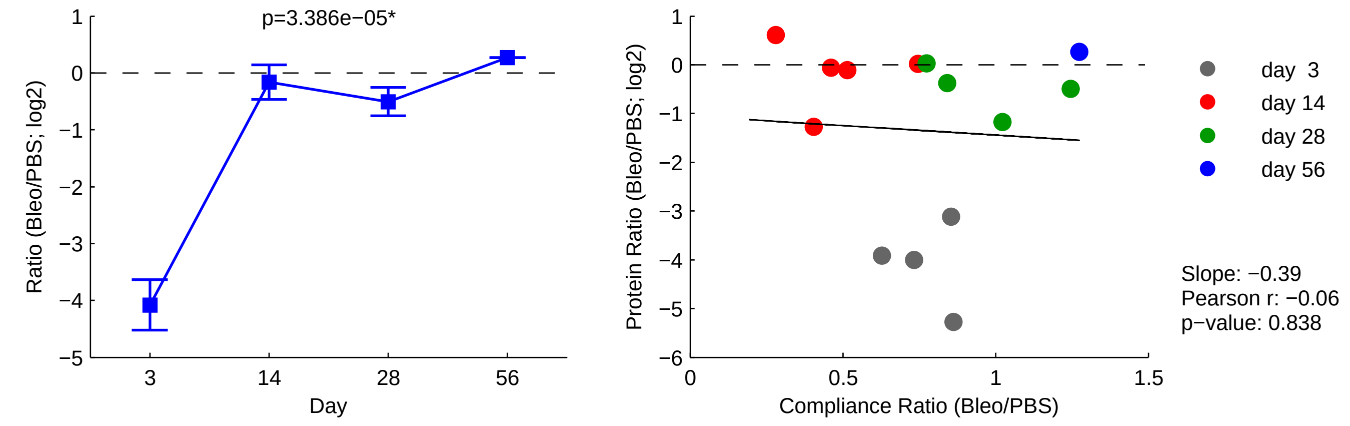

O88492 − Plin4 (id: 2183)

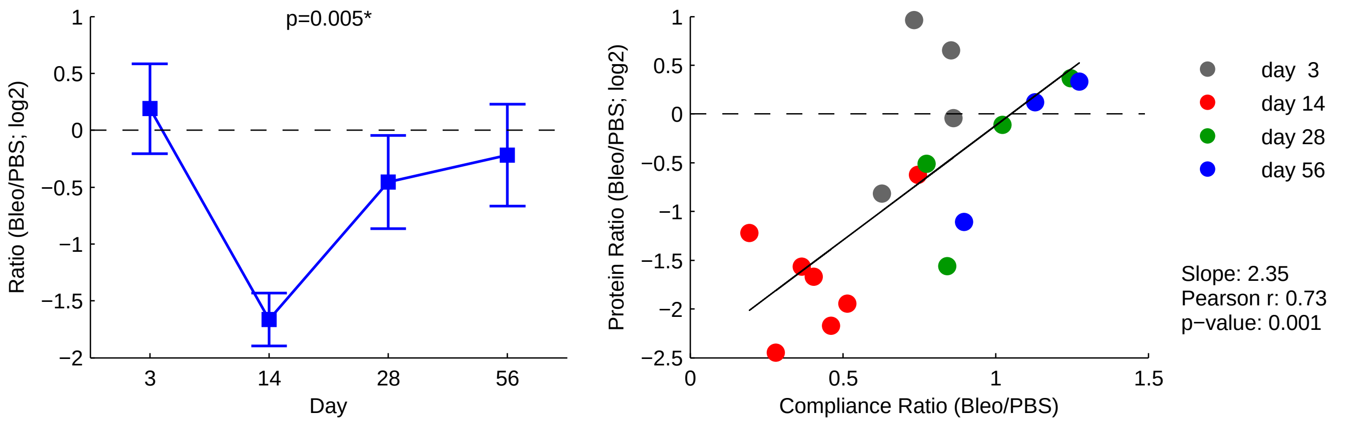

O88502 − Pde8a (id: 2184)

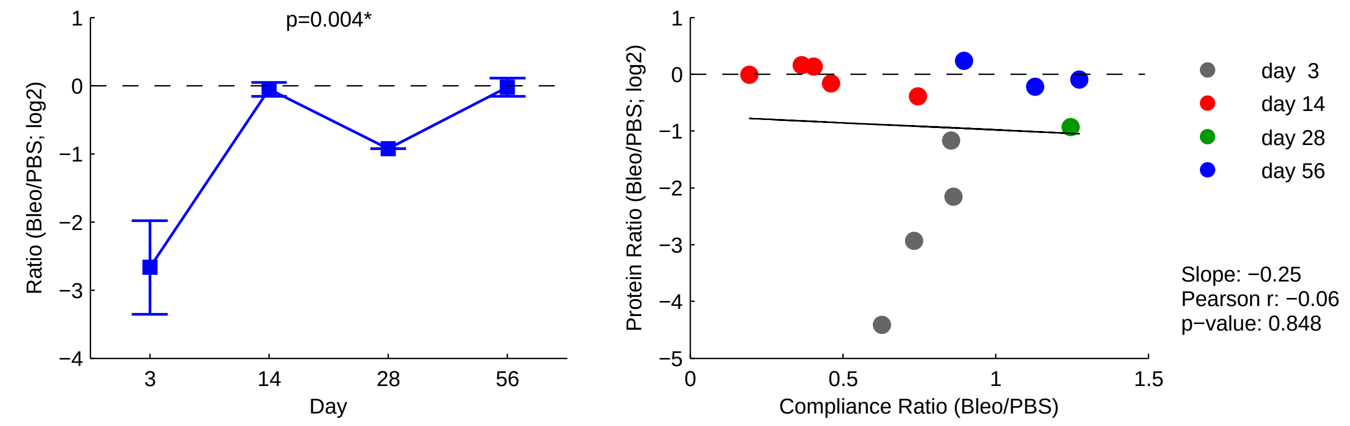

O88508 − Dnmt3a (id: 2185)

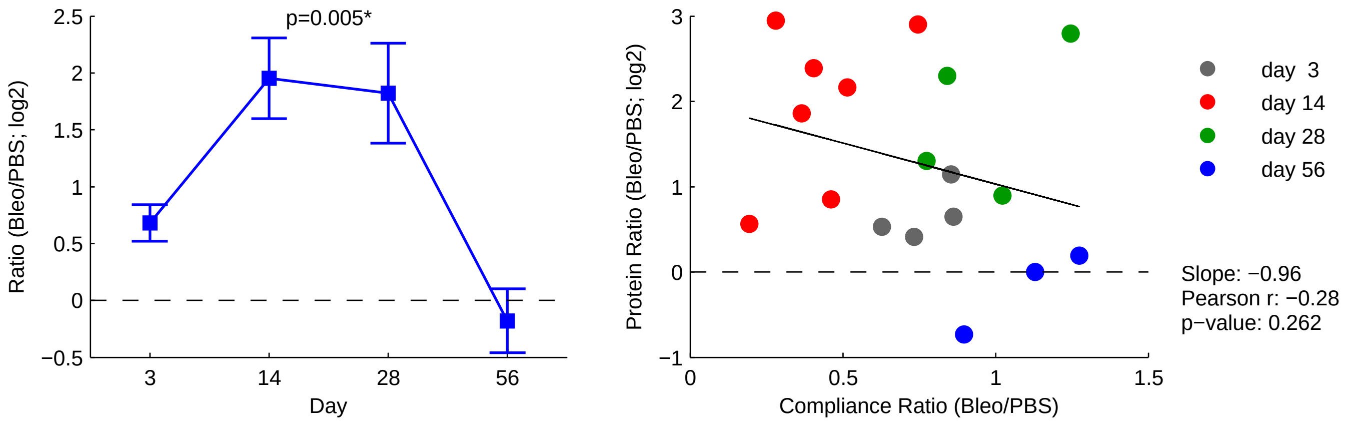

O88520 − Shoc2 (id: 2186)

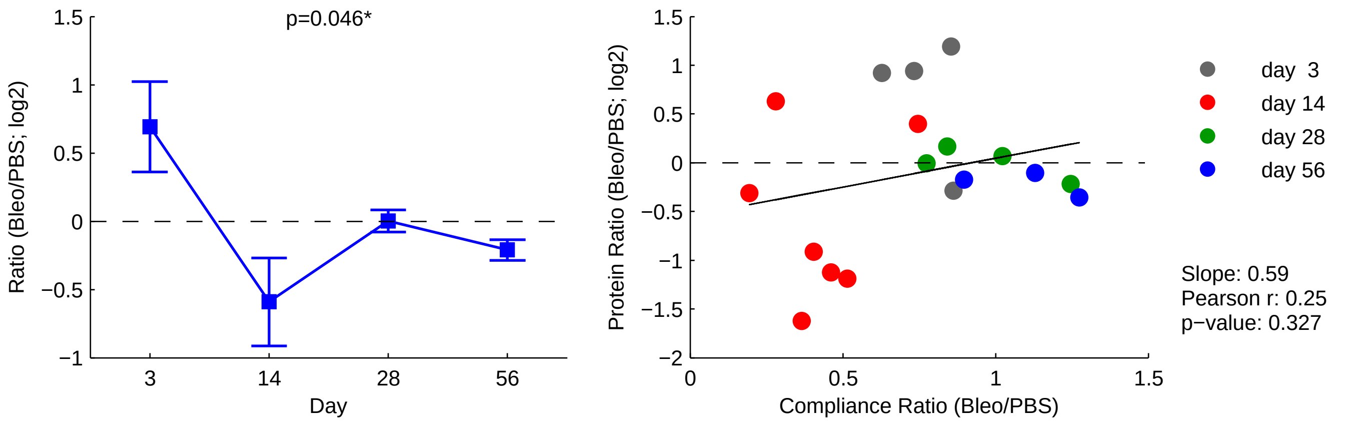

O88531 − Ppt1 (id: 2187)

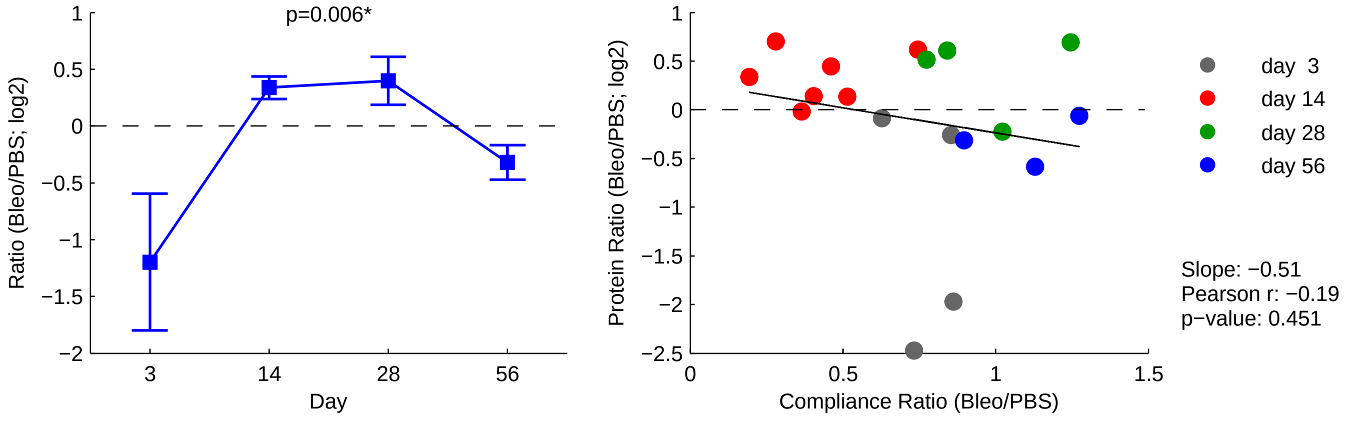

O88532 − Zfr (id: 2188)

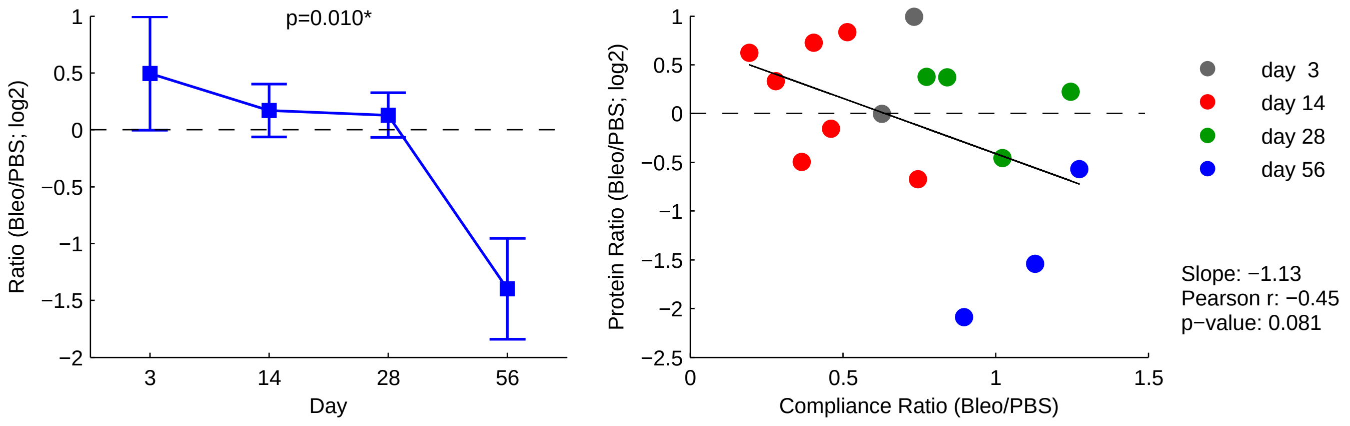

O88543 − Cops3 (id: 2189)

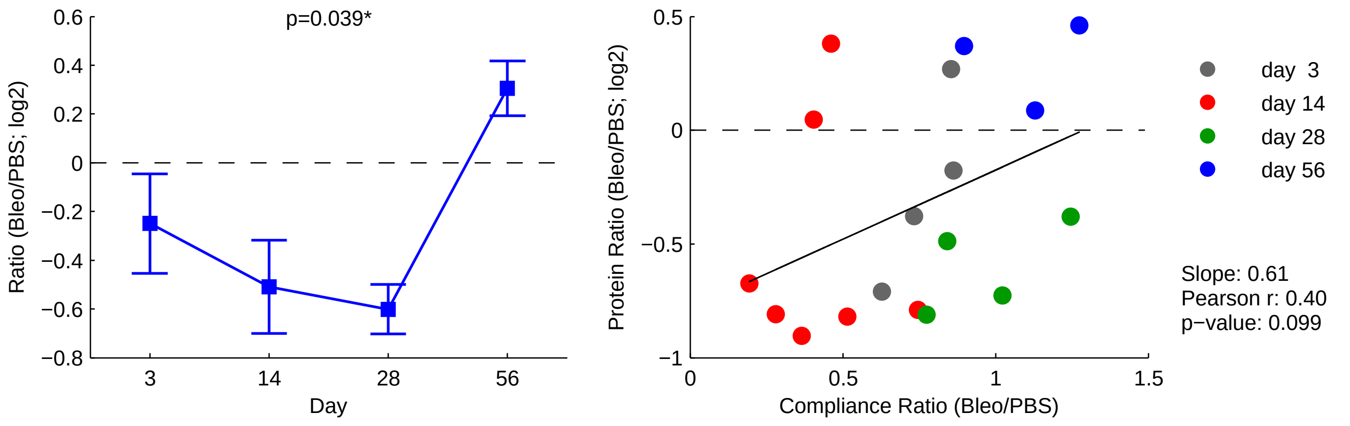

O88544 − Cops4 (id: 2190)

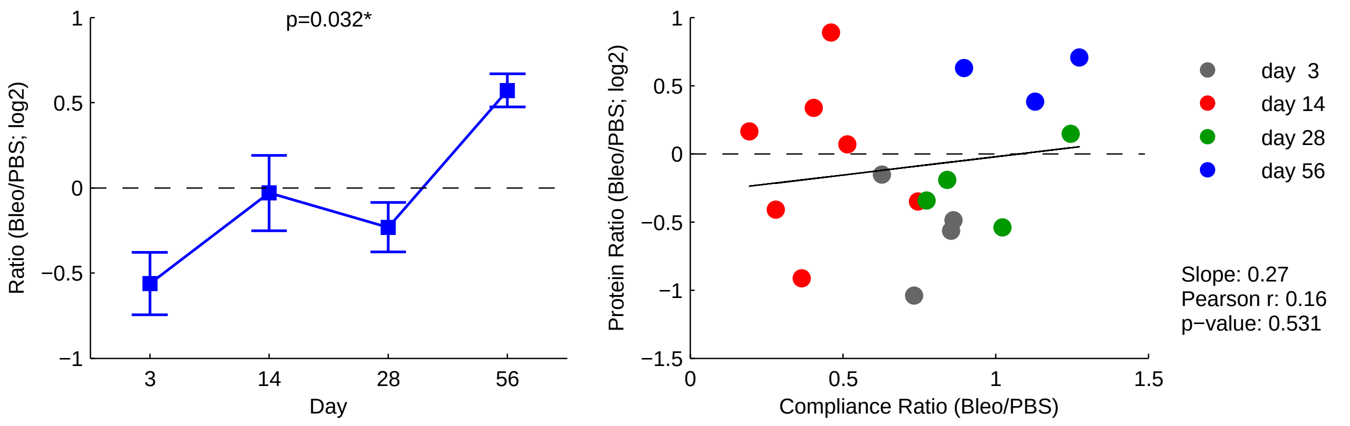

O88587−2 − Comt (id: 2195)

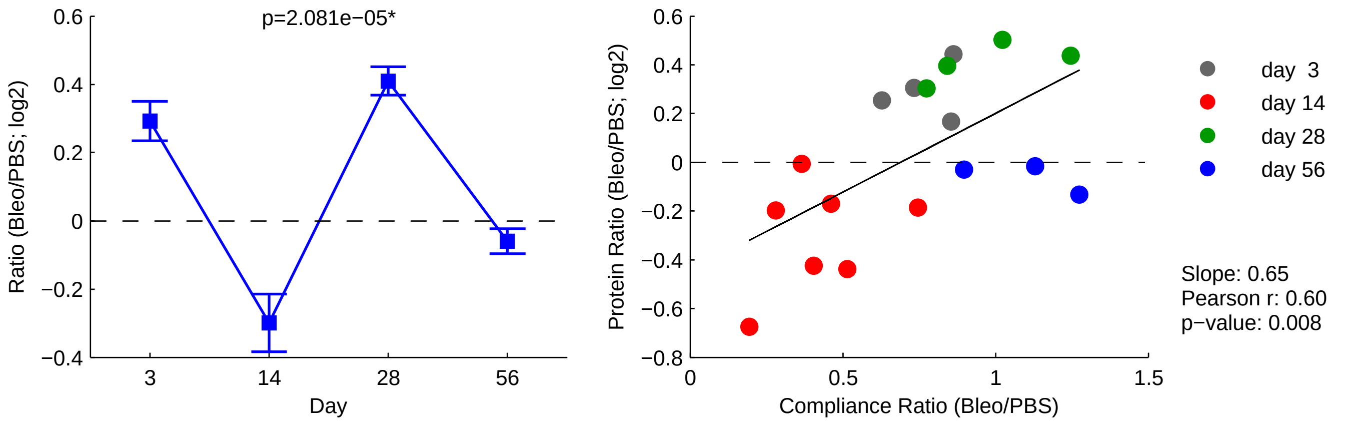

O88630 − Gosr1 (id: 2198)

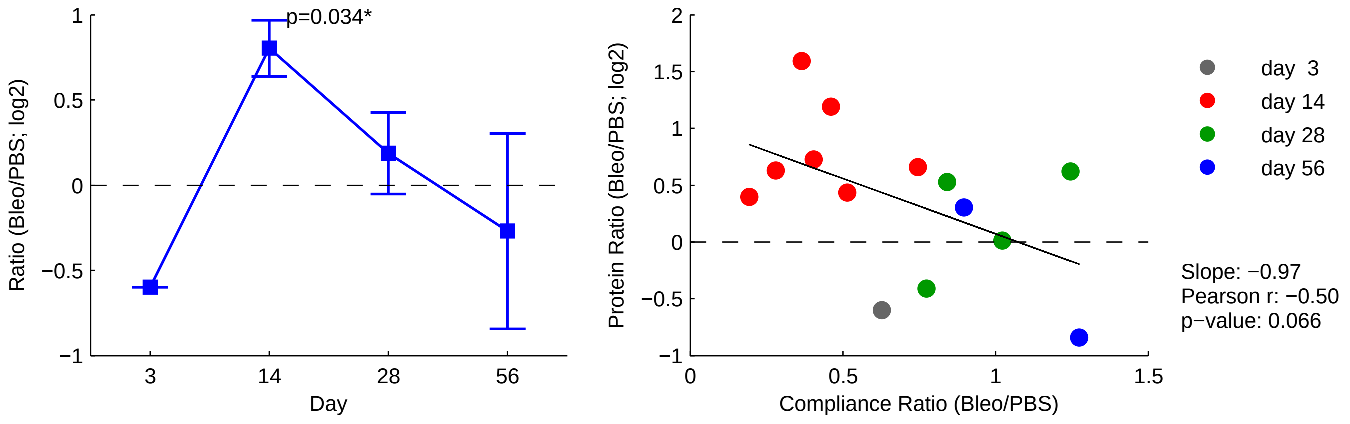

O88653 − Lamtor3 (id: 2200)

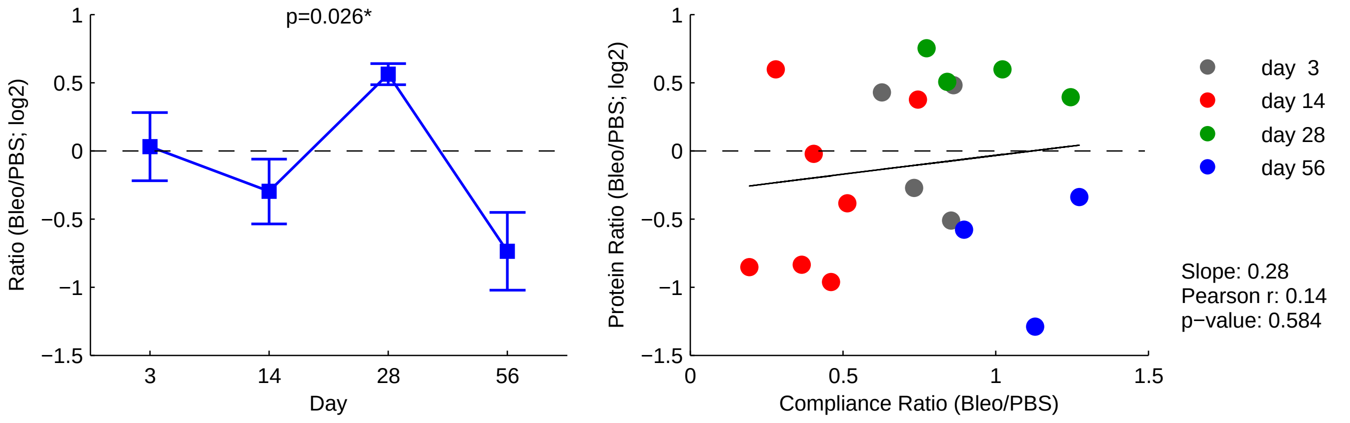

O88665 − Brd7 (id: 2201)

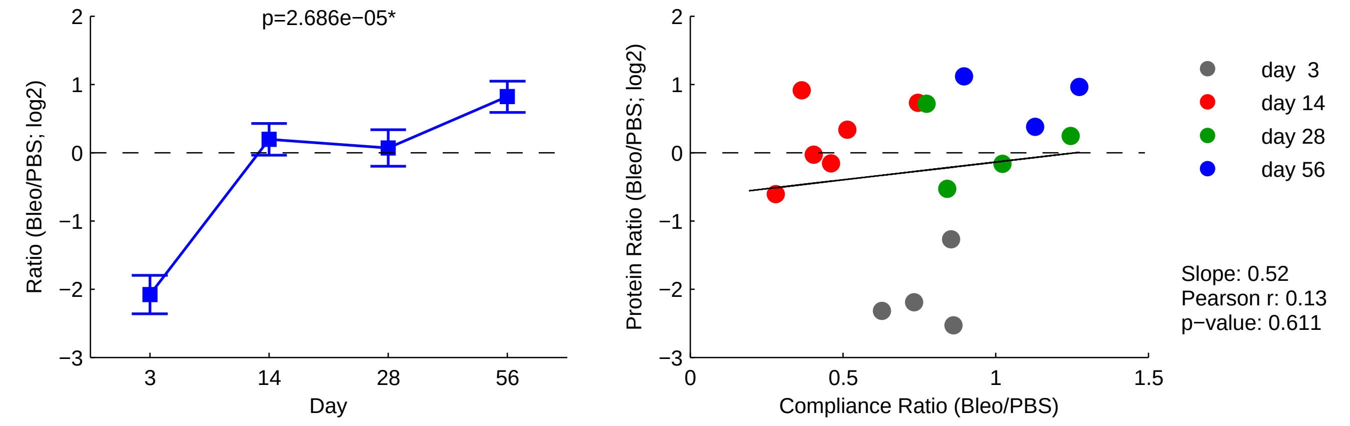

O88668 − Creg1 (id: 2202)

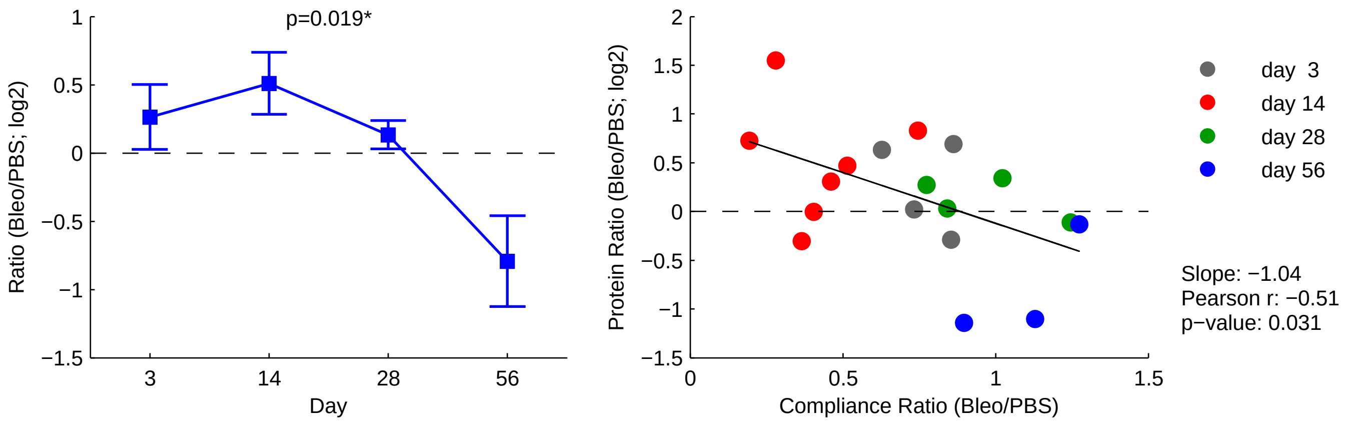

O88685 − Psmc3 (id: 2204)

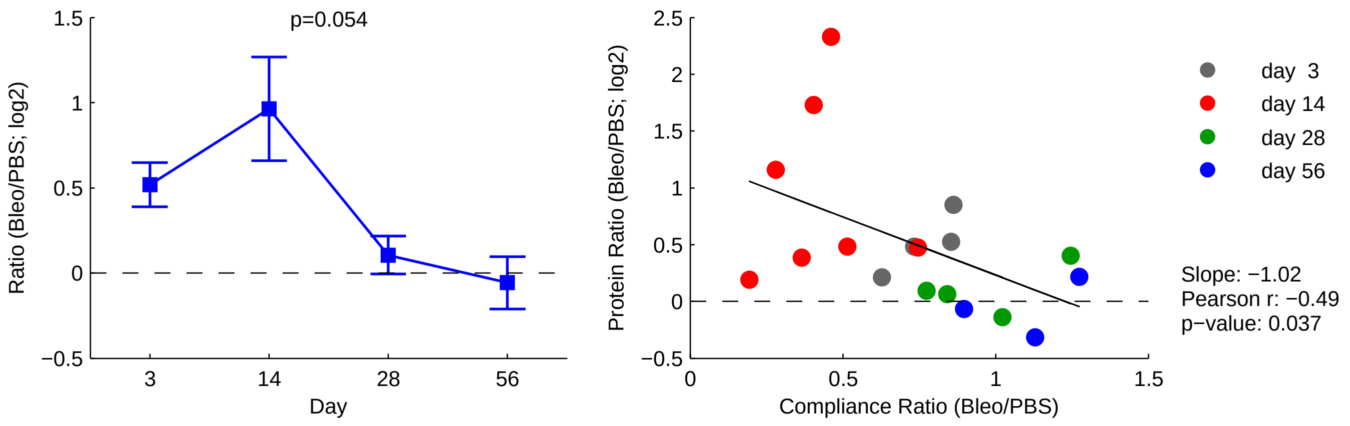

O88697 − Stk16 (id: 2207)

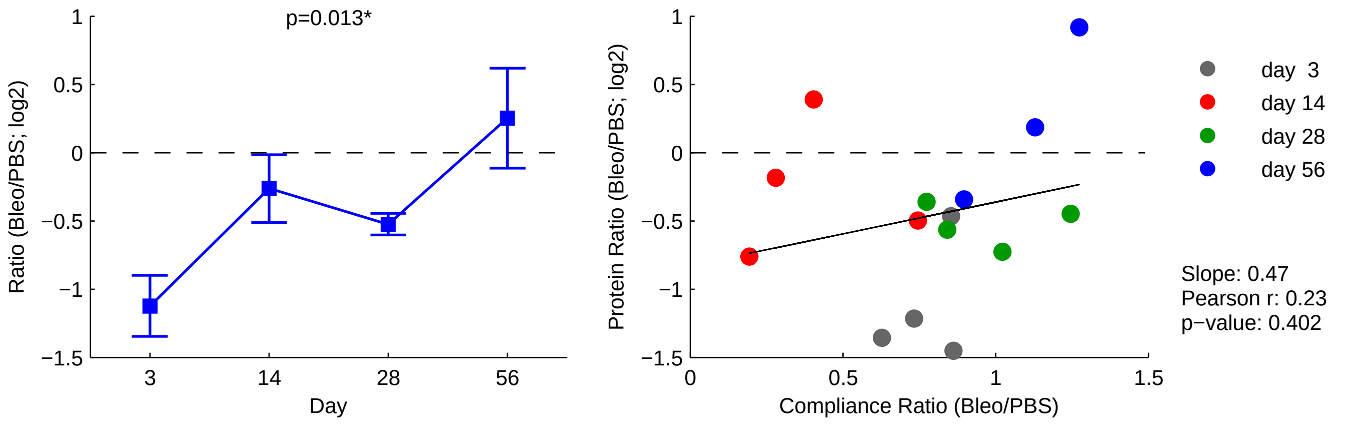

O88735 − Map7 (id: 2210)

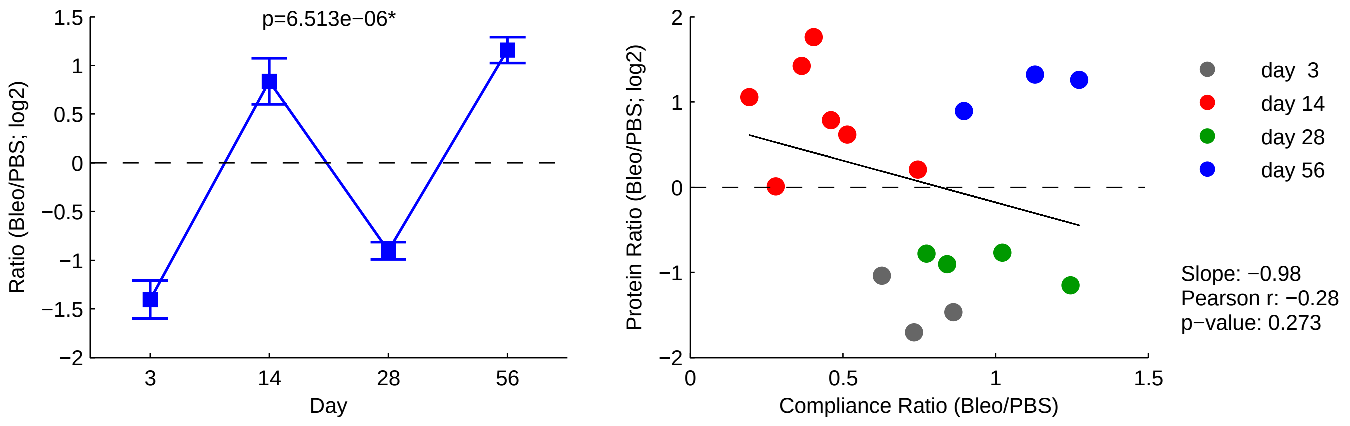

O88736 − Hsd17b7 (id: 2211)

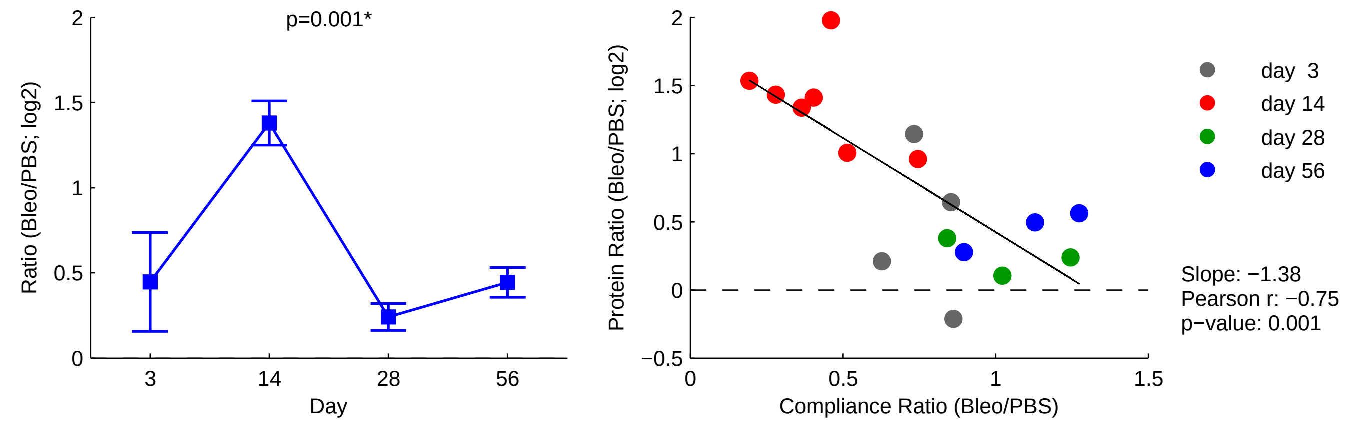

O88746 − Tom1 (id: 2212)

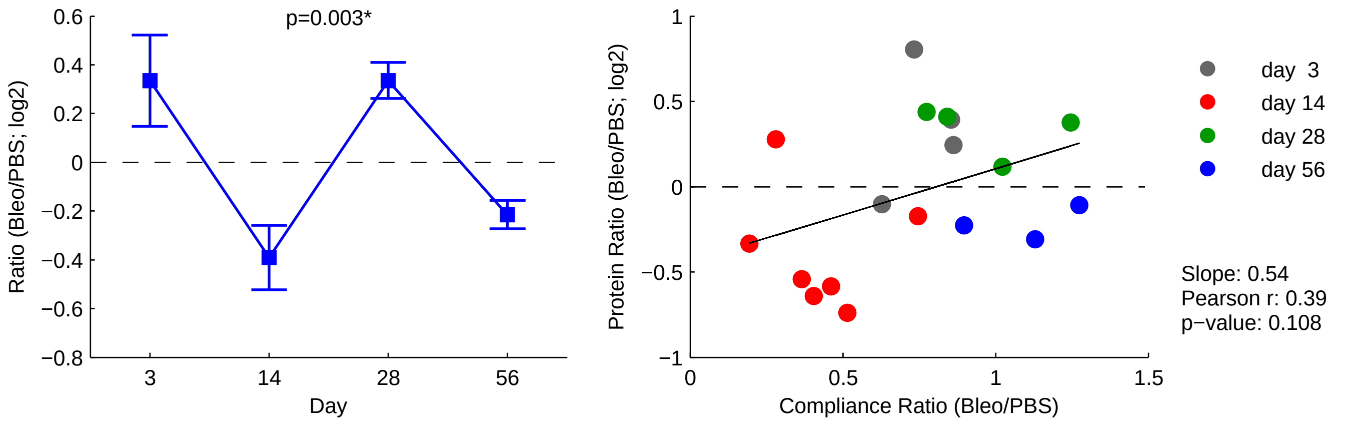

O88792 − F11r (id: 2214)

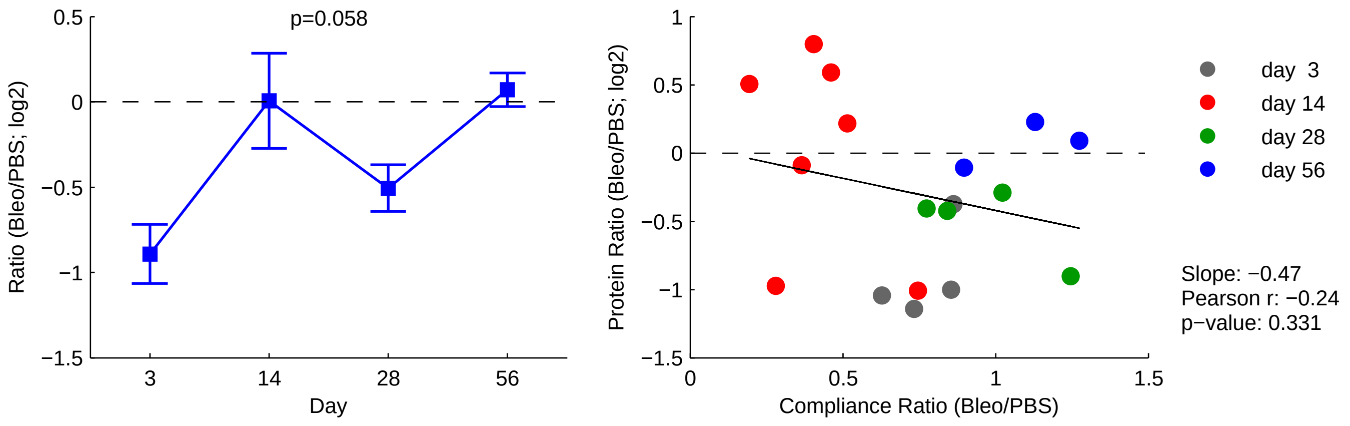

O88811 − Stam2 (id: 2216)

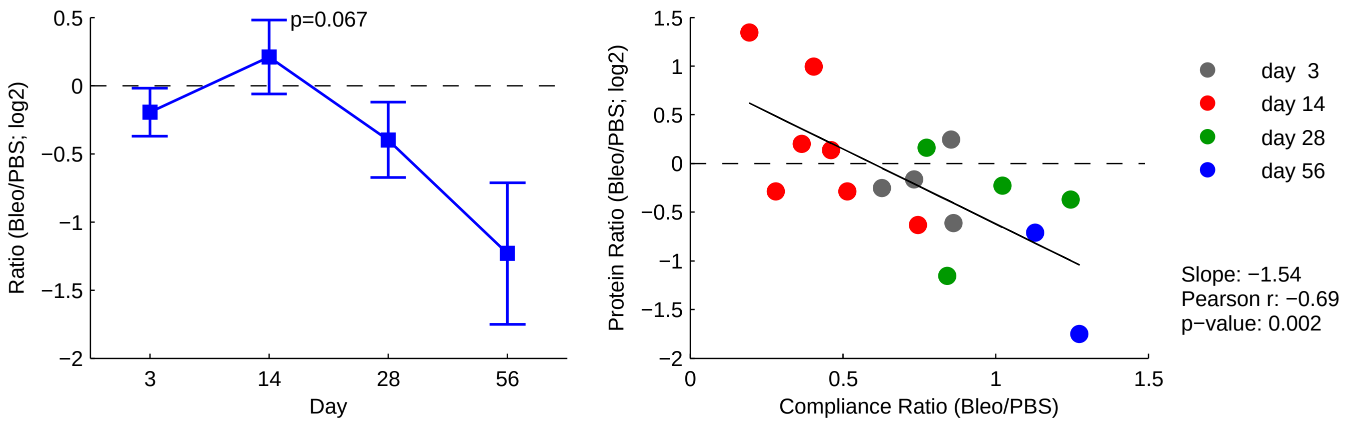

O88844 − Idh1 (id: 2220)

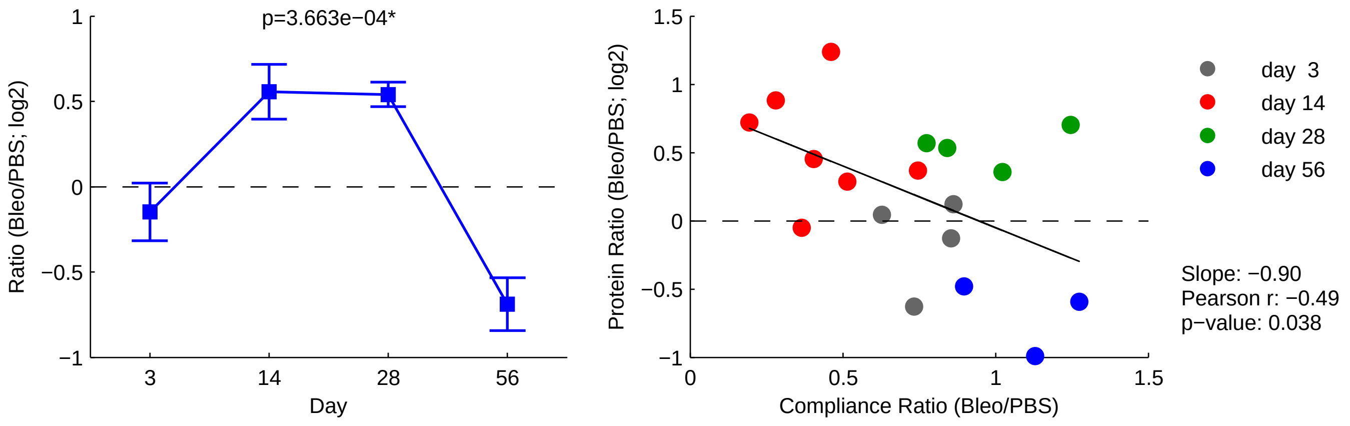

O88851 − Rbbp9 (id: 2222)

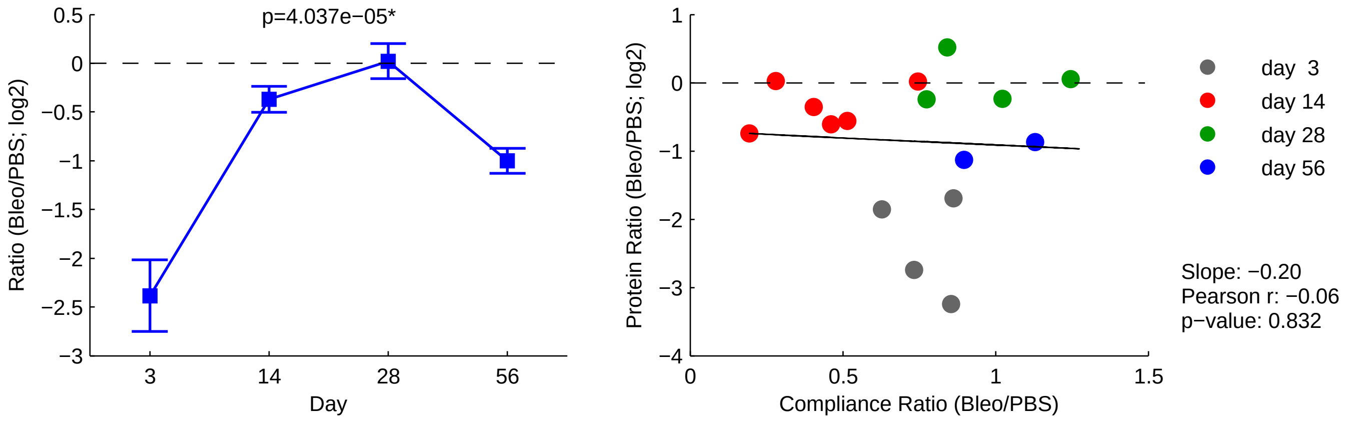

O88856 − Tpst2 (id: 2223)

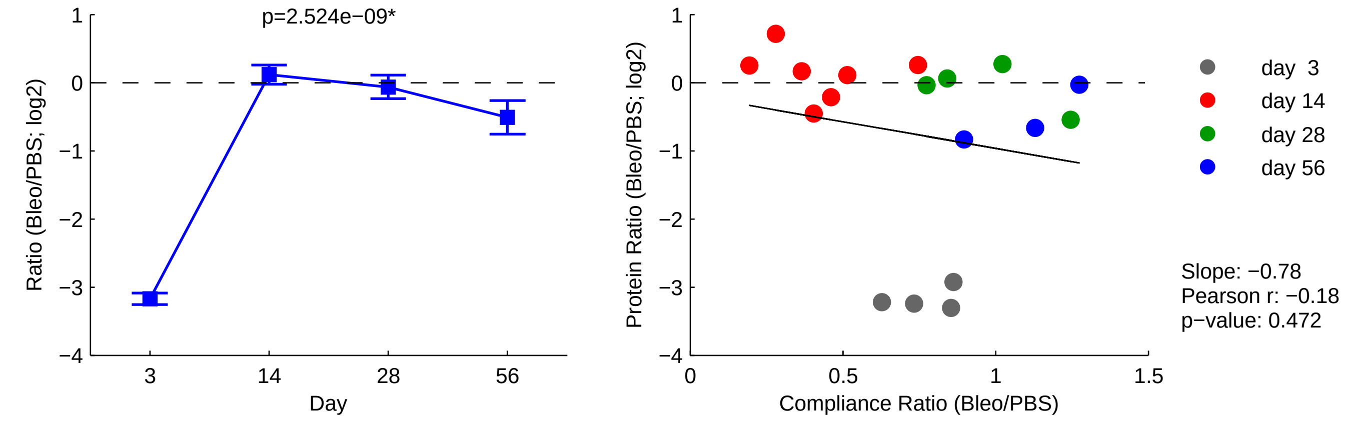

O88874 − Ccnk (id: 2224)

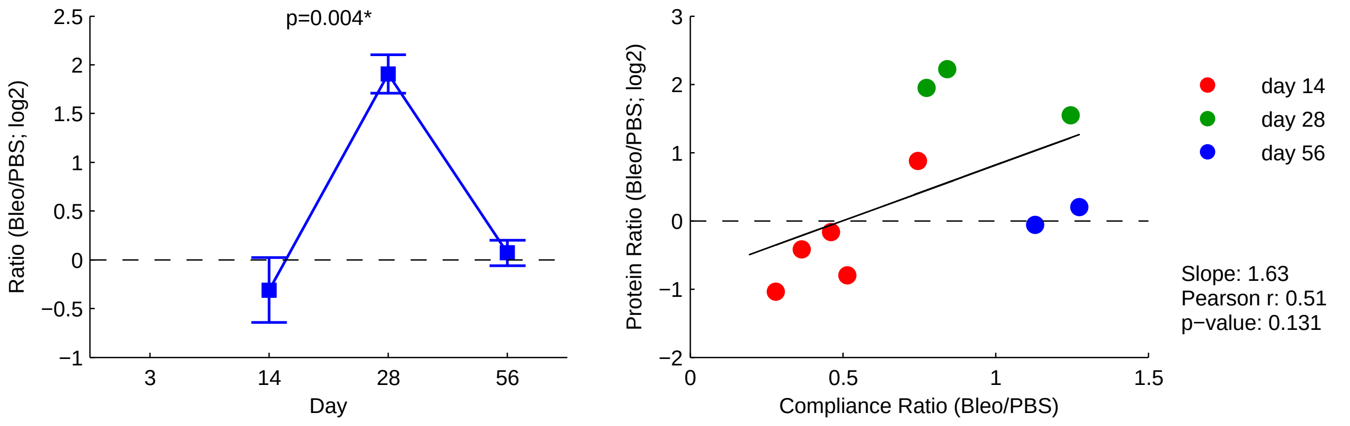

O88876 − Dhrs3 (id: 2225)

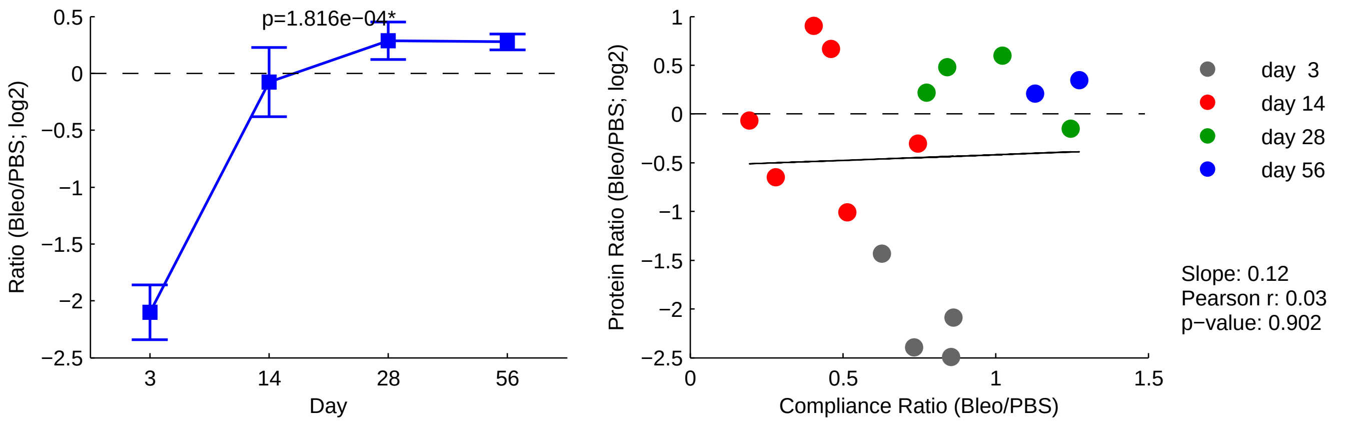

O88952 − Lin7c (id: 2231)

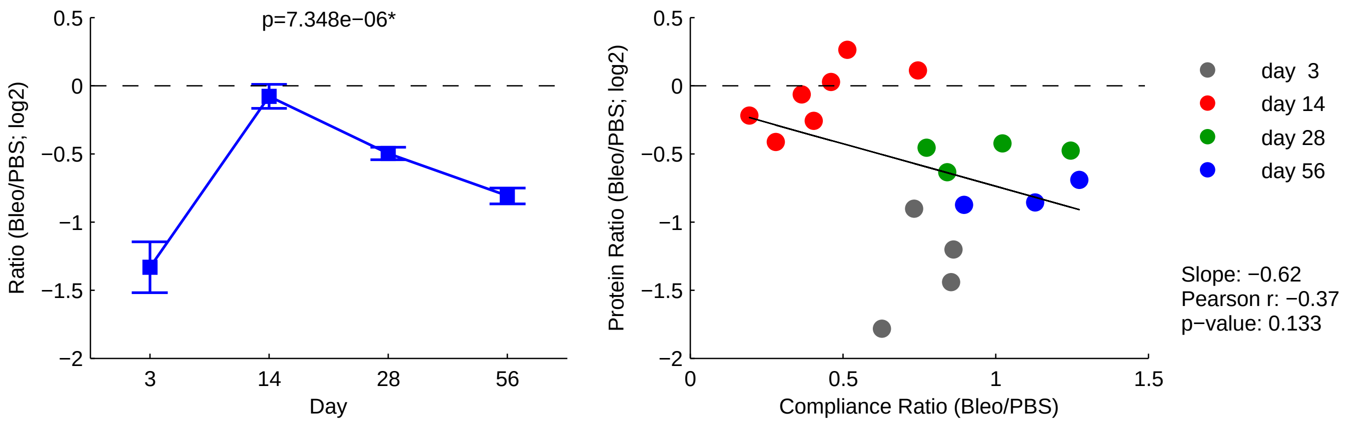

O88967 − Yme1l1 (id: 2233)

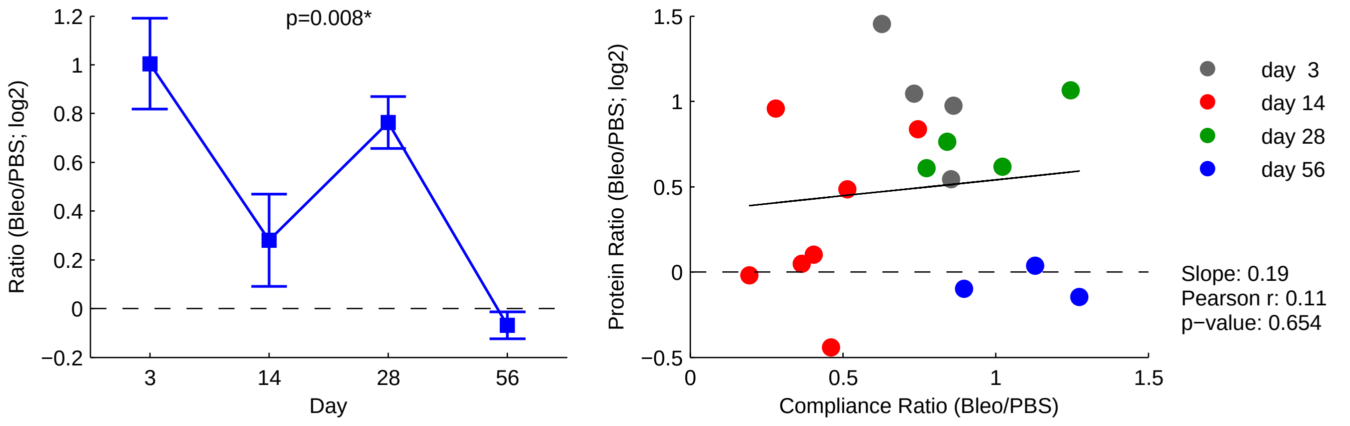

O88968 − Tcn2 (id: 2234)

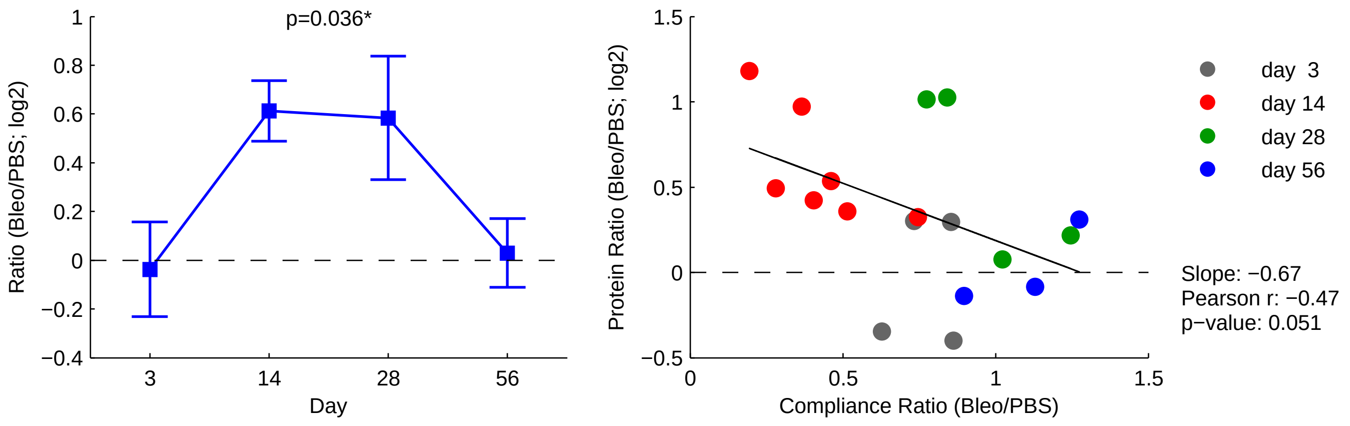

O88986 − Gcat (id: 2236)

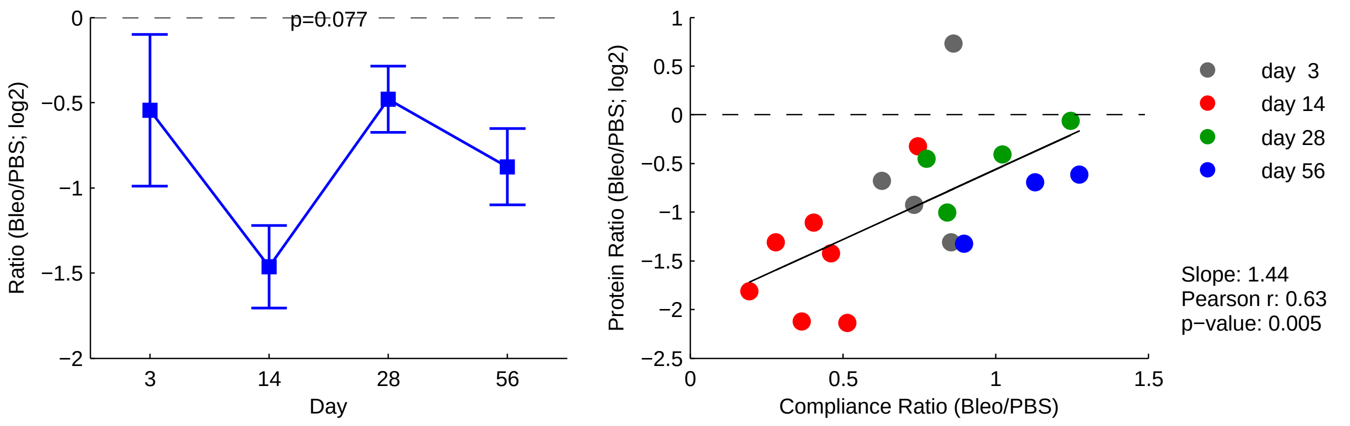

O88990 − Actn3 (id: 2237)

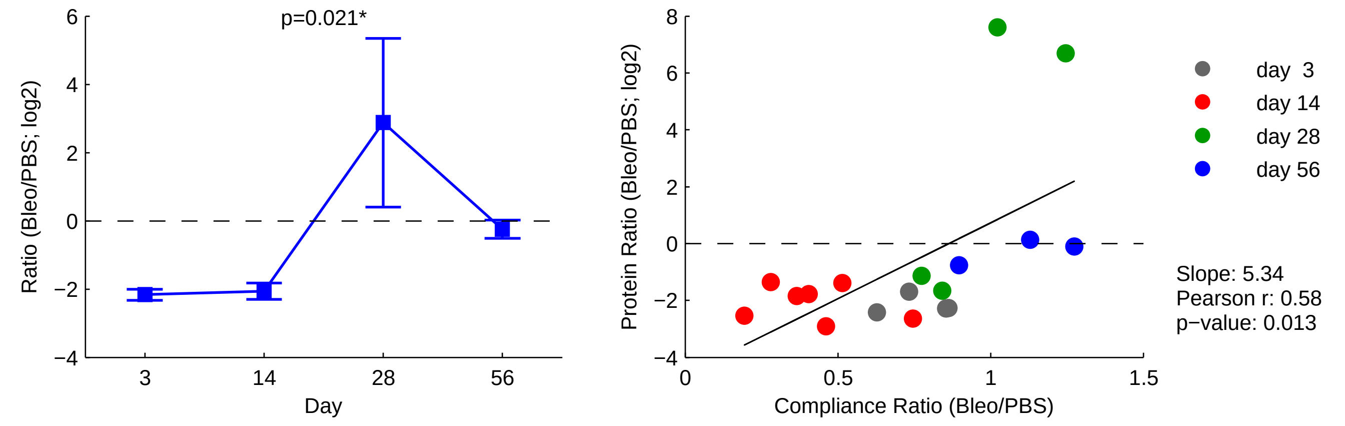

O89001 − Cpd (id: 2238)

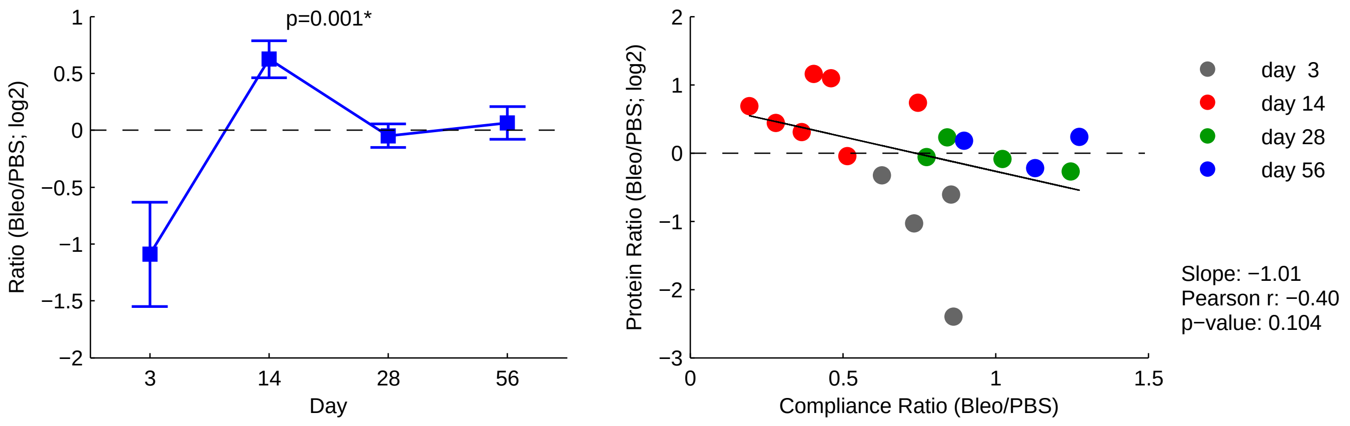

O89016 − Abcd4 (id: 2239)

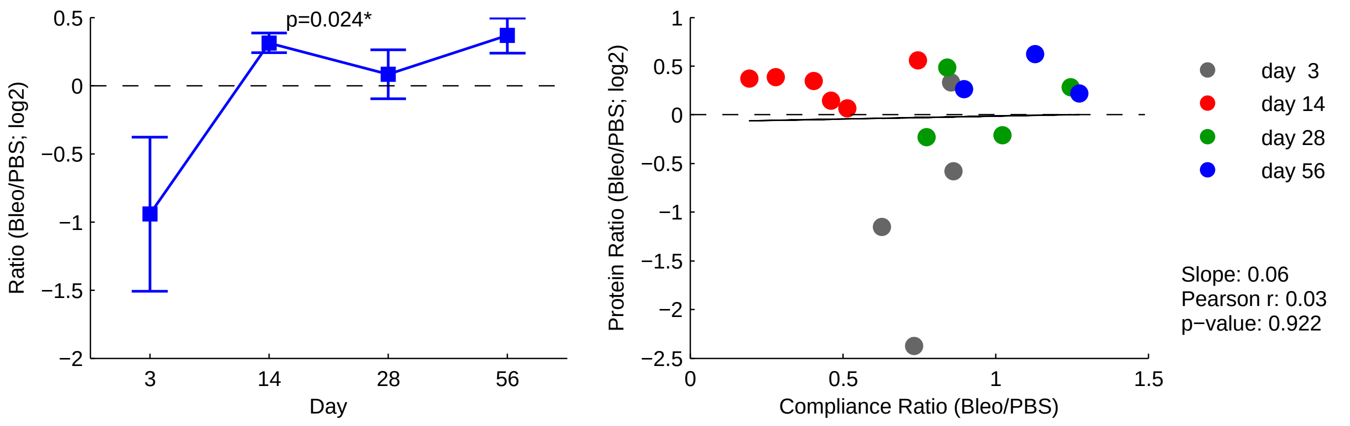

O89017 − Lgmn (id: 2240)

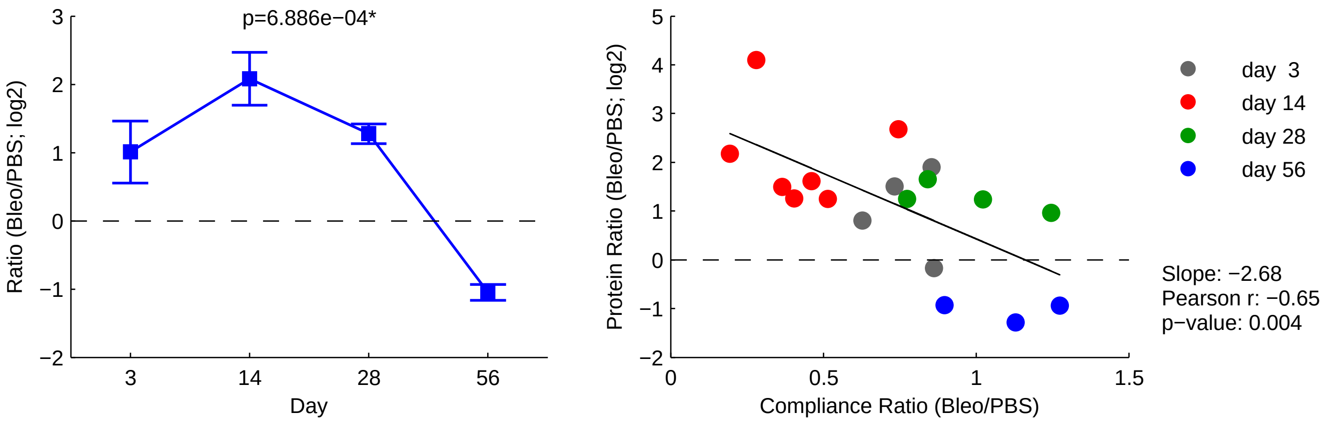

O89023 − Tpp1 (id: 2242)

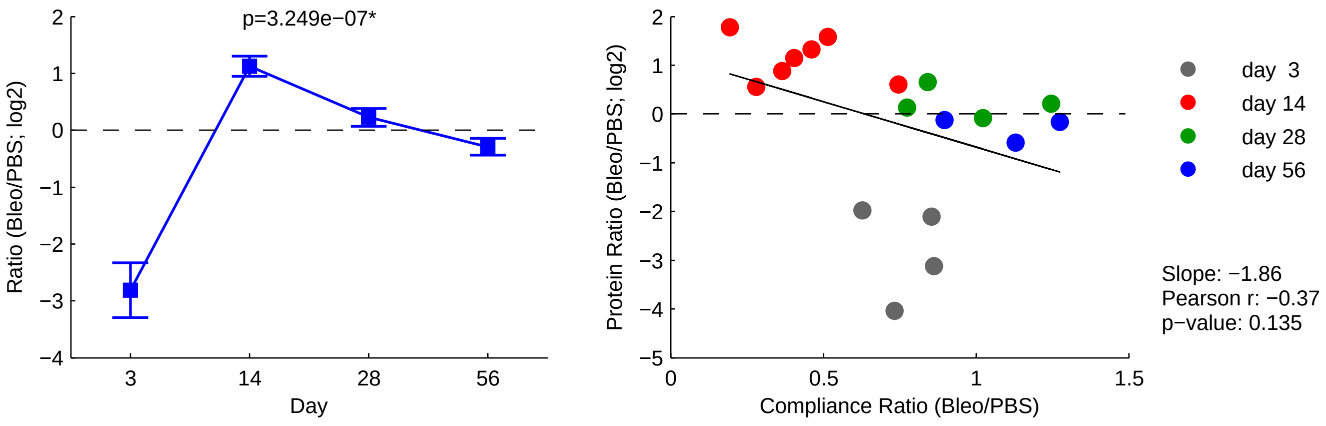

O89051 − Itm2b (id: 2244)

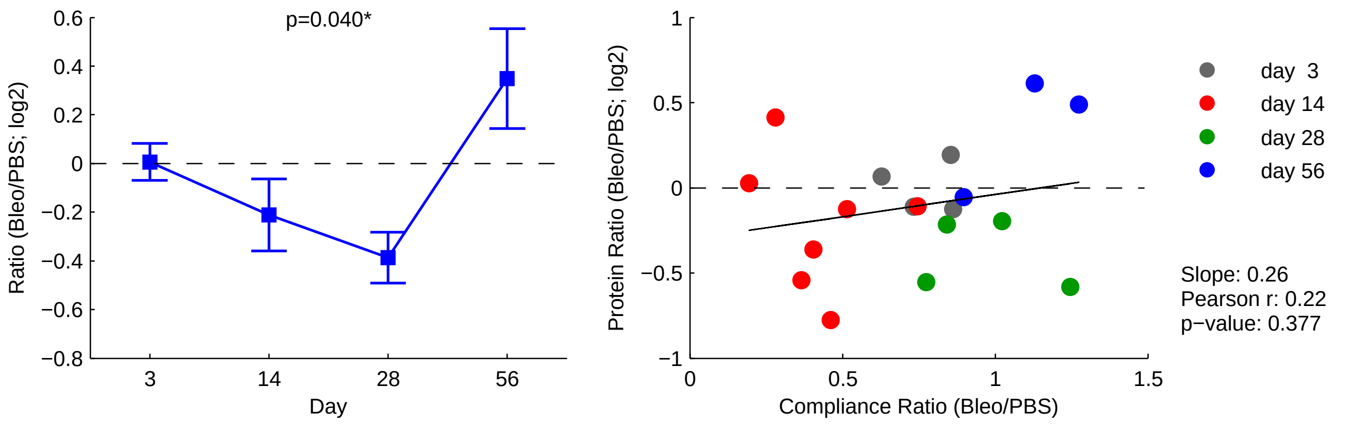

O89079 − Cope (id: 2246)

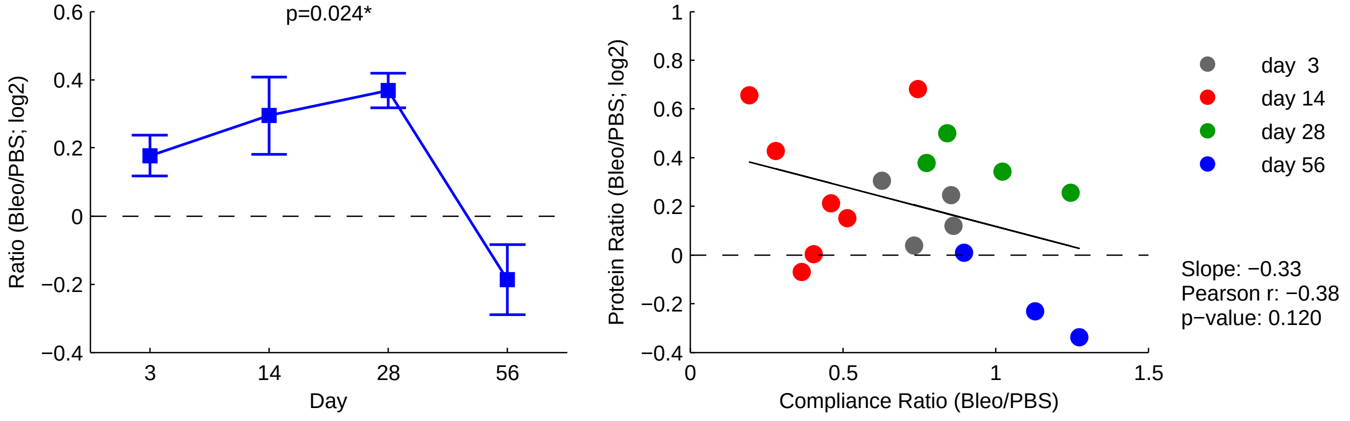

O89086 − Rbm3 (id: 2247)

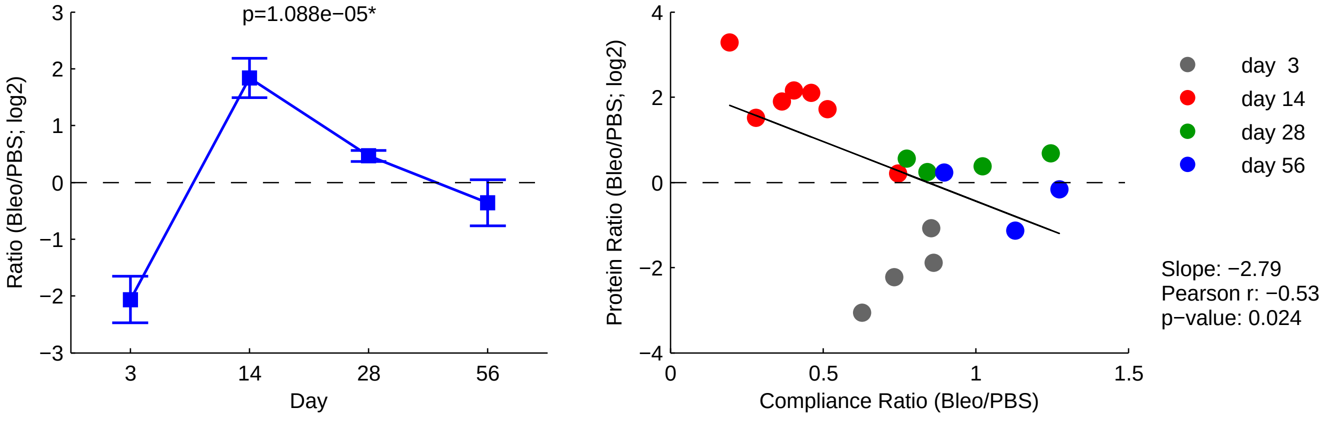

O89103 − Cd93 (id: 2249)

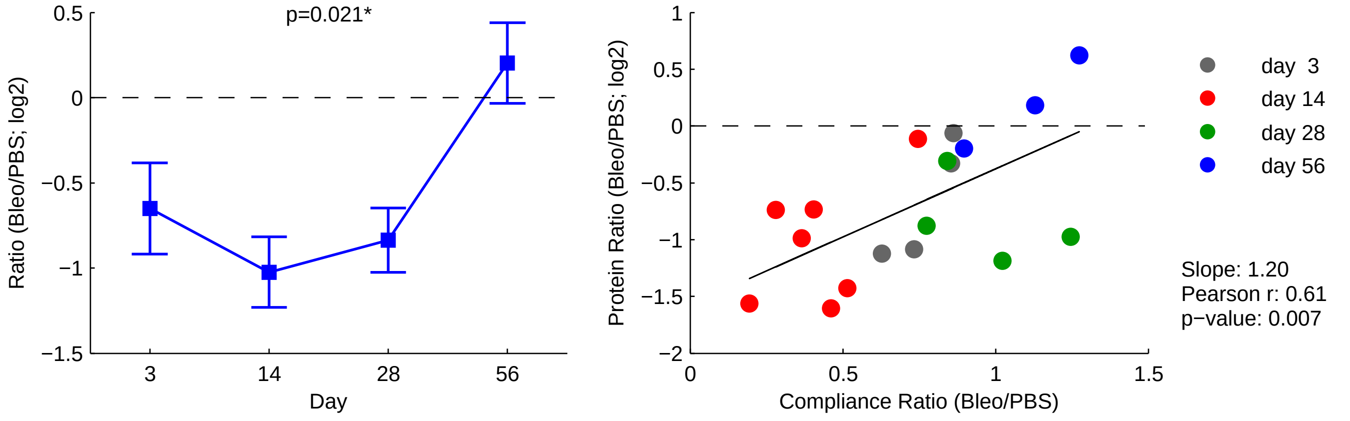

O89112 − Lancl1 (id: 2252)

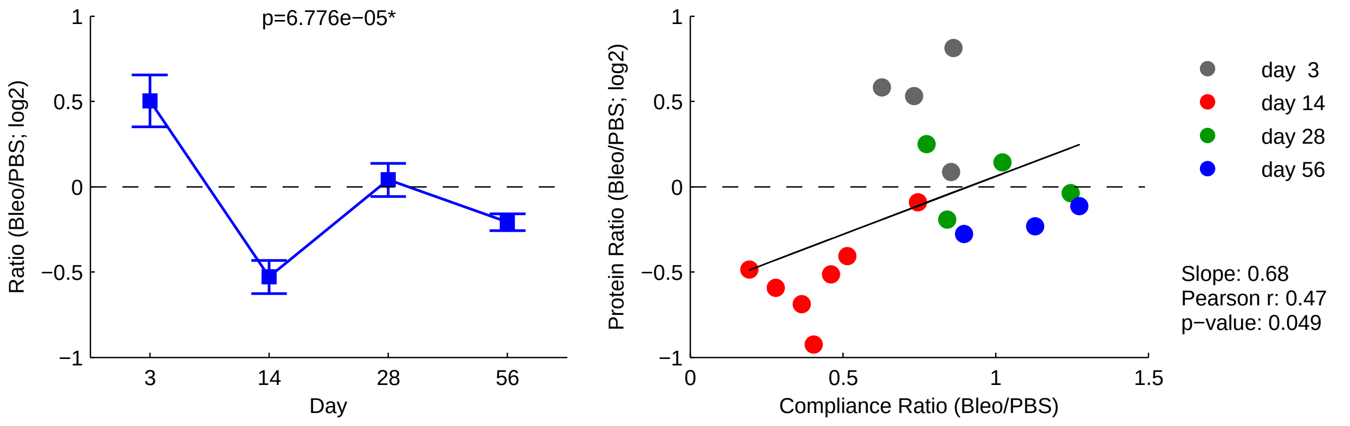

O89116 − Vti1a (id: 2253)

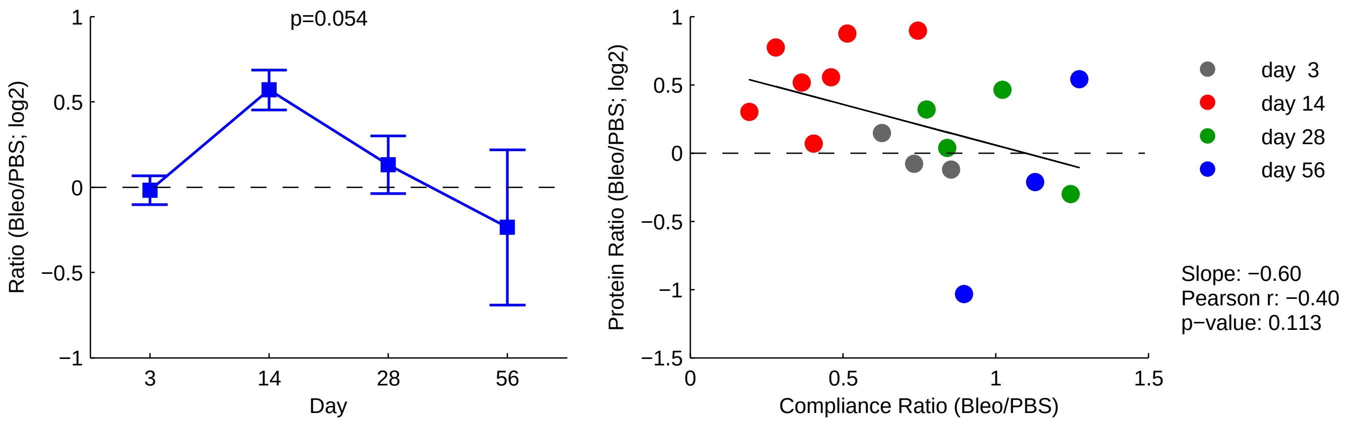

P00329 − Adh1 (id: 2256)

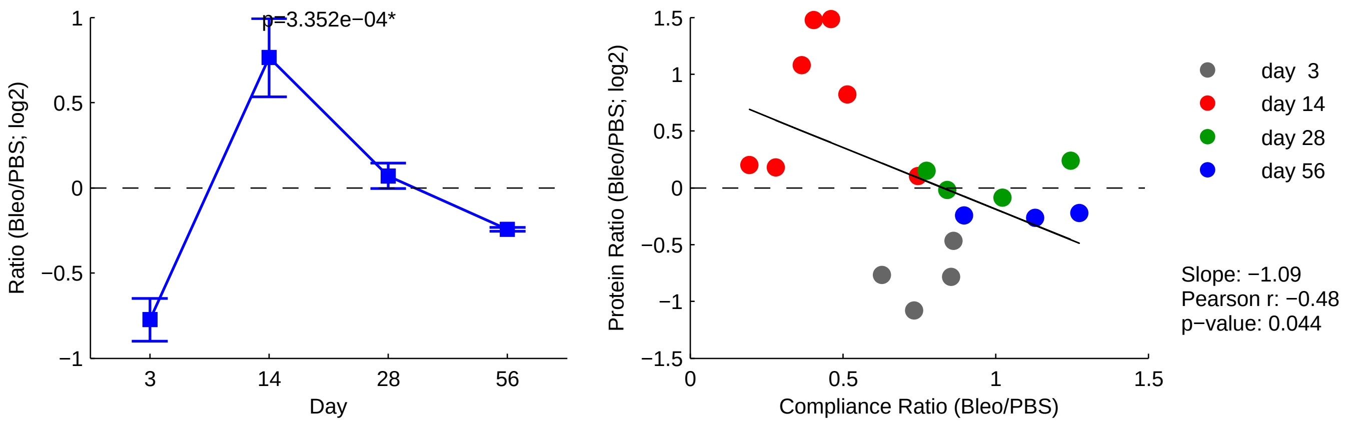

Q7JCZ1 − mt−Co2 (id: 2259)

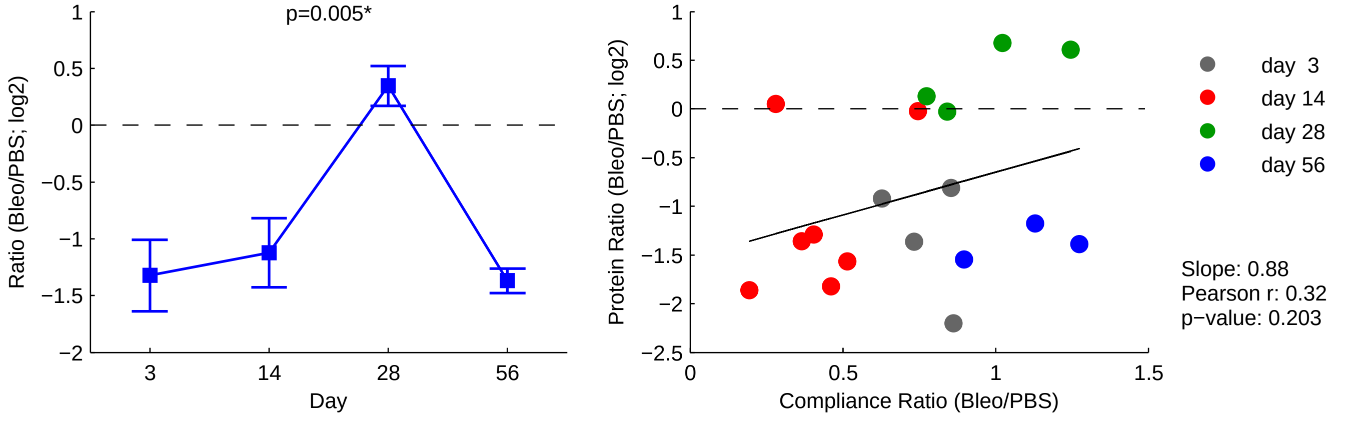

P00920 − Ca2 (id: 2262)

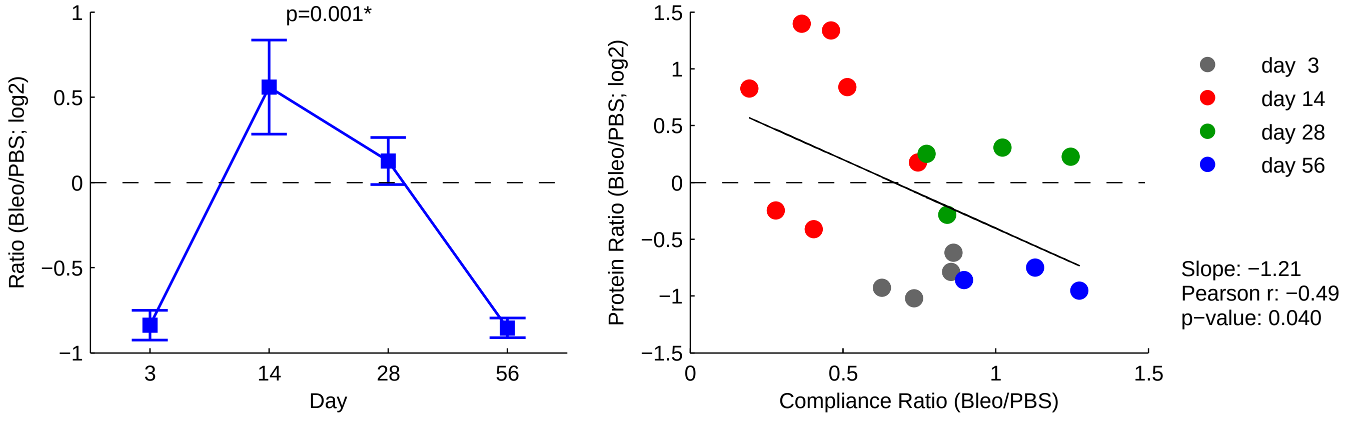

P01027 − C3 (id: 2263)

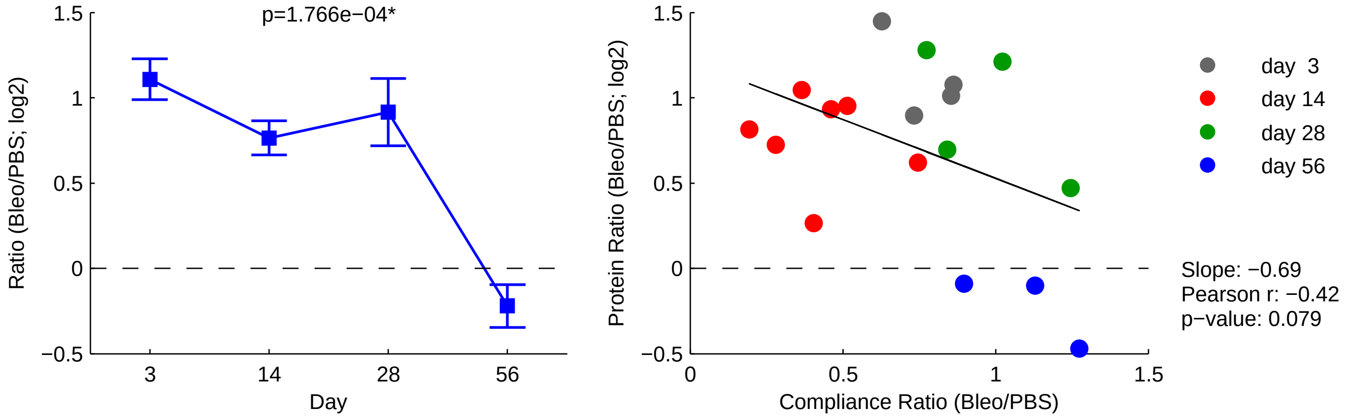

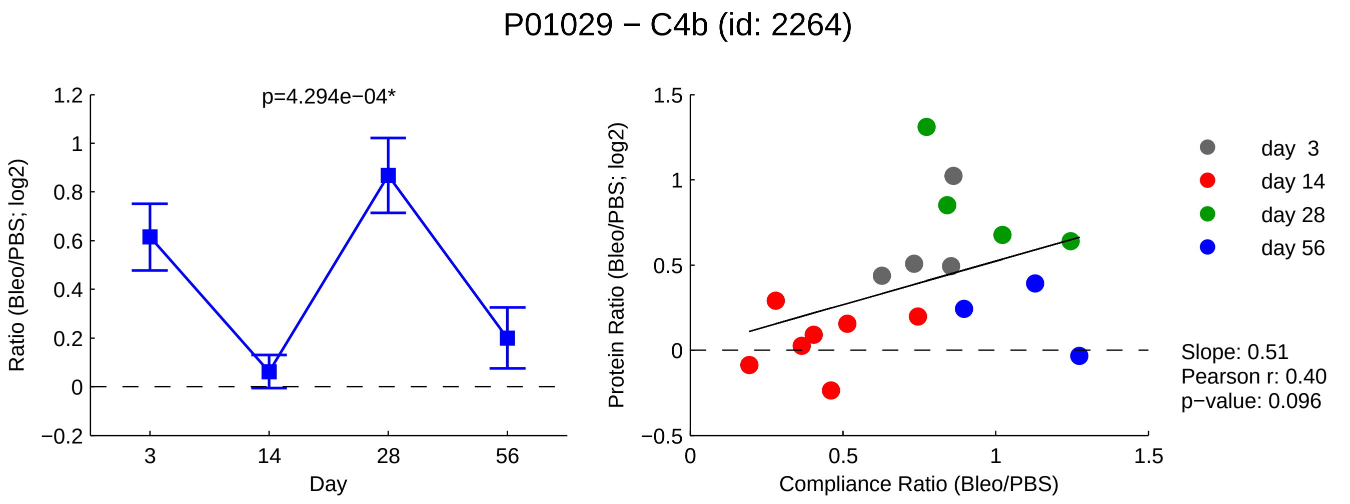
P01029 − C4b (id: 2264)
p=4.294e−04*
Ratio (Bleo/PBS; log2)
Day
Protein Ratio (Bleo/PBS; log2)
Compliance Ratio (Bleo/PBS)
day 3
day 14
day 28
day 56
Slope: 0.51
Pearson r: 0.40
p−value: 0.096

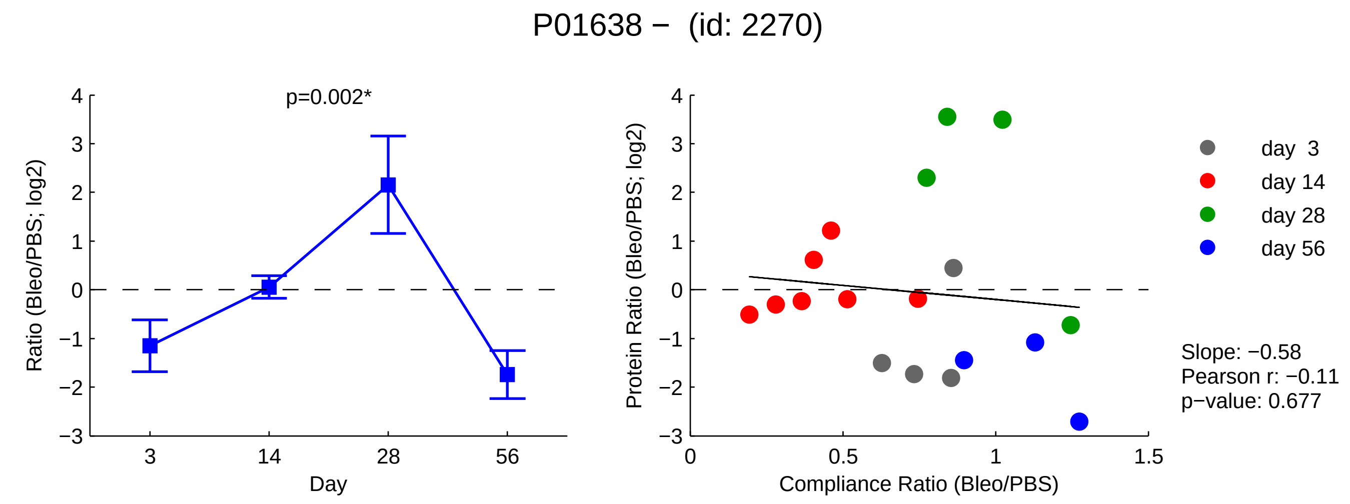
P01638 − (id: 2270)
p=0.002*
Ratio (Bleo/PBS; log2)
Day
Protein Ratio (Bleo/PBS; log2)
Compliance Ratio (Bleo/PBS)
day 3
day 14
day 28
day 56
Slope: −0.58
Pearson r: −0.11
p−value: 0.677

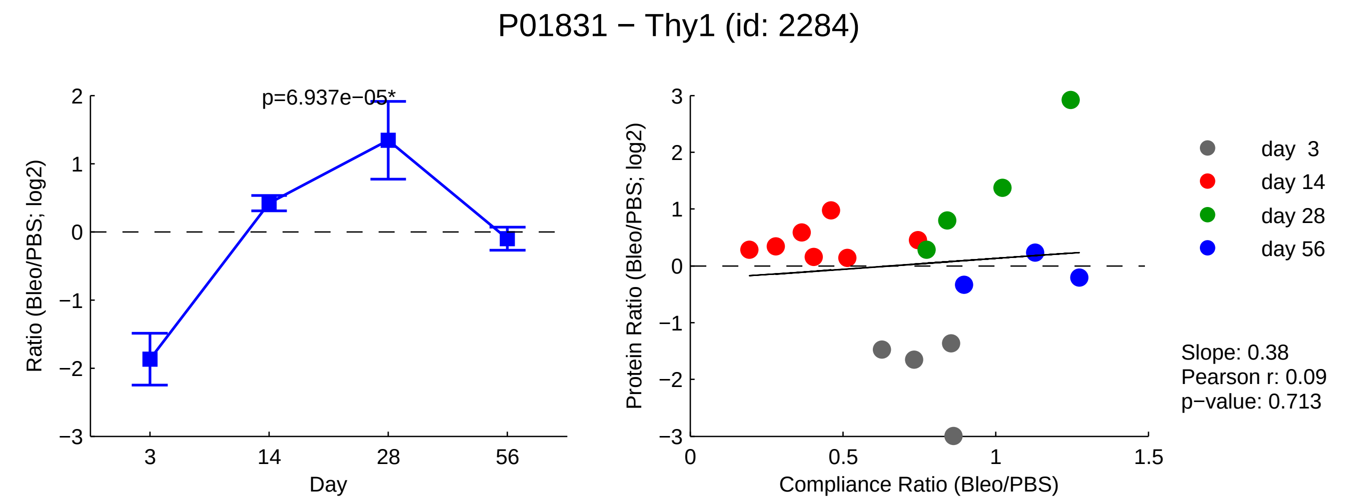
P01831 − Thy1 (id: 2284)
p=6.937e−05*
Ratio (Bleo/PBS; log2)
Day
Protein Ratio (Bleo/PBS; log2)
Compliance Ratio (Bleo/PBS)
day 3
day 14
day 28
day 56
Slope: 0.38
Pearson r: 0.09
p−value: 0.713

P01844 − Iglc2 (id: 2287)

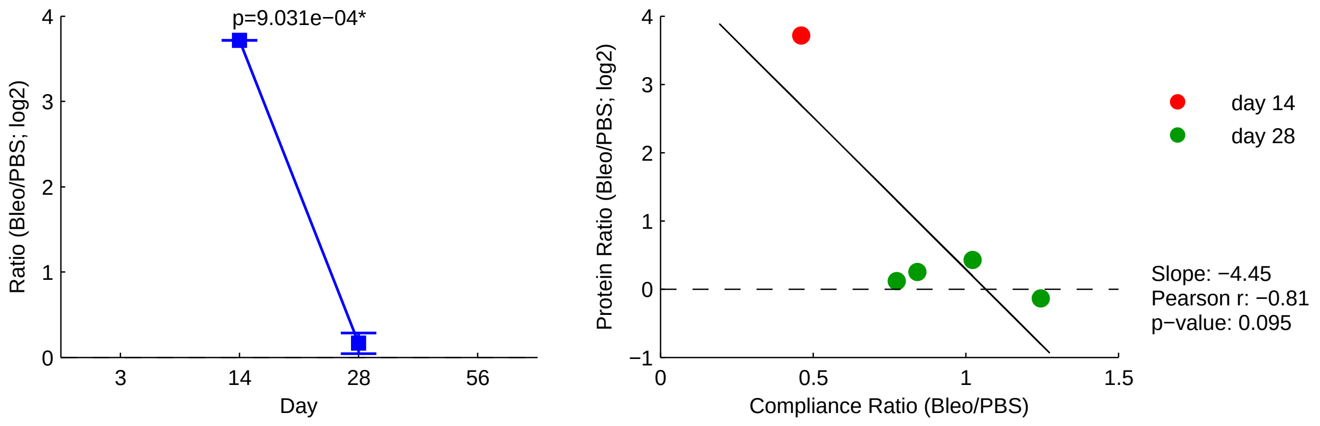

P01872 − Igh−6 (id: 2292)

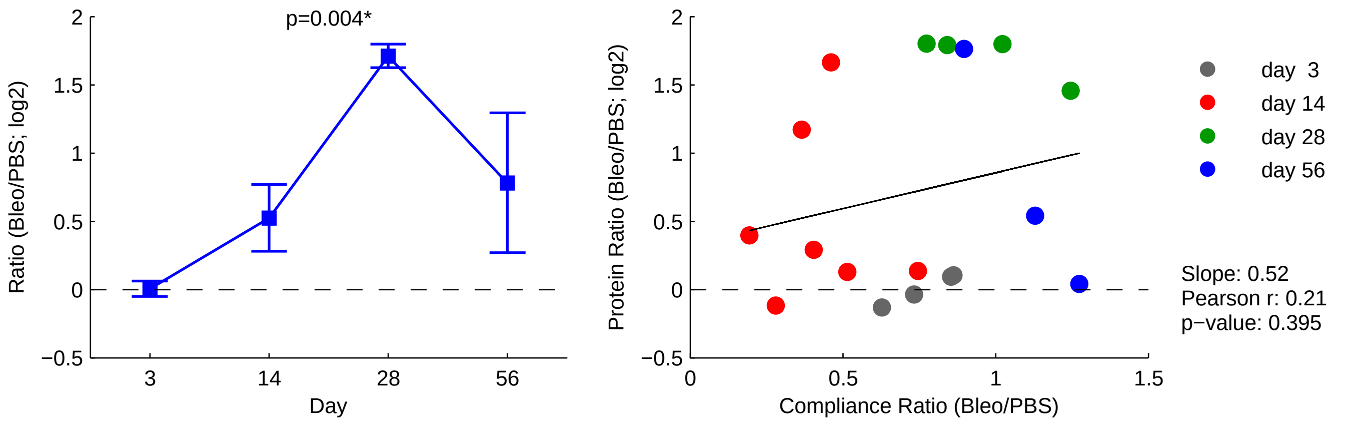

P01878 − (id: 2293)

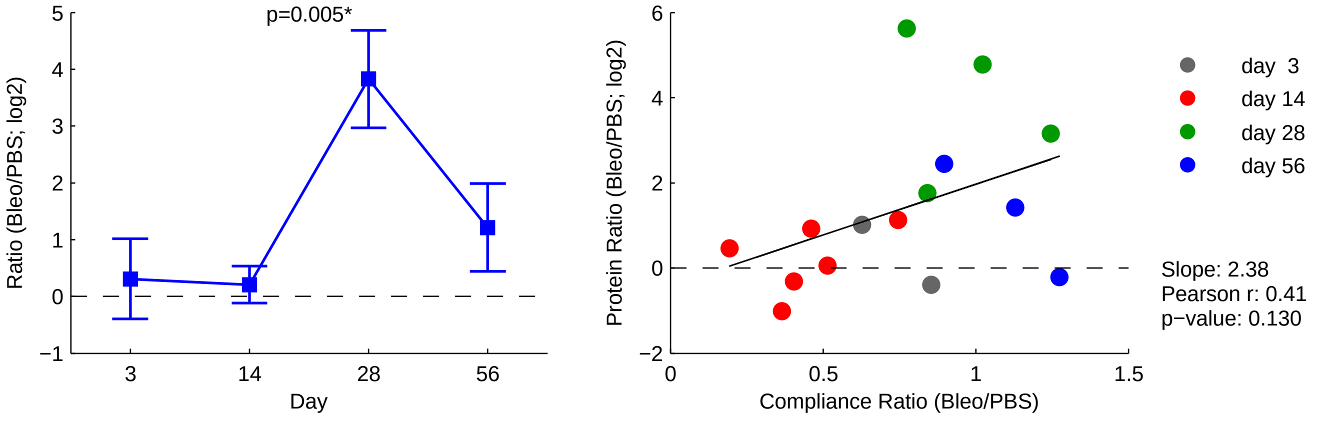

P01887 − B2m (id: 2295)

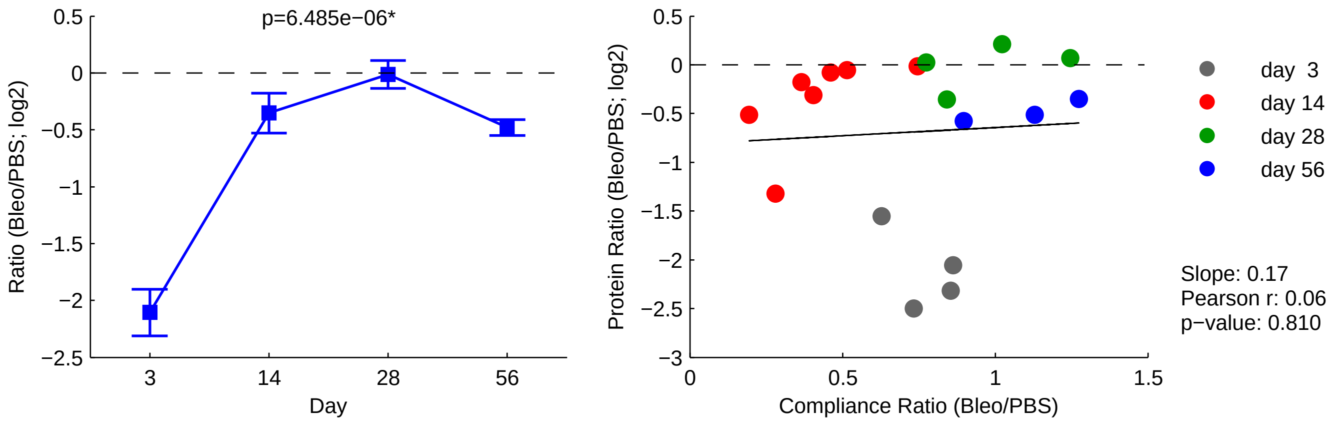

P01899 − H2−D1 (id: 2297)

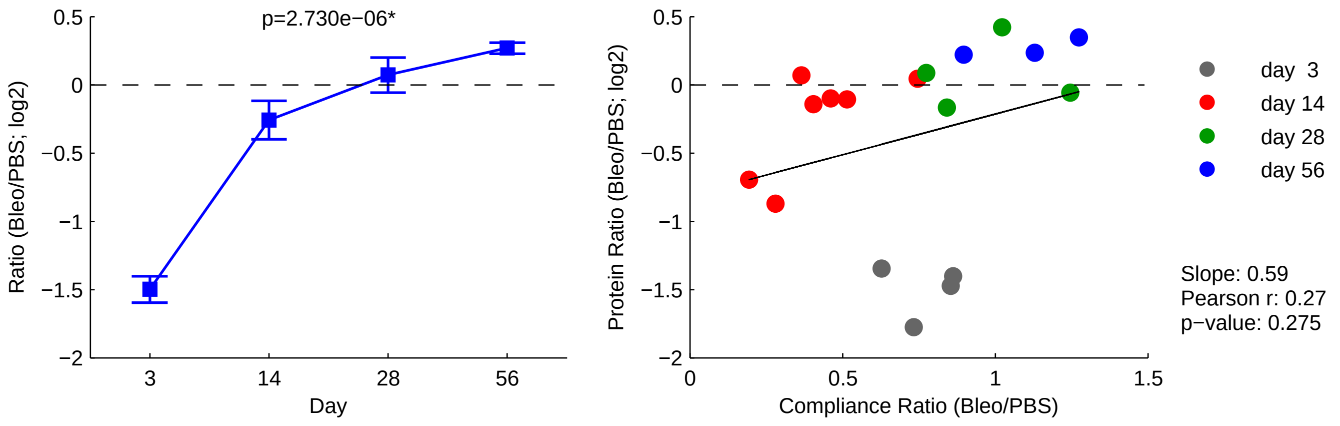

P01901 − H2−K1 (id: 2298)

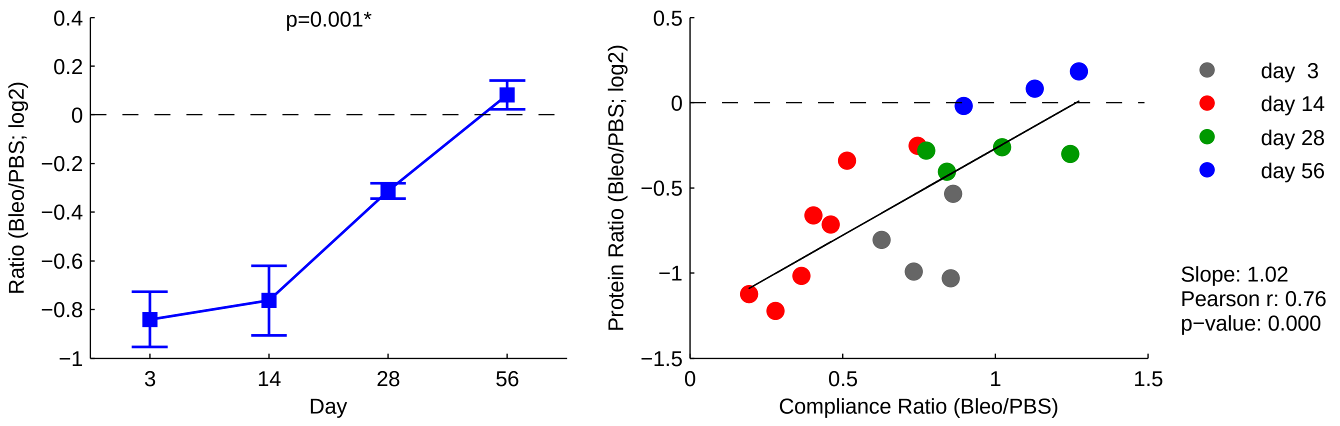

P02463 − Col4a1 (id: 2303)

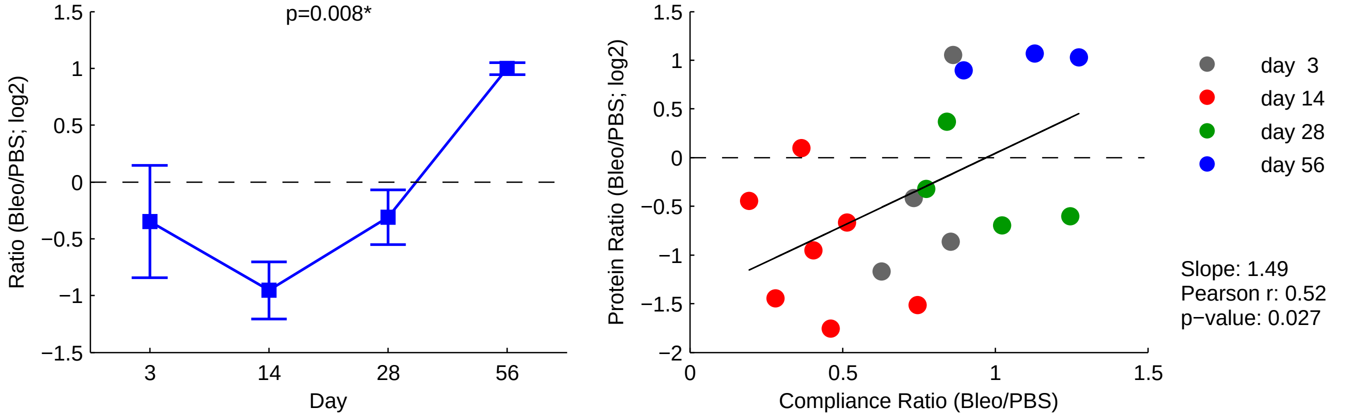

P03888 − Mtnd1 (id: 2304)

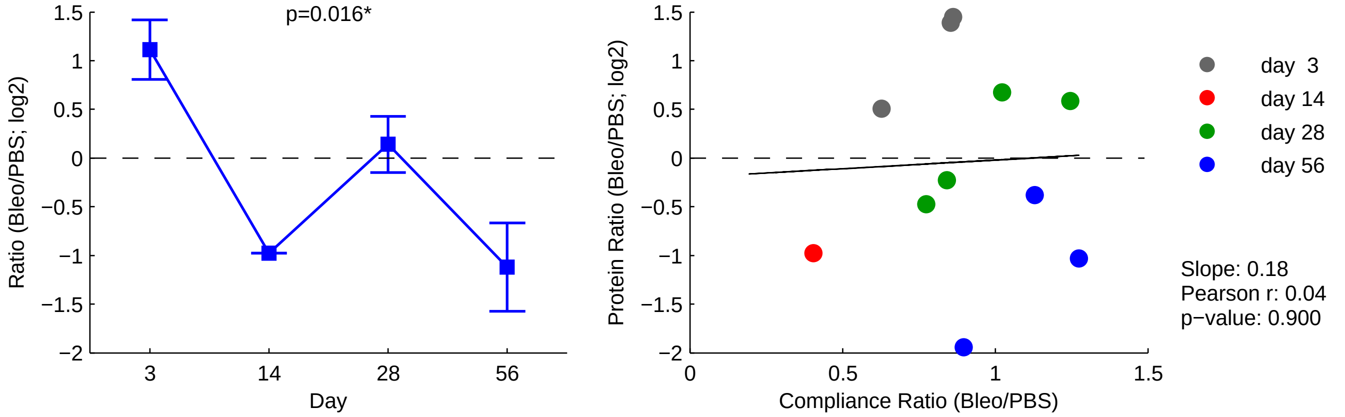

Q9MD59 − mt−Nd2 (id: 2305)

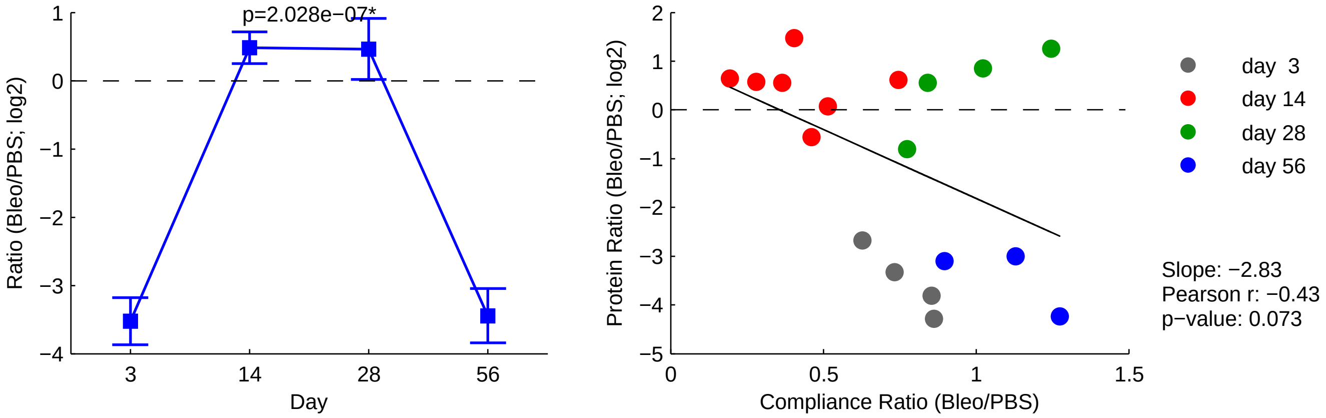

Q7JCZ0 − mt−Atp8 (id: 2308)

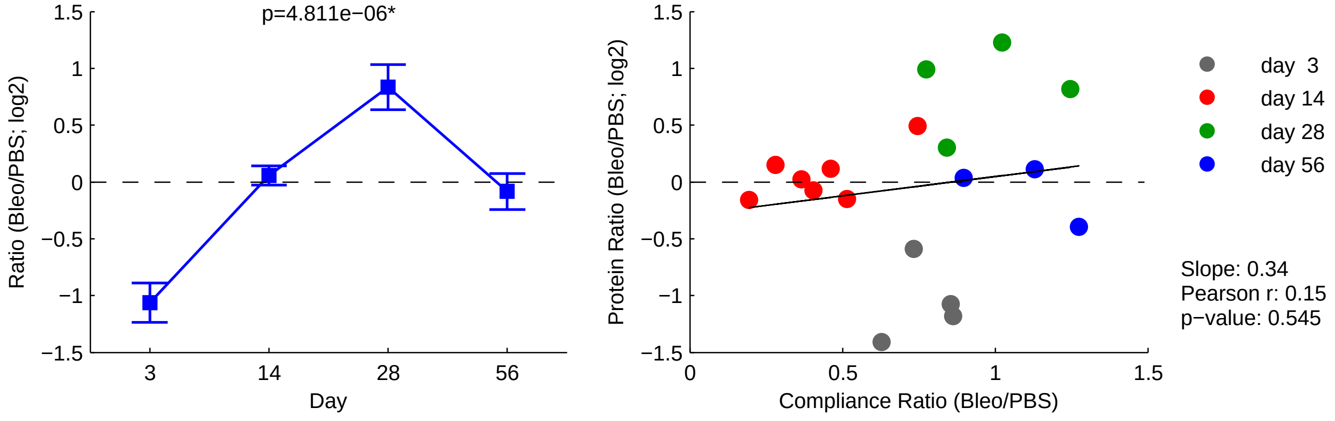

P03953−2 − Cfd (id: 2309)

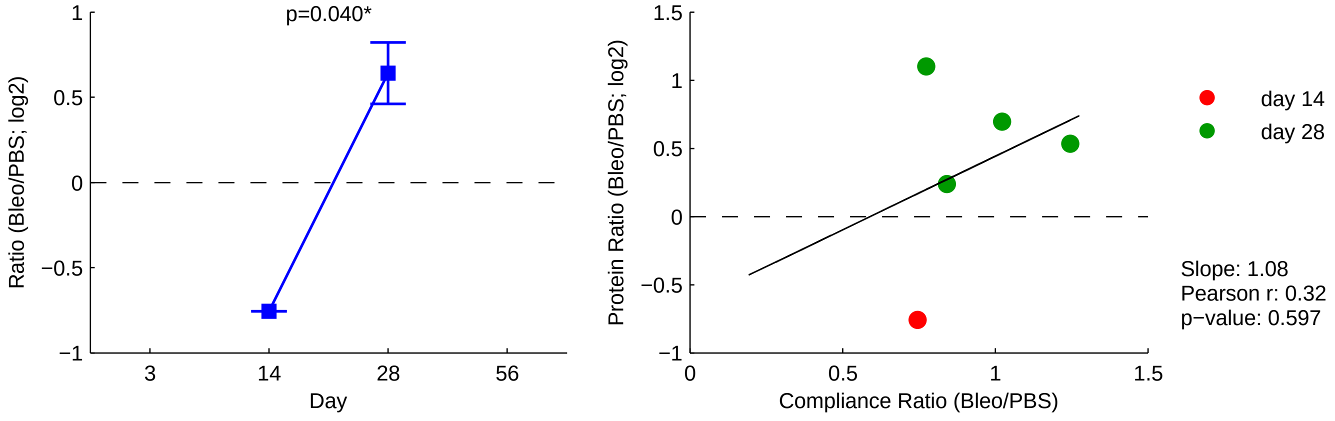

P04117 − Fabp4 (id: 2314)

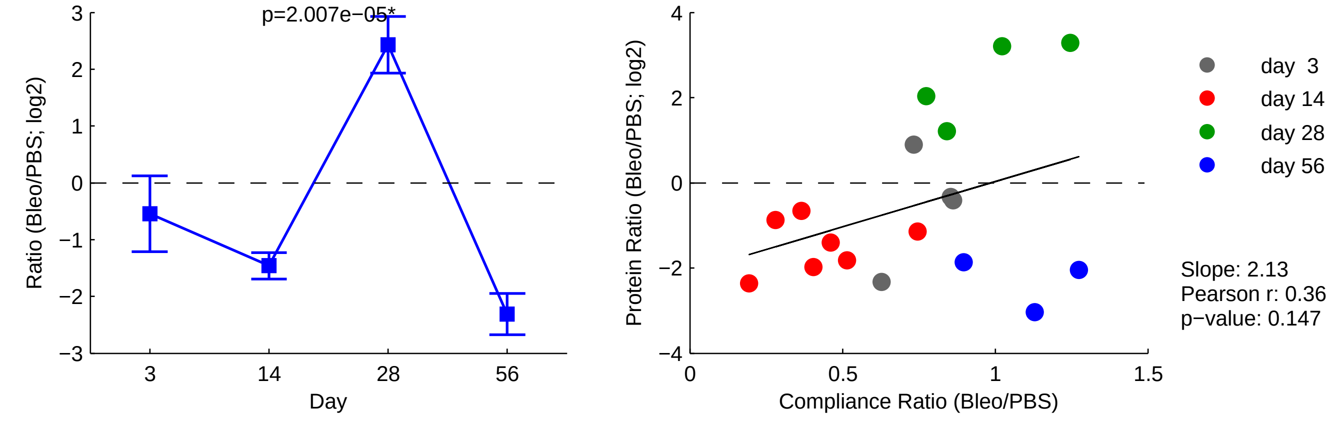

P04223 − H2−K1 (id: 2317)

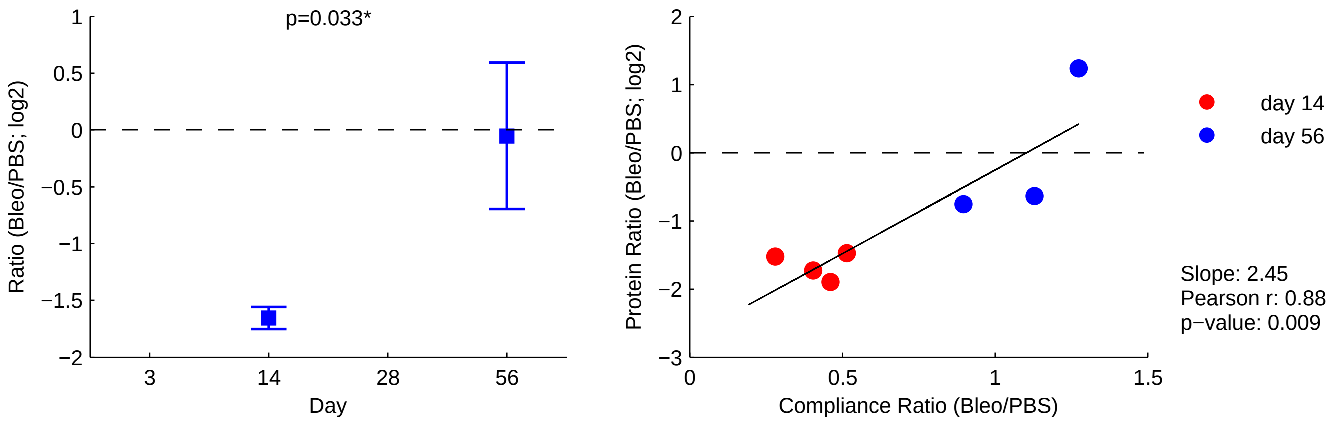

P04441−2 − Cd74 (id: 2320)

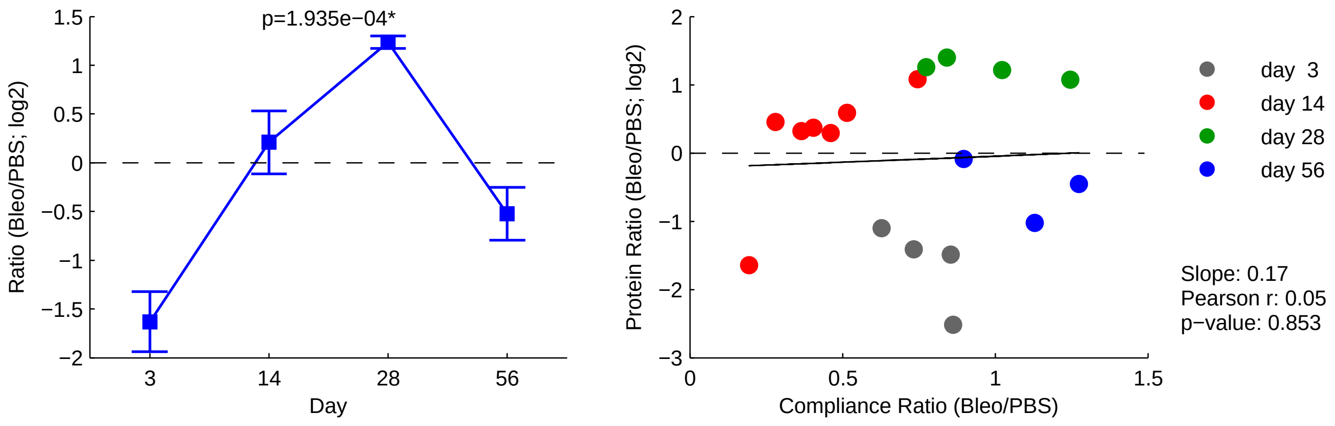

P04627 − Araf (id: 2321)

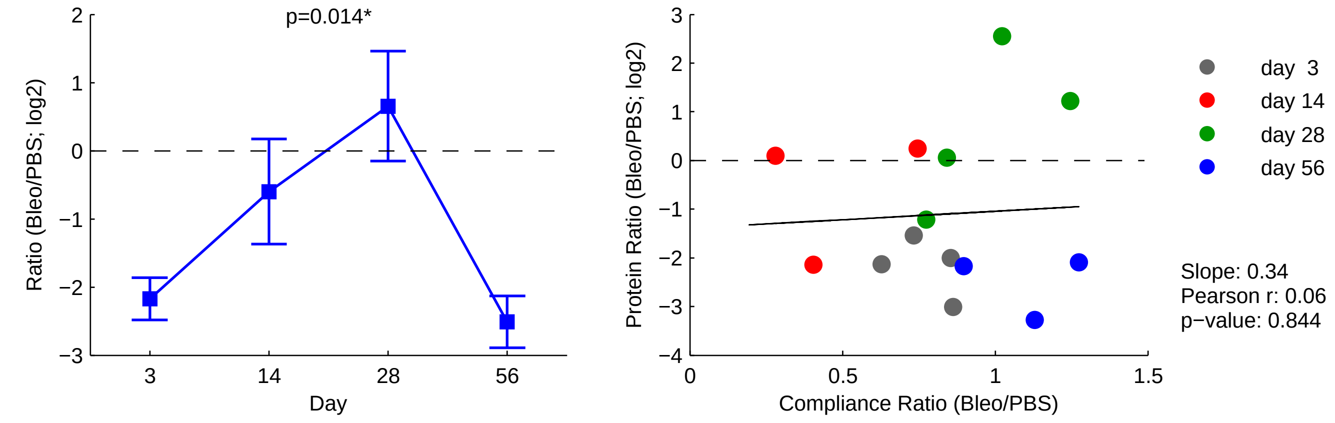

P04925 − Prnp (id: 2324)

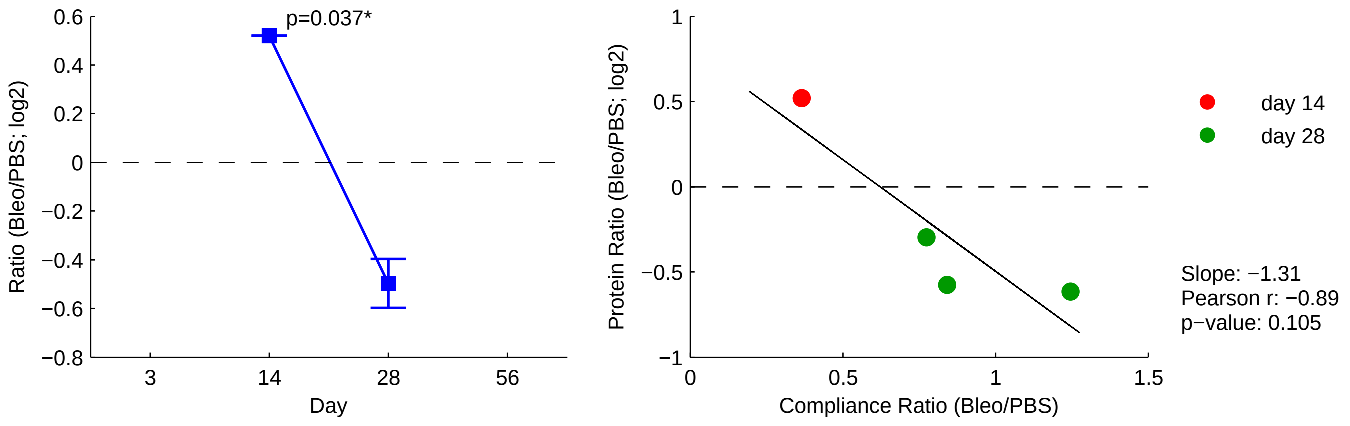

P05201 − Got1 (id: 2329)

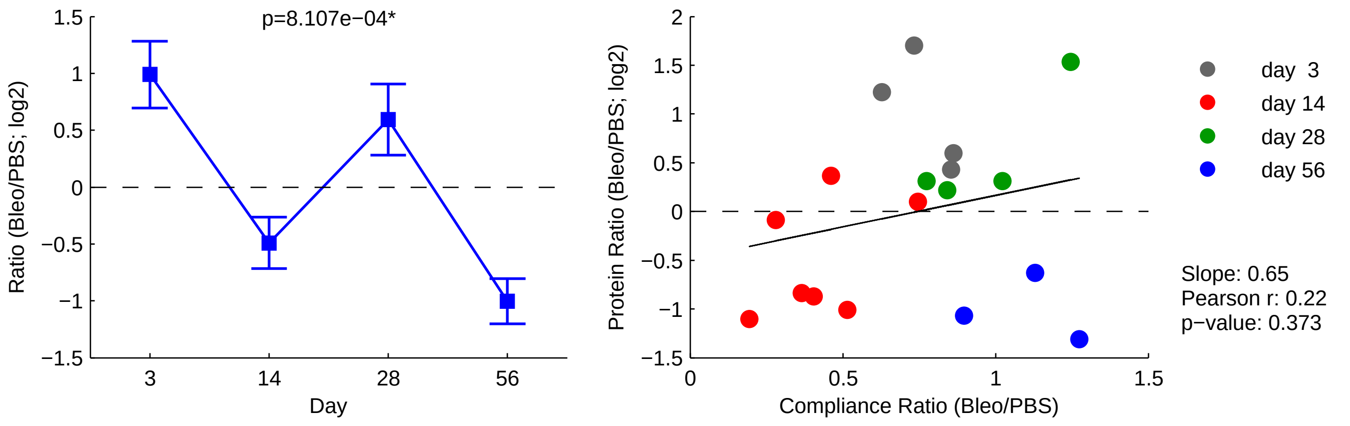

P05202 − Got2 (id: 2330)

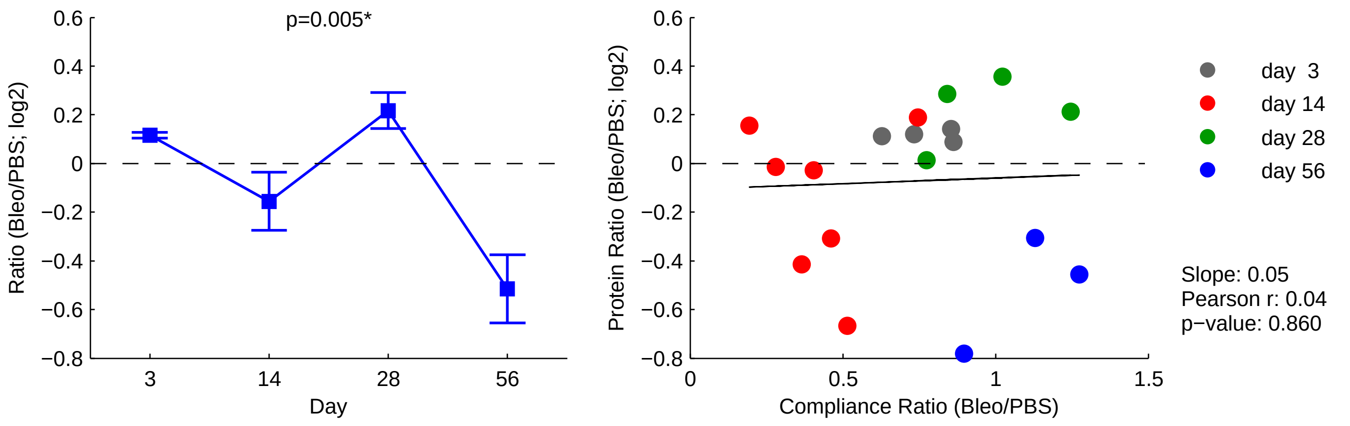

P05213 − Tuba1b (id: 2332)

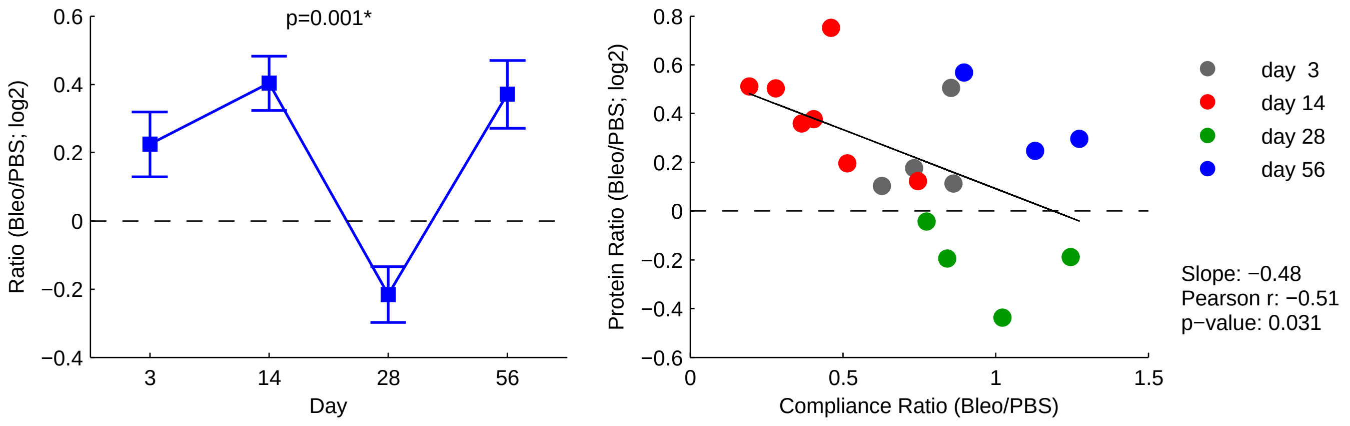

P05977 − Myl1 (id: 2337)

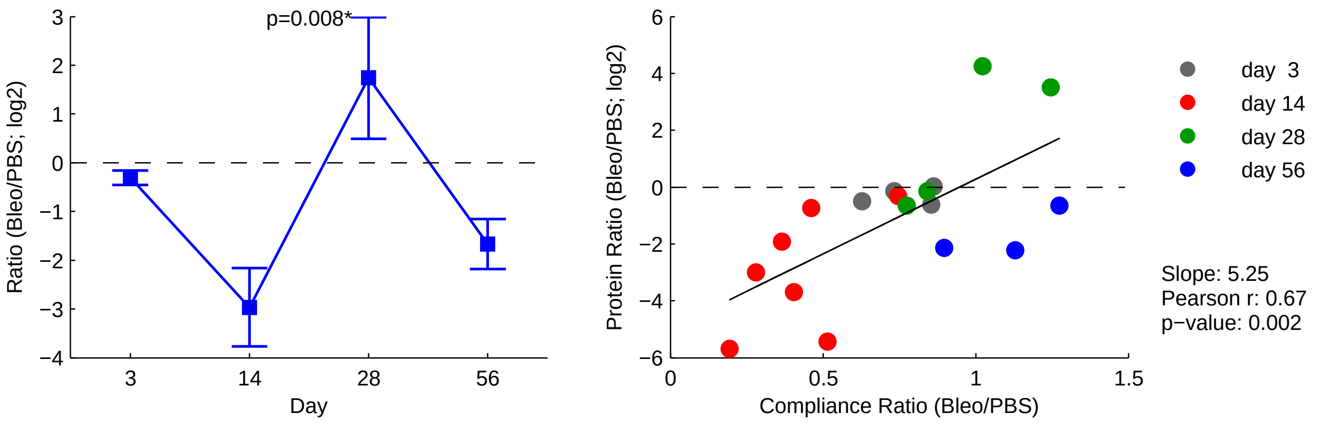

P06151 − Ldha (id: 2339)

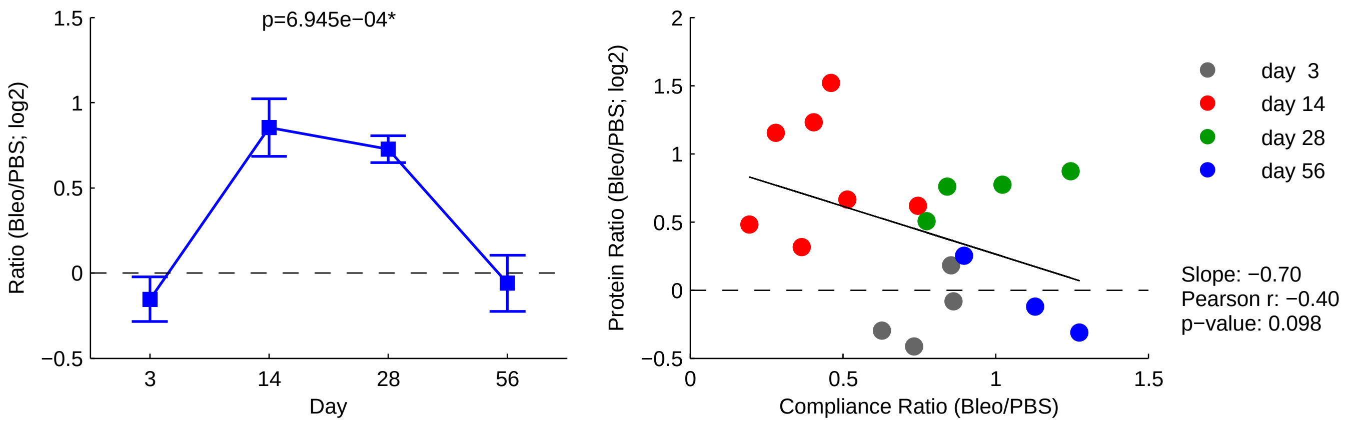

P06330 − (id: 2341)

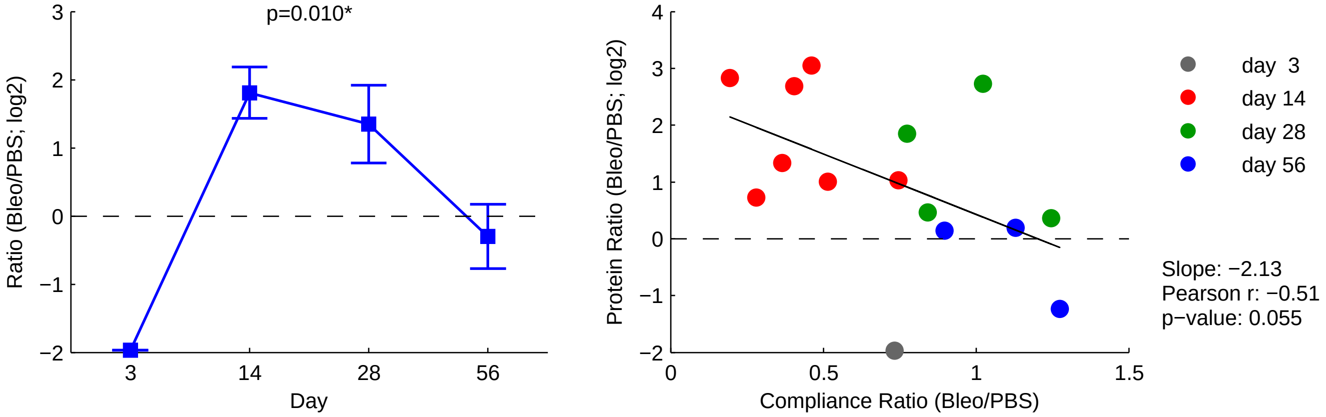

P06684 − C5 (id: 2345)

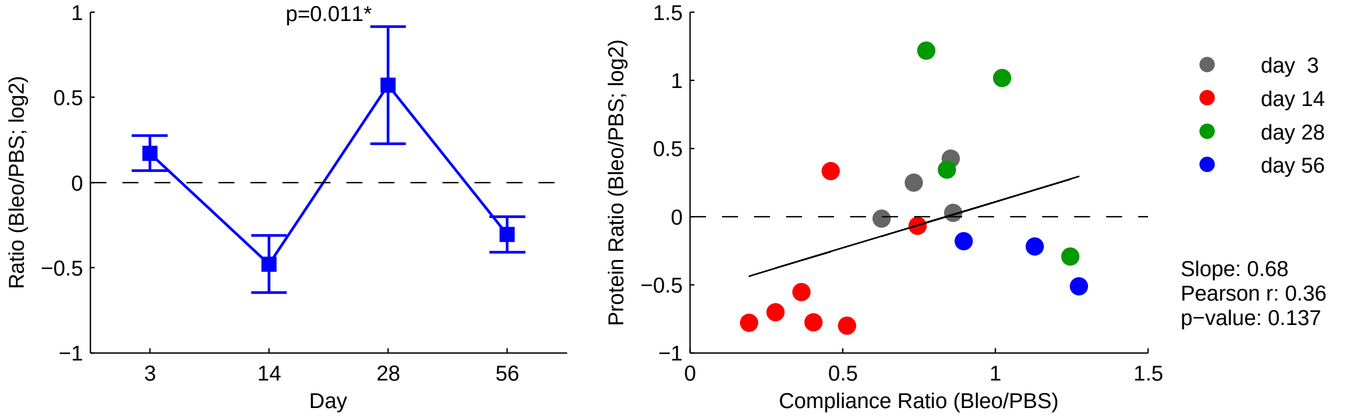

P06728 − Apoa4 (id: 2346)

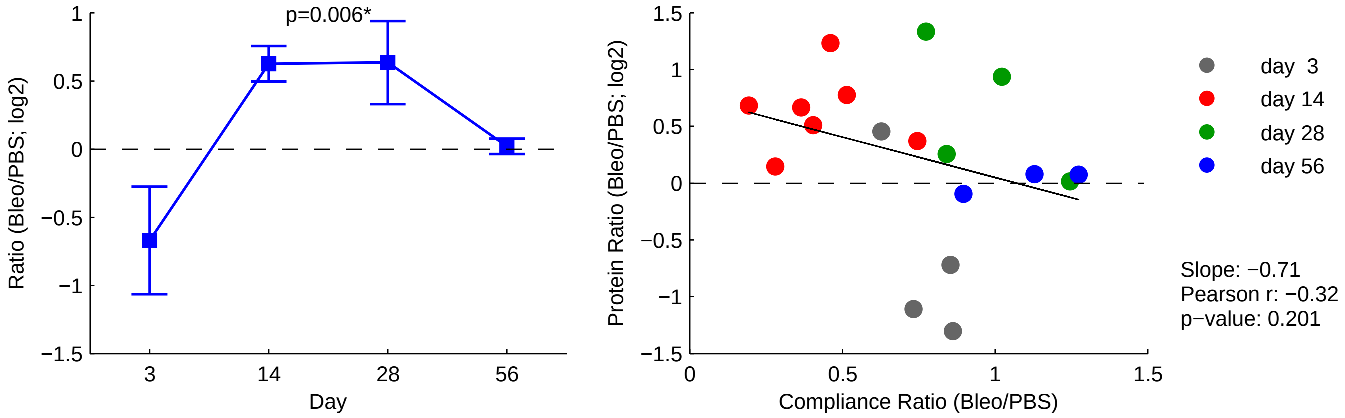

P06745 − Gpi (id: 2347)

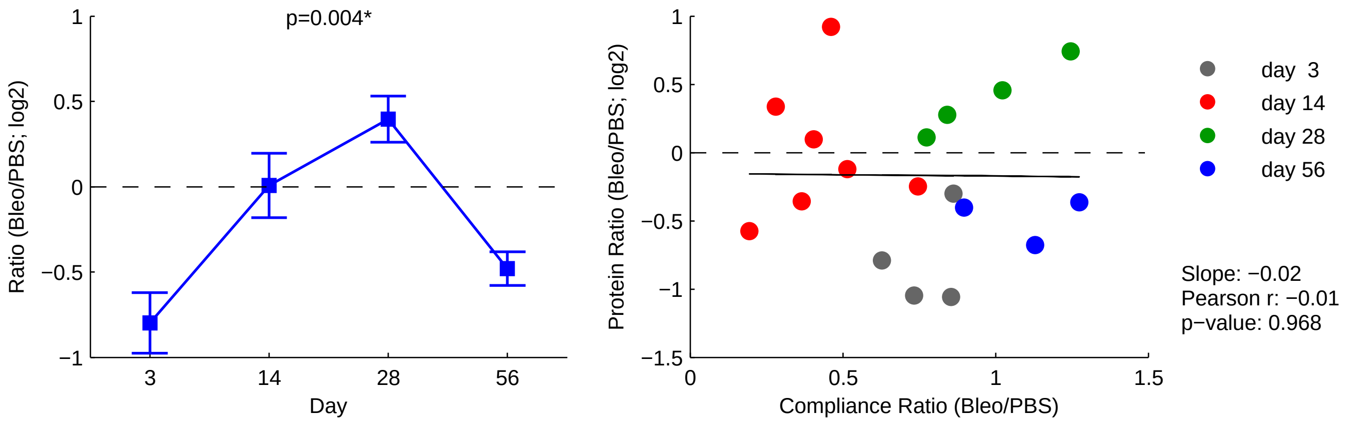

P06797 − Ctsl1 (id: 2348)

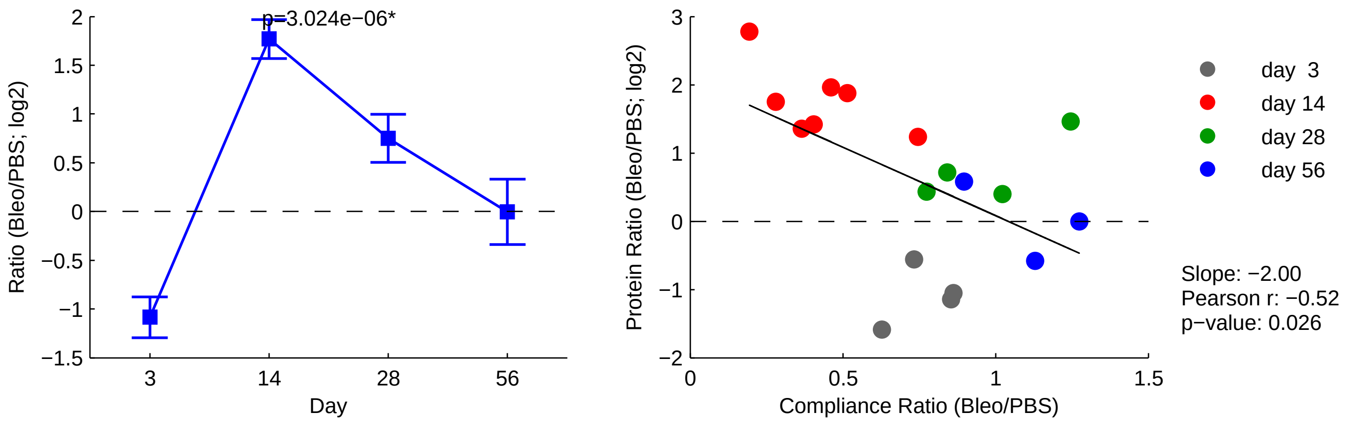

P06800−3 − Ptprc (id: 2349)

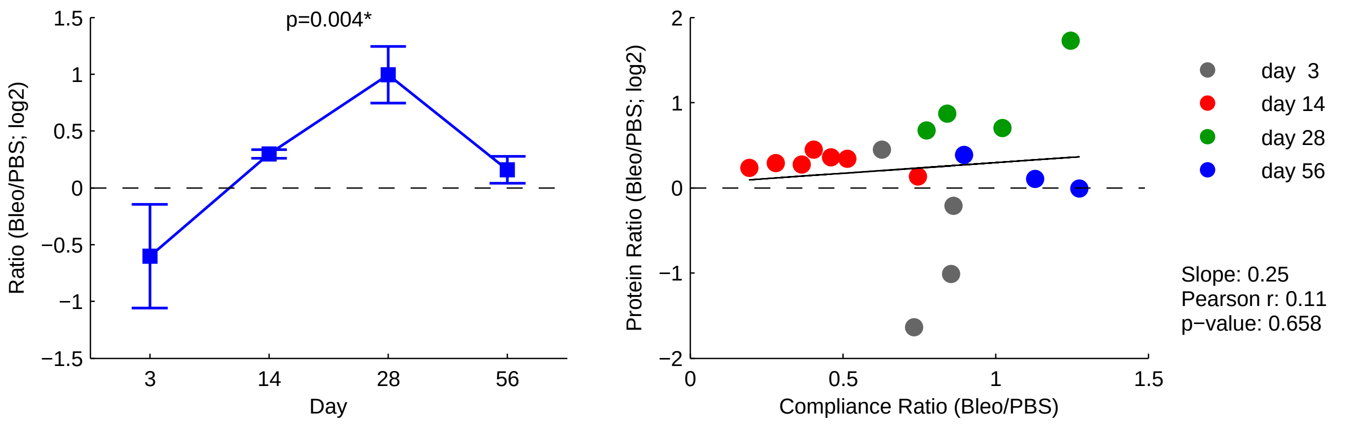

P06801 − Me1 (id: 2350)

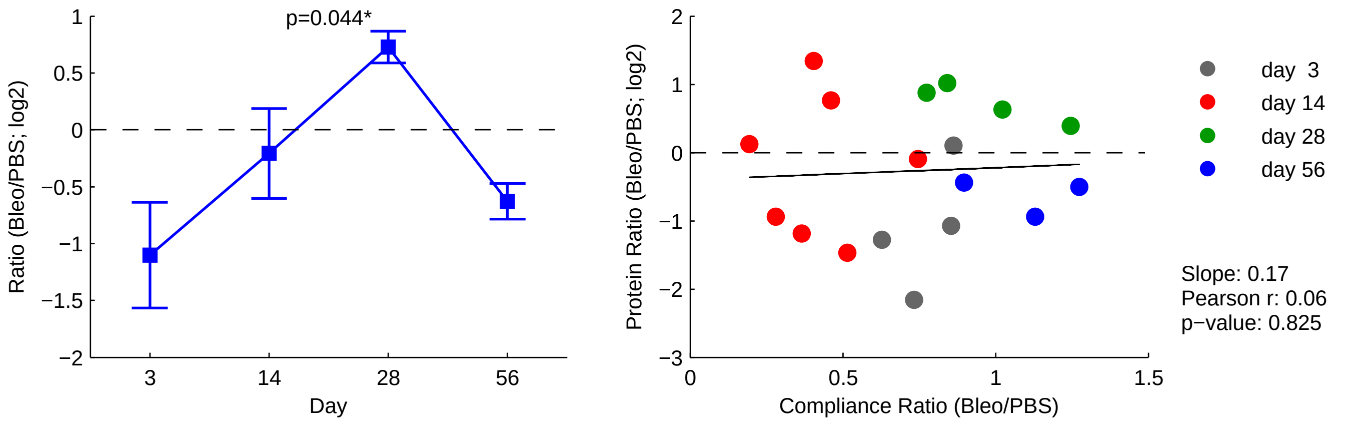

P06837 − Gap43 (id: 2351)

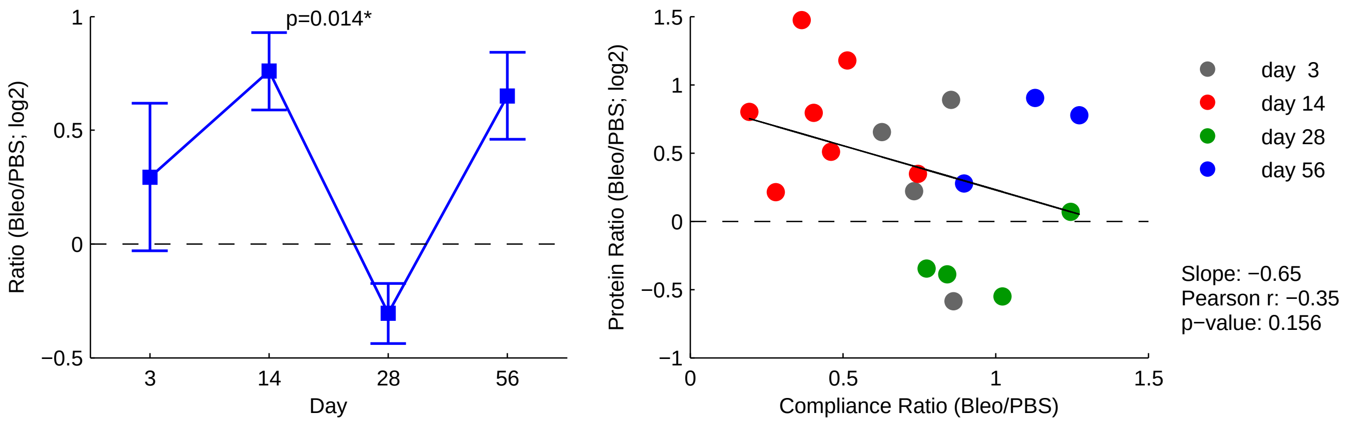

P07091 − S100a4 (id: 2353)

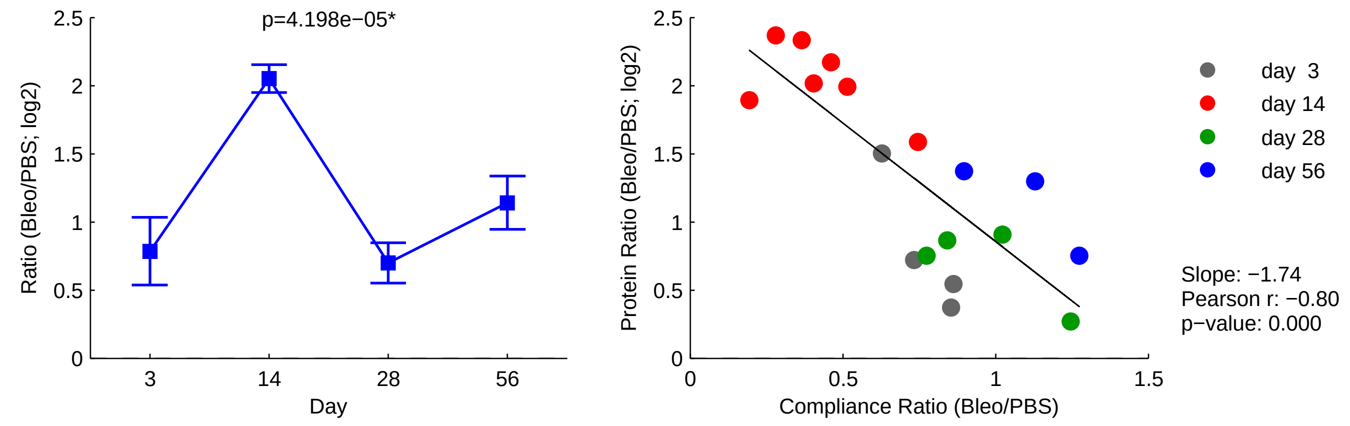

Q5NCU4 − Sparc (id: 2354)

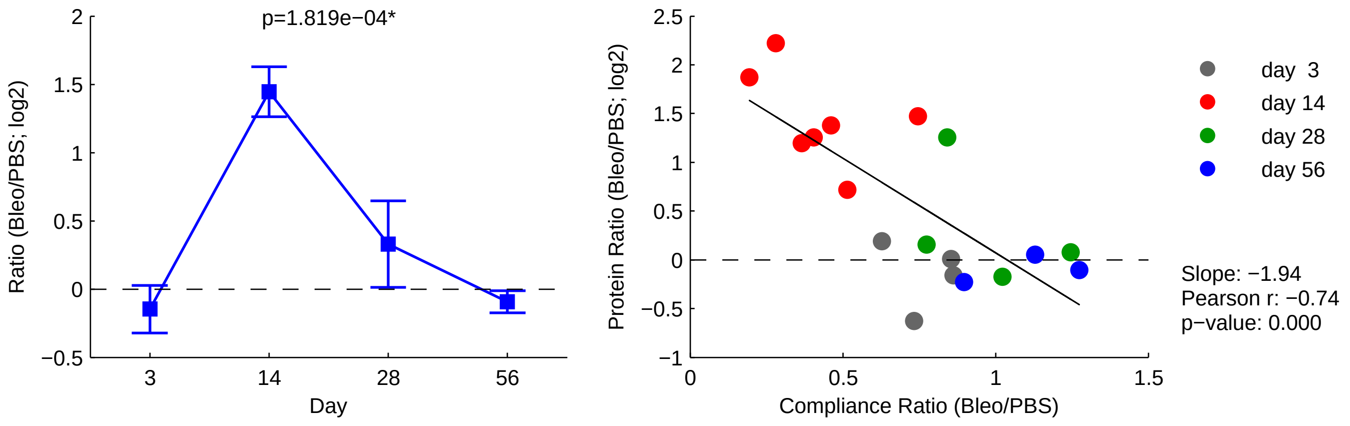

P07309 − Ttr (id: 2355)

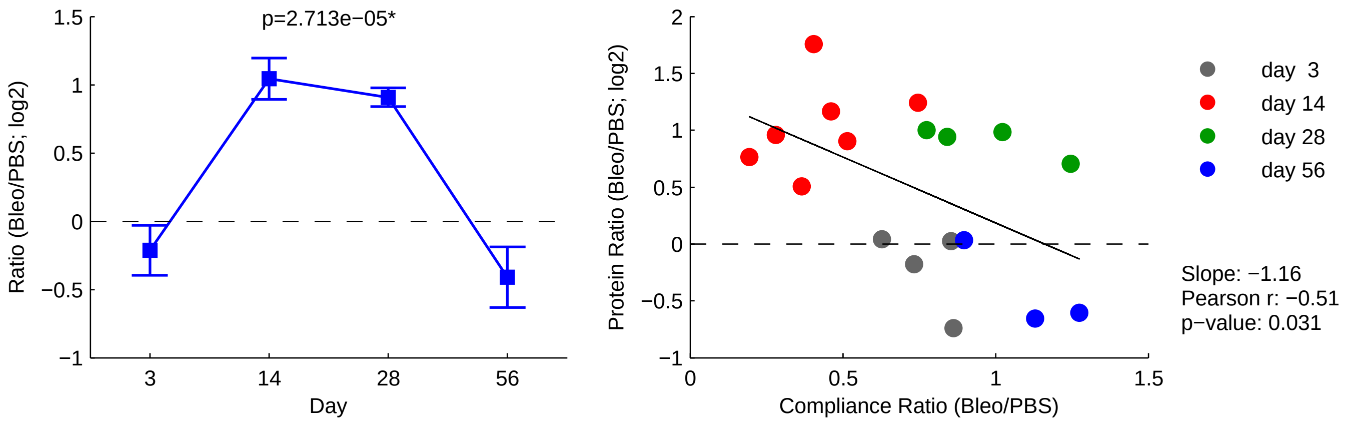

P07724 − Alb (id: 2358)

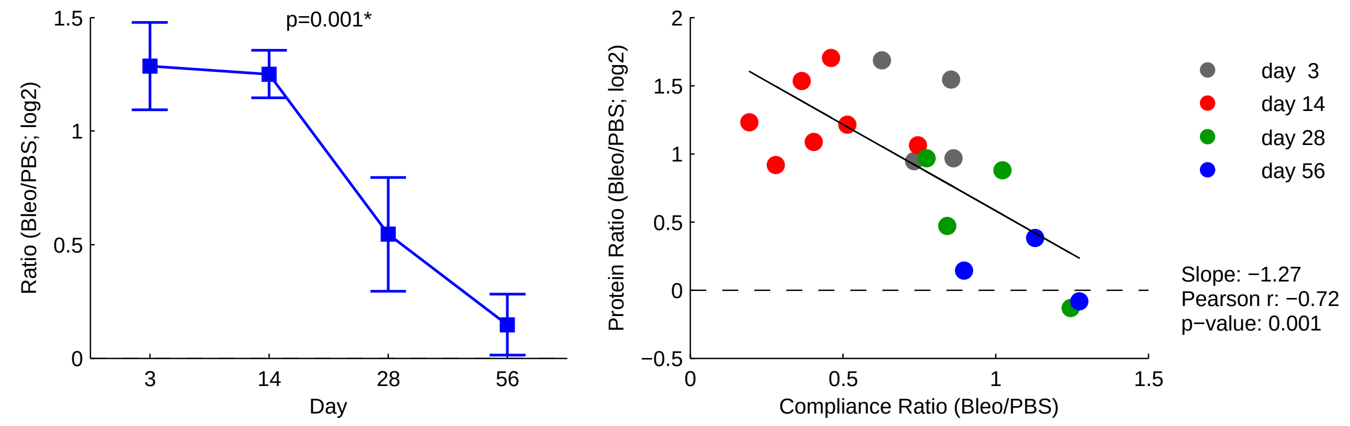

P07742 − Rrm1 (id: 2359)

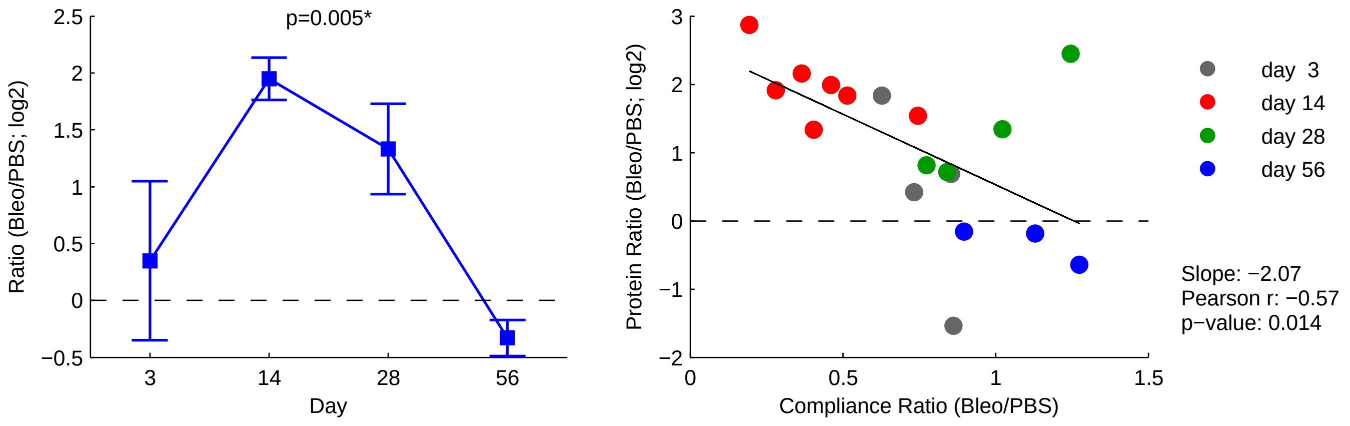

P07758 − Serpina1a (id: 2360)

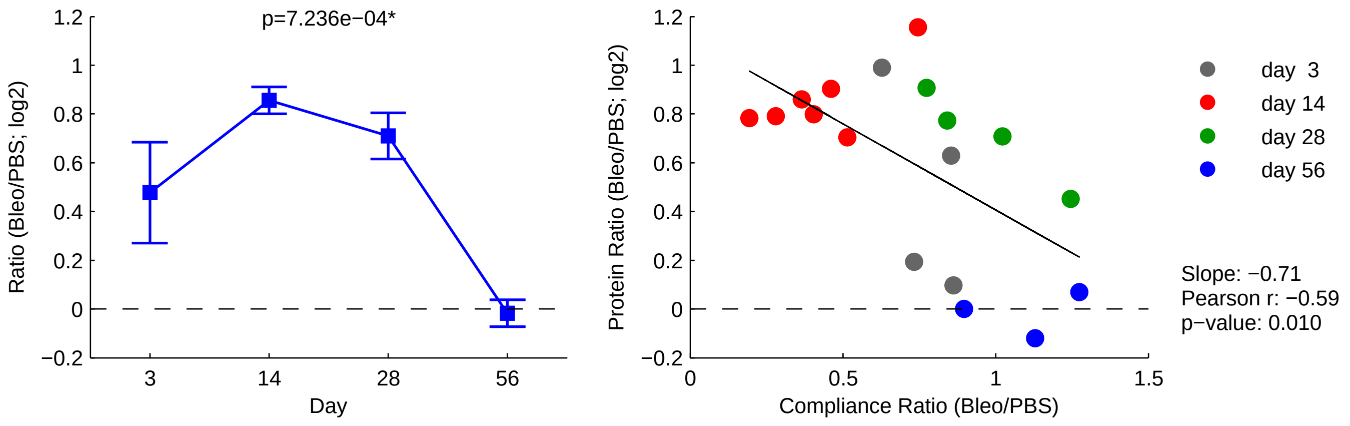

P07759 − Serpina3k (id: 2361)

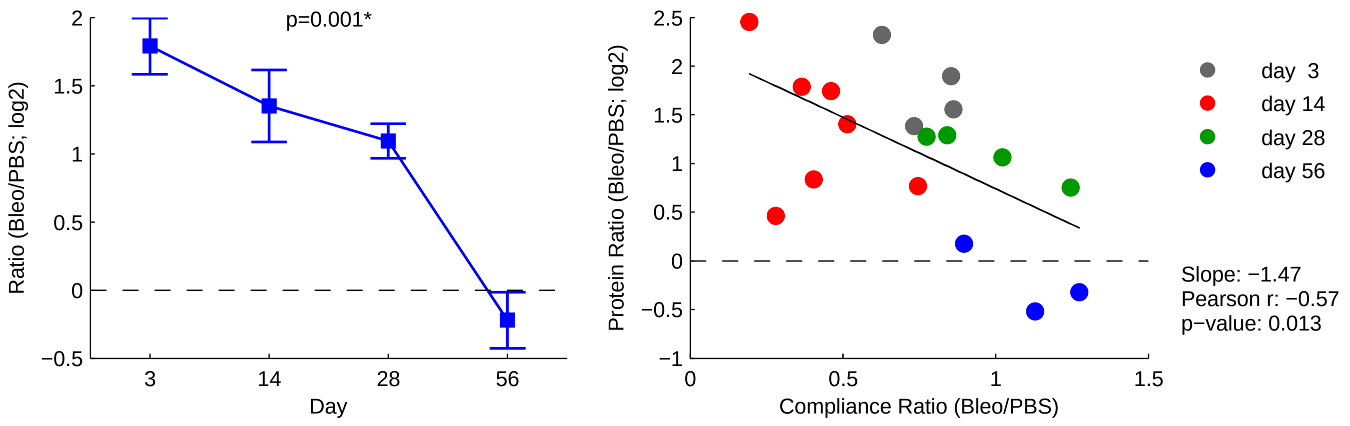

P07901 − Hsp90aa1 (id: 2362)

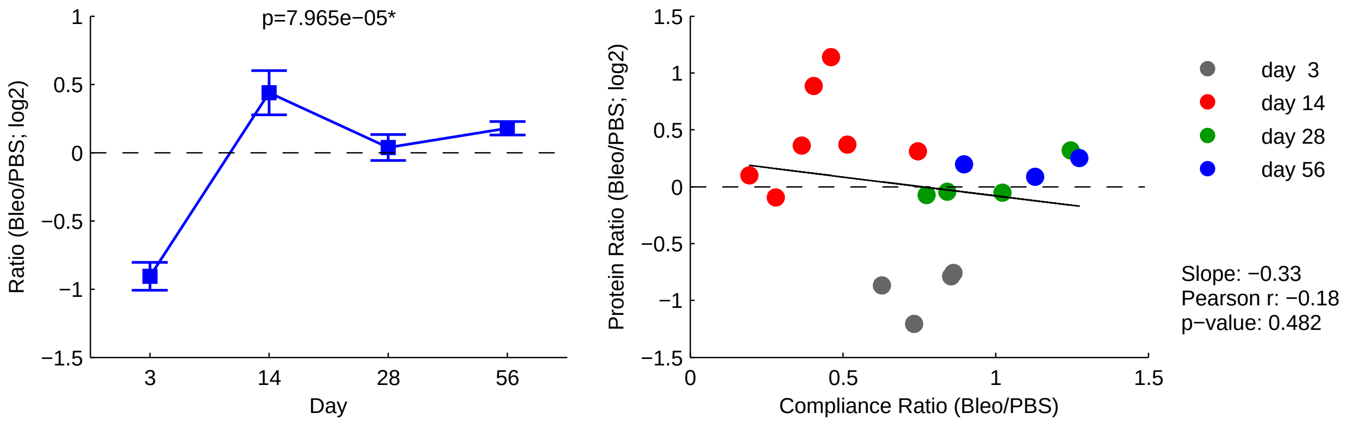

P08003 − Pdia4 (id: 2364)

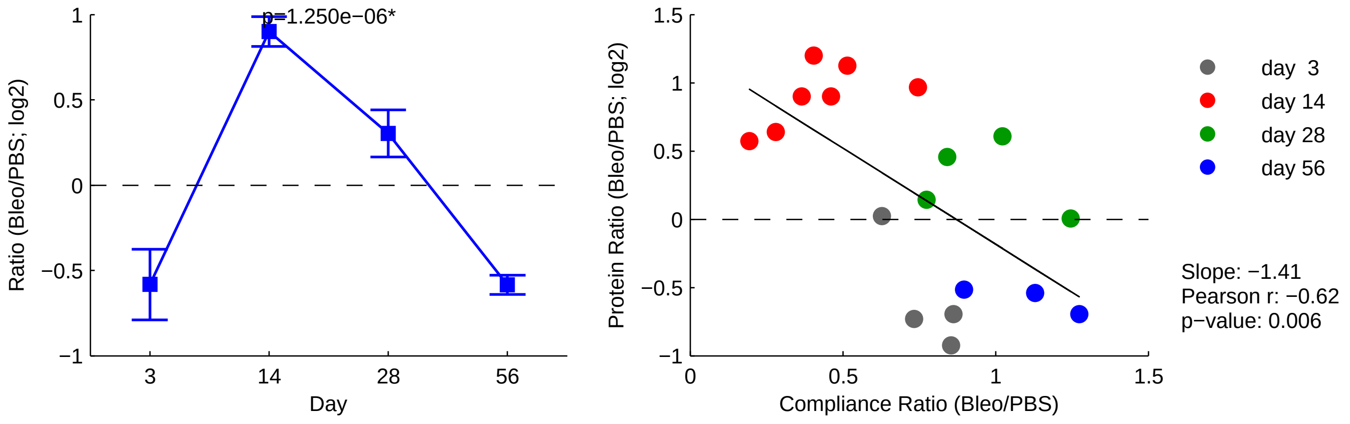

P08074 − Cbr2 (id: 2368)

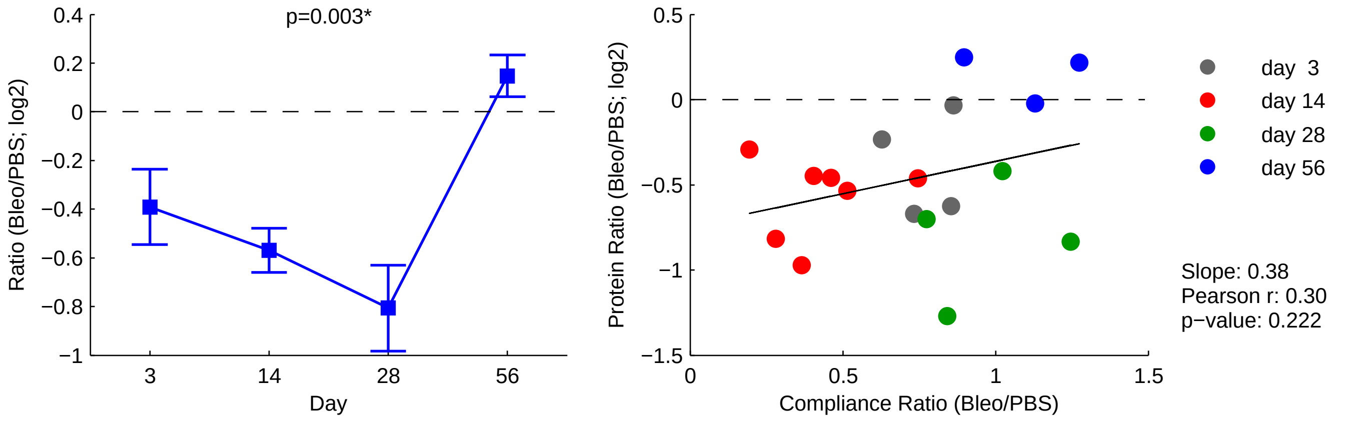

P08113 − Hsp90b1 (id: 2369)

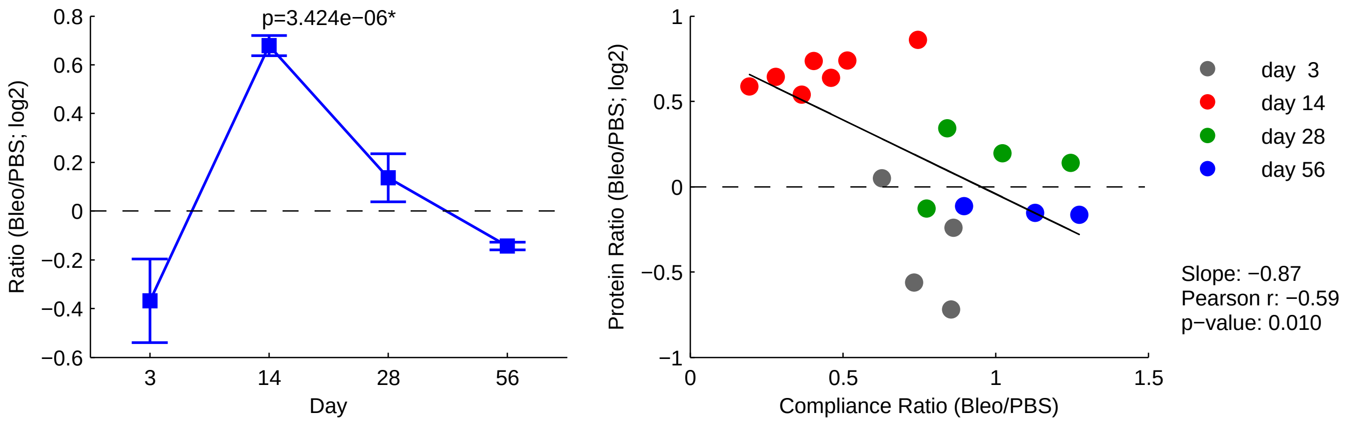

P08122 − Col4a2 (id: 2371)

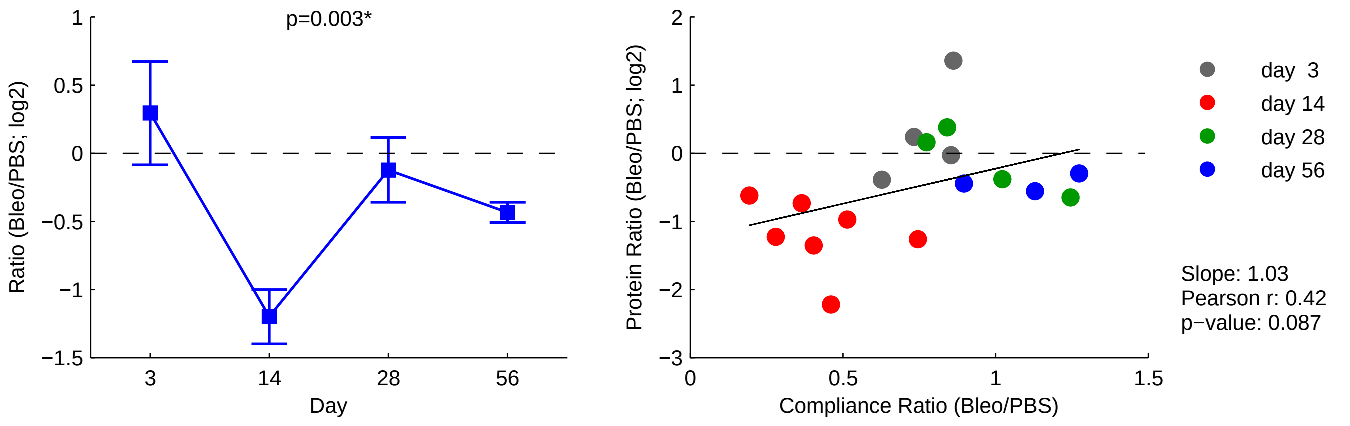

P08207 − S100a10 (id: 2372)

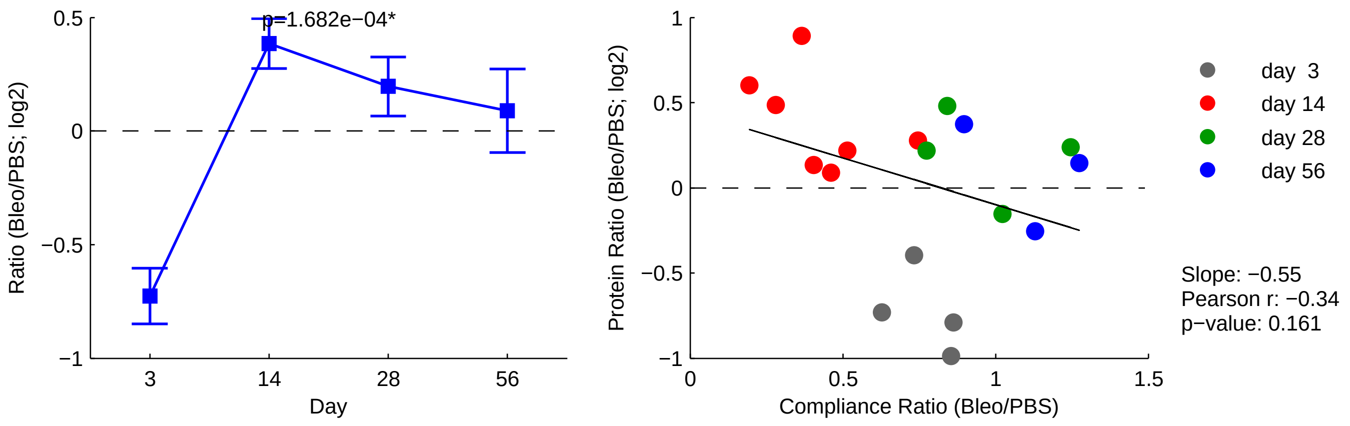

P08226 − Apoe (id: 2373)

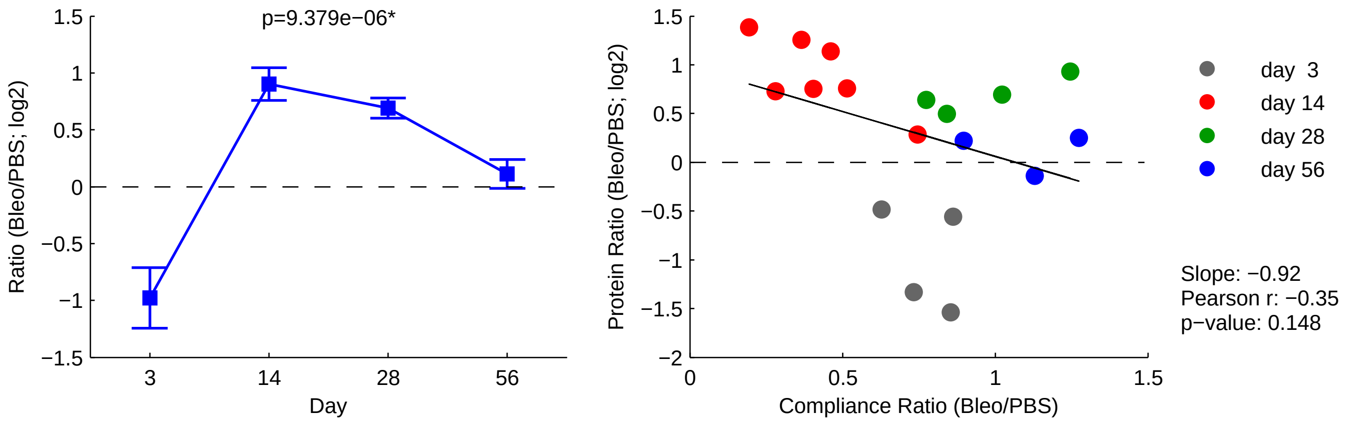

P08249 − Mdh2 (id: 2375)

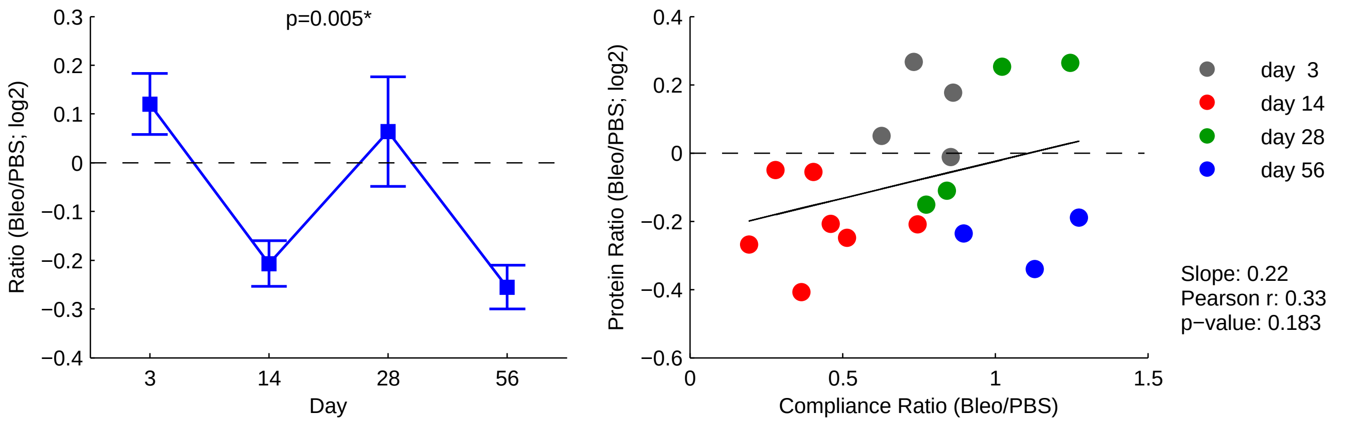

P08553 − Nefm (id: 2379)

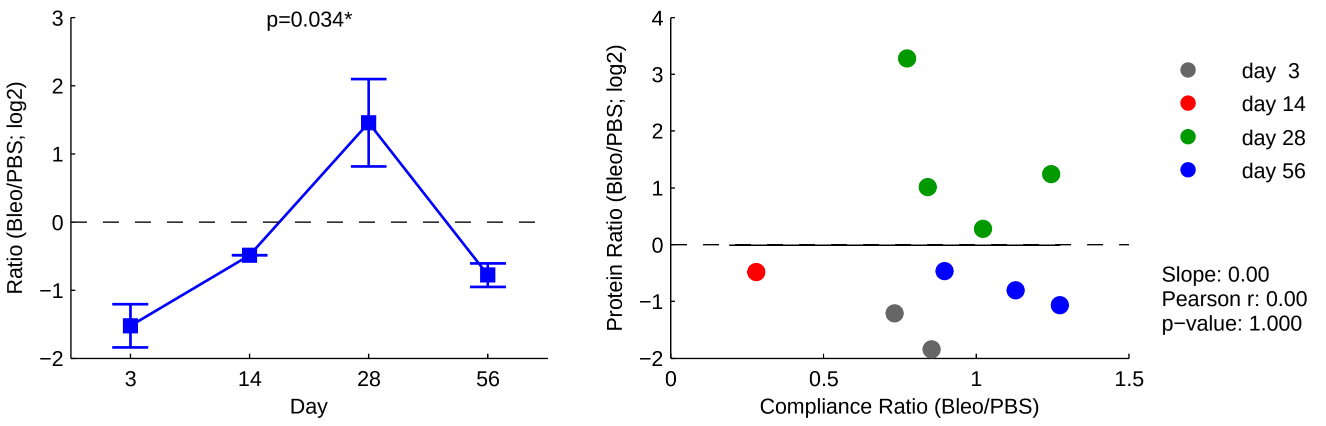

P08752 − Gnai2 (id: 2381)

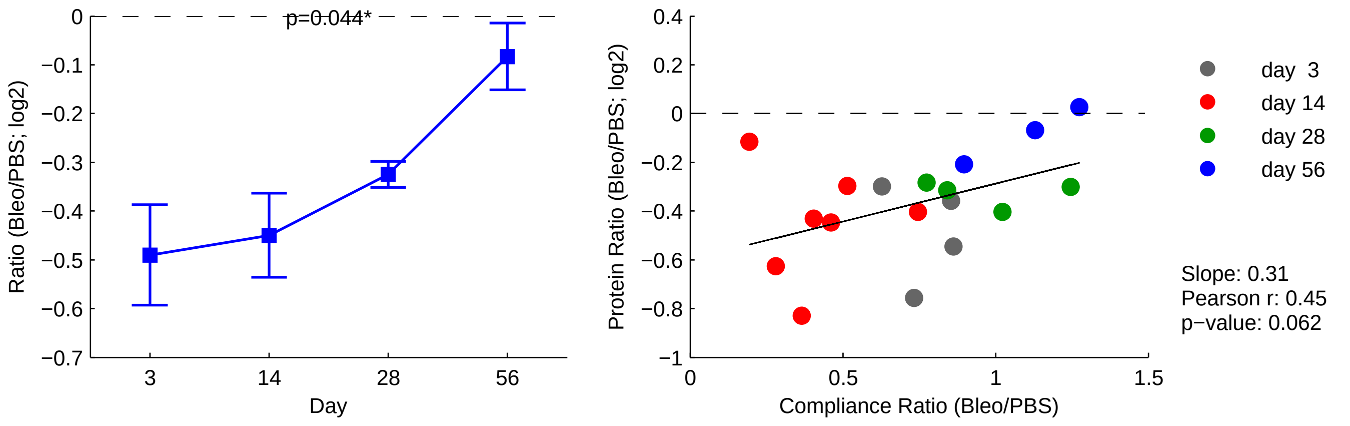

P08905 − Lyz2 (id: 2383)

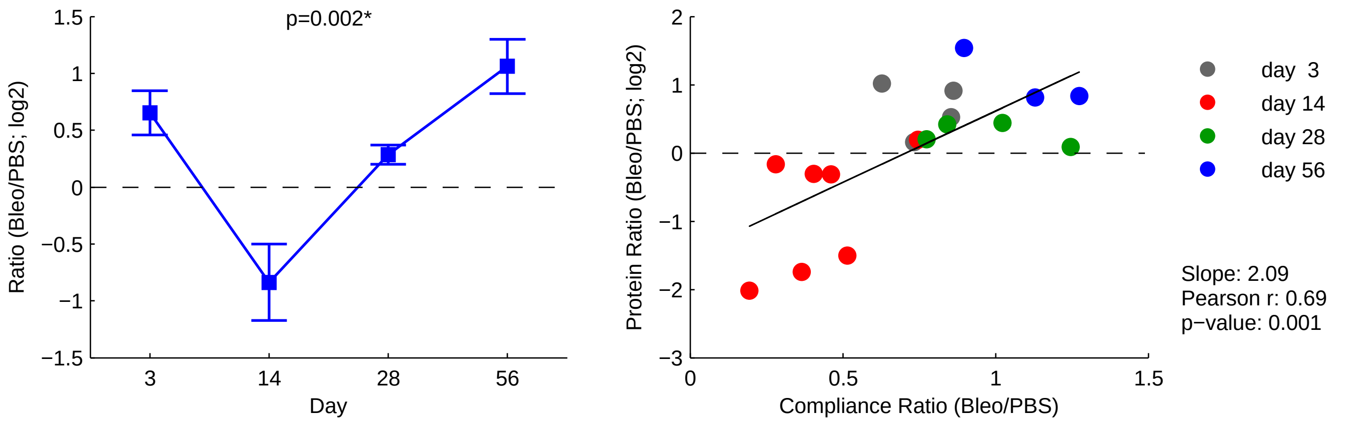

P09021 − Hoxa5 (id: 2384)

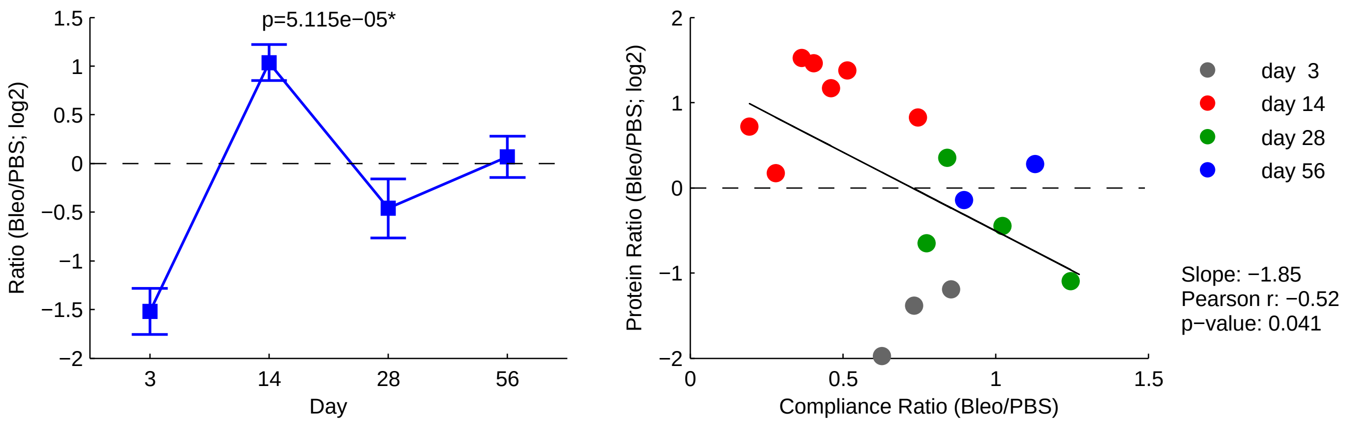

P09055 − Itgb1 (id: 2385)

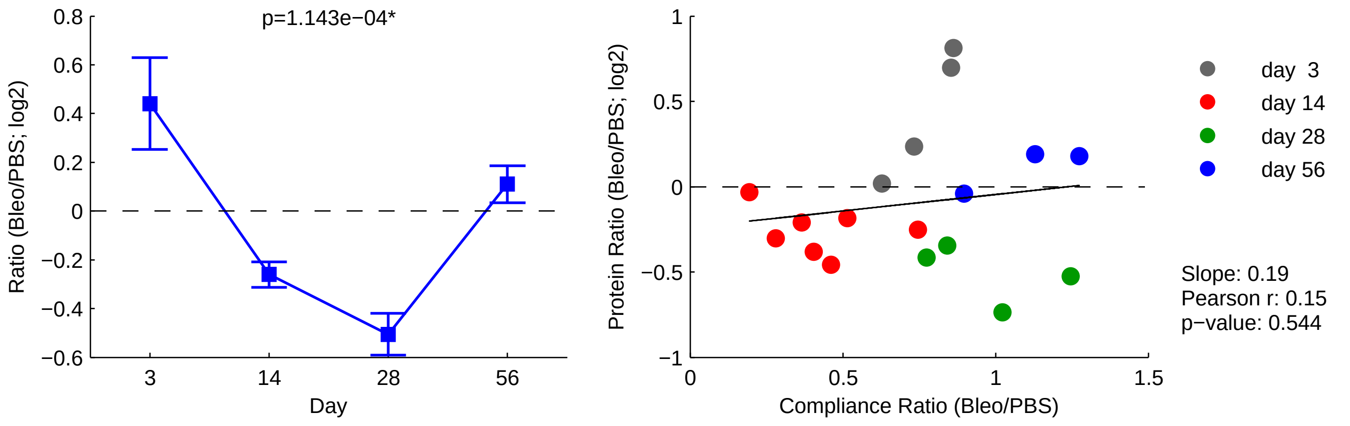

P09103 − P4hb (id: 2386)

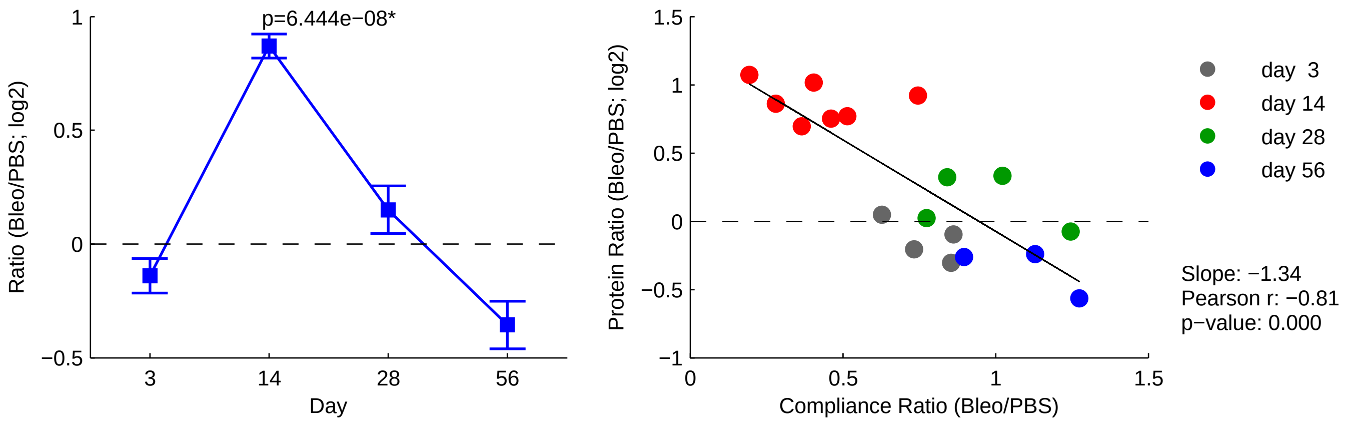

P09411 − Pgk1 (id: 2388)

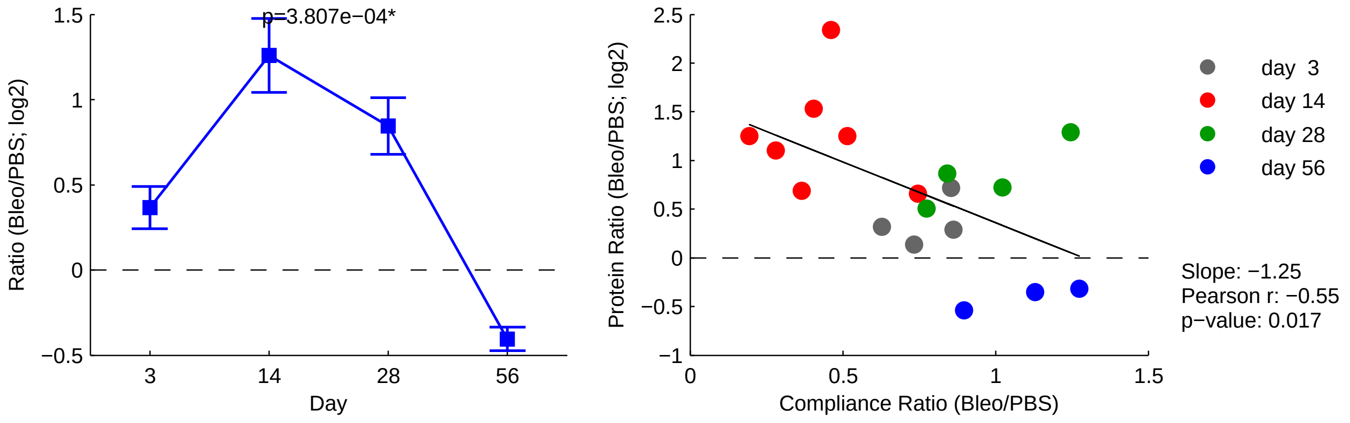

P09470 − Ace (id: 2389)

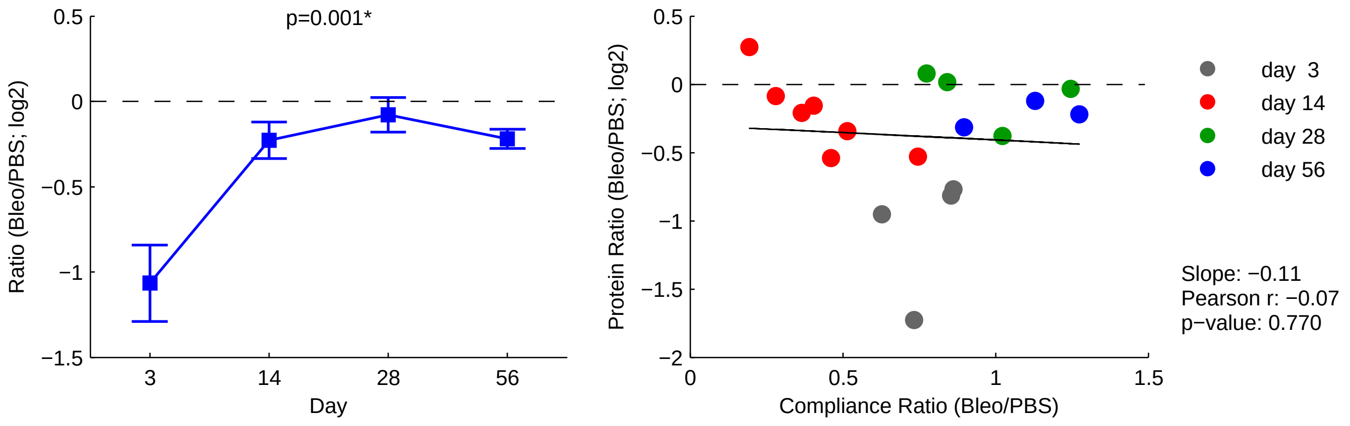

P09528 − Fth1 (id: 2390)

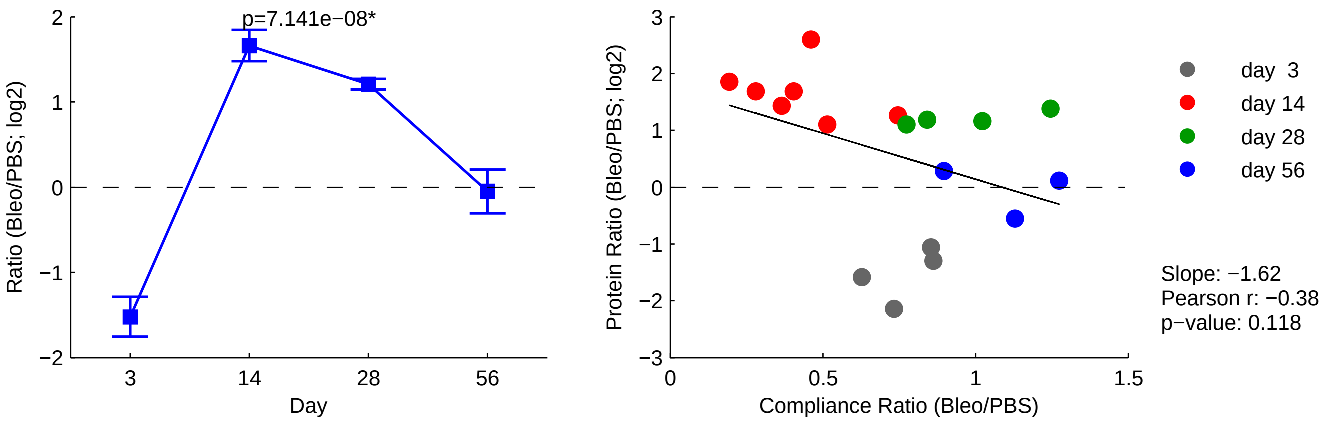

P09542 − Myl3 (id: 2391)

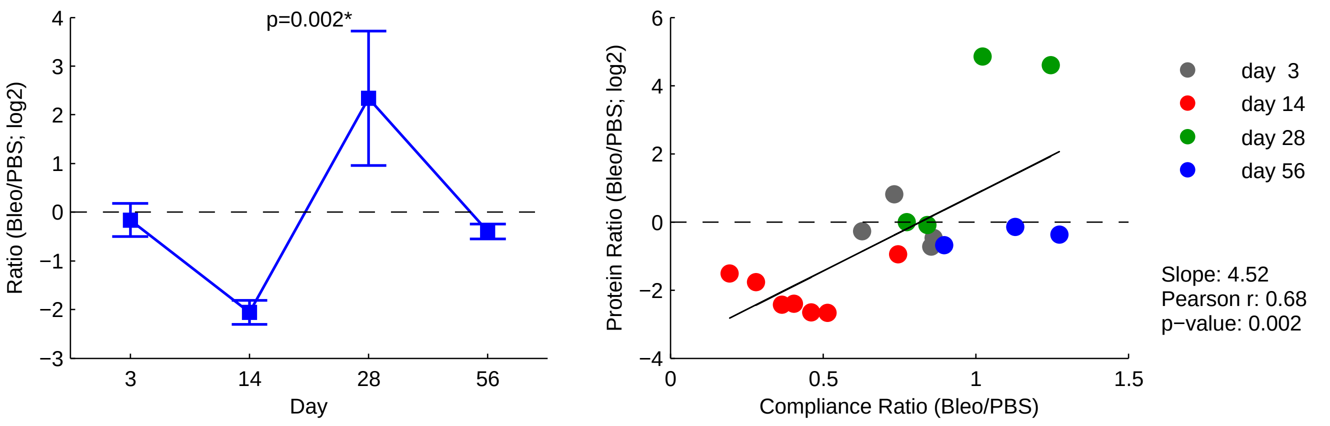

P09581 − Csf1r (id: 2392)

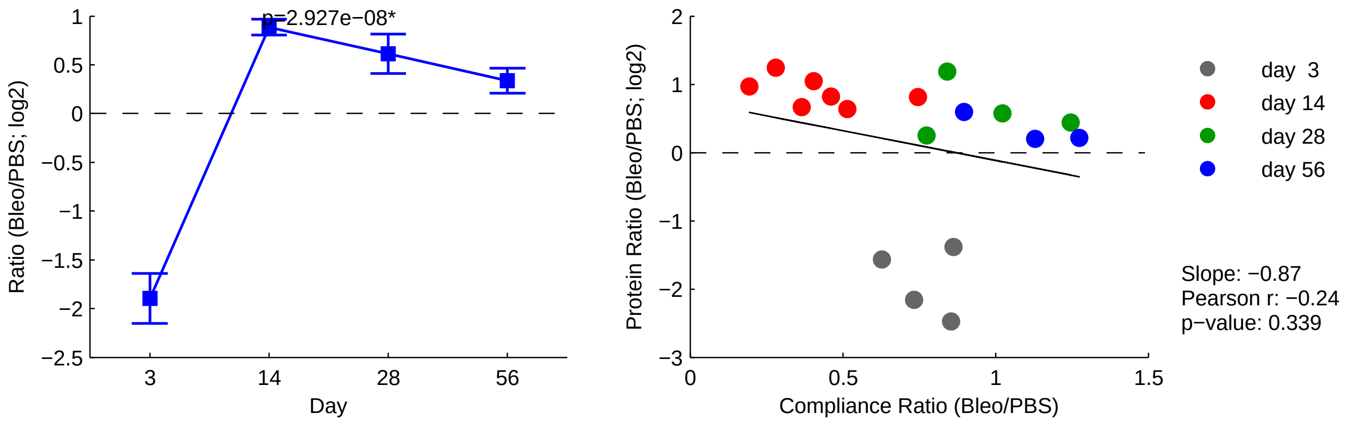

P09671 − Sod2 (id: 2393)

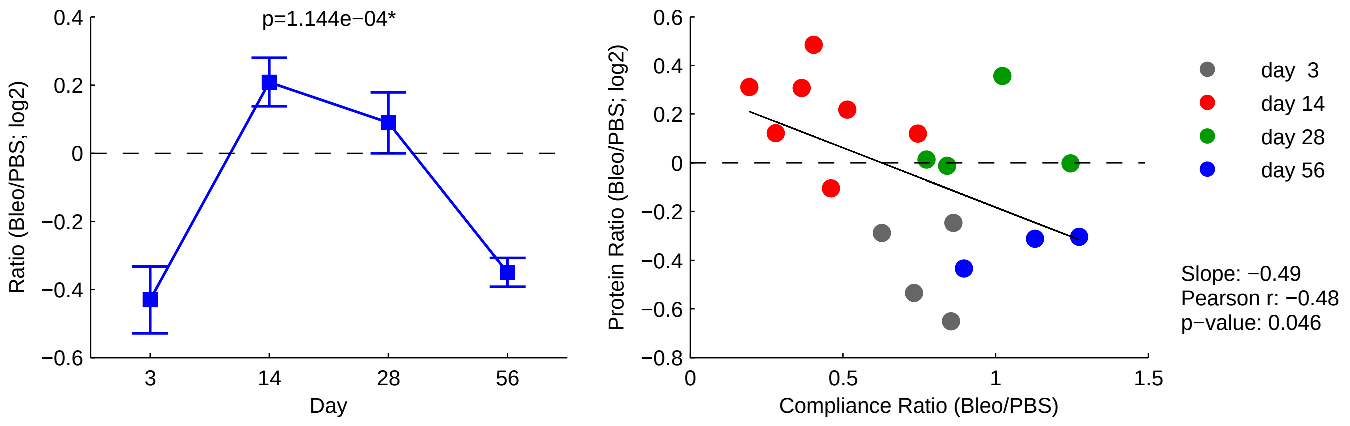

P09803 − Cdh1 (id: 2394)

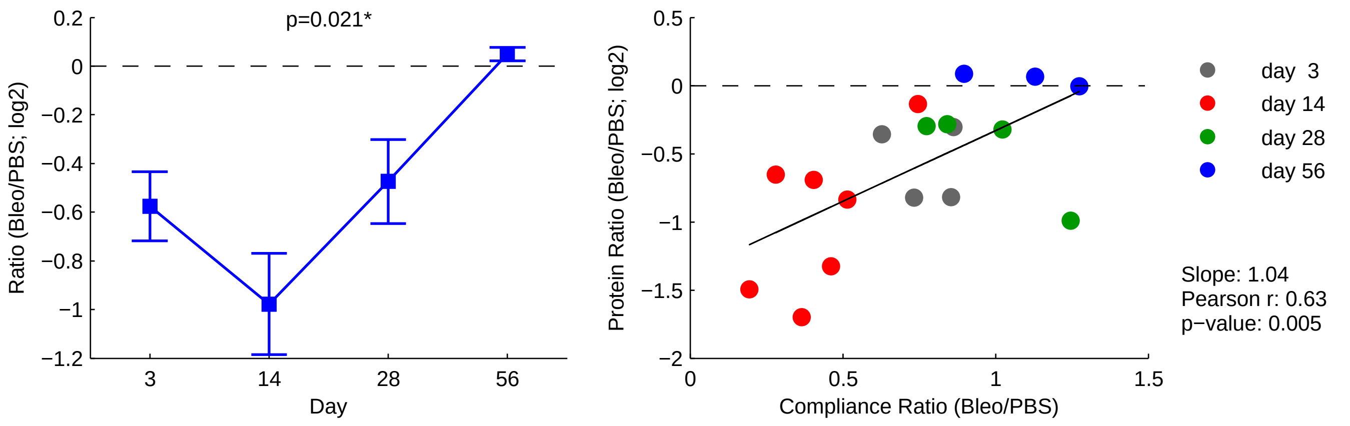

P09813 − Apoa2 (id: 2395)

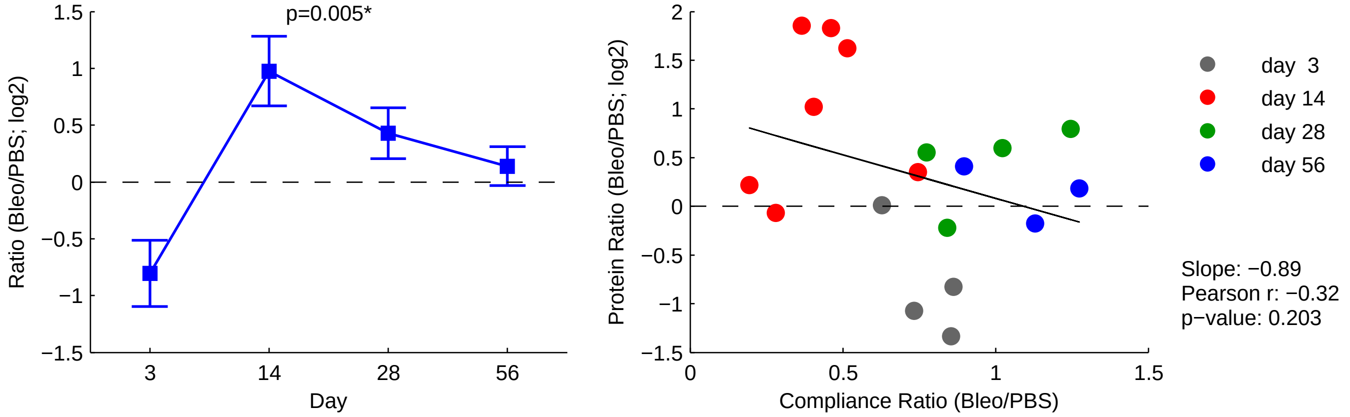

P0C0A3 − Chmp6 (id: 2397)

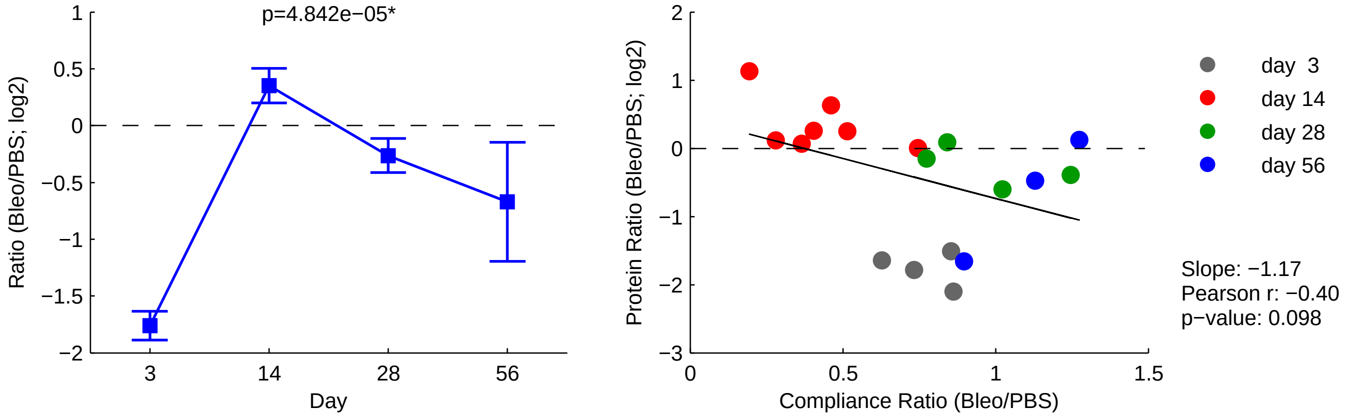

P0C605 − Prkg1 (id: 2399)

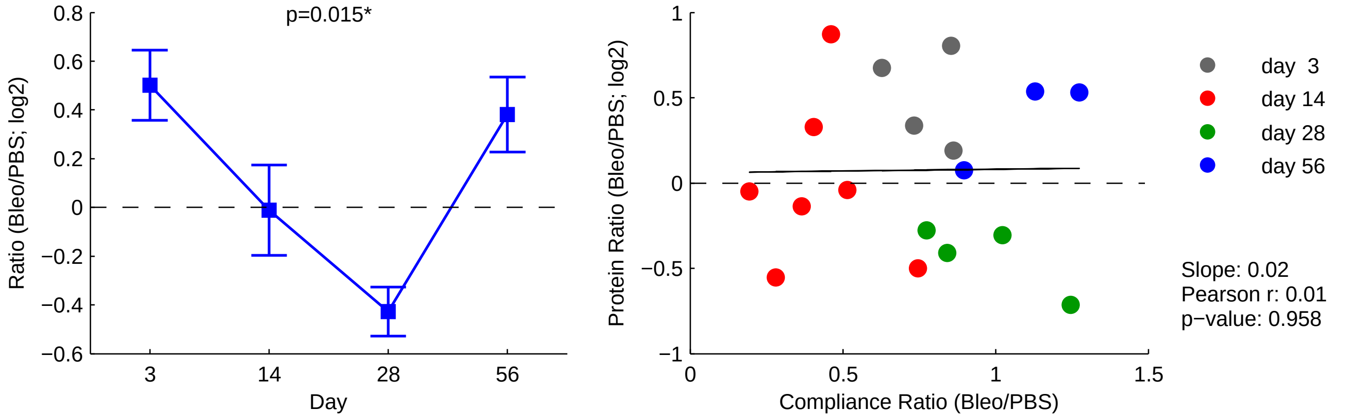

P0CW03 − Ly6c2 (id: 2402)

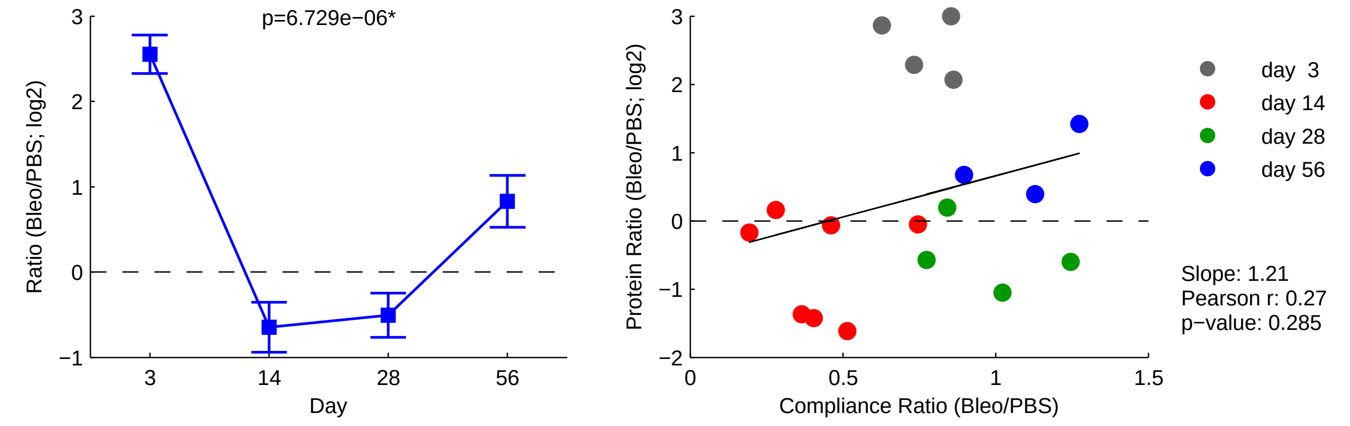

P0DJE0 − Pet100 (id: 2403)

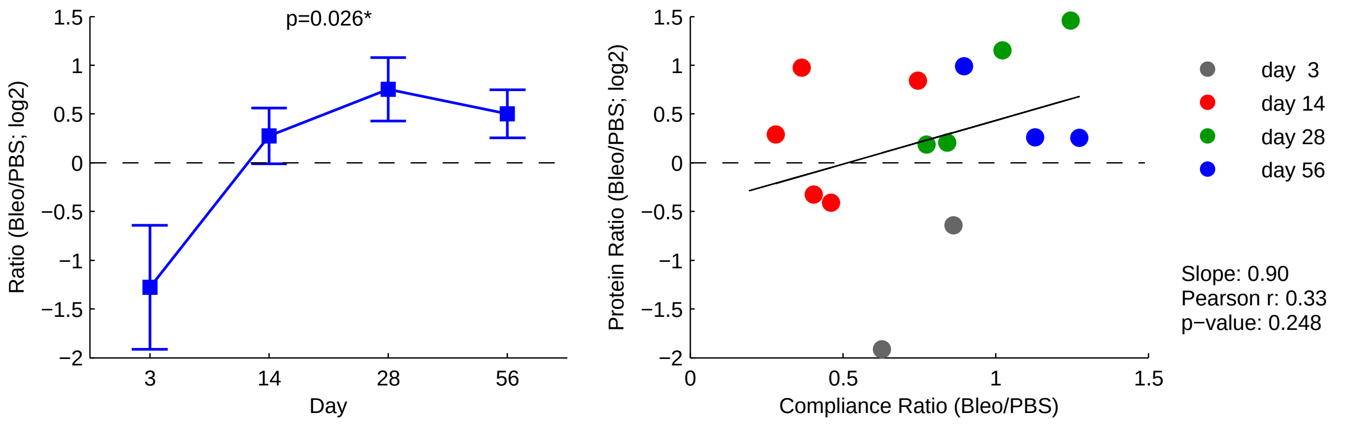

P10107 − Anxa1 (id: 2405)

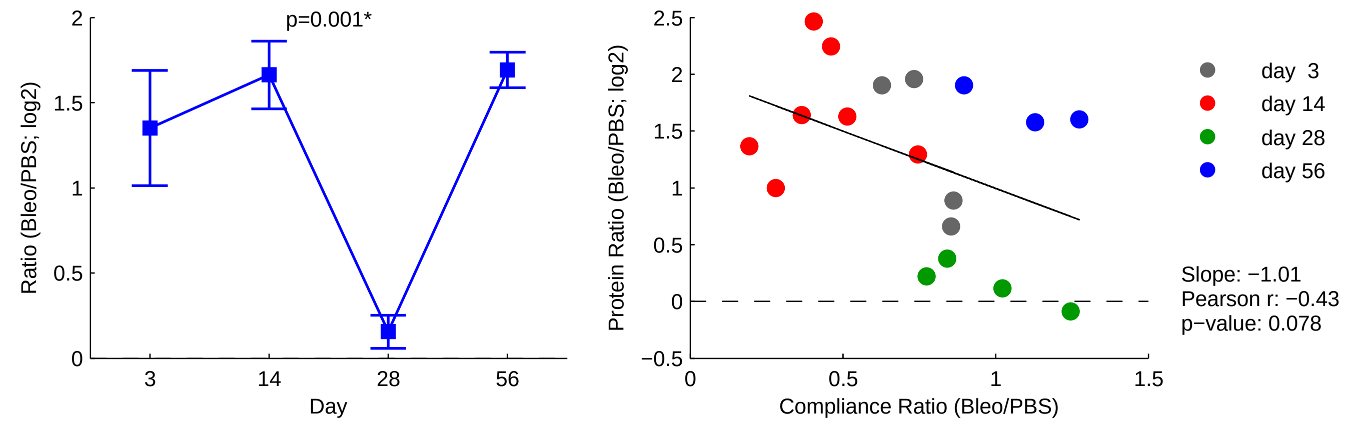

P10126 − Eef1a1 (id: 2406)

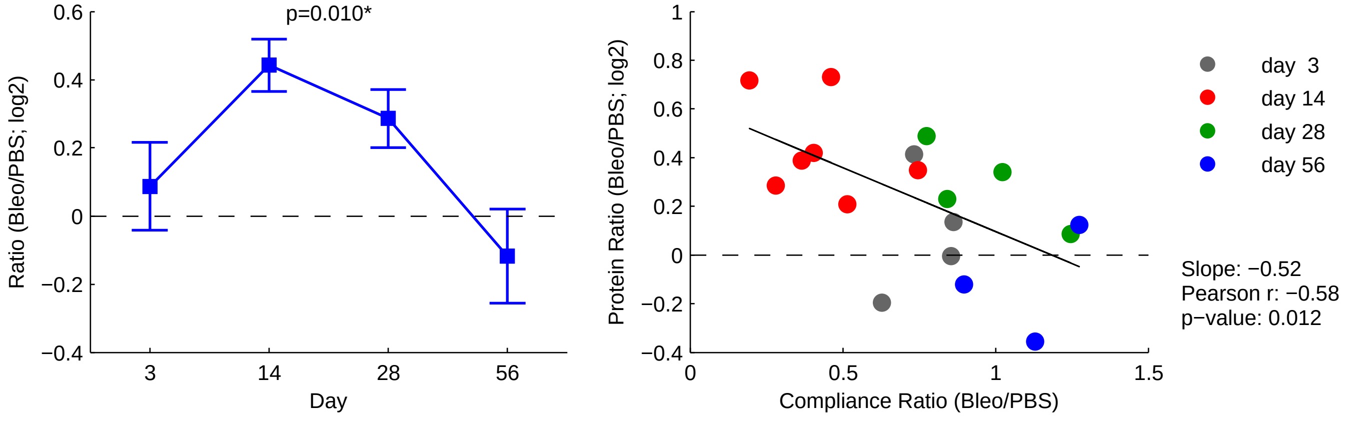

P10493 − Nid1 (id: 2410)

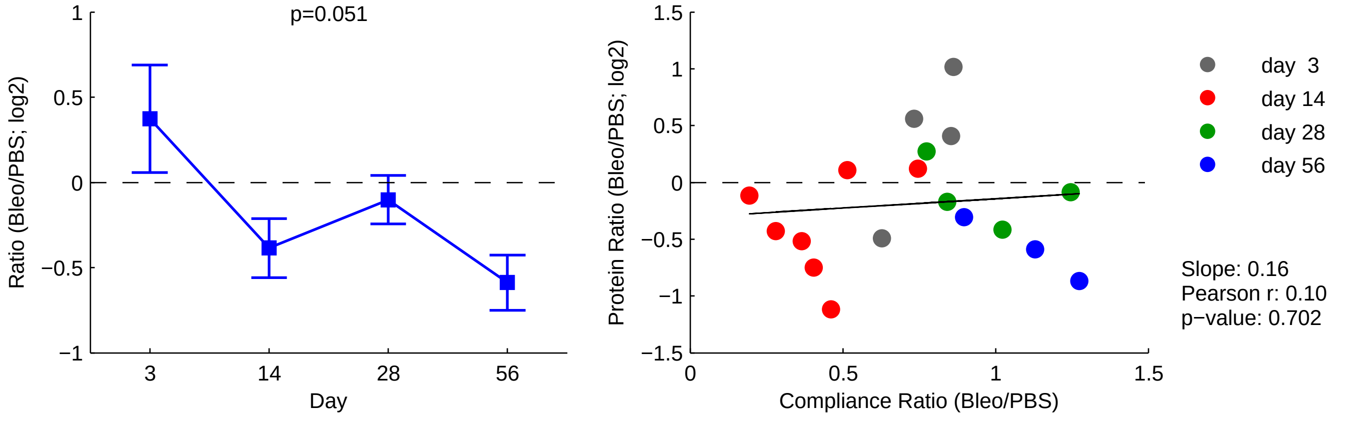

P10518 − Alad (id: 2411)

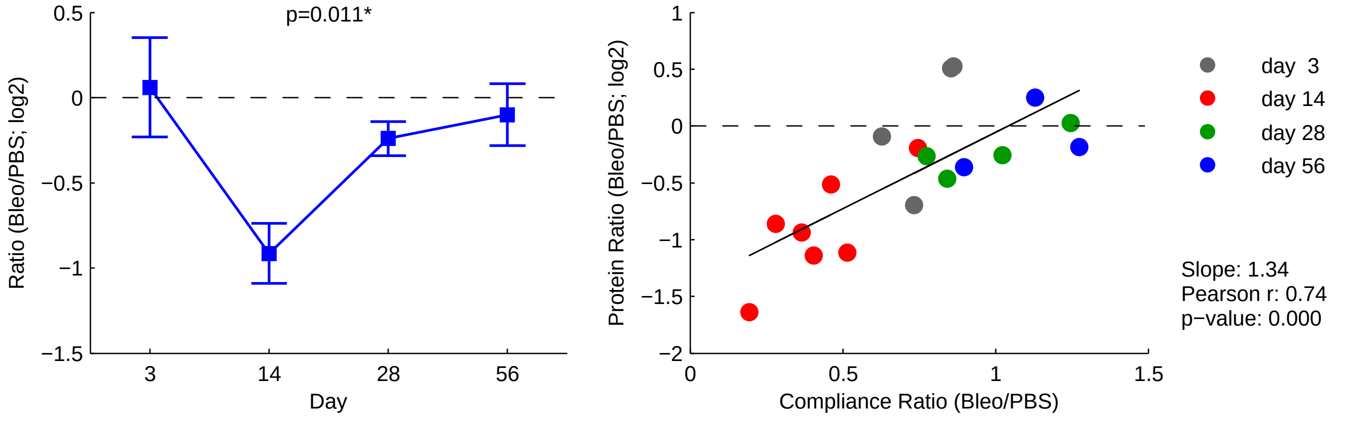

P10605 − Ctsb (id: 2412)

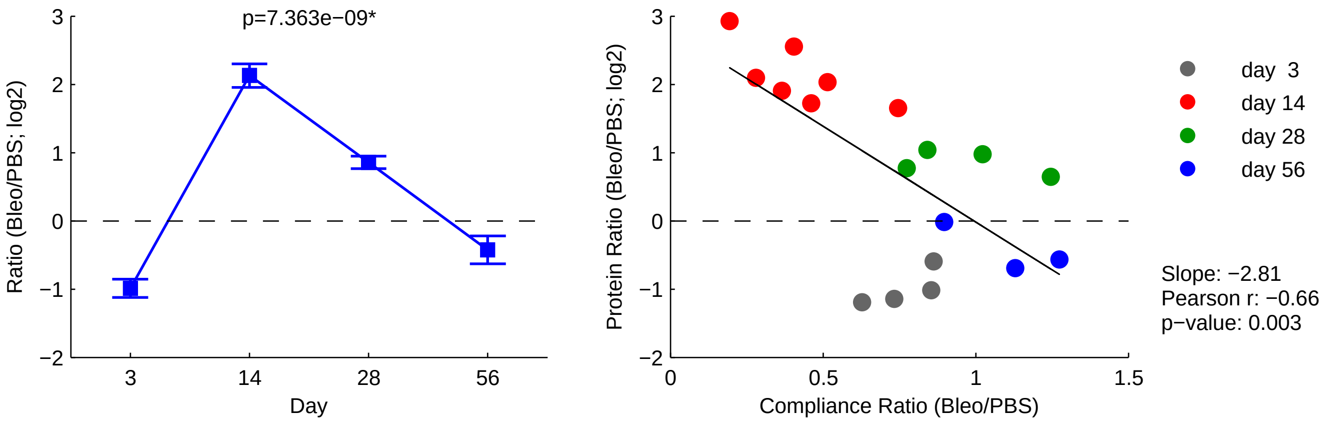

P10639 − Txn (id: 2415)

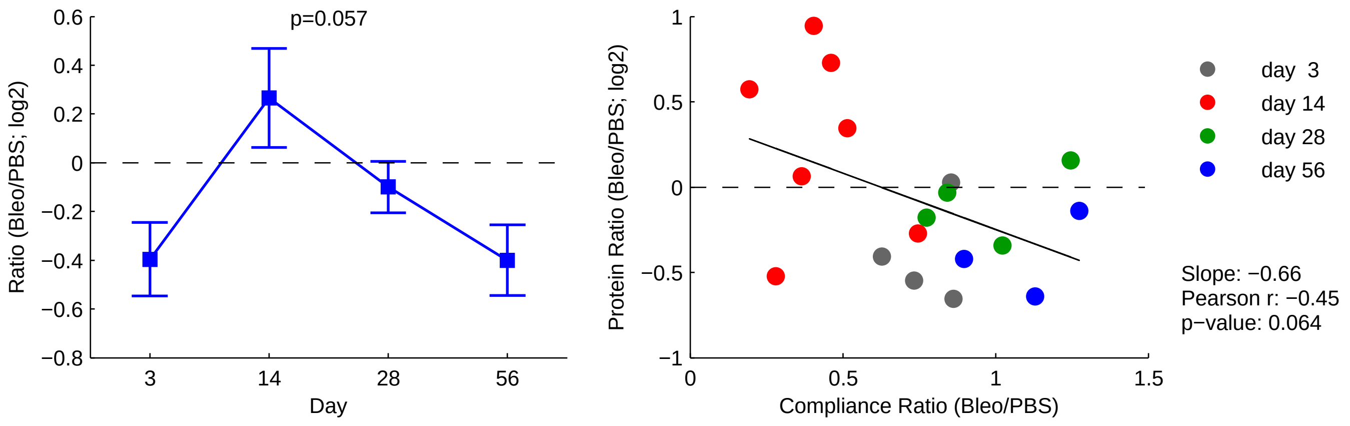

P10649 − Gstm1 (id: 2416)

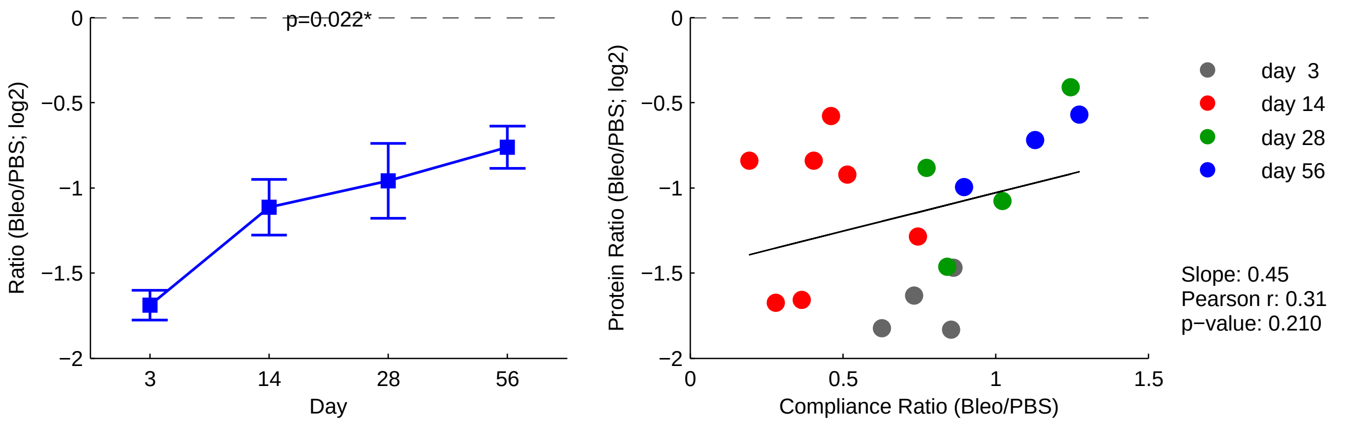

P10833 − Rras (id: 2419)

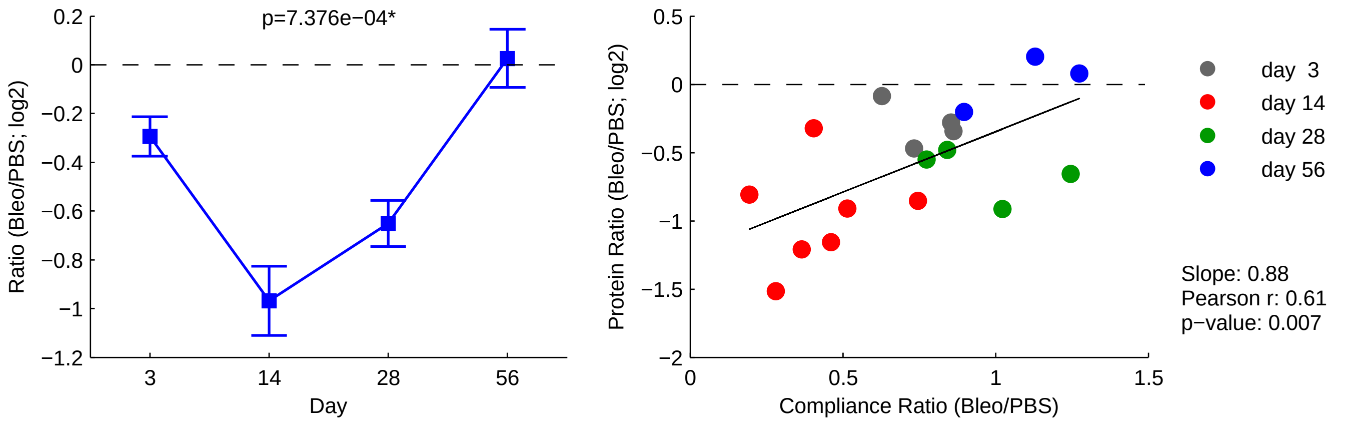

P10852 − Slc3a2 (id: 2420)

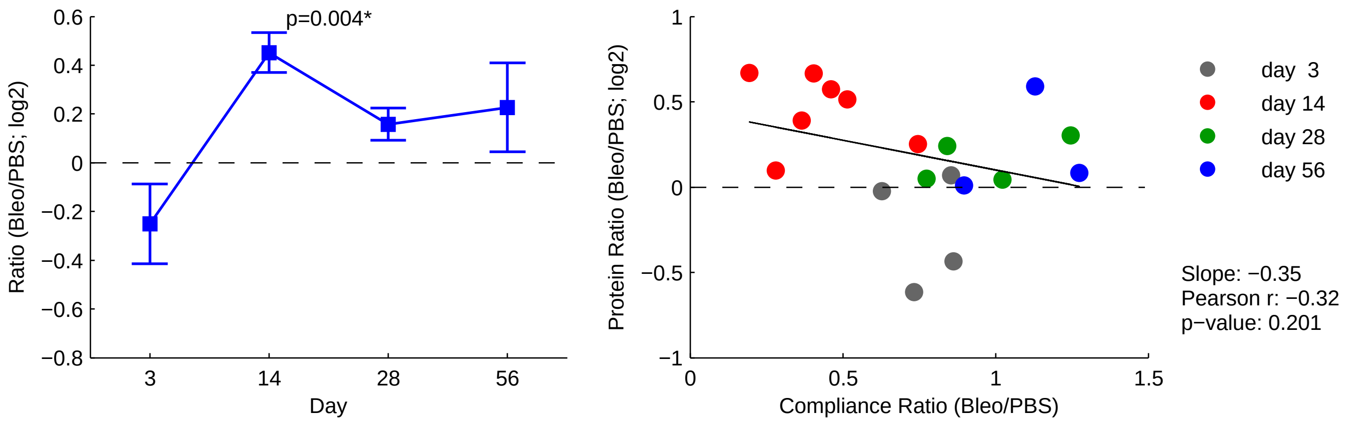

P10853 − Hist1h2bf (id: 2421)

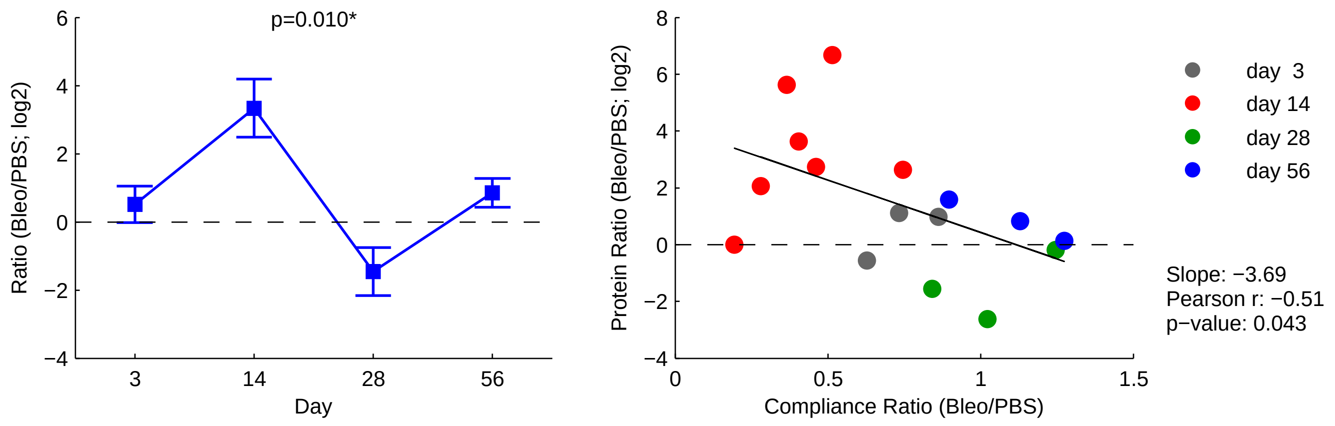

P10922 − H1f0 (id: 2422)

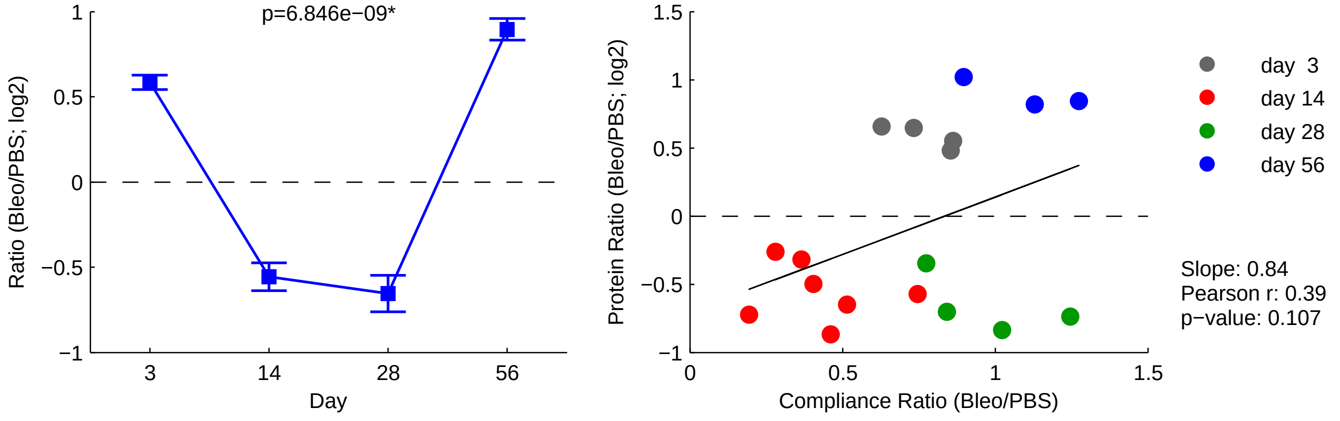

P11031 − Sub1 (id: 2423)

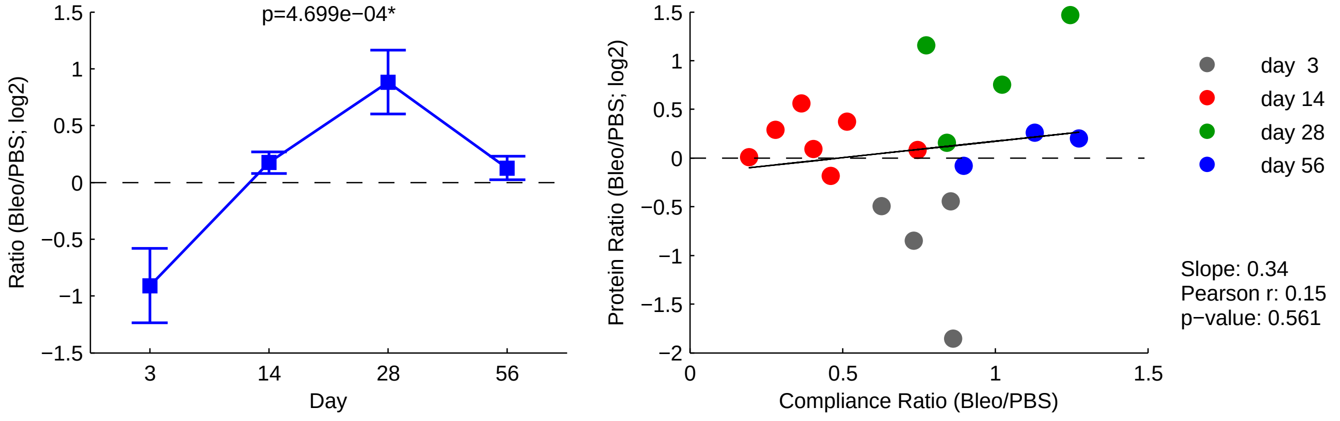

P11032−2 − Gzma (id: 2424)

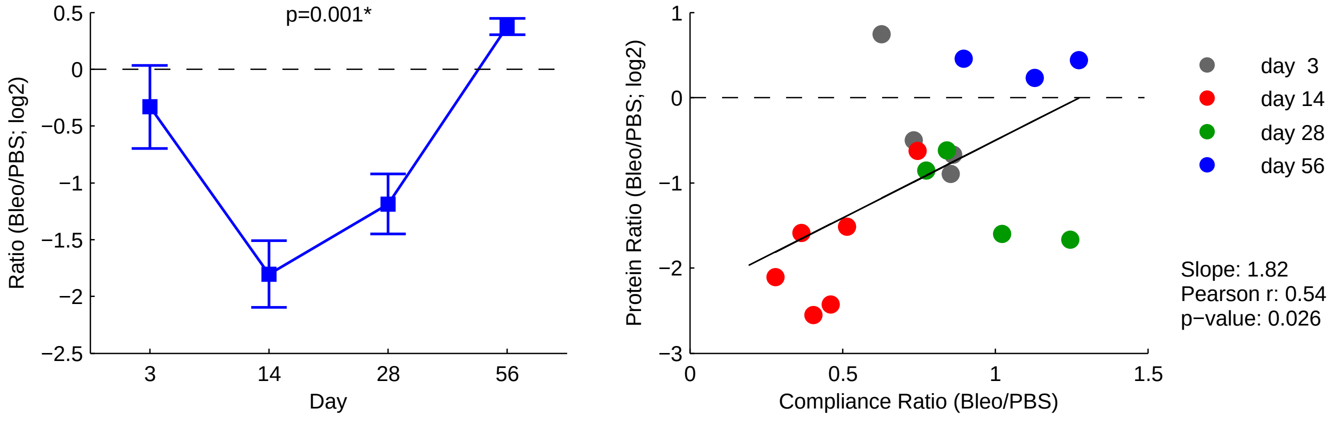

P11152 − Lpl (id: 2426)

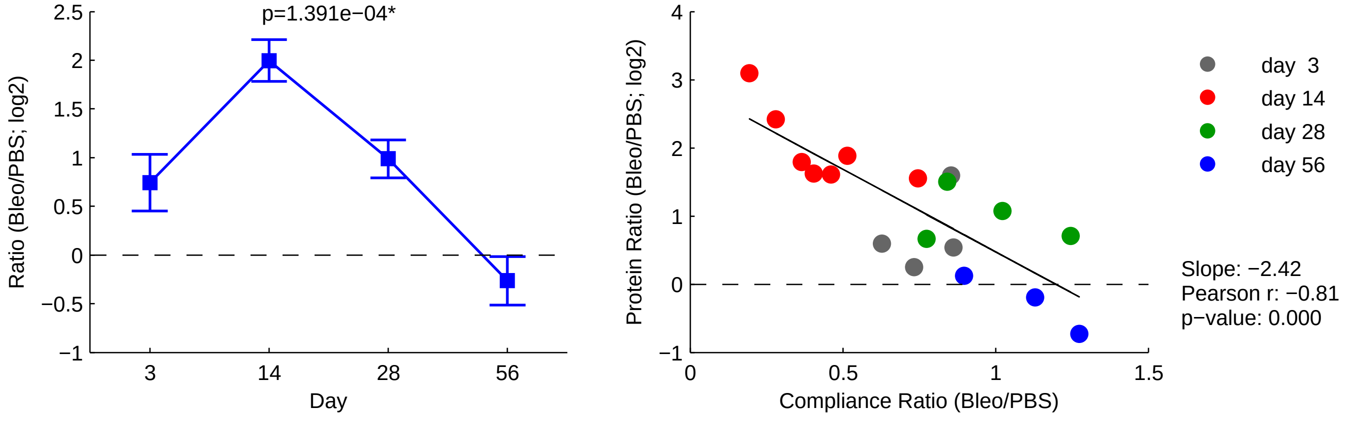

P11214 − Plat (id: 2427)

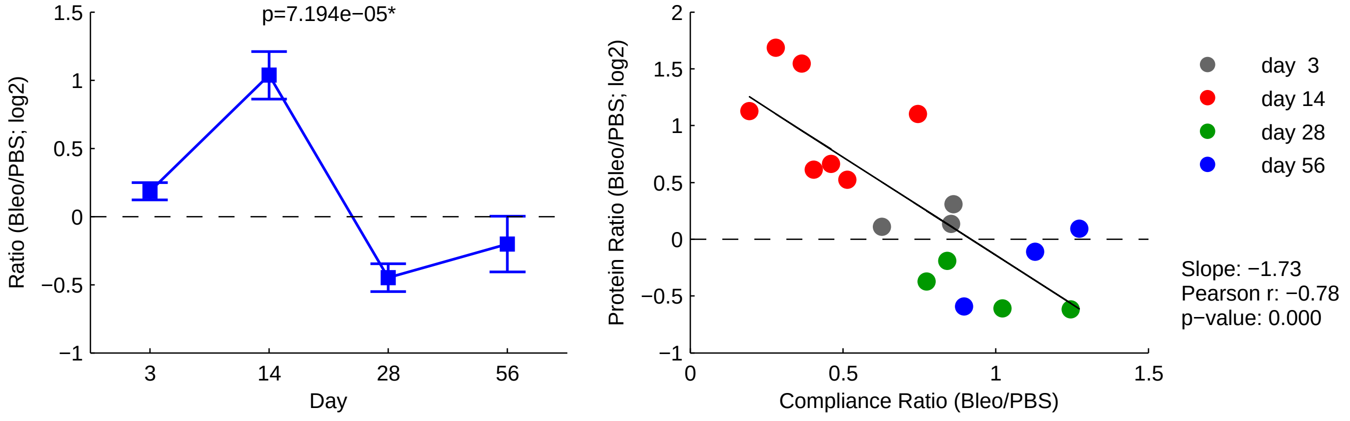

P11247 − Mpo (id: 2428)

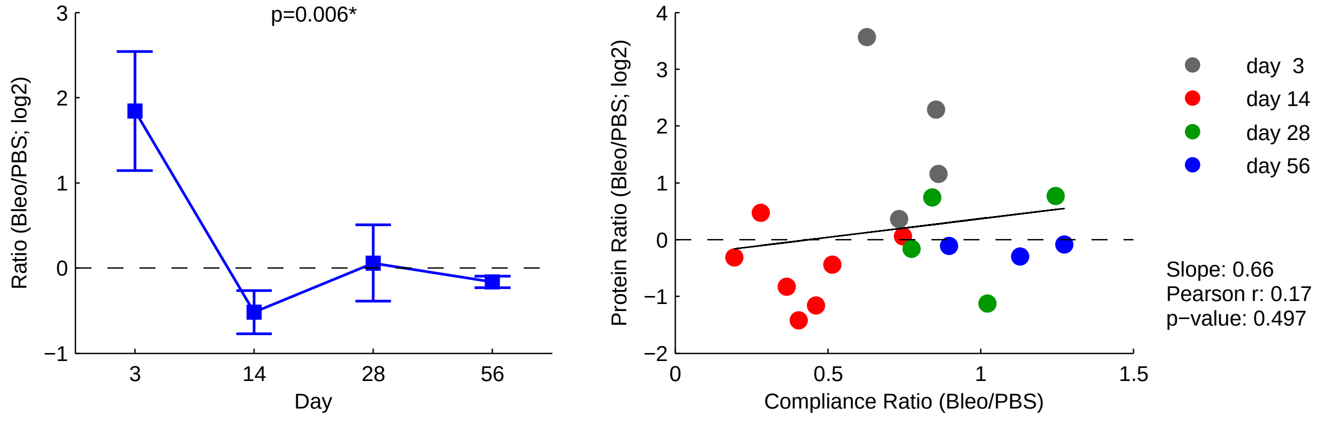

P11276 − Fn1 (id: 2429)

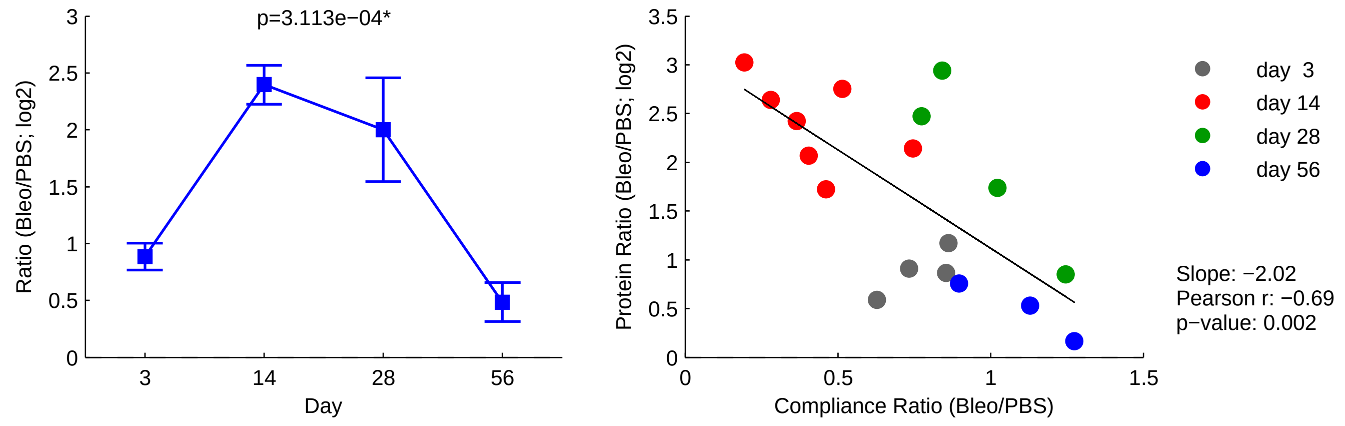

P11438 − Lamp1 (id: 2431)

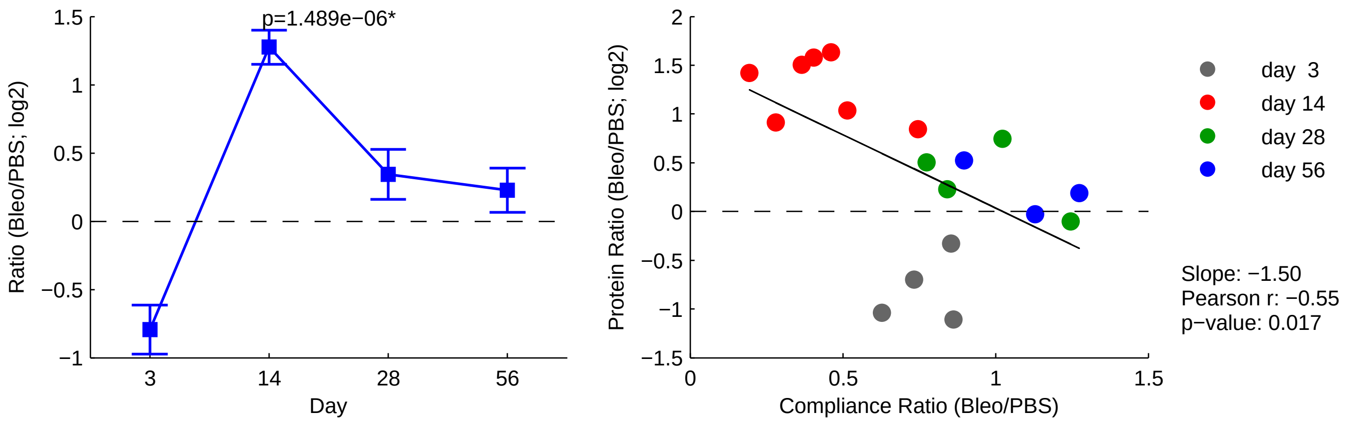

P11499 − Hsp90ab1 (id: 2433)

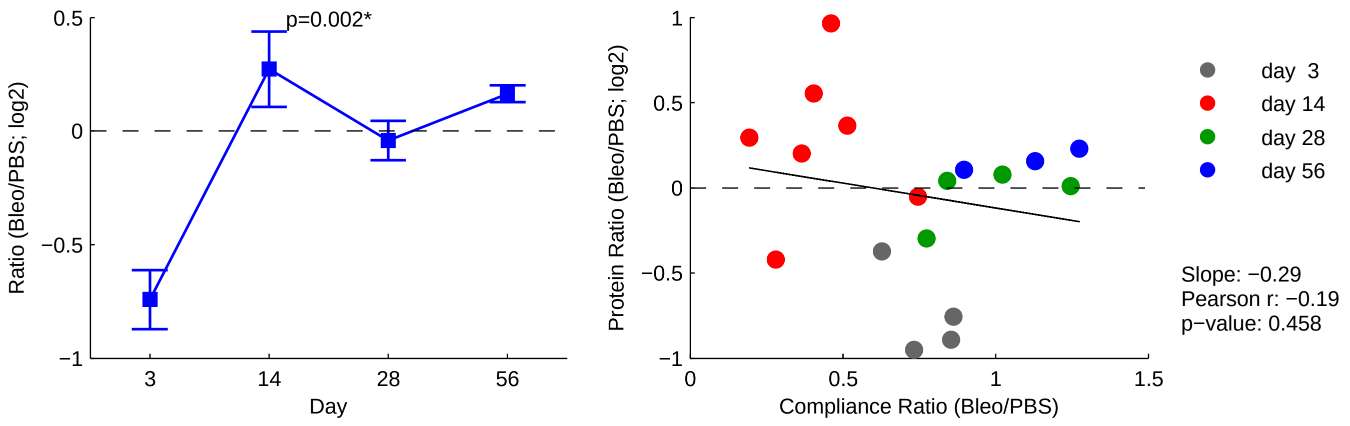

P11531 − Dmd (id: 2434)

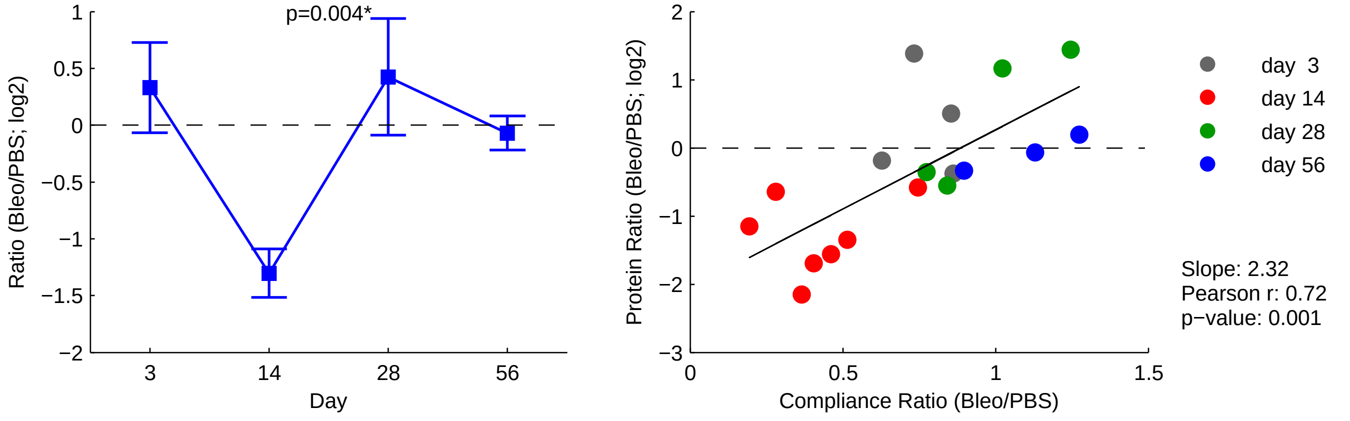

P11609 − Cd1d1 (id: 2435)

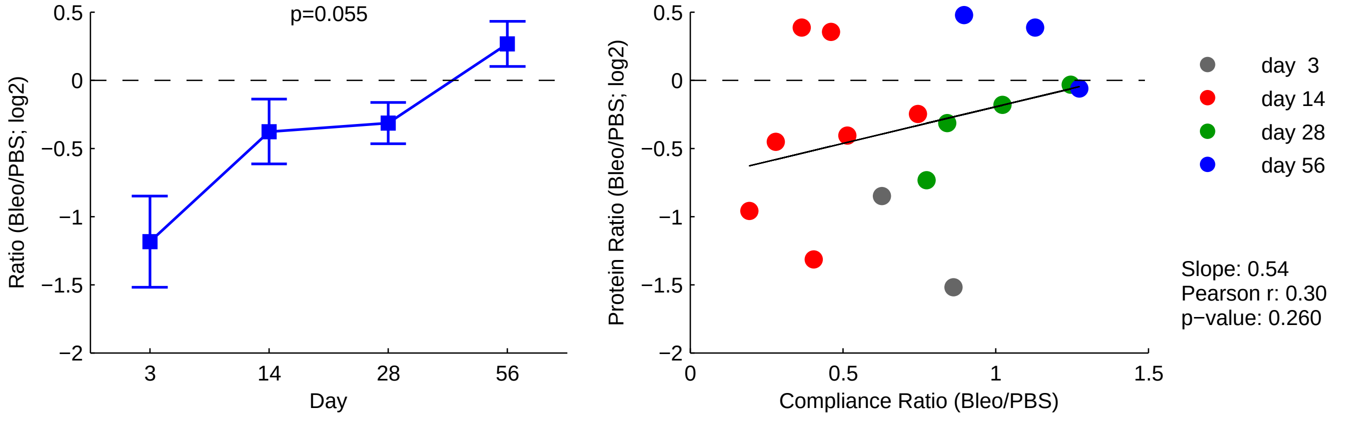

P11672 − Lcn2 (id: 2436)

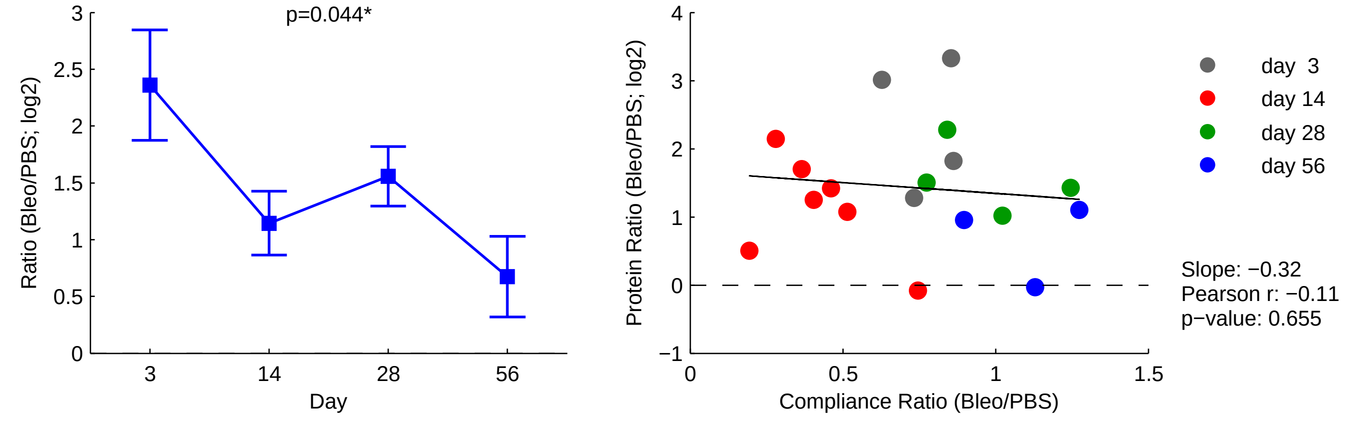

P11679 − Krt8 (id: 2437)

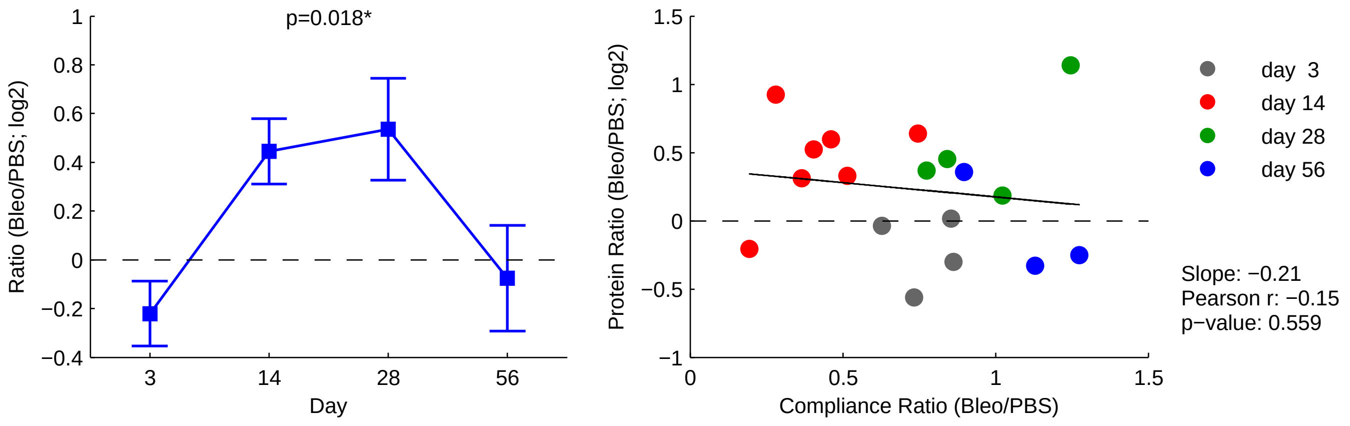

P11688 − Itga5 (id: 2439)

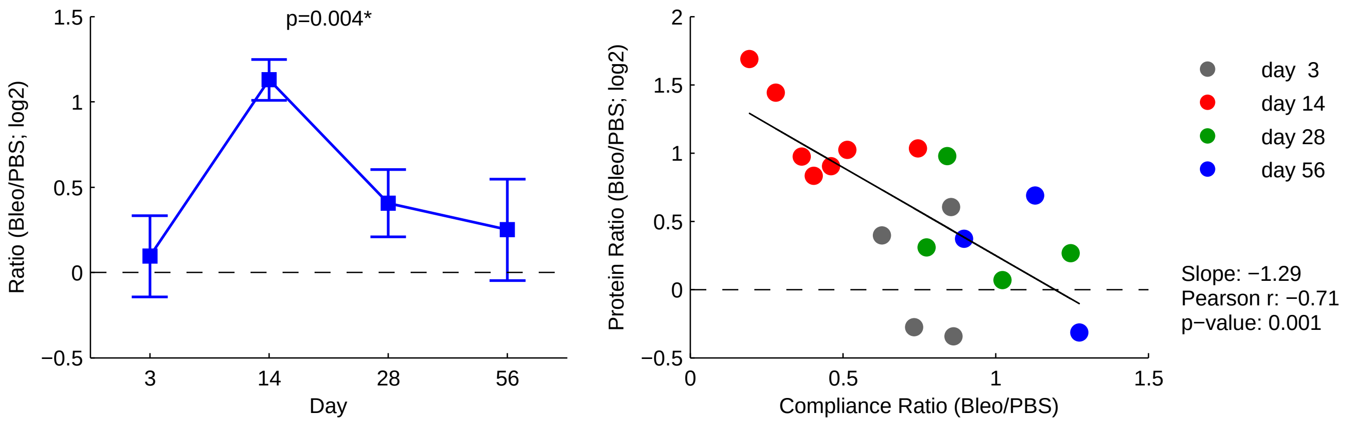

Q542I8 − Itgb2 (id: 2441)

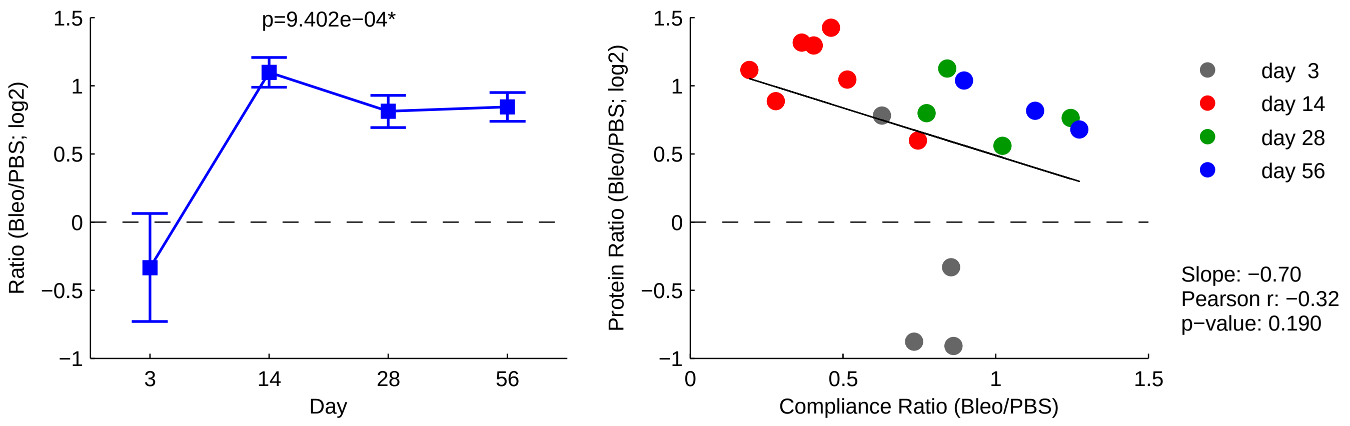

P11881−8 − Itpr1 (id: 2442)

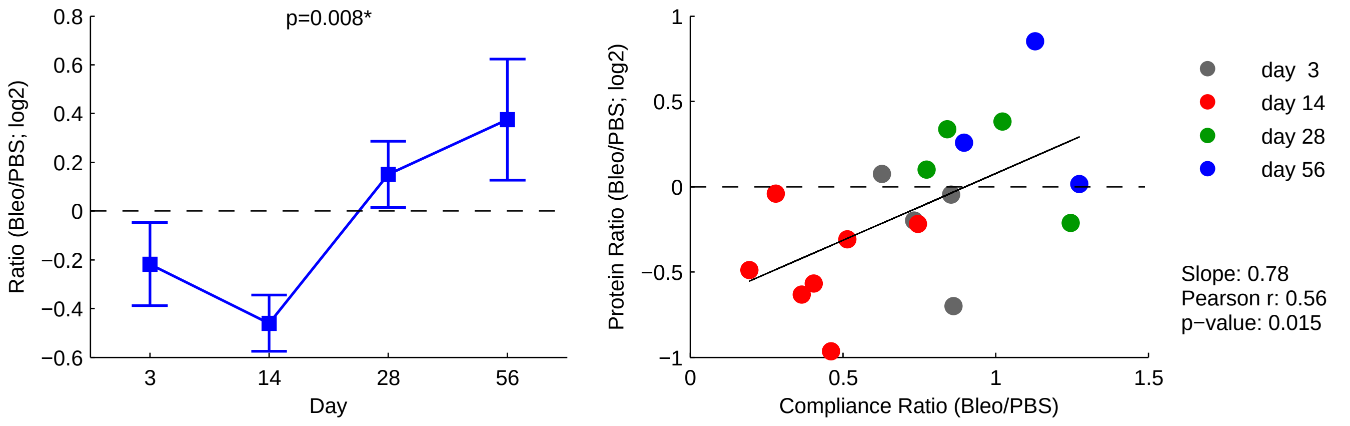

P11928 − Oas1a (id: 2444)

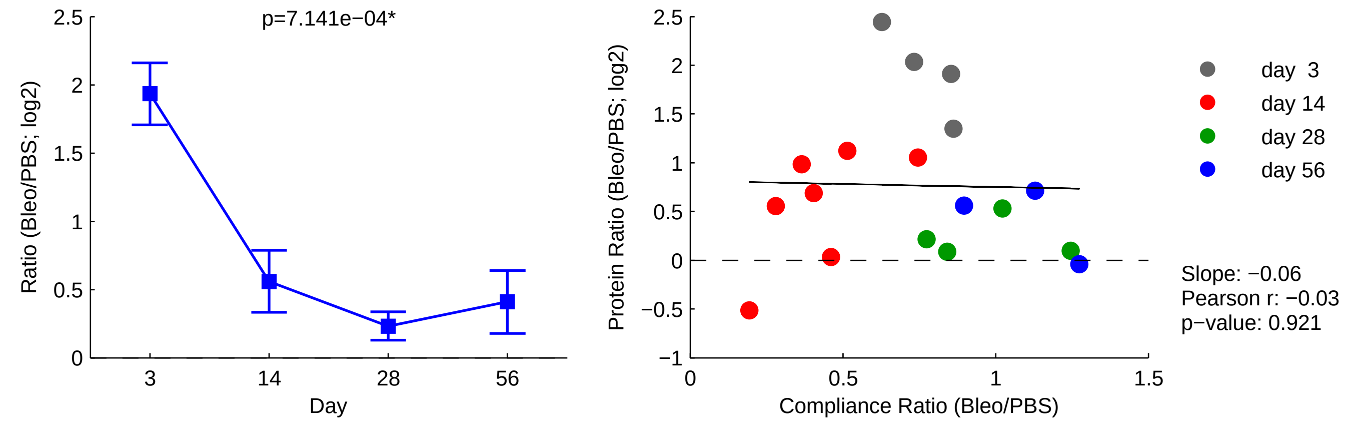

P11930 − Nudt19 (id: 2445)

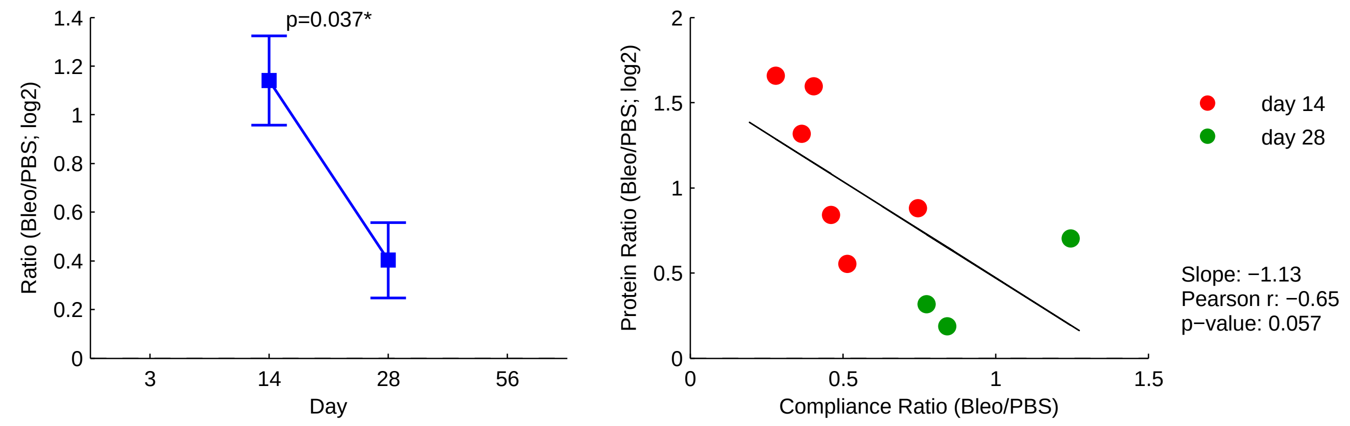

P12023 − App (id: 2448)

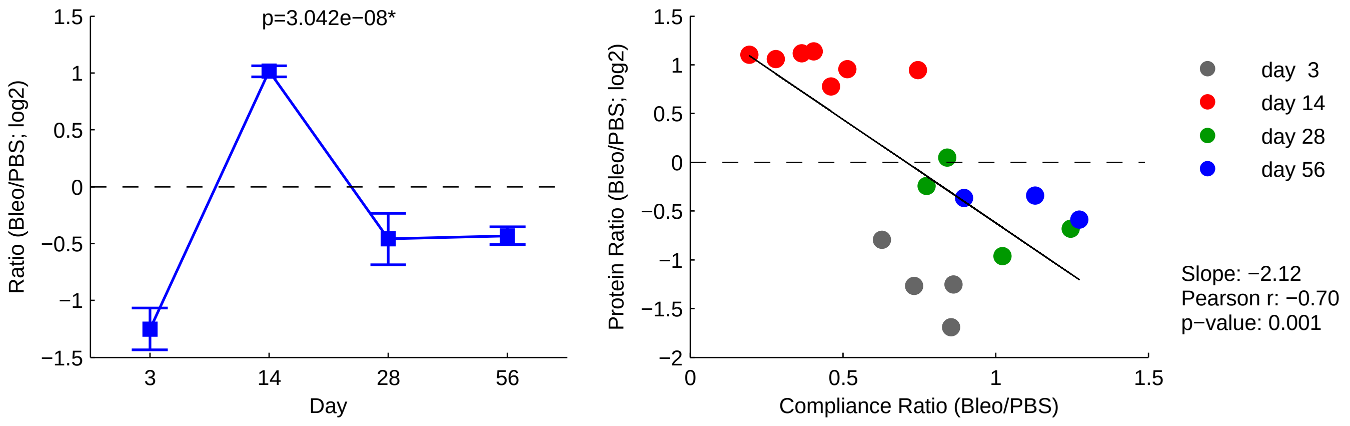

P12242 − Ucp1 (id: 2449)

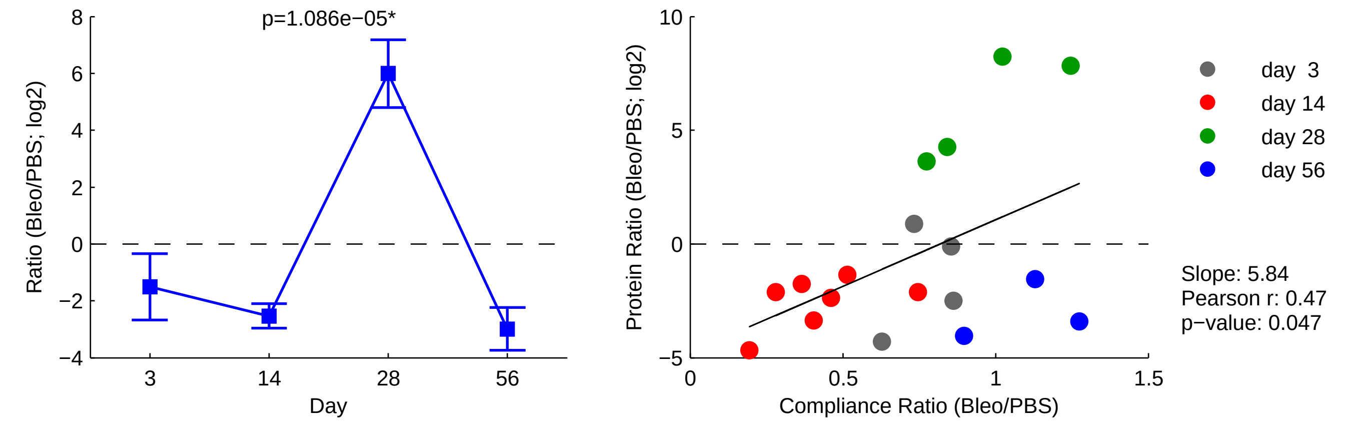

P12265 − Gusb (id: 2450)

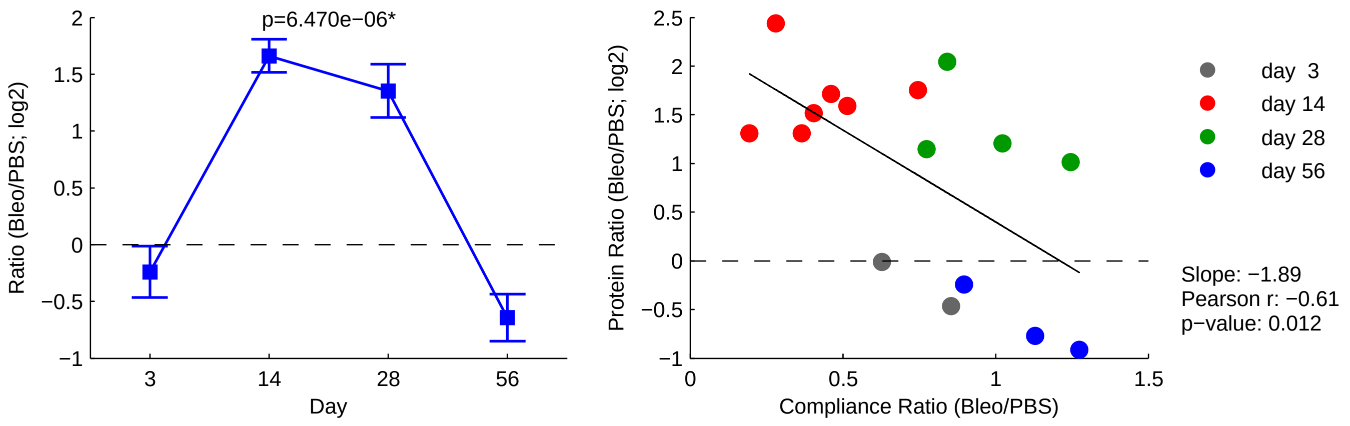

P12382 − Pfkl (id: 2451)

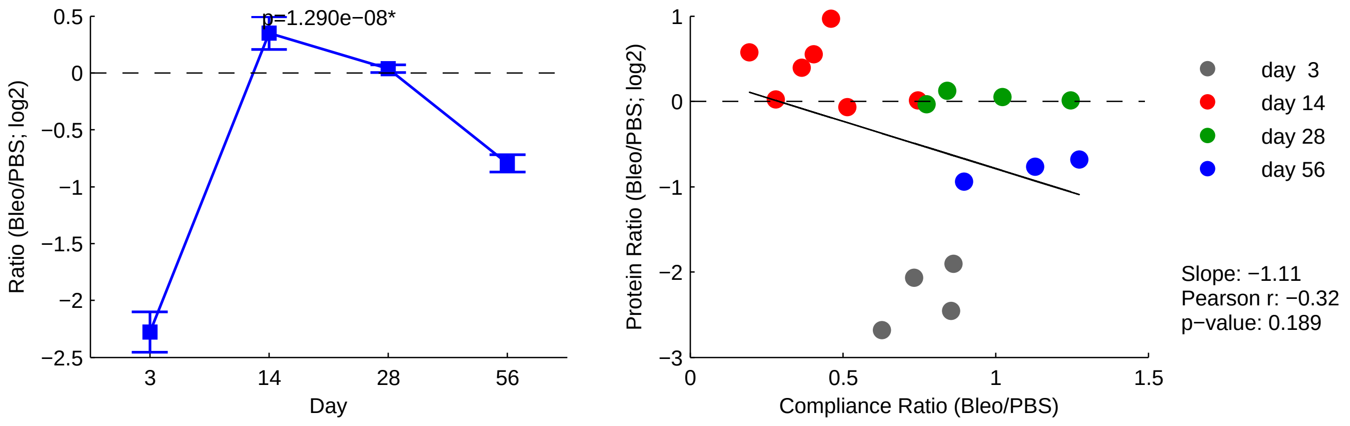

P12399 − Ctla2a (id: 2453)

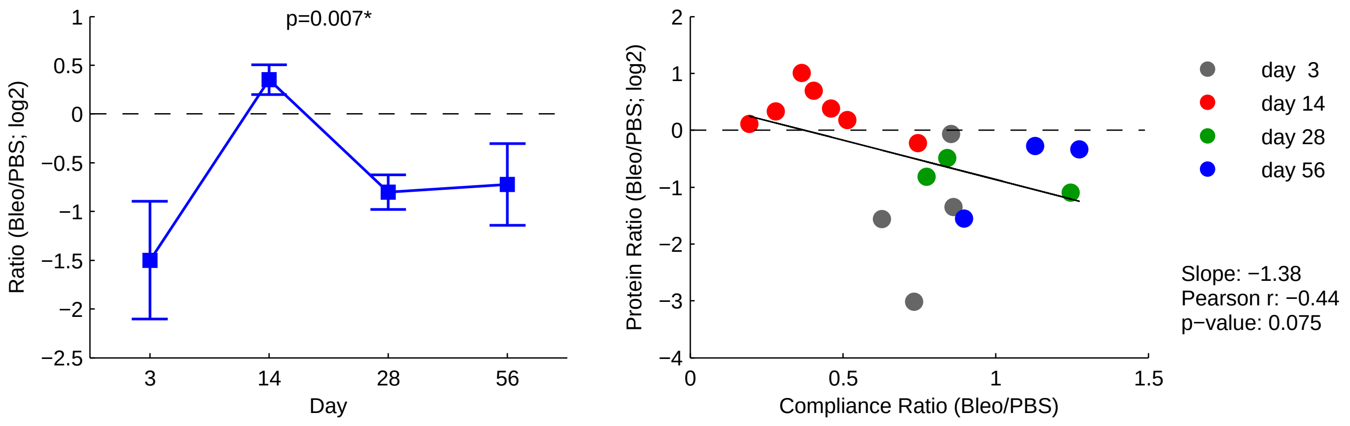

P12787 − Cox5a (id: 2456)

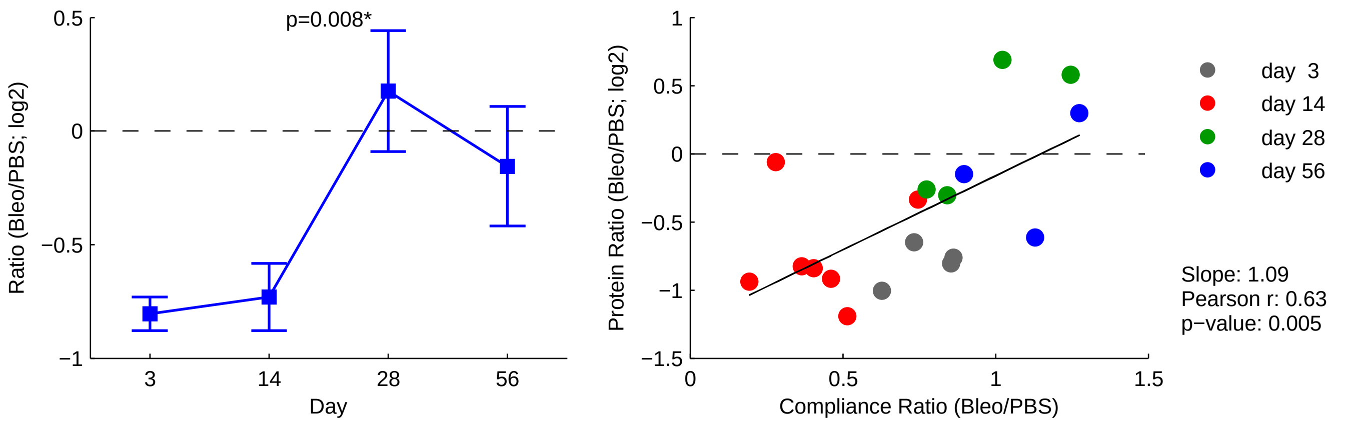

P12815 − Pdcd6 (id: 2459)

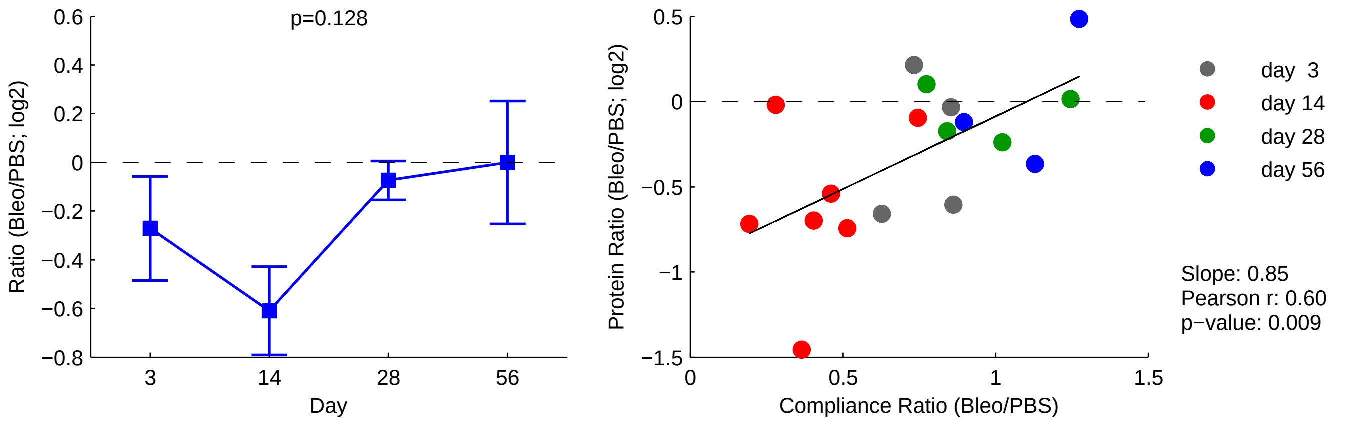

P12970 − Rpl7a (id: 2460)

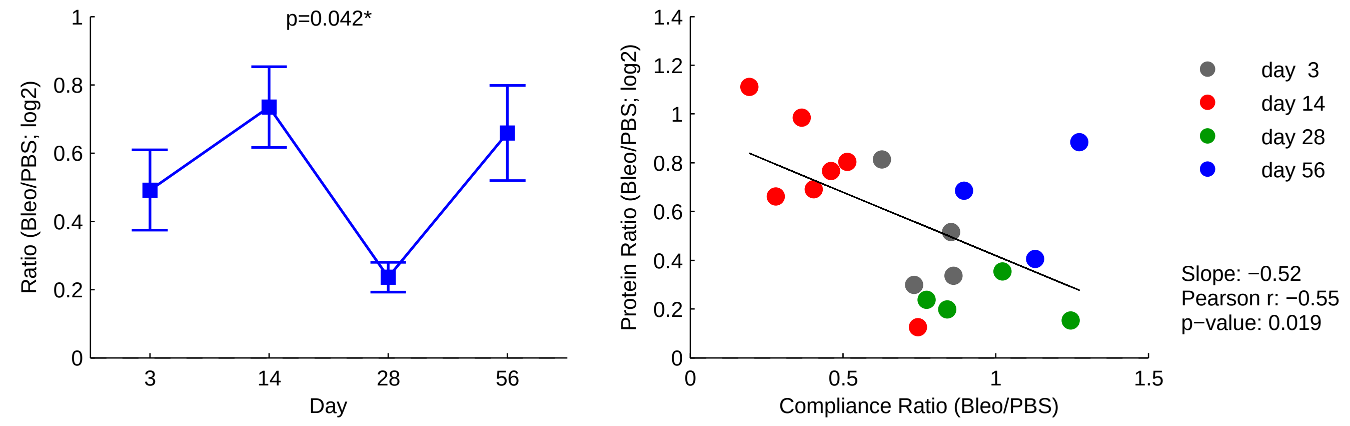

P13020−2 − (id: 2462)

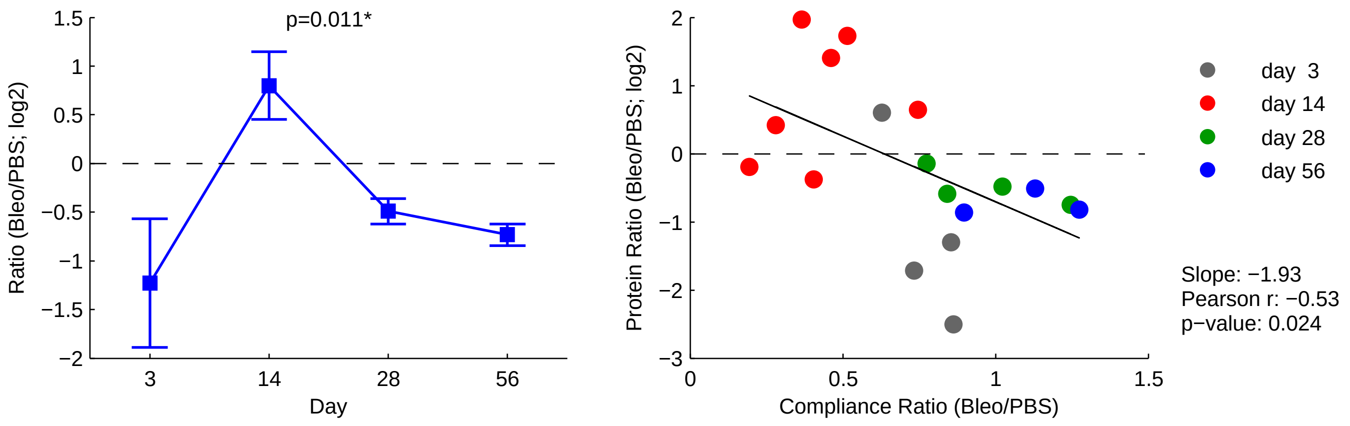

P13516 − Scd1 (id: 2468)

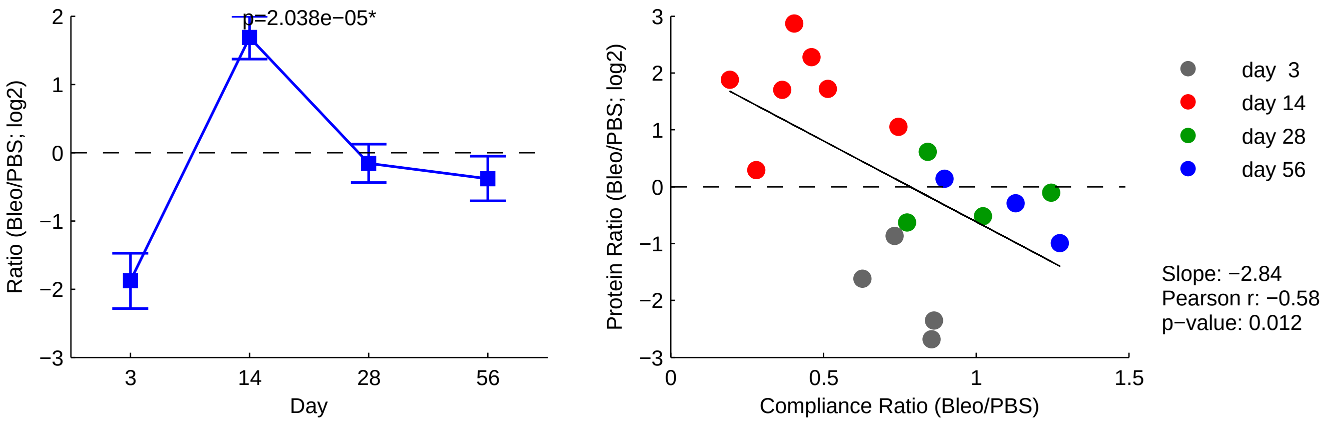

P13541 − Myh3 (id: 2469)

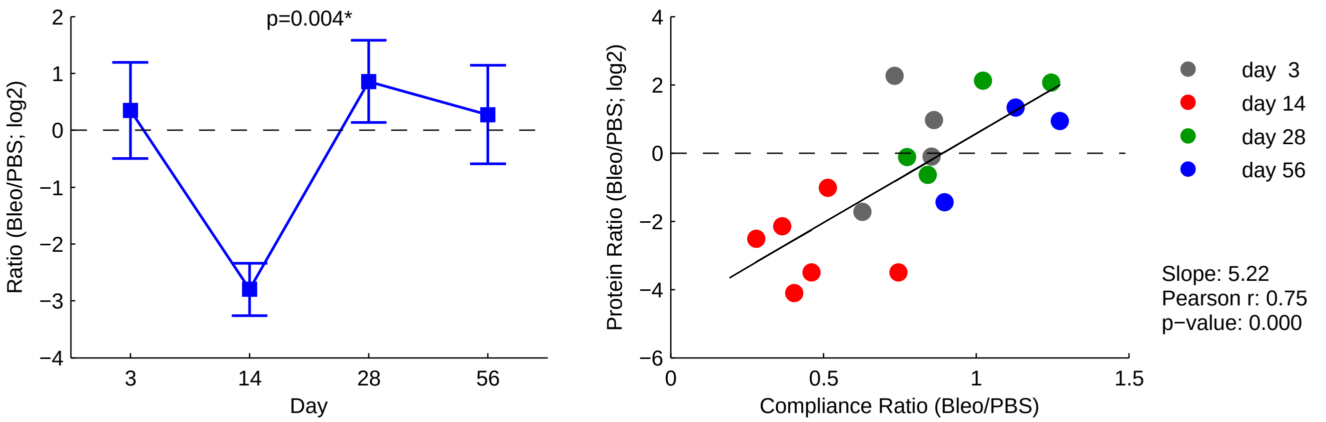

P13542 − Myh8 (id: 2470)

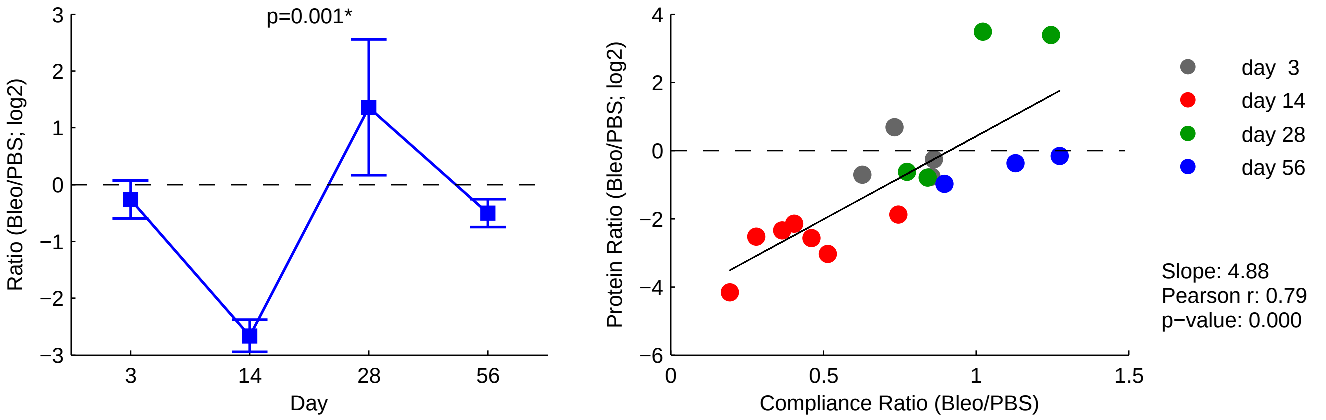

P13597−2 − Icam1 (id: 2471)

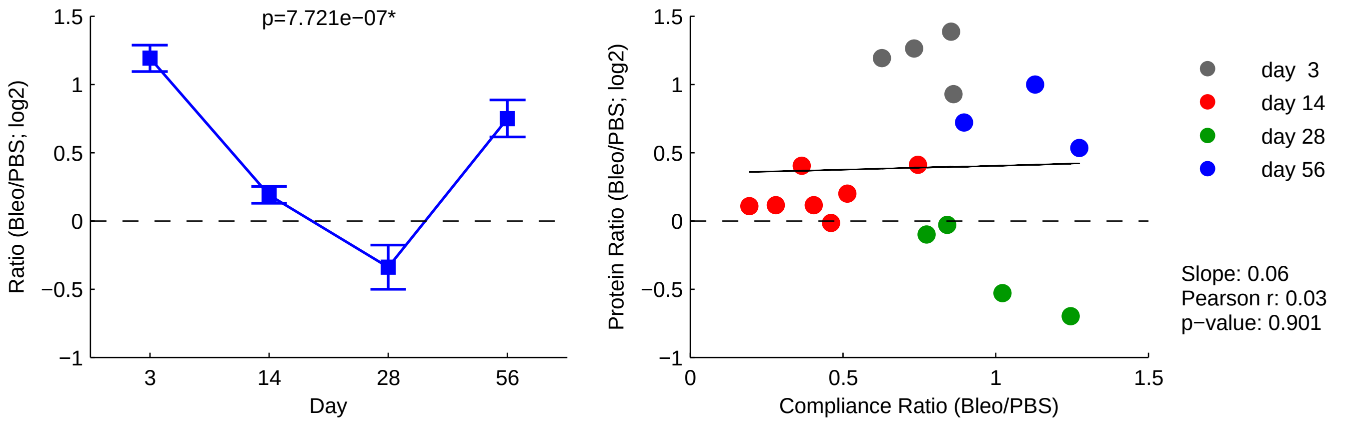

P13634 − Ca1 (id: 2473)

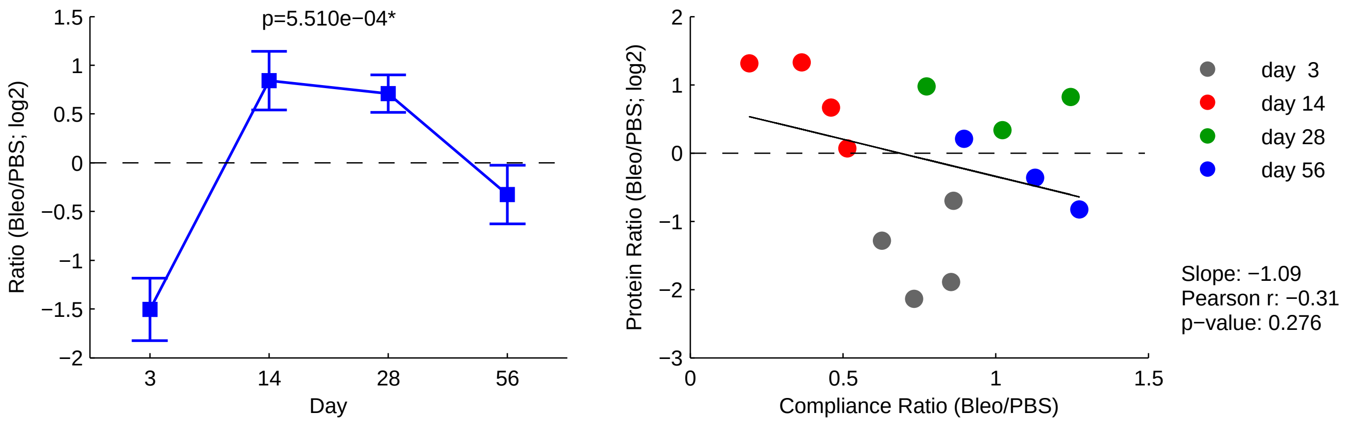

P13707 − Gpd1 (id: 2474)

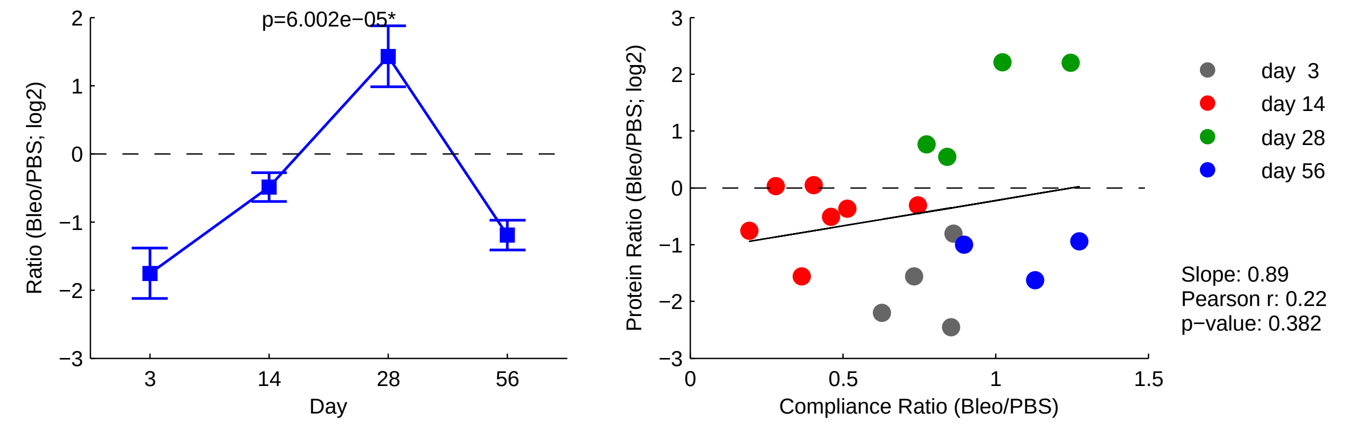

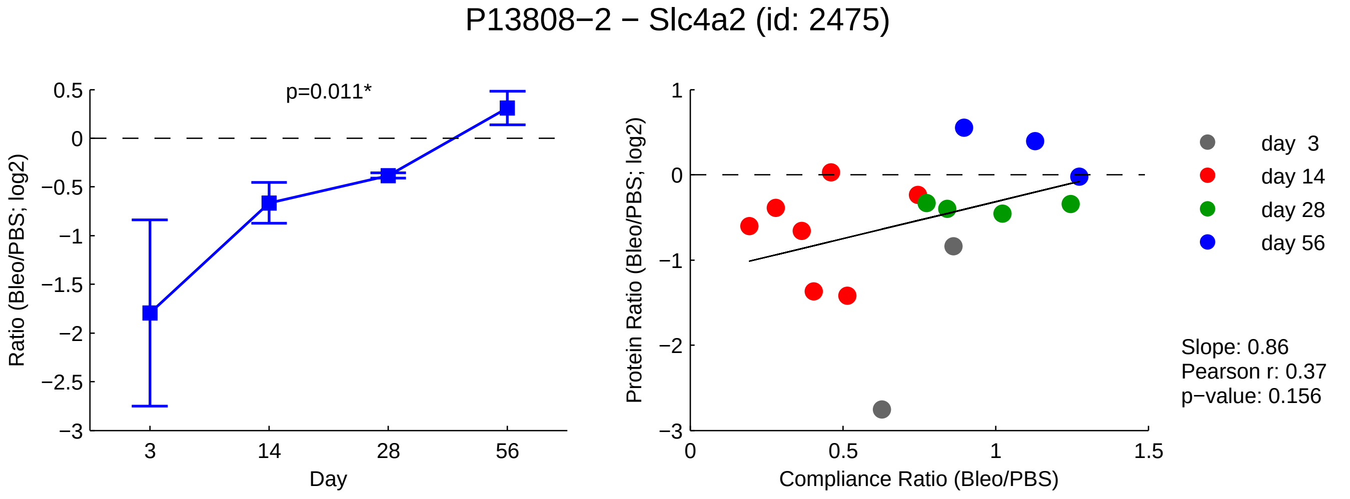
P13808−2 − Slc4a2 (id: 2475)
p=0.011*
Ratio (Bleo/PBS; log2)
Day
Protein Ratio (Bleo/PBS; log2)
Compliance Ratio (Bleo/PBS)
day 3
day 14
day 28
day 56
Slope: 0.86
Pearson r: 0.37
p−value: 0.156

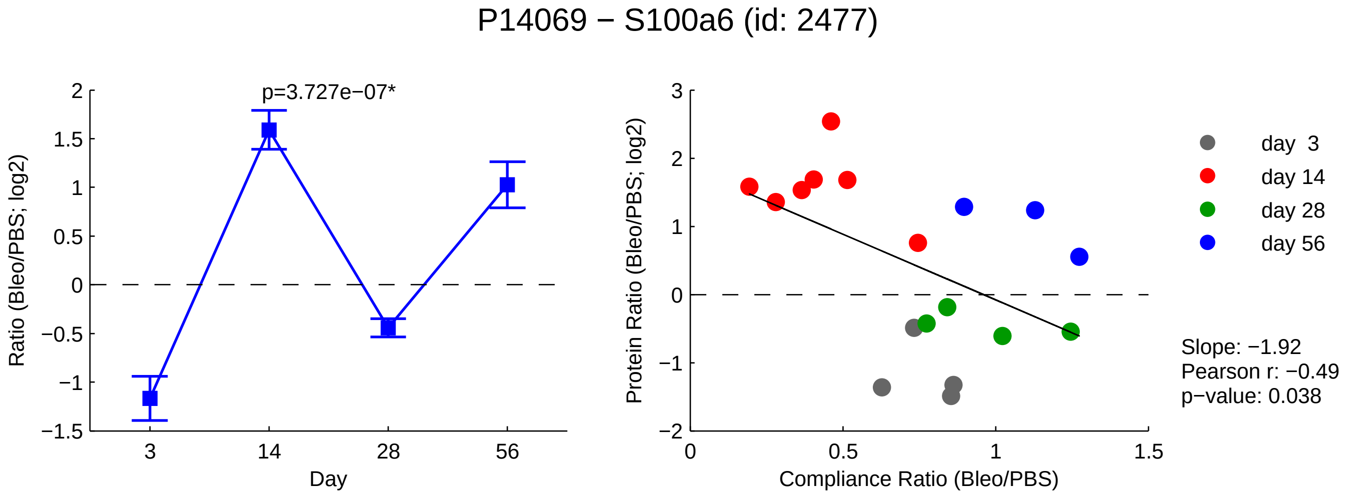
P14069 − S100a6 (id: 2477)
p=3.727e−07*
Ratio (Bleo/PBS; log2)
Day
Protein Ratio (Bleo/PBS; log2)
Compliance Ratio (Bleo/PBS)
day 3
day 14
day 28
day 56
Slope: −1.92
Pearson r: −0.49
p−value: 0.038

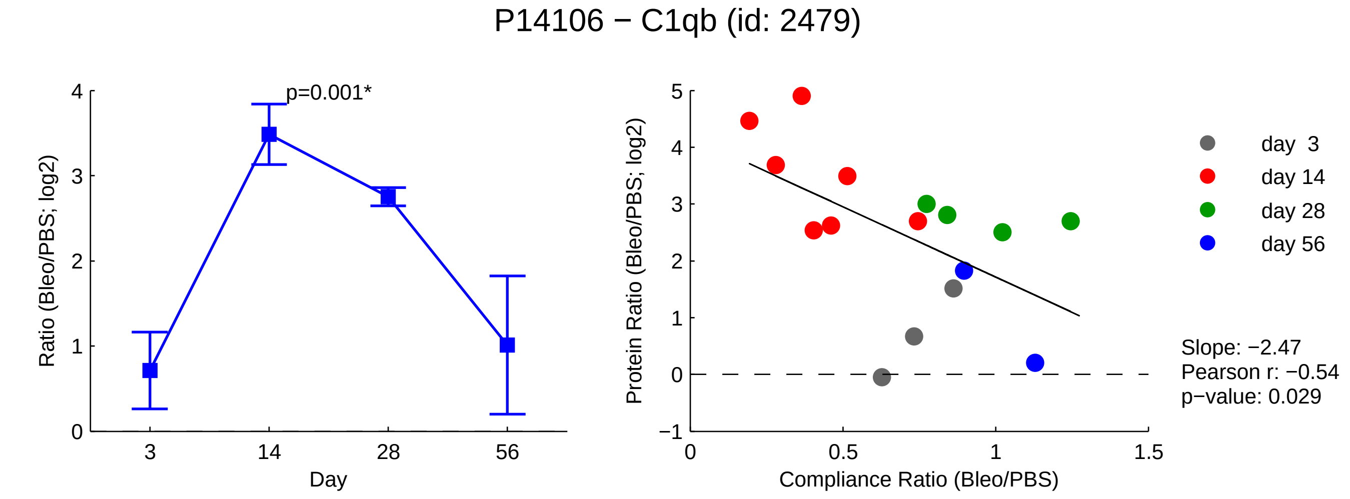
P14106 − C1qb (id: 2479)
p=0.001*
Ratio (Bleo/PBS; log2)
Day
Protein Ratio (Bleo/PBS; log2)
Compliance Ratio (Bleo/PBS)
day 3
day 14
day 28
day 56
Slope: −2.47
Pearson r: −0.54
p−value: 0.029

P14115 − Rpl27a (id: 2480)

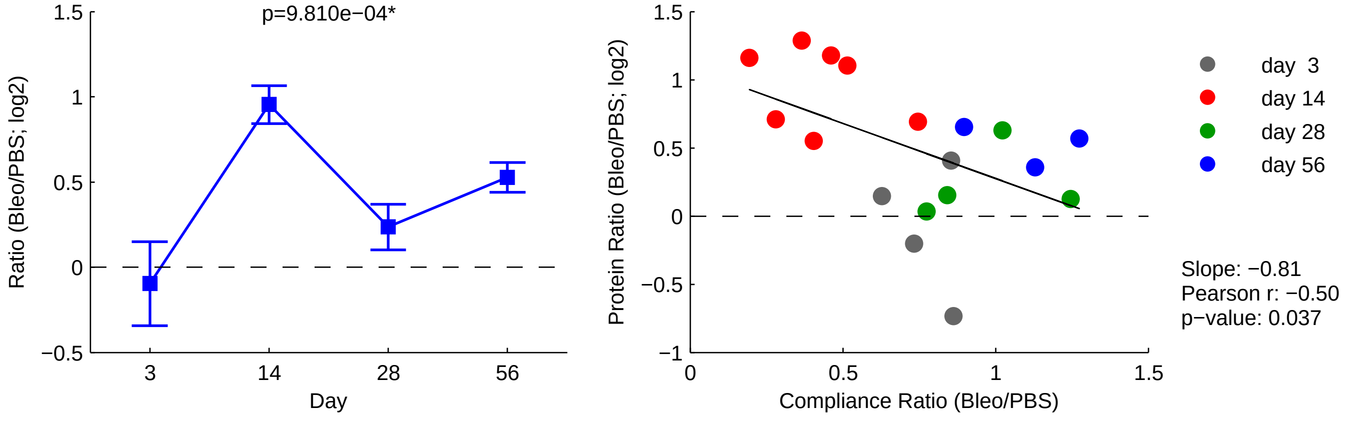

P14131 − Rps16 (id: 2481)

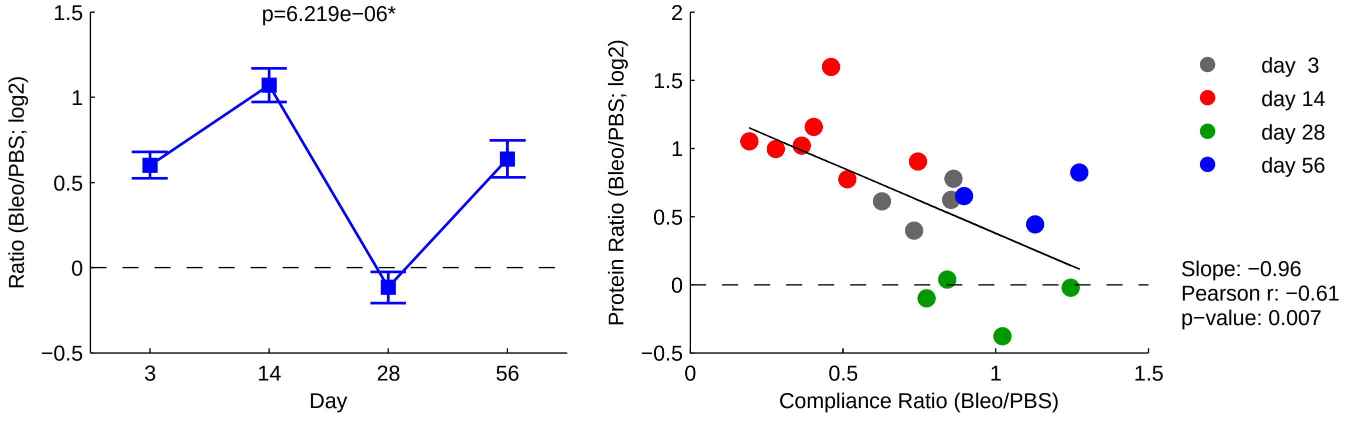

P14148 − Rpl7 (id: 2482)

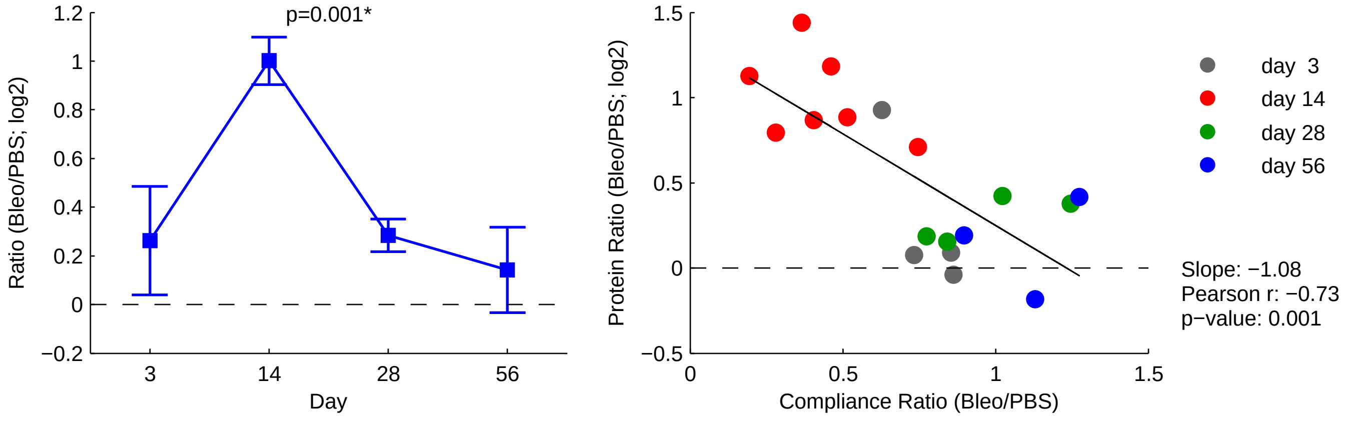

P14152 − Mdh1 (id: 2483)

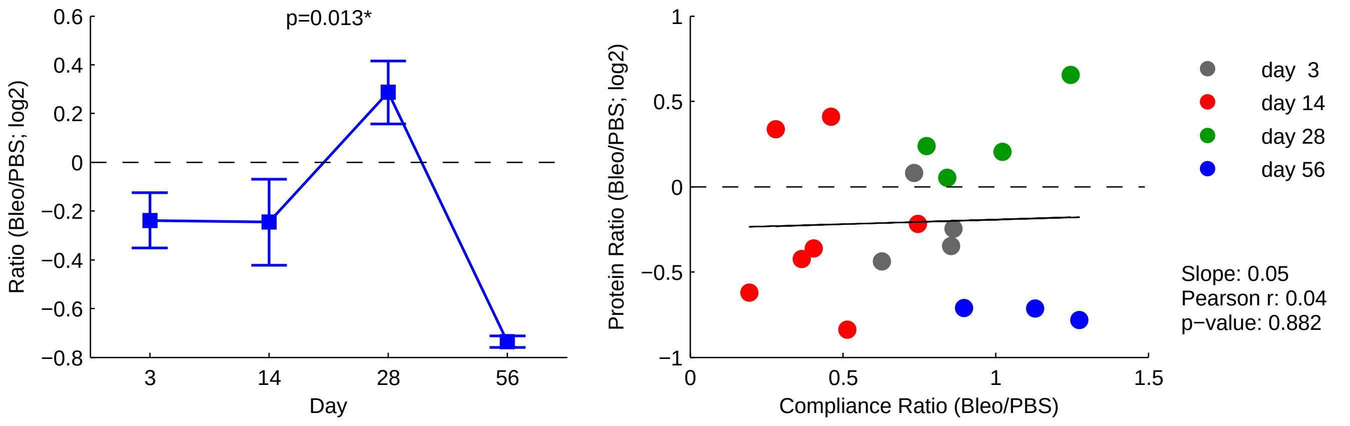

P14206 − Rpsa (id: 2484)

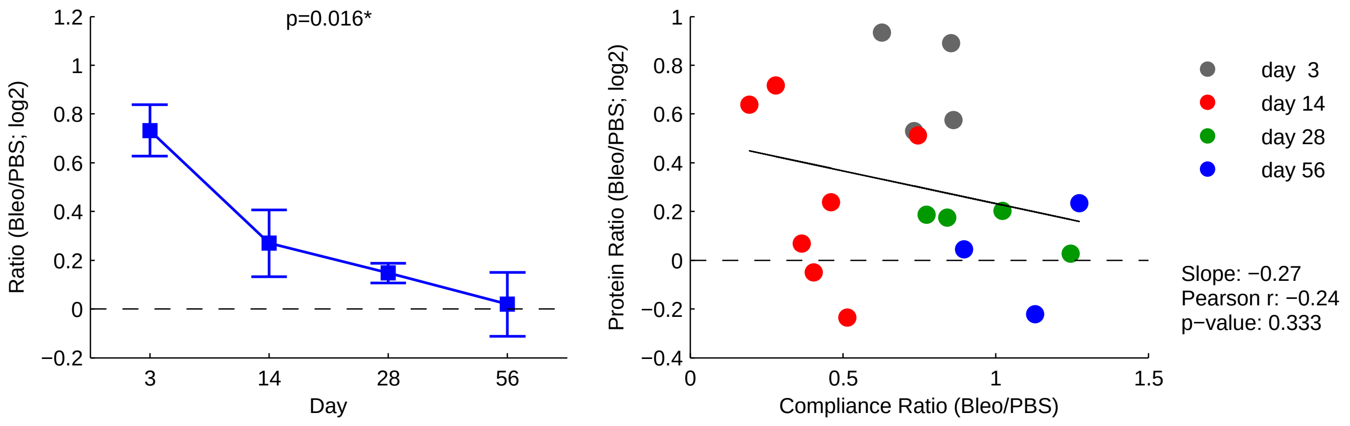

P14211 − Calr (id: 2485)

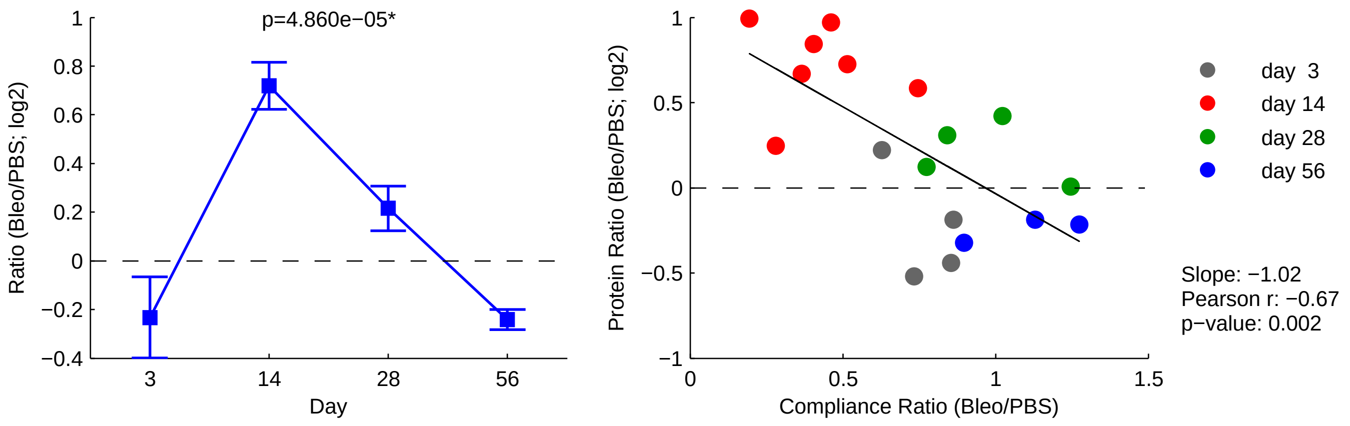

P14234 − Fgr (id: 2487)

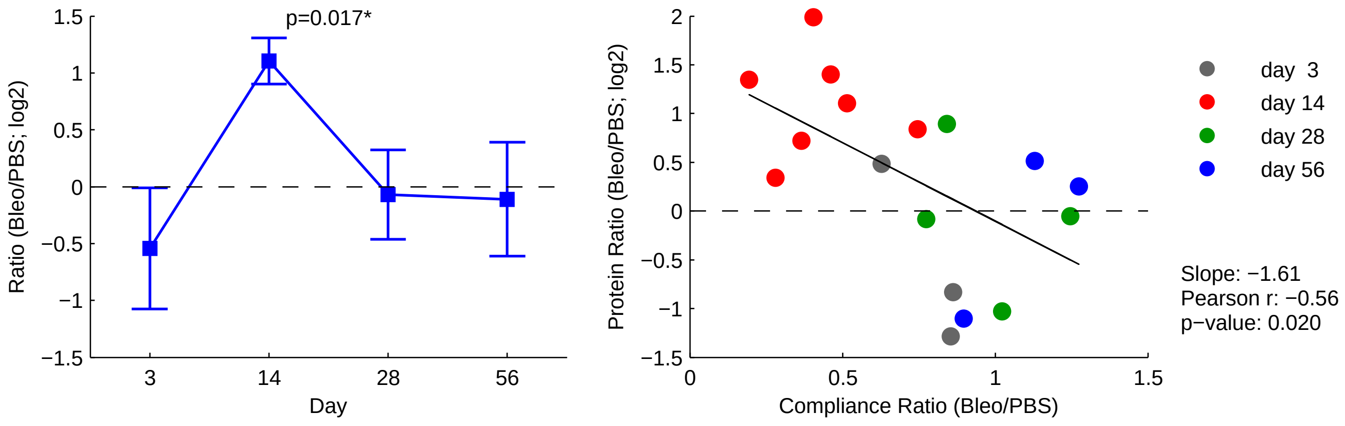

P14434 − H2−Aa (id: 2489)

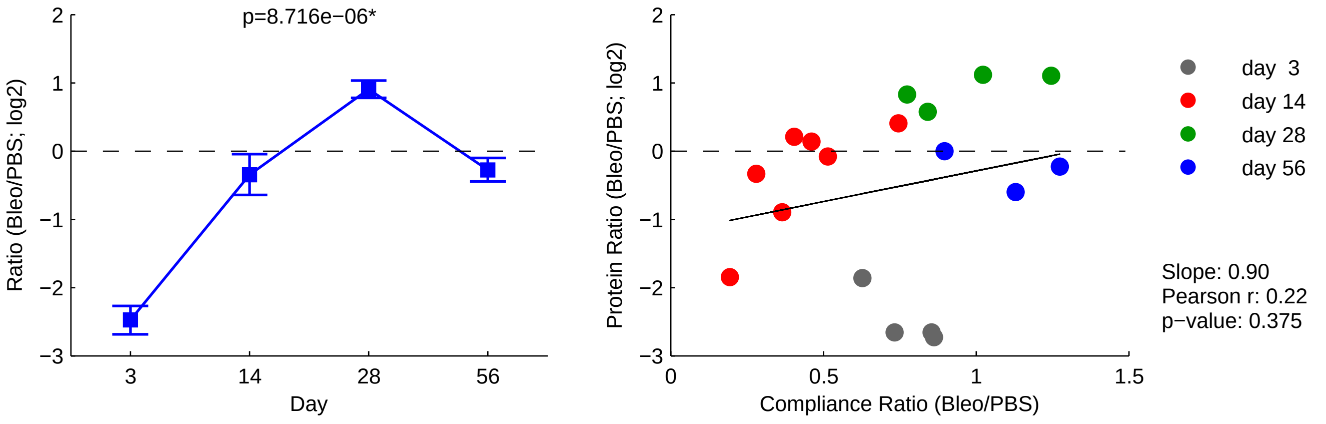

P14483 − H2−Ab1 (id: 2490)

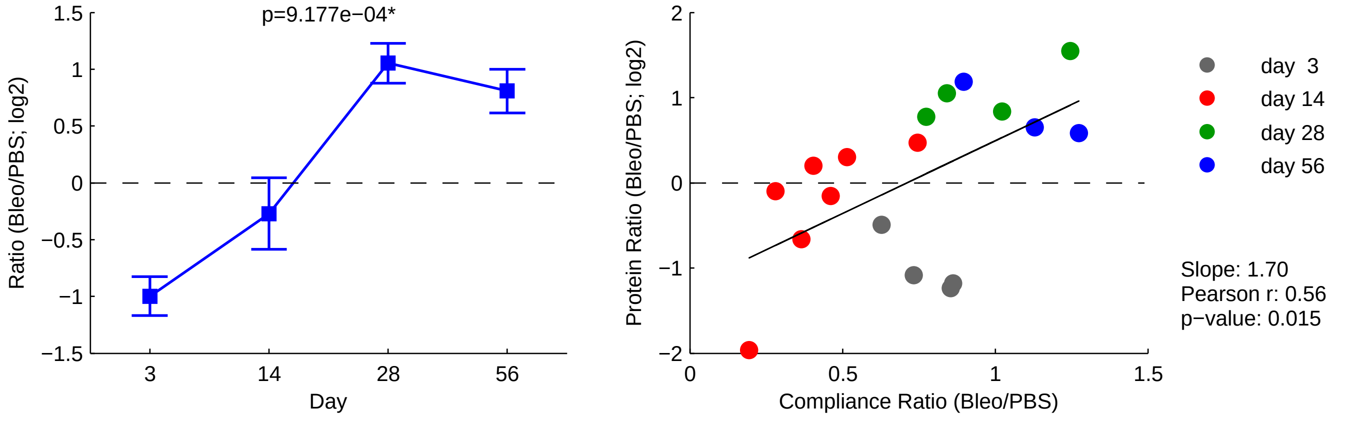

P14576 − Srp54 (id: 2491)

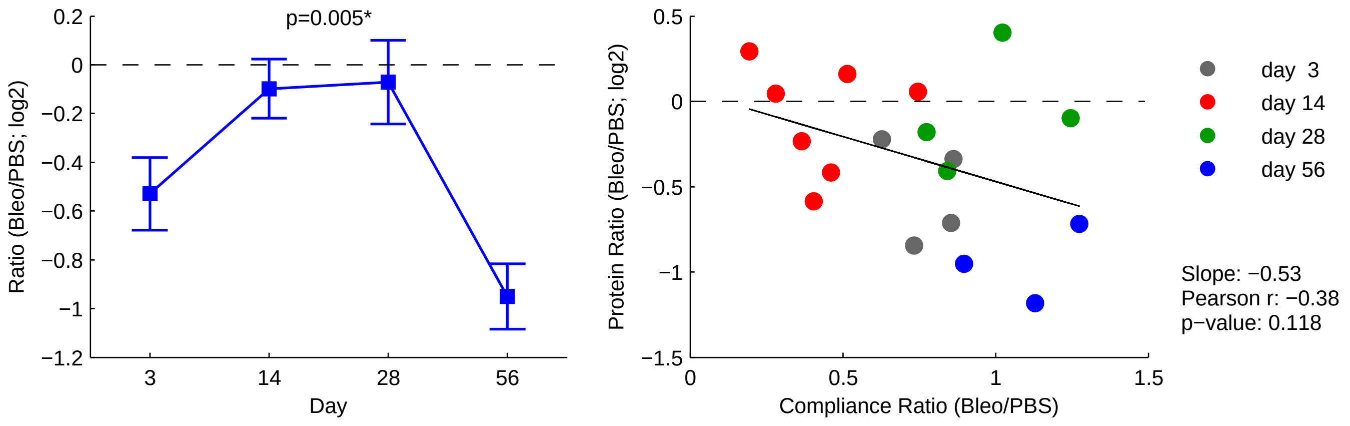

P14602 − Hspb1 (id: 2492)

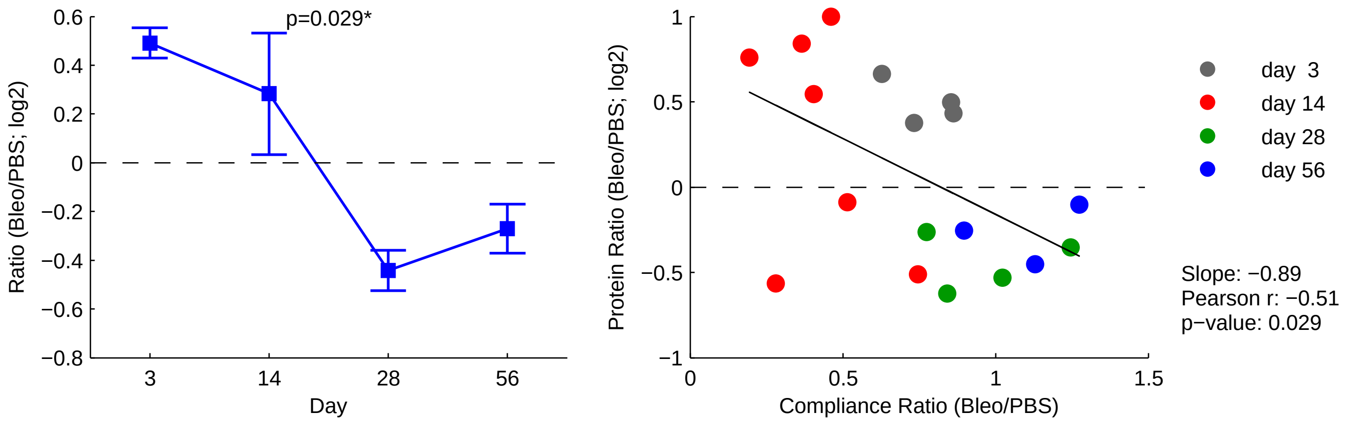

P14685 − Psmd3 (id: 2493)

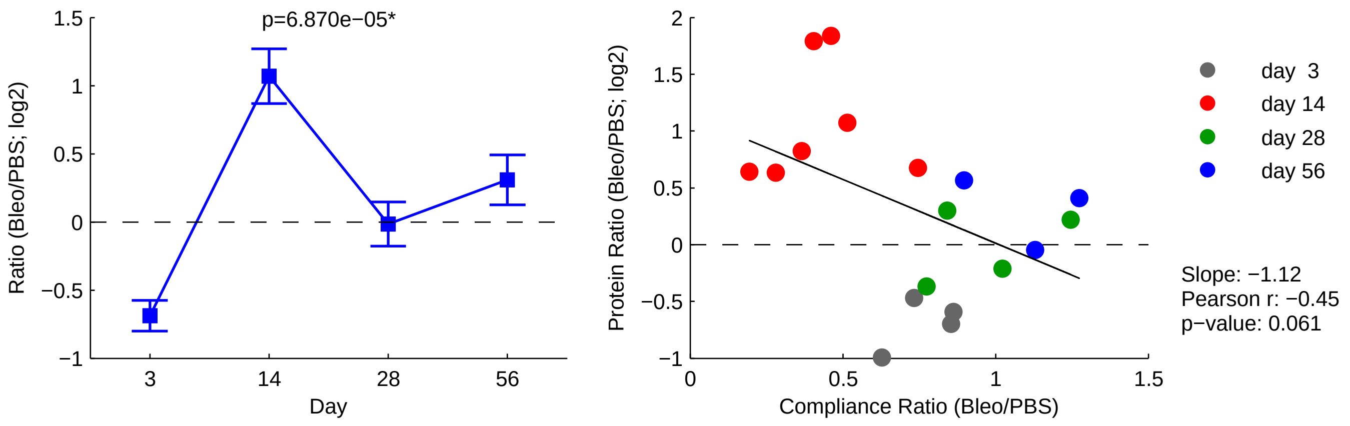

P14824 − Anxa6 (id: 2495)

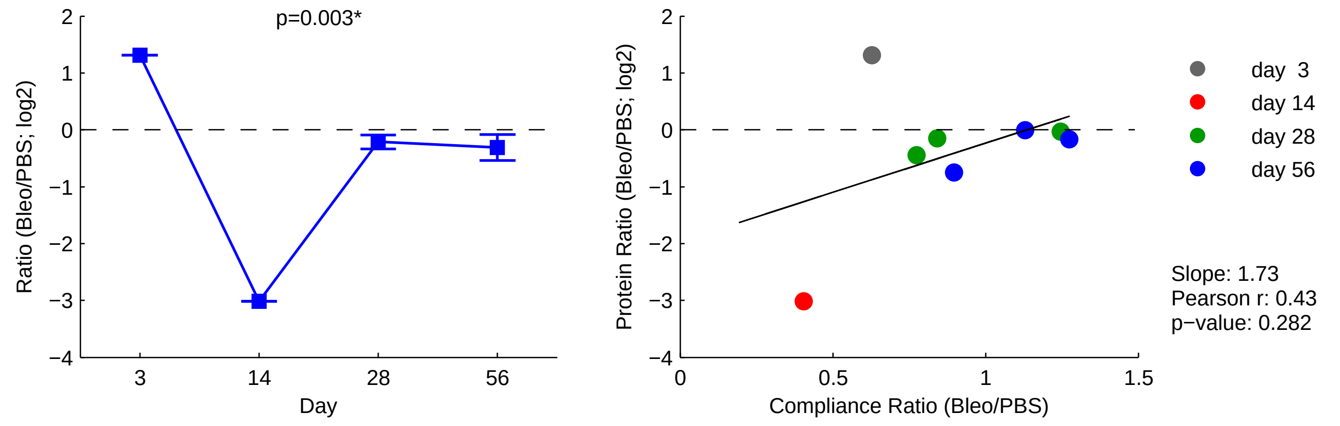

P14869 − Rplp0 (id: 2496)

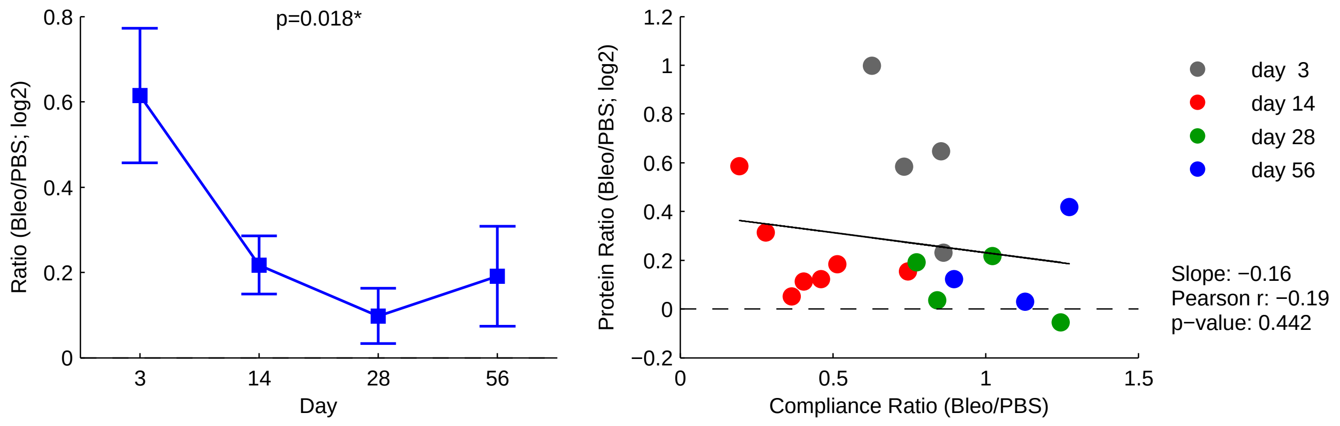

P14873 − Map1b (id: 2497)

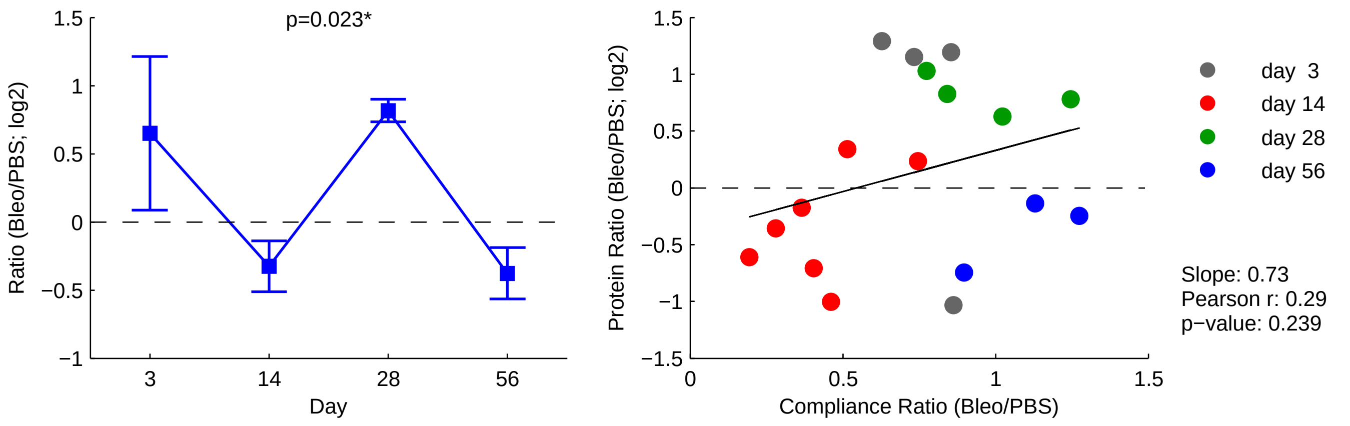

P14901 − Hmox1 (id: 2498)

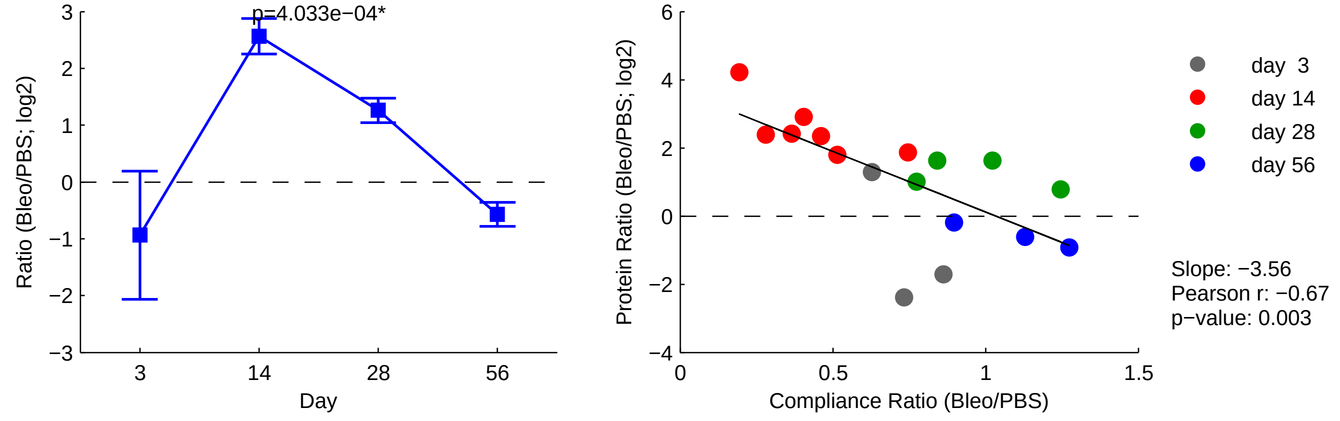

P15066 − Jund (id: 2499)

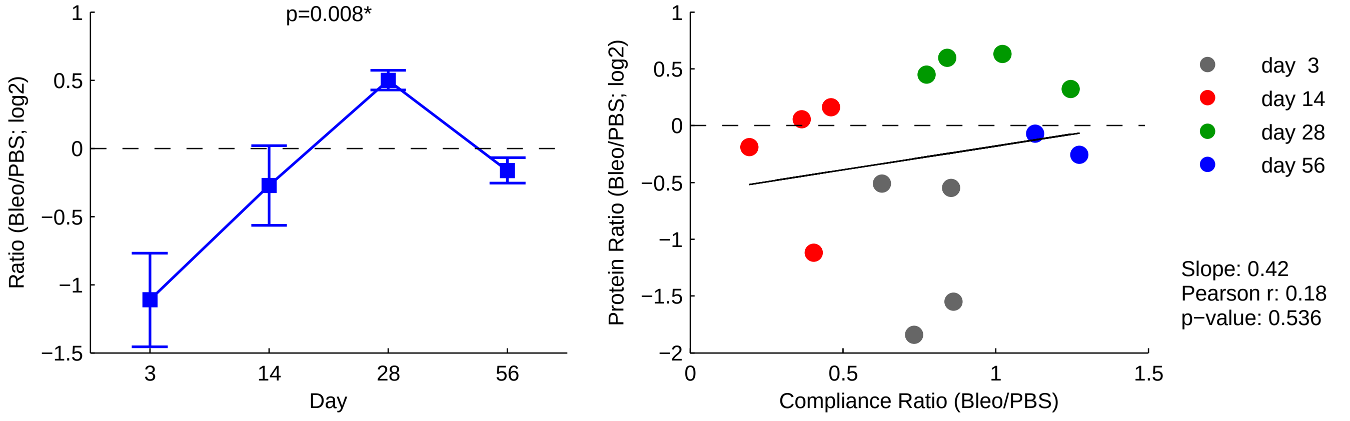

P15089 − Cpa3 (id: 2500)

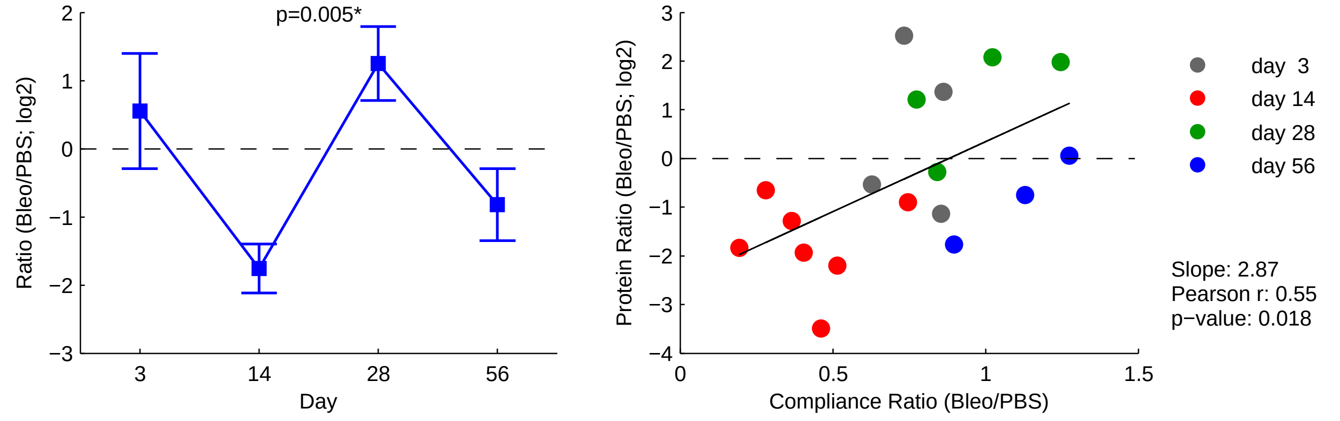

P15092−2 − Ifi204 (id: 2501)

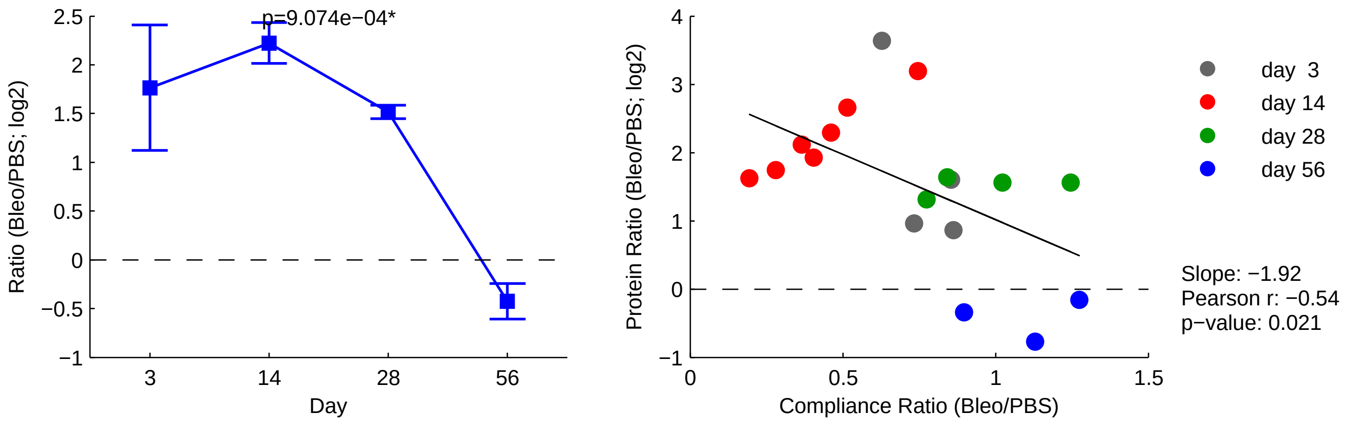

P15306 − Thbd (id: 2506)

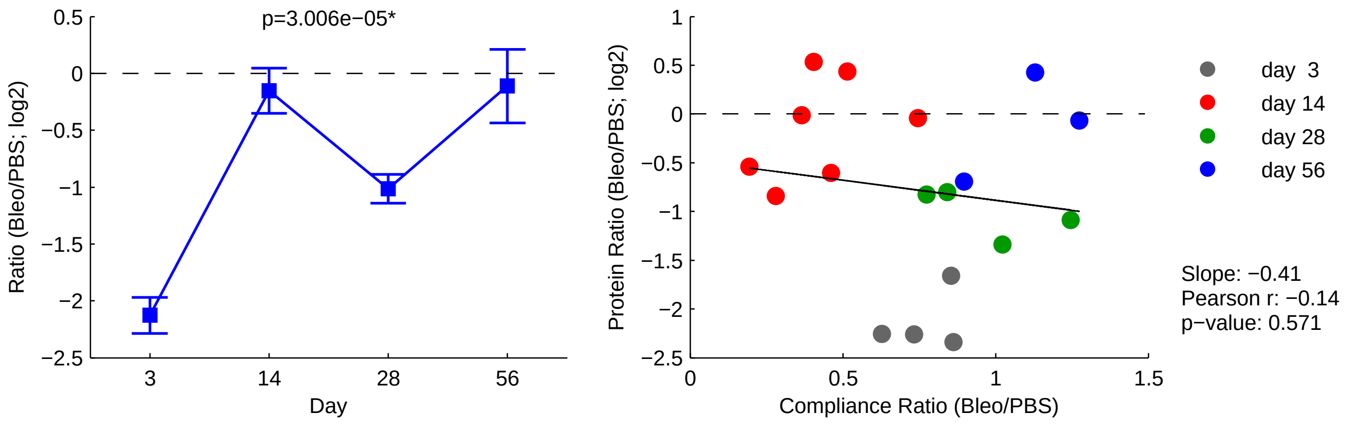

P15532 − Nme1 (id: 2508)

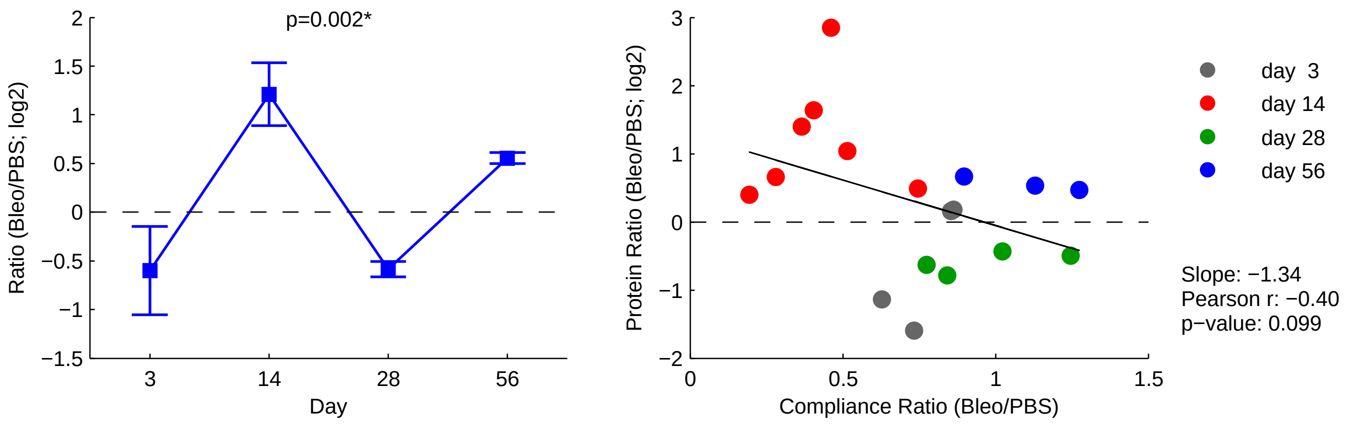

P15655 − Fgf2 (id: 2512)

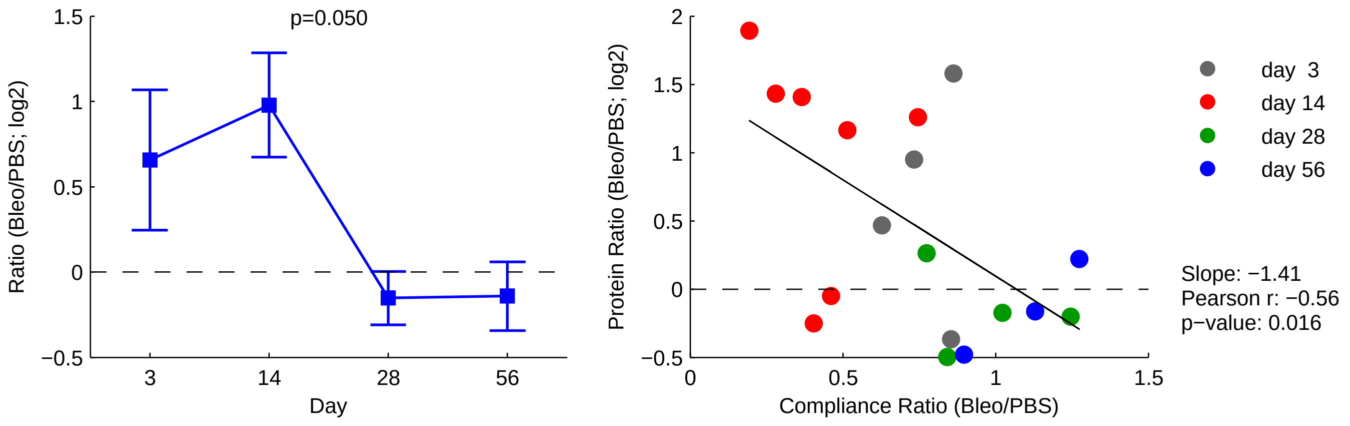

P15864 − Hist1h1c (id: 2513)

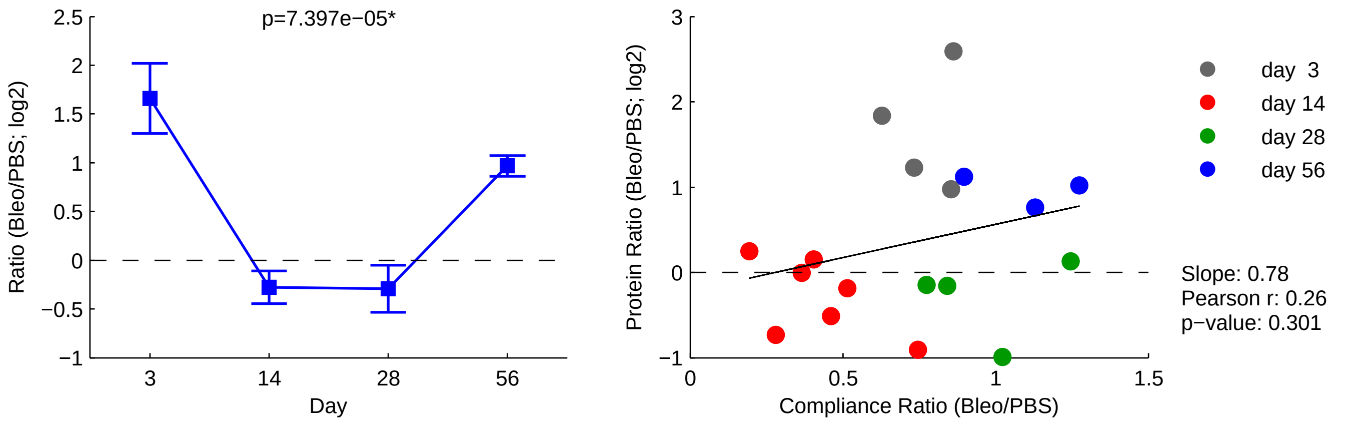

P16015 − Ca3 (id: 2516)

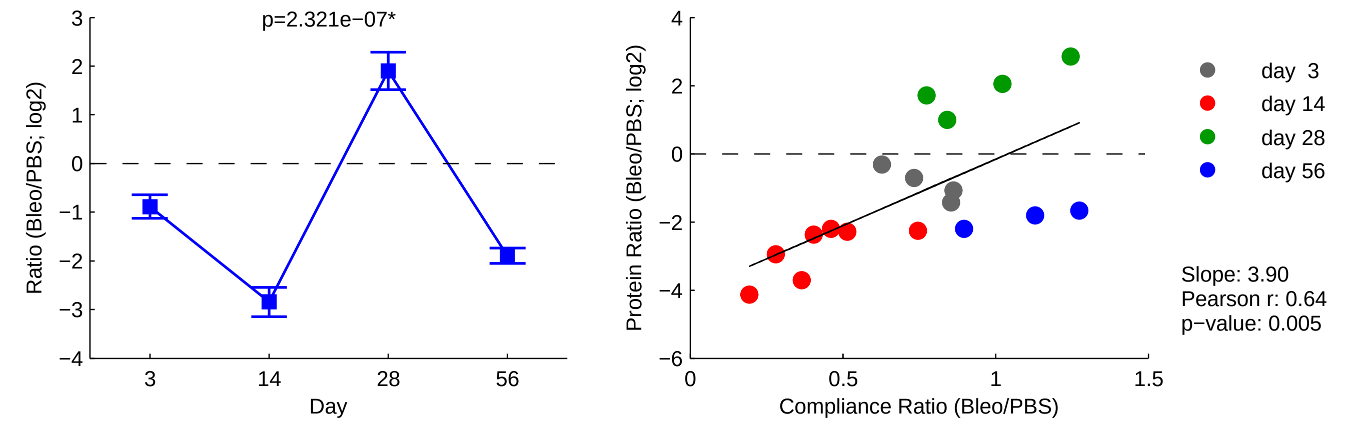

P16045 − Lgals1 (id: 2517)

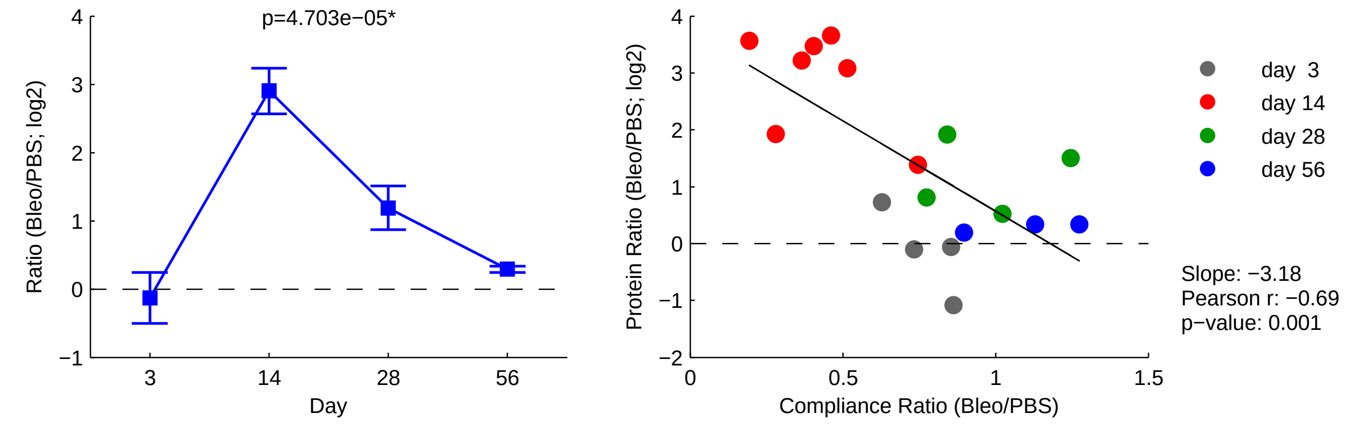

P16054 − Prkce (id: 2518)

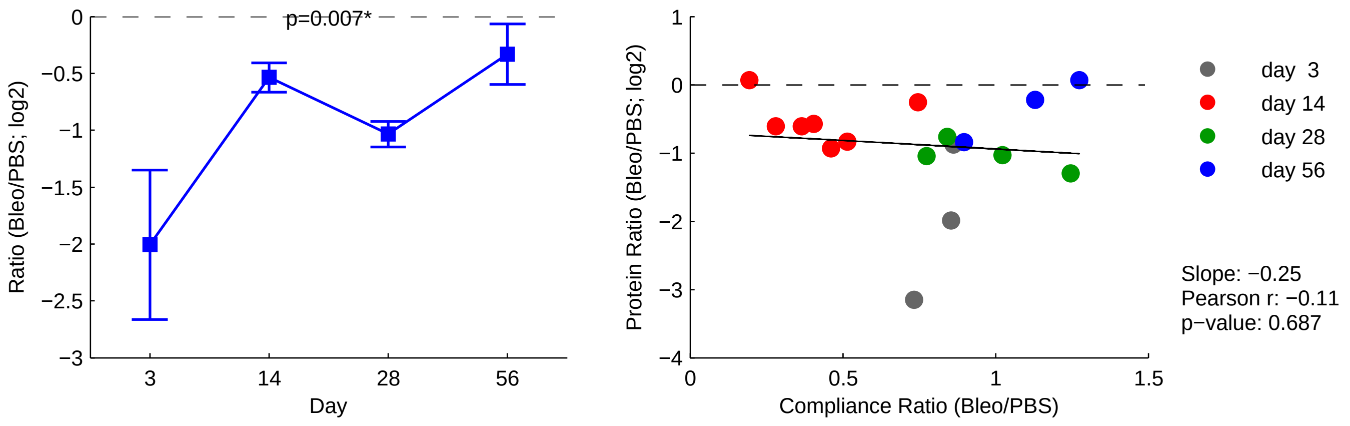

Q8C253 − Lgals3 (id: 2519)

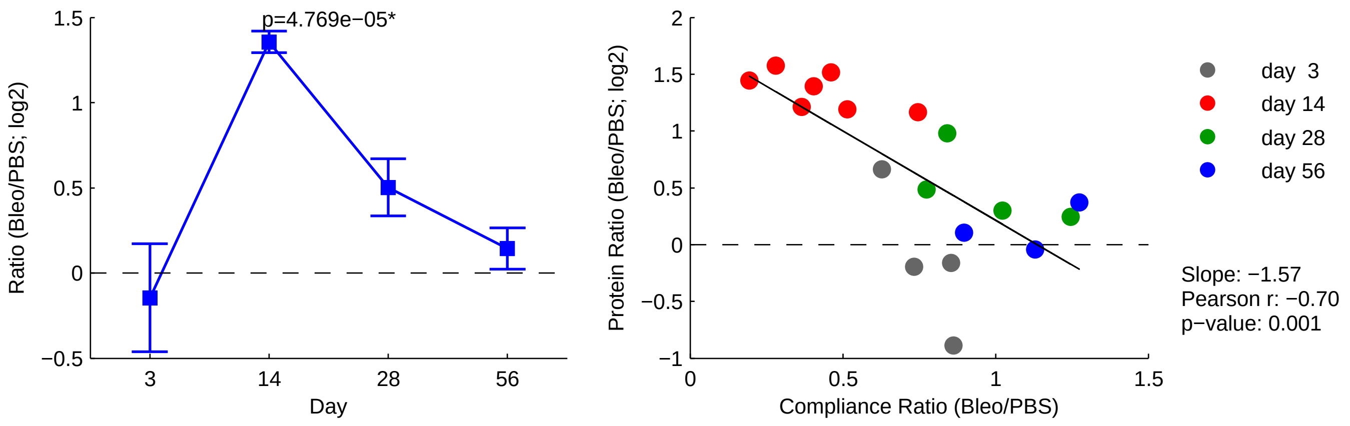

P16254 − Srp14 (id: 2521)

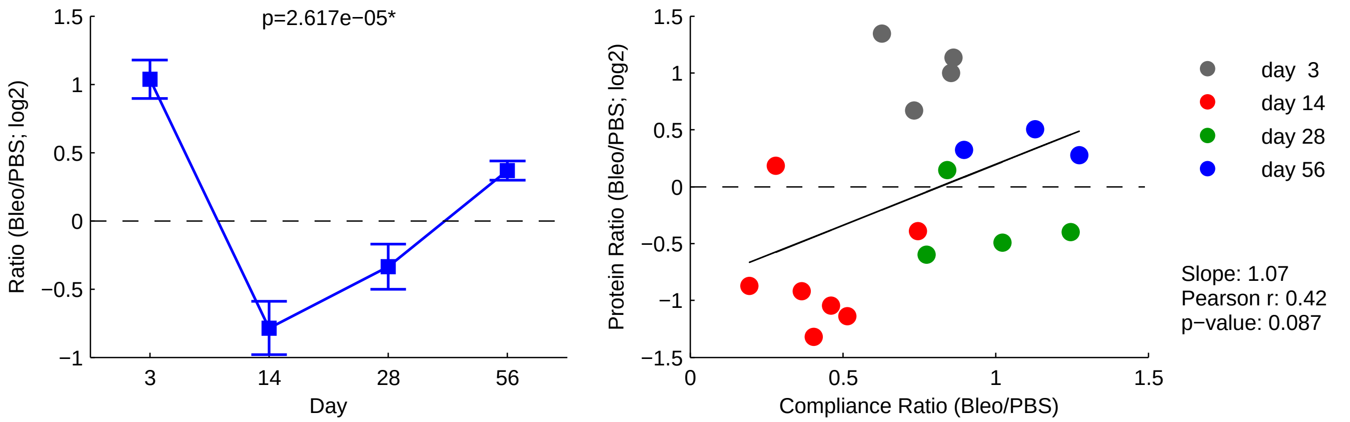

P16330 − Cnp (id: 2522)

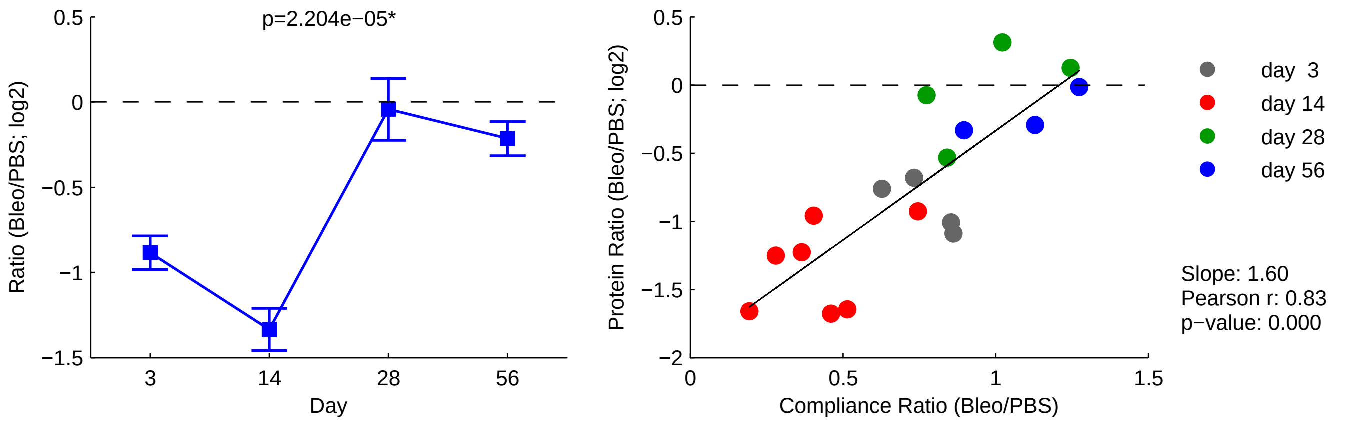

P16332 − Mut (id: 2523)

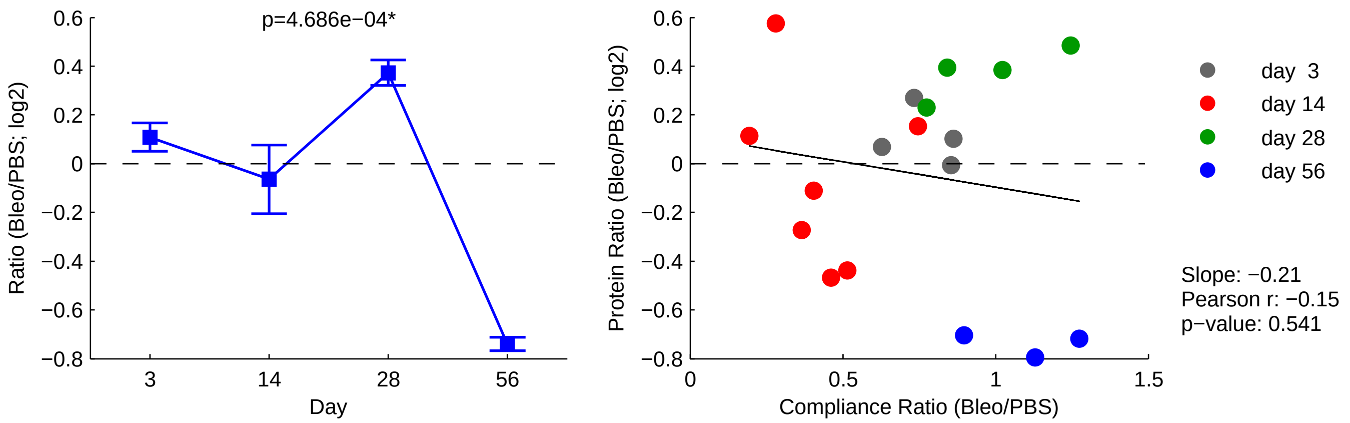

P16406 − Enpep (id: 2524)

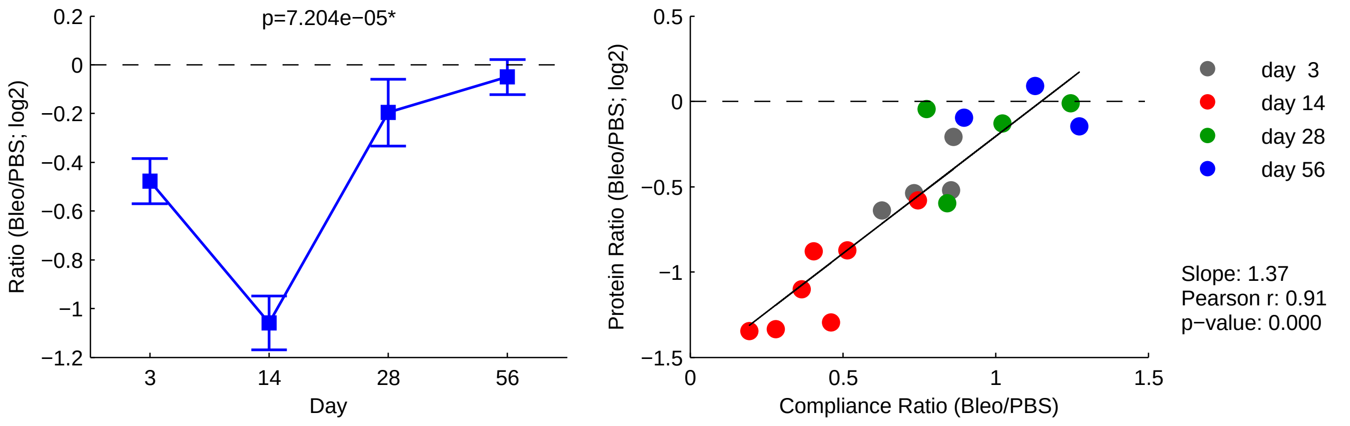

P16460 − Ass1 (id: 2525)

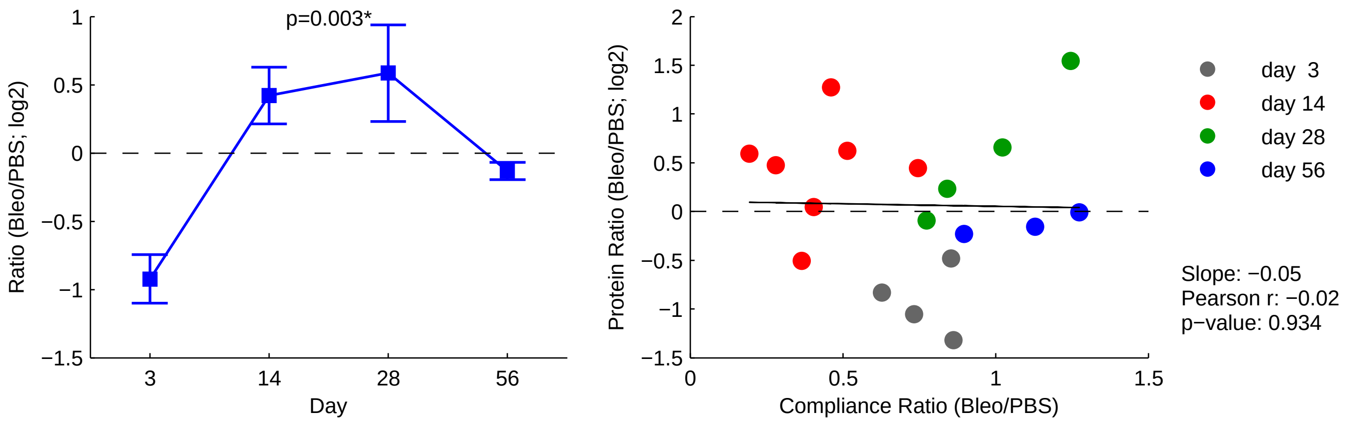

P16546 − Sptan1 (id: 2526)

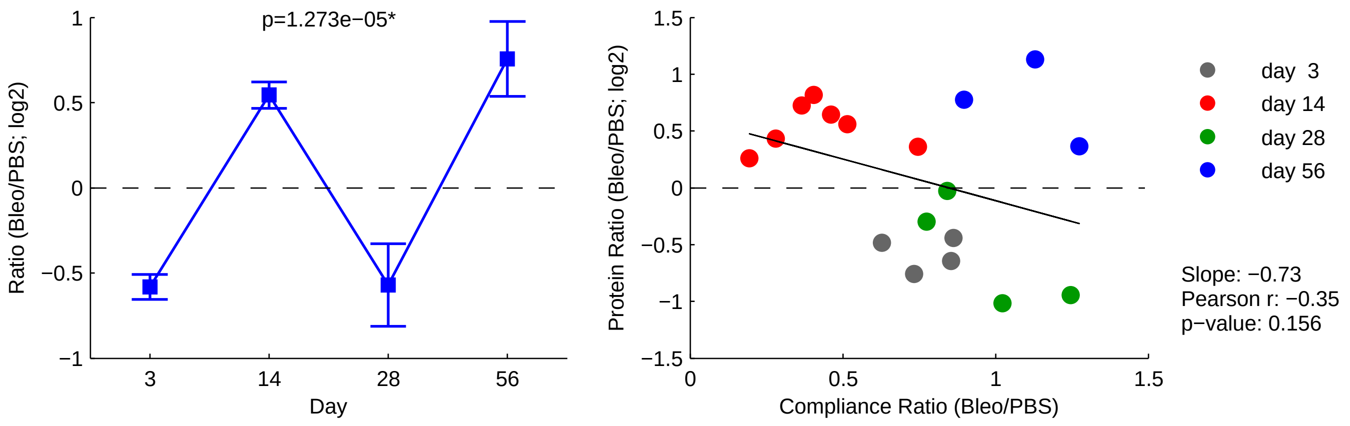

P16858 − Gapdh (id: 2528)

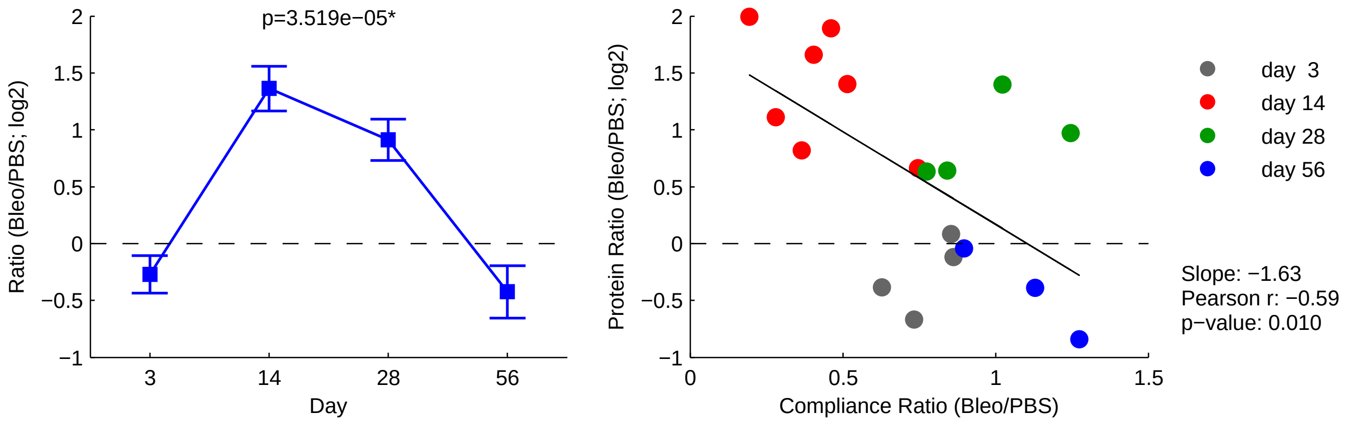

P17095 − Hmga1 (id: 2533)

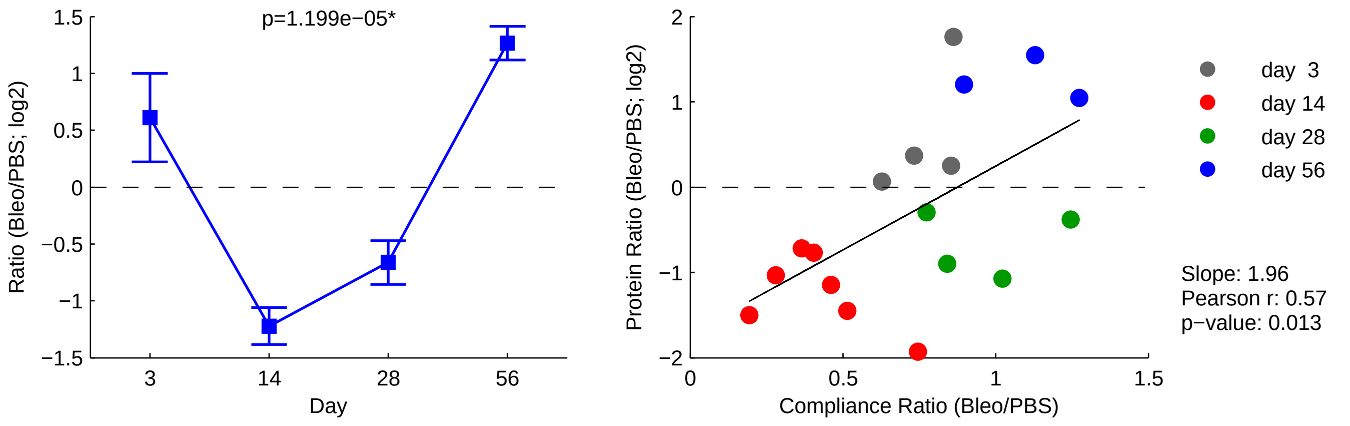

P17095−1 − (id: 2534)

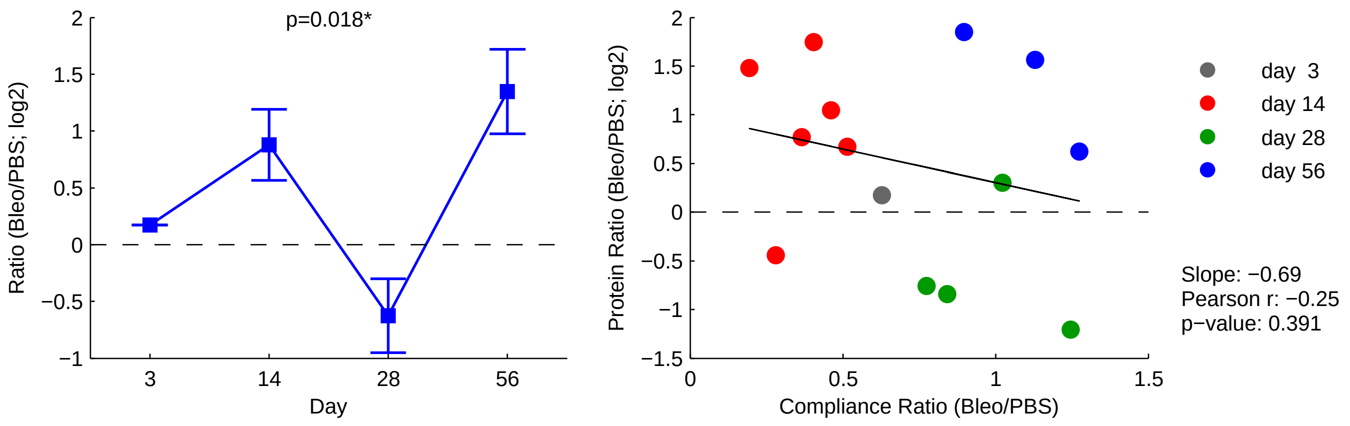

P17141 − Zfp37 (id: 2536)

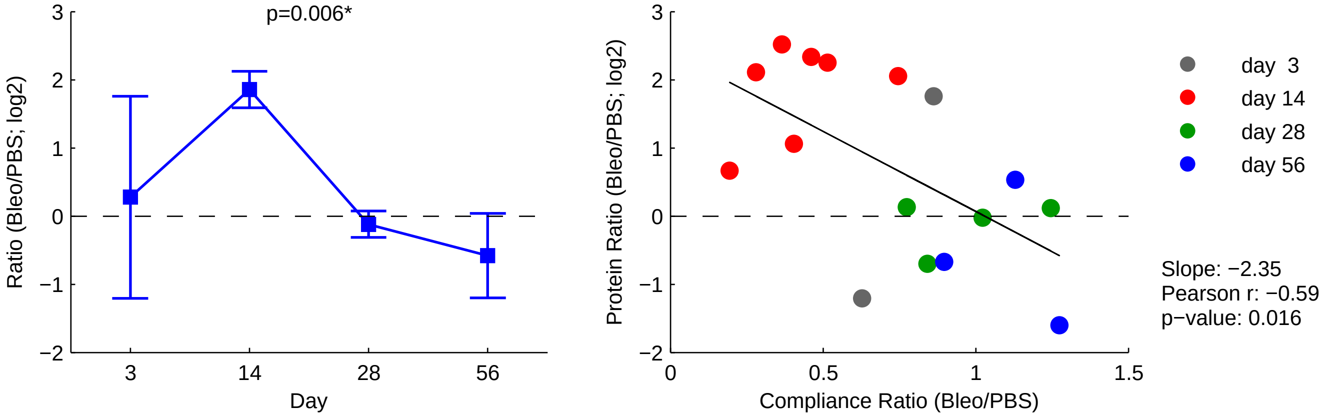

P17156 − Hspa2 (id: 2537)

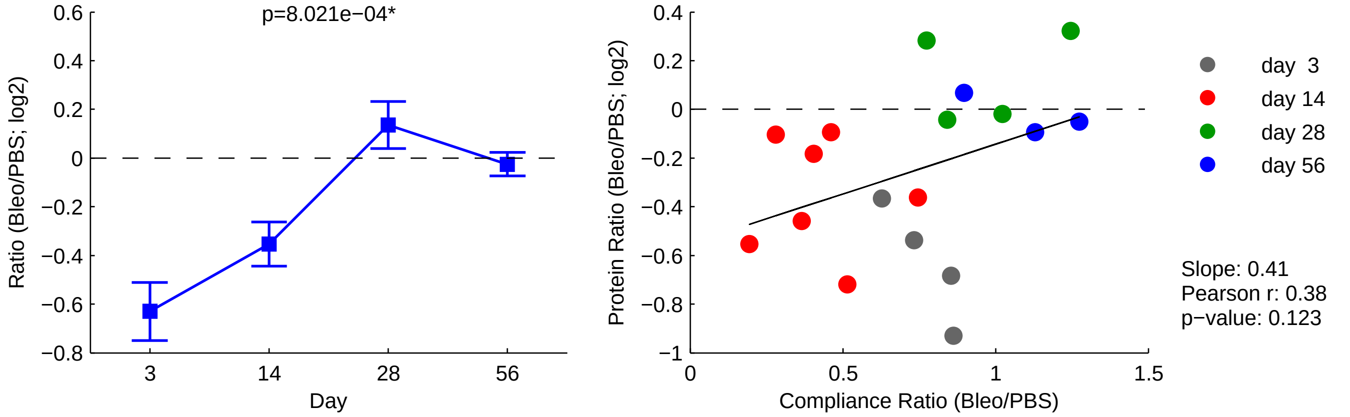

P17182 − Eno1 (id: 2538)

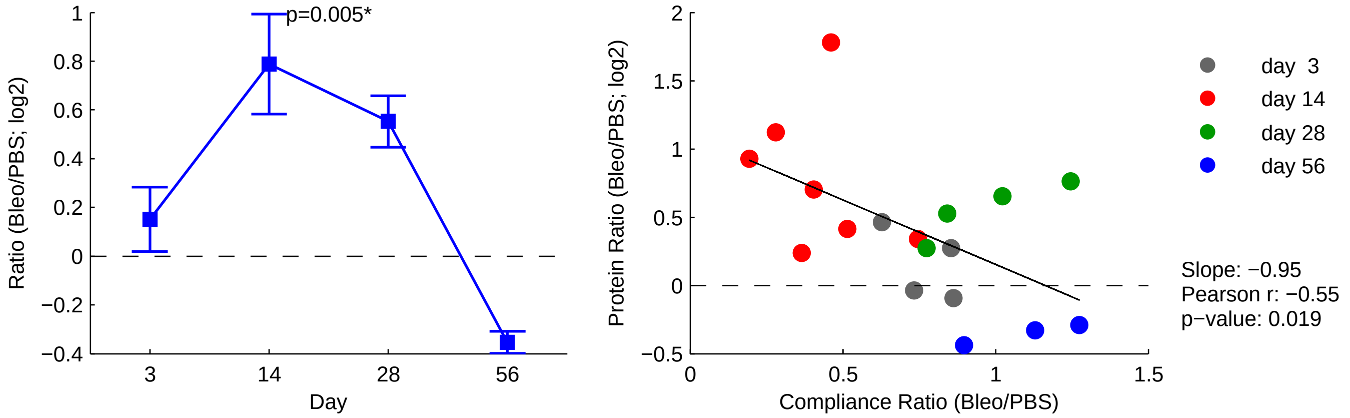

P17427 − Ap2a2 (id: 2540)

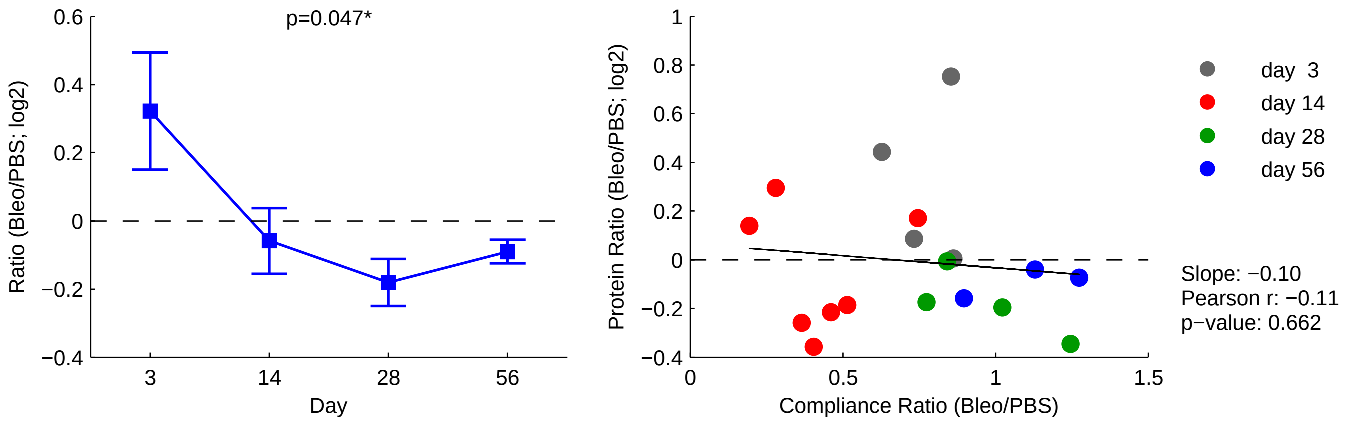

P17439 − Gba (id: 2542)

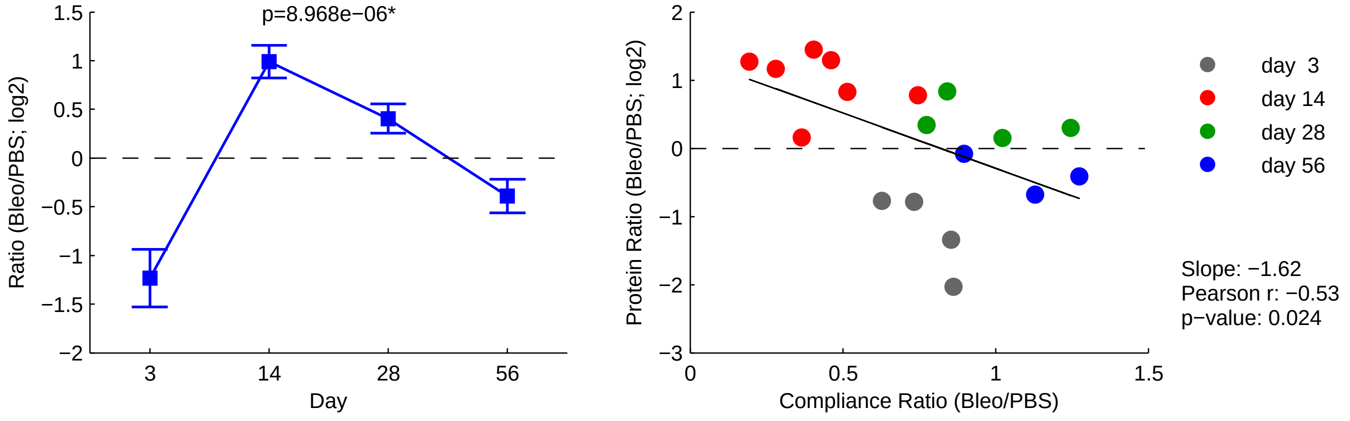

P17563 − Selenbp1 (id: 2543)

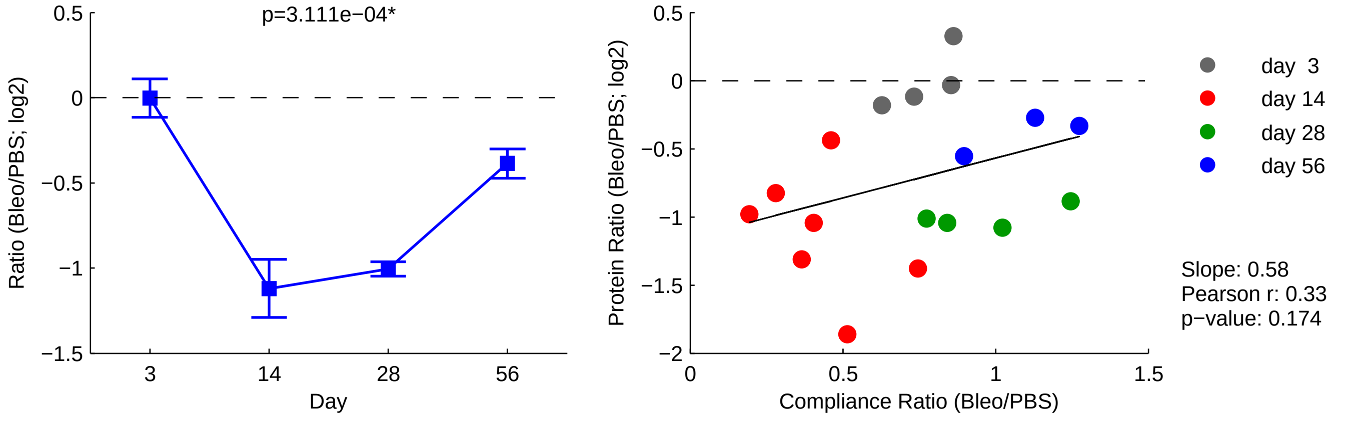

P17665 − Cox7c (id: 2544)

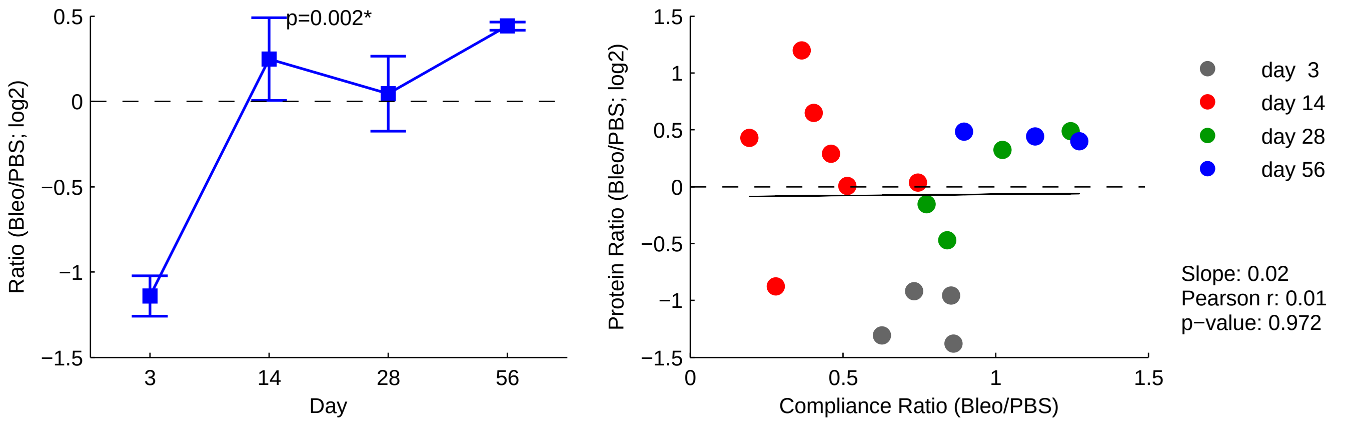

P17710−3 − (id: 2546)

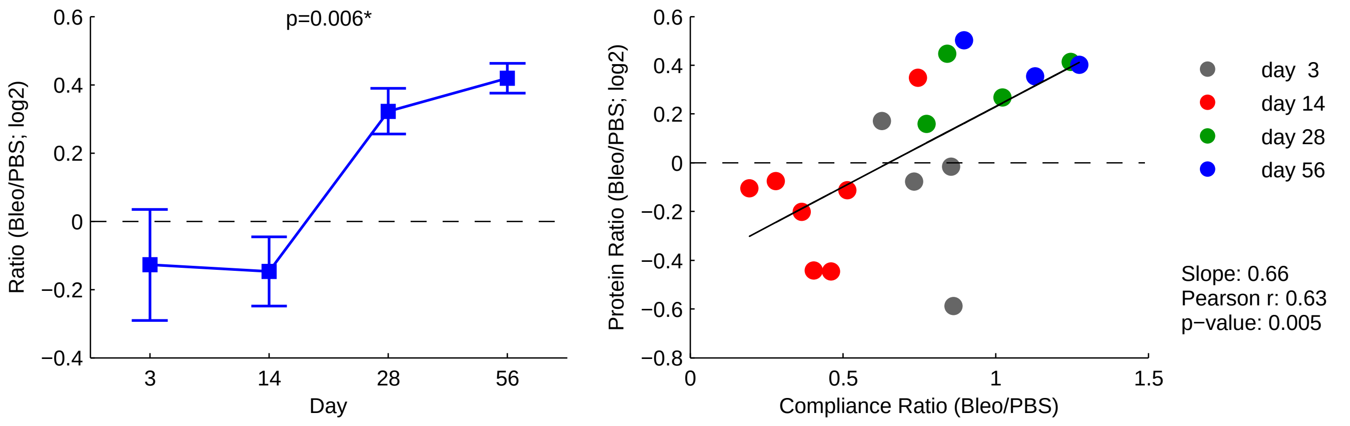

P17742 − Ppia (id: 2547)

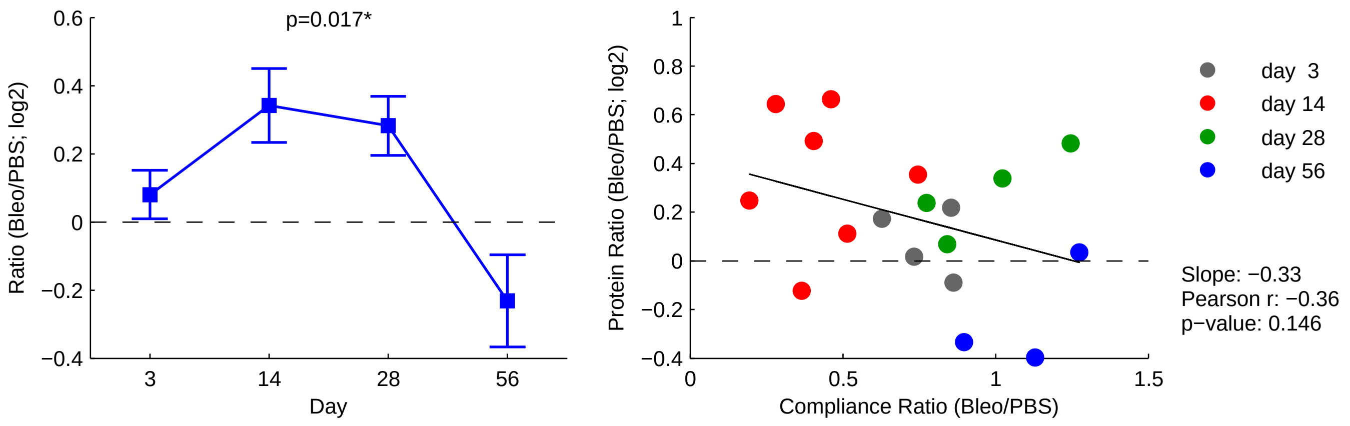

P17751 − Tpi1 (id: 2548)

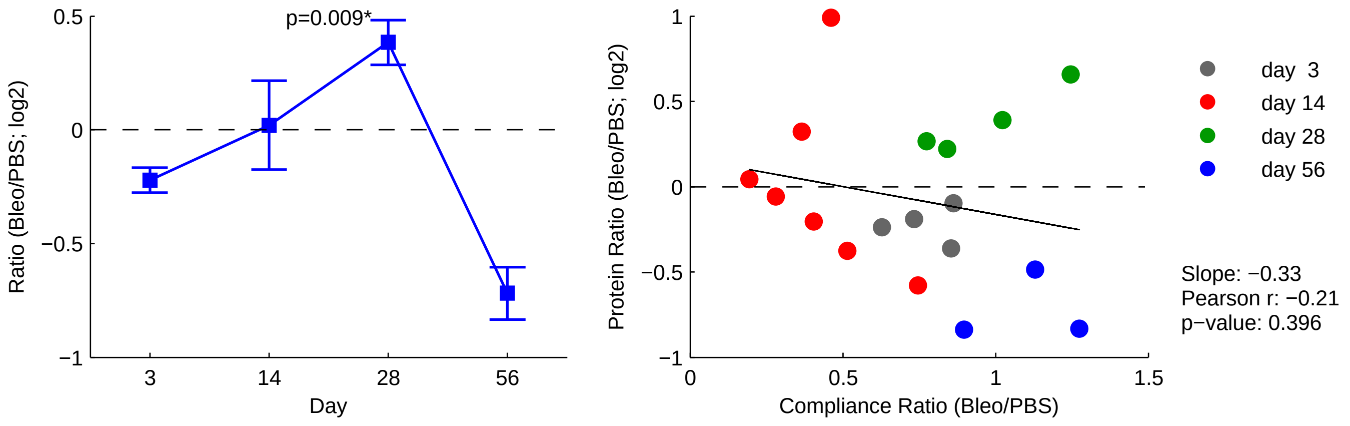

P17918 − Pcna (id: 2551)

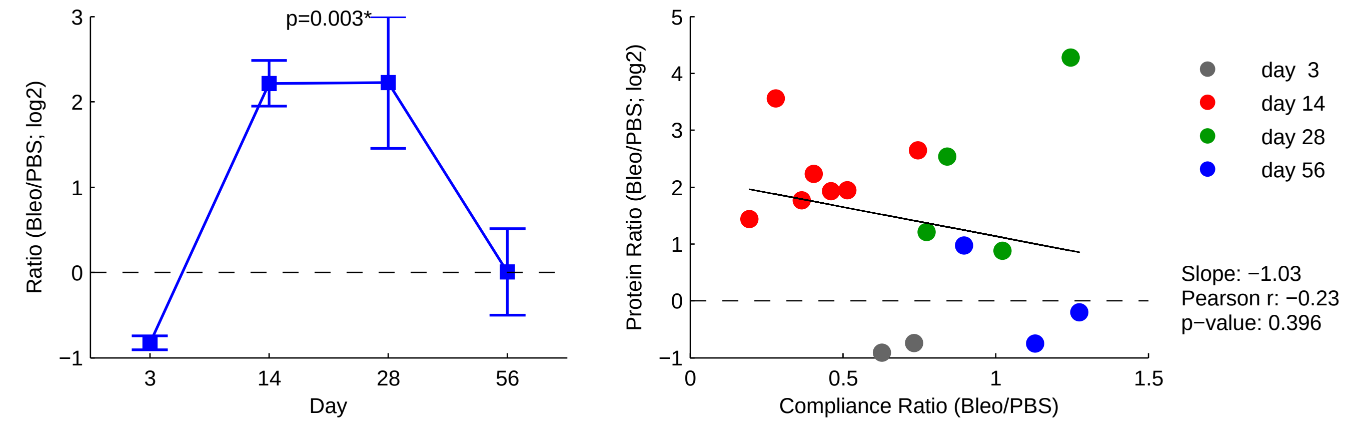

P18242 − Ctsd (id: 2556)

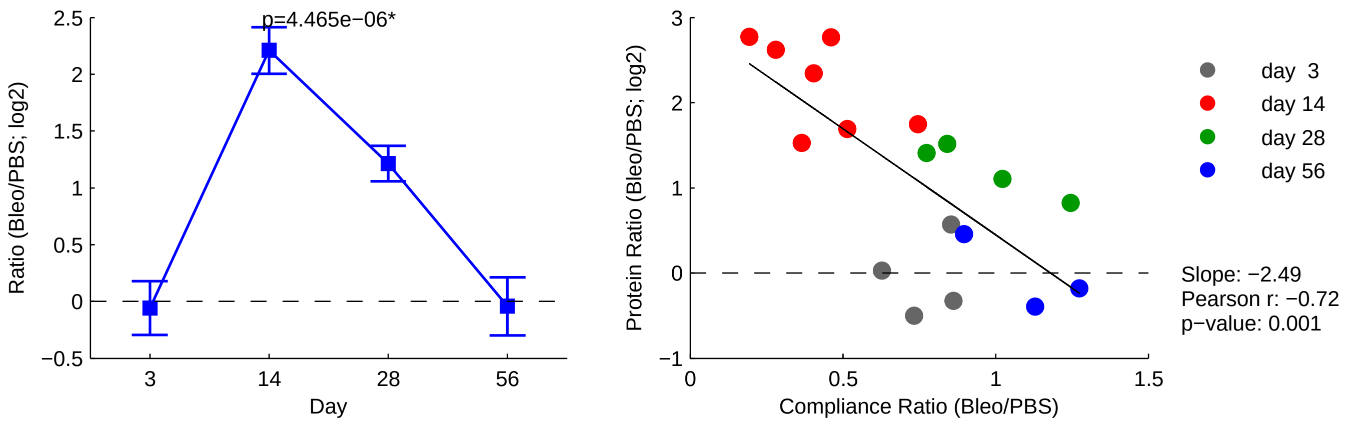

P18293 − Npr1 (id: 2557)

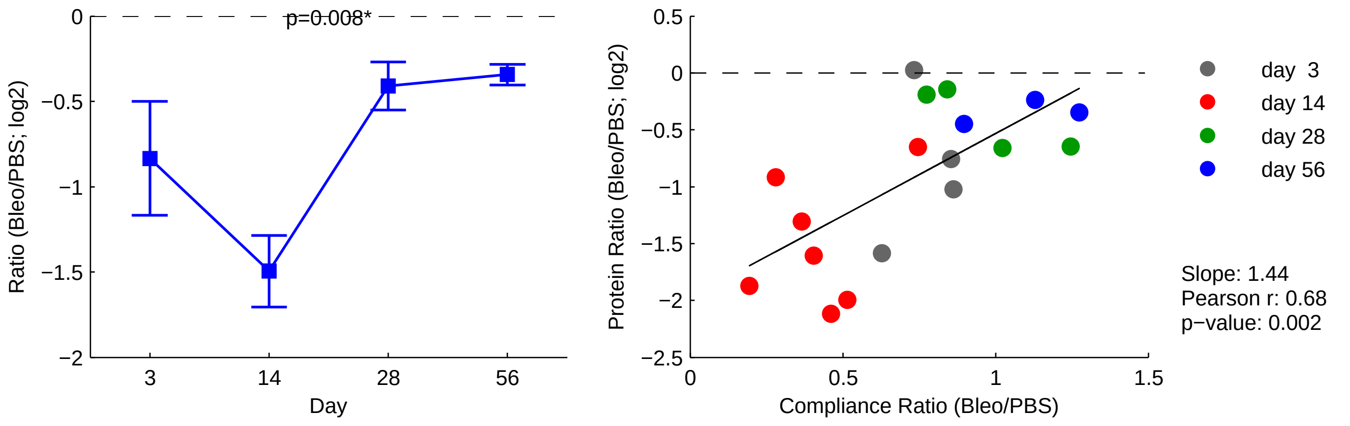

P18406 − Cyr61 (id: 2558)

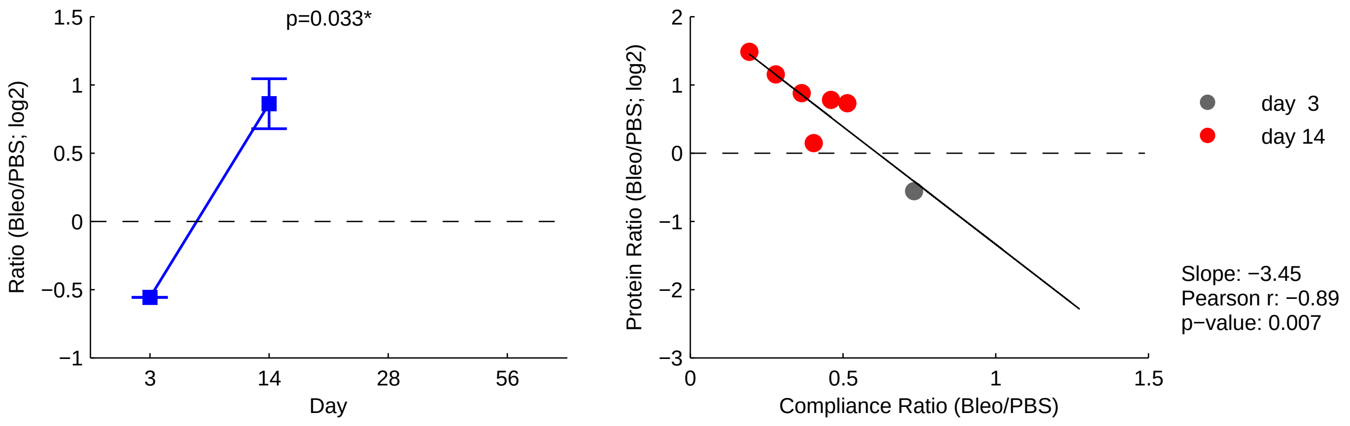

P18528 − (id: 2562)

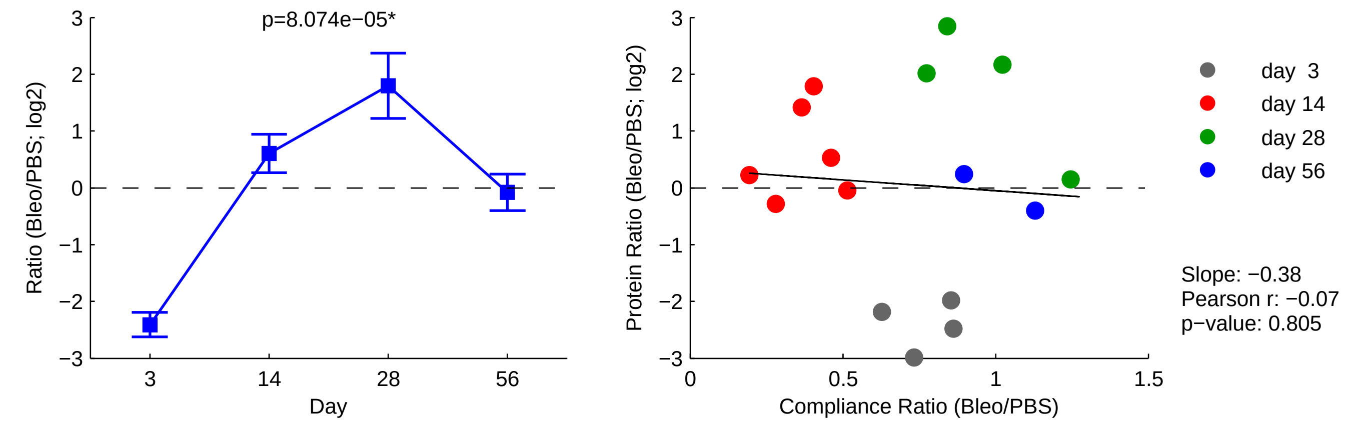

P18654 − Rps6ka3 (id: 2565)

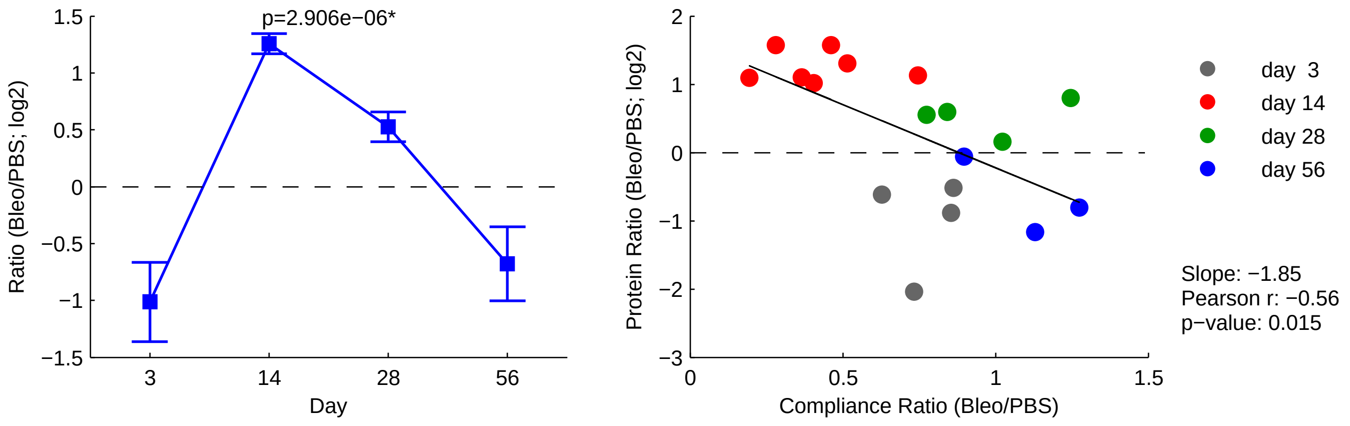

P18760 − Cfl1 (id: 2566)

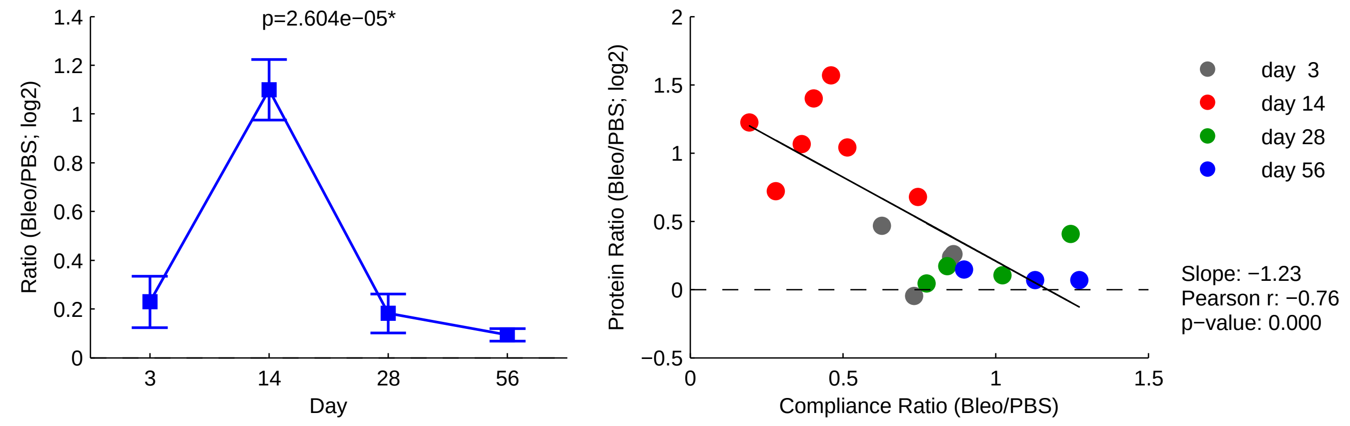

P18828−2 − Sdc1 (id: 2567)

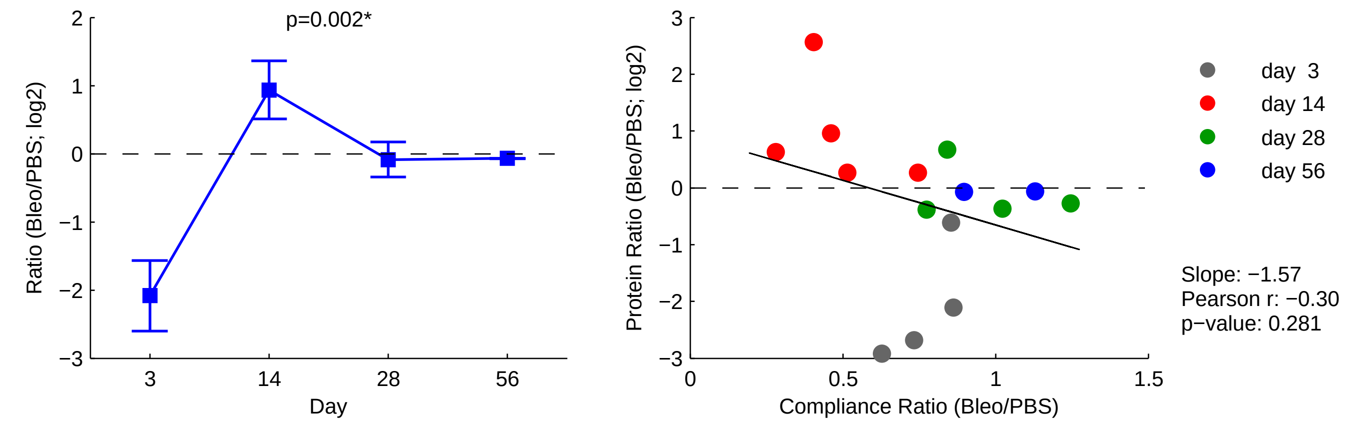

P18872−2 − Gnao1 (id: 2569)

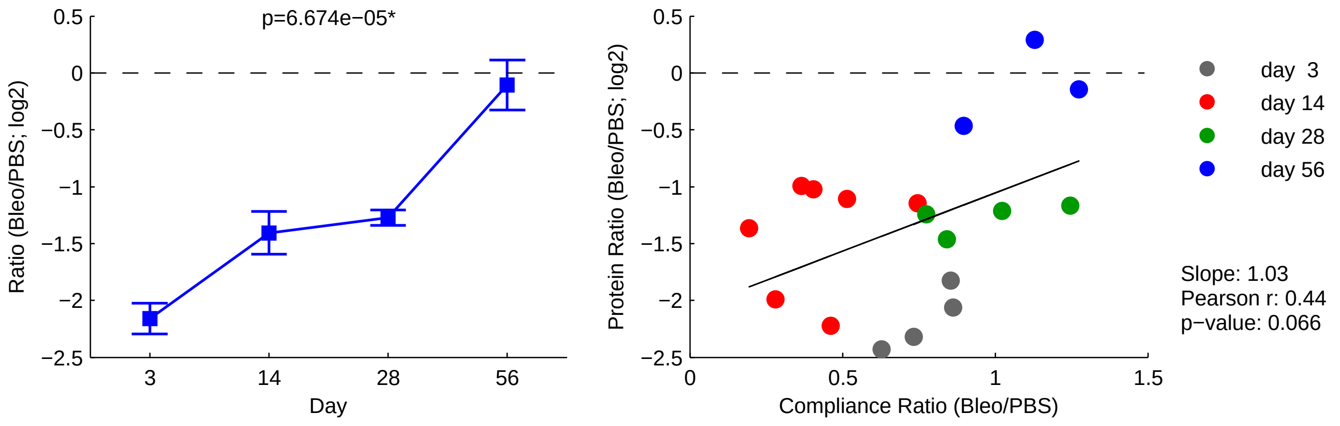

P19096 − Fasn (id: 2570)

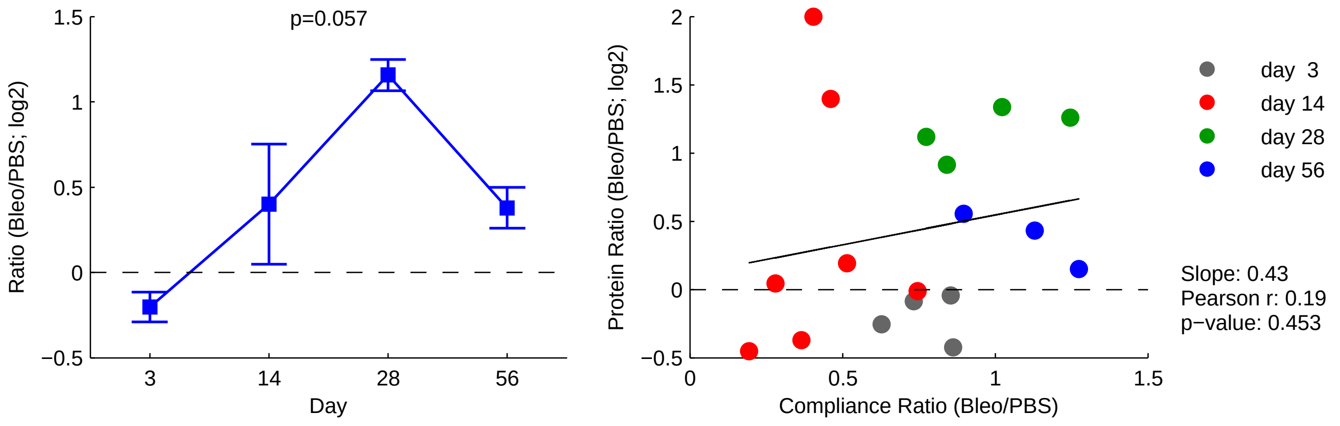

P19123 − Tnnc1 (id: 2571)

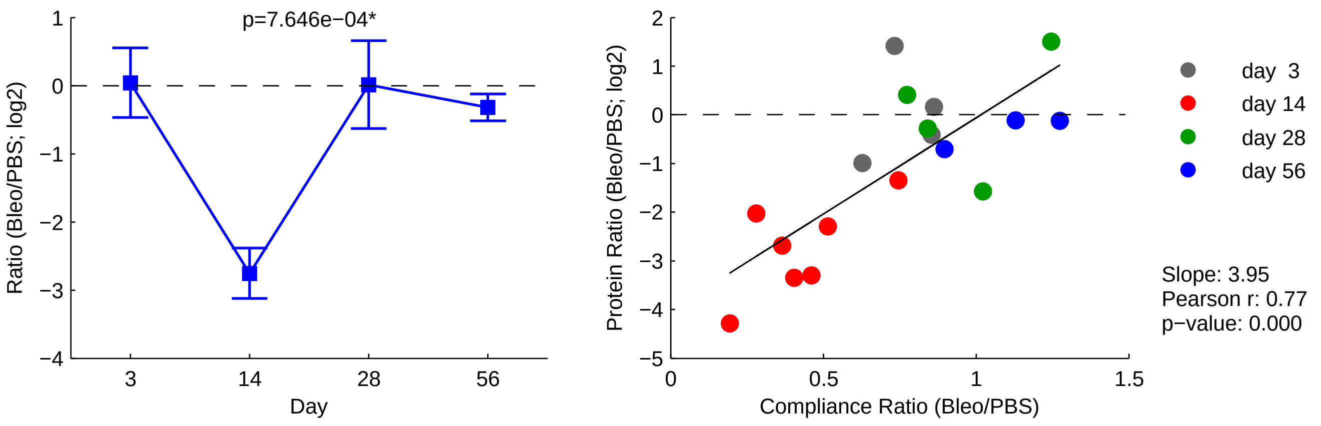

P19253 − Rpl13a (id: 2575)

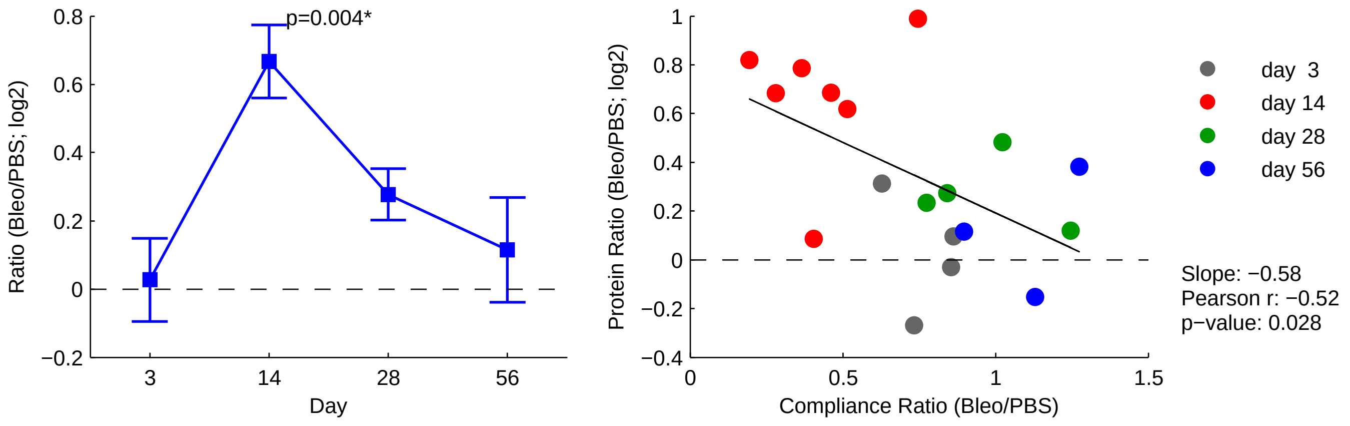

P19324 − Serpinh1 (id: 2576)

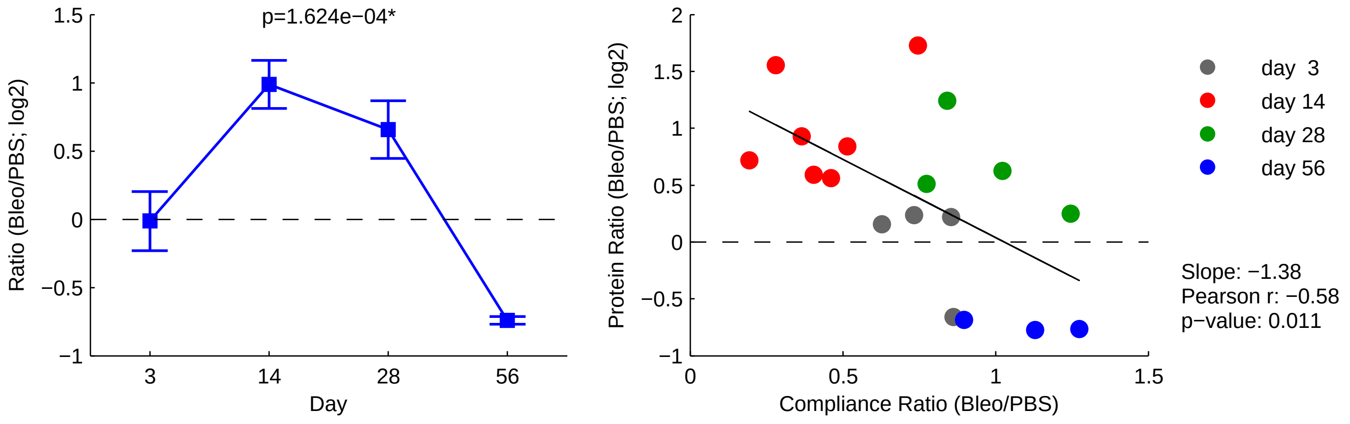

P19437 − Ms4a1 (id: 2577)

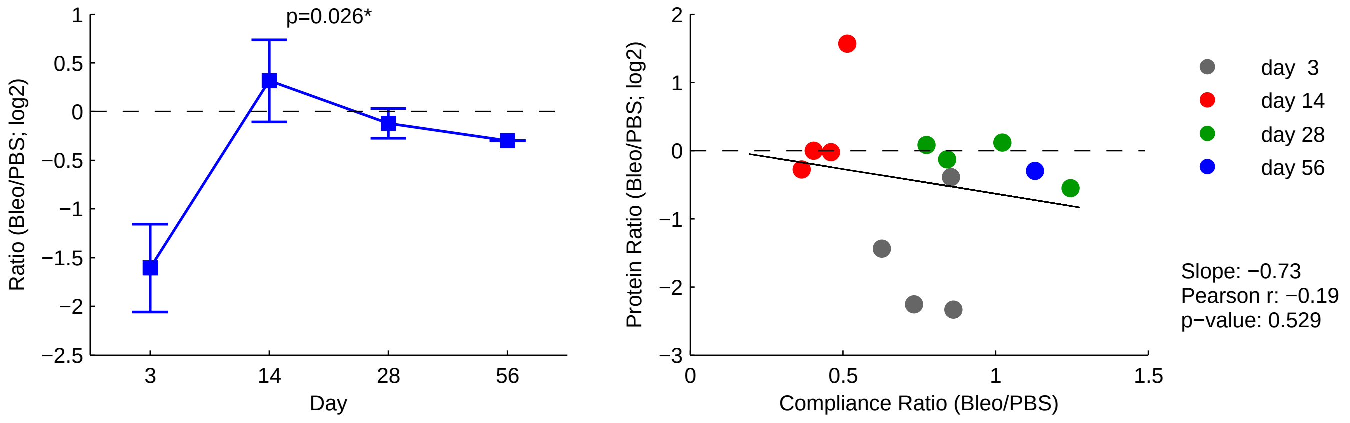

P19536 − Cox5b (id: 2578)

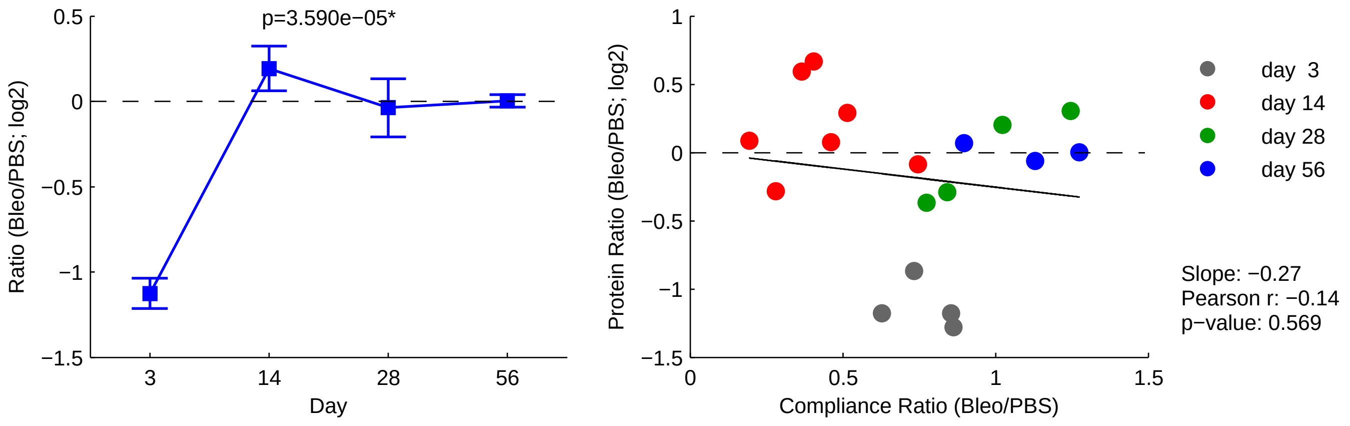

P19783 − Cox4i1 (id: 2580)

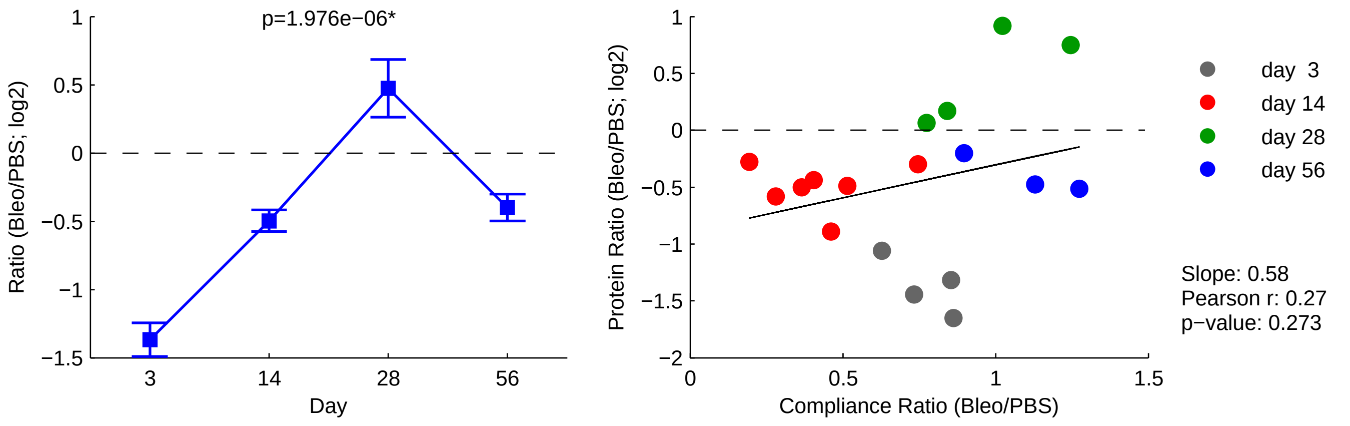

P19788 − Mgp (id: 2581)

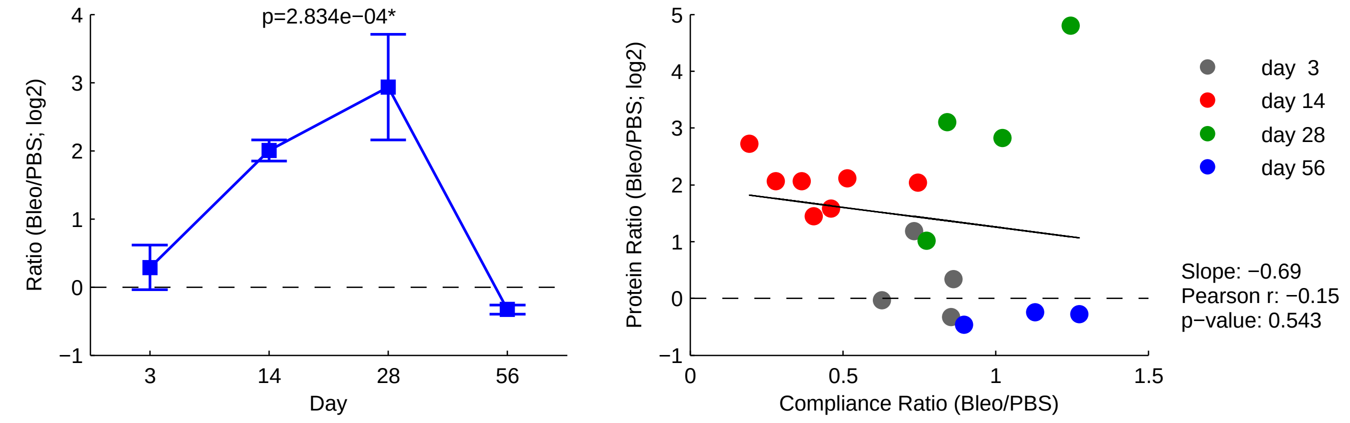

P19973 − Lsp1 (id: 2582)

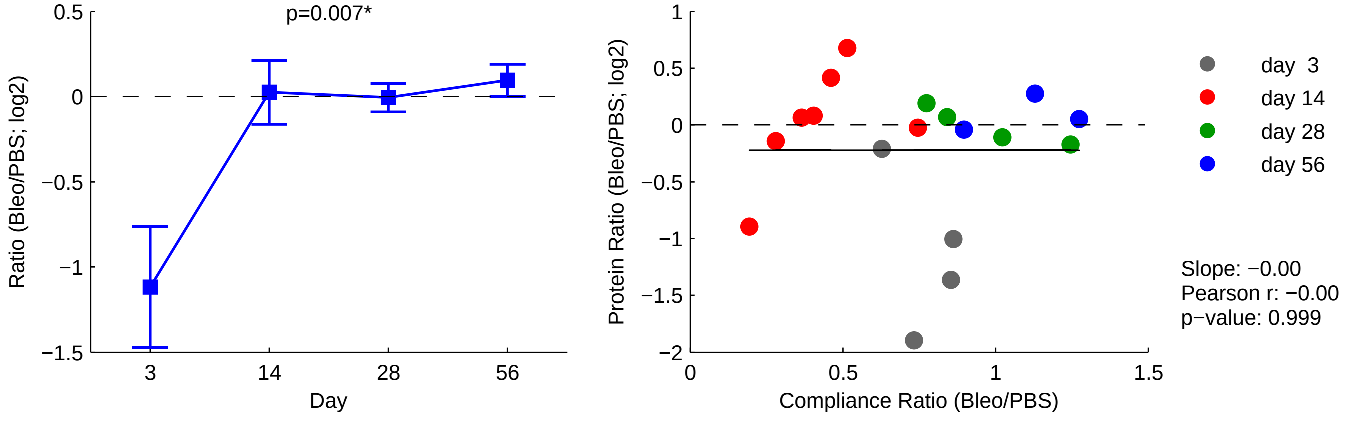

P20029 − Hspa5 (id: 2583)

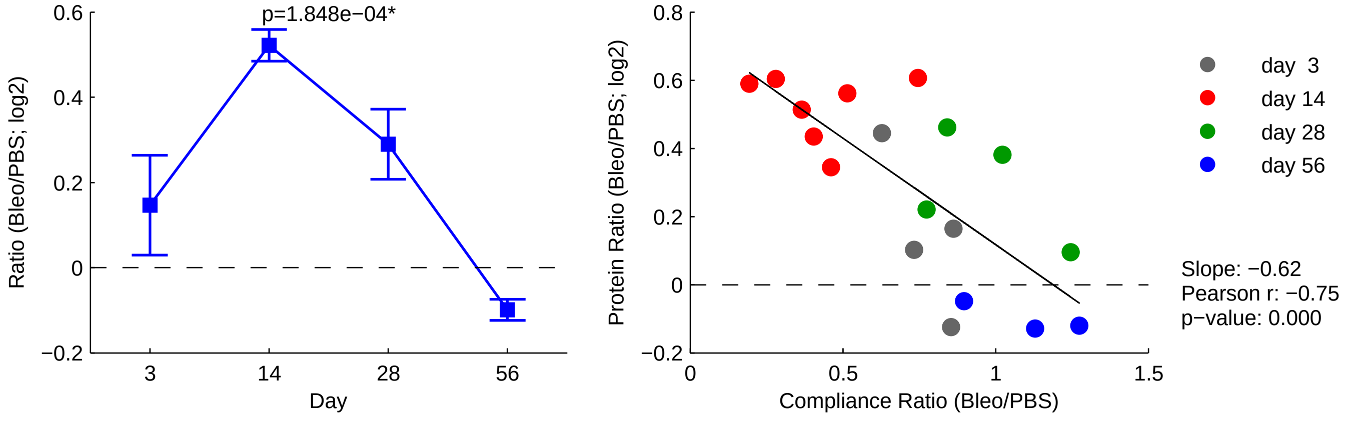

P20060 − Hexb (id: 2584)

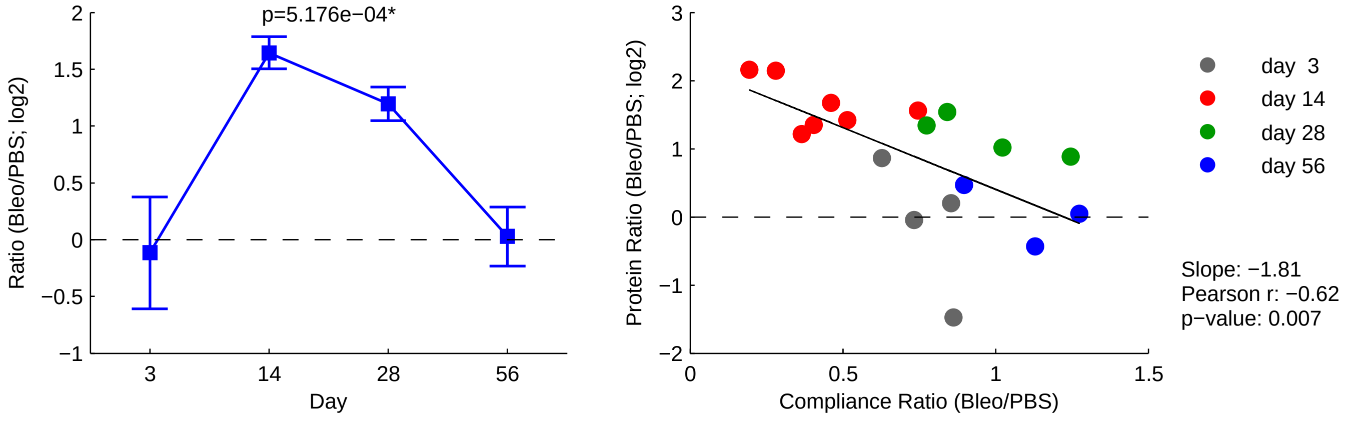

P20065−2 − Tmsb4x (id: 2585)

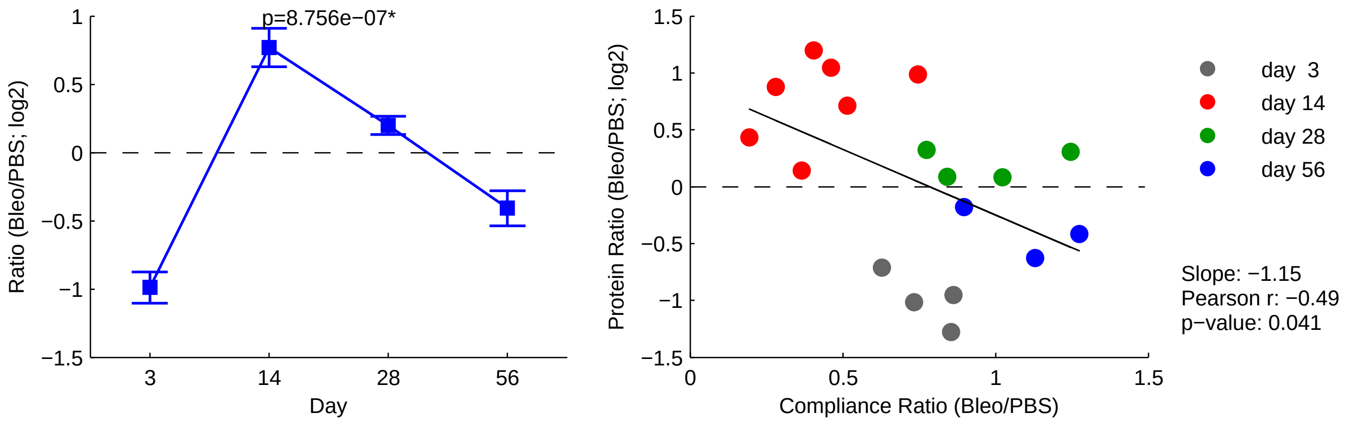

P20108 − Prdx3 (id: 2586)

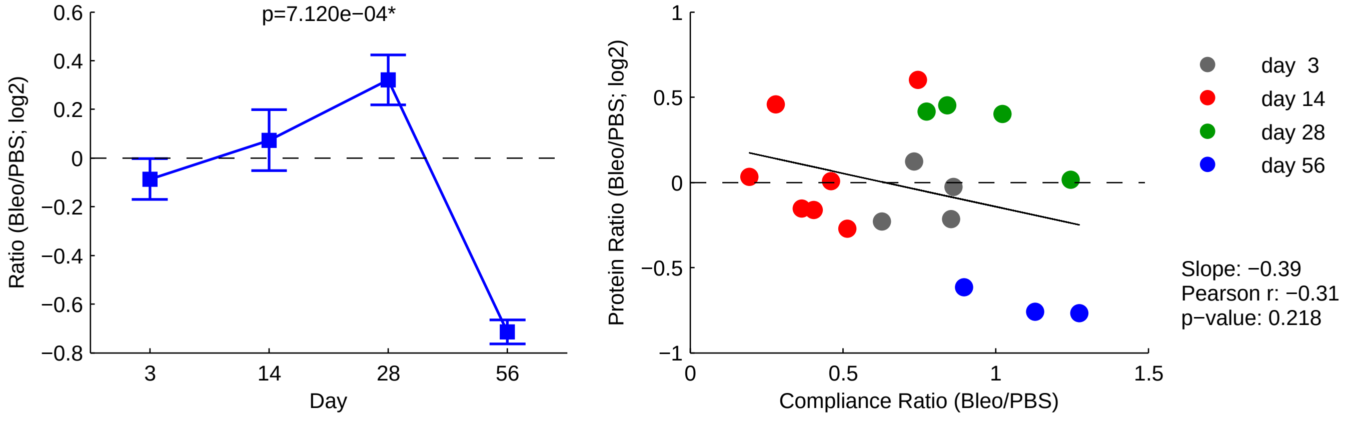

P20352 − F3 (id: 2588)

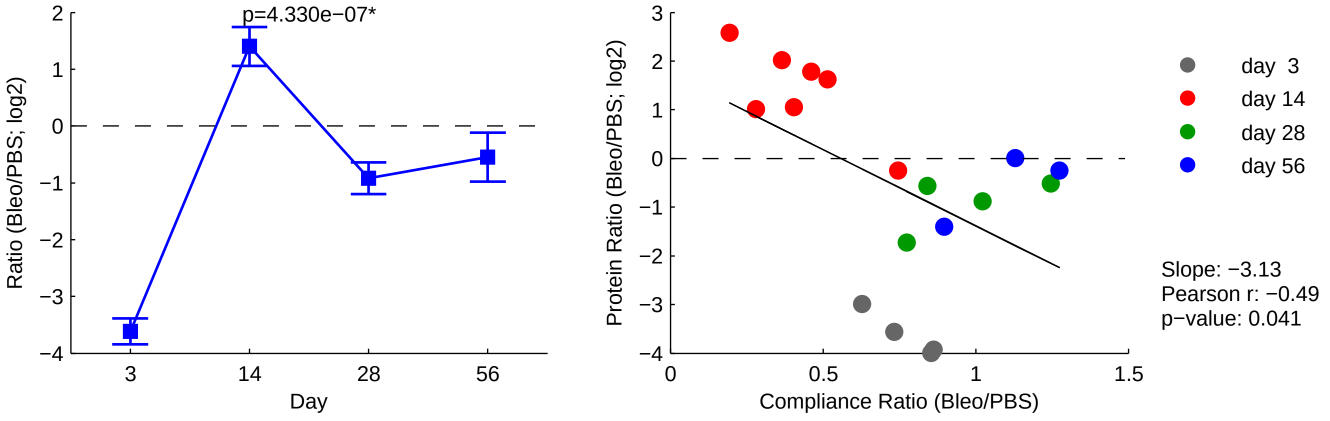

P20491 − Fcer1g (id: 2590)

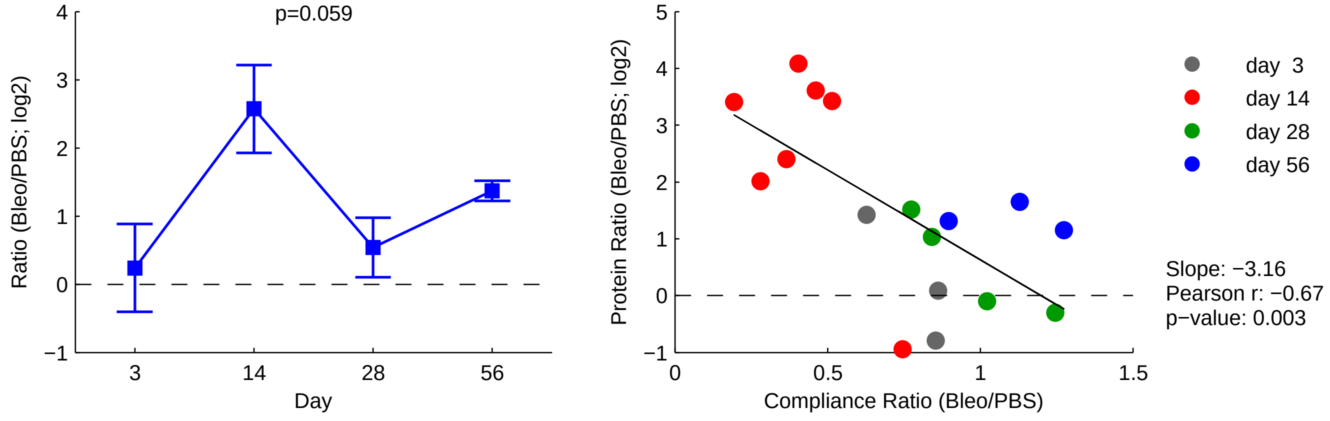

P20722 − Bmp6 (id: 2592)

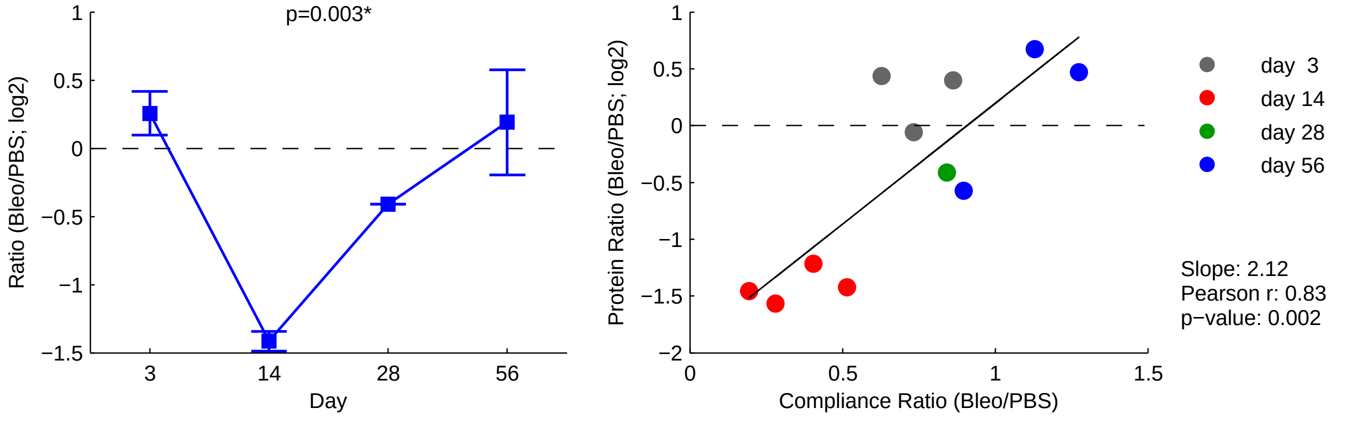

P20801 − Tnnc2 (id: 2593)

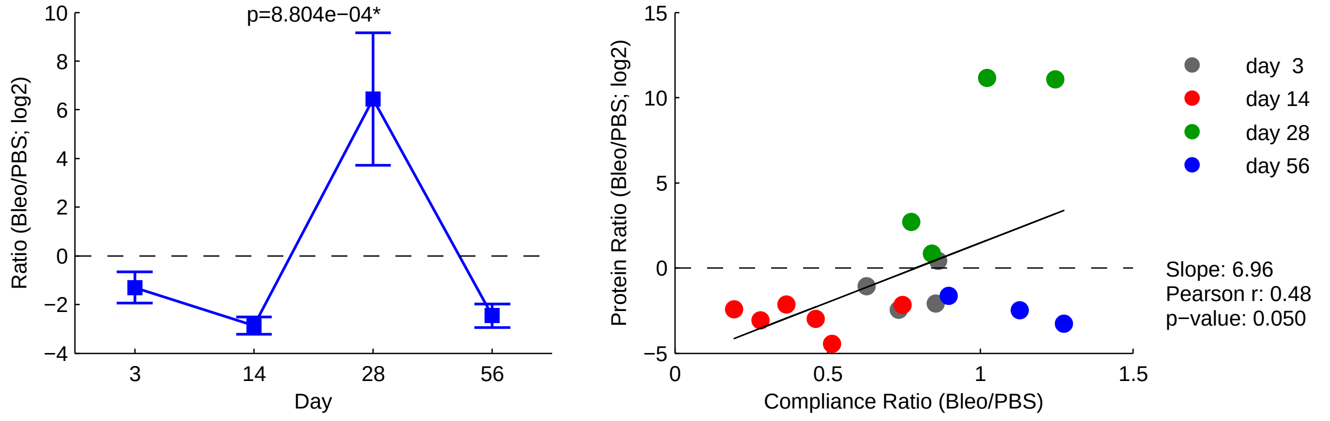

P20918 − Plg (id: 2594)

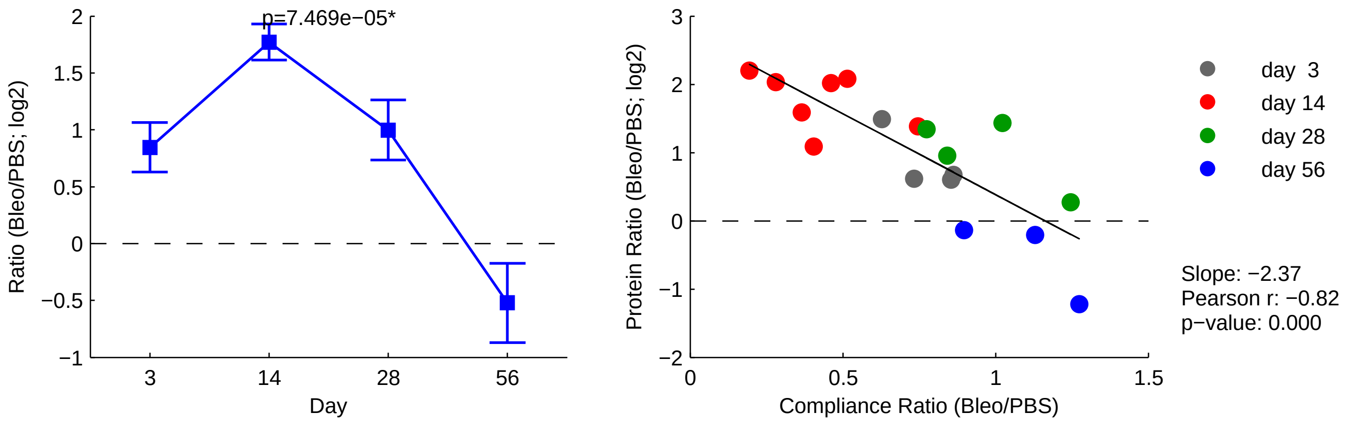

P21107 − Tpm3 (id: 2596)

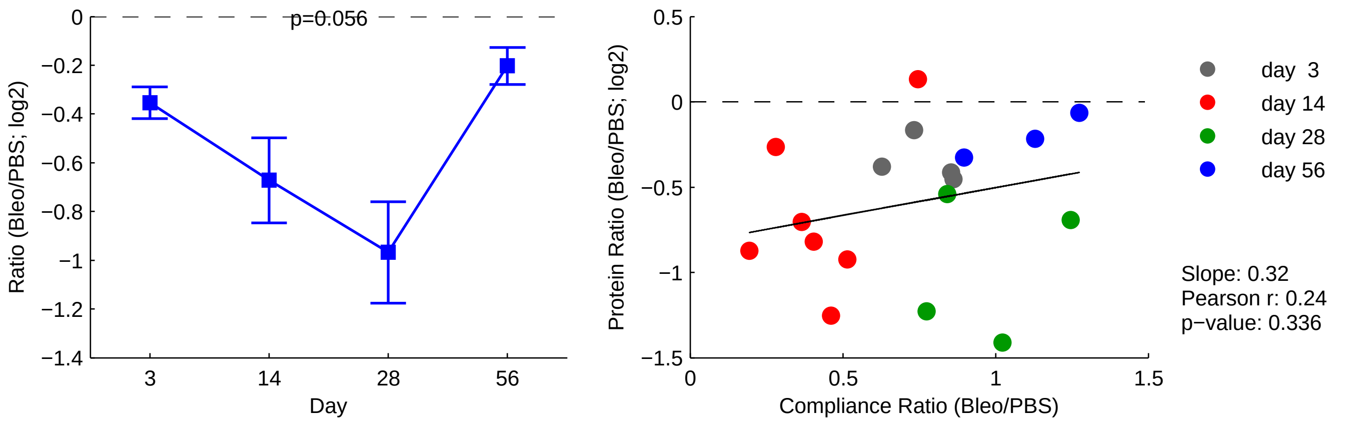

P21107−2 − (id: 2597)

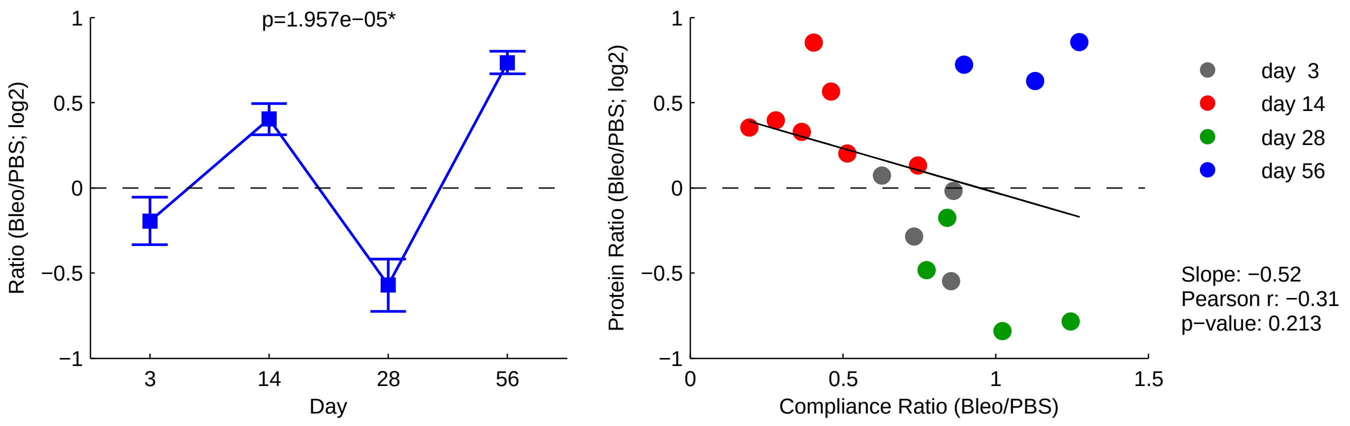

P21278 − Gna11 (id: 2599)

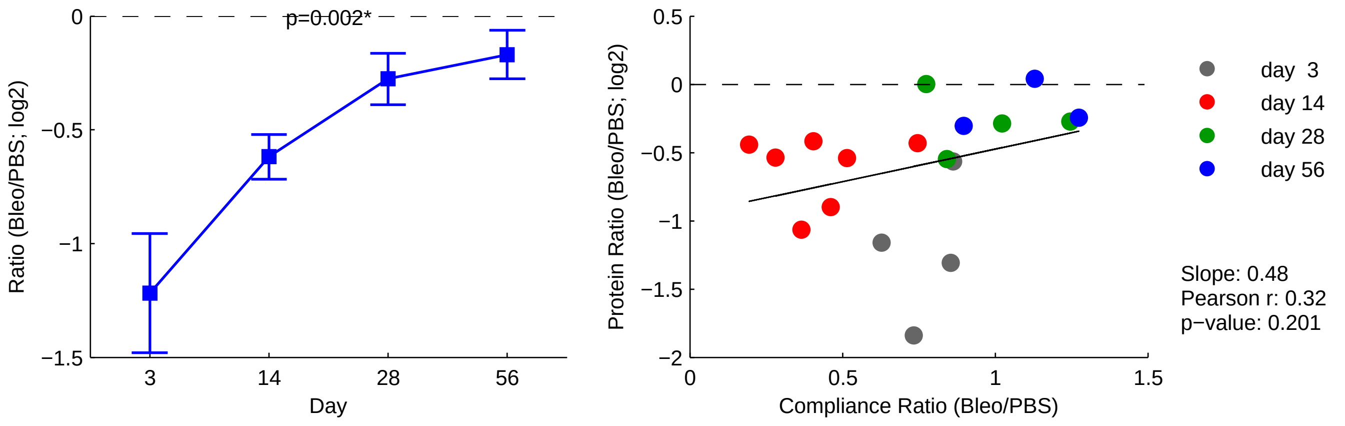

P21279 − Gnaq (id: 2600)

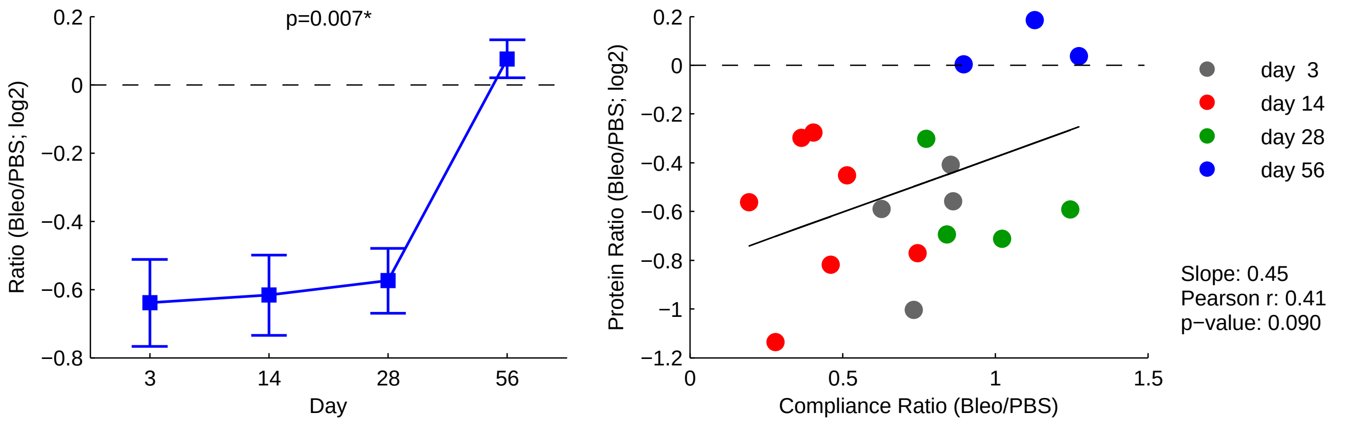

P21447 − Abcb1a (id: 2601)

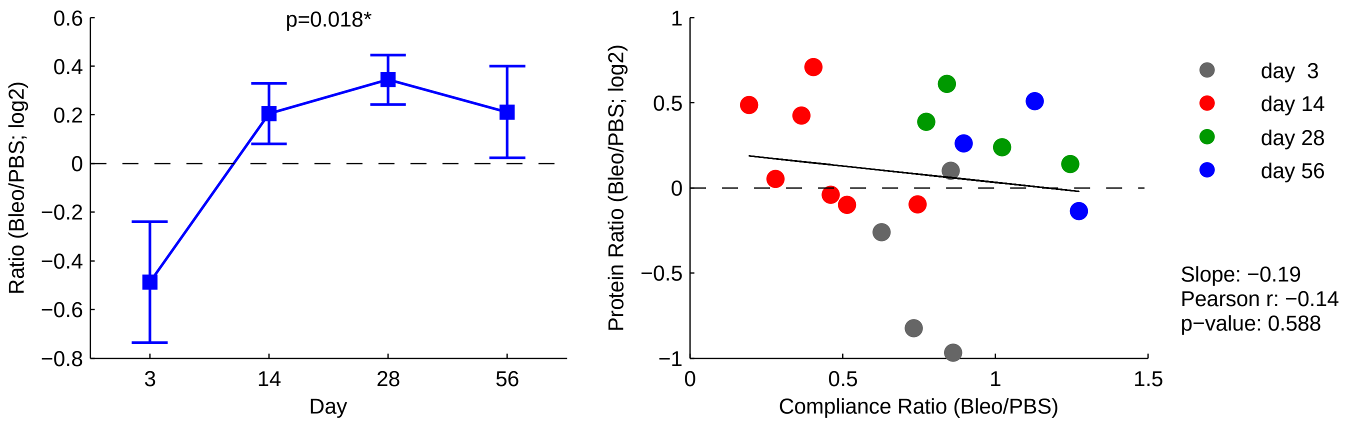

P21460 − Cst3 (id: 2602)

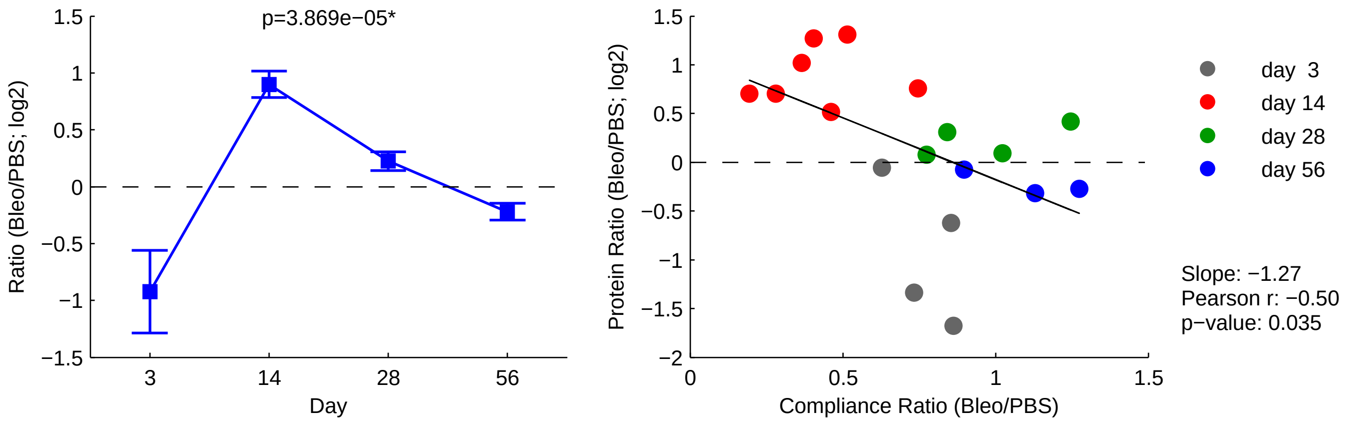

P21550 − Eno3 (id: 2603)

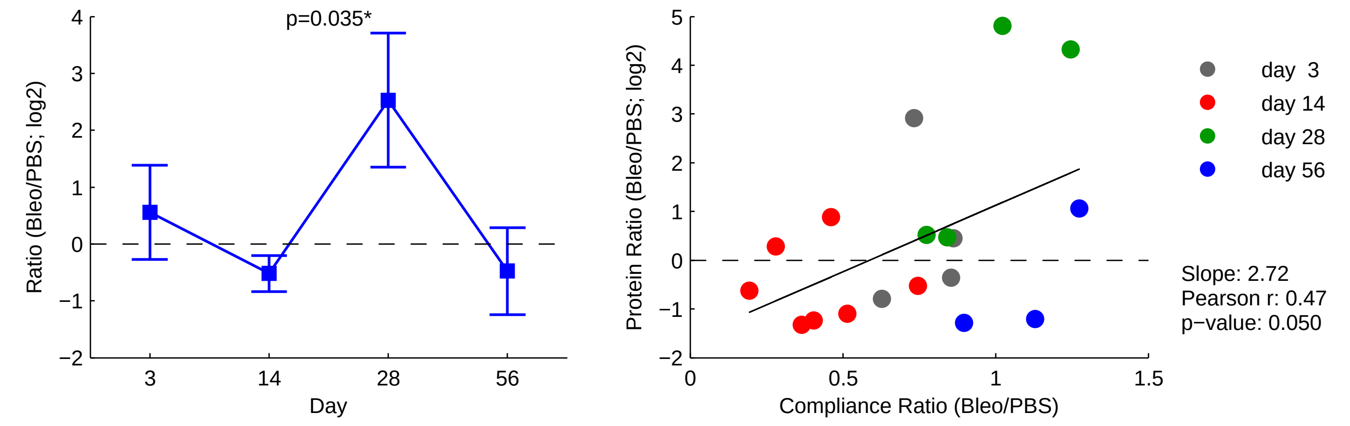

P21614 − Gc (id: 2605)

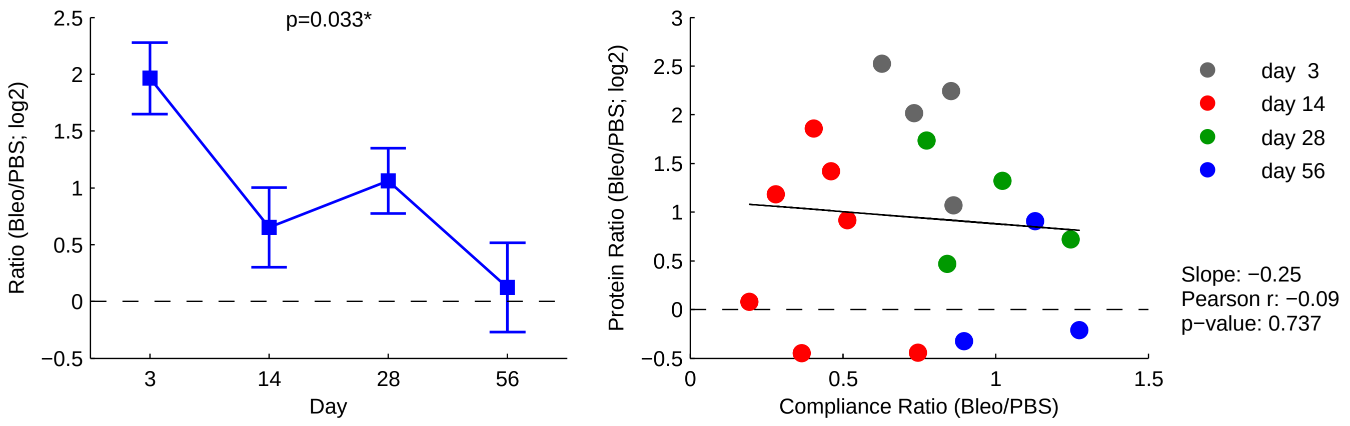

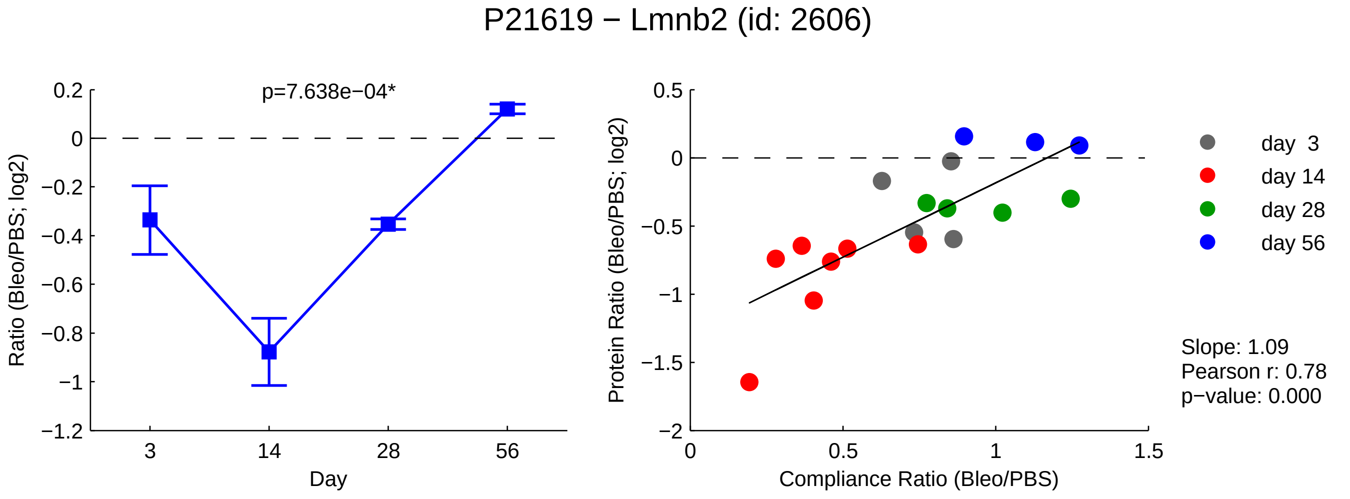
P21619 − Lmnb2 (id: 2606)
p=7.638e−04*
Ratio (Bleo/PBS; log2)
Day
Protein Ratio (Bleo/PBS; log2)
Compliance Ratio (Bleo/PBS)
day 3
day 14
day 28
day 56
Slope: 1.09
Pearson r: 0.78
p−value: 0.000

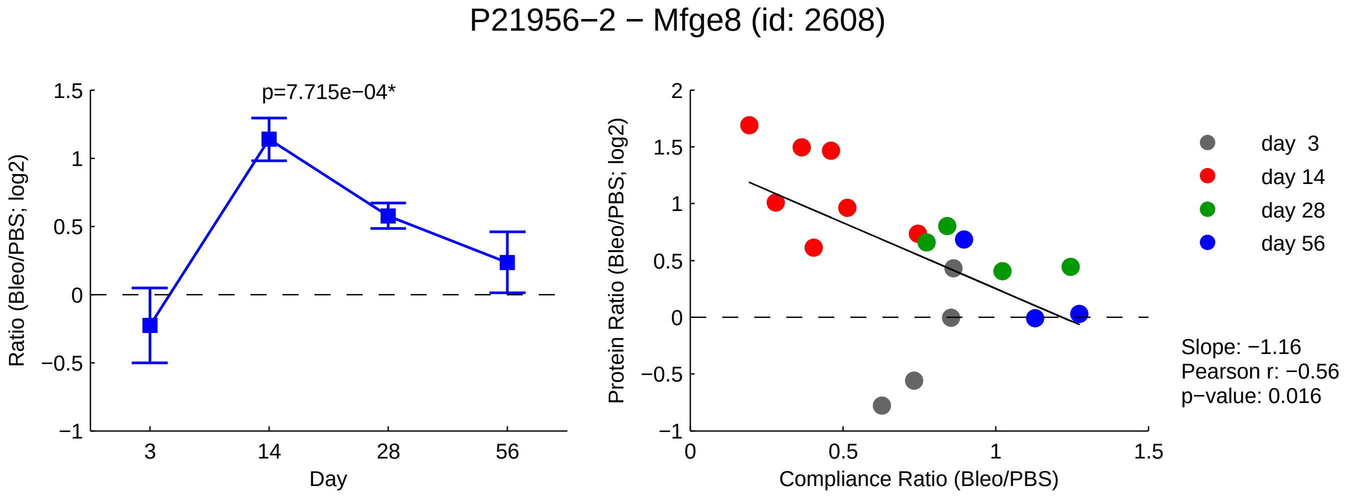
P21956−2 − Mfge8 (id: 2608)
p=7.715e−04*
Ratio (Bleo/PBS; log2)
Day
Protein Ratio (Bleo/PBS; log2)
Compliance Ratio (Bleo/PBS)
day 3
day 14
day 28
day 56
Slope: −1.16
Pearson r: −0.56
p−value: 0.016

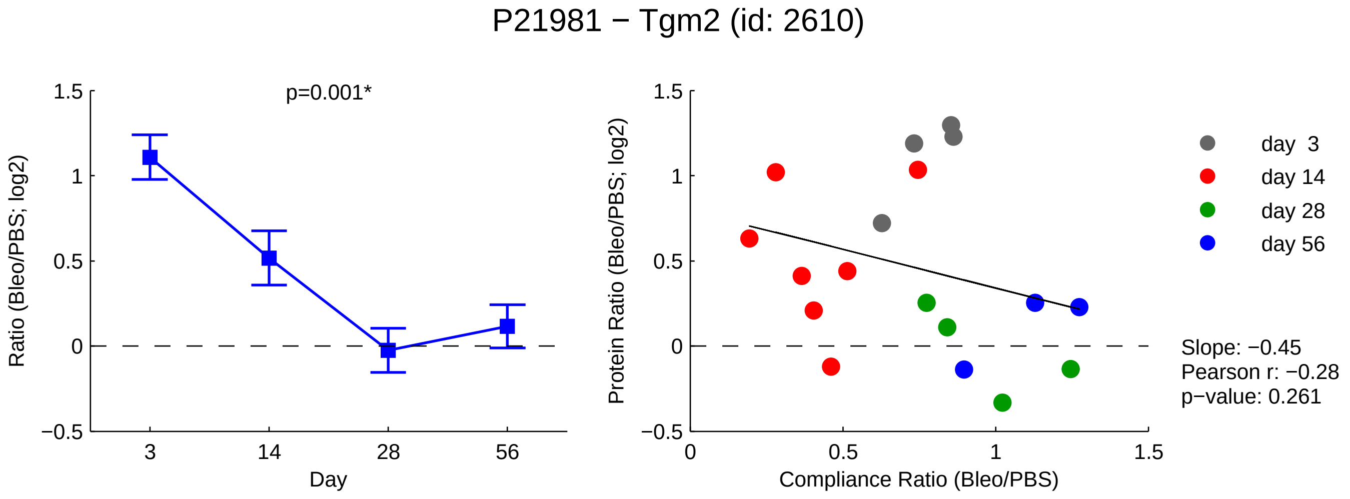
P21981 − Tgm2 (id: 2610)
p=0.001*
Ratio (Bleo/PBS; log2)
Day
Protein Ratio (Bleo/PBS; log2)
Compliance Ratio (Bleo/PBS)
day 3
day 14
day 28
day 56
Slope: −0.45
Pearson r: −0.28
p−value: 0.261

P22366 − Myd88 (id: 2612)

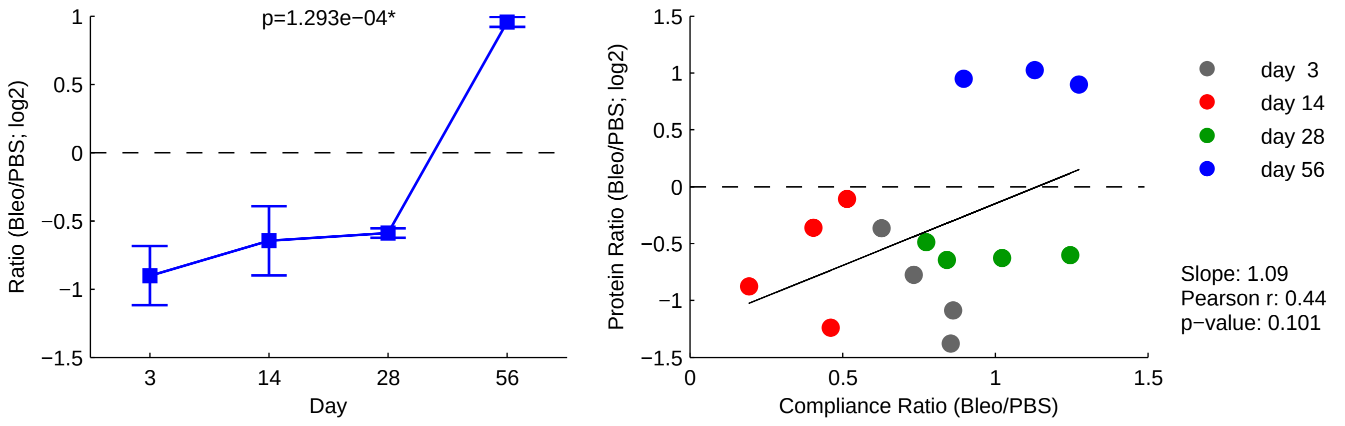

P22599 − Serpina1b (id: 2614)

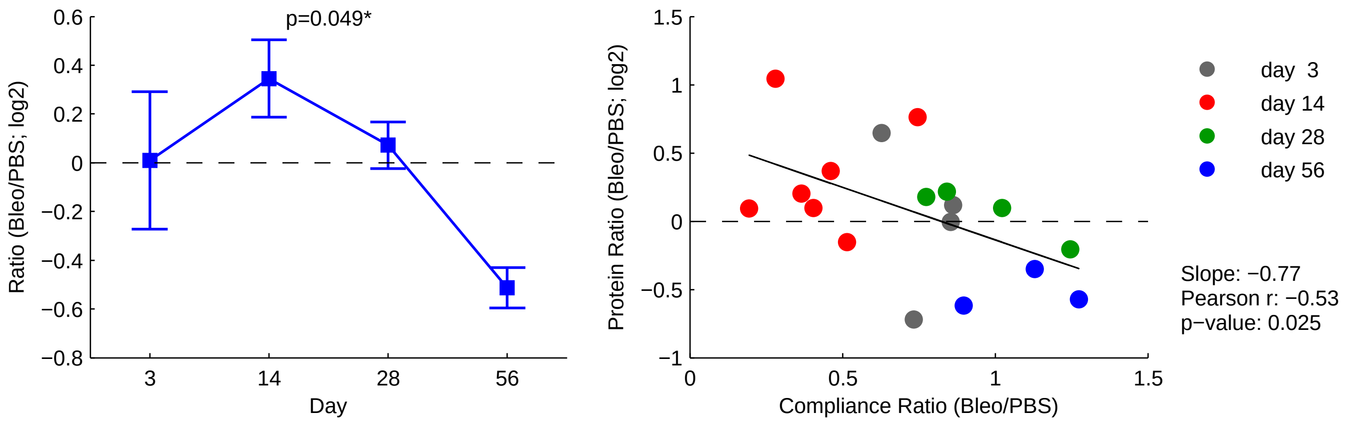

P22682 − Cbl (id: 2616)

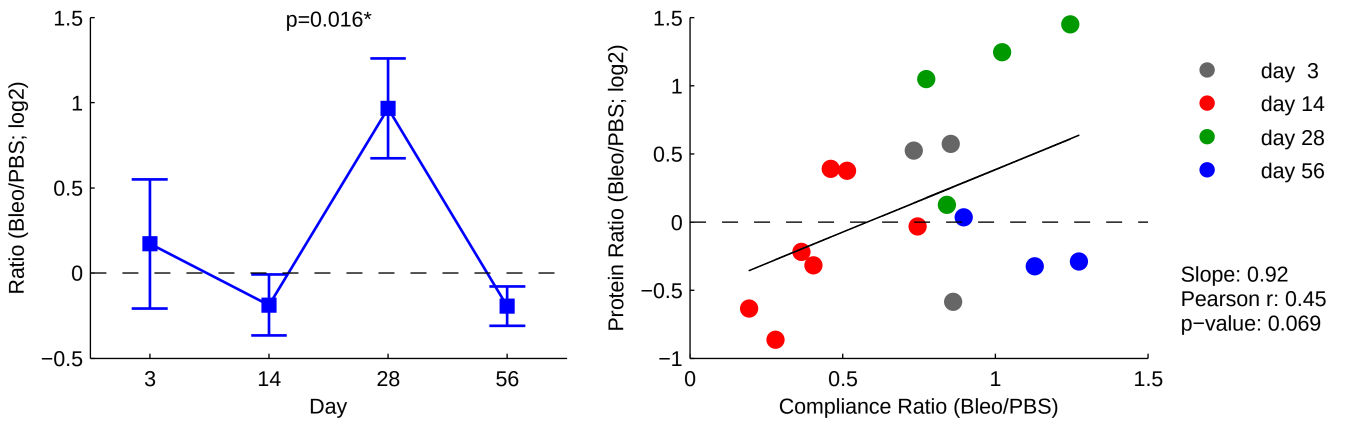

Q8CGP6 − Hist1h2ah (id: 2618)

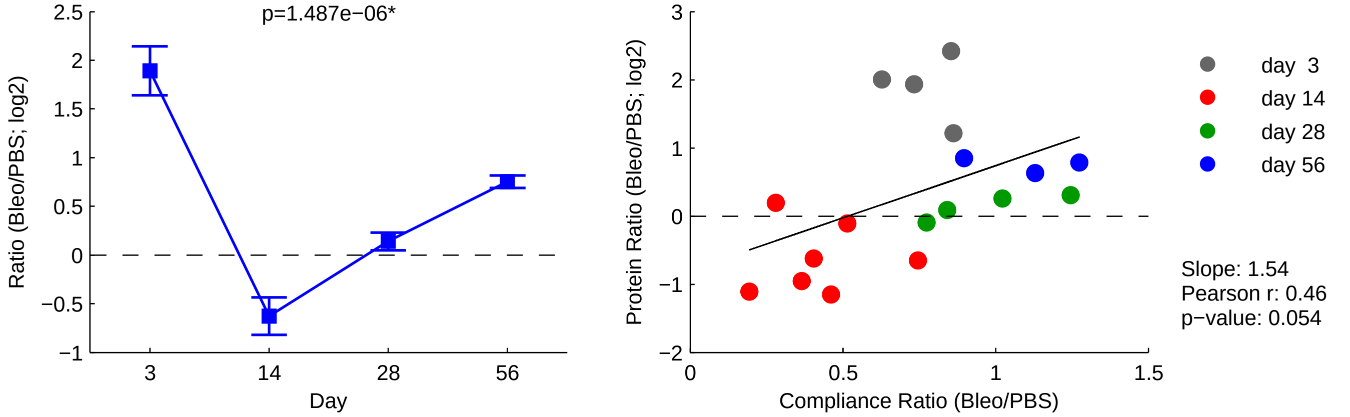

P22907−2 − Hmbs (id: 2620)

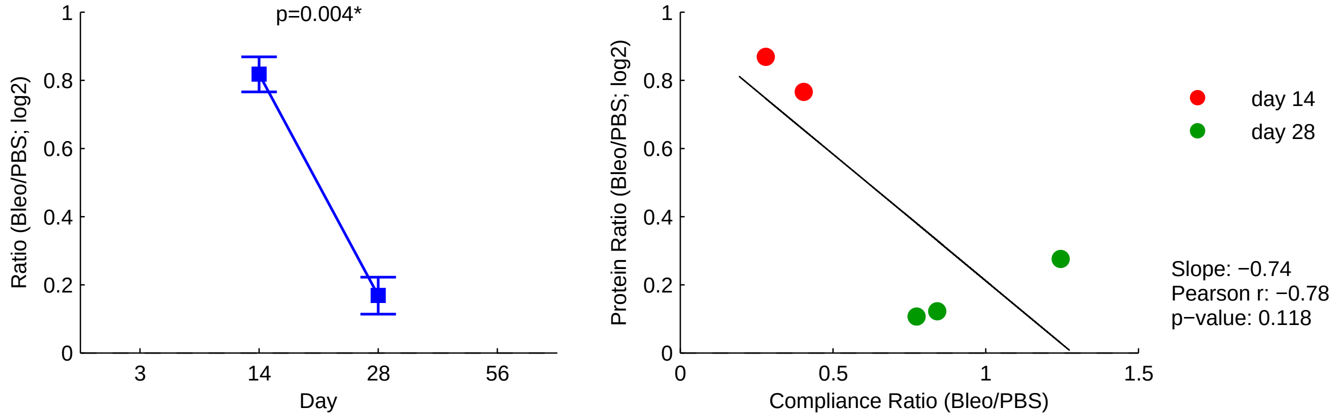

P23116 − Eif3a (id: 2621)

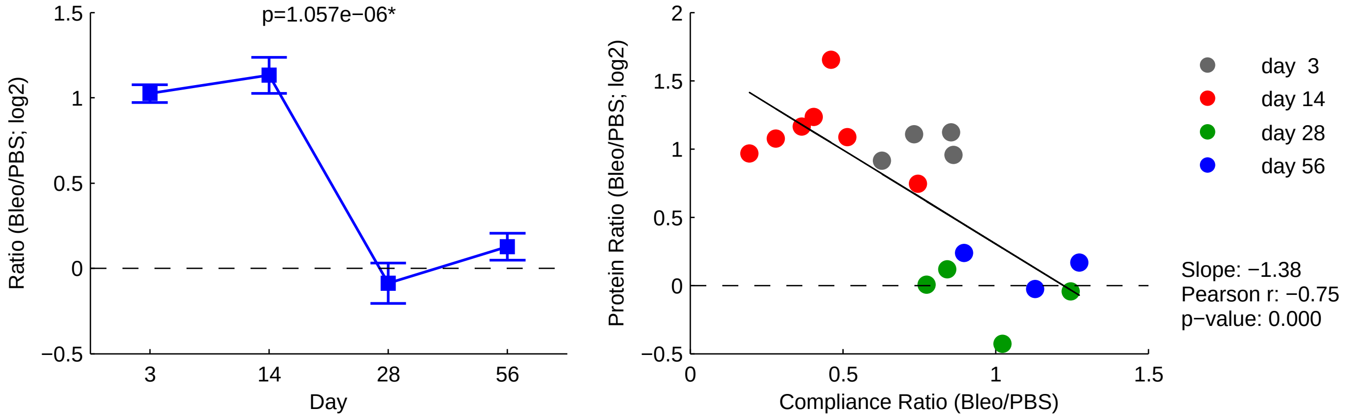

P23242 − Gja1 (id: 2622)

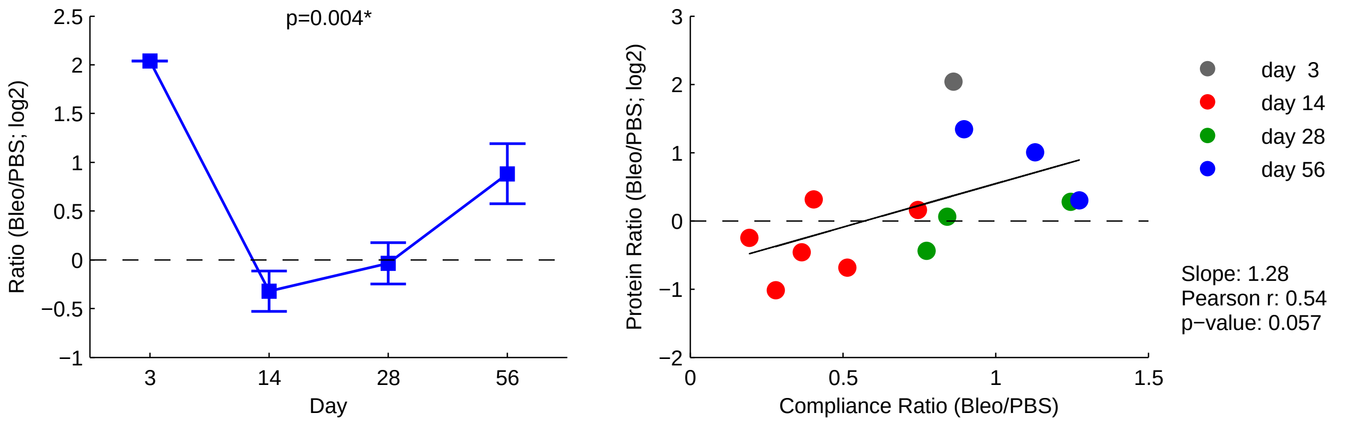

Q543K9 − Pnp (id: 2627)

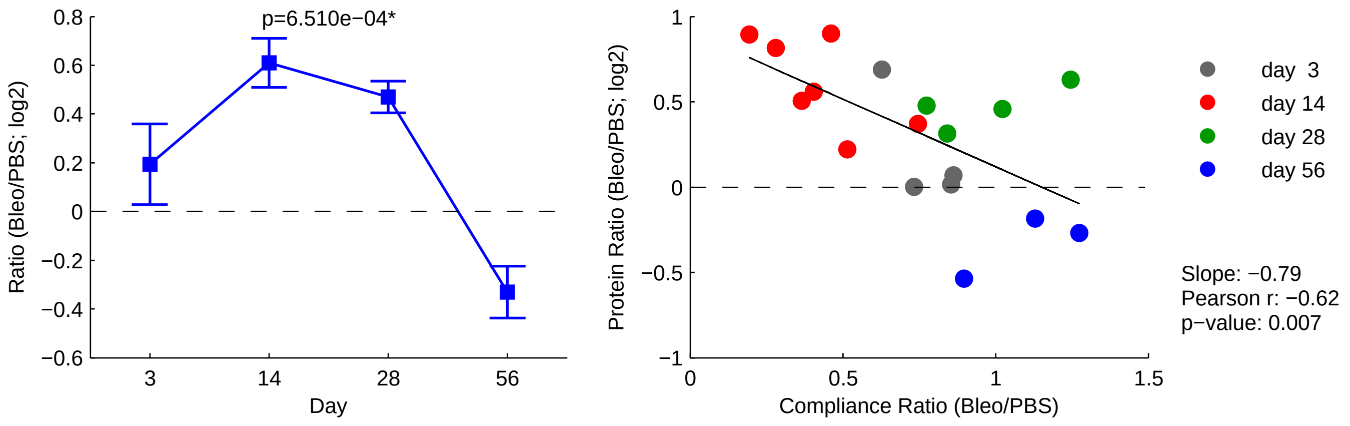

P23780 − Glb1 (id: 2629)

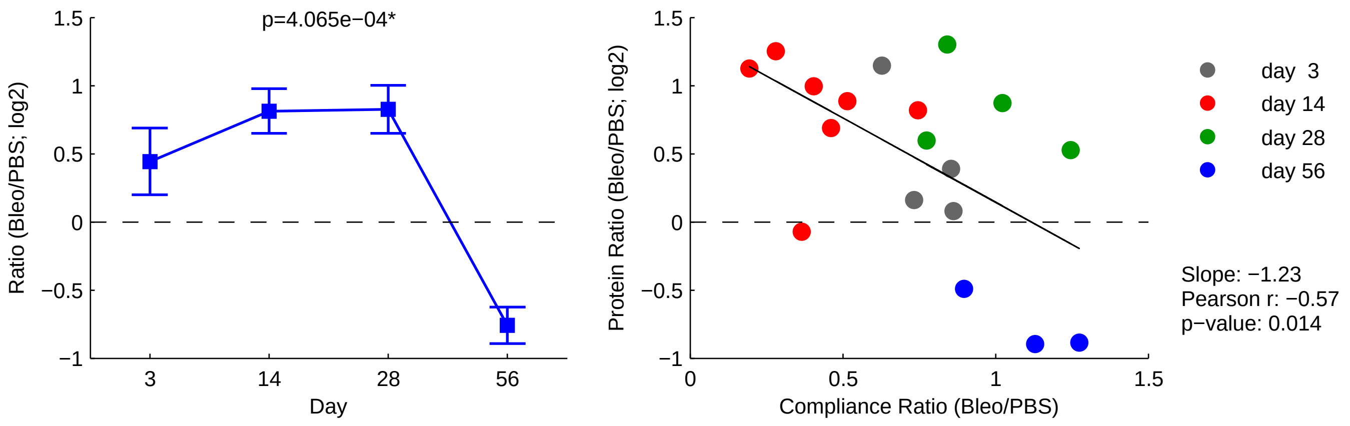

P23881 − Tcea3 (id: 2630)

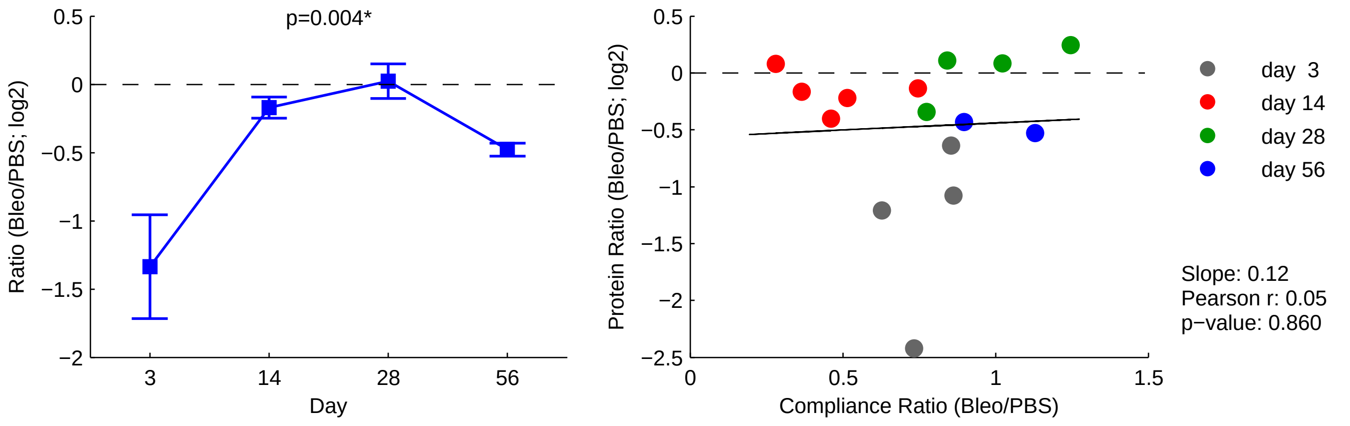

P23927 − Cryab (id: 2631)

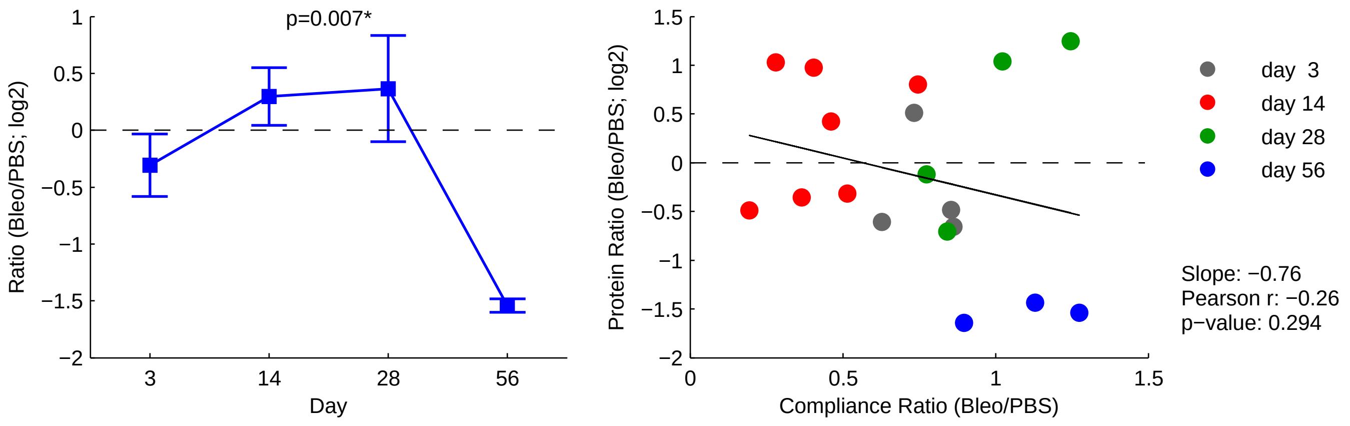

P23953 − Ces1c (id: 2633)

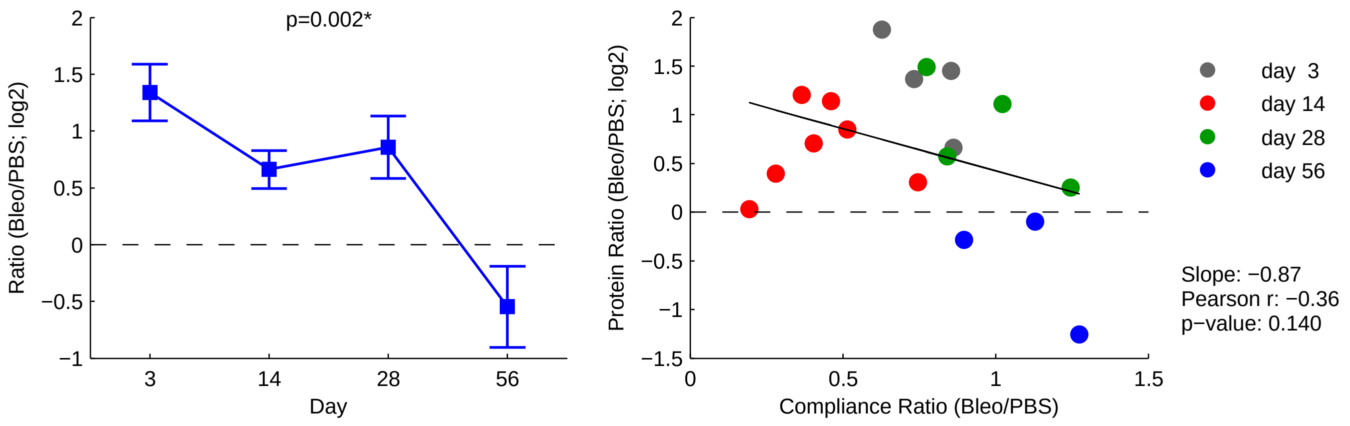

P24270 − Cat (id: 2634)

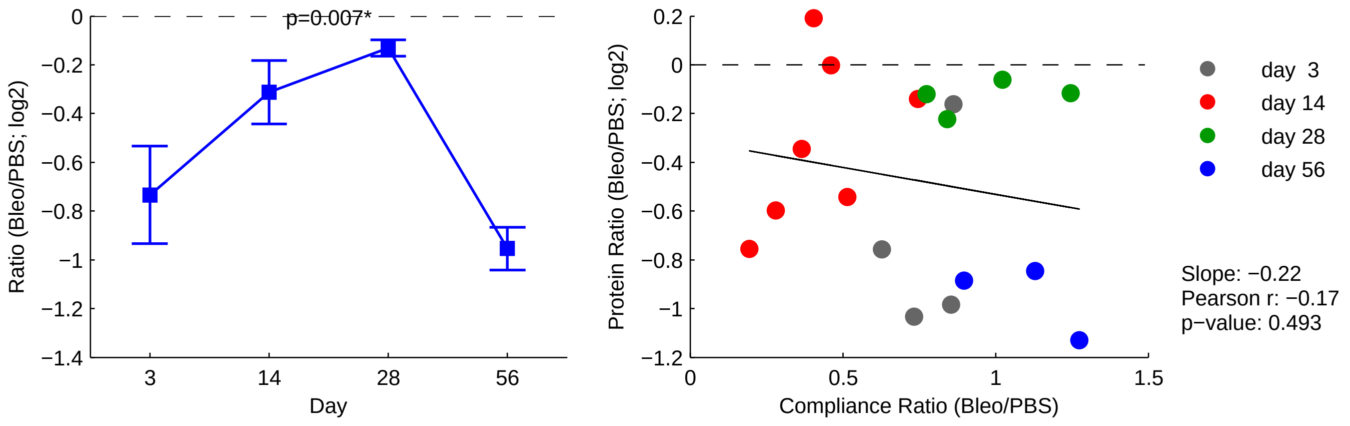

P24472 − Gsta4 (id: 2636)

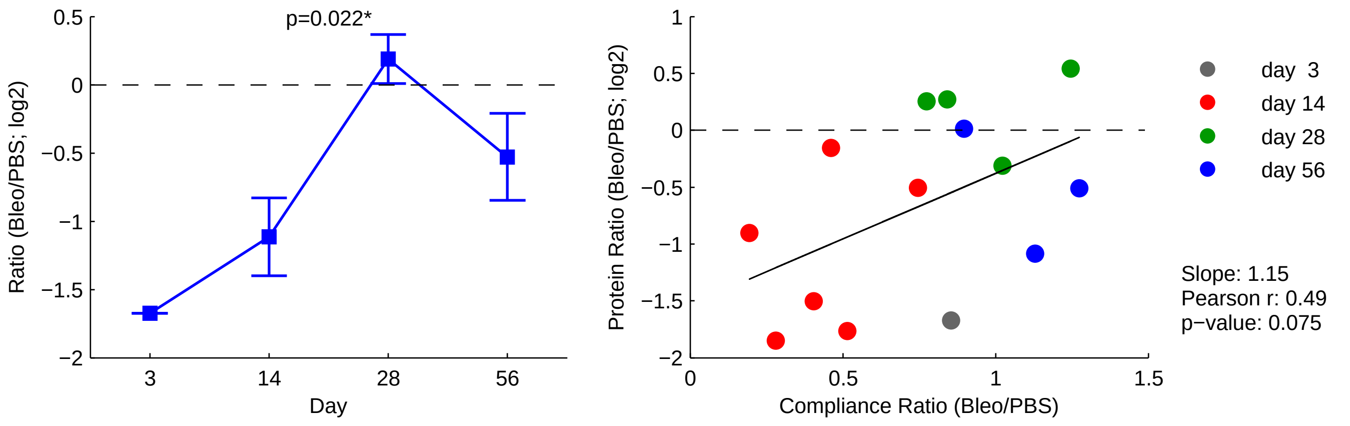

P24547 − Impdh2 (id: 2639)

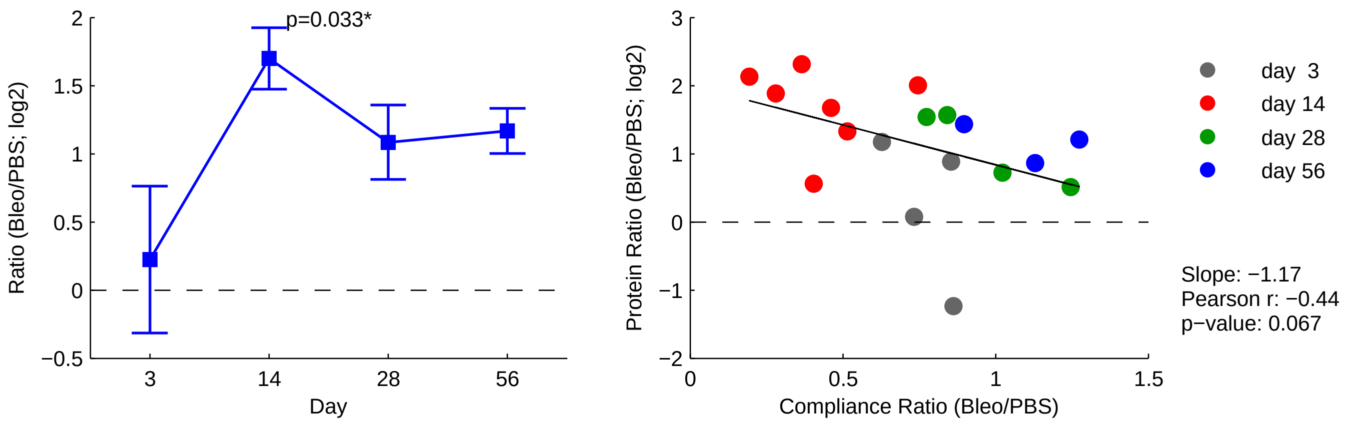

P24638 − Acp2 (id: 2641)

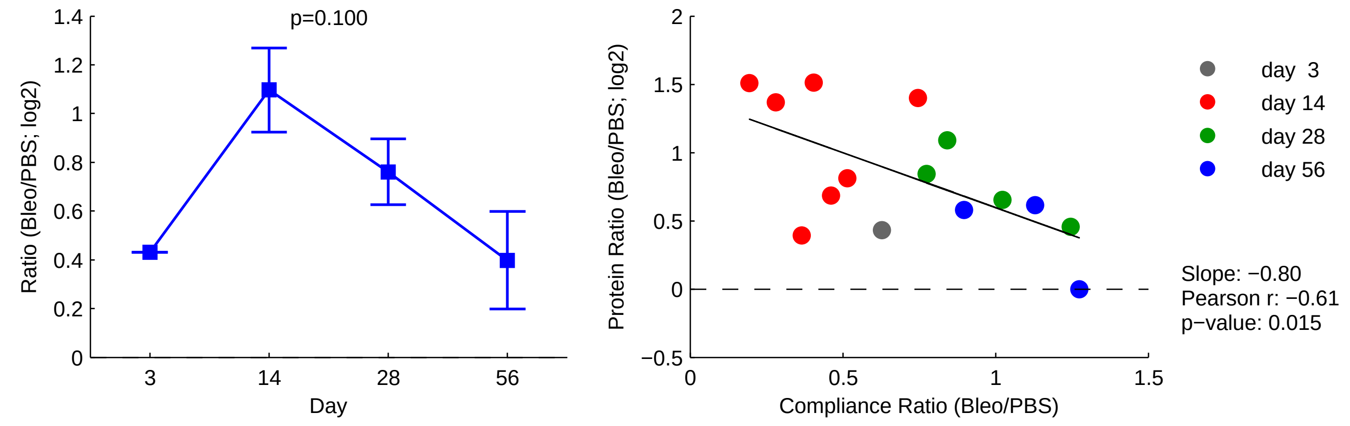

P25206 − Mcm3 (id: 2645)

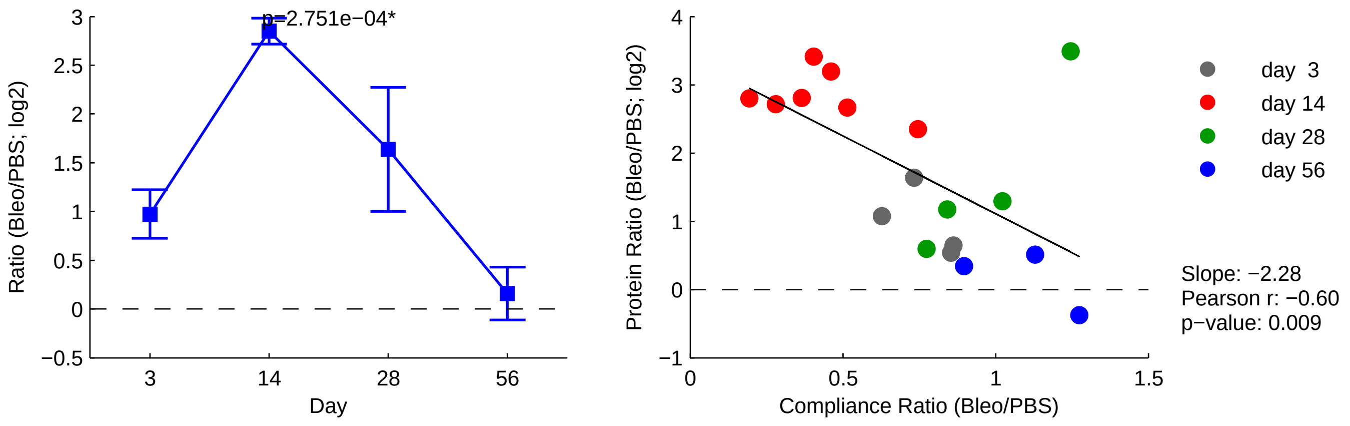

P25444 − Rps2 (id: 2647)

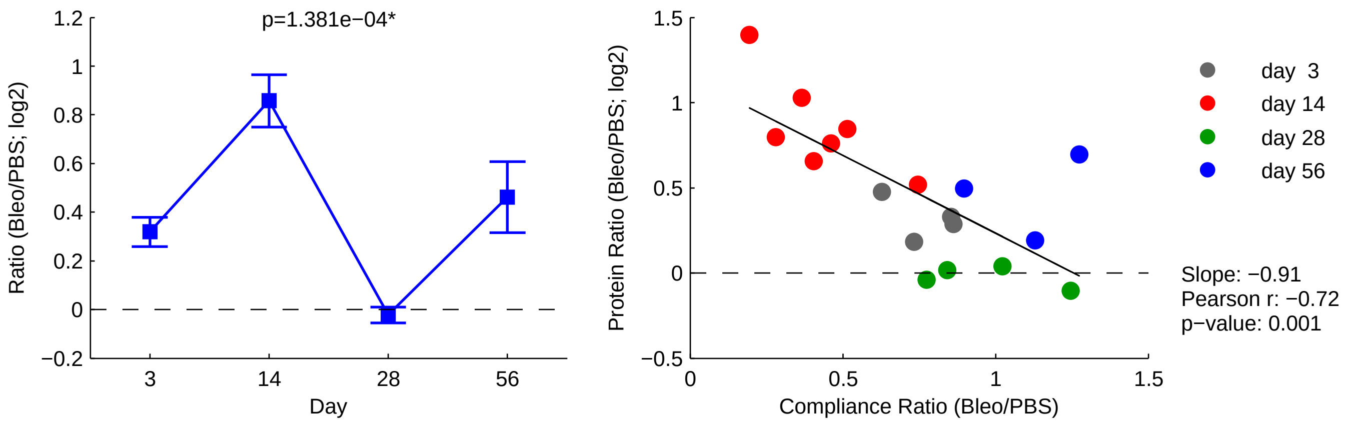

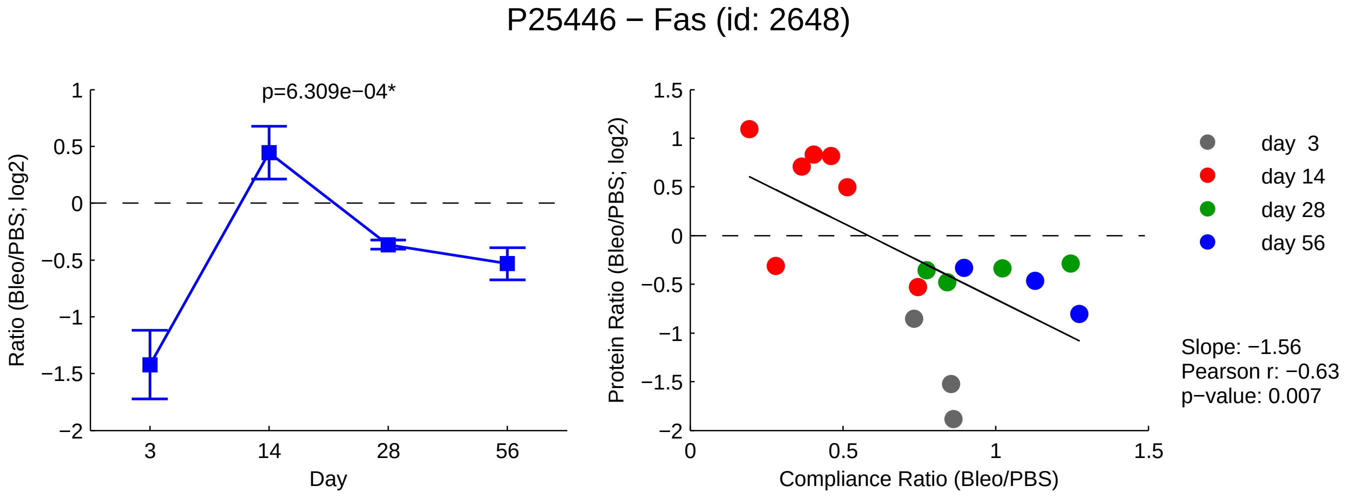
P25446 − Fas (id: 2648)
p=6.309e−04*
Ratio (Bleo/PBS; log2)
Day
Protein Ratio (Bleo/PBS; log2)
Compliance Ratio (Bleo/PBS)
day 3
day 14
day 28
day 56
Slope: −1.56
Pearson r: −0.63
p−value: 0.007

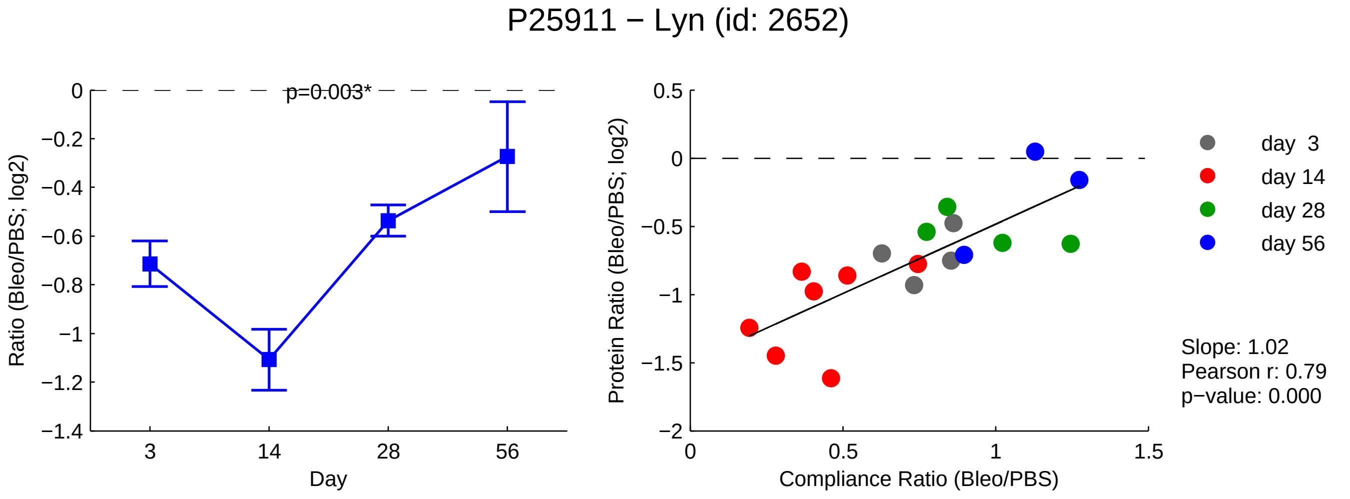
P25911 − Lyn (id: 2652)
p=0.003*
Ratio (Bleo/PBS; log2)
Day
Protein Ratio (Bleo/PBS; log2)
Compliance Ratio (Bleo/PBS)
day 3
day 14
day 28
day 56
Slope: 1.02
Pearson r: 0.79
p−value: 0.000

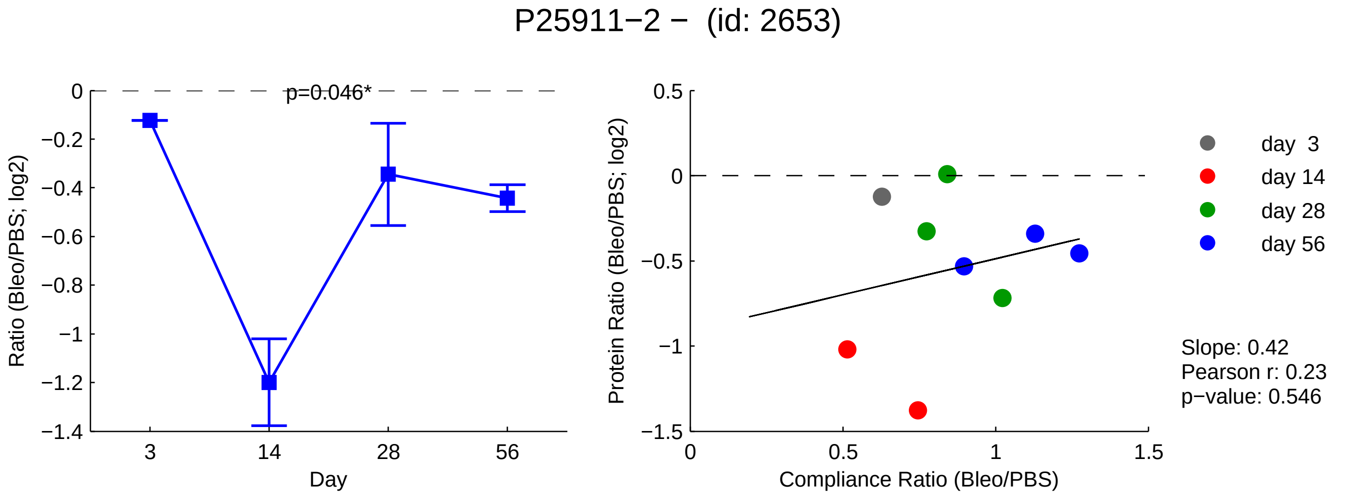
P25911−2 − (id: 2653)
p=0.046*
Ratio (Bleo/PBS; log2)
Day
Protein Ratio (Bleo/PBS; log2)
Compliance Ratio (Bleo/PBS)
day 3
day 14
day 28
day 56
Slope: 0.42
Pearson r: 0.23
p−value: 0.546

P25916 − Bmi1 (id: 2654)

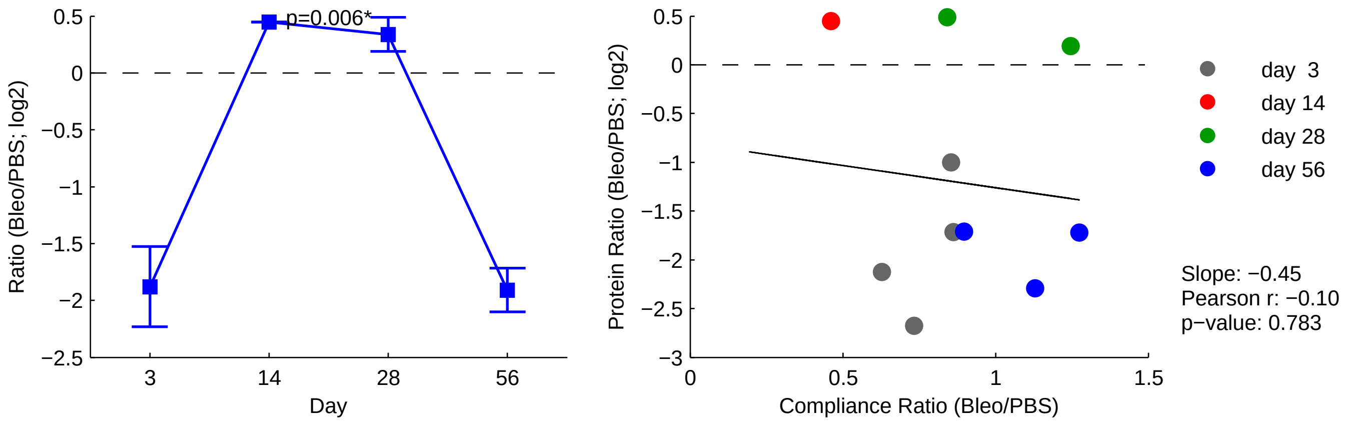

P26011 − Itgb7 (id: 2655)

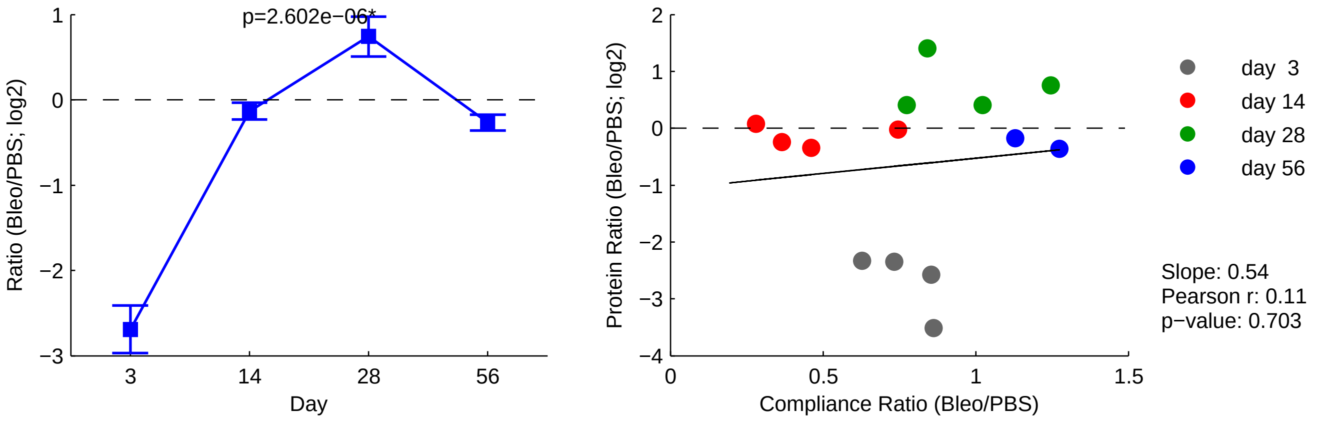

P26039 − Tln1 (id: 2656)

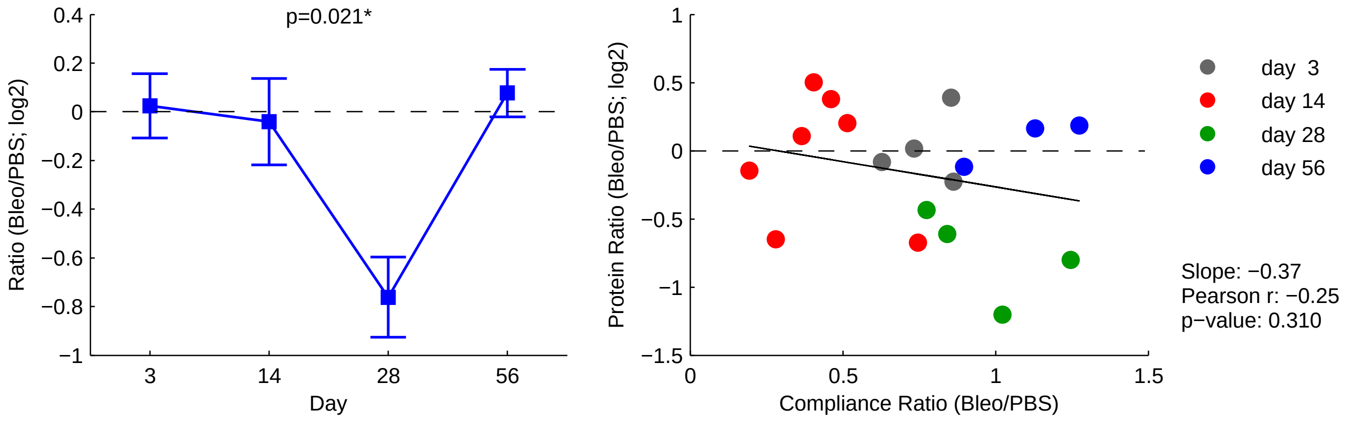

P26040 − Ezr (id: 2657)

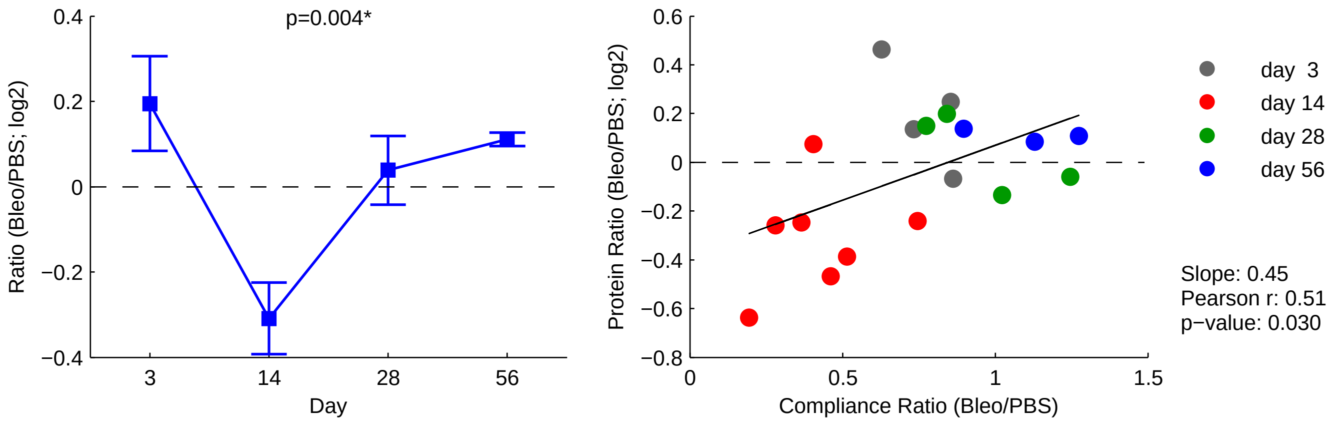

P26041 − Msn (id: 2658)

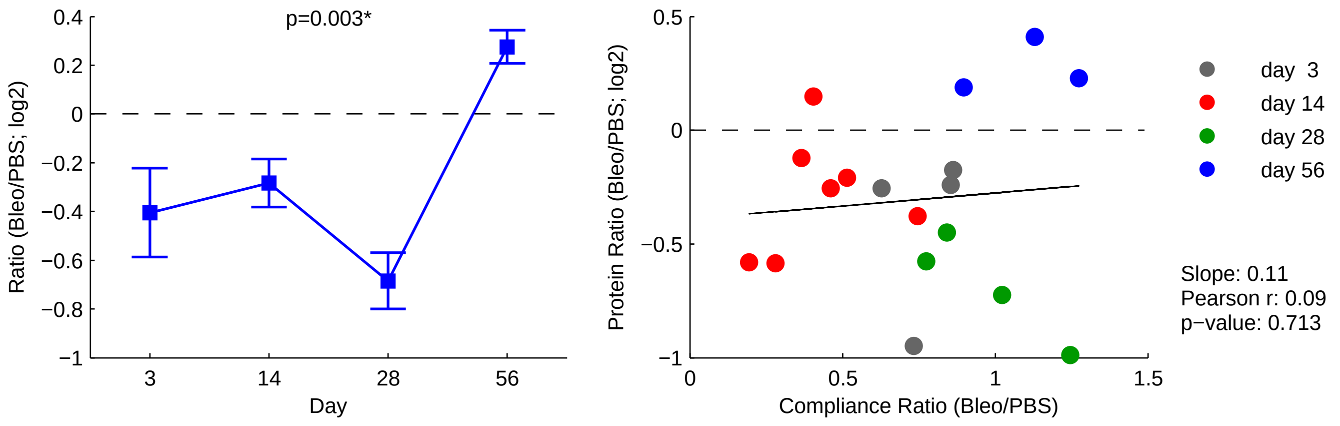

P26043 − Rdx (id: 2659)

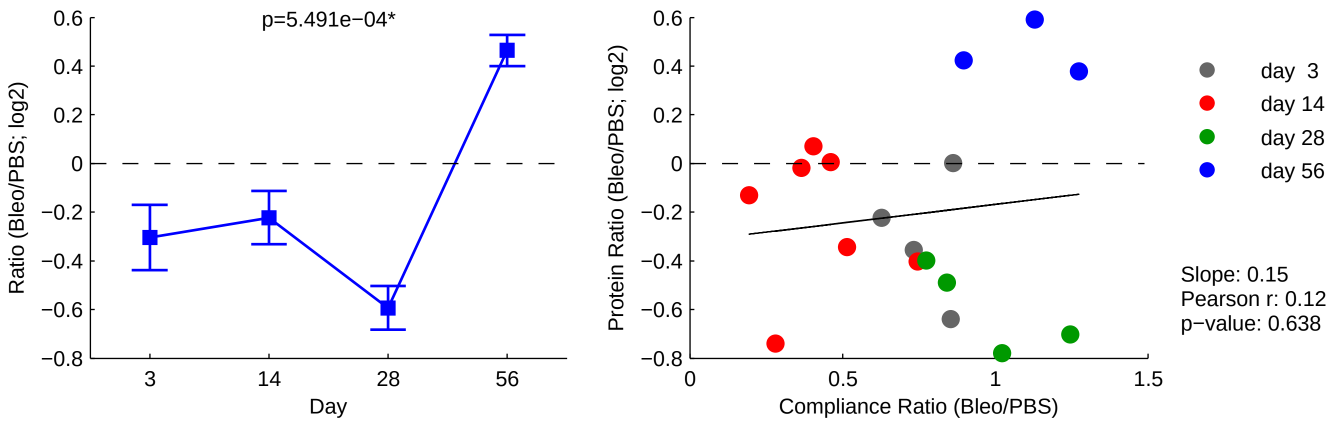

P26187 − Mgmt (id: 2661)

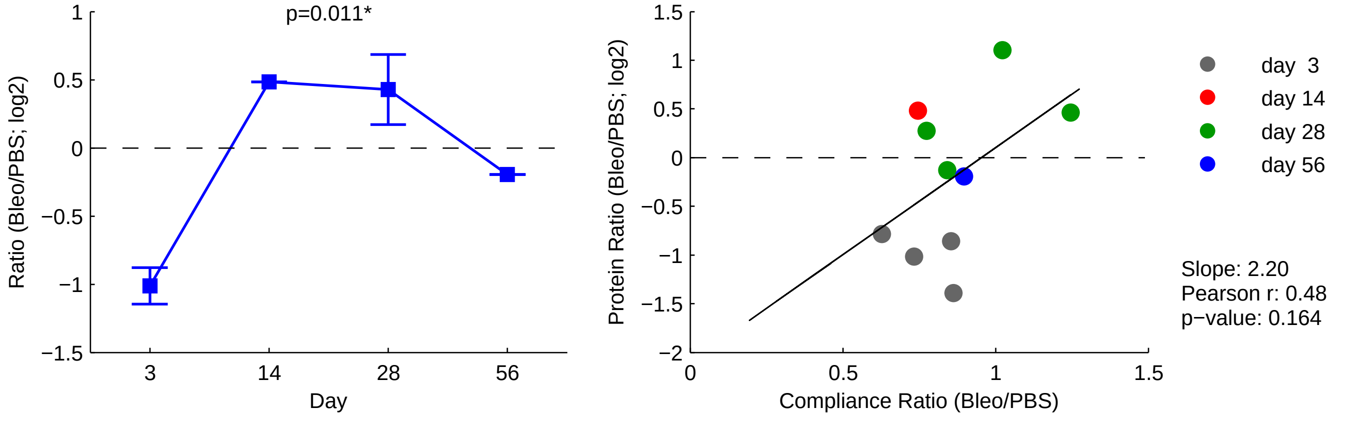

P26350 − Ptma (id: 2664)

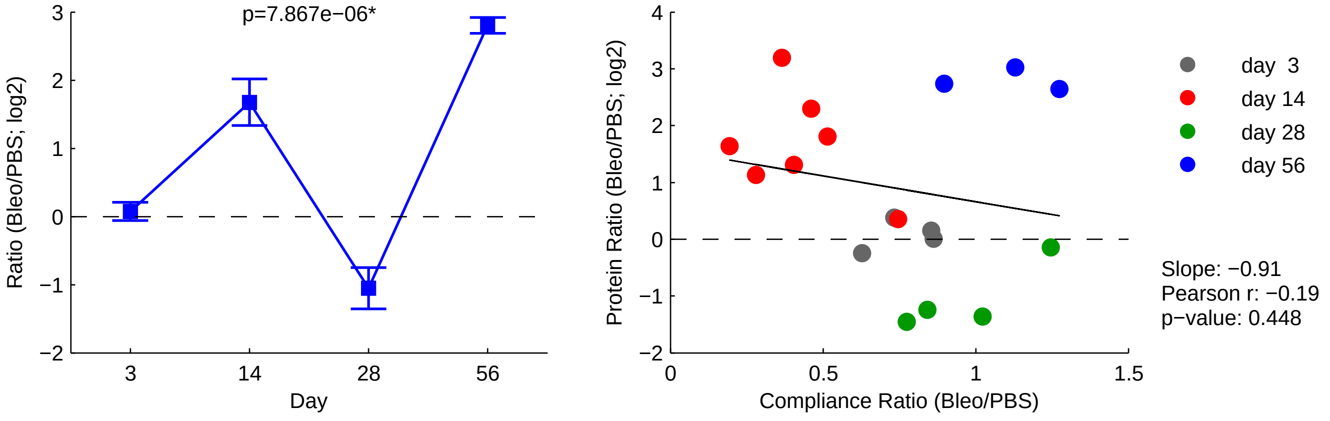

P26369 − U2af2 (id: 2665)

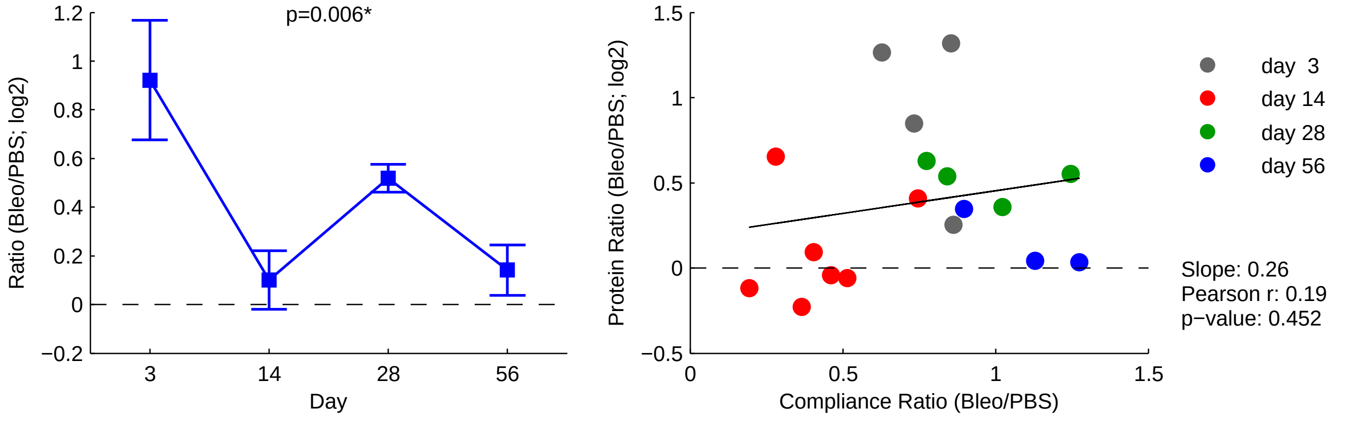

P26443 − Glud1 (id: 2666)

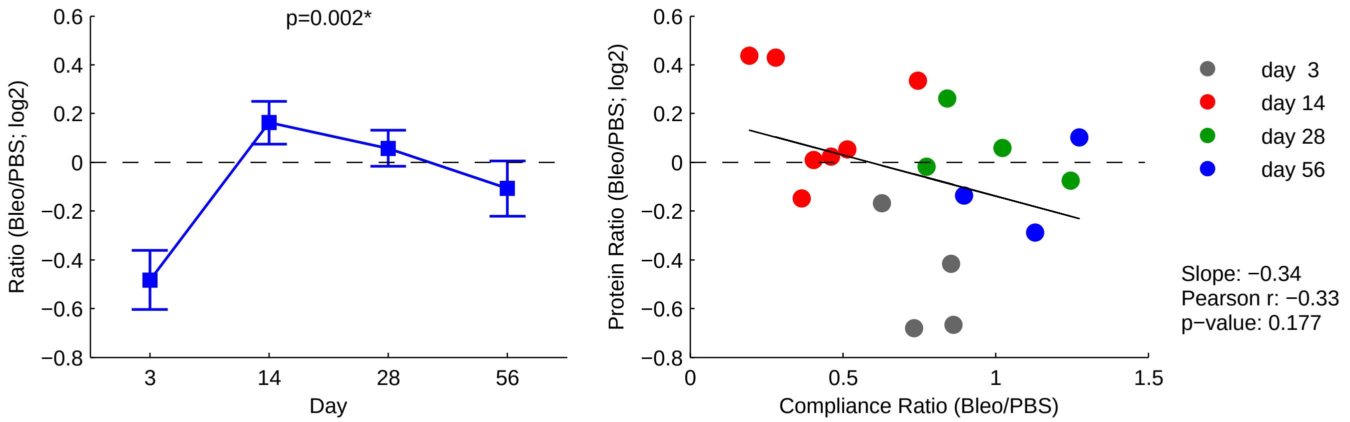

P26516 − Psmd7 (id: 2668)

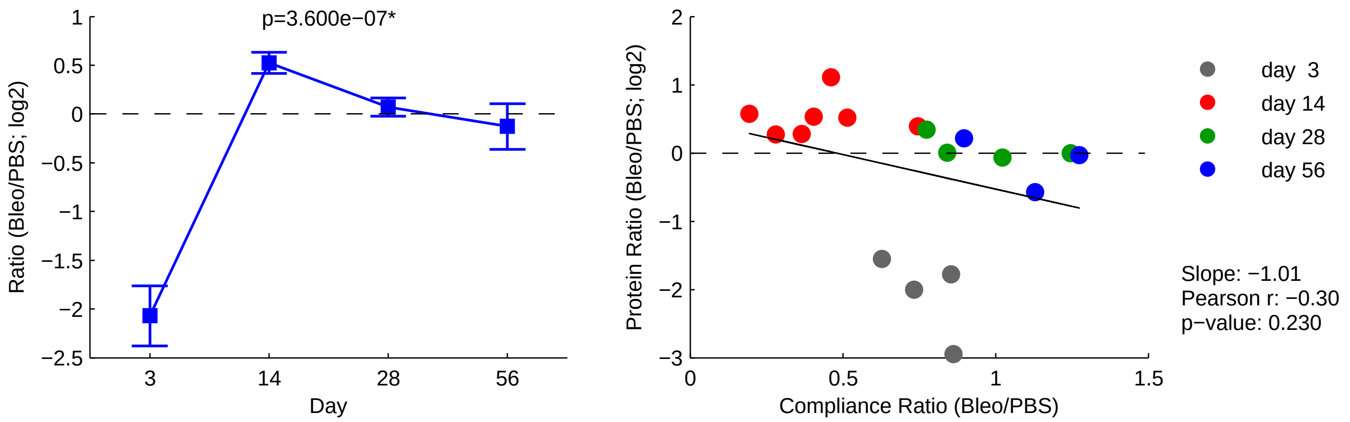

P26638 − Sars (id: 2670)

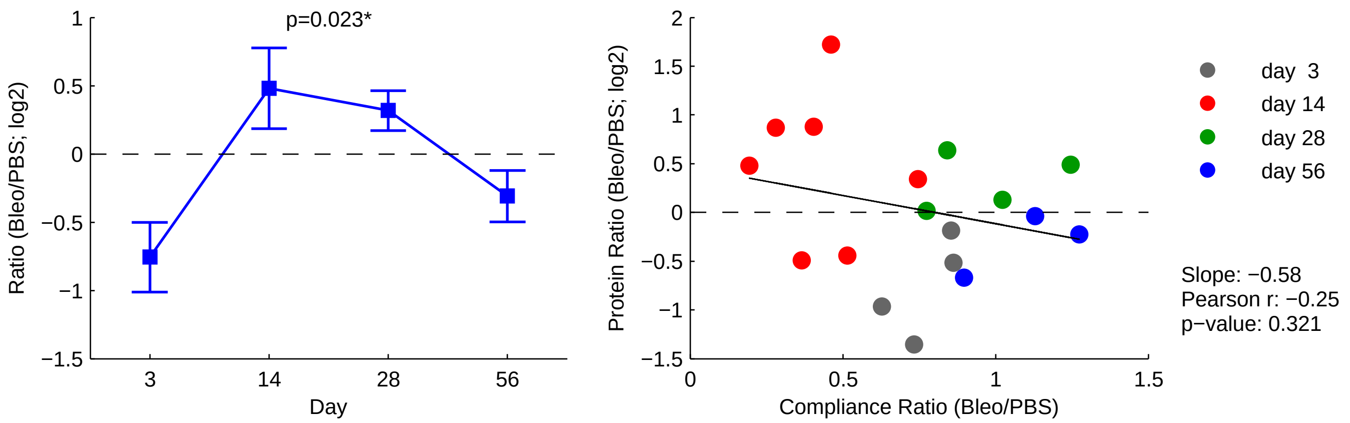

P26645 − Marcks (id: 2671)

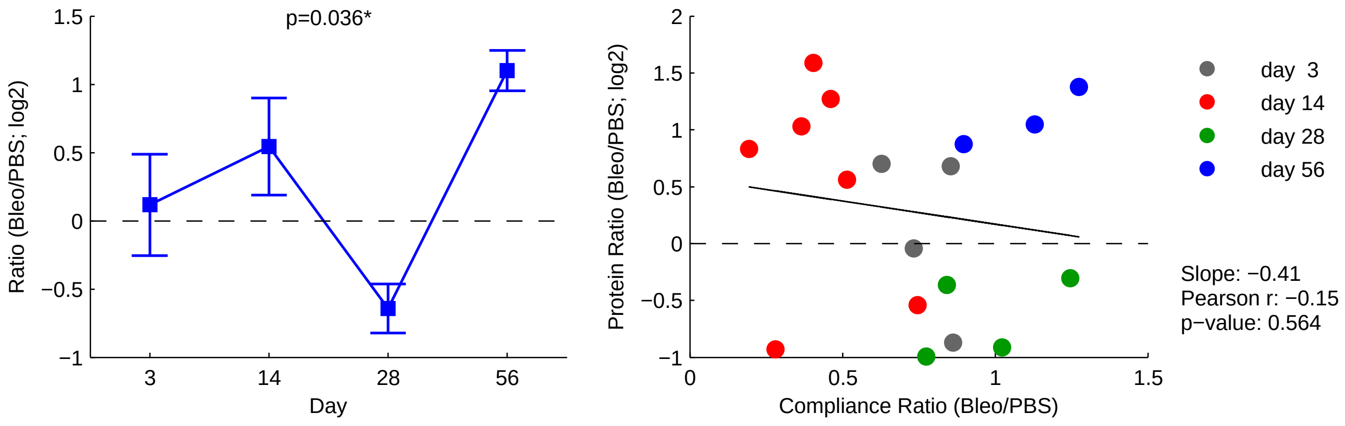

P26883 − Fkbp1a (id: 2672)

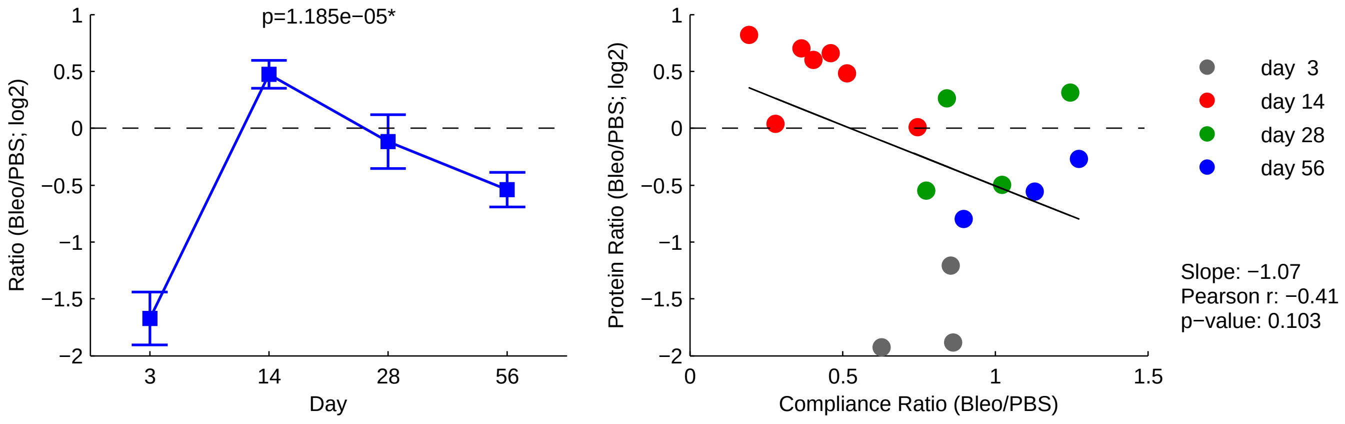

P27005 − S100a8 (id: 2674)

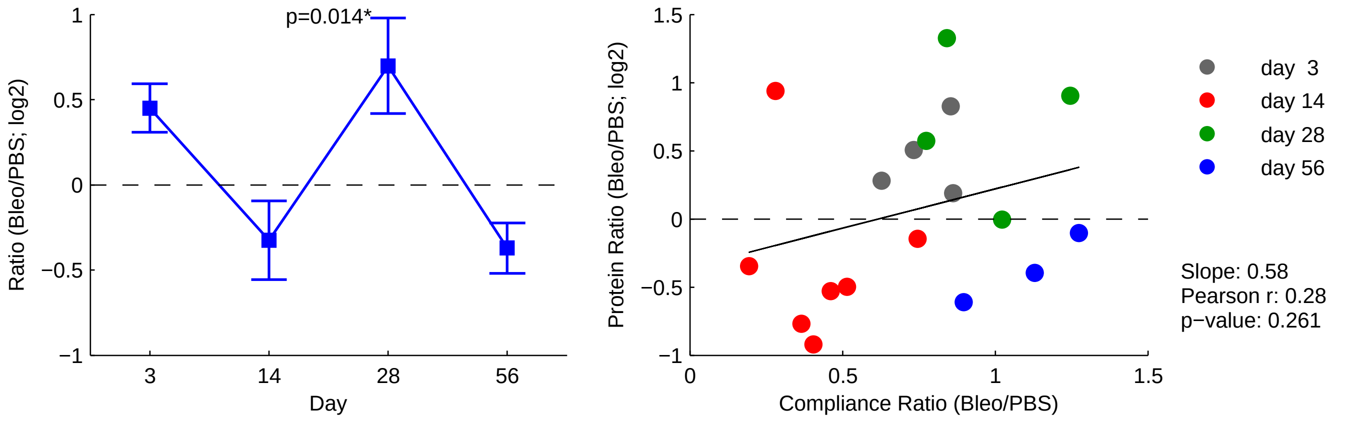

P27046 − Man2a1 (id: 2675)

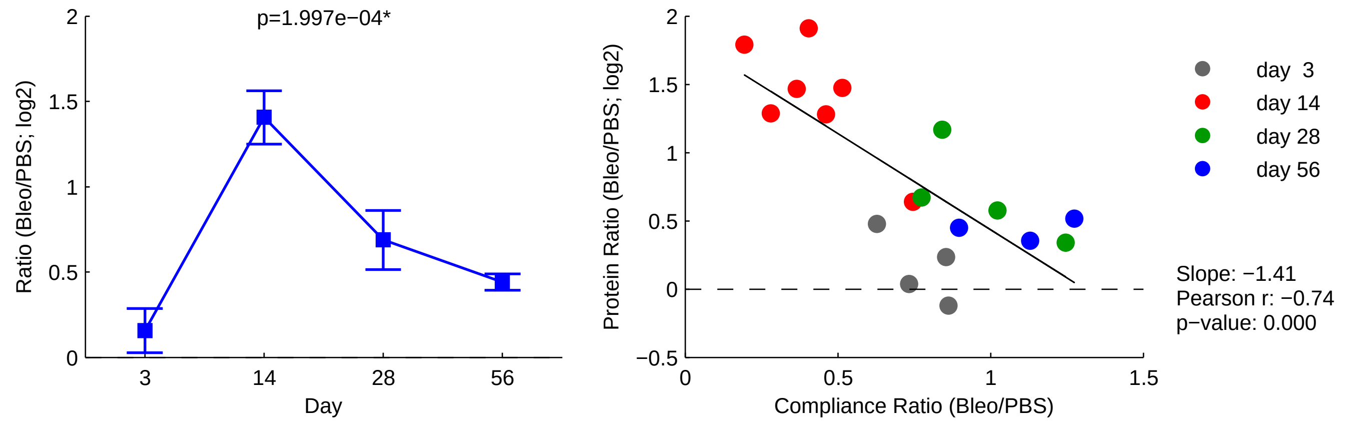

P27048 − Snrpb (id: 2676)

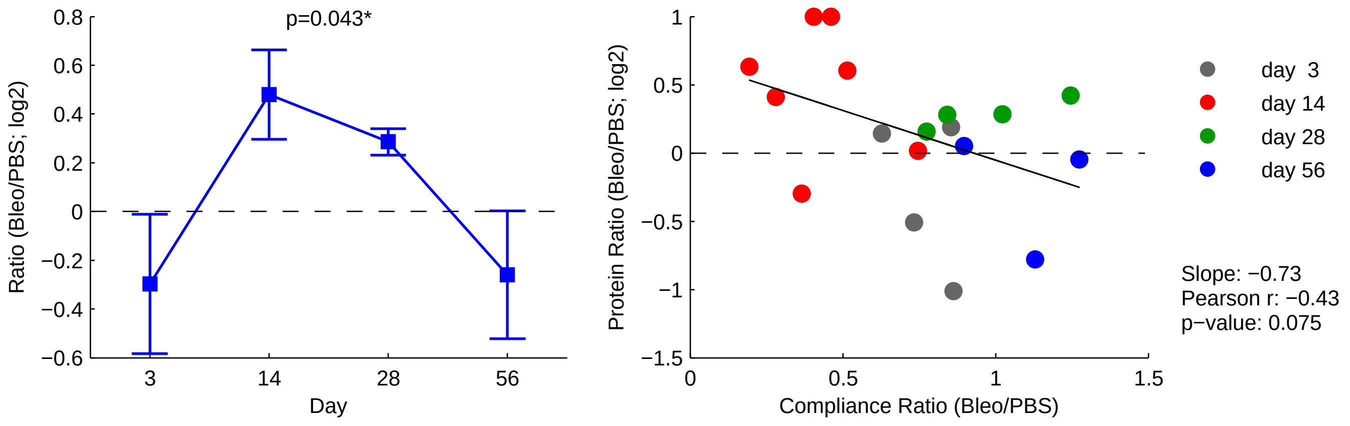

P27512 − Cd40 (id: 2679)

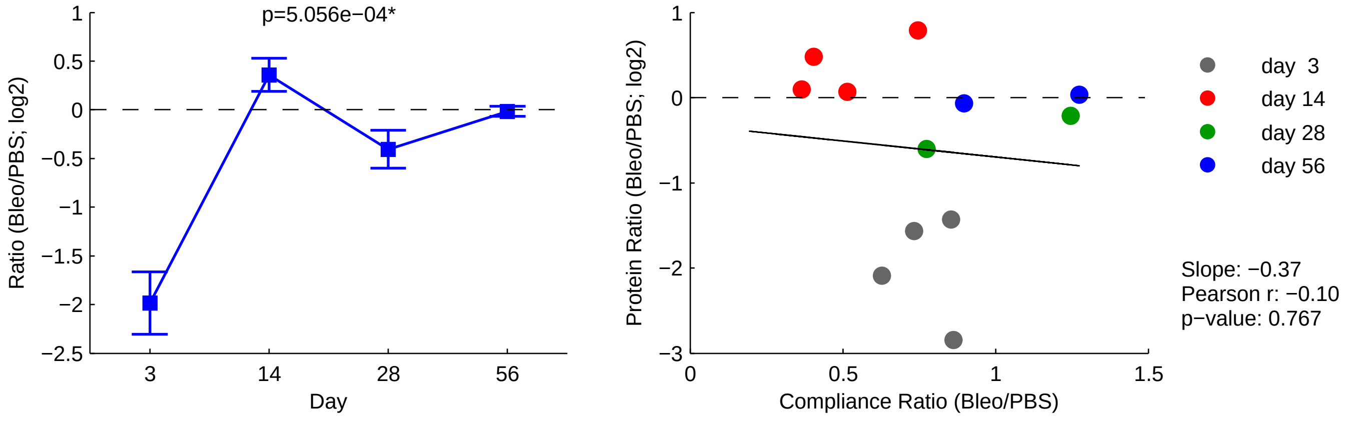

P27546−2 − Map4 (id: 2680)

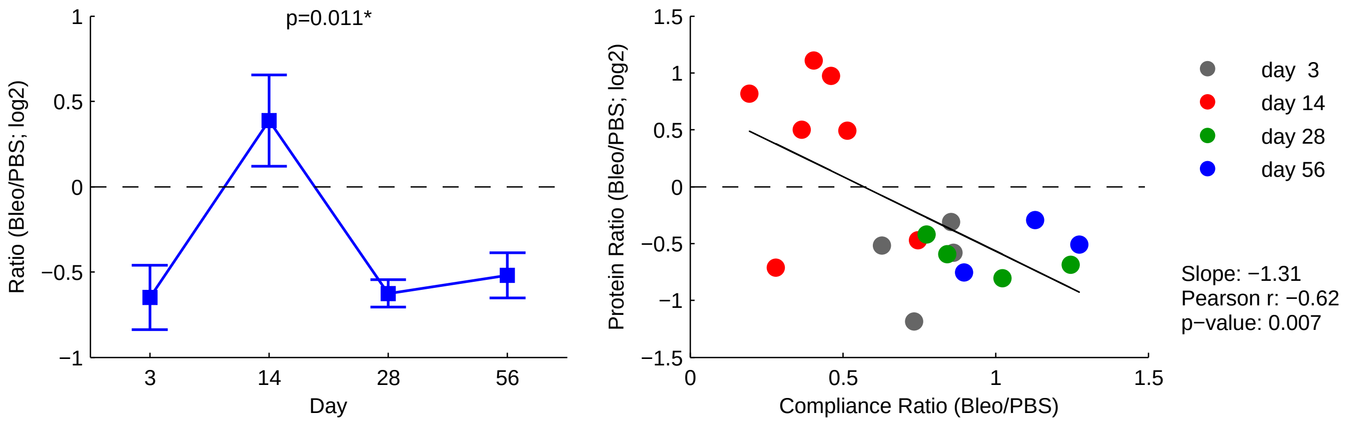

P27601 − Gna13 (id: 2682)

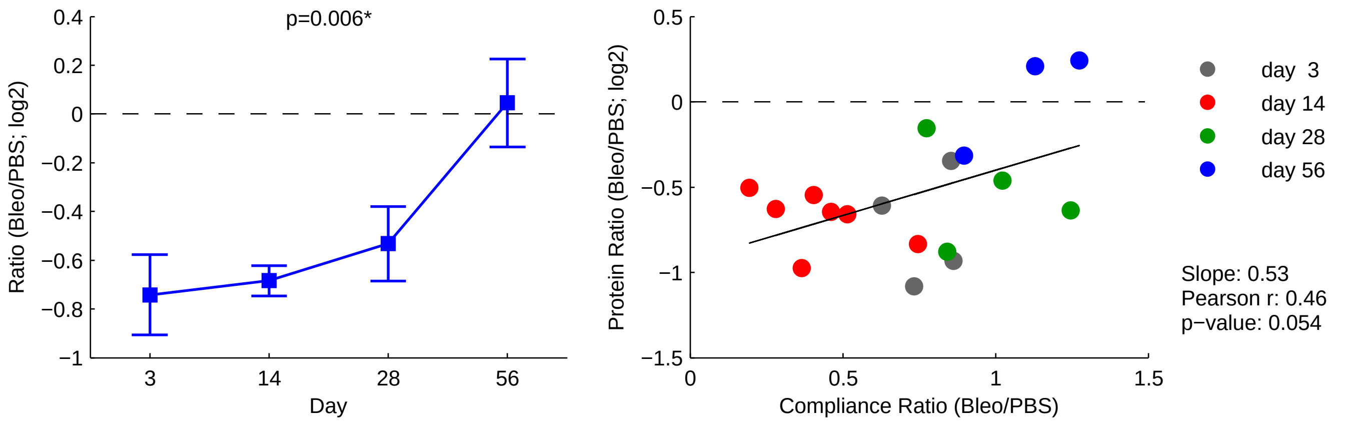

P27612 − Plaa (id: 2683)

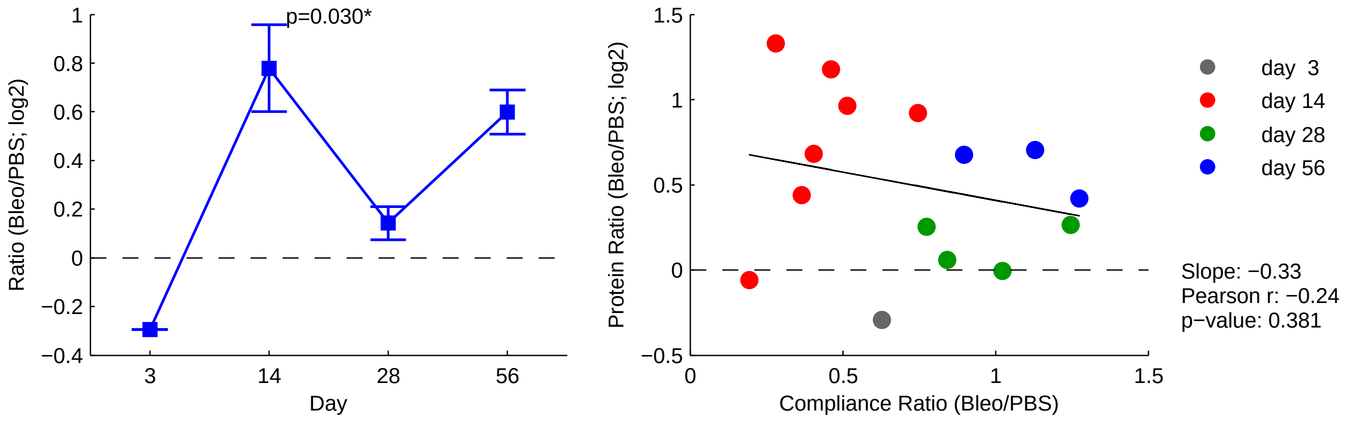

P27659 − Rpl3 (id: 2686)

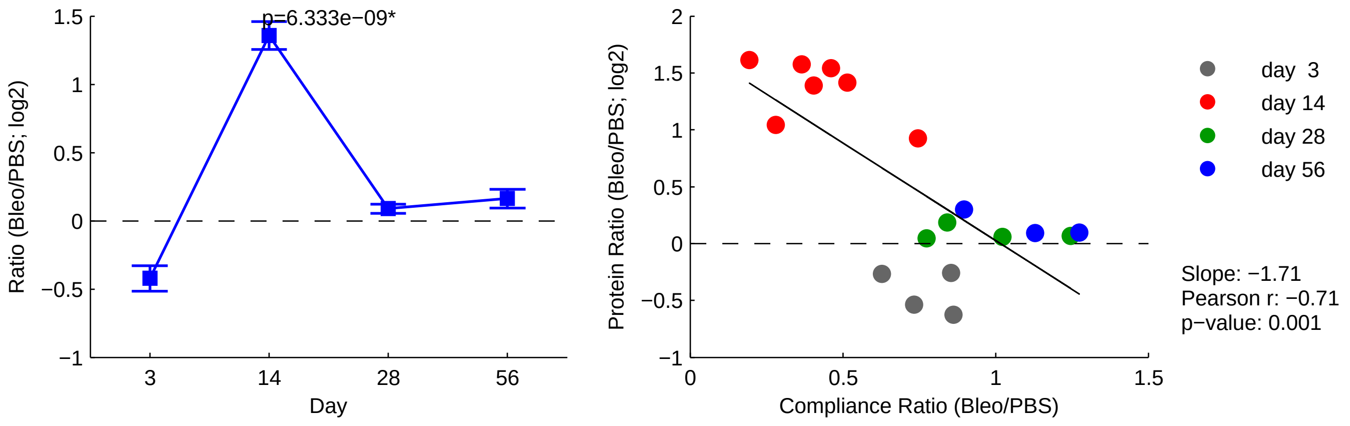

P27661 − H2afx (id: 2687)

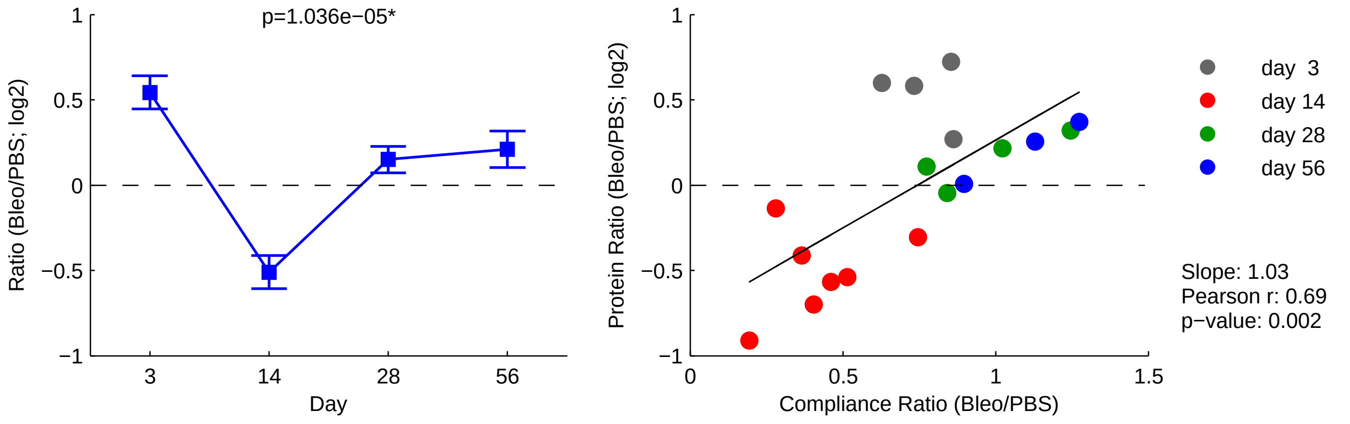

P27773 − Pdia3 (id: 2688)

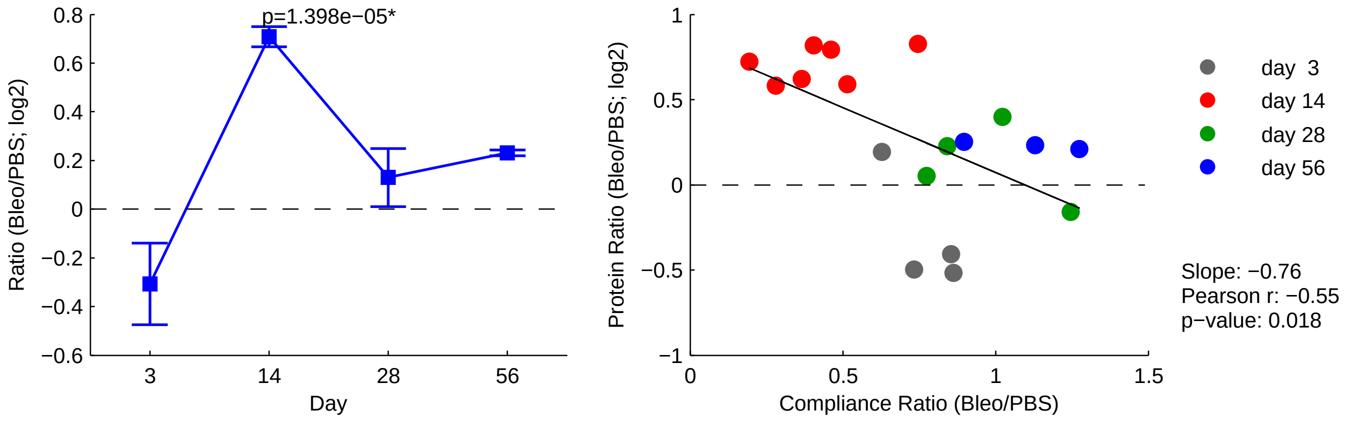

P27808 − Mgat1 (id: 2689)

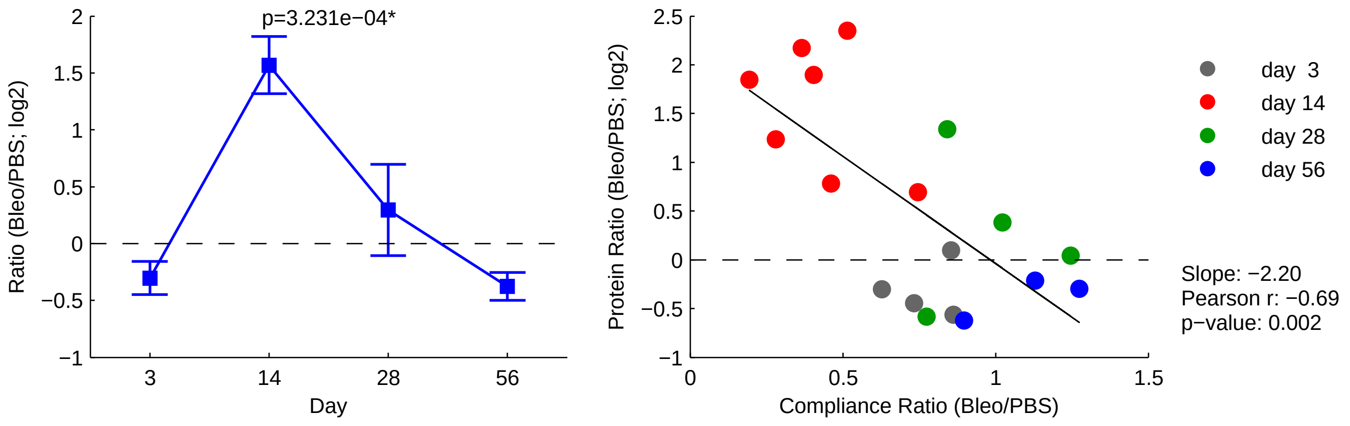

P28033 − Cebpb (id: 2691)

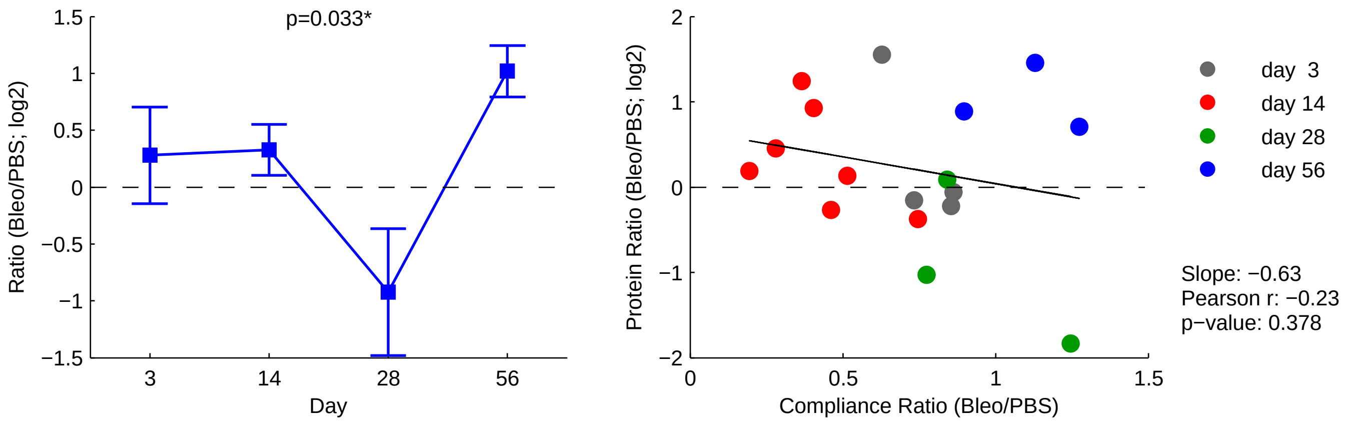

P28063 − Psmb8 (id: 2692)

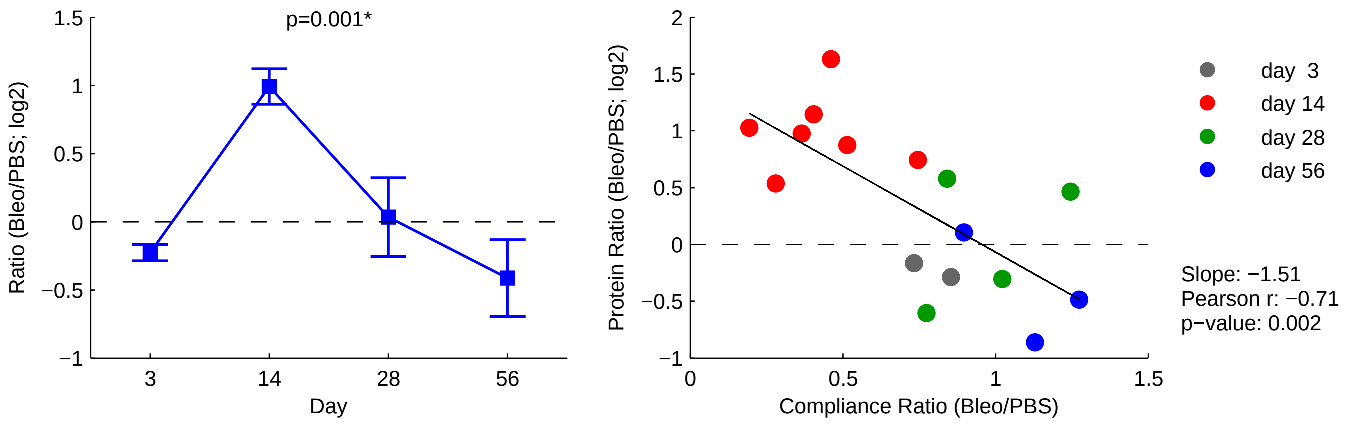

P28271 − Aco1 (id: 2693)

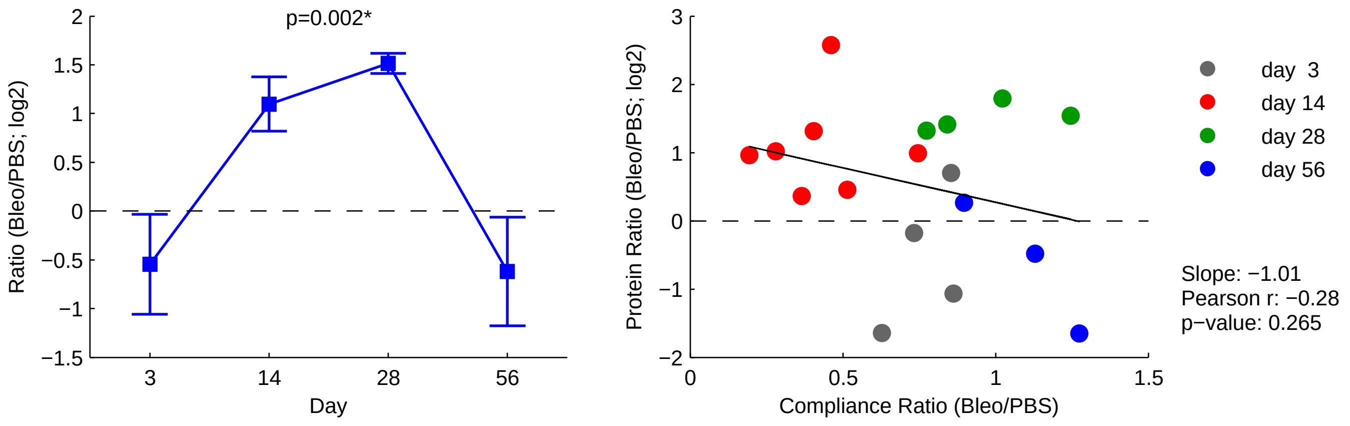

P28293 − Ctsg (id: 2694)

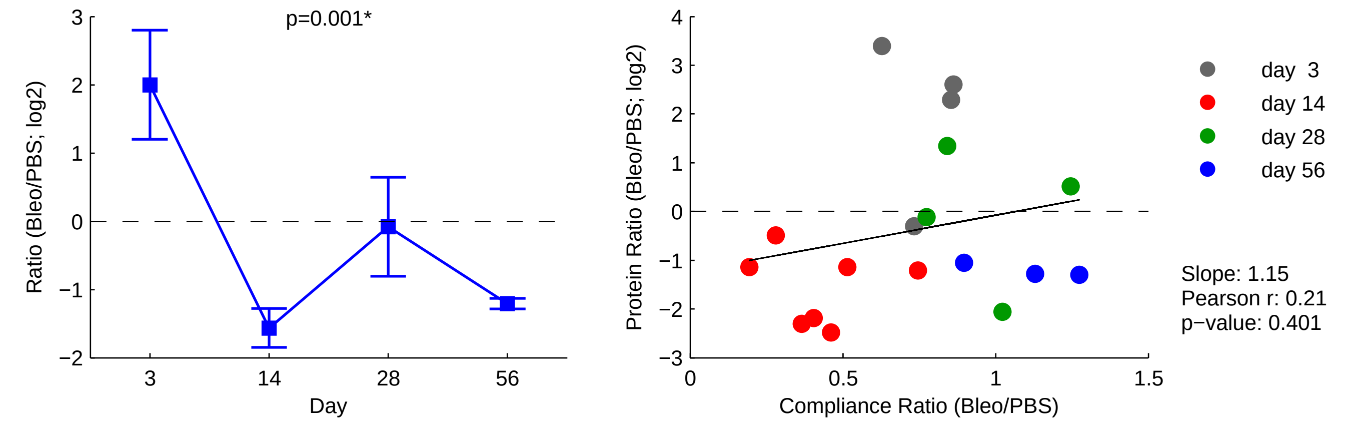

P28352 − Apex1 (id: 2696)

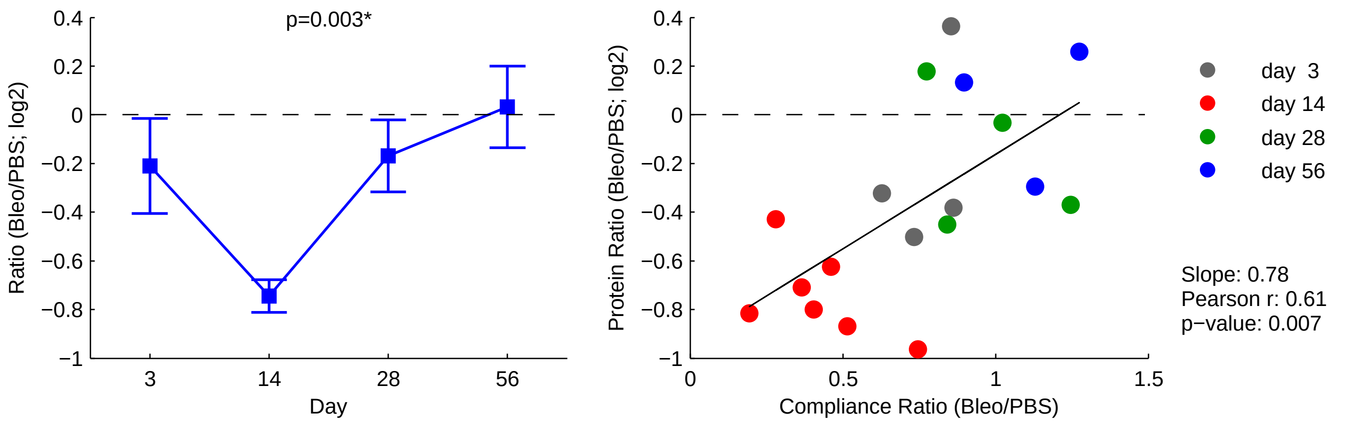

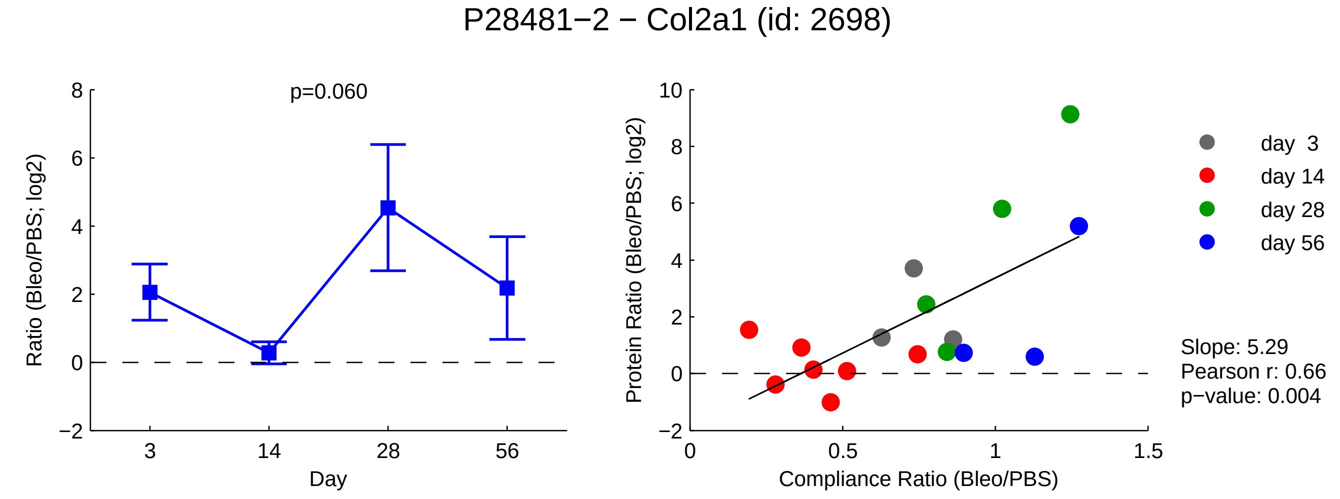

P28481−2 − Col2a1 (id: 2698)
p=0.060
Ratio (Bleo/PBS; log2)
Day
Protein Ratio (Bleo/PBS; log2)
Compliance Ratio (Bleo/PBS)
day 3
day 14
day 28
day 56
Slope: 5.29
Pearson r: 0.66
p−value: 0.004

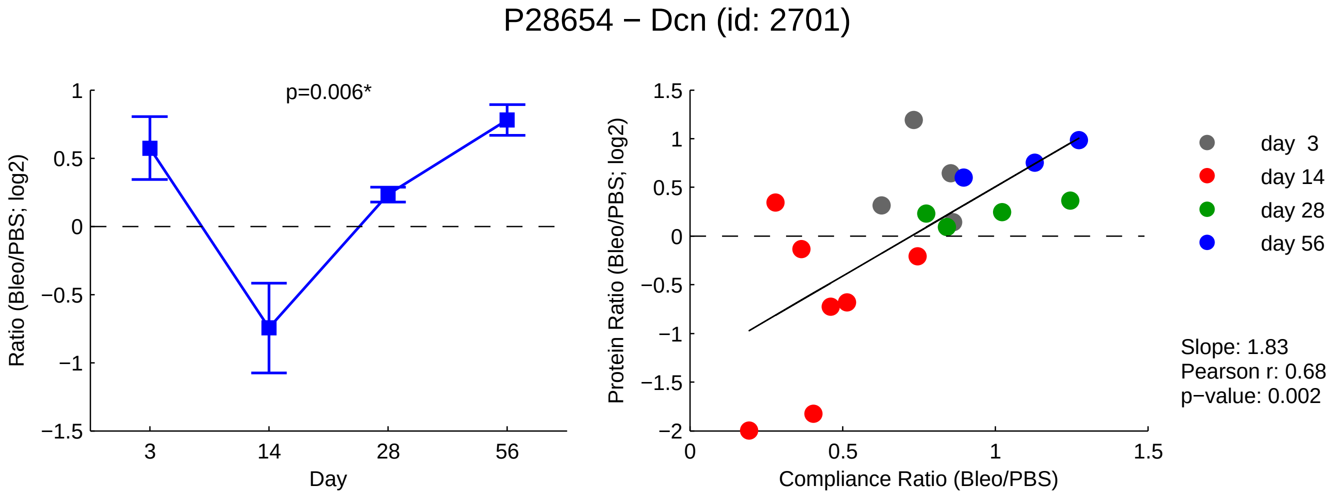

P28654 − Dcn (id: 2701)
p=0.006*
Ratio (Bleo/PBS; log2)
Day
Protein Ratio (Bleo/PBS; log2)
Compliance Ratio (Bleo/PBS)
day 3
day 14
day 28
day 56
Slope: 1.83
Pearson r: 0.68
p−value: 0.002

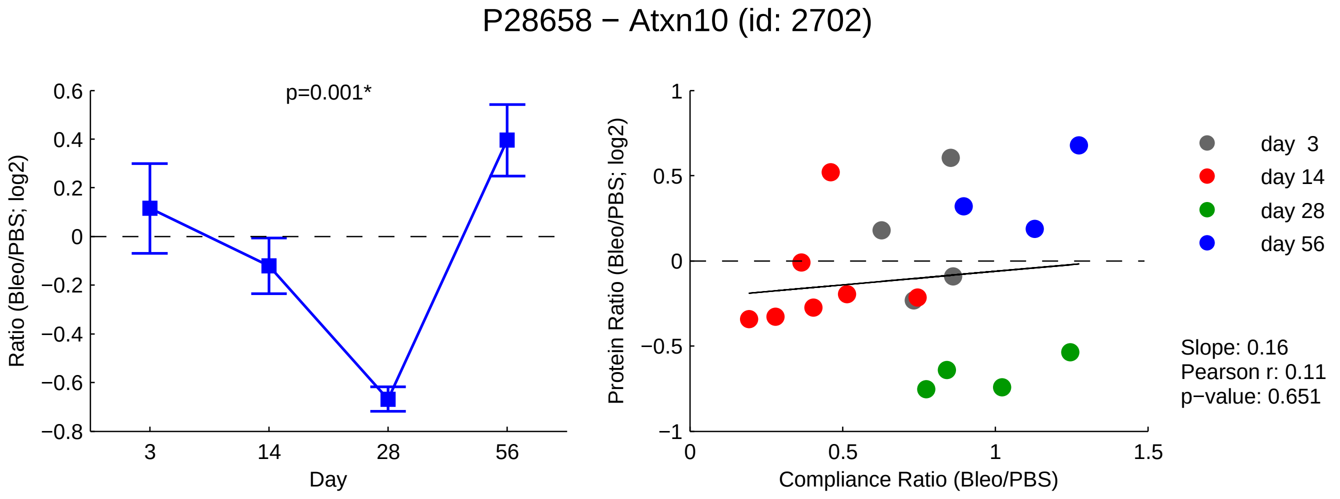

P28658 − Atxn10 (id: 2702)
p=0.001*
Ratio (Bleo/PBS; log2)
Day
Protein Ratio (Bleo/PBS; log2)
Compliance Ratio (Bleo/PBS)
day 3
day 14
day 28
day 56
Slope: 0.16
Pearson r: 0.11
p−value: 0.651

P28667 − Marcksl1 (id: 2706)

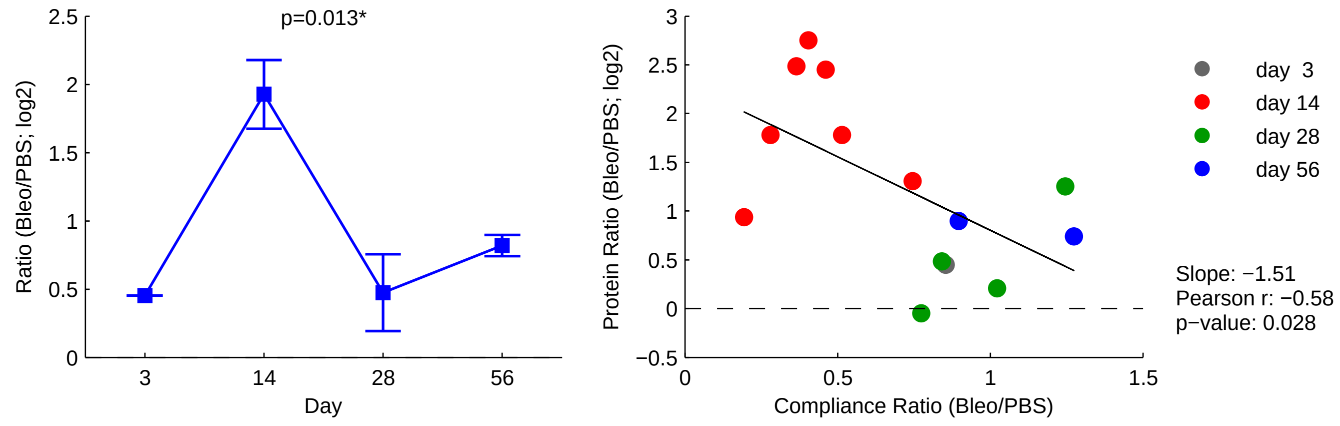

P28843 − Dpp4 (id: 2709)

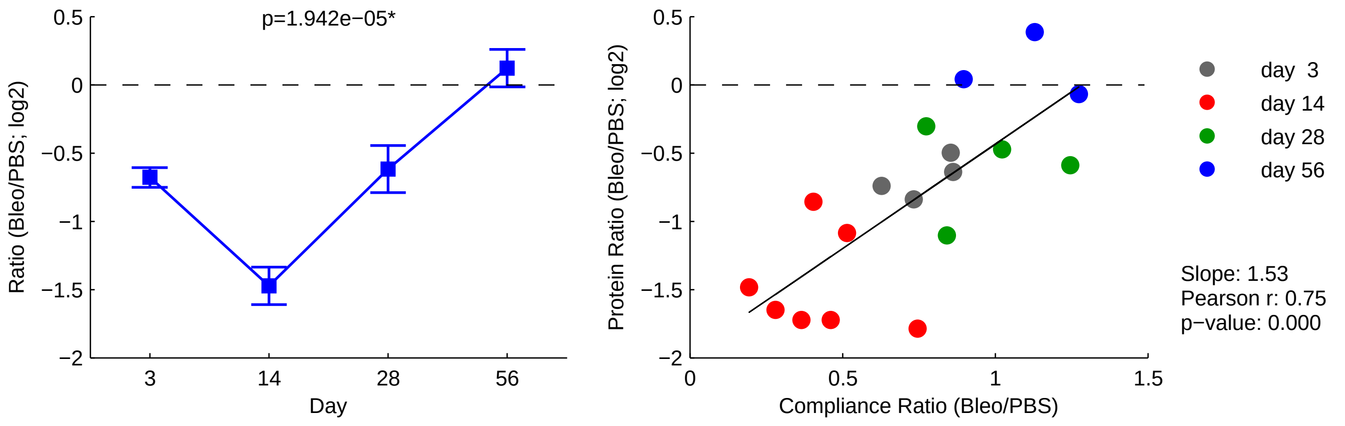

P28862 − Mmp3 (id: 2710)

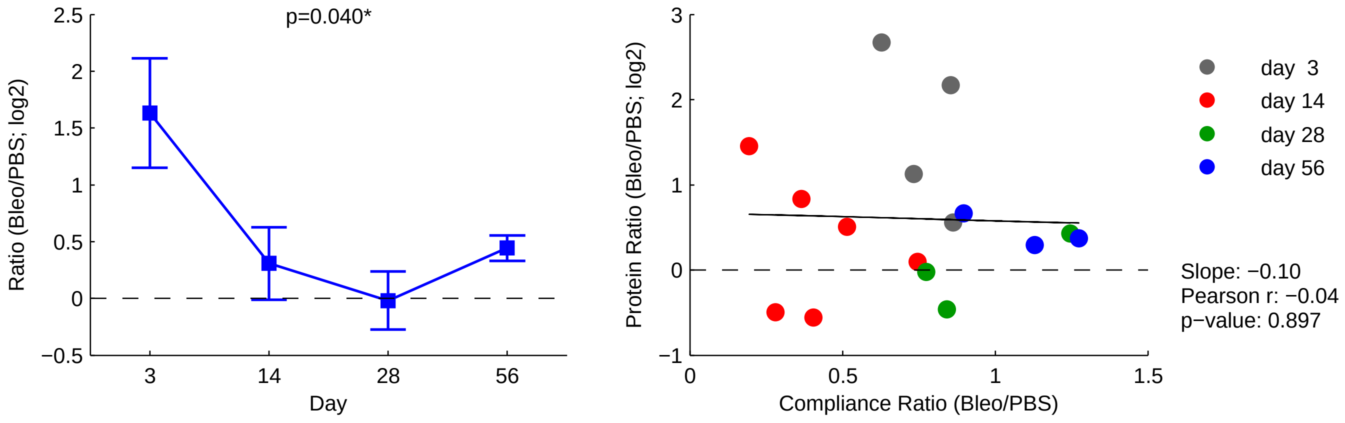

P28867 − Prkcd (id: 2711)

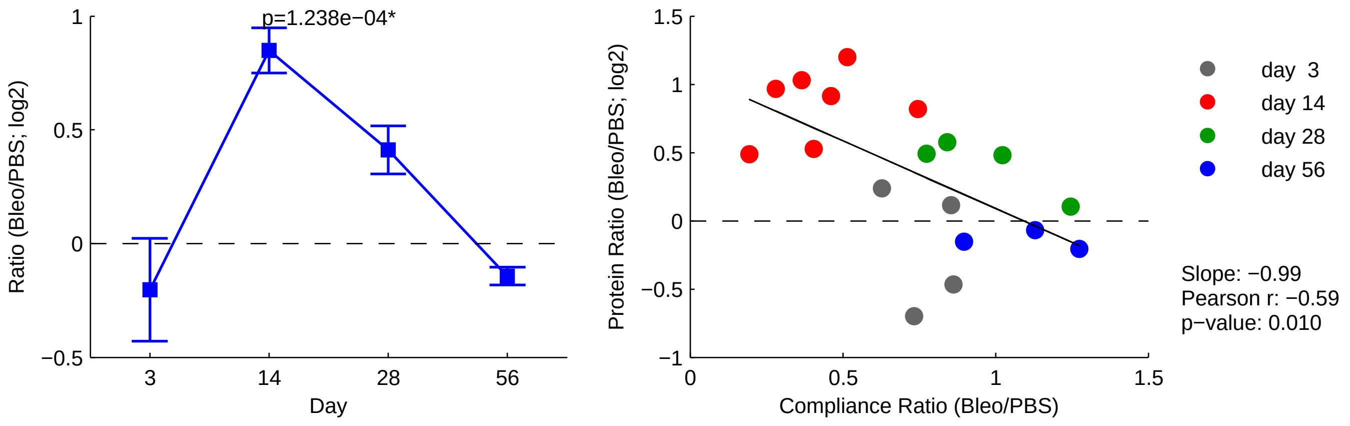

P29341 − Pabpc1 (id: 2713)

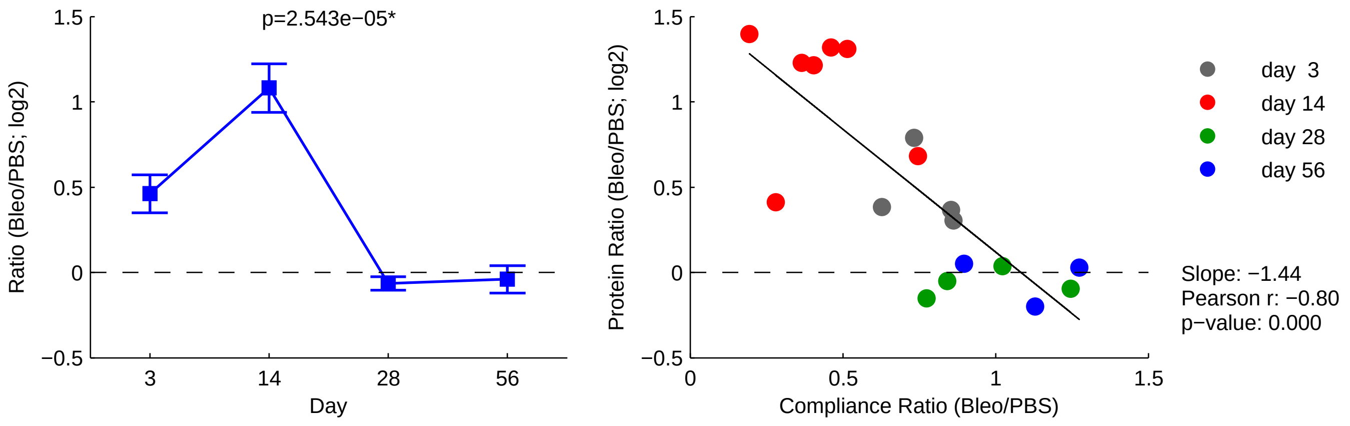

P29416 − Hexa (id: 2716)

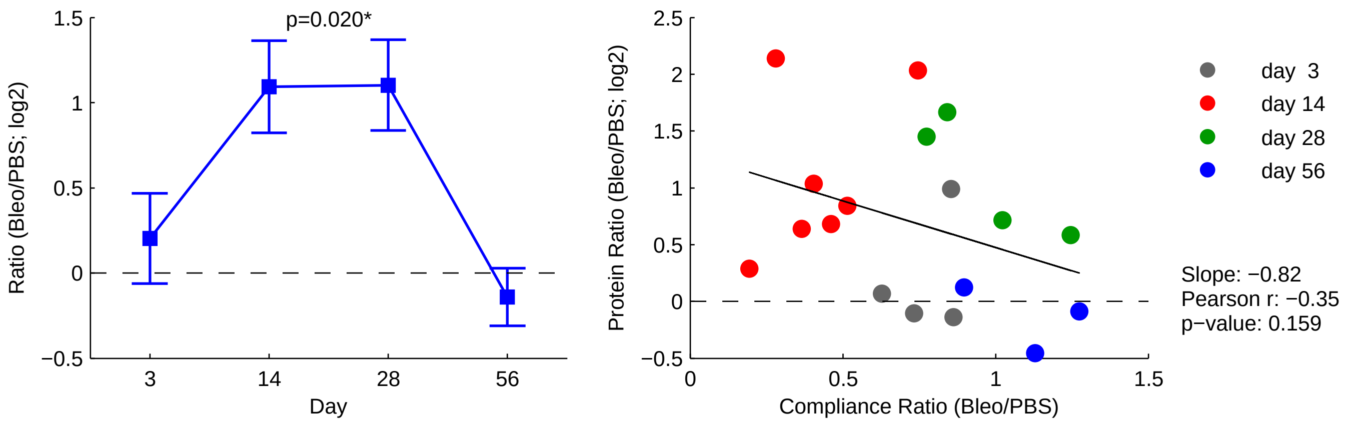

Q3UPN1 − Vcam1 (id: 2718)

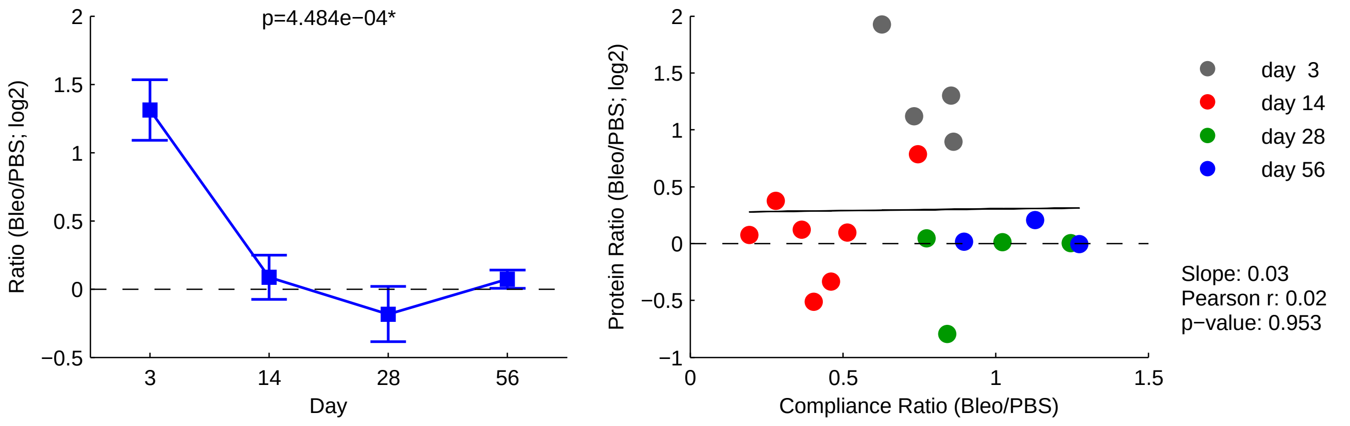

P29595 − Nedd8 (id: 2719)

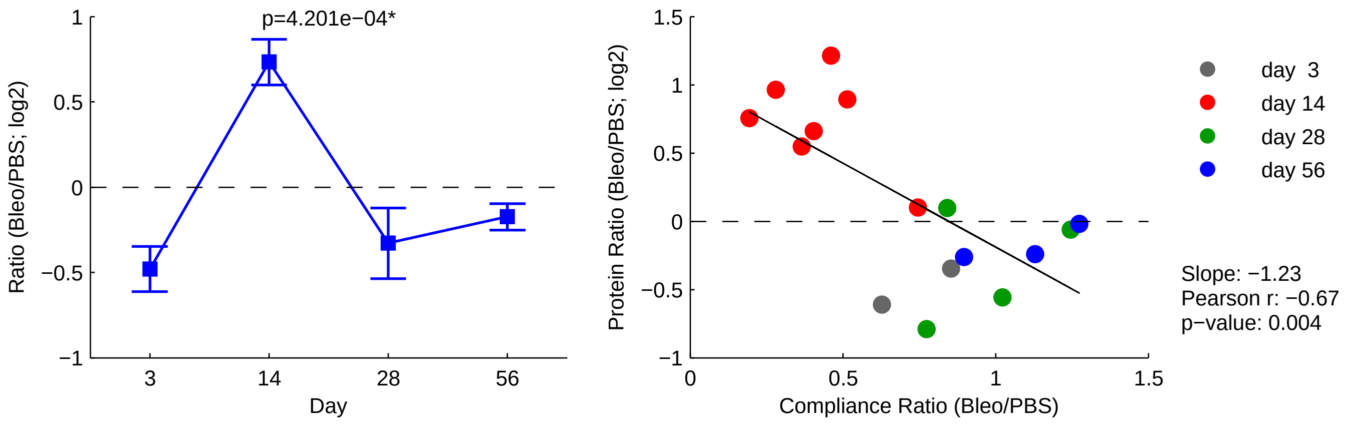

P29758 − Oat (id: 2721)

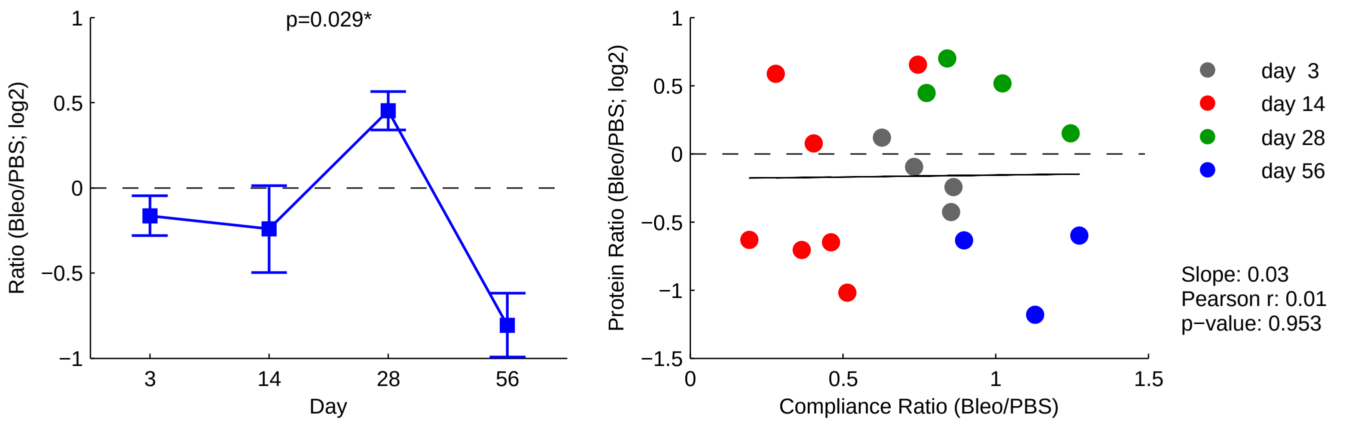

P30115 − Gsta3 (id: 2724)

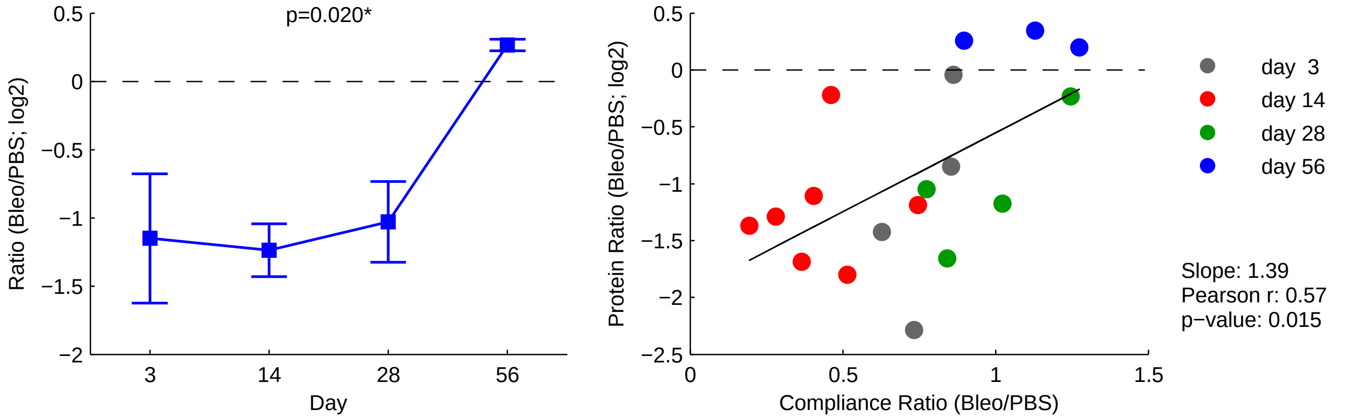

P30275 − Ckmt1 (id: 2726)

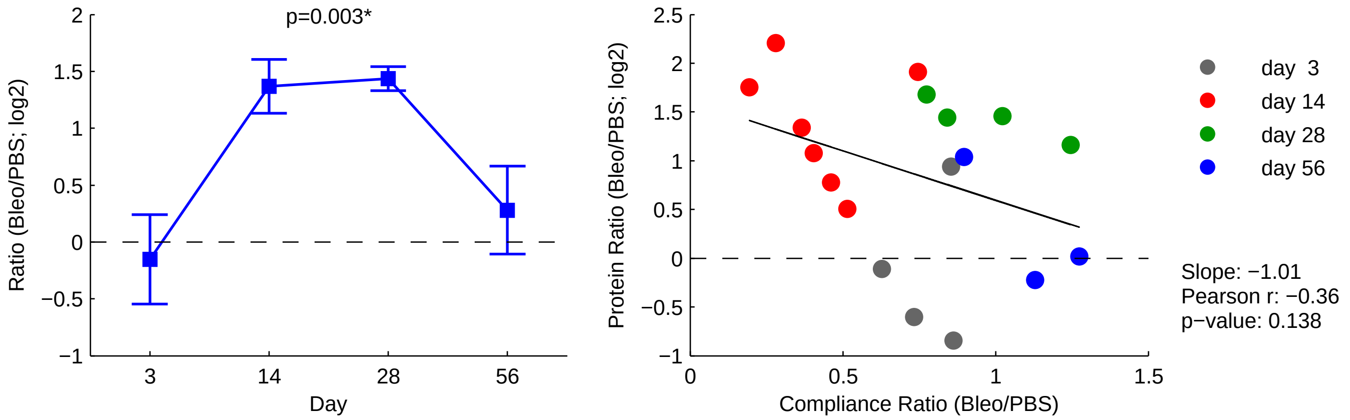

P30412 − Ppic (id: 2729)

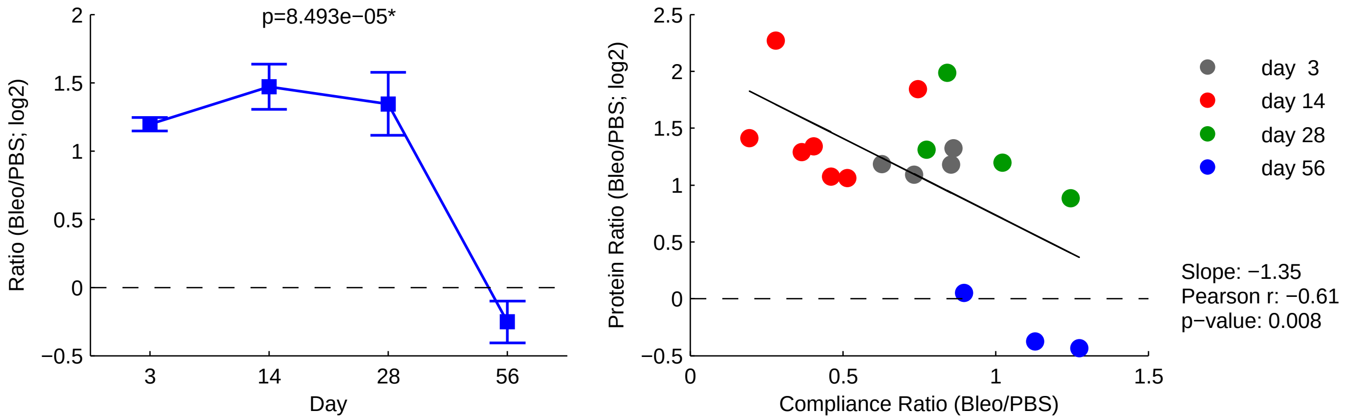

P30677 − Gna14 (id: 2732)

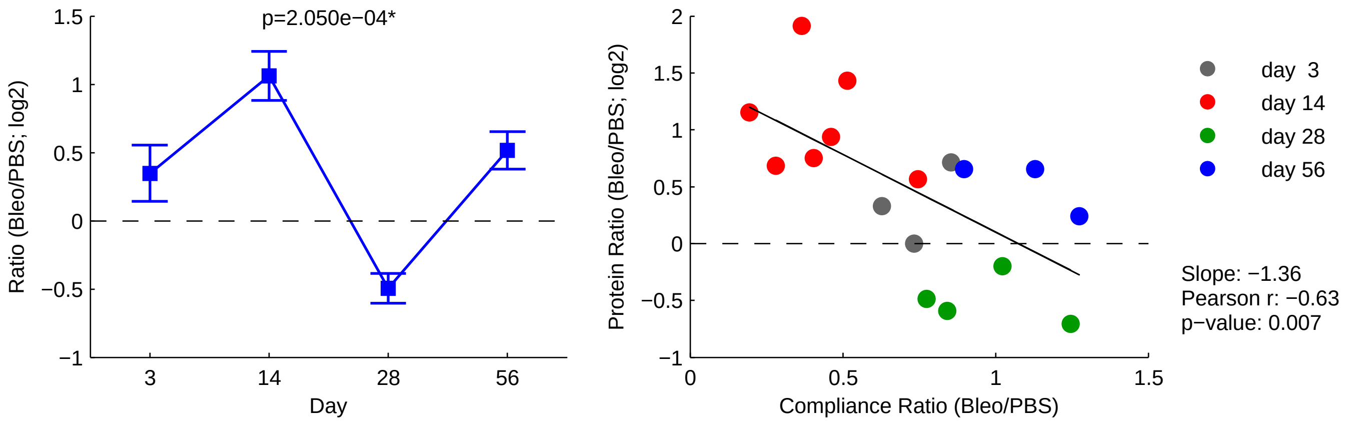

P30681 − Hmgb2 (id: 2733)

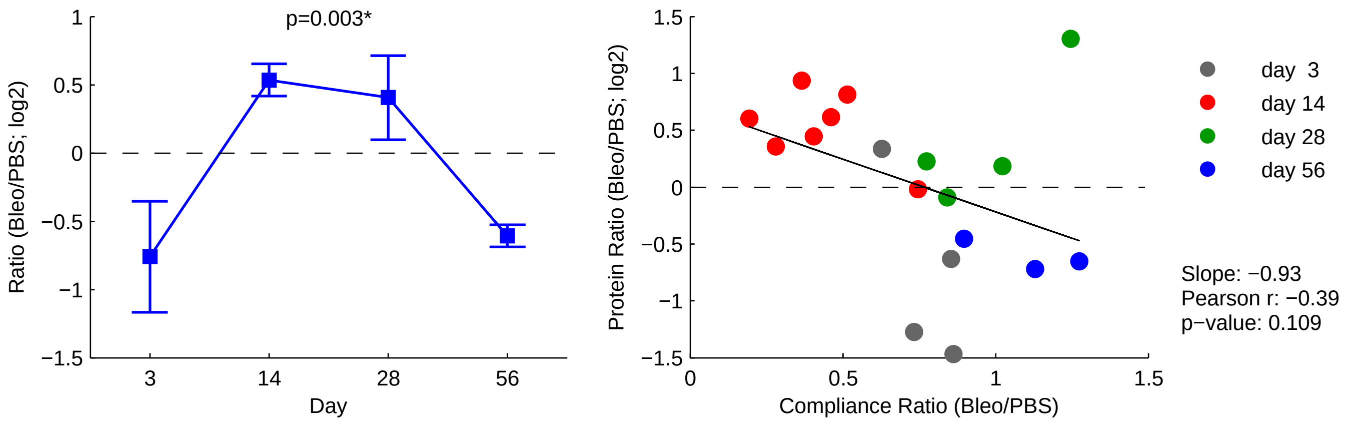

P30999−2 − Ctnnd1 (id: 2735)

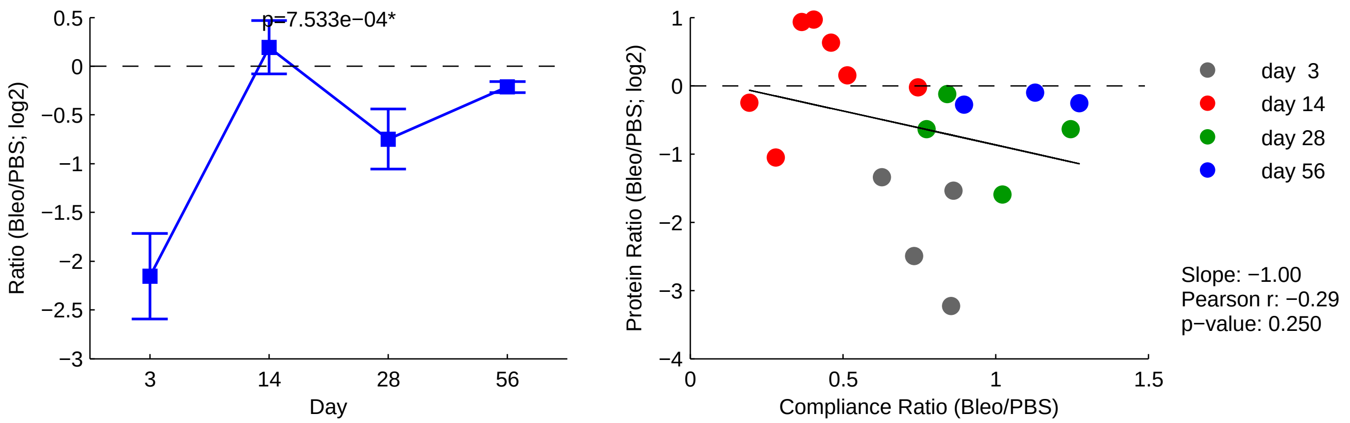

P31230 − Aimp1 (id: 2737)

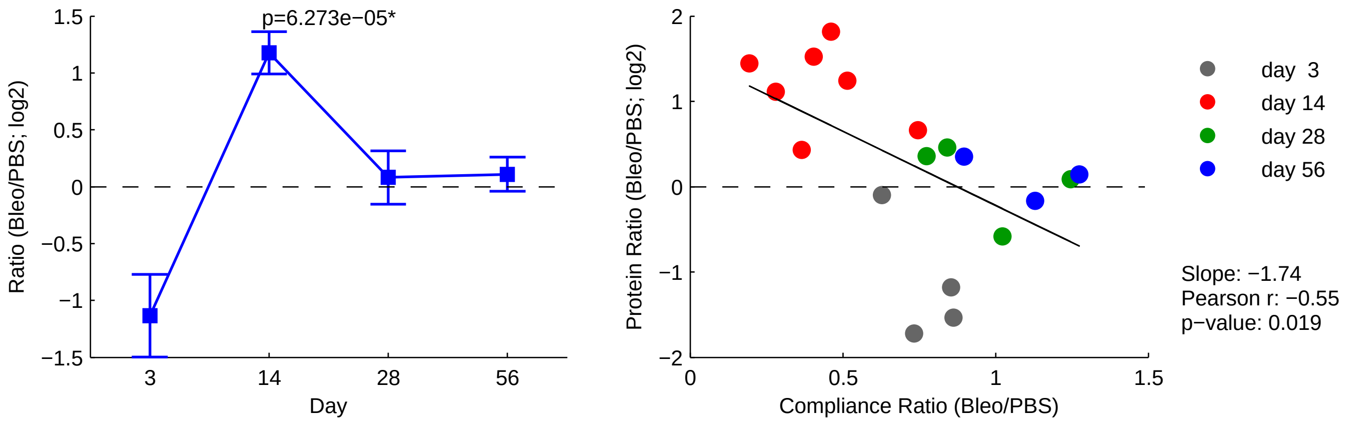

P31324 − Prkar2b (id: 2738)

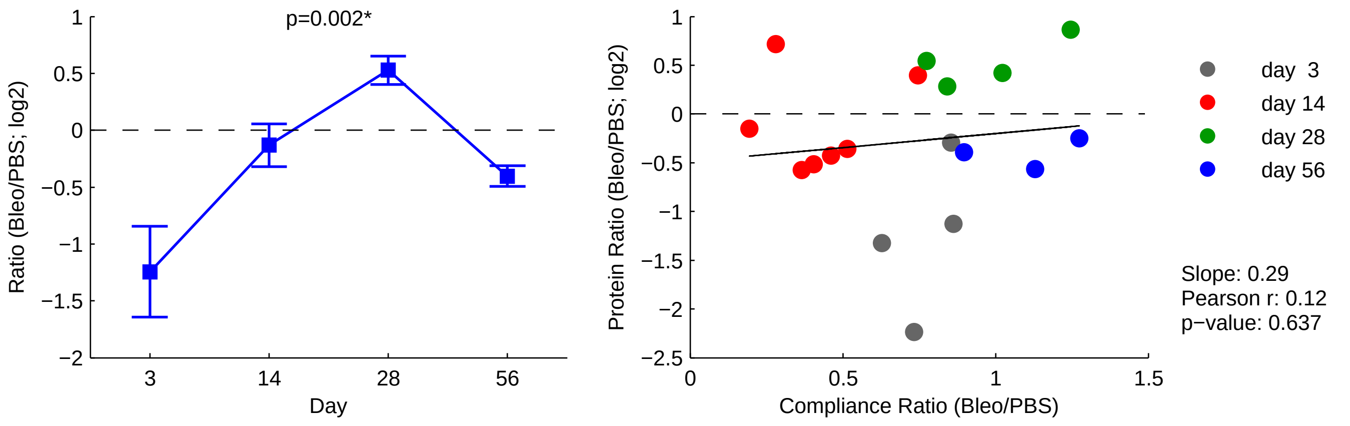

P31428 − Dpep1 (id: 2739)

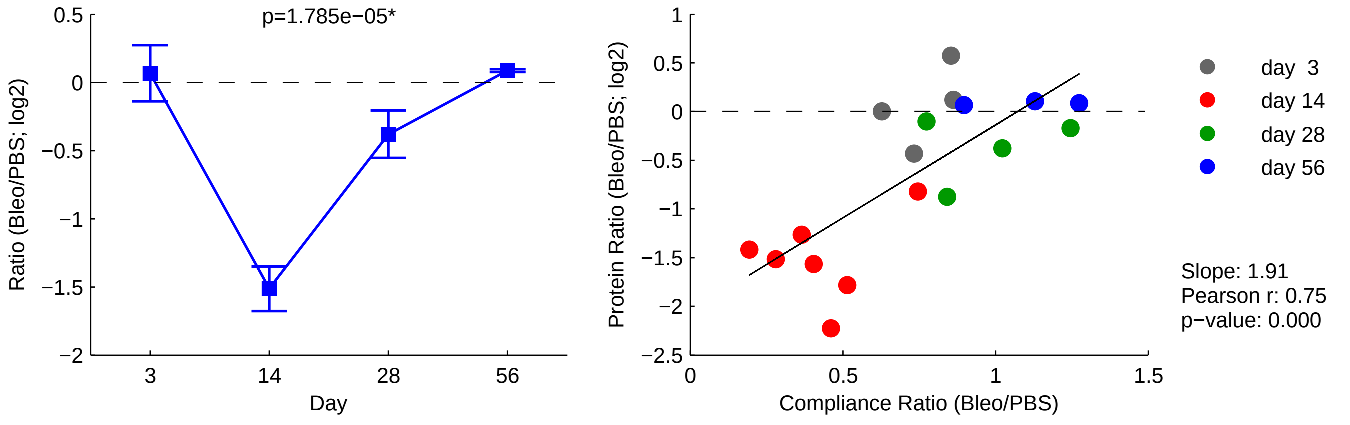

P31786 − Dbi (id: 2742)

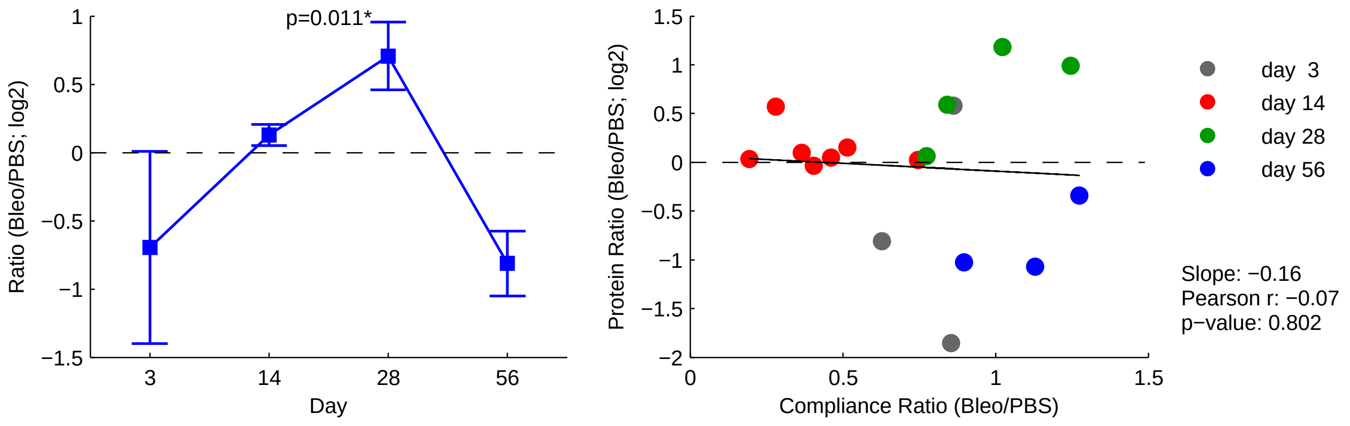

P31938 − Map2k1 (id: 2745)

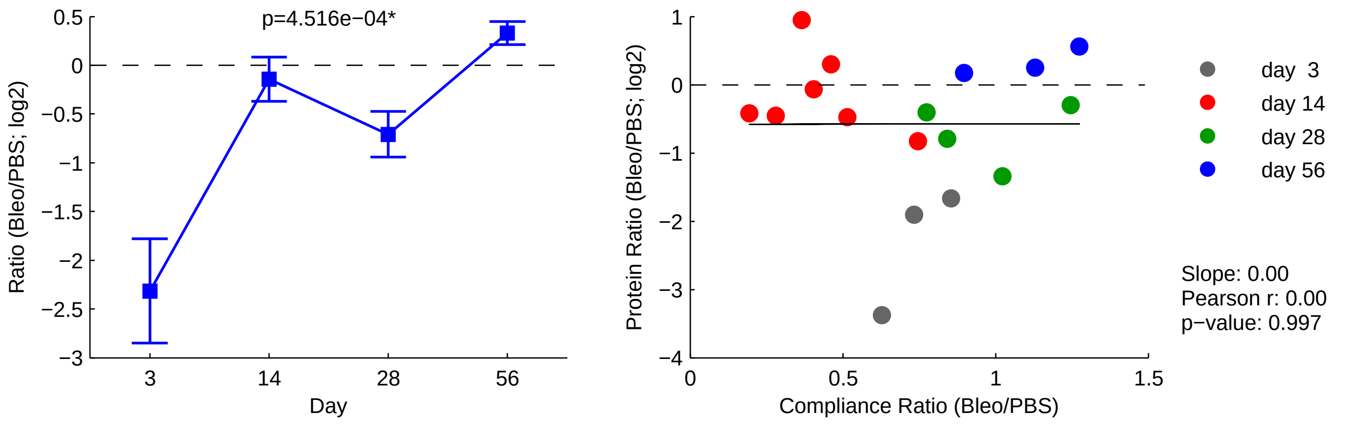

P32020 − Scp2 (id: 2746)

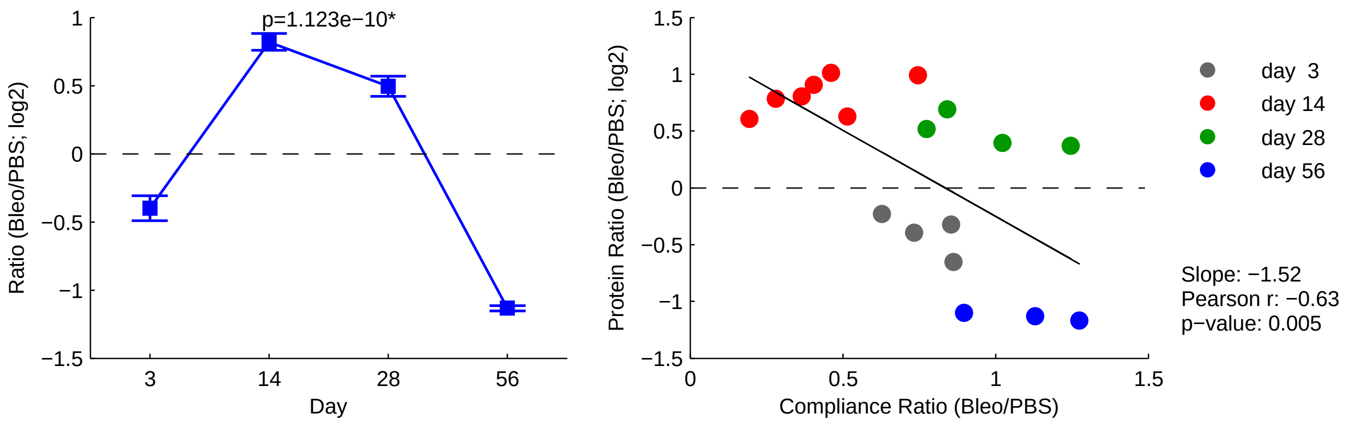

P32067 − Ssb (id: 2748)

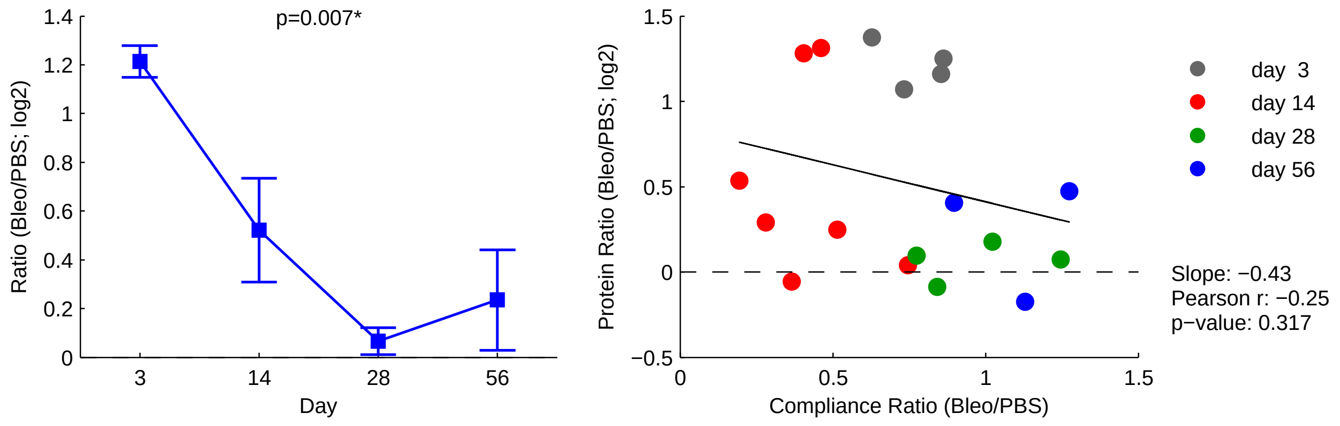

P32261 − Serpinc1 (id: 2750)

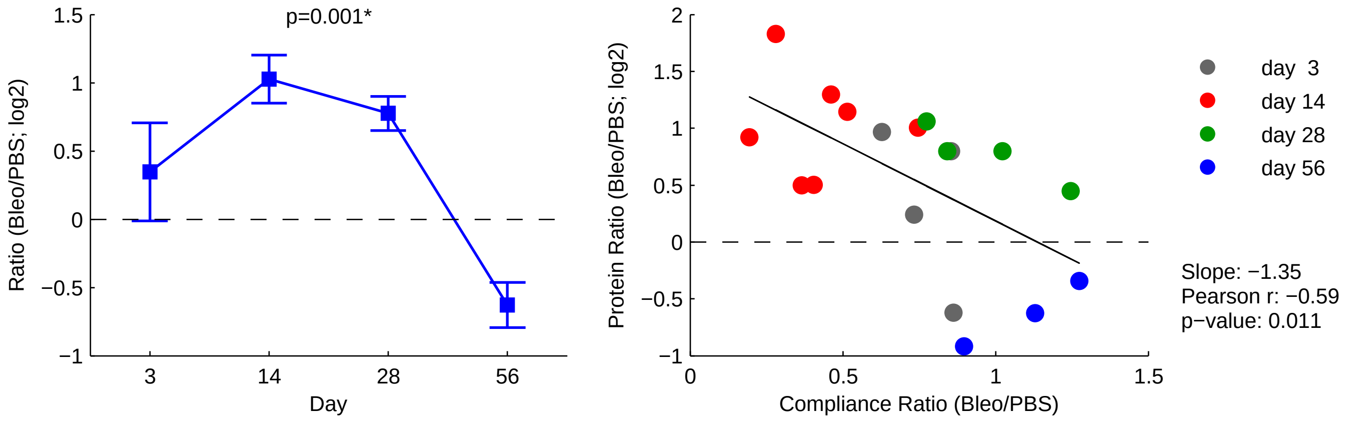

P32921−2 − Wars (id: 2754)

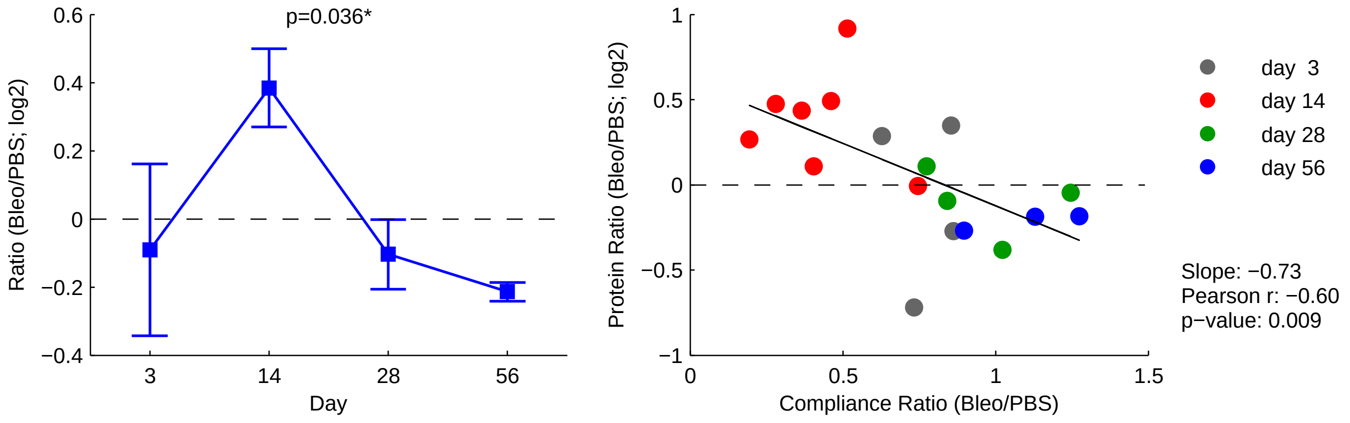

P33267 − Cyp2f2 (id: 2757)

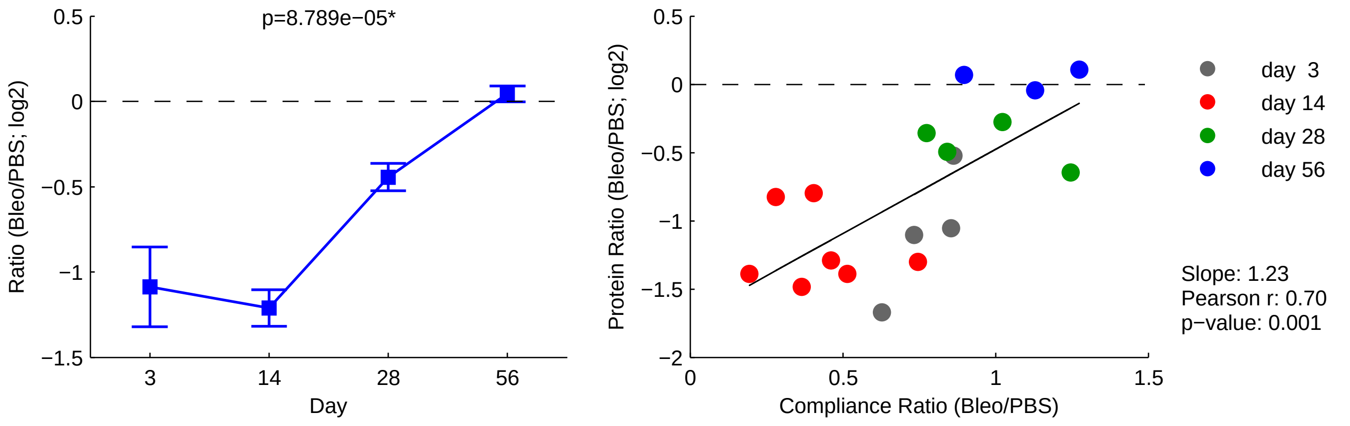

P34022 − Ranbp1 (id: 2760)

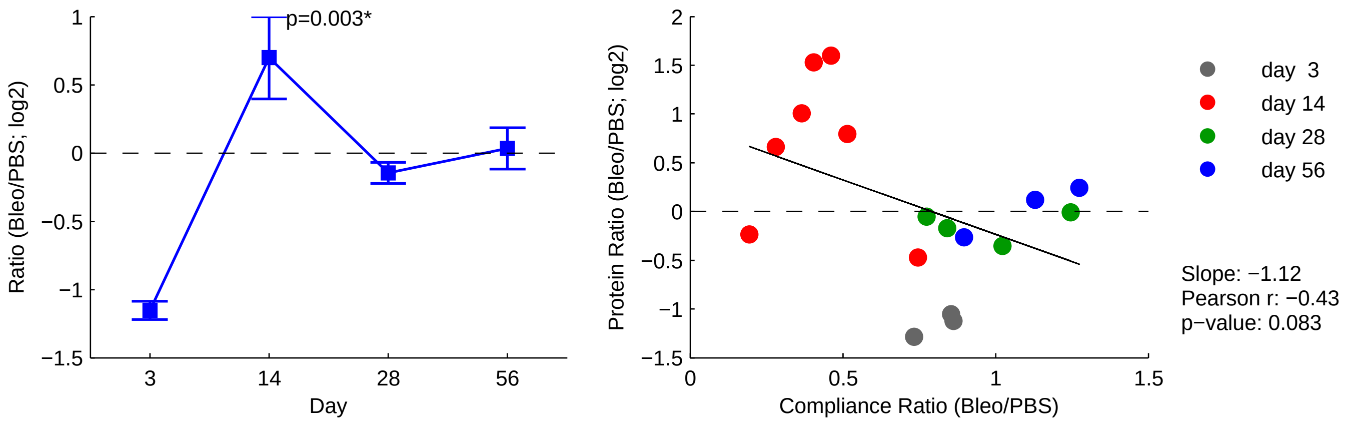

P34152−3 − Ptk2 (id: 2762)

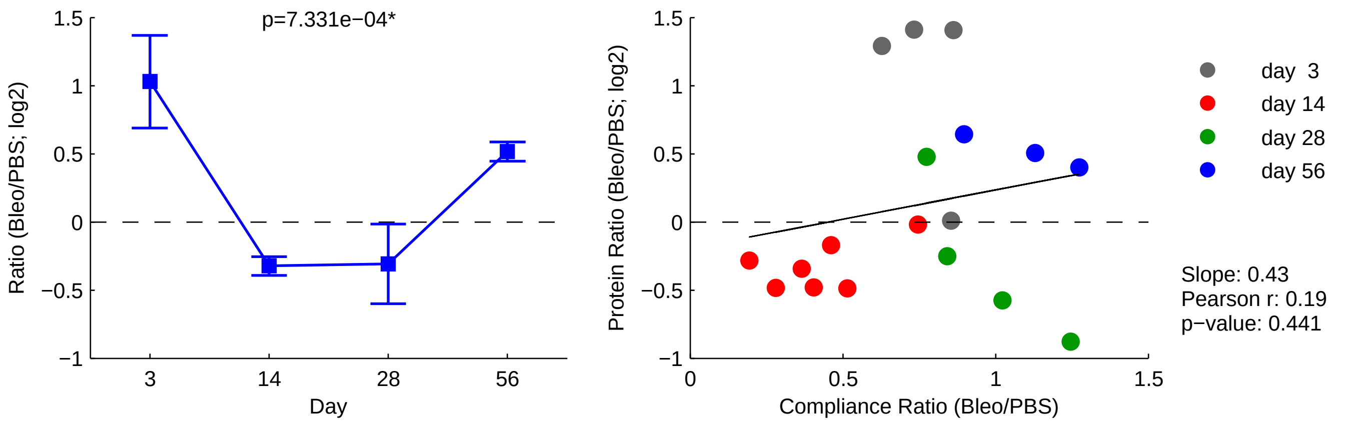

P34914 − Ephx2 (id: 2764)

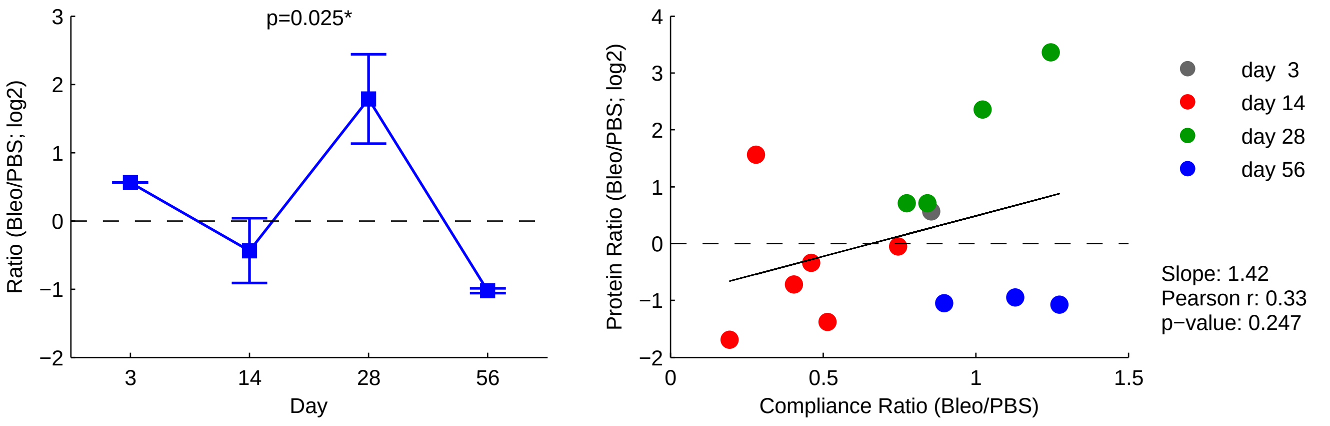

P34928 − Apoc1 (id: 2765)

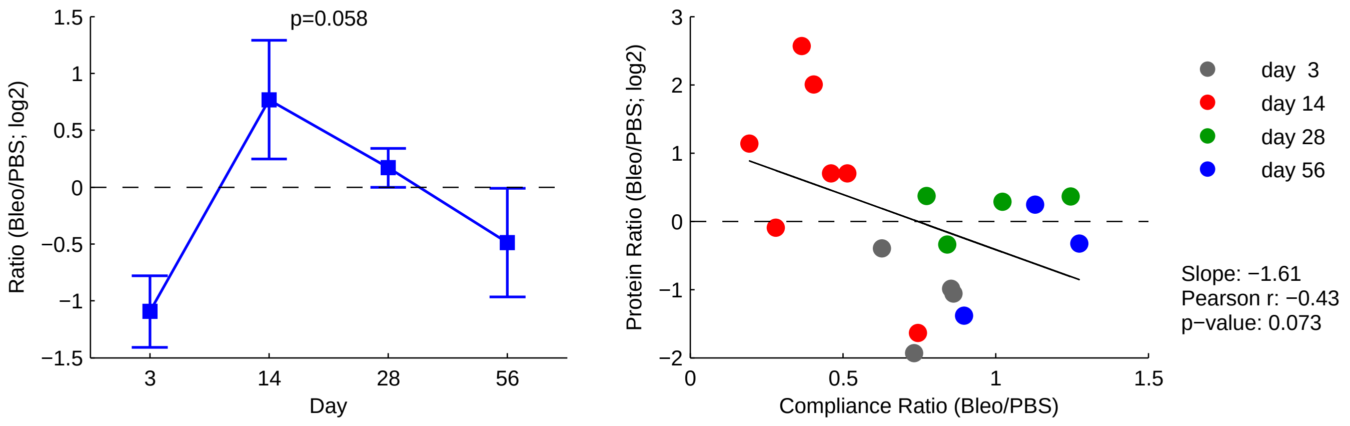

P35235 − Ptpn11 (id: 2768)

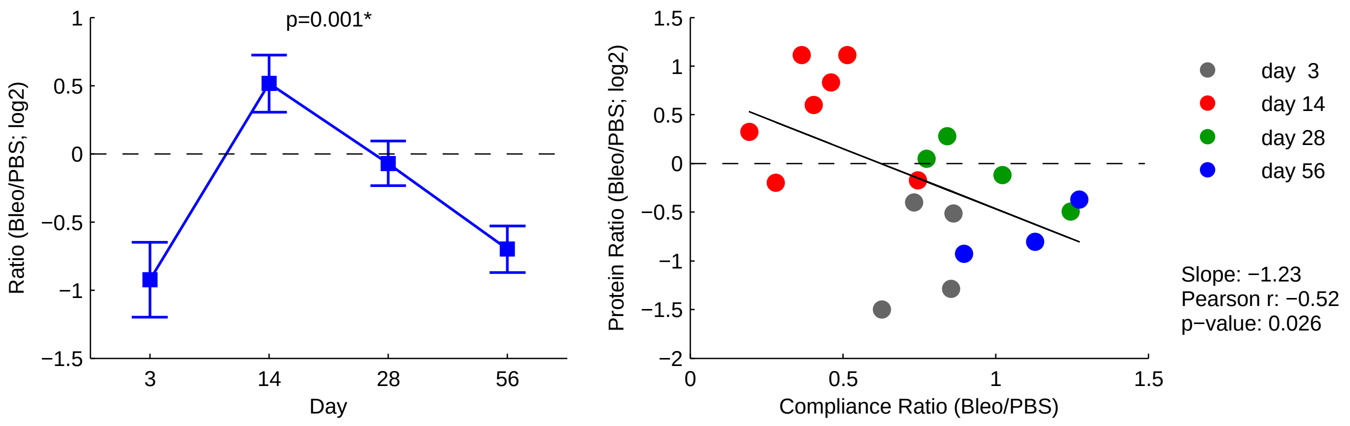

P35276 − Rab3d (id: 2770)

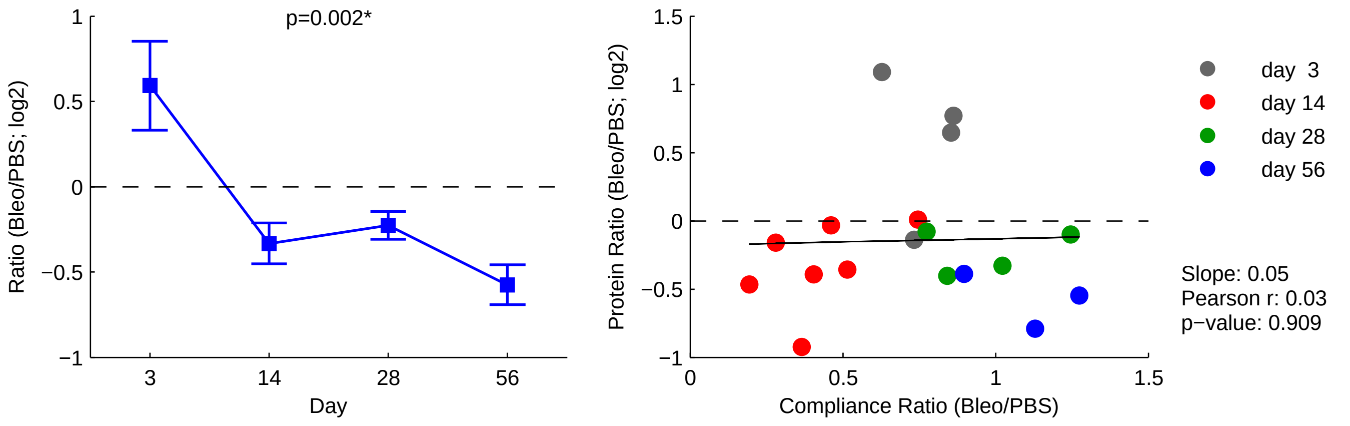

P35279 − Rab6a (id: 2772)

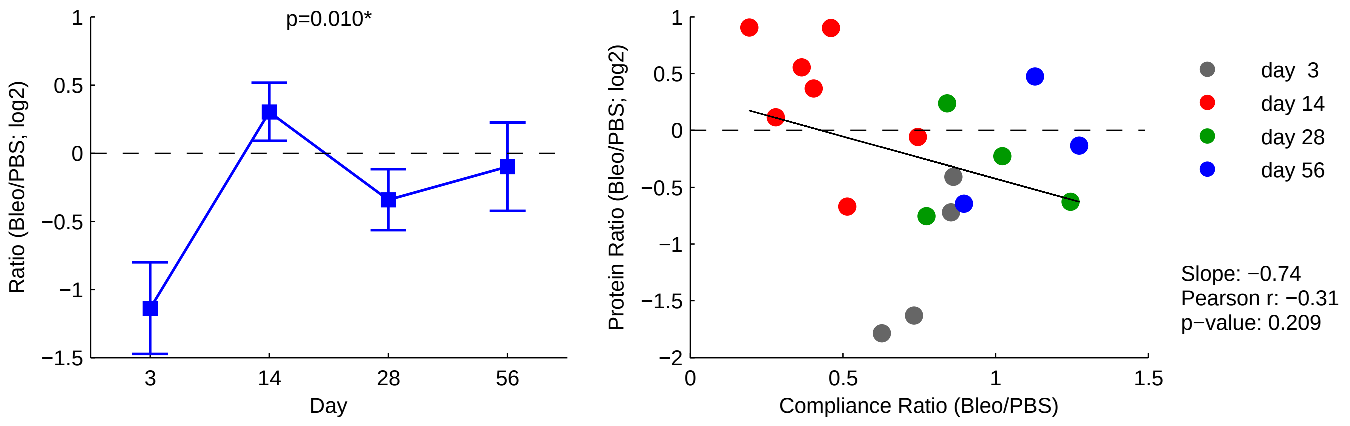

P35285 − Rab22a (id: 2775)

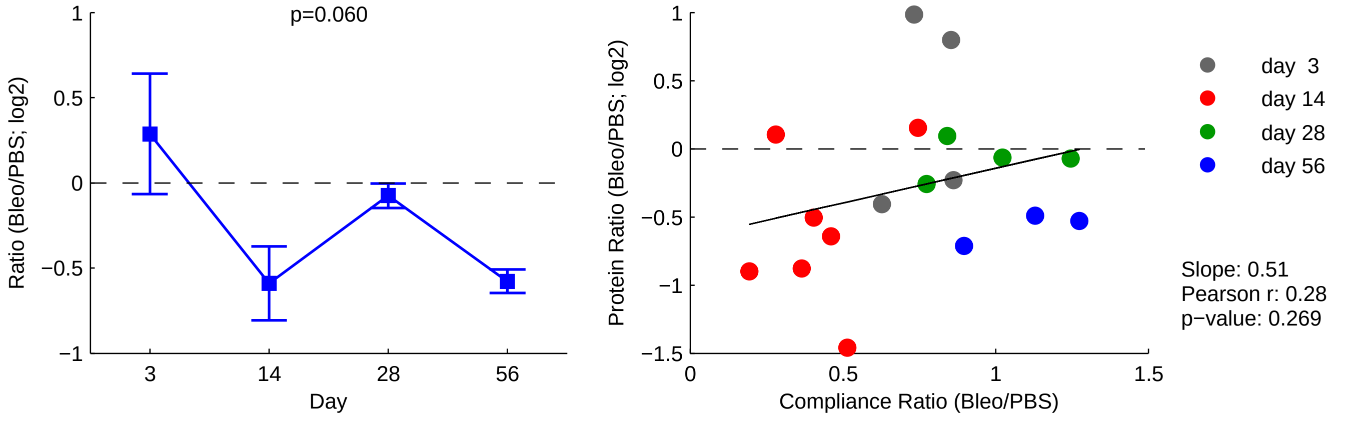

P35290 − Rab24 (id: 2777)

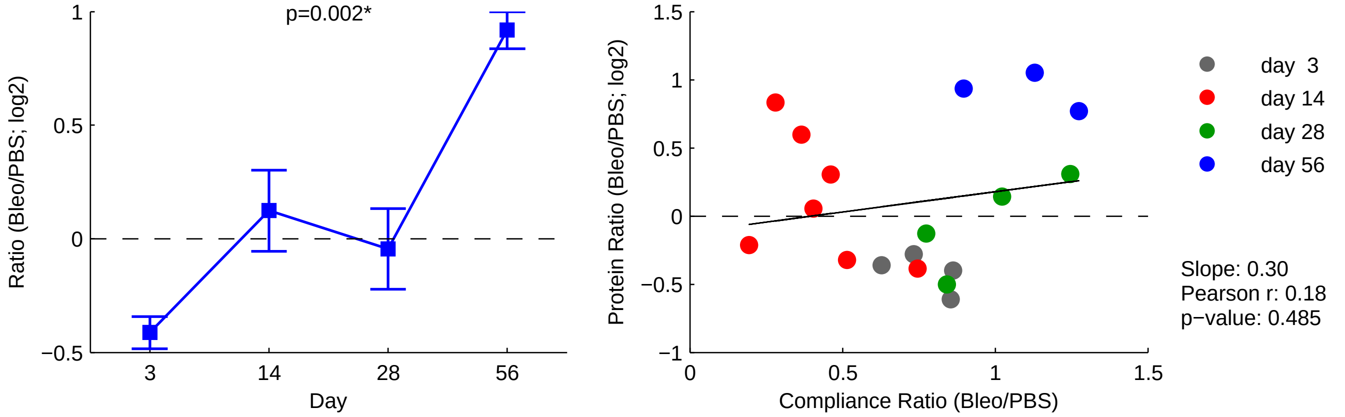

P35486 − Pdha1 (id: 2786)

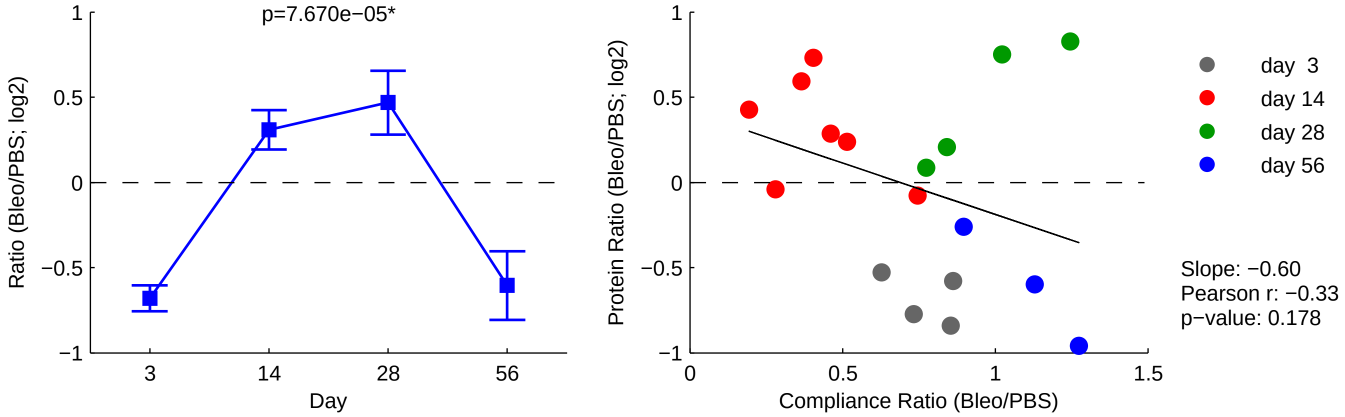

P35505 − Fah (id: 2788)

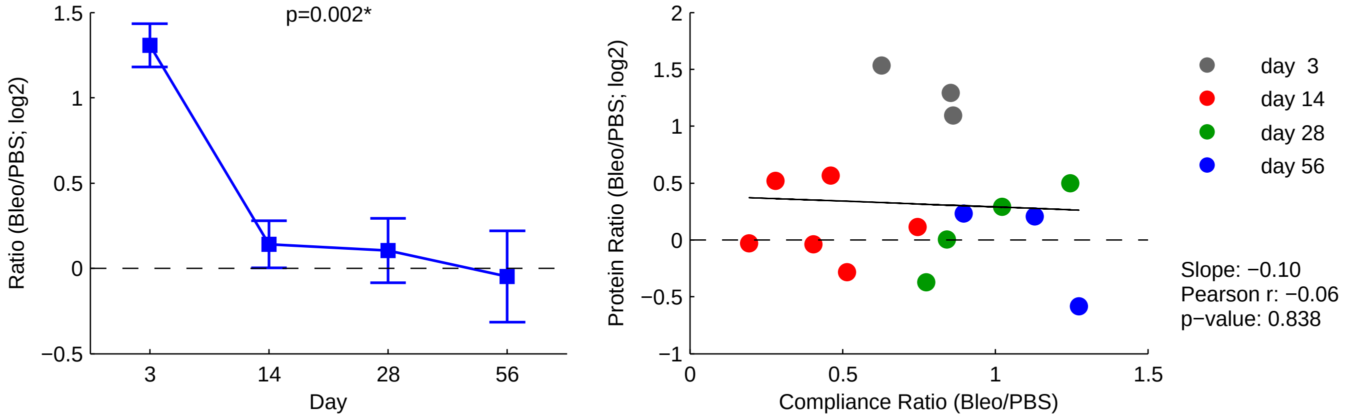

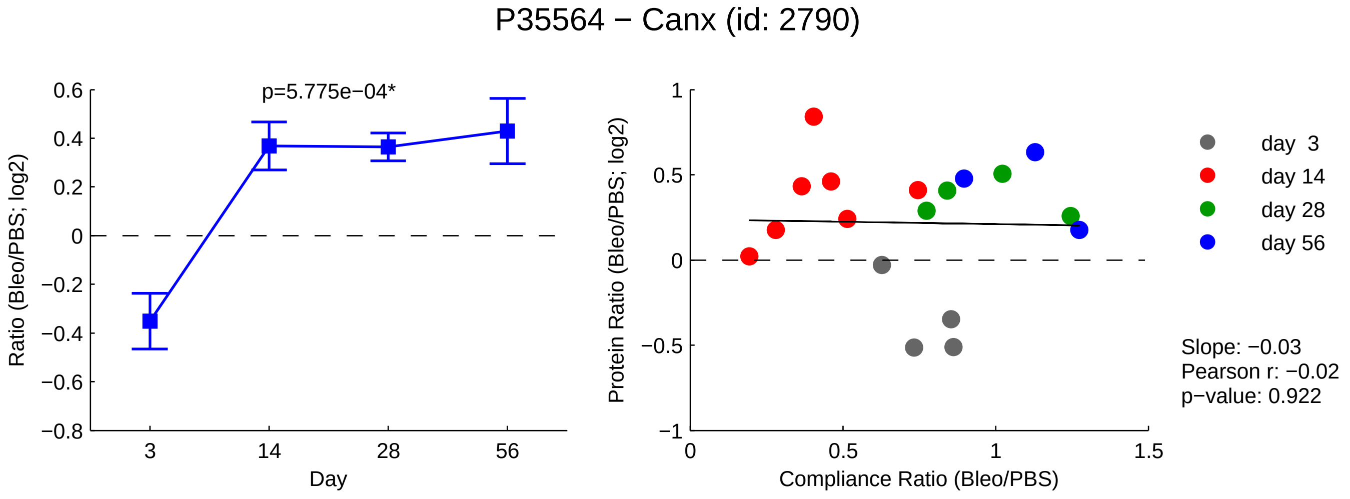
P35564 − Canx (id: 2790)
p=5.775e−04*
Ratio (Bleo/PBS; log2)
Day
Protein Ratio (Bleo/PBS; log2)
Compliance Ratio (Bleo/PBS)
day 3
day 14
day 28
day 56
Slope: −0.03
Pearson r: −0.02
p−value: 0.922

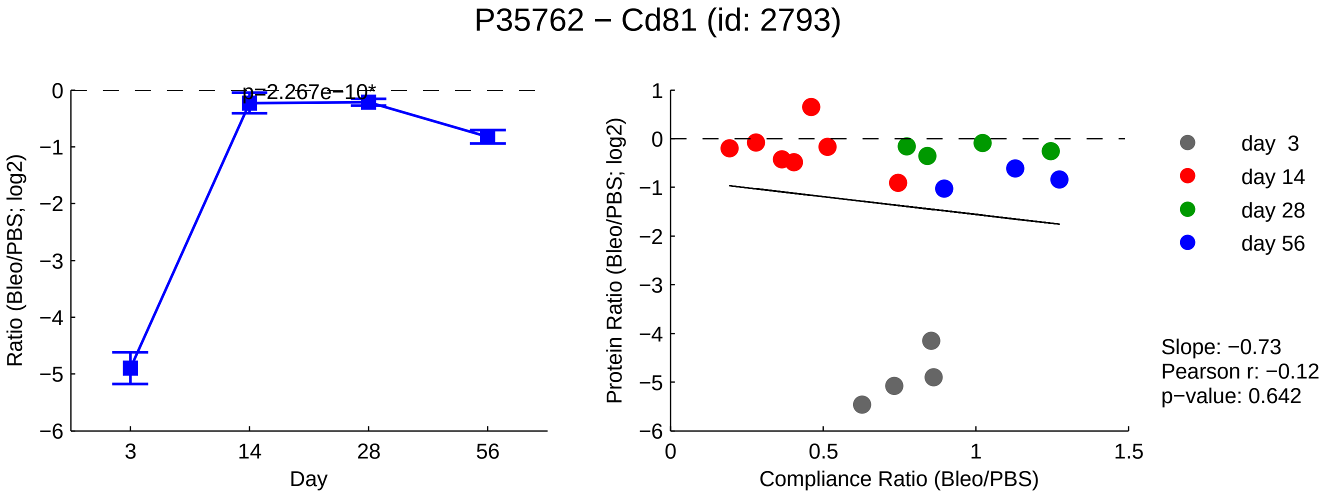
P35762 − Cd81 (id: 2793)
p=2.267e−10*
Ratio (Bleo/PBS; log2)
Day
Protein Ratio (Bleo/PBS; log2)
Compliance Ratio (Bleo/PBS)
day 3
day 14
day 28
day 56
Slope: −0.73
Pearson r: −0.12
p−value: 0.642

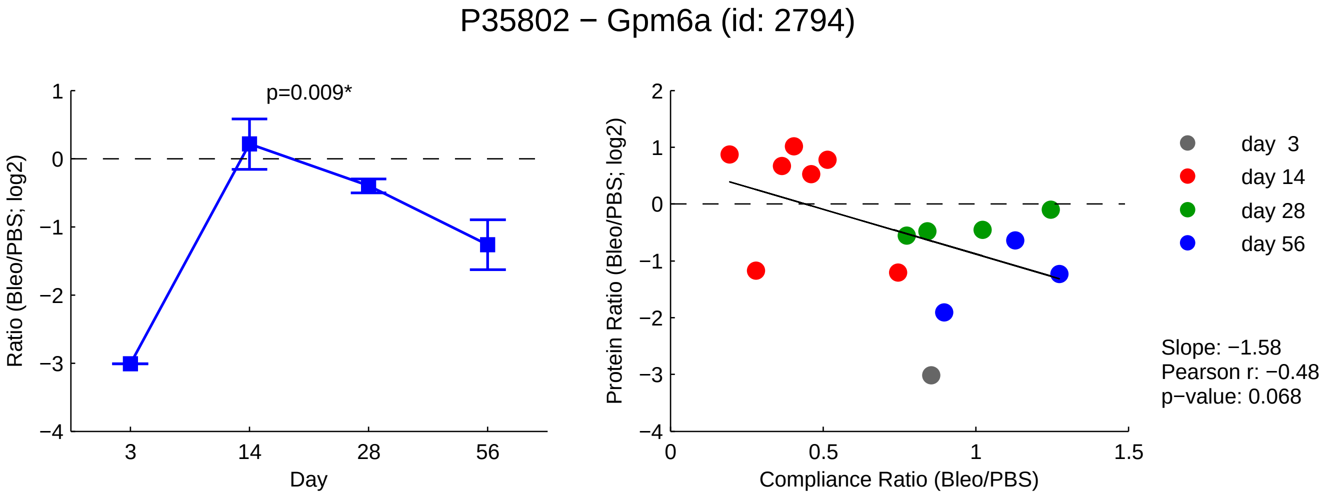
P35802 − Gpm6a (id: 2794)
p=0.009*
Ratio (Bleo/PBS; log2)
Day
Protein Ratio (Bleo/PBS; log2)
Compliance Ratio (Bleo/PBS)
day 3
day 14
day 28
day 56
Slope: −1.58
Pearson r: −0.48
p−value: 0.068

P35821 − Ptpn1 (id: 2795)

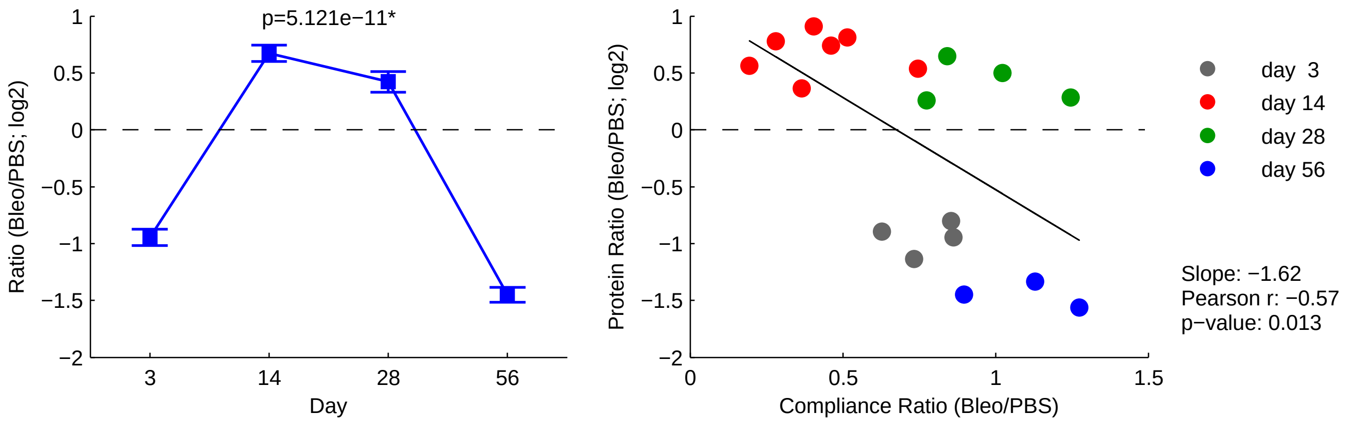

P35831 − Ptpn12 (id: 2797)

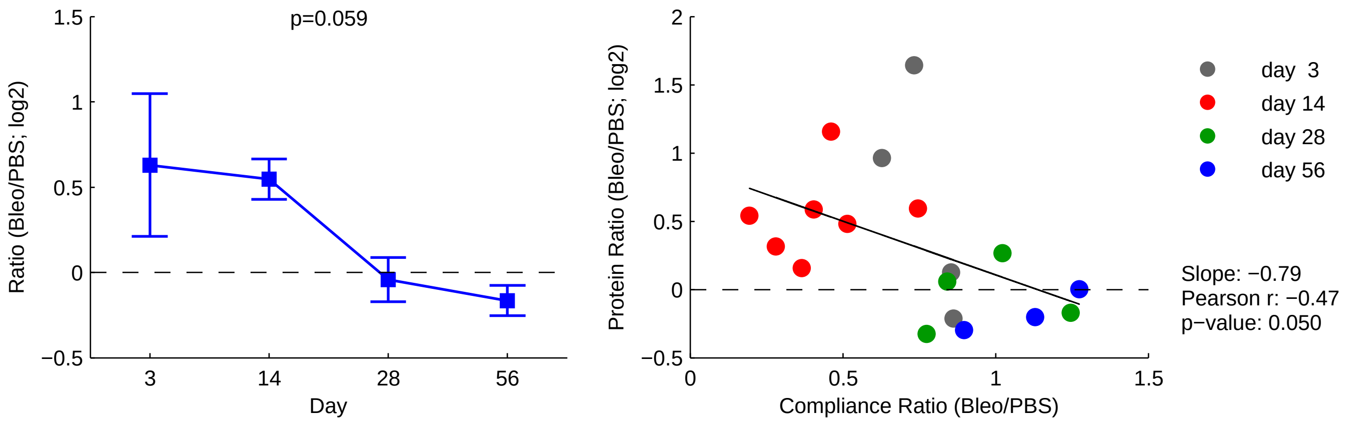

Q8VCD0 − Kdr (id: 2799)

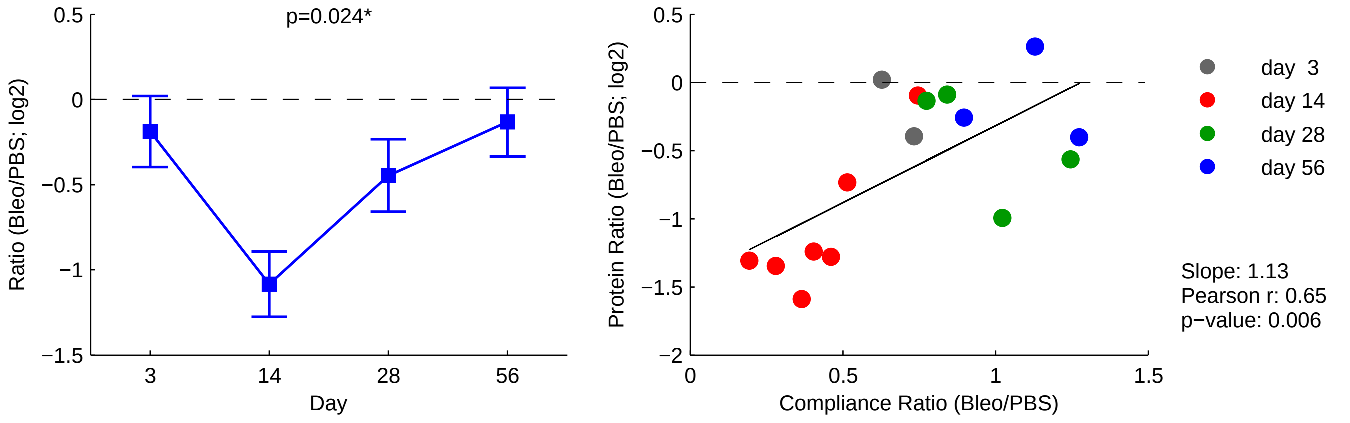

P35951 − Ldlr (id: 2800)

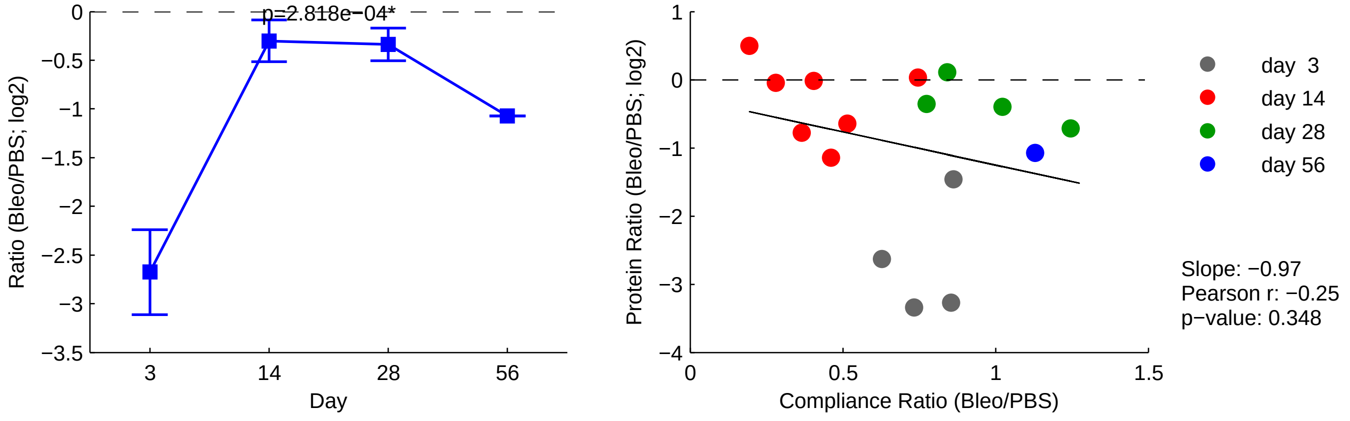

P35979 − Rpl12 (id: 2802)

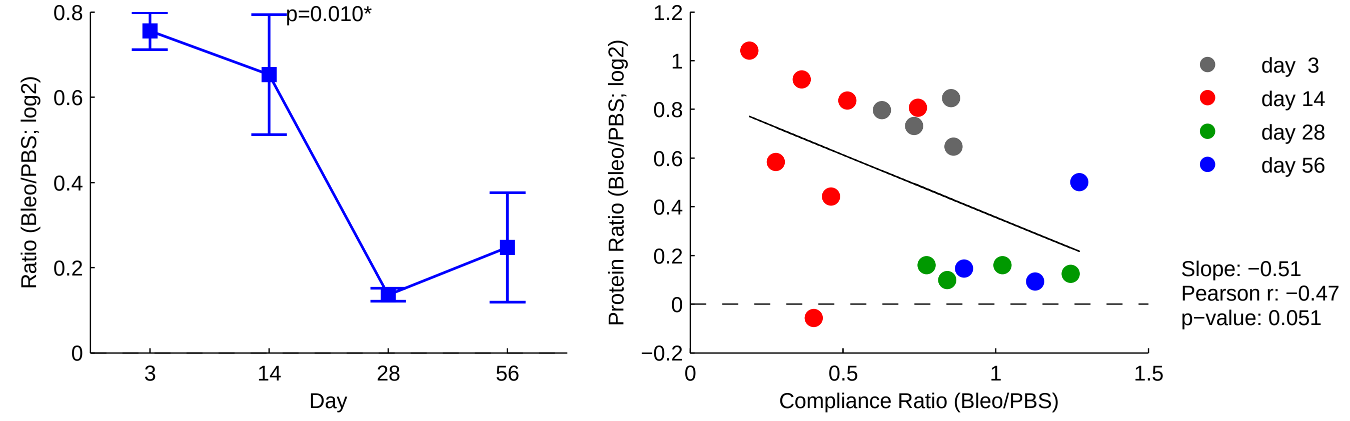

P35980 − Rpl18 (id: 2803)

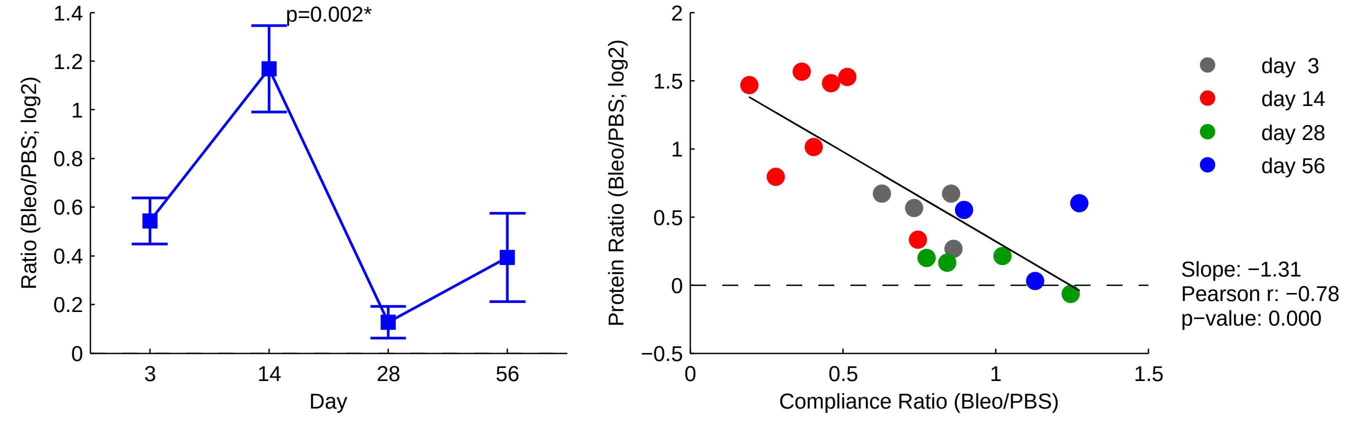

P36371 − Tap2 (id: 2805)

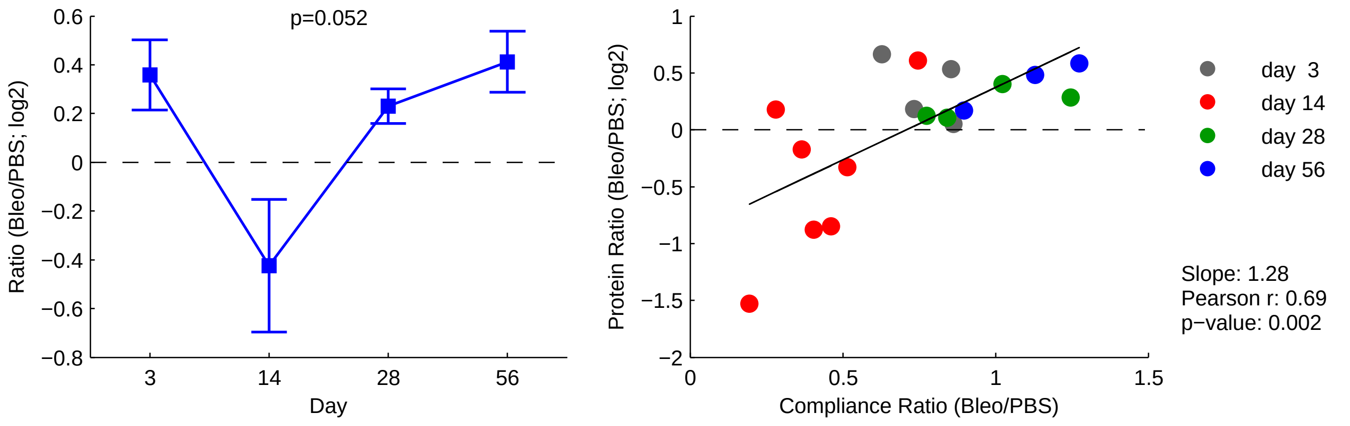

P36423 − Tbxas1 (id: 2806)

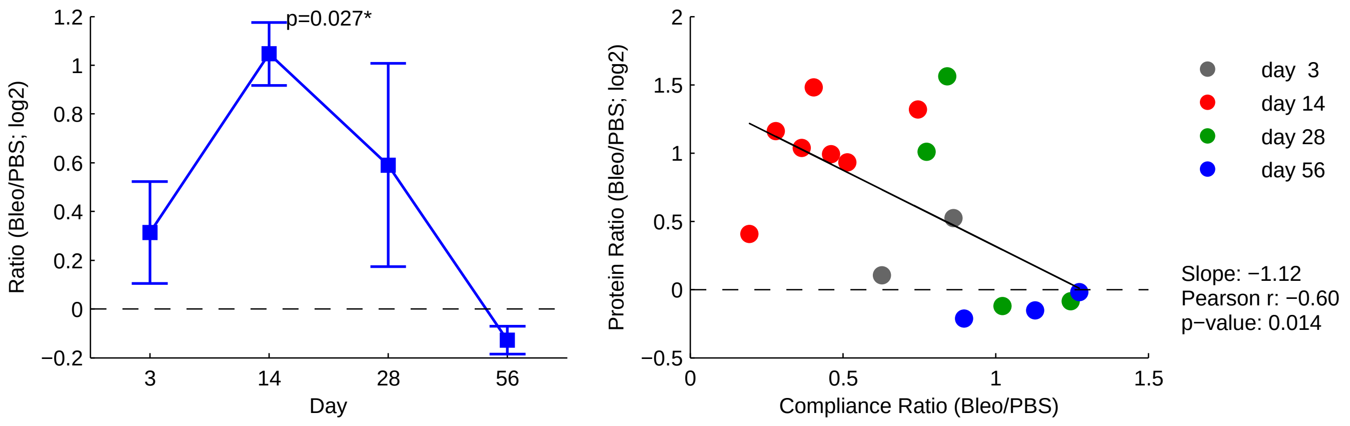

P36552 − Cpox (id: 2807)

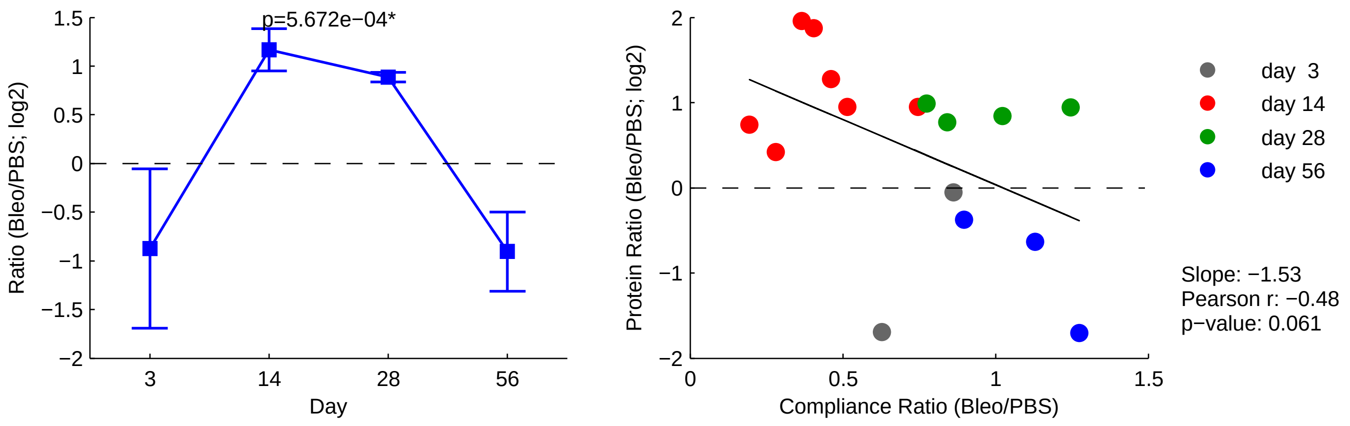

P37040 − Por (id: 2809)

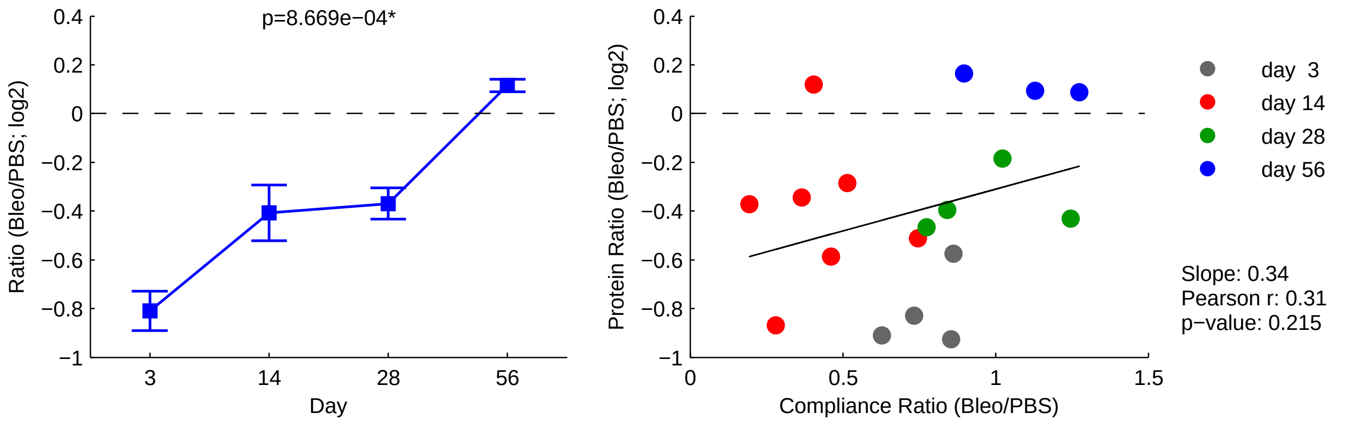

P37804 − Tagln (id: 2810)

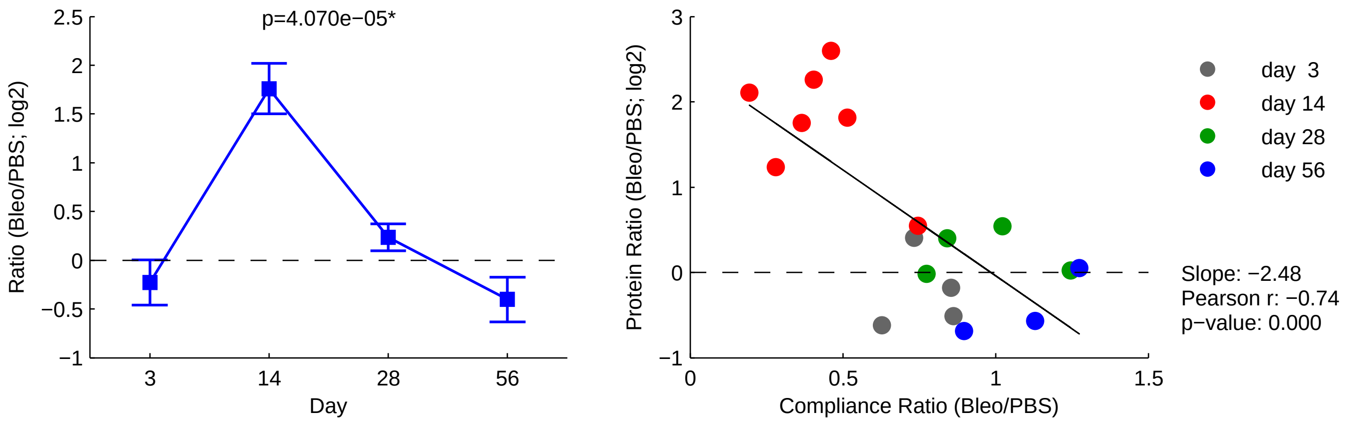

P37889−2 − Fbln2 (id: 2811)

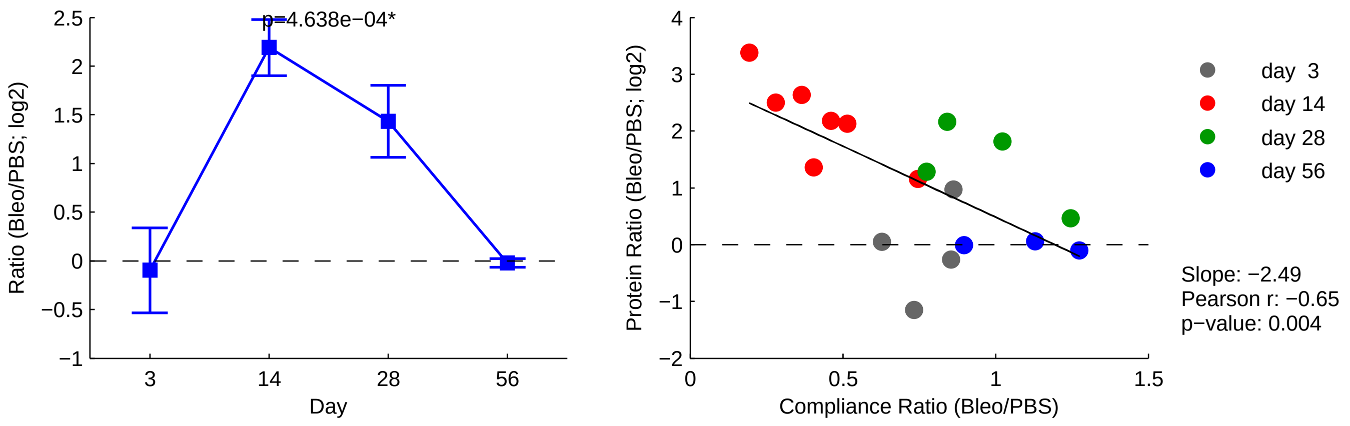

P38060 − Hmgcl (id: 2812)

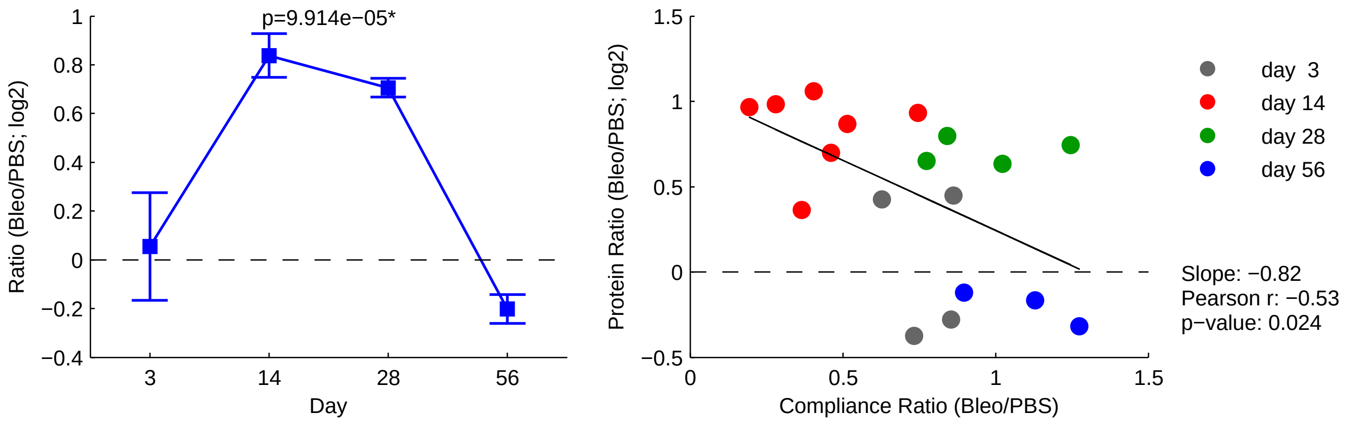

P38647 − Hspa9 (id: 2813)

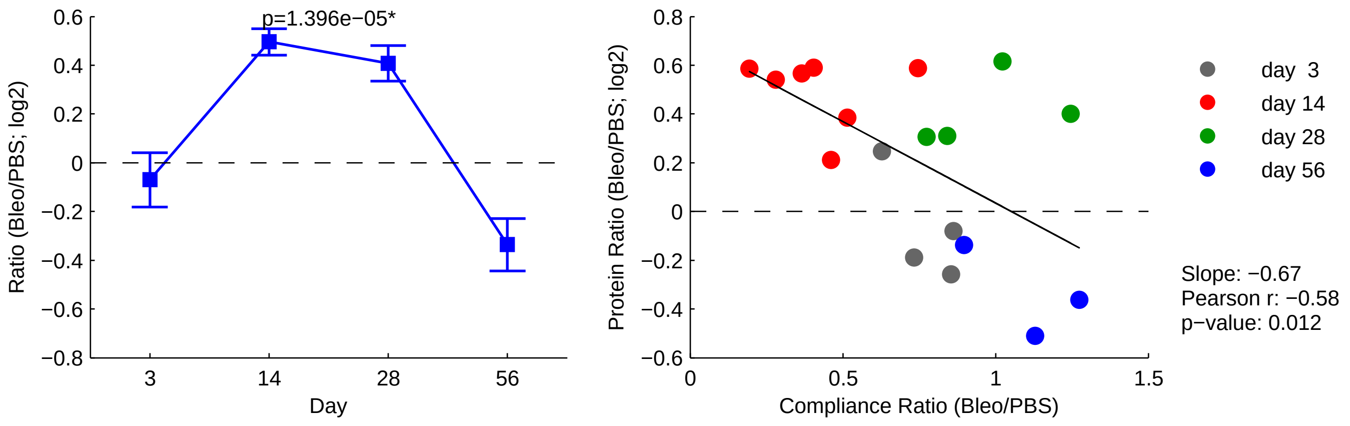

Q3T9X3 − Dnm2 (id: 2816)

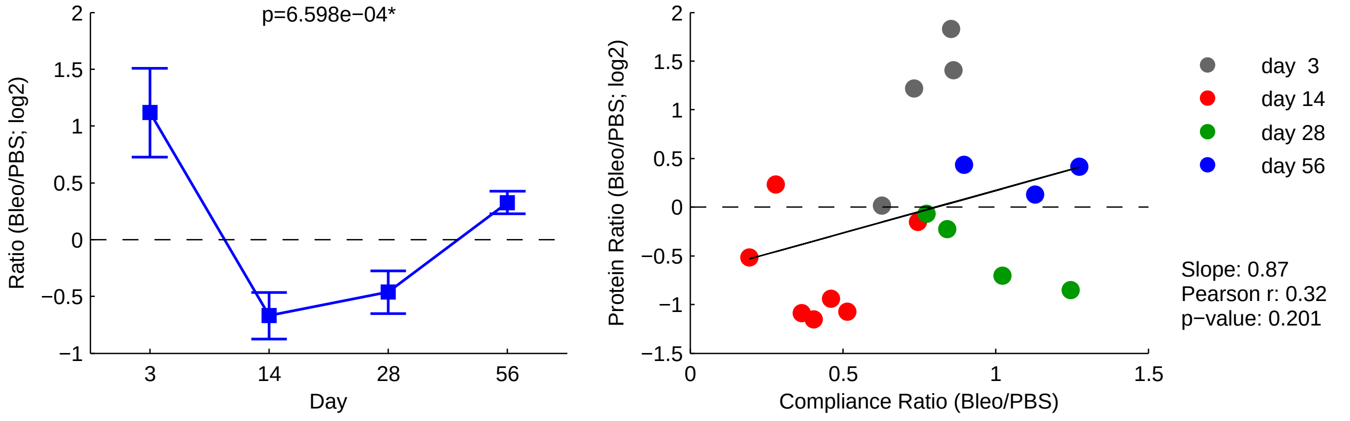

P39098−2 − Man1a2 (id: 2817)

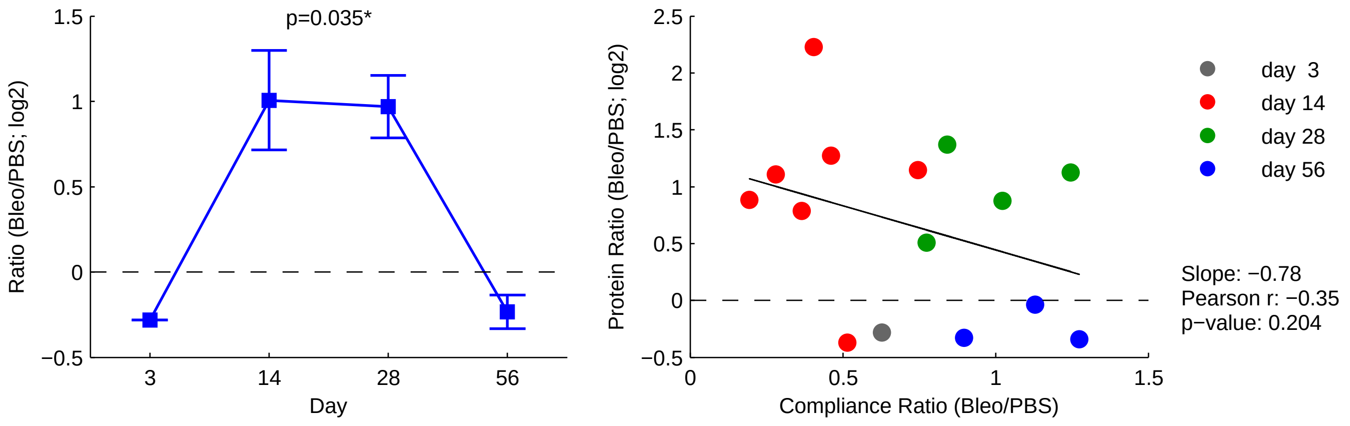

P39655 − Alox12 (id: 2821)

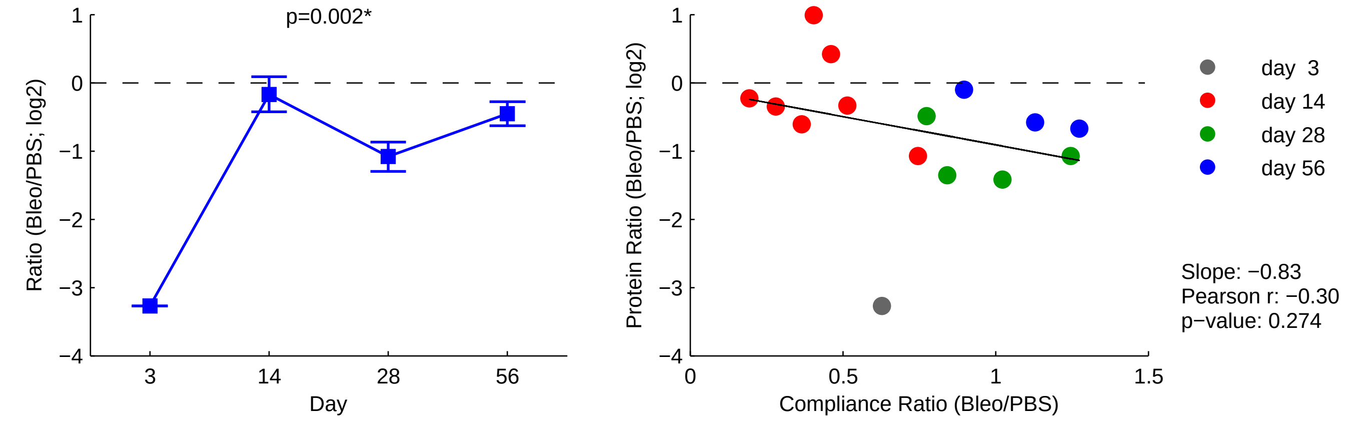

P39688 − Fyn (id: 2822)

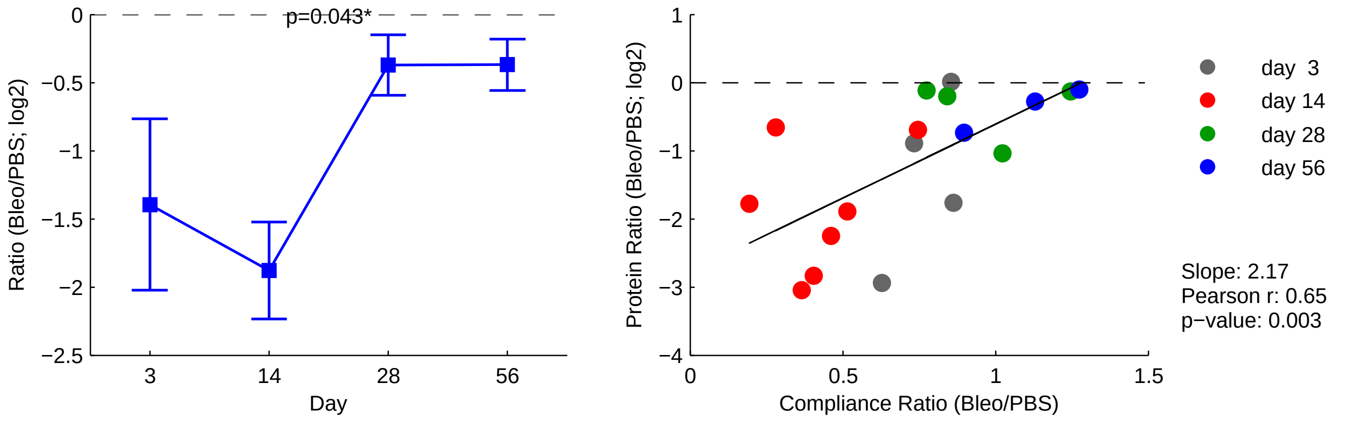

P39876 − Timp3 (id: 2825)

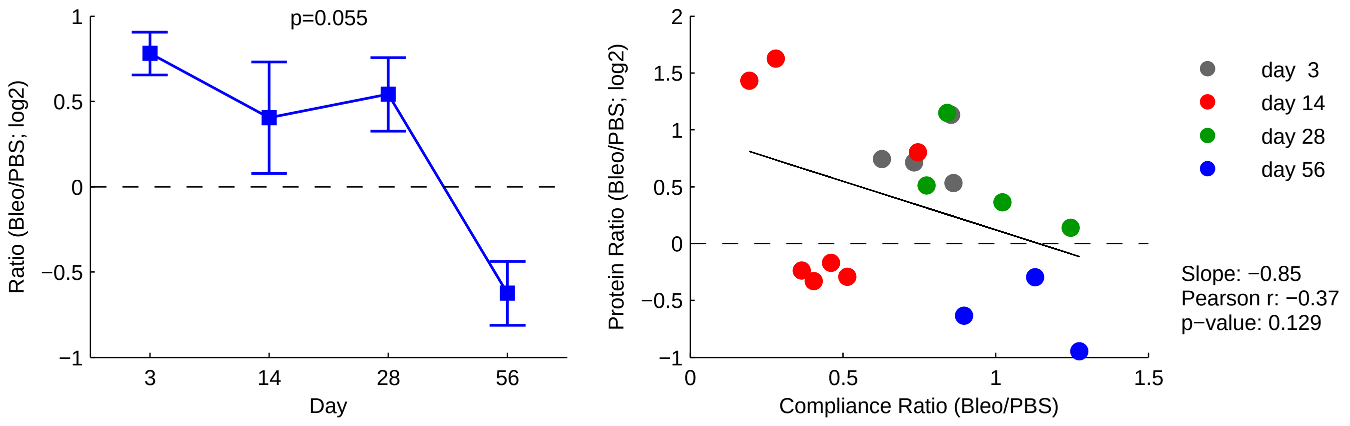

P40124 − Cap1 (id: 2826)

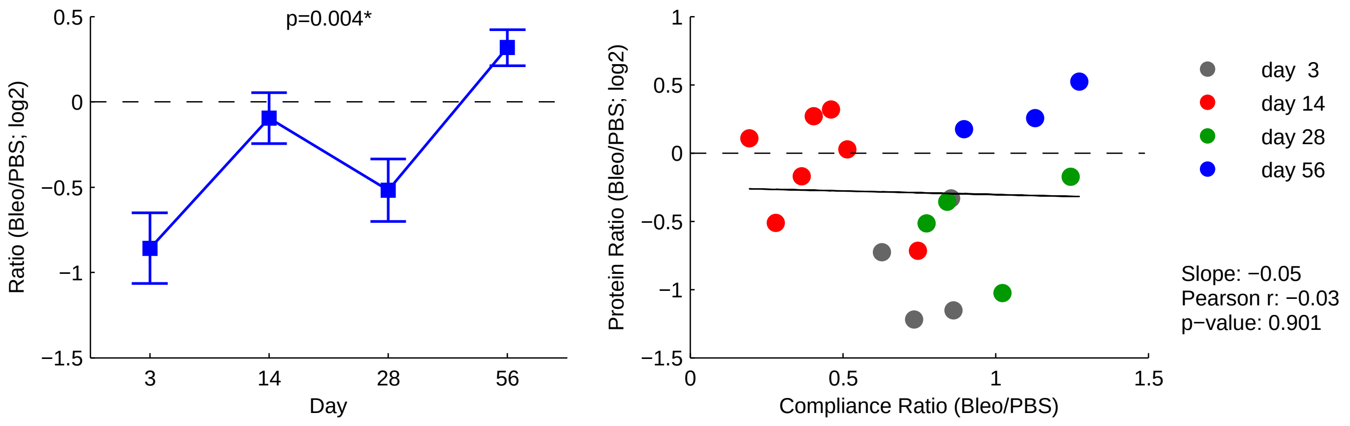

P40142 − Tkt (id: 2827)

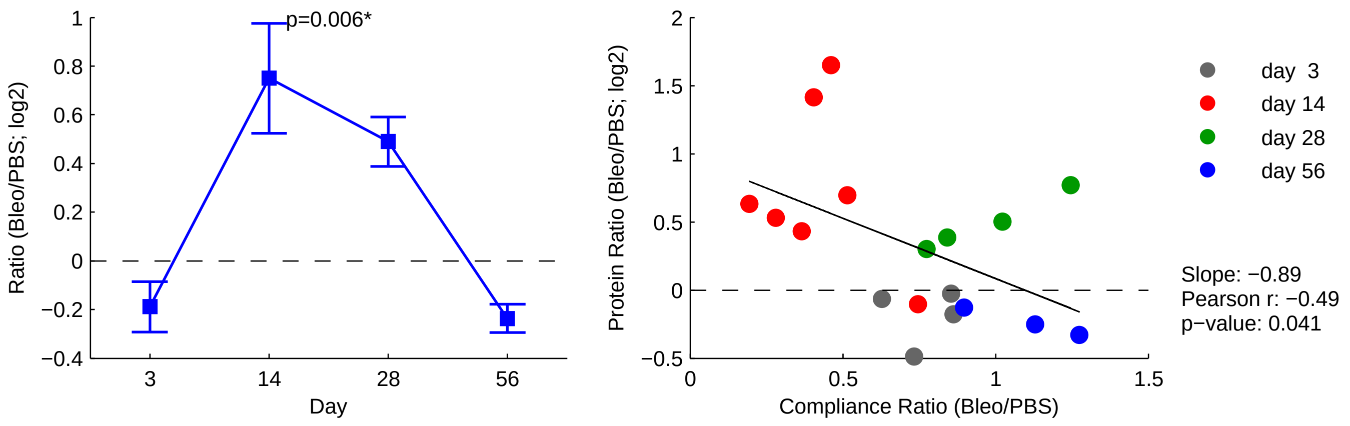

P40201 − Chd1 (id: 2828)

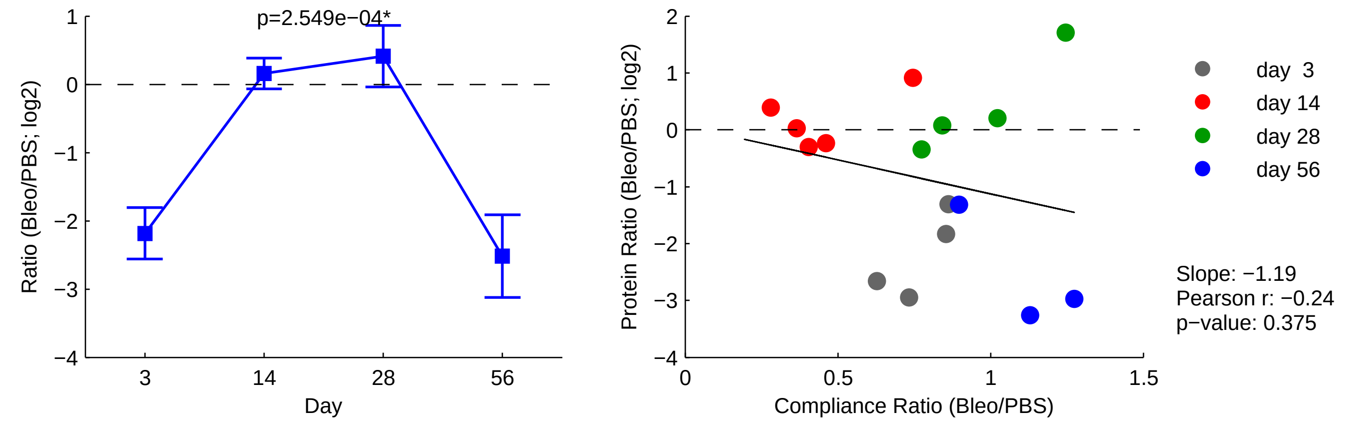

P40237 − Cd82 (id: 2829)

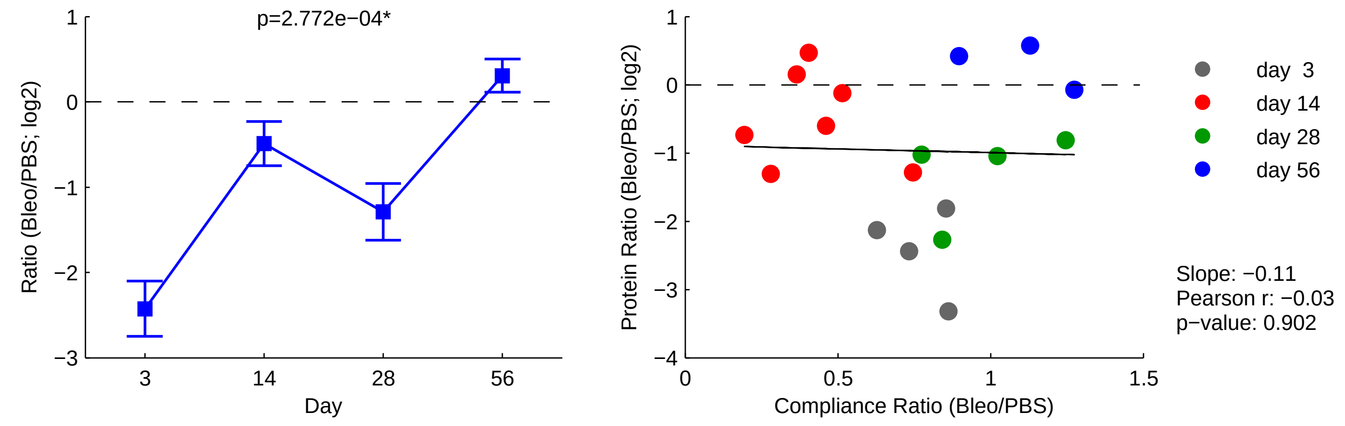

P40240 − Cd9 (id: 2830)

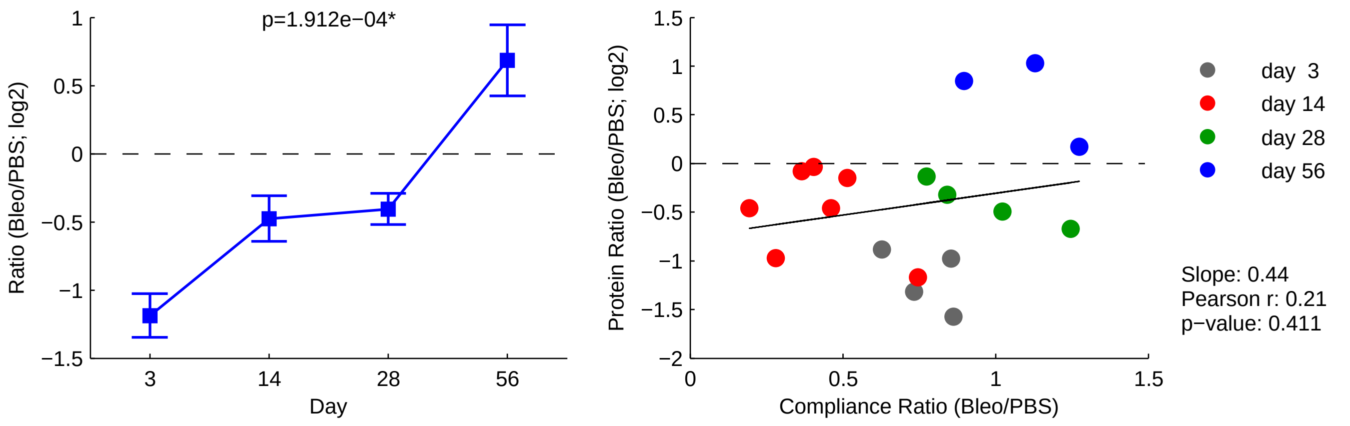

P40336 − Vps26a (id: 2831)

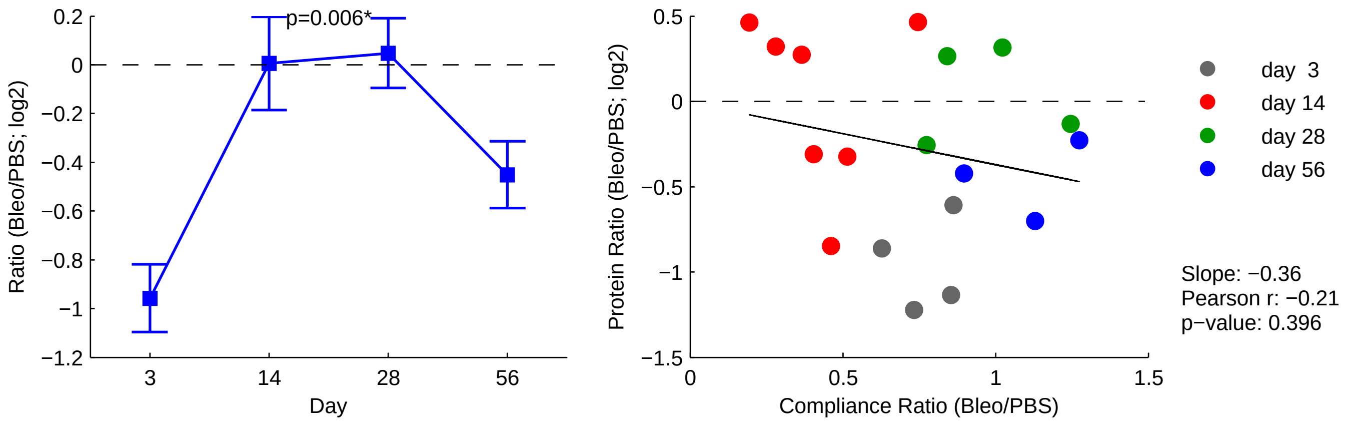

P40630−2 − Tfam (id: 2833)

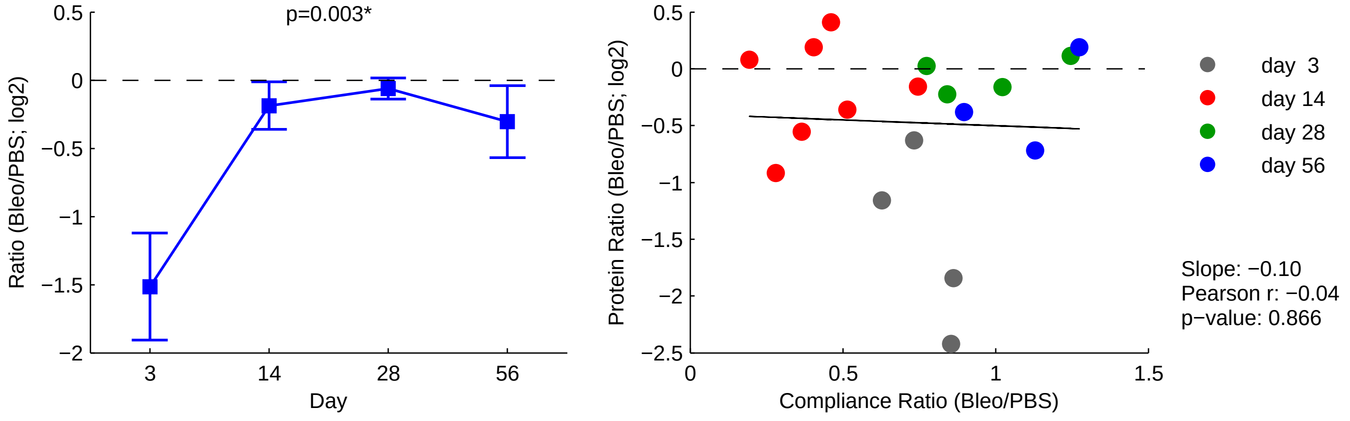

P40936 − Inmt (id: 2834)

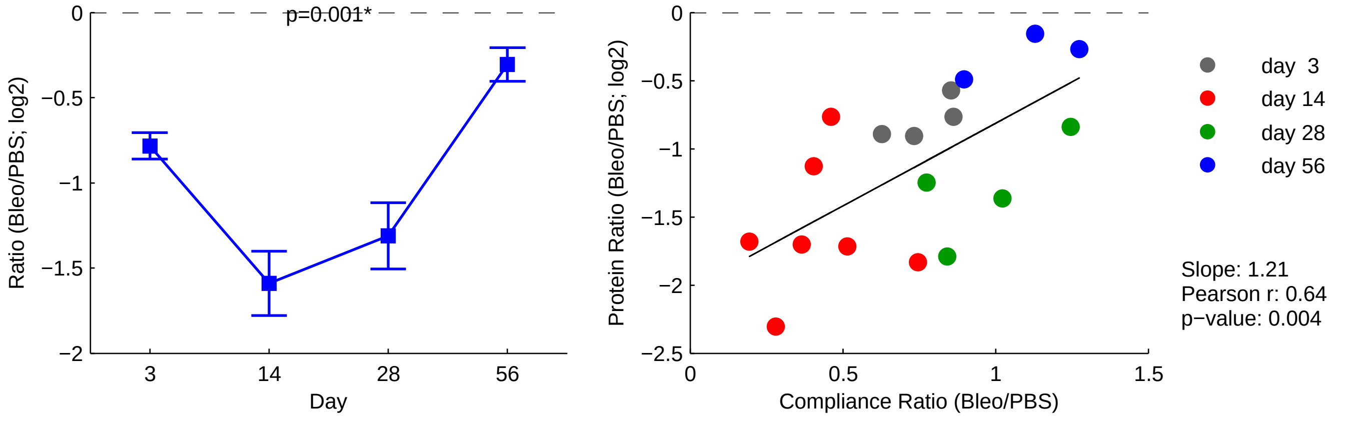

P41105 − Rpl28 (id: 2835)

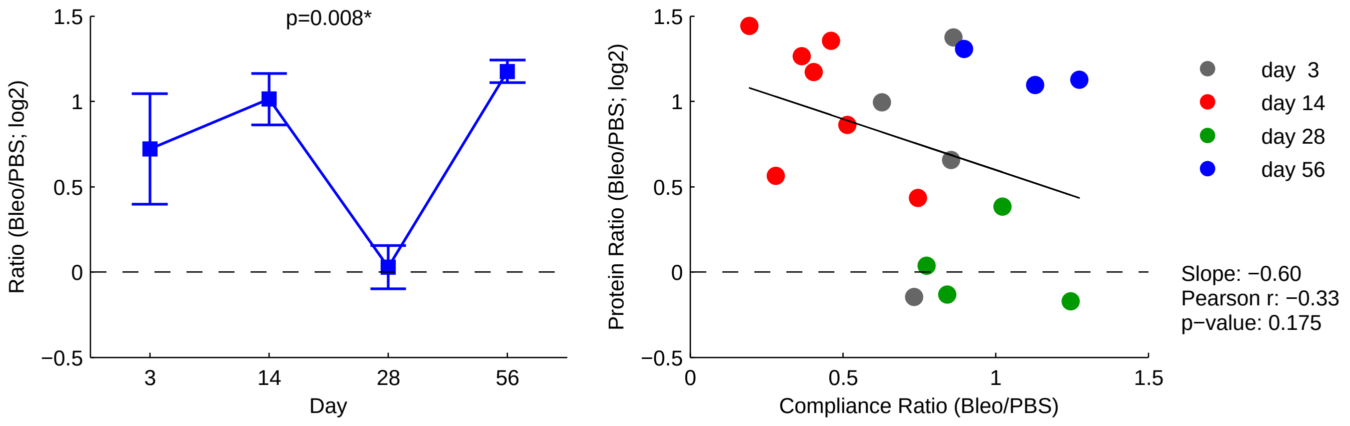

P41216 − Acsl1 (id: 2836)

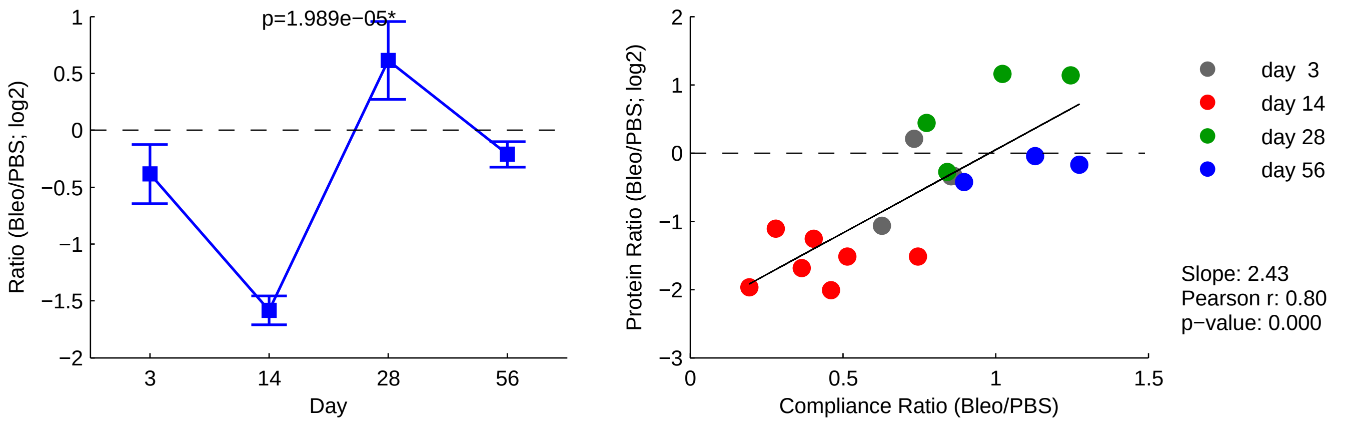

P41241 − Csk (id: 2838)

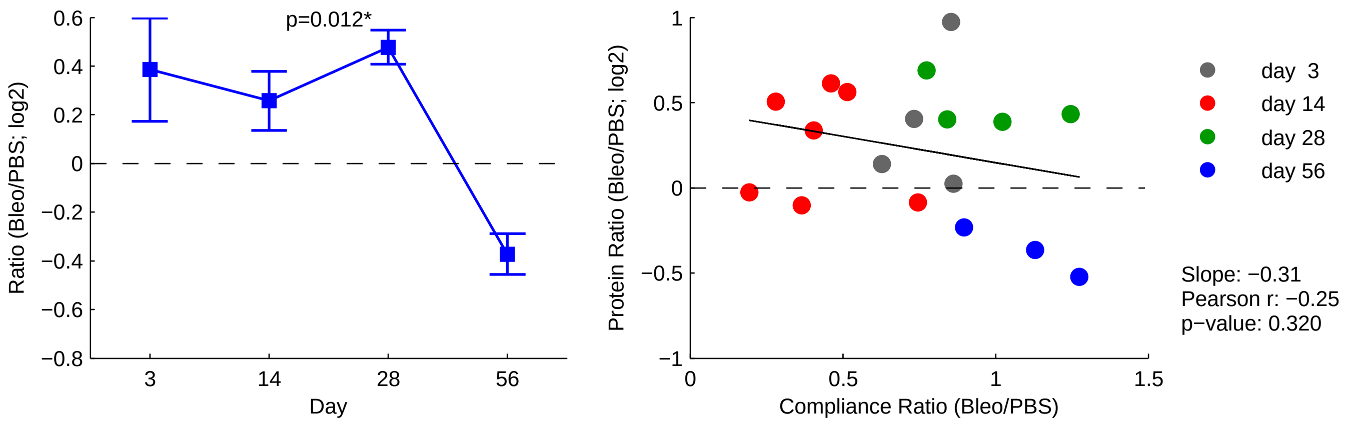

P41245 − Mmp9 (id: 2839)

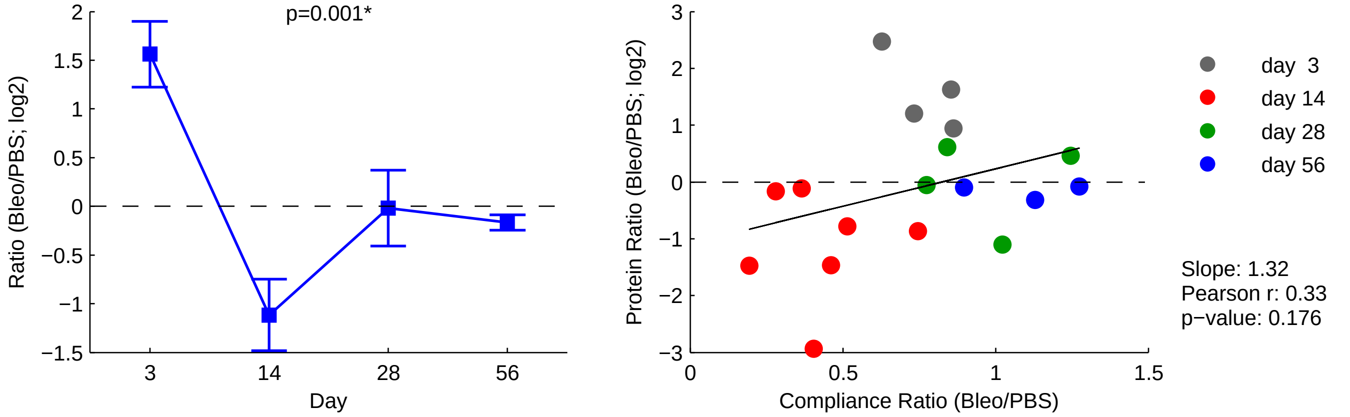

P42125 − Eci1 (id: 2843)

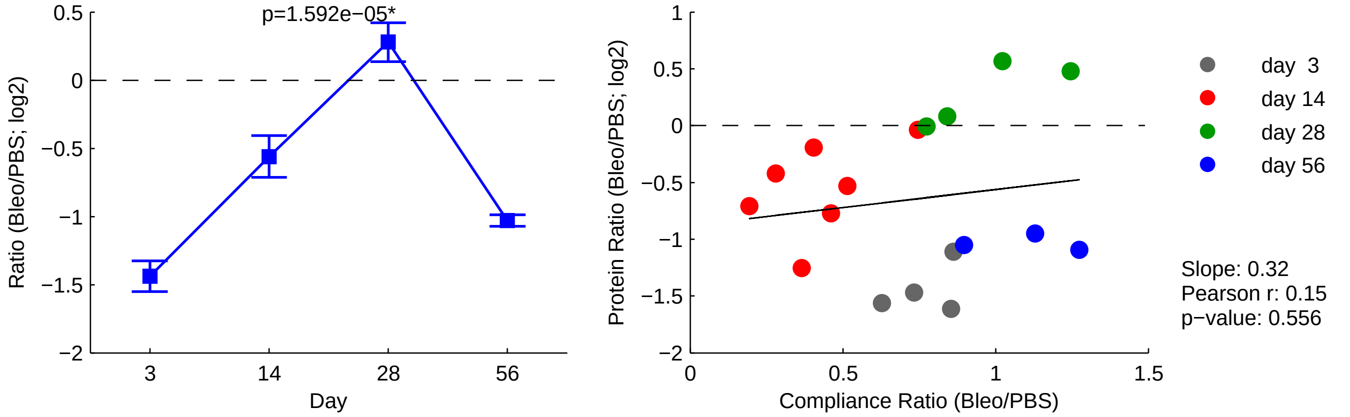

P42128 − Foxk1 (id: 2844)

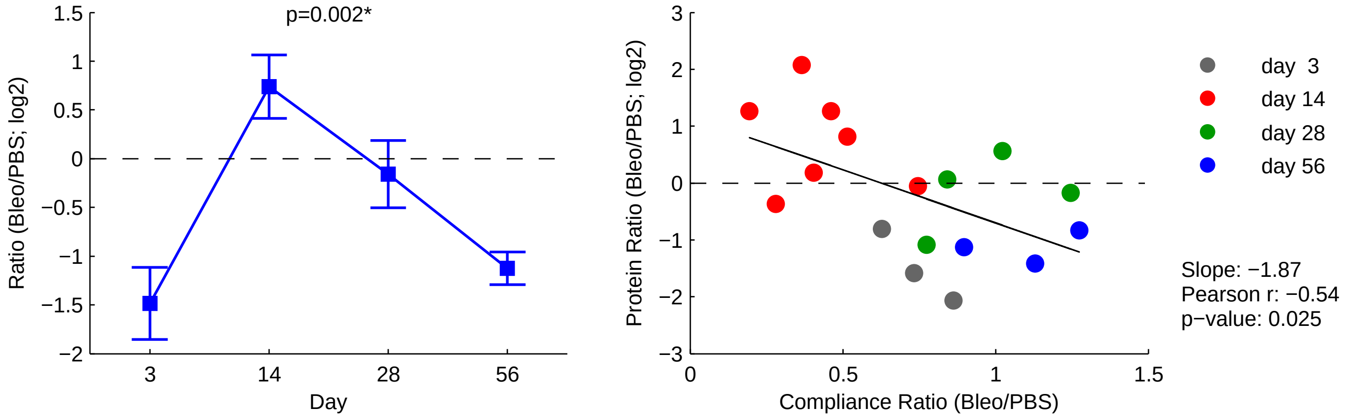

P42232 − Stat5b (id: 2849)

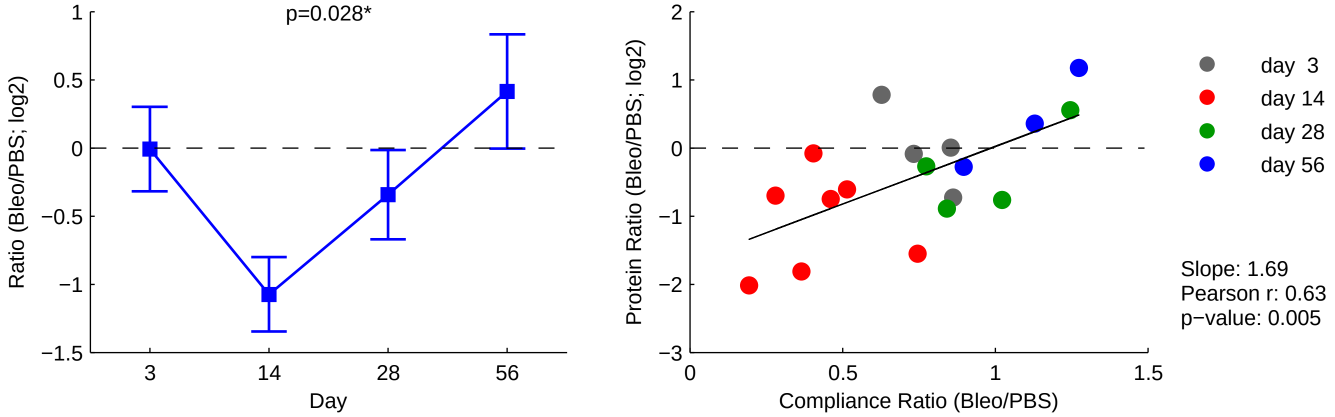

P42567 − Eps15 (id: 2851)

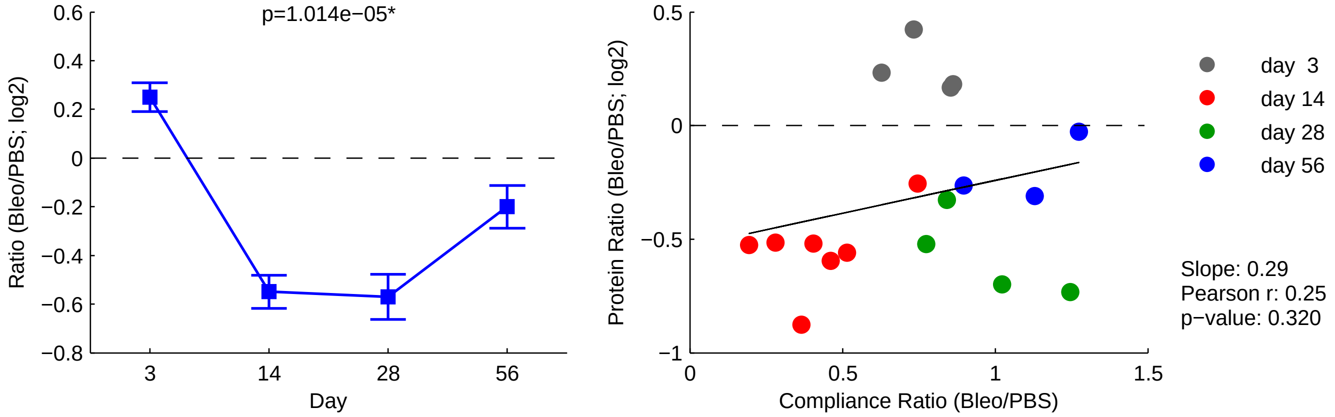

P42669 − Pura (id: 2852)

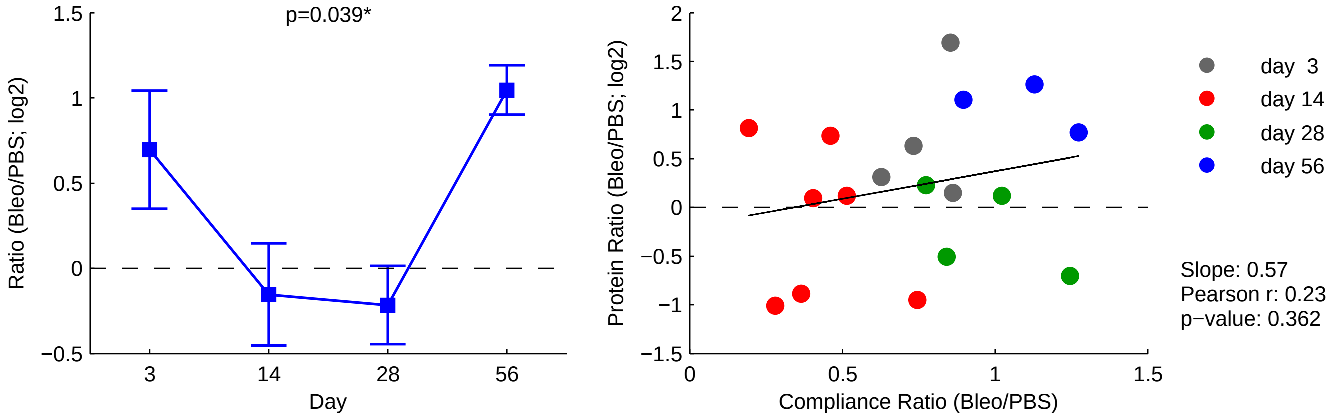

P42703 − Lifr (id: 2853)

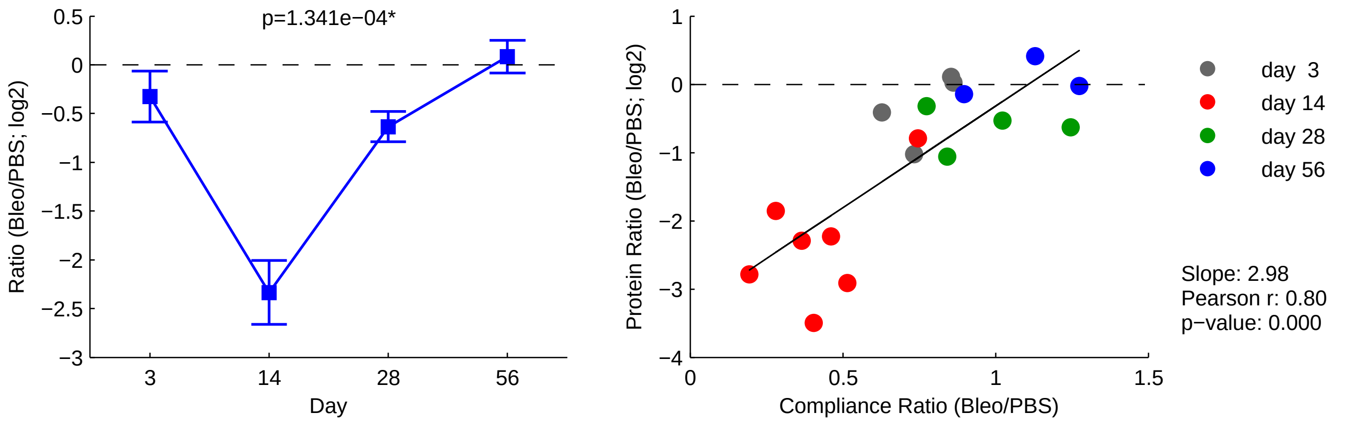

P43023 − Cox6a2 (id: 2856)

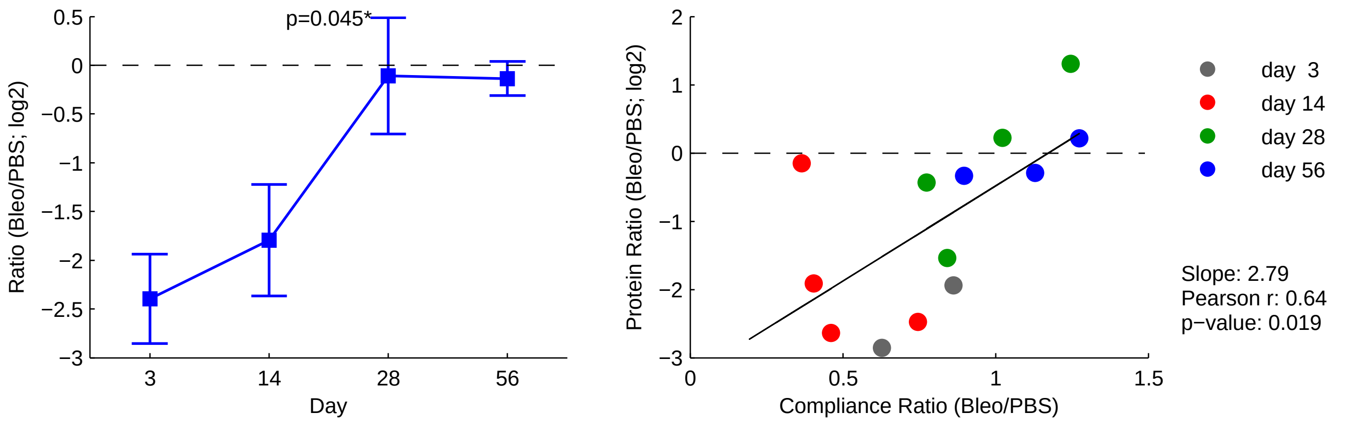

P43024 − Cox6a1 (id: 2857)

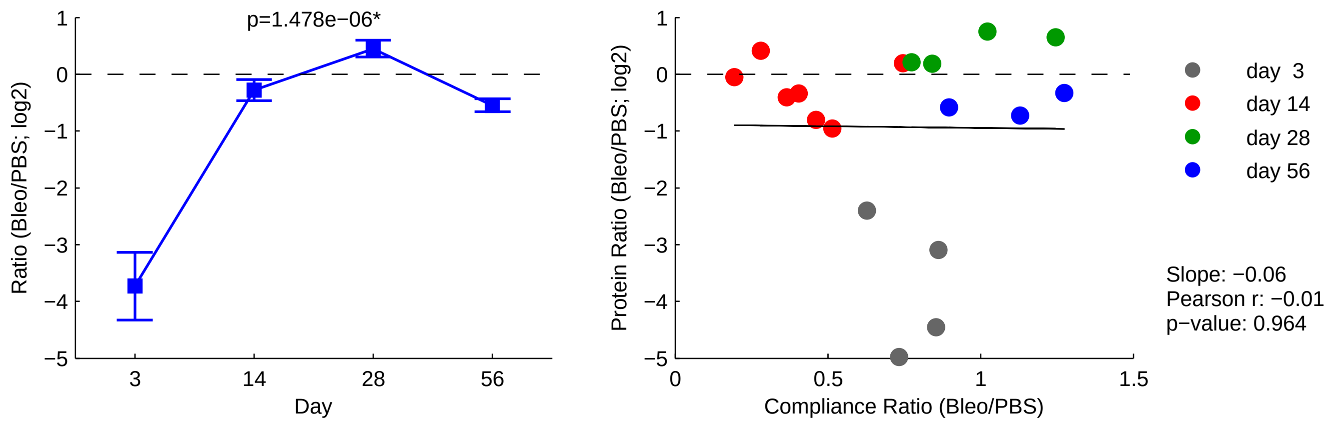

P43247 − Msh2 (id: 2859)

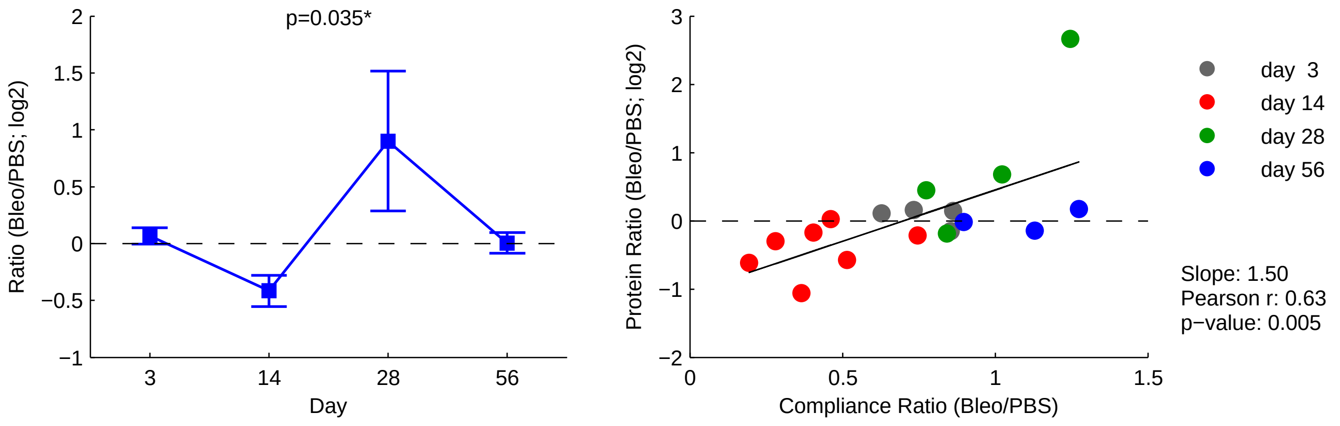

P43274 − Hist1h1e (id: 2860)

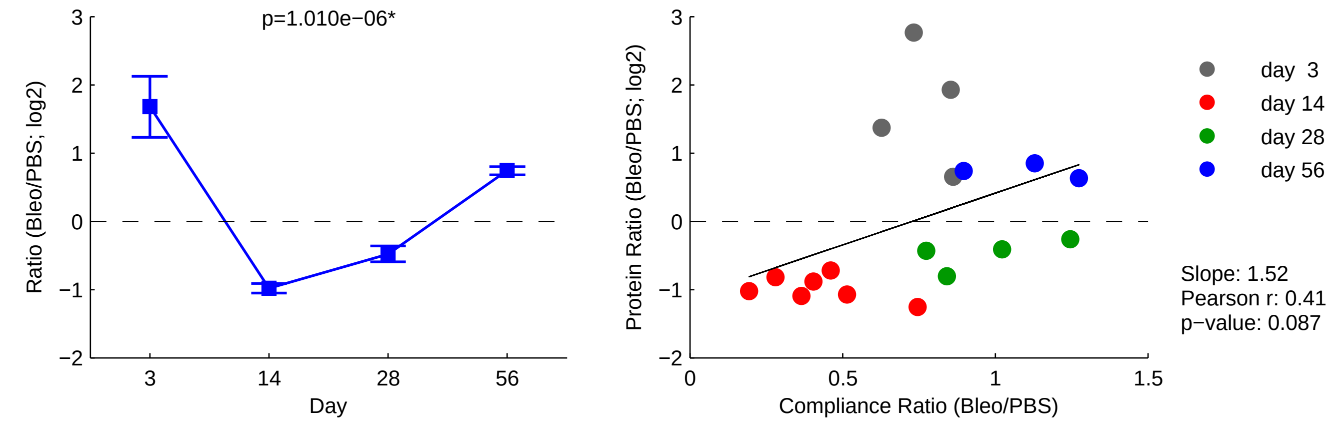

P43275 − Hist1h1a (id: 2861)

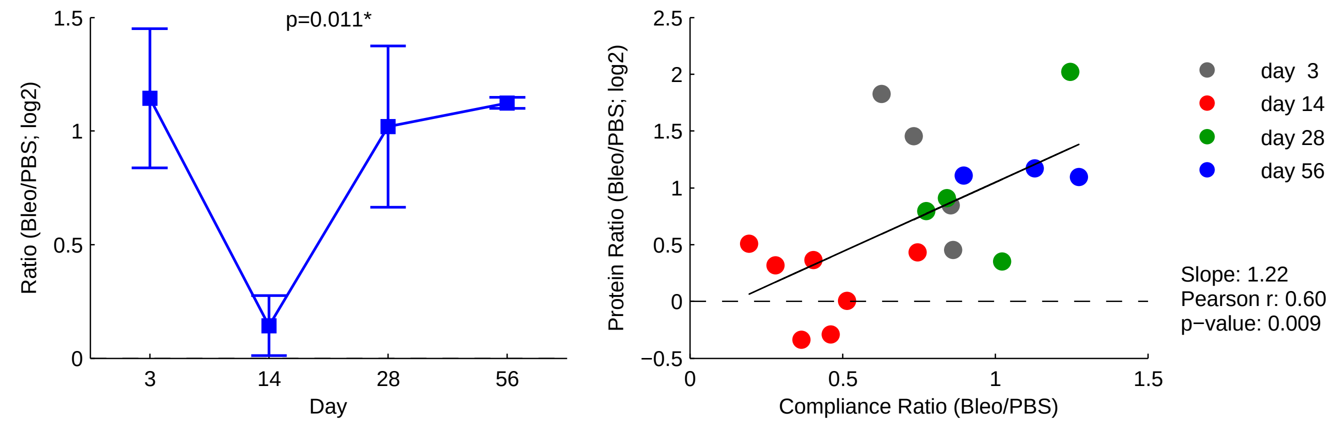

P43276 − Hist1h1b (id: 2862)

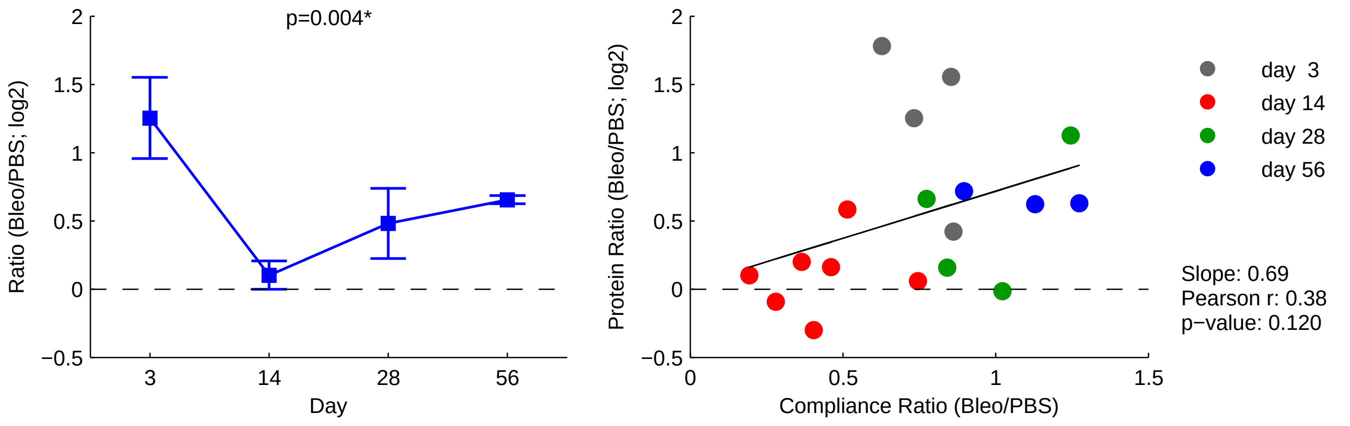

P43277 − Hist1h1d (id: 2863)

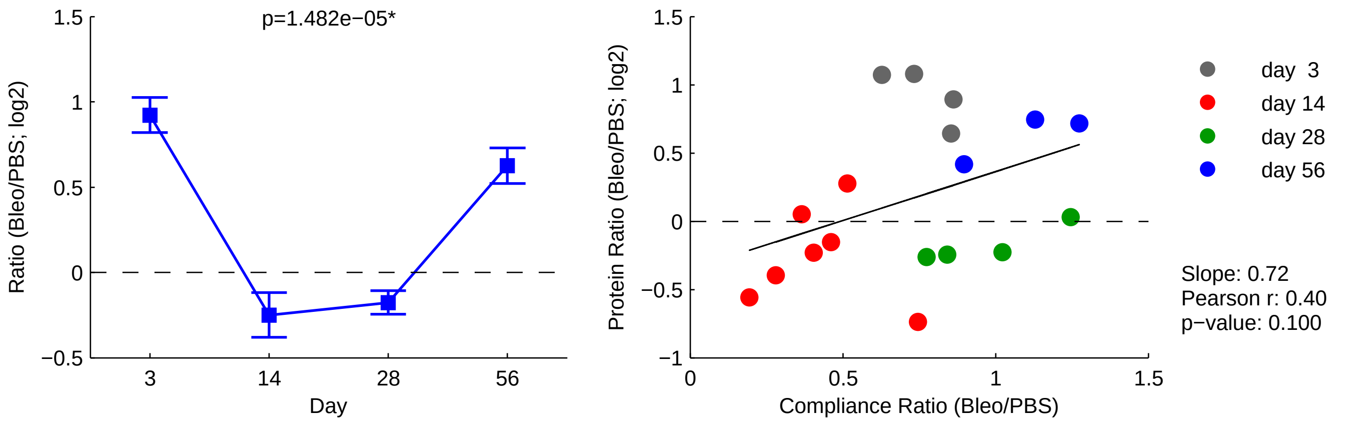

P43406 − Itgav (id: 2865)

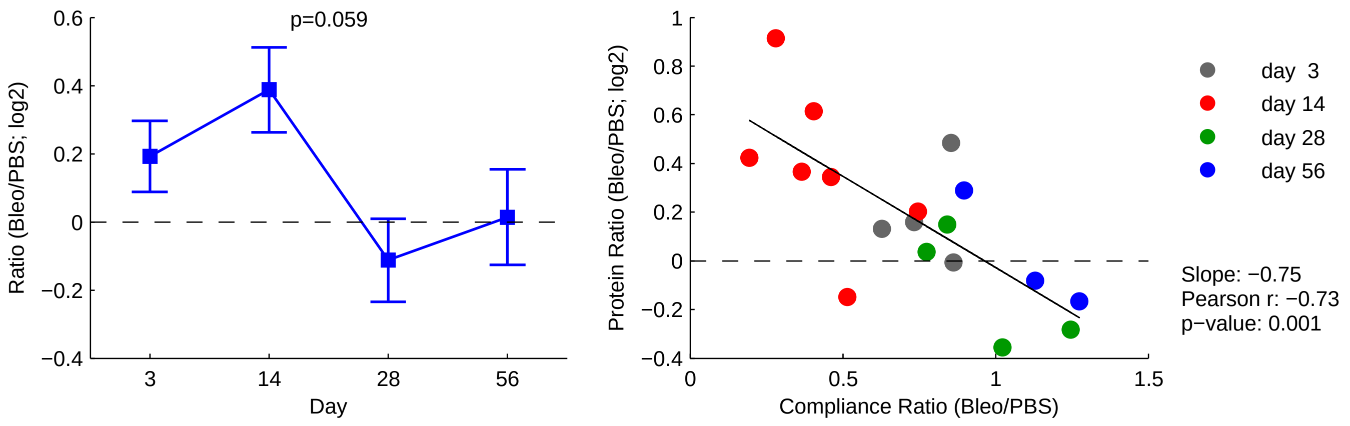

P43883 − Plin2 (id: 2867)

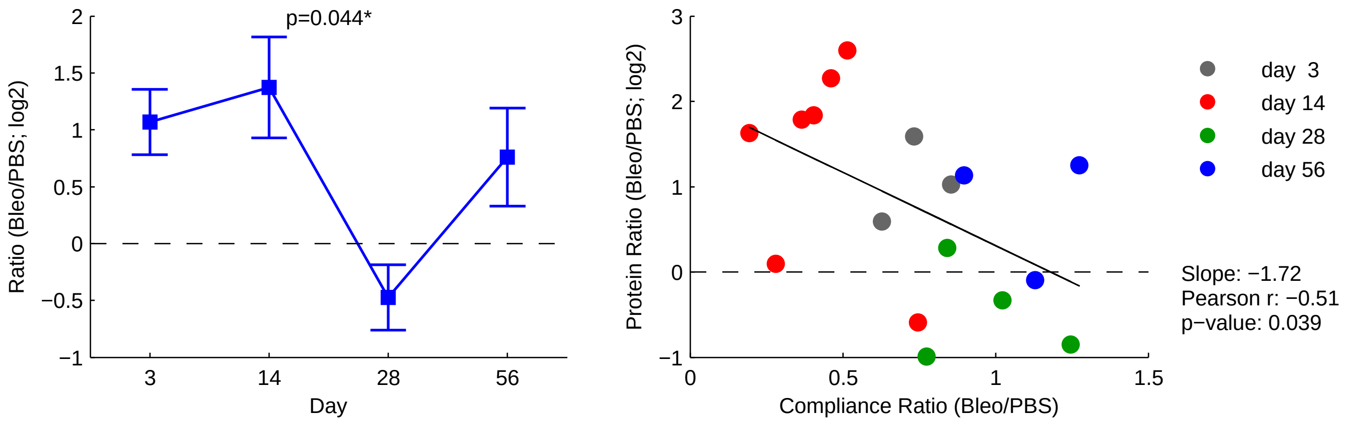

P45376 − Akr1b1 (id: 2868)

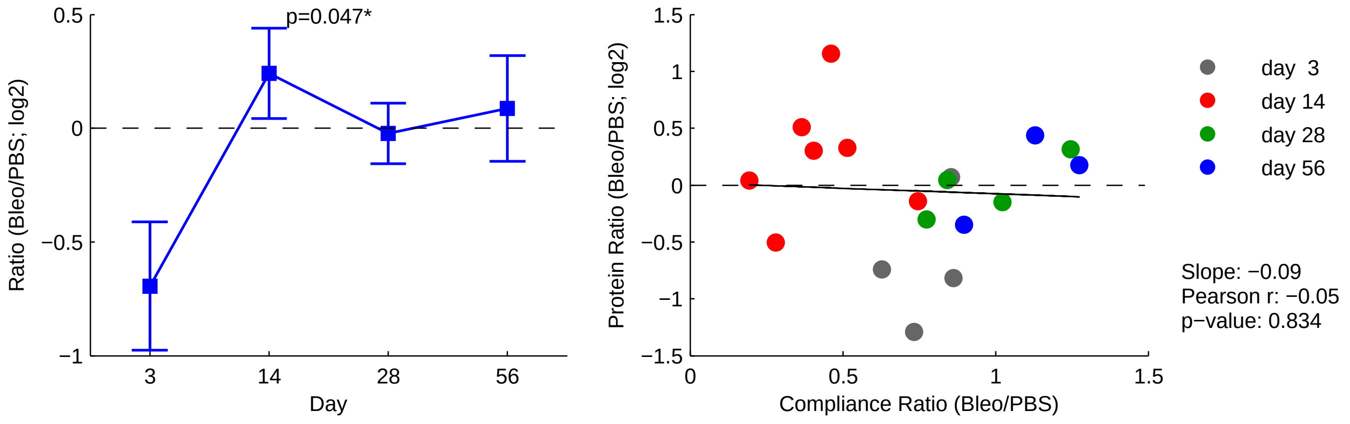

P45377 − Akr1b8 (id: 2869)

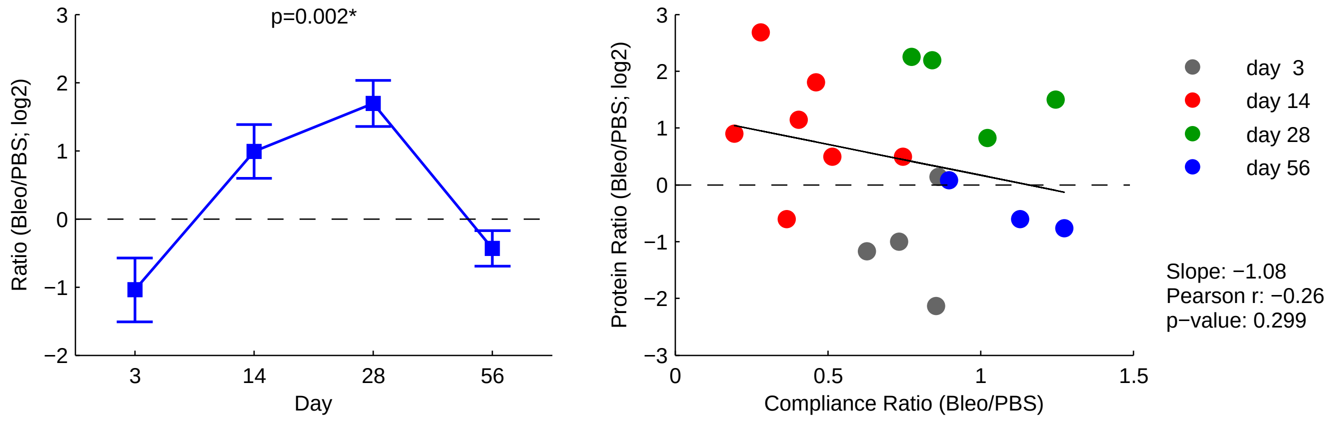

P45700 − Man1a1 (id: 2871)

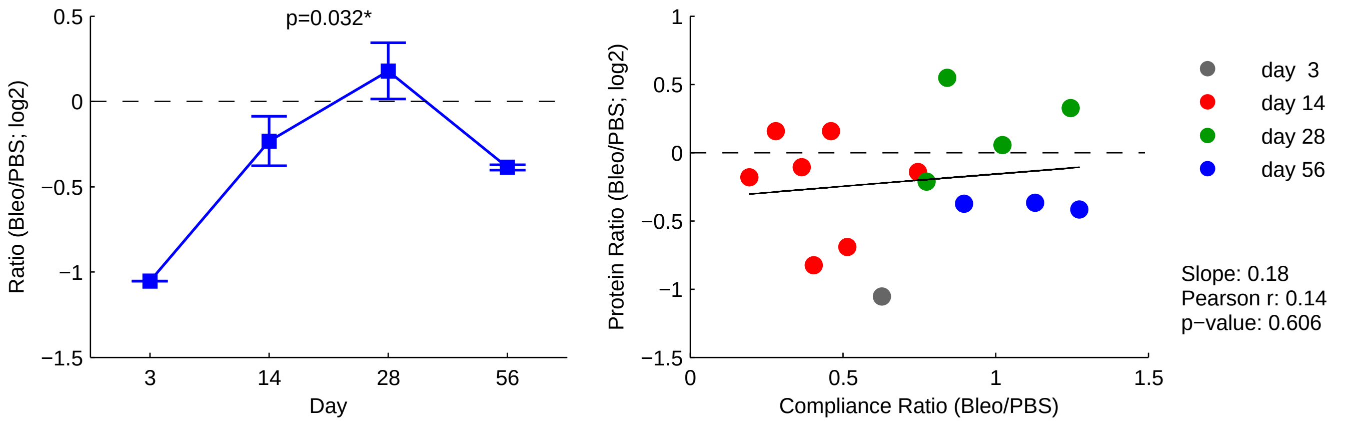

P45952 − Acadm (id: 2873)

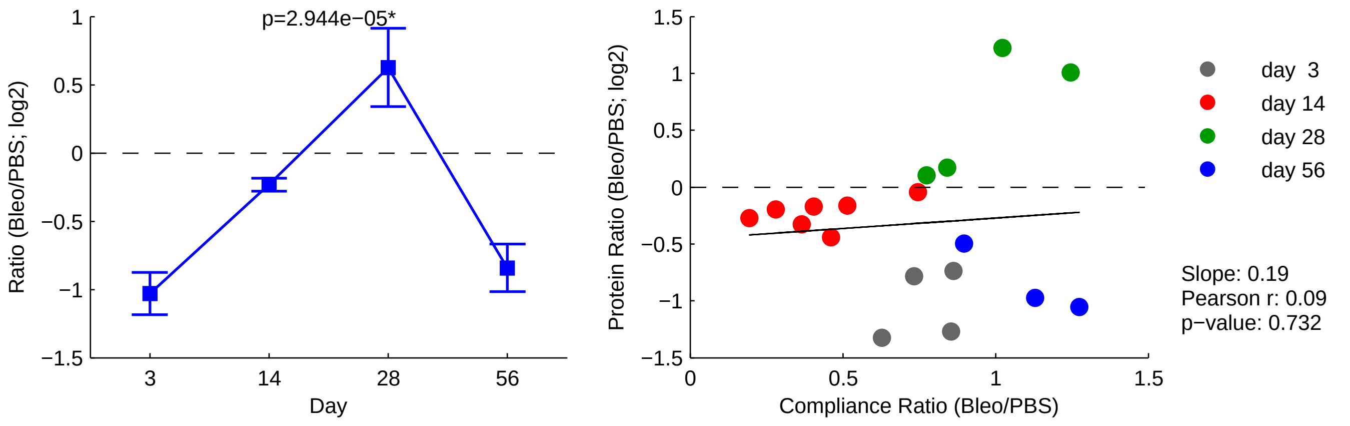

P46061 − Rangap1 (id: 2874)

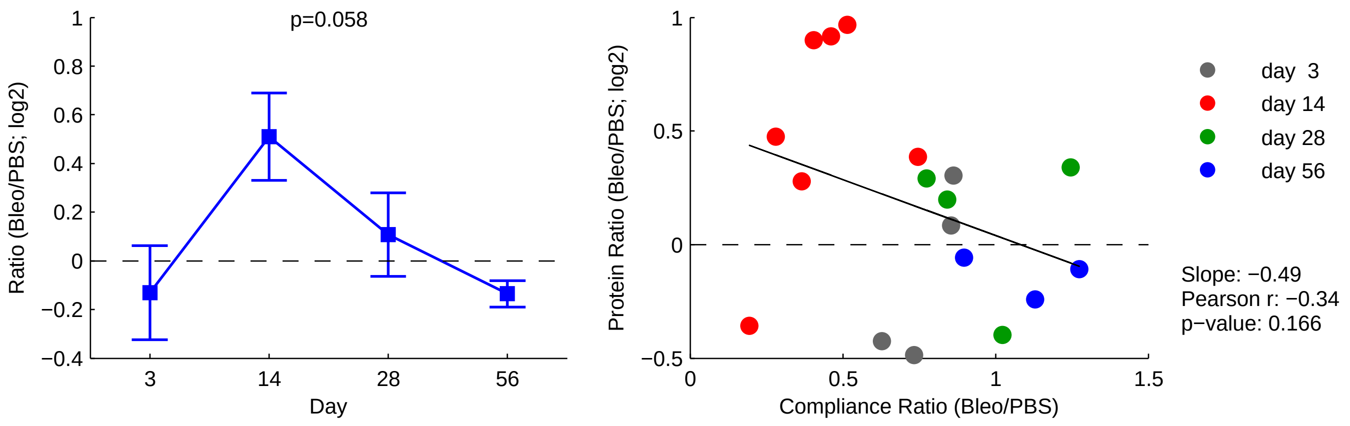

P46412 − Gpx3 (id: 2875)

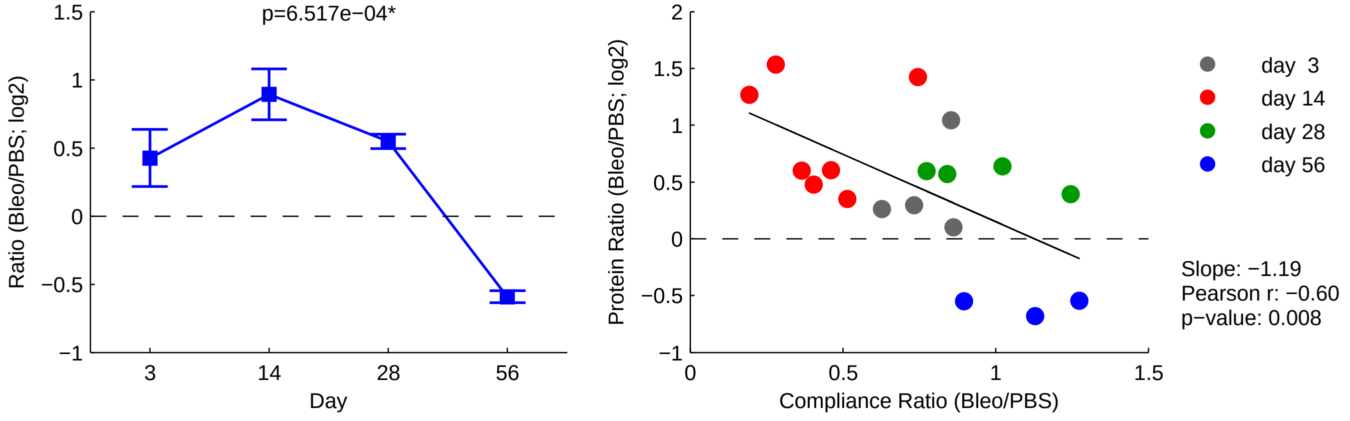

P46460 − Nsf (id: 2877)

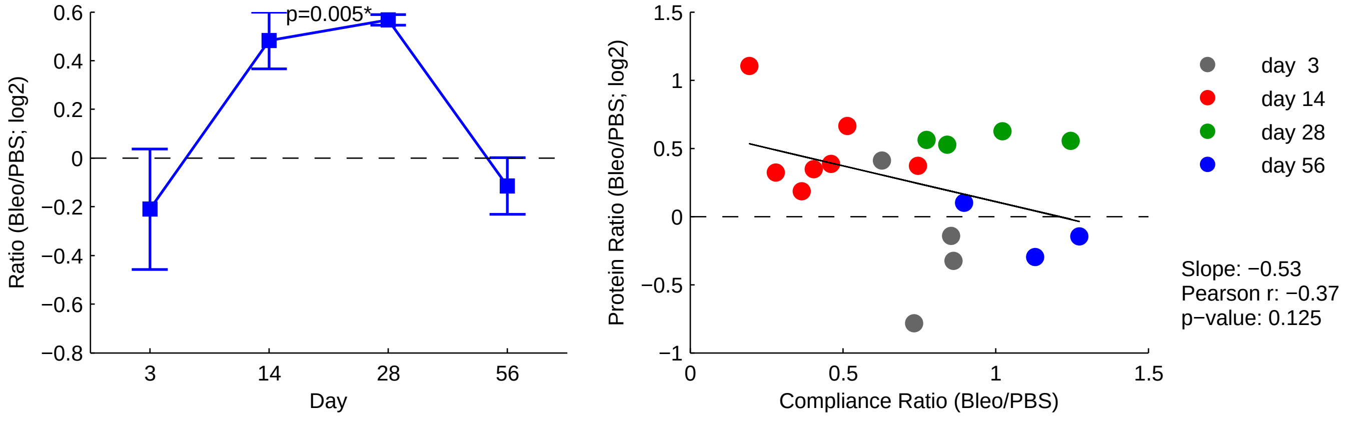

P46471 − Psmc2 (id: 2879)

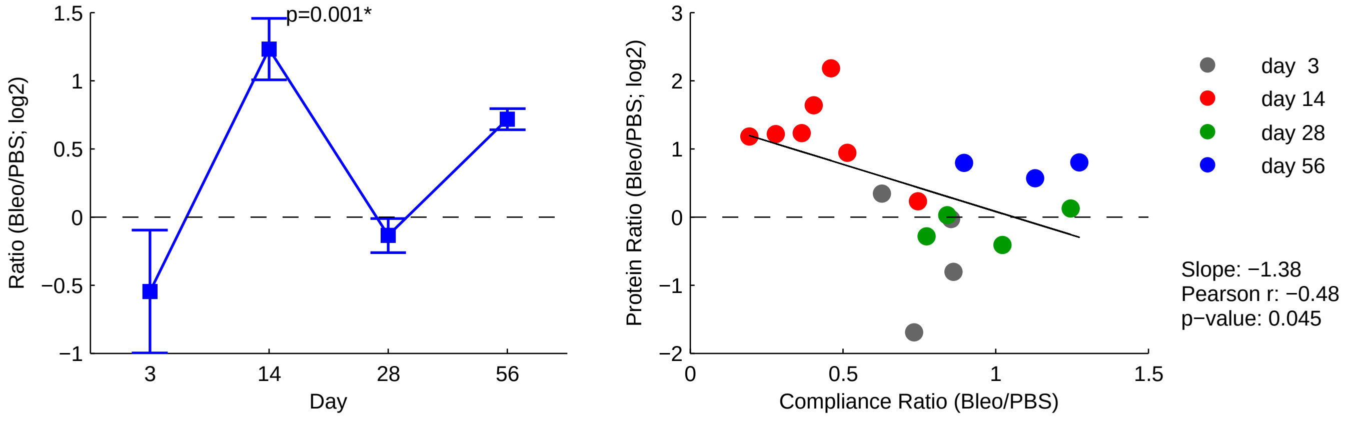

P46638 − Rab11b (id: 2880)

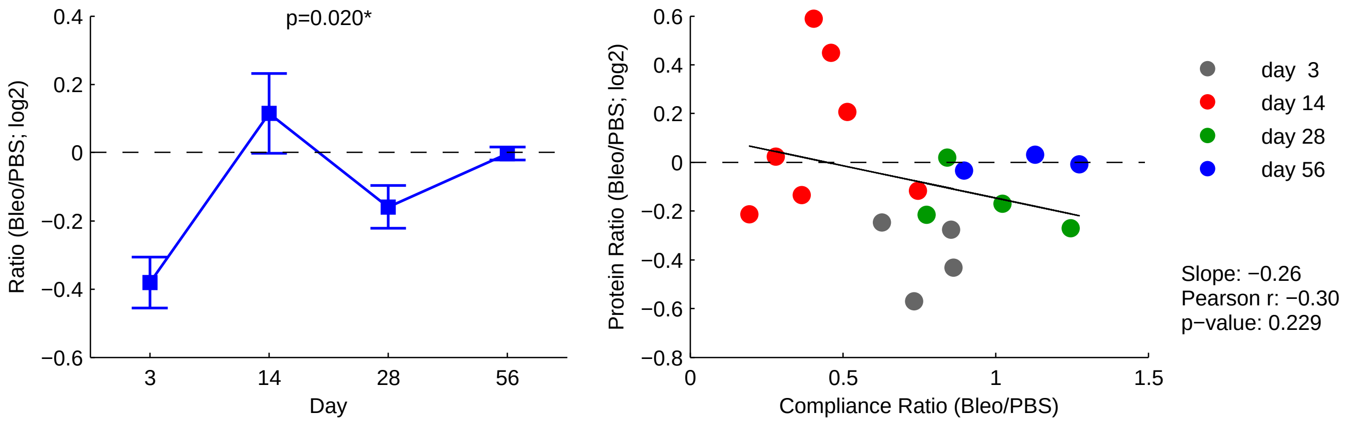

P46656 − Fdx1 (id: 2881)

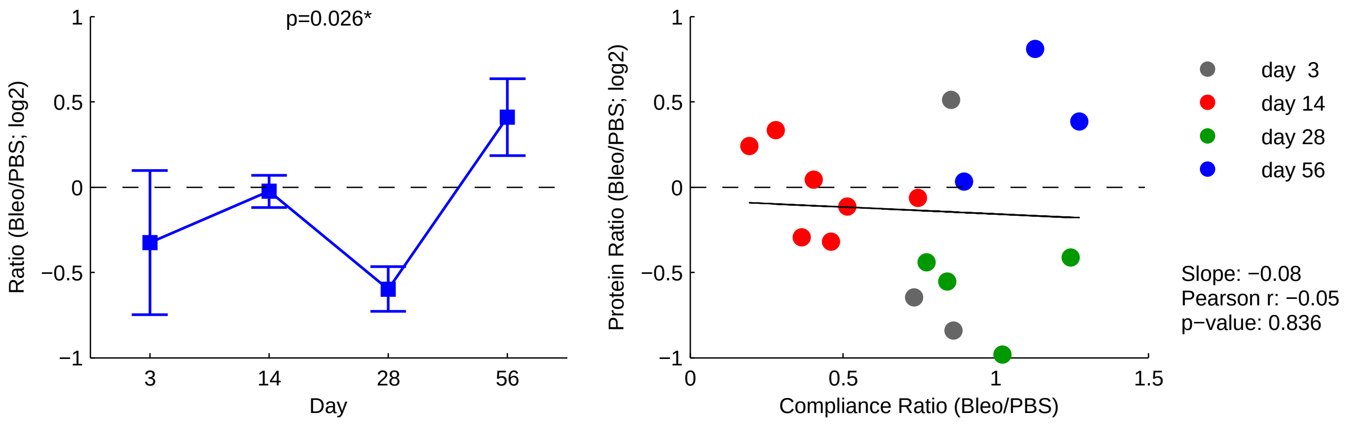

P46662−2 − Nf2 (id: 2882)

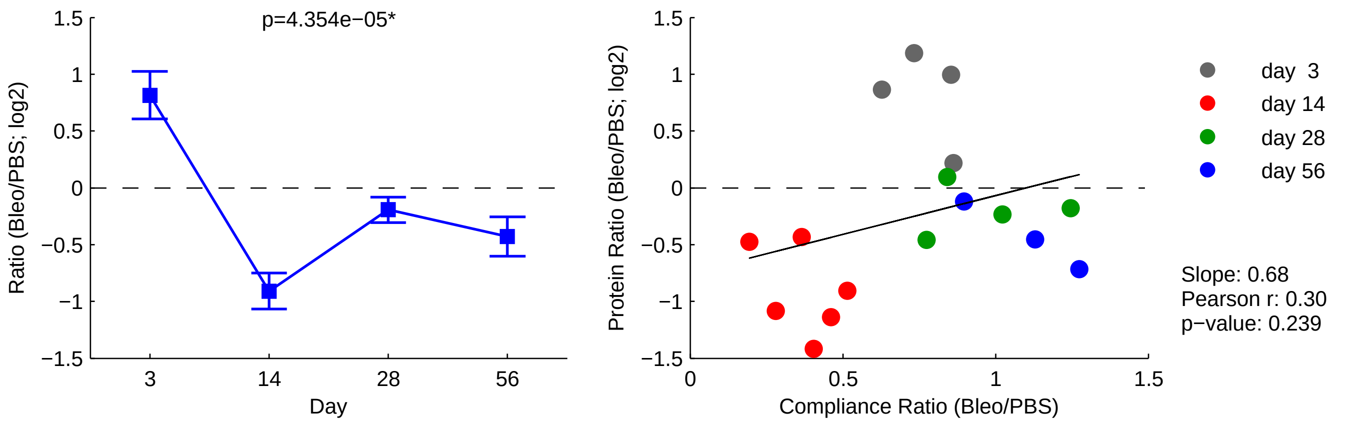

P46664 − Adss (id: 2883)

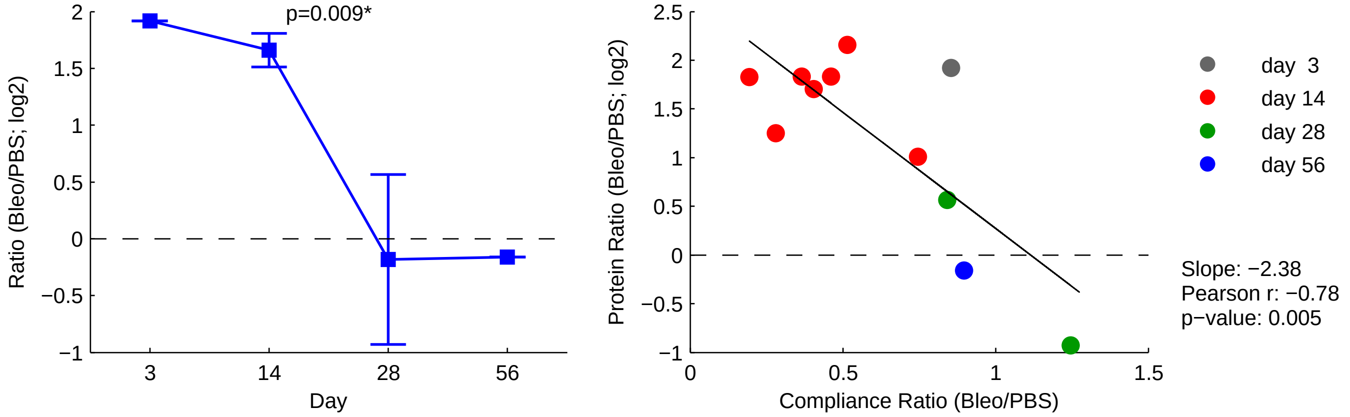

P46735 − Myo1b (id: 2884)

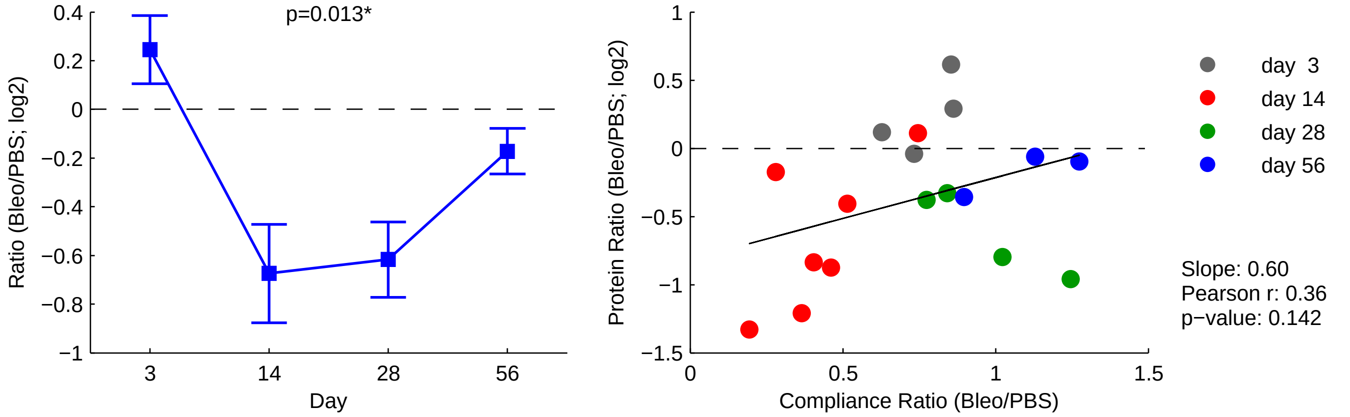

P46737 − Brcc3 (id: 2885)

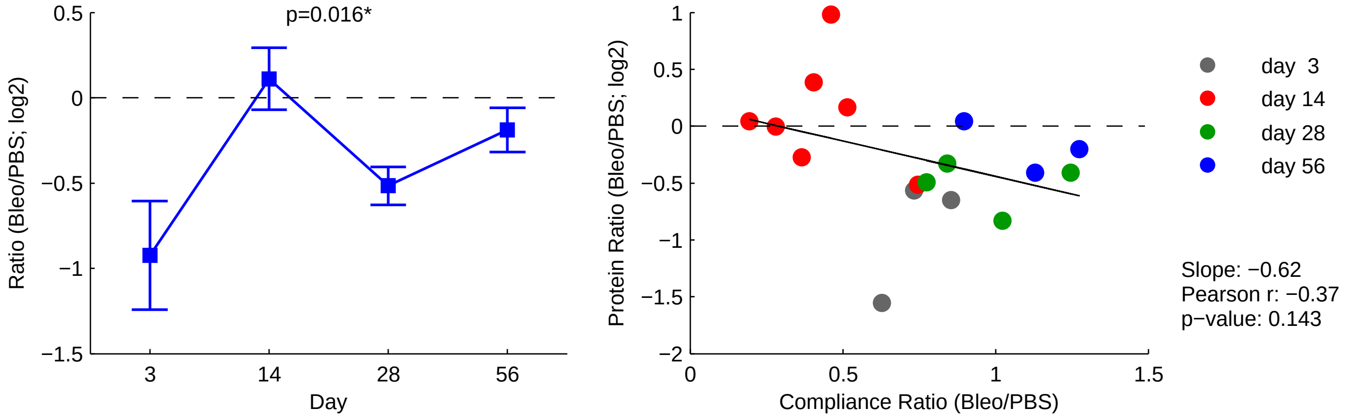

P47713 − Pla2g4a (id: 2891)

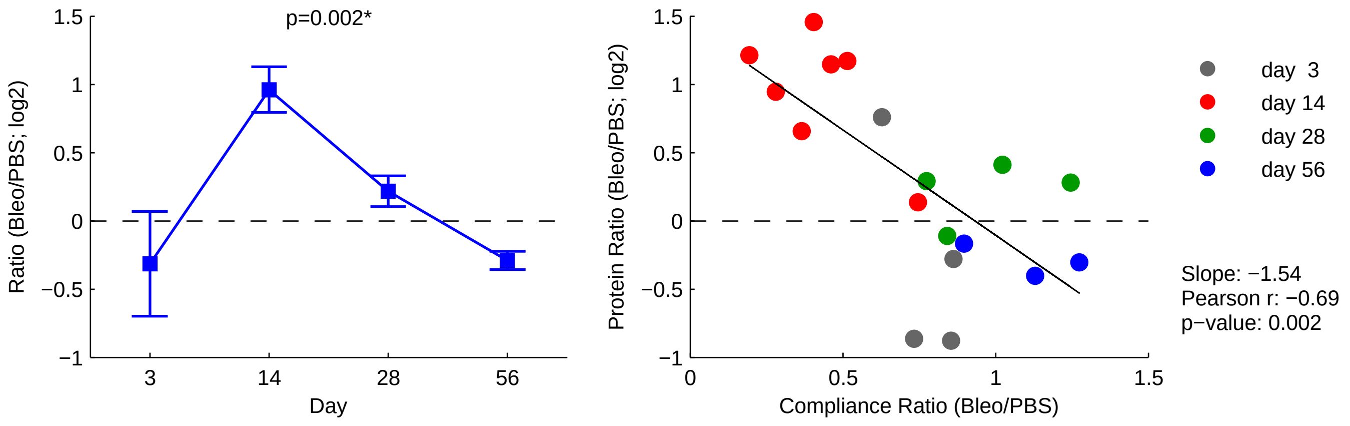

P47738 − Aldh2 (id: 2892)

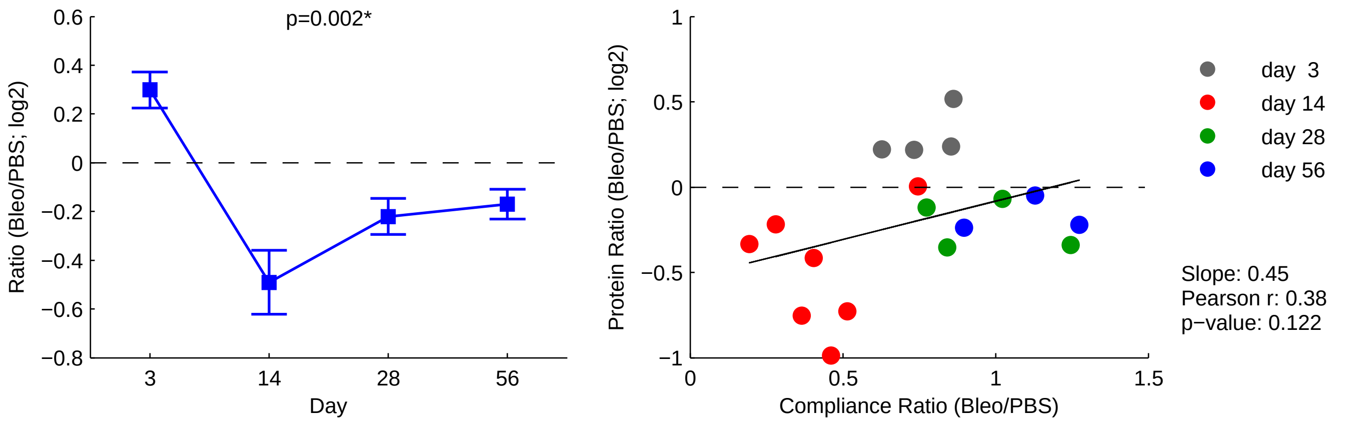

P47791−2 − Gsr (id: 2897)

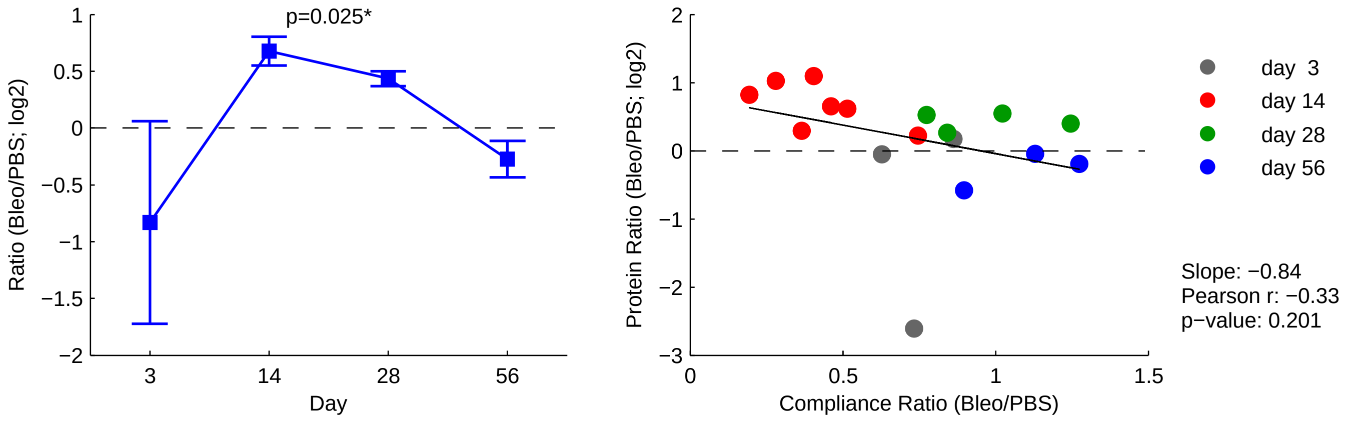

P47809 − Map2k4 (id: 2898)

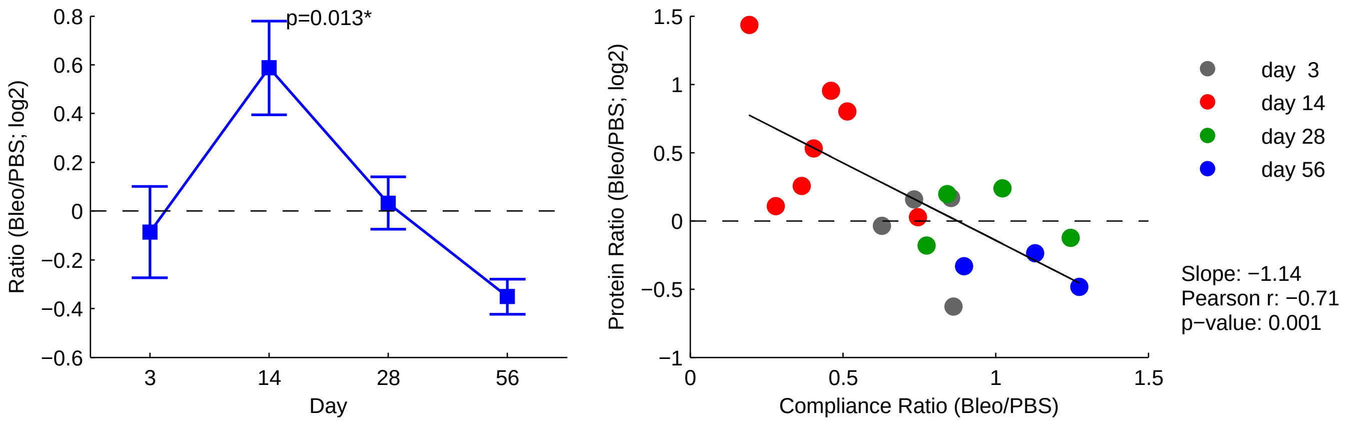

P47856−2 − Gfpt1 (id: 2900)

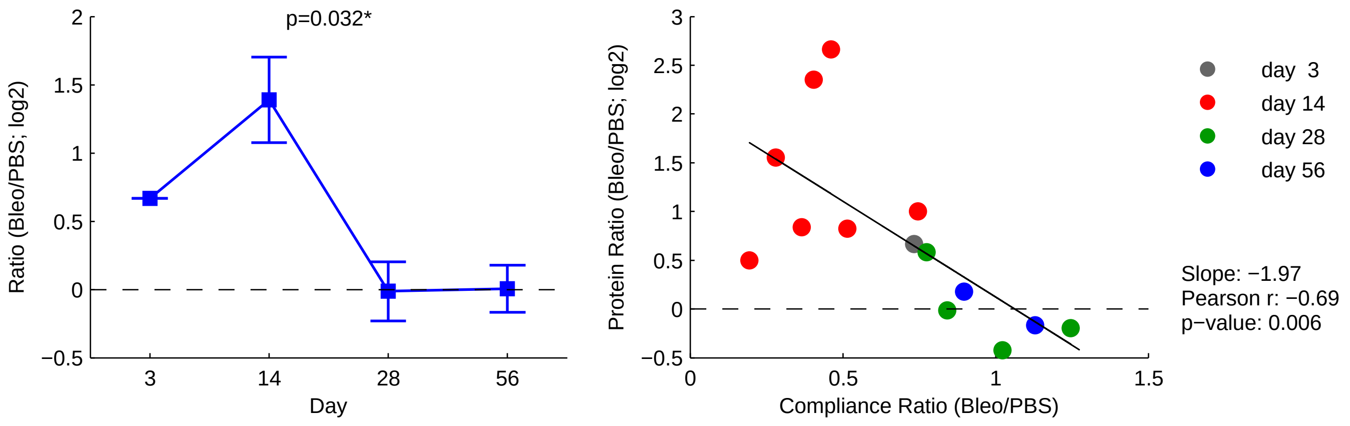

P47877 − Igfbp2 (id: 2902)

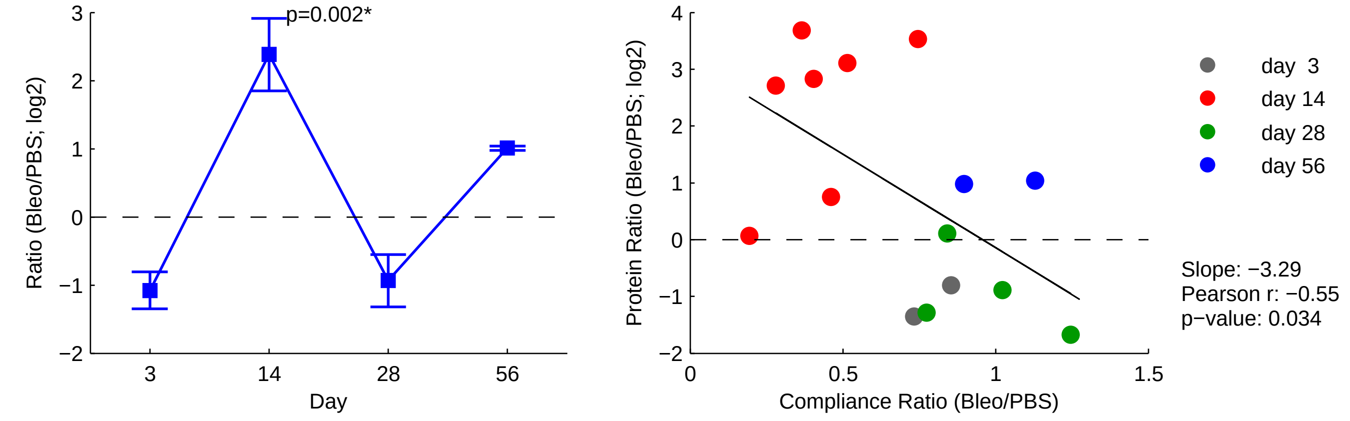

P47880 − Igfbp6 (id: 2903)

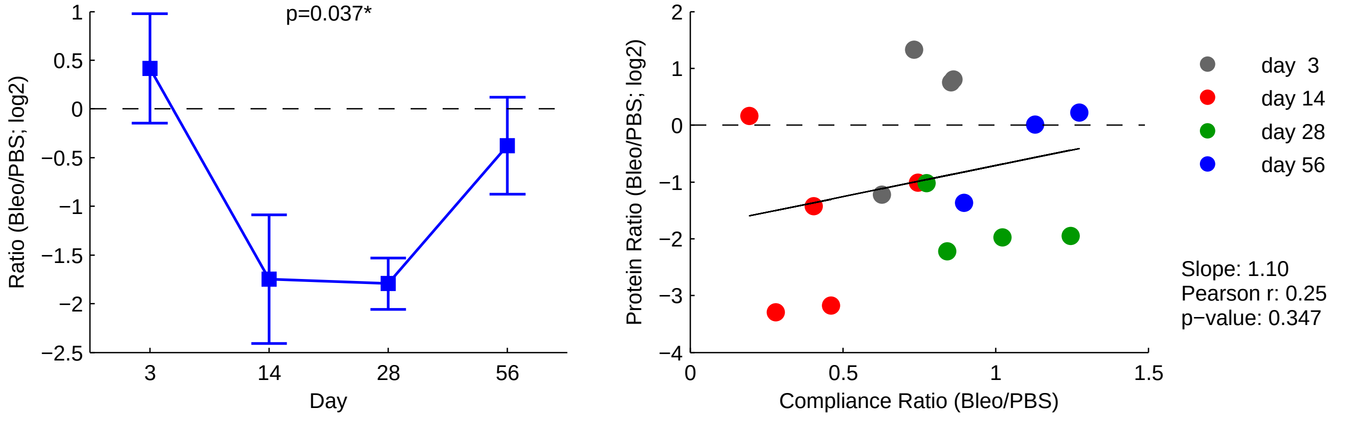

P47911 − Rpl6 (id: 2904)

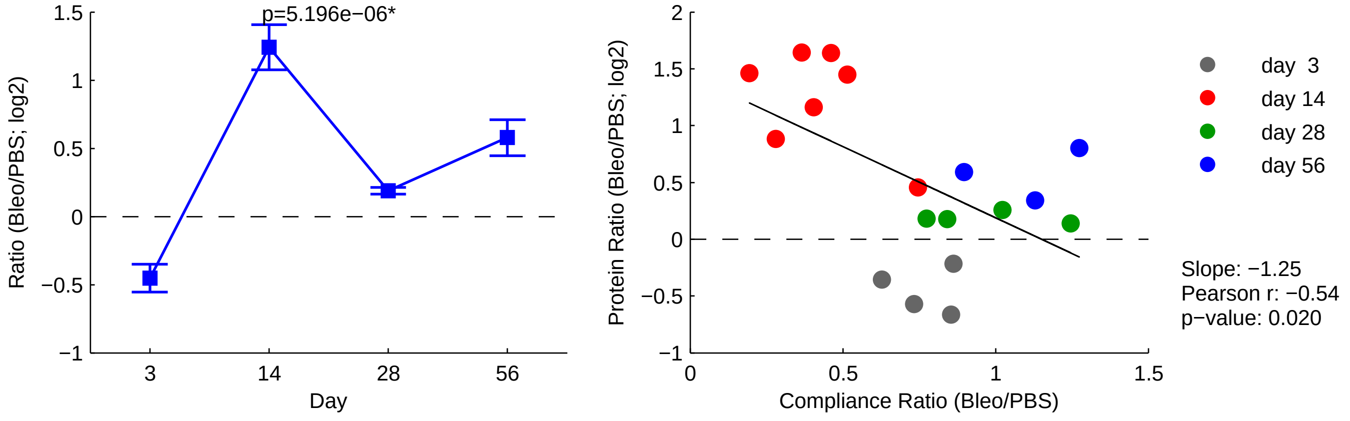

P47941 − Crkl (id: 2905)

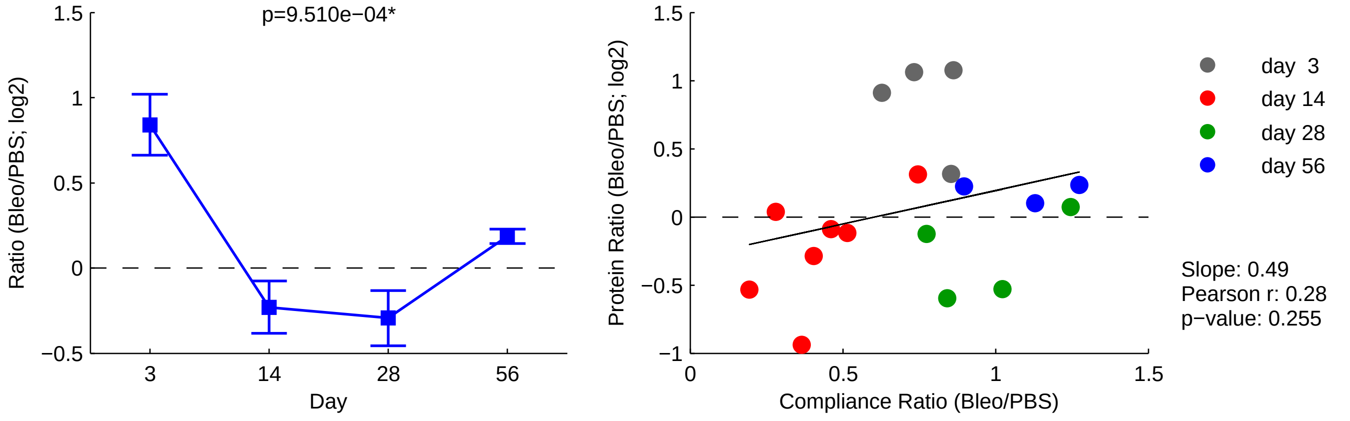

P47955 − Rplp1 (id: 2906)

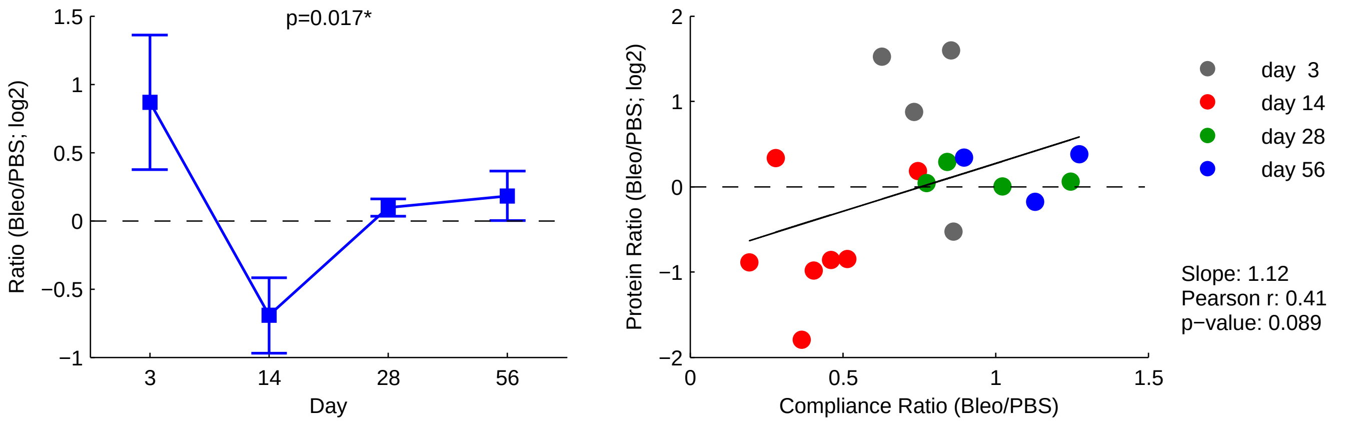

P47962 − Rpl5 (id: 2907)

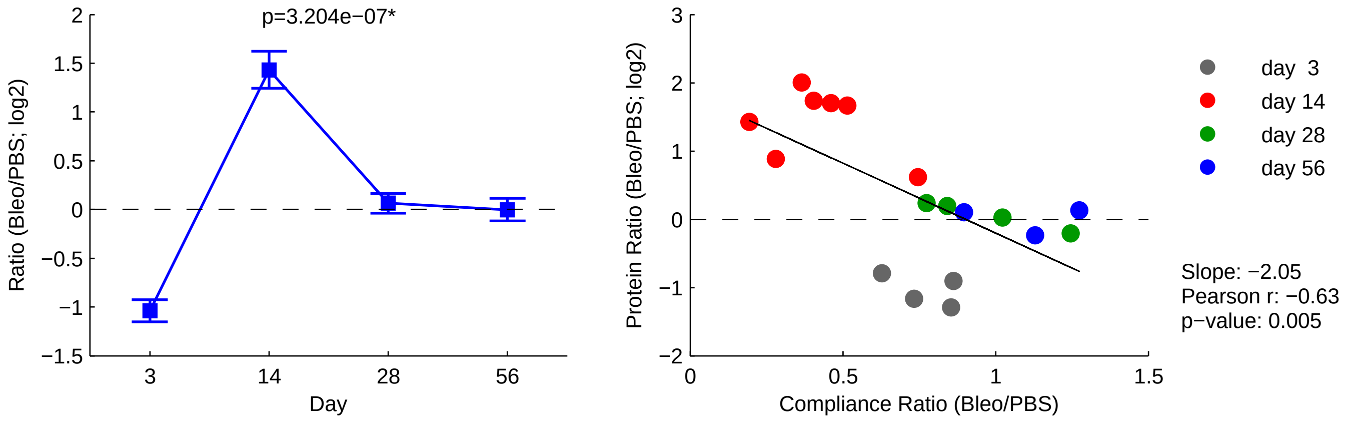

P47963 − Rpl13 (id: 2908)

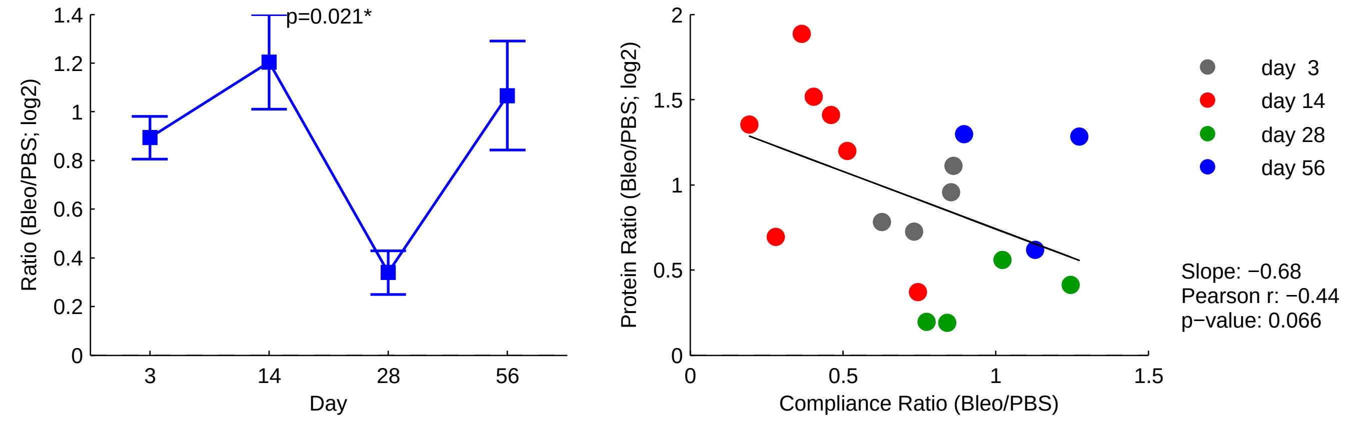

P48025 − Syk (id: 2911)

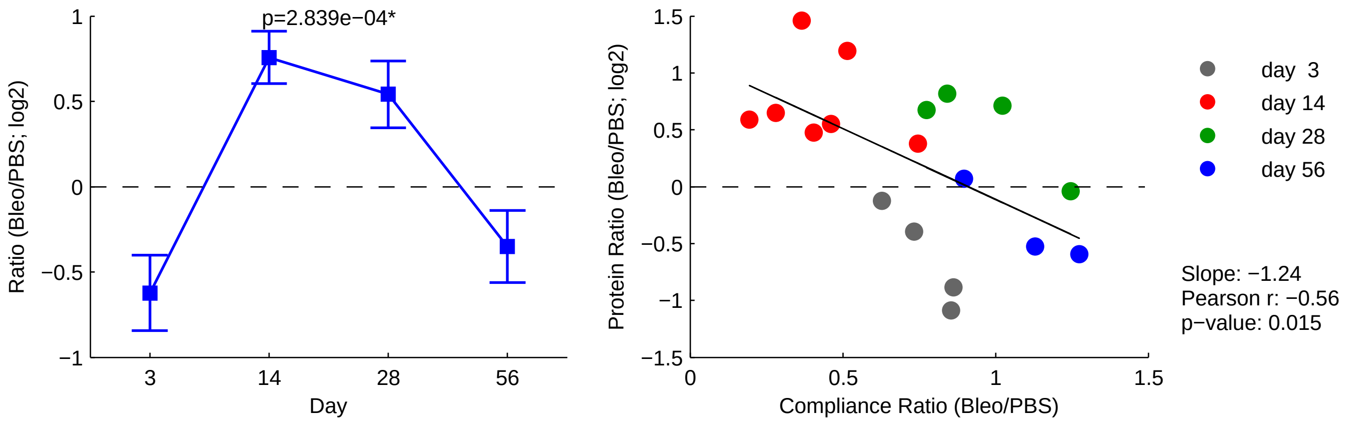

P48036 − Anxa5 (id: 2912)

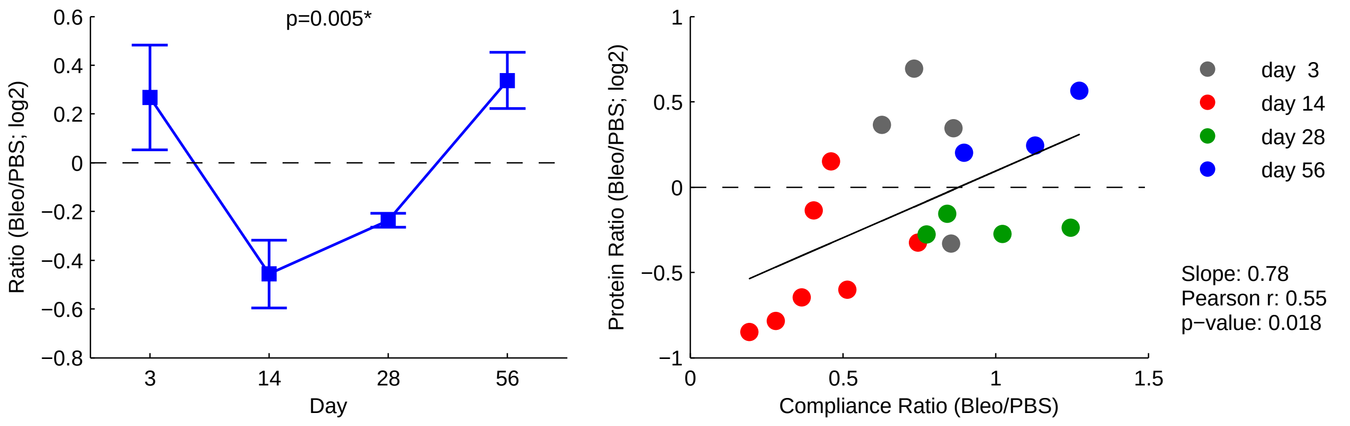

P48302 − Ednrb (id: 2913)

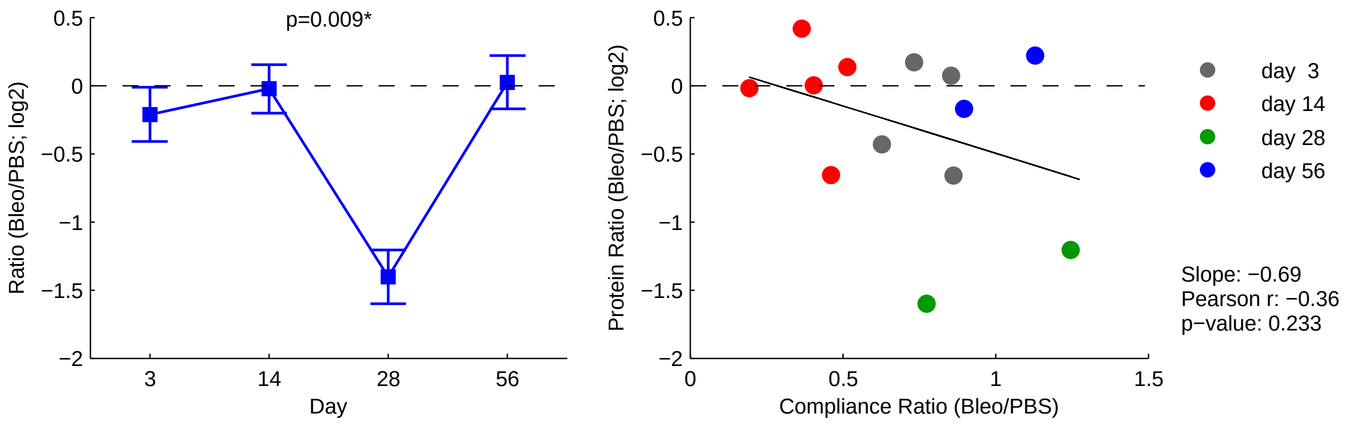

P48356−3 − Lepr (id: 2915)

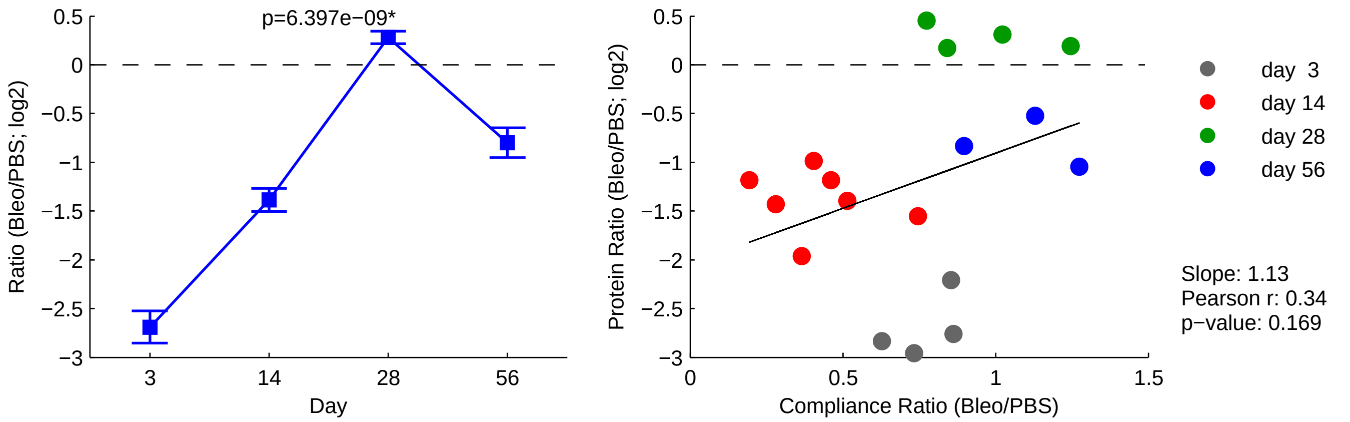

P48410 − Abcd1 (id: 2917)

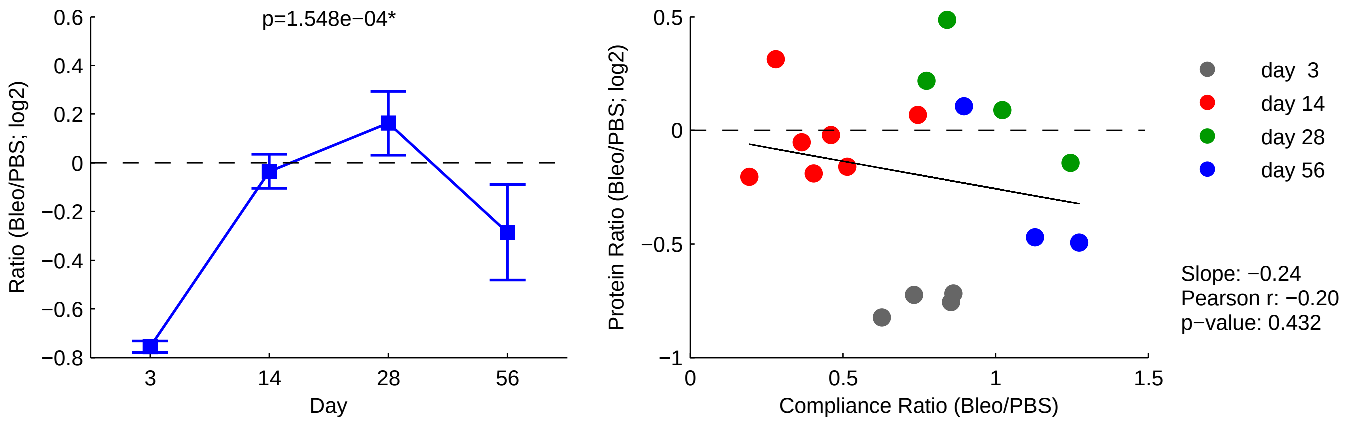

P48428 − Tbca (id: 2918)

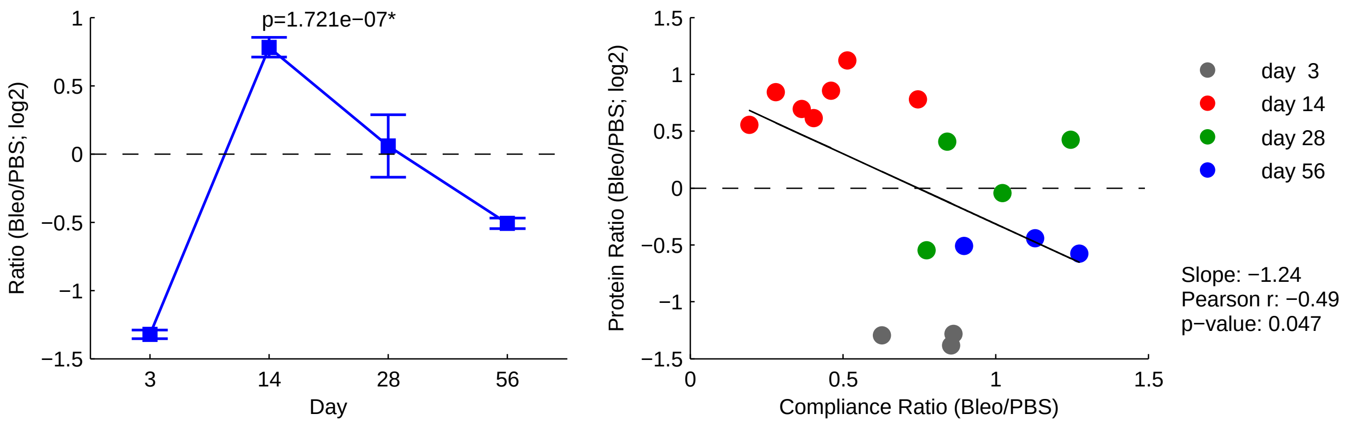

Q8BLF6 − Idua (id: 2919)

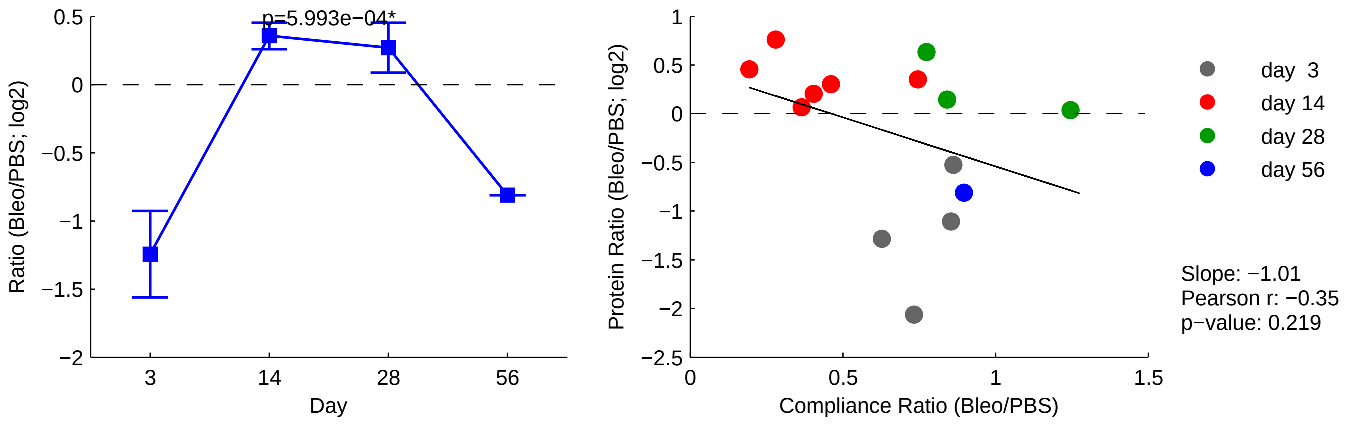

P48678 − Lmna (id: 2921)

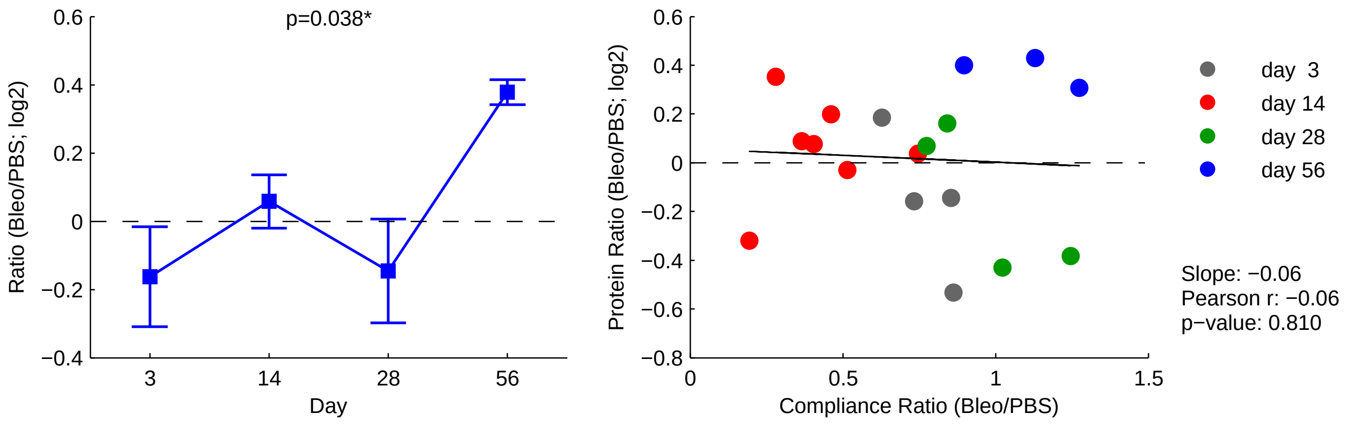

P48758 − Cbr1 (id: 2924)

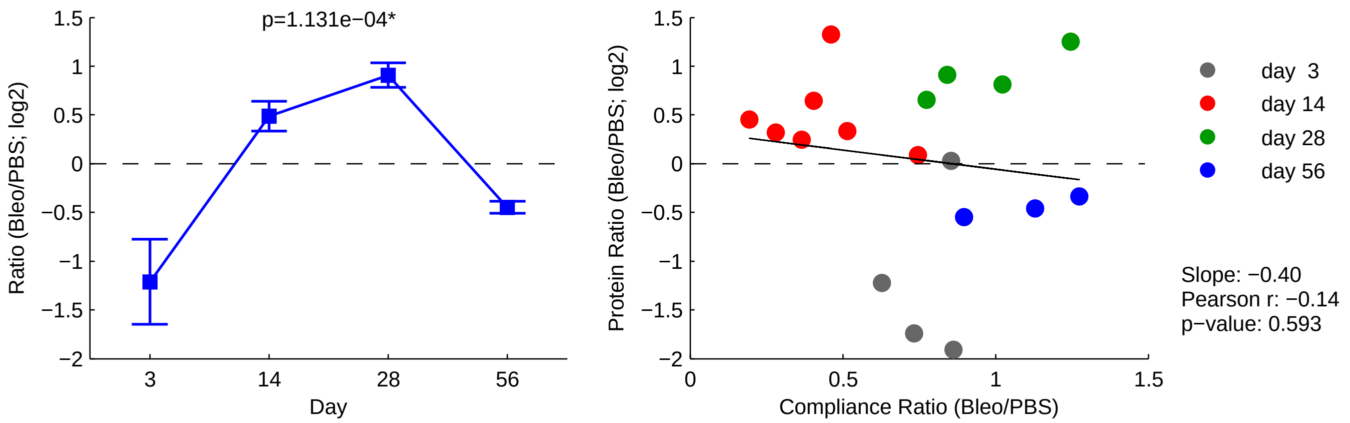

P48759 − Ptx3 (id: 2925)

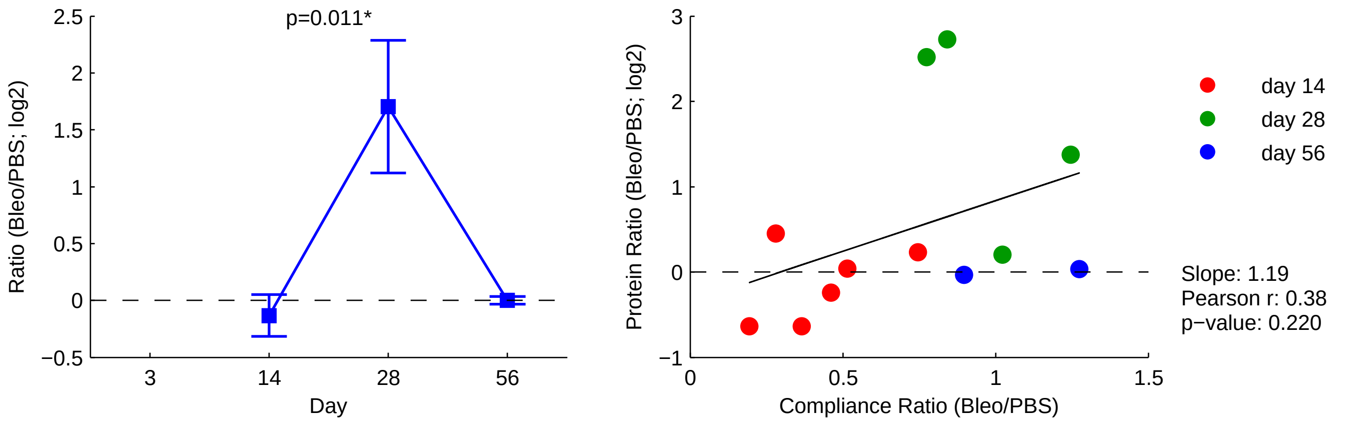

P48771 − Cox7a2 (id: 2926)

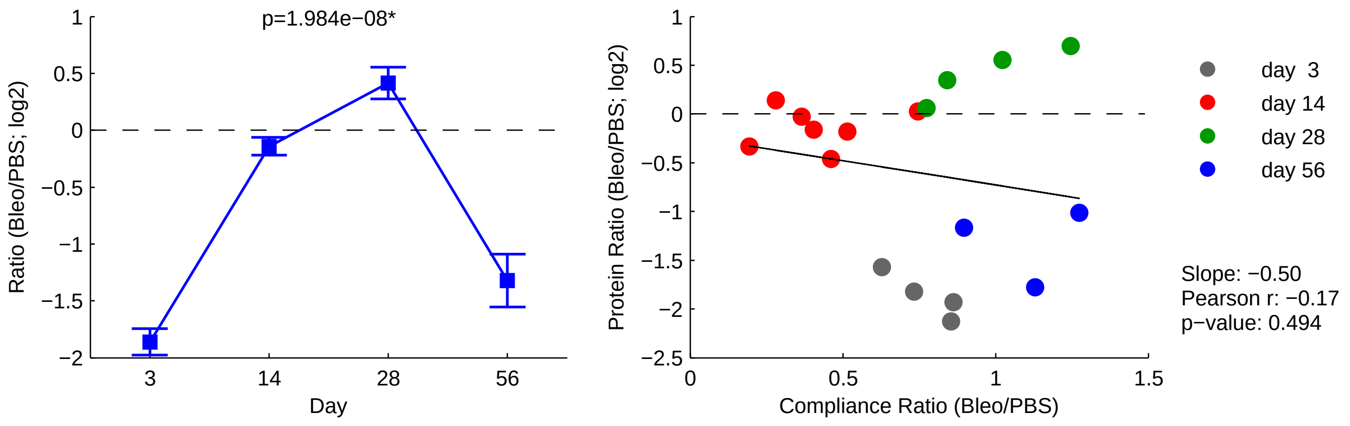

P48772 − Cox8b (id: 2927)

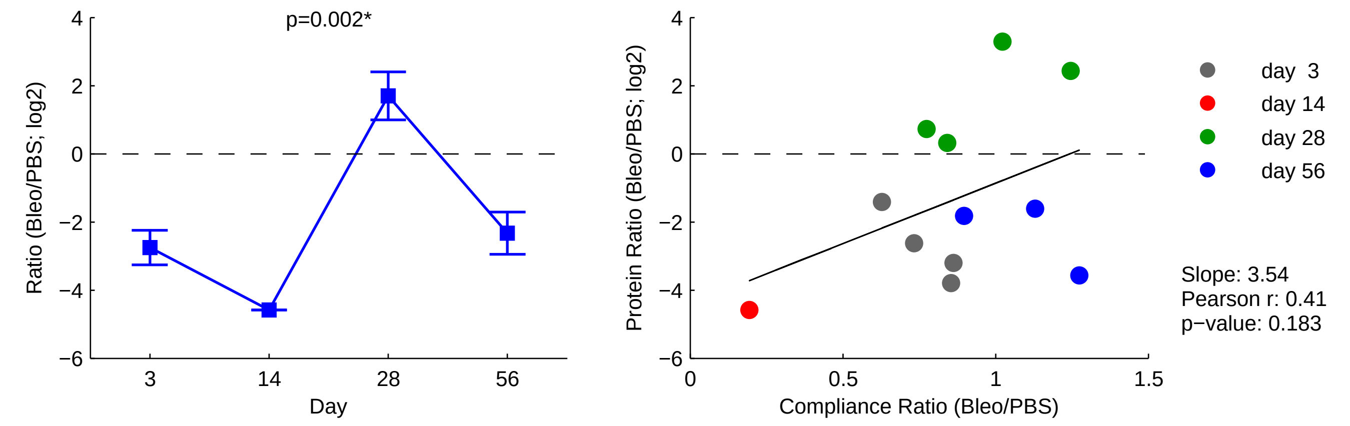

P48787 − Tnni3 (id: 2928)

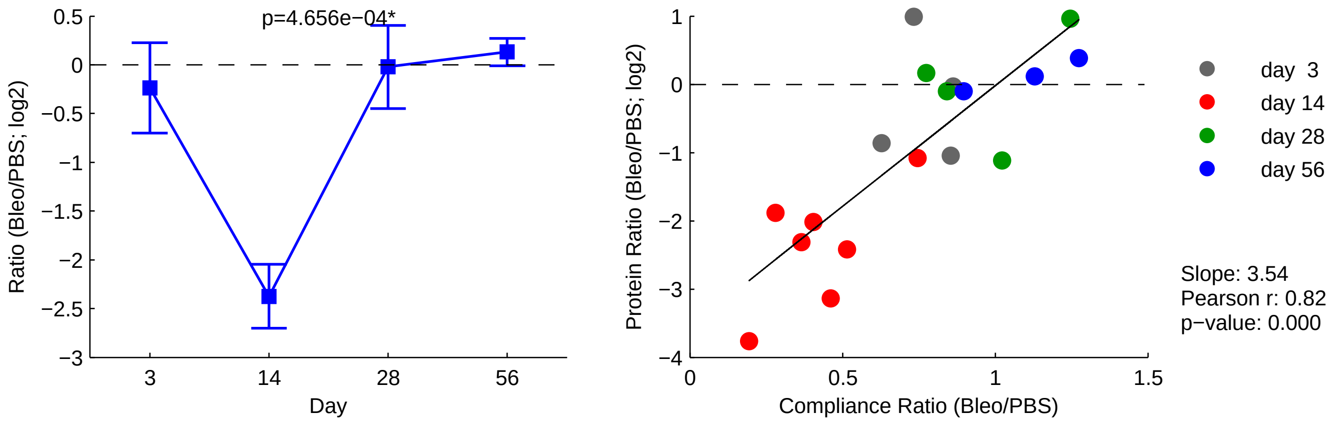

P48962 − Slc25a4 (id: 2929)

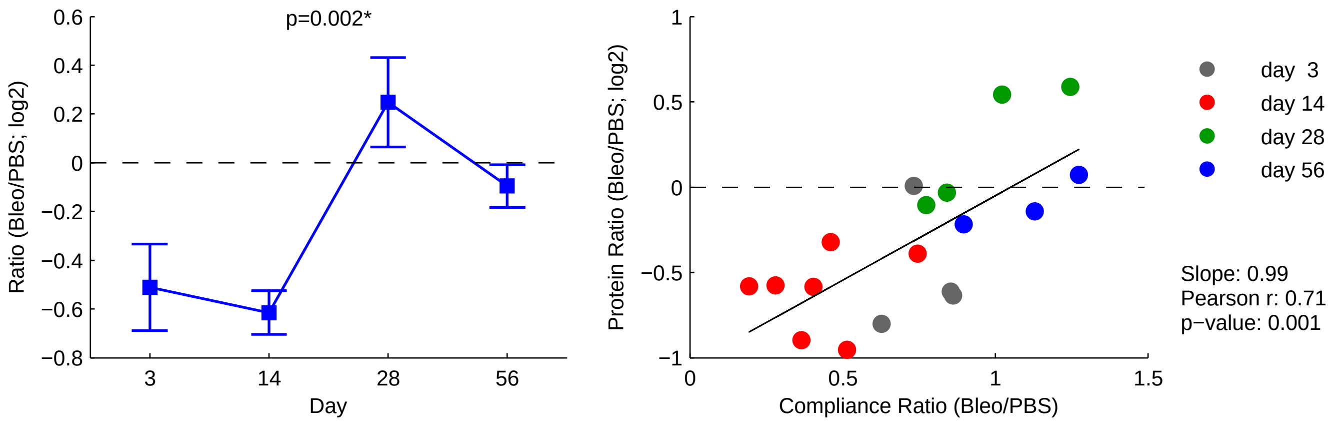

P49135 − Ercc3 (id: 2933)

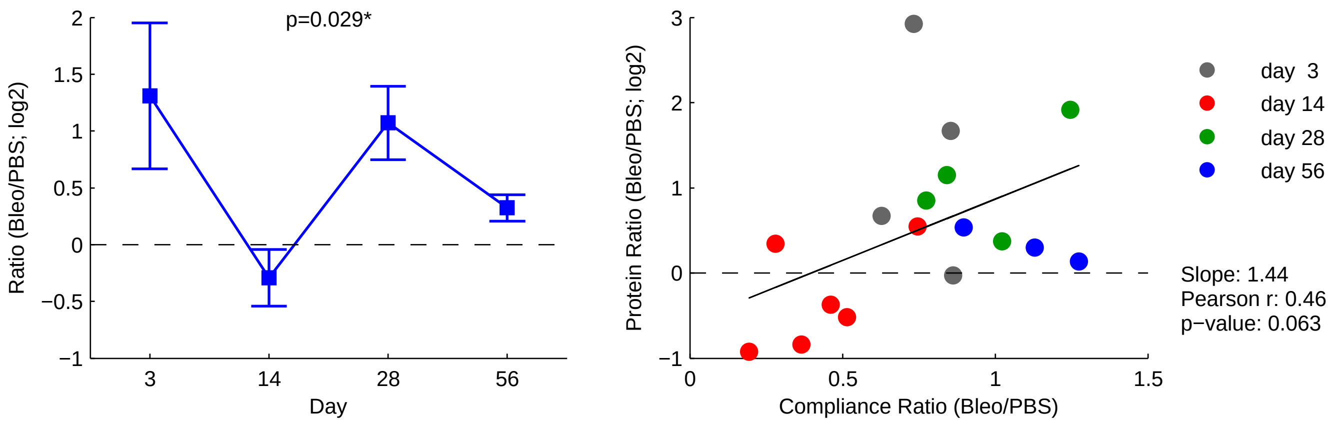

P49138 − Mapkapk2 (id: 2934)

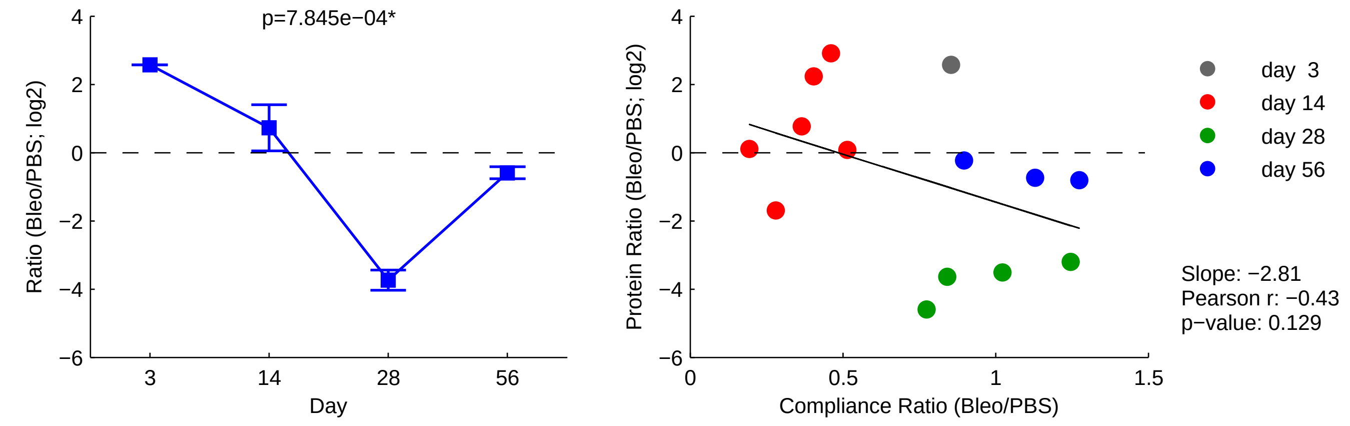

P49312 − Hnrnpa1 (id: 2939)

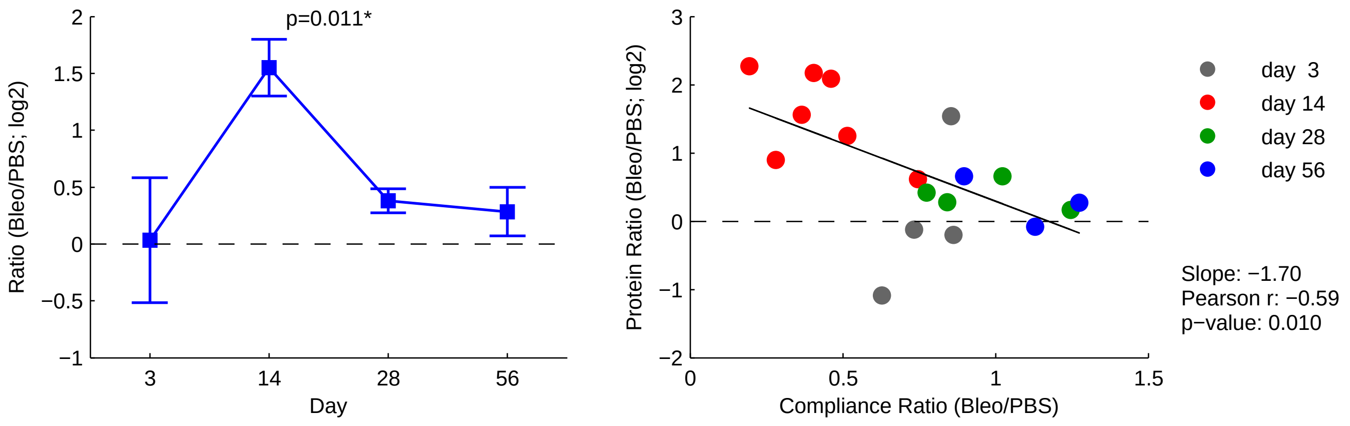

P49443 − Ppm1a (id: 2941)

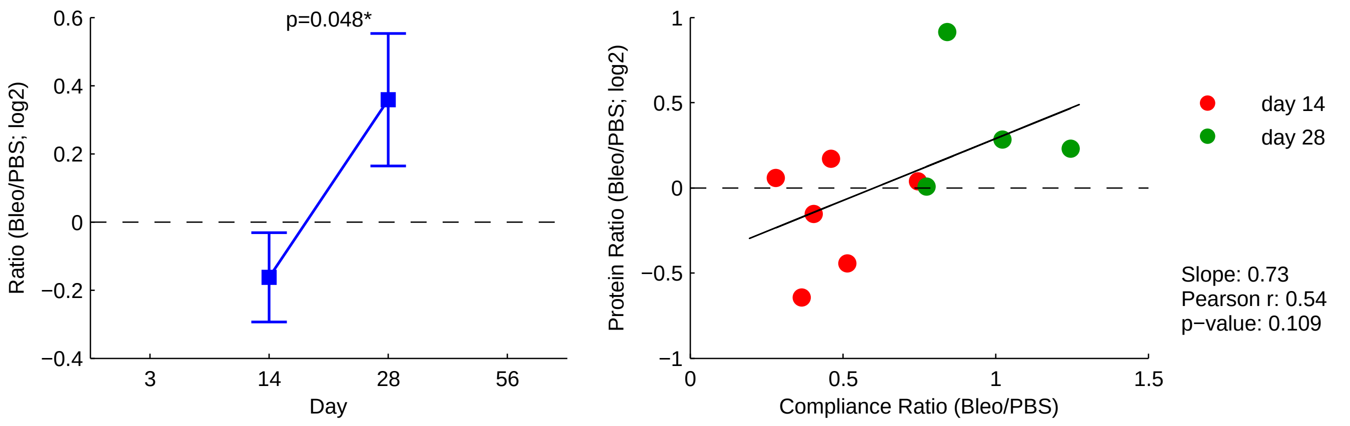

P49446−3 − Ptpre (id: 2942)

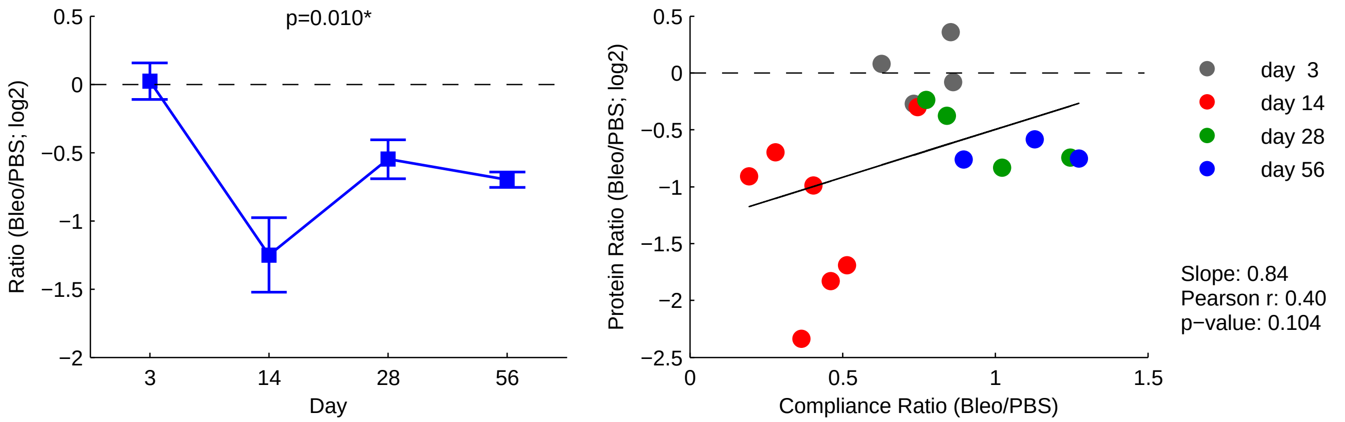

P49710 − Hcls1 (id: 2946)

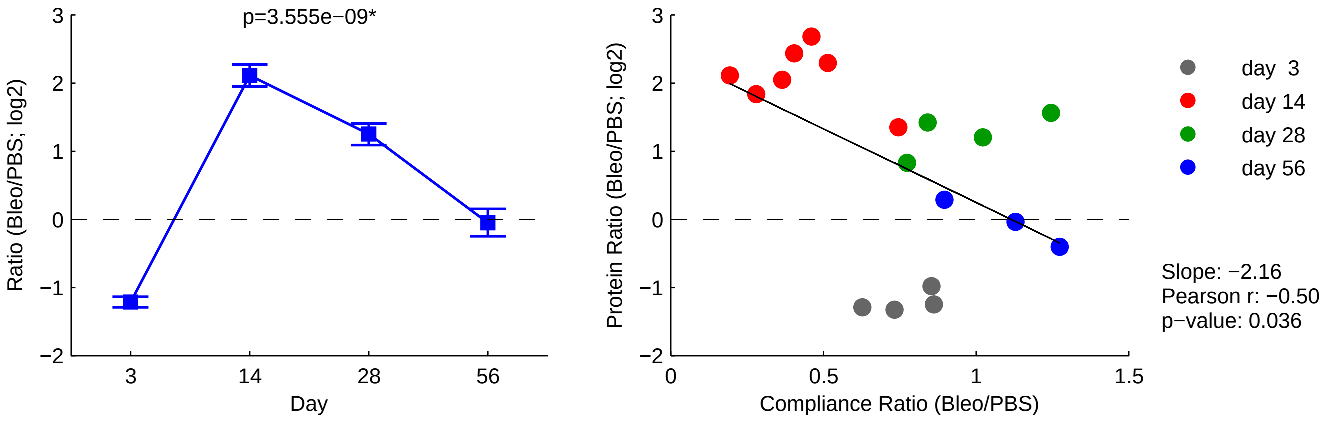

P49813 − Tmod1 (id: 2951)

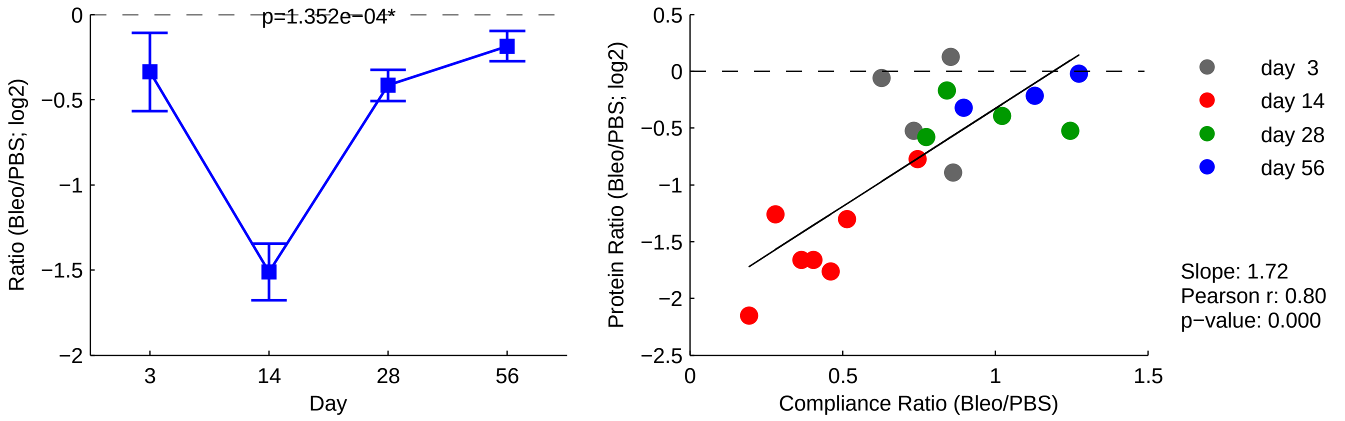

P49817 − Cav1 (id: 2952)

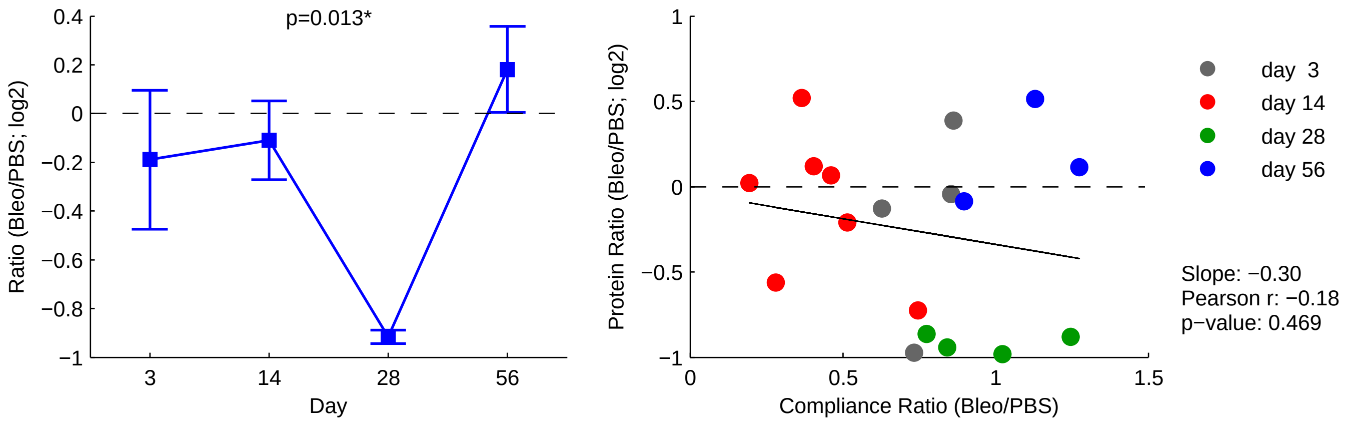

P49817−2 − Cav1 (id: 2953)

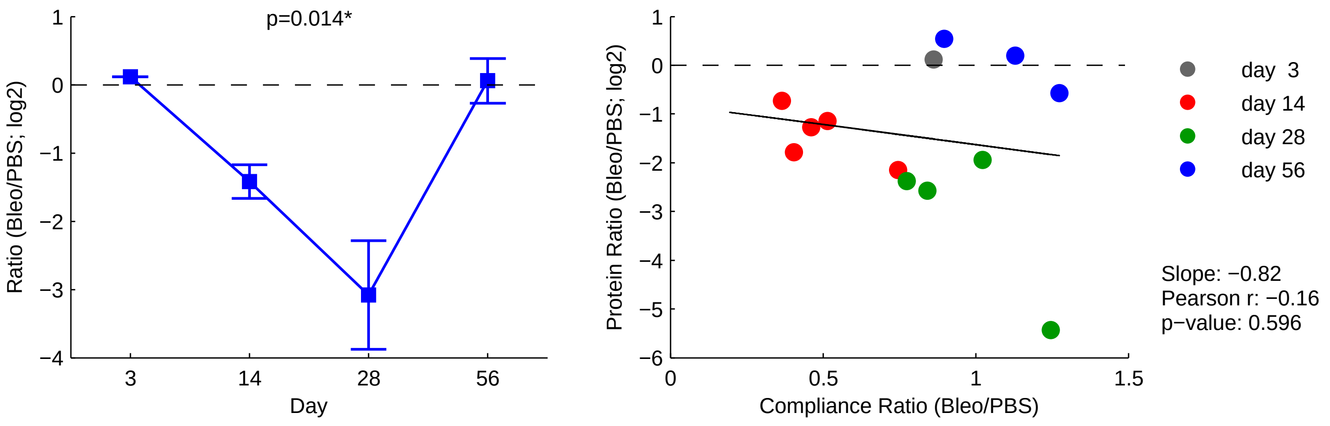

P49935 − Ctsh (id: 2954)

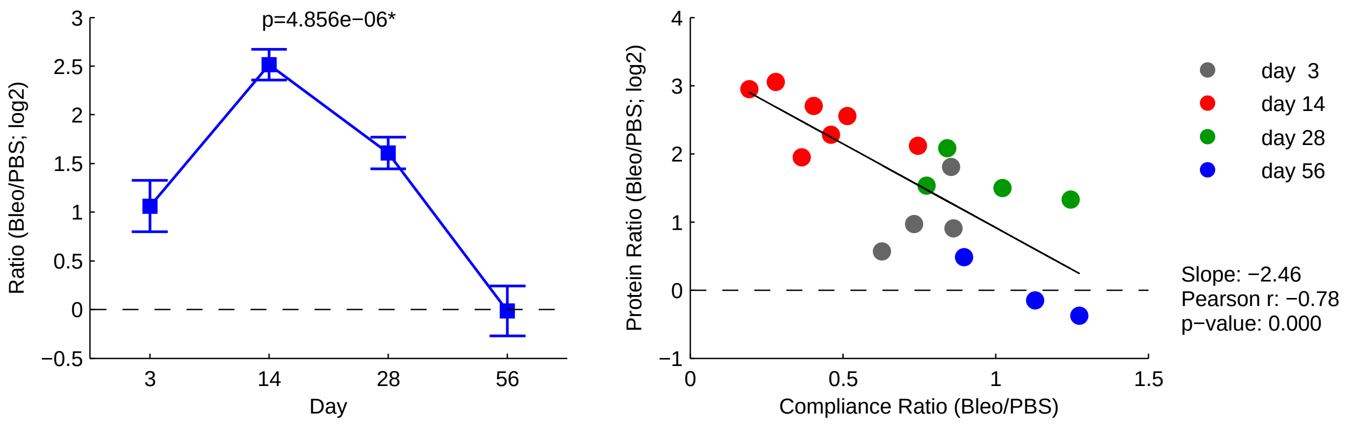

P50172 − Hsd11b1 (id: 2959)

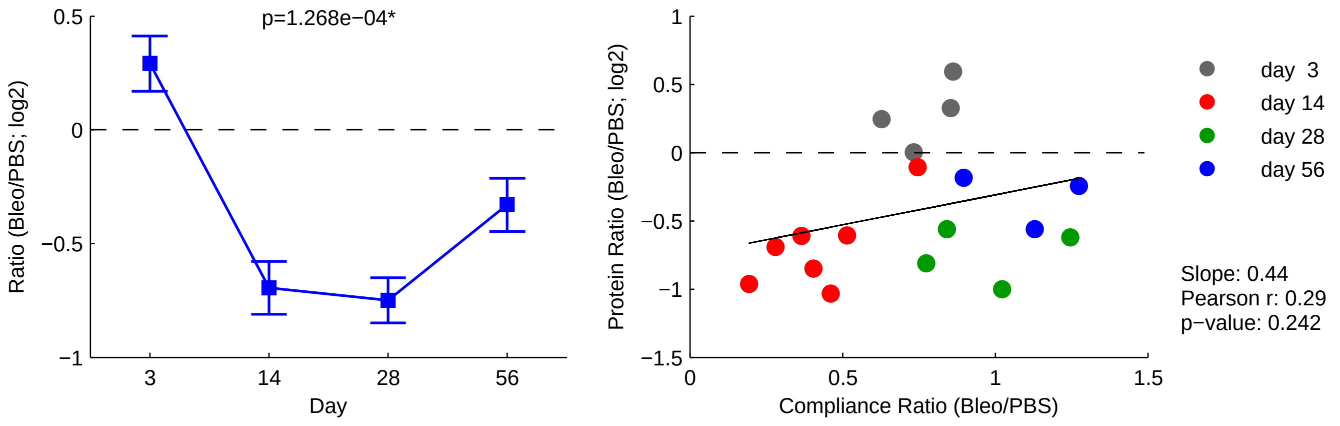

P50220 − Nkx2−1 (id: 2960)

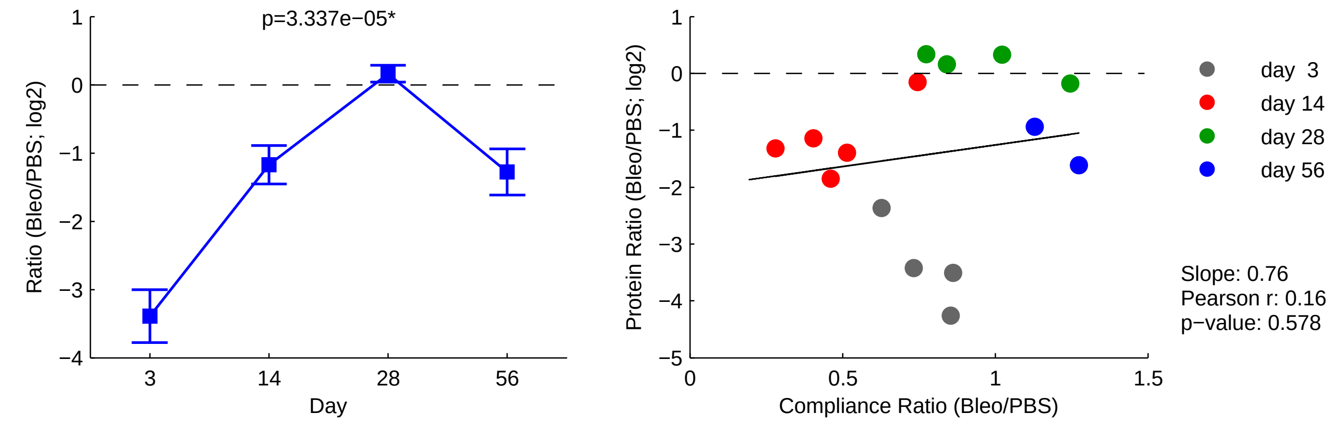

P50285 − Fmo1 (id: 2963)

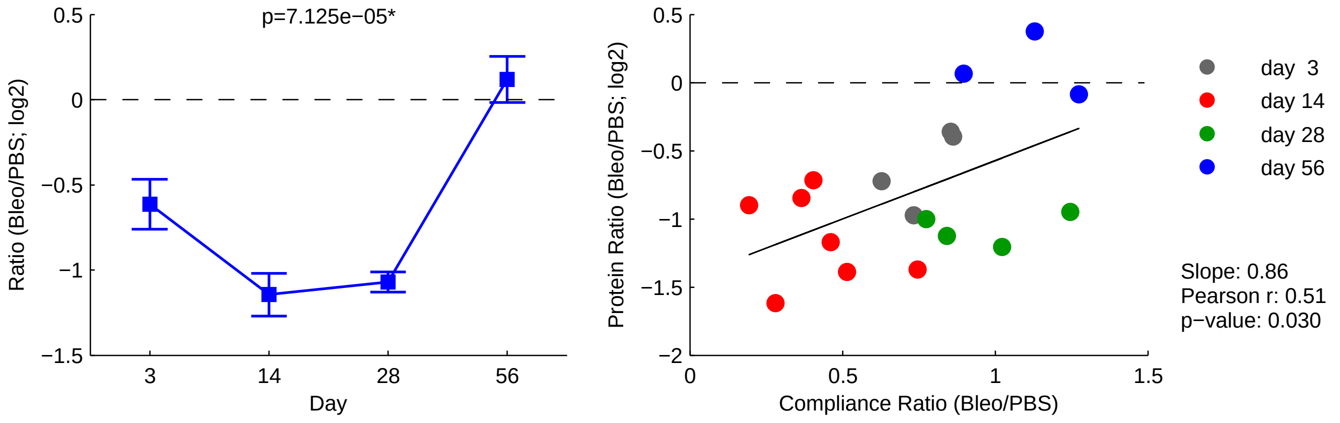

P50404 − Sftpd (id: 2965)

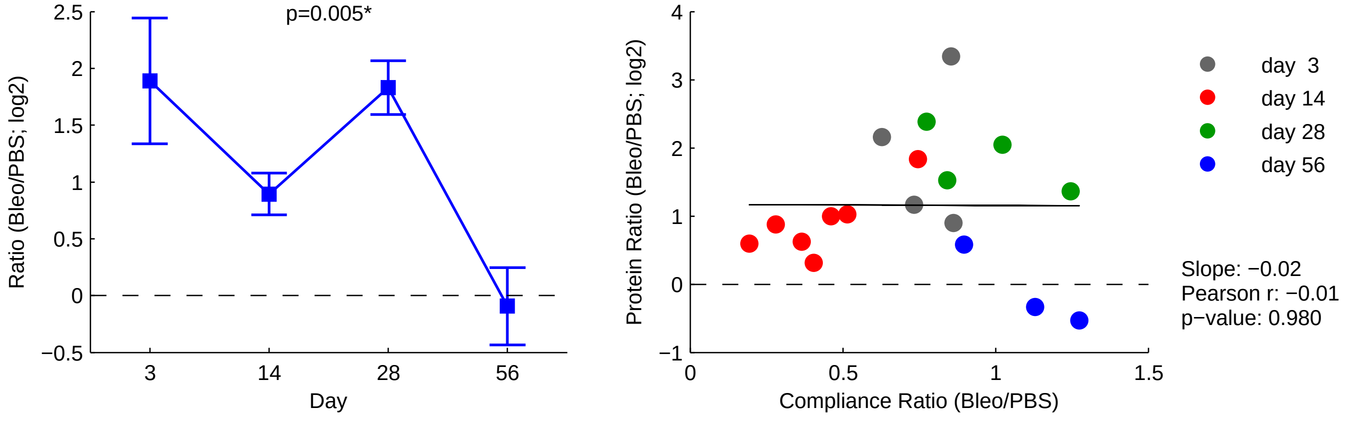

P50427 − Sts (id: 2966)

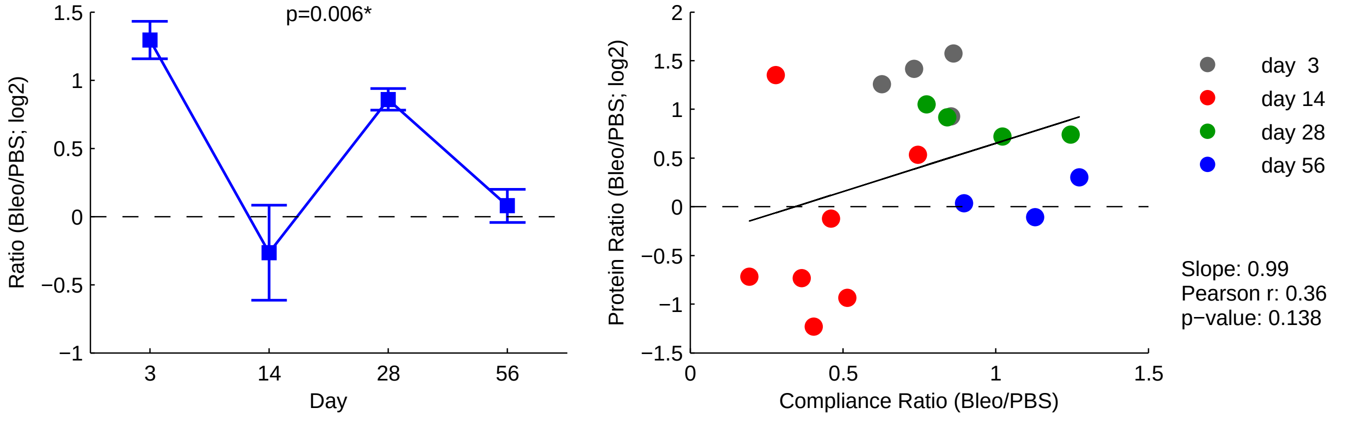

P50429 − Arsb (id: 2968)

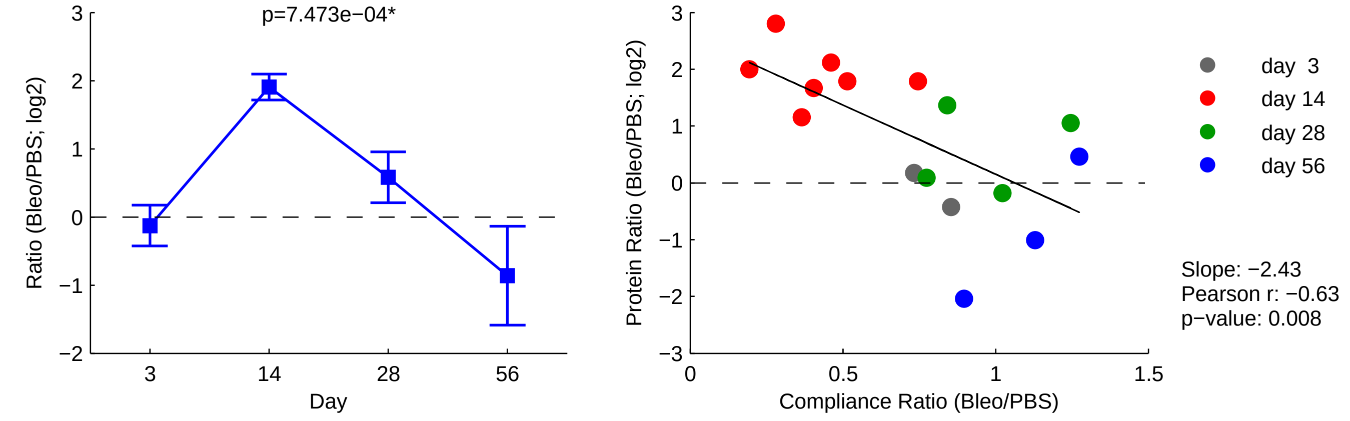

P50462 − Csrp3 (id: 2969)

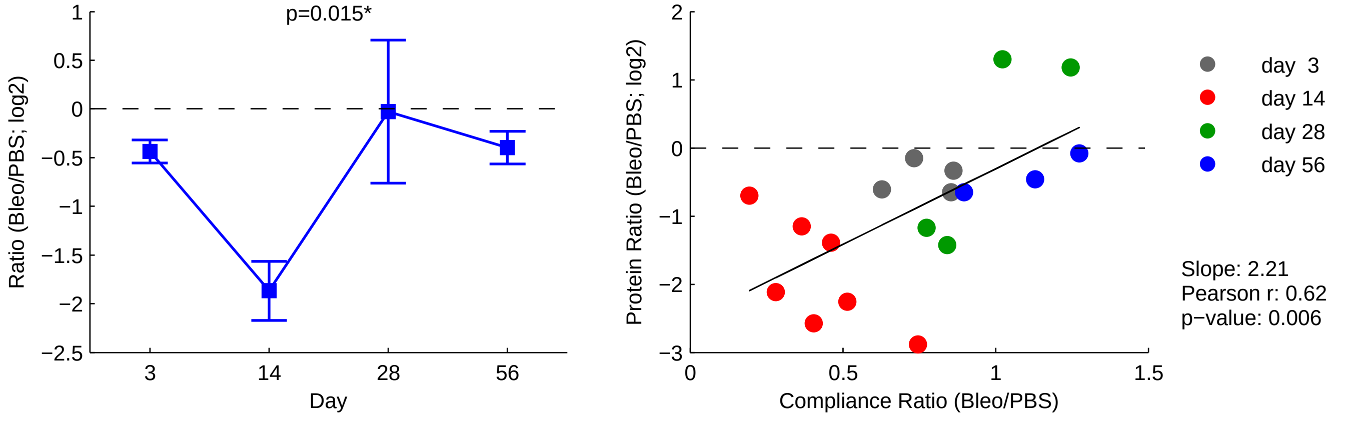

P50516 − Atp6v1a (id: 2970)

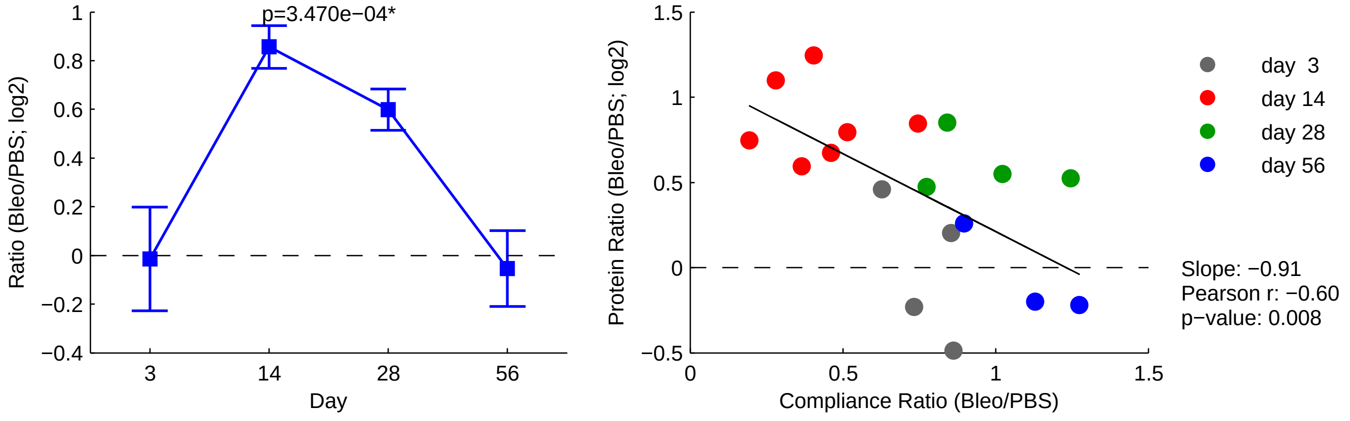

P50518 − Atp6v1e1 (id: 2971)

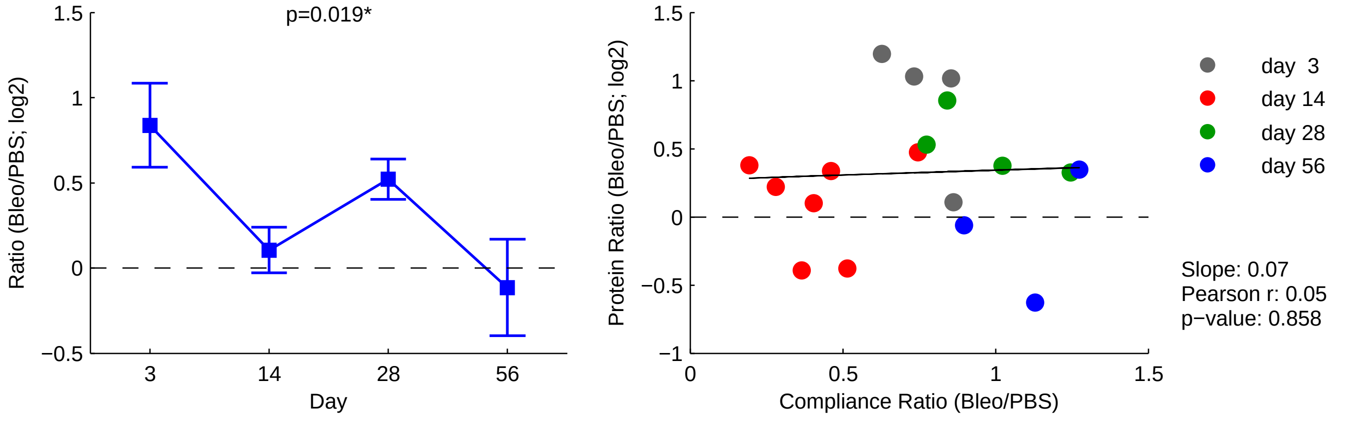

P50543 − S100a11 (id: 2972)

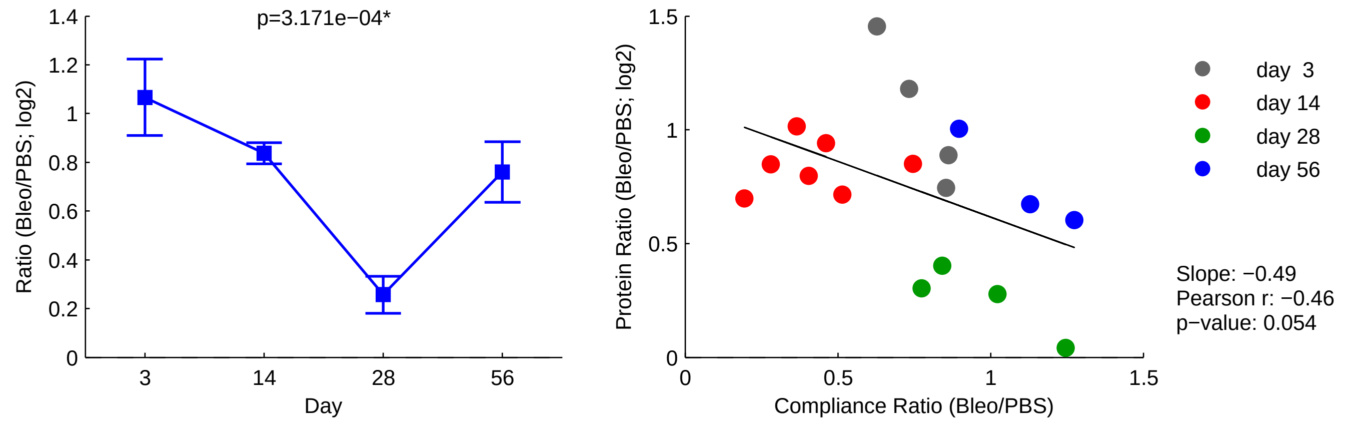

P50544 − Acadvl (id: 2973)

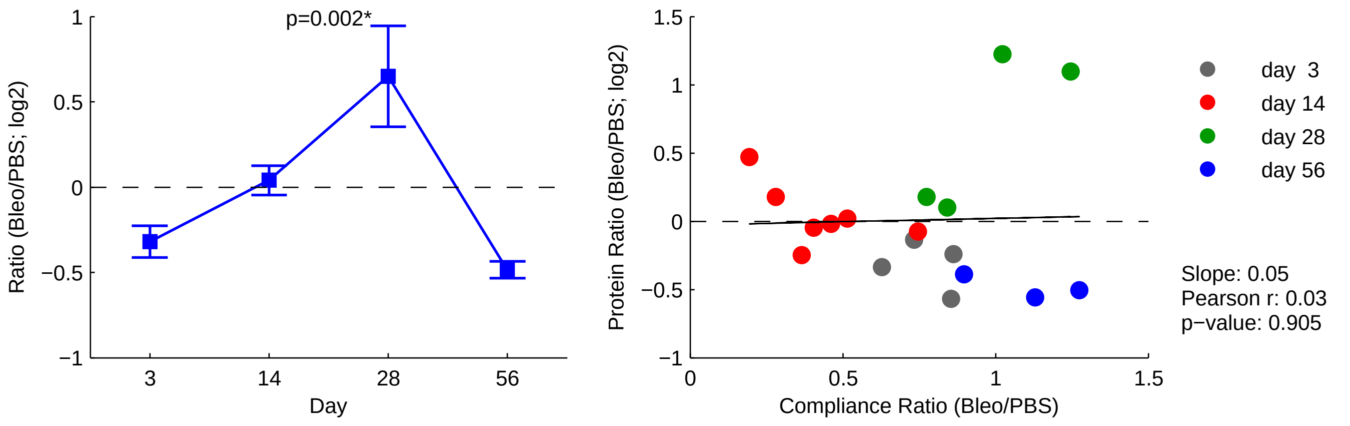

P50580 − Pa2g4 (id: 2974)

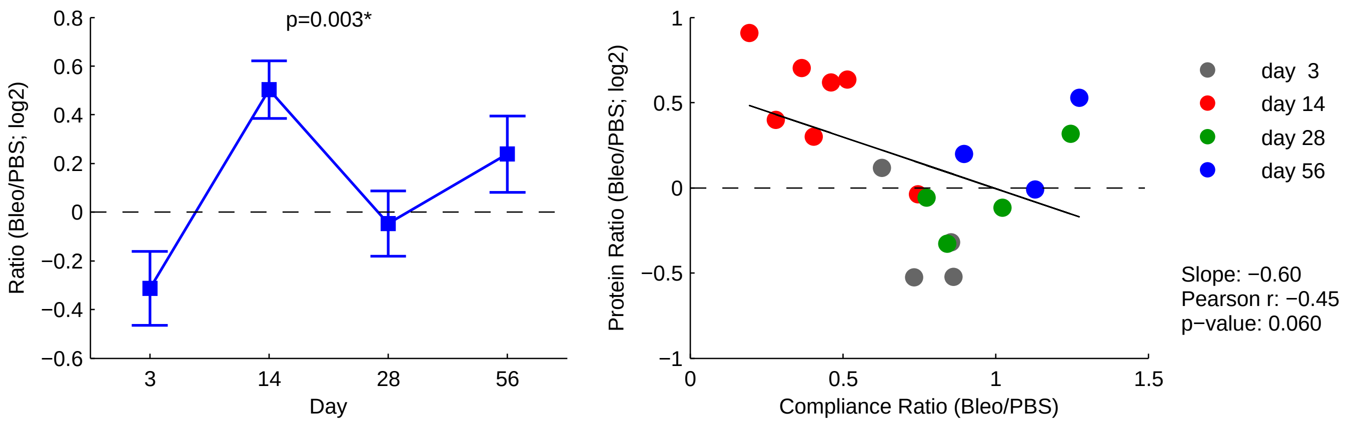

P50592 − Tnfsf10 (id: 2975)

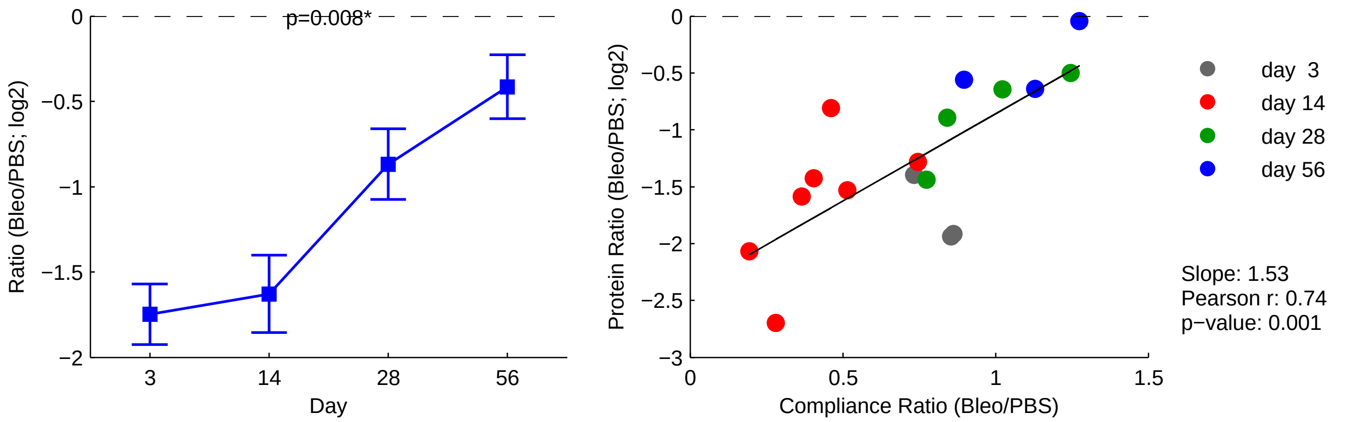

P50608 − Fmod (id: 2976)

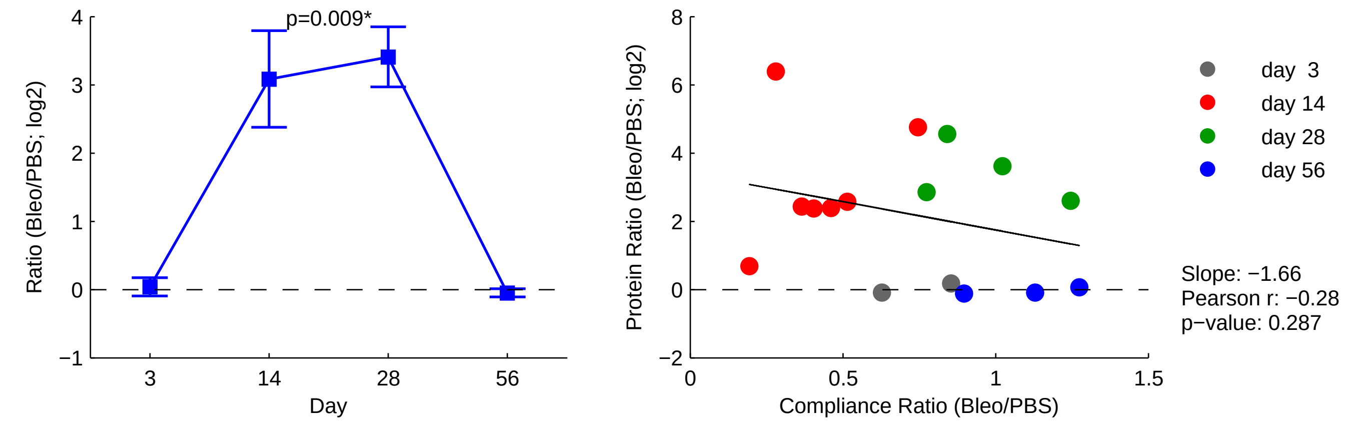

Q8CE80 − Cast (id: 2979)

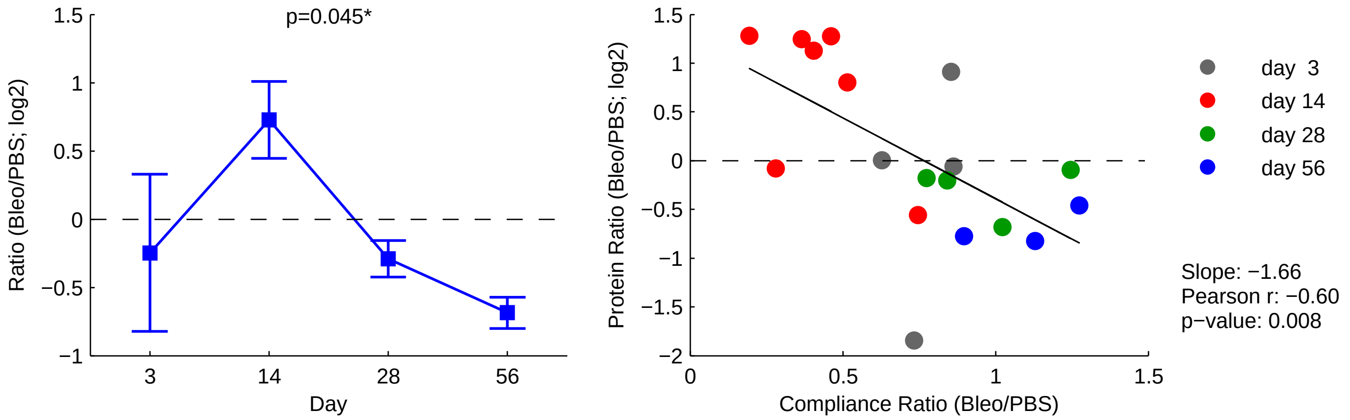

P51174 − Acadl (id: 2982)

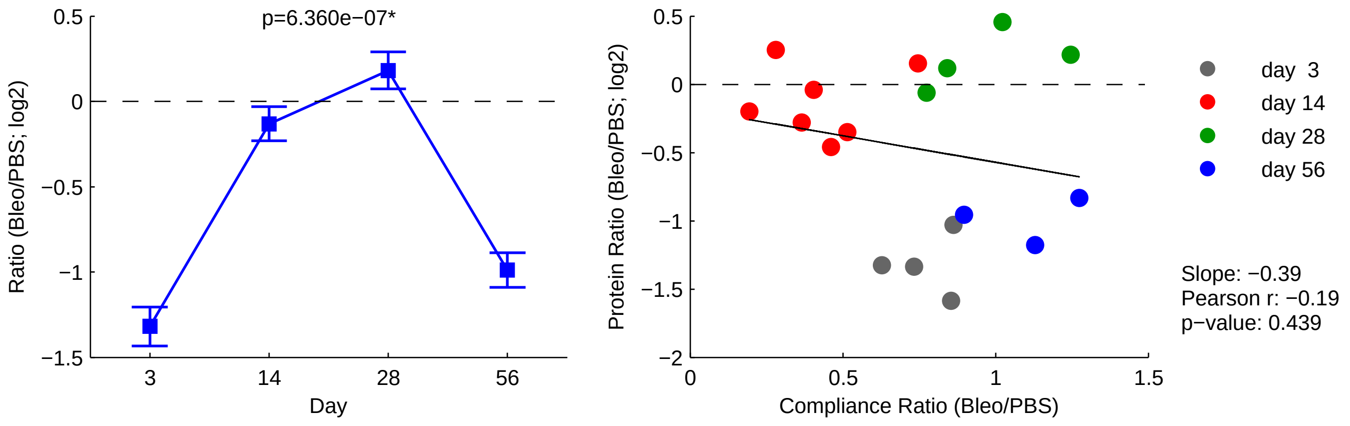

P51175 − Ppox (id: 2983)

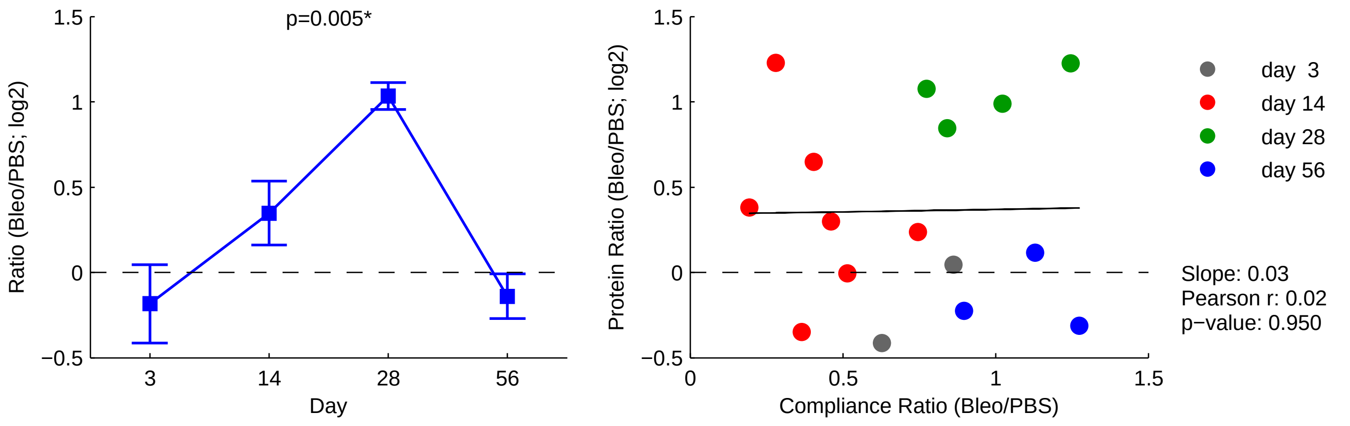

P51432 − Plcb3 (id: 2985)

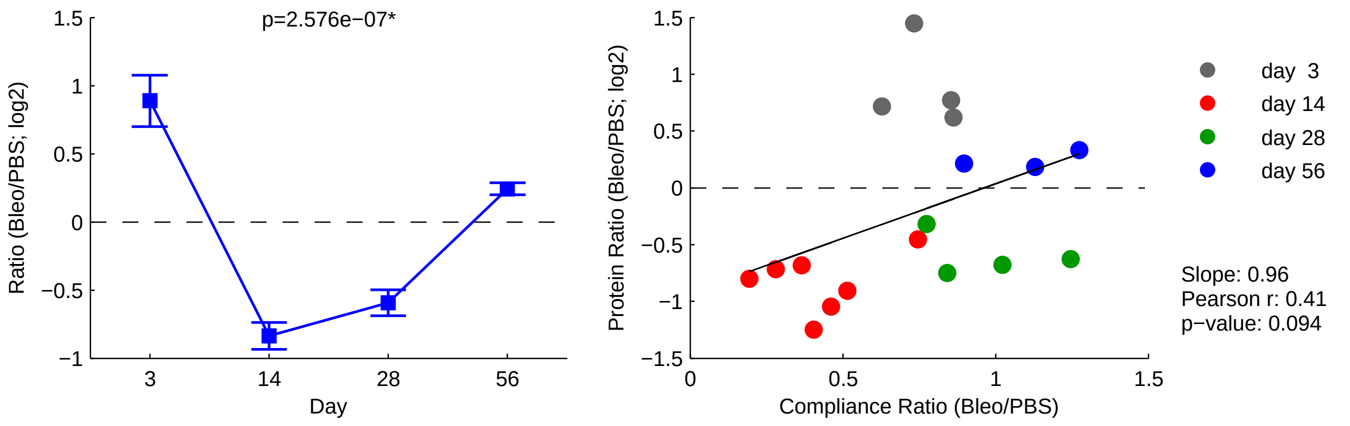

P51437 − Camp (id: 2986)

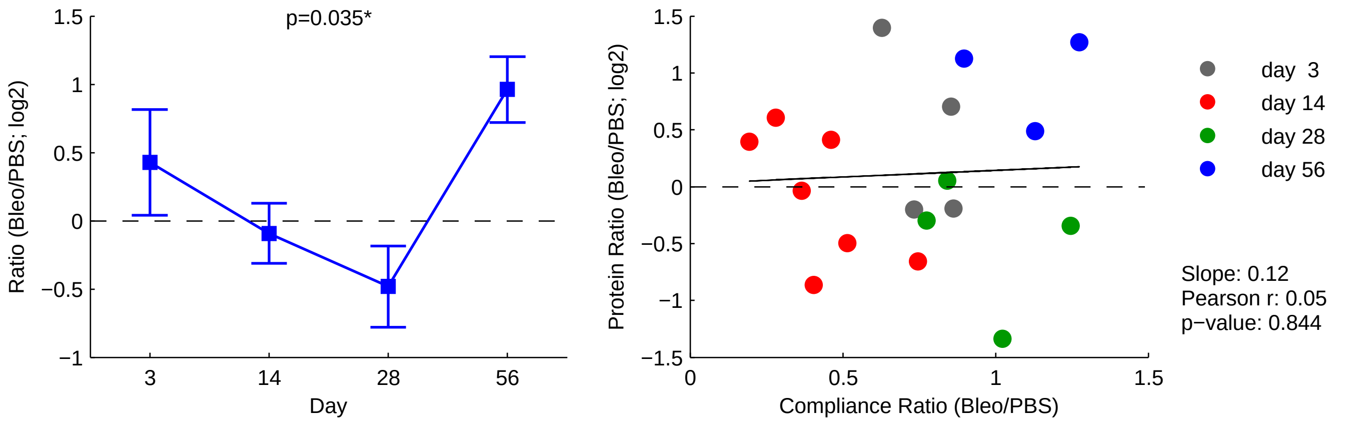

P51660 − Hsd17b4 (id: 2990)

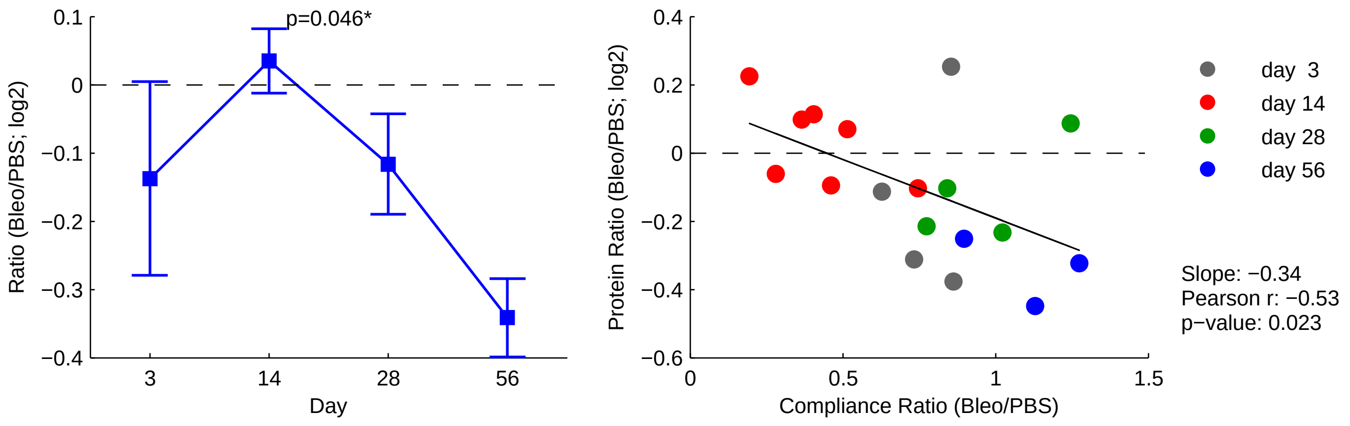

P51807 − Dynlt1 (id: 2995)

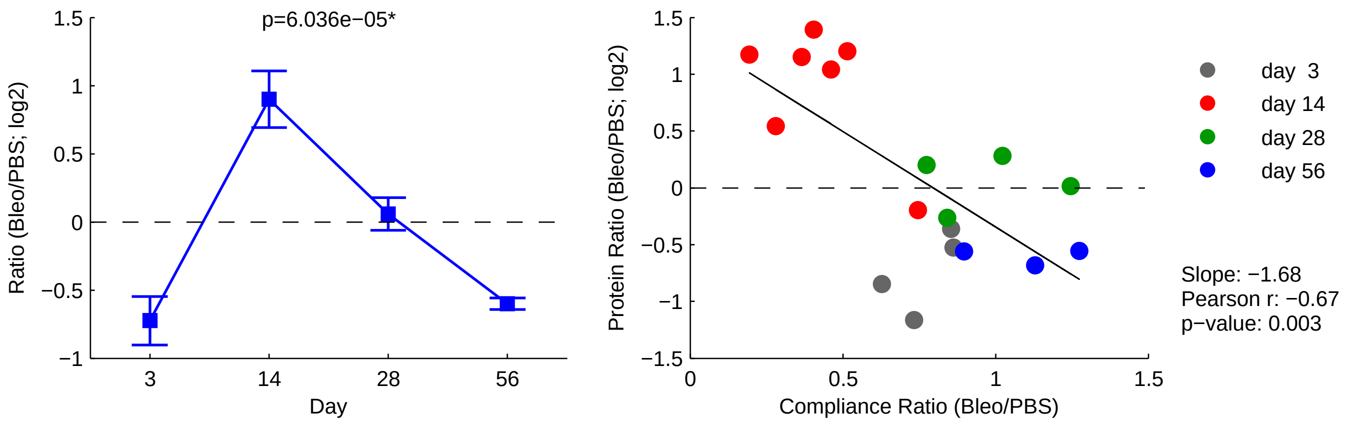

P51829 − Adcy7 (id: 2996)

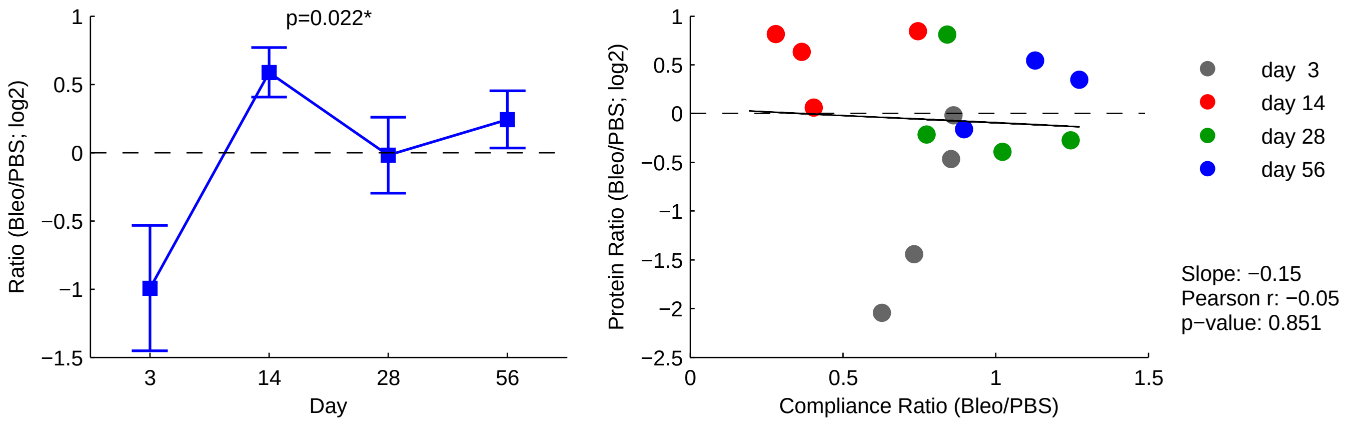

P51855 − Gss (id: 2998)

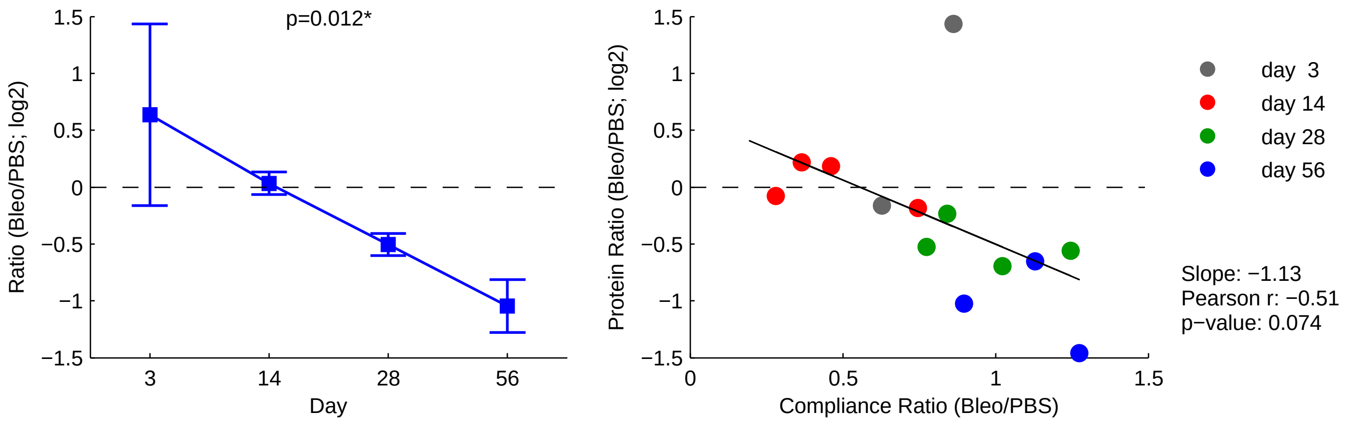

P51859 − Hdgf (id: 2999)

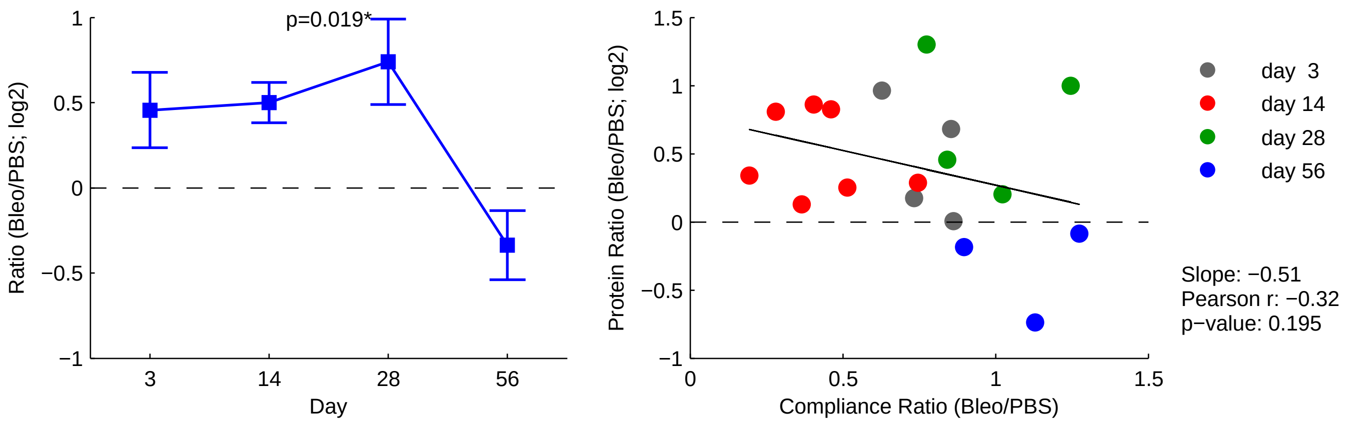

P51885 − Lum (id: 3002)

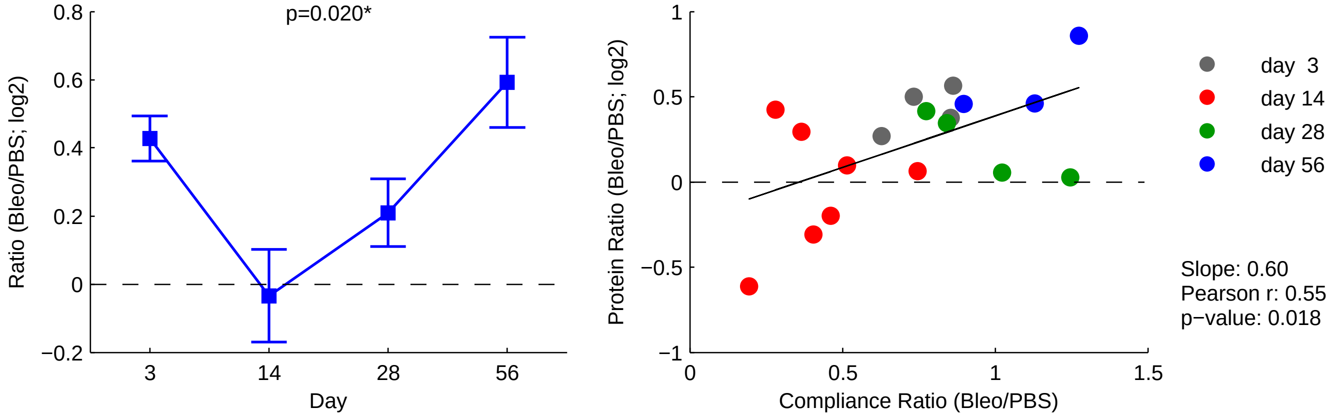

P52196 − Tst (id: 3007)

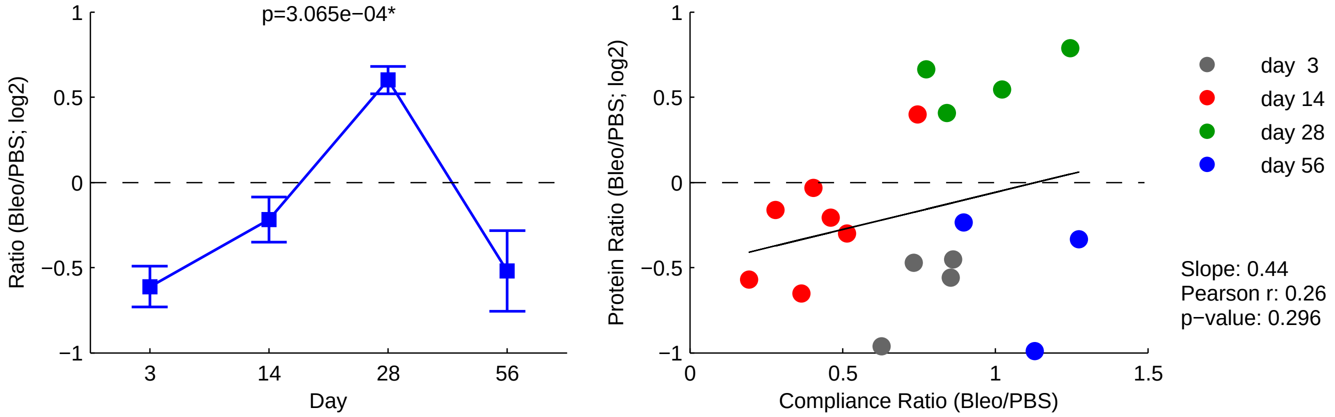

P52430 − Pon1 (id: 3009)

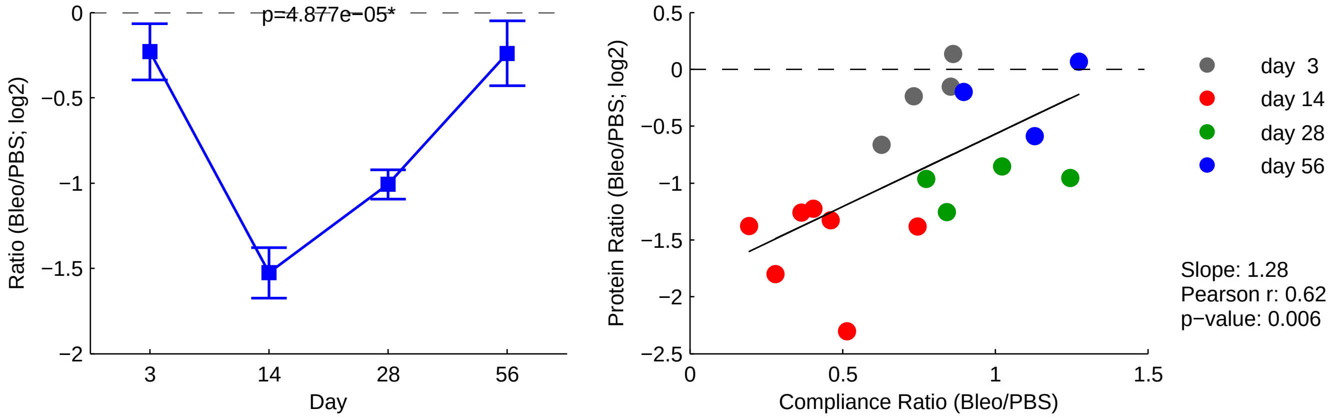

P52431 − Pold1 (id: 3010)

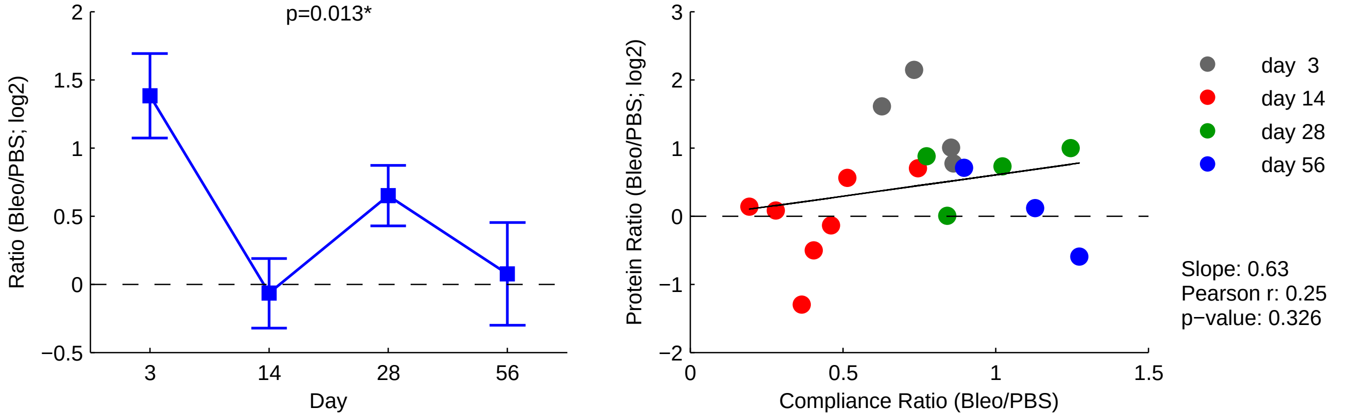

P52479 − Usp10 (id: 3011)

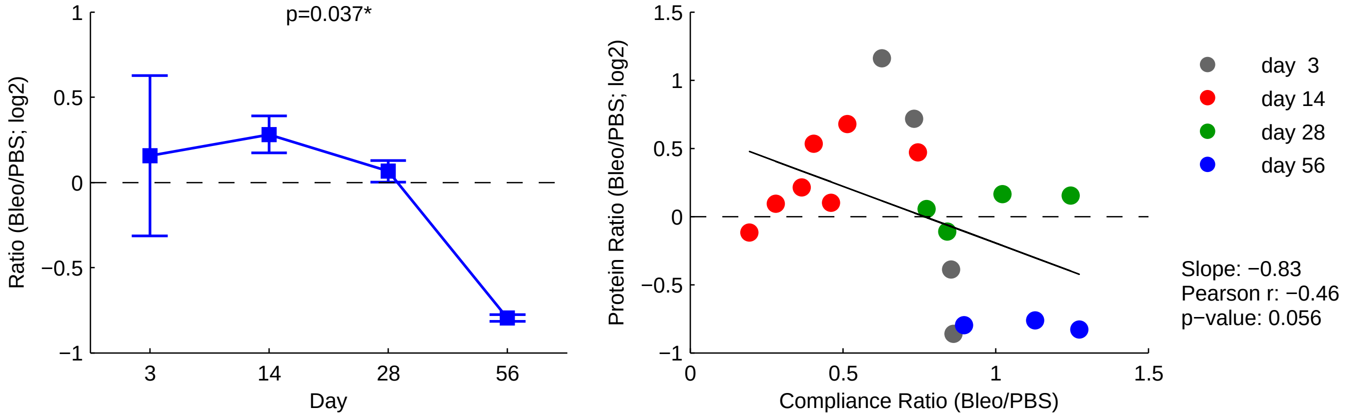

P52480 − Pkm (id: 3012)

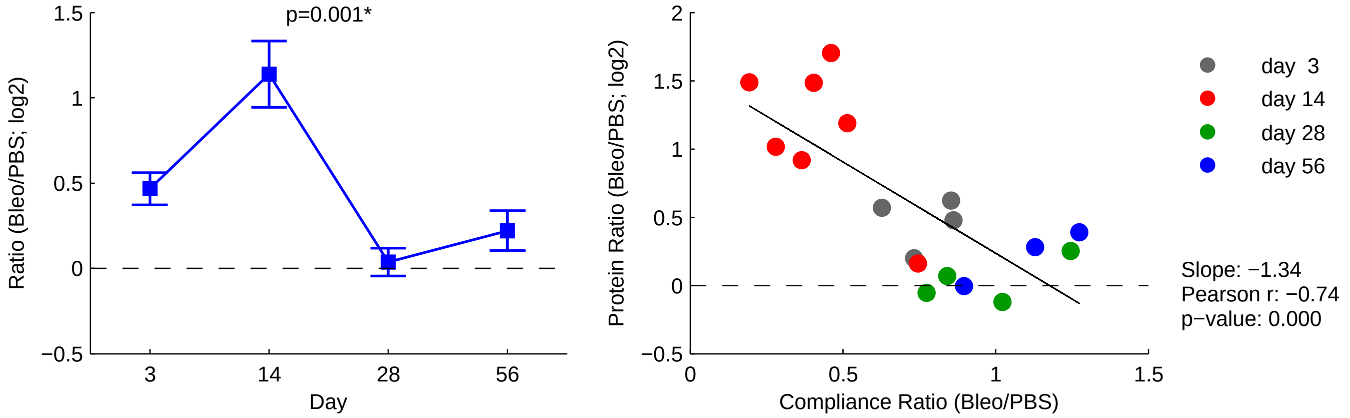

P52480−2 − (id: 3013)

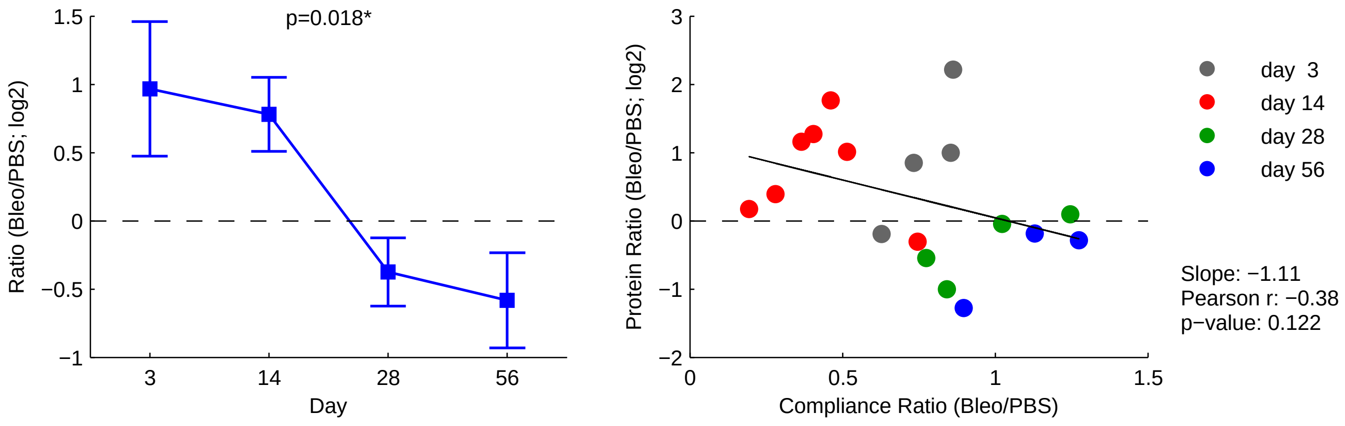

P52503 − Ndufs6 (id: 3014)

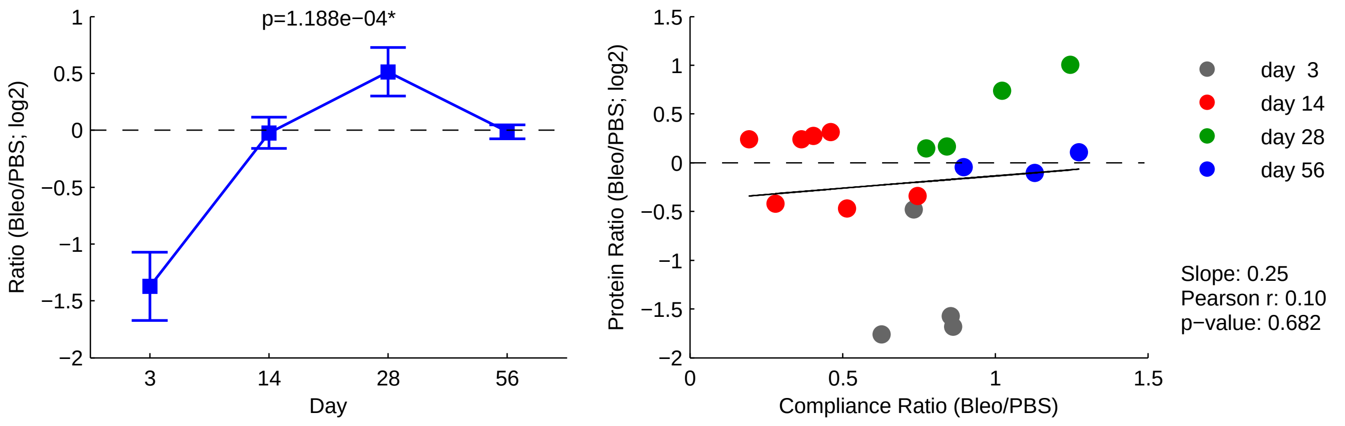

P52633 − Stat6 (id: 3016)

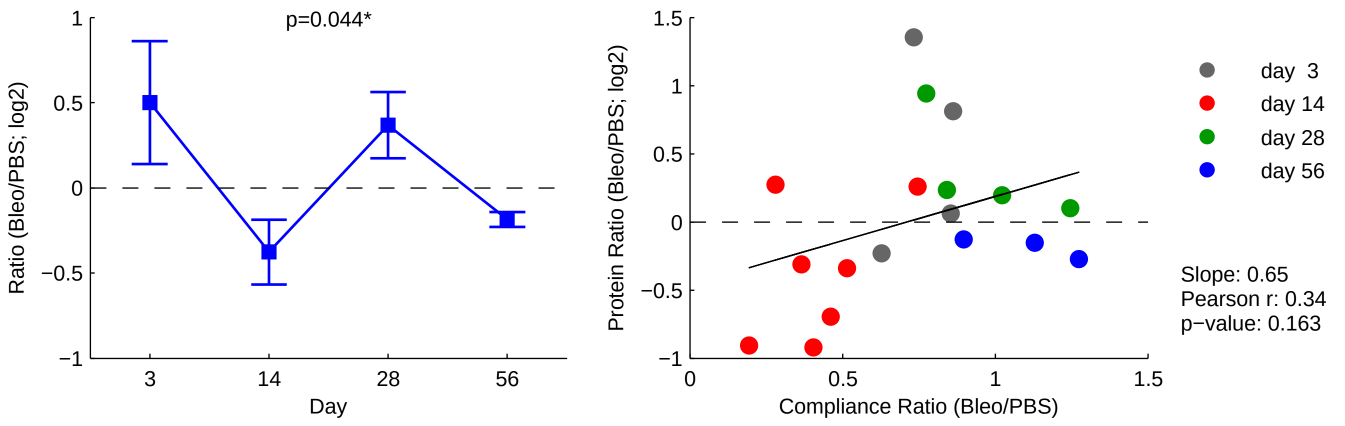

P52795 − Efnb1 (id: 3019)

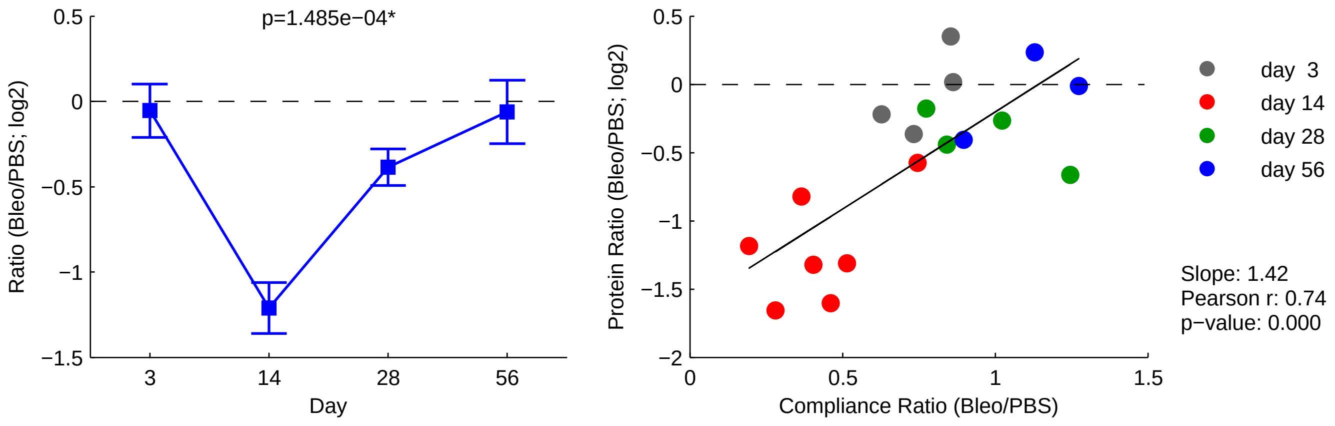

P52800 − Efnb2 (id: 3020)

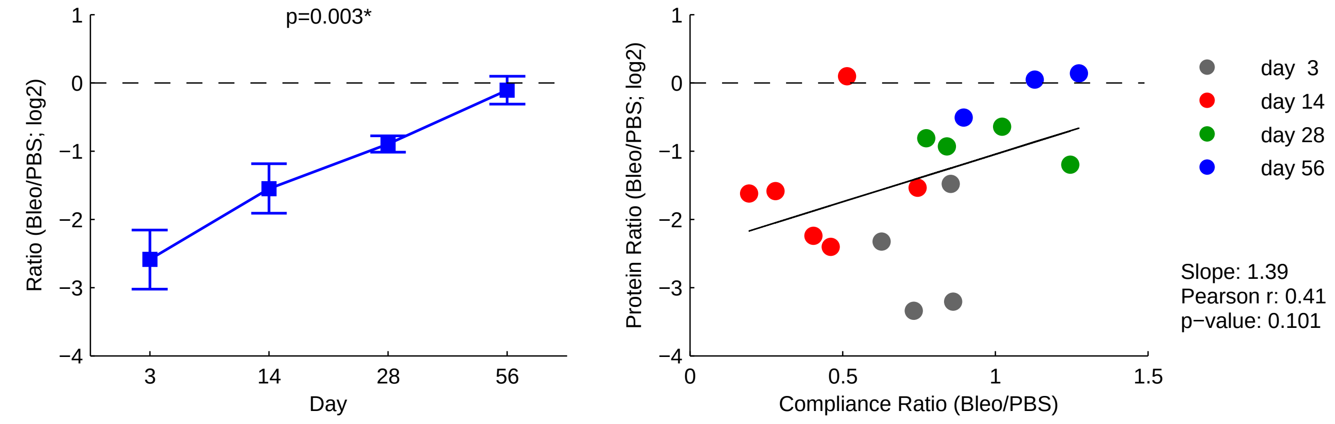

P52825 − Cpt2 (id: 3021)

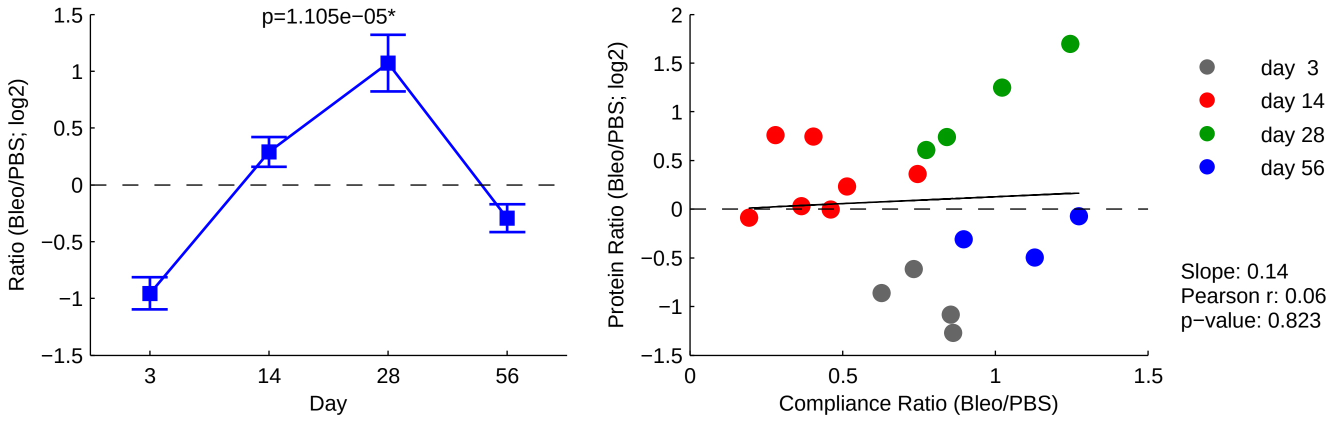

P52875 − Tmem165 (id: 3022)

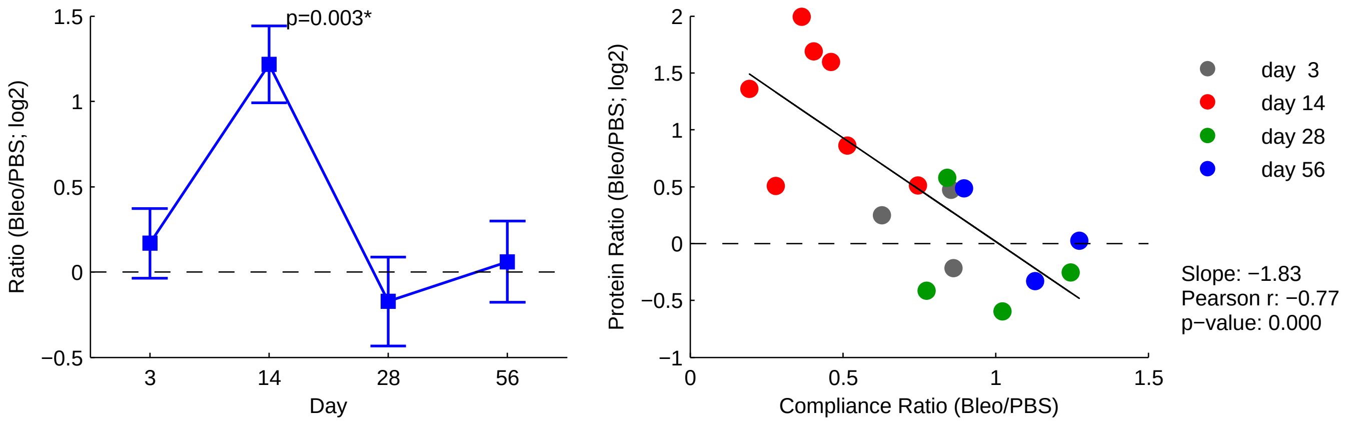

P53395 − Dbt (id: 3024)

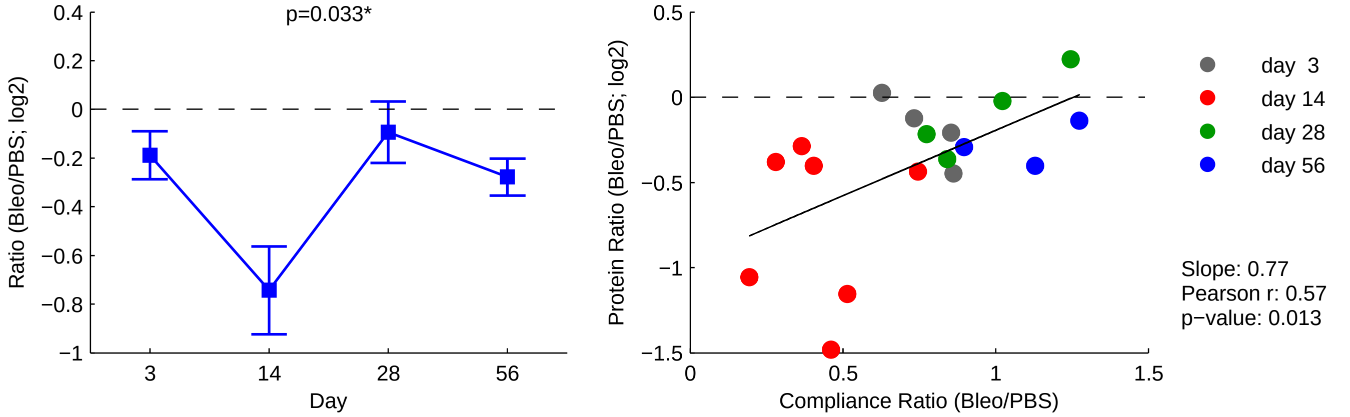

P53690 − Mmp14 (id: 3027)

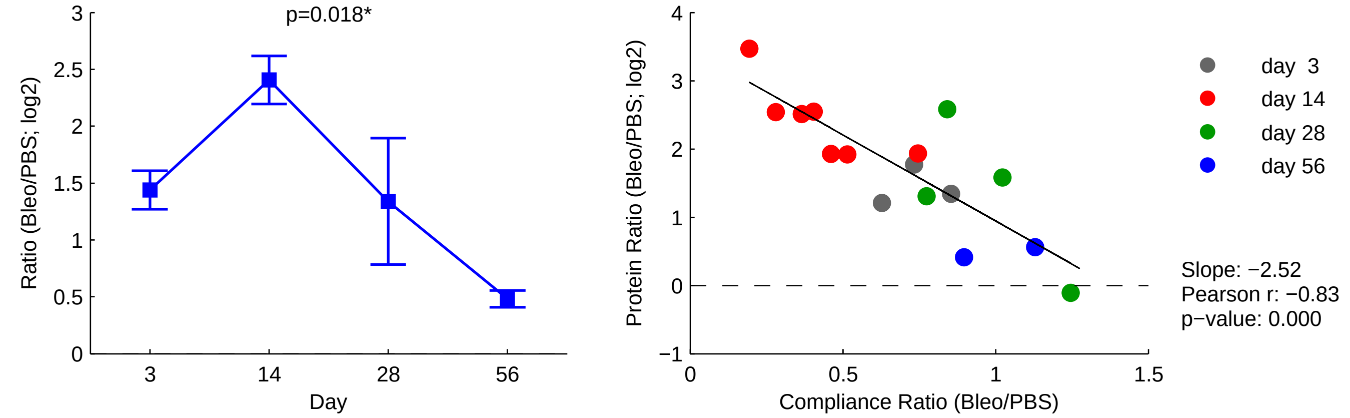

P53702 − Hccs (id: 3028)

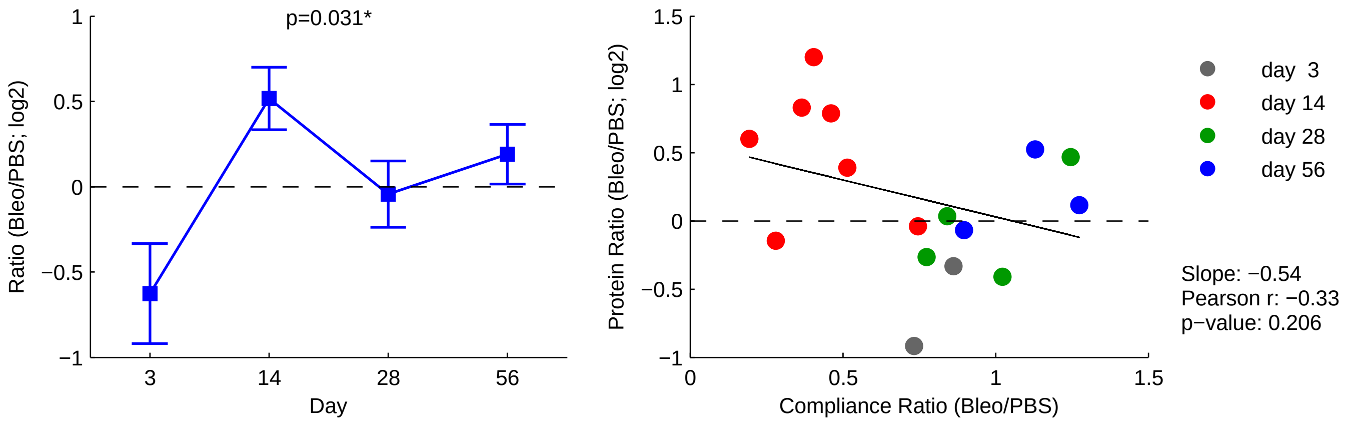

P53810 − Pitpna (id: 3030)

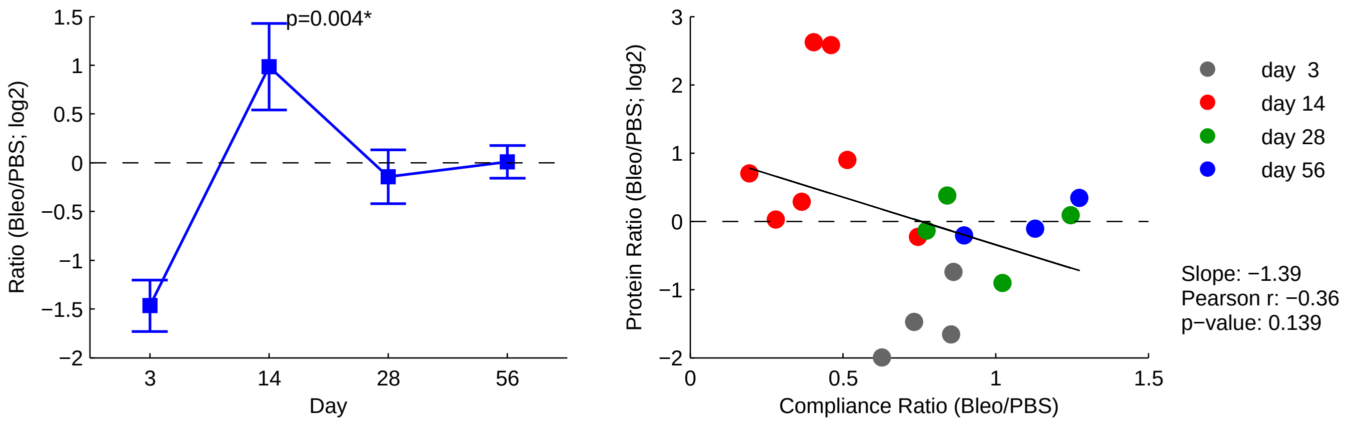

P53811 − Pitpnb (id: 3031)

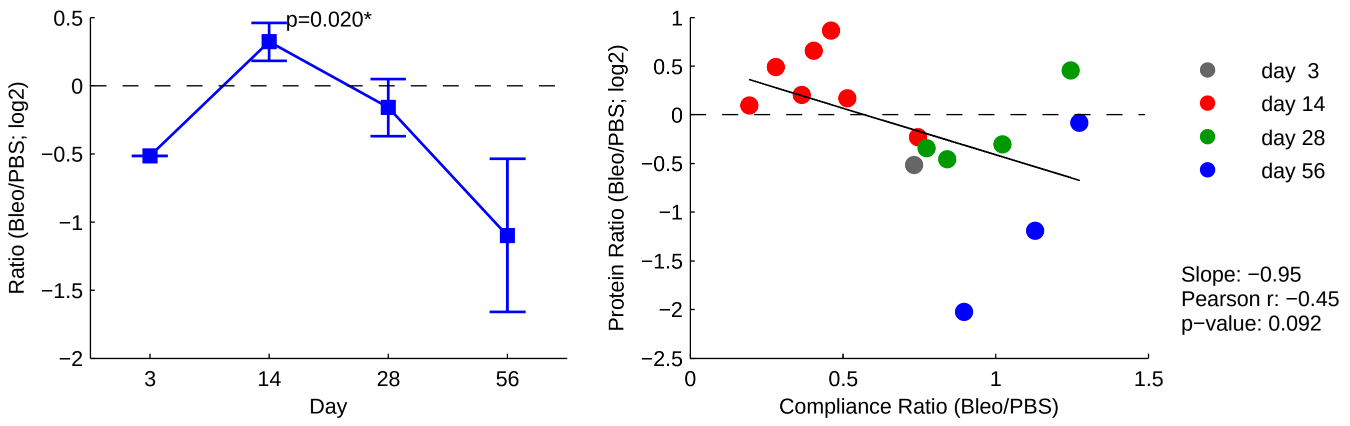

P53994 − Rab2a (id: 3033)

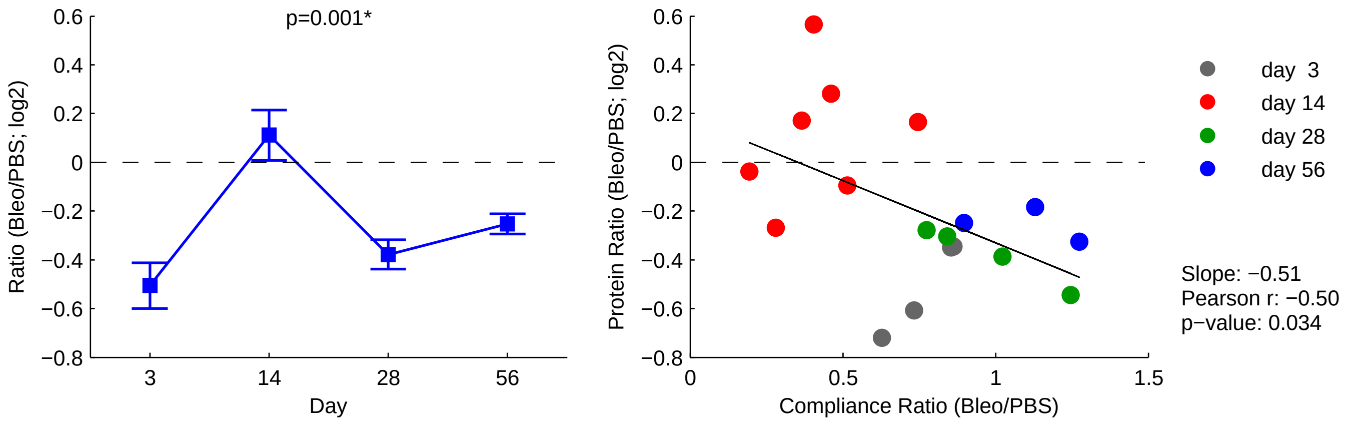

P54071 − Idh2 (id: 3036)

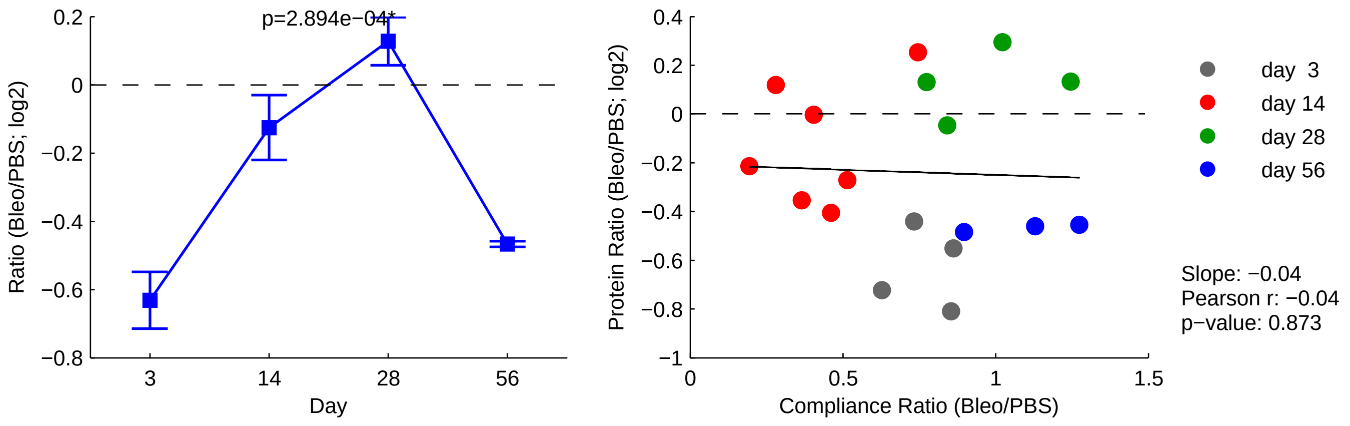

P54116 − Stom (id: 3038)

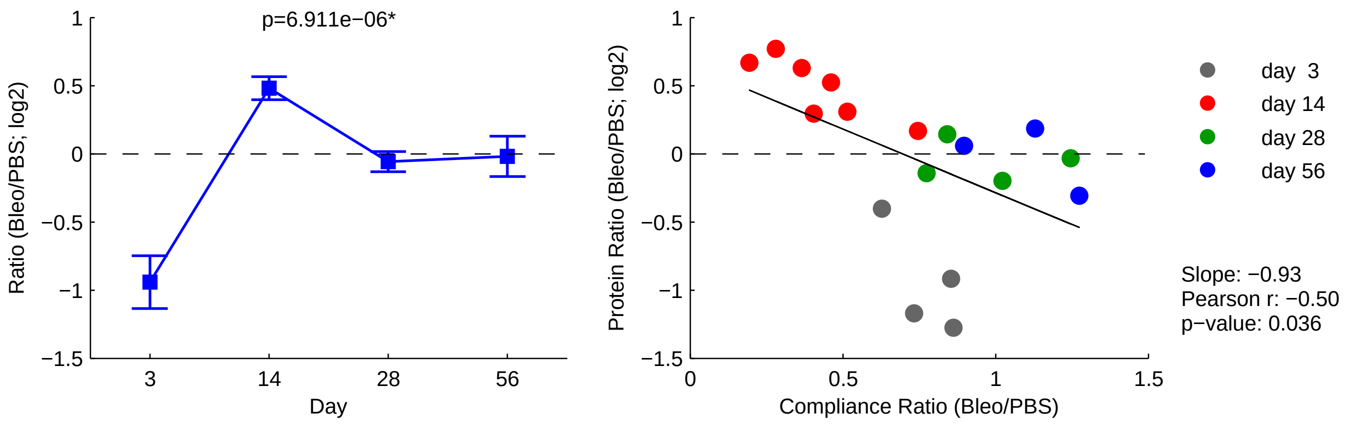

P54320 − Eln (id: 3041)

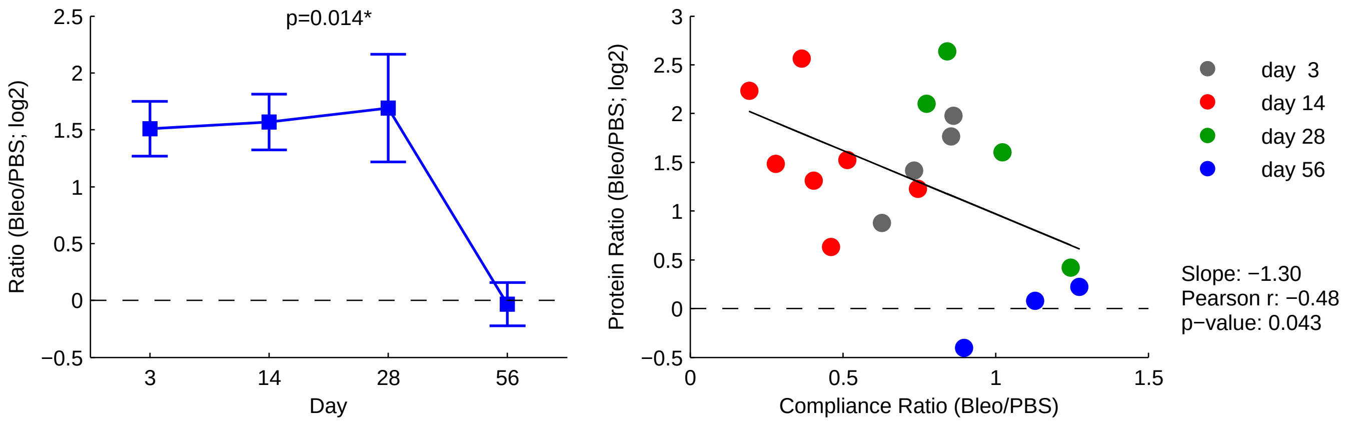

Q8CAP3 − Rad23a (id: 3042)

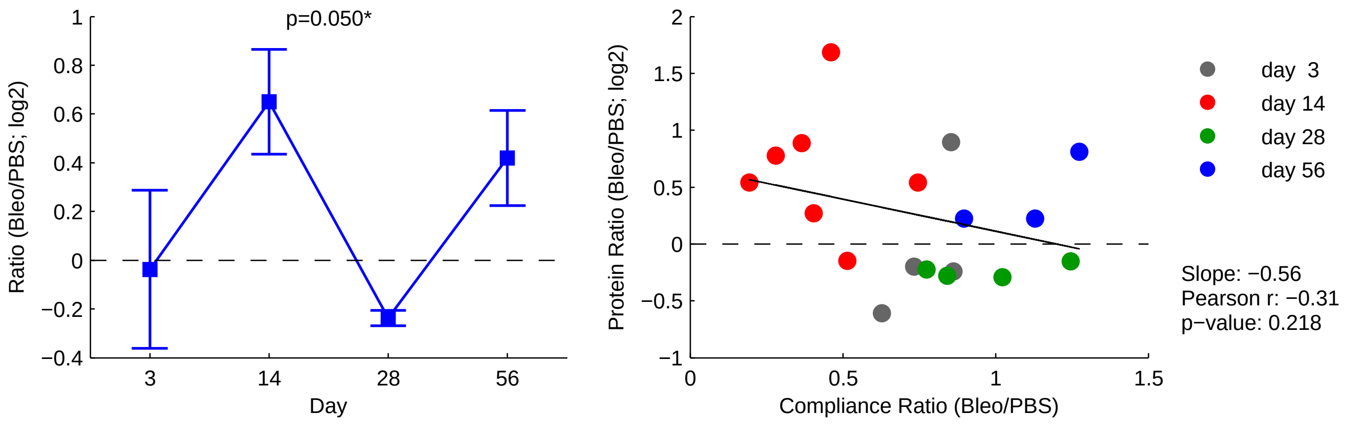

P54728 − Rad23b (id: 3043)

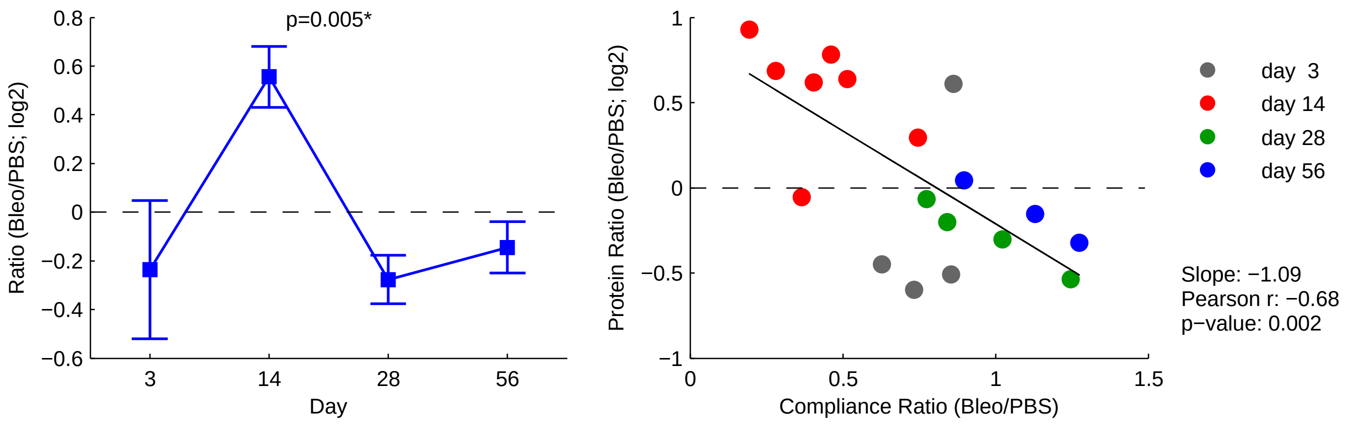

P54729 − Nub1 (id: 3044)

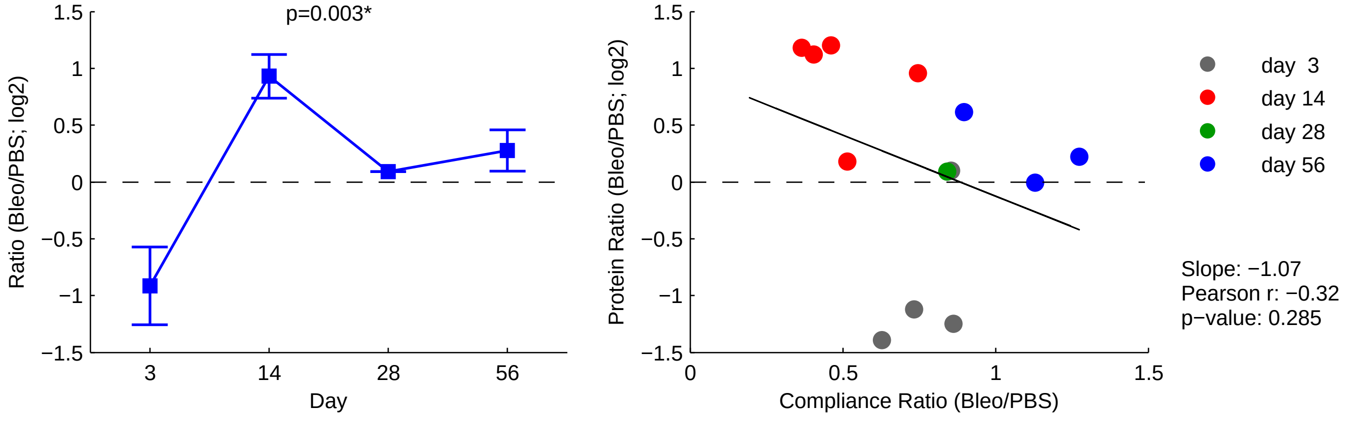

P54731 − Faf1 (id: 3045)

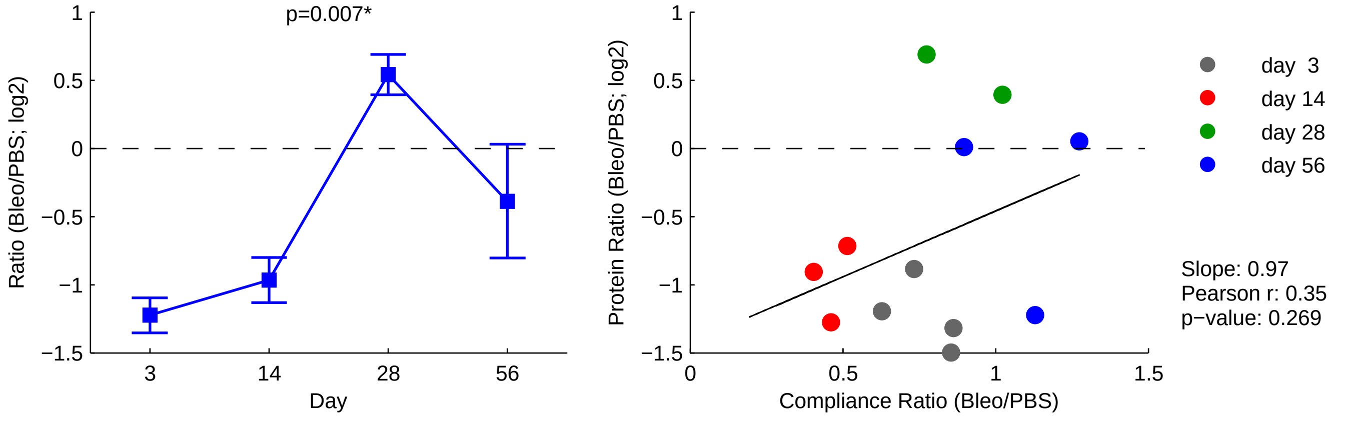

P54751 − St3gal1 (id: 3046)

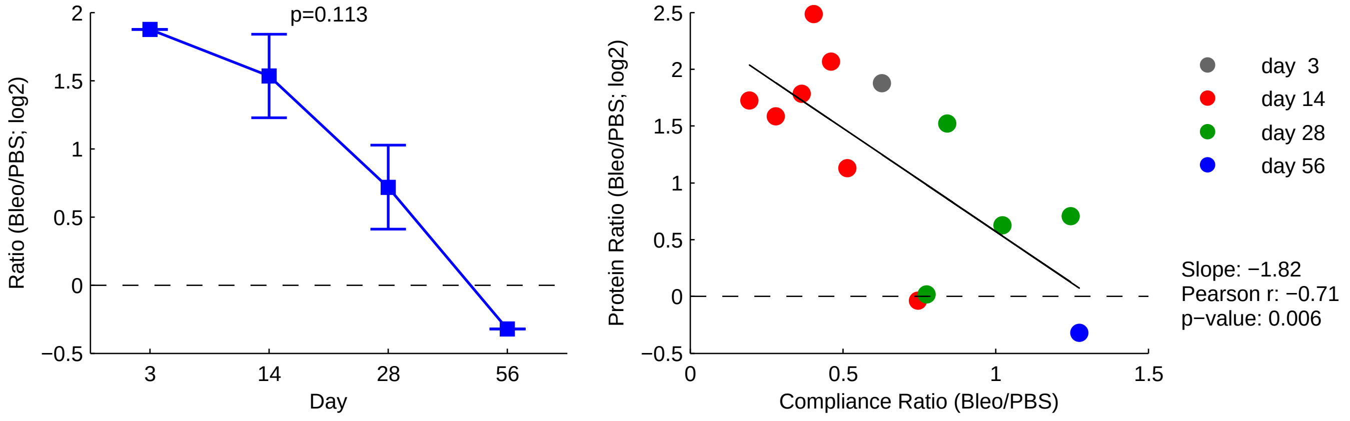

P54775 − Psmc4 (id: 3048)

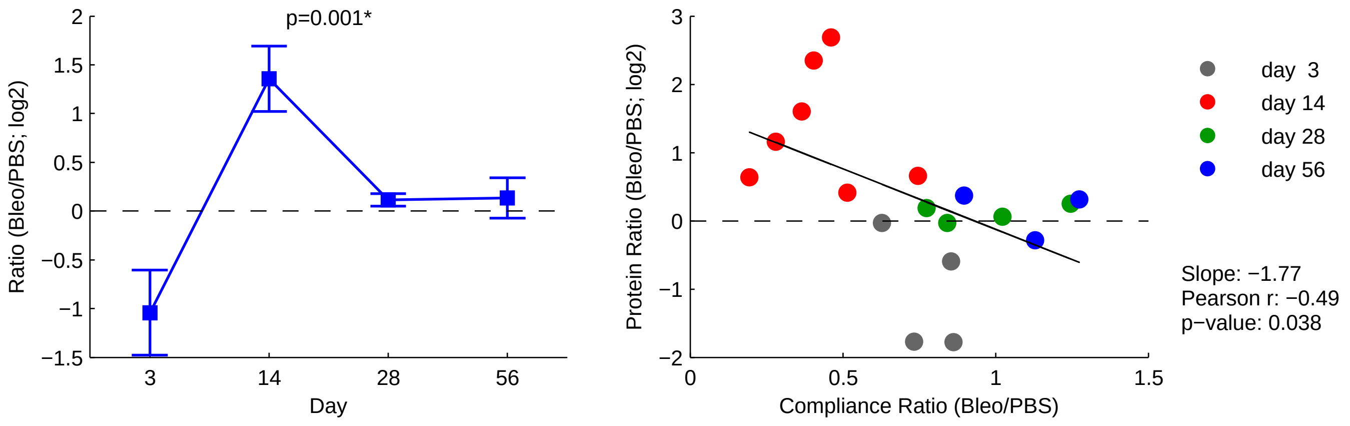

P54823 − Ddx6 (id: 3052)

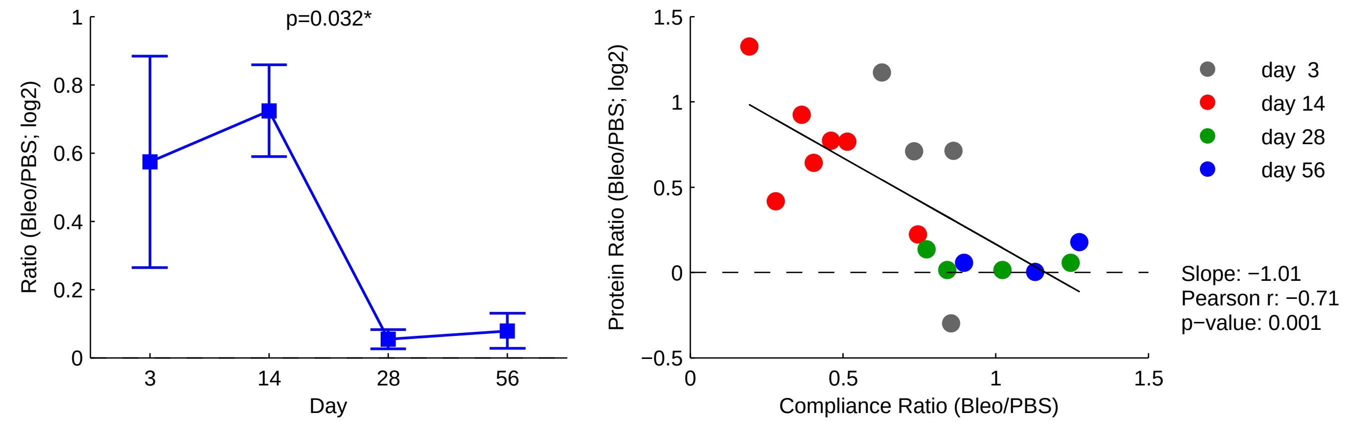

P54923 − Adprh (id: 3054)

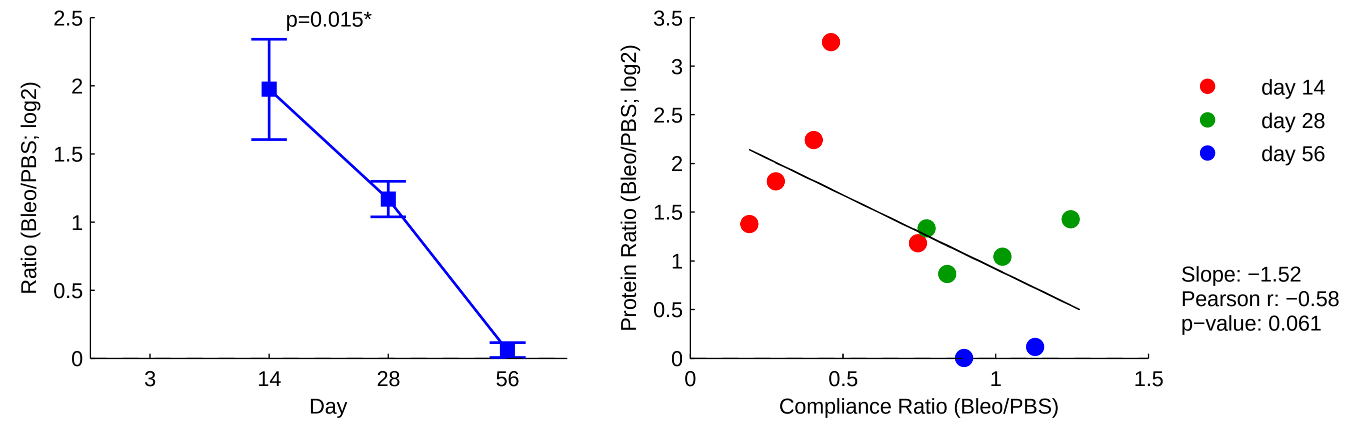

P55065 − Pltp (id: 3056)

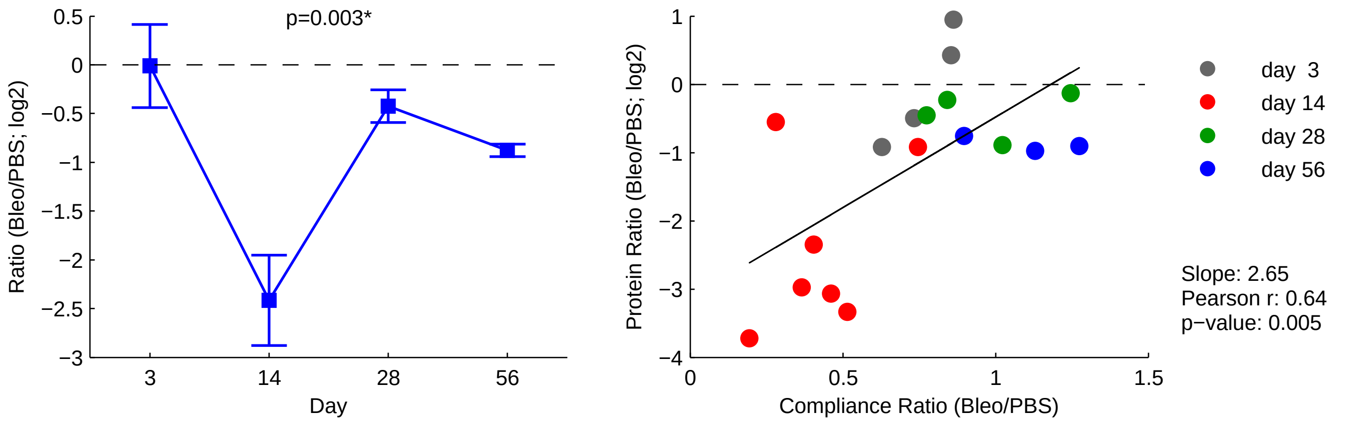

P55096 − Abcd3 (id: 3057)

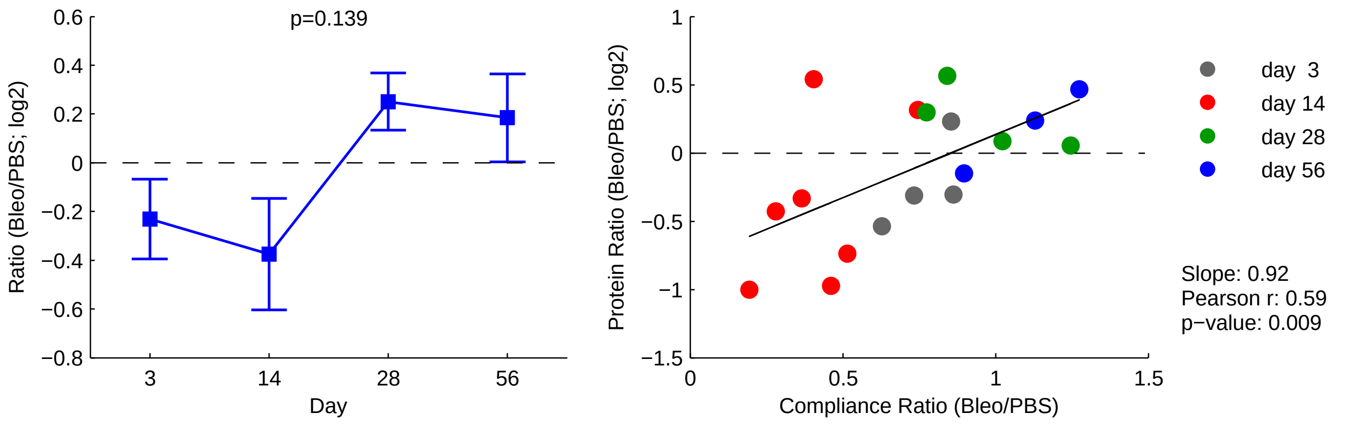

P55097 − Ctsk (id: 3058)

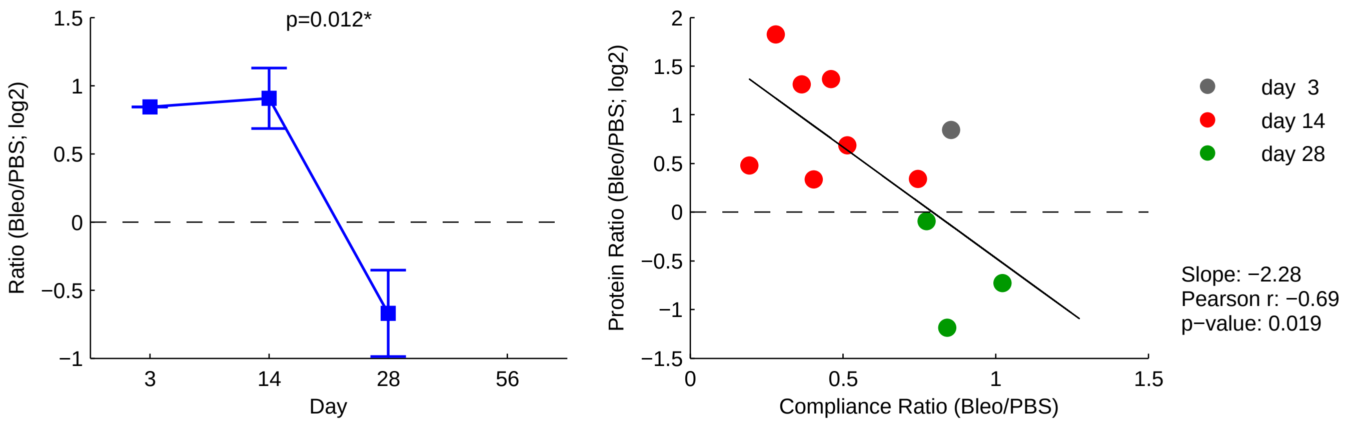

P55200−2 − Mll (id: 3059)

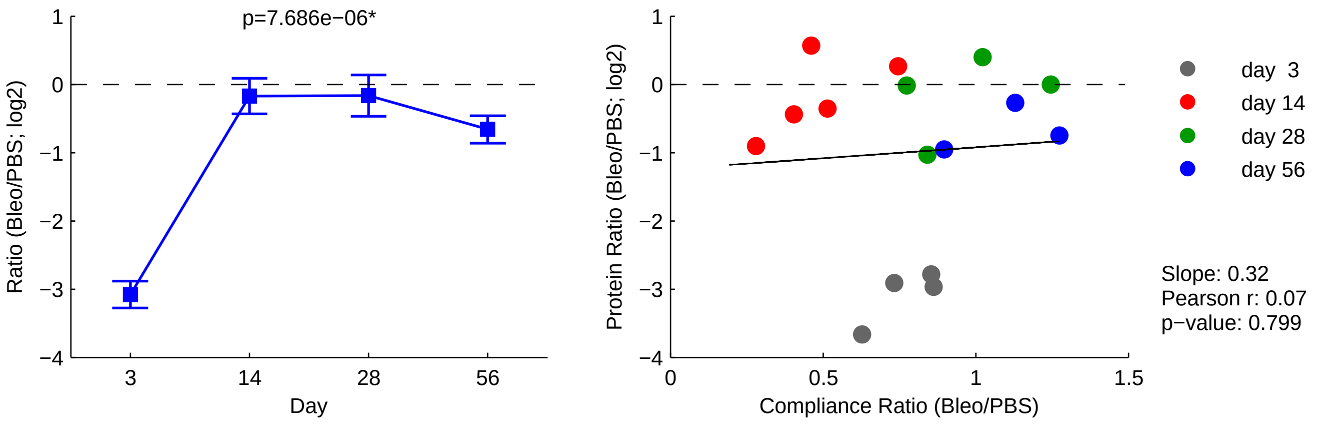

P55264 − Adk (id: 3061)

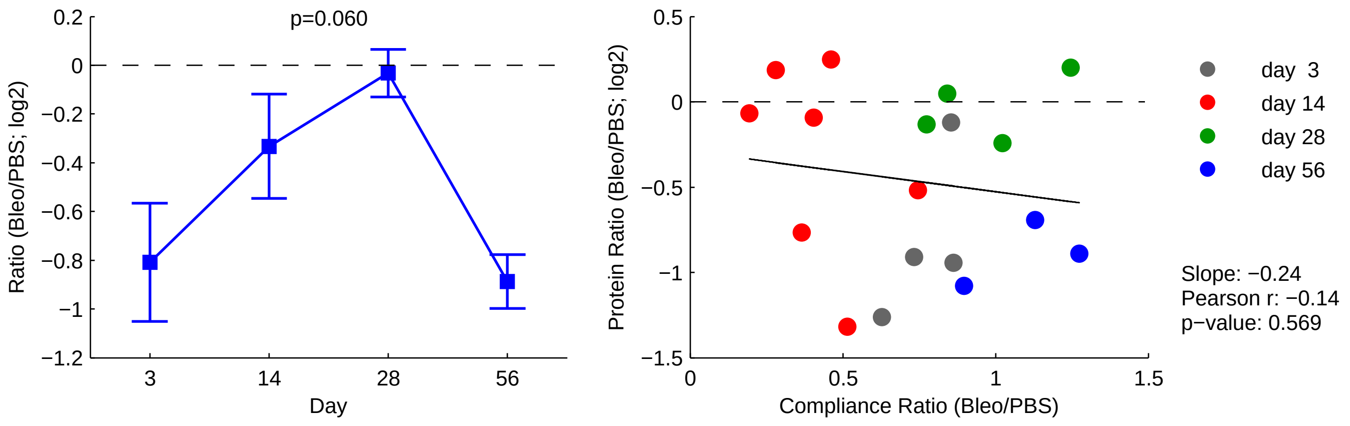

P55264−2 − (id: 3062)

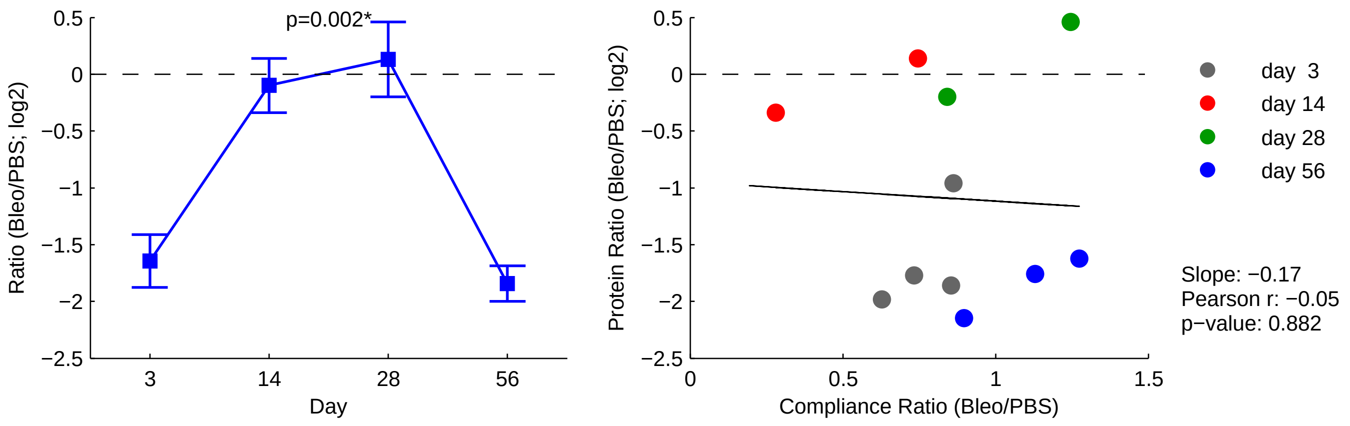

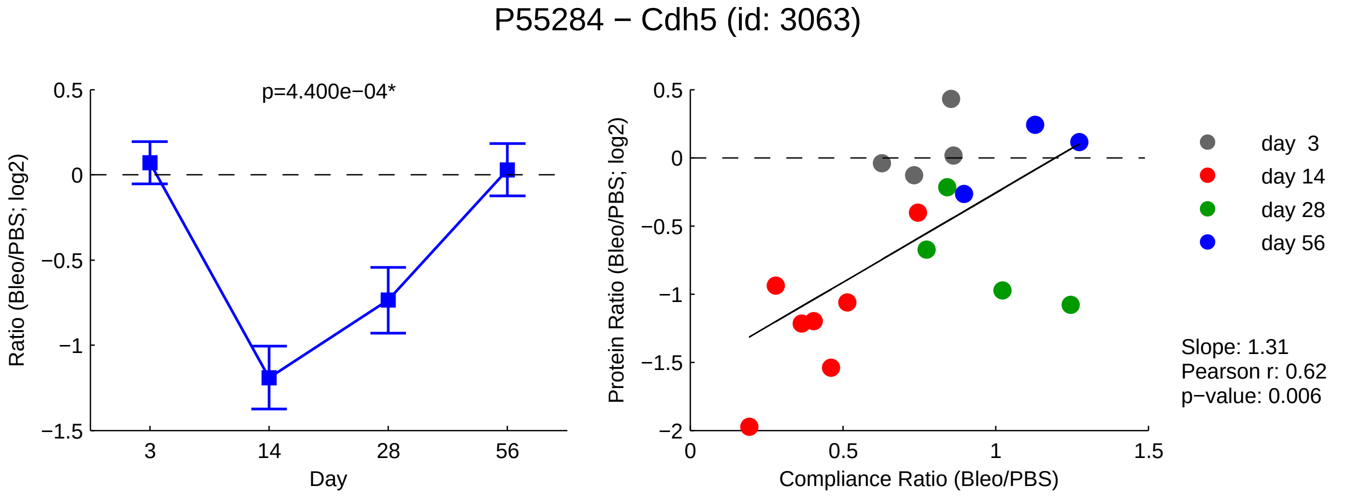

P55284 − Cdh5 (id: 3063)
p=4.400e−04*
Ratio (Bleo/PBS; log2)
Day
Protein Ratio (Bleo/PBS; log2)
Compliance Ratio (Bleo/PBS)
day 3
day 14
day 28
day 56
Slope: 1.31
Pearson r: 0.62
p−value: 0.006

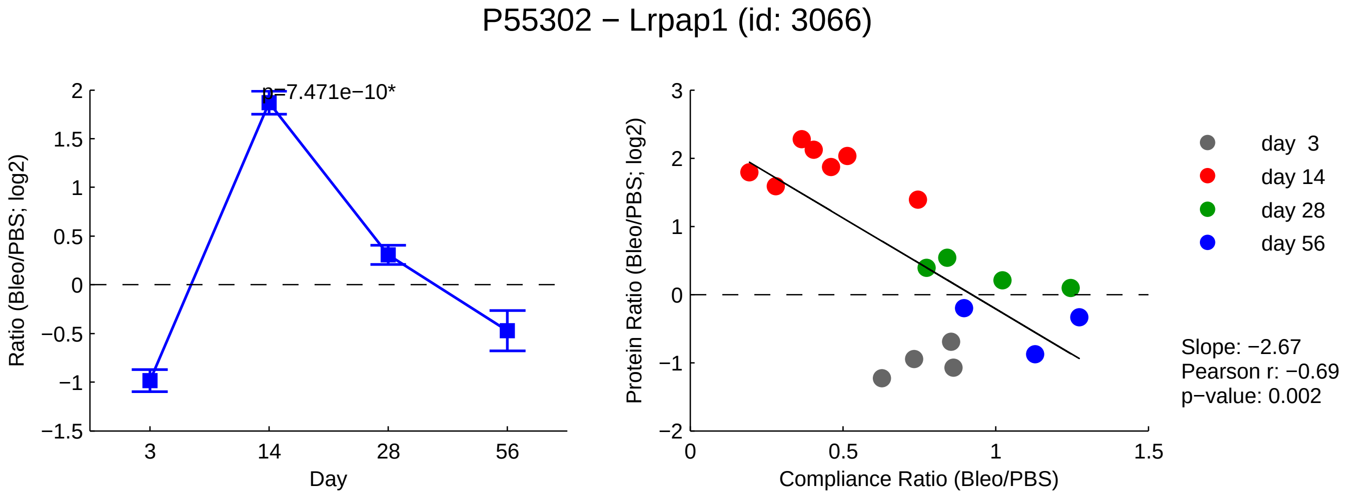

P55302 − Lrpap1 (id: 3066)
p=7.471e−10*
Ratio (Bleo/PBS; log2)
Day
Protein Ratio (Bleo/PBS; log2)
Compliance Ratio (Bleo/PBS)
day 3
day 14
day 28
day 56
Slope: −2.67
Pearson r: −0.69
p−value: 0.002

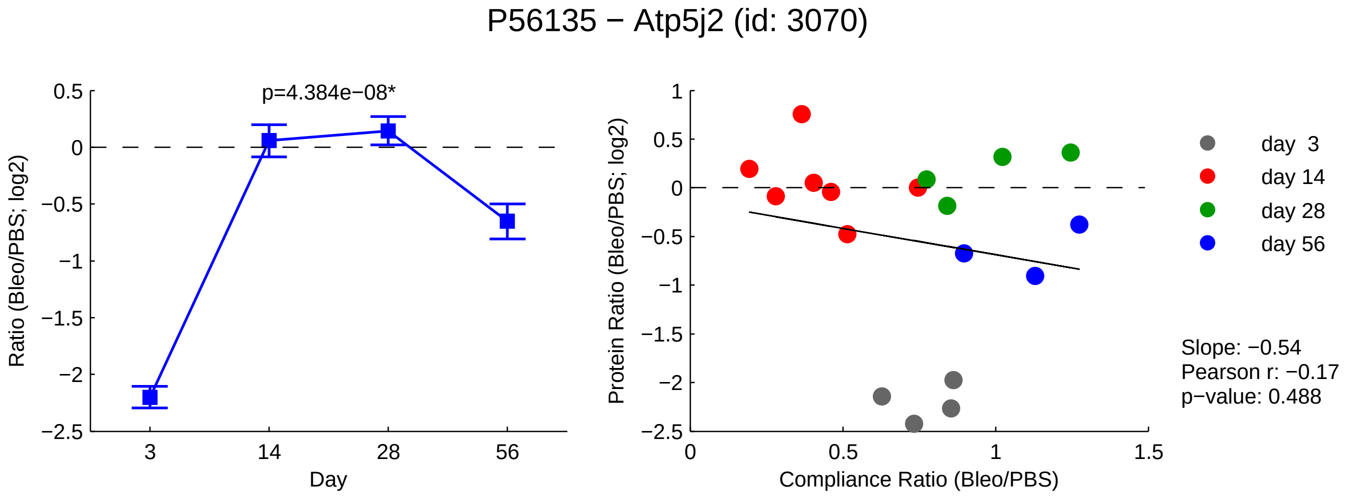

P56135 − Atp5j2 (id: 3070)
p=4.384e−08*
Ratio (Bleo/PBS; log2)
Day
Protein Ratio (Bleo/PBS; log2)
Compliance Ratio (Bleo/PBS)
day 3
day 14
day 28
day 56
Slope: −0.54
Pearson r: −0.17
p−value: 0.488

P56382 − Atp5e (id: 3078)

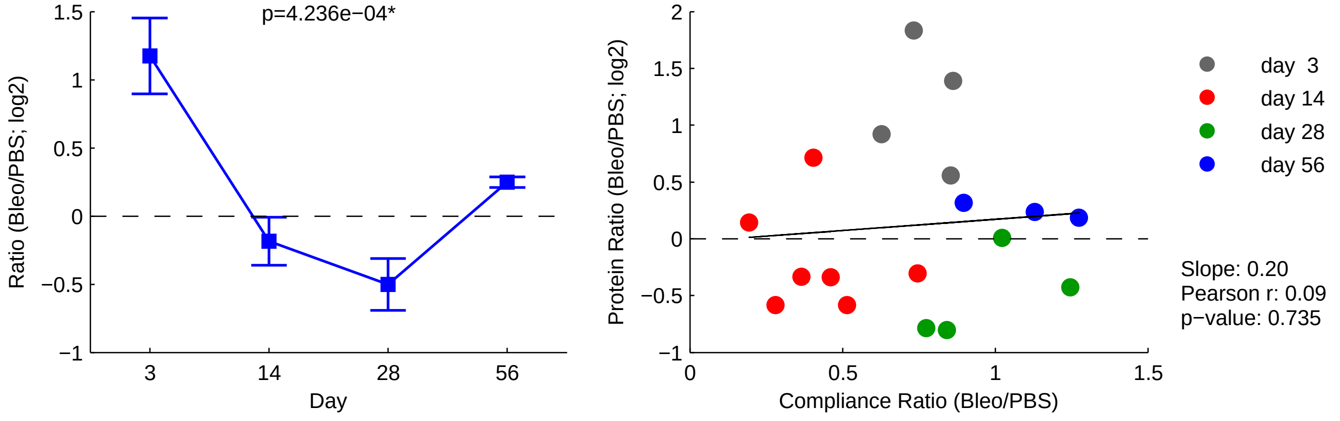

P56387 − Dynlt3 (id: 3079)

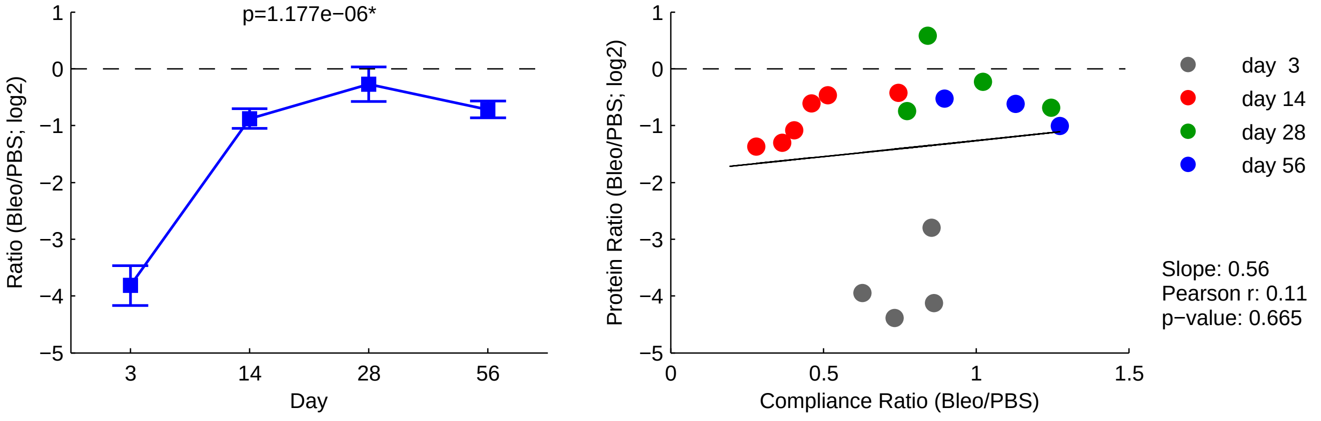

P56391 − Cox6b1 (id: 3080)

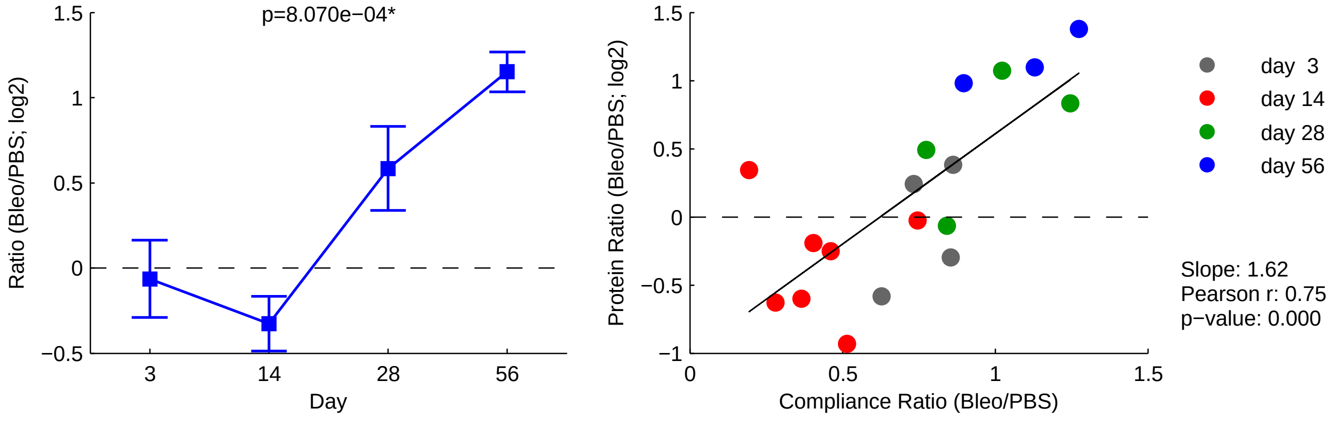

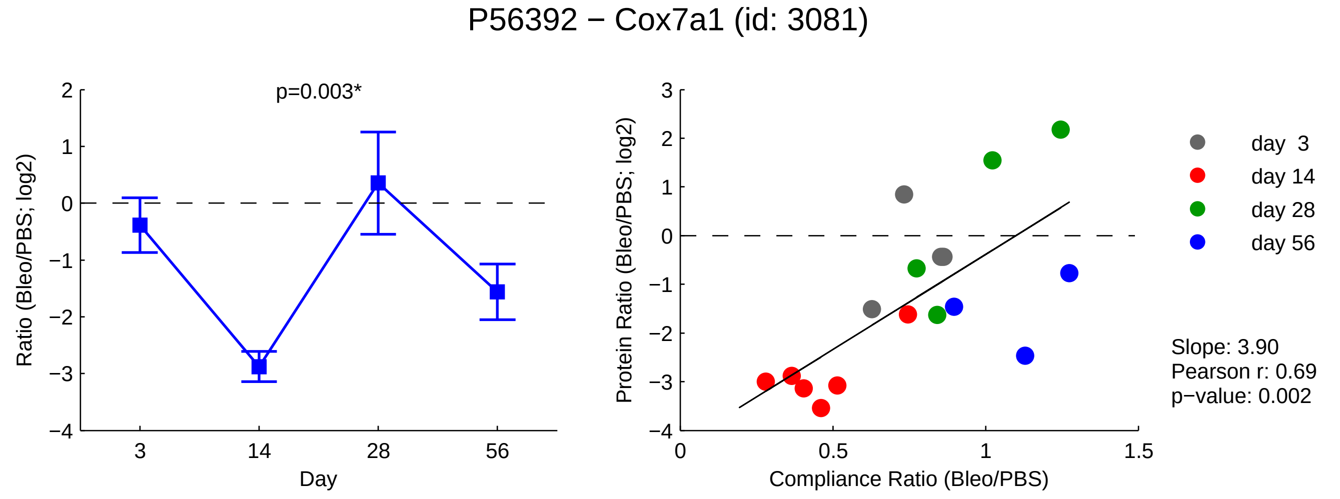
P56392 − Cox7a1 (id: 3081)
p=0.003*
Ratio (Bleo/PBS; log2)
Day
Protein Ratio (Bleo/PBS; log2)
Compliance Ratio (Bleo/PBS)
day 3
day 14
day 28
day 56
Slope: 3.90
Pearson r: 0.69
p−value: 0.002

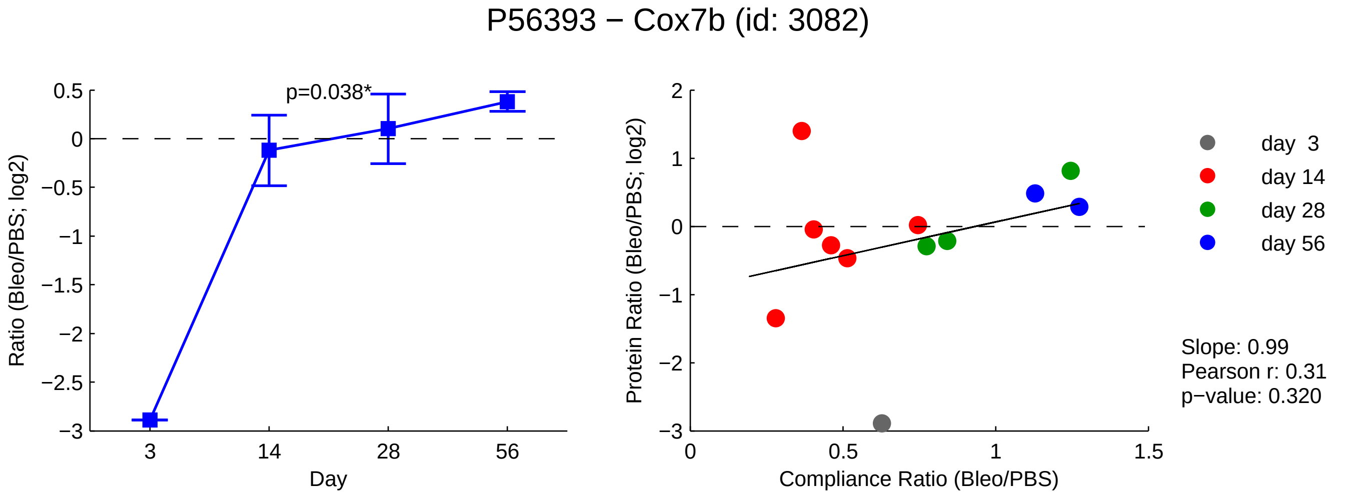
P56393 − Cox7b (id: 3082)
p=0.038*
Ratio (Bleo/PBS; log2)
Day
Protein Ratio (Bleo/PBS; log2)
Compliance Ratio (Bleo/PBS)
day 3
day 14
day 28
day 56
Slope: 0.99
Pearson r: 0.31
p−value: 0.320

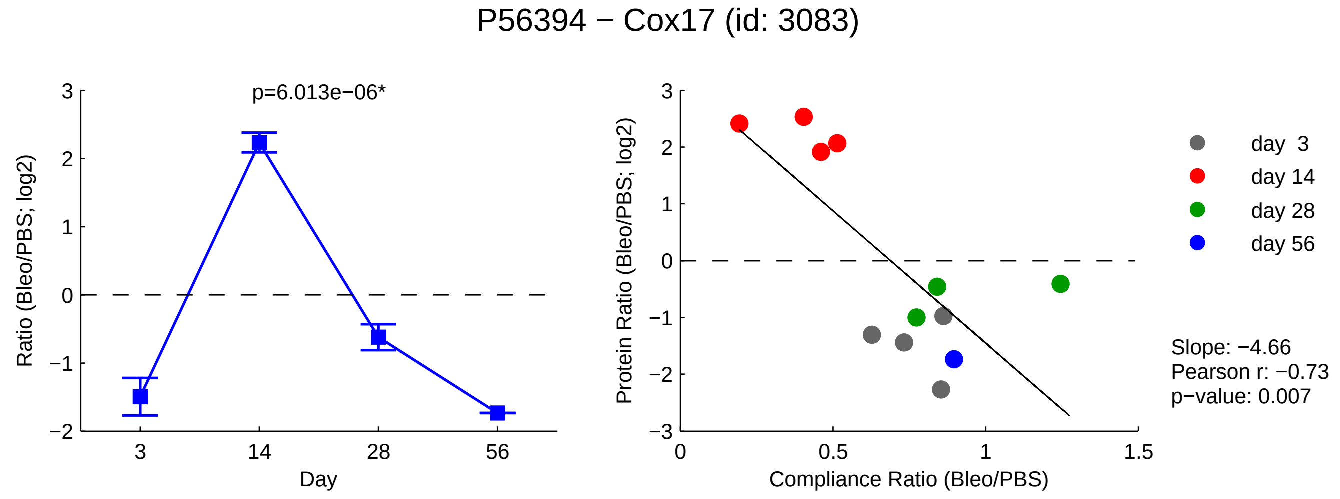
P56394 − Cox17 (id: 3083)
p=6.013e−06*
Ratio (Bleo/PBS; log2)
Day
Protein Ratio (Bleo/PBS; log2)
Compliance Ratio (Bleo/PBS)
day 3
day 14
day 28
day 56
Slope: −4.66
Pearson r: −0.73
p−value: 0.007

P56480 − Atp5b (id: 3085)

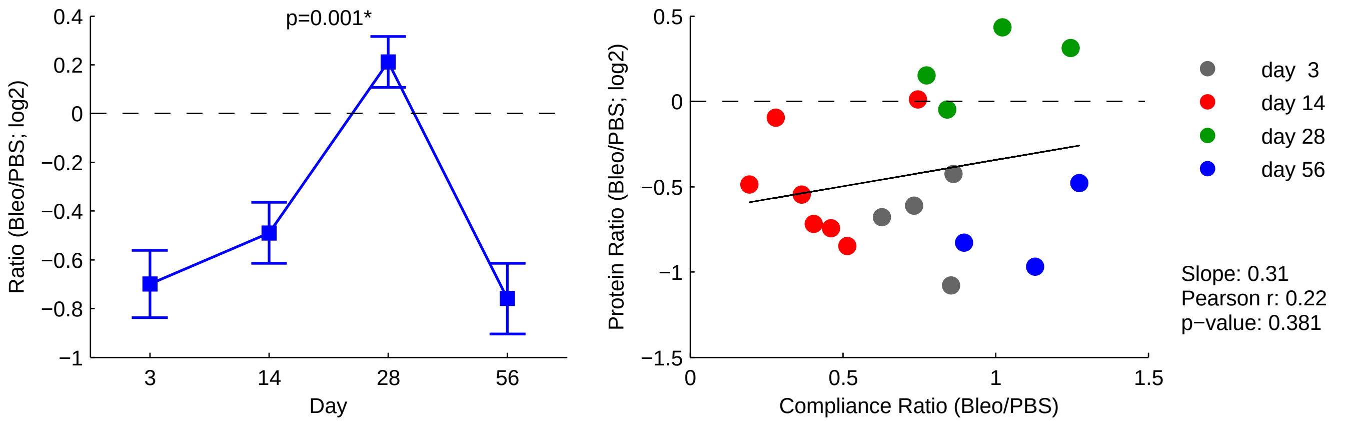

P56501 − Ucp3 (id: 3086)

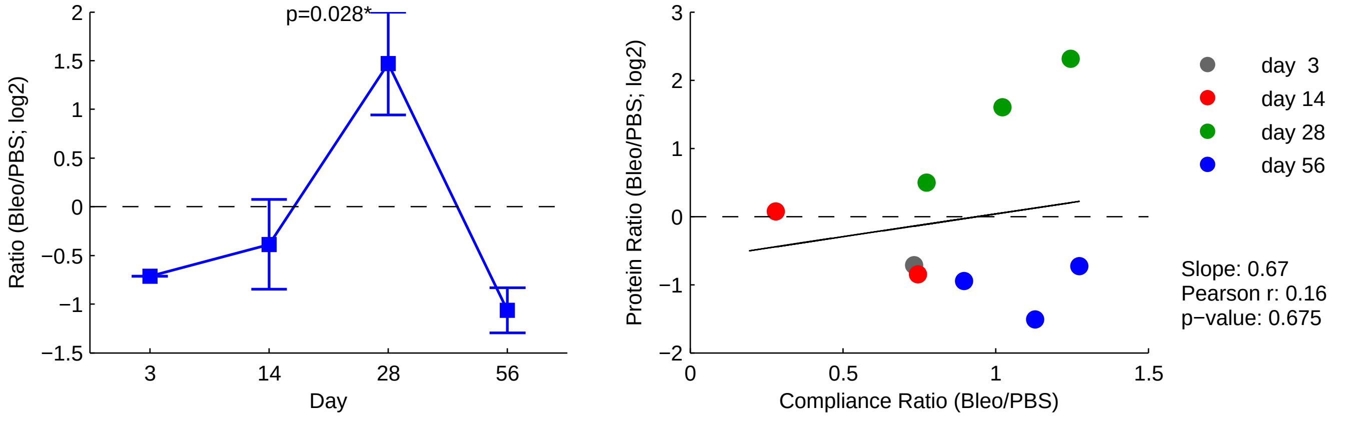

P56528 − Cd38 (id: 3087)

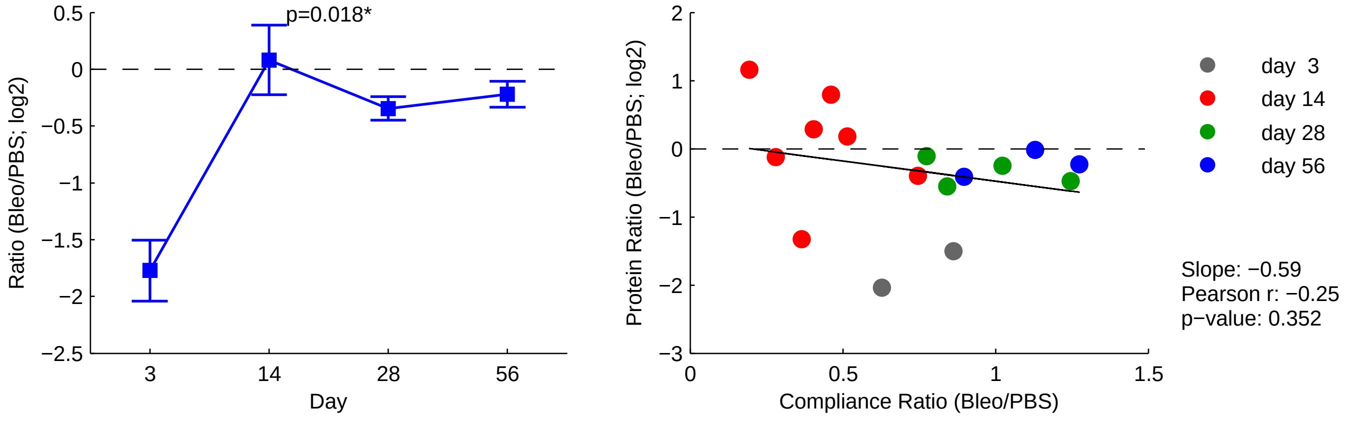

Q91YZ2 − Ctbp2 (id: 3088)

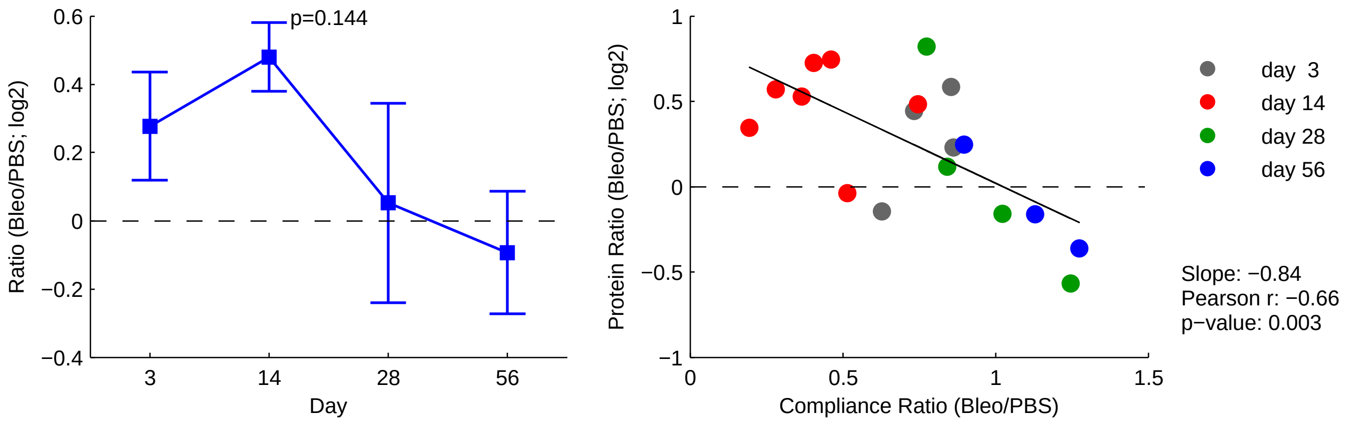

P56564 − Slc1a3 (id: 3089)

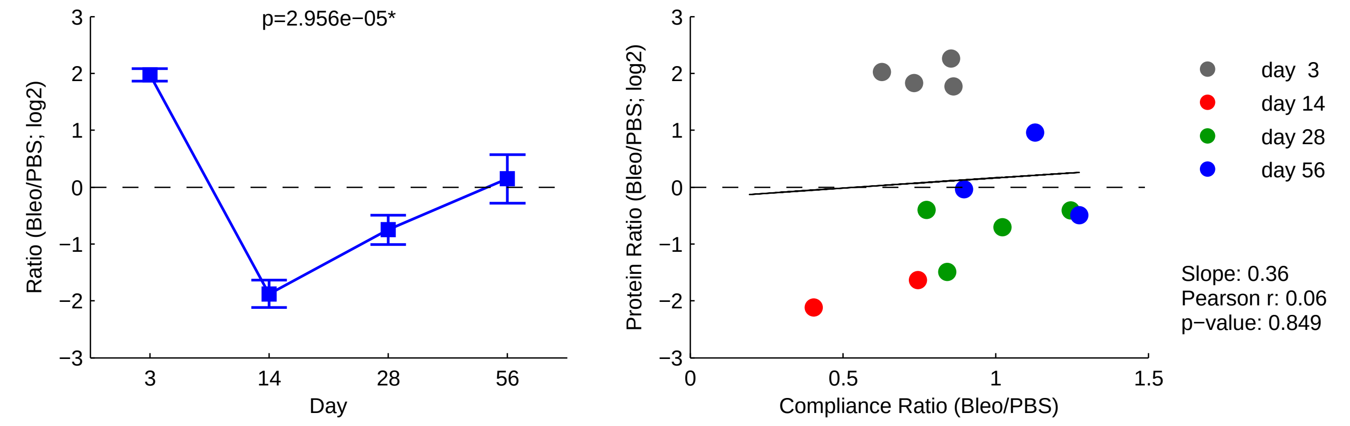

P56857−3 − Cldn18 (id: 3094)

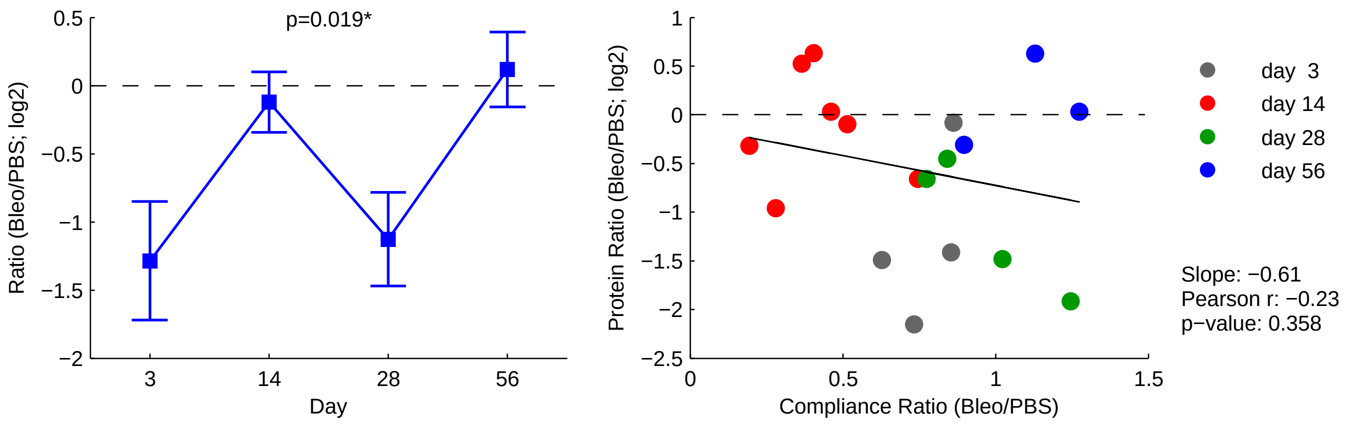

Q8CFQ9 − Fus (id: 3096)

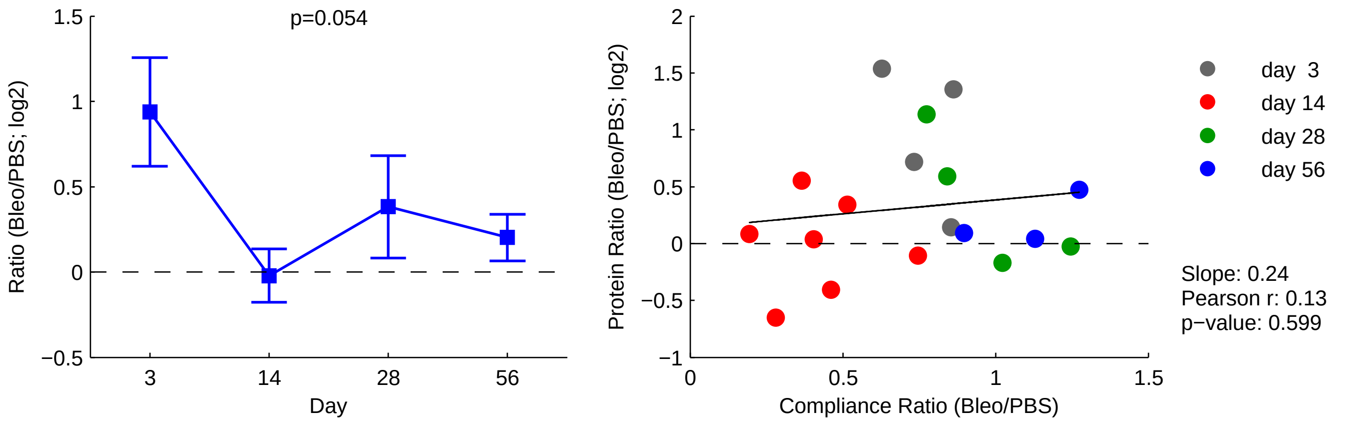

P57746 − Atp6v1d (id: 3101)

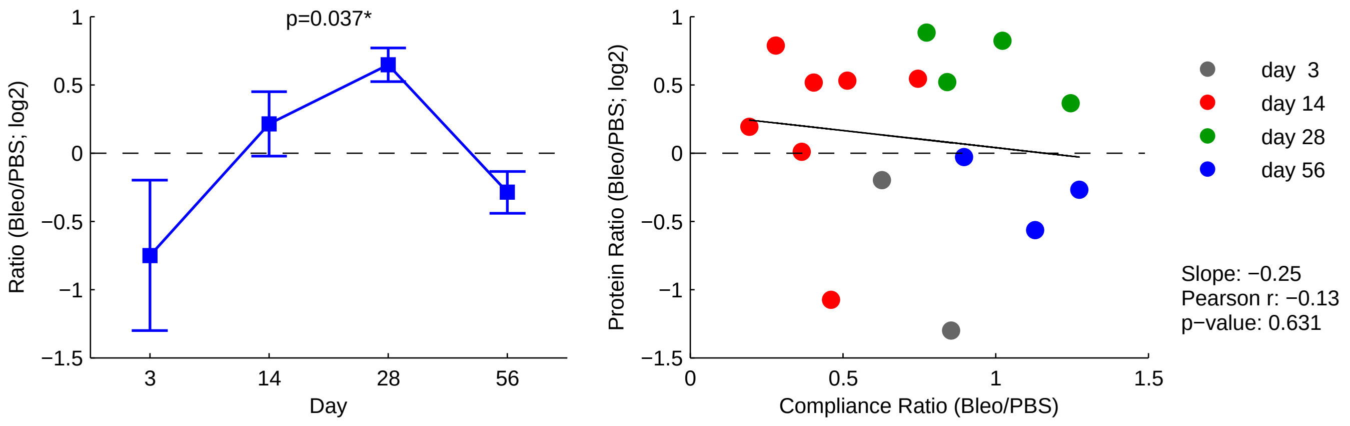

P57759 − Erp29 (id: 3102)

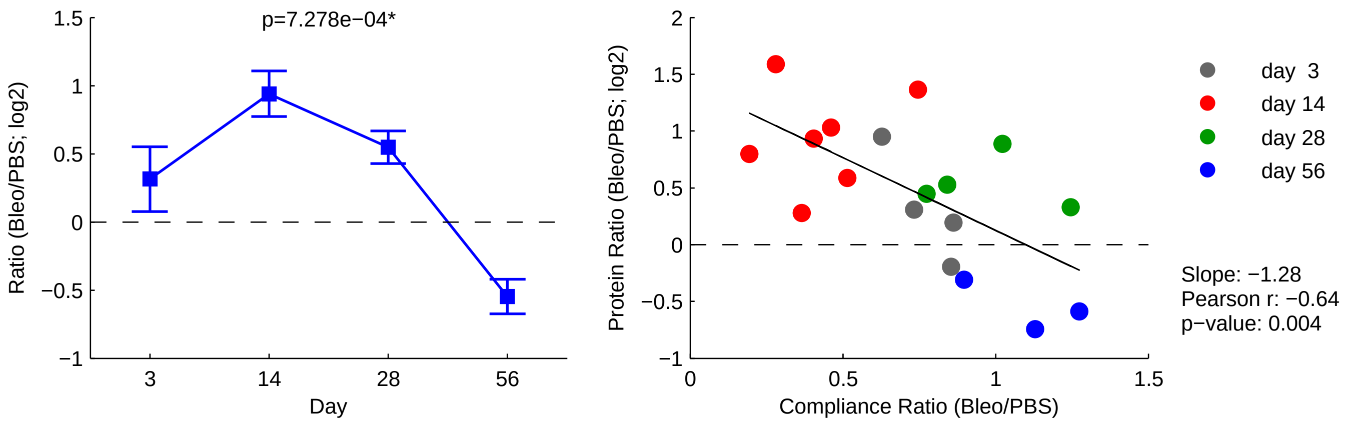

P57780 − Actn4 (id: 3103)

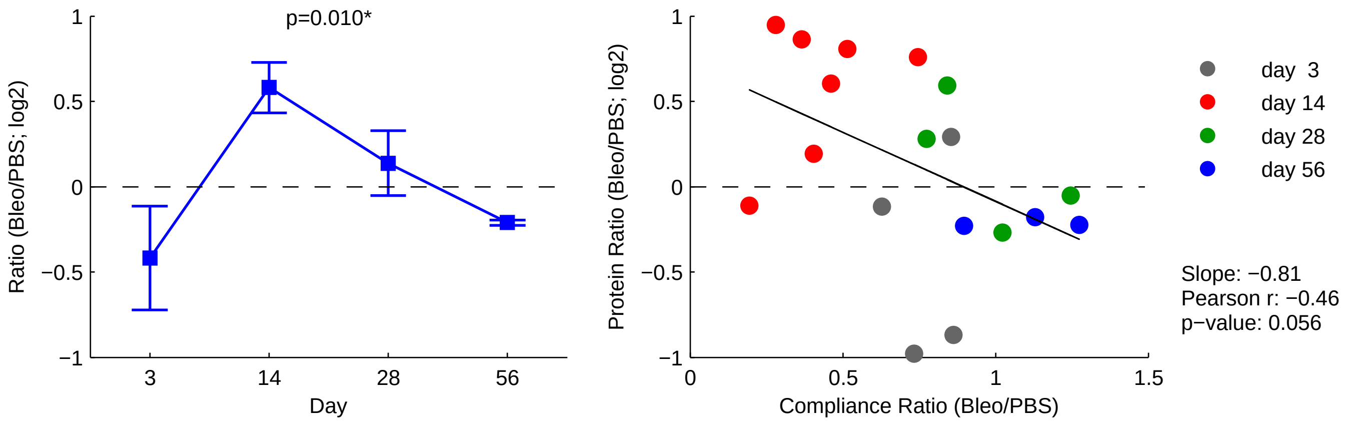

P58021 − Tm9sf2 (id: 3105)

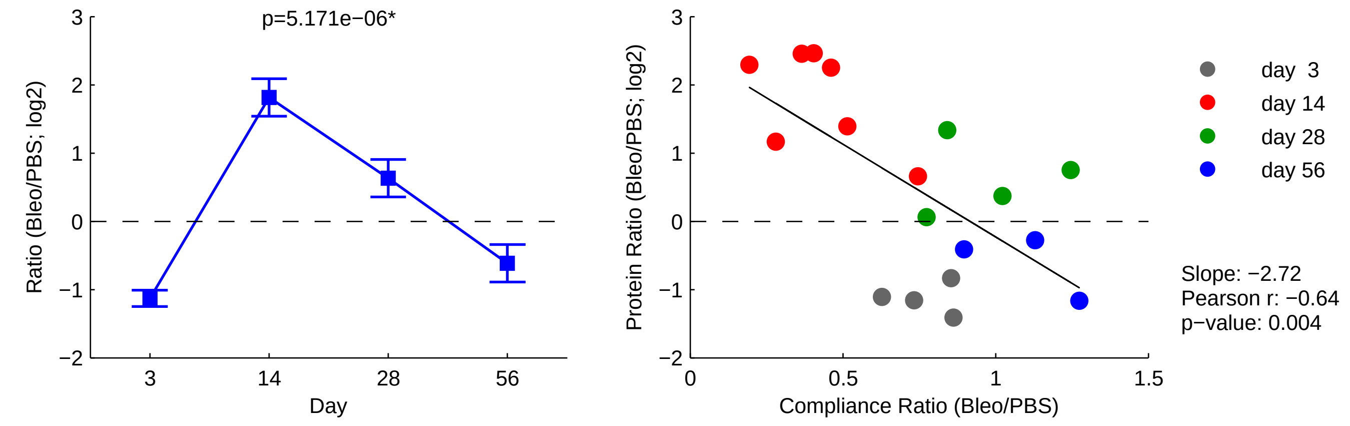

P58022 − Loxl2 (id: 3106)

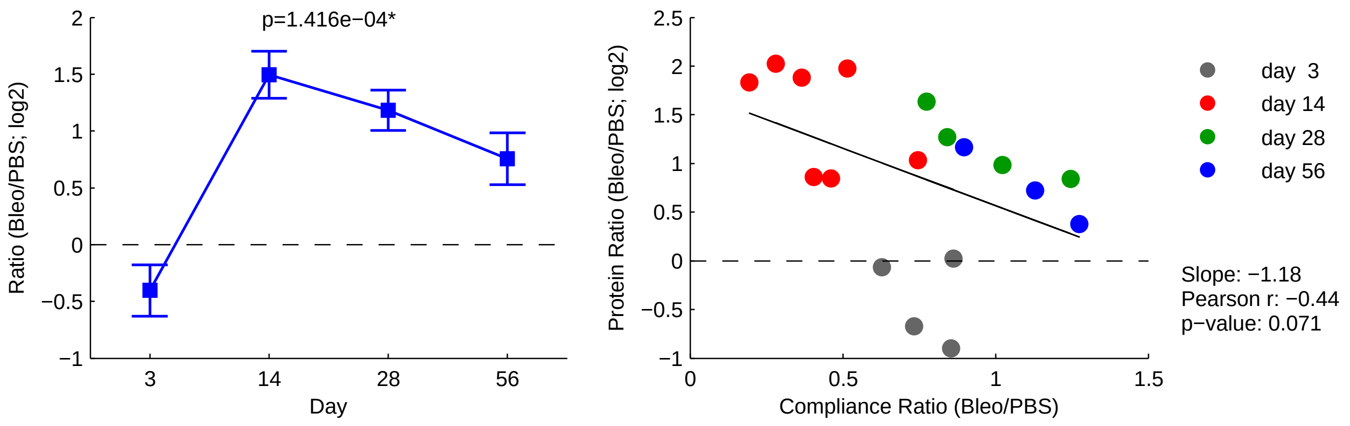

P58064 − Mrps6 (id: 3107)

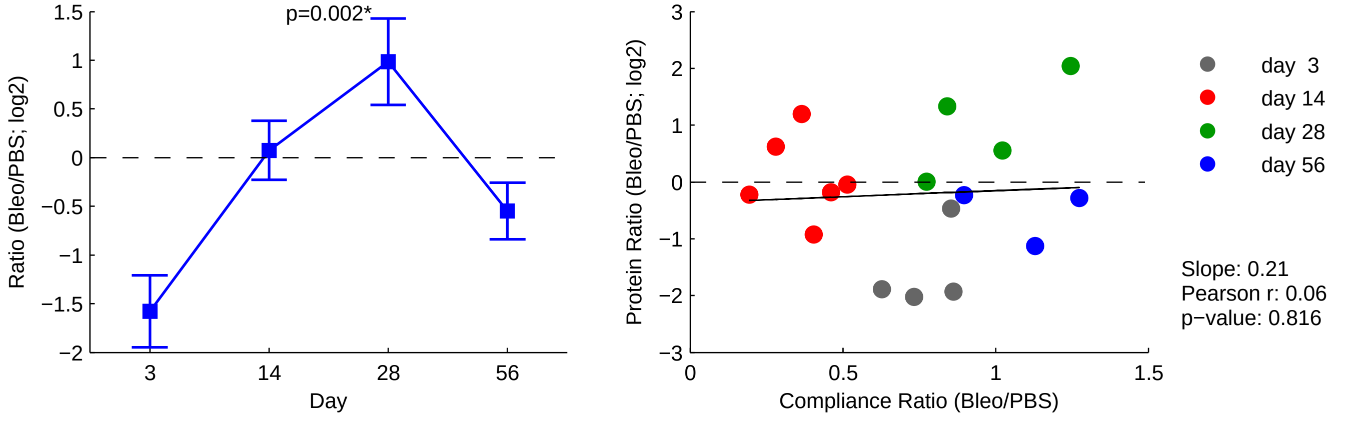

P58196 − Plscr4 (id: 3110)

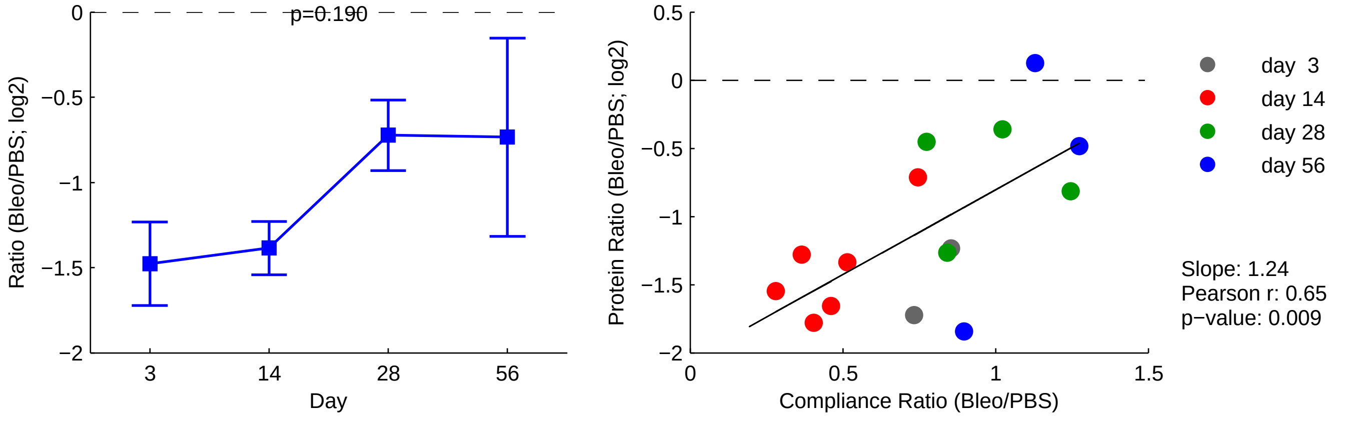

P58252 − Eef2 (id: 3111)

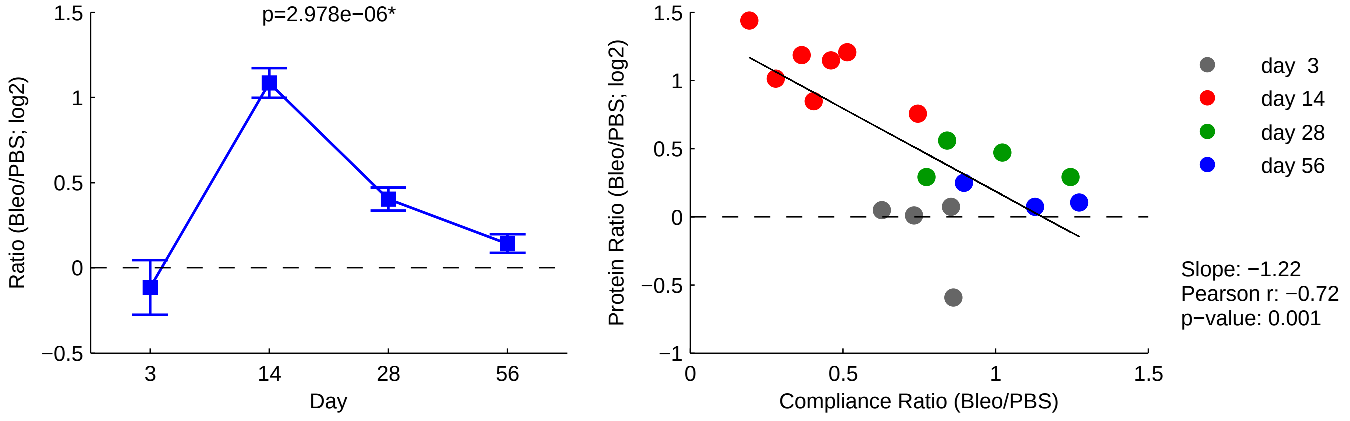

P58281−2 − Opa1 (id: 3112)

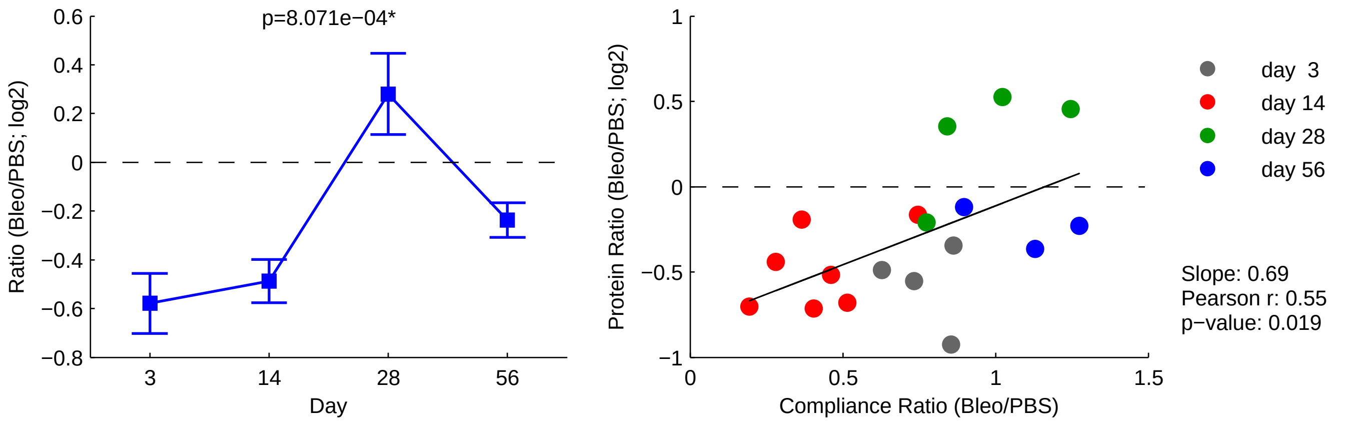

P58404−2 − Strn4 (id: 3115)

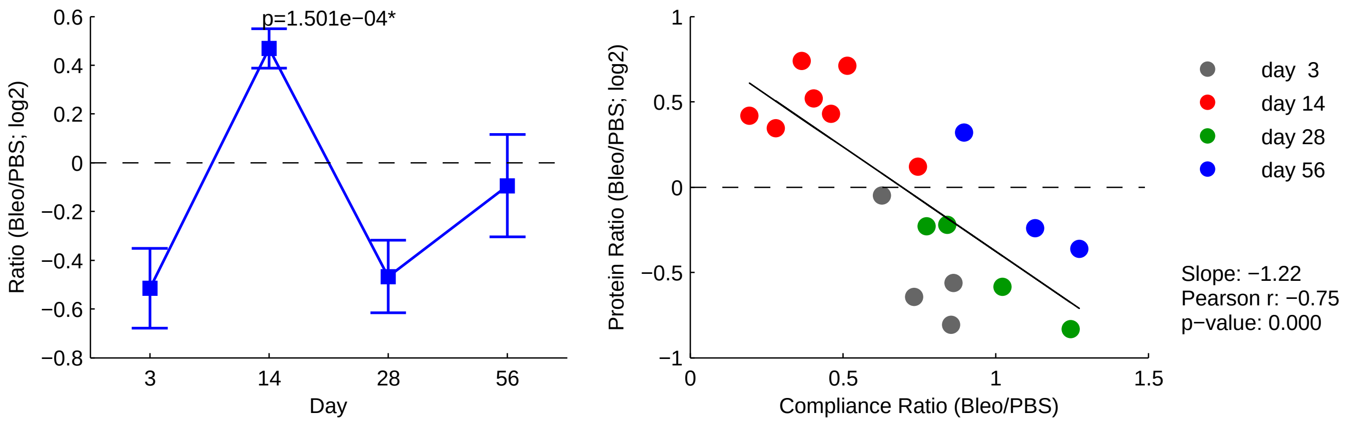

P58465 − Ctdspl (id: 3116)

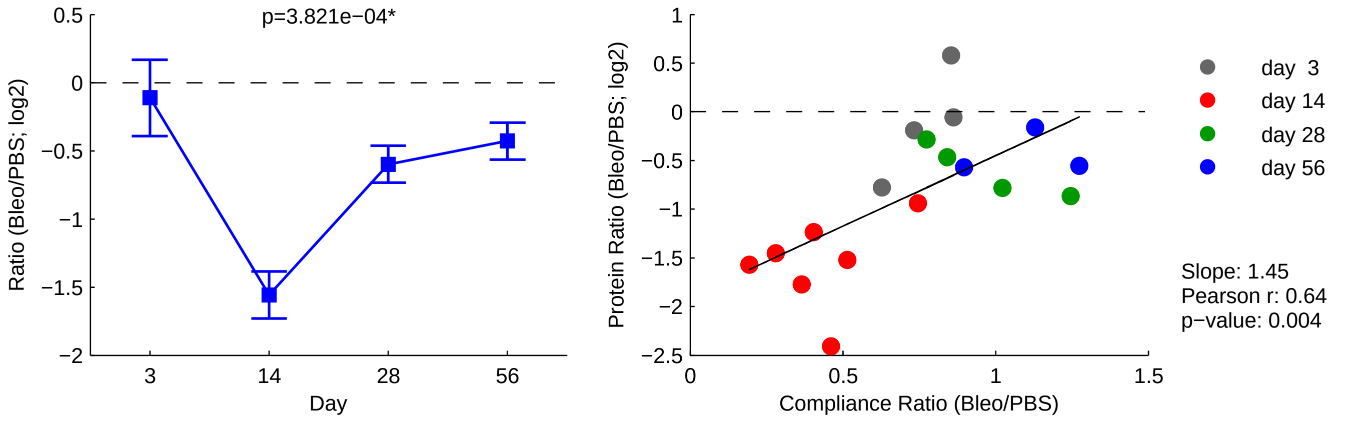

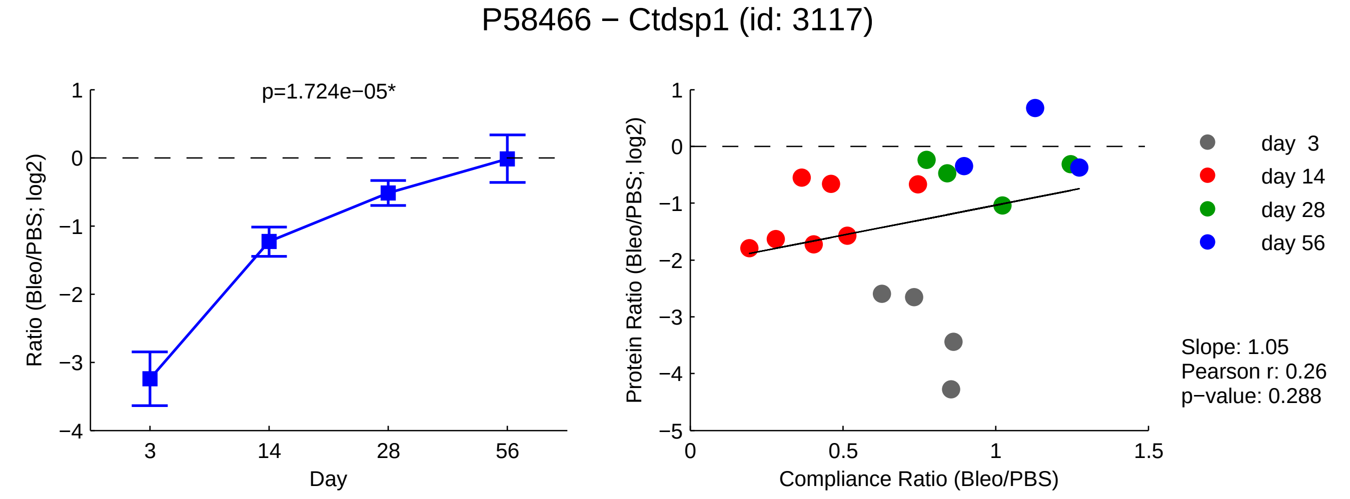
P58466 − Ctdsp1 (id: 3117)
p=1.724e−05*
Ratio (Bleo/PBS; log2)
Day
Protein Ratio (Bleo/PBS; log2)
Compliance Ratio (Bleo/PBS)
day 3
day 14
day 28
day 56
Slope: 1.05
Pearson r: 0.26
p−value: 0.288

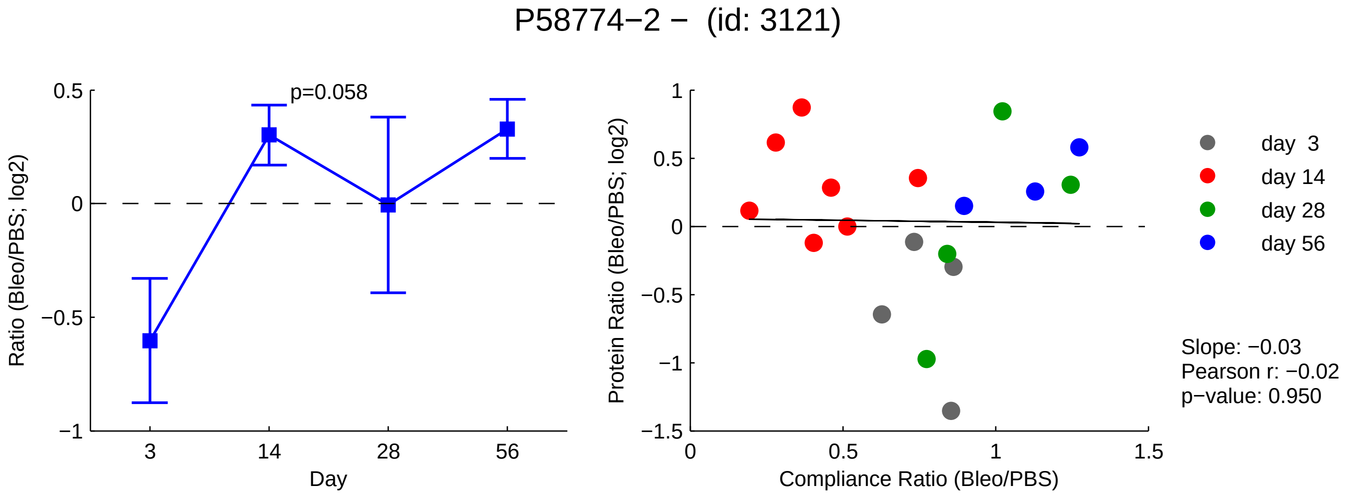
P58774−2 − (id: 3121)
p=0.058
Ratio (Bleo/PBS; log2)
Day
Protein Ratio (Bleo/PBS; log2)
Compliance Ratio (Bleo/PBS)
day 3
day 14
day 28
day 56
Slope: −0.03
Pearson r: −0.02
p−value: 0.950

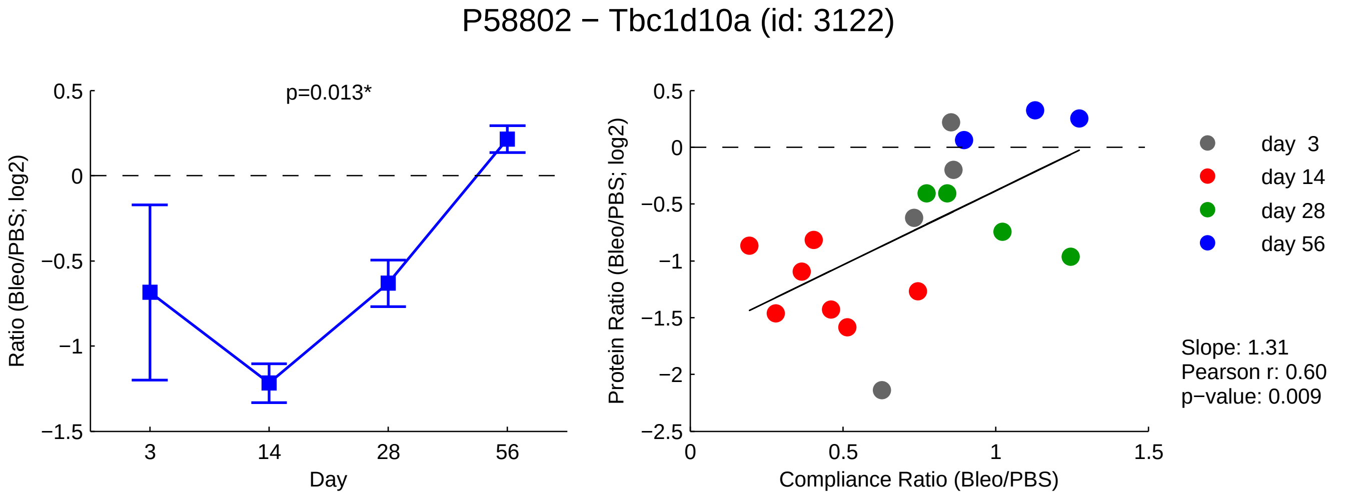
P58802 − Tbc1d10a (id: 3122)
p=0.013*
Ratio (Bleo/PBS; log2)
Day
Protein Ratio (Bleo/PBS; log2)
Compliance Ratio (Bleo/PBS)
day 3
day 14
day 28
day 56
Slope: 1.31
Pearson r: 0.60
p−value: 0.009

P58854 − Tubgcp3 (id: 3123)

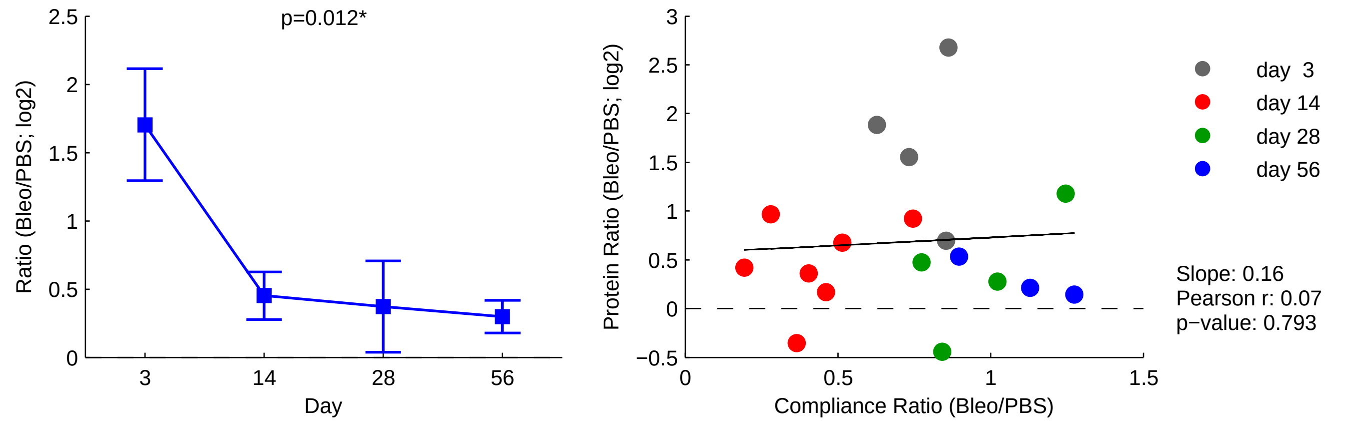

P58871 − Tnks1bp1 (id: 3124)

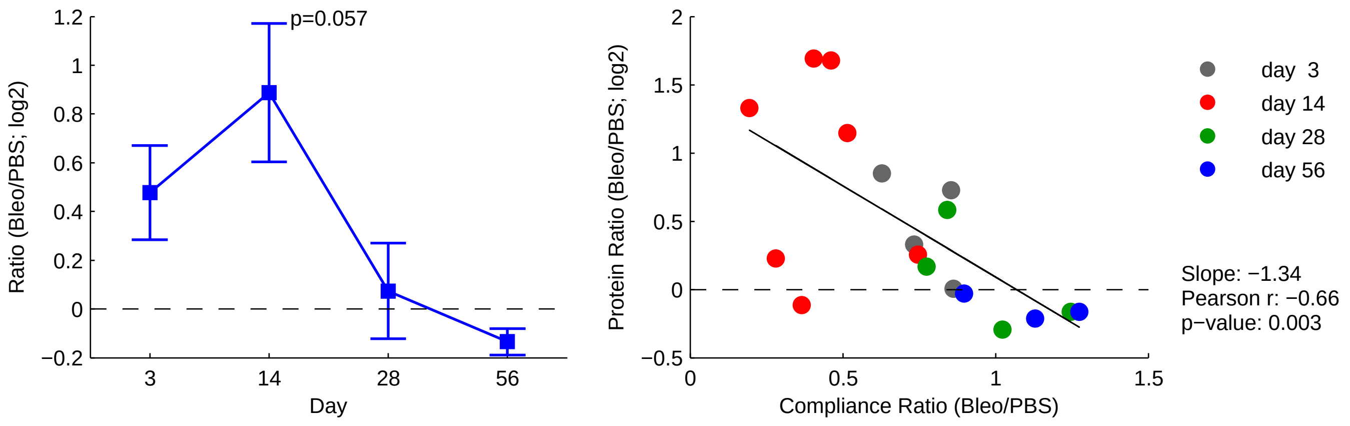

P59017 − Bcl2l13 (id: 3127)

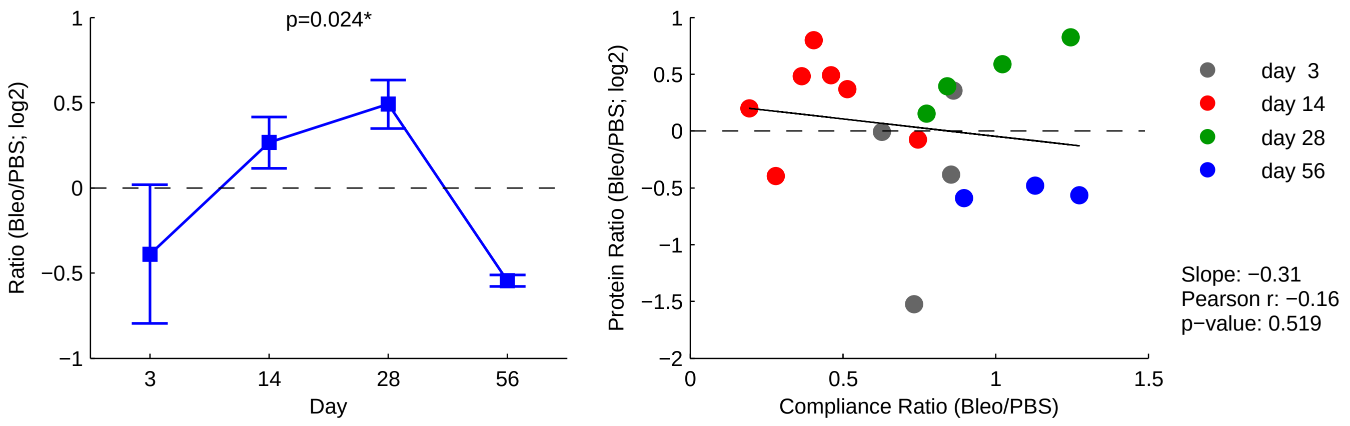

P59114 − Pcif1 (id: 3130)

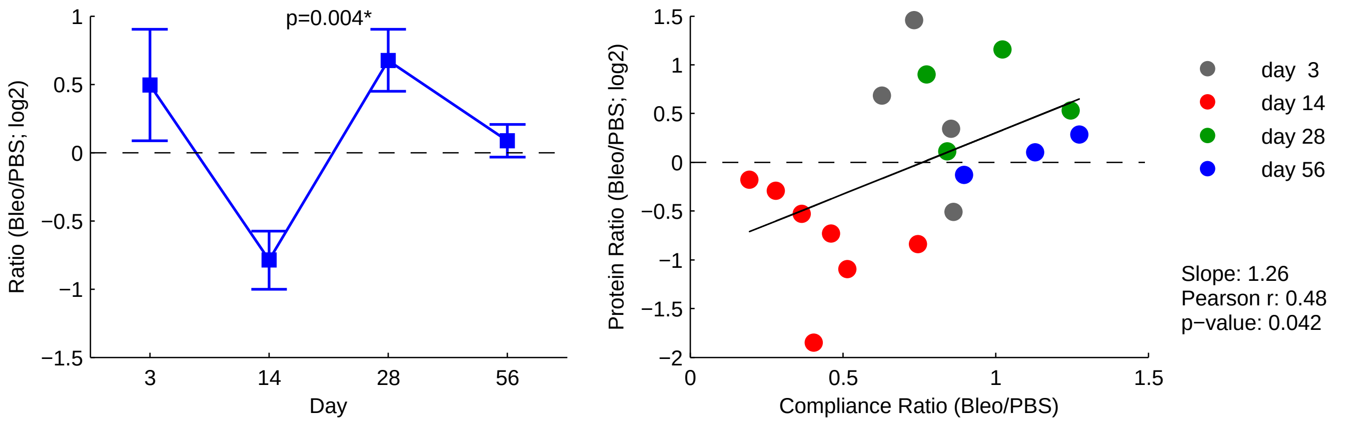

P59222 − Scarf2 (id: 3131)

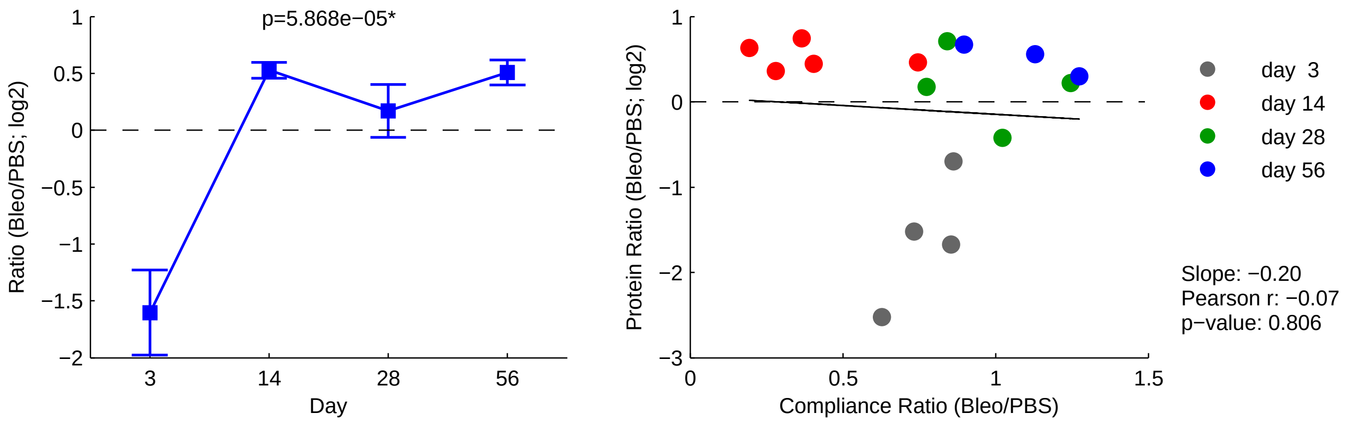

P59235 − Nup43 (id: 3132)

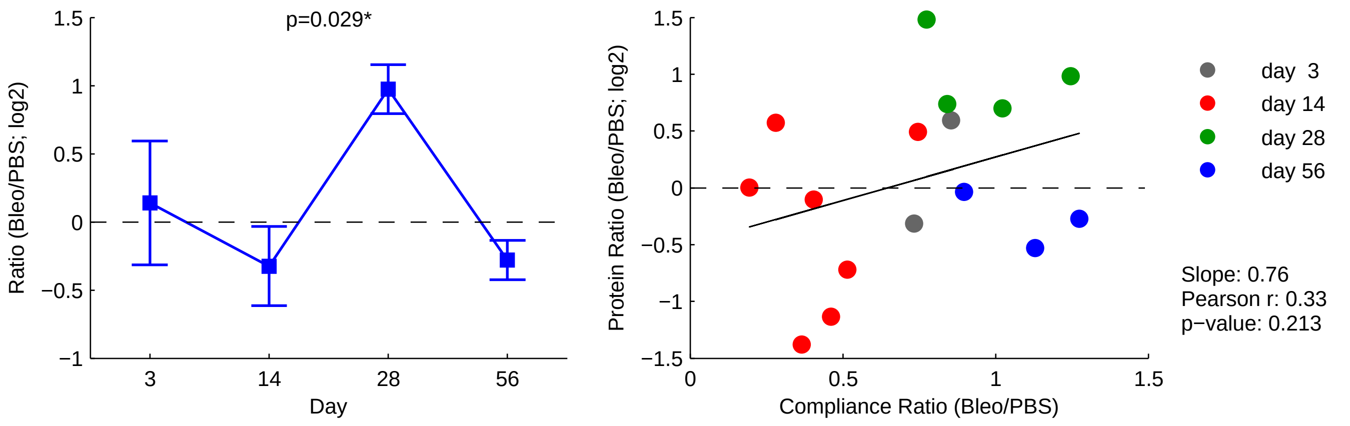

P59240 − Nphp4 (id: 3133)

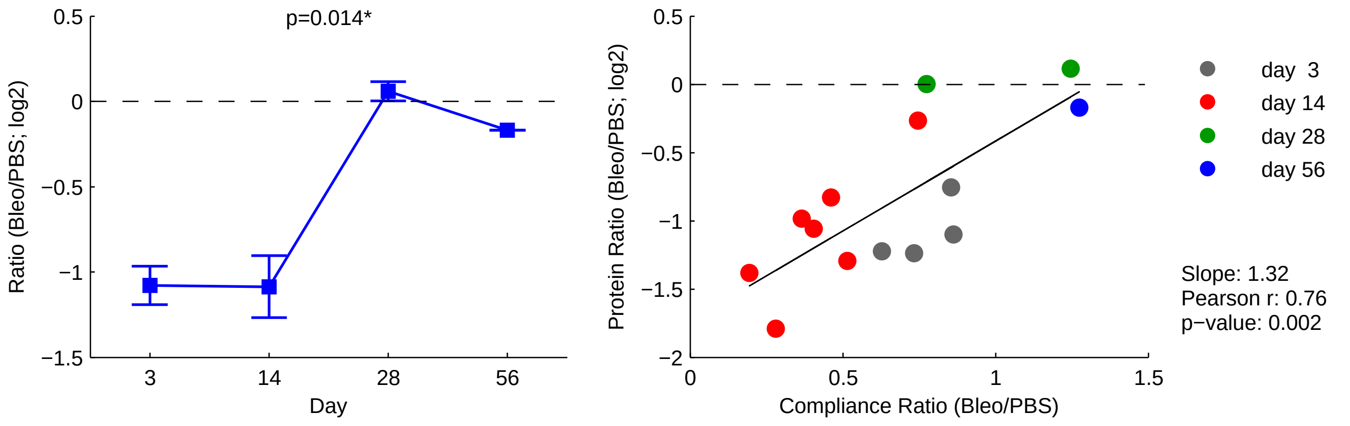

P59279 − Rab2b (id: 3136)

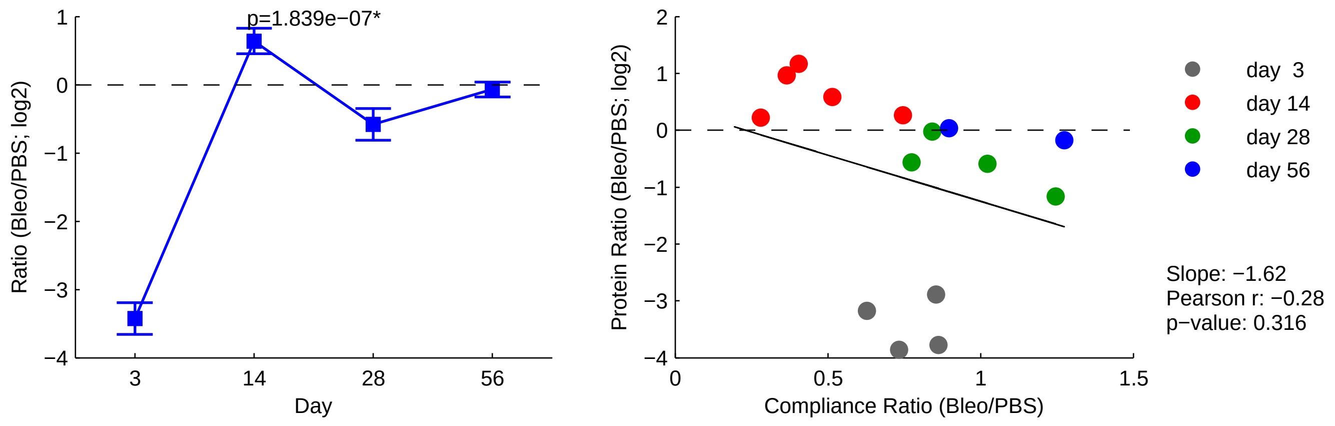

P59325 − Eif5 (id: 3137)

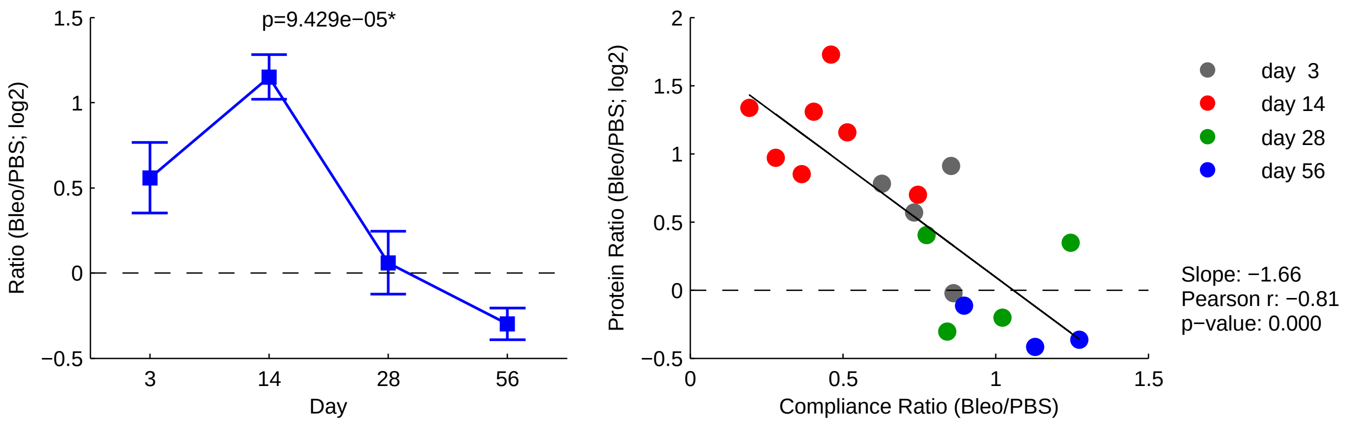

P59383 − Lrrn4 (id: 3138)

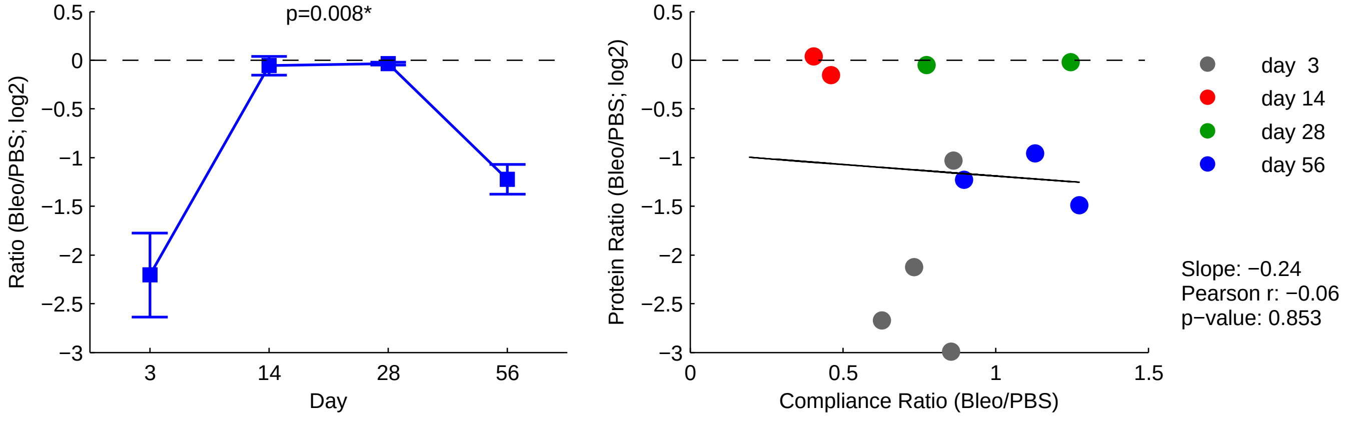

P59672 − Anks1a (id: 3141)

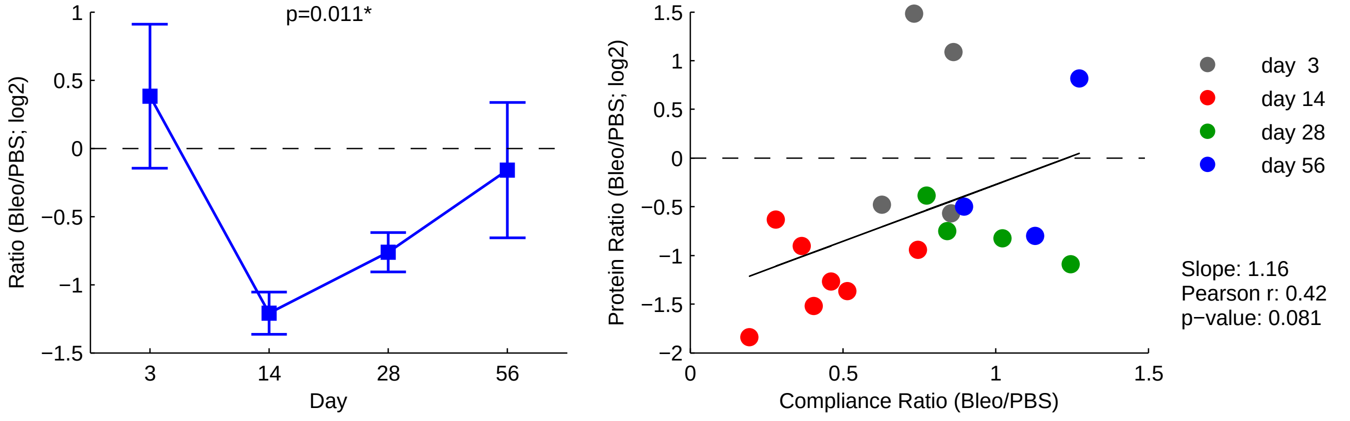

P59708 − Sf3b14 (id: 3142)

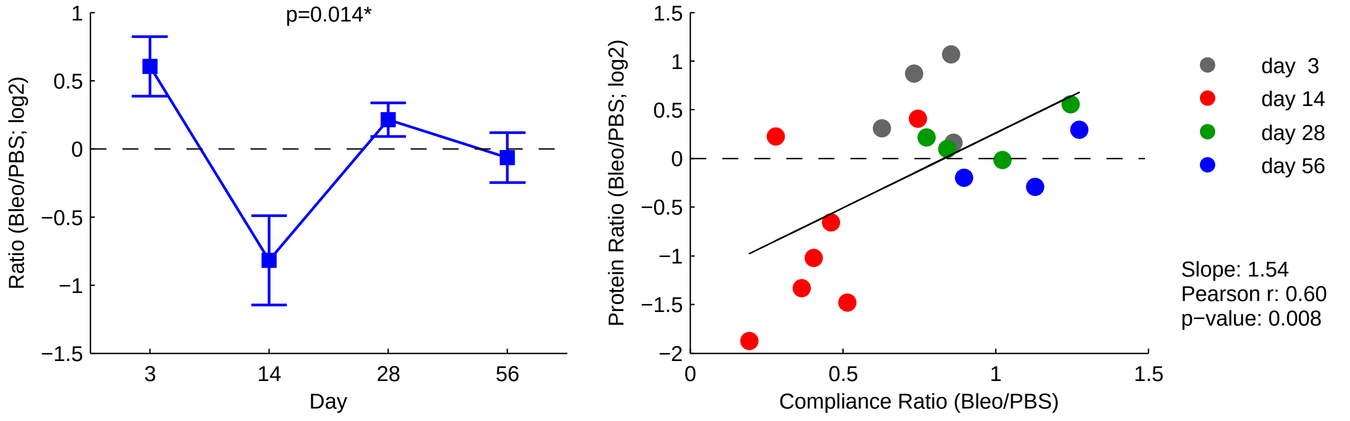

P59764 − Dock4 (id: 3144)

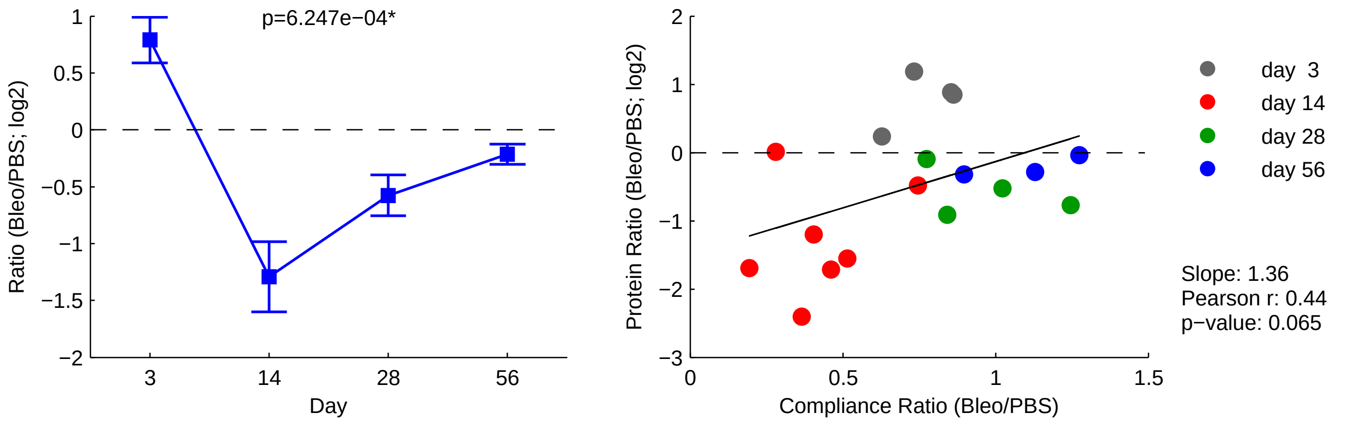

P60060 − Sec61g (id: 3147)

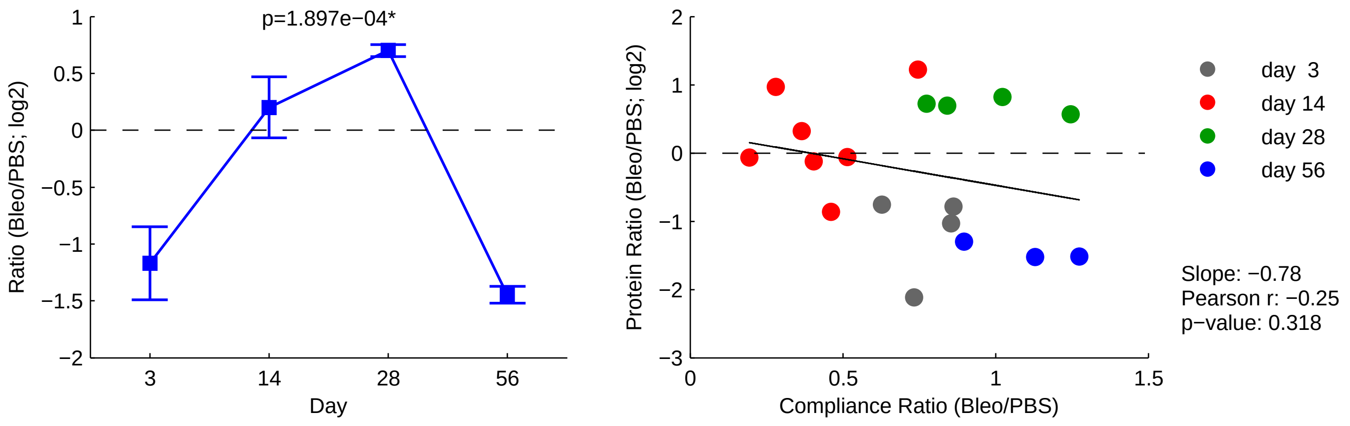

P60122 − Ruvbl1 (id: 3148)

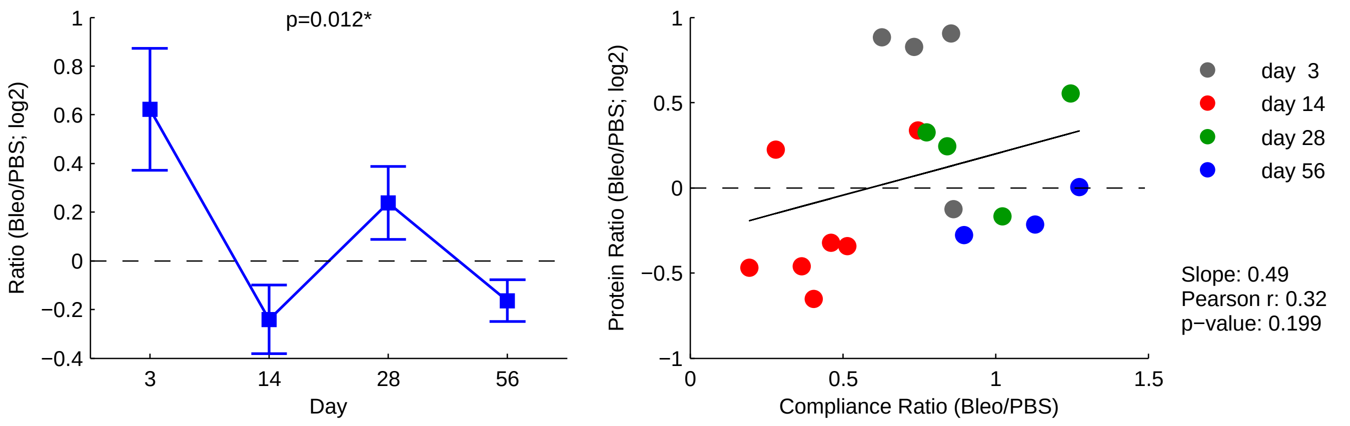

P60229 − Eif3e (id: 3149)

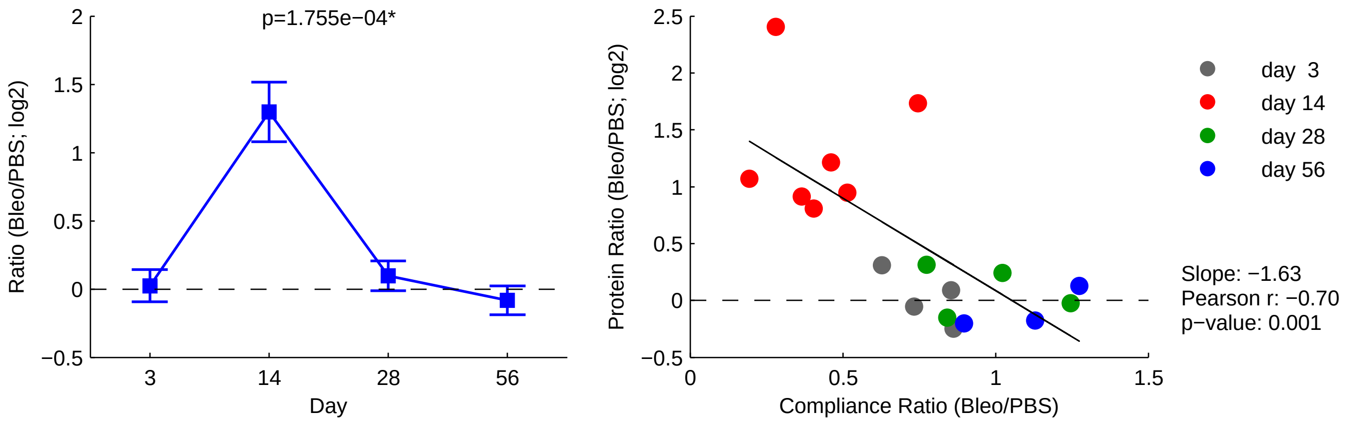

P60335 − Pcbp1 (id: 3150)

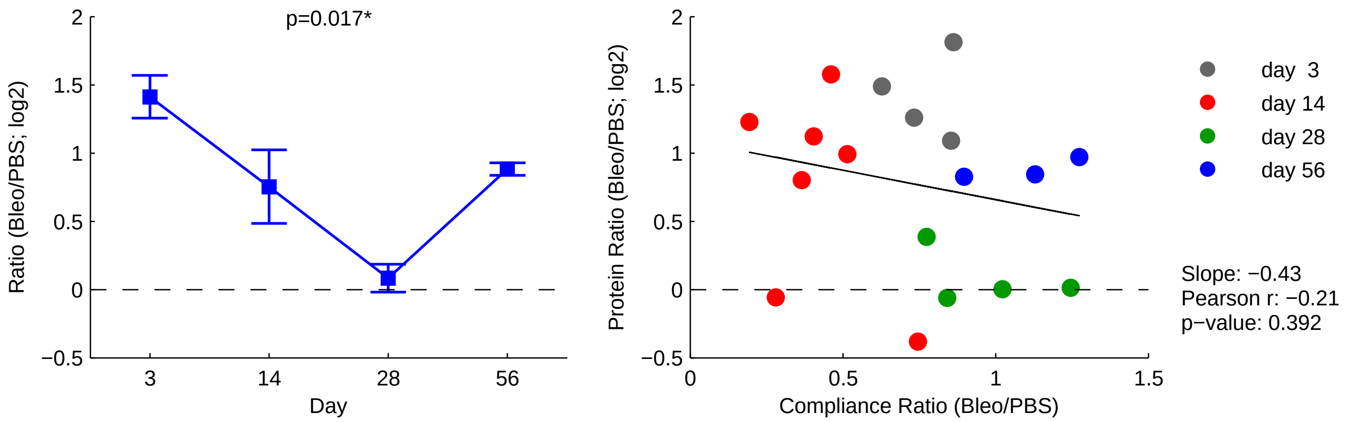

P60670 − Nploc4 (id: 3154)

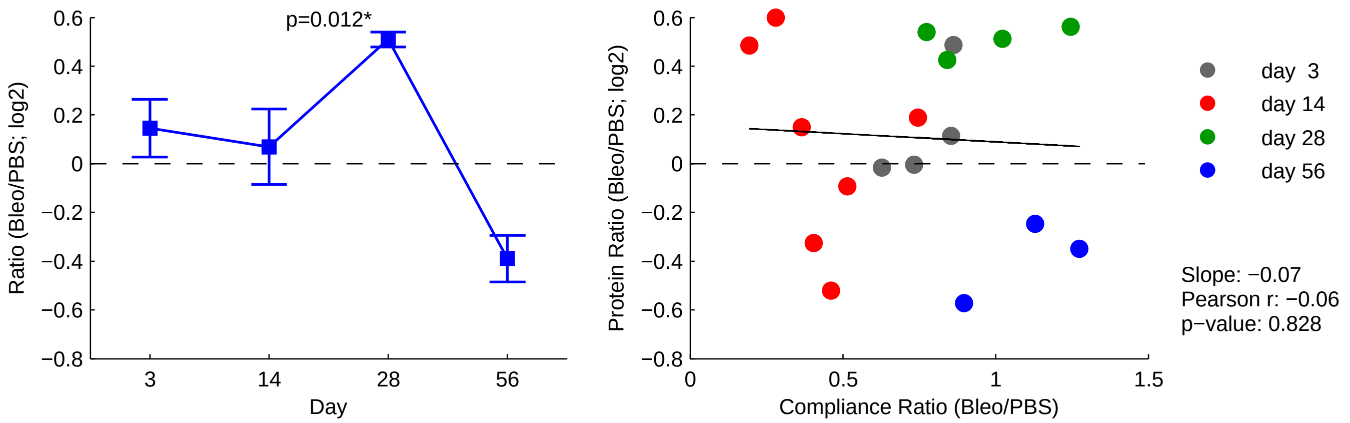

P60762−2 − Morf4l1 (id: 3155)

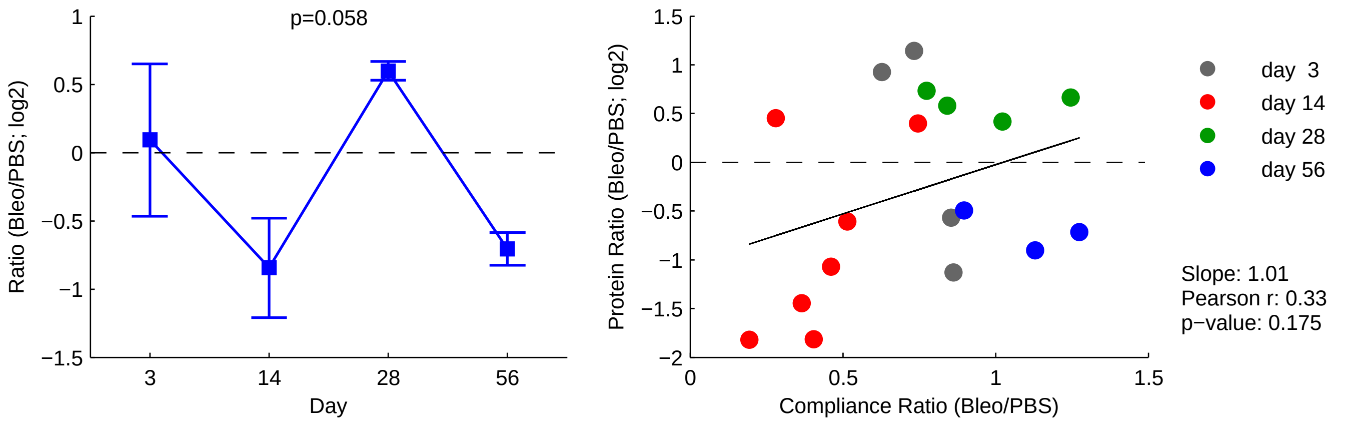

P60766 − Cdc42 (id: 3156)

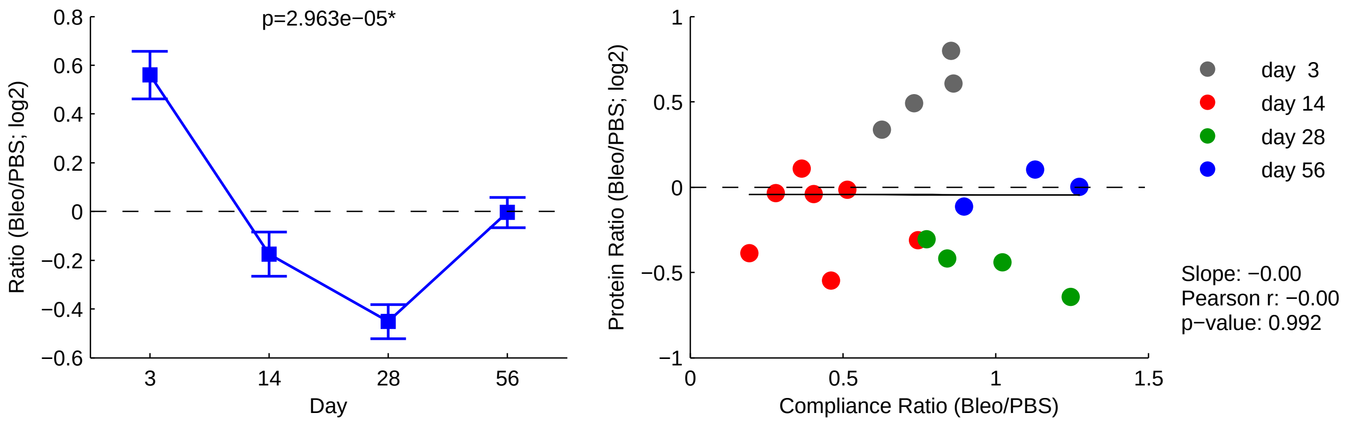

P60824 − Cirbp (id: 3158)

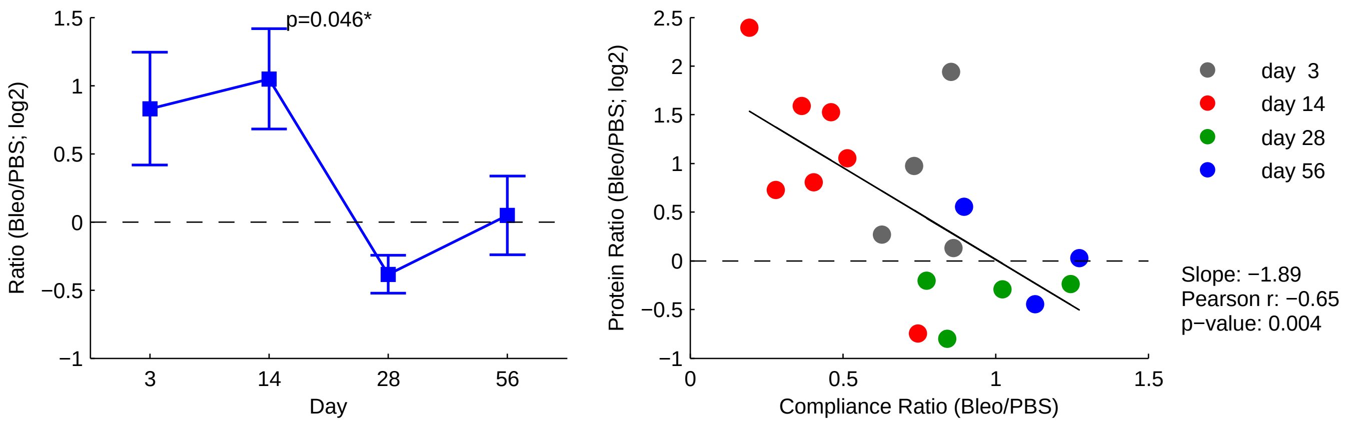

P60843 − Eif4a1 (id: 3159)

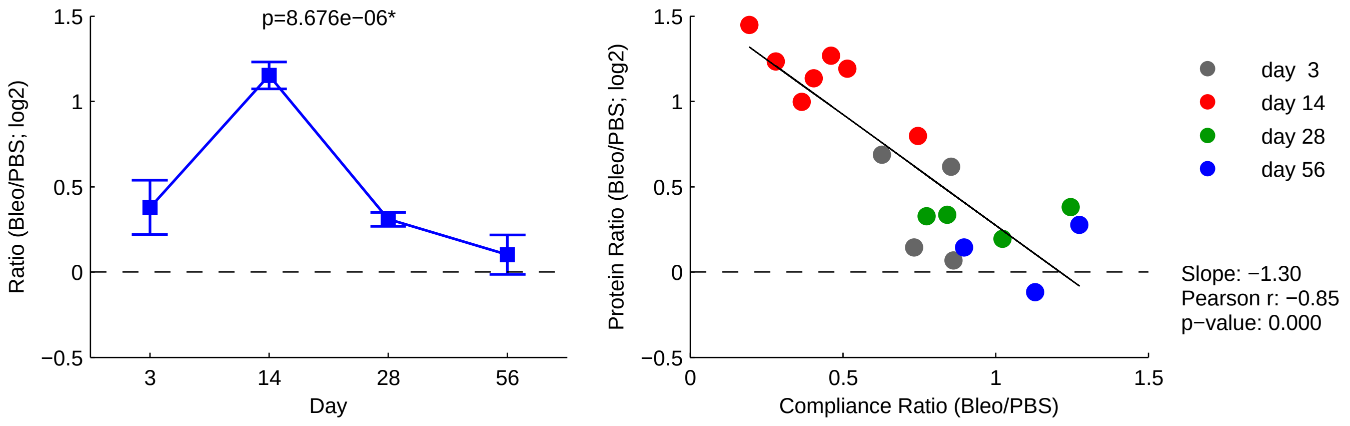

P60867 − Rps20 (id: 3160)

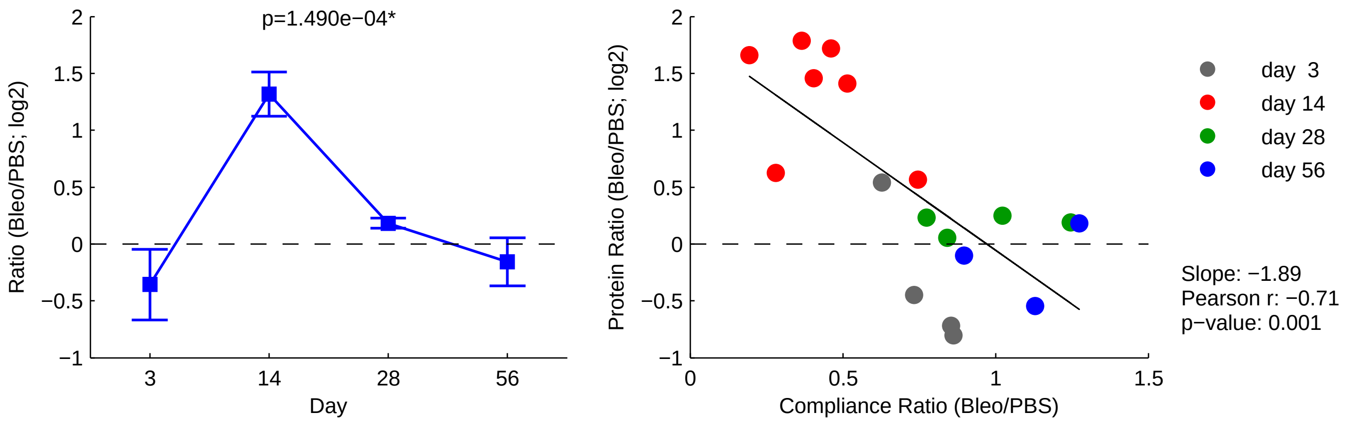

P61021 − Rab5b (id: 3162)

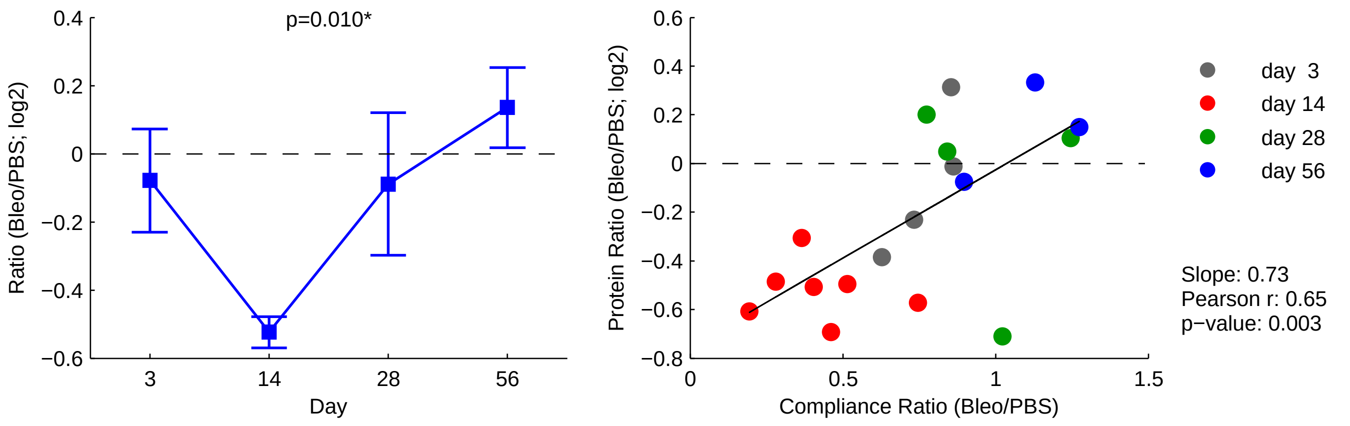

P61028 − Rab8b (id: 3164)

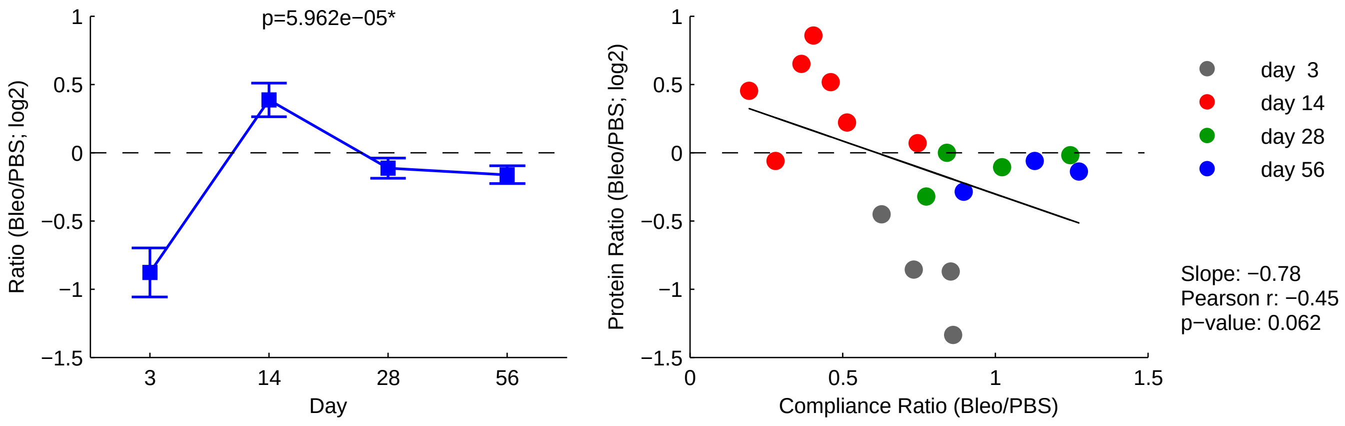

P61087 − Ube2k (id: 3167)

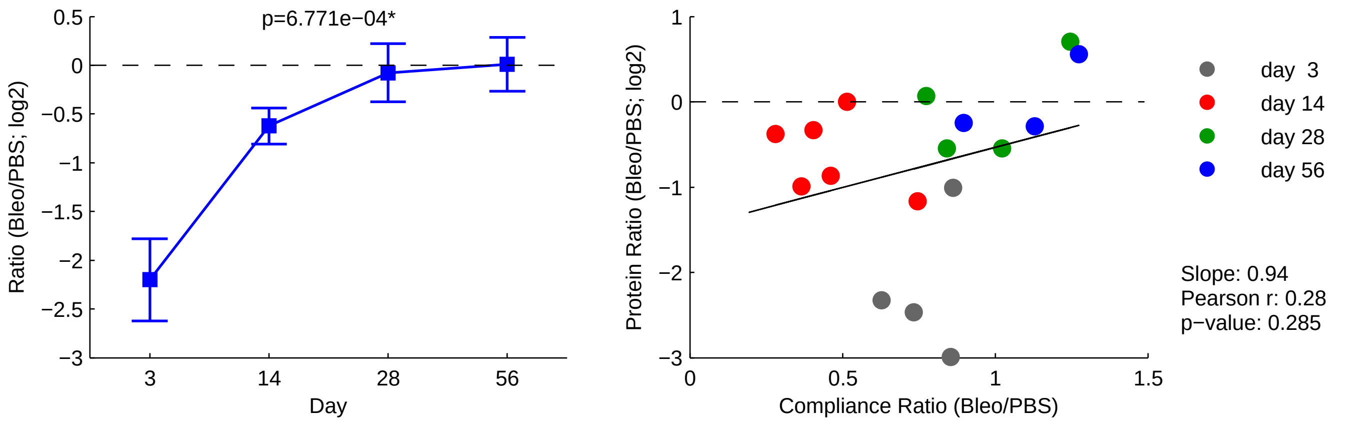

P61089 − Ube2n (id: 3168)

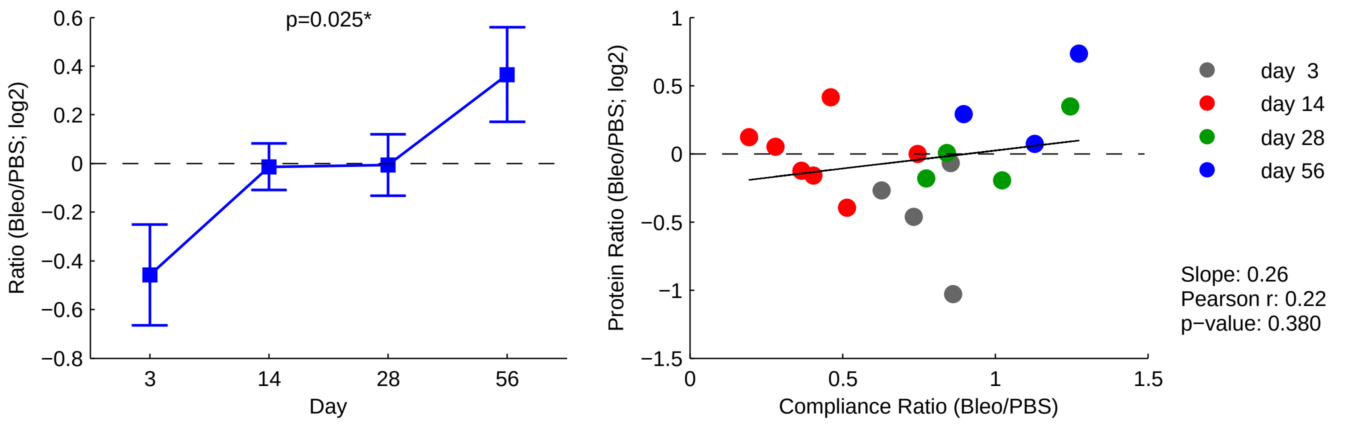

P61161 − Actr2 (id: 3170)

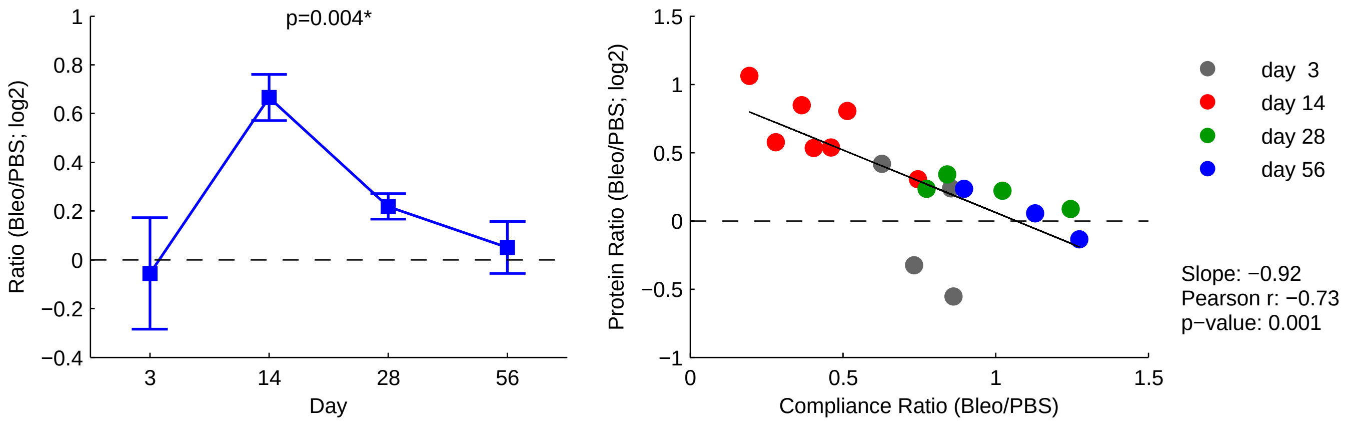

P61164 − Actr1a (id: 3171)

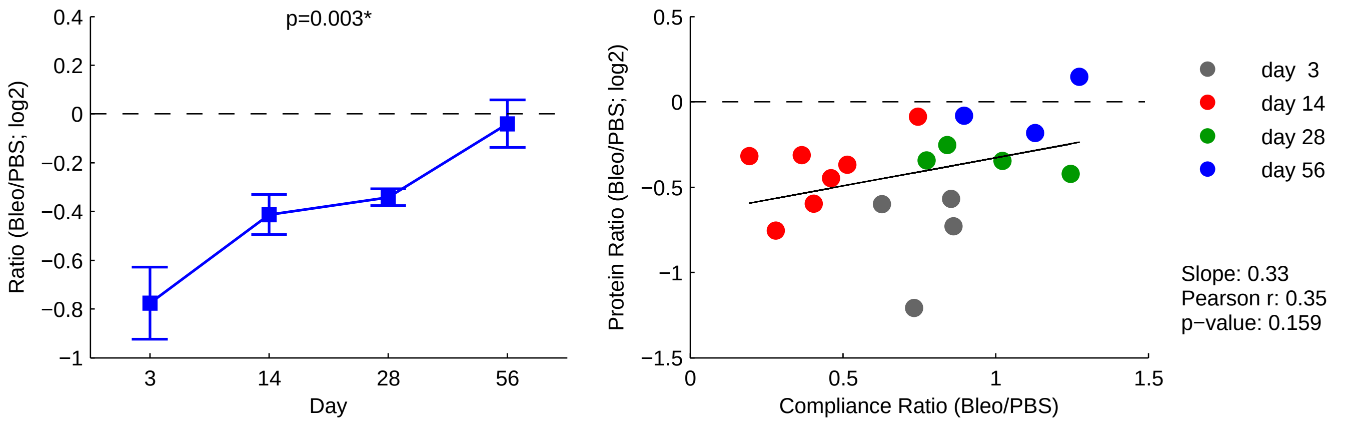

P61202 − Cops2 (id: 3172)

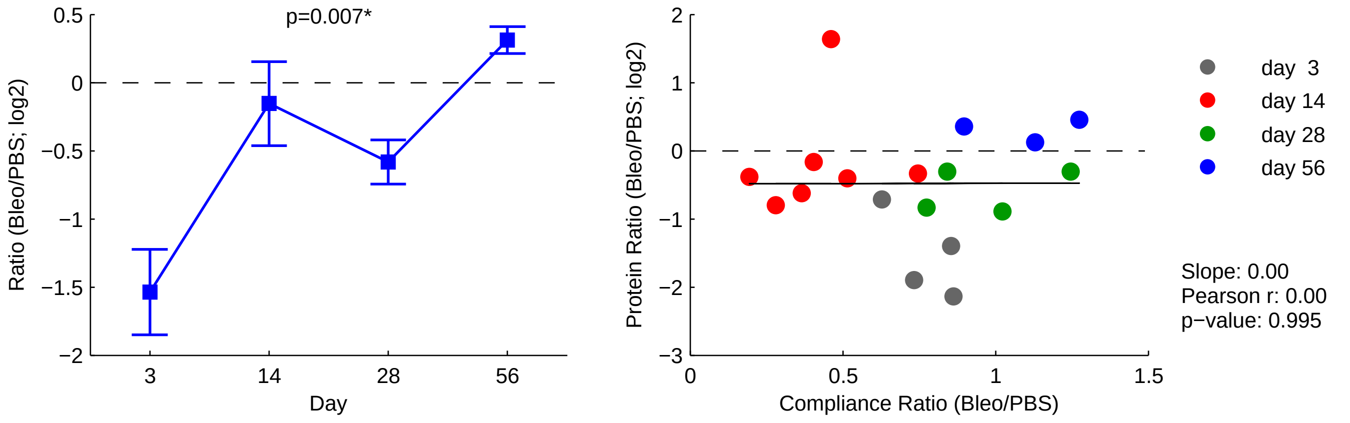

P61211 − Arl1 (id: 3174)

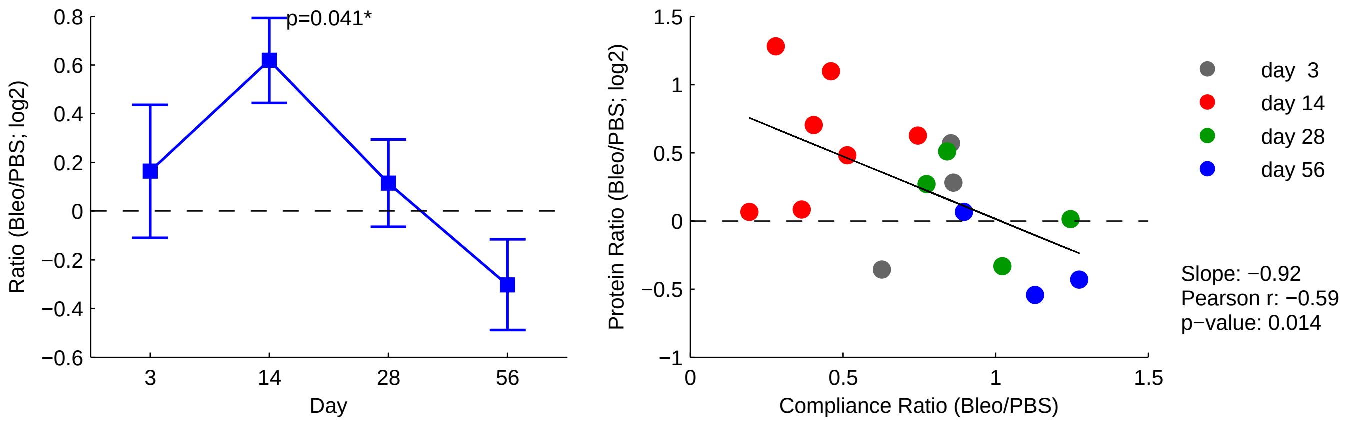

P61222 − Abce1 (id: 3176)

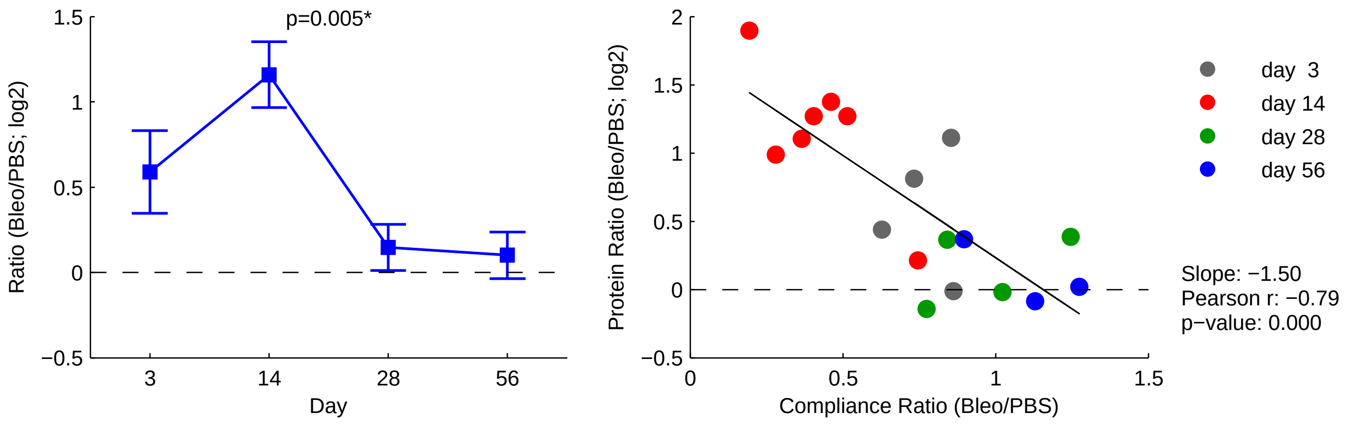

P61226 − Rap2b (id: 3177)

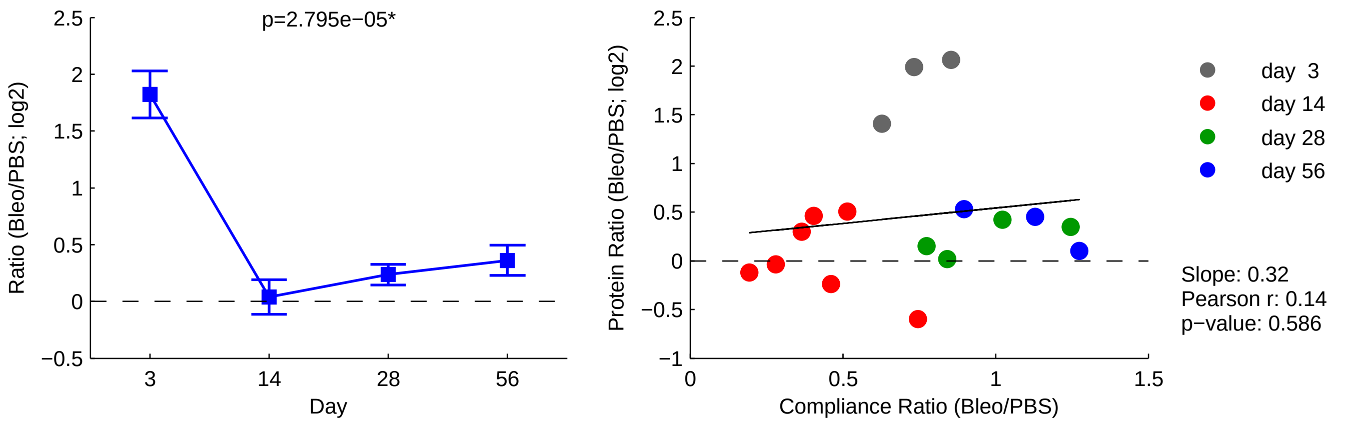

P61255 − Rpl26 (id: 3178)

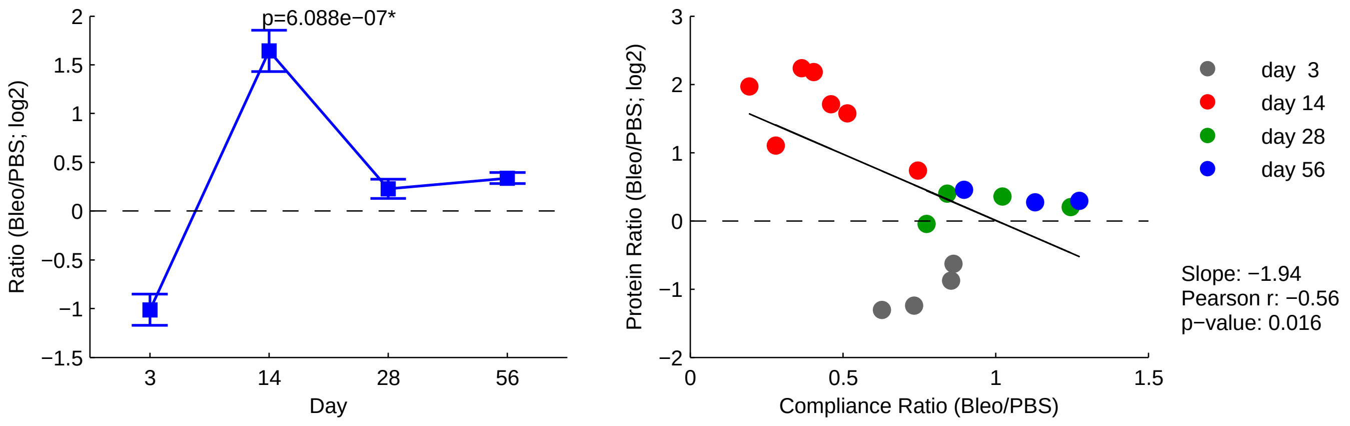

P61294 − Rab6b (id: 3180)

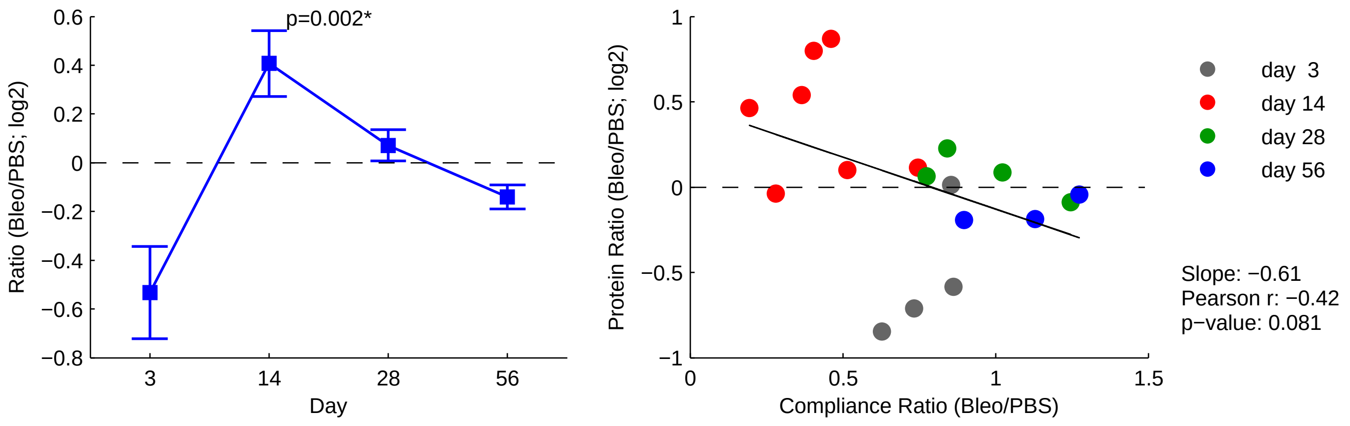

P61358 − Rpl27 (id: 3182)

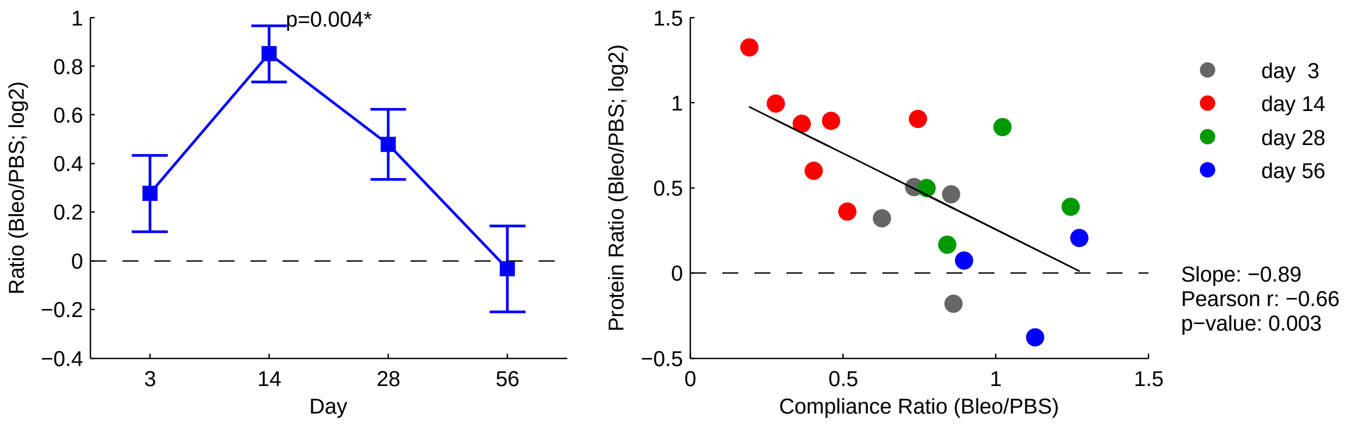

P61514 − Rpl37a (id: 3185)

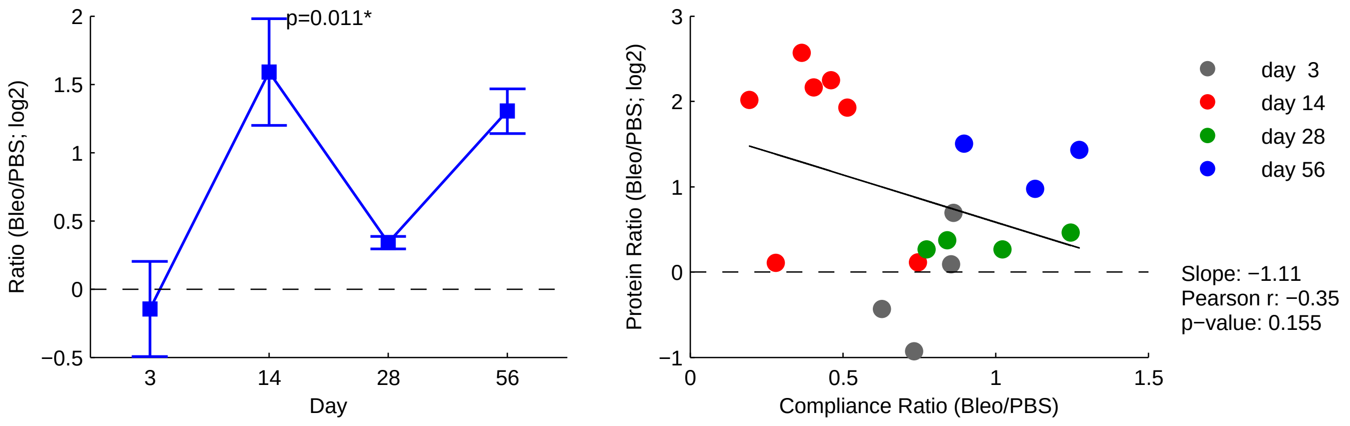

P61600 − Naa20 (id: 3186)

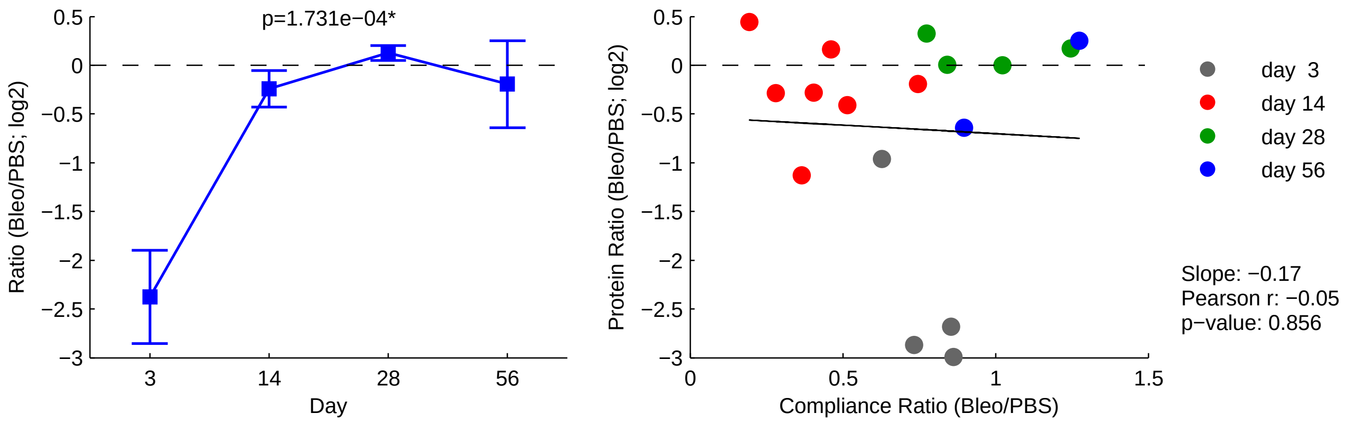

P61750 − Arf4 (id: 3188)

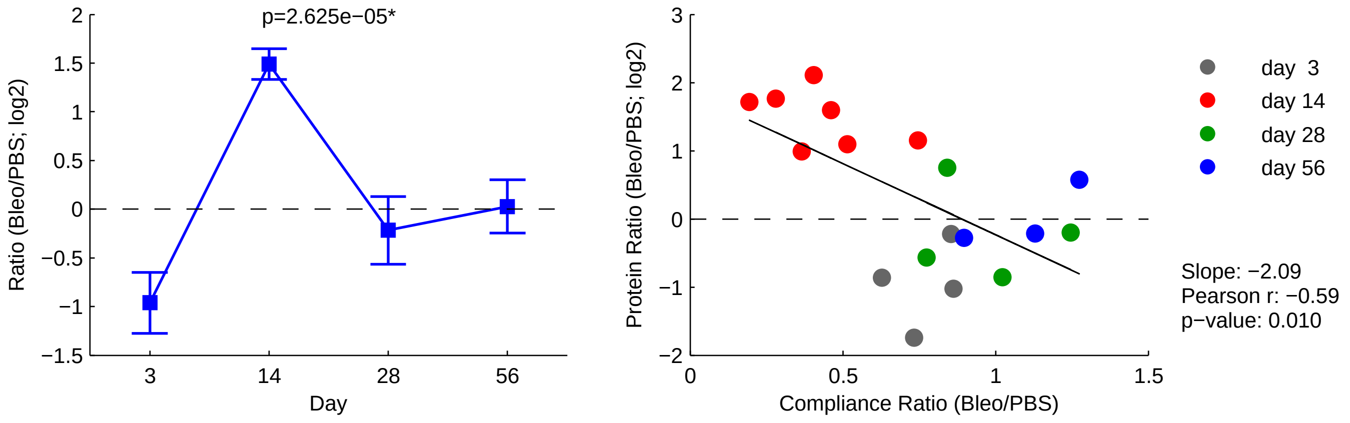

P61759 − Vbp1 (id: 3189)

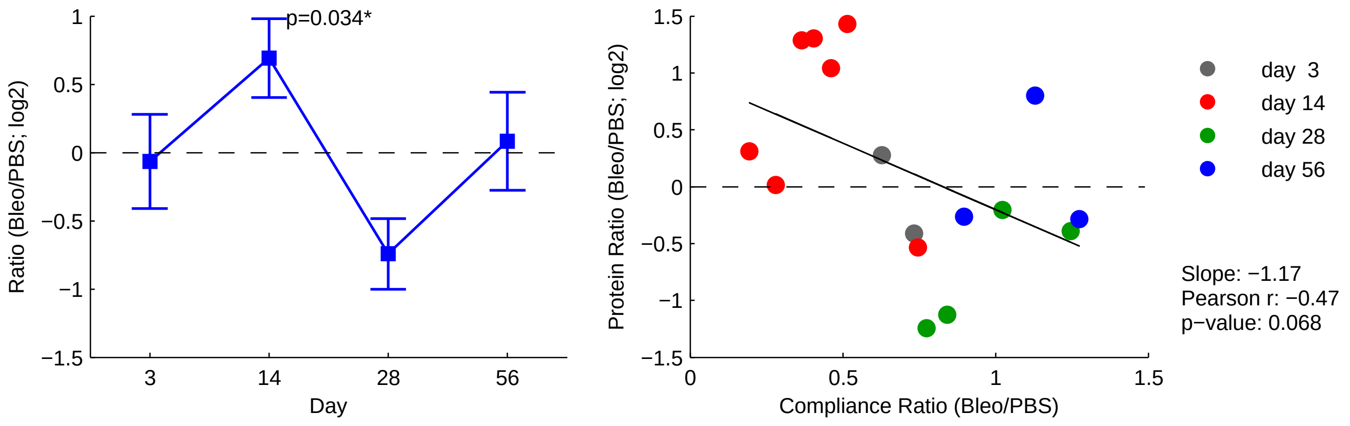

P61922 − Abat (id: 3192)

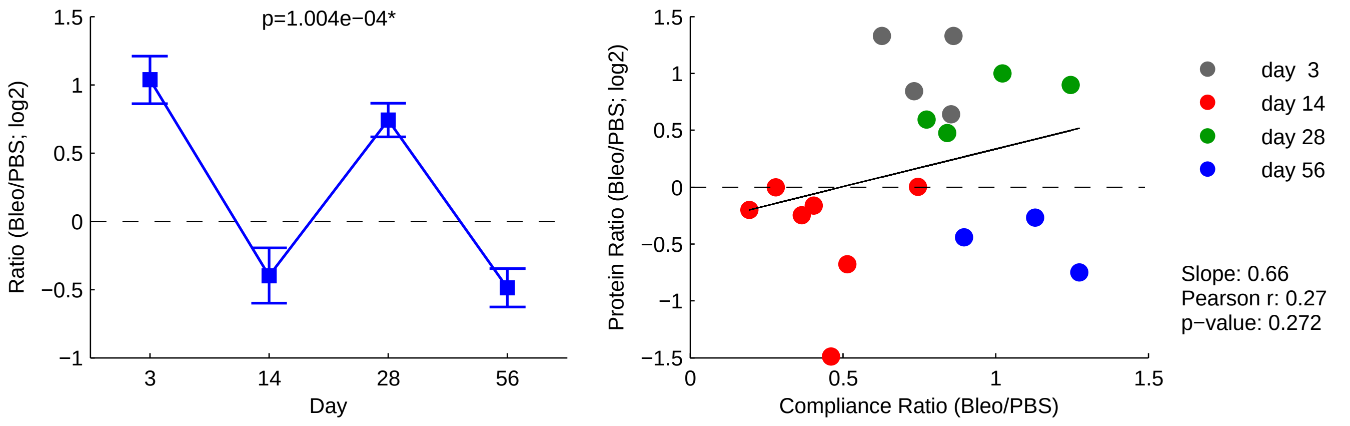

P61924 − Copz1 (id: 3193)

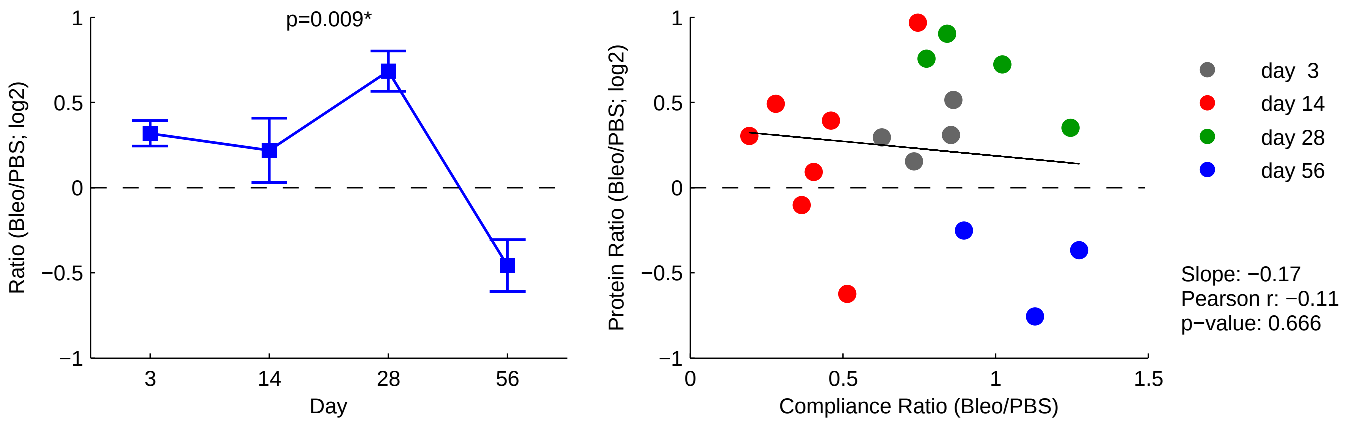

P61953 − Gng11 (id: 3194)

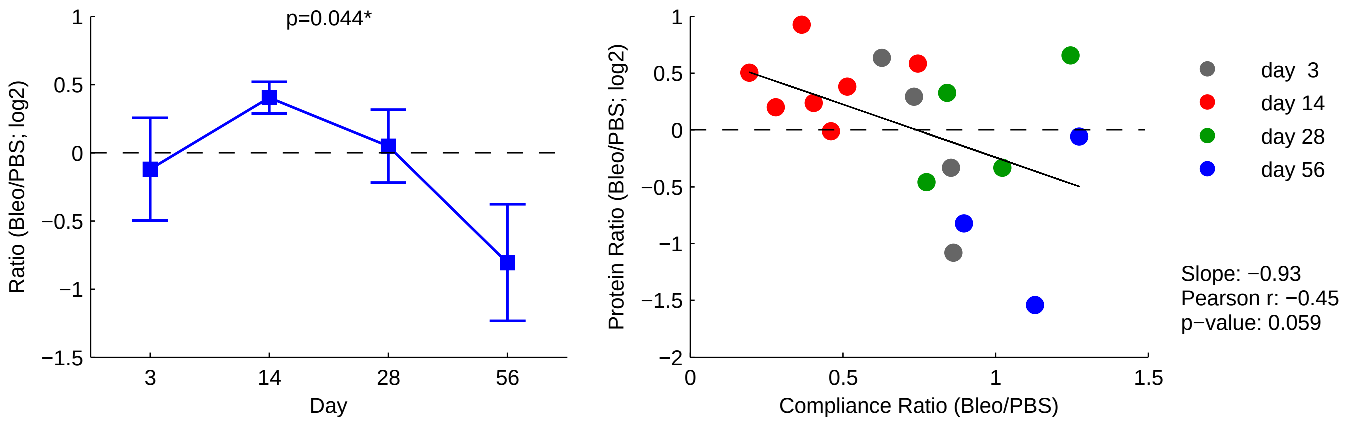

P61979−2 − Hnrnpk (id: 3199)

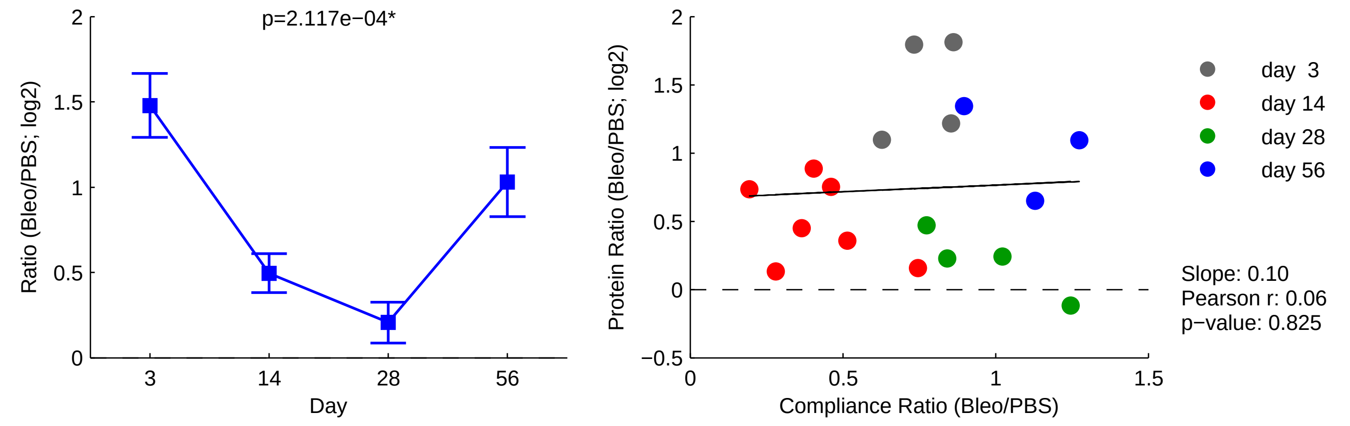

P61982 − Ywhag (id: 3200)

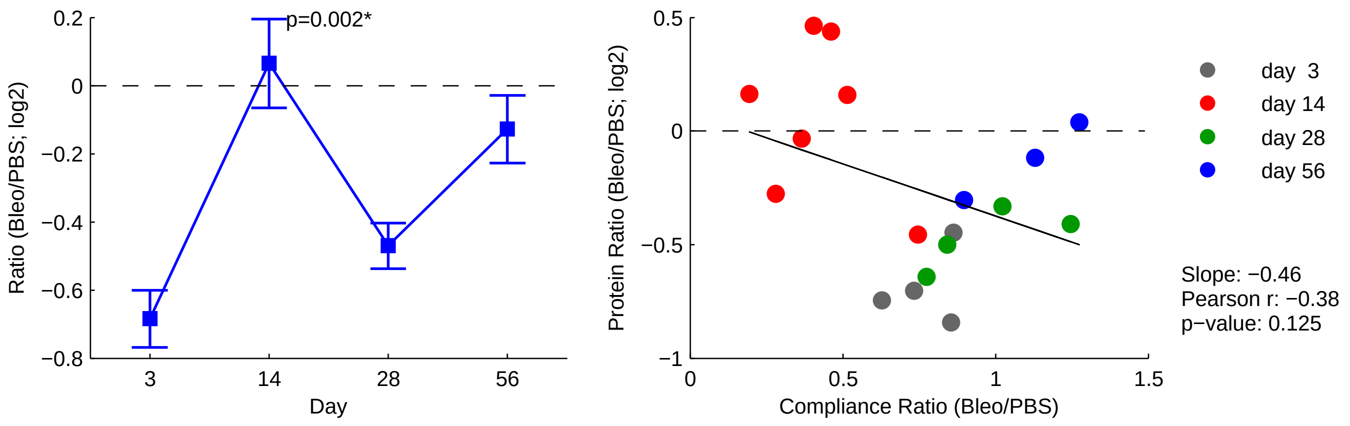

P62046 − Lrch1 (id: 3201)

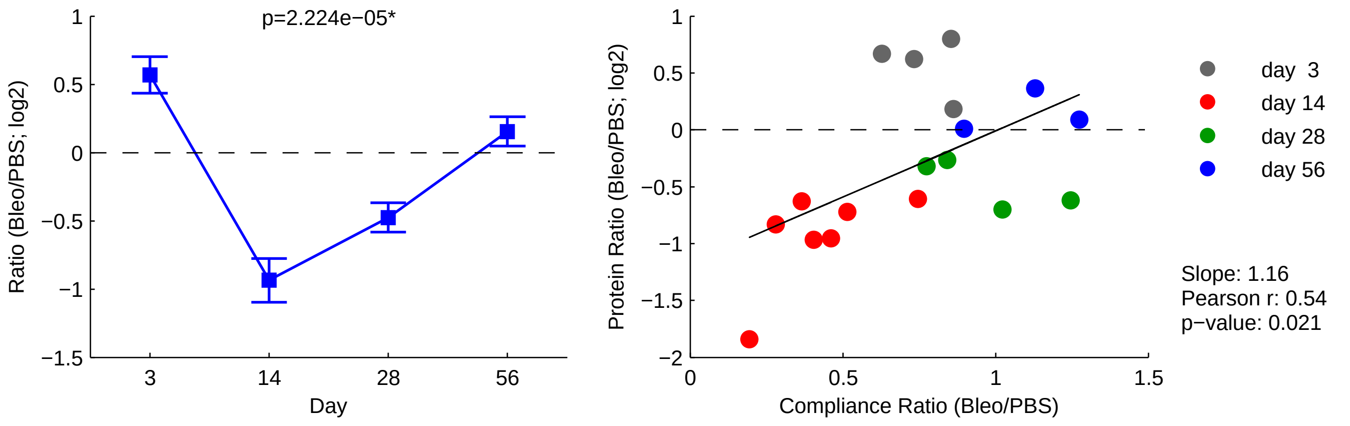

P62071 − Rras2 (id: 3202)

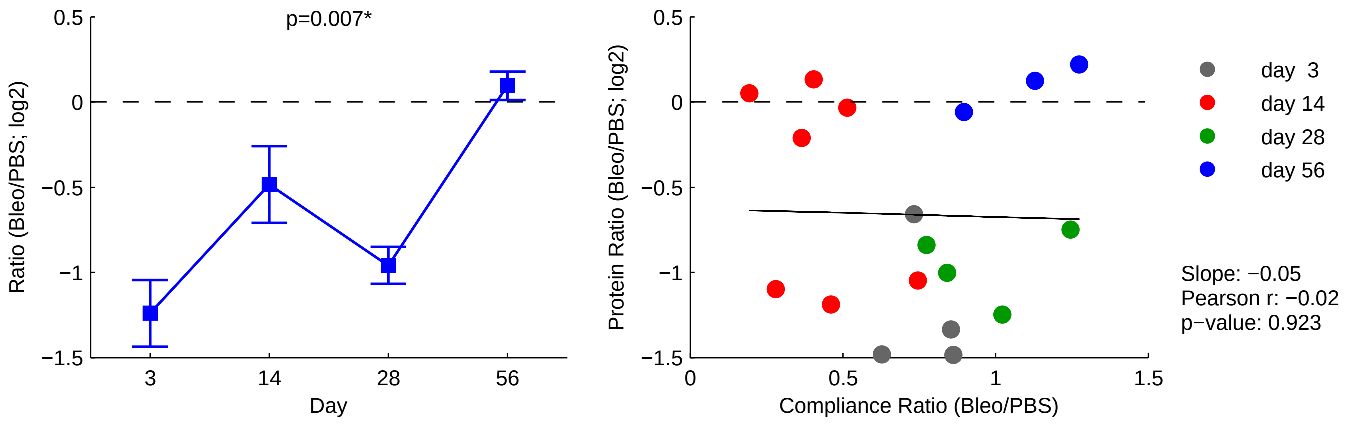

P62073 − Timm10 (id: 3203)

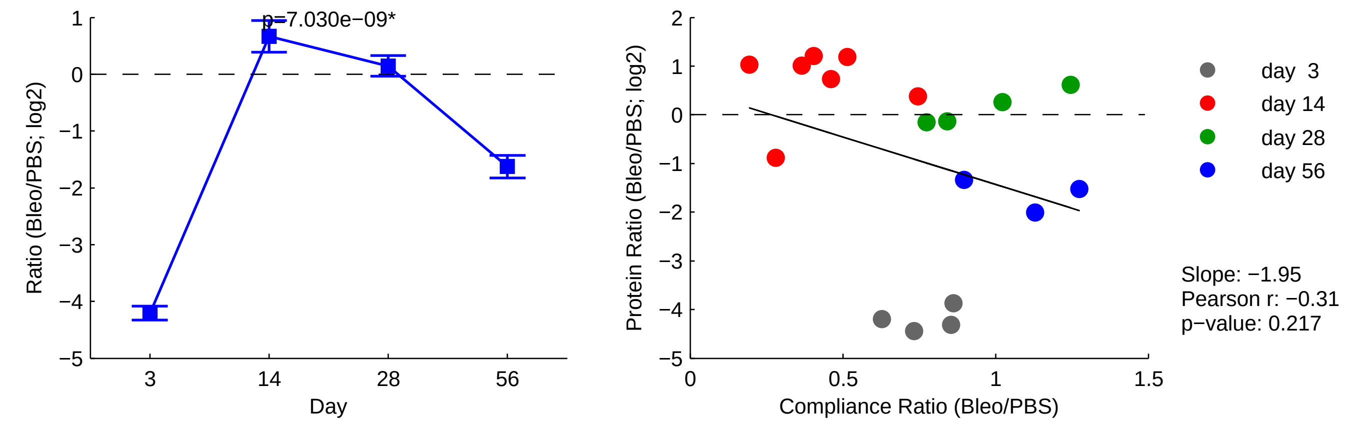

P62077 − Timm8b (id: 3205)

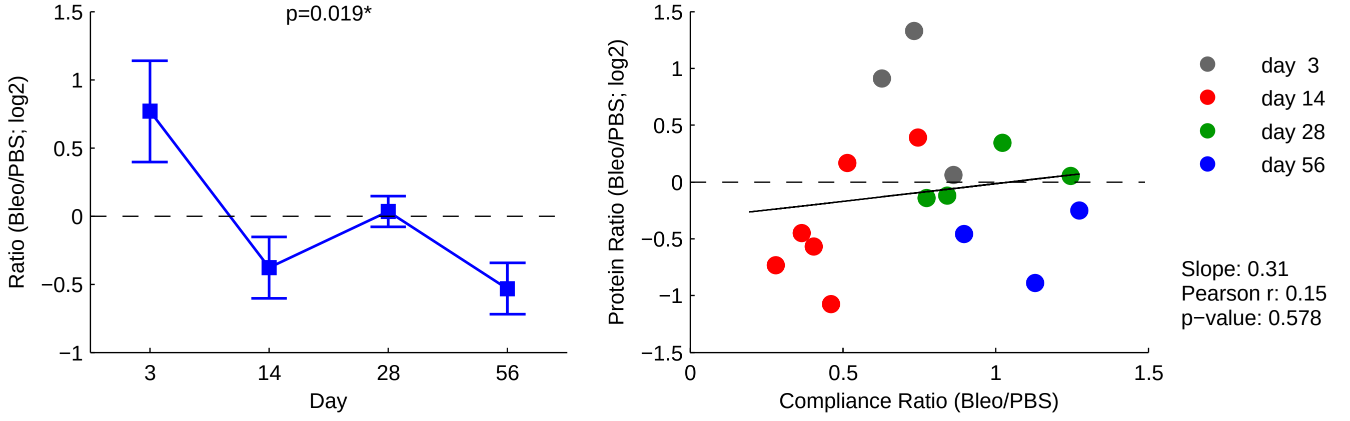

P62082 − Rps7 (id: 3206)

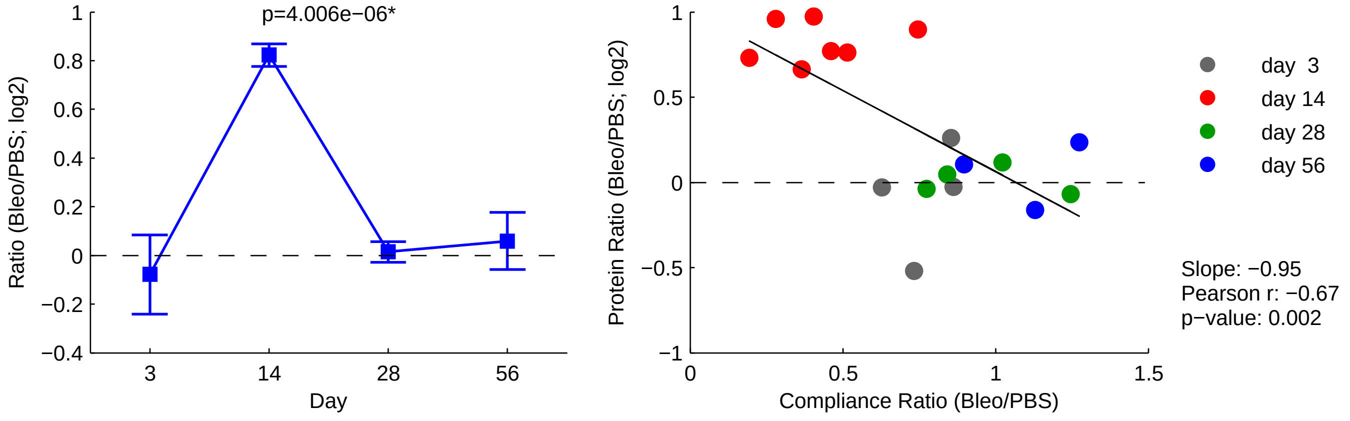

P62141 − Ppp1cb (id: 3208)

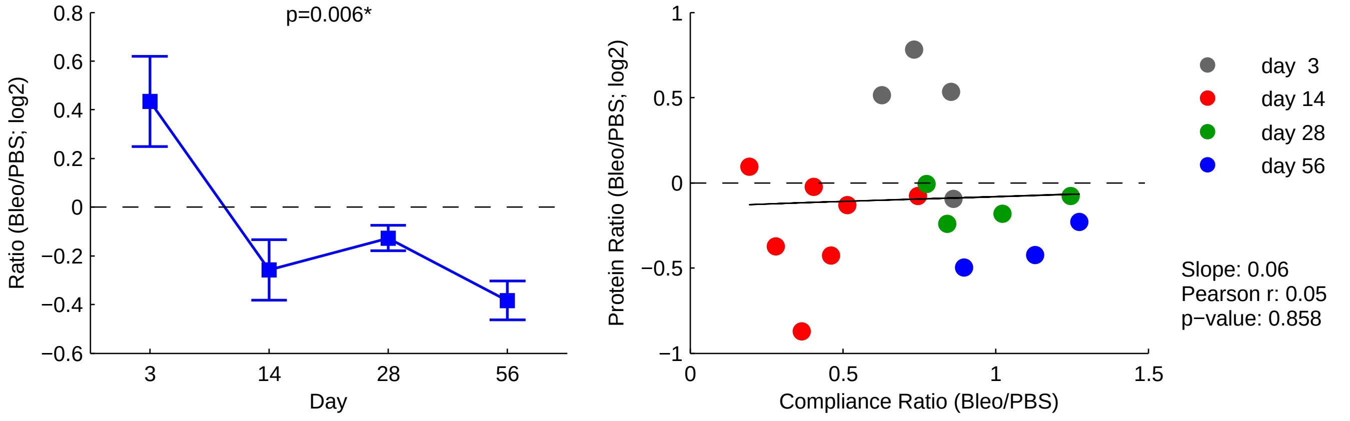

P62192 − Psmc1 (id: 3209)

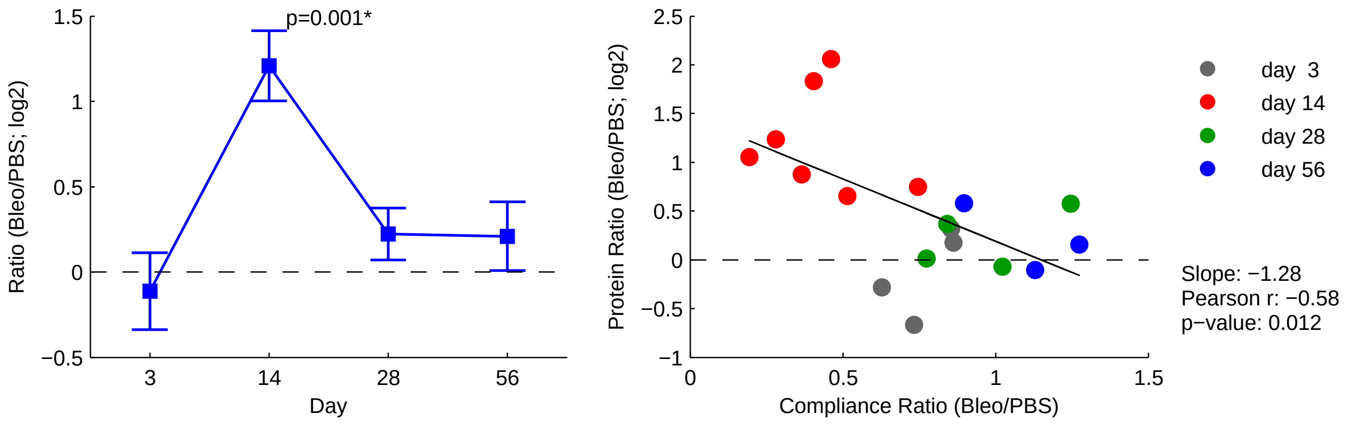

P62196 − Psmc5 (id: 3210)

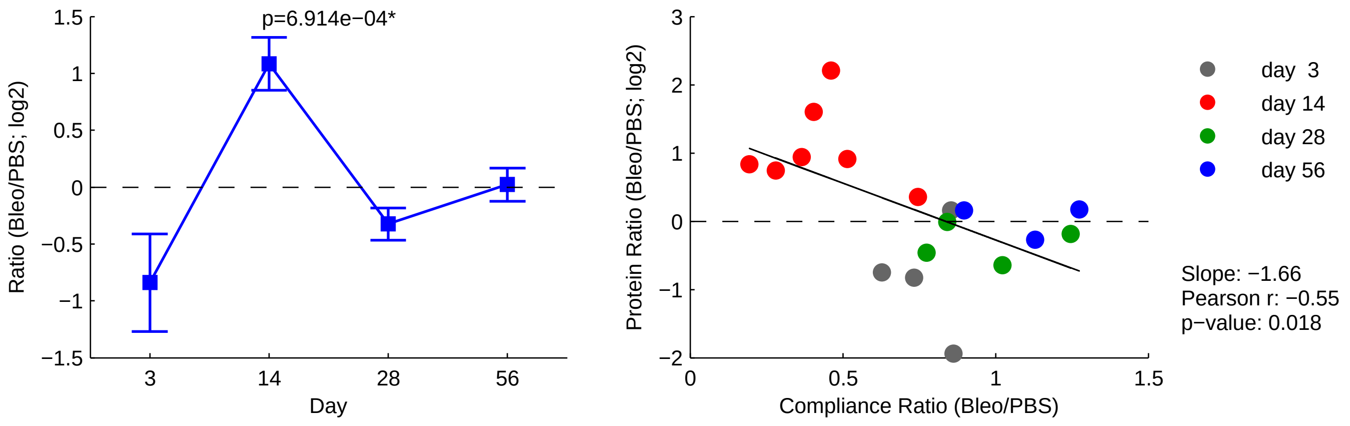

P62245 − Rps15a (id: 3213)

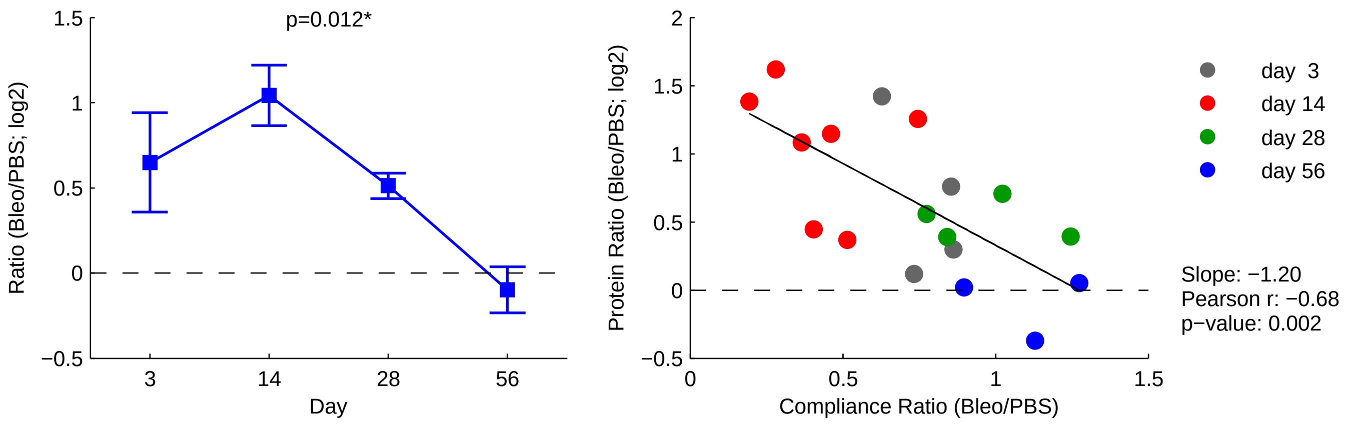

P62259 − Ywhae (id: 3215)

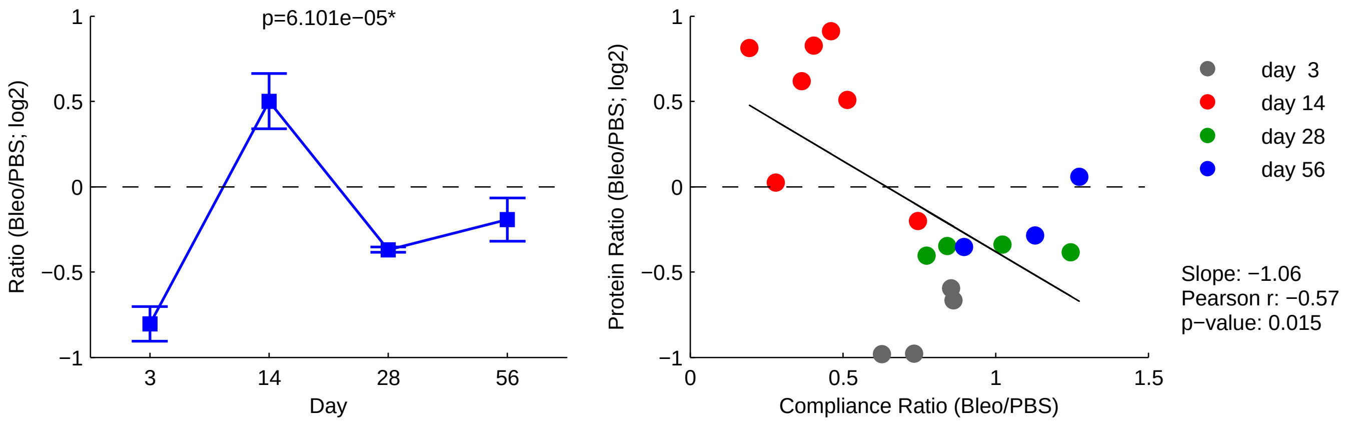

P62264 − Rps14 (id: 3216)

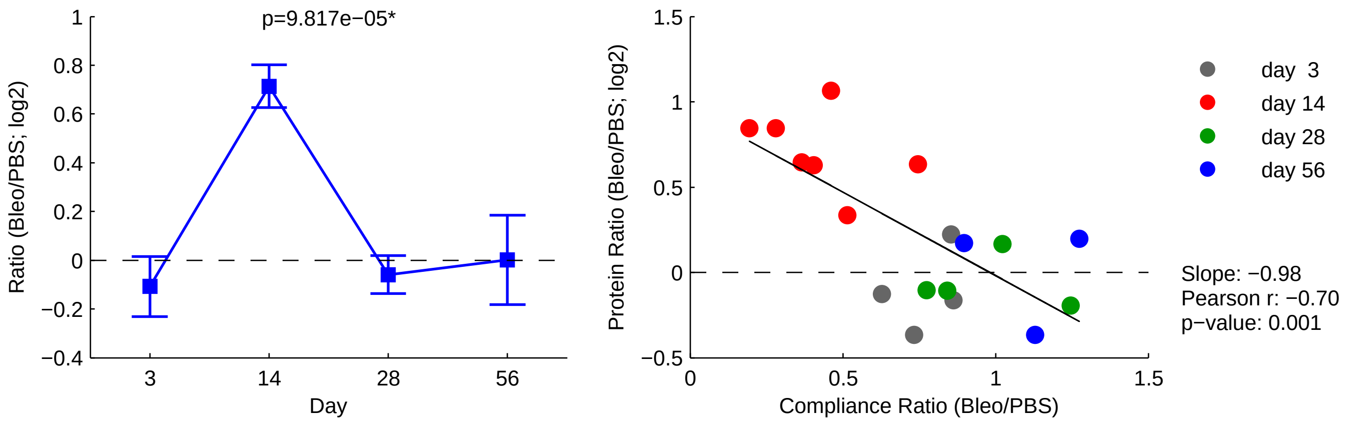

P62267 − Rps23 (id: 3217)

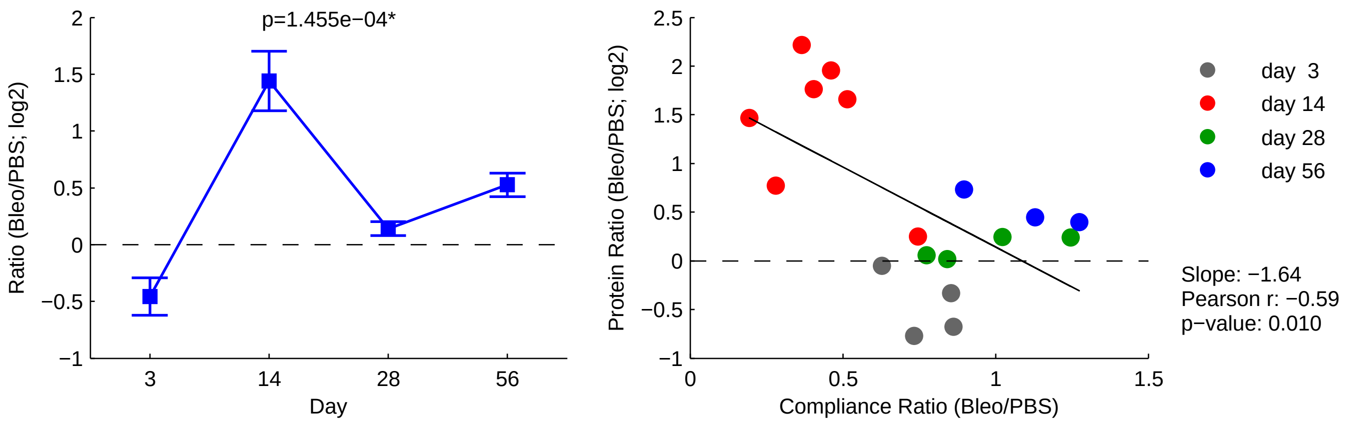

P62274 − Rps29 (id: 3218)

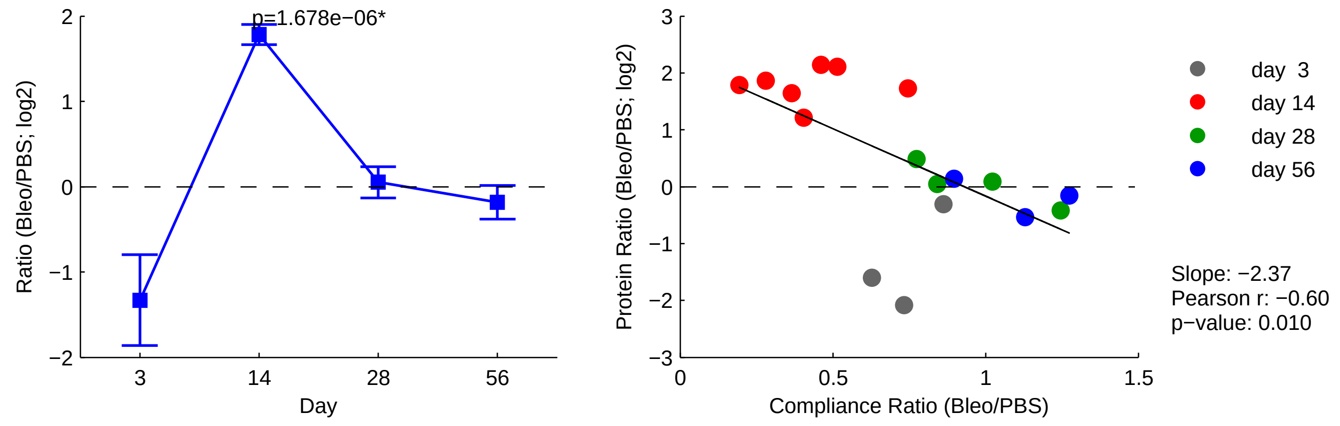

P62281 − Rps11 (id: 3219)

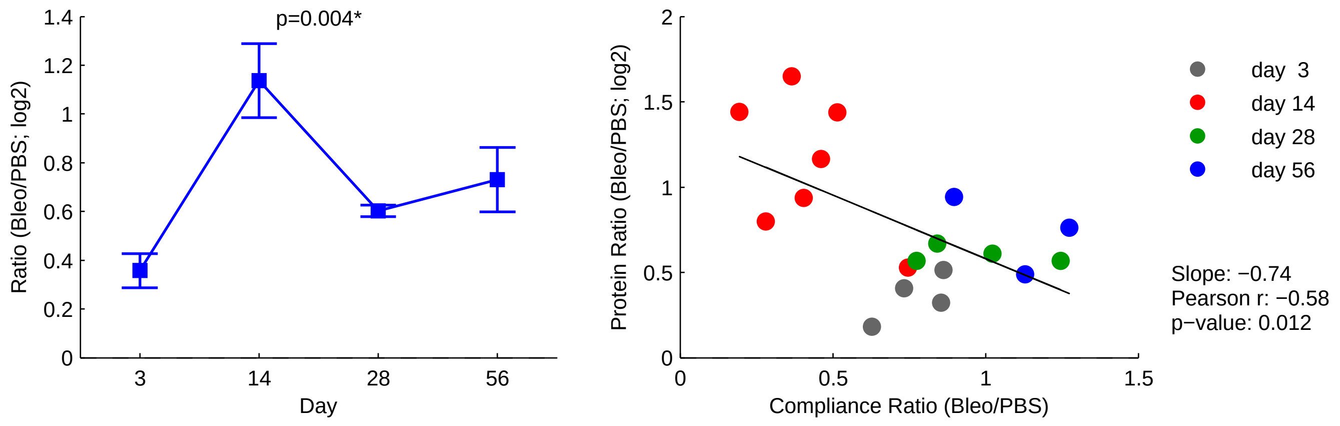

P62305 − Snrpe (id: 3221)

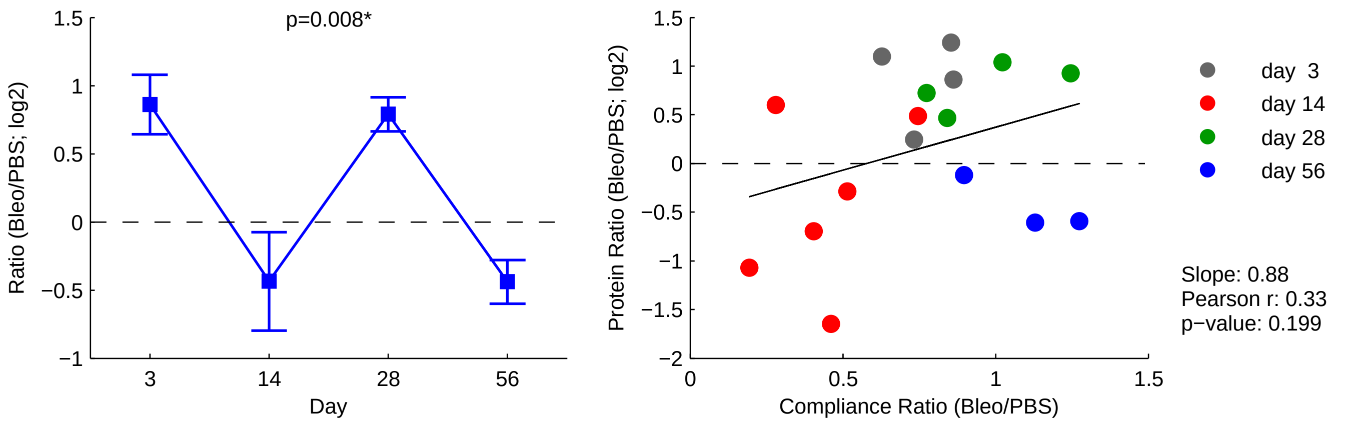

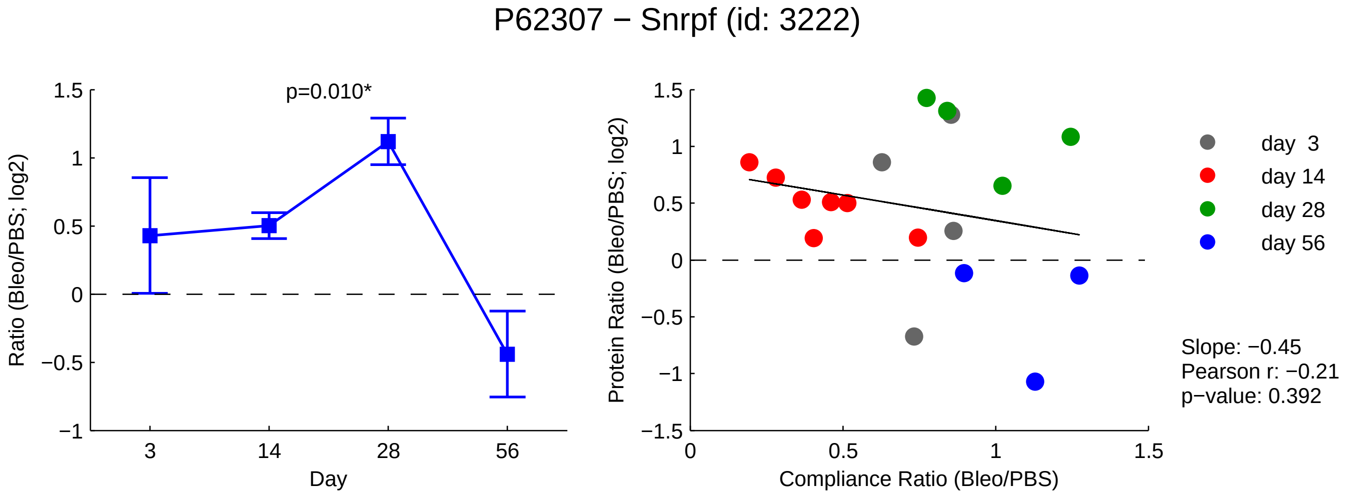

P62307 − Snrpf (id: 3222)
p=0.010*
Ratio (Bleo/PBS; log2)
Day
Protein Ratio (Bleo/PBS; log2)
Compliance Ratio (Bleo/PBS)
day 3
day 14
day 28
day 56
Slope: −0.45
Pearson r: −0.21
p−value: 0.392

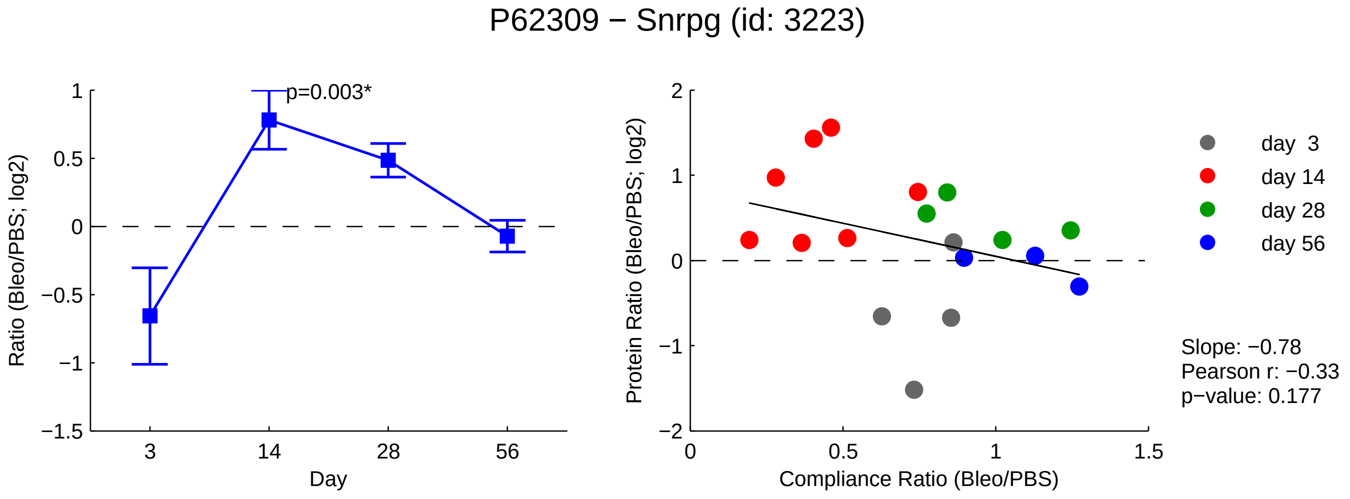

P62309 − Snrpg (id: 3223)
p=0.003*
Ratio (Bleo/PBS; log2)
Day
Protein Ratio (Bleo/PBS; log2)
Compliance Ratio (Bleo/PBS)
day 3
day 14
day 28
day 56
Slope: −0.78
Pearson r: −0.33
p−value: 0.177

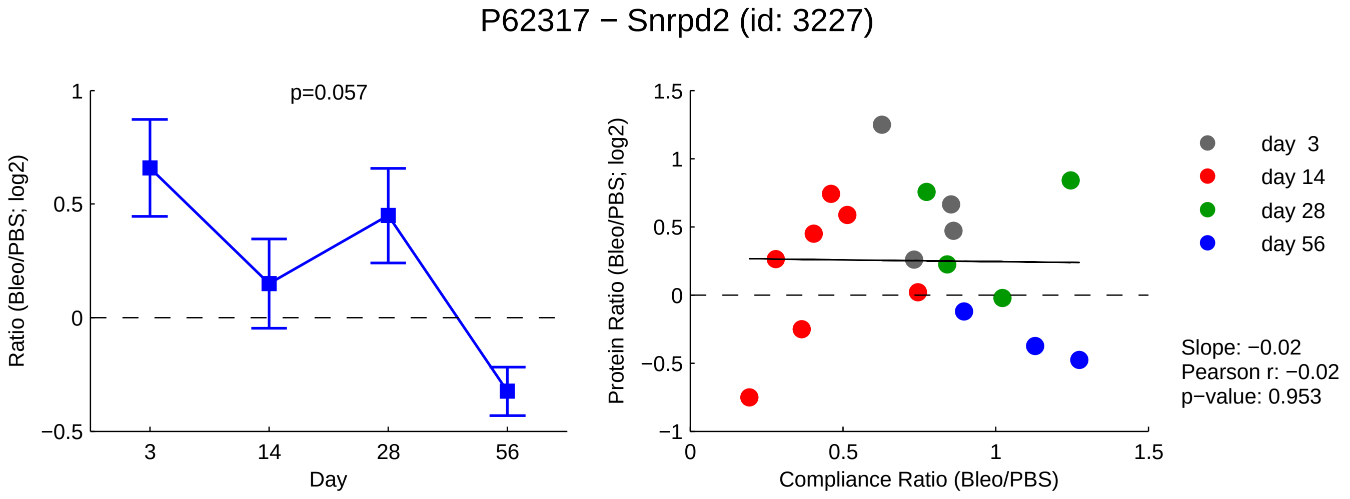

P62317 − Snrpd2 (id: 3227)
p=0.057
Ratio (Bleo/PBS; log2)
Day
Protein Ratio (Bleo/PBS; log2)
Compliance Ratio (Bleo/PBS)
day 3
day 14
day 28
day 56
Slope: −0.02
Pearson r: −0.02
p−value: 0.953

P62320 − Snrpd3 (id: 3228)

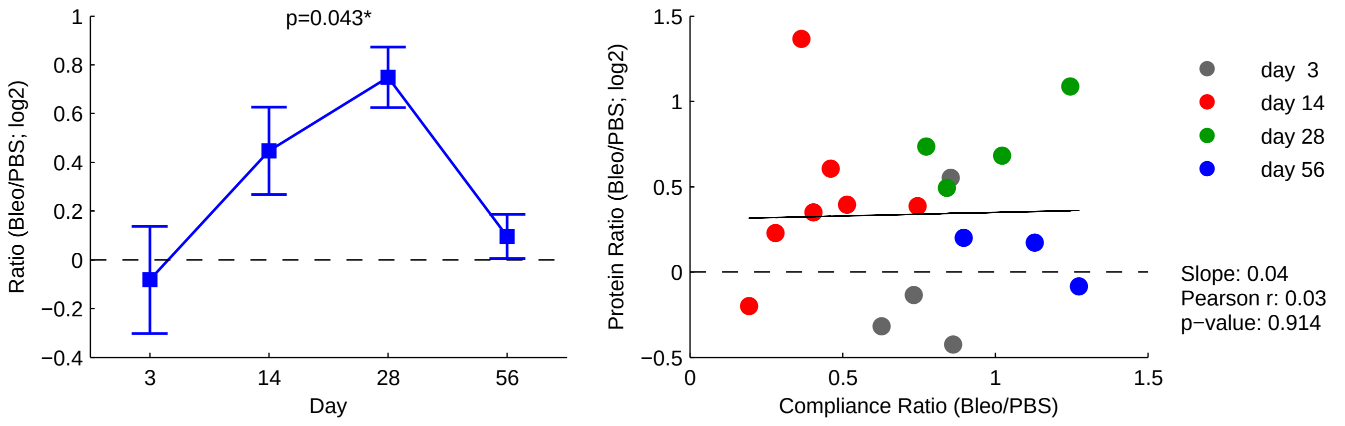

P62322 − Lsm5 (id: 3229)

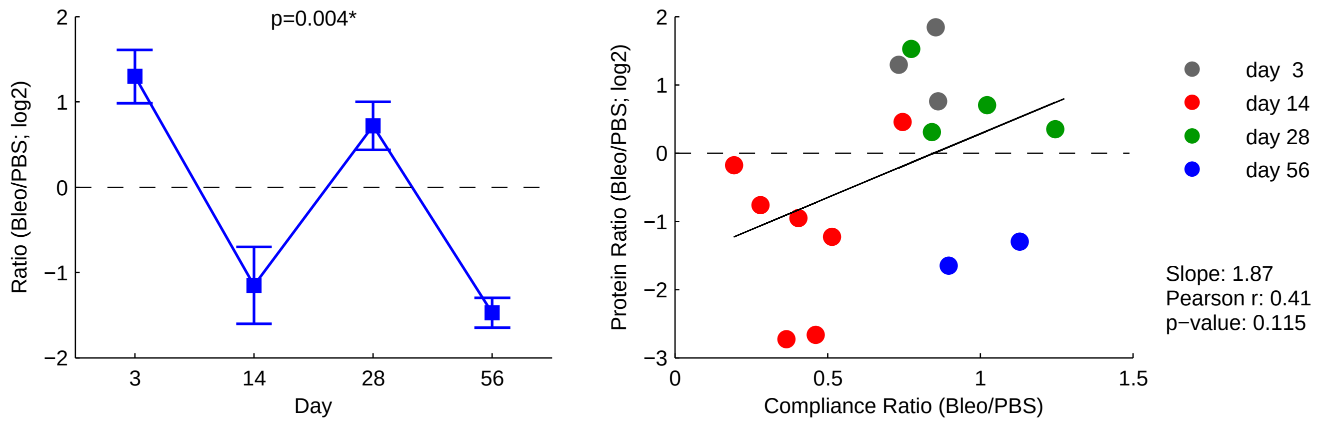

P62331 − Arf6 (id: 3230)

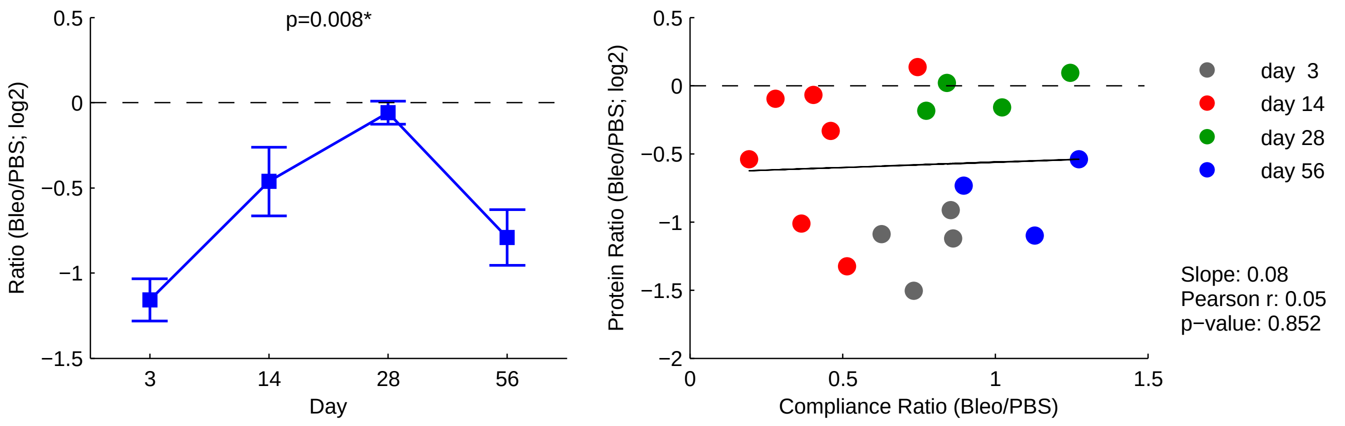

P62334 − Psmc6 (id: 3231)

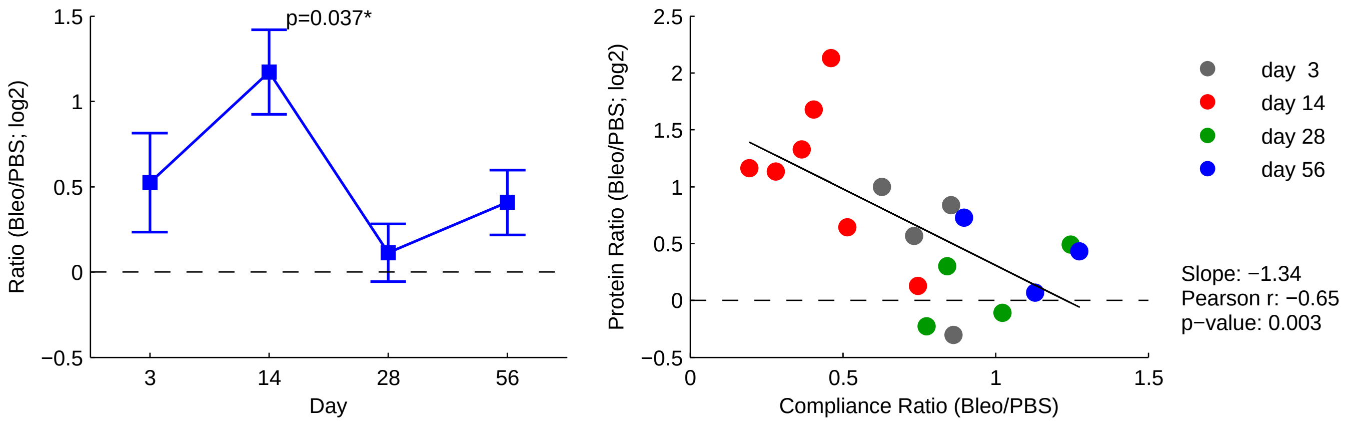

P62342 − Selt (id: 3233)

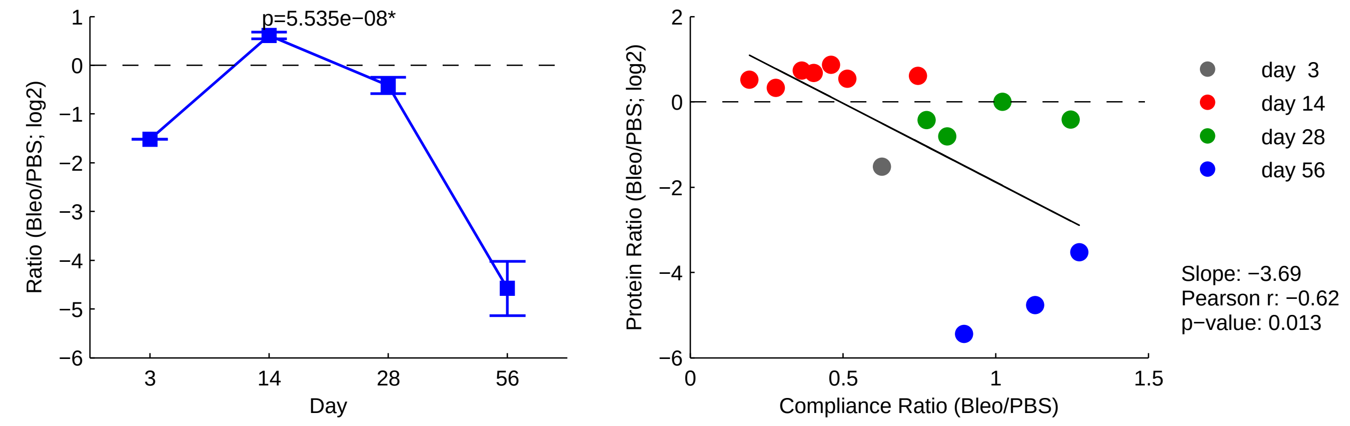

P62488 − Polr2g (id: 3236)

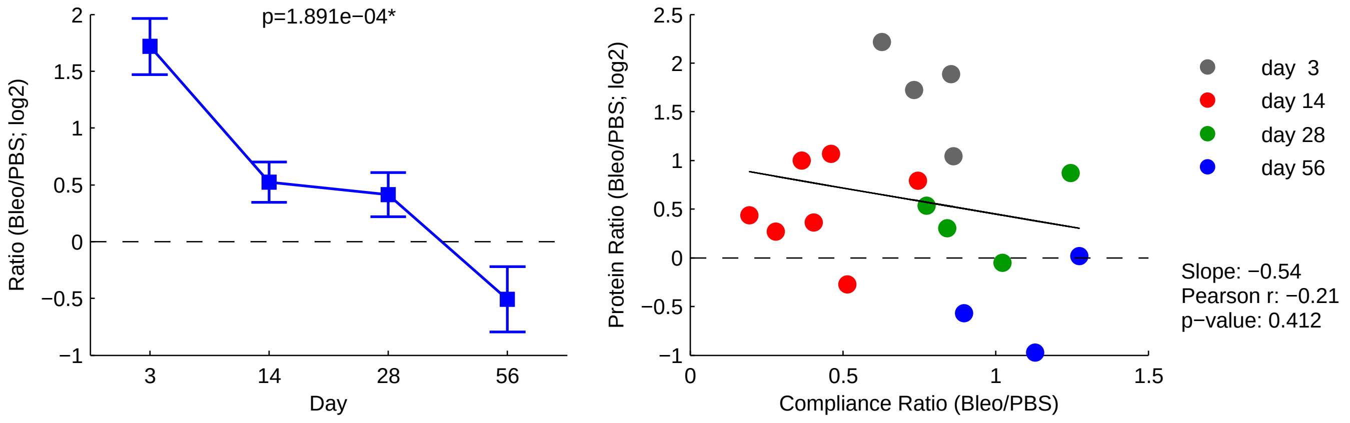

P62627 − Dynlrb1 (id: 3238)

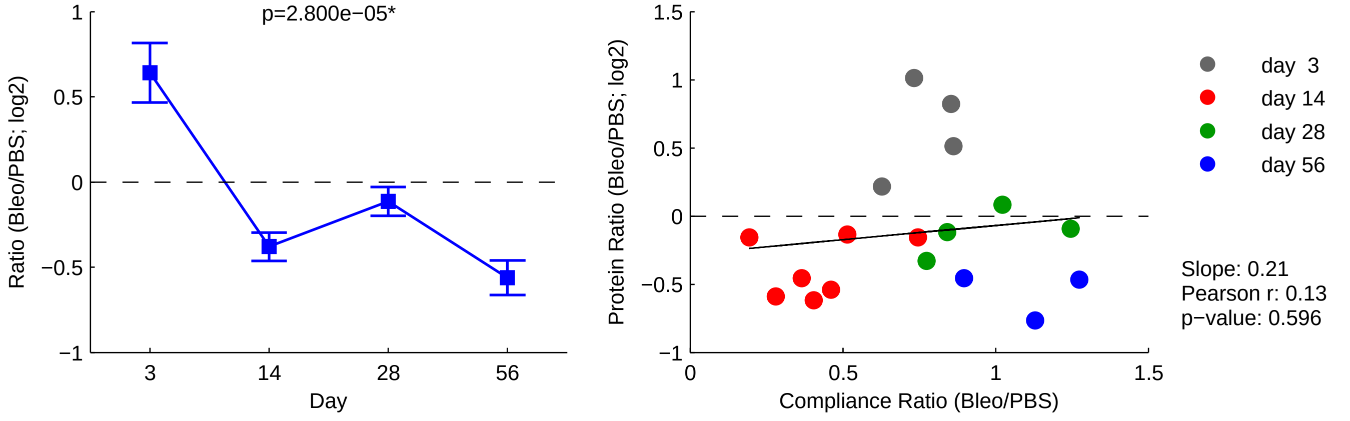

P62631 − Eef1a2 (id: 3239)

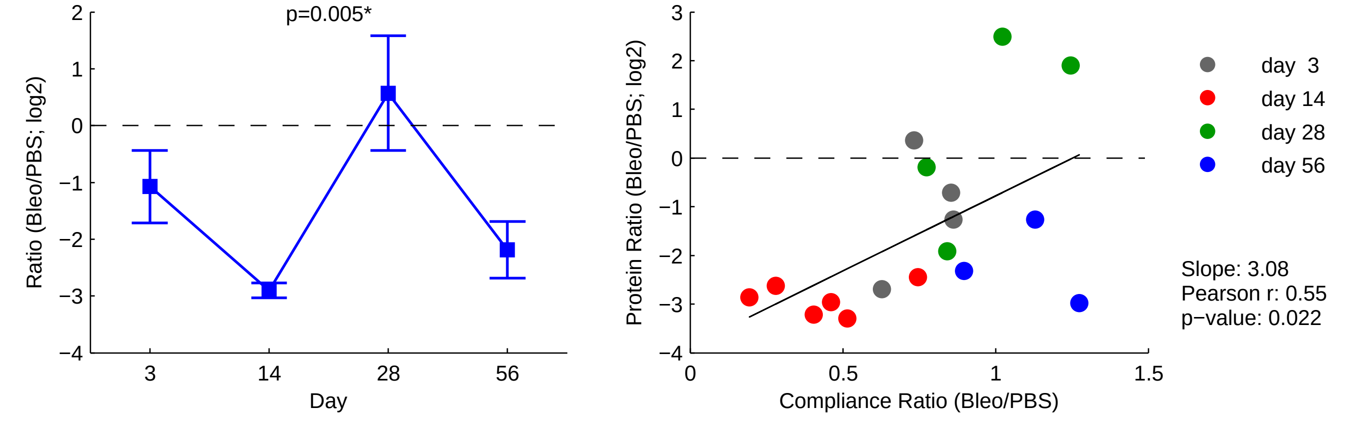

P62702 − Rps4x (id: 3240)

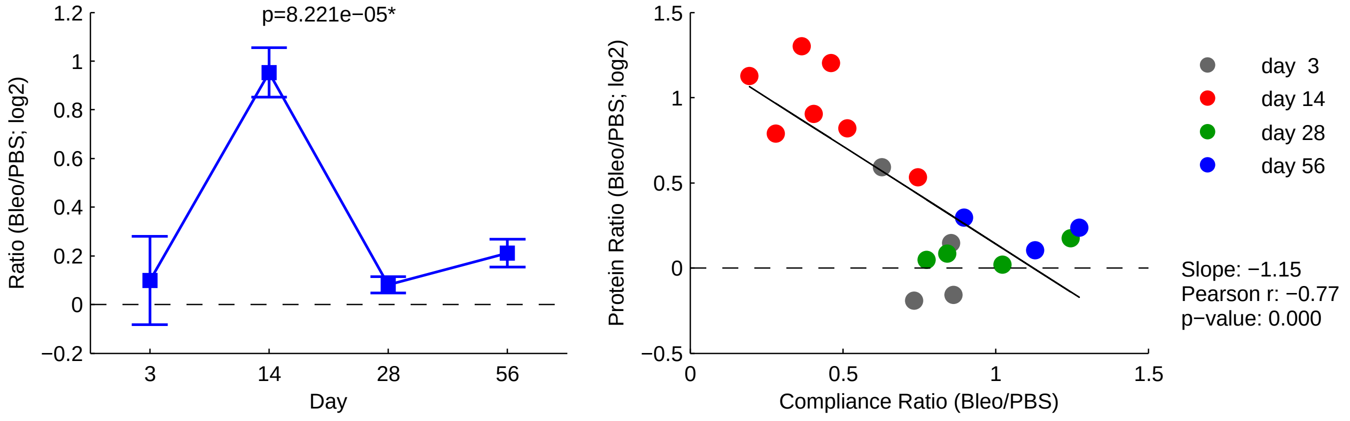

P62717 − Rpl18a (id: 3242)

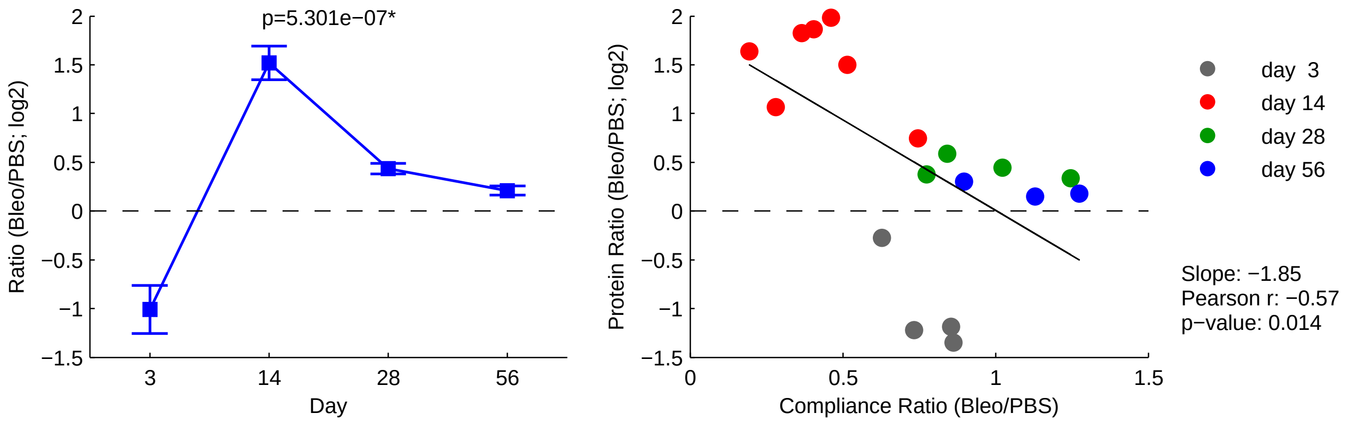

P62737 − Acta2 (id: 3243)

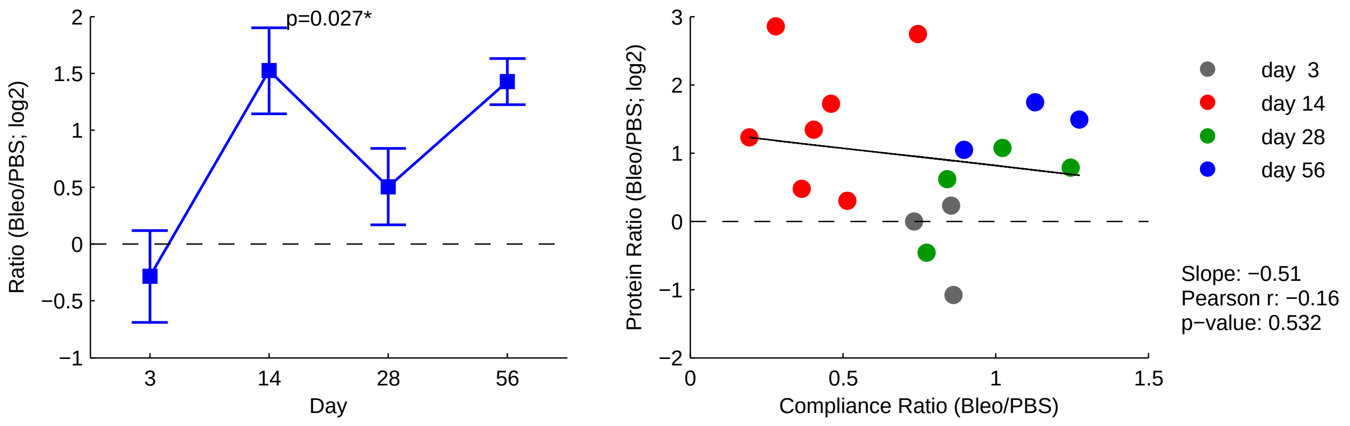

P62743 − Ap2s1 (id: 3244)

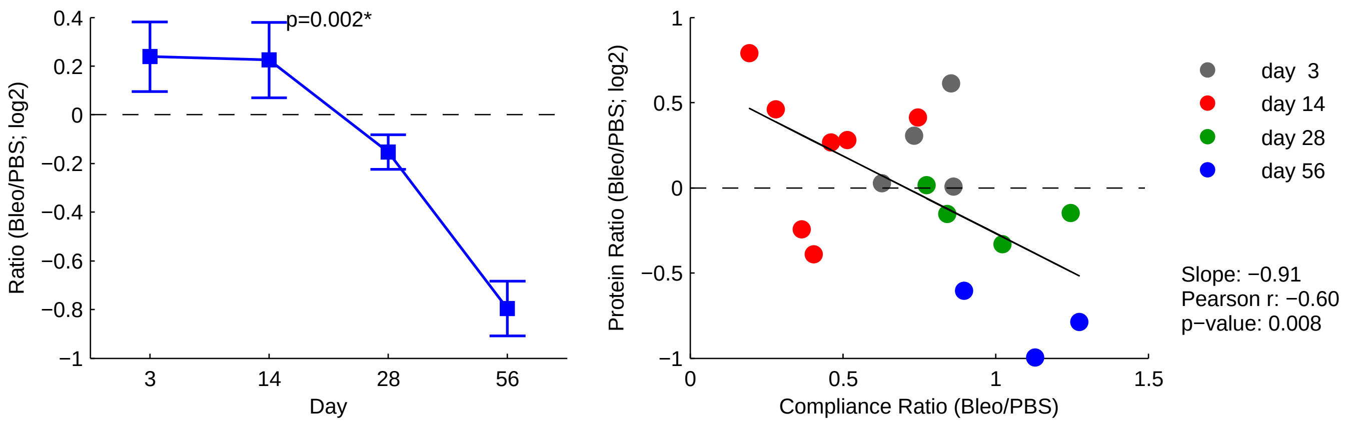

P62746 − Rhob (id: 3245)

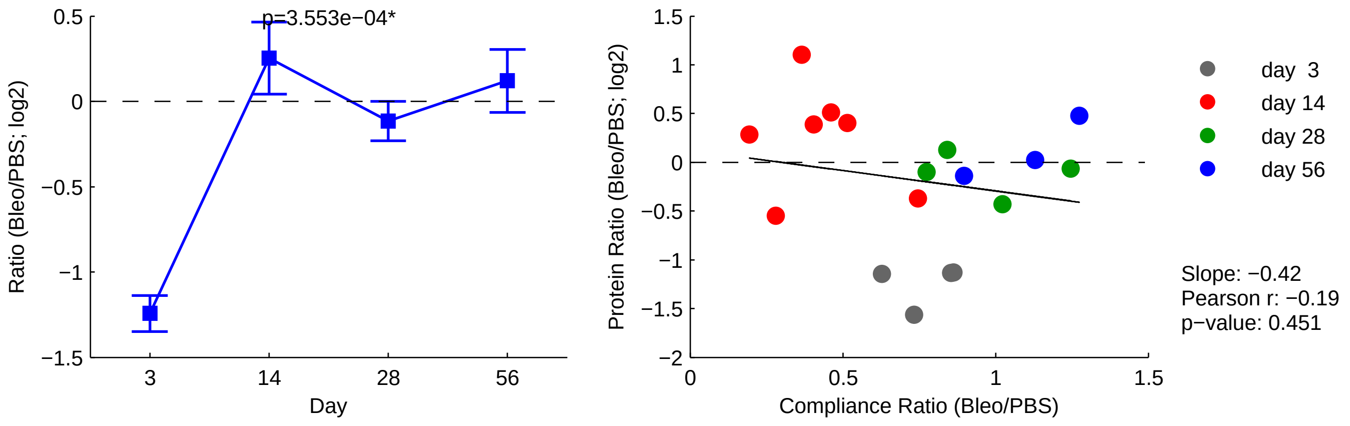

P62748 − Hpcal1 (id: 3246)

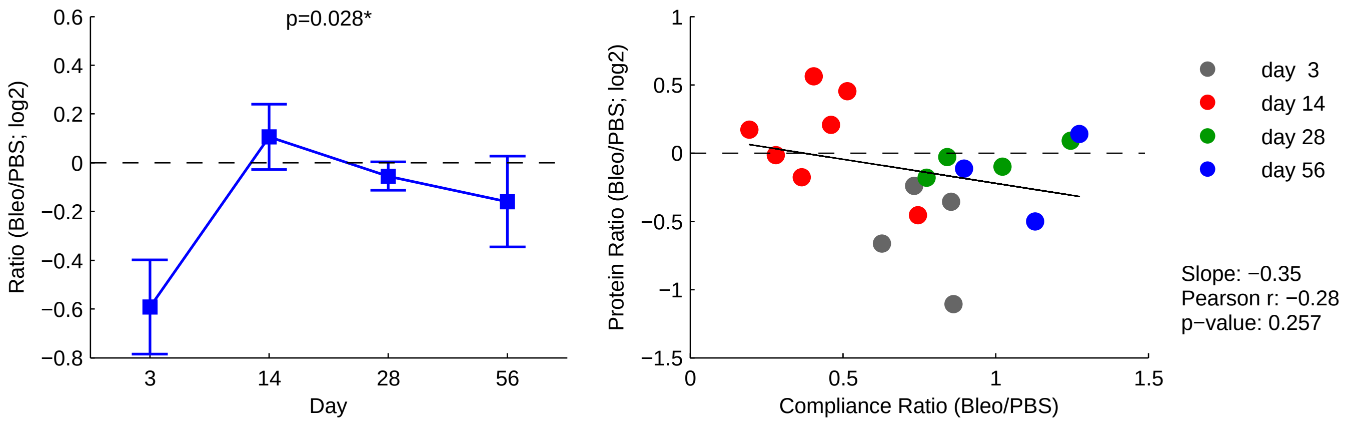

P62754 − Rps6 (id: 3247)

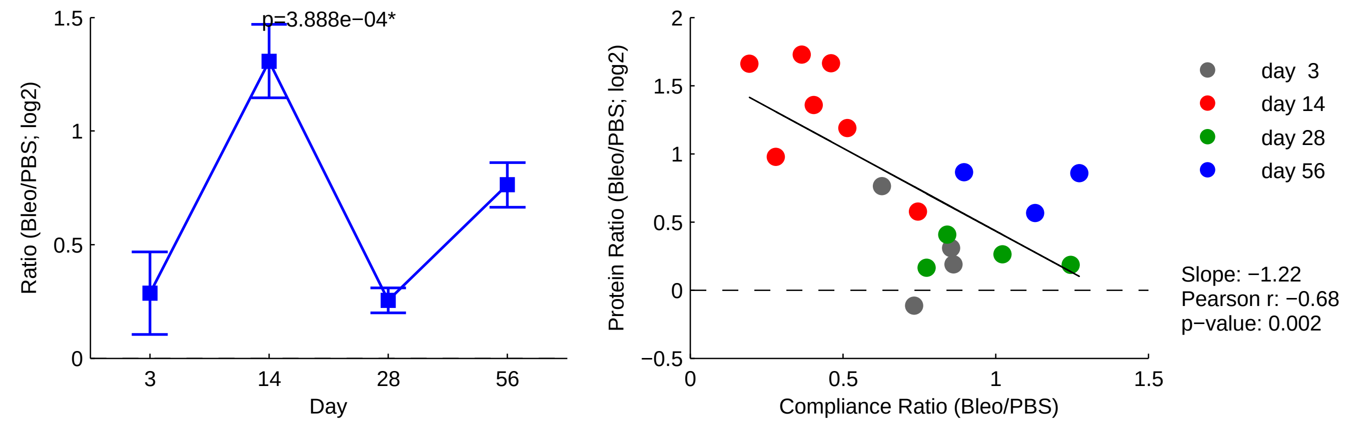

P62774 − Mtpn (id: 3249)

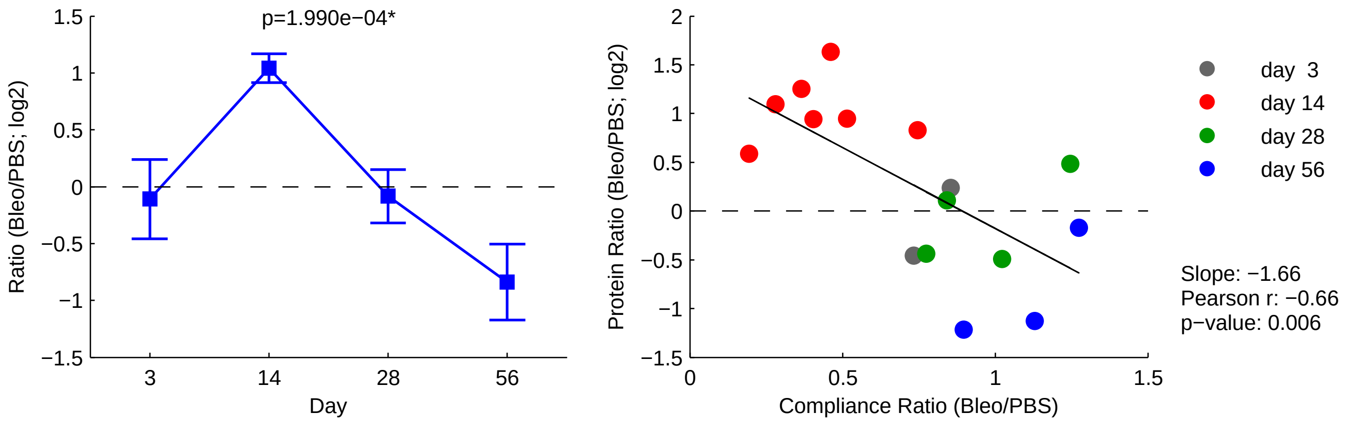

P62806 − Hist1h4a (id: 3250)

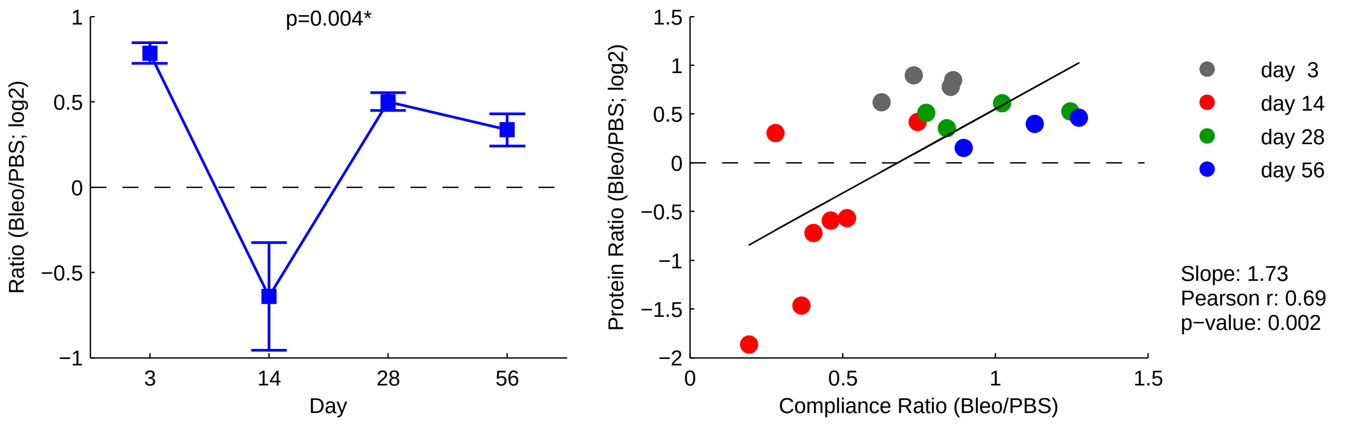

P62814 − Atp6v1b2 (id: 3251)

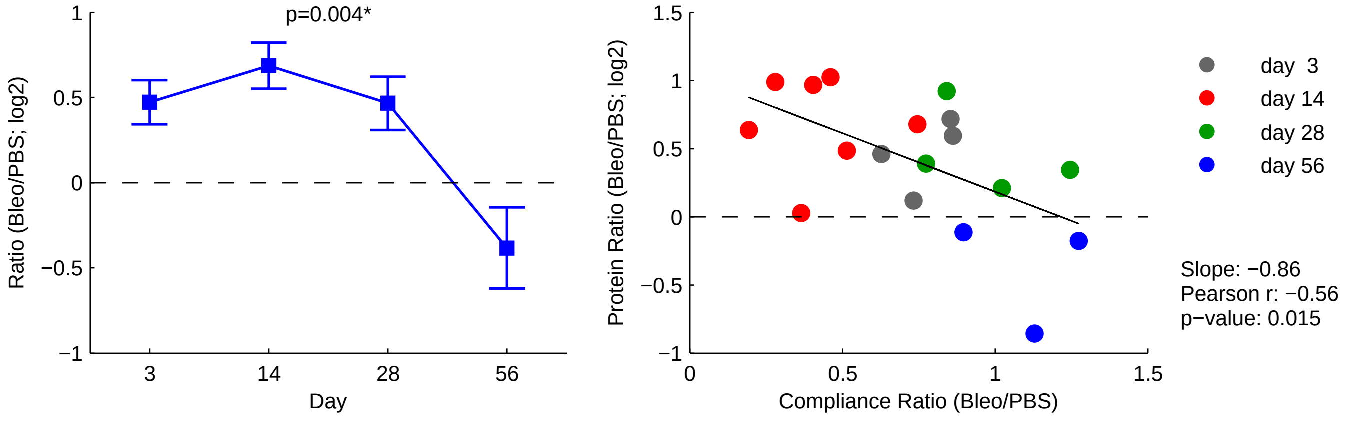

P62830 − Rpl23 (id: 3254)

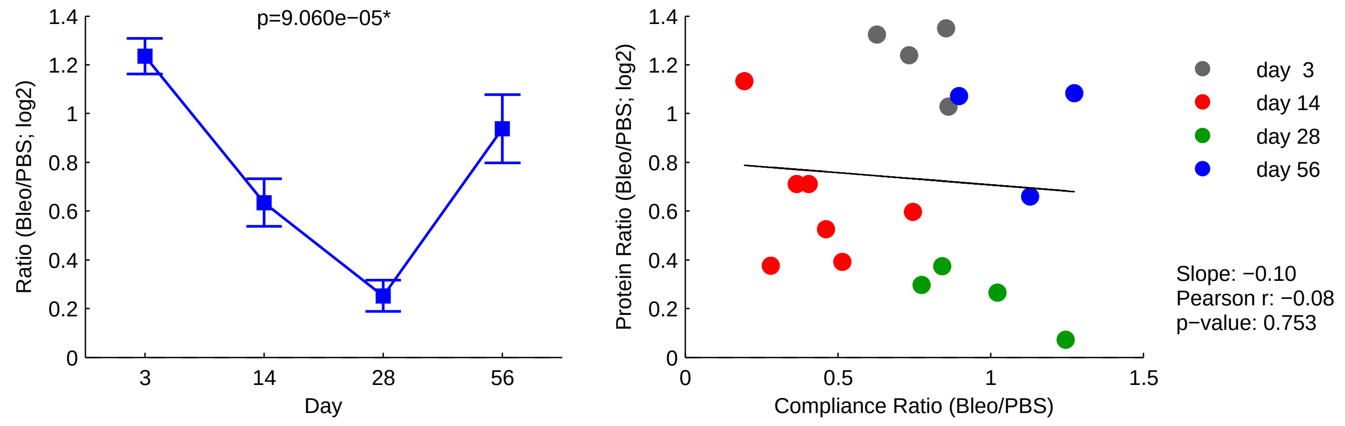

P62835 − Rap1a (id: 3255)

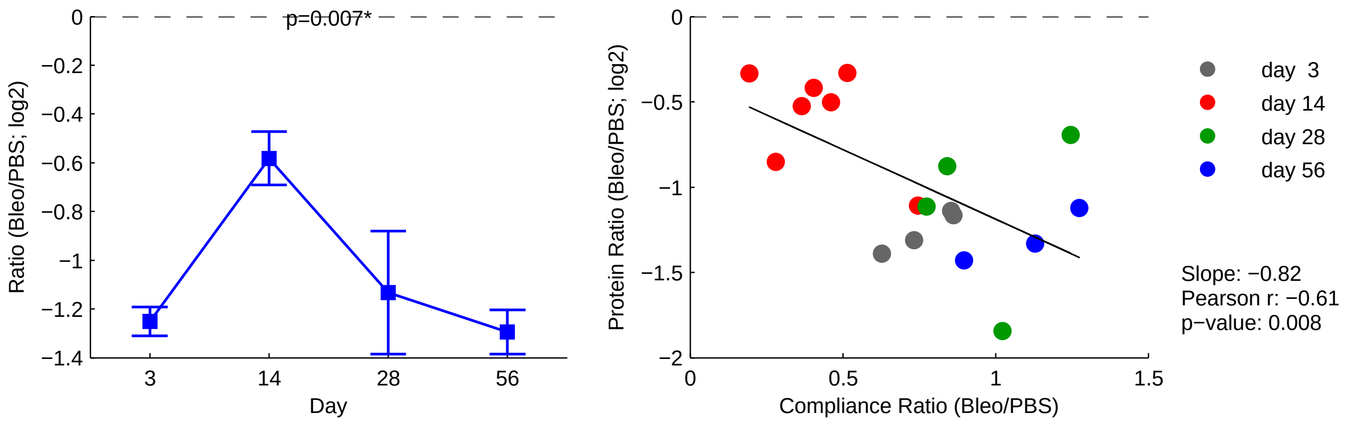

P62843 − Rps15 (id: 3256)

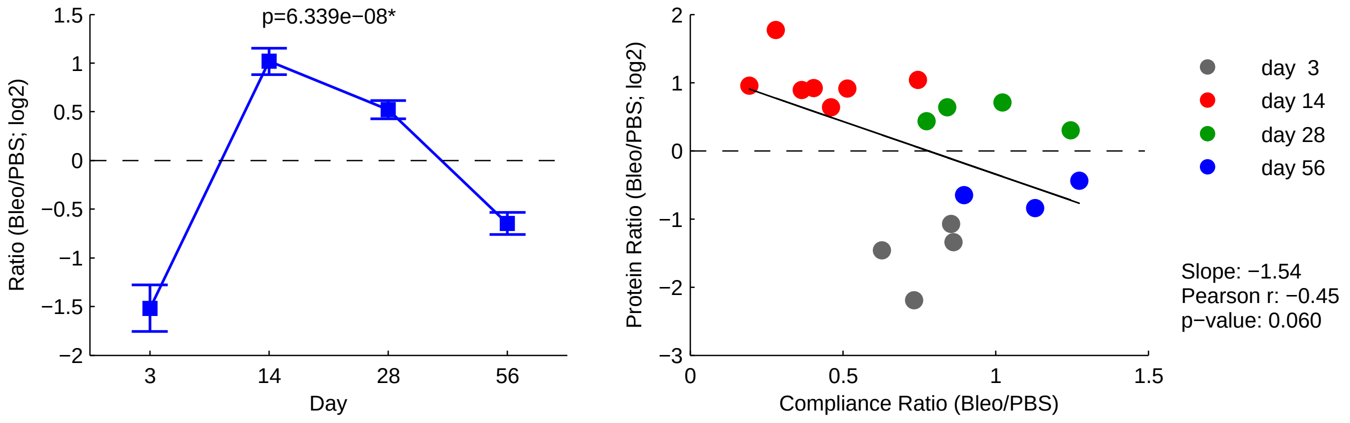

P62849−2 − Rps24 (id: 3257)

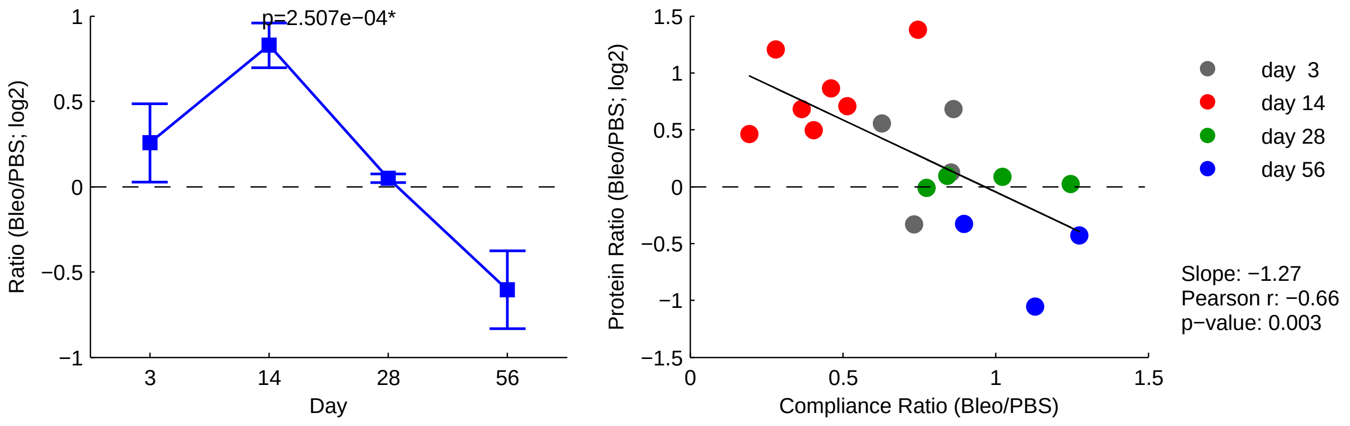

P62852 − Rps25 (id: 3258)

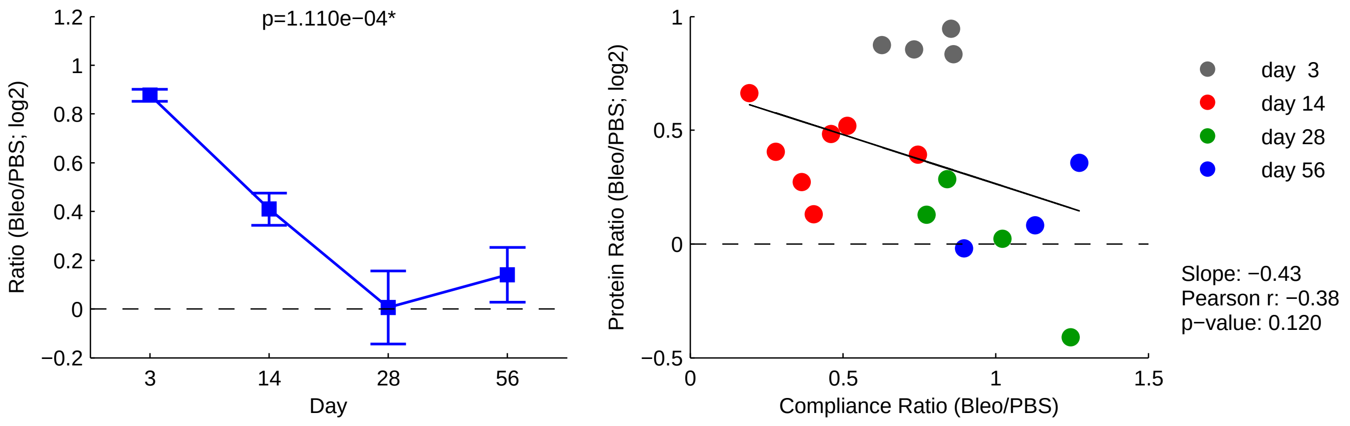

P62855 − Rps26 (id: 3259)

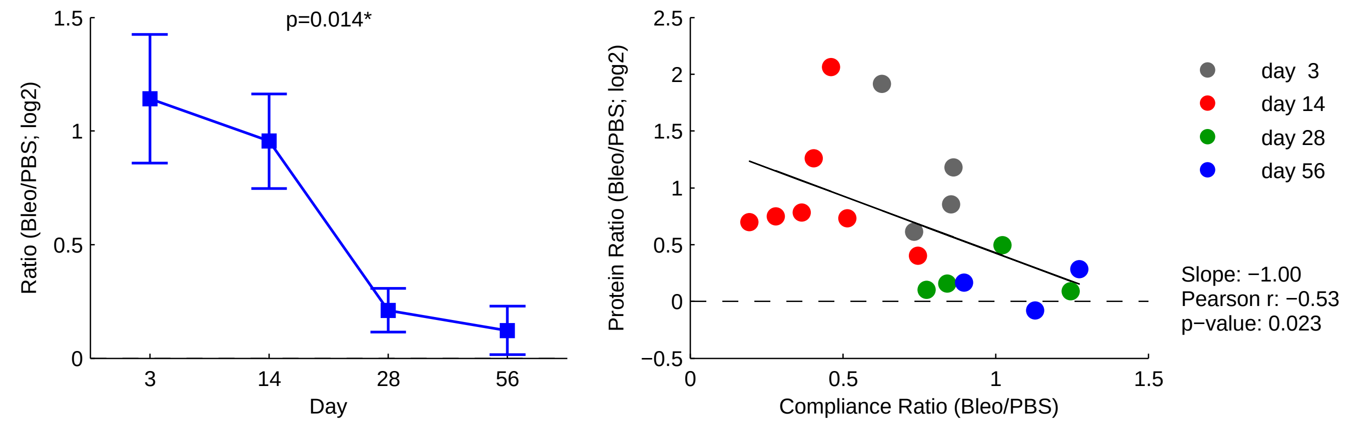

P62869 − Tceb2 (id: 3262)

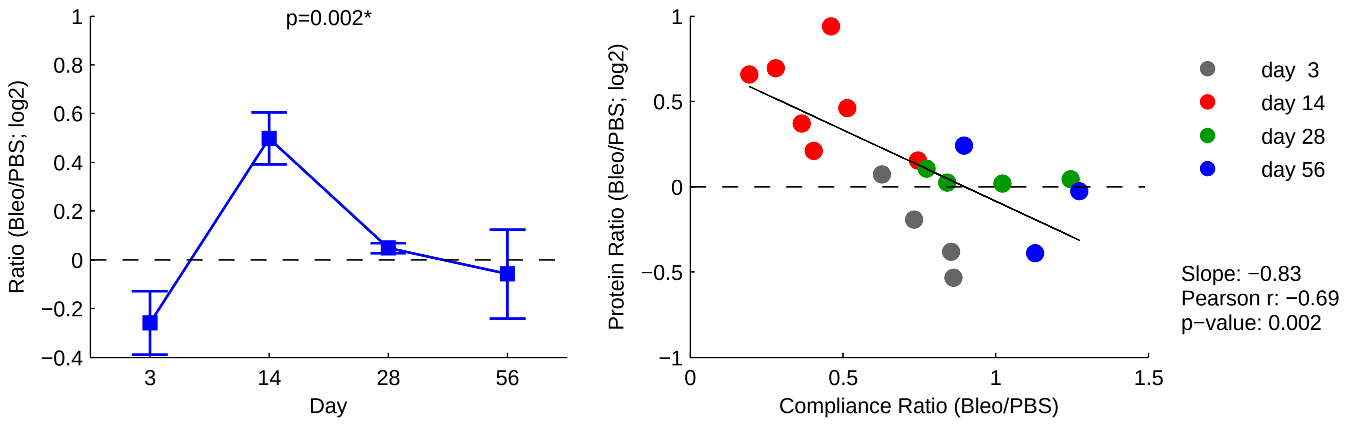

P62874 − Gnb1 (id: 3263)

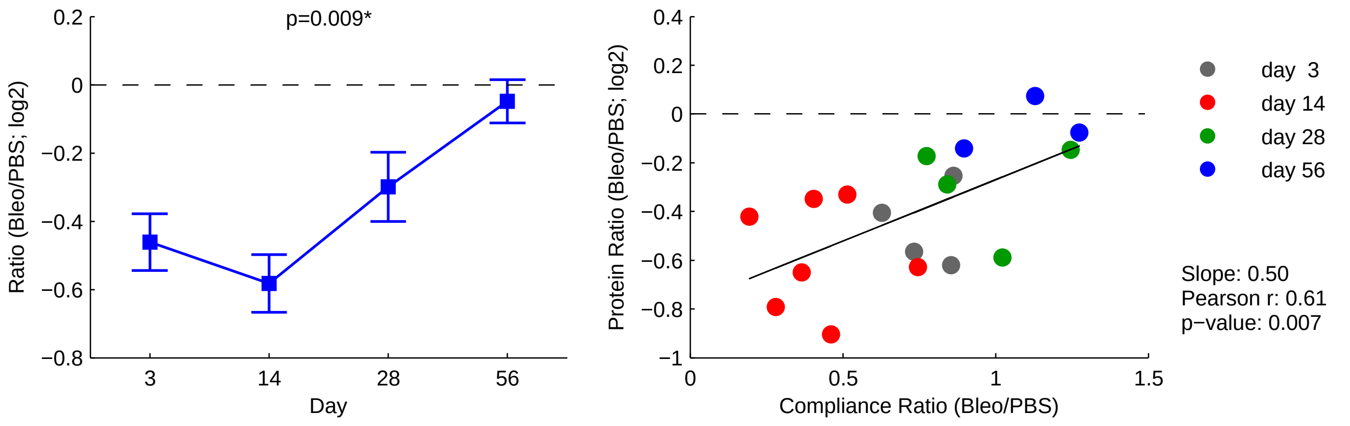

P62876 − Polr2l (id: 3264)

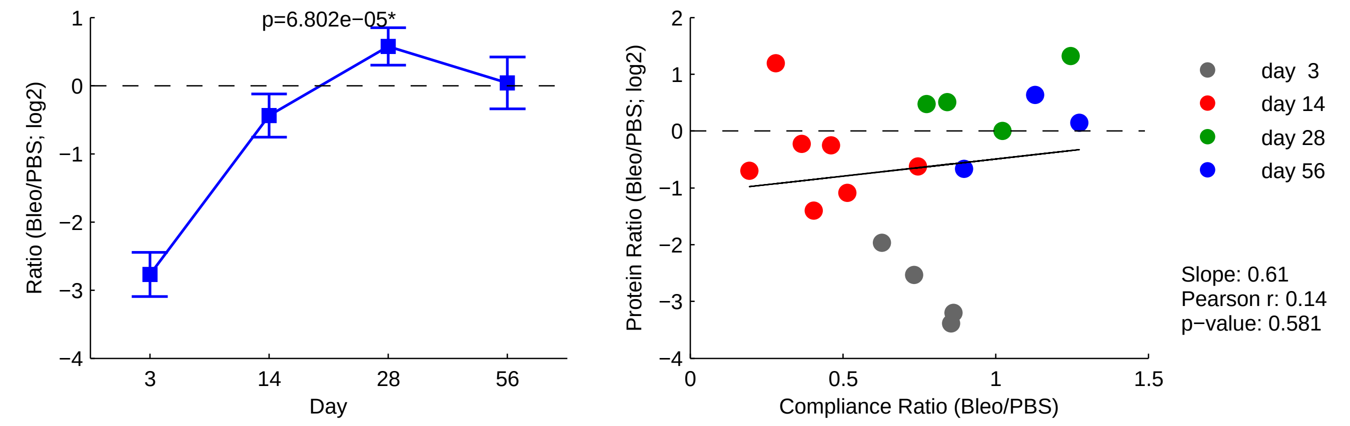

P62880 − Gnb2 (id: 3266)

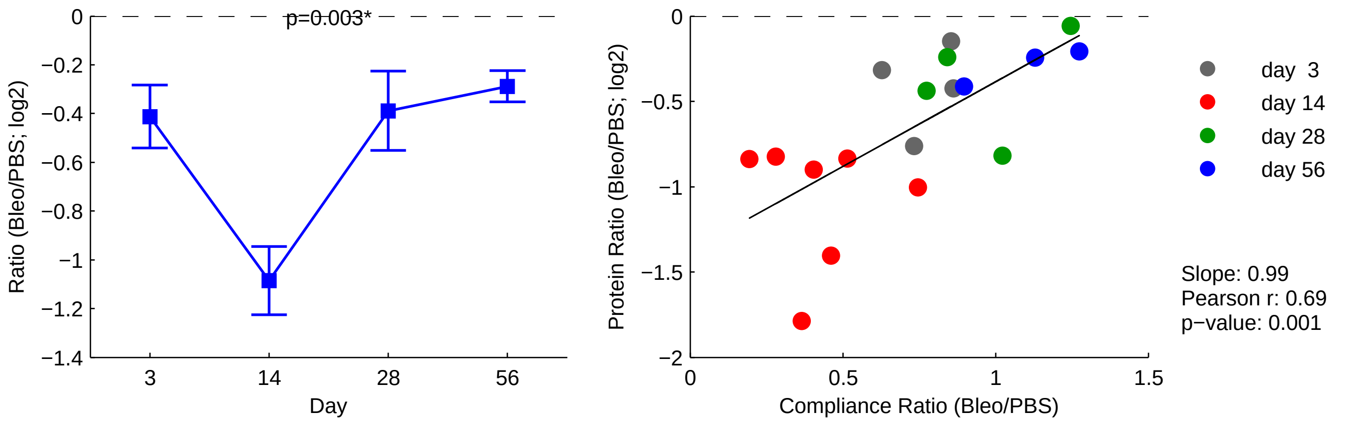

P62881−2 − Gnb5 (id: 3267)

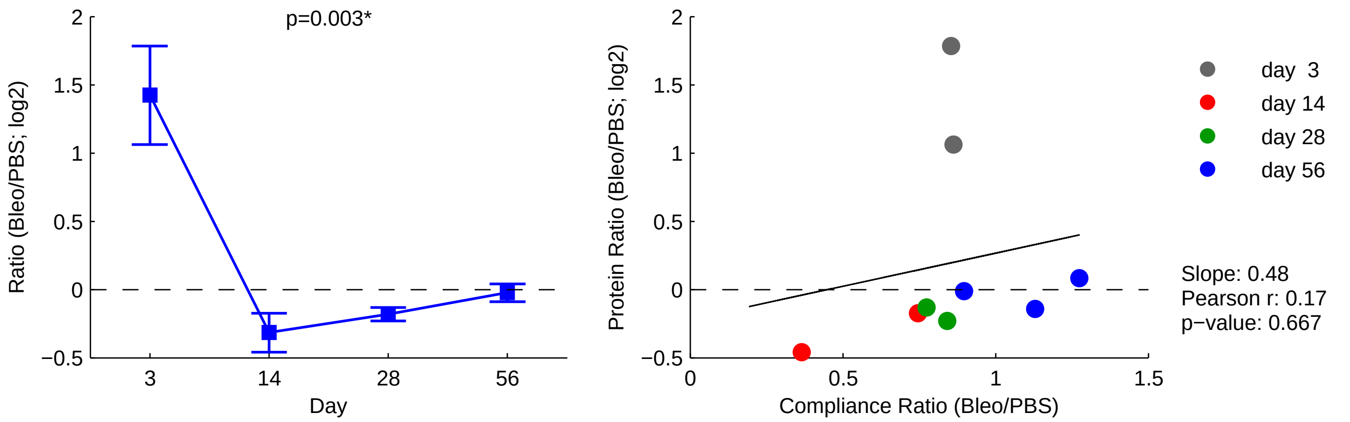

P62889 − Rpl30 (id: 3268)

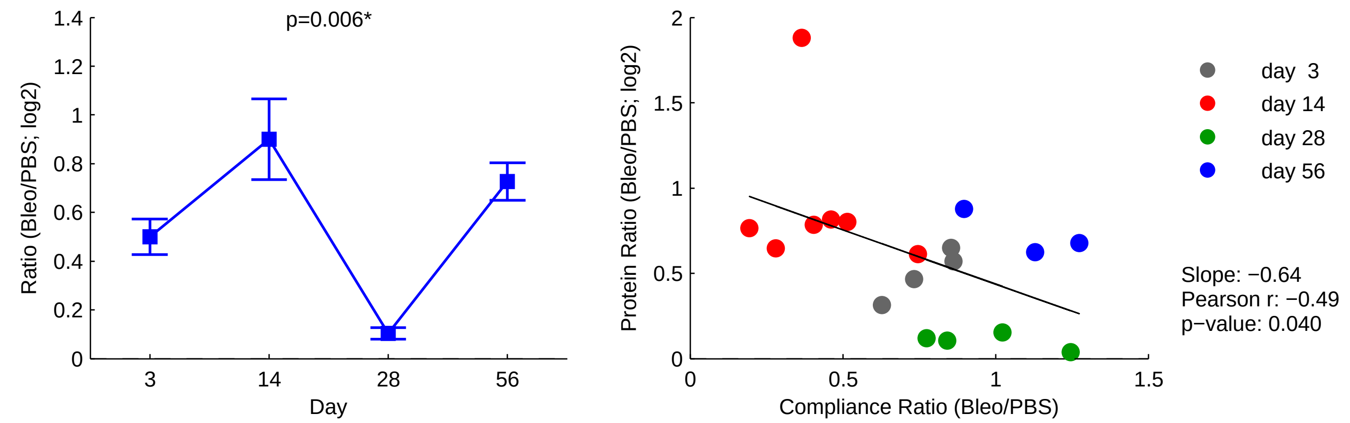

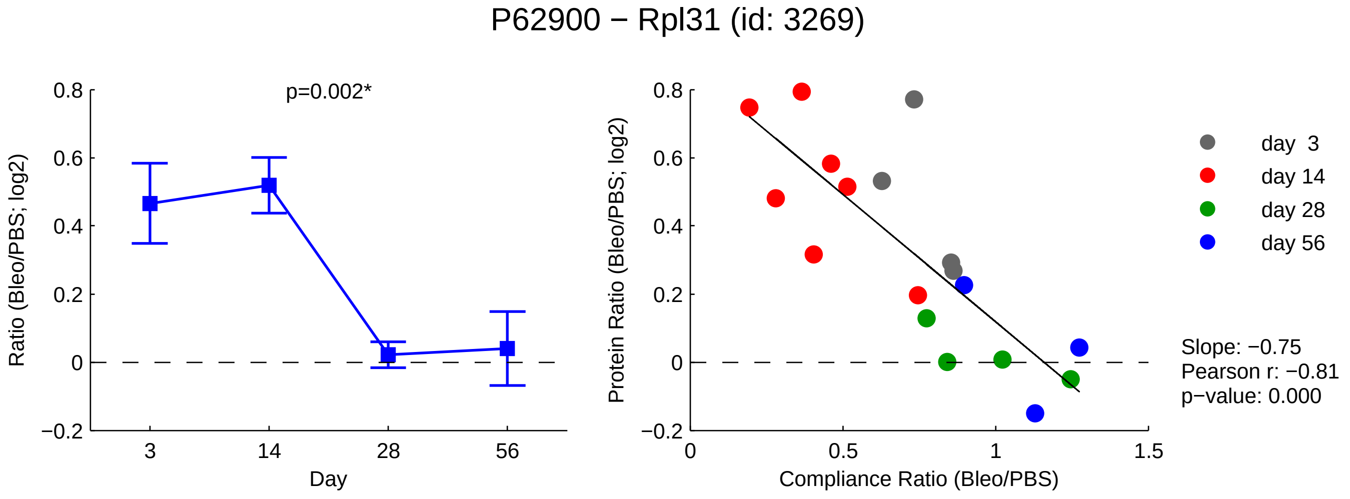
P62900 − Rpl31 (id: 3269)
p=0.002*
Ratio (Bleo/PBS; log2)
Day
Protein Ratio (Bleo/PBS; log2)
Compliance Ratio (Bleo/PBS)
day 3
day 14
day 28
day 56
Slope: −0.75
Pearson r: −0.81
p−value: 0.000

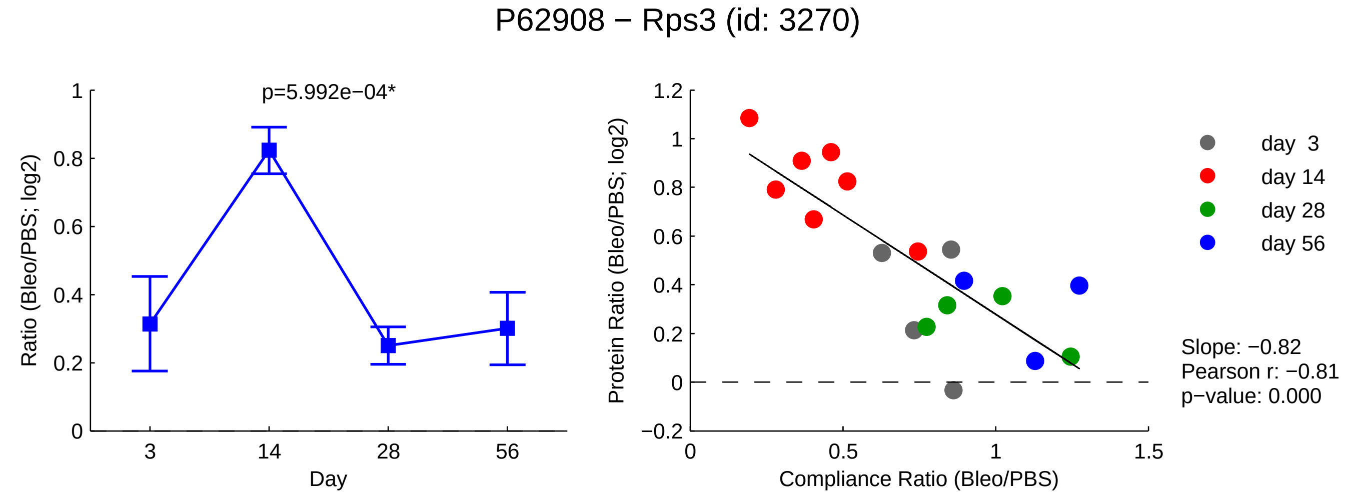
P62908 − Rps3 (id: 3270)
p=5.992e−04*
Ratio (Bleo/PBS; log2)
Day
Protein Ratio (Bleo/PBS; log2)
Compliance Ratio (Bleo/PBS)
day 3
day 14
day 28
day 56
Slope: −0.82
Pearson r: −0.81
p−value: 0.000

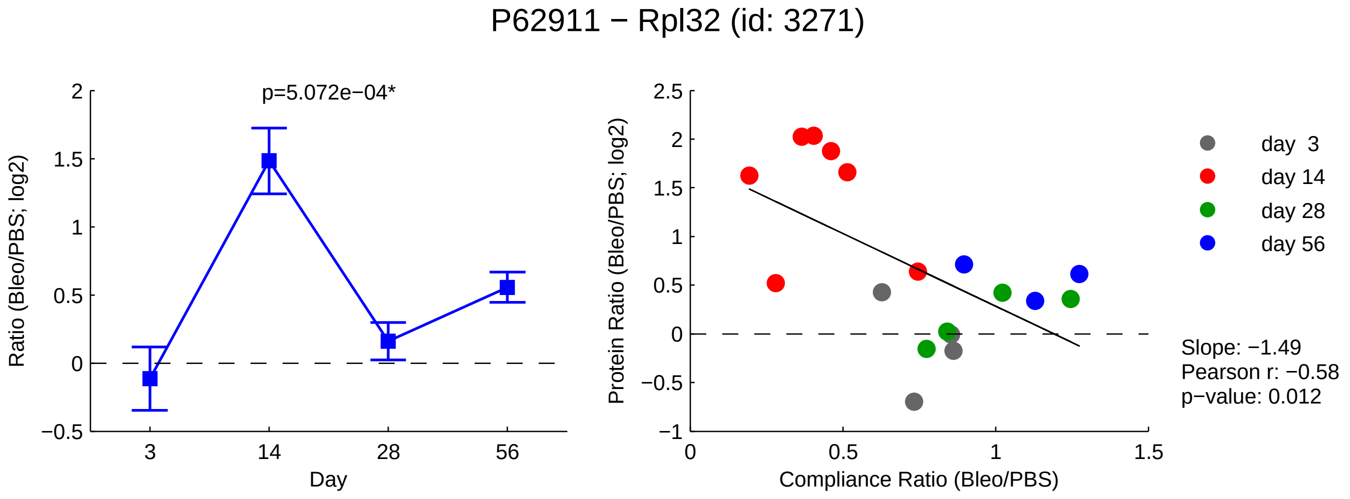
P62911 − Rpl32 (id: 3271)
p=5.072e−04*
Ratio (Bleo/PBS; log2)
Day
Protein Ratio (Bleo/PBS; log2)
Compliance Ratio (Bleo/PBS)
day 3
day 14
day 28
day 56
Slope: −1.49
Pearson r: −0.58
p−value: 0.012

P62915 − Gtf2b (id: 3272)

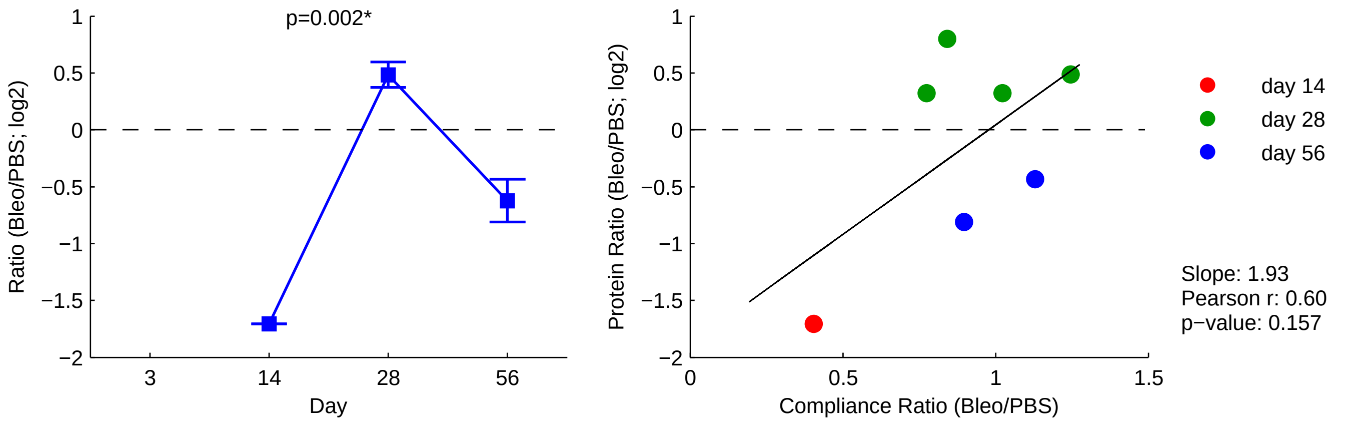

P62918 − Rpl8 (id: 3273)

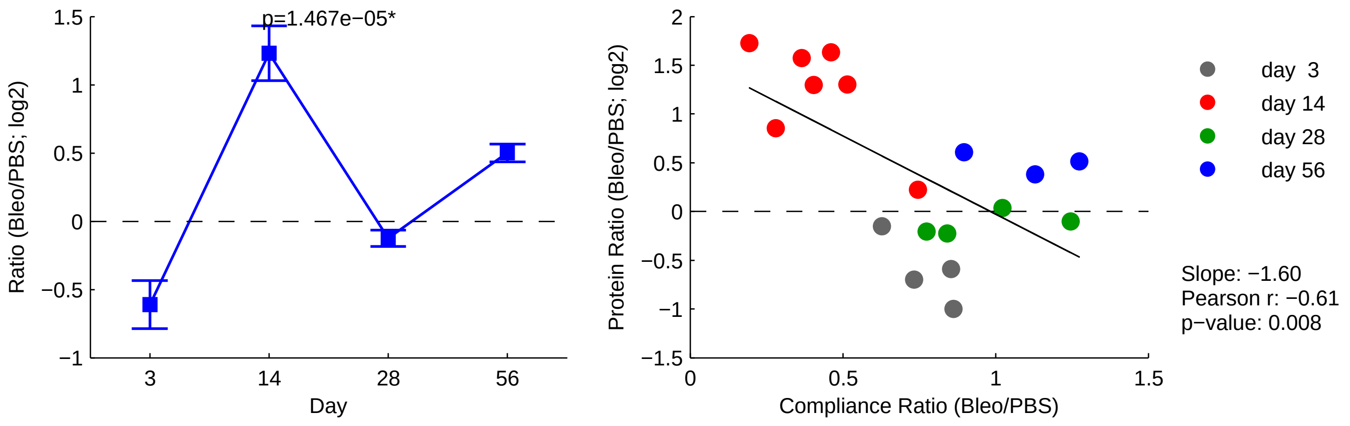

P62962 − Pfn1 (id: 3275)

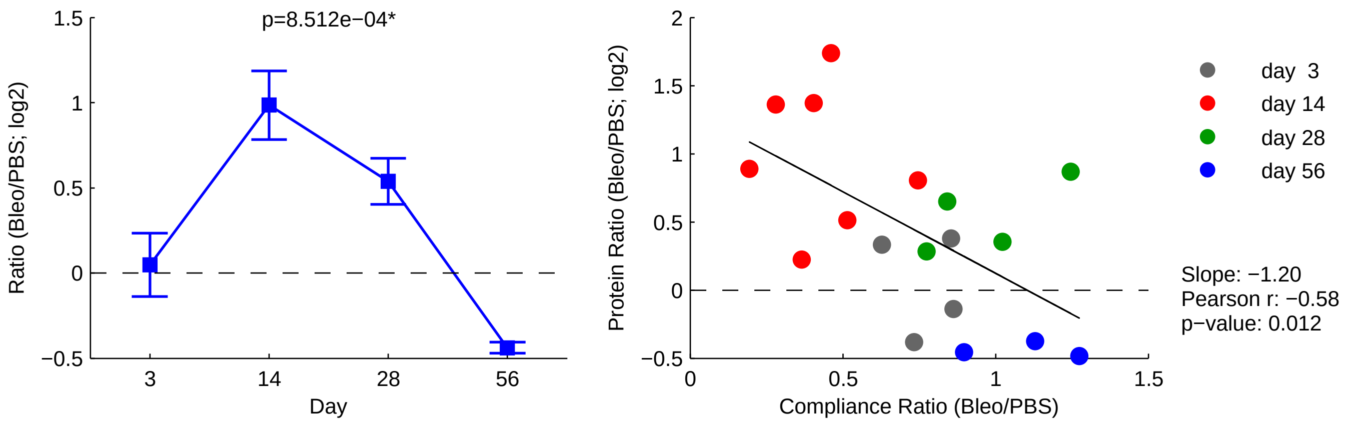

P62983 − Rps27a (id: 3276)

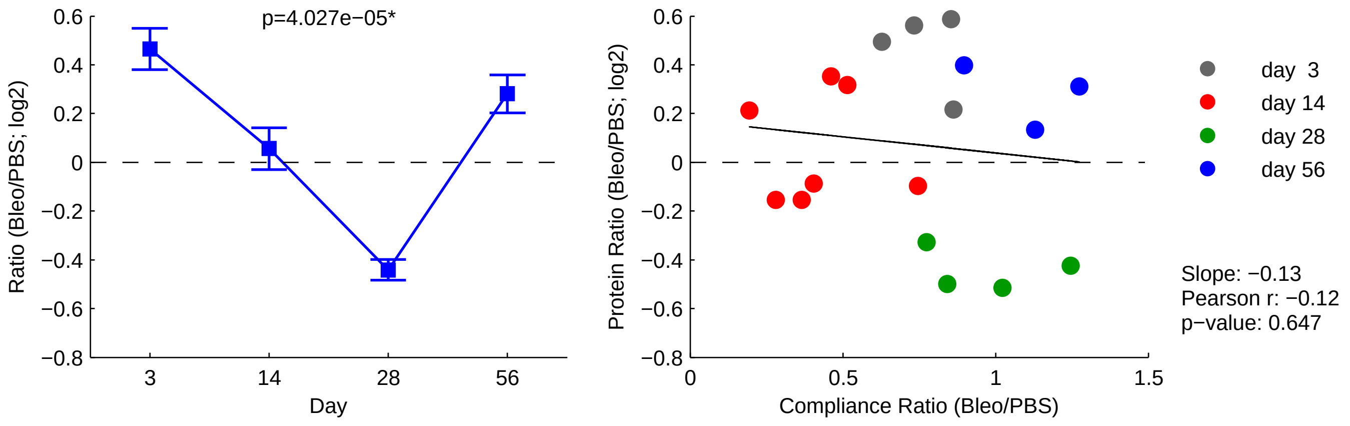

P62996 − Tra2b (id: 3277)

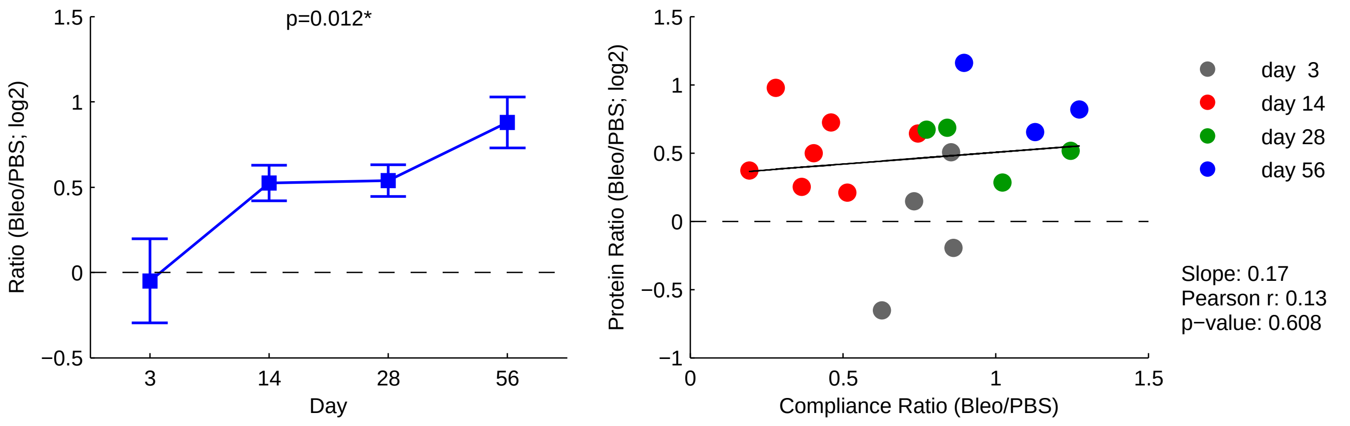

P63005 − Pafah1b1 (id: 3279)

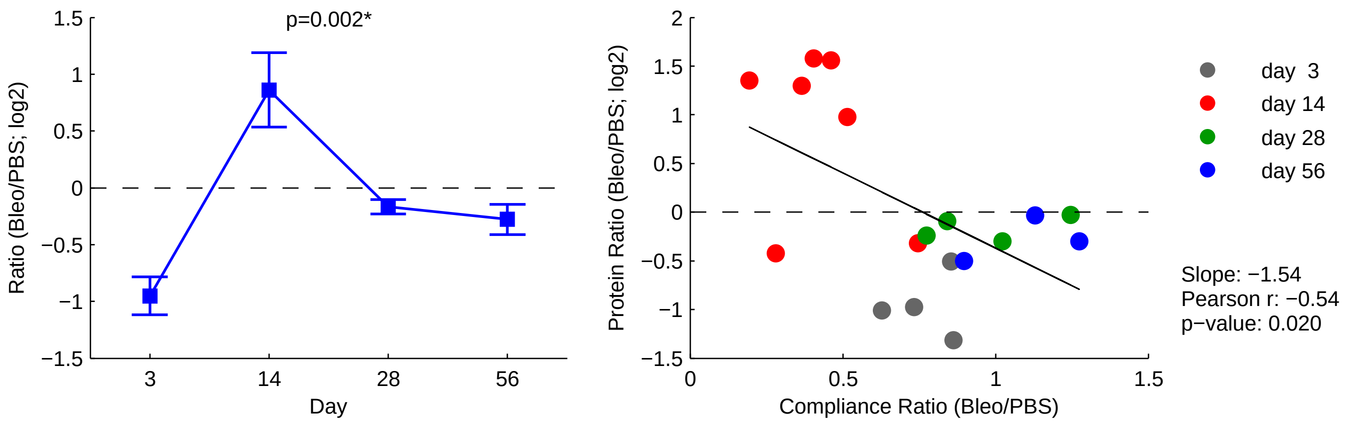

P63011 − Rab3a (id: 3280)

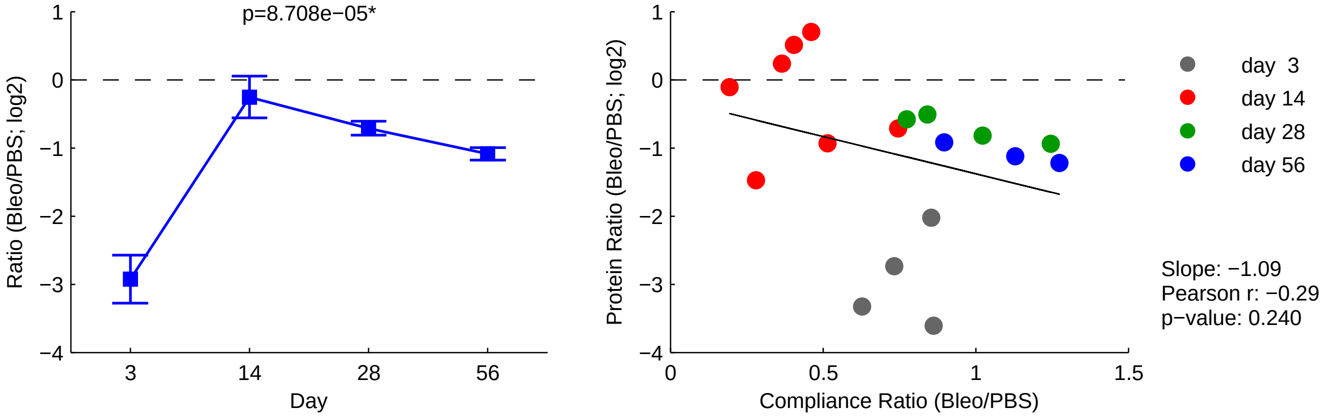

P63017 − Hspa8 (id: 3281)

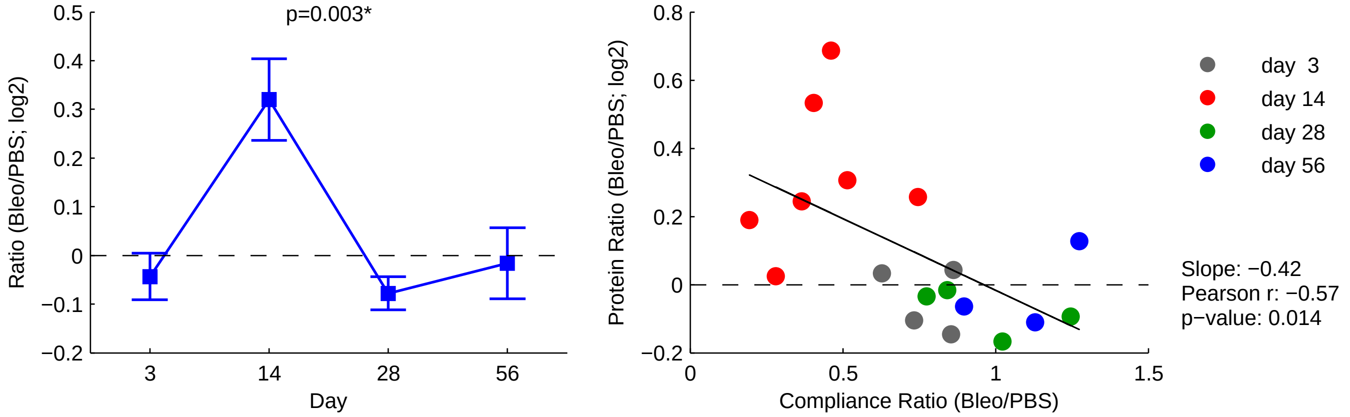

P63028 − Tpt1 (id: 3283)

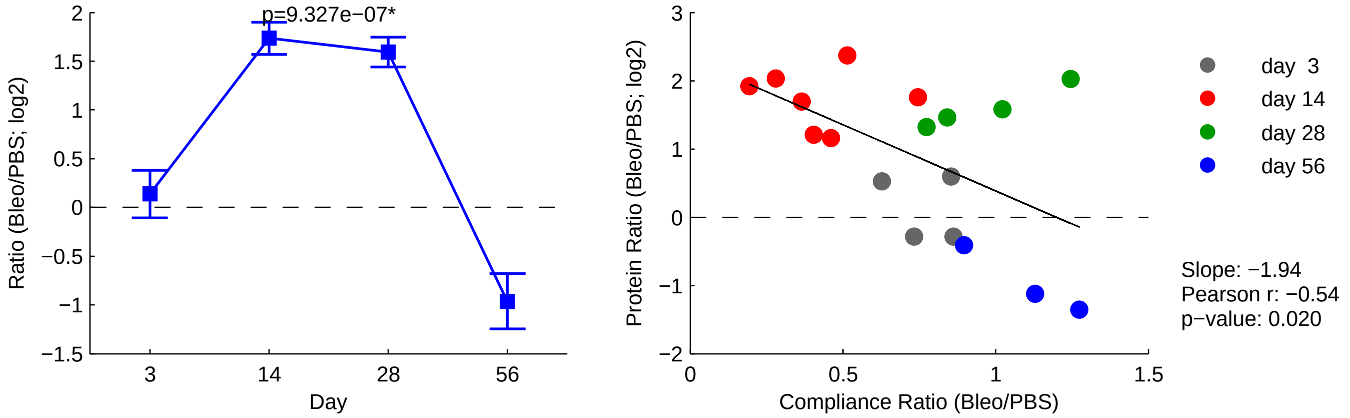

P63030 − Mpc1 (id: 3284)

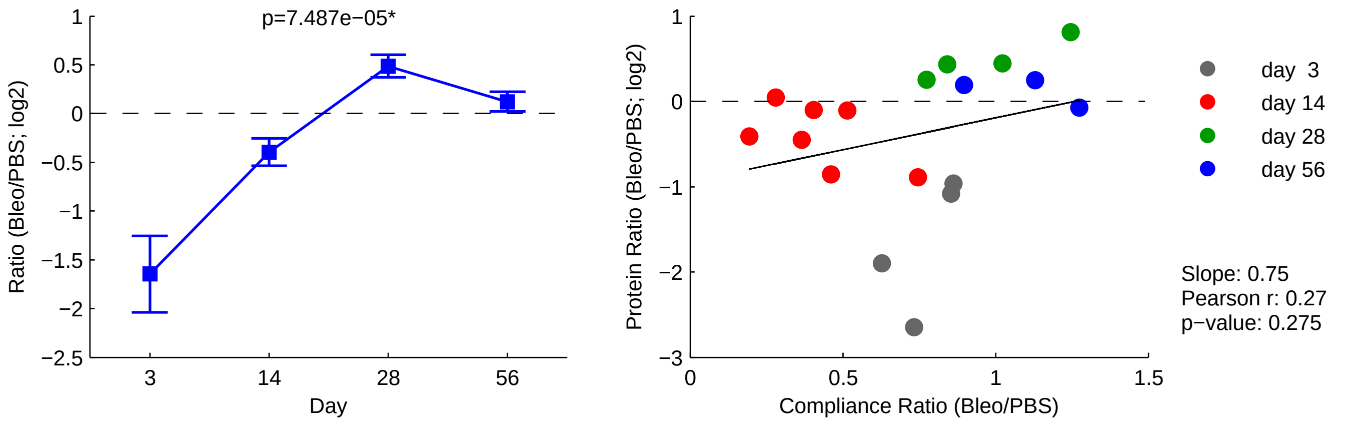

P63038 − Hspd1 (id: 3286)

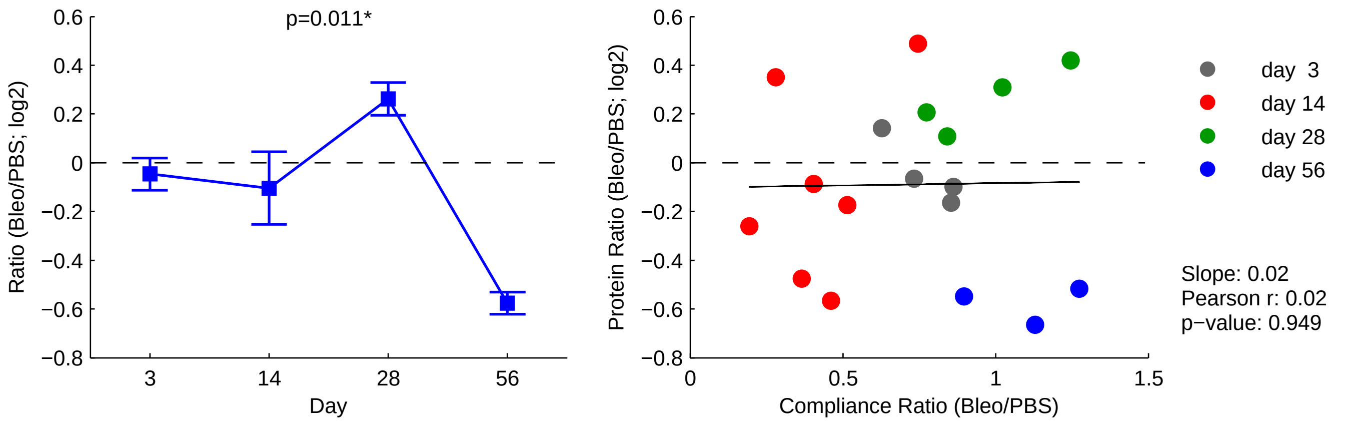

P63073 − Eif4e (id: 3288)

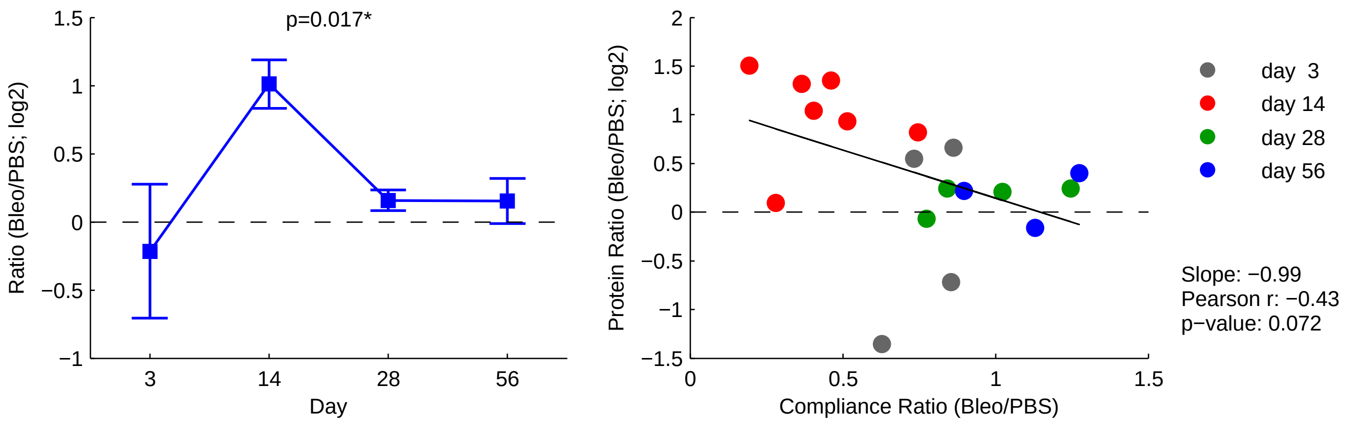

P63085 − Mapk1 (id: 3289)

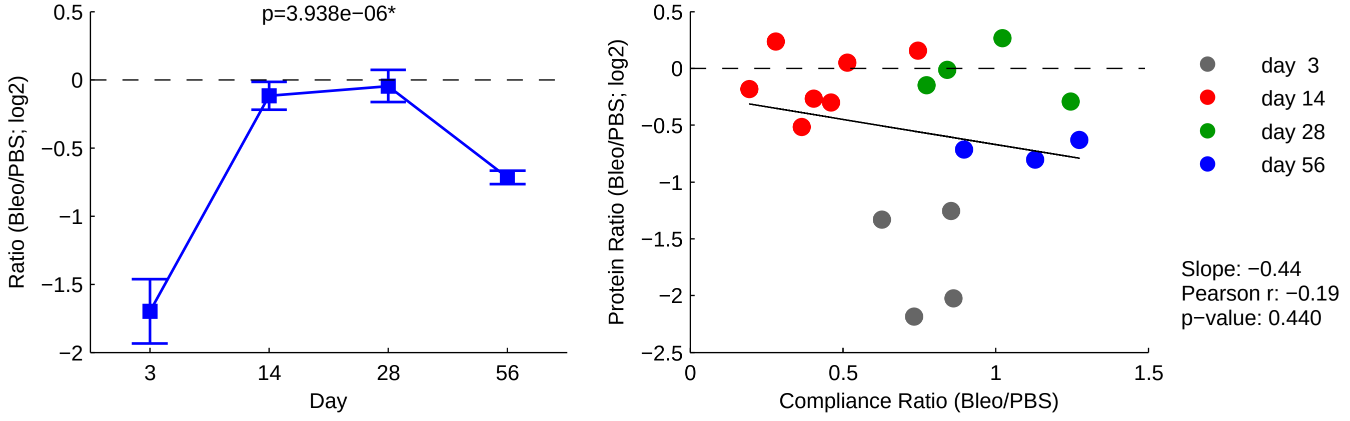

P63087−2 − Ppp1cc (id: 3290)

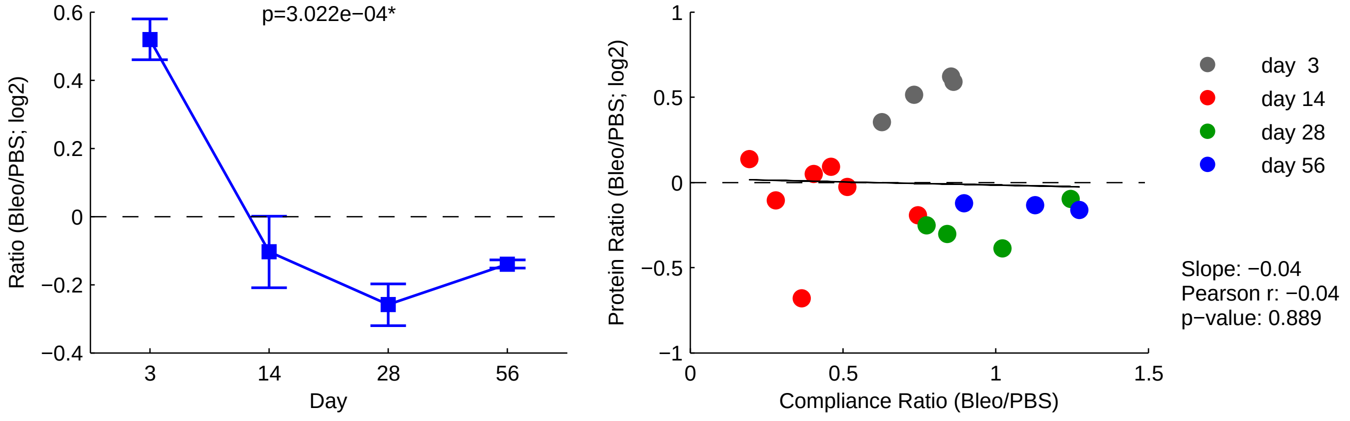

P63094 − Gnas (id: 3291)

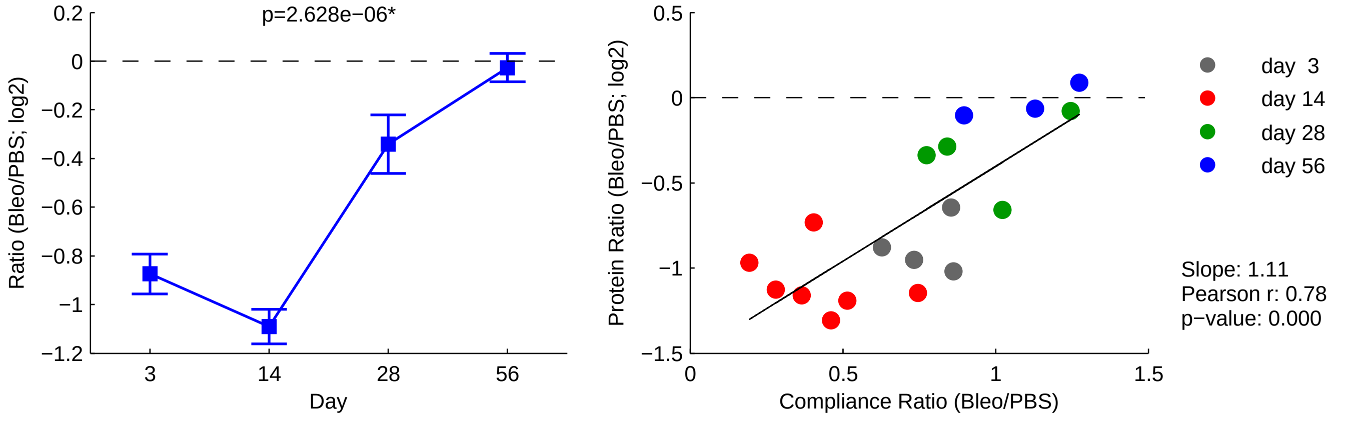

P63101 − Ywhaz (id: 3293)

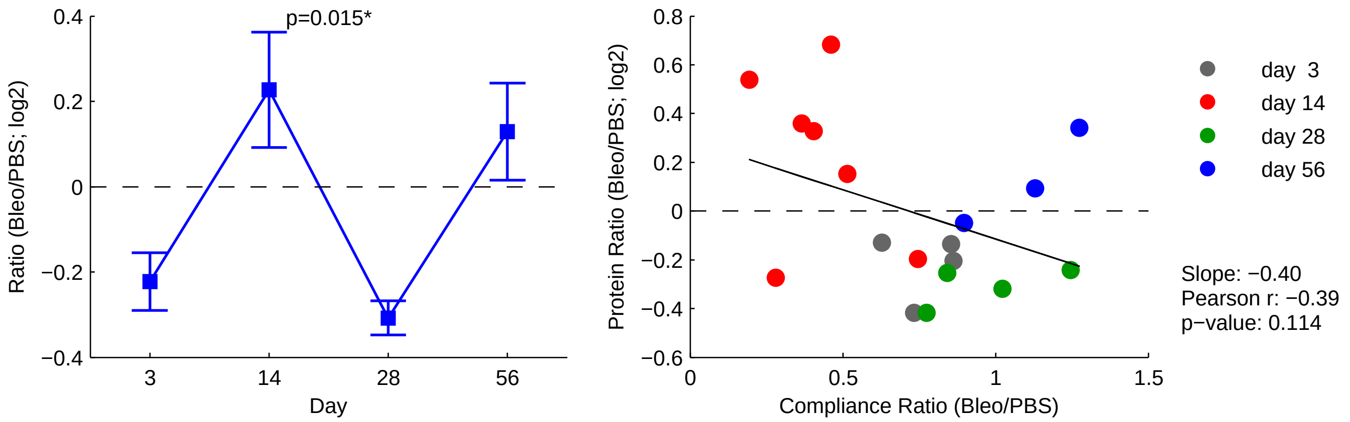

P63139 − Nfyb (id: 3294)

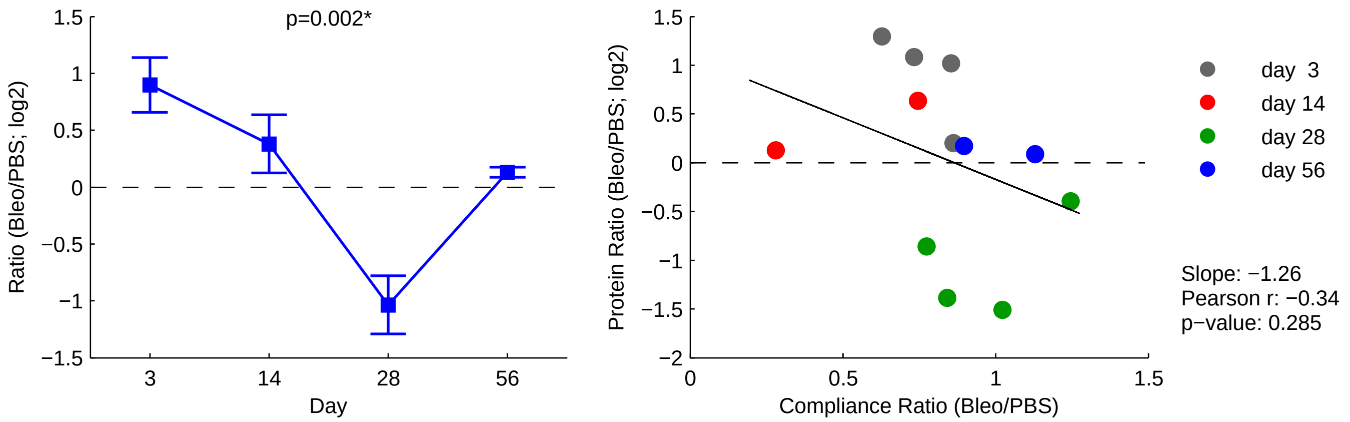

P63158 − Hmgb1 (id: 3296)

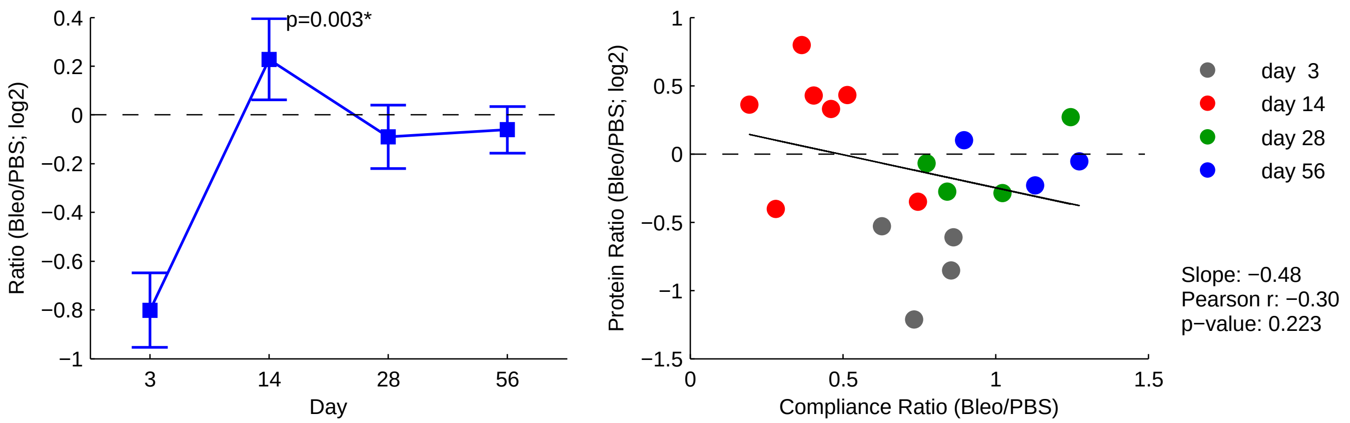

P63166 − Sumo1 (id: 3297)

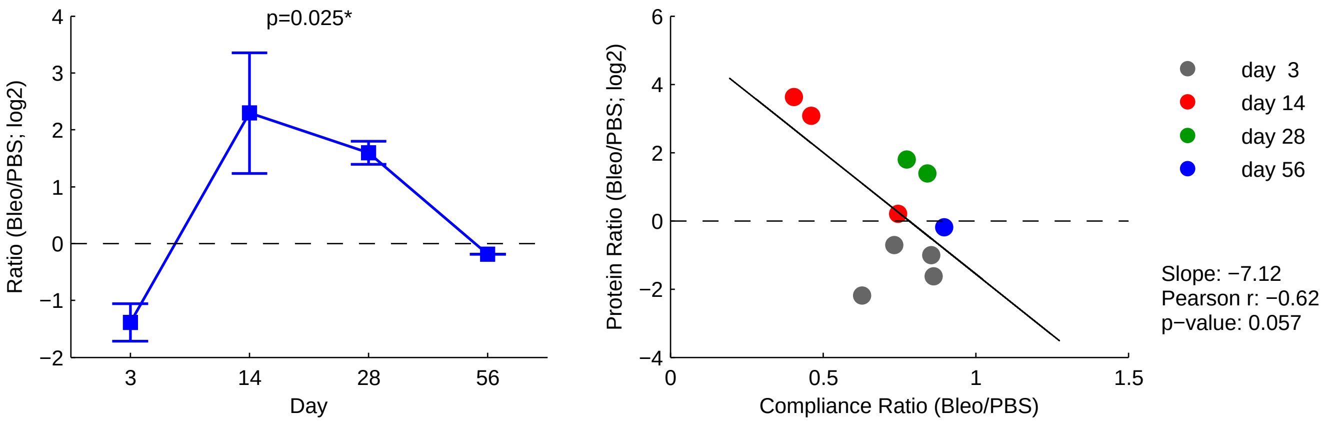

P63168 − Dynll1 (id: 3298)

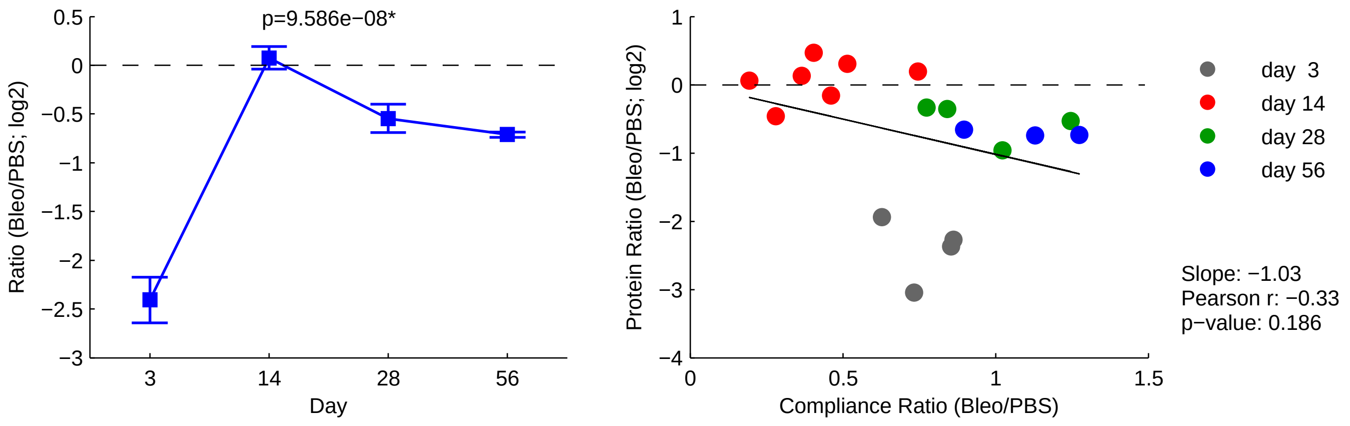

P63213 − Gng2 (id: 3299)

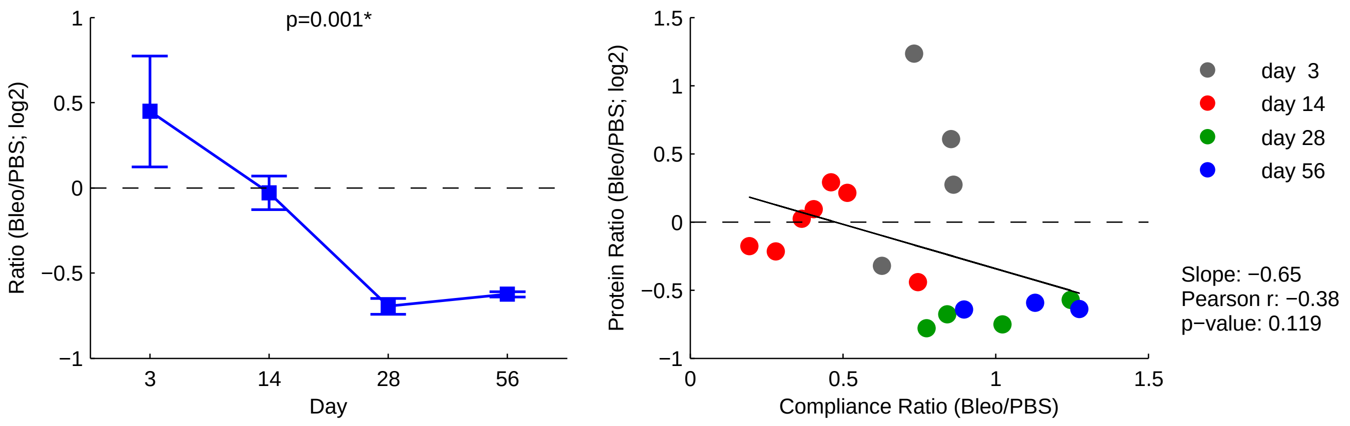

P63242 − Eif5a (id: 3300)

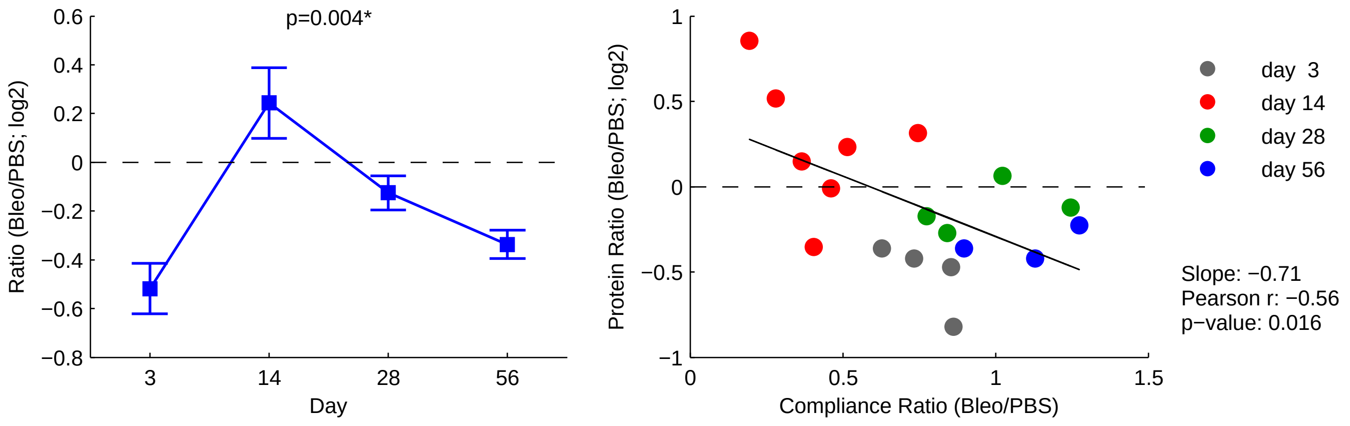

P63254 − Crip1 (id: 3301)

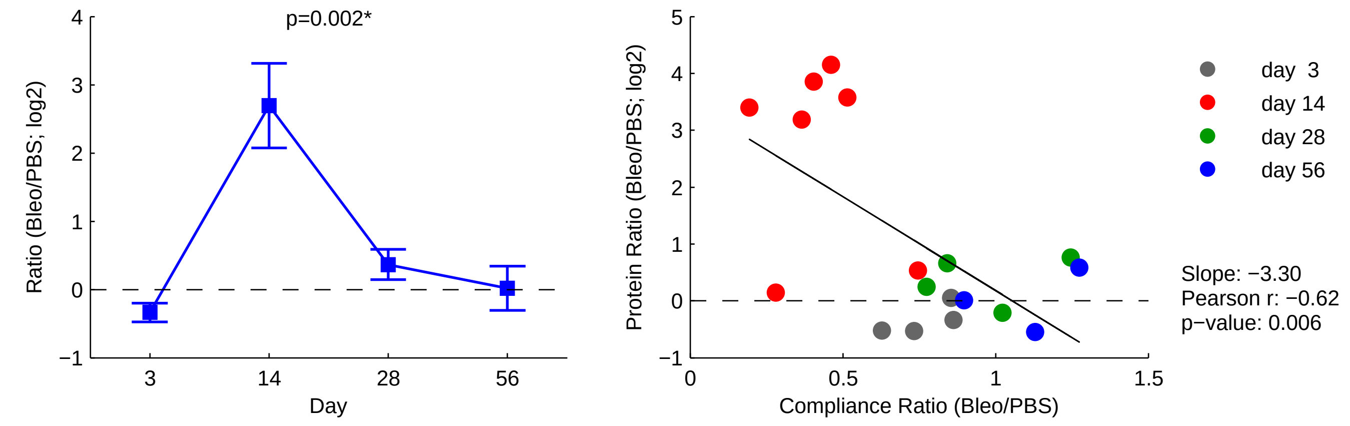

P63276 − Rps17 (id: 3304)

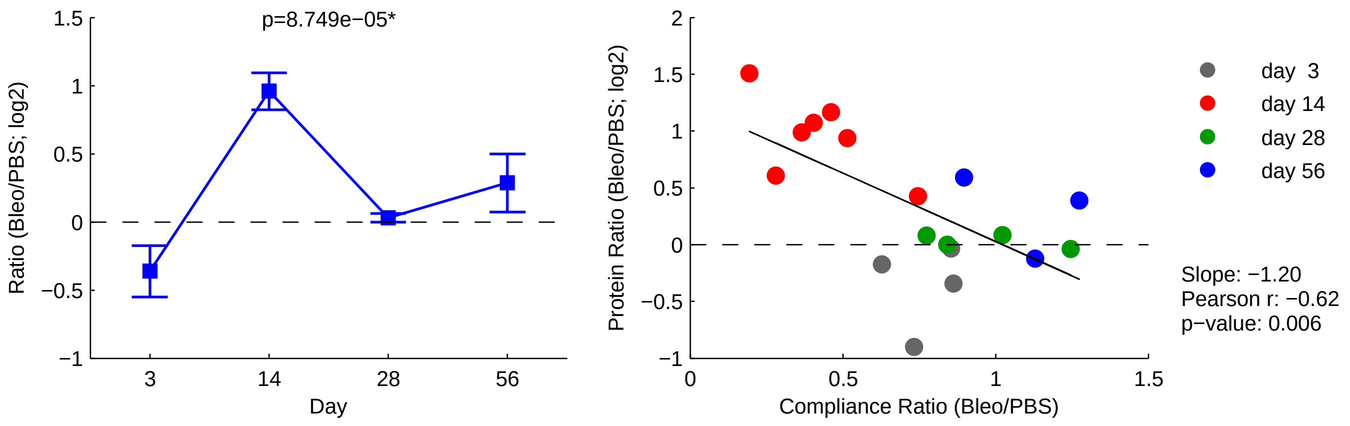

P63325 − Rps10 (id: 3307)

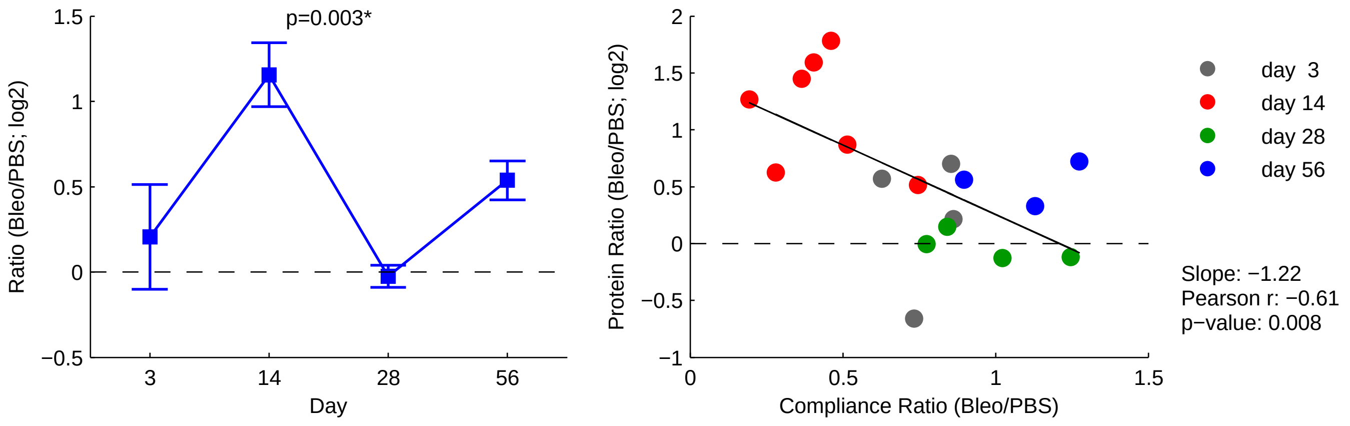

P67778 − Phb (id: 3310)

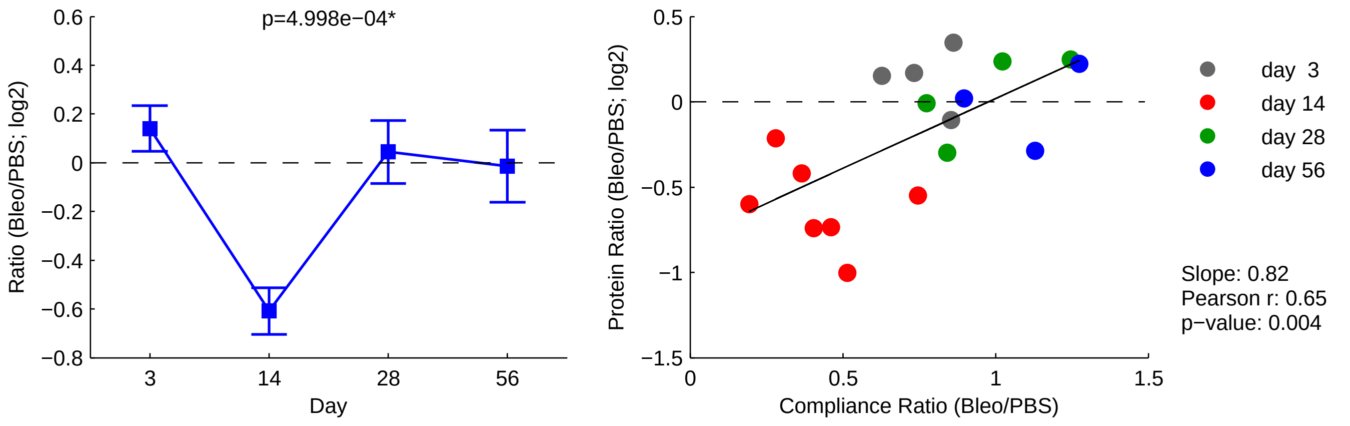

P67871 − Csnk2b (id: 3311)

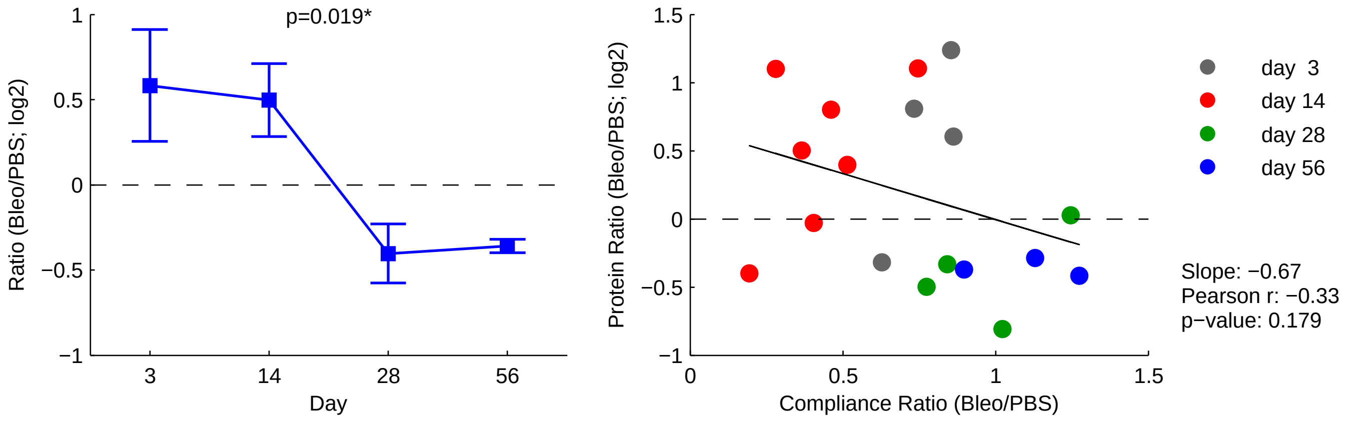

P68033 − Actc1 (id: 3313)

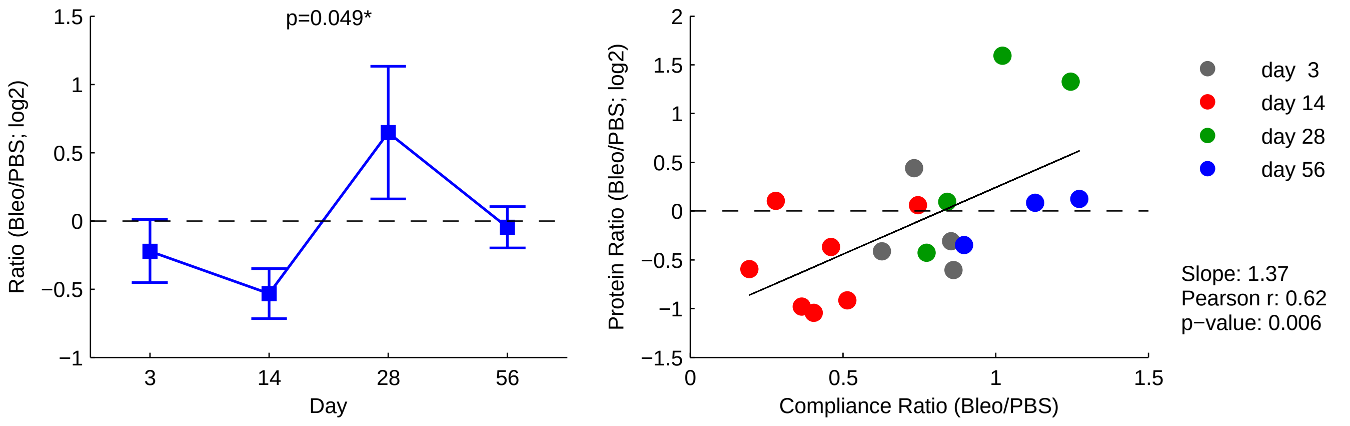

P68134 − Acta1 (id: 3316)

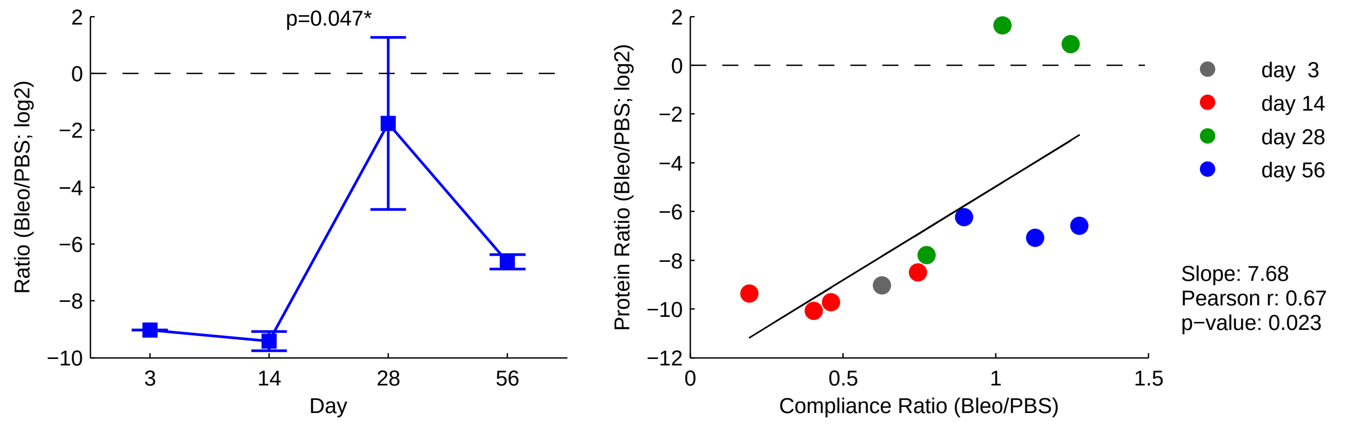

P68254 − Ywhaq (id: 3318)

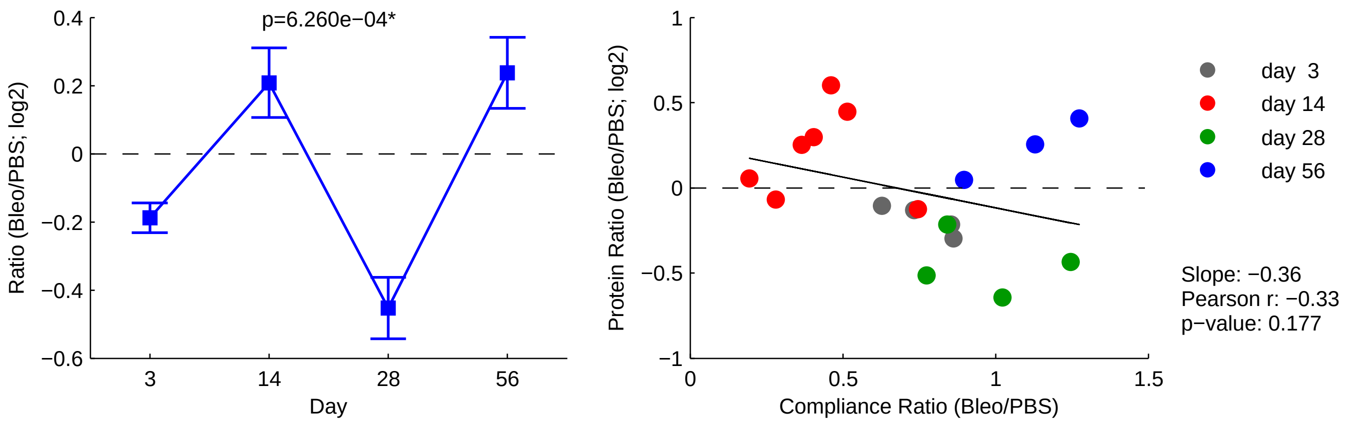

P68368 − Tuba4a (id: 3319)

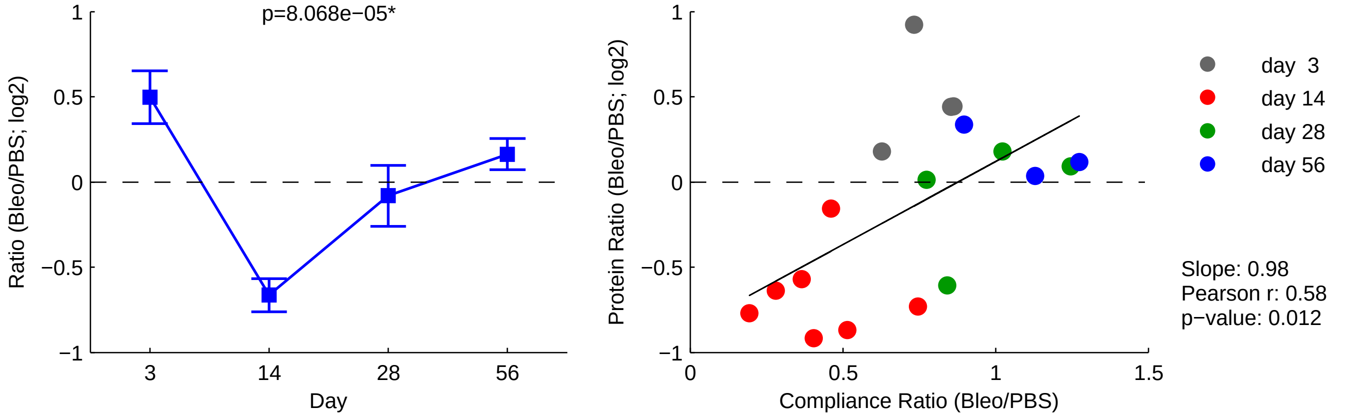

P68369 − Tuba1a (id: 3320)

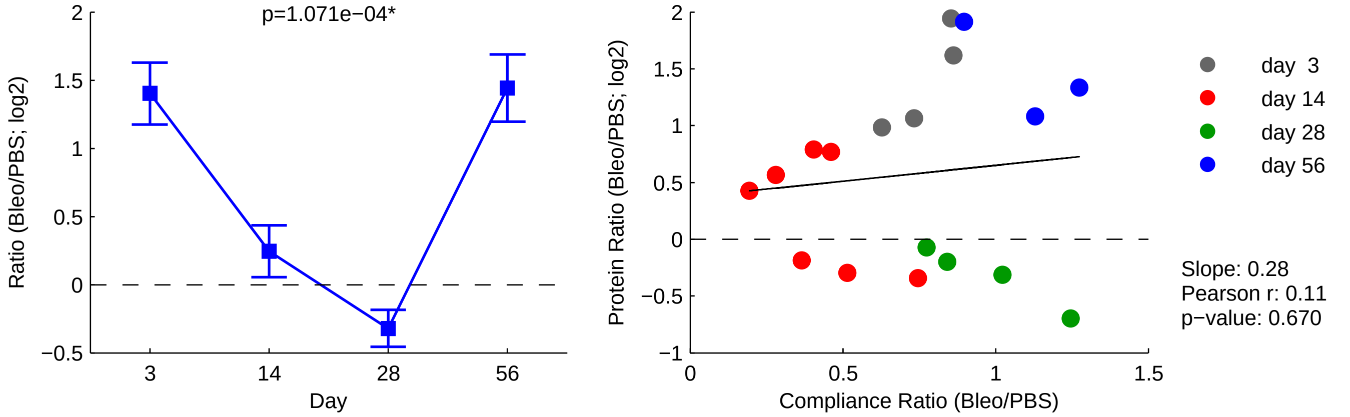

P68372 − Tubb4b (id: 3321)

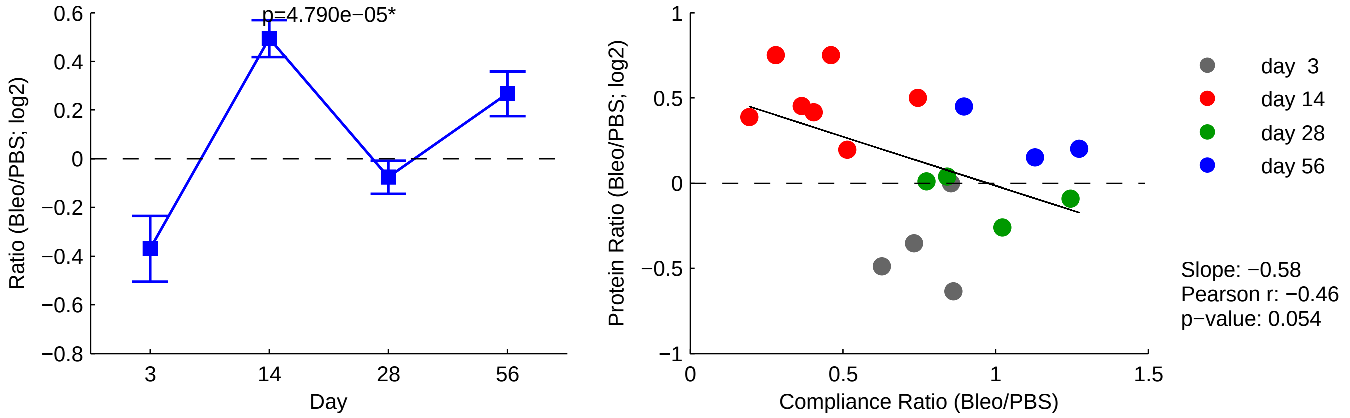

P68404−2 − Prkcb (id: 3323)

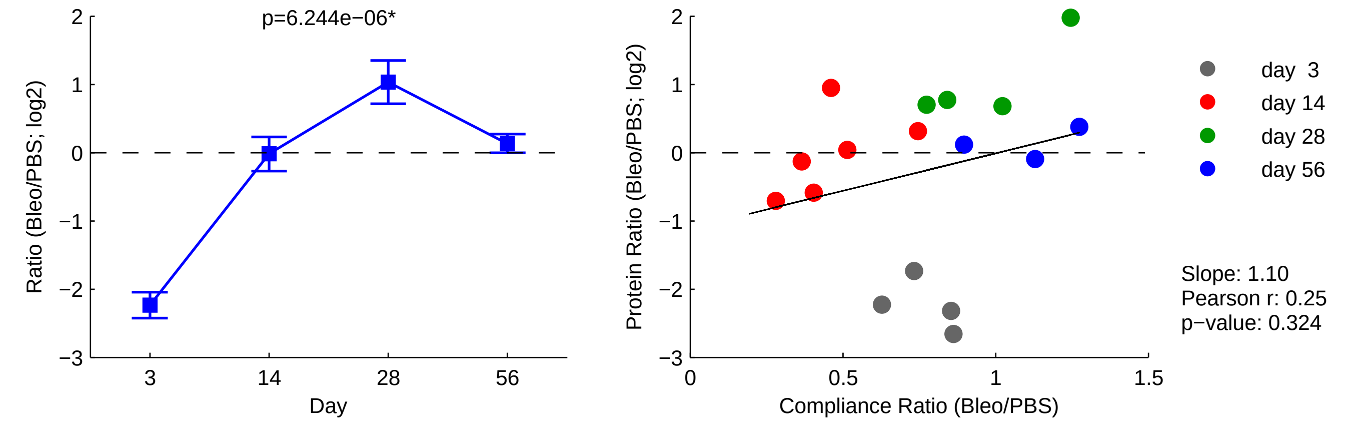

P68510 − Ywhah (id: 3325)

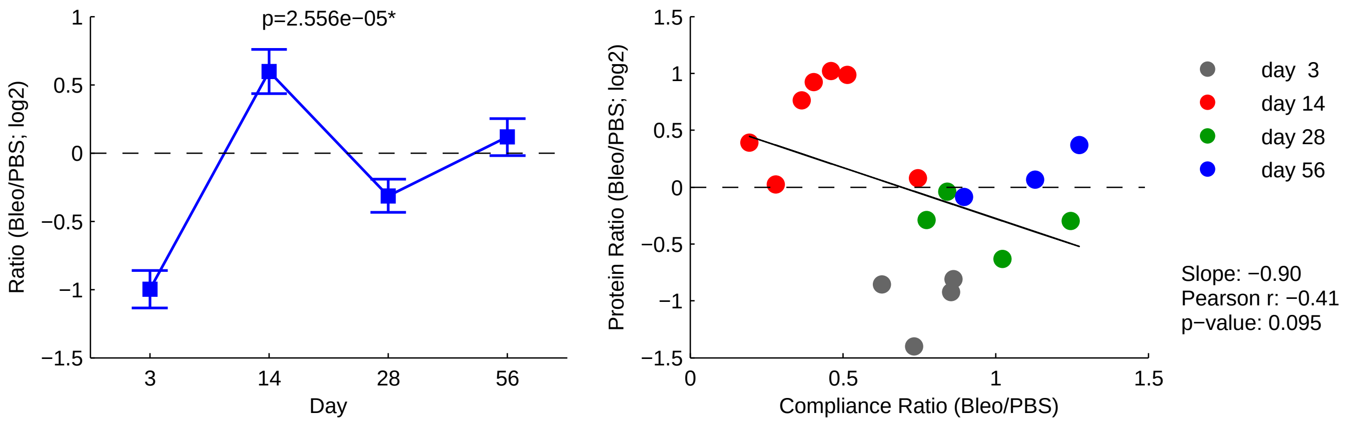

P70122 − Sbds (id: 3327)

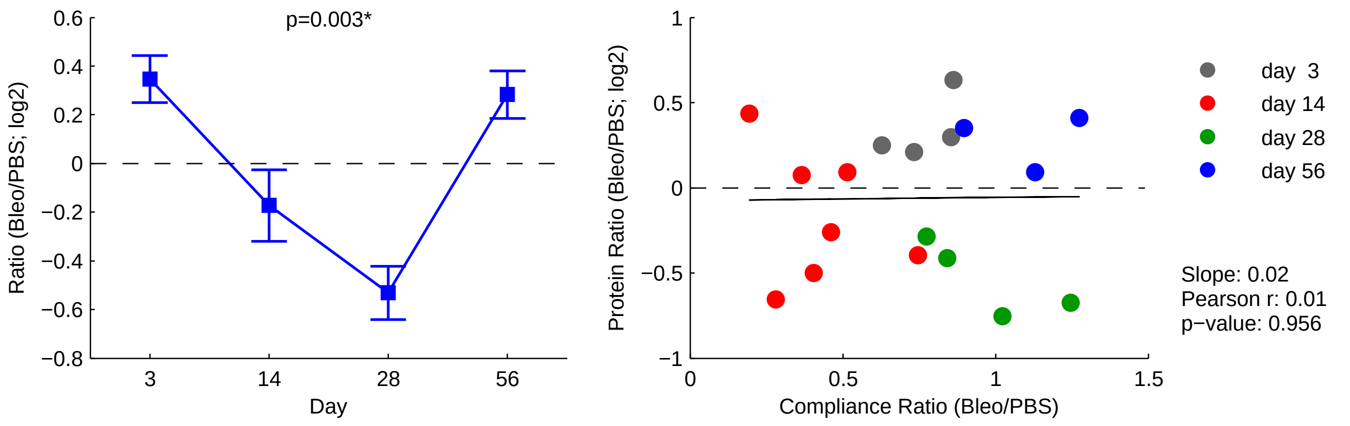

P70195 − Psmb7 (id: 3333)

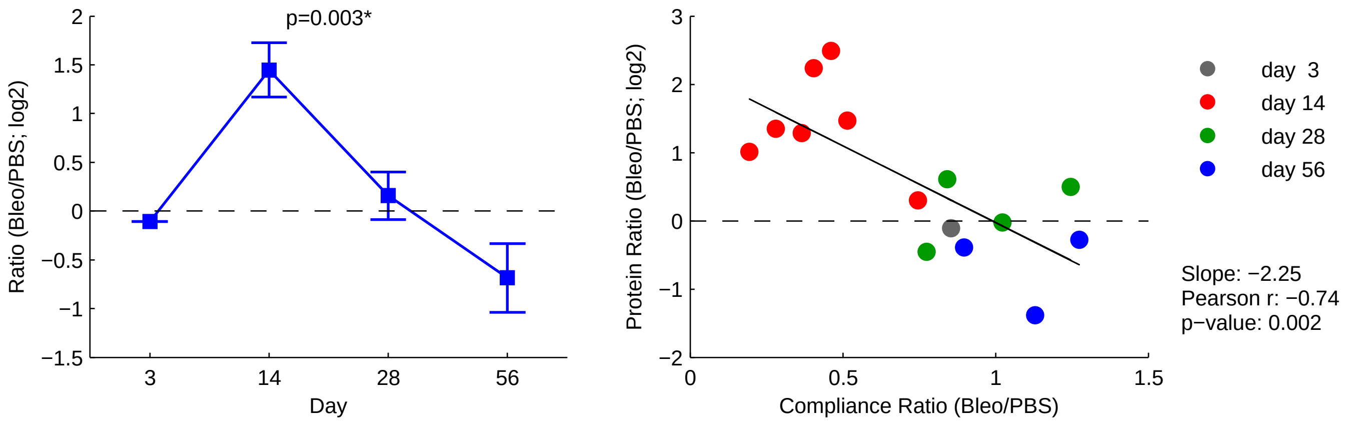

P70202 − Lxn (id: 3335)

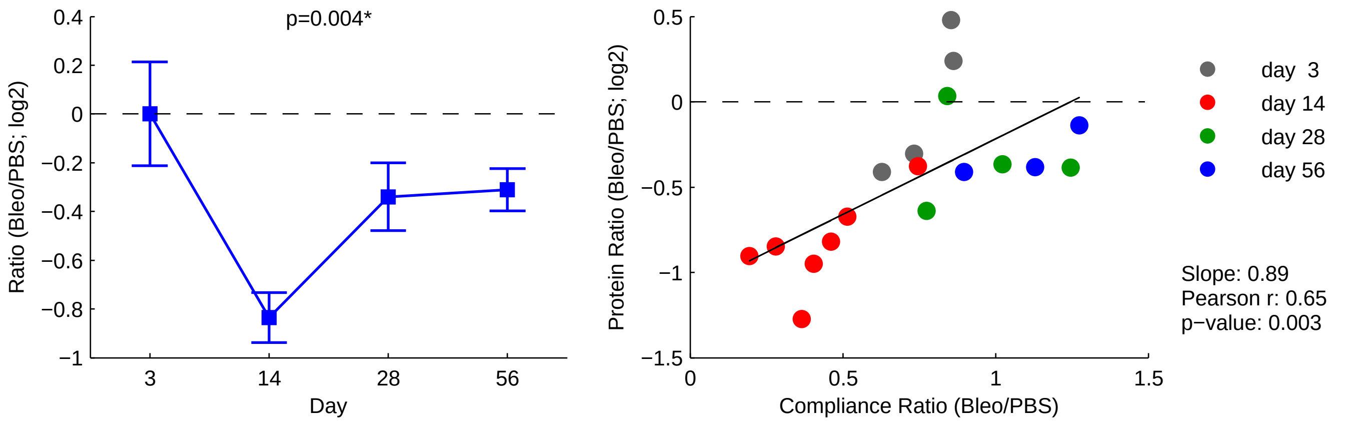

P70227 − Itpr3 (id: 3339)

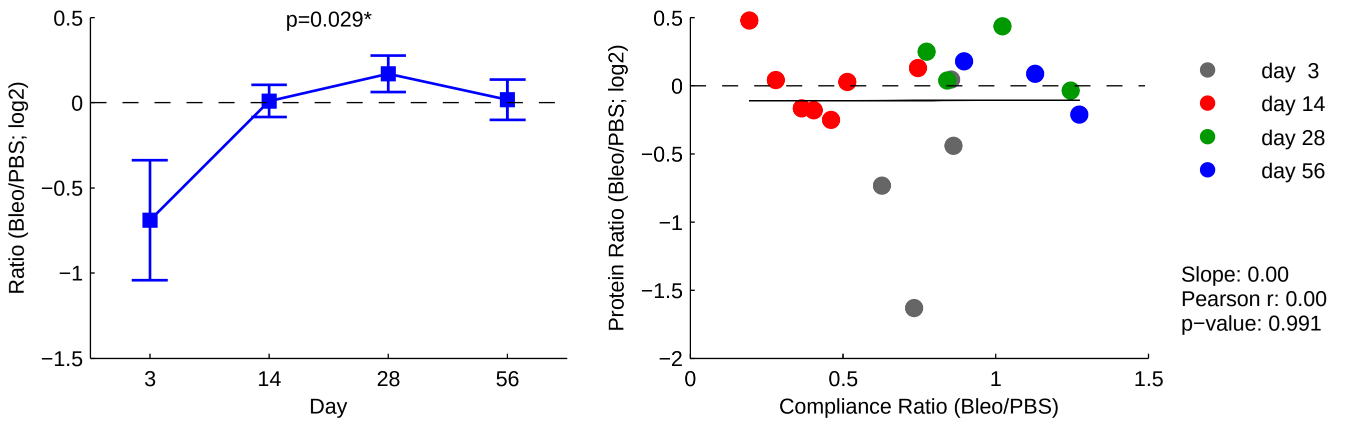

P70245 − Ebp (id: 3341)

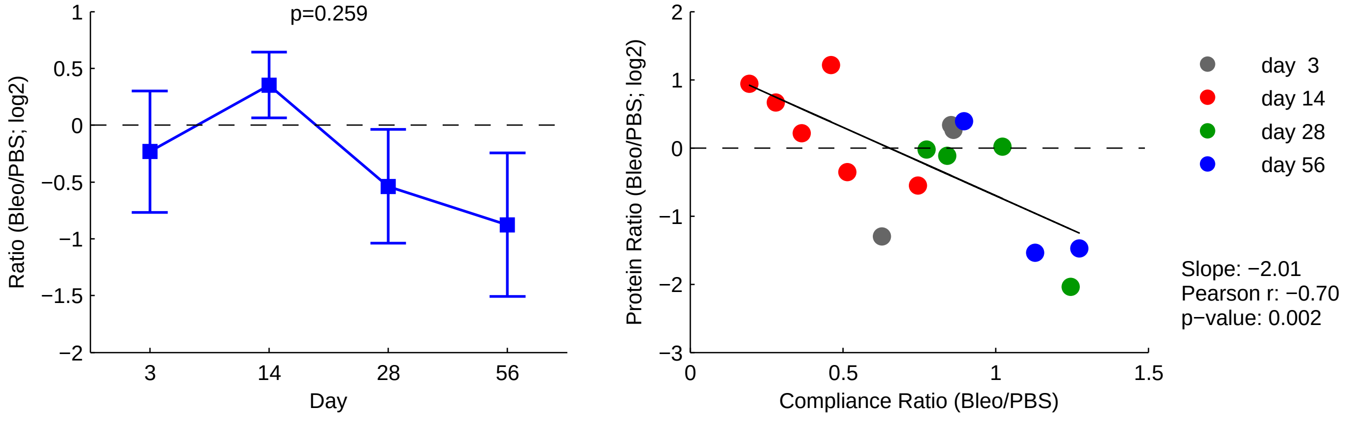

P70268 − Pkn1 (id: 3346)

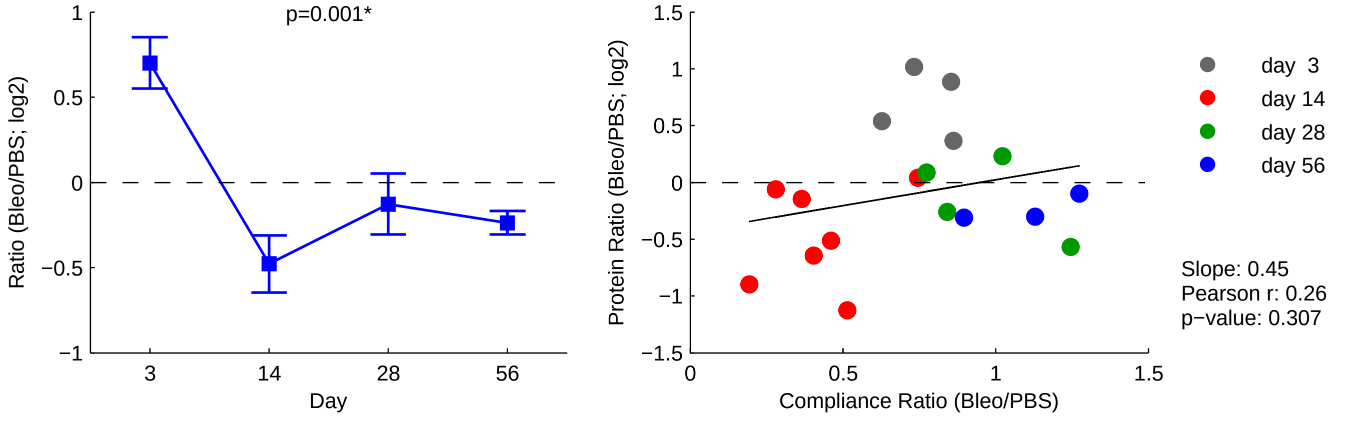

P70271 − Pdlim4 (id: 3347)

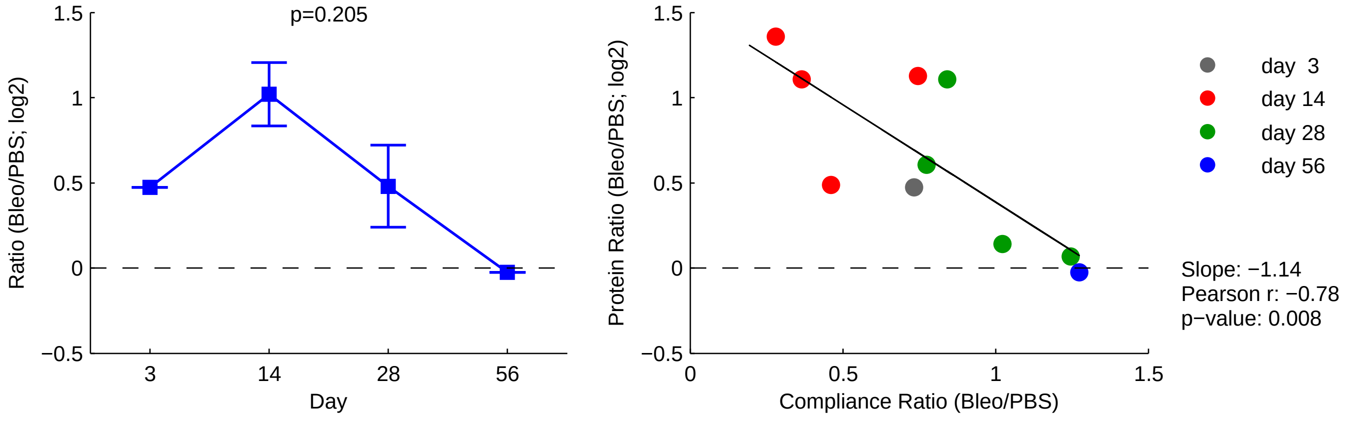

P70275 − Sema3e (id: 3349)

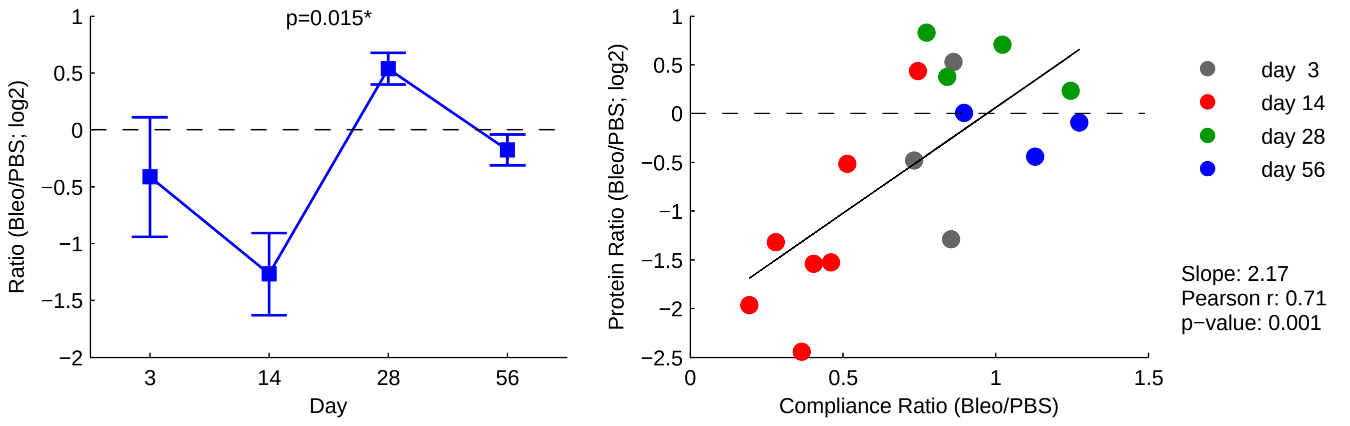

P70290 − Mpp1 (id: 3354)

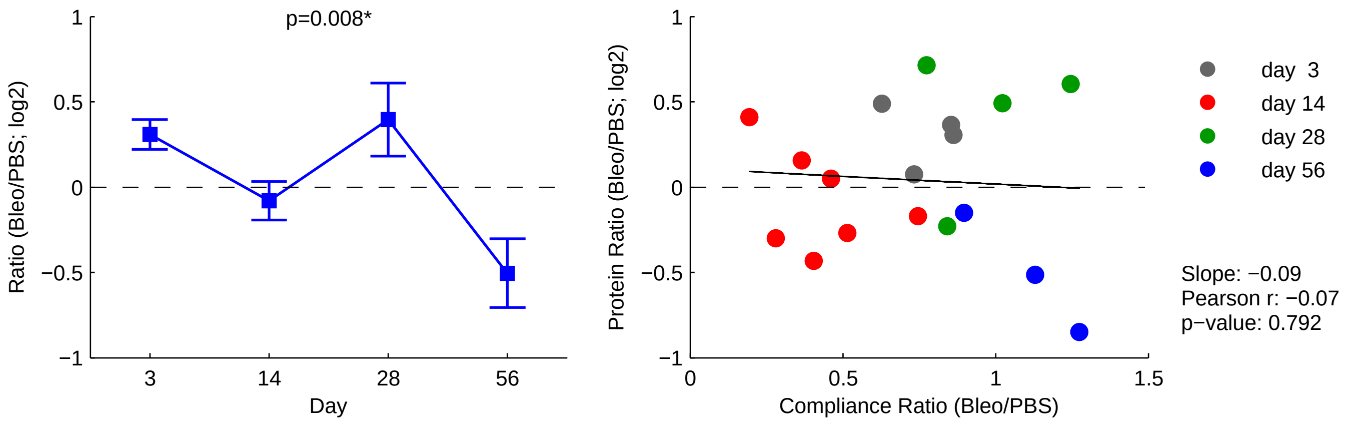

P70295 − Aup1 (id: 3355)

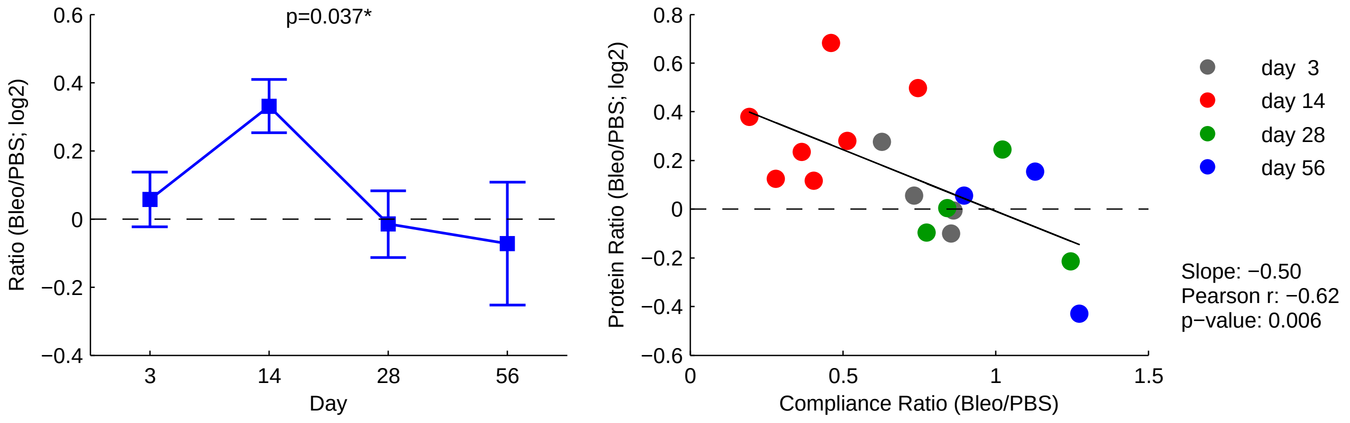

P70296 − Pebp1 (id: 3356)

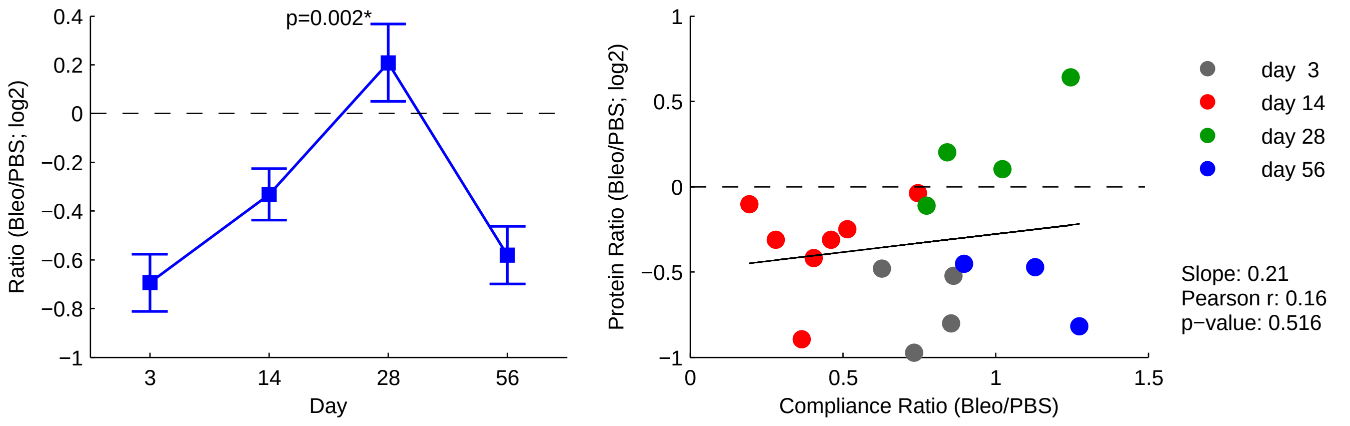

Q3UGN9 − Stam (id: 3357)

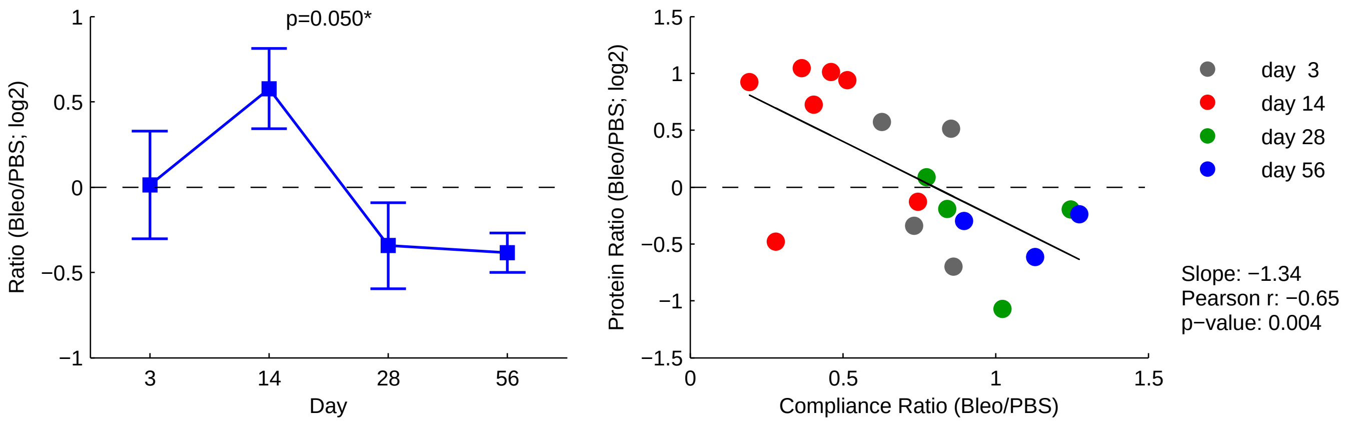

P70302 − Stim1 (id: 3358)

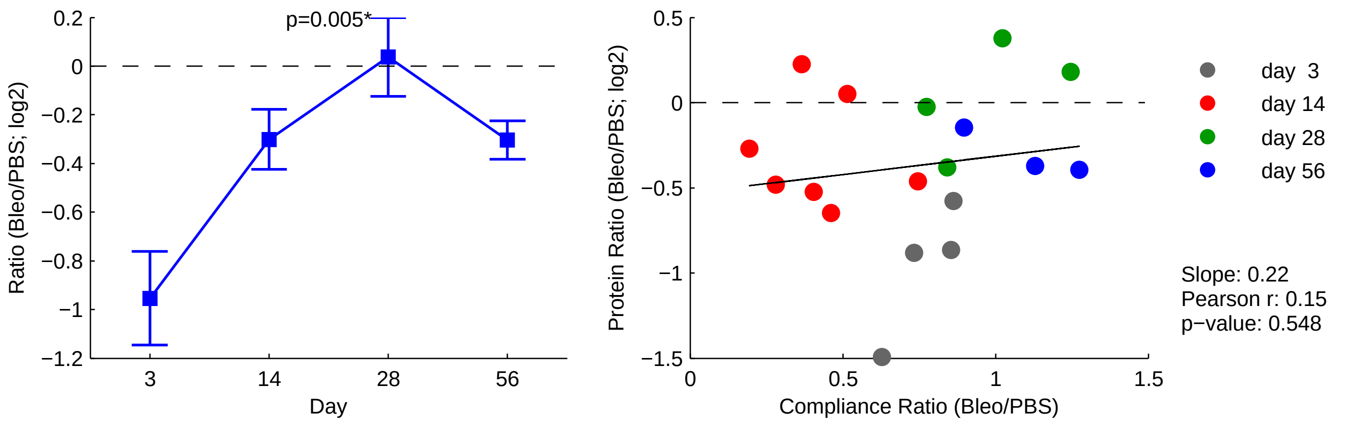

P70303 − Ctps2 (id: 3359)

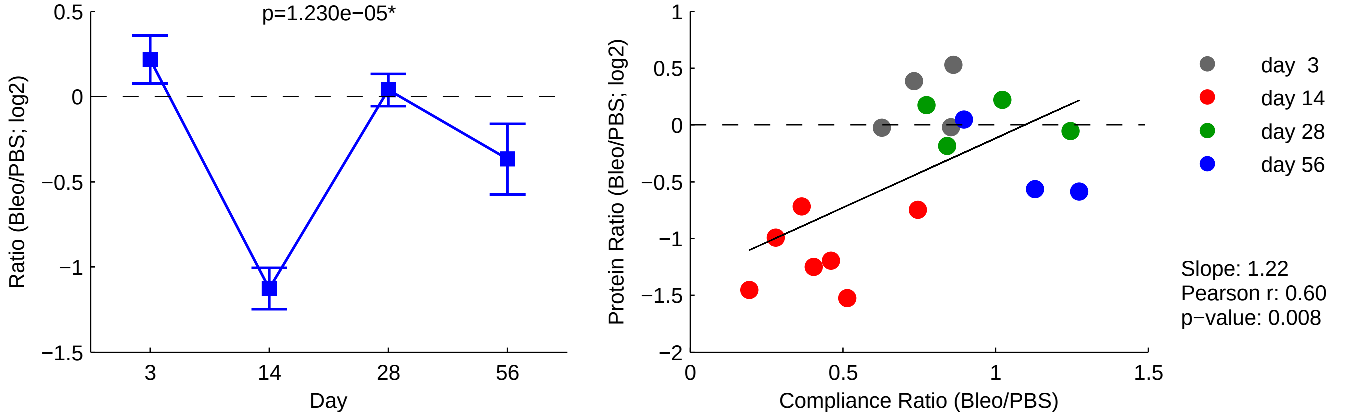

P70333 − Hnrnph2 (id: 3364)

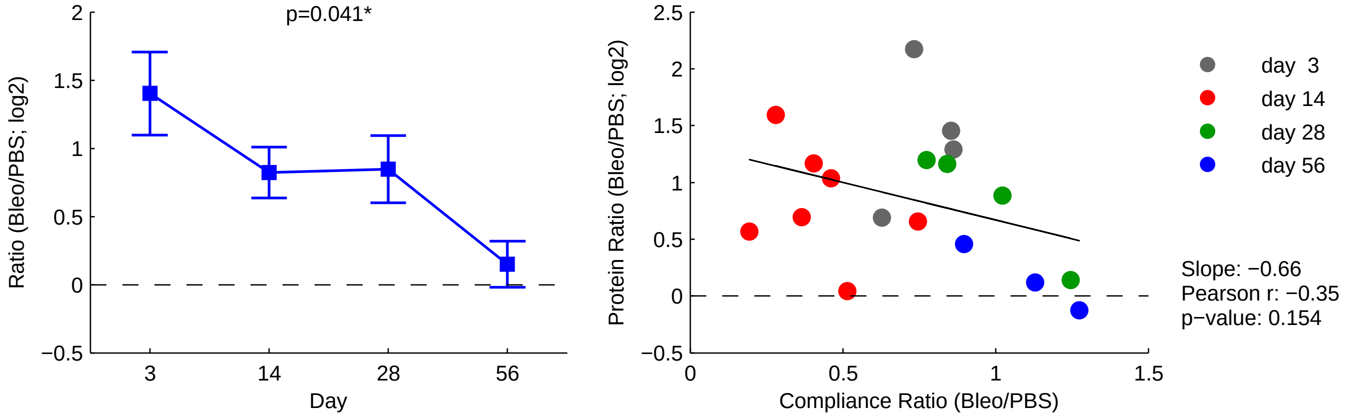

Q8C3Y6 − Smad1 (id: 3366)

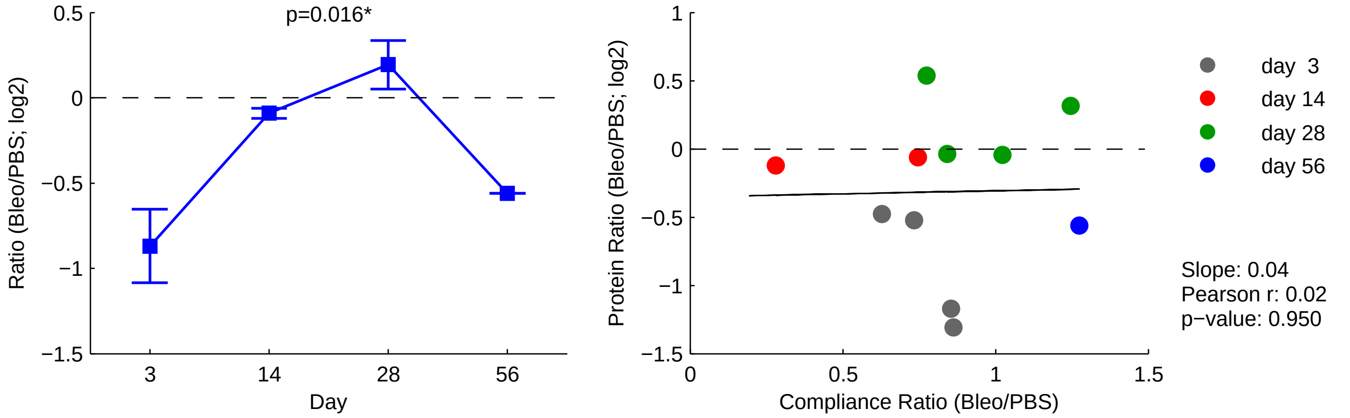

P70362 − Ufd1l (id: 3370)

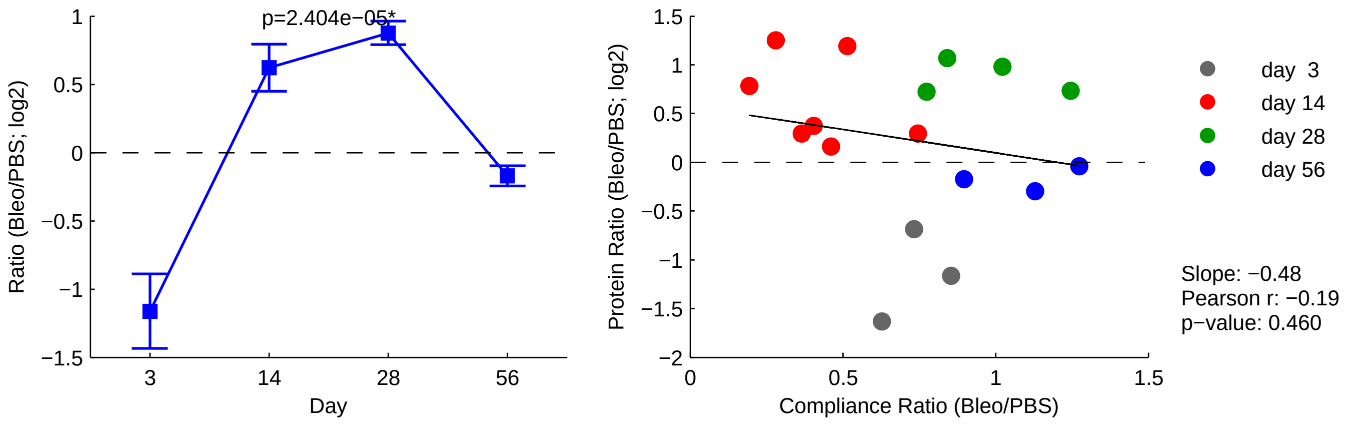

P70387 − Hfe (id: 3373)

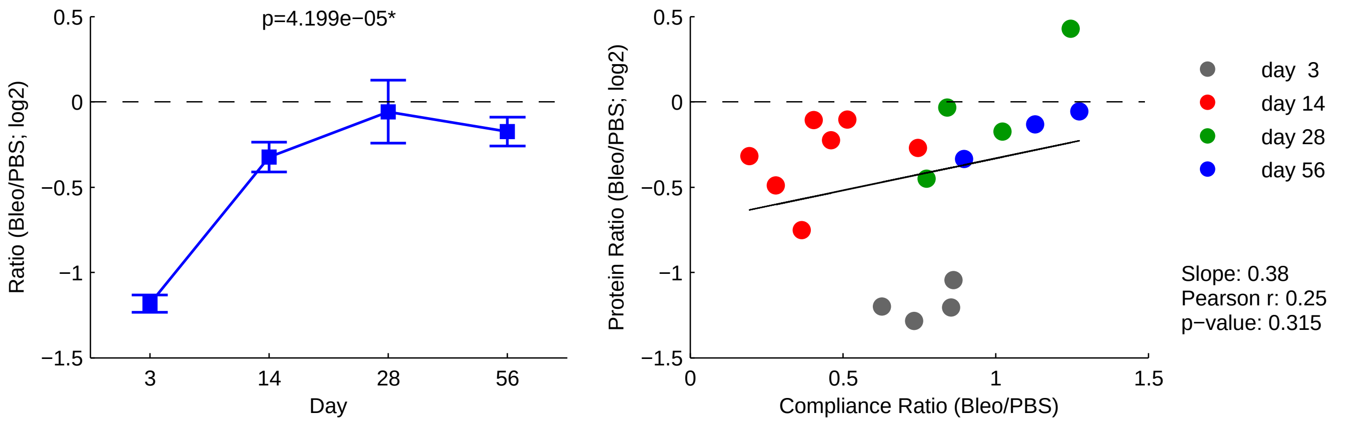

Q4FE56 − Usp9x (id: 3377)

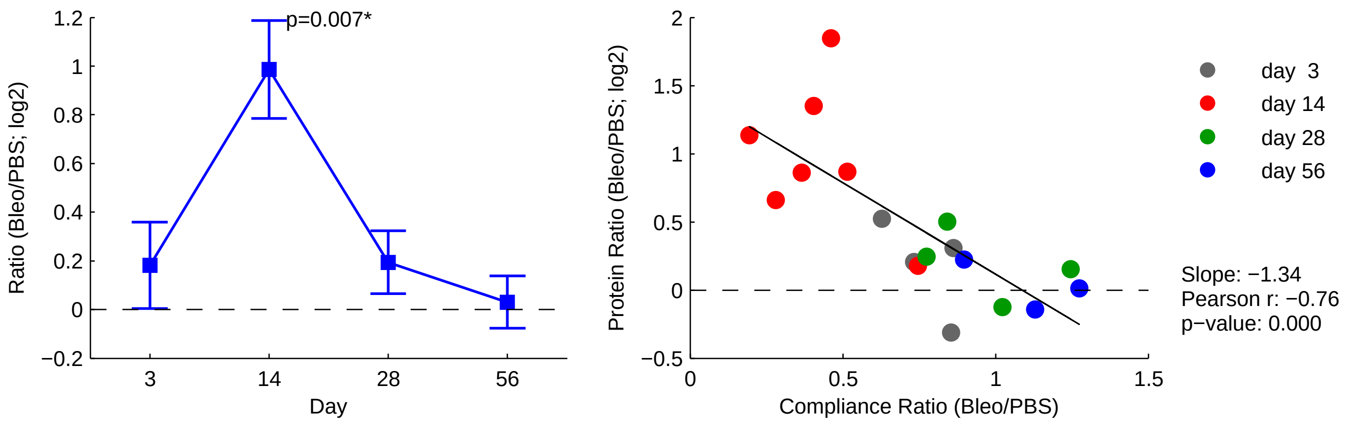

P70402 − Mybph (id: 3378)

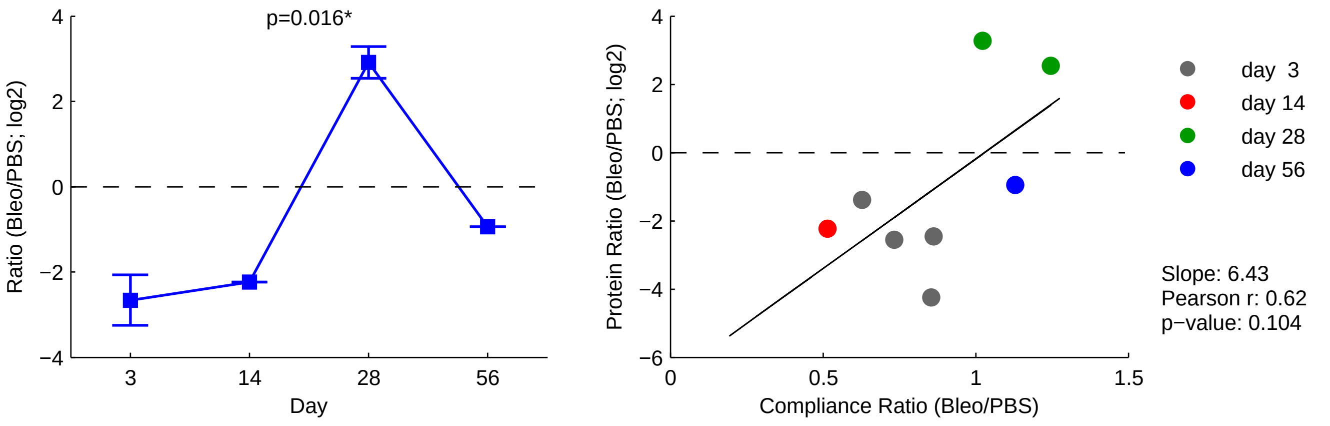

P70404 − Idh3g (id: 3379)

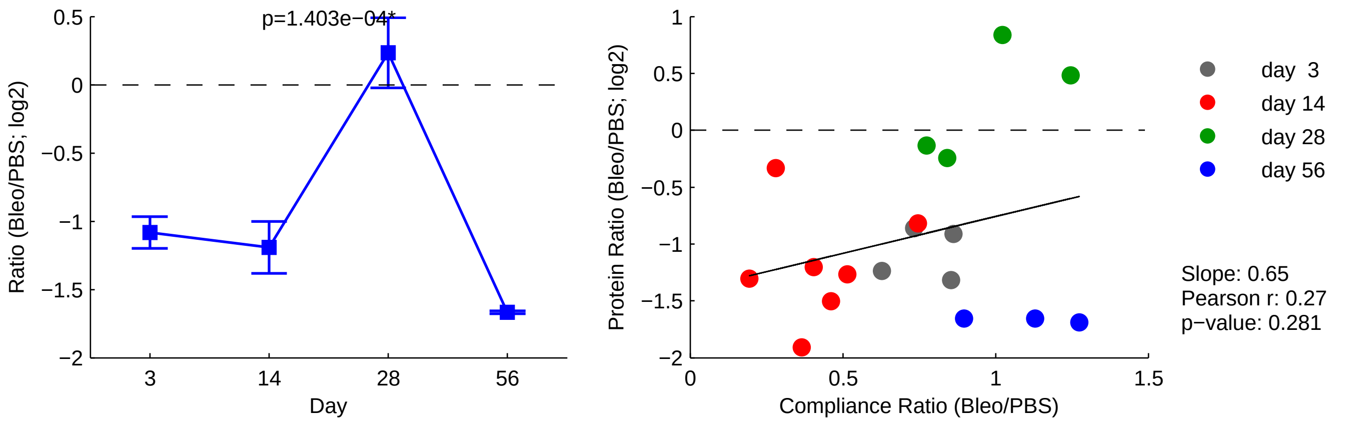

P70419 − Galnt3 (id: 3381)

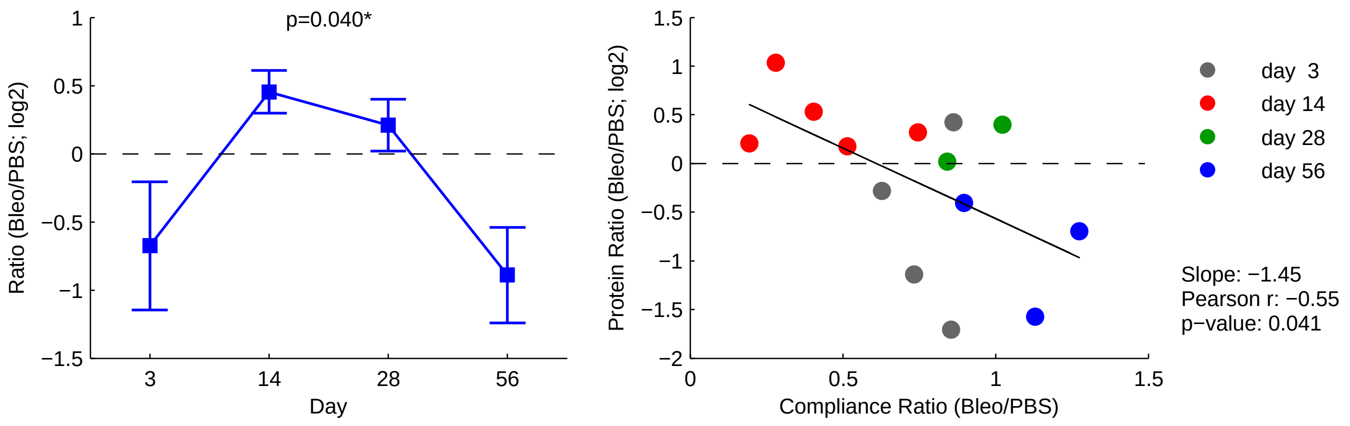

P70428 − Ext2 (id: 3383)

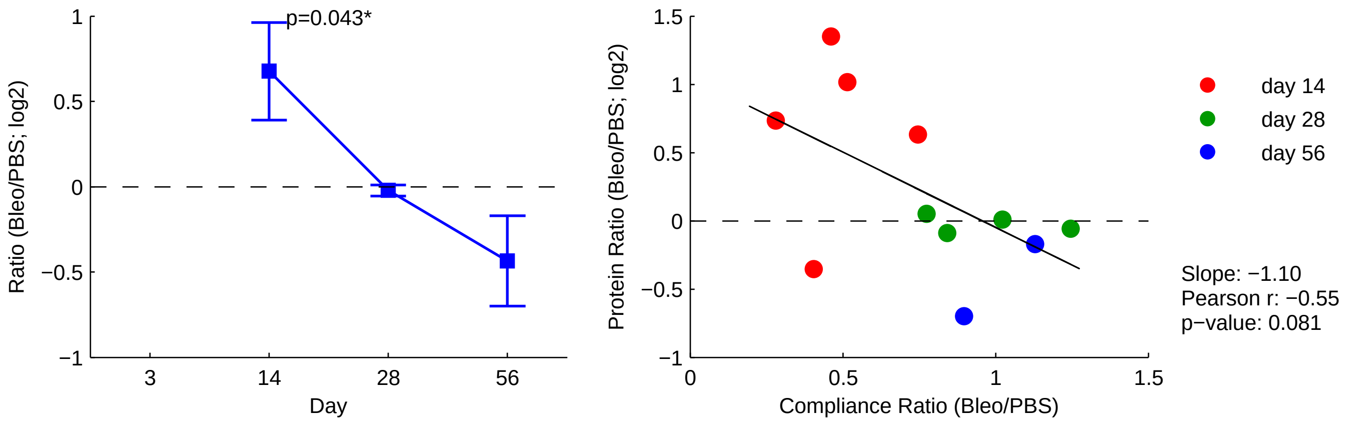

P70441 − Slc9a3r1 (id: 3385)

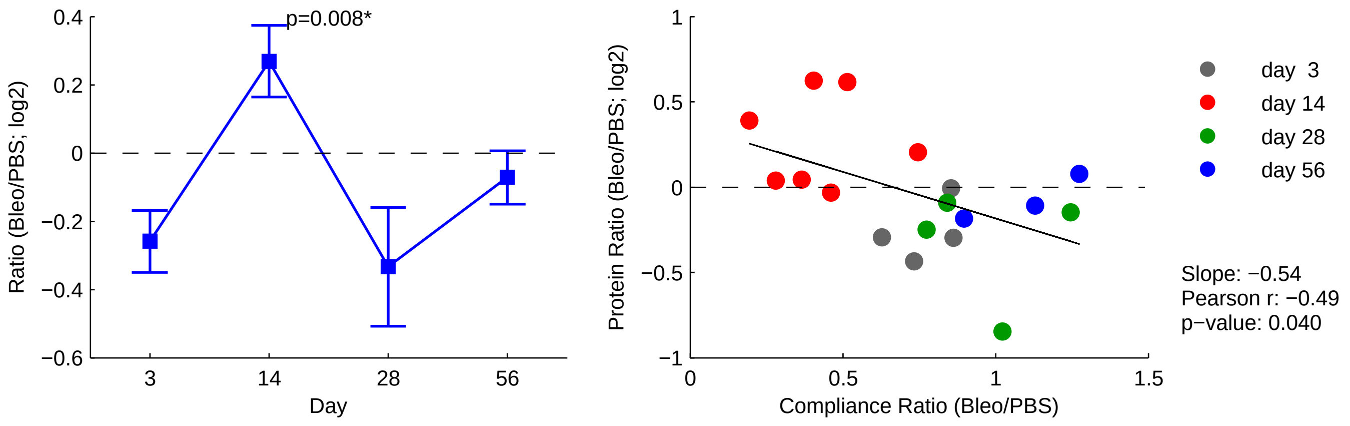

P70451−5 − Fer (id: 3387)

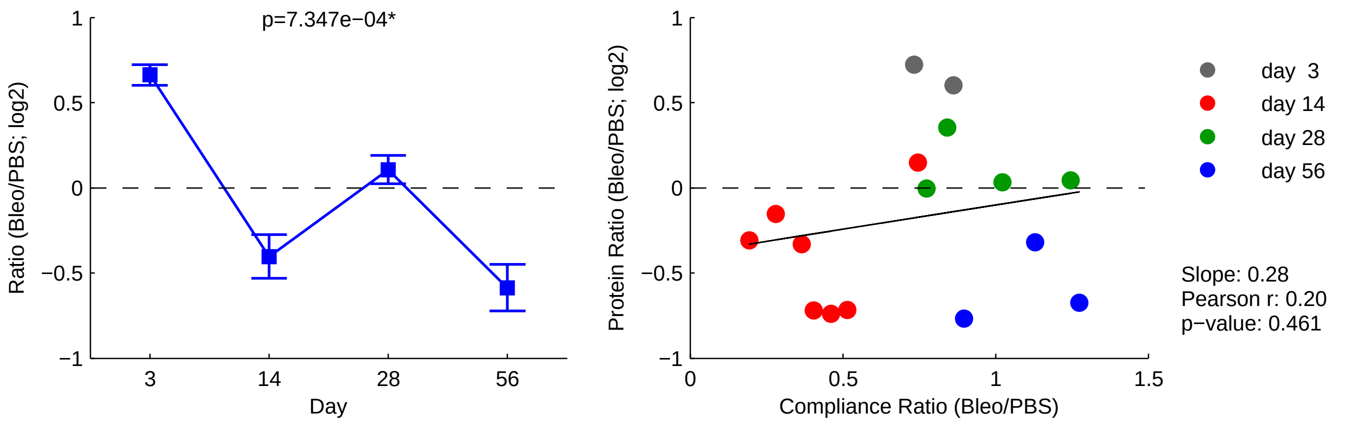

P70452 − Stx4 (id: 3388)

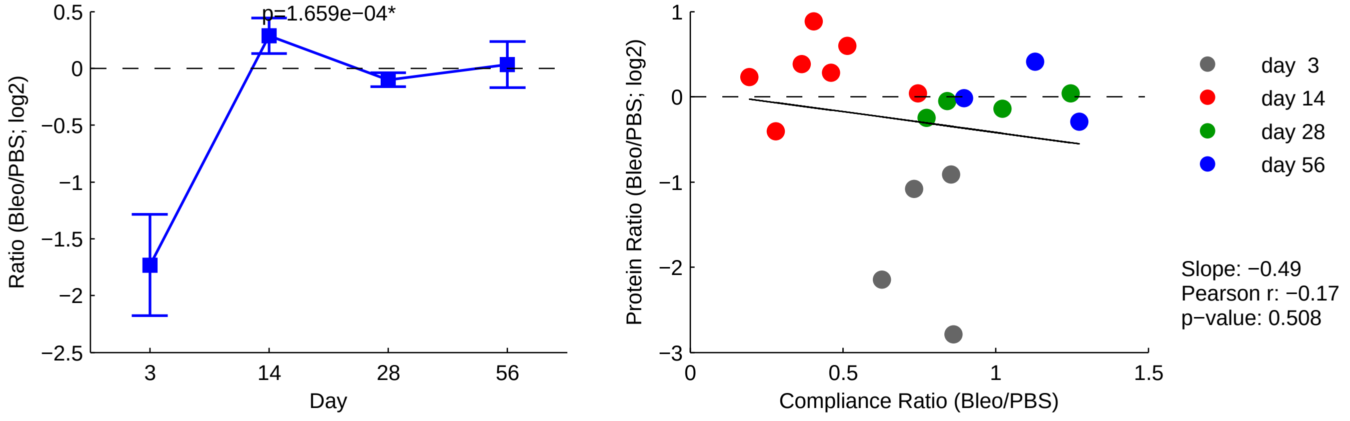

P70460 − Vasp (id: 3389)

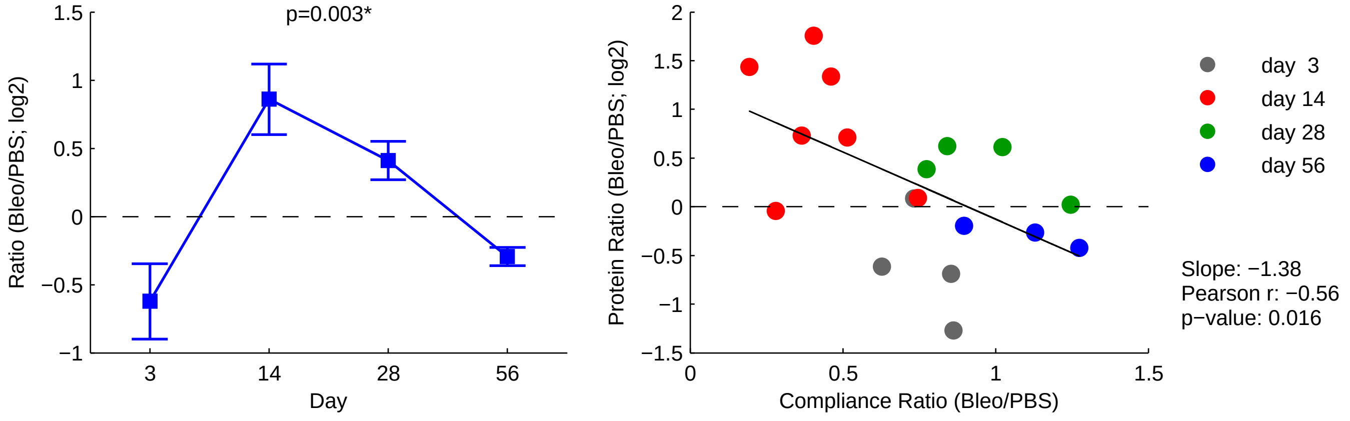

P70658−2 − Cxcr4 (id: 3390)

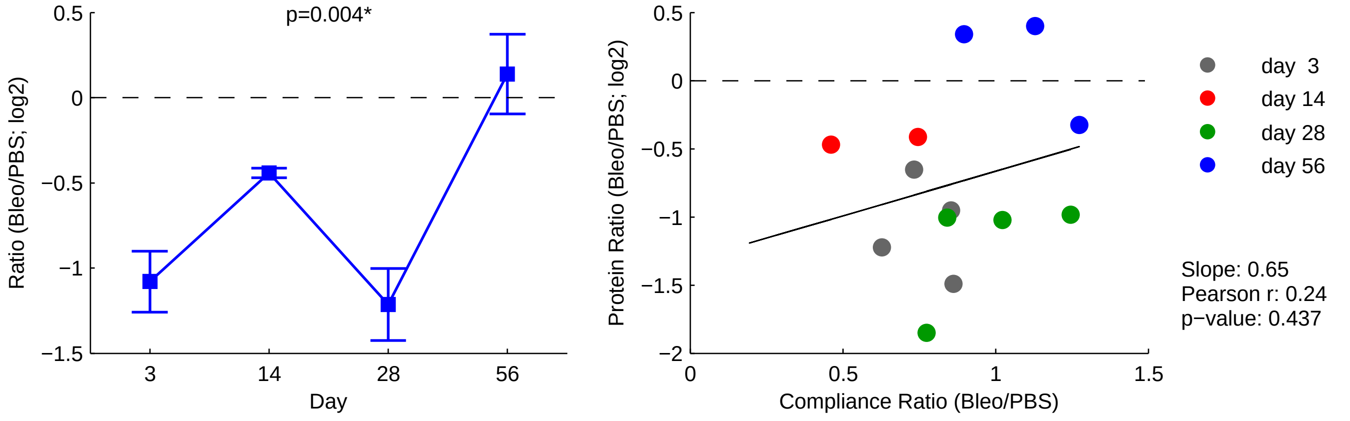

P70663 − Sparcl1 (id: 3391)

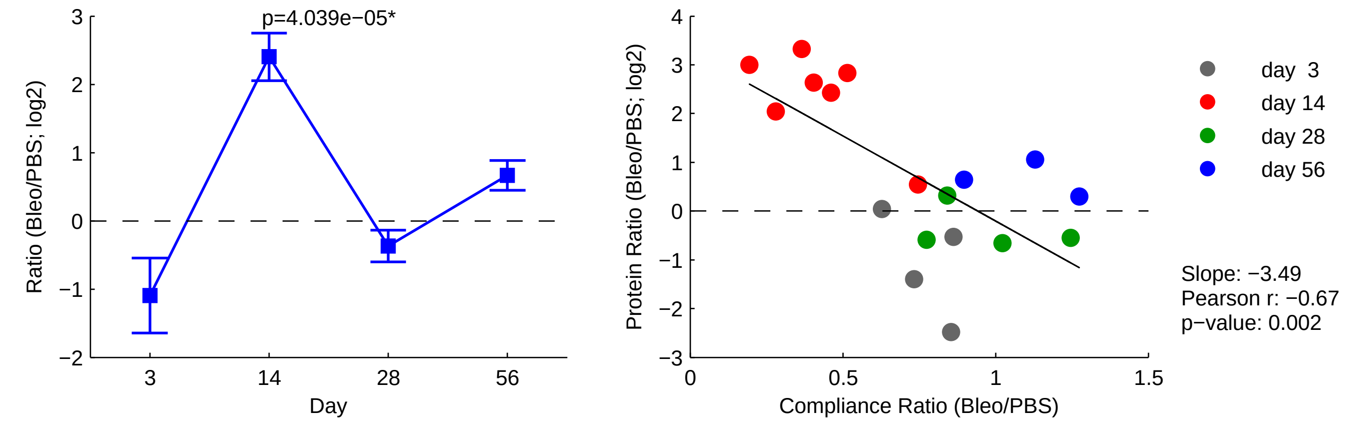

P70669 − Phex (id: 3392)

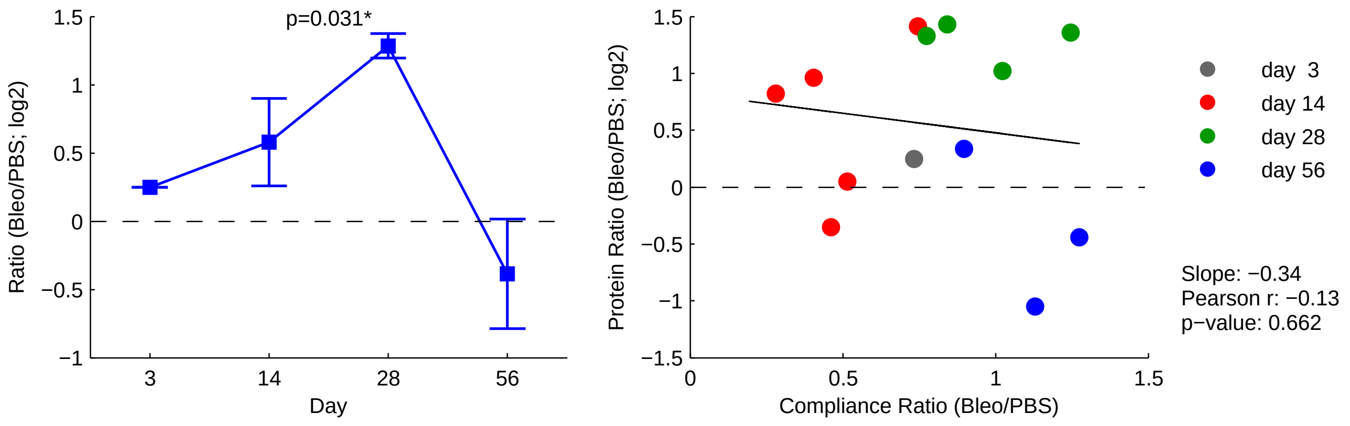

P70670 − Naca (id: 3393)

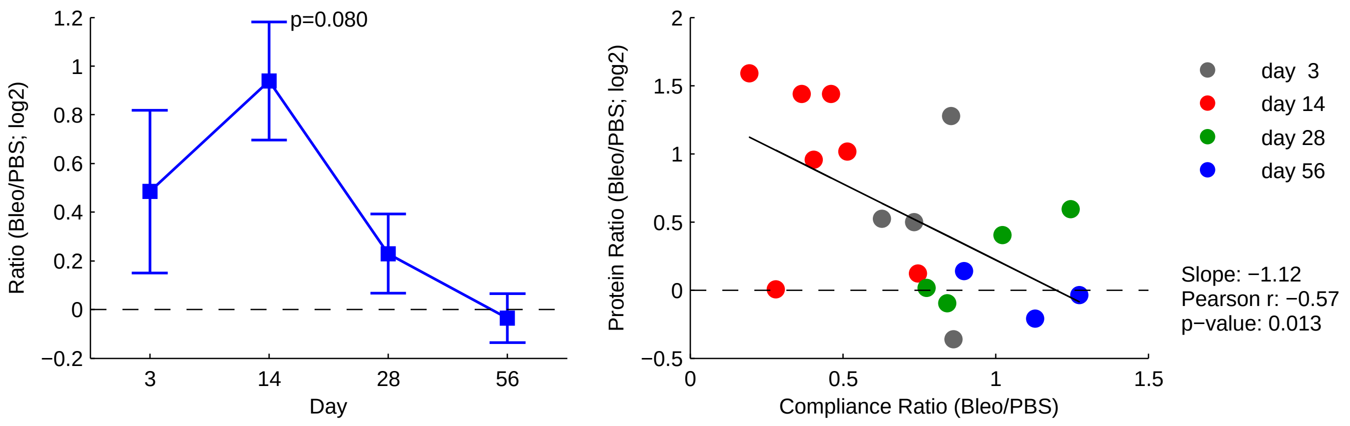

P70671 − Irf3 (id: 3394)

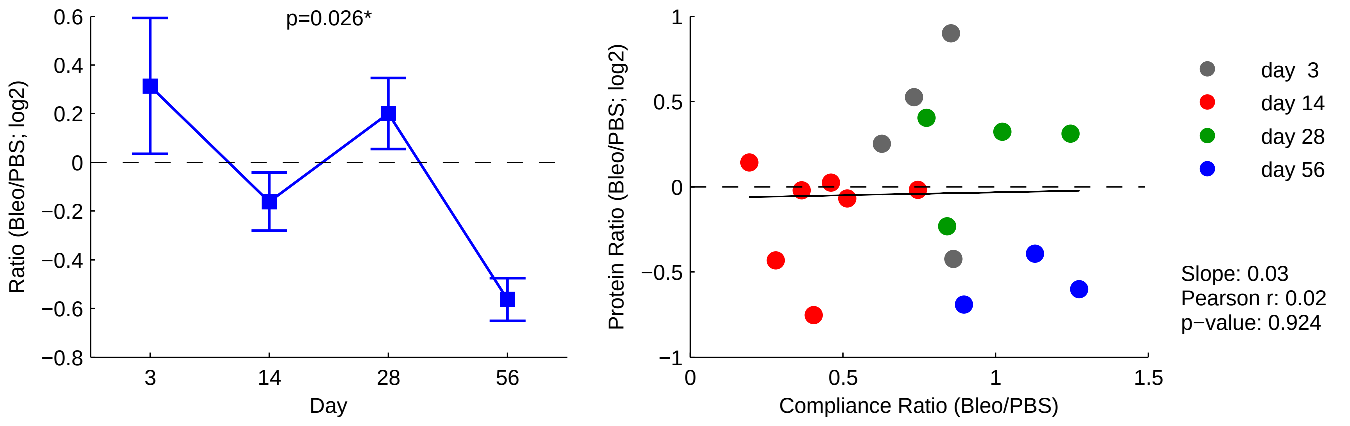

P70698 − Ctps1 (id: 3397)

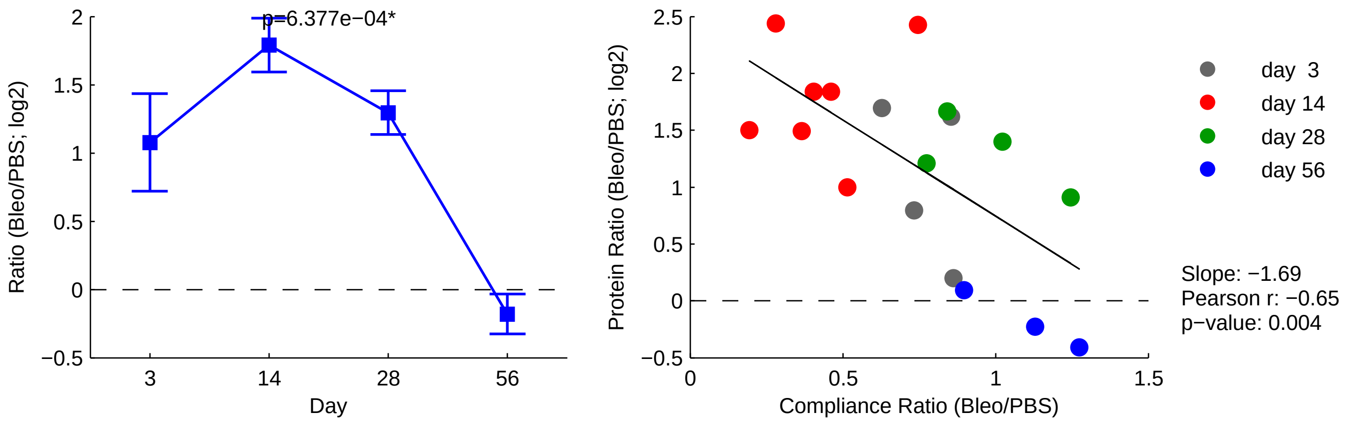

P70699 − Gaa (id: 3398)

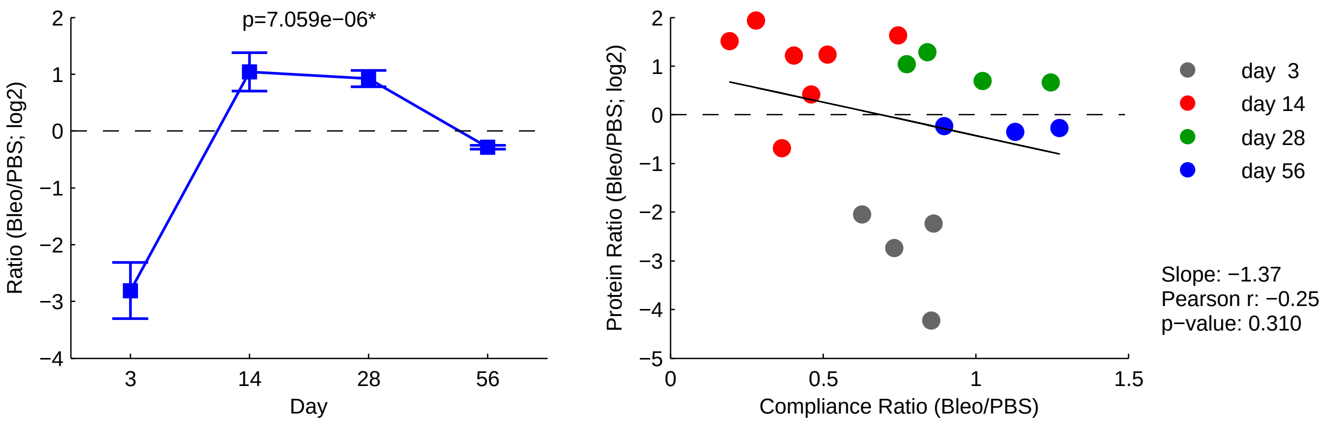

P80316 − Cct5 (id: 3404)

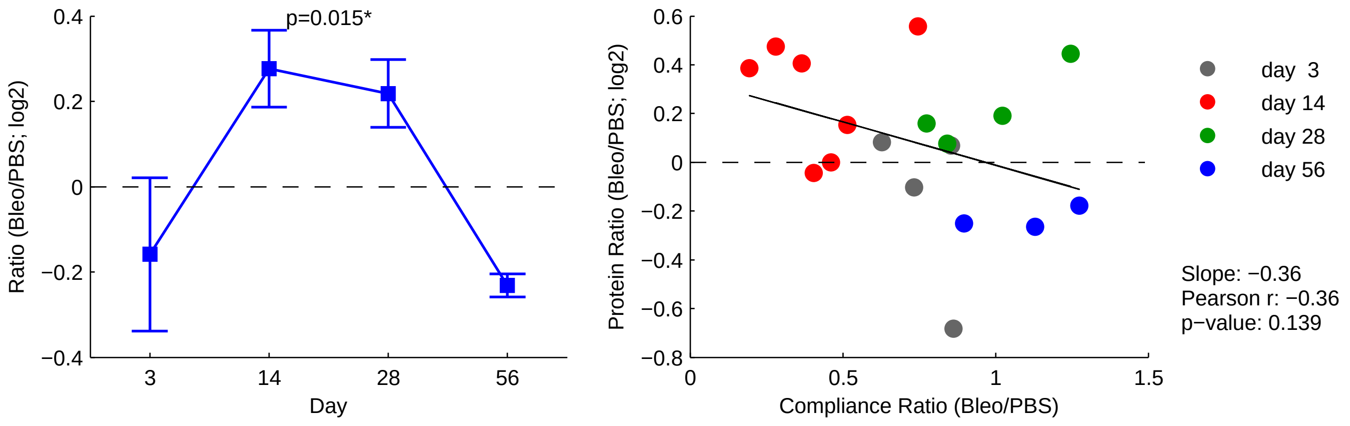

P80317 − Cct6a (id: 3405)

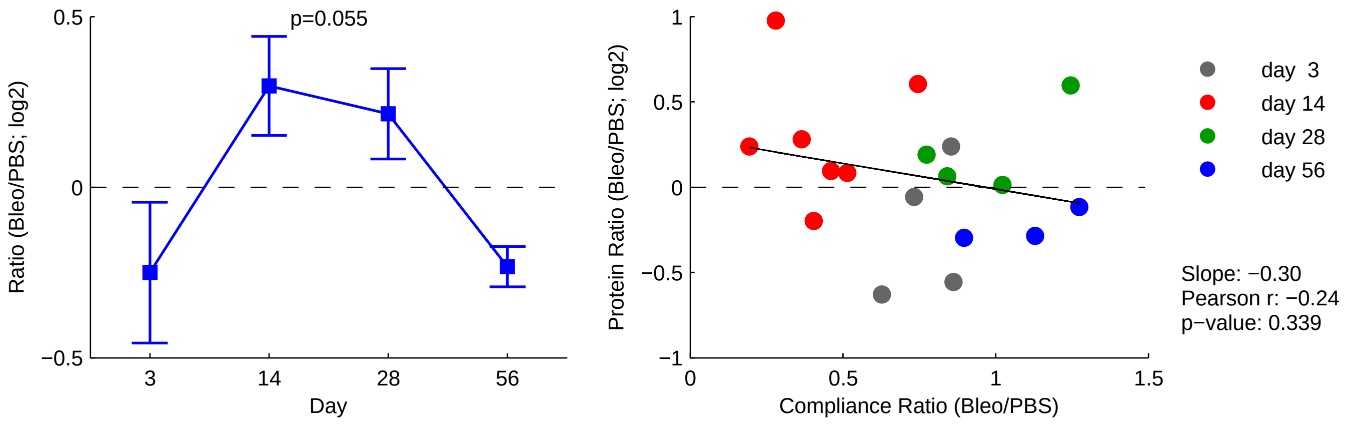

P81117 − Nucb2 (id: 3408)

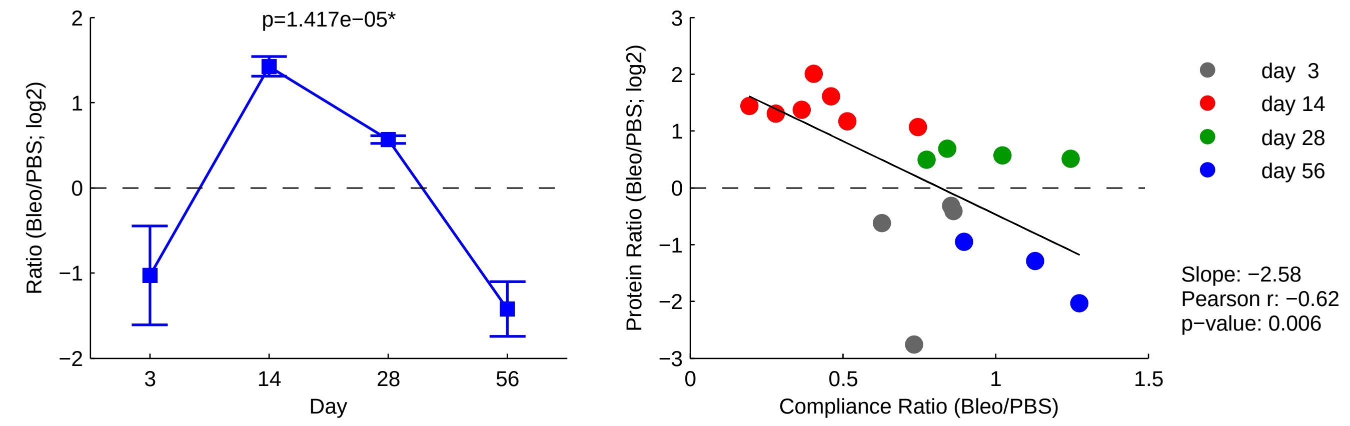

P82198 − Tgfbi (id: 3411)

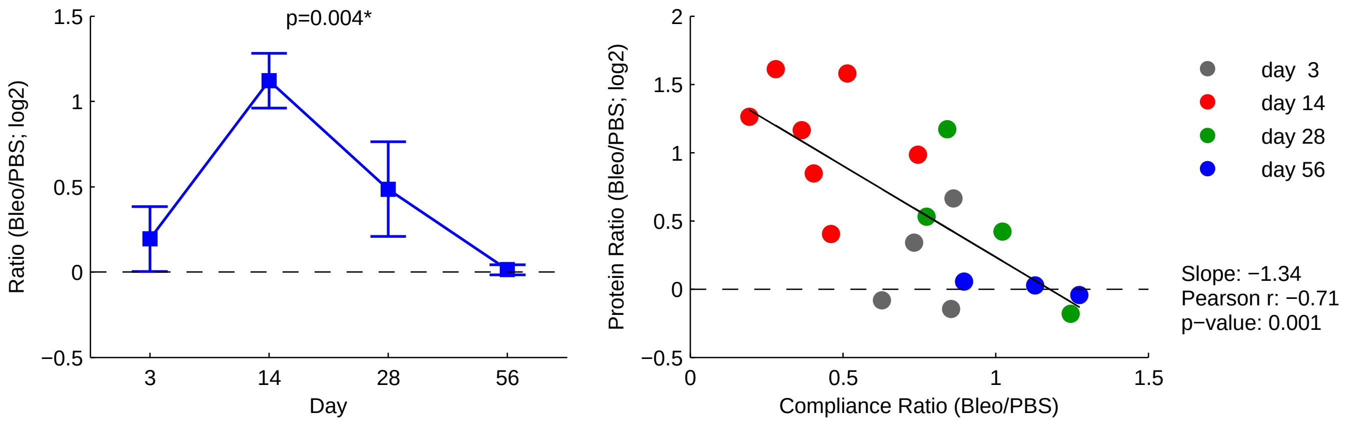

P82347 − Sgcd (id: 3413)

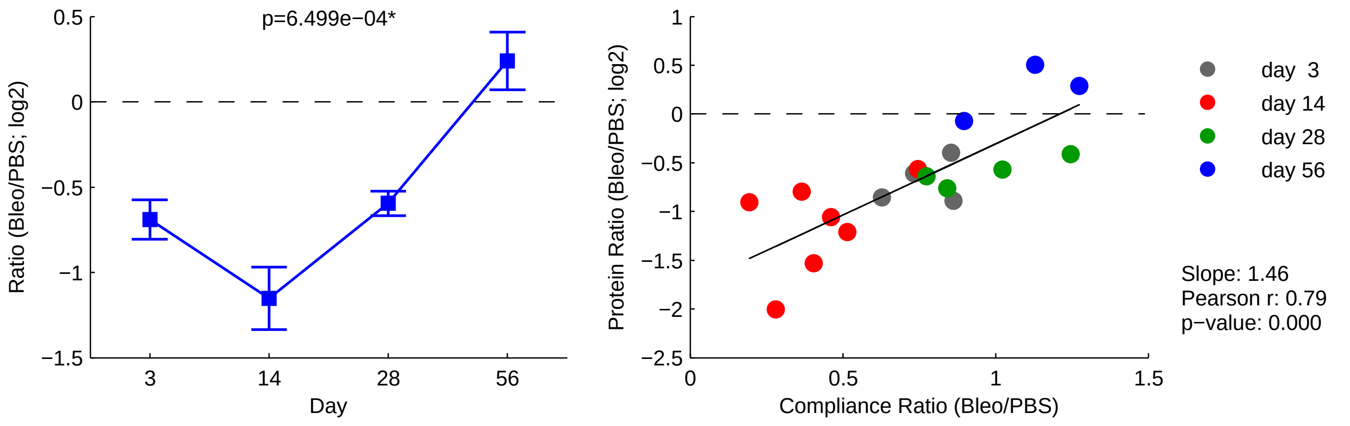

P83741−4 − Wnk1 (id: 3417)

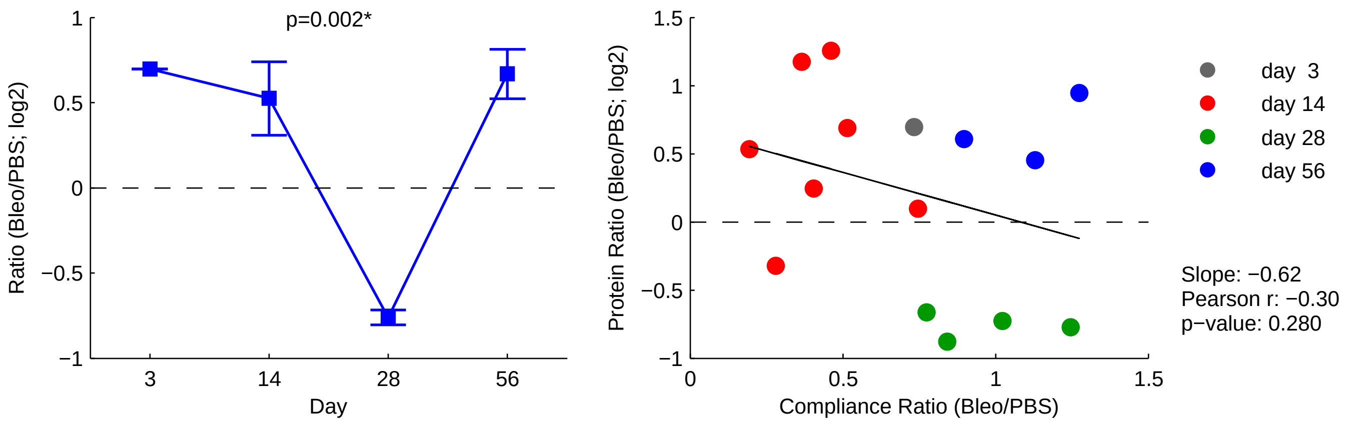

P83870 − Phf5a (id: 3418)

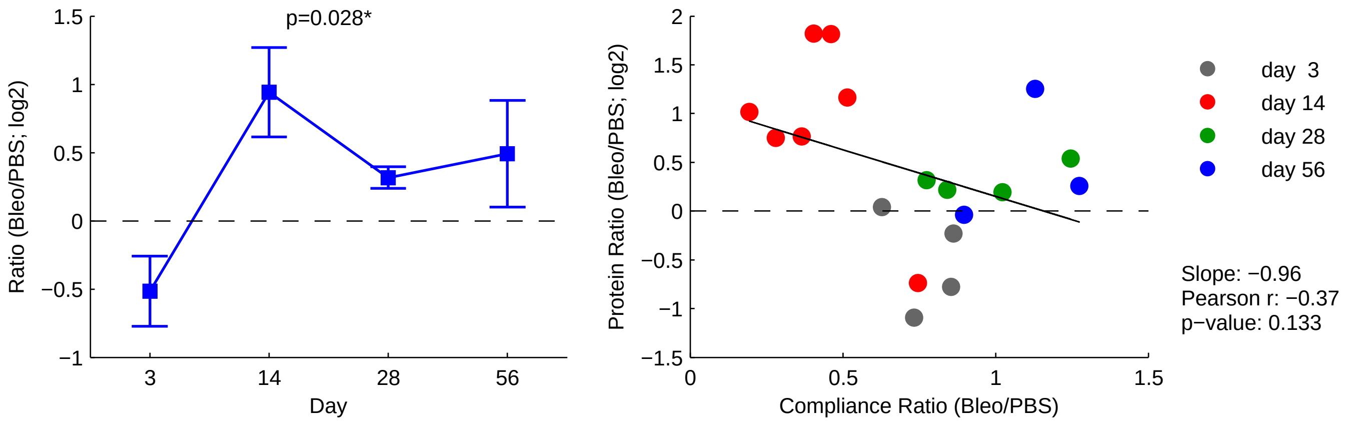

P83882 − Rpl36a (id: 3420)

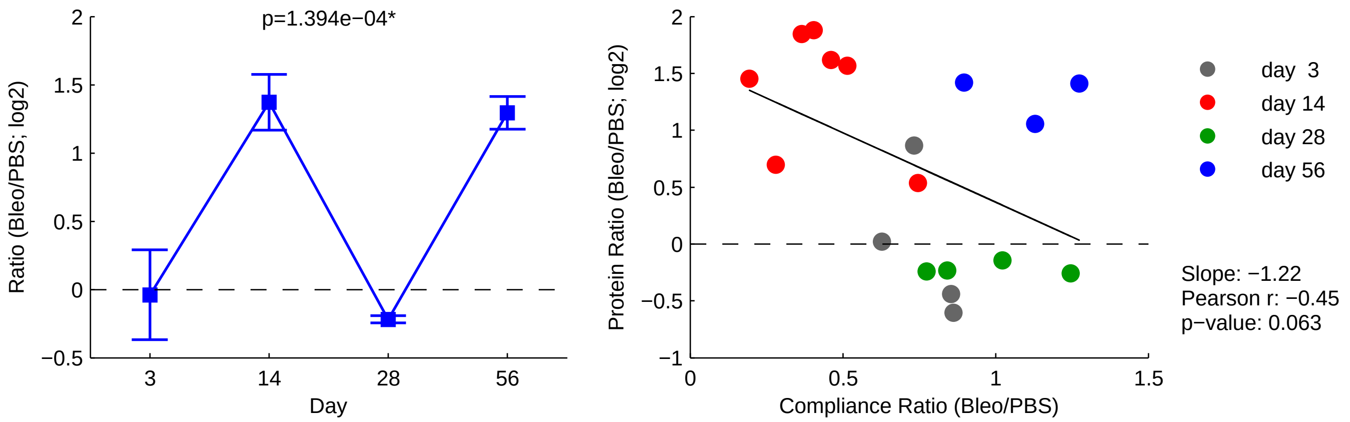

P83887 − Tubg1 (id: 3421)

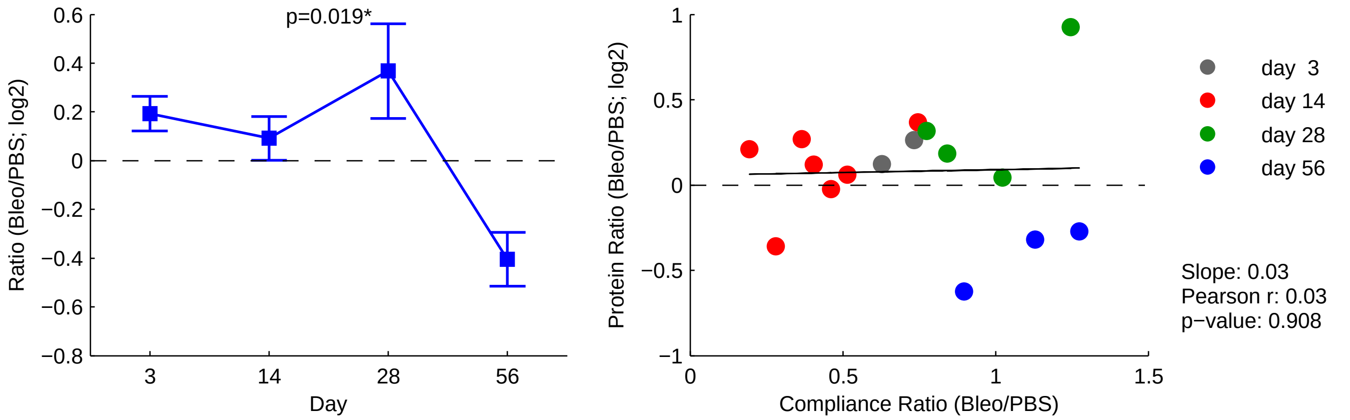

P83917 − Cbx1 (id: 3422)

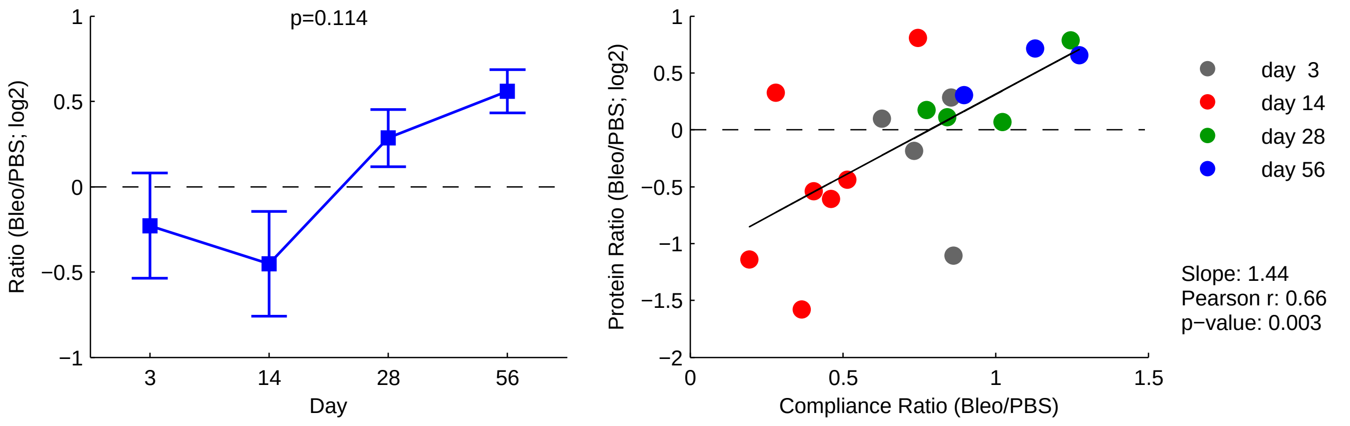

P83940 − Tceb1 (id: 3423)

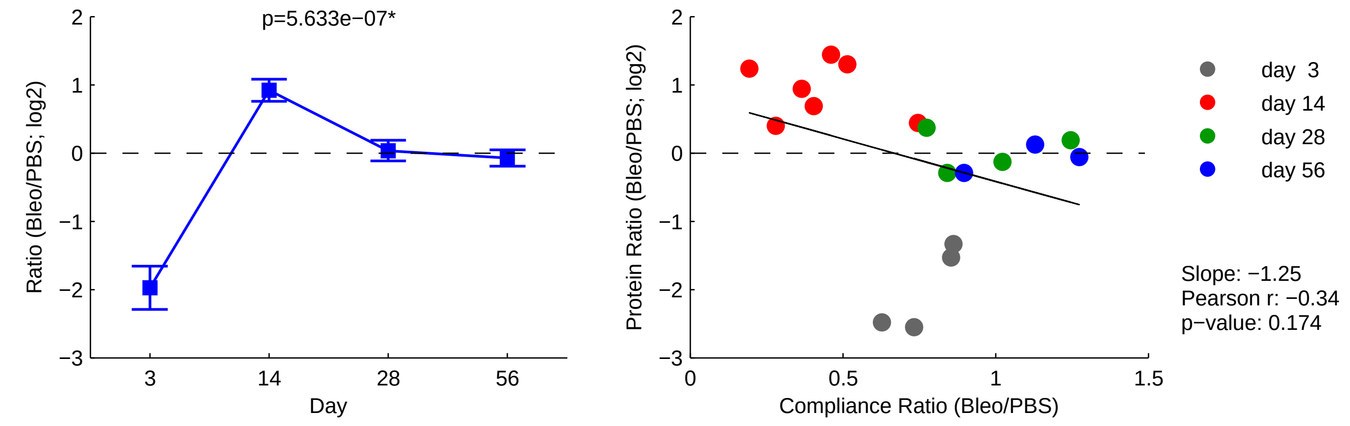

P84078 − Arf1 (id: 3424)

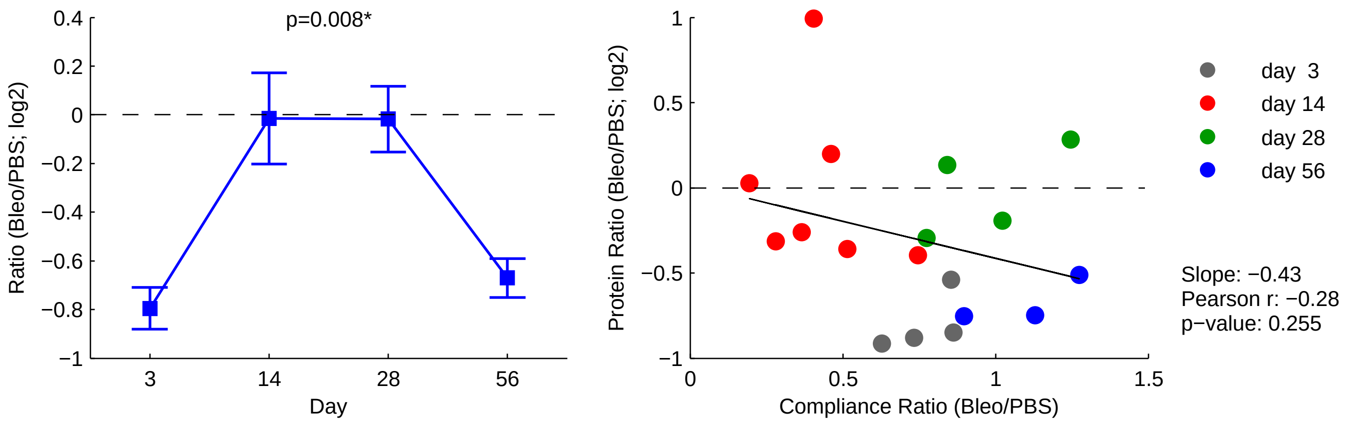

P84084 − Arf5 (id: 3425)

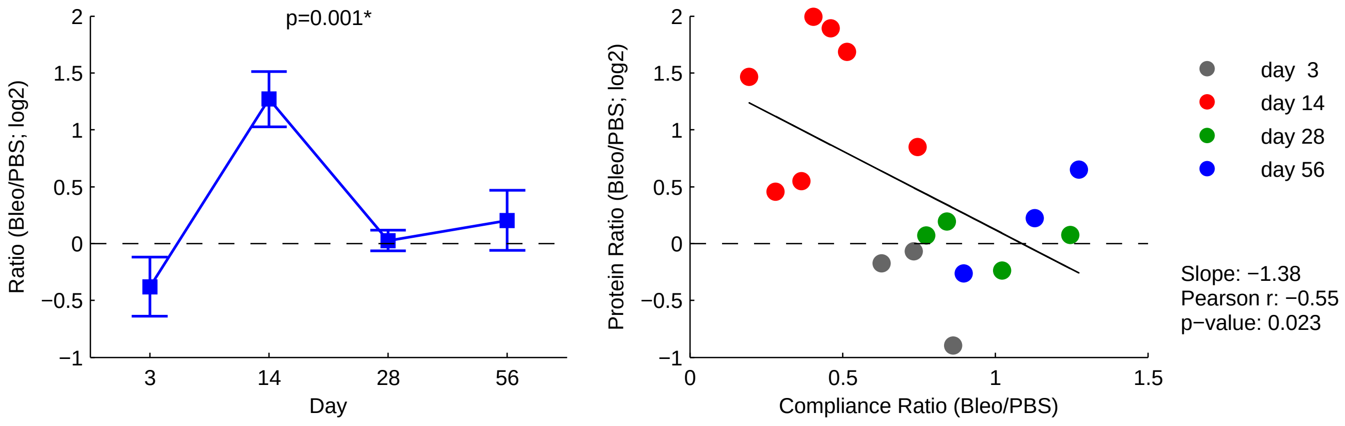

P84089 − Erh (id: 3426)

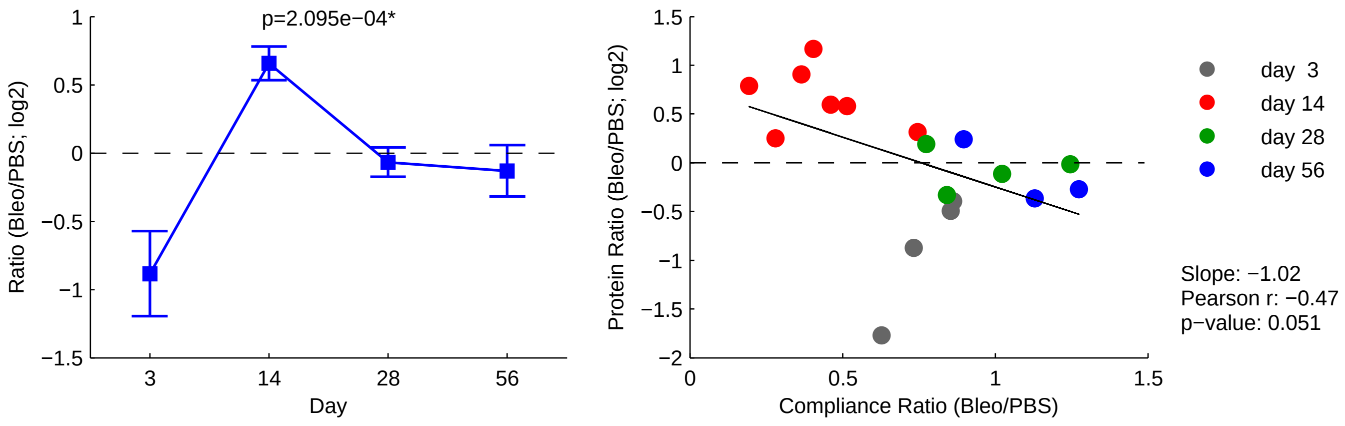

Q3TWV4 − Ap2m1 (id: 3427)

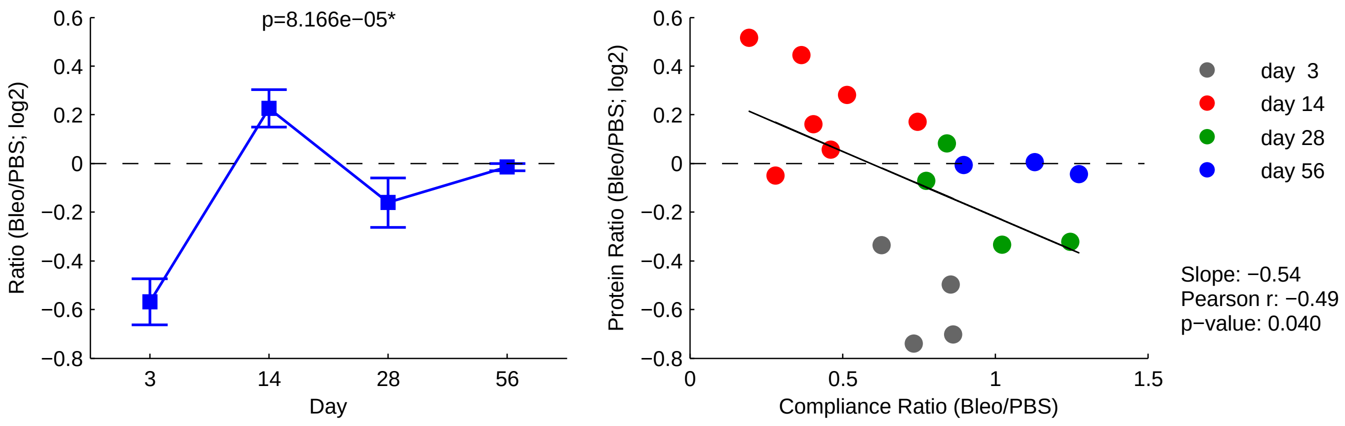

P84096 − Rhog (id: 3428)

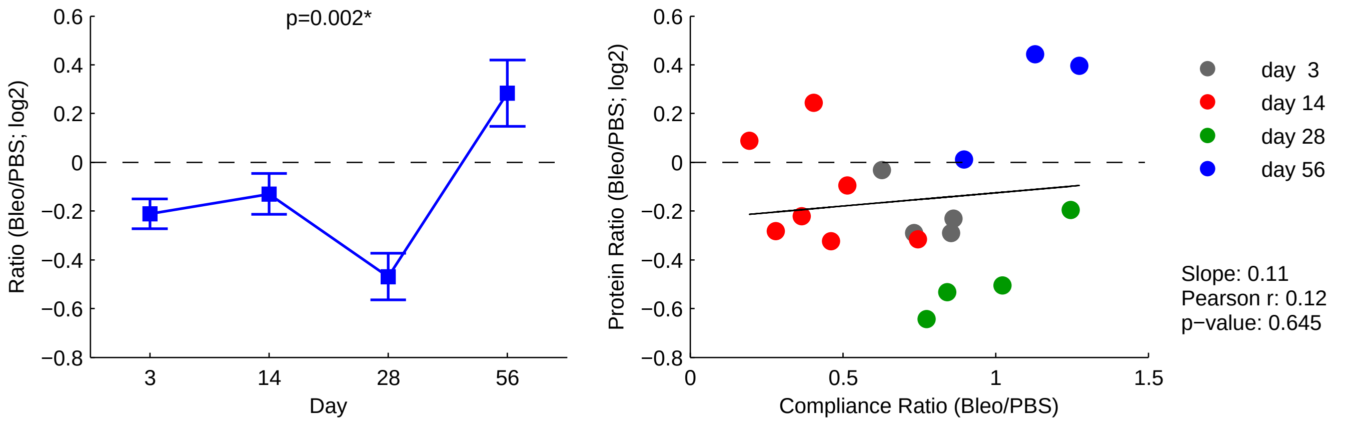

P85094 − Isoc2a (id: 3432)

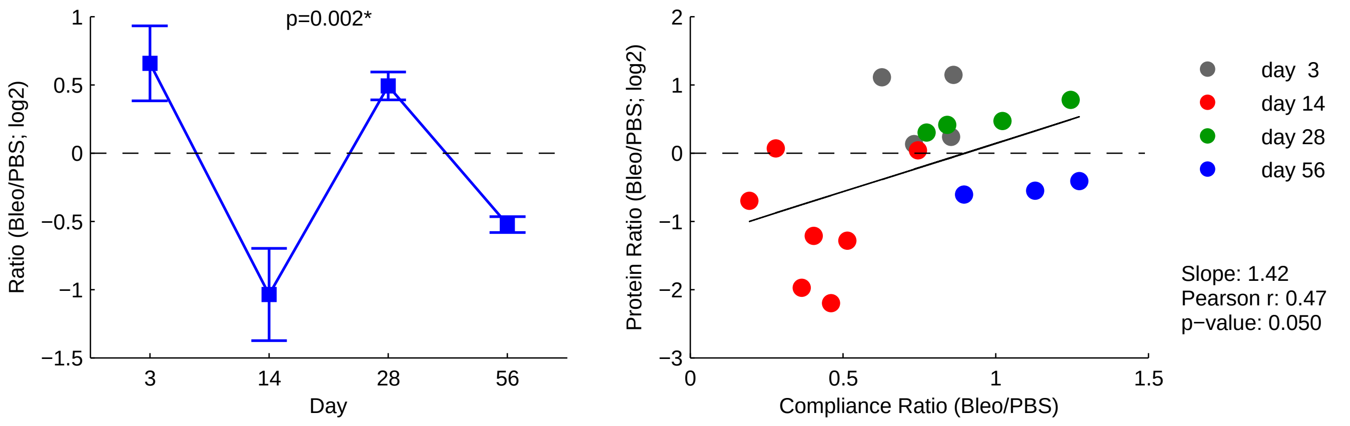

P97290 − Serping1 (id: 3434)

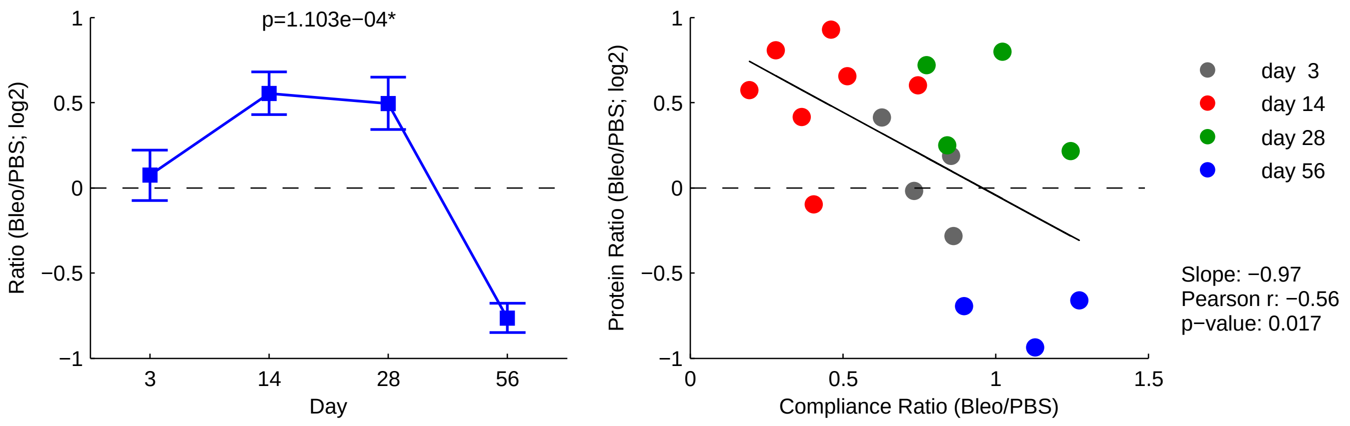

P97314 − Csrp2 (id: 3442)

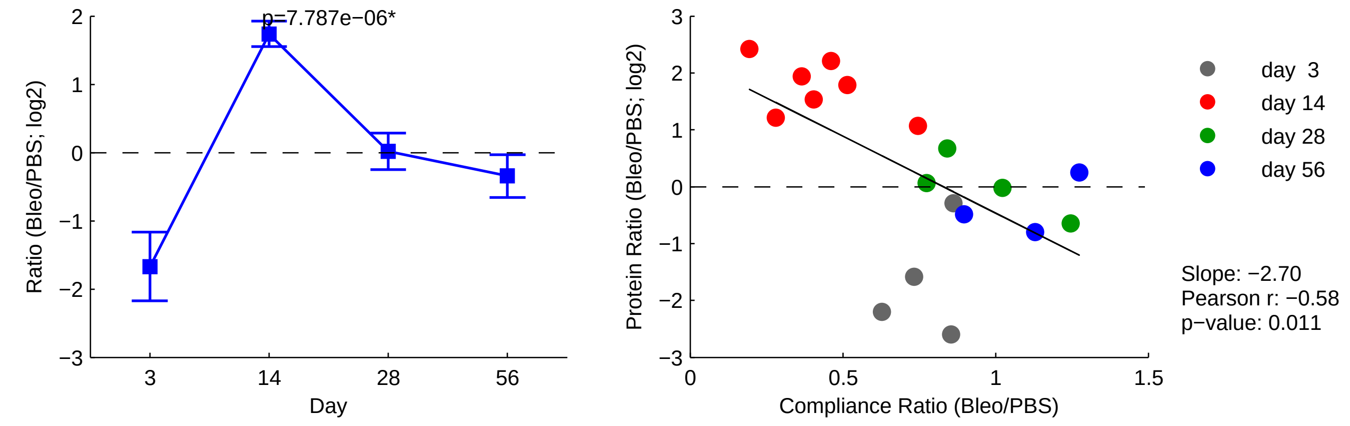

P97315 − Csrp1 (id: 3443)

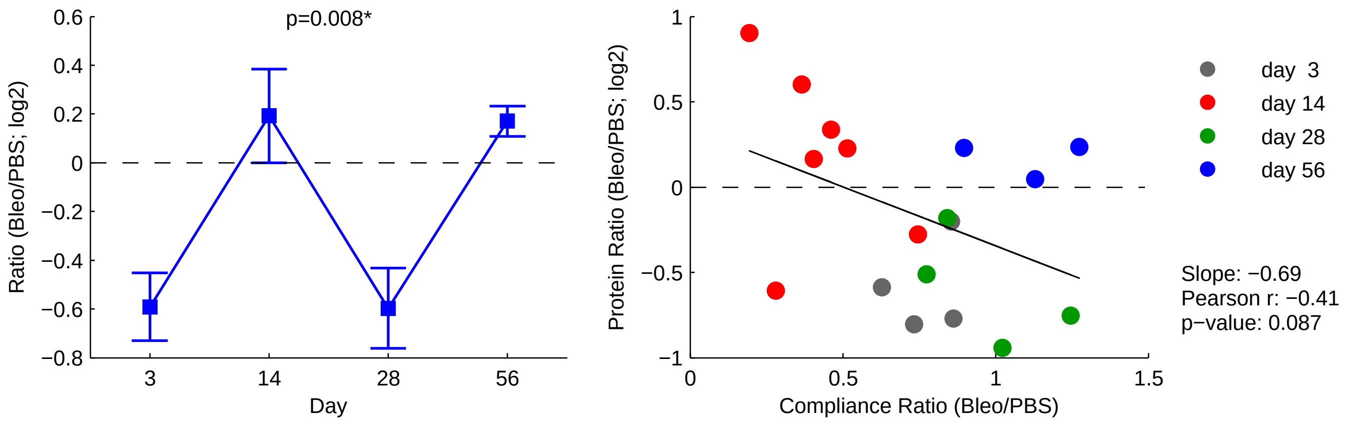

P97333 − Nrp1 (id: 3444)

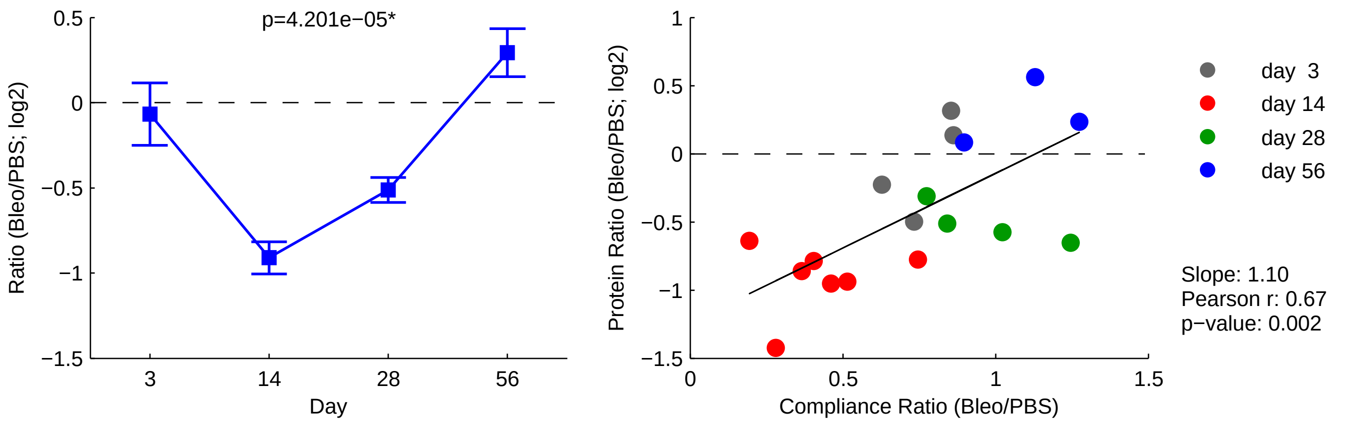

P97351 − Rps3a (id: 3449)

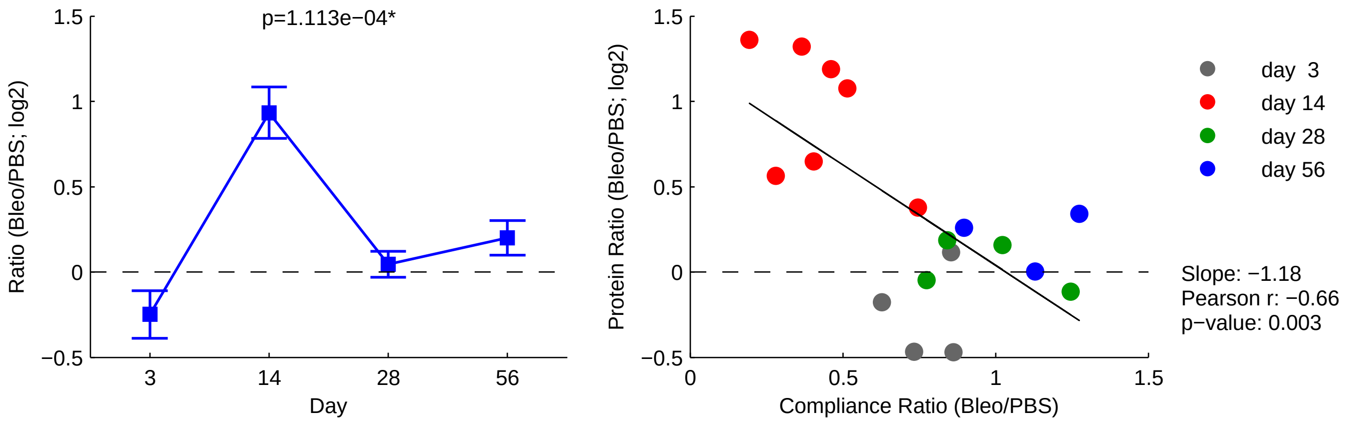

P97360 − Etv6 (id: 3451)

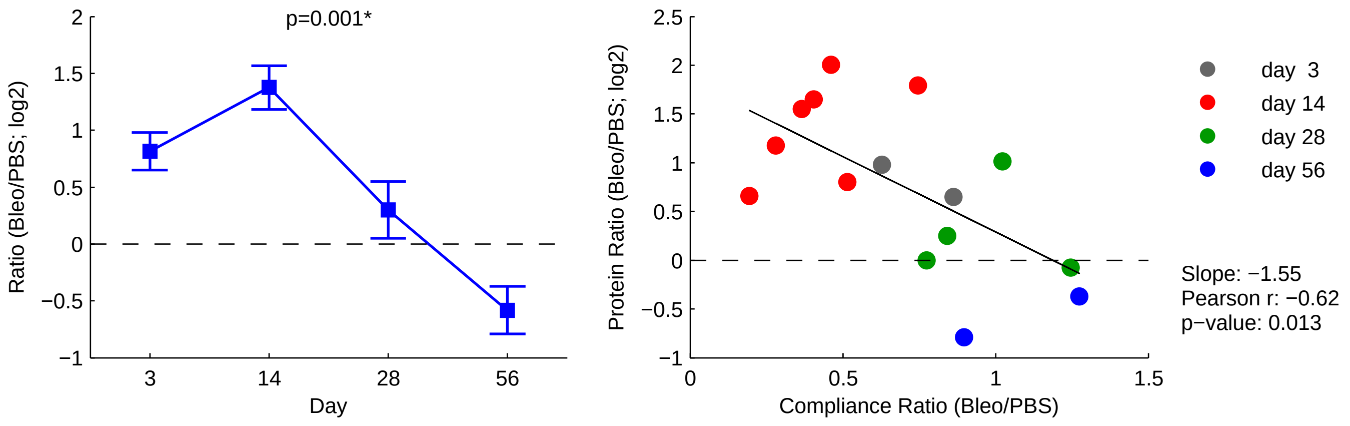

P97361 − Bpifa1 (id: 3452)

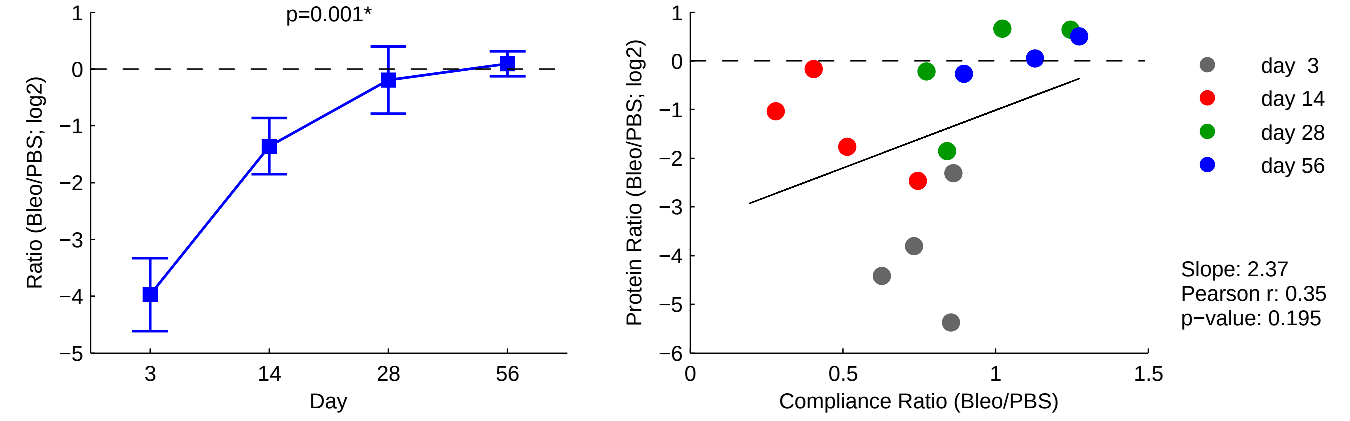

P97363 − Sptlc2 (id: 3453)

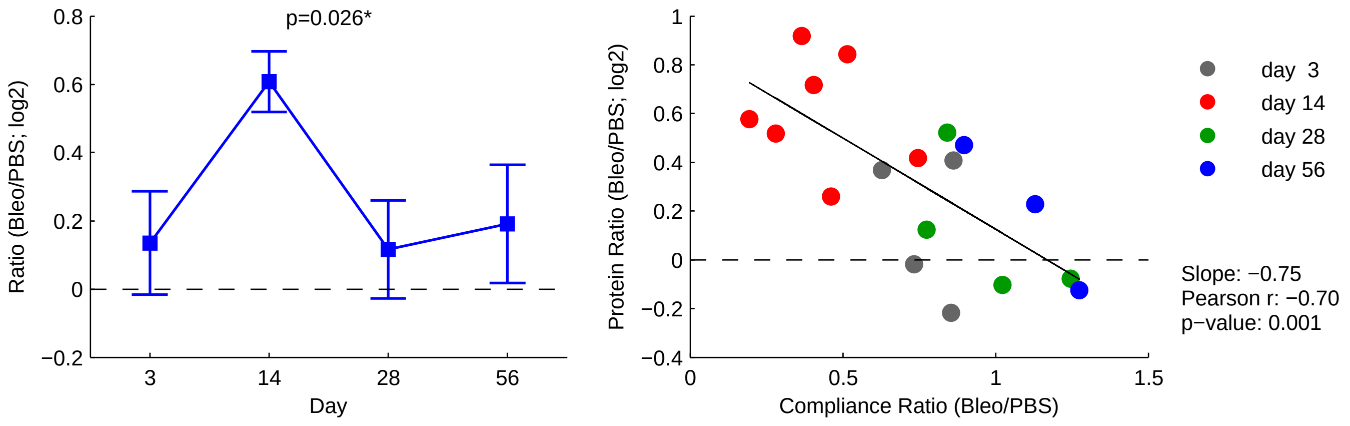

P97364 − Sephs2 (id: 3454)

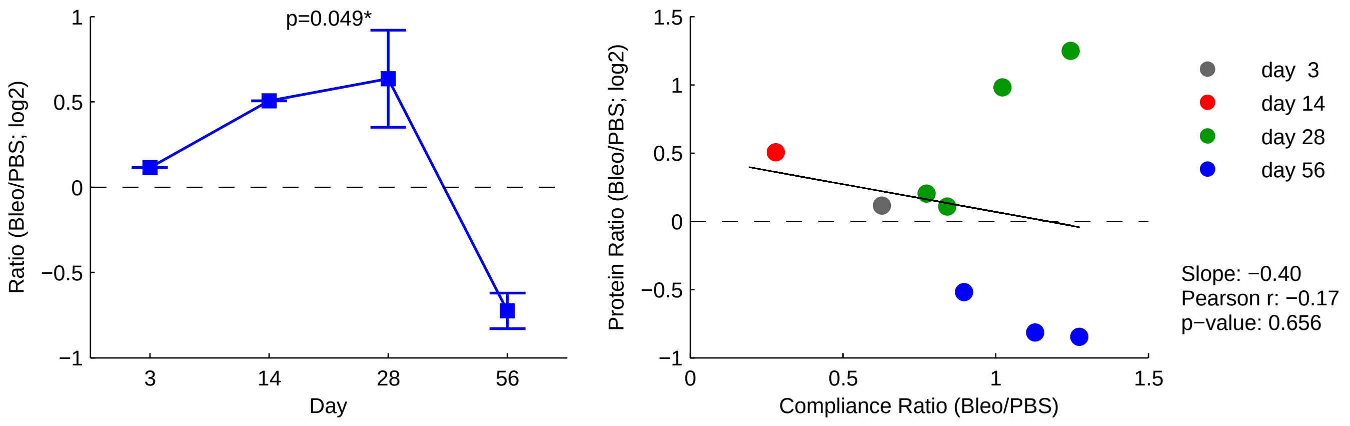

P97369 − Ncf4 (id: 3455)

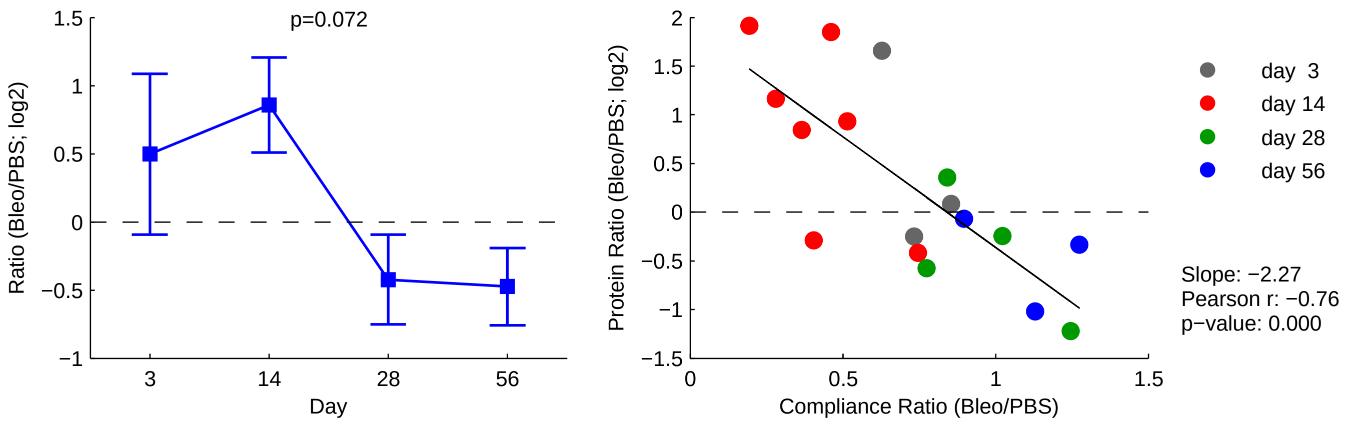

P97370 − Atp1b3 (id: 3456)

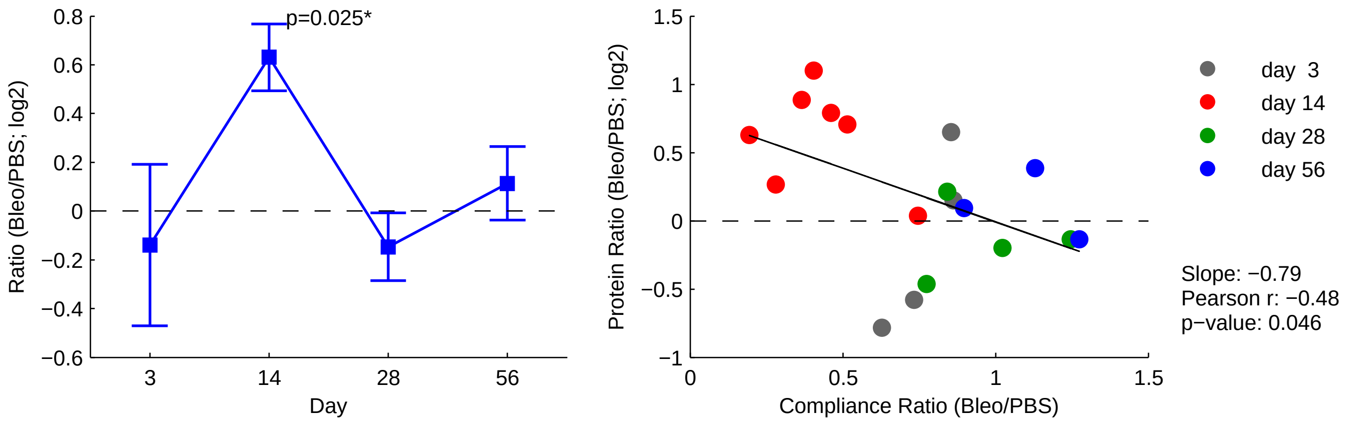

P97372 − Psme2 (id: 3457)

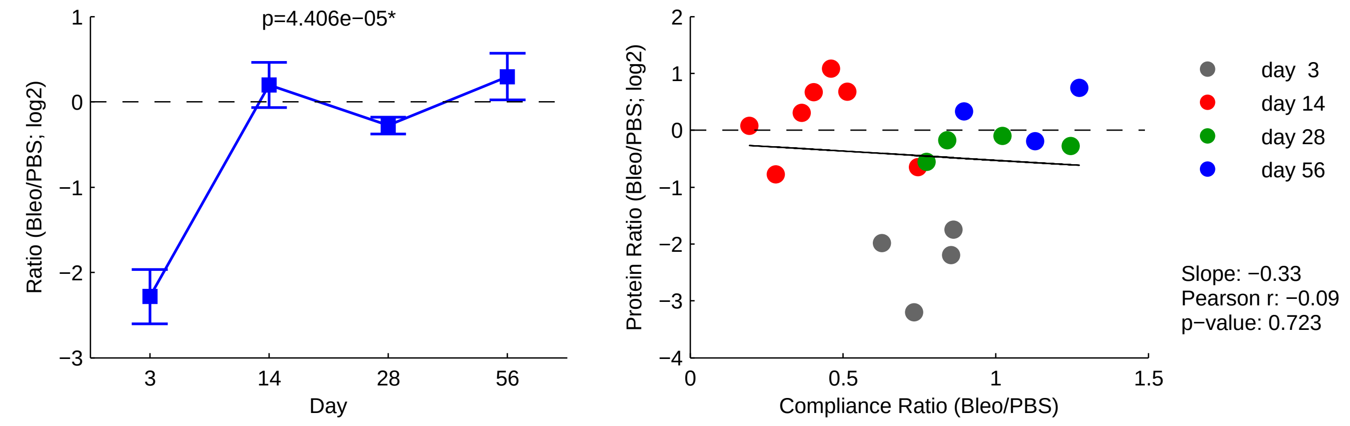

P97377−2 − Cdk2 (id: 3459)

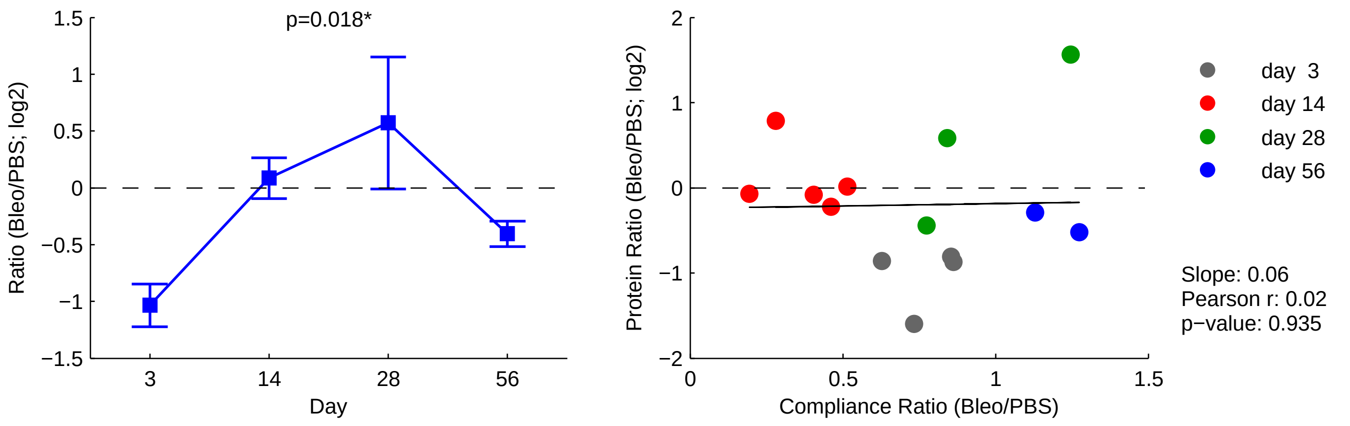

P97379 − G3bp2 (id: 3460)

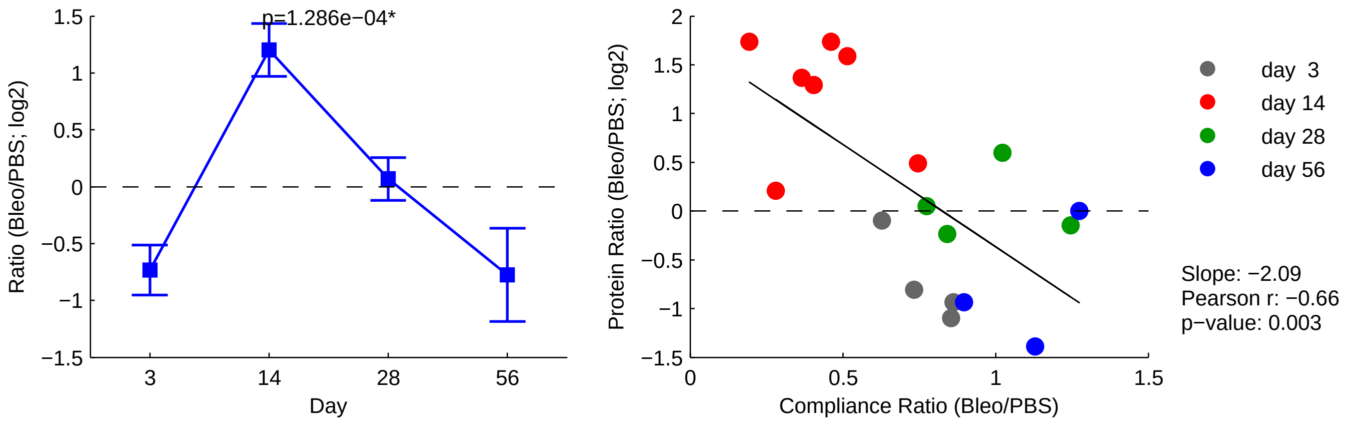

P97384 − Anxa11 (id: 3461)

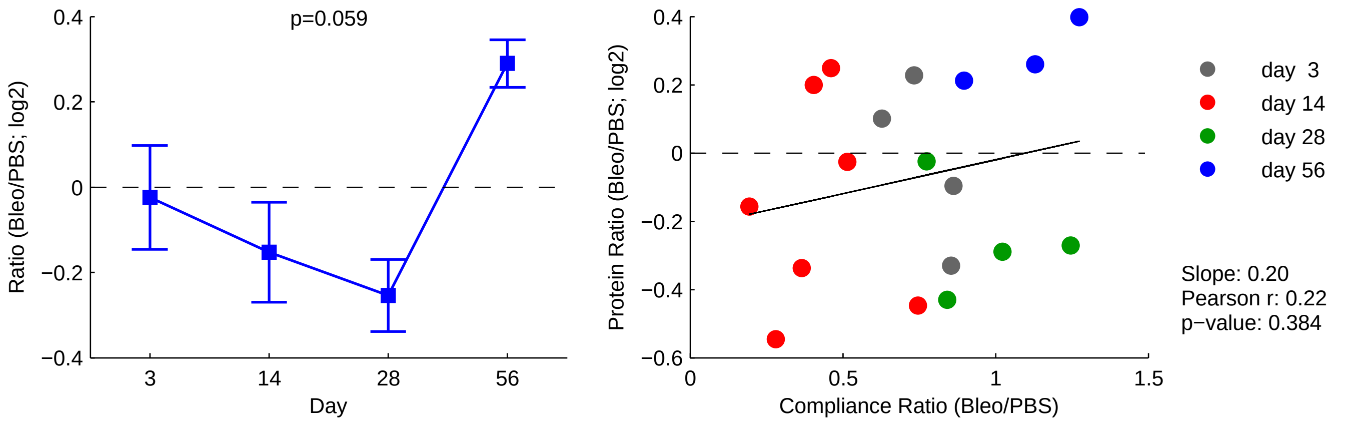

P97390 − Vps45 (id: 3462)

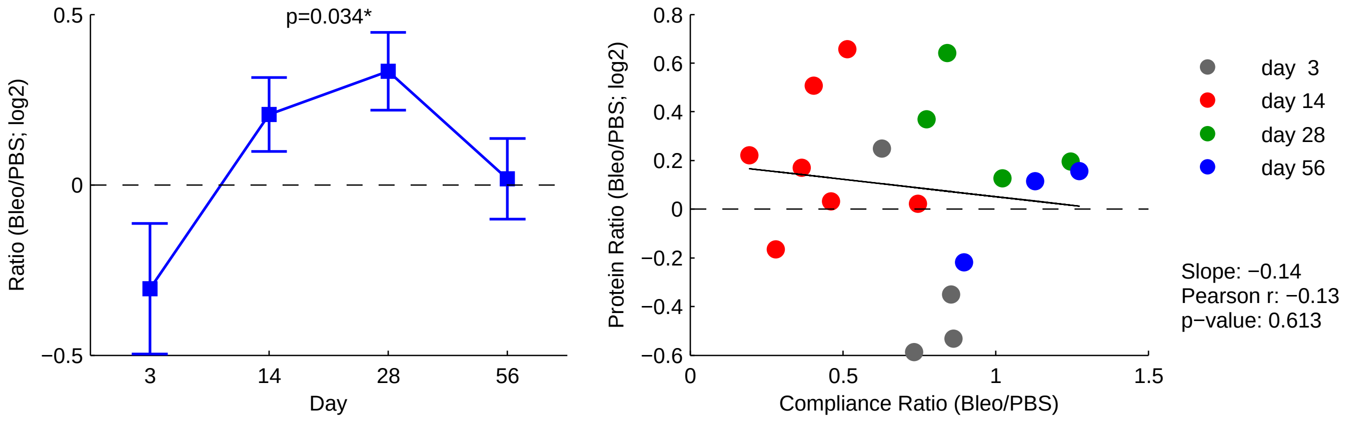

P97434 − Mprip (id: 3467)

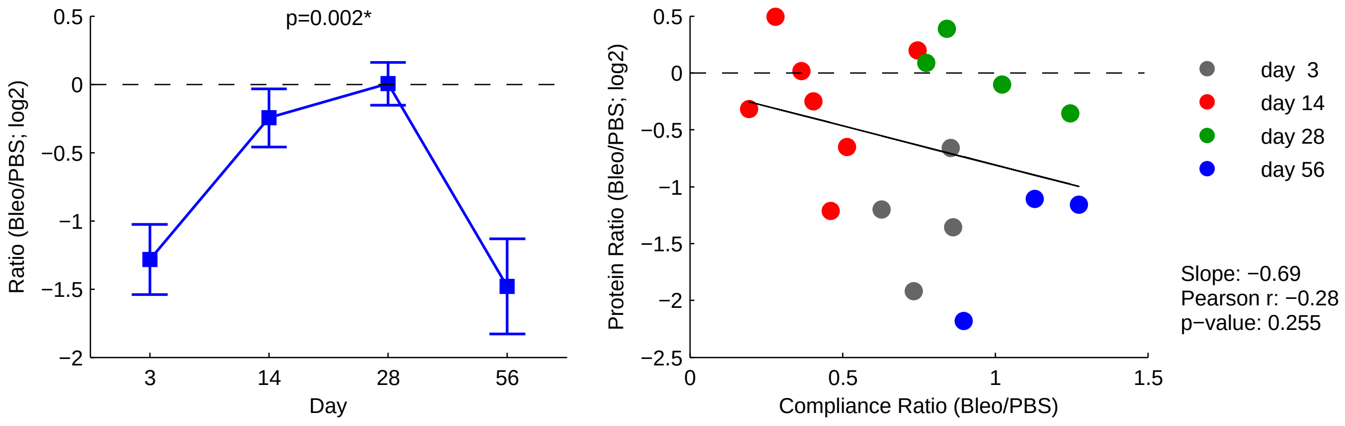

P97443 − Smyd1 (id: 3468)

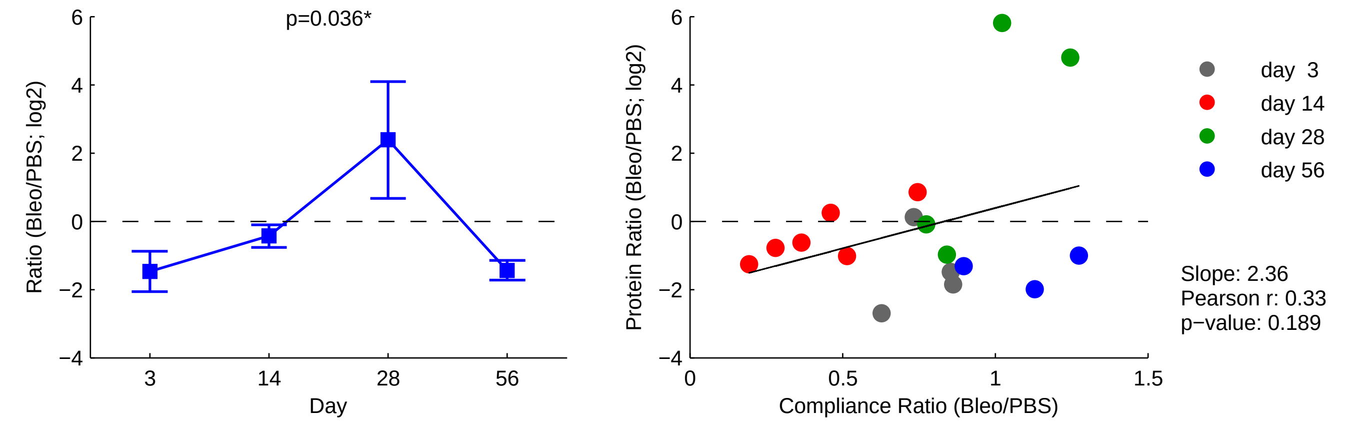

P97449 − Anpep (id: 3470)

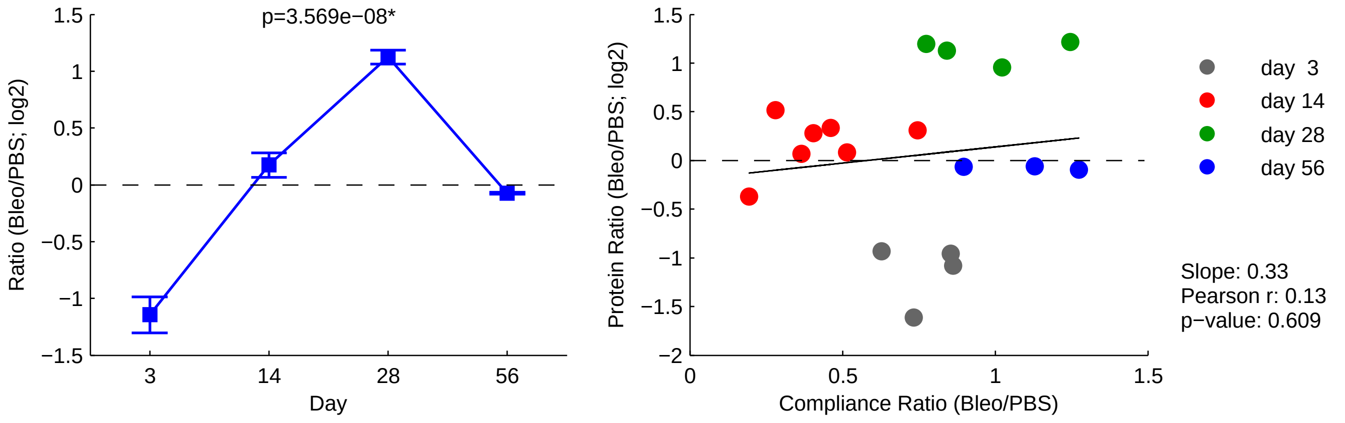

P97452 − Bop1 (id: 3472)

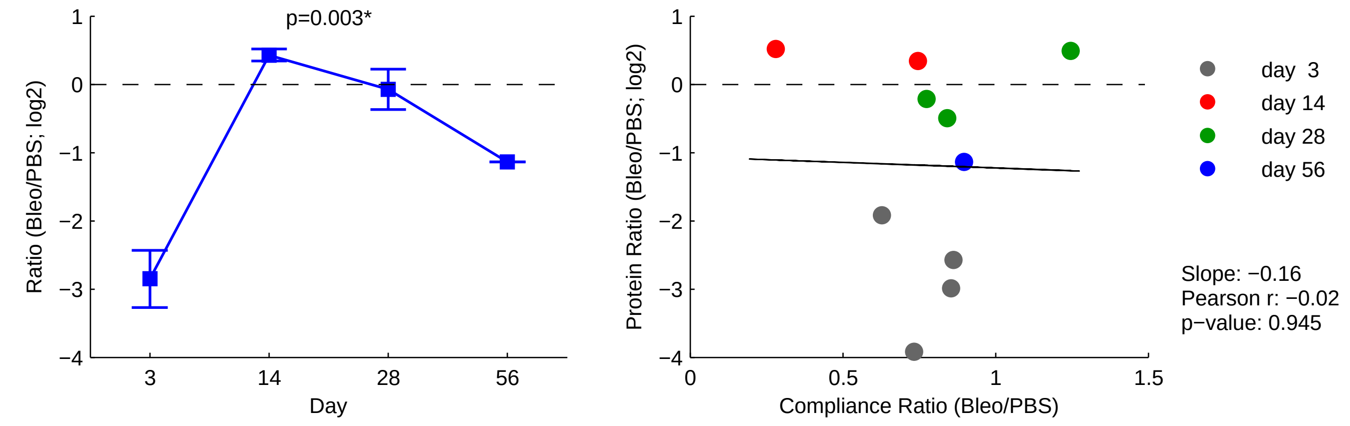

P97454 − Smad5 (id: 3473)

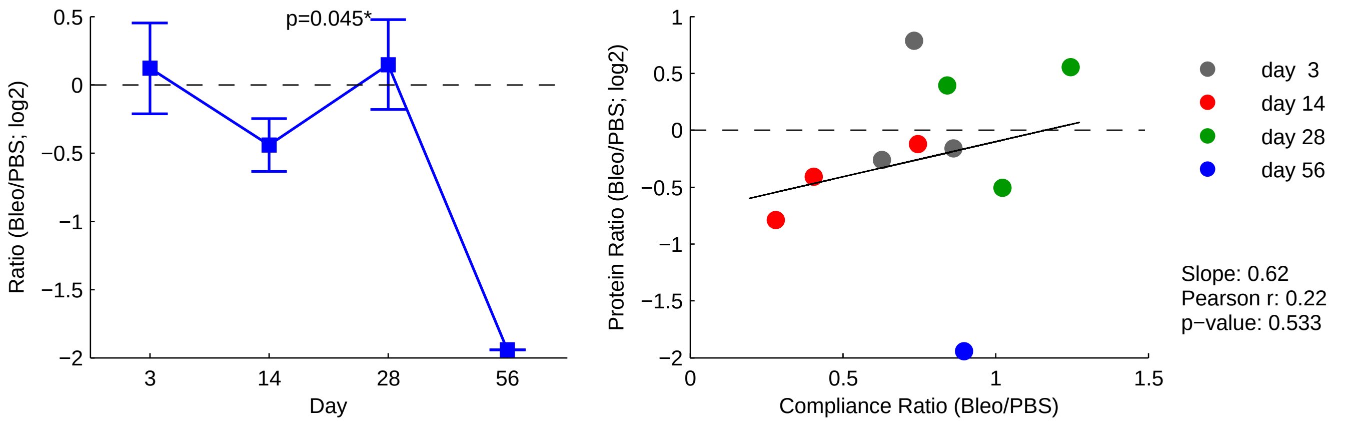

P97457 − Mylpf (id: 3474)

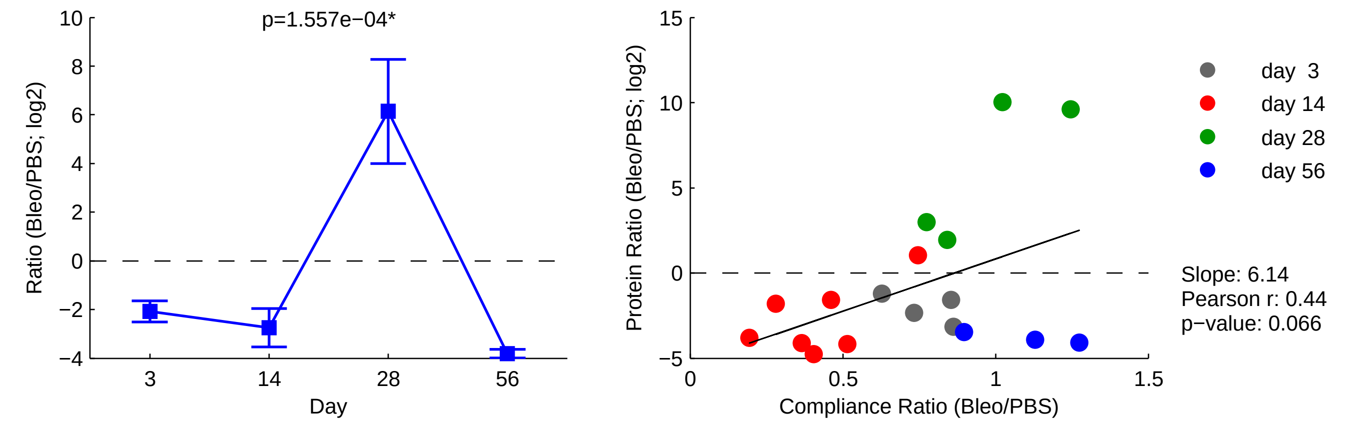

P97465 − Dok1 (id: 3476)

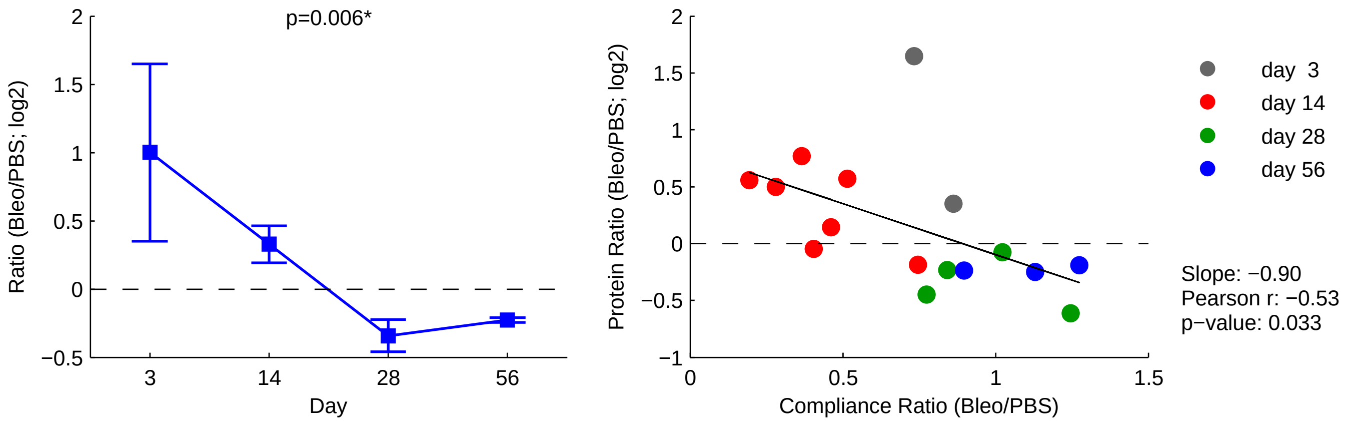

P97468 − Cmklr1 (id: 3477)

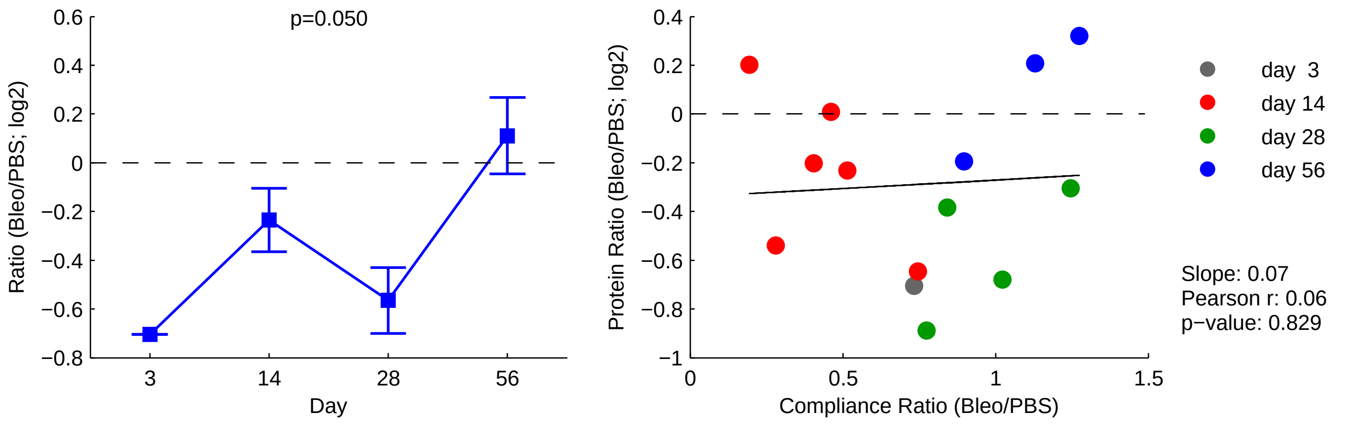

P97478 − Coq7 (id: 3480)

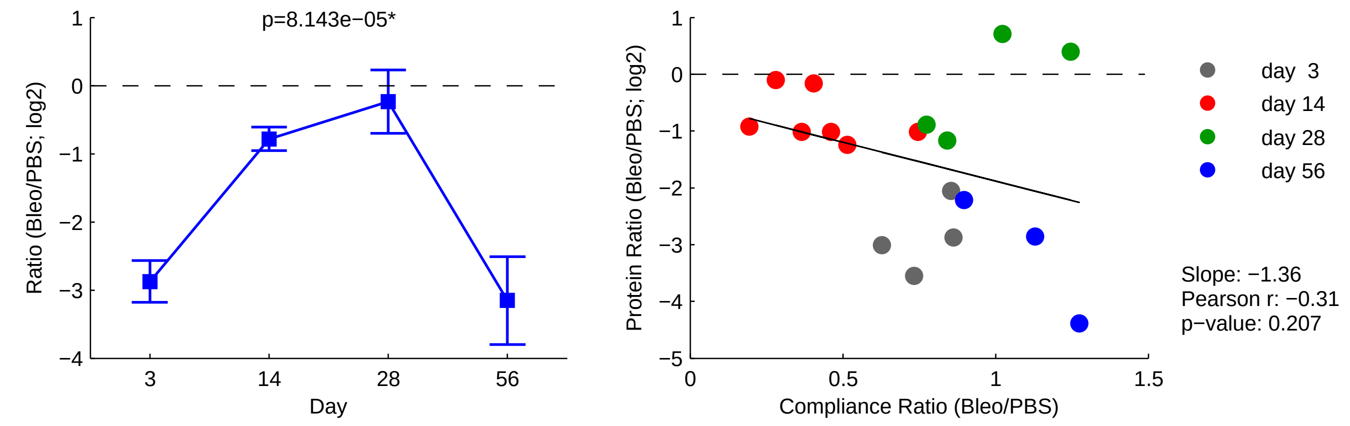

P97479−2 − Myo7a (id: 3481)

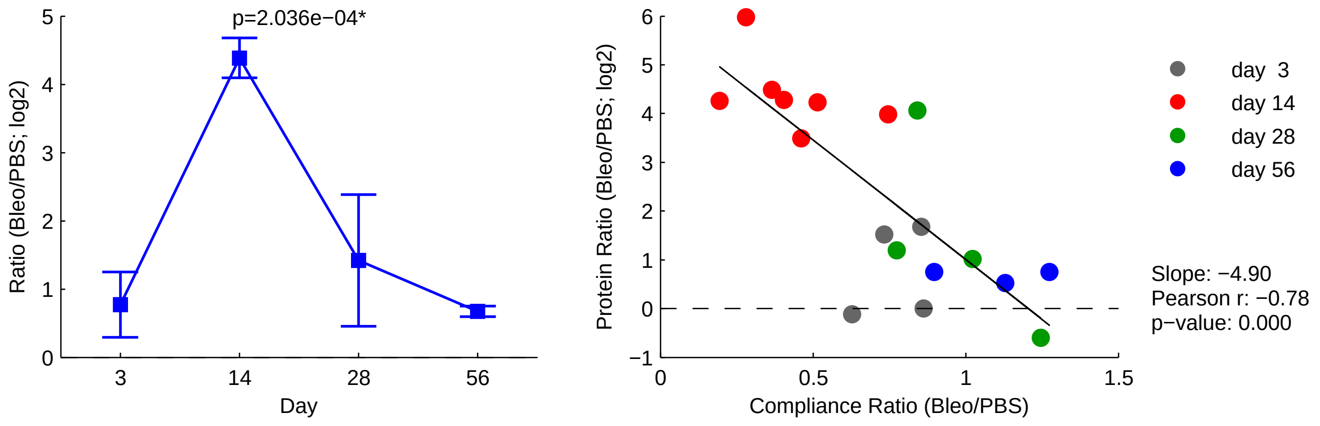

P97494 − Gclc (id: 3485)

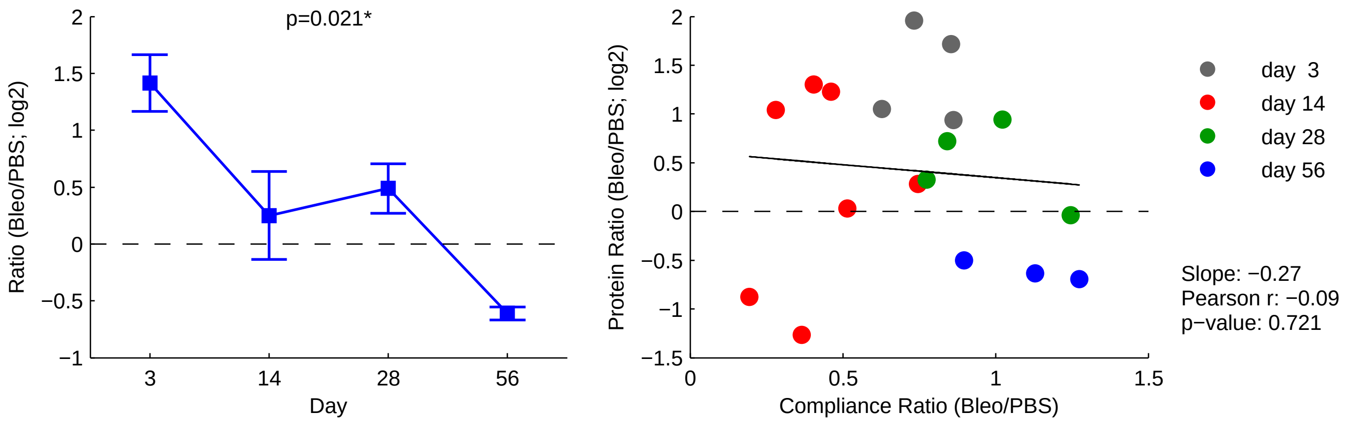

P97496−2 − Smarcc1 (id: 3486)

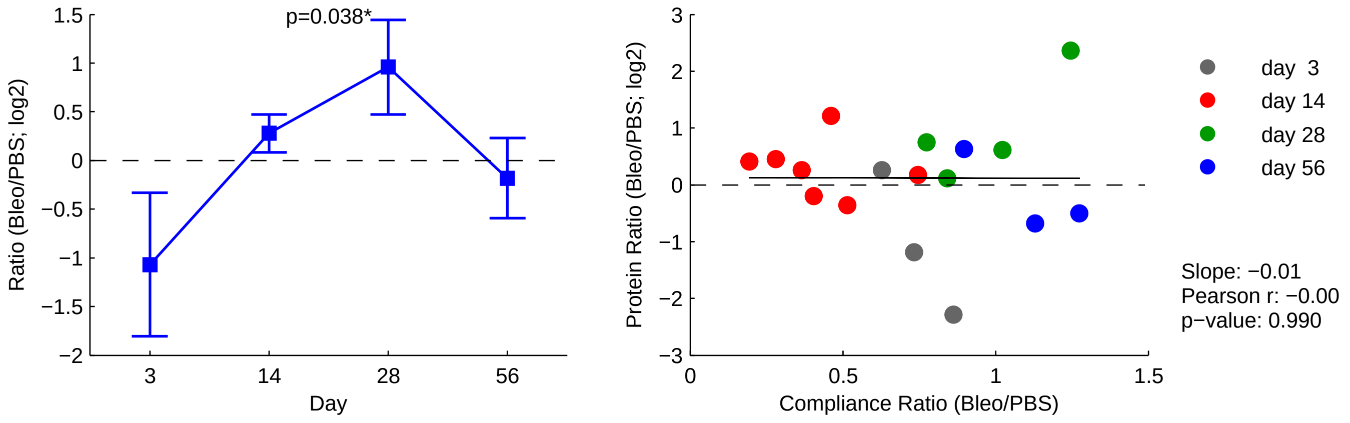

P97501 − Fmo3 (id: 3488)

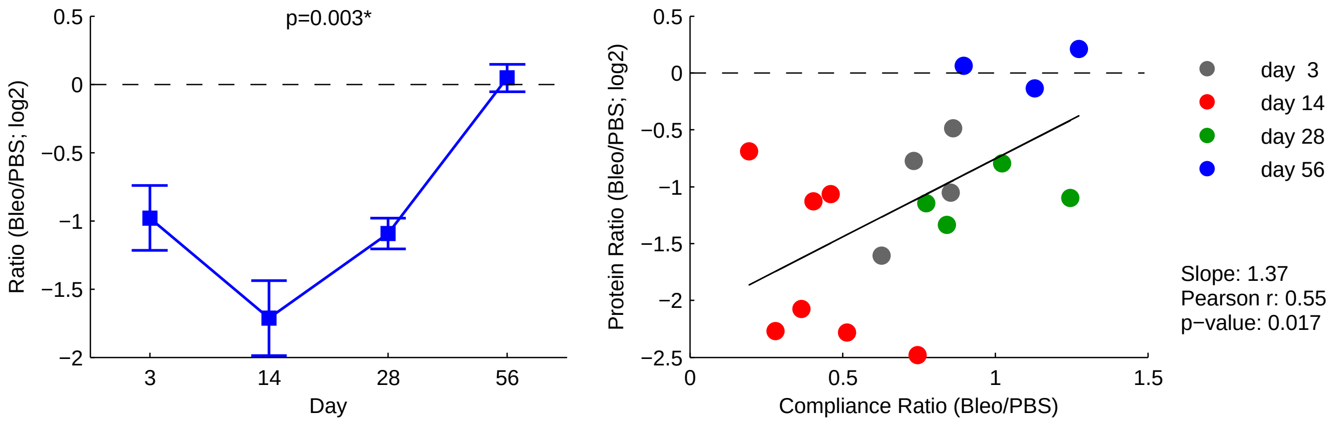

P97742 − Cpt1a (id: 3489)

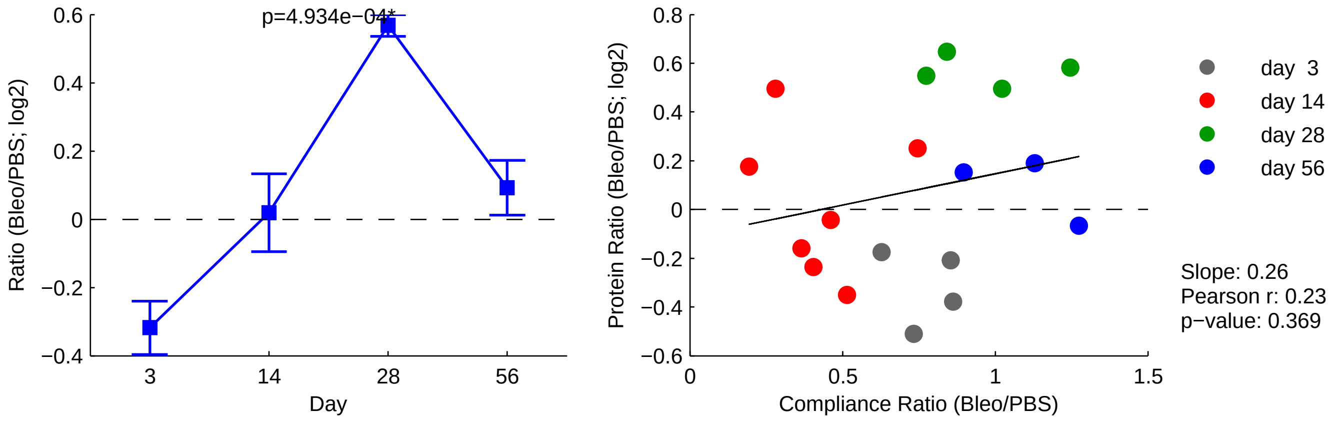

P97765 − Wbp2 (id: 3490)

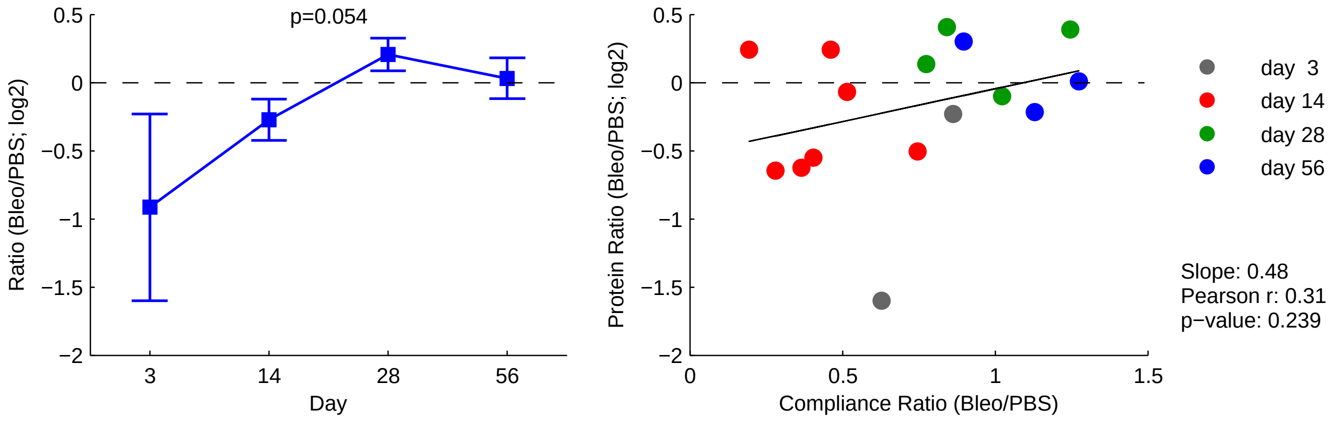

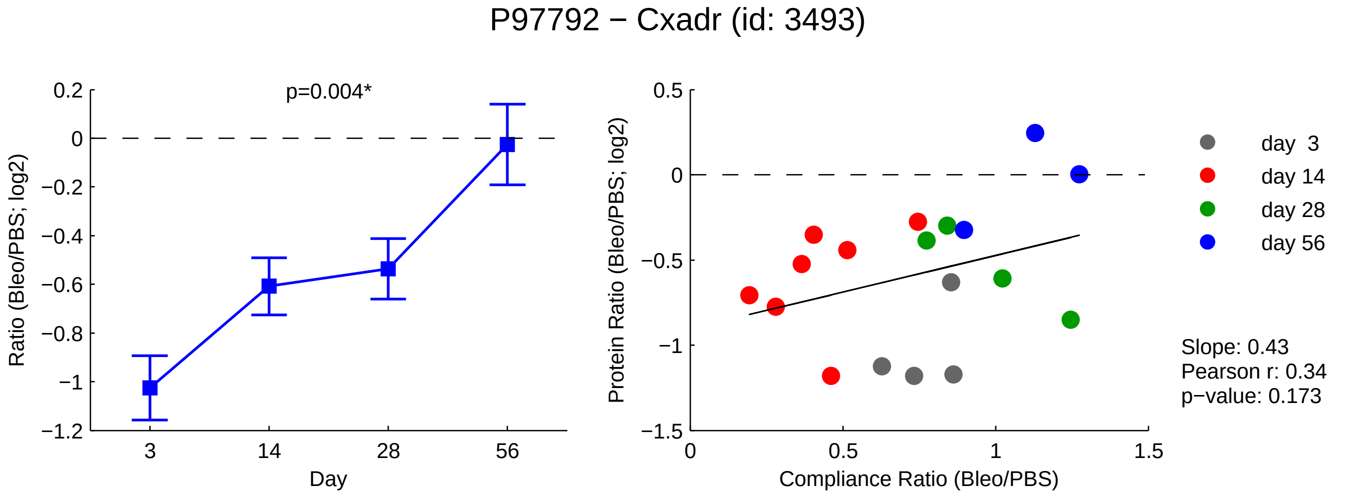
P97792 − Cxadr (id: 3493)
p=0.004*
Ratio (Bleo/PBS; log2)
Day
Protein Ratio (Bleo/PBS; log2)
Compliance Ratio (Bleo/PBS)
day 3
day 14
day 28
day 56
Slope: 0.43
Pearson r: 0.34
p−value: 0.173

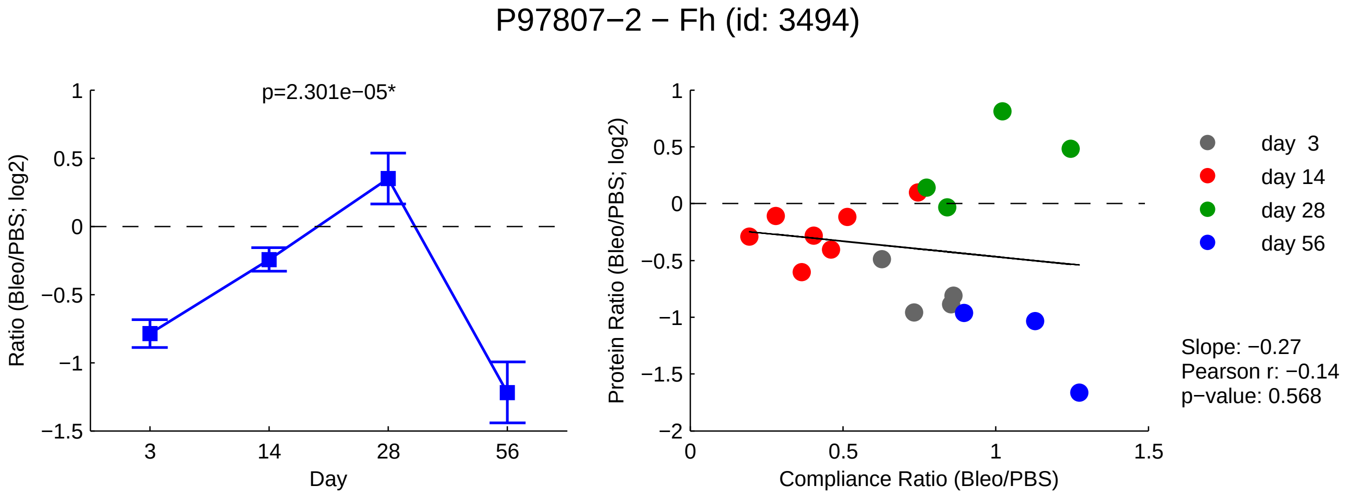
P97807−2 − Fh (id: 3494)
p=2.301e−05*
Ratio (Bleo/PBS; log2)
Day
Protein Ratio (Bleo/PBS; log2)
Compliance Ratio (Bleo/PBS)
day 3
day 14
day 28
day 56
Slope: −0.27
Pearson r: −0.14
p−value: 0.568

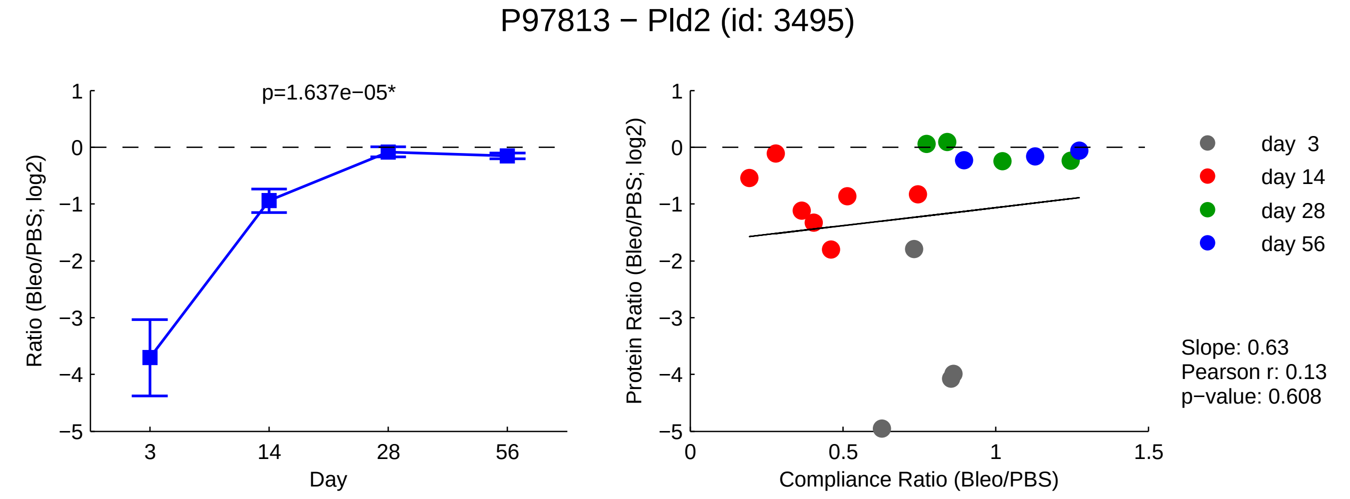
P97813 − Pld2 (id: 3495)
p=1.637e−05*
Ratio (Bleo/PBS; log2)
Day
Protein Ratio (Bleo/PBS; log2)
Compliance Ratio (Bleo/PBS)
day 3
day 14
day 28
day 56
Slope: 0.63
Pearson r: 0.13
p−value: 0.608

P97823 − Lypla1 (id: 3499)

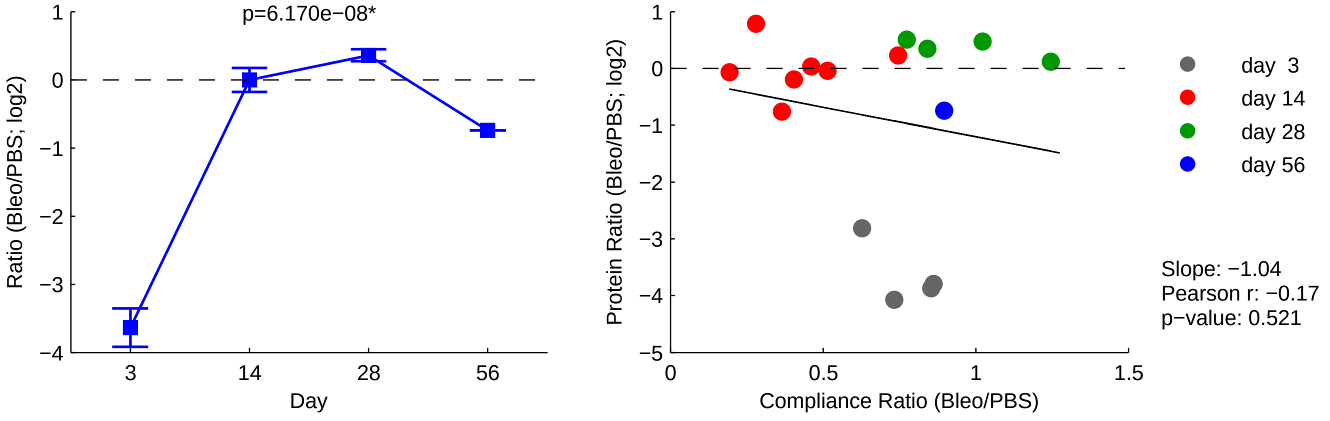

P97855 − G3bp1 (id: 3501)

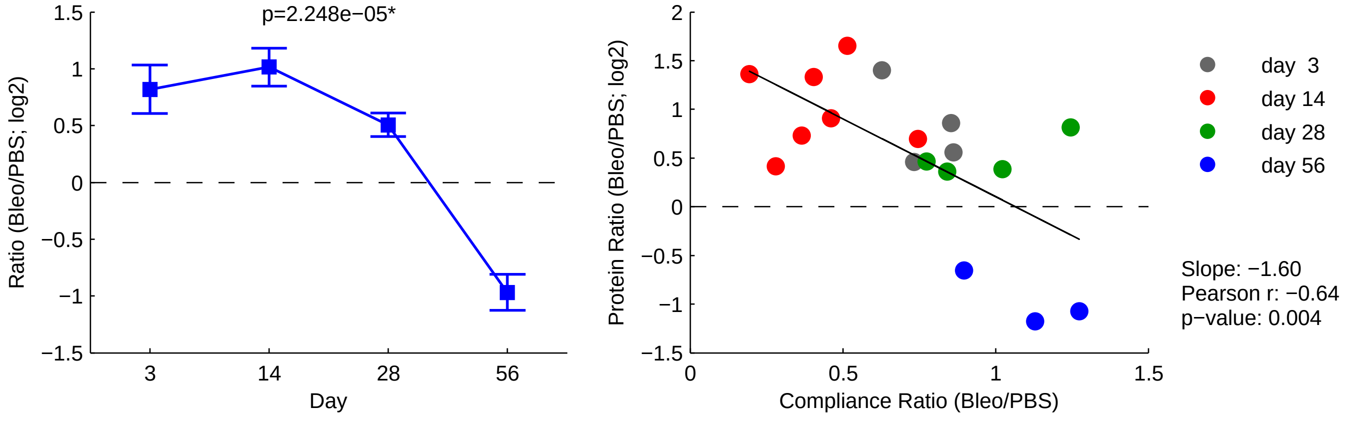

P97864 − Casp7 (id: 3502)

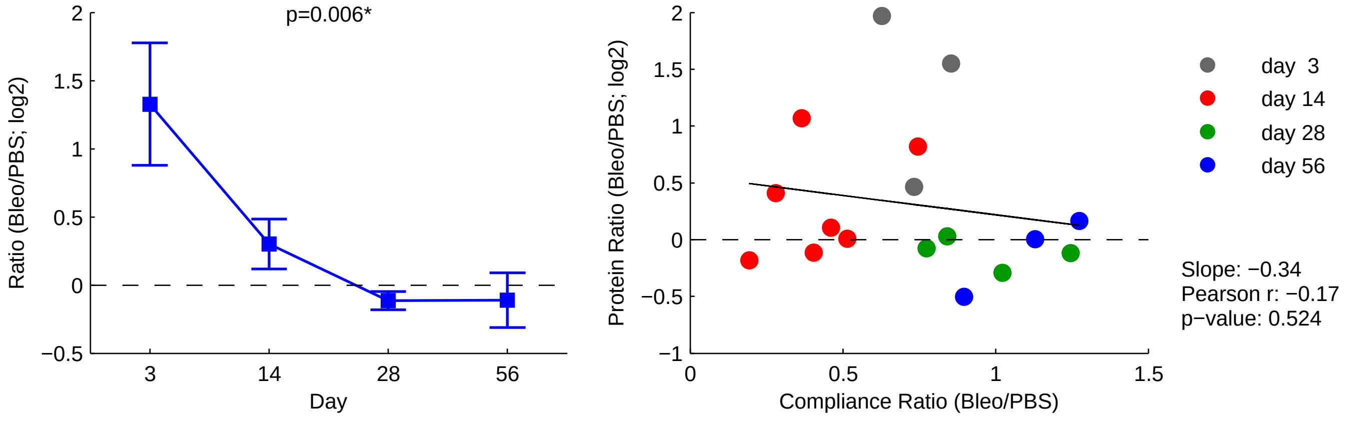

P97868−2 − Rbbp6 (id: 3503)

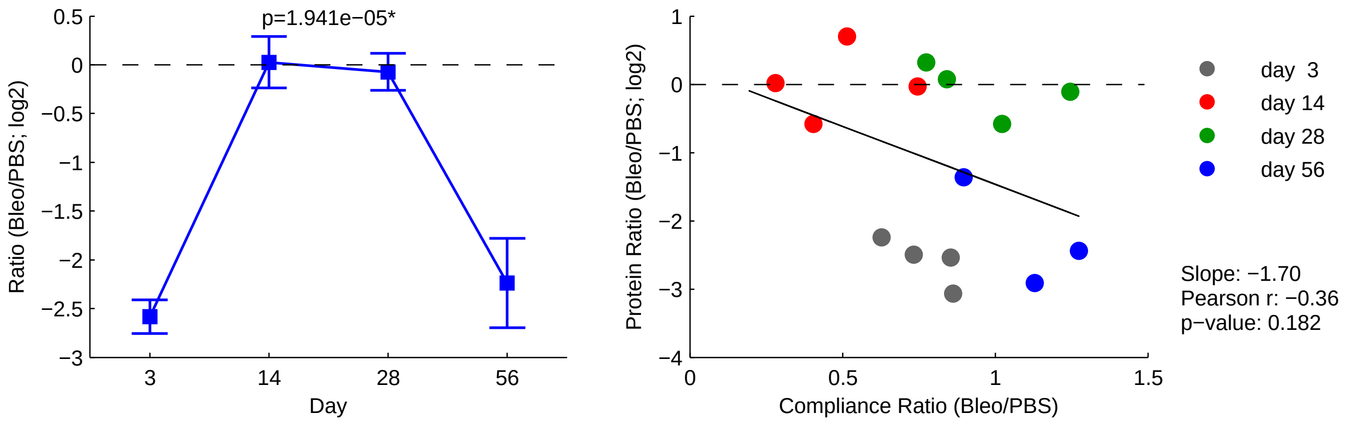

P97872 − Fmo5 (id: 3504)

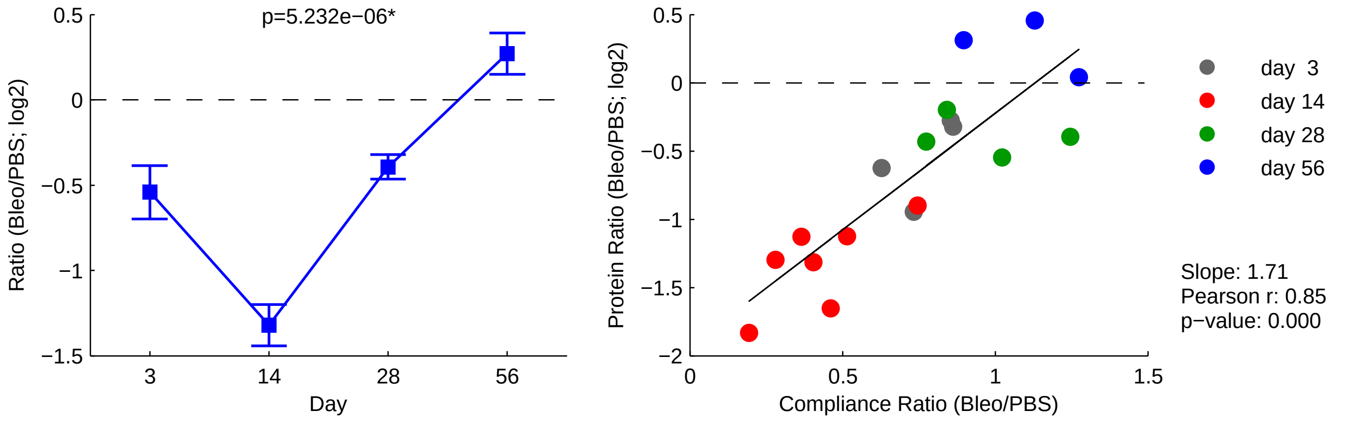

P97873 − Loxl1 (id: 3505)

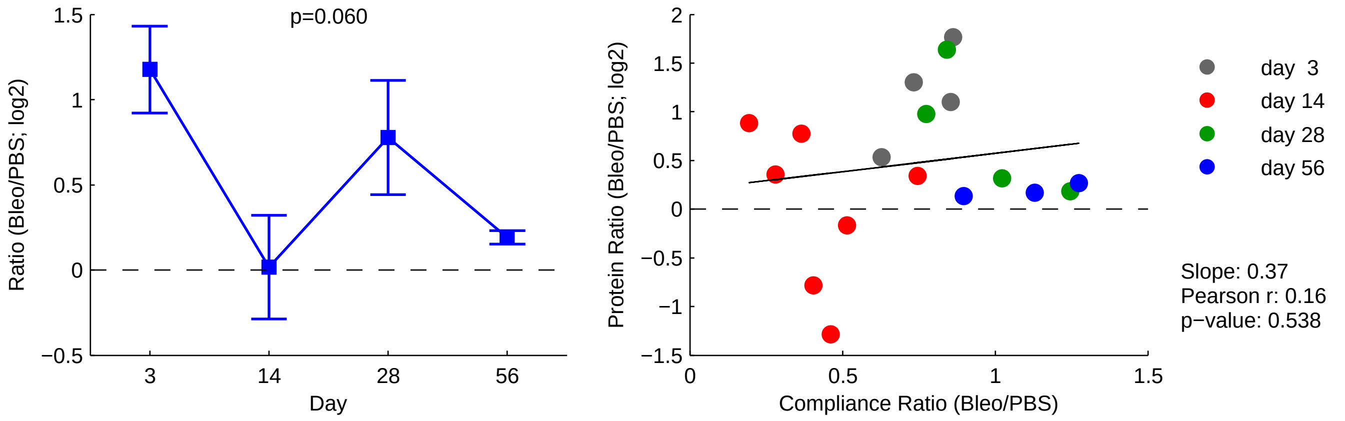

P97927 − Lama4 (id: 3506)

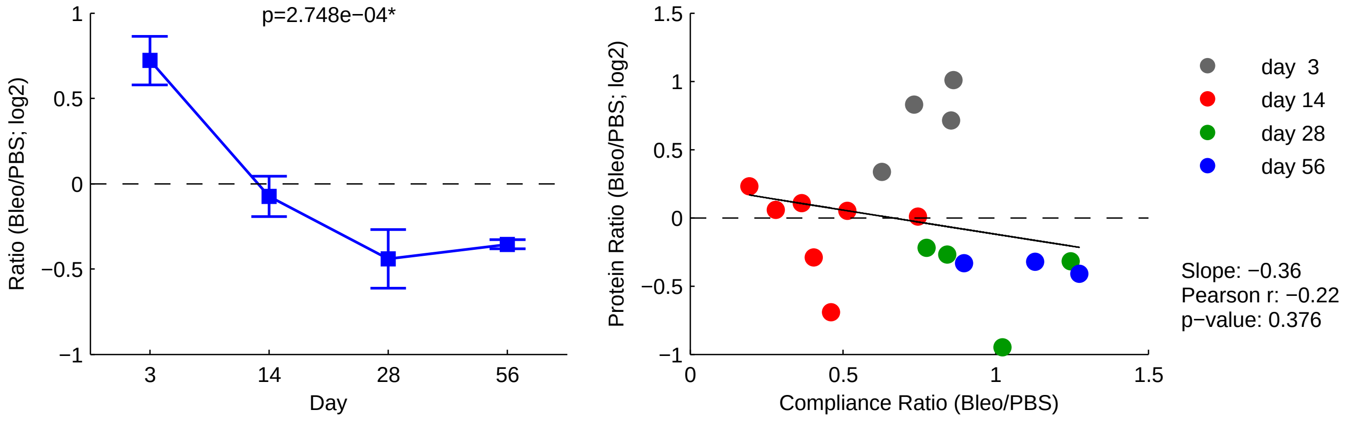

P97930 − Dtymk (id: 3507)

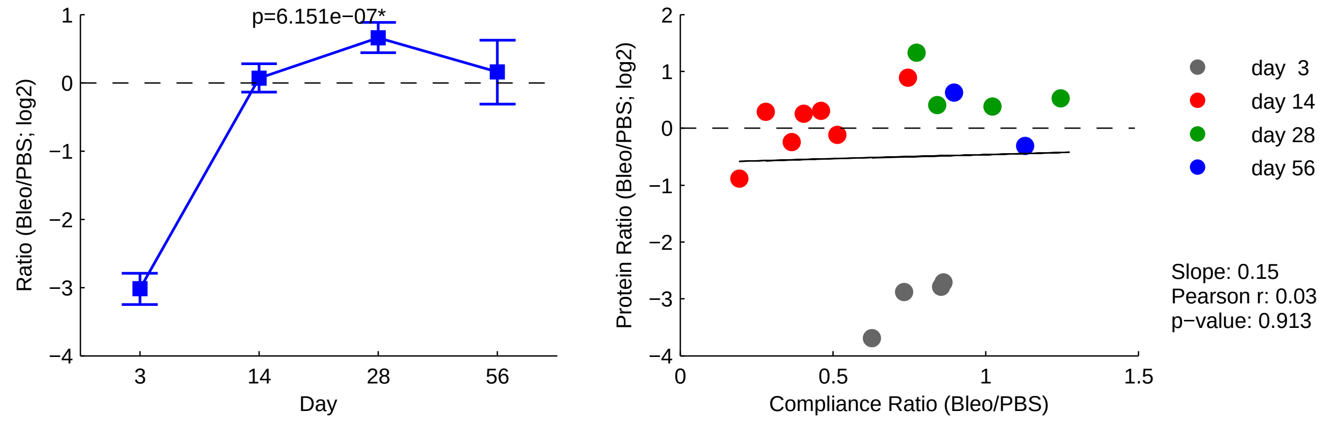

P98086 − C1qa (id: 3510)

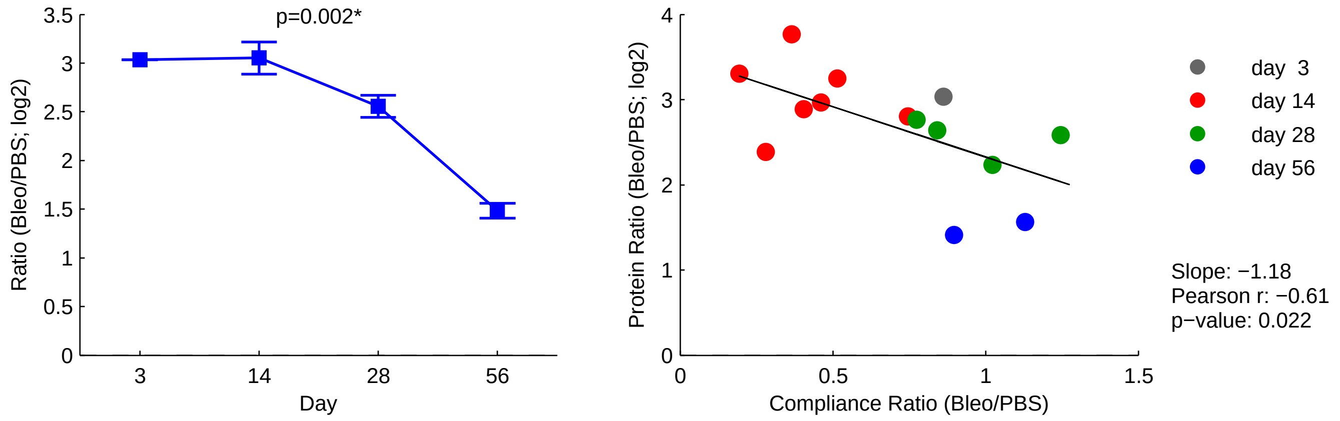

P98191 − Cds1 (id: 3512)

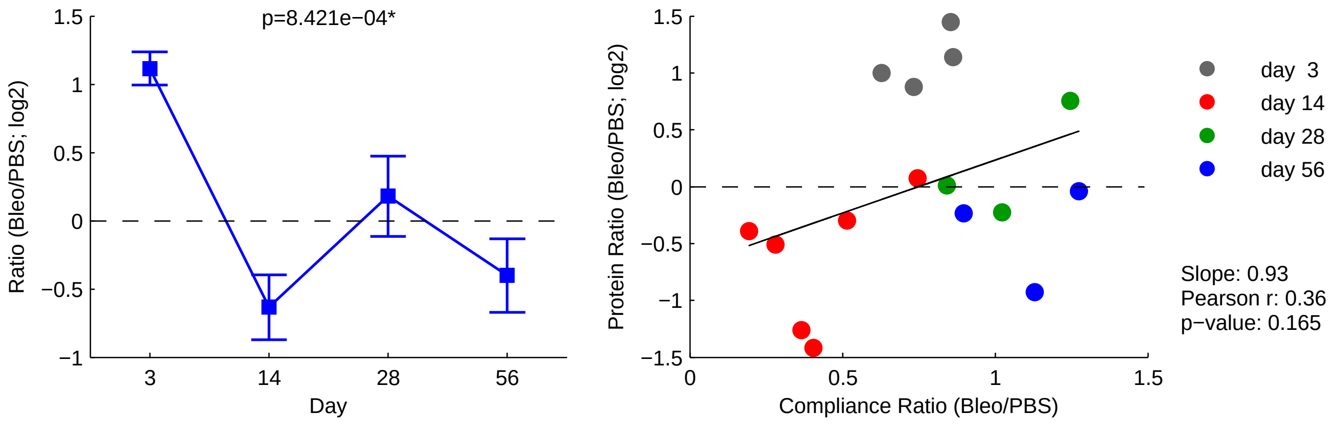

P98192 − Gnpat (id: 3513)

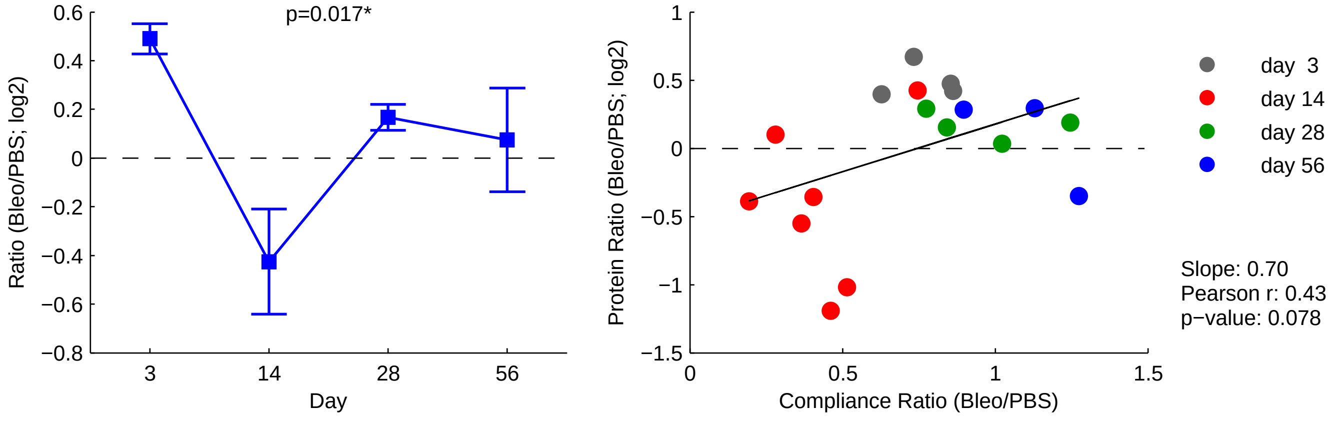

P98203−4 − Arvcf (id: 3514)

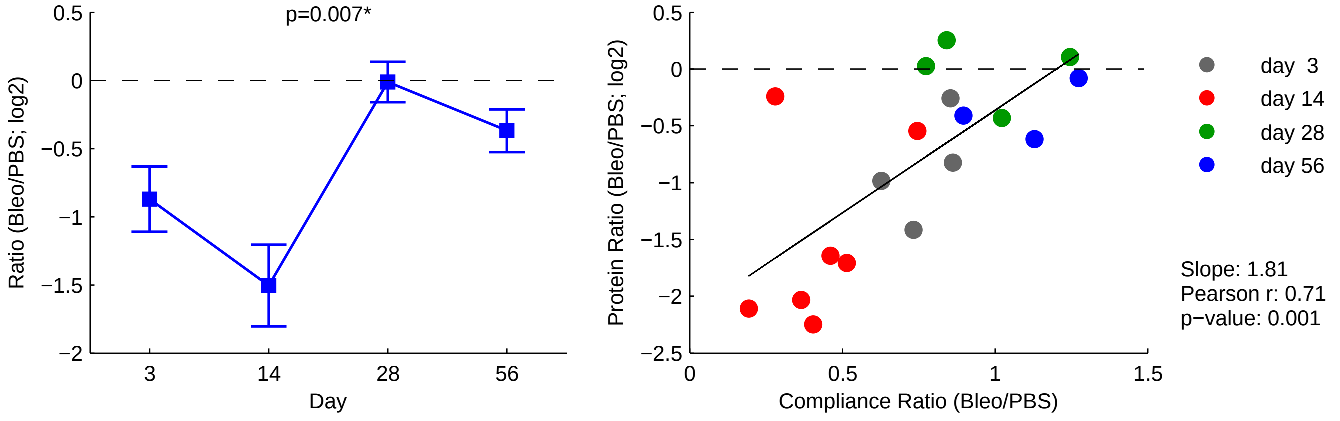

P99024 − Tubb5 (id: 3515)

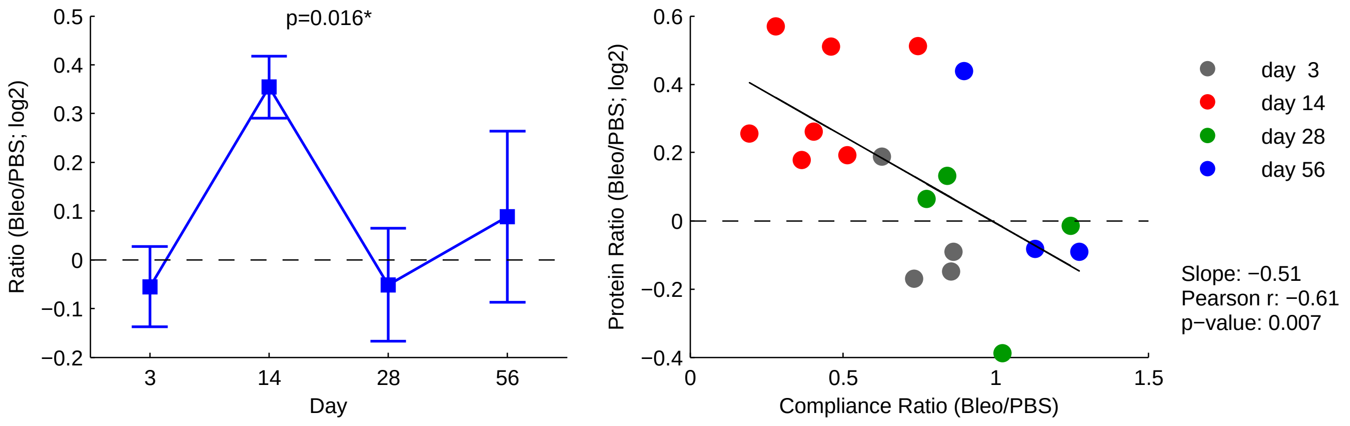

P99026 − Psmb4 (id: 3516)

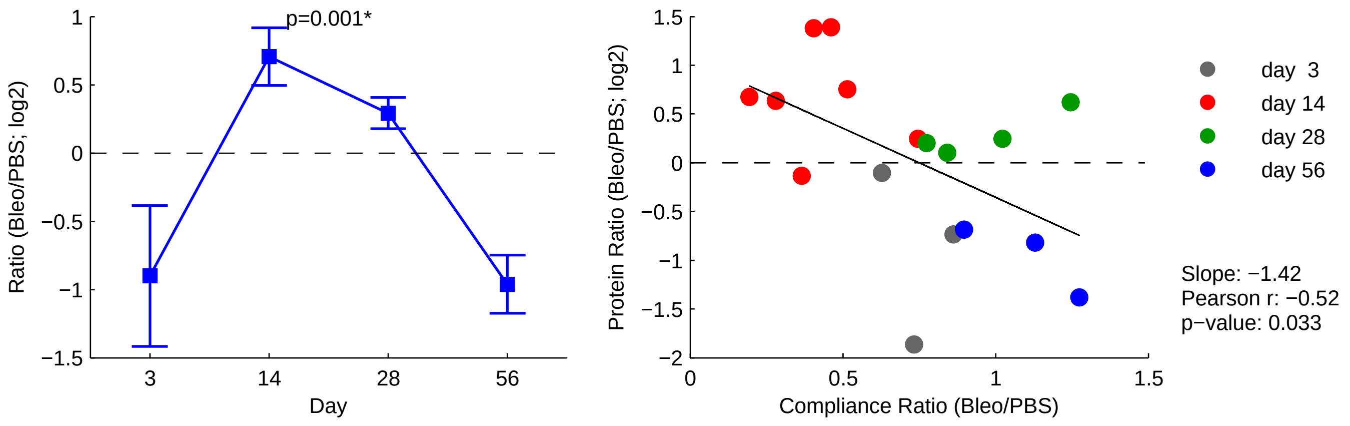

P99027 − Rplp2 (id: 3517)

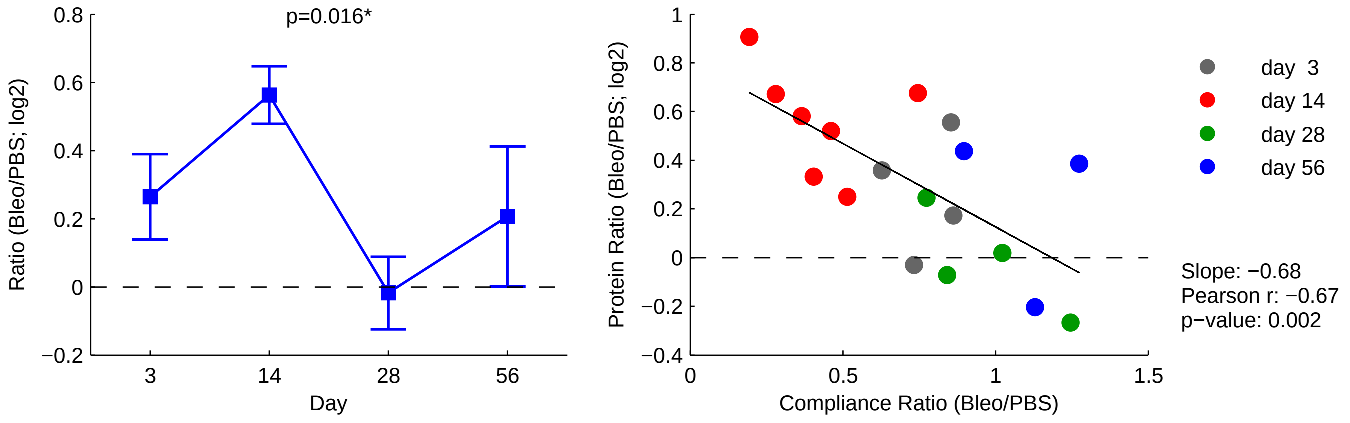

P99028 − Uqcrh (id: 3518)

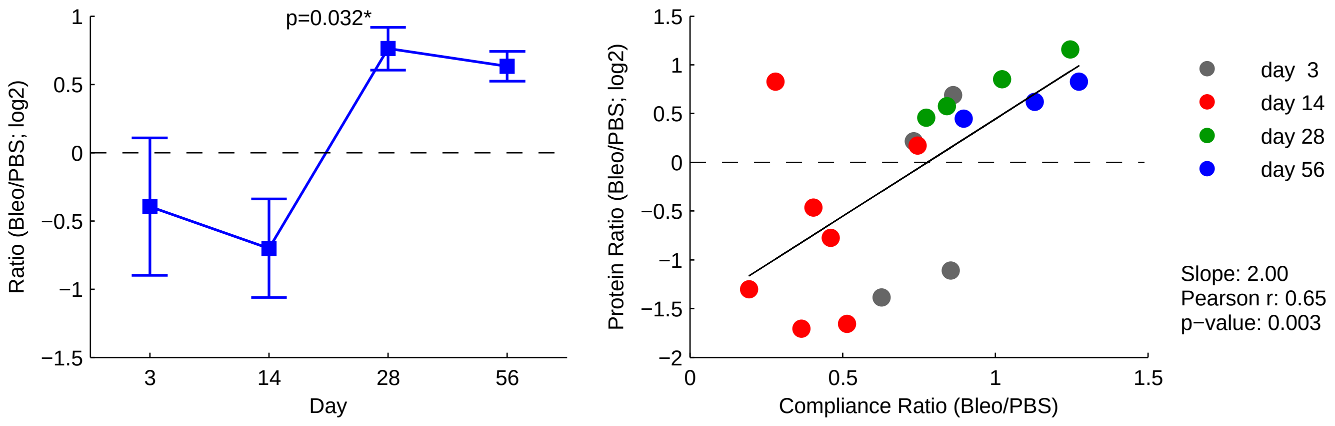

P99029−2 − Prdx5 (id: 3519)

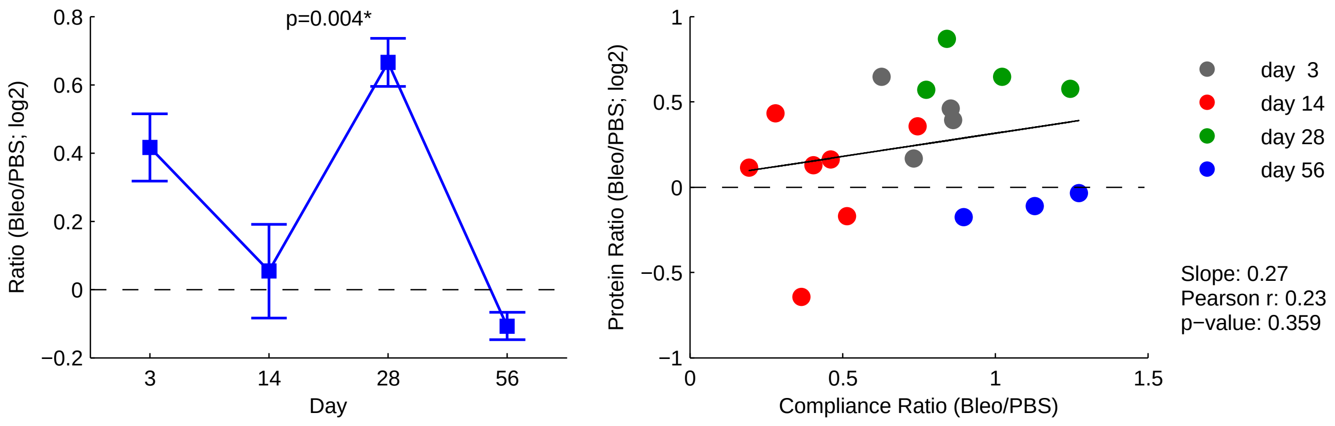

Q00422 − Gabpa (id: 3522)

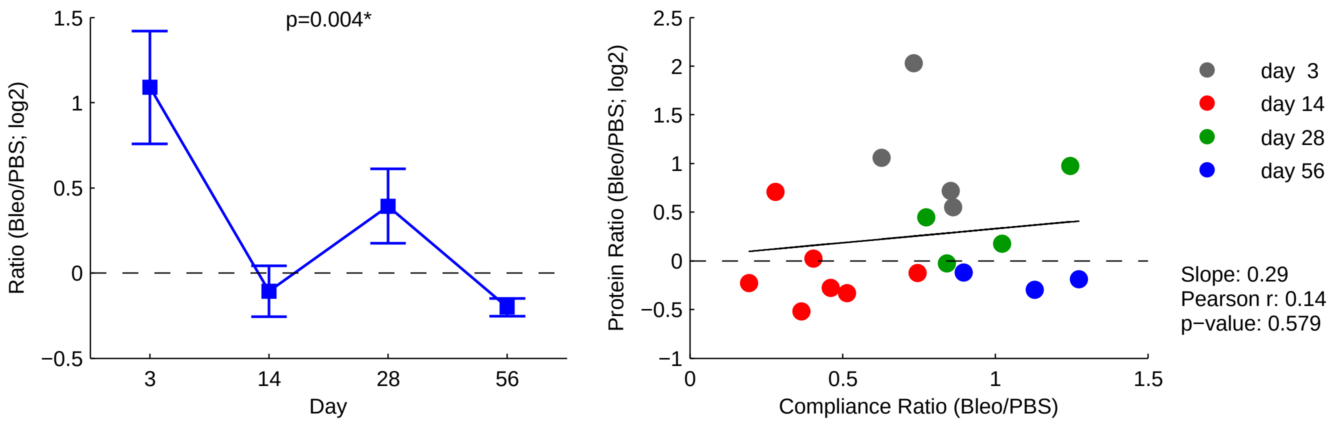

Q00519 − Xdh (id: 3523)

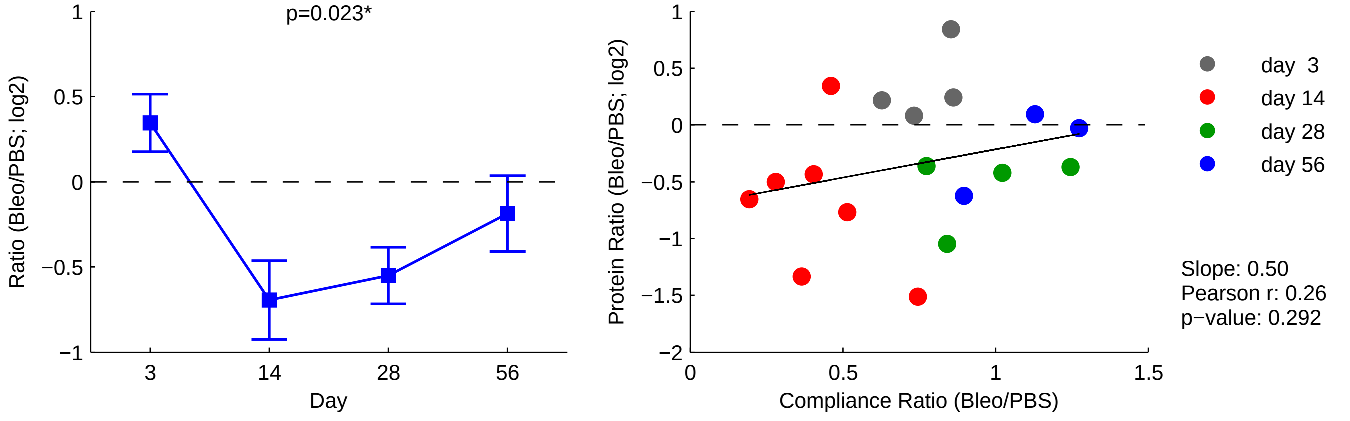

Q00612 − G6pdx (id: 3526)

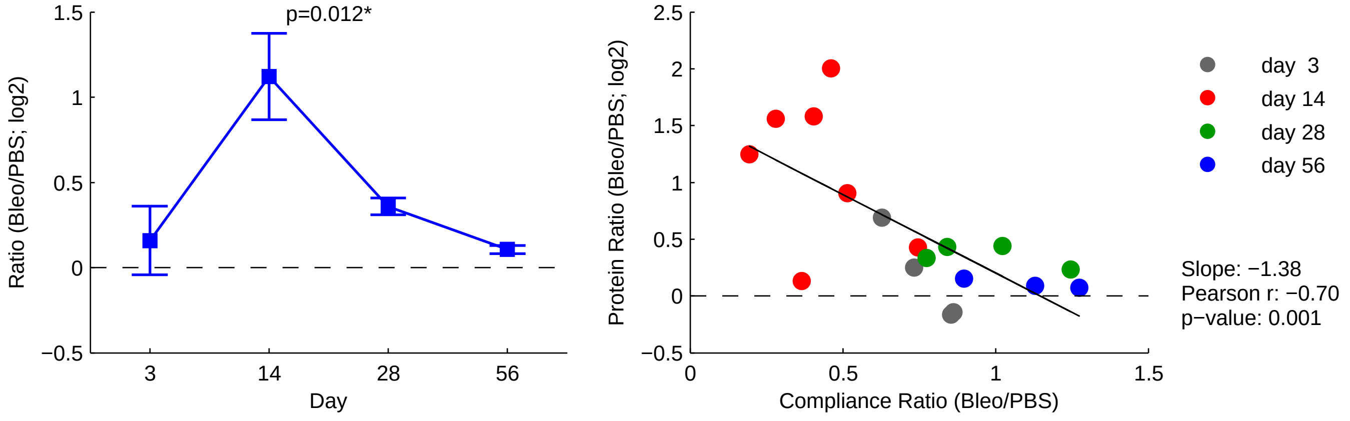

Q00623 − Apoa1 (id: 3527)

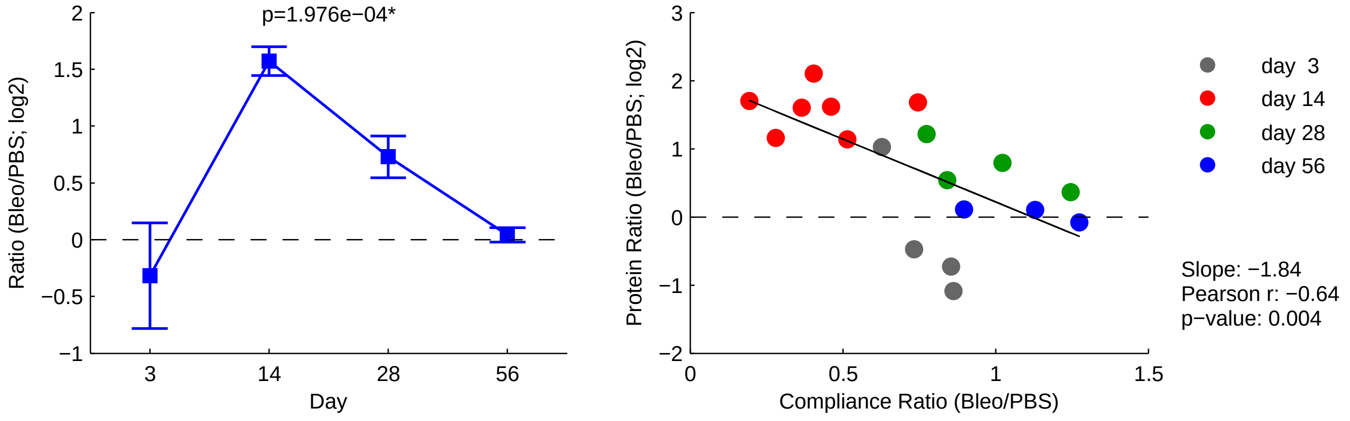

Q792F9 − Itga4 (id: 3528)

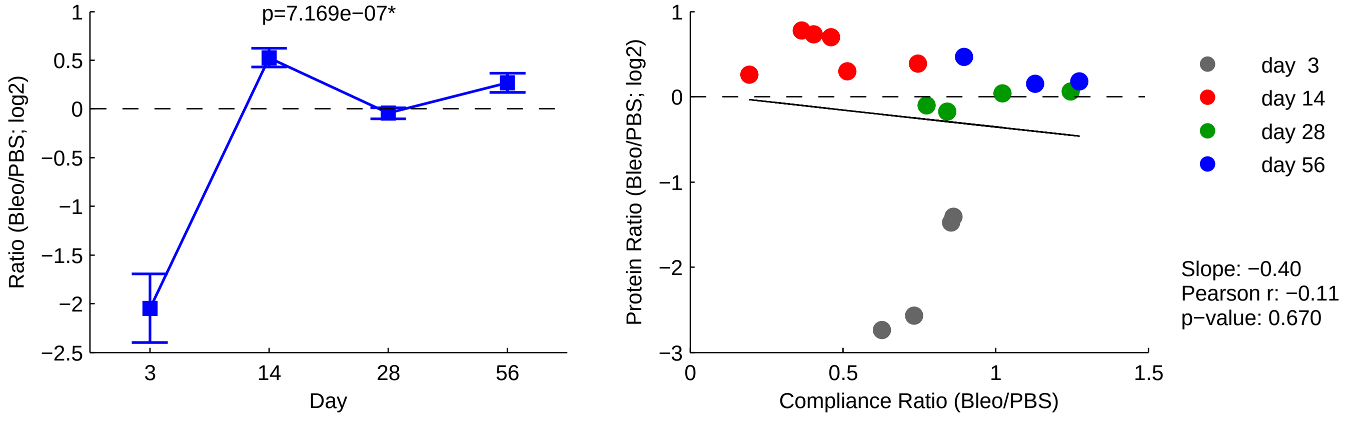

Q00896 − Serpina1c (id: 3530)

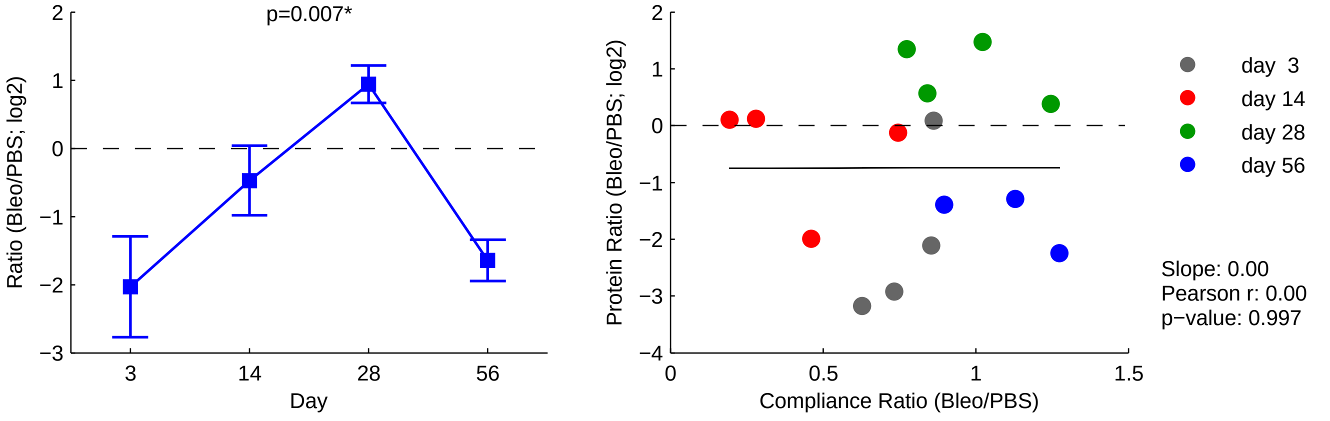

Q00897 − Serpina1d (id: 3531)

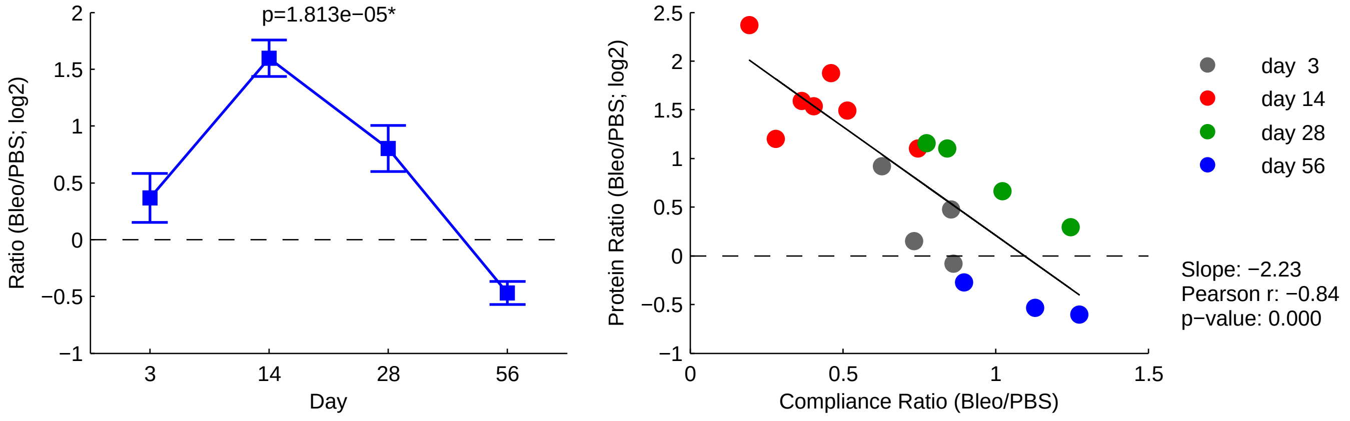

Q00899 − Yy1 (id: 3533)

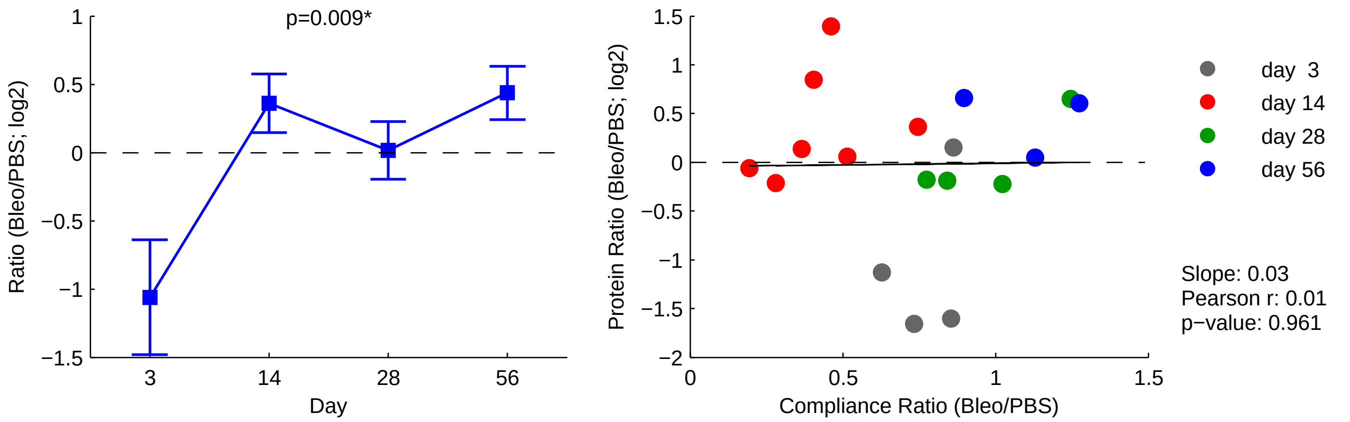

Q00915 − Rbp1 (id: 3534)

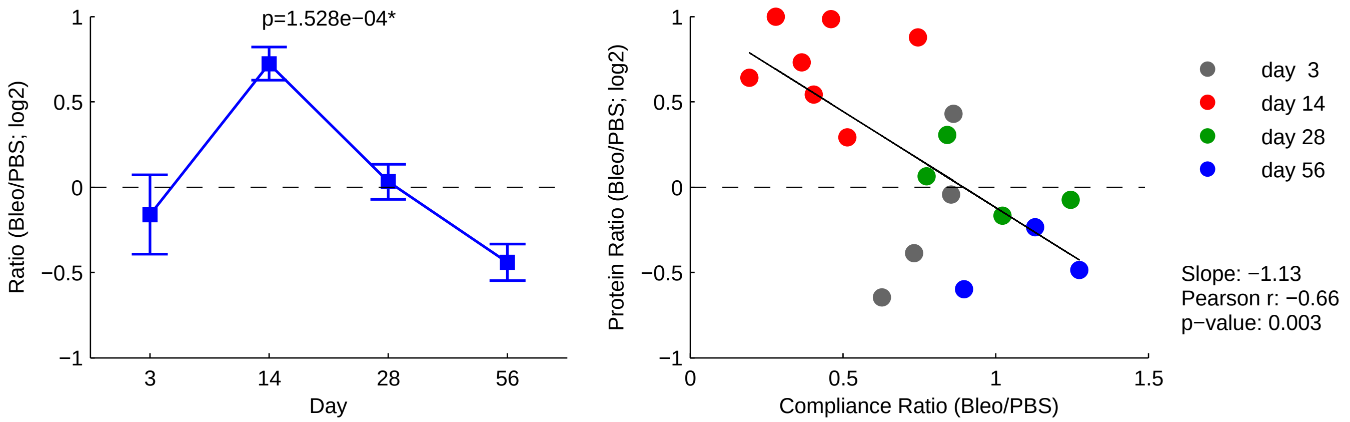

Q62347 − Creb1 (id: 3538)

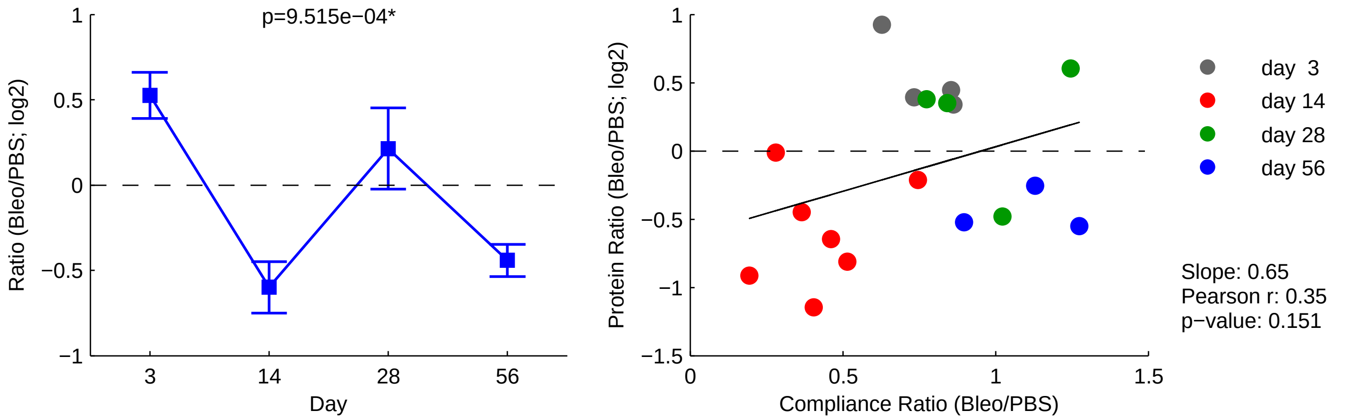

Q01279 − Egfr (id: 3541)

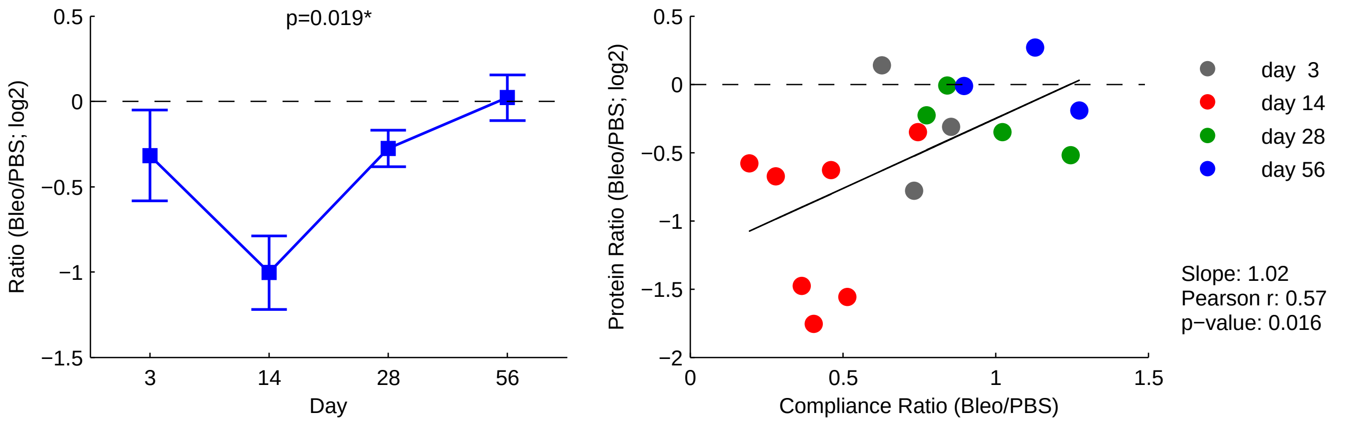

Q01853 − Vcp (id: 3546)

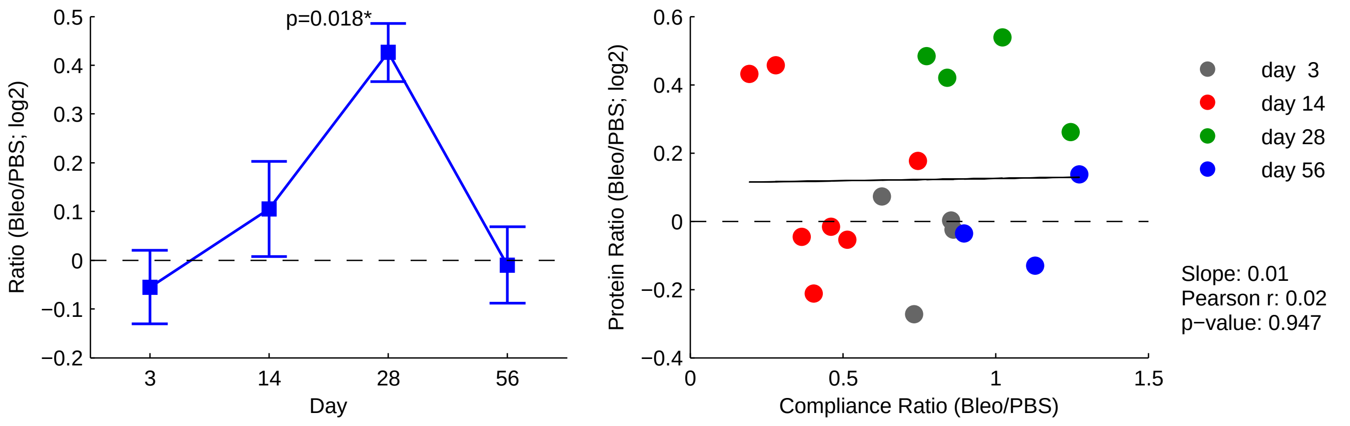

Q02013 − Aqp1 (id: 3547)

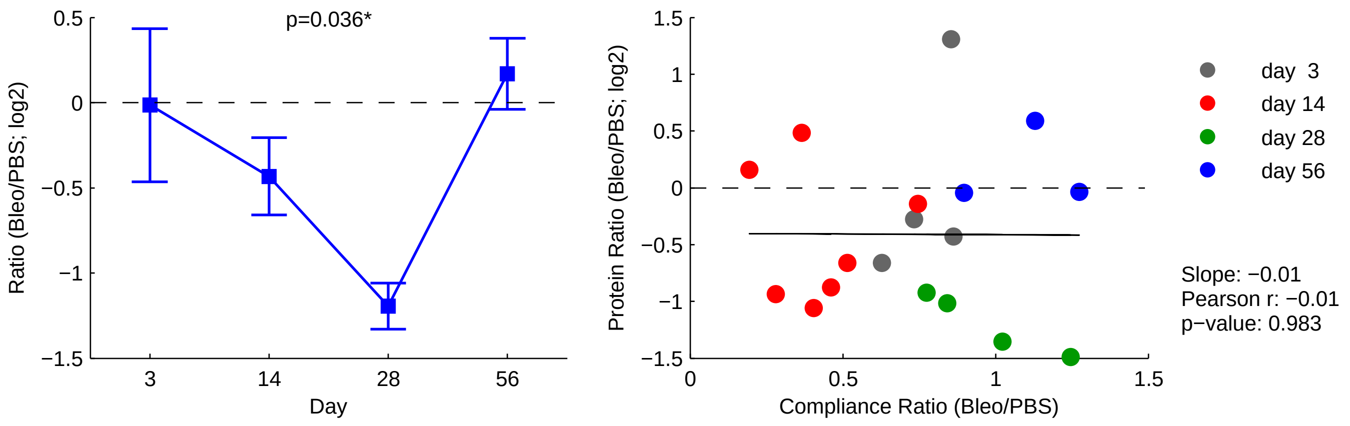

Q02053 − Uba1 (id: 3548)

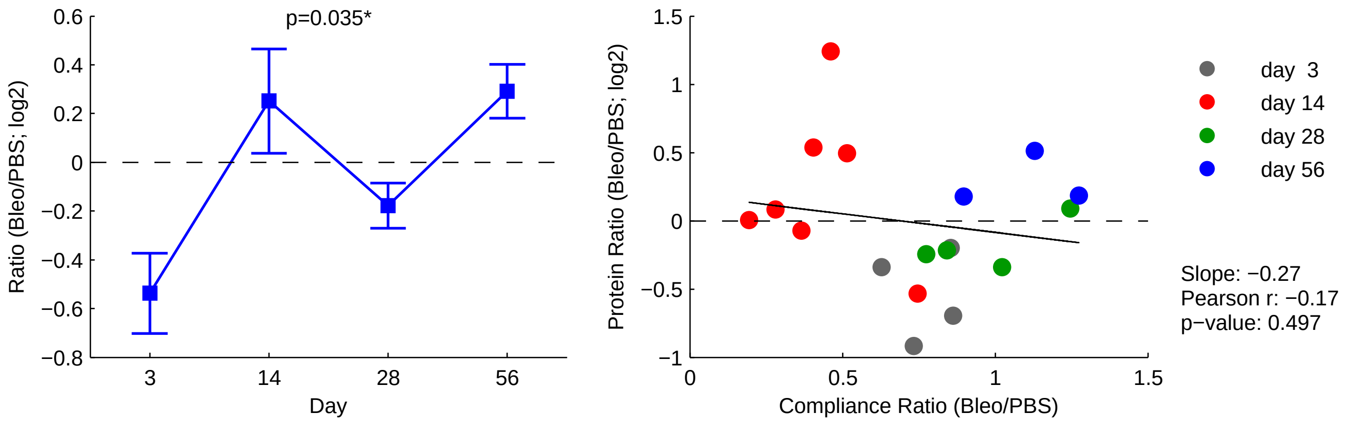

Q02257 − Jup (id: 3552)

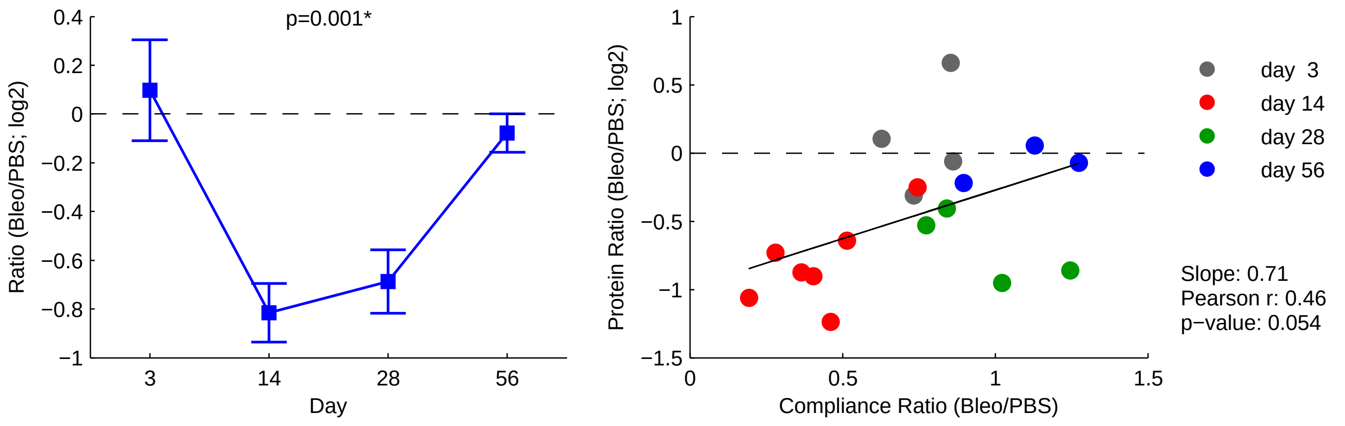

Q02384 − Sos2 (id: 3553)

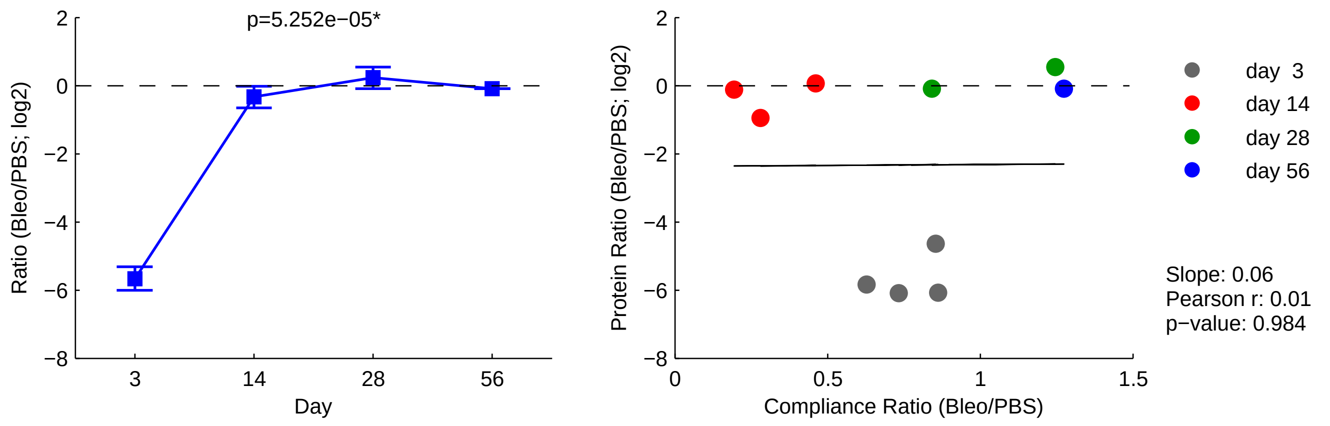

Q02566 − Myh6 (id: 3555)

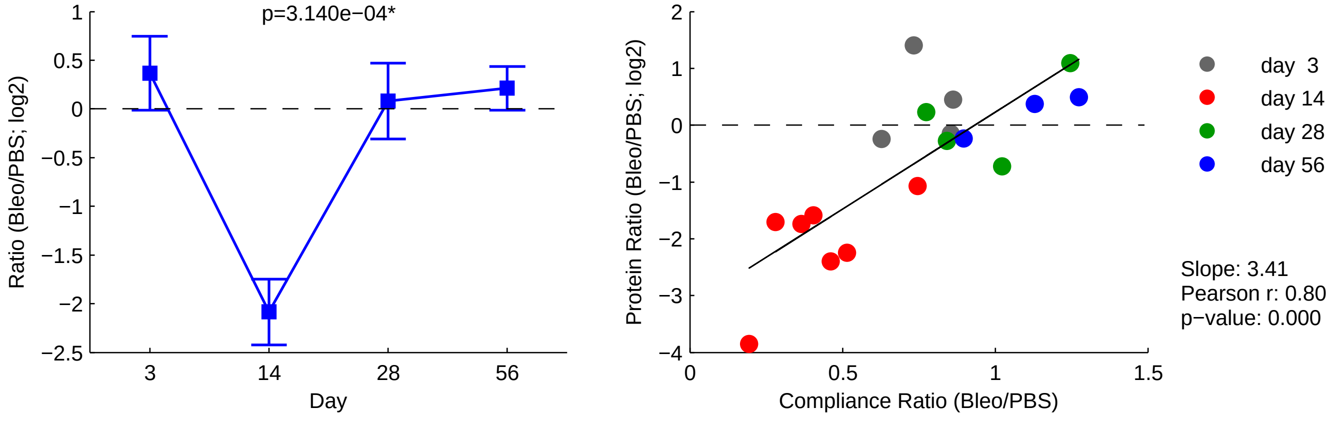

Q02614 − Sap30bp (id: 3556)

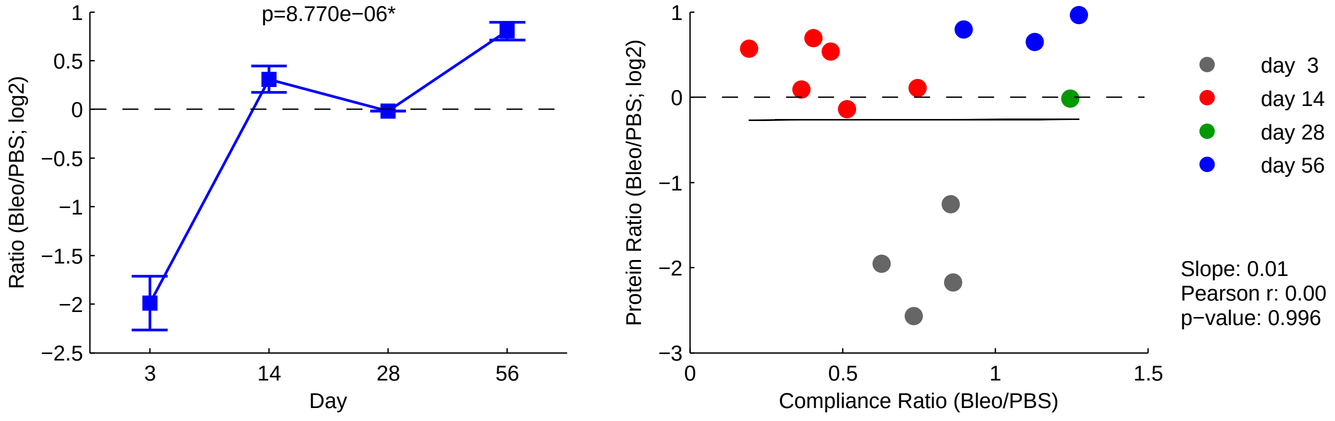

Q02780−3 − Nfia (id: 3557)

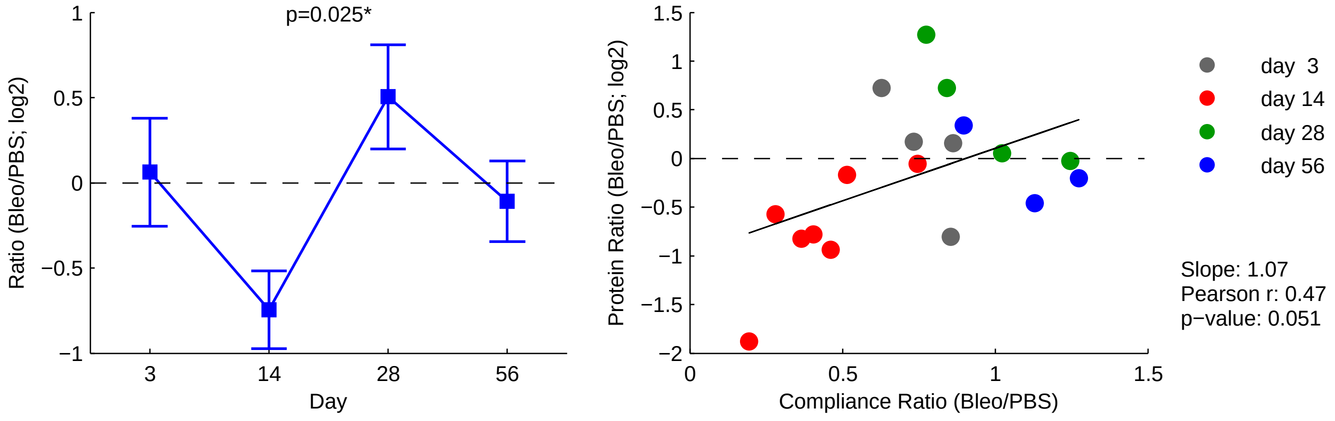

Q02788 − Col6a2 (id: 3558)

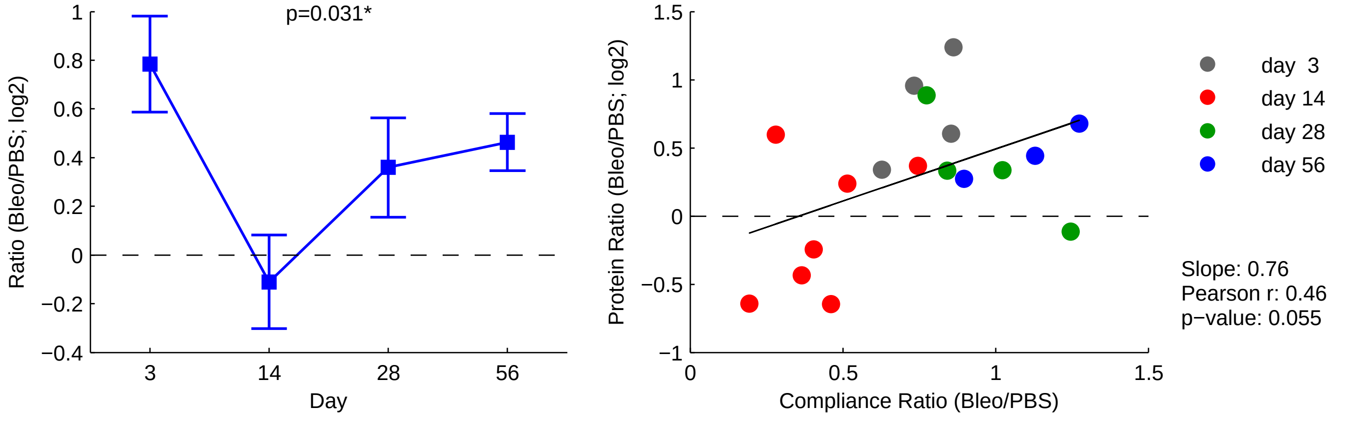

Q02819 − Nucb1 (id: 3559)

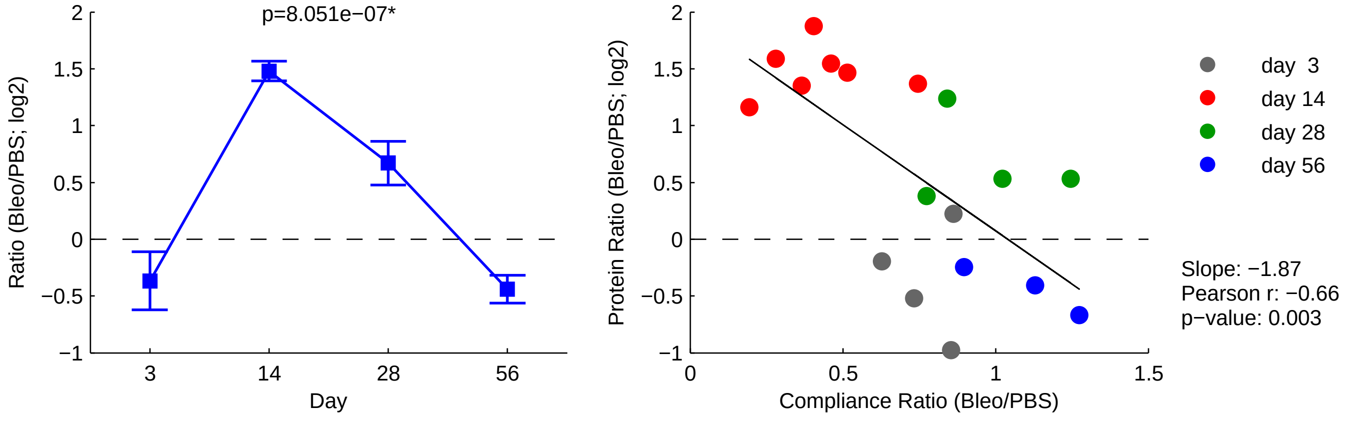

Q02956 − Prkcz (id: 3560)

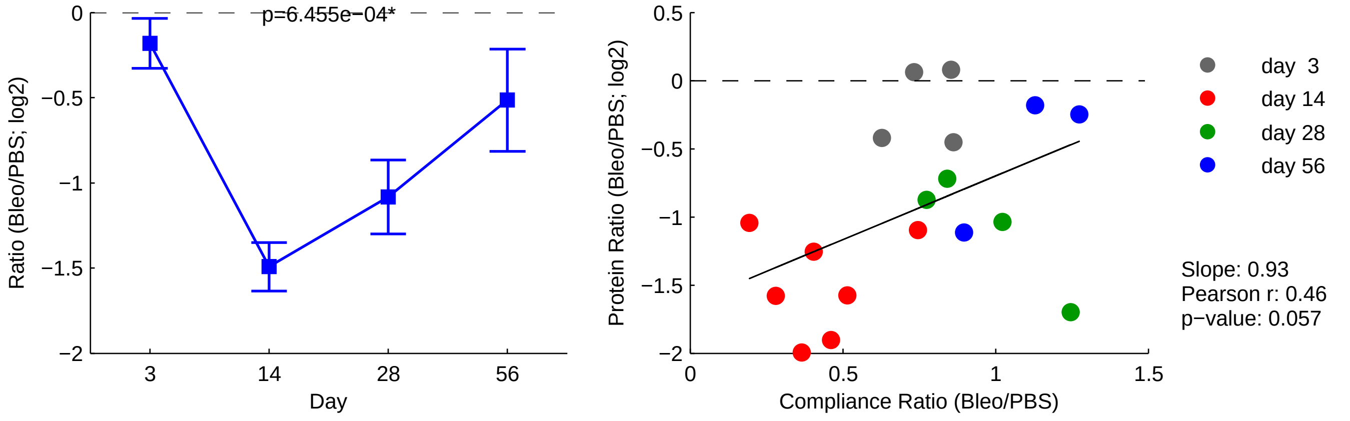

Q03137−2 − Epha4 (id: 3561)

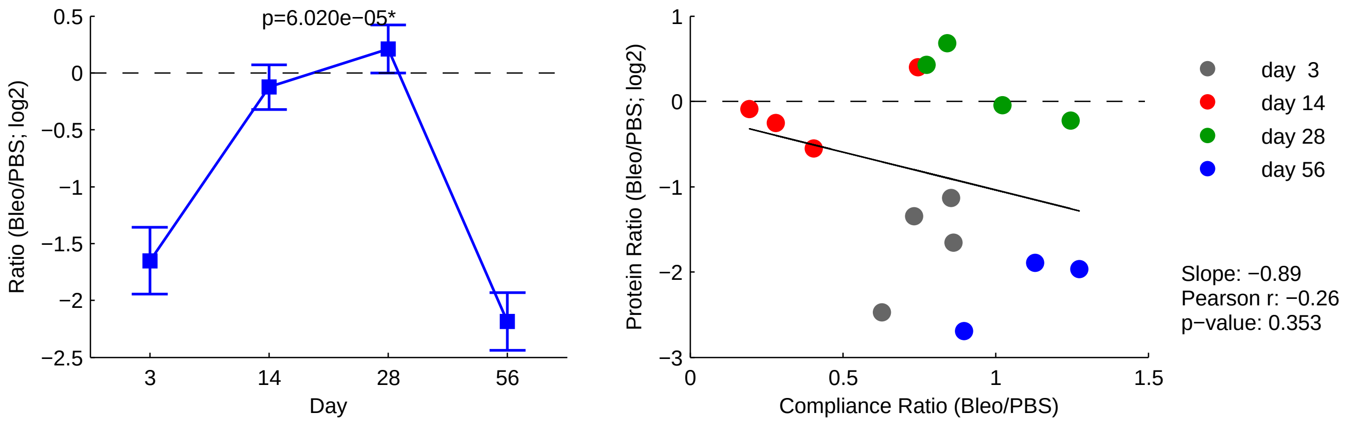

Q03141−3 − Mark3 (id: 3562)

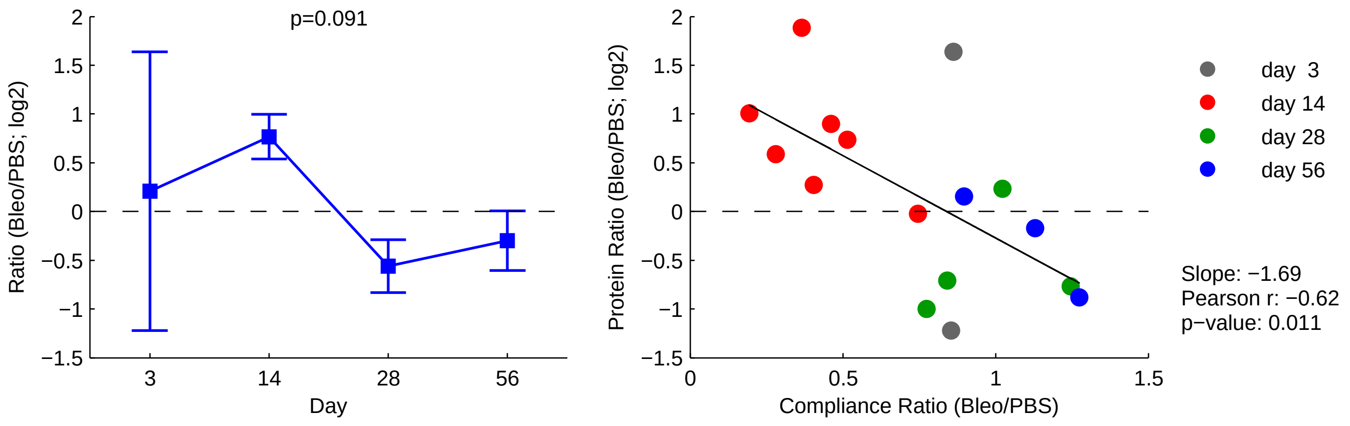

Q03265 − Atp5a1 (id: 3565)

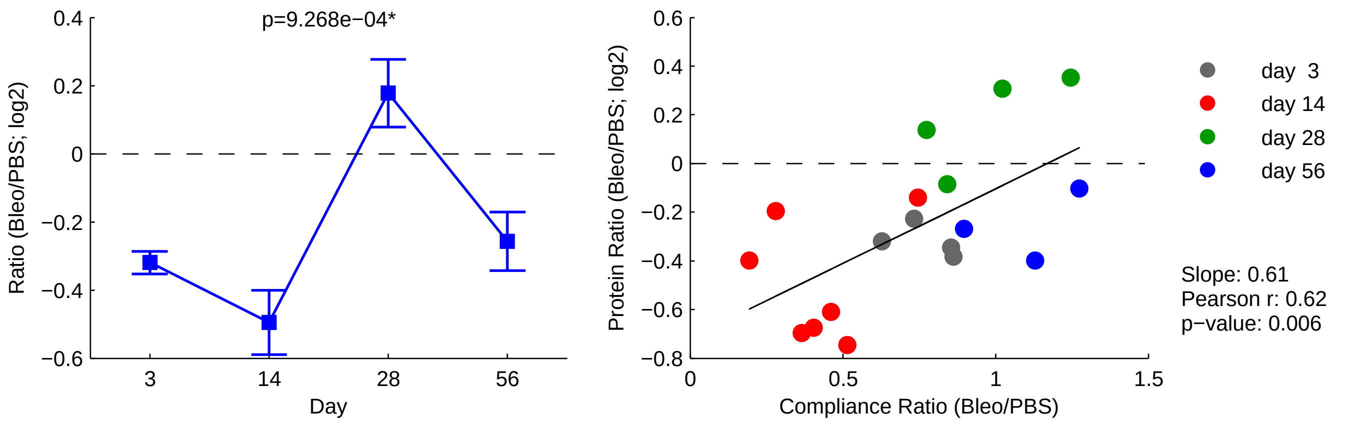

Q03958 − Pfdn6 (id: 3571)

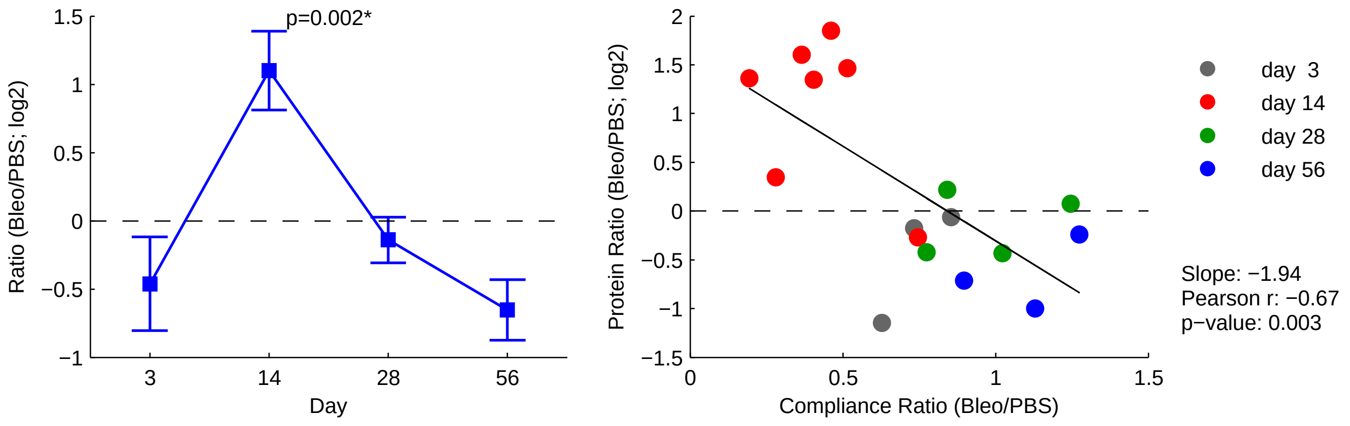

Q03963 − Eif2ak2 (id: 3572)

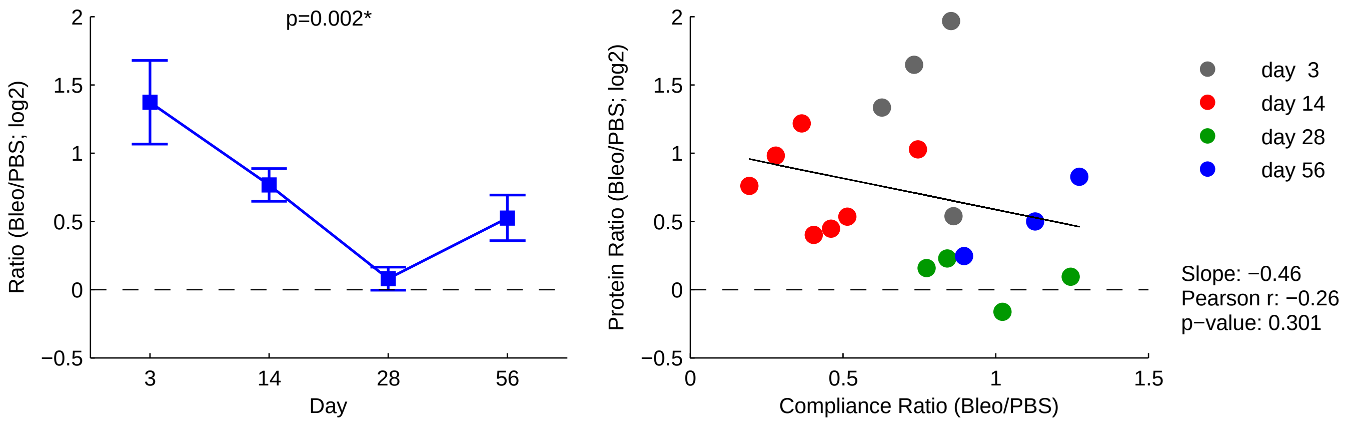

Q04447 − Ckb (id: 3574)

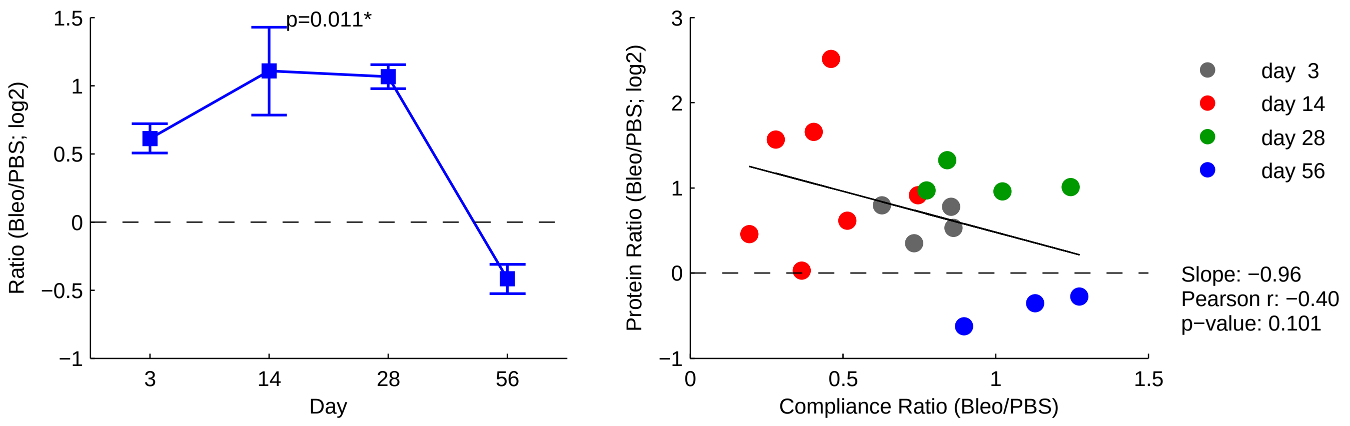

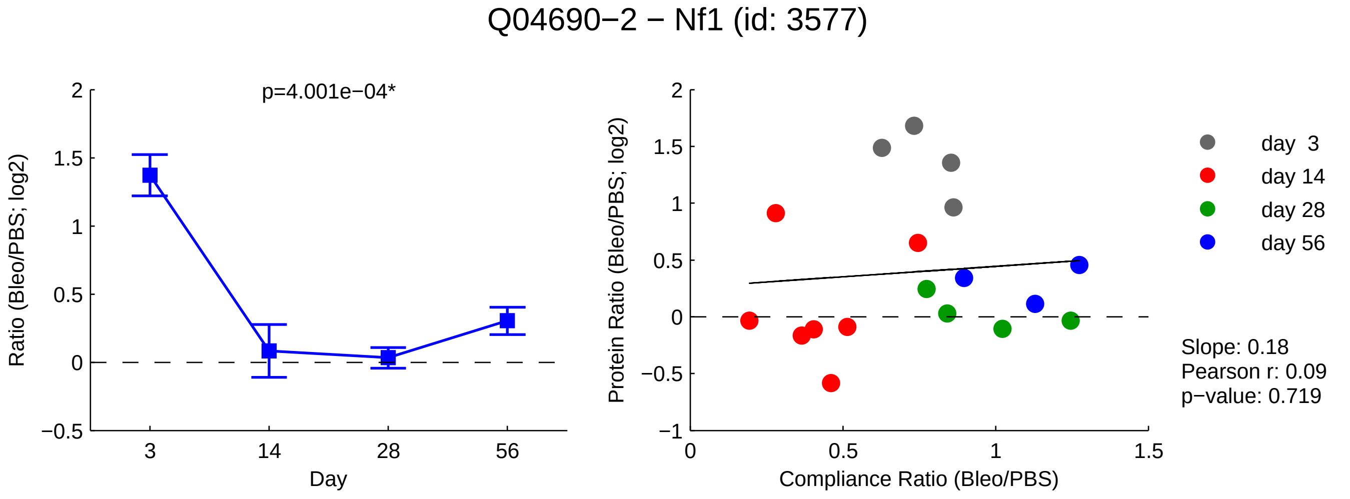
Q04690−2 − Nf1 (id: 3577)
p=4.001e−04*
Ratio (Bleo/PBS; log2)
Day
Protein Ratio (Bleo/PBS; log2)
Compliance Ratio (Bleo/PBS)
day 3
day 14
day 28
day 56
Slope: 0.18
Pearson r: 0.09
p−value: 0.719

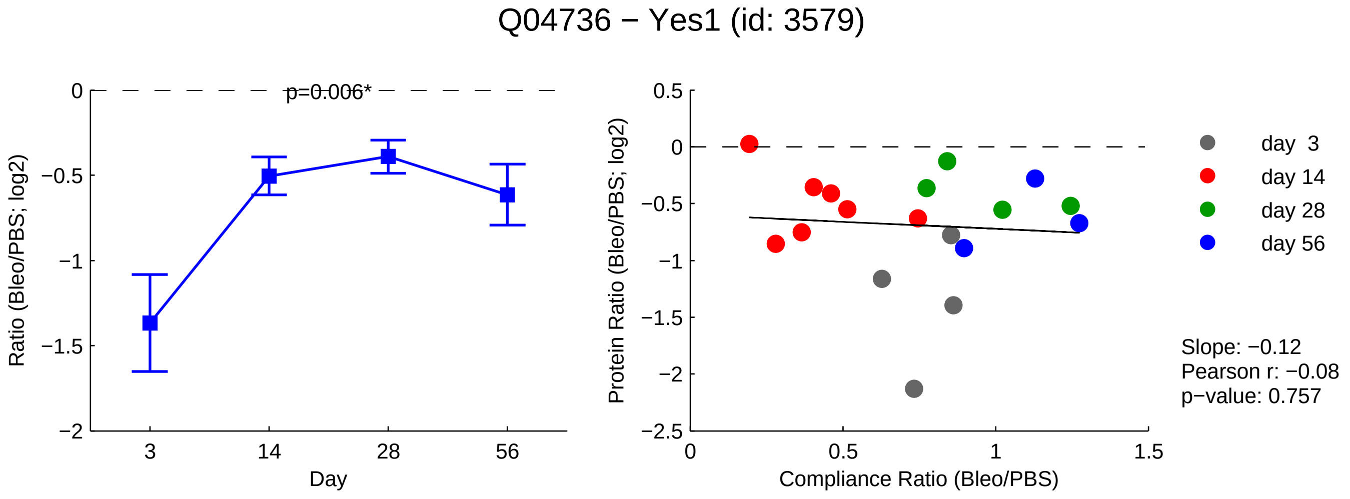
Q04736 − Yes1 (id: 3579)
p=0.006*
Ratio (Bleo/PBS; log2)
Day
Protein Ratio (Bleo/PBS; log2)
Compliance Ratio (Bleo/PBS)
day 3
day 14
day 28
day 56
Slope: −0.12
Pearson r: −0.08
p−value: 0.757

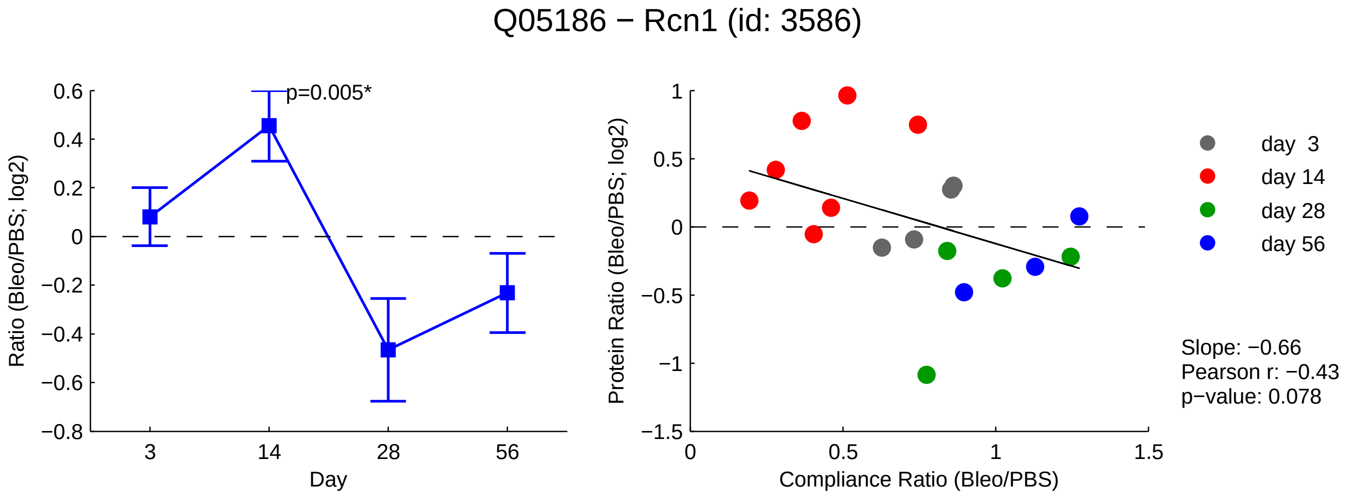
Q05186 − Rcn1 (id: 3586)
p=0.005*
Ratio (Bleo/PBS; log2)
Day
Protein Ratio (Bleo/PBS; log2)
Compliance Ratio (Bleo/PBS)
day 3
day 14
day 28
day 56
Slope: −0.66
Pearson r: −0.43
p−value: 0.078

Q05306 − Col10a1 (id: 3587)

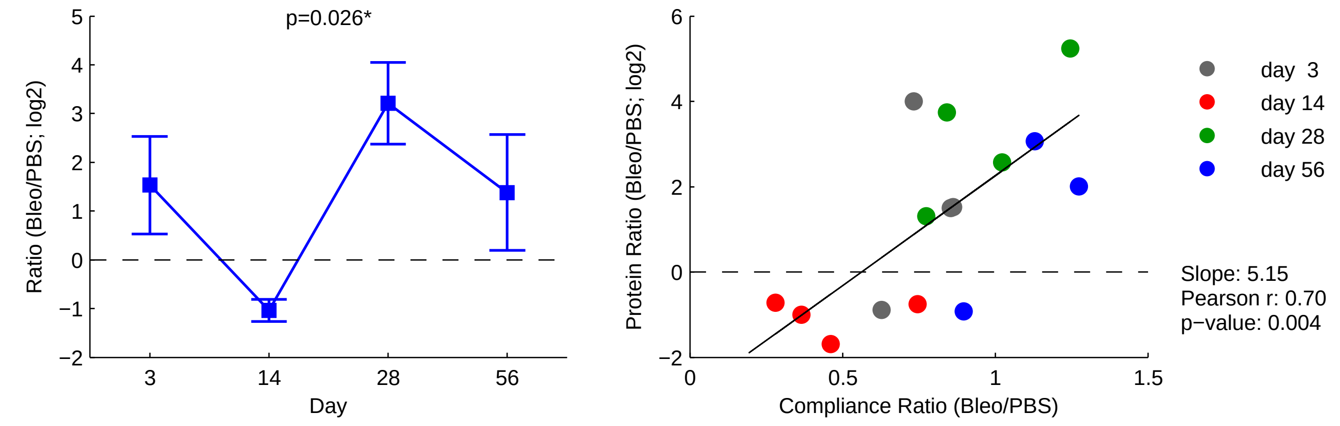

Q05BC3−2 − Eml1 (id: 3592)

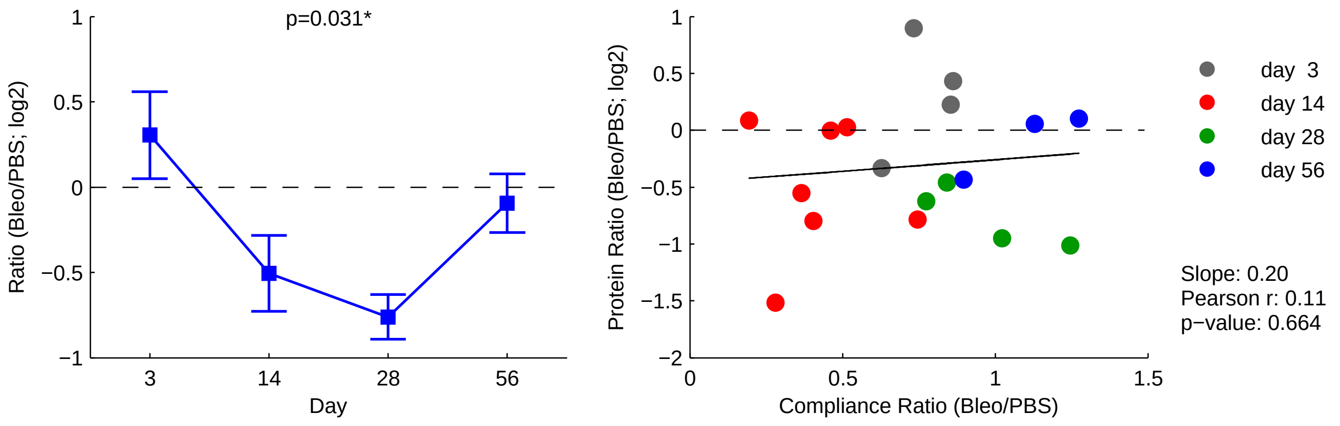

Q05C51 − Rnaseh2a (id: 3593)

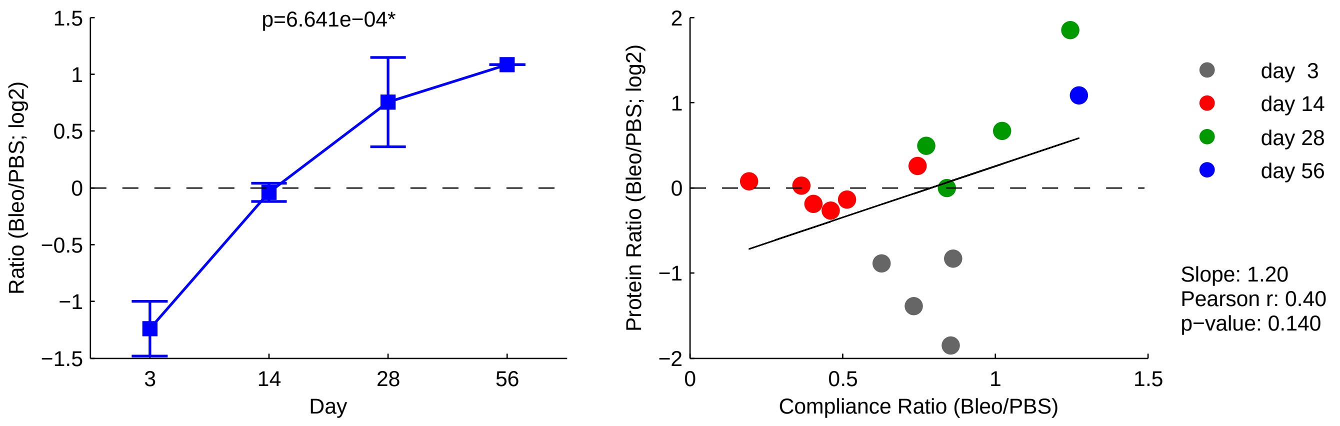

Q05D44 − Eif5b (id: 3595)

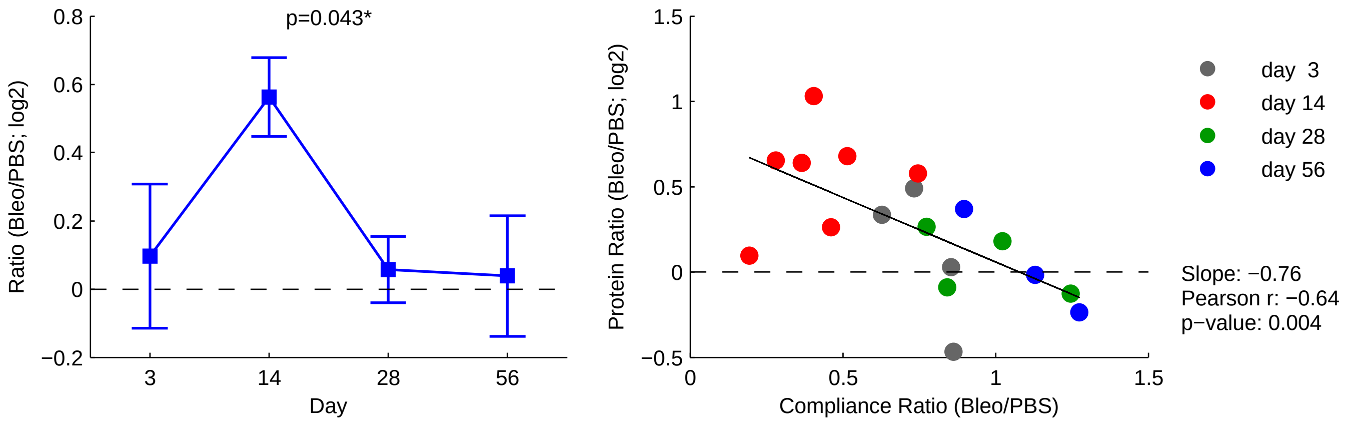

Q06138 − Cab39 (id: 3596)

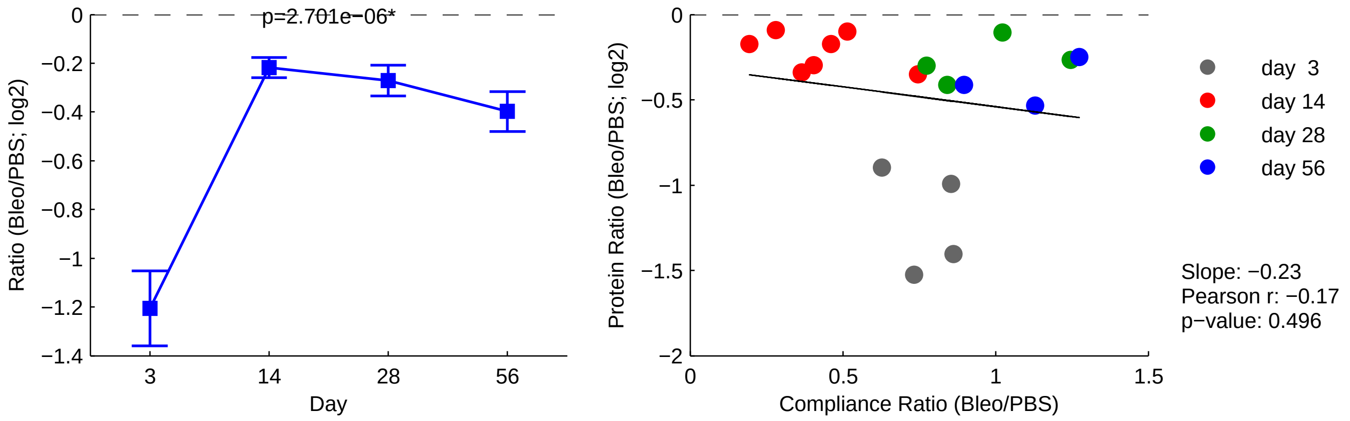

Q06180−2 − Ptpn2 (id: 3597)

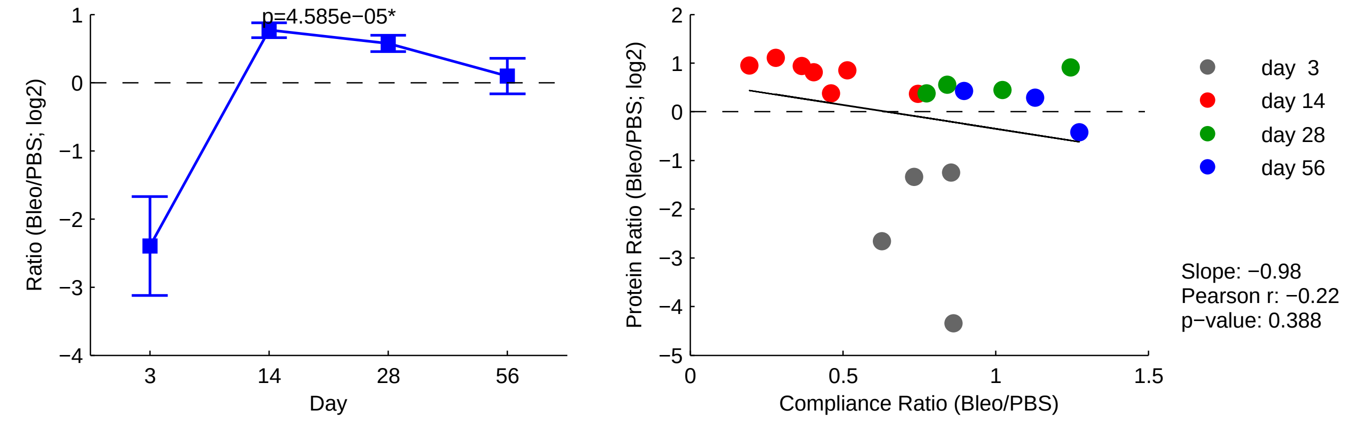

Q06185 − Atp5i (id: 3598)

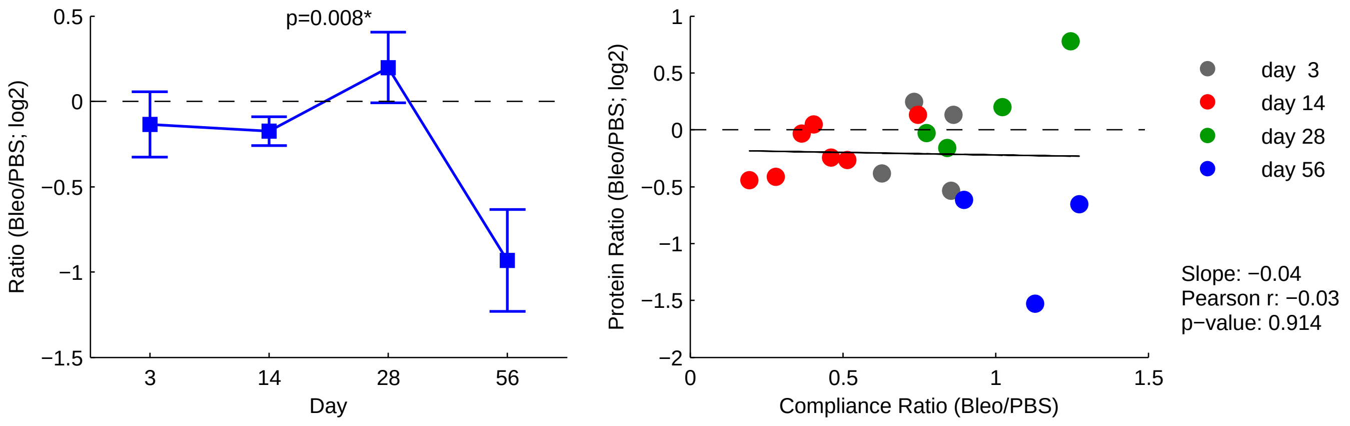

Q06318 − Scgb1a1 (id: 3599)

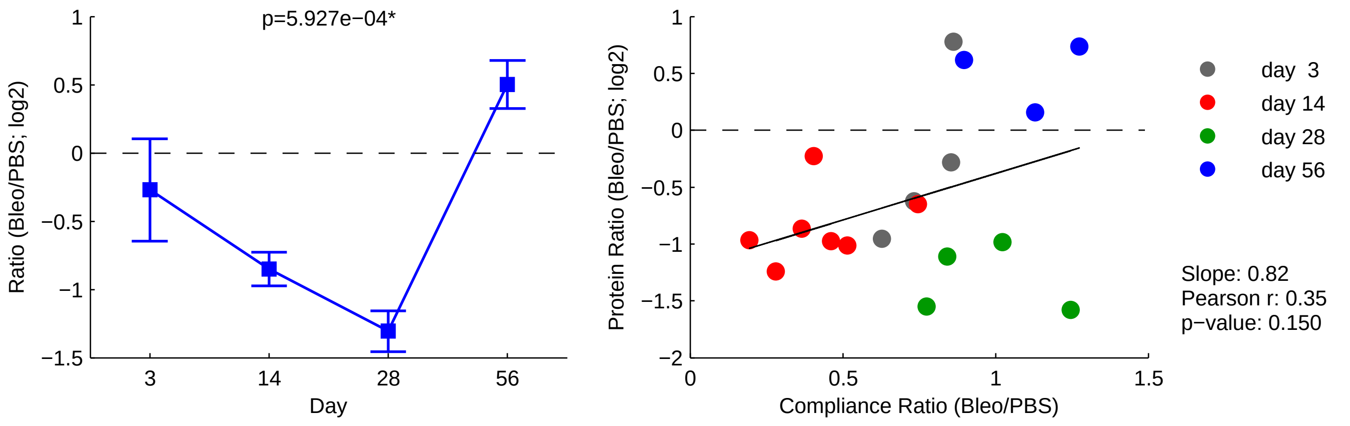

Q06335−2 − Aplp2 (id: 3600)

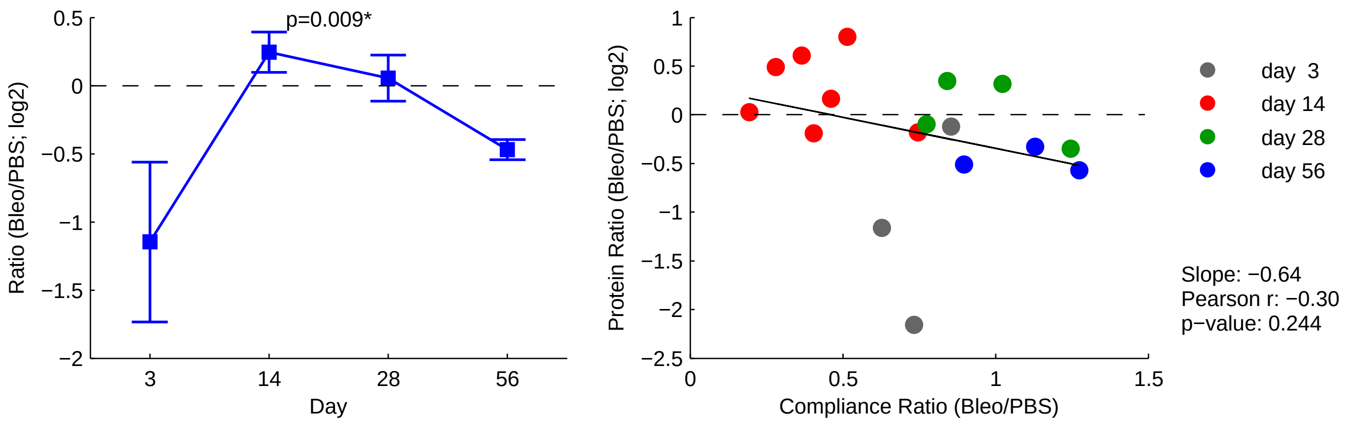

Q06806 − Tie1 (id: 3602)

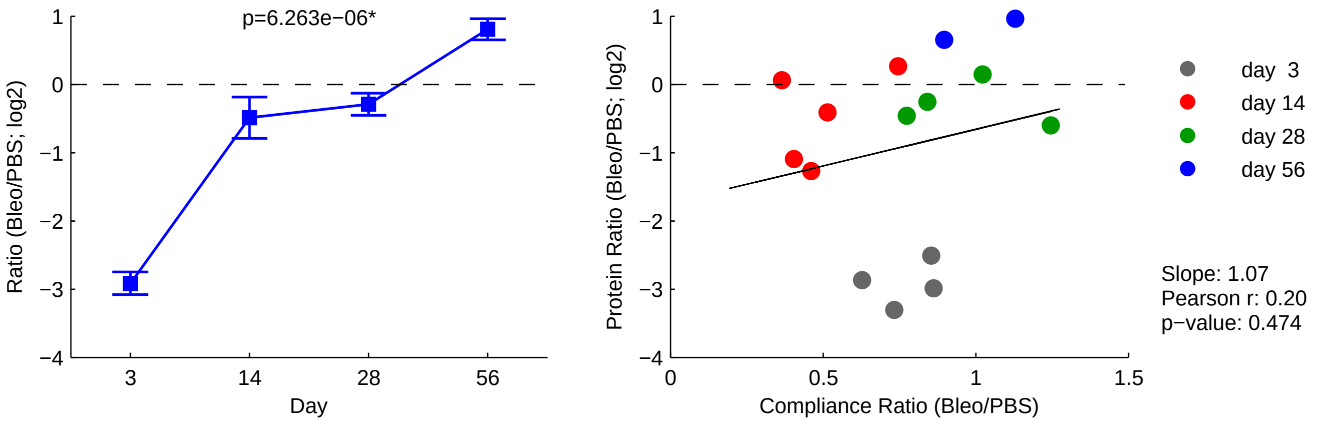

Q07076 − Anxa7 (id: 3604)

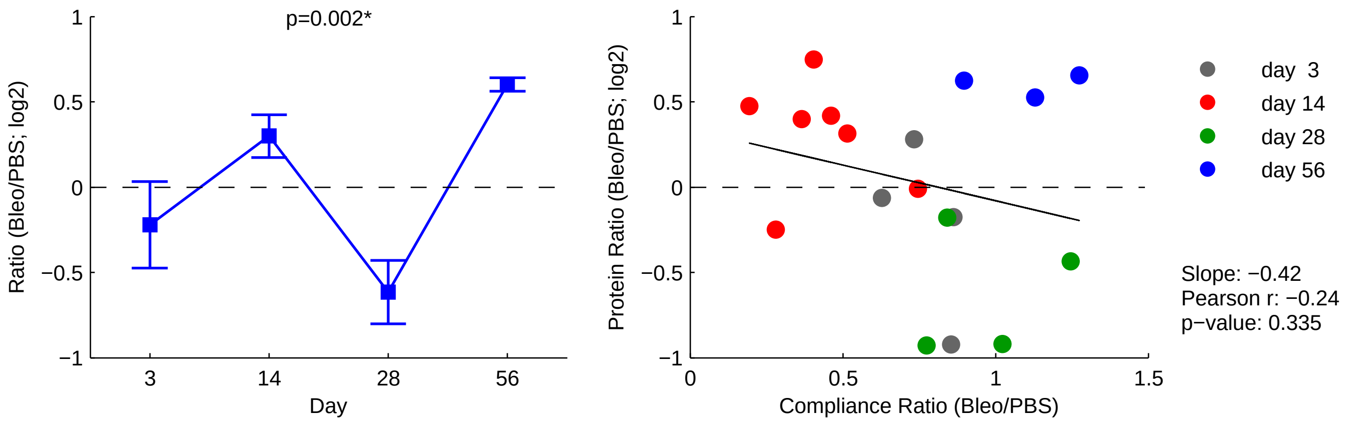

Q07113 − Igf2r (id: 3606)

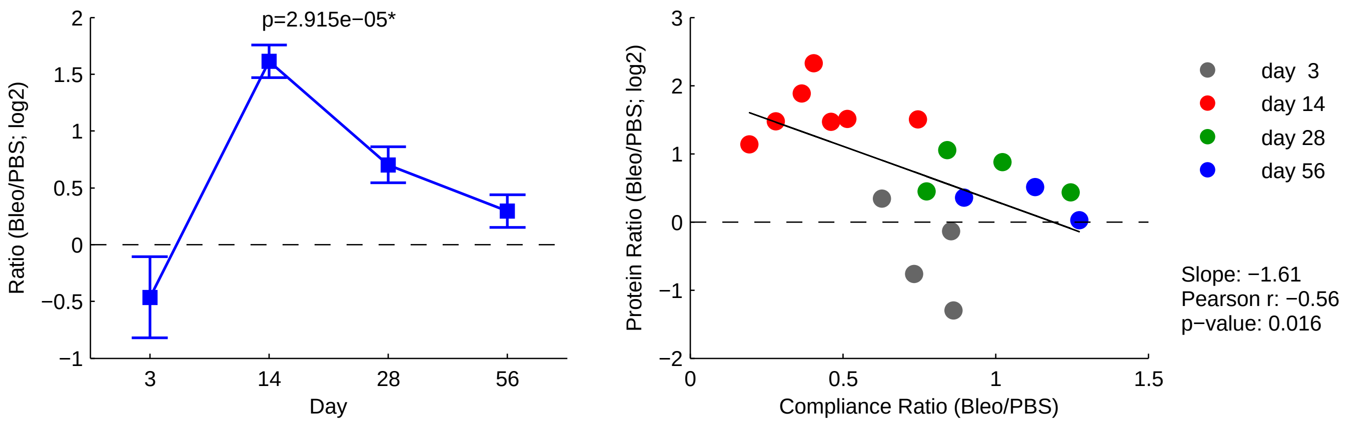

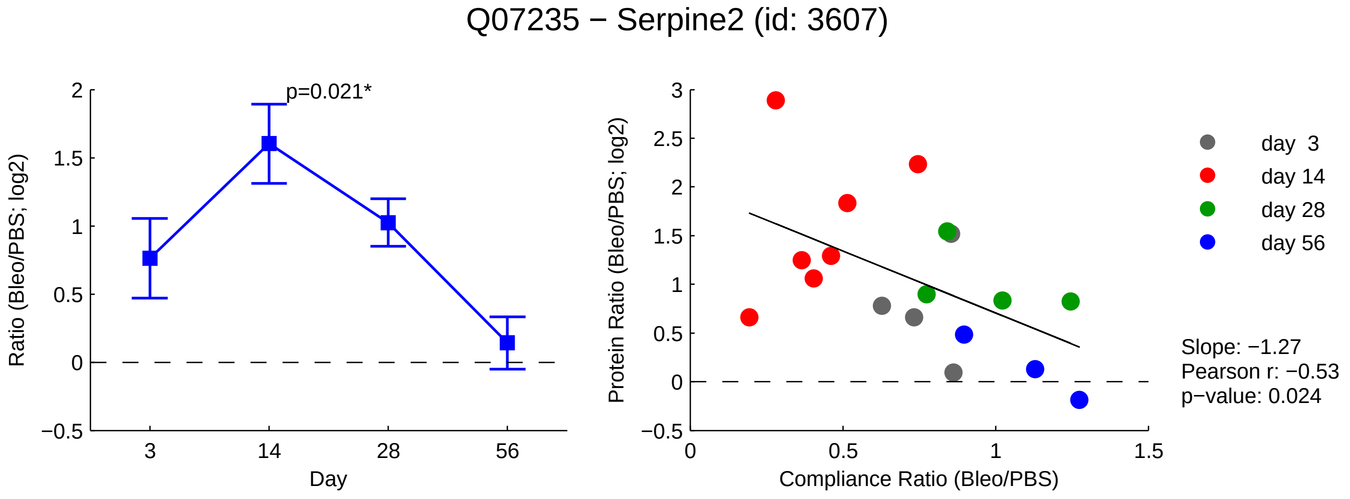
Q07235 − Serpine2 (id: 3607)
p=0.021*
Ratio (Bleo/PBS; log2)
Day
Protein Ratio (Bleo/PBS; log2)
Compliance Ratio (Bleo/PBS)
day 3
day 14
day 28
day 56
Slope: −1.27
Pearson r: −0.53
p−value: 0.024

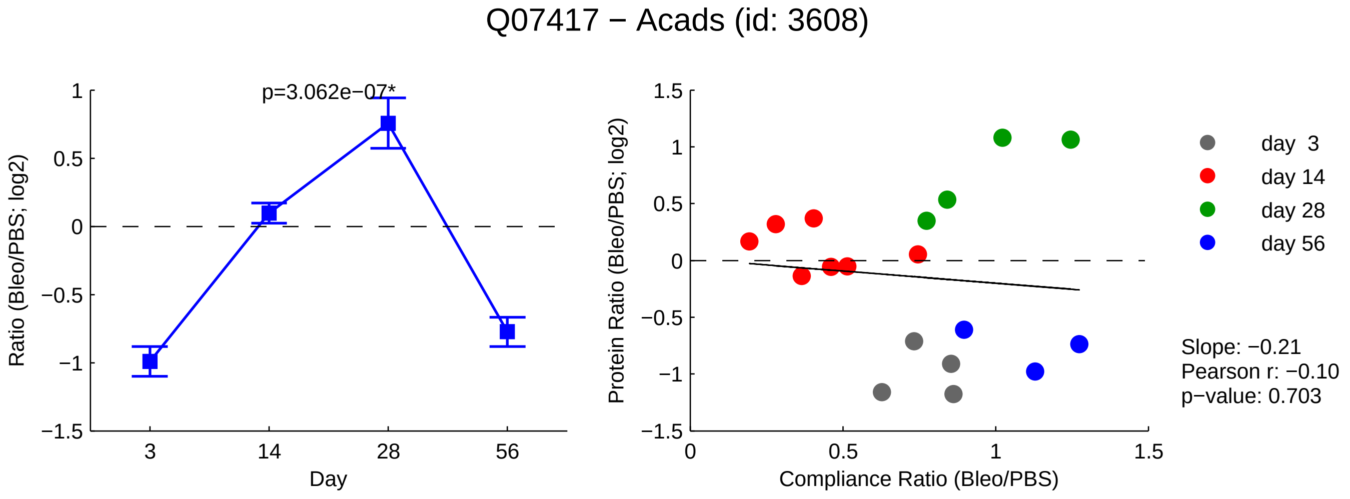
Q07417 − Acads (id: 3608)
p=3.062e−07*
Ratio (Bleo/PBS; log2)
Day
Protein Ratio (Bleo/PBS; log2)
Compliance Ratio (Bleo/PBS)
day 3
day 14
day 28
day 56
Slope: −0.21
Pearson r: −0.10
p−value: 0.703

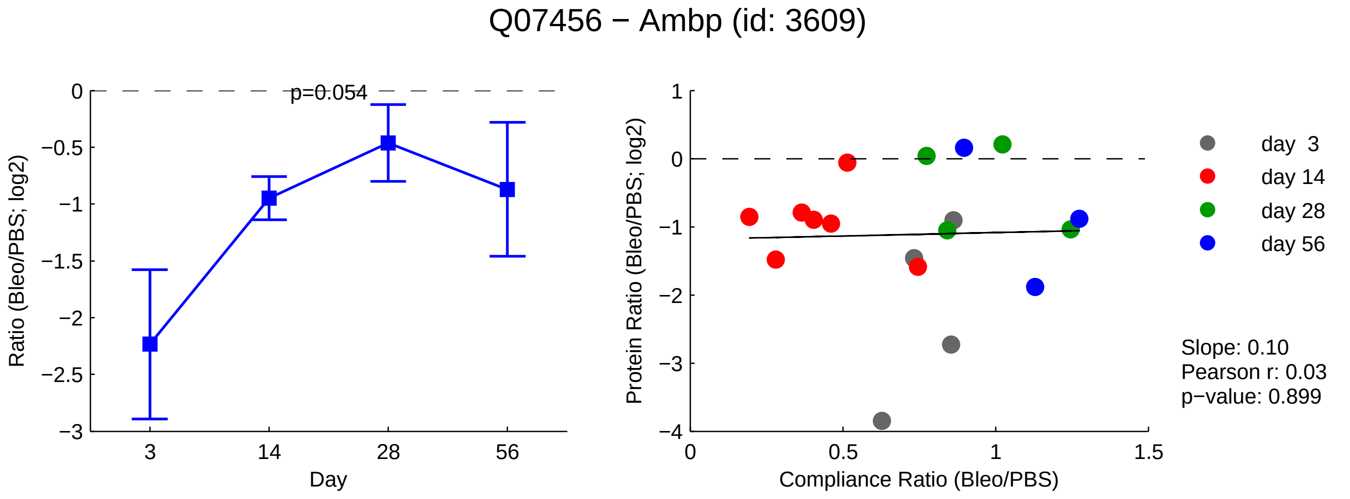
Q07456 − Ambp (id: 3609)
p=0.054
Ratio (Bleo/PBS; log2)
Day
Protein Ratio (Bleo/PBS; log2)
Compliance Ratio (Bleo/PBS)
day 3
day 14
day 28
day 56
Slope: 0.10
Pearson r: 0.03
p−value: 0.899

Q07797 − Lgals3bp (id: 3611)

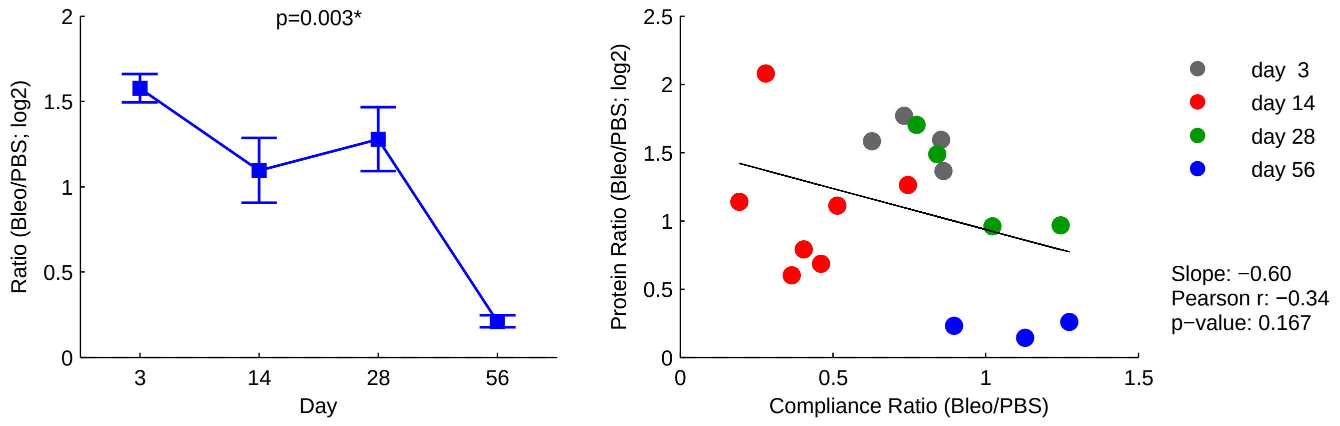

Q07813 − Bax (id: 3612)

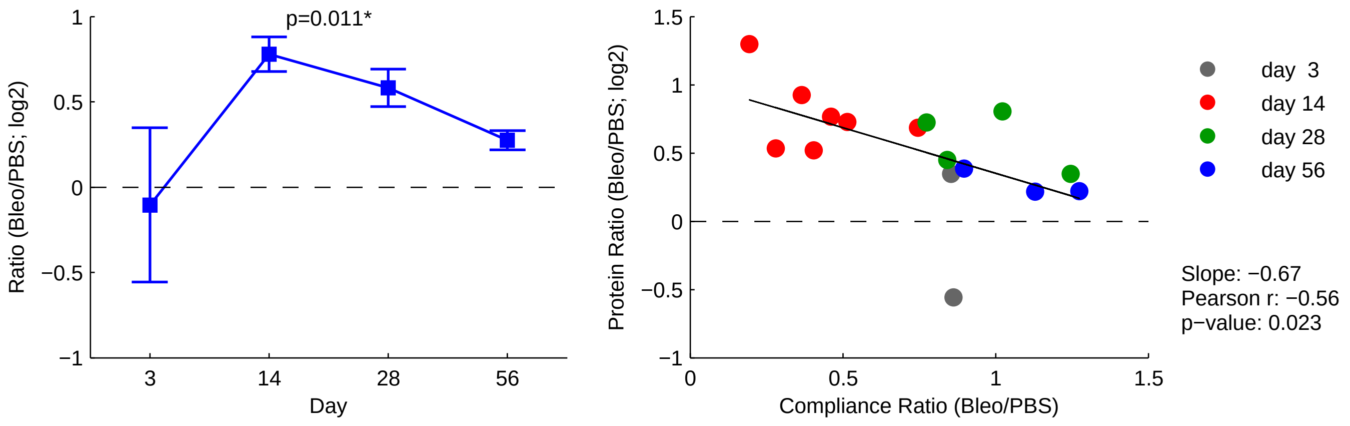

Q07968 − F13b (id: 3614)

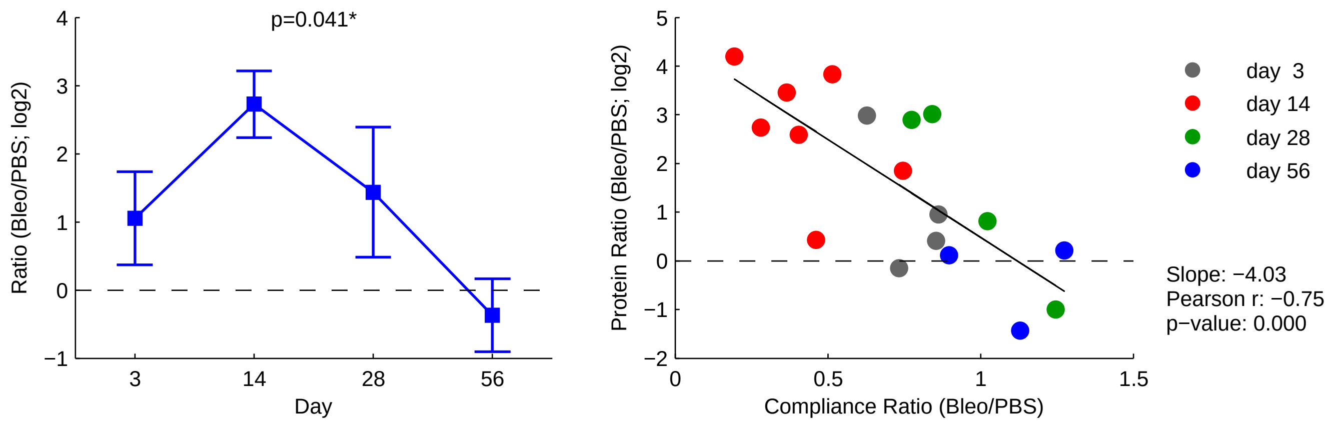

Q08091 − Cnn1 (id: 3616)

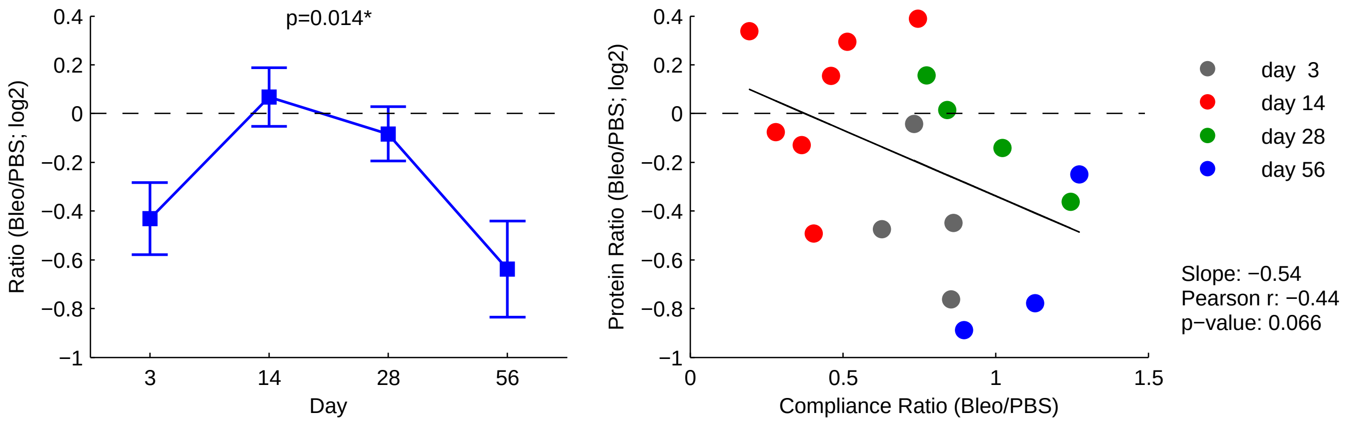

Q08093 − Cnn2 (id: 3617)

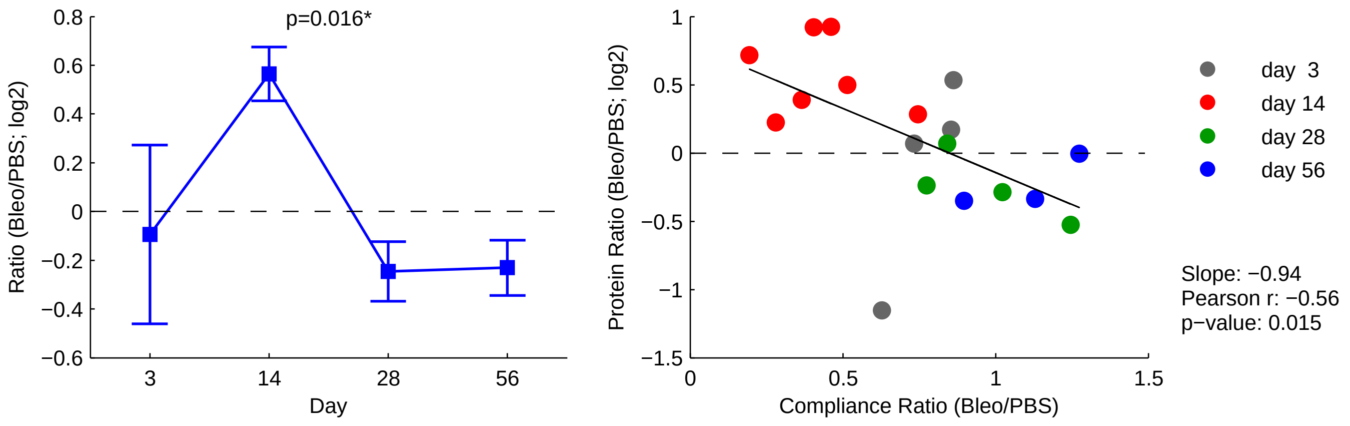

Q08481 − Pecam1 (id: 3621)

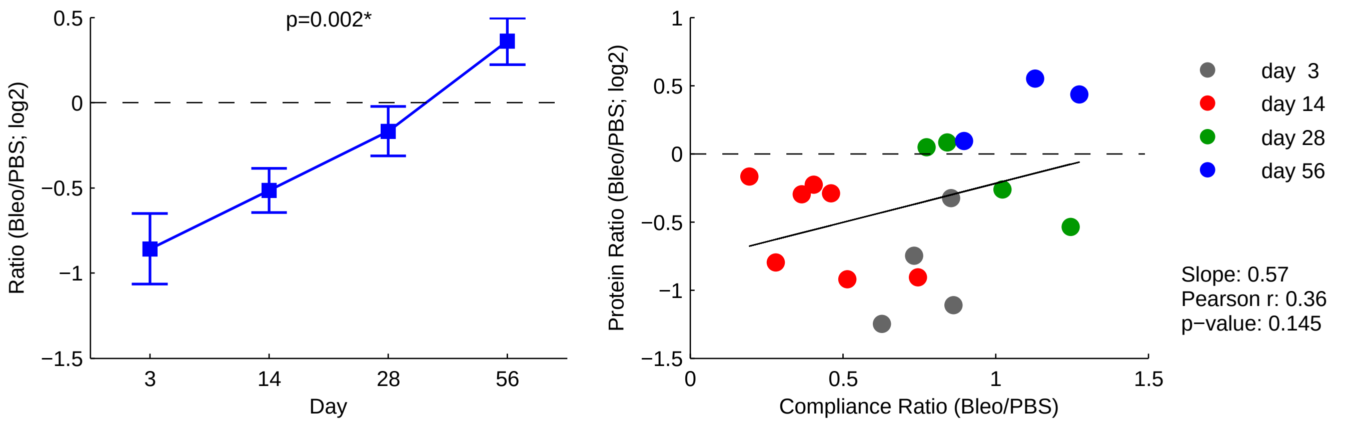

Q08509 − Eps8 (id: 3623)

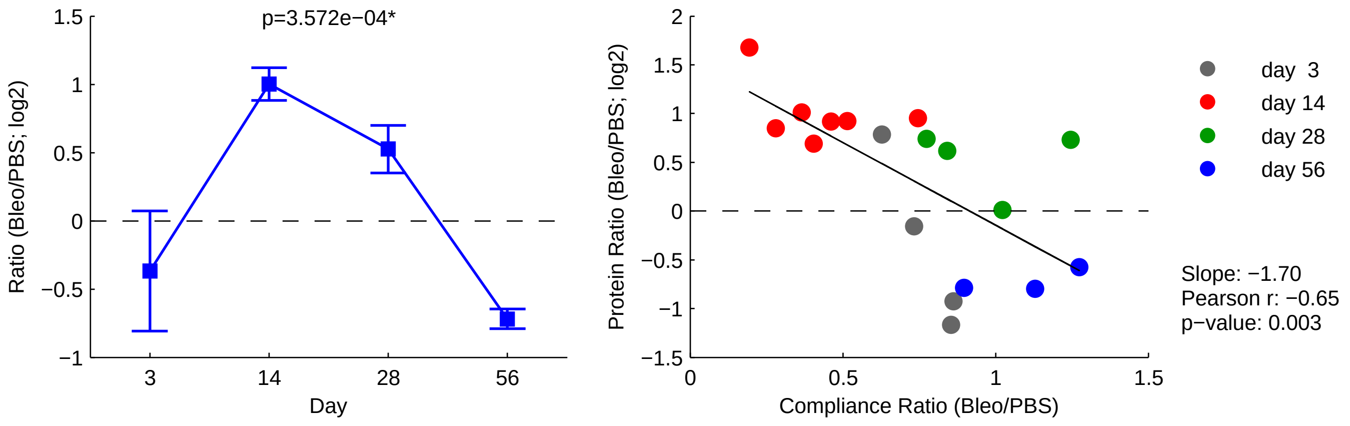

Q08857 − Cd36 (id: 3628)

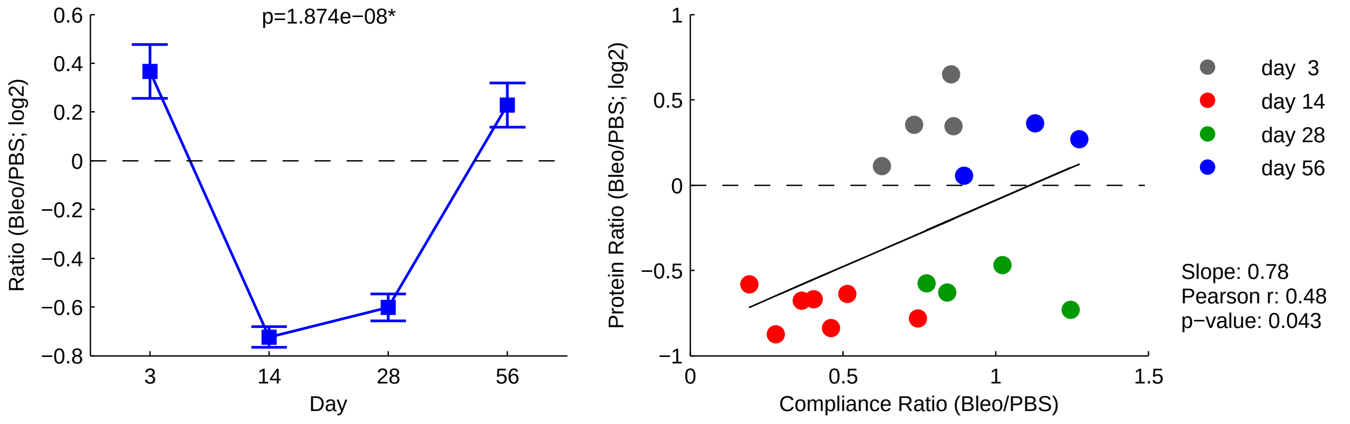

Q09200 − B4galnt1 (id: 3633)

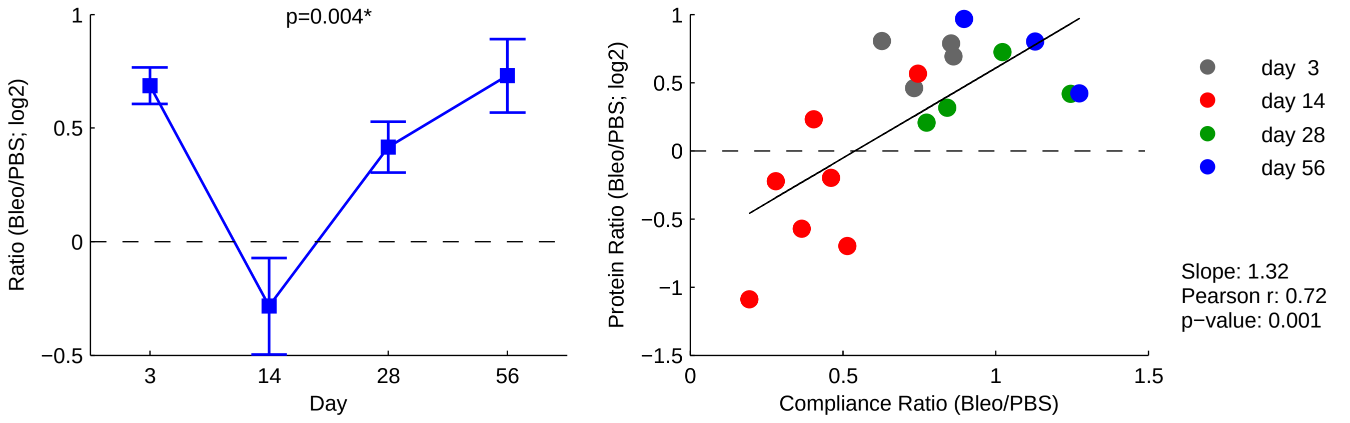

Q0KL02−3 − Trio (id: 3635)

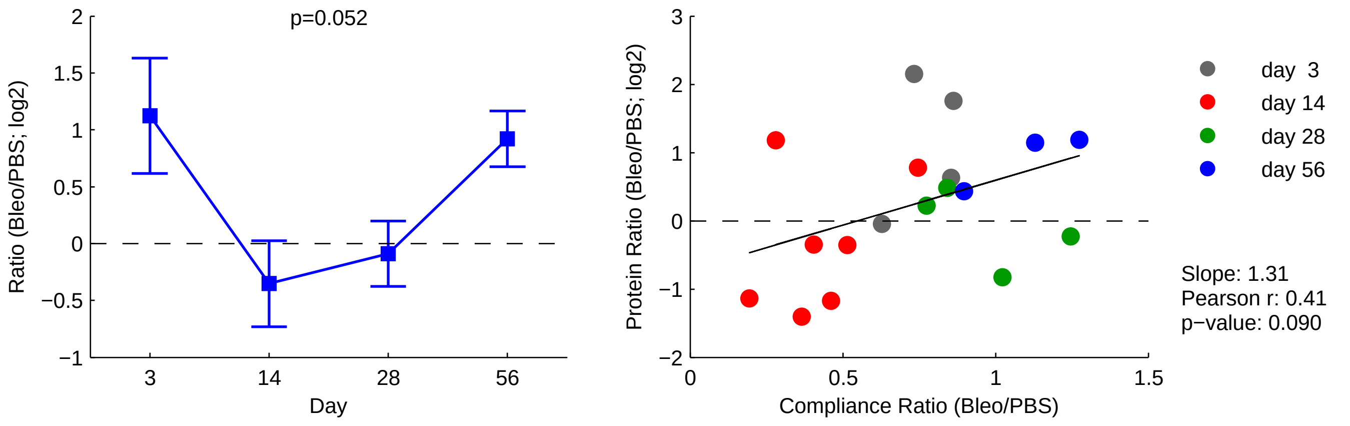

Q0P557 − Spata18 (id: 3636)

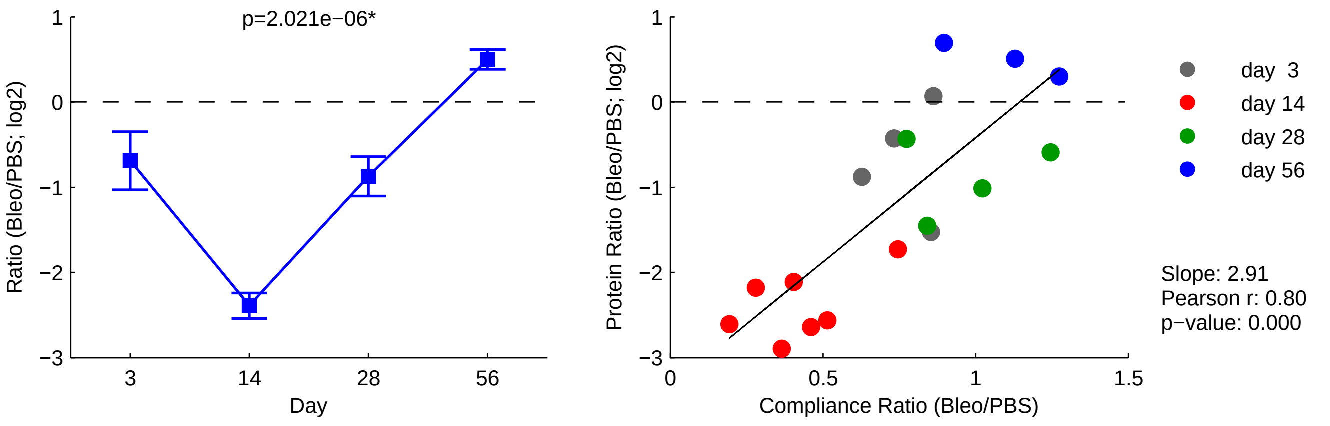

Q0VF55 − Atp2b3 (id: 3644)

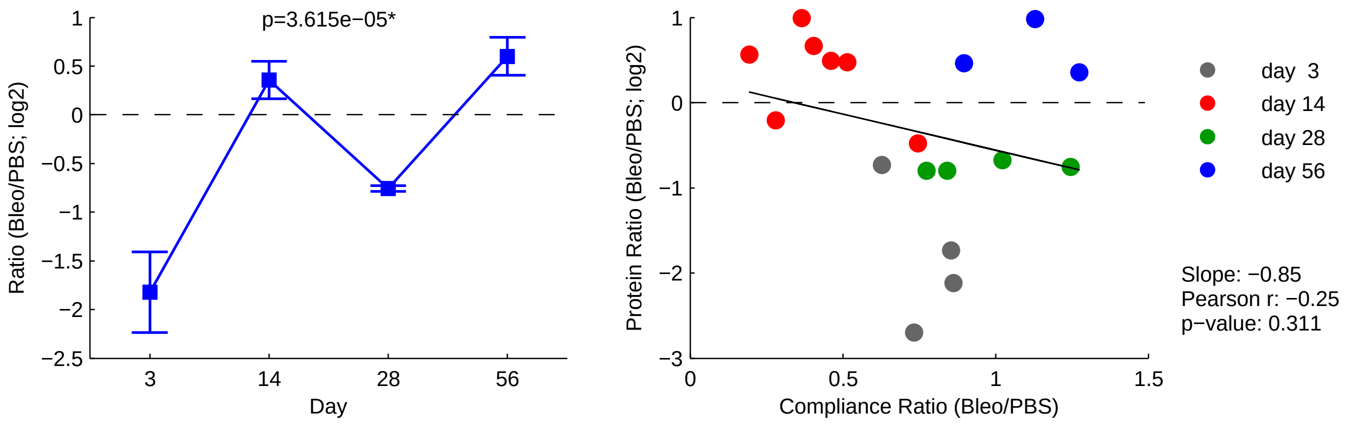

Q149L6 − Dnajb14 (id: 3654)

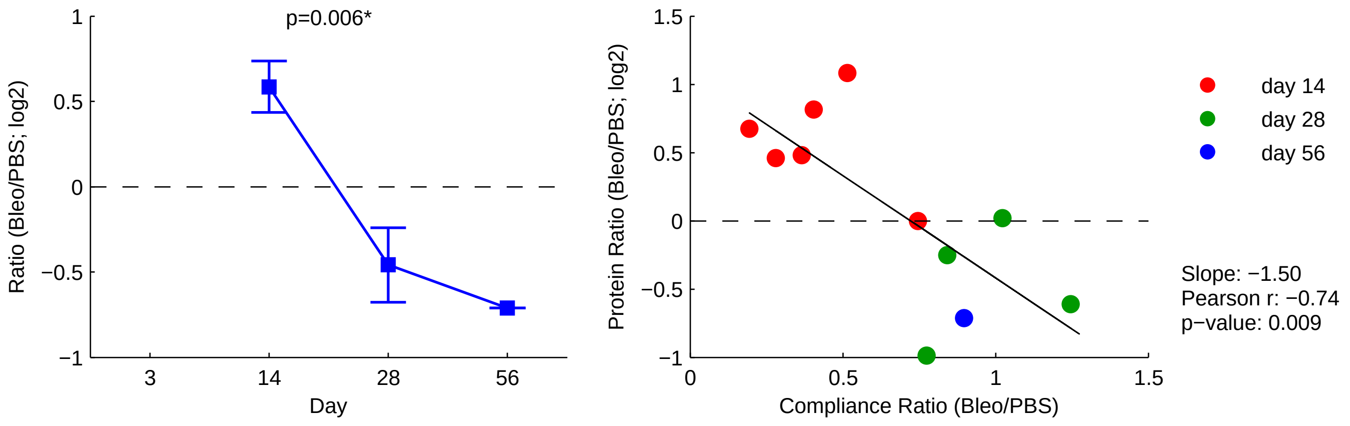

Q14B65 − Cep95 (id: 3660)

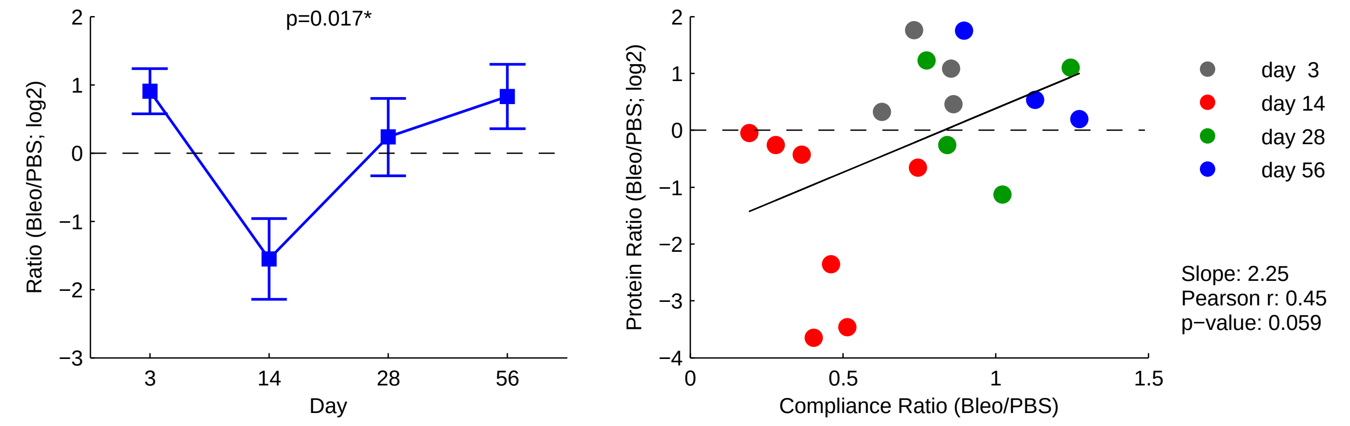

Q14BI5 − Myom2 (id: 3661)

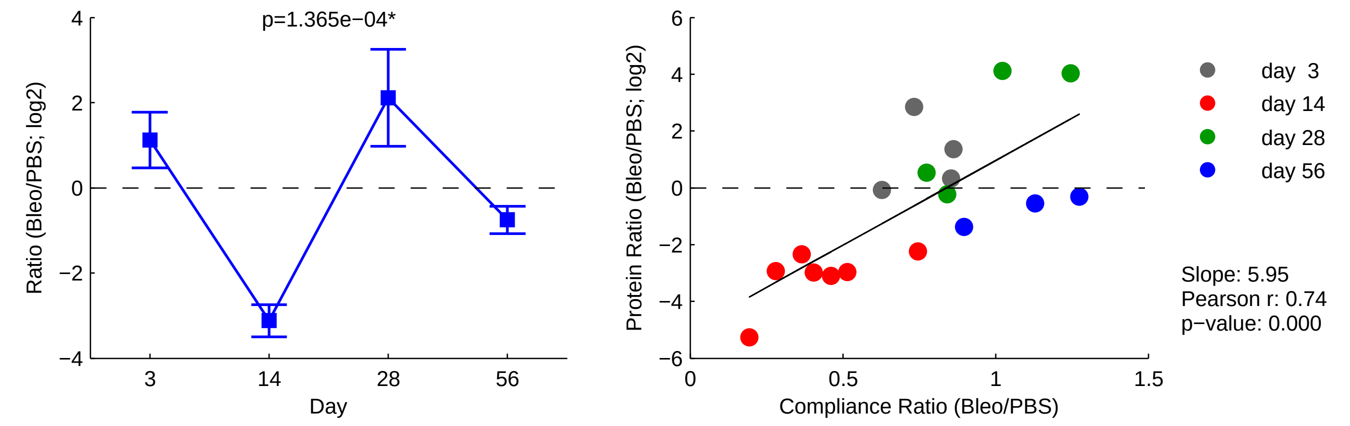

Q14C51 − Ptcd3 (id: 3663)

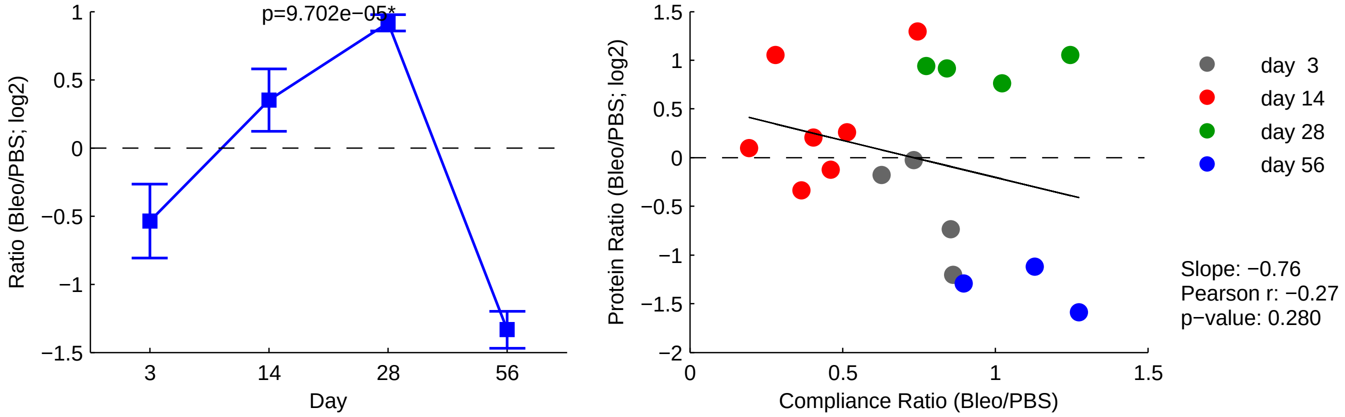

Q14DH7 − Acss3 (id: 3665)

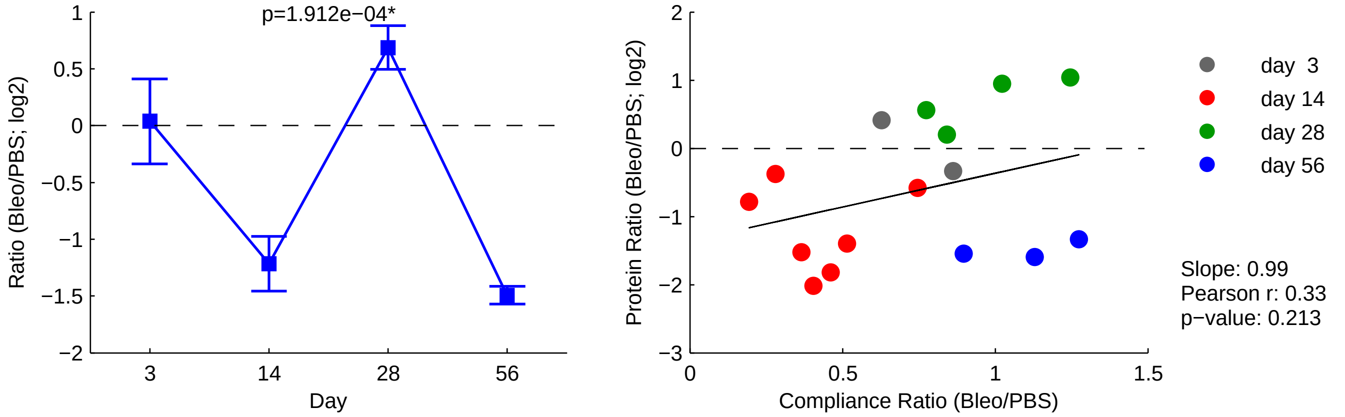

Q19LI2 − A1bg (id: 3666)

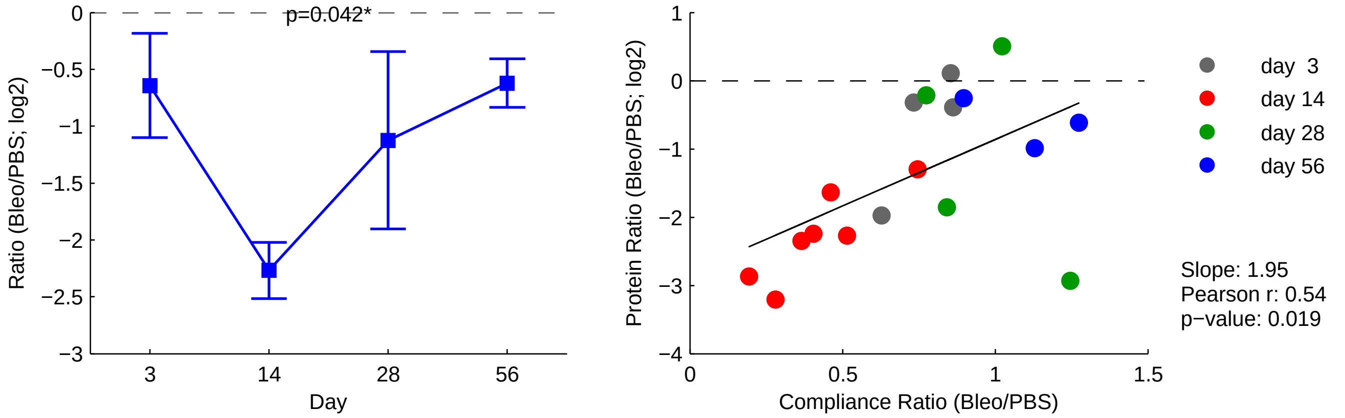

Q2EMV9 − Parp14 (id: 3671)

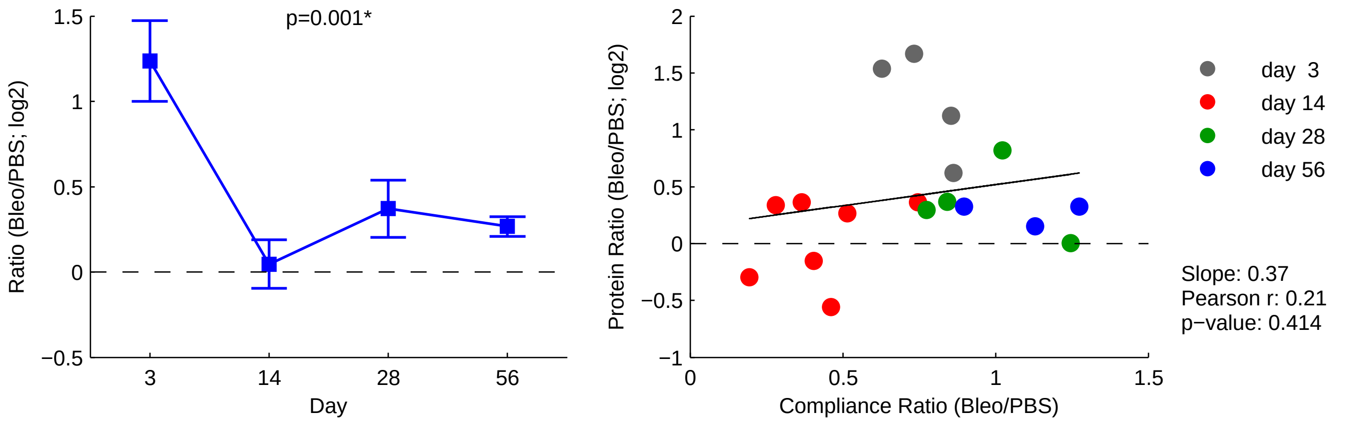

Q2HXL6 − Edem3 (id: 3672)

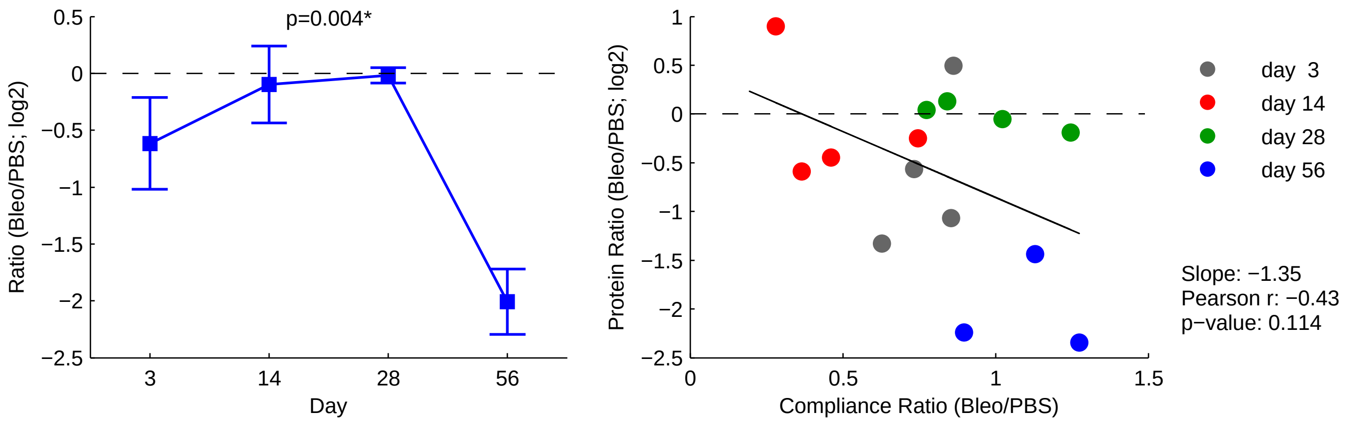

Q2TPA8 − Hsdl2 (id: 3678)

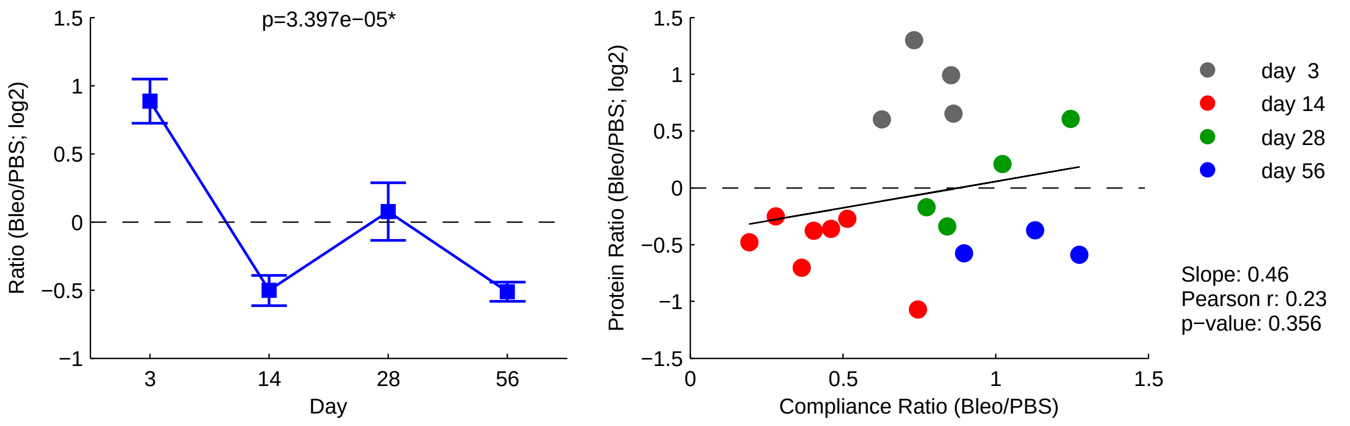

Q2UY11 − Col28a1 (id: 3680)

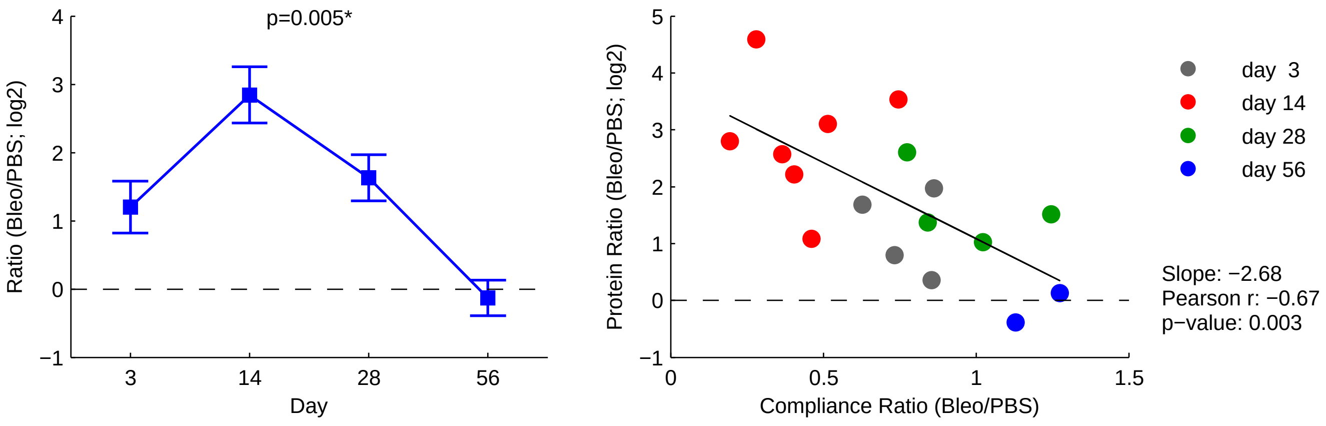

Q2VPQ9 − Meaf6 (id: 3682)

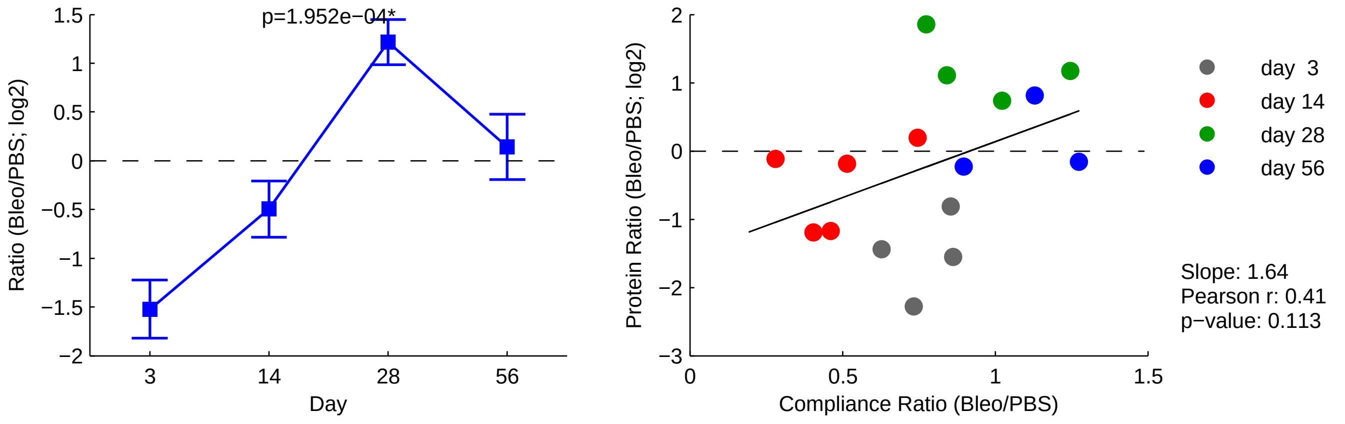

Q31094 − H2−DMb1 (id: 3684)

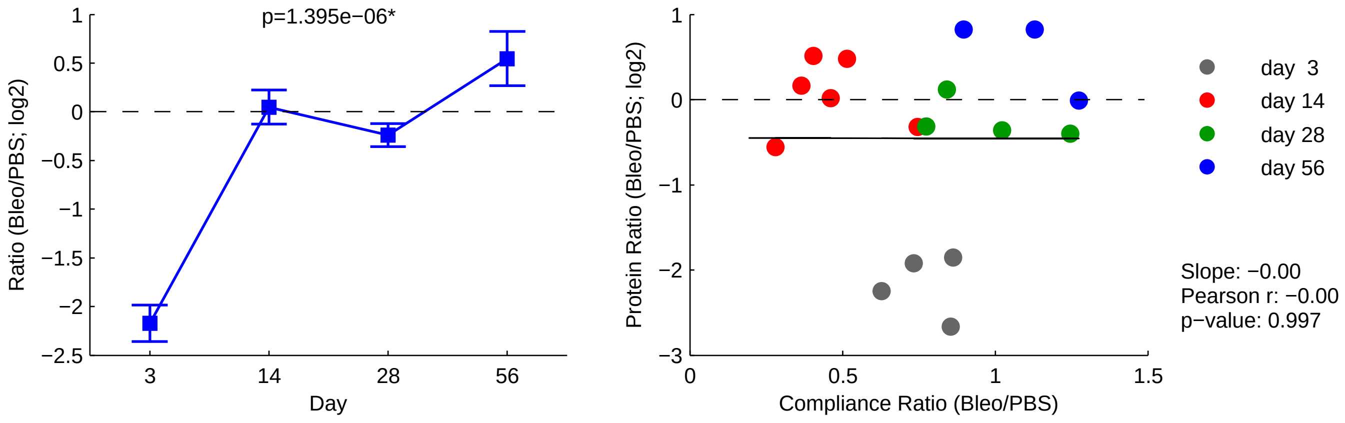

Q31125 − Slc39a7 (id: 3686)

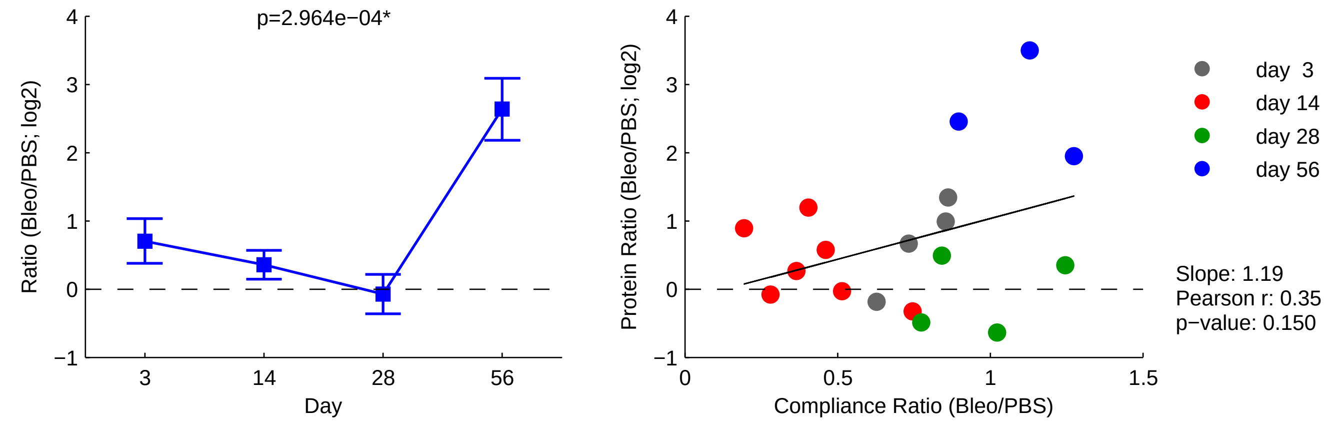

Q31621 − H2−DMa (id: 3687)

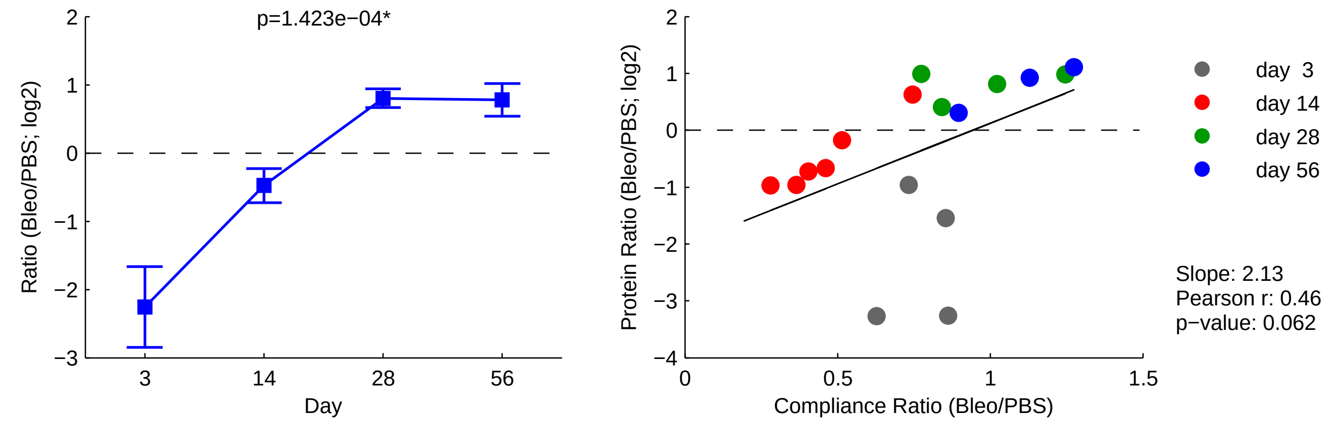

Q3B7Z2 − Osbp (id: 3690)

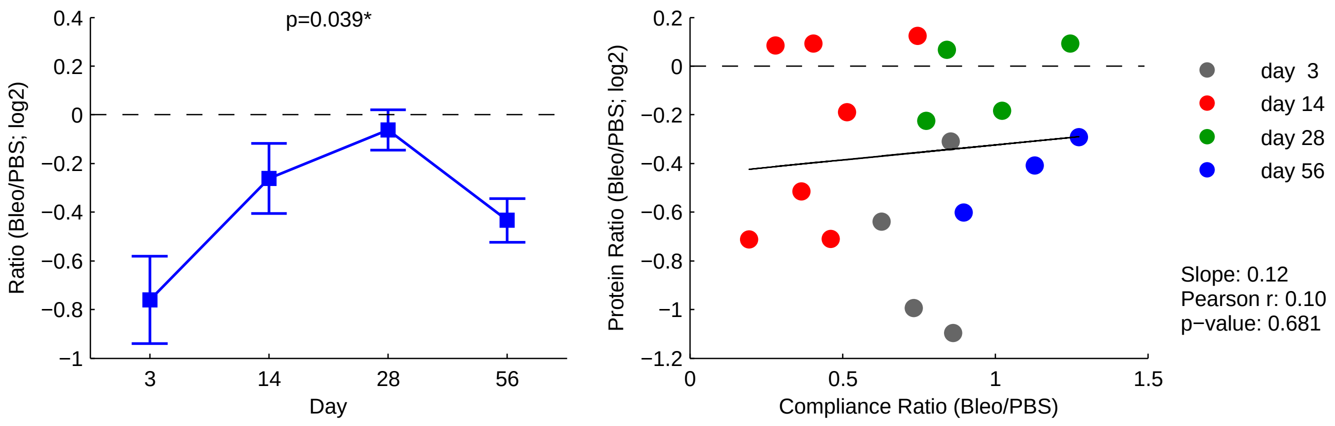

Q3LAC4 − Prex2 (id: 3692)

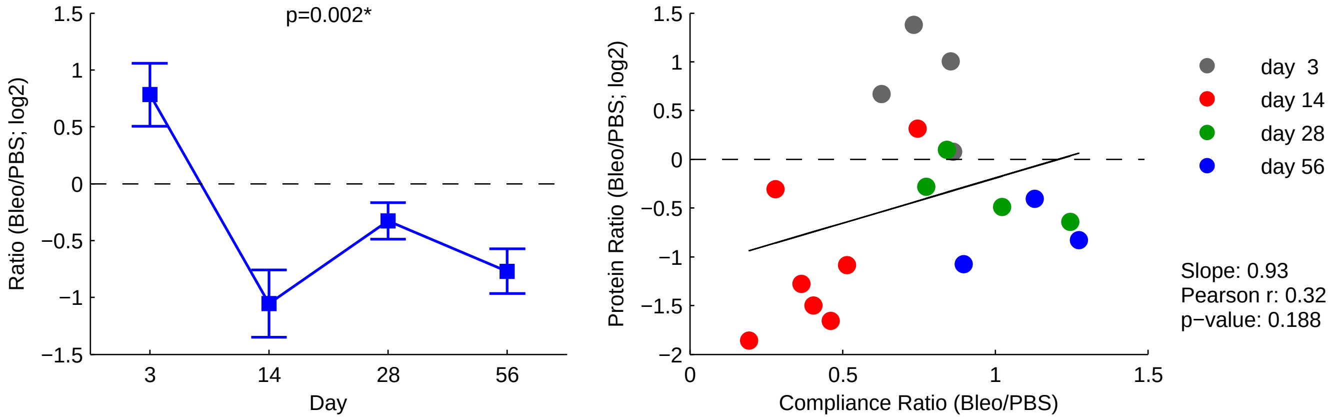

Q3T9T1 − Tmcc2 (id: 3693)

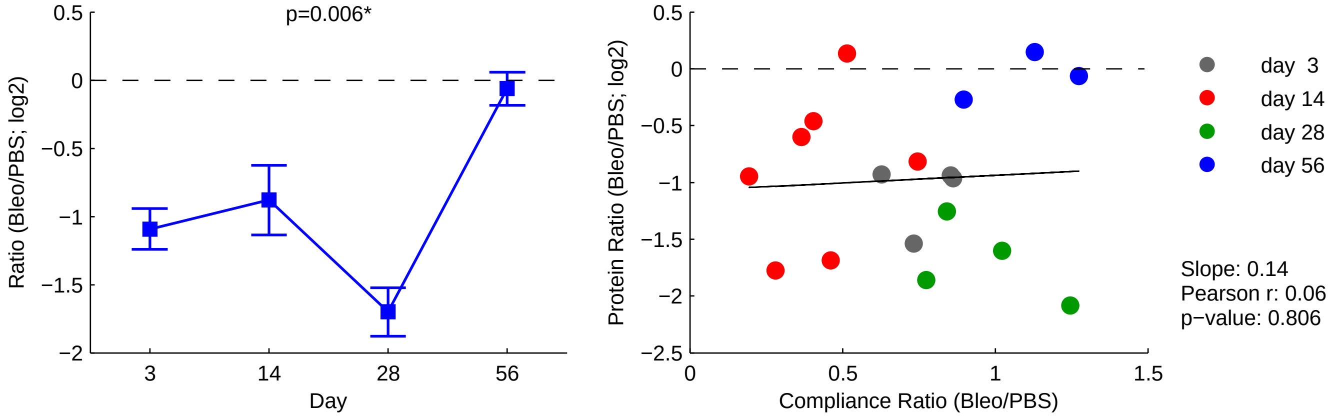

Q3TAA8 − Pigu (id: 3696)

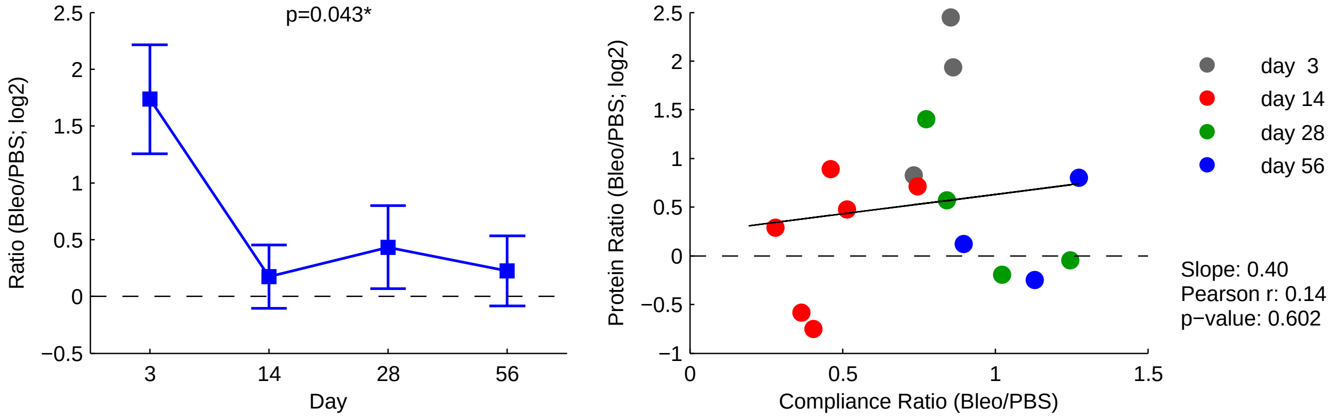

Q80WC7−2 − Agfg2 (id: 3701)

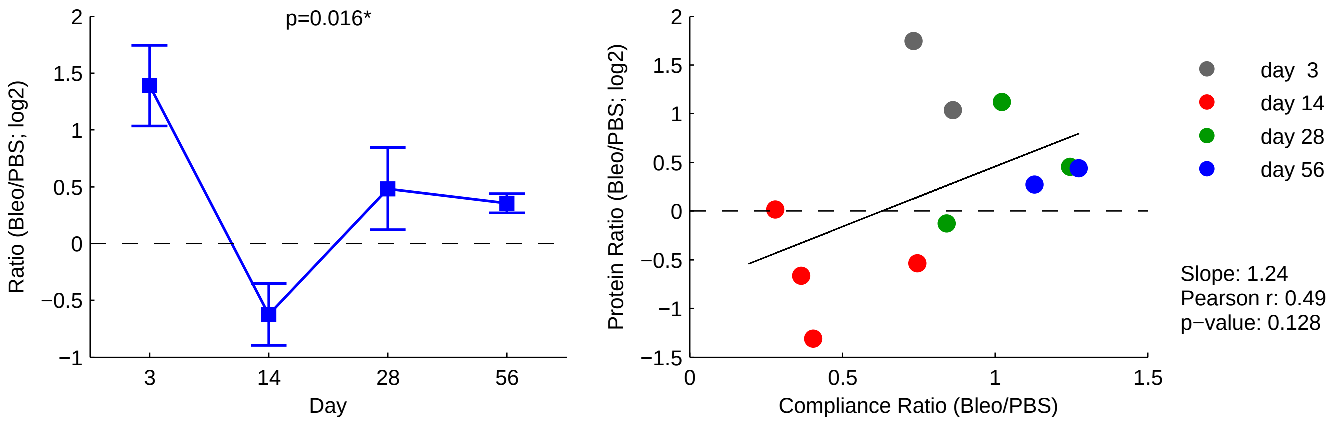

Q3TBW2 − Mrpl10 (id: 3702)

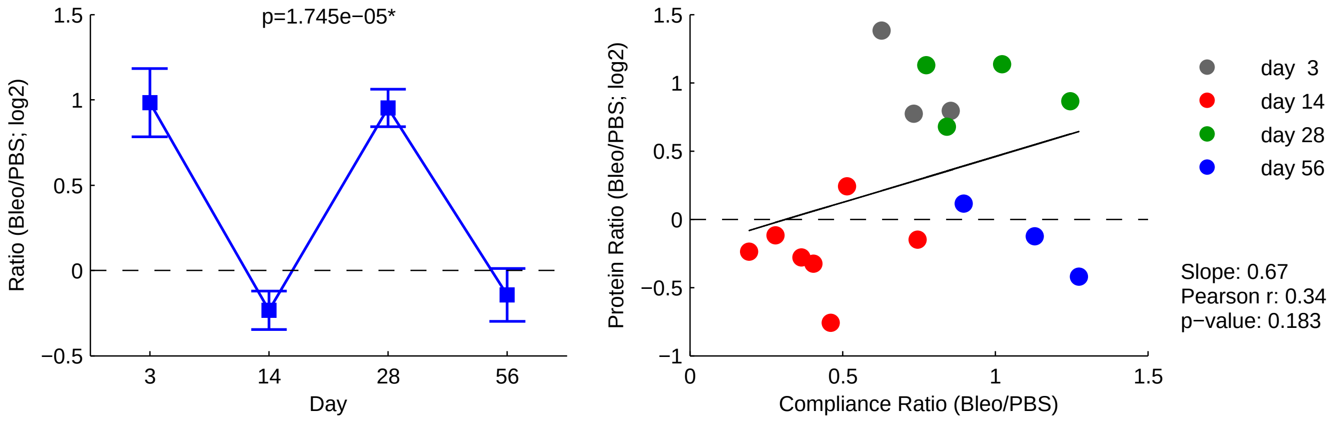

Q3TC72 − Fahd2 (id: 3705)

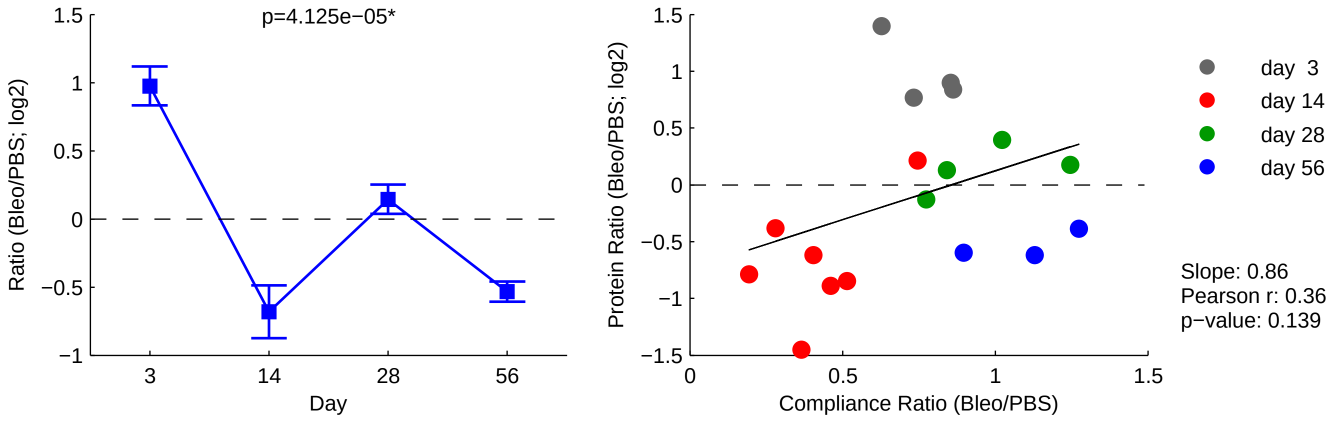

Q3TCJ1 − Fam175b (id: 3708)

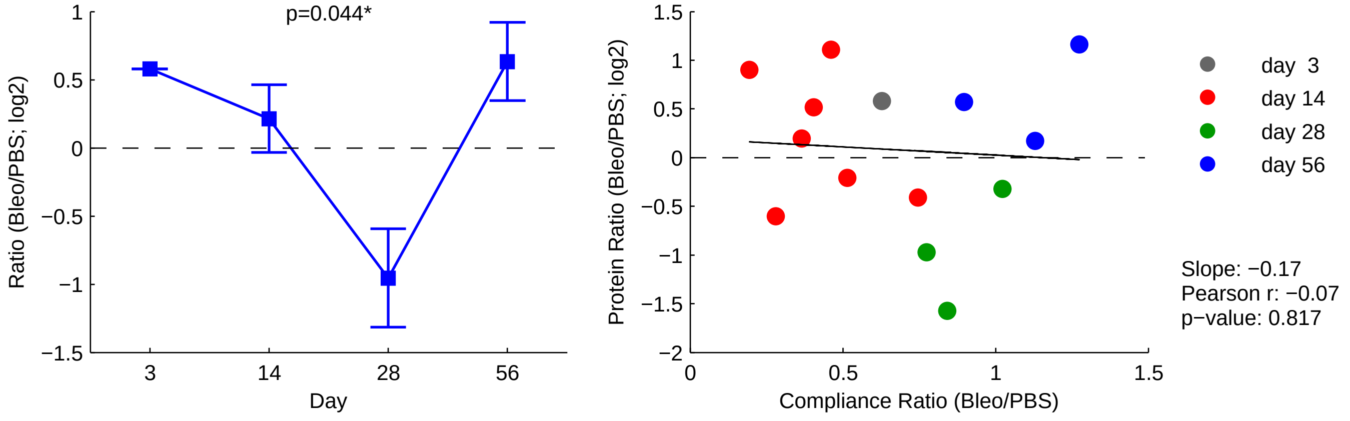

Q3TCN2 − Plbd2 (id: 3710)

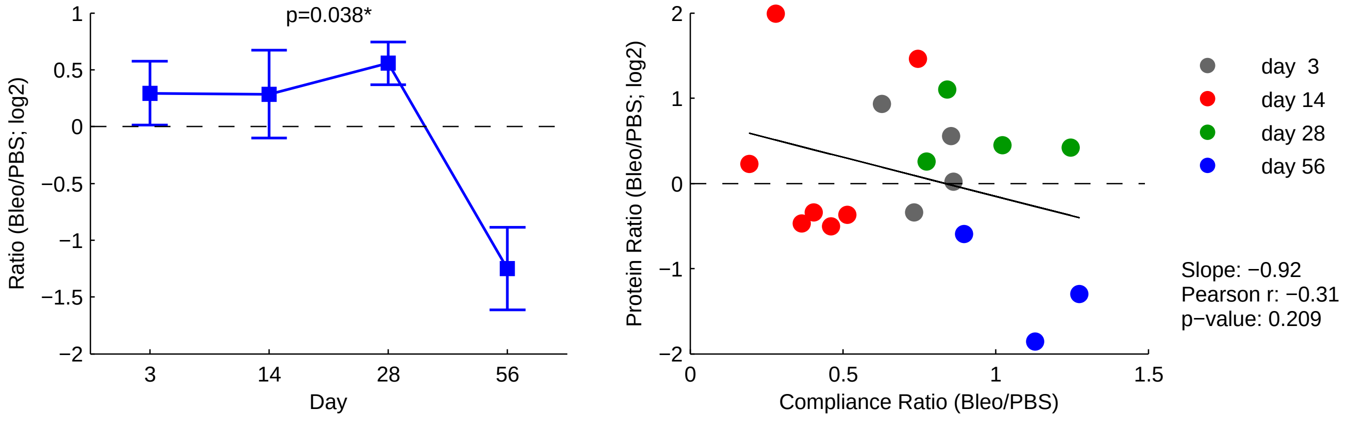

Q9R233−2 − Tapbp (id: 3711)

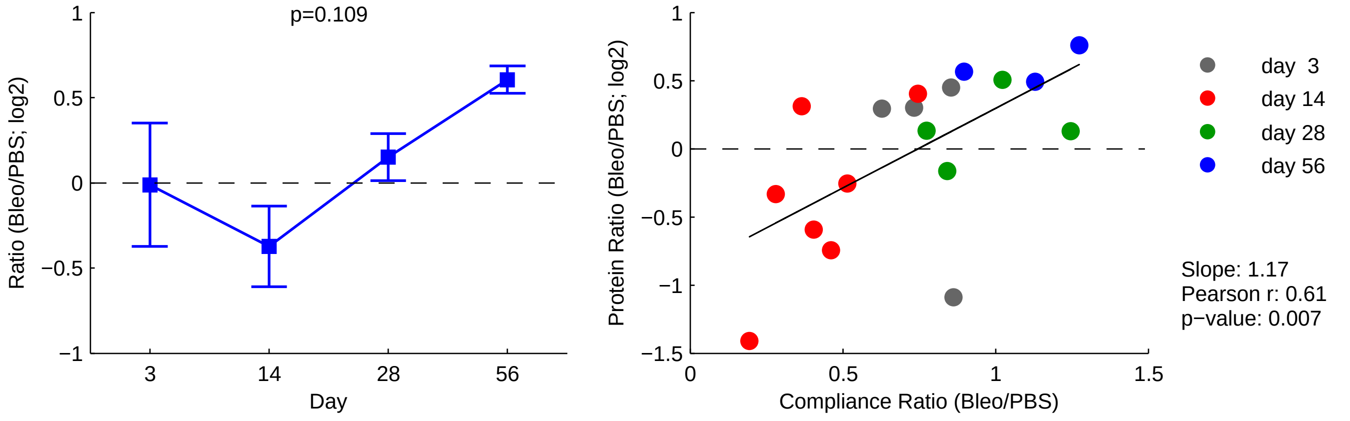

Q3TDD9 − Ppp1r21 (id: 3713)

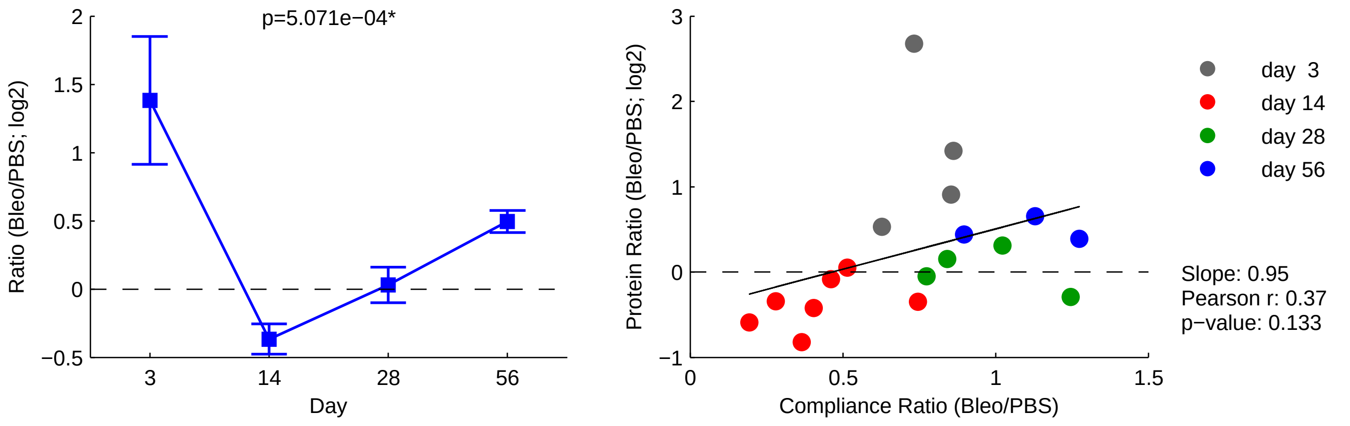

Q3TDQ1 − Stt3b (id: 3715)

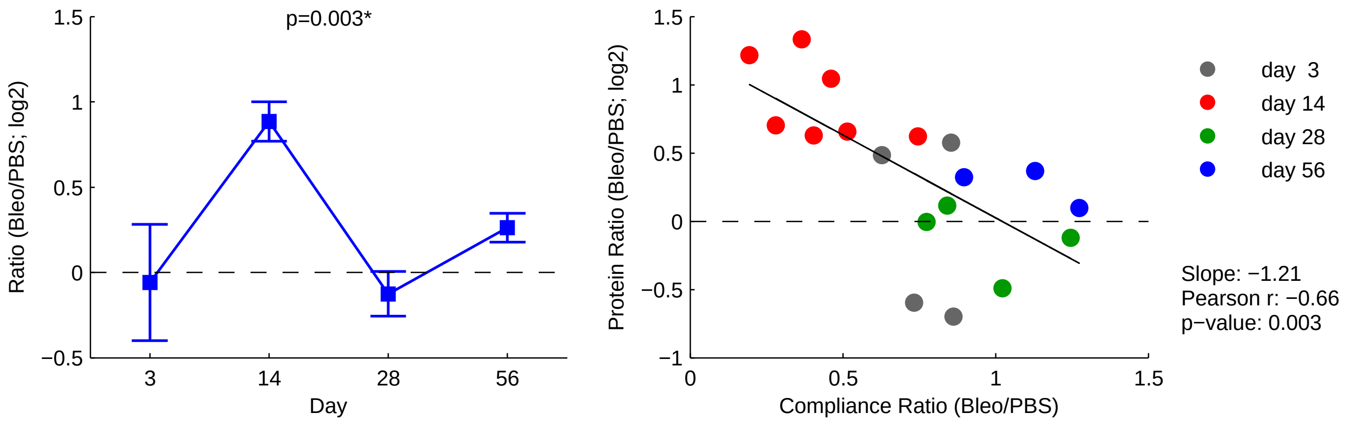

Q3TDT0 − Trim3 (id: 3716)

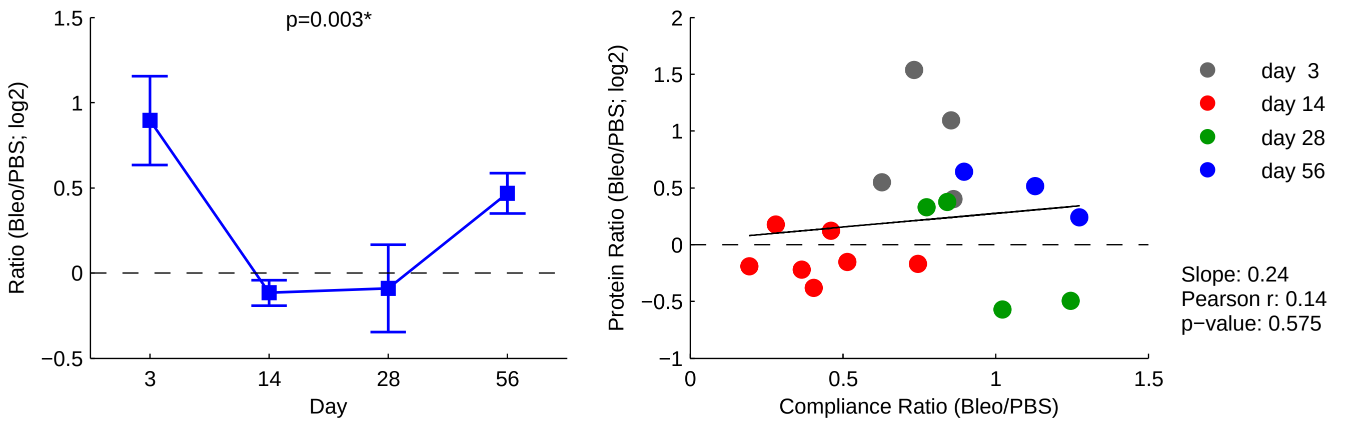

Q3TEA8 − Hp1bp3 (id: 3718)

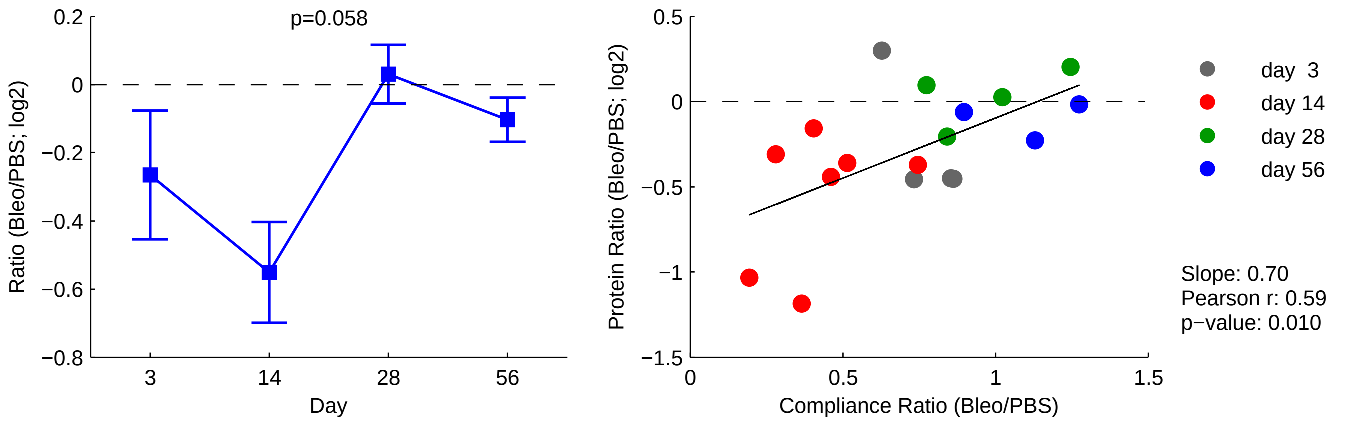

Q3TFD2 − Lpcat1 (id: 3719)

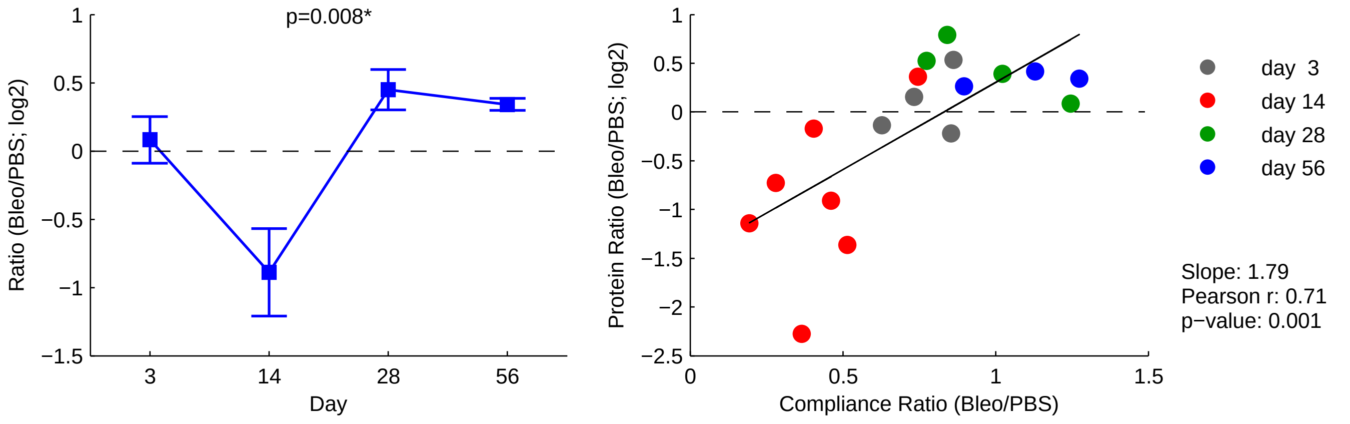

Q3TGG2 − Atxn2l (id: 3723)

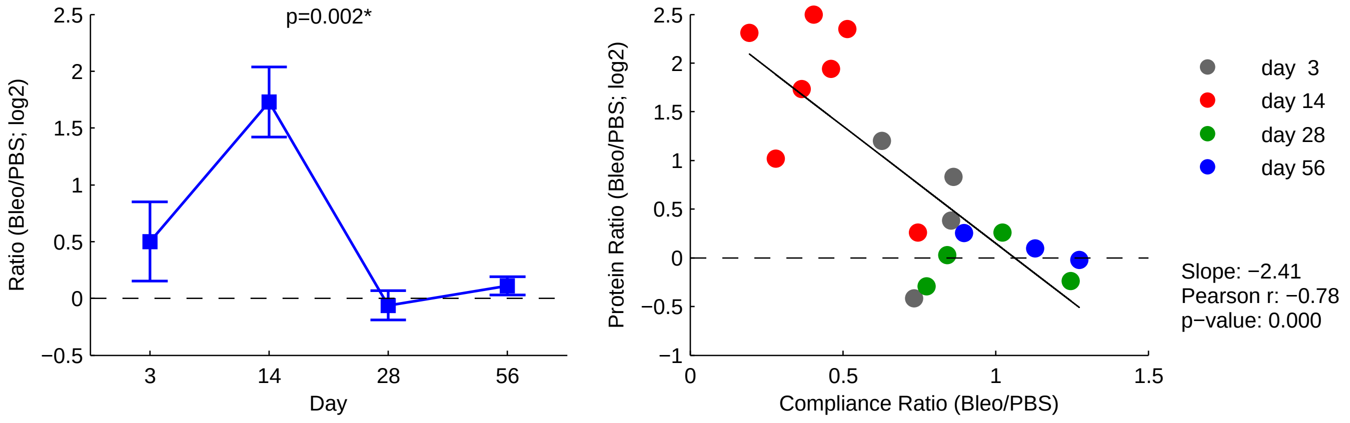

Q3TGW2 − Eepd1 (id: 3725)

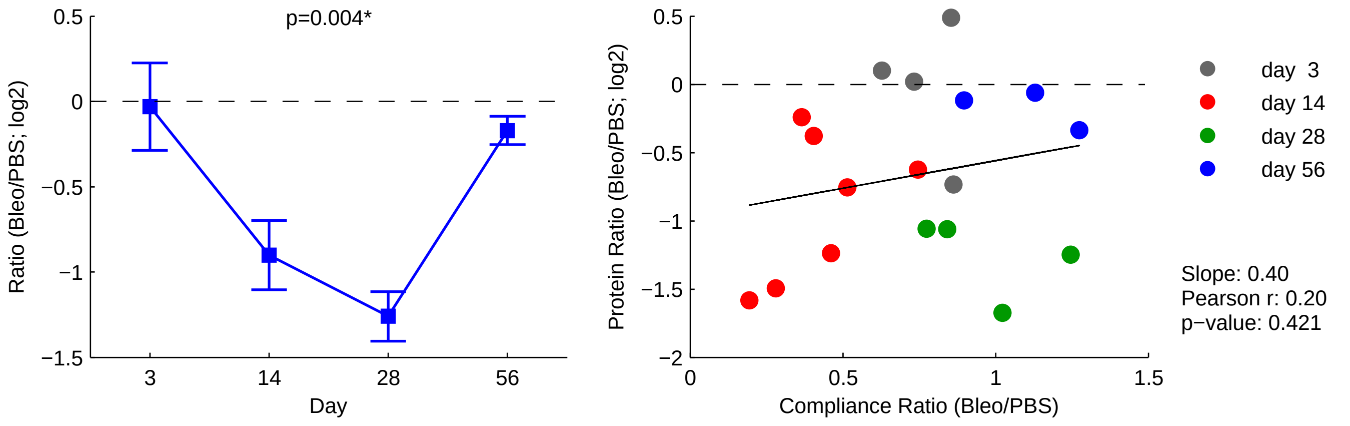

Q3TGY1 − Rsrc1 (id: 3726)

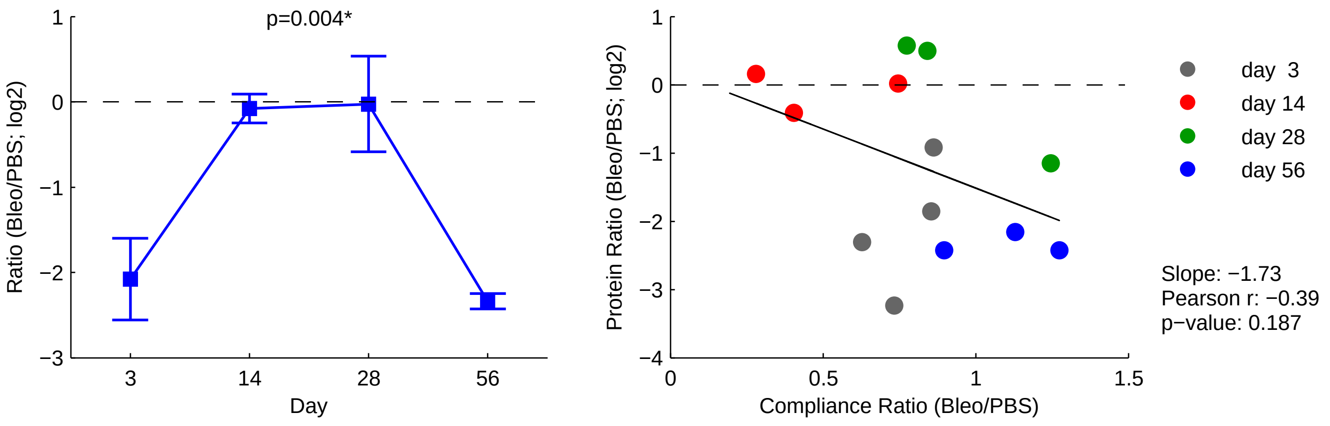

Q3THE2 − Myl12b (id: 3727)

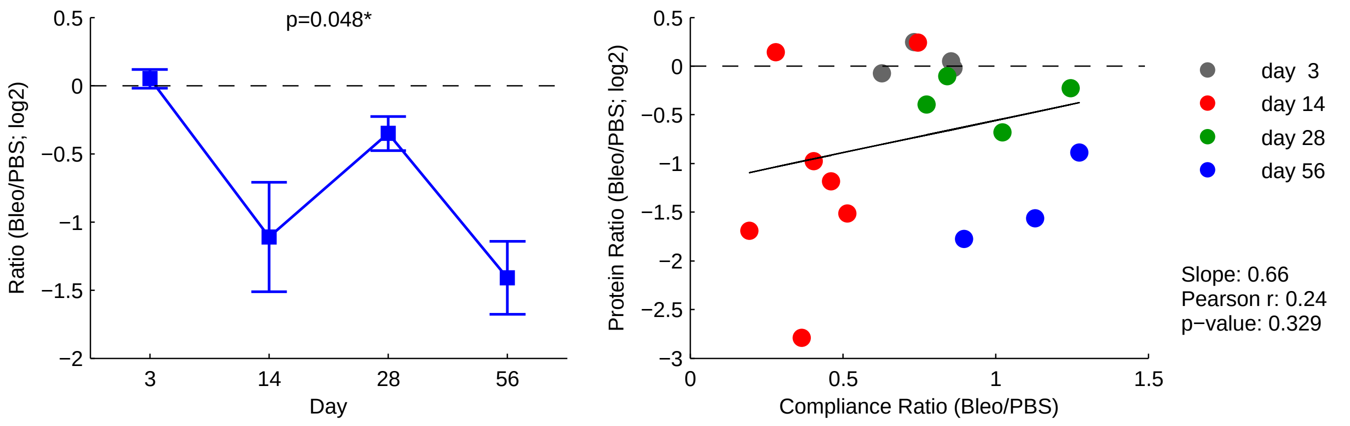

Q3THK7 − Gmps (id: 3729)

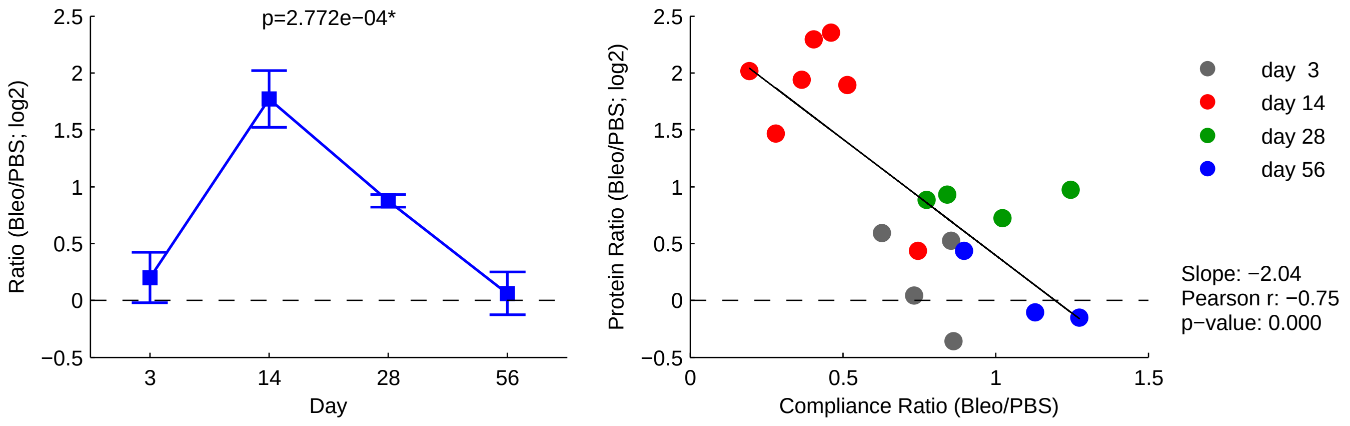

Q3THS6 − Mat2a (id: 3730)

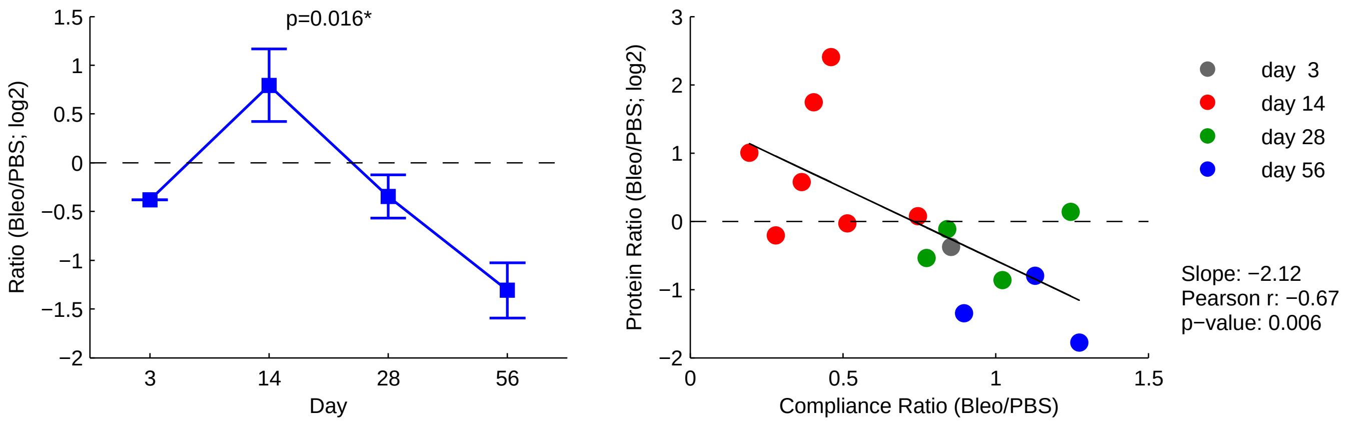

Q3TIR3 − Ric8a (id: 3731)

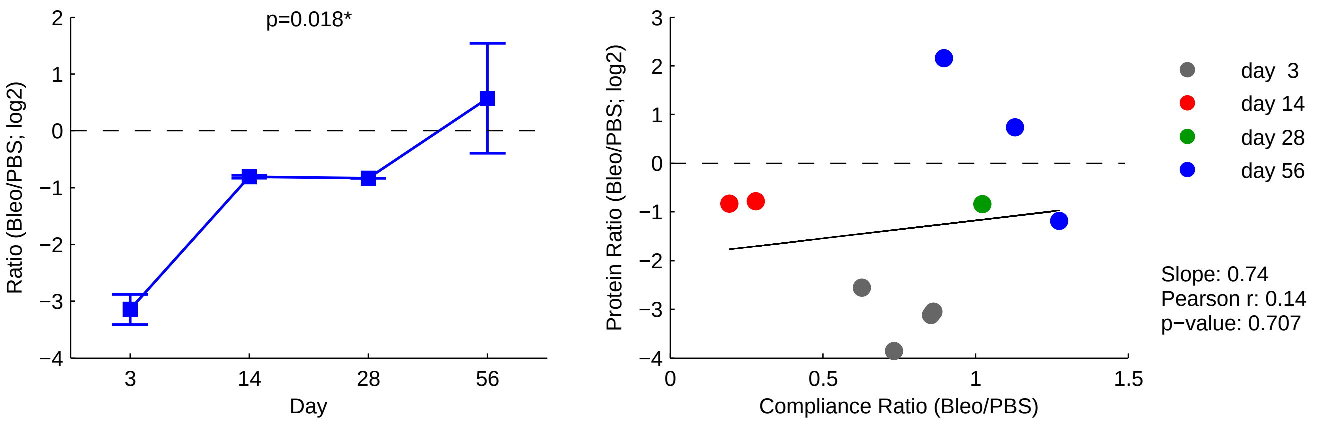

Q3TIX9 − Usp39 (id: 3735)

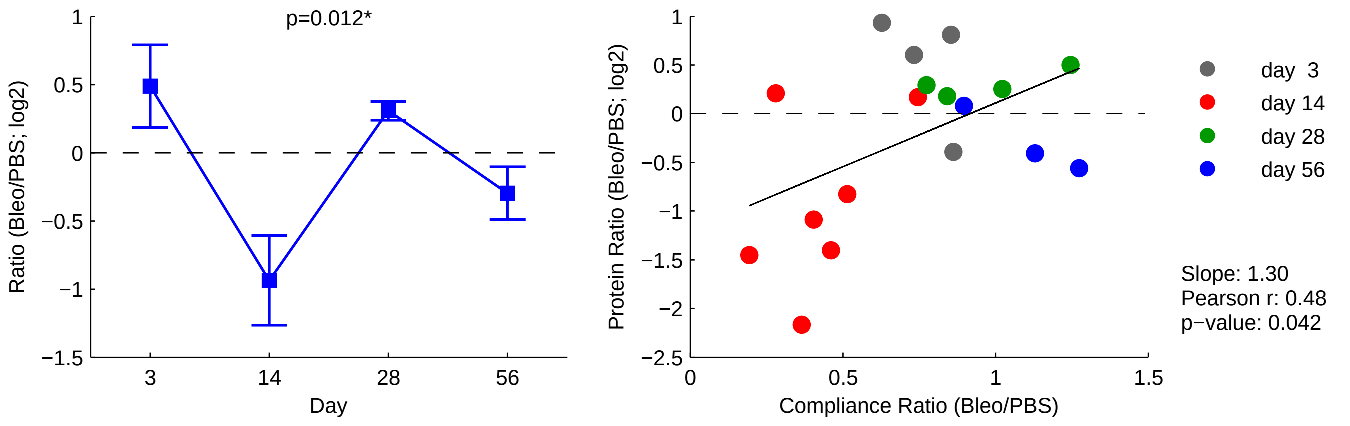

Q3TJ55 − Stx2 (id: 3736)

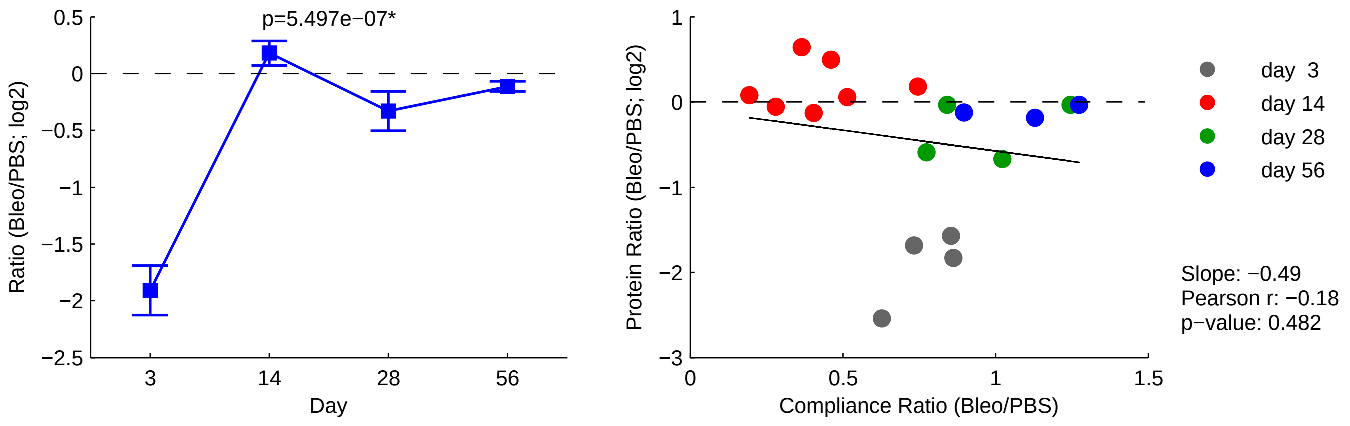

Q3TJD7 − Pdlim7 (id: 3737)

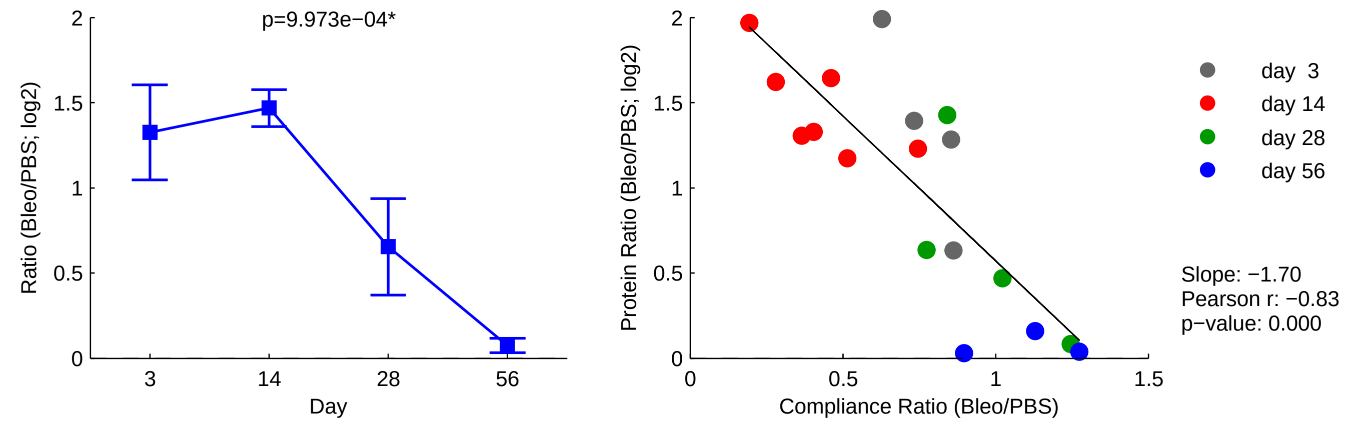

Q3TLH4−5 − Prrc2c (id: 3745)

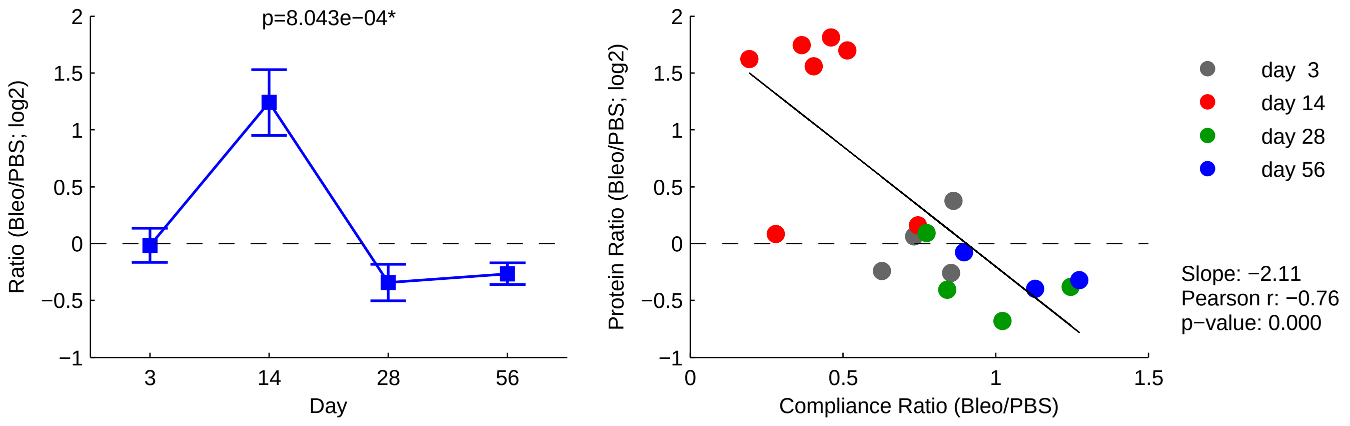

Q3TLP5 − Echdc2 (id: 3746)

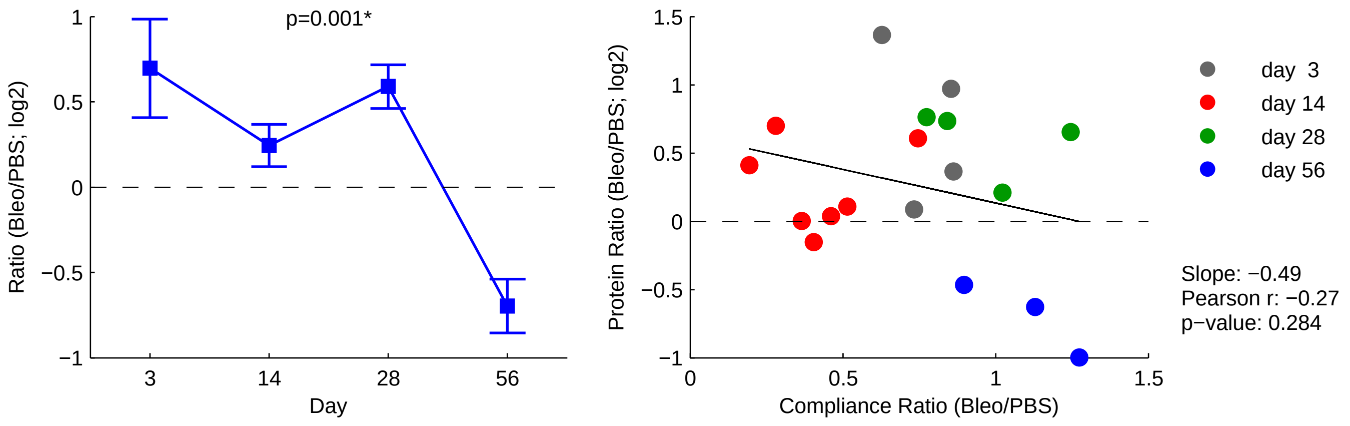

Q3TLQ0 − Map2 (id: 3747)

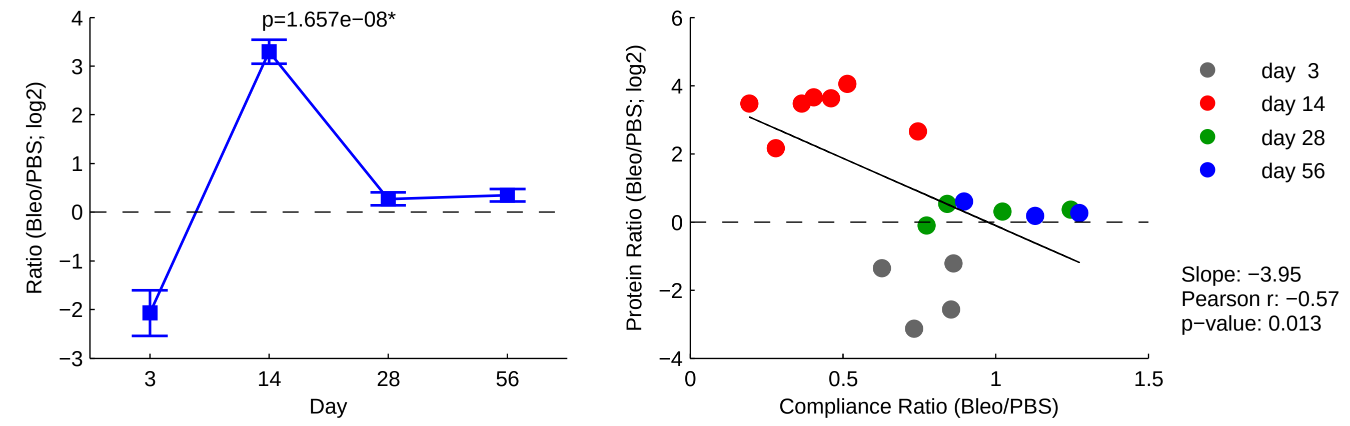

Q3TNN4 − Slc25a44 (id: 3749)

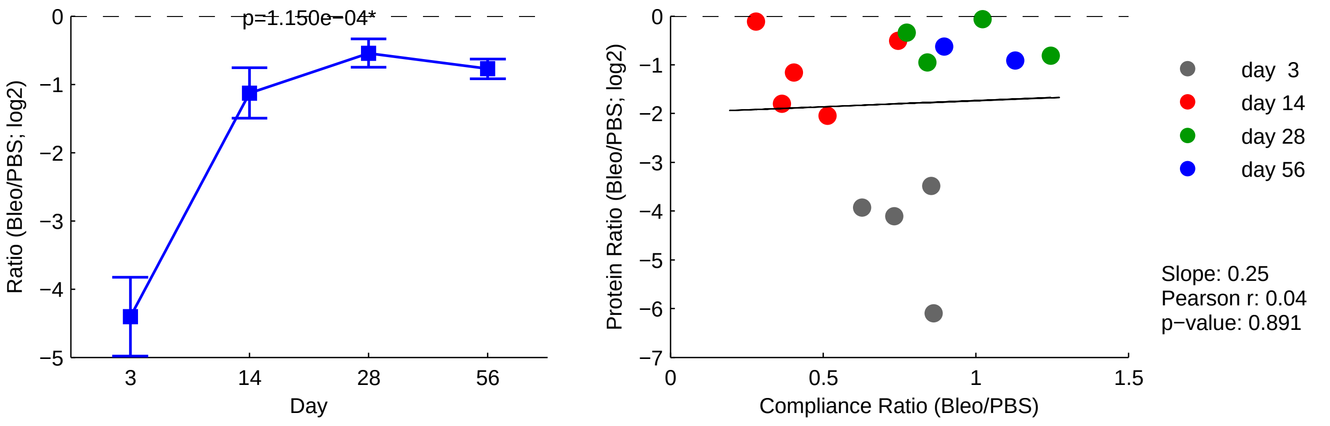

Q3TMP1 − Gtf3c3 (id: 3752)

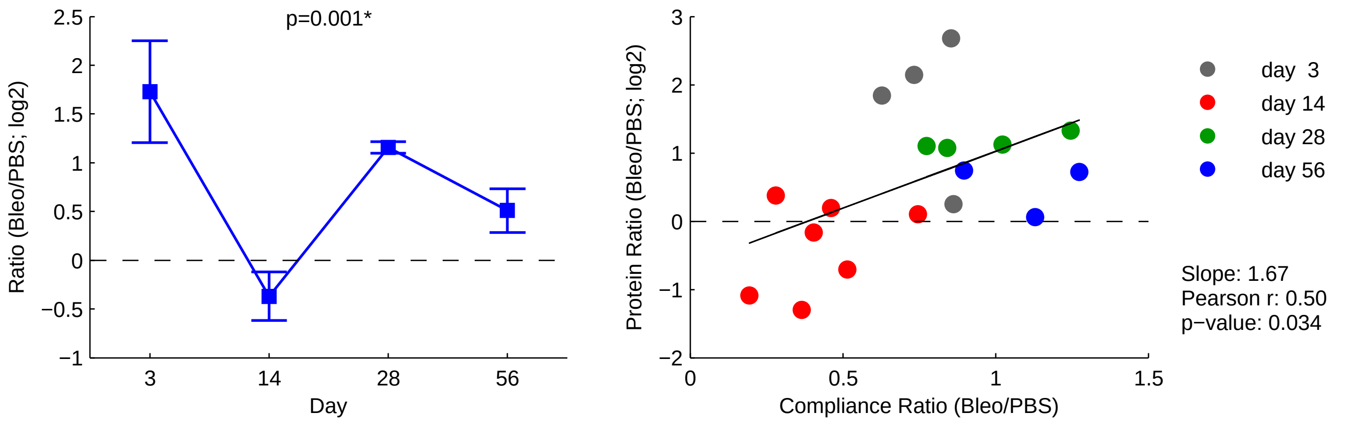

Q3TMW1 − Ccdc102a (id: 3754)

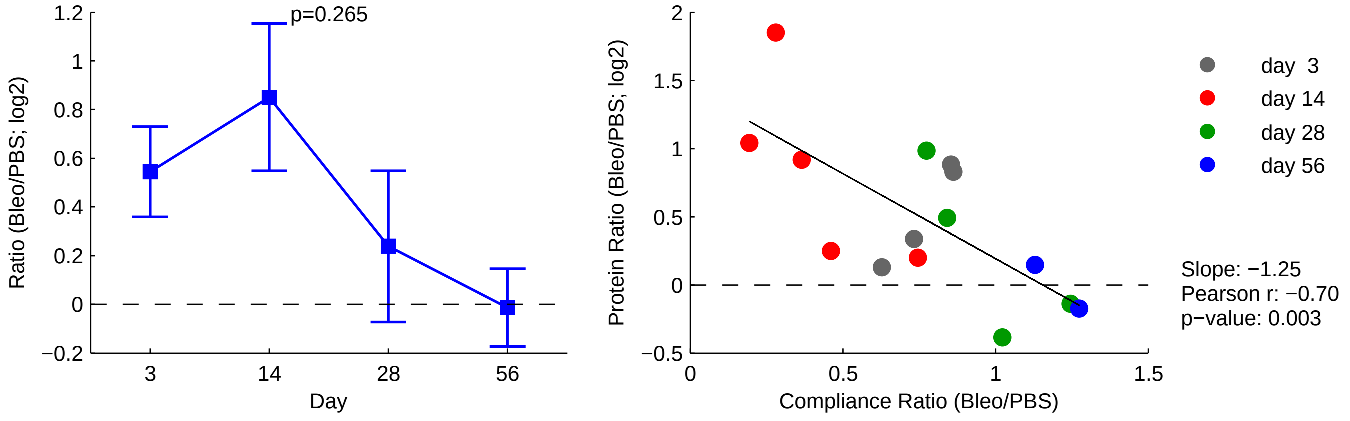

Q80ZP8 − Manf (id: 3755)

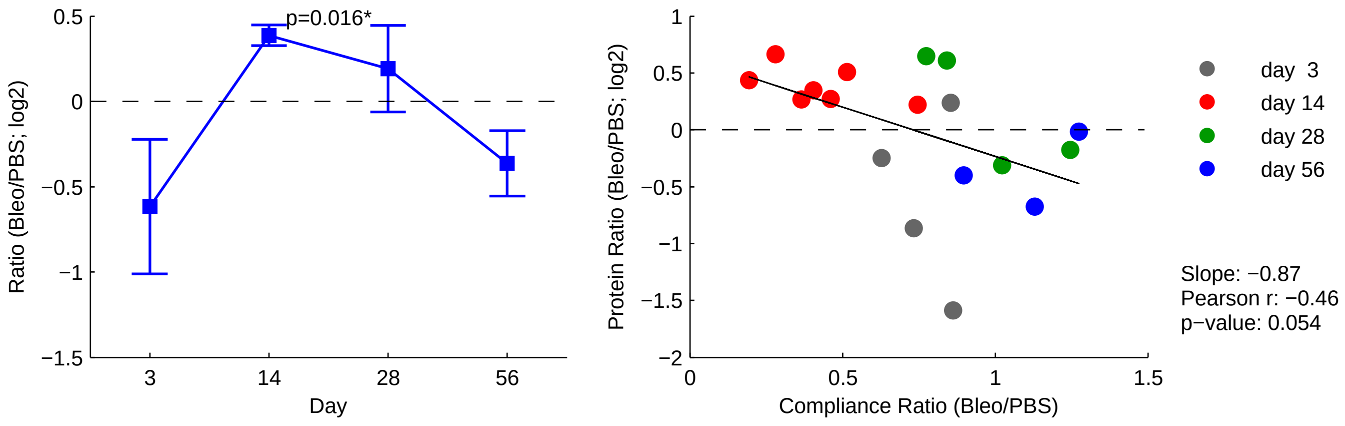

Q3TNL8−2 − Itprip (id: 3757)

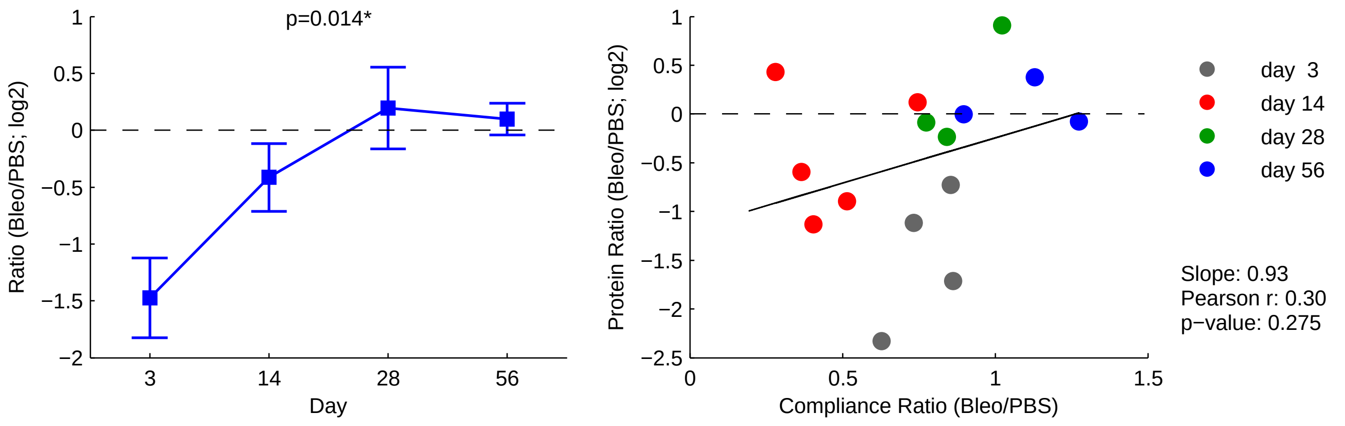

Q9WUZ9 − Entpd5 (id: 3763)

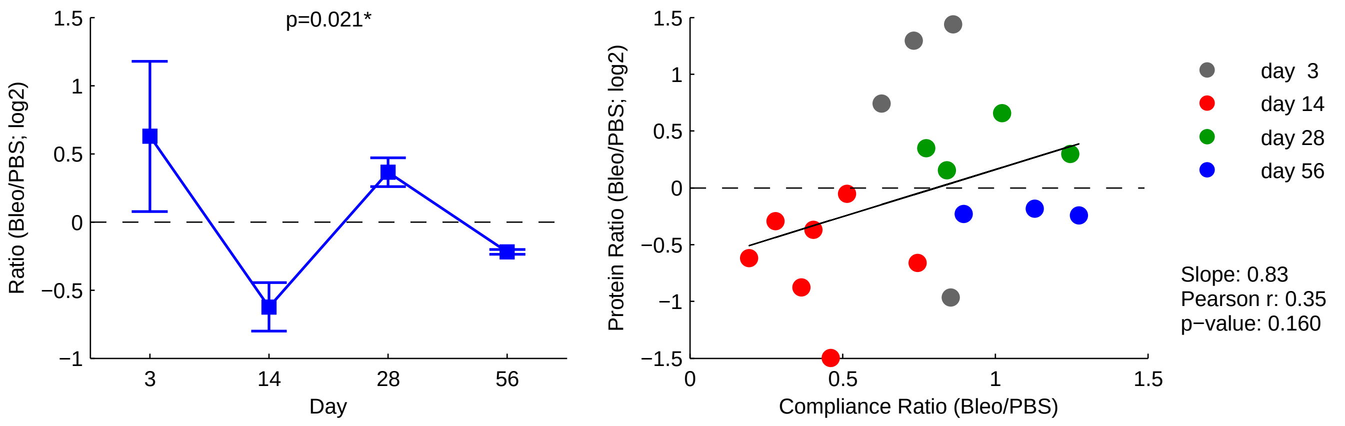

Q3TRM4−2 − Pnpla6 (id: 3766)

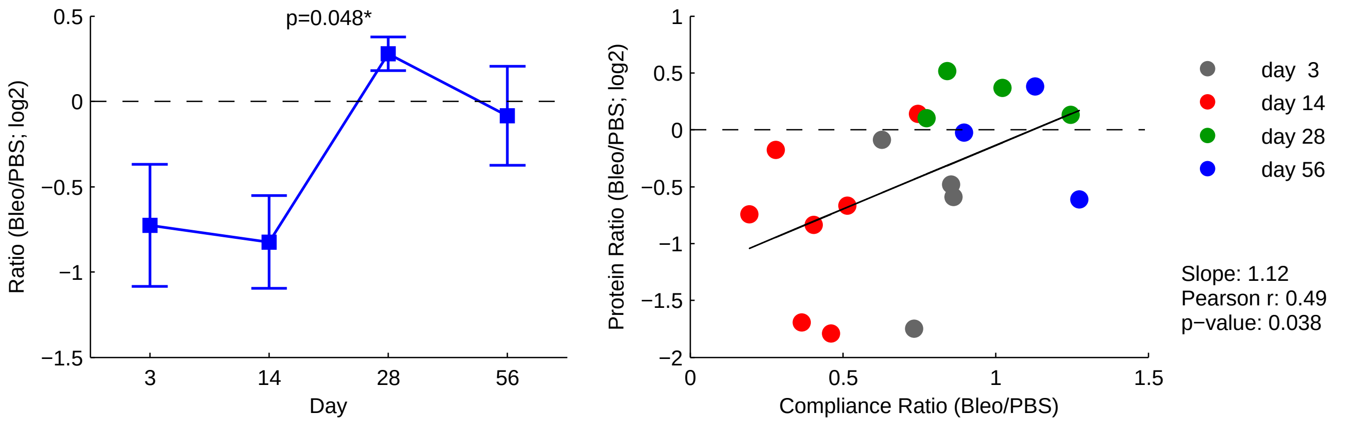

Q3TSG4 − Alkbh5 (id: 3767)

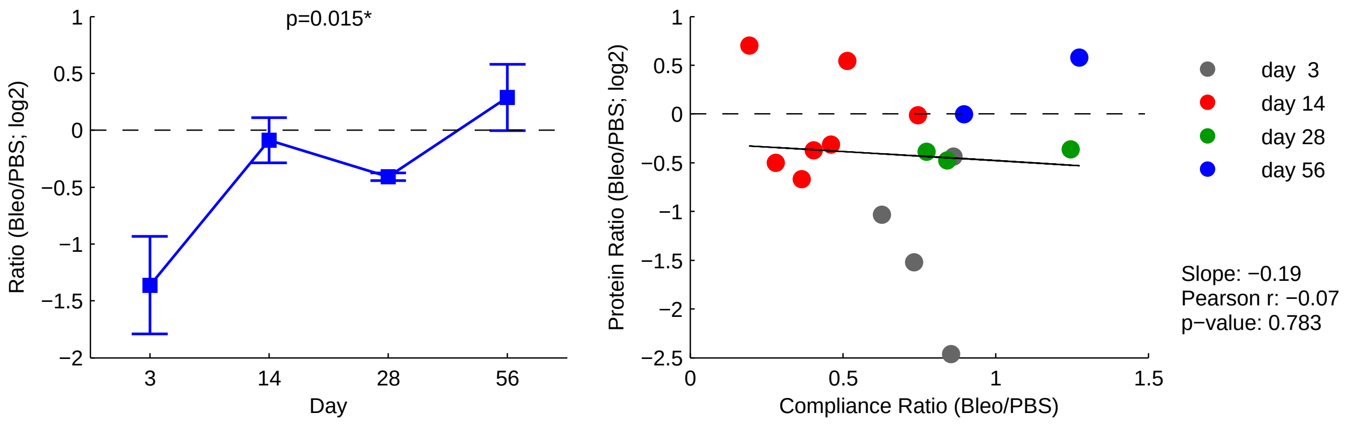

Q3TSR0 − Extl2 (id: 3768)

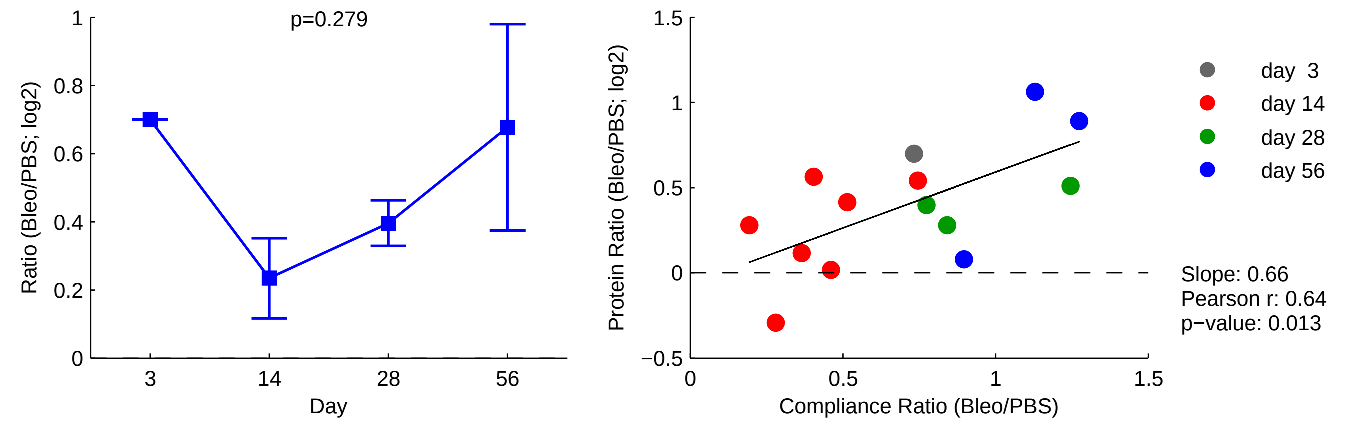

Q3TVI8 − Pbxip1 (id: 3775)

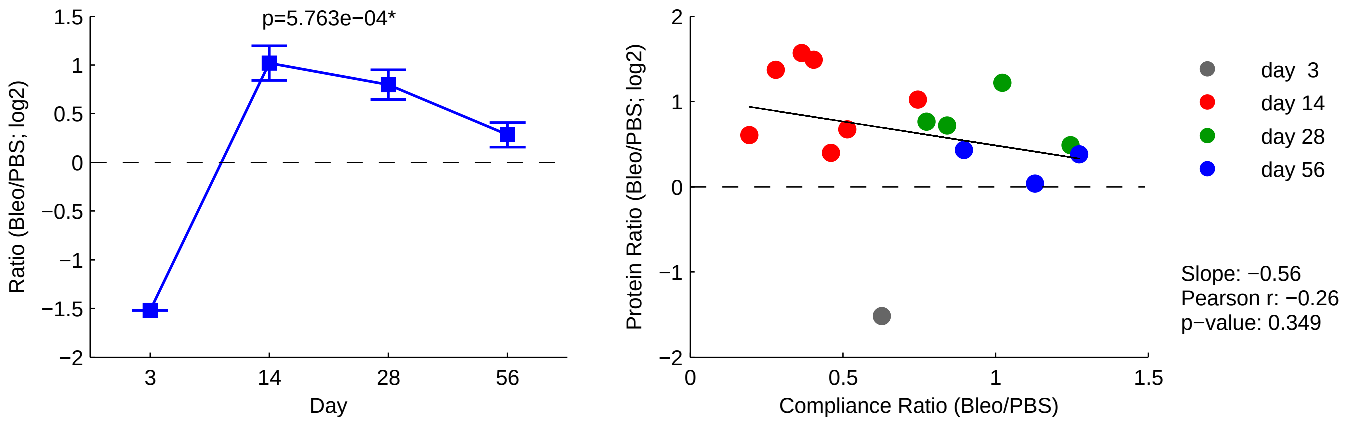

Q3TVP5 − Fam105a (id: 3776)

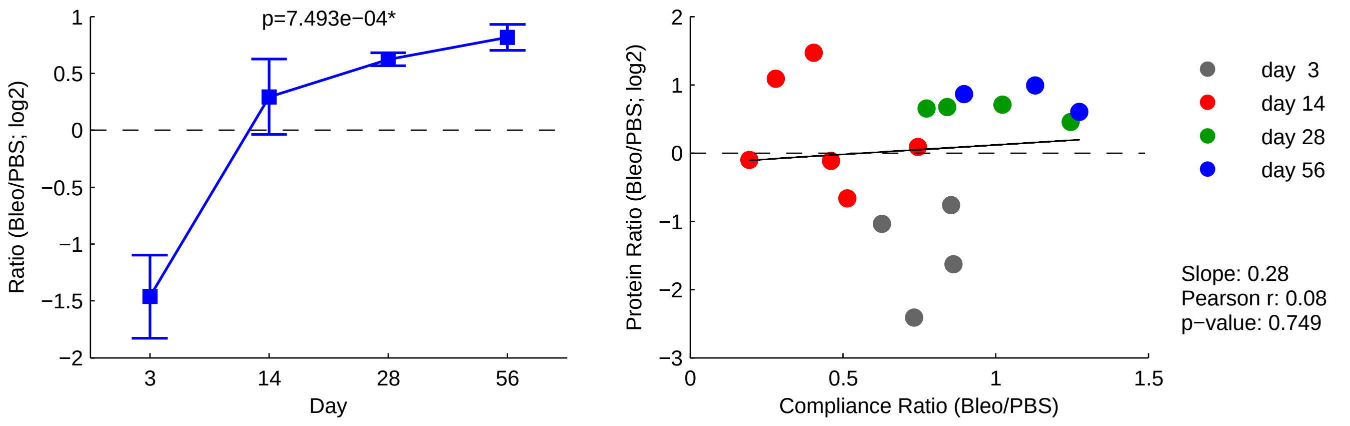

Q922L6 − Nelfcd (id: 3778)

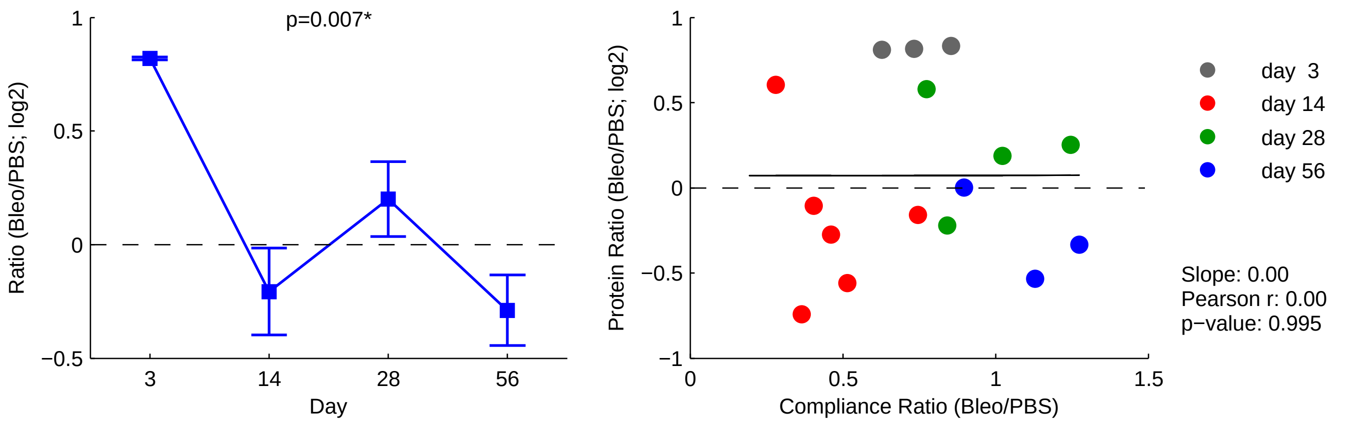

Q3TWI9 − Tmem63b (id: 3780)

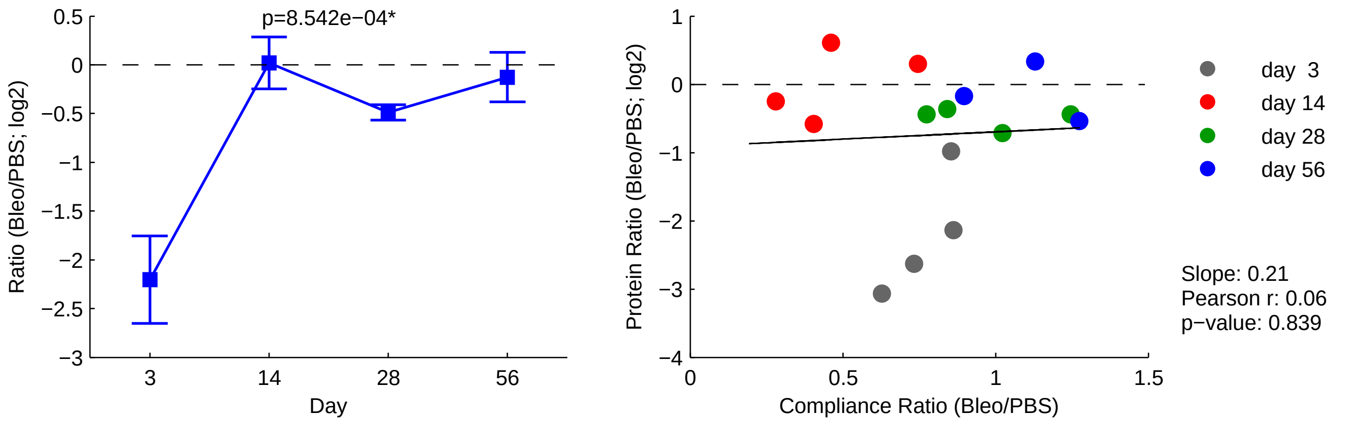

Q3TXS7 − Psmd1 (id: 3782)

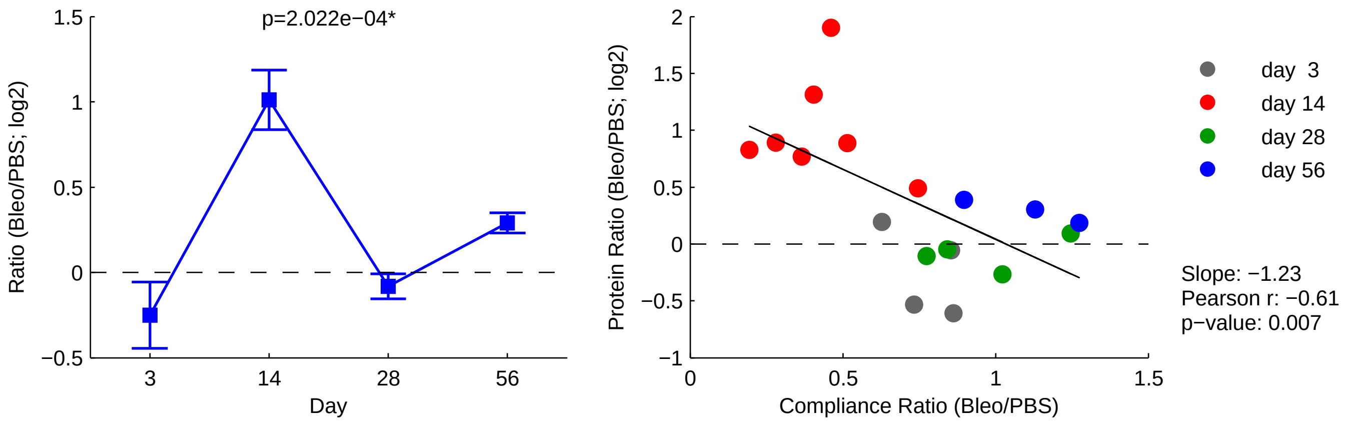

Q3TXT3−2 − Inip (id: 3783)

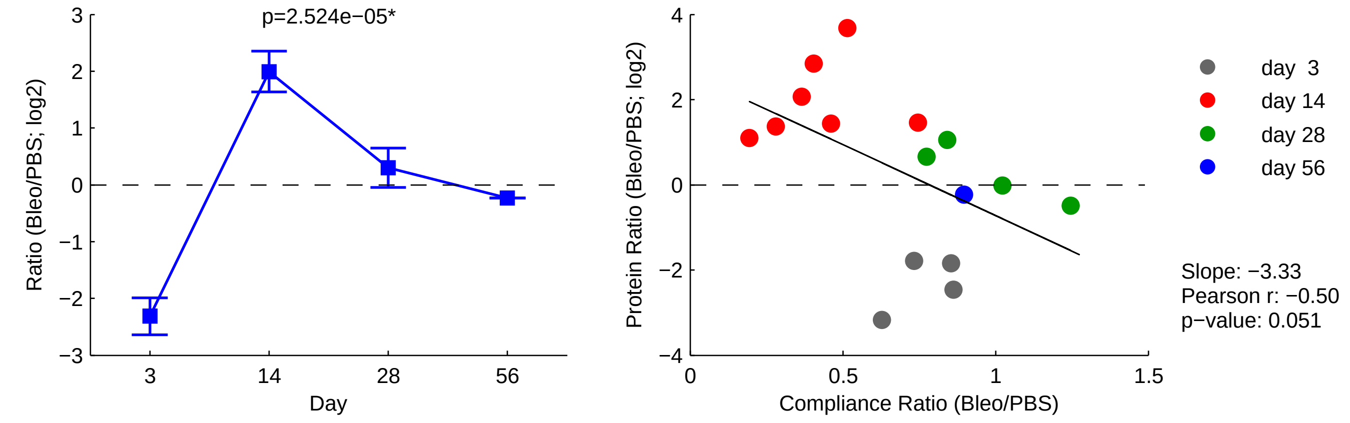

Q921E2 − Rab31 (id: 3784)

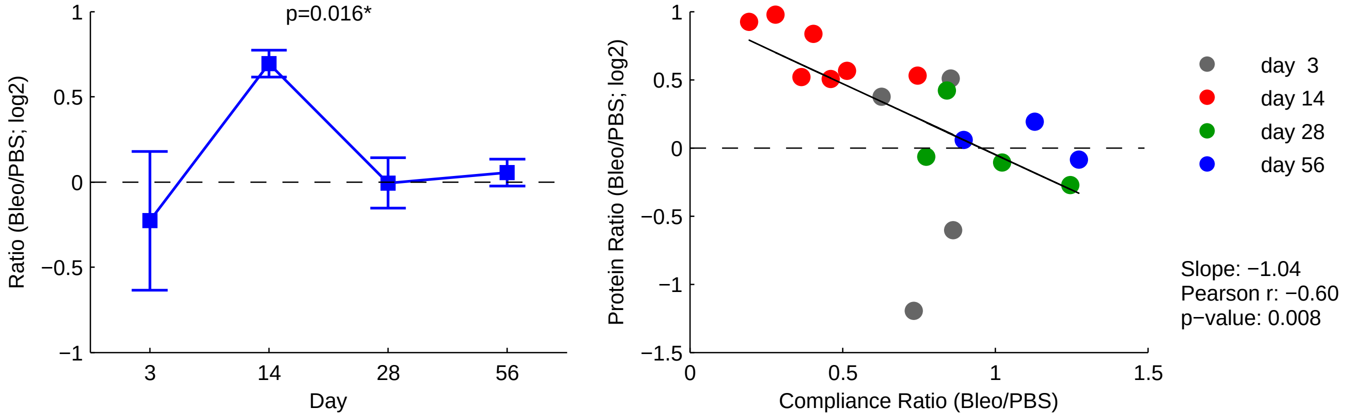

Q3TYA4 − Stxbp6 (id: 3787)

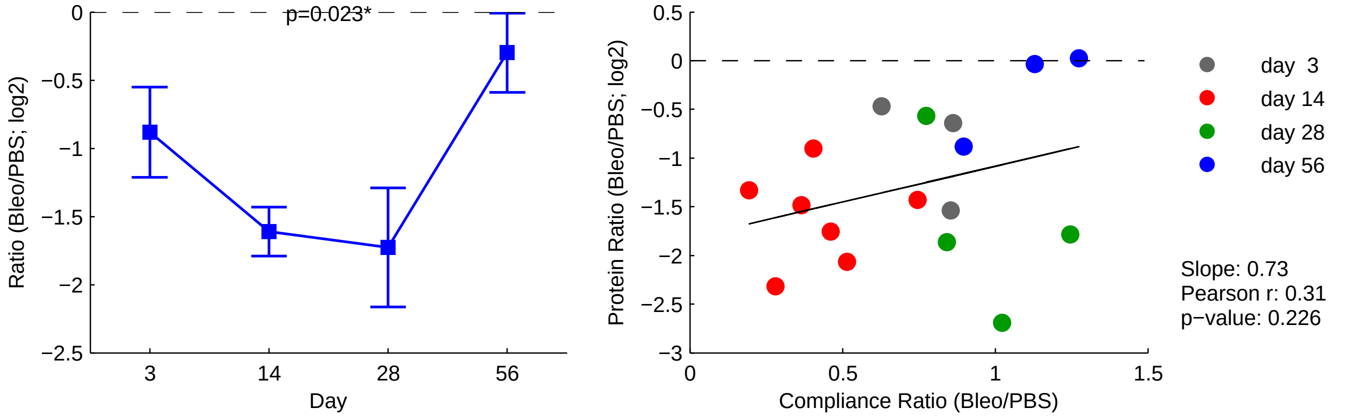

Q3TYD6 − Lmtk2 (id: 3789)

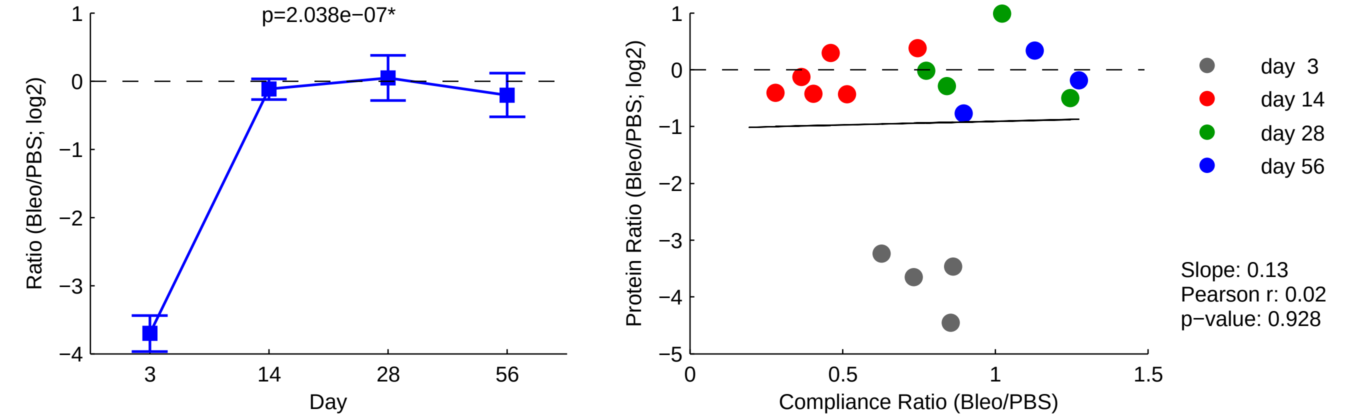

Q3TZX8−3 − Nol9 (id: 3795)

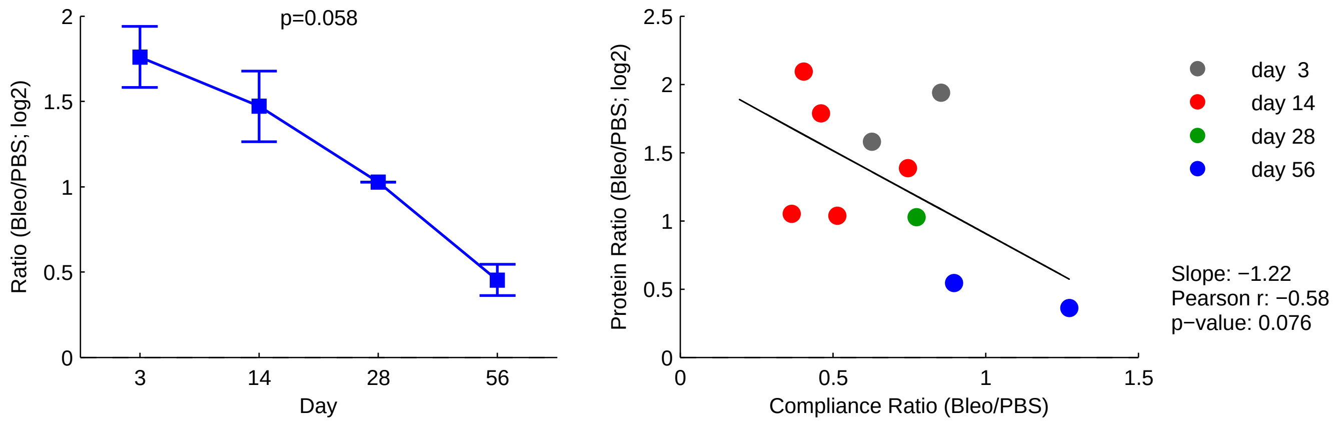

Q3TZZ7 − Esyt2 (id: 3796)

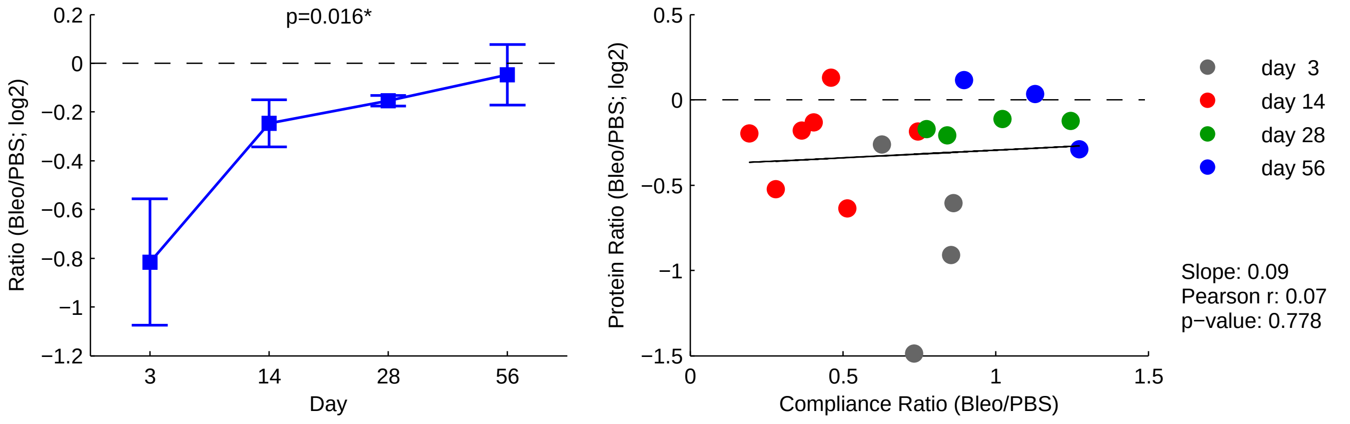

Q3U0J8 − Tbc1d2b (id: 3797)

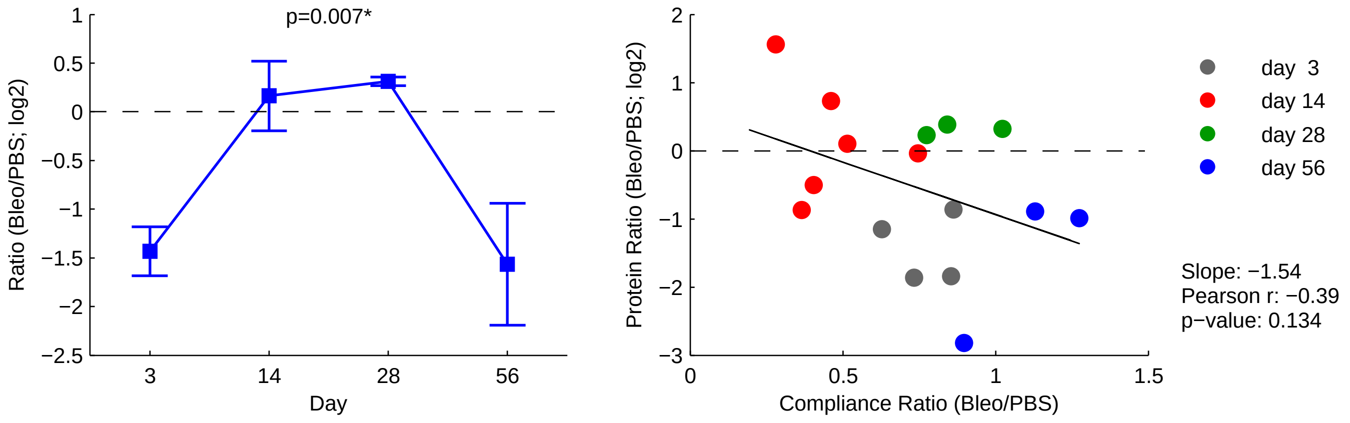

Q3U0M1−2 − Trappc9 (id: 3798)

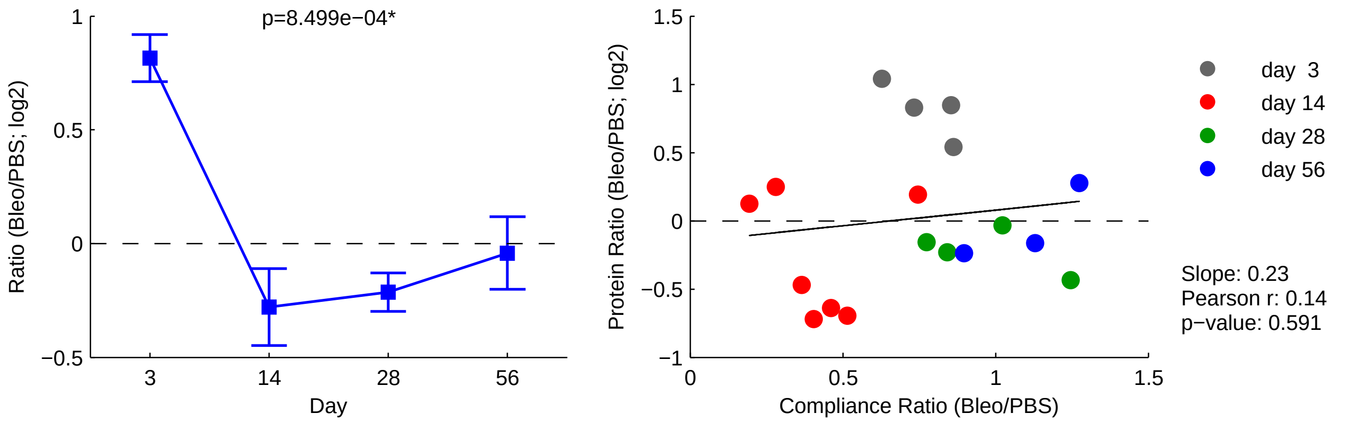

Q3U0S6 − Rasip1 (id: 3799)

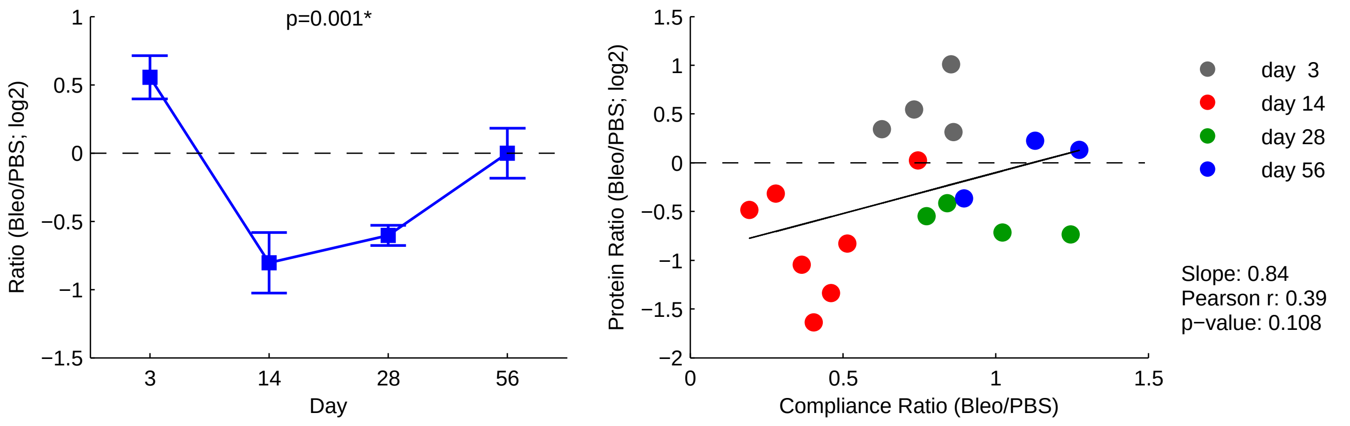

Q3U0V2 − Tradd (id: 3801)

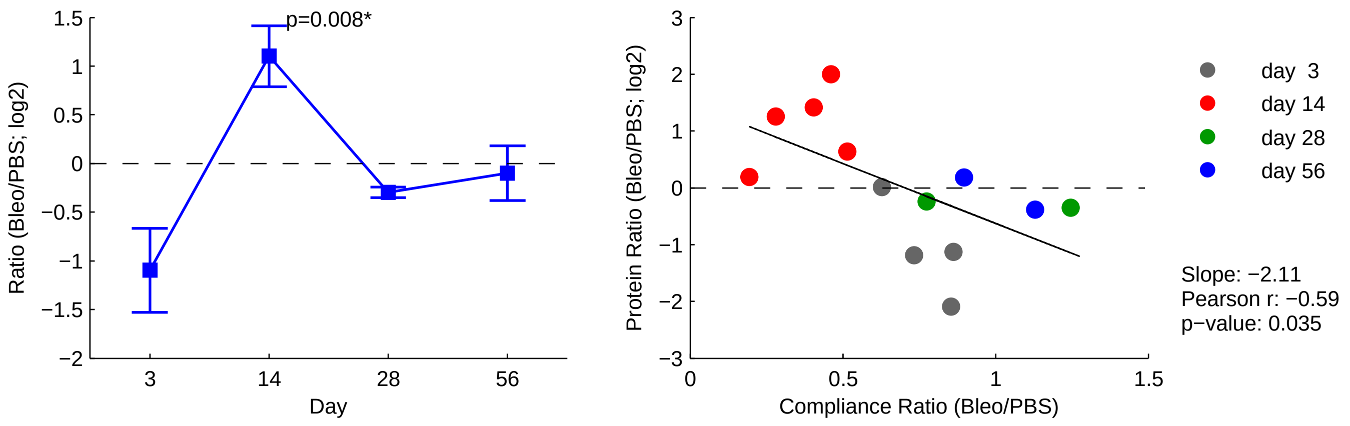

Q9CYH2 − Fam213a (id: 3802)

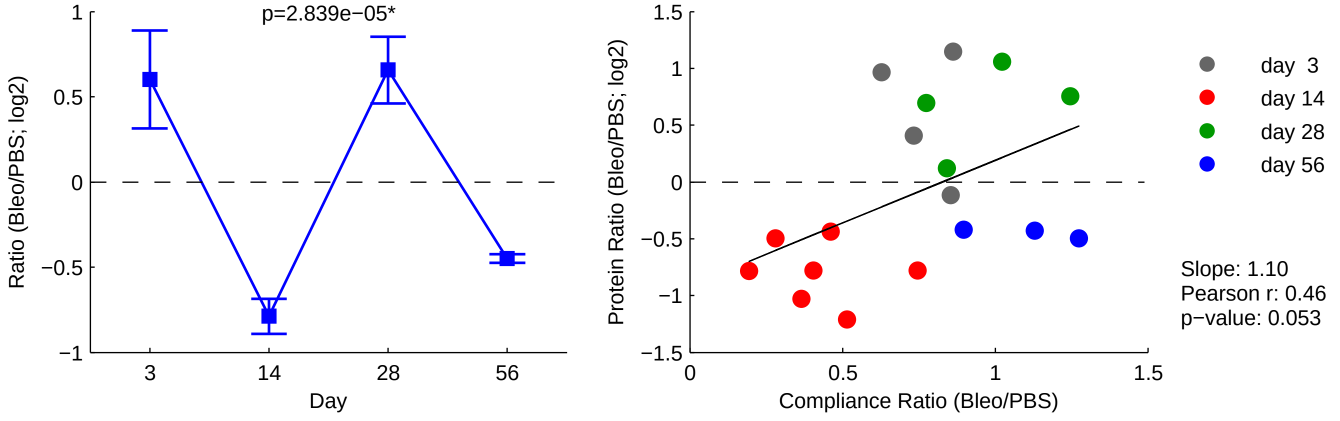

Q3U186 − Rars2 (id: 3803)

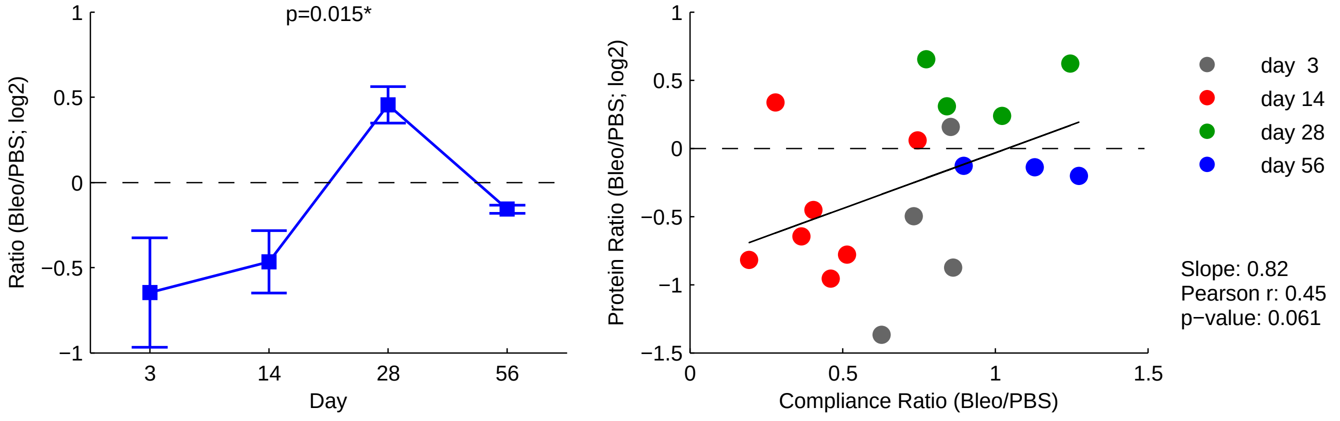

Q3U1T3 − Brms1l (id: 3809)

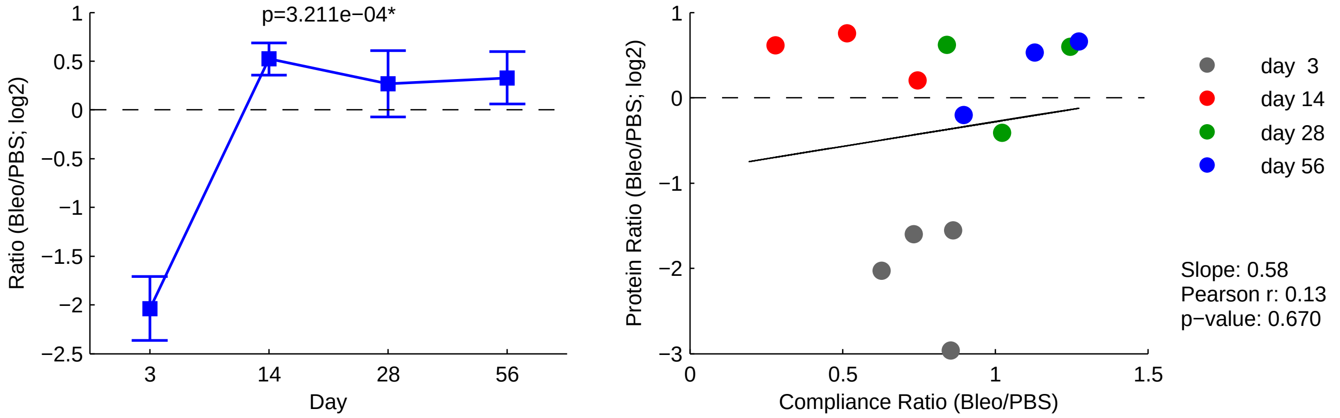

Q3U1U4 − Itgam (id: 3810)

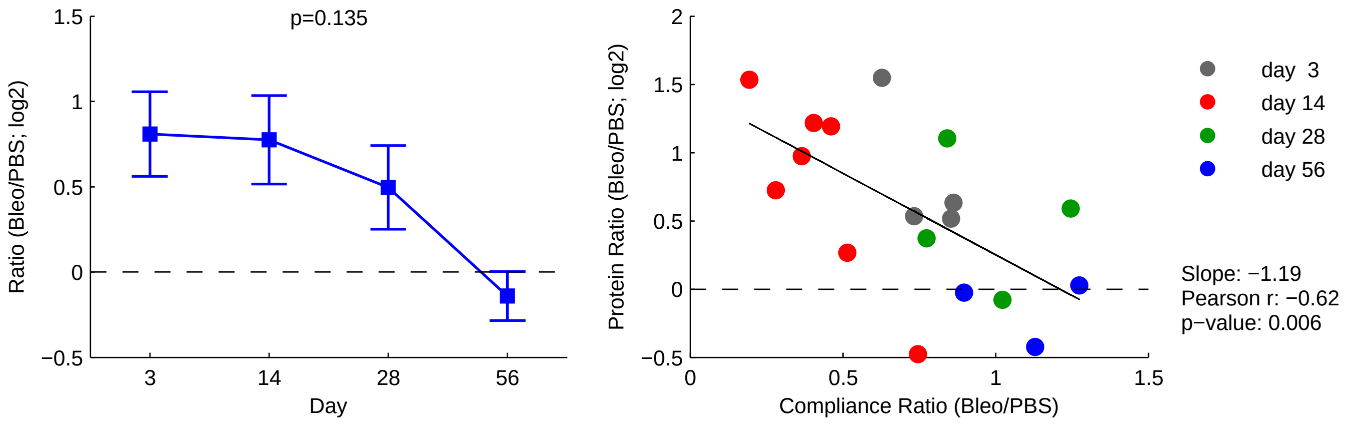

Q3U1Z5 − Gpsm3 (id: 3812)

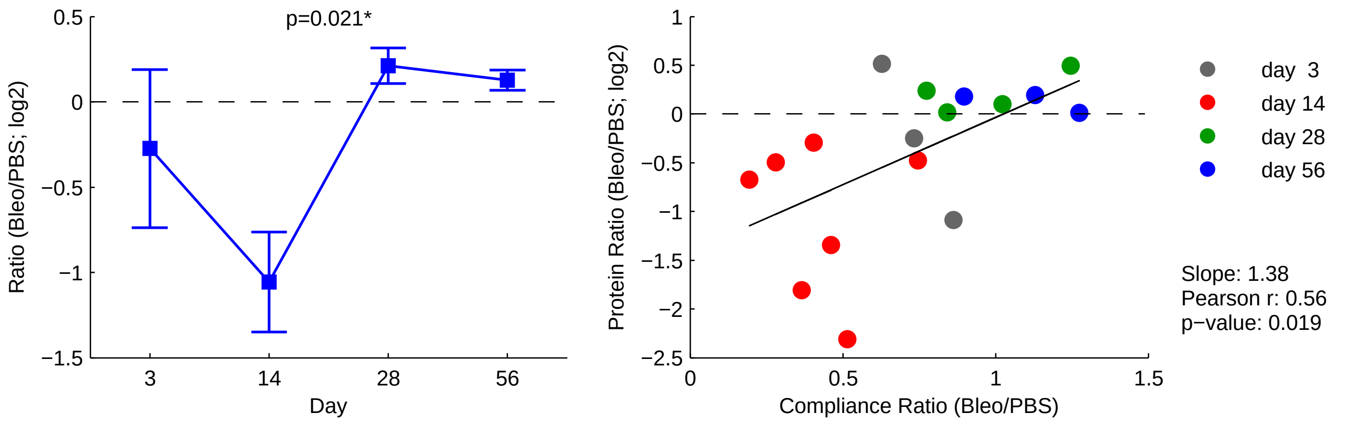

Q3U213 − Serac1 (id: 3813)

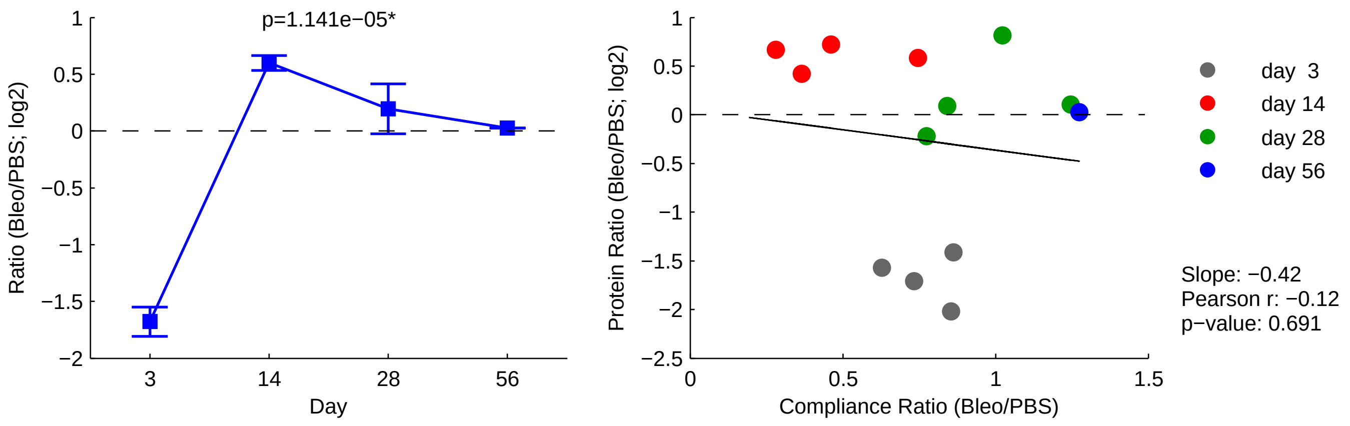

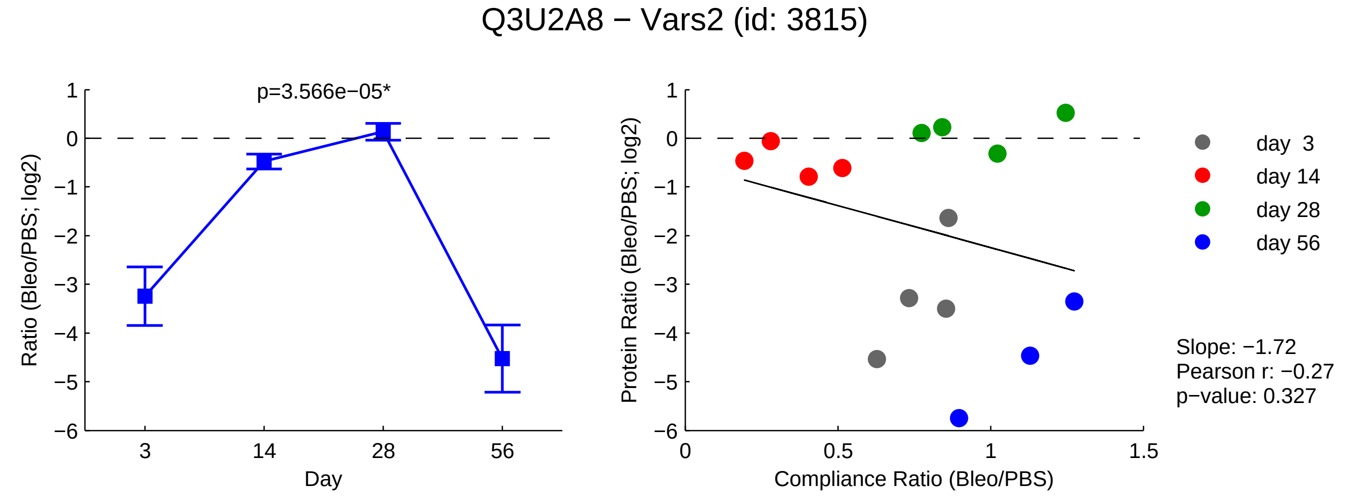
Q3U2A8 − Vars2 (id: 3815)
p=3.566e−05*
Ratio (Bleo/PBS; log2)
Day
Protein Ratio (Bleo/PBS; log2)
Compliance Ratio (Bleo/PBS)
day 3
day 14
day 28
day 56
Slope: −1.72
Pearson r: −0.27
p−value: 0.327

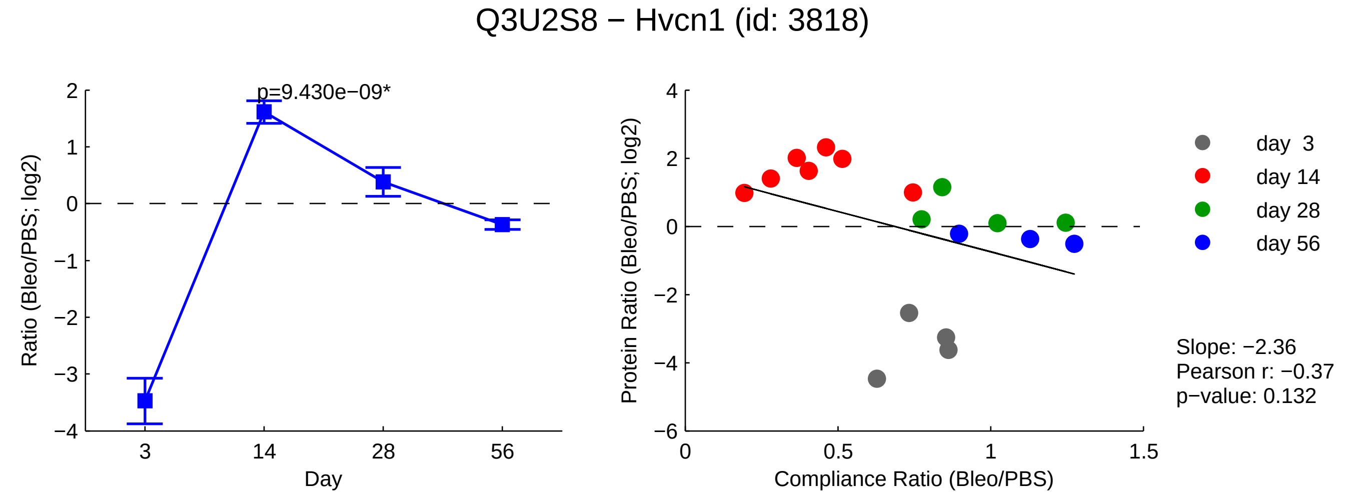
Q3U2S8 − Hvcn1 (id: 3818)
p=9.430e−09*
Ratio (Bleo/PBS; log2)
Day
Protein Ratio (Bleo/PBS; log2)
Compliance Ratio (Bleo/PBS)
day 3
day 14
day 28
day 56
Slope: −2.36
Pearson r: −0.37
p−value: 0.132

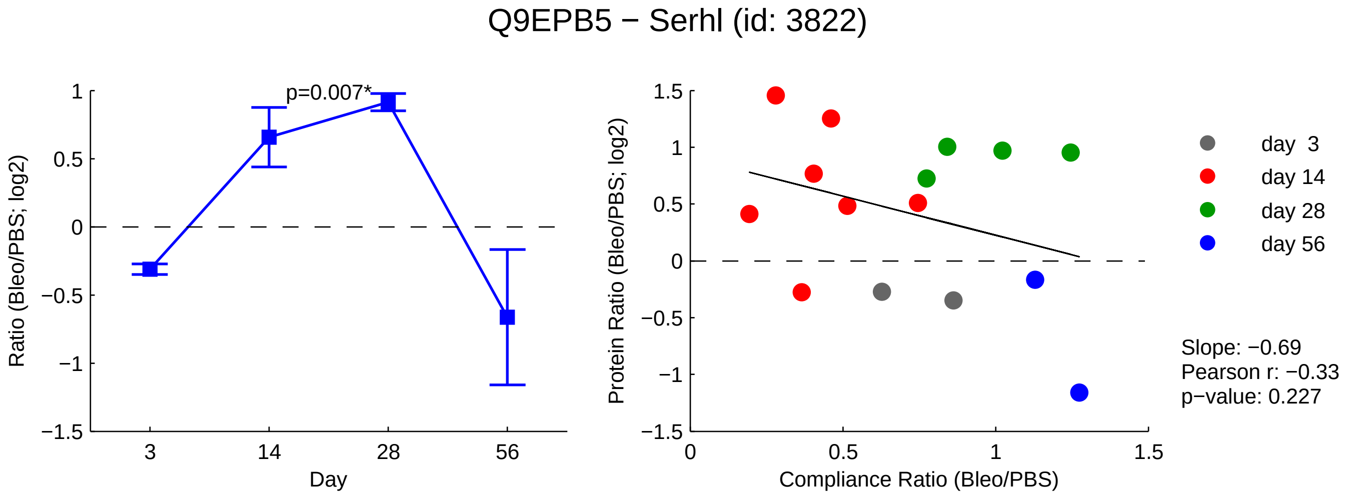
Q9EPB5 − Serhl (id: 3822)
p=0.007*
Ratio (Bleo/PBS; log2)
Day
Protein Ratio (Bleo/PBS; log2)
Compliance Ratio (Bleo/PBS)
day 3
day 14
day 28
day 56
Slope: −0.69
Pearson r: −0.33
p−value: 0.227

Q3U3J1 − Bckdha (id: 3823)

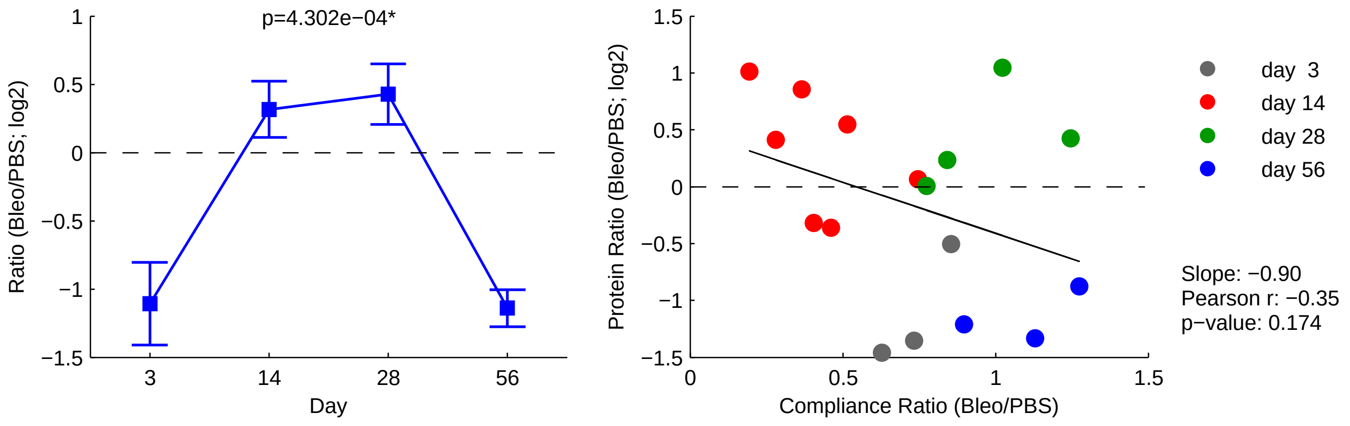

Q3U3R4−3 − Lmf1 (id: 3825)

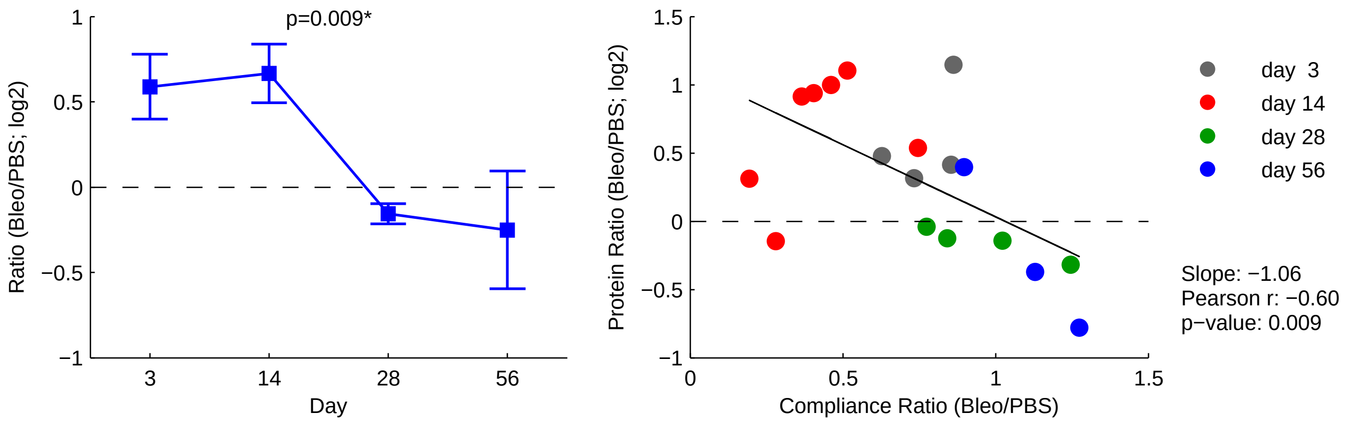

Q3U422 − Ndufv3 (id: 3827)

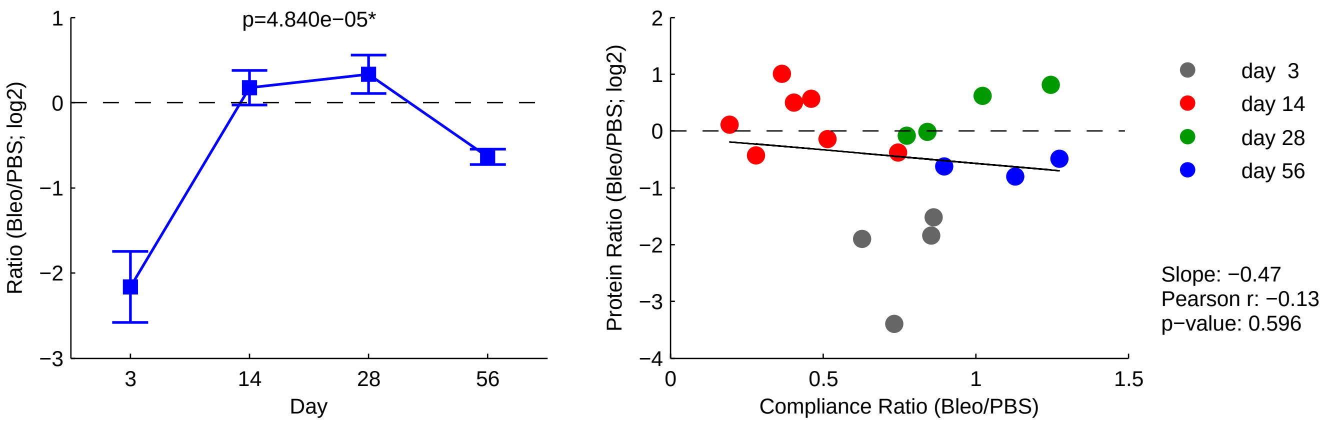

Q3U5C8 − Arhgef16 (id: 3833)

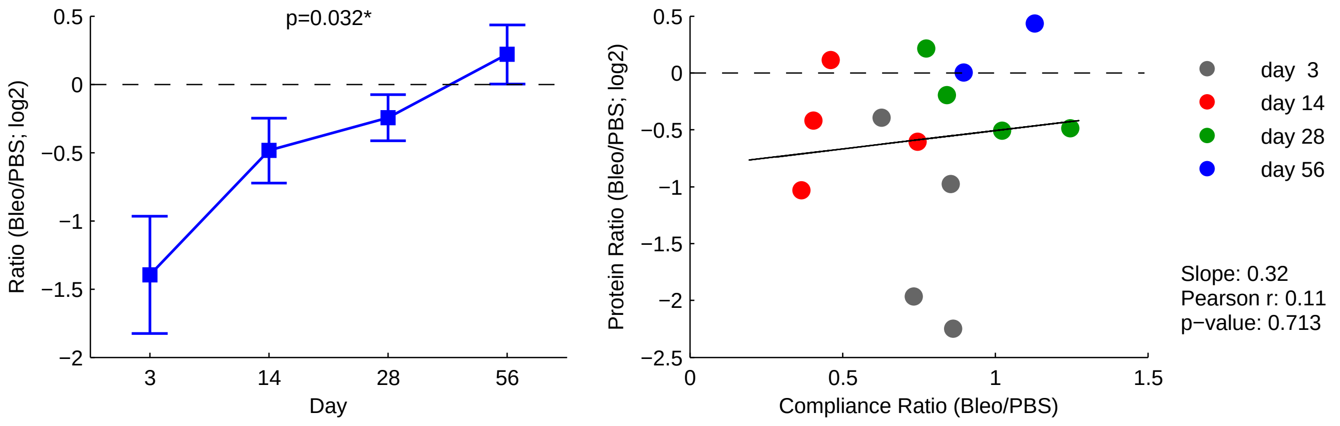

Q3U5Q7 − Cmpk2 (id: 3835)

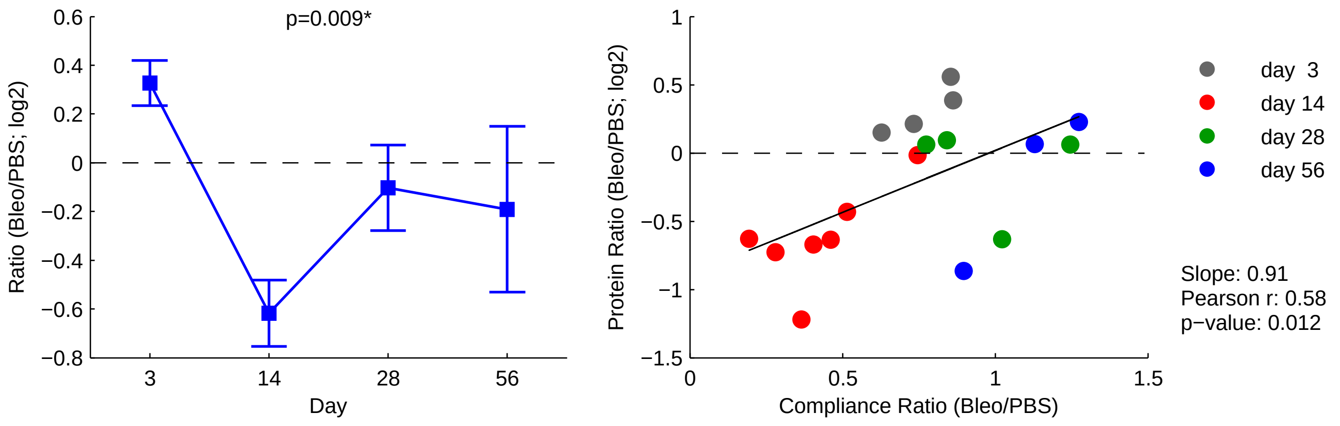

Q3U5V8 − Stx11 (id: 3836)

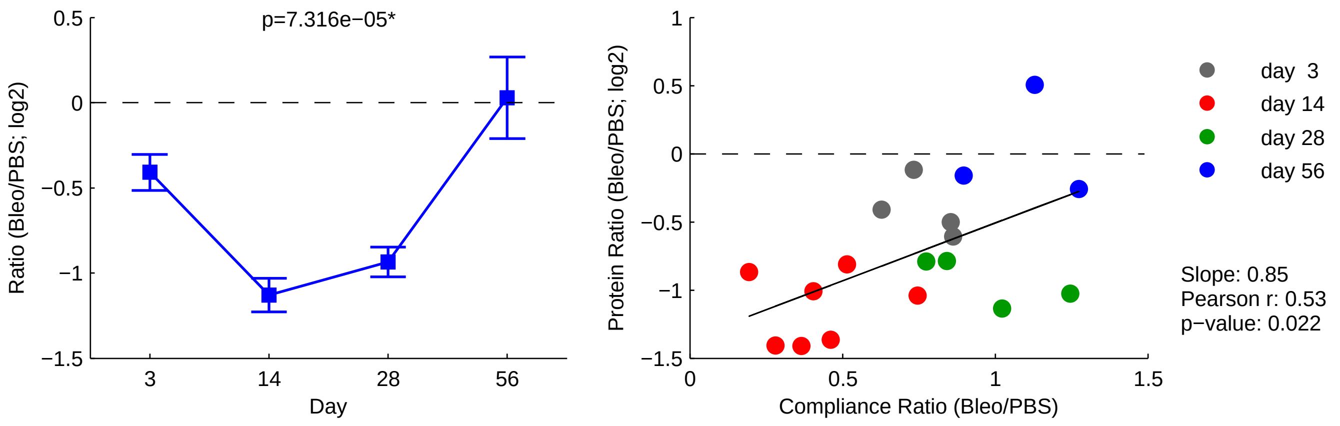

Q3U6K5 − Spata6 (id: 3837)

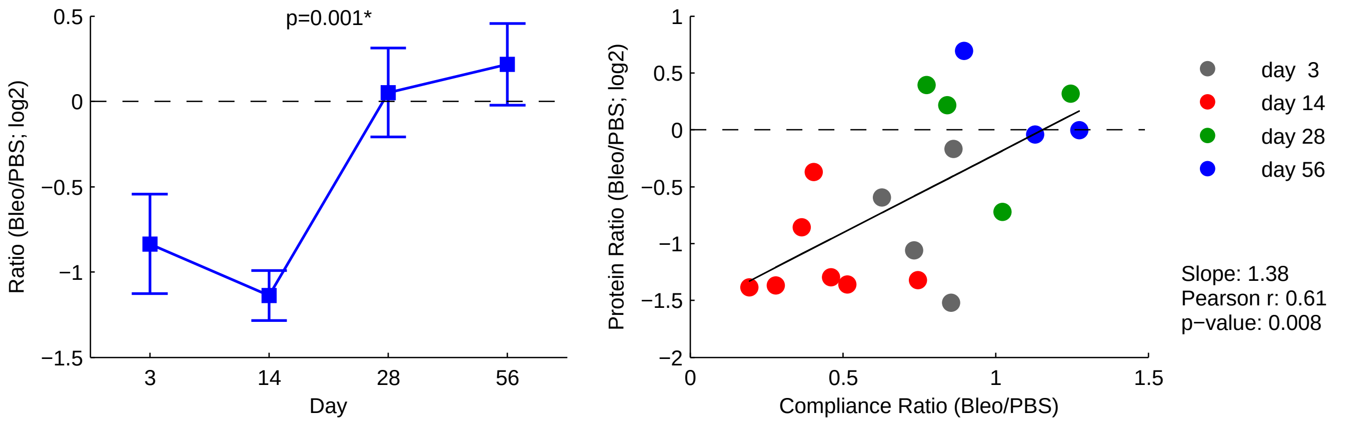

Q3U741 − Ddx17 (id: 3839)

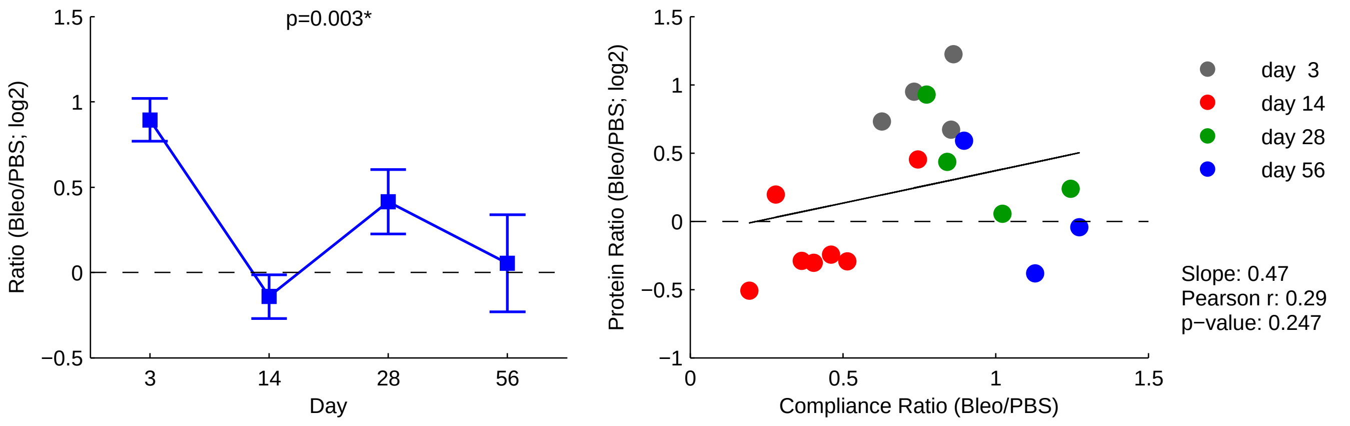

Q3U7R1 − Esyt1 (id: 3841)

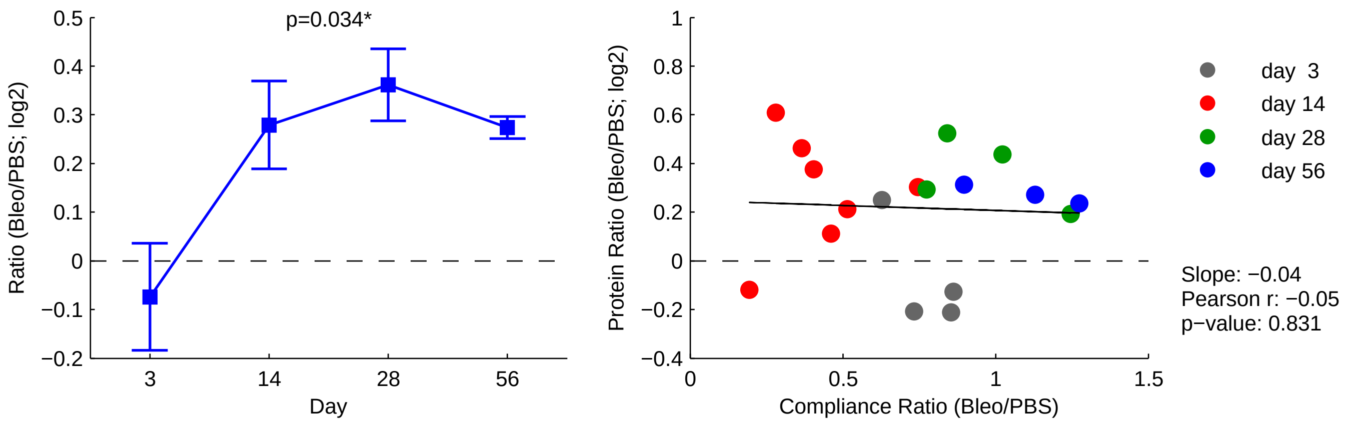

Q3UBG2 − Pid1 (id: 3851)

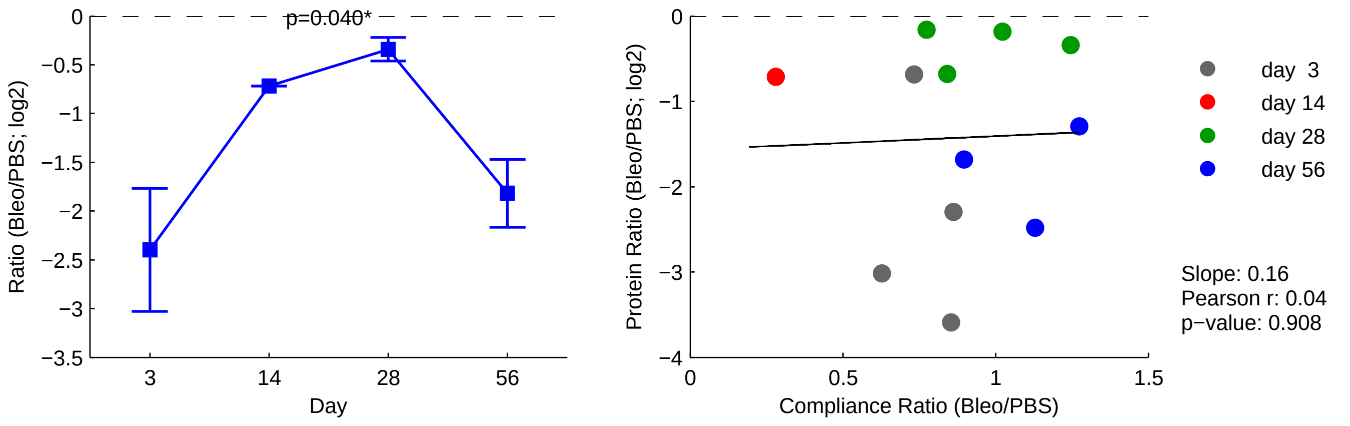

Q3UDE2 − Ttll12 (id: 3853)

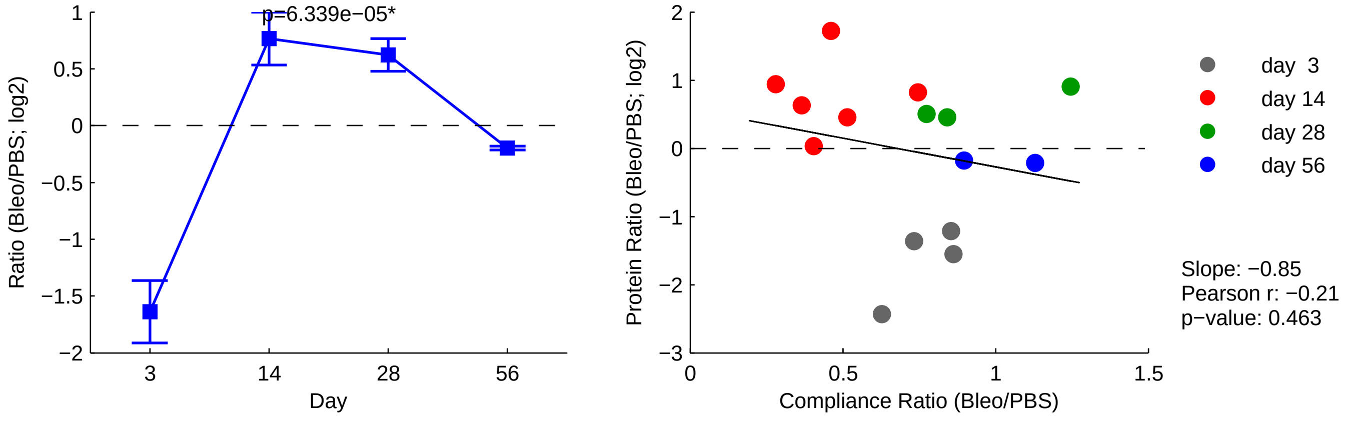

Q3UF95 − Bag6 (id: 3857)

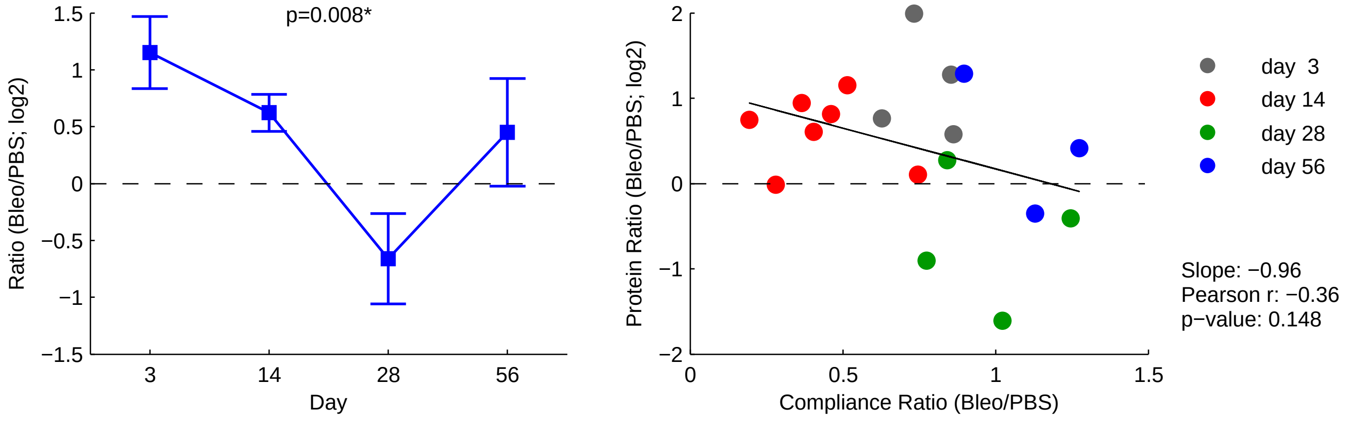

Q3UFK8 − Frmd8 (id: 3858)

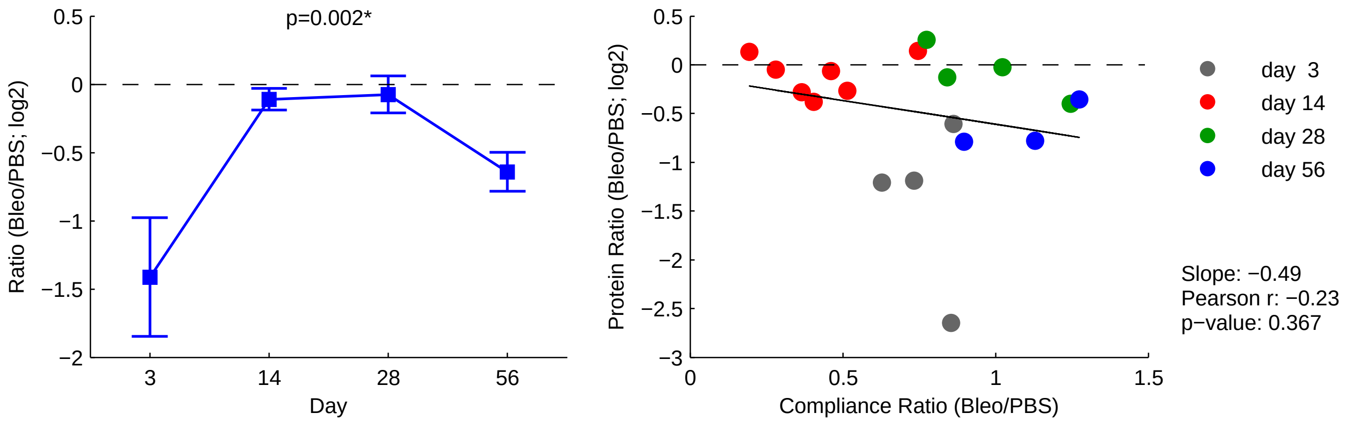

Q9DA80 − Rsph3b (id: 3860)

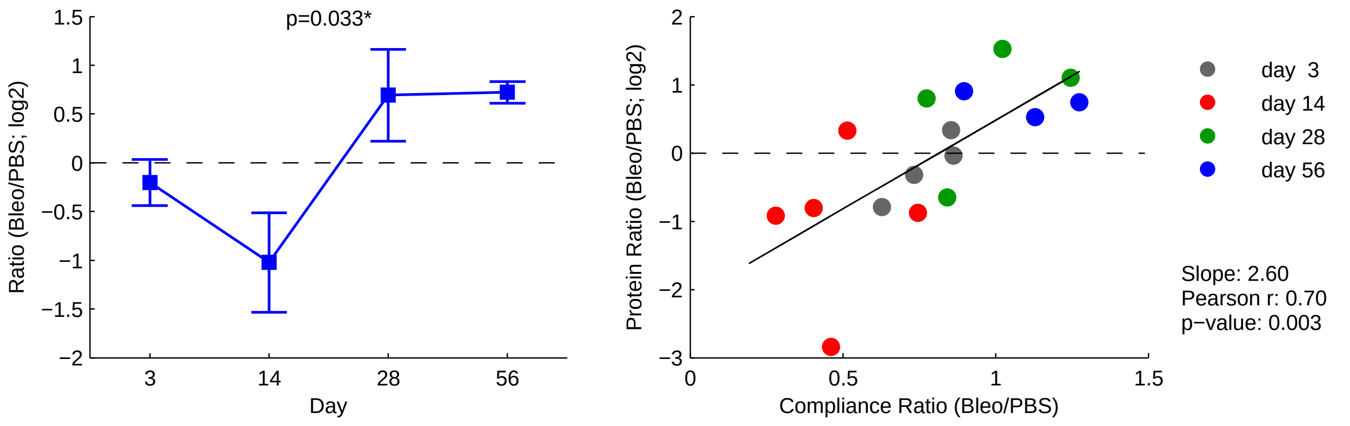

Q3UFY8 − Trmt10c (id: 3861)

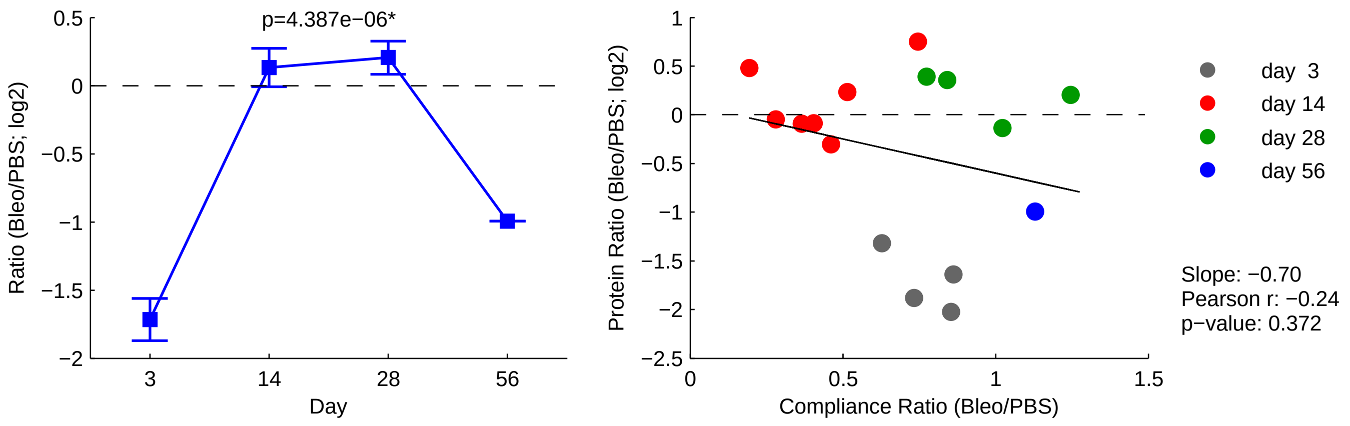

Q9JII5−2 − Dazap1 (id: 3862)

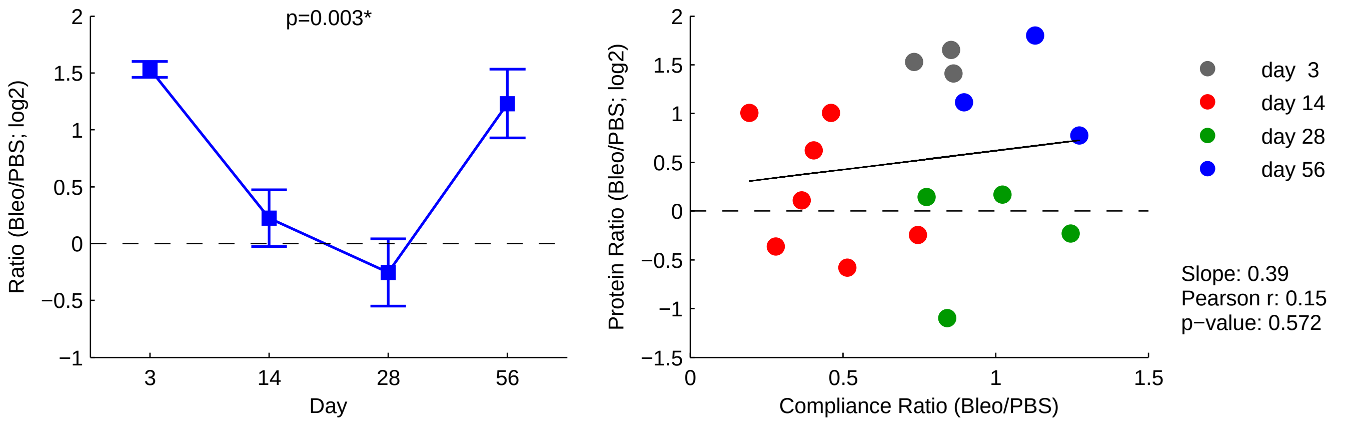

Q3UGC7 − Eif3j1 (id: 3863)

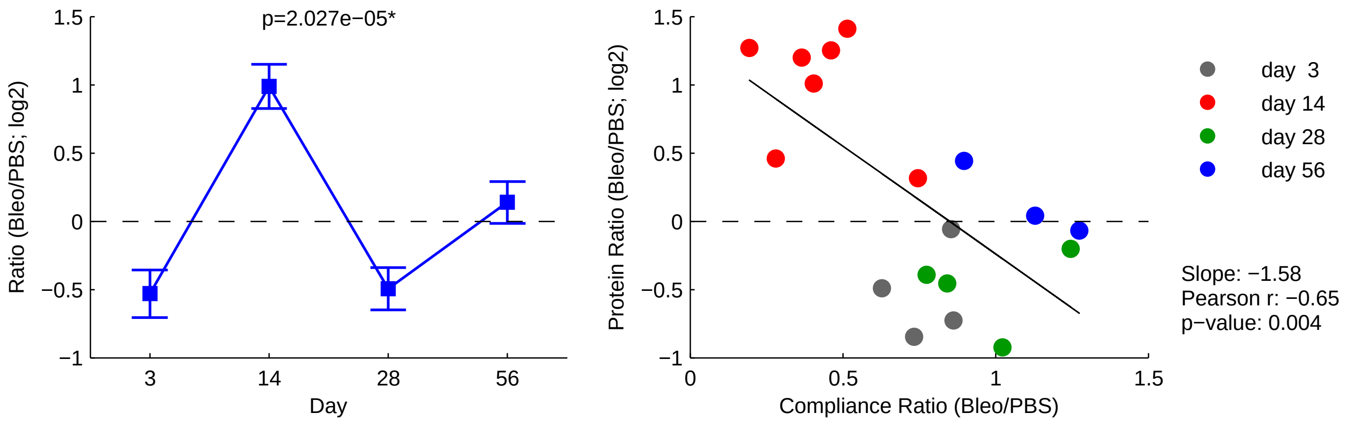

Q3UGF1−3 − Wdr19 (id: 3864)

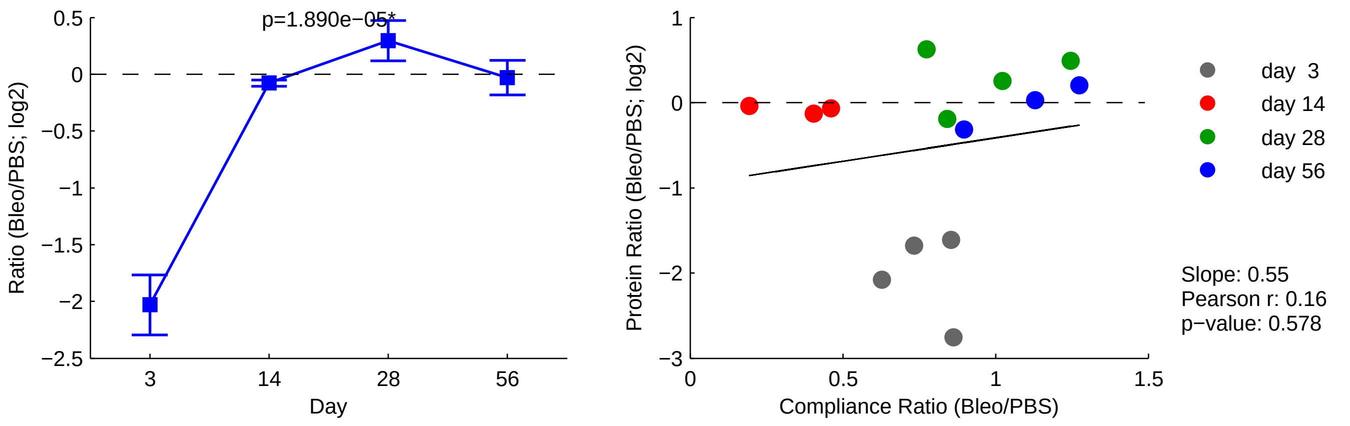

Q3UGR5 − Hdhd2 (id: 3865)

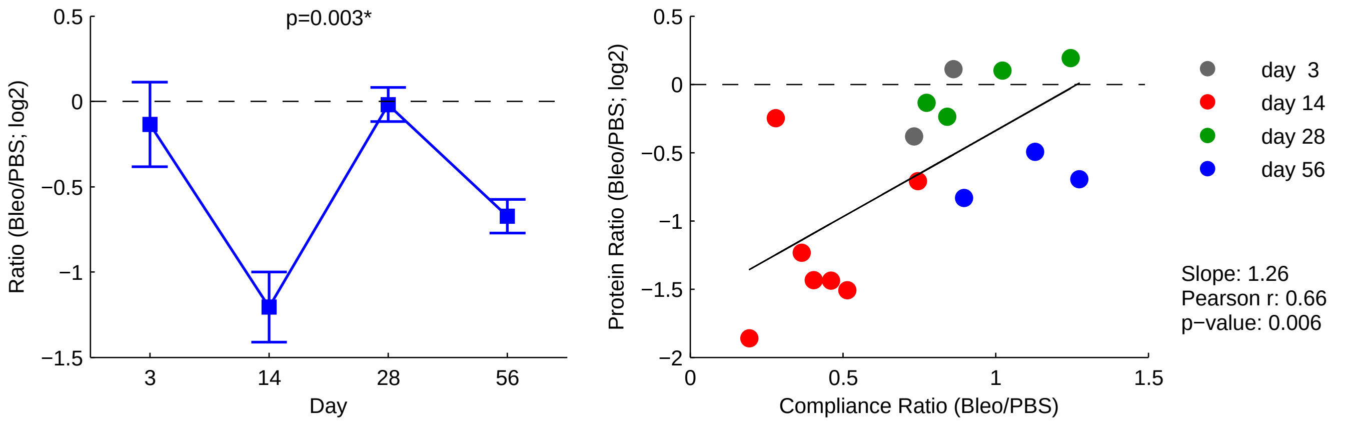

Q91ZZ2 − Rapgef1 (id: 3868)

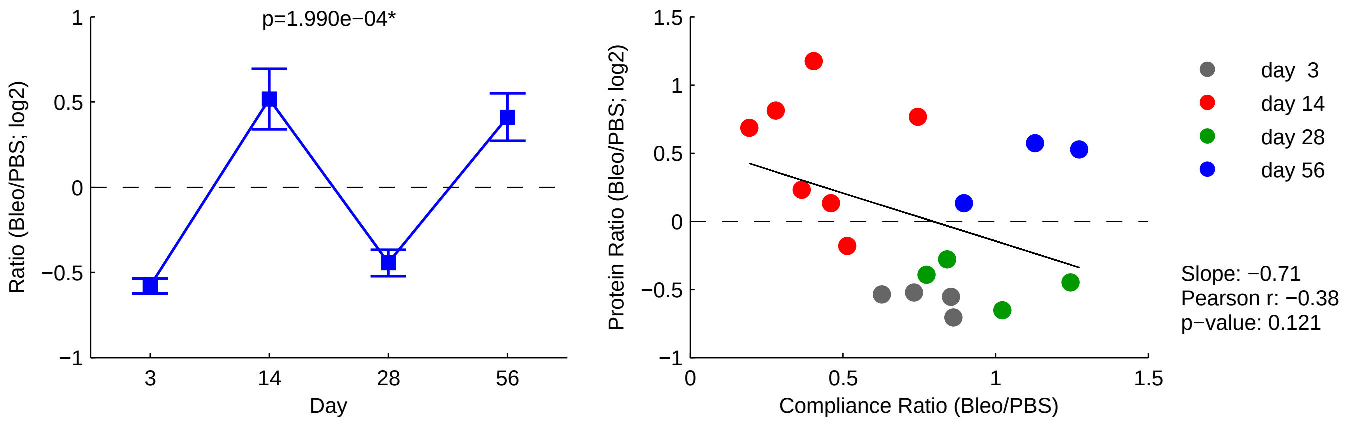

Q3UH60 − Dip2b (id: 3871)

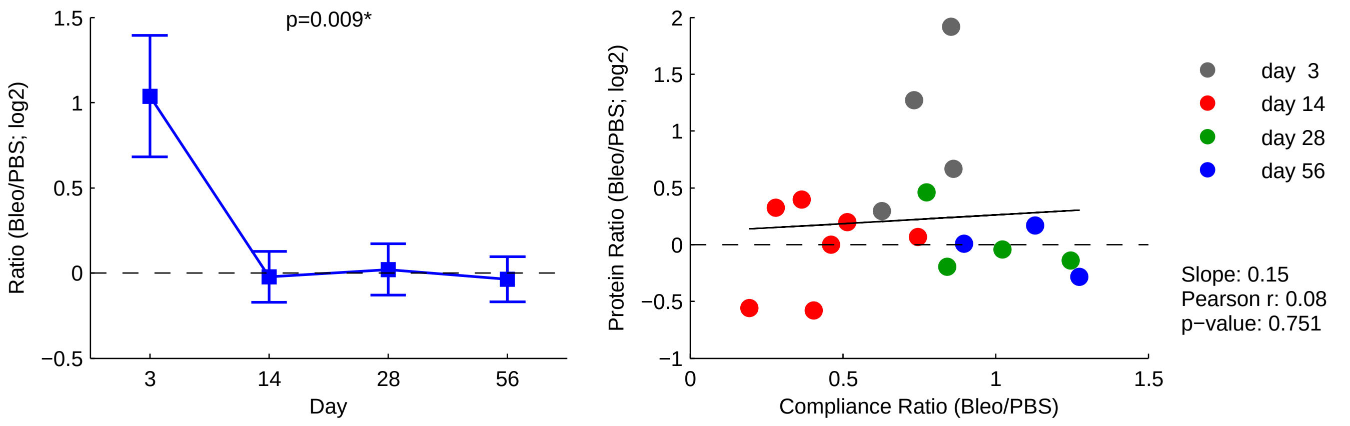

Q3UH93 − Plxnd1 (id: 3872)

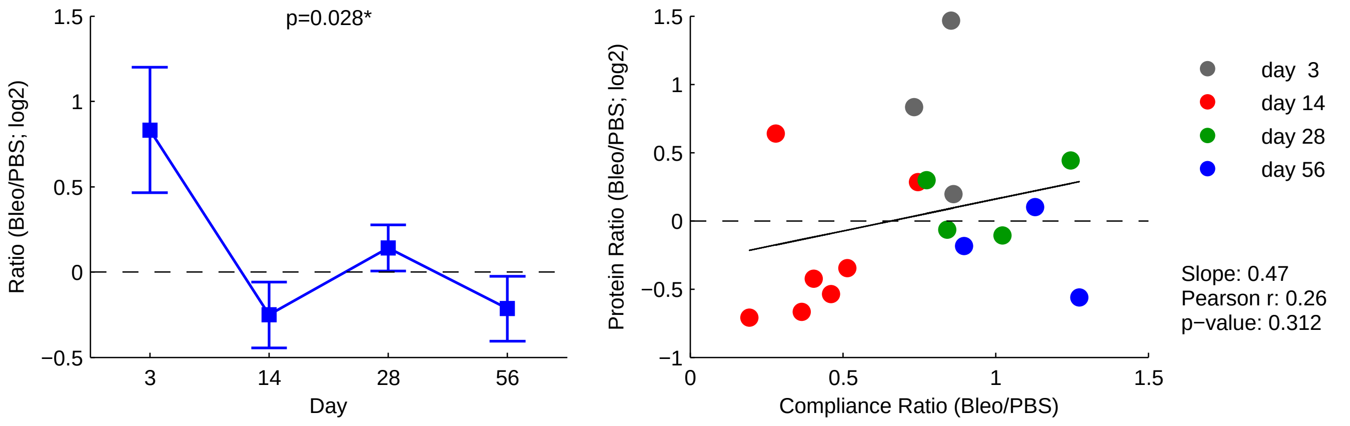

Q3UHB1 − Nt5dc3 (id: 3874)

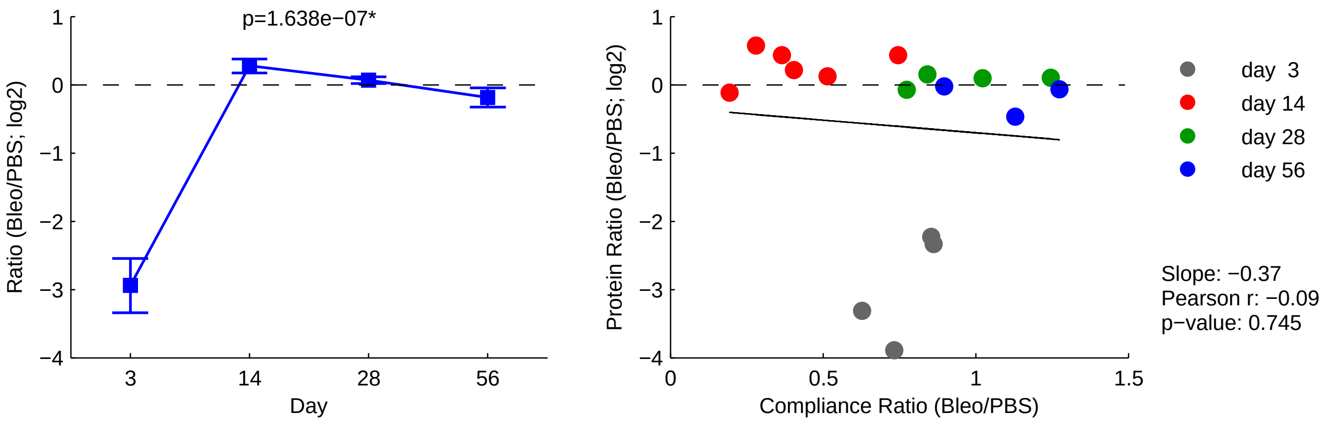

Q3UHG5 − Tspan7 (id: 3879)

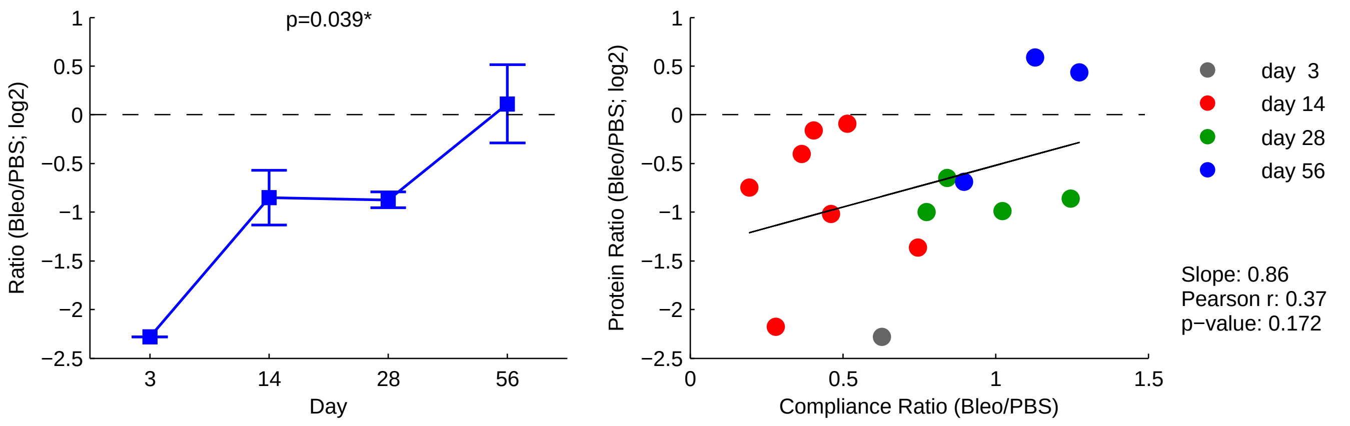

Q3UHN9 − Ndst1 (id: 3885)

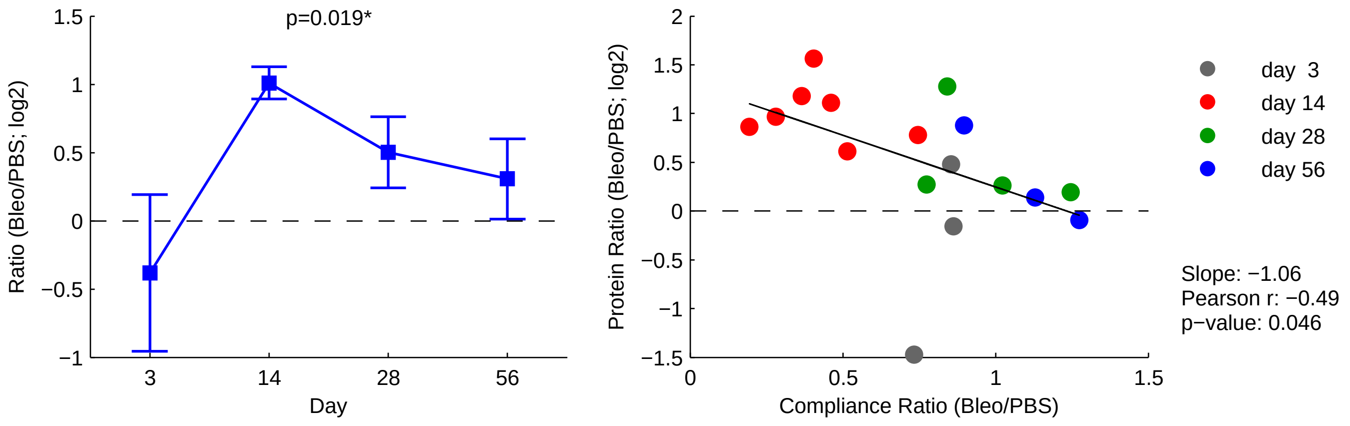

Q3UHQ6 − Dopey2 (id: 3886)

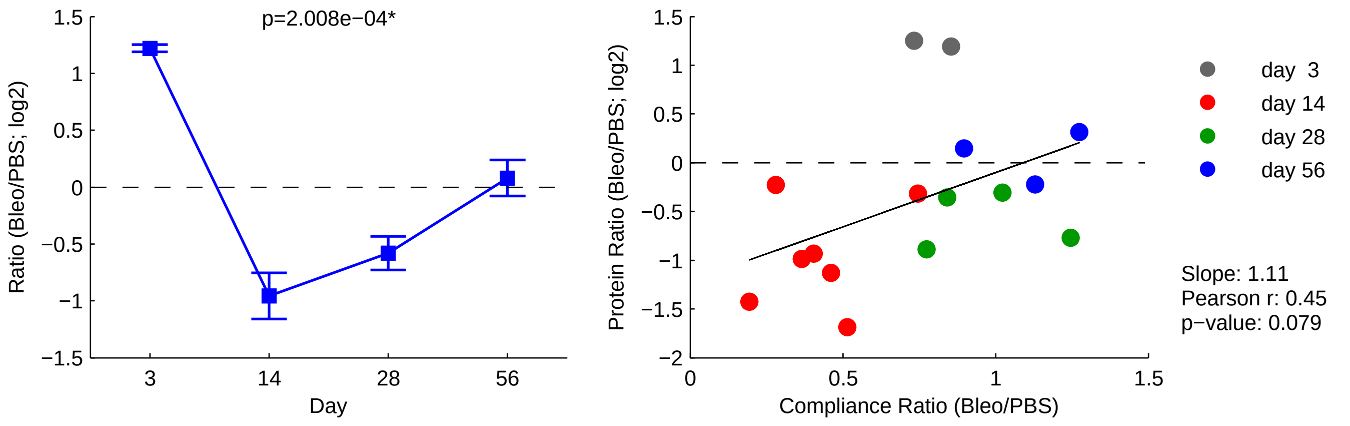

Q3UHX2 − Pdap1 (id: 3888)

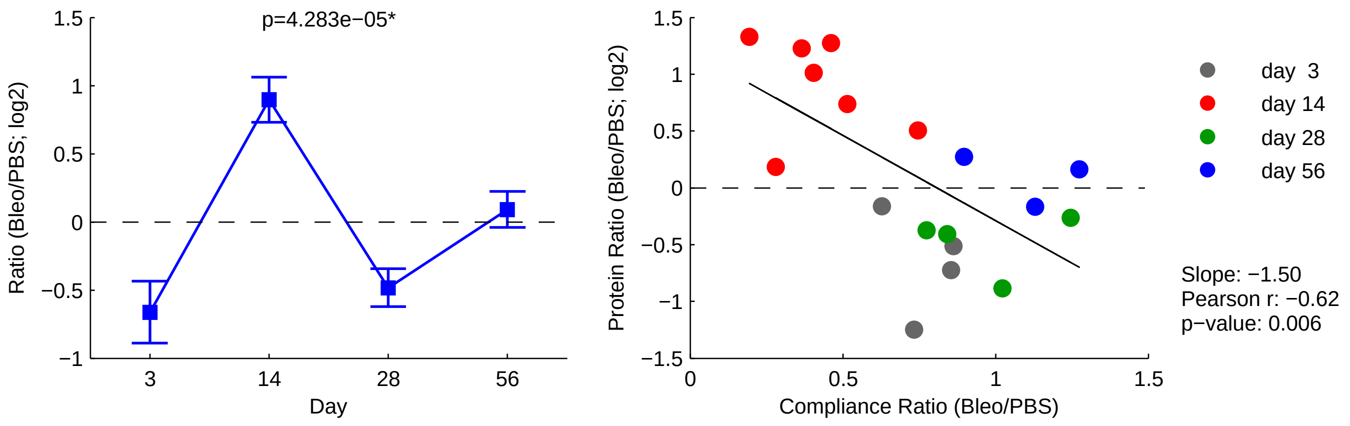

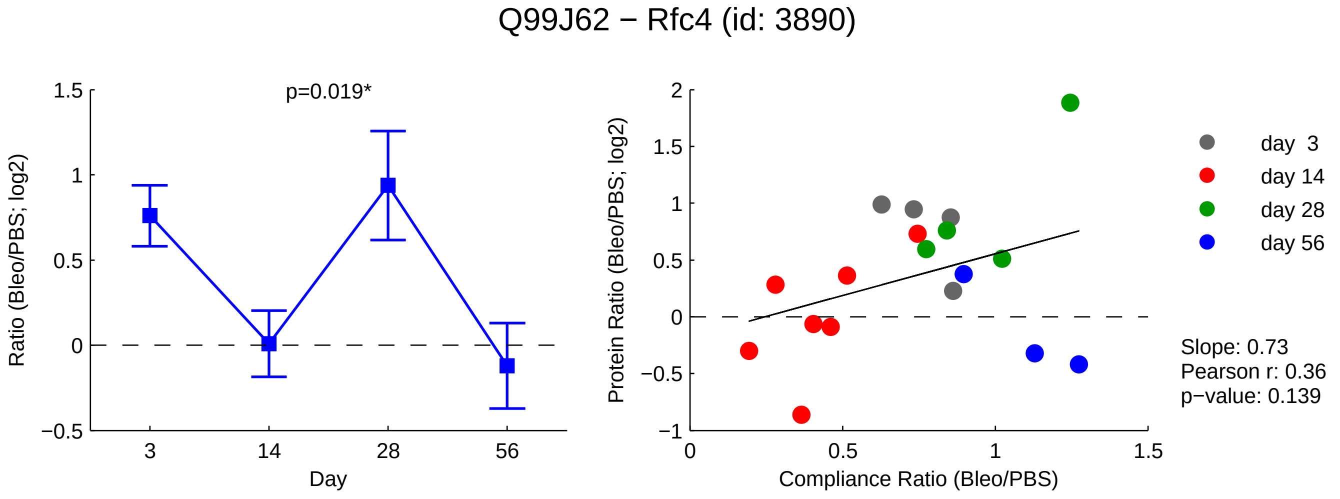
Q99J62 − Rfc4 (id: 3890)
p=0.019*
Ratio (Bleo/PBS; log2)
Day
Protein Ratio (Bleo/PBS; log2)
Compliance Ratio (Bleo/PBS)
day 3
day 14
day 28
day 56
Slope: 0.73
Pearson r: 0.36
p−value: 0.139

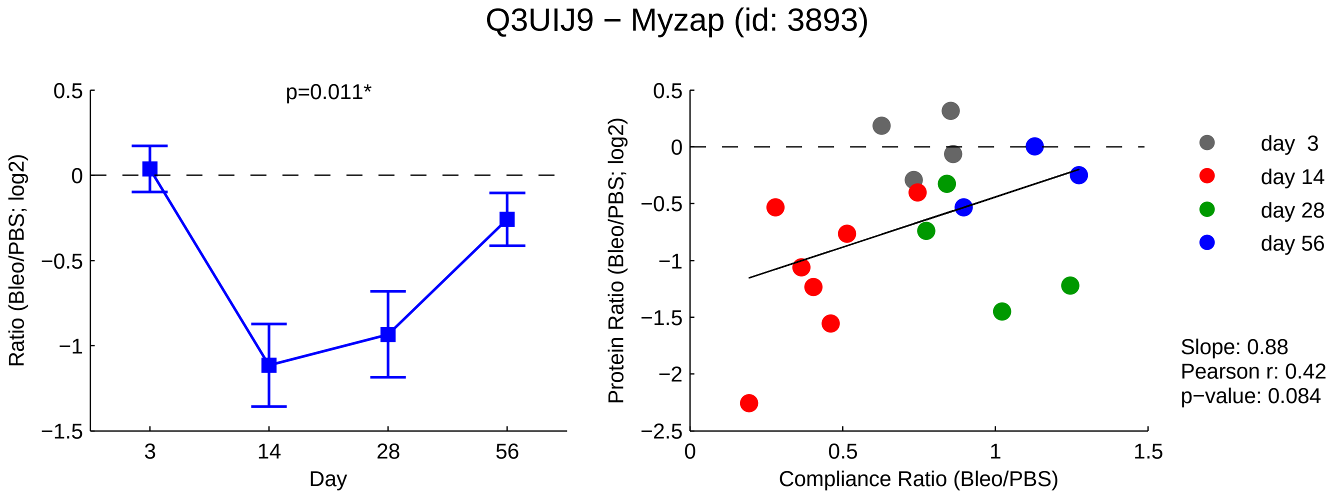
Q3UIJ9 − Myzap (id: 3893)
p=0.011*
Ratio (Bleo/PBS; log2)
Day
Protein Ratio (Bleo/PBS; log2)
Compliance Ratio (Bleo/PBS)
day 3
day 14
day 28
day 56
Slope: 0.88
Pearson r: 0.42
p−value: 0.084

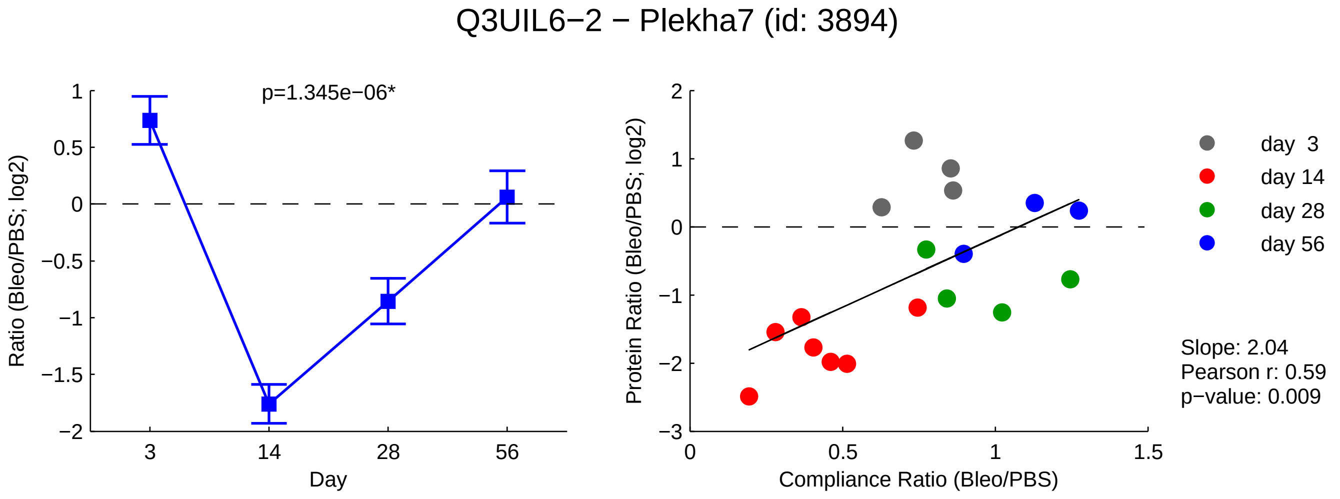
Q3UIL6−2 − Plekha7 (id: 3894)
p=1.345e−06*
Ratio (Bleo/PBS; log2)
Day
Protein Ratio (Bleo/PBS; log2)
Compliance Ratio (Bleo/PBS)
day 3
day 14
day 28
day 56
Slope: 2.04
Pearson r: 0.59
p−value: 0.009

Q3UIR3 − Dtx3l (id: 3895)

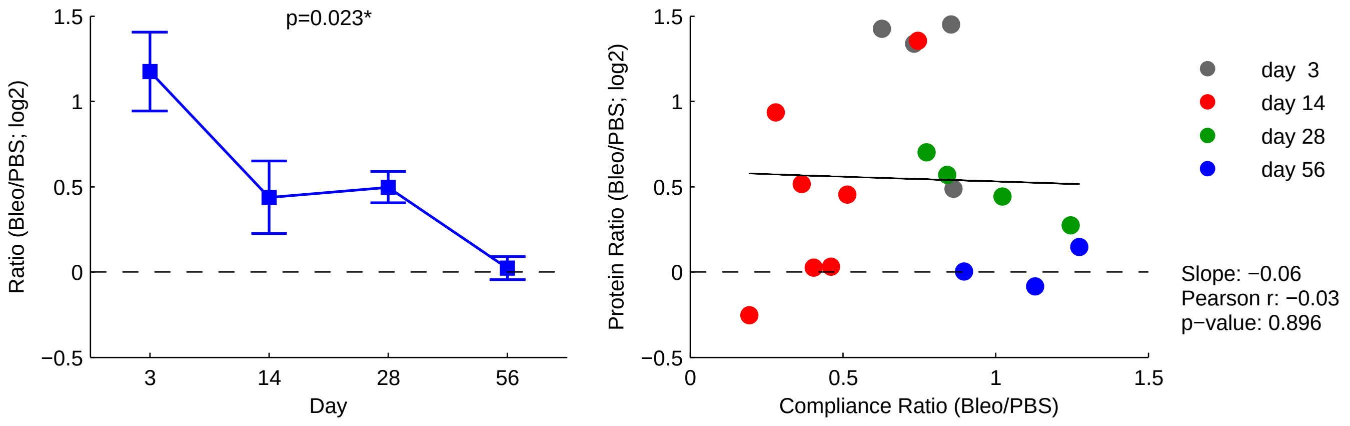

Q3UIU2 − Ndufb6 (id: 3896)

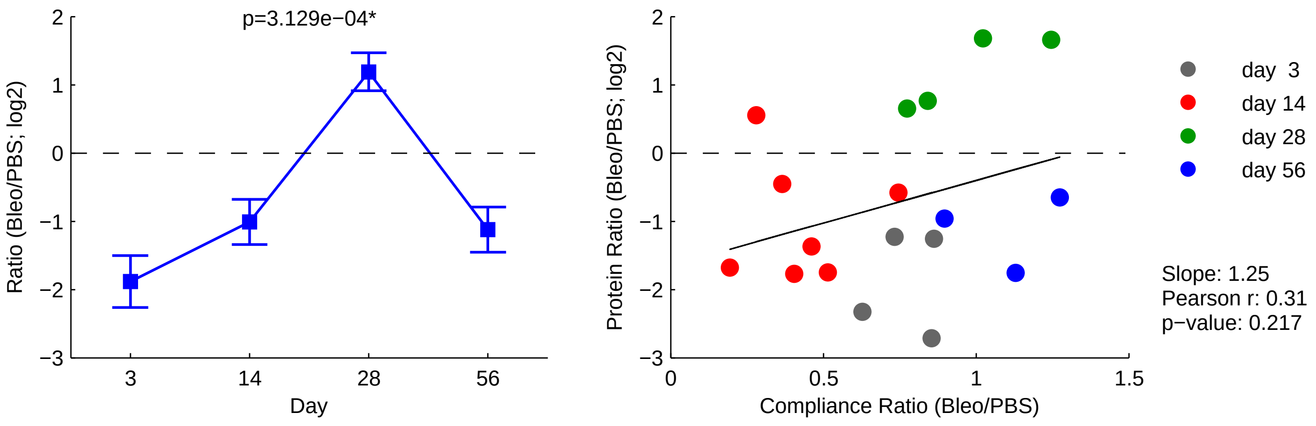

Q3UJU9 − Rmdn3 (id: 3899)

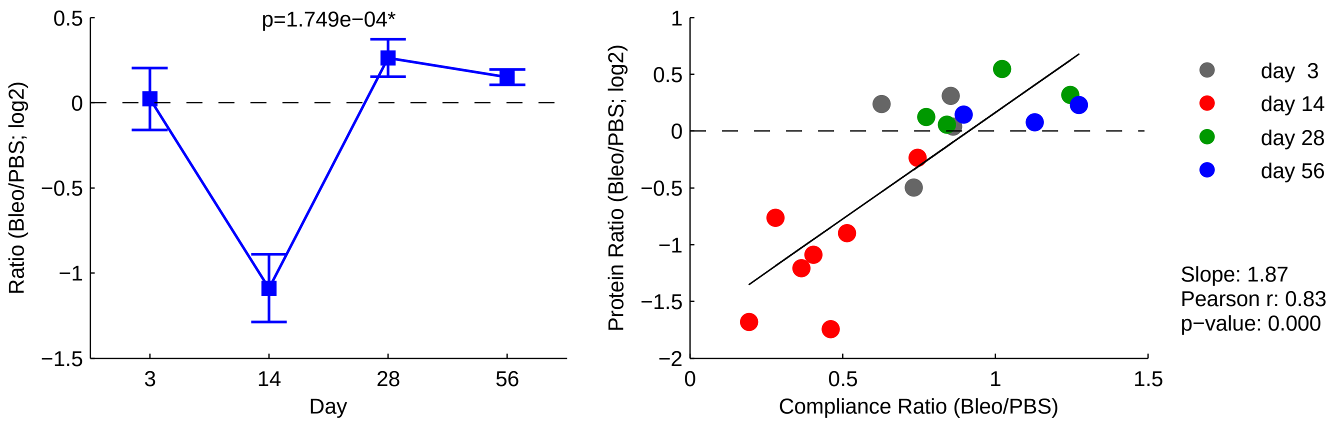

Q3UKC1 − Tax1bp1 (id: 3902)

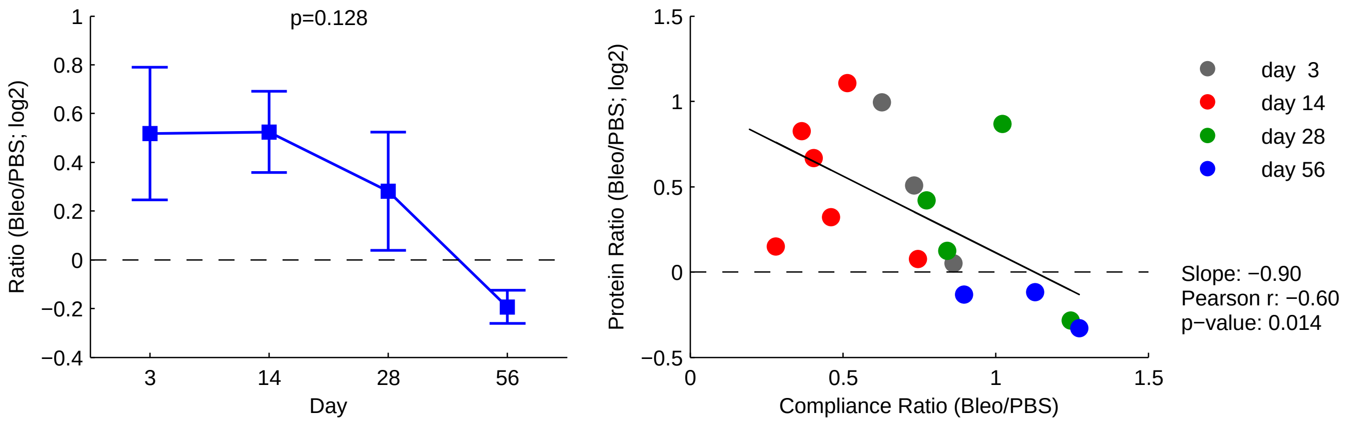

Q3UL36 − Arglu1 (id: 3906)

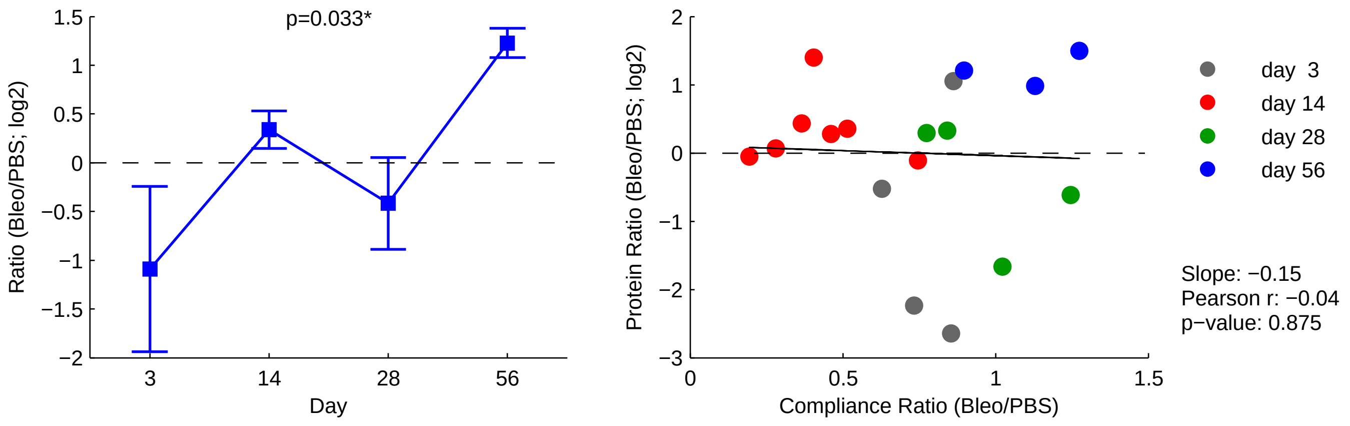

Q3ULB0 − Rbm6 (id: 3907)

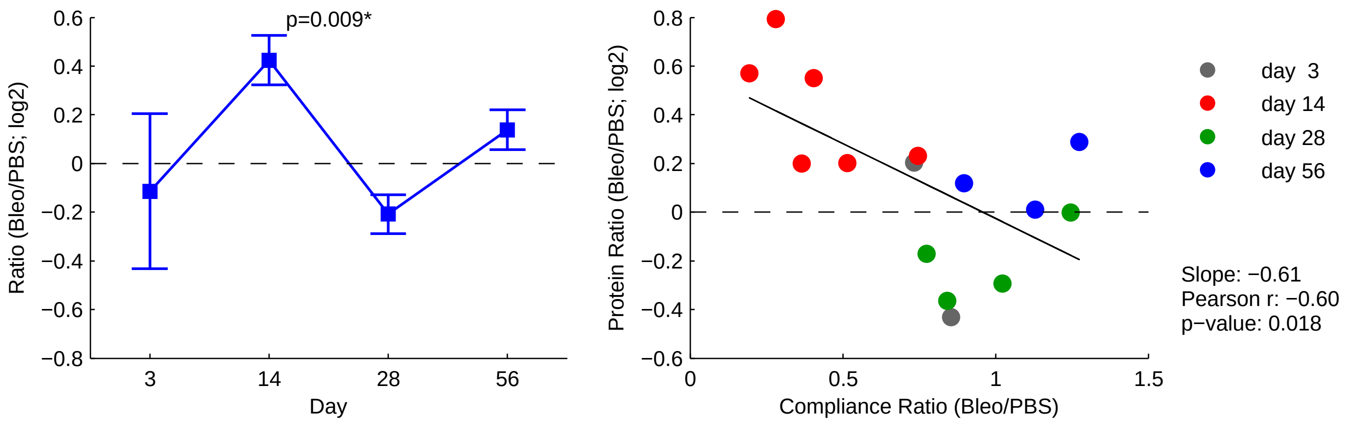

Q3ULD5 − Mccc2 (id: 3908)

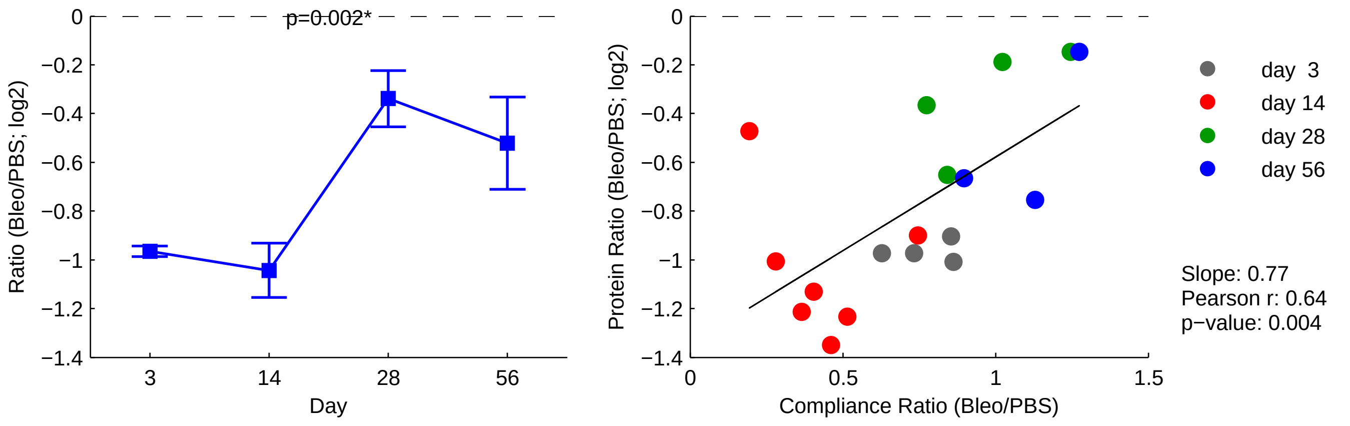

Q3ULJ0−2 − Gpd1l (id: 3909)

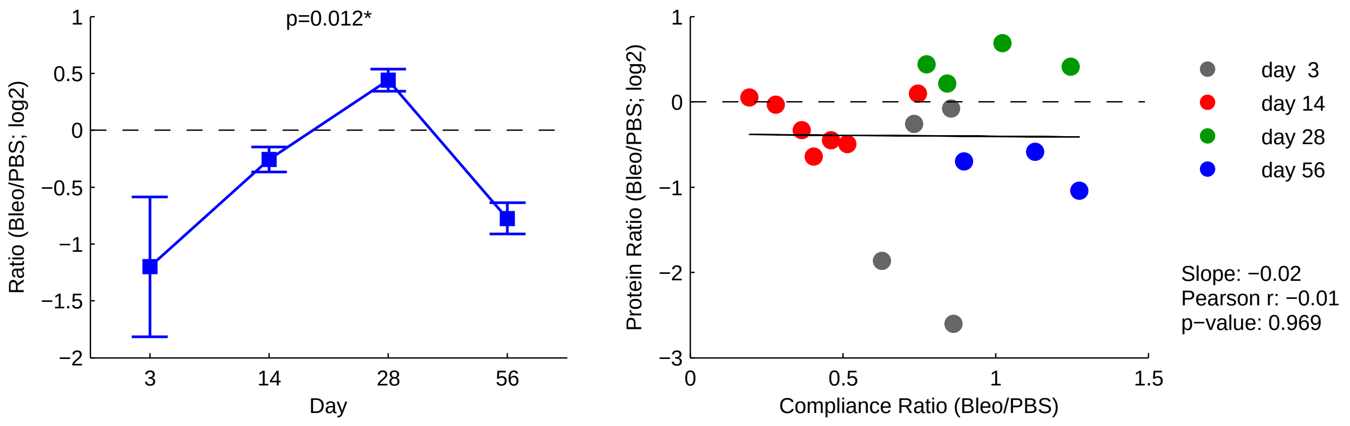

Q3ULL6 − Upf3b (id: 3910)

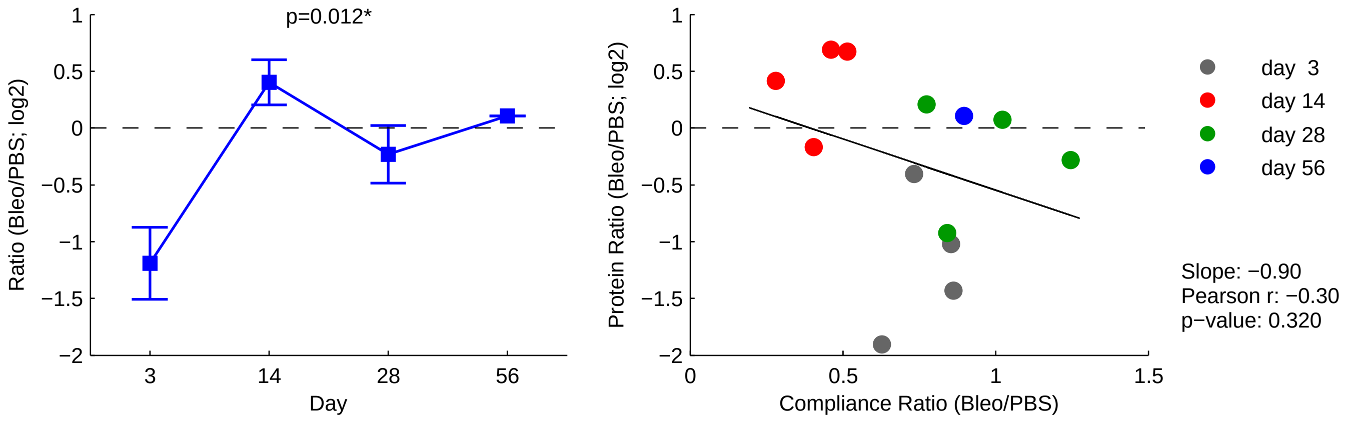

Q3UM29 − Cog7 (id: 3912)

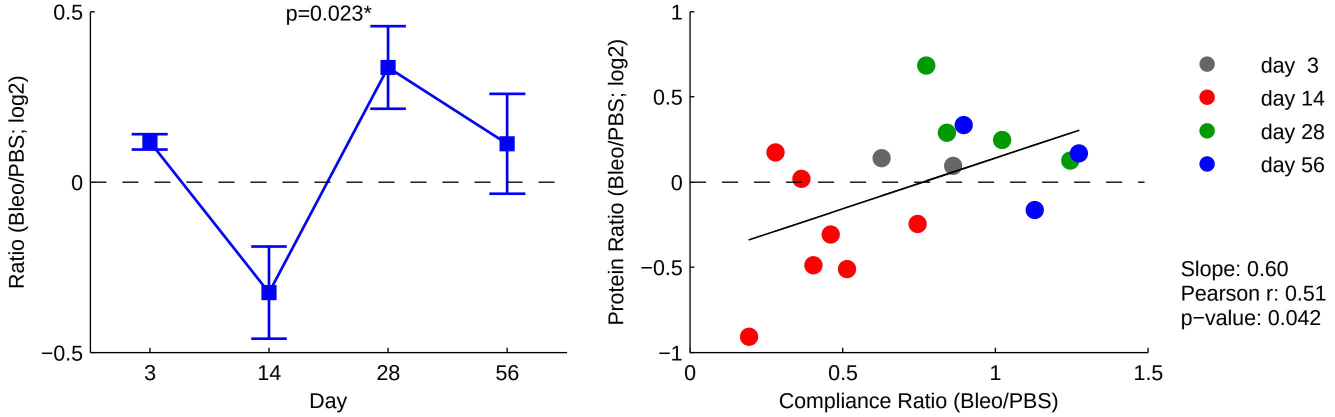

Q3UM45 − Ppp1r7 (id: 3913)

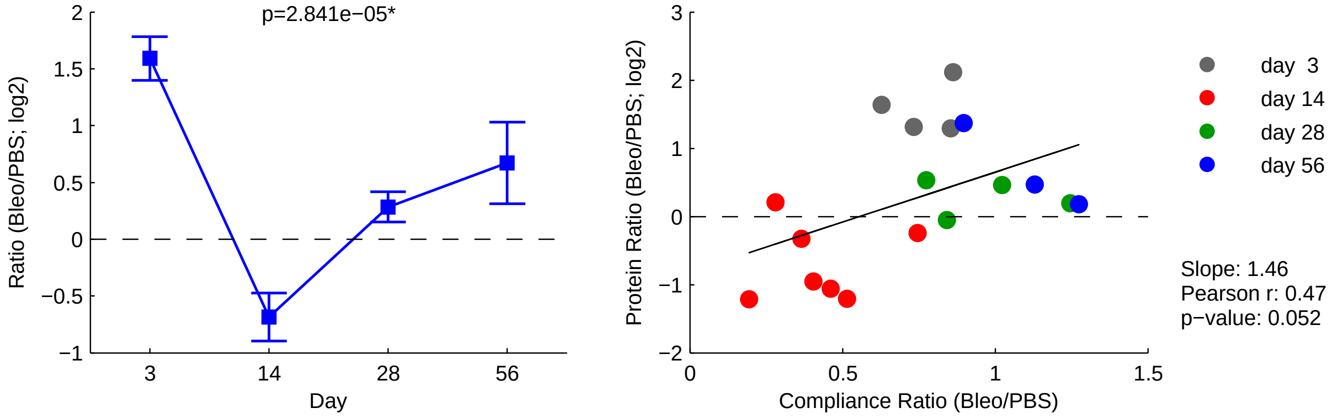

Q3UMB9 − Kiaa1033 (id: 3915)

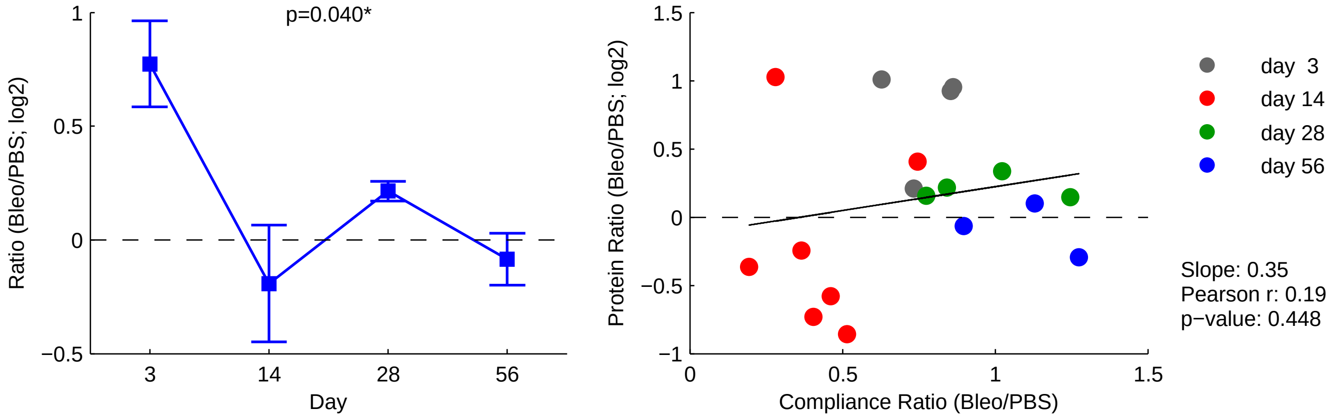

Q3UMR5 − Mcu (id: 3917)

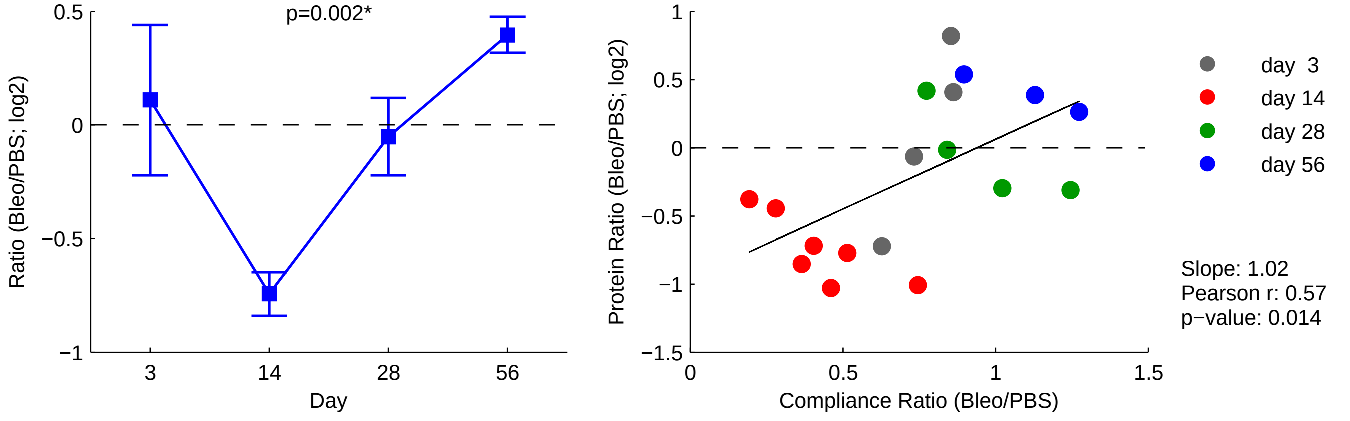

Q3UMU9−2 − Hdgfrp2 (id: 3919)

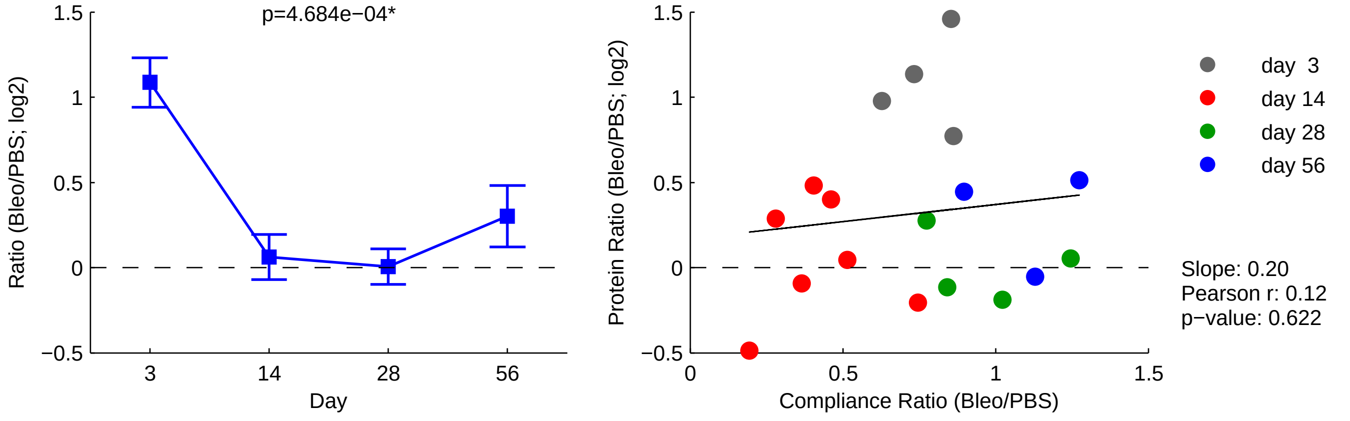

Q3UMW8 − Cln5 (id: 3920)

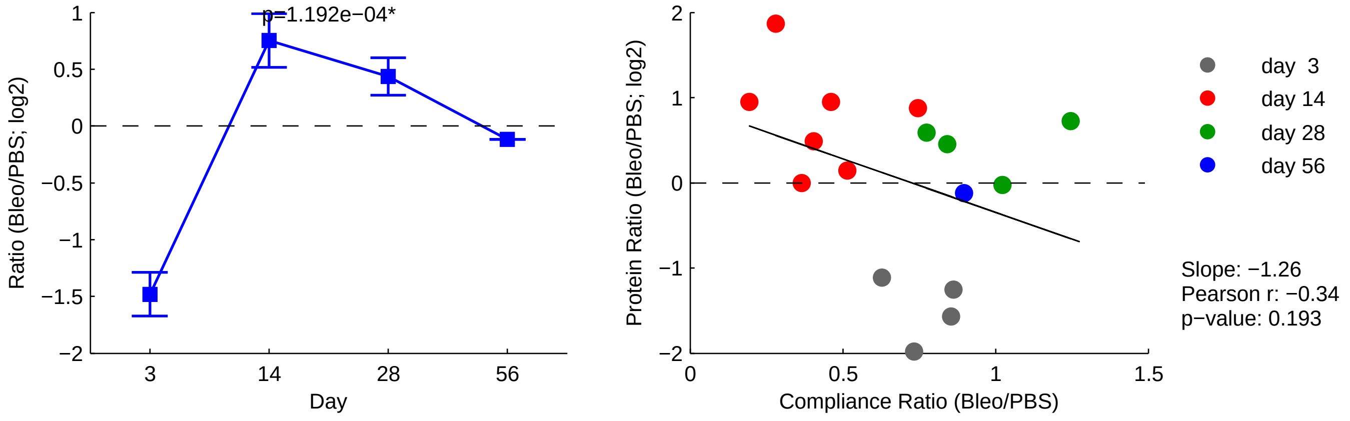

Q3UN02 − Lclat1 (id: 3921)

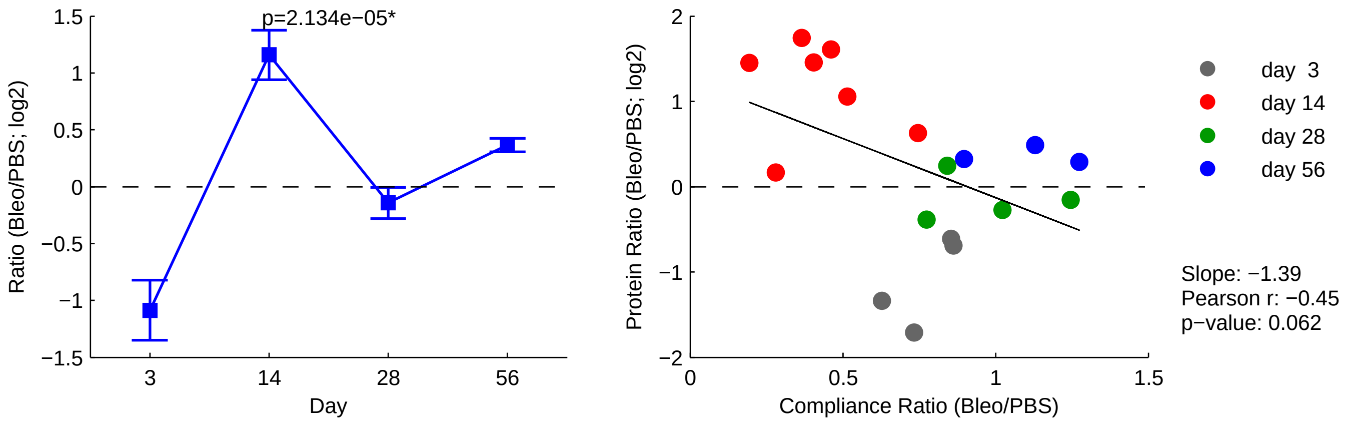

Q3UN04 − Usp30 (id: 3922)

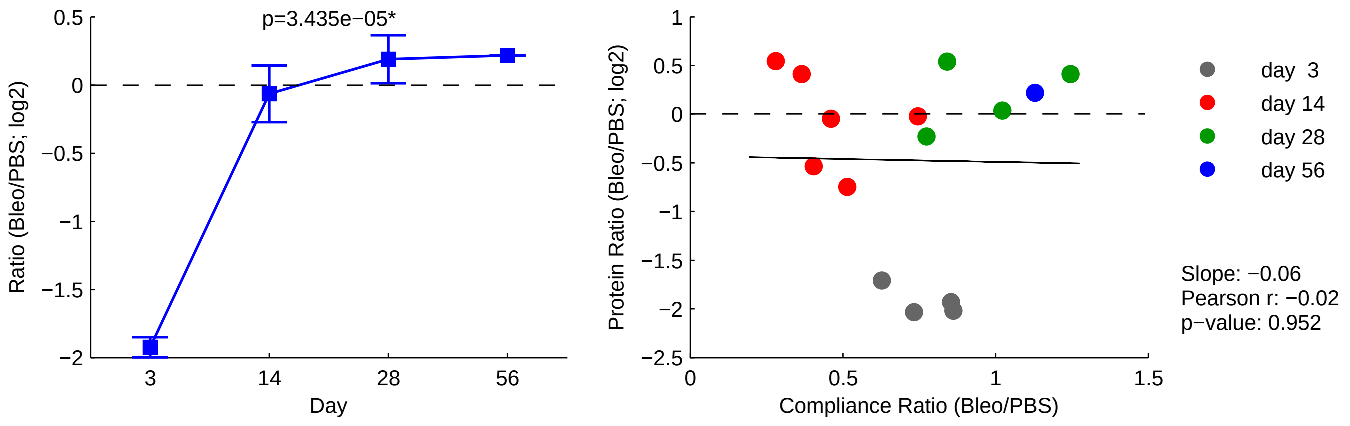

Q3UN88 − Mcpt4 (id: 3923)

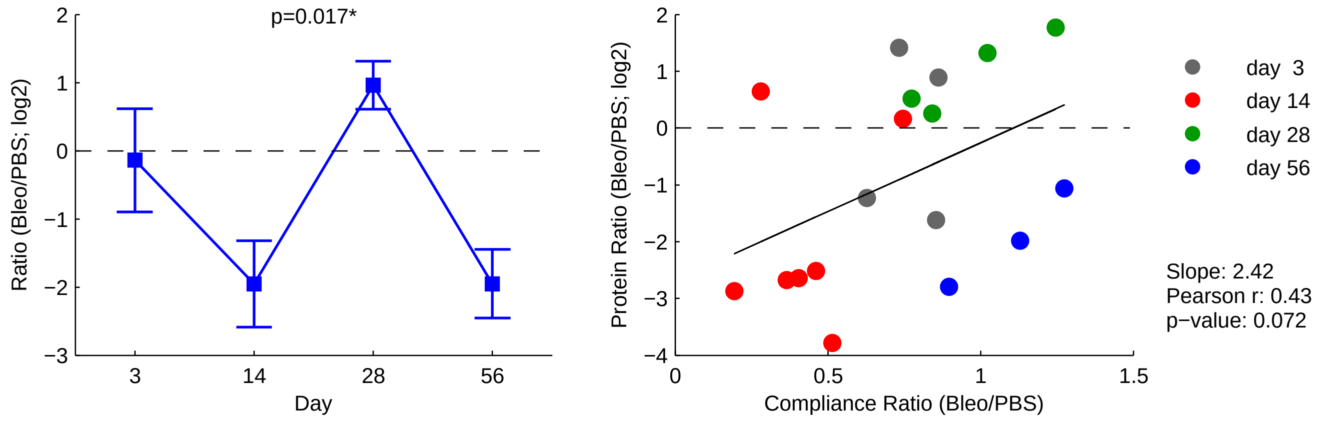

Q3UNZ8 − (id: 3925)

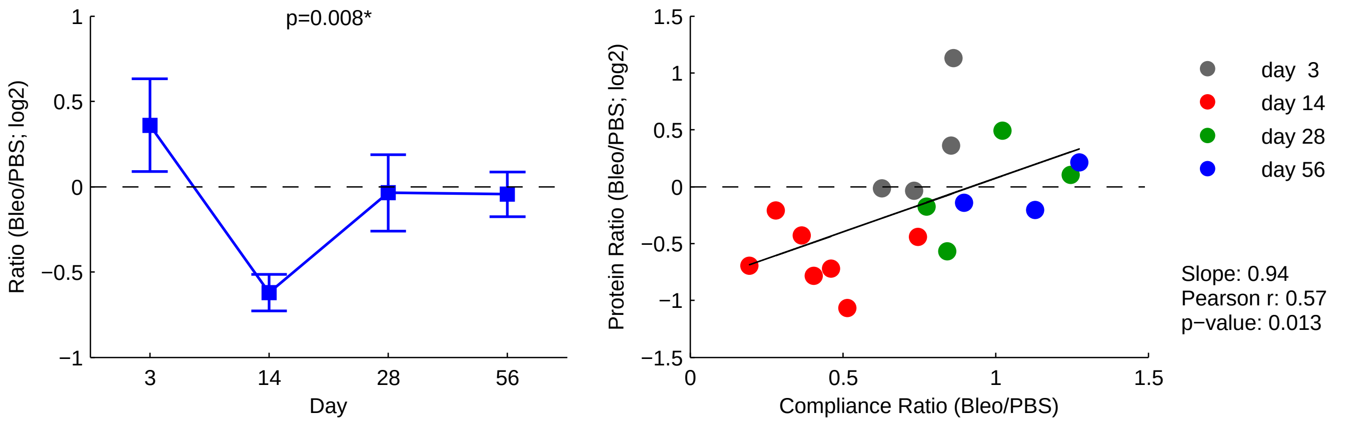

Q3UPH1 − Prrc1 (id: 3927)

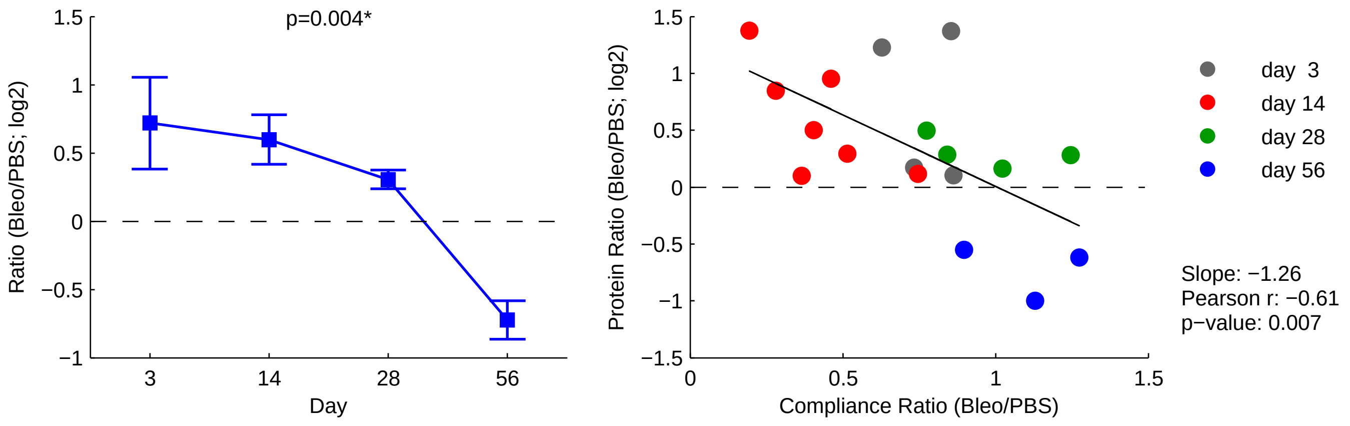

Q3UPL0 − Sec31a (id: 3930)

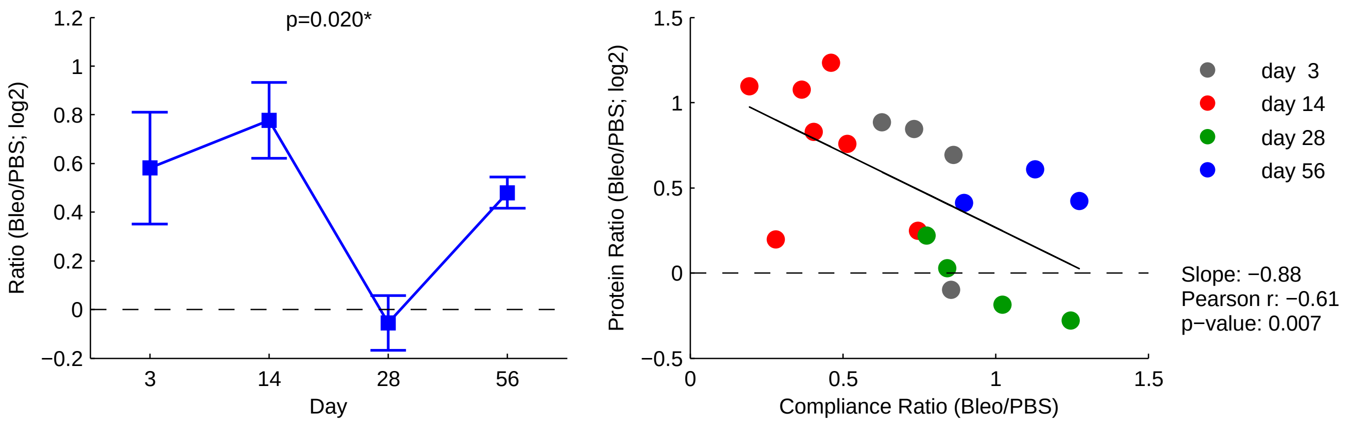

Q3UQA7 − Selh (id: 3934)

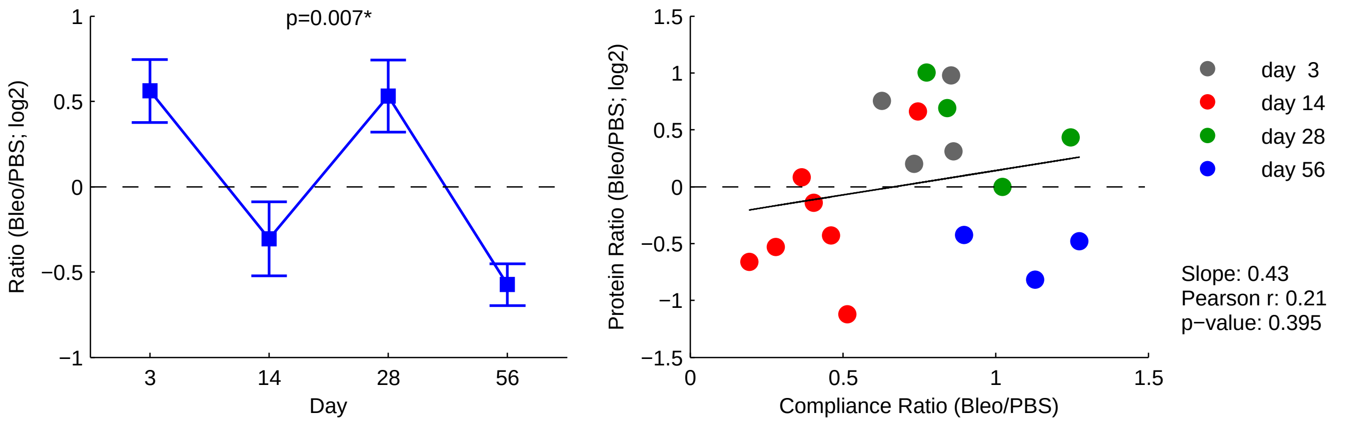

Q3UQN2 − Fcho2 (id: 3935)

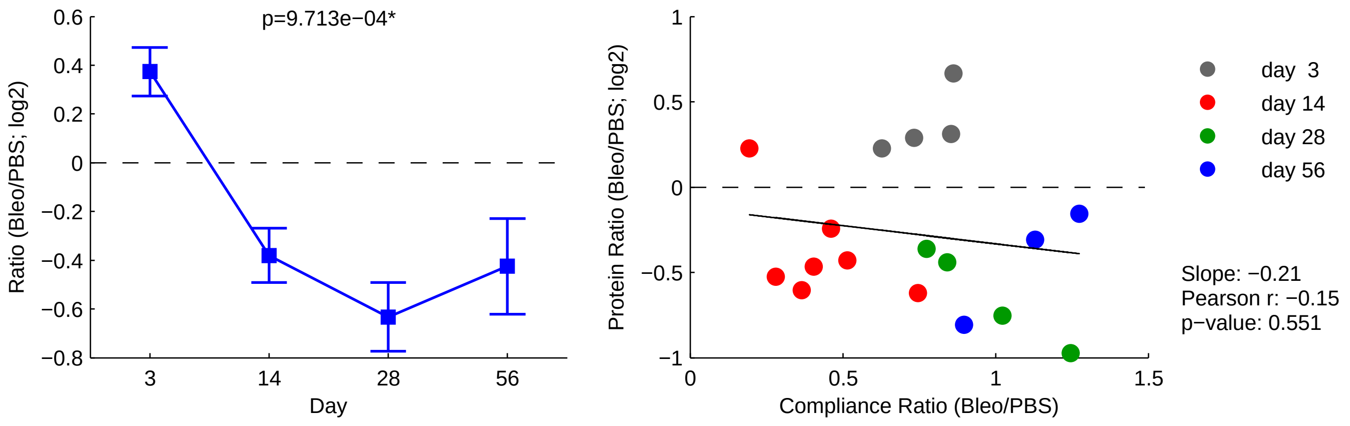

Q3UR70 − Tgfbrap1 (id: 3937)

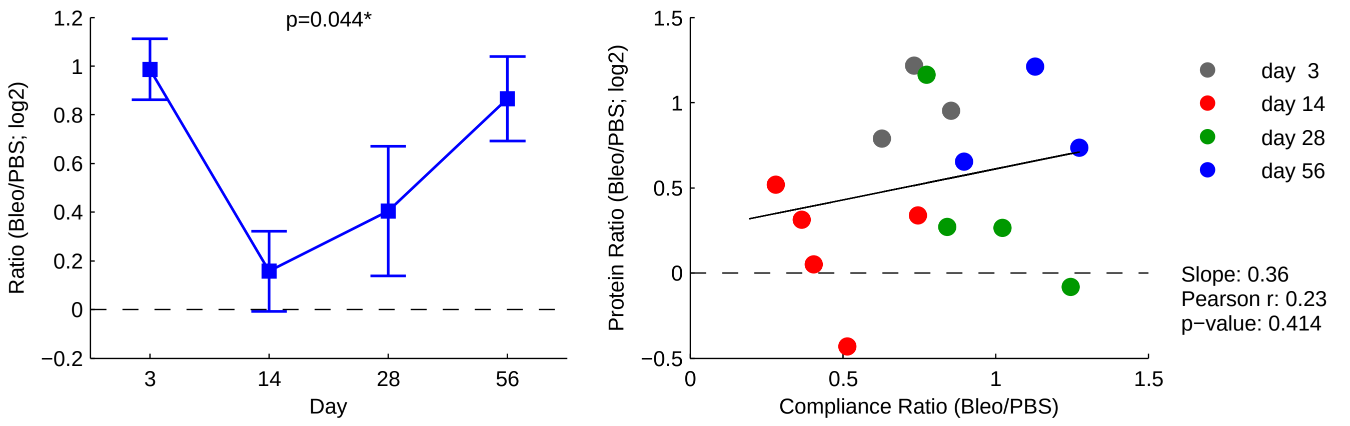

Q3URE1 − Acsf3 (id: 3939)

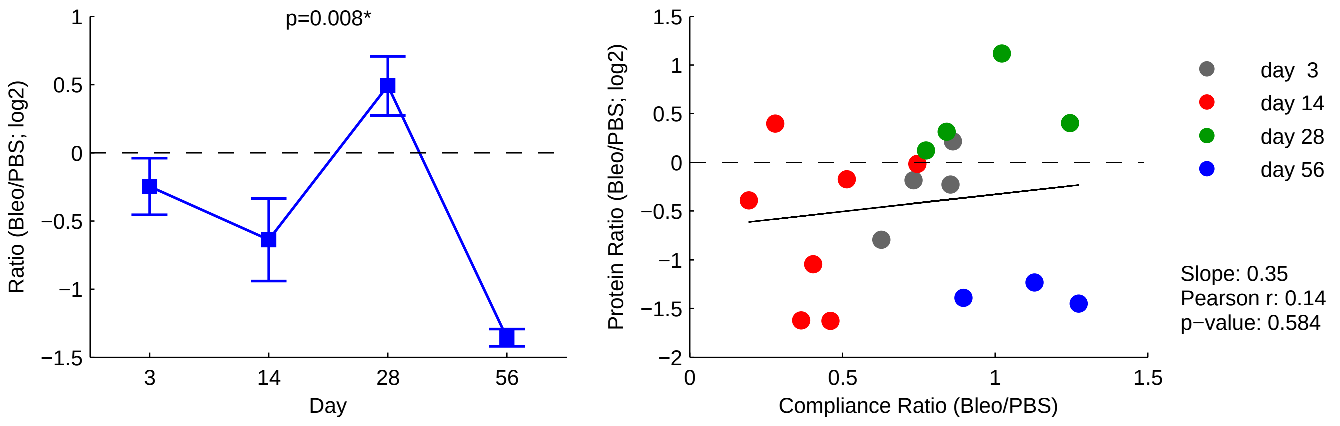

Q3URS9−2 − Ccdc51 (id: 3943)

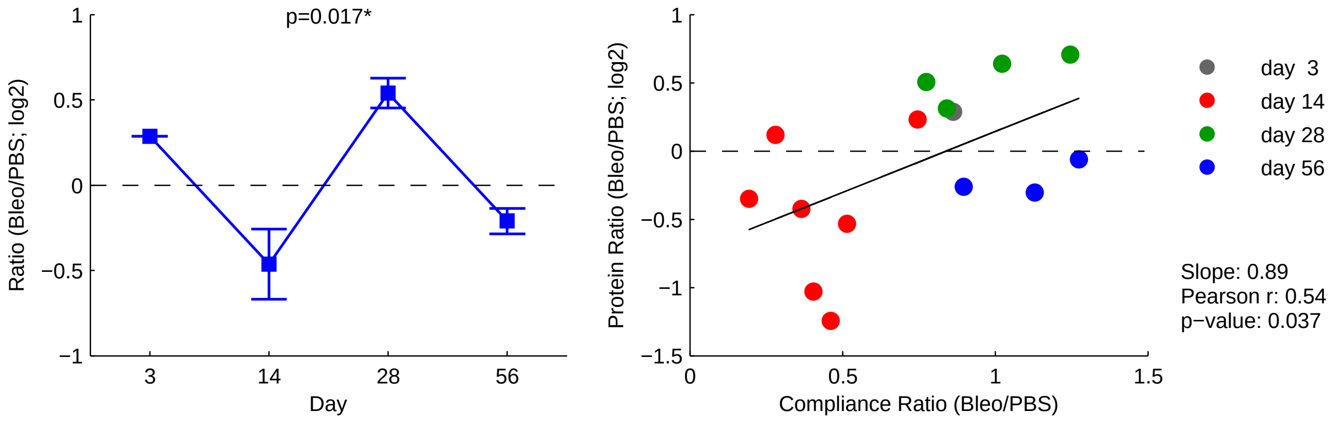

Q3USS3 − Drc1 (id: 3946)

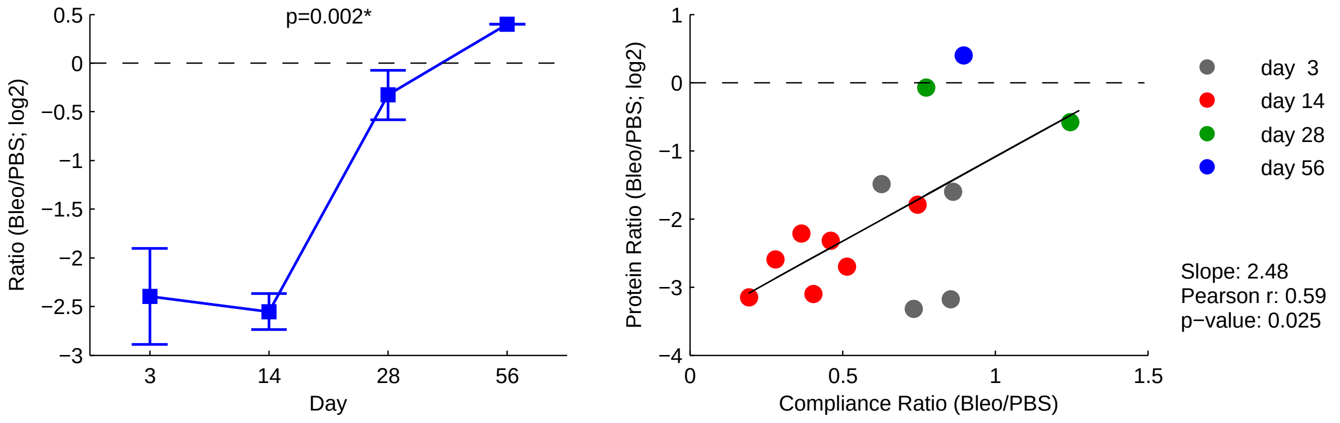

Q3UTJ2−2 − Sorbs2 (id: 3947)

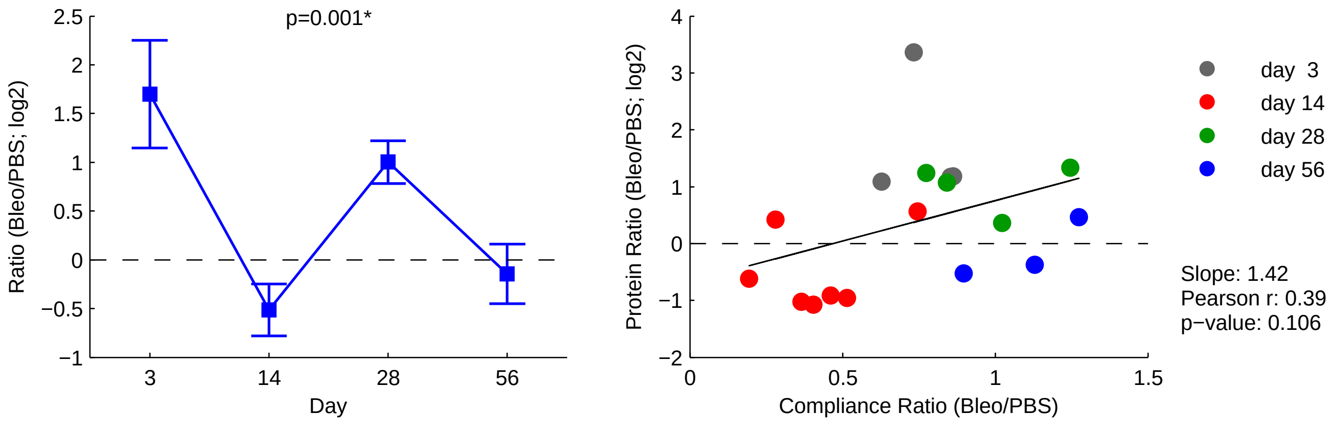

Q3UU56 − Gtf3c2 (id: 3951)

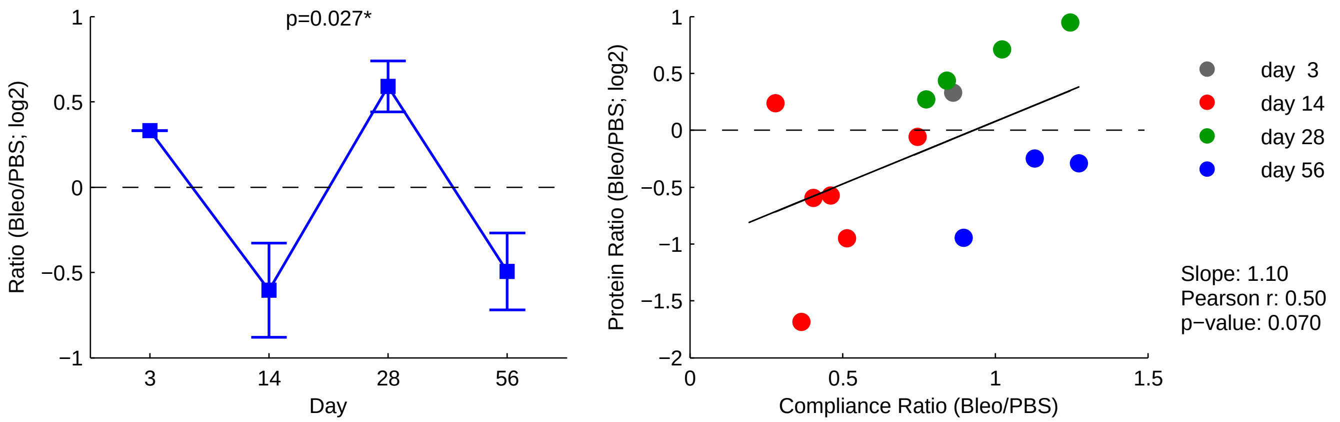

Q3UUI3 − Them4 (id: 3952)

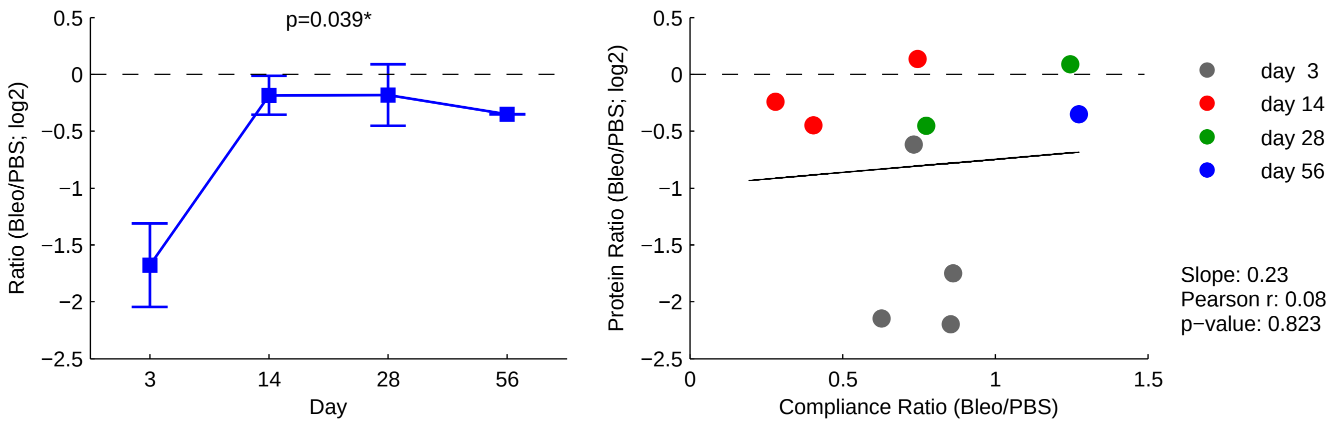

Q61458 − Ccnh (id: 3954)

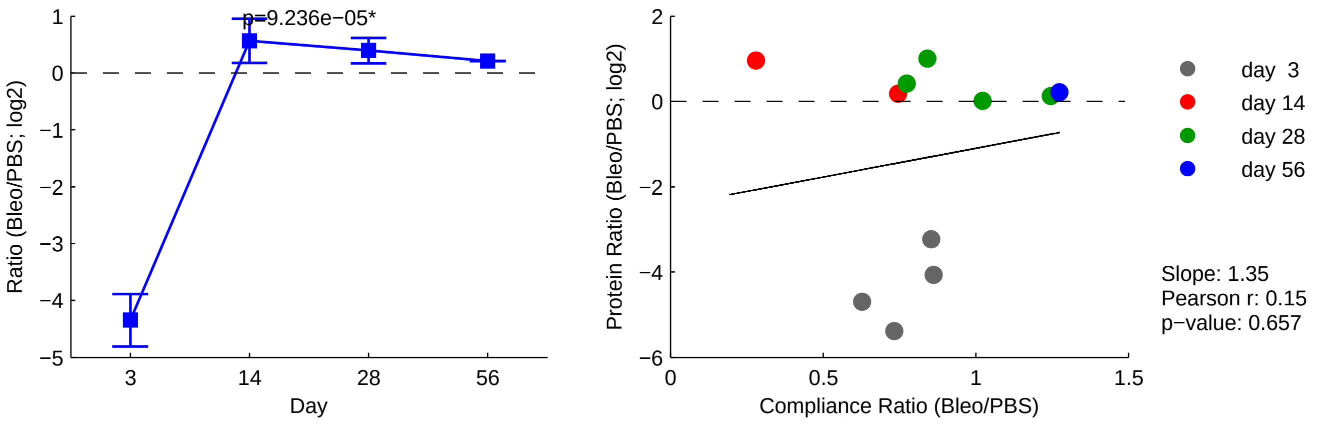

Q3UVG3 − Fam91a1 (id: 3959)

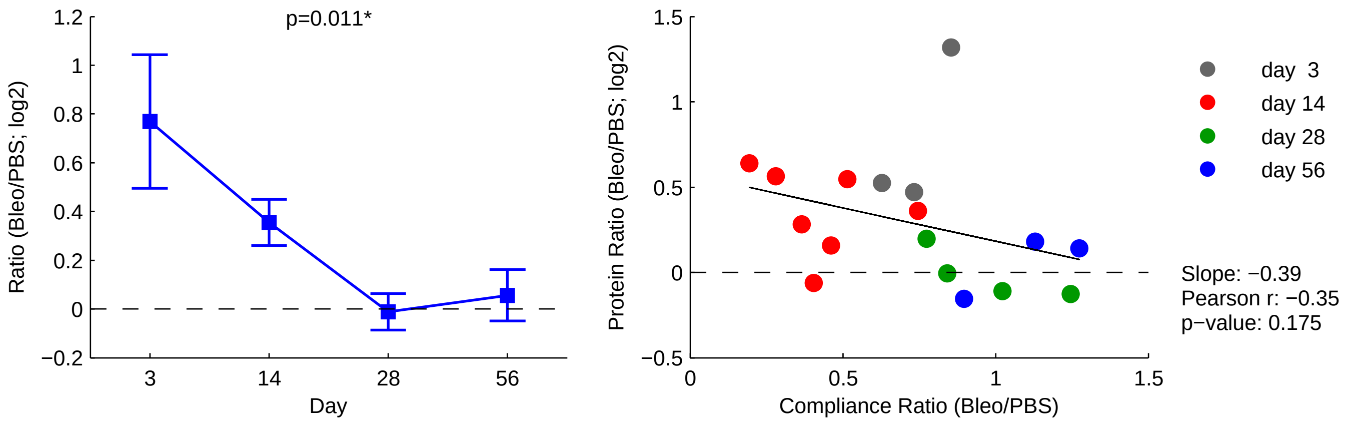

Q3UVK0 − Ermp1 (id: 3960)

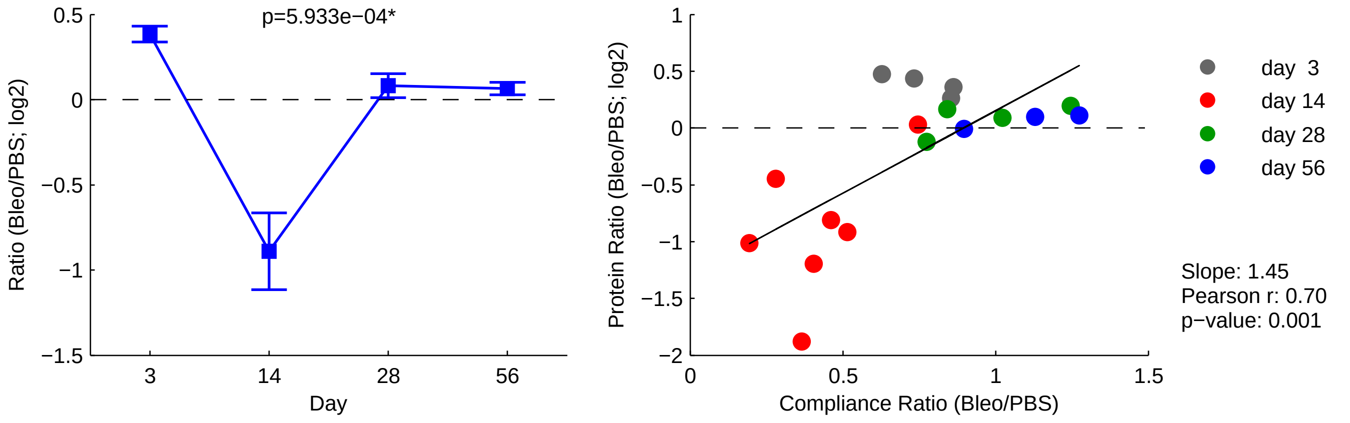

Q3UW53 − Fam129a (id: 3962)

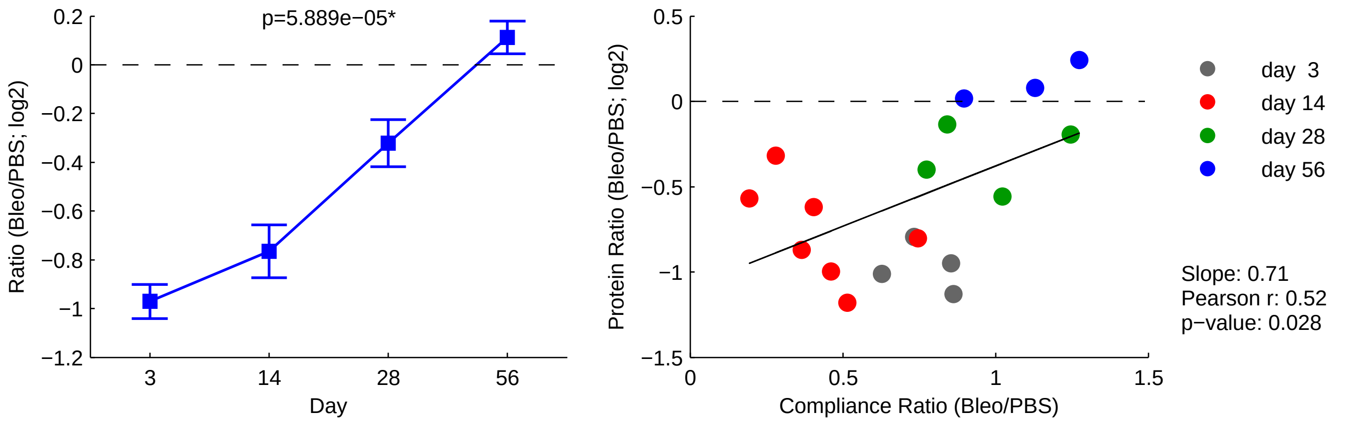

Q99J99 − Mpst (id: 3963)

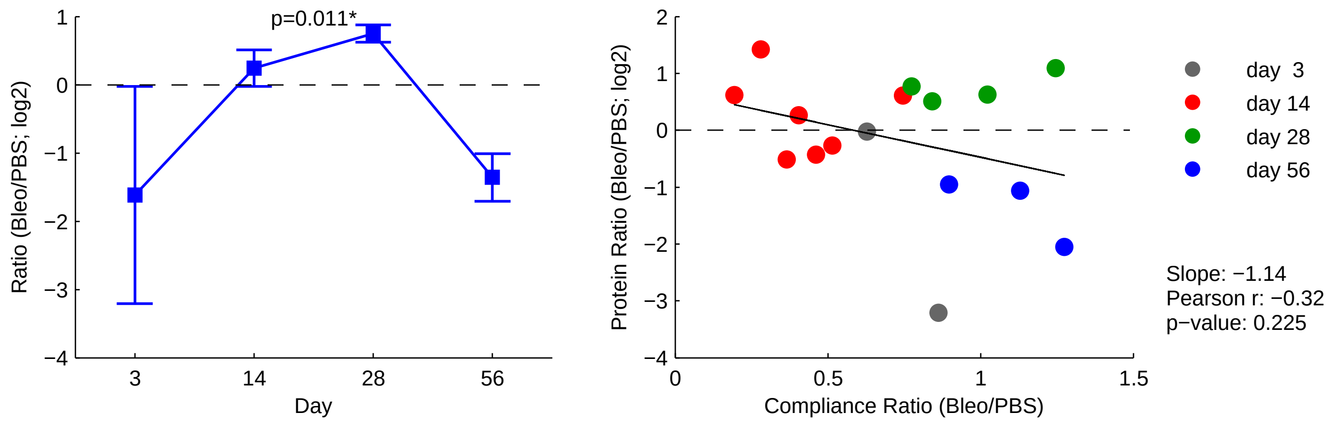

Q3UXG7 − Pofut1 (id: 3968)

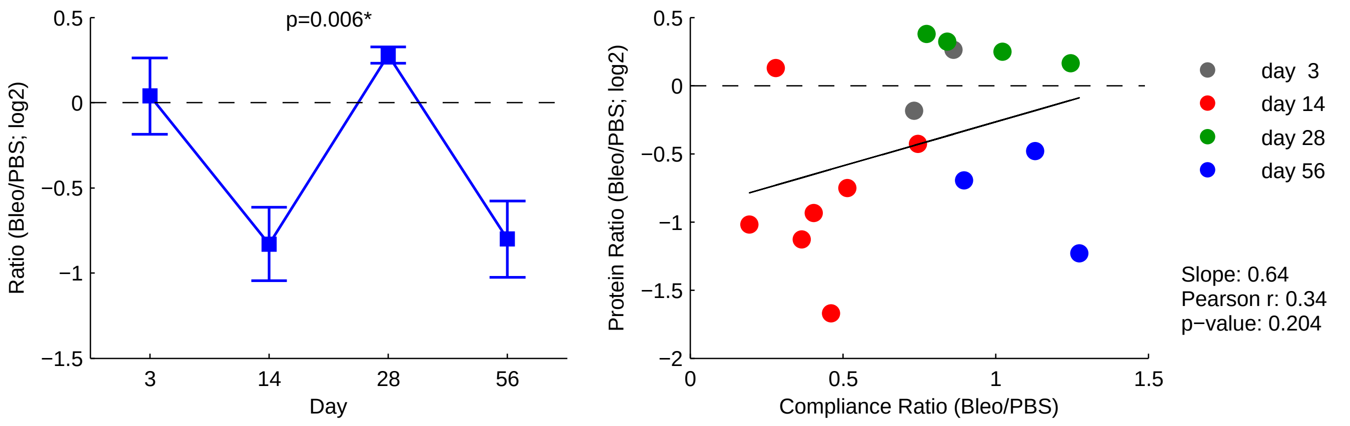

Q3UZ39 − Lrrfip1 (id: 3972)

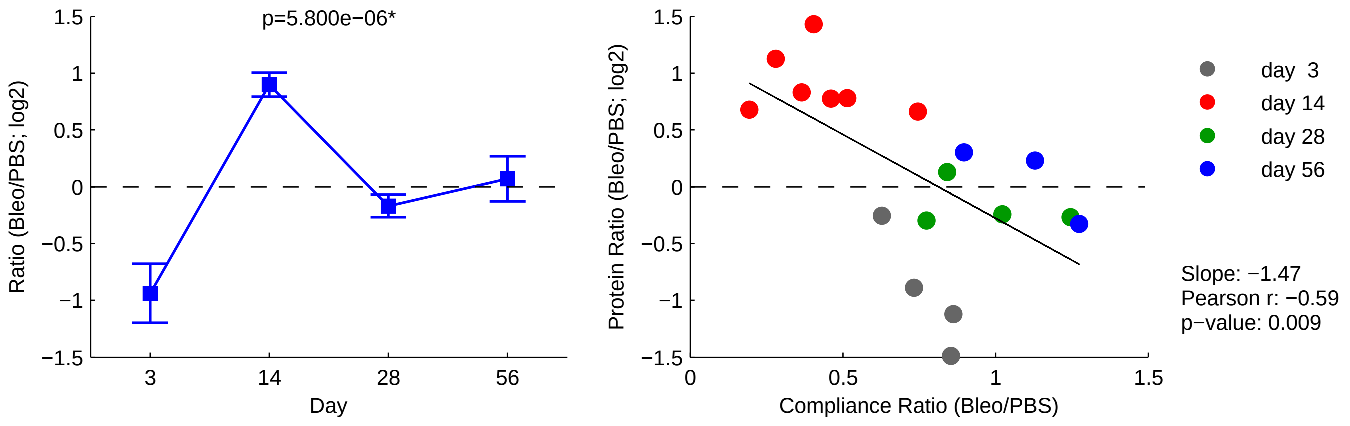

Q80TL1 − Adcy2 (id: 3984)

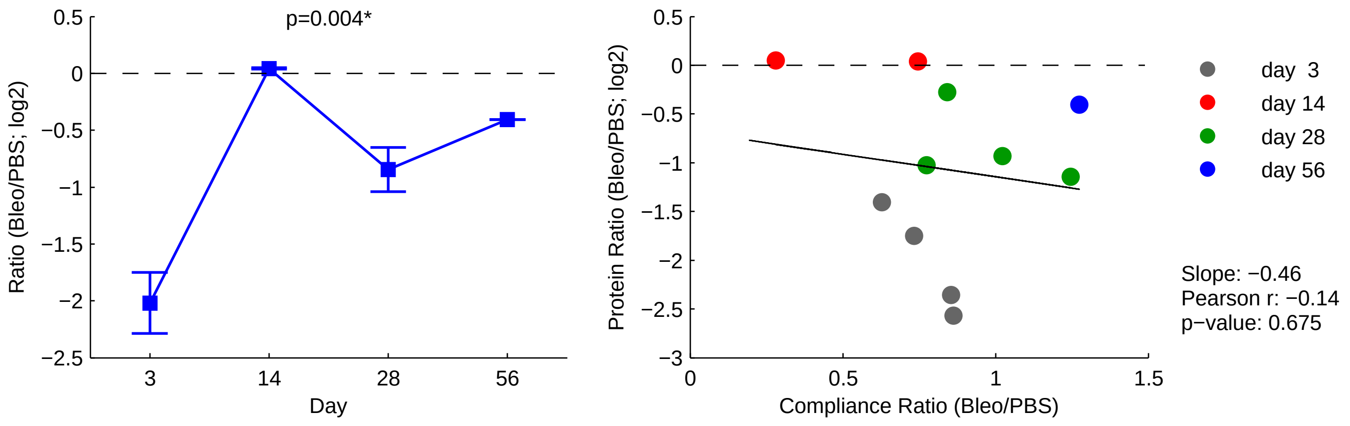

Q3V384 − Lace1 (id: 3988)

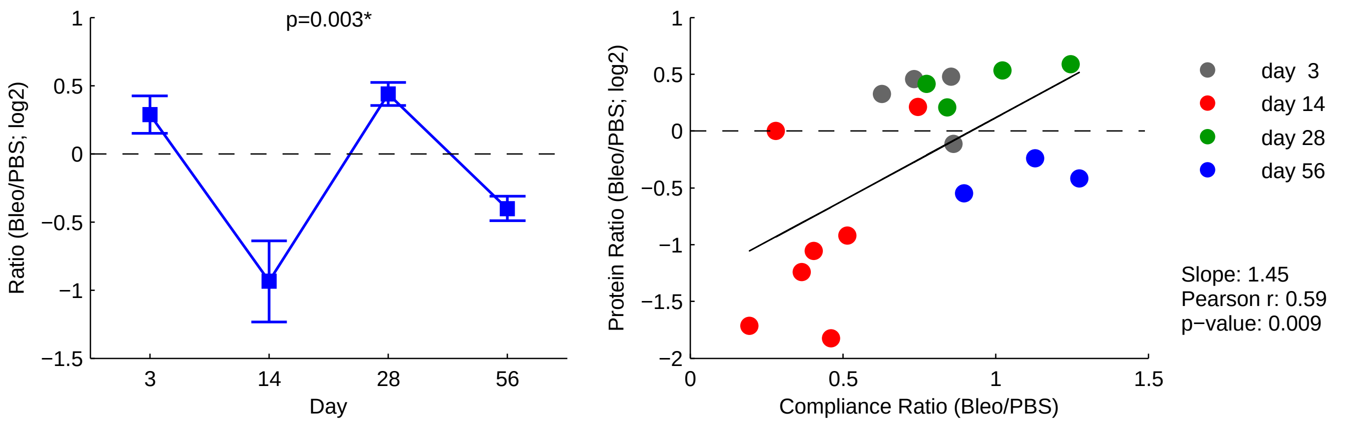

Q3V3N7 − Bbs1 (id: 3990)

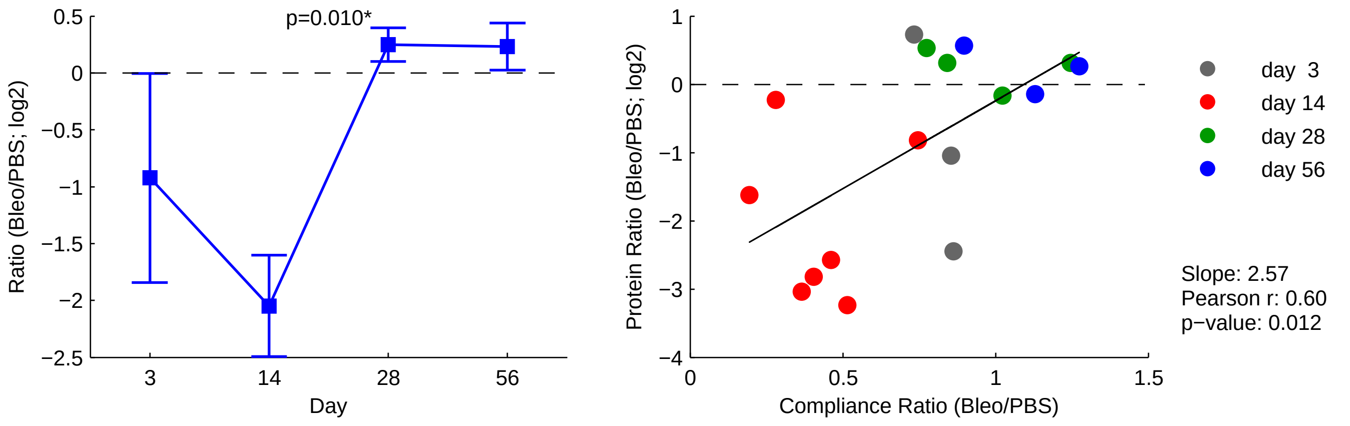

Q3V3R1 − Mthfd1l (id: 3991)

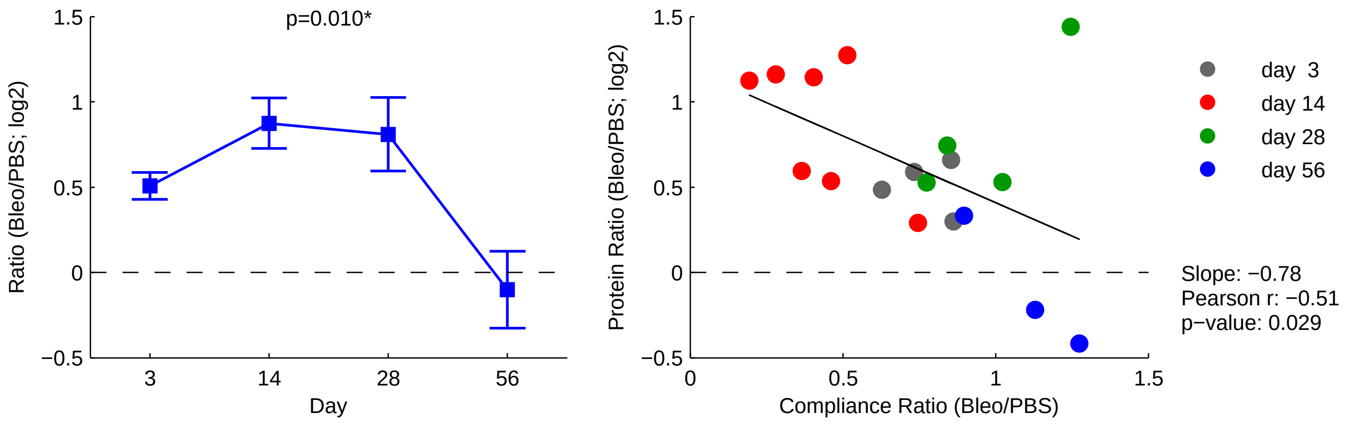

Q3V3R4 − Itga1 (id: 3992)

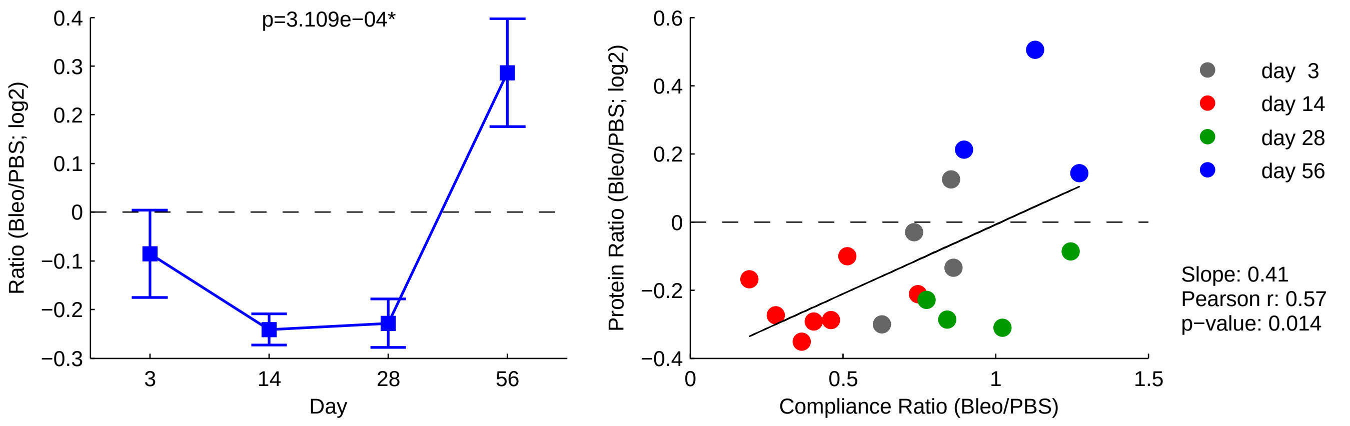

Q3V4B5 − Commd6 (id: 3995)

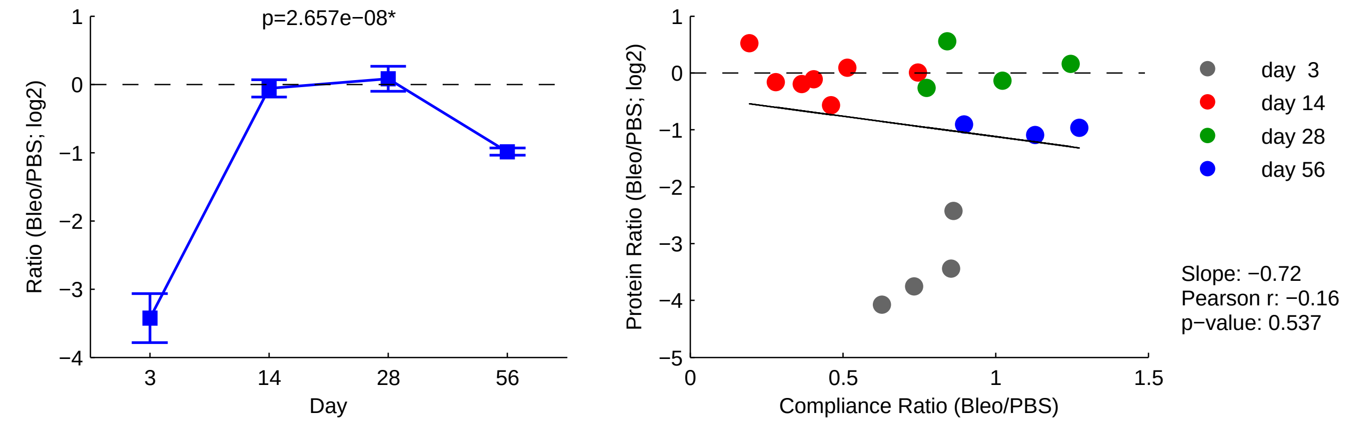

Q45VK7 − Dync2h1 (id: 3996)

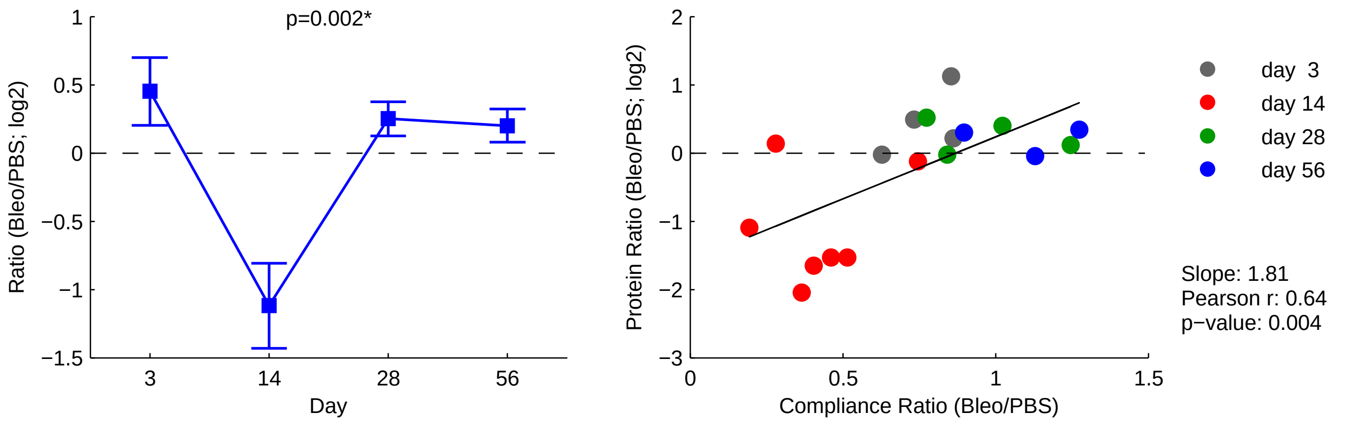

Q499E4−2 − Dzip1l (id: 3997)

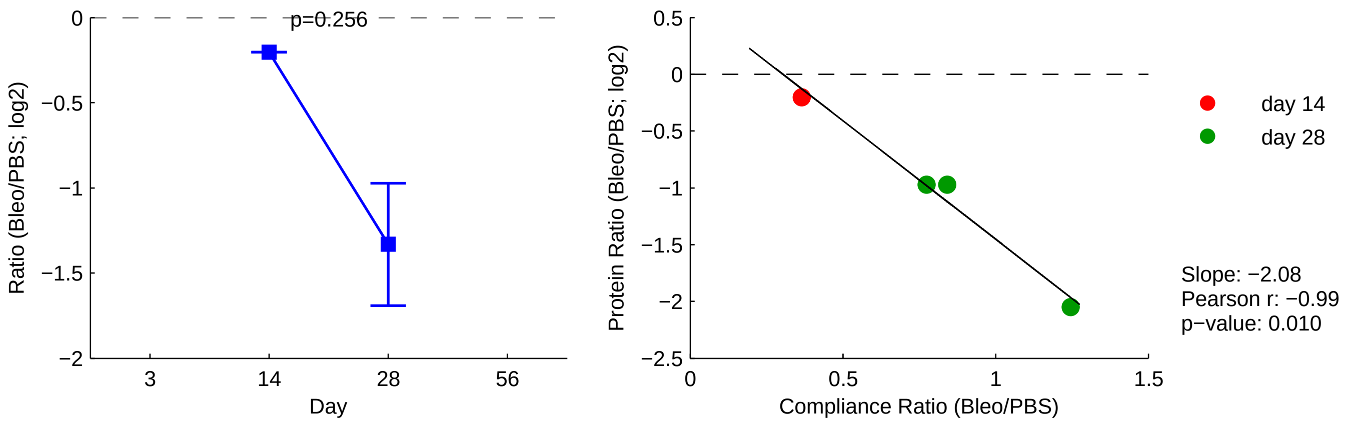

Q4ACU6 − Shank3 (id: 4000)

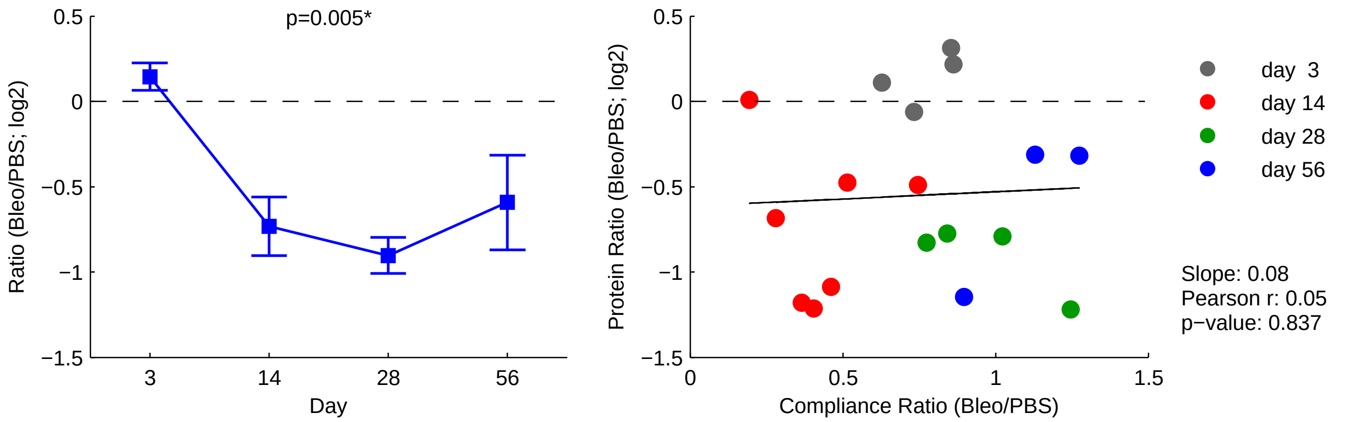

Q4FZC9 − Syne3 (id: 4003)

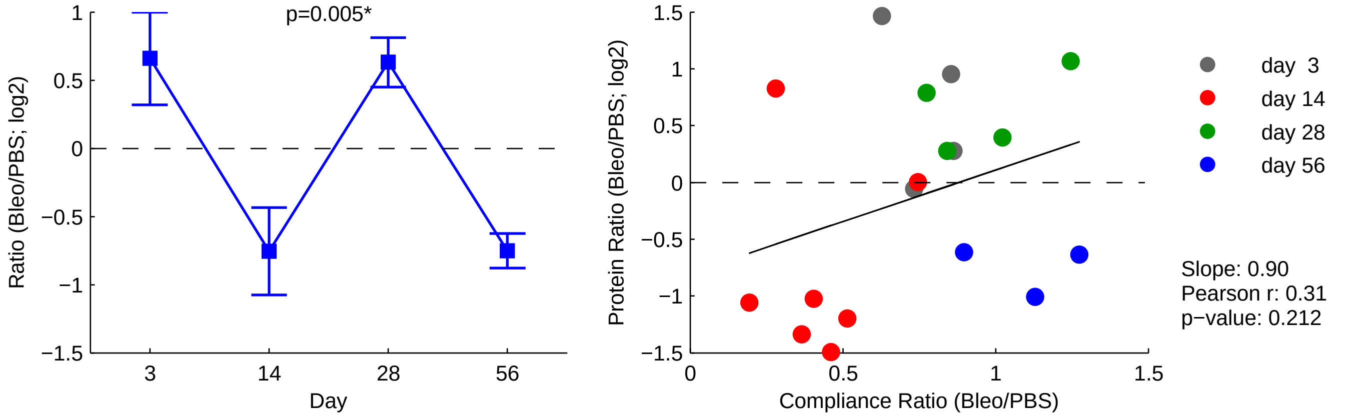

Q4FZG9 − Ndufa4l2 (id: 4004)

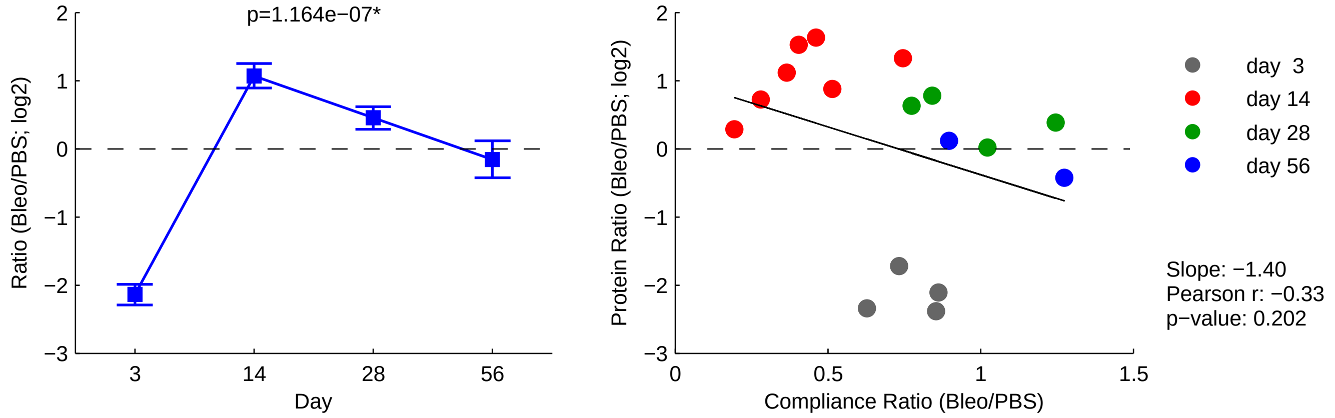

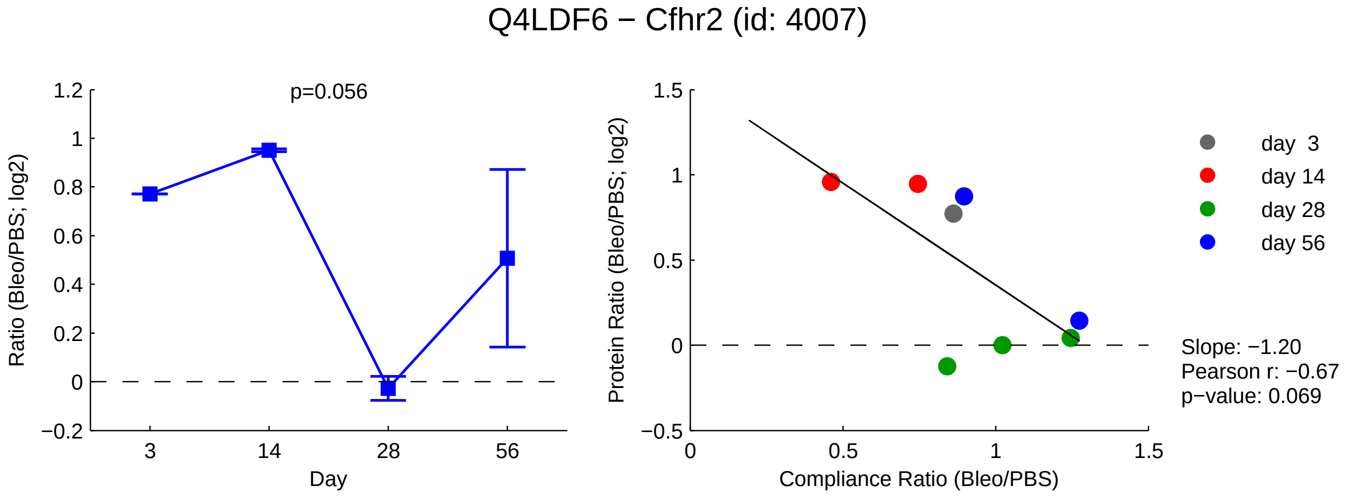
Q4LDF6 − Cfhr2 (id: 4007)
p=0.056
Ratio (Bleo/PBS; log2)
Day
Protein Ratio (Bleo/PBS; log2)
Compliance Ratio (Bleo/PBS)
day 3
day 14
day 28
day 56
Slope: −1.20
Pearson r: −0.67
p−value: 0.069

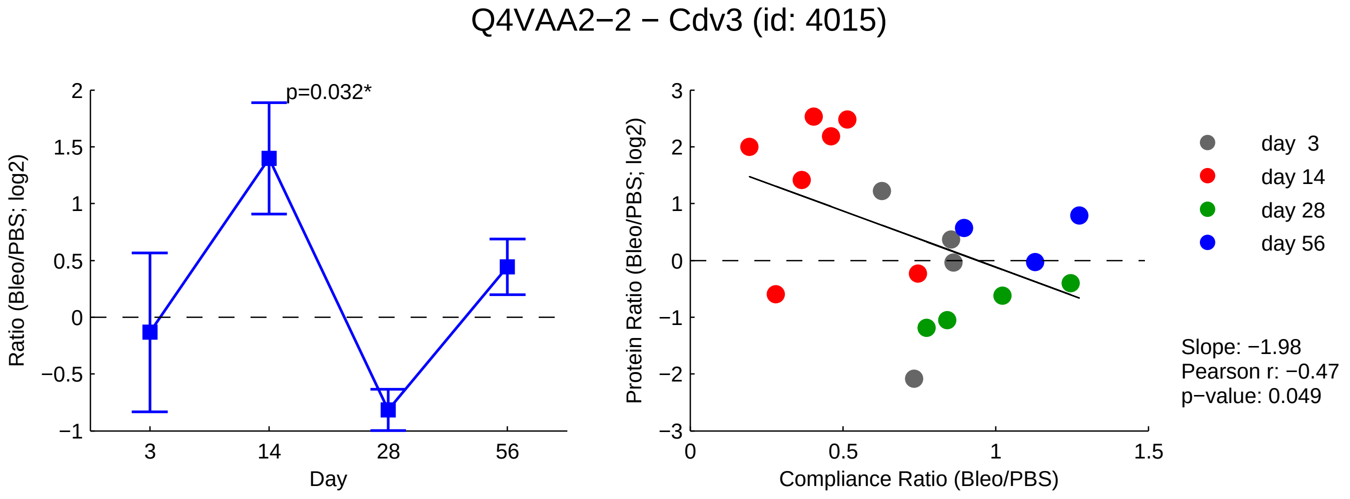
Q4VAA2−2 − Cdv3 (id: 4015)
p=0.032*
Ratio (Bleo/PBS; log2)
Day
Protein Ratio (Bleo/PBS; log2)
Compliance Ratio (Bleo/PBS)
day 3
day 14
day 28
day 56
Slope: −1.98
Pearson r: −0.47
p−value: 0.049

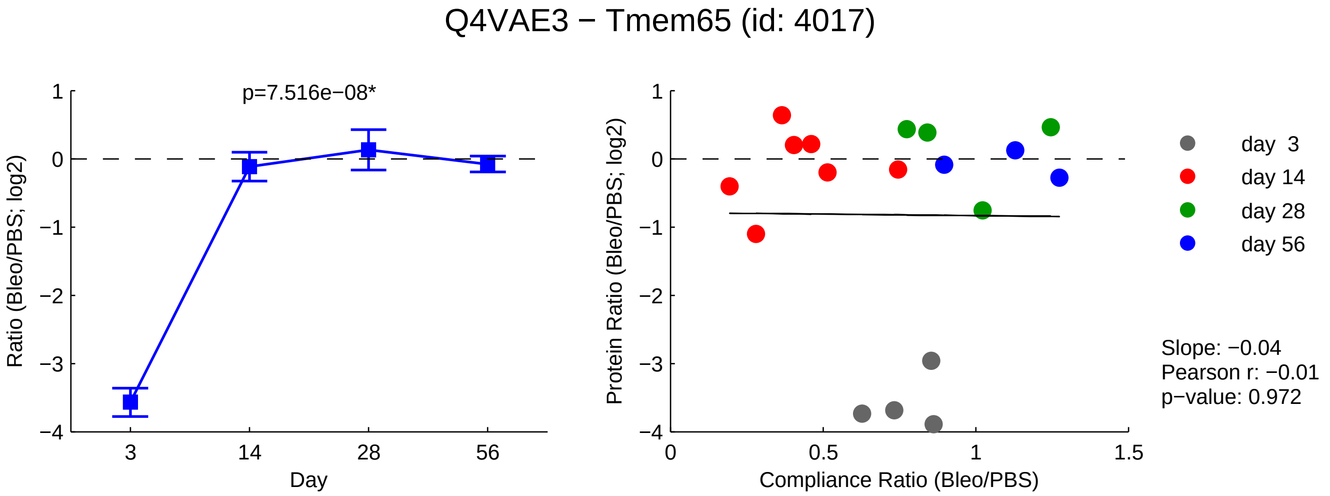
Q4VAE3 − Tmem65 (id: 4017)
p=7.516e−08*
Ratio (Bleo/PBS; log2)
Day
Protein Ratio (Bleo/PBS; log2)
Compliance Ratio (Bleo/PBS)
day 3
day 14
day 28
day 56
Slope: −0.04
Pearson r: −0.01
p−value: 0.972

Q4VBE8 − Wdr18 (id: 4020)

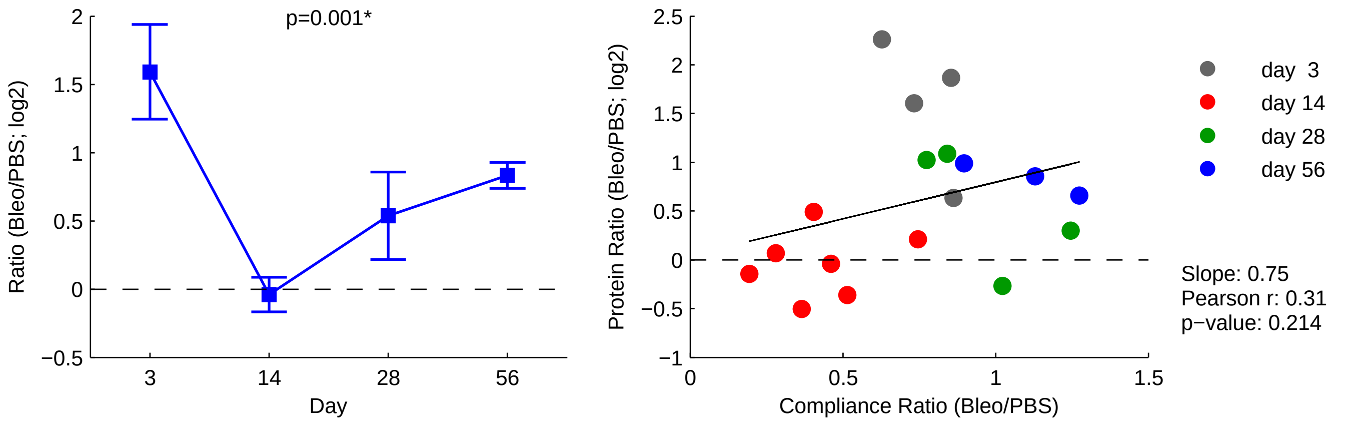

Q501J7 − Phactr4 (id: 4024)

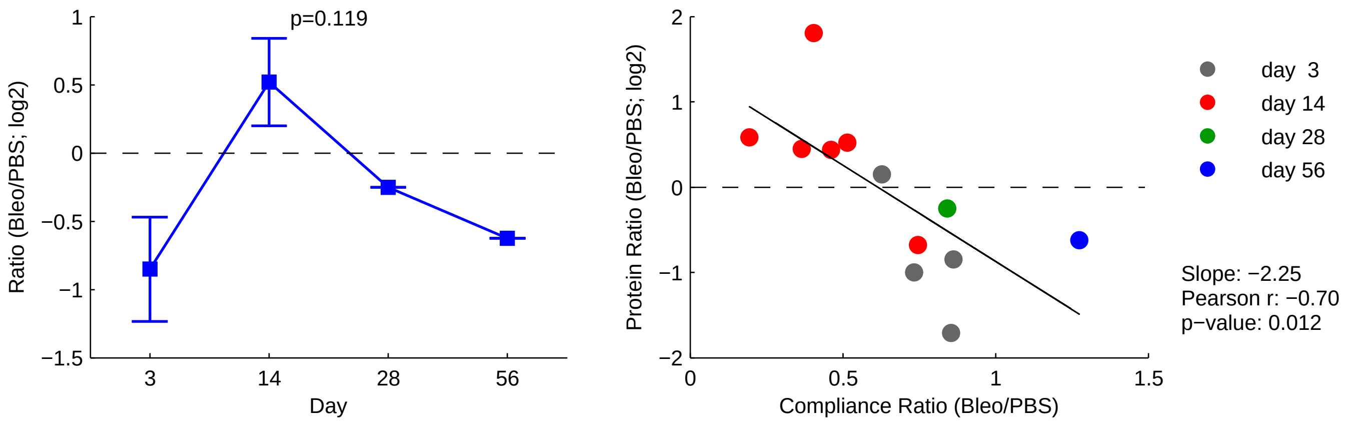

Q505D7 − Opa3 (id: 4026)

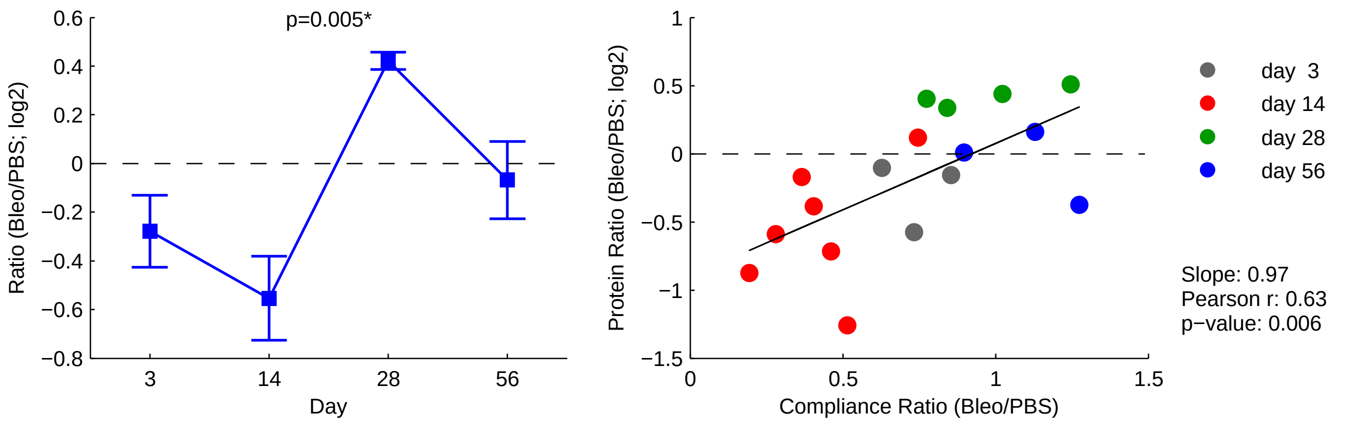

Q505F5 − Lrrc47 (id: 4028)

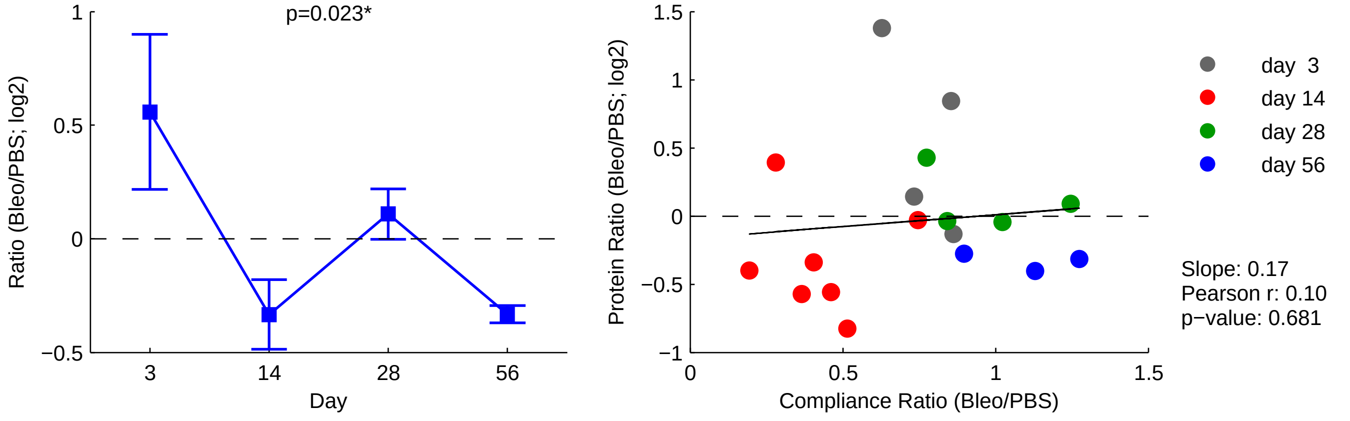

Q542V3 − Srsf4 (id: 4030)

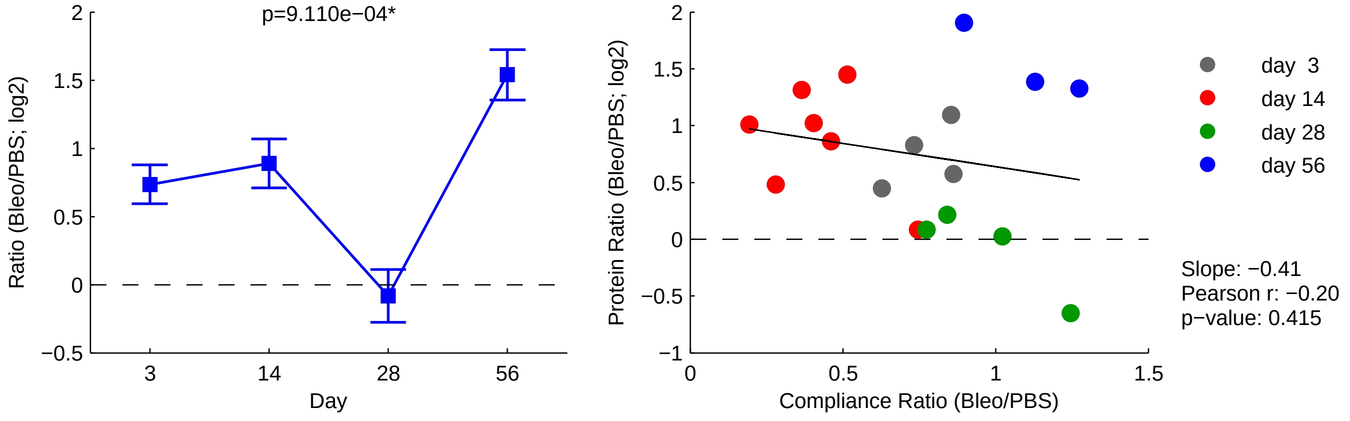

Q544X6 − Fech (id: 4032)

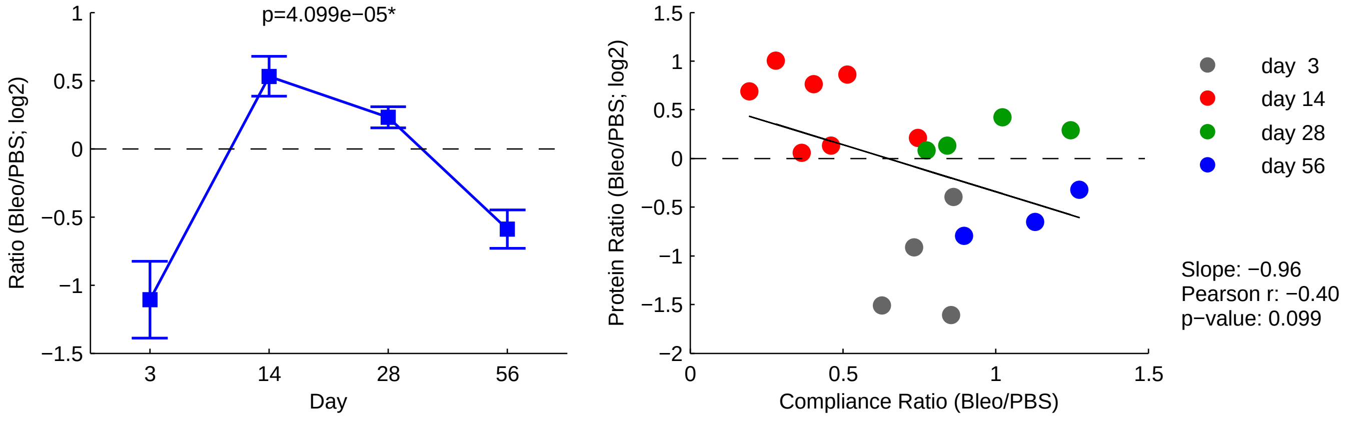

Q569Z6 − Thrap3 (id: 4035)

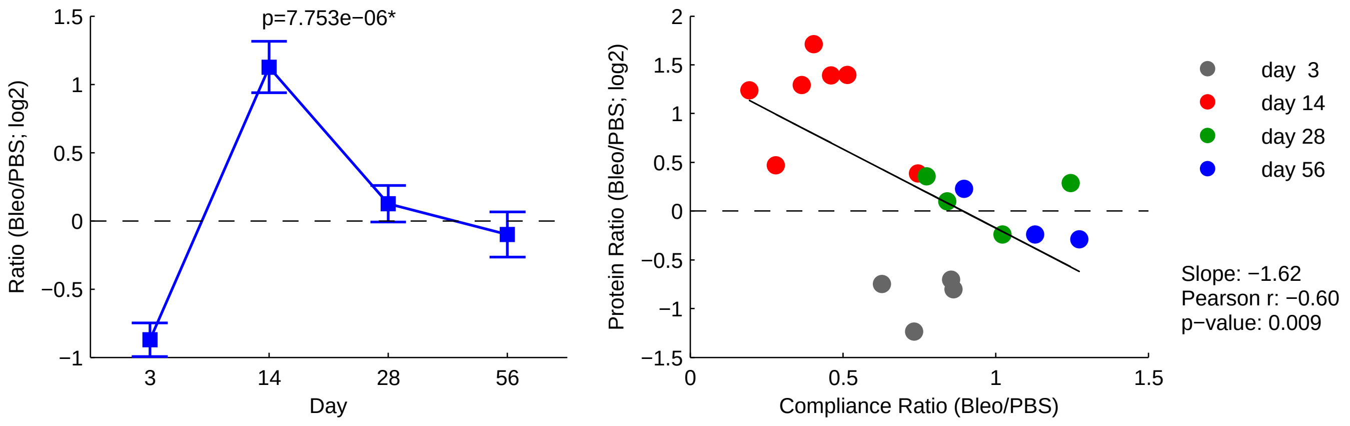

Q56A06 − Tmtc2 (id: 4036)

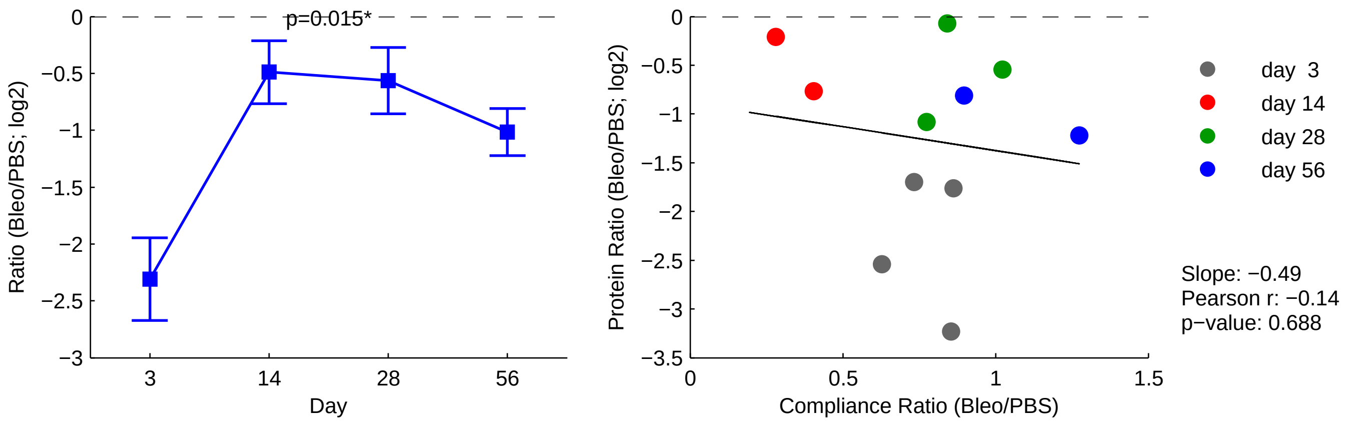

Q571E4 − Galns (id: 4040)

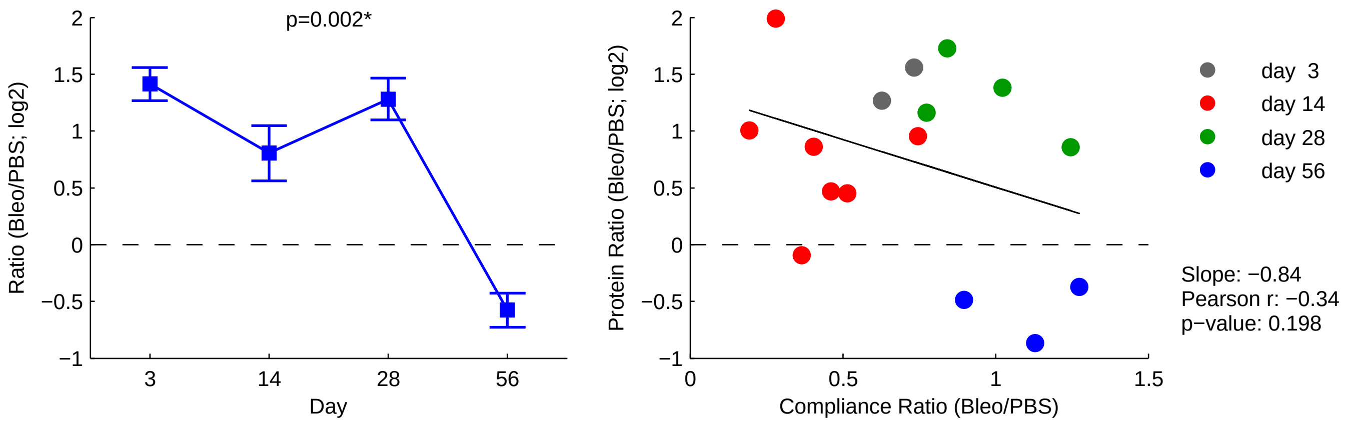

Q59IW9 − Abi3bp (id: 4043)

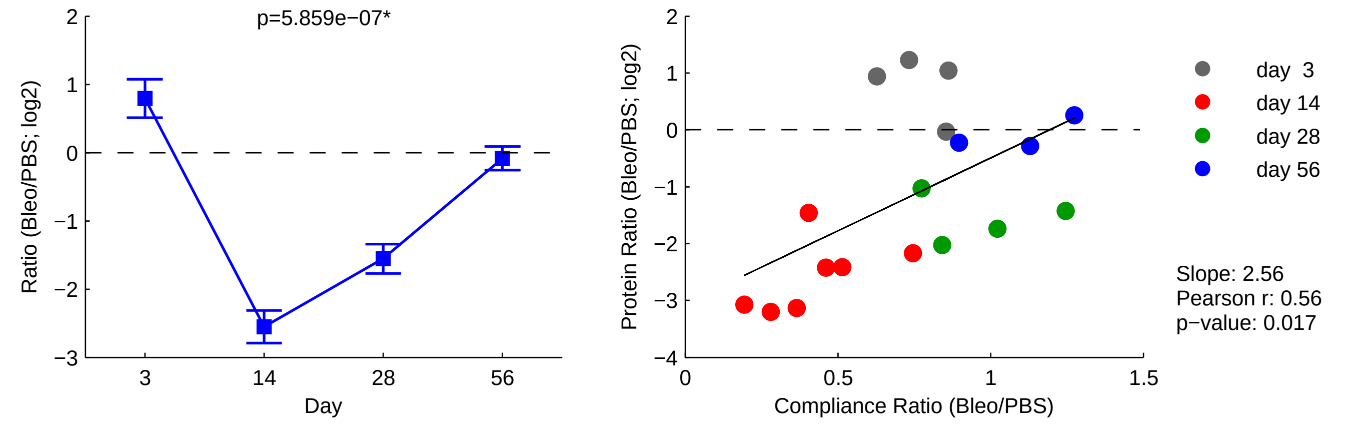

Q59J78 − Ndufaf2 (id: 4044)

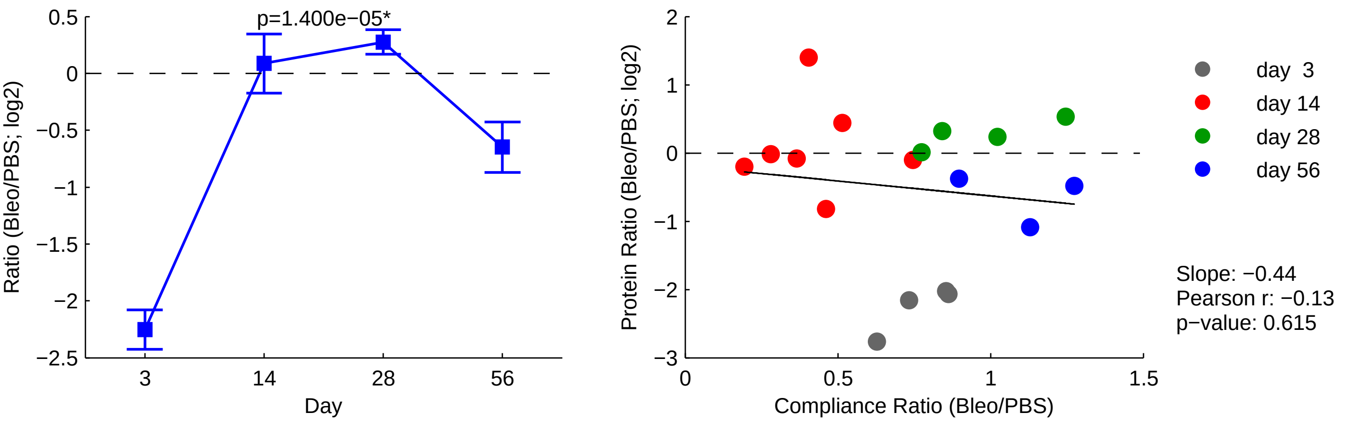

Q59J91 − Galnt18 (id: 4045)

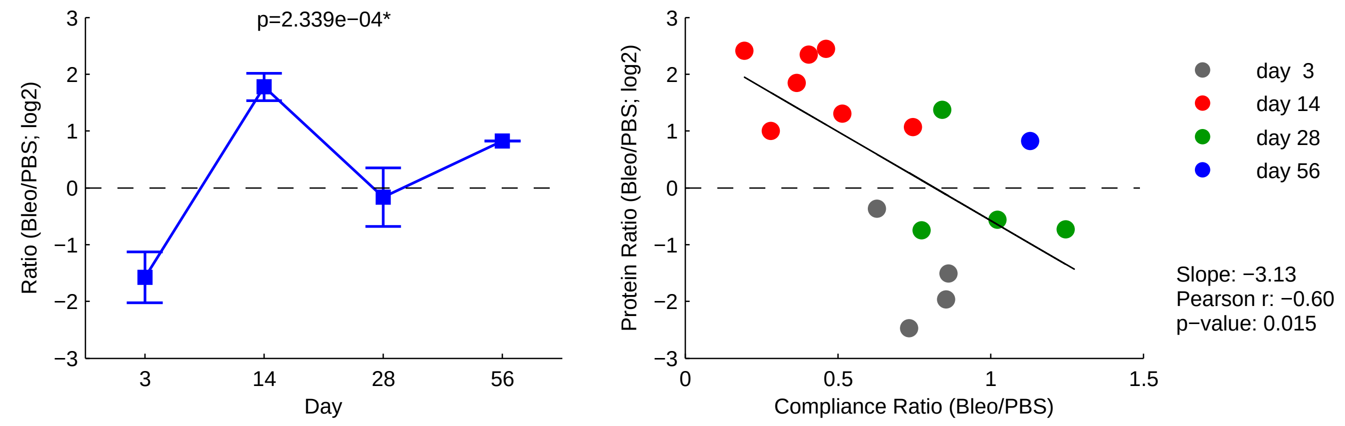

Q5DTU0 − Afap1l2 (id: 4051)

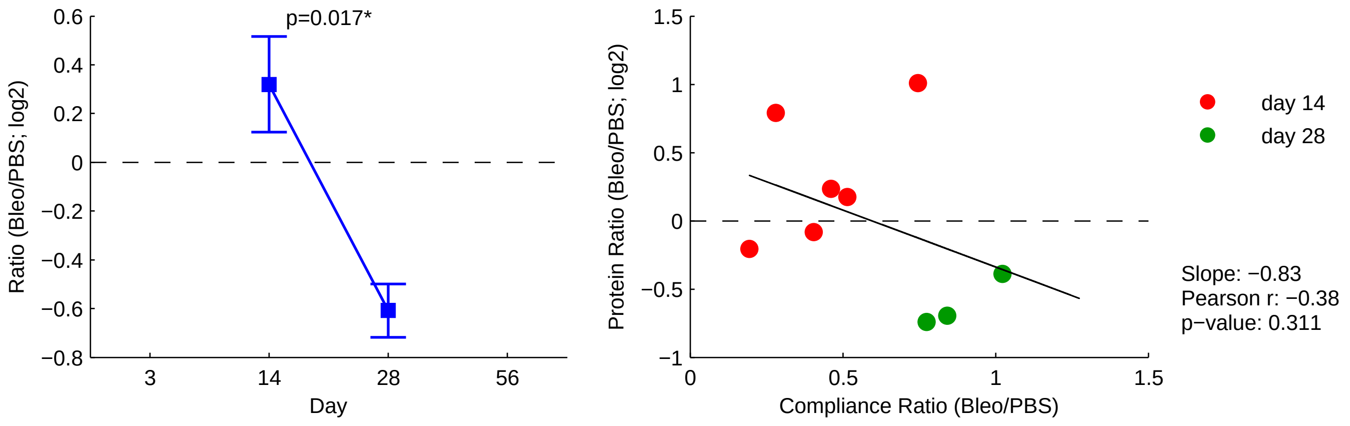

Q5DTX6 − Jcad (id: 4052)

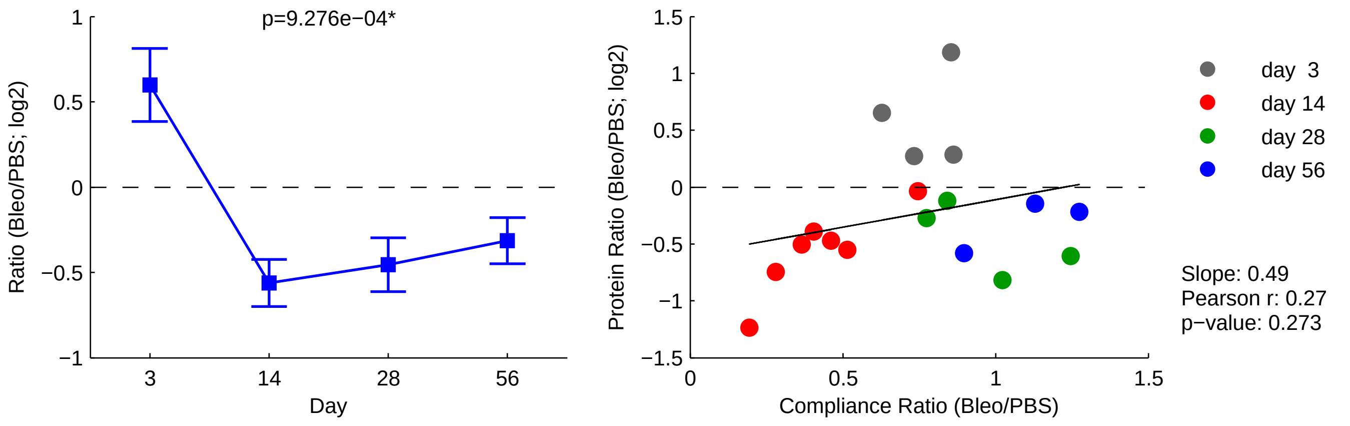

Q5DU00−2 − Dcdc2 (id: 4053)

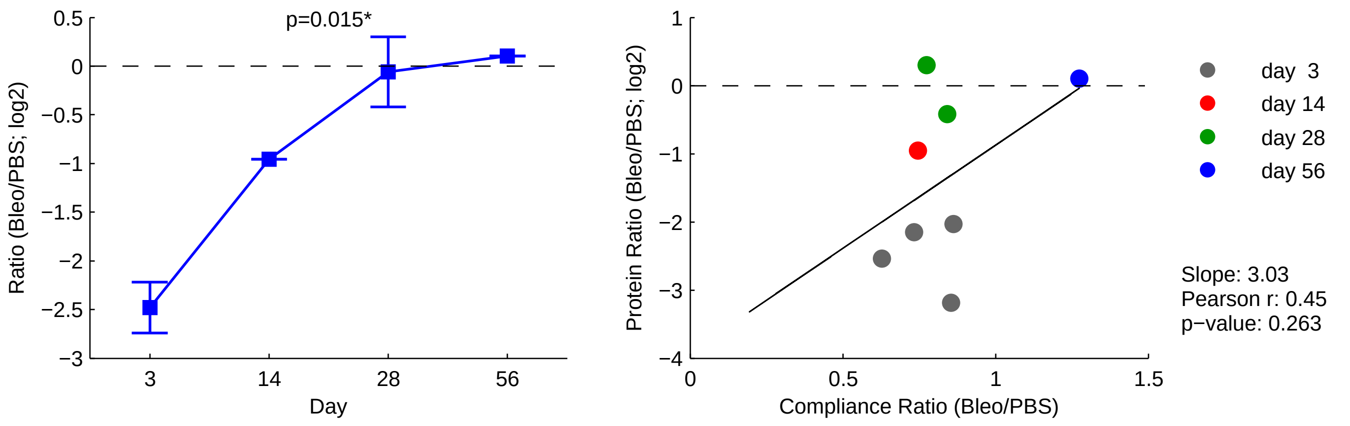

Q5EBG6 − Hspb6 (id: 4056)

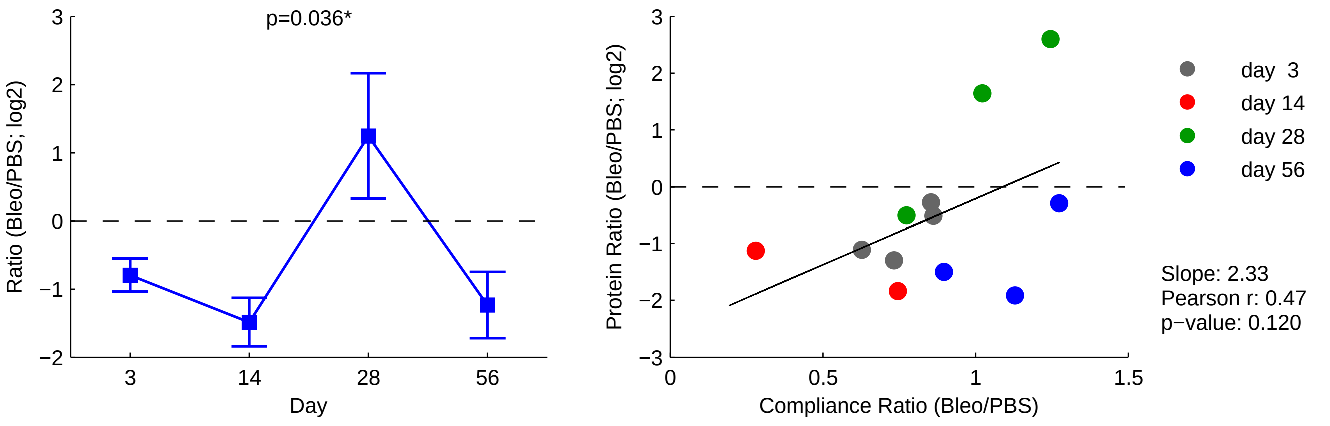

Q5F258 − Git1 (id: 4061)

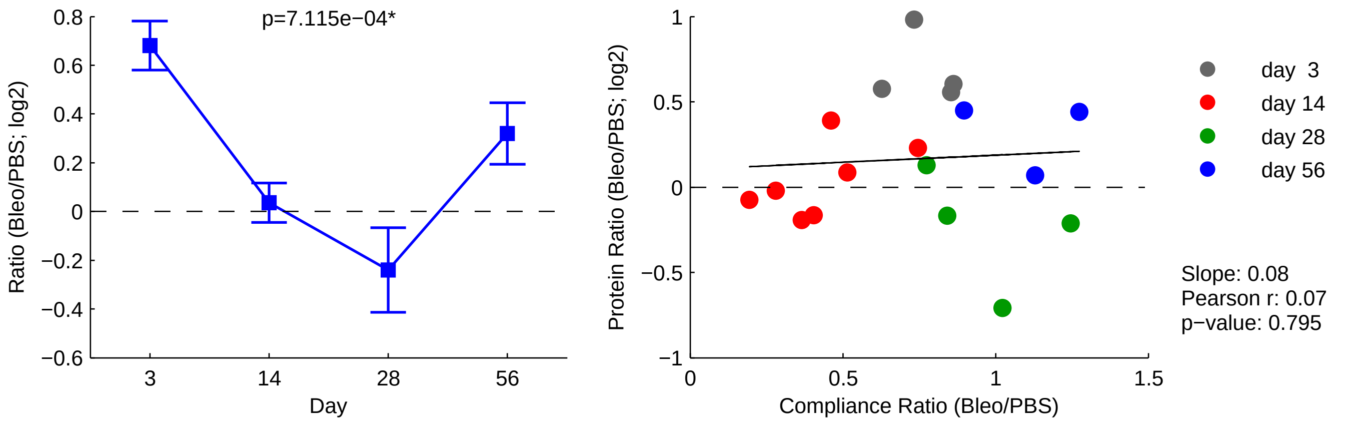

Q5F2E7 − Nufip2 (id: 4064)

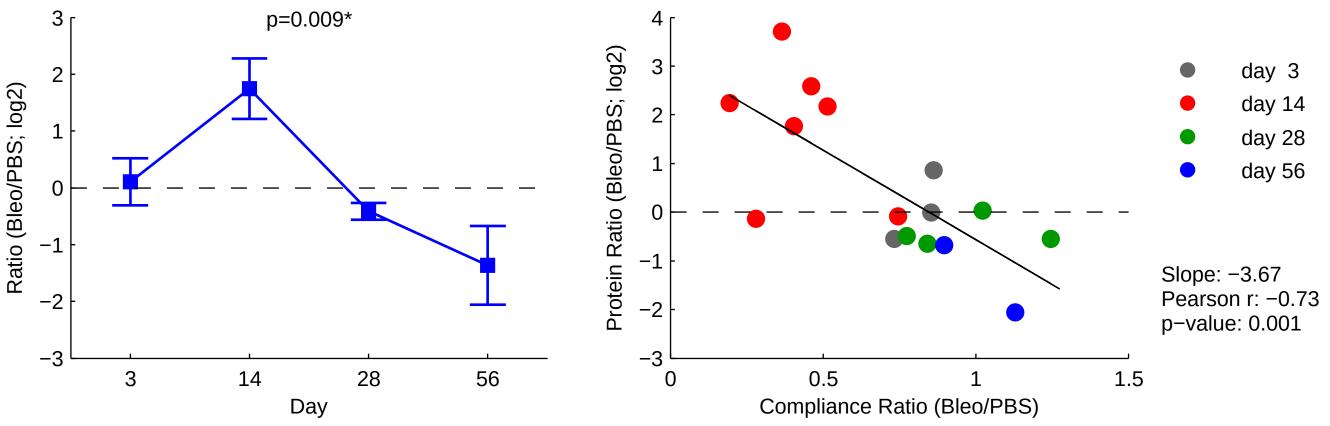

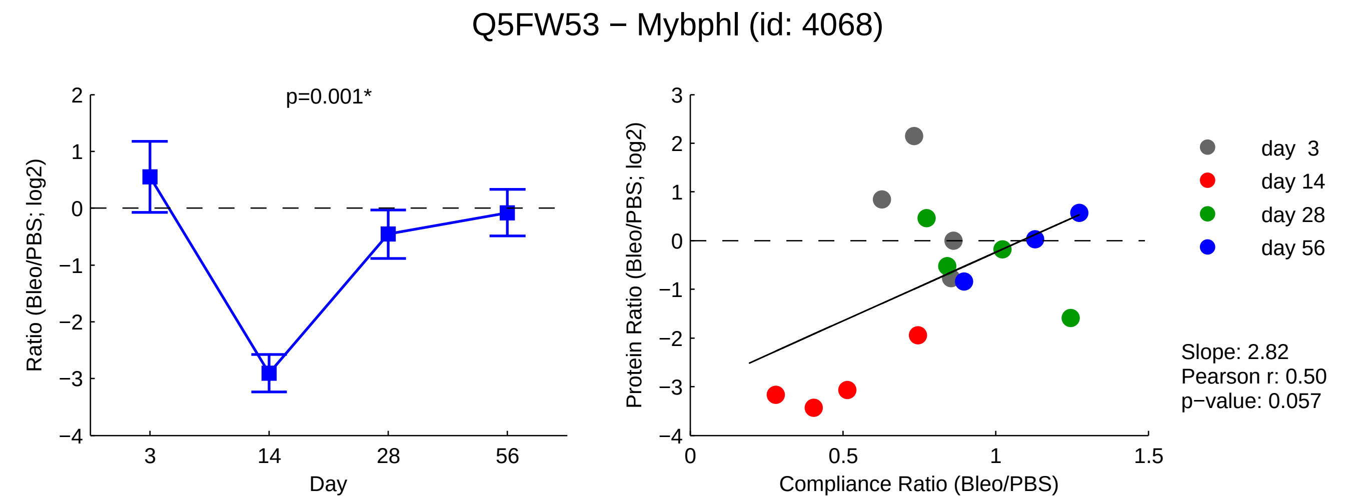

Q5FW53 − Mybphl (id: 4068)
p=0.001*
Ratio (Bleo/PBS; log2)
Day
3
14
28
56
Protein Ratio (Bleo/PBS; log2)
Compliance Ratio (Bleo/PBS)
day 3
day 14
day 28
day 56
Slope: 2.82
Pearson r: 0.50
p−value: 0.057

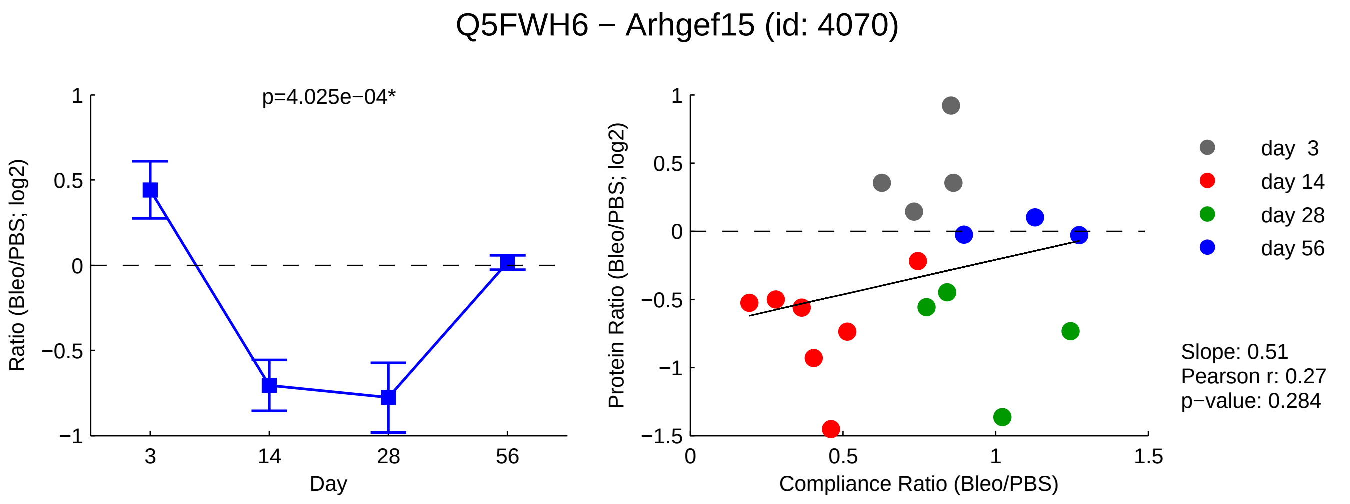

Q5FWH6 − Arhgef15 (id: 4070)
p=4.025e−04*
Ratio (Bleo/PBS; log2)
Day
3
14
28
56
Protein Ratio (Bleo/PBS; log2)
Compliance Ratio (Bleo/PBS)
day 3
day 14
day 28
day 56
Slope: 0.51
Pearson r: 0.27
p−value: 0.284

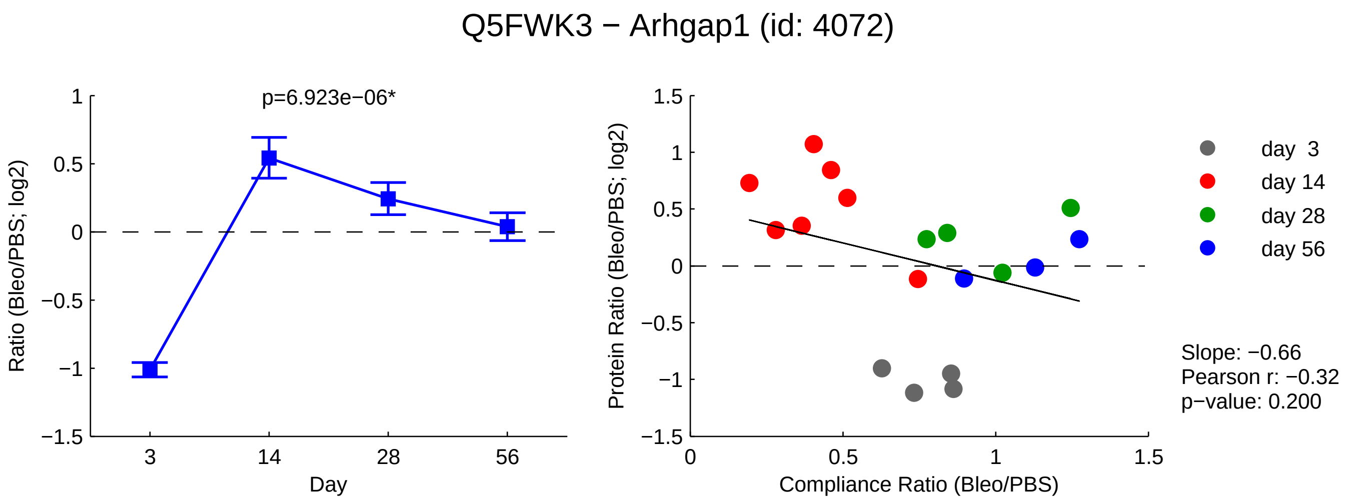

Q5FWK3 − Arhgap1 (id: 4072)
p=6.923e−06*
Ratio (Bleo/PBS; log2)
Day
3
14
28
56
Protein Ratio (Bleo/PBS; log2)
Compliance Ratio (Bleo/PBS)
day 3
day 14
day 28
day 56
Slope: −0.66
Pearson r: −0.32
p−value: 0.200

Q5H8C4−2 − Vps13a (id: 4074)

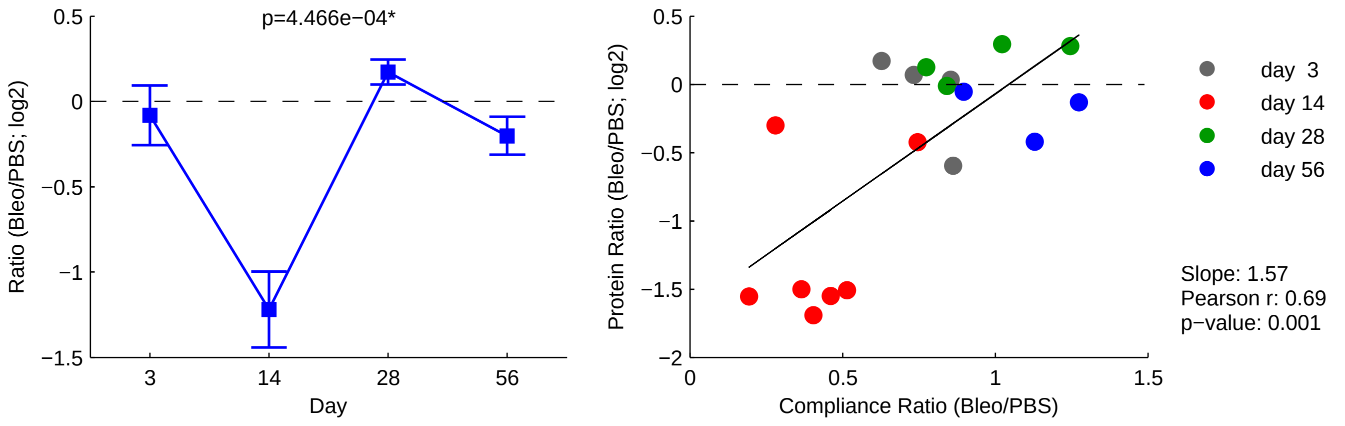

Q5HZI9 − Slc25a51 (id: 4076)

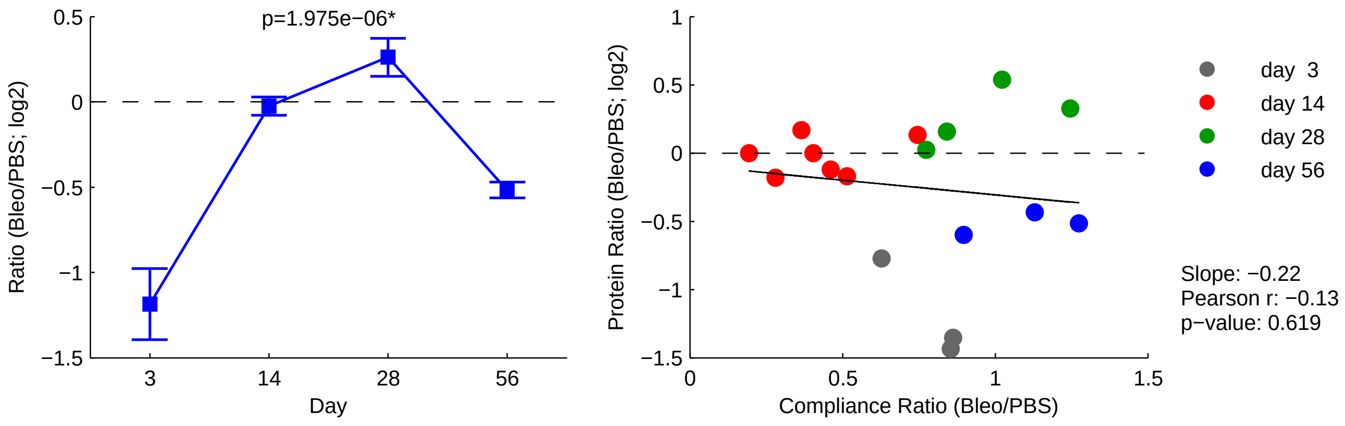

Q5IRJ6 − Slc30a9 (id: 4080)

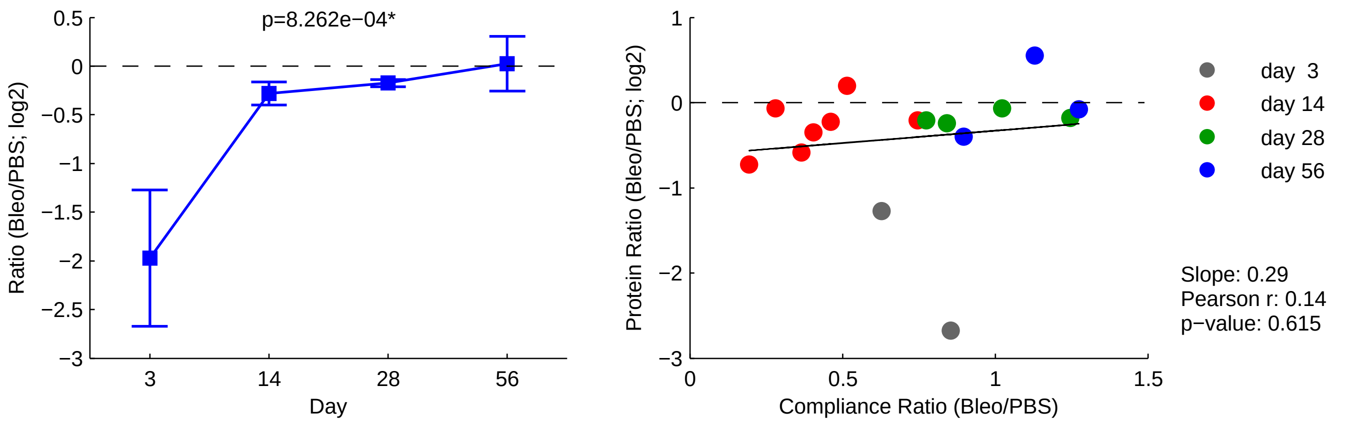

Q5KU39 − Vps41 (id: 4081)

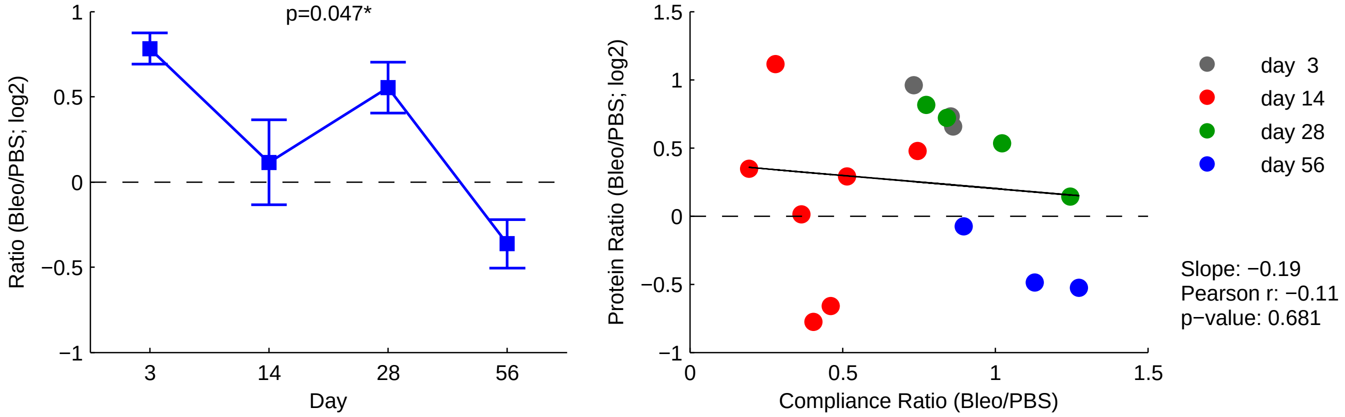

Q5M8M2 − Odf3b (id: 4082)

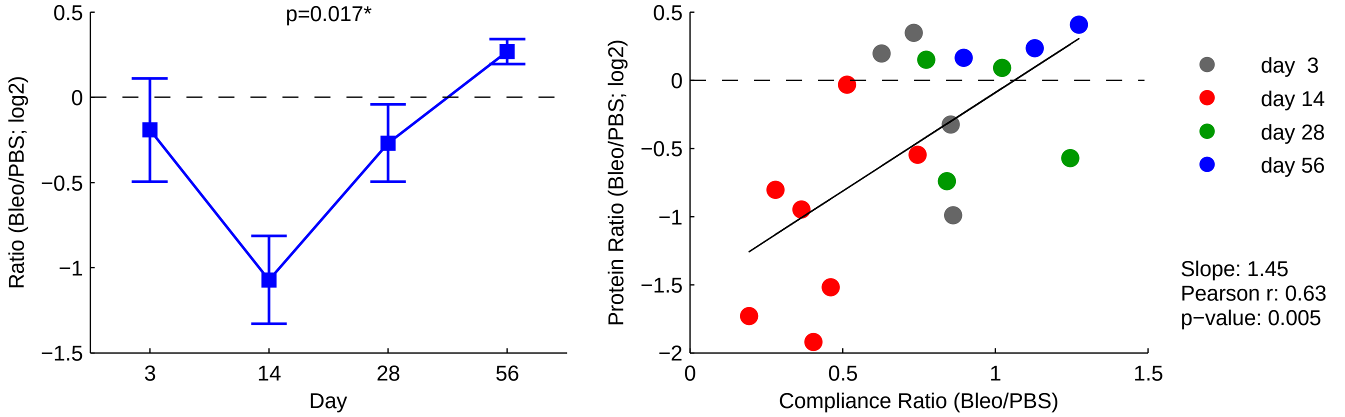

Q5M8N0 − Cnrip1 (id: 4083)

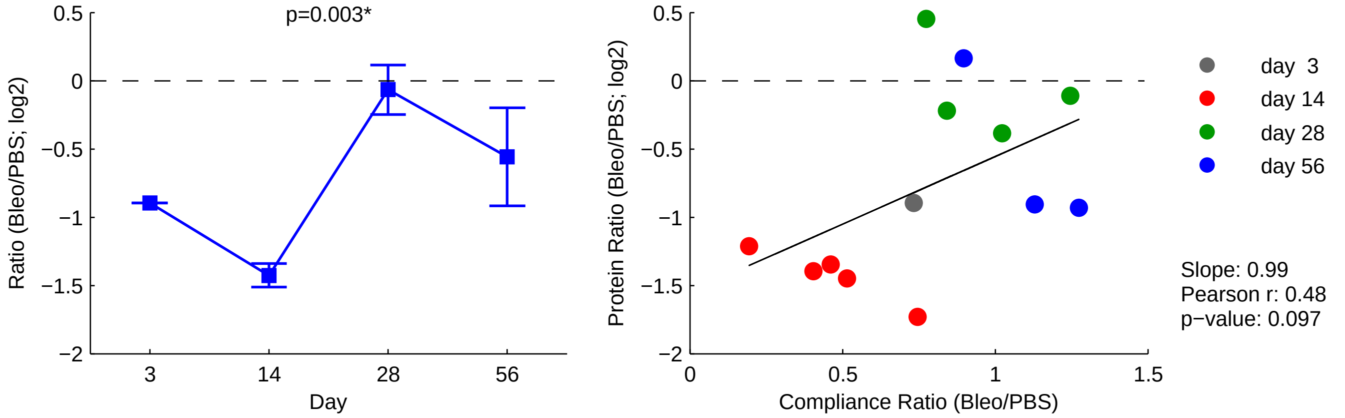

Q5M8N4−2 − Sdr39u1 (id: 4084)

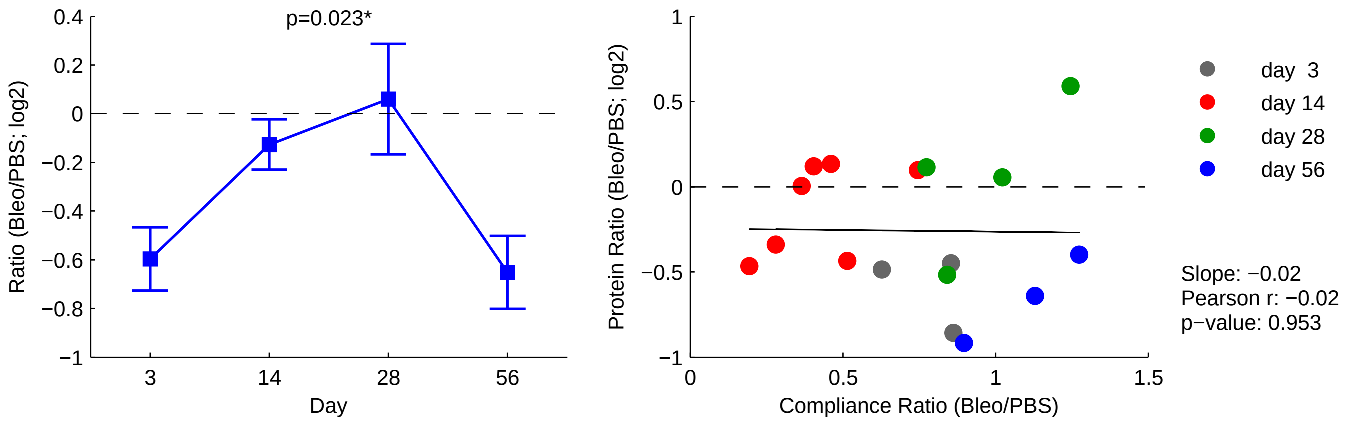

Q5NCE8 − Mrs2 (id: 4088)

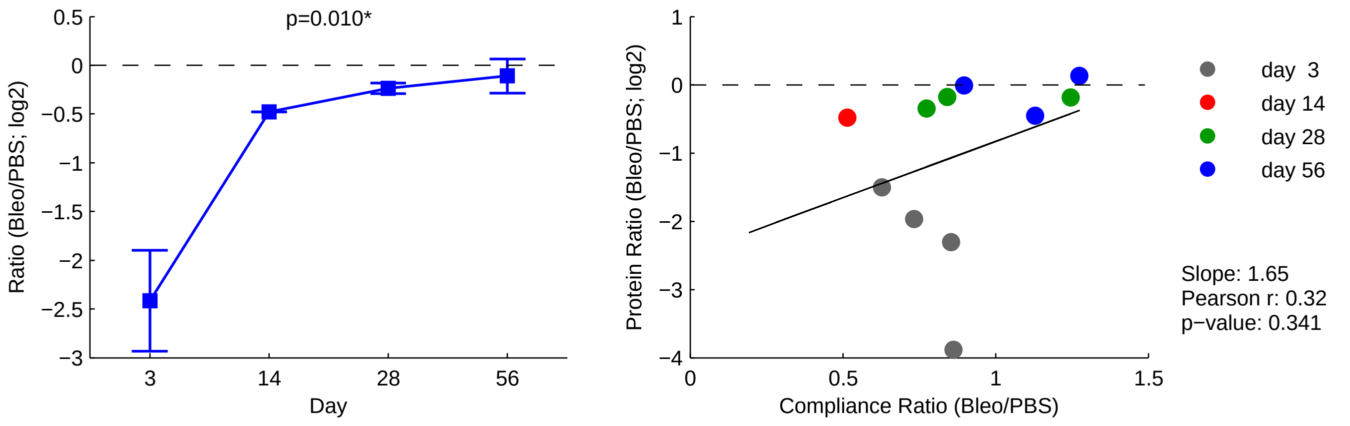

Q5PRF0 − Heatr5a (id: 4095)

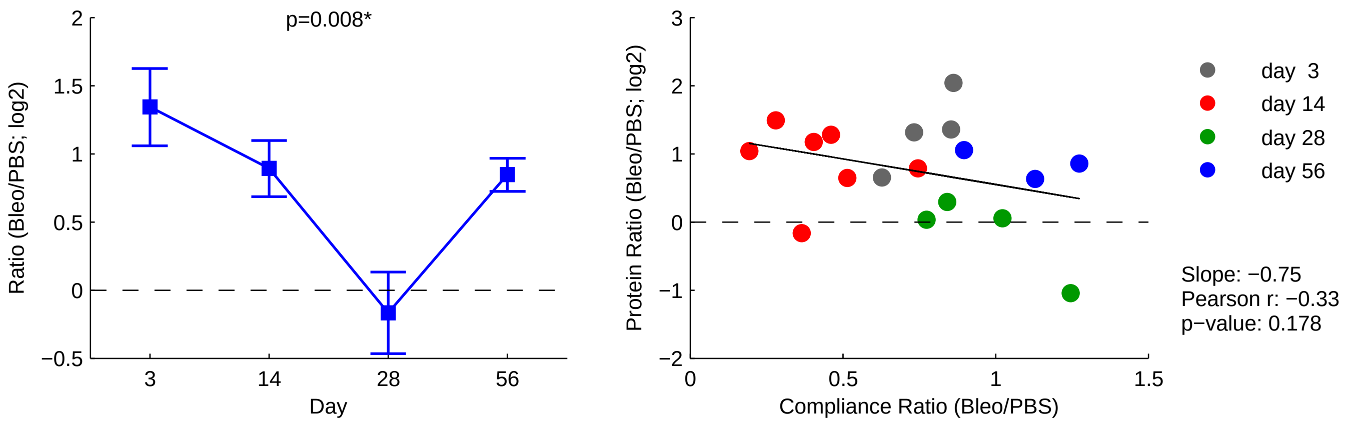

Q5RL20 − Mrpl43 (id: 4102)

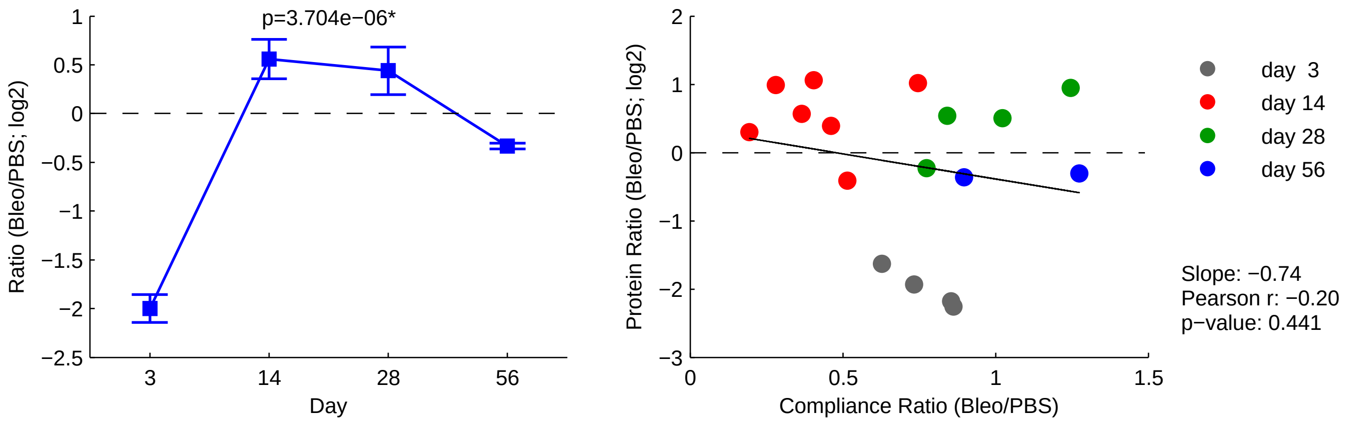

Q5S006 − Lrrk2 (id: 4105)

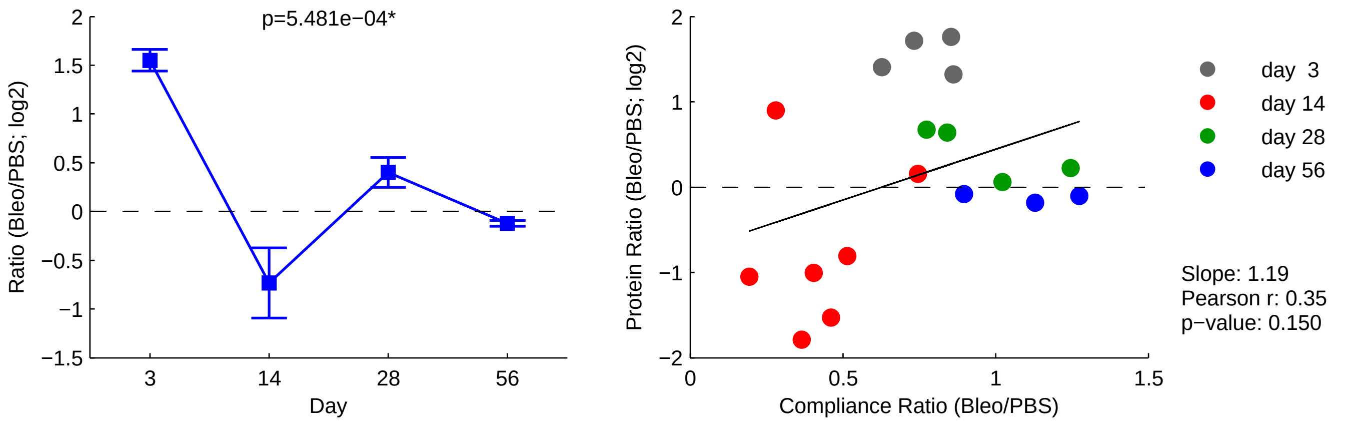

Q5SPW0−2 − Vps54 (id: 4108)

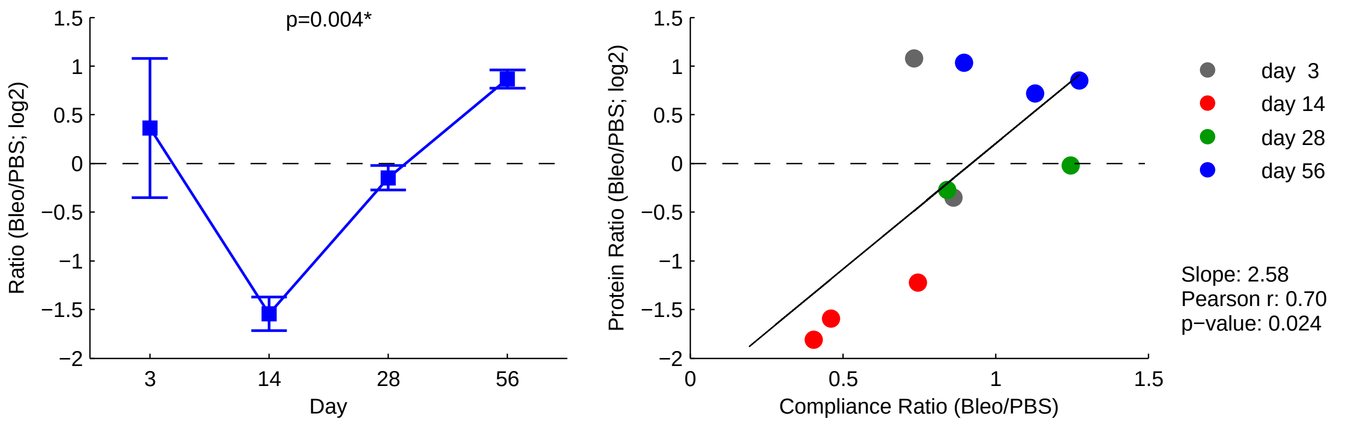

Q9EQ61 − Pes1 (id: 4109)

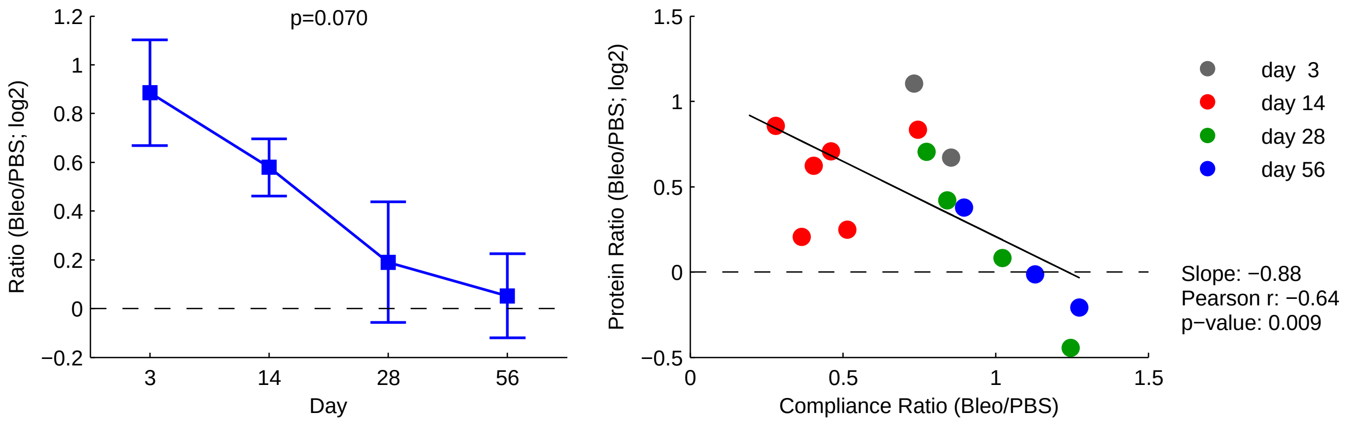

Q5SQ27 − Sec14l3 (id: 4110)

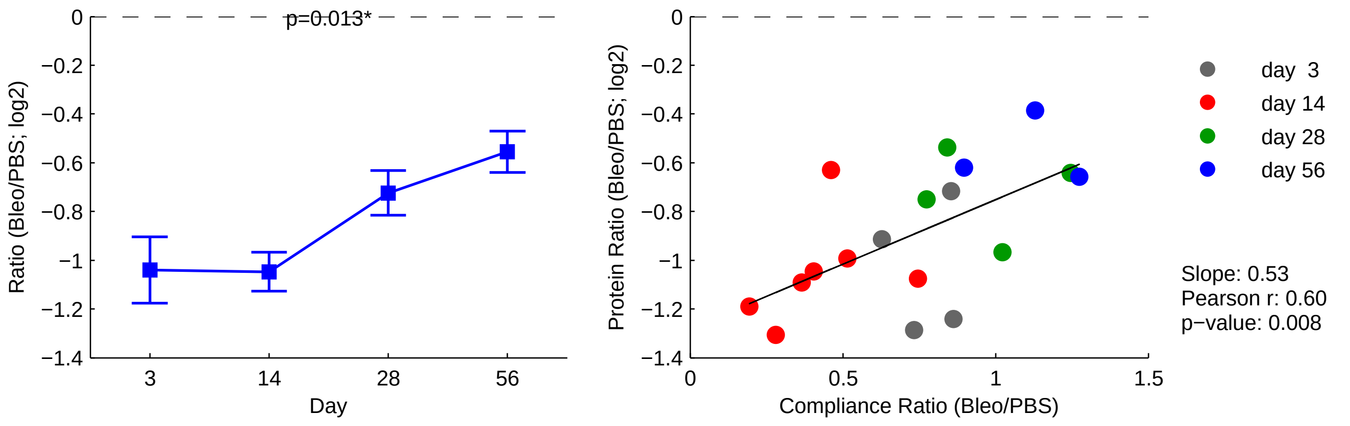

Q5SQX6 − Cyfip2 (id: 4112)

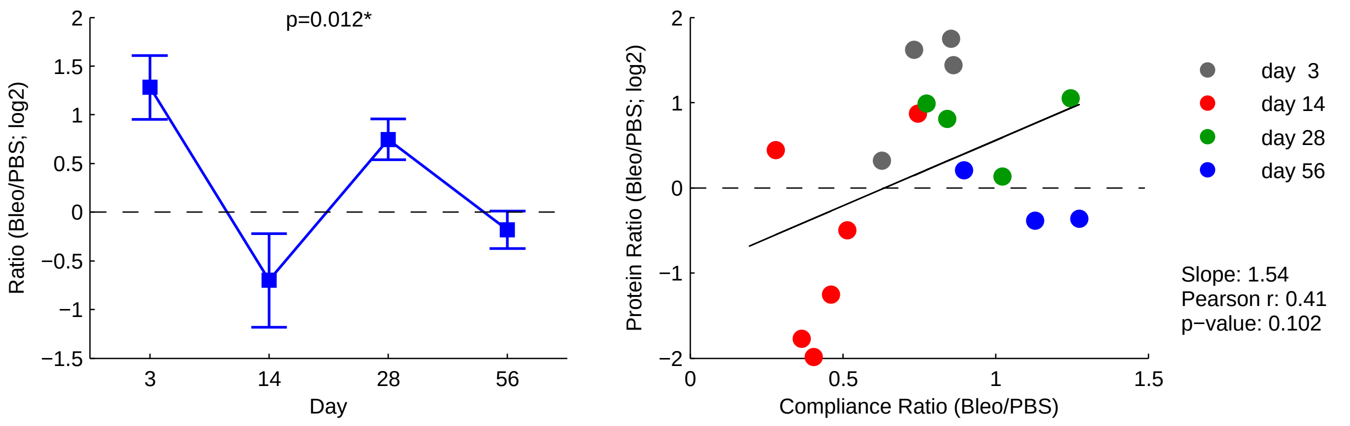

Q5SRX1 − Tom1l2 (id: 4113)

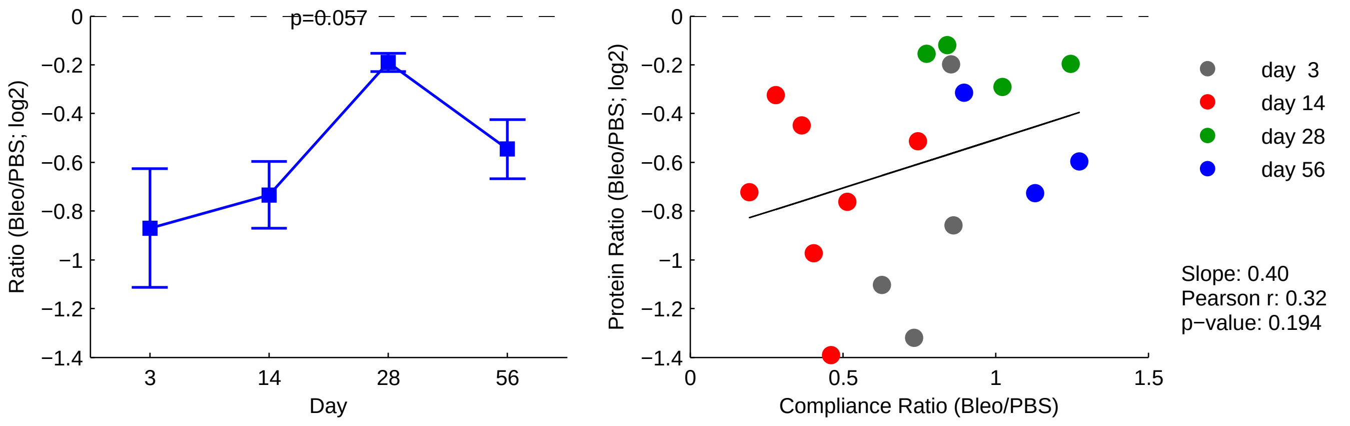

Q5SS80 − Dhrs13 (id: 4116)

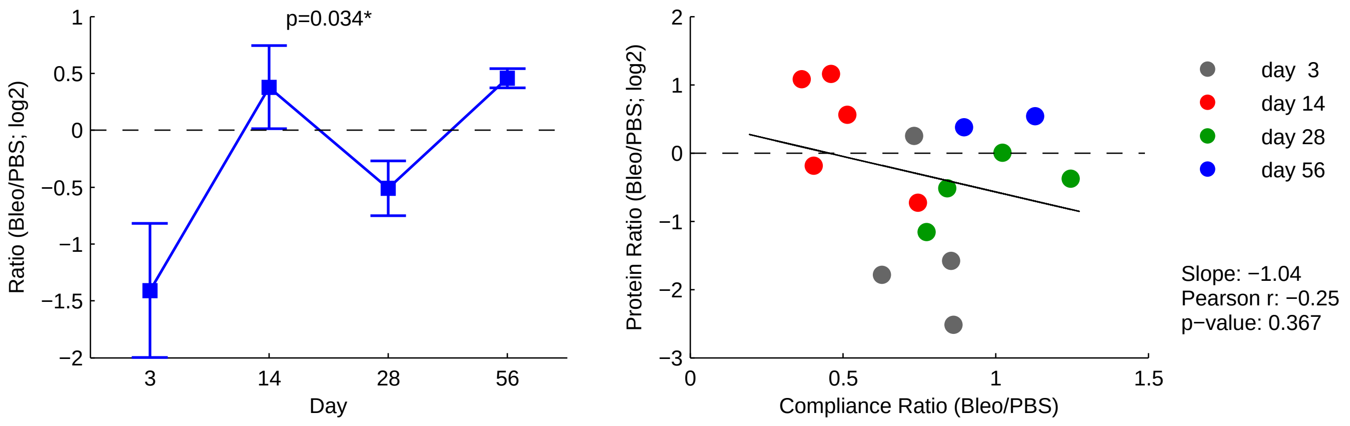

Q5SSM3−2 − Arhgap44 (id: 4123)

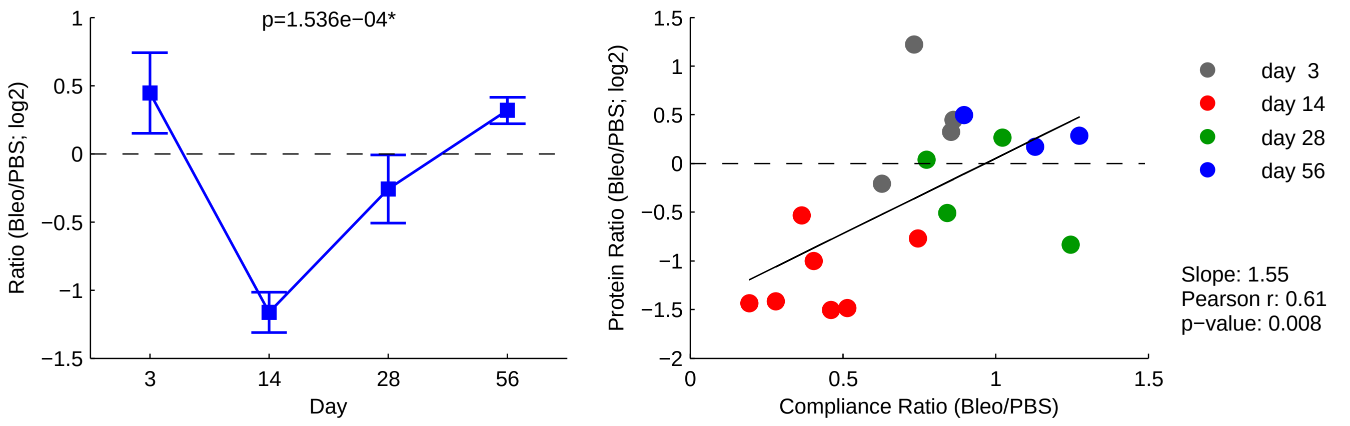

Q5SSZ5 − Tns3 (id: 4124)

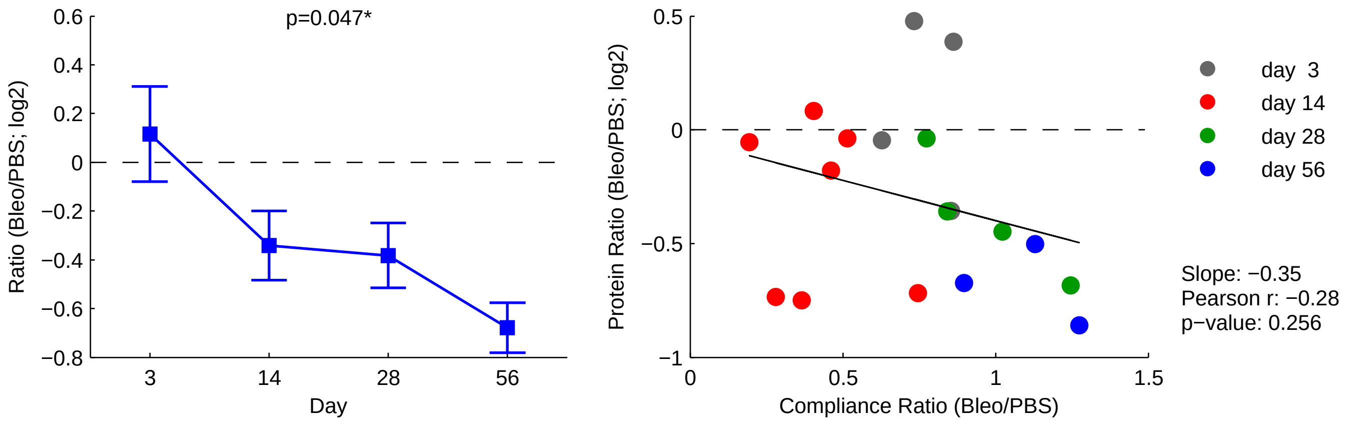

Q5SUF2 − Luc7l3 (id: 4128)

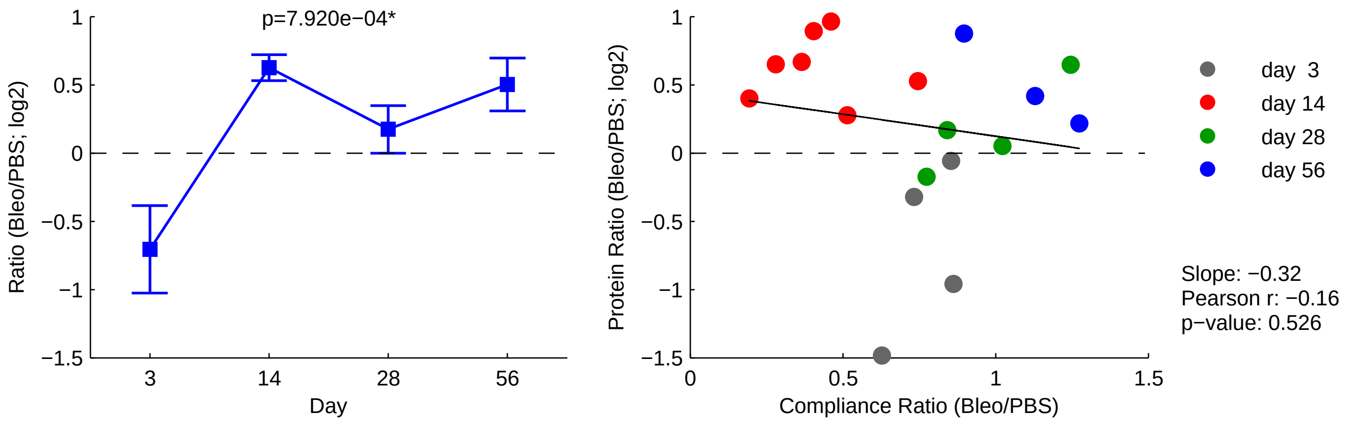

Q5SUH6 − Clint1 (id: 4129)

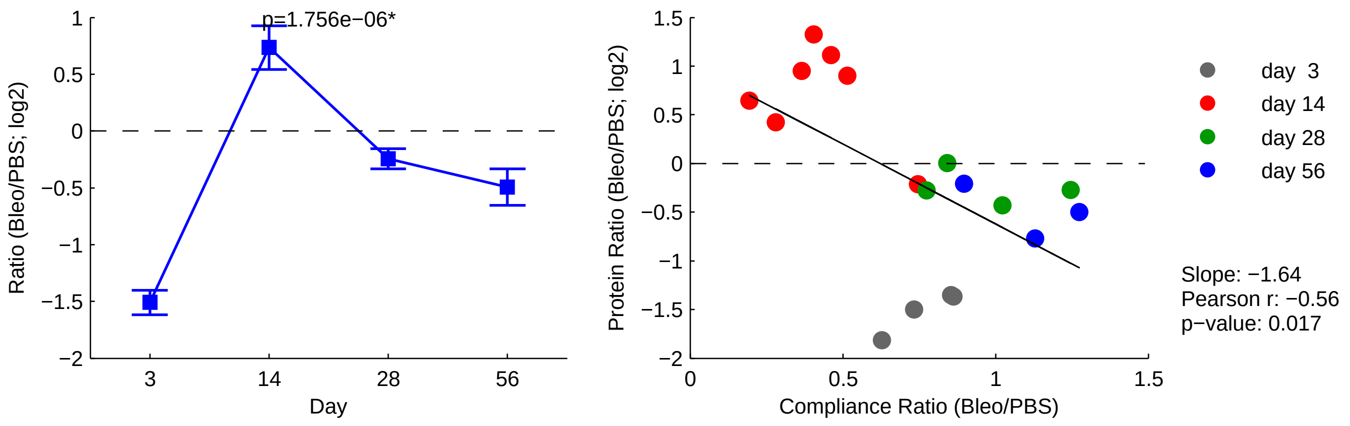

Q5SUT0 − Ewsr1 (id: 4133)

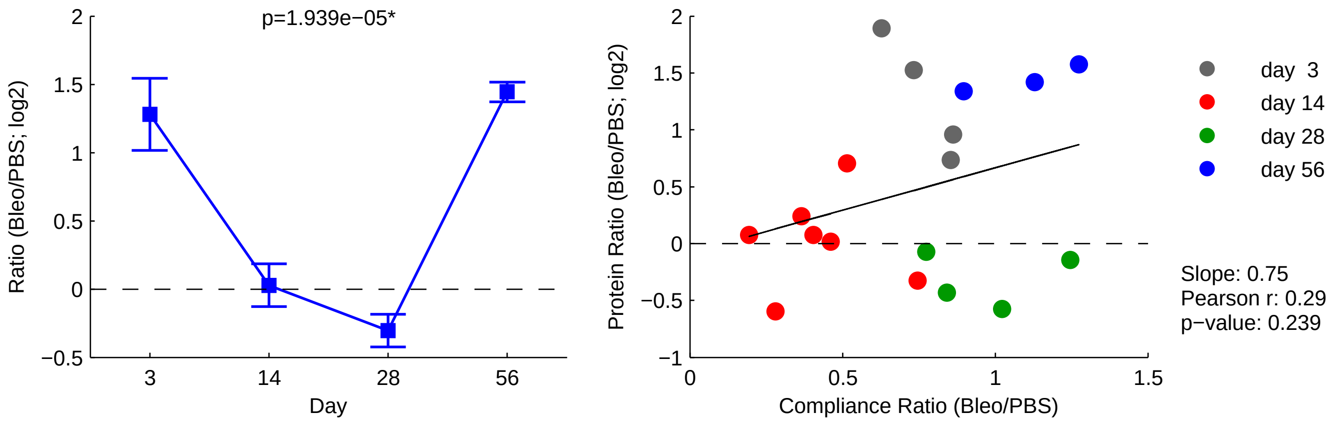

Q5SVR0−2 − Tbc1d9b (id: 4141)

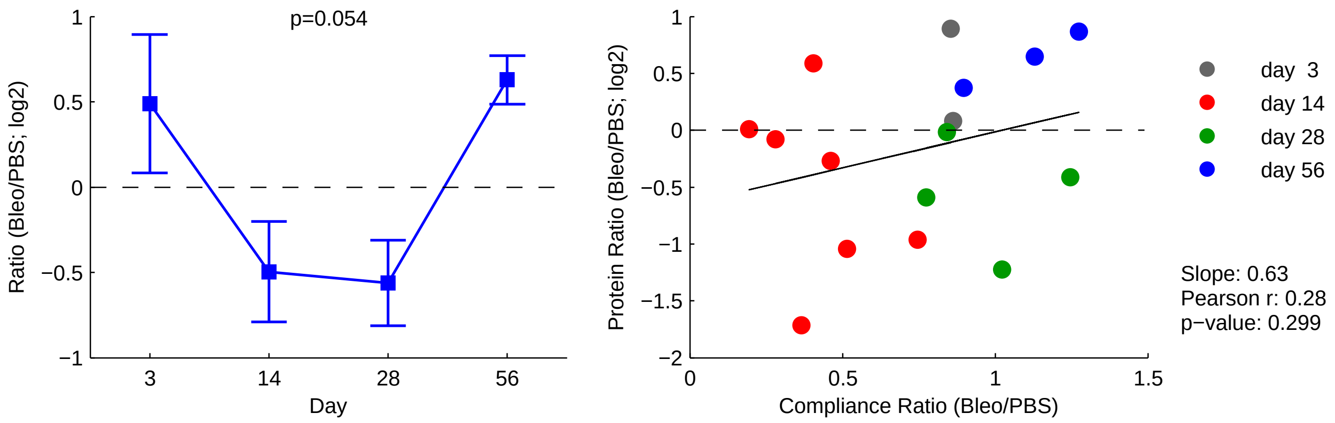

Q5SVT2 − Trim16 (id: 4142)

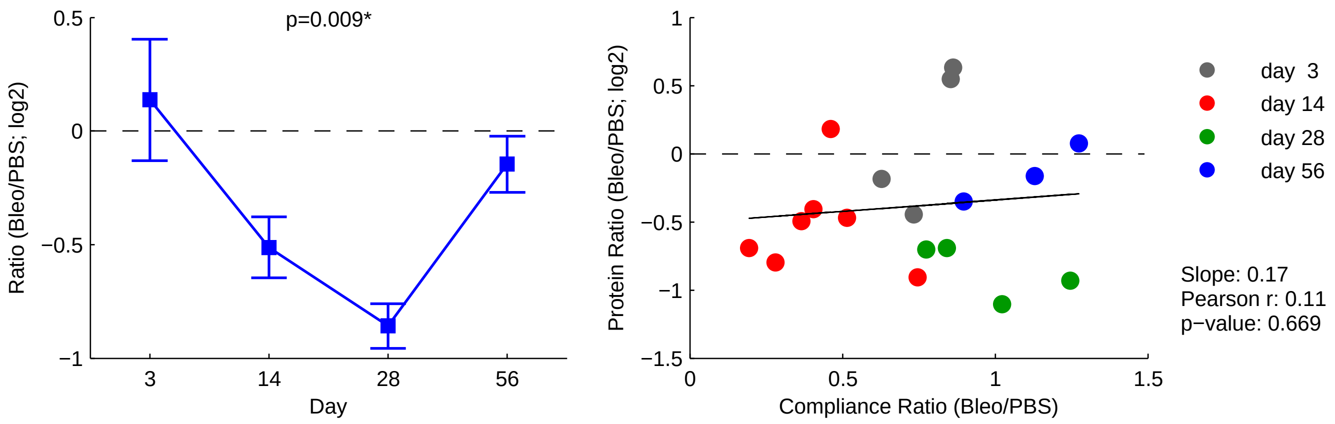

Q8VEE4 − Rpa1 (id: 4146)

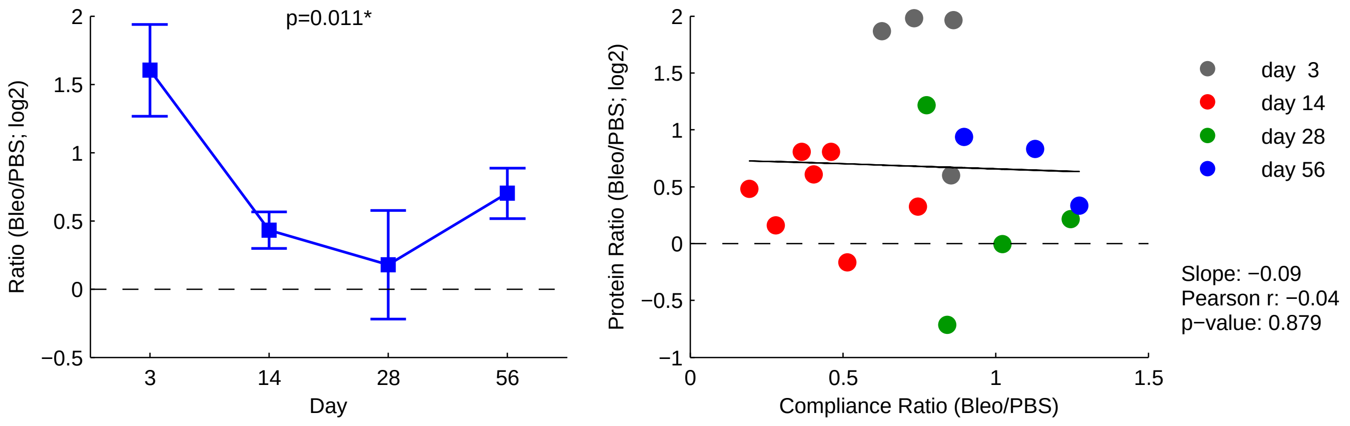

Q5SWT3 − Slc25a35 (id: 4147)

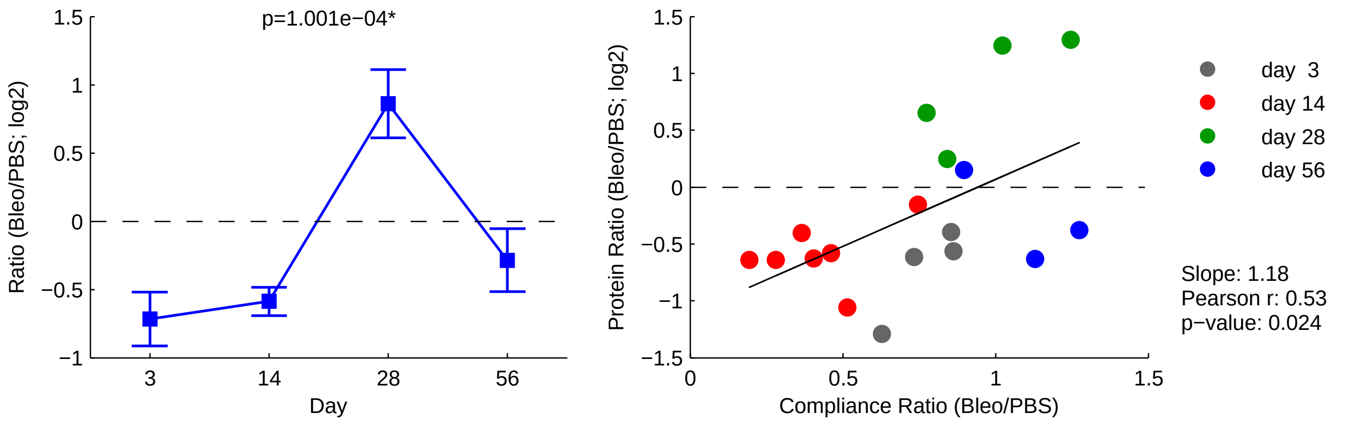

Q5SX40 − Myh1 (id: 4152)

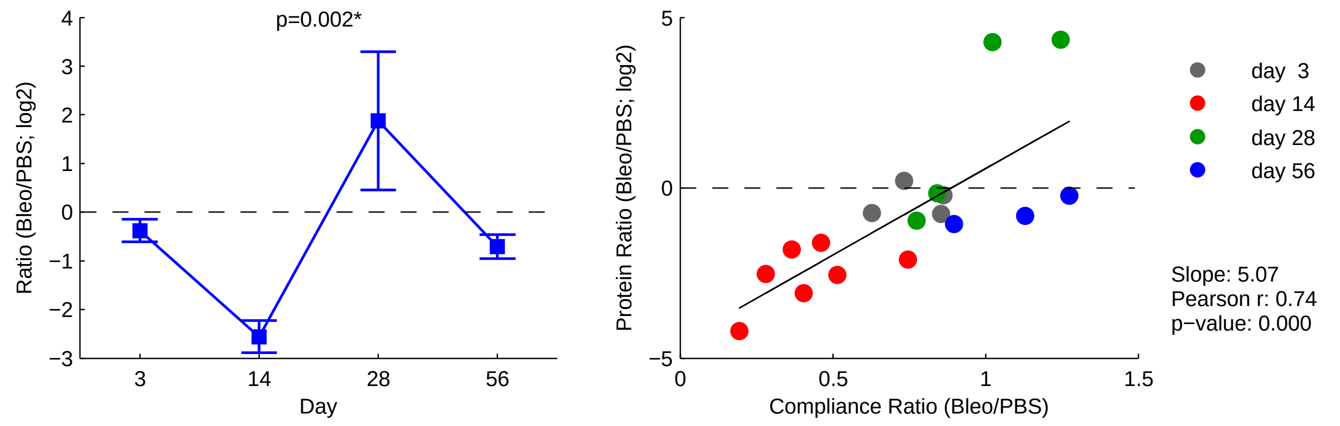

Q60716 − P4ha2 (id: 4154)

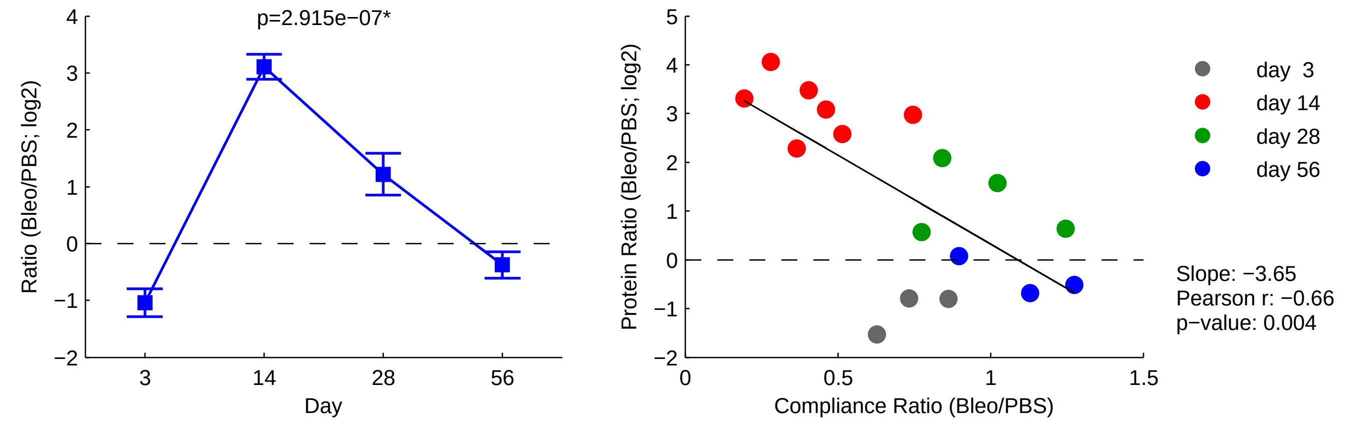

Q68FD5 − Cltc (id: 4157)

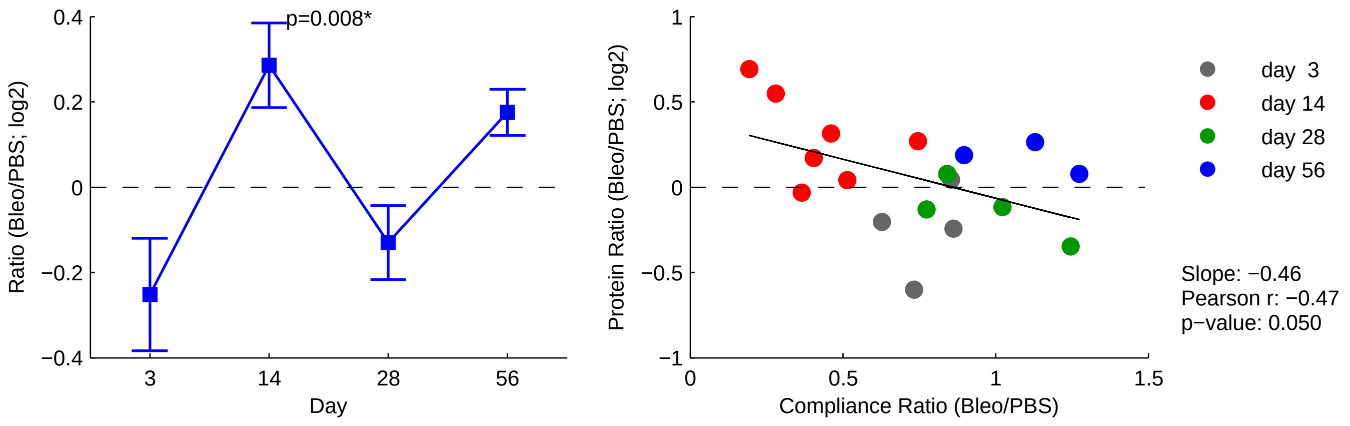

Q5SXY1−3 − Specc1 (id: 4158)

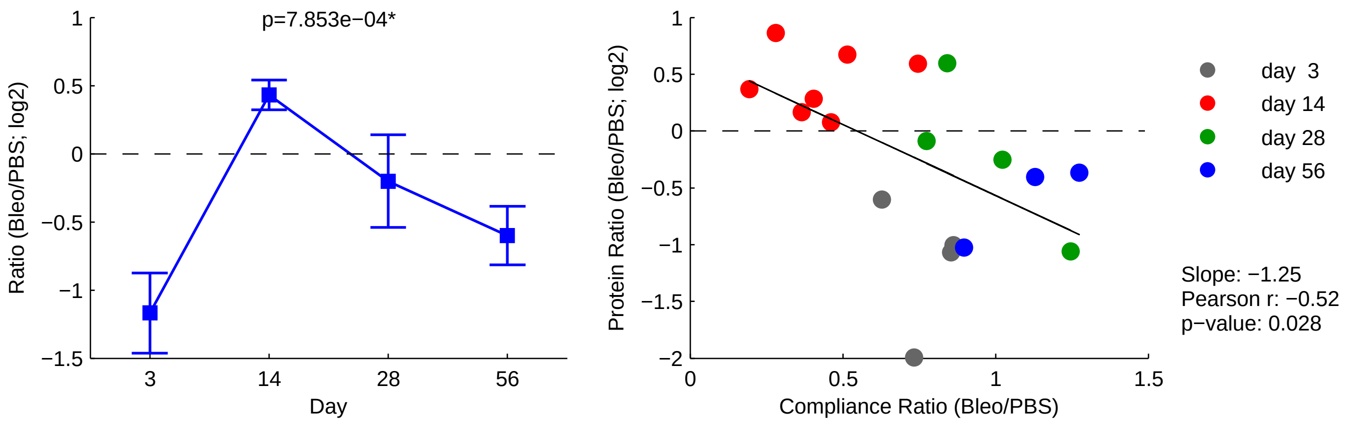

Q5SYD0 − Myo1d (id: 4159)

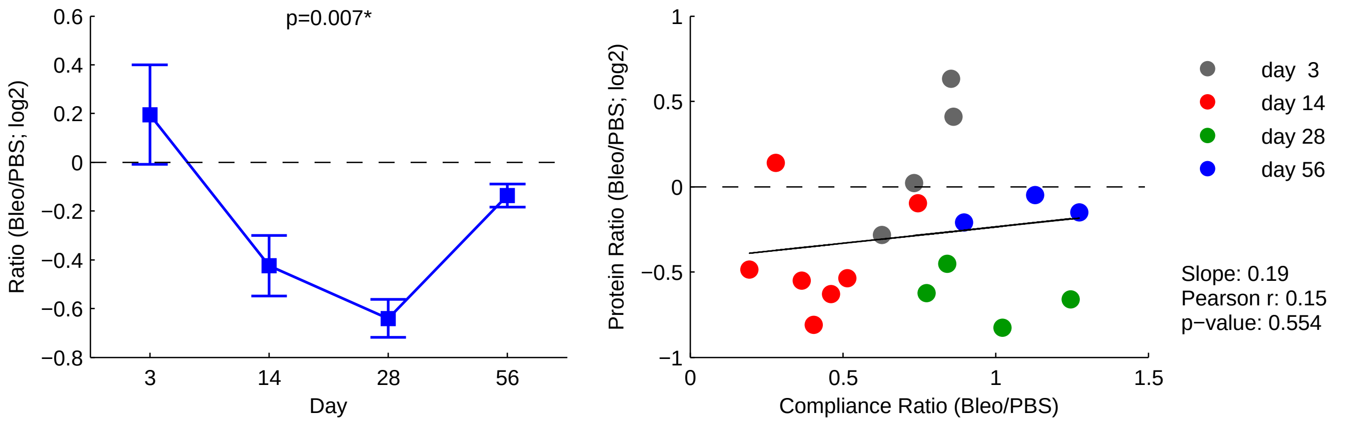

Q5SYL3 − Kiaa0100 (id: 4160)

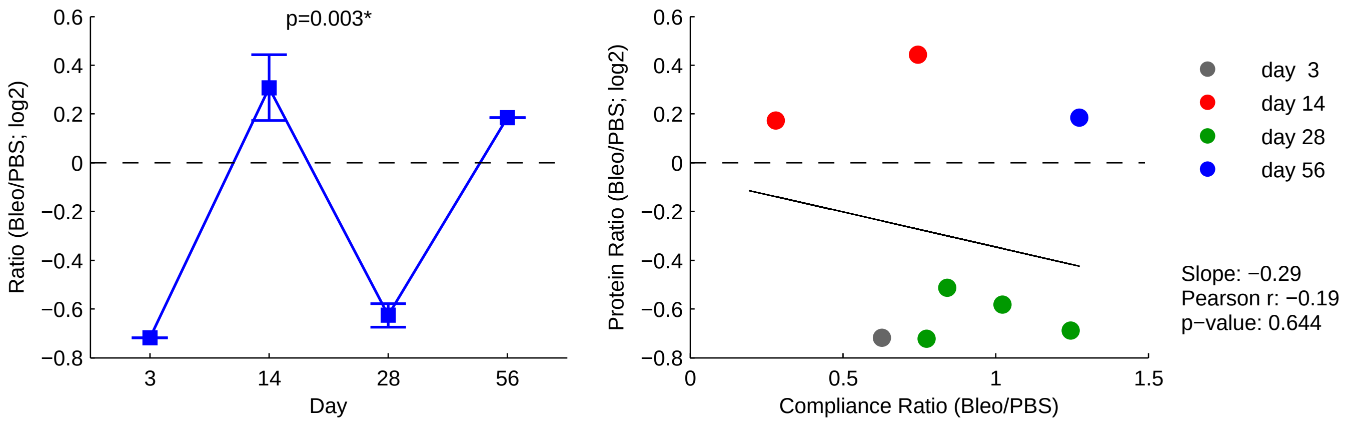

Q5U458 − Dnajc11 (id: 4163)

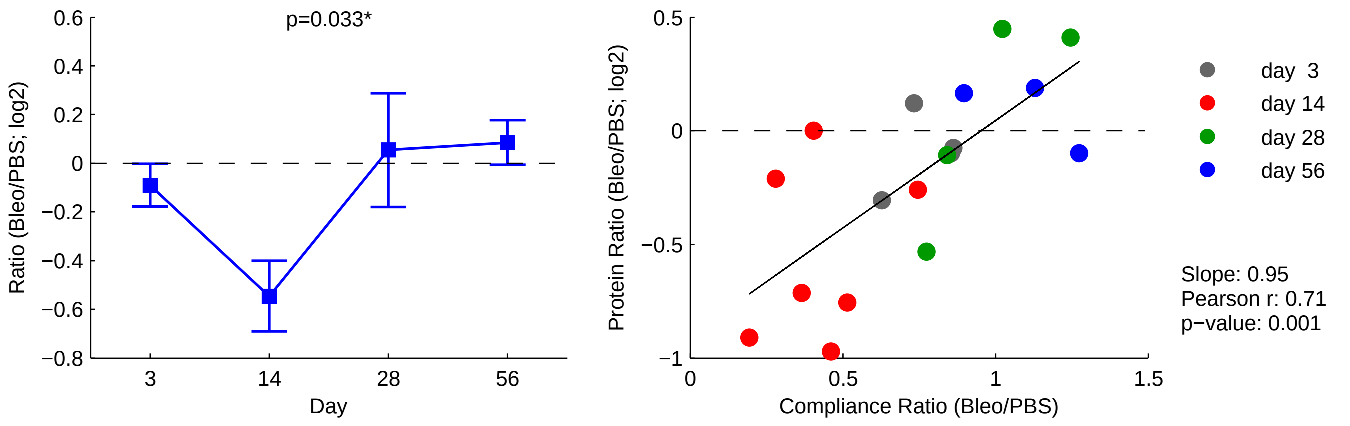

Q5XF89 − Atp13a3 (id: 4169)

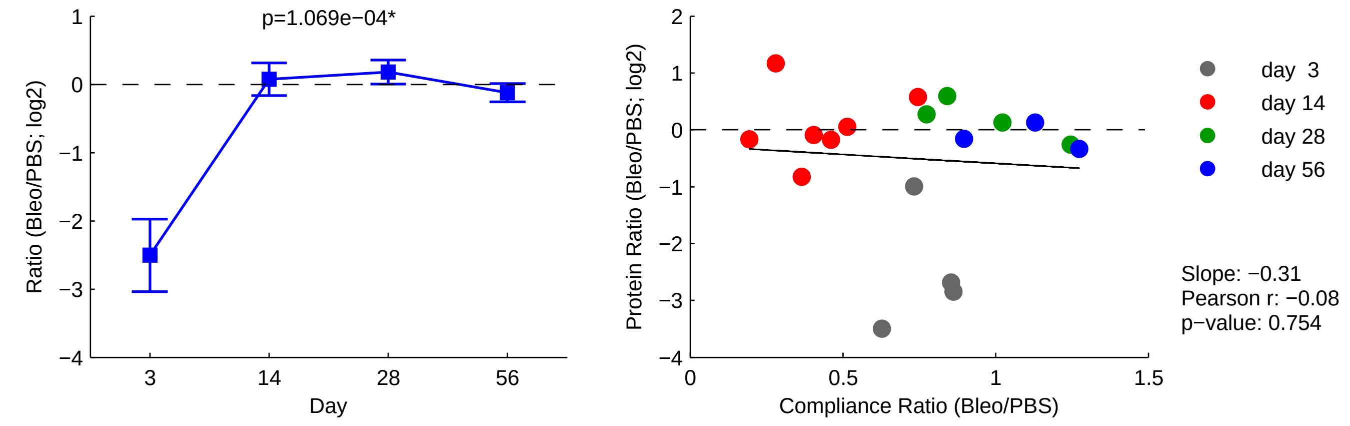

Q5XG73−2 − Acbd5 (id: 4171)

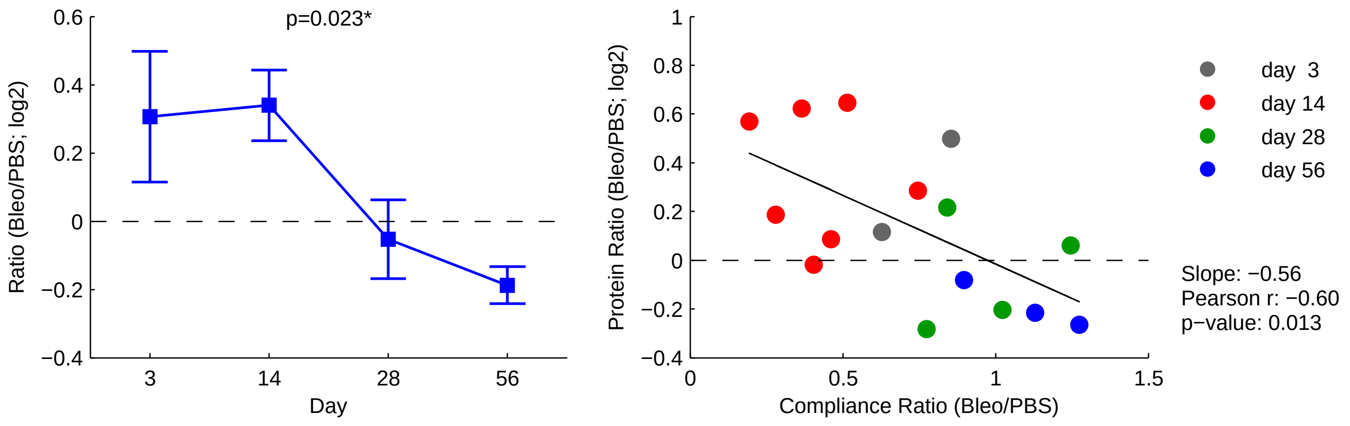

Q5XJF6 − Rpl10a (id: 4173)

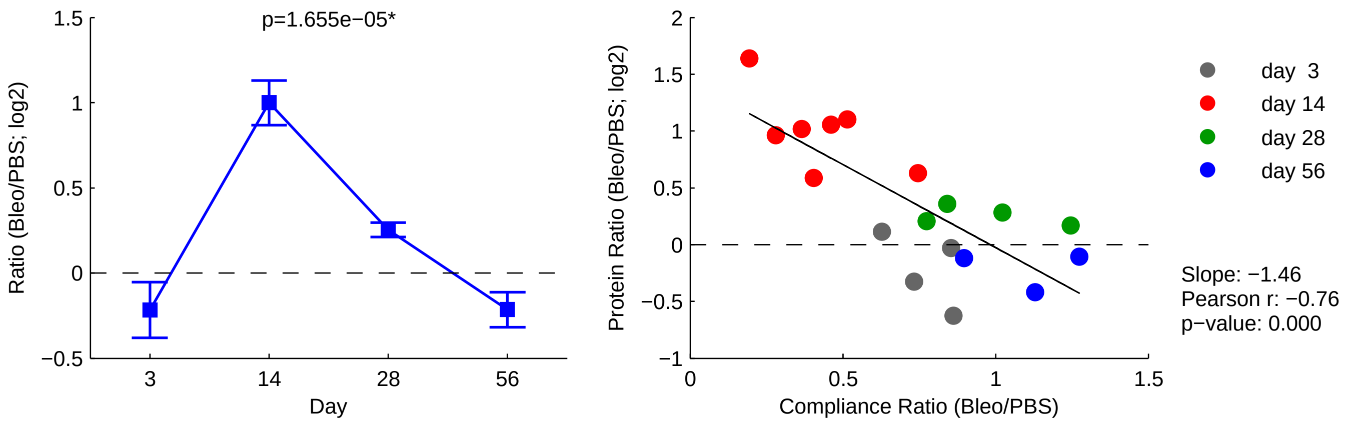

Q5XJY4 − Parl (id: 4174)

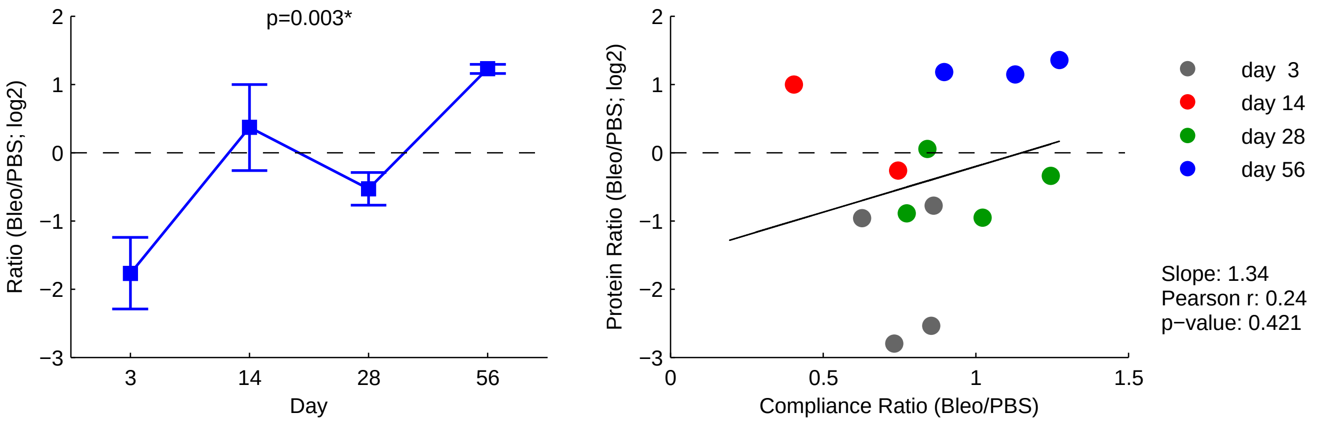

Q5XJY5 − Arcn1 (id: 4175)

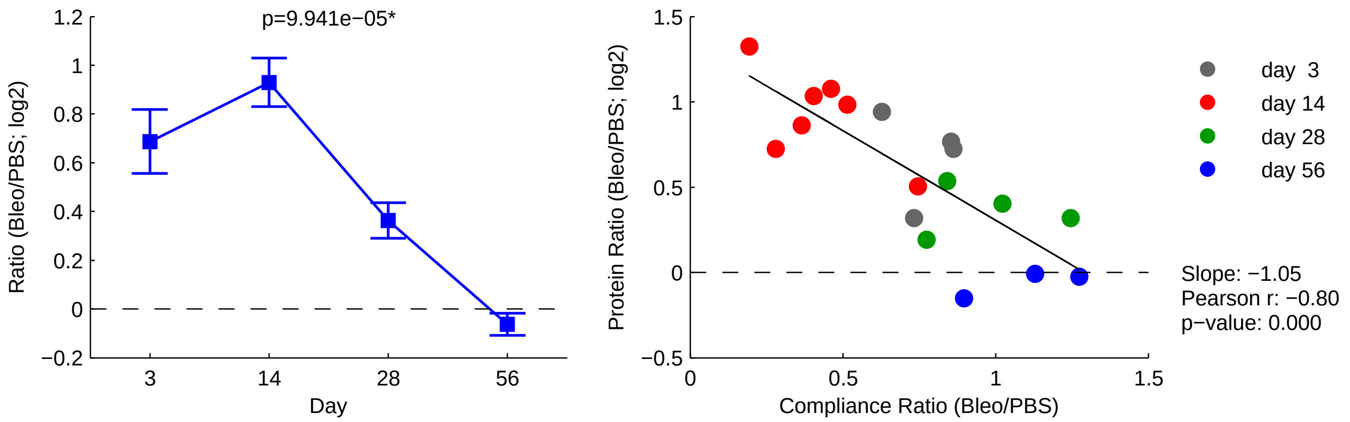

Q5XKN4 − Jagn1 (id: 4177)

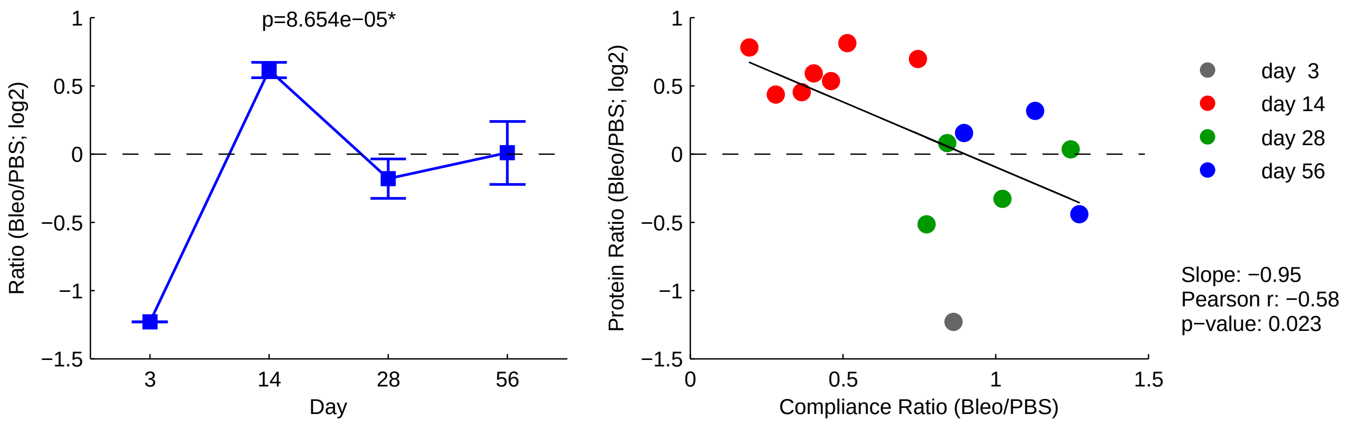

Q60520 − Sin3a (id: 4179)

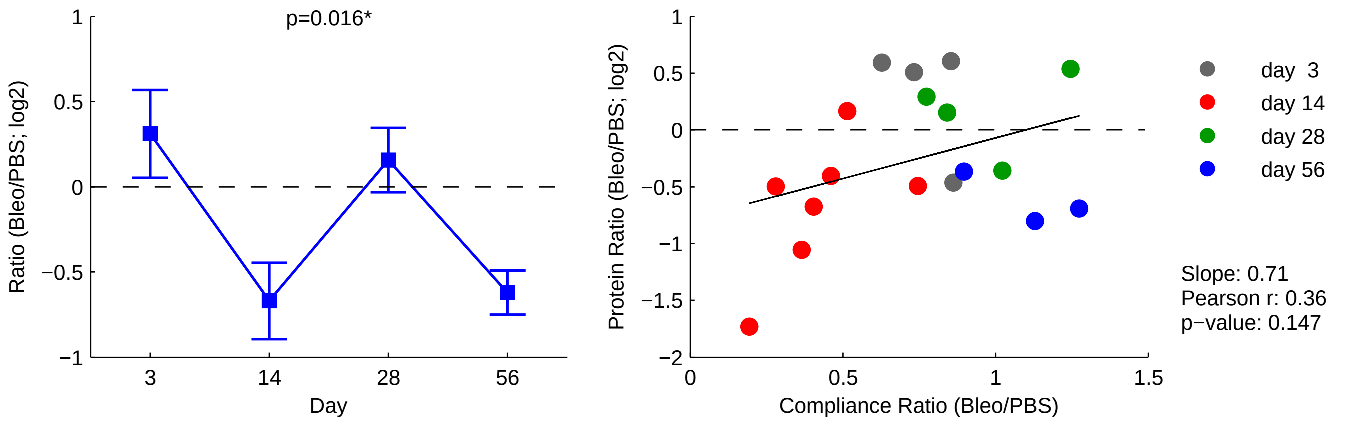

Q60597 − Ogdh (id: 4183)

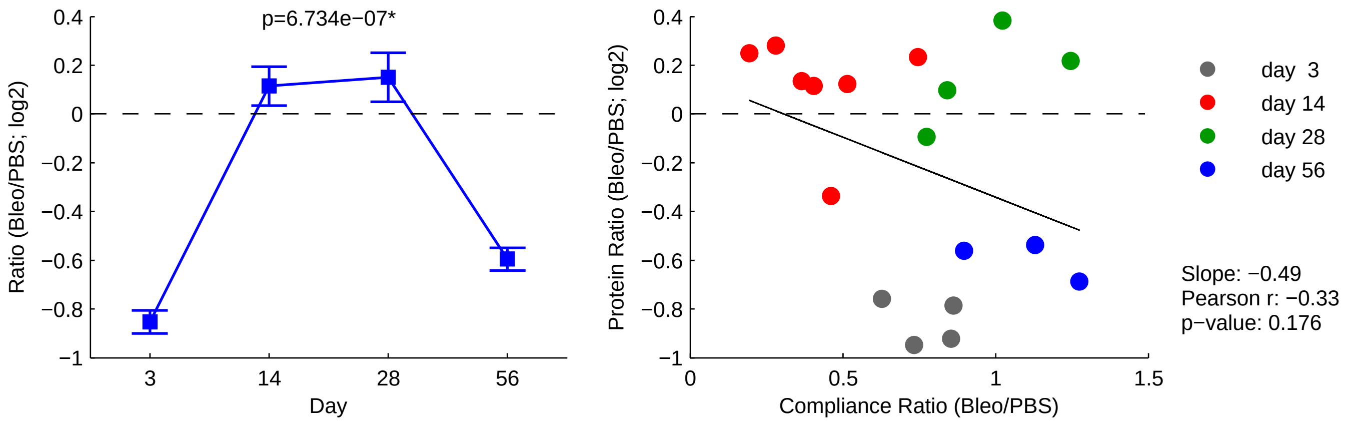

Q60598 − Cttn (id: 4184)

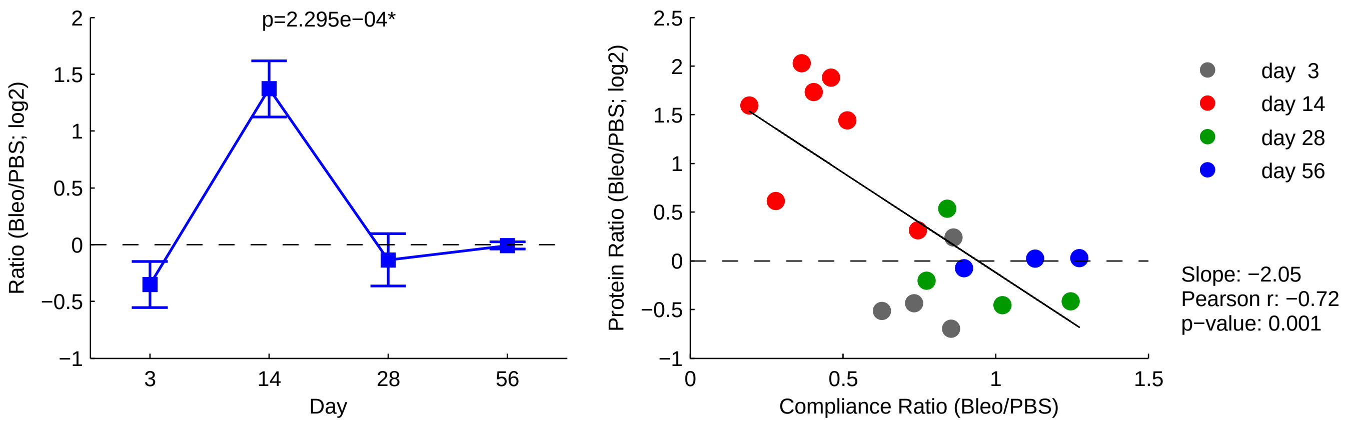

Q60631 − Grb2 (id: 4188)

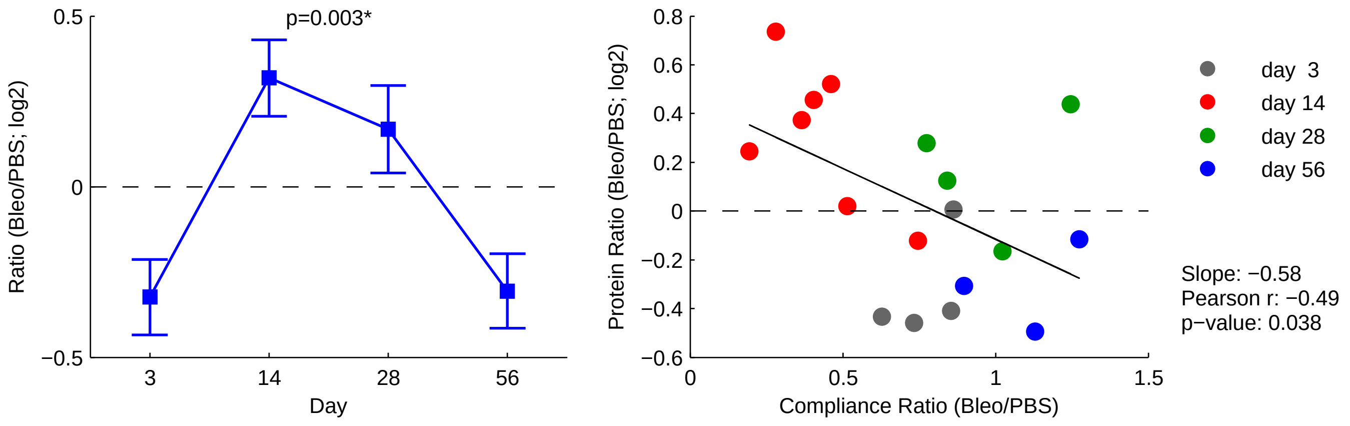

Q60668−3 − Hnrnpd (id: 4190)

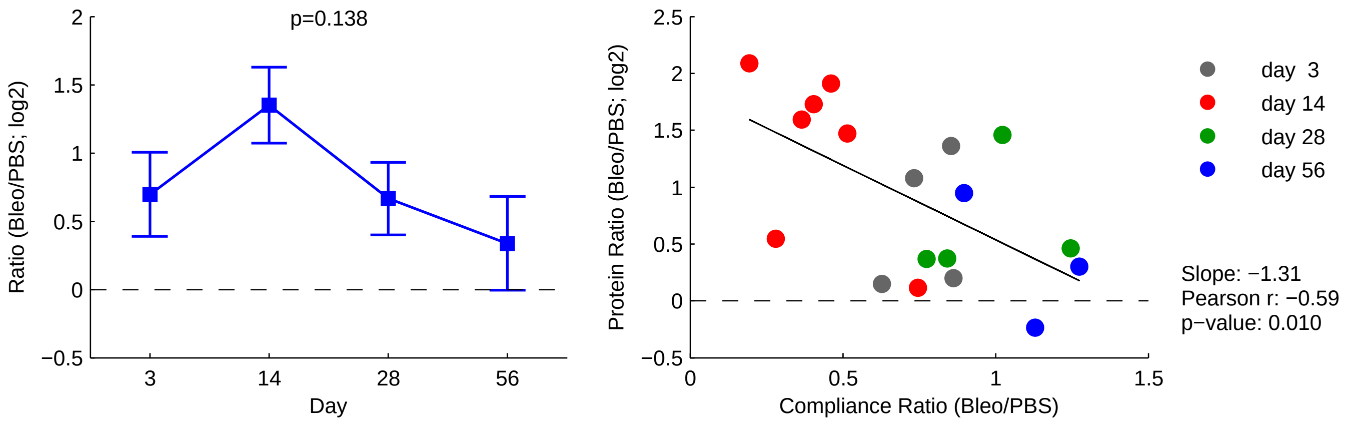

Q60714 − Slc27a1 (id: 4196)

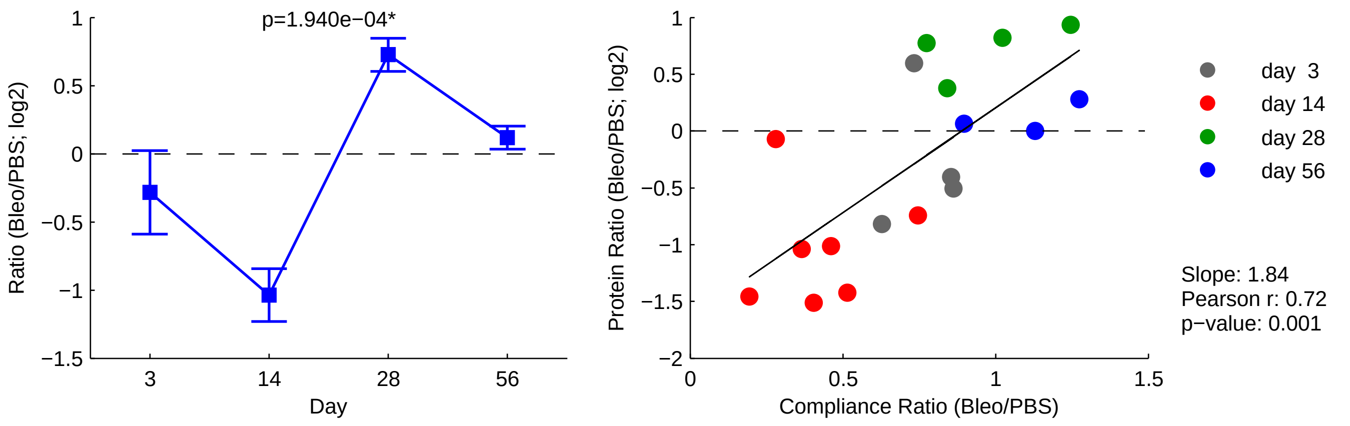

Q60715−2 − P4ha1 (id: 4198)

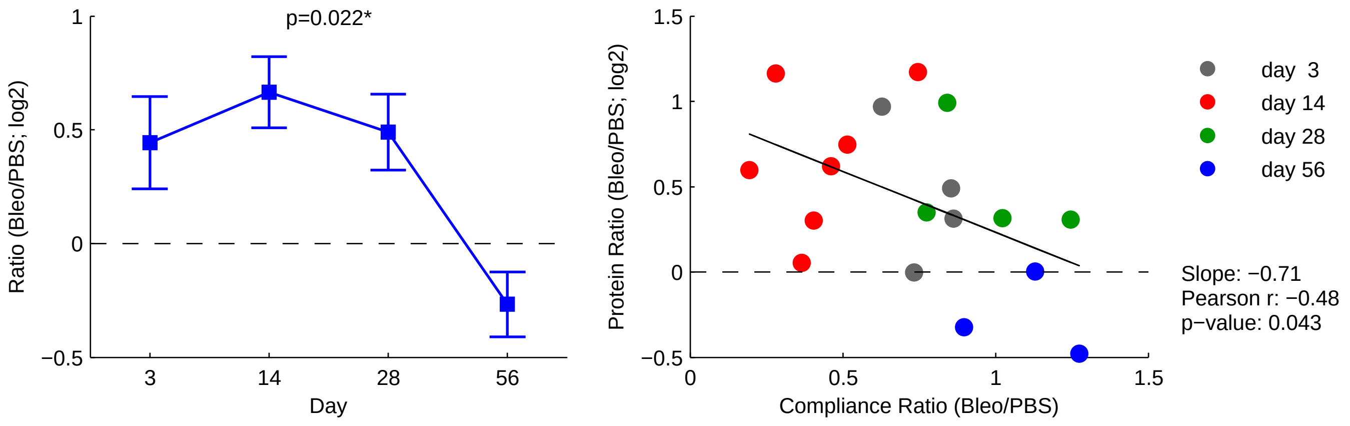

Q60749 − Khdrbs1 (id: 4201)

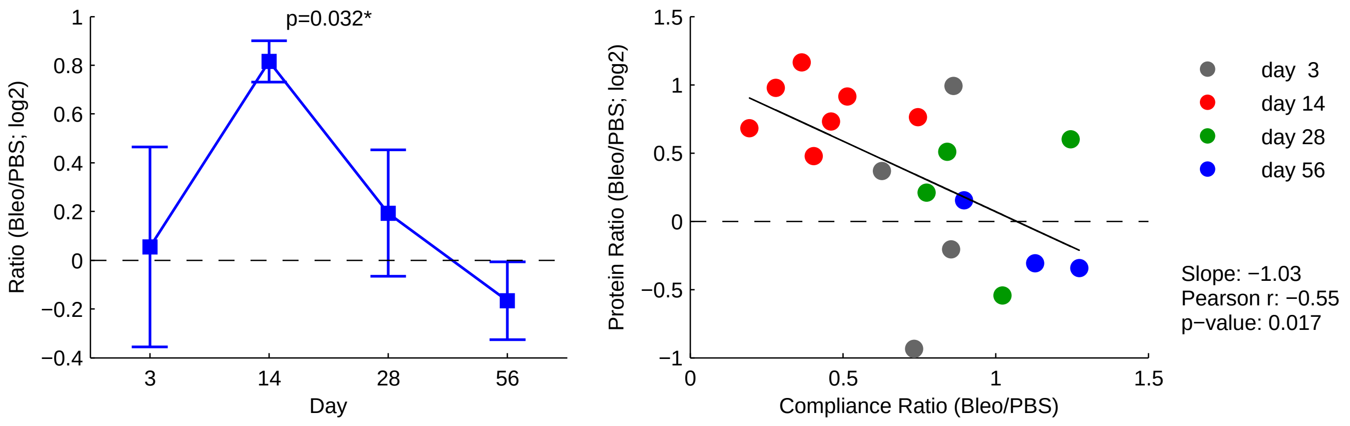

Q60766−2 − Irgm1 (id: 4206)

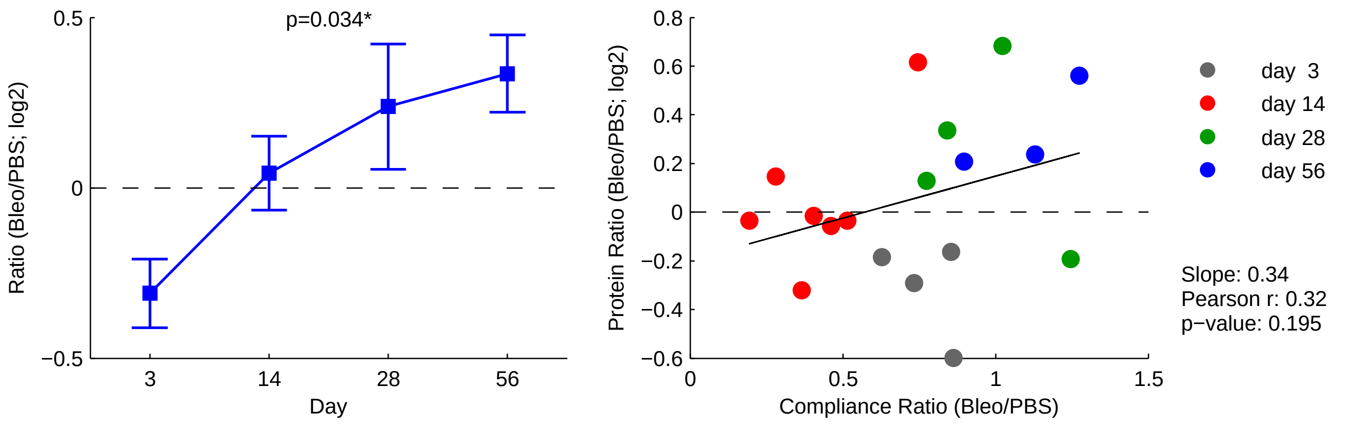

Q60770 − Stxbp3 (id: 4208)

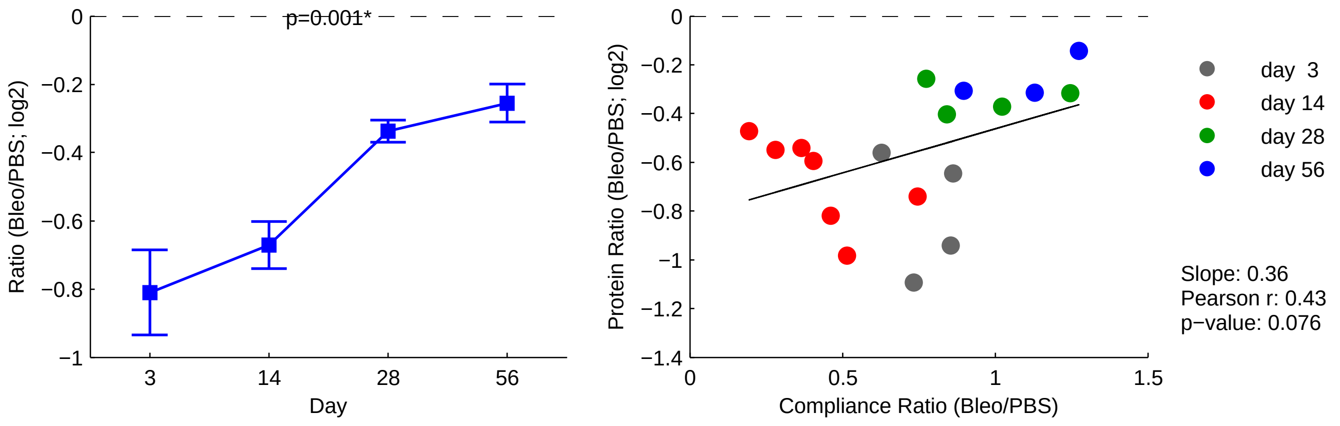

Q60779 − Gas8 (id: 4209)

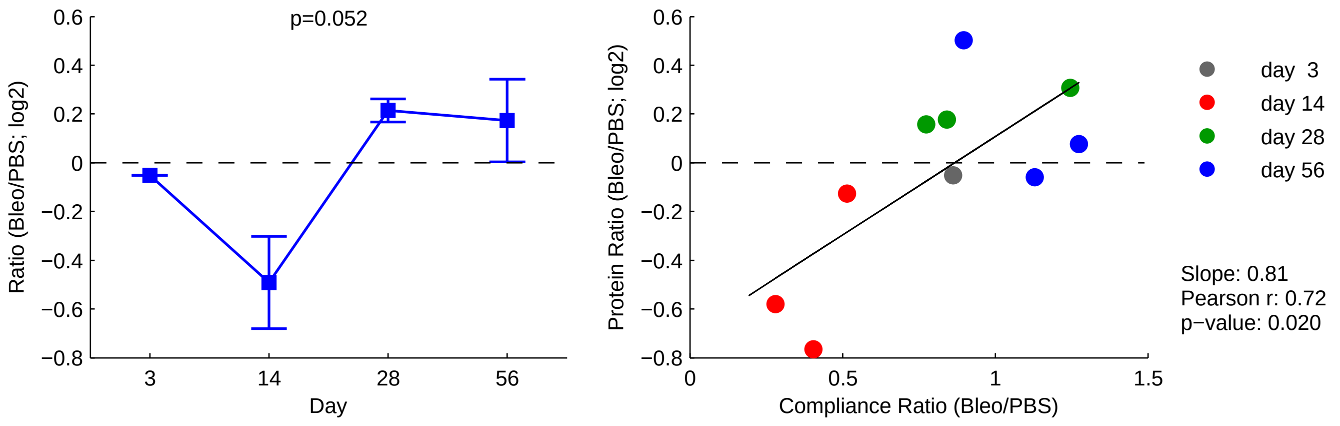

Q60790 − Rasa3 (id: 4210)

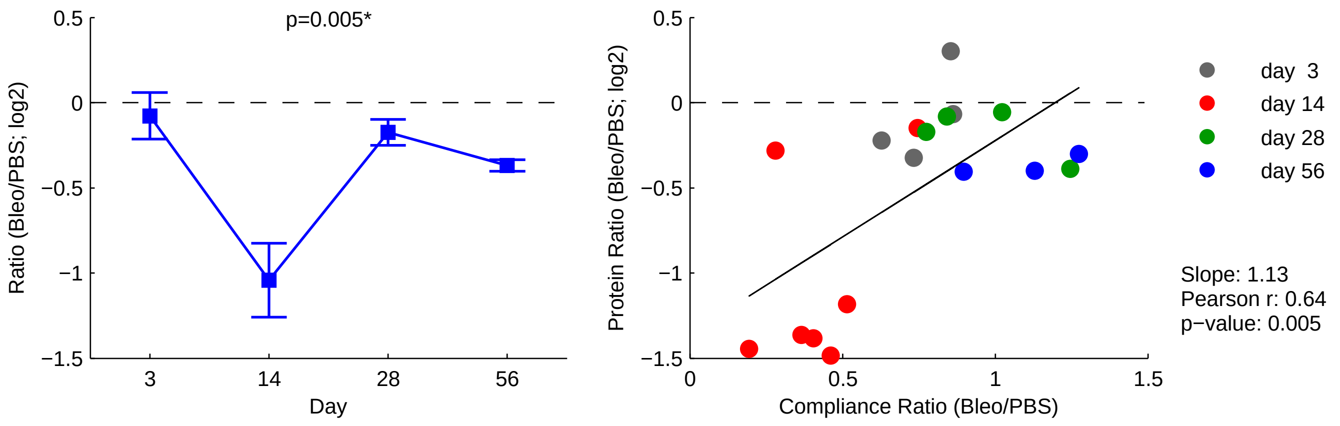

Q60809 − Cnot7 (id: 4212)

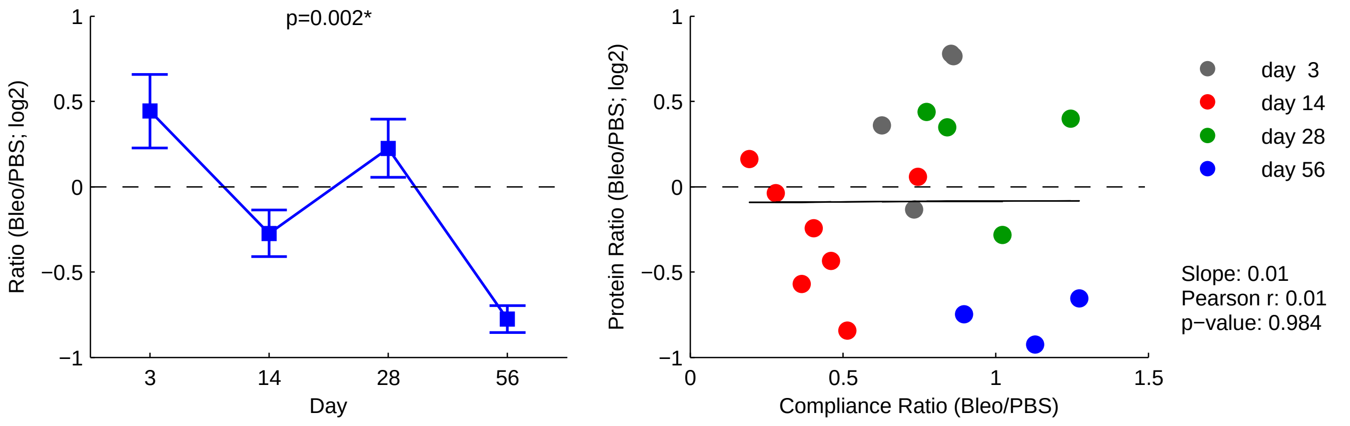

Q60854 − Serpinb6 (id: 4215)

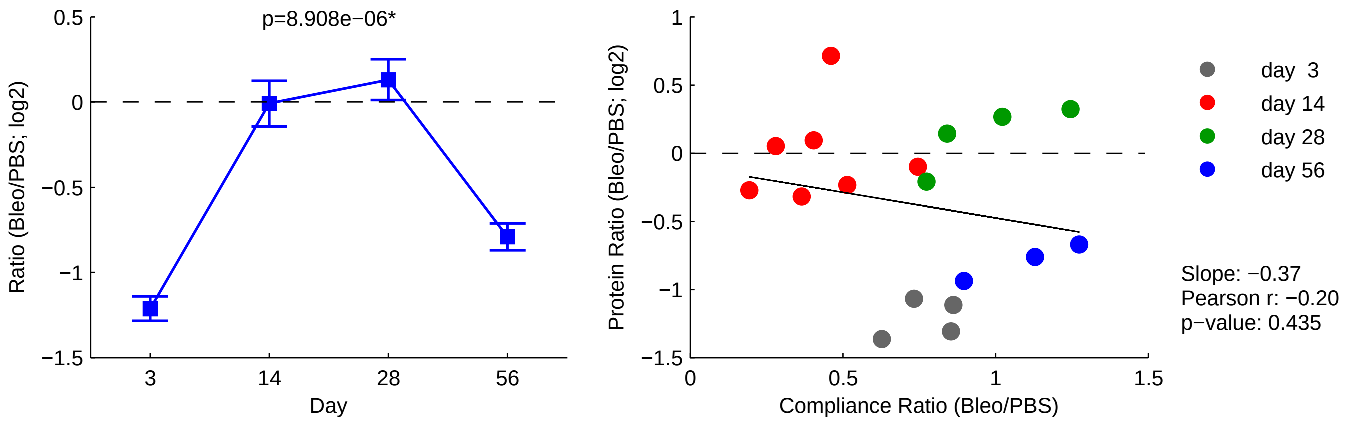

Q60864 − Stip1 (id: 4217)

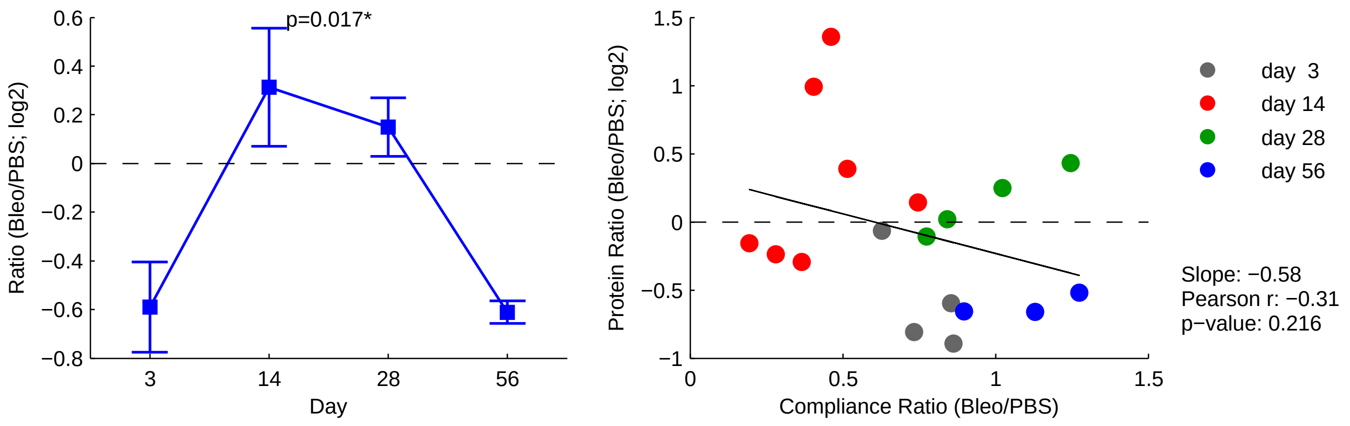

Q60865 − Caprin1 (id: 4218)

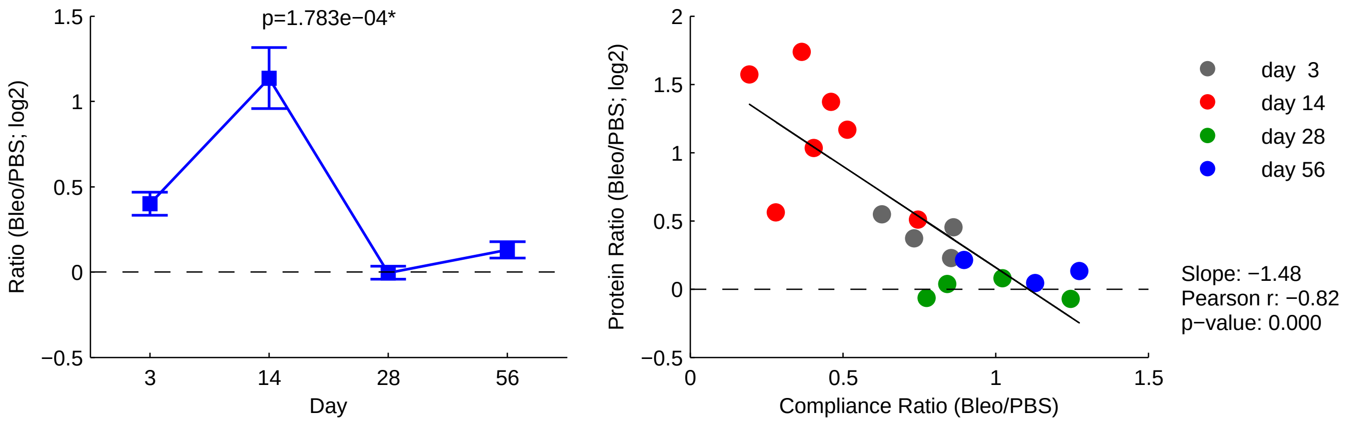

Q60932−2 − Vdac1 (id: 4222)

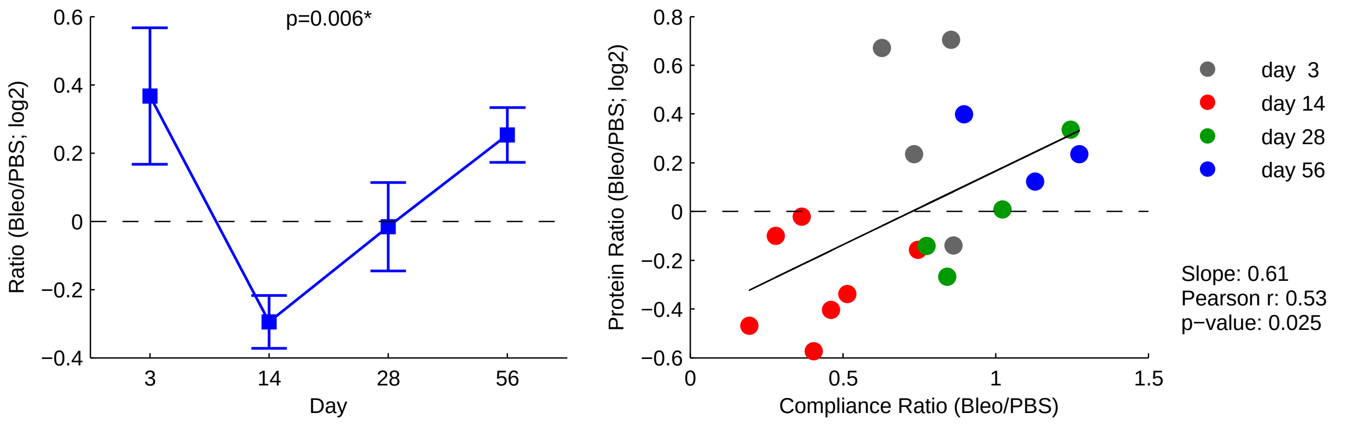

Q60936 − Adck3 (id: 4224)

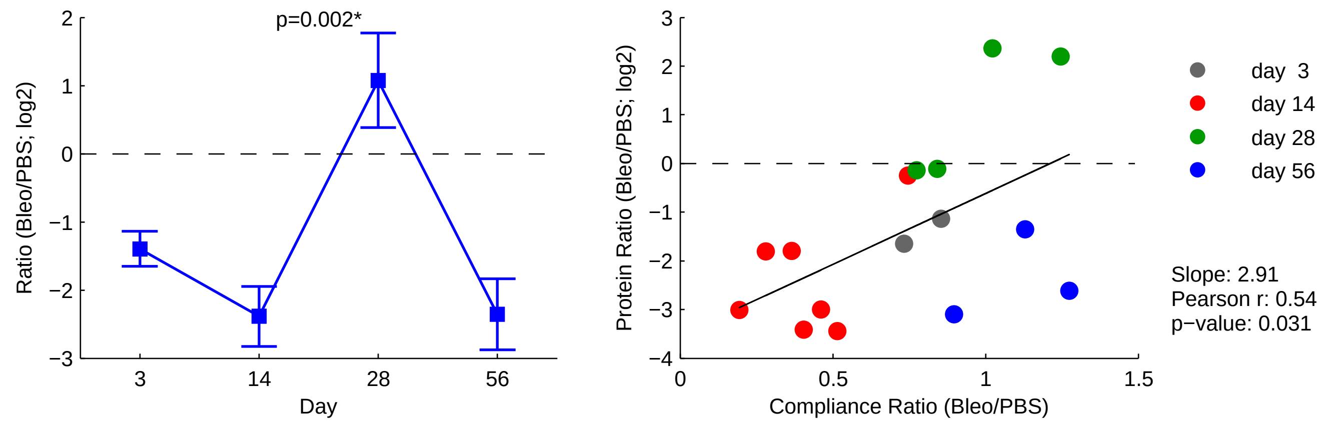

Q60953 − Pml (id: 4226)

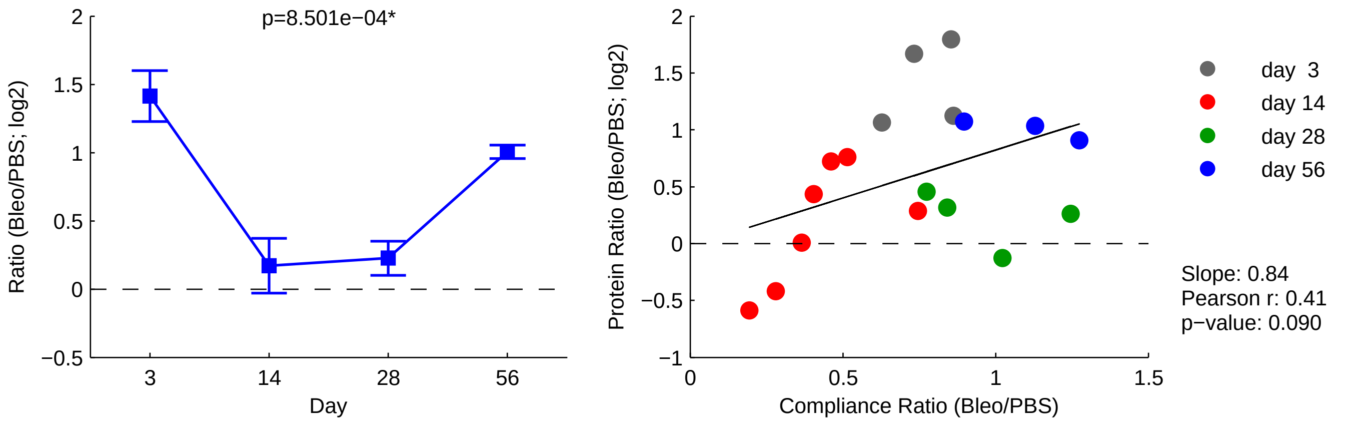

Q60972 − Rbbp4 (id: 4231)

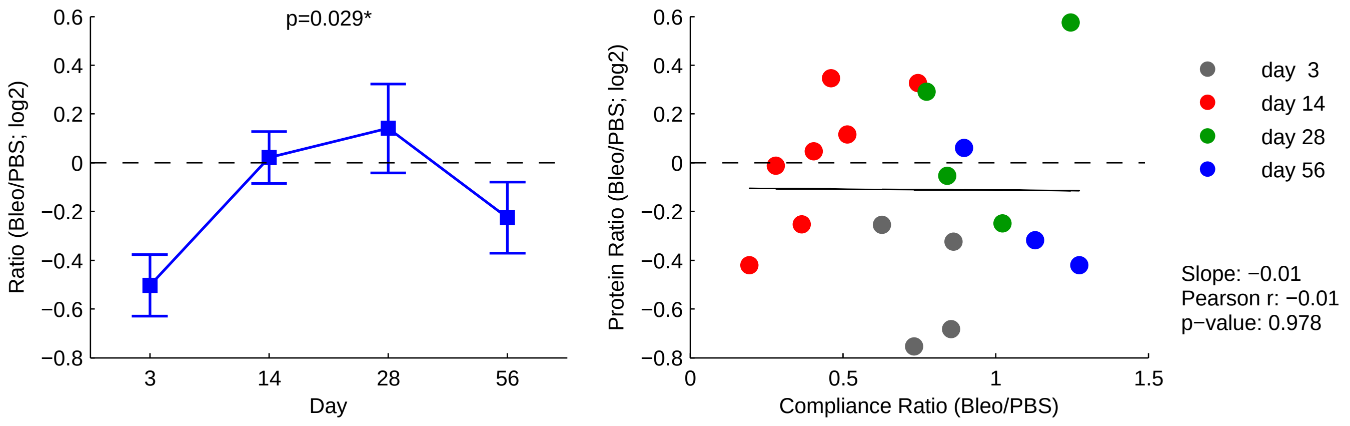

Q61001 − Lama5 (id: 4238)

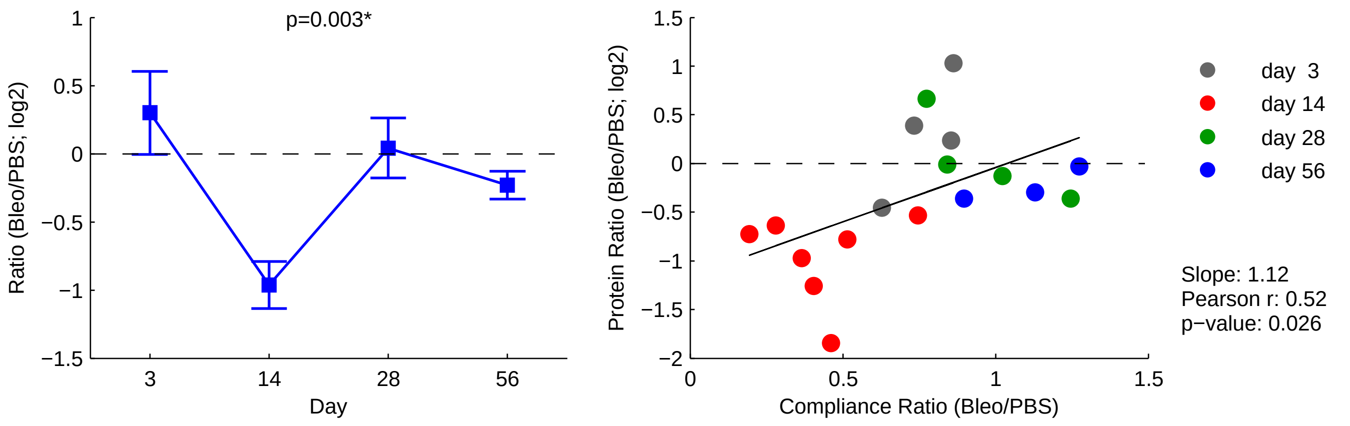

Q61009 − Scarb1 (id: 4239)

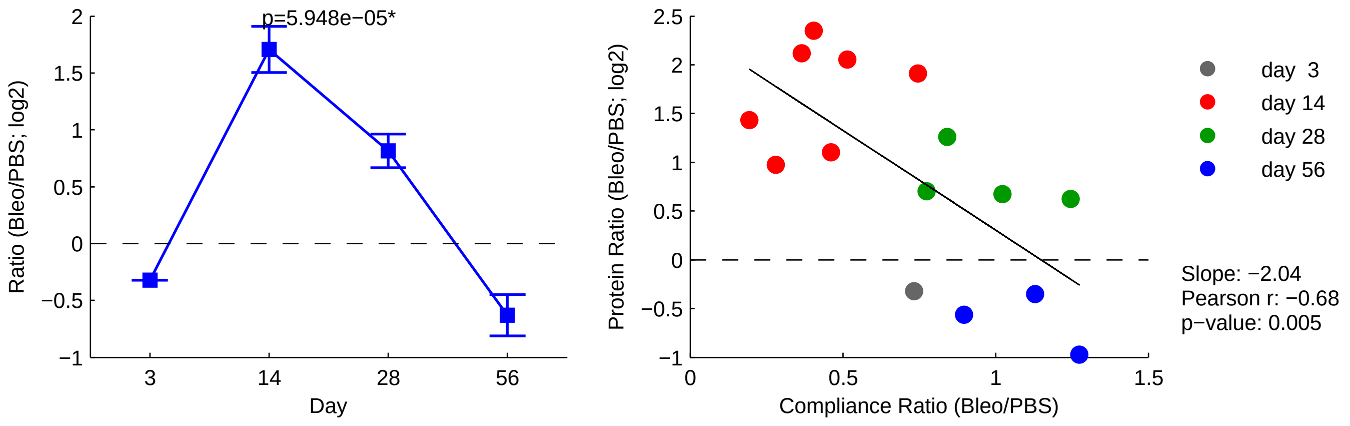

Q61017 − Gngt2 (id: 4241)

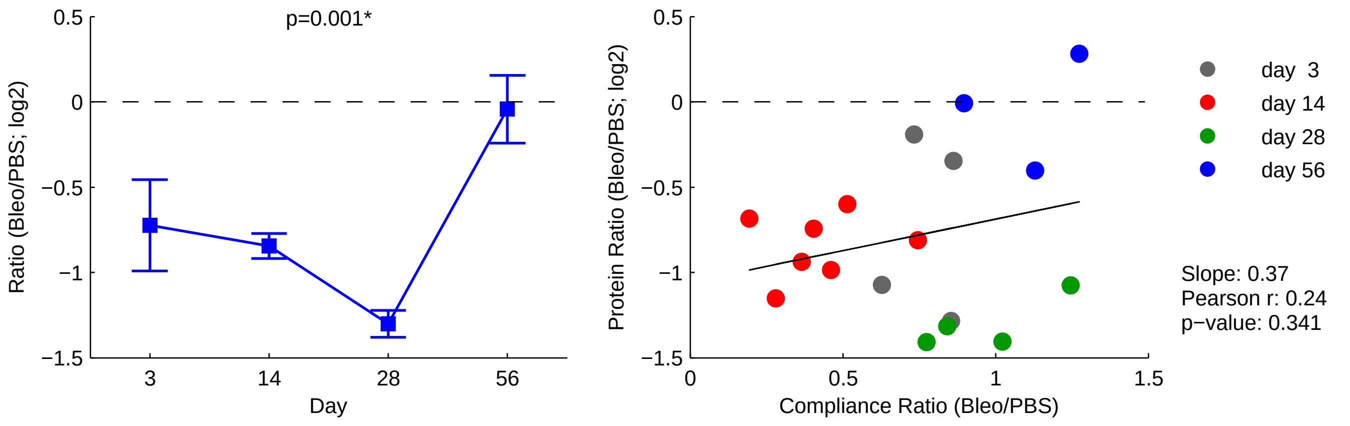

Q61025 − Ift20 (id: 4242)

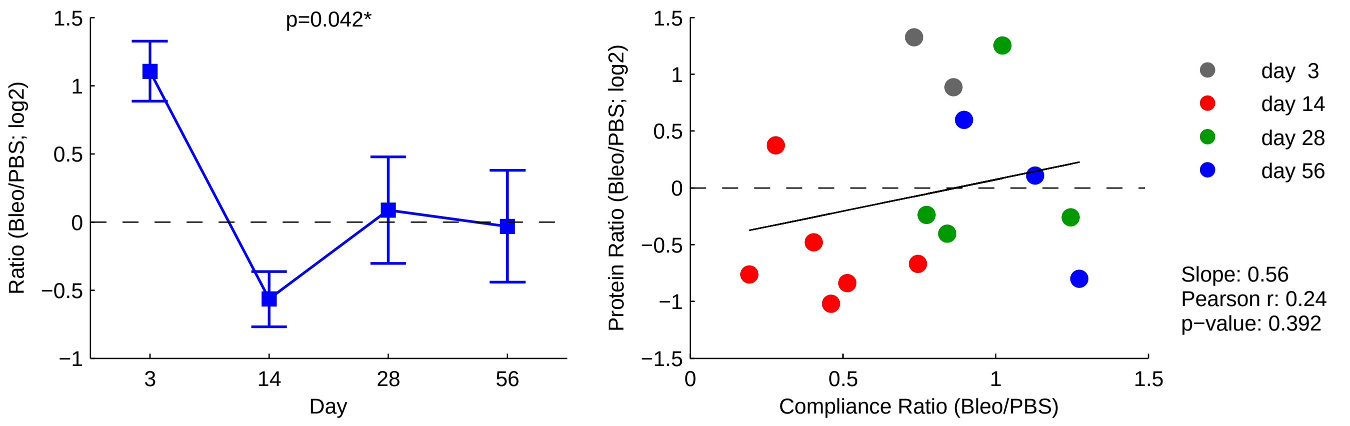

Q61029 − Tmpo (id: 4243)

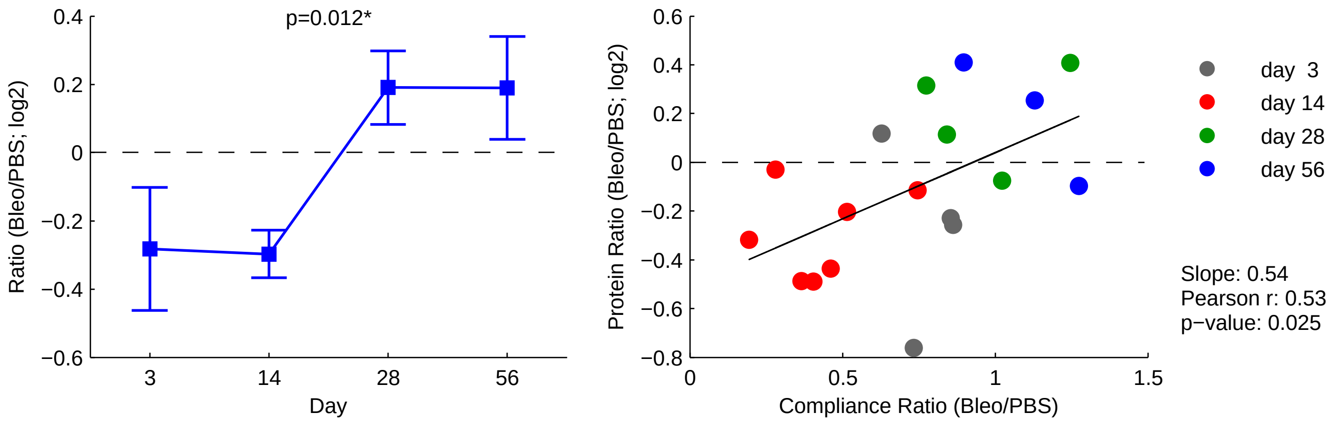

Q61029−3 − (id: 4244)

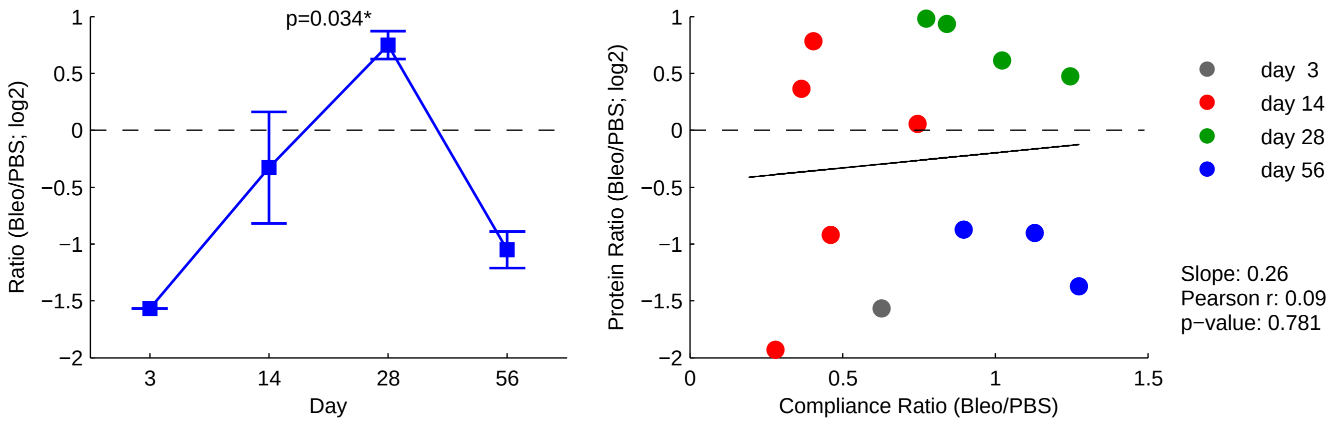

Q61033 − Tmpo (id: 4245)

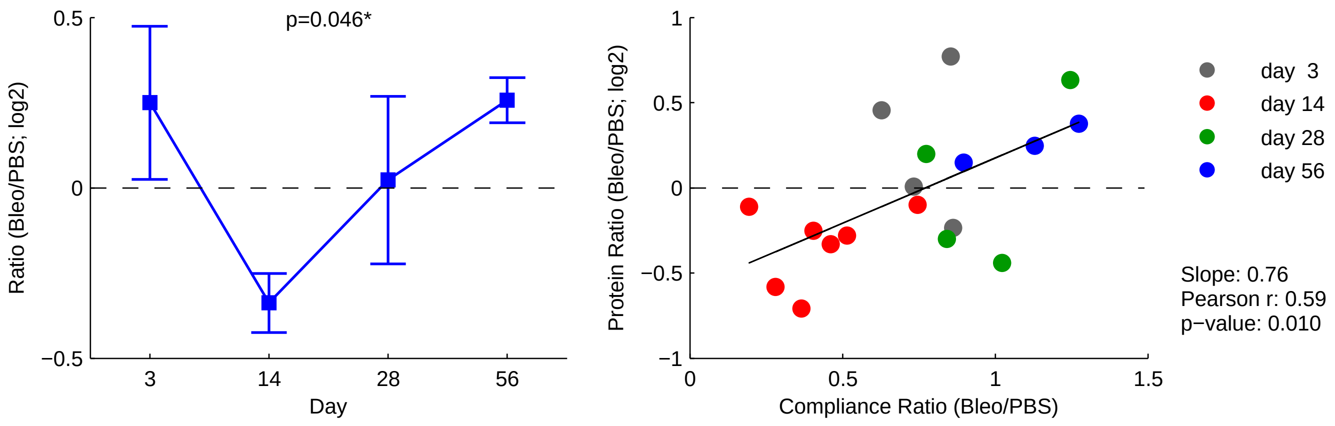

Q61069 − Usf1 (id: 4248)

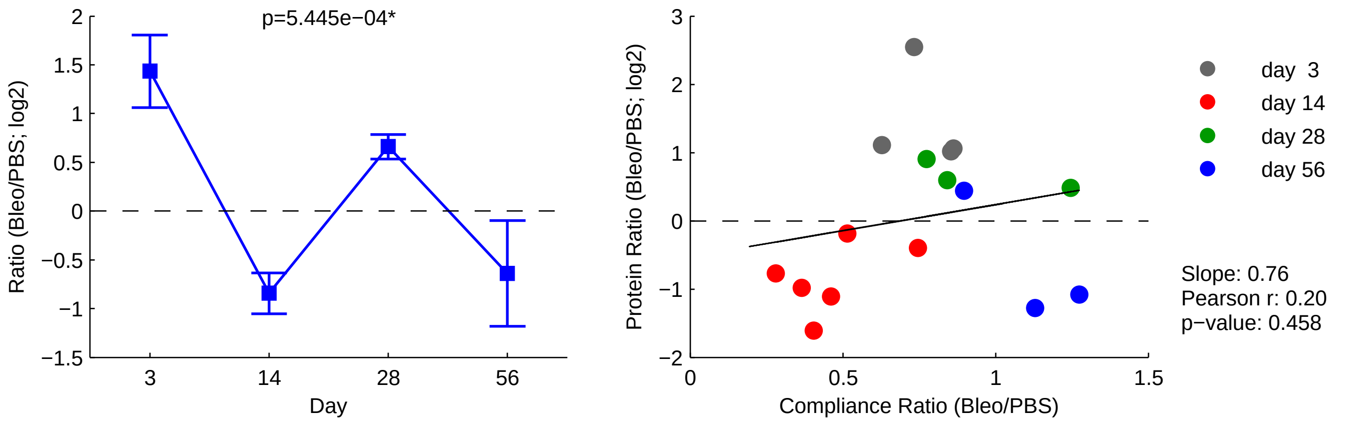

Q61070 − Ei24 (id: 4249)

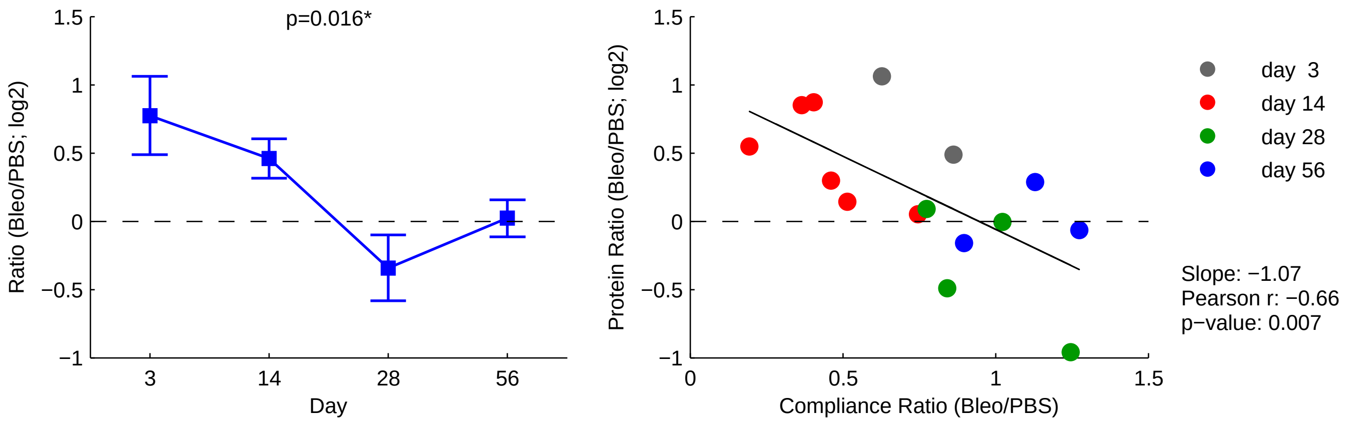

Q61074 − Ppm1g (id: 4250)

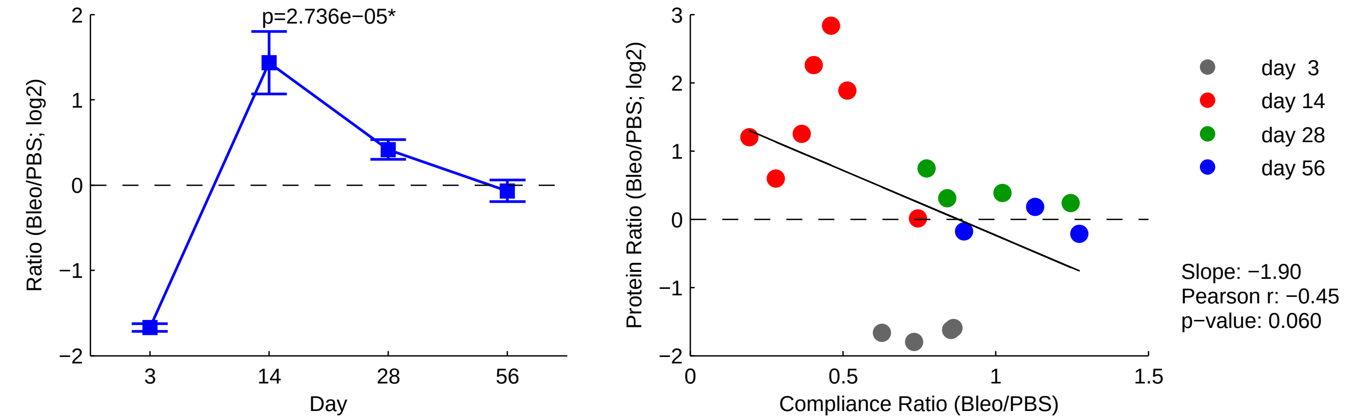

Q61081 − Cdc37 (id: 4252)

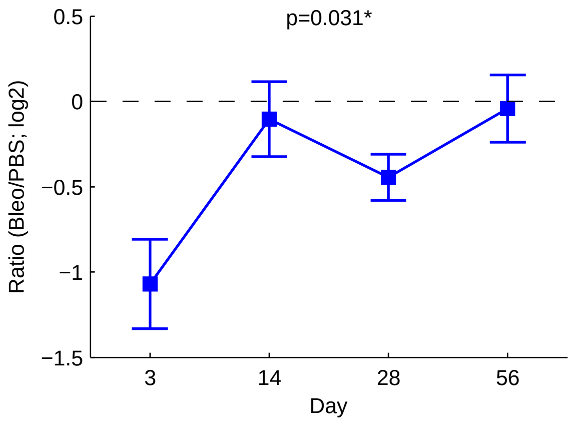

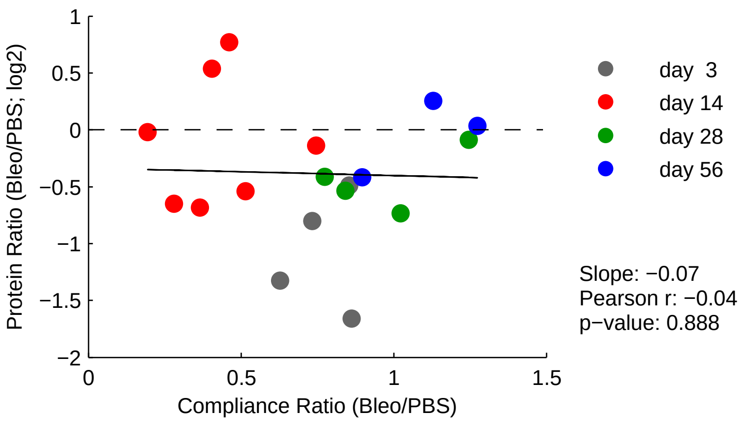

Q61087 − Lamb3 (id: 4253)

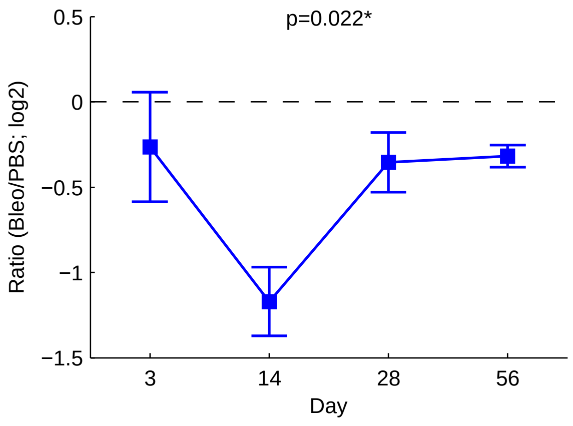

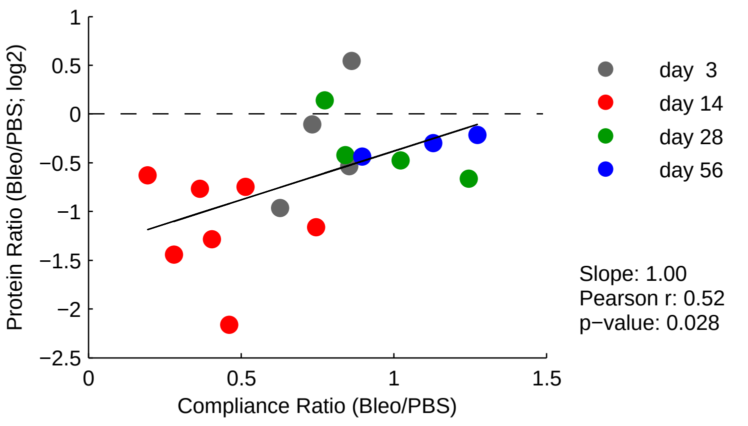

Q61093 − Cybb (id: 4257)

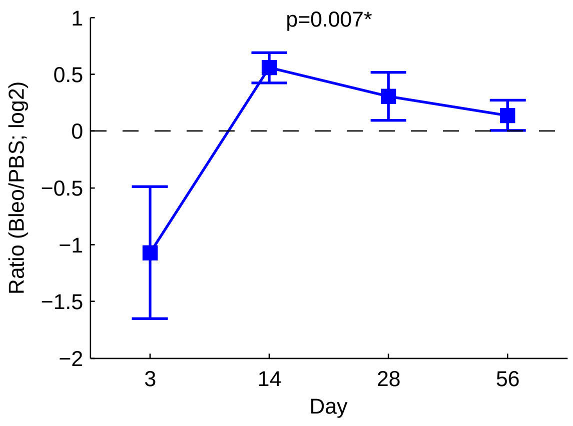

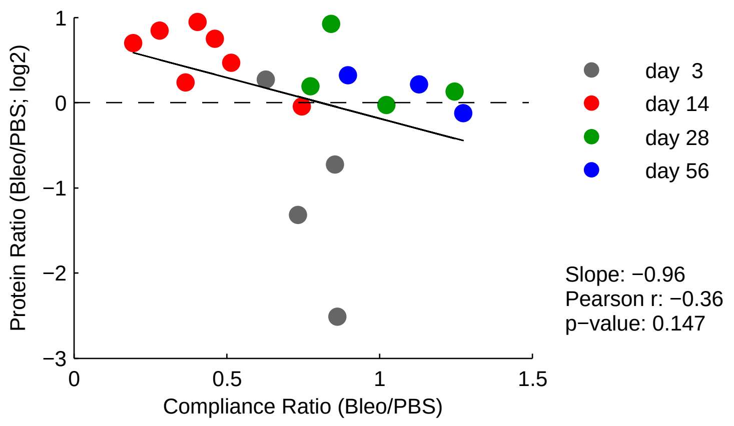

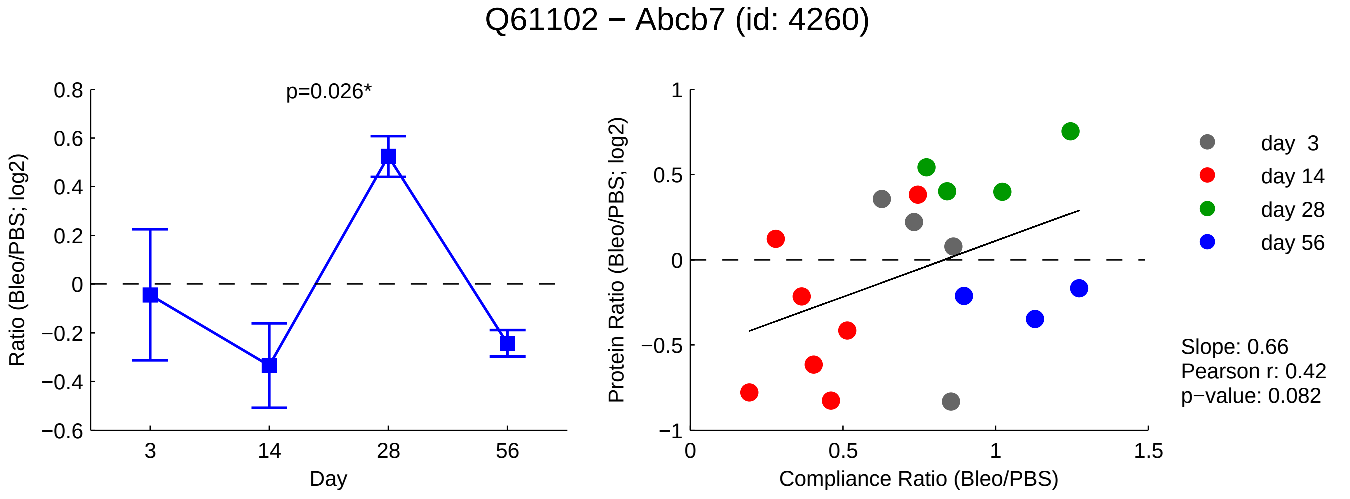
Q61102 − Abcb7 (id: 4260)
p=0.026*
Ratio (Bleo/PBS; log2)
Day
Protein Ratio (Bleo/PBS; log2)
Compliance Ratio (Bleo/PBS)
day 3
day 14
day 28
day 56
Slope: 0.66
Pearson r: 0.42
p−value: 0.082

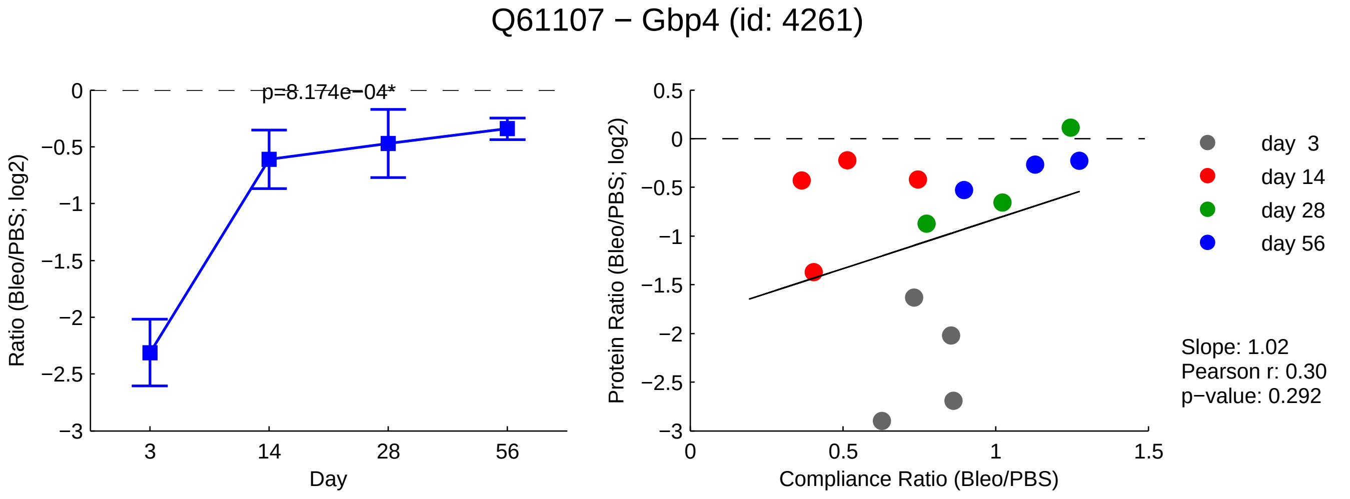
Q61107 − Gbp4 (id: 4261)
p=8.174e−04*
Ratio (Bleo/PBS; log2)
Day
Protein Ratio (Bleo/PBS; log2)
Compliance Ratio (Bleo/PBS)
day 3
day 14
day 28
day 56
Slope: 1.02
Pearson r: 0.30
p−value: 0.292

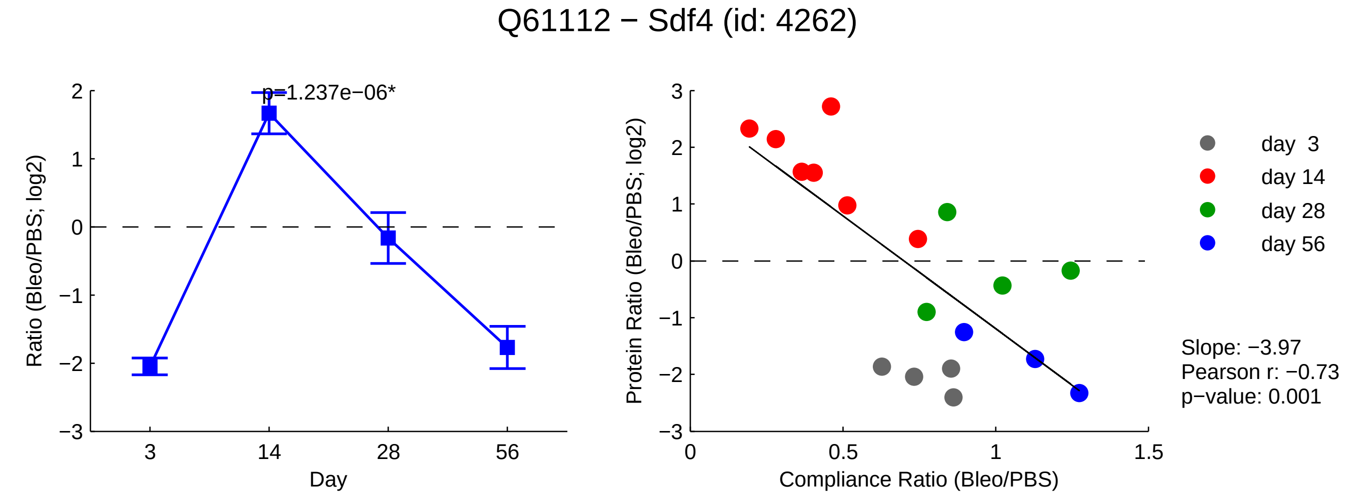
Q61112 − Sdf4 (id: 4262)
p=1.237e−06*
Ratio (Bleo/PBS; log2)
Day
Protein Ratio (Bleo/PBS; log2)
Compliance Ratio (Bleo/PBS)
day 3
day 14
day 28
day 56
Slope: −3.97
Pearson r: −0.73
p−value: 0.001

Q61129 − Cfi (id: 4264)

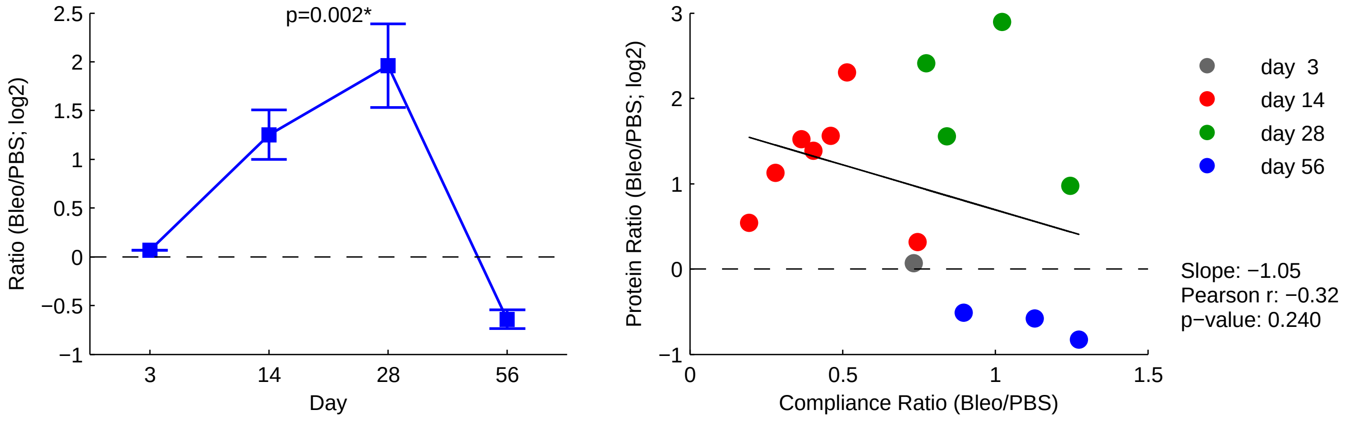

Q61133 − Gstt2 (id: 4265)

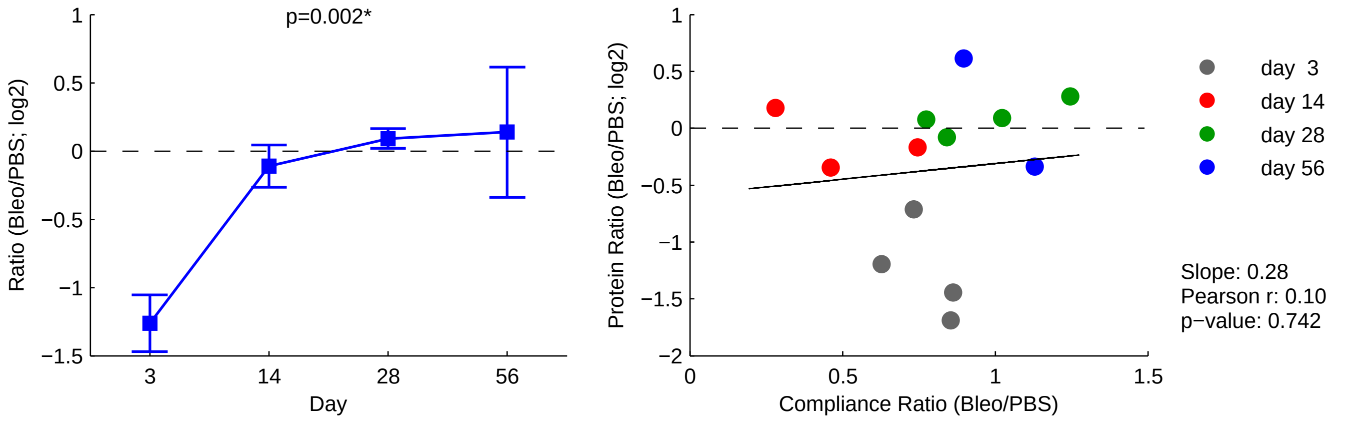

Q61140−2 − Bcar1 (id: 4267)

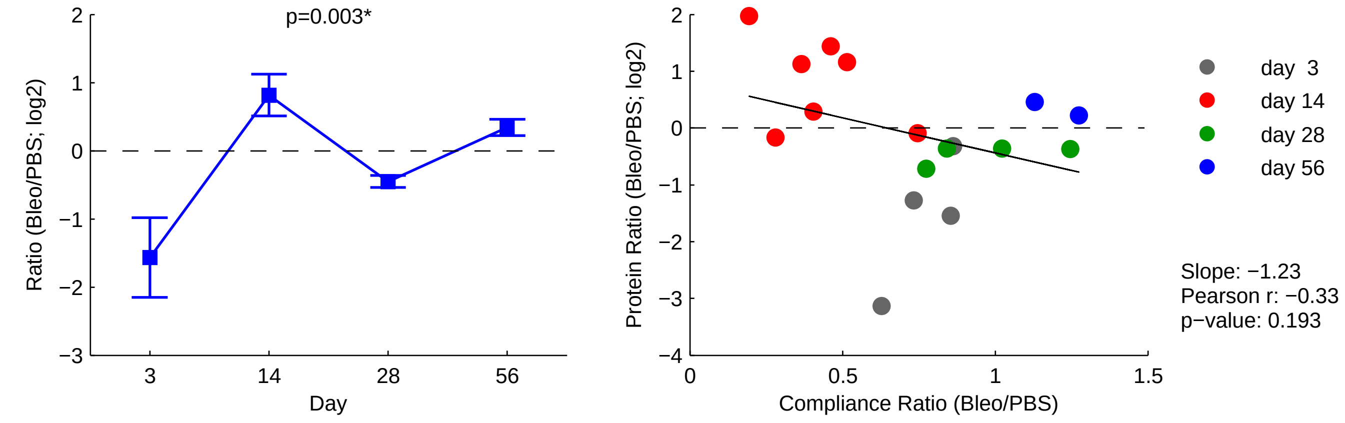

Q61143 − Trpc6 (id: 4269)

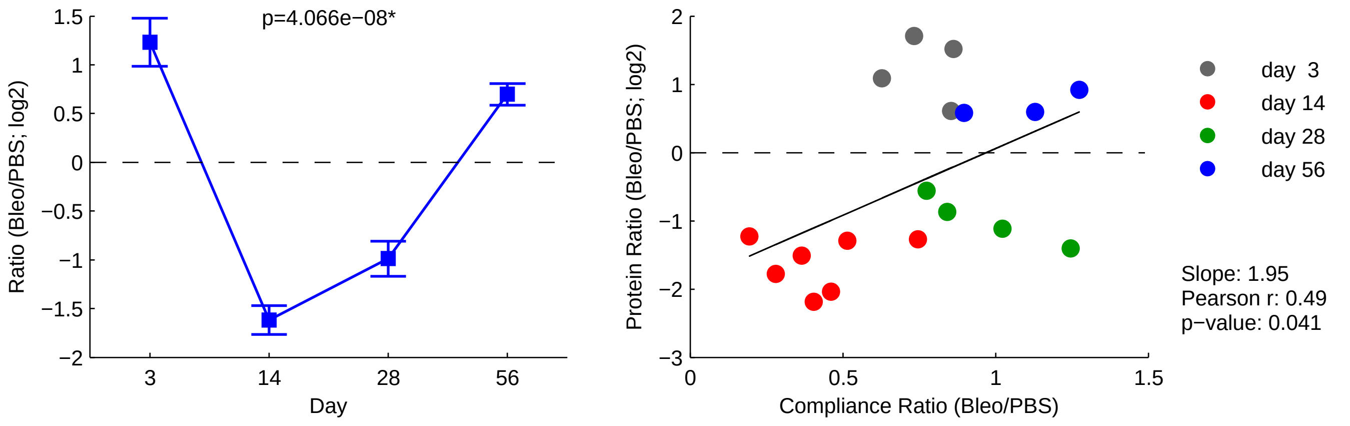

Q61151 − Ppp2r5e (id: 4270)

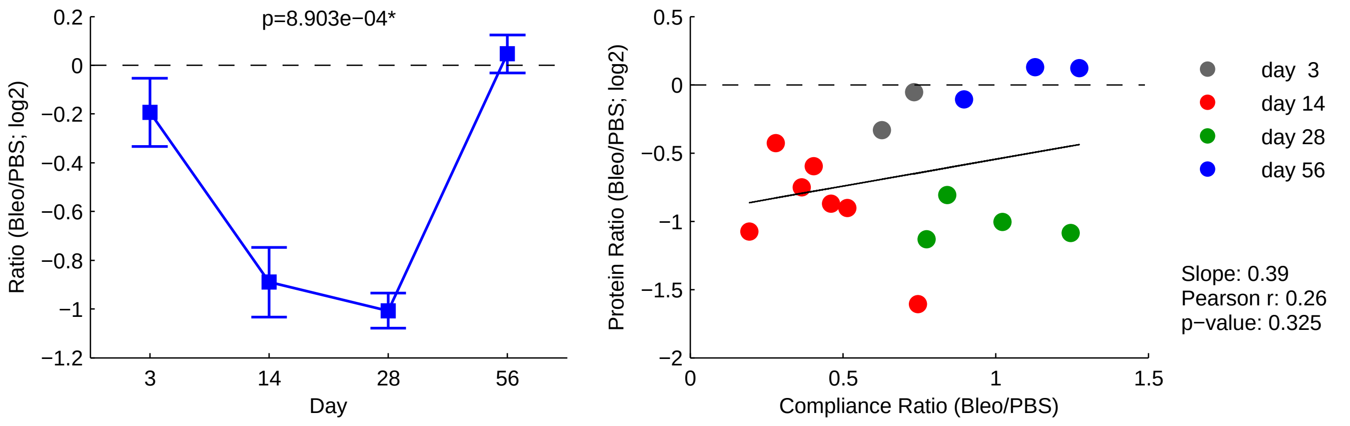

Q61161 − Map4k2 (id: 4273)

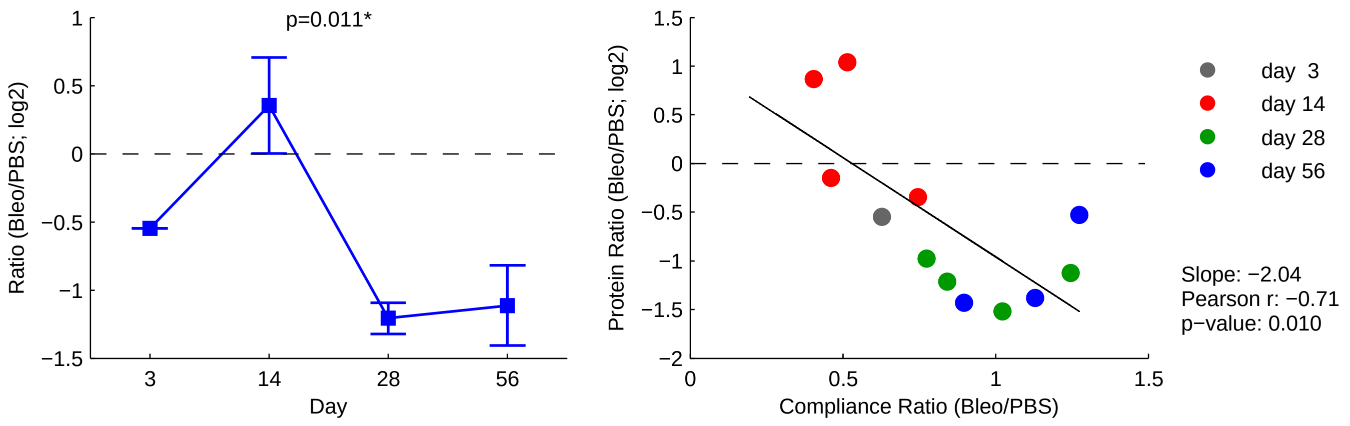

Q61164 − Ctcf (id: 4274)

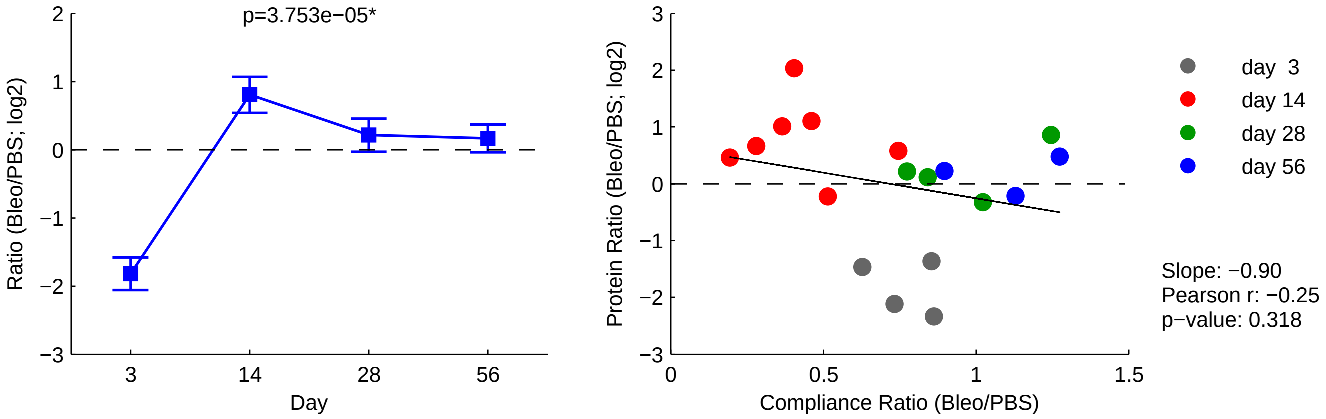

Q61165 − Slc9a1 (id: 4275)

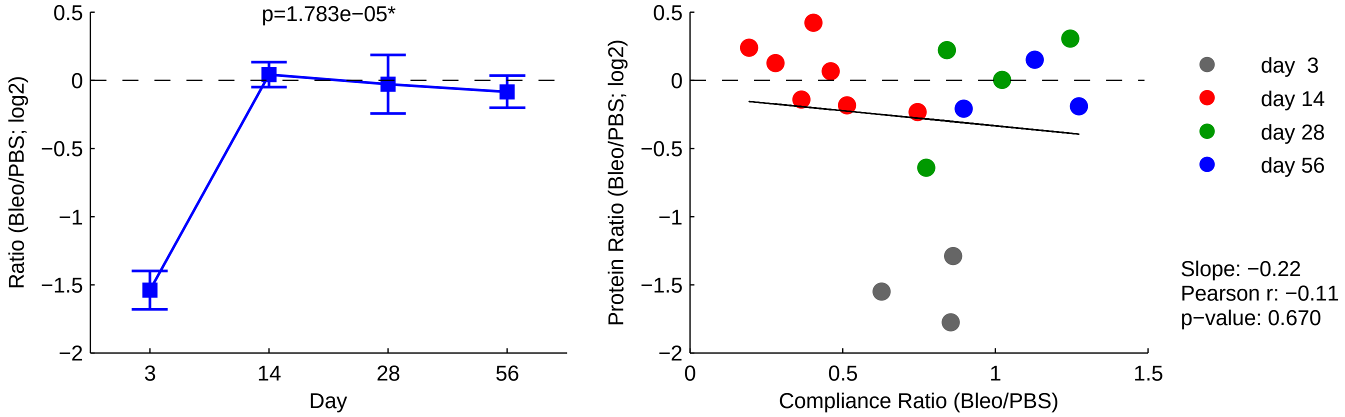

Q61166 − Mapre1 (id: 4276)

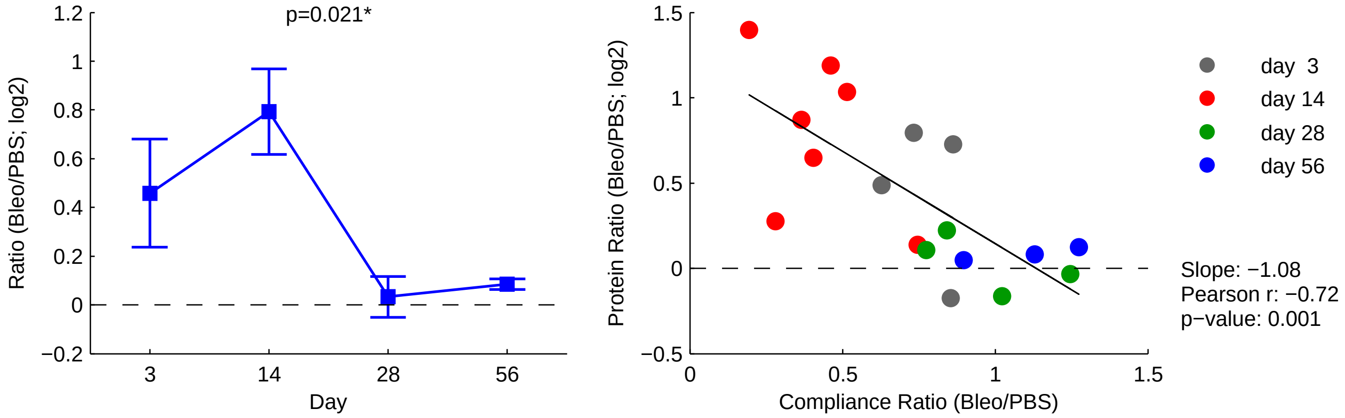

Q61176 − Arg1 (id: 4279)

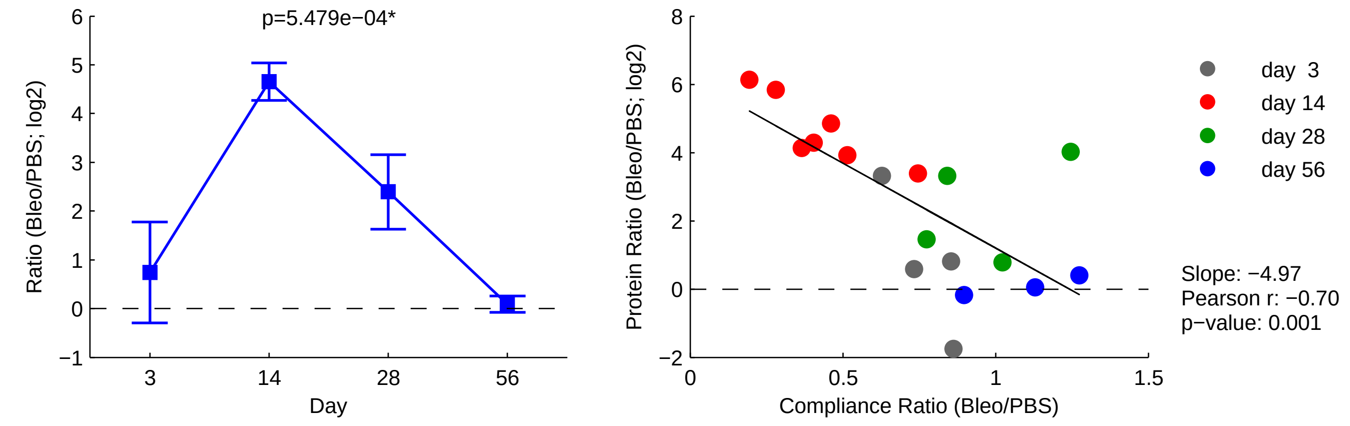

Q61187 − Tsg101 (id: 4280)

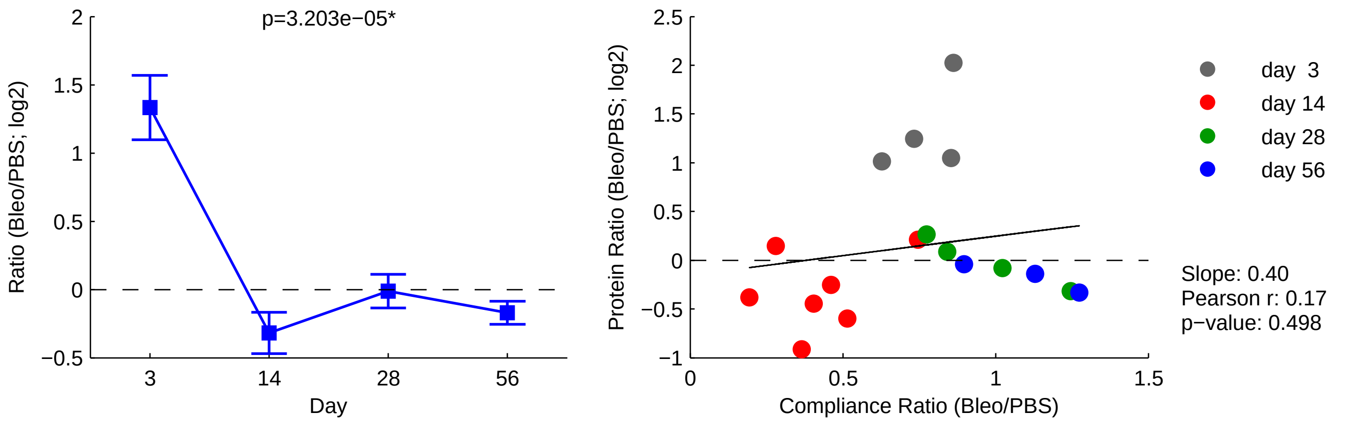

Q61191 − Hcfc1 (id: 4281)

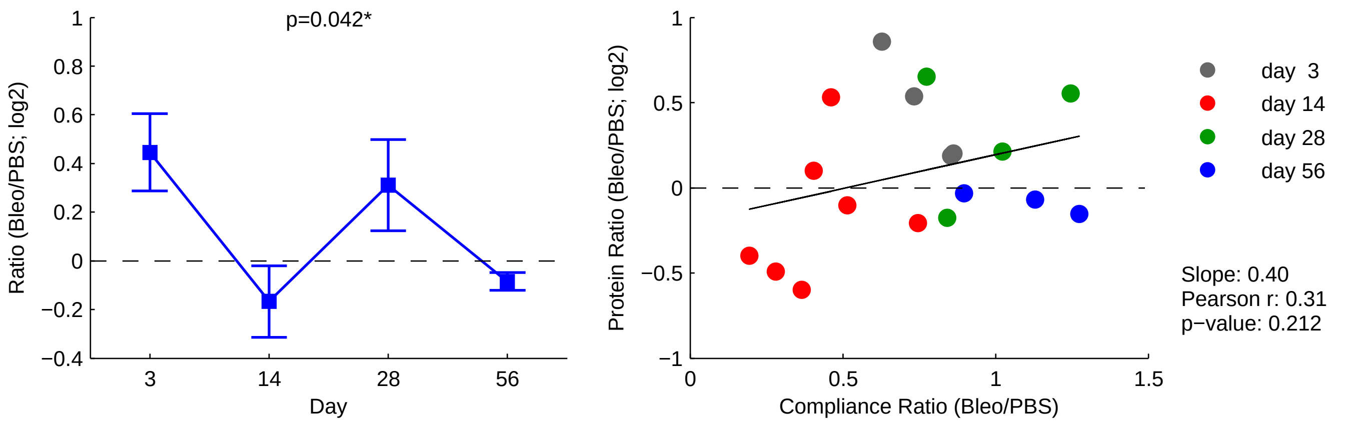

Q61206 − Pafah1b2 (id: 4283)

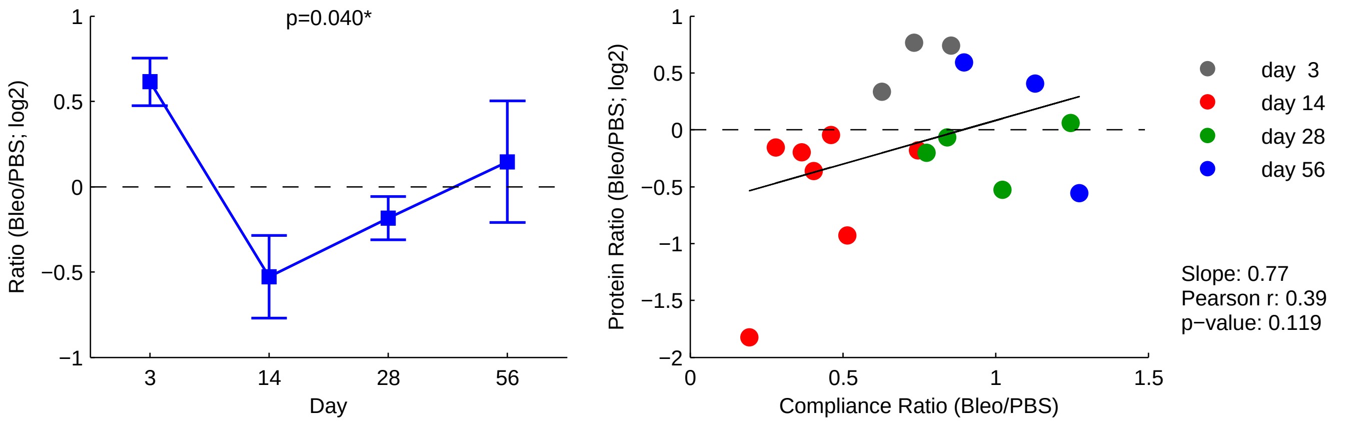

Q61211 − Eif2d (id: 4284)

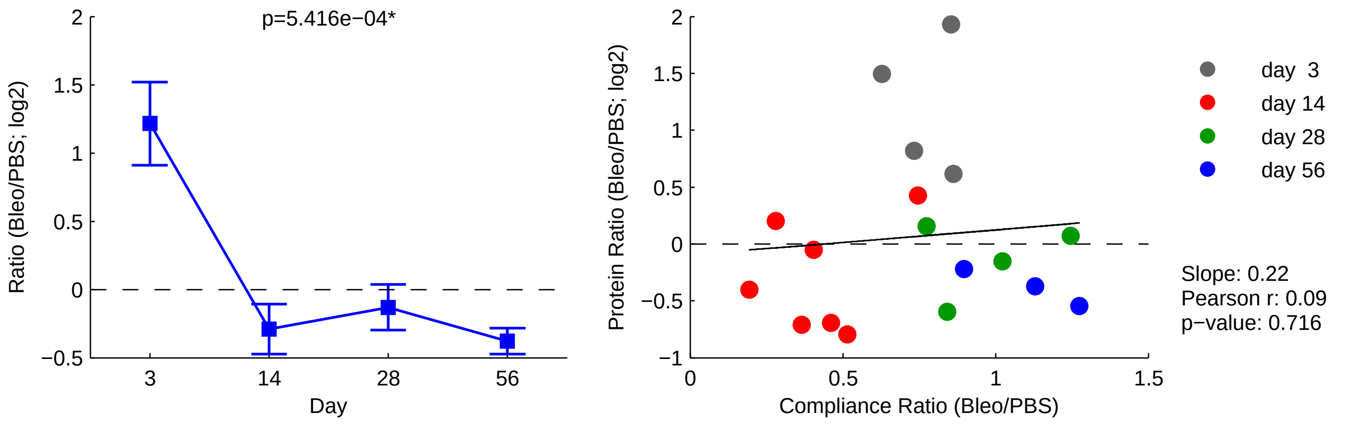

Q61233 − Lcp1 (id: 4286)

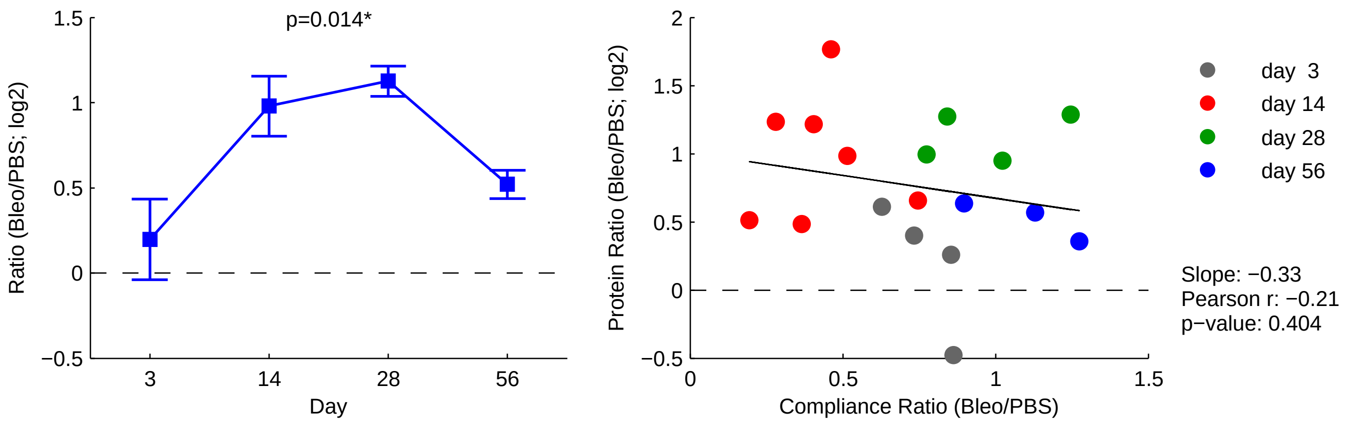

Q61235 − Sntb2 (id: 4287)

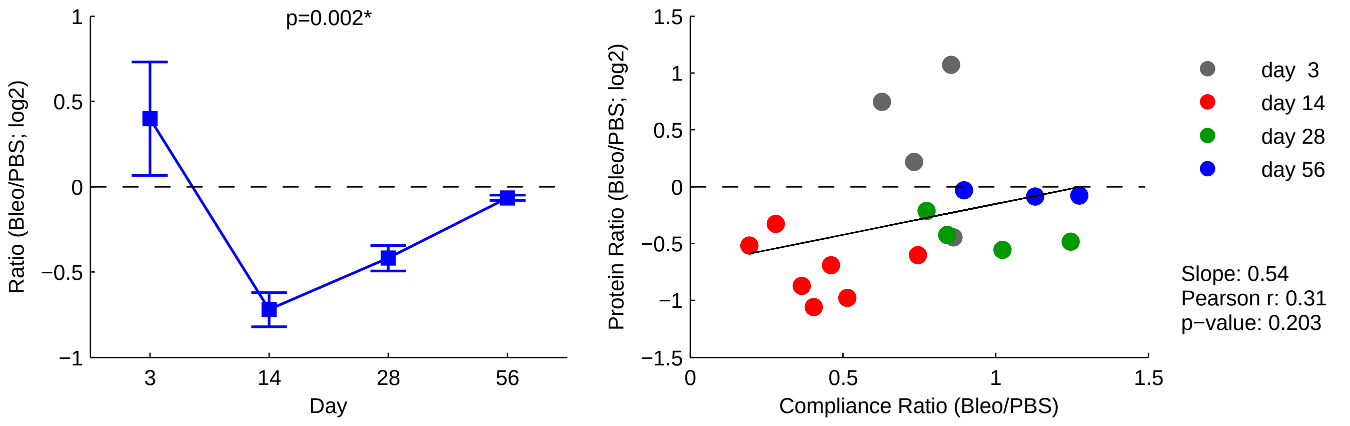

Q61245−2 − Col11a1 (id: 4289)

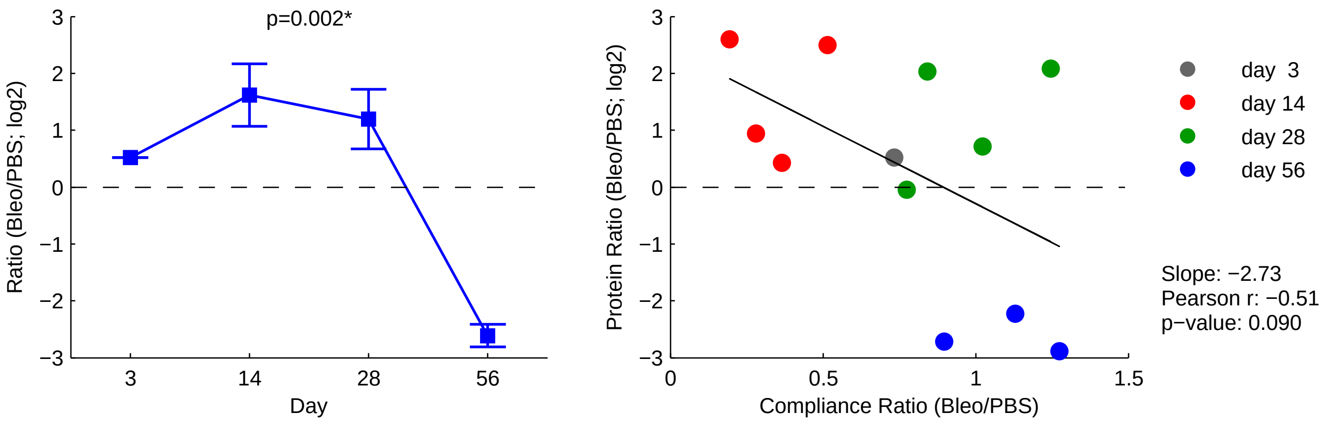

Q61247 − Serpinf2 (id: 4290)

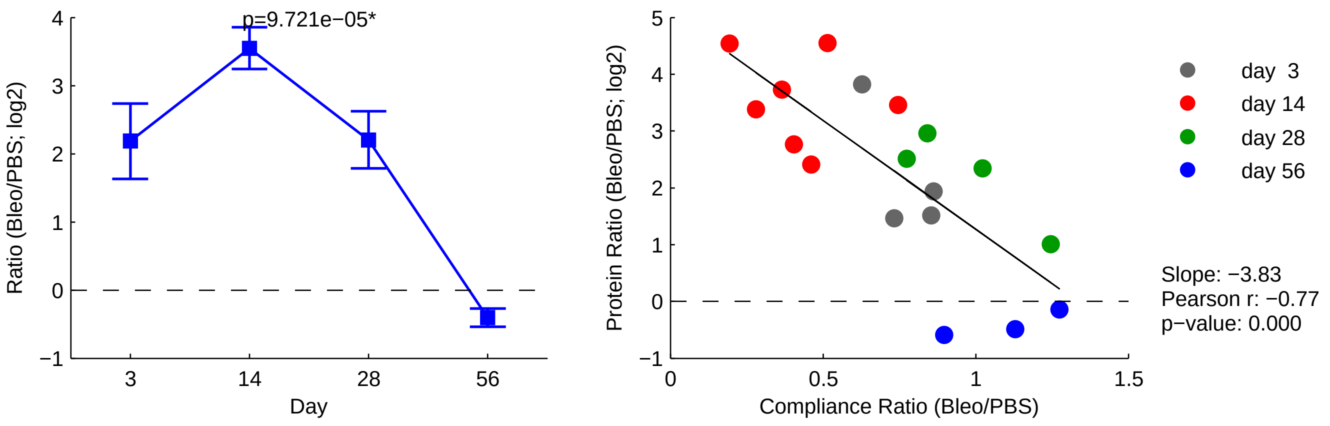

Q61249 − Igbp1 (id: 4291)

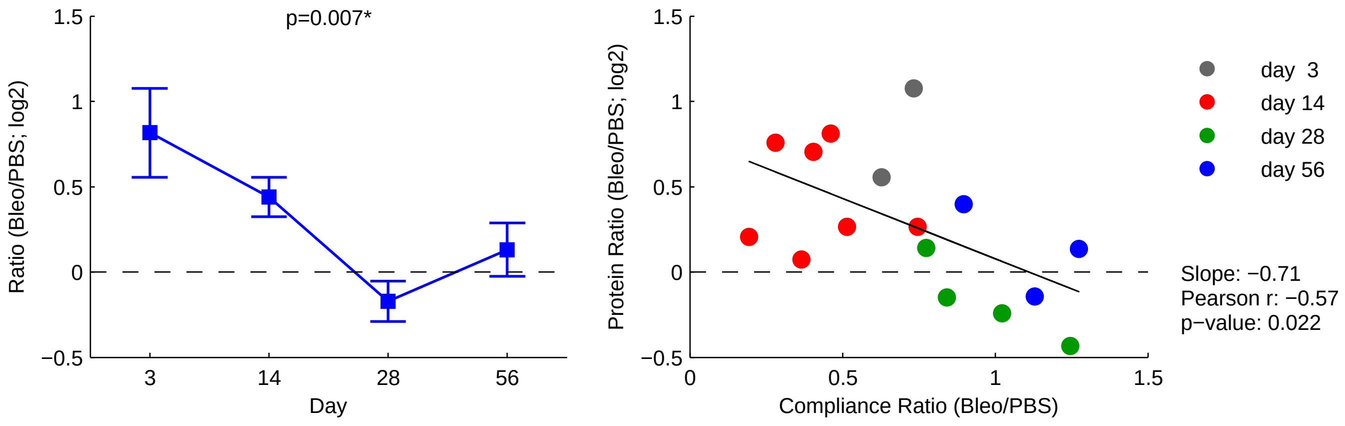

Q61263 − Soat1 (id: 4292)

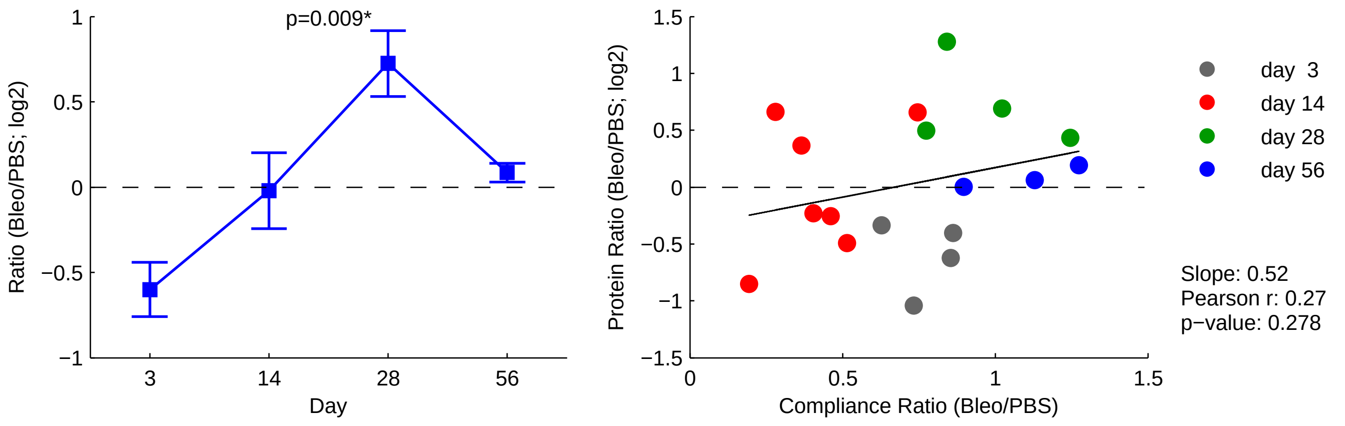

Q61288 − Acvrl1 (id: 4298)

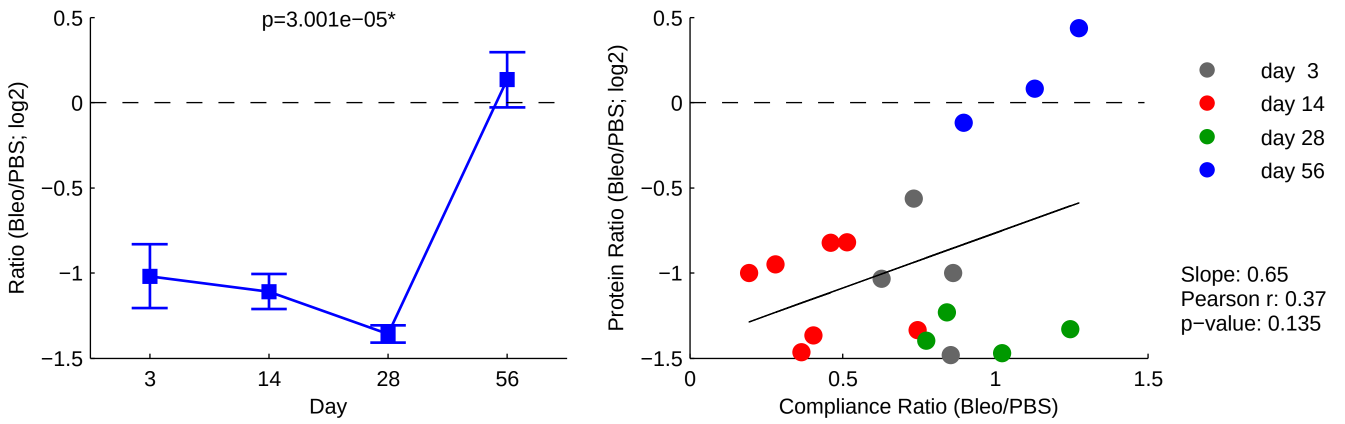

Q61292 − Lamb2 (id: 4299)

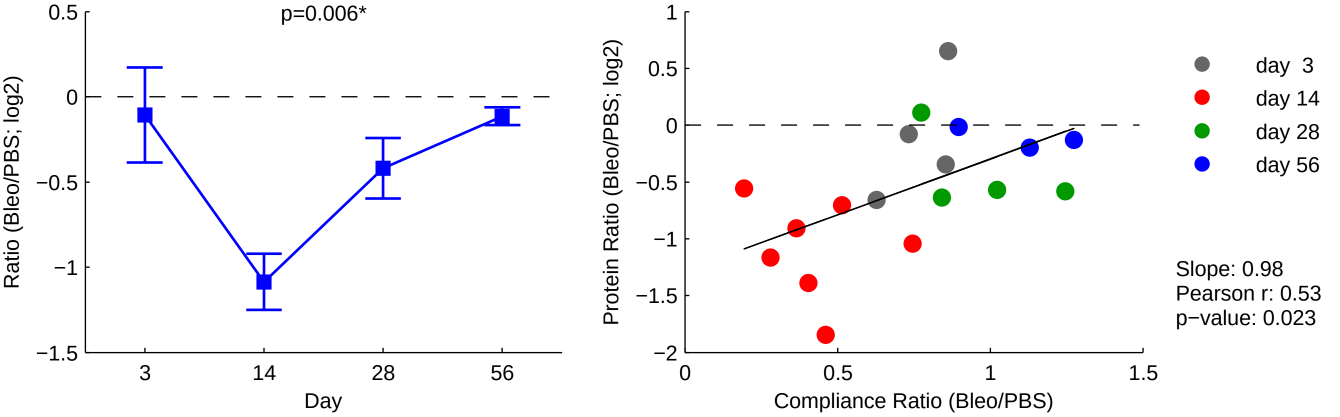

Q61334 − Bcap29 (id: 4300)

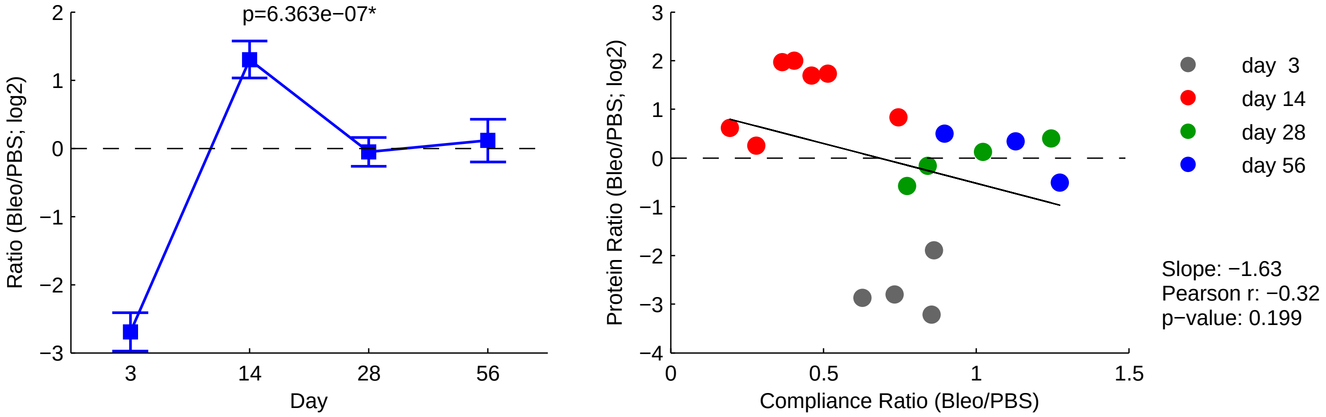

Q61335 − Bcap31 (id: 4301)

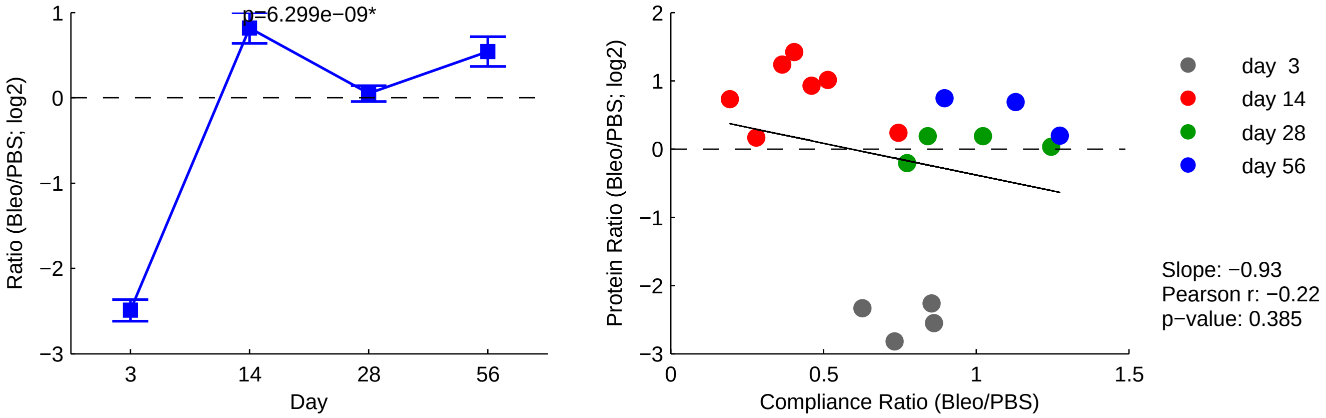

Q61391 − Mme (id: 4304)

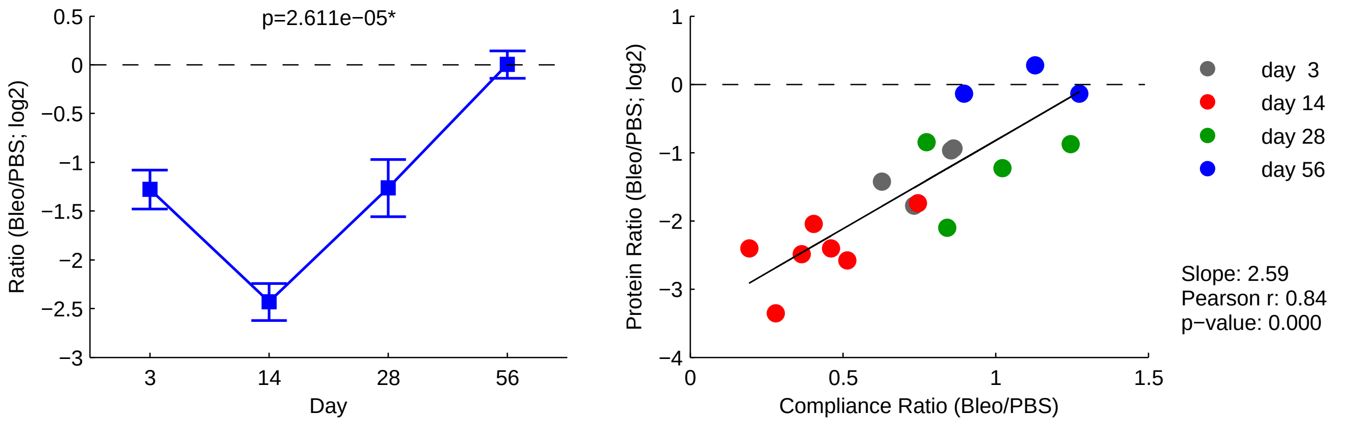

Q61398 − Pcolce (id: 4305)

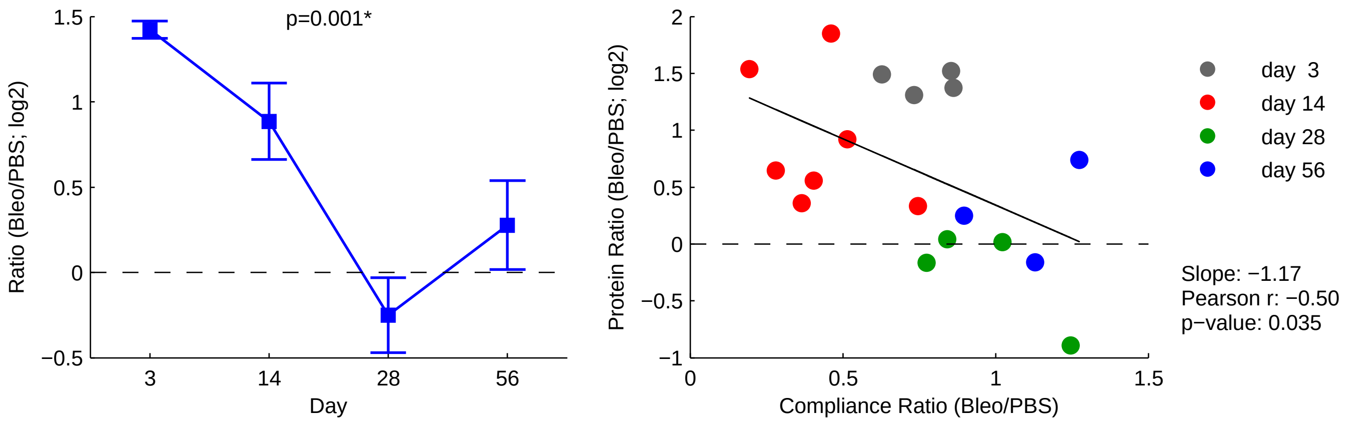

Q61418 − Clcn4 (id: 4307)

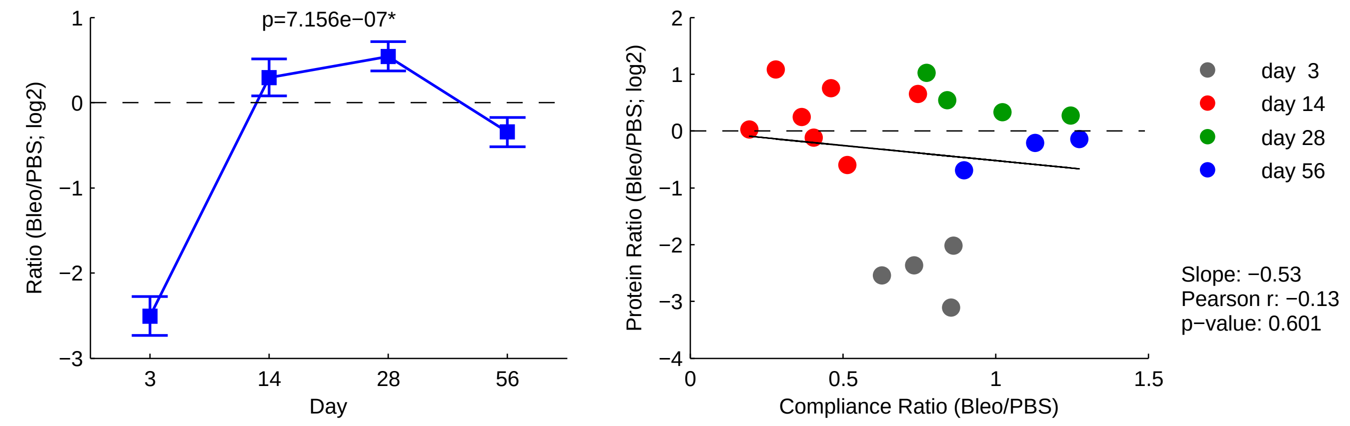

Q61425 − Hadh (id: 4308)

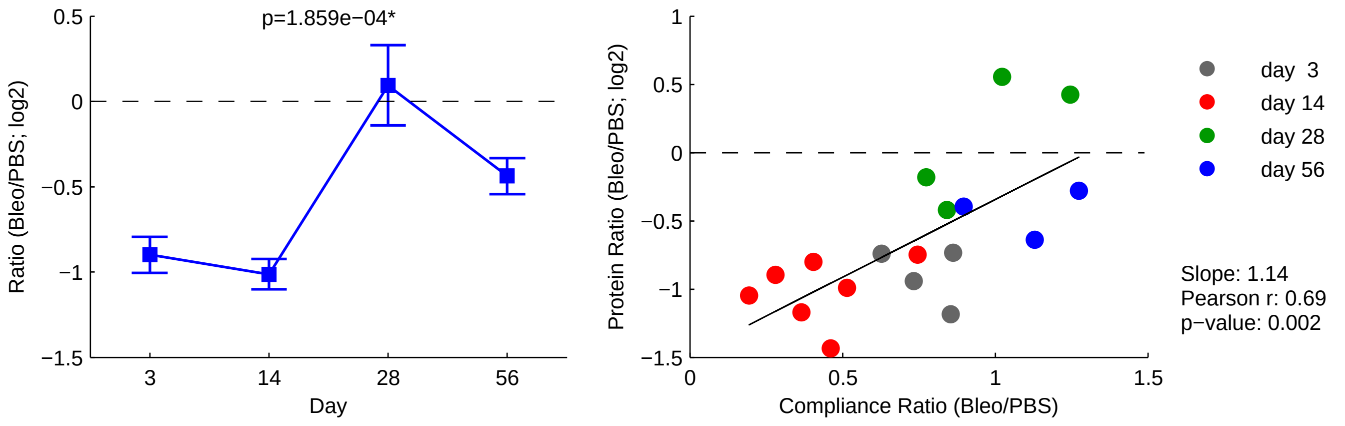

Q61462−2 − Cyba (id: 4309)

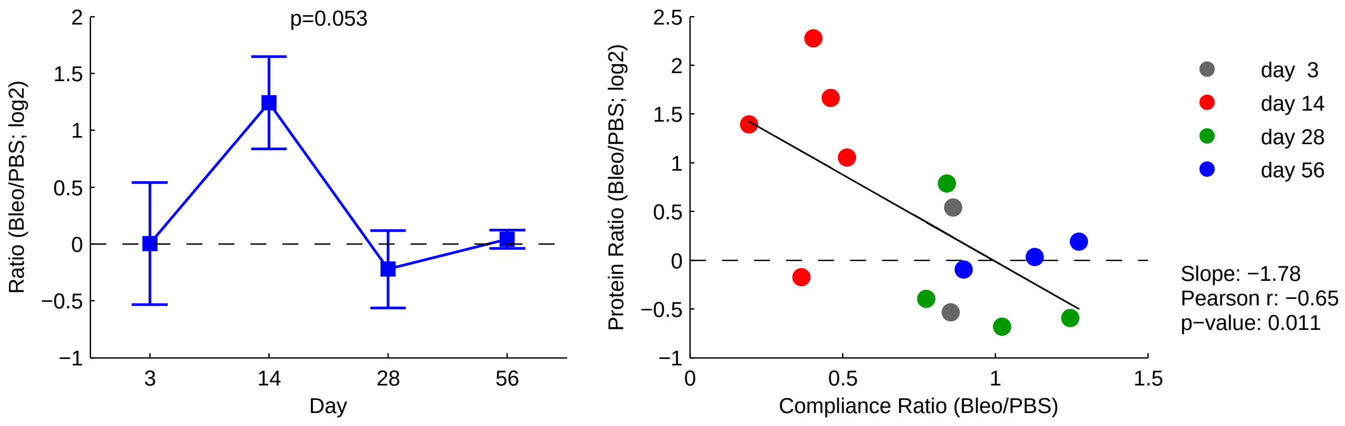

Q61469 − Ppap2a (id: 4312)

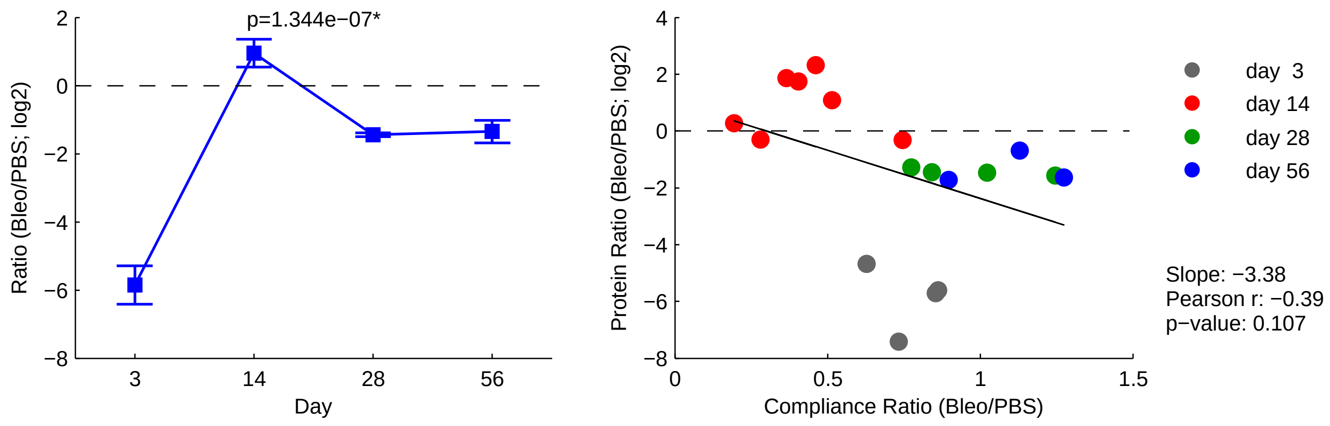

Q61503 − Nt5e (id: 4315)

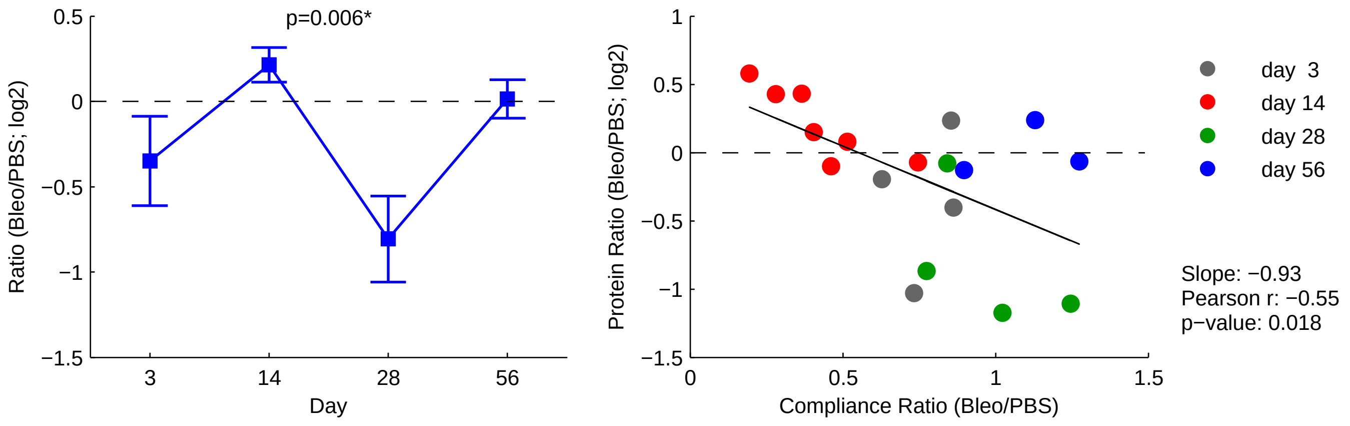

Q61508 − Ecm1 (id: 4316)

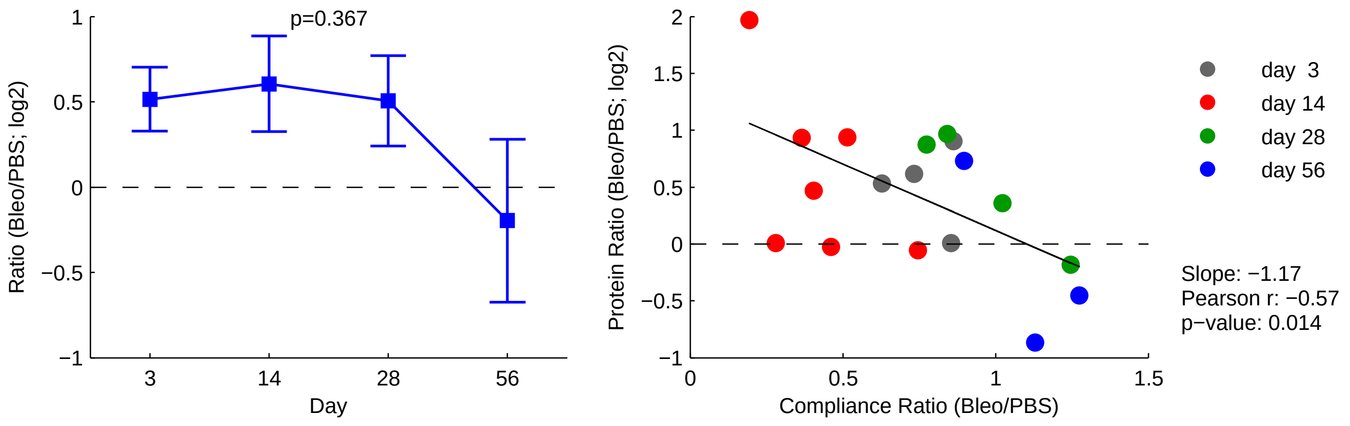

Q61510 − Trim25 (id: 4317)

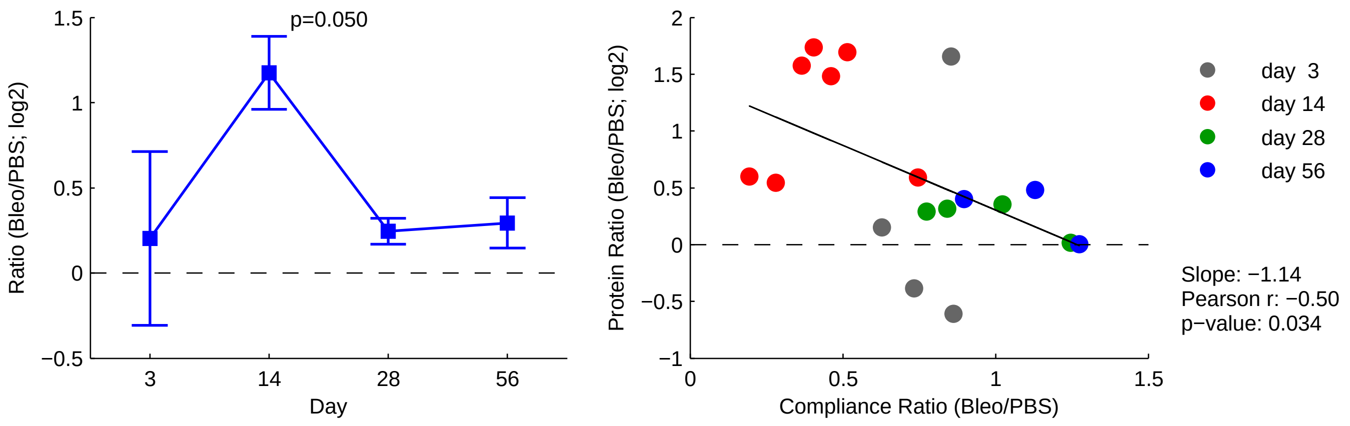

Q61542 − Stard3 (id: 4318)

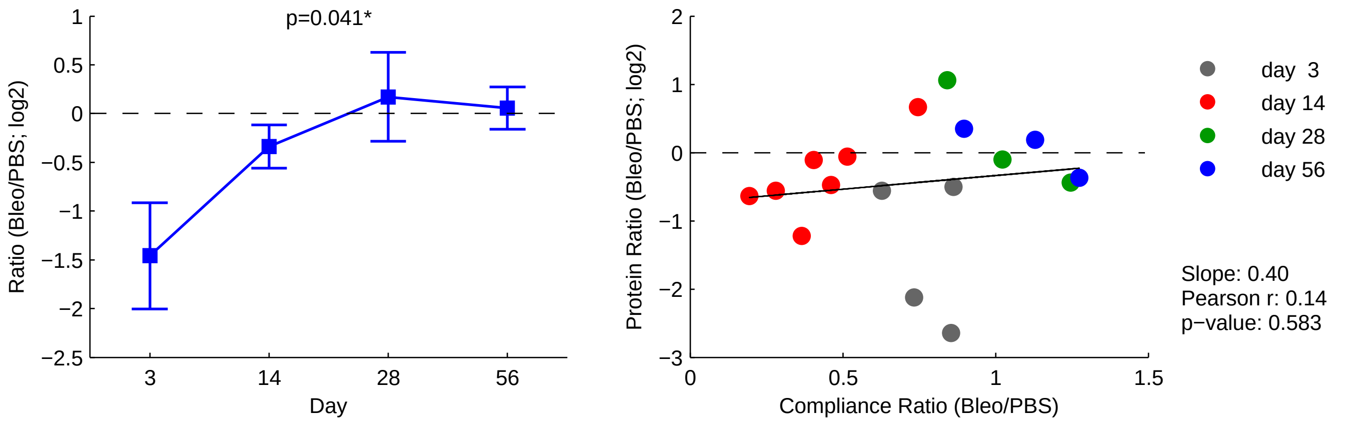

Q61550 − Rad21 (id: 4320)

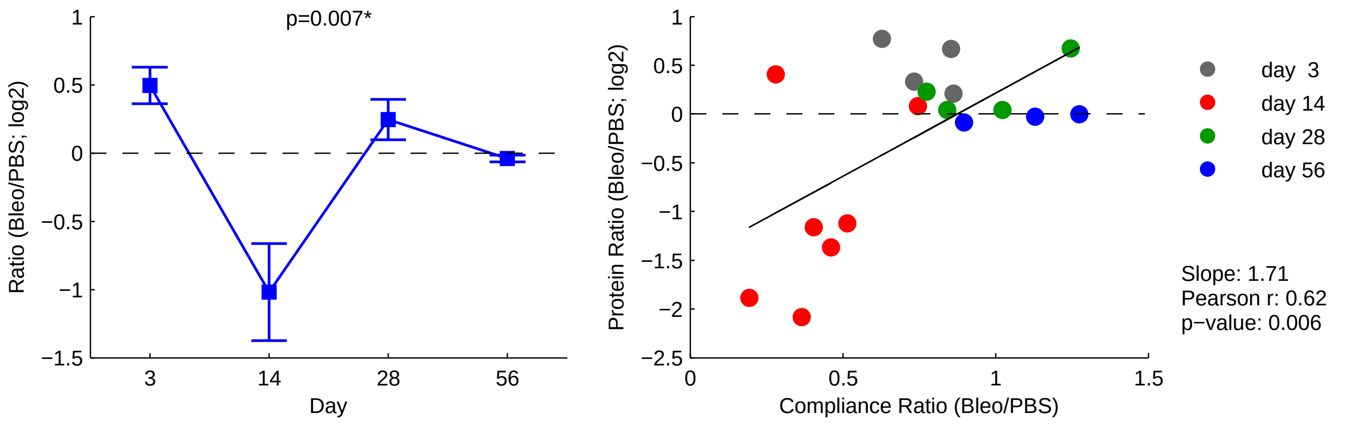

Q61559 − Fcgrt (id: 4323)

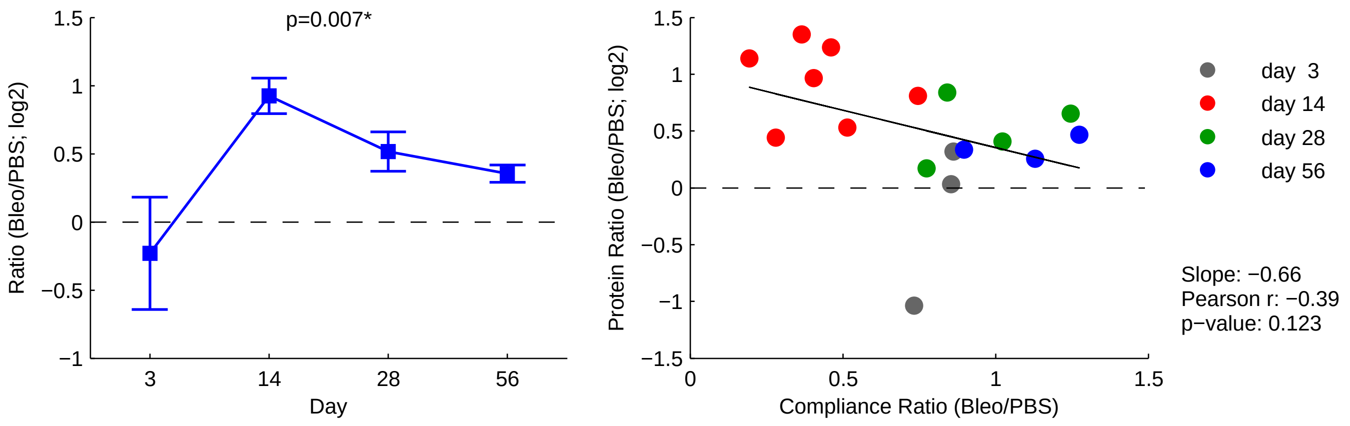

Q61576 − Fkbp10 (id: 4324)

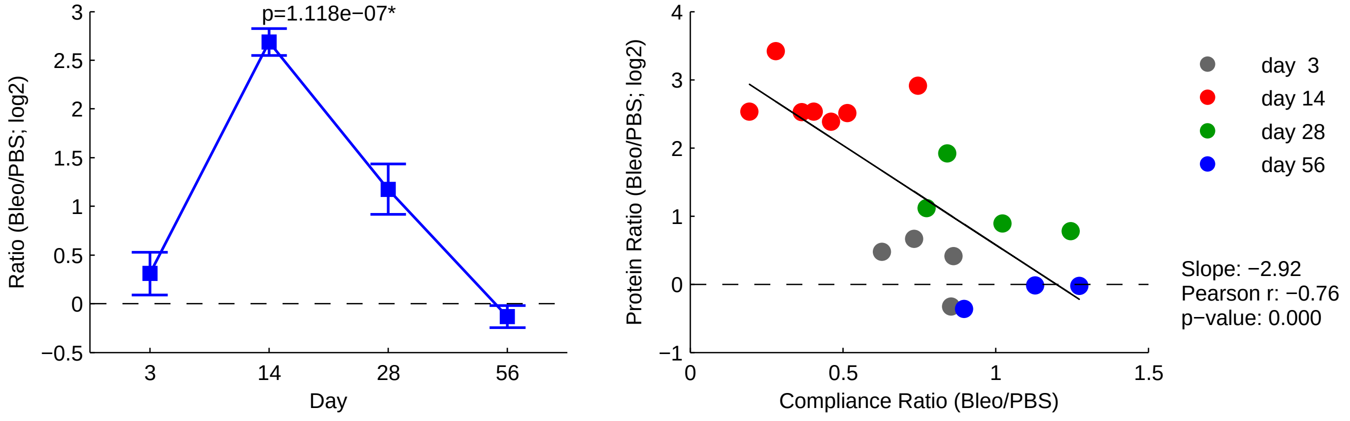

Q61578 − Fdxr (id: 4325)

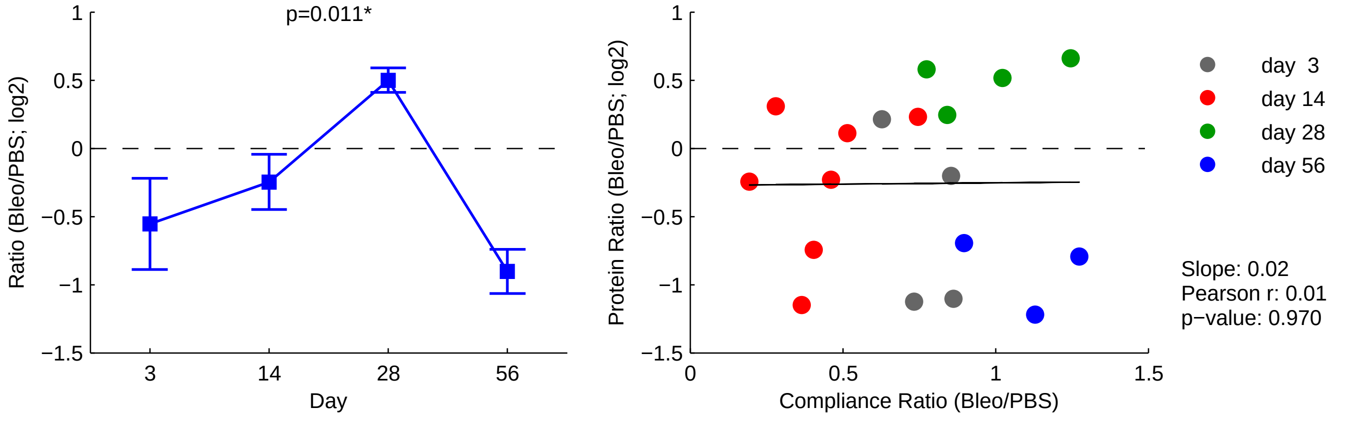

Q61584−4 − Fxr1 (id: 4326)

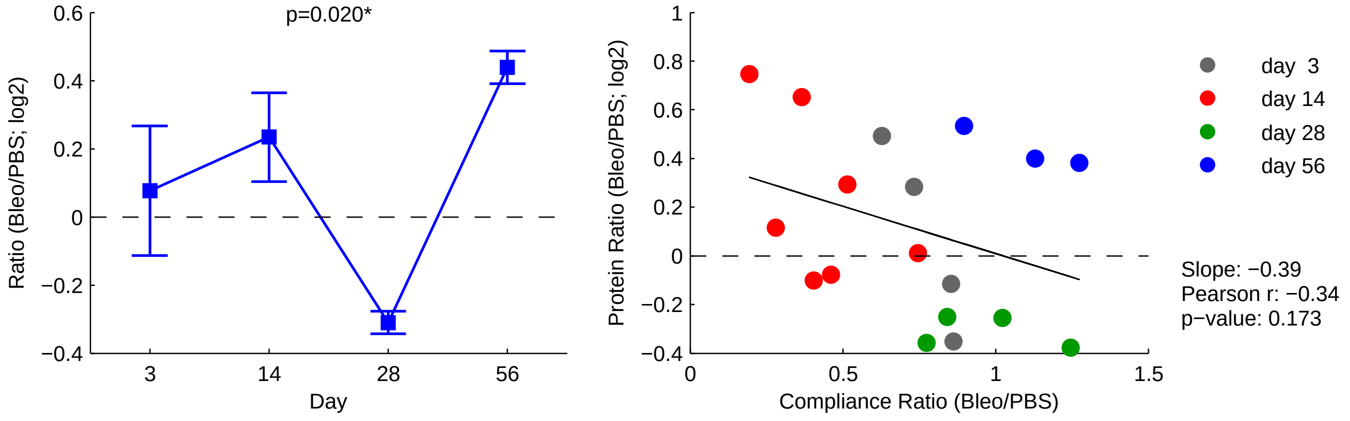

Q61586 − Gpam (id: 4328)

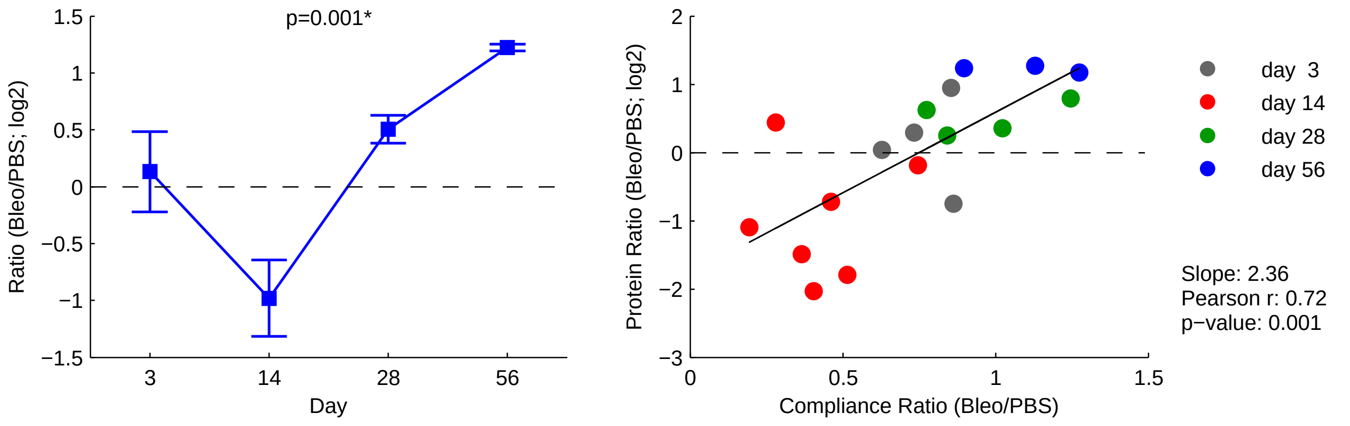

Q61598 − Gdi2 (id: 4332)

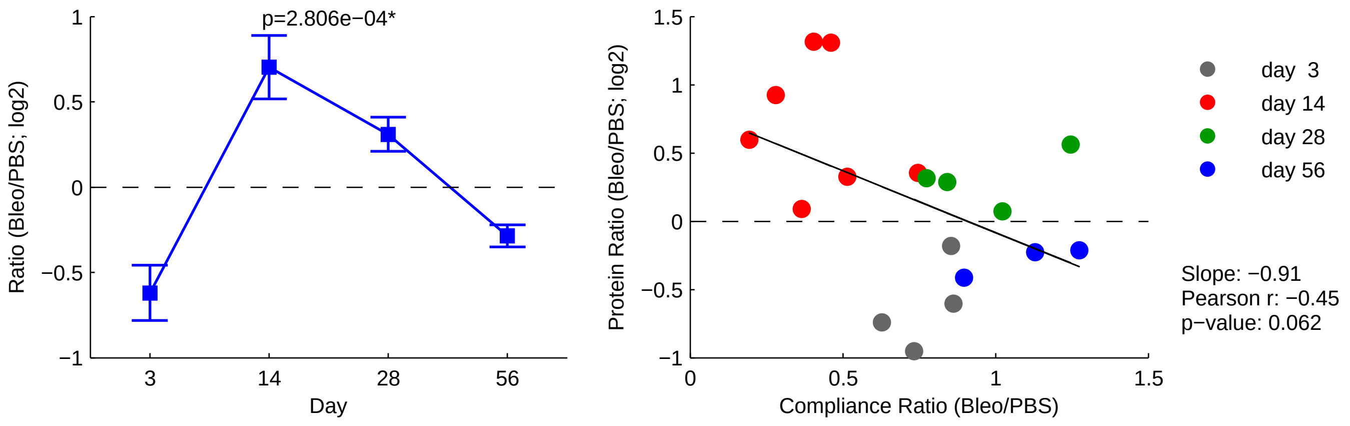

Q61635 − Ifi47 (id: 4336)

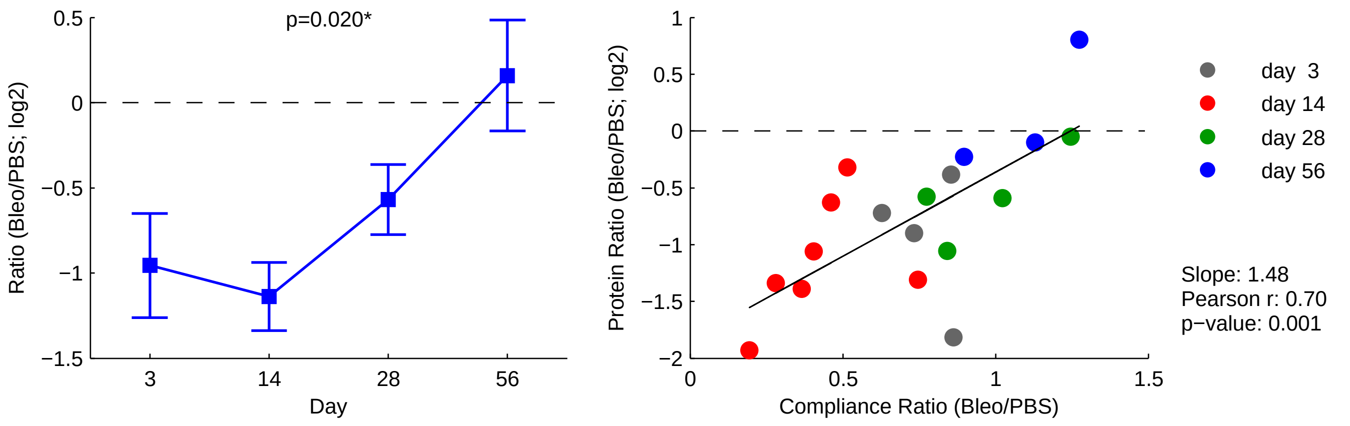

Q61646 − Hp (id: 4337)

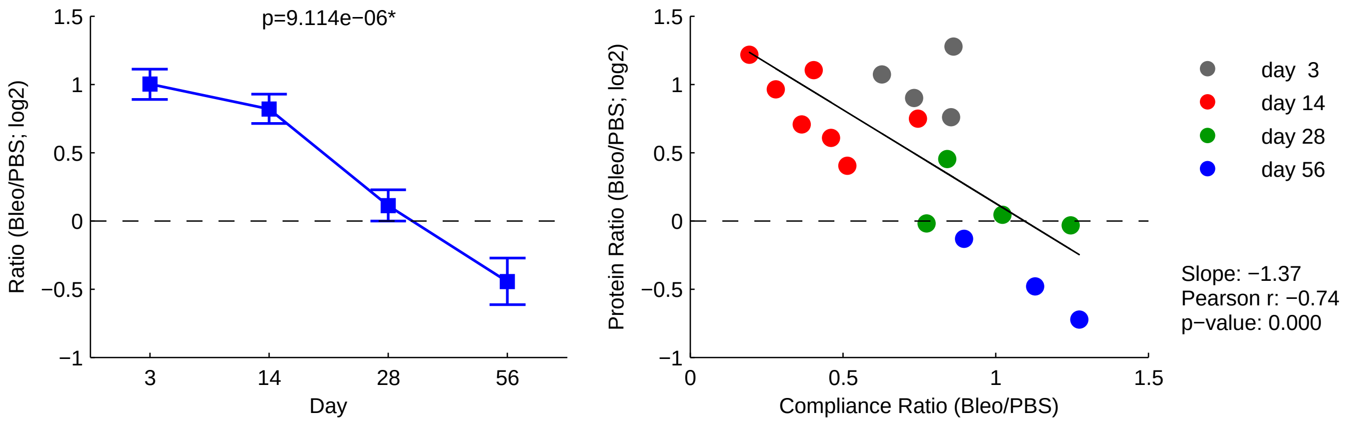

Q61655 − Ddx19a (id: 4338)

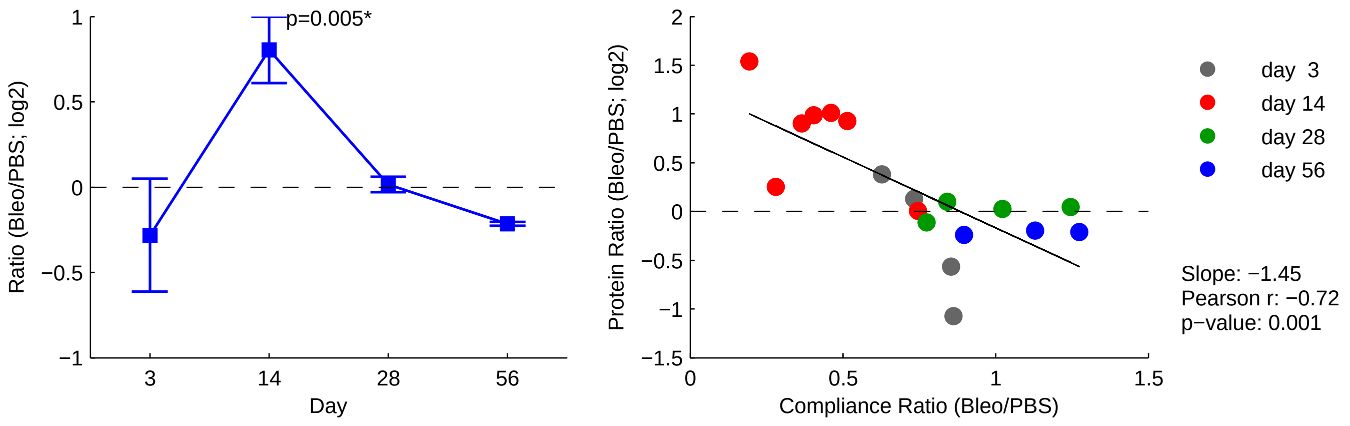

Q61656 − Ddx5 (id: 4339)

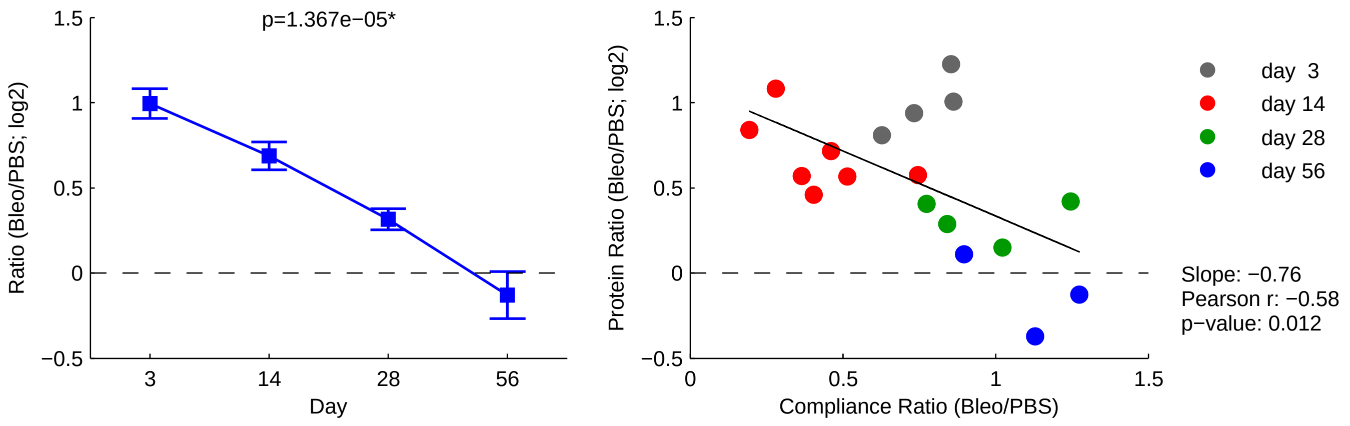

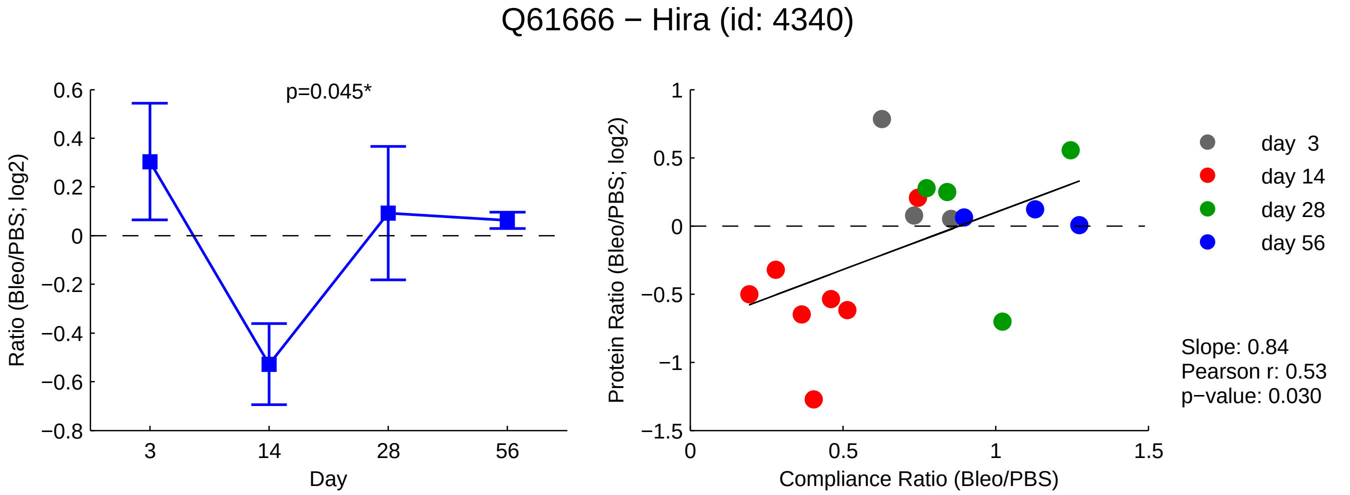
Q61666 − Hira (id: 4340)
p=0.045*
Ratio (Bleo/PBS; log2)
Day
Protein Ratio (Bleo/PBS; log2)
Compliance Ratio (Bleo/PBS)
day 3
day 14
day 28
day 56
Slope: 0.84
Pearson r: 0.53
p−value: 0.030

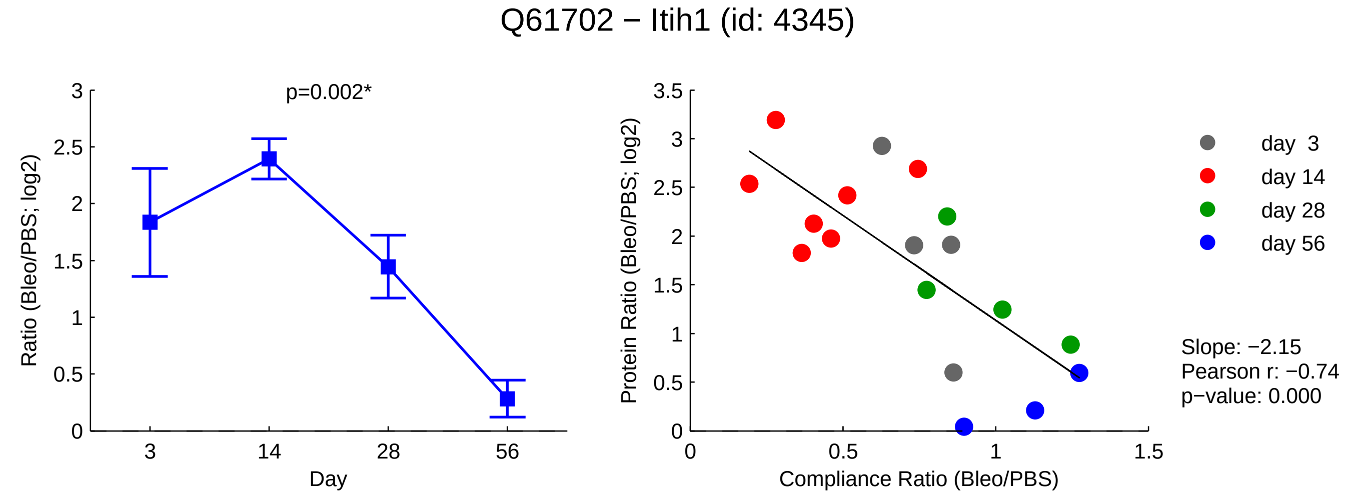
Q61702 − Itih1 (id: 4345)
p=0.002*
Ratio (Bleo/PBS; log2)
Day
Protein Ratio (Bleo/PBS; log2)
Compliance Ratio (Bleo/PBS)
day 3
day 14
day 28
day 56
Slope: −2.15
Pearson r: −0.74
p−value: 0.000

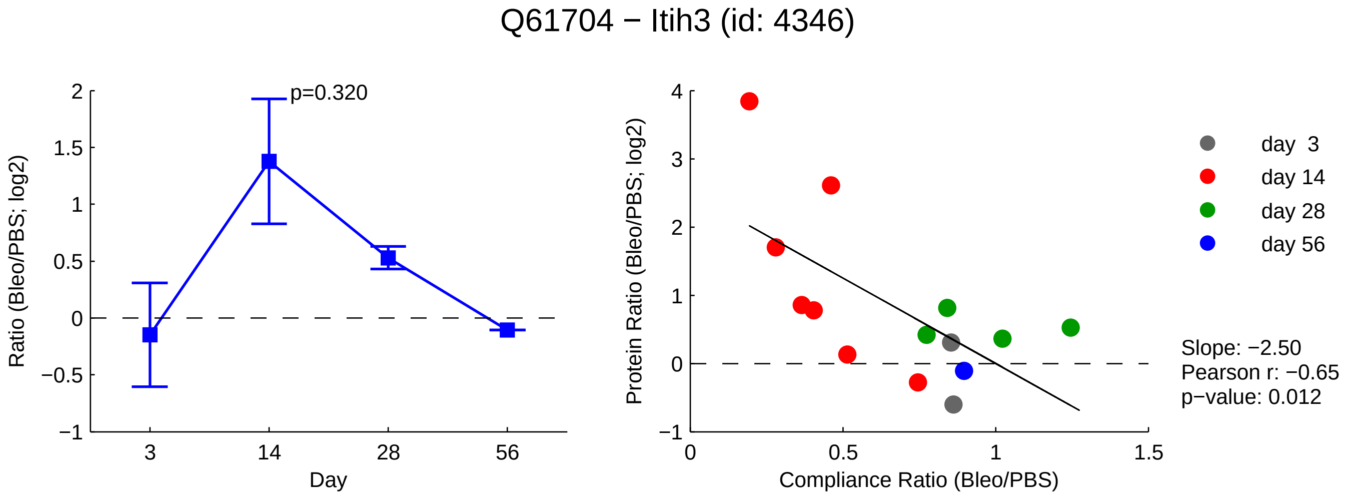
Q61704 − Itih3 (id: 4346)
p=0.320
Ratio (Bleo/PBS; log2)
Day
Protein Ratio (Bleo/PBS; log2)
Compliance Ratio (Bleo/PBS)
day 3
day 14
day 28
day 56
Slope: −2.50
Pearson r: −0.65
p−value: 0.012

Q61712 − Dnajc1 (id: 4347)

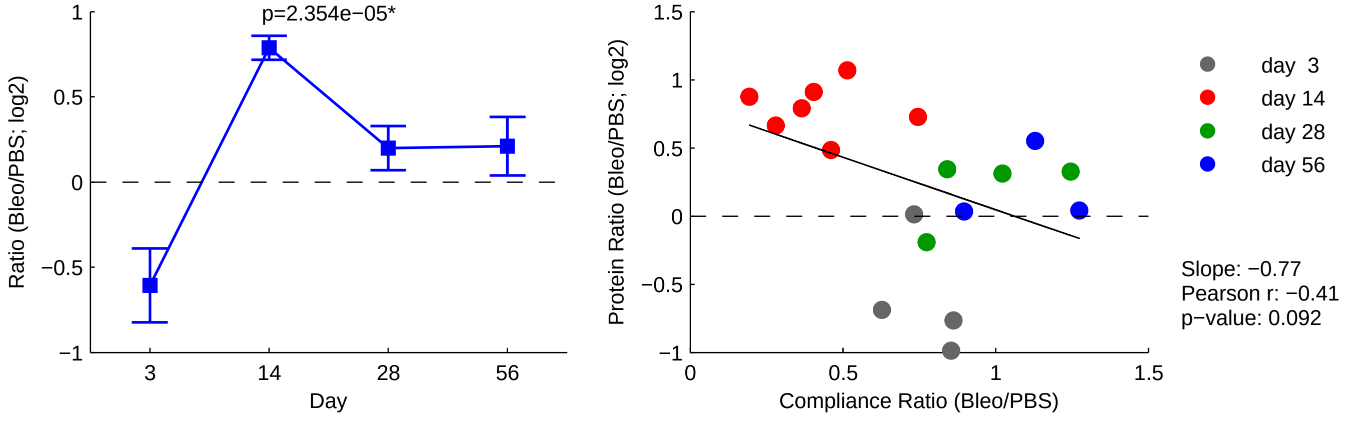

Q61733 − Mrps31 (id: 4349)

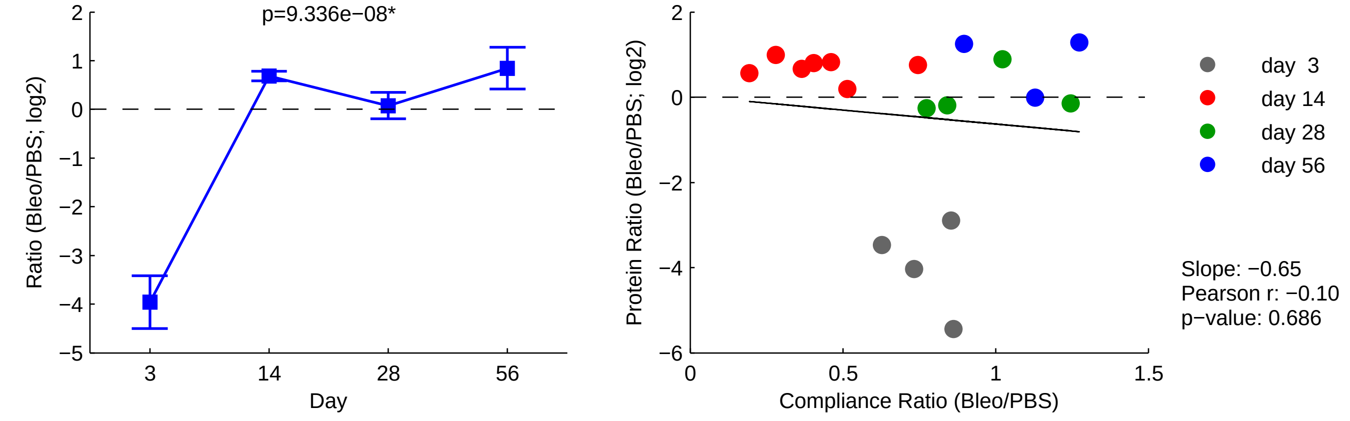

Q61735 − Cd47 (id: 4350)

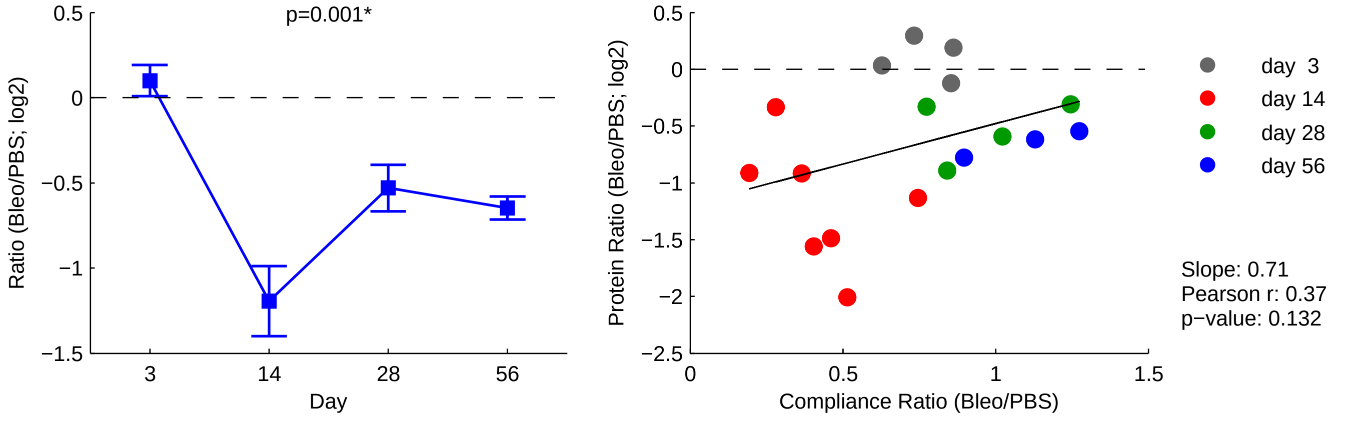

Q61739 − Itga6 (id: 4352)

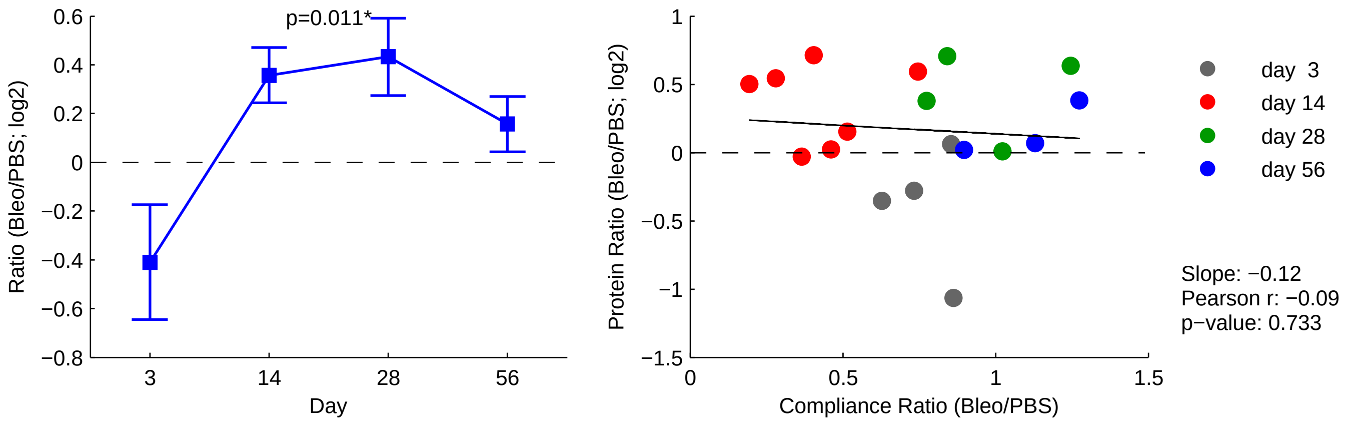

Q61753 − Phgdh (id: 4353)

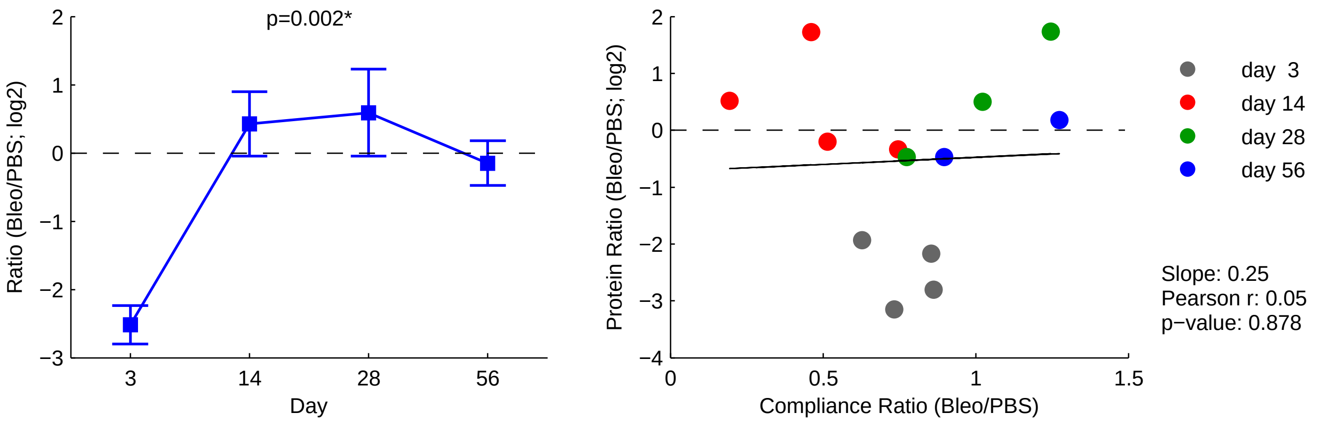

Q61768 − Kif5b (id: 4354)

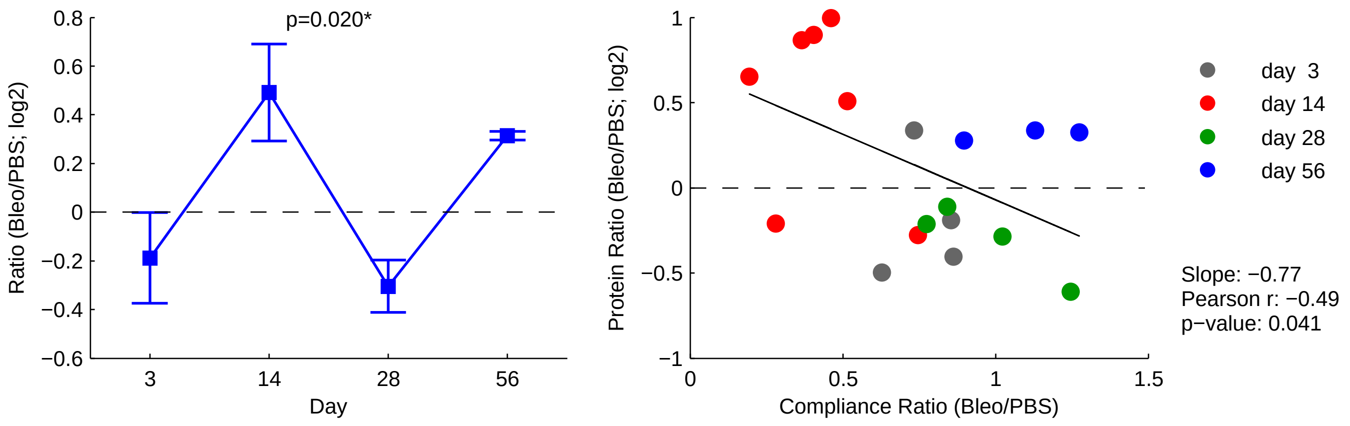

Q61792 − Lasp1 (id: 4358)

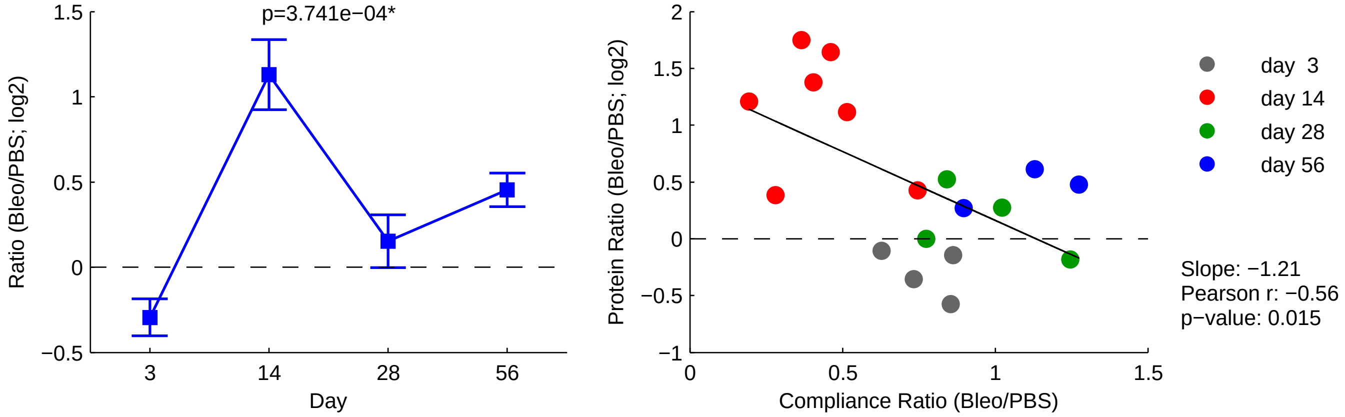

Q61818−2 − Rai1 (id: 4360)

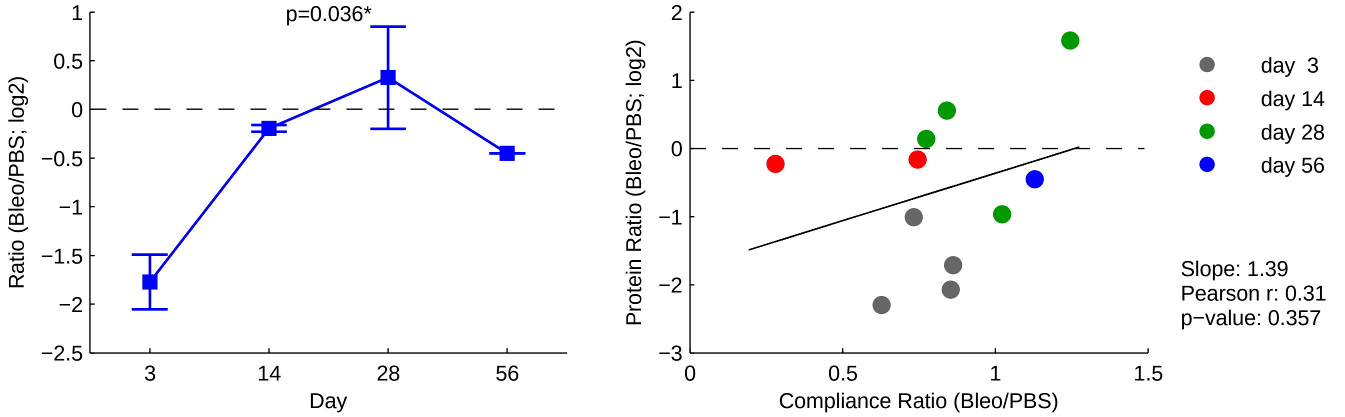

Q61827 − Mafk (id: 4362)

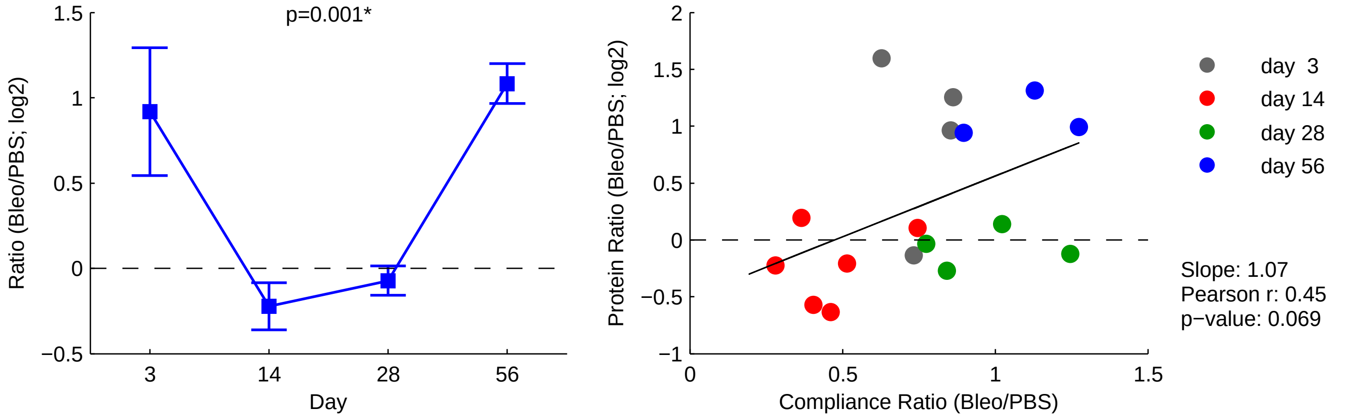

Q61830 − Mrc1 (id: 4363)

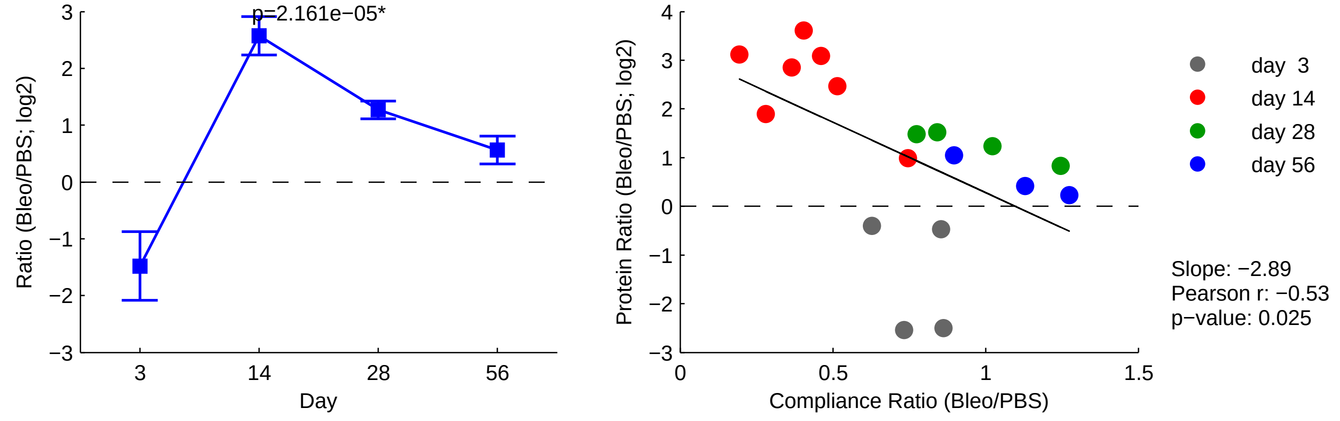

Q61879 − Myh10 (id: 4366)

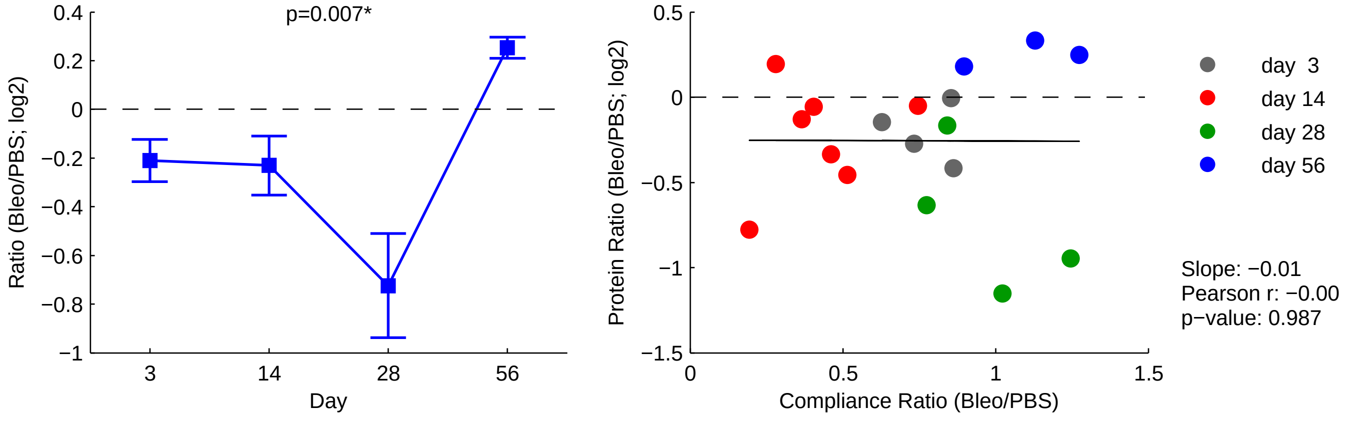

Q61937 − Npm1 (id: 4368)

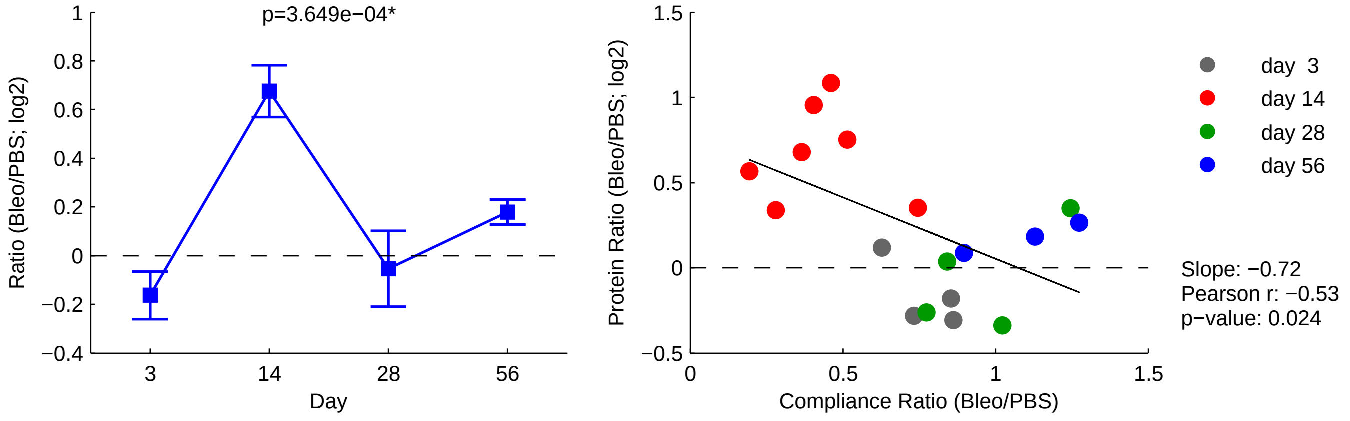

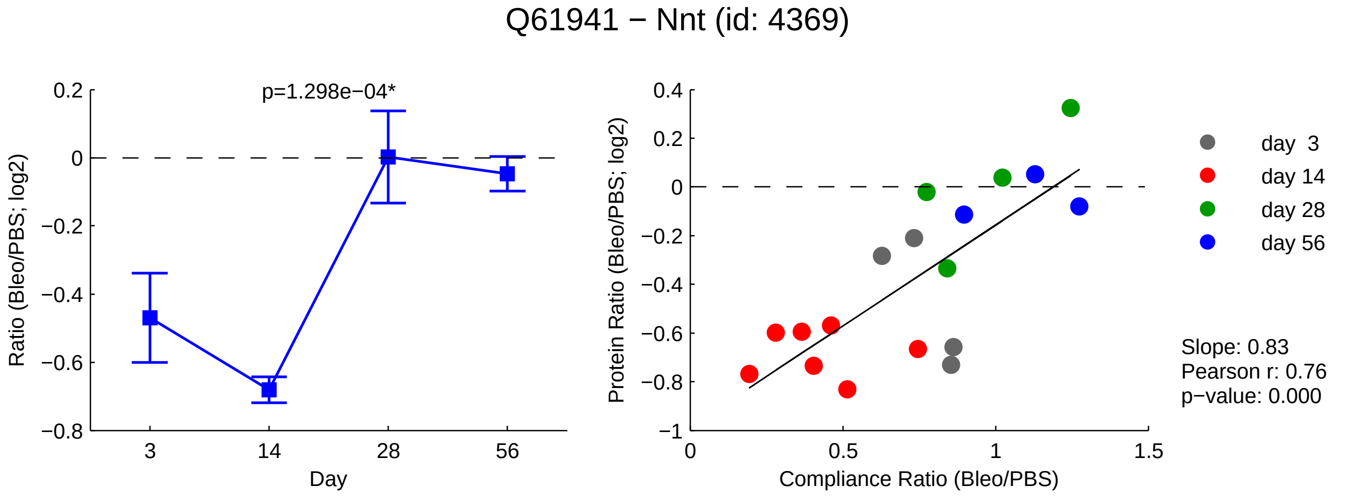
Q61941 − Nnt (id: 4369)
p=1.298e−04*
Ratio (Bleo/PBS; log2)
0.2
0
−0.2
−0.4
−0.6
−0.8
3
14
28
56
Day
Protein Ratio (Bleo/PBS; log2)
0.4
0.2
0
−0.2
−0.4
−0.6
−0.8
−1
0
0.5
1
1.5
Compliance Ratio (Bleo/PBS)
day 3
day 14
day 28
day 56
Slope: 0.83
Pearson r: 0.76
p−value: 0.000

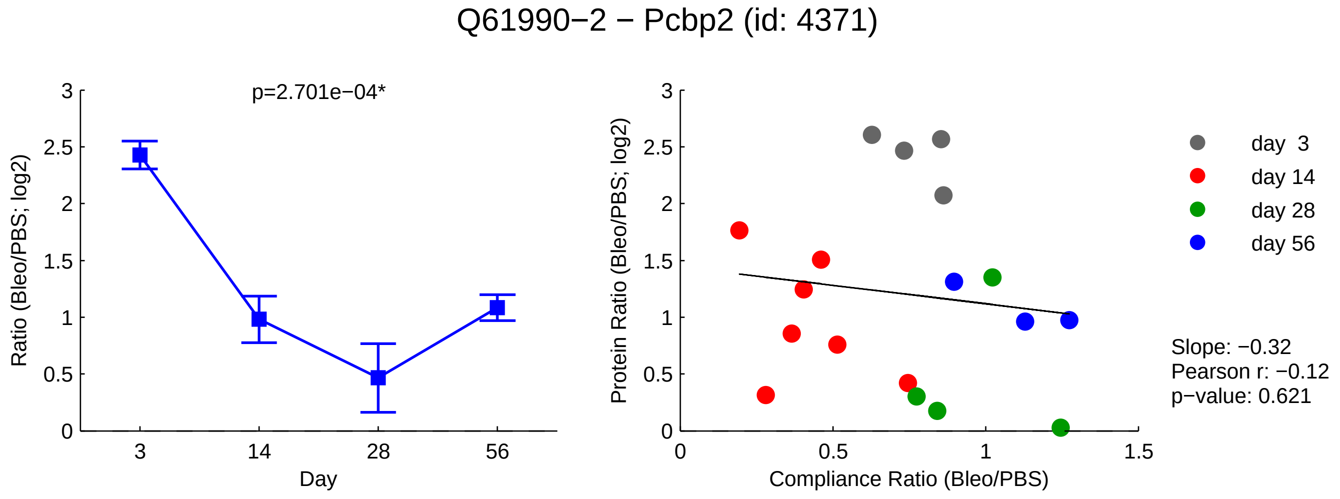
Q61990−2 − Pcbp2 (id: 4371)
p=2.701e−04*
Ratio (Bleo/PBS; log2)
3
2.5
2
1.5
1
0.5
0
3
14
28
56
Day
Protein Ratio (Bleo/PBS; log2)
3
2.5
2
1.5
1
0.5
0
0
0.5
1
1.5
Compliance Ratio (Bleo/PBS)
day 3
day 14
day 28
day 56
Slope: −0.32
Pearson r: −0.12
p−value: 0.621

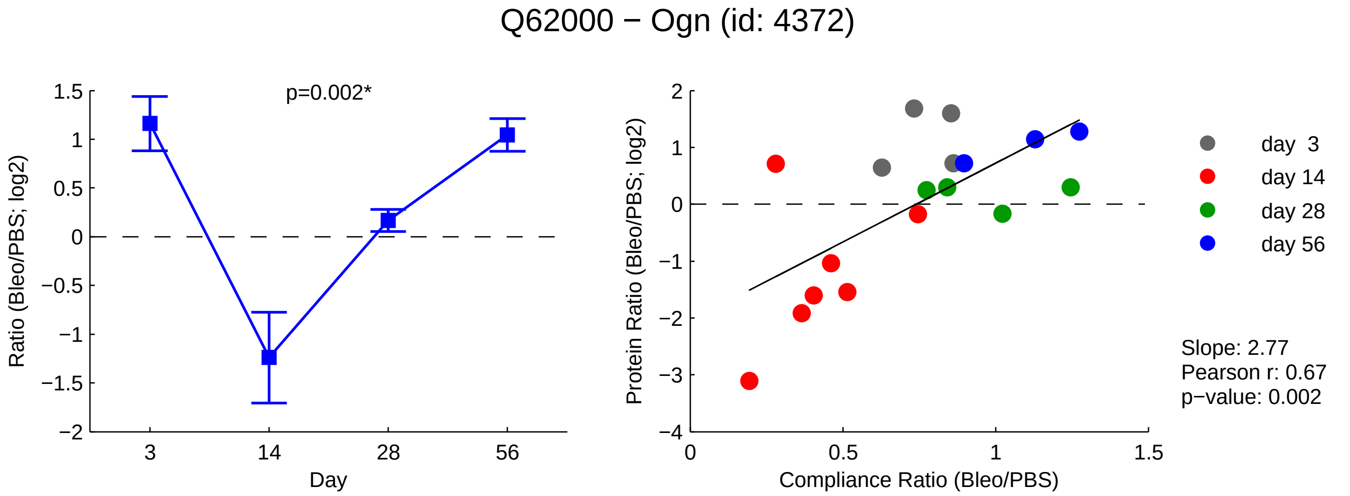
Q62000 − Ogn (id: 4372)
p=0.002*
Ratio (Bleo/PBS; log2)
1.5
1
0.5
0
−0.5
−1
−1.5
−2
3
14
28
56
Day
Protein Ratio (Bleo/PBS; log2)
2
1
0
−1
−2
−3
−4
0
0.5
1
1.5
Compliance Ratio (Bleo/PBS)
day 3
day 14
day 28
day 56
Slope: 2.77
Pearson r: 0.67
p−value: 0.002

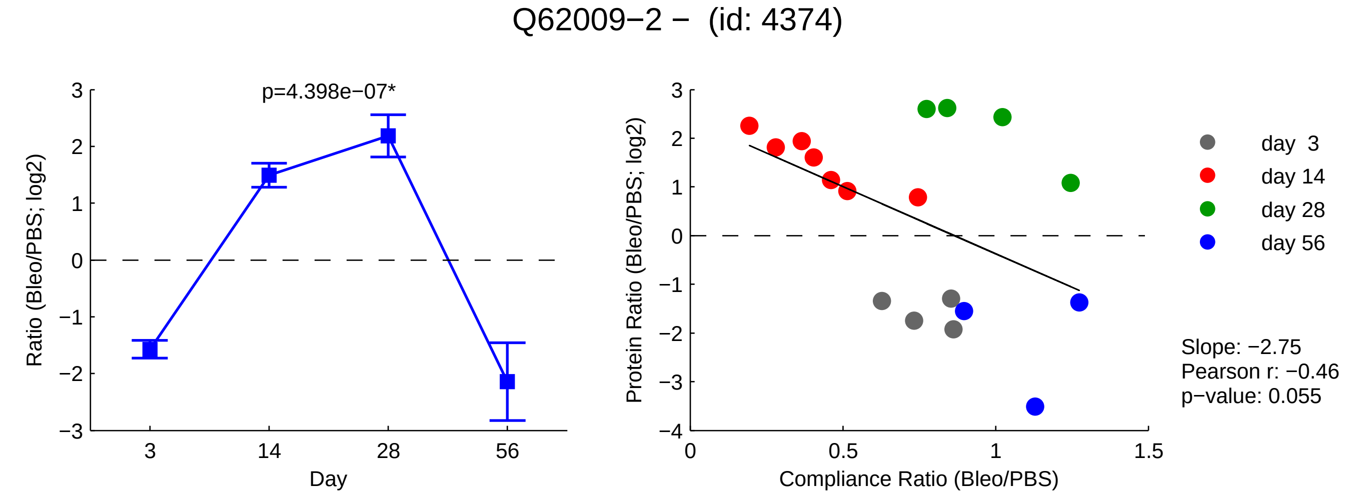
Q62009−2 − (id: 4374)
p=4.398e−07*
Ratio (Bleo/PBS; log2)
Day
Protein Ratio (Bleo/PBS; log2)
Compliance Ratio (Bleo/PBS)
day 3
day 14
day 28
day 56
Slope: −2.75
Pearson r: −0.46
p−value: 0.055

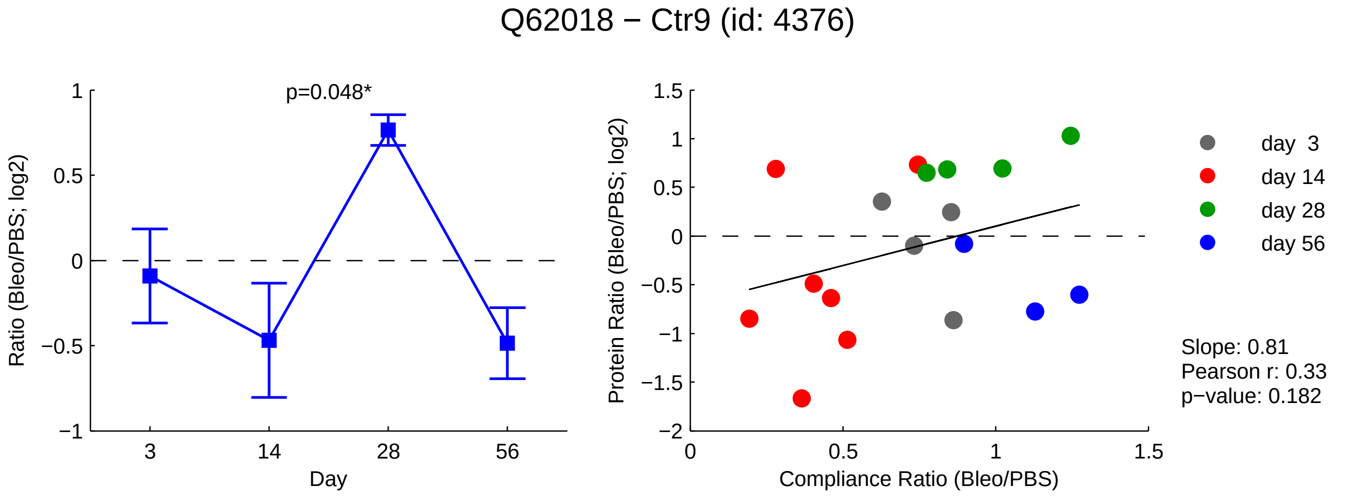
Q62018 − Ctr9 (id: 4376)
p=0.048*
Ratio (Bleo/PBS; log2)
Day
Protein Ratio (Bleo/PBS; log2)
Compliance Ratio (Bleo/PBS)
day 3
day 14
day 28
day 56
Slope: 0.81
Pearson r: 0.33
p−value: 0.182

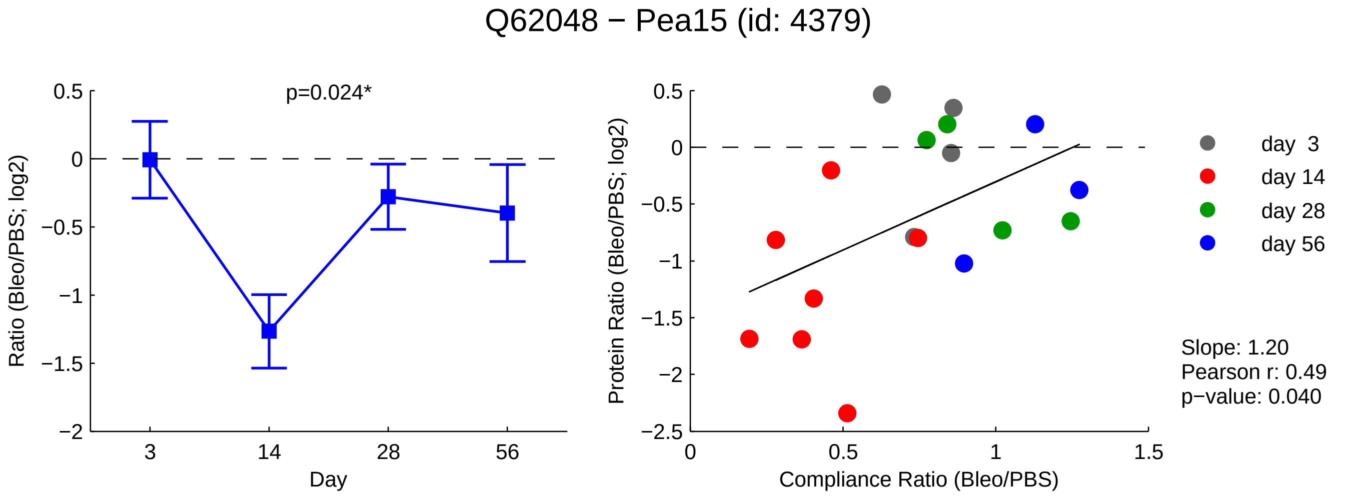
Q62048 − Pea15 (id: 4379)
p=0.024*
Ratio (Bleo/PBS; log2)
Day
Protein Ratio (Bleo/PBS; log2)
Compliance Ratio (Bleo/PBS)
day 3
day 14
day 28
day 56
Slope: 1.20
Pearson r: 0.49
p−value: 0.040

Q62074 − Prkci (id: 4381)

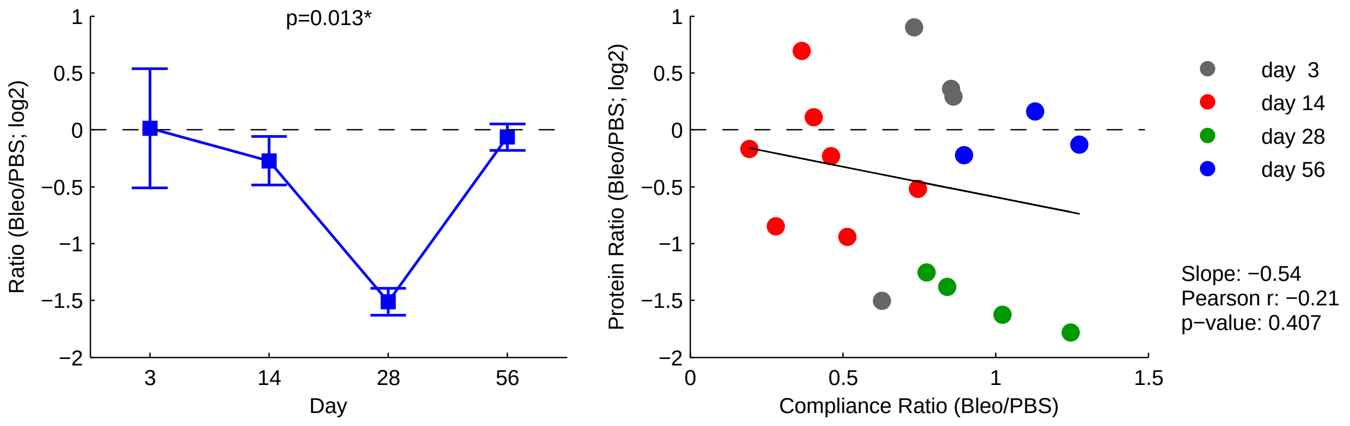

Q62086 − Pon2 (id: 4383)

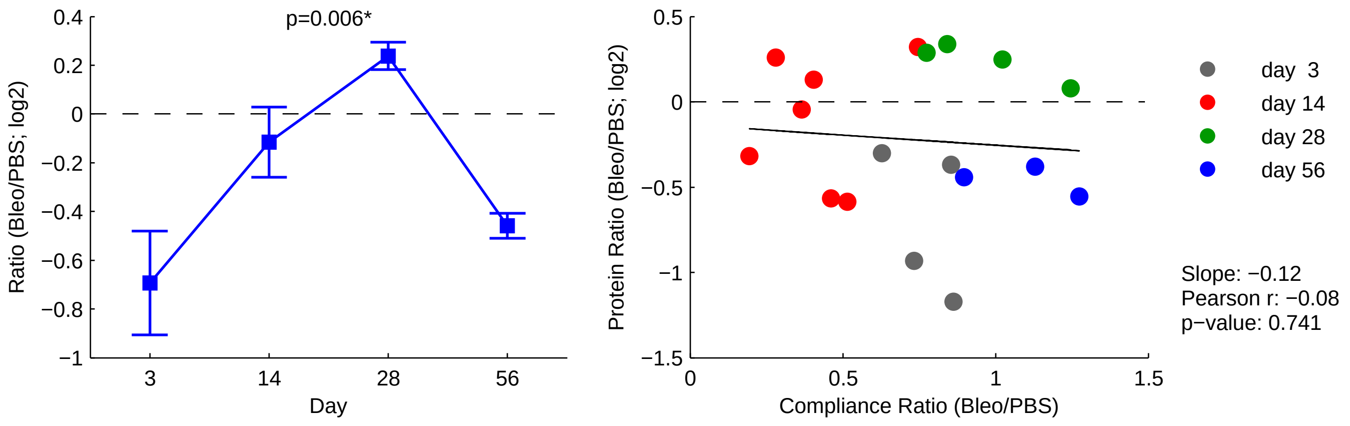

Q62087 − Pon3 (id: 4384)

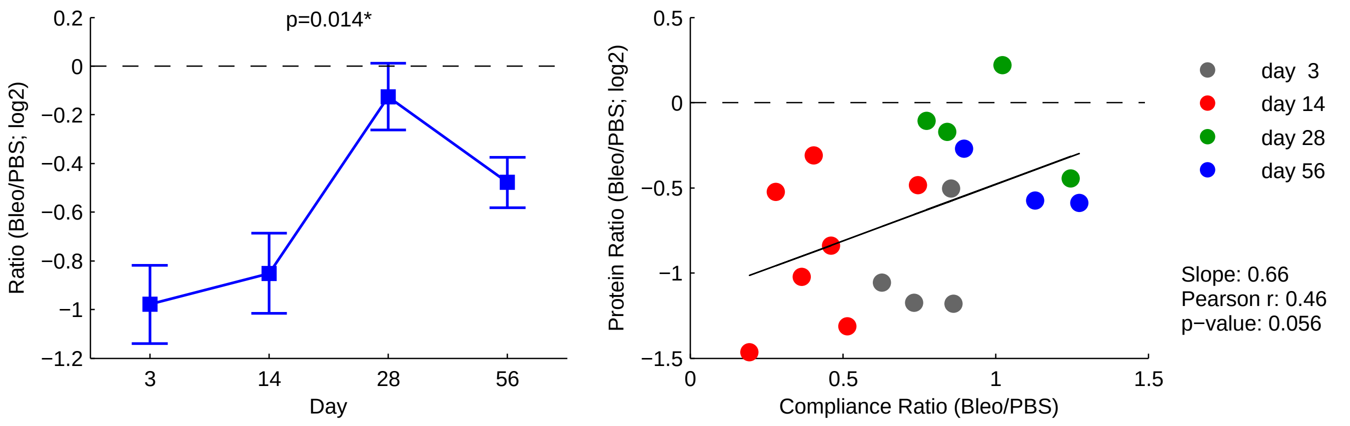

Q62095 − Ddx3y (id: 4386)

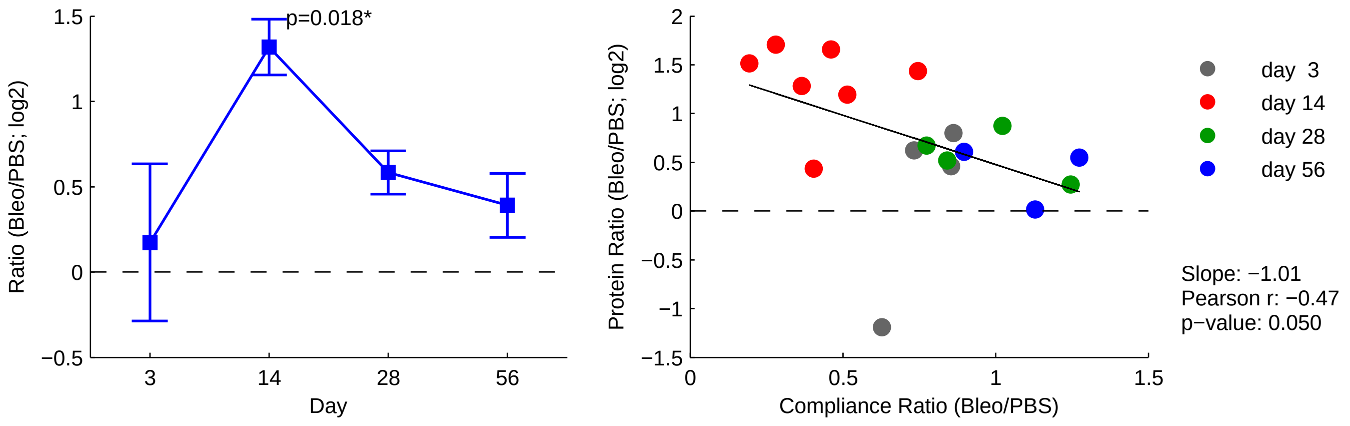

Q62159 − Rhoc (id: 4391)

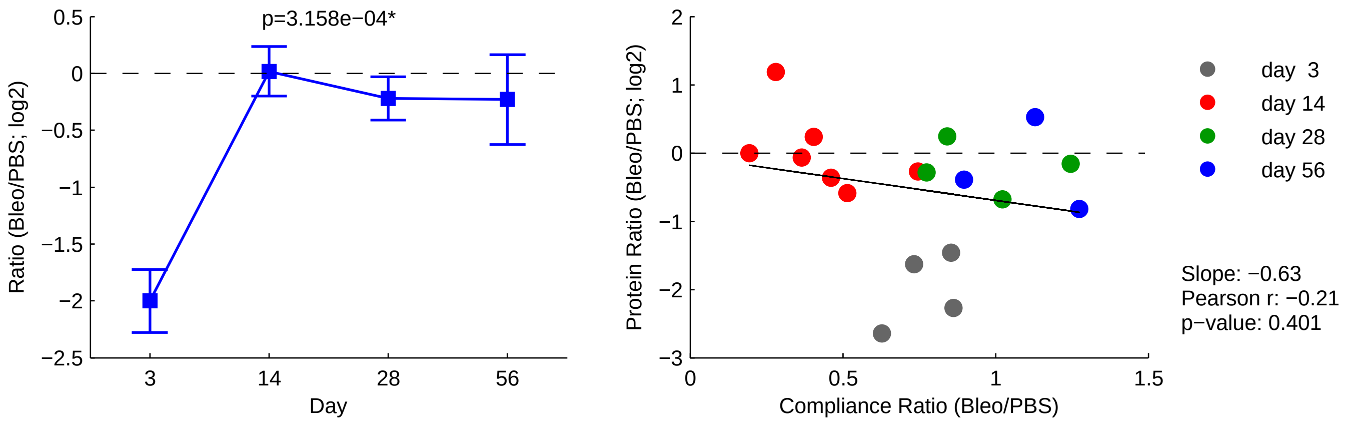

Q62165 − Dag1 (id: 4392)

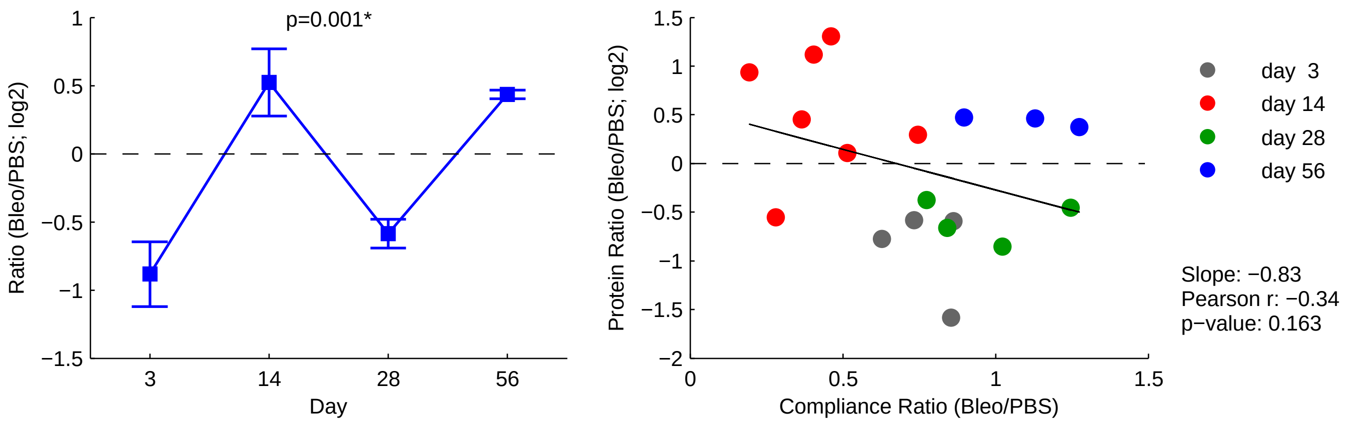

Q62167 − Ddx3x (id: 4393)

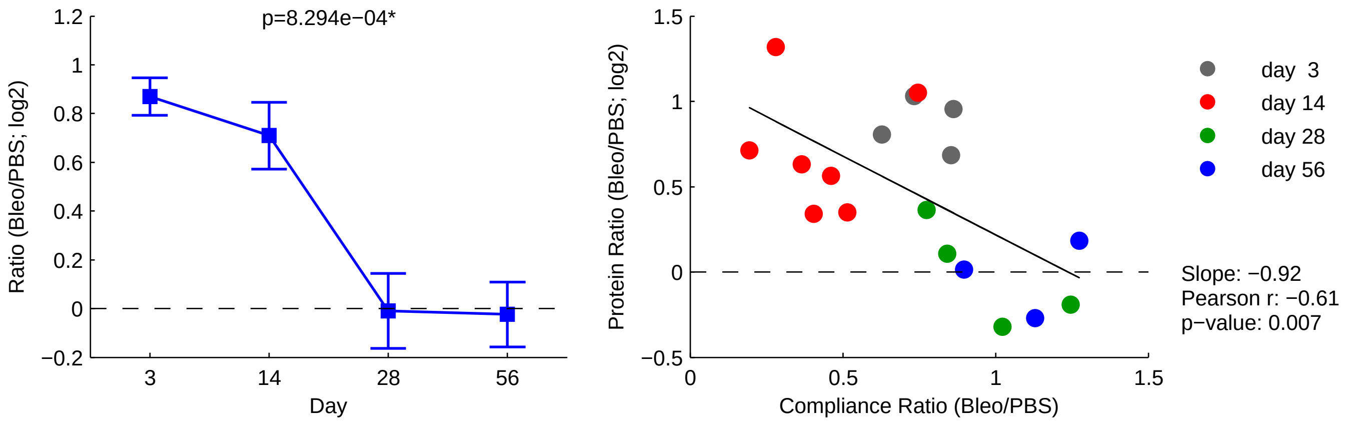

Q62172 − Ralbp1 (id: 4394)

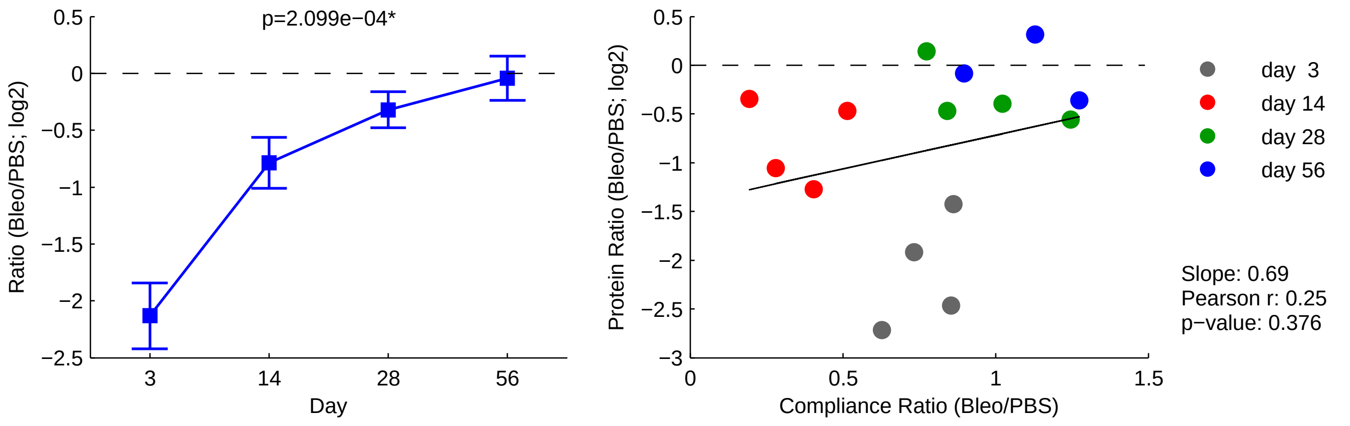

Q62181 − Sema3c (id: 4396)

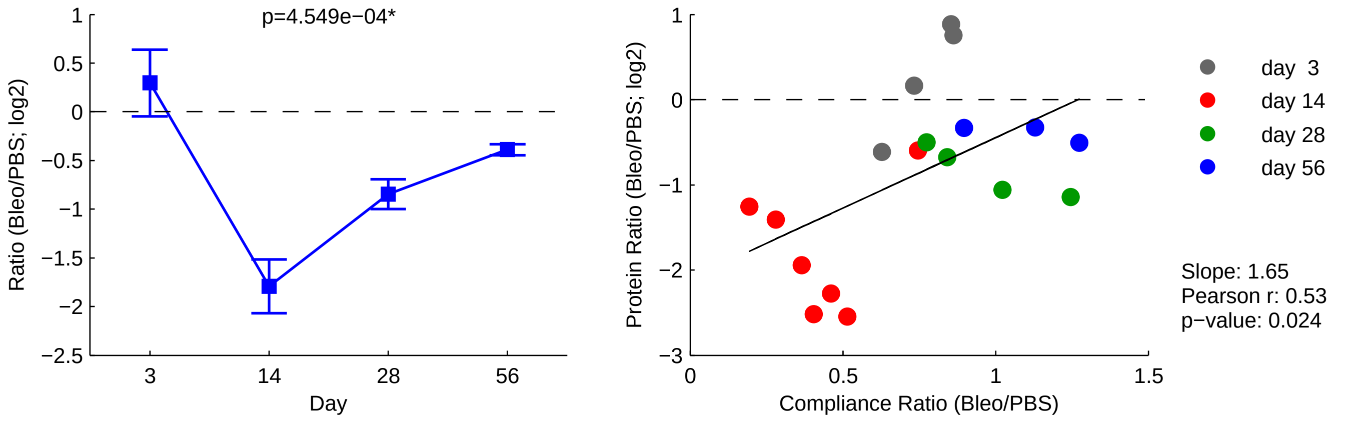

Q62186 − Ssr4 (id: 4397)

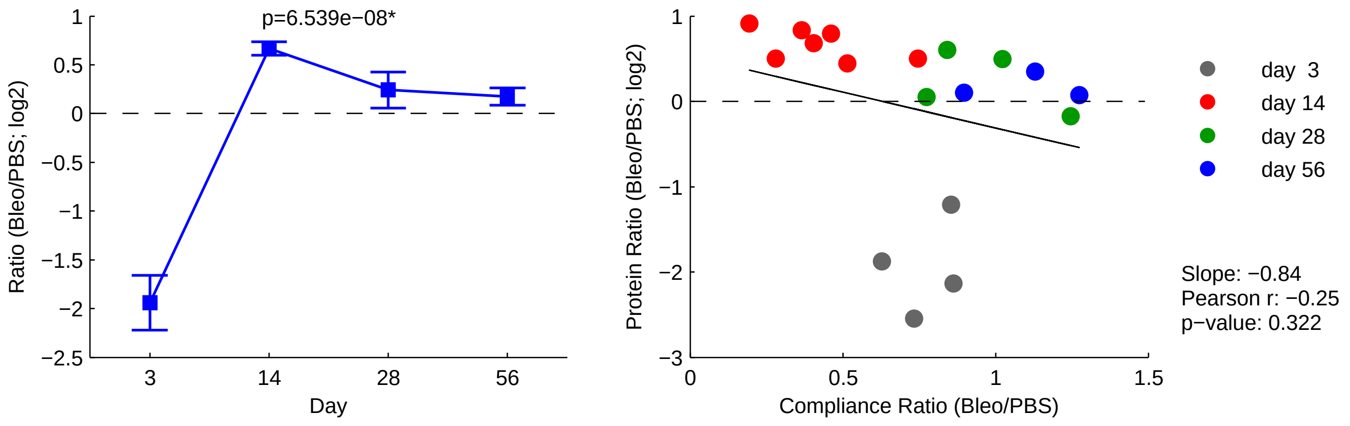

Q62192 − Cd180 (id: 4399)

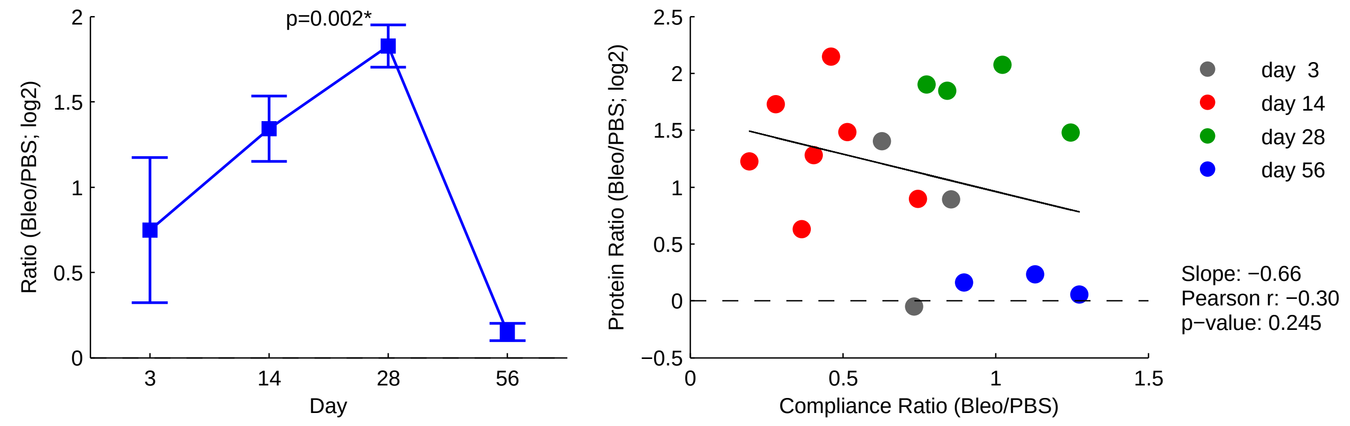

Q62234−2 − Myom1 (id: 4401)

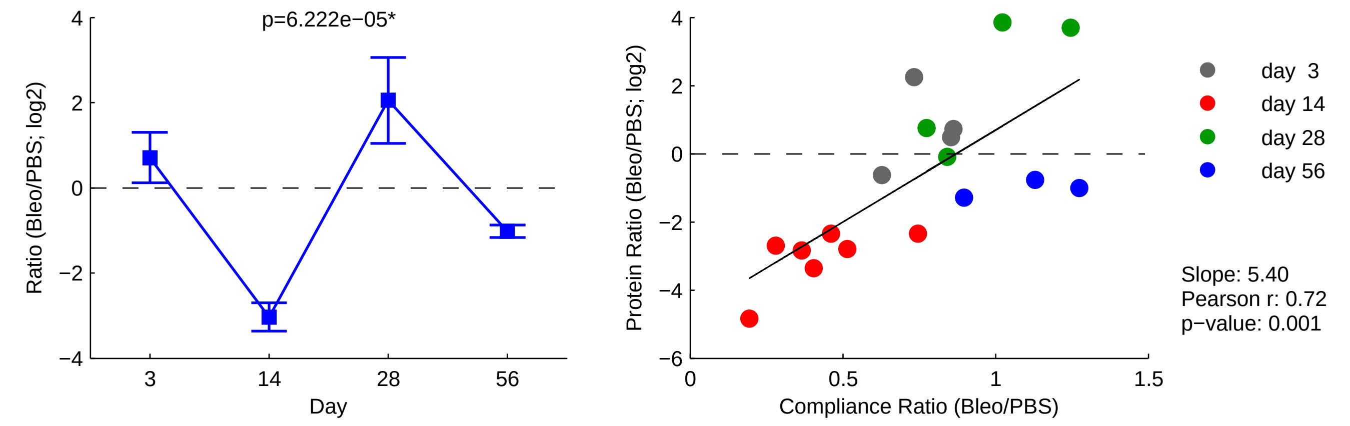

Q62241 − Snrpc (id: 4402)

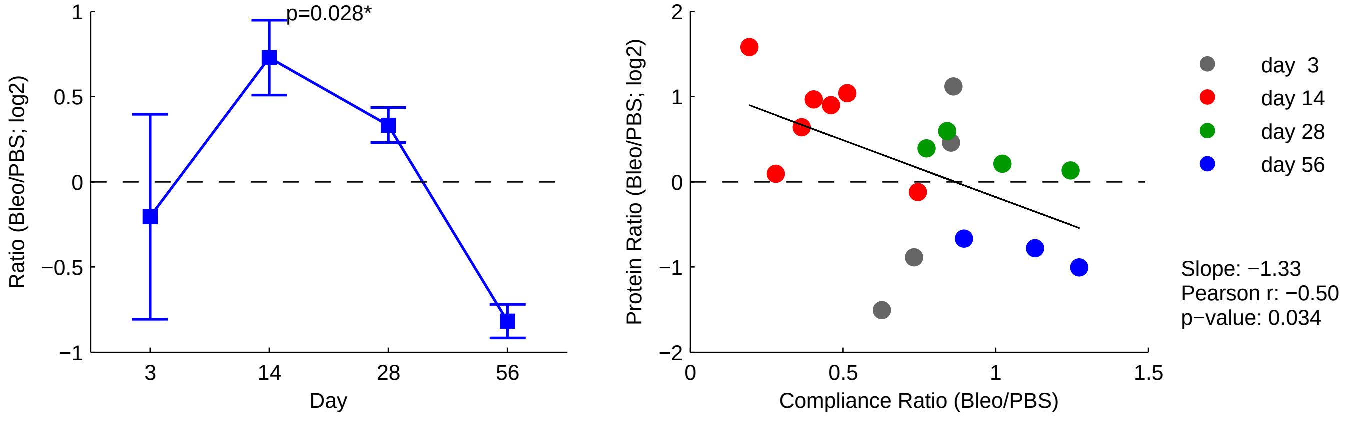

Q8BKL1 − Ss18 (id: 4406)

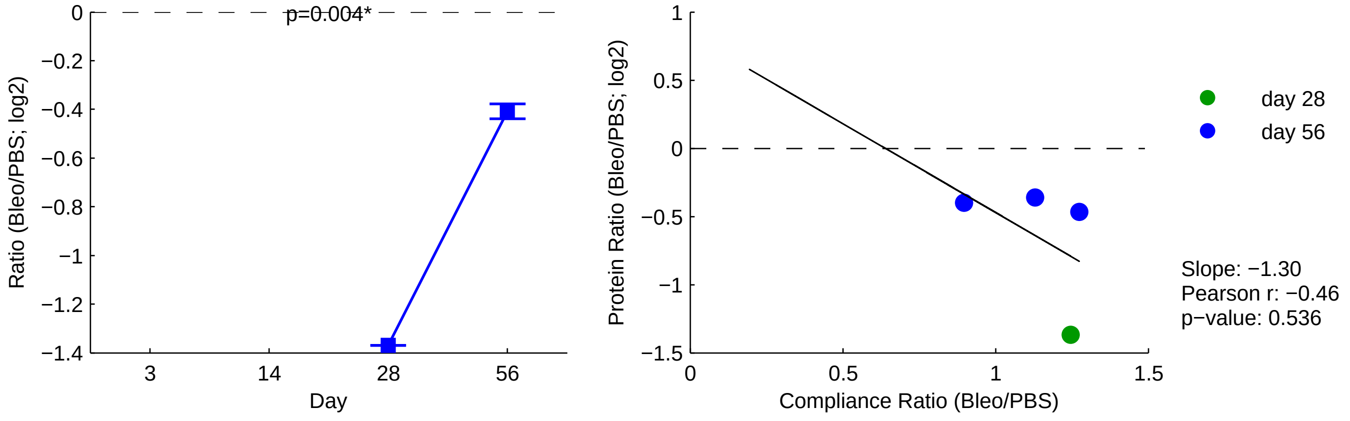

Q62293 − Tgtp (id: 4407)

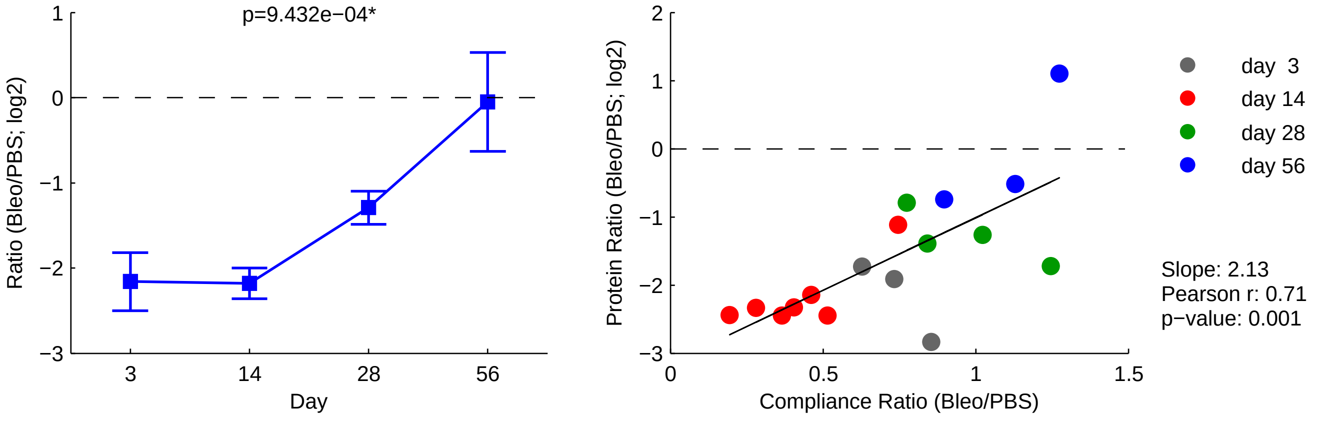

Q62312−2 − Tgfbr2 (id: 4408)

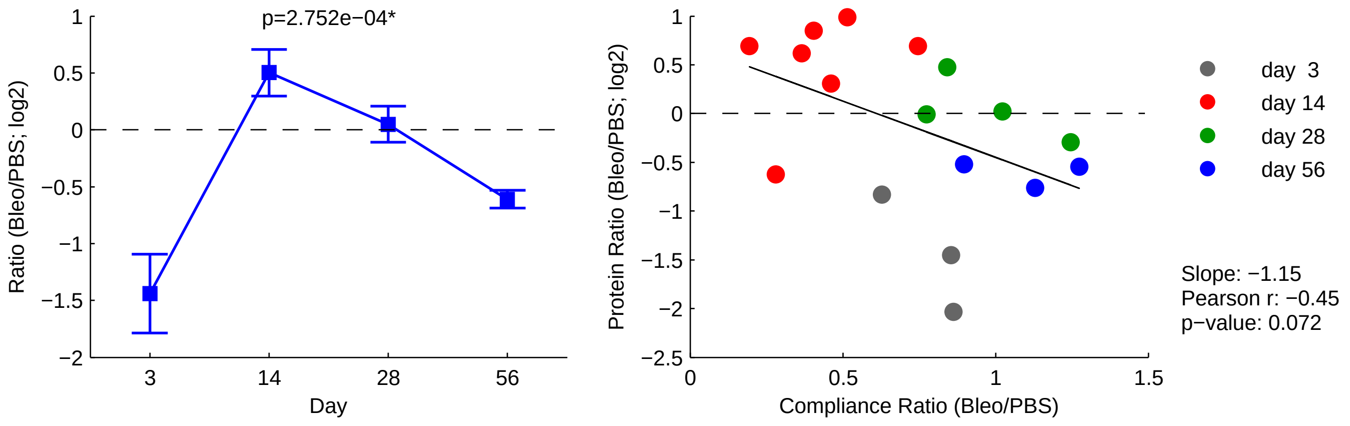

Q62318 − Trim28 (id: 4410)

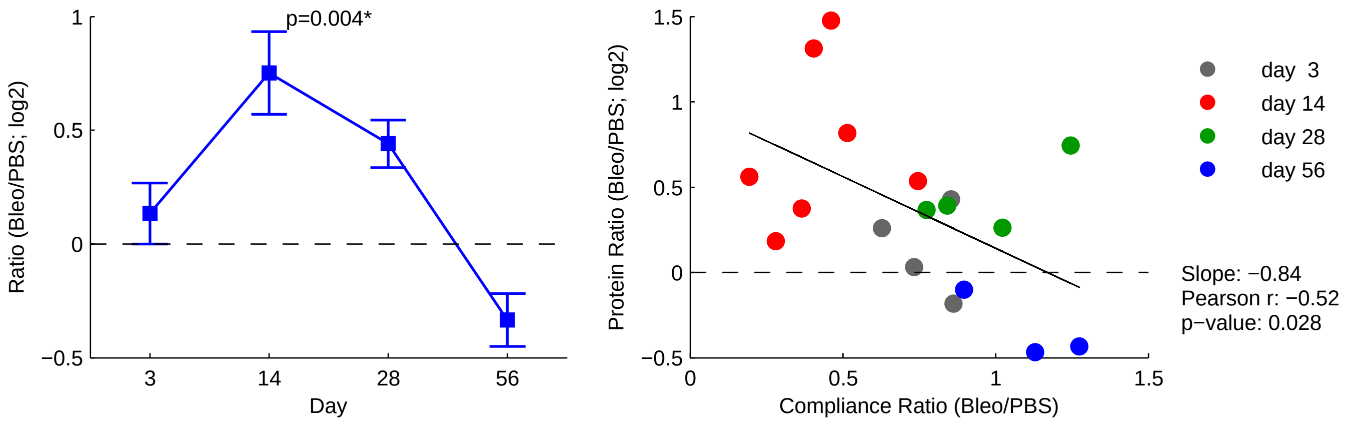

Q62348 − Tsn (id: 4411)

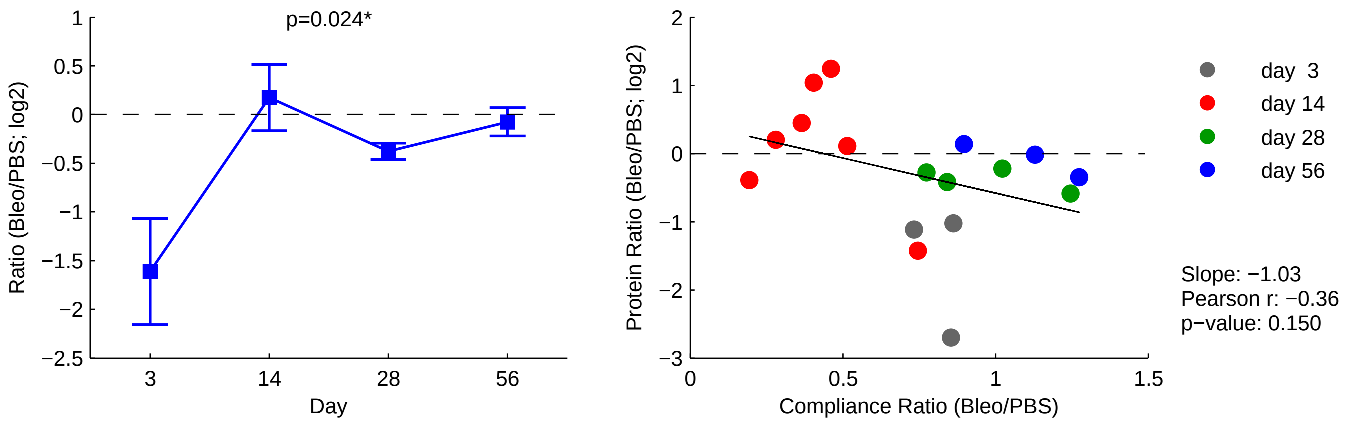

Q62351 − Tfrc (id: 4412)

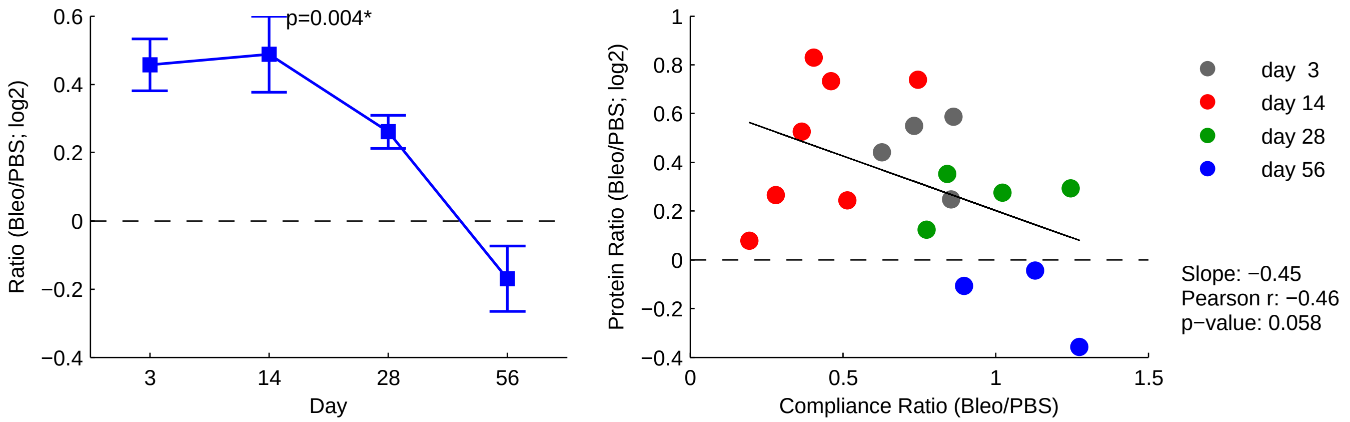

Q62356 − Fstl1 (id: 4413)

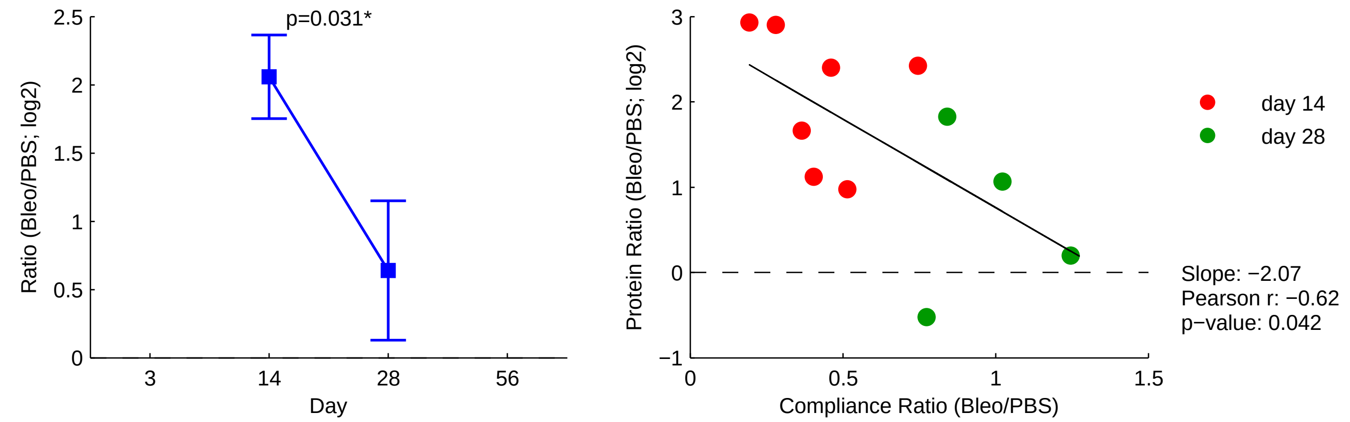

Q62419 − Sh3gl1 (id: 4421)

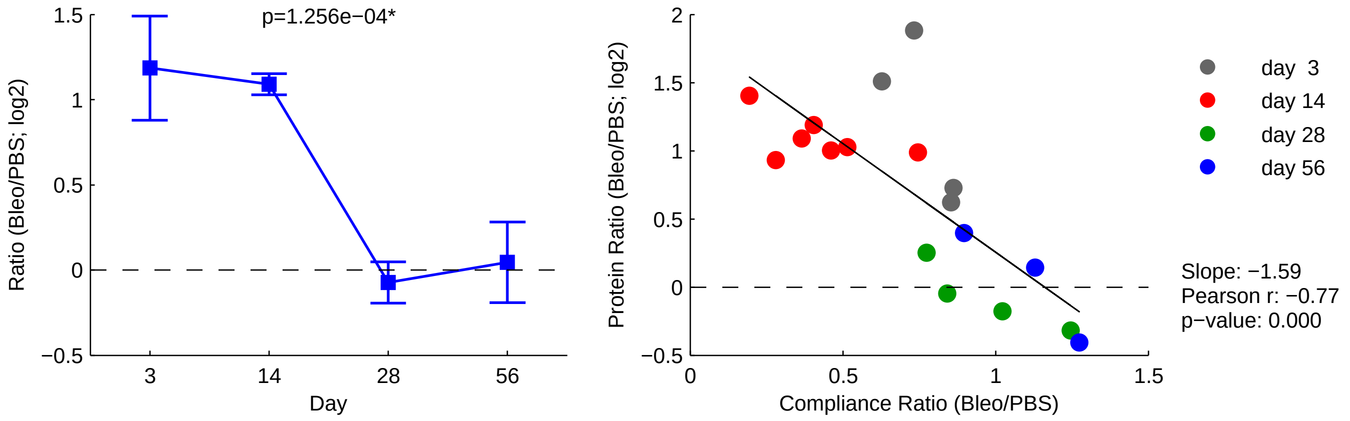

Q62422 − Ostf1 (id: 4422)

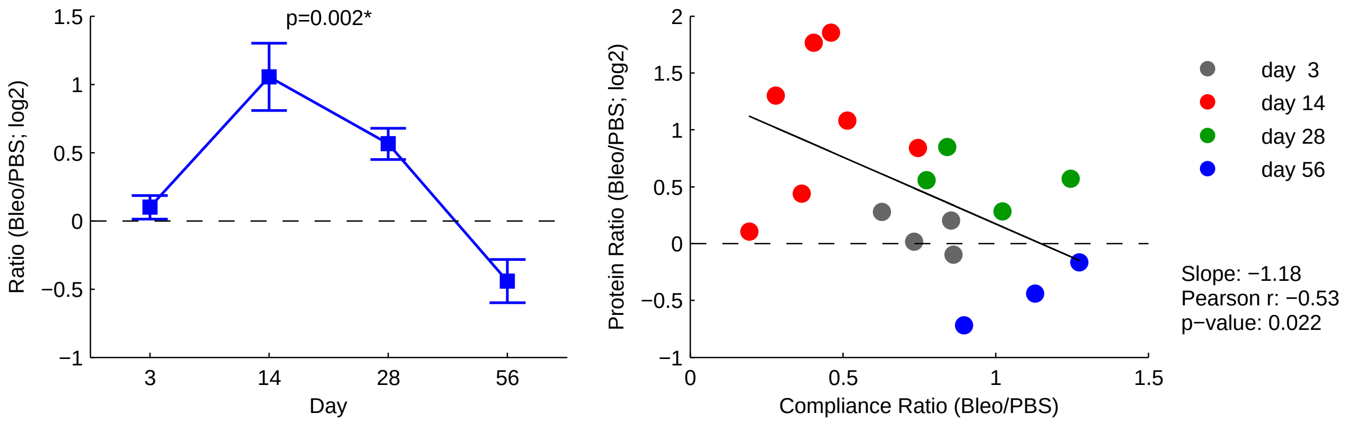

Q62425 − Ndufa4 (id: 4423)

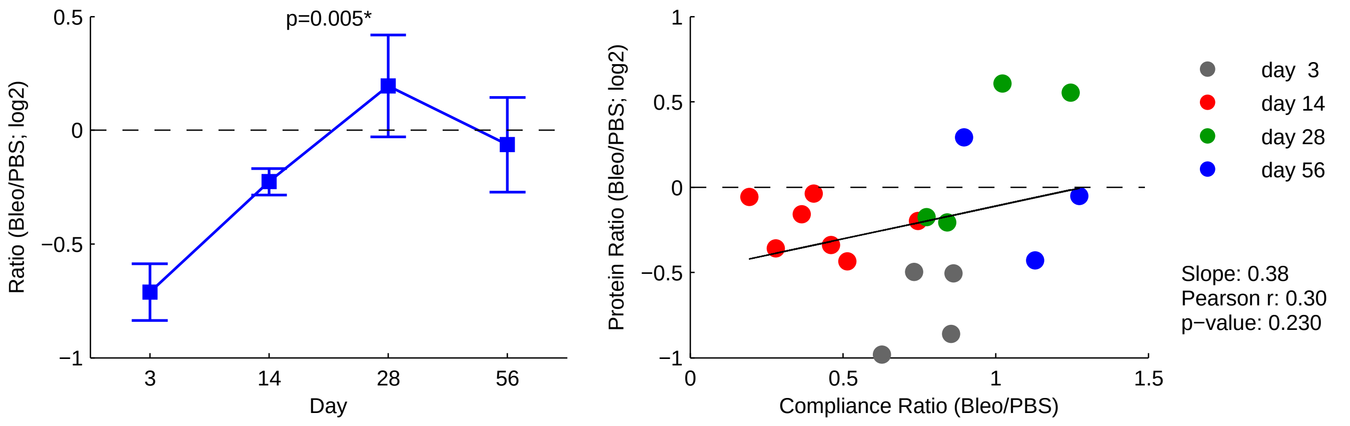

Q62426 − Cstb (id: 4424)

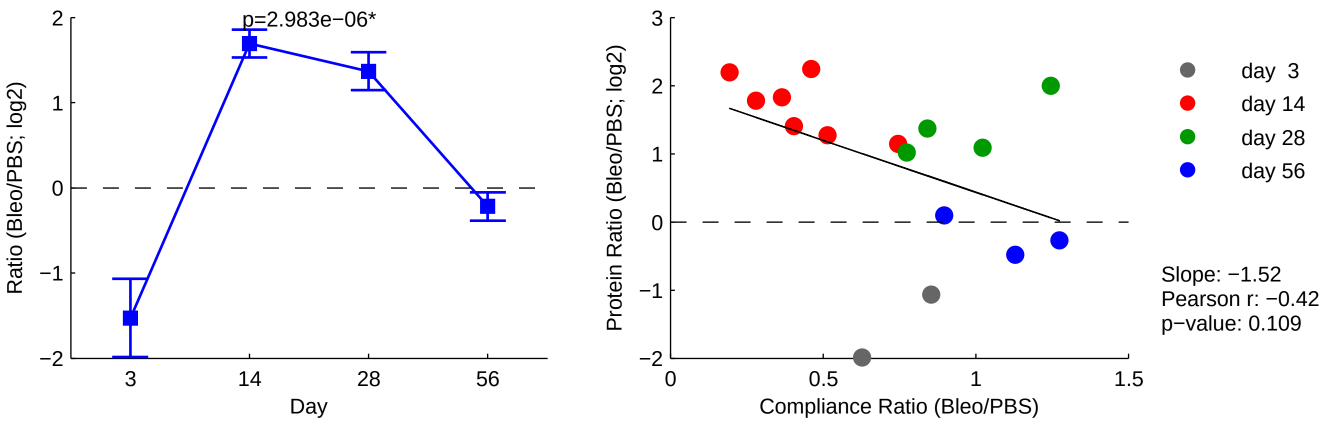

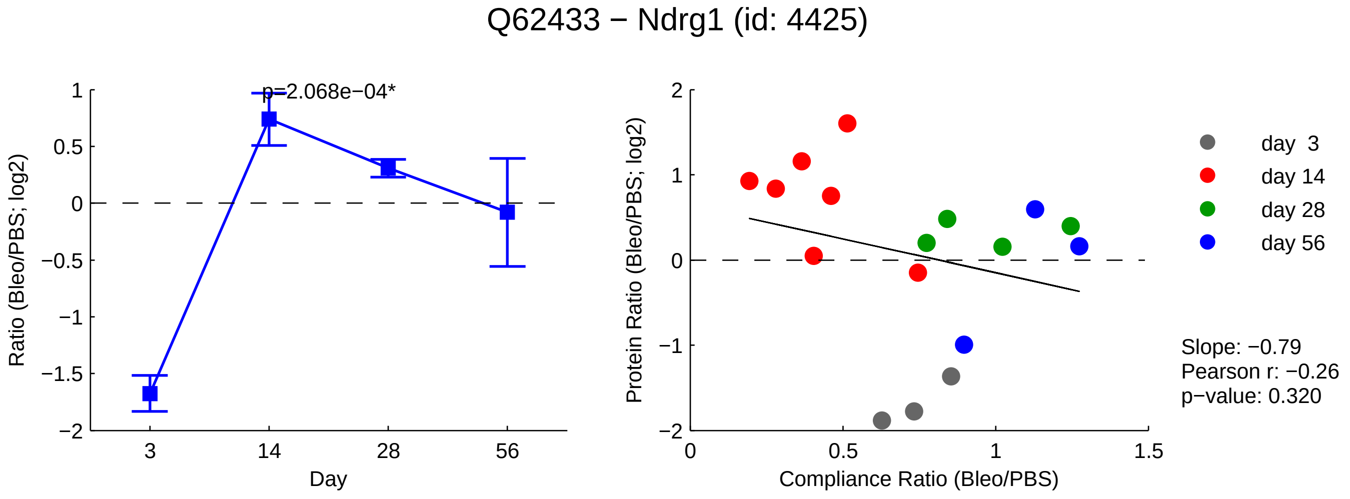
Q62433 − Ndrg1 (id: 4425)
p=2.068e−04*
Ratio (Bleo/PBS; log2)
Day
Protein Ratio (Bleo/PBS; log2)
Compliance Ratio (Bleo/PBS)
day 3
day 14
day 28
day 56
Slope: −0.79
Pearson r: −0.26
p−value: 0.320

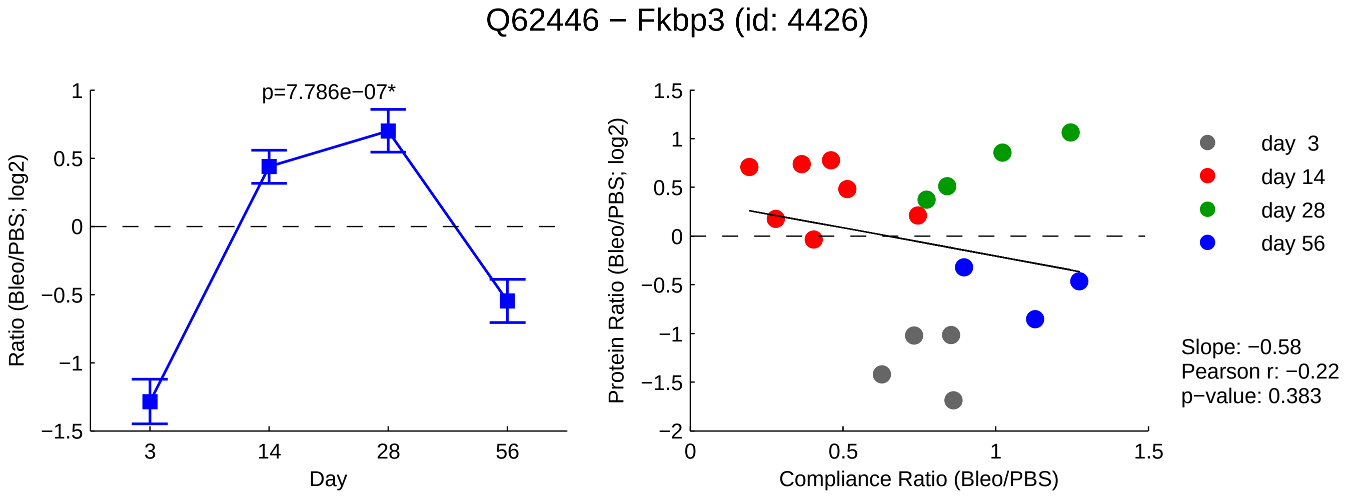
Q62446 − Fkbp3 (id: 4426)
p=7.786e−07*
Ratio (Bleo/PBS; log2)
Day
Protein Ratio (Bleo/PBS; log2)
Compliance Ratio (Bleo/PBS)
day 3
day 14
day 28
day 56
Slope: −0.58
Pearson r: −0.22
p−value: 0.383

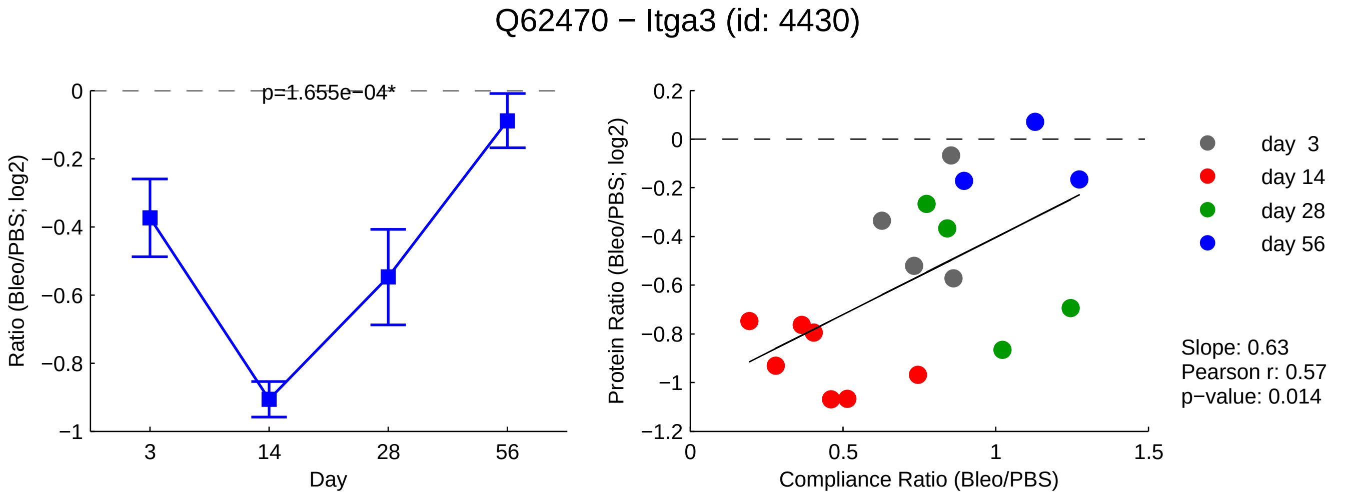
Q62470 − Itga3 (id: 4430)
p=1.655e−04*
Ratio (Bleo/PBS; log2)
Day
Protein Ratio (Bleo/PBS; log2)
Compliance Ratio (Bleo/PBS)
day 3
day 14
day 28
day 56
Slope: 0.63
Pearson r: 0.57
p−value: 0.014

Q62523 − Zyx (id: 4432)

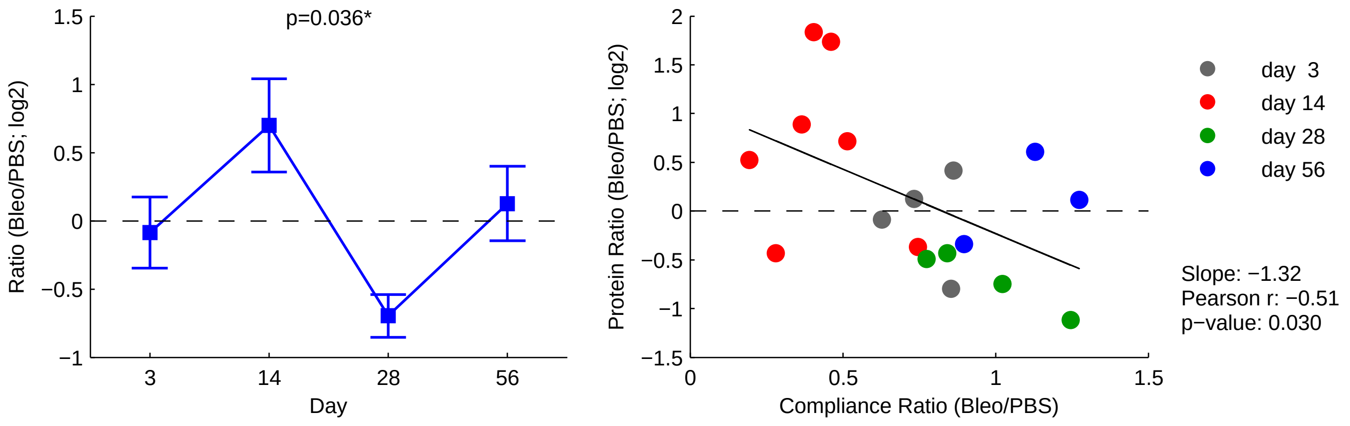

Q63739 − Ptp4a1 (id: 4434)

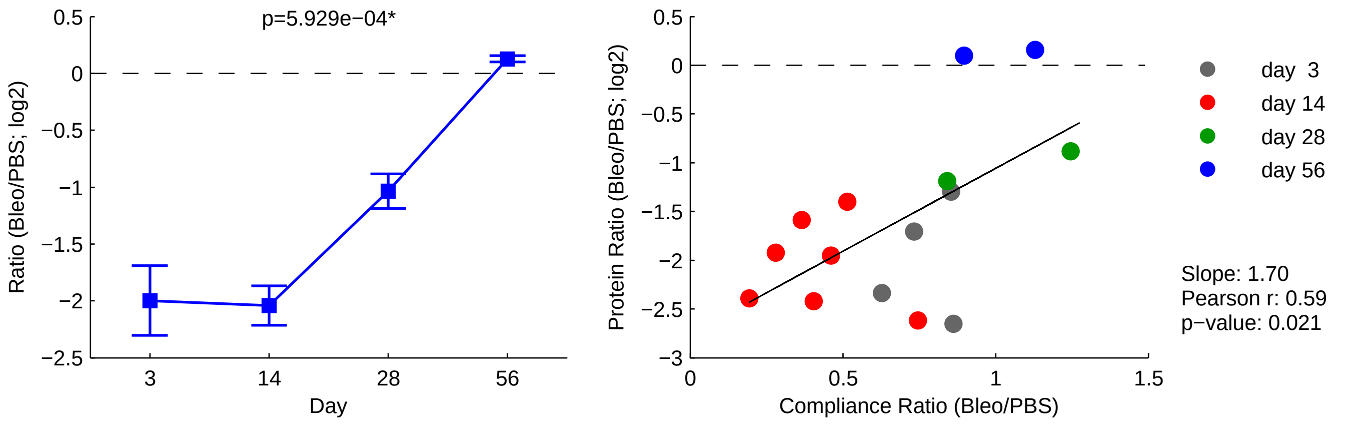

Q63810−2 − Ppp3r1 (id: 4435)

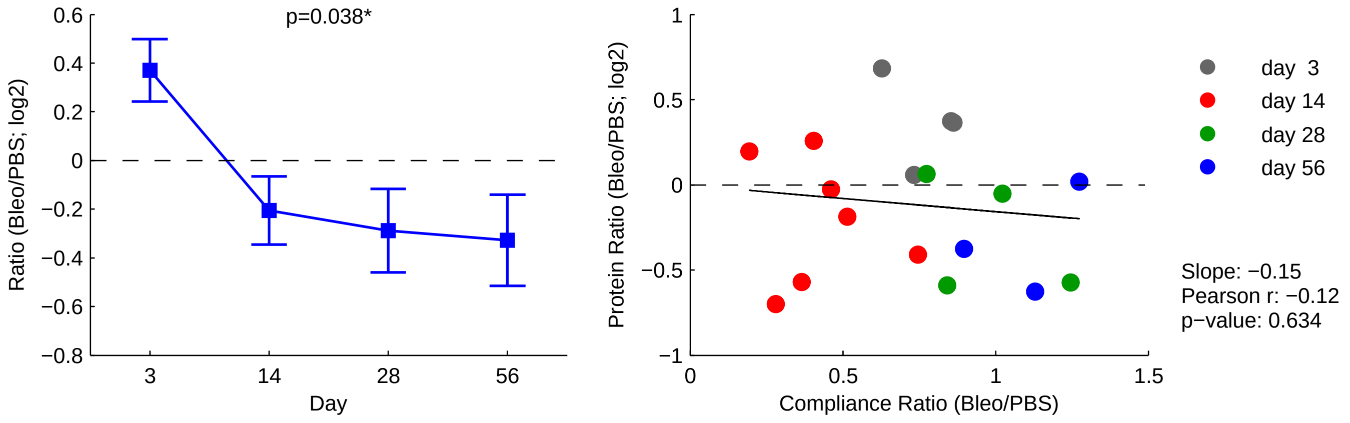

Q63829 − Commd3 (id: 4436)

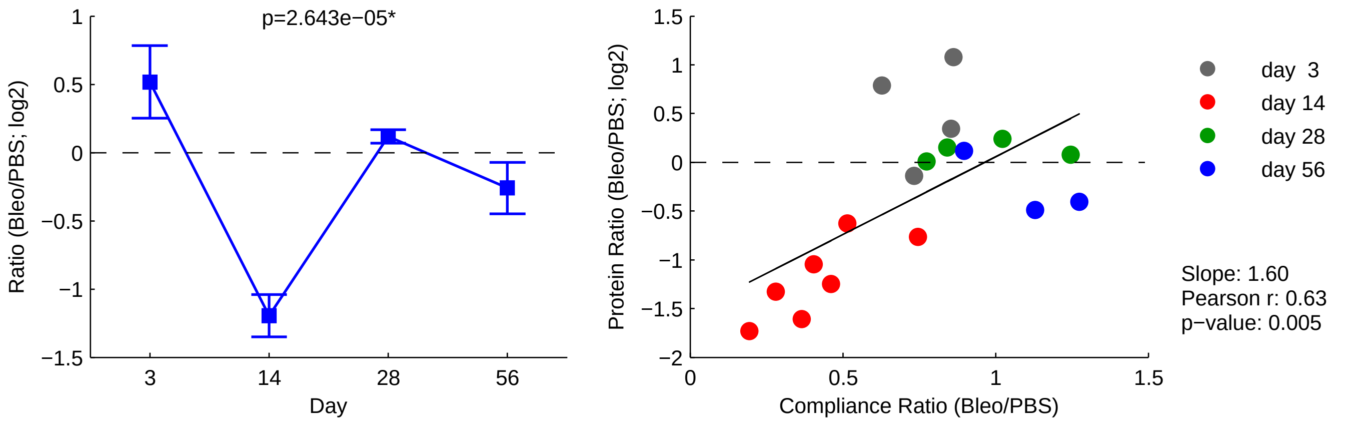

Q63844 − Mapk3 (id: 4437)

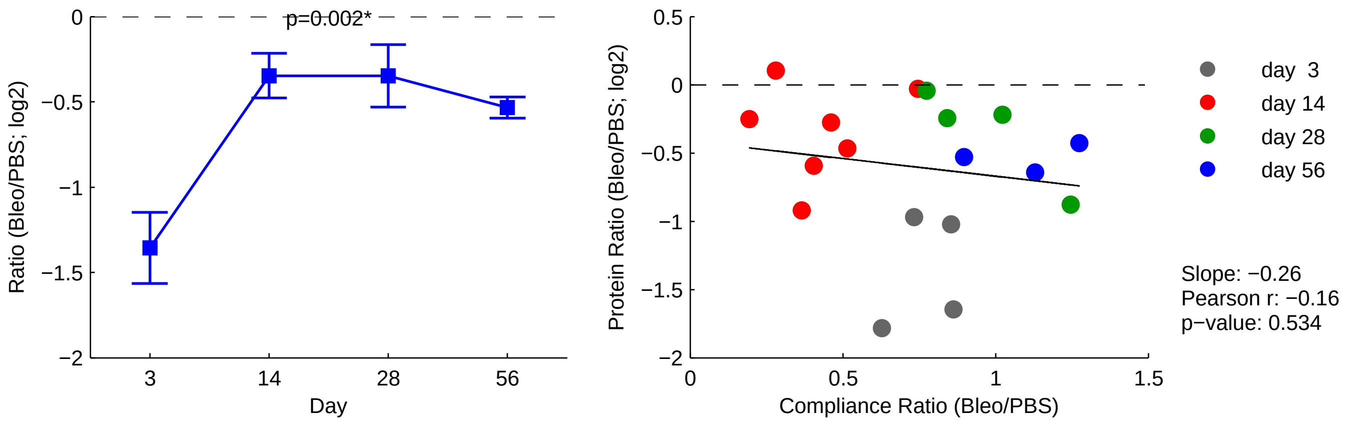

Q63918 − Sdpr (id: 4441)

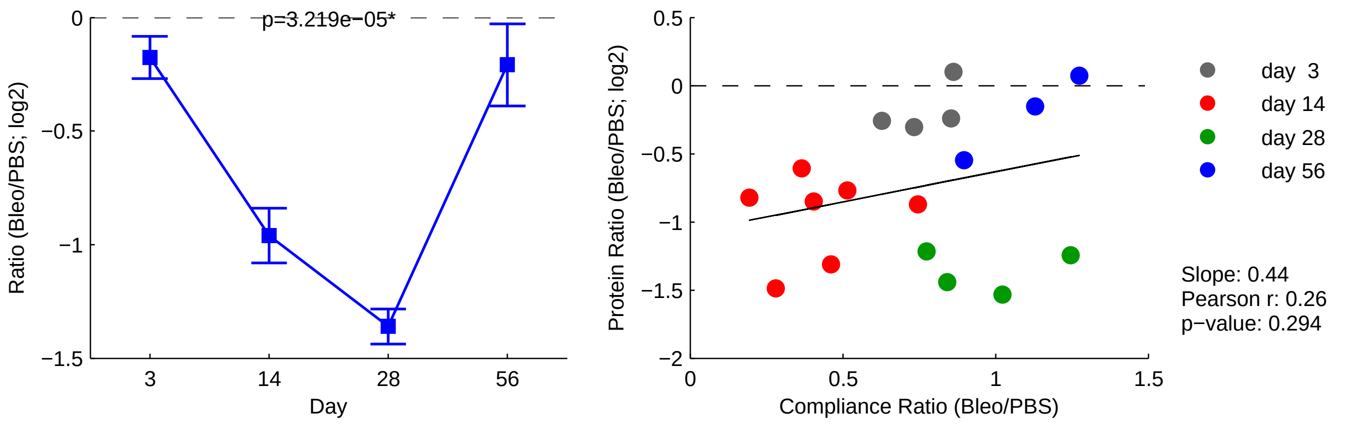

Q91YS7 − Map2k2 (id: 4442)

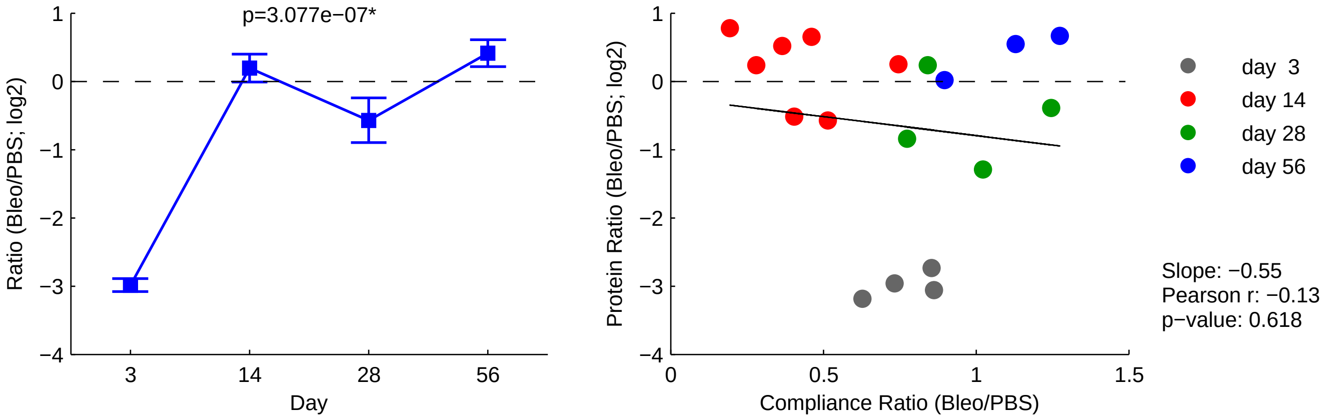

Q921S6 − Mef2d (id: 4443)

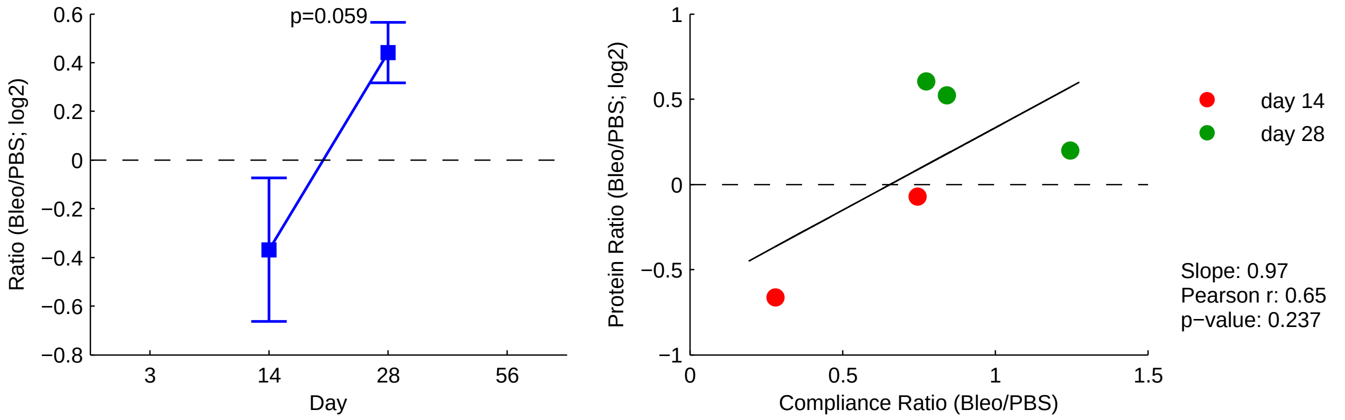

Q63ZW6 − Col4a5 (id: 4444)

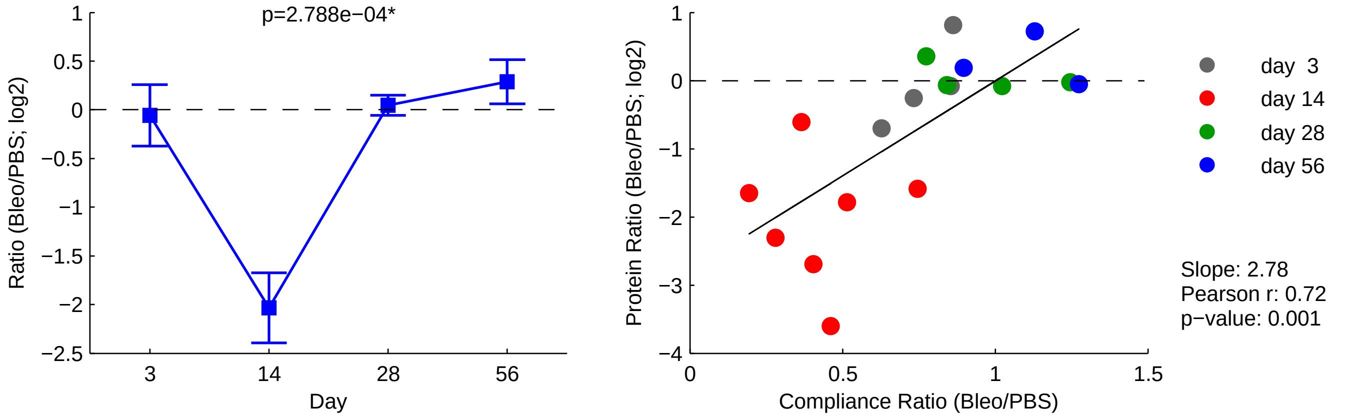

Q63ZW7 − Inadl (id: 4445)

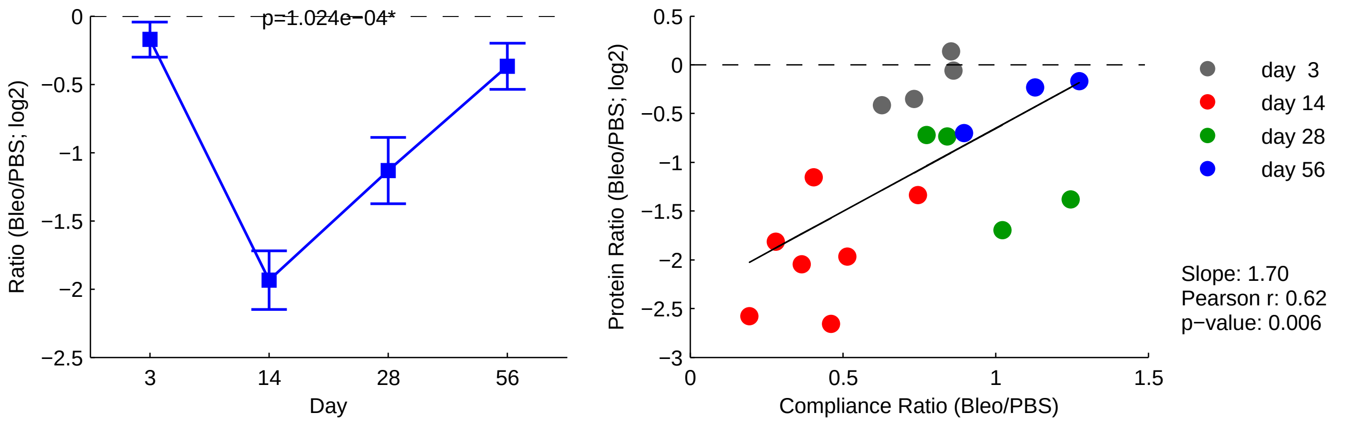

Q64010 − Crk (id: 4446)

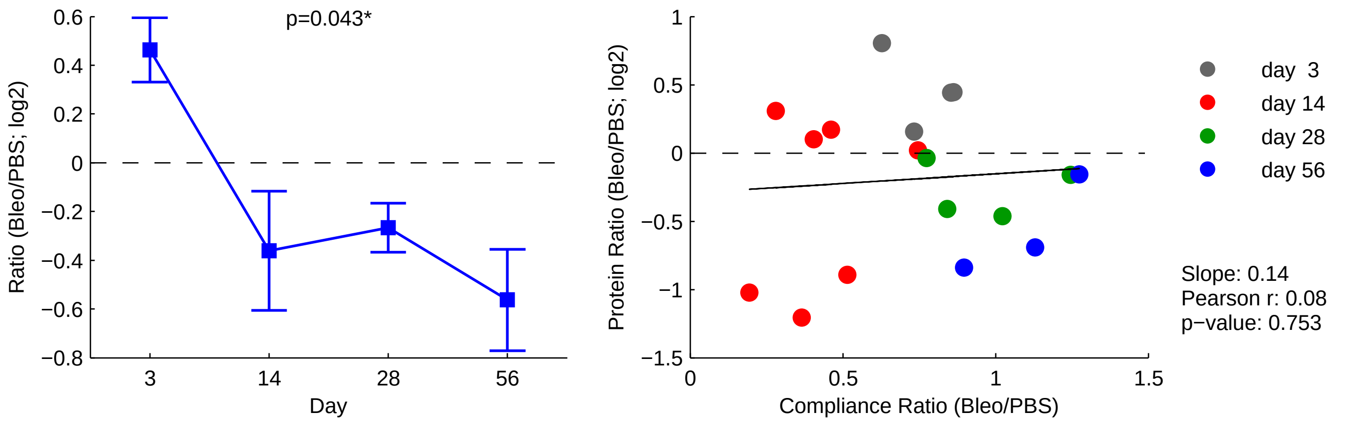

Q640N1 − Aebp1 (id: 4448)

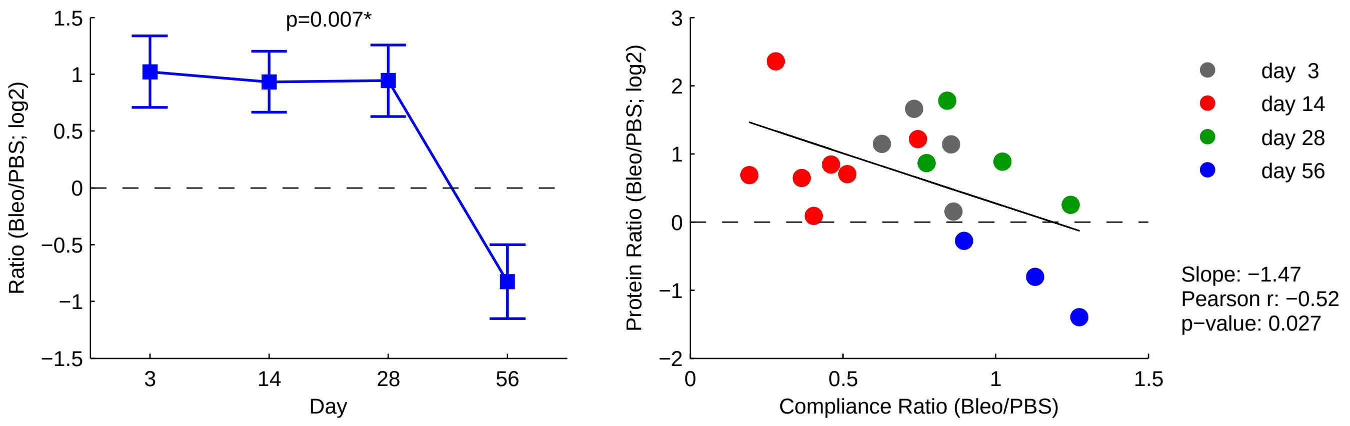

Q640N2 − Arl13b (id: 4449)

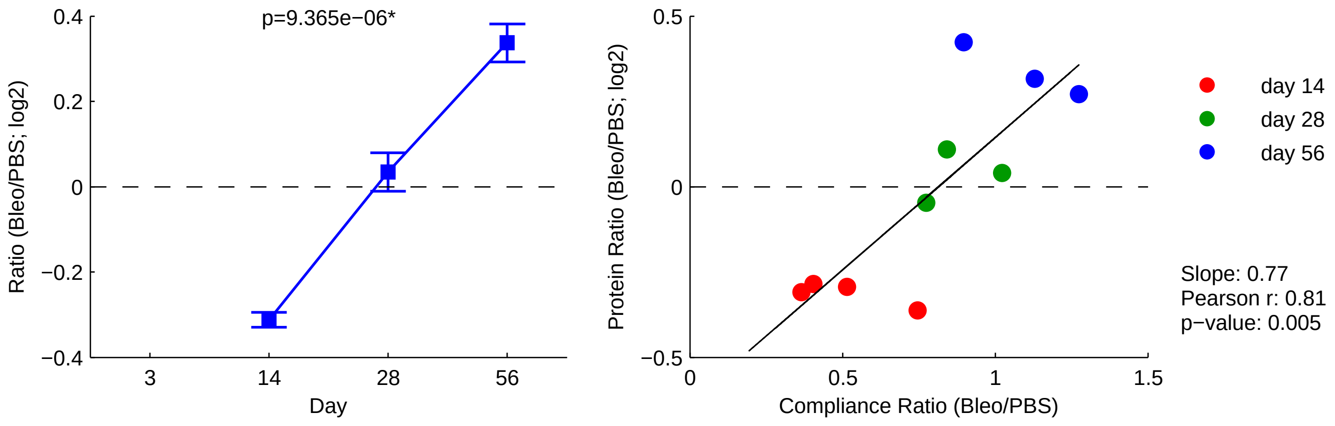

Q64105 − Spr (id: 4450)

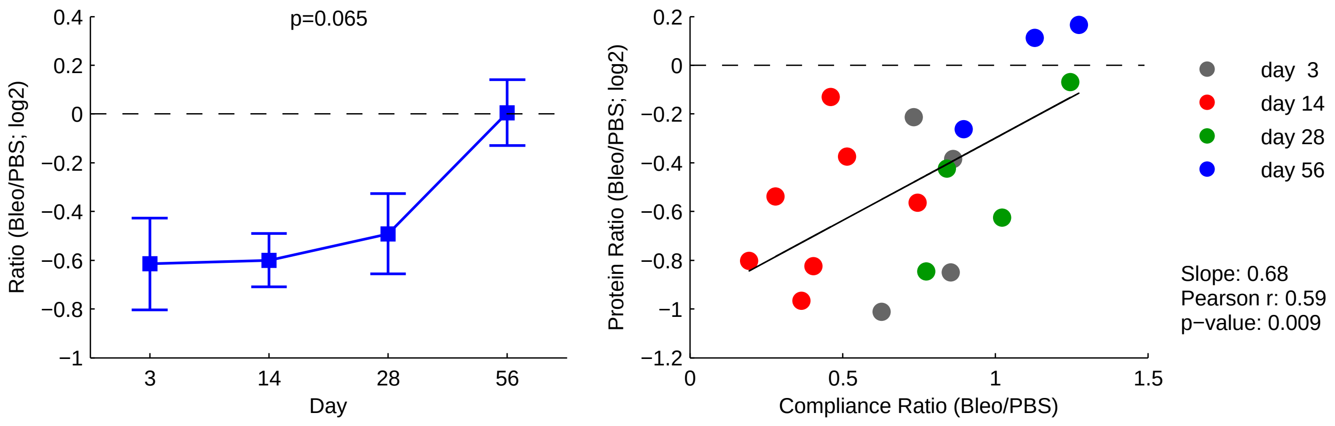

Q64133 − Maoa (id: 4452)

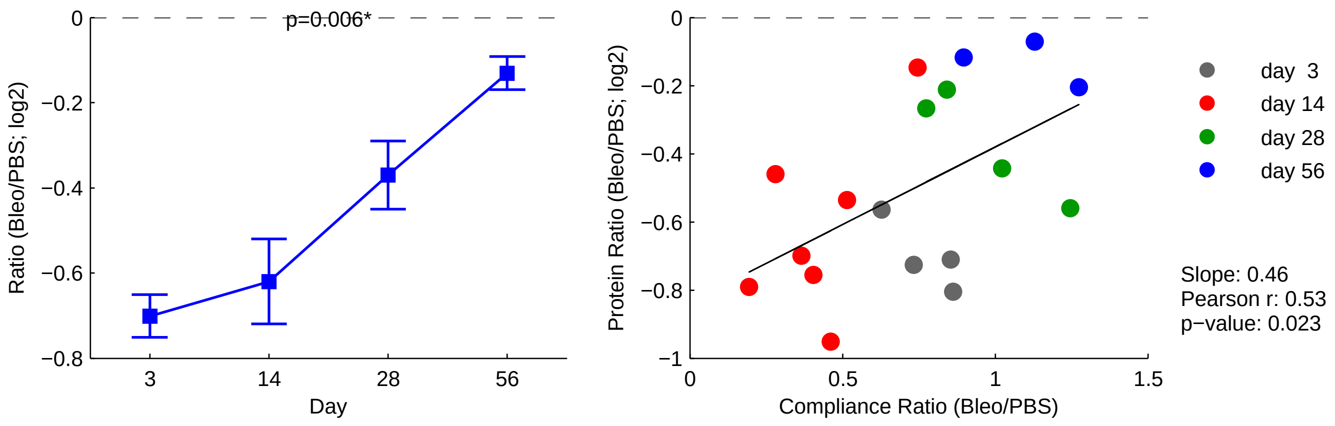

Q64152−2 − Btf3 (id: 4454)

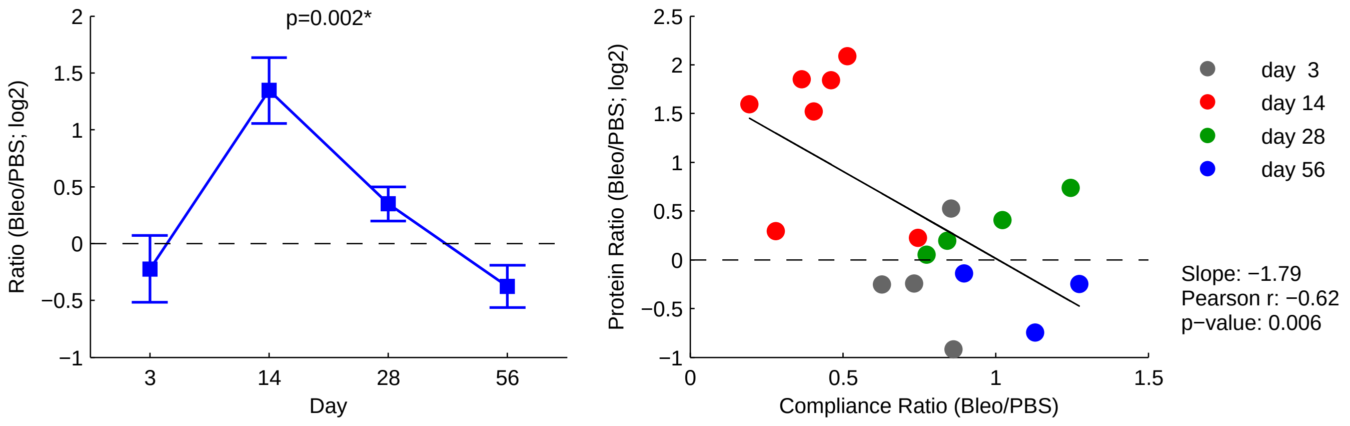

Q64191 − Aga (id: 4455)

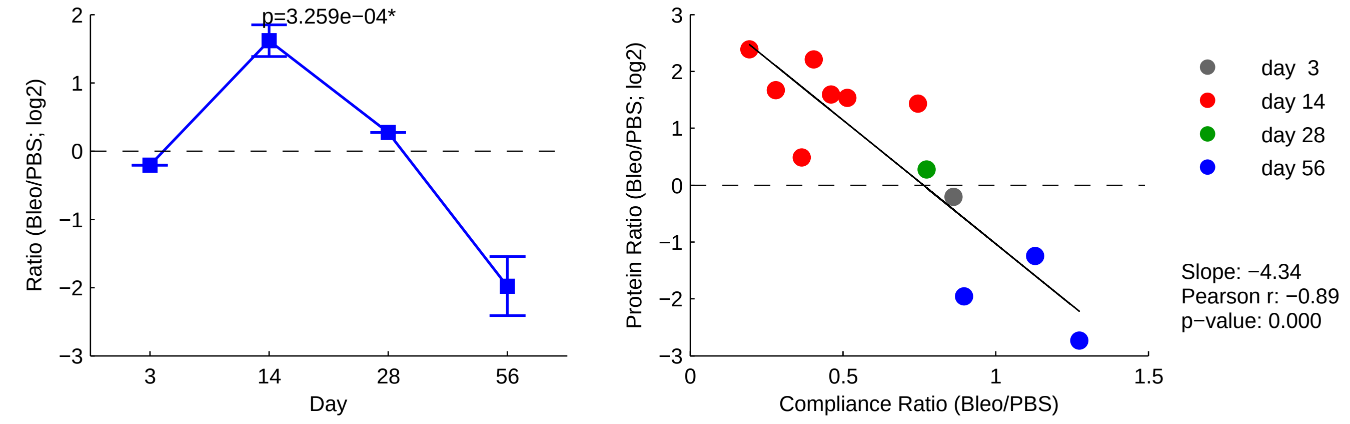

Q64277 − Bst1 (id: 4458)

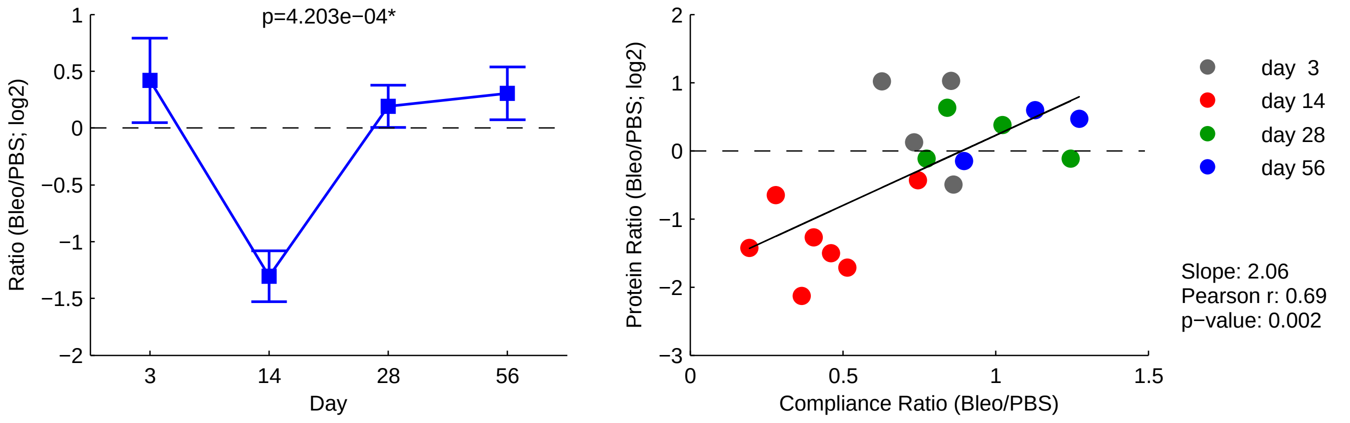

Q64282 − Ifit1 (id: 4460)

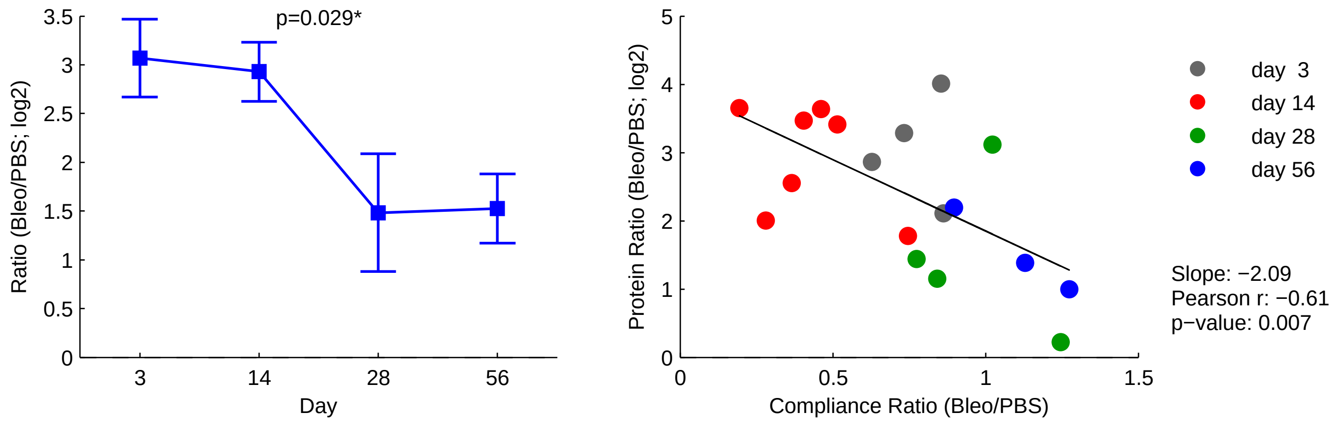

Q64310 − Surf4 (id: 4461)

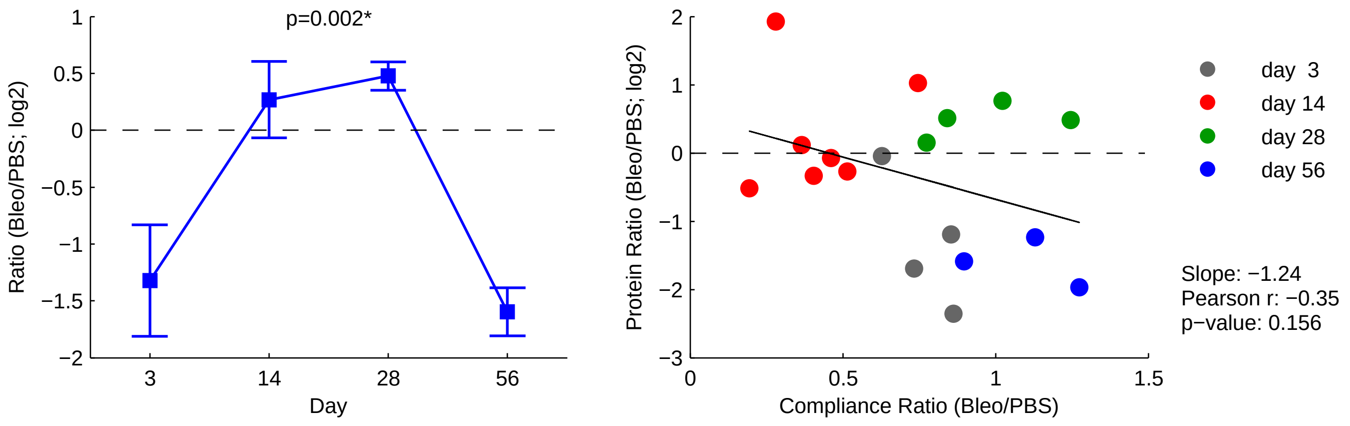

Q64314 − Cd34 (id: 4462)

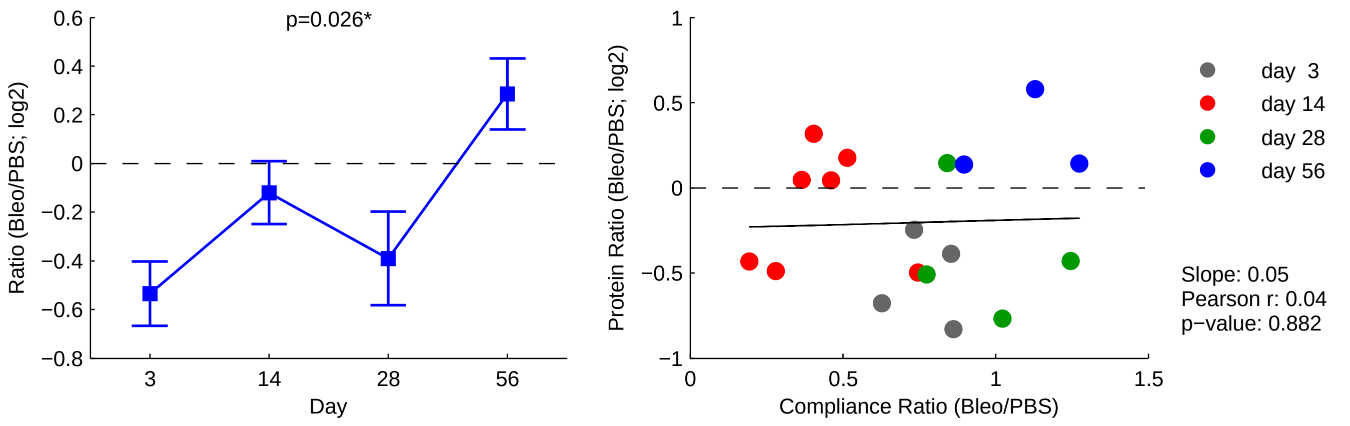

Q64337−2 − Sqstm1 (id: 4468)

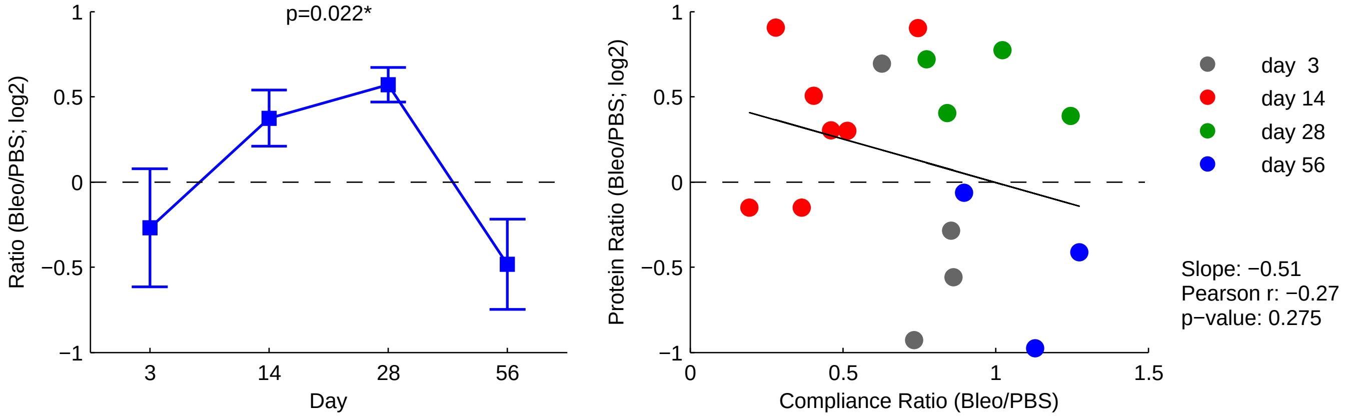

Q64339 − Isg15 (id: 4469)

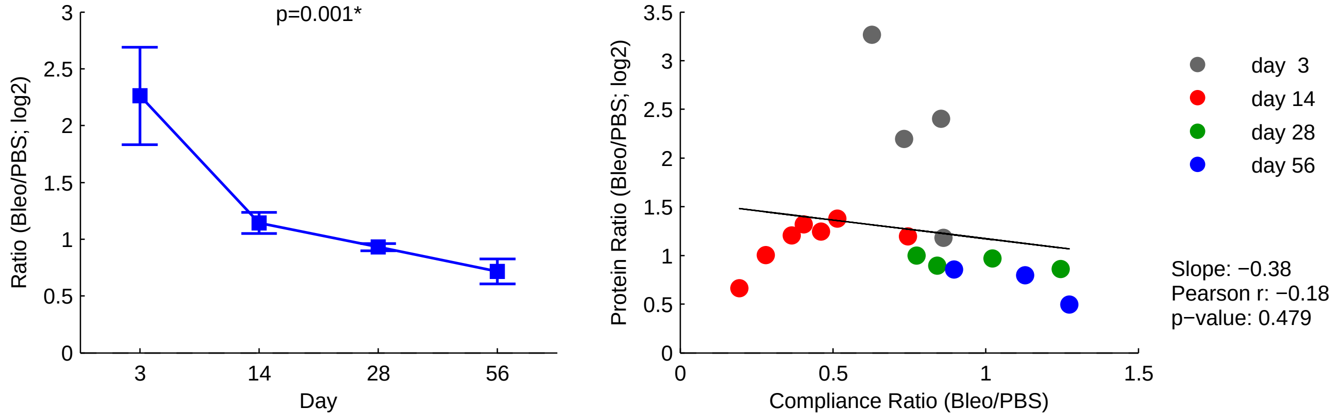

Q64373−2 − Bcl2l1 (id: 4473)

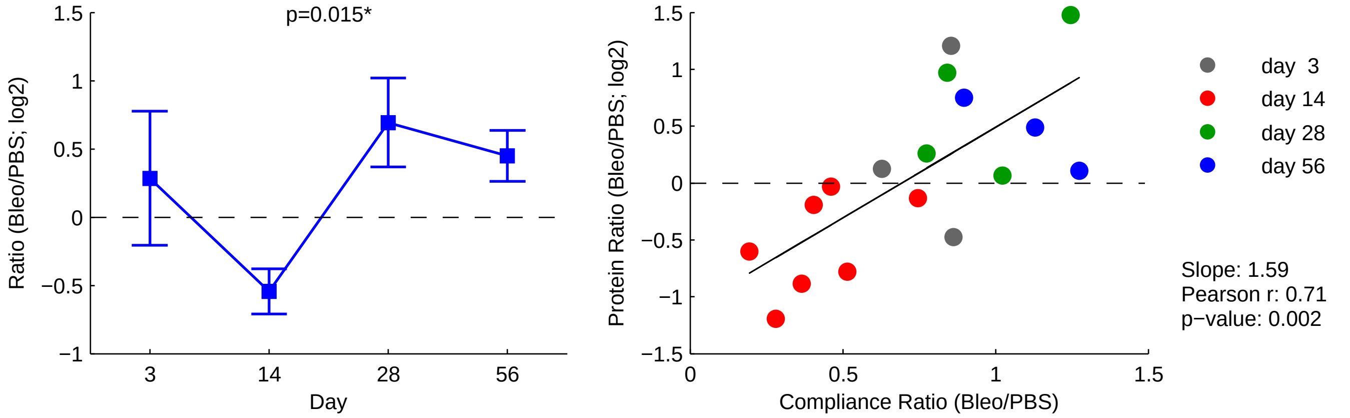

Q64378 − Fkbp5 (id: 4475)

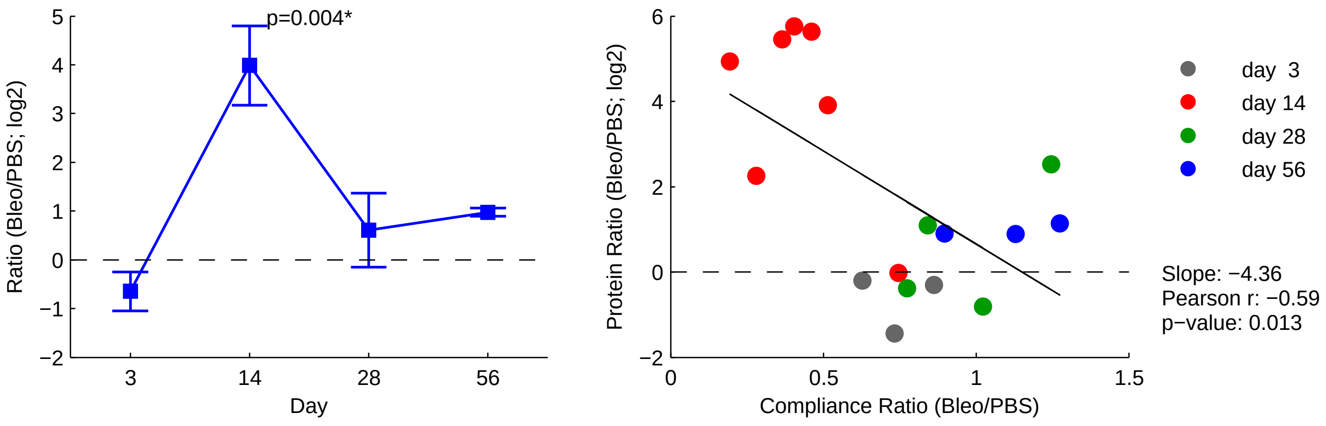

Q64435 − Ugt1a6 (id: 4477)

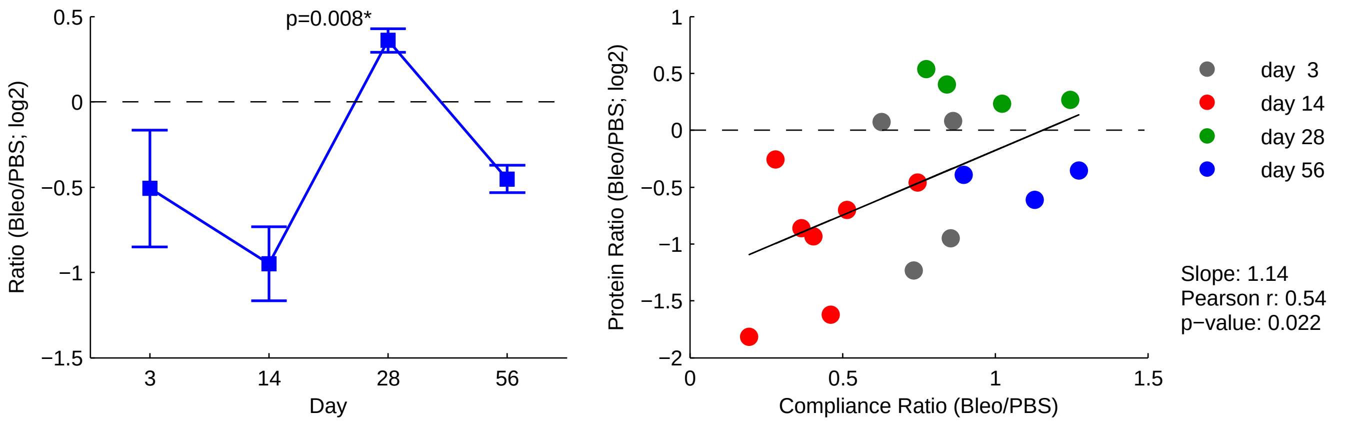

Q64437 − Adh7 (id: 4478)

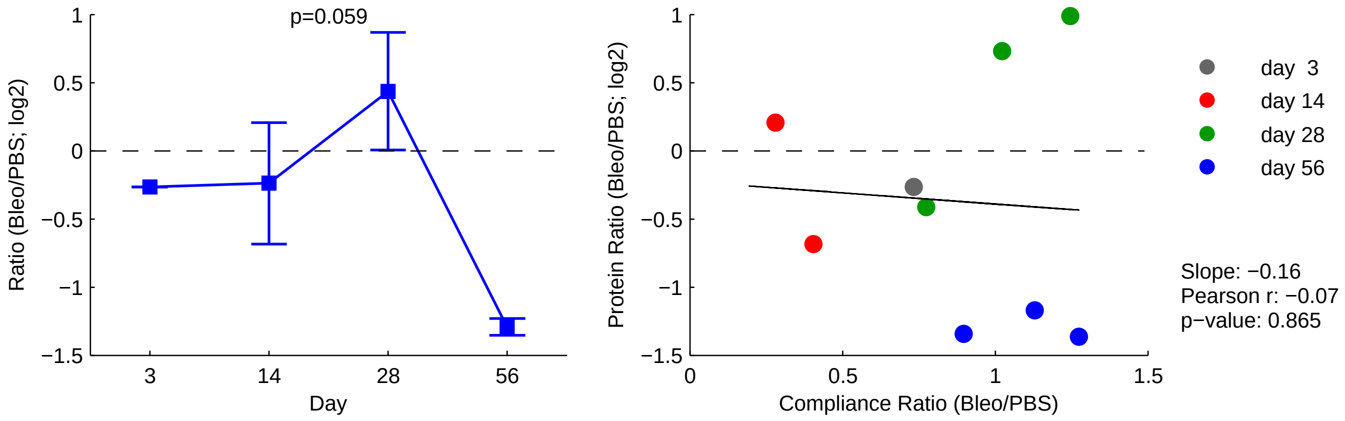

Q64442 − Sord (id: 4479)

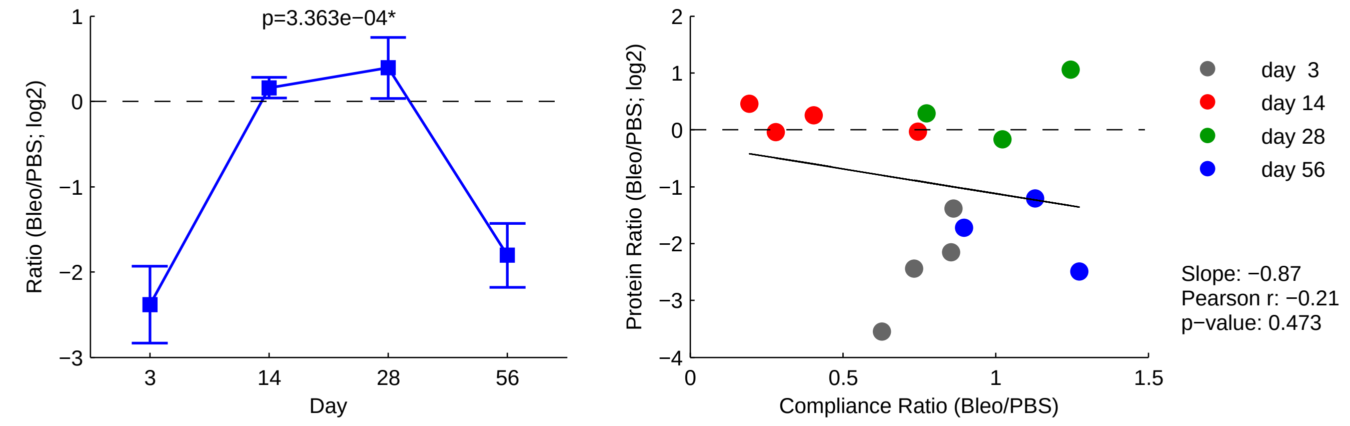

Q64444 − Ca4 (id: 4480)

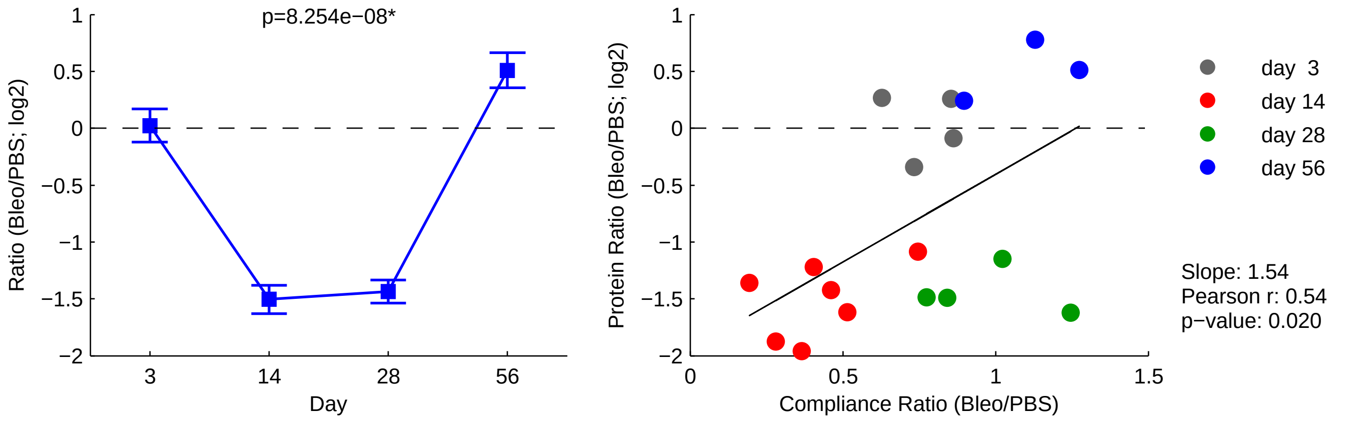

Q64449 − Mrc2 (id: 4481)

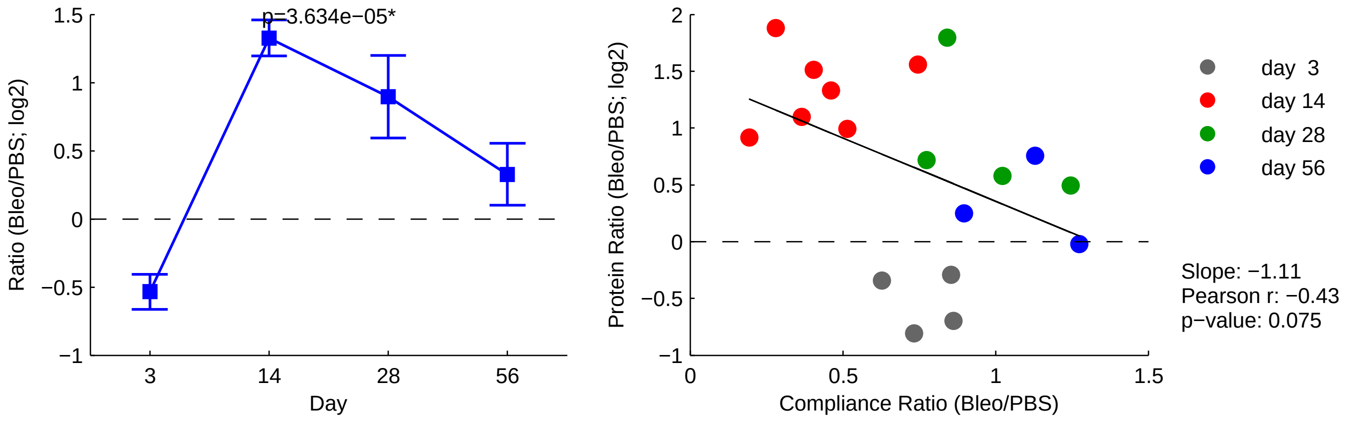

Q64462 − Cyp4b1 (id: 4482)

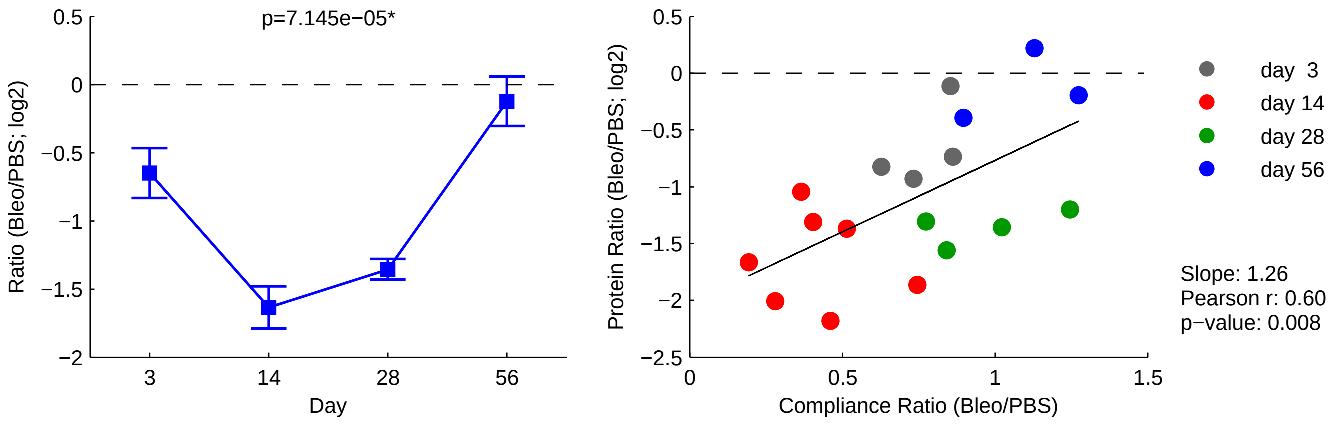

Q64464 − Cyp3a13 (id: 4483)

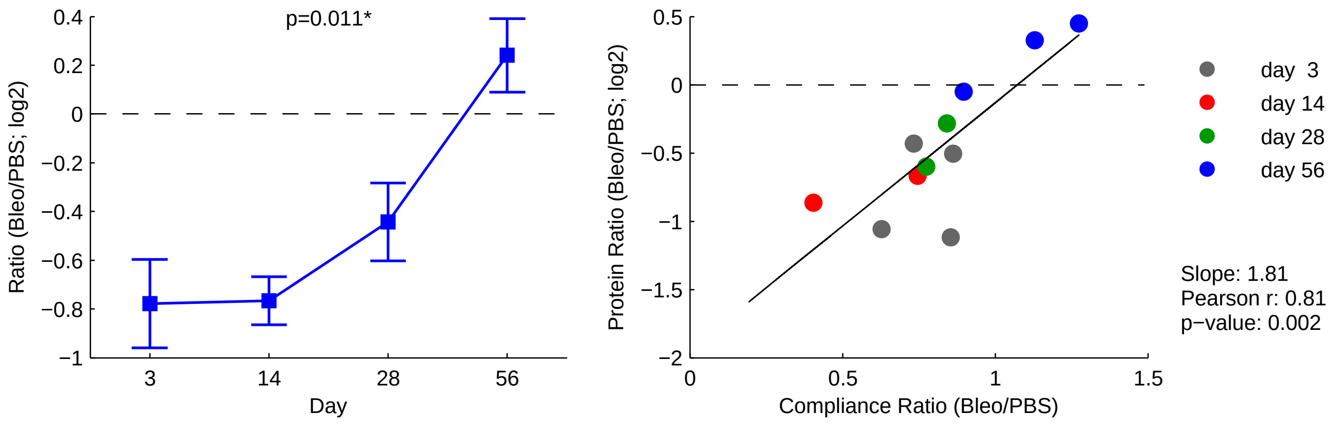

Q64471 − Gstt1 (id: 4484)

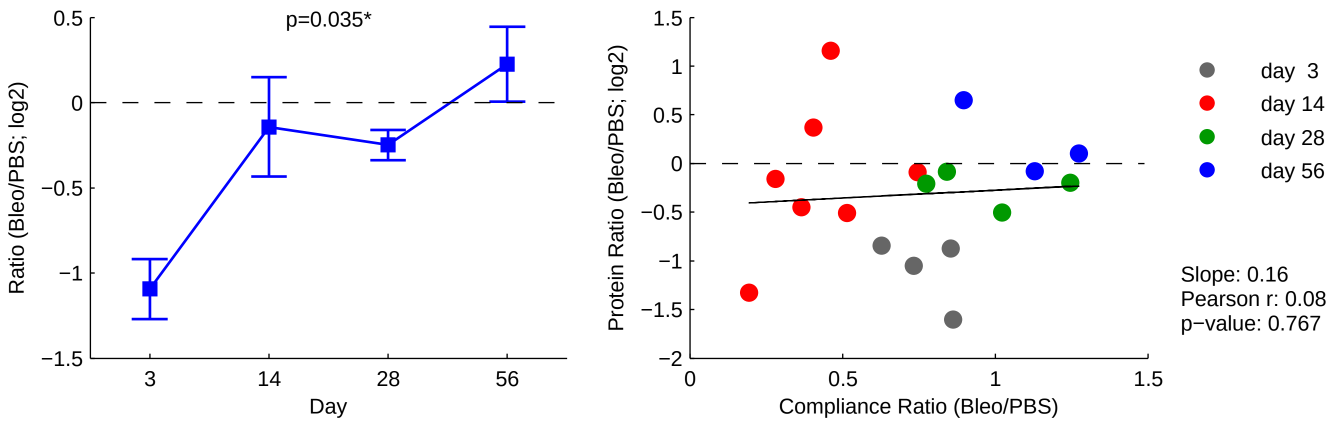

Q64514−2 − Tpp2 (id: 4488)

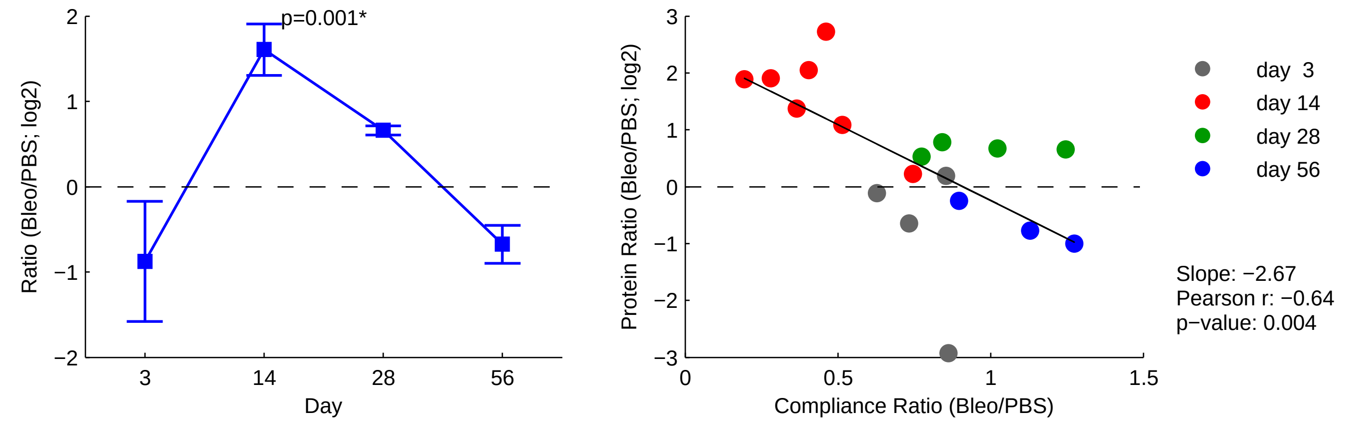

Q64521 − Gpd2 (id: 4491)

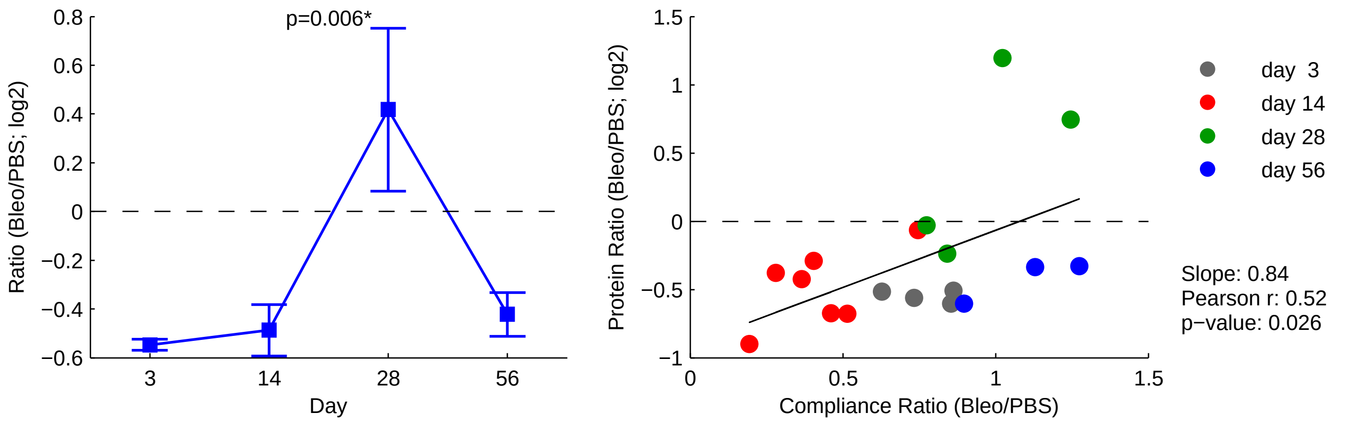

Q64674 − Srm (id: 4496)

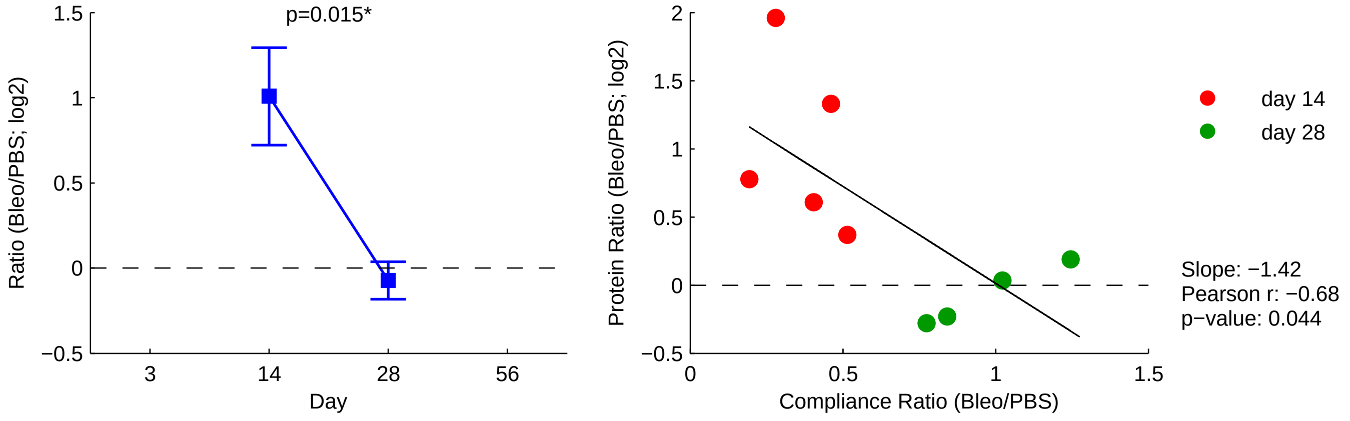

Q64695 − Procr (id: 4497)

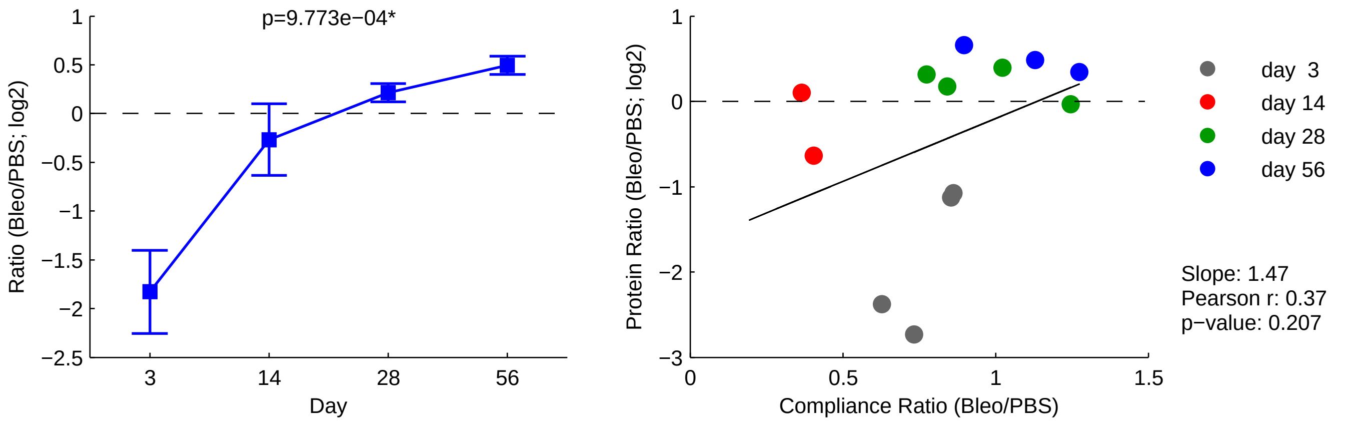

Q64727 − Vcl (id: 4500)

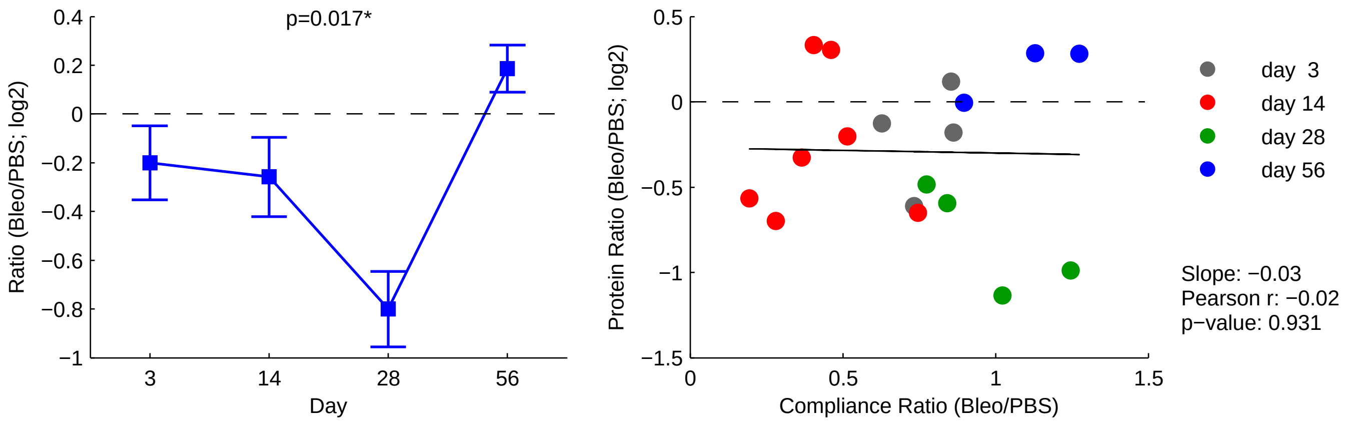

Q64735−2 − Cr1l (id: 4501)

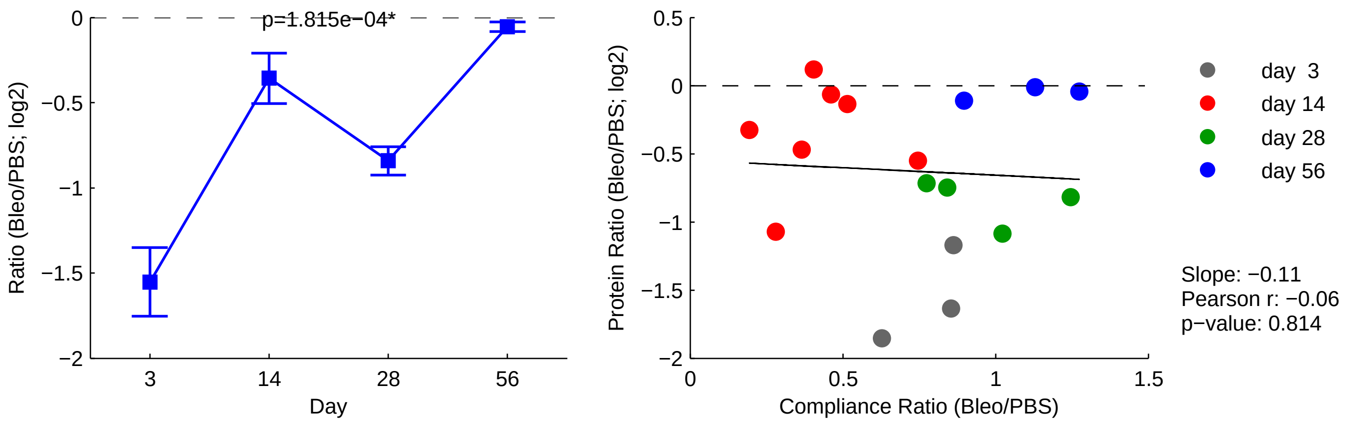

Q65CL1 − Ctnna3 (id: 4505)

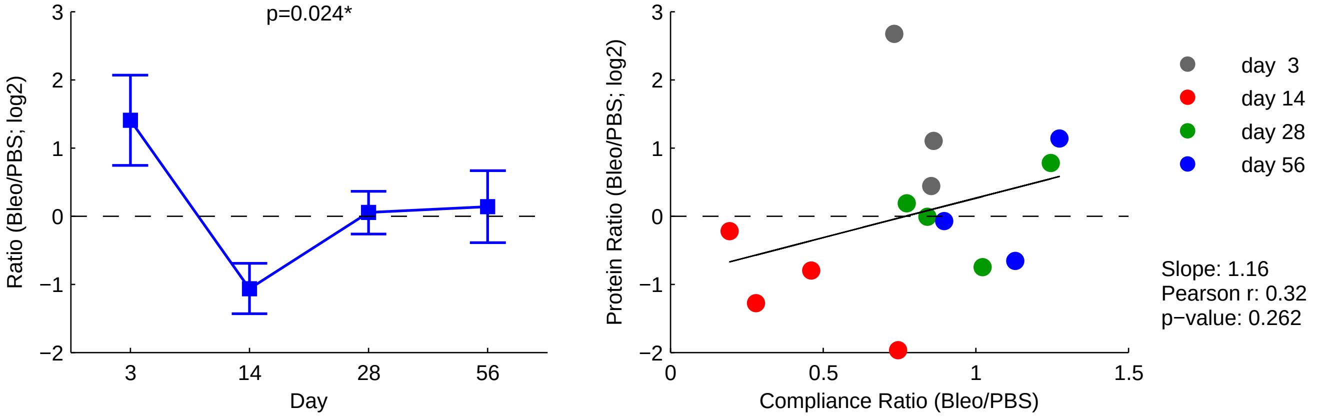

Q66GT5 − Ptpmt1 (id: 4506)

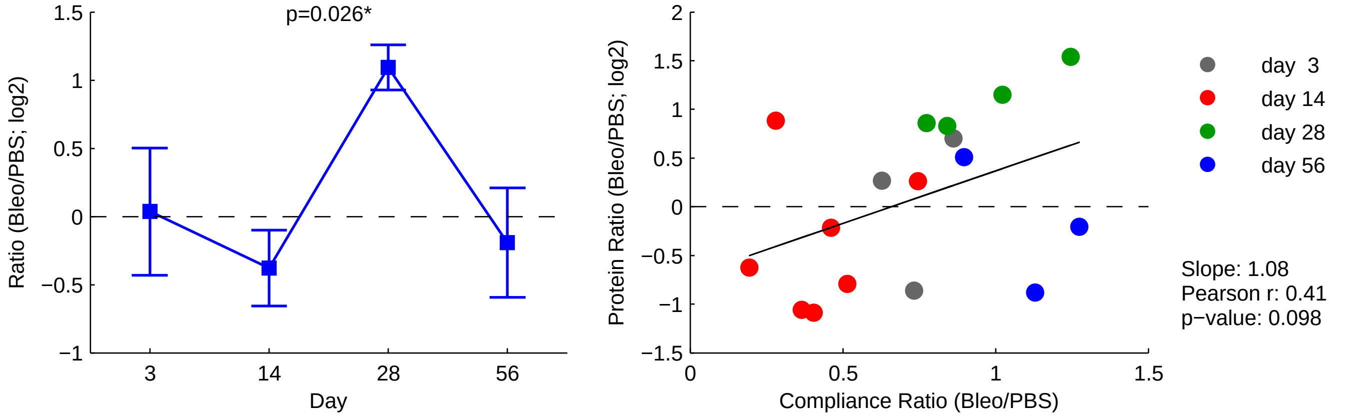

Q66JX5−2 − Fgfr1op (id: 4509)

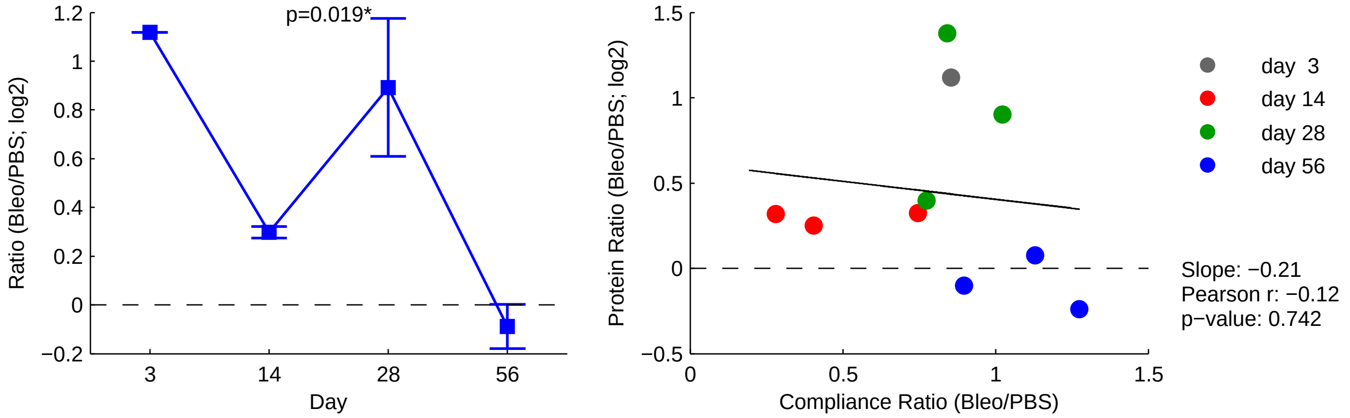

Q66K08 − Cilp (id: 4510)

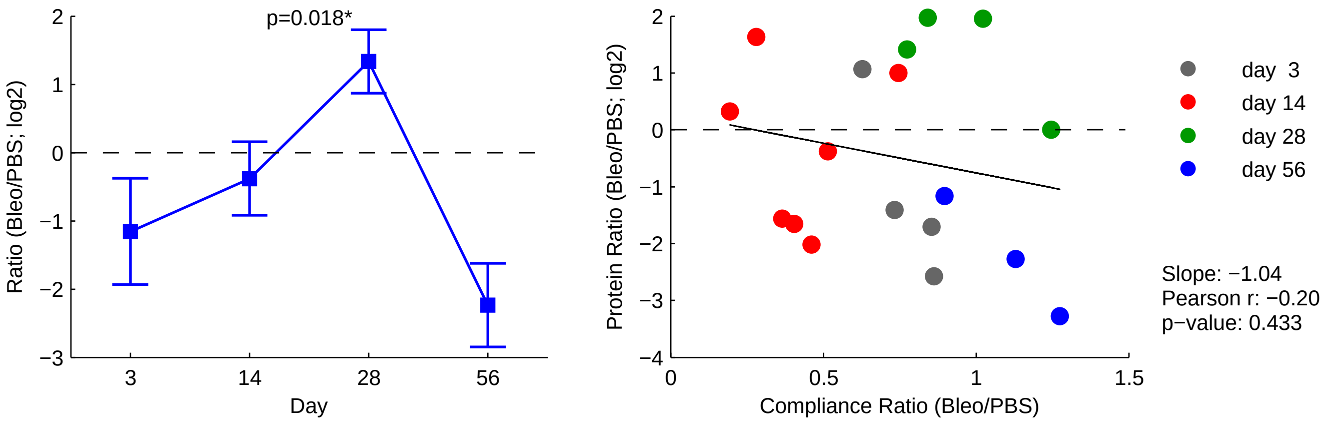

Q68FE6 − Fam65a (id: 4513)

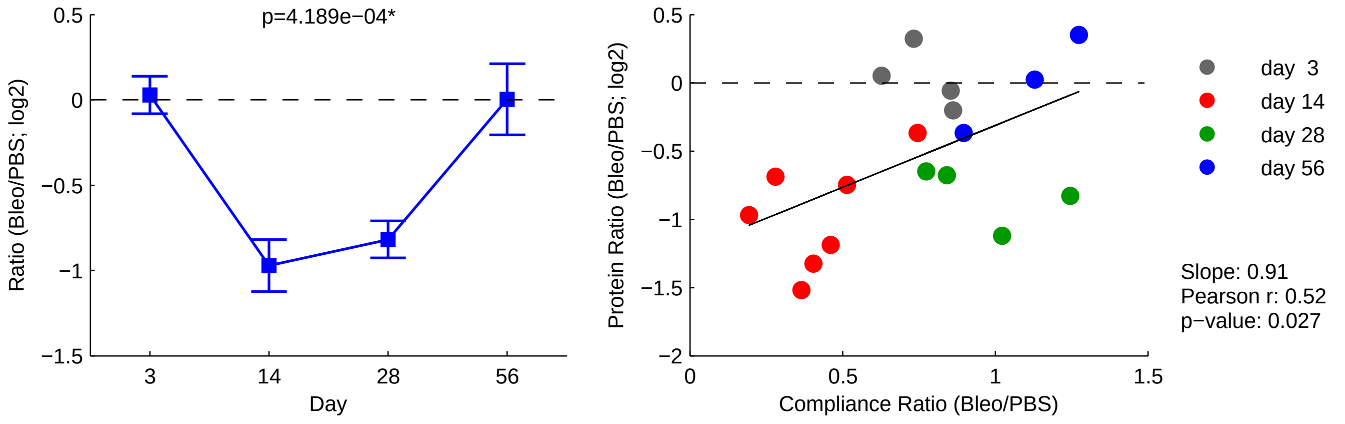

Q69Z99 − Znf512 (id: 4519)

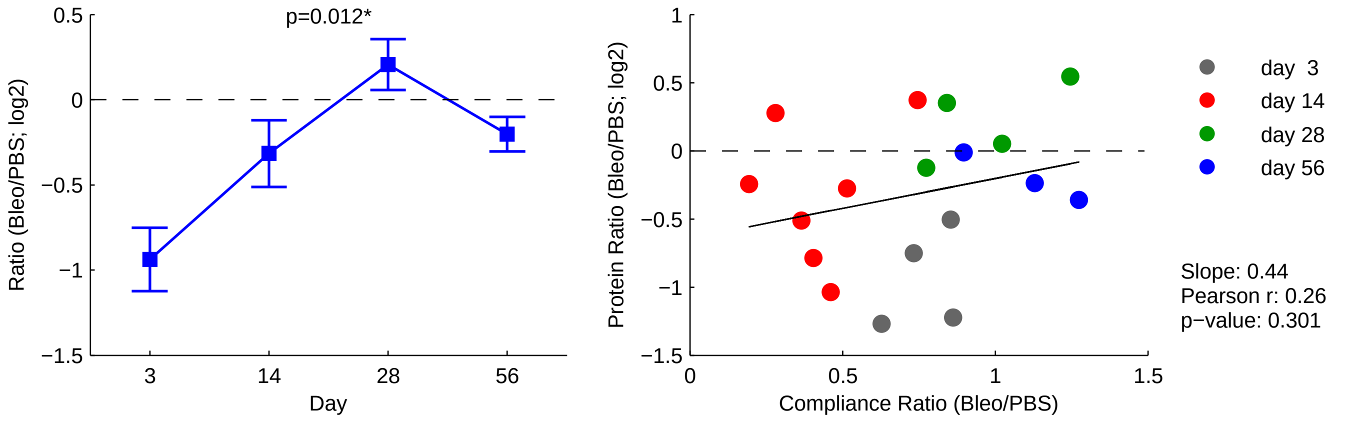

Q69ZN7 − Myof (id: 4523)

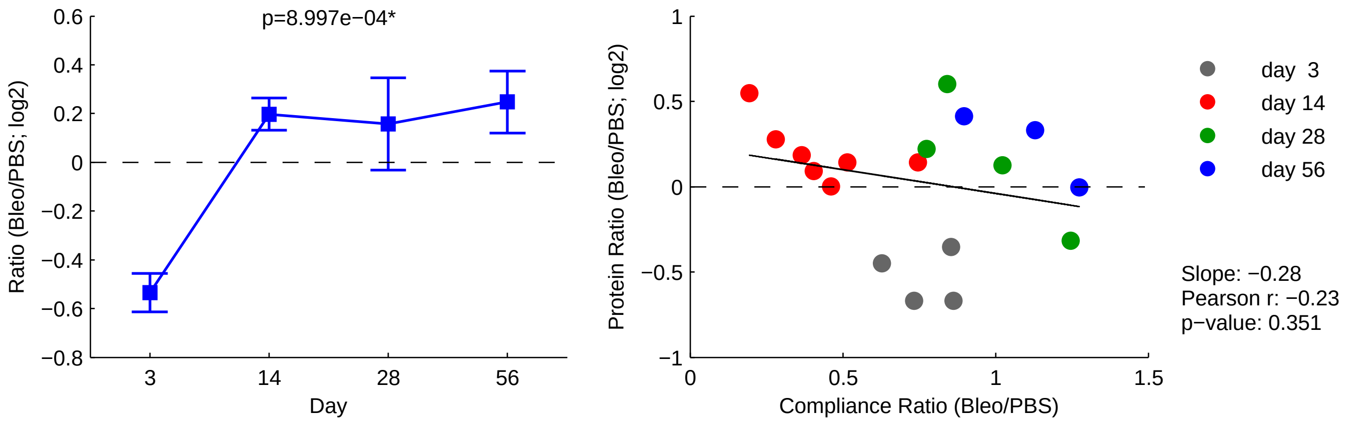

Q69ZQ2 − Isy1 (id: 4525)

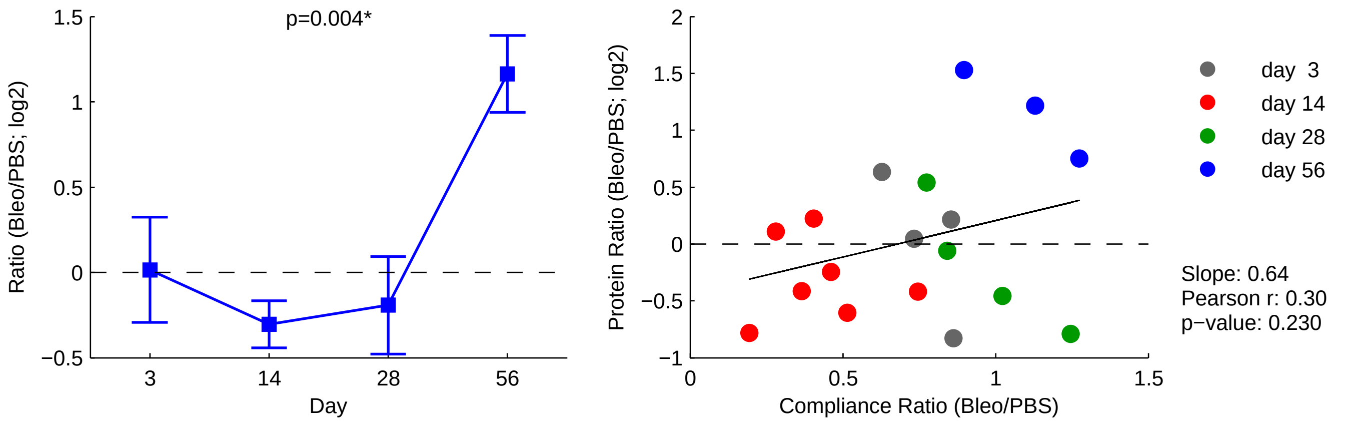

Q69ZR9−2 − Fam208a (id: 4526)

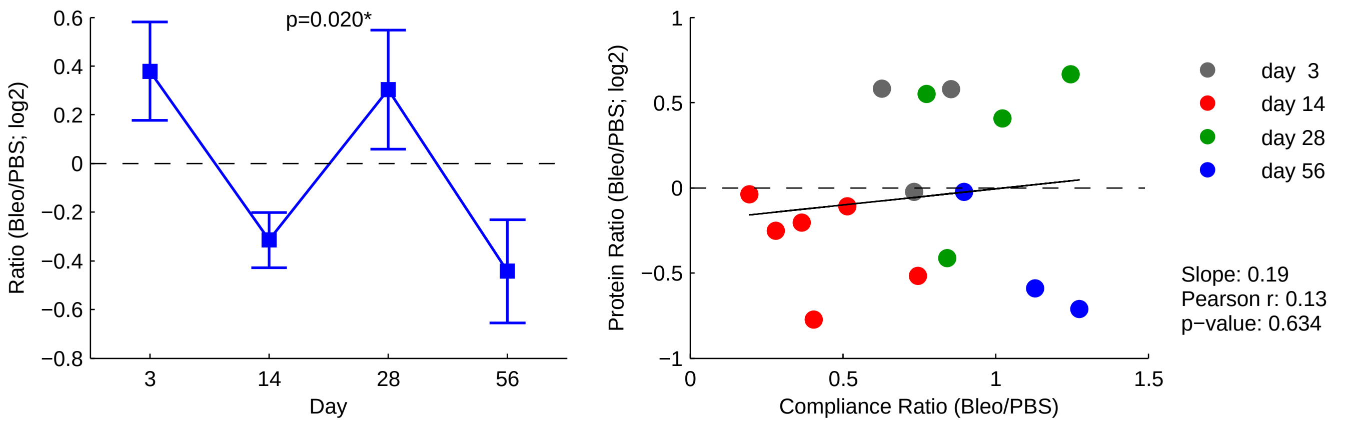

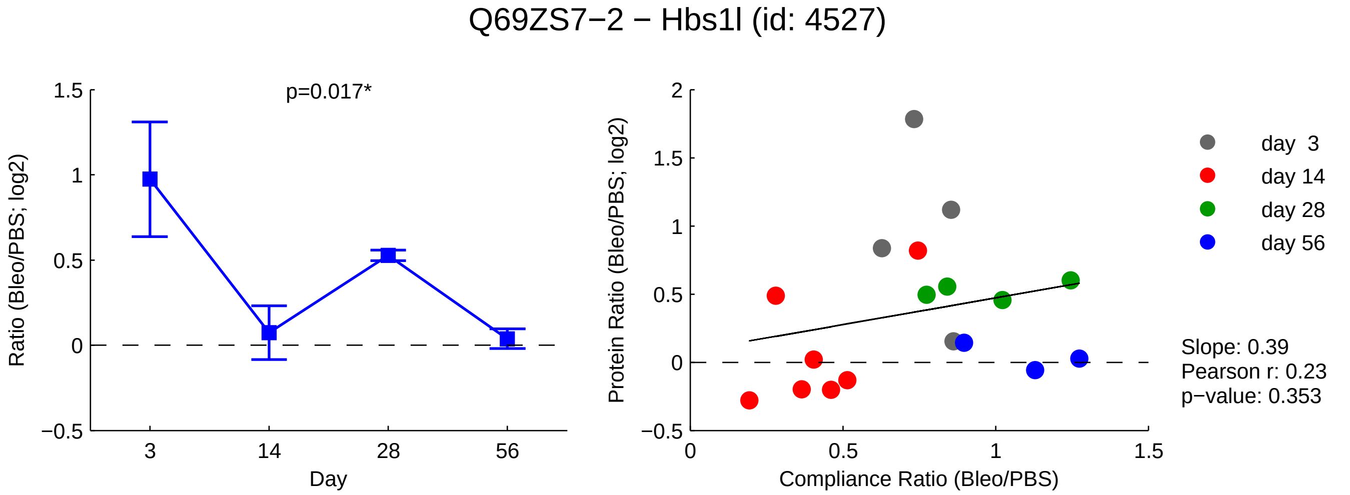
Q69ZS7−2 − Hbs1l (id: 4527)
p=0.017*
Ratio (Bleo/PBS; log2)
Day
Protein Ratio (Bleo/PBS; log2)
Compliance Ratio (Bleo/PBS)
day 3
day 14
day 28
day 56
Slope: 0.39
Pearson r: 0.23
p−value: 0.353

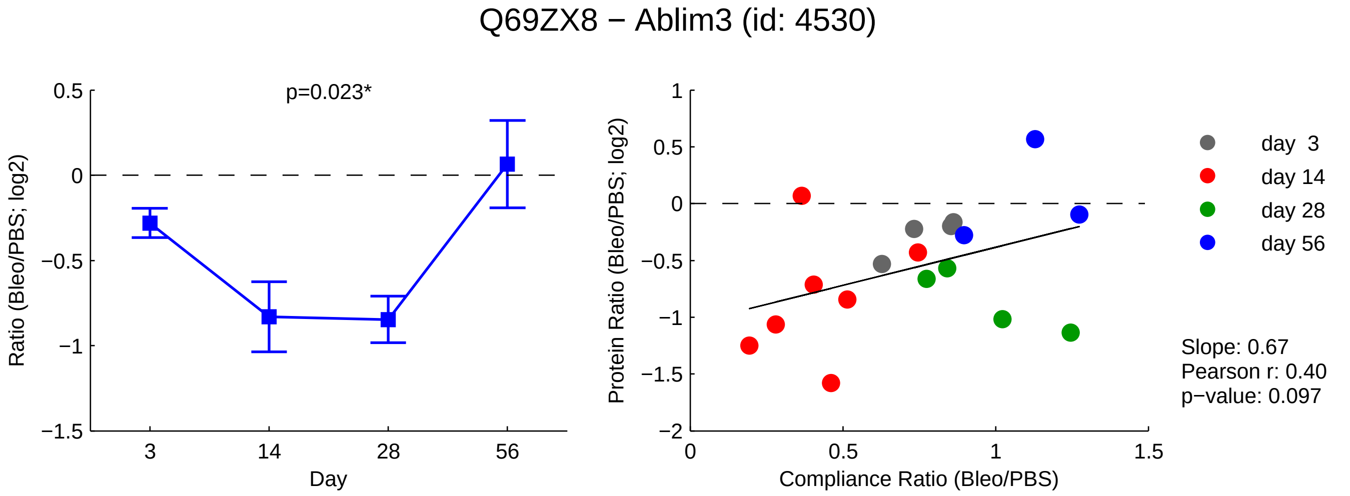
Q69ZX8 − Ablim3 (id: 4530)
p=0.023*
Ratio (Bleo/PBS; log2)
Day
Protein Ratio (Bleo/PBS; log2)
Compliance Ratio (Bleo/PBS)
day 3
day 14
day 28
day 56
Slope: 0.67
Pearson r: 0.40
p−value: 0.097

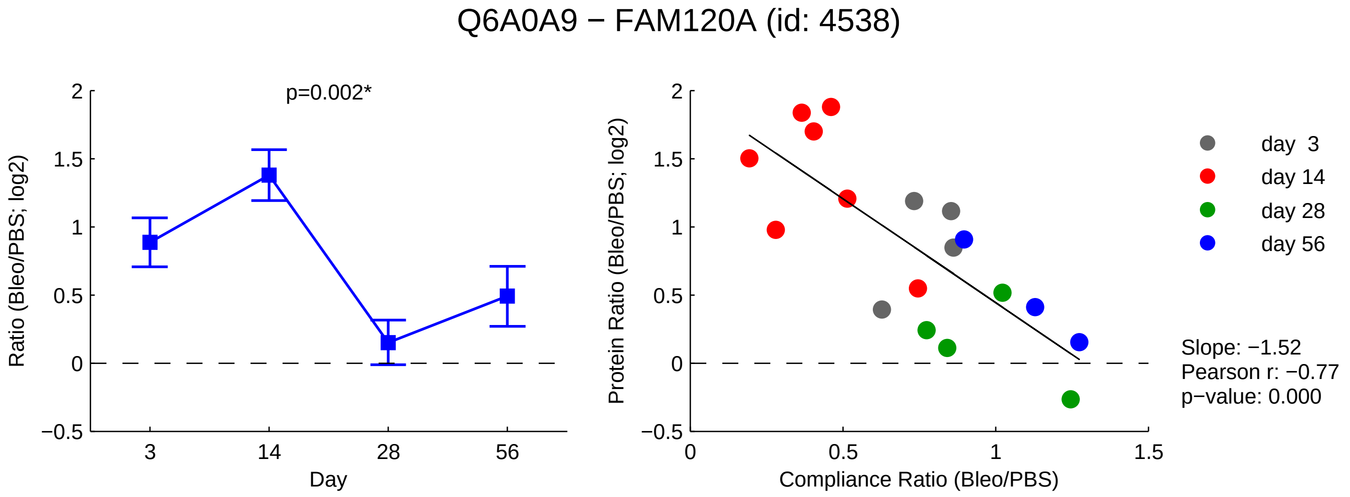
Q6A0A9 − FAM120A (id: 4538)
p=0.002*
Ratio (Bleo/PBS; log2)
Day
Protein Ratio (Bleo/PBS; log2)
Compliance Ratio (Bleo/PBS)
day 3
day 14
day 28
day 56
Slope: −1.52
Pearson r: −0.77
p−value: 0.000

Q6A0D4 − Rftn1 (id: 4539)

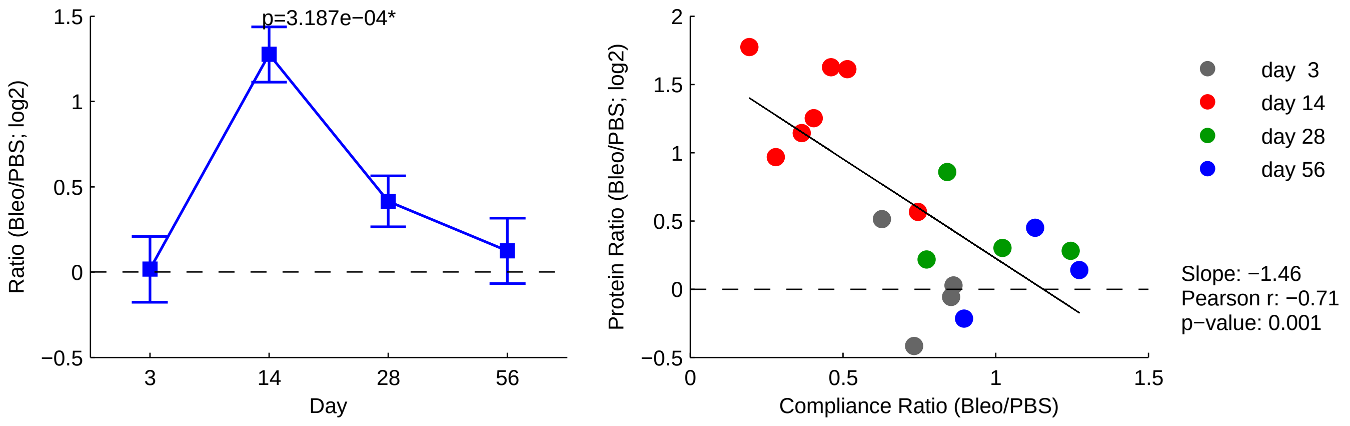

Q6DIA2 − Exoc3l4 (id: 4545)

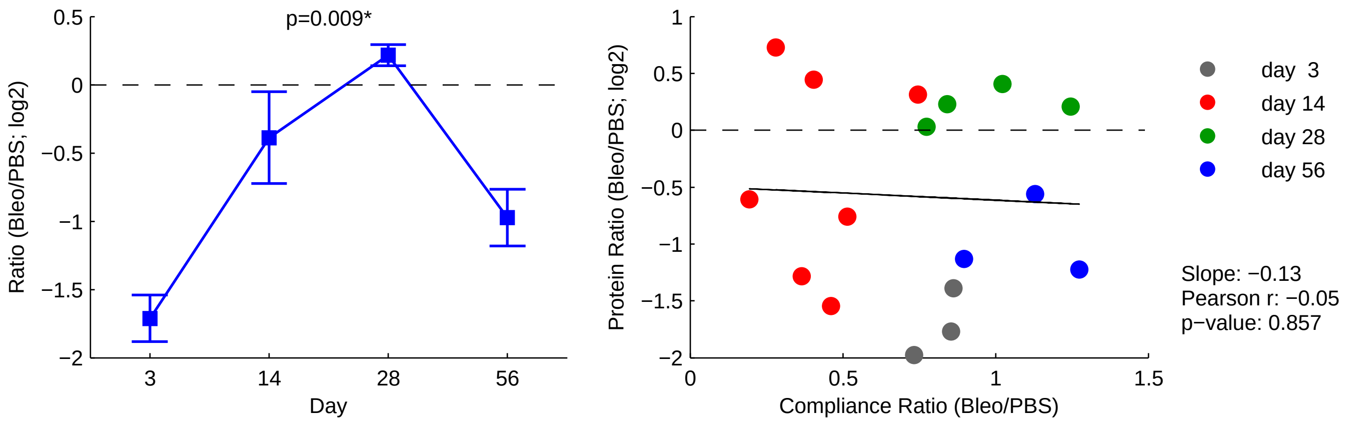

Q6DID7 − Wls (id: 4547)

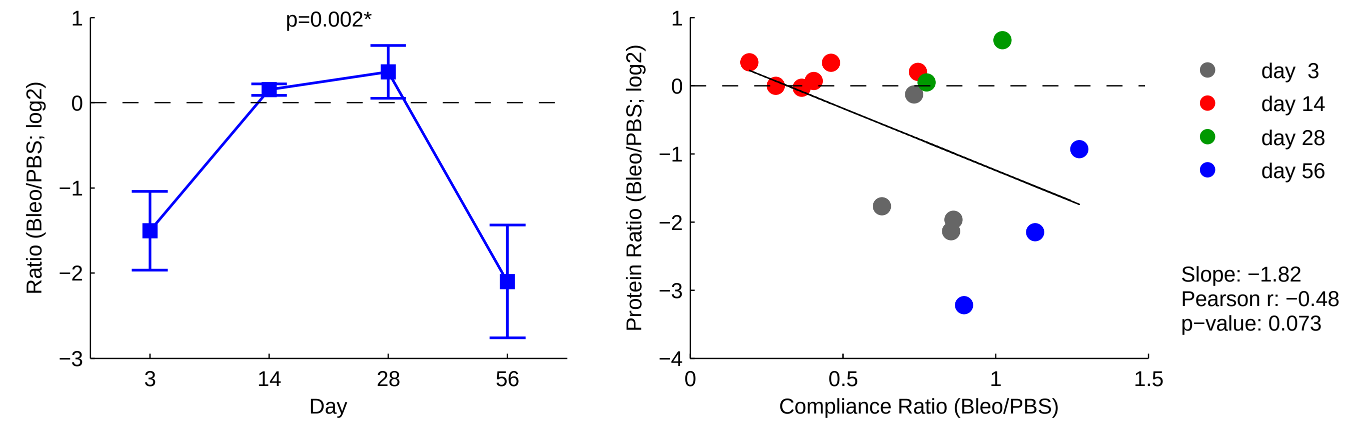

Q6DYE8 − Enpp3 (id: 4549)

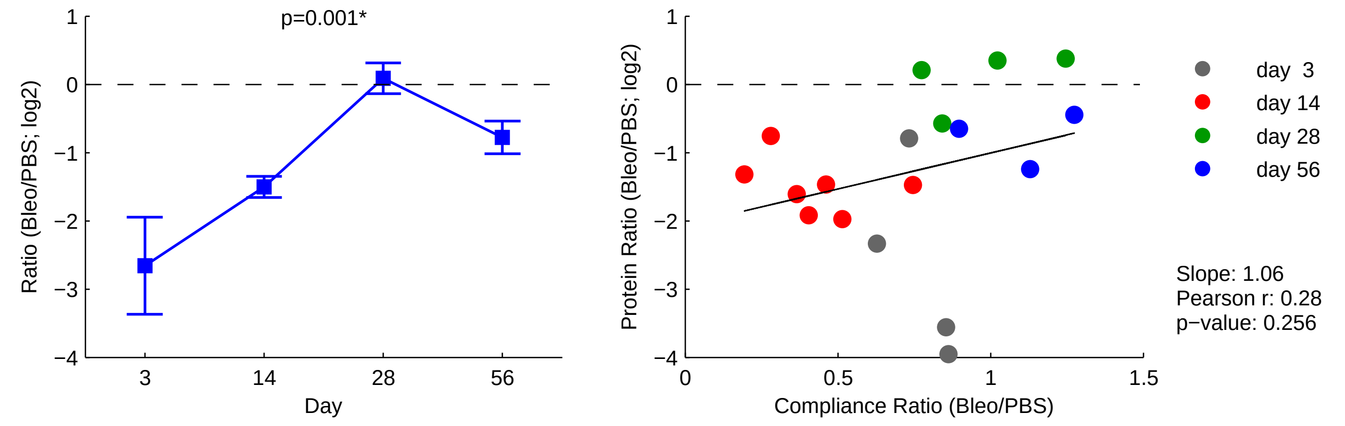

Q6GT24 − Prdx6 (id: 4556)

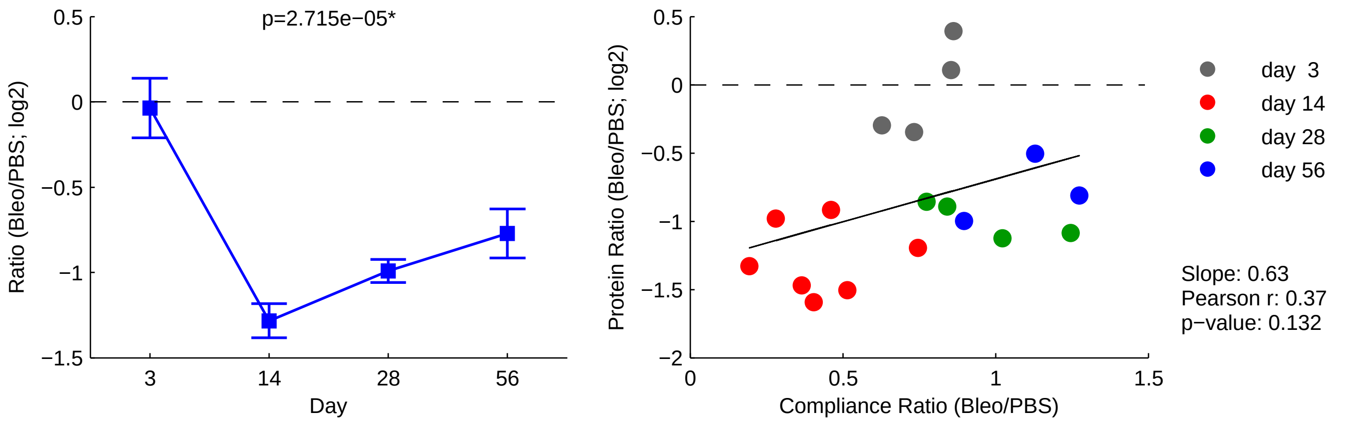

Q6GV12 − Kdsr (id: 4558)

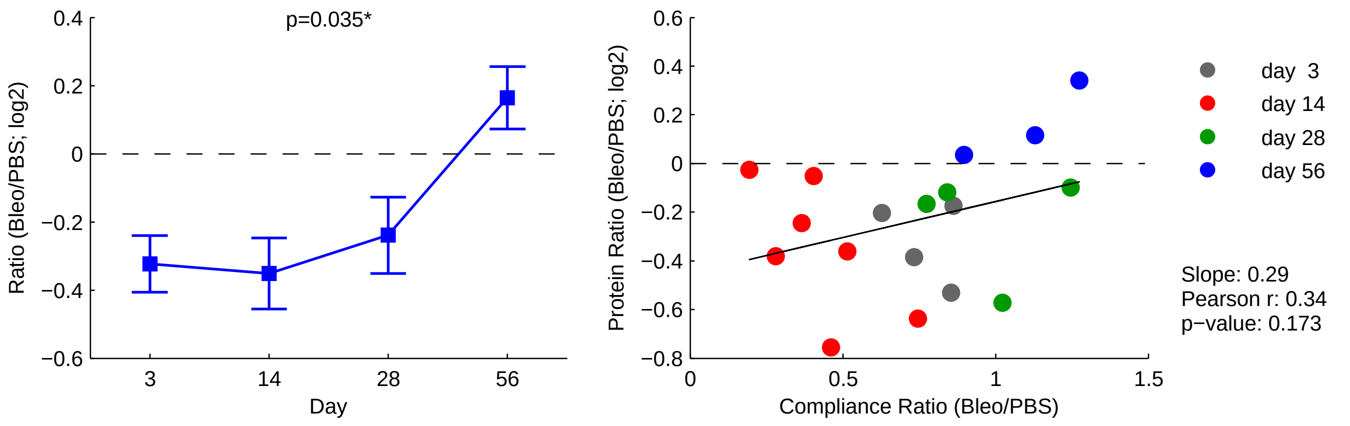

Q6GYP7 − Ralgapa1 (id: 4559)

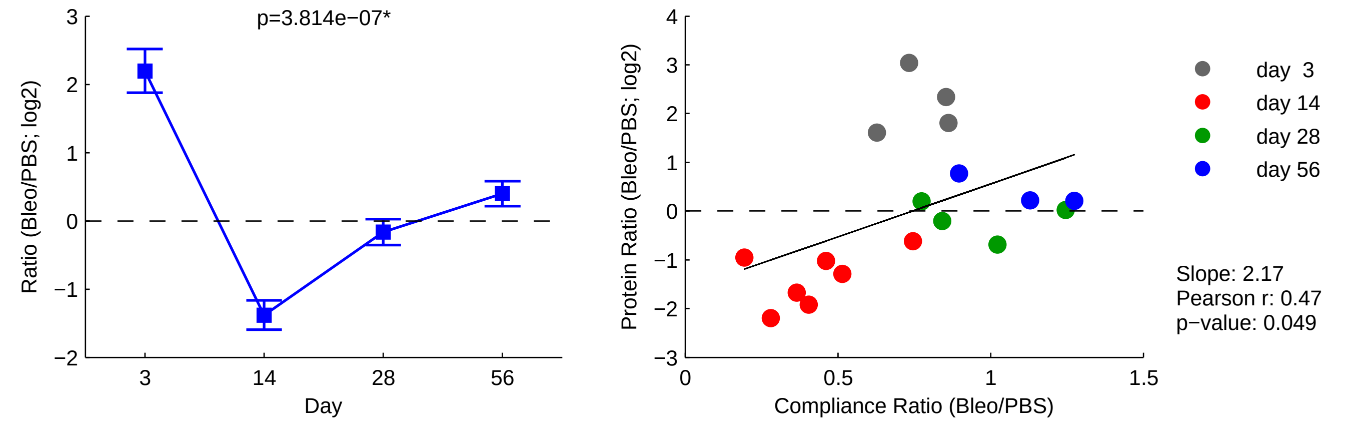

Q6IRU2 − Tpm4 (id: 4563)

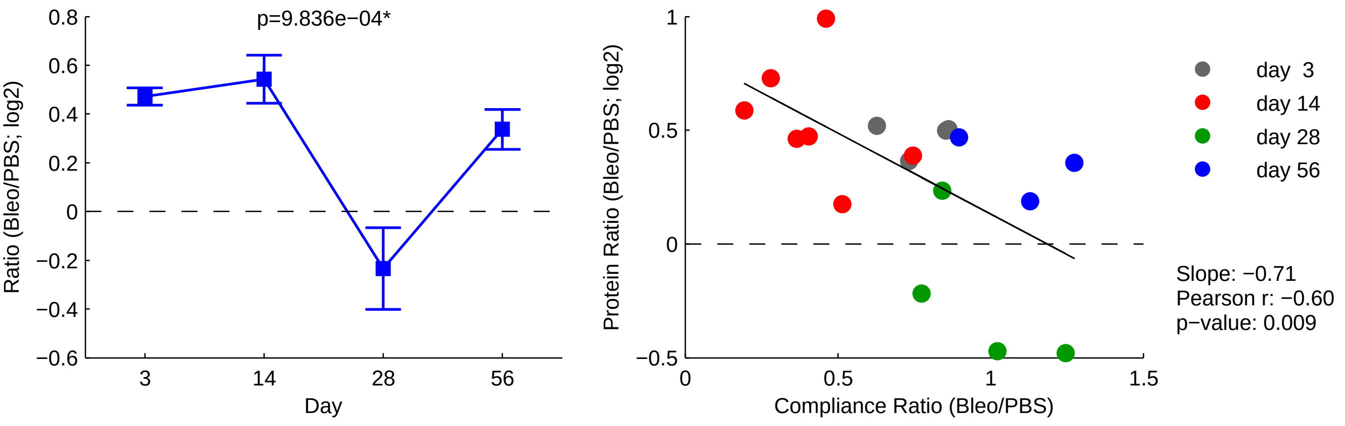

Q6IRU5−2 − Cltb (id: 4564)

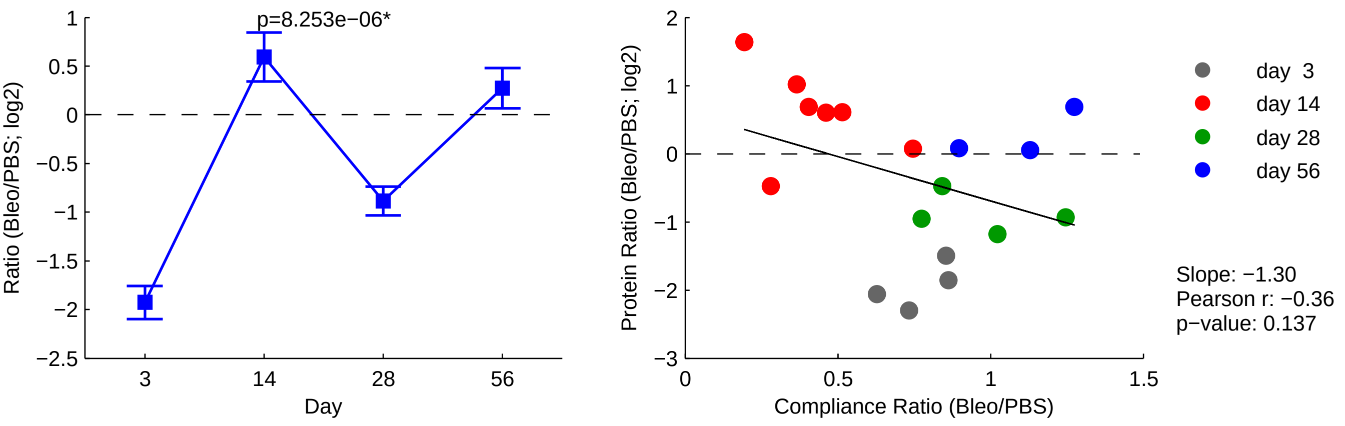

Q6IRU7 − Cep78 (id: 4565)

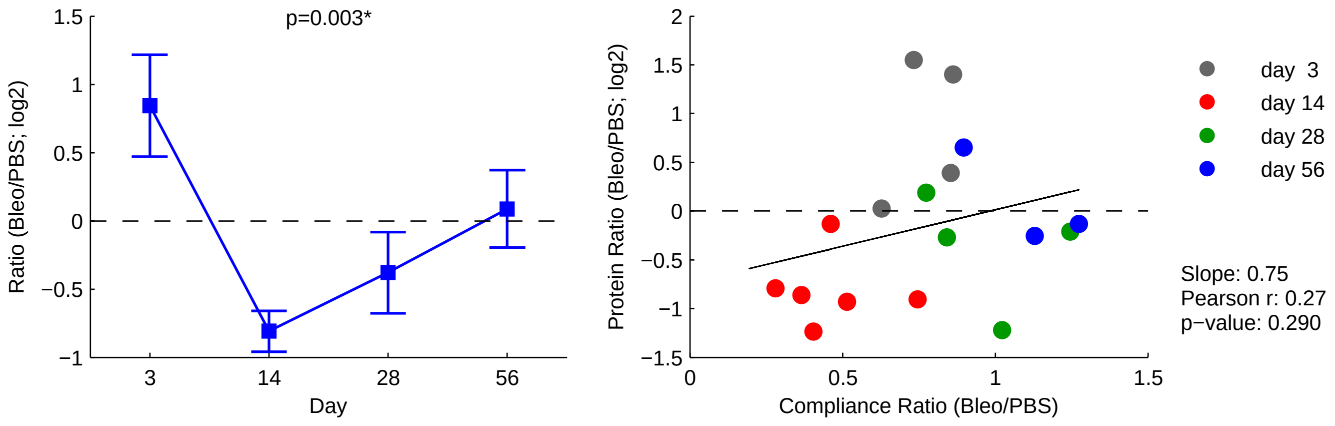

Q6KAR6 − Exoc3 (id: 4566)

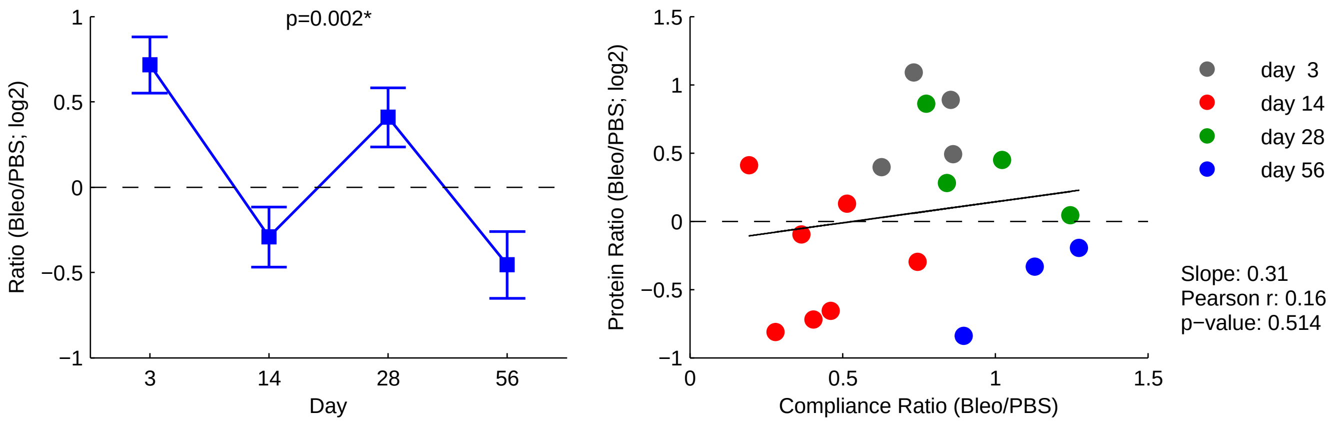

Q6NS46 − Pdcd11 (id: 4568)

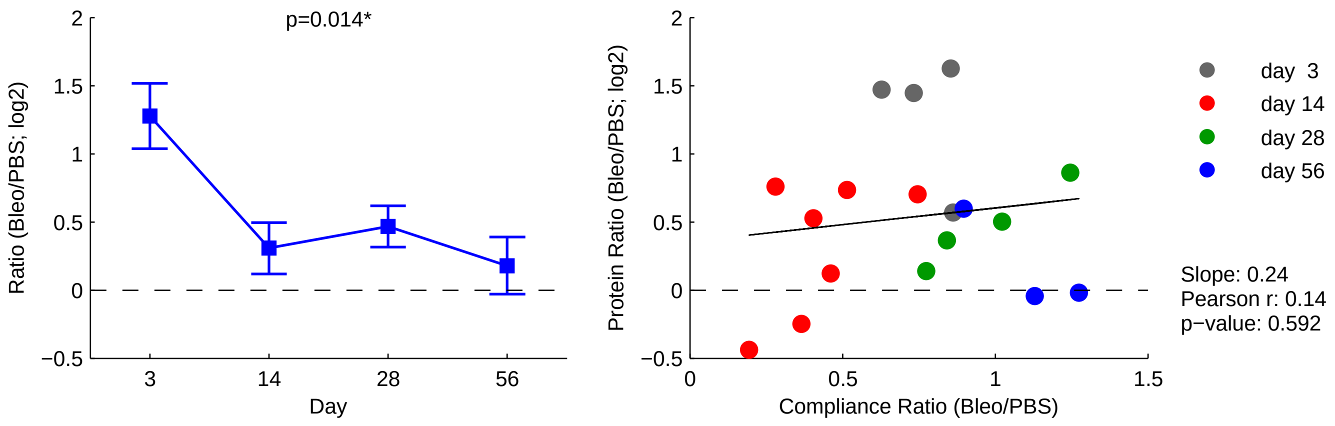

Q6NVD0 − Frem2 (id: 4578)

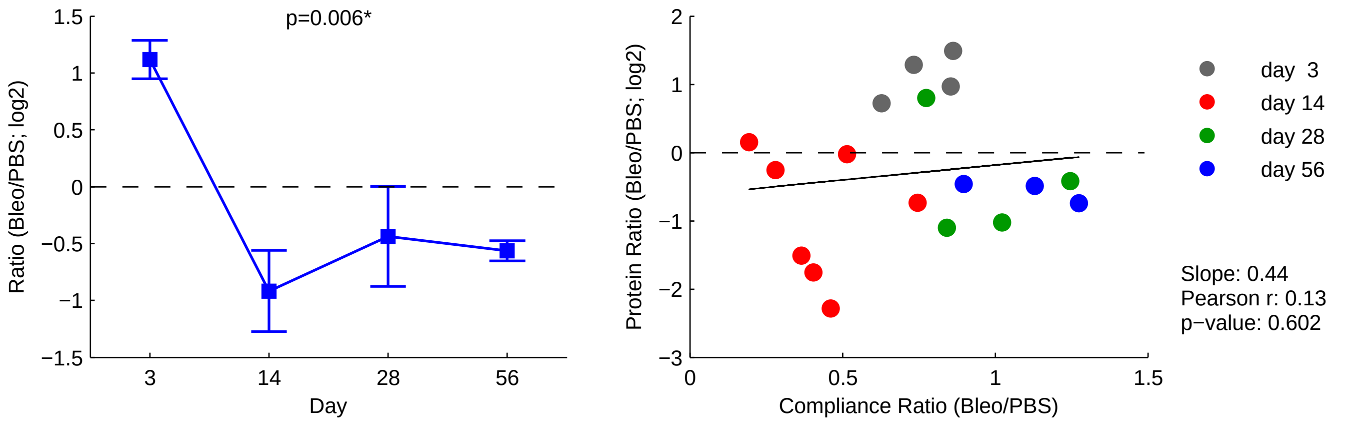

Q6NVF2 − Pld1 (id: 4581)

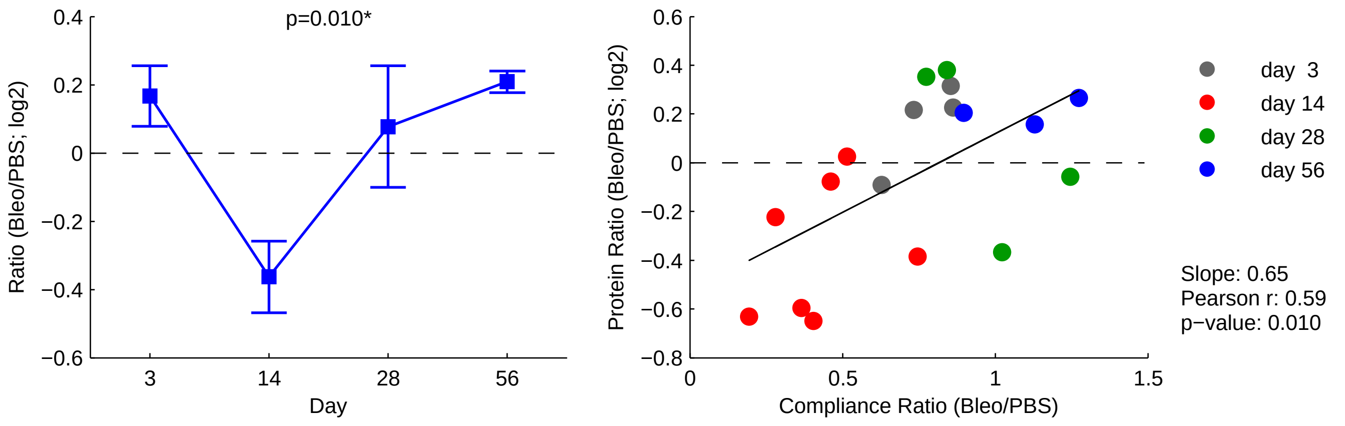

Q6NWW9 − Fndc3b (id: 4584)

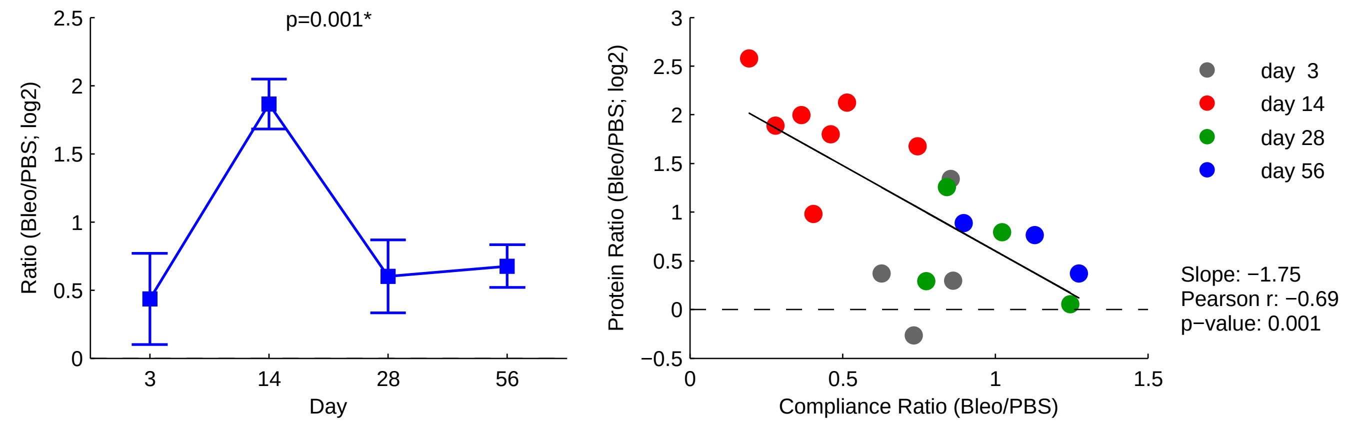

Q6NXI6−2 − Rprd2 (id: 4586)

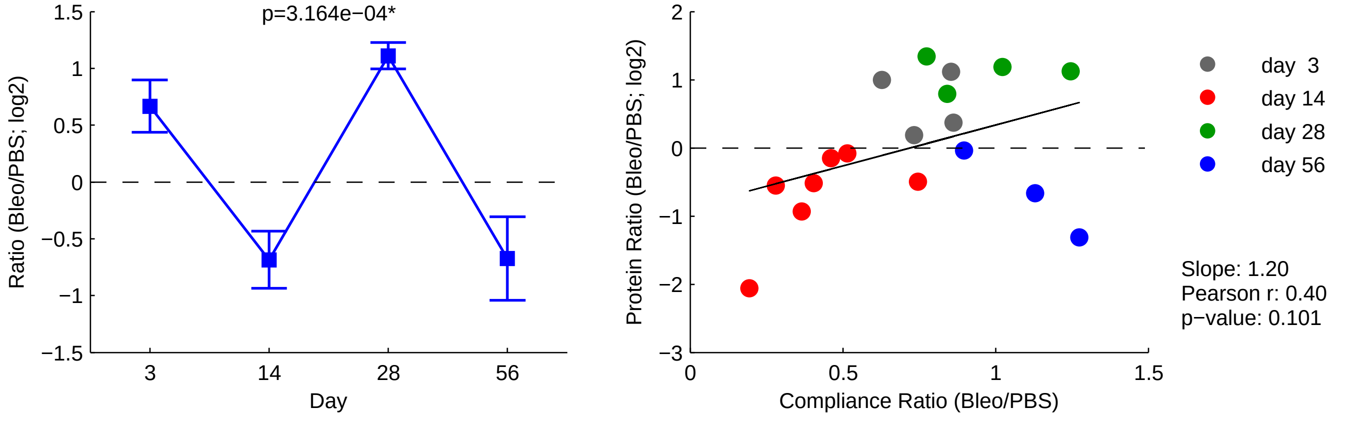

Q6NXL1 − Sec24d (id: 4588)

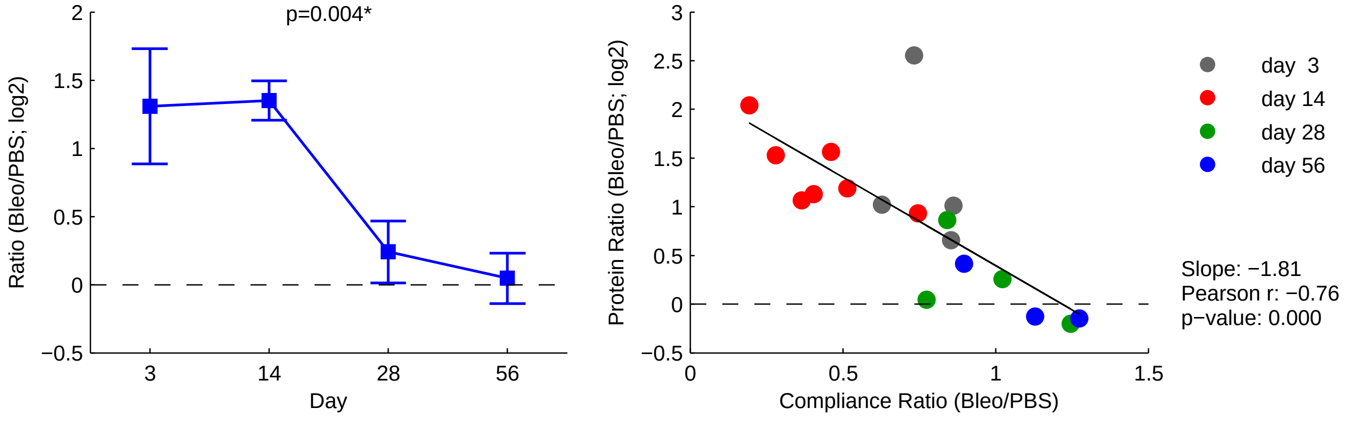

Q6NXM2 − Rcbtb1 (id: 4589)

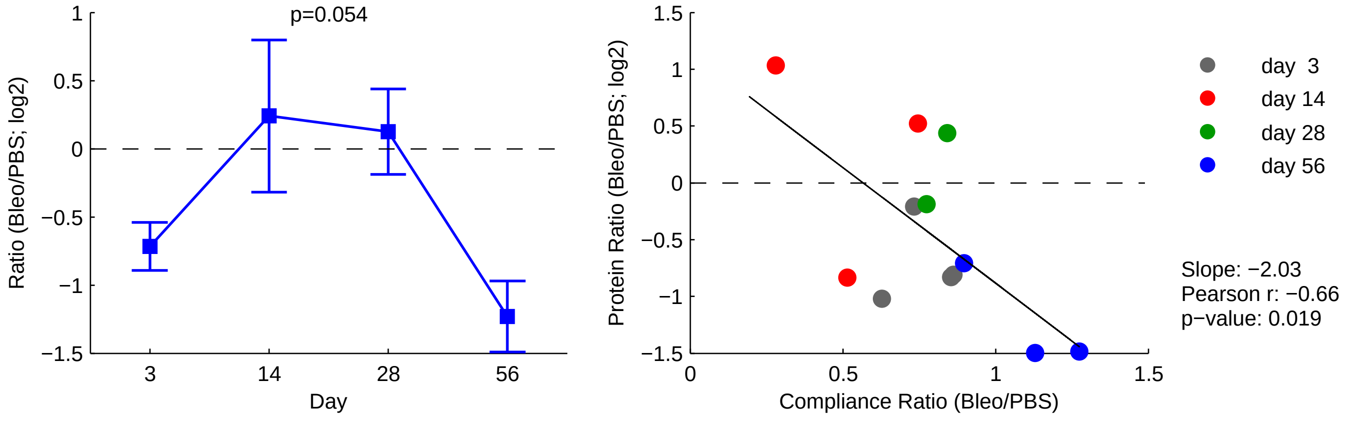

Q6NY15 − Tsga10 (id: 4591)

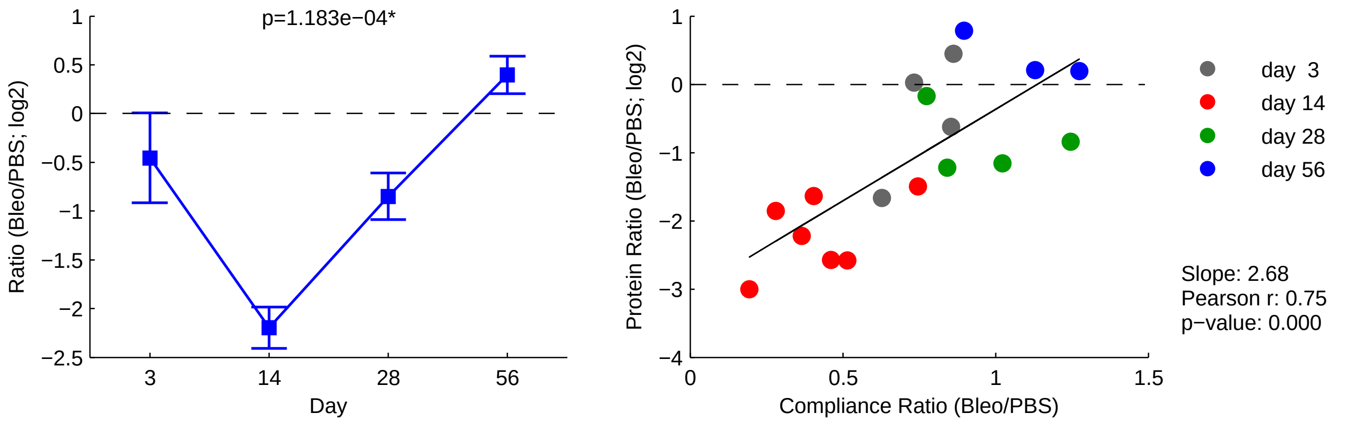

Q6NZB0 − Dnajc8 (id: 4592)

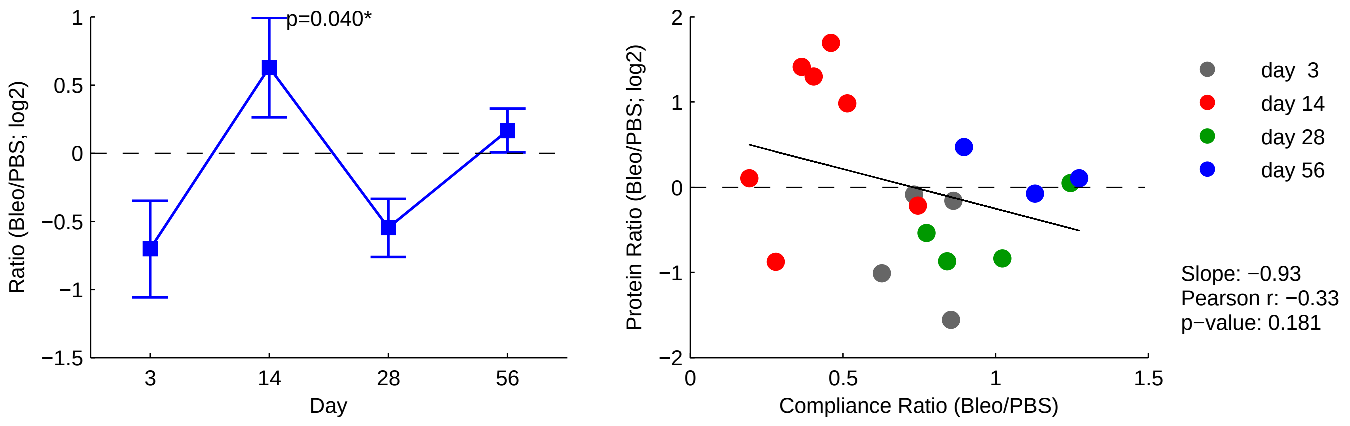

Q6NZD2 − Snx1 (id: 4593)

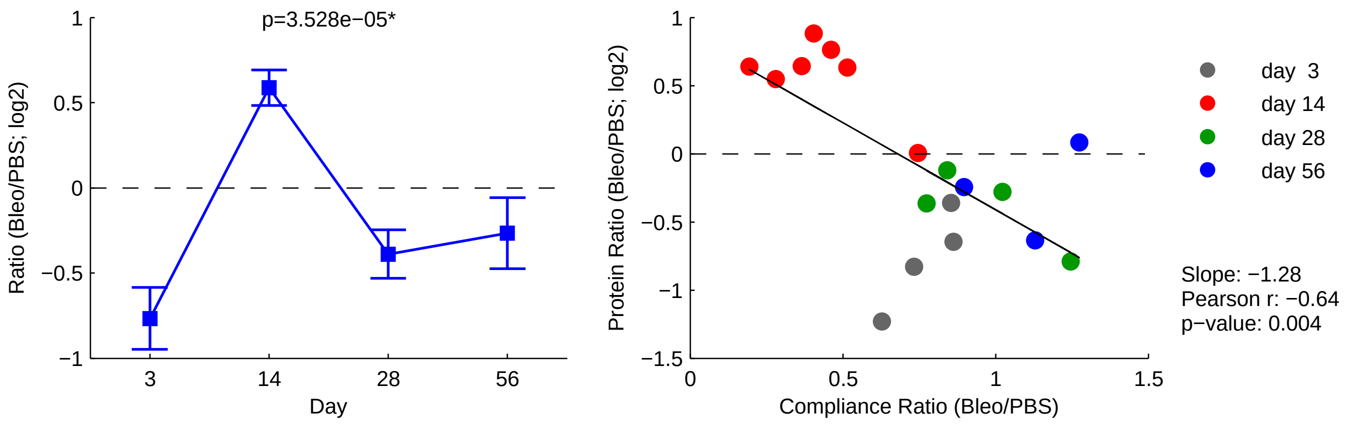

Q6NZR5 − Skiv2l (id: 4599)

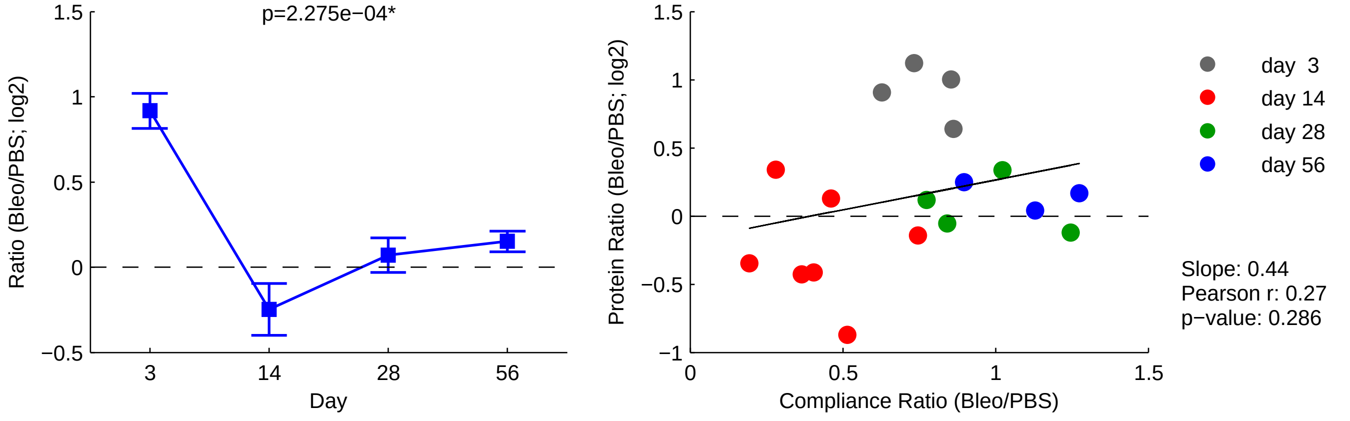

Q6P069−2 − Sri (id: 4600)

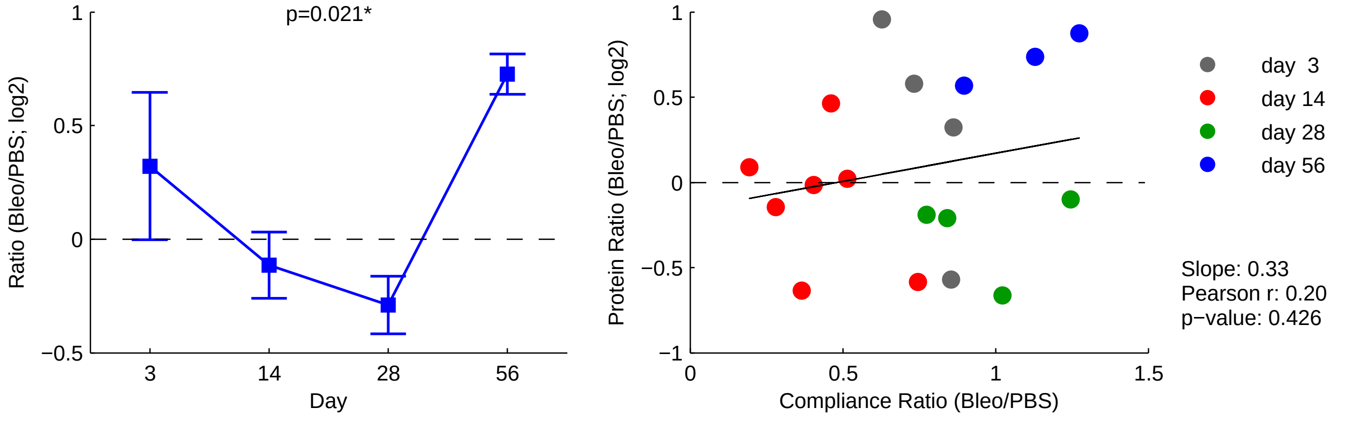

Q6P1B1 − Xpnpep1 (id: 4601)

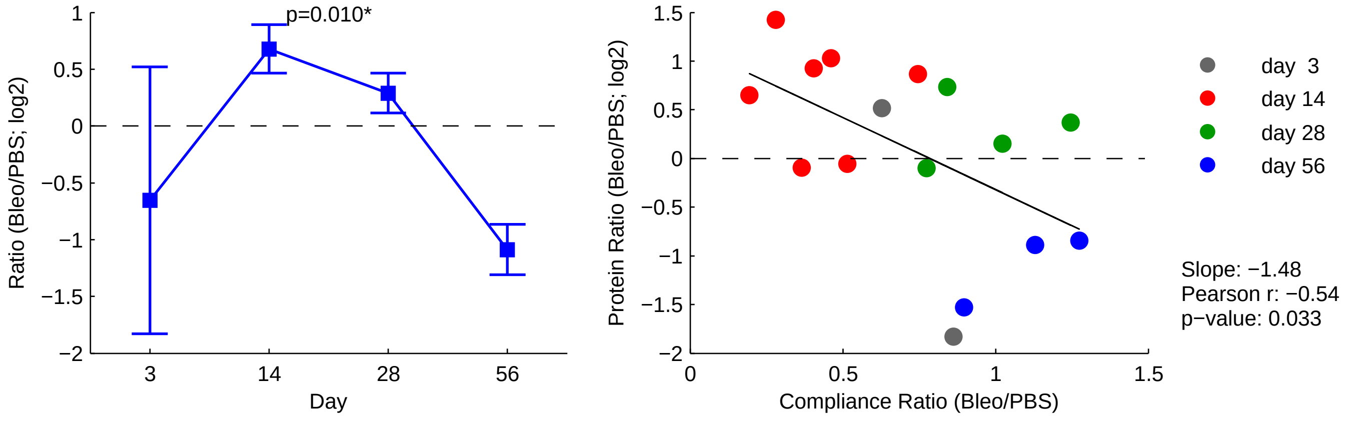

Q6P1E8 − Efcab6 (id: 4602)

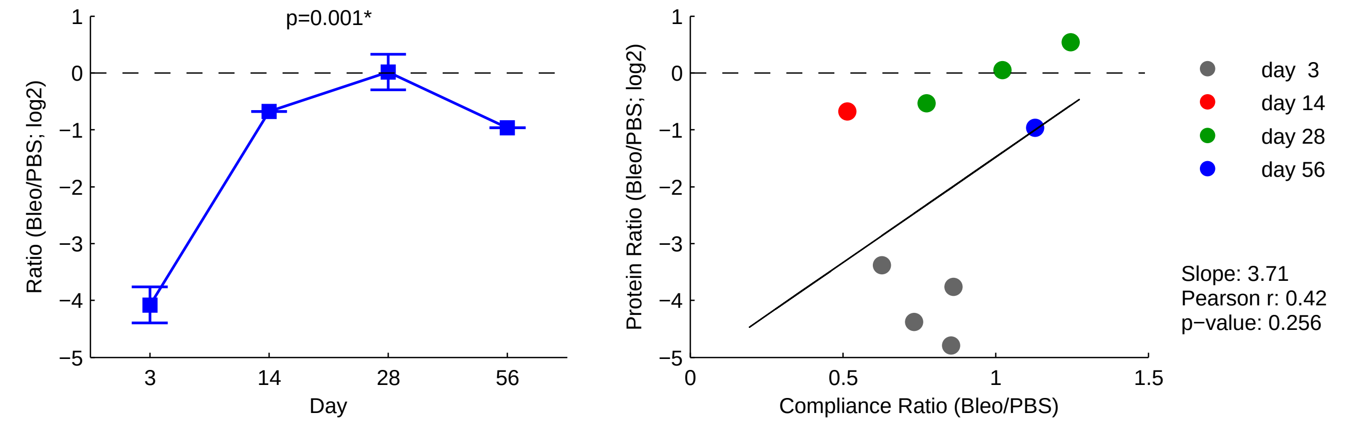

Q9WVL6 − Extl3 (id: 4605)

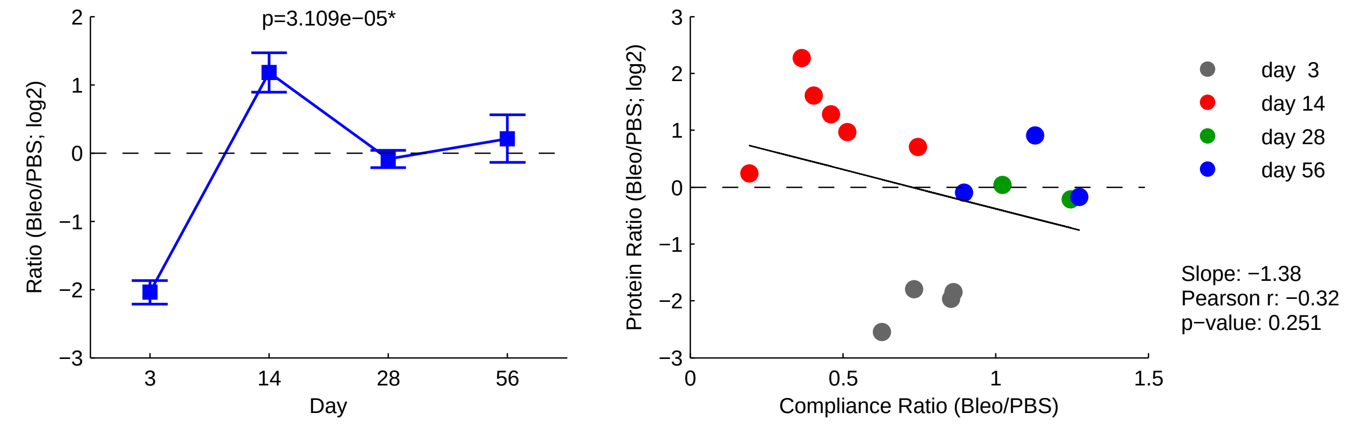

Q6P1H6 − Ankle2 (id: 4606)

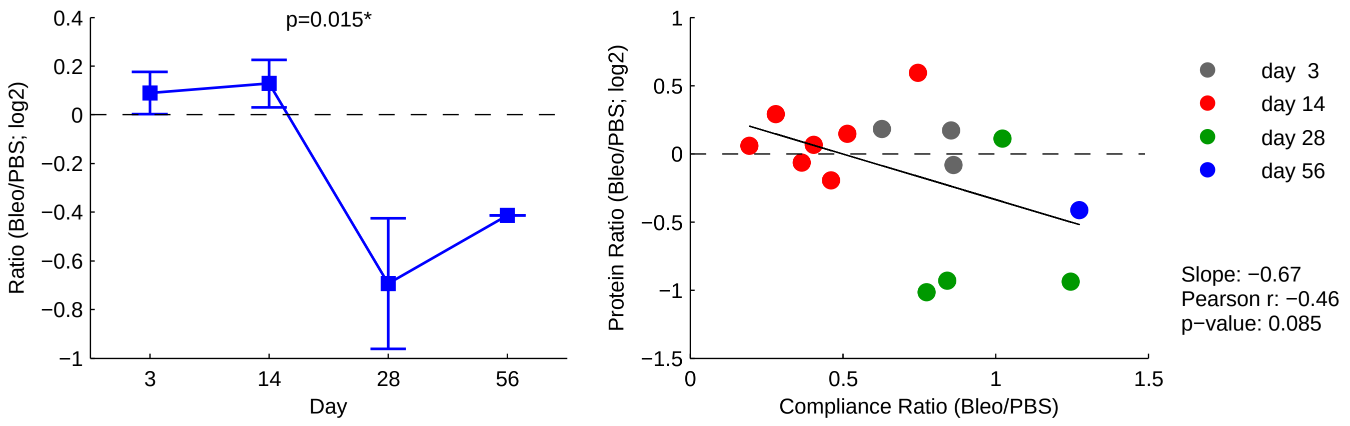

Q6P2B1 − Tnpo3 (id: 4609)

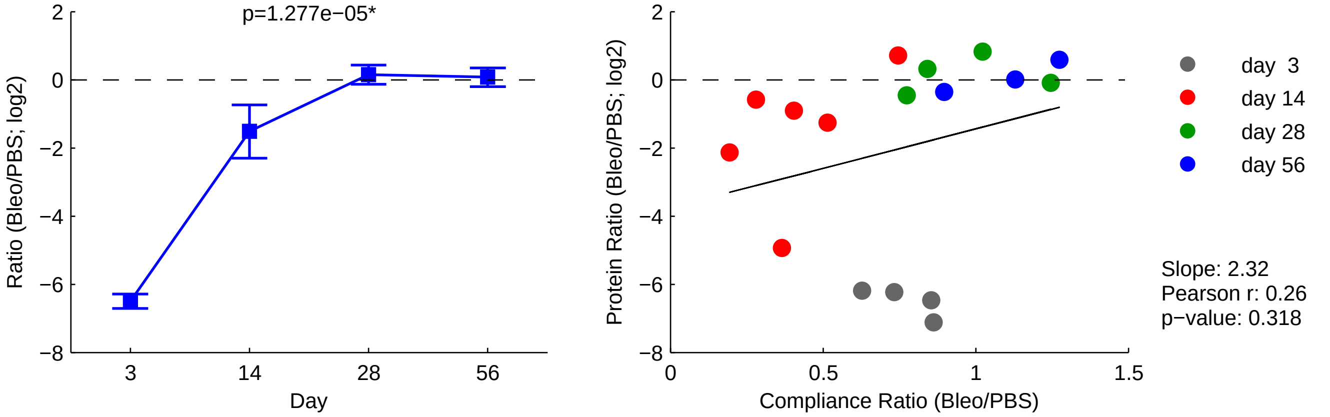

Q6P2L7 − Casc4 (id: 4611)

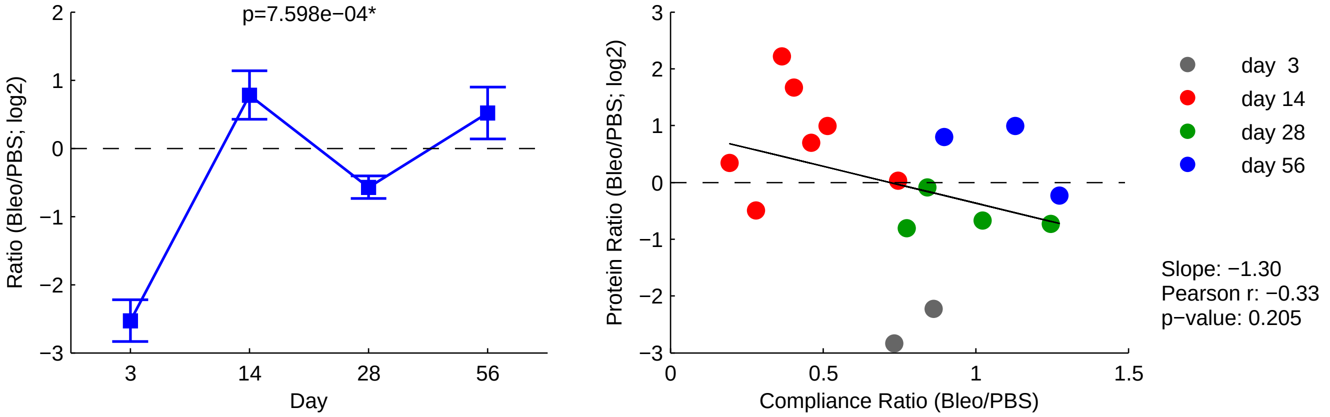

Q6P3D0 − Nudt16 (id: 4614)

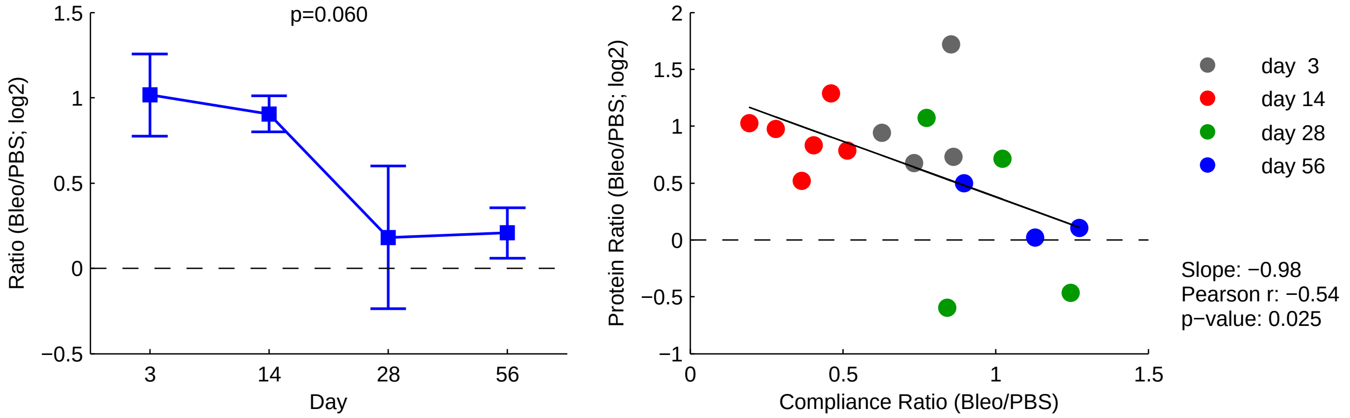

Q6P4T2 − Snrnp200 (id: 4617)

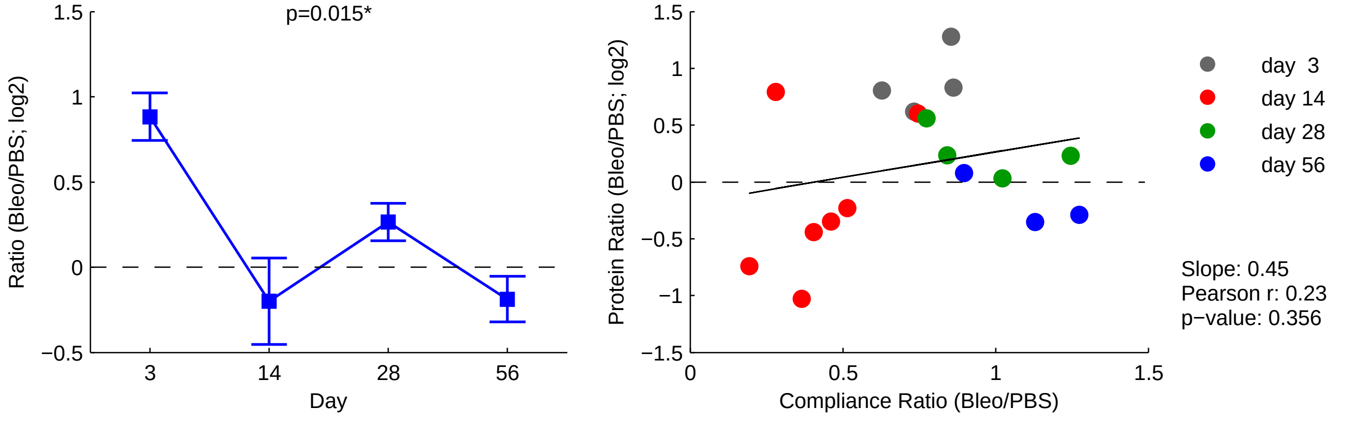

Q6P549 − Inppl1 (id: 4619)

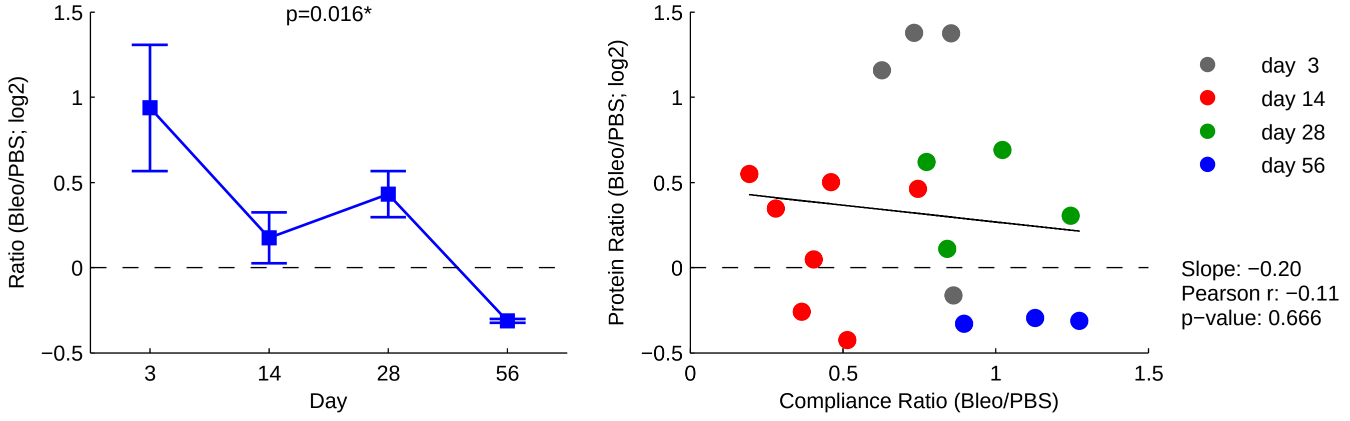

Q6P5B0 − Rrp12 (id: 4620)

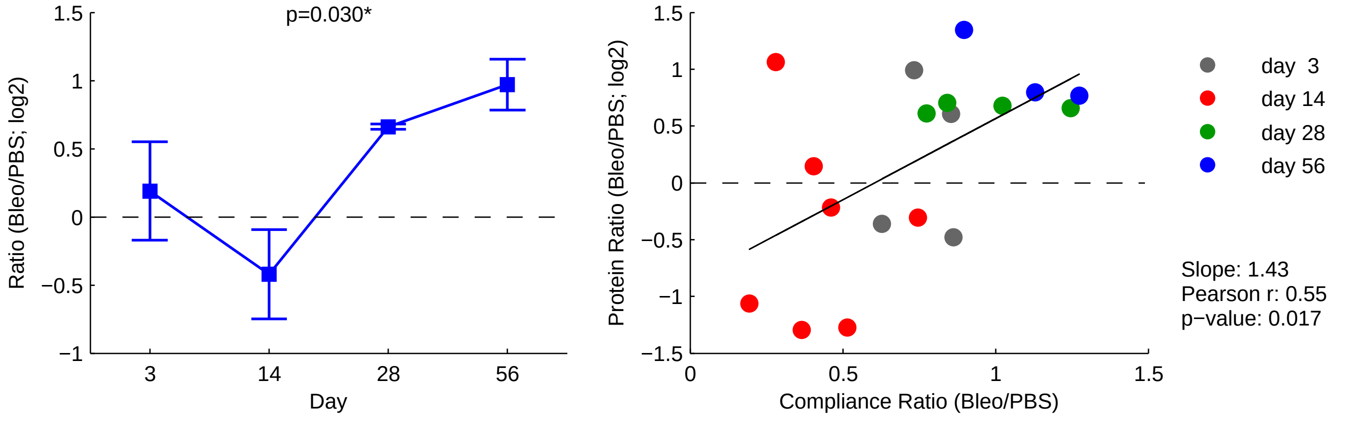

Q6P5B5 − Fxr2 (id: 4621)

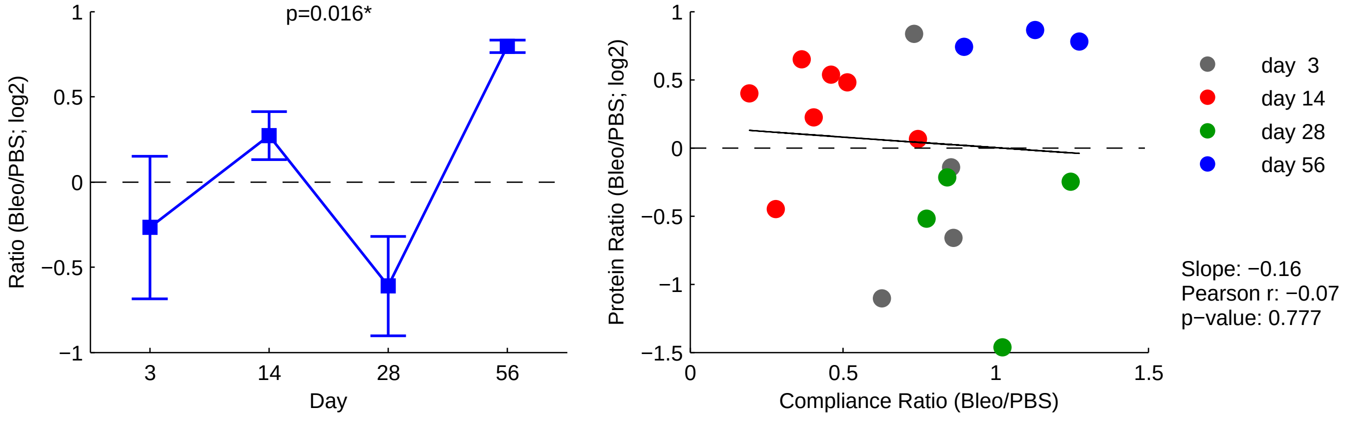

Q6P5D3−2 − Dhx57 (id: 4622)

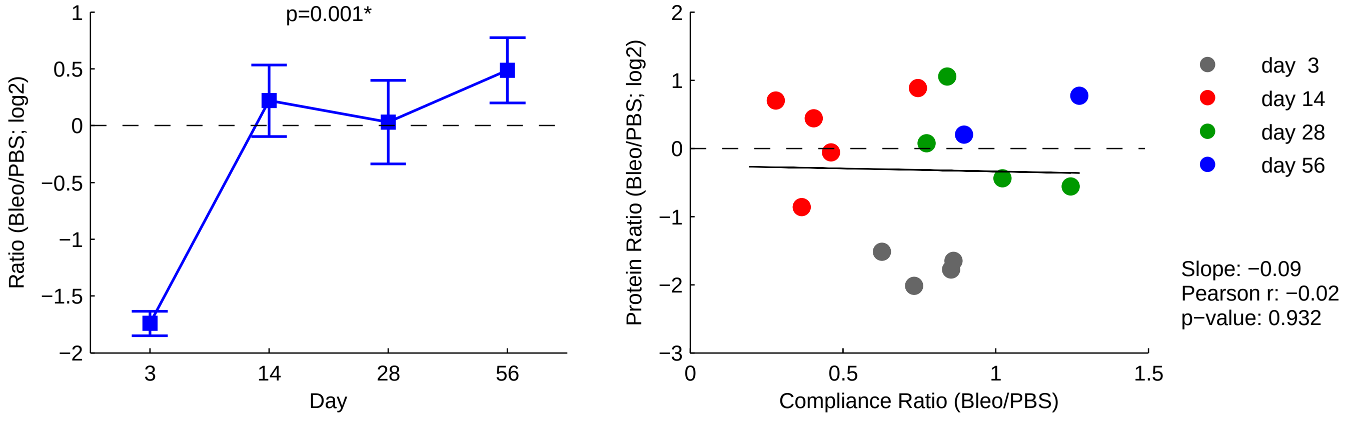

Q6P5D4 − Cep135 (id: 4623)

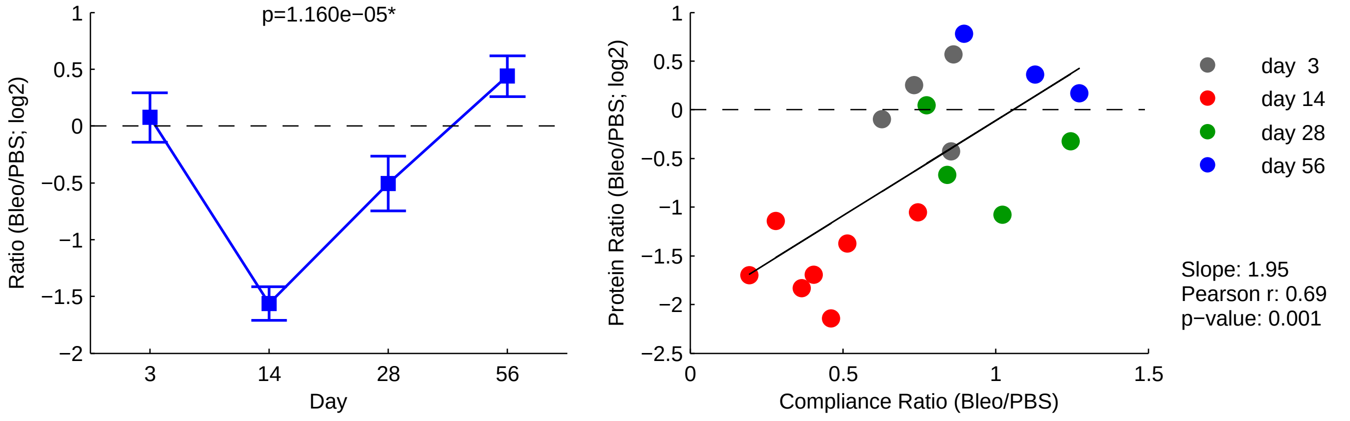

Q6P5E4 − Uggt1 (id: 4625)

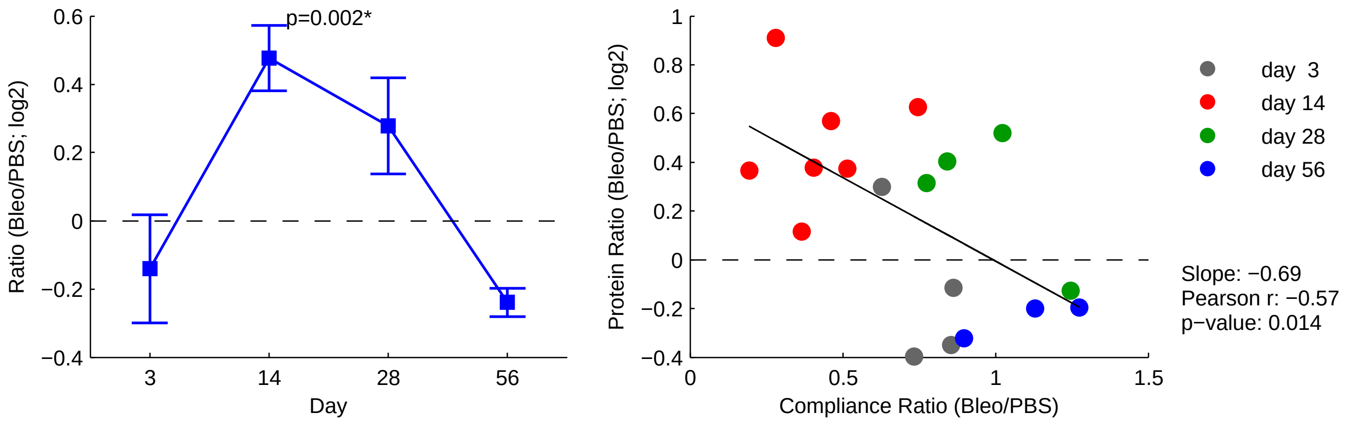

Q6P5F9 − Xpo1 (id: 4629)

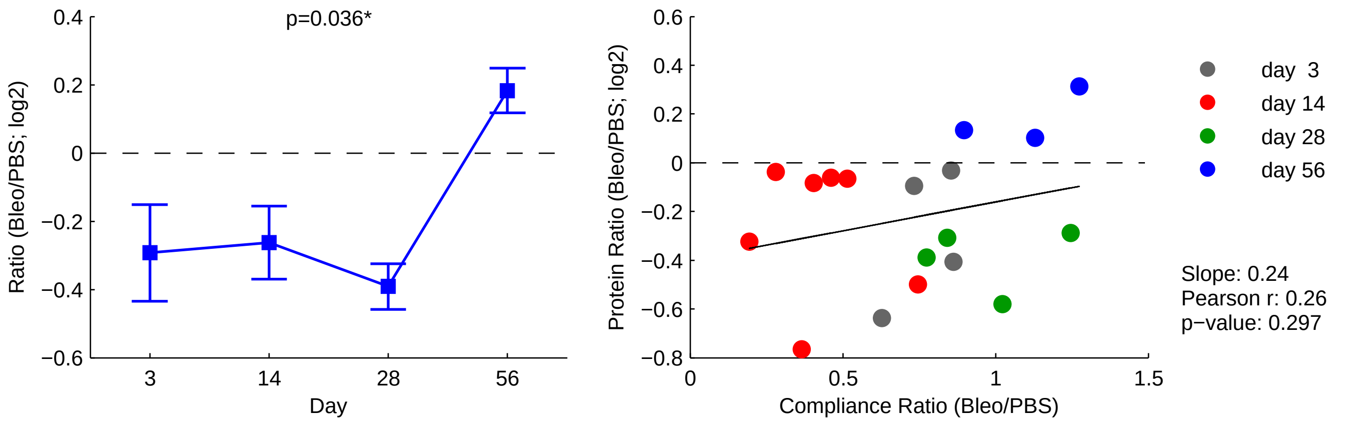

Q6P5H2 − Nes (id: 4630)

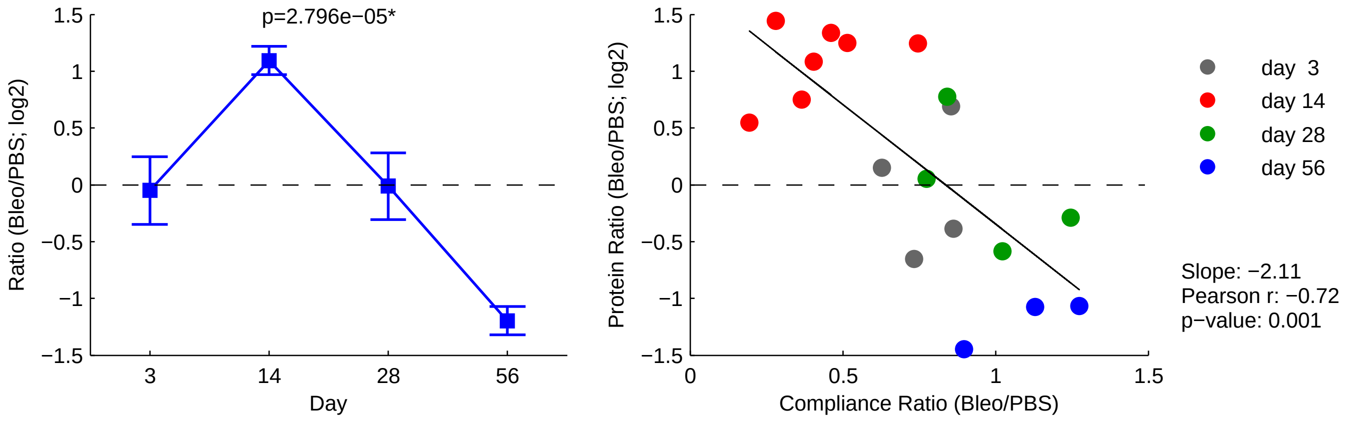

Q6P6I8 − Sirpa (id: 4631)

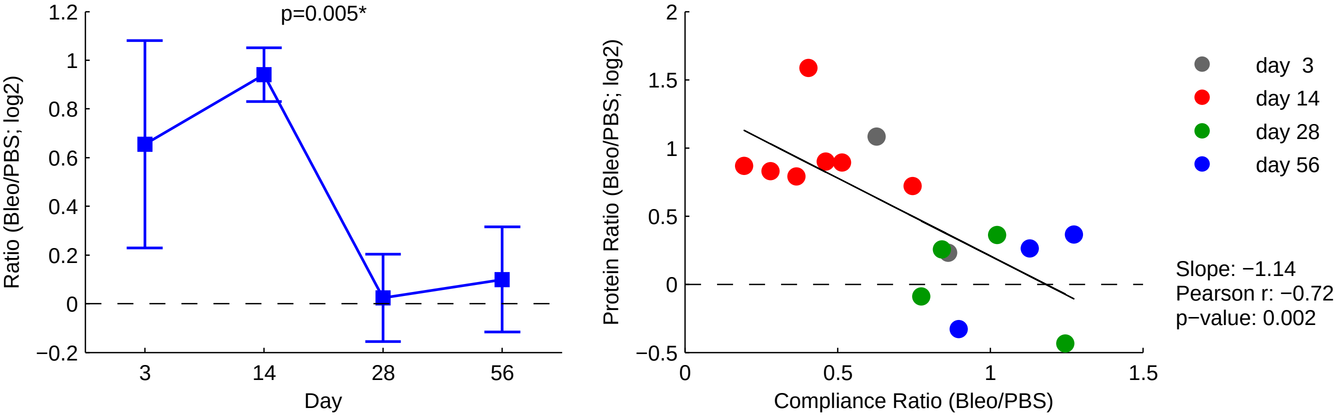

Q6P6L5 − Mybpc1 (id: 4633)

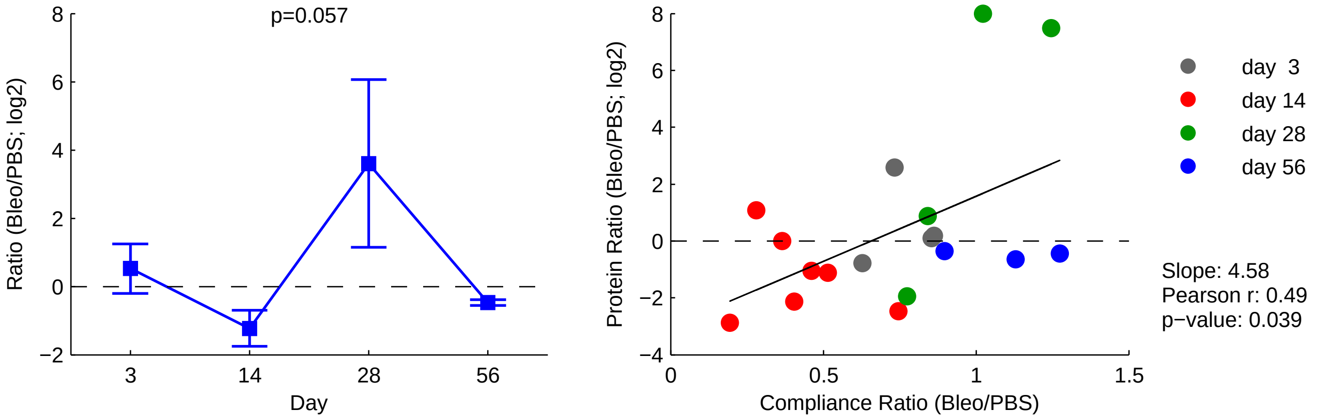

Q6P8I4 − Pcnp (id: 4637)

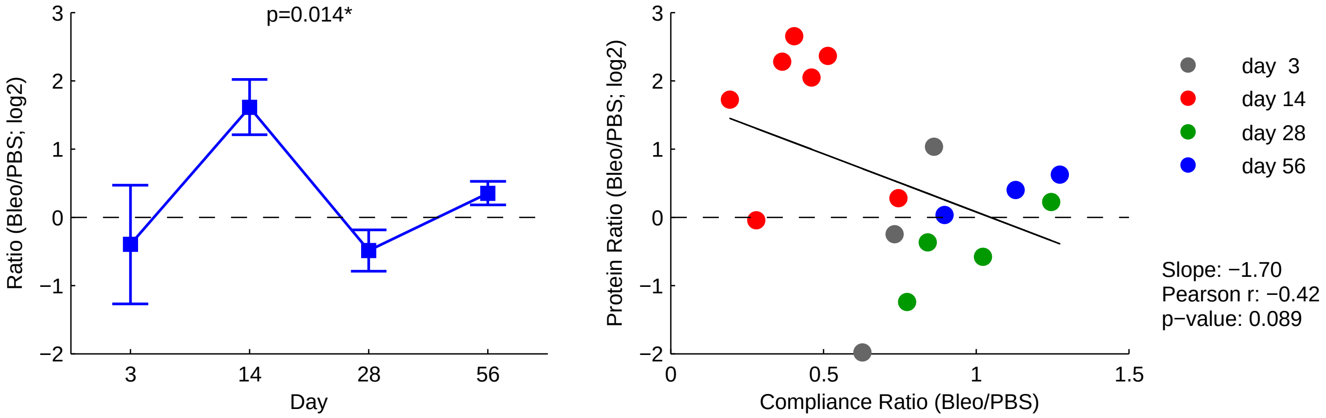

Q6P8J7 − Ckmt2 (id: 4639)

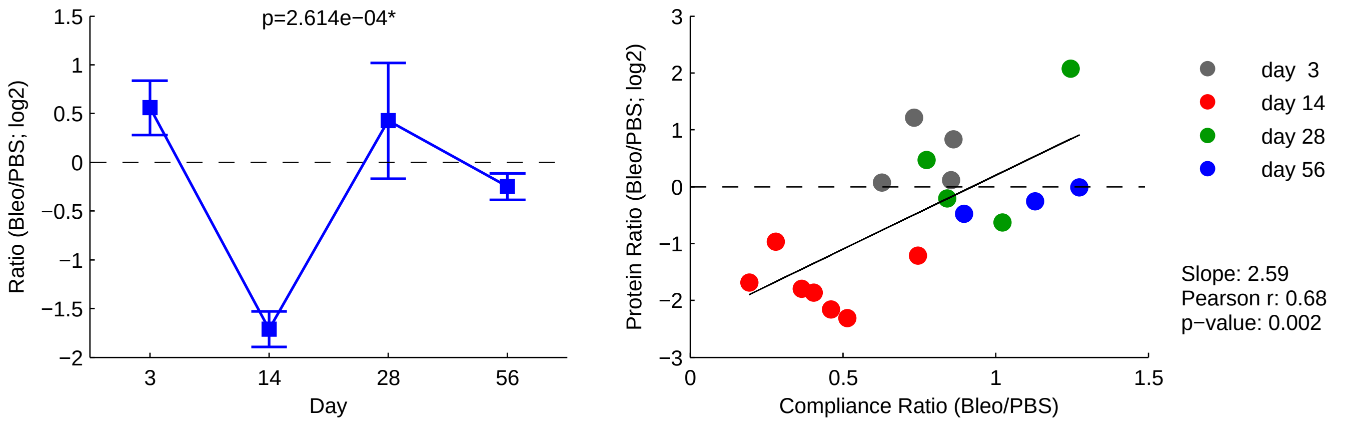

Q6P8N8 − Clpx (id: 4640)

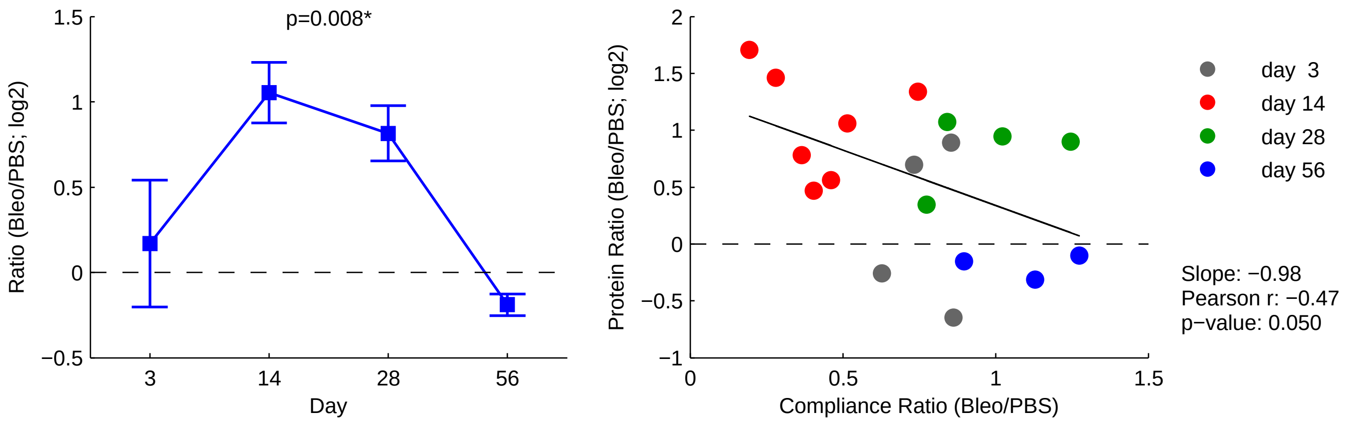

Q6P8P8 − Sftpc (id: 4641)

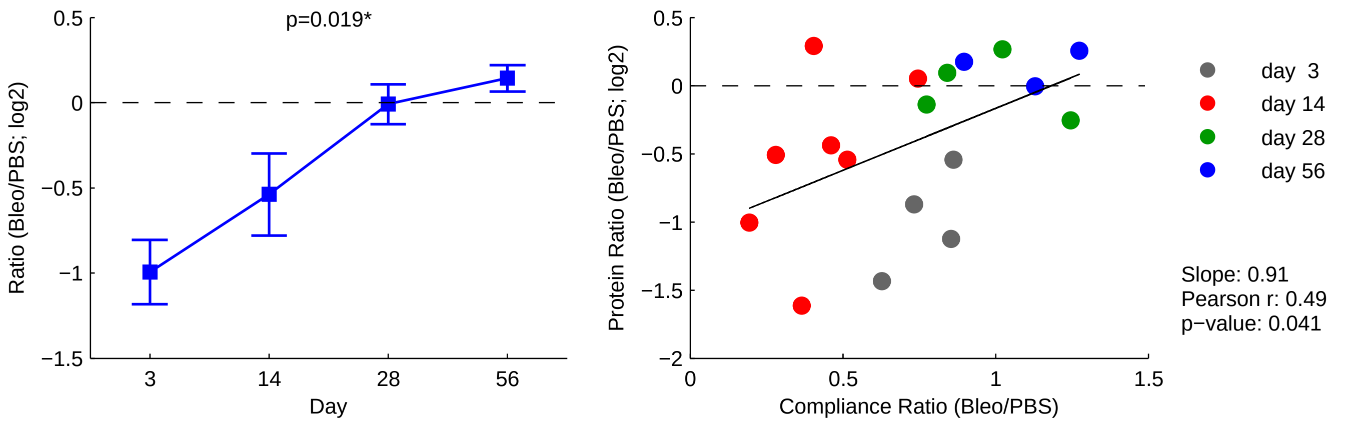

Q6P8X1 − Snx6 (id: 4642)

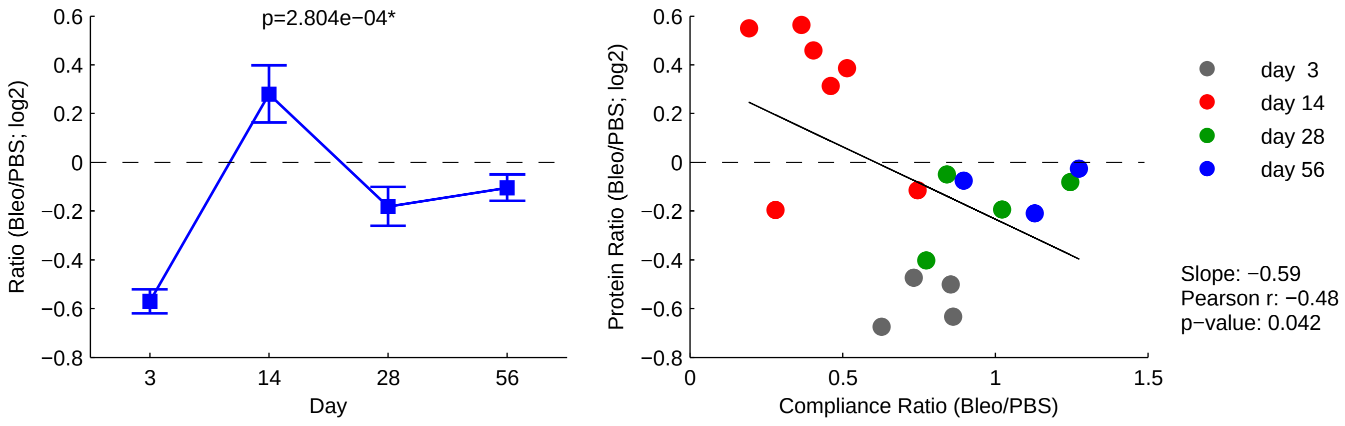

Q6P9J5 − Kank4 (id: 4643)

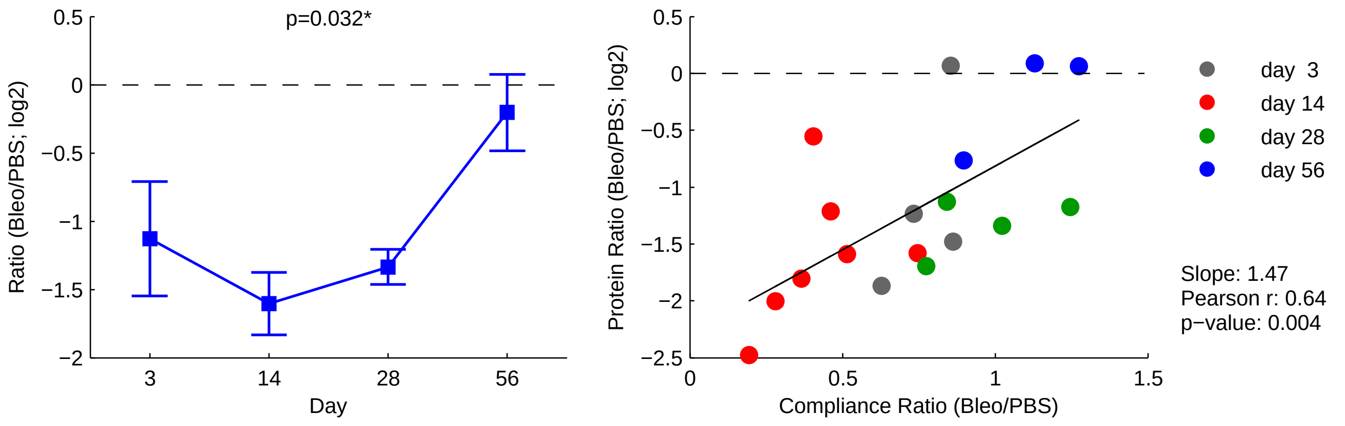

Q6P9J9 − Ano6 (id: 4644)

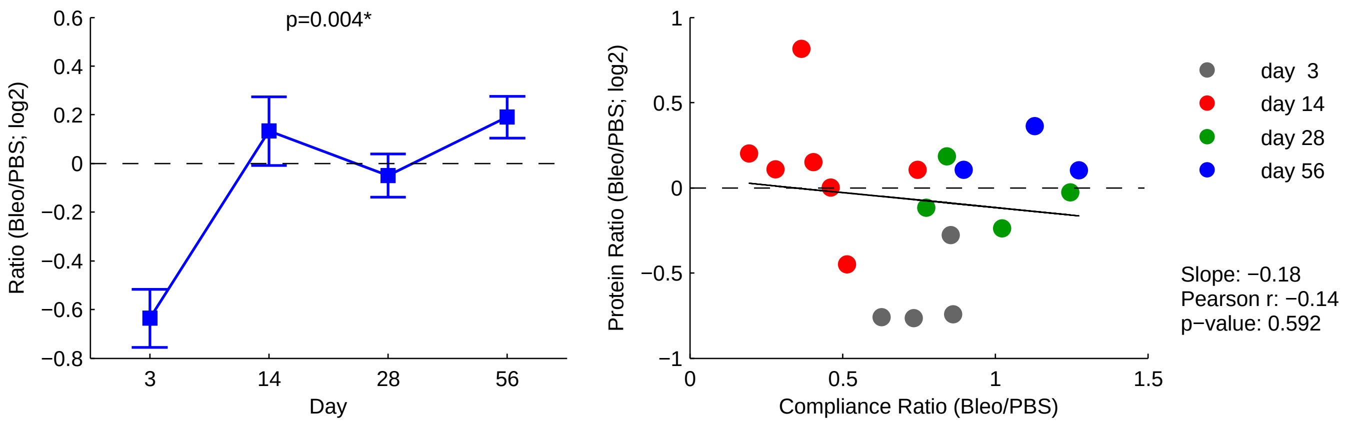

Q6P9Q4 − Fhod1 (id: 4646)

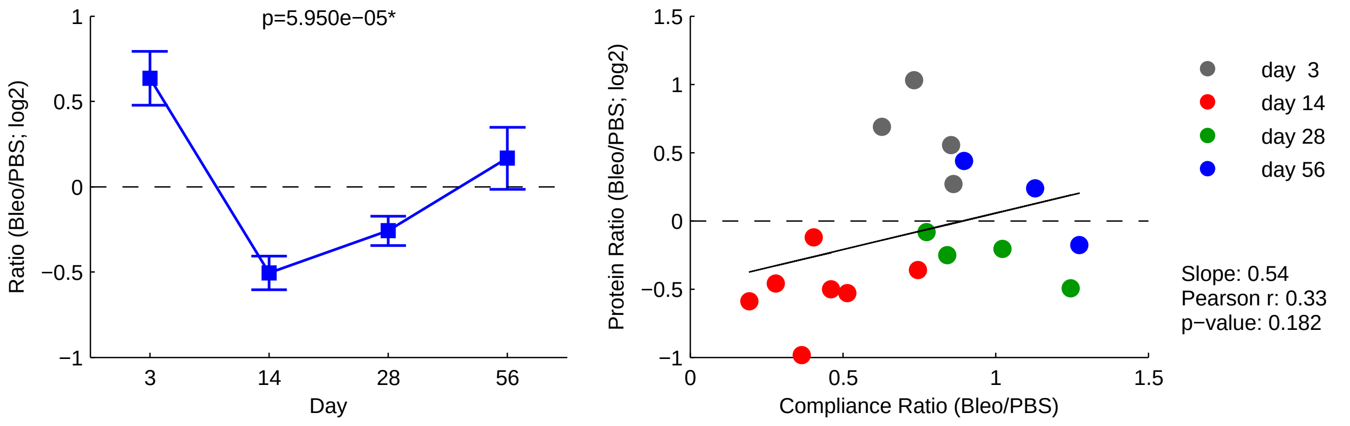

Q6P9Q6 − Fkbp15 (id: 4647)

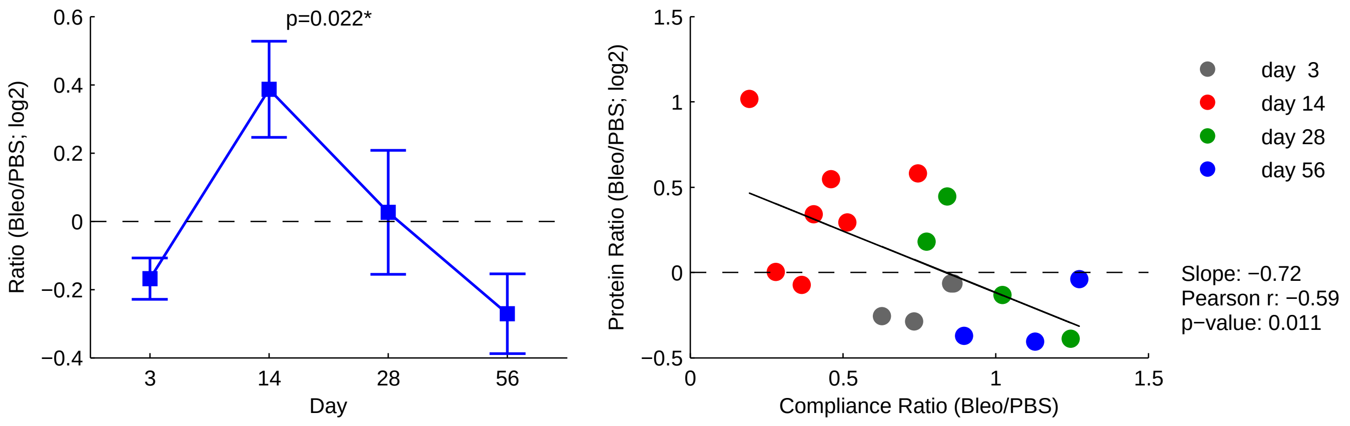

Q6P9R2 − Oxsr1 (id: 4649)

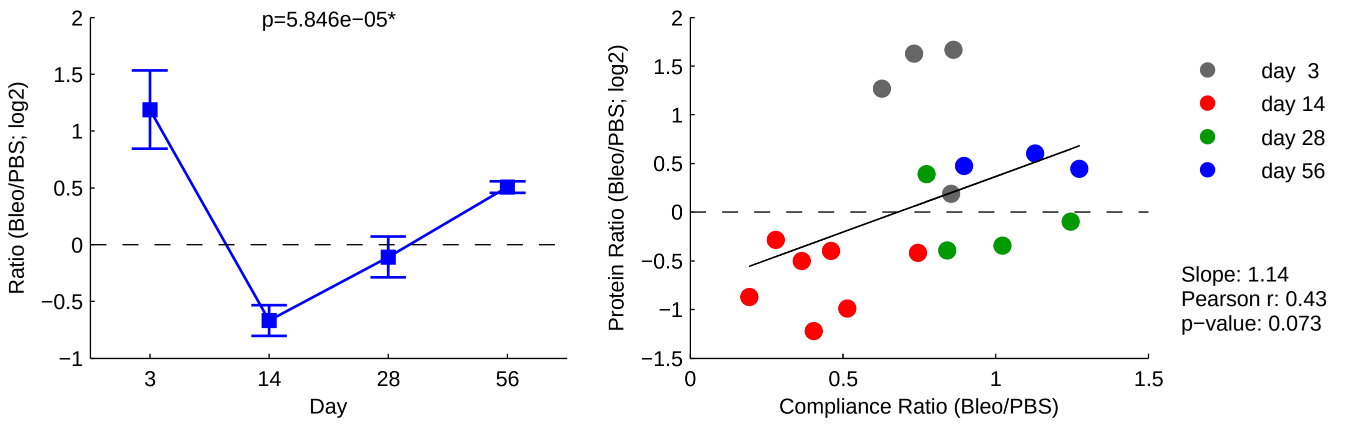

Q6P9R4 − Arhgef18 (id: 4650)

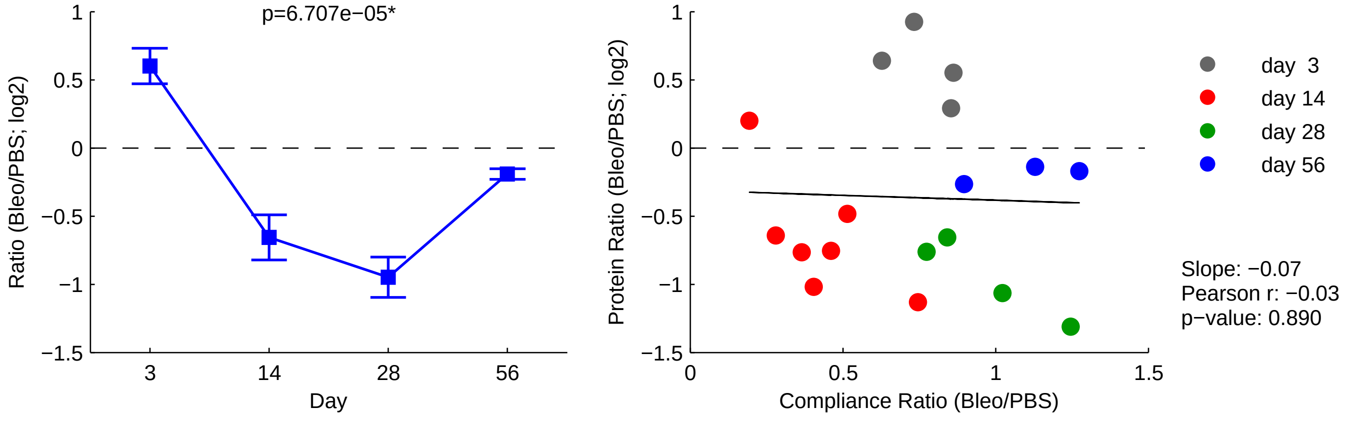

Q6PAV2−2 − Herc4 (id: 4660)

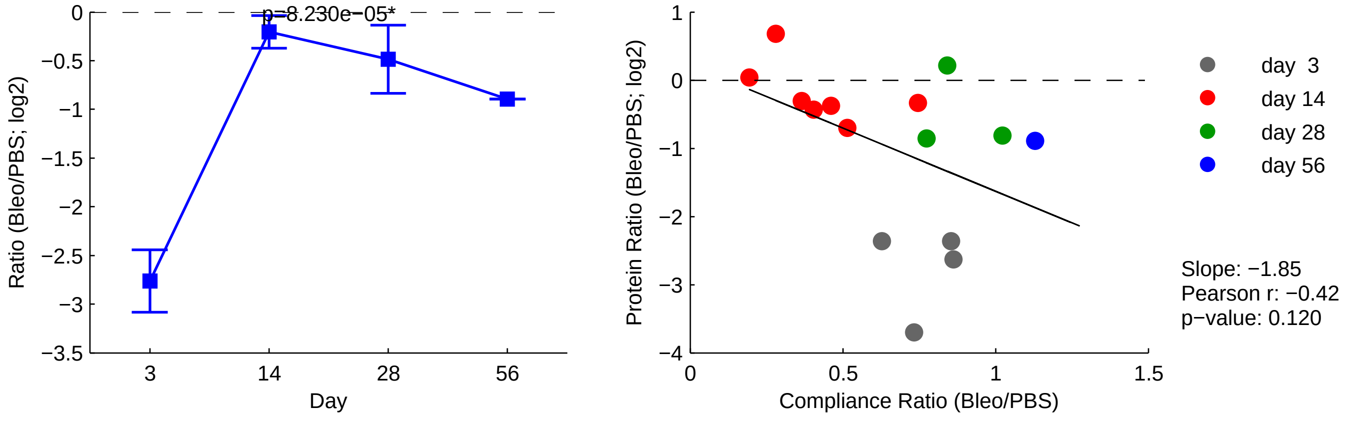

Q6PB44−2 − Ptpn23 (id: 4661)

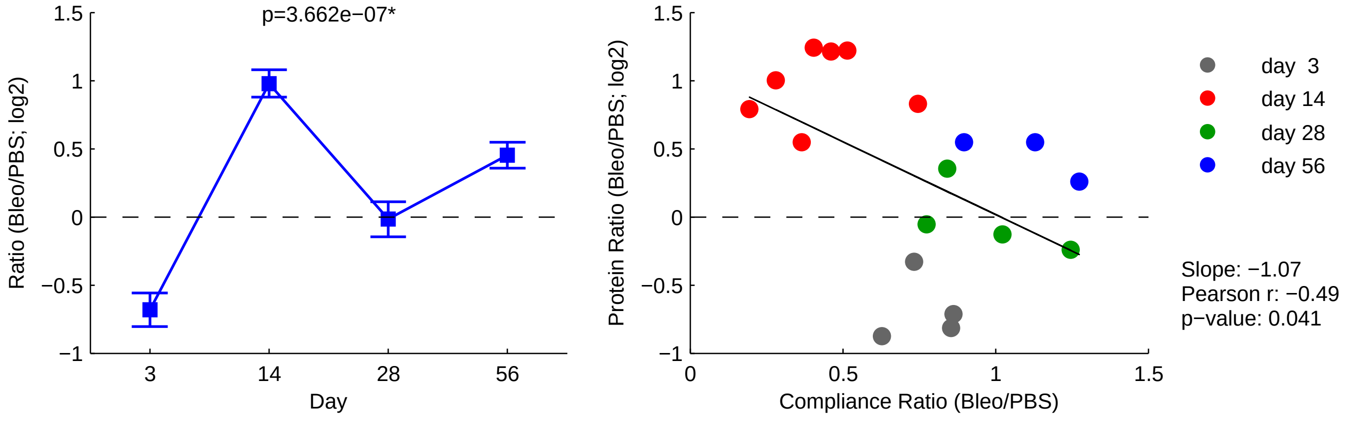

Q6PB66 − Lrpprc (id: 4662)

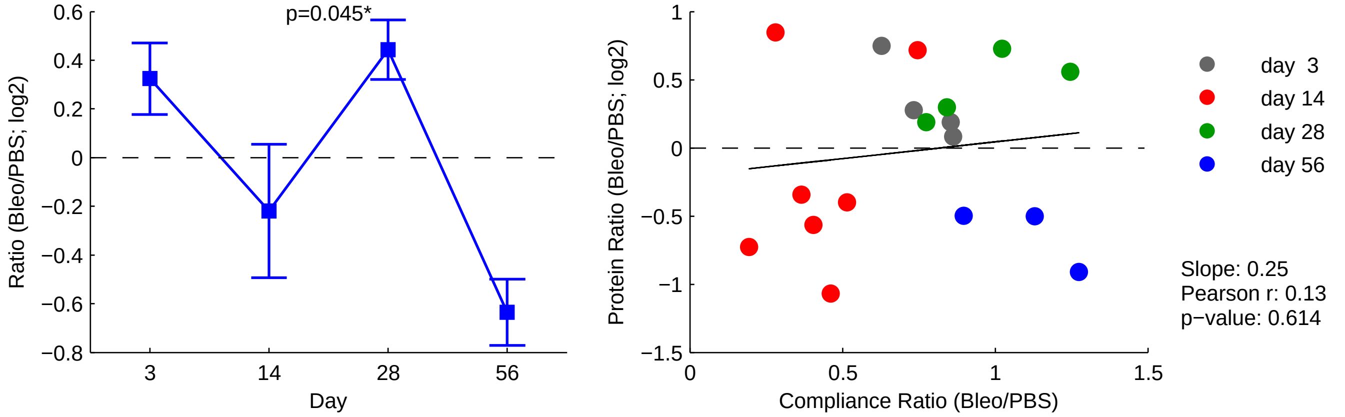

Q6PB93 − Galnt2 (id: 4663)

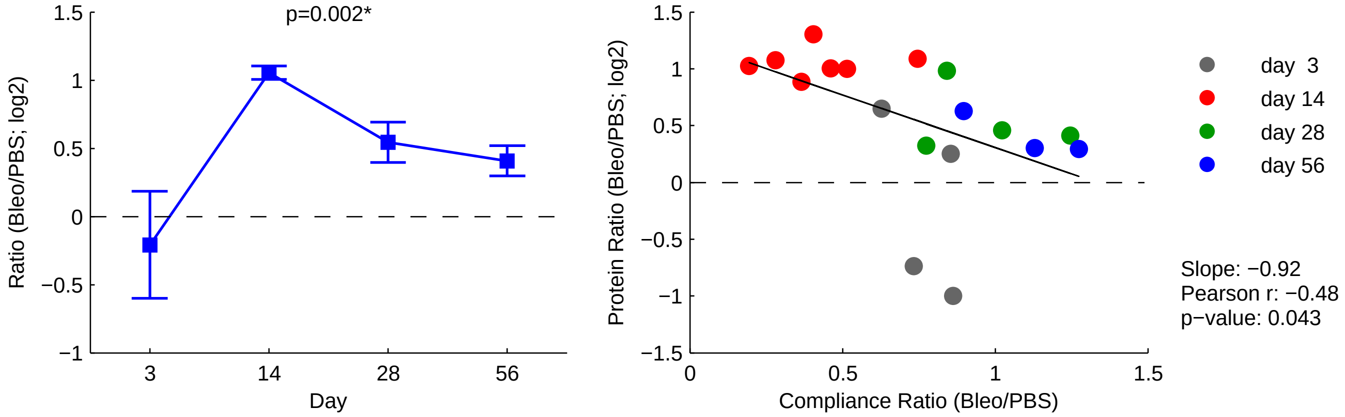

Q6PCM2−2 − Ints6 (id: 4664)

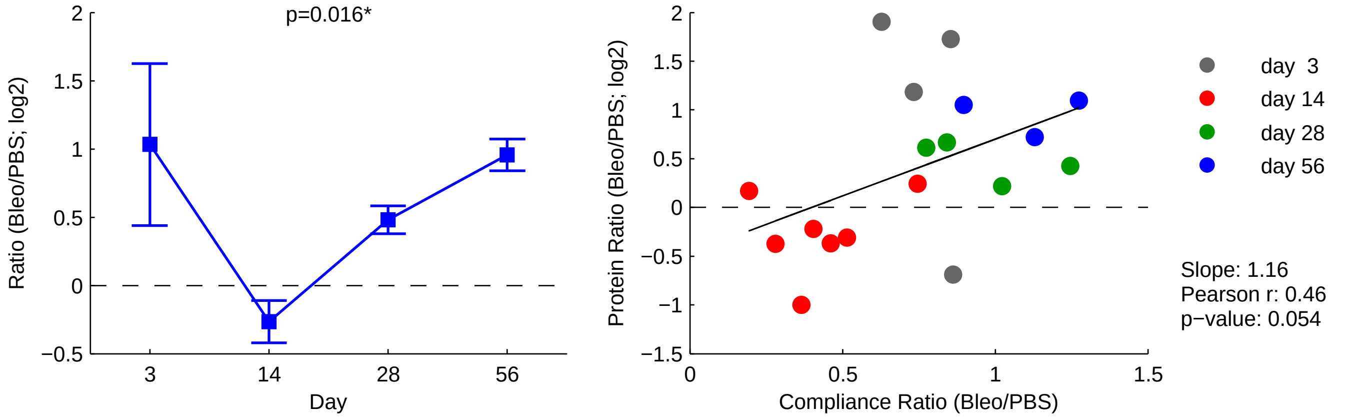

Q6PCP5−2 − (id: 4667)

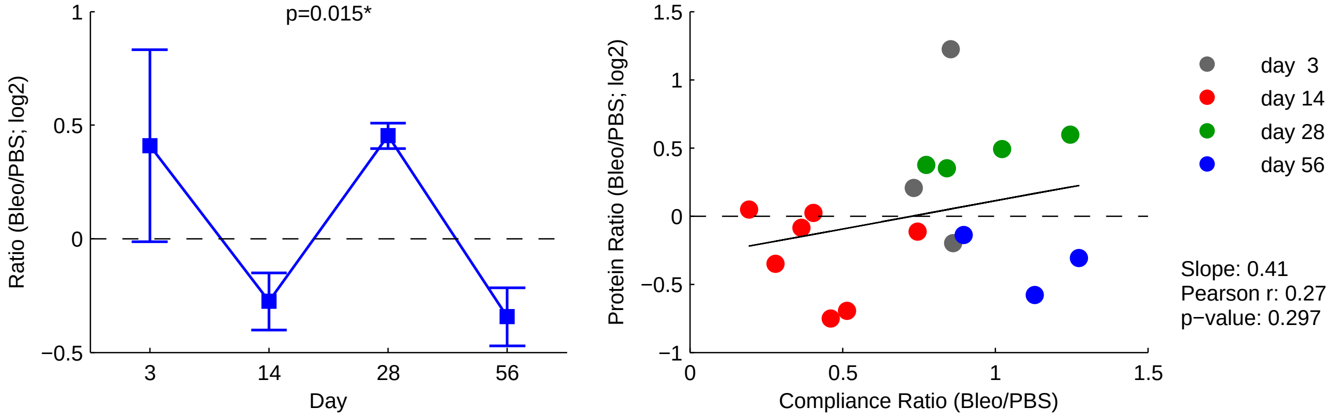

Q6PD03 − Ppp2r5a (id: 4669)

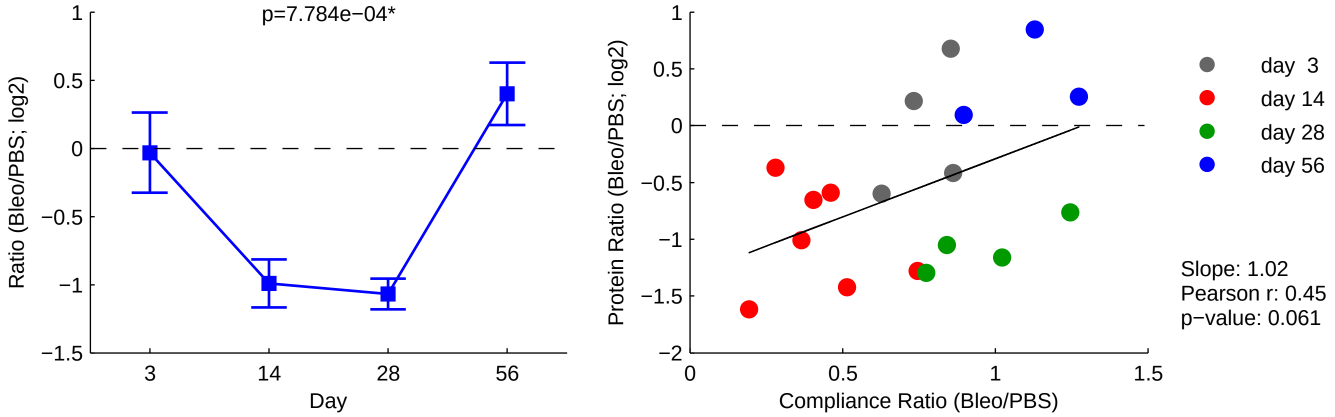

Q6PD26 − Pigs (id: 4673)

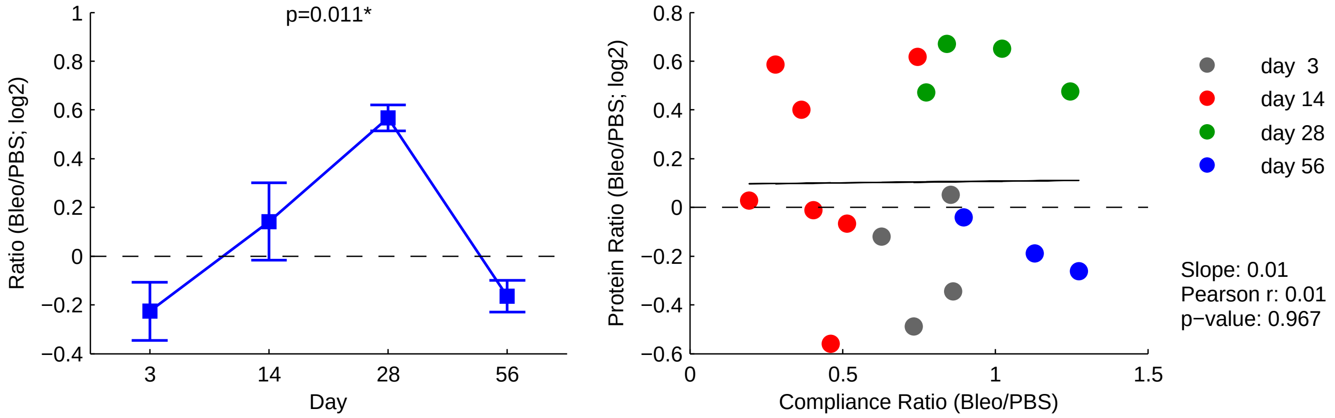

Q6PDI5−2 − Ecm29 (id: 4676)

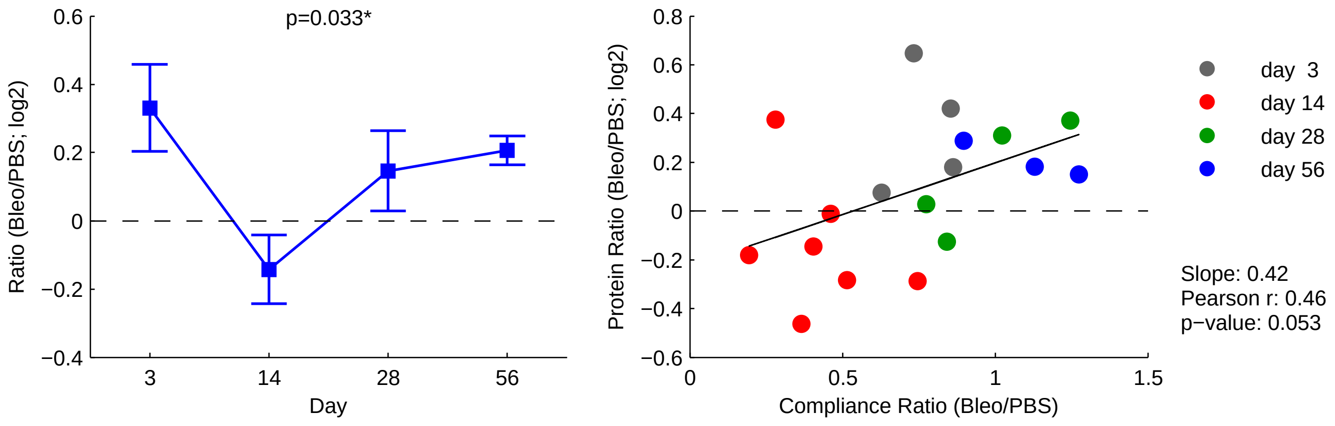

Q6PDI6 − Fam63b (id: 4677)

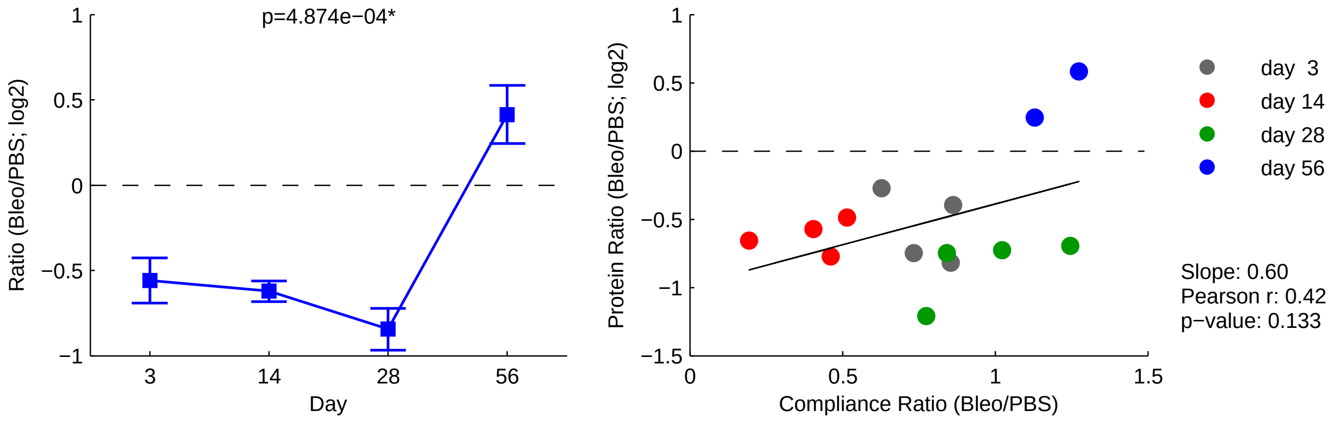

Q6PDL0 − Dync1li2 (id: 4679)

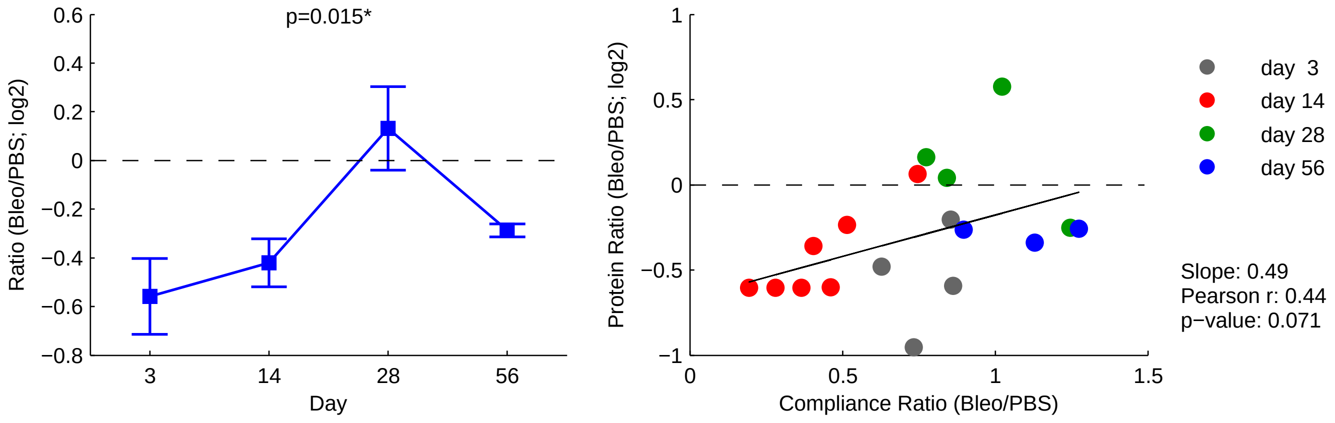

Q6PE54 − Dhx40 (id: 4684)

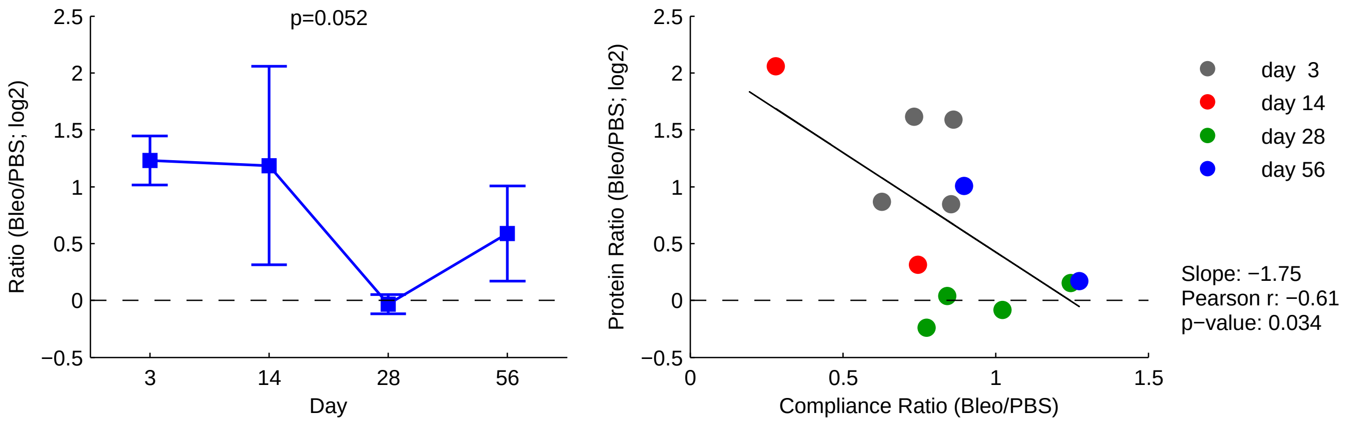

Q6PF96 − Etfdh (id: 4688)

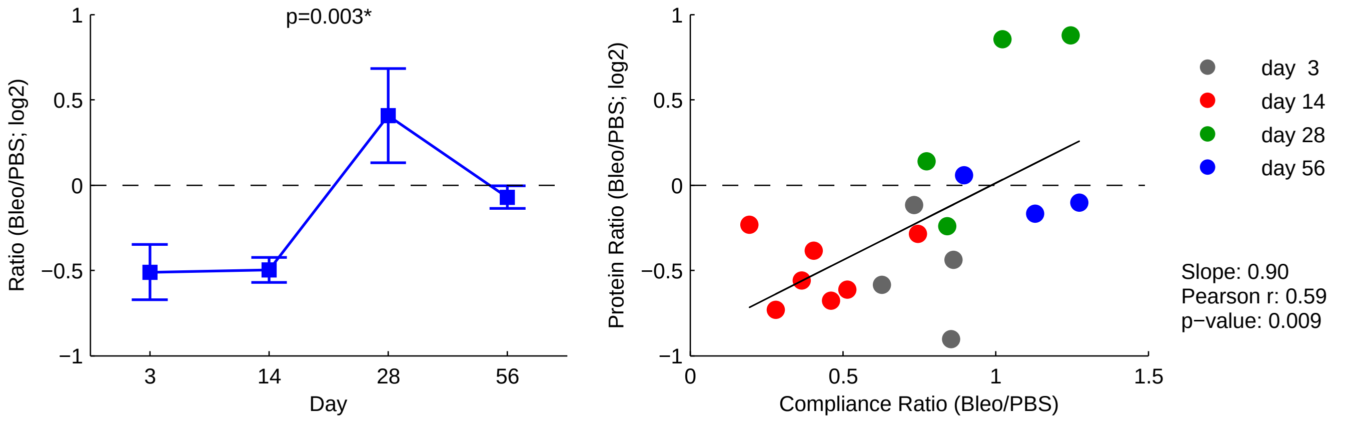

Q6PGB6−2 − Naa50 (id: 4691)

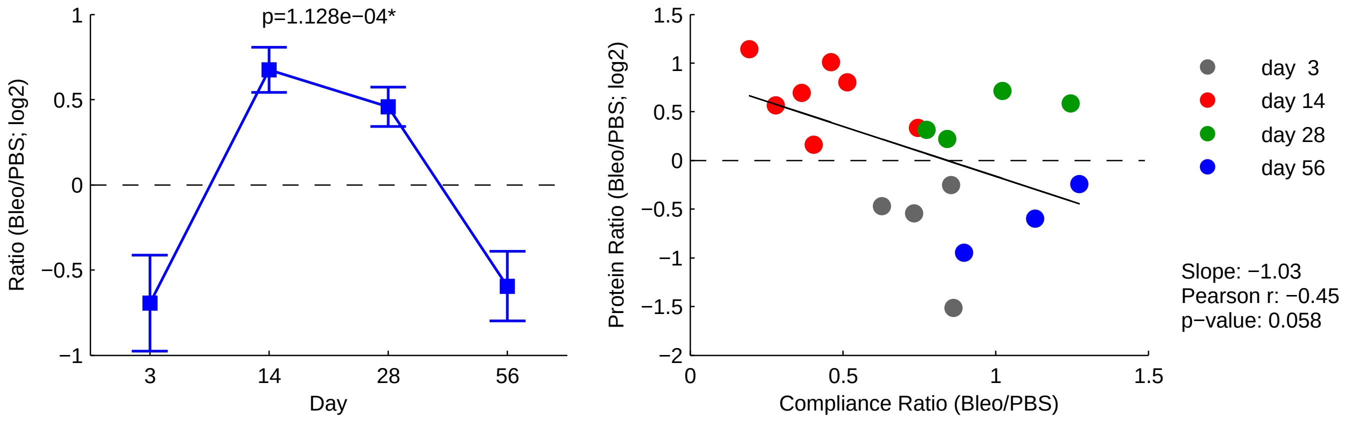

Q6PGL7 − Fam21 (id: 4698)

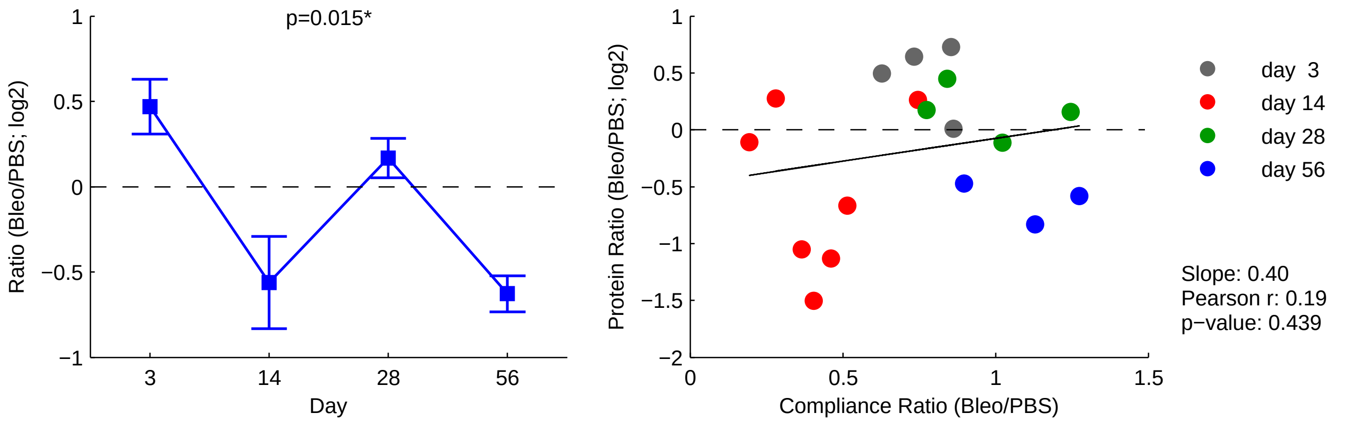

Q6PHU5 − Sort1 (id: 4702)

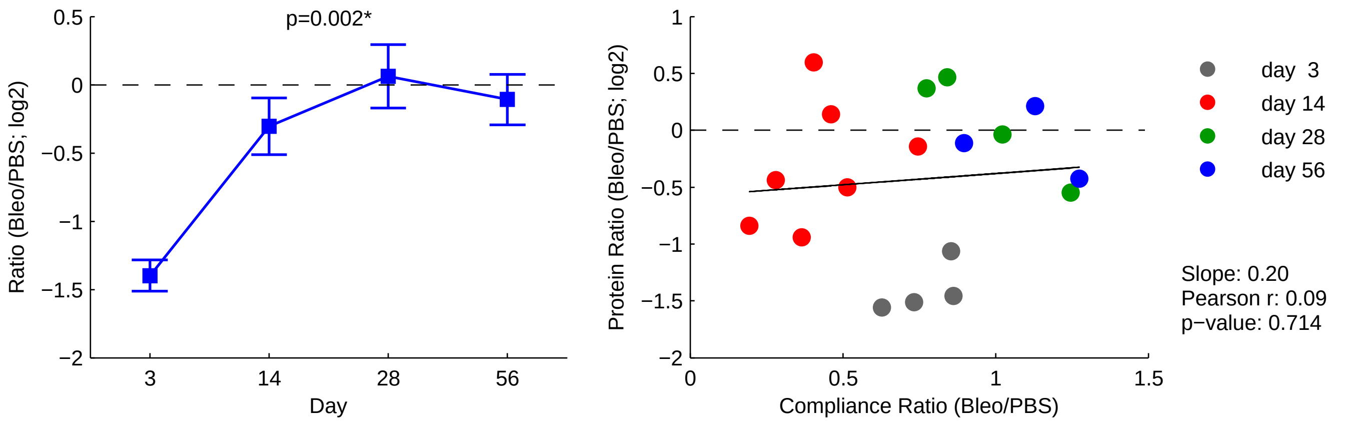

Q6PHZ2 − Camk2d (id: 4703)

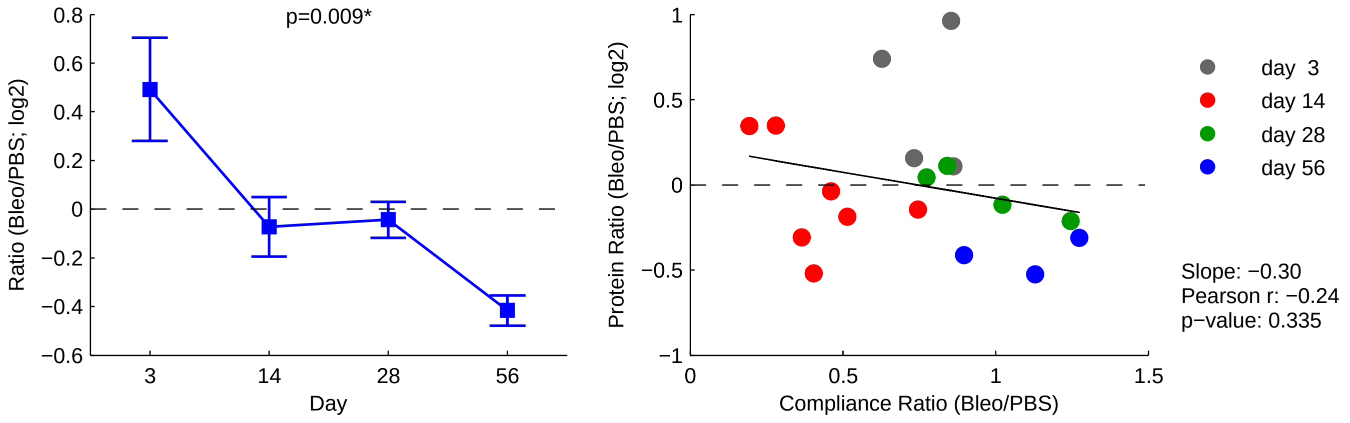

Q6PIP5 − Nudcd1 (id: 4706)

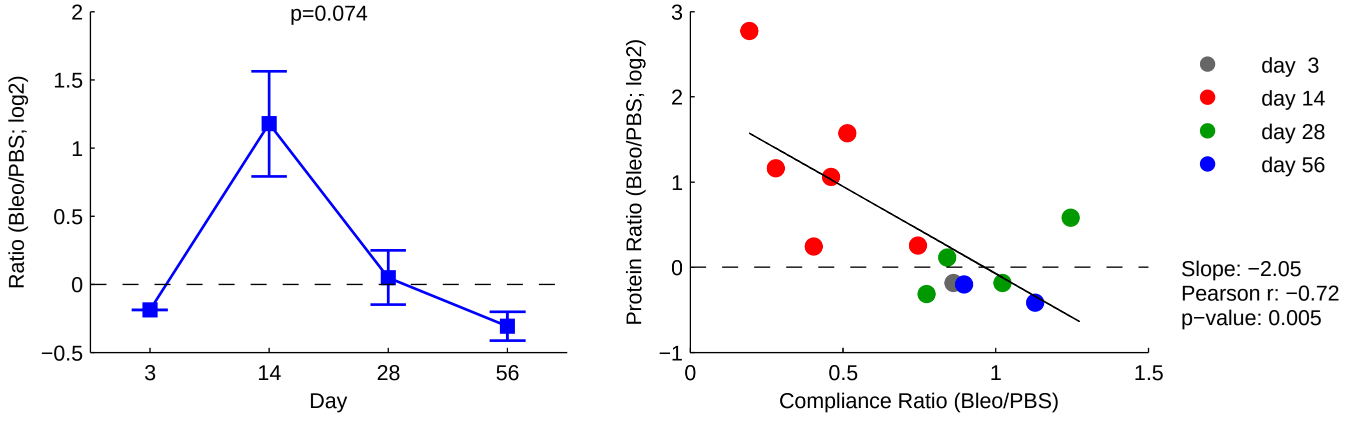

Q6PIU9 − (id: 4707)

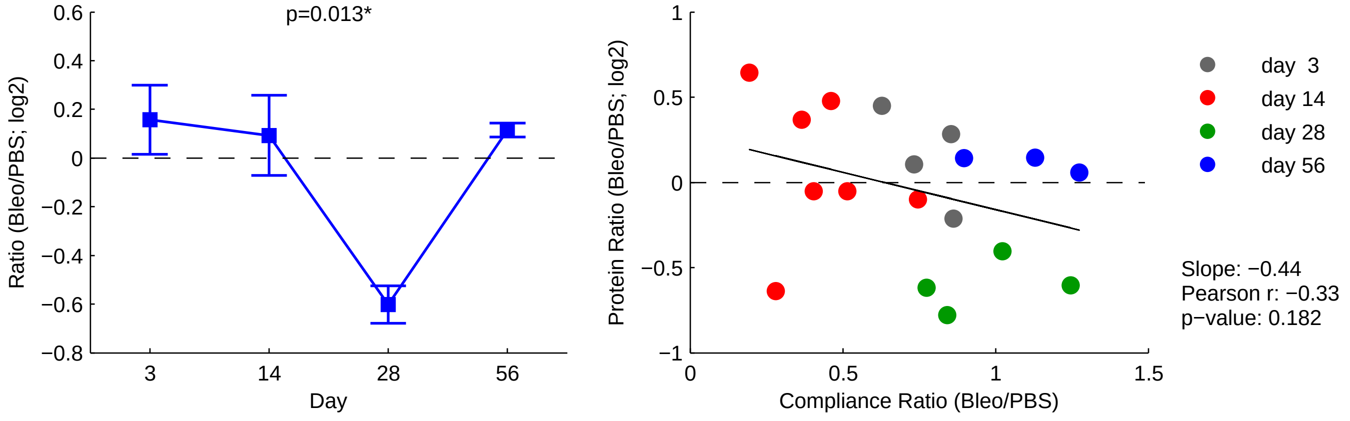

Q6PNC0 − Dmxl1 (id: 4709)

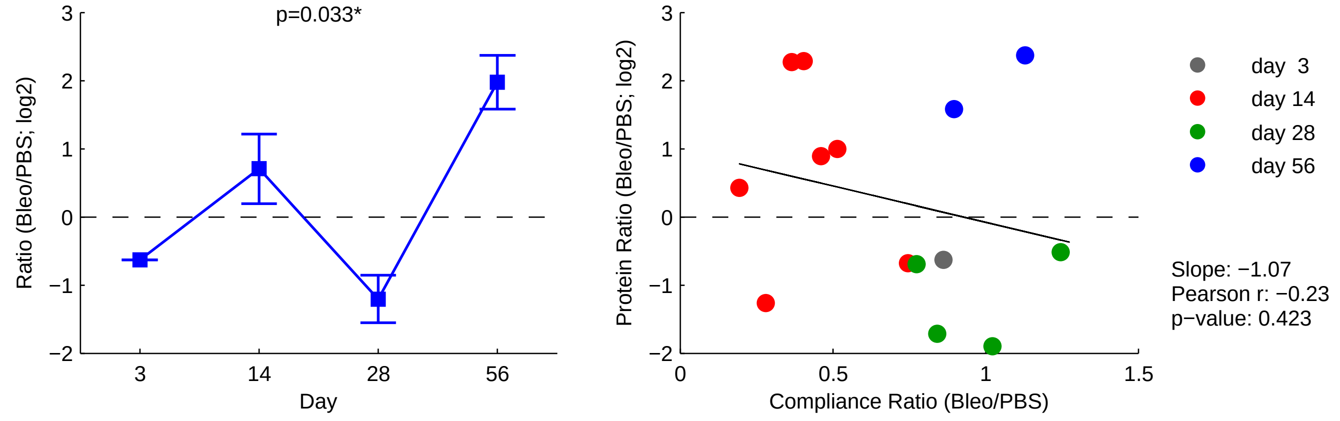

Q6Q899 − Ddx58 (id: 4713)

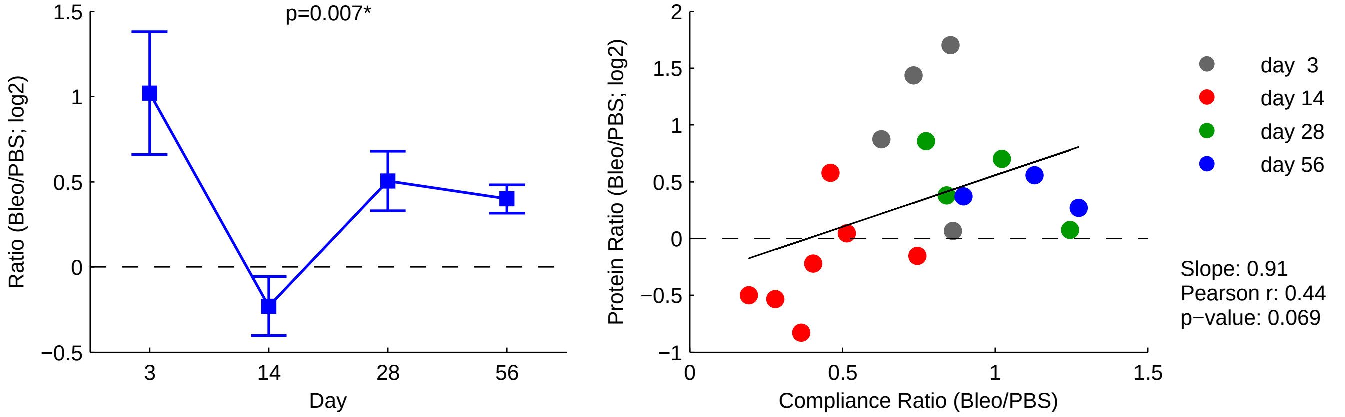

Q6R891 − Ppp1r9b (id: 4717)

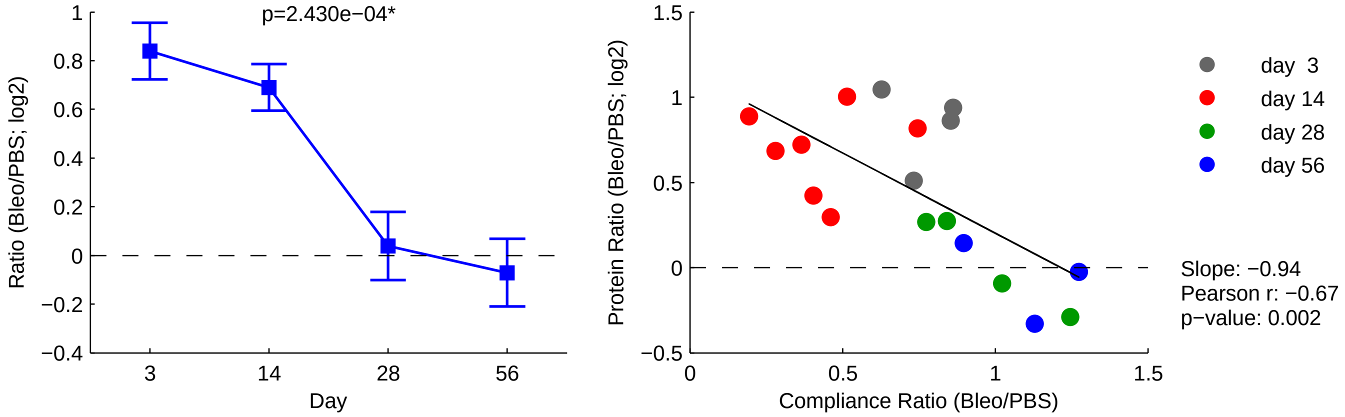

Q6RHR9 − Magi1 (id: 4718)

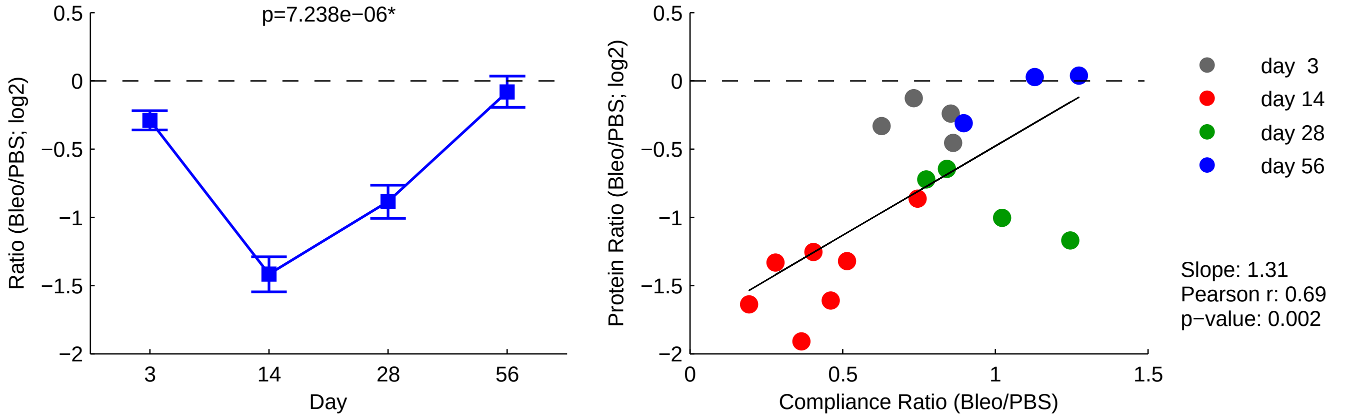

Q6URW6−2 − Myh14 (id: 4728)

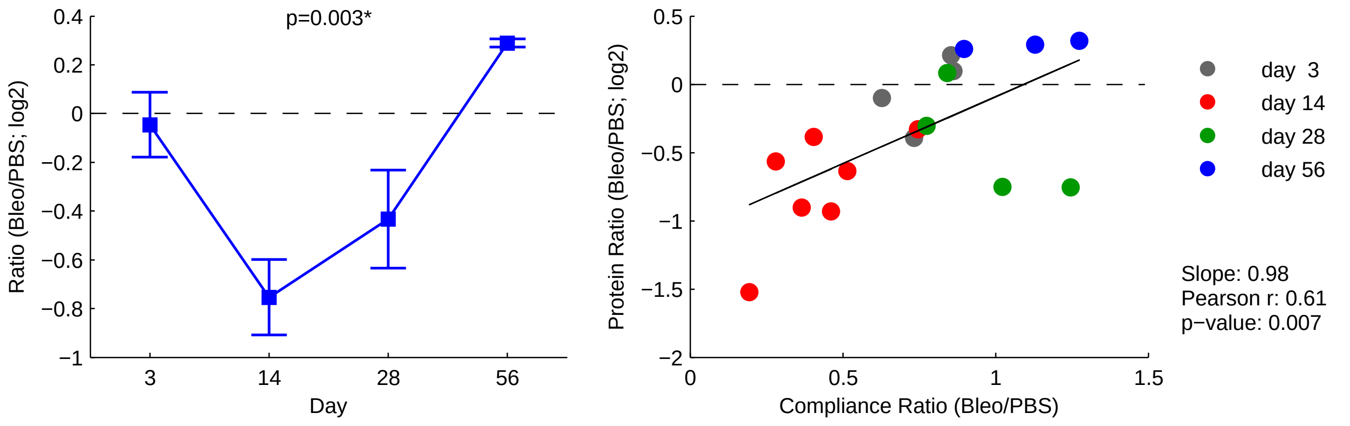

Q6VN19 − Ranbp10 (id: 4731)

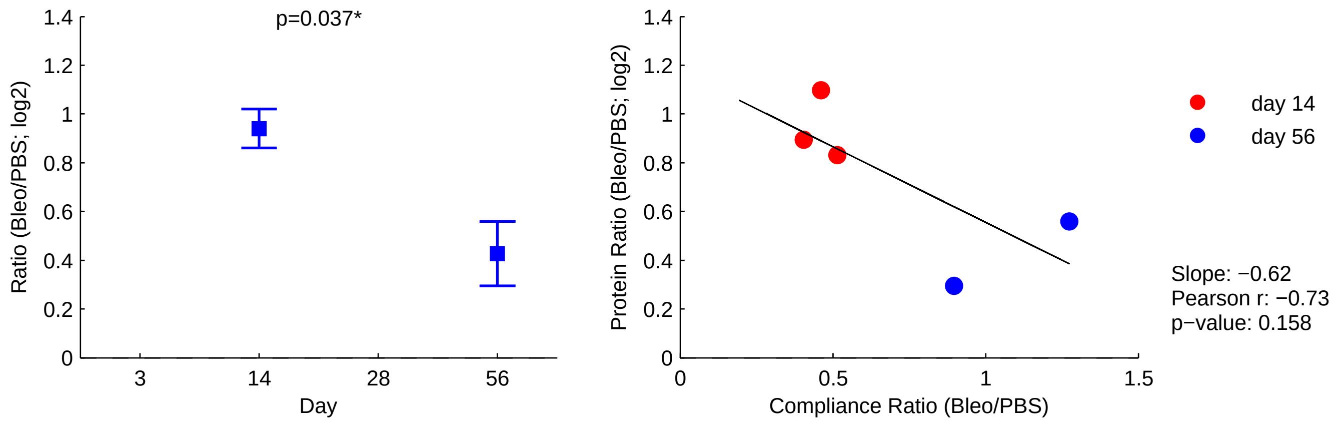

Q6VVW5 − Npr2 (id: 4732)

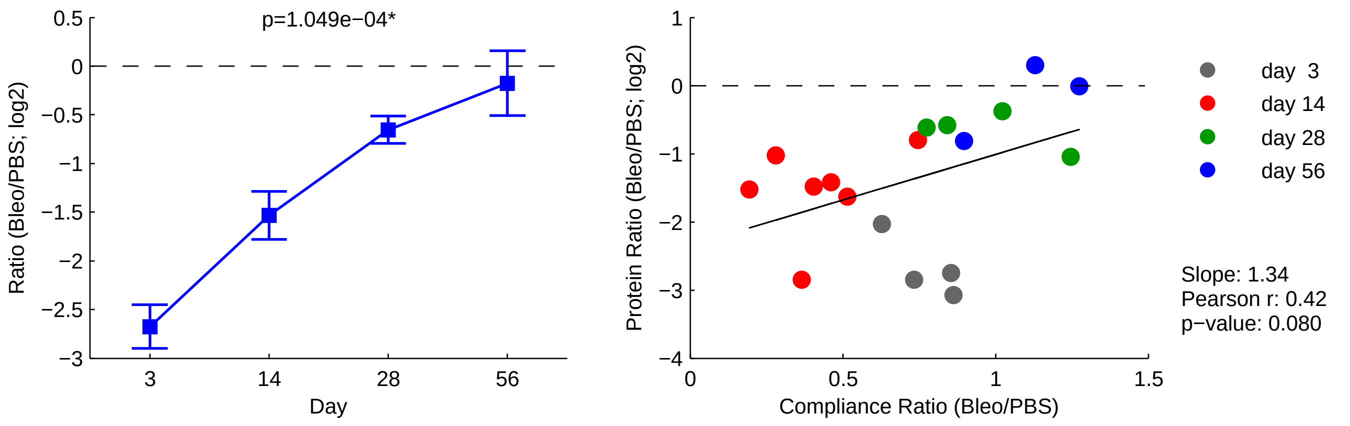

Q6WVG3 − Kctd12 (id: 4736)

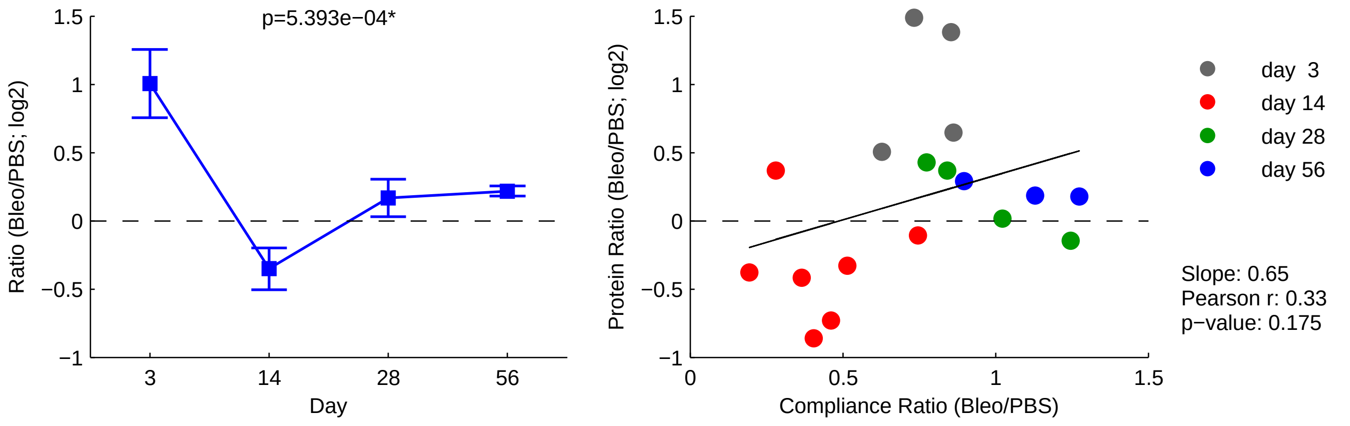

Q6XLQ8 − Calu (id: 4737)

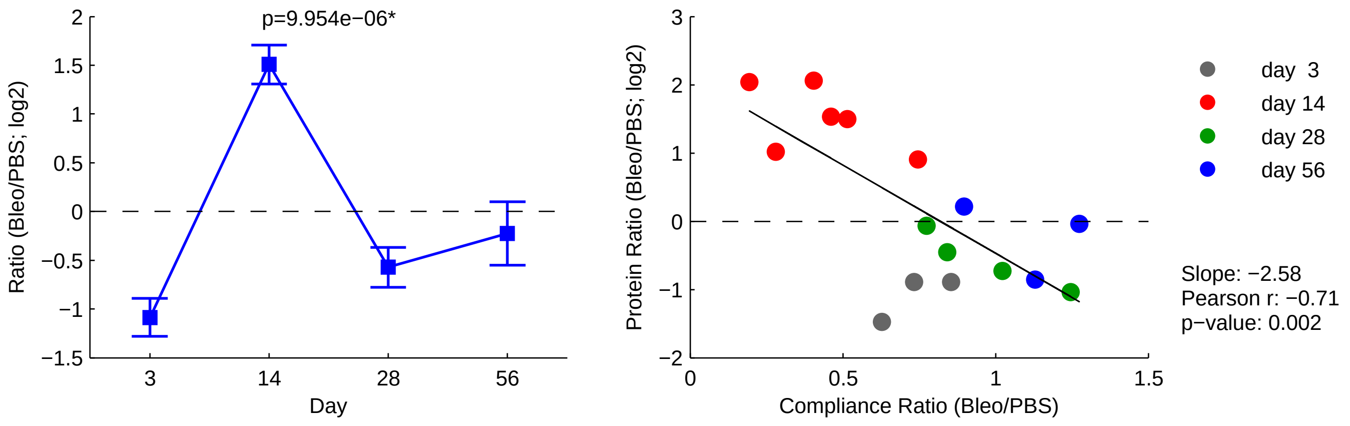

Q6ZPF4 − Fmnl3 (id: 4741)

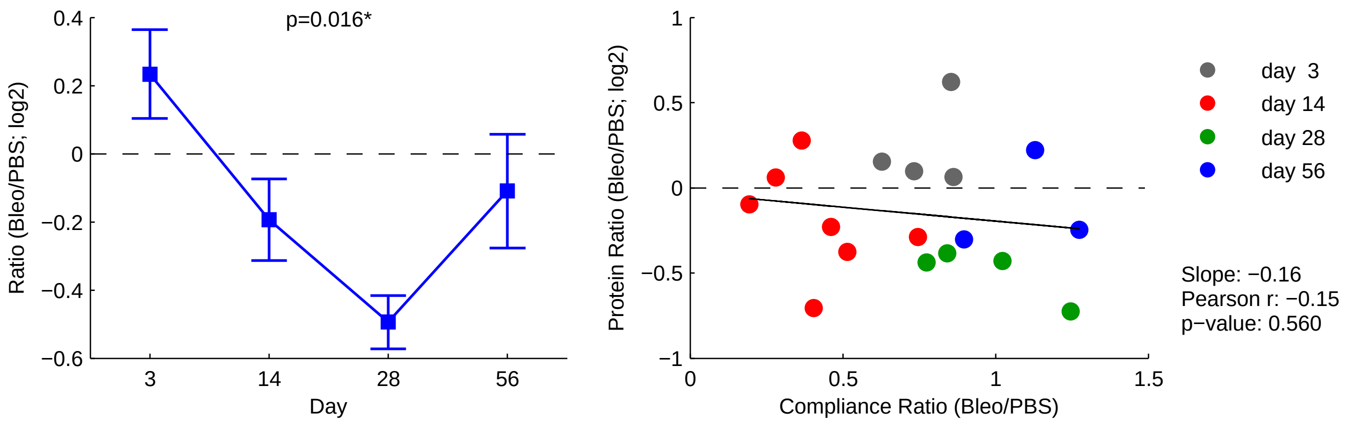

Q6ZPJ0 − Tex2 (id: 4742)

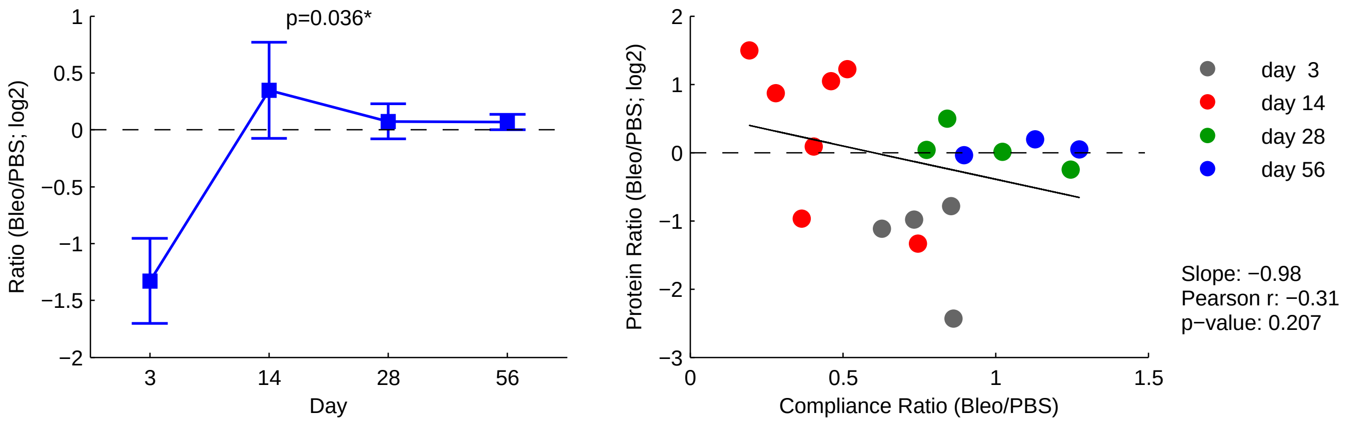

Q6ZPS6 − Ankib1 (id: 4746)

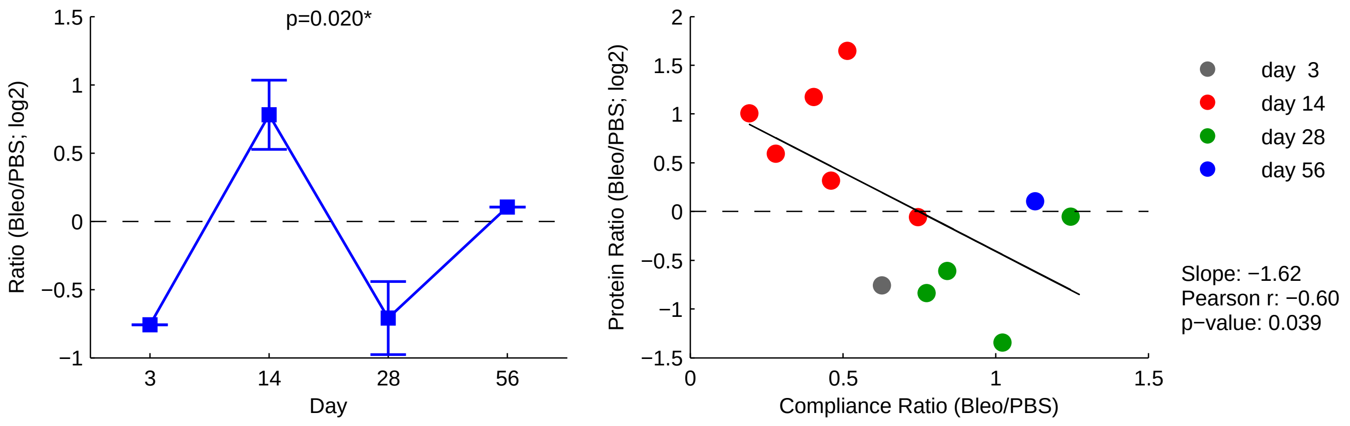

Q6ZQ08 − Cnot1 (id: 4749)

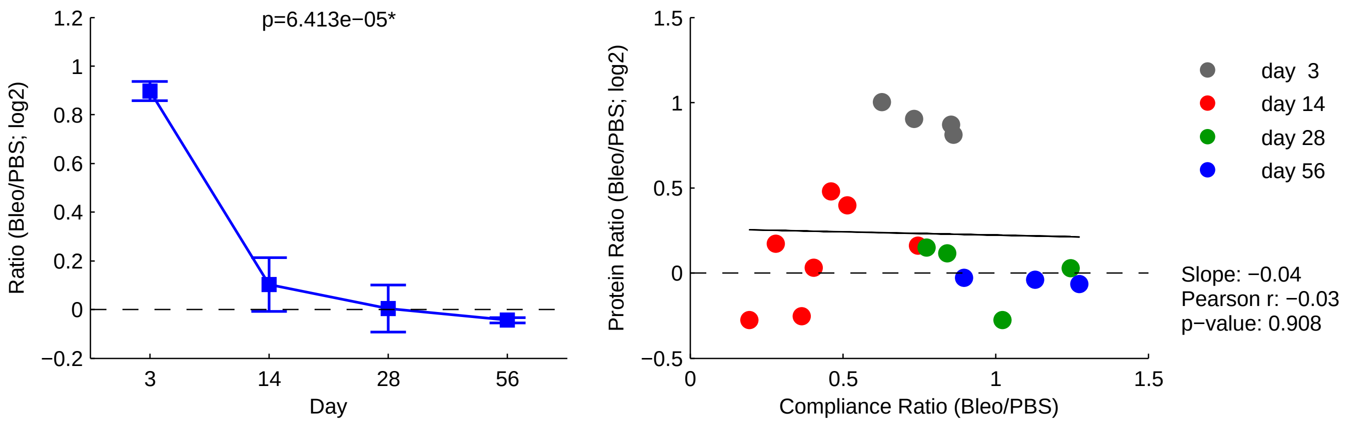

Q6ZQ29−2 − Taok2 (id: 4751)

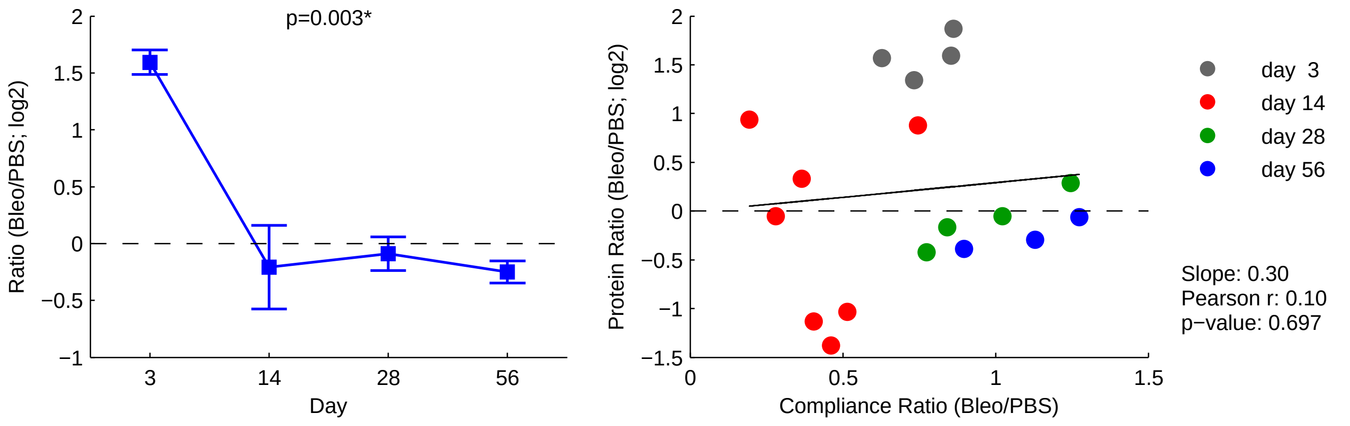

Q6ZQ73 − Cand2 (id: 4753)

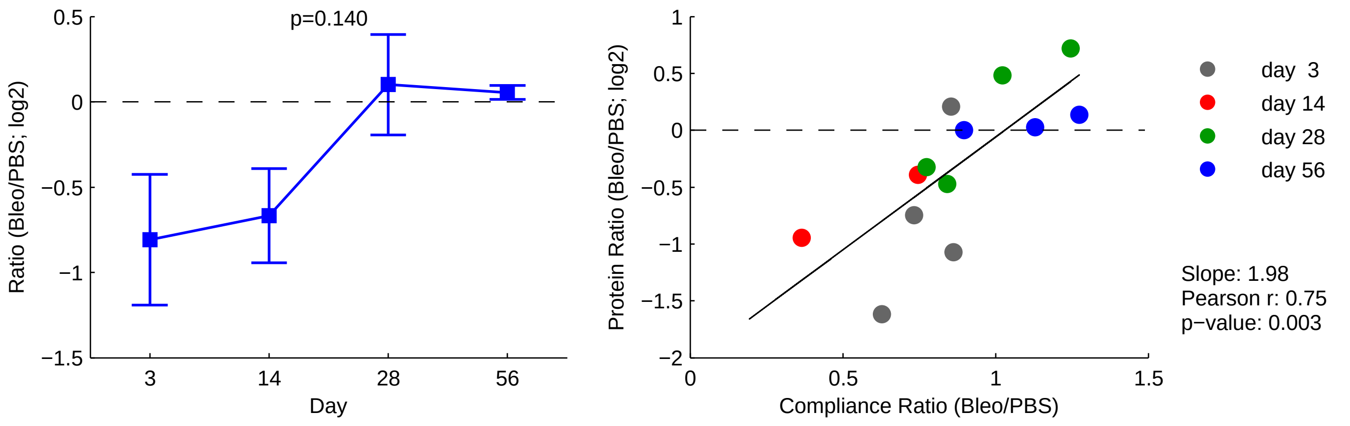

Q6ZQI3 − Mlec (id: 4755)

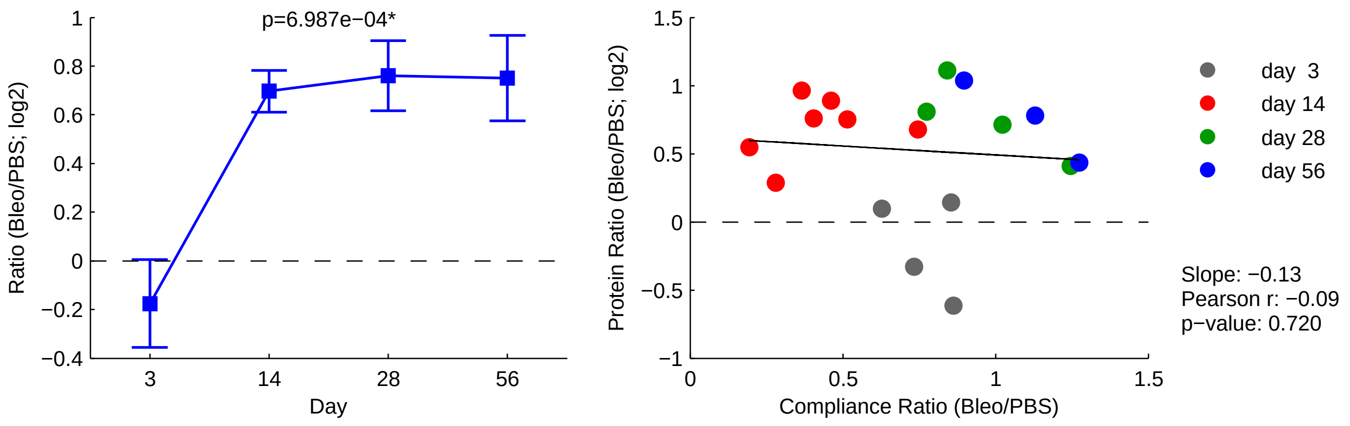

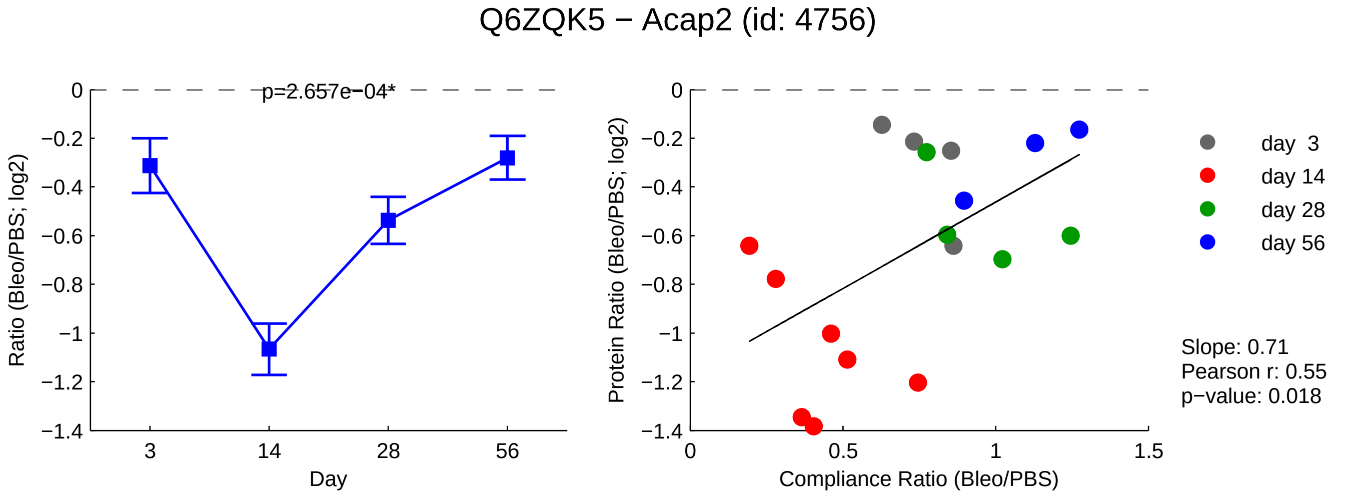
Q6ZQK5 − Acap2 (id: 4756)
p=2.657e−04*
Ratio (Bleo/PBS; log2)
Day
Protein Ratio (Bleo/PBS; log2)
Compliance Ratio (Bleo/PBS)
day 3
day 14
day 28
day 56
Slope: 0.71
Pearson r: 0.55
p−value: 0.018

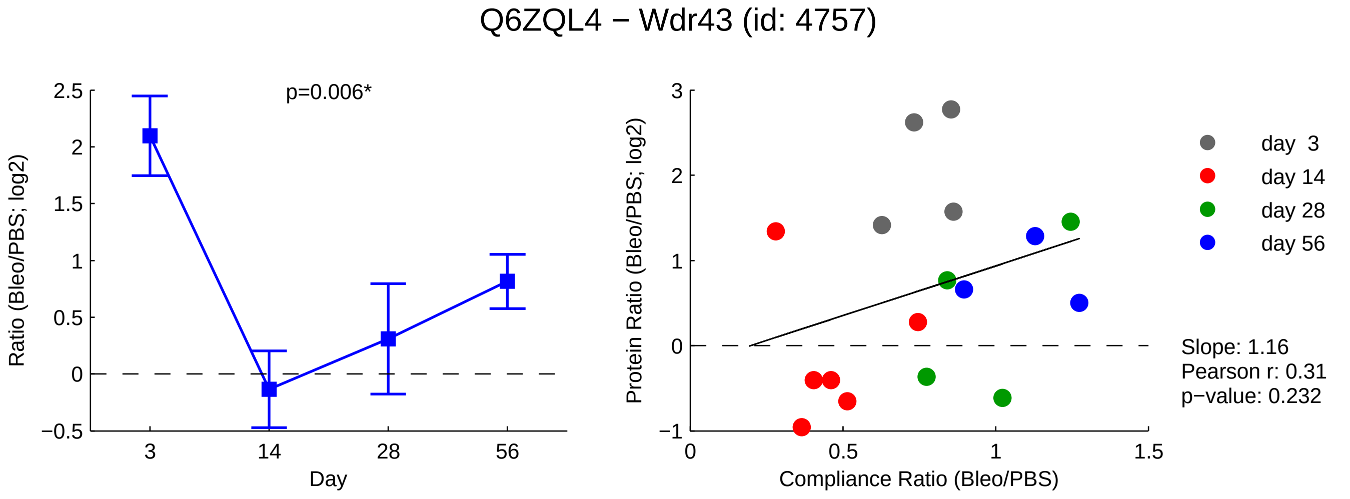
Q6ZQL4 − Wdr43 (id: 4757)
p=0.006*
Ratio (Bleo/PBS; log2)
Day
Protein Ratio (Bleo/PBS; log2)
Compliance Ratio (Bleo/PBS)
day 3
day 14
day 28
day 56
Slope: 1.16
Pearson r: 0.31
p−value: 0.232

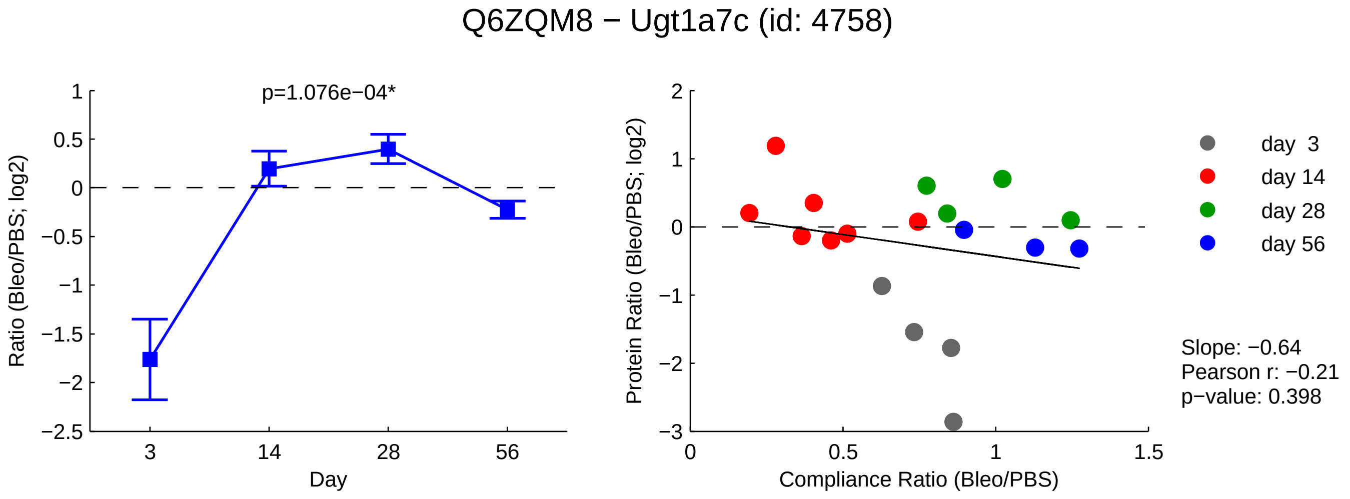
Q6ZQM8 − Ugt1a7c (id: 4758)
p=1.076e−04*
Ratio (Bleo/PBS; log2)
Day
Protein Ratio (Bleo/PBS; log2)
Compliance Ratio (Bleo/PBS)
day 3
day 14
day 28
day 56
Slope: −0.64
Pearson r: −0.21
p−value: 0.398

Q6ZWN5 − Rps9 (id: 4760)

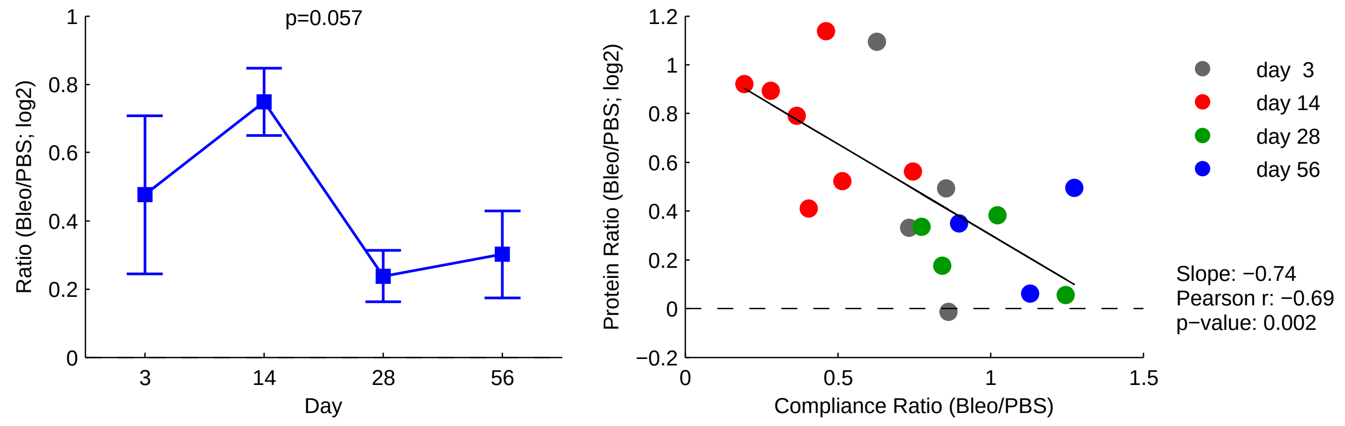

Q6ZWQ7 − Spcs3 (id: 4761)

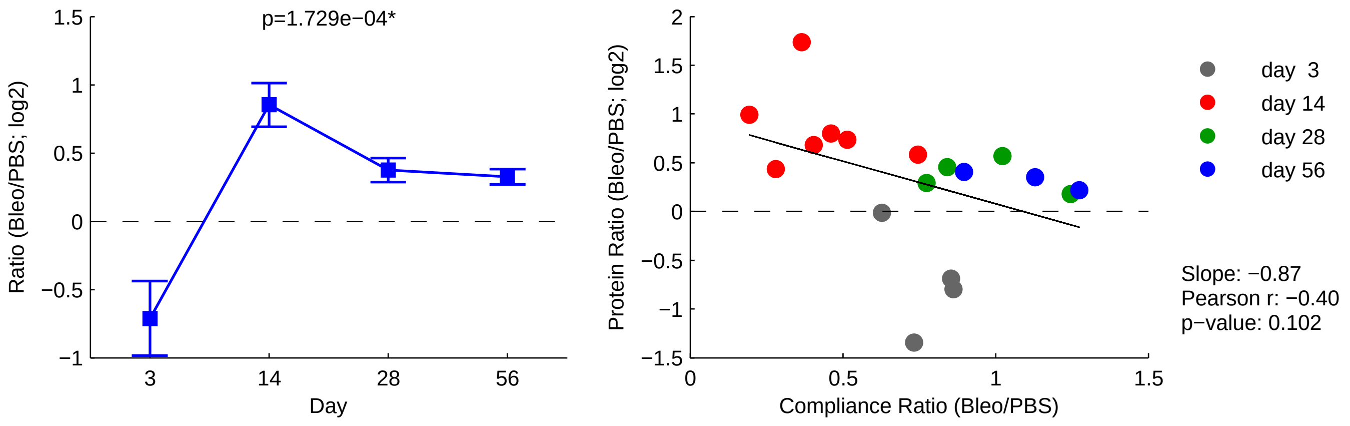

Q6ZWR6−4 − Syne1 (id: 4763)

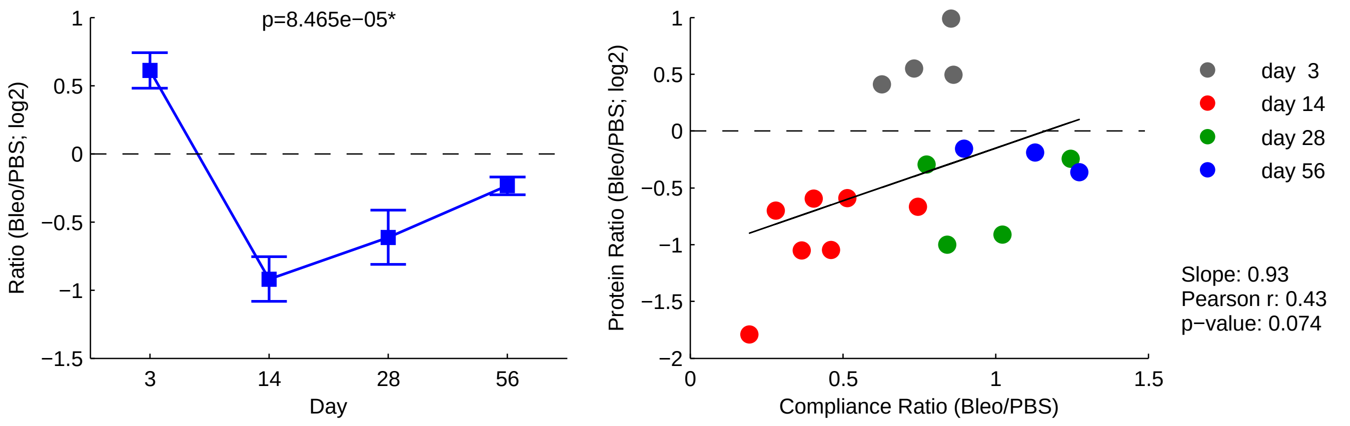

Q6ZWU9 − Rps27 (id: 4765)

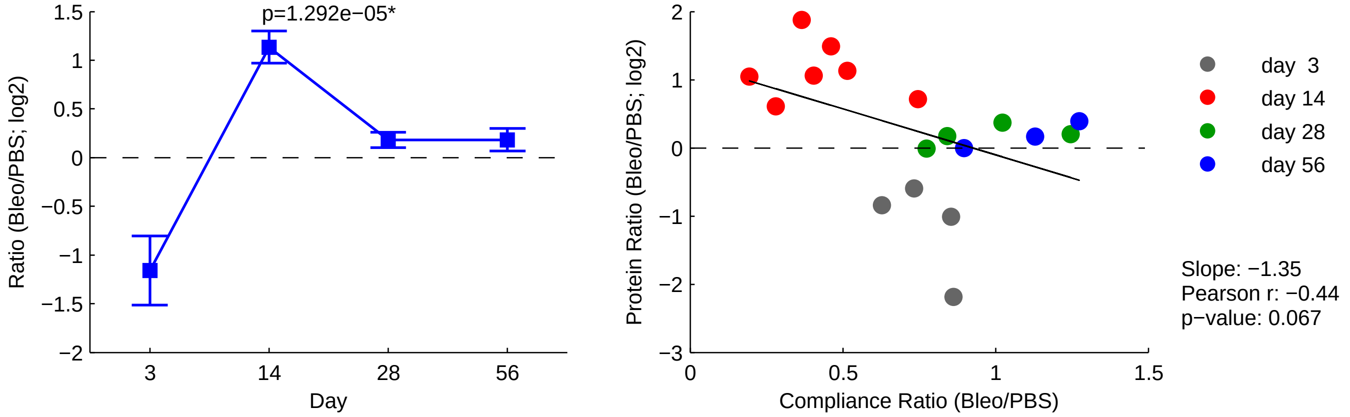

Q6ZWV3 − Rpl10 (id: 4766)

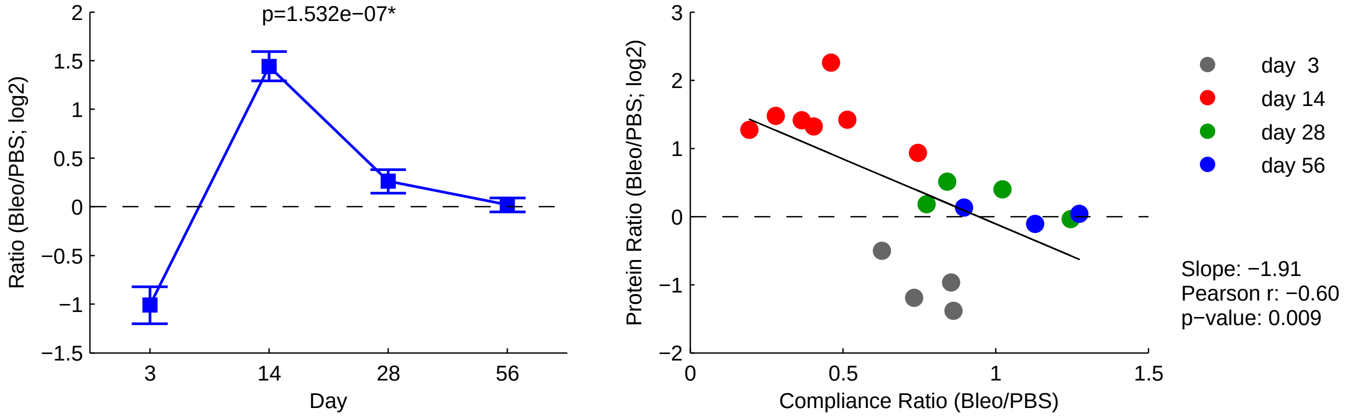

Q6ZWV7 − Rpl35 (id: 4767)

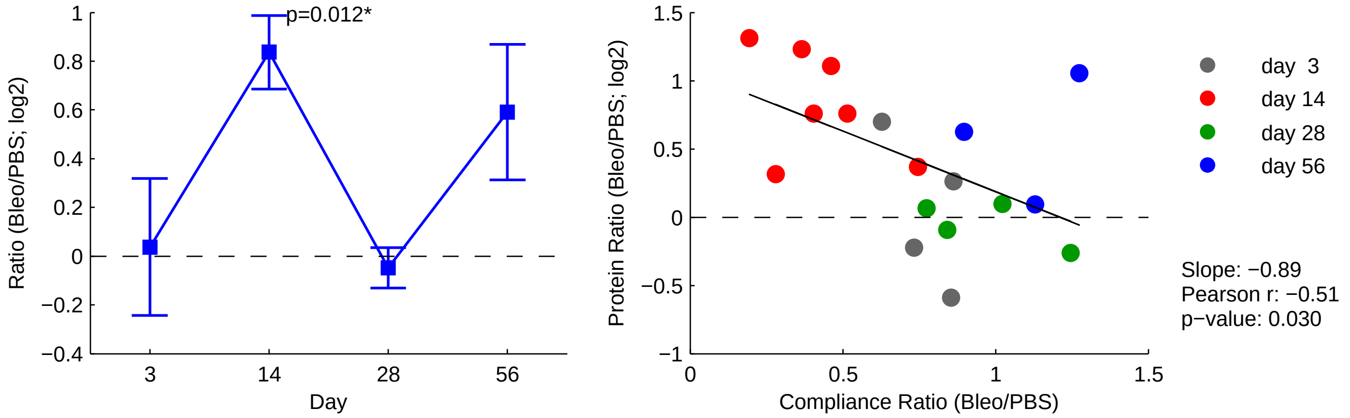

Q6ZWX6 − Eif2s1 (id: 4768)

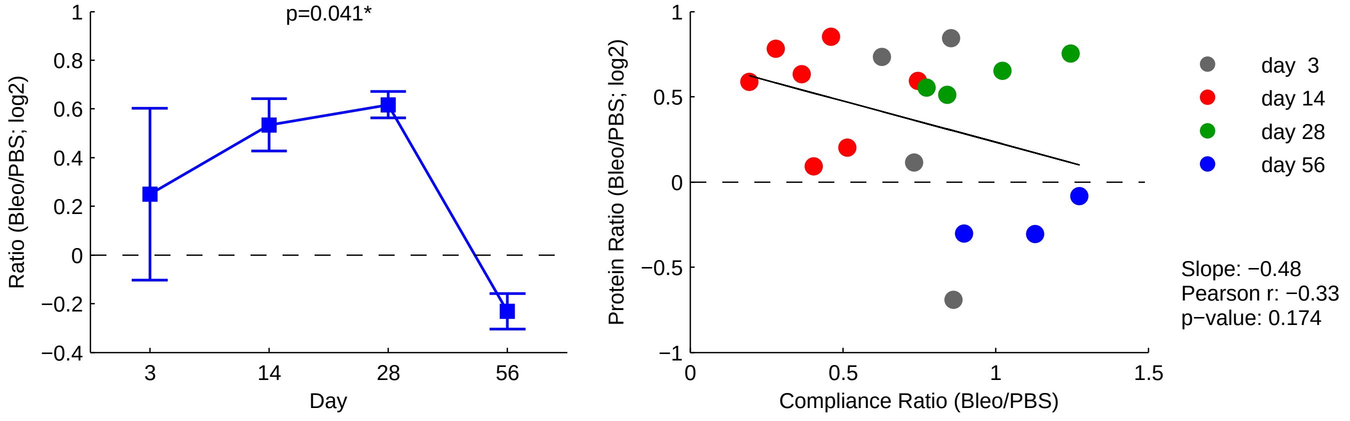

Q6ZWY3 − Rps27l (id: 4769)

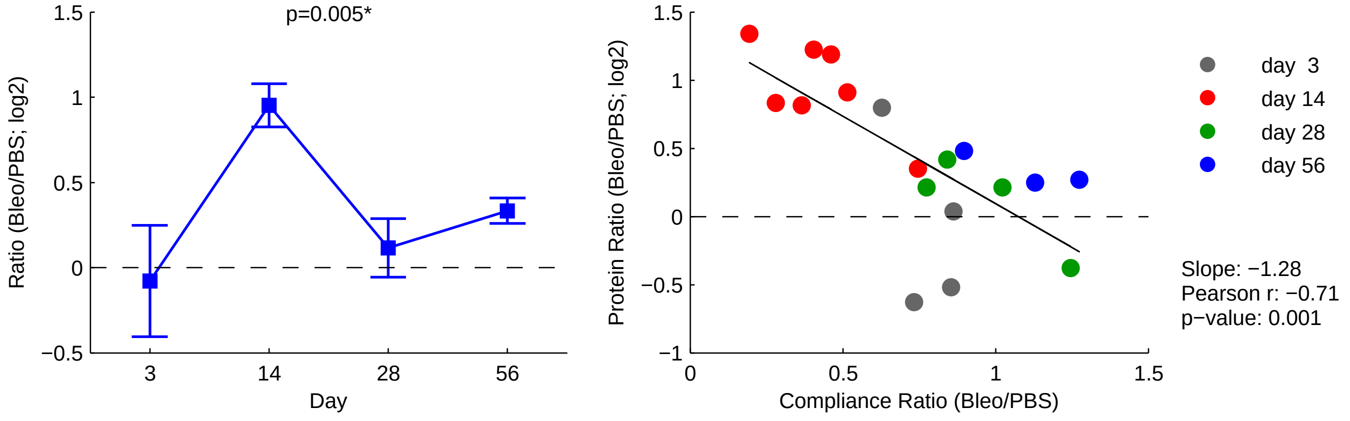

Q6ZWY8 − Tmsb10 (id: 4770)

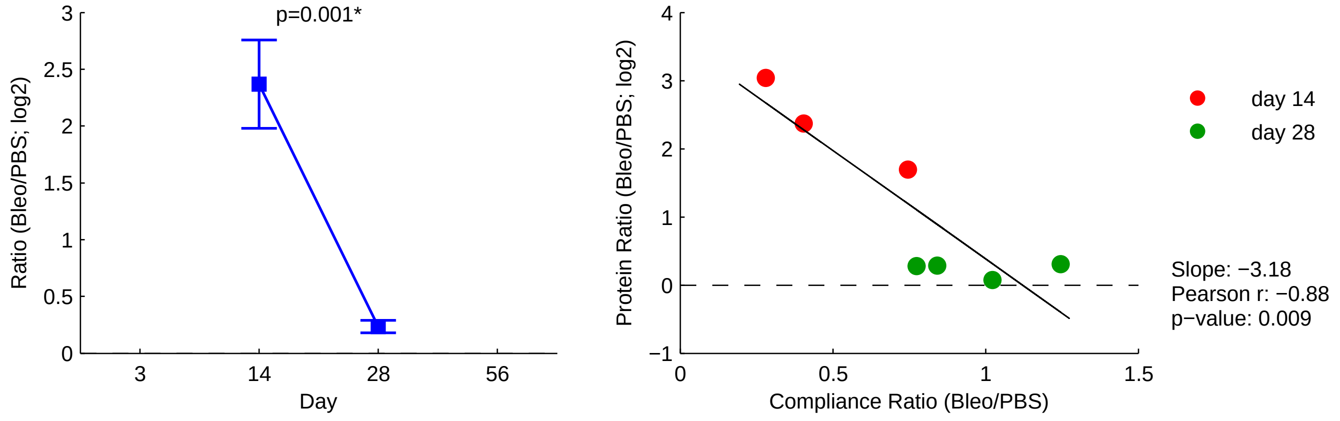

Q6ZWZ6 − Rps12 (id: 4771)

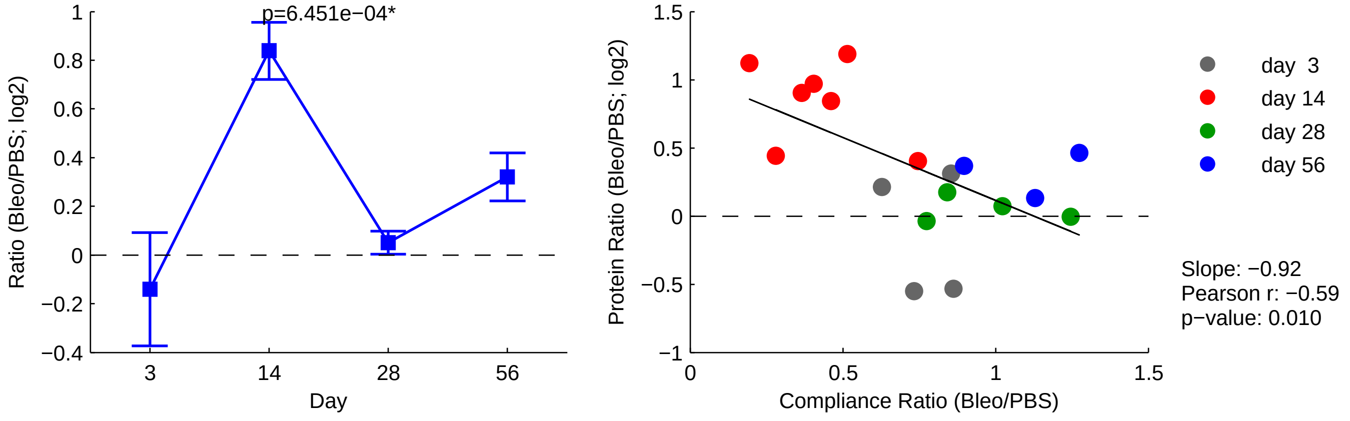

Q9CPR4 − Rpl17 (id: 4772)

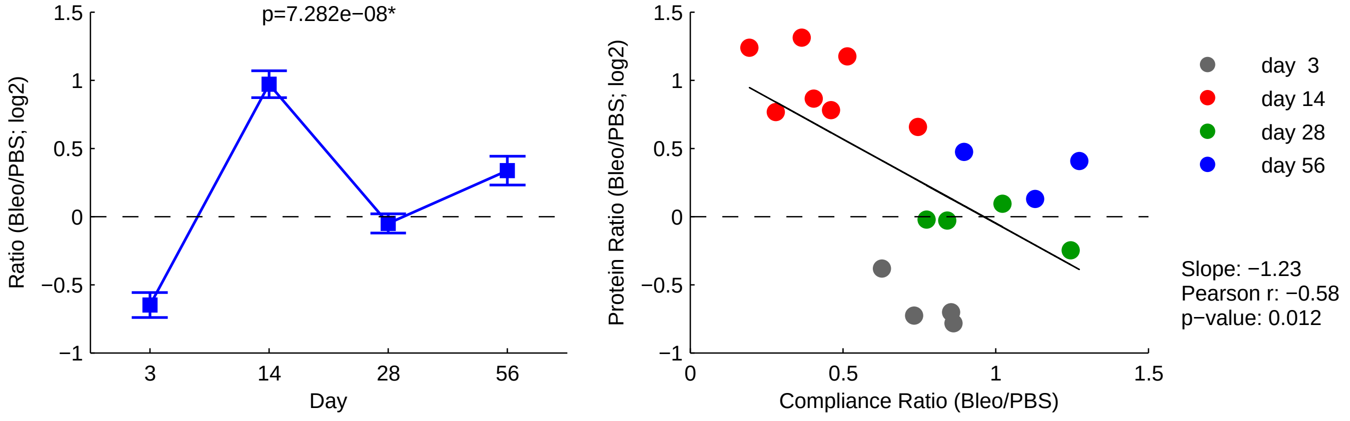

Q70IV5−2 − Synm (id: 4774)

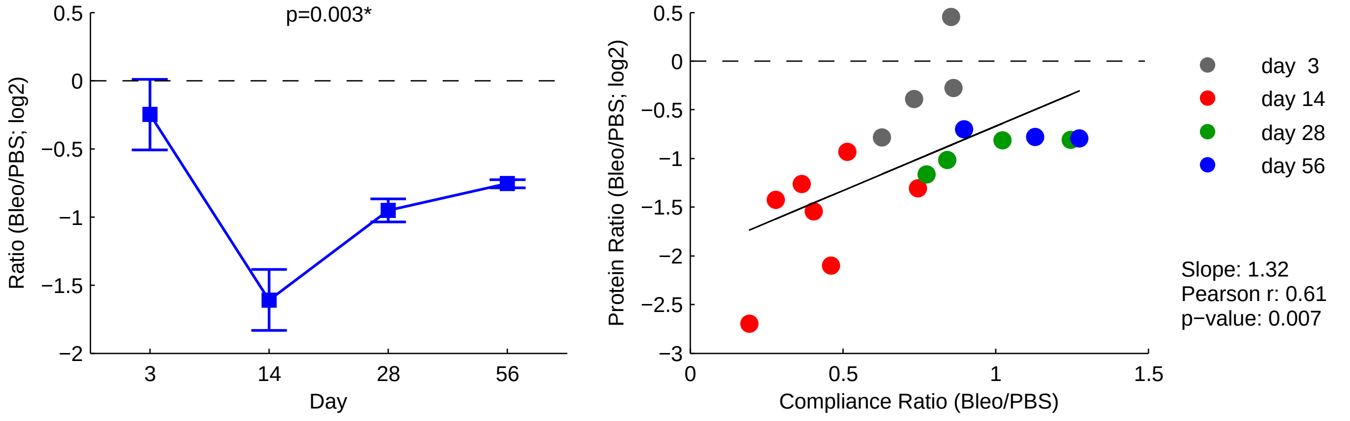

Q70KY4 − Foxo6 (id: 4775)

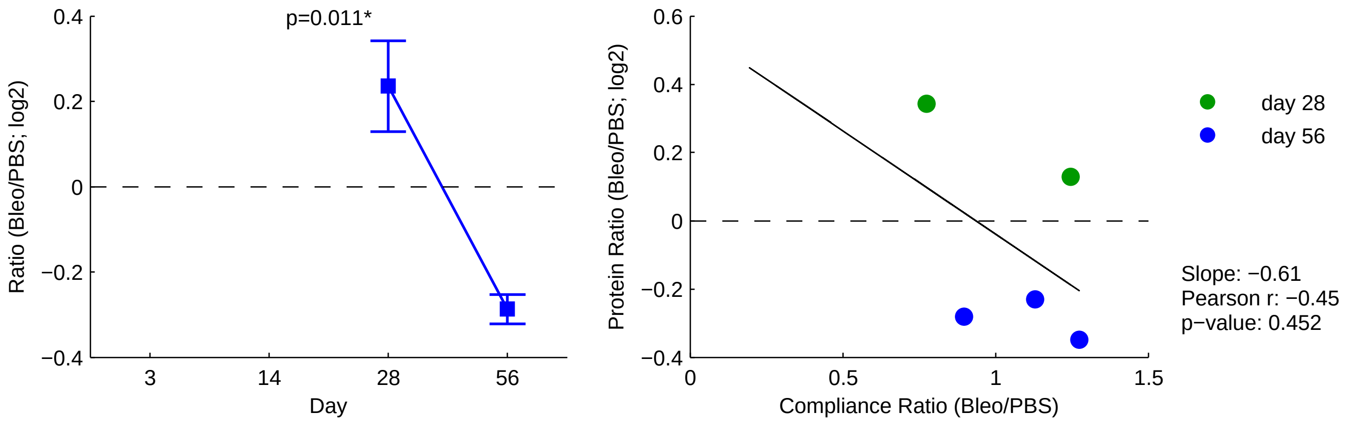

Q71FD7 − Fblim1 (id: 4777)

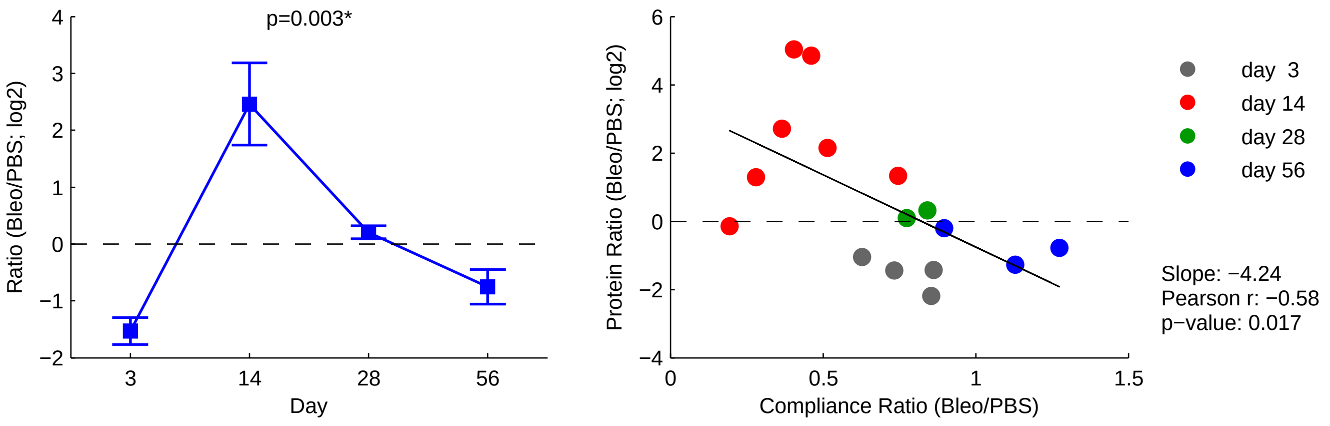

Q71RI9−2 − Ccbl2 (id: 4778)

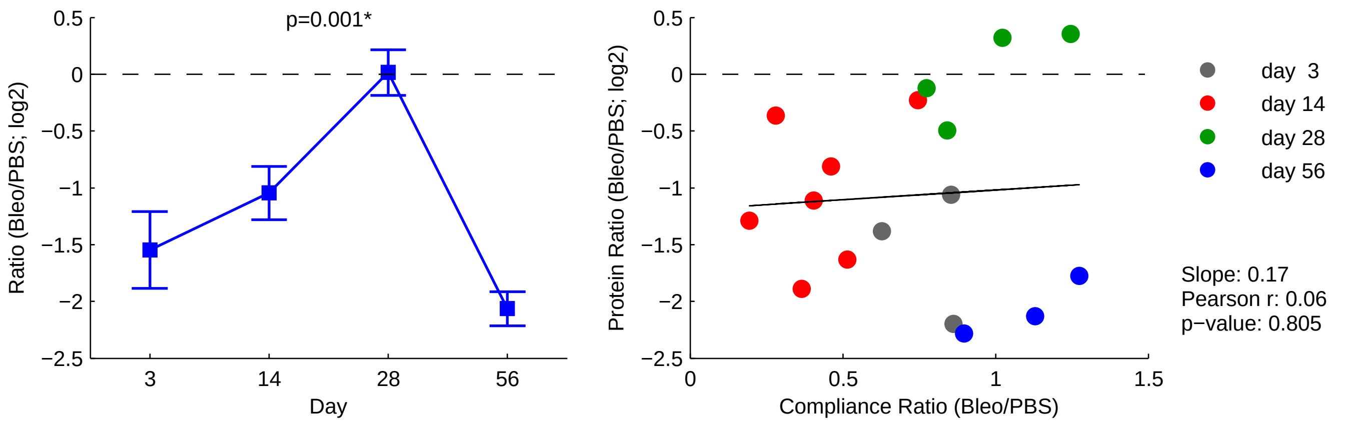

Q75N62 − Gimap8 (id: 4779)

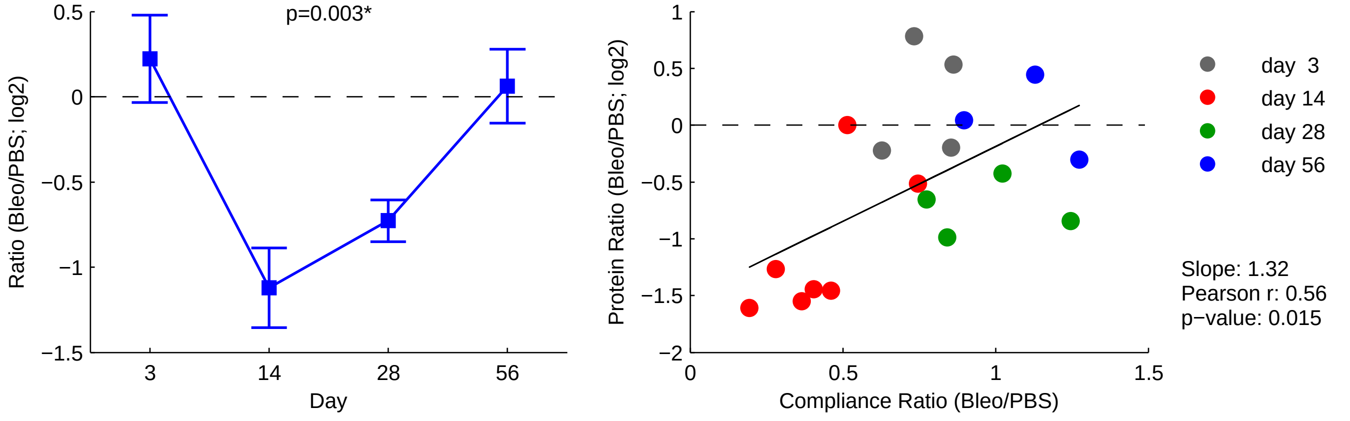

Q78IK2 − Usmg5 (id: 4783)

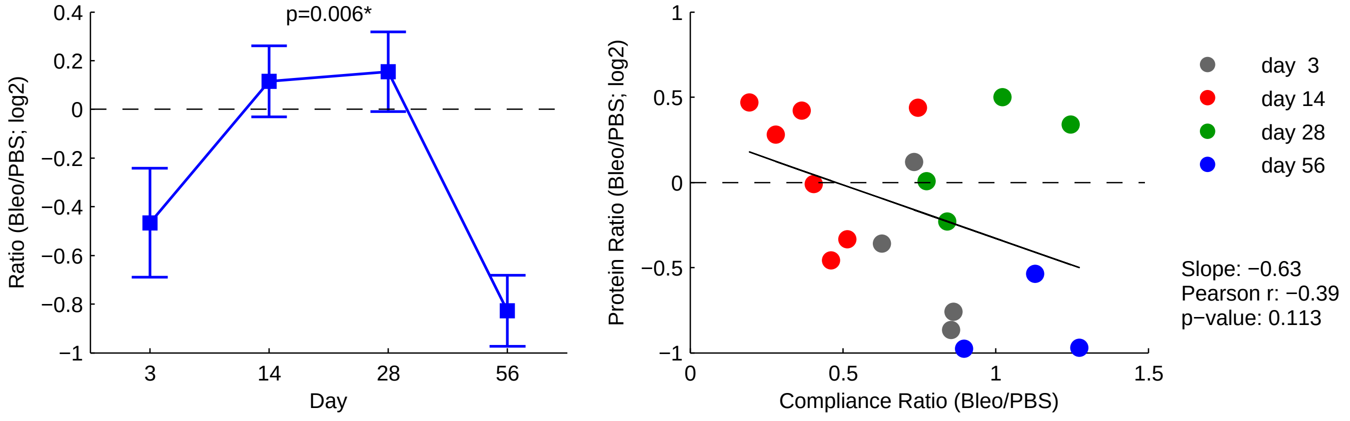

Q78IK4 − Apool (id: 4784)

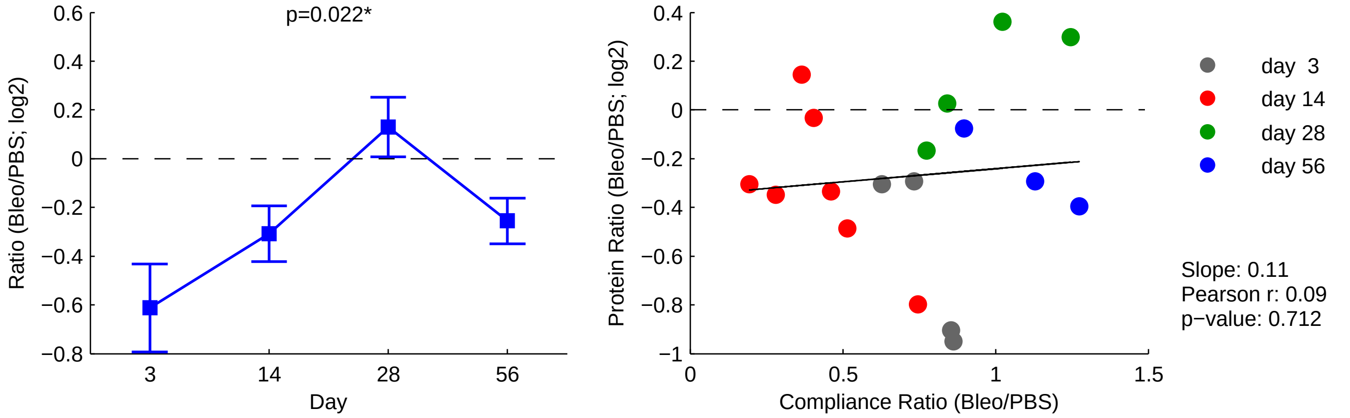

Q78IQ7 − Slc39a4 (id: 4785)

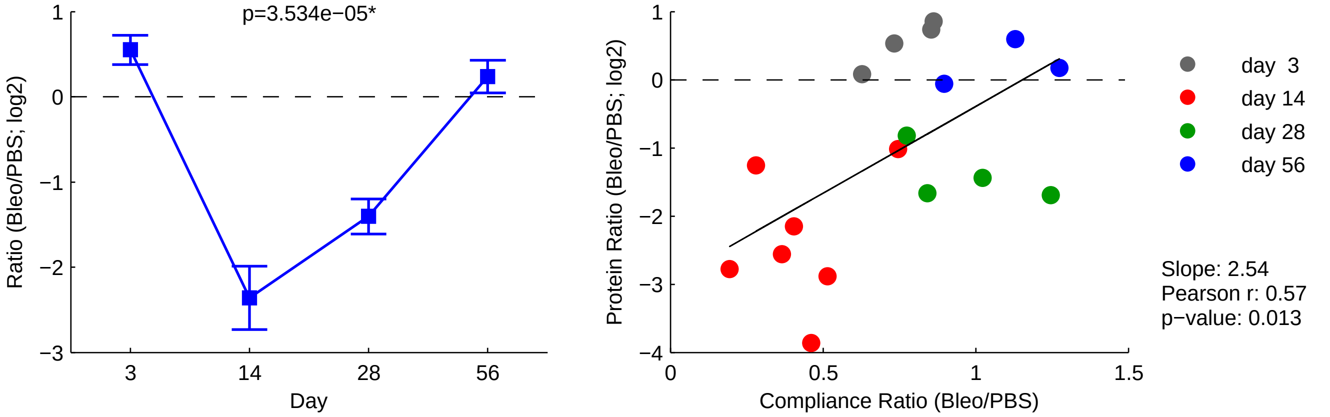

Q78IS1 − Tmed3 (id: 4786)

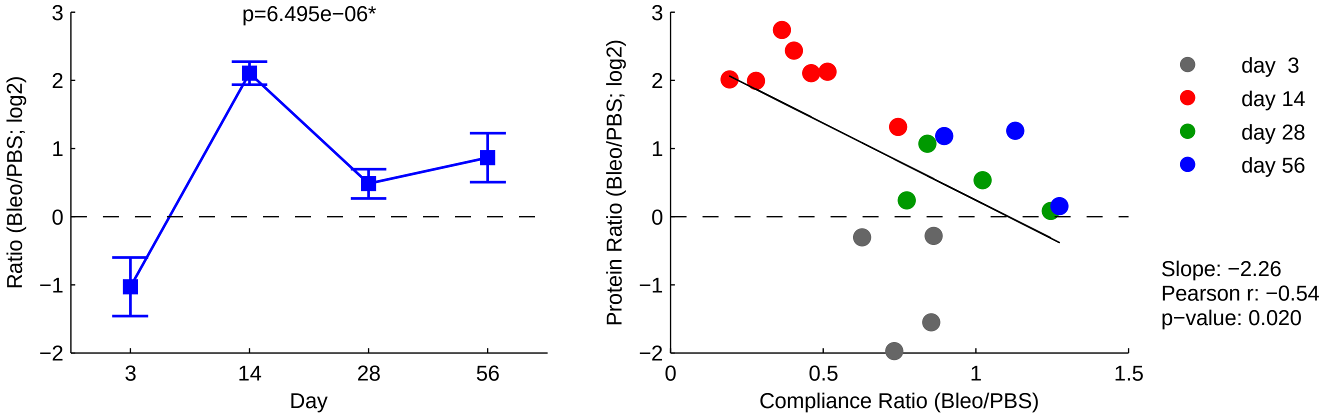

Q78J03 − Msrb2 (id: 4787)

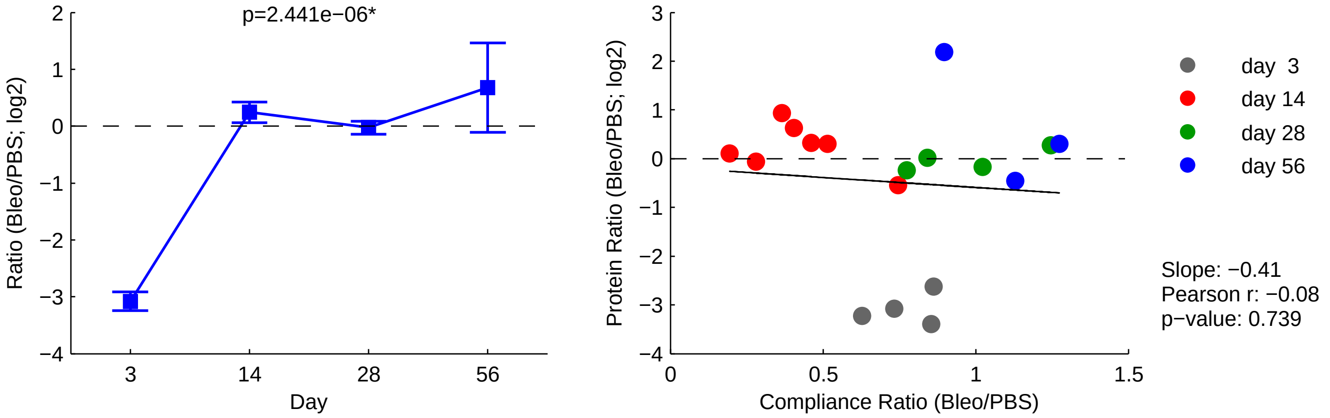

Q78PG9 − Ccdc25 (id: 4790)

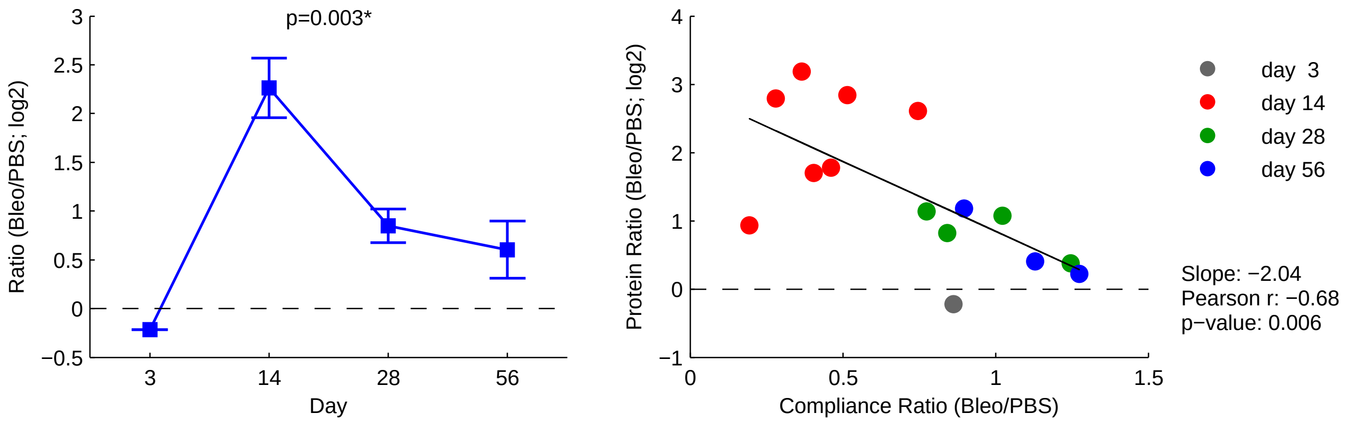

Q78ZA7 − Nap1l4 (id: 4795)

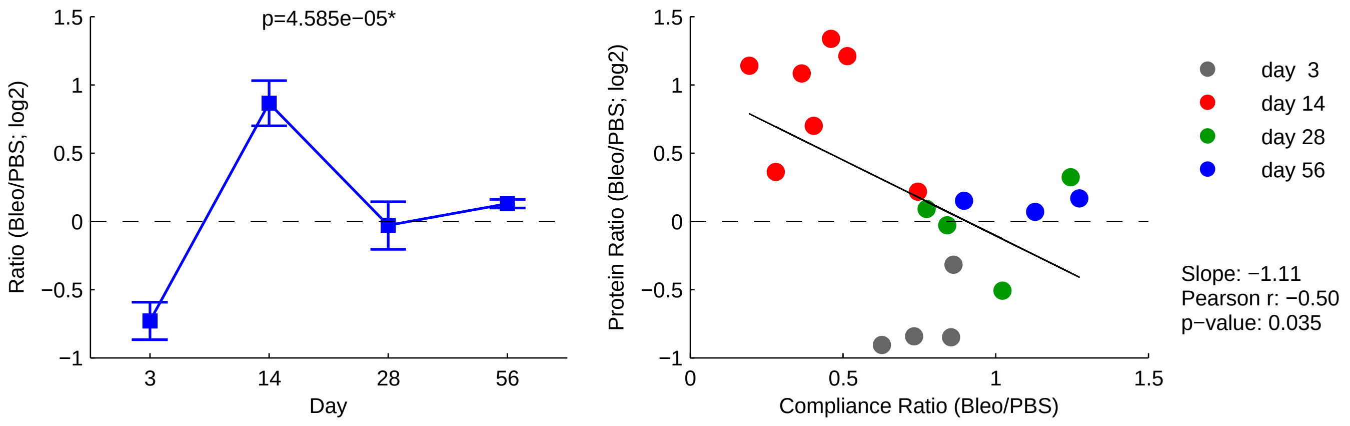

Q78ZM0 − Snx3 (id: 4796)

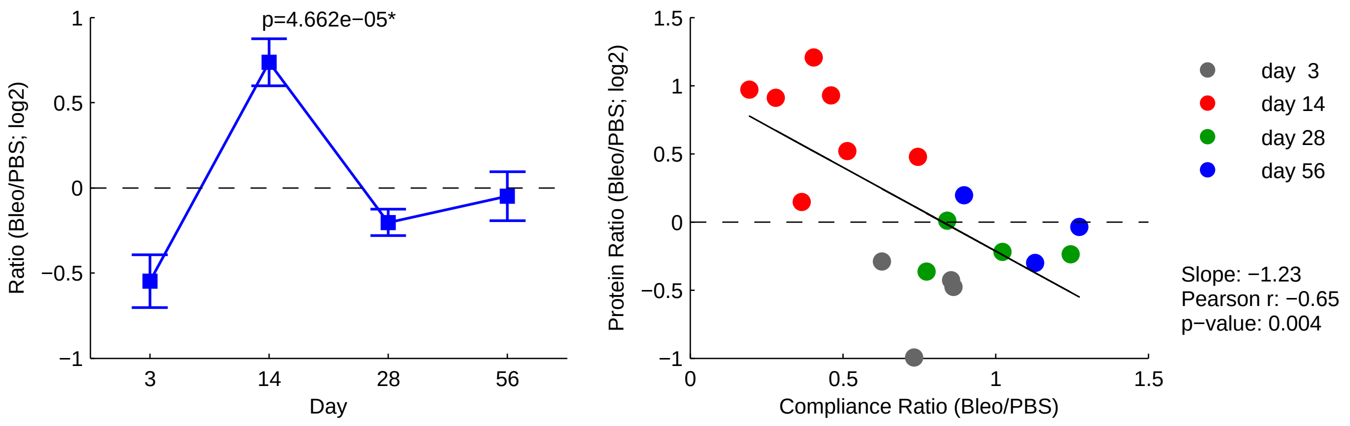

Q791T5−2 − Mtch1 (id: 4797)

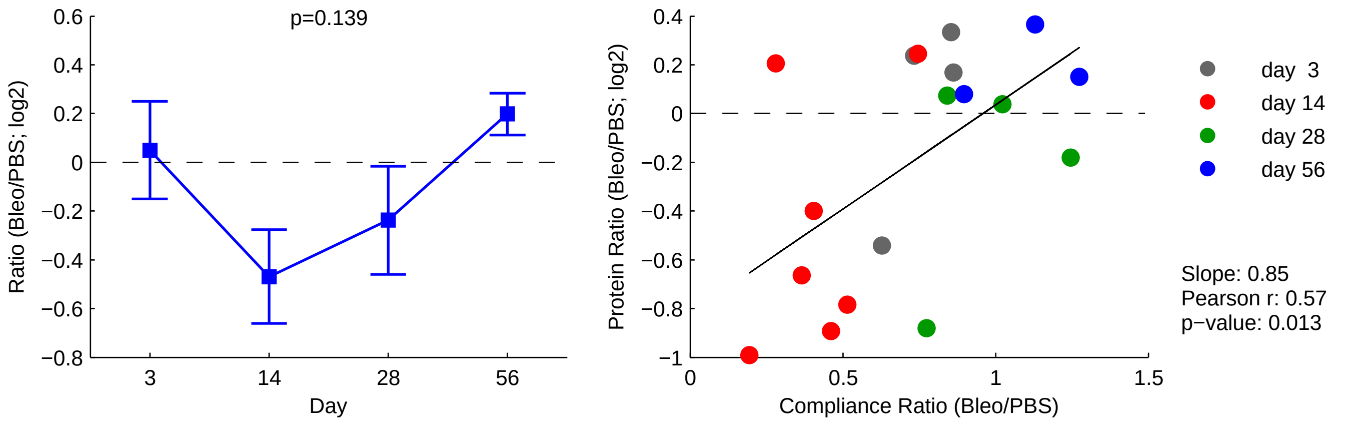

Q7M6Y3−5 − (id: 4801)

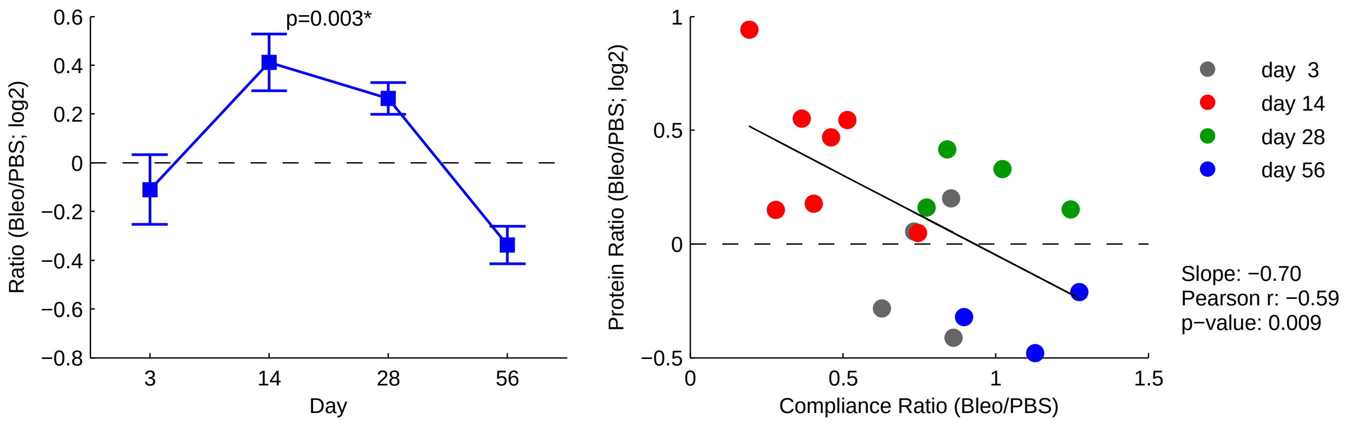

Q7M759 − Abhd17b (id: 4803)

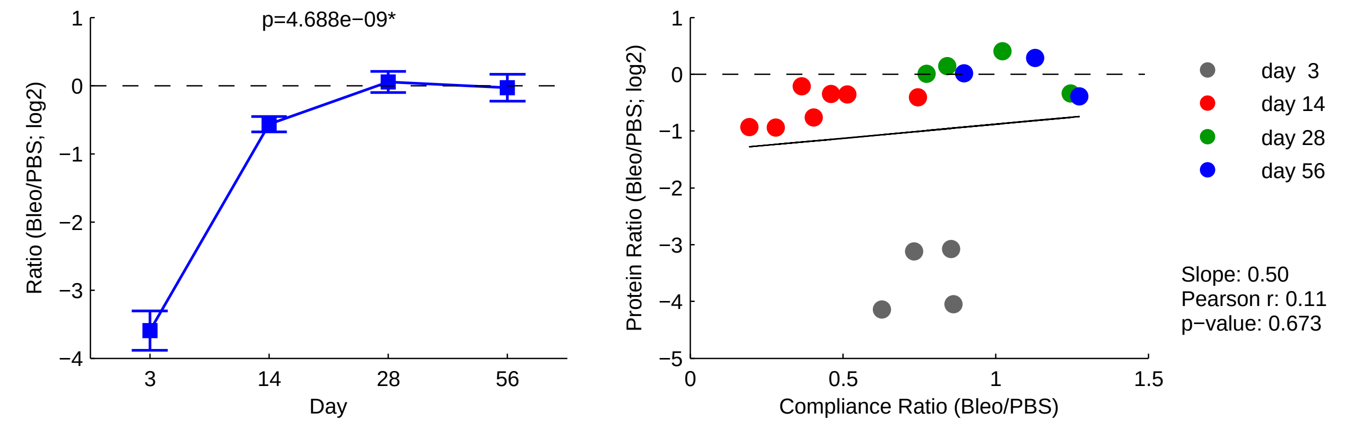

Q7TMF3 − Ndufa12 (id: 4807)

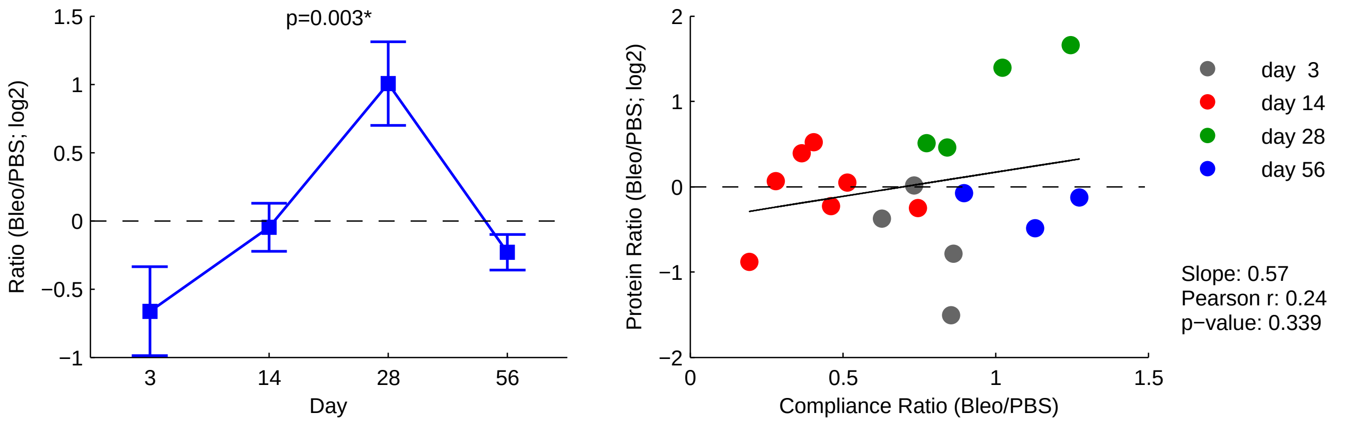

Q7TMG8 − Gbas (id: 4808)

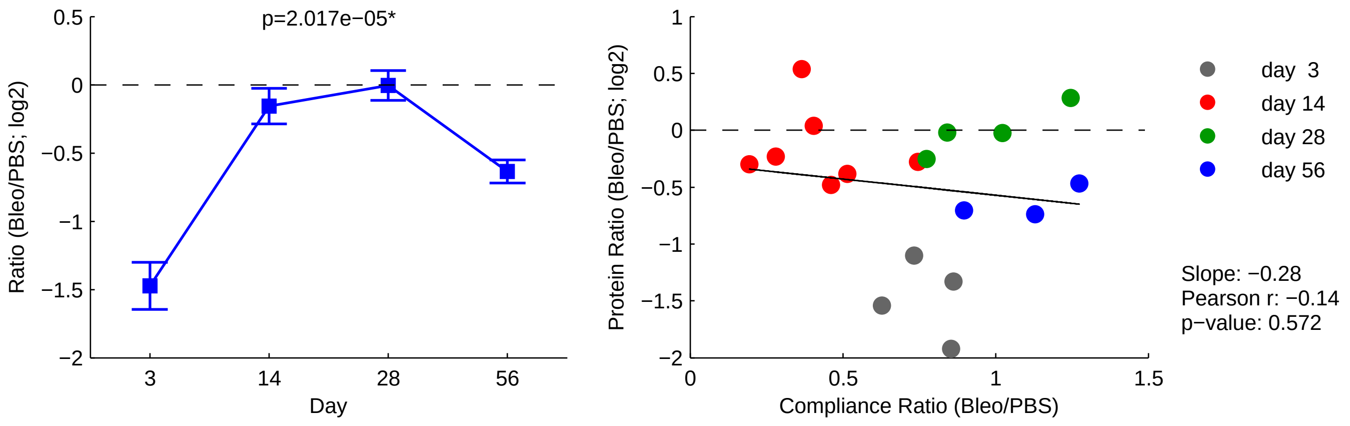

Q7TMK9−2 − Syncrip (id: 4811)

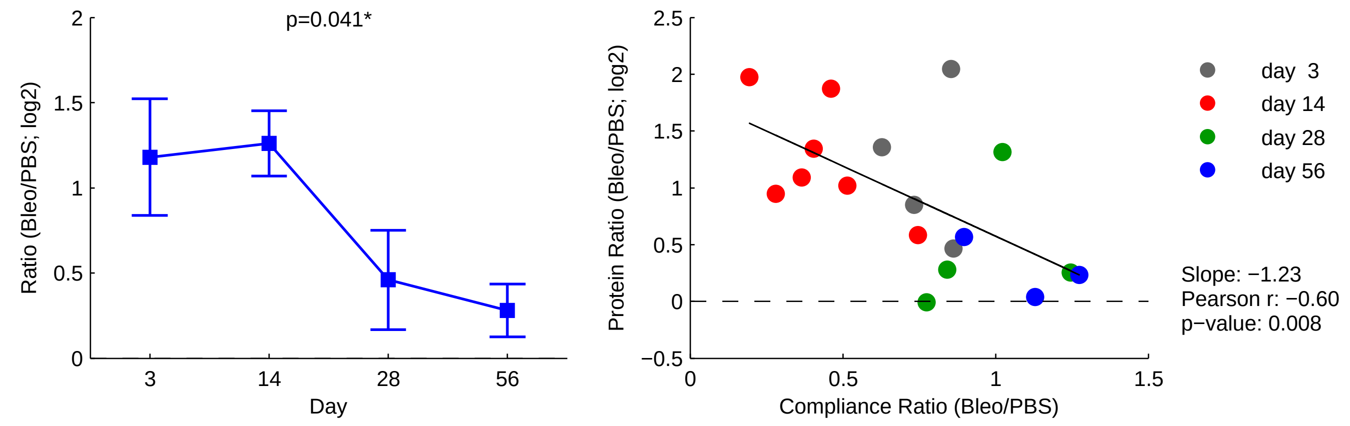

Q7TMM9 − Tubb2a (id: 4813)

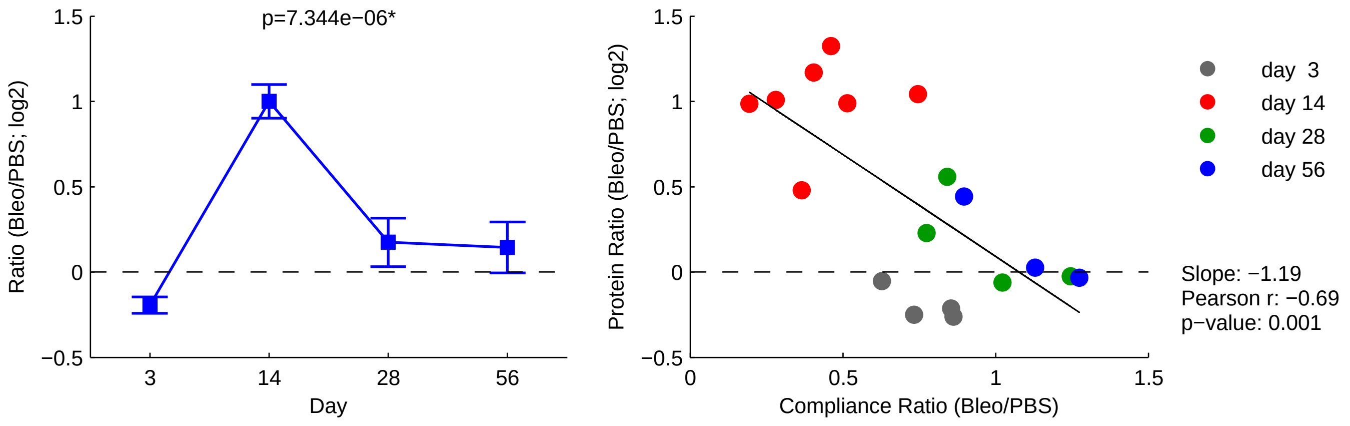

Q7TMR0 − Prcp (id: 4815)

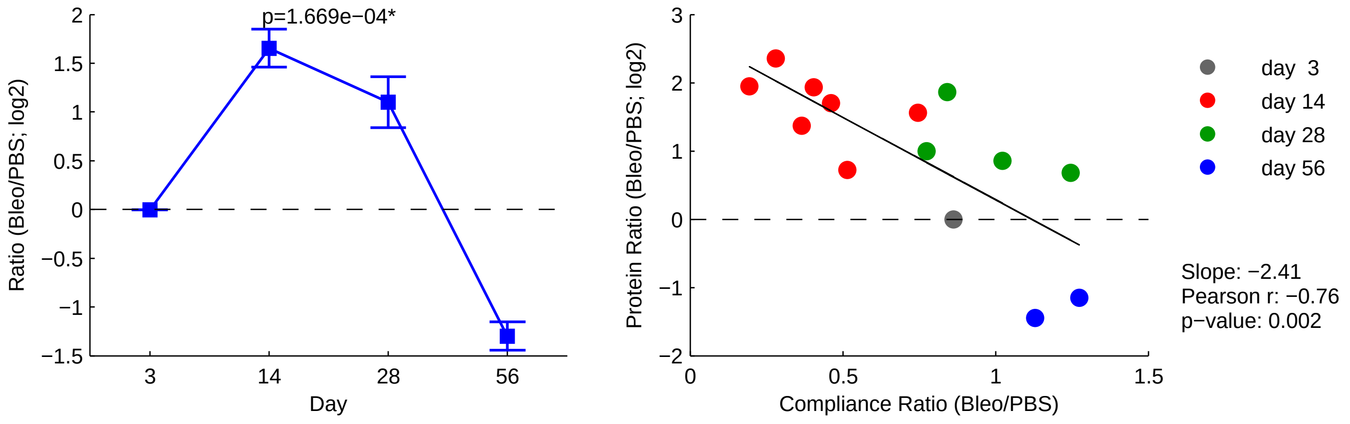

Q7TN29−2 − Smap2 (id: 4821)

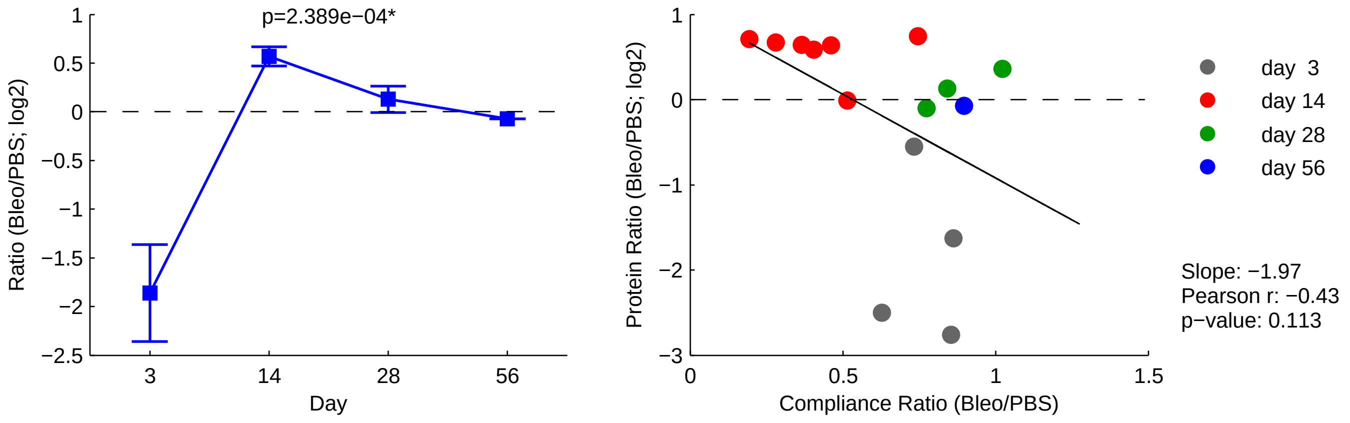

Q7TNC4−2 − Luc7l2 (id: 4824)

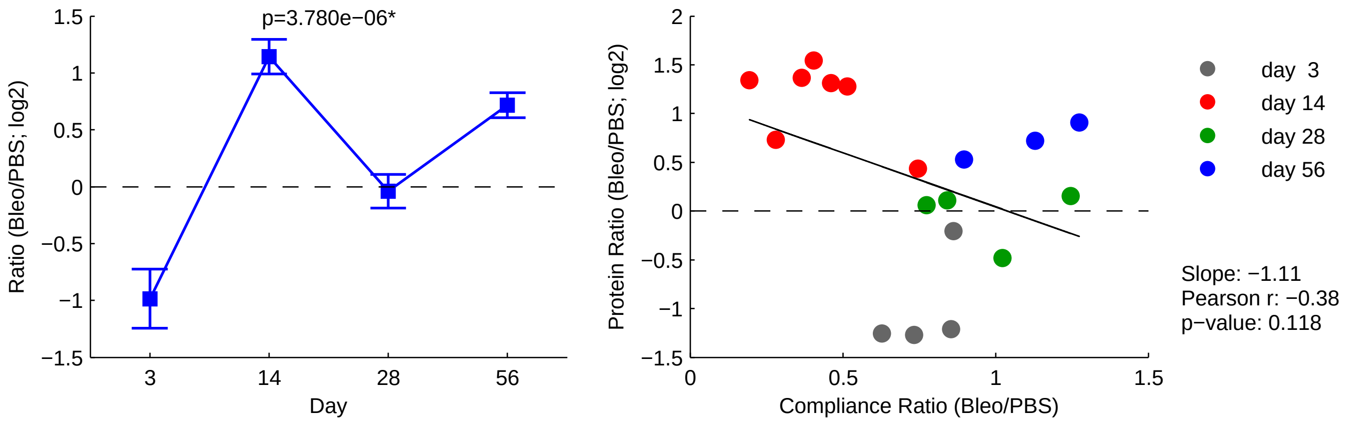

Q7TNG5 − Eml2 (id: 4827)

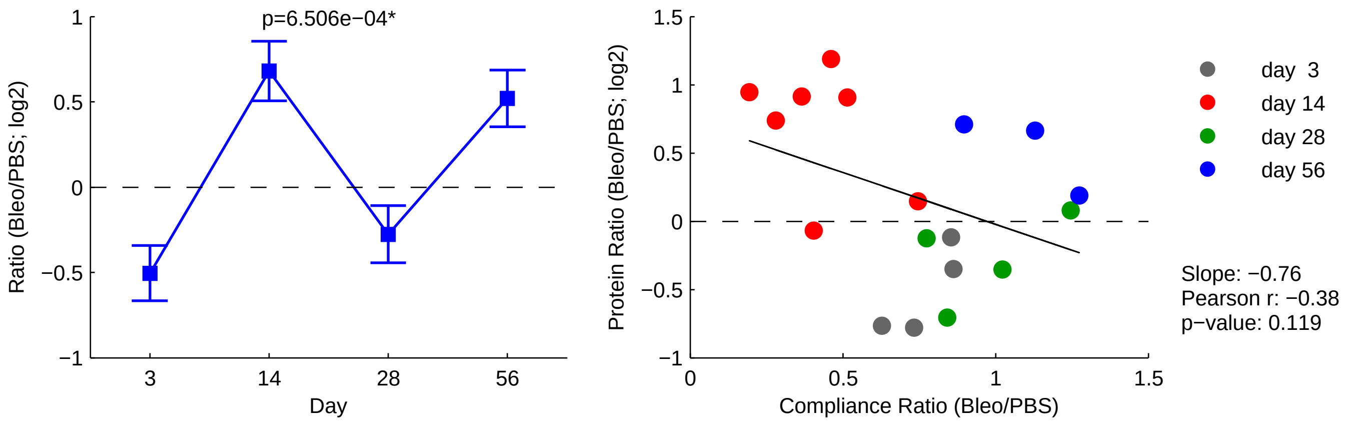

Q7TPM9 − Mtmr10 (id: 4834)

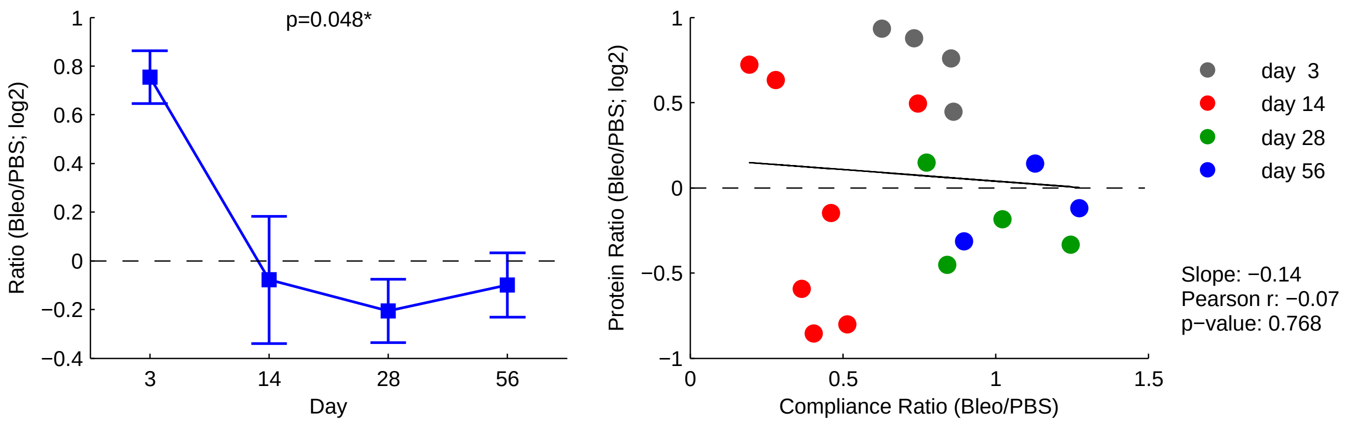

Q7TPN9 − Prr14 (id: 4835)

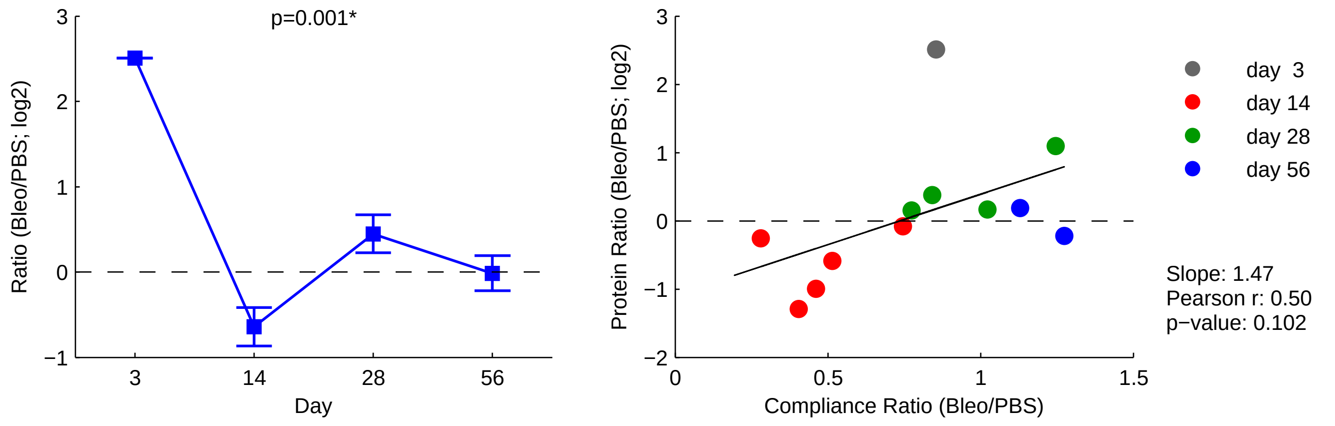

Q7TPV4 − Mybbp1a (id: 4838)

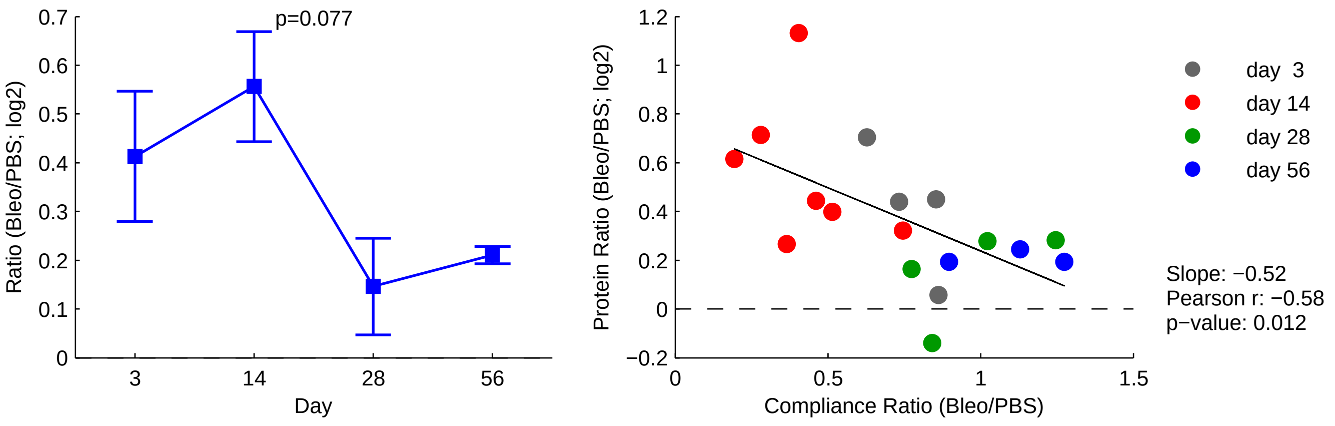

Q7TPW1 − Nexn (id: 4839)

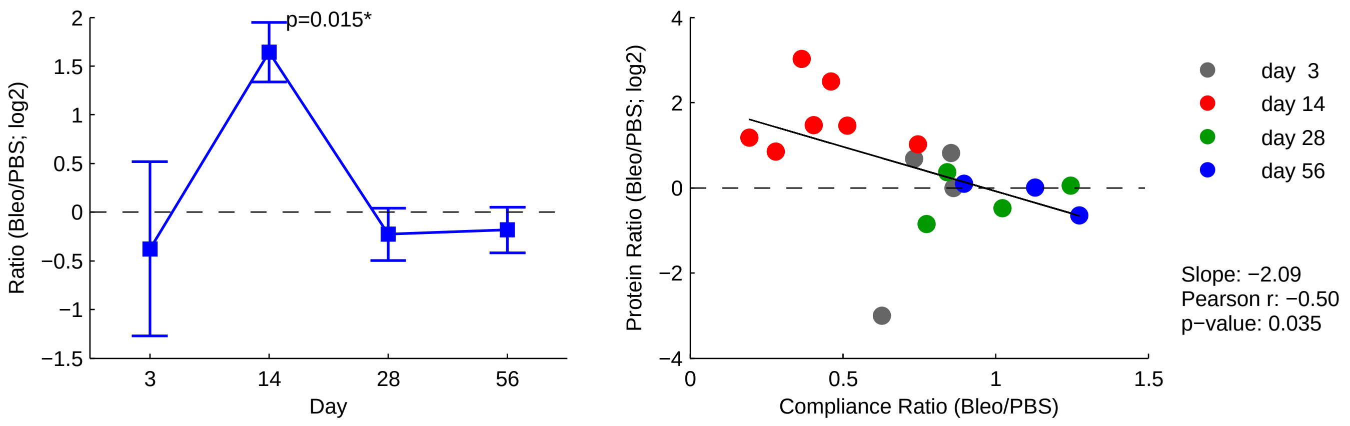

Q7TQ48 − Srl (id: 4840)

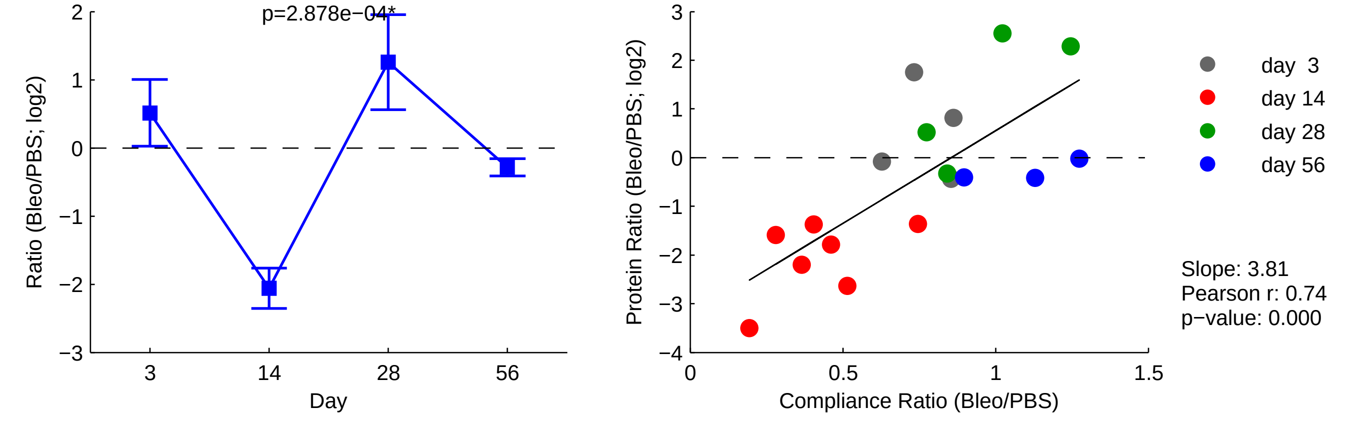

Q7TQ62 − Podn (id: 4841)

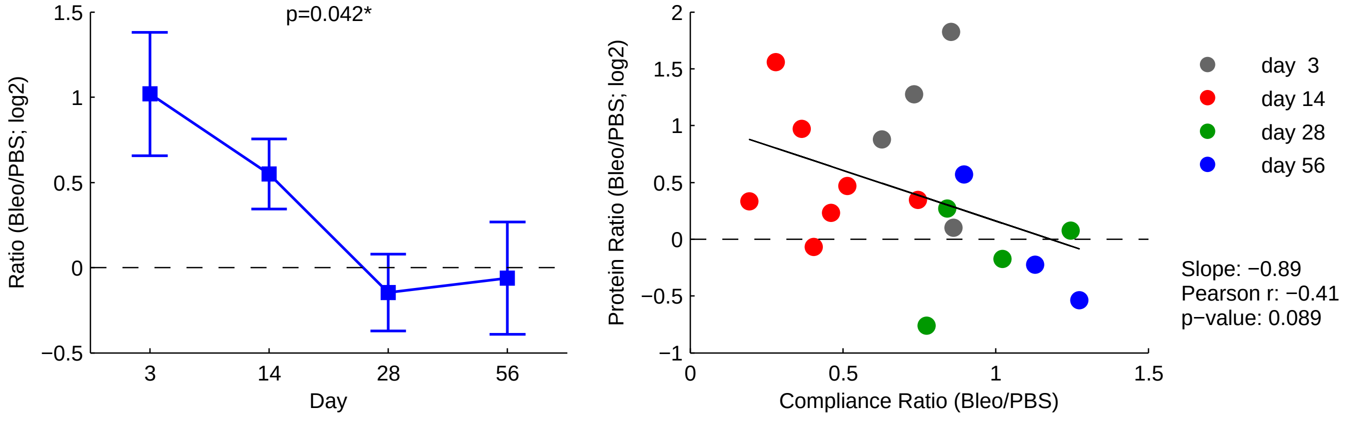

Q7TQD2 − Tppp (id: 4843)

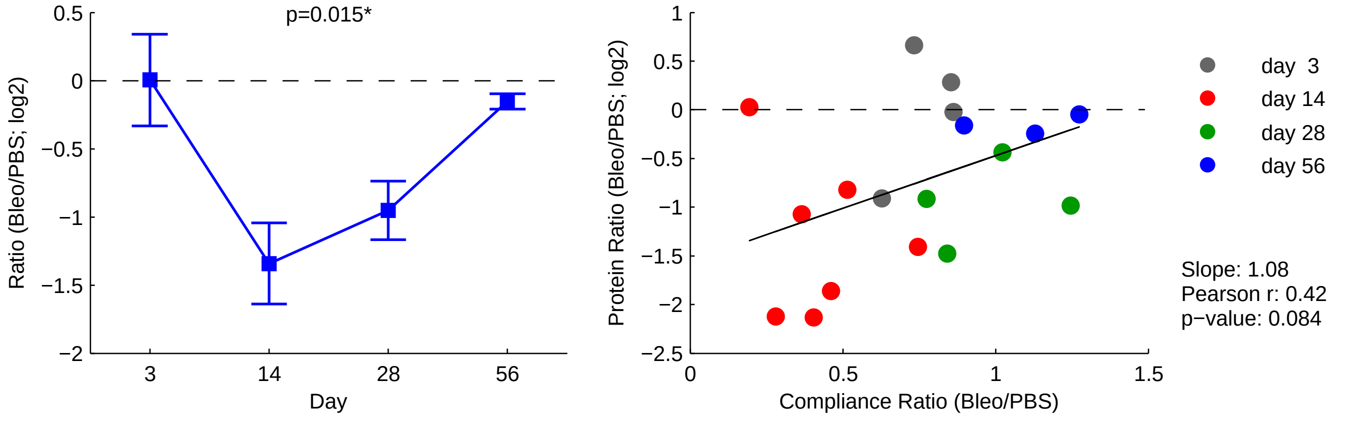

Q7TQF7 − Amph (id: 4845)

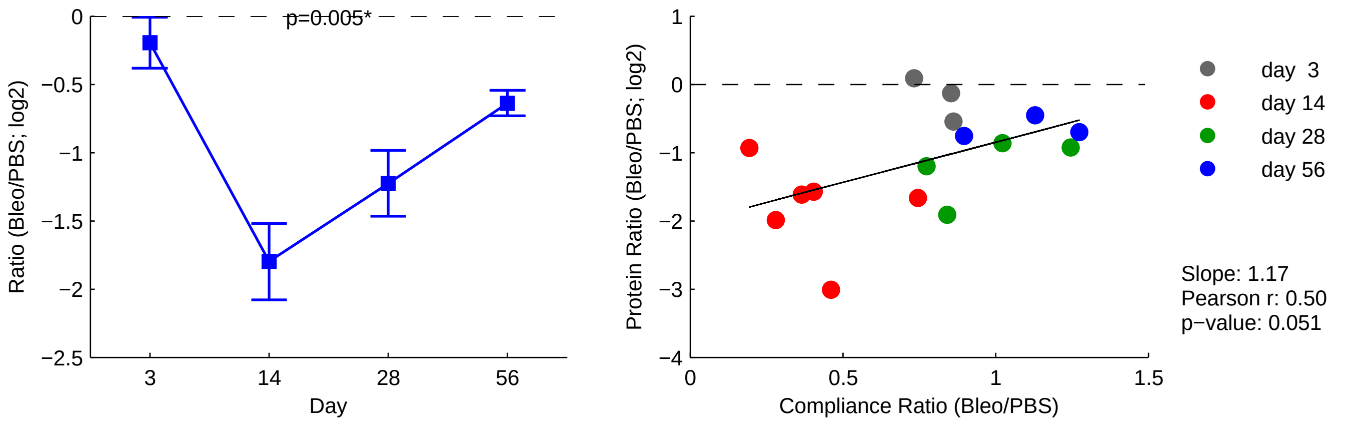

Q7TQK1 − Ints7 (id: 4849)

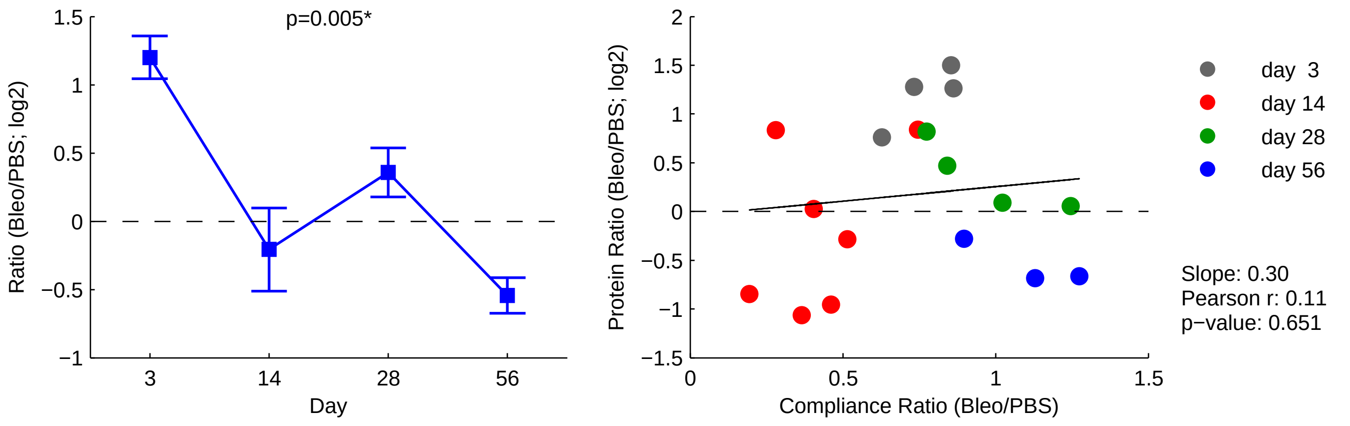

Q7TSC1 − Prrc2a (id: 4853)

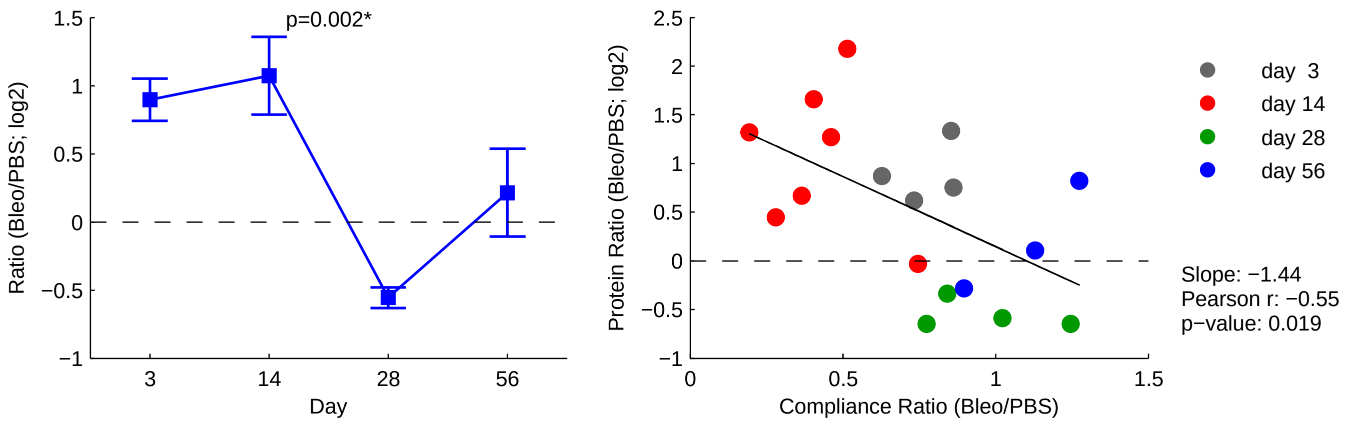

Q7TSG1 − Cep120 (id: 4858)

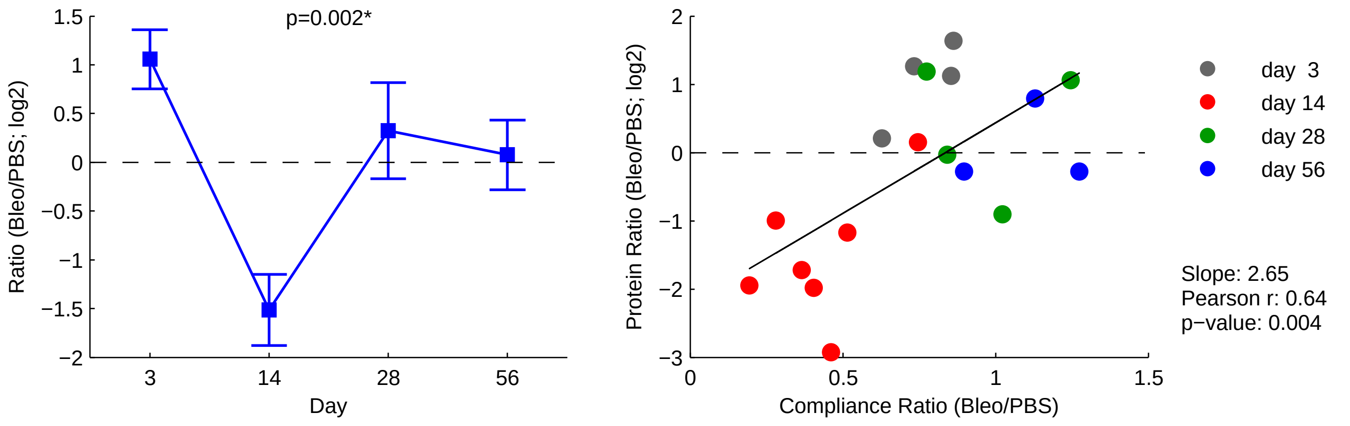

Q7TSJ2 − Map6 (id: 4861)

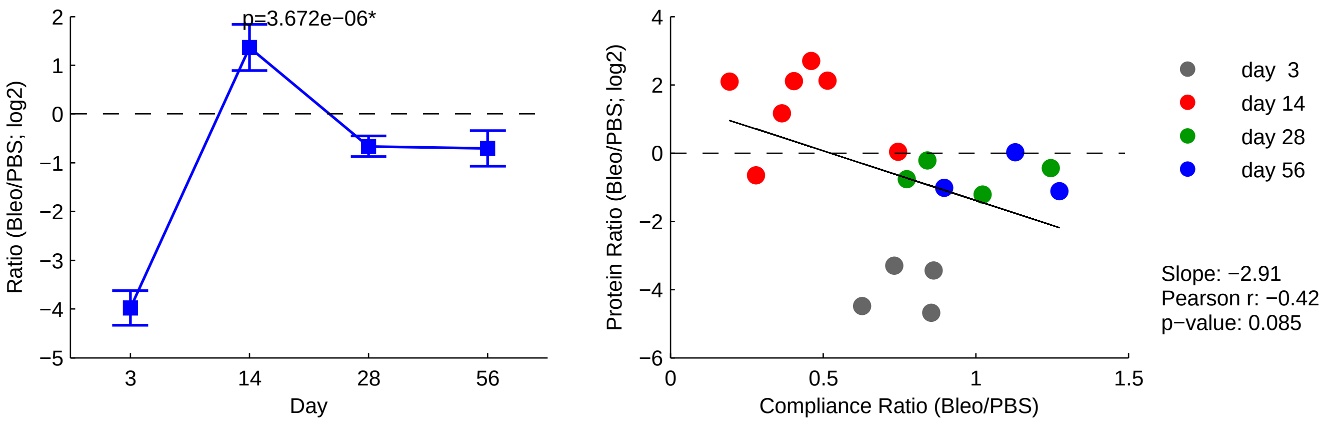

Q7TSQ8 − Pdpr (id: 4863)

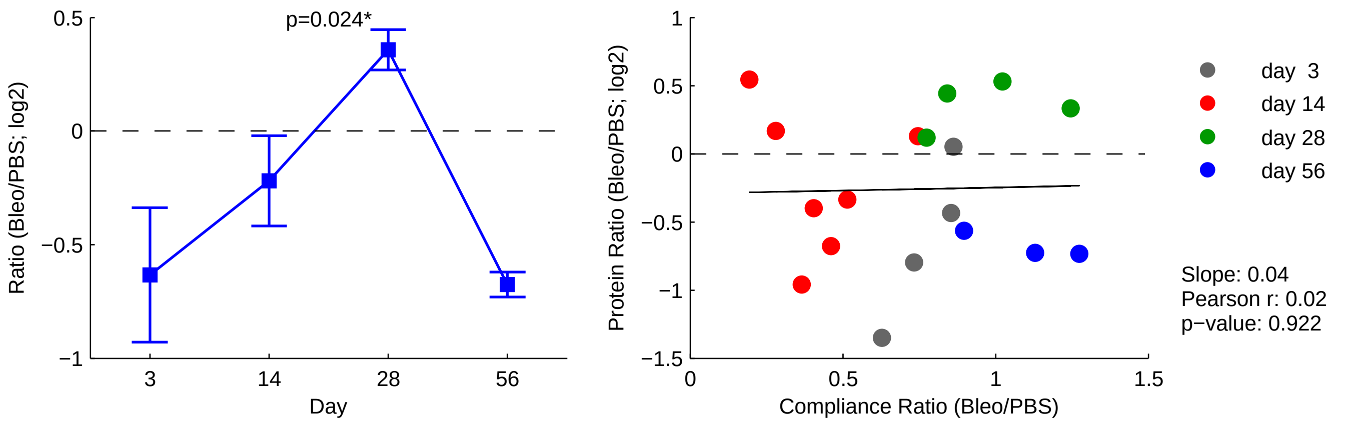

Q7TST5 − Lamp3 (id: 4864)

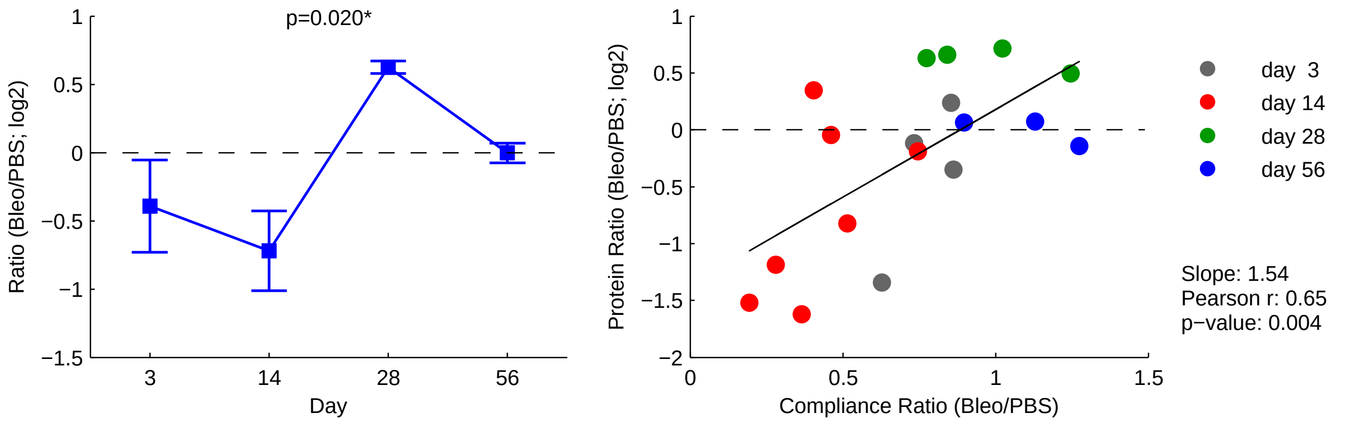

Q7TT00−3 − Supt20h (id: 4867)

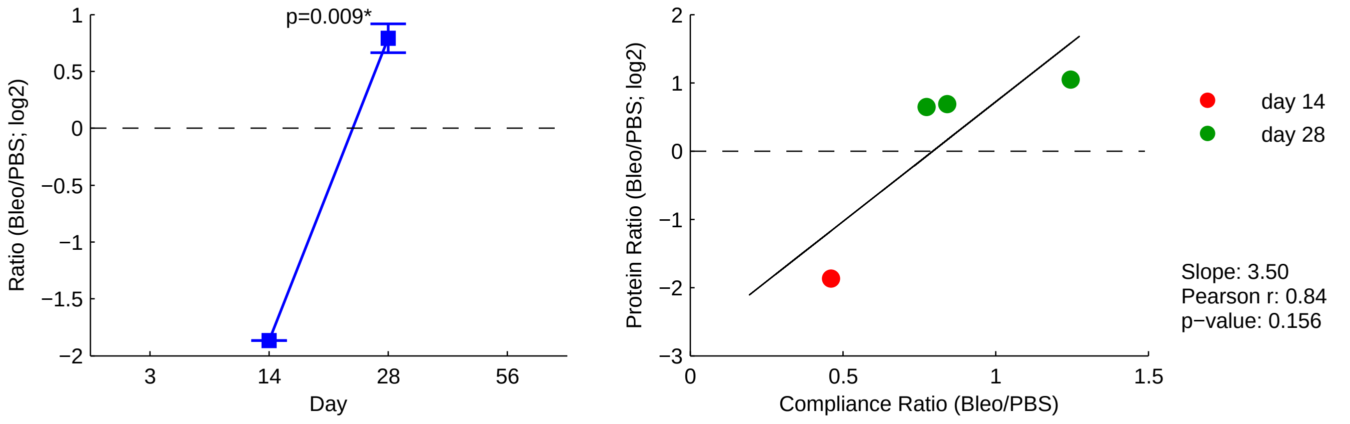

Q7TT21 − Tsc2 (id: 4868)

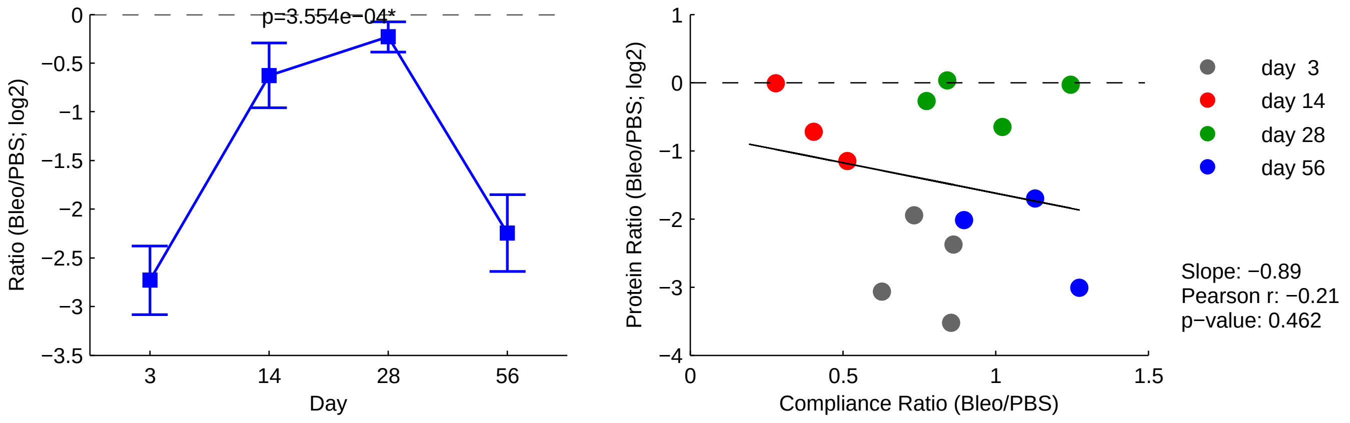

Q7TT50 − Cdc42bpb (id: 4871)

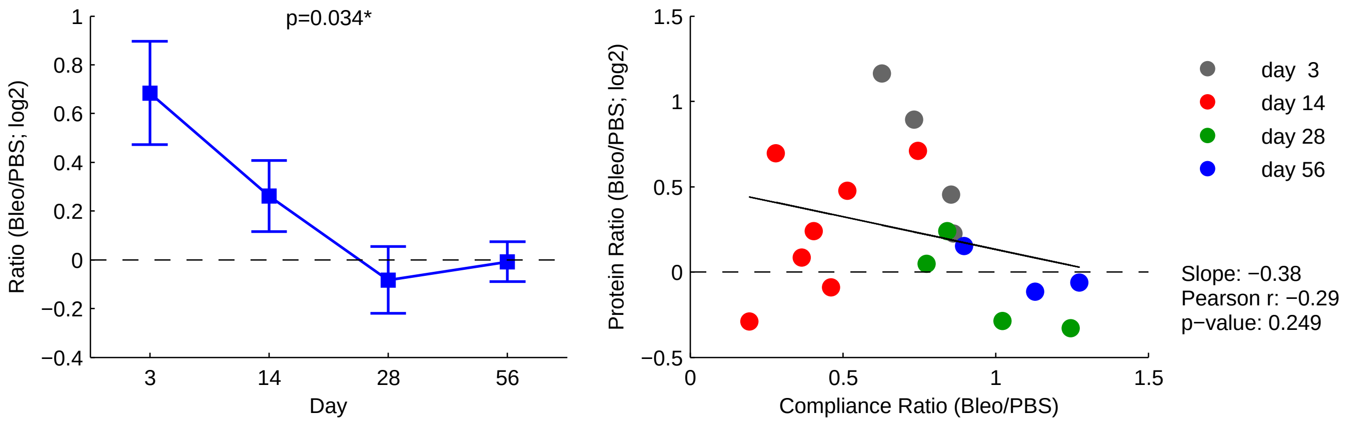

Q80SY3 − Atp6v0d2 (id: 4873)

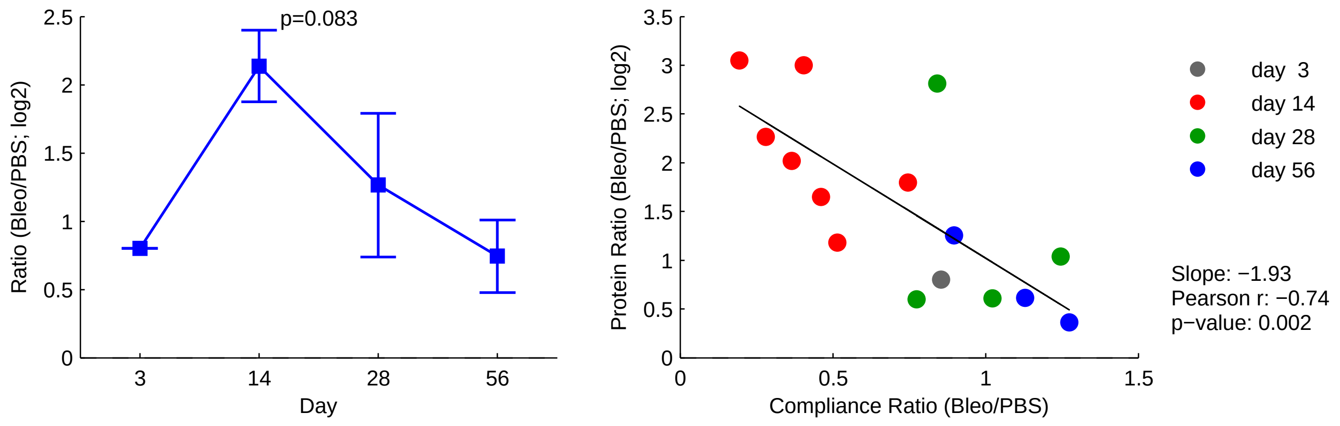

Q80SZ7 − Gng5 (id: 4875)

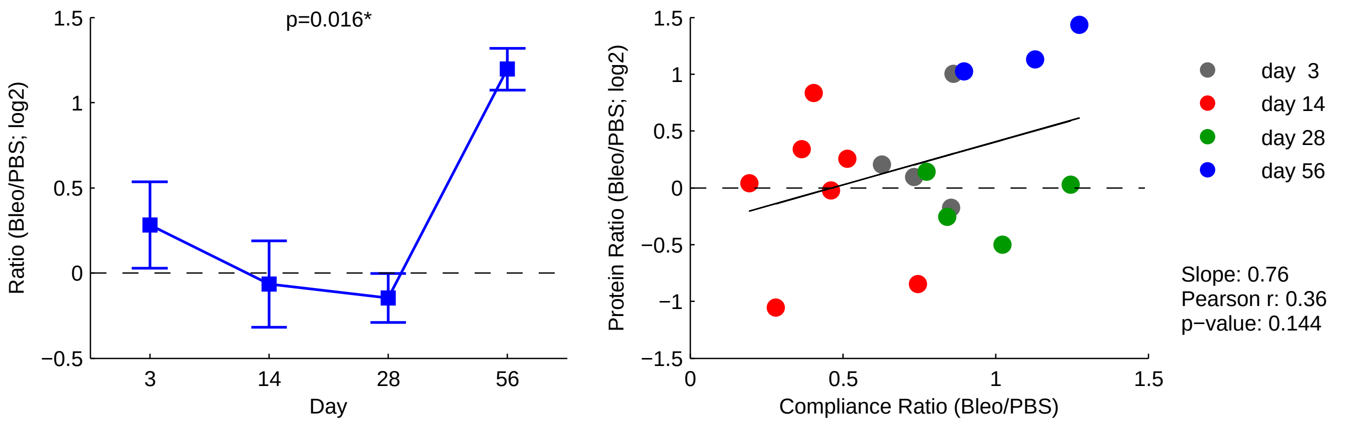

Q80T06 − Eef1d (id: 4876)

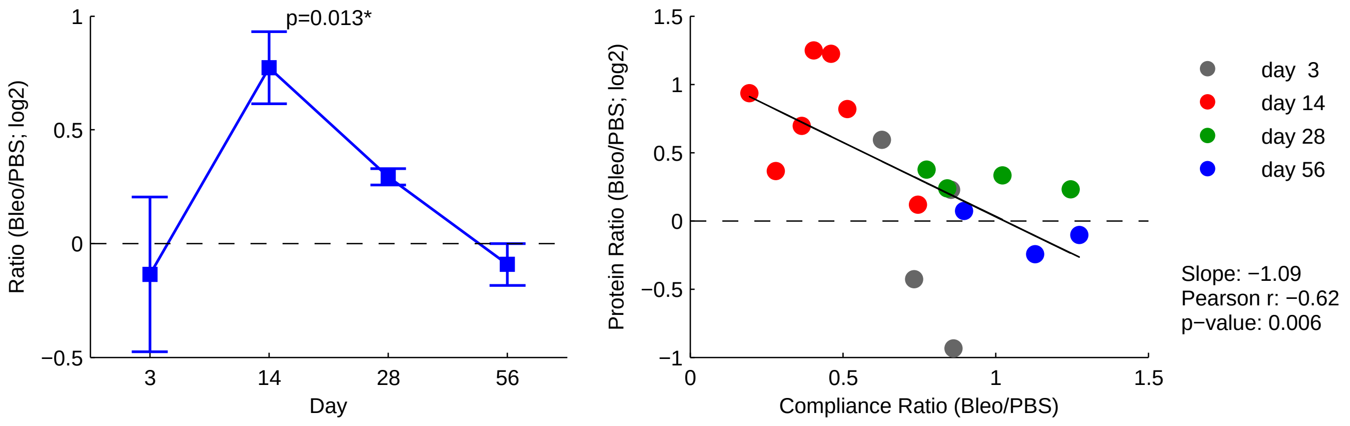

Q80T14 − Fras1 (id: 4877)

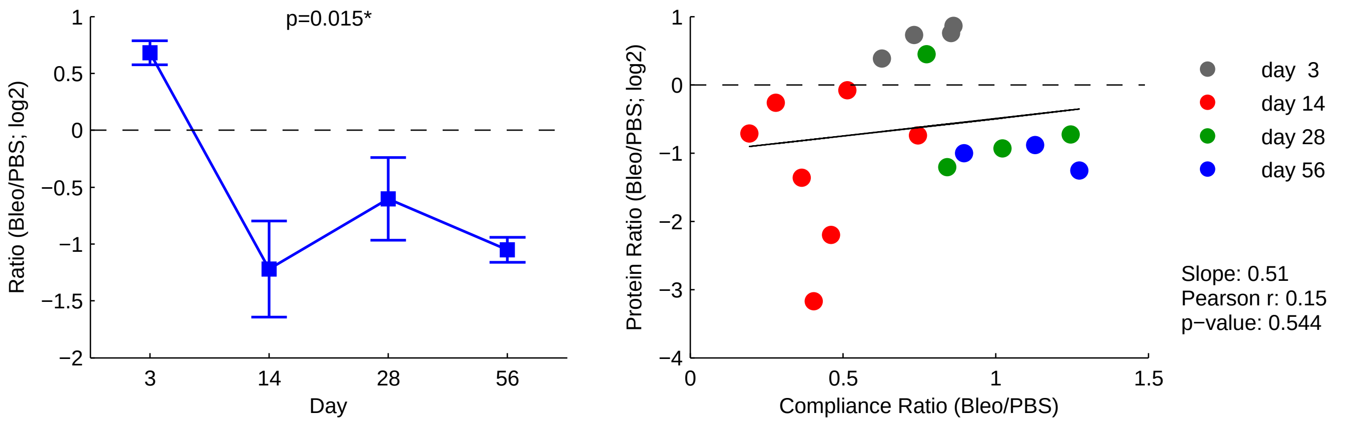

Q80T21 − Adamtsl4 (id: 4878)

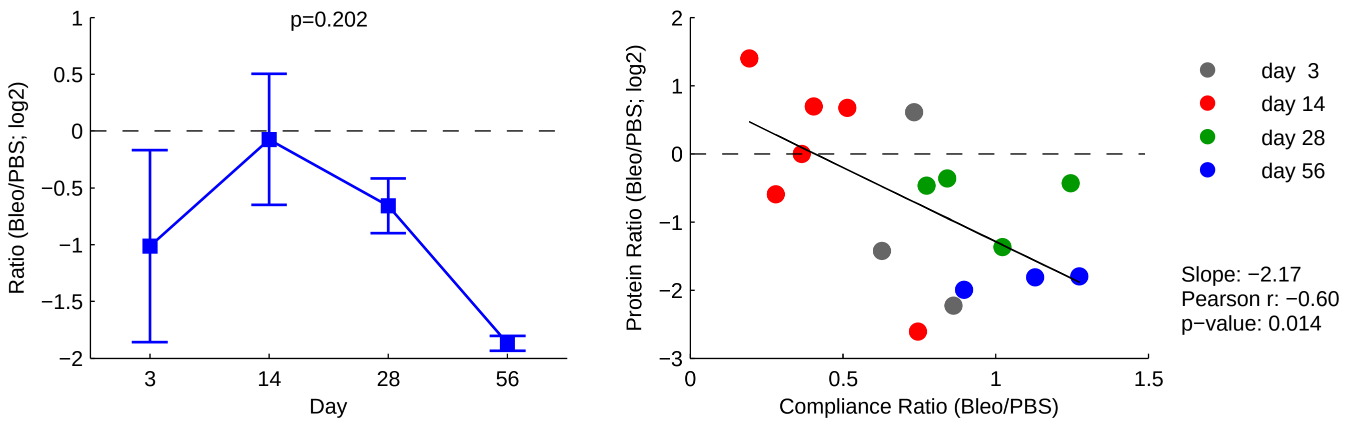

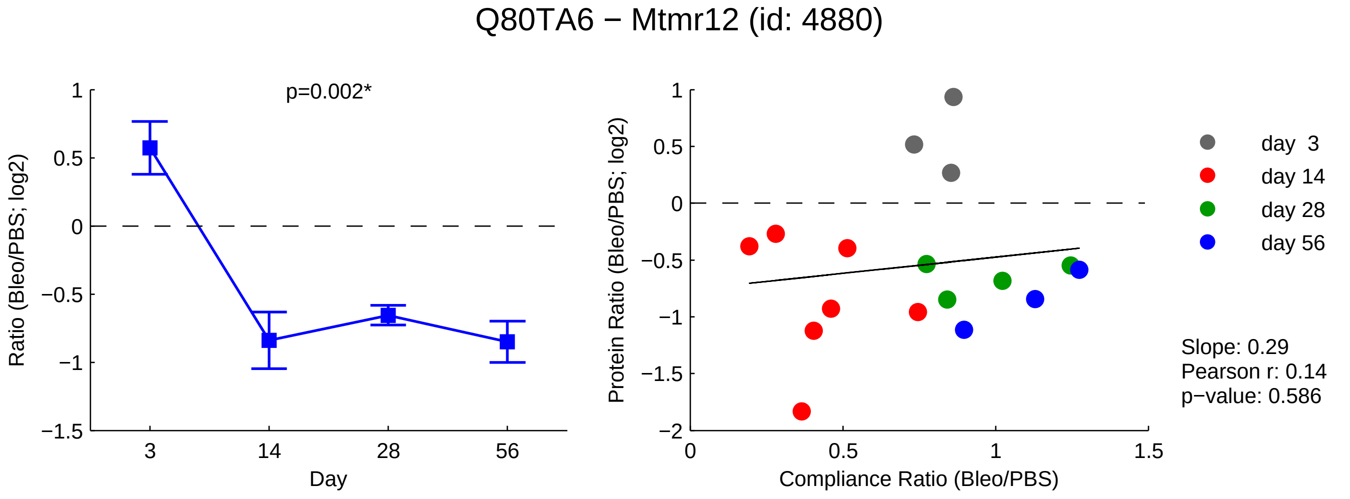

Q80TA6 − Mtmr12 (id: 4880)
p=0.002*
Ratio (Bleo/PBS; log2)
Day
Protein Ratio (Bleo/PBS; log2)
Compliance Ratio (Bleo/PBS)
day 3
day 14
day 28
day 56
Slope: 0.29
Pearson r: 0.14
p−value: 0.586

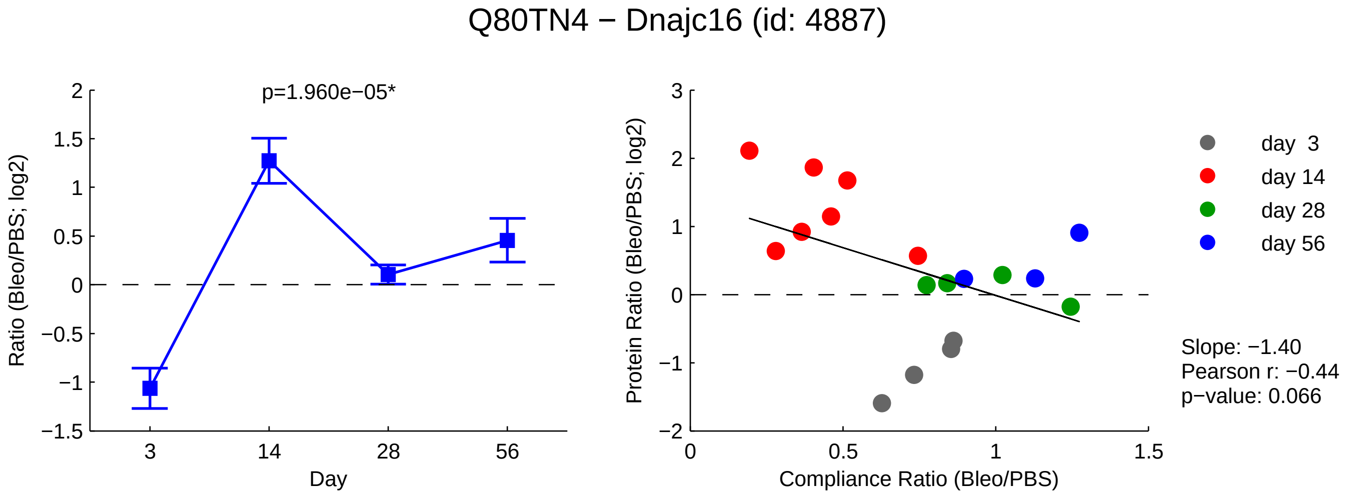

Q80TN4 − Dnajc16 (id: 4887)
p=1.960e−05*
Ratio (Bleo/PBS; log2)
Day
Protein Ratio (Bleo/PBS; log2)
Compliance Ratio (Bleo/PBS)
day 3
day 14
day 28
day 56
Slope: −1.40
Pearson r: −0.44
p−value: 0.066

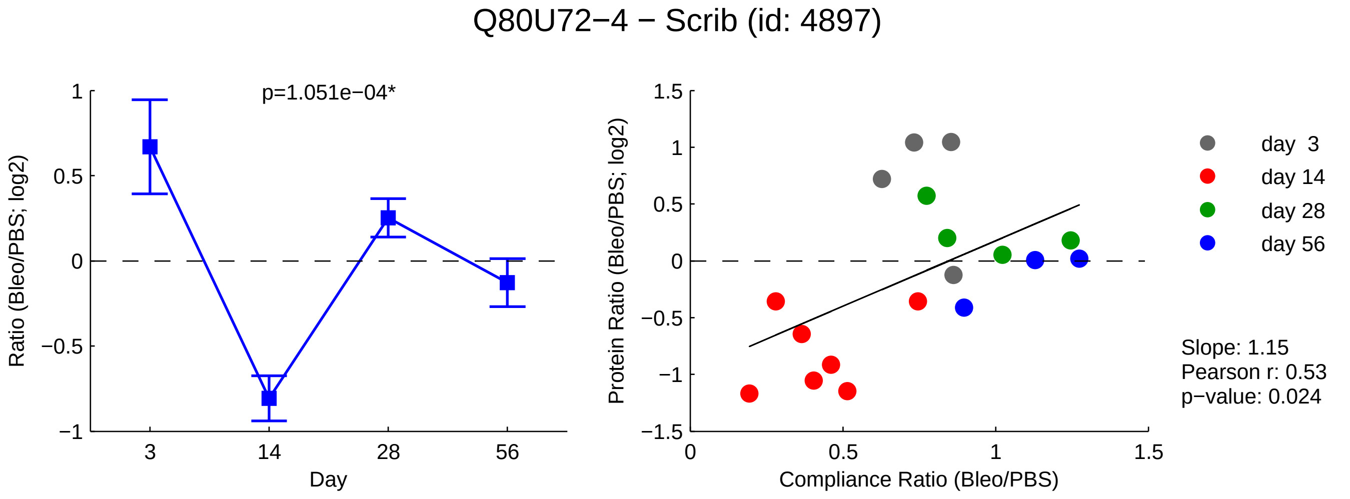

Q80U72−4 − Scrib (id: 4897)
p=1.051e−04*
Ratio (Bleo/PBS; log2)
Day
Protein Ratio (Bleo/PBS; log2)
Compliance Ratio (Bleo/PBS)
day 3
day 14
day 28
day 56
Slope: 1.15
Pearson r: 0.53
p−value: 0.024

Q80UG5−3 − Sept9 (id: 4903)

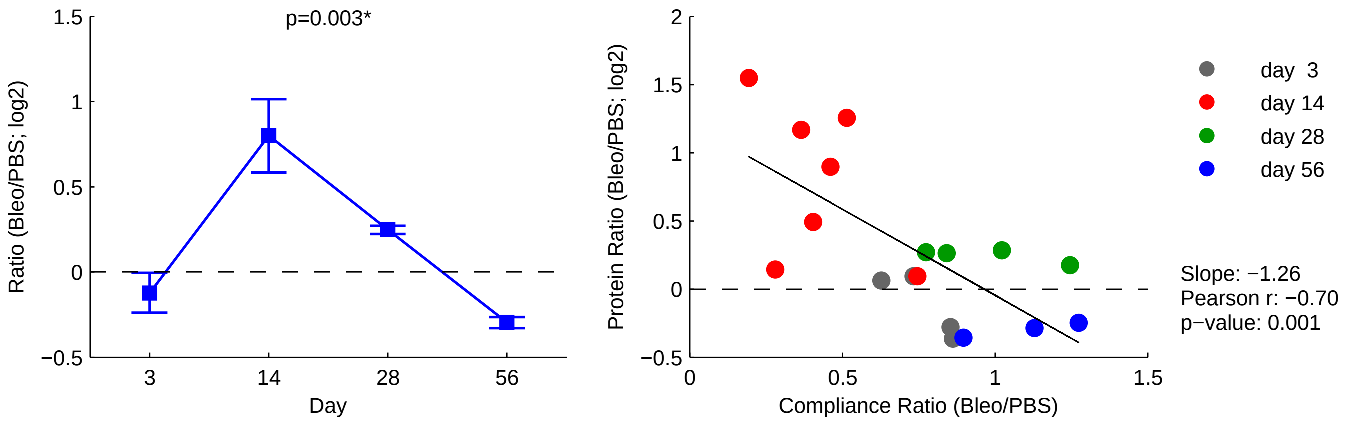

Q80UK0 − Sestd1 (id: 4905)

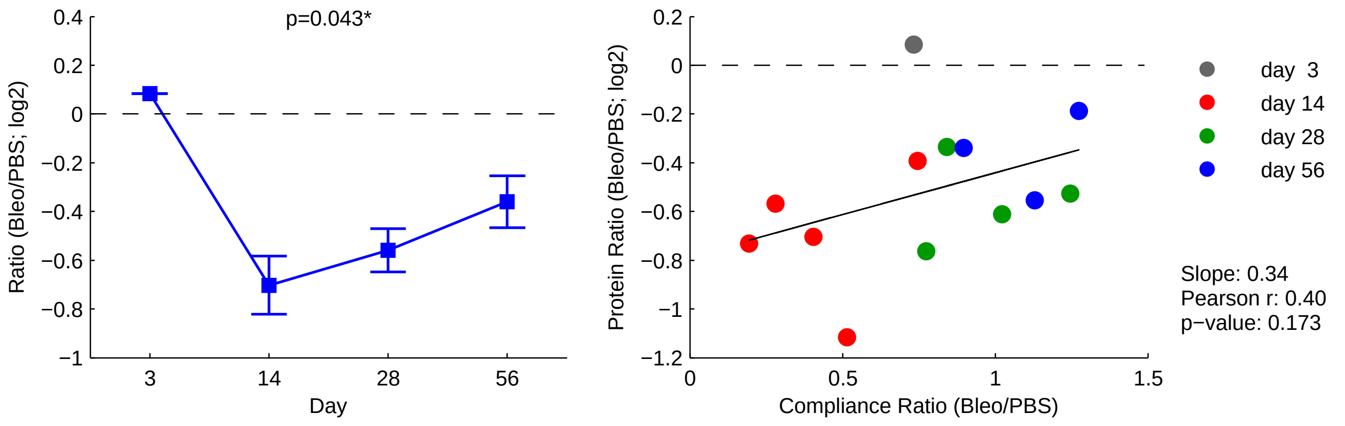

Q80UM7 − Mogs (id: 4907)

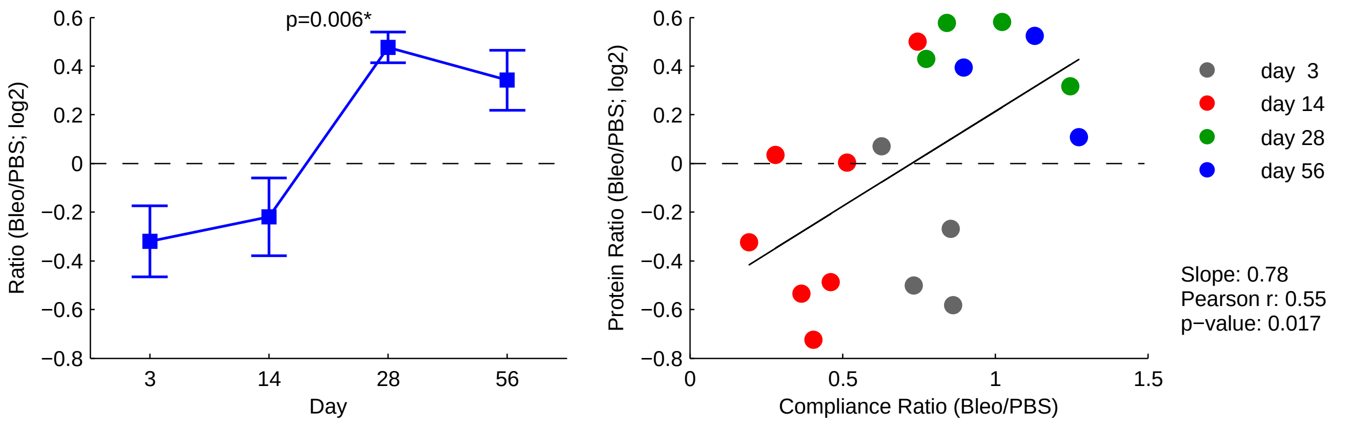

Q80UR4 − Prss34 (id: 4910)

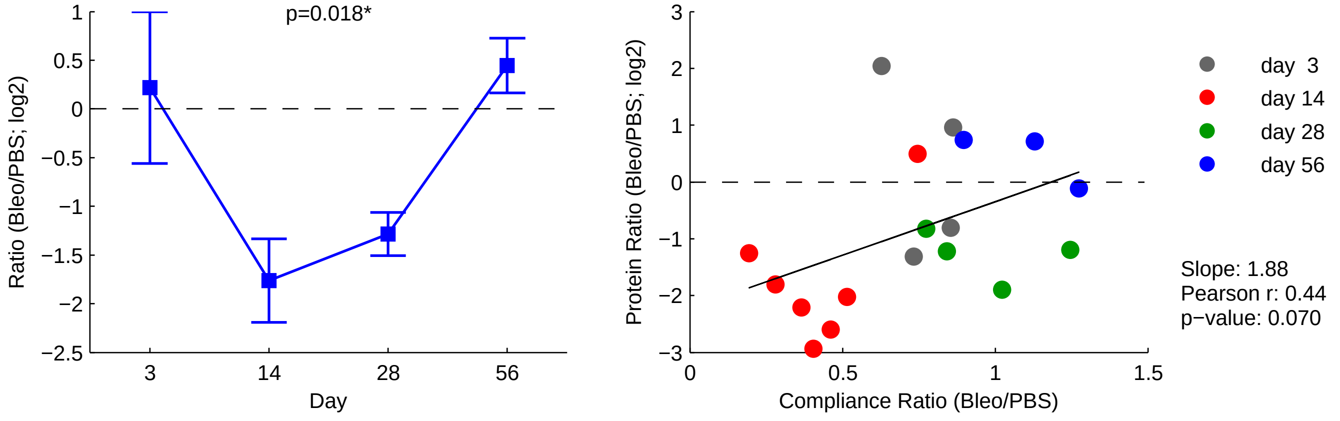

Q80UU9 − Pgrmc2 (id: 4912)

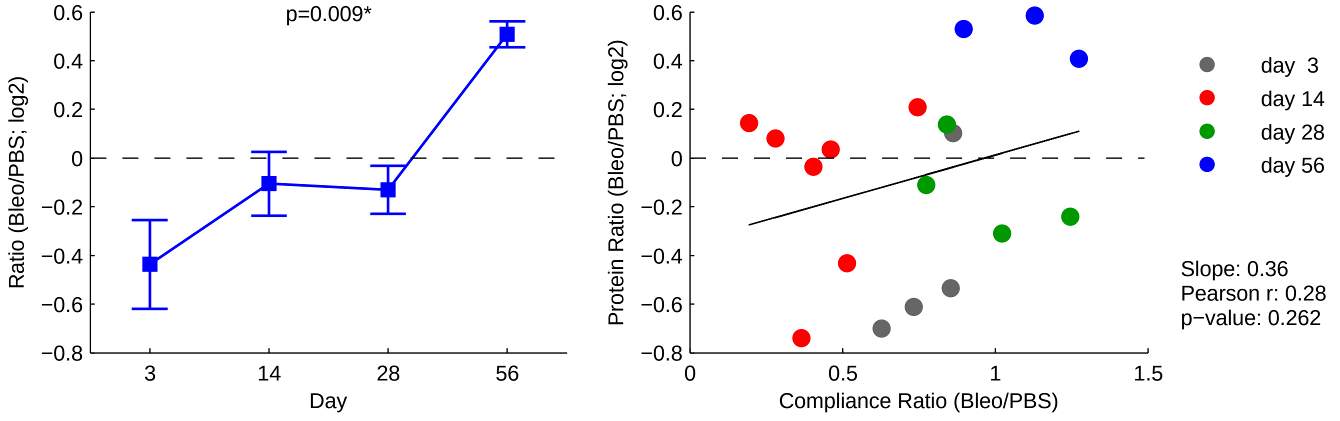

Q80UW5 − Cdc42bpg (id: 4913)

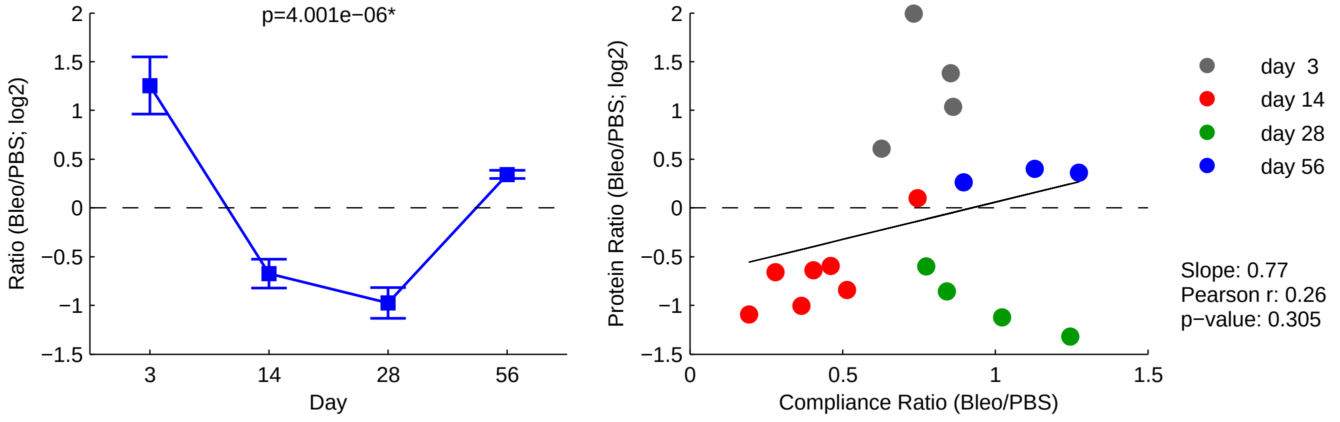

Q80UY2 − Kcmf1 (id: 4916)

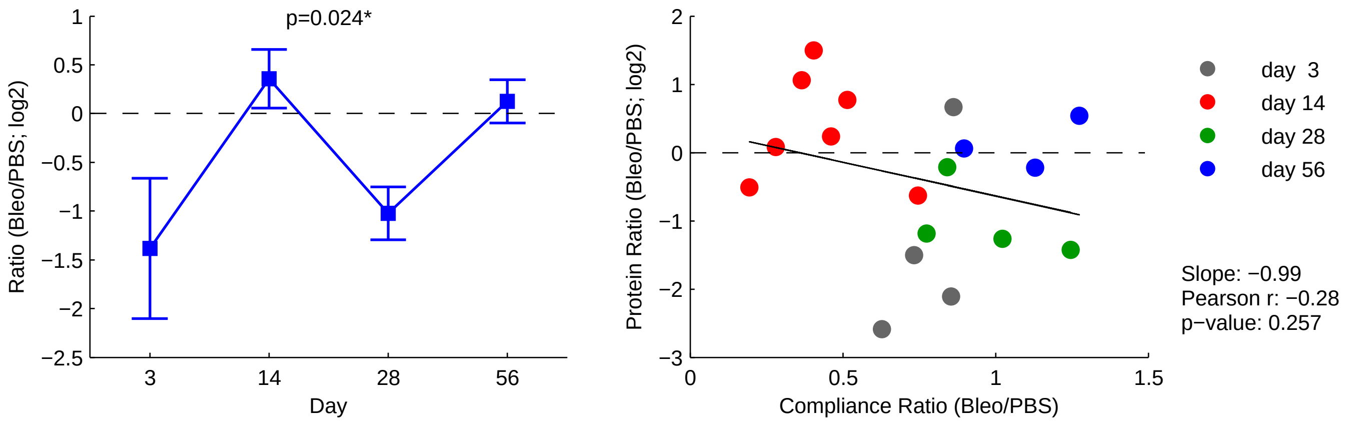

Q80V26 − Impad1 (id: 4917)

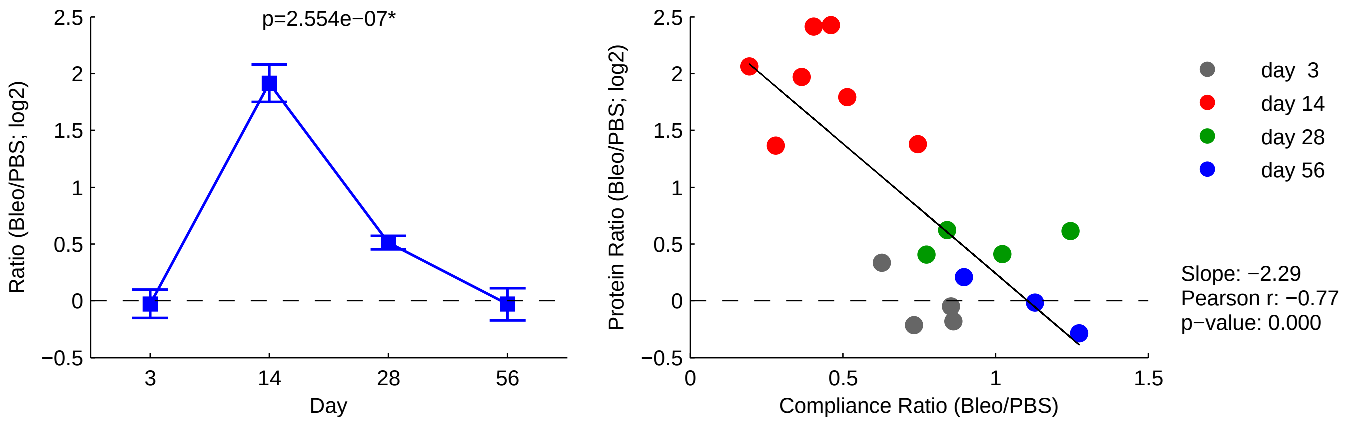

Q80V53 − Chst14 (id: 4919)

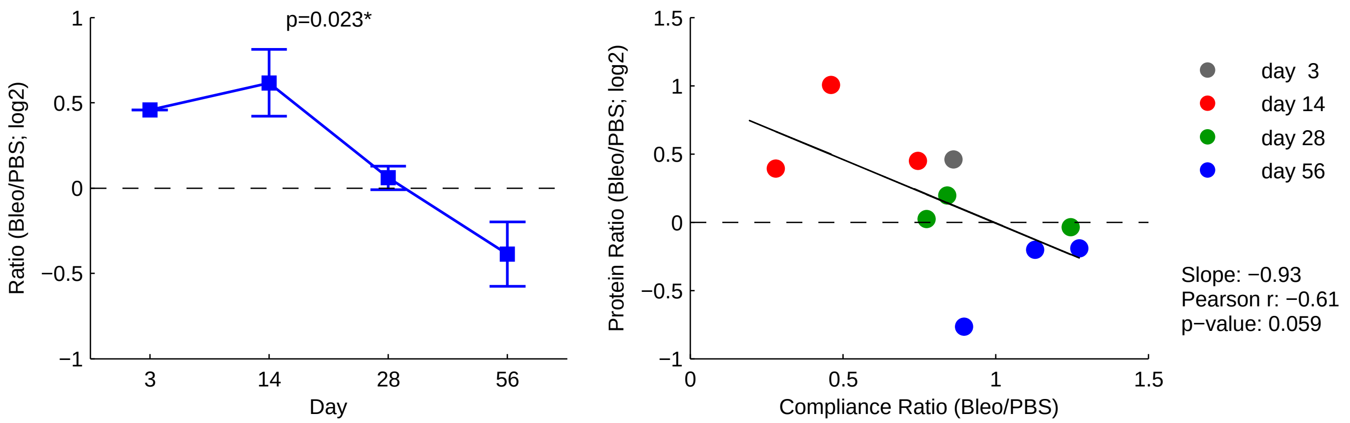

Q80V70 − Megf6 (id: 4920)

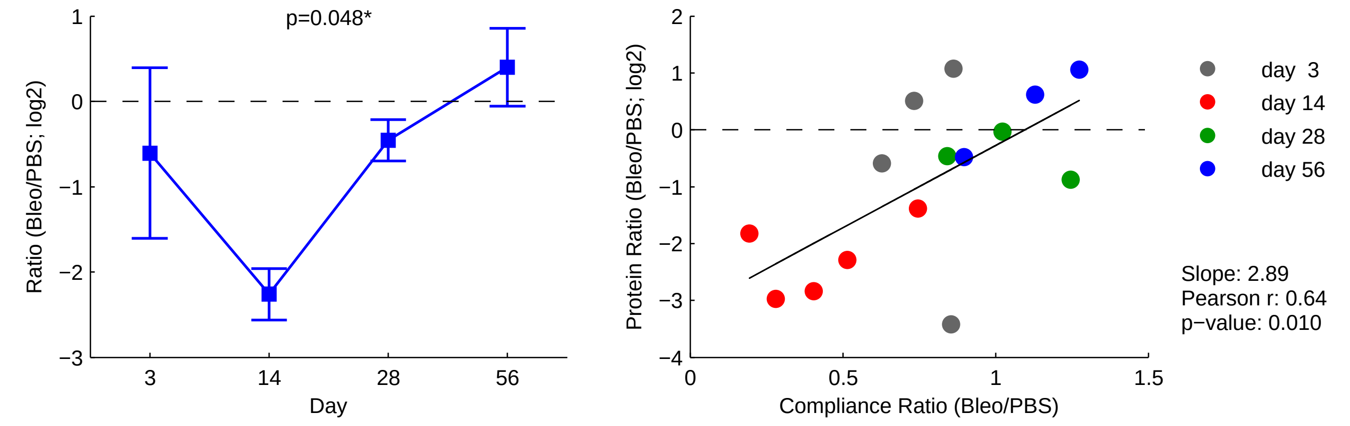

Q80VP0 − Tecpr1 (id: 4929)

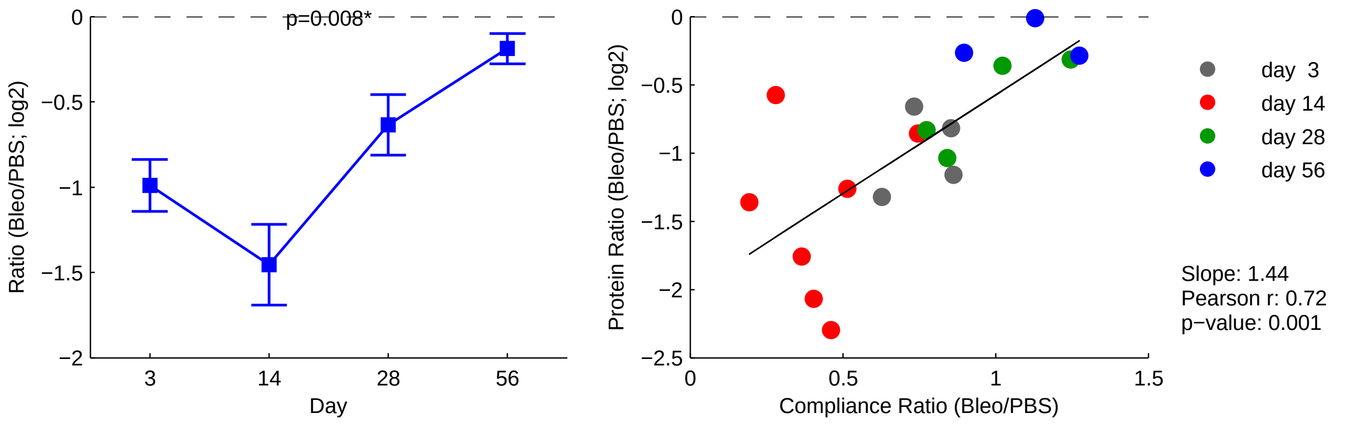

Q80VQ0 − Aldh3b1 (id: 4932)

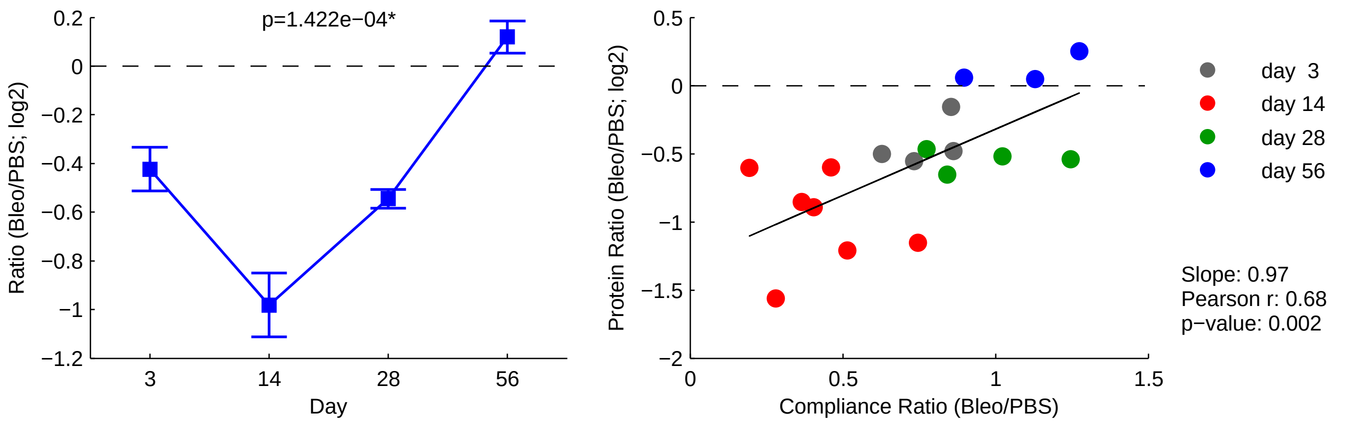

Q80VQ1 − Lrrc1 (id: 4933)

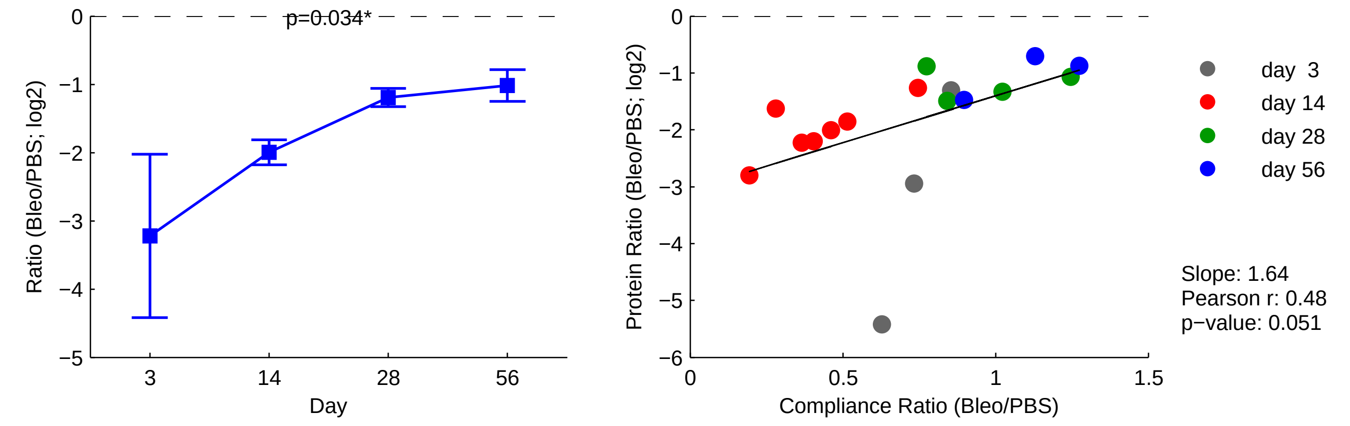

Q80W54 − Zmpste24 (id: 4936)

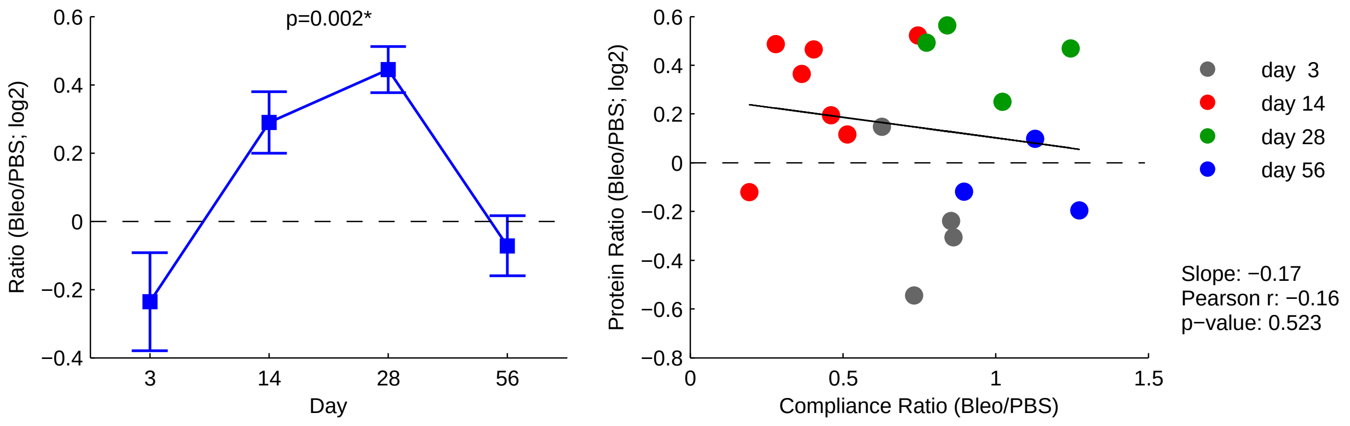

Q80WQ2 − Vac14 (id: 4942)

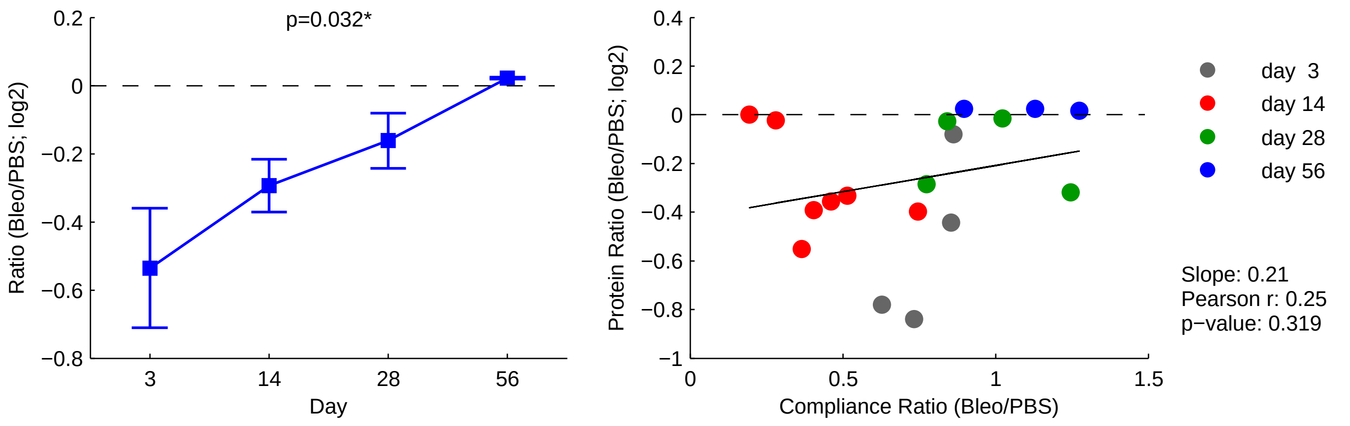

Q80X32 − (id: 4948)

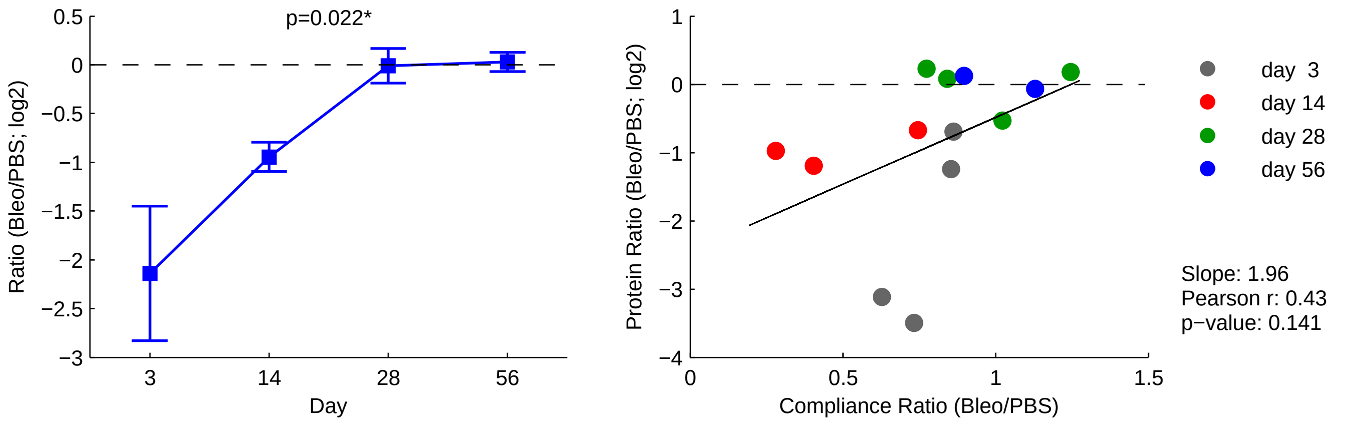

Q80X41−2 − Vrk1 (id: 4949)

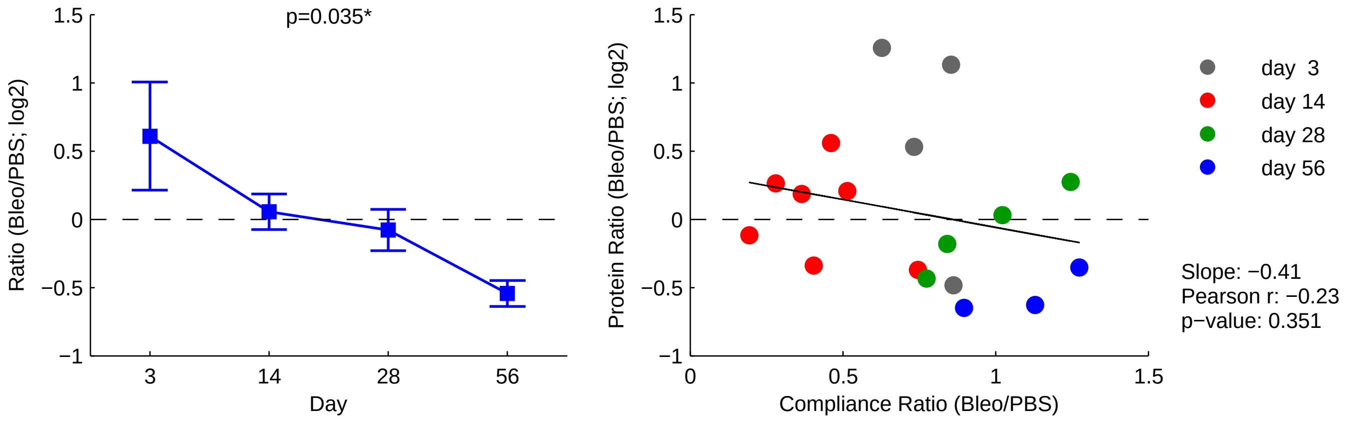

Q80X50−3 − Ubap2l (id: 4950)

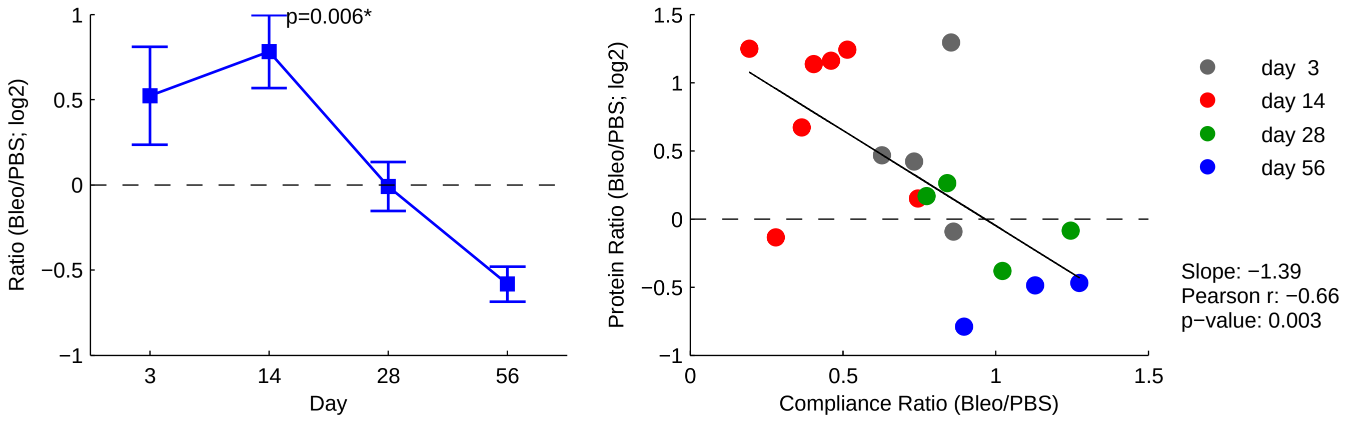

Q80X73 − Pelo (id: 4952)

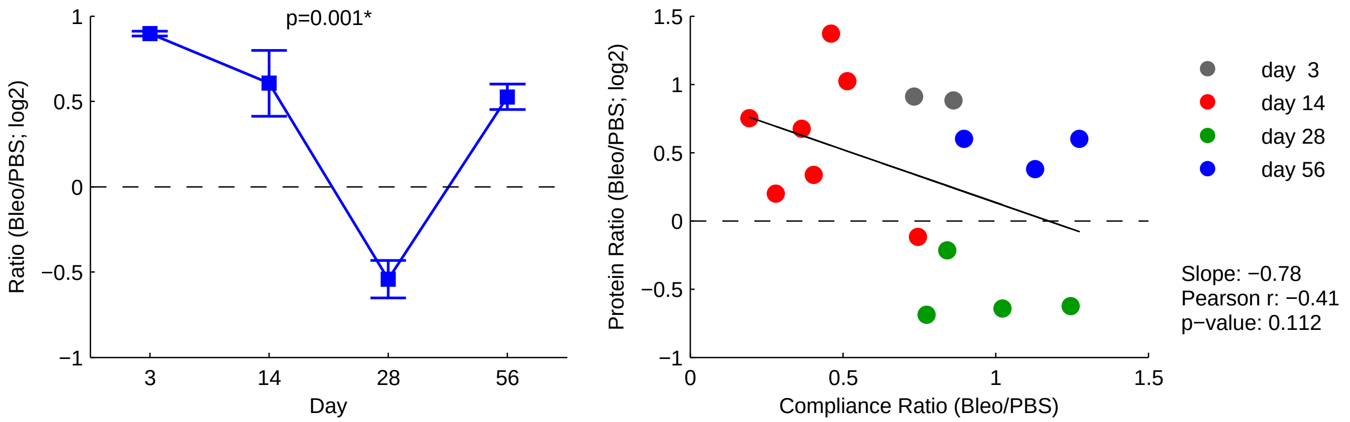

Q80X80 − C2cd2l (id: 4953)

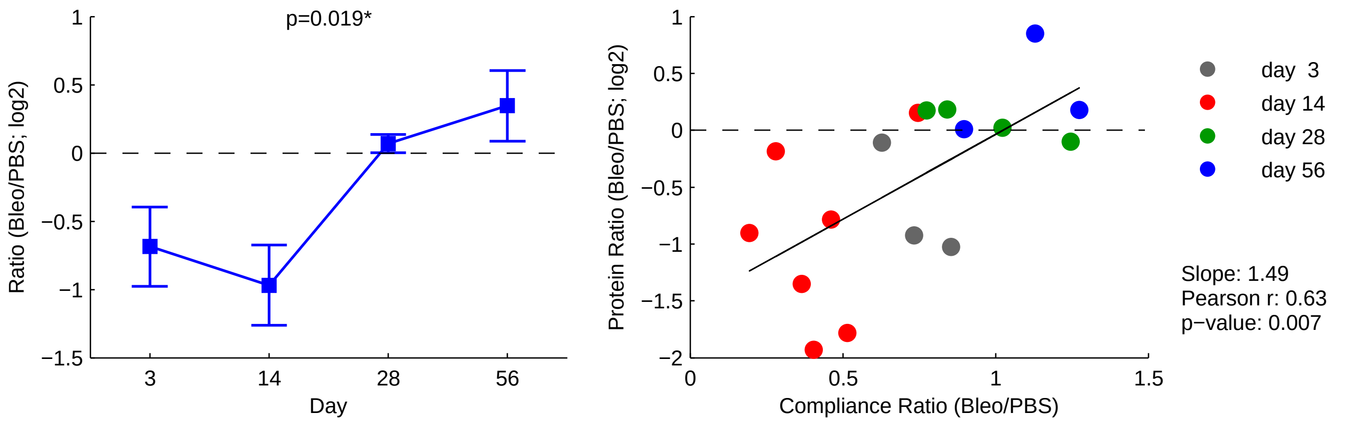

Q80X90 − Flnb (id: 4955)

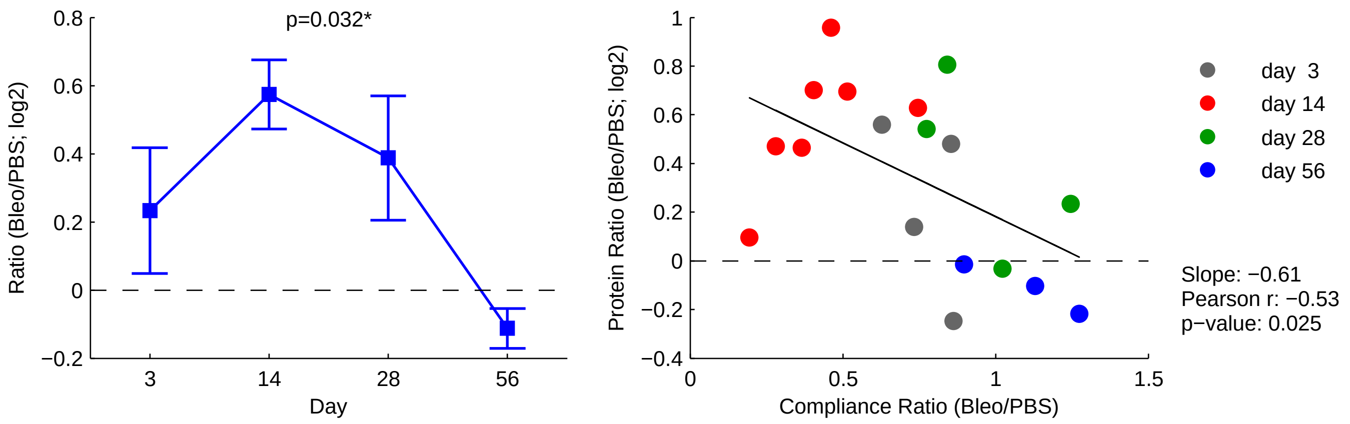

Q80X95 − Rraga (id: 4956)

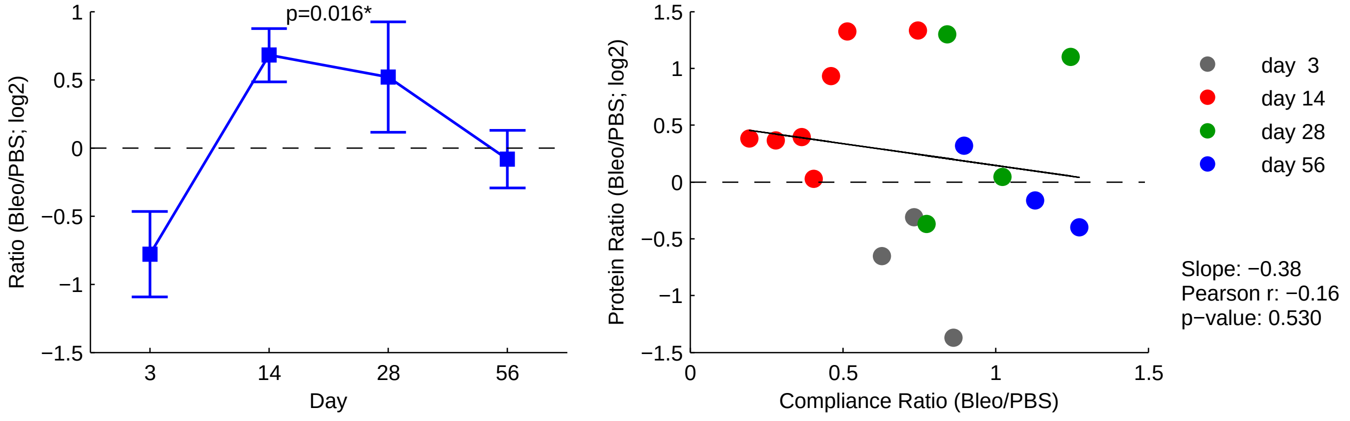

Q80X98 − Dhx38 (id: 4957)

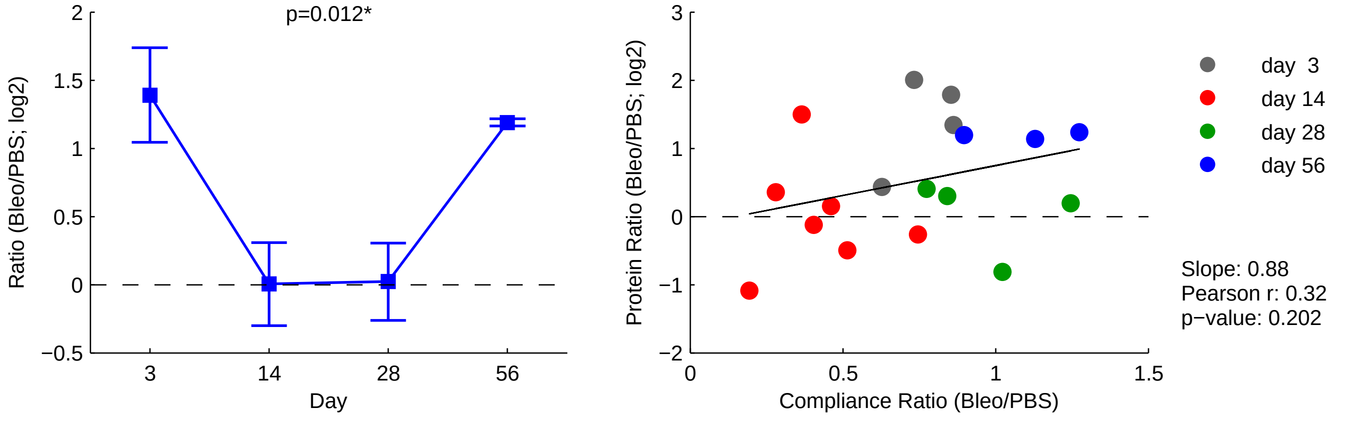

Q80XB4−4 − Nrap (id: 4958)

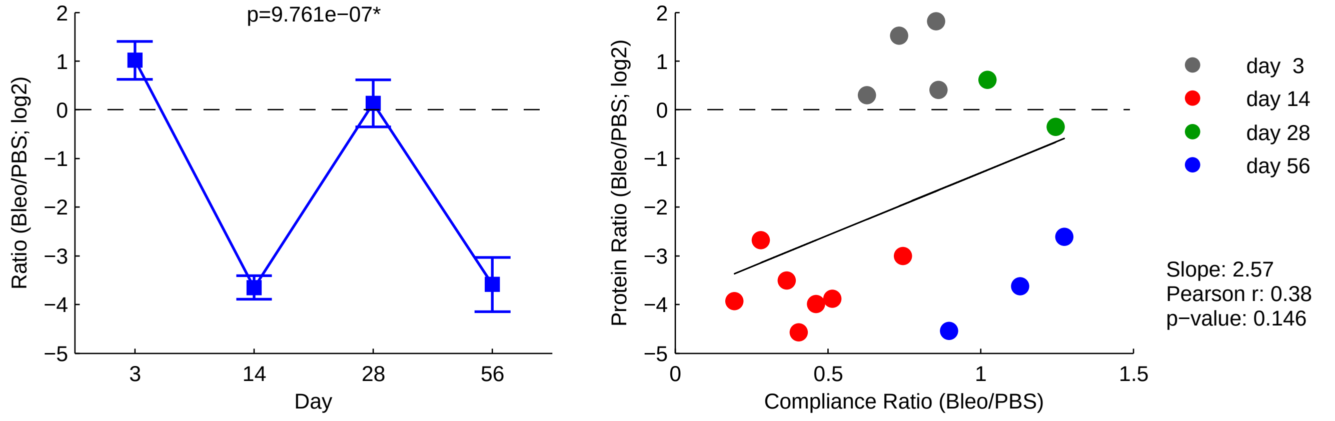

Q80XI4 − Pip4k2b (id: 4961)

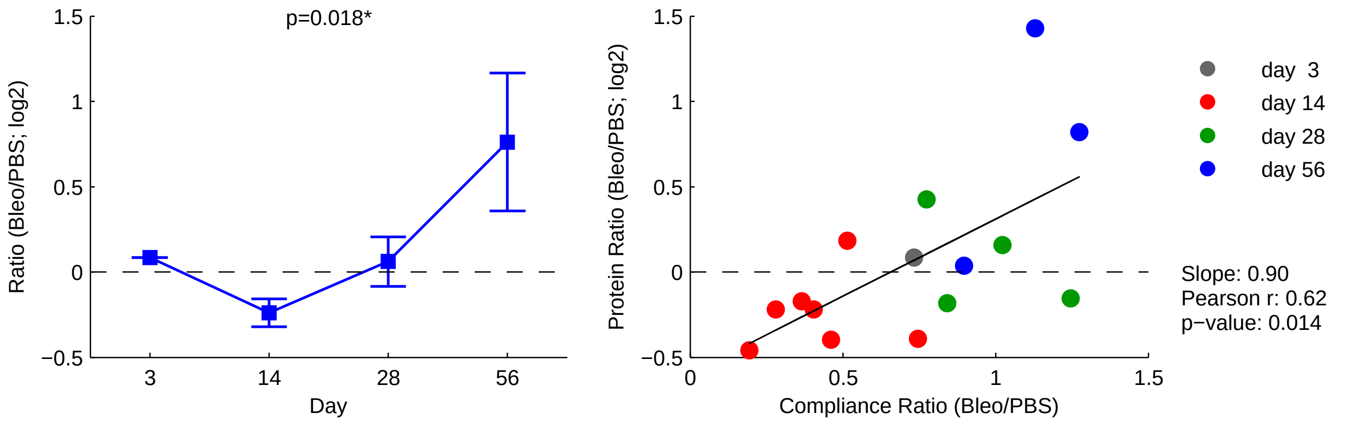

Q80XN0 − Bdh1 (id: 4965)

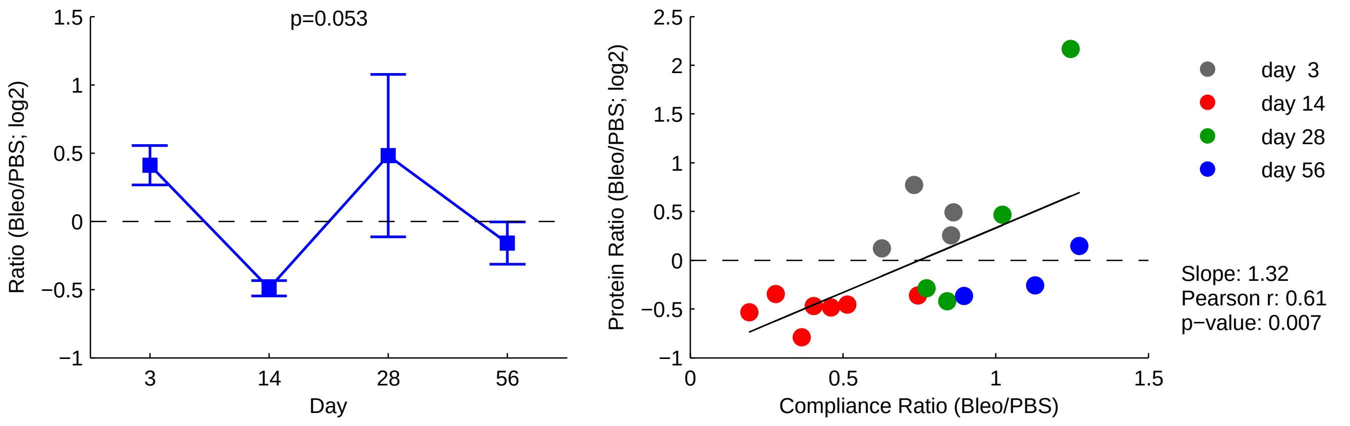

Q80XP8 − Fam76b (id: 4966)

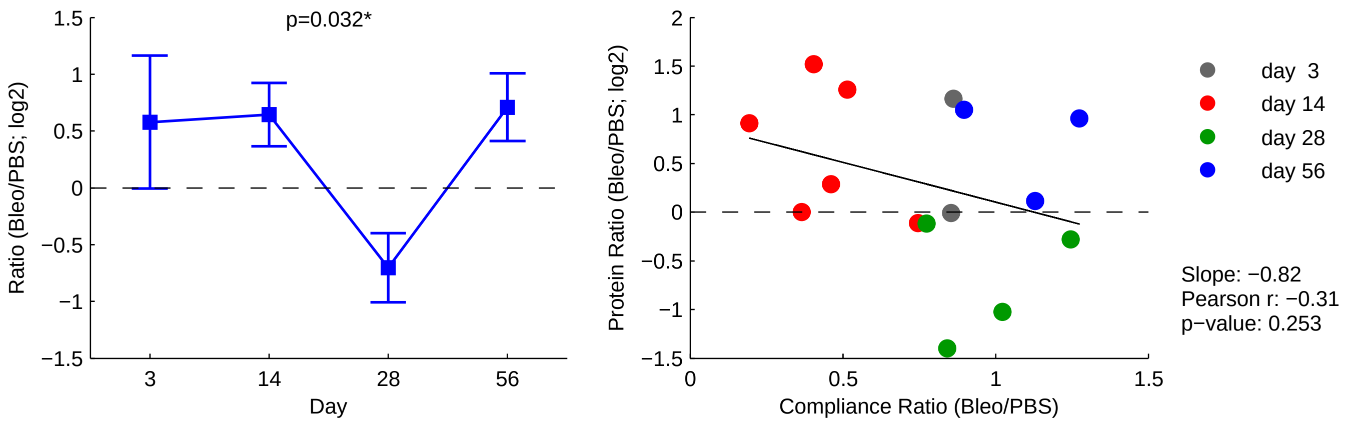

Q80XQ2 − Tbc1d5 (id: 4967)

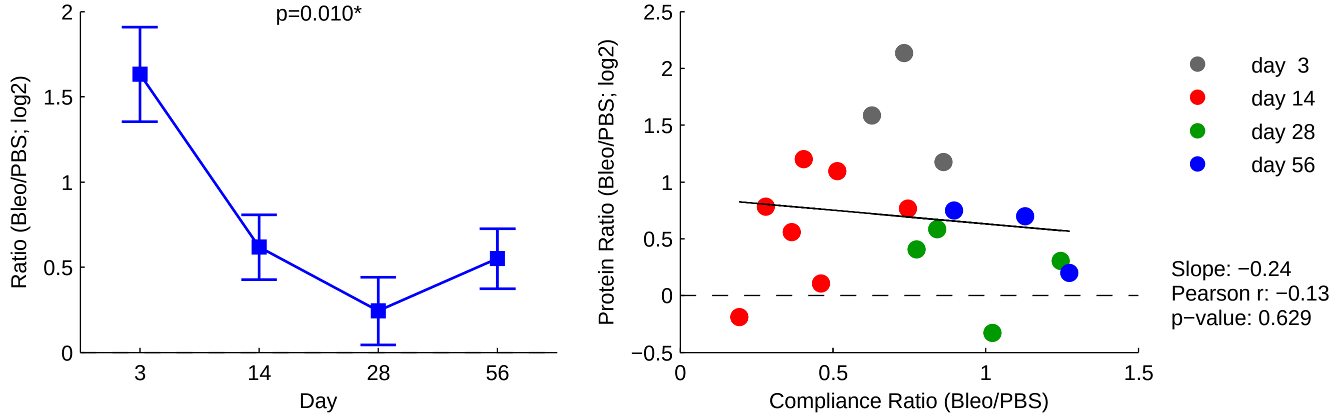

Q80XU3 − Nucks1 (id: 4968)

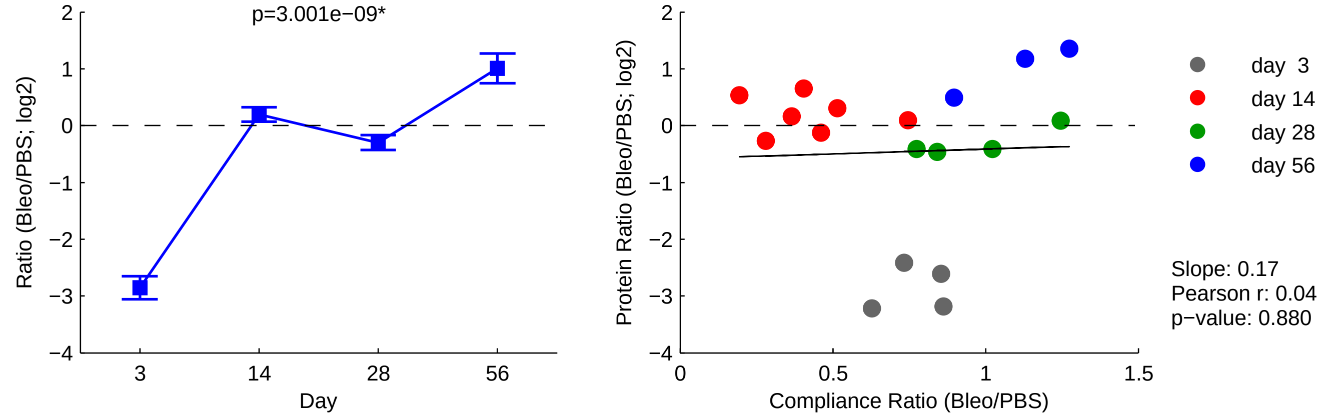

Q80Y17 − Llgl1 (id: 4970)

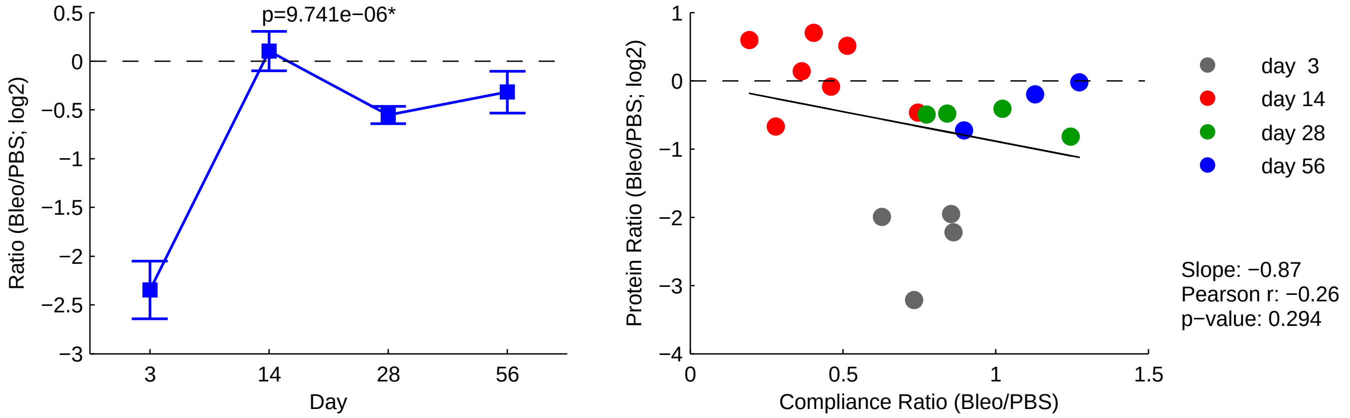

Q80Y24 − Prickle2 (id: 4971)

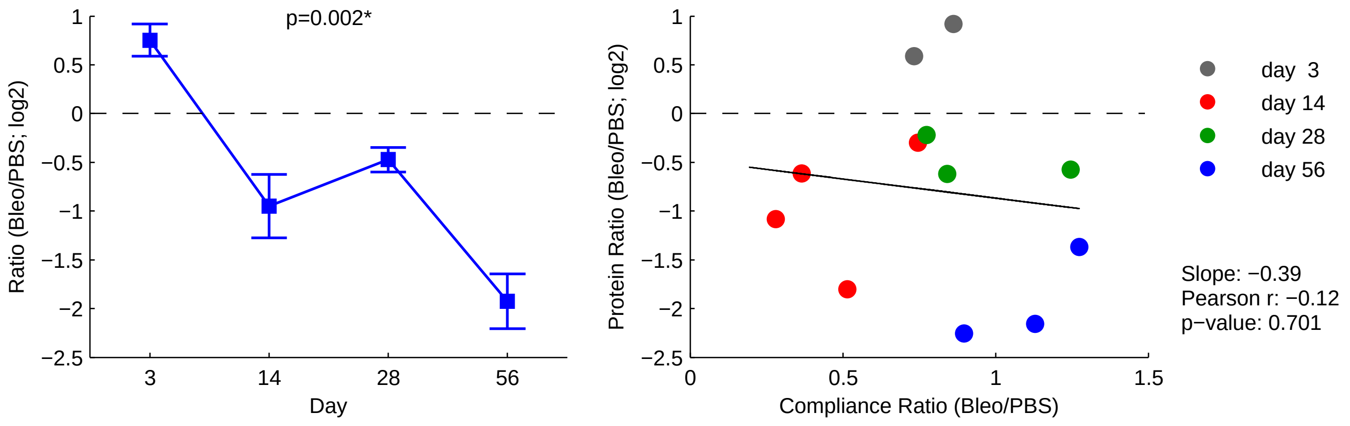

Q80Y56 − Zfyve20 (id: 4975)

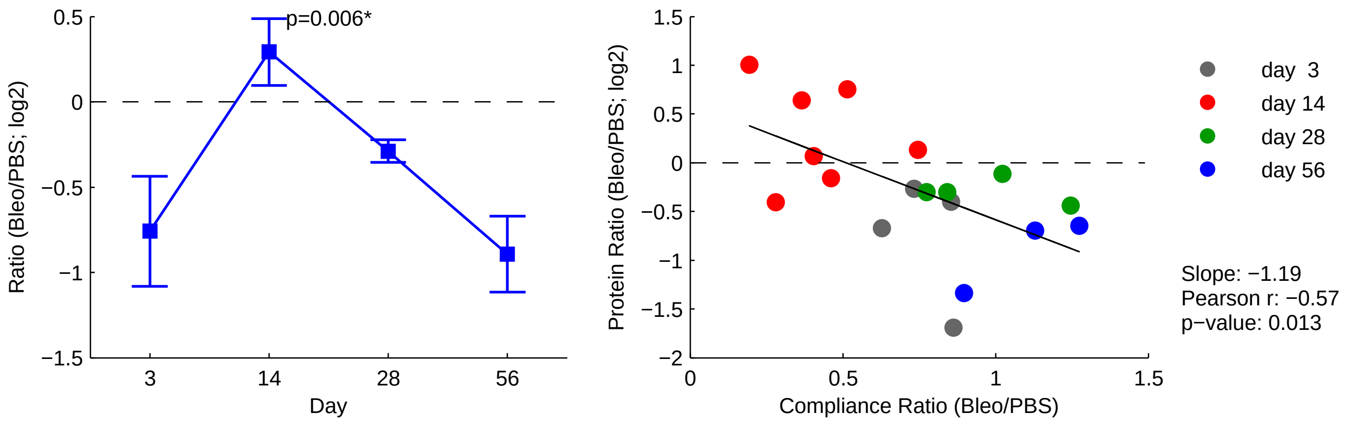

Q80Y75 − Dnajb13 (id: 4976)

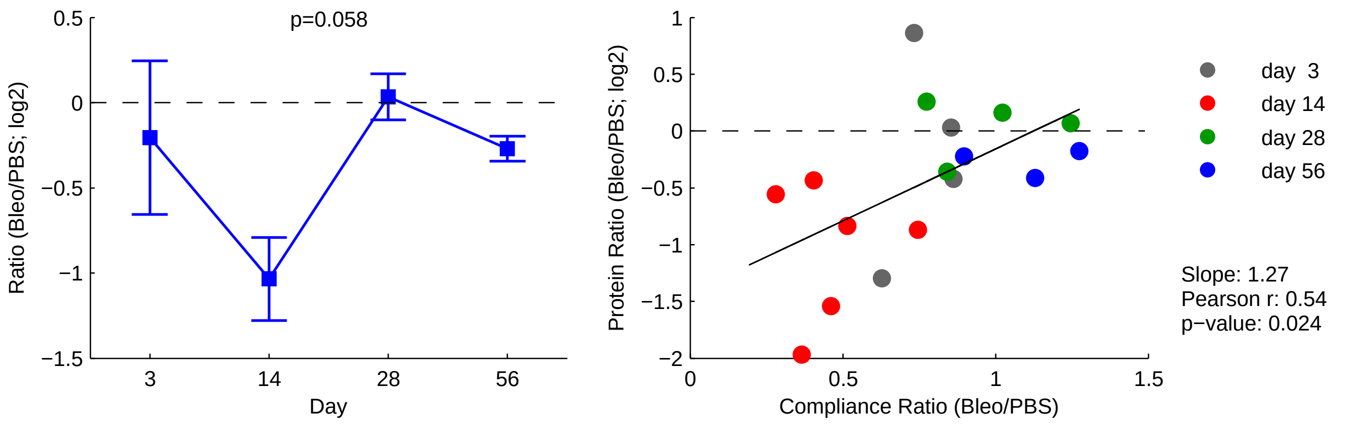

Q80YD1 − Supv3l1 (id: 4979)

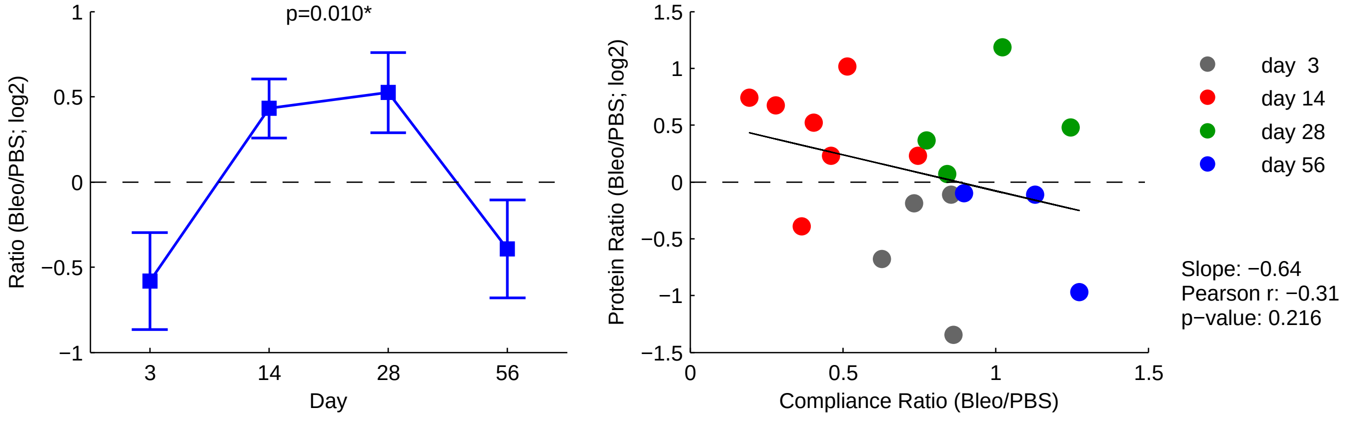

Q80YQ1 − Thbs1 (id: 4982)

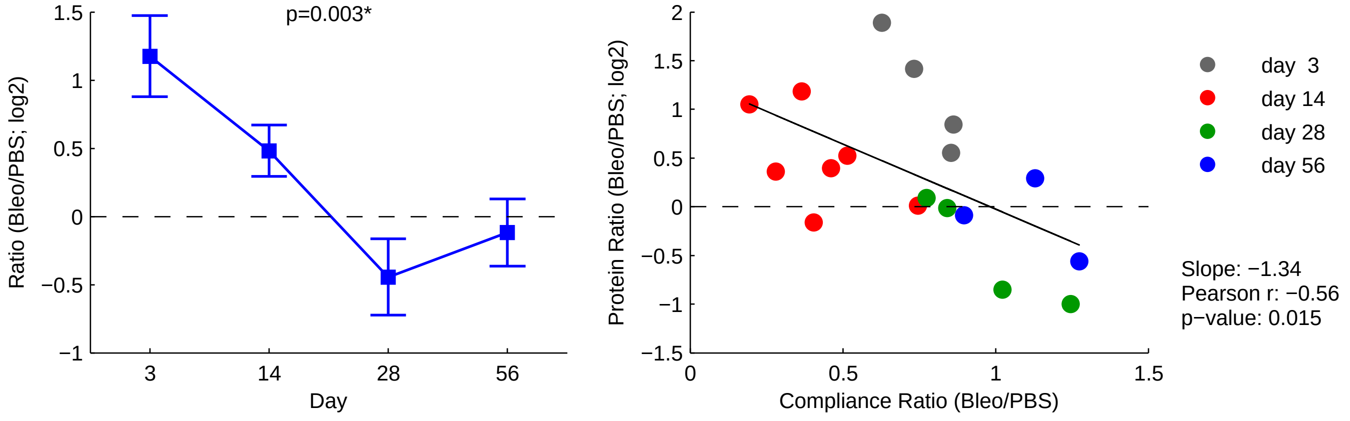

Q80YR4−2 − Znf598 (id: 4983)

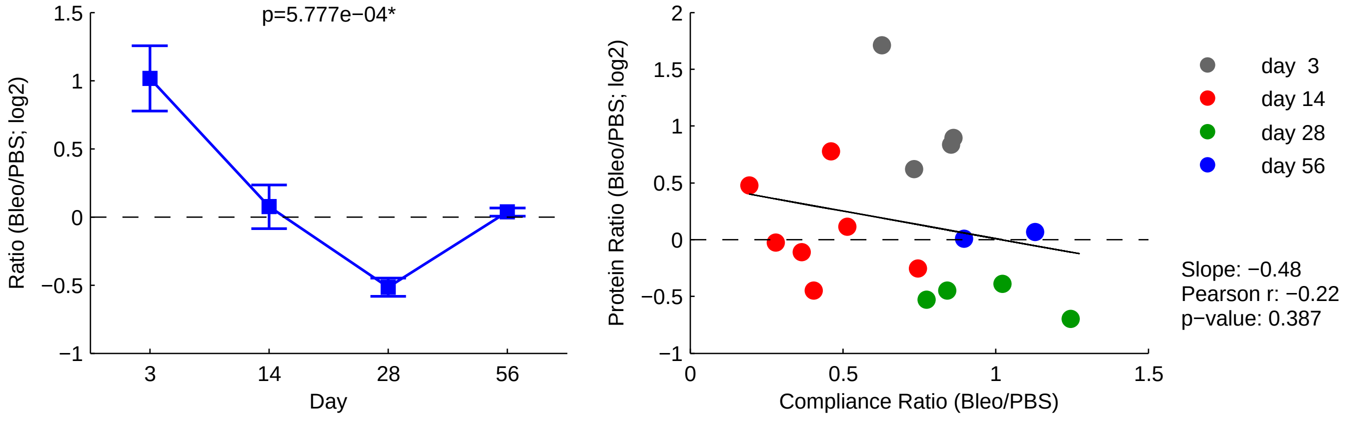

Q80YX1 − Tnc (id: 4990)

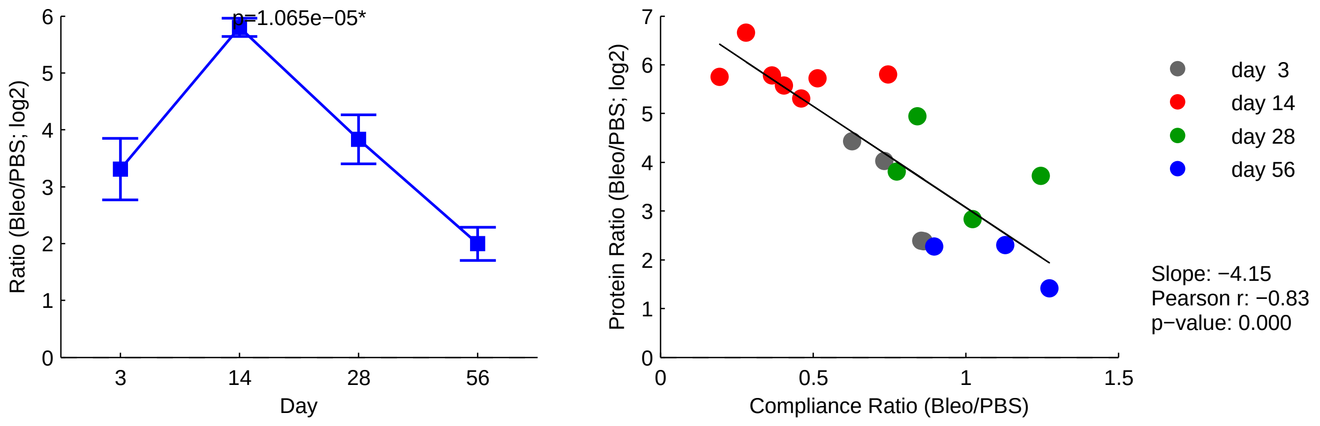

Q80Z96 − Vangl1 (id: 4993)

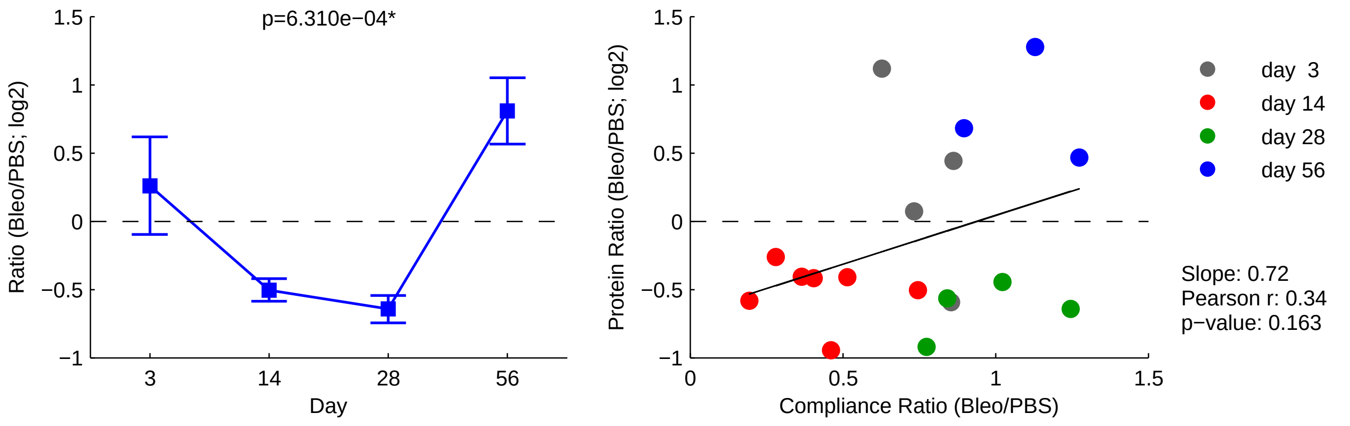

Q80ZS3 − Mrps26 (id: 4998)

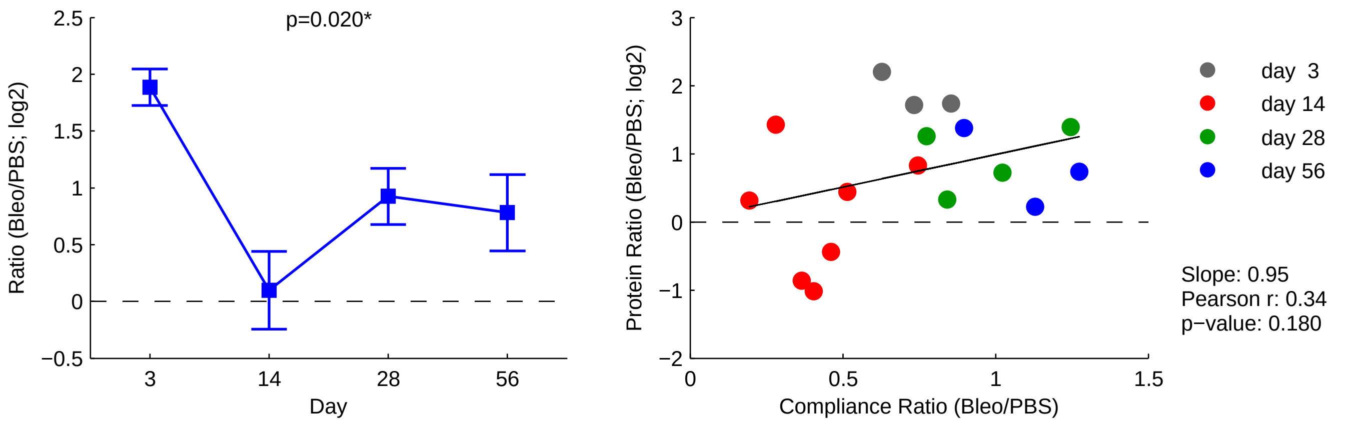

Q80ZU0 − Arl5a (id: 4999)

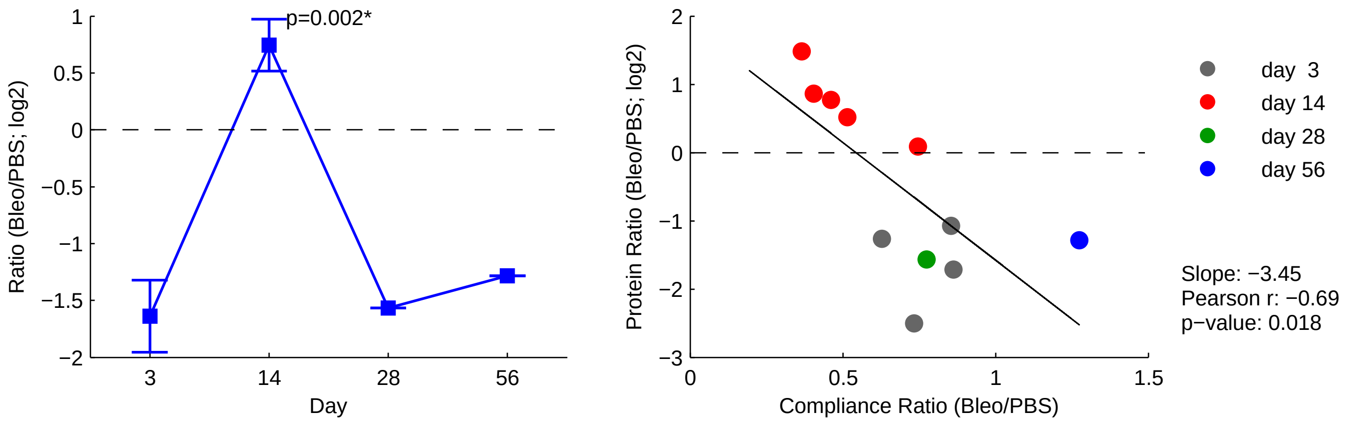

Q80ZW2 − Them6 (id: 5002)

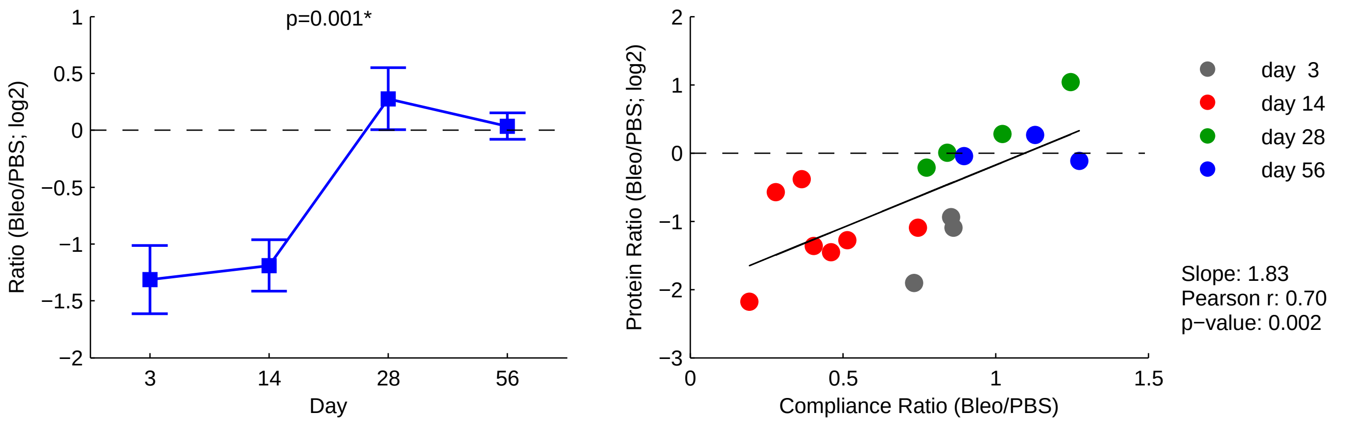

Q80ZX0 − Sec24b (id: 5003)

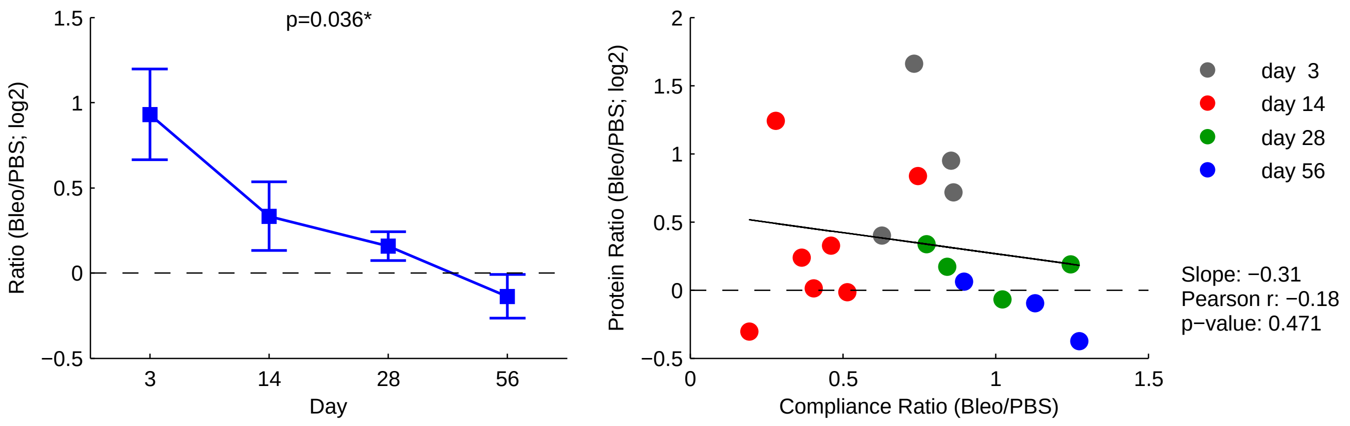

Q810A7 − Ddx42 (id: 5004)

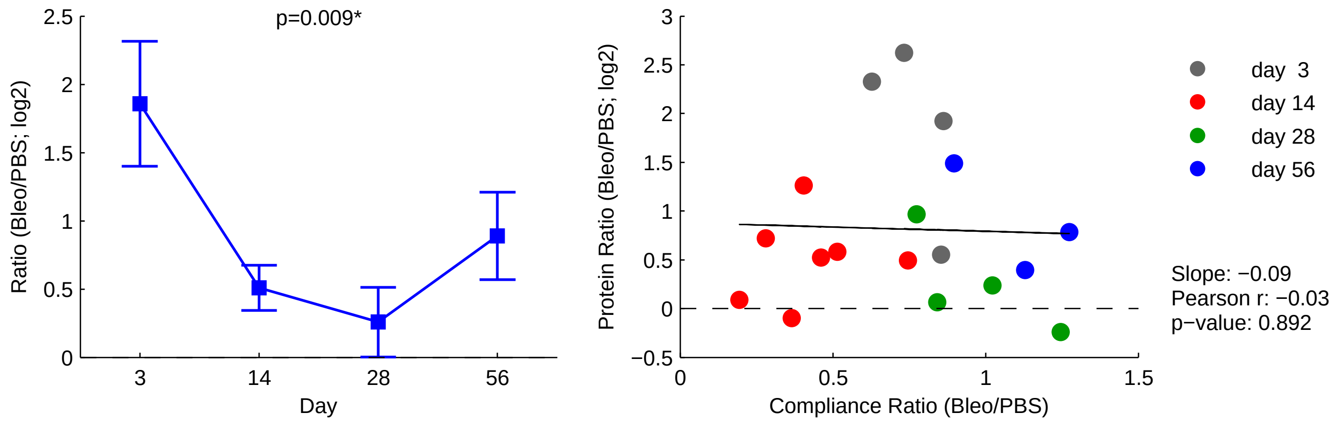

Q810Q5 − Nmes1 (id: 5009)

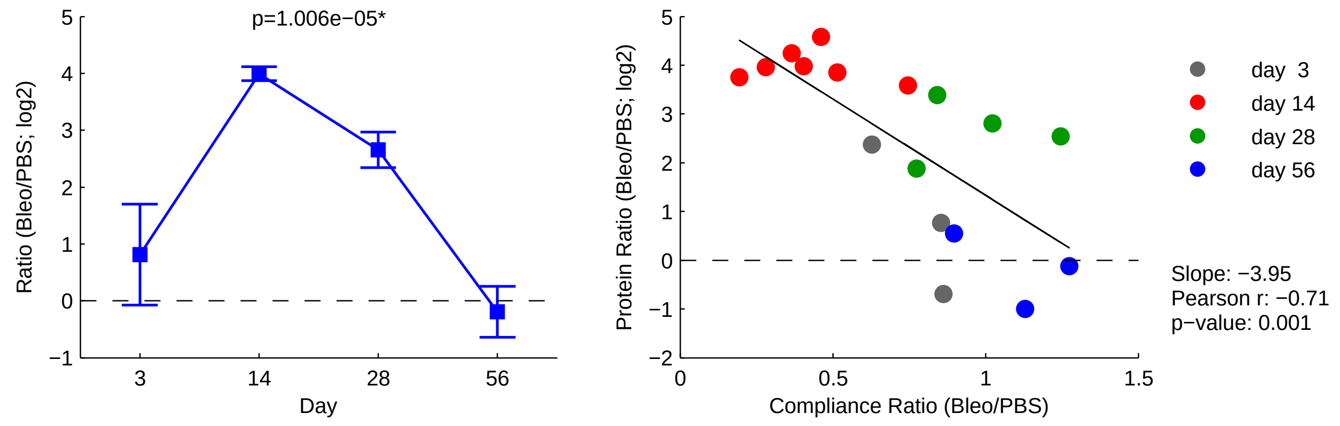

Q810S1 − Ccdc109b (id: 5010)

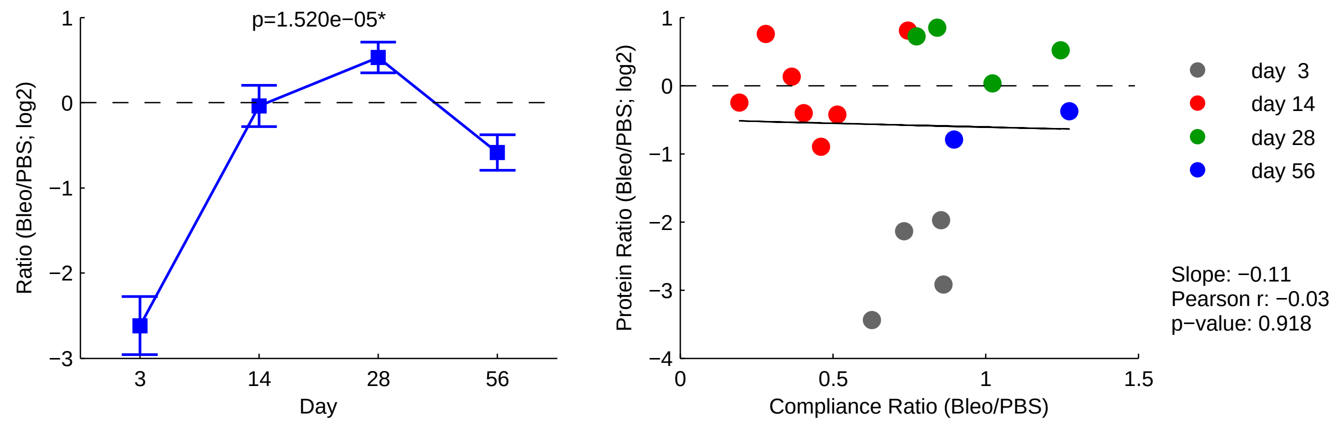

Q810V0 − Mphosph10 (id: 5013)

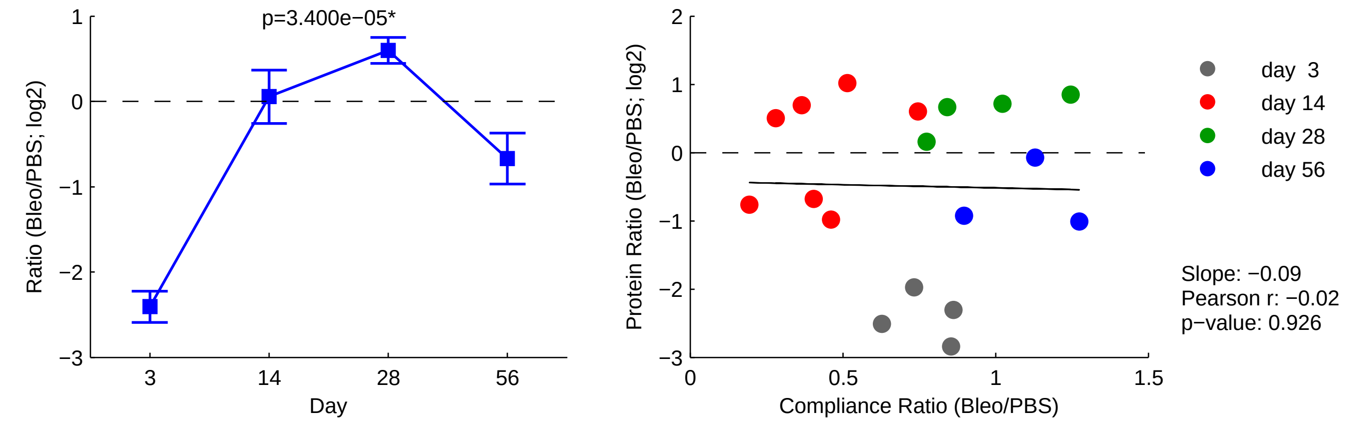

Q811S7 − Ubp1 (id: 5016)

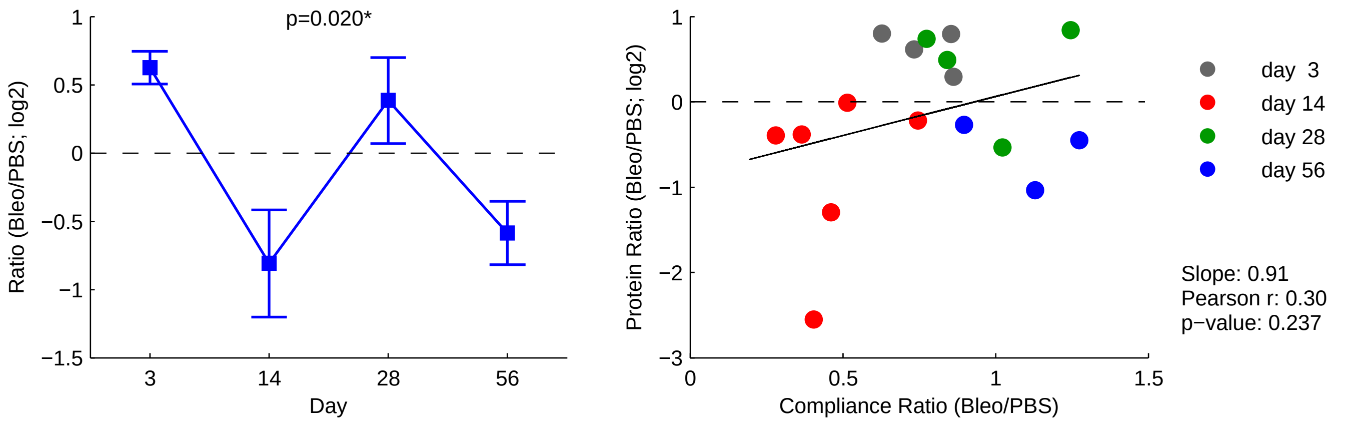

Q8BFP9 − Pdk1 (id: 5021)

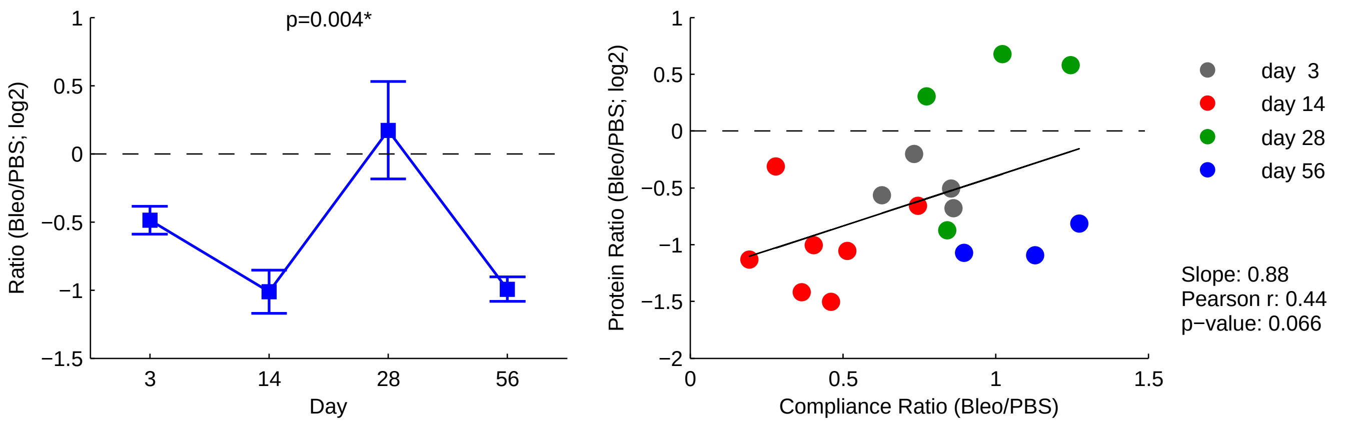

Q8BFQ8 − Pddc1 (id: 5023)

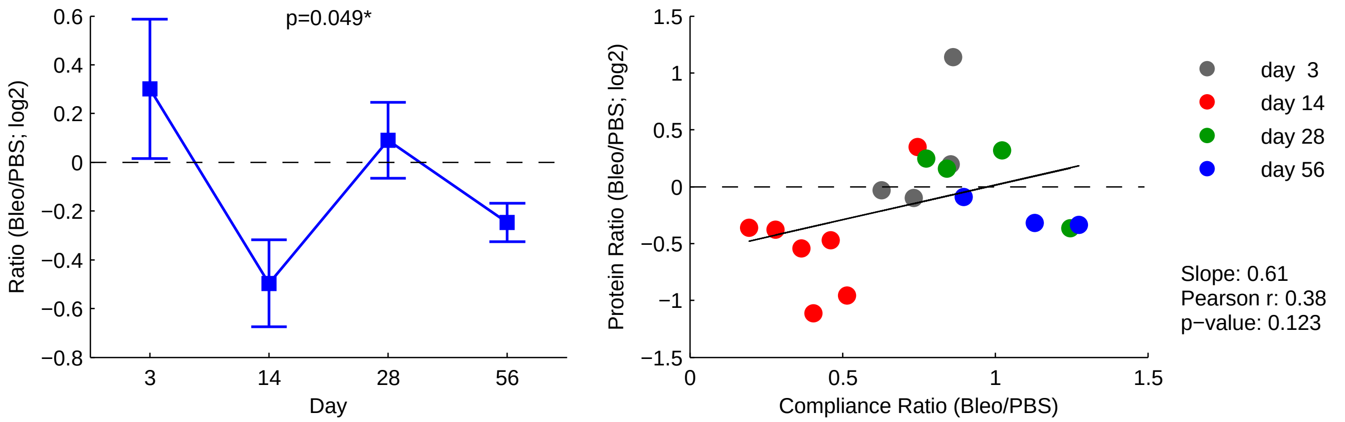

Q8BFR4 − Gns (id: 5025)

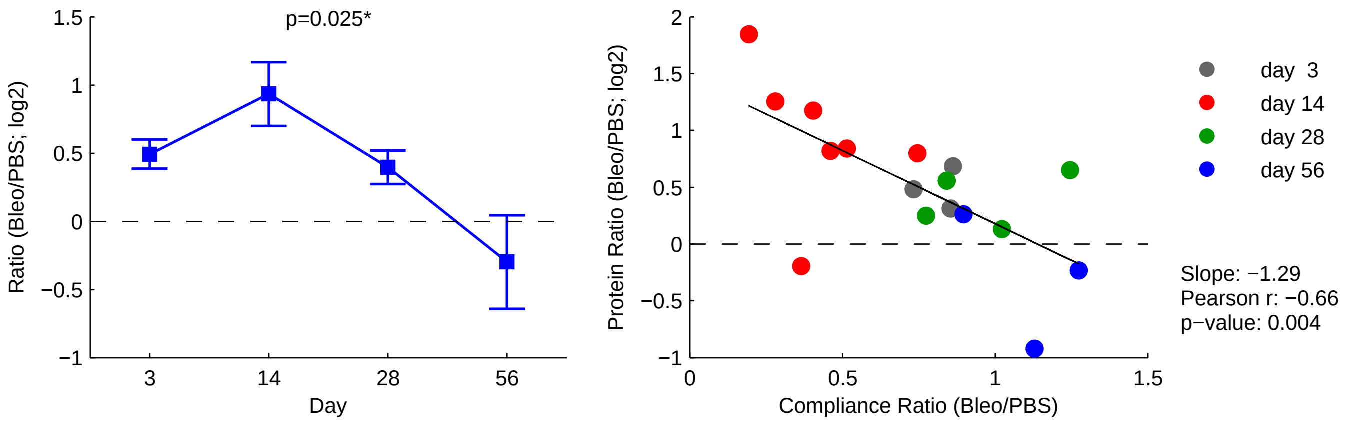

Q8BFR5 − Tufm (id: 5026)

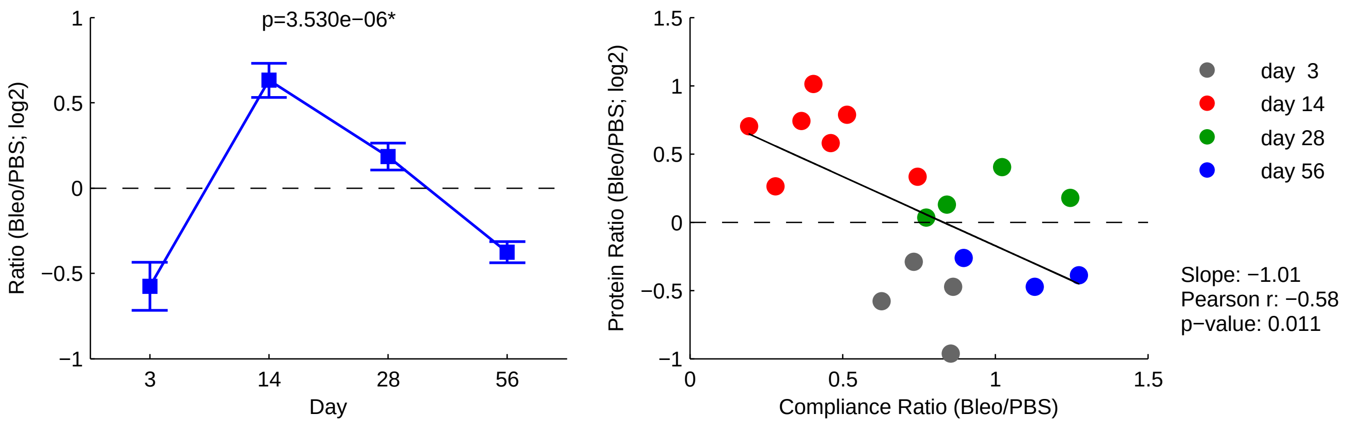

Q8BFW7 − Lpp (id: 5031)

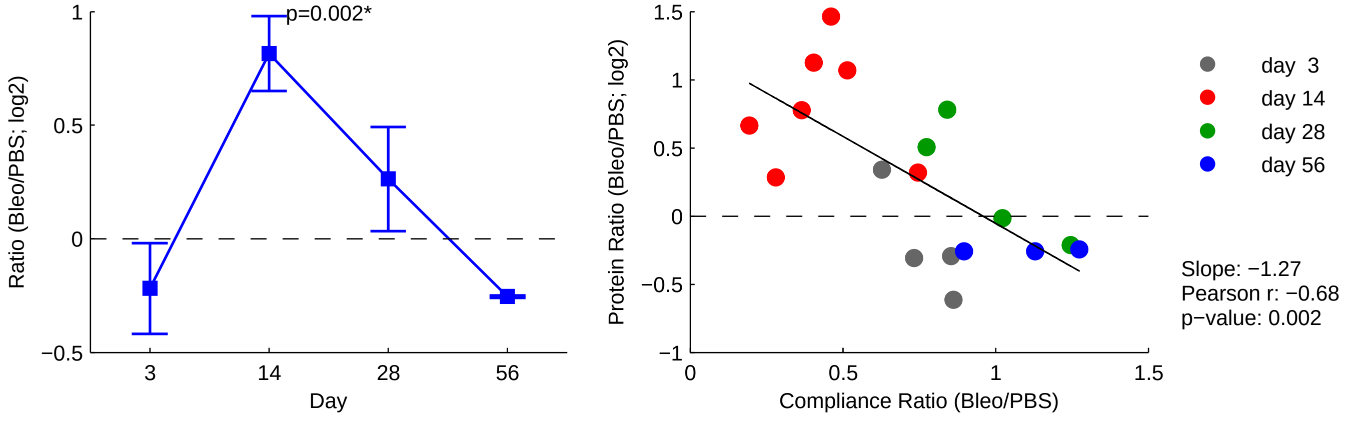

Q8BFY6 − Pef1 (id: 5032)

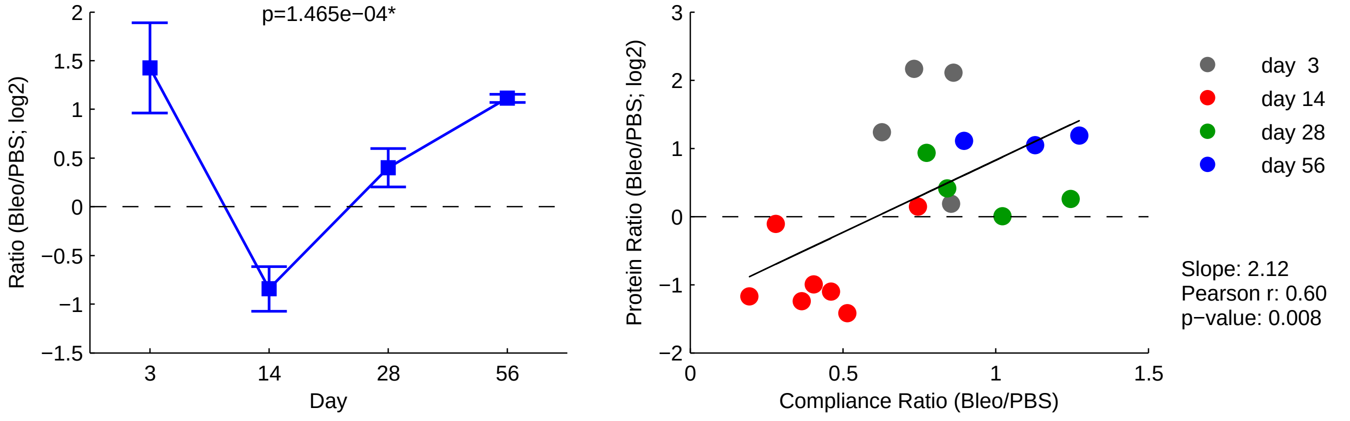

Q8BFZ9 − Erlin2 (id: 5035)

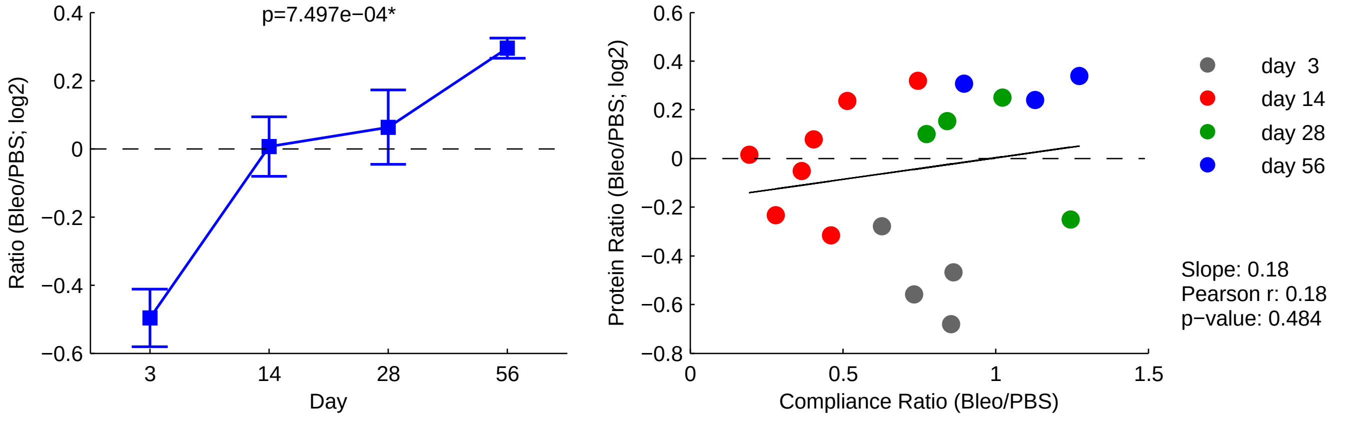

Q8BG07−2 − Pld4 (id: 5036)

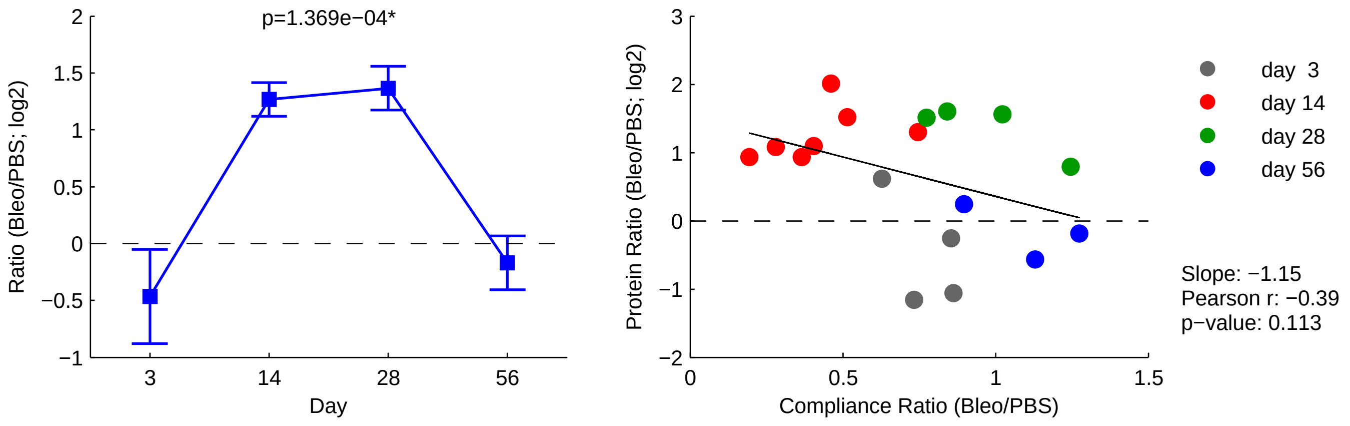

Q8BG15−6 − Ctdspl2 (id: 5038)

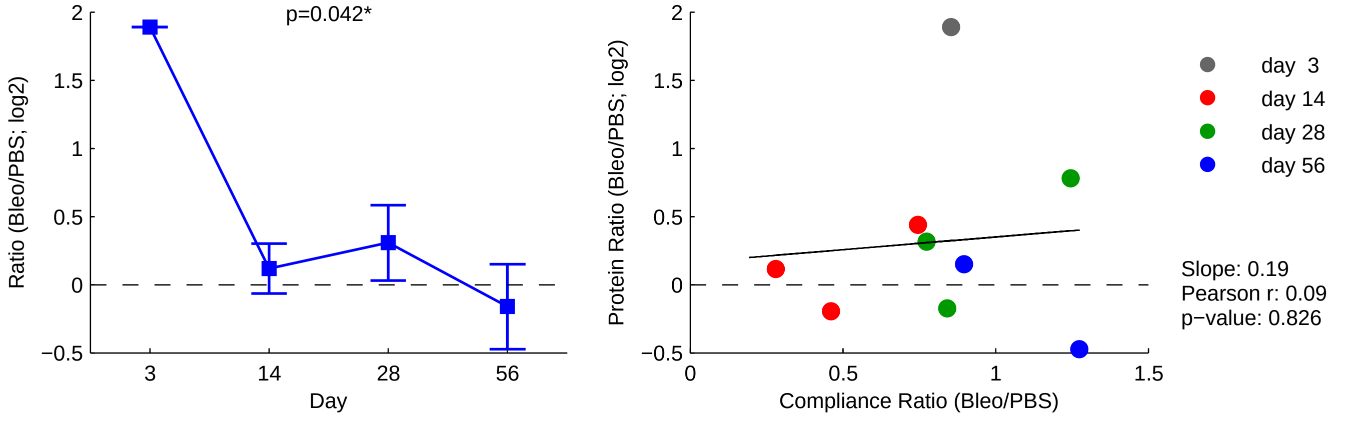

Q8BG32 − Psmd11 (id: 5040)

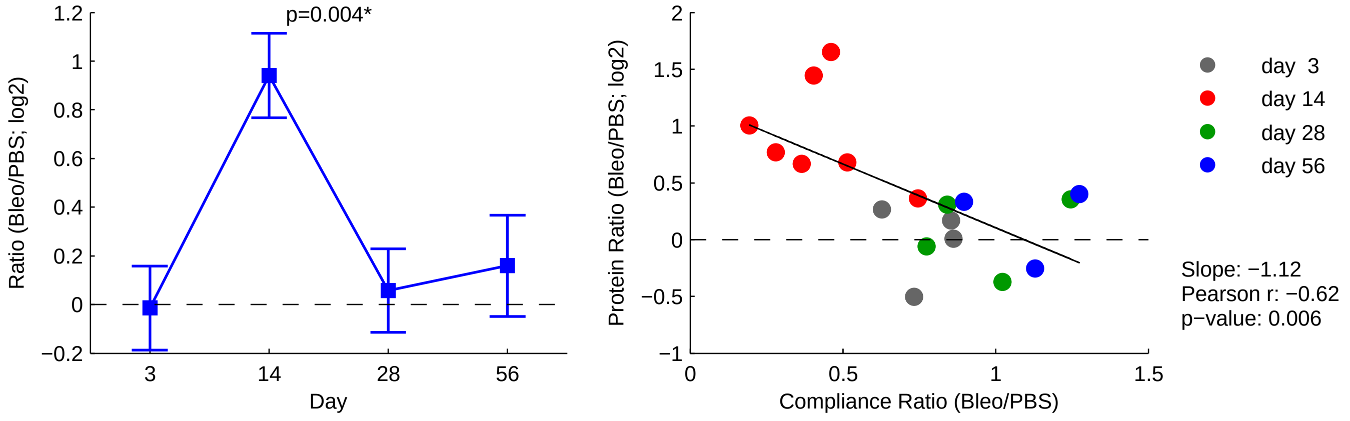

Q8BG60−2 − Txnip (id: 5044)

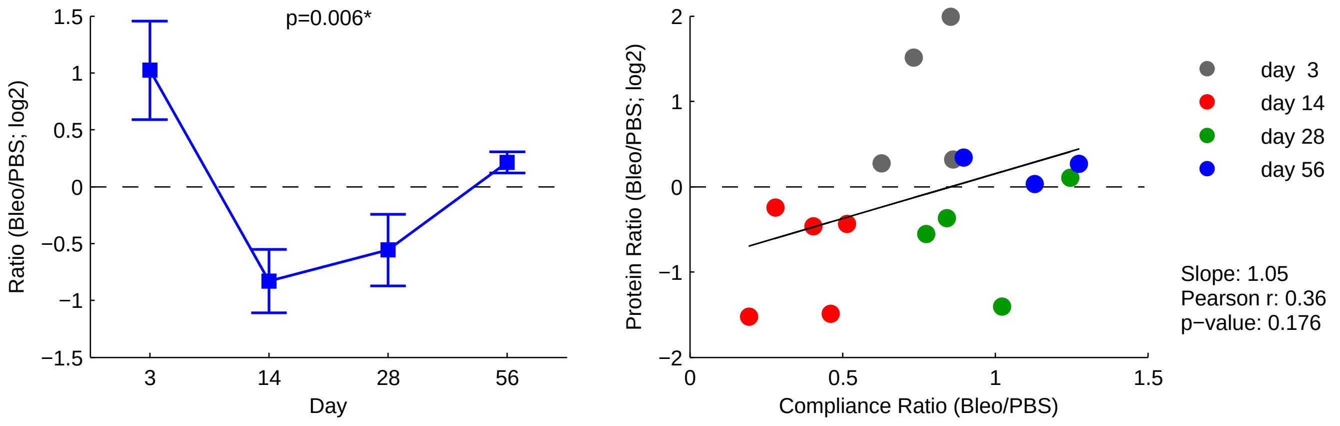

Q8BG67 − Efr3a (id: 5045)

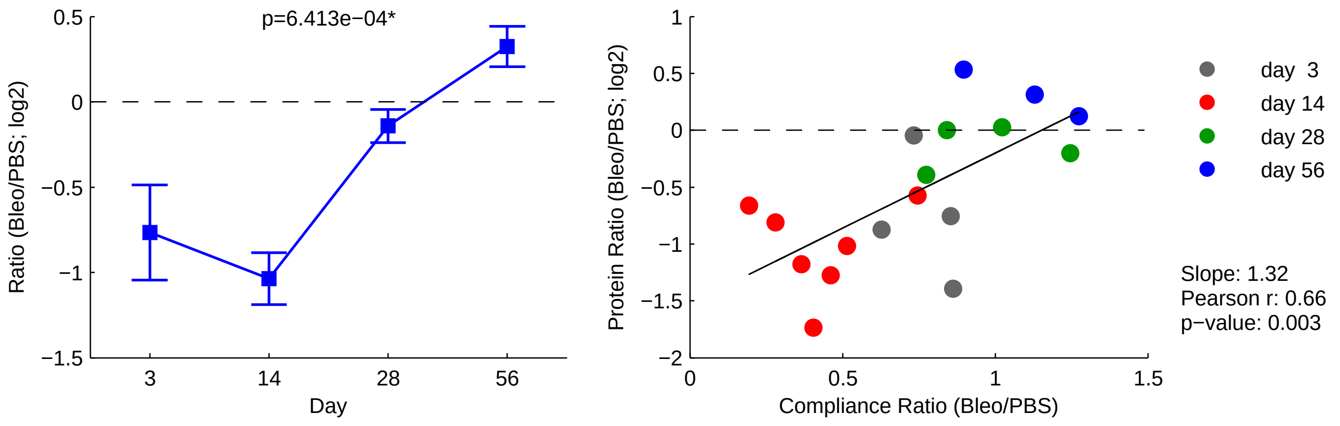

Q8BG73 − Sh3bgrl2 (id: 5046)

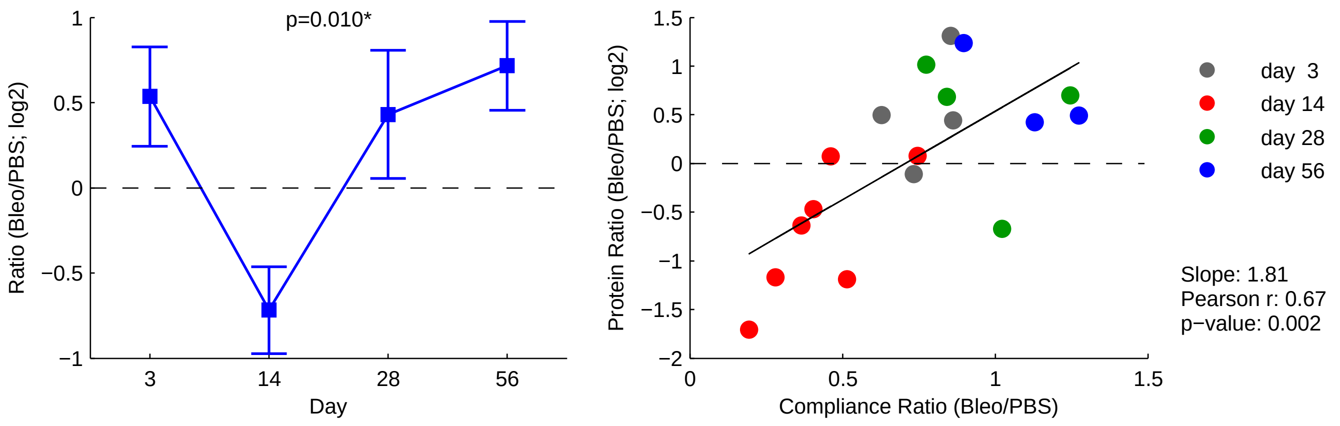

Q8BG94 − Commd7 (id: 5048)

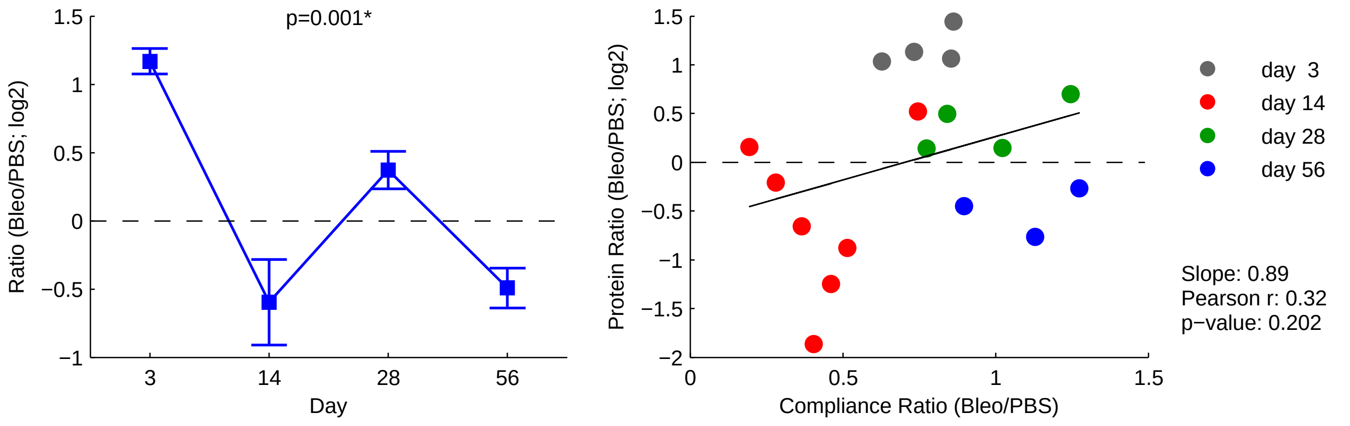

Q8BG95 − Ppp1r12b (id: 5049)

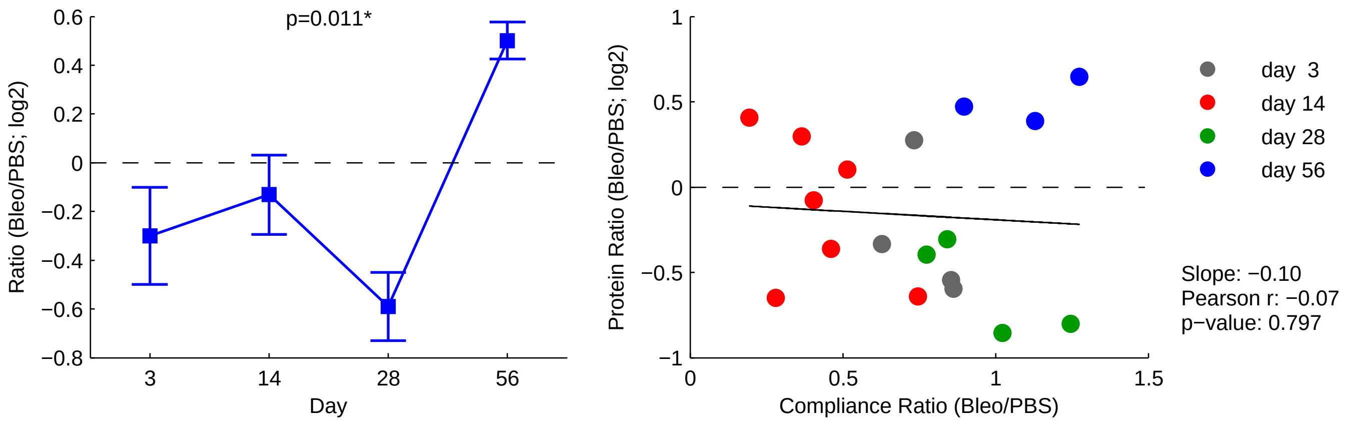

Q8BGB5 − Limd2 (id: 5053)

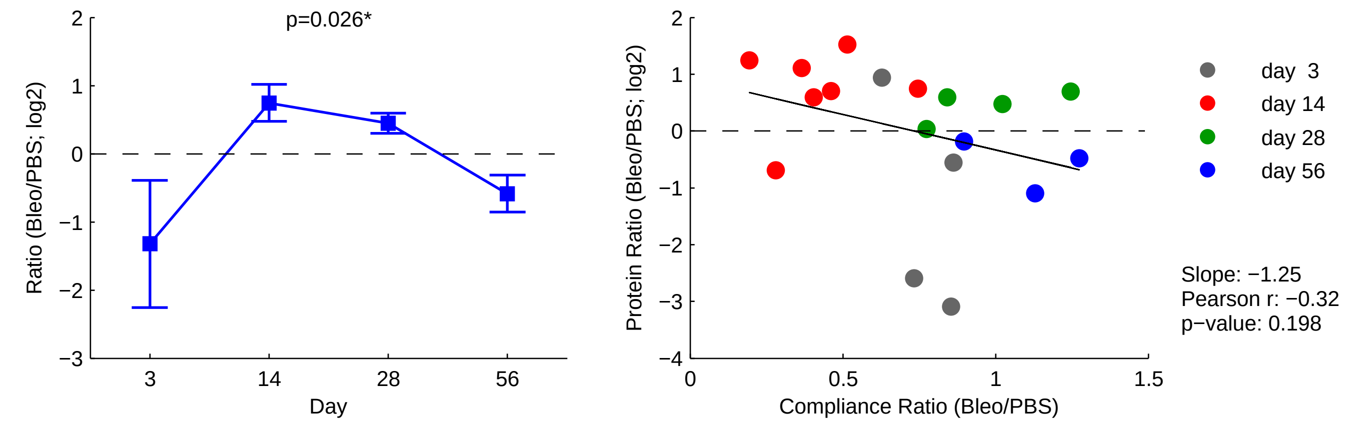

Q8BGC0 − Htatsf1 (id: 5056)

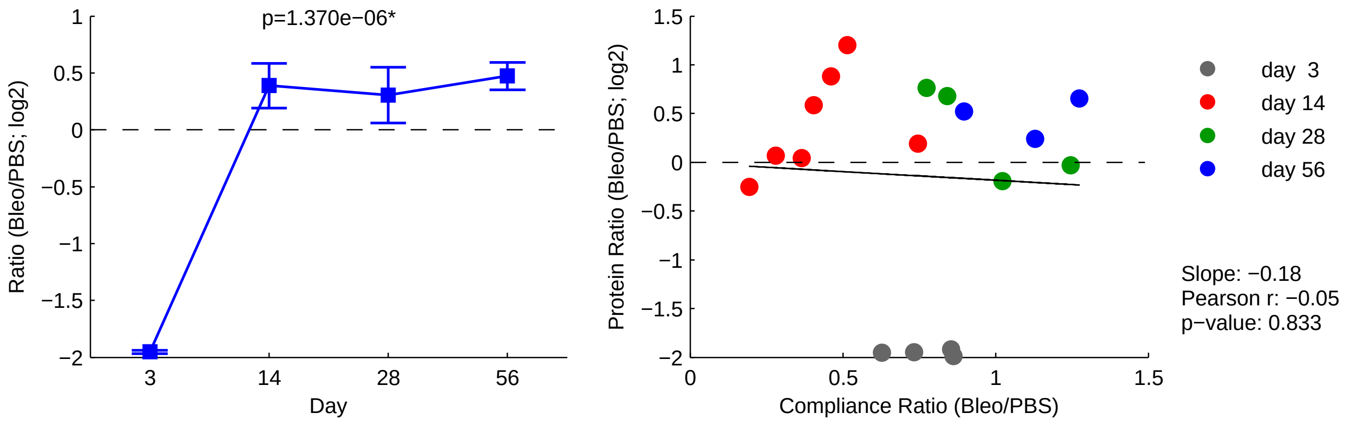

Q8BGC4 − Zadh2 (id: 5057)

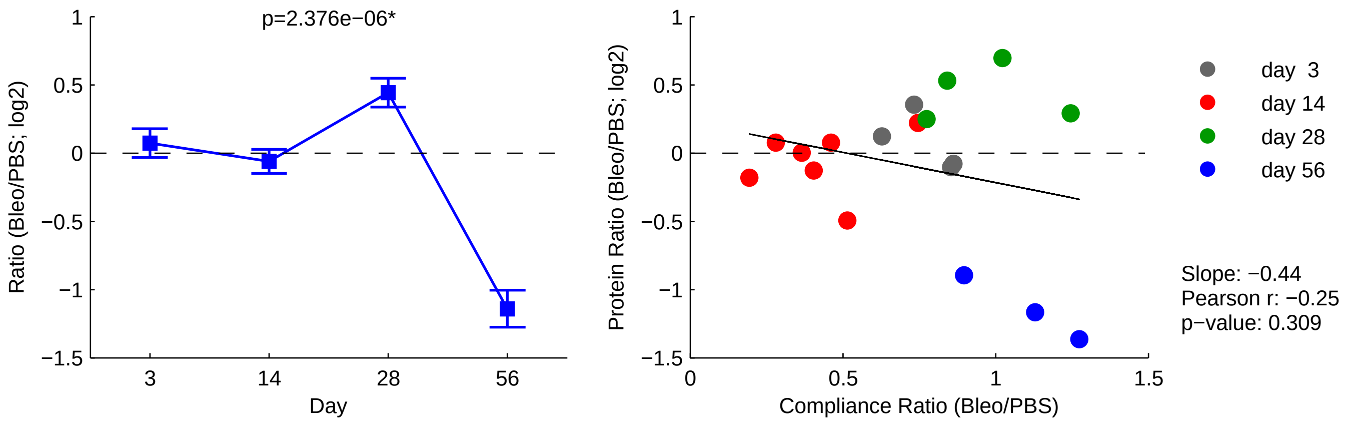

Q8BGD4 − Slco4c1 (id: 5059)

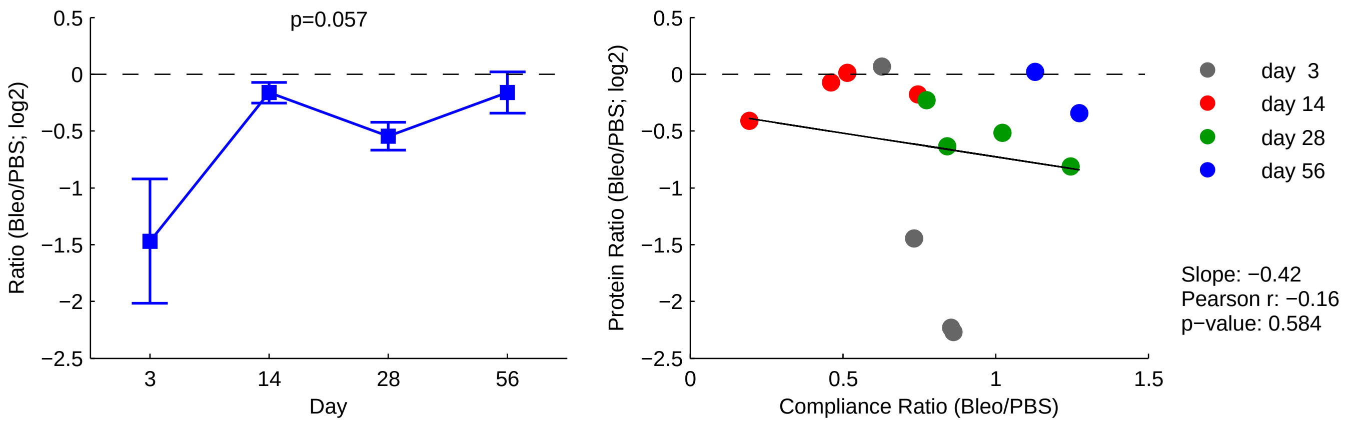

Q8BGD9 − Eif4b (id: 5063)

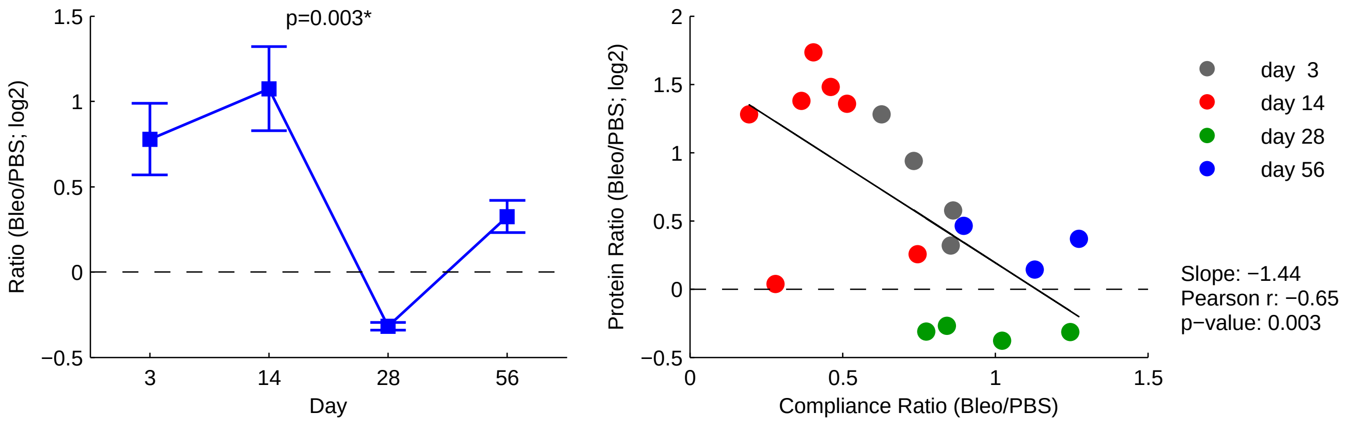

Q8BGF6 − Elmod2 (id: 5064)

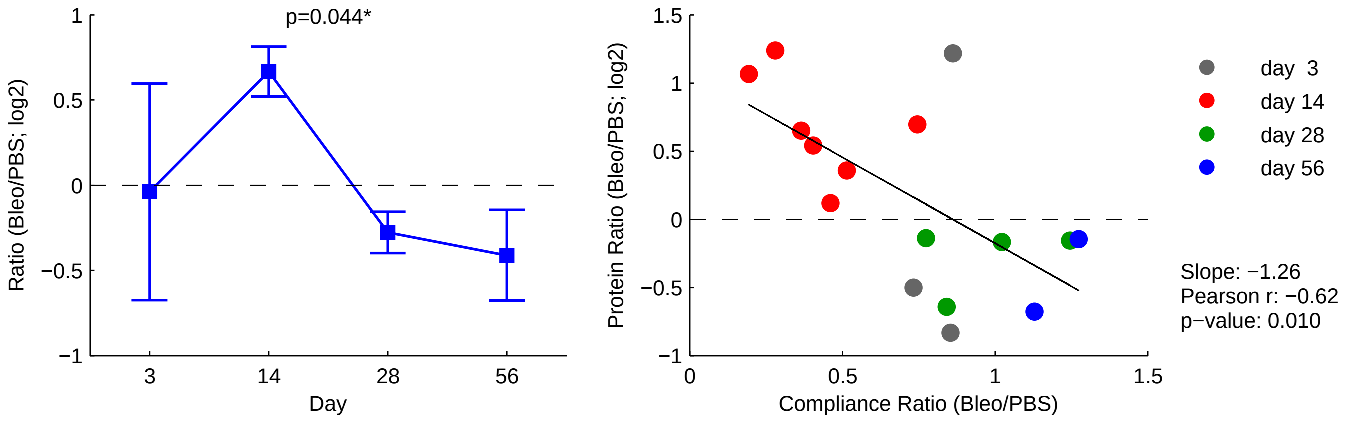

Q8BGH2 − Samm50 (id: 5067)

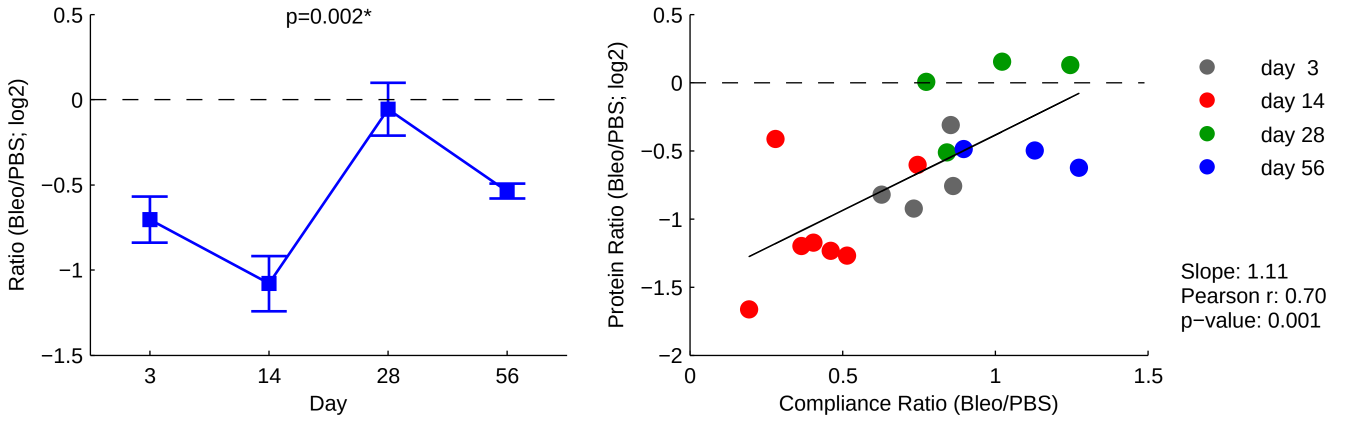

Q8BGH7 − Cdc42se2 (id: 5069)

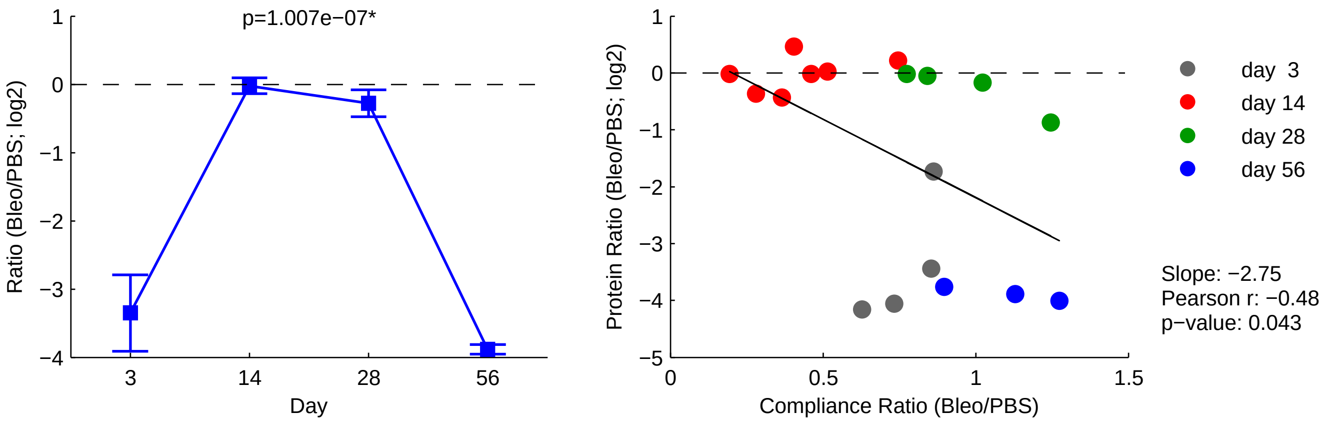

Q8BGK6 − Slc7a6 (id: 5071)

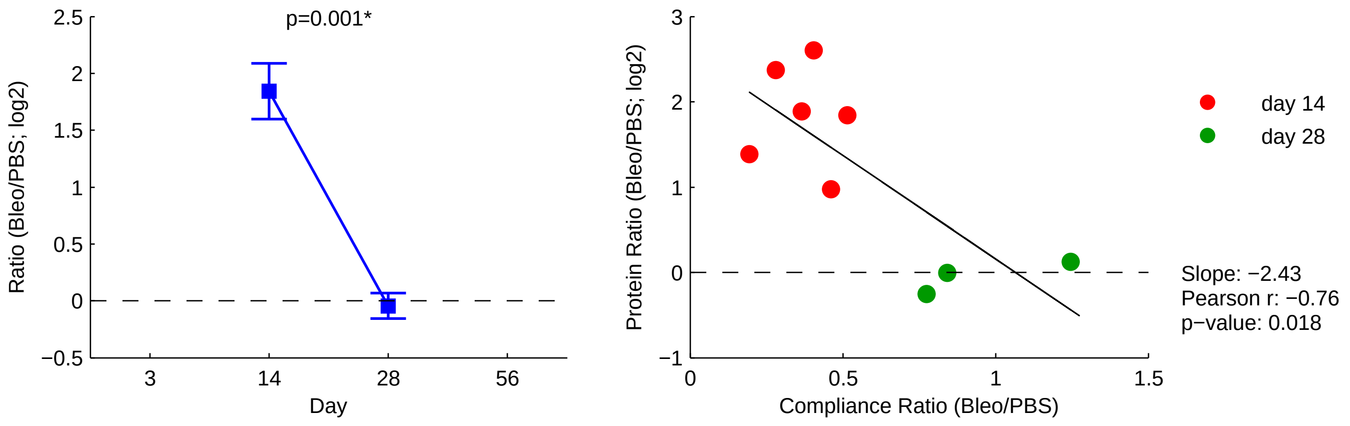

Q8BGP6−2 − Slc25a40 (id: 5073)

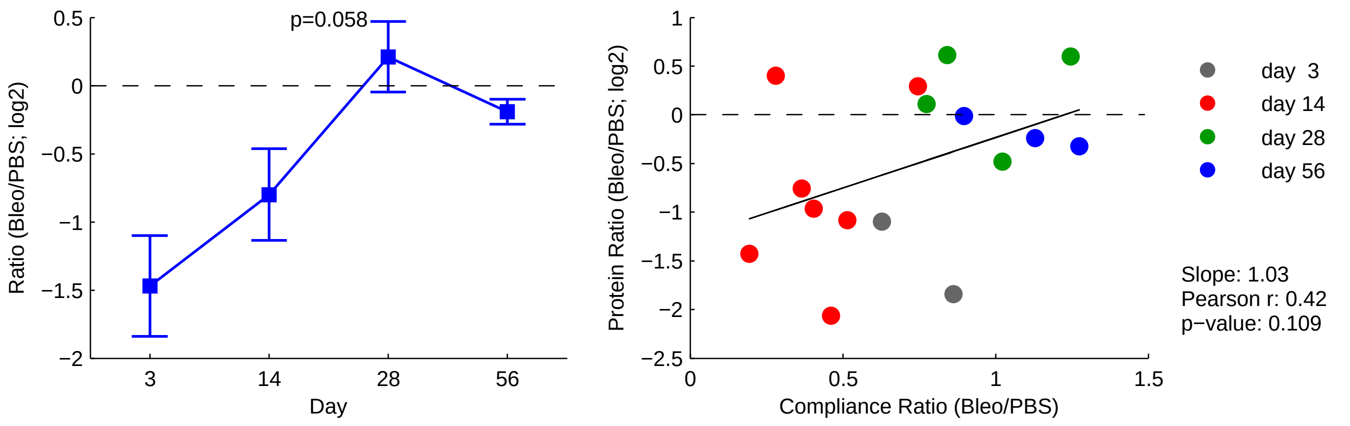

Q8BGQ1 − Vipas39 (id: 5074)

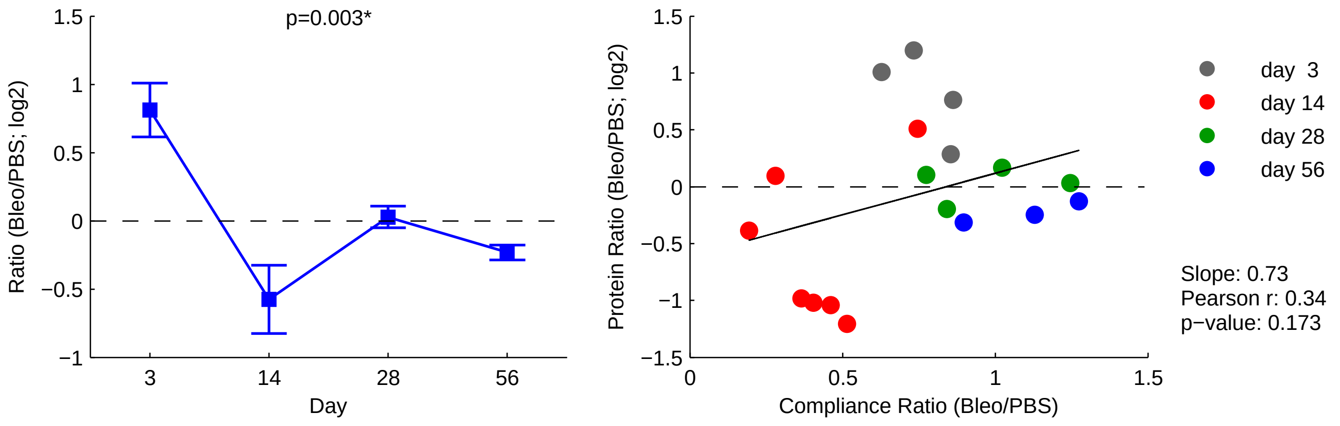

Q8BGR6 − Arl15 (id: 5077)

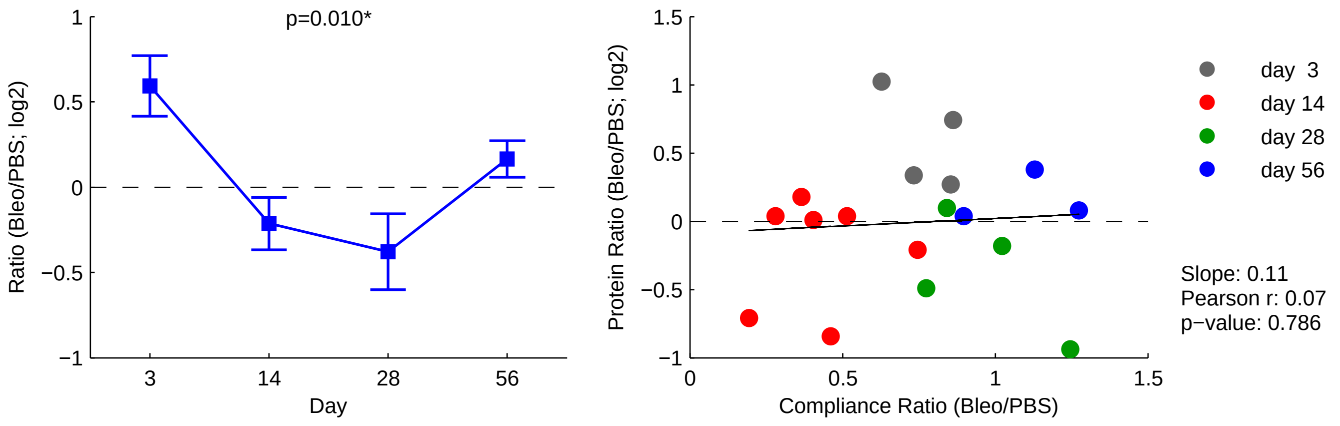

Q8BGS1 − Epb41l5 (id: 5080)

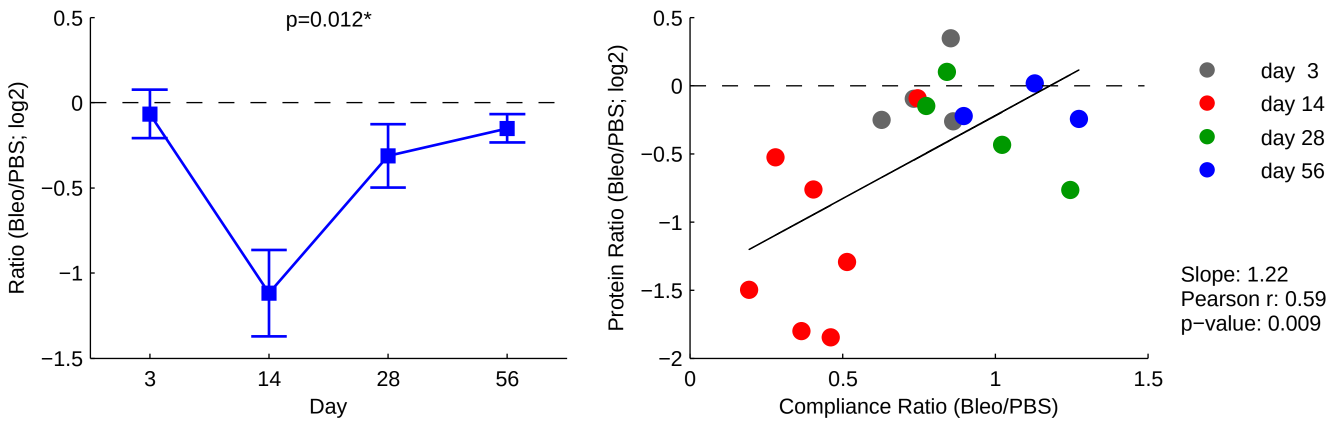

Q8BGS7−2 − Cept1 (id: 5082)

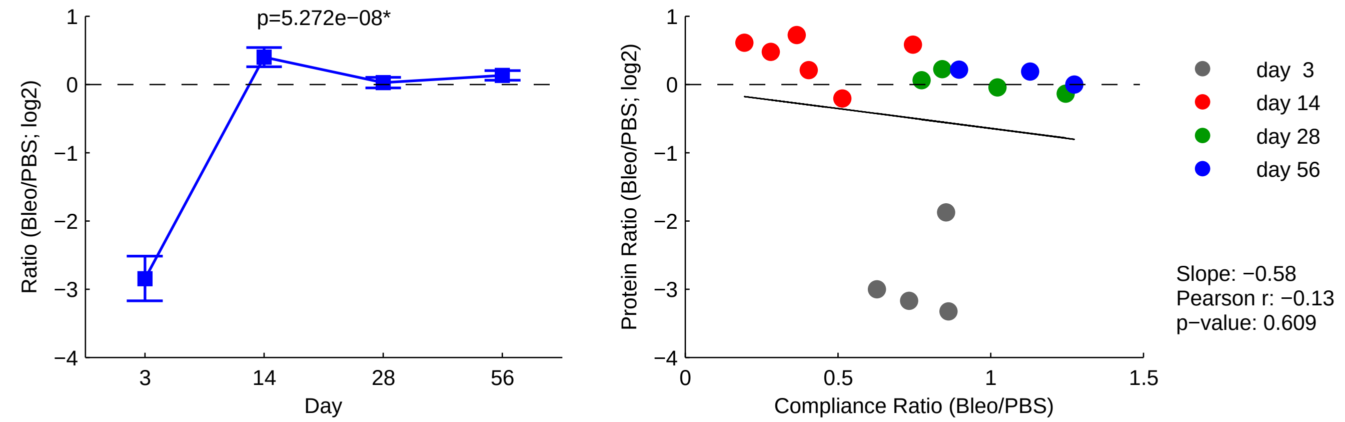

Q8BGT1 − Flrt3 (id: 5083)

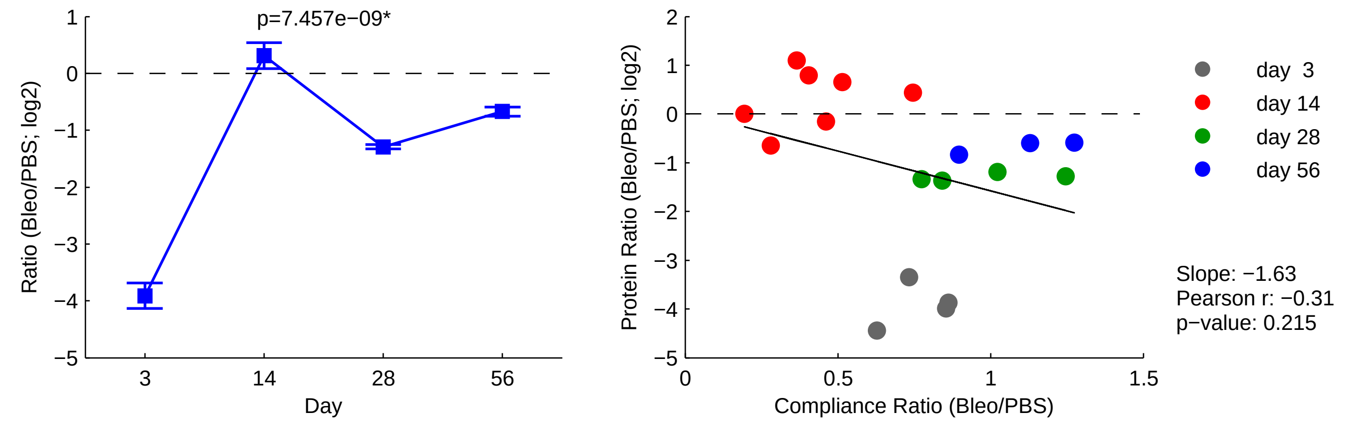

Q8BGU5 − Ccny (id: 5087)

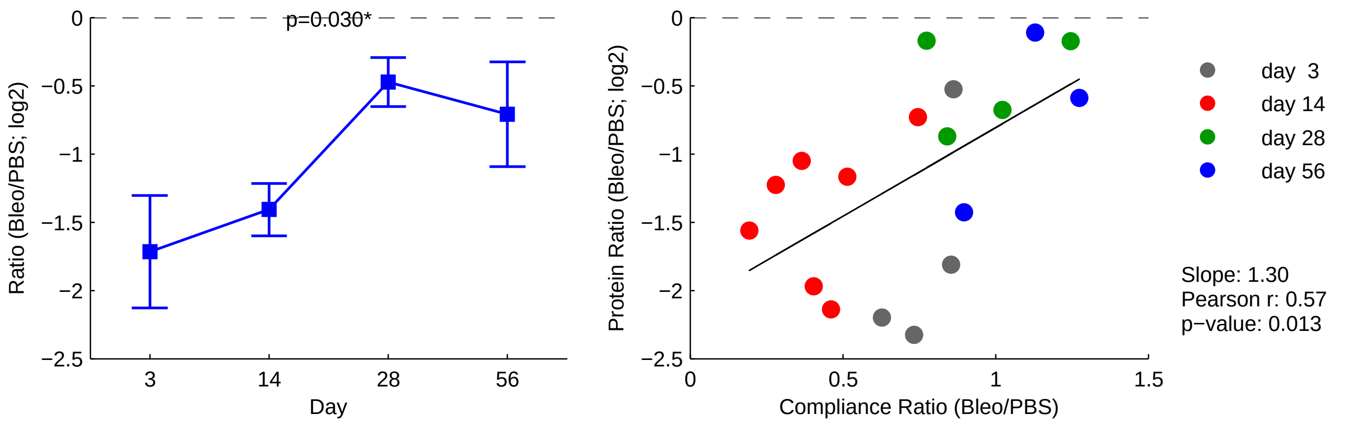

Q8BGV3 − Tacstd2 (id: 5089)

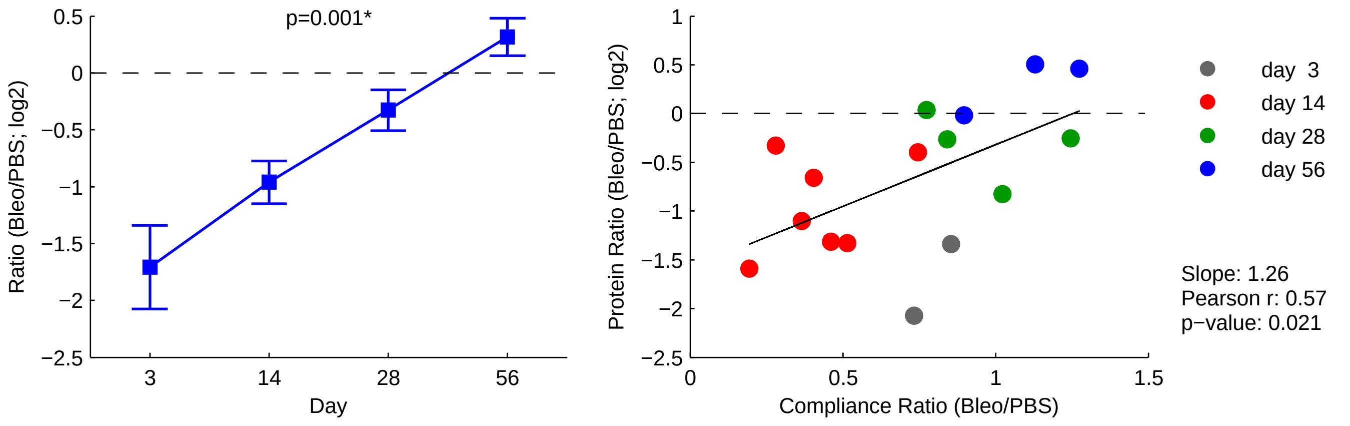

Q8BGX2 − (id: 5091)

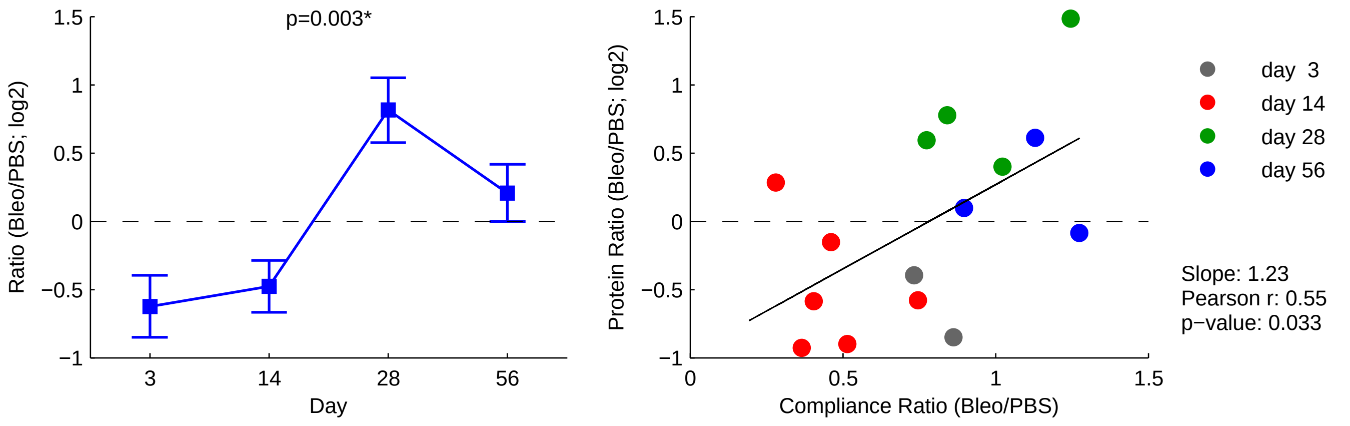

Q8BH04 − Pck2 (id: 5096)

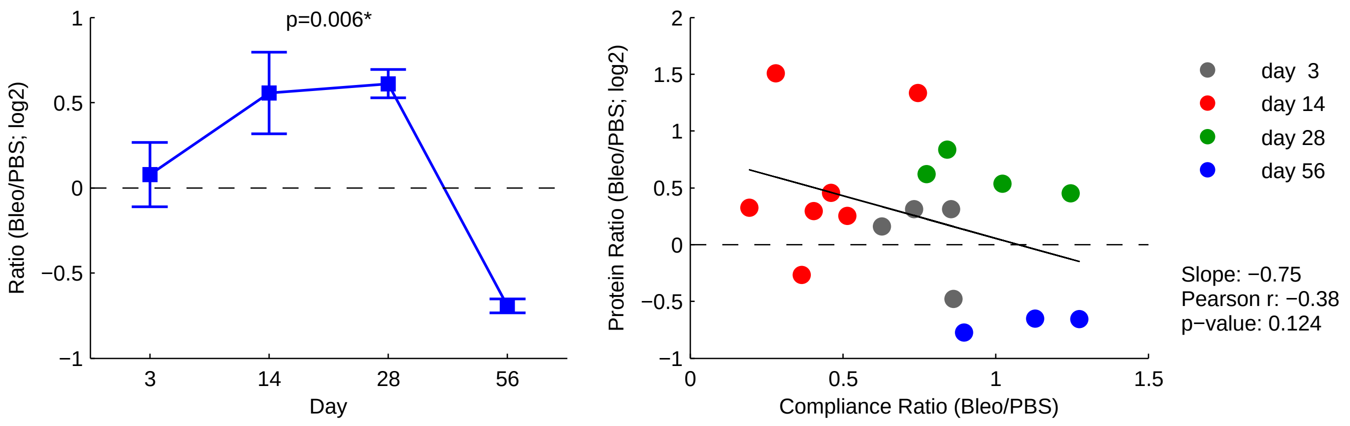

Q8BH40 − Stx7 (id: 5101)

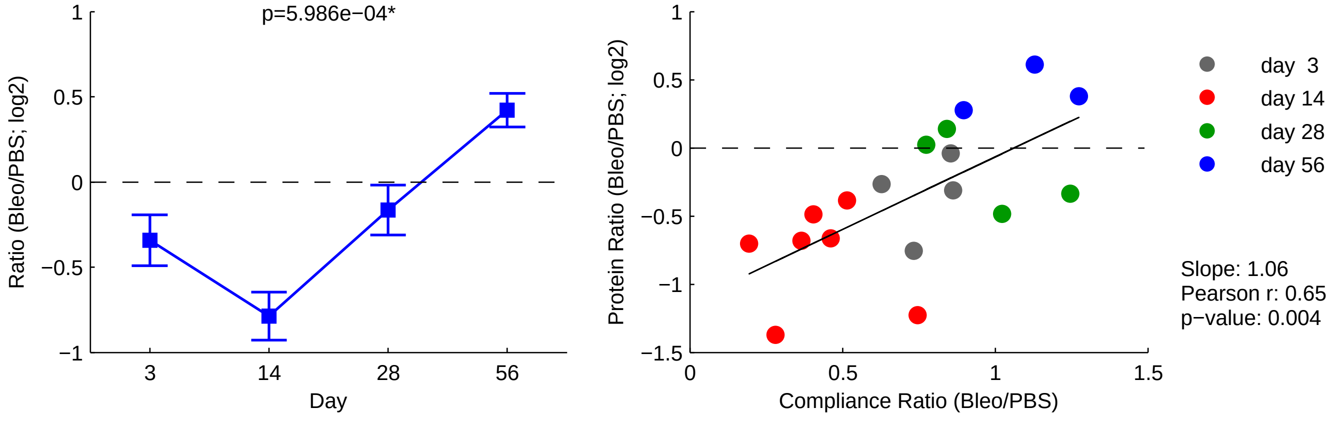

Q8BH55 − Thnsl1 (id: 5105)

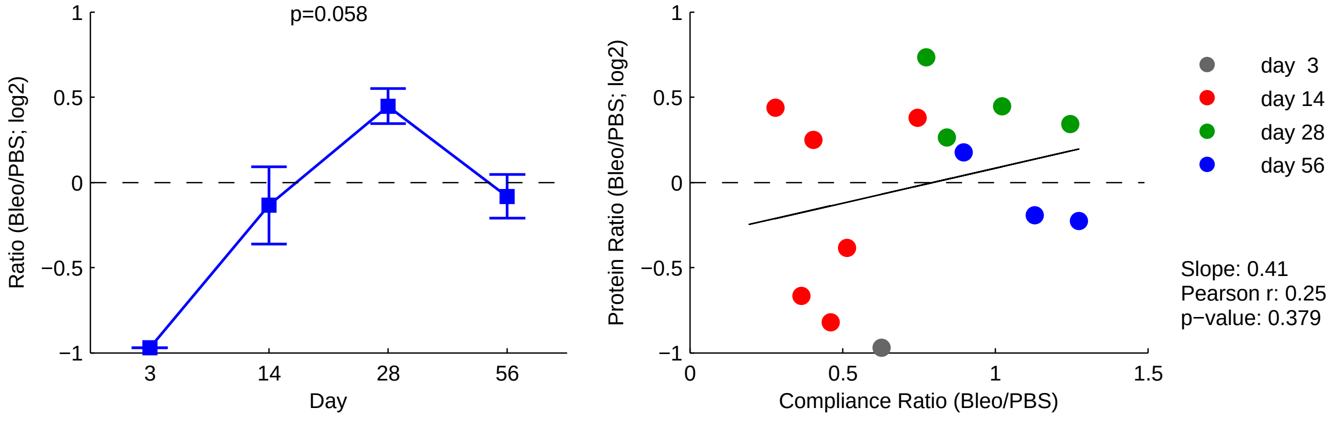

Q8BH59 − Slc25a12 (id: 5108)

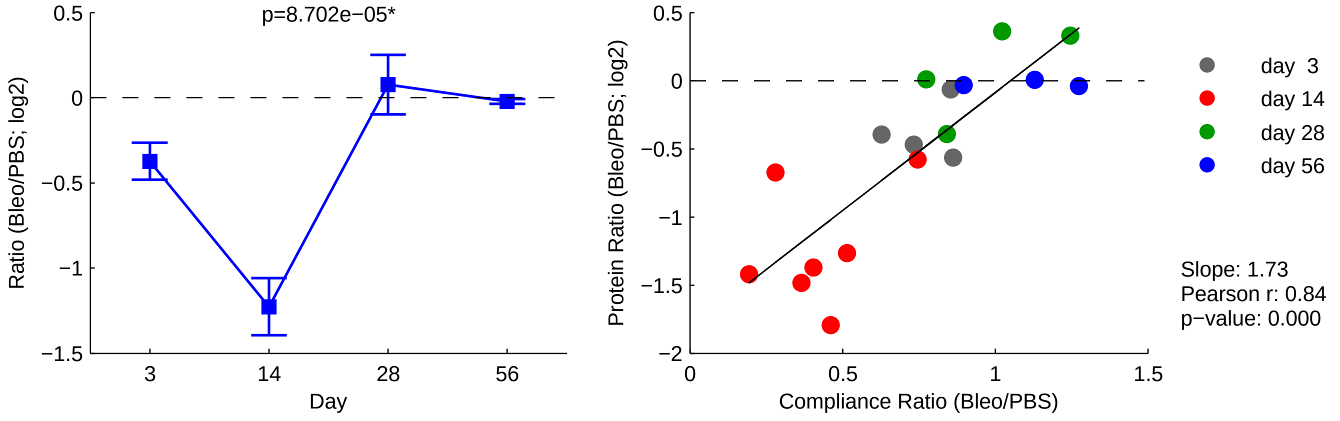

Q8BH61 − F13a1 (id: 5109)

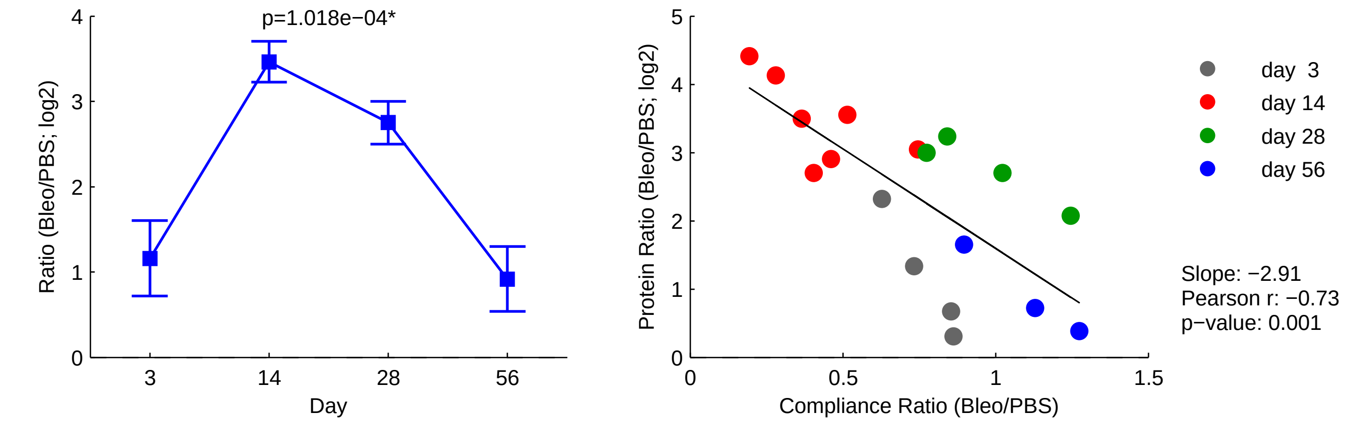

Q8BH64 − Ehd2 (id: 5110)

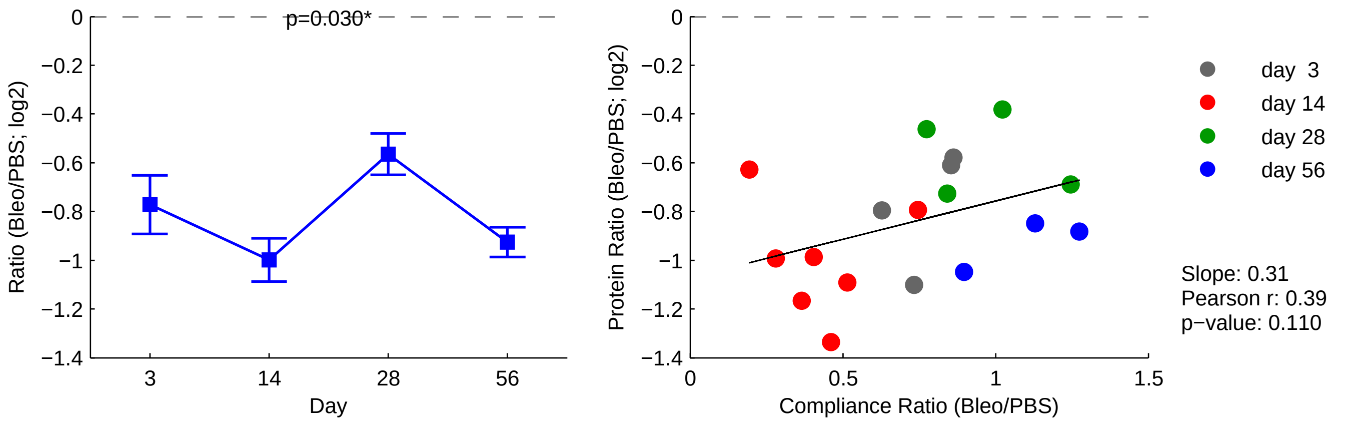

Q8BH65 − Dennd6a (id: 5111)

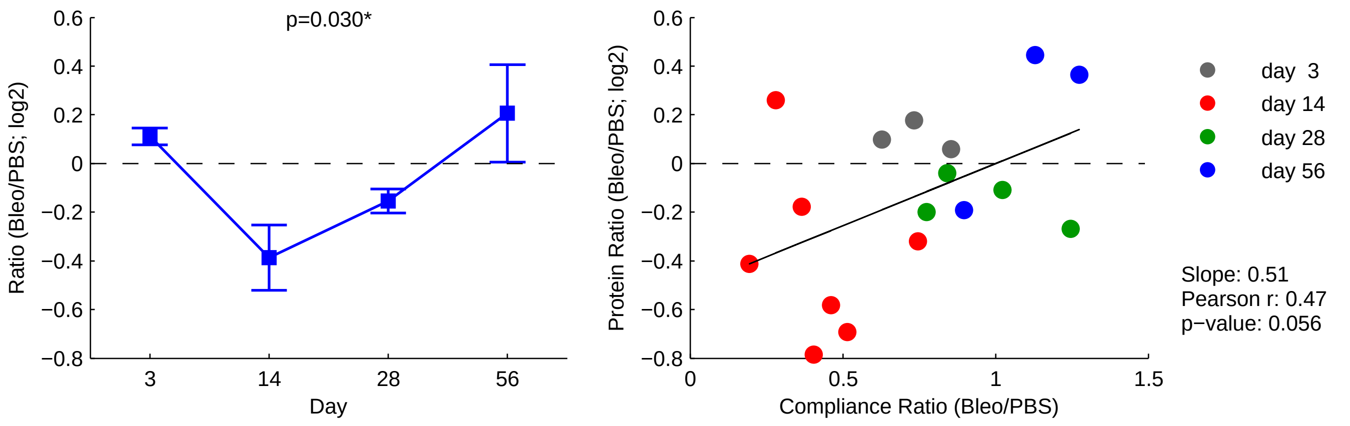

Q8BH69 − Sephs1 (id: 5113)

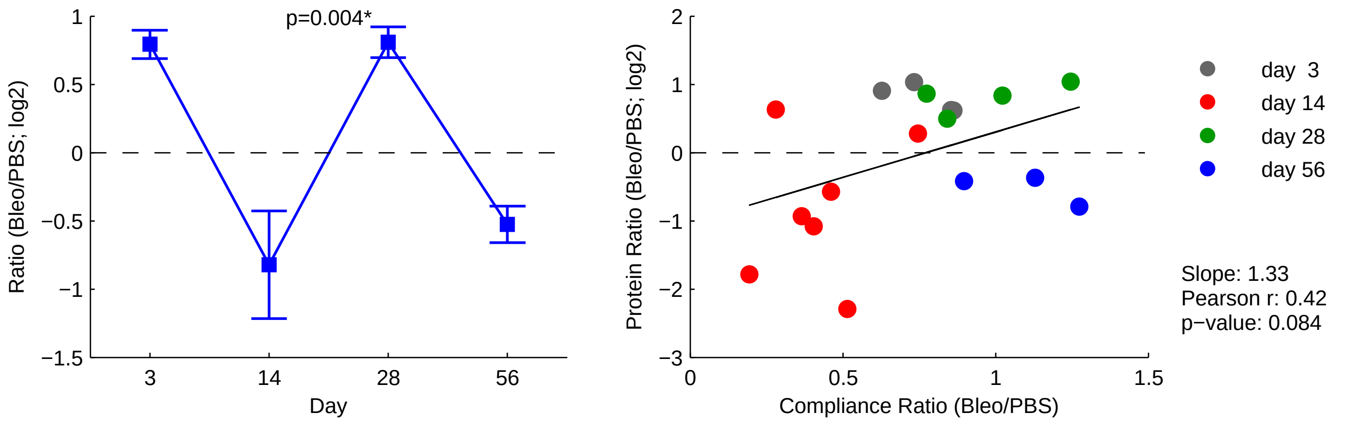

Q8BH73 − Qpctl (id: 5114)

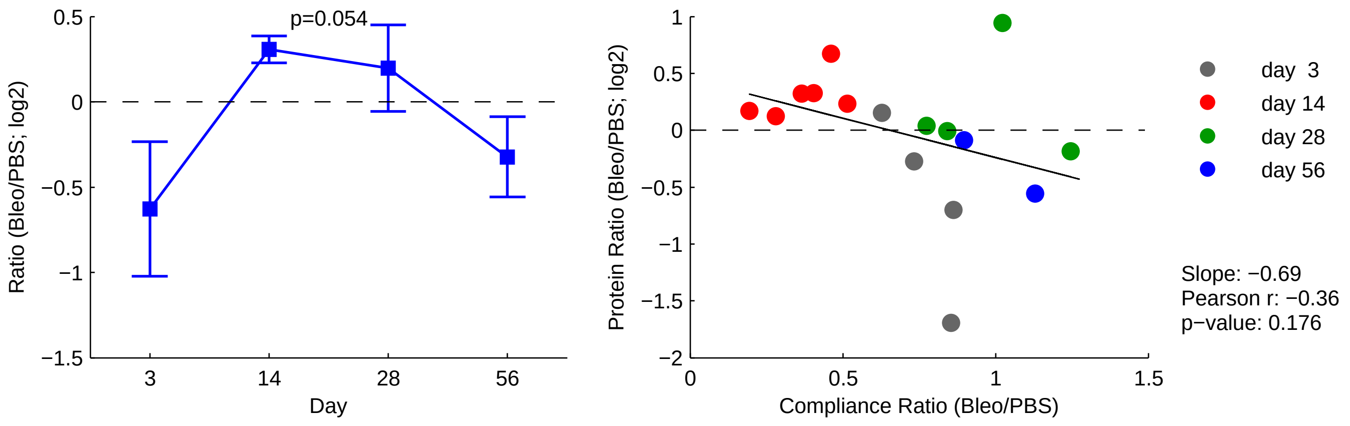

Q8BH79−2 − Ano10 (id: 5117)

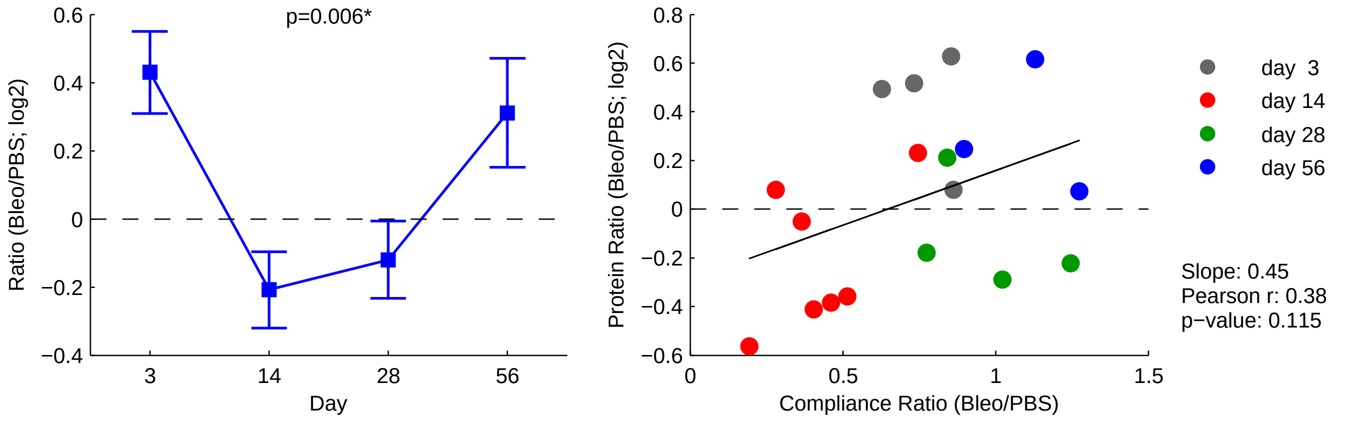

Q9QY76 − Vapb (id: 5118)

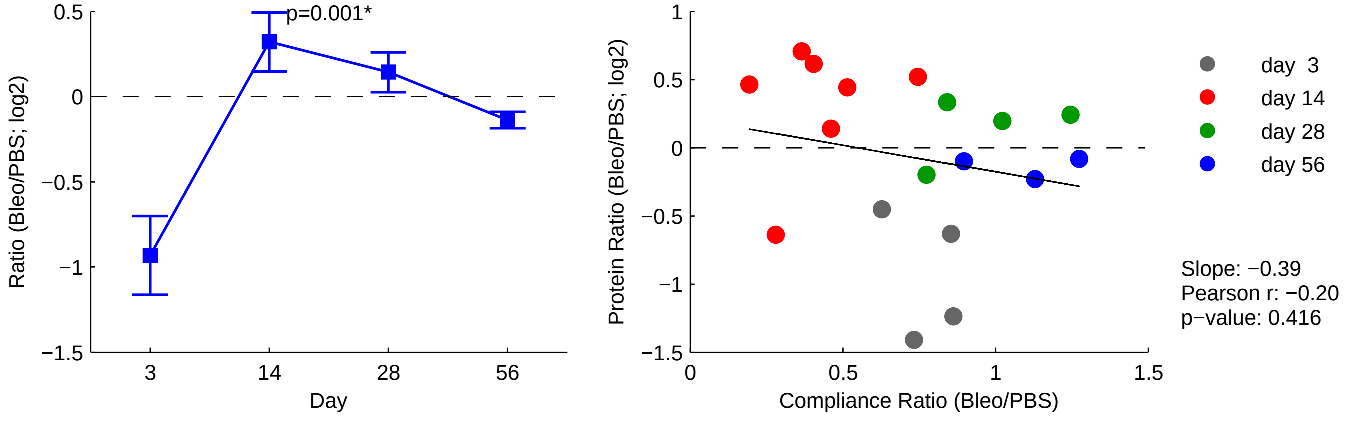

Q8BH95 − Echs1 (id: 5120)

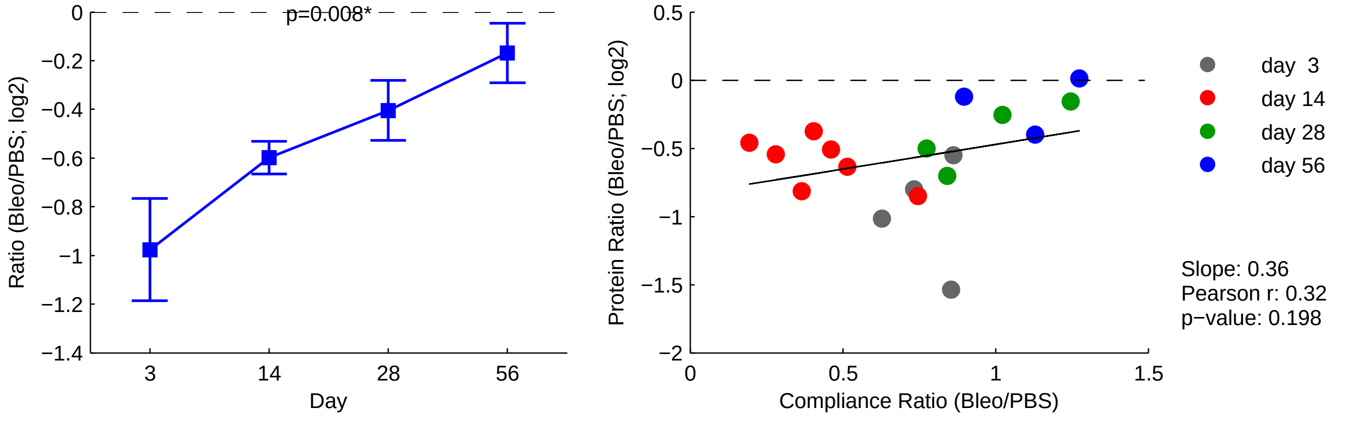

Q8BHB0 − Nod1 (id: 5124)

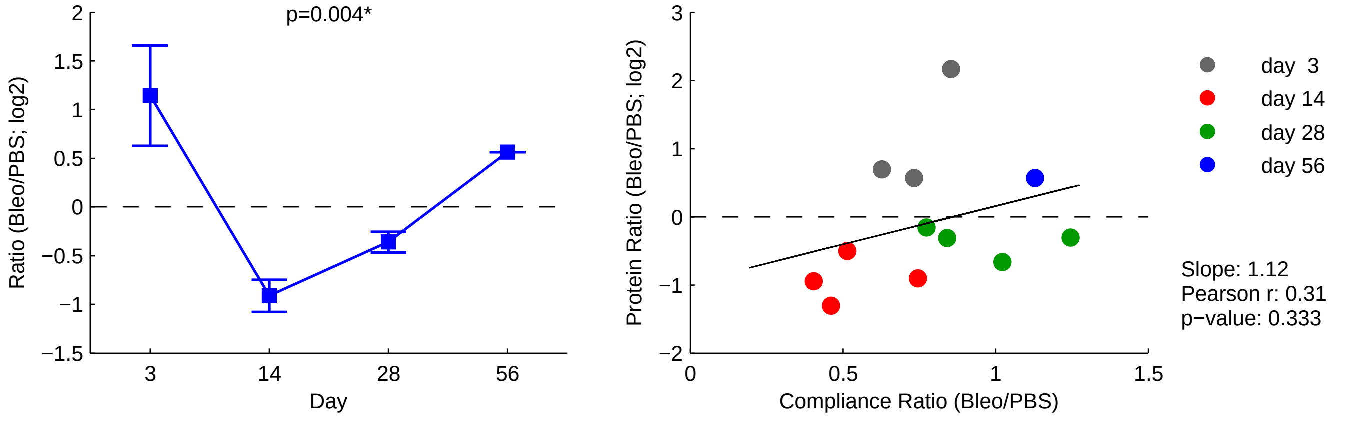

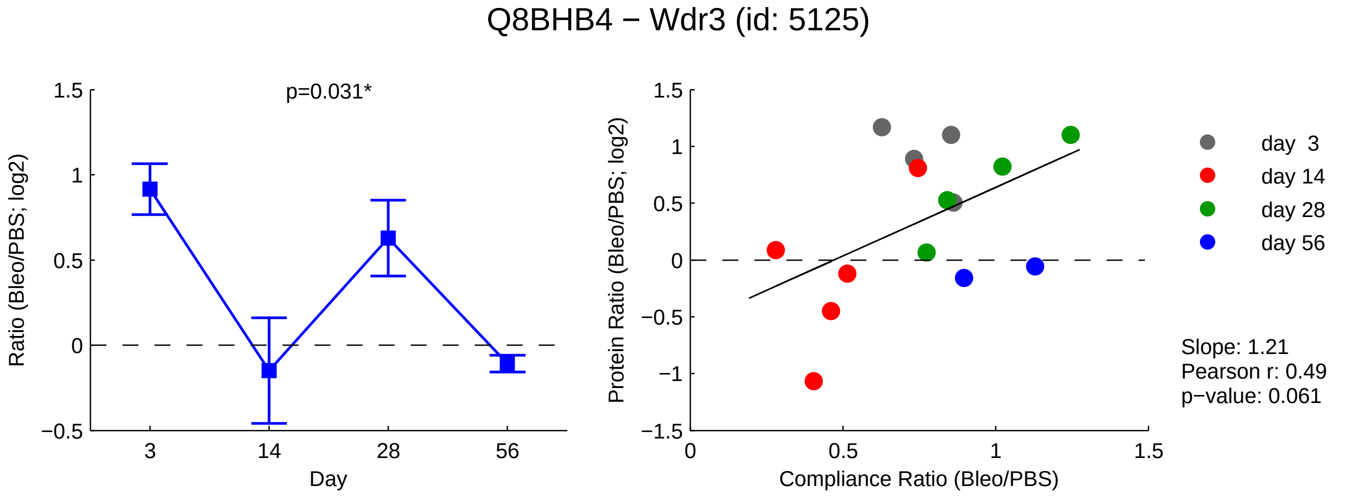
Q8BHB4 − Wdr3 (id: 5125)
p=0.031*
Ratio (Bleo/PBS; log2)
Day
Protein Ratio (Bleo/PBS; log2)
Compliance Ratio (Bleo/PBS)
day 3
day 14
day 28
day 56
Slope: 1.21
Pearson r: 0.49
p−value: 0.061

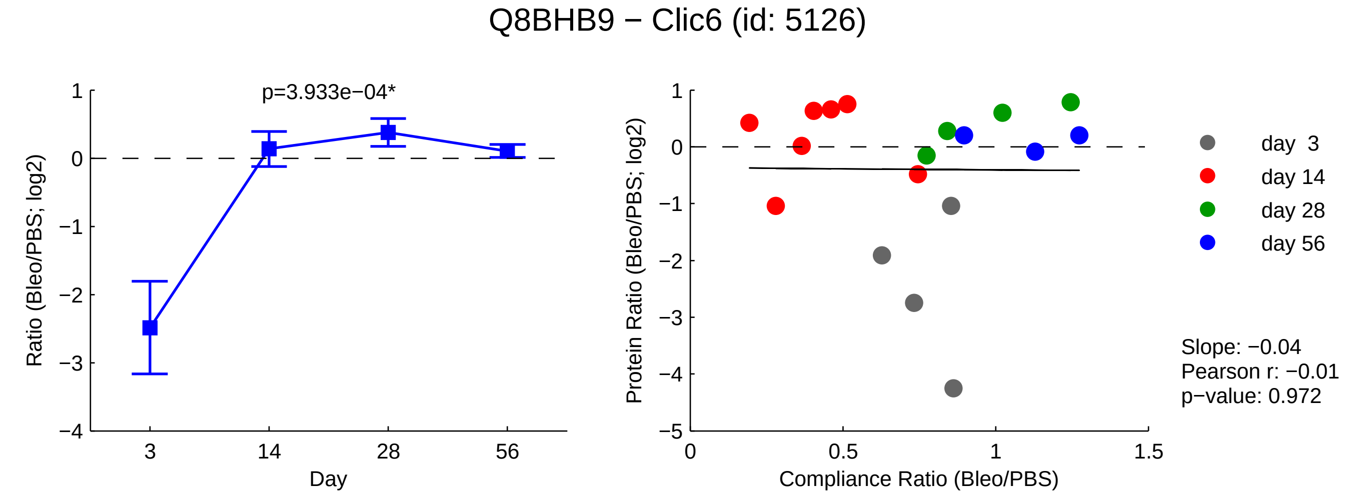
Q8BHB9 − Clic6 (id: 5126)
p=3.933e−04*
Ratio (Bleo/PBS; log2)
Day
Protein Ratio (Bleo/PBS; log2)
Compliance Ratio (Bleo/PBS)
day 3
day 14
day 28
day 56
Slope: −0.04
Pearson r: −0.01
p−value: 0.972

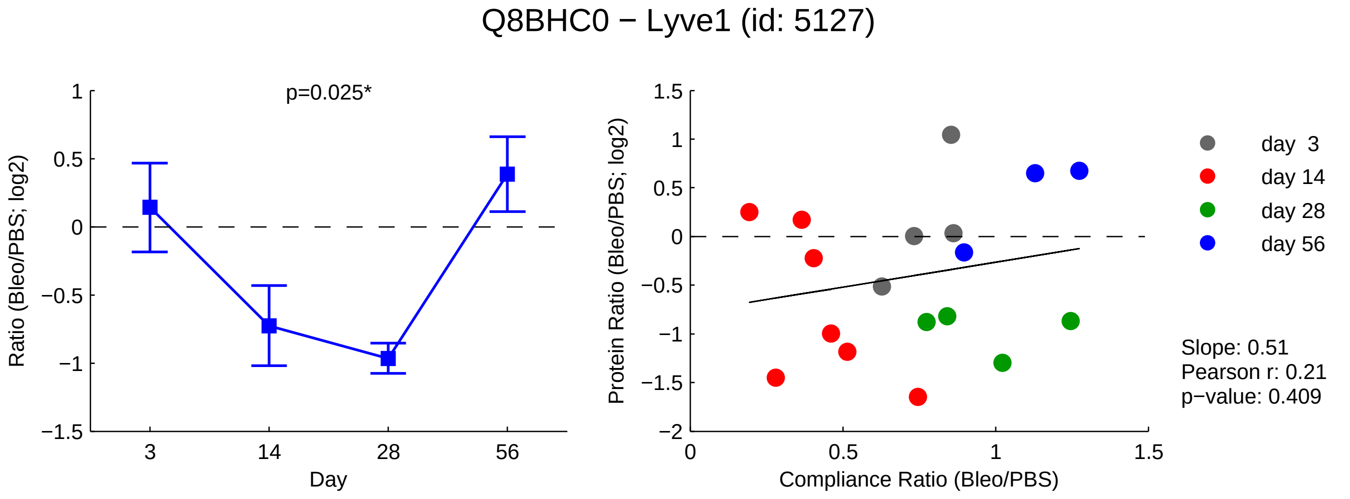
Q8BHC0 − Lyve1 (id: 5127)
p=0.025*
Ratio (Bleo/PBS; log2)
Day
Protein Ratio (Bleo/PBS; log2)
Compliance Ratio (Bleo/PBS)
day 3
day 14
day 28
day 56
Slope: 0.51
Pearson r: 0.21
p−value: 0.409

Q8BHC4 − Dcakd (id: 5128)

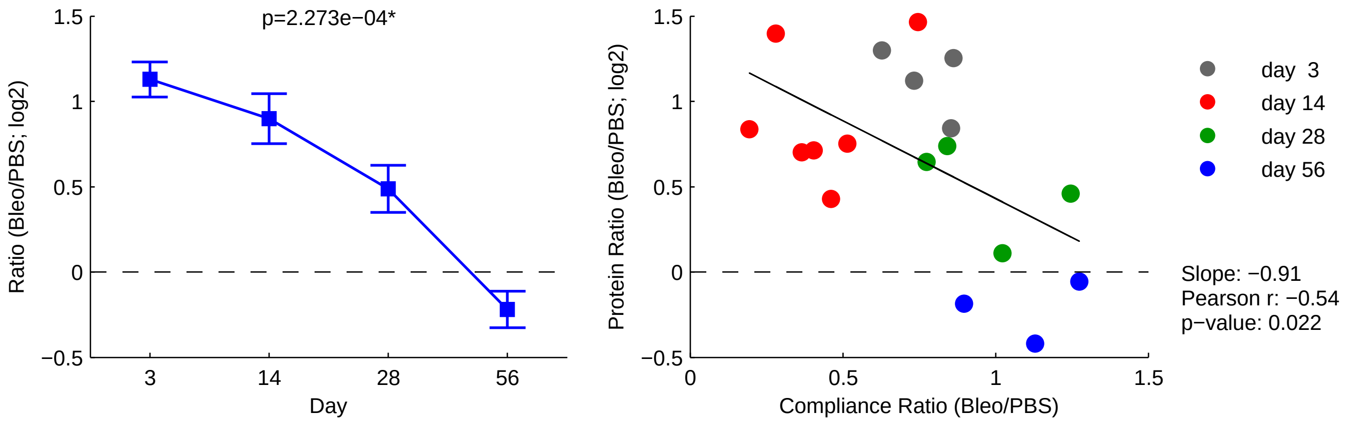

Q8BHC9 − Fut11 (id: 5129)

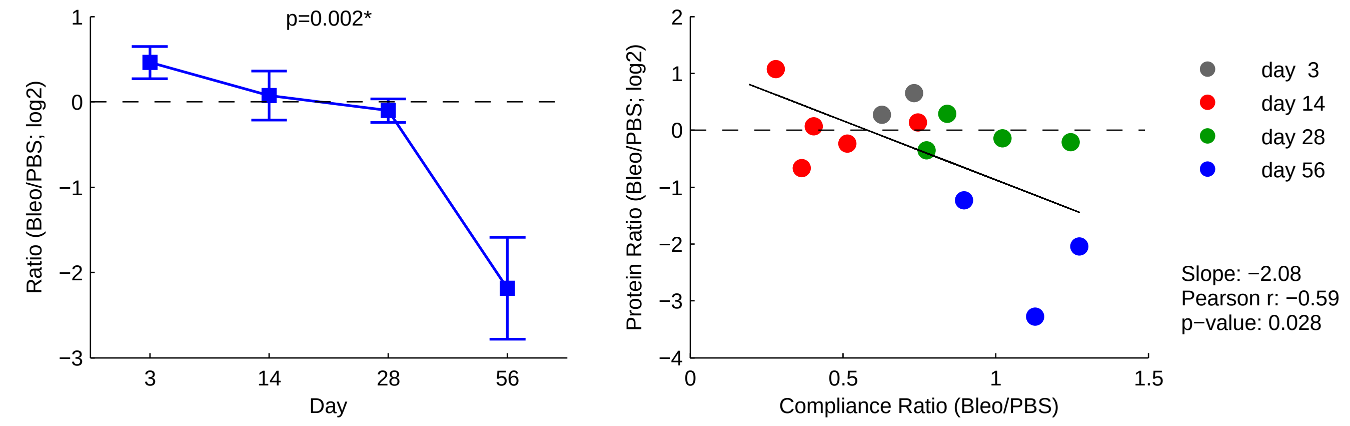

Q8BHD0 − Rab39a (id: 5130)

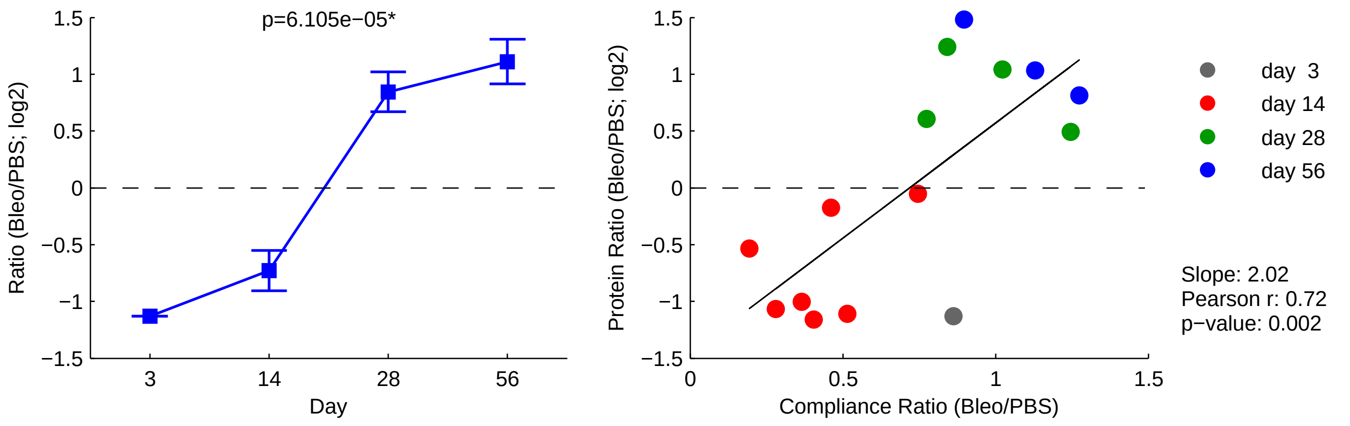

Q8BHE5 − Bmp3 (id: 5131)

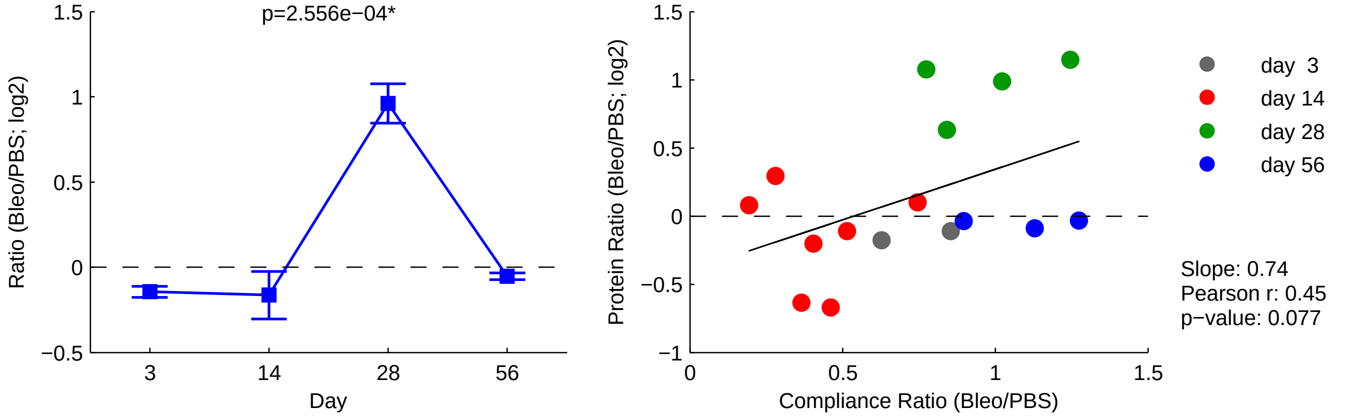

Q8BHE8 − (id: 5132)

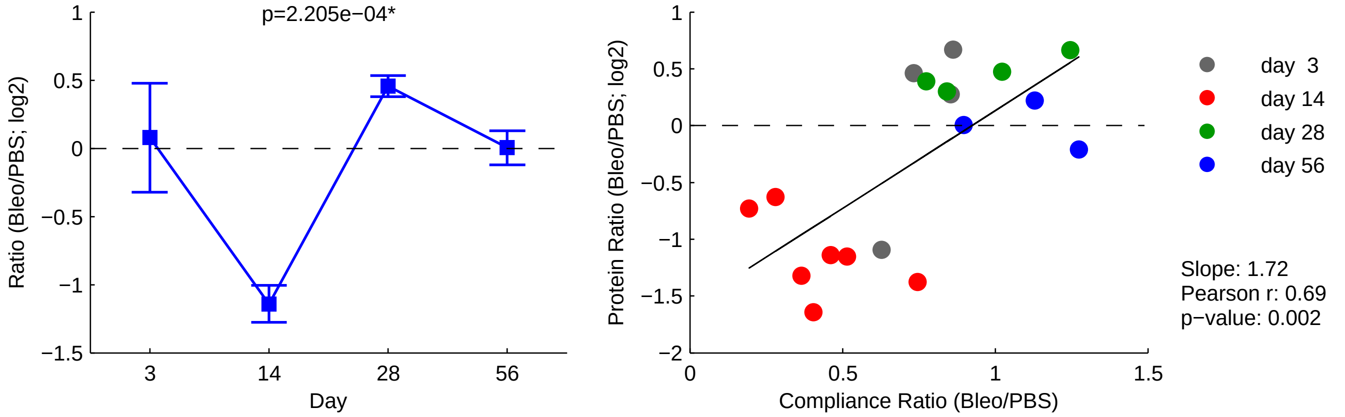

Q8BHF7 − Pgs1 (id: 5133)

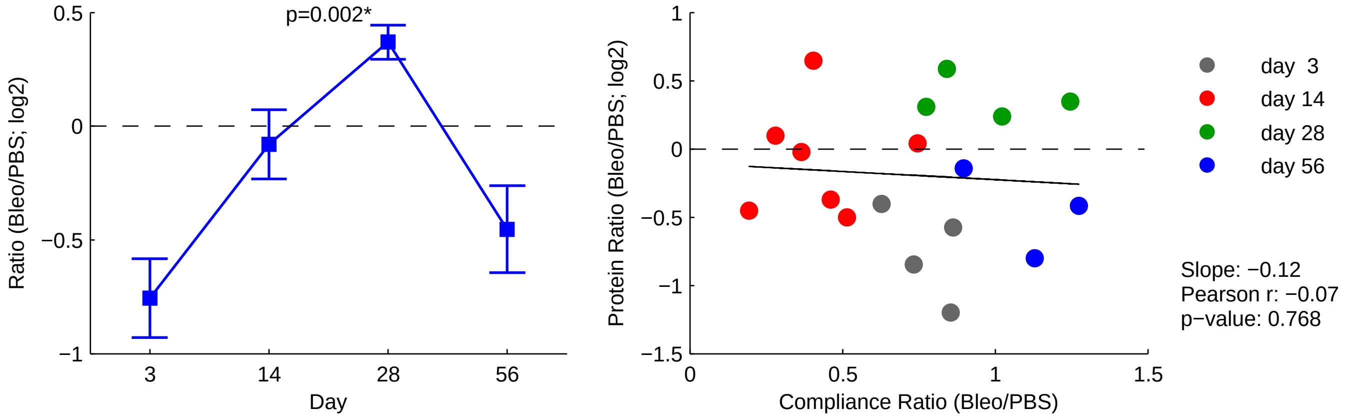

Q8BHG9 − Cggbp1 (id: 5134)

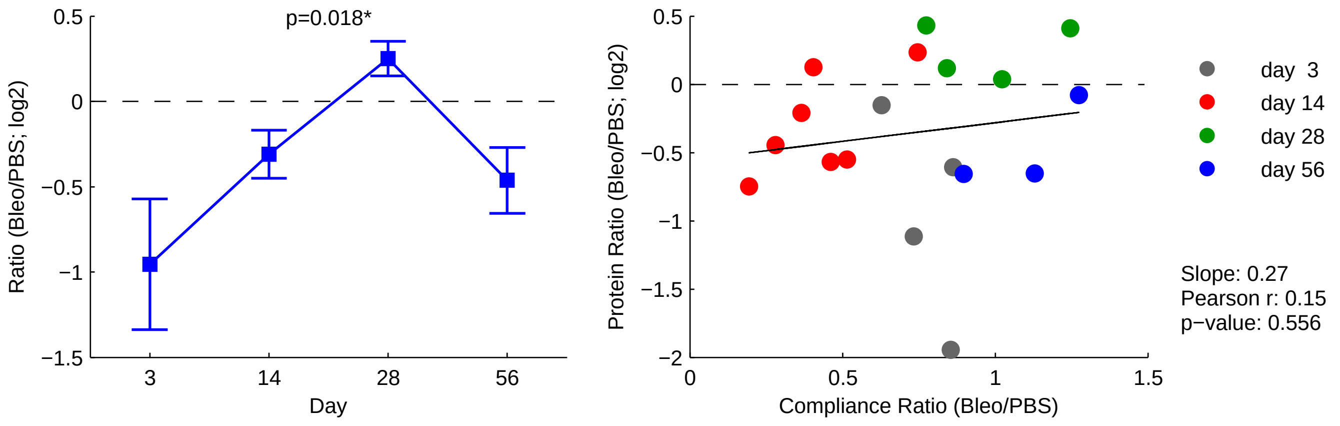

Q8BHK3−2 − Slc36a2 (id: 5137)

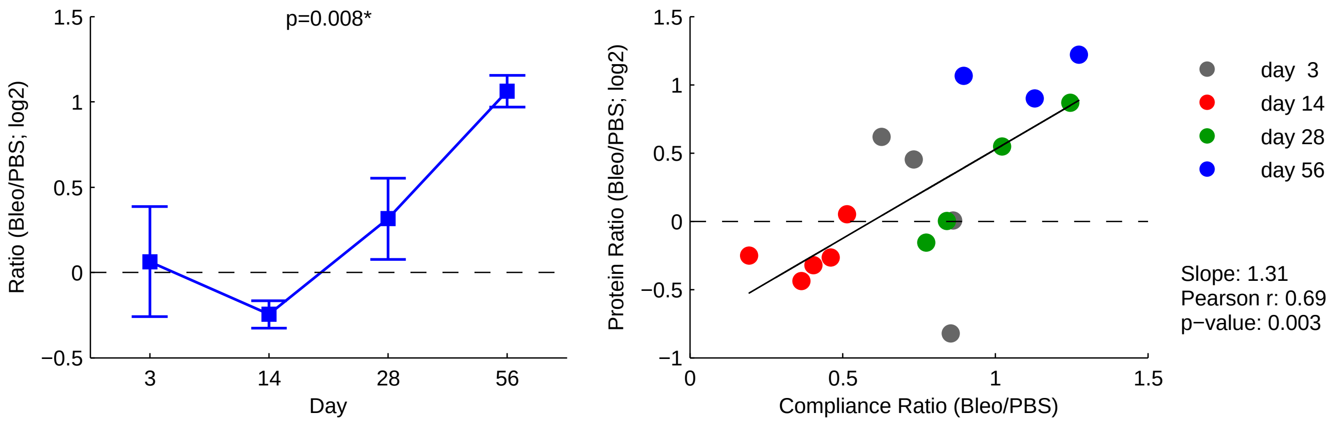

Q8BHL3 − Tbc1d10b (id: 5138)

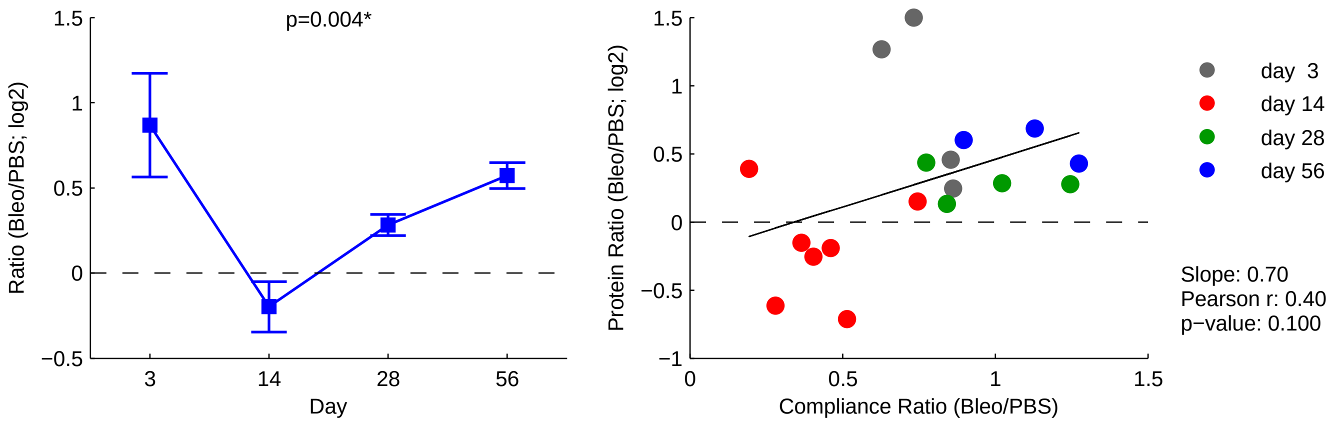

Q8BHN0 − Ppm1l (id: 5140)

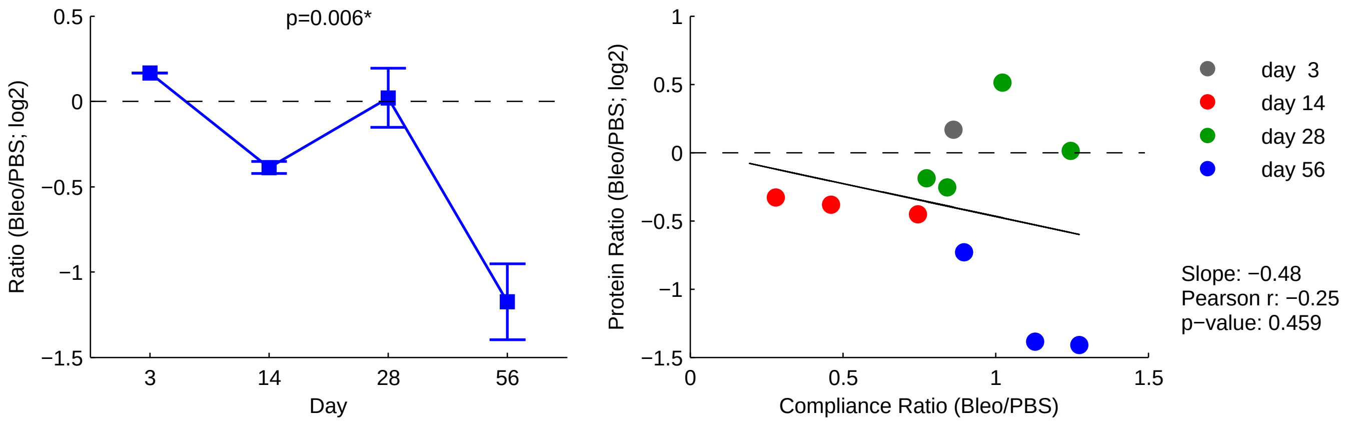

Q8BHN1−3 − Txlng (id: 5141)

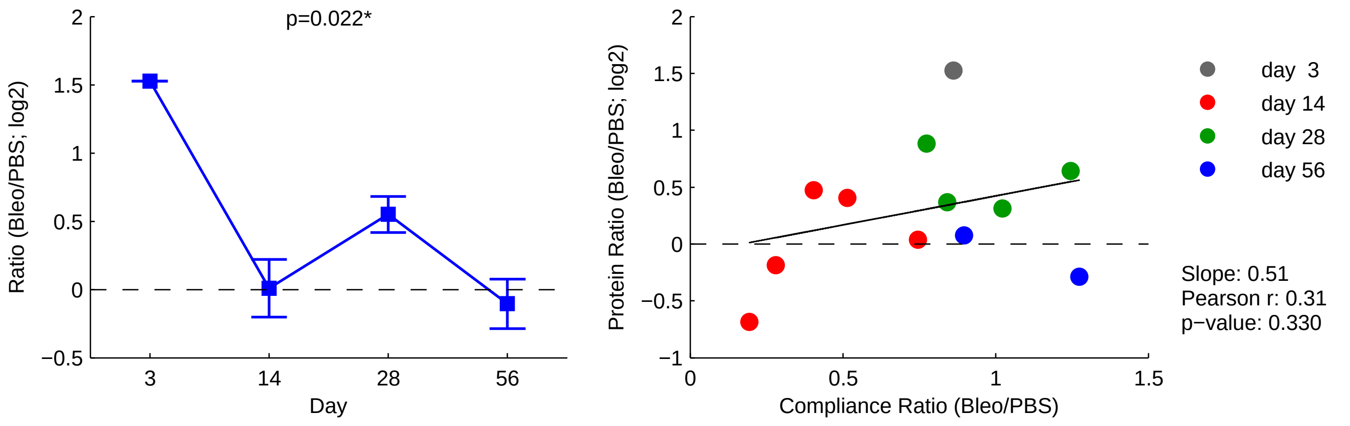

Q8BHN3−2 − (id: 5143)

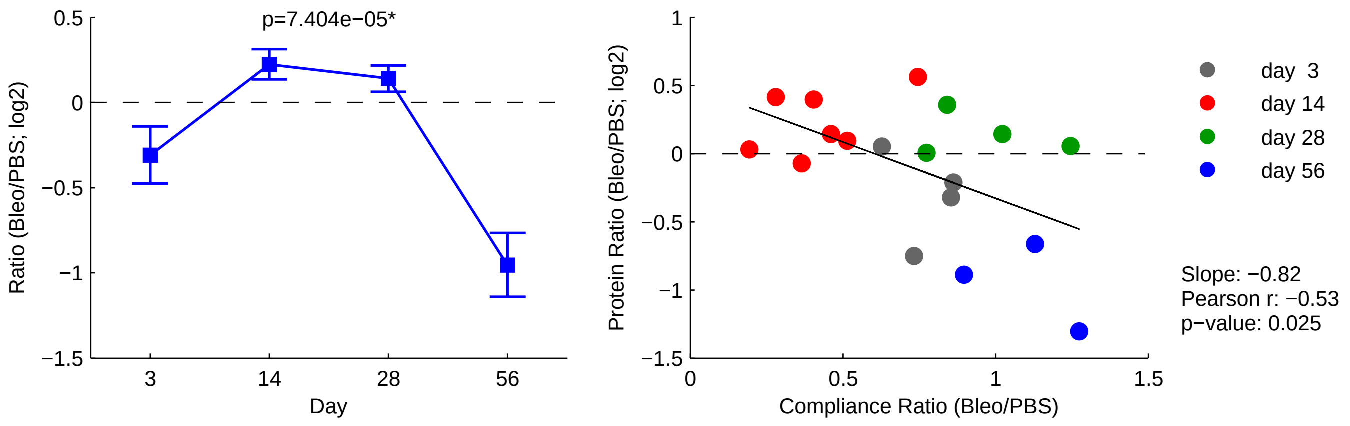

Q8BHT6 − B3galtl (id: 5147)

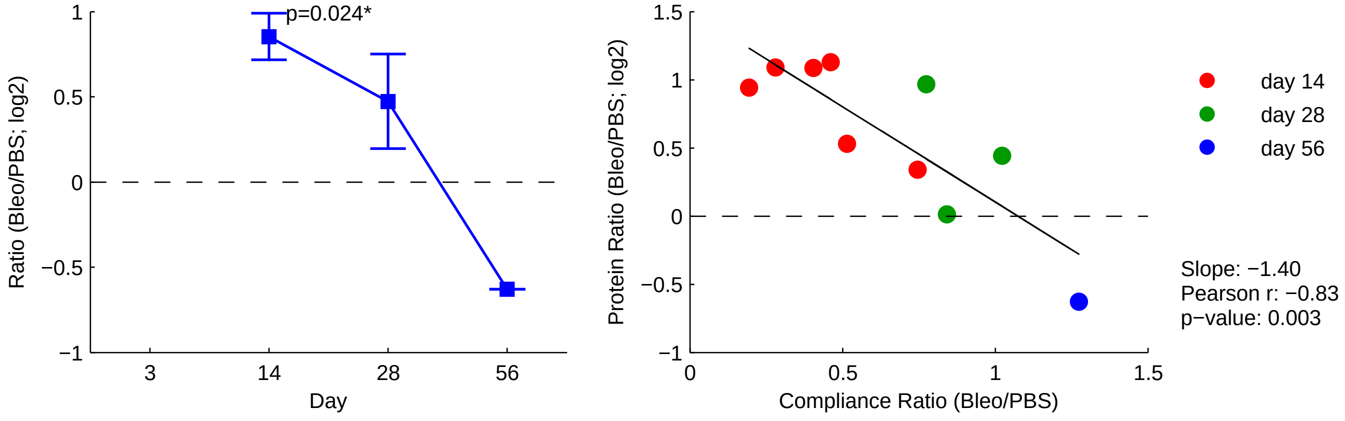

Q8BHY3 − Ano1 (id: 5151)

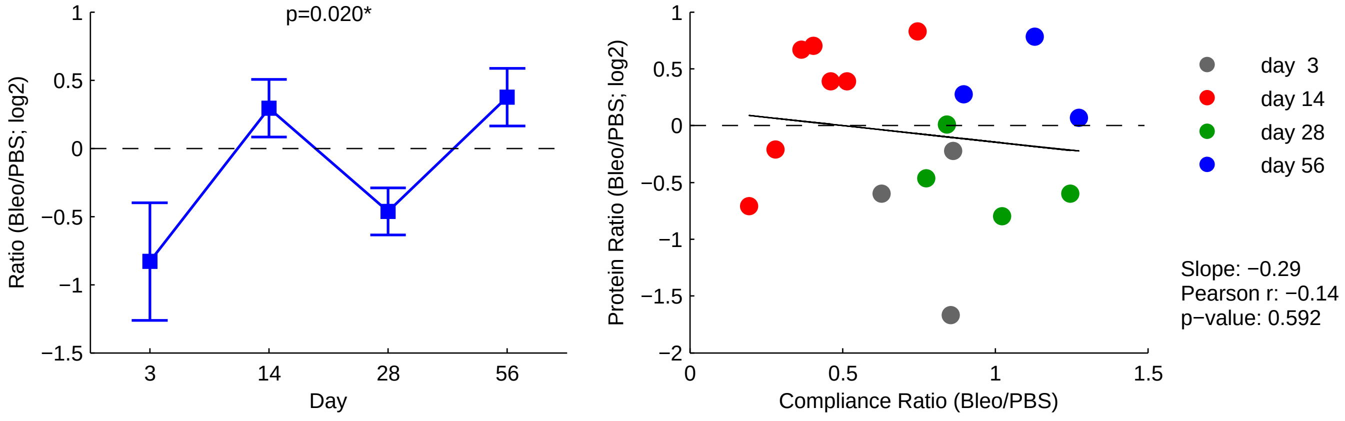

Q8BHZ0 − Fam49a (id: 5152)

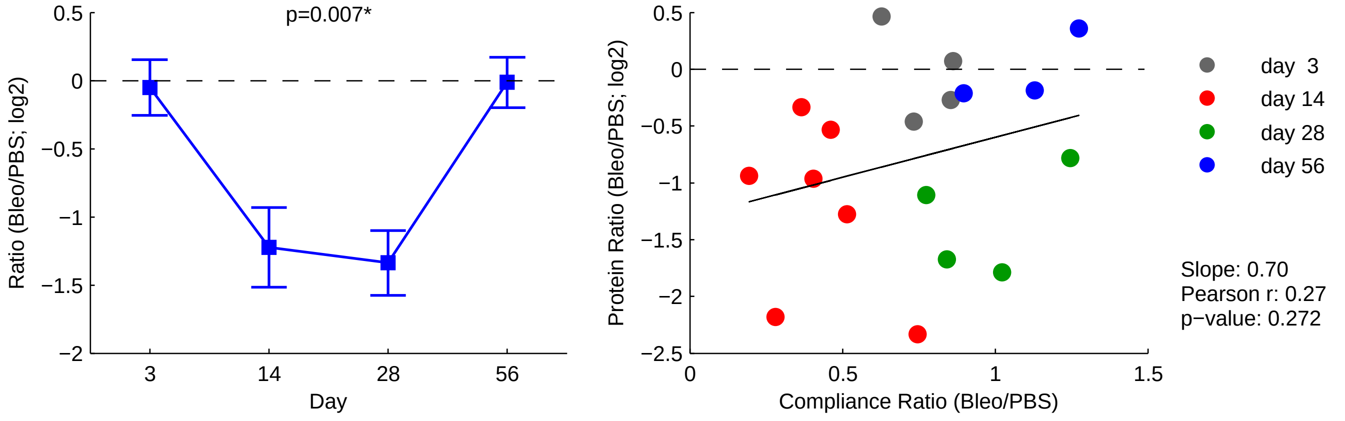

Q8BI08 − Mal2 (id: 5155)

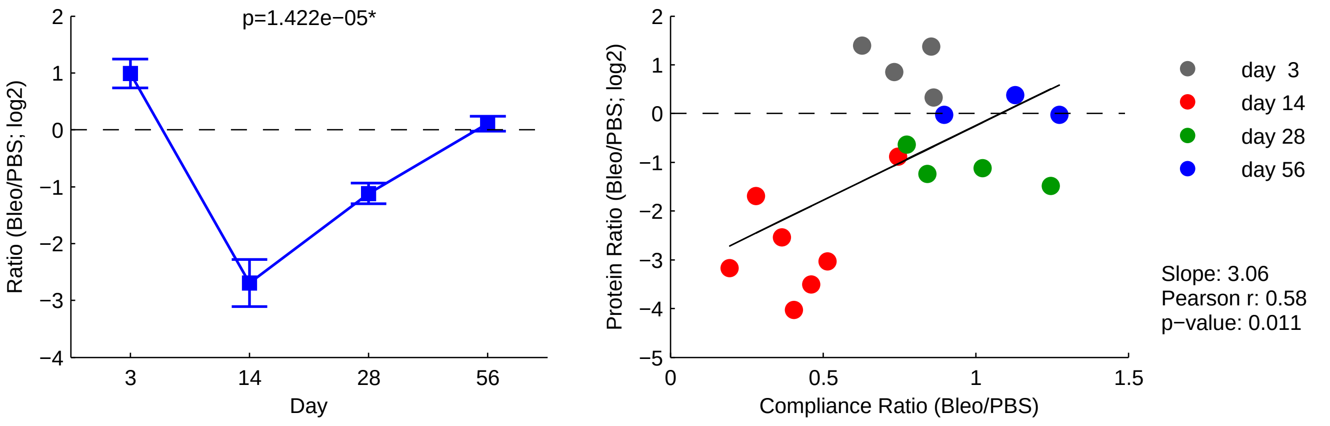

Q8BI22 − Cep128 (id: 5156)

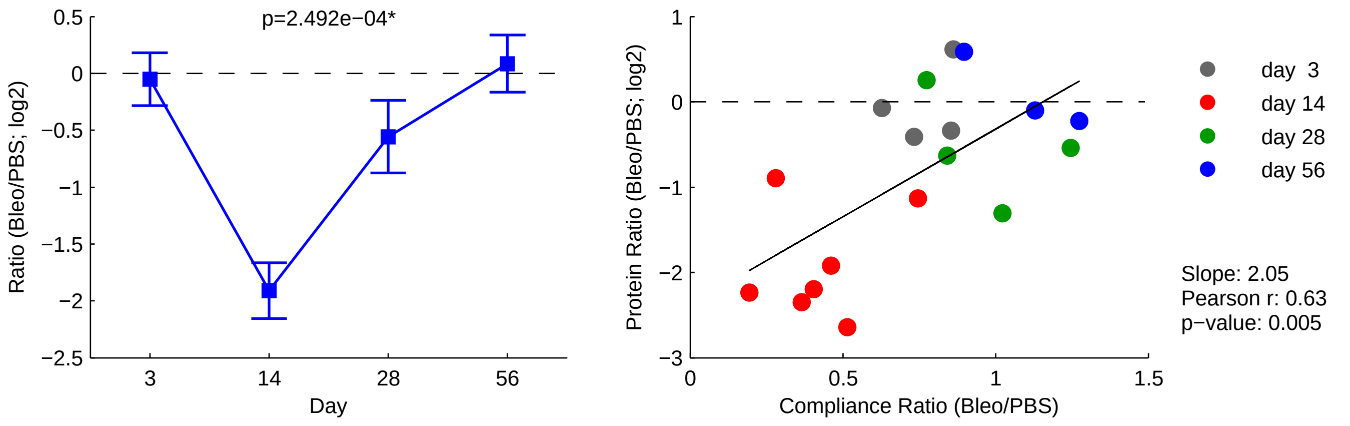

Q8BI29 − Sarg (id: 5157)

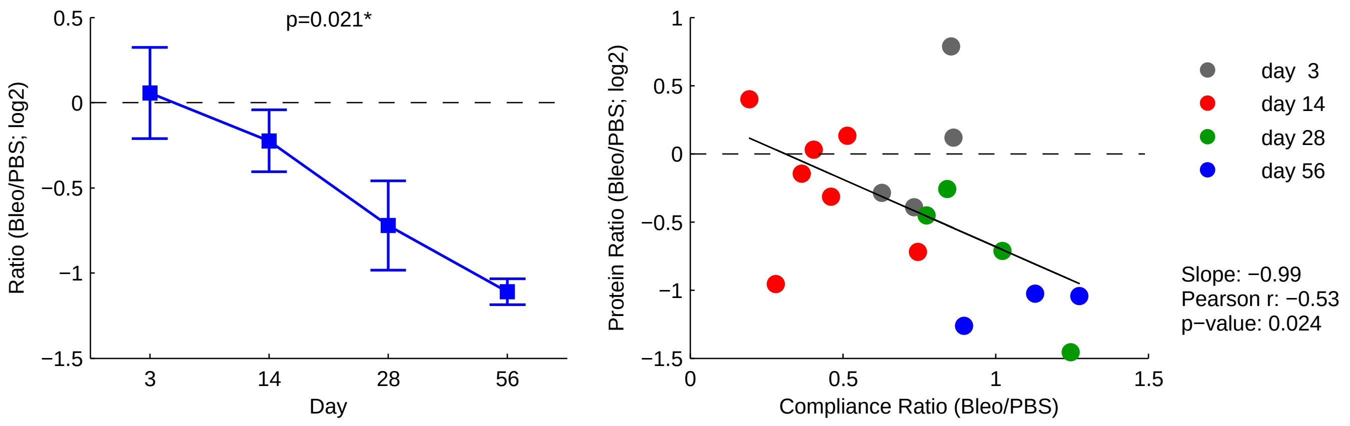

Q8BI72 − Cdkn2aip (id: 5158)

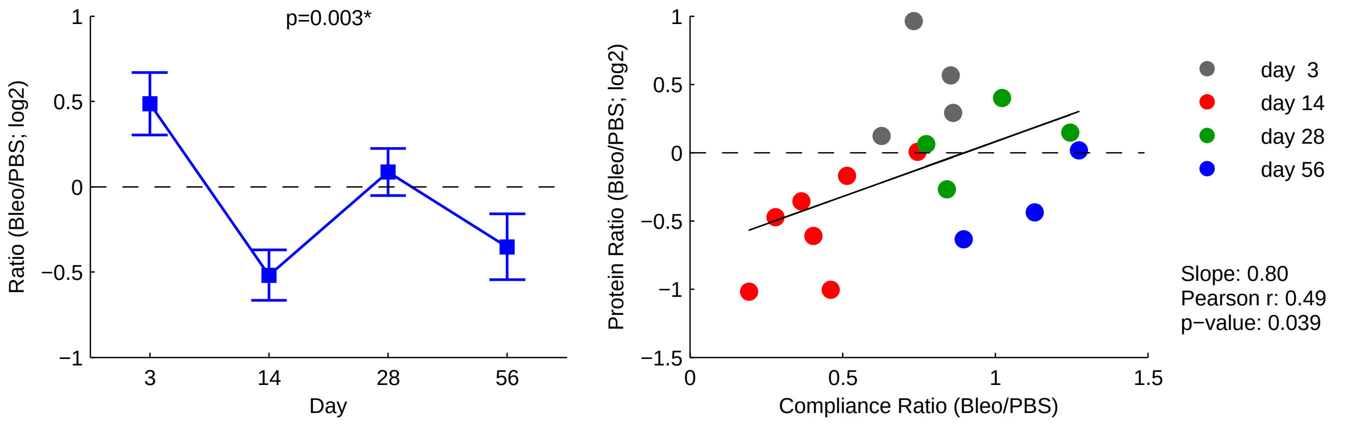

Q8BI79 − Ccdc40 (id: 5159)

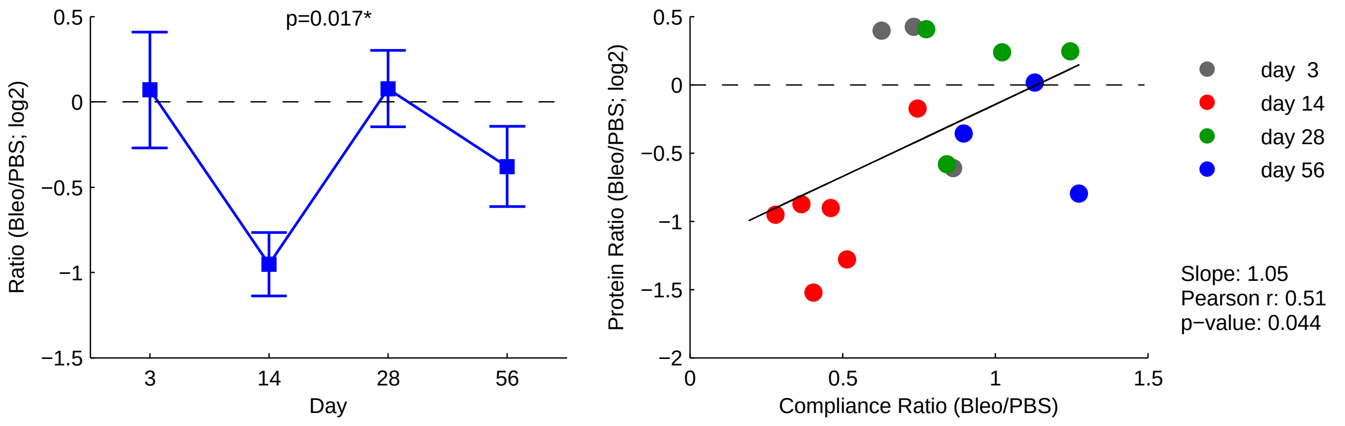

Q8BI84 − Mia3 (id: 5160)

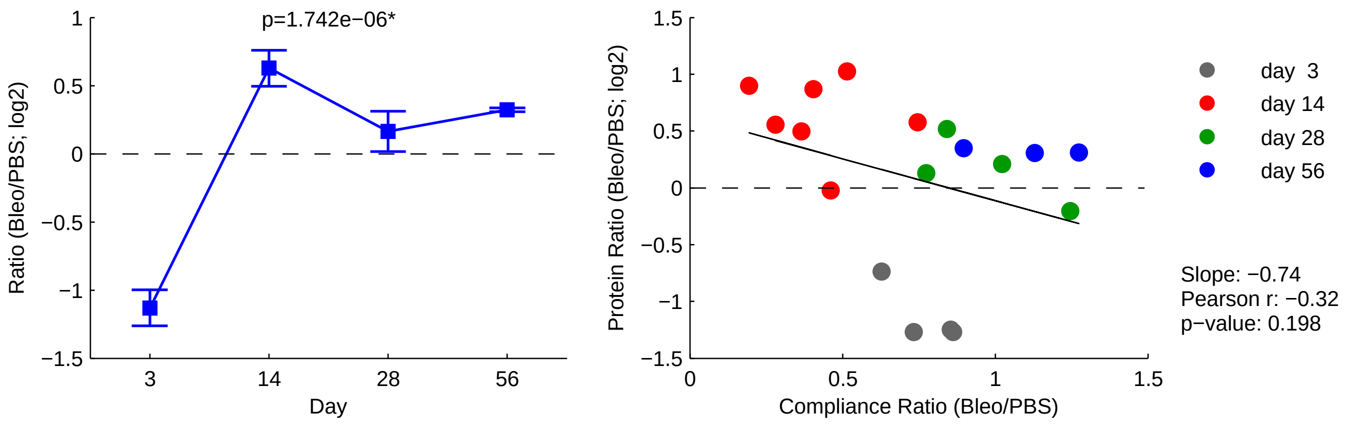

Q8BIG7 − Comtd1 (id: 5163)

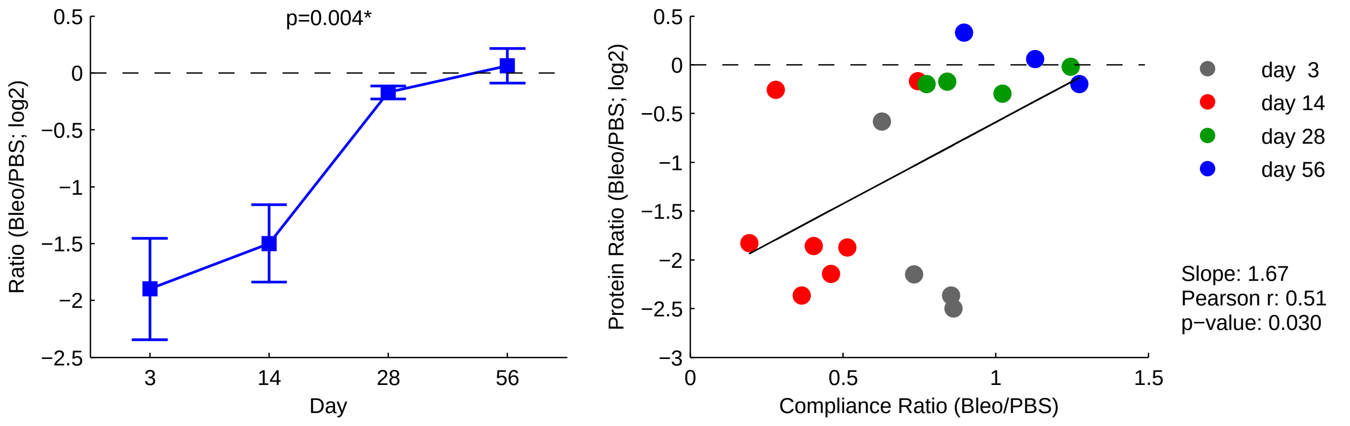

Q8BIJ6 − Iars2 (id: 5164)

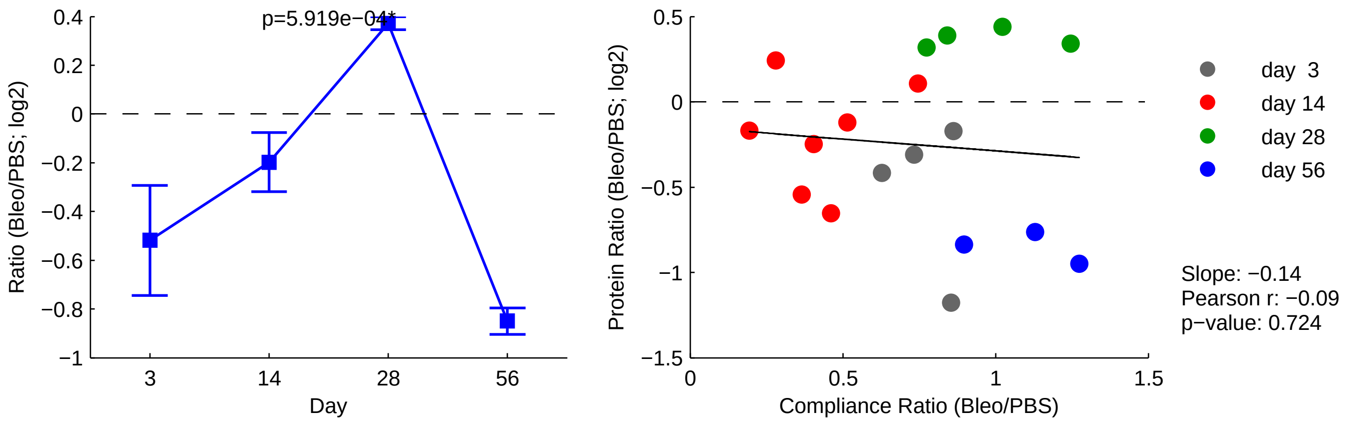

Q8BIJ7 − Rufy1 (id: 5165)

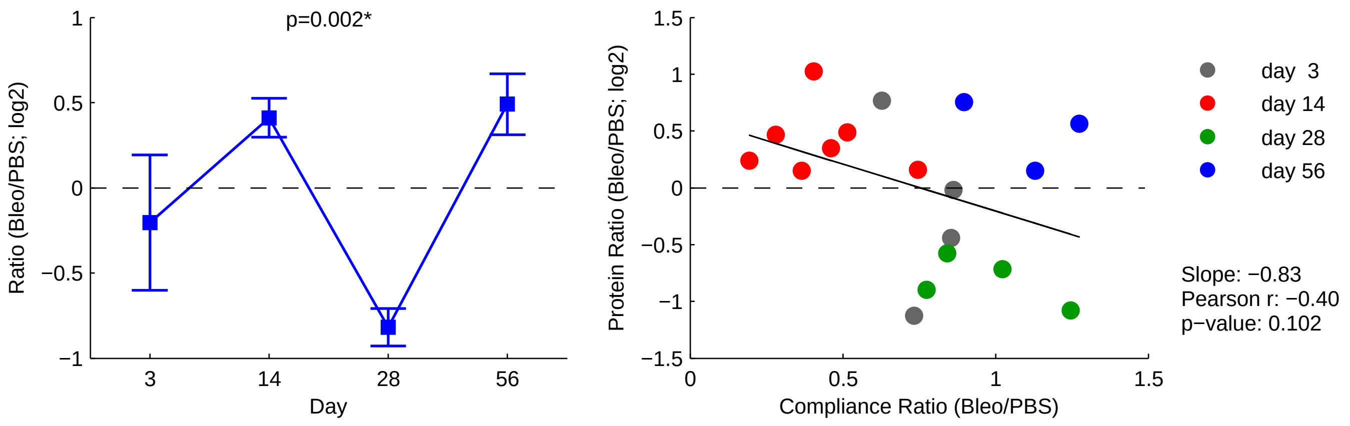

Q8BIP0 − Dars2 (id: 5166)

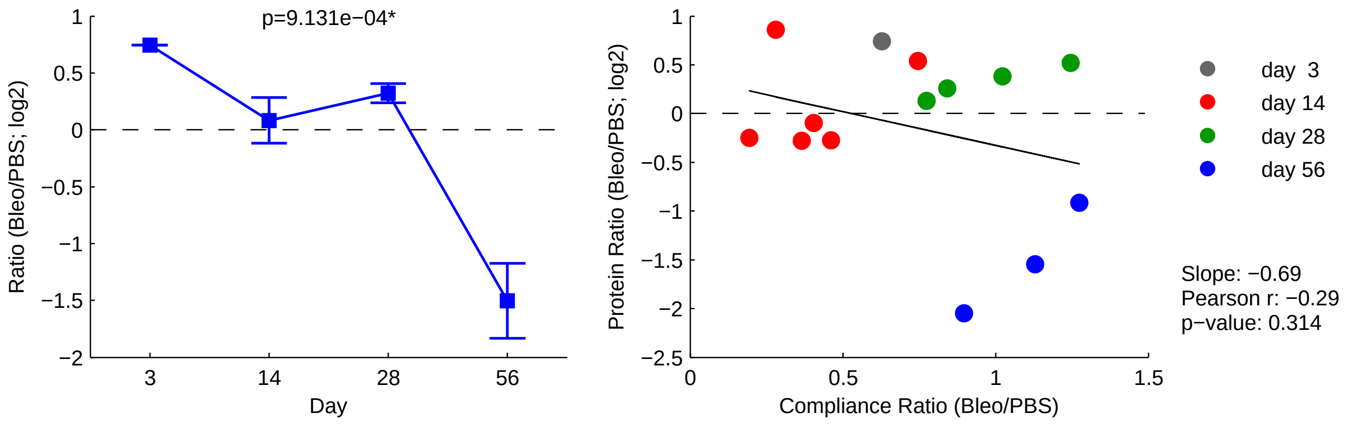

Q8BJ05 − Zc3h14 (id: 5172)

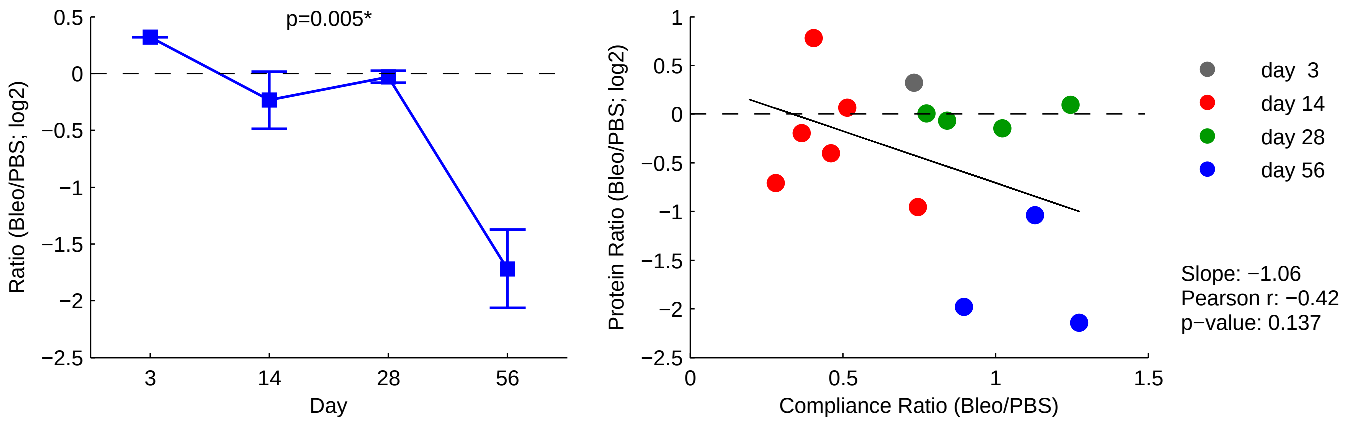

Q8BJ64 − Chdh (id: 5176)

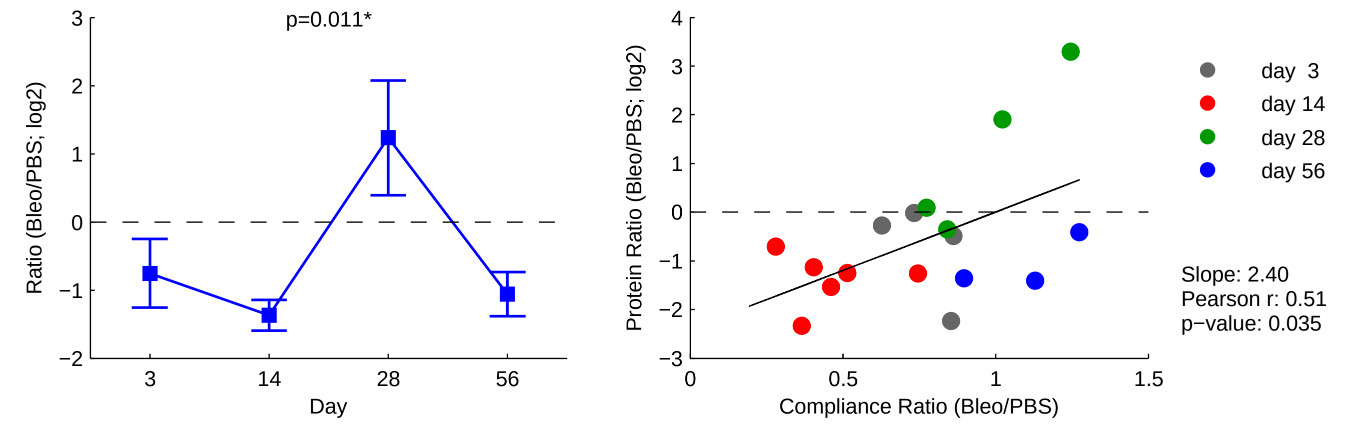

Q8BJ71 − Nup93 (id: 5177)

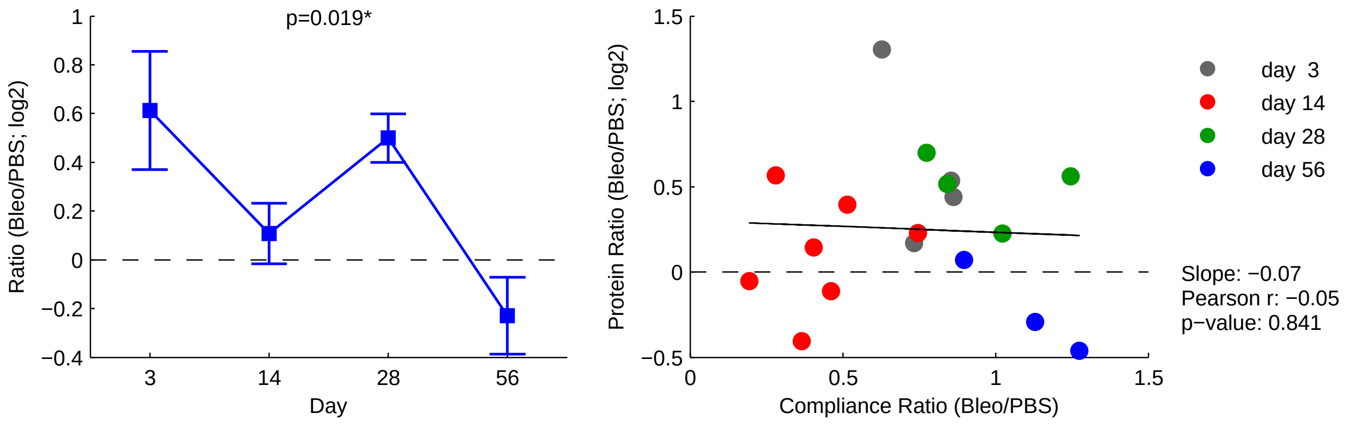

Q8BJD1 − Itih5 (id: 5178)

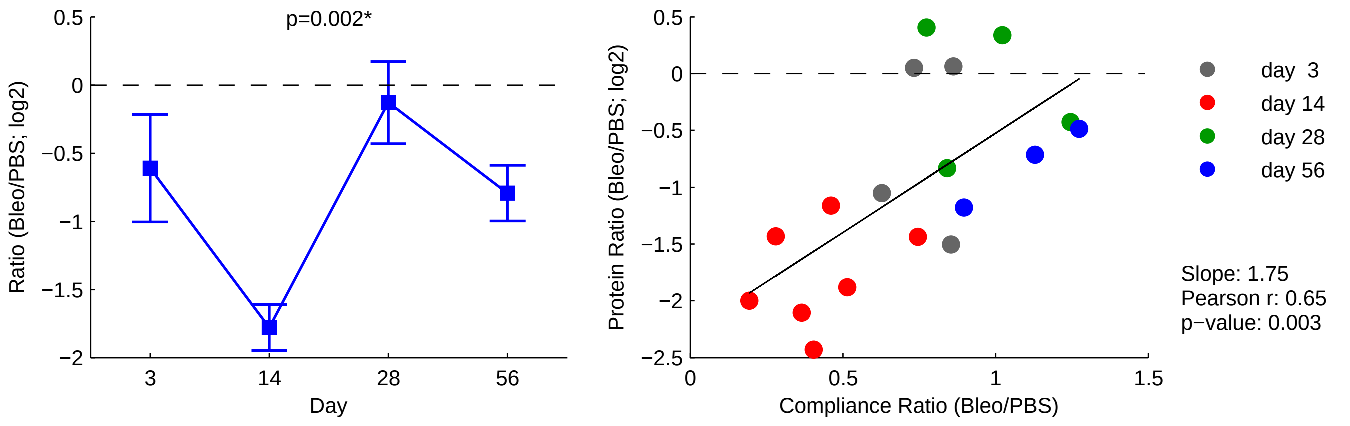

Q8BJU0−2 − Sgta (id: 5184)

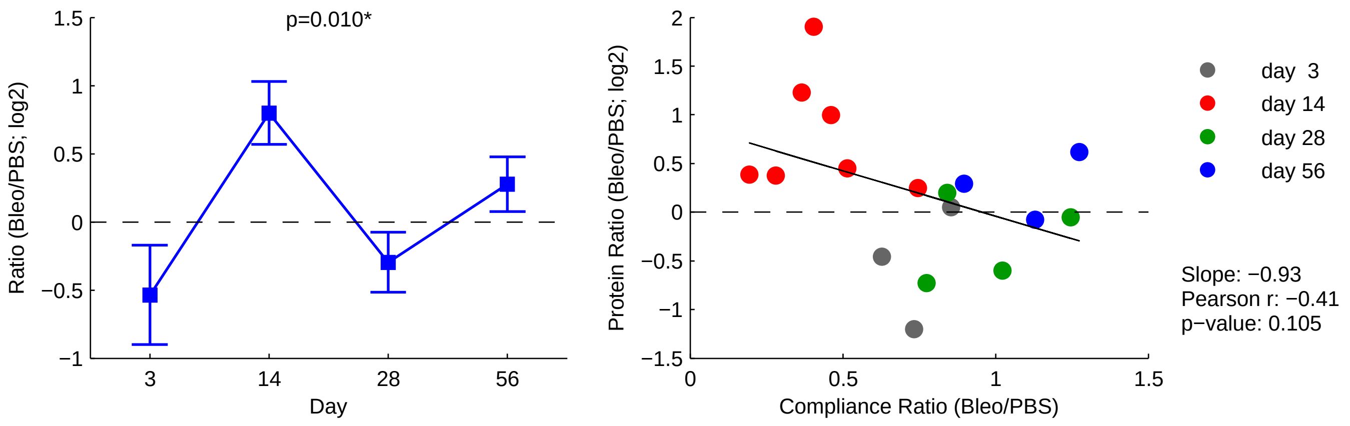

Q8BJW6 − Eif2a (id: 5188)

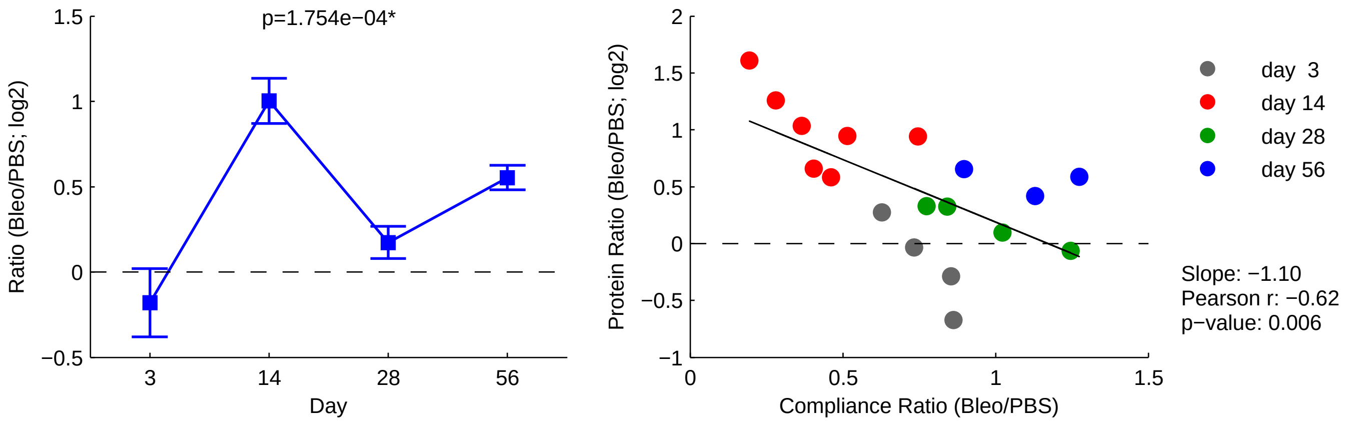

Q8BJY1 − Psmd5 (id: 5189)

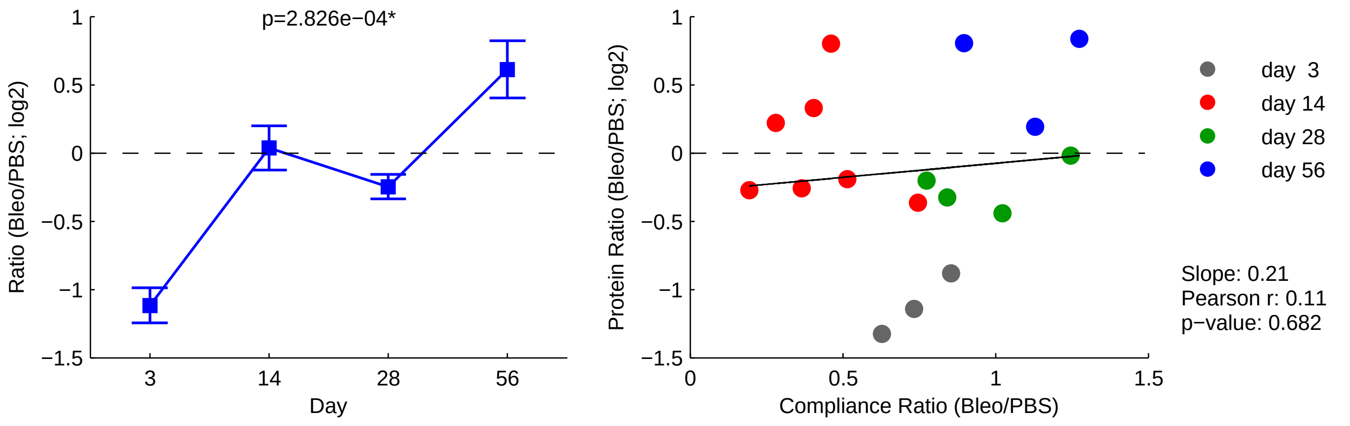

Q8BJZ4 − Mrps35 (id: 5190)

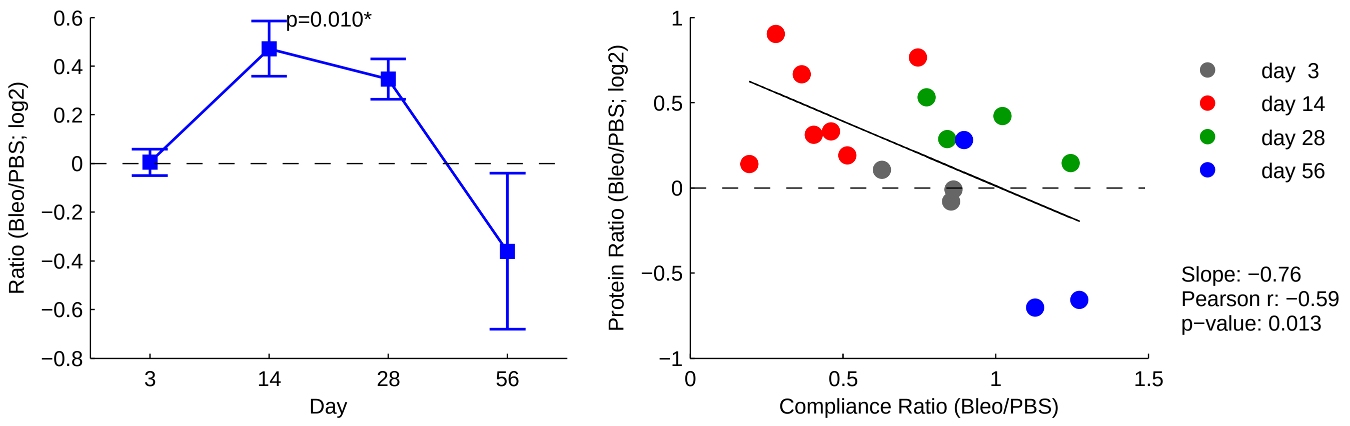

Q8BK03−2 − Fam73b (id: 5191)

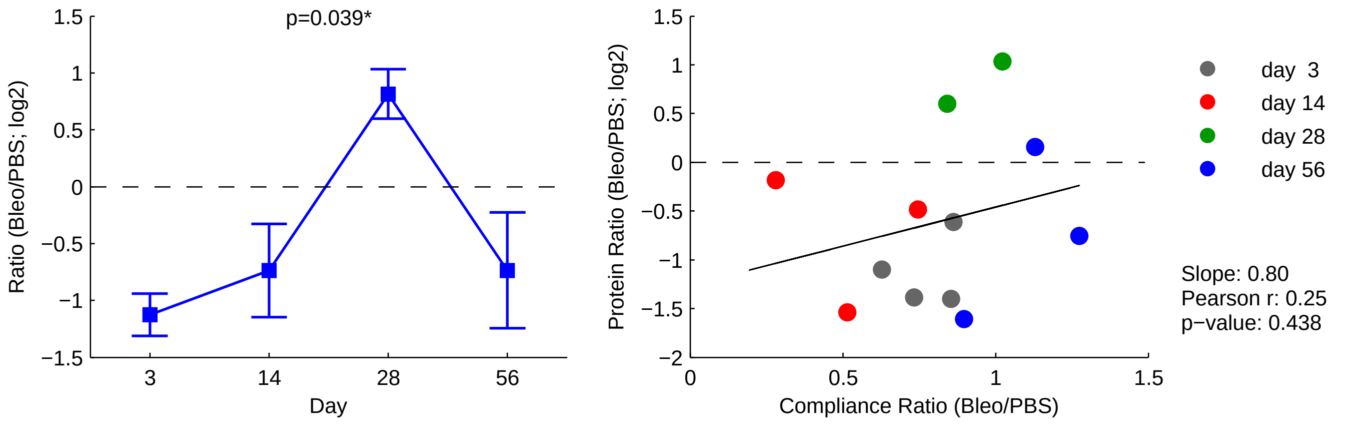

Q8BK62 − Olfml3 (id: 5195)

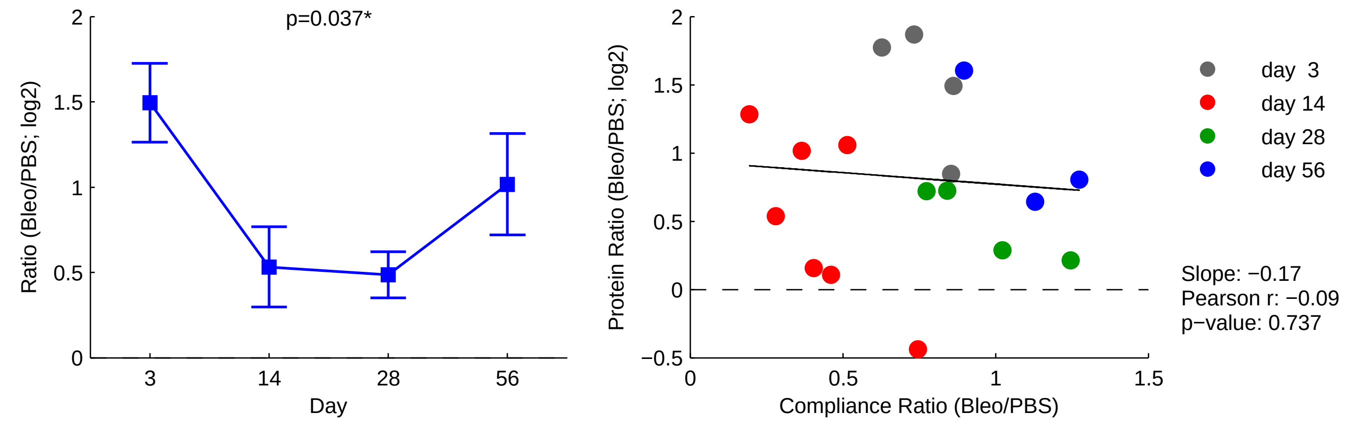

Q8BK64 − Ahsa1 (id: 5197)

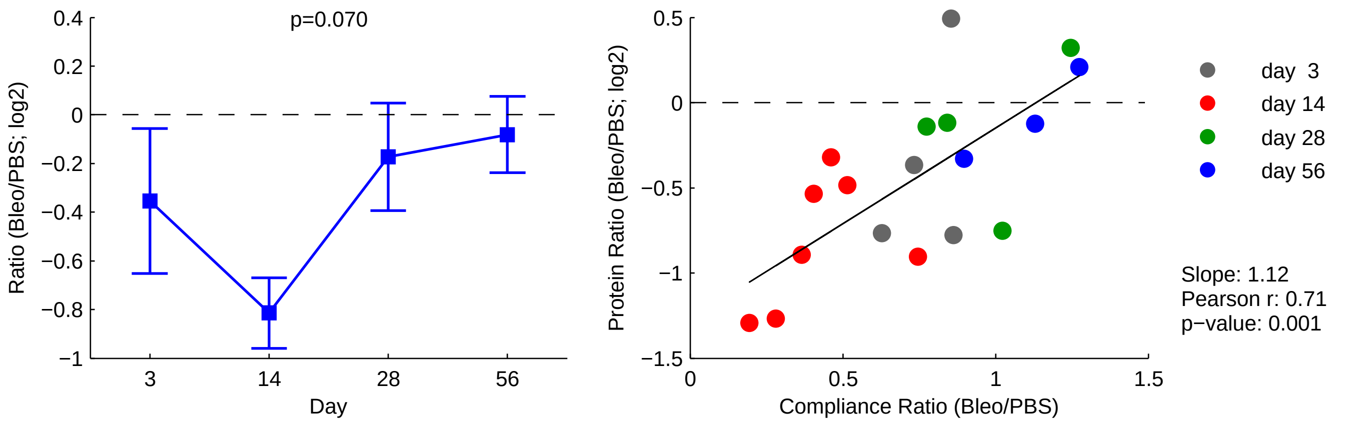

Q8BK67 − Rcc2 (id: 5198)

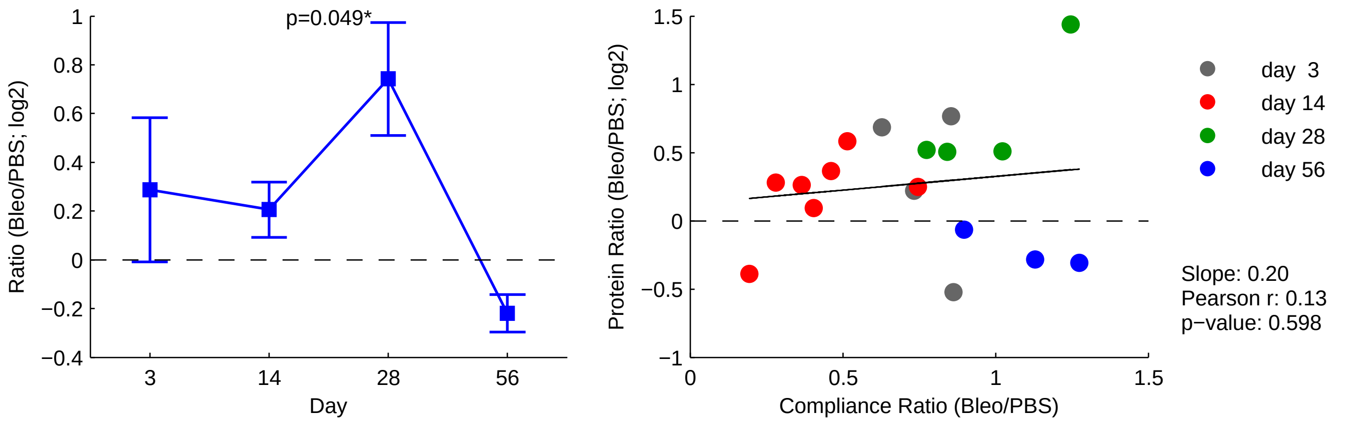

Q8BK72 − Mrps27 (id: 5199)

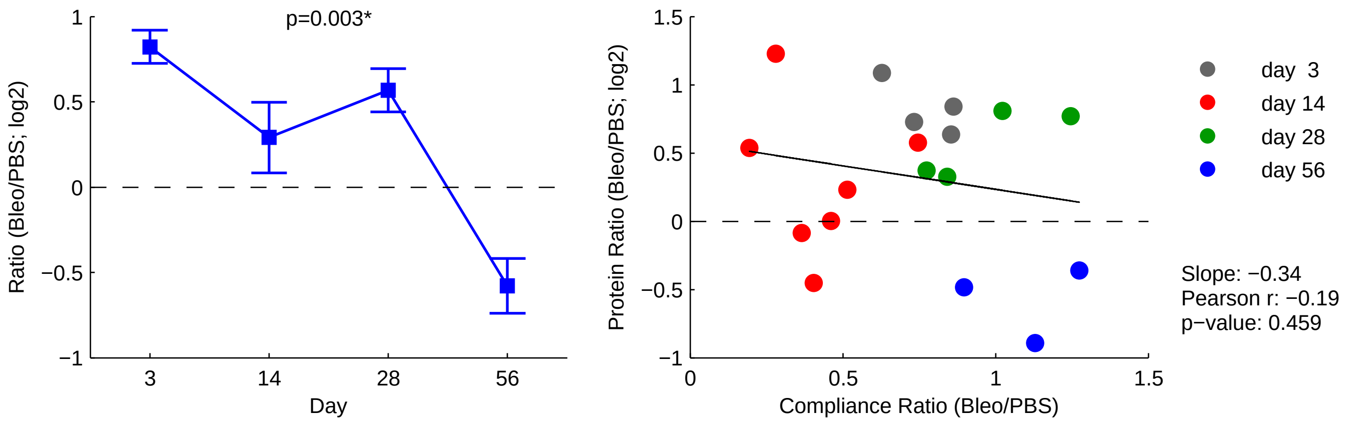

Q8BKC5 − Ipo5 (id: 5200)

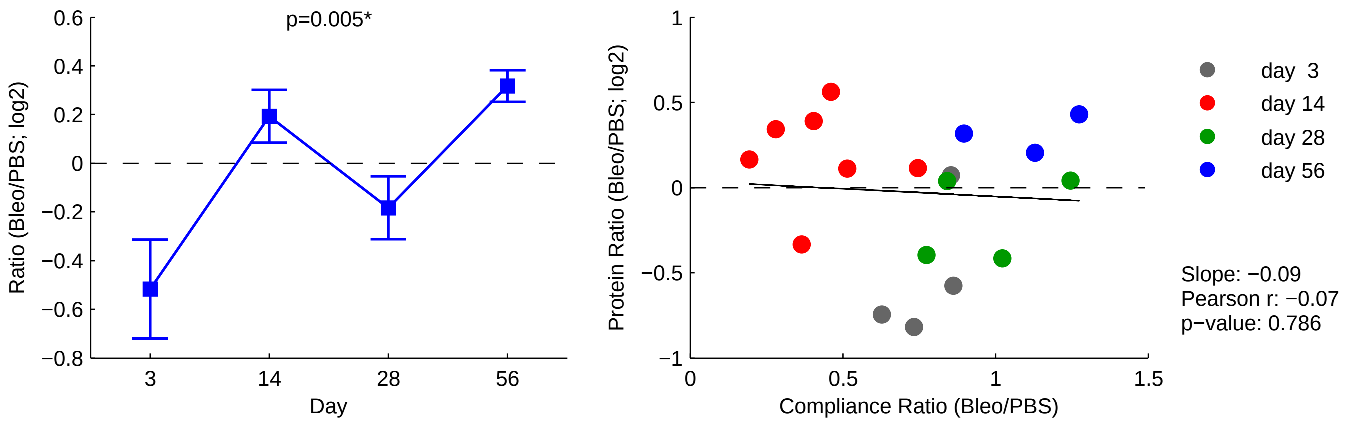

Q8BKE6 − Cyp20a1 (id: 5201)

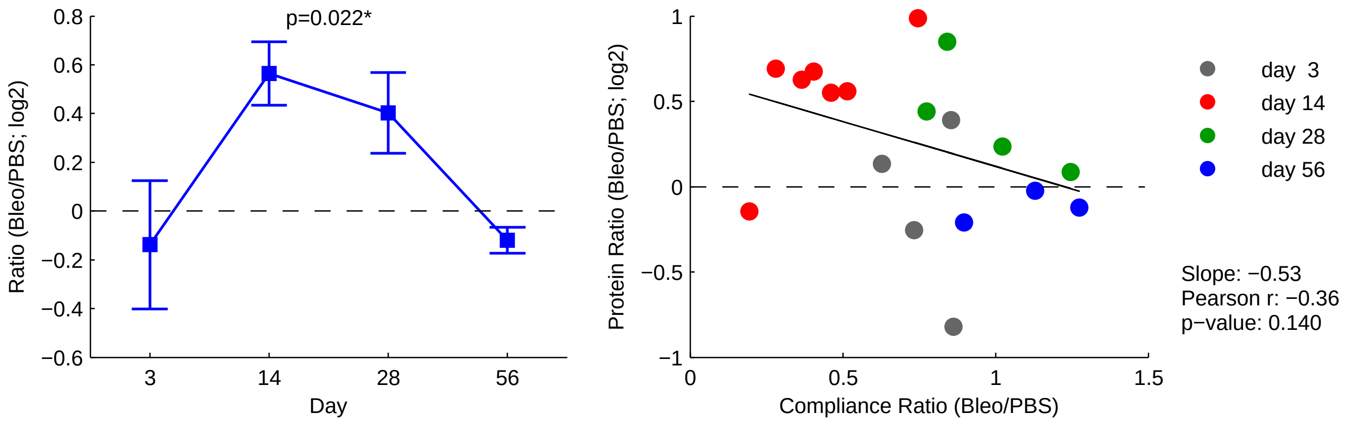

Q8BKG3 − Ptk7 (id: 5203)

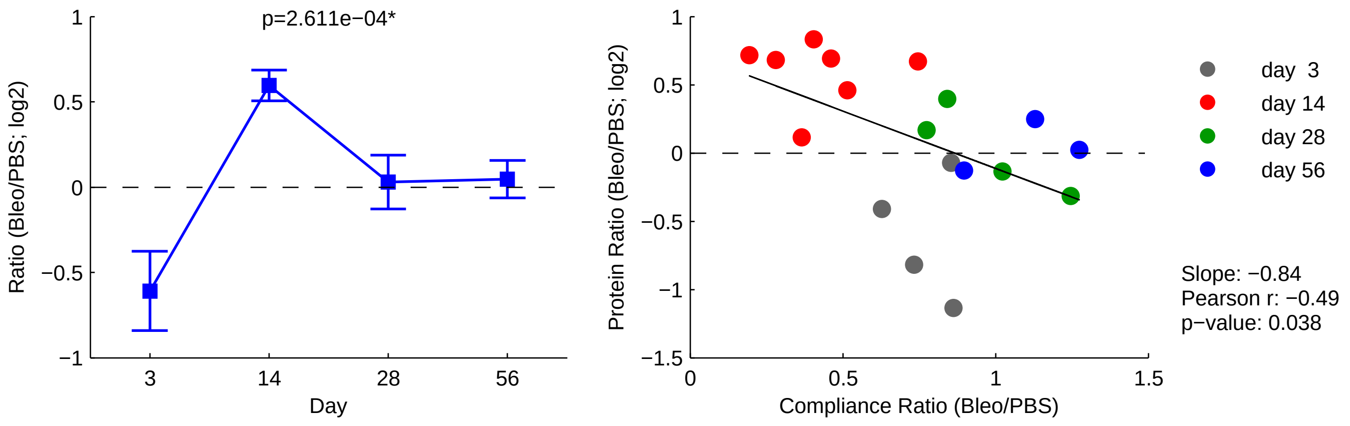

Q8BKJ9 − Sirt7 (id: 5204)

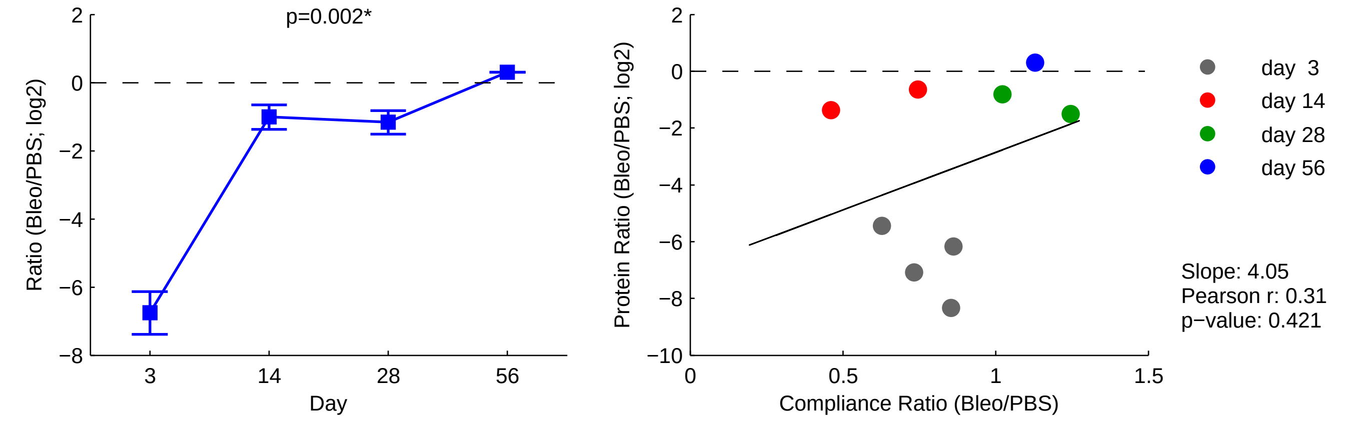

Q8BKU8−2 − Tmem87b (id: 5210)

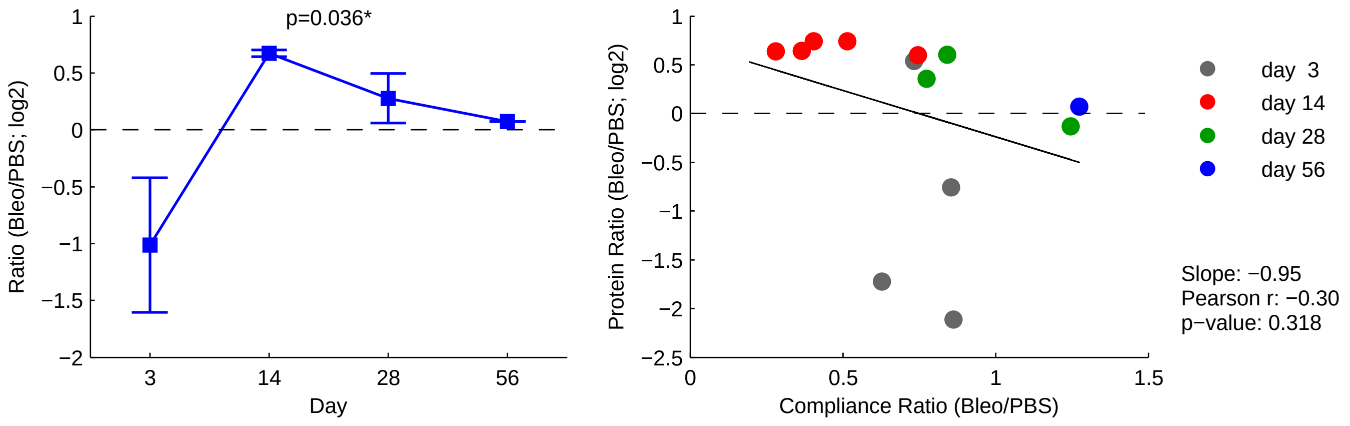

Q8BKZ9 − Pdhx (id: 5214)

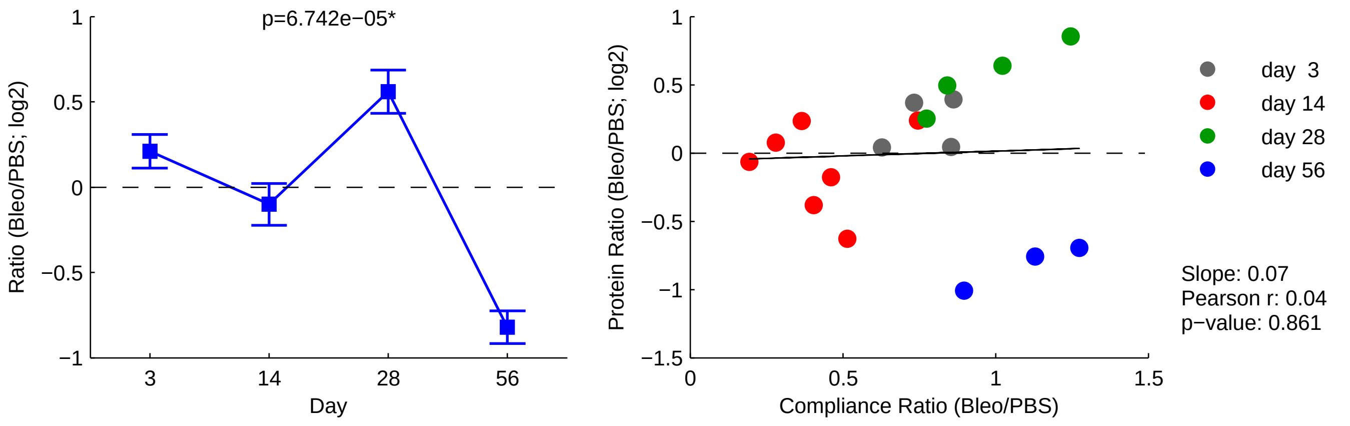

Q8BL66 − Eea1 (id: 5217)

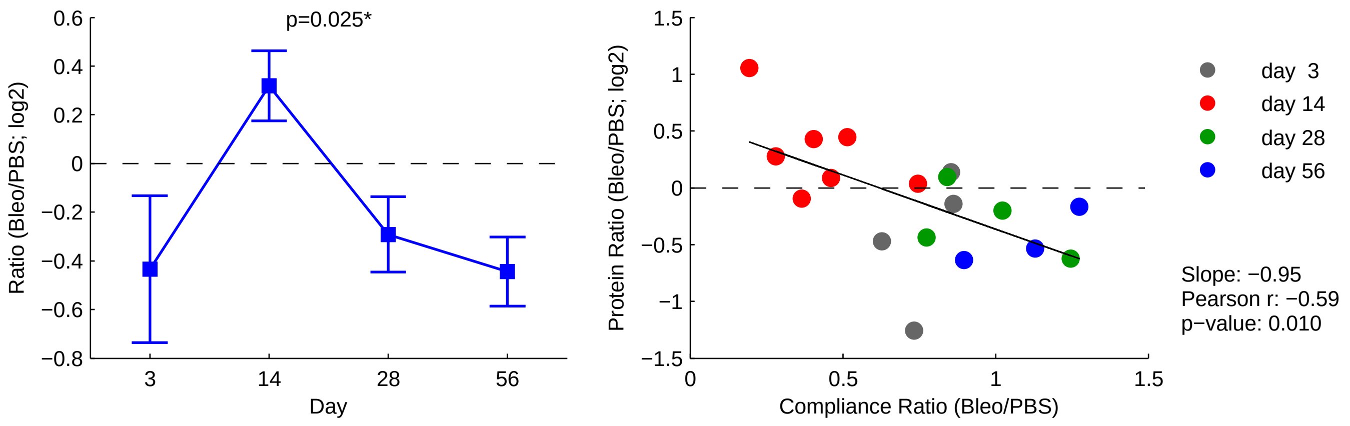

Q8BLX7 − Col16a1 (id: 5225)

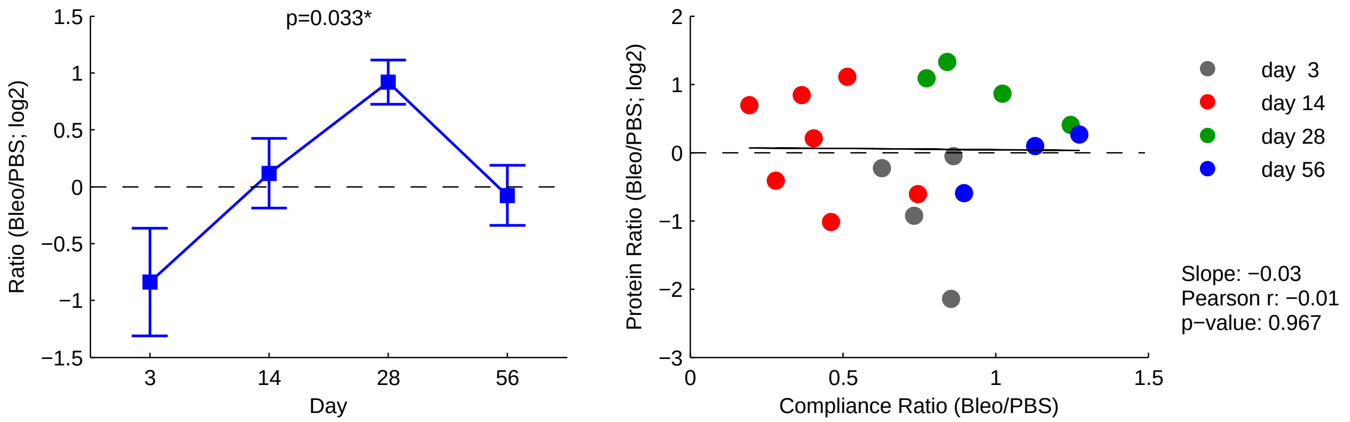

Q8BM39 − Prpf18 (id: 5226)

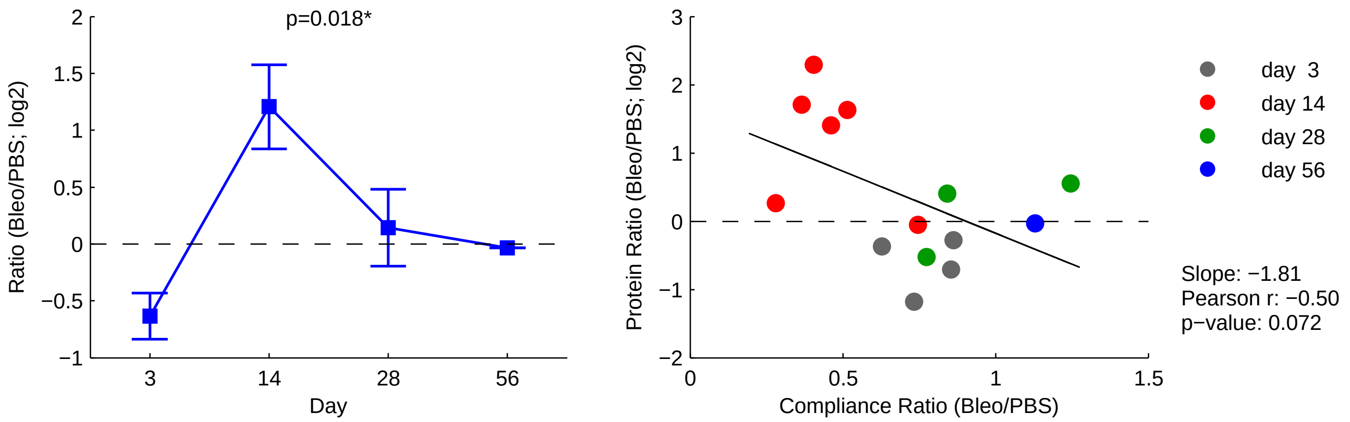

Q8BM55 − Tmem214 (id: 5228)

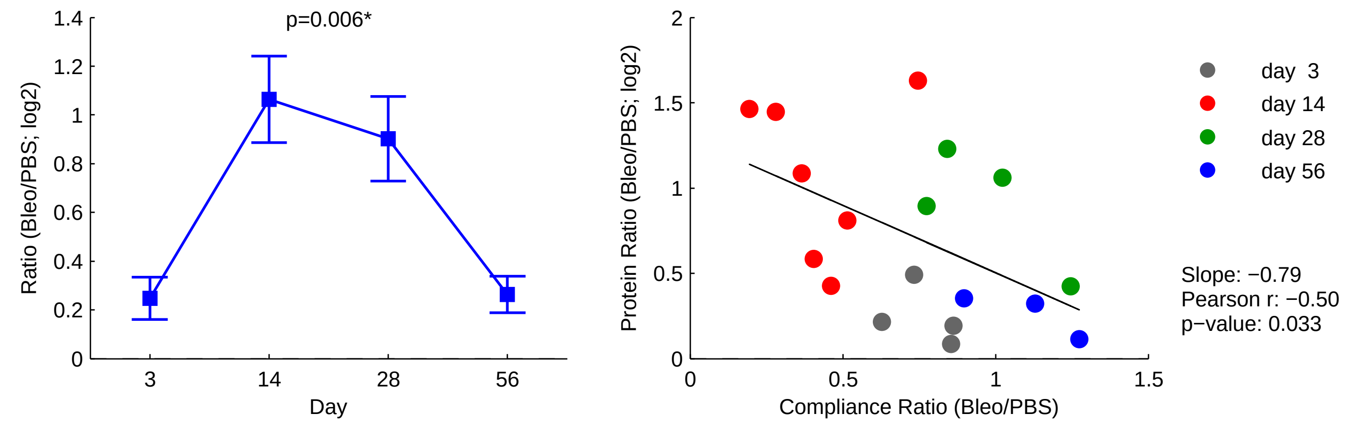

Q8BM72 − Hspa13 (id: 5229)

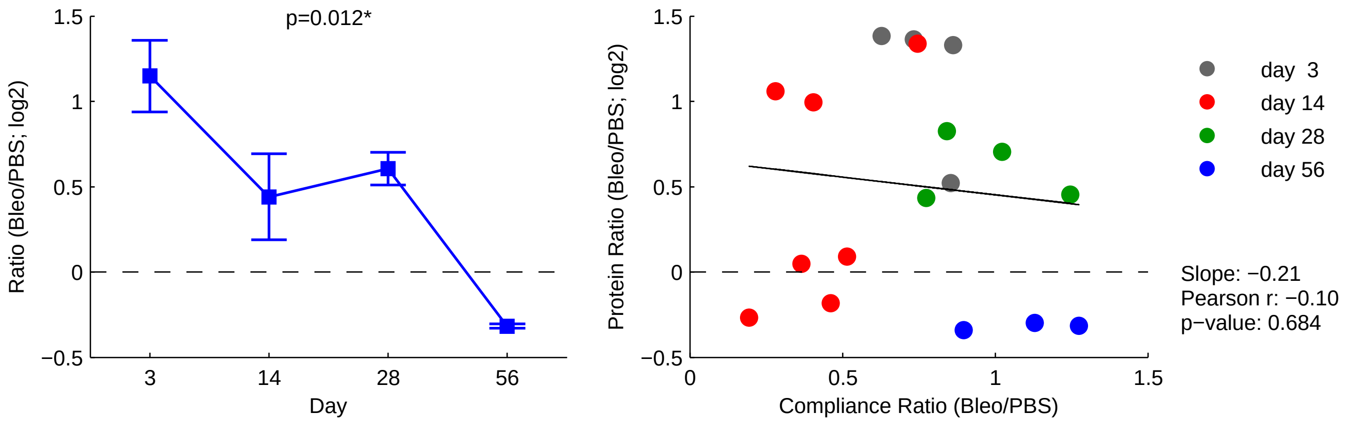

Q8BMA6 − Srp68 (id: 5231)

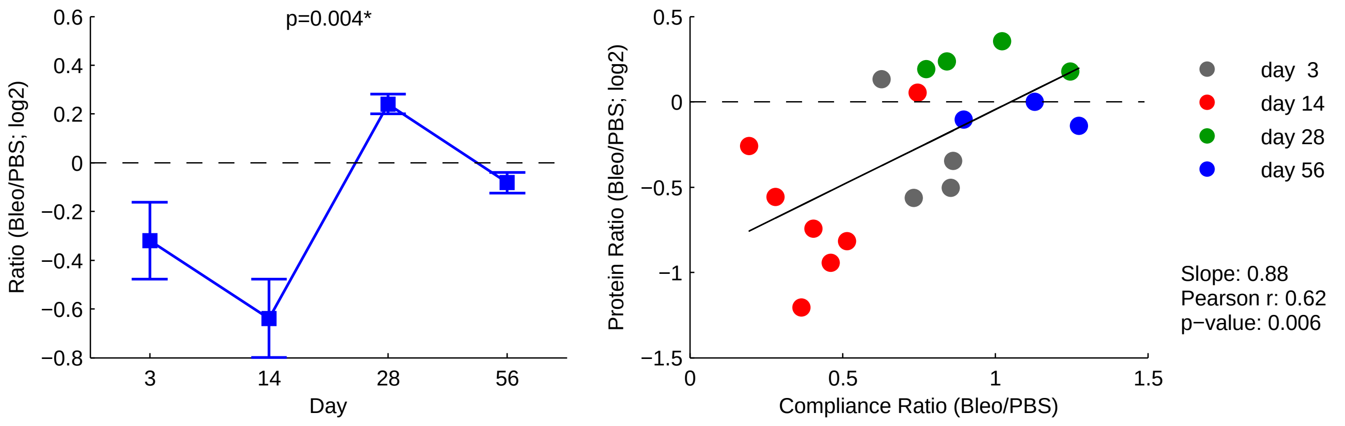

Q8BMC4 − Nop9 (id: 5234)

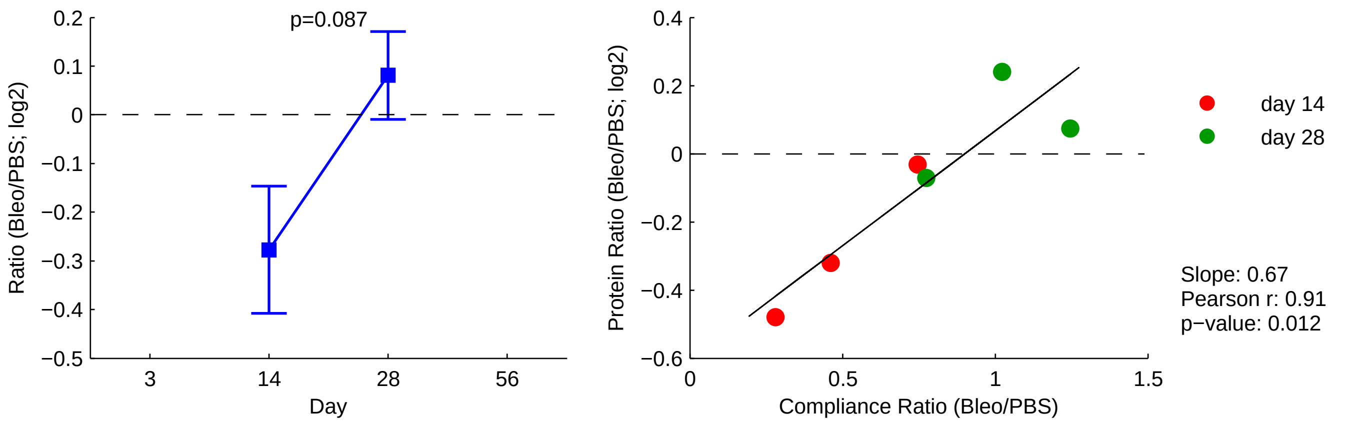

Q8BMD8 − Slc25a24 (id: 5236)

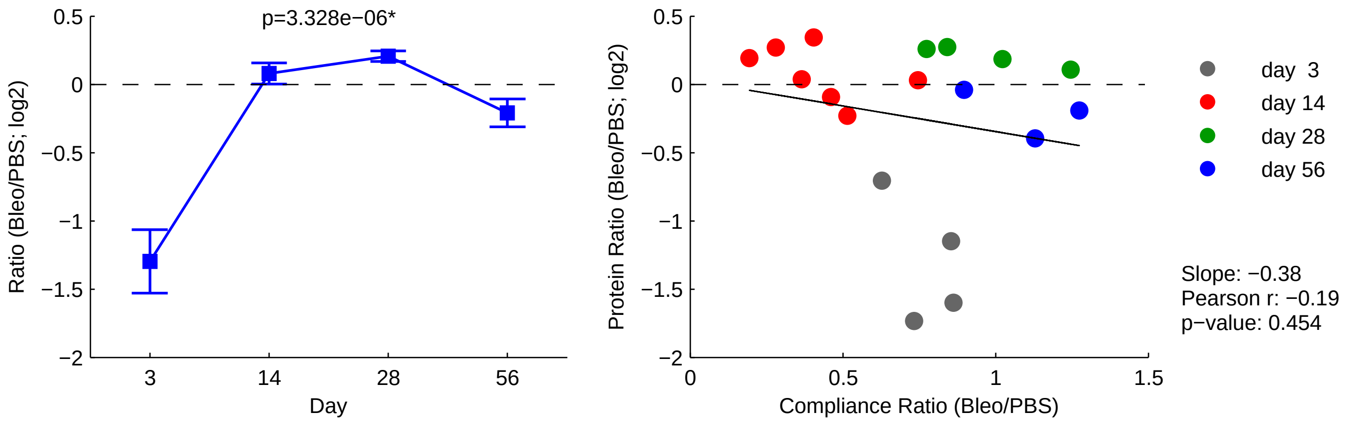

Q8BMF4 − Dlat (id: 5238)

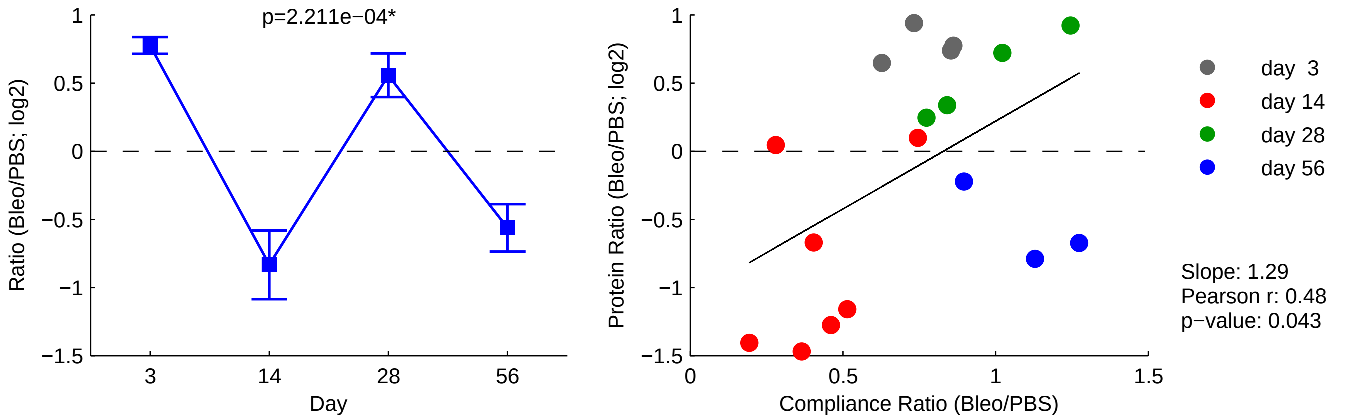

Q8BMG8 − Slc25a32 (id: 5239)

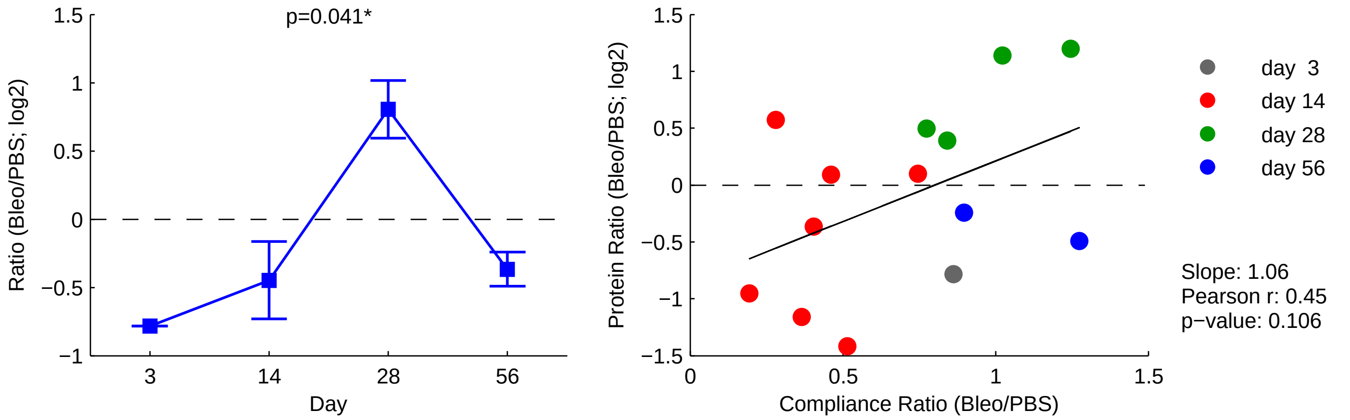

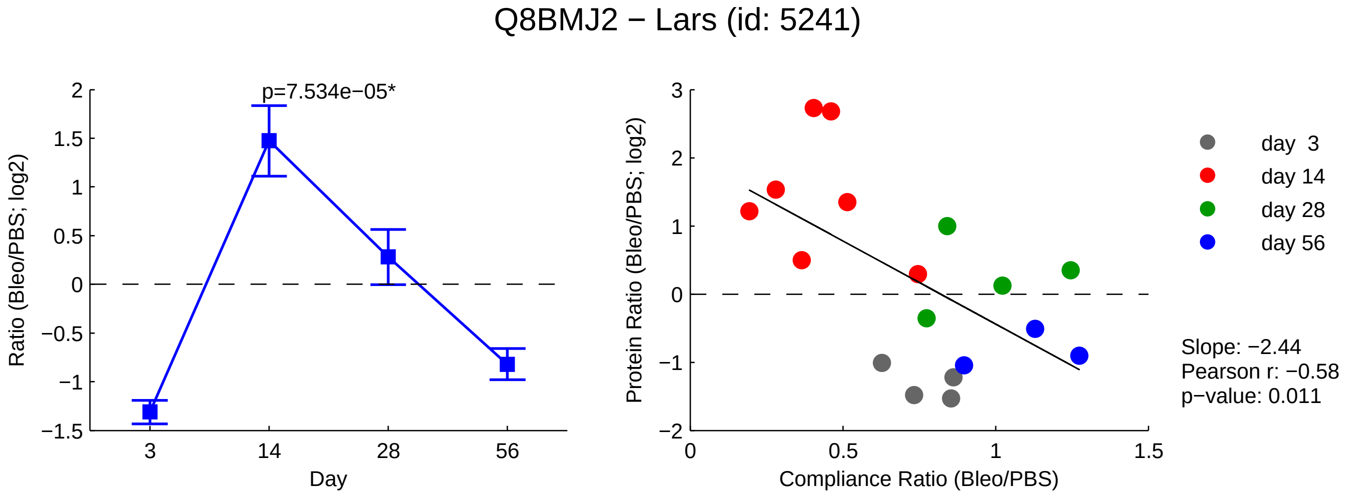
Q8BMJ2 − Lars (id: 5241)
p=7.534e−05*
Ratio (Bleo/PBS; log2)
Day
Protein Ratio (Bleo/PBS; log2)
Compliance Ratio (Bleo/PBS)
day 3
day 14
day 28
day 56
Slope: −2.44
Pearson r: −0.58
p−value: 0.011

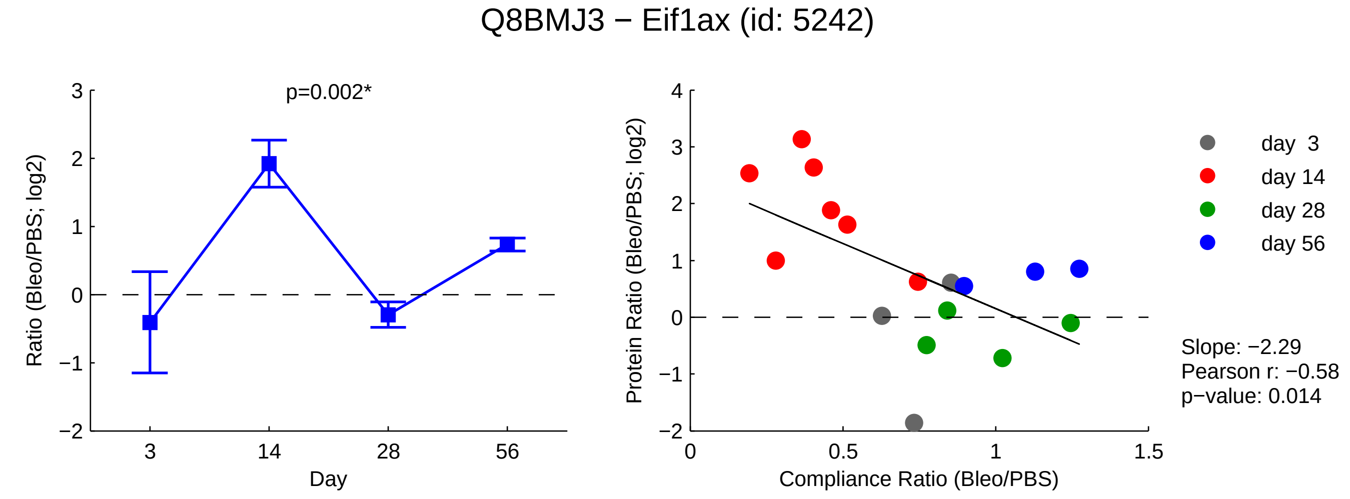
Q8BMJ3 − Eif1ax (id: 5242)
p=0.002*
Ratio (Bleo/PBS; log2)
Day
Protein Ratio (Bleo/PBS; log2)
Compliance Ratio (Bleo/PBS)
day 3
day 14
day 28
day 56
Slope: −2.29
Pearson r: −0.58
p−value: 0.014

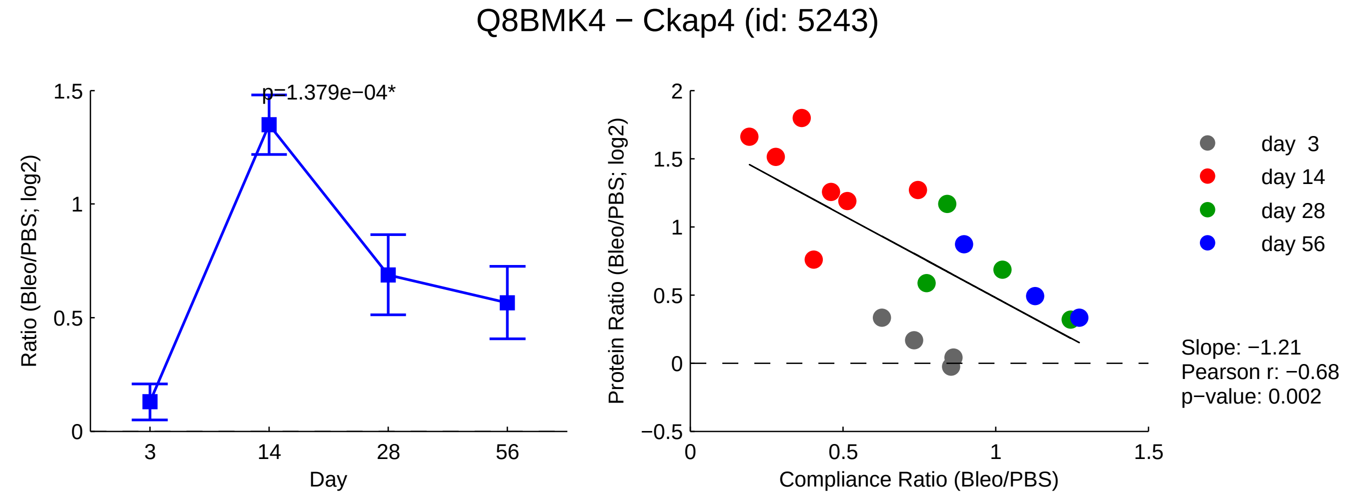
Q8BMK4 − Ckap4 (id: 5243)
p=1.379e−04*
Ratio (Bleo/PBS; log2)
Day
Protein Ratio (Bleo/PBS; log2)
Compliance Ratio (Bleo/PBS)
day 3
day 14
day 28
day 56
Slope: −1.21
Pearson r: −0.68
p−value: 0.002

Q8BML9 − Qars (id: 5246)

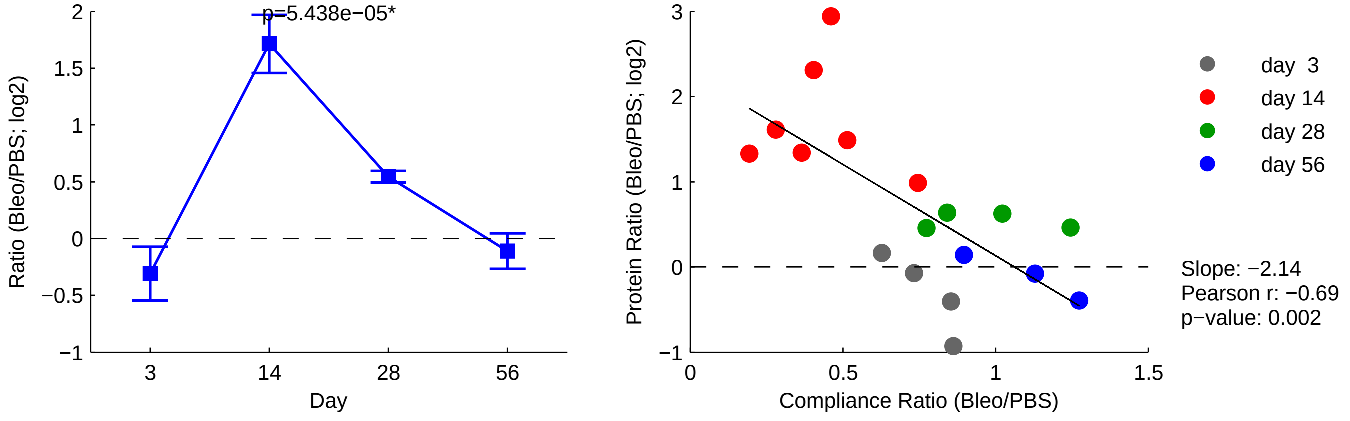

Q8BMP6 − Acbd3 (id: 5247)

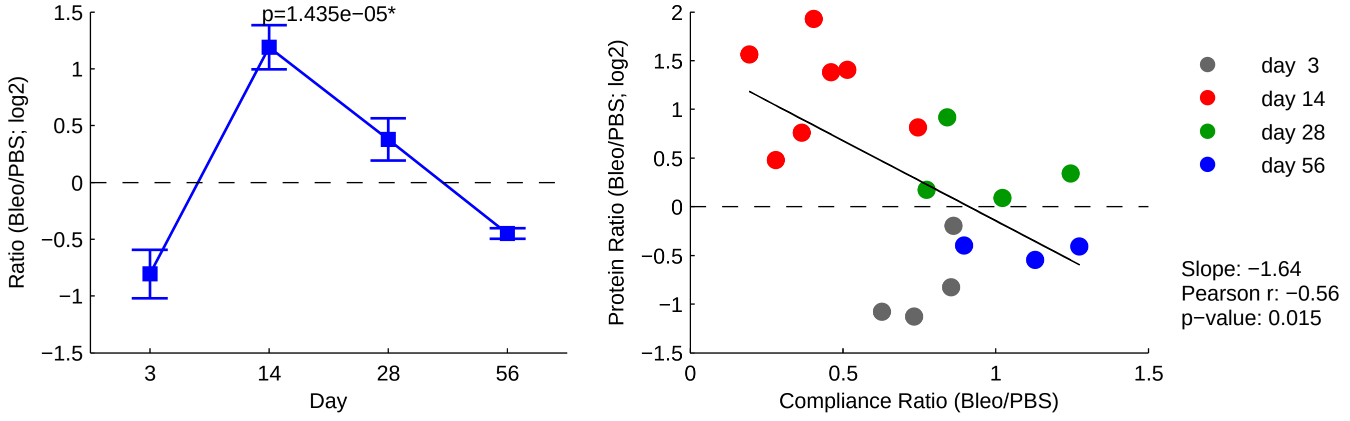

Q8BMQ2 − Gtf3c4 (id: 5248)

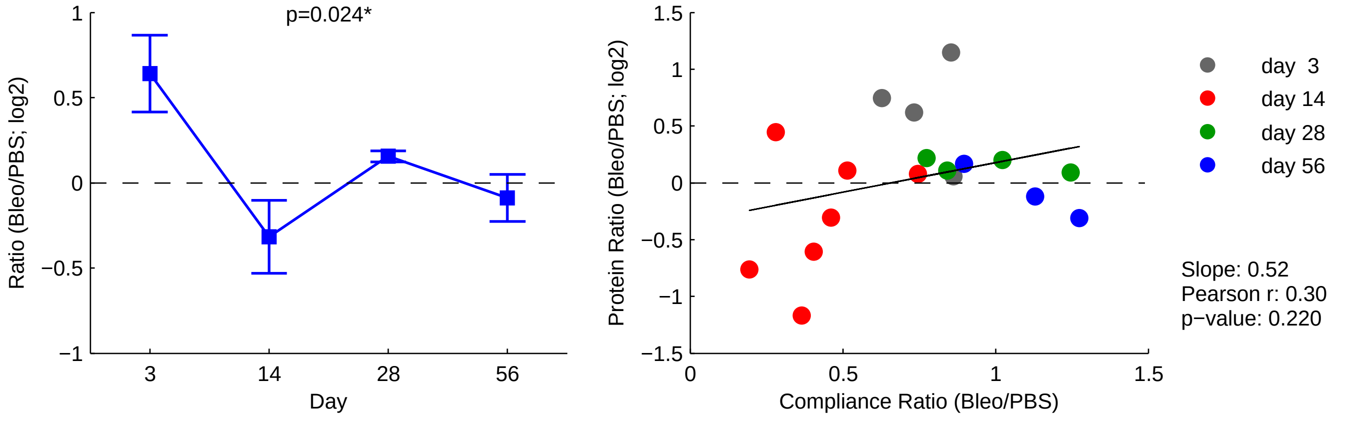

Q8BMS1 − Hadha (id: 5250)

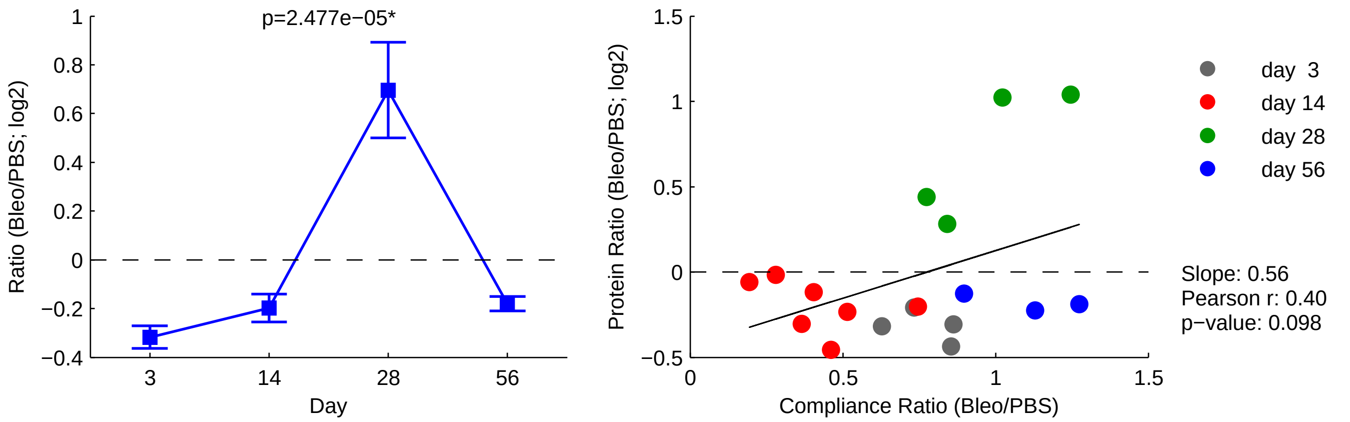

Q8BMS4 − Coq3 (id: 5252)

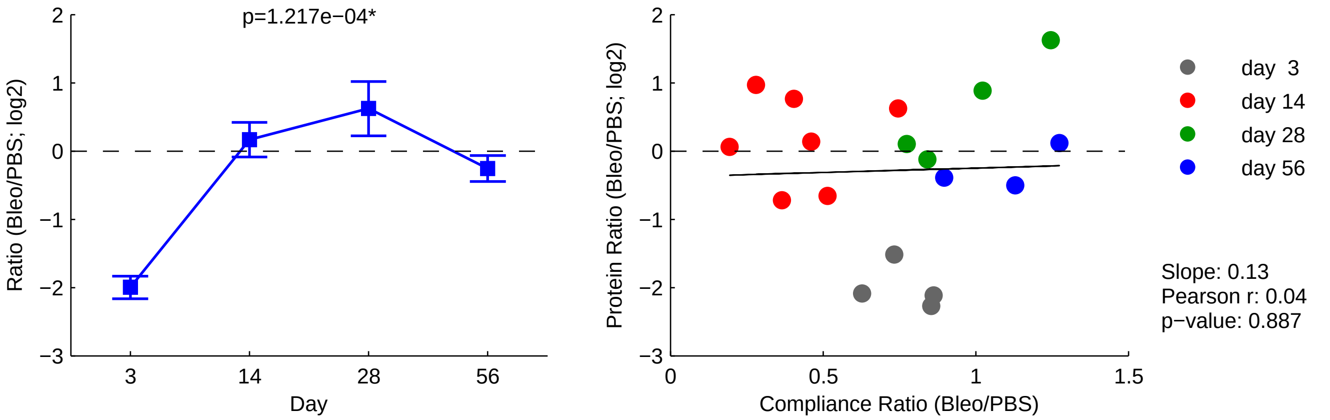

Q8BND5−3 − Qsox1 (id: 5258)

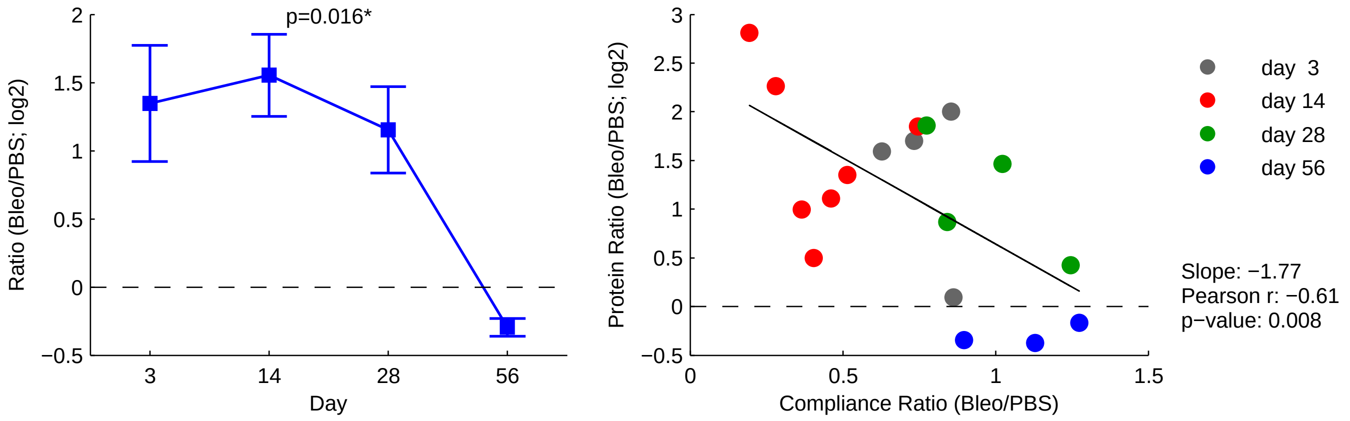

Q8BNW9 − Kbtbd11 (id: 5260)

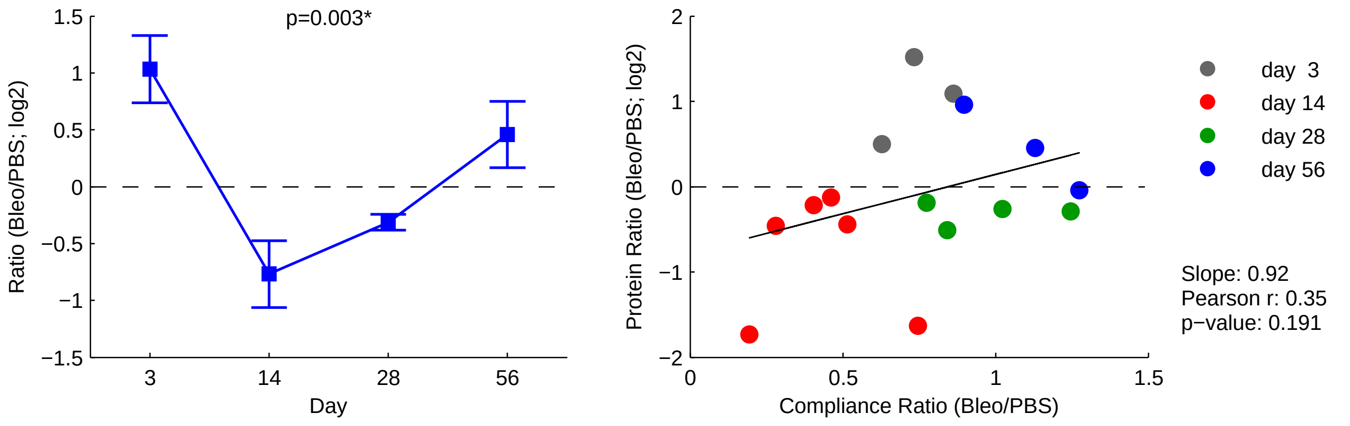

Q8BP40 − Acp6 (id: 5263)

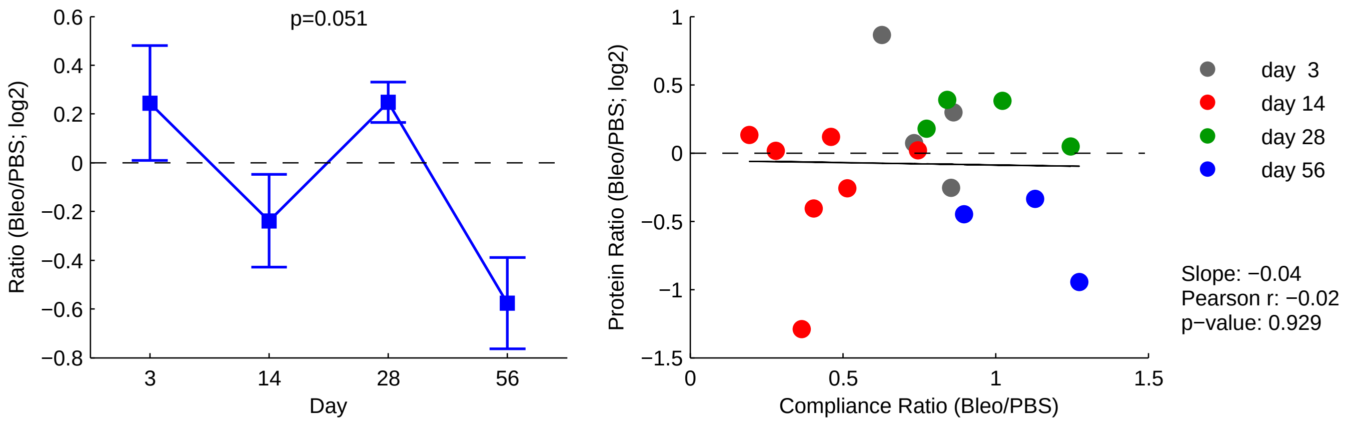

Q8BP43 − Tpm1 (id: 5264)

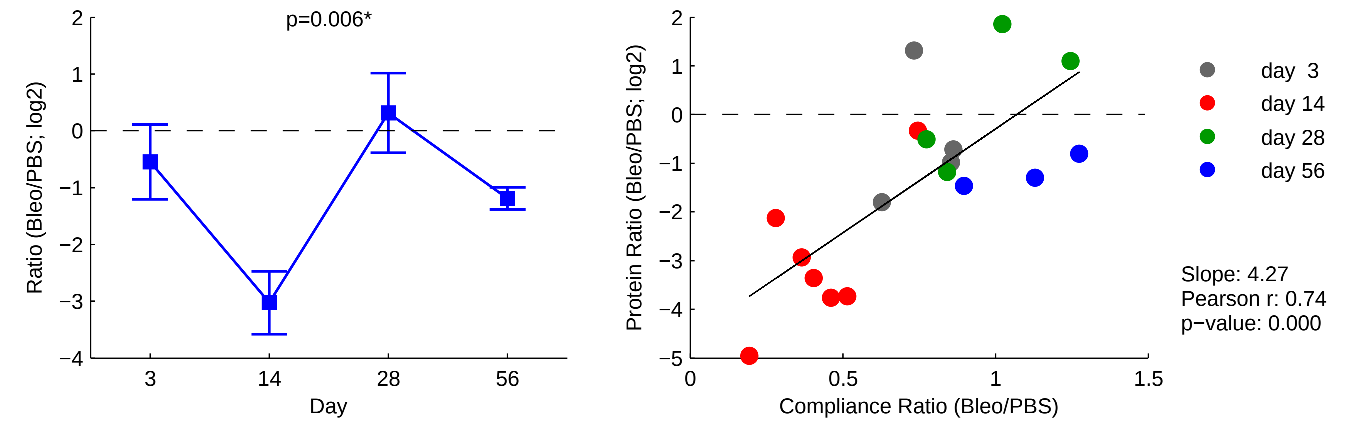

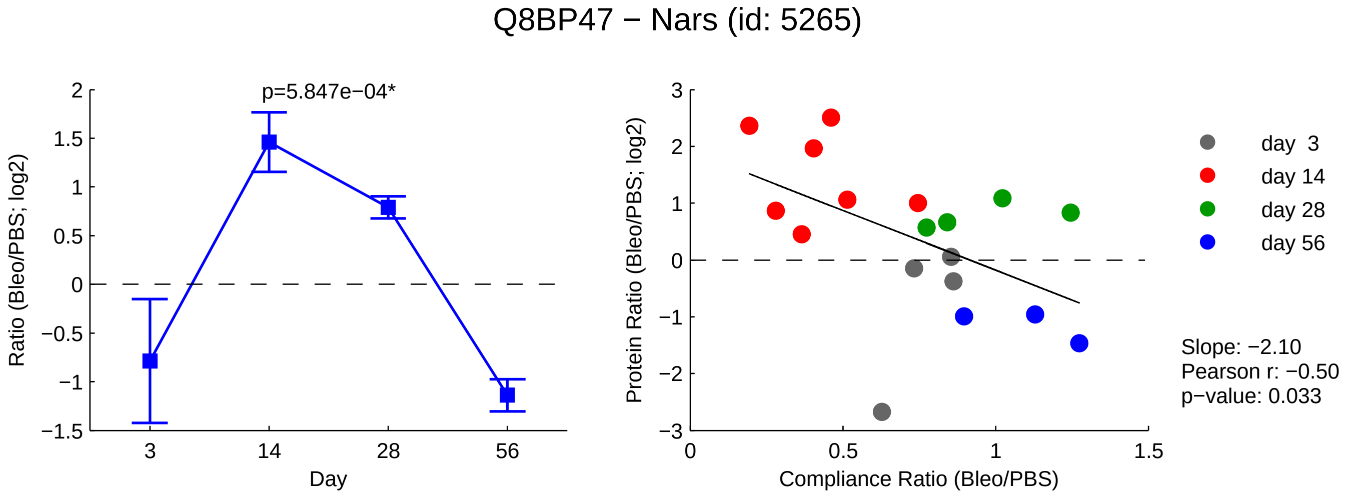
Q8BP47 − Nars (id: 5265)
p=5.847e−04*
Ratio (Bleo/PBS; log2)
Day
Protein Ratio (Bleo/PBS; log2)
Compliance Ratio (Bleo/PBS)
day 3
day 14
day 28
day 56
Slope: −2.10
Pearson r: −0.50
p−value: 0.033

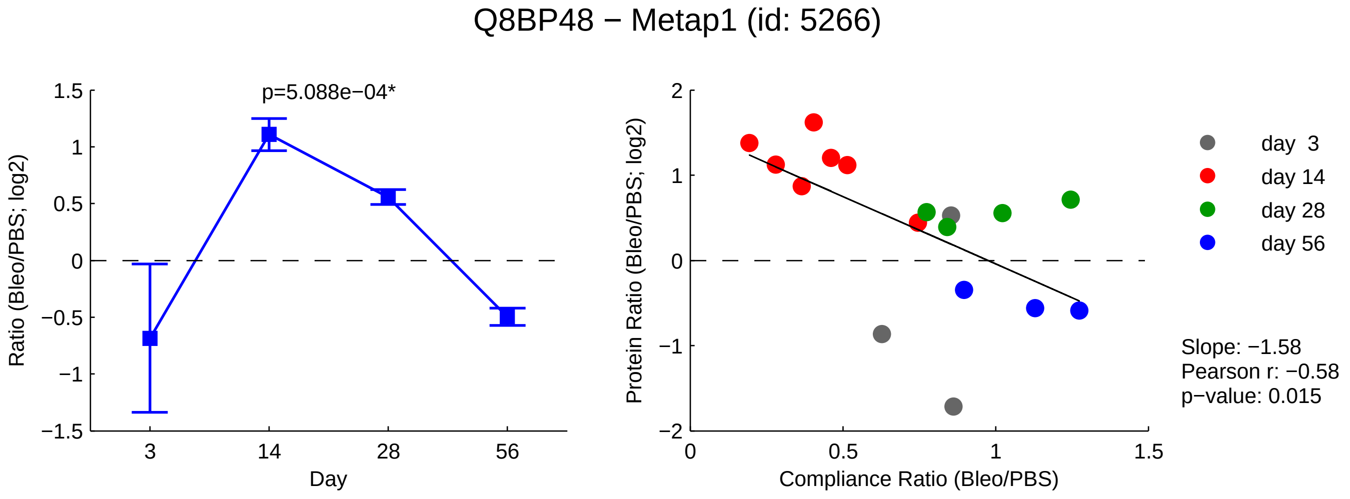
Q8BP48 − Metap1 (id: 5266)
p=5.088e−04*
Ratio (Bleo/PBS; log2)
Day
Protein Ratio (Bleo/PBS; log2)
Compliance Ratio (Bleo/PBS)
day 3
day 14
day 28
day 56
Slope: −1.58
Pearson r: −0.58
p−value: 0.015

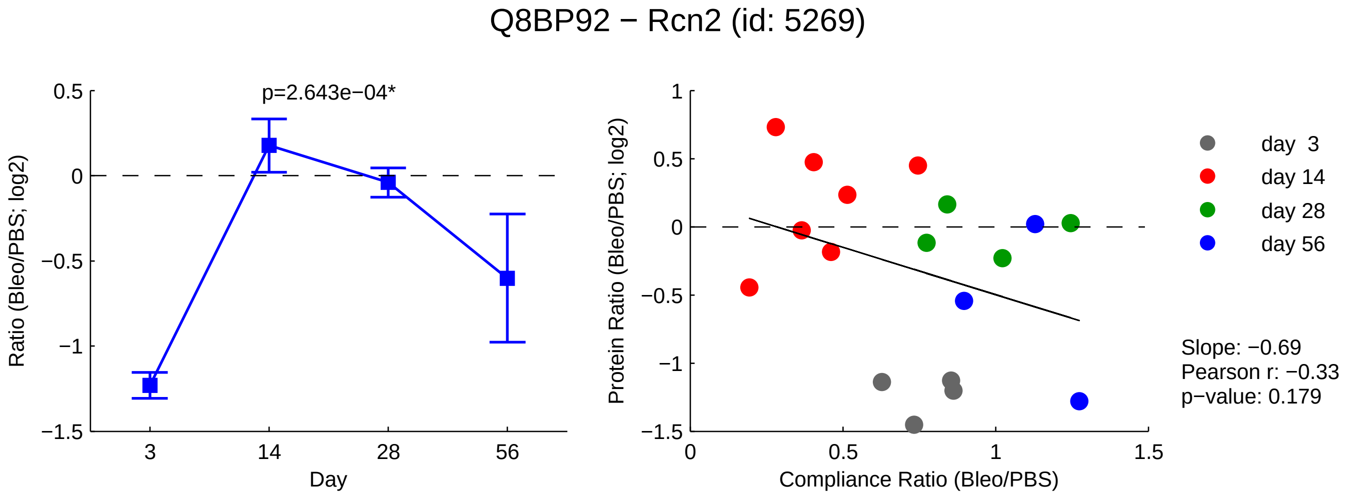
Q8BP92 − Rcn2 (id: 5269)
p=2.643e−04*
Ratio (Bleo/PBS; log2)
Day
Protein Ratio (Bleo/PBS; log2)
Compliance Ratio (Bleo/PBS)
day 3
day 14
day 28
day 56
Slope: −0.69
Pearson r: −0.33
p−value: 0.179

Q8BPB5 − Efemp1 (id: 5271)

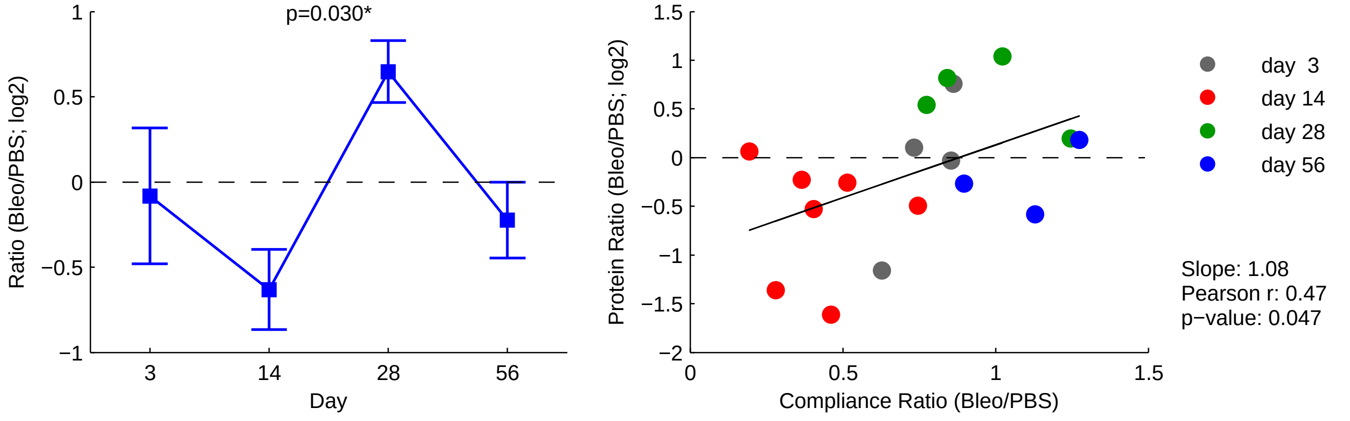

Q8BPS4 − Gpr180 (id: 5274)

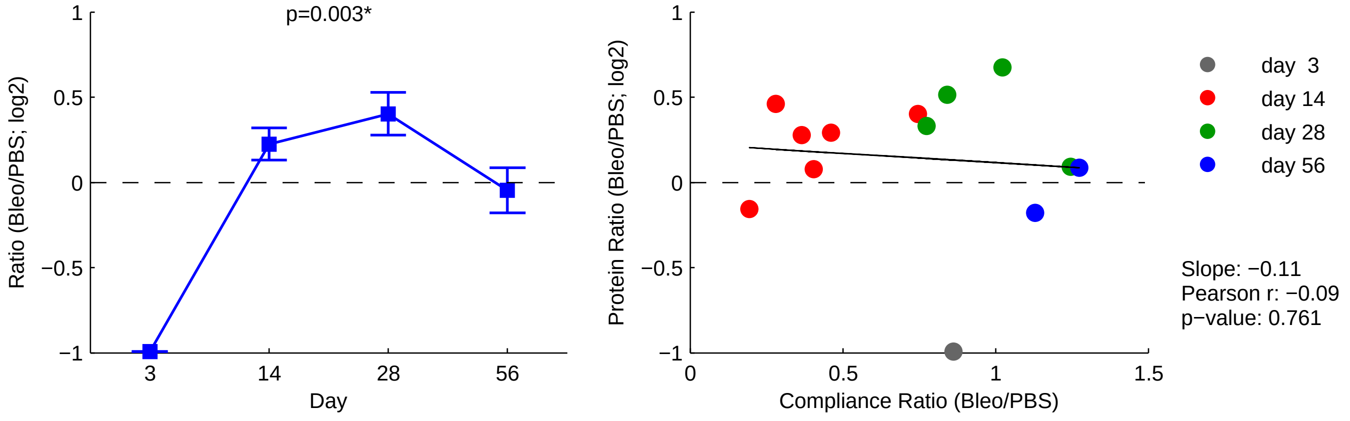

Q8BPU7 − Elmo1 (id: 5275)

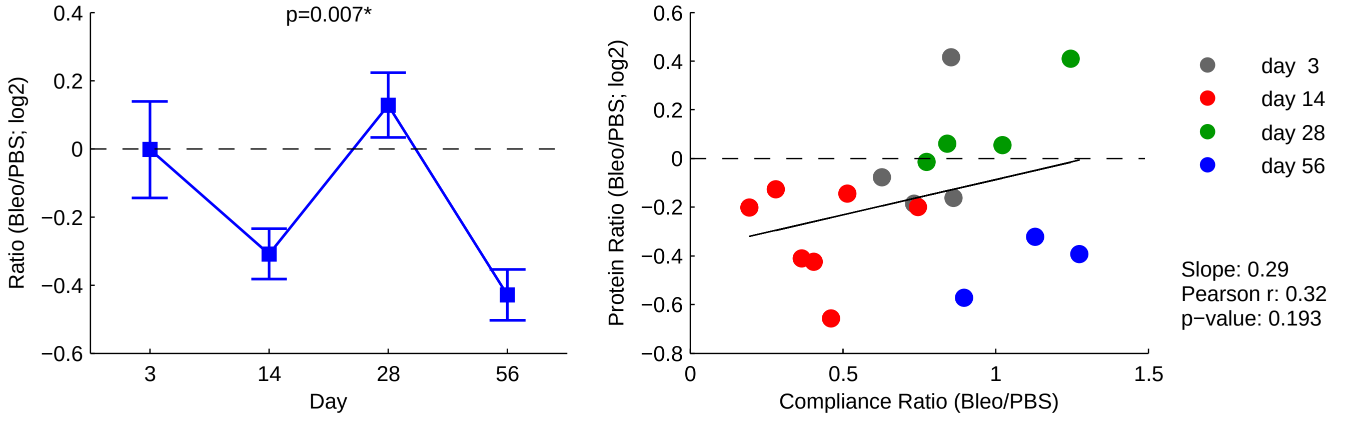

Q8BQ30 − Ppp1r18 (id: 5276)

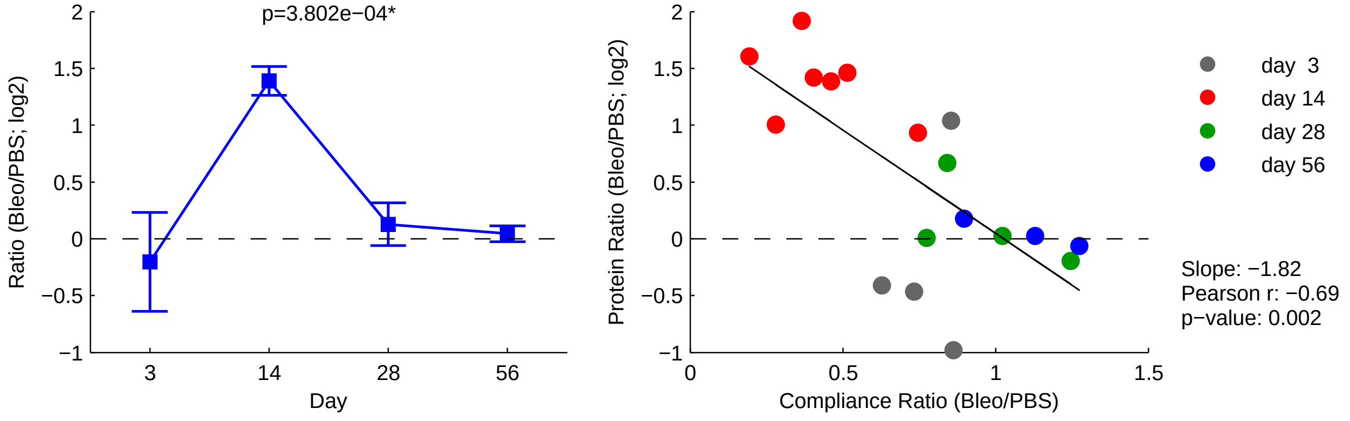

Q8BQ47 − Cnpy4 (id: 5278)

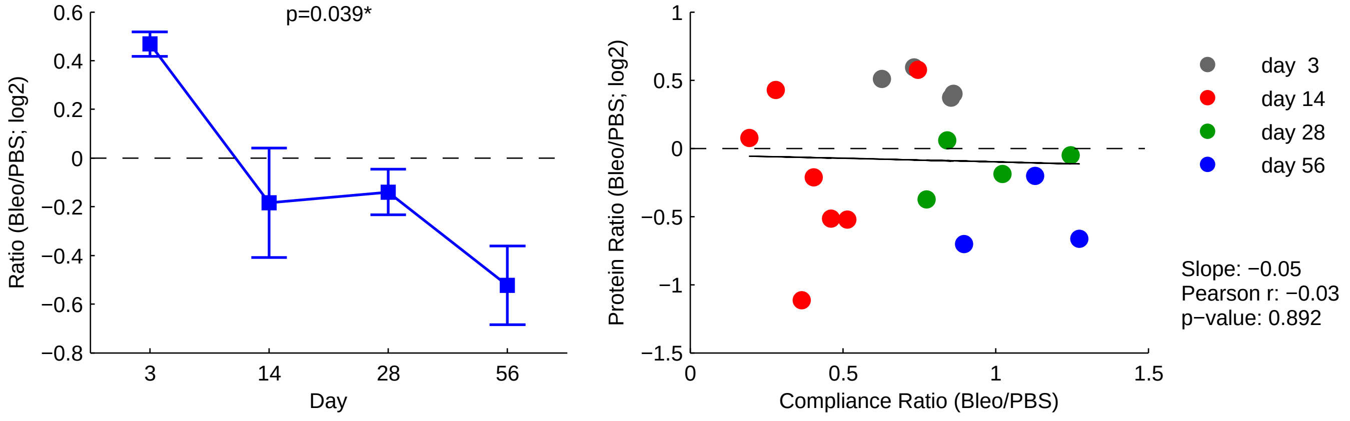

Q8BR65 − Suds3 (id: 5281)

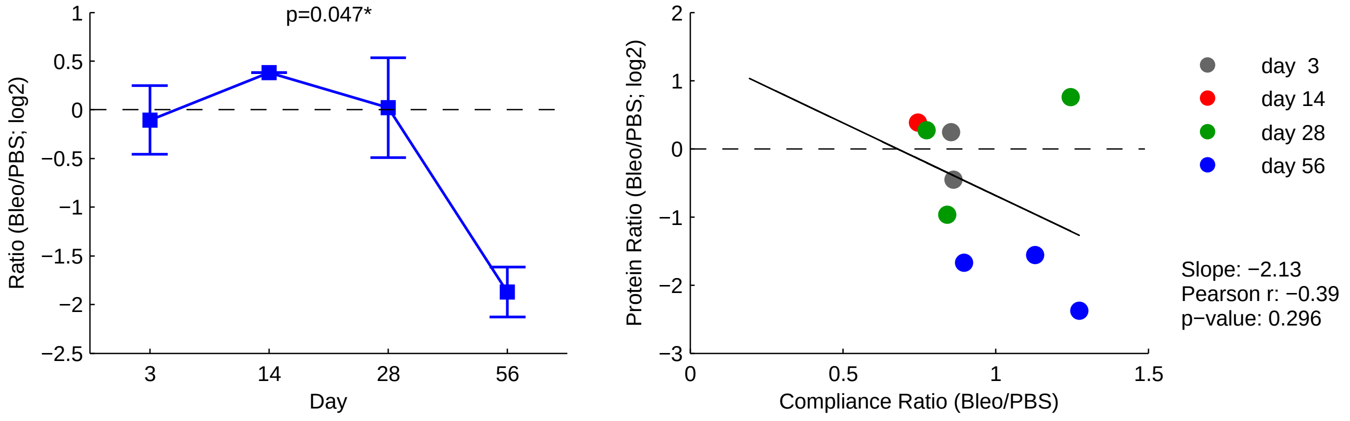

Q8BR90 − (id: 5282)

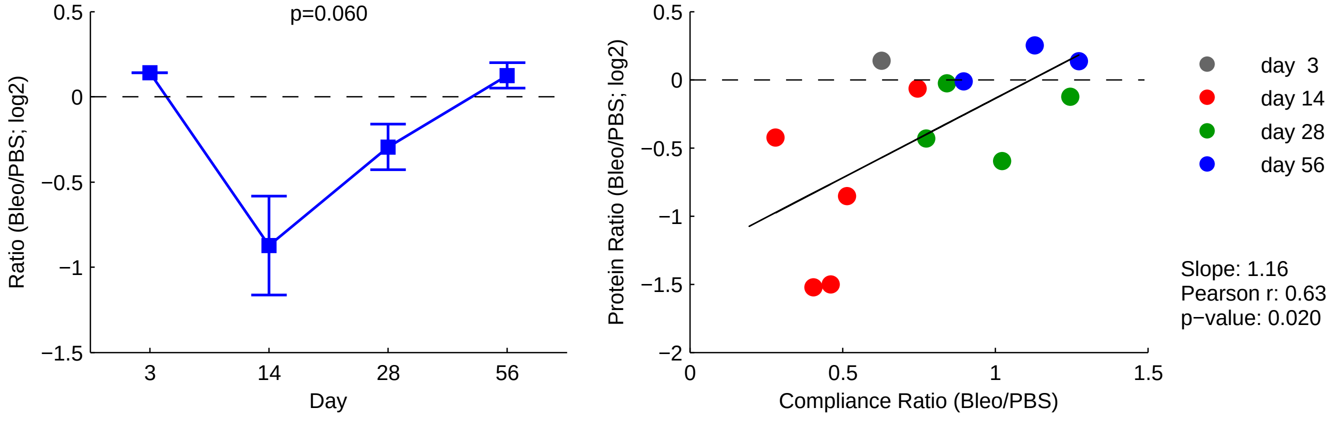

Q8BRF7 − Scfd1 (id: 5285)

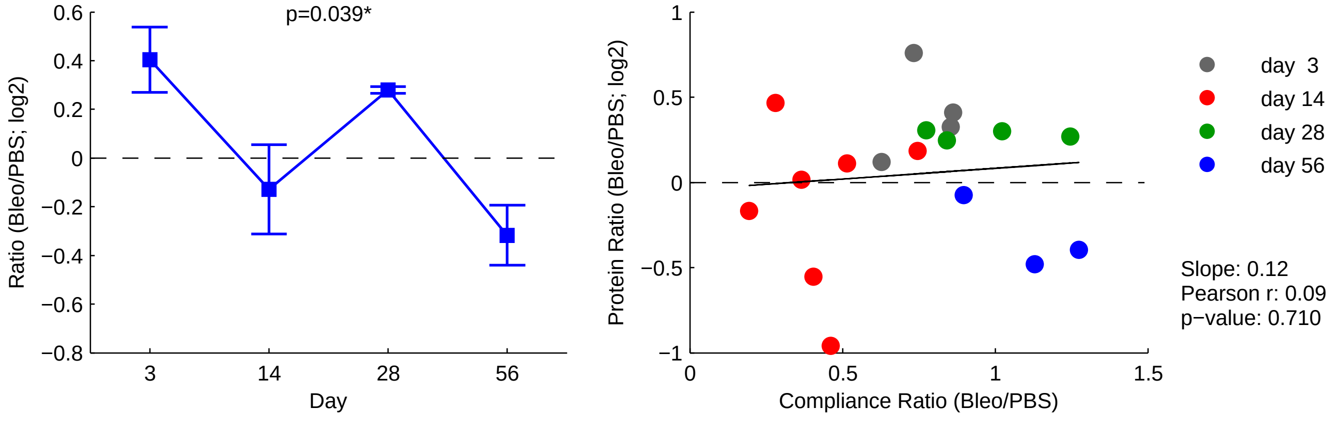

Q8BRK8 − Prkaa2 (id: 5287)

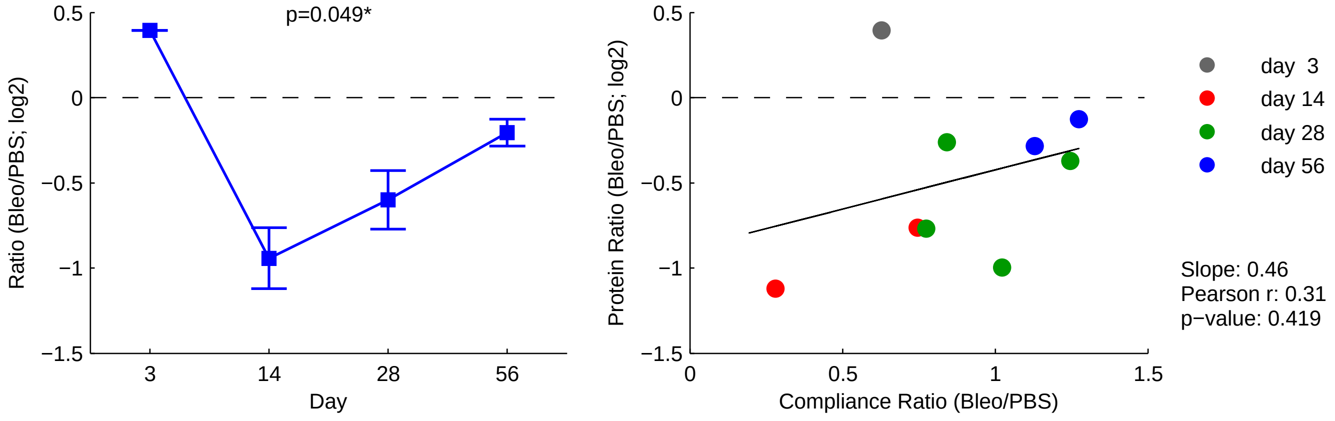

Q8BRT1−5 − (id: 5289)

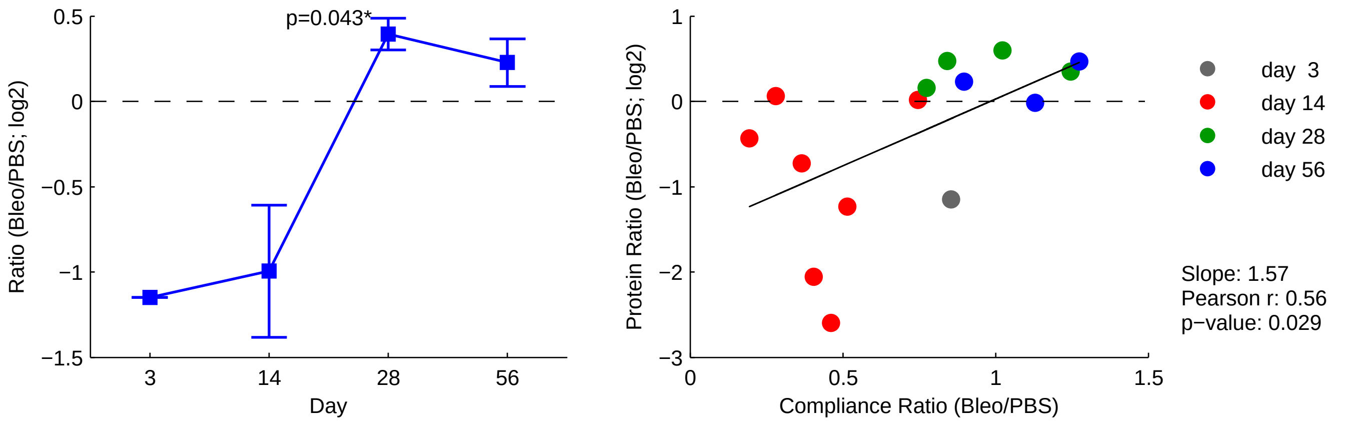

Q8BSE0 − Rmdn2 (id: 5298)

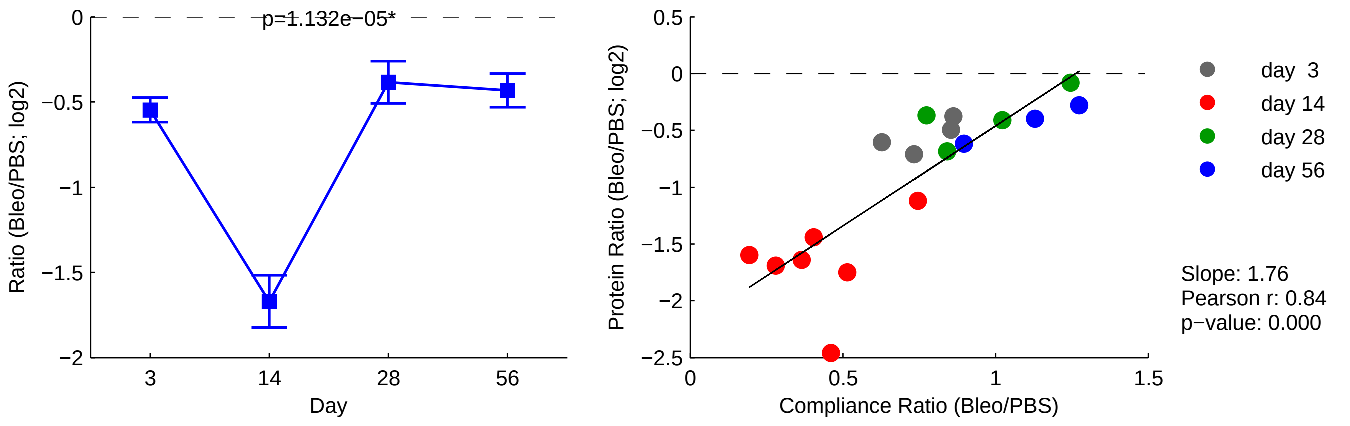

Q8BTE5 − 1110001A16Rik (id: 5304)

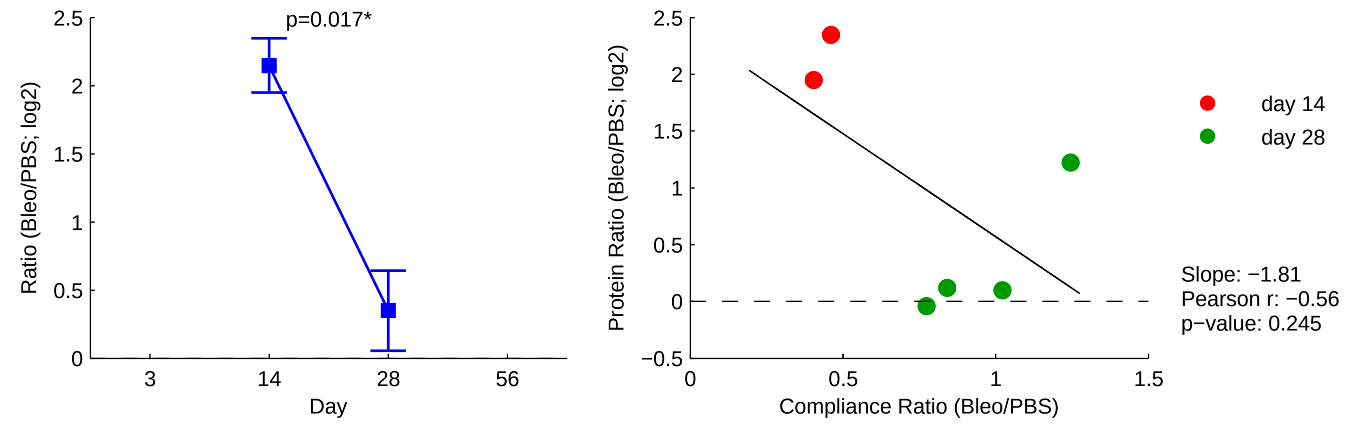

Q8BTI9 − Pik3cb (id: 5307)

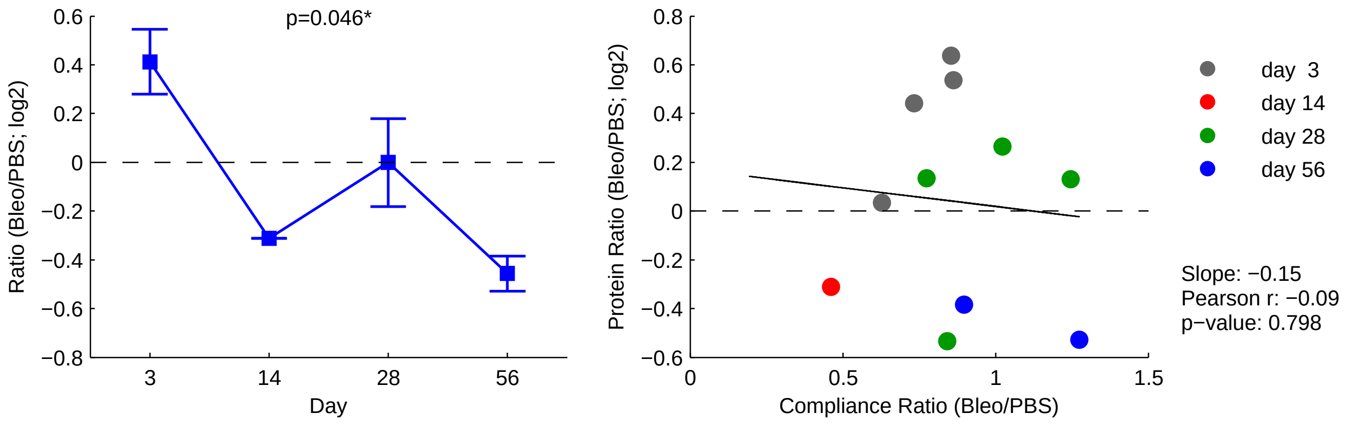

Q8BTJ4−2 − Enpp4 (id: 5308)

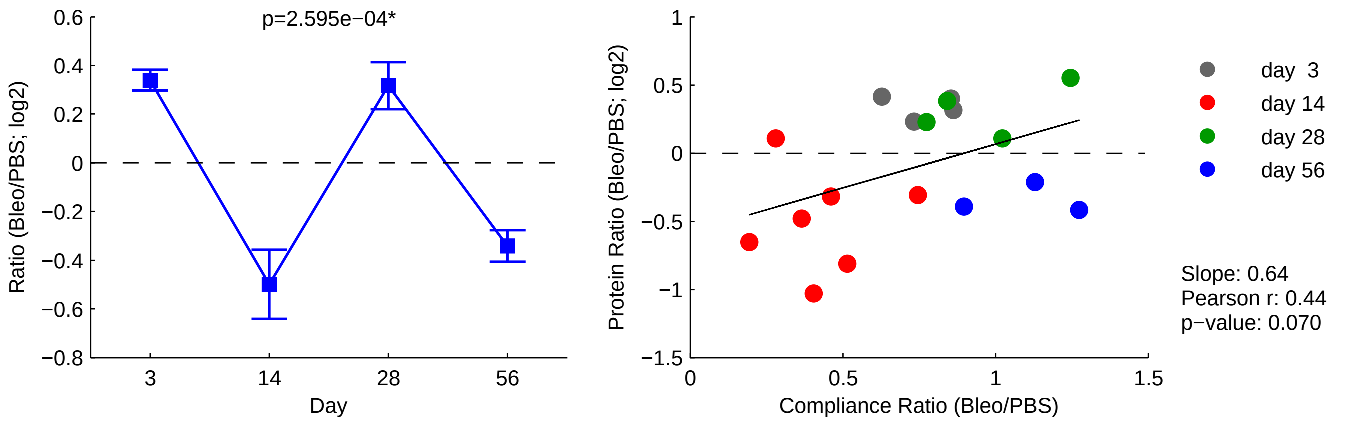

Q8BTS3 − Gbp9 (id: 5310)

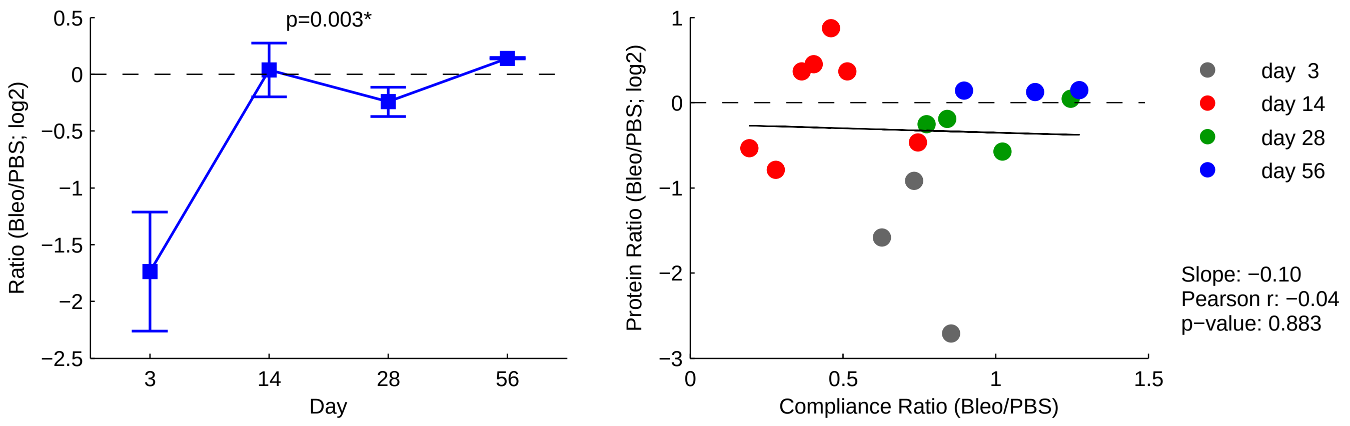

Q8BTS4 − Nup54 (id: 5311)

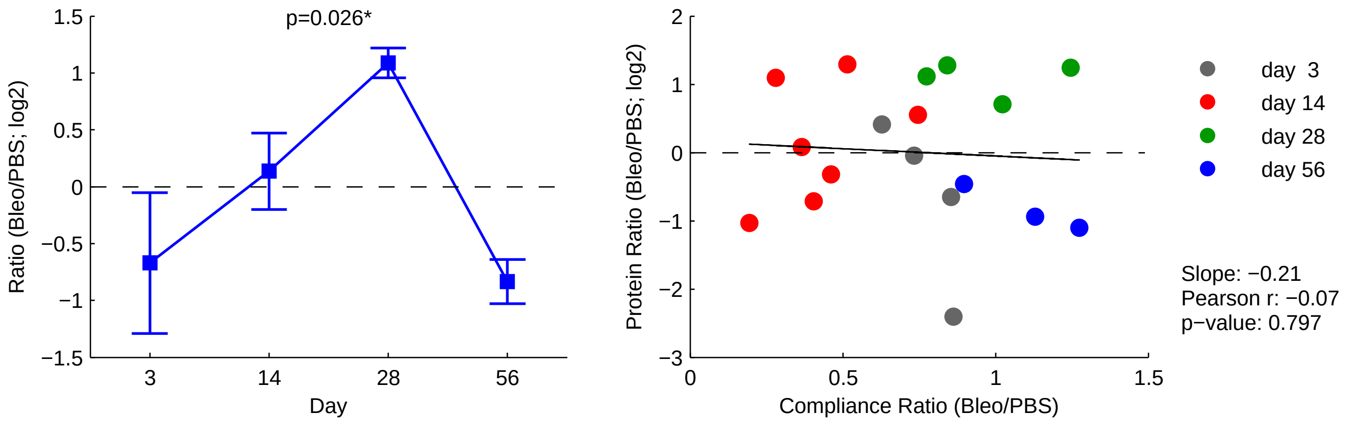

Q8BTV1 − Tusc3 (id: 5313)

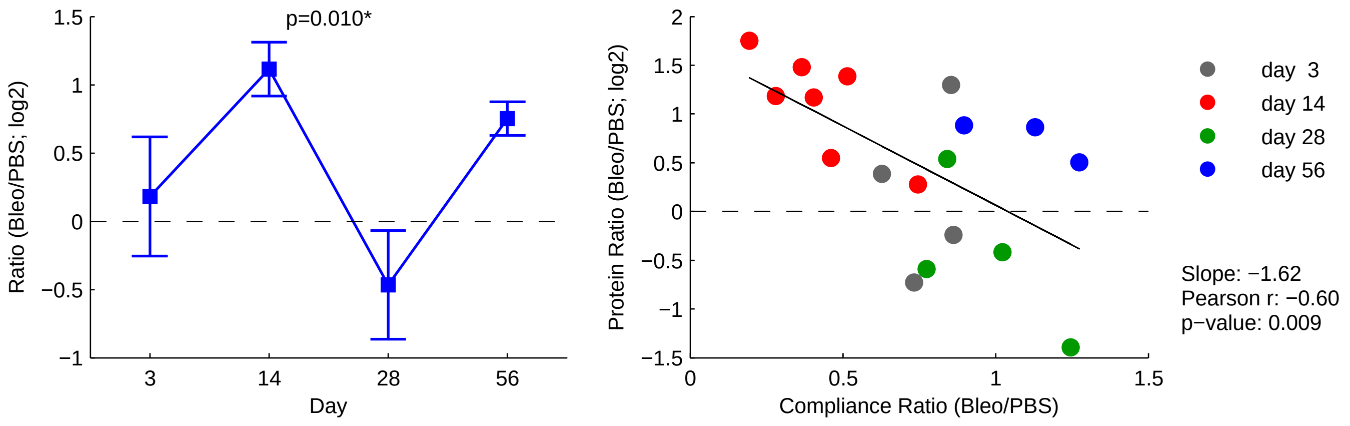

Q8BTW3 − Exosc6 (id: 5315)

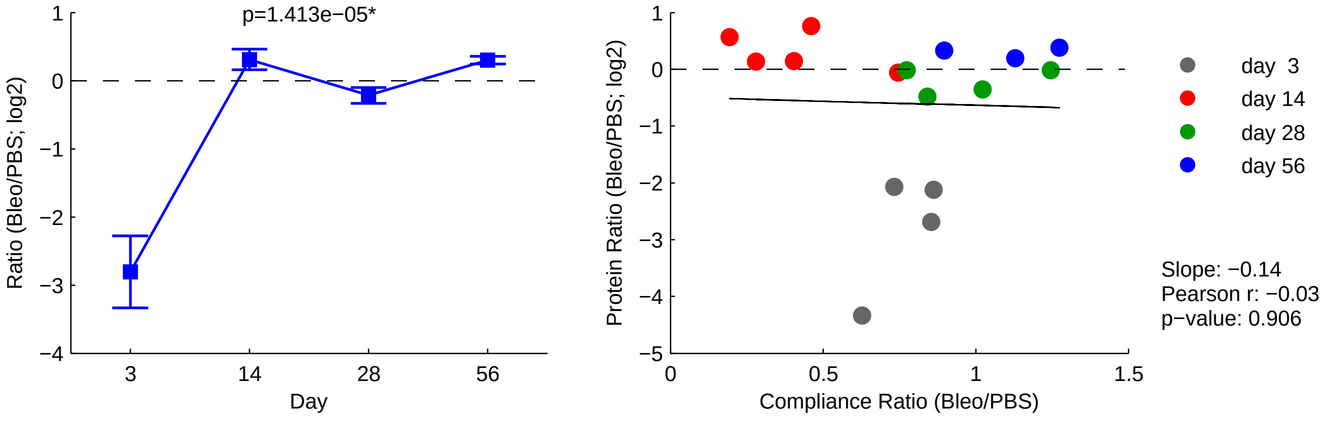

Q8BTW9 − Pak4 (id: 5316)

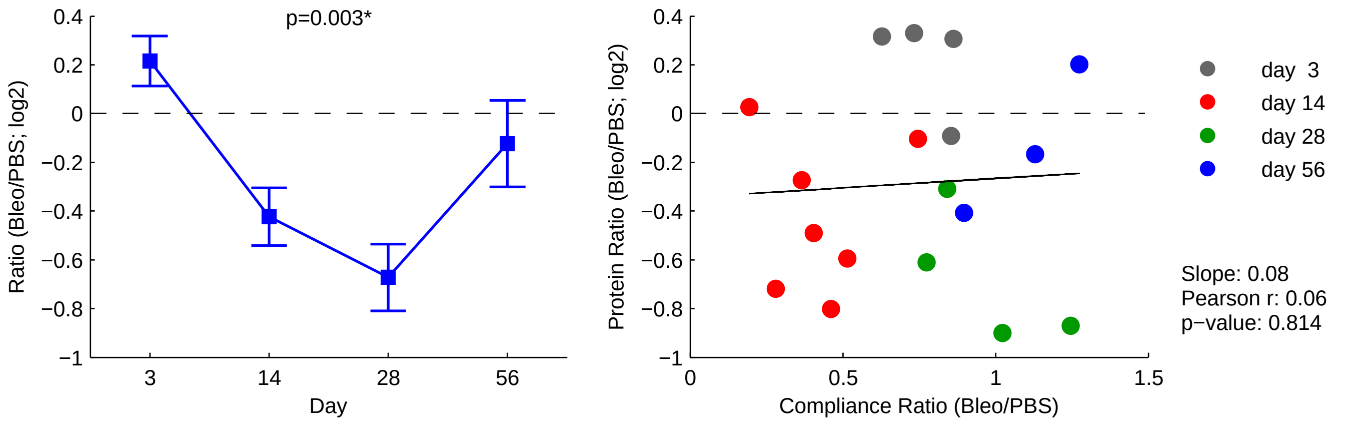

Q8BTZ5 − Ankrd46 (id: 5321)

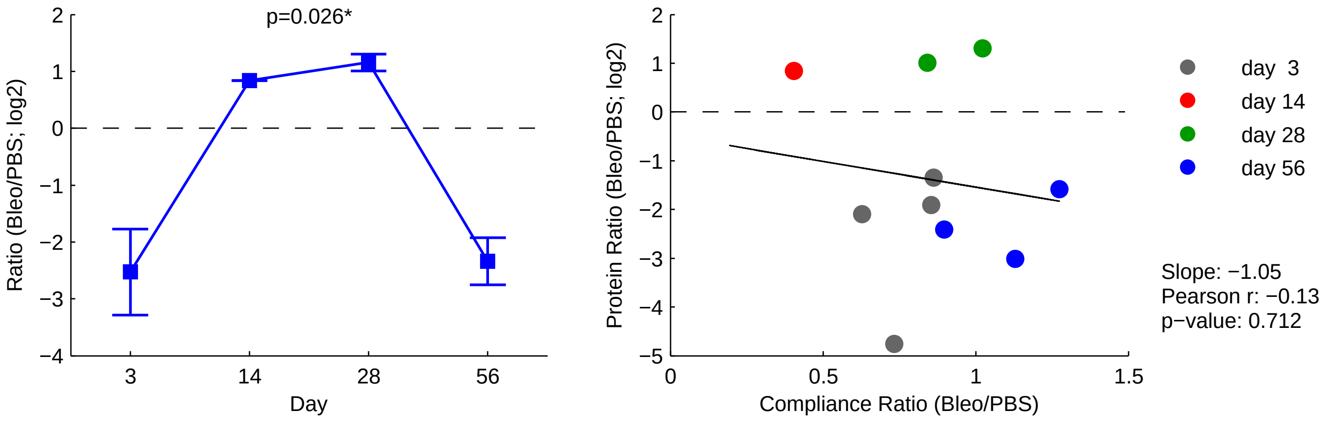

Q8BTZ7 − Gmppb (id: 5322)

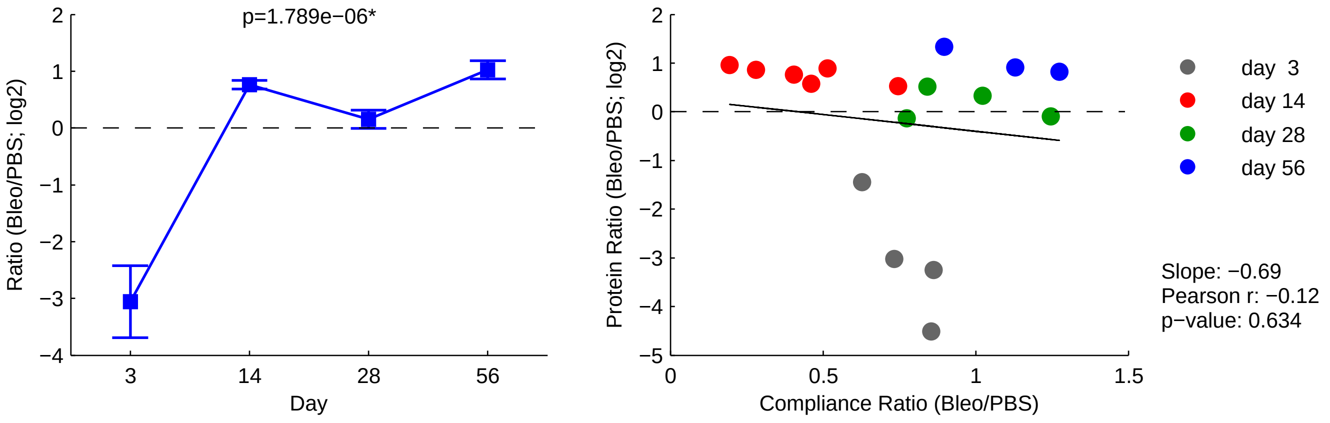

Q8BU14 − Sec62 (id: 5325)

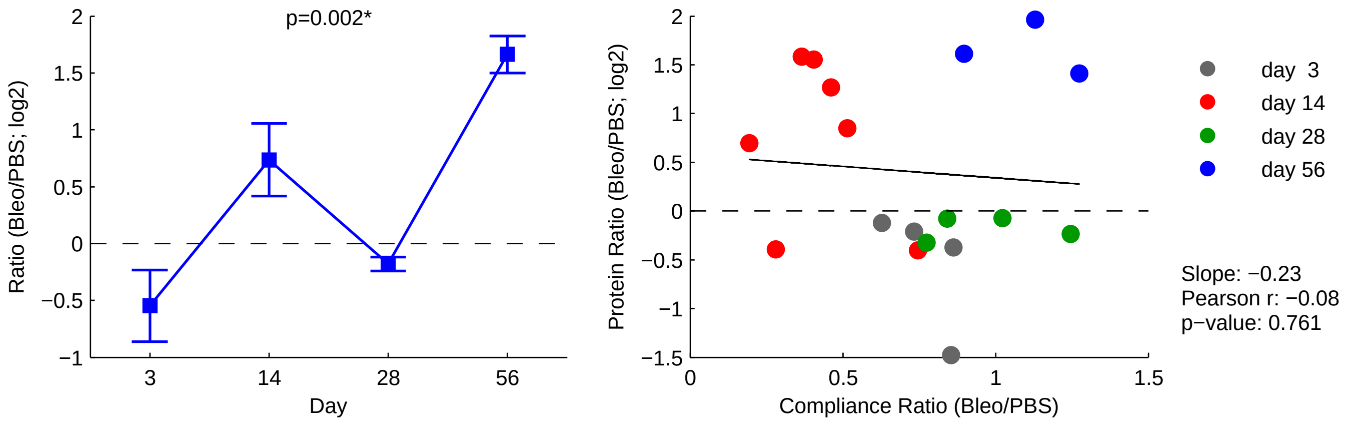

Q8BU30 − Iars (id: 5327)

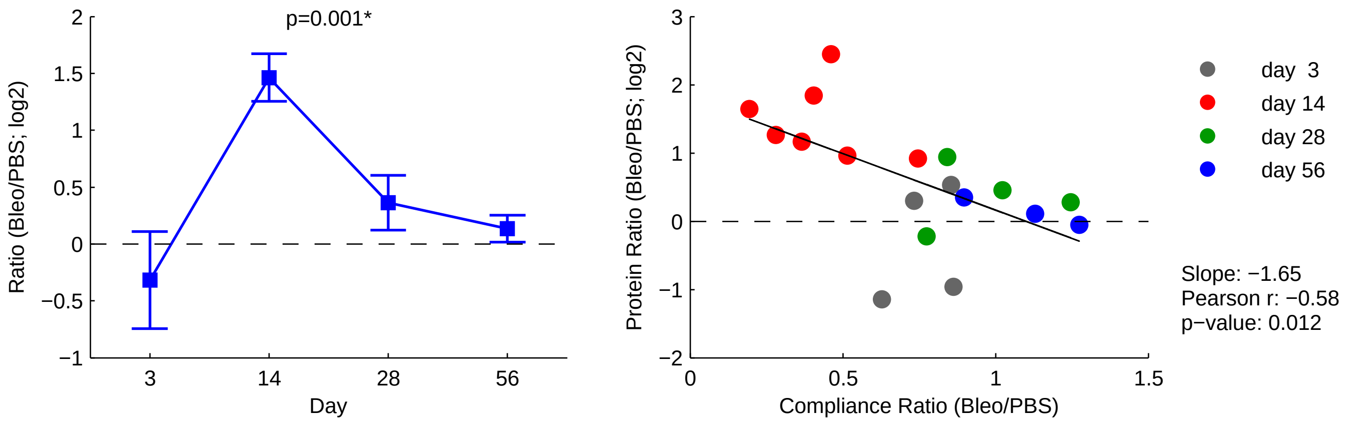

Q8BU31 − Rap2c (id: 5328)

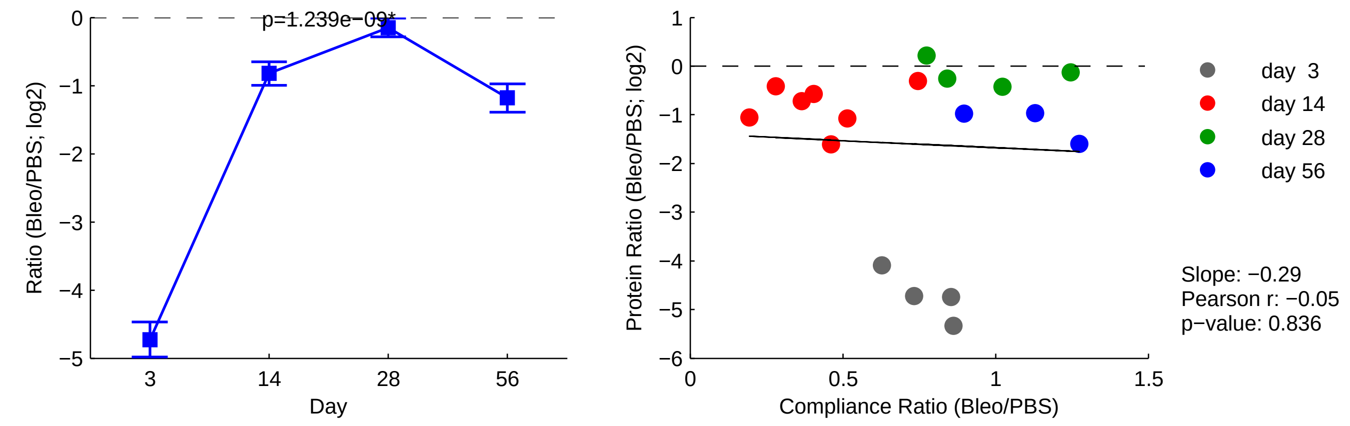

Q8BU33 − Ilvbl (id: 5329)

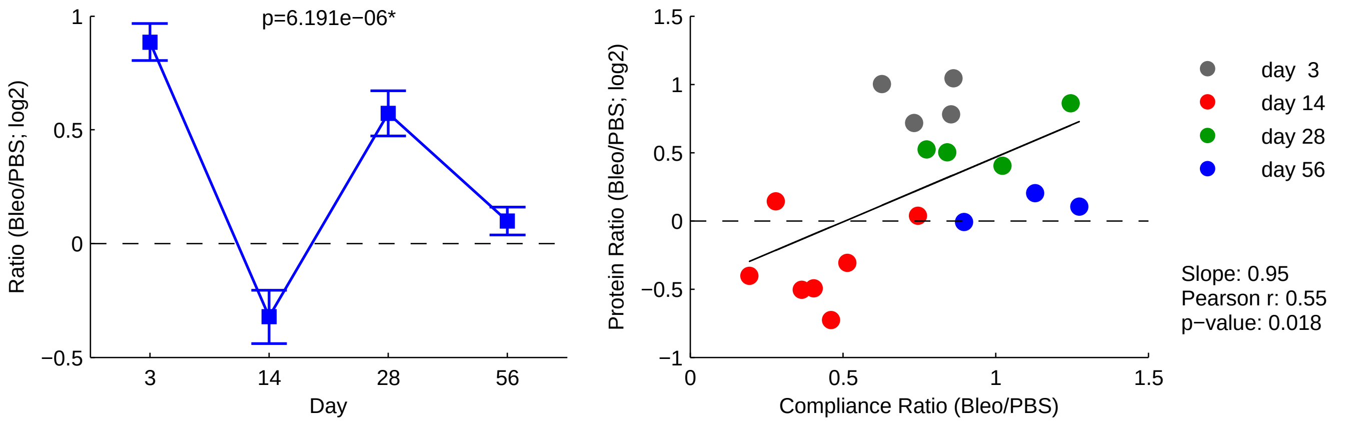

Q8BU88 − Mrpl22 (id: 5331)

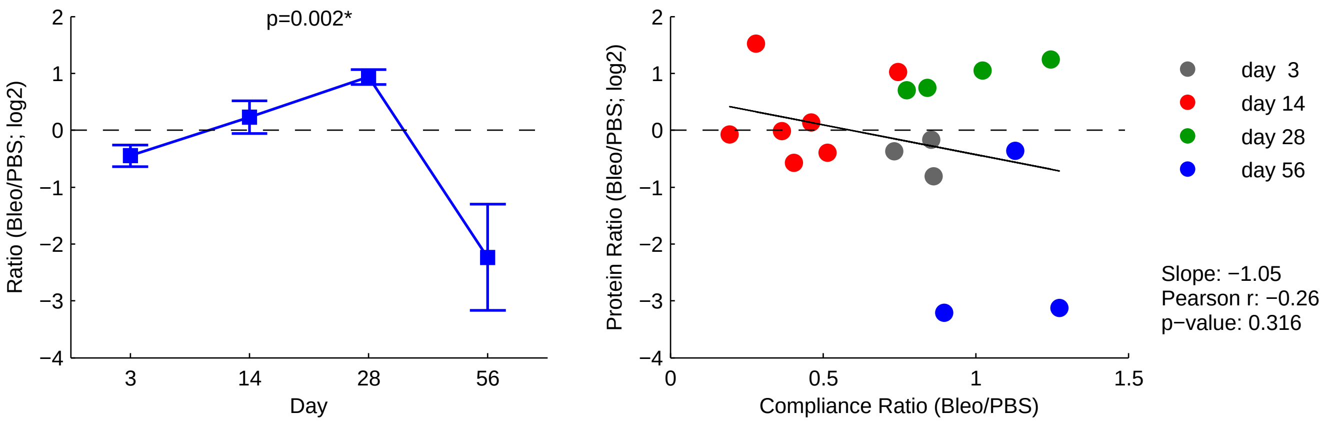

Q8BUE4 − Aifm2 (id: 5332)

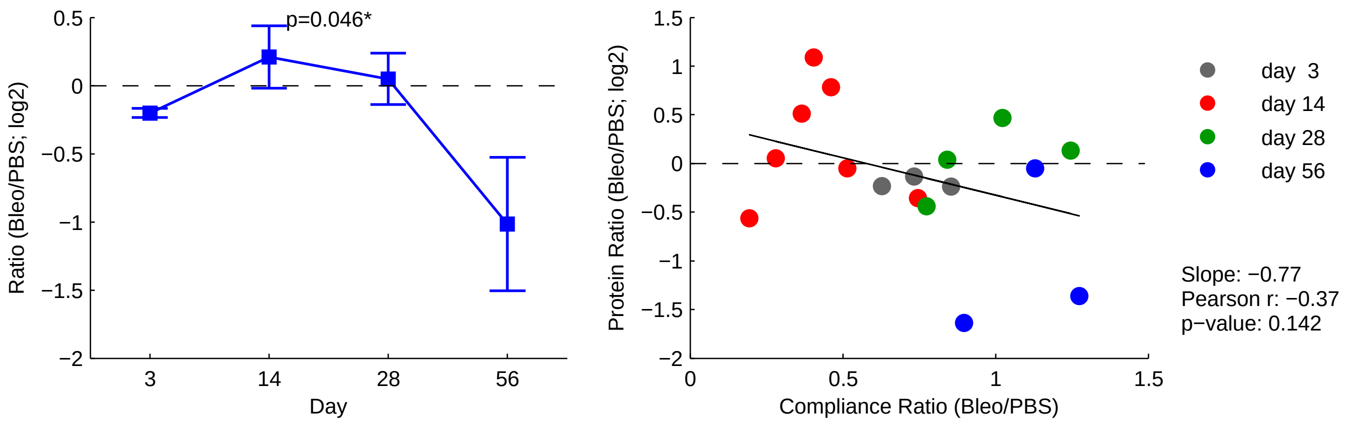

Q8BUV8 − Gpr107 (id: 5341)

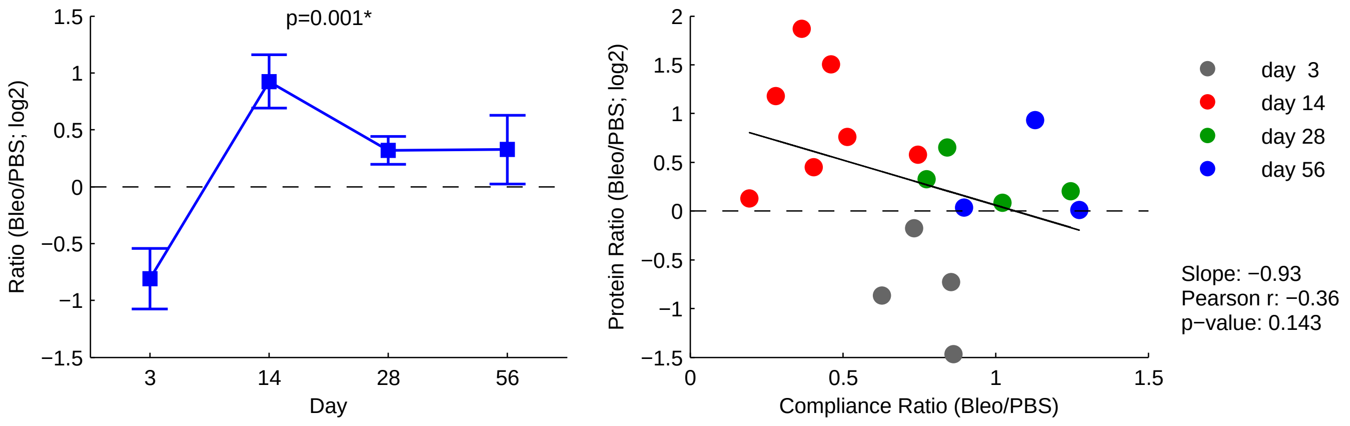

Q8BUY5 − Timmdc1 (id: 5342)

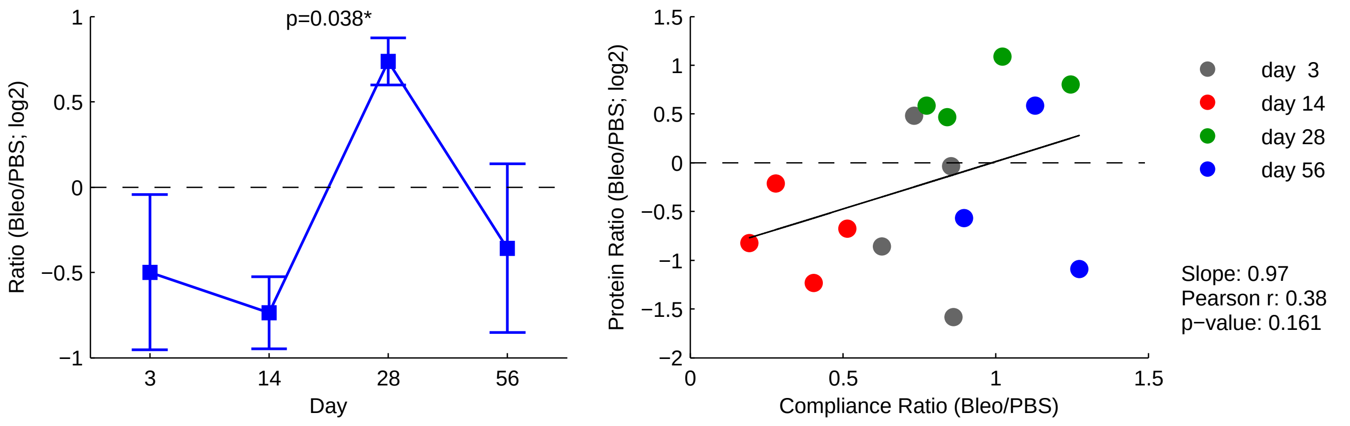

Q8BVE3 − Atp6v1h (id: 5347)

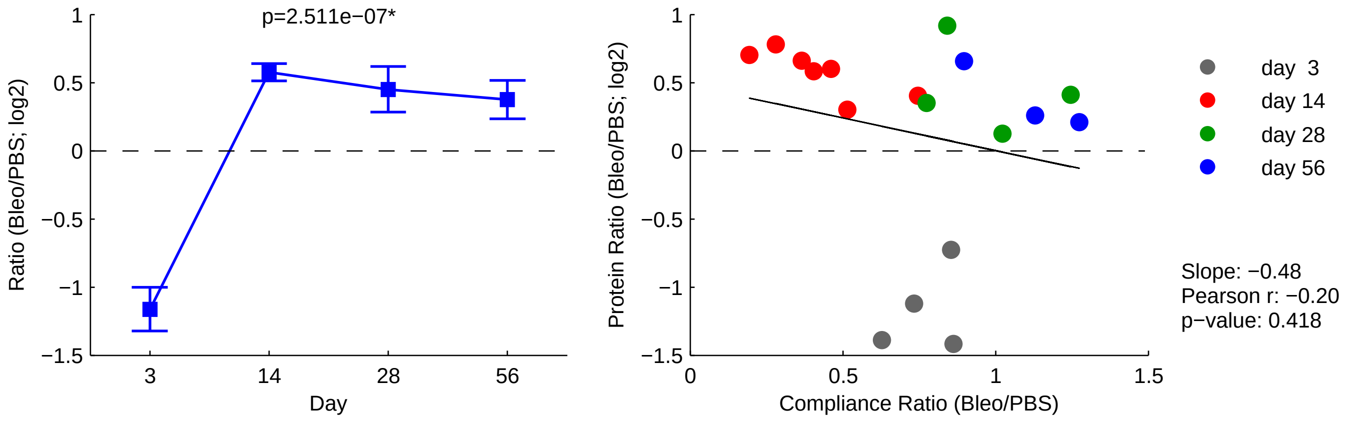

Q8BVF2 − Pdcl3 (id: 5349)

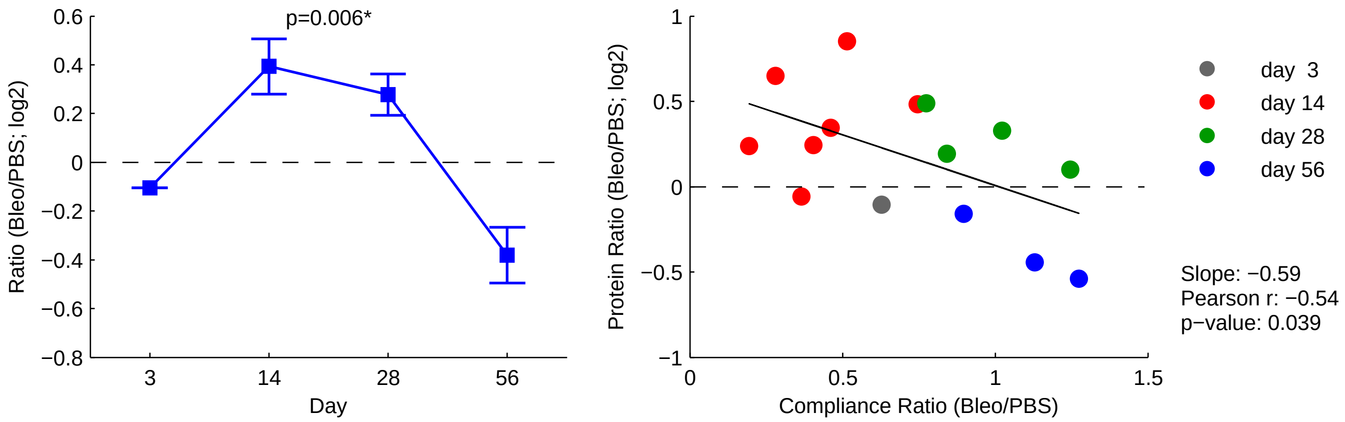

Q8BVK9 − Sp110 (id: 5353)

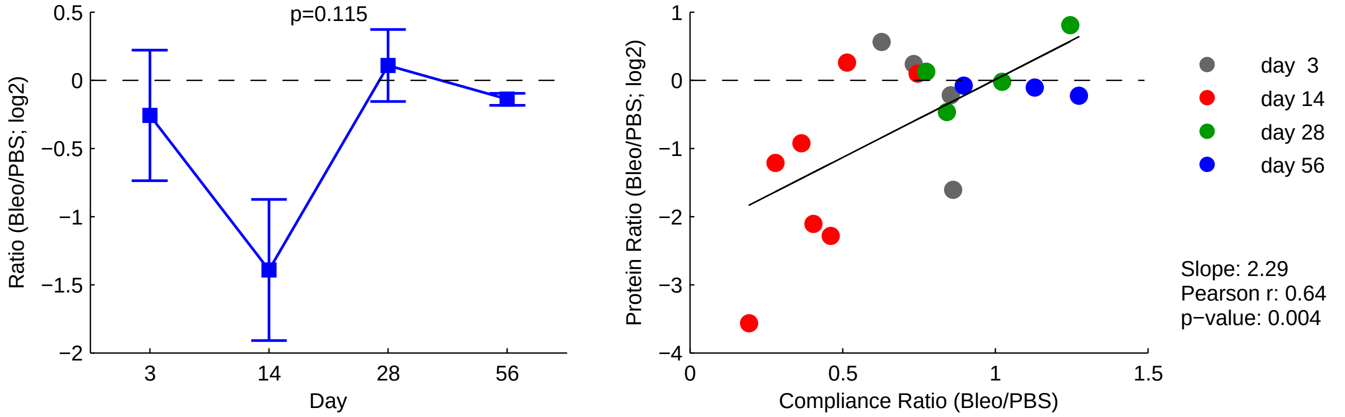

Q8BVL3 − Snx17 (id: 5354)

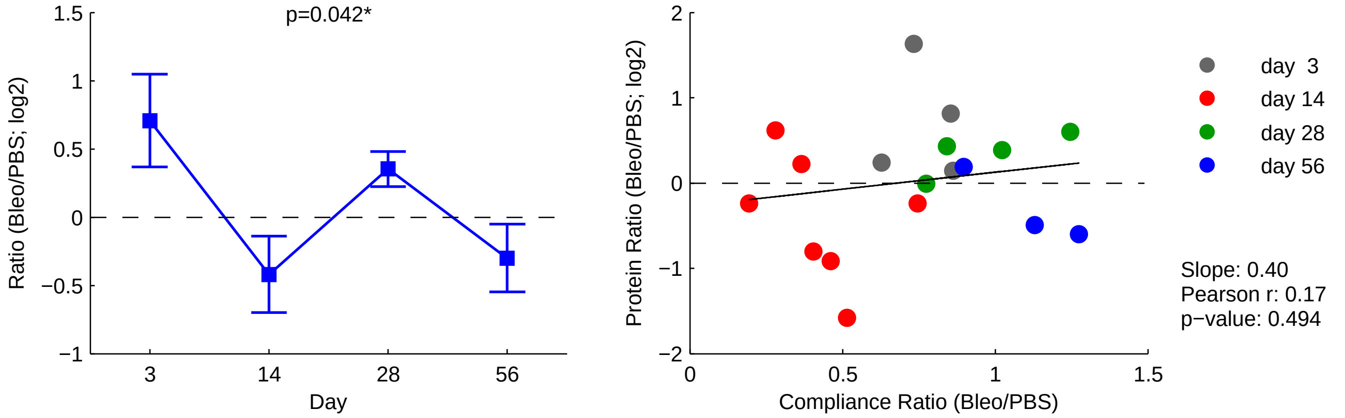

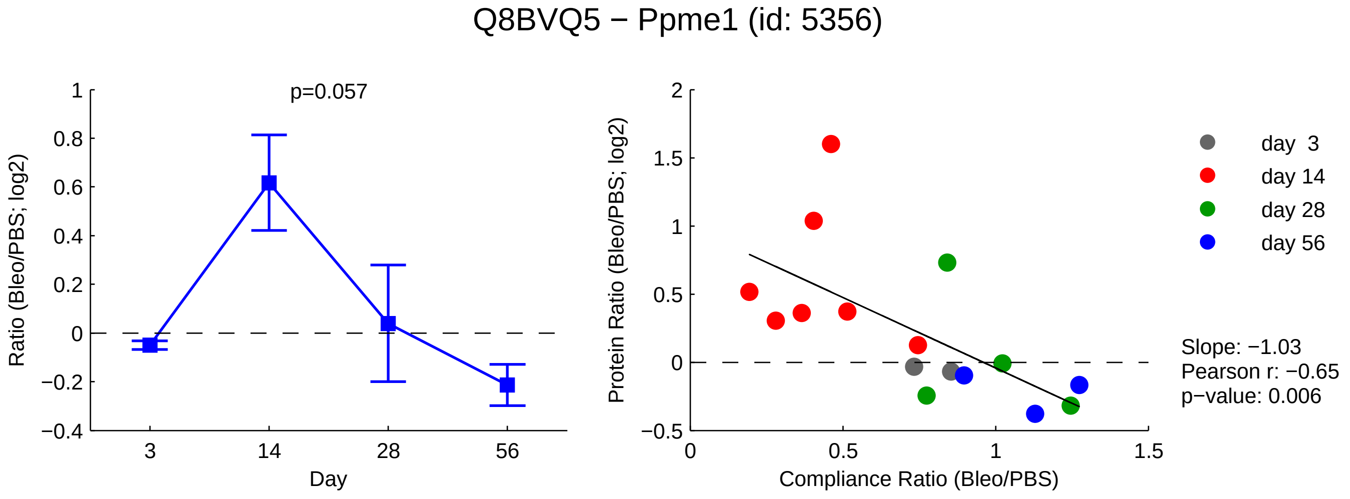
Q8BVQ5 − Ppme1 (id: 5356)
p=0.057
Ratio (Bleo/PBS; log2)
Day
Protein Ratio (Bleo/PBS; log2)
Compliance Ratio (Bleo/PBS)
day 3
day 14
day 28
day 56
Slope: −1.03
Pearson r: −0.65
p−value: 0.006

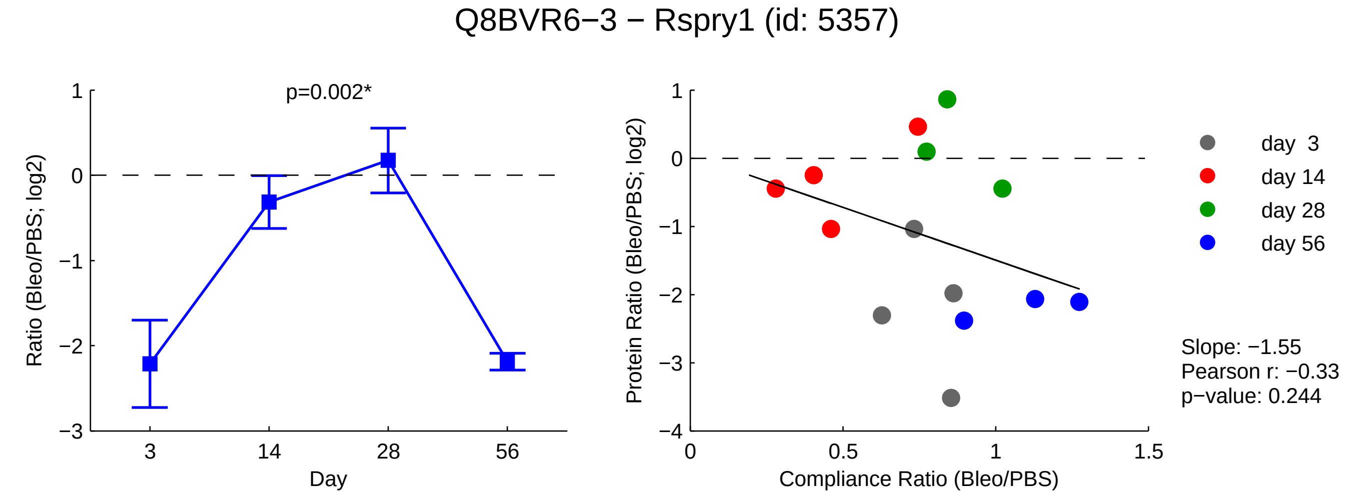
Q8BVR6−3 − Rspry1 (id: 5357)
p=0.002*
Ratio (Bleo/PBS; log2)
Day
Protein Ratio (Bleo/PBS; log2)
Compliance Ratio (Bleo/PBS)
day 3
day 14
day 28
day 56
Slope: −1.55
Pearson r: −0.33
p−value: 0.244

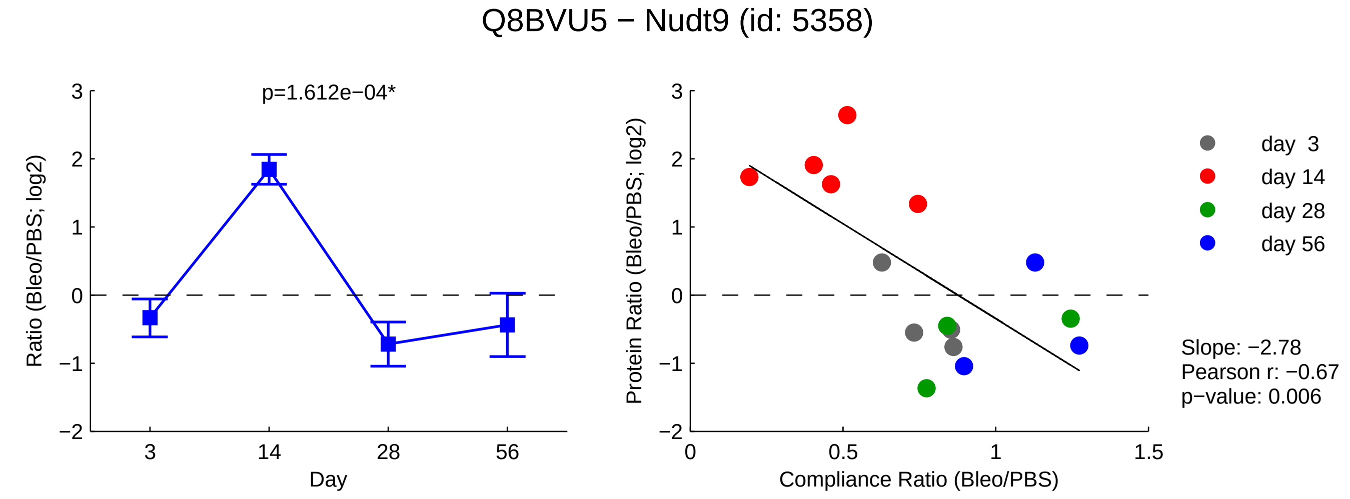
Q8BVU5 − Nudt9 (id: 5358)
p=1.612e−04*
Ratio (Bleo/PBS; log2)
Day
Protein Ratio (Bleo/PBS; log2)
Compliance Ratio (Bleo/PBS)
day 3
day 14
day 28
day 56
Slope: −2.78
Pearson r: −0.67
p−value: 0.006

Q8BW75 − Maob (id: 5364)

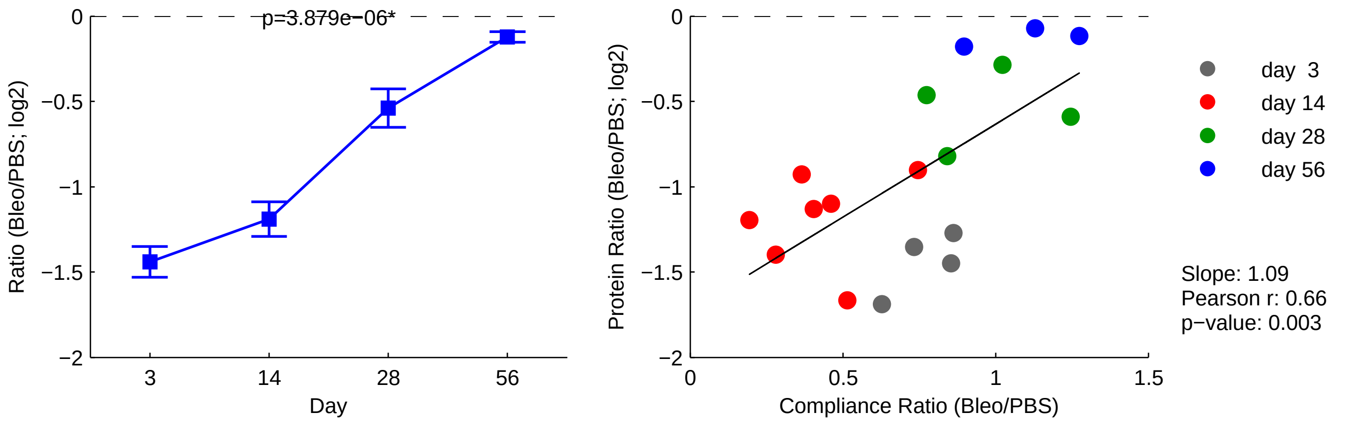

Q8BWG8 − Arrb1 (id: 5368)

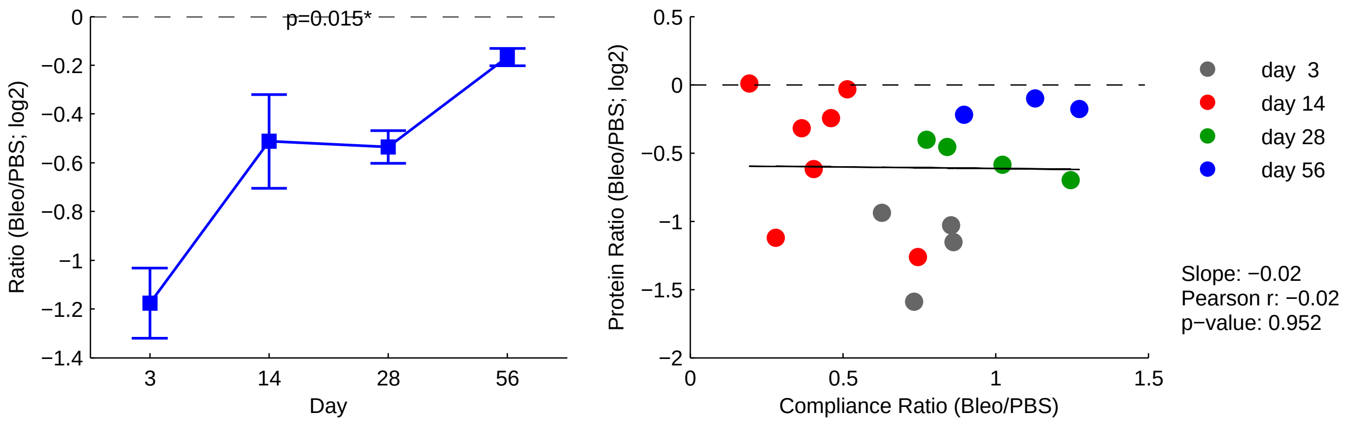

Q8BWG9 − Orai1 (id: 5370)

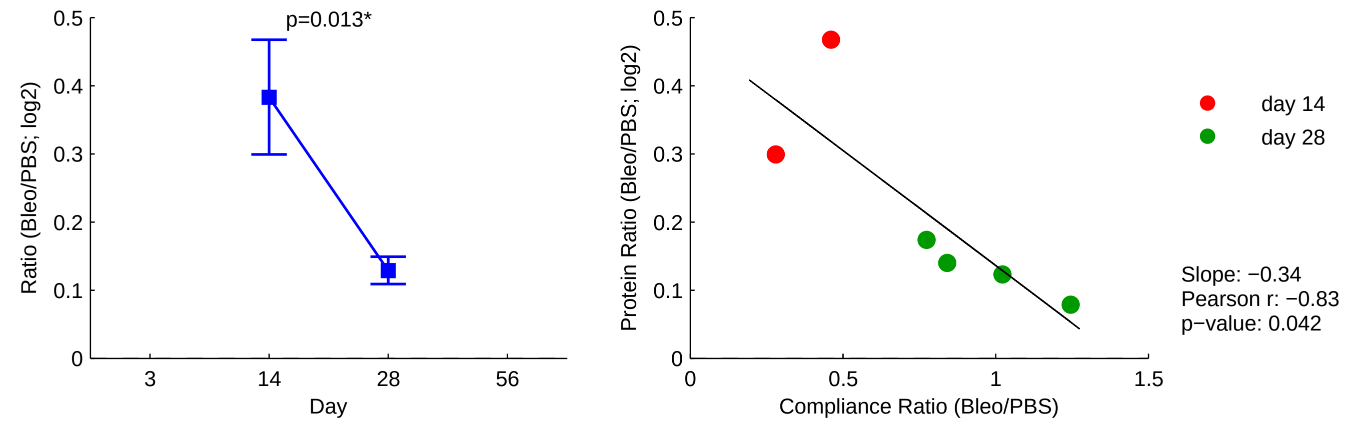

Q8BWM0 − Ptges2 (id: 5372)

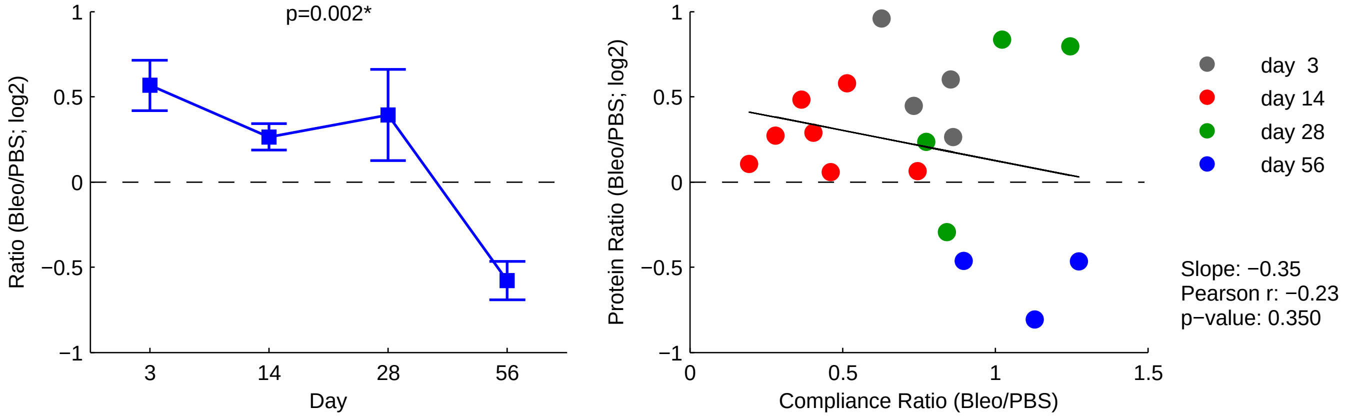

Q8BWT1 − Acaa2 (id: 5376)

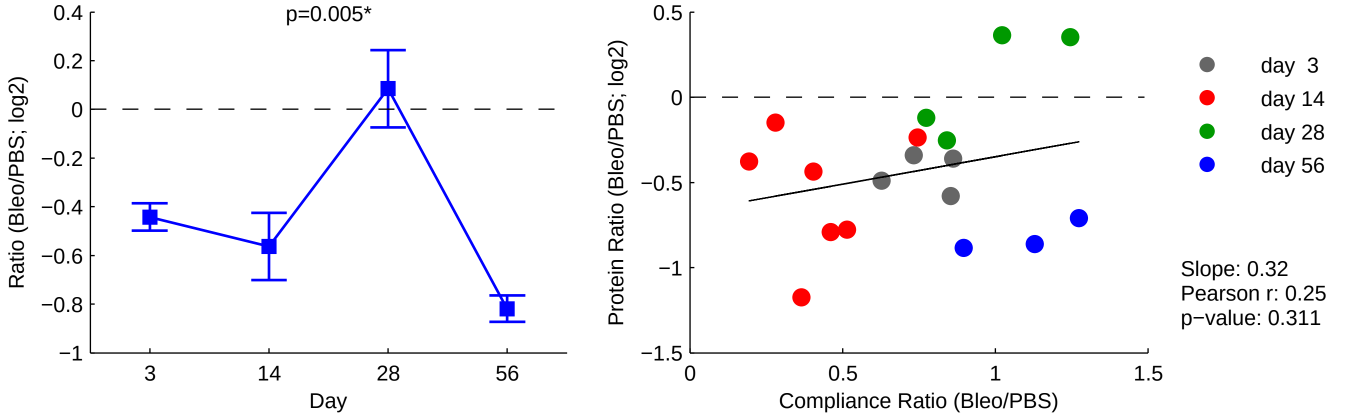

Q8BWW9 − Pkn2 (id: 5378)

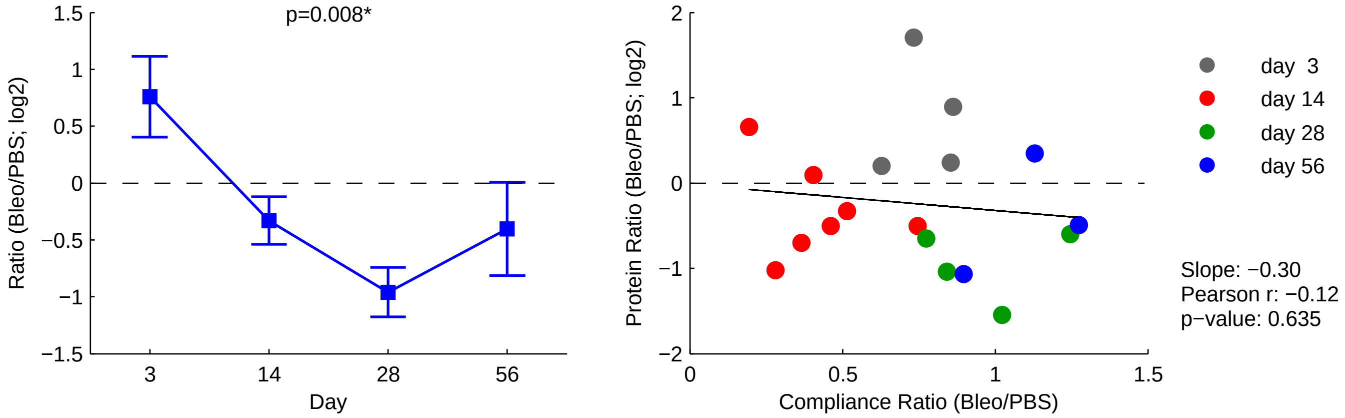

Q8BWY2 − Clec1a (id: 5379)

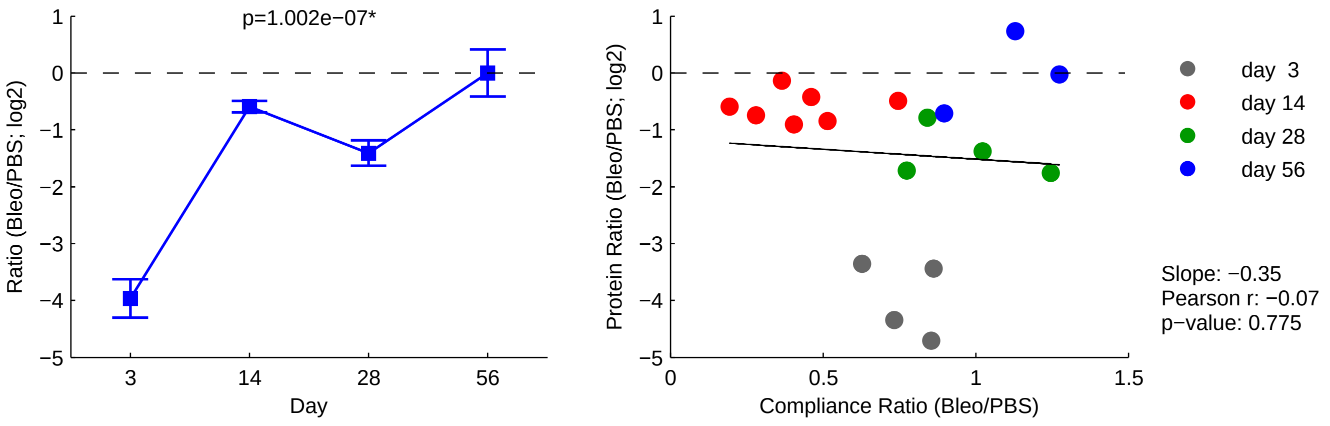

Q8BWY3 − Etf1 (id: 5380)

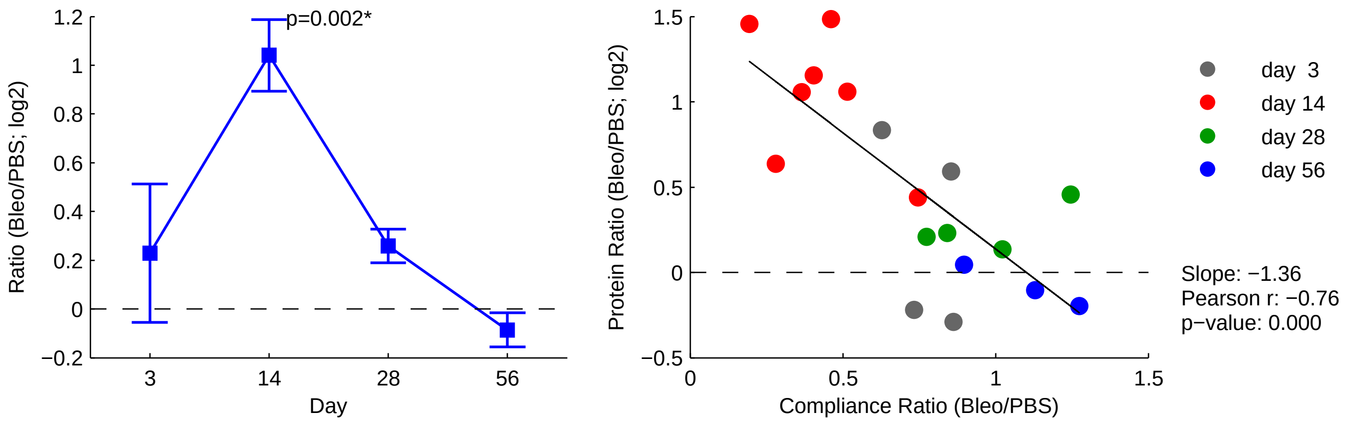

Q8BWZ3 − Naa25 (id: 5381)

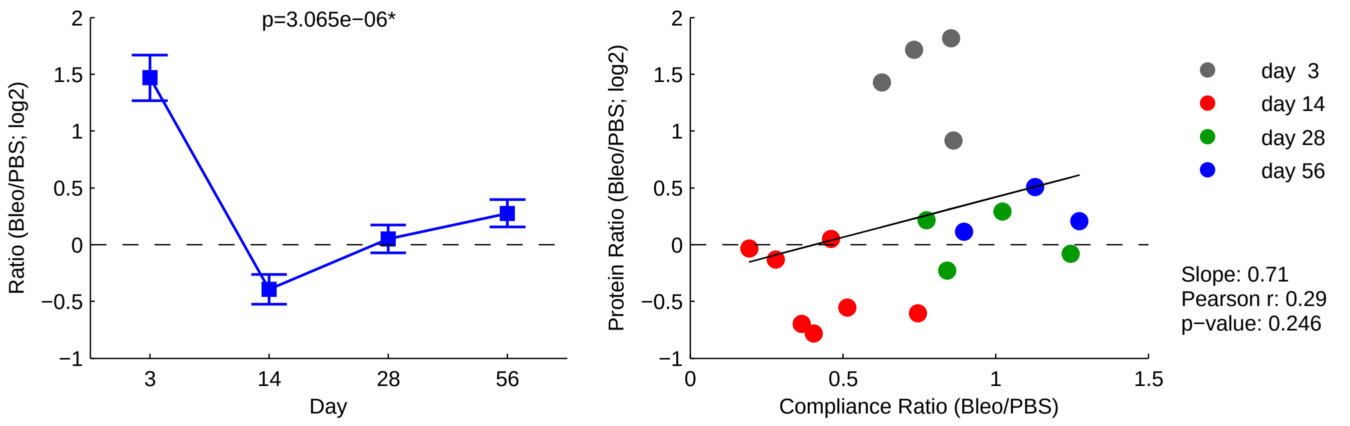

Q8BX10−2 − Pgam5 (id: 5384)

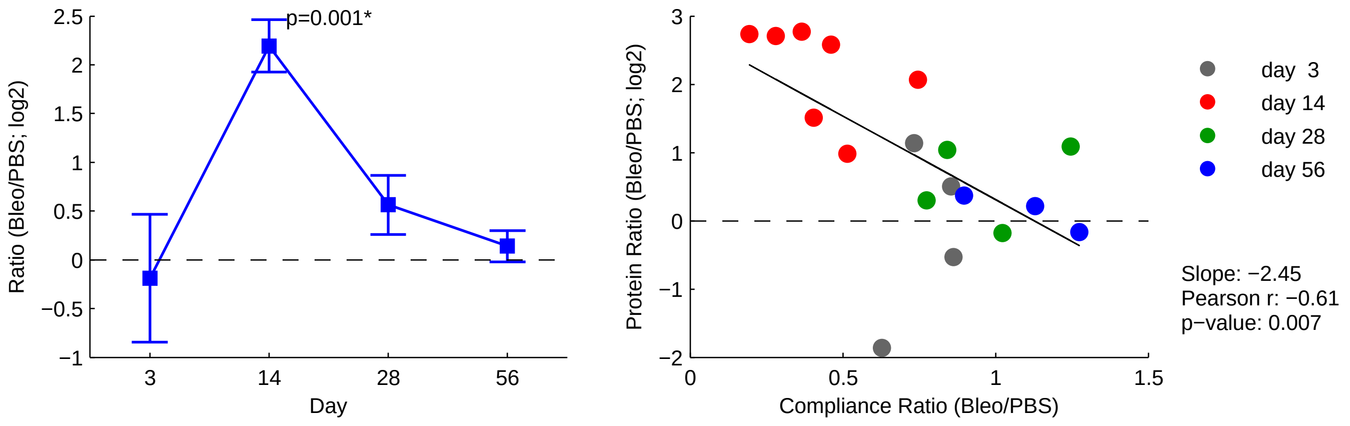

Q8BX70 − Vps13c (id: 5388)

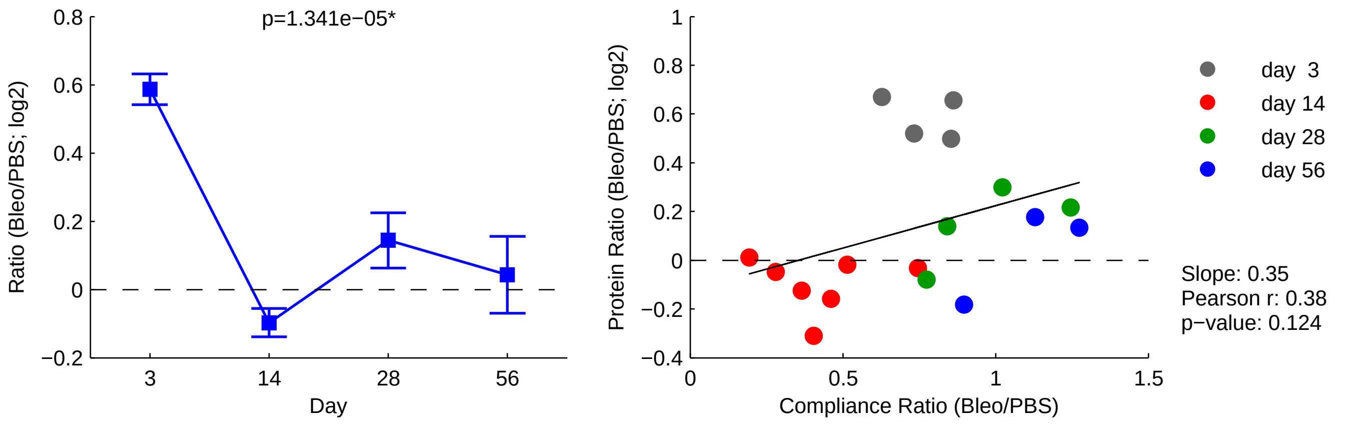

Q8BX80 − Engase (id: 5389)

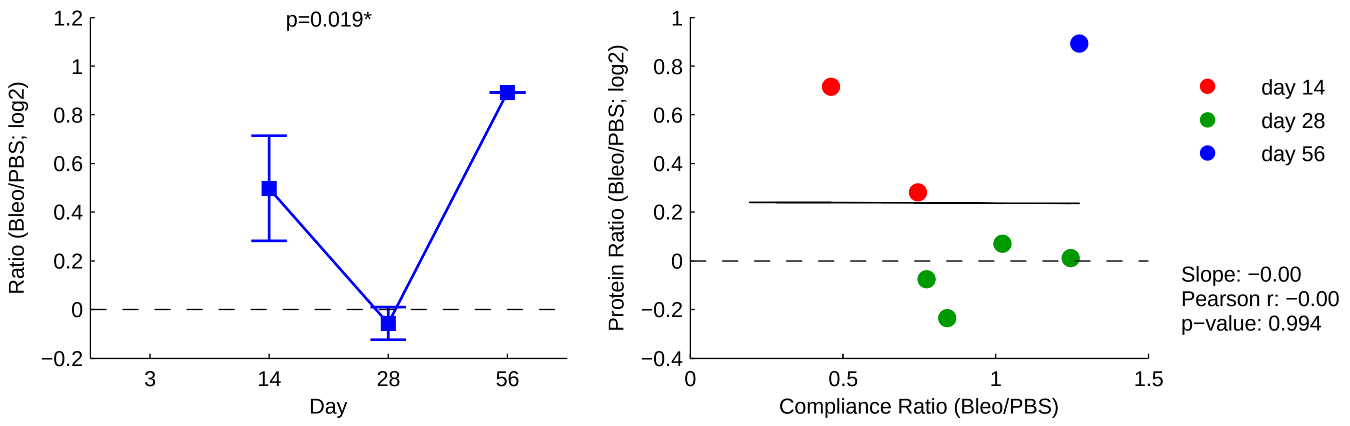

Q8BX94 − Osbpl2 (id: 5390)

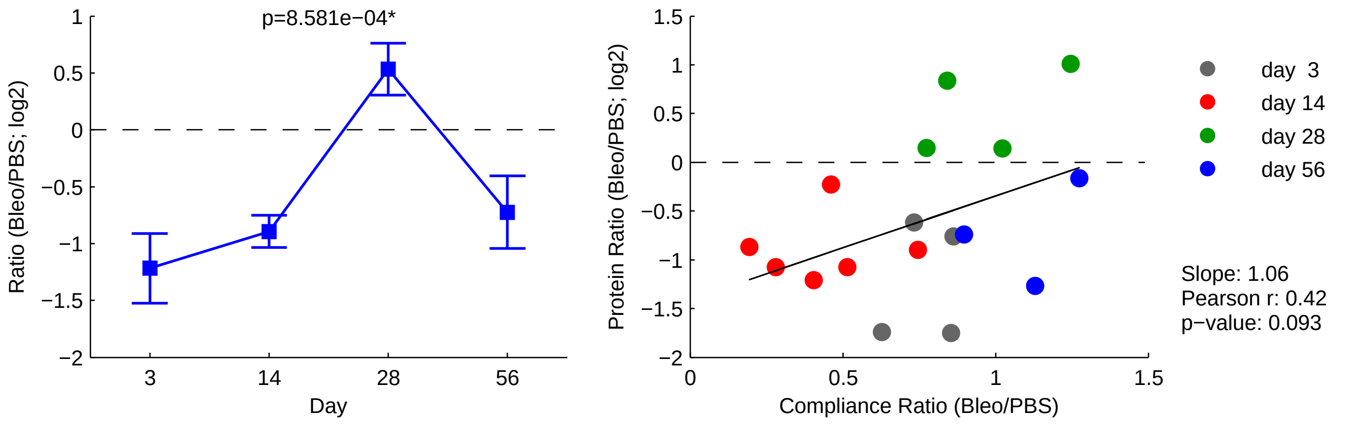

Q8BXA5 − Clptm1l (id: 5391)

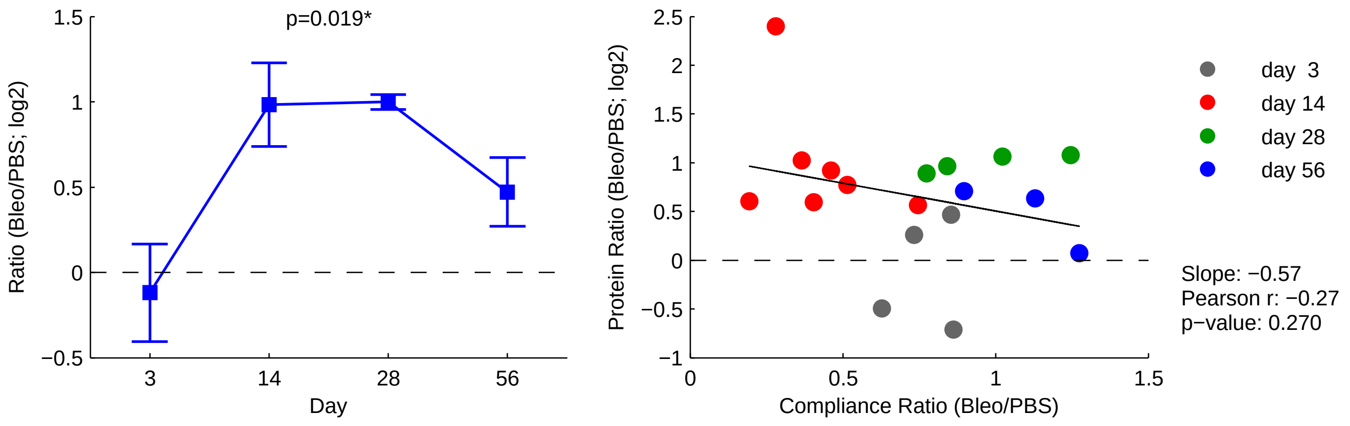

Q8BXC6 − Commd2 (id: 5392)

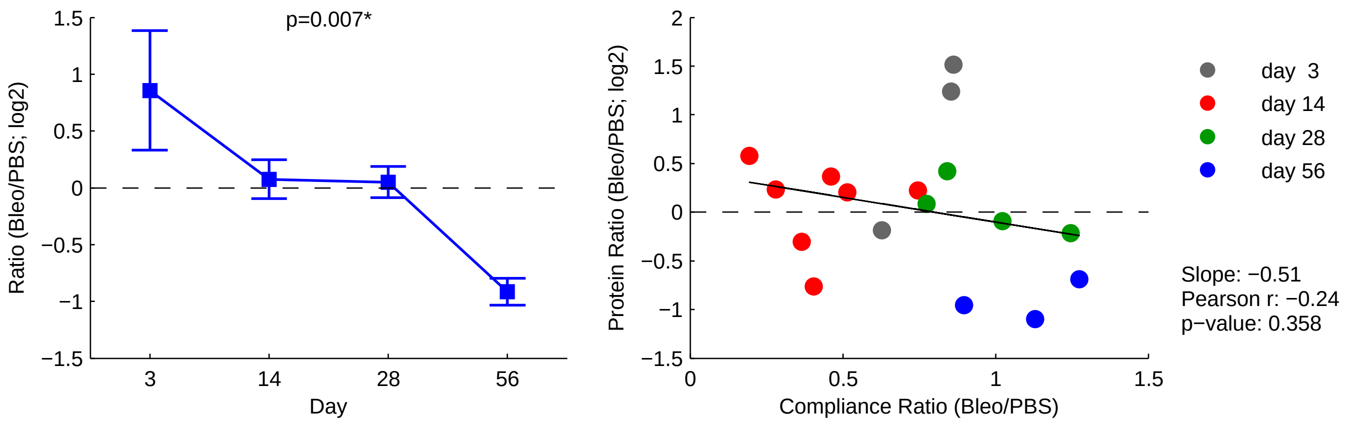

Q8BXK9 − Clic5 (id: 5395)

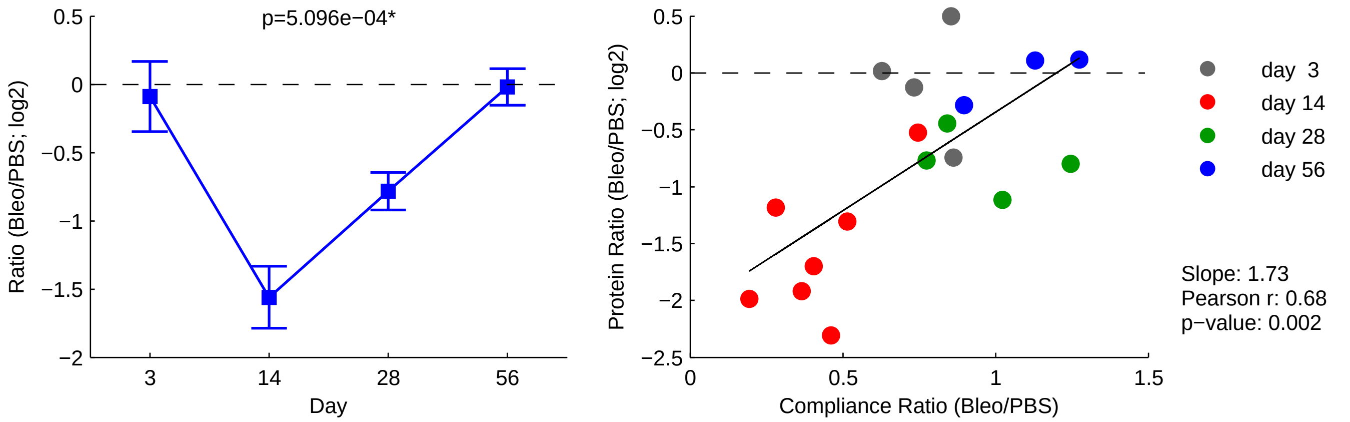

Q8BXZ1 − Tmx3 (id: 5400)

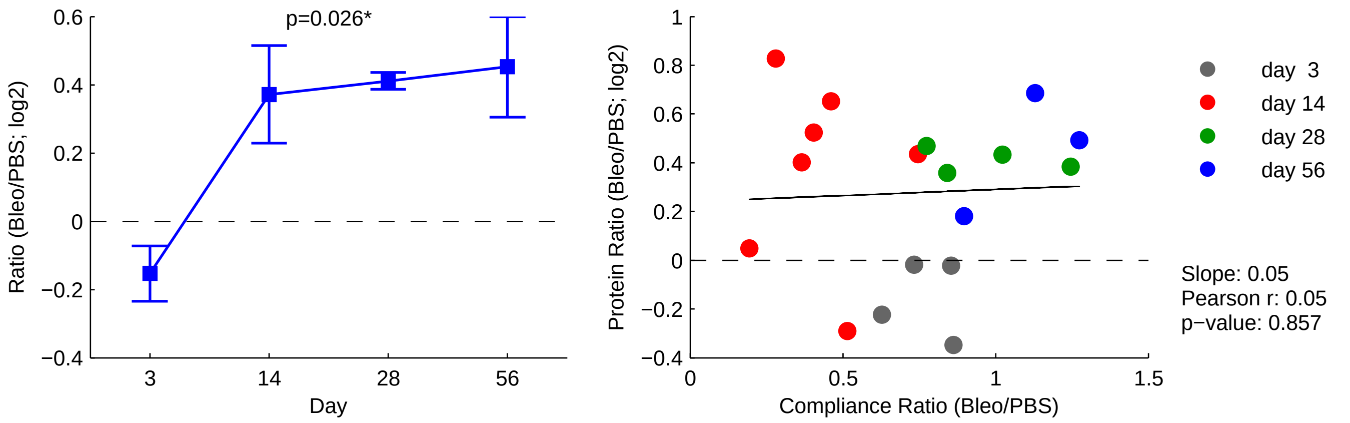

Q8BY89 − Slc44a2 (id: 5402)

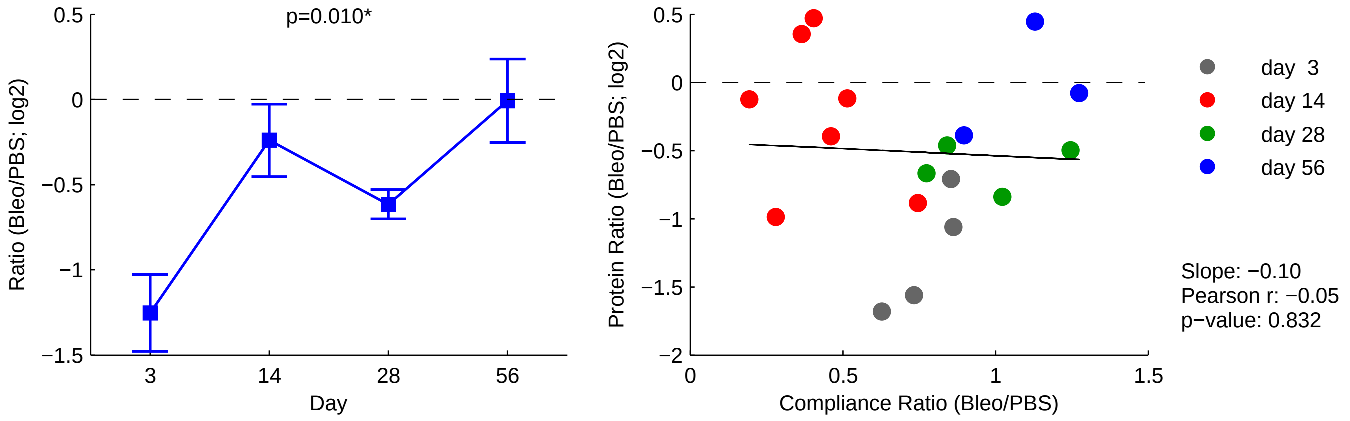

Q8BYH7 − Tbc1d17 (id: 5406)

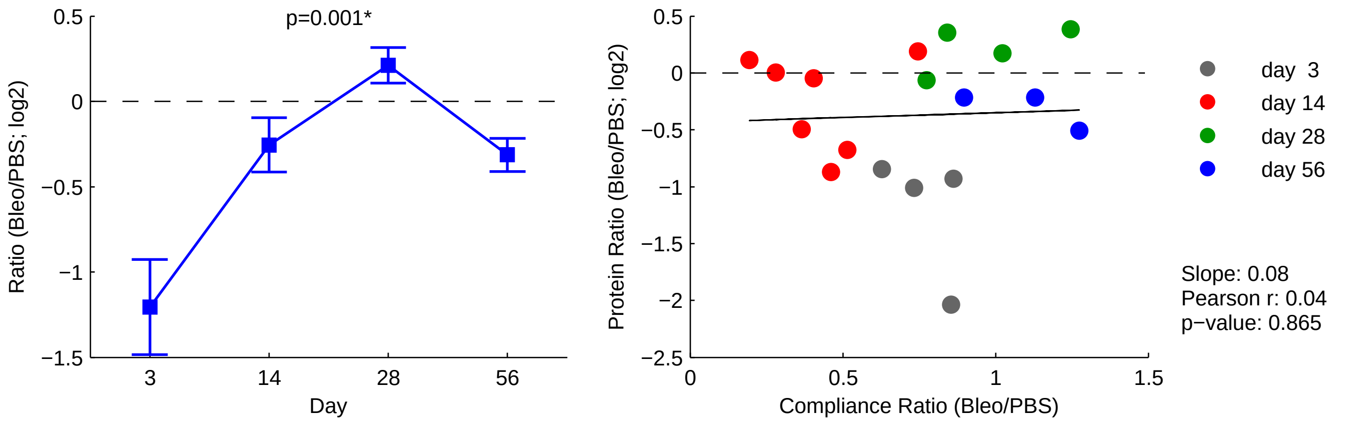

Q8BYI6−2 − Lpcat2 (id: 5407)

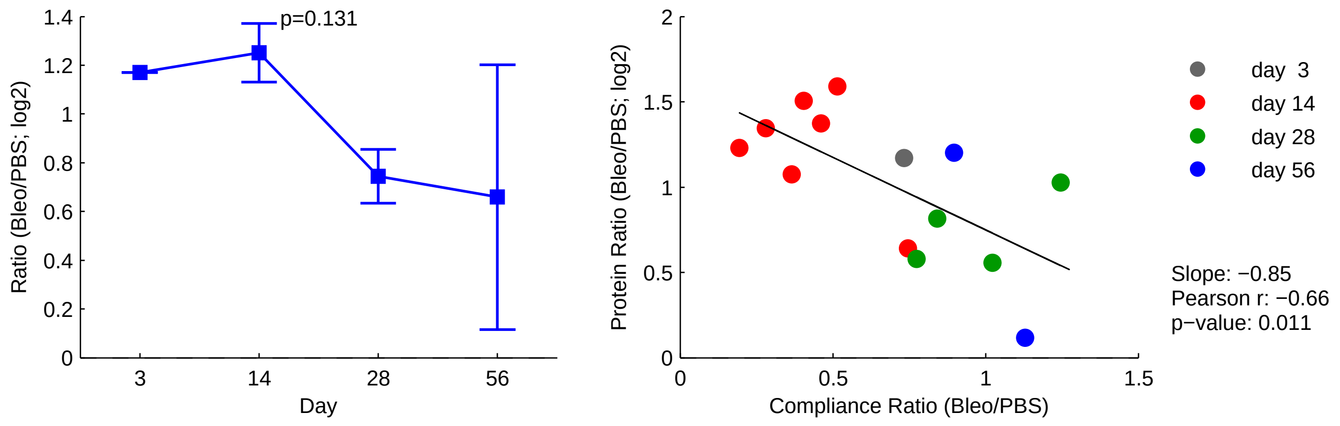

Q8BYK6 − Ythdf3 (id: 5409)

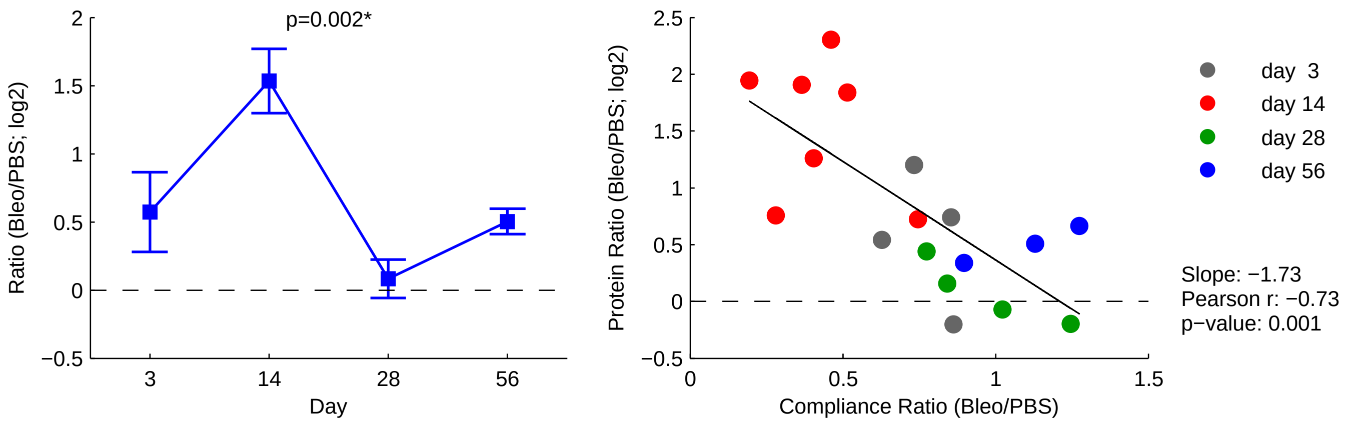

Q8BYL4 − Yars2 (id: 5410)

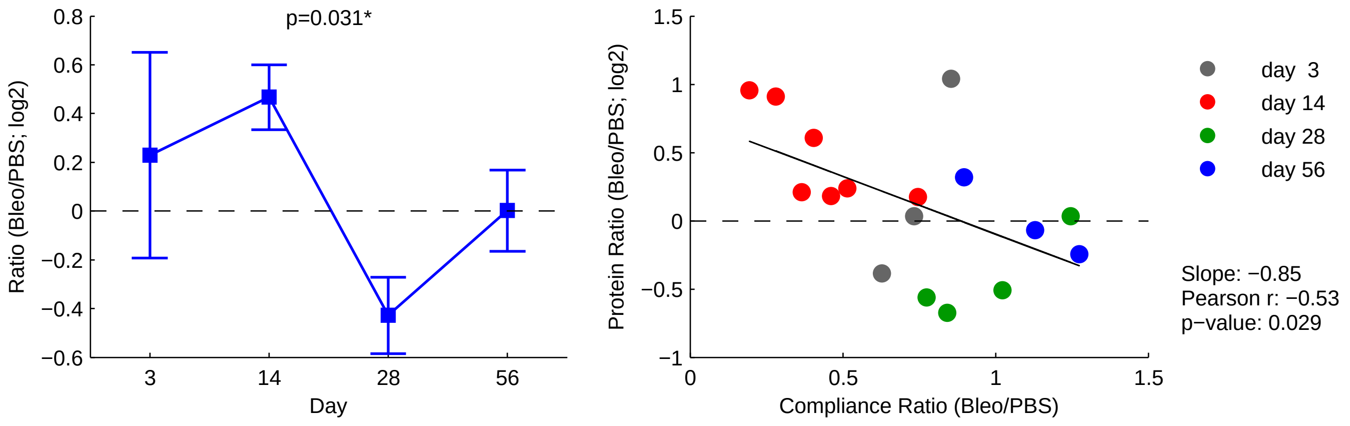

Q8BYM7 − Rsph4a (id: 5411)

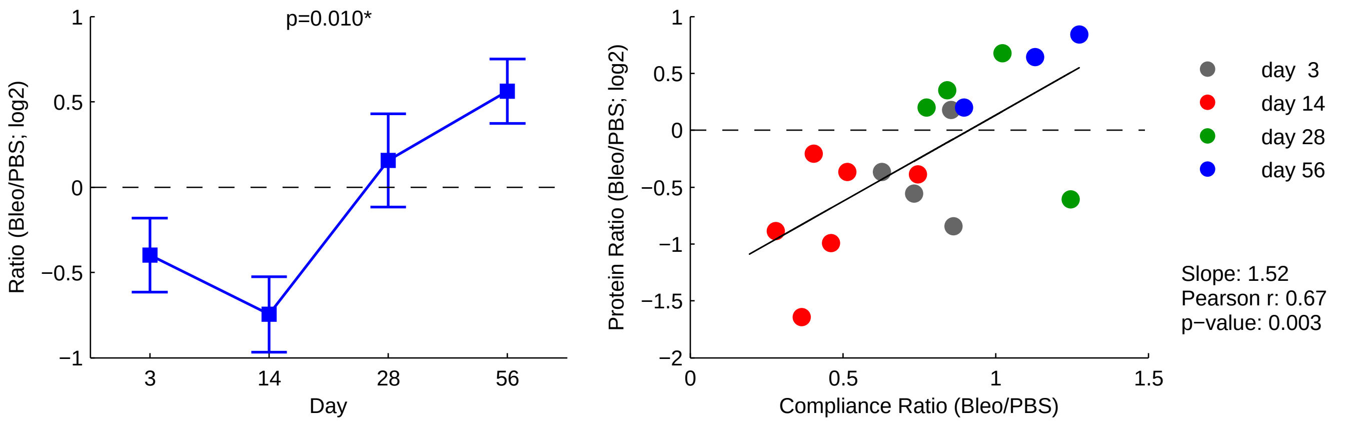

Q8BYU6 − Tor1aip2 (id: 5414)

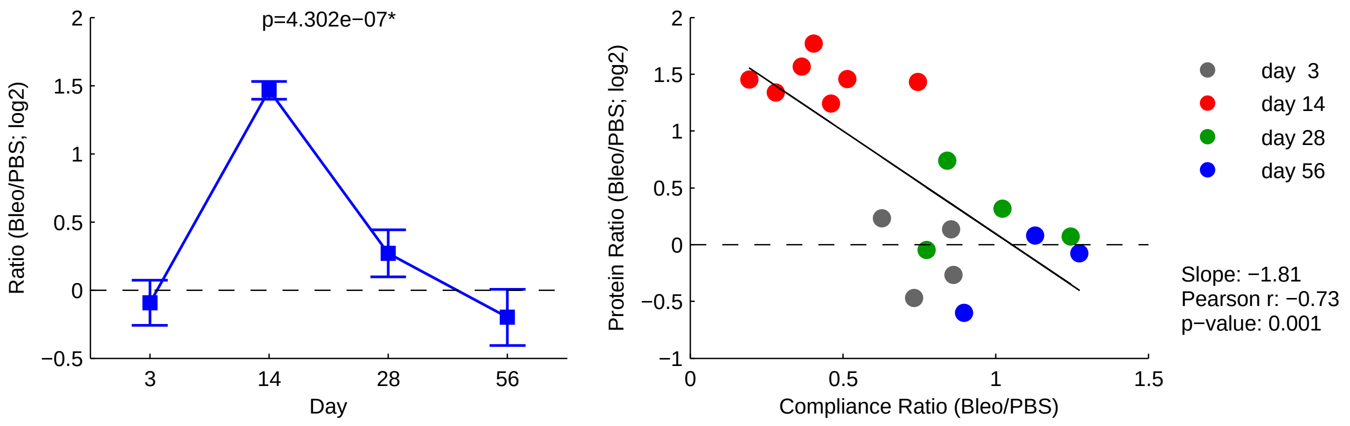

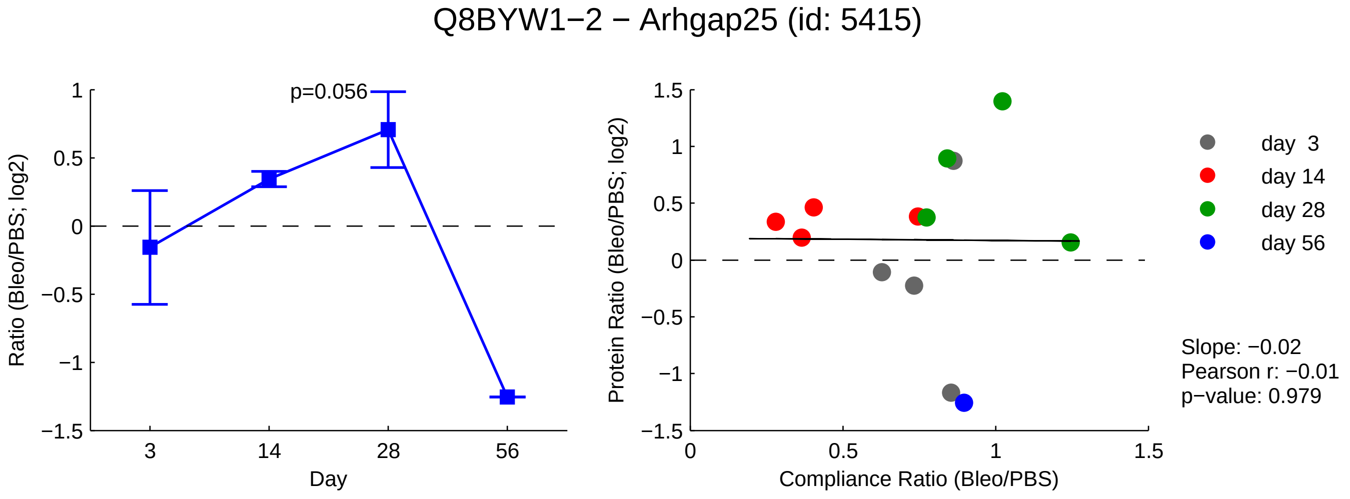
Q8BYW1−2 − Arhgap25 (id: 5415)
p=0.056
Ratio (Bleo/PBS; log2)
Day
Protein Ratio (Bleo/PBS; log2)
Compliance Ratio (Bleo/PBS)
day 3
day 14
day 28
day 56
Slope: −0.02
Pearson r: −0.01
p−value: 0.979

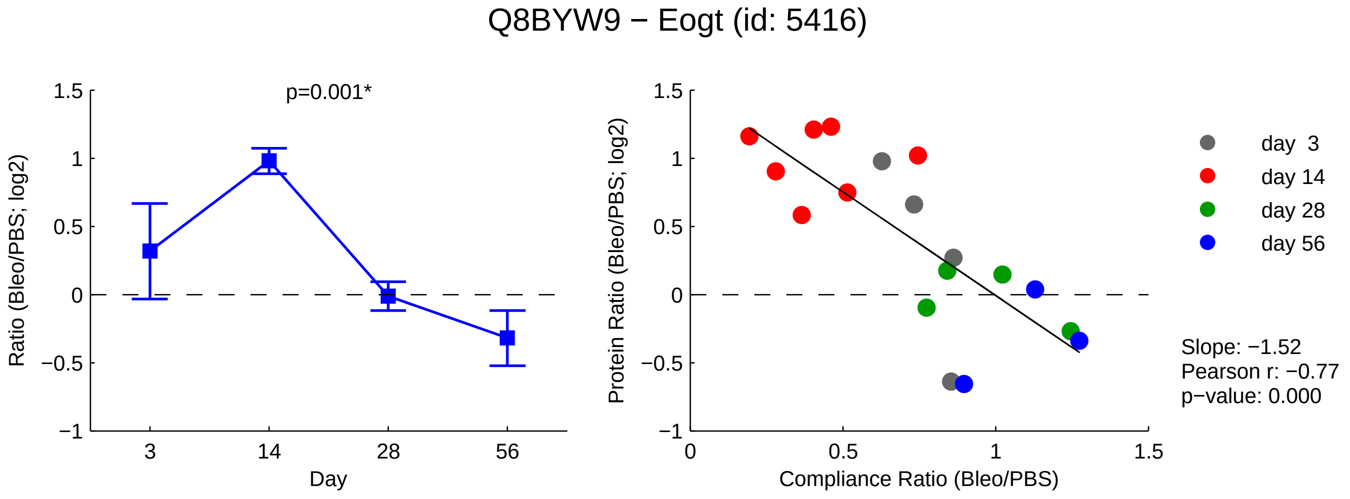
Q8BYW9 − Eogt (id: 5416)
p=0.001*
Ratio (Bleo/PBS; log2)
Day
Protein Ratio (Bleo/PBS; log2)
Compliance Ratio (Bleo/PBS)
day 3
day 14
day 28
day 56
Slope: −1.52
Pearson r: −0.77
p−value: 0.000

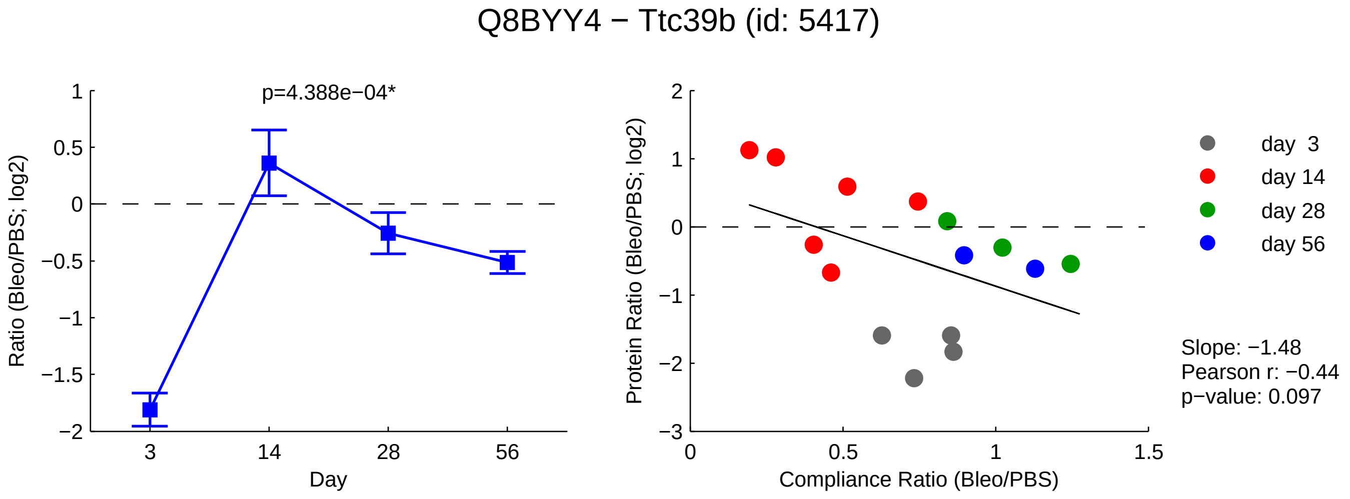
Q8BYY4 − Ttc39b (id: 5417)
p=4.388e−04*
Ratio (Bleo/PBS; log2)
Day
Protein Ratio (Bleo/PBS; log2)
Compliance Ratio (Bleo/PBS)
day 3
day 14
day 28
day 56
Slope: −1.48
Pearson r: −0.44
p−value: 0.097

Q8BYZ7 − Elmo3 (id: 5419)

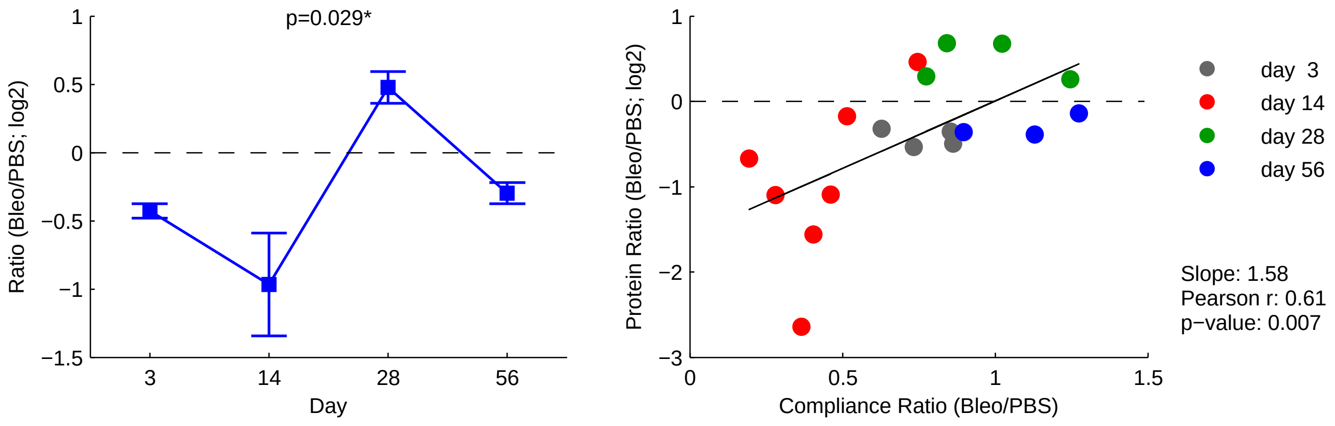

Q8BZ03 − Prkd2 (id: 5420)

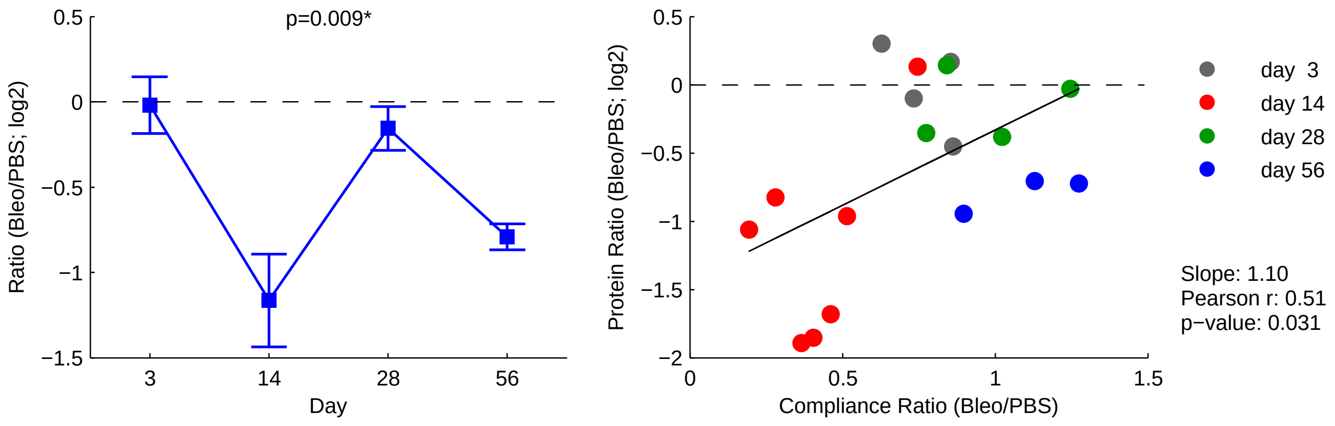

Q8BZ36−2 − Rint1 (id: 5422)

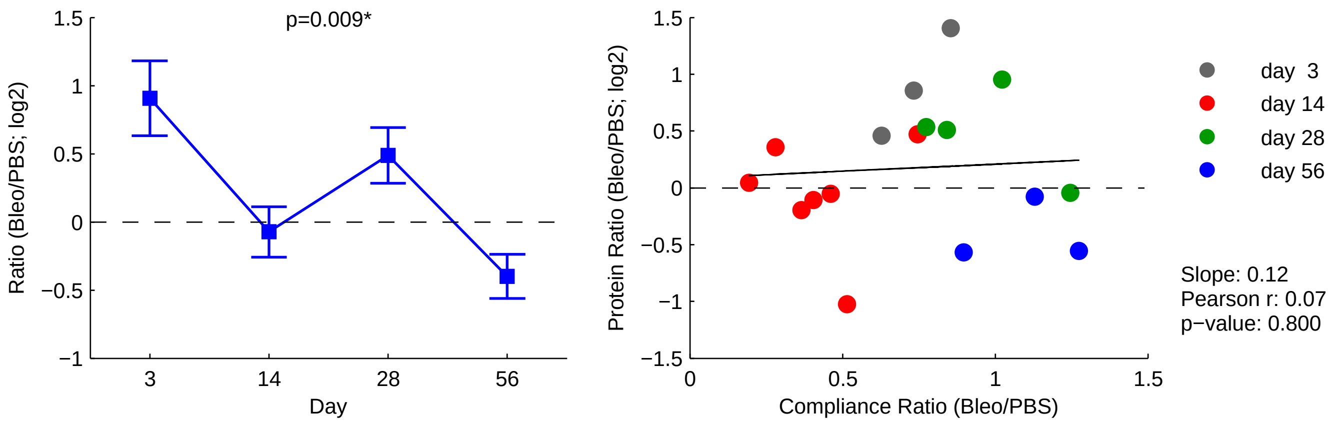

Q8BZ60 − Ston2 (id: 5424)

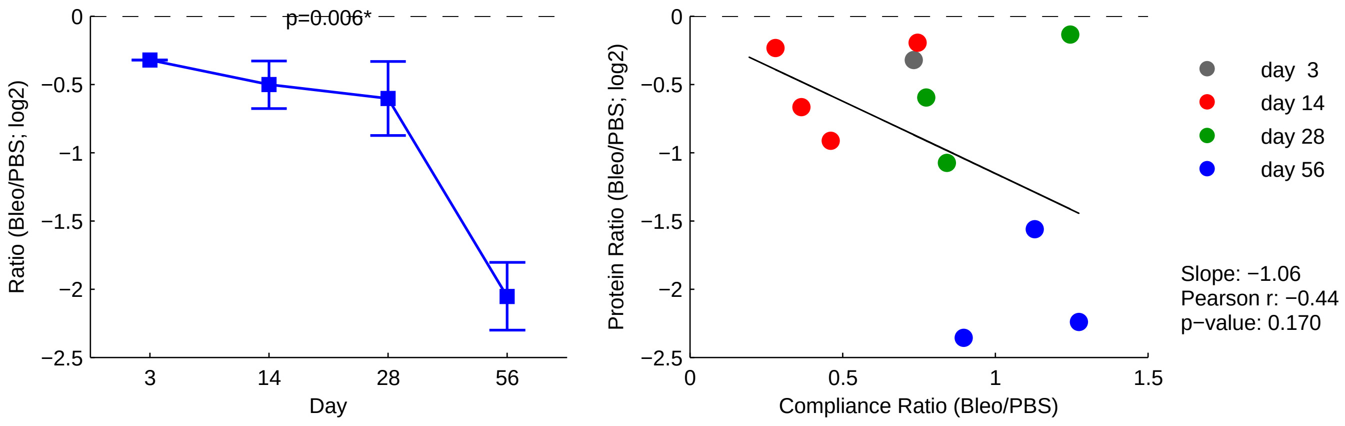

Q8BZF8 − Pgm5 (id: 5426)

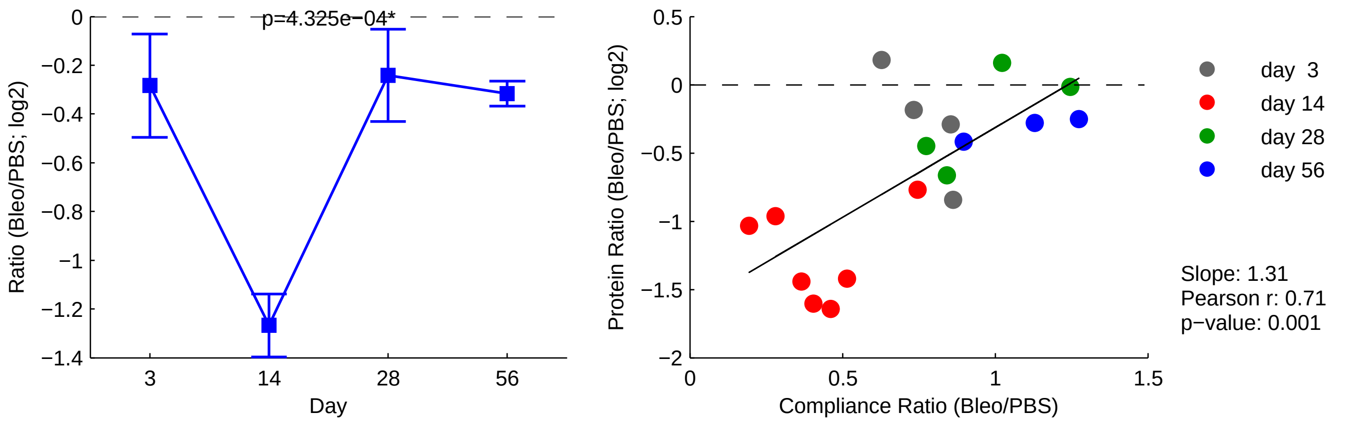

Q8BZI0 − Afap1l1 (id: 5427)

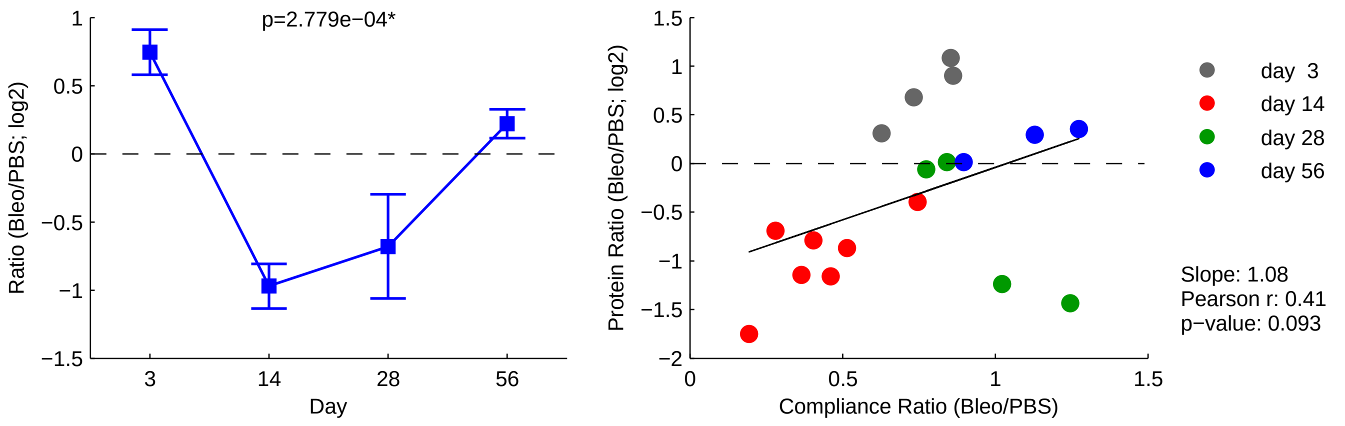

Q8BZR9 − (id: 5429)

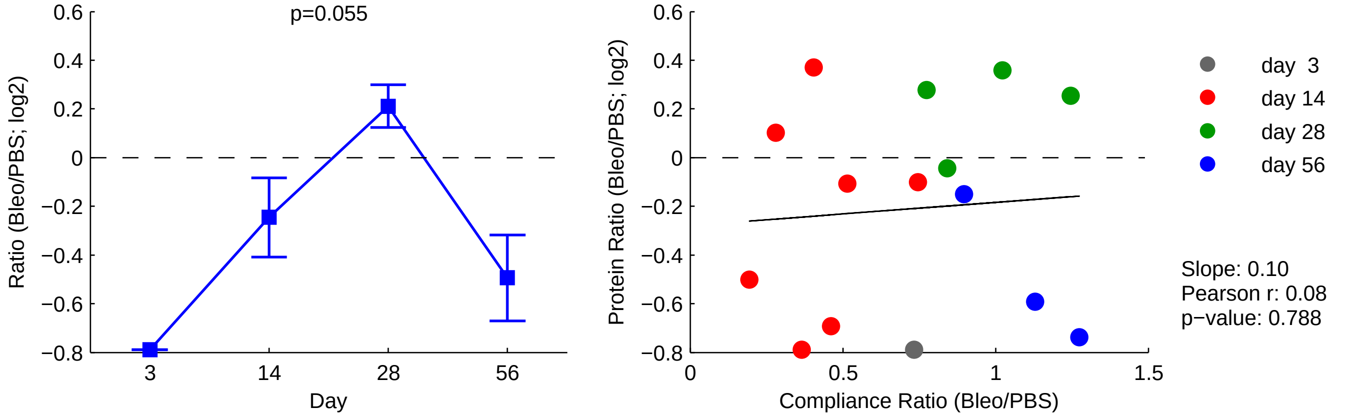

Q8BZW8 − Nhlrc2 (id: 5430)

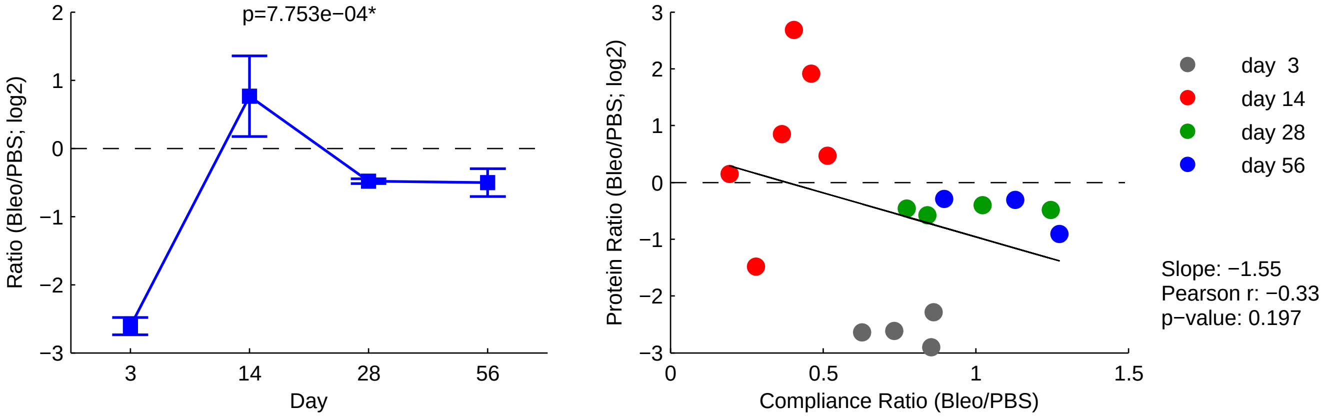

Q8C052 − Map1s (id: 5435)

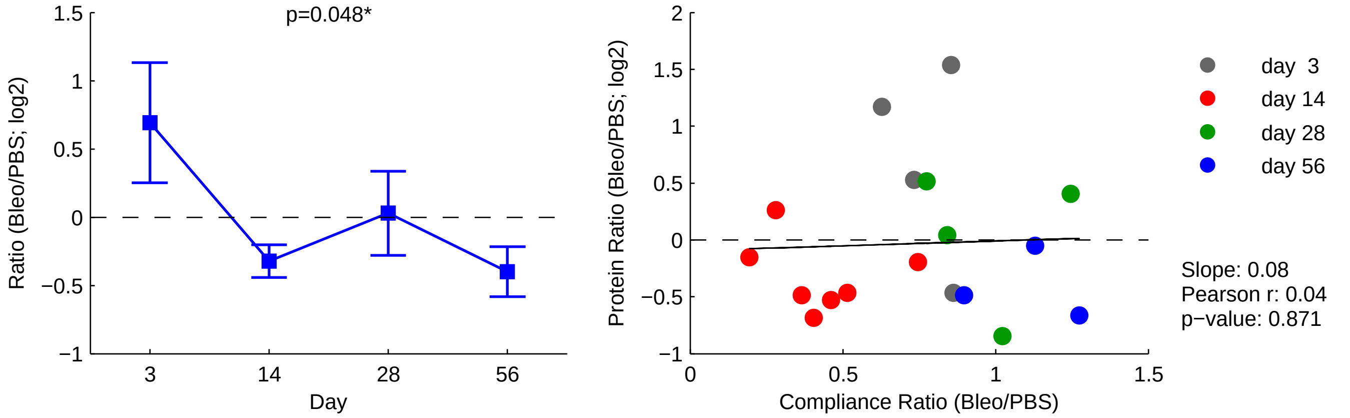

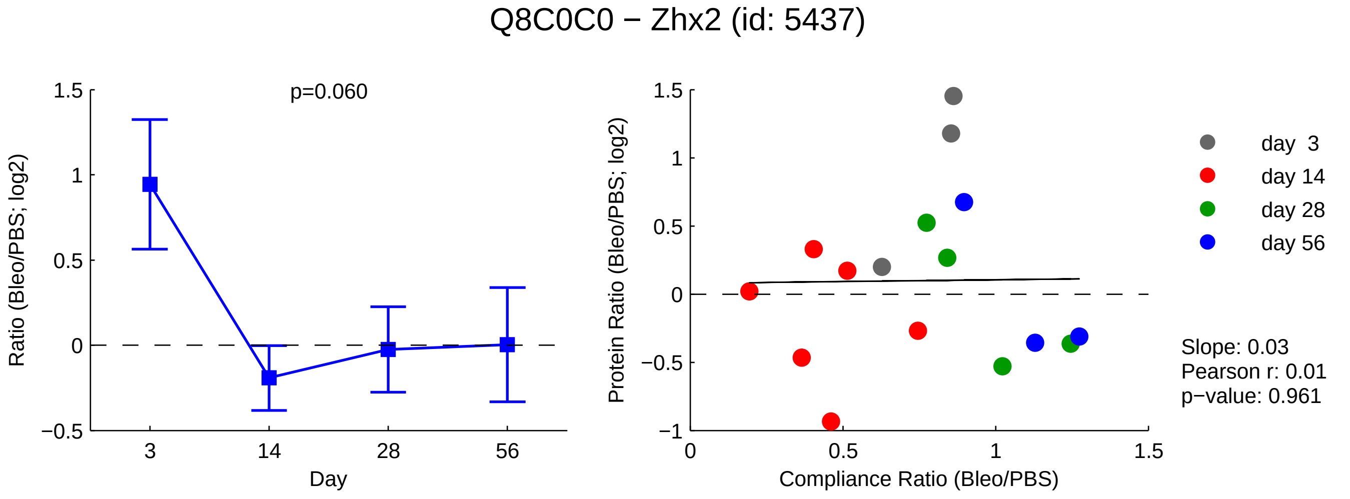
Q8C0C0 − Zhx2 (id: 5437)
p=0.060
Ratio (Bleo/PBS; log2)
Day
Protein Ratio (Bleo/PBS; log2)
Compliance Ratio (Bleo/PBS)
day 3
day 14
day 28
day 56
Slope: 0.03
Pearson r: 0.01
p−value: 0.961

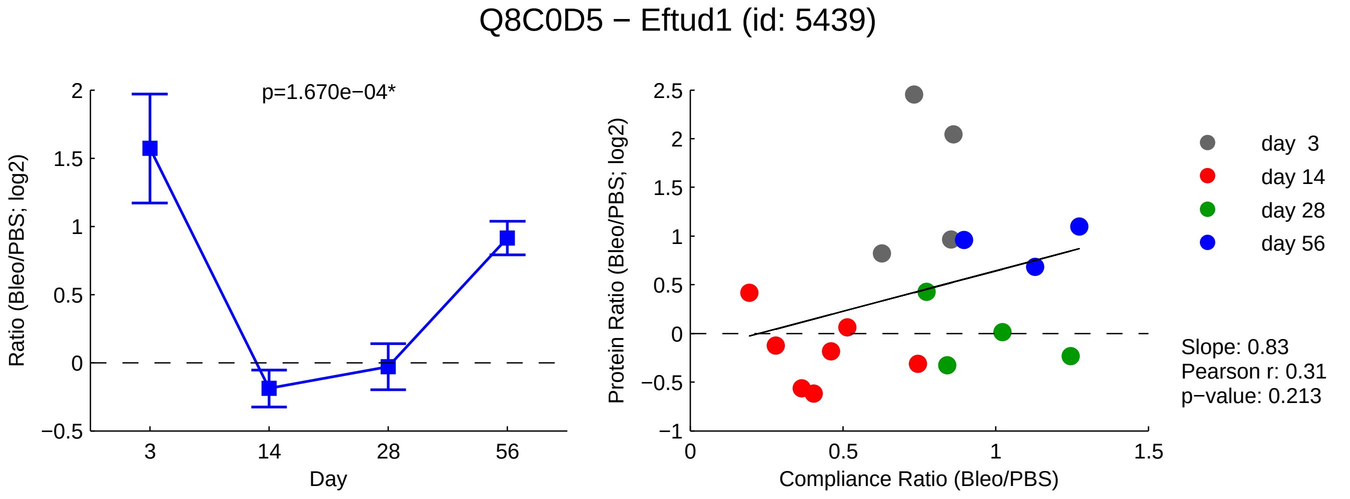
Q8C0D5 − Eftud1 (id: 5439)
p=1.670e−04*
Ratio (Bleo/PBS; log2)
Day
Protein Ratio (Bleo/PBS; log2)
Compliance Ratio (Bleo/PBS)
day 3
day 14
day 28
day 56
Slope: 0.83
Pearson r: 0.31
p−value: 0.213

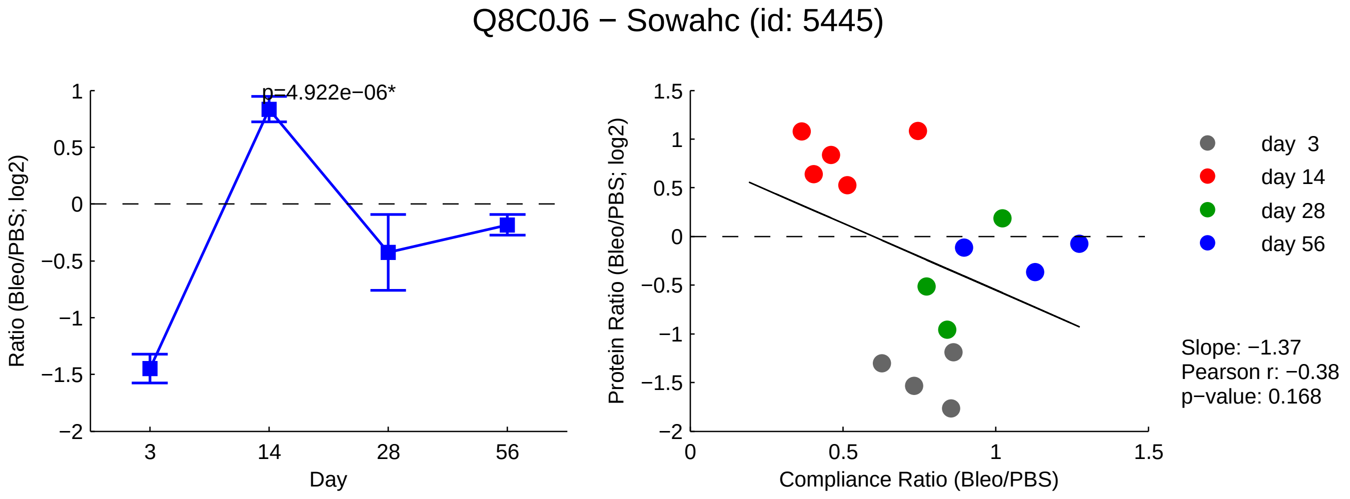
Q8C0J6 − Sowahc (id: 5445)
p=4.922e−06*
Ratio (Bleo/PBS; log2)
Day
Protein Ratio (Bleo/PBS; log2)
Compliance Ratio (Bleo/PBS)
day 3
day 14
day 28
day 56
Slope: −1.37
Pearson r: −0.38
p−value: 0.168

Q8C0K5 − Slc25a16 (id: 5446)

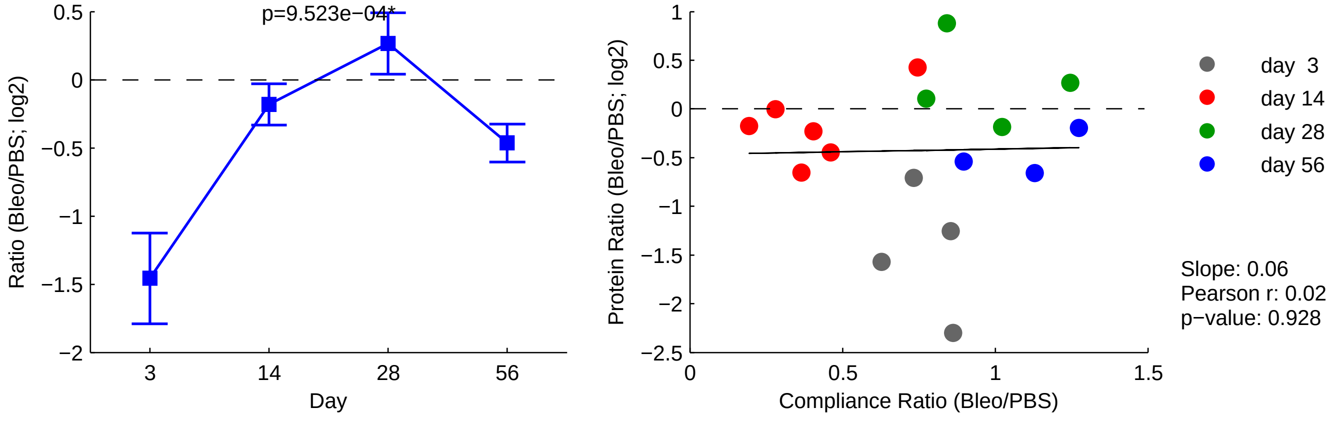

Q8C0K8 − Uvrag (id: 5447)

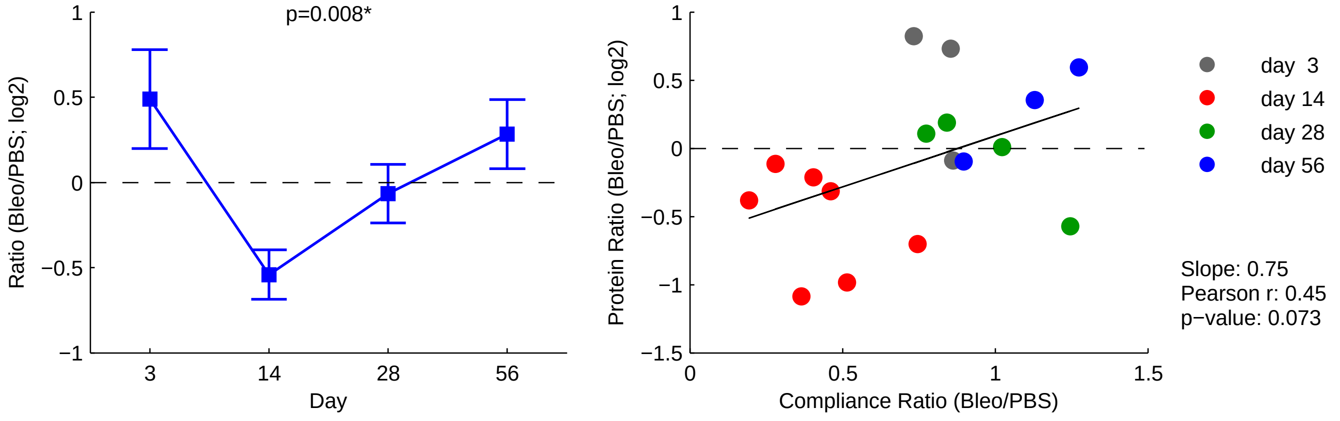

Q8C0L0 − Tmx4 (id: 5448)

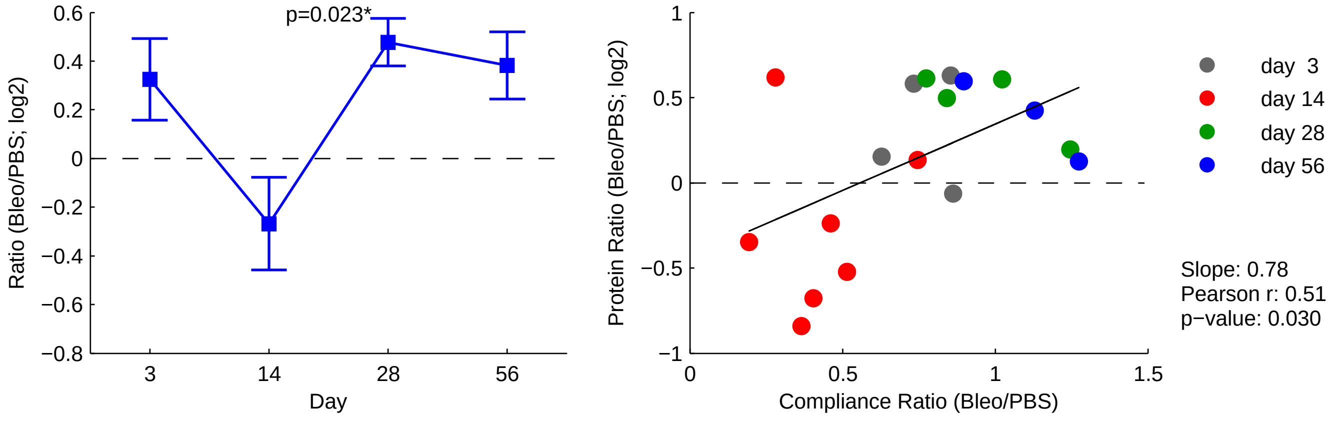

Q8C0N2 − Agpat9 (id: 5452)

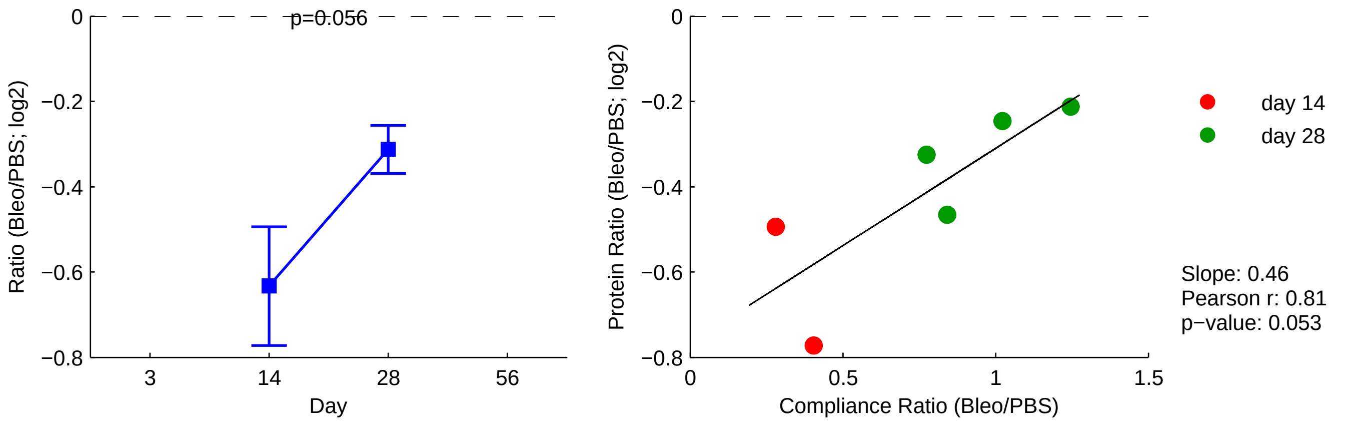

Q8C0S4 − Ttc21a (id: 5455)

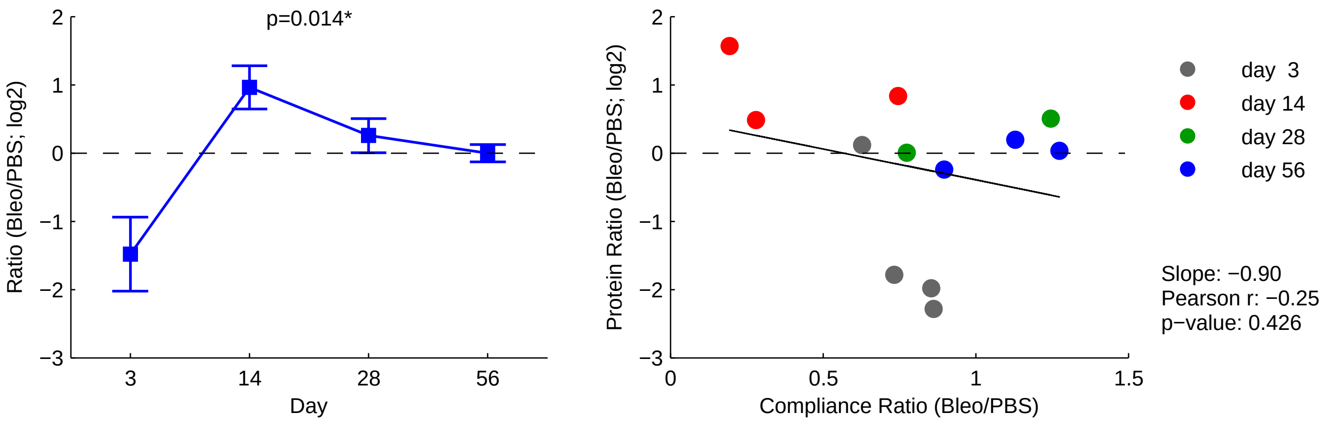

Q8C0Z1 − Itfg3 (id: 5459)

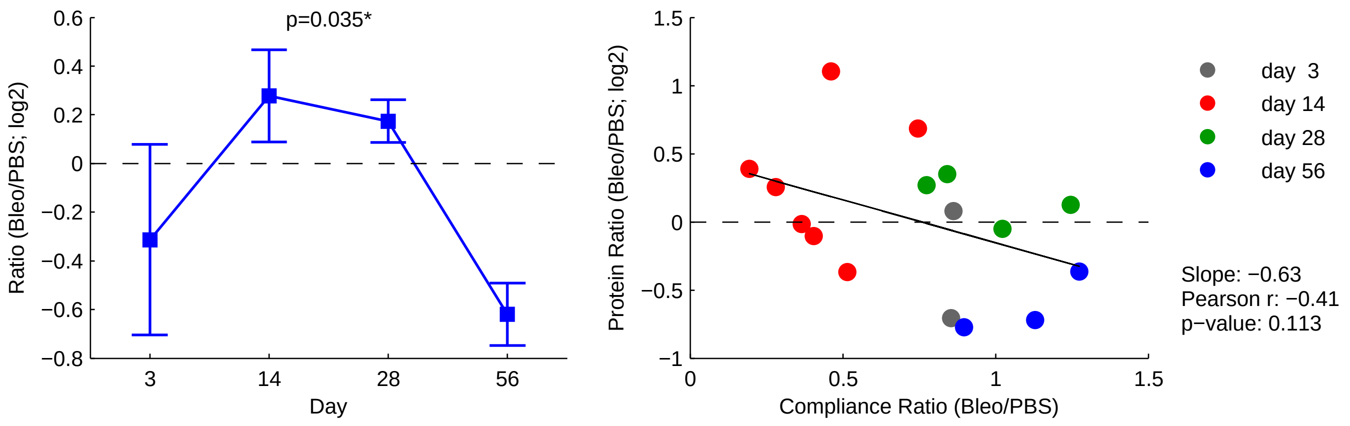

Q8C119 − Ndnf (id: 5461)

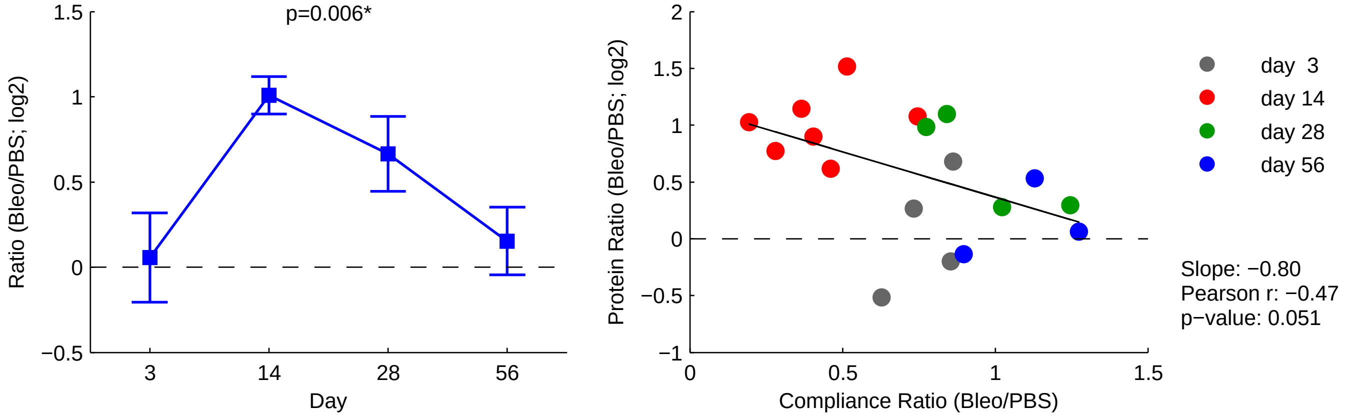

Q8C129 − Lnpep (id: 5462)

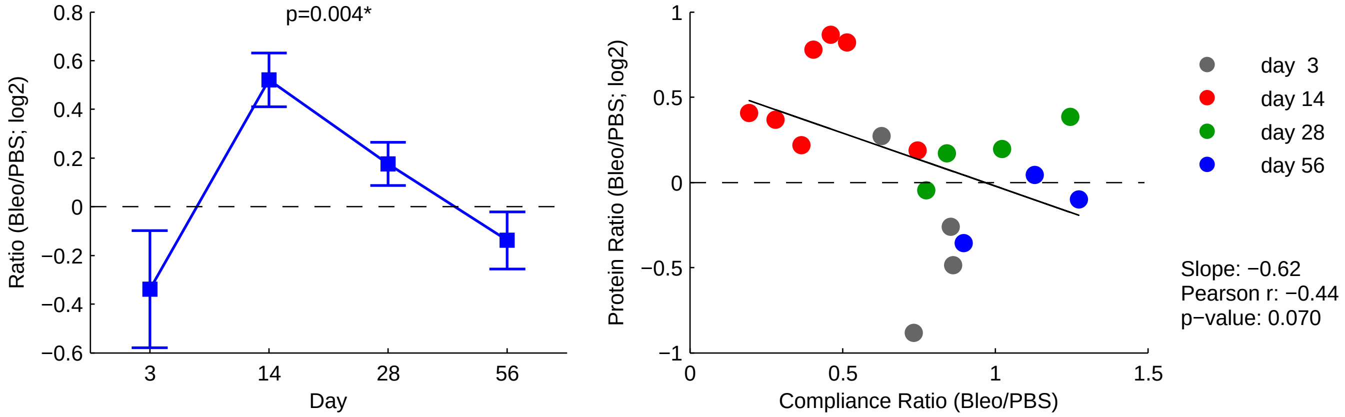

Q8C142 − Ldlrap1 (id: 5463)

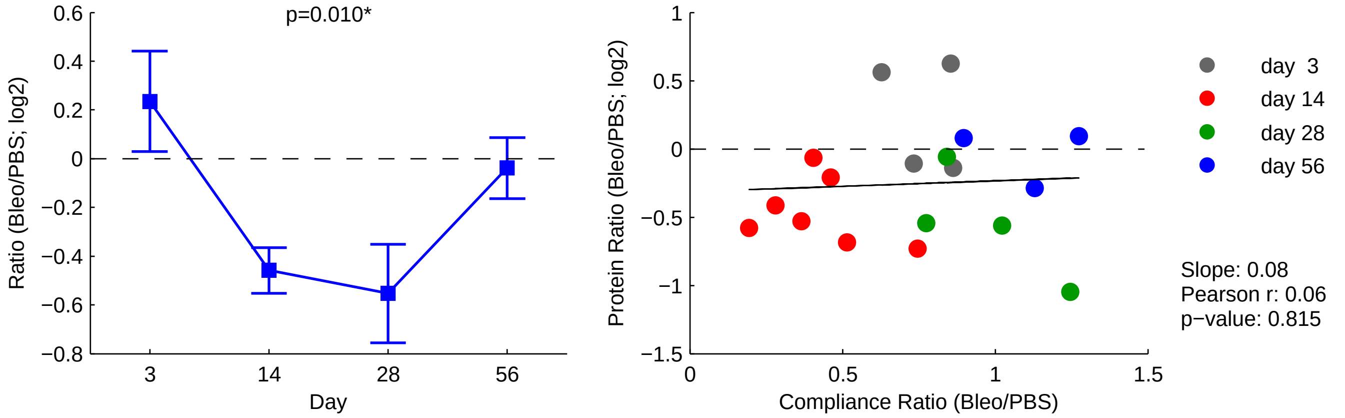

Q8C147 − Dock8 (id: 5464)

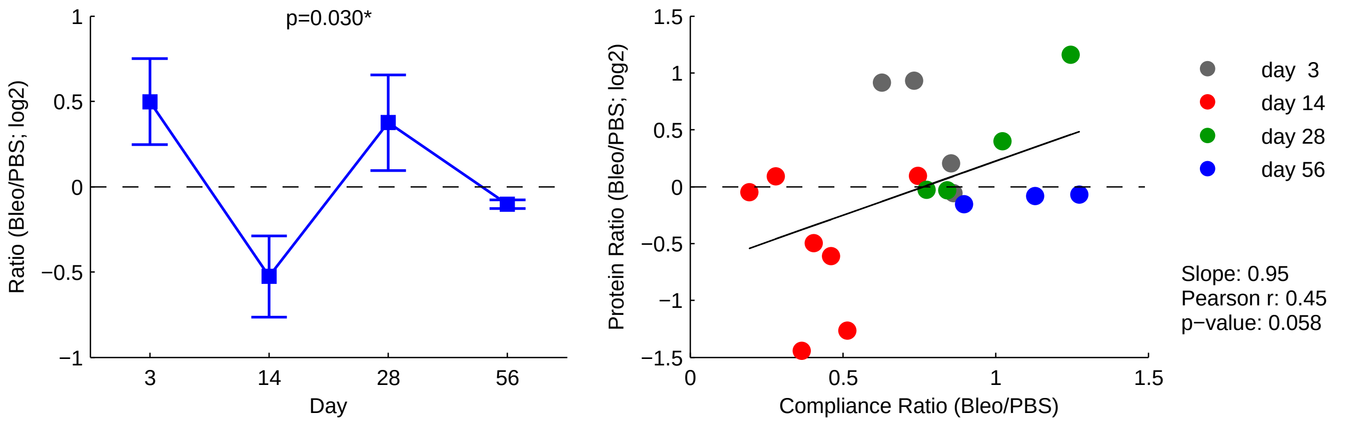

Q8C181−2 − (id: 5472)

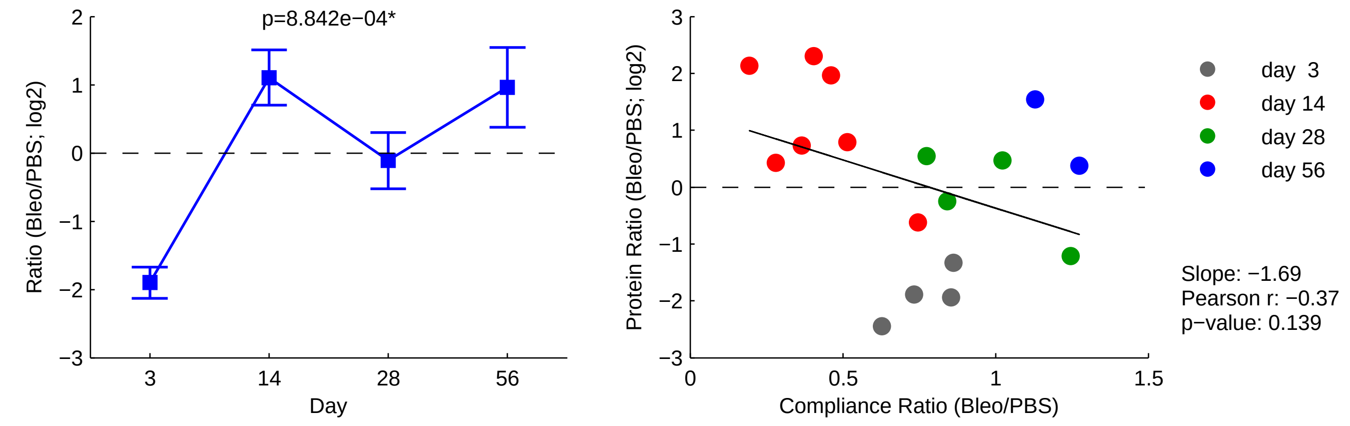

Q8C1E7 − Tmem120a (id: 5477)

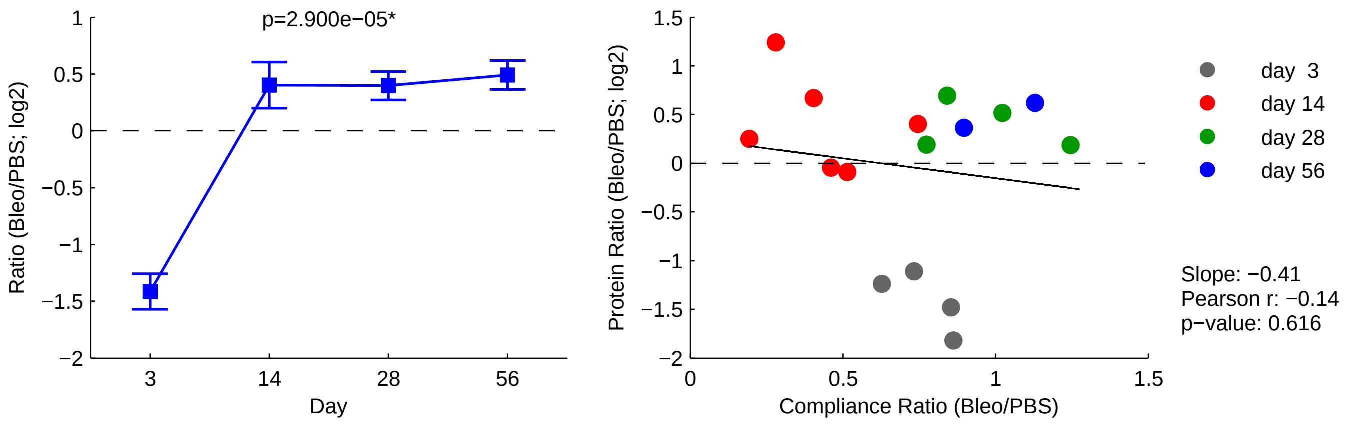

Q8C1Q6 − Smim4 (id: 5479)

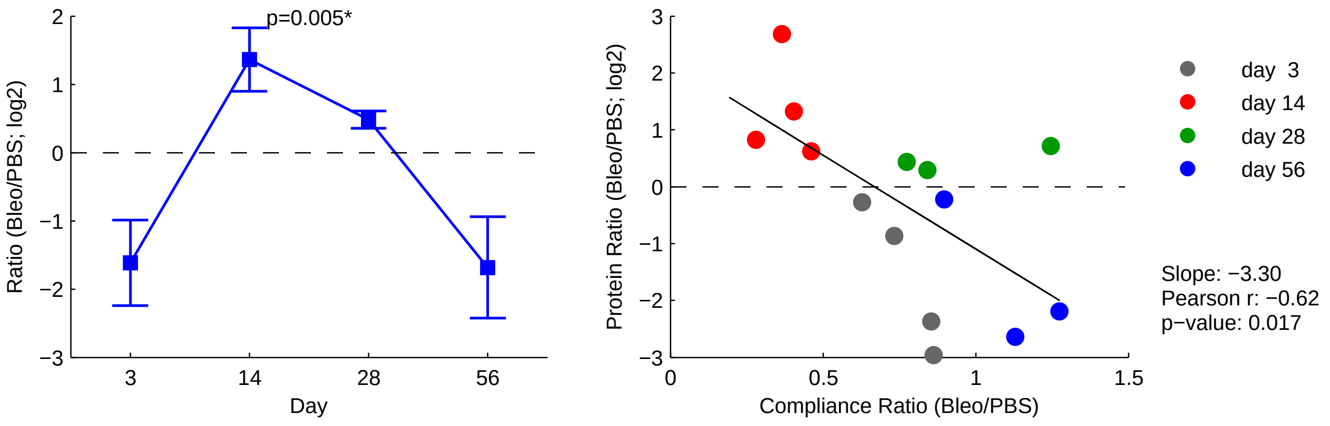

Q8C2E7 − Kiaa0196 (id: 5484)

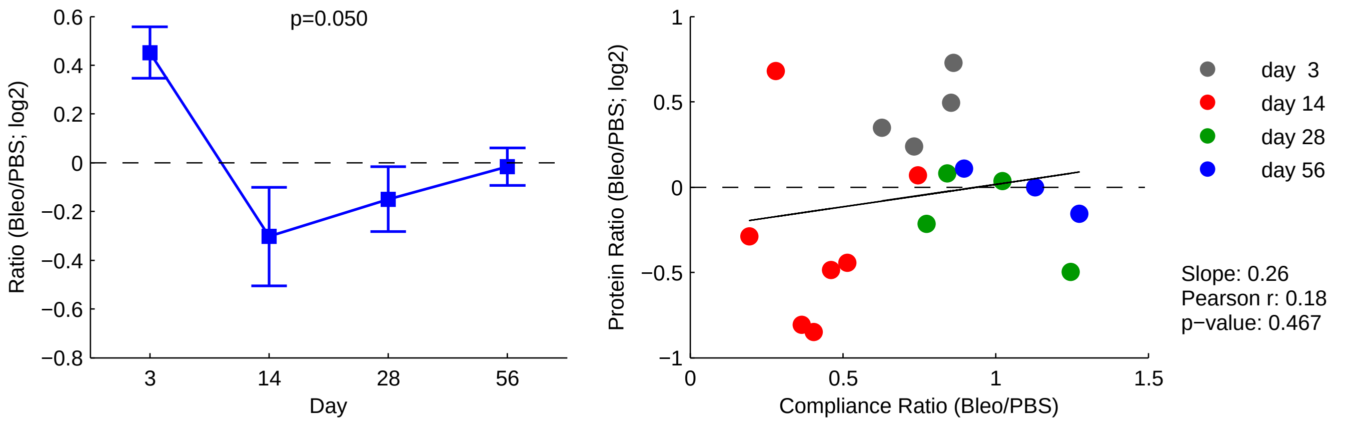

Q8C2Q3 − Rbm14 (id: 5486)

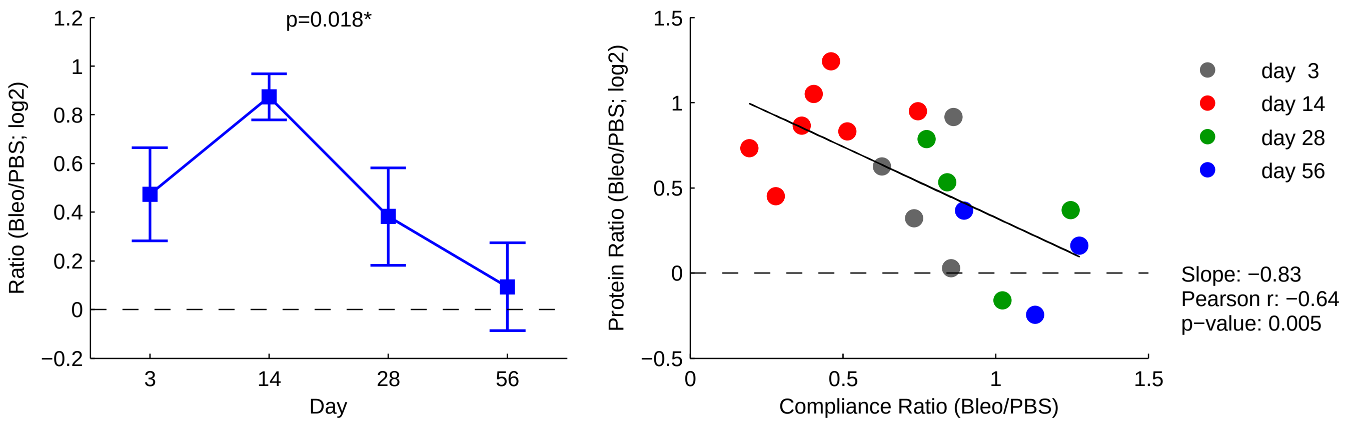

Q8C2Q8 − Atp5c1 (id: 5487)

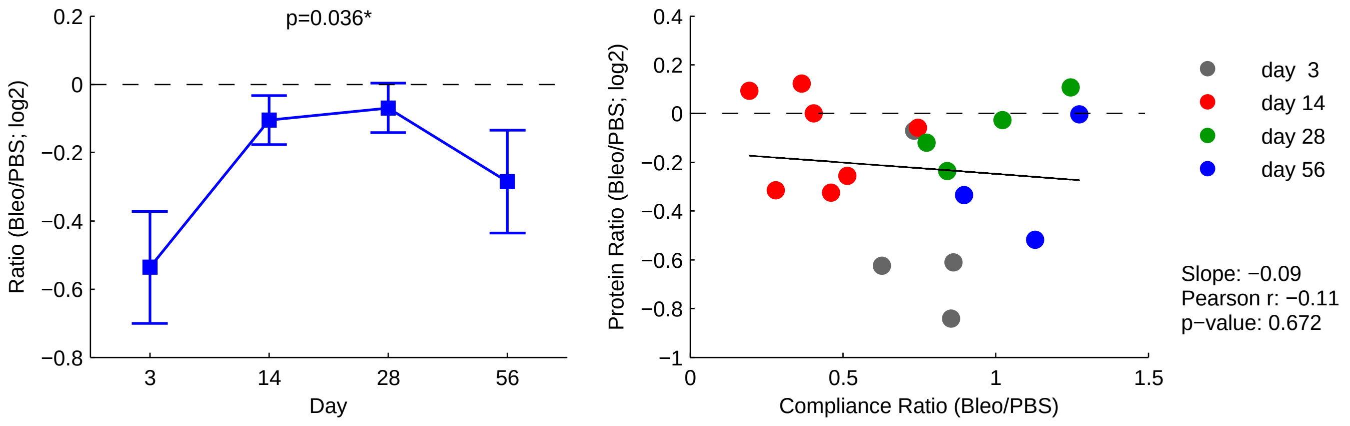

Q8C3J5 − Dock2 (id: 5489)

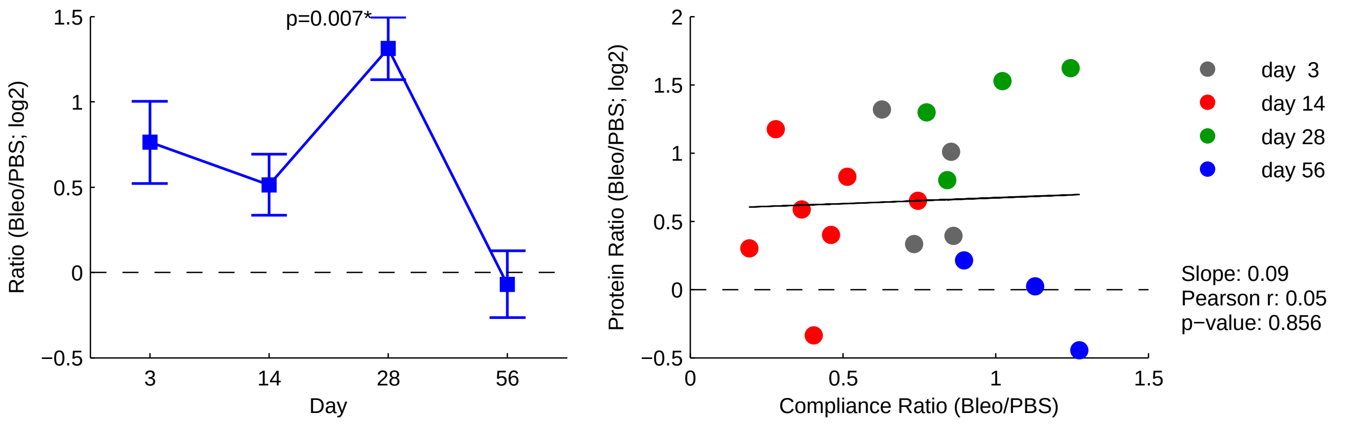

Q8C3X2 − Ccdc90b (id: 5493)

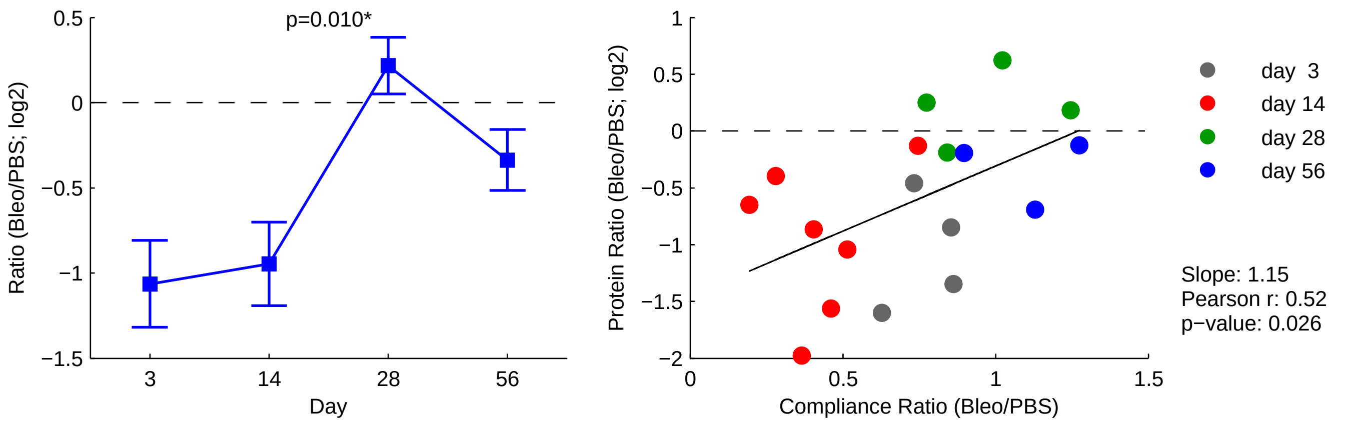

Q8C3X4 − Guf1 (id: 5494)

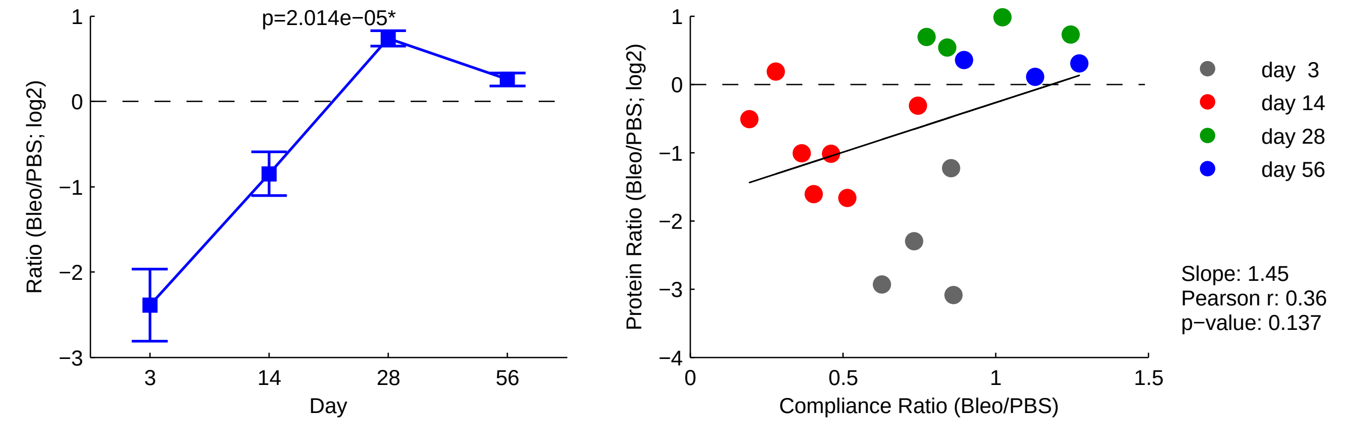

Q8C3X8 − Lmf2 (id: 5495)

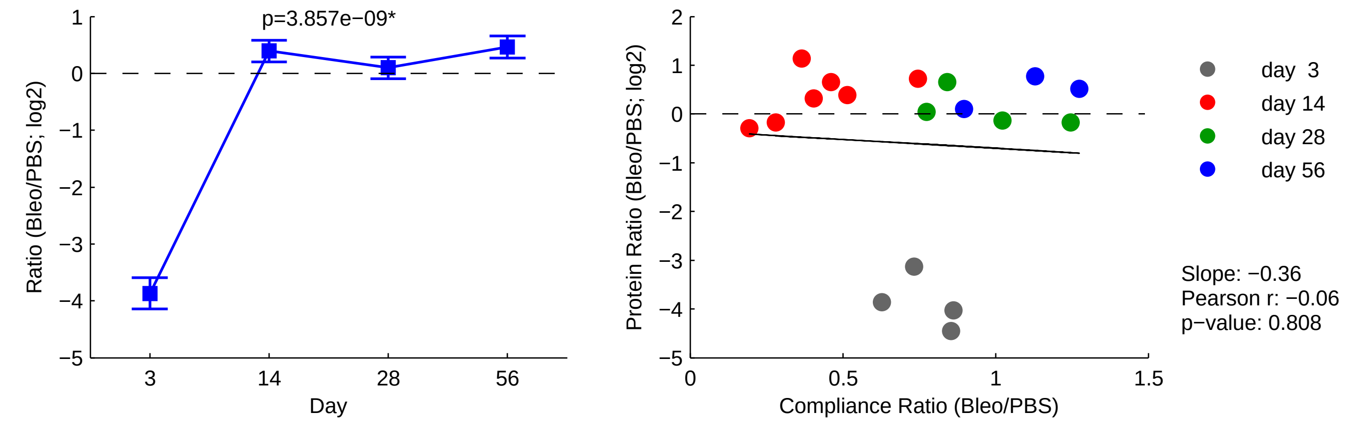

Q8C405 − Sp100 (id: 5496)

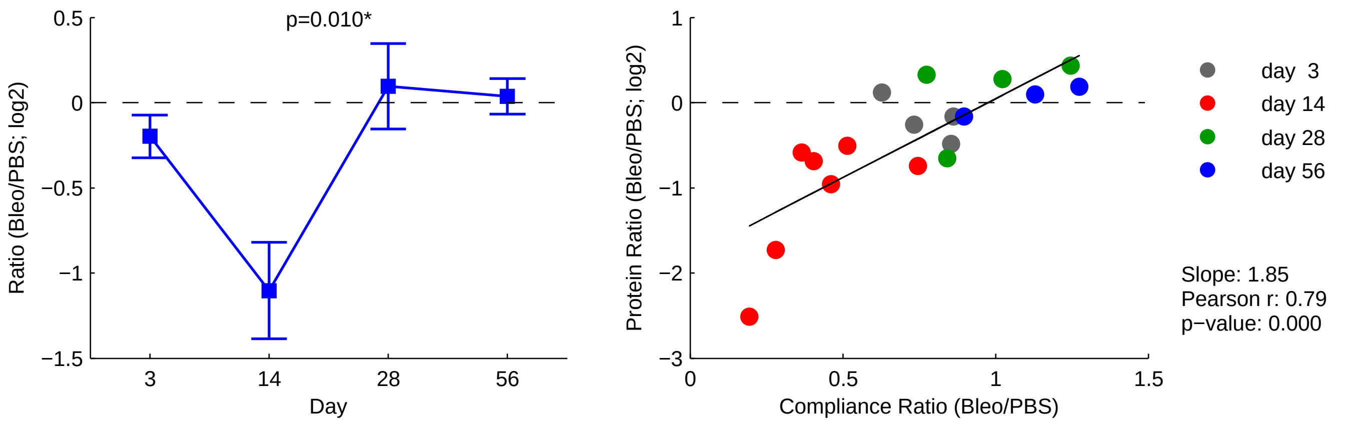

Q9QYI4 − Dnajb12 (id: 5499)

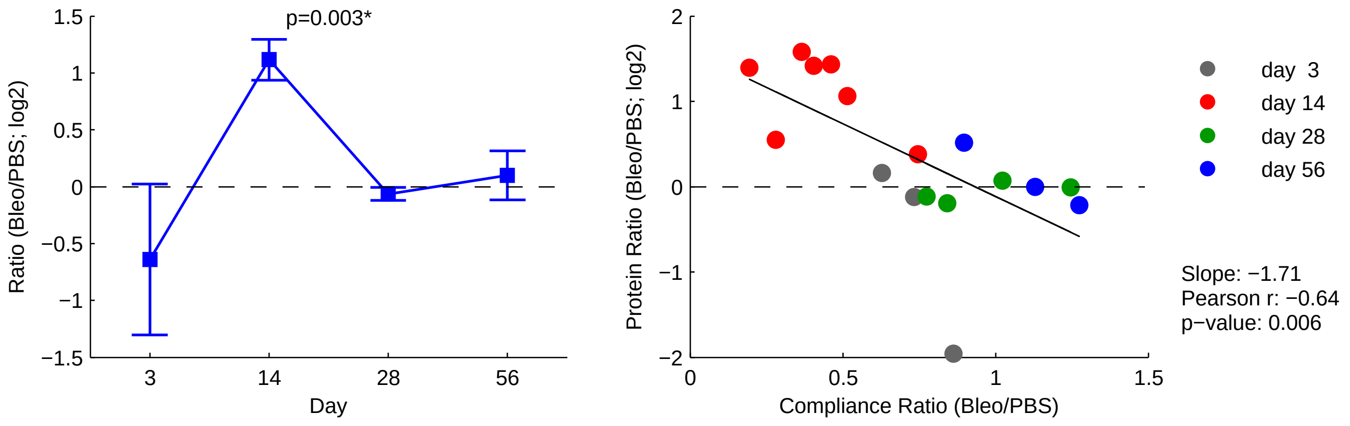

Q8C4J7 − Tbl3 (id: 5500)

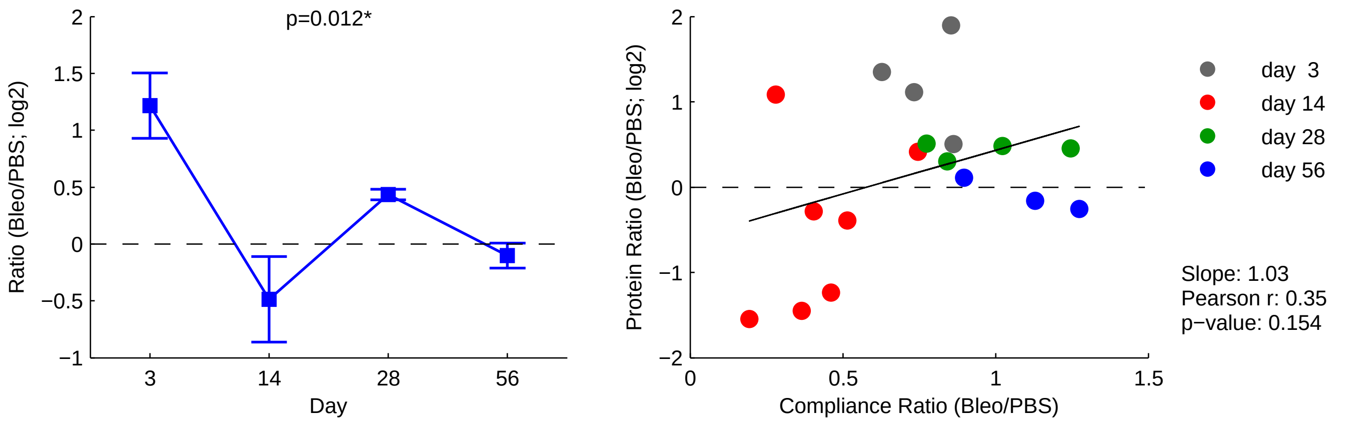

Q8C4Q6 − Aida (id: 5502)

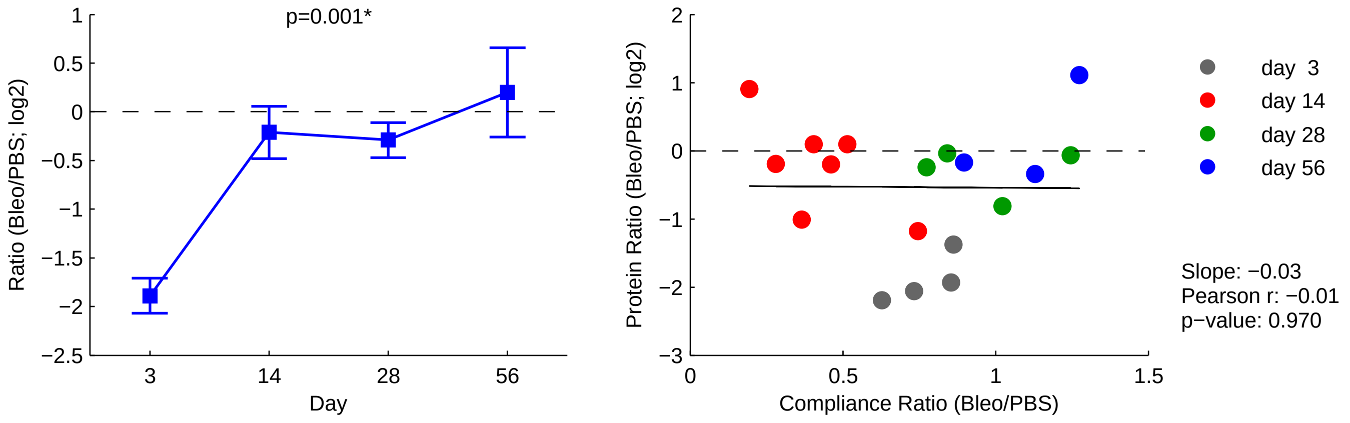

Q8C4X2 − Csnk1g3 (id: 5504)

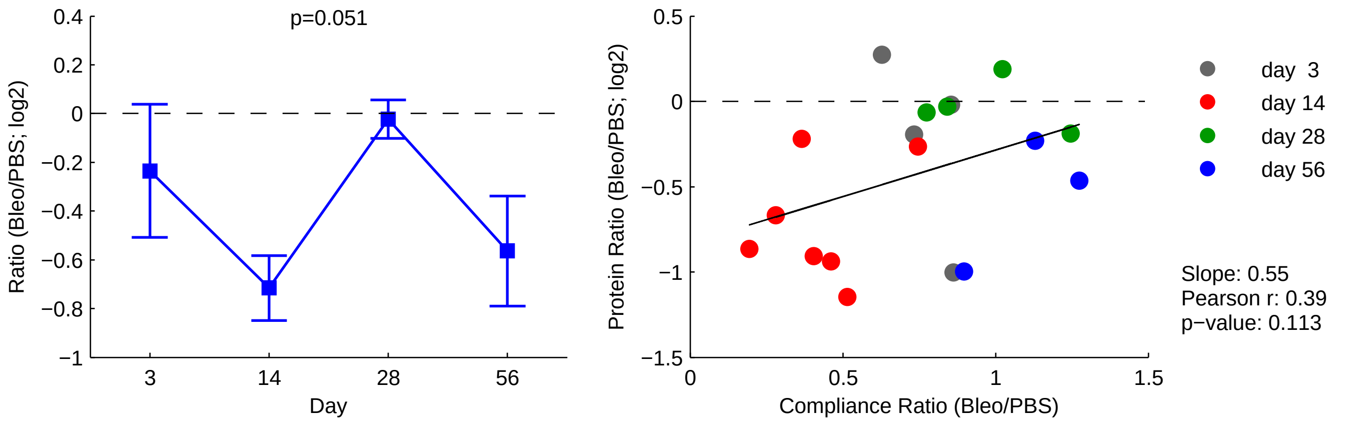

Q8C4Y3 − Nelfb (id: 5505)

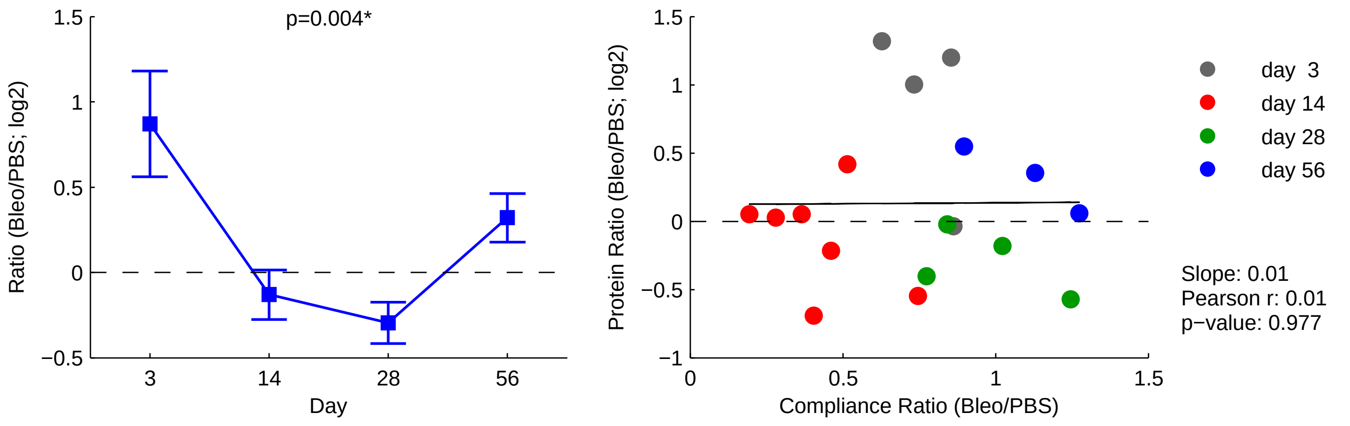

Q8C522 − Endod1 (id: 5507)

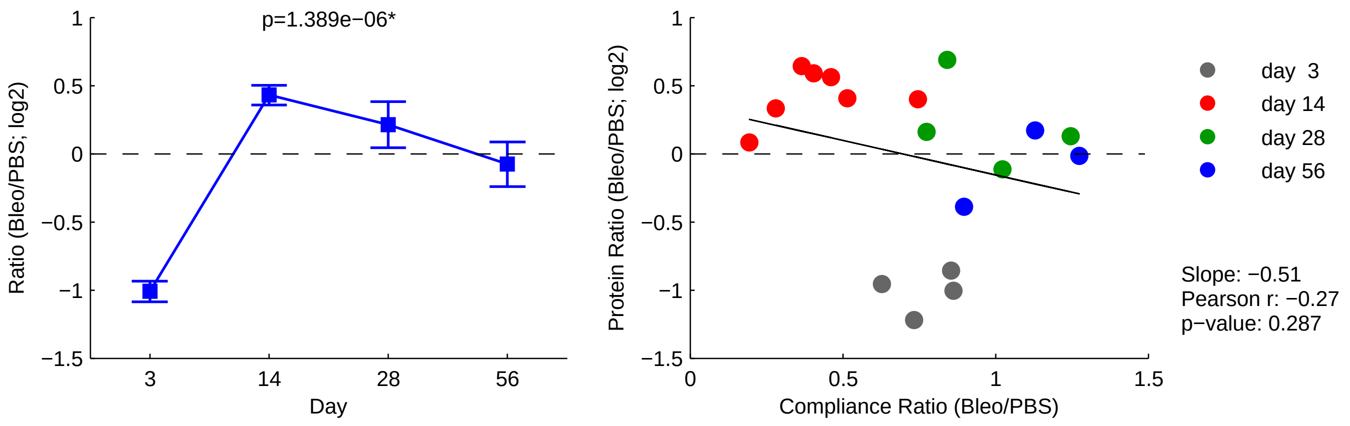

Q8C547 − Heatr5b (id: 5508)

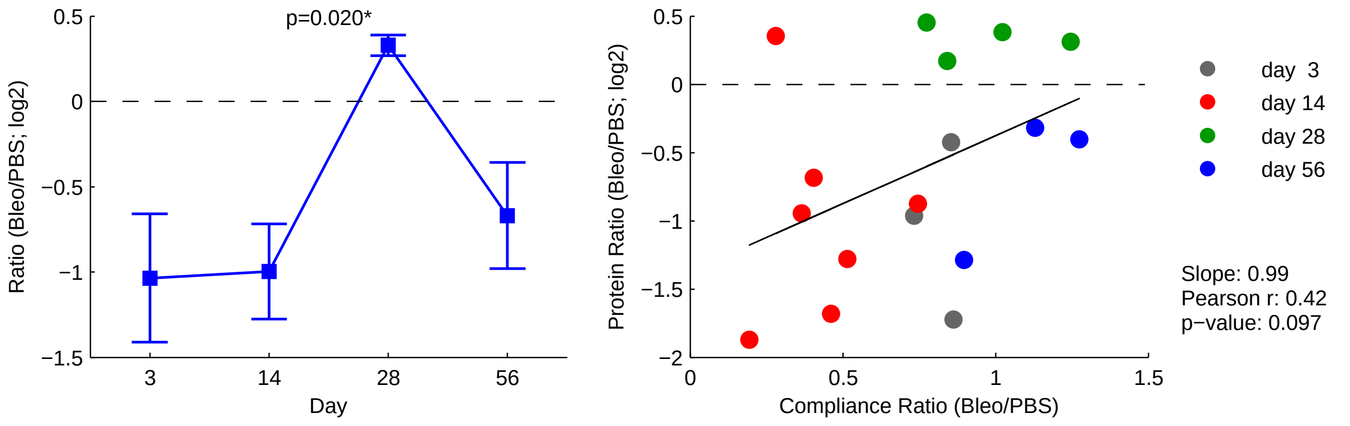

Q8C561−2 − Lmbrd2 (id: 5509)

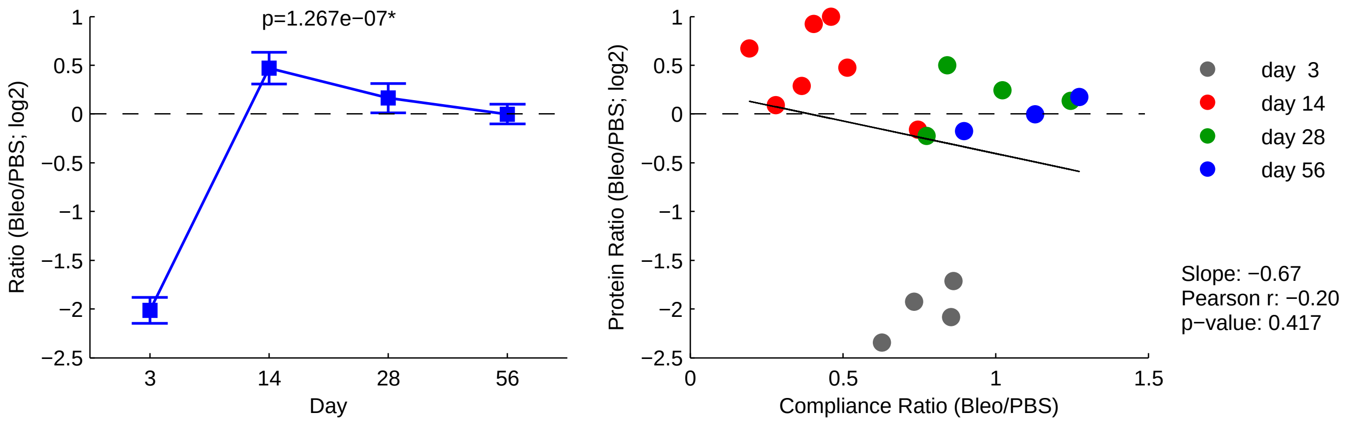

Q8C570 − Rae1 (id: 5511)

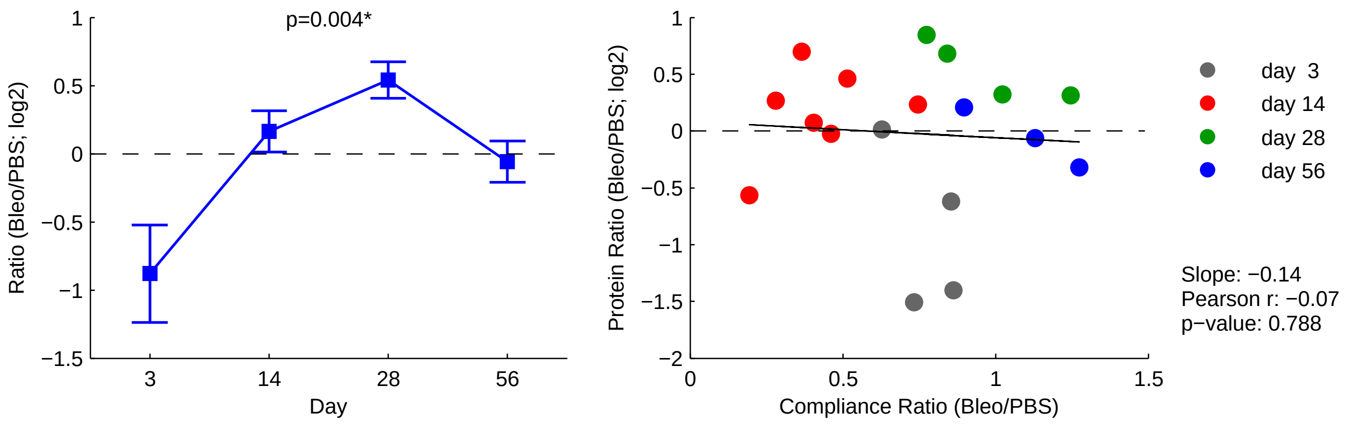

Q8C5H8−2 − Nadkd1 (id: 5512)

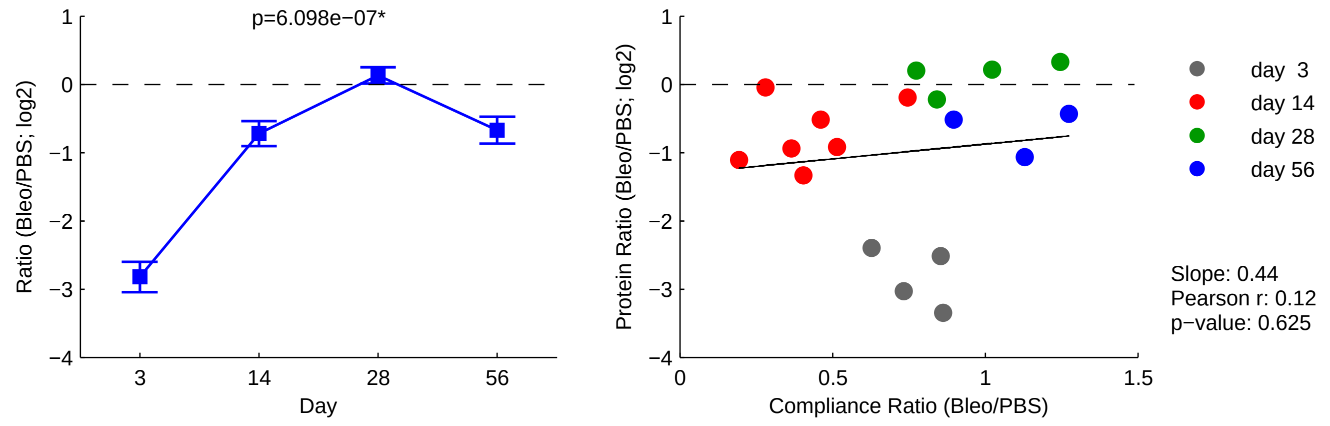

Q8C5V8 − 4932418E24Rik (id: 5517)

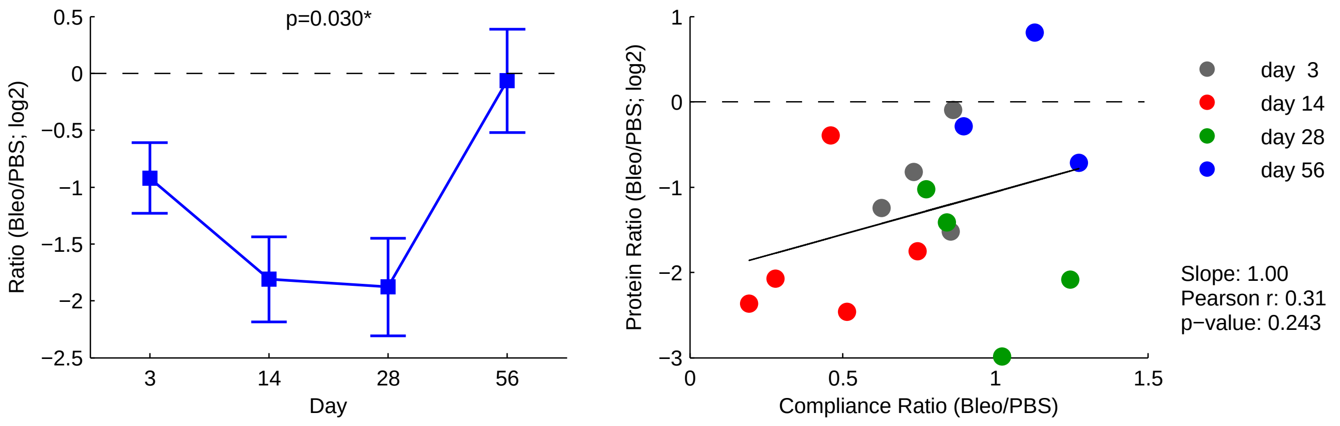

Q8C6B0 − Mettl7a1 (id: 5522)

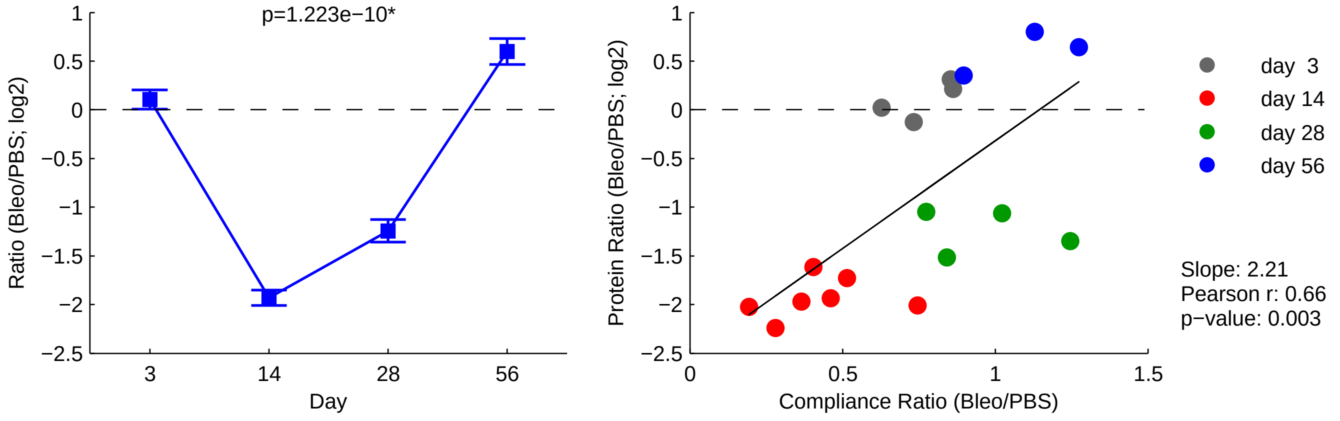

Q8C6I2 − Sdhaf2 (id: 5523)

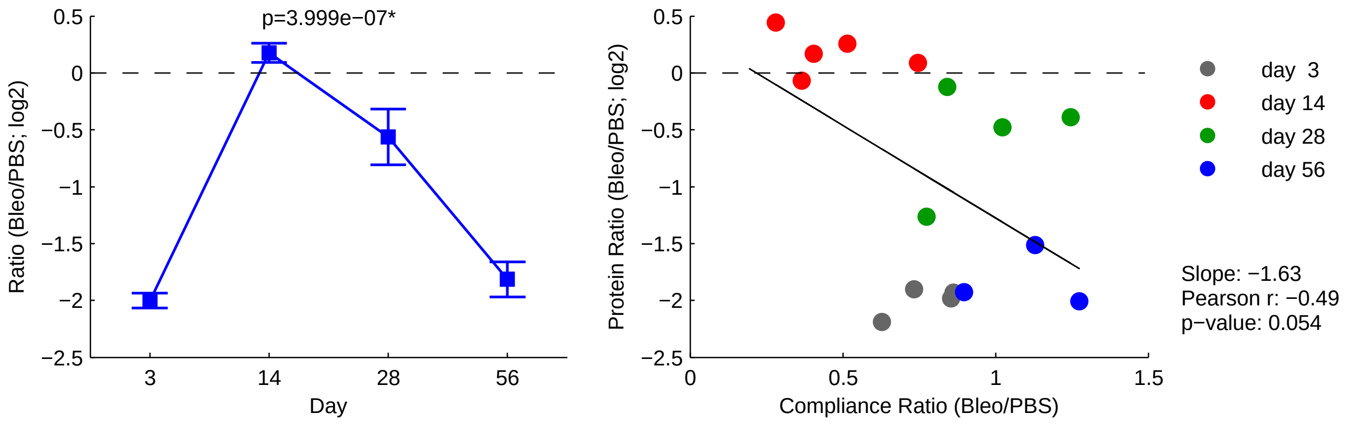

Q8C6I5 − Nfs1 (id: 5524)

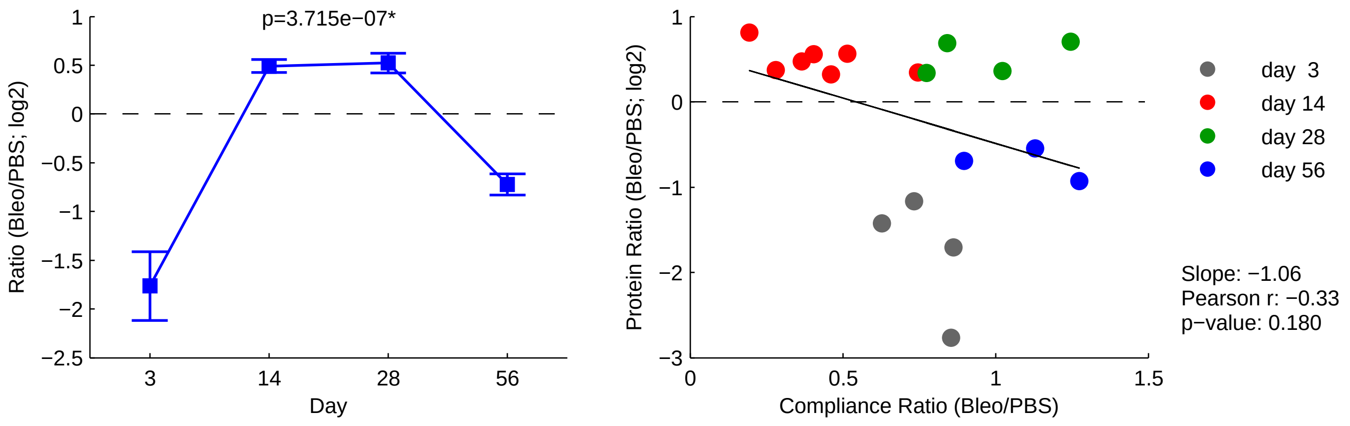

Q8C729 − Fam126b (id: 5525)

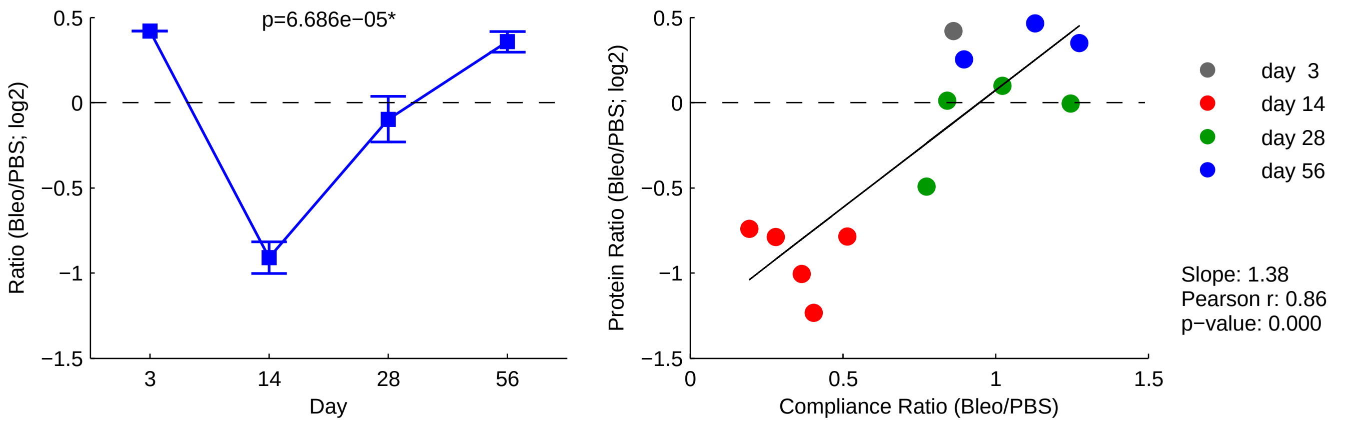

Q8C754 − Vps52 (id: 5527)

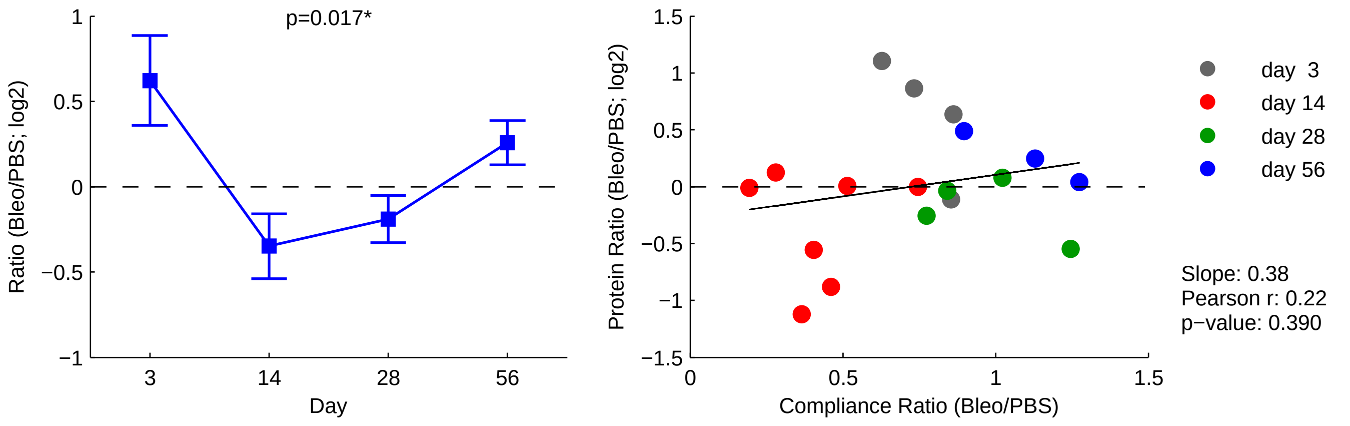

Q8C7B4 − Ggt5 (id: 5529)

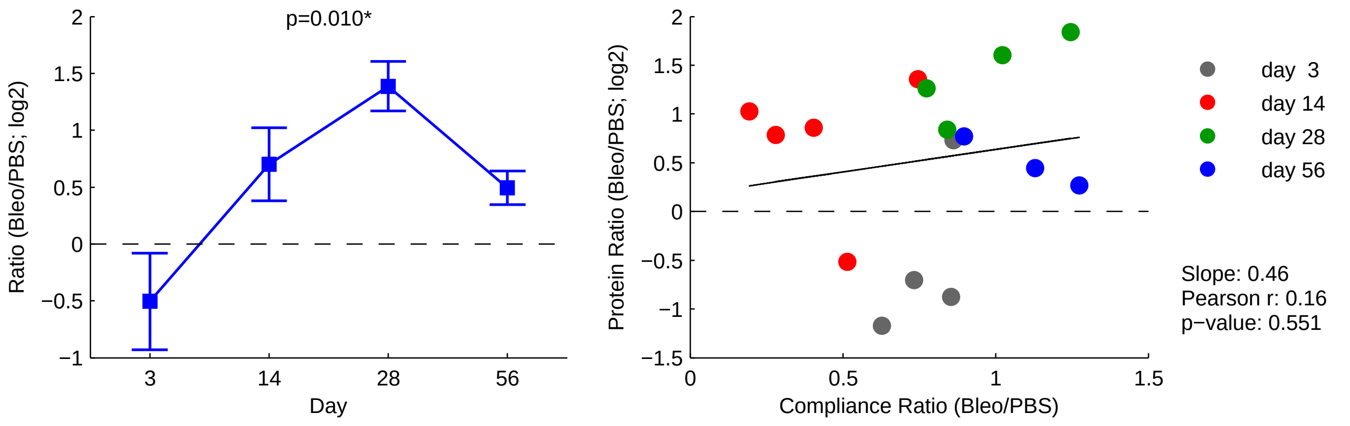

Q8C7E7 − Stbd1 (id: 5530)

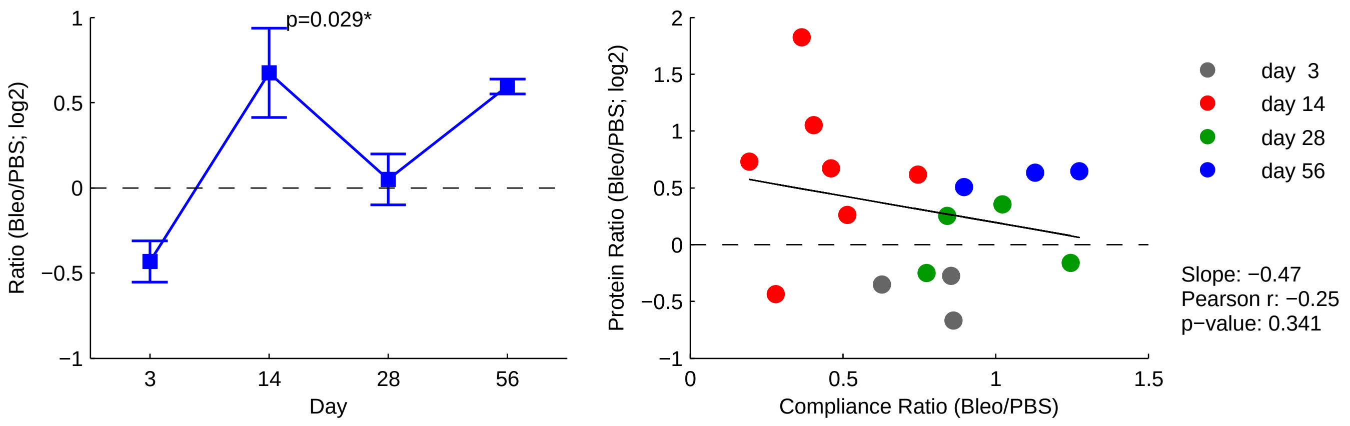

Q8C7H1 − Mmaa (id: 5532)

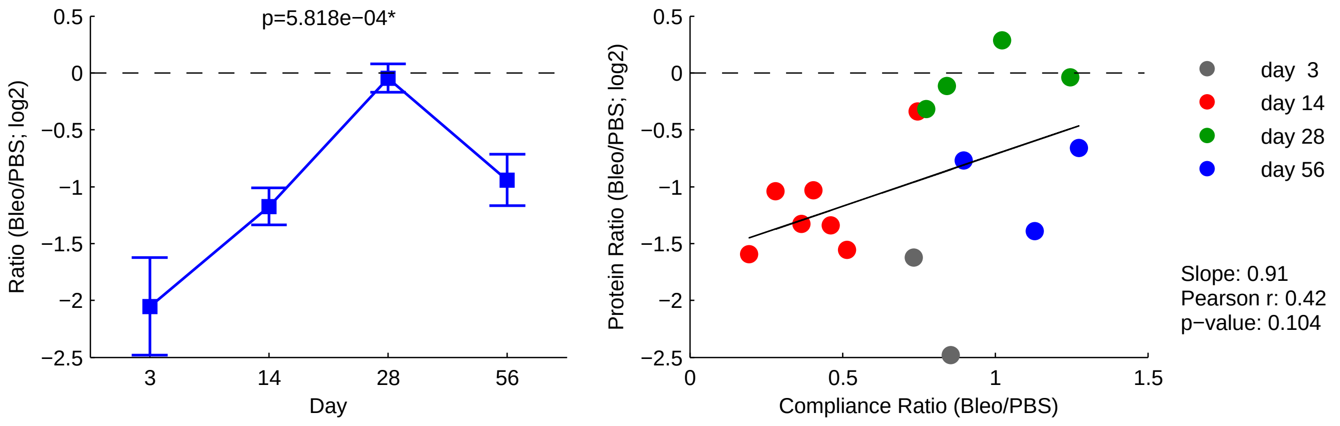

Q8C7J6 − Kctd12b (id: 5533)

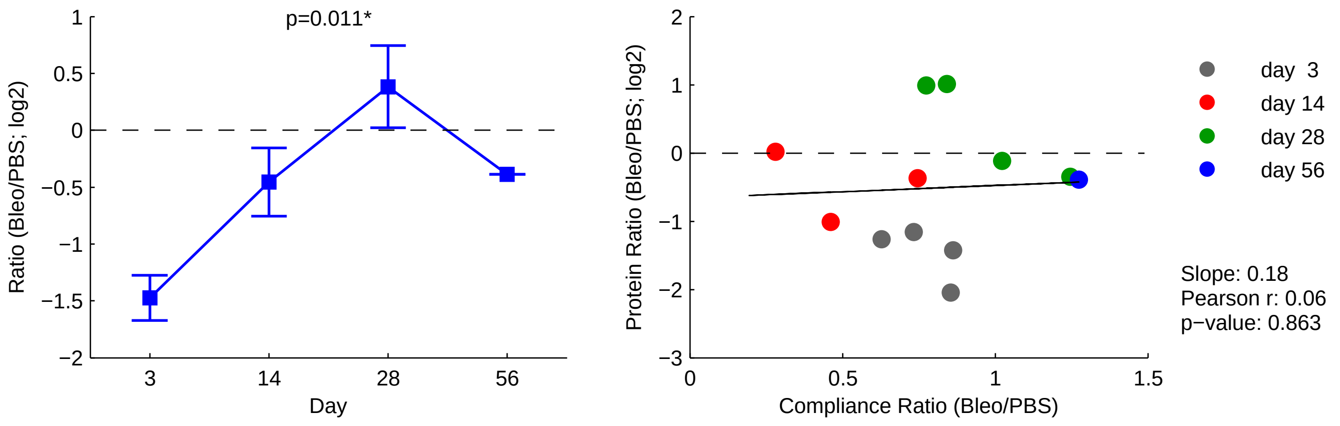

Q8C7K6 − Pcyox1l (id: 5534)

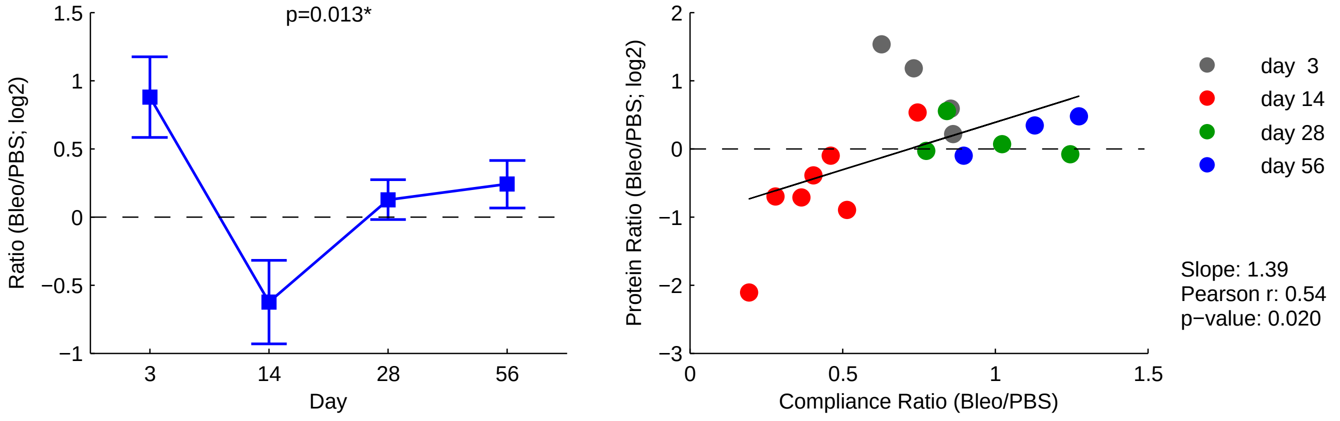

Q8C804 − Spice1 (id: 5539)

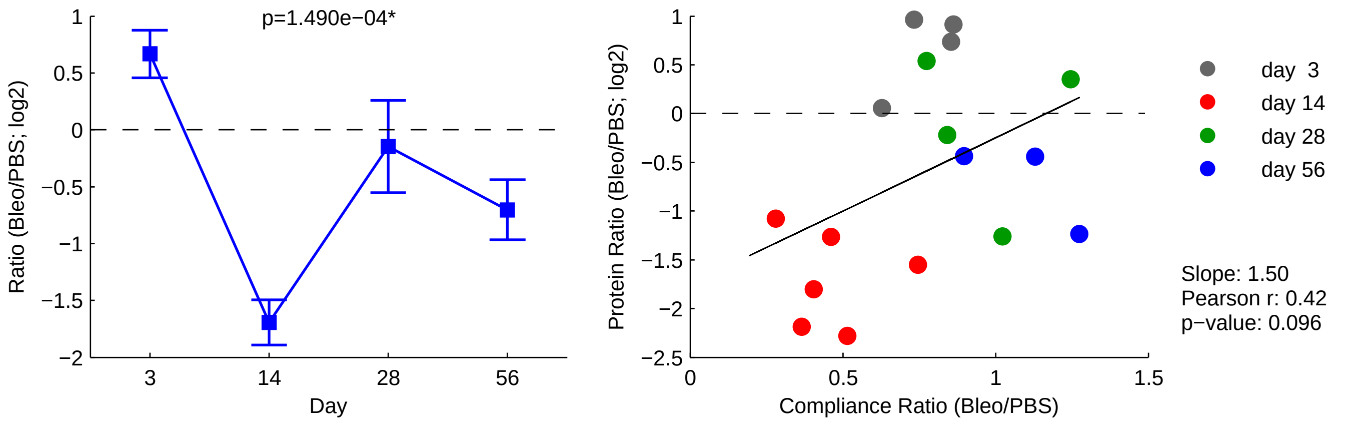

Q8C845 − Efhd2 (id: 5541)

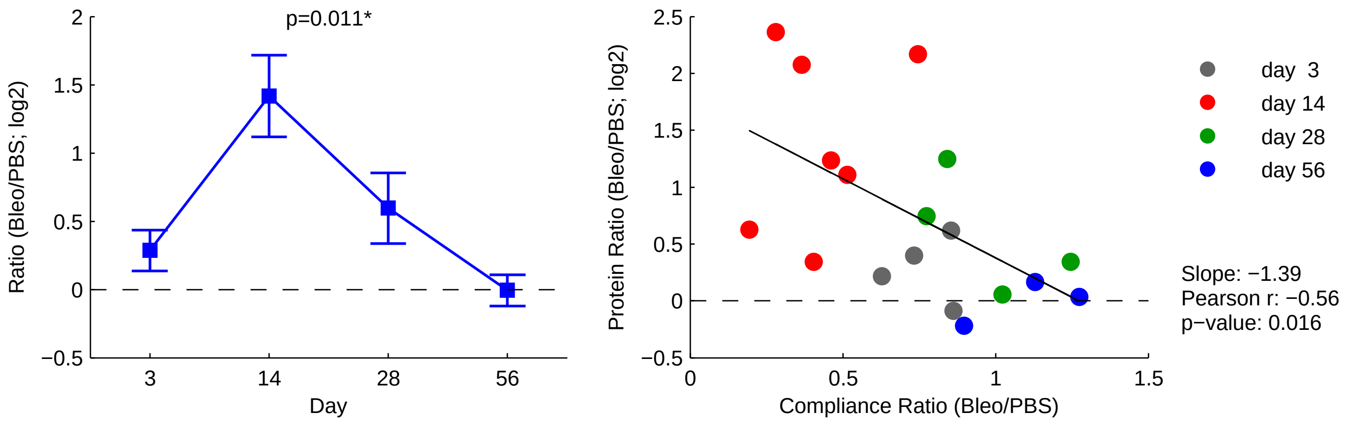

Q8C8R3 − Ank2 (id: 5544)

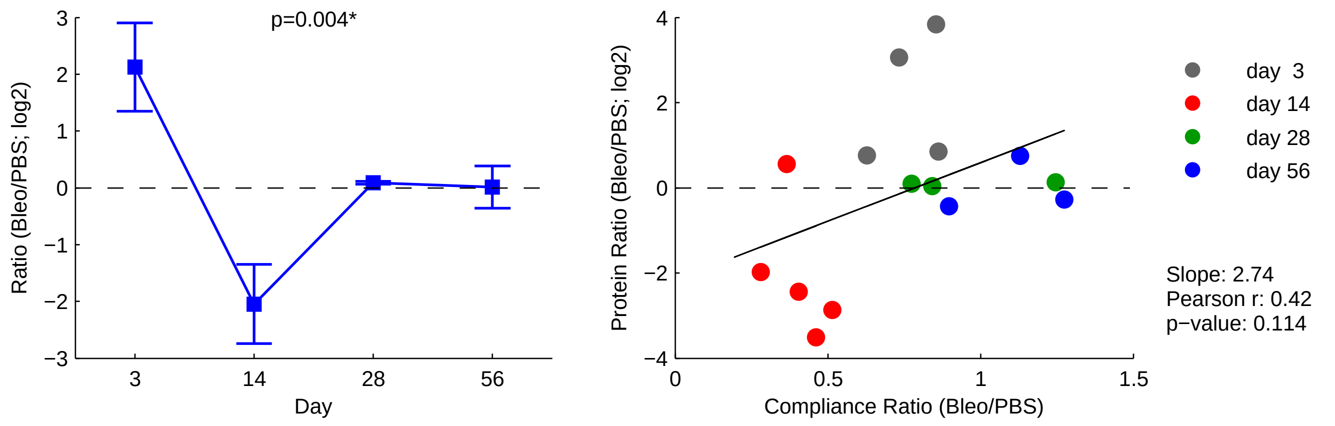

Q8C8U0 − Ppfibp1 (id: 5545)

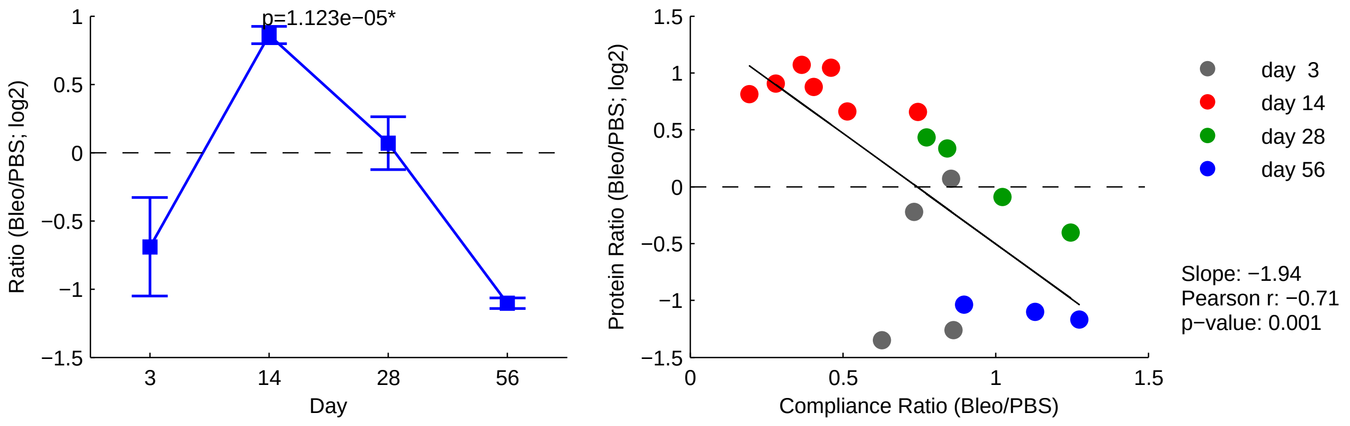

Q8CAG6 − Plek (id: 5553)

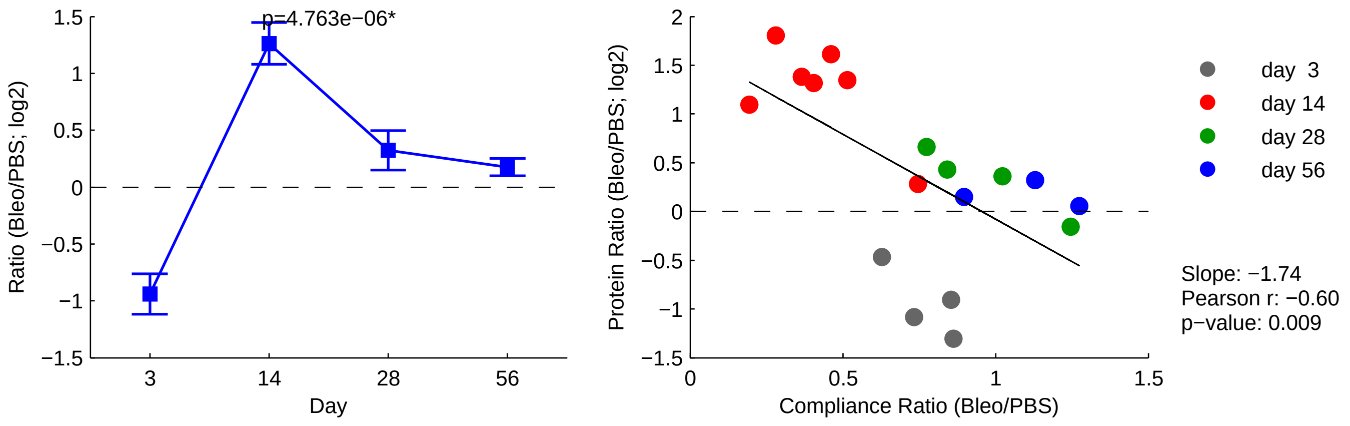

Q8CAK1 − Iba57 (id: 5554)

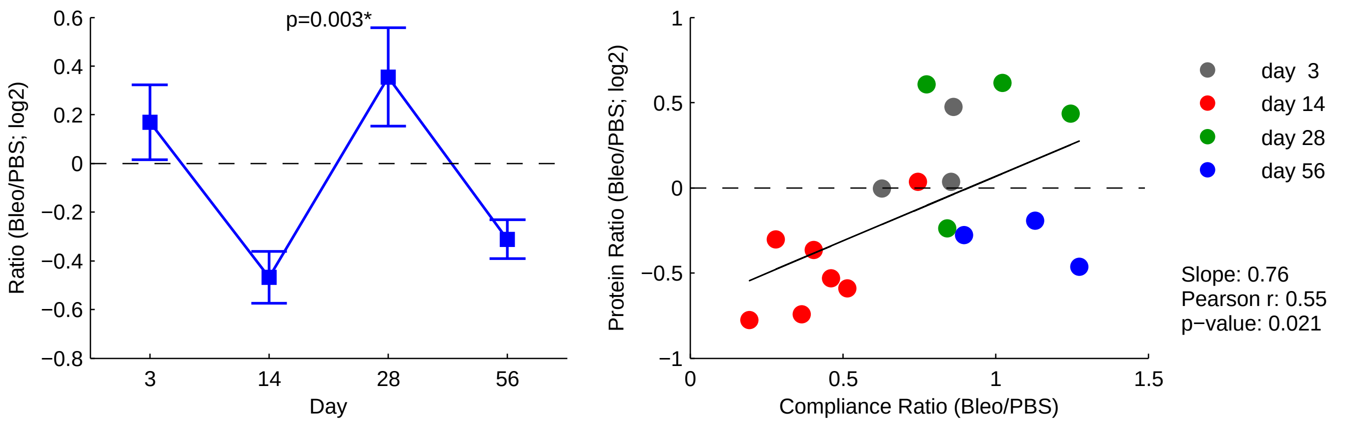

Q8CAQ8−2 − (id: 5556)

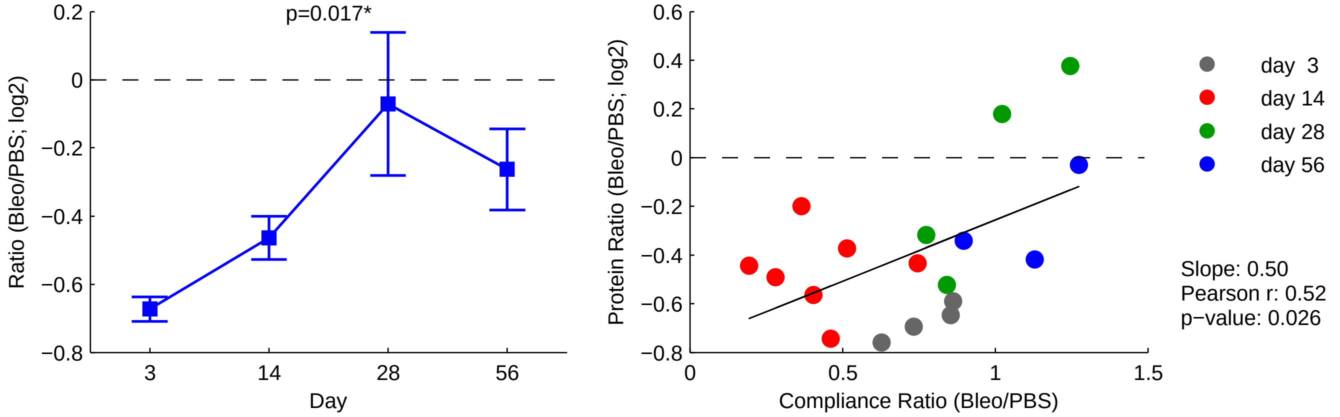

Q8CAS9−2 − Parp9 (id: 5558)

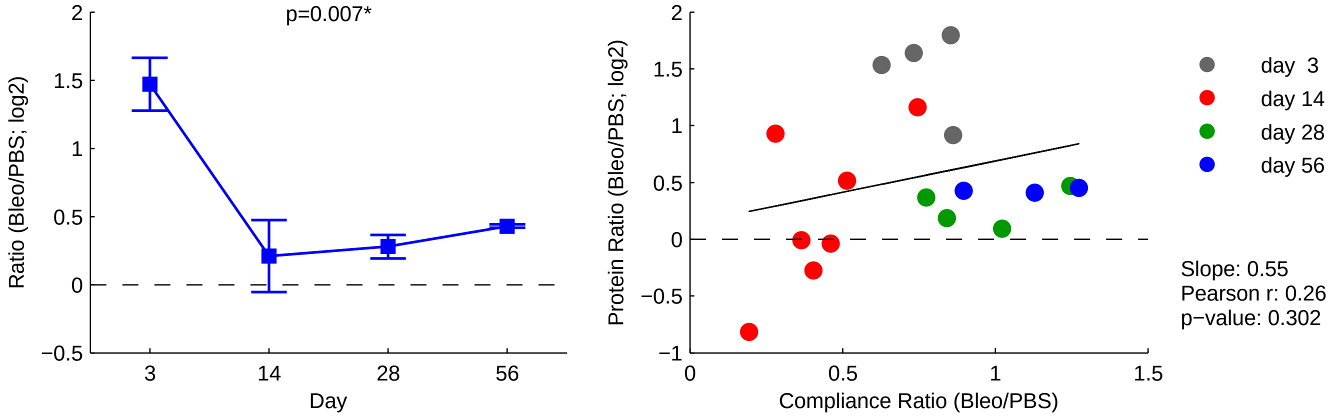

Q8CBA2 − Slfn5 (id: 5564)

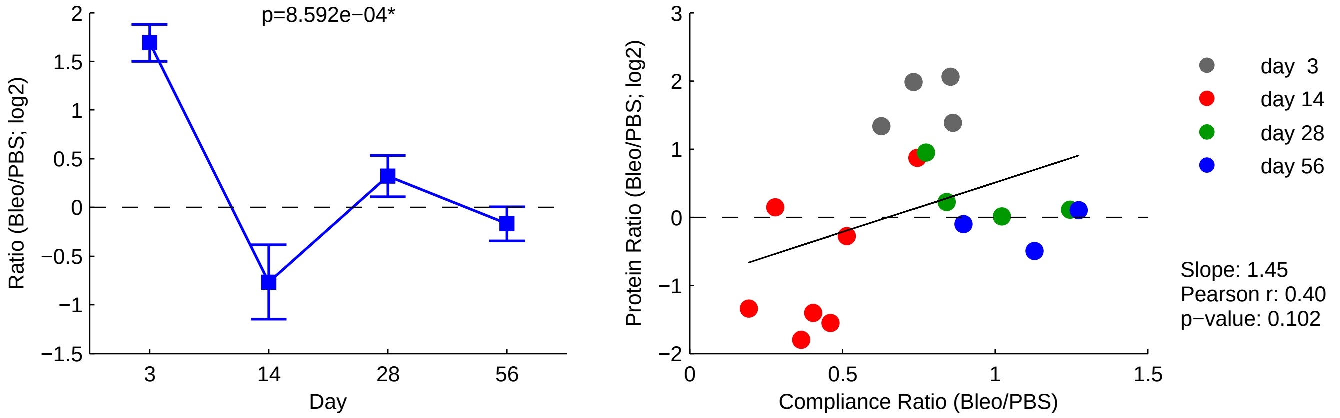

Q8CBC4 − Cnst (id: 5565)

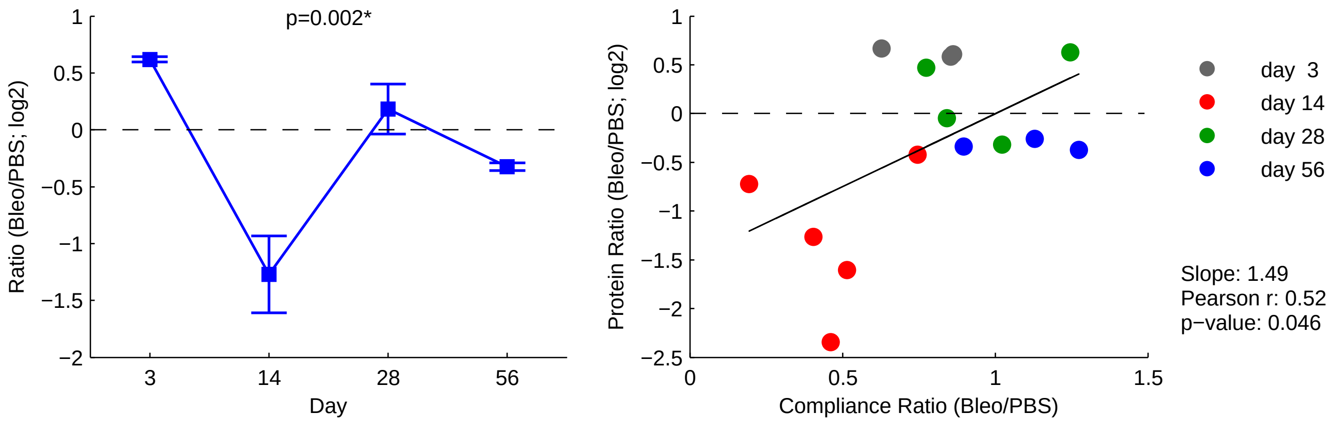

Q8CBQ5 − Pi4k2b (id: 5567)

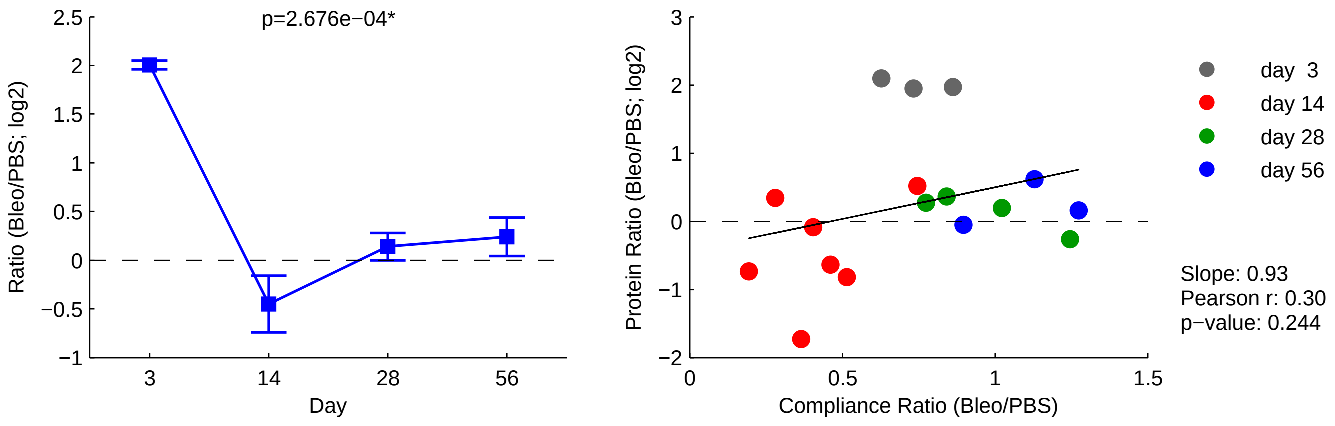

Q8CC88 − Vwa8 (id: 5574)

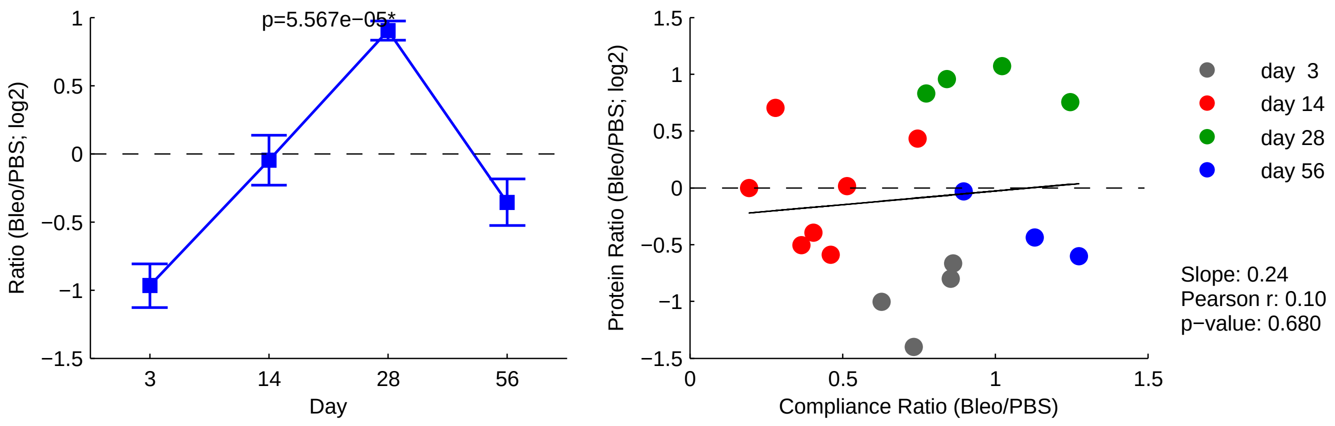

Q8CCH2 − Nhlrc3 (id: 5577)

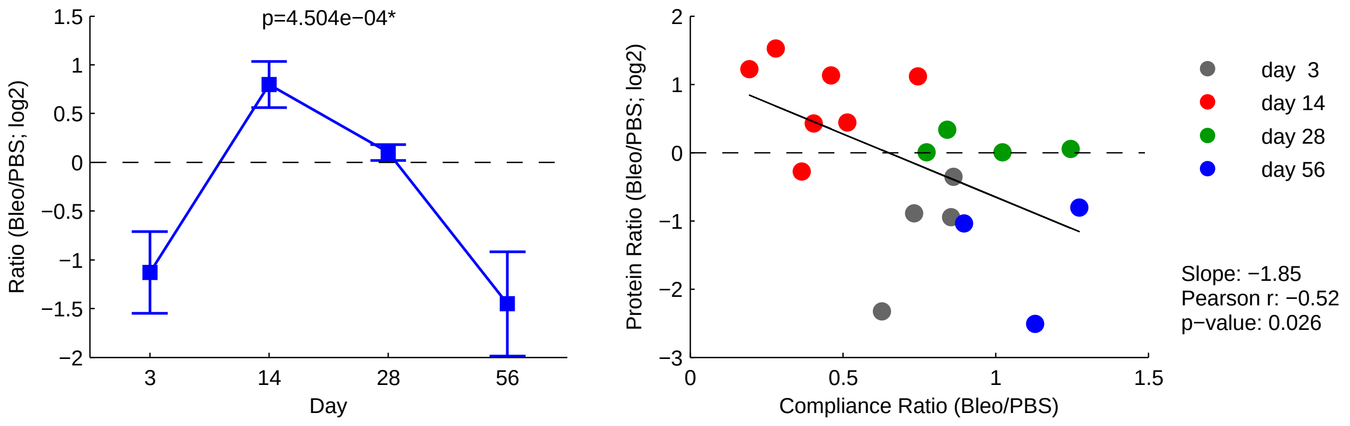

Q8CD10 − Micu2 (id: 5584)

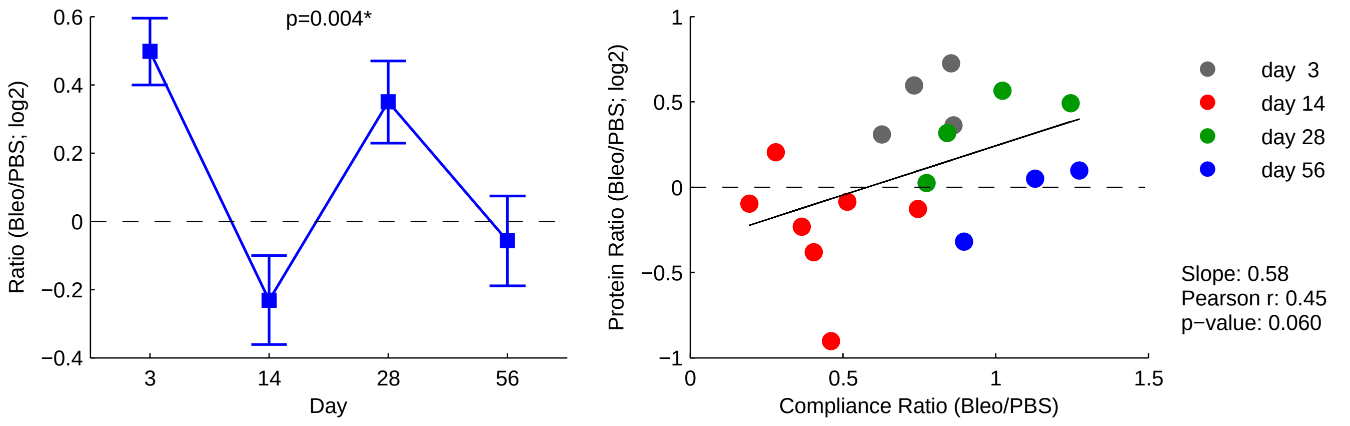

Q8CDG3 − Vcpip1 (id: 5588)

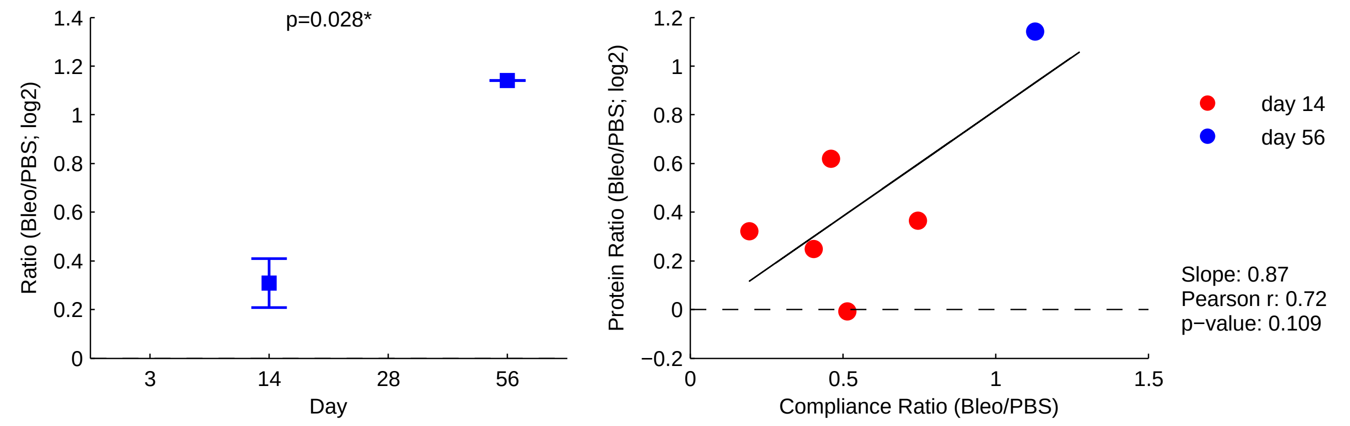

Q8CDI9 − Lipe (id: 5590)

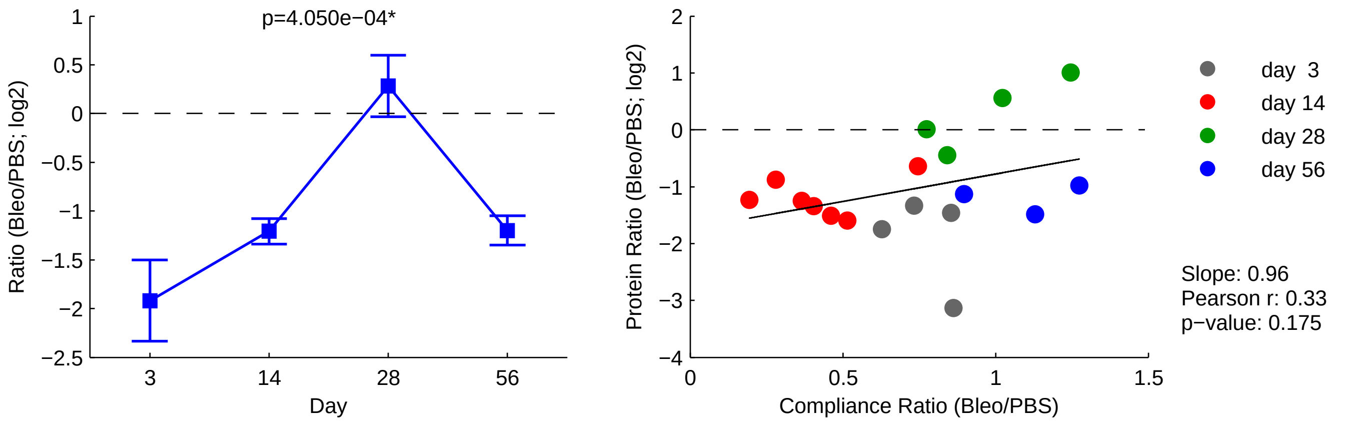

Q8CDJ8 − Ston1 (id: 5592)

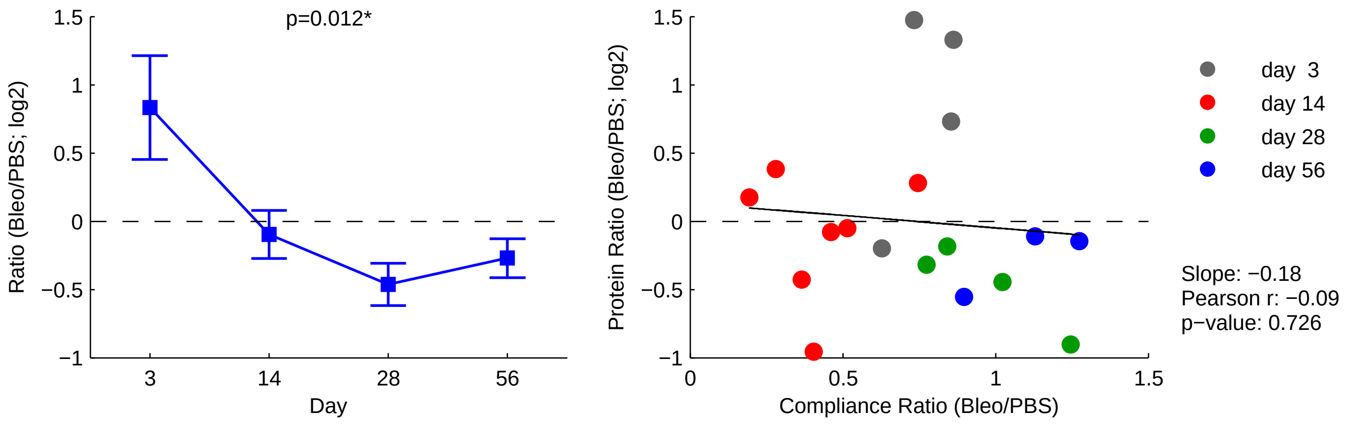

Q8CDN6 − Txnl1 (id: 5594)

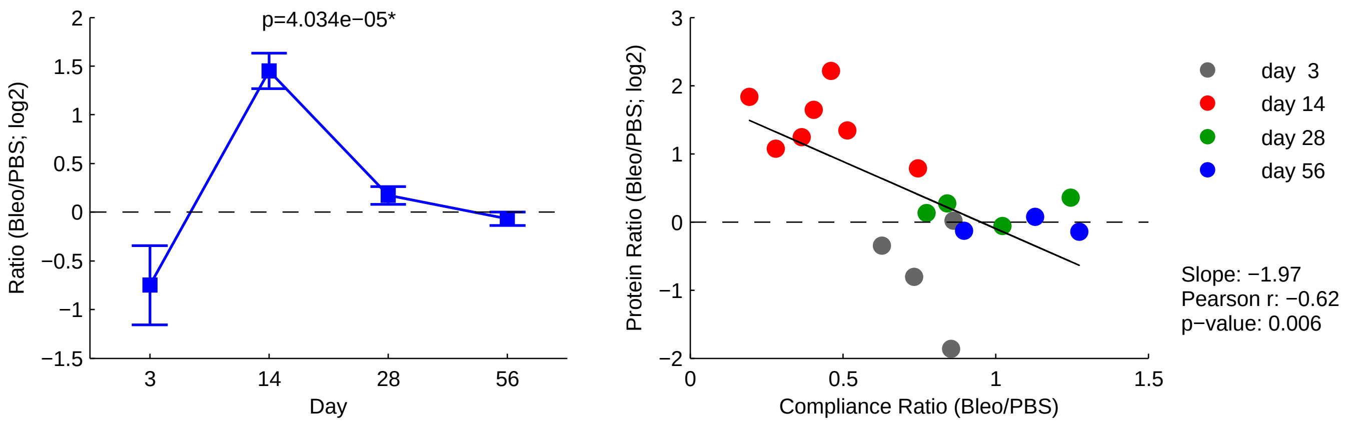

Q8CEE7 − Rdh13 (id: 5604)

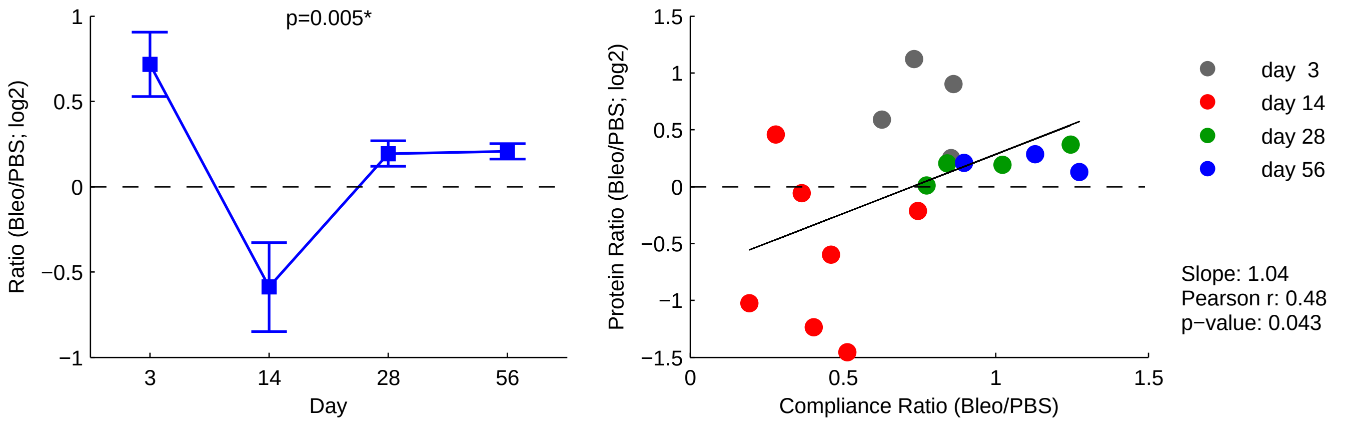

Q8CFA2 − Amt (id: 5607)

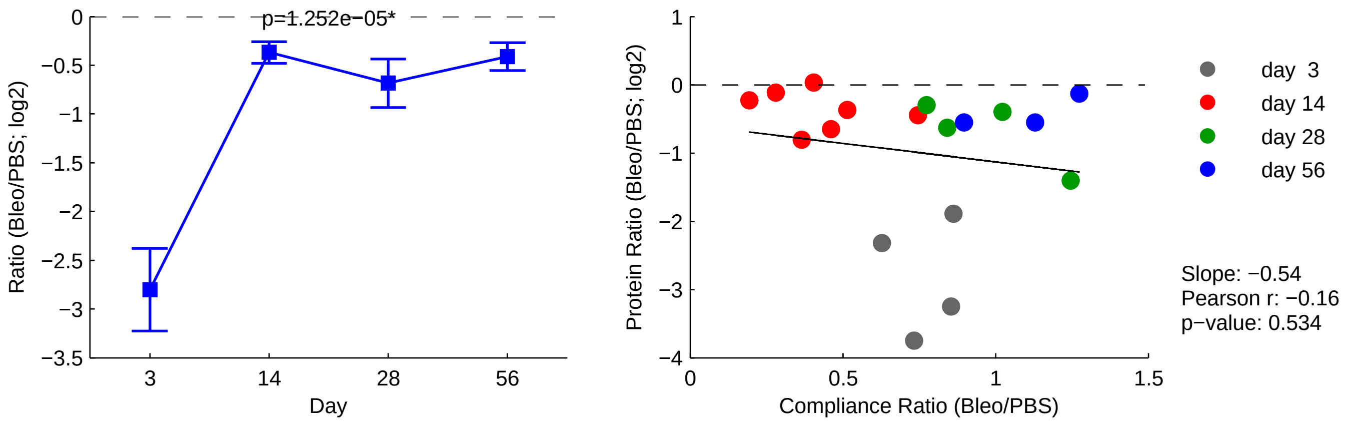

Q8CFB8 − Parp3 (id: 5609)

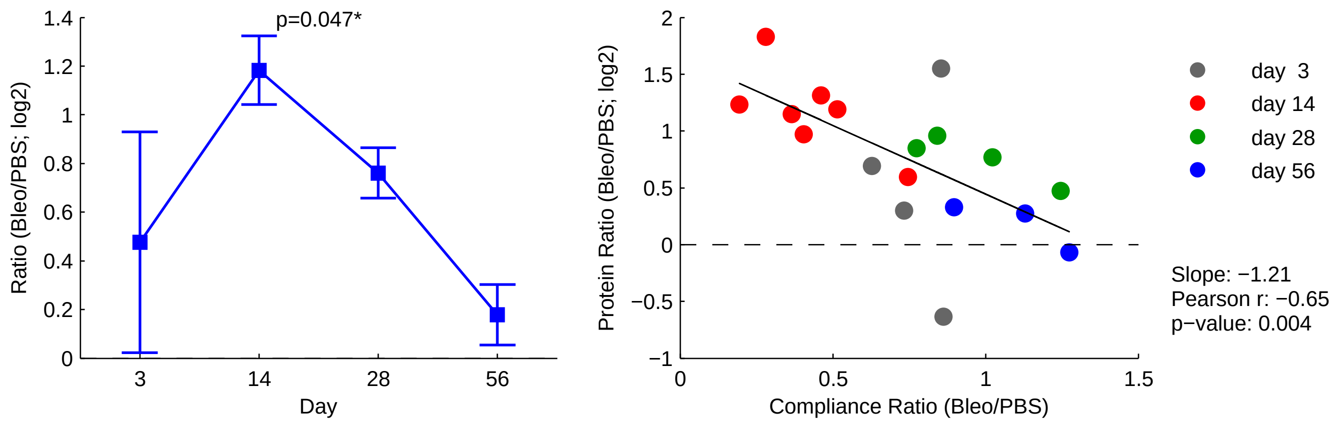

Q8CFE2 − (id: 5612)

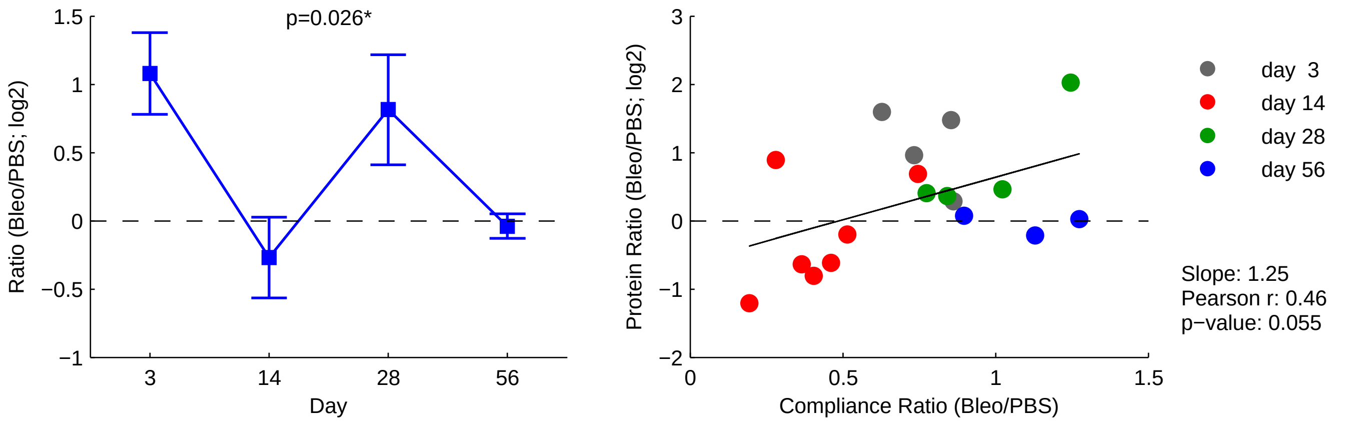

Q8CFJ7 − Slc25a45 (id: 5615)

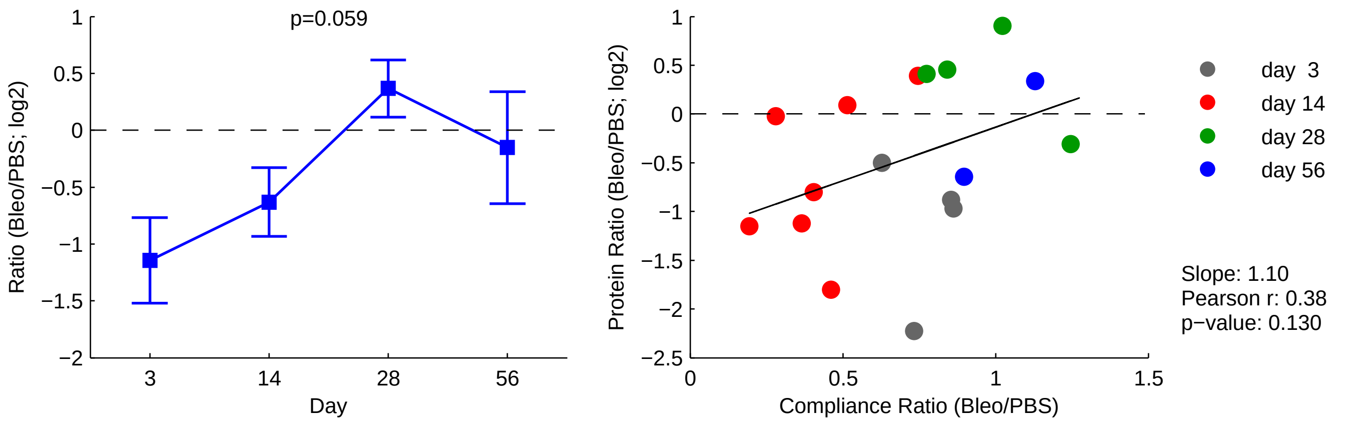

Q8CFX3 − Pcdh1 (id: 5618)

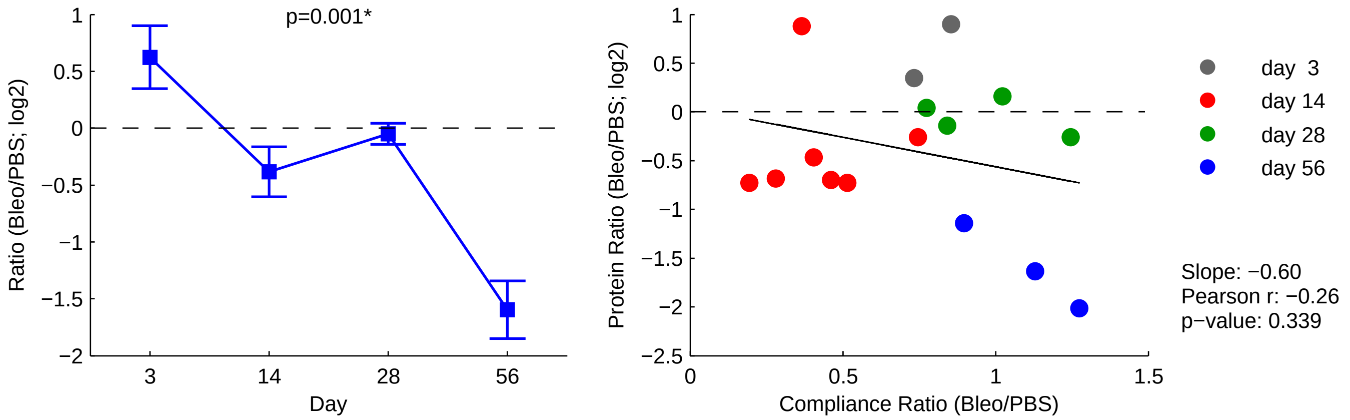

Q8CFZ4 − Gpc3 (id: 5619)

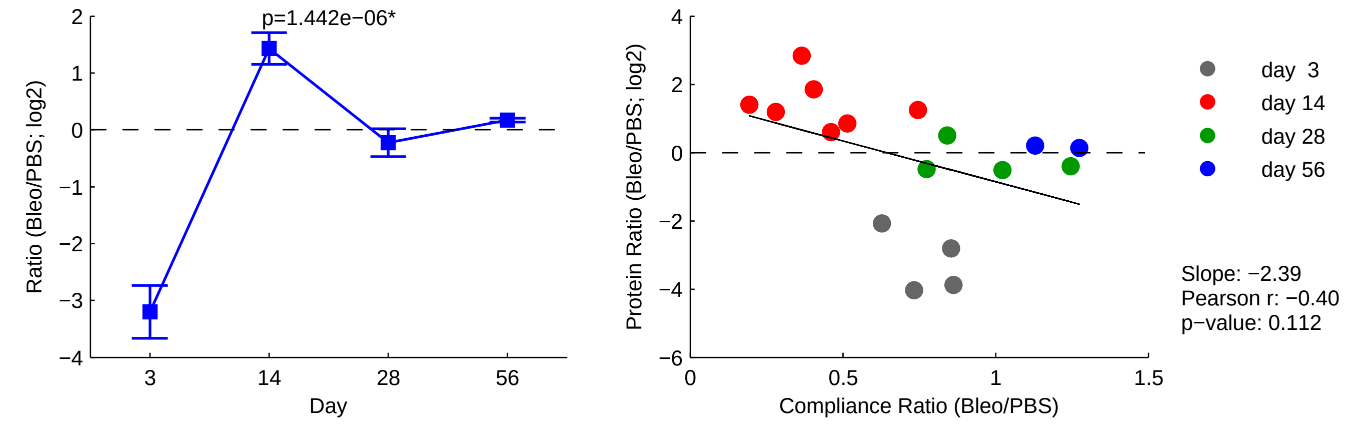

Q8CG48 − Smc2 (id: 5624)

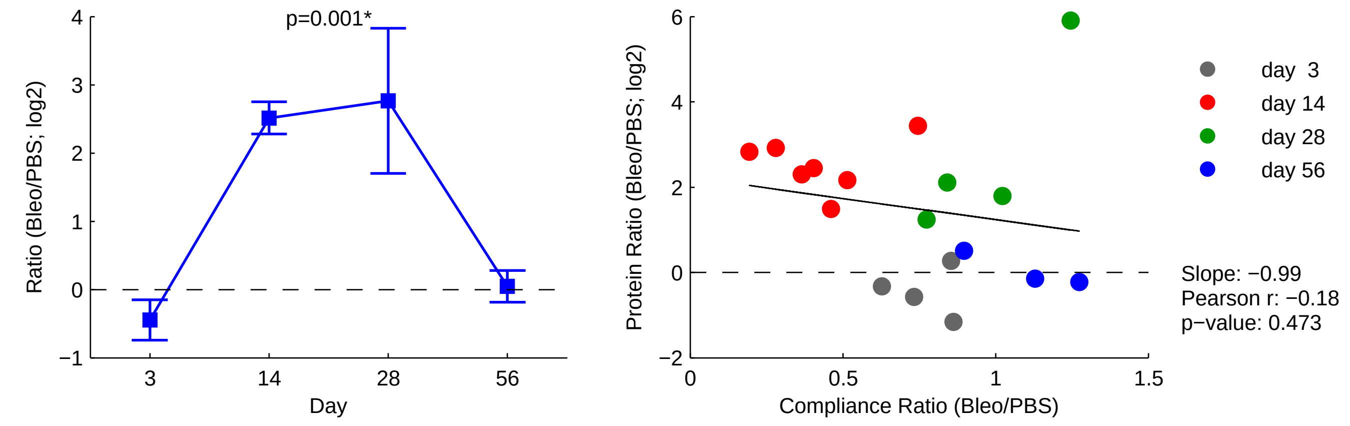

Q8CG72 − Adprhl2 (id: 5627)

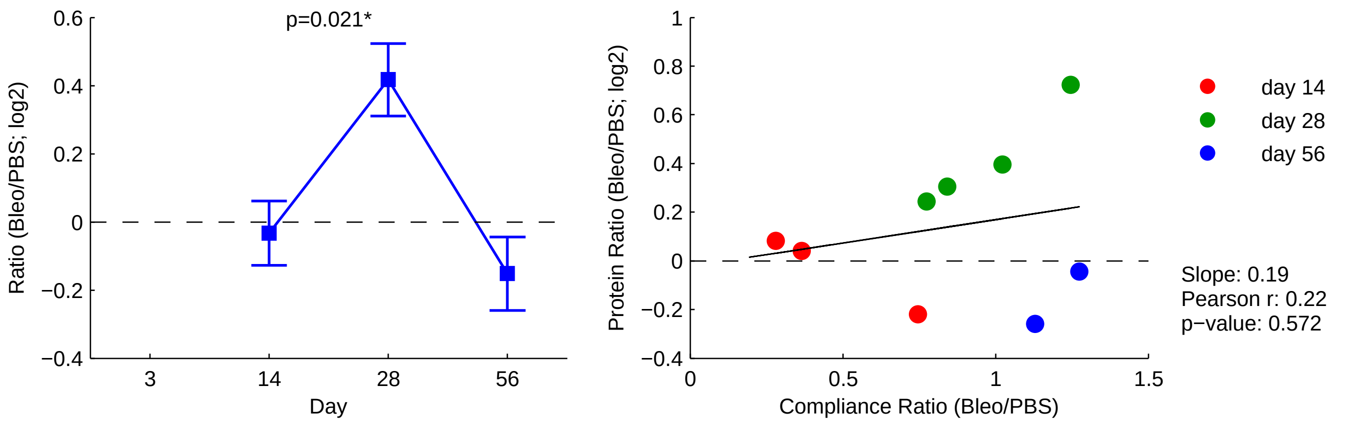

Q8CG73 − Rpgrip1l (id: 5628)

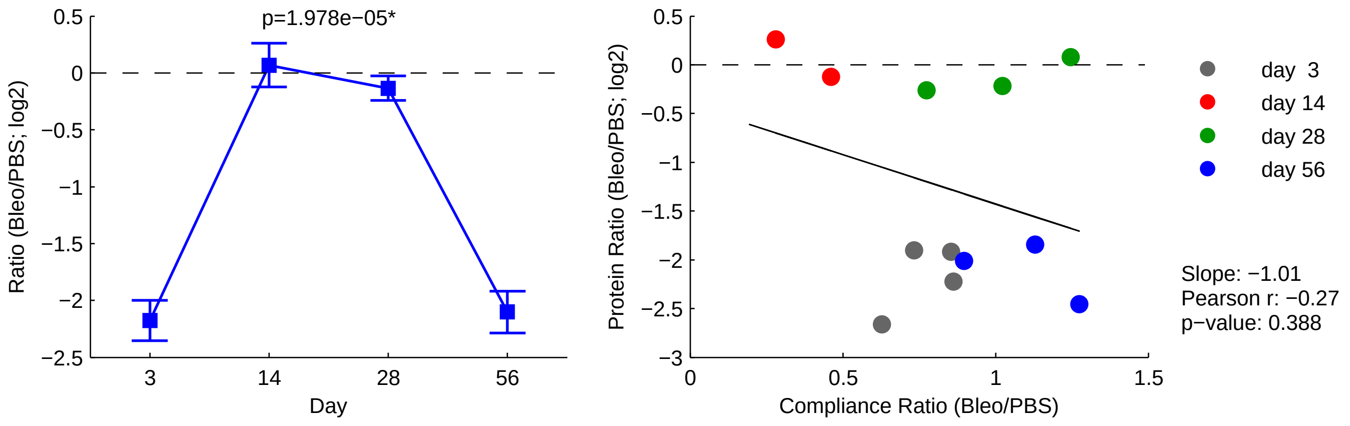

Q8CGB3 − Uaca (id: 5633)

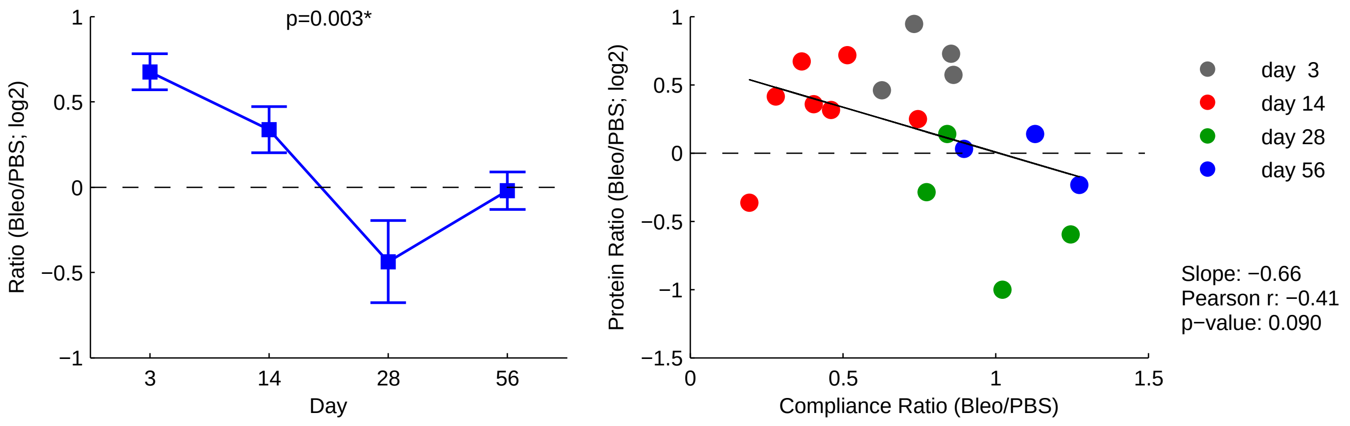

Q8CGB6−2 − Tenc1 (id: 5634)

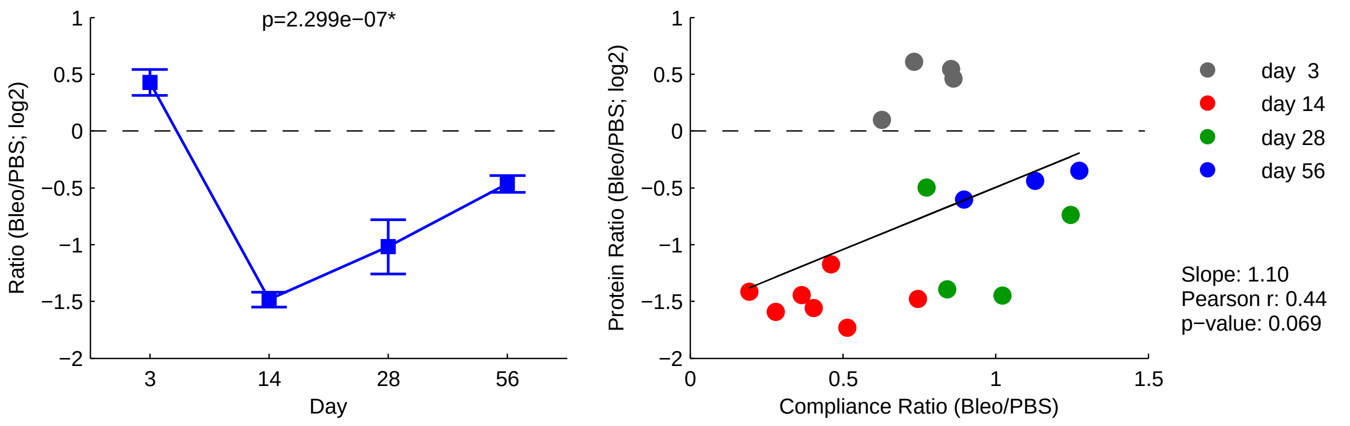

Q8CGC6 − Rbm28 (id: 5635)

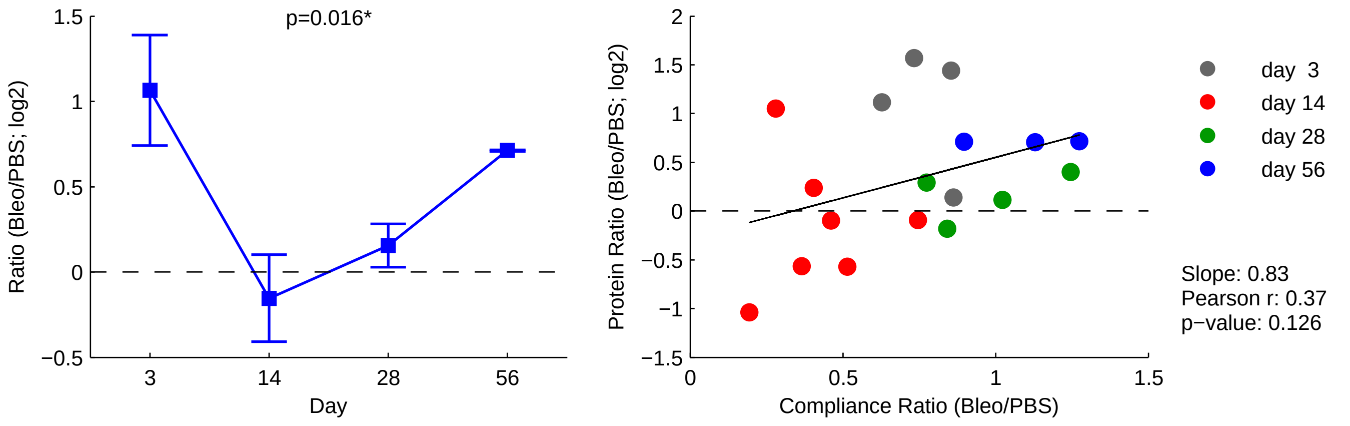

Q8CGC7 − Eprs (id: 5636)

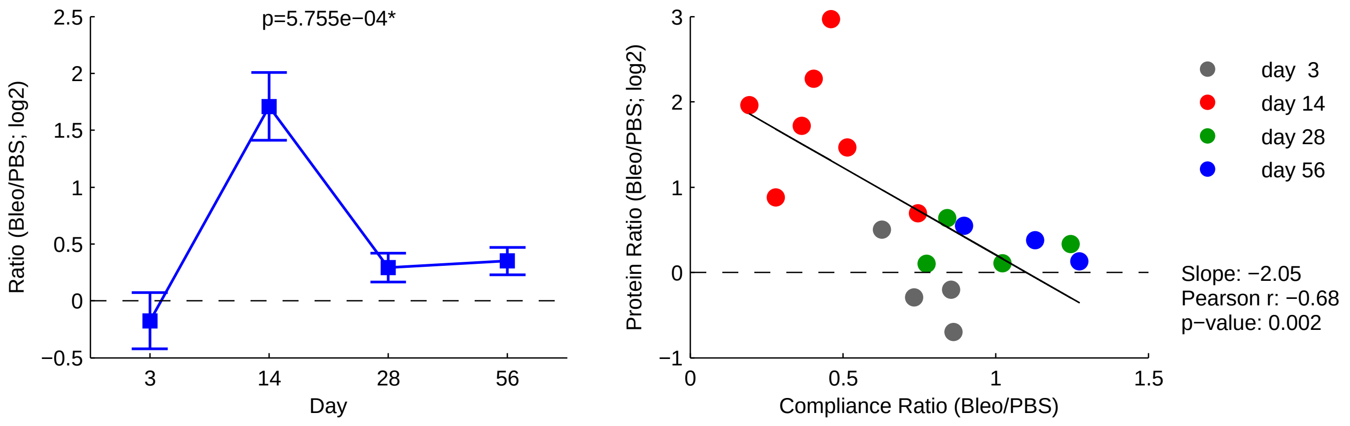

Q8CGF1 − Arhgap29 (id: 5638)

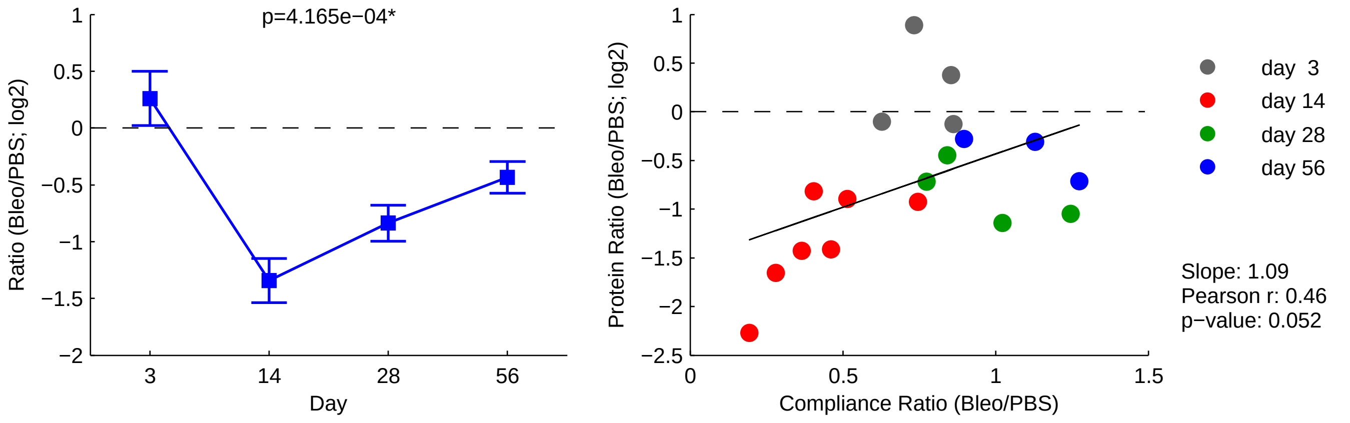

Q8CGF7 − Tcerg1 (id: 5640)

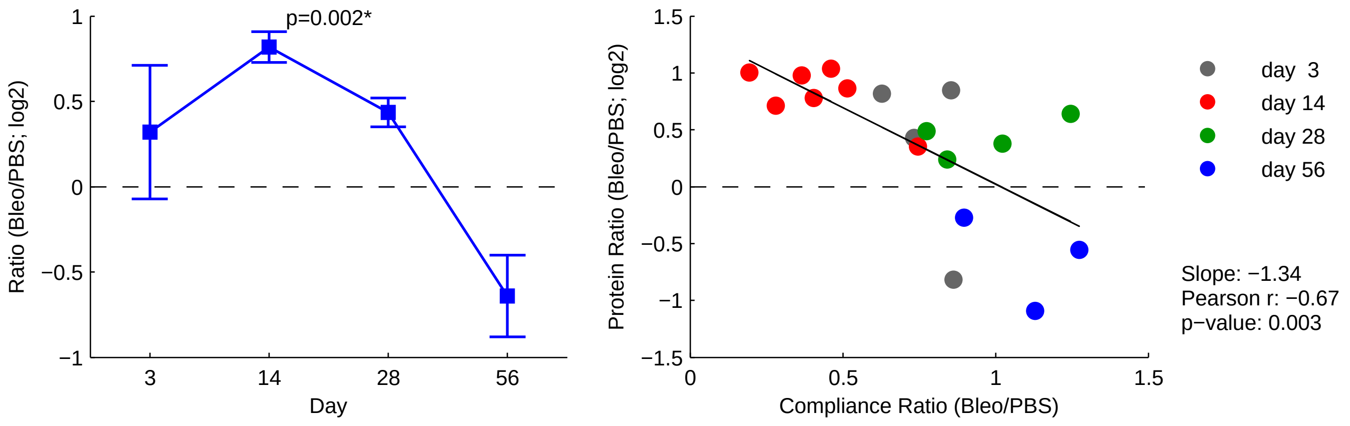

Q8CGK3 − Lonp1 (id: 5641)

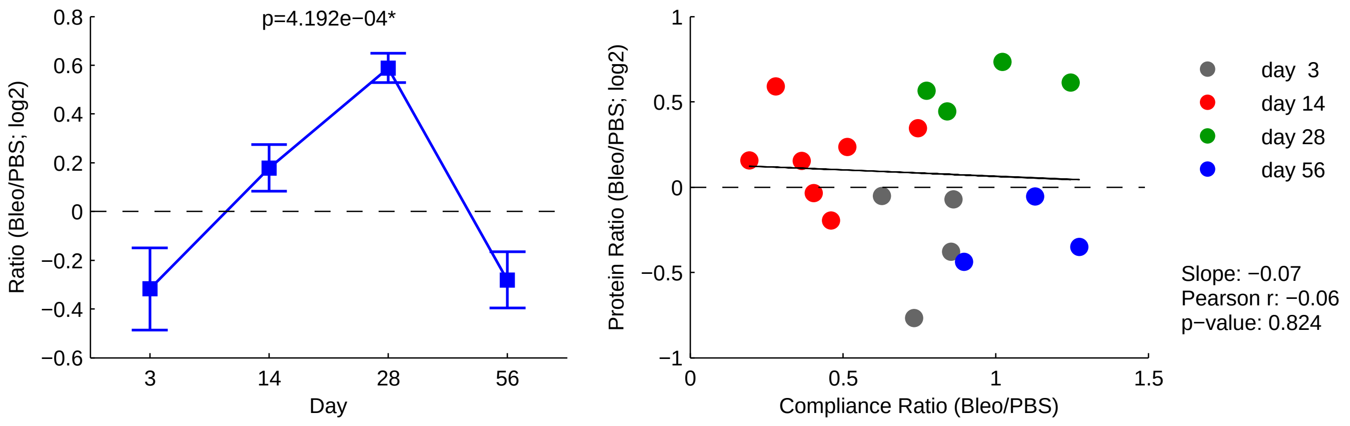

Q8CGN5 − Plin1 (id: 5643)

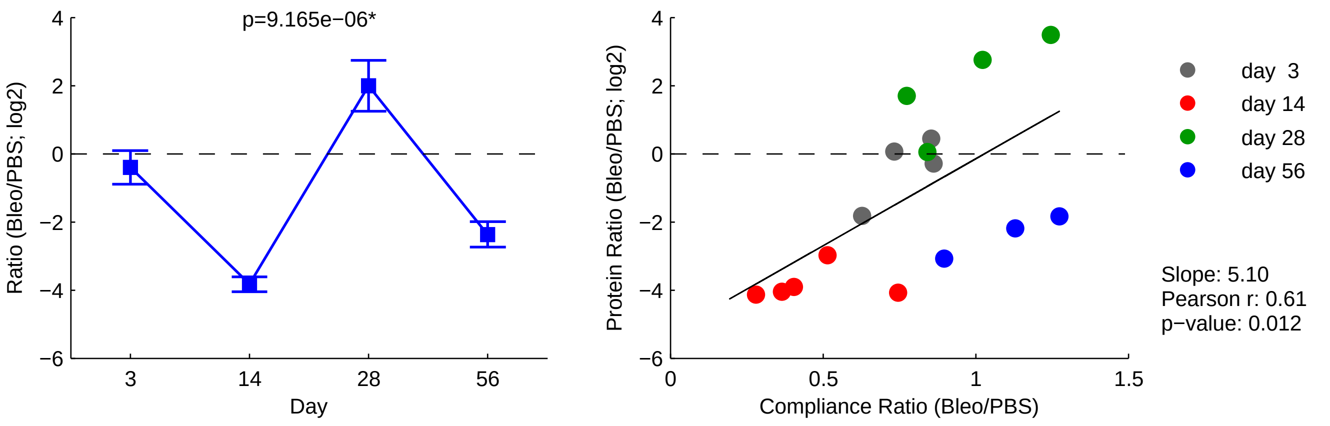

Q8CH18 − Ccar1 (id: 5648)

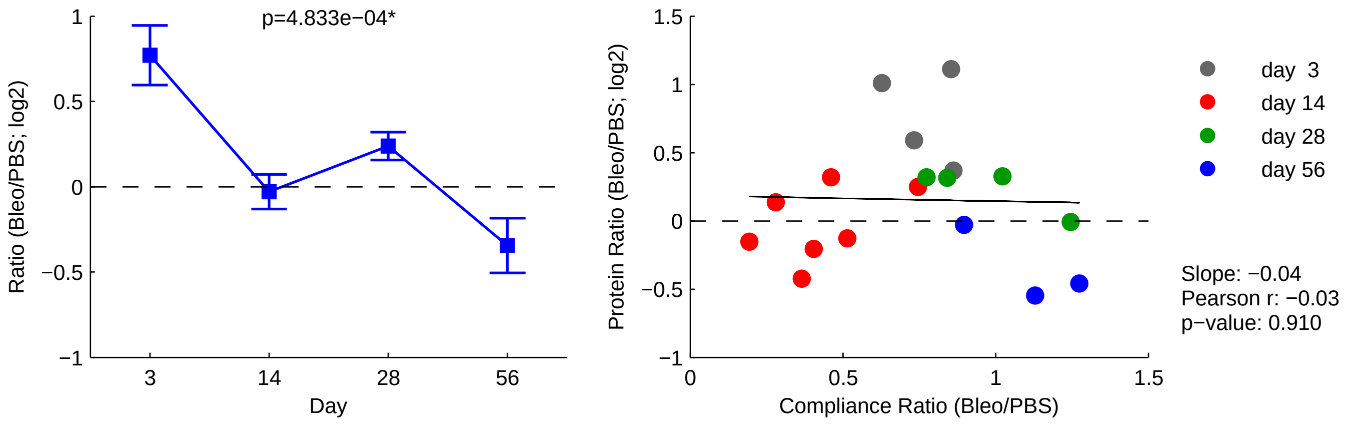

Q8CH72 − Trim32 (id: 5650)

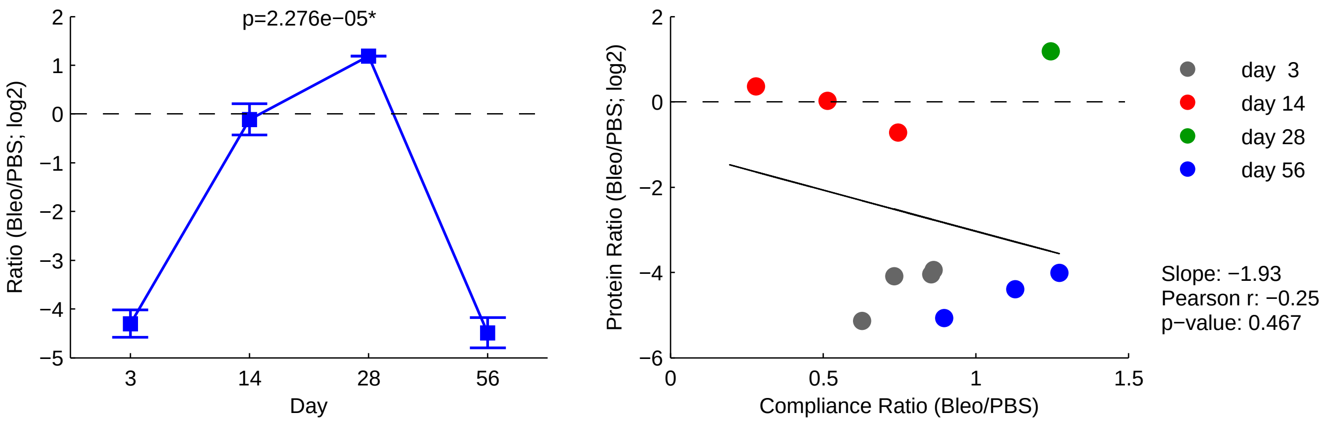

Q8CHP5−2 − Wibg (id: 5655)

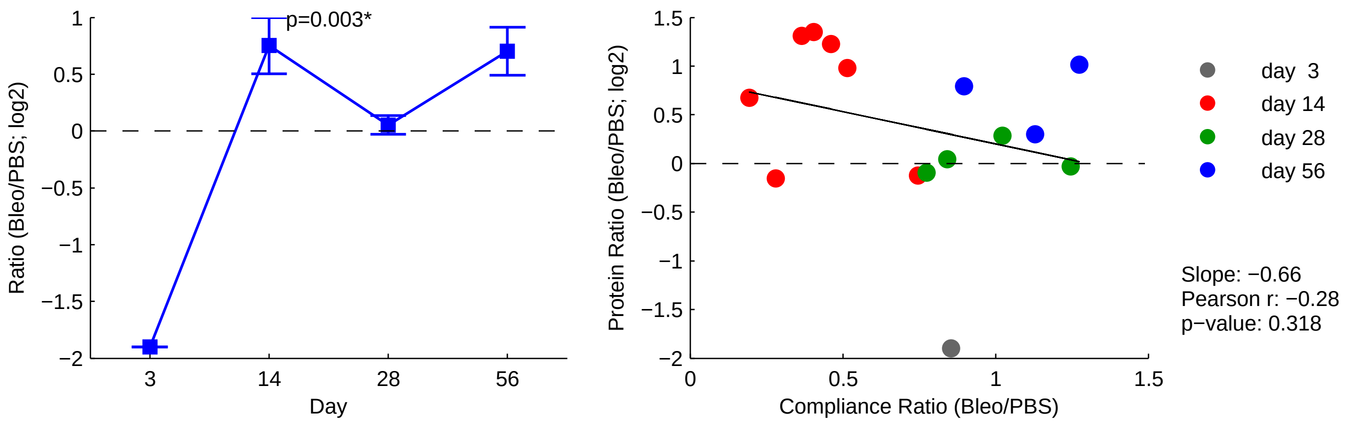

Q8CHS7 − Dhrs7c (id: 5657)

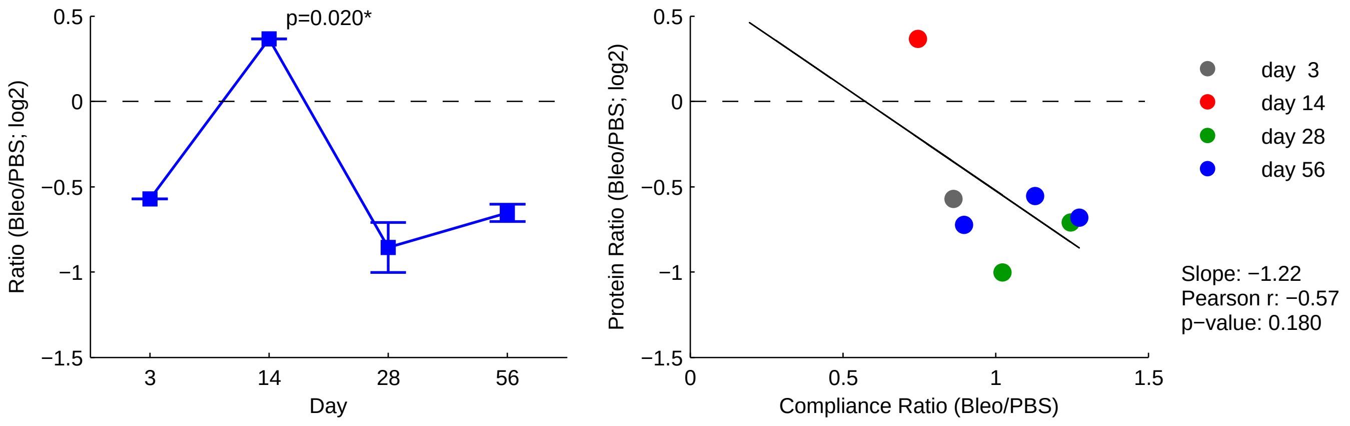

Q8CHT0 − Aldh4a1 (id: 5659)

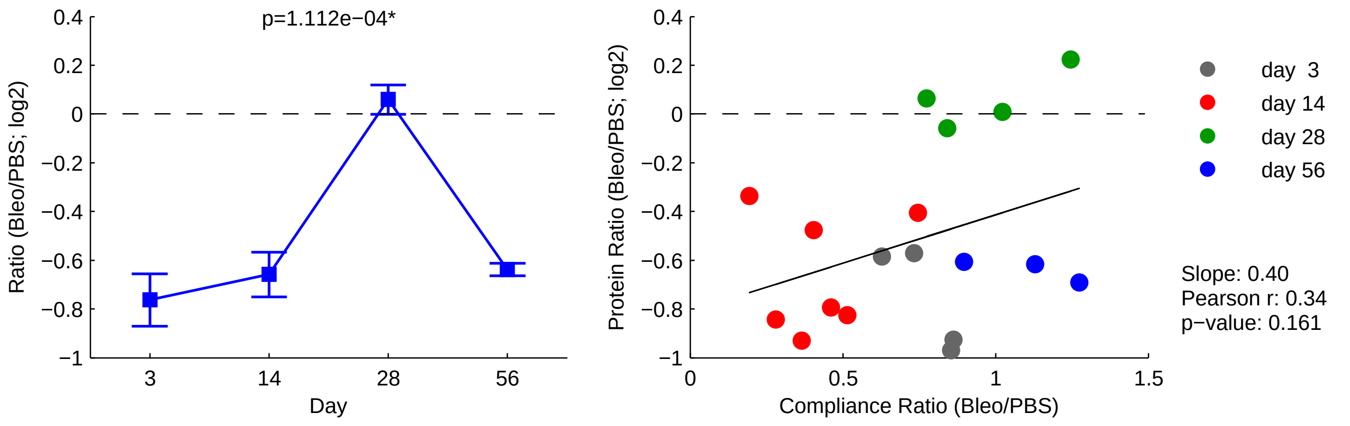

Q8CHY3 − Dym (id: 5663)

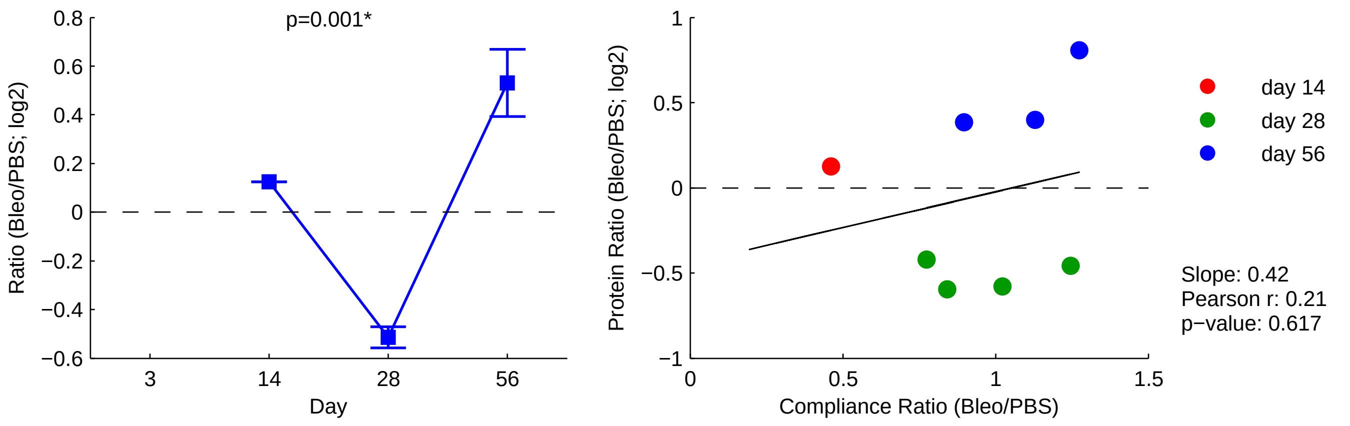

Q8CI08−2 − Slain2 (id: 5664)

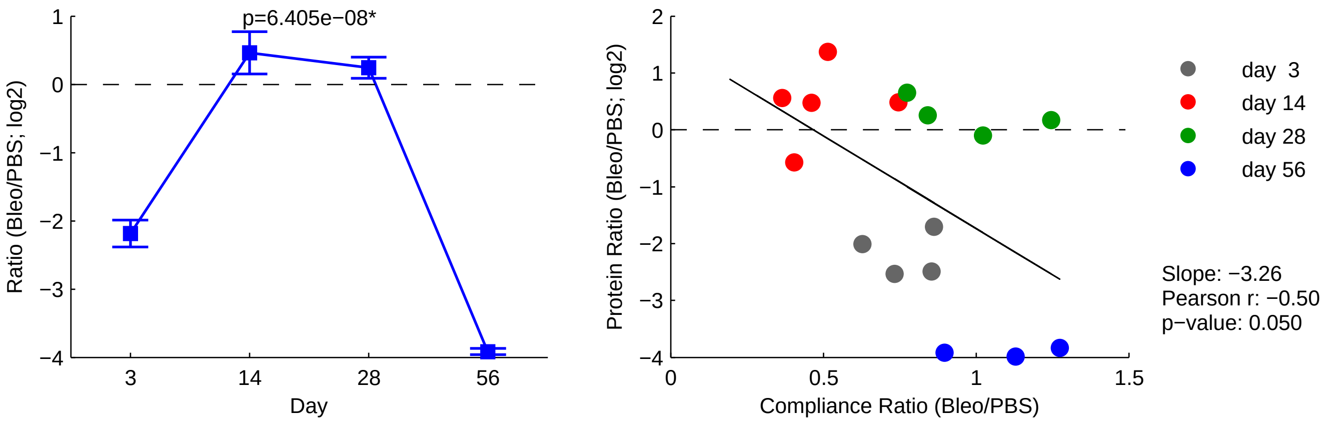

Q8CI11−2 − Gnl3 (id: 5665)

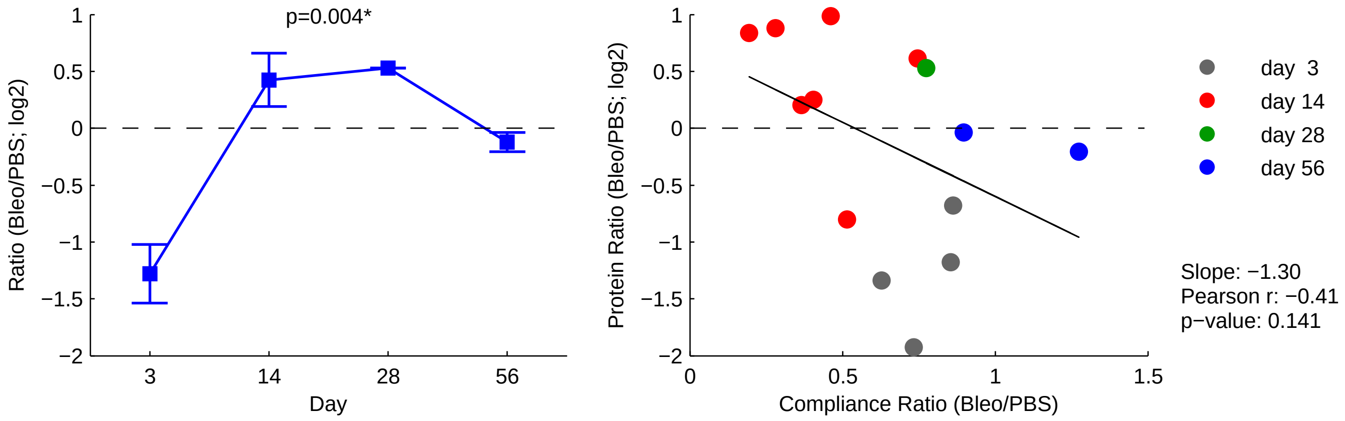

Q8CI32 − Bag5 (id: 5666)

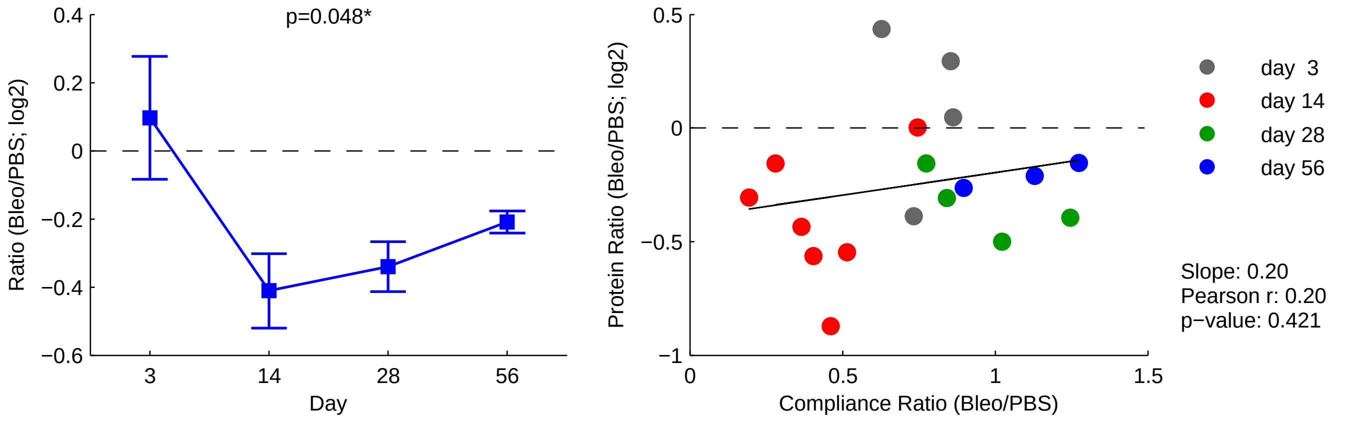

Q8CI33 − Cwf19l1 (id: 5667)

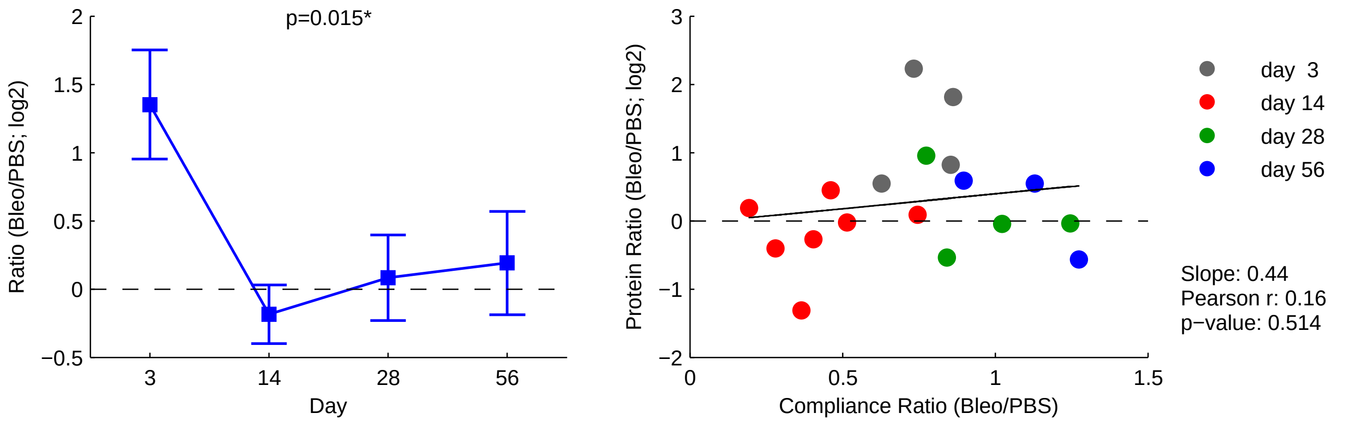

Q8CI51 − Pdlim5 (id: 5669)

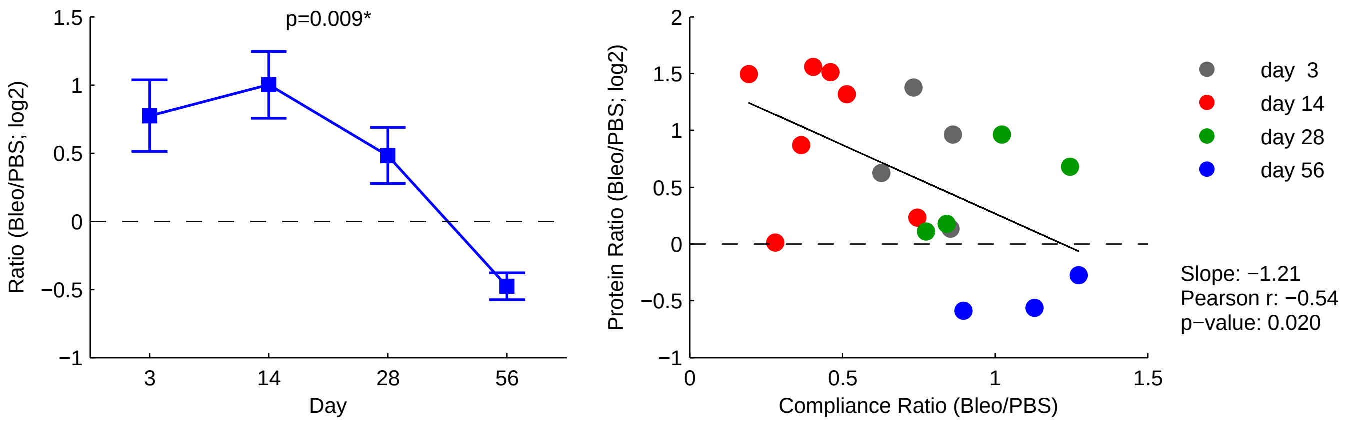

Q8CI78 − Rmnd1 (id: 5674)

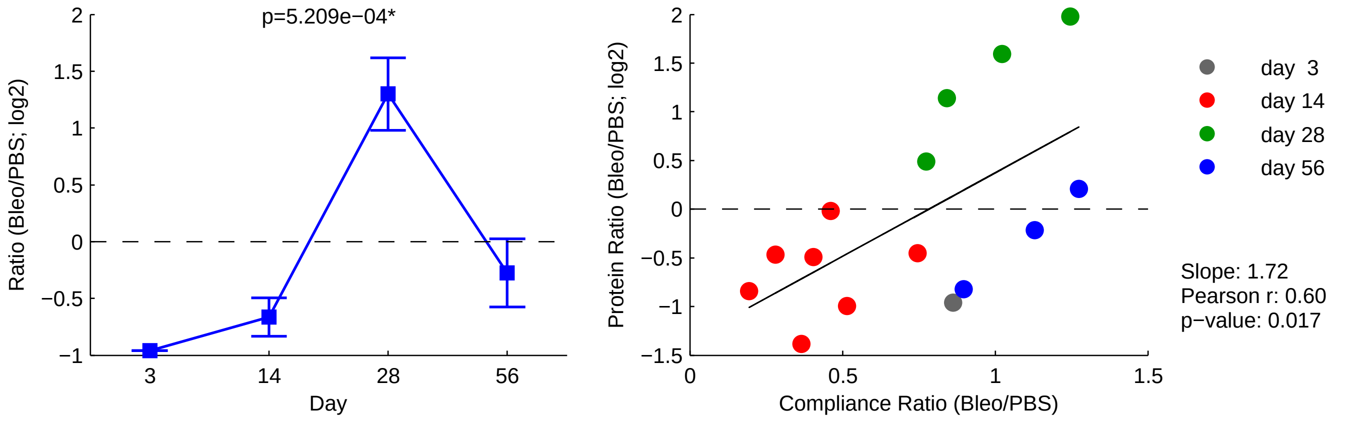

Q8CIE6 − Copa (id: 5677)

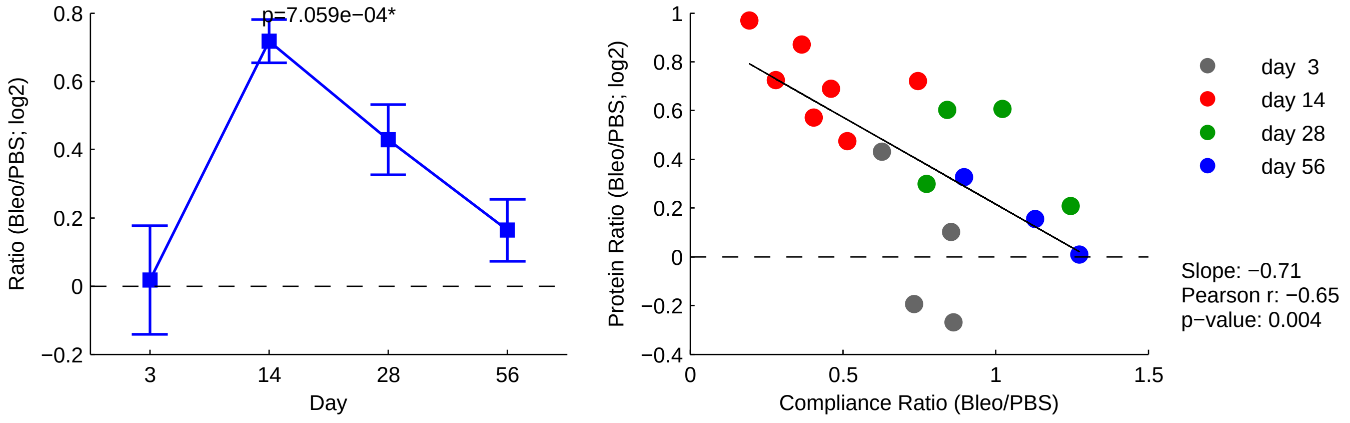

Q8CIF4 − Btd (id: 5678)

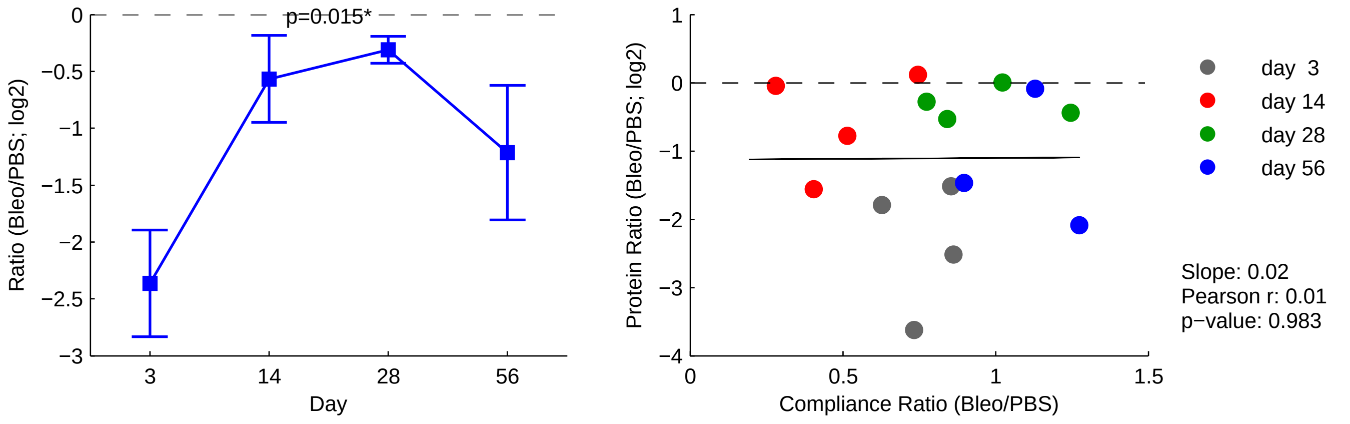

Q8CIF6 − Sidt2 (id: 5679)

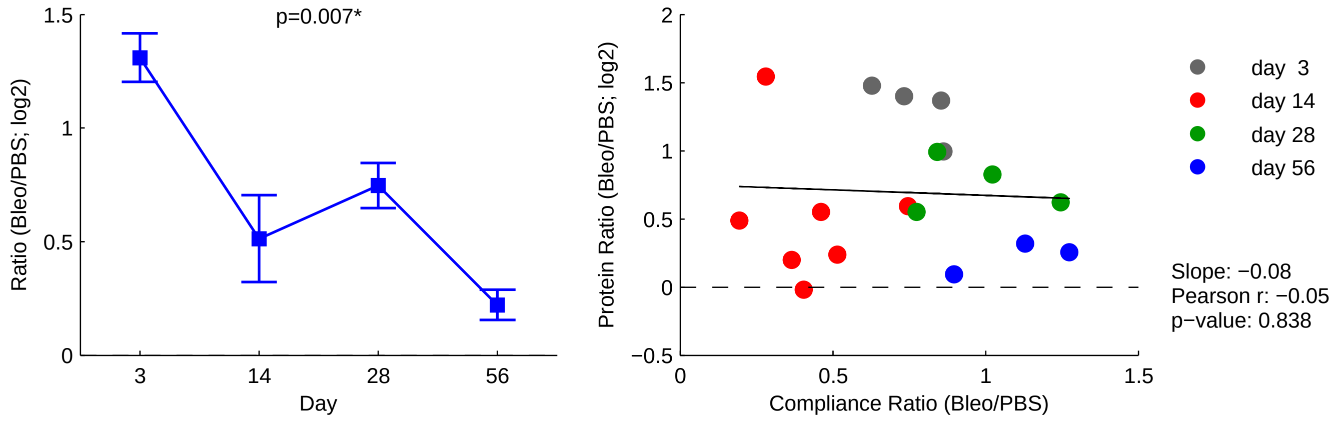

Q8CIH5 − Plcg2 (id: 5681)

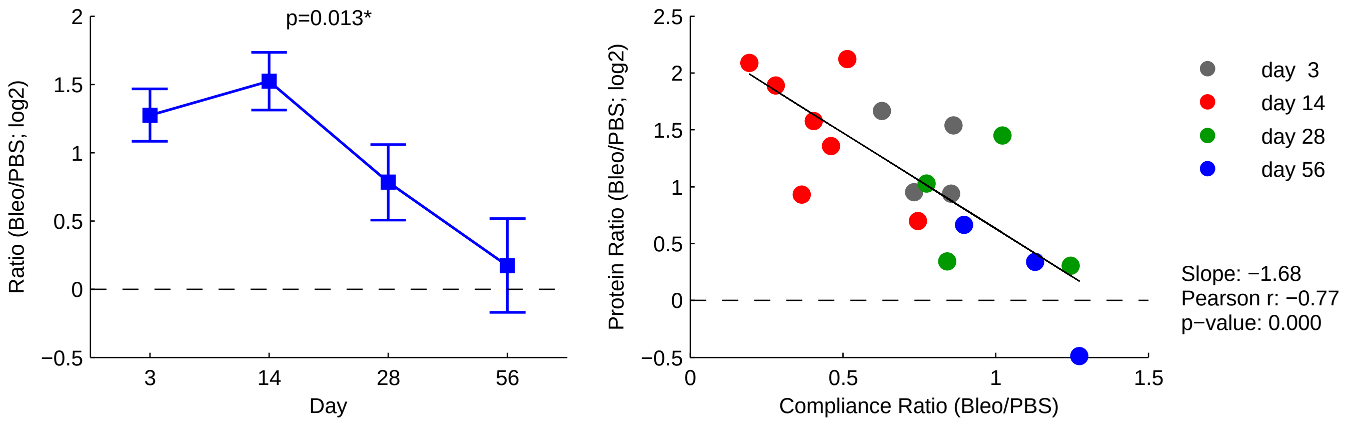

Q8CIM1 − Lrrc45 (id: 5683)

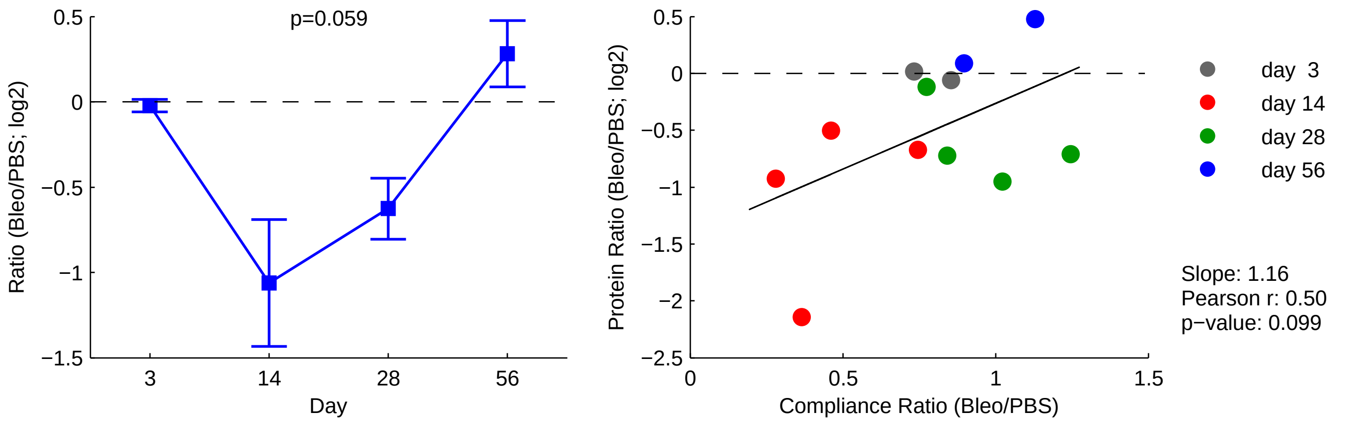

Q8CIM8 − Ints4 (id: 5684)

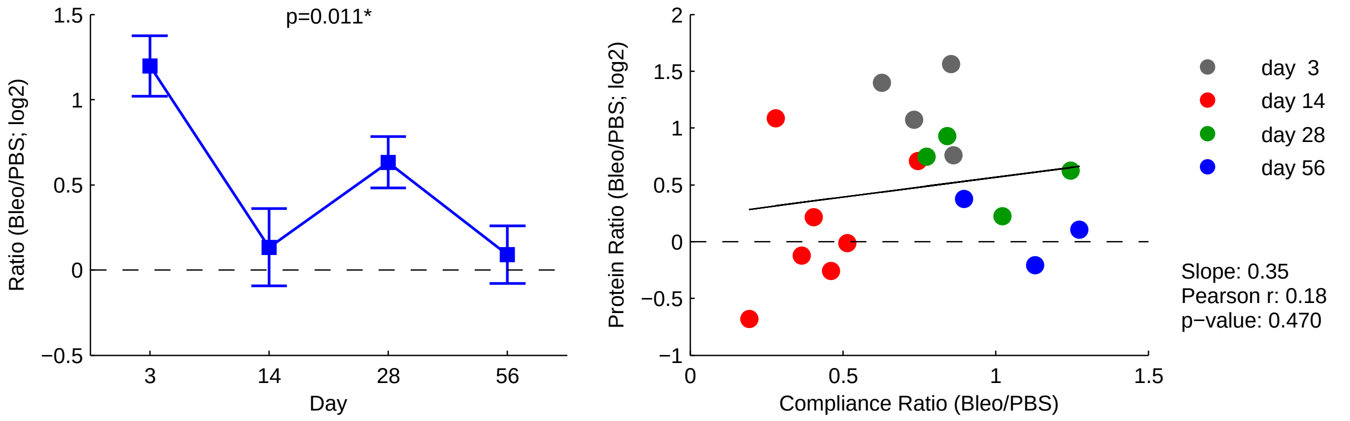

Q8CJ40−2 − (id: 5688)

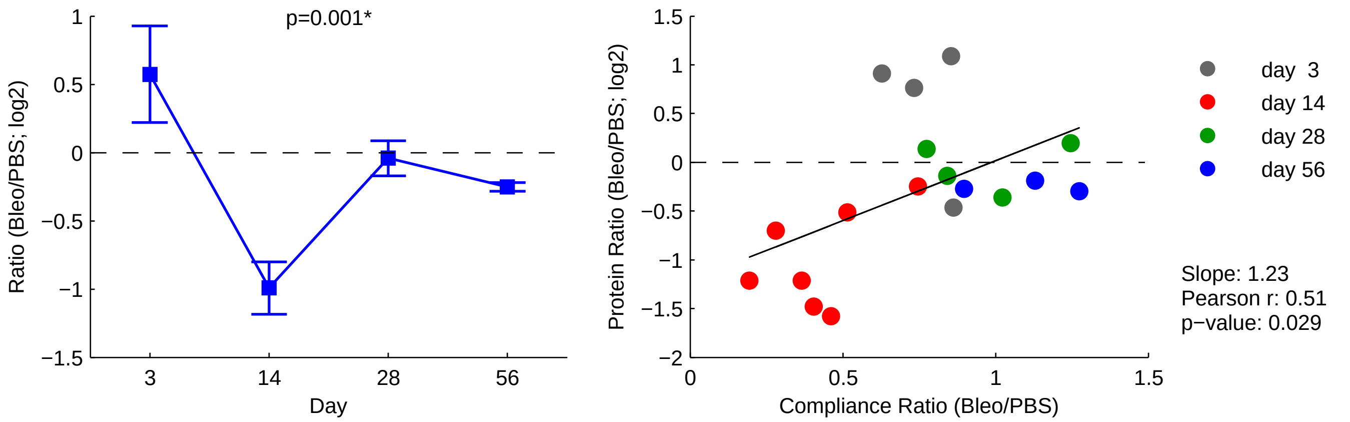

Q8CJC6 − Scgb3a1 (id: 5692)

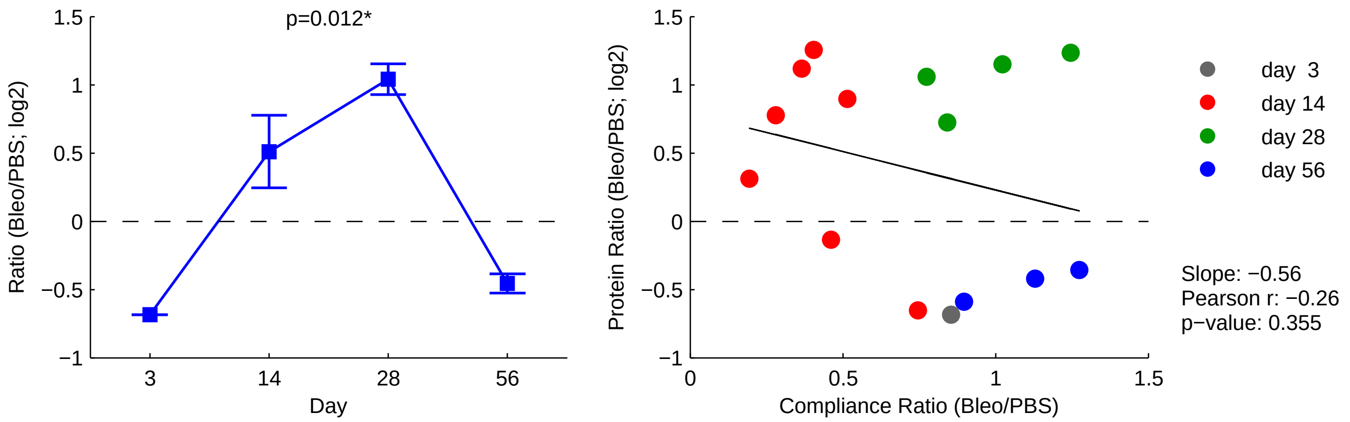

Q8CJF7 − Ahctf1 (id: 5693)

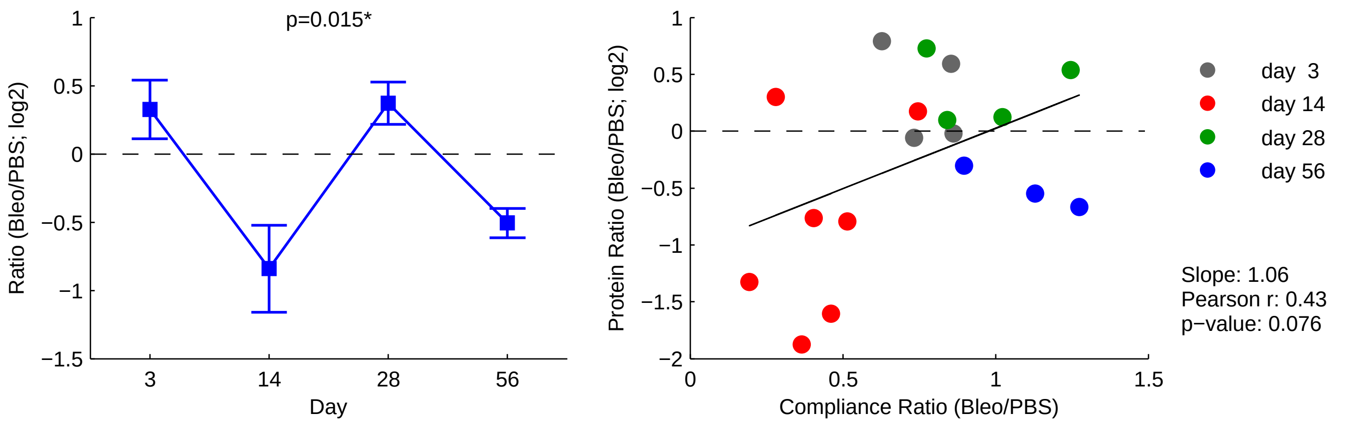

Q8CJG1 − Eif2c1 (id: 5696)

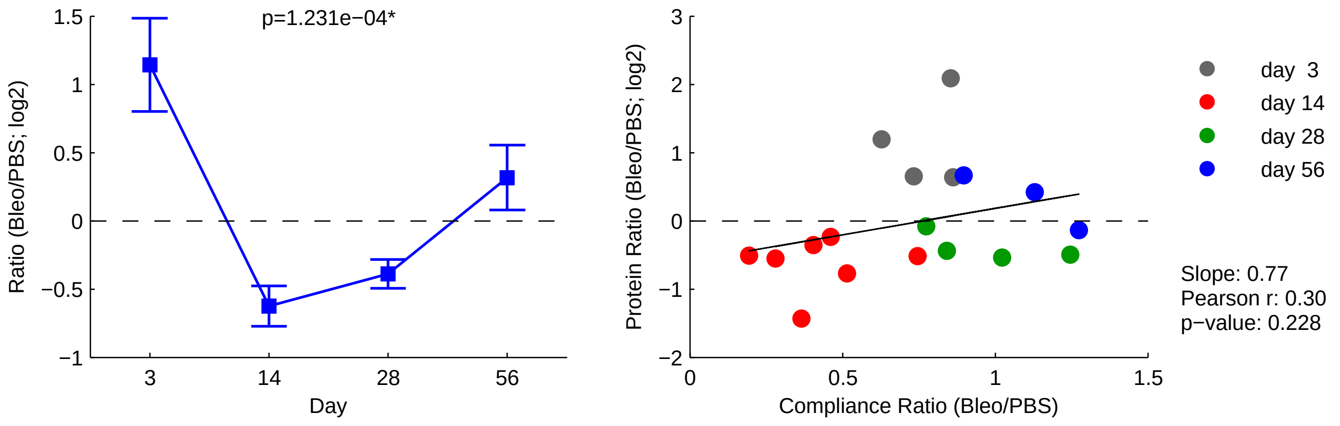

Q8CJH3 − Plxnb1 (id: 5697)

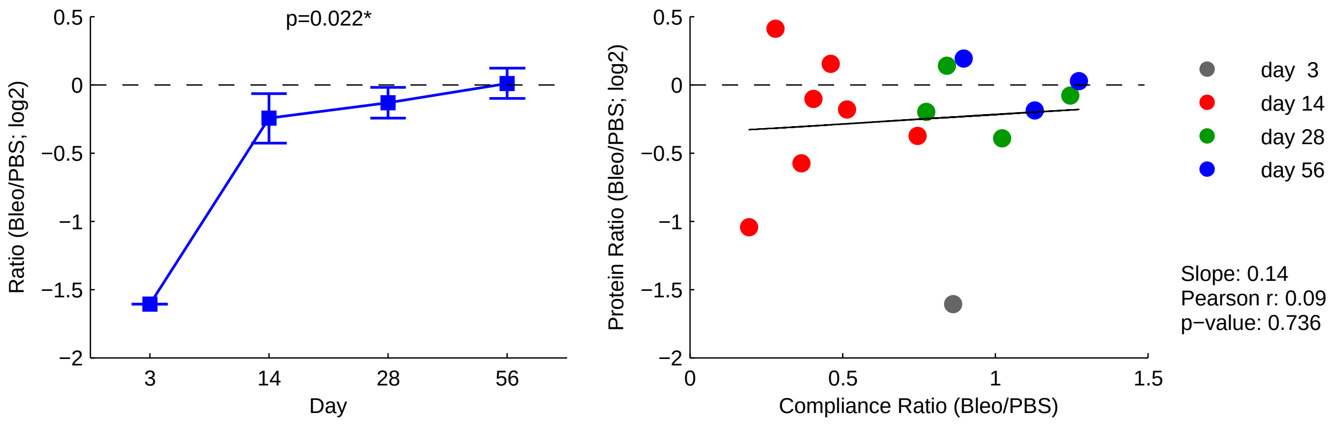

Q8JZM7 − Cdc73 (id: 5703)

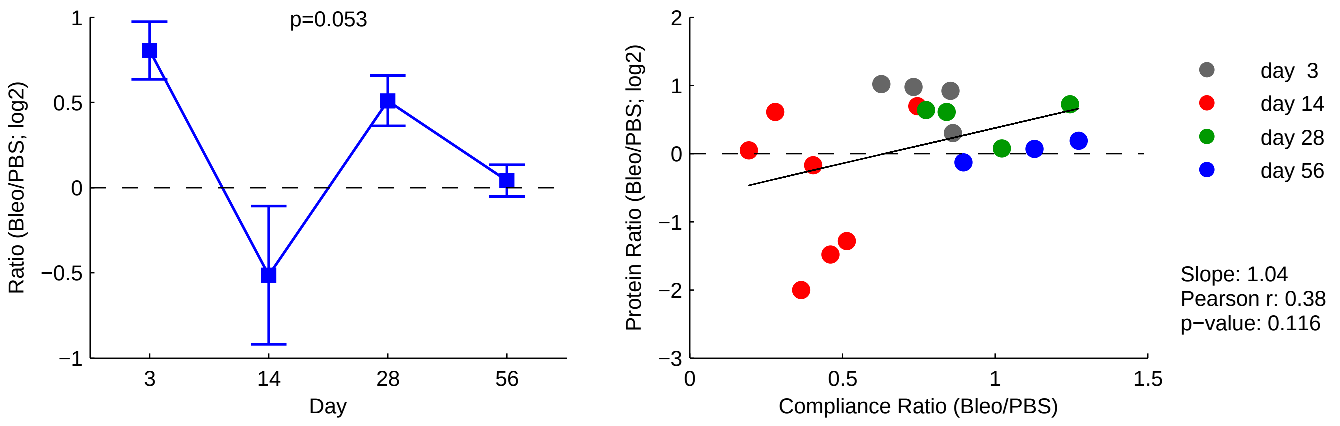

Q8JZN5 − Acad9 (id: 5704)

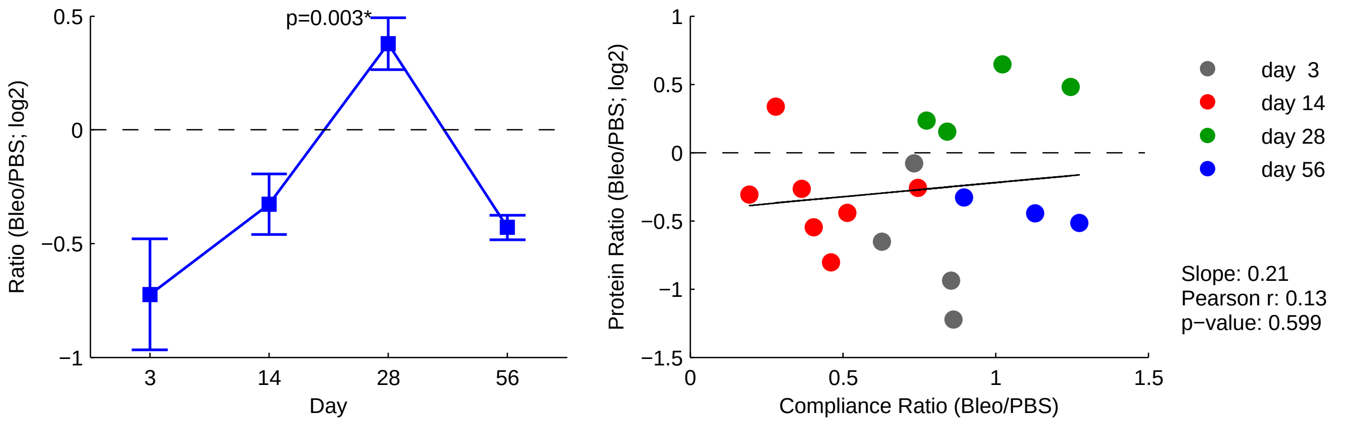

Q8JZQ2 − Afg3l2 (id: 5707)

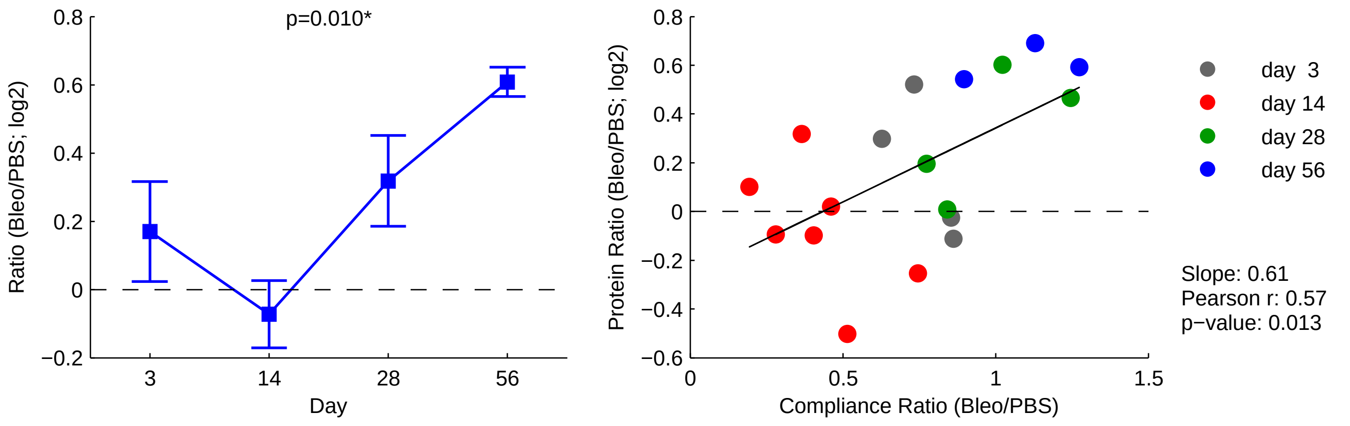

Q8JZQ9 − Eif3b (id: 5708)

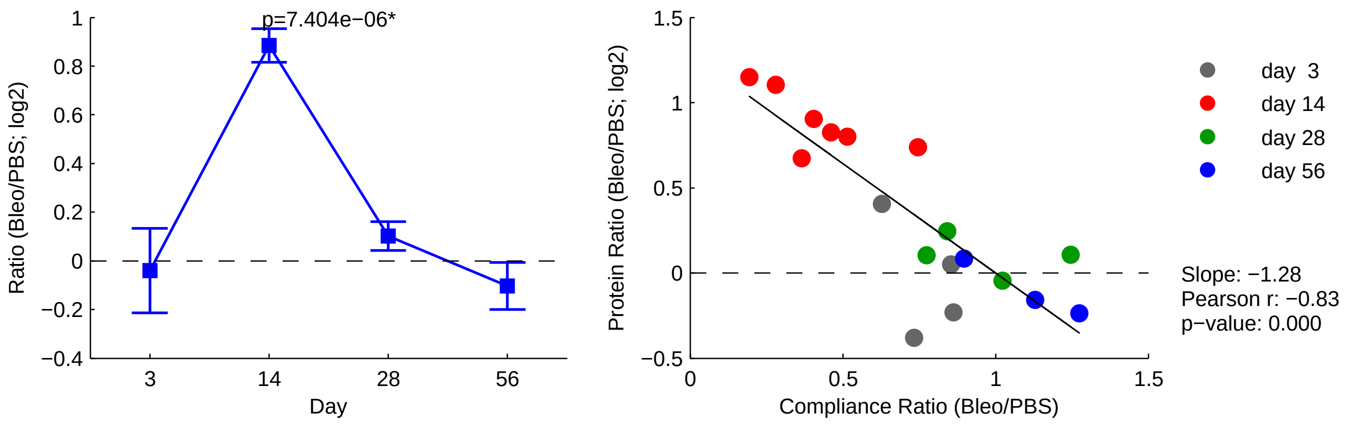

Q8JZR0 − Acsl5 (id: 5709)

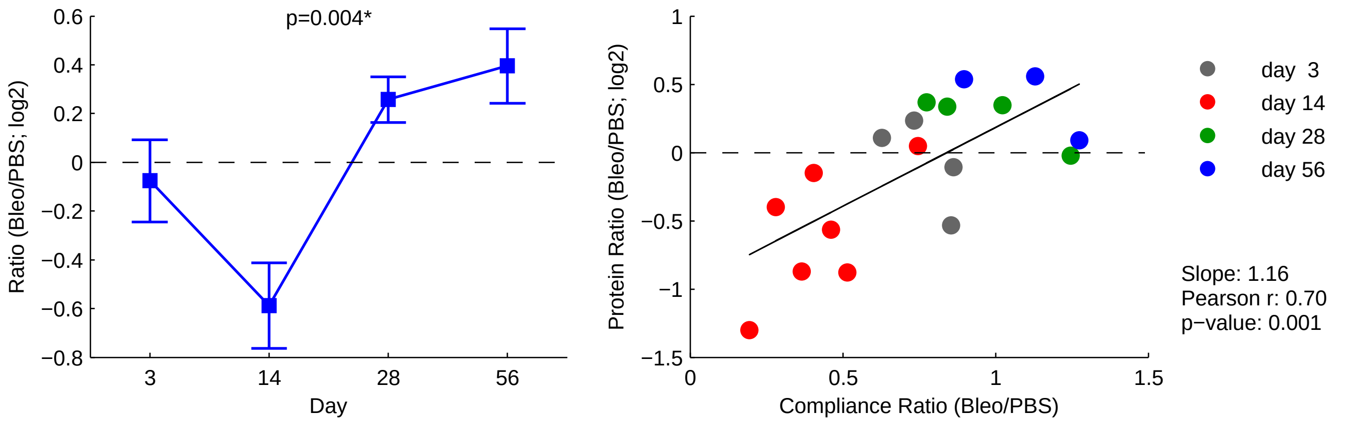

Q8JZU2 − Slc25a1 (id: 5711)

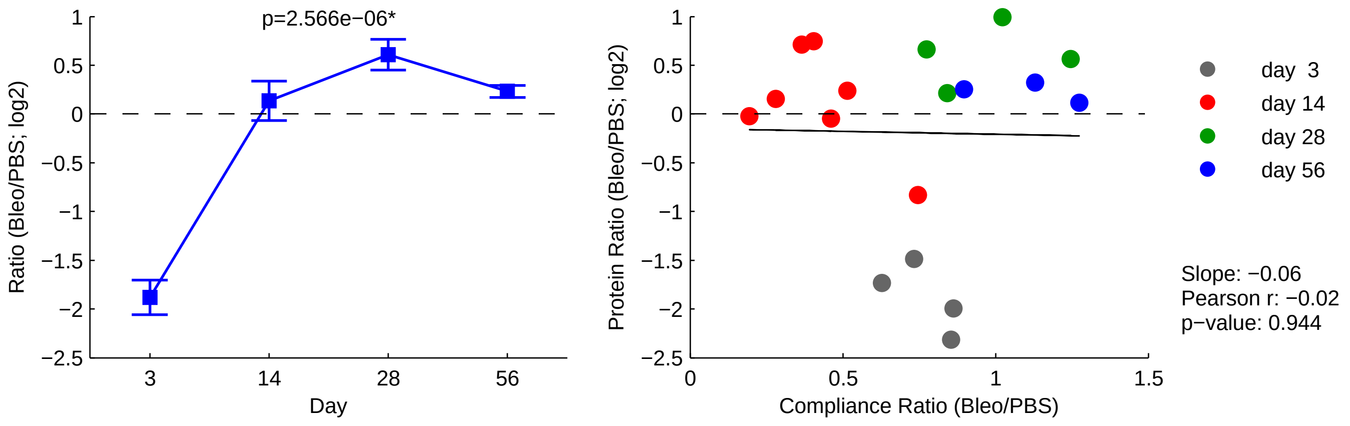

Q8JZX3−2 − Poc1a (id: 5713)

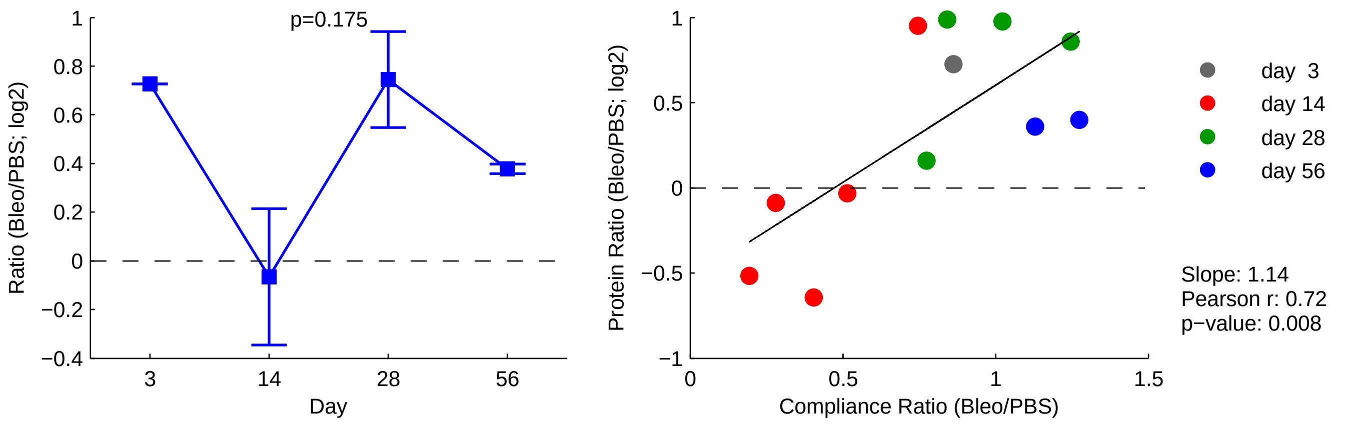

Q8JZX5 − Lrrc57 (id: 5715)

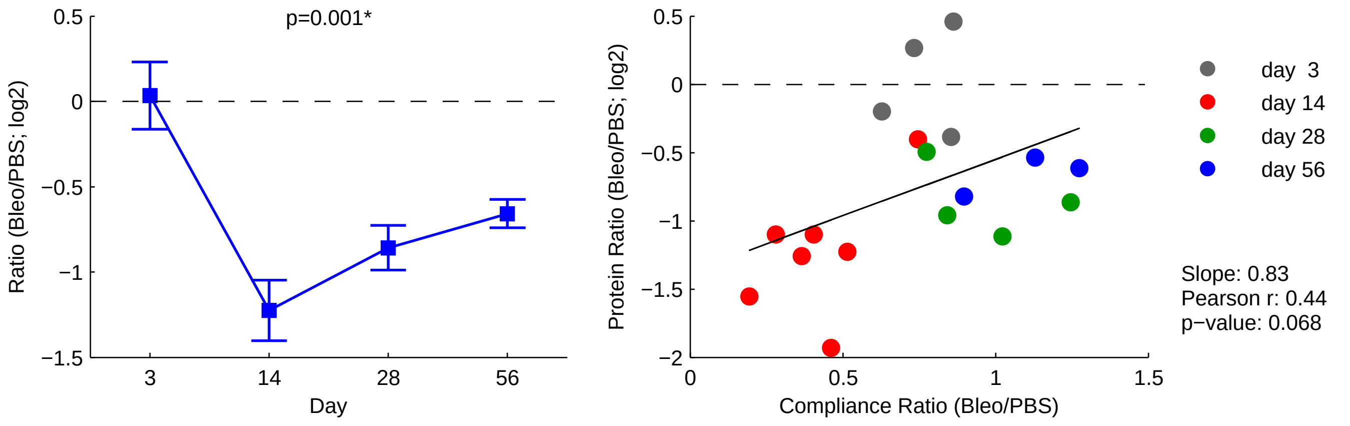

Q8K010 − Oplah (id: 5720)

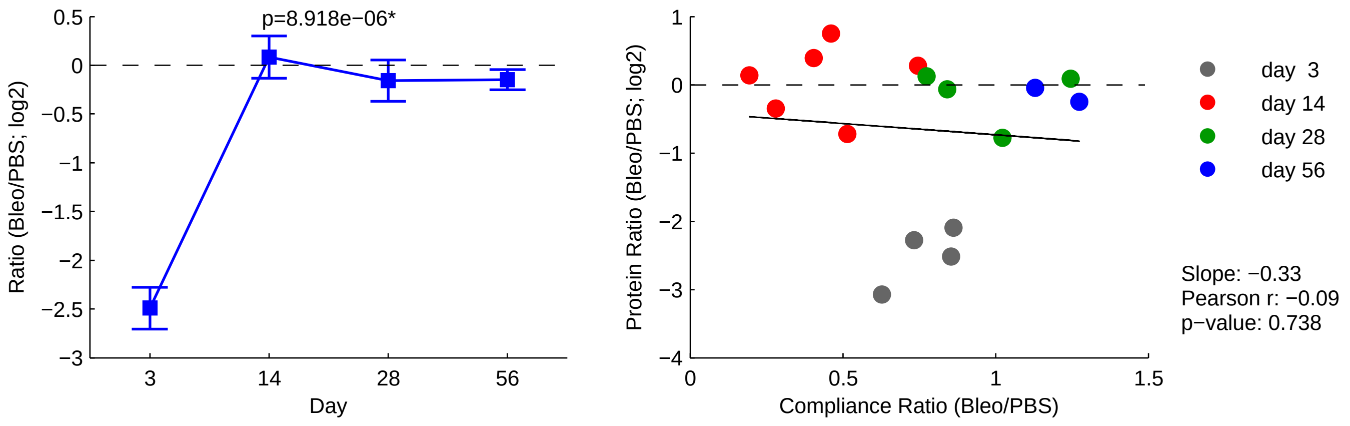

Q8K019−2 − Bclaf1 (id: 5722)

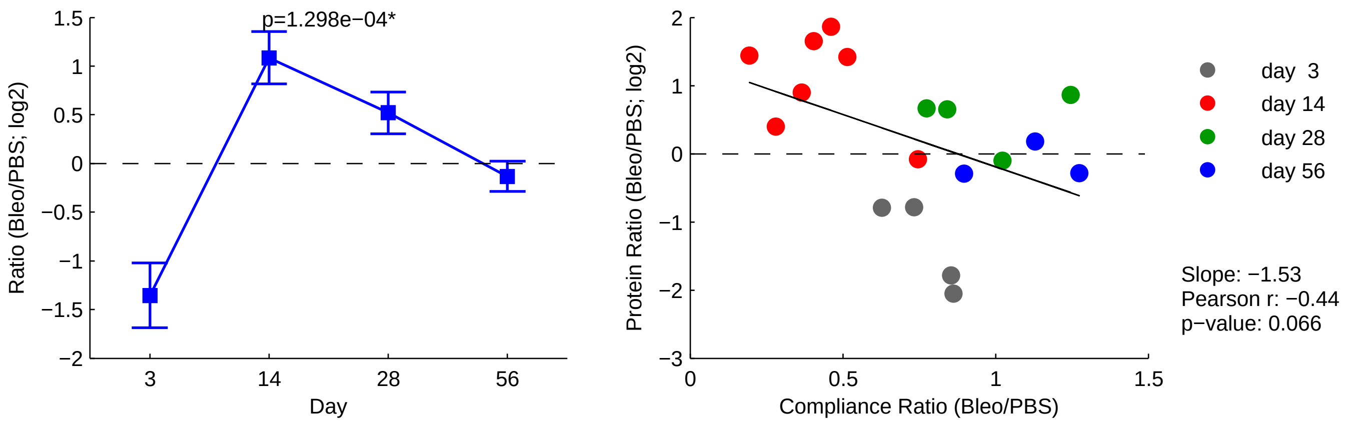

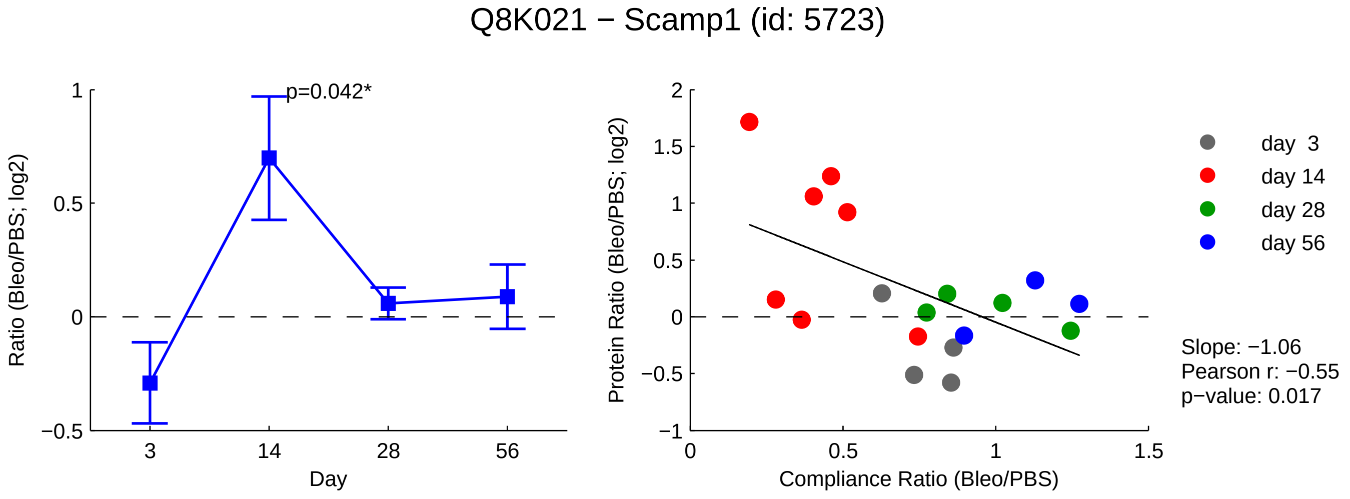
Q8K021 − Scamp1 (id: 5723)
p=0.042*
Ratio (Bleo/PBS; log2)
Day
Protein Ratio (Bleo/PBS; log2)
Compliance Ratio (Bleo/PBS)
day 3
day 14
day 28
day 56
Slope: −1.06
Pearson r: −0.55
p−value: 0.017

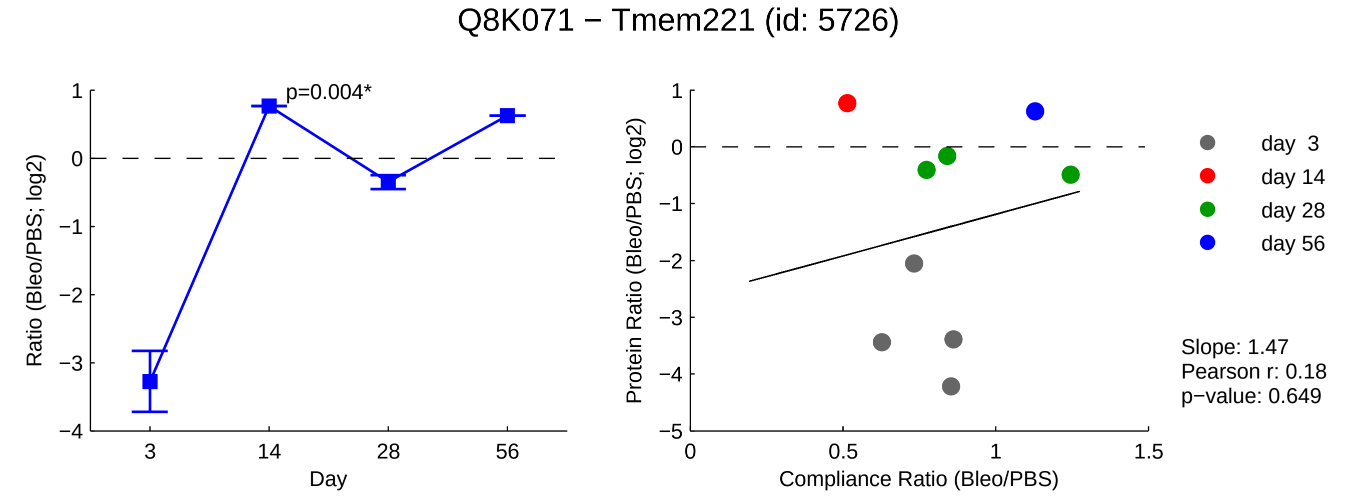
Q8K071 − Tmem221 (id: 5726)
p=0.004*
Ratio (Bleo/PBS; log2)
Day
Protein Ratio (Bleo/PBS; log2)
Compliance Ratio (Bleo/PBS)
day 3
day 14
day 28
day 56
Slope: 1.47
Pearson r: 0.18
p−value: 0.649

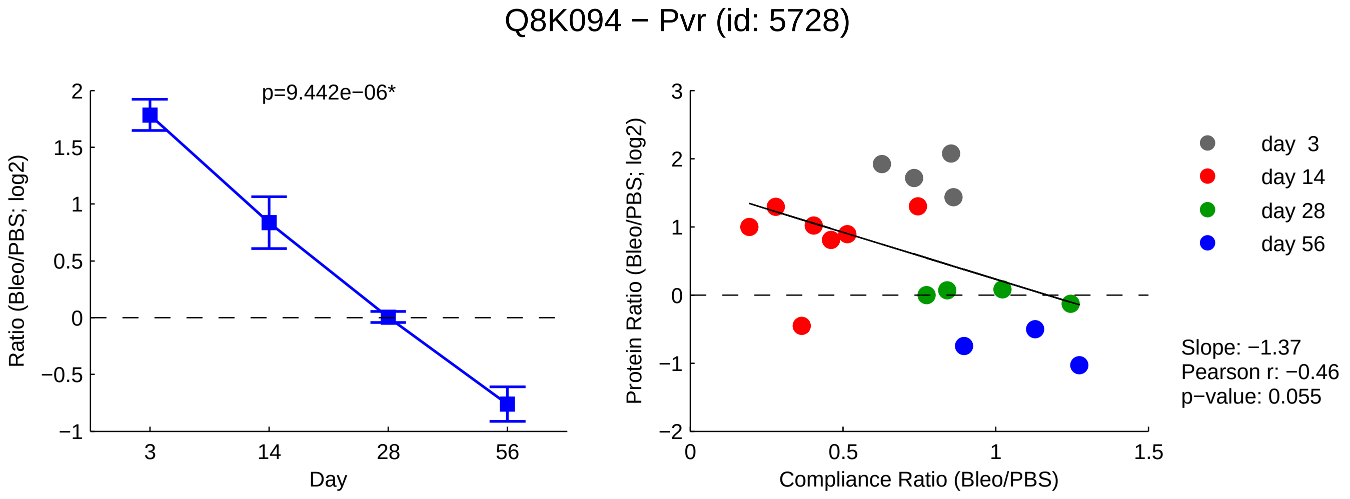
Q8K094 − Pvr (id: 5728)
p=9.442e−06*
Ratio (Bleo/PBS; log2)
Day
Protein Ratio (Bleo/PBS; log2)
Compliance Ratio (Bleo/PBS)
day 3
day 14
day 28
day 56
Slope: −1.37
Pearson r: −0.46
p−value: 0.055

Q8K0C4 − Cyp51a1 (id: 5730)

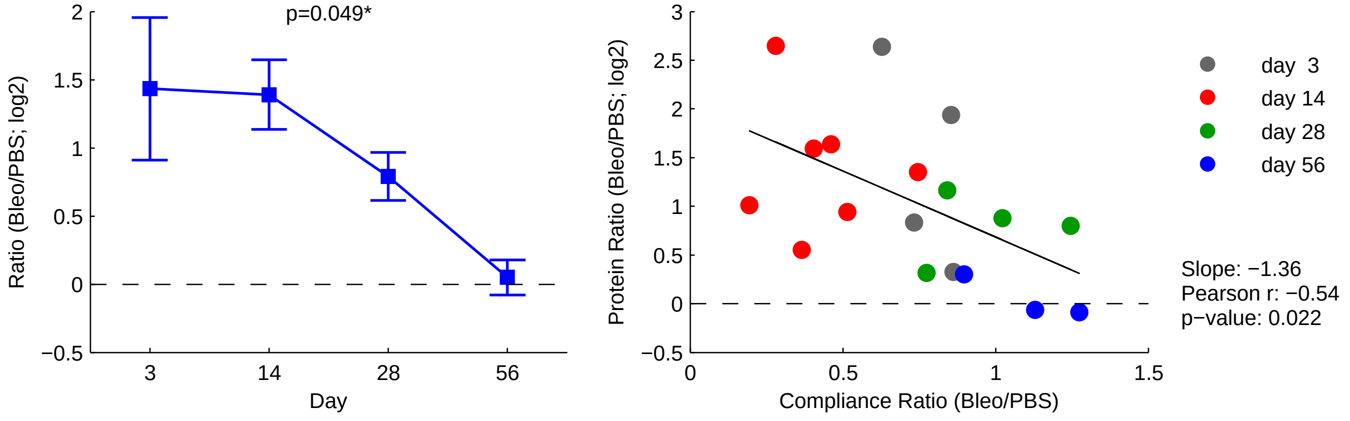

Q8K0D0 − Cdk17 (id: 5732)

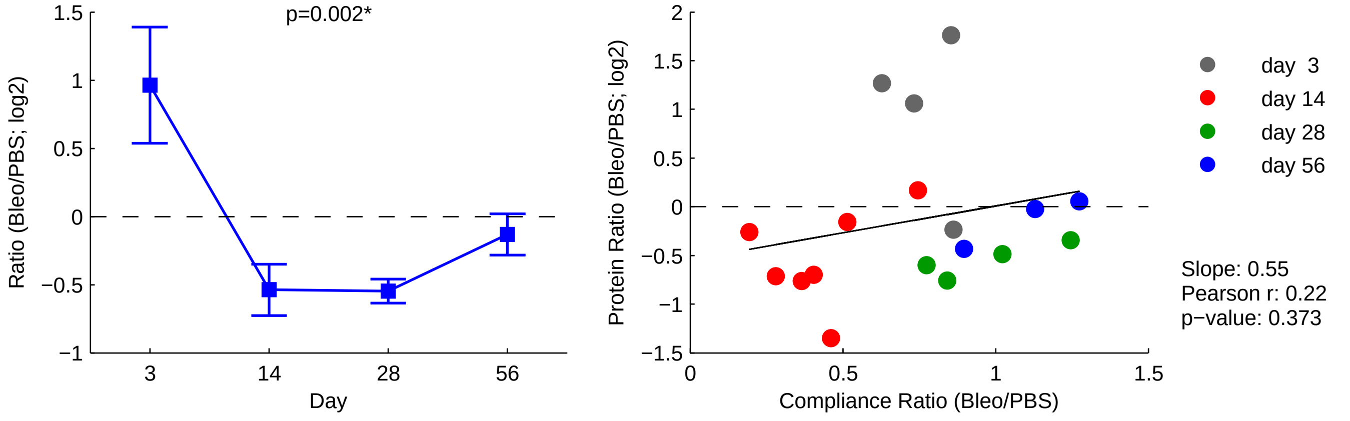

Q8K0D5 − Gfm1 (id: 5733)

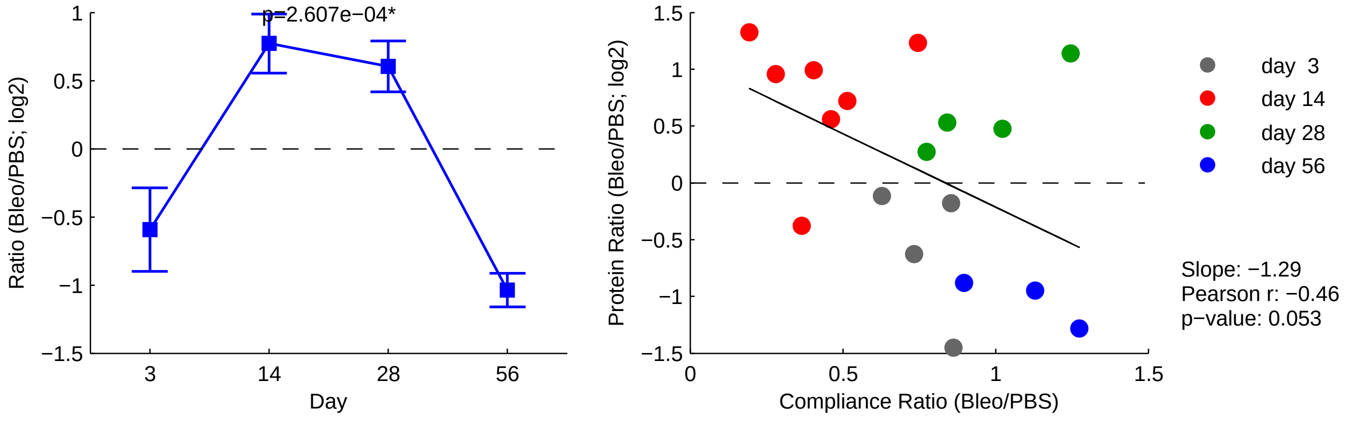

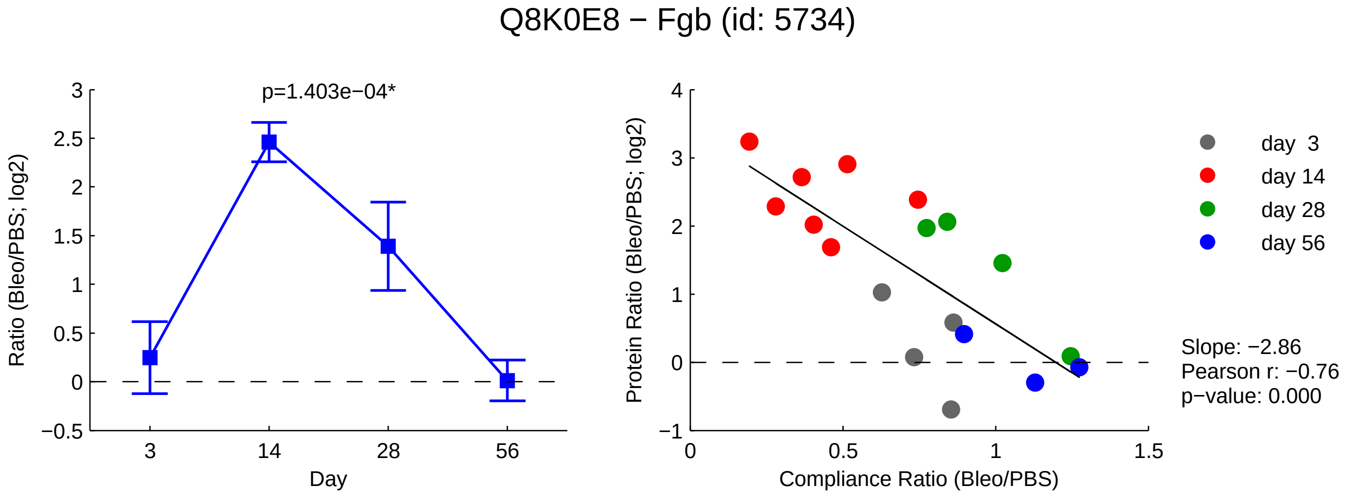
Q8K0E8 − Fgb (id: 5734)
p=1.403e−04*
Ratio (Bleo/PBS; log2)
Day
Protein Ratio (Bleo/PBS; log2)
Compliance Ratio (Bleo/PBS)
day 3
day 14
day 28
day 56
Slope: −2.86
Pearson r: −0.76
p−value: 0.000

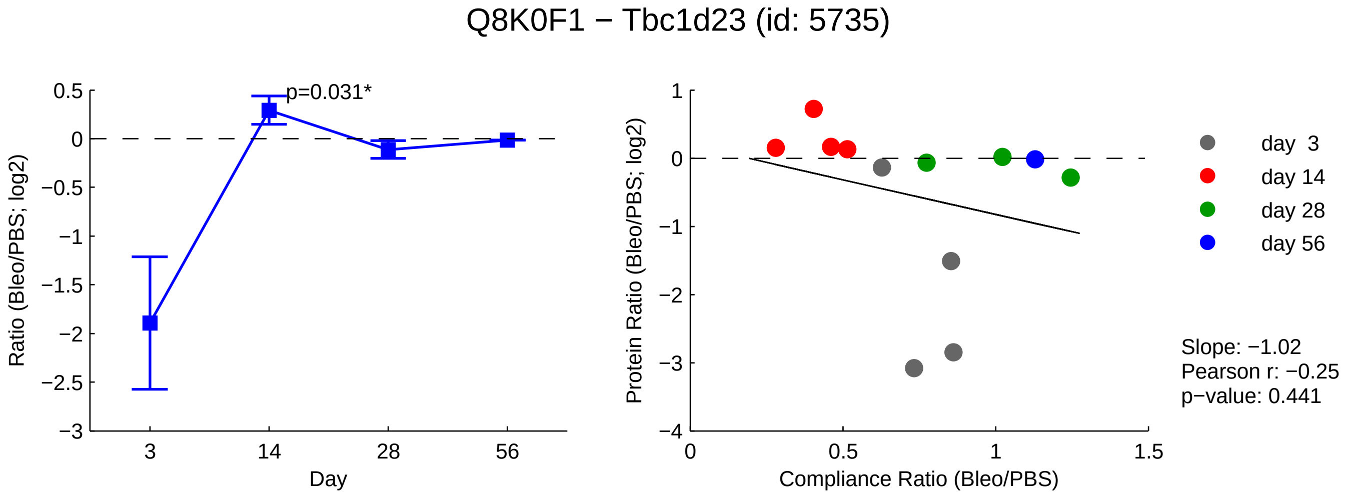
Q8K0F1 − Tbc1d23 (id: 5735)
p=0.031*
Ratio (Bleo/PBS; log2)
Day
Protein Ratio (Bleo/PBS; log2)
Compliance Ratio (Bleo/PBS)
day 3
day 14
day 28
day 56
Slope: −1.02
Pearson r: −0.25
p−value: 0.441

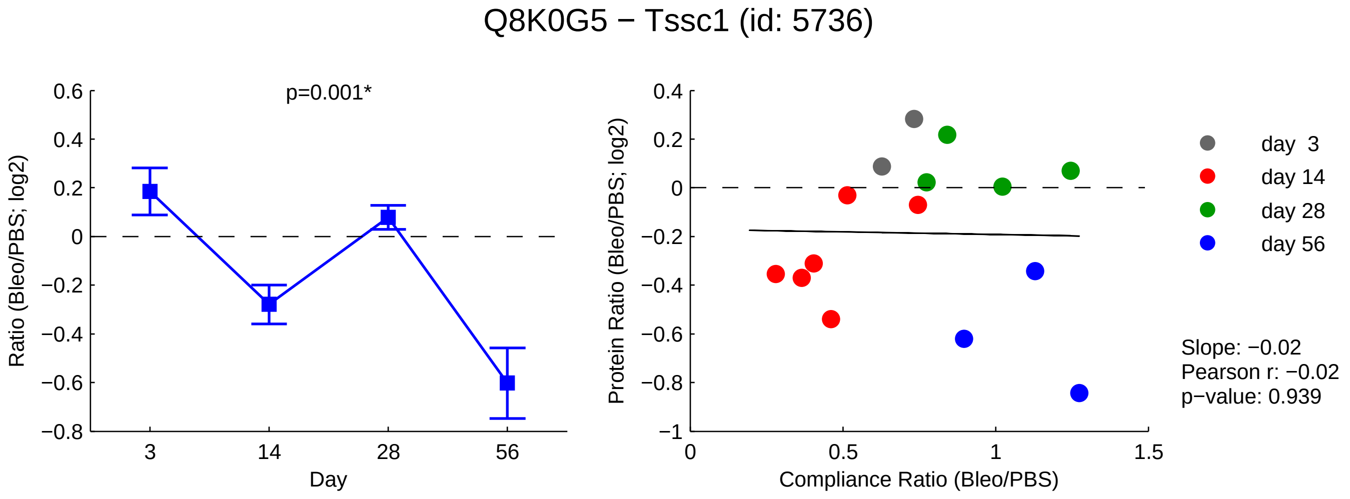
Q8K0G5 − Tssc1 (id: 5736)
p=0.001*
Ratio (Bleo/PBS; log2)
Day
Protein Ratio (Bleo/PBS; log2)
Compliance Ratio (Bleo/PBS)
day 3
day 14
day 28
day 56
Slope: −0.02
Pearson r: −0.02
p−value: 0.939

Q8K0H5 − Taf10 (id: 5737)

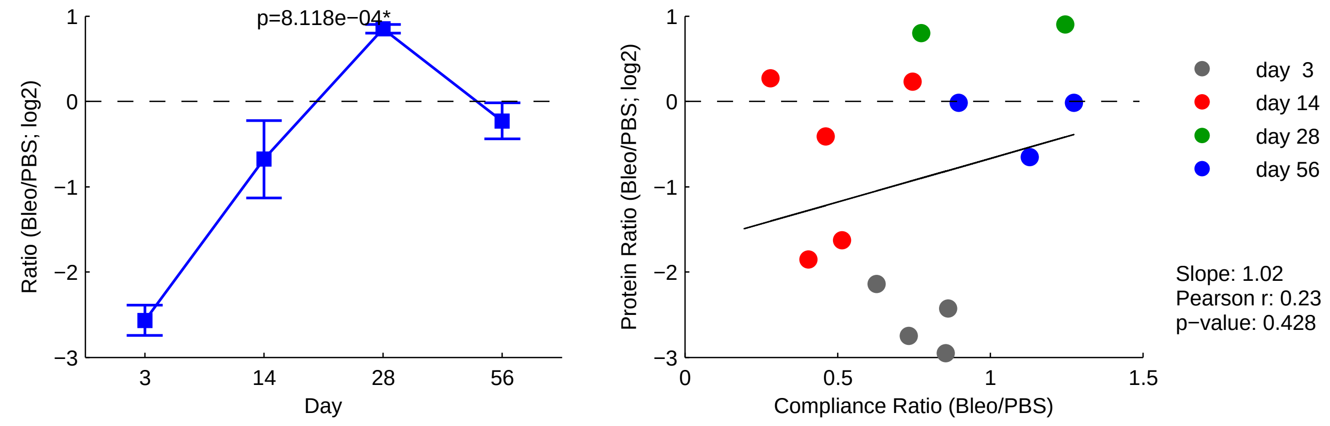

Q8K0L9−2 − Zbtb20 (id: 5738)

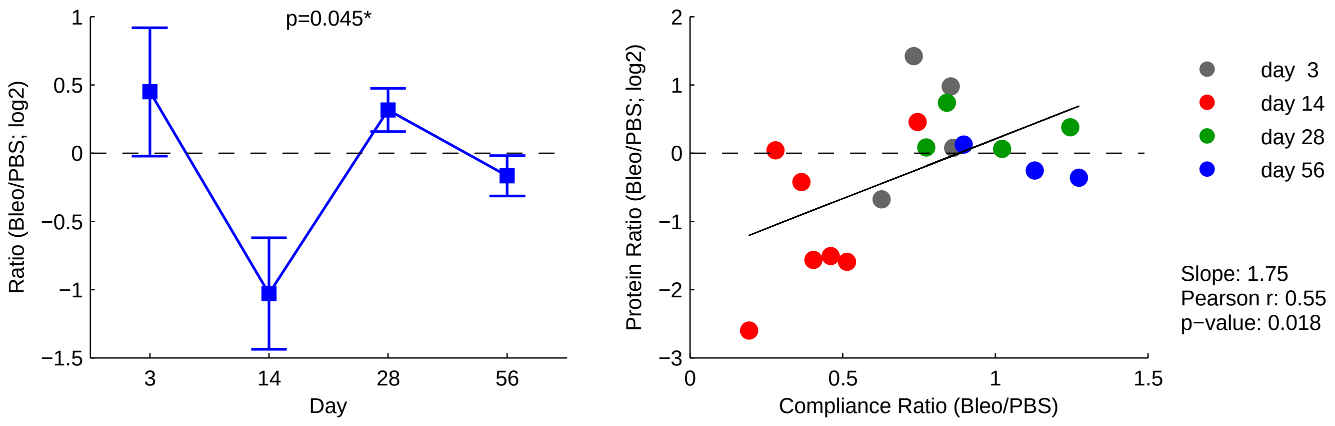

Q8K0P3 − Kiaa1609 (id: 5739)

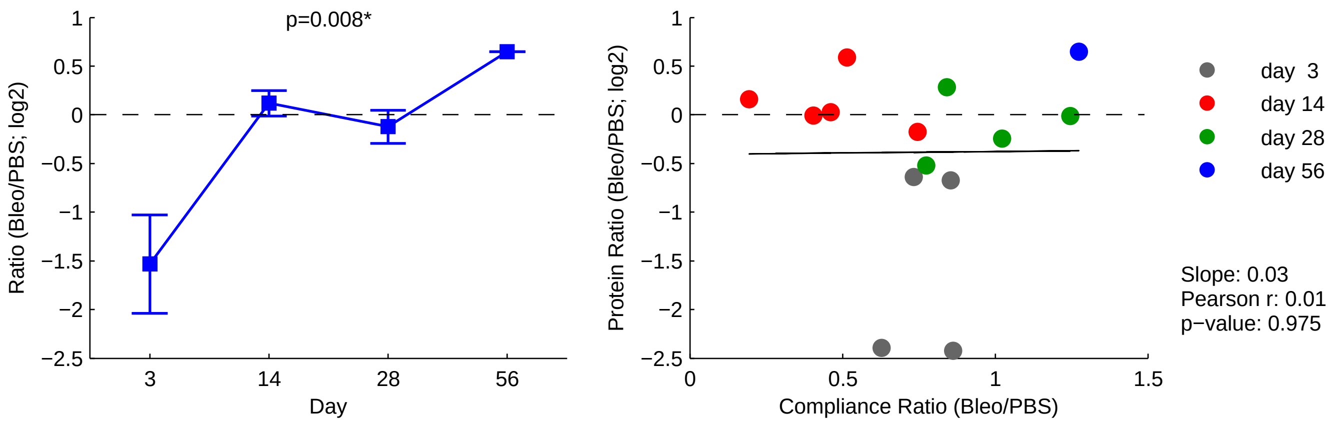

Q8K0Q5 − Arhgap18 (id: 5740)

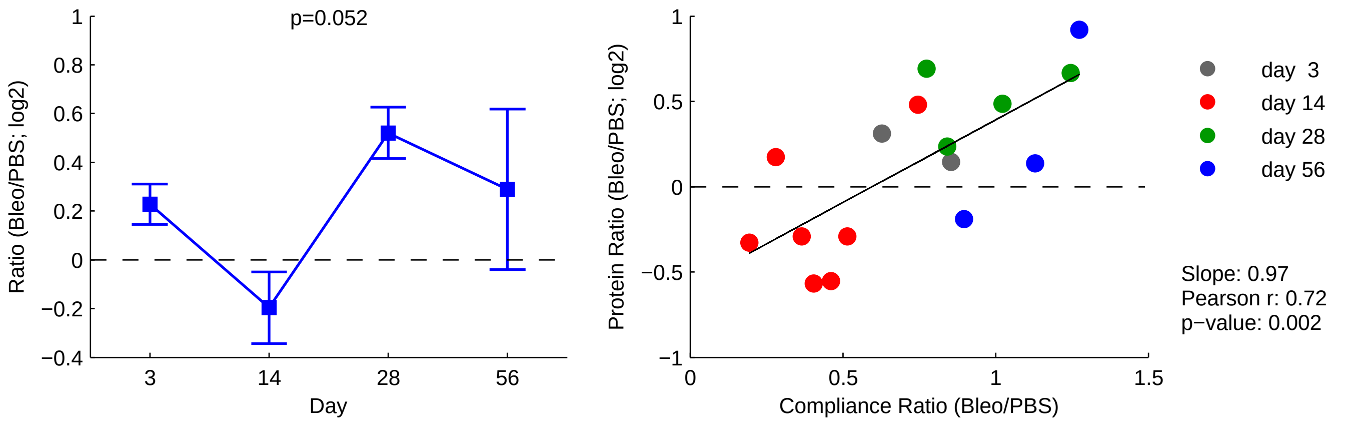

Q8K0T0 − Rtn1 (id: 5741)

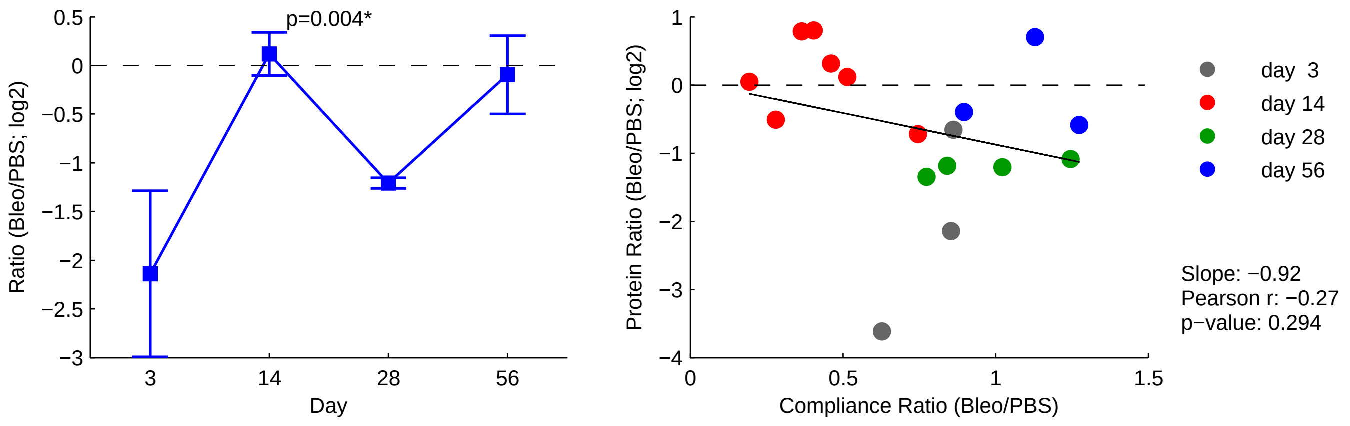

Q8K0U4 − Hspa12a (id: 5743)

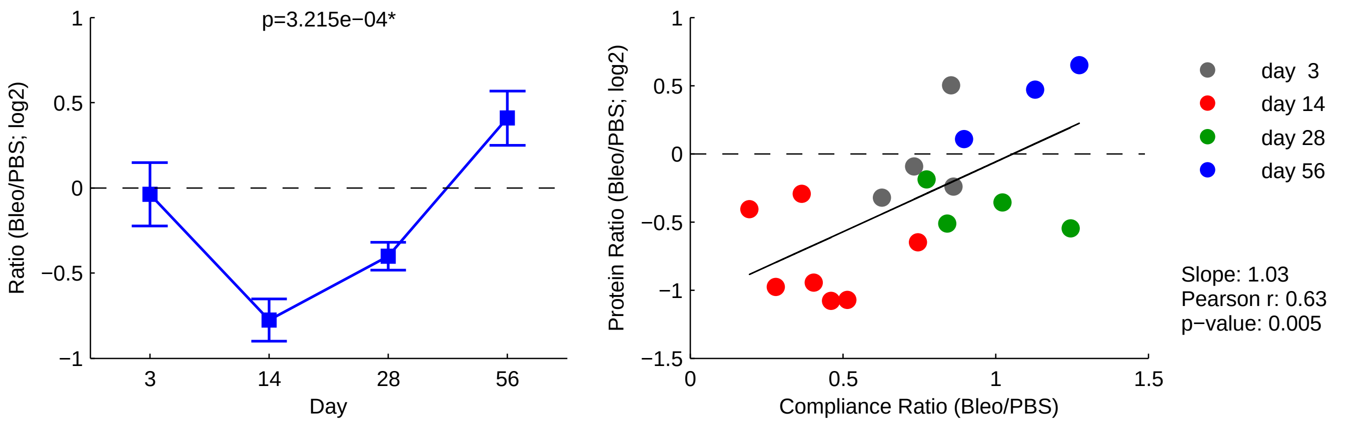

Q8K0Z7 − Taco1 (id: 5746)

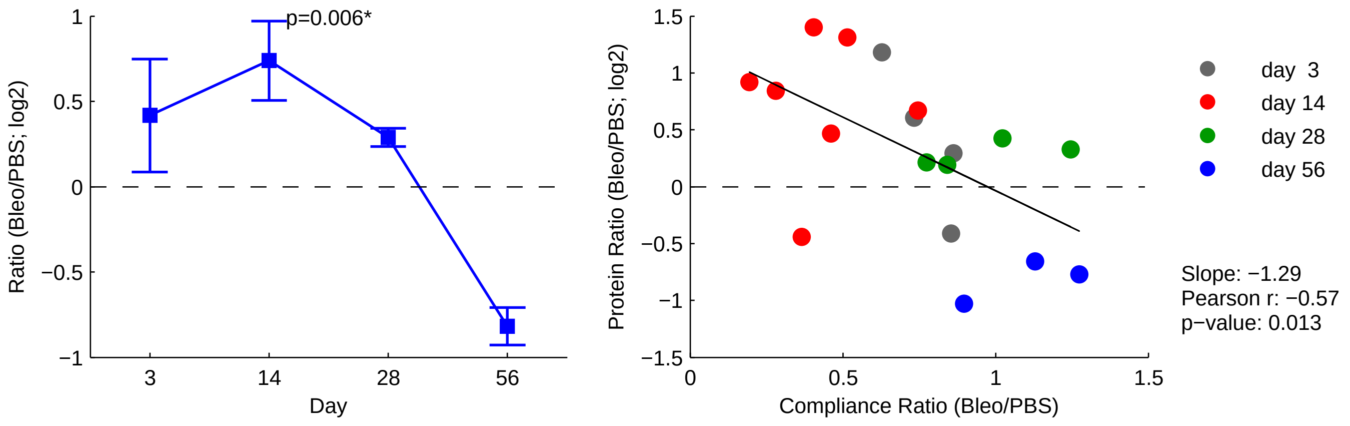

Q8K124 − Plekho2 (id: 5748)

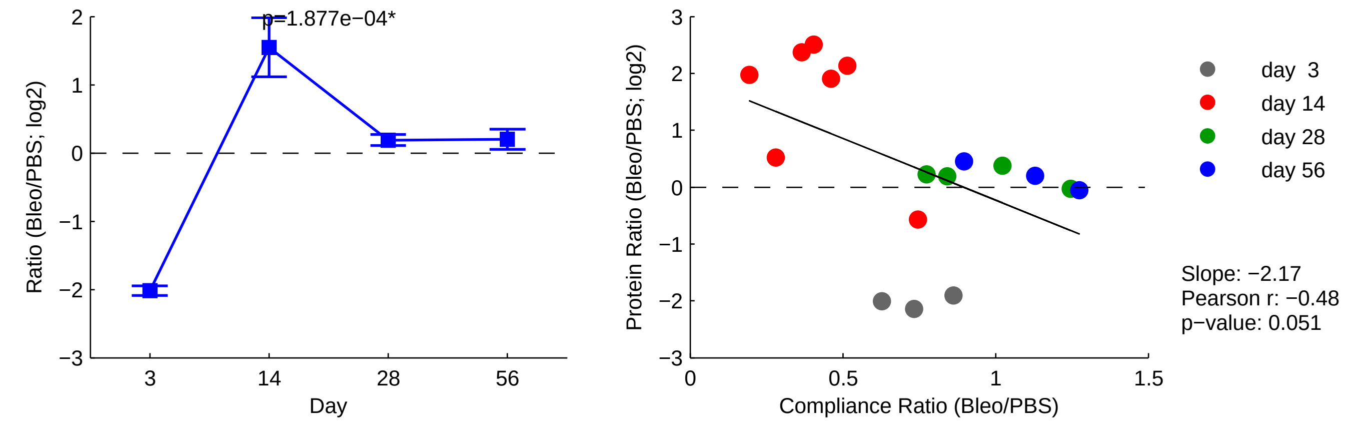

Q8K135 − Kiaa0319l (id: 5749)

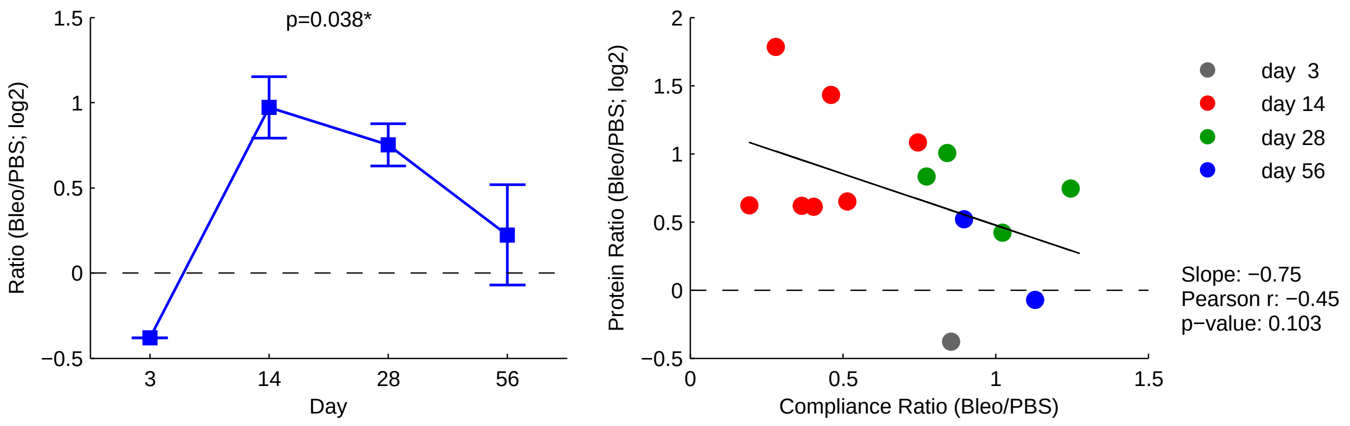

Q8K183 − Pdxk (id: 5750)

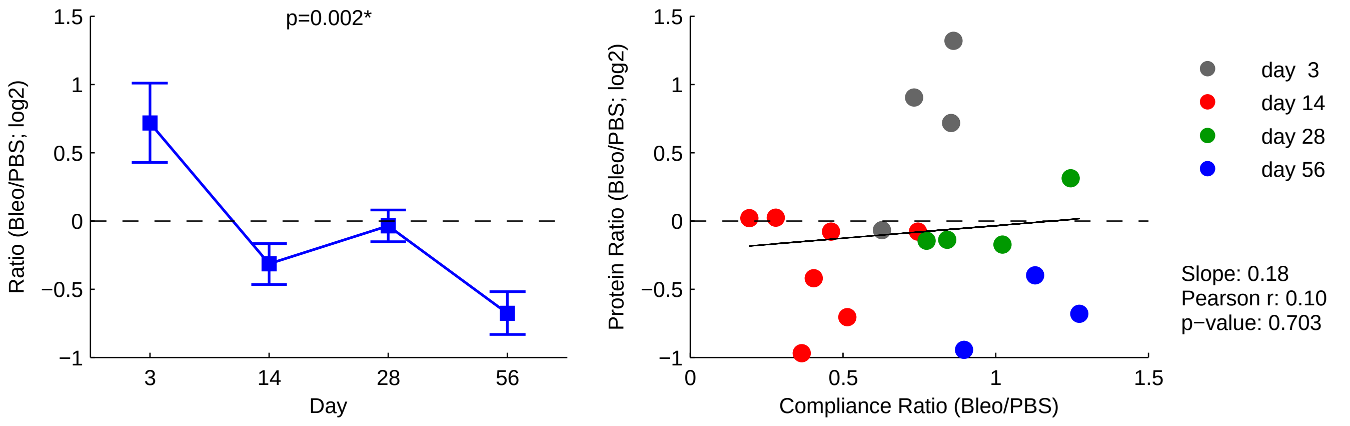

Q8K1A6 − Cc2d1a (id: 5753)

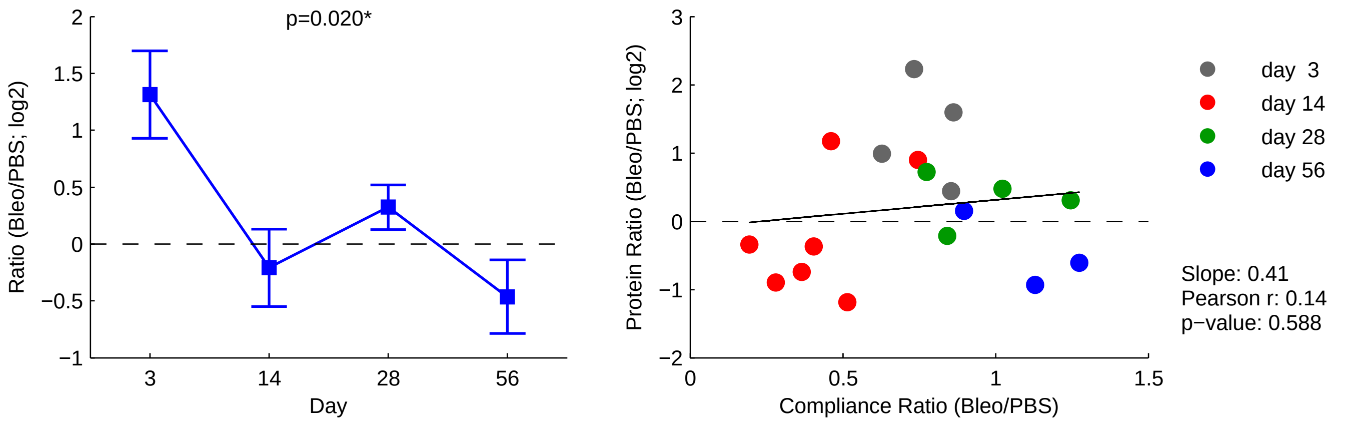

Q8K1J6 − Trnt1 (id: 5759)

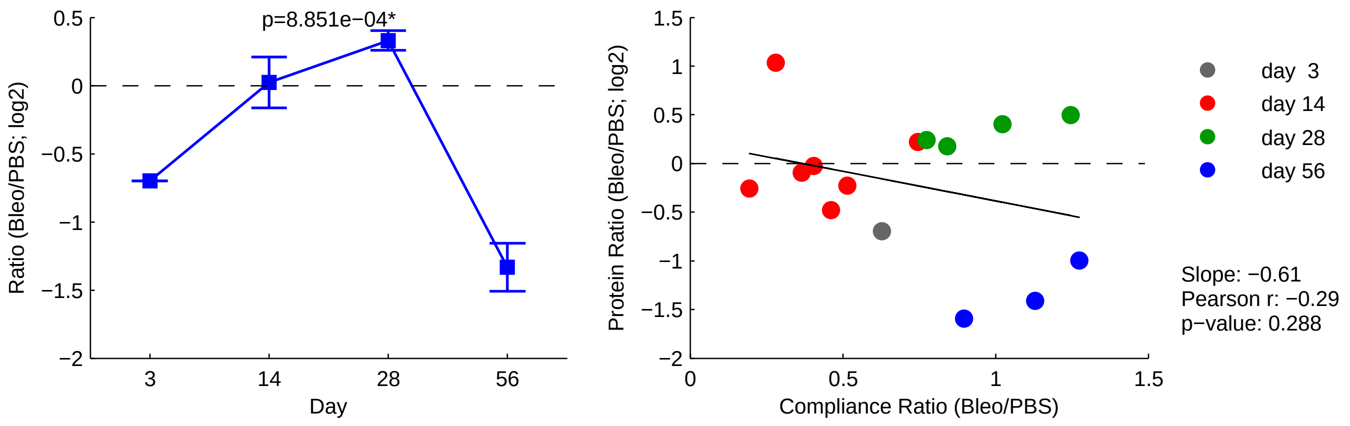

Q8K1N2 − Phldb2 (id: 5764)

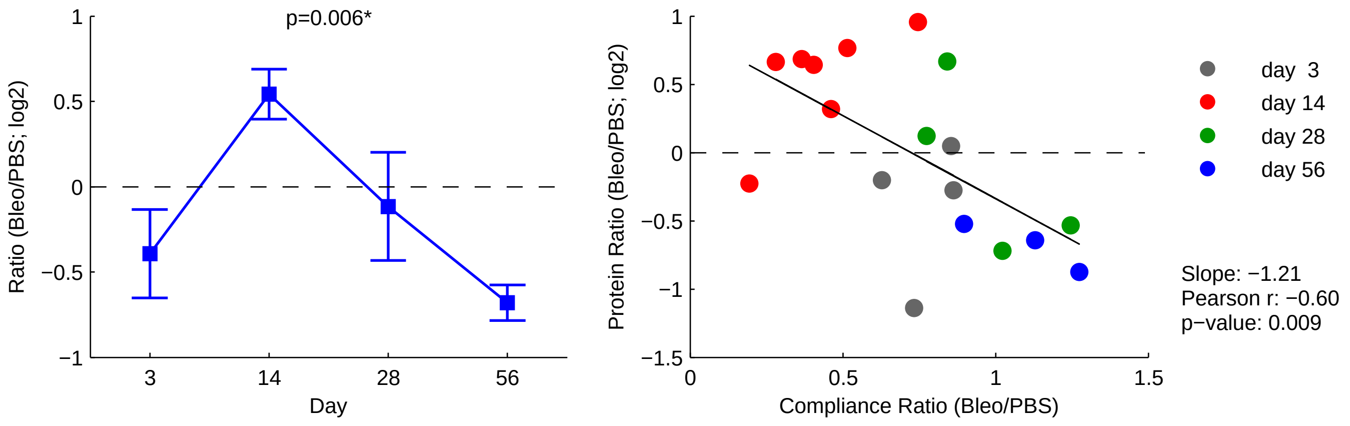

Q8K1R3 − Pnpt1 (id: 5766)

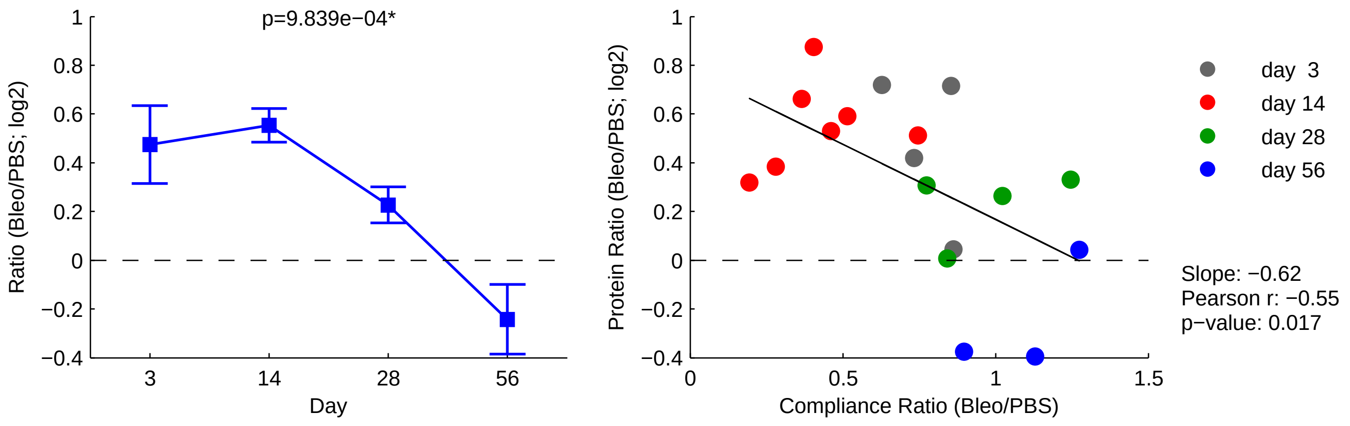

Q8K1R7 − Nek9 (id: 5767)

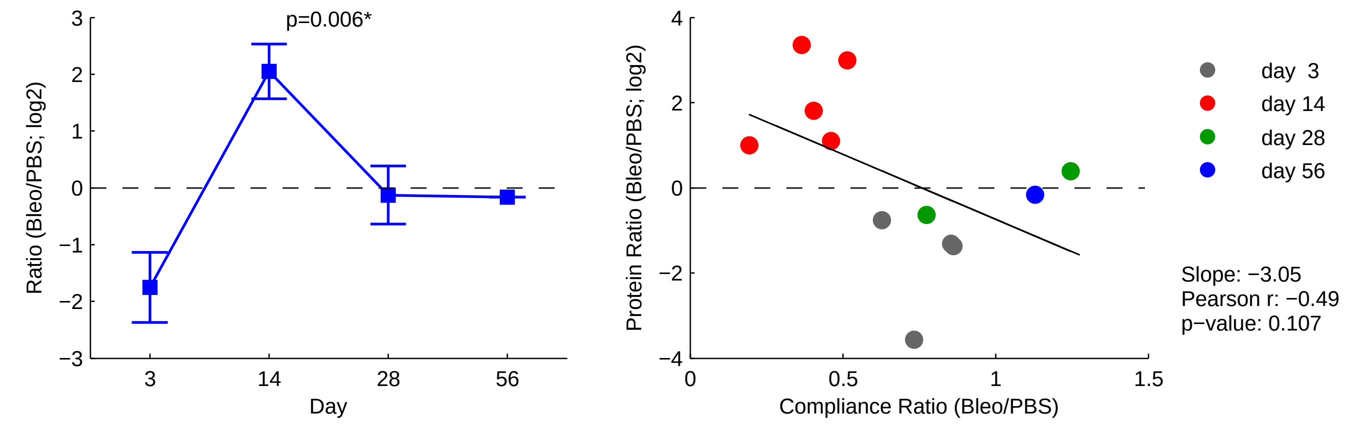

Q8K1T1 − Lrrc25 (id: 5769)

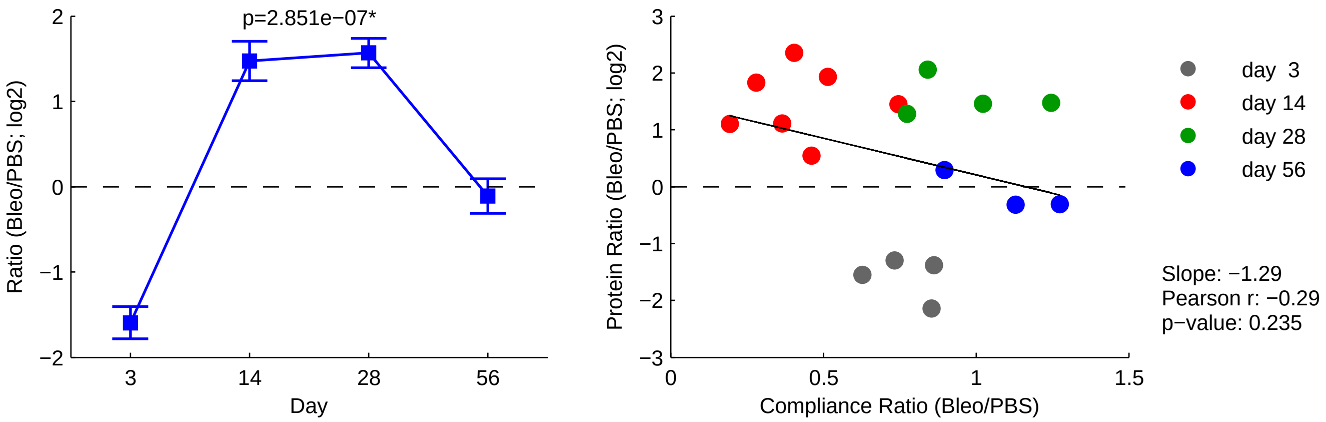

Q8K1Z0 − Coq9 (id: 5771)

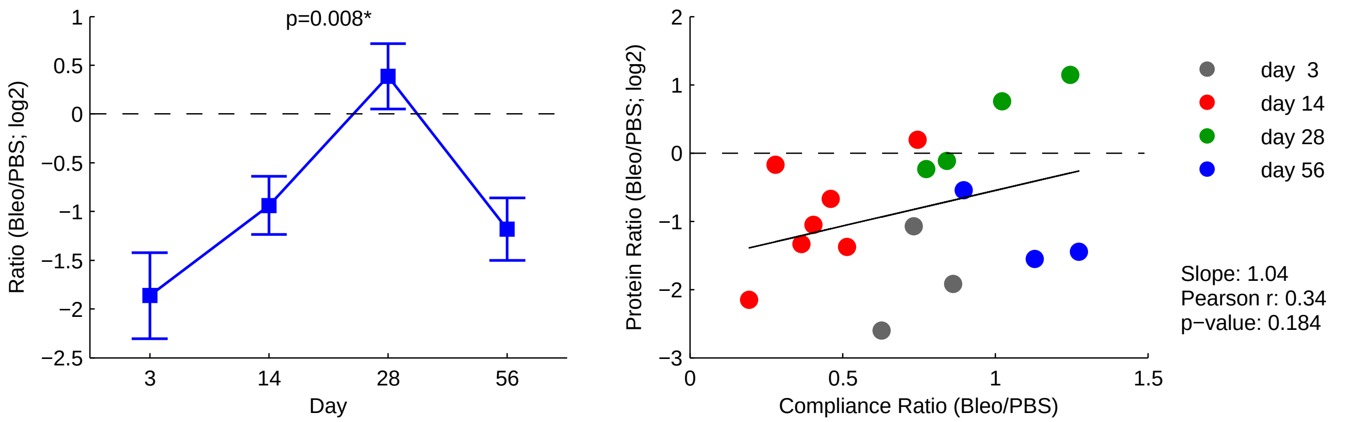

Q8K212 − Pacs1 (id: 5775)

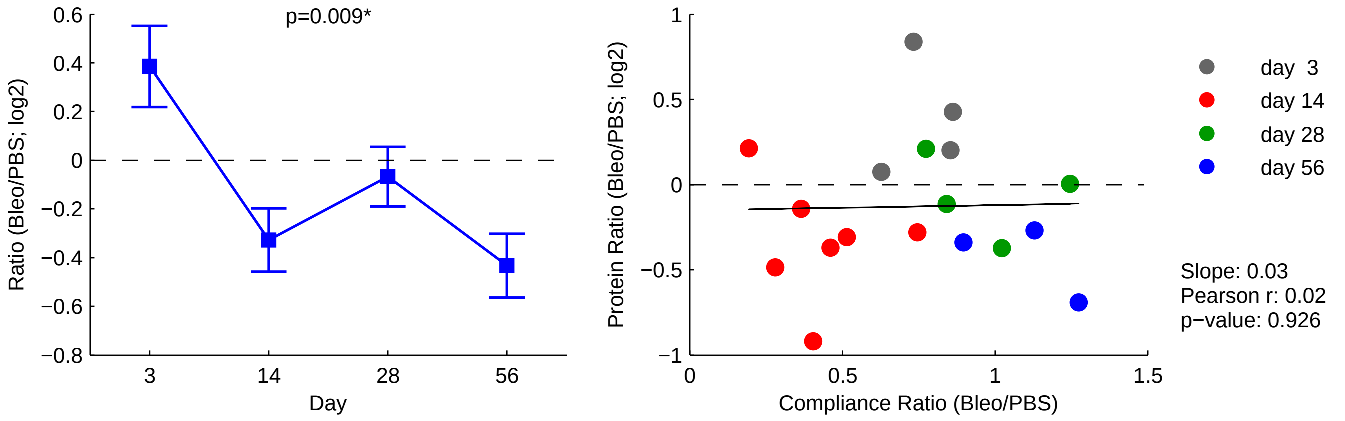

Q8K215 − Lyrm4 (id: 5776)

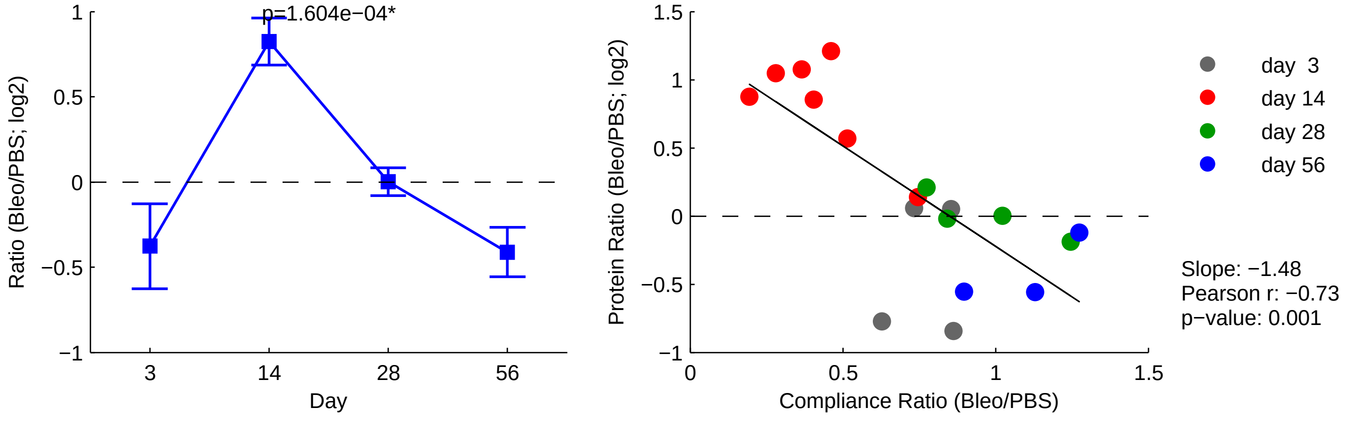

Q8K221 − Arfip2 (id: 5777)

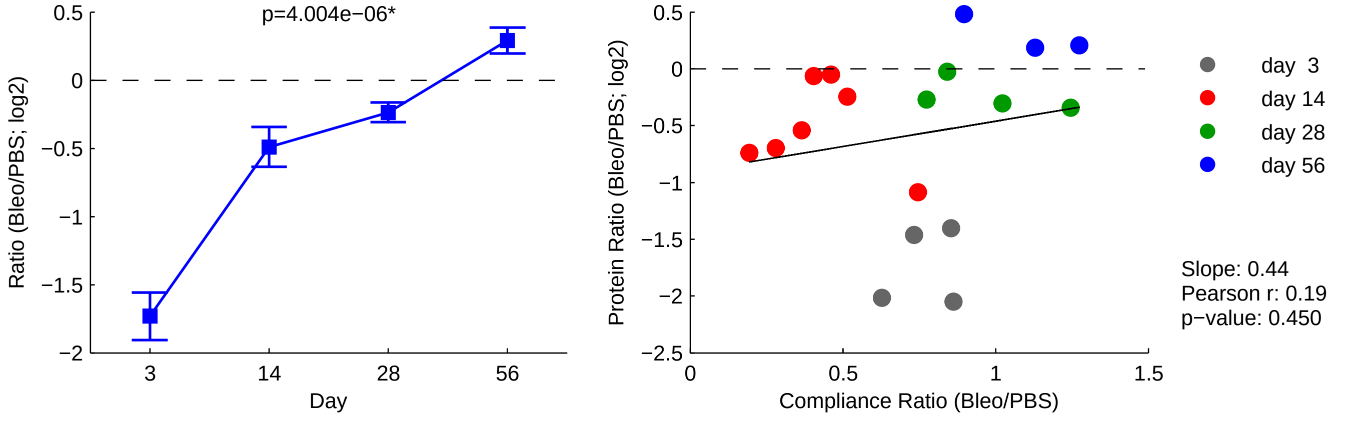

Q8K224 − Nat10 (id: 5778)

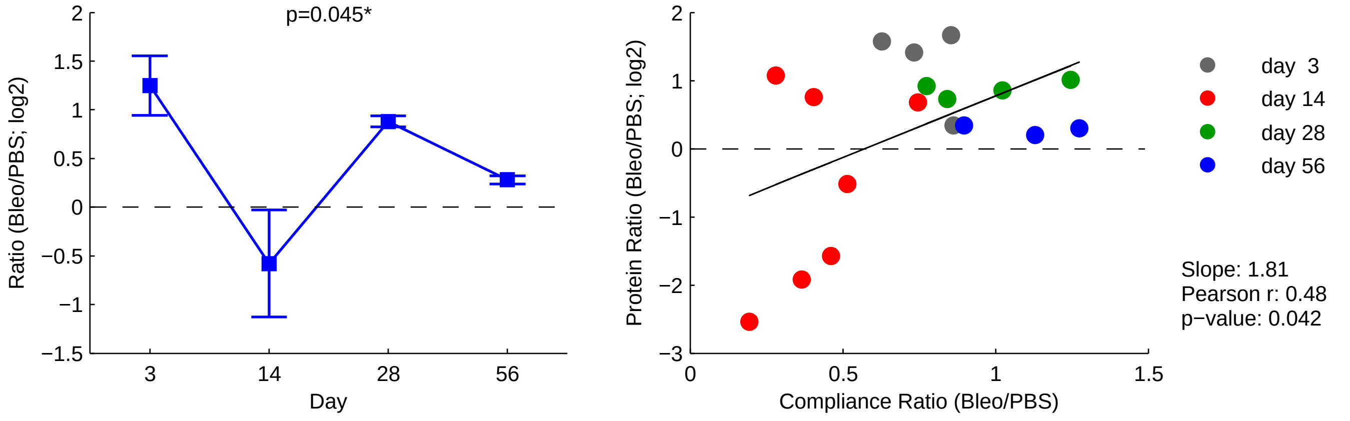

Q8K262 − Plac9 (id: 5779)

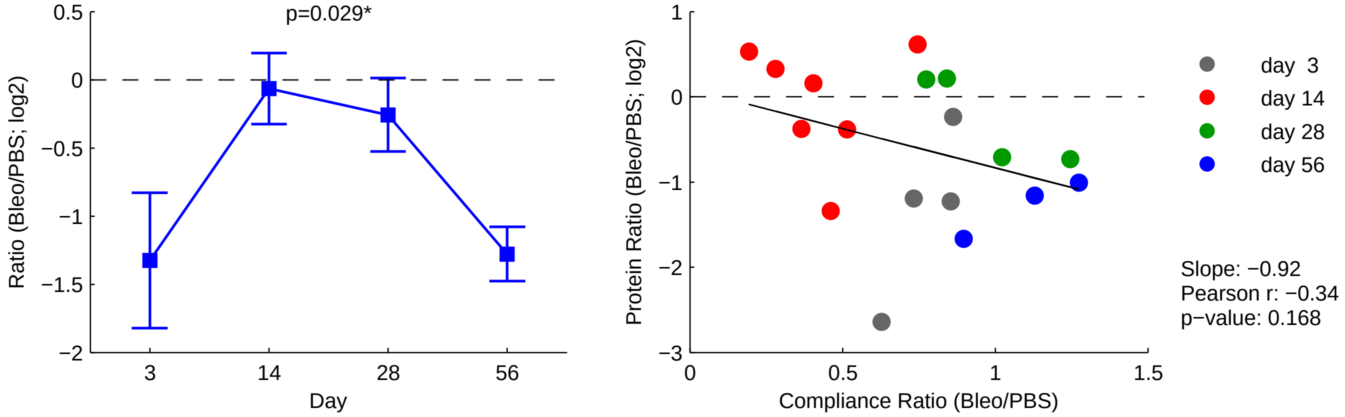

Q8K274 − Fn3krp (id: 5782)

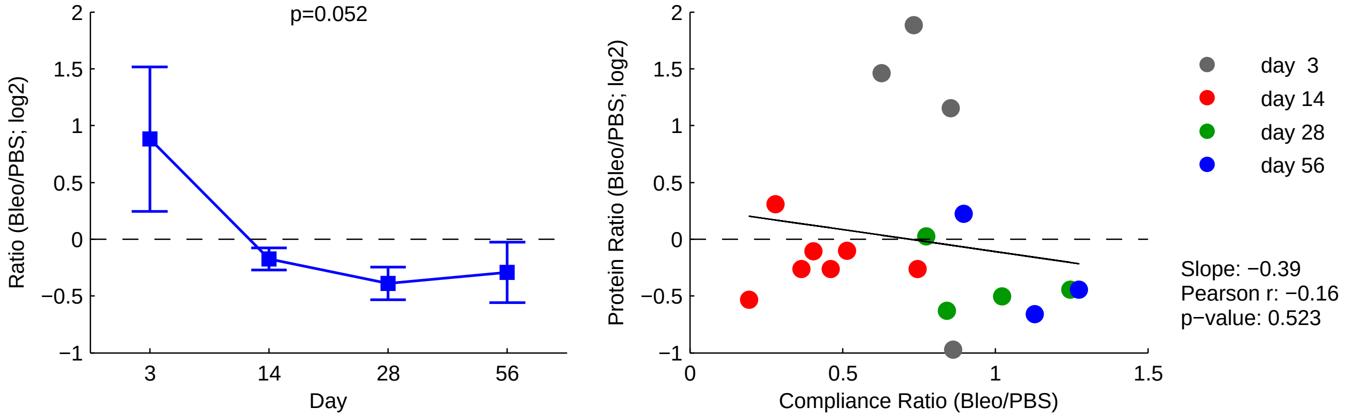

Q8K284 − Gtf3c1 (id: 5783)

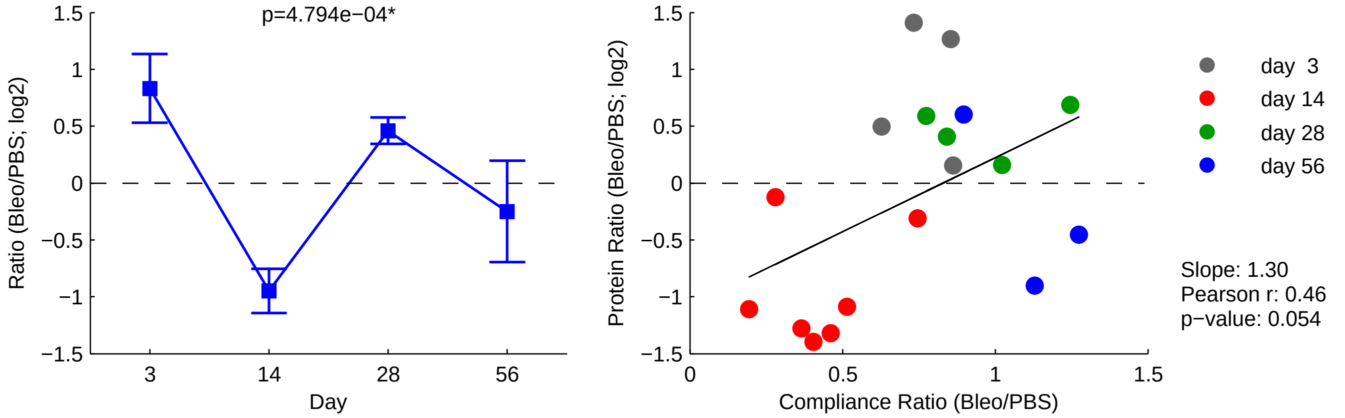

Q8K297 − Colgalt1 (id: 5784)

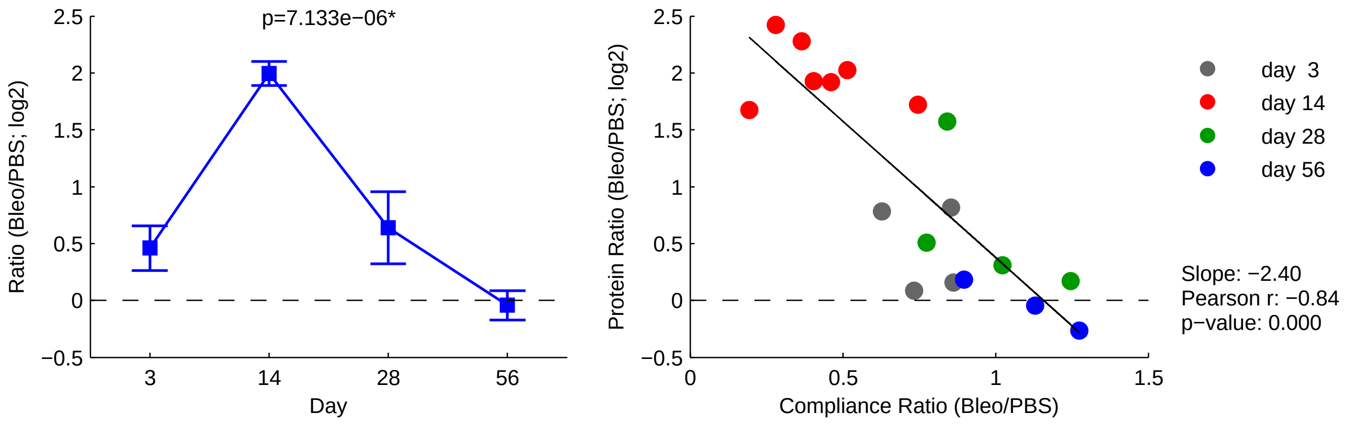

Q8K2A7 − Ints10 (id: 5786)

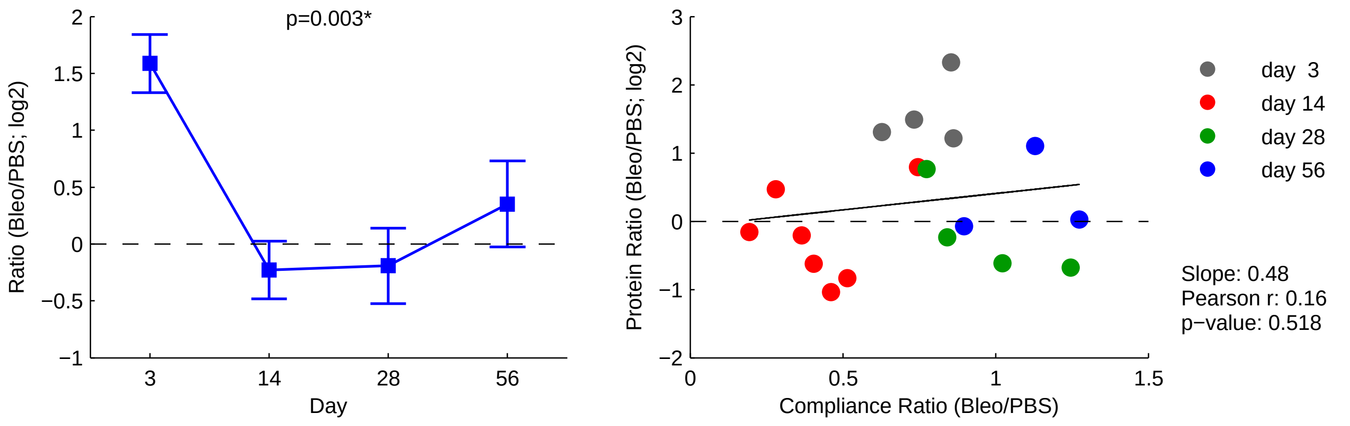

Q8K2B3 − Sdha (id: 5788)

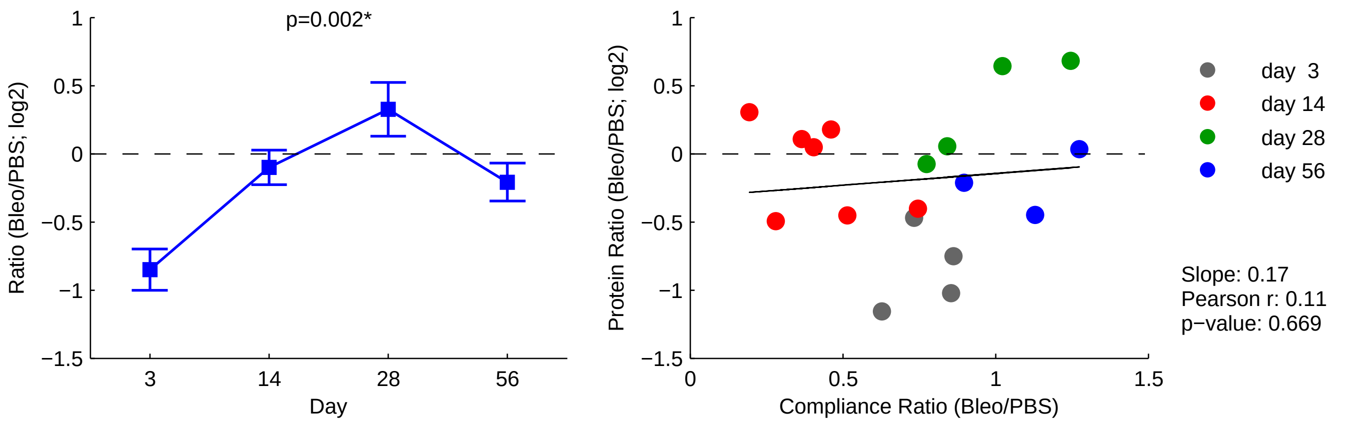

Q8K2C6 − Sirt5 (id: 5789)

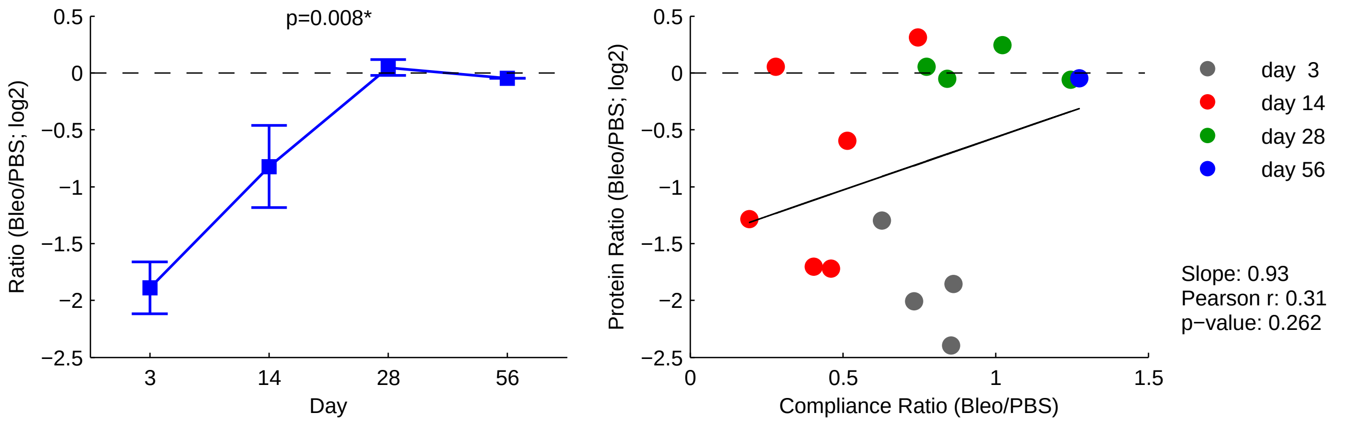

Q8K2C7−2 − Os9 (id: 5790)

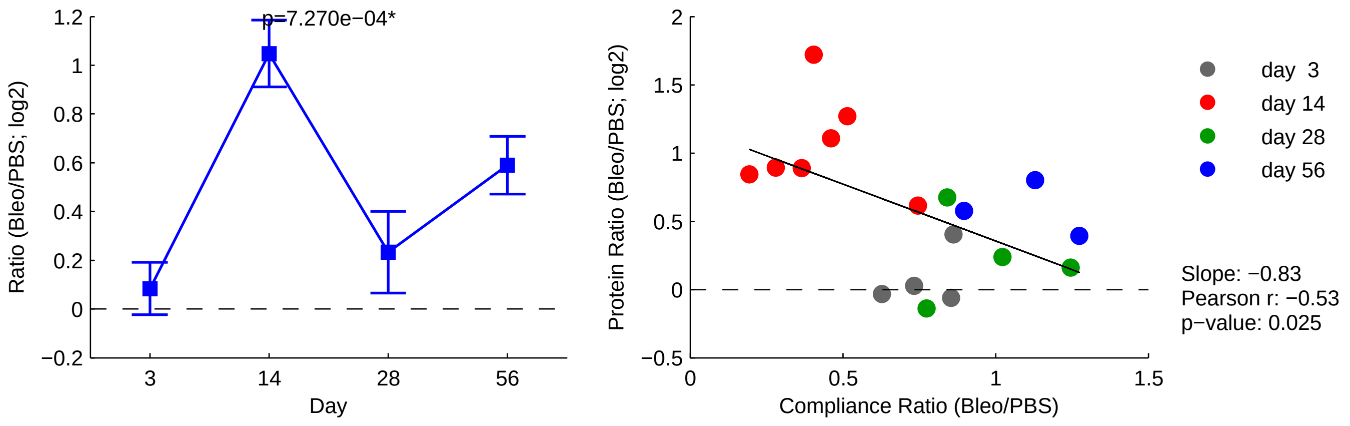

Q8K2C9 − ptplad1 (id: 5792)

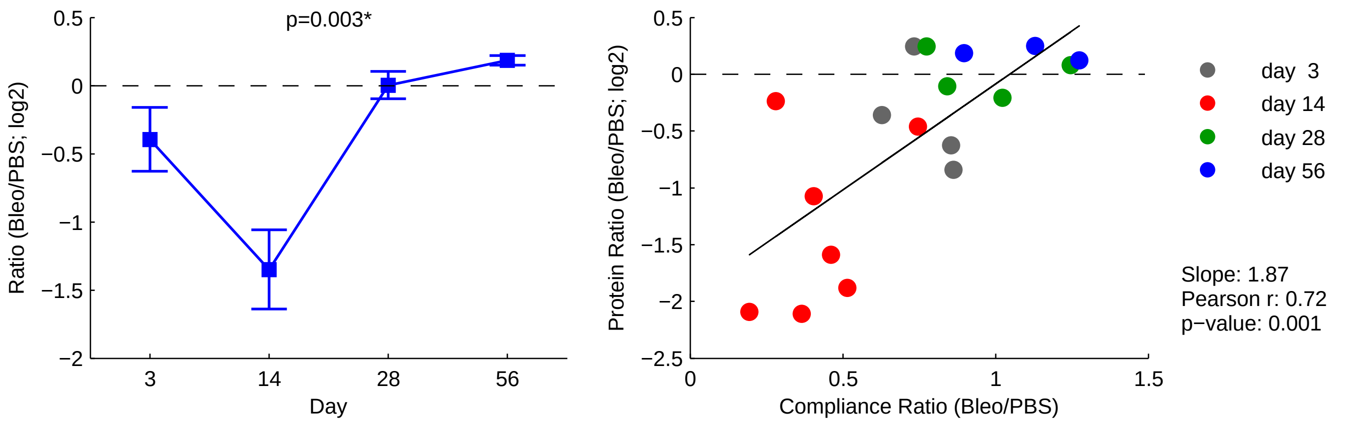

Q9JI19 − Fibp (id: 5794)

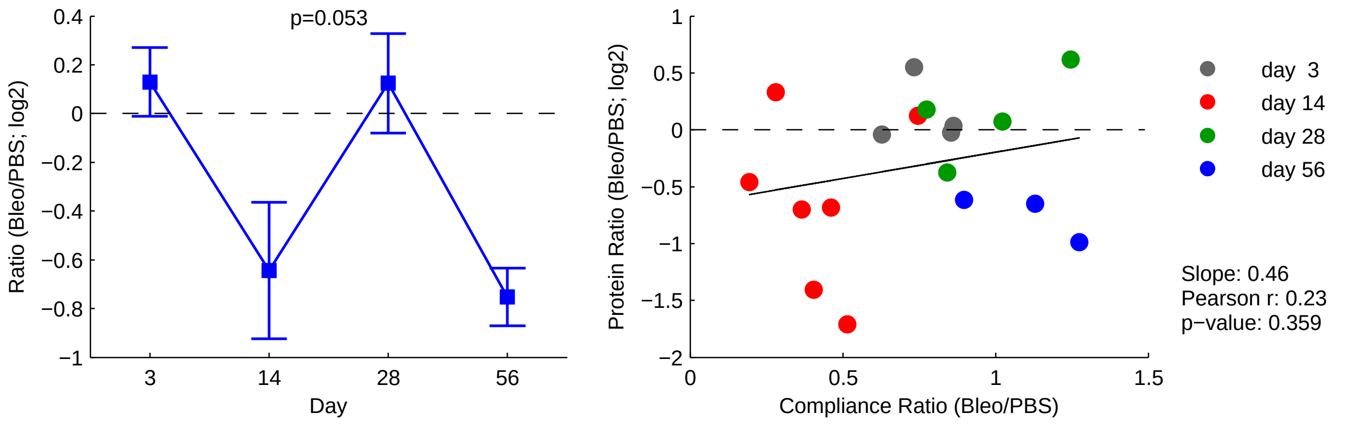

Q8K2F0 − Brd3 (id: 5795)

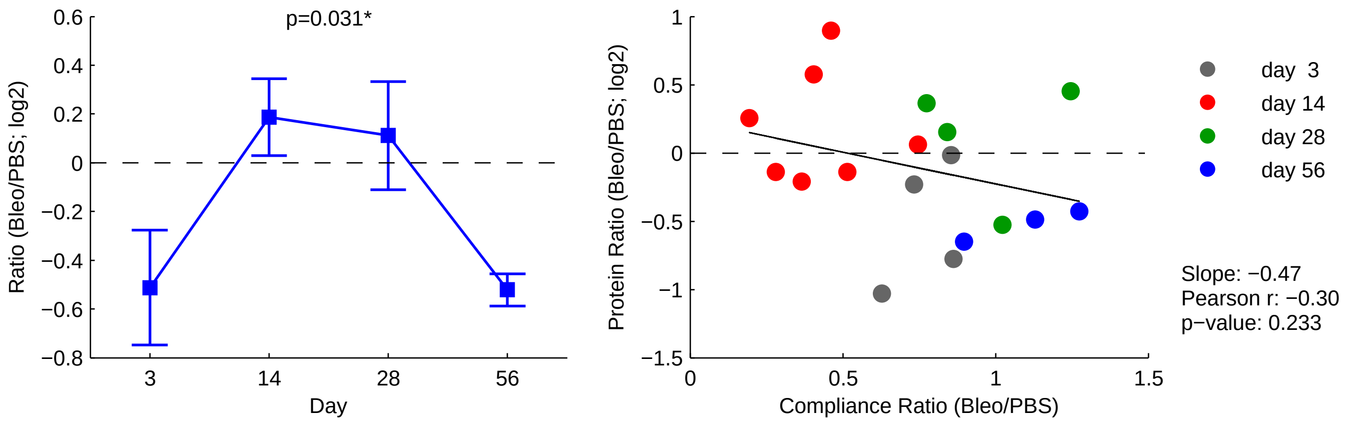

Q8K2F8 − Lsm14a (id: 5796)

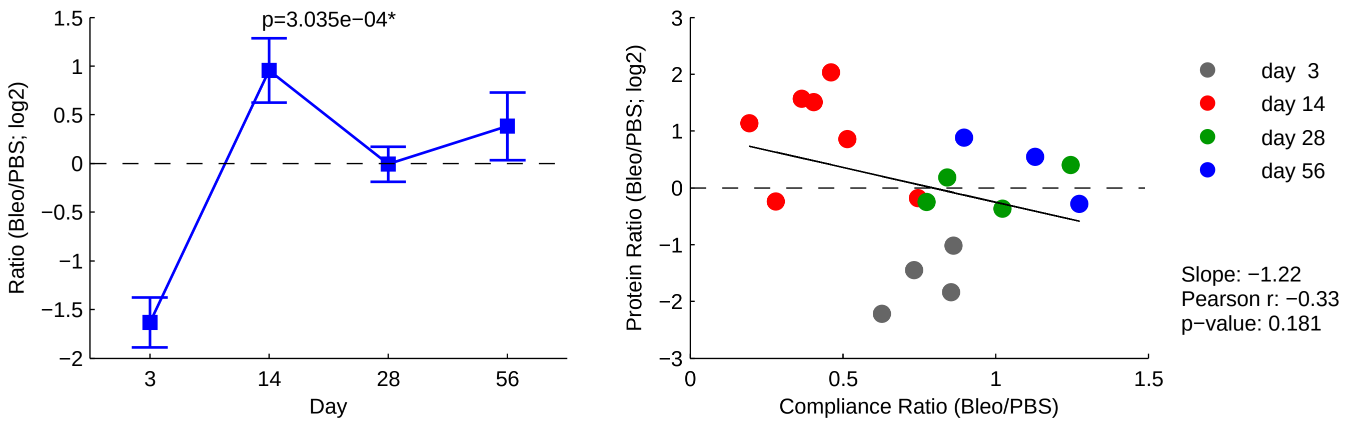

Q8K2H2 − Otud6b (id: 5798)

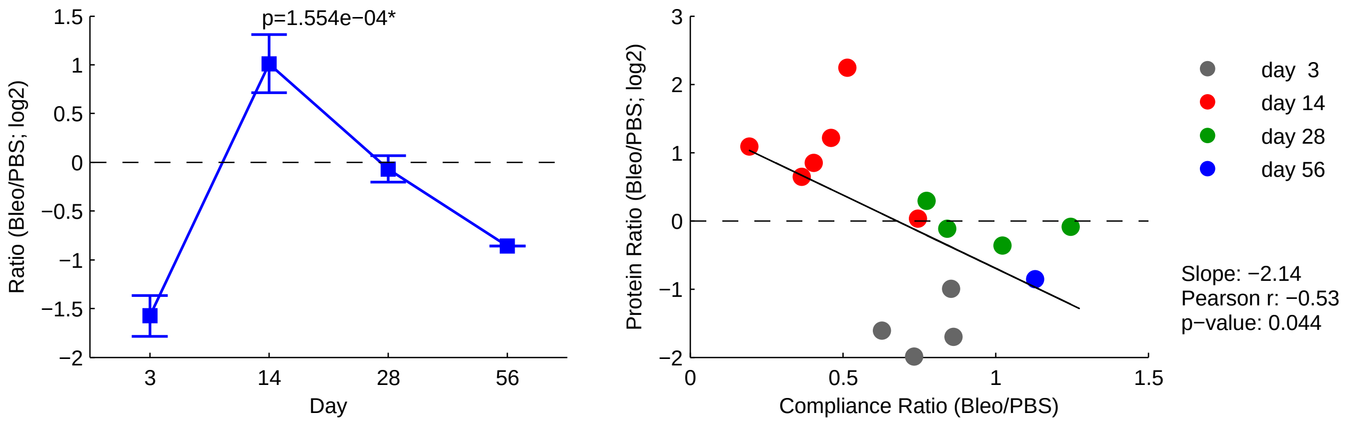

Q8K2I3 − Fmo2 (id: 5800)

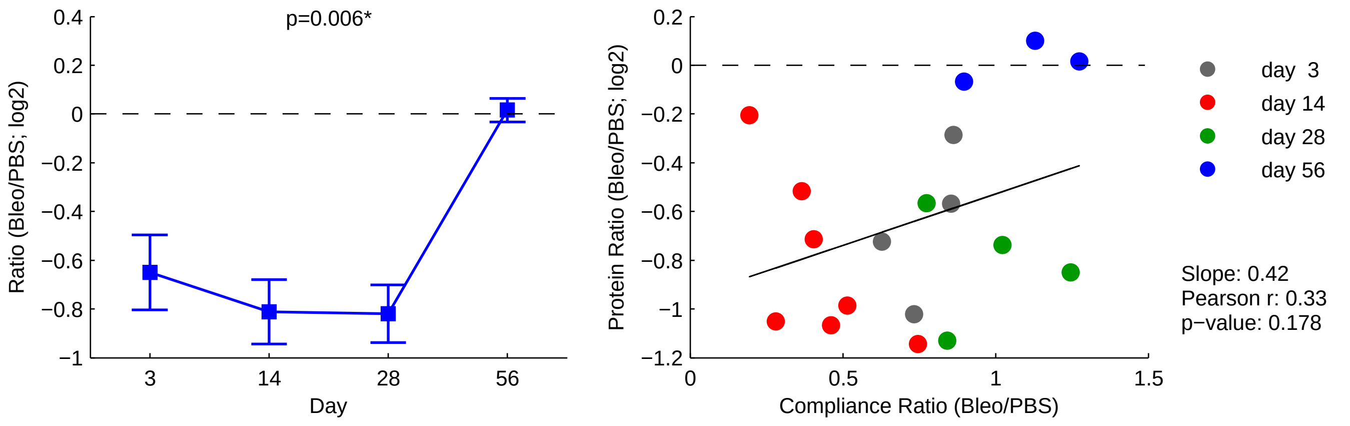

Q8K2K6−3 − Agfg1 (id: 5804)

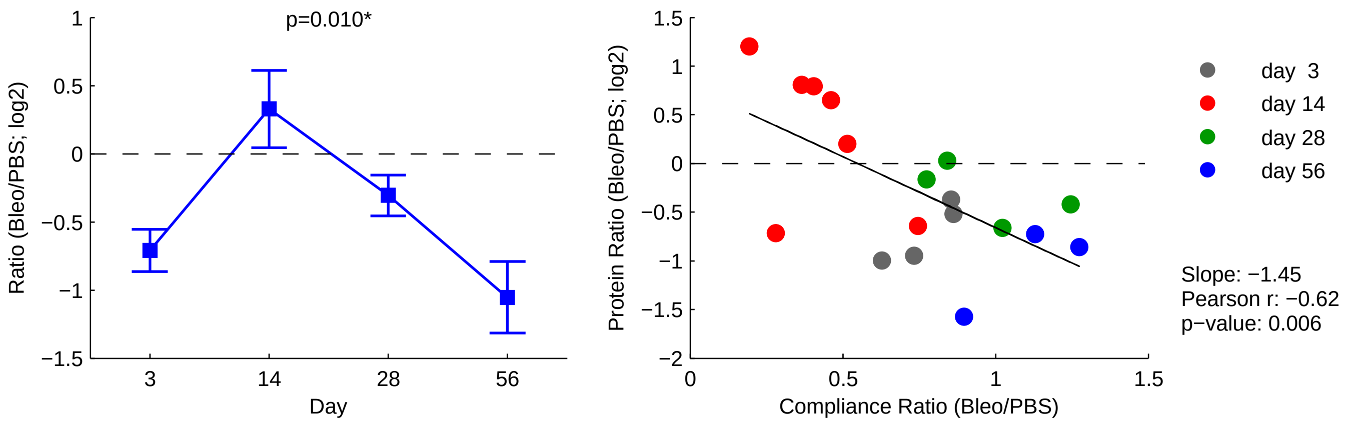

Q8K2L8 − Trappc12 (id: 5805)

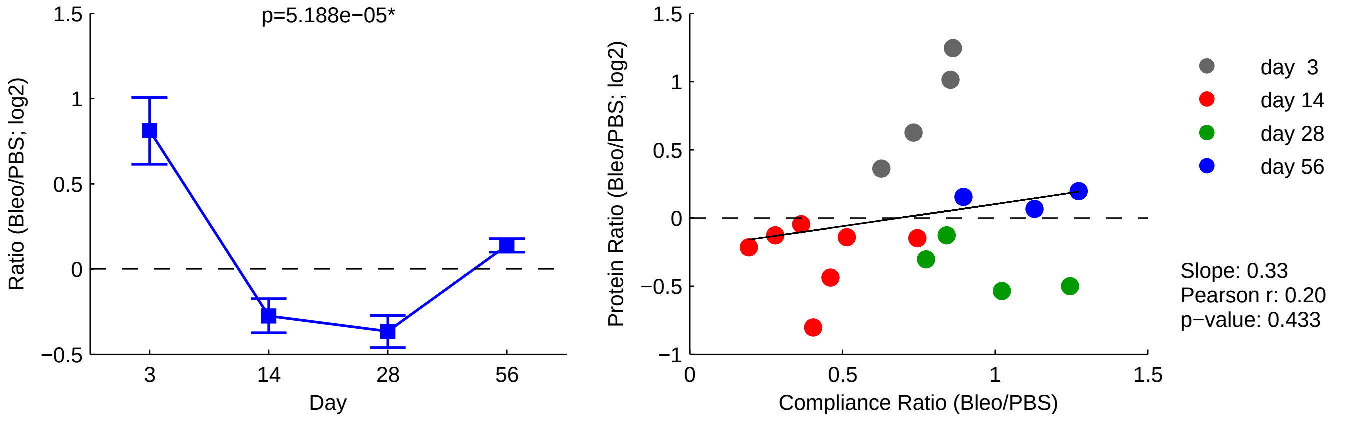

Q8K2Q0 − Commd9 (id: 5807)

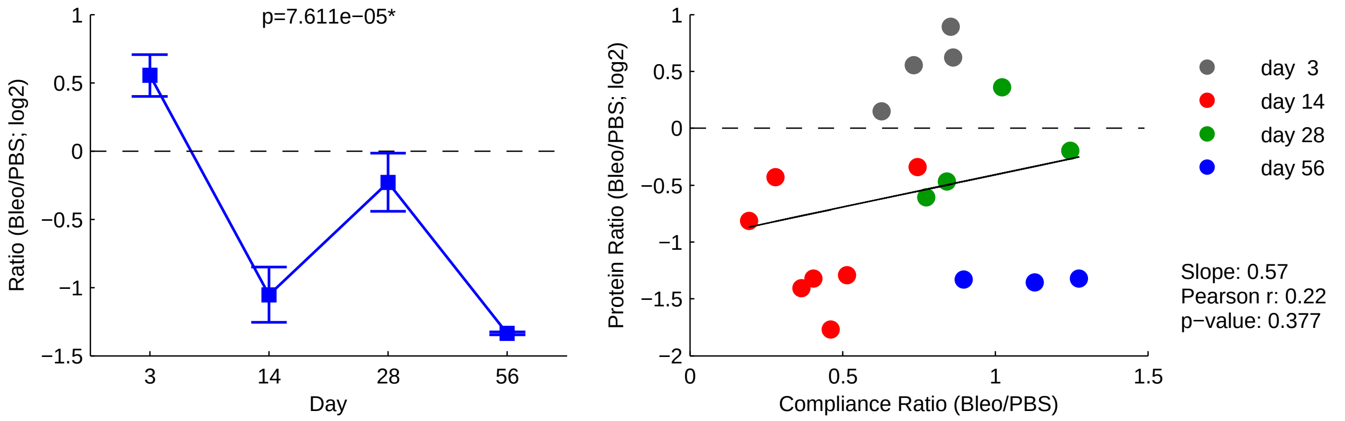

Q8K2Q7 − Brox (id: 5808)

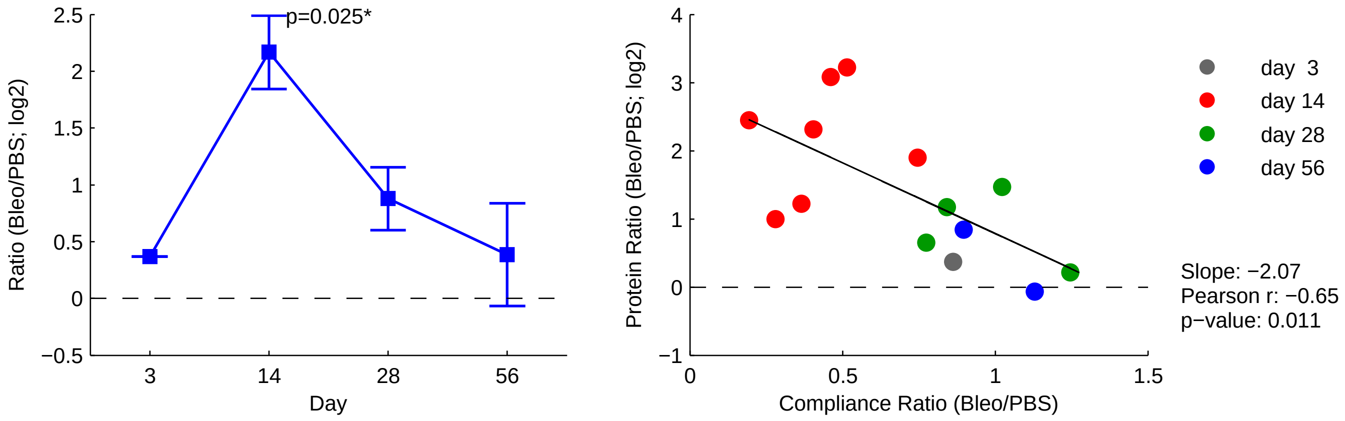

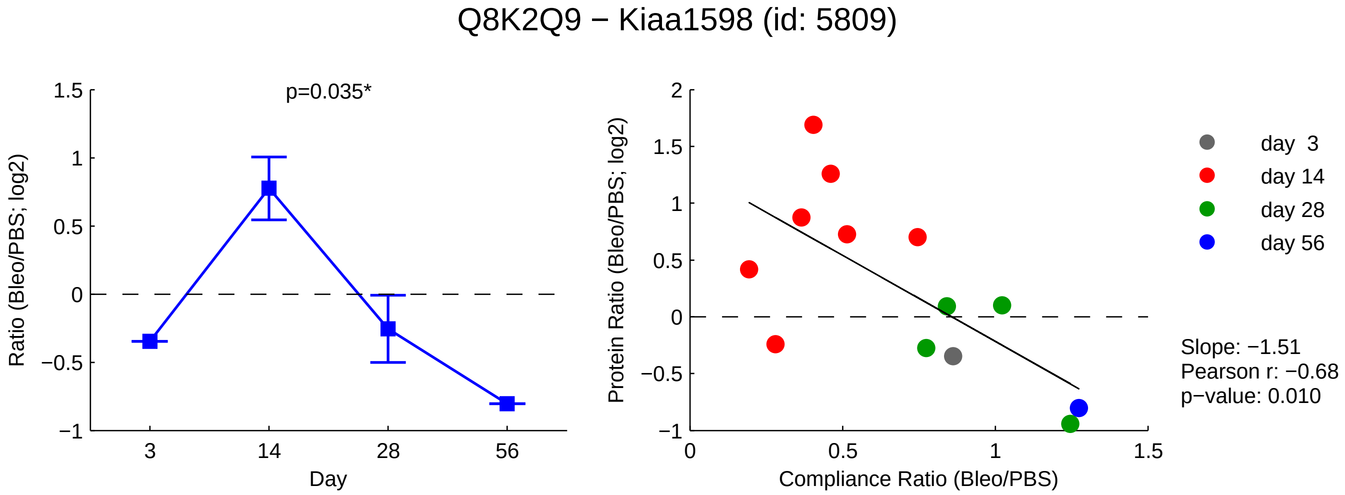
Q8K2Q9 − Kiaa1598 (id: 5809)
p=0.035*
Ratio (Bleo/PBS; log2)
Day
Protein Ratio (Bleo/PBS; log2)
Compliance Ratio (Bleo/PBS)
day 3
day 14
day 28
day 56
Slope: −1.51
Pearson r: −0.68
p−value: 0.010

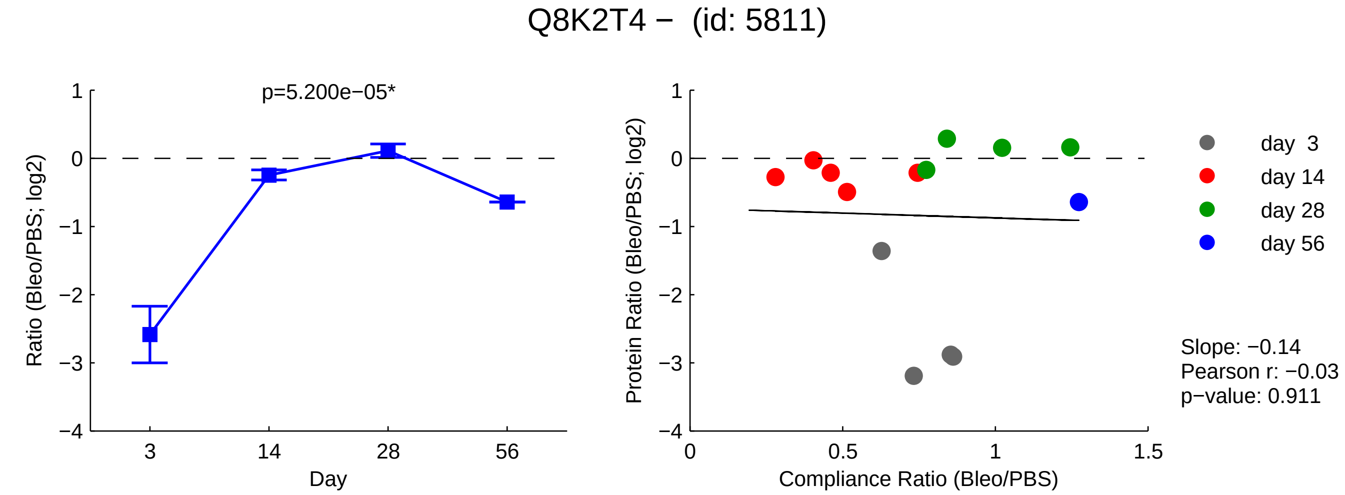
Q8K2T4 − (id: 5811)
p=5.200e−05*
Ratio (Bleo/PBS; log2)
Day
Protein Ratio (Bleo/PBS; log2)
Compliance Ratio (Bleo/PBS)
day 3
day 14
day 28
day 56
Slope: −0.14
Pearson r: −0.03
p−value: 0.911

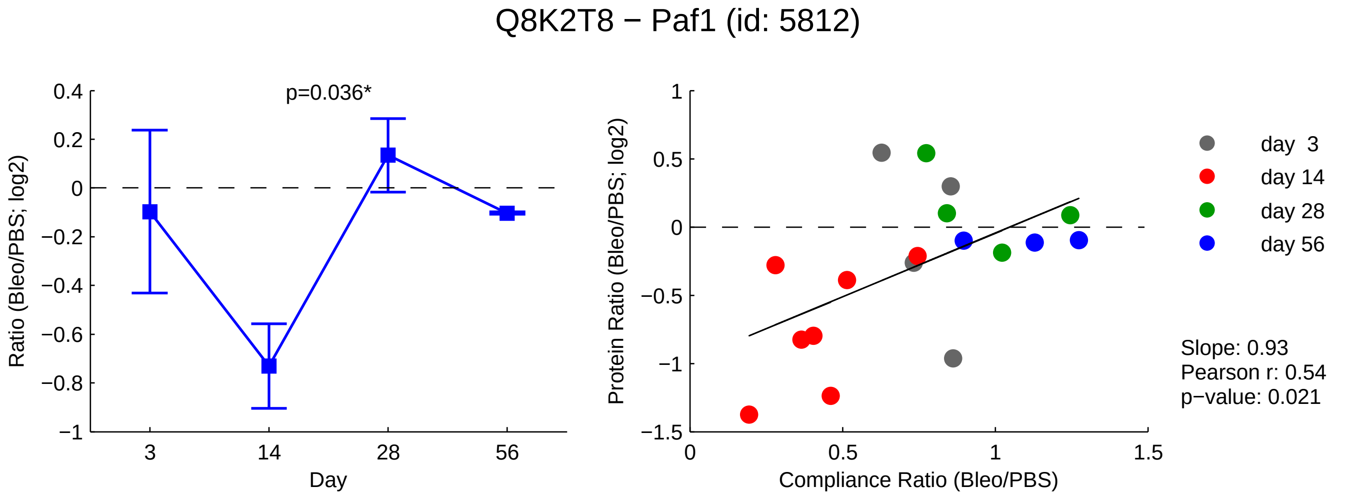
Q8K2T8 − Paf1 (id: 5812)
p=0.036*
Ratio (Bleo/PBS; log2)
Day
Protein Ratio (Bleo/PBS; log2)
Compliance Ratio (Bleo/PBS)
day 3
day 14
day 28
day 56
Slope: 0.93
Pearson r: 0.54
p−value: 0.021

Q8K2X3 − Obfc1 (id: 5814)

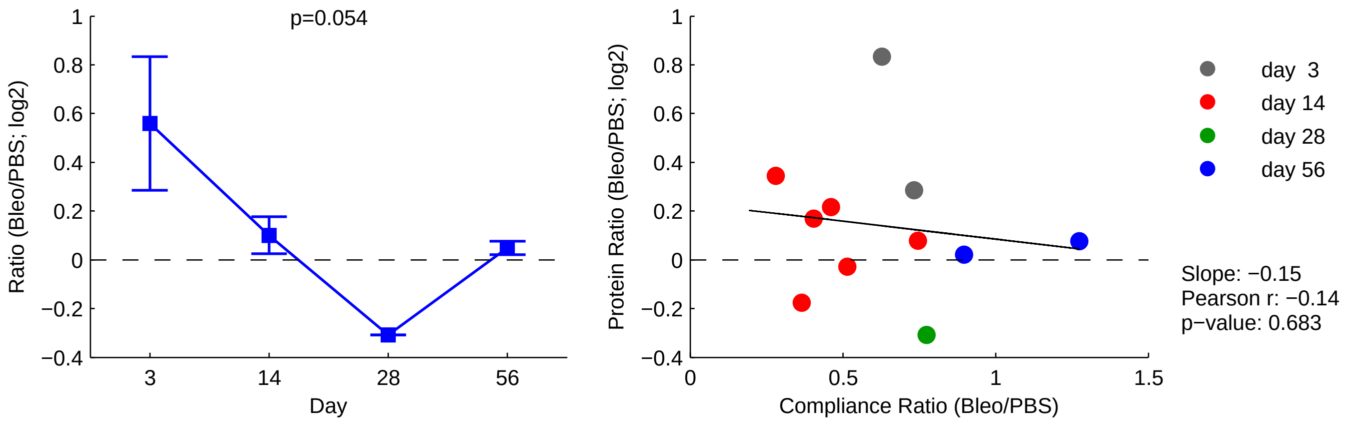

Q8K2Y3 − Eva1b (id: 5816)

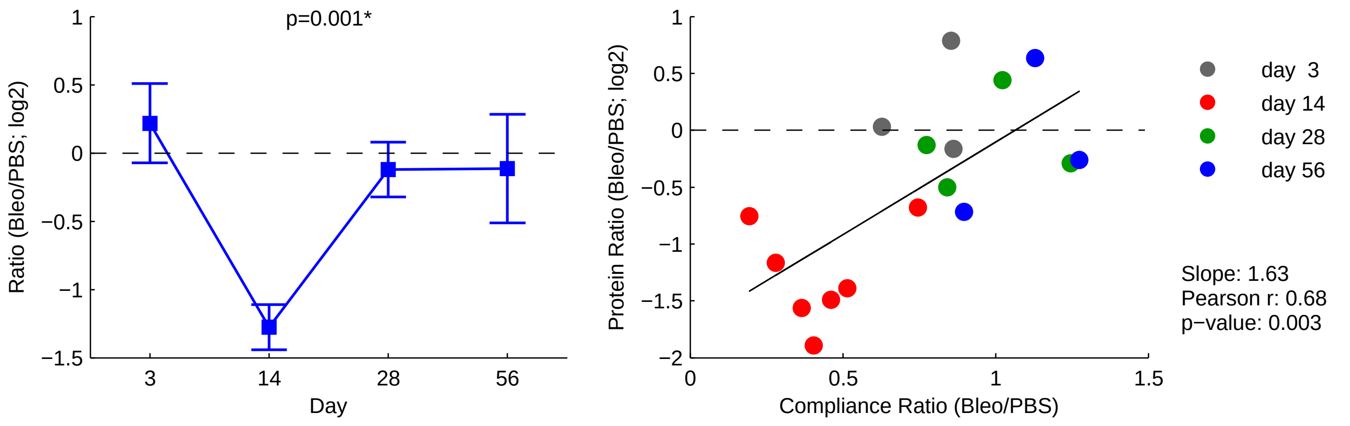

Q8K2Y7 − Mrpl47 (id: 5817)

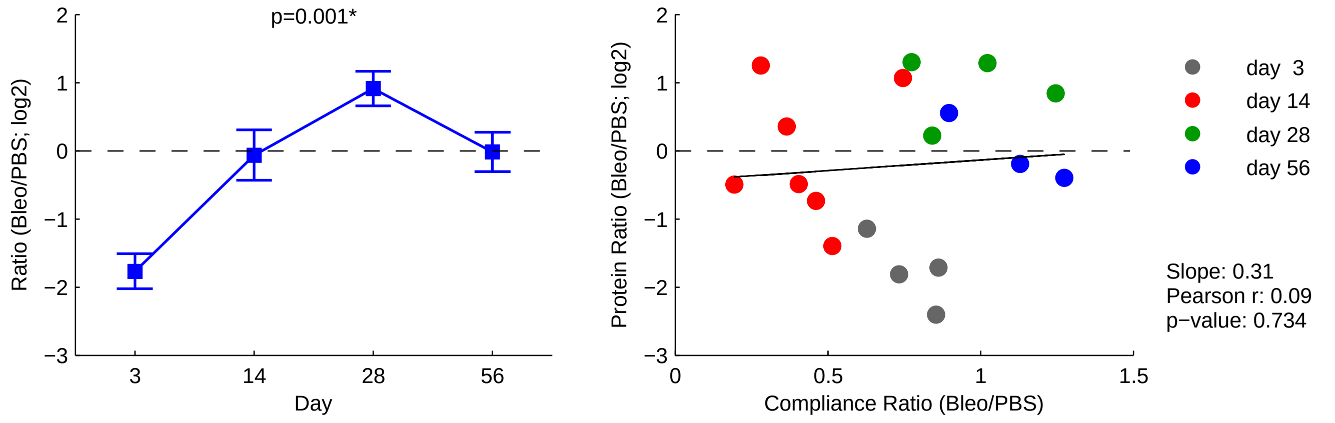

Q8K301 − Ddx52 (id: 5819)

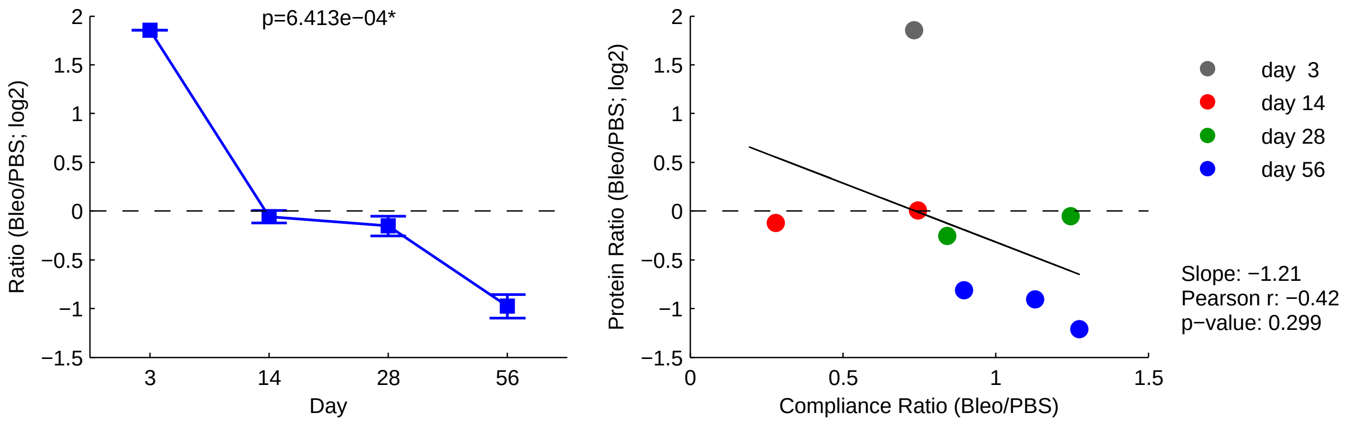

Q8K310 − Matr3 (id: 5821)

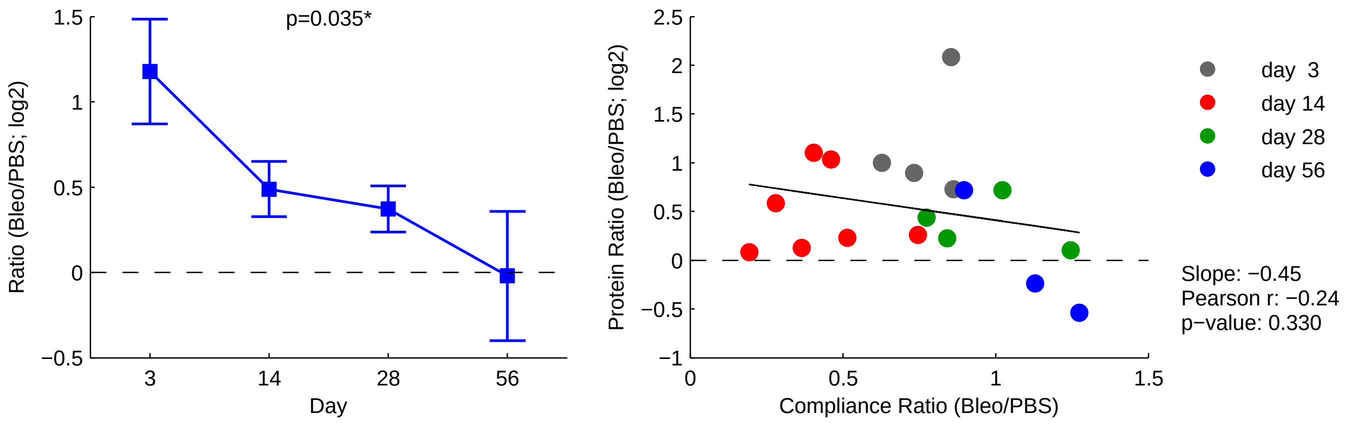

Q8K337−3 − Inpp5b (id: 5823)

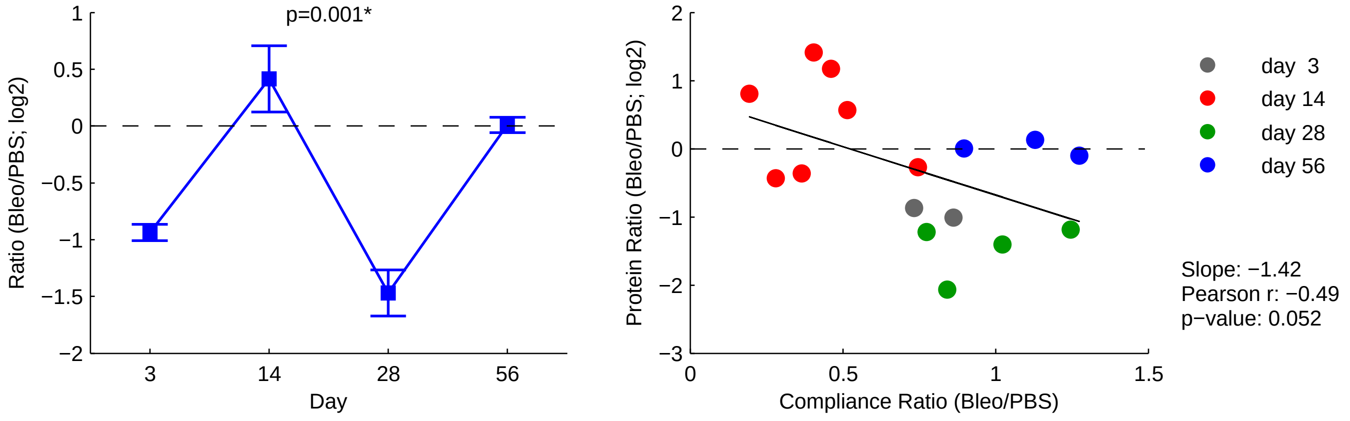

Q8K363 − Ddx18 (id: 5826)

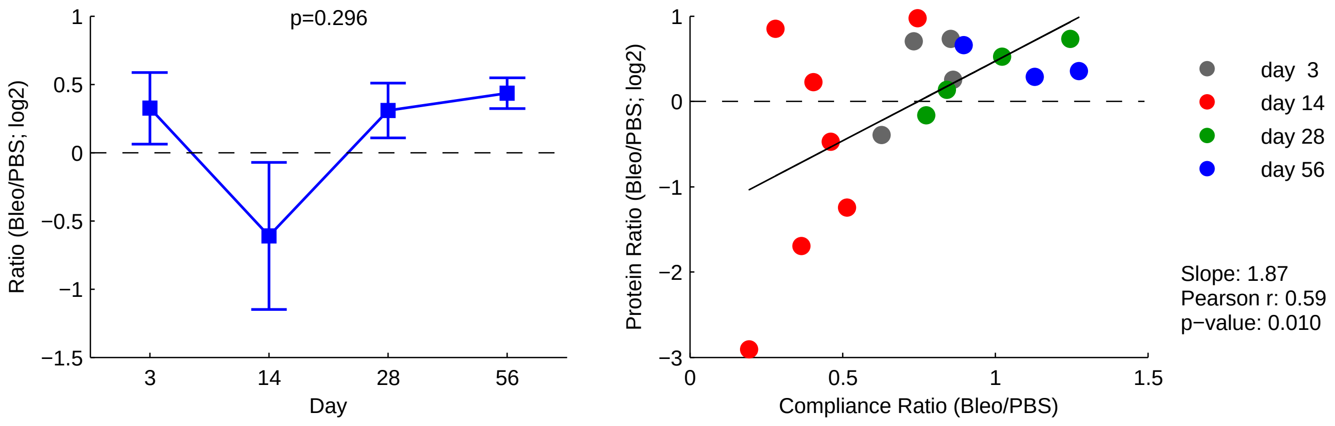

Q8K370 − Acad10 (id: 5827)

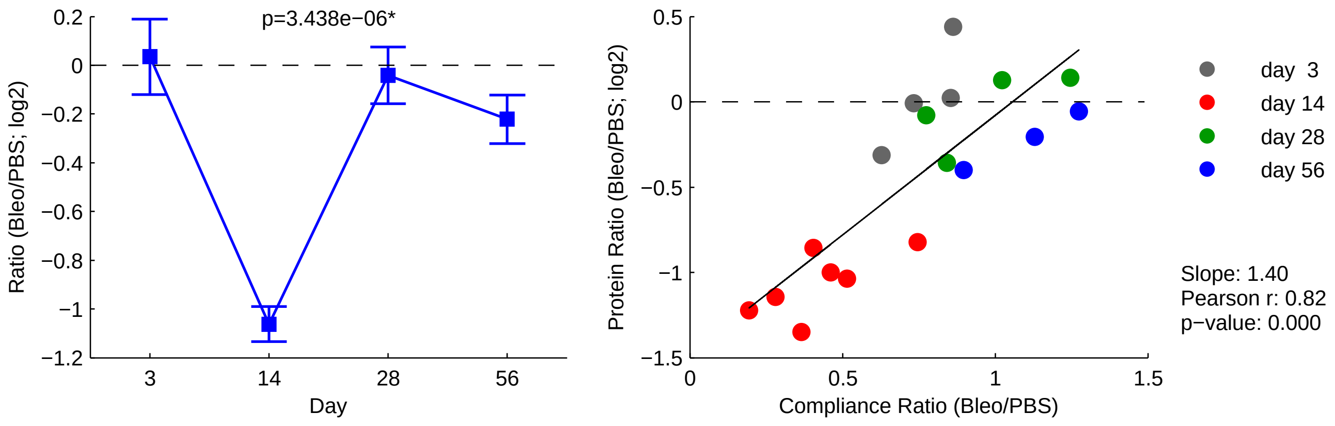

Q8K389 − Cdk5rap2 (id: 5830)

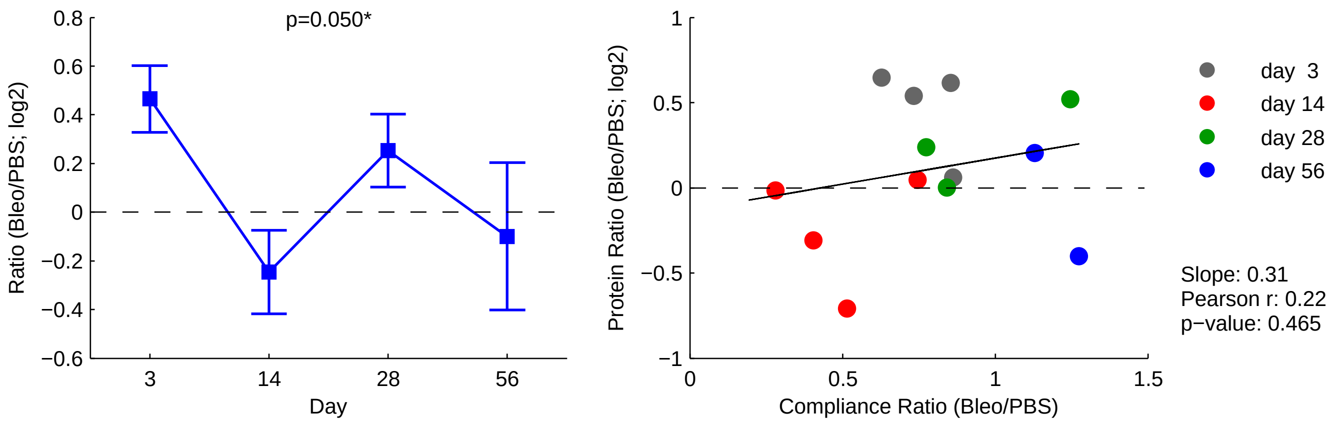

Q8K3A0 − Hscb (id: 5831)

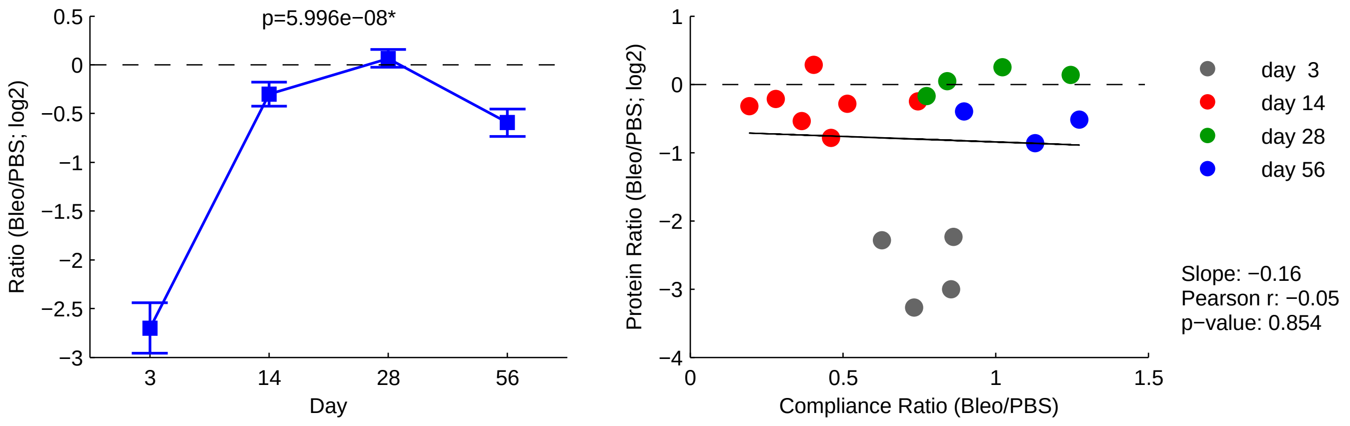

Q8K3G5 − Vrk3 (id: 5835)

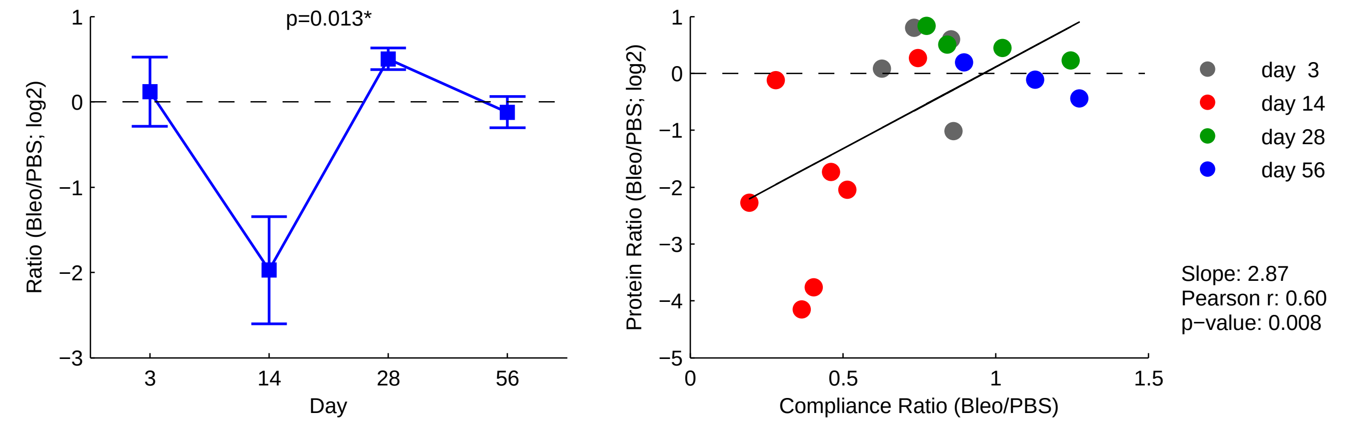

Q8K3G9 − Appl2 (id: 5836)

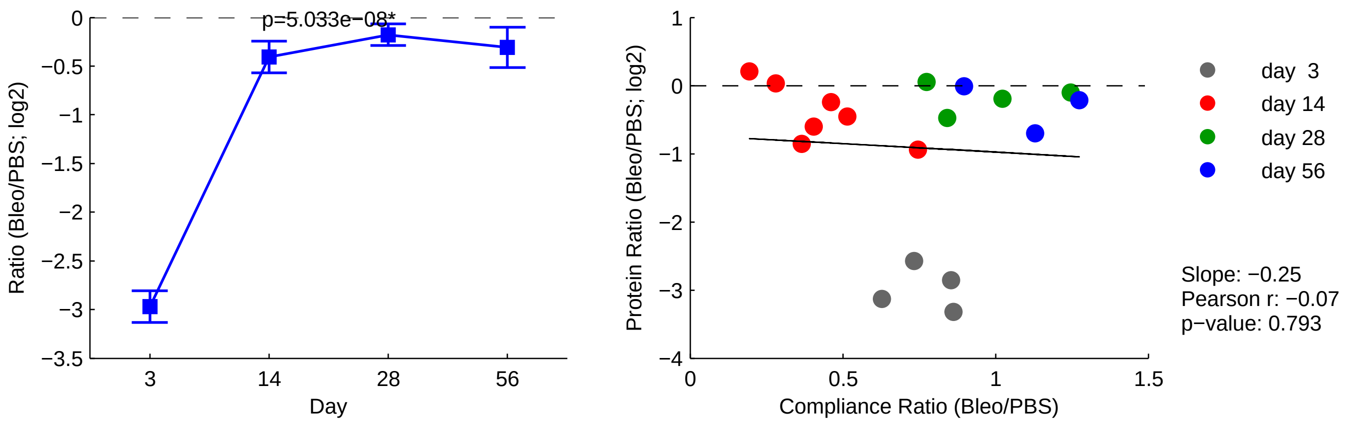

Q8K3J1 − Ndufs8 (id: 5839)

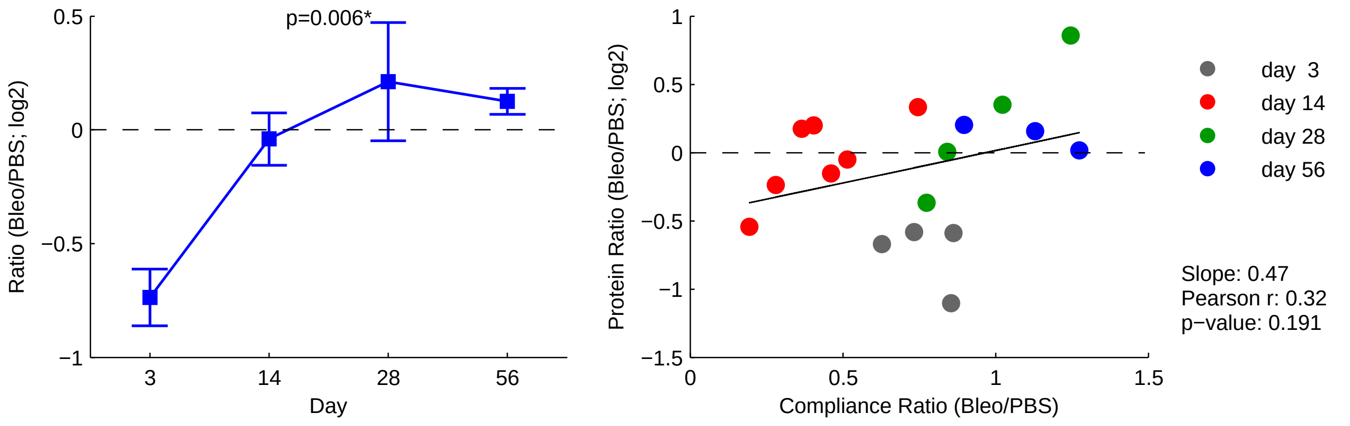

Q8K3K7 − Agpat2 (id: 5841)

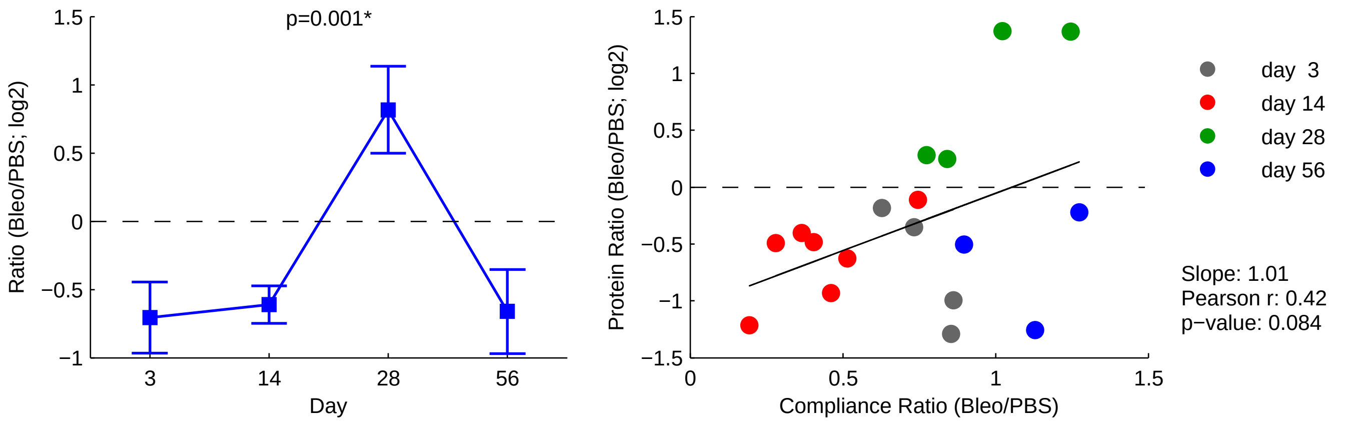

Q8K409 − Polb (id: 5847)

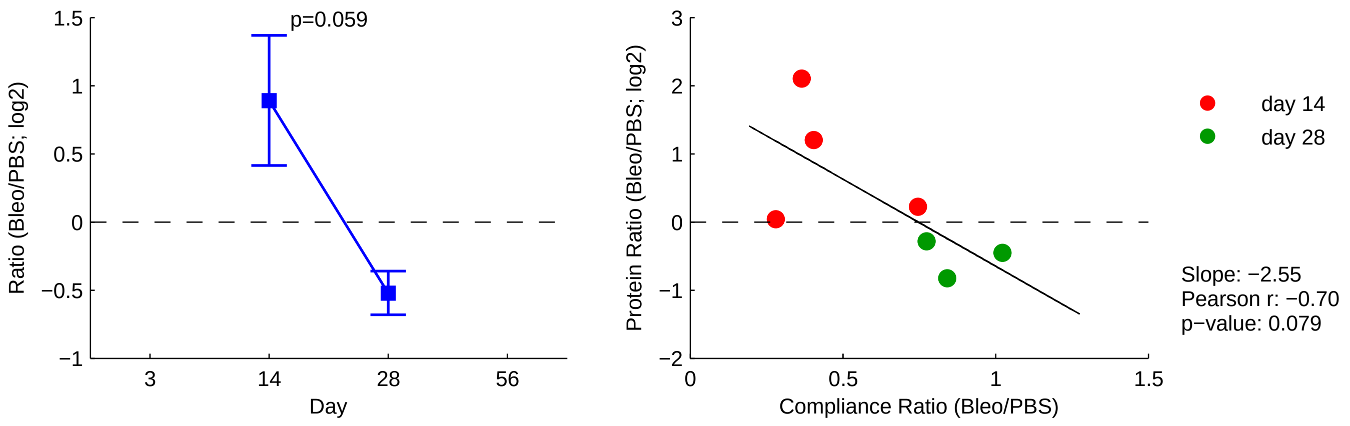

Q8K411−2 − Pitrm1 (id: 5848)

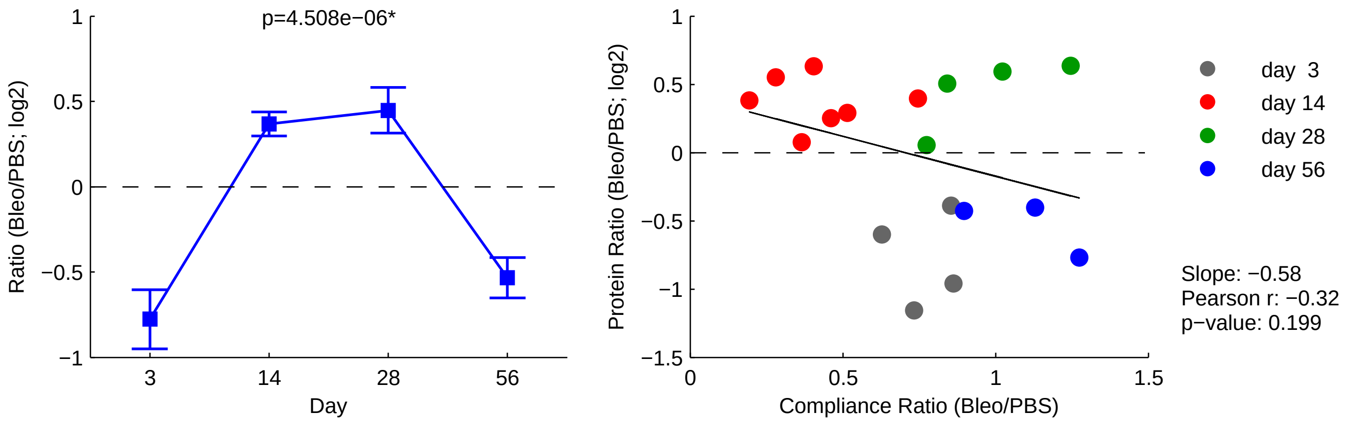

Q8K426 − Retnlg (id: 5849)

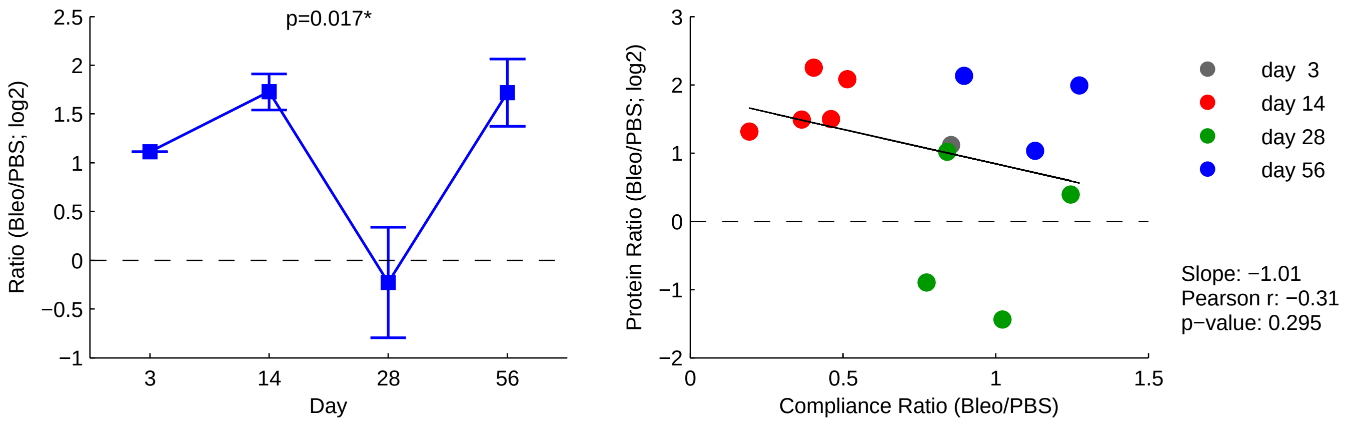

Q8K440−2 − Abca8b (id: 5850)

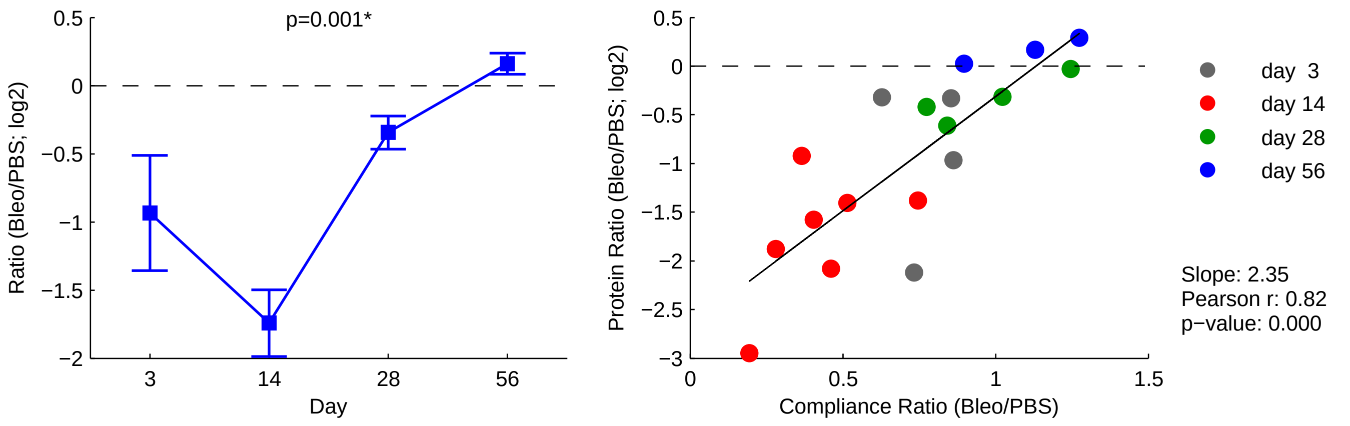

Q8K448 − Abca5 (id: 5852)

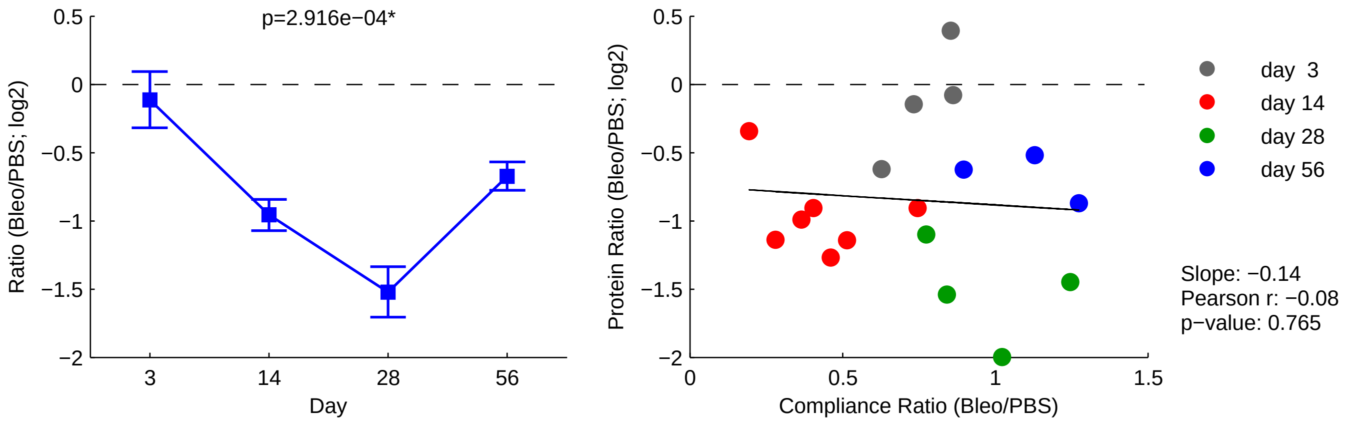

Q8K449 − Abca9 (id: 5853)

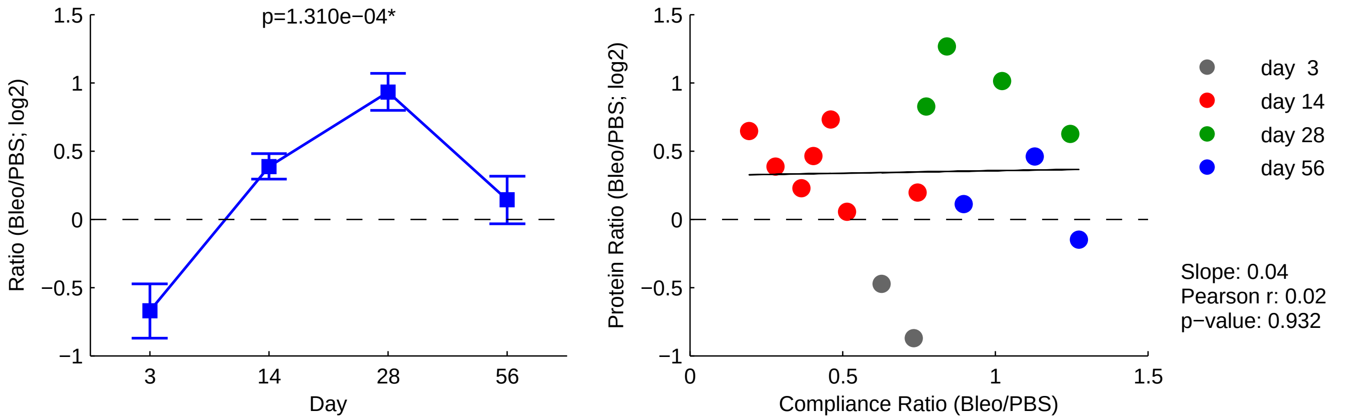

Q8K479 − C1qtnf5 (id: 5855)

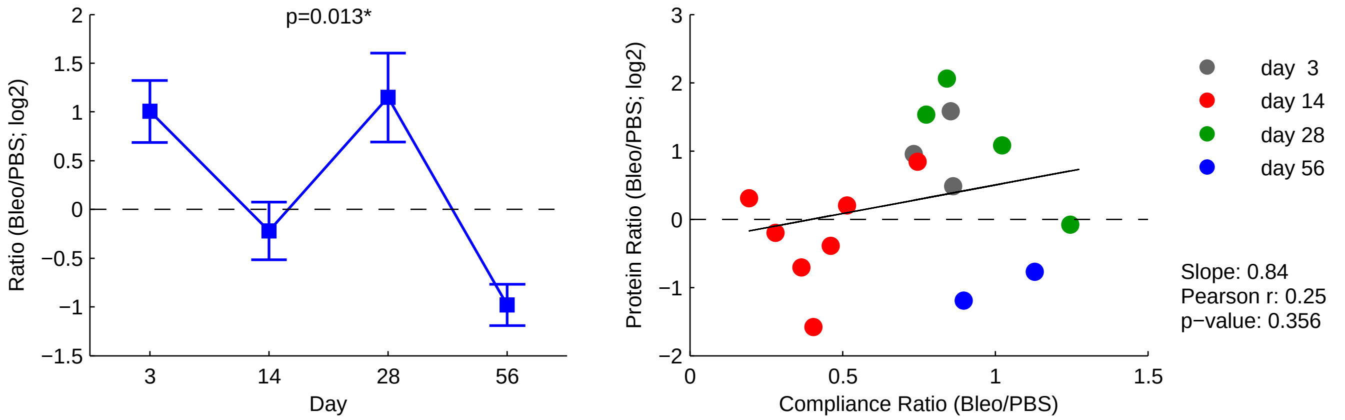

Q8K482 − Emilin2 (id: 5856)

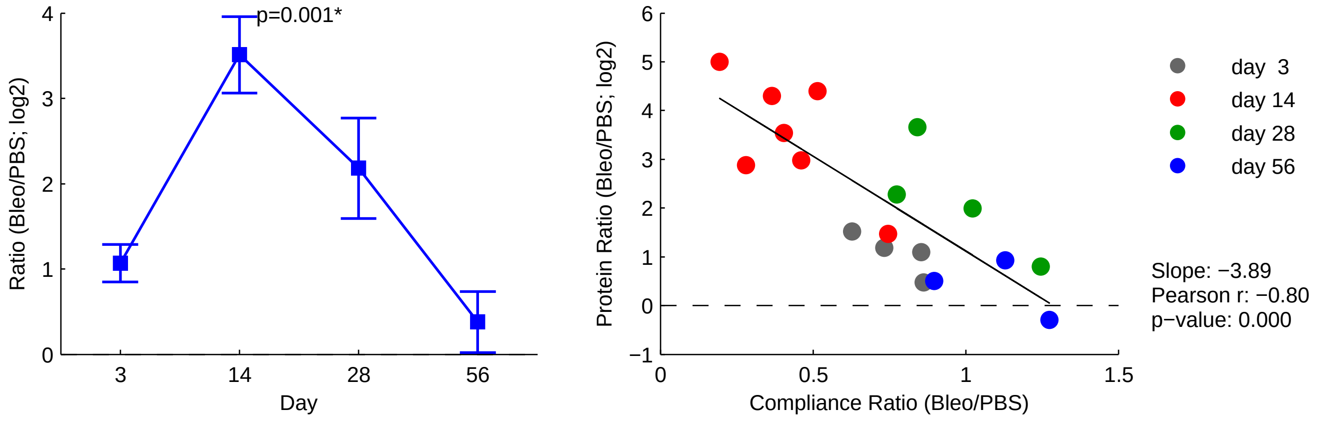

Q8K4F5 − Abhd11 (id: 5858)

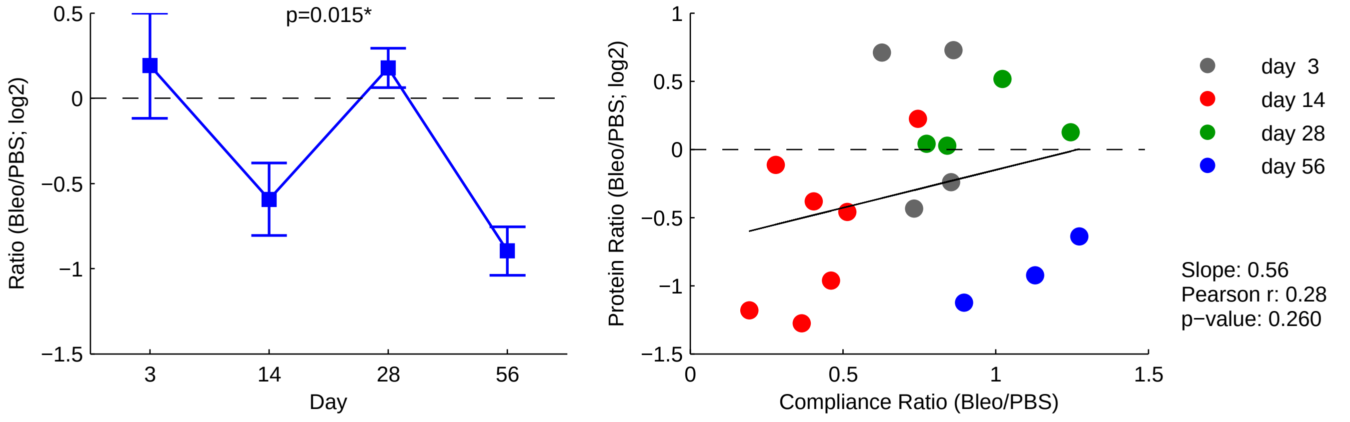

Q8K4L4 − Pof1b (id: 5861)

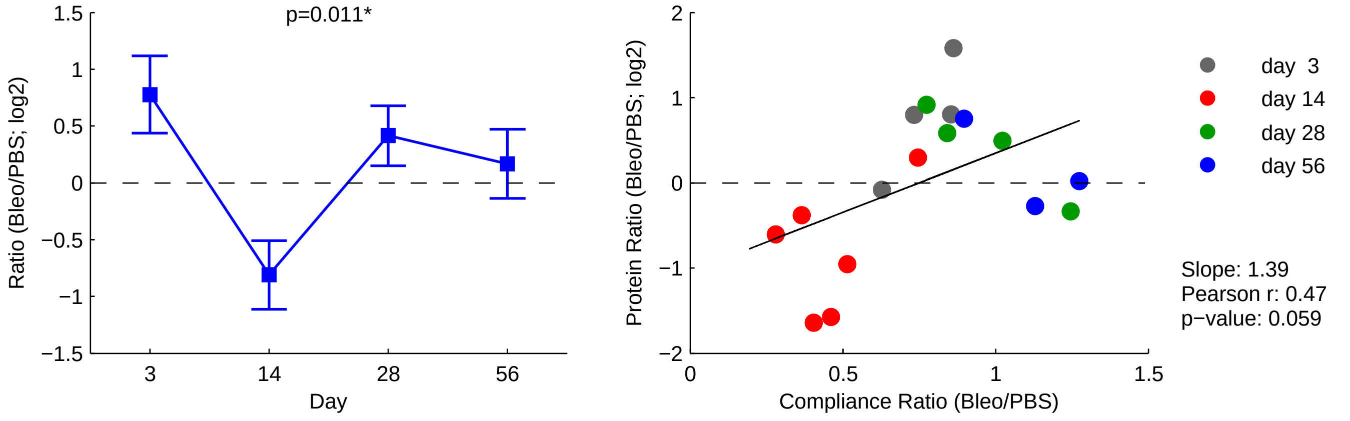

Q8K4Q8 − Colec12 (id: 5866)

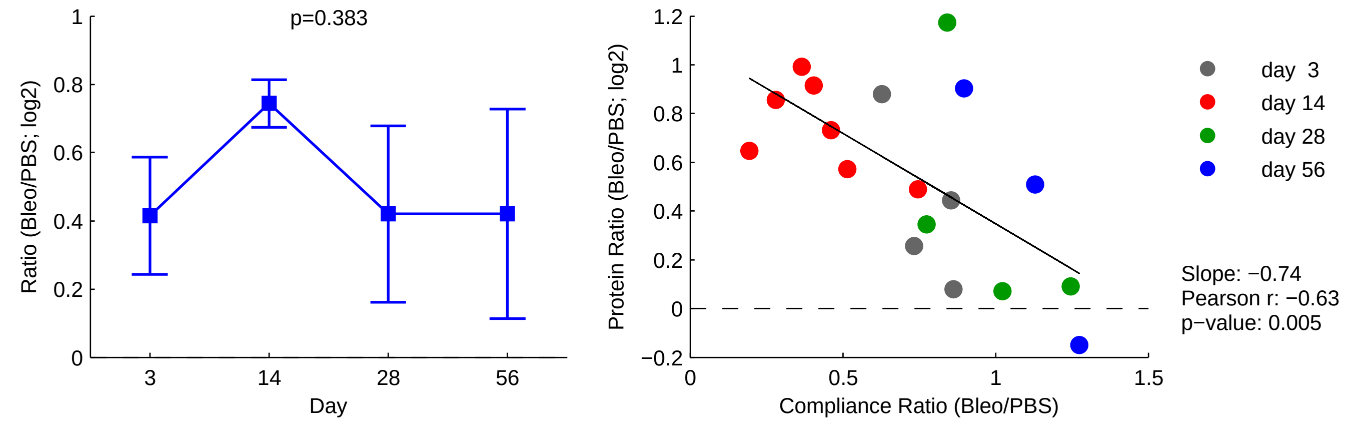

Q8K4R4−2 − Pitpnc1 (id: 5867)

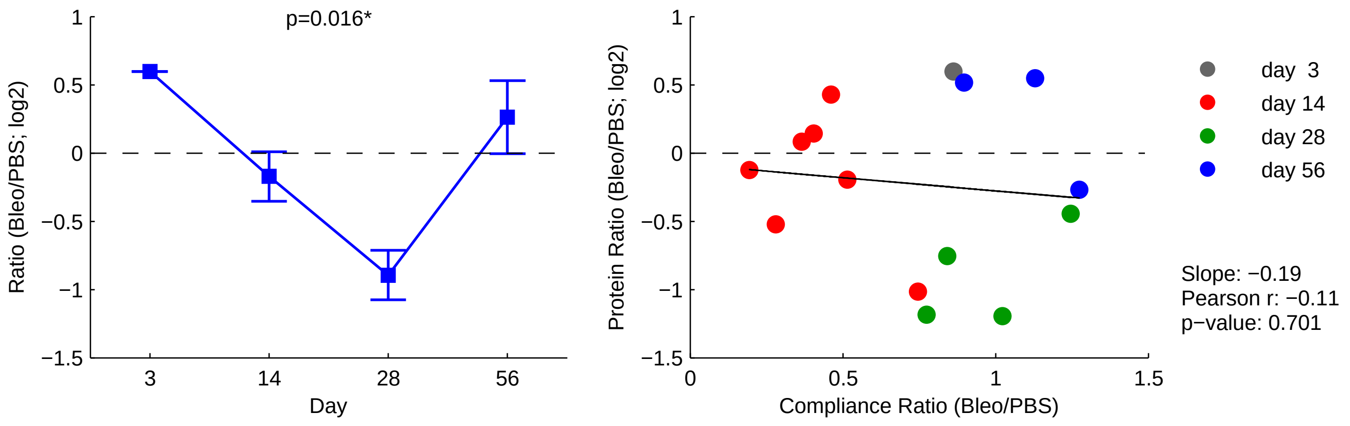

Q8K4X7 − Agpat4 (id: 5868)

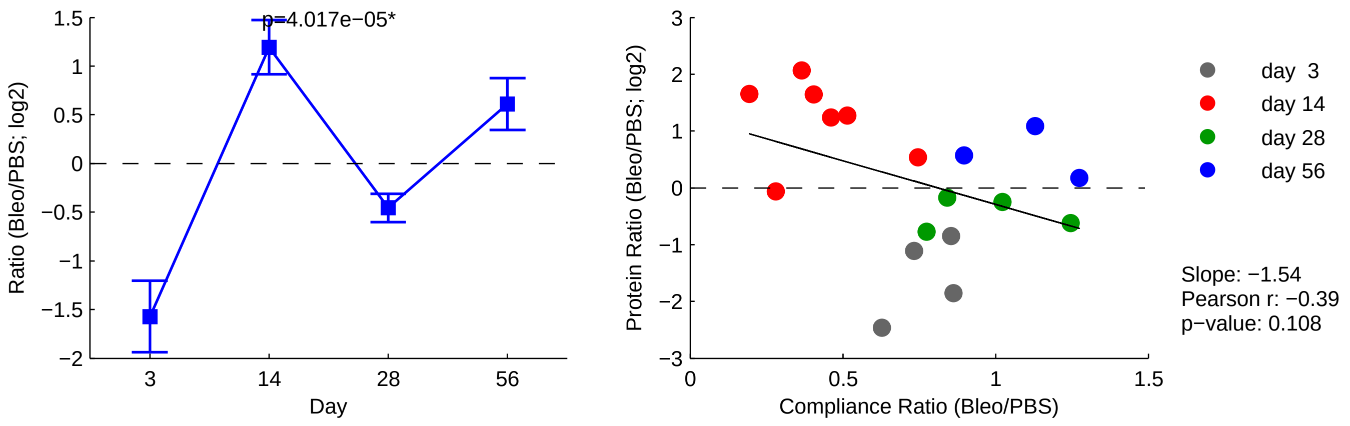

Q8K4Z3 − Apoa1bp (id: 5869)

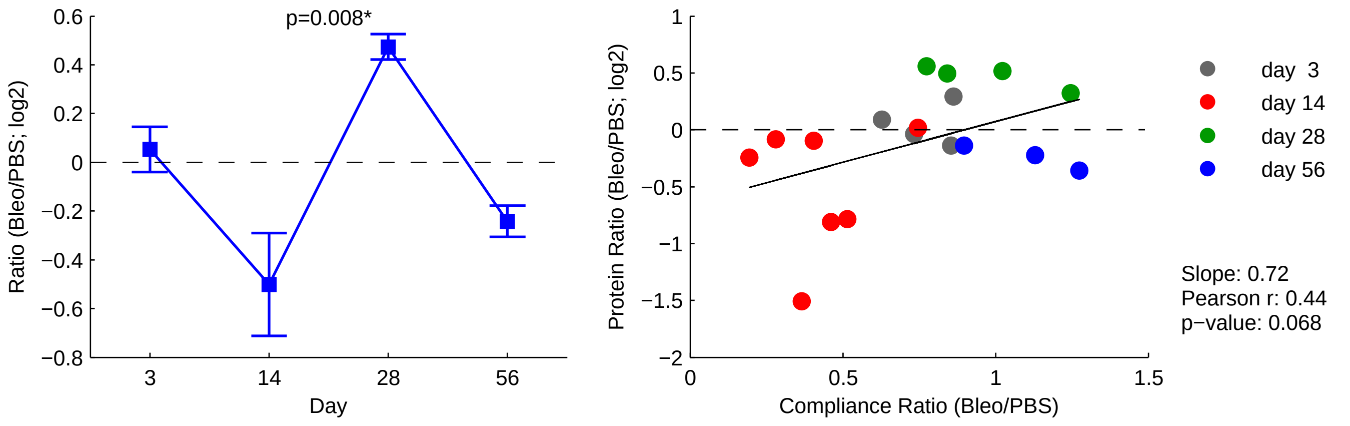

Q8K4Z5 − Sf3a1 (id: 5870)

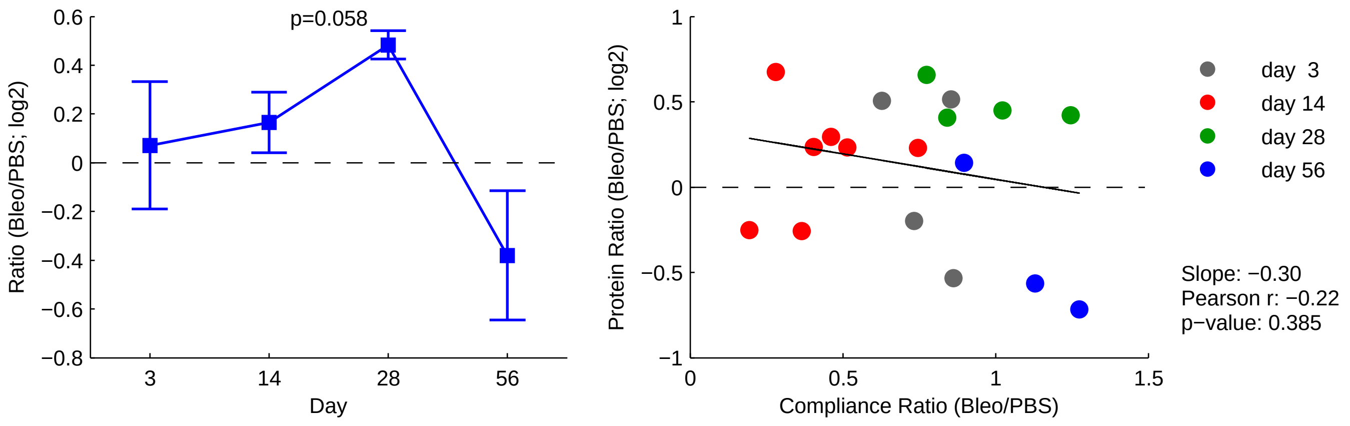

Q8K5B2 − Mcfd2 (id: 5872)

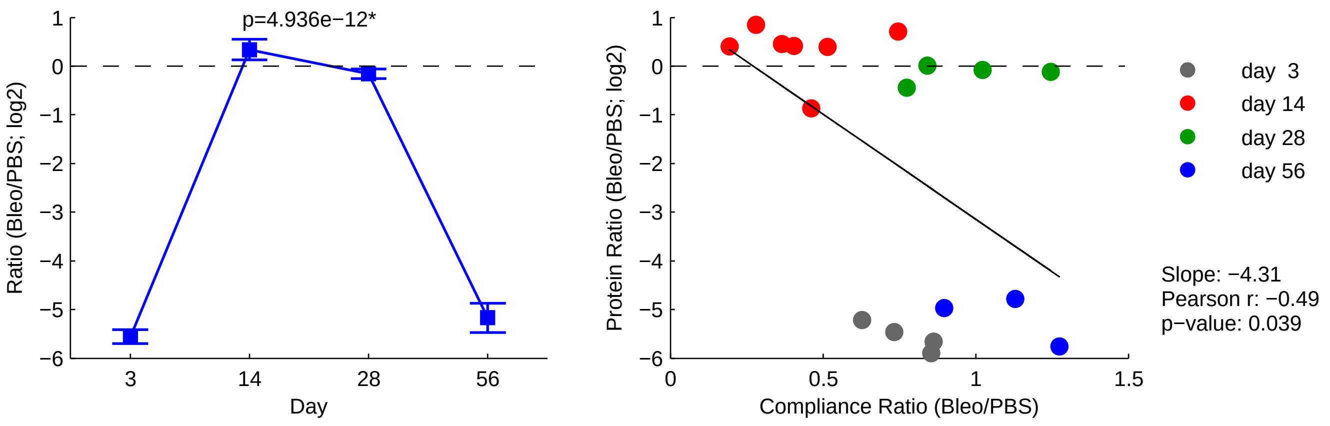

Q8QZS1 − Hibch (id: 5874)

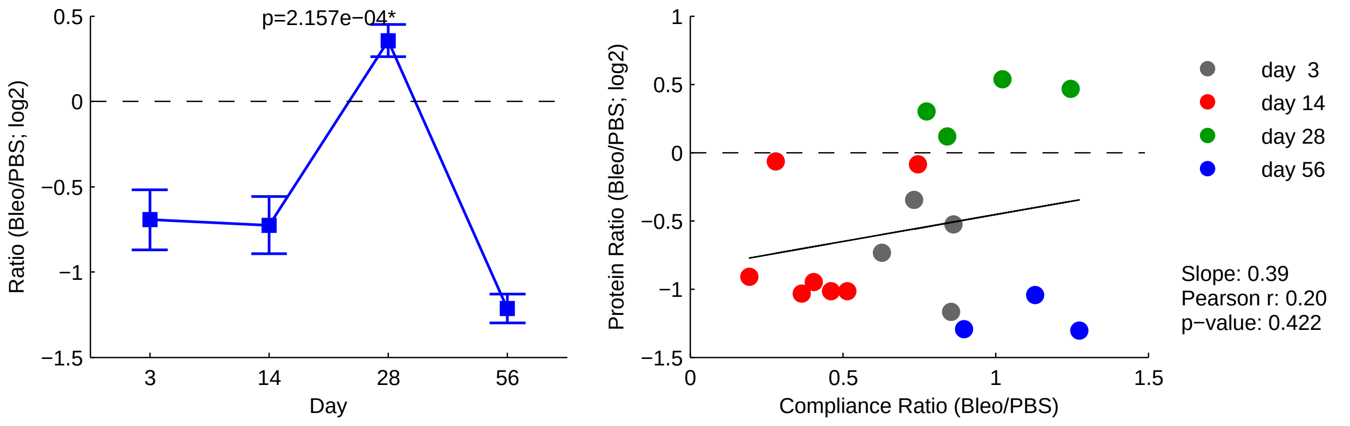

Q8QZT1 − Acat1 (id: 5875)

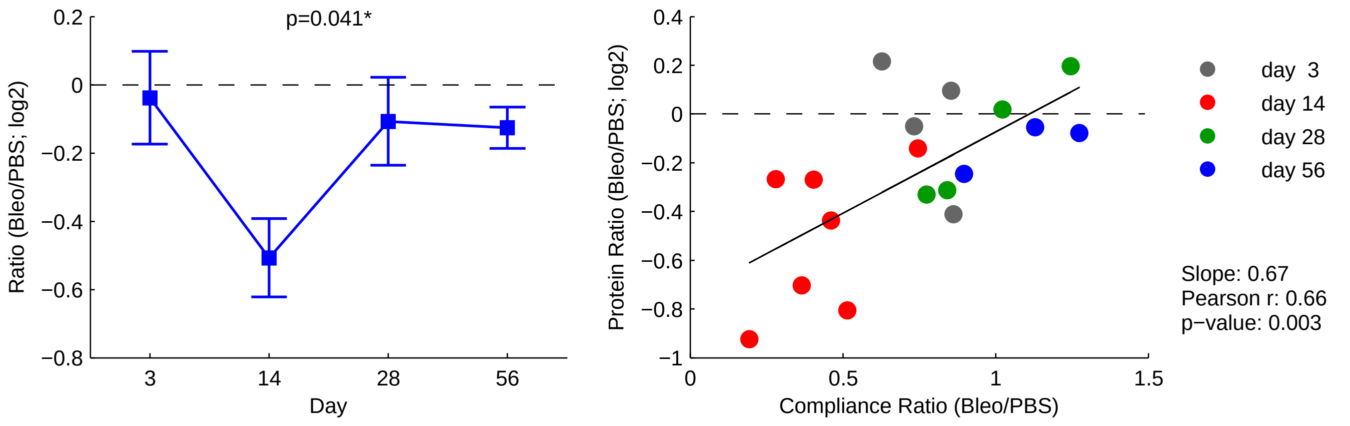

Q8QZY1 − Eif3l (id: 5879)

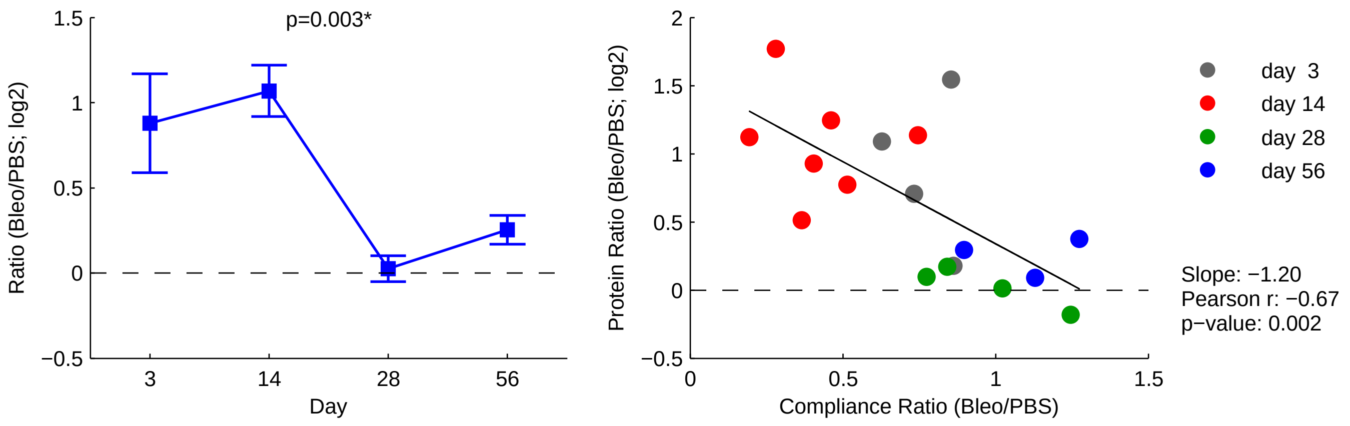

Q8R016 − Blmh (id: 5885)

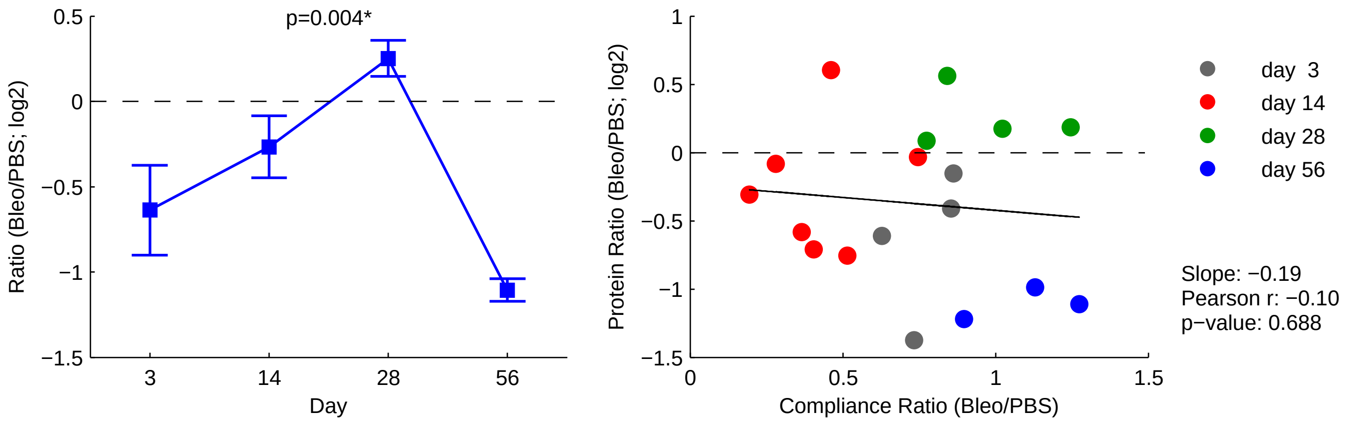

Q8R050−2 − Gspt1 (id: 5887)

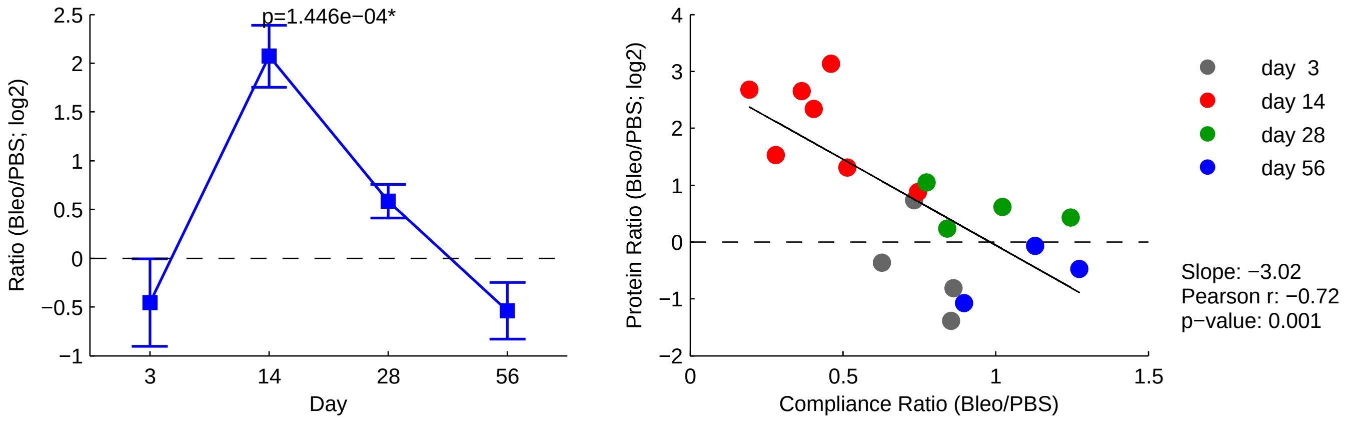

Q8R0A0 − Gtf2f2 (id: 5890)

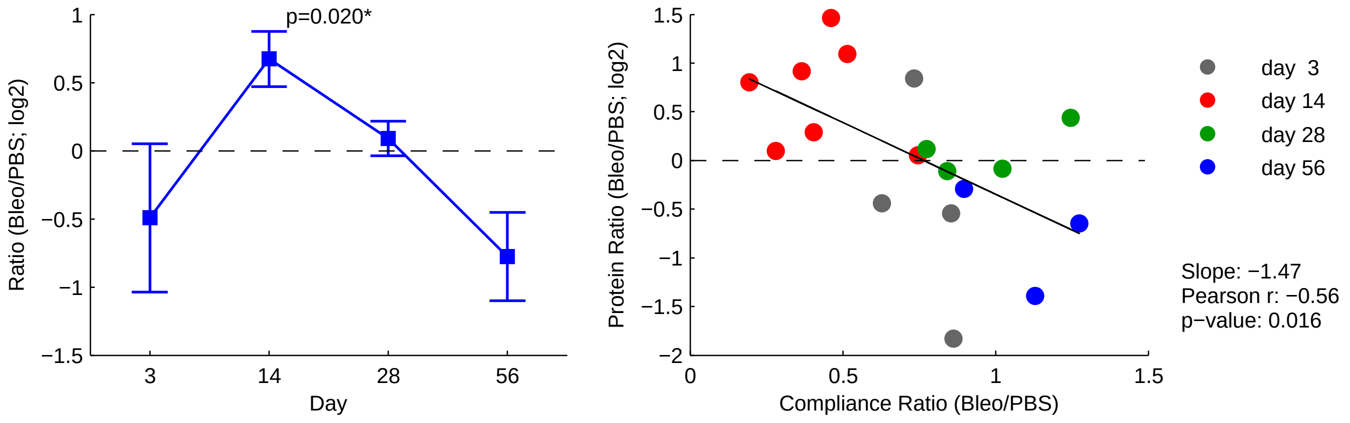

Q8R0F8 − Fahd1 (id: 5894)

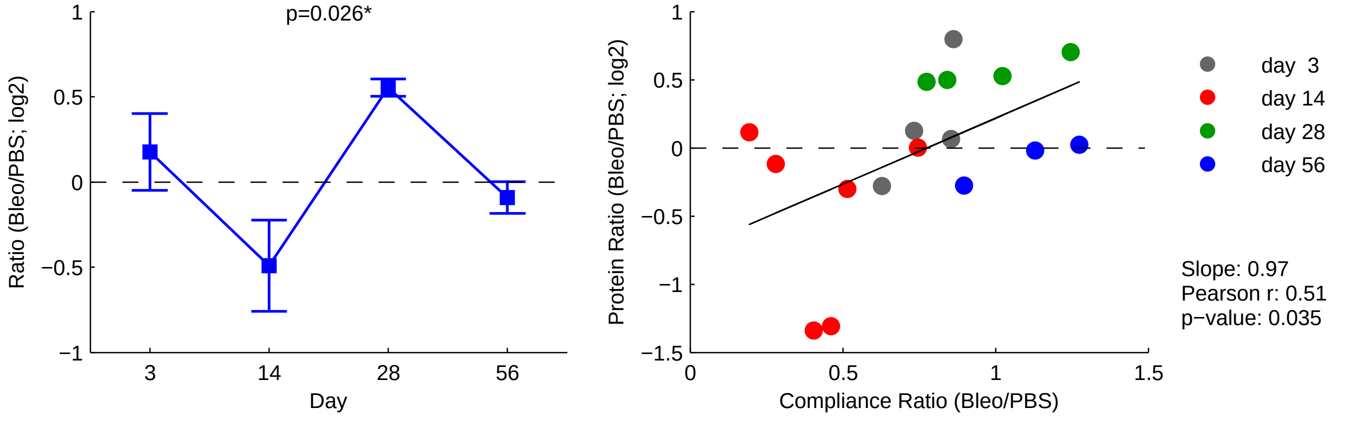

Q8R0F9 − Sec14l4 (id: 5895)

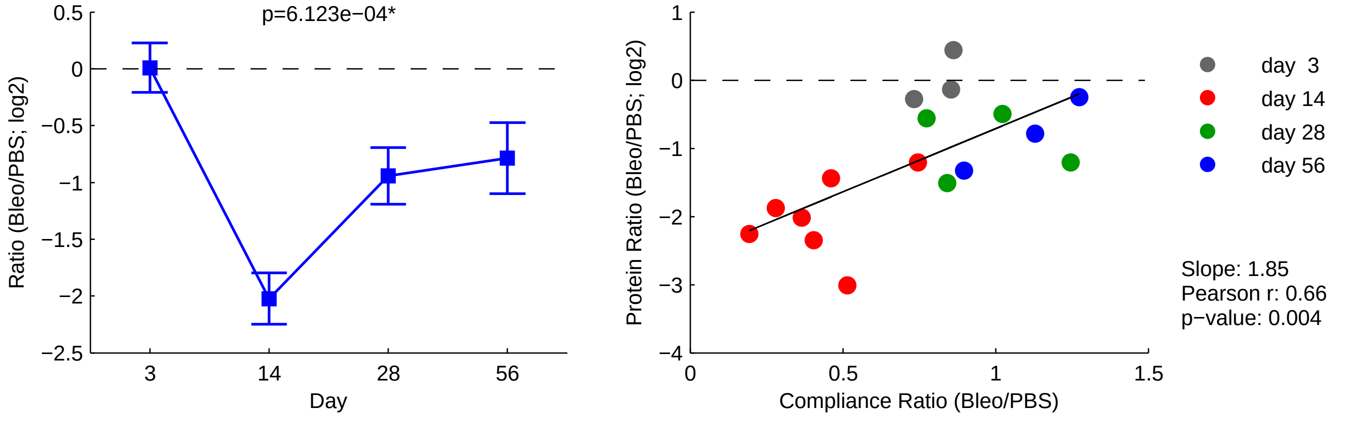

Q8R0H9 − Gga1 (id: 5897)

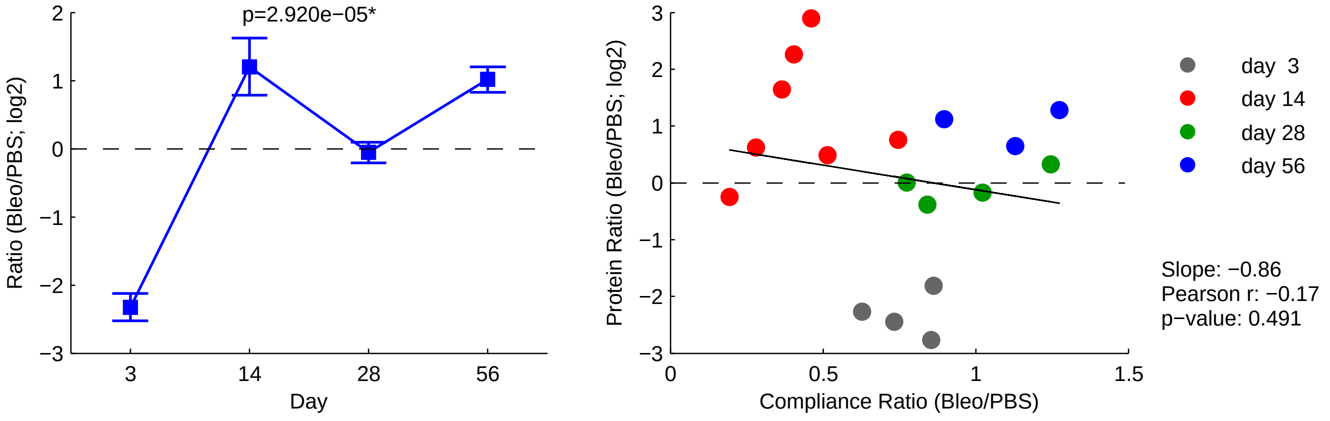

Q8R0L9−2 − Tada3 (id: 5900)

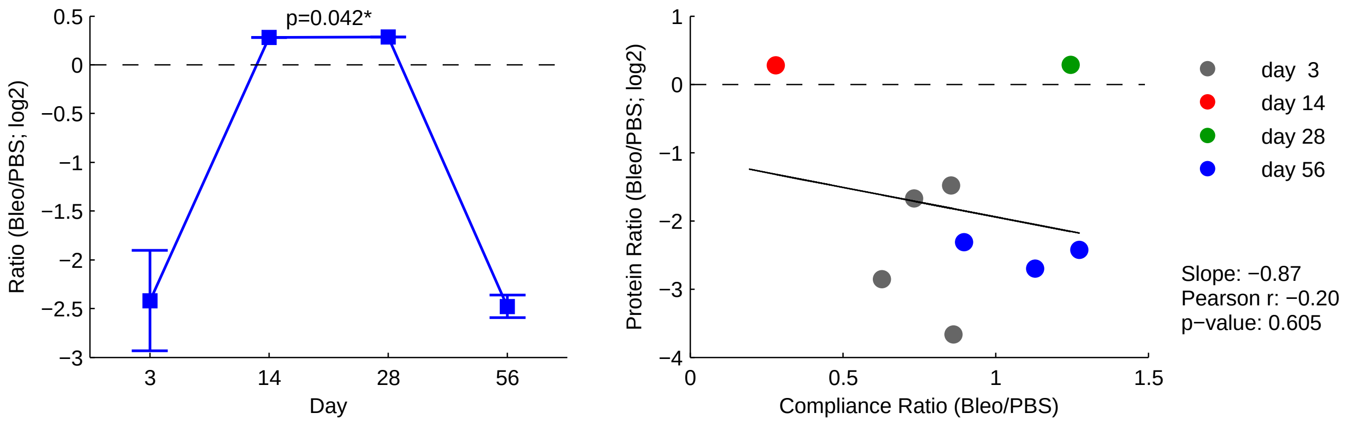

Q8R0N6 − Adhfe1 (id: 5901)

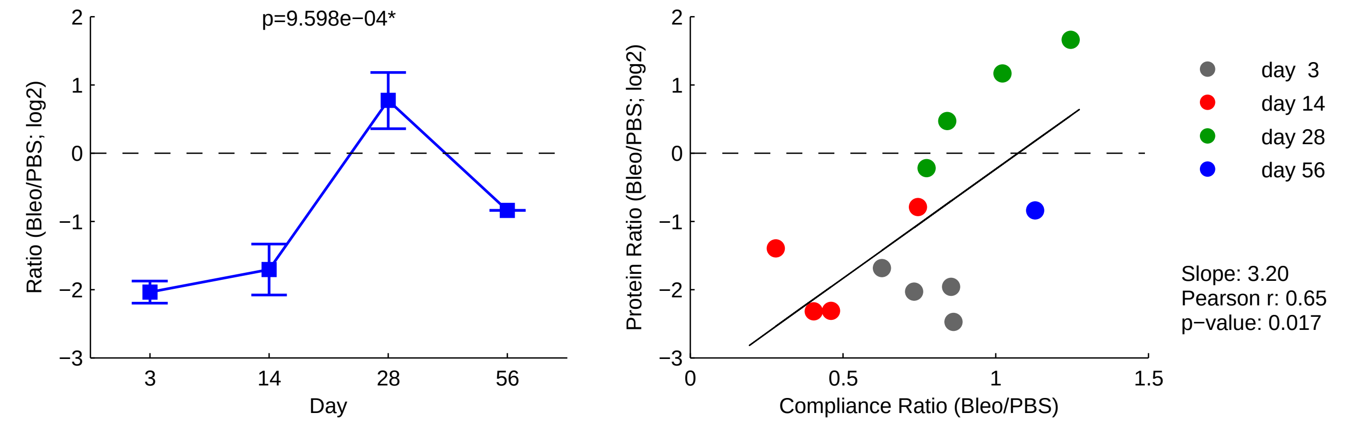

Q8R0X7 − Sgpl1 (id: 5905)

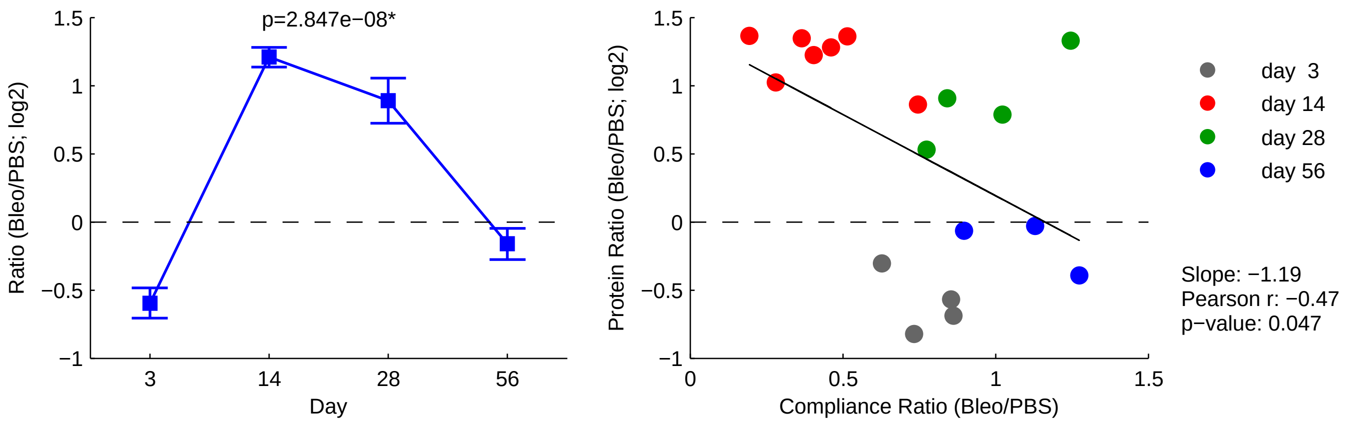

Q8R0Y8 − Slc25a42 (id: 5907)

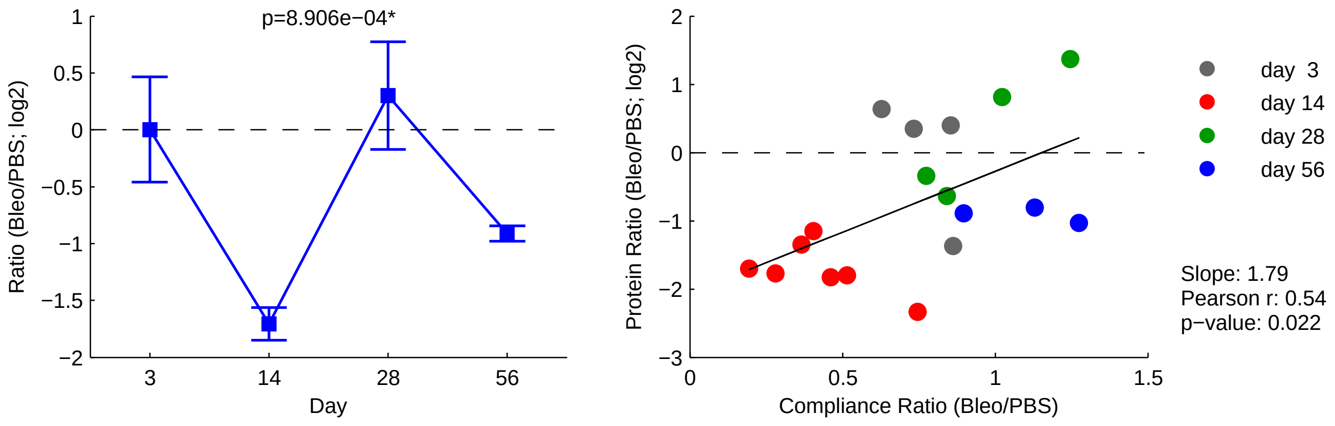

Q8R127 − Sccpdh (id: 5911)

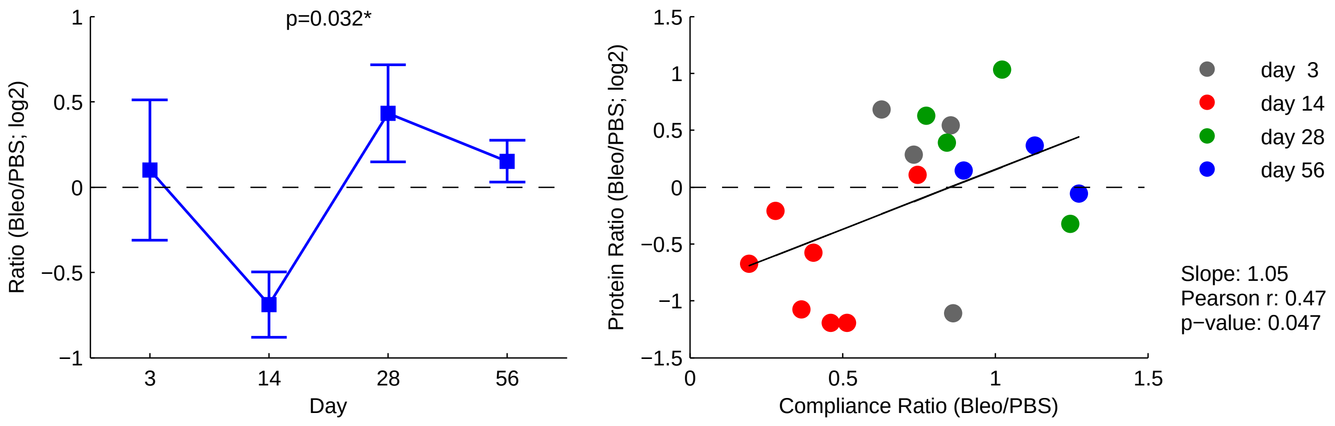

Q8R138 − Tmem119 (id: 5912)

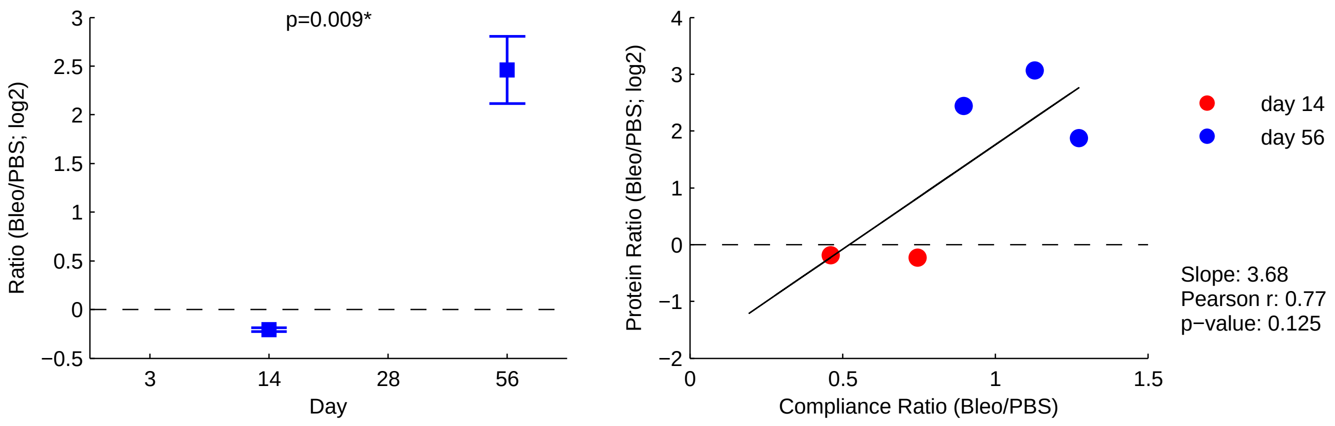

Q8R151 − Znfx1 (id: 5915)

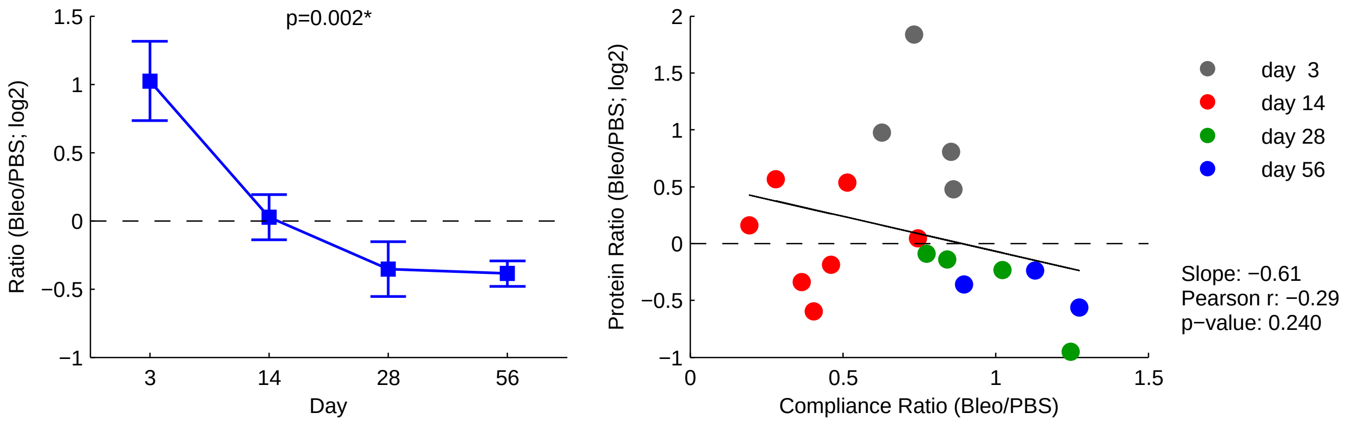

Q8R164 − Bphl (id: 5916)

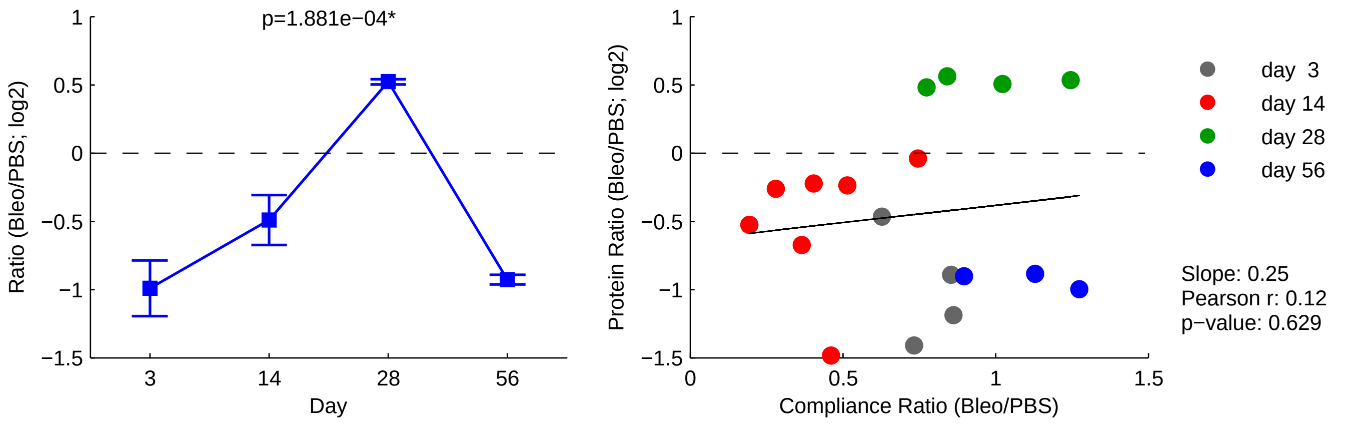

Q8R1B4 − Eif3c (id: 5919)

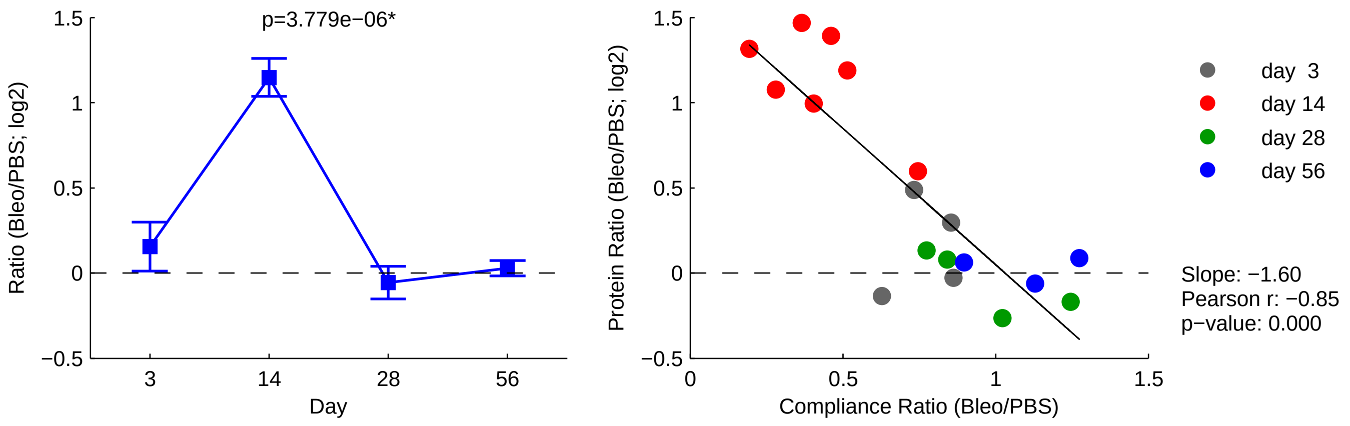

Q8R1F1 − Fam129b (id: 5921)

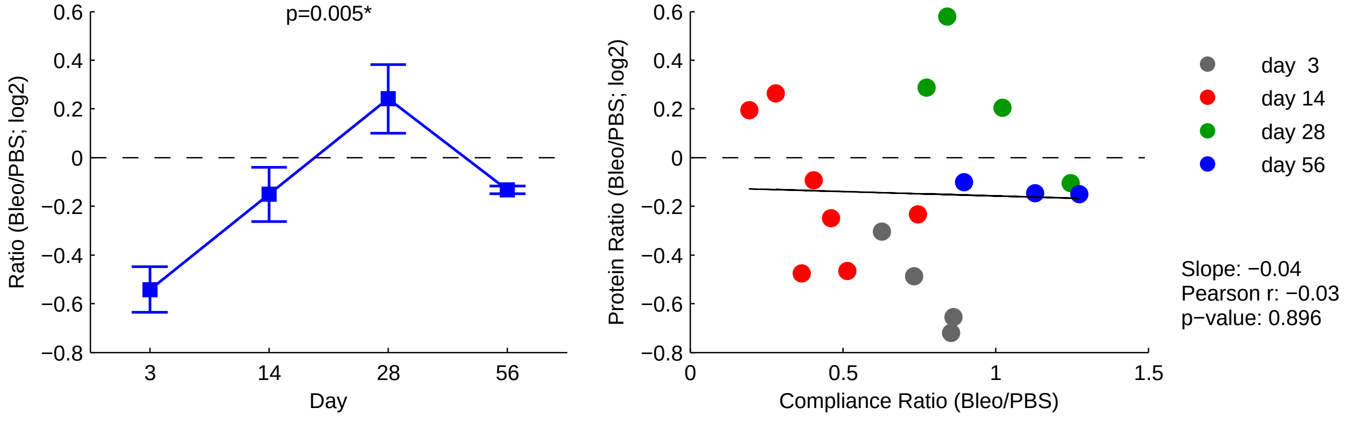

Q8R1G6 − Pdlim2 (id: 5924)

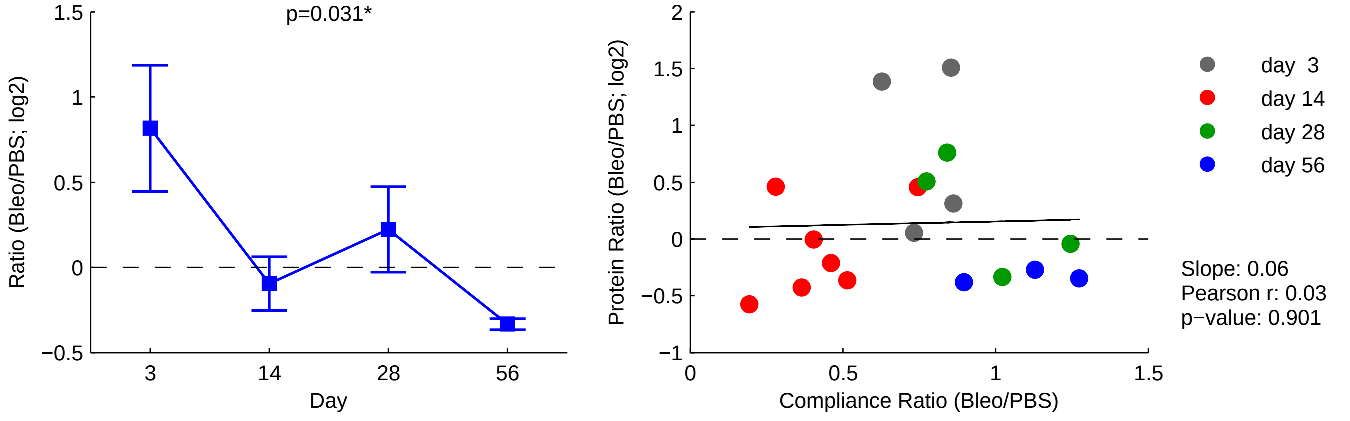

Q8R1H0 − Hopx (id: 5925)

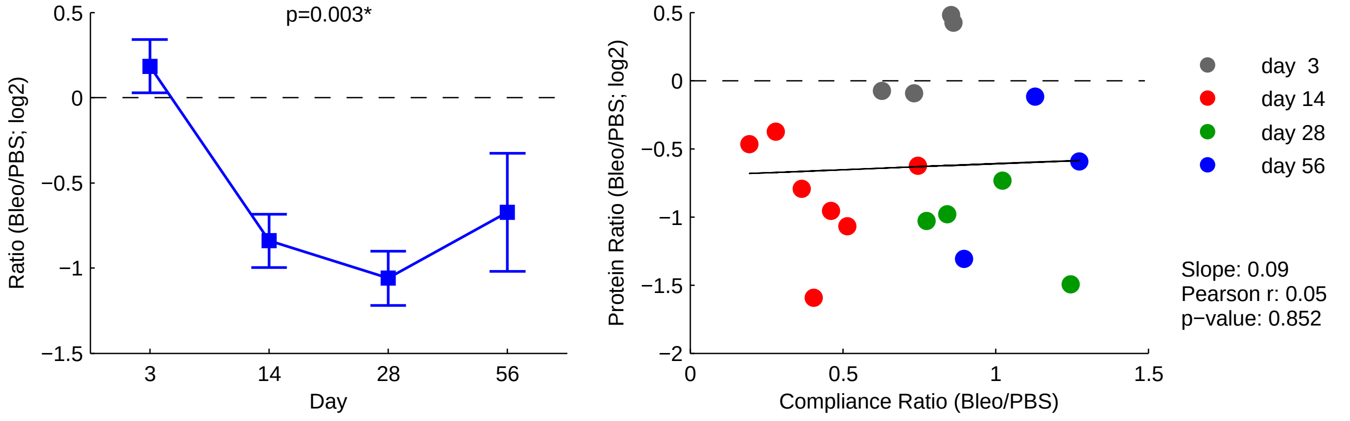

Q8R1I1 − Uqcr10 (id: 5926)

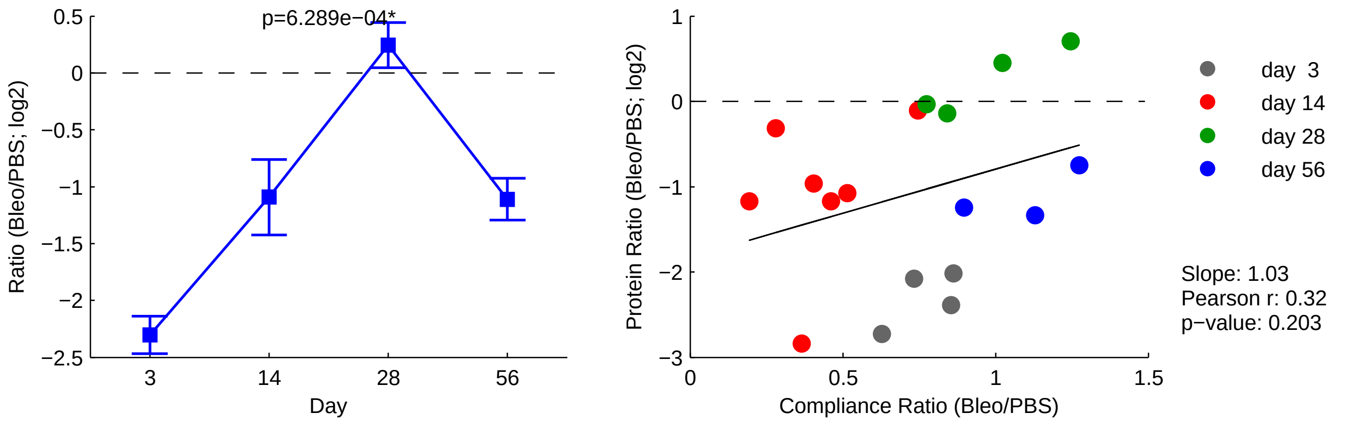

Q8R1J9 − Tor2a (id: 5927)

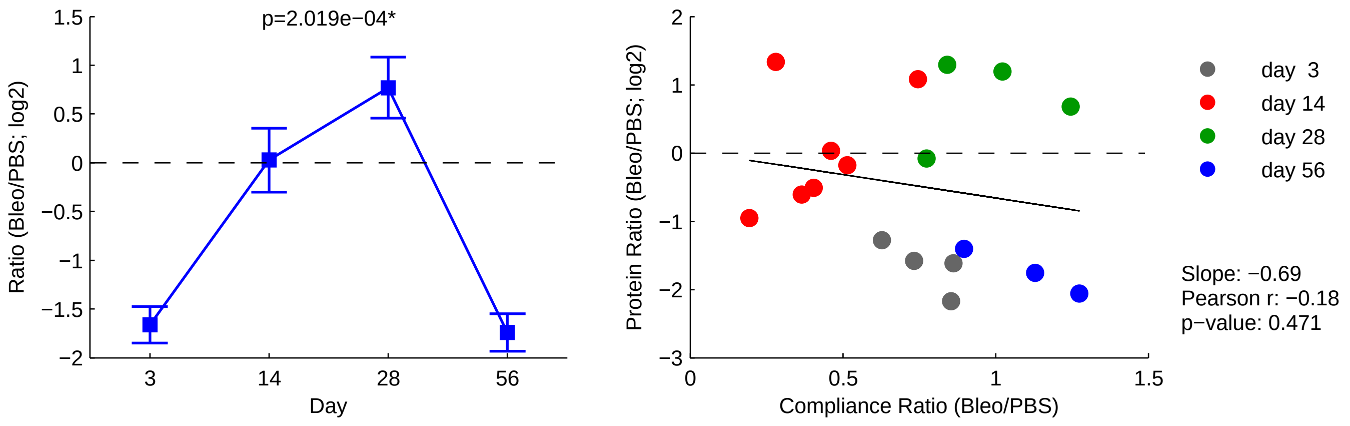

Q8R1K1 − Ubac2 (id: 5928)

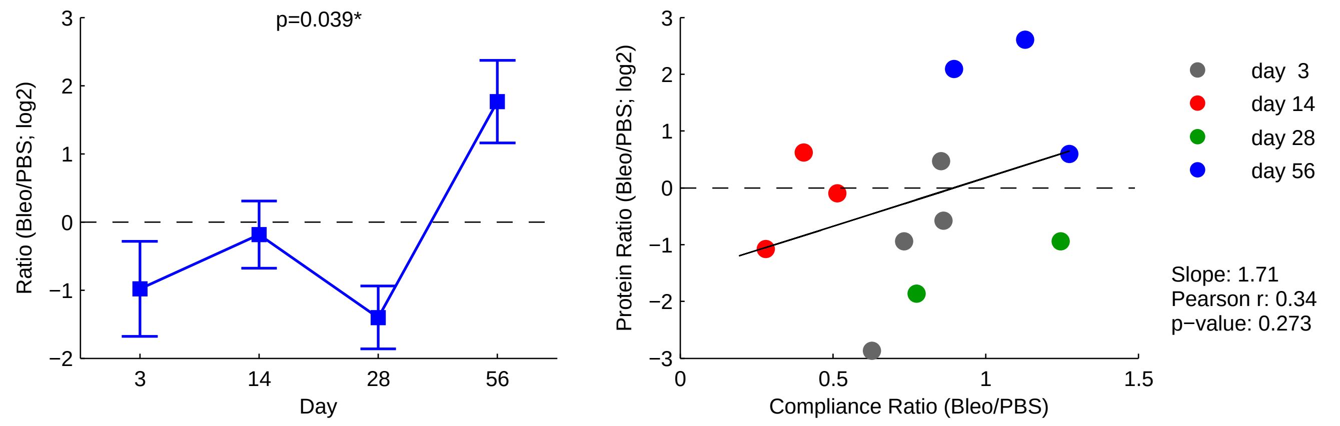

Q8R1T1 − Chmp7 (id: 5933)

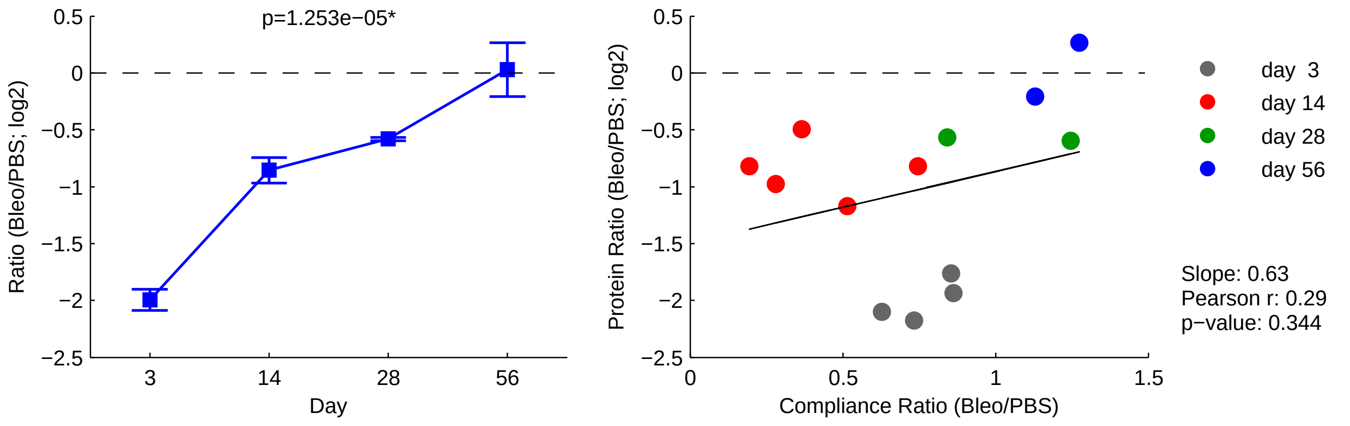

Q8R1V4 − Tmed4 (id: 5934)

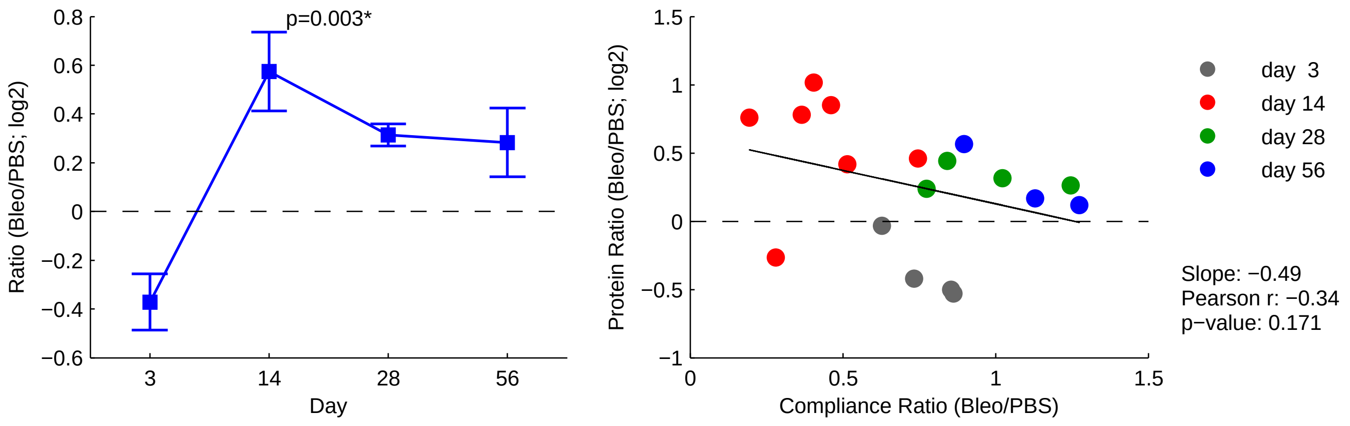

Q8R2K3 − Ssbp1 (id: 5939)

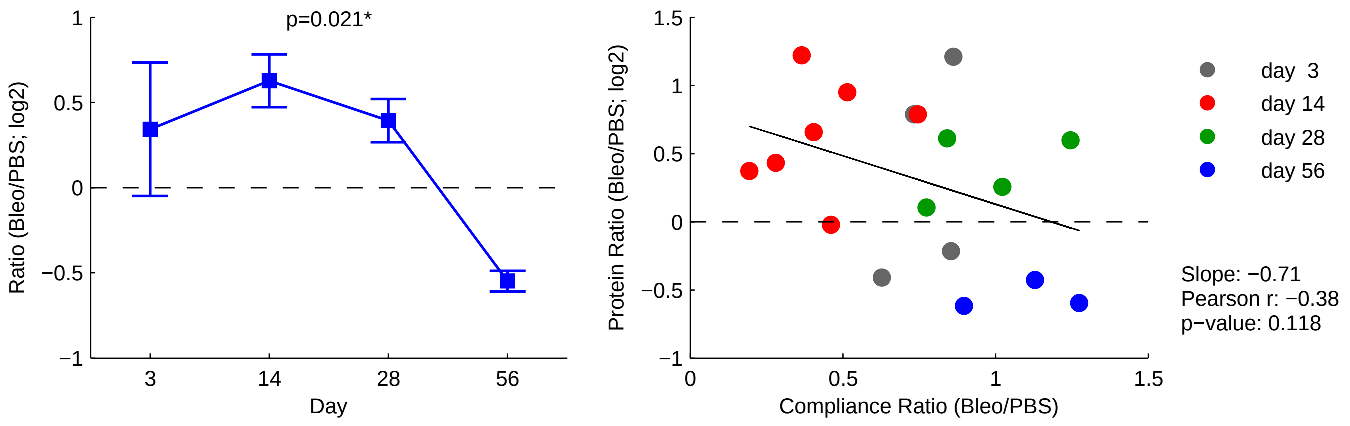

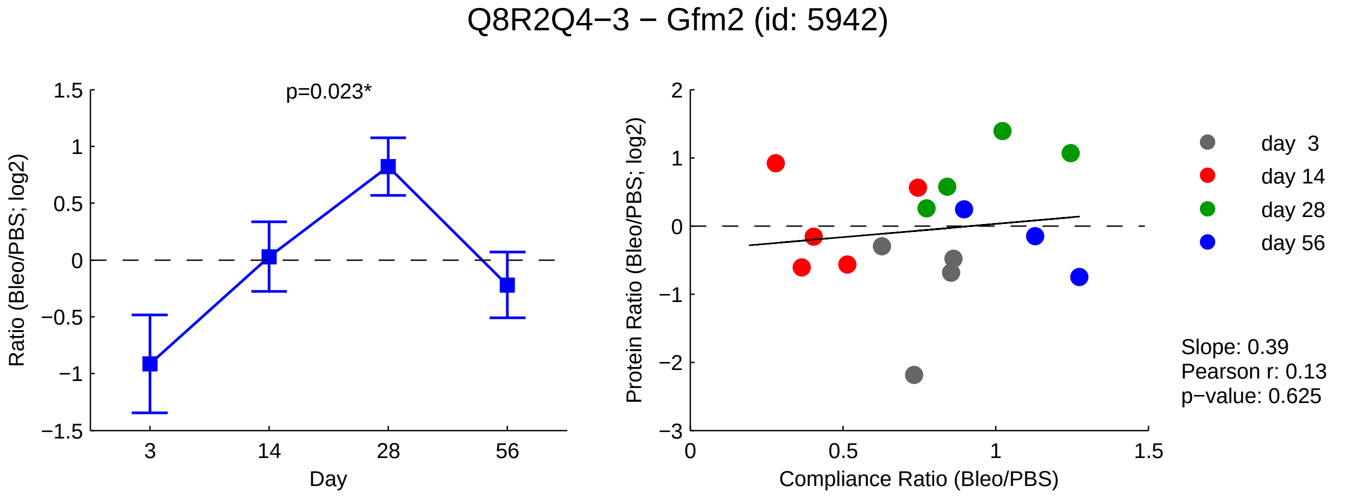
Q8R2Q4−3 − Gfm2 (id: 5942)
p=0.023*
Ratio (Bleo/PBS; log2)
Day
Protein Ratio (Bleo/PBS; log2)
Compliance Ratio (Bleo/PBS)
day 3
day 14
day 28
day 56
Slope: 0.39
Pearson r: 0.13
p−value: 0.625

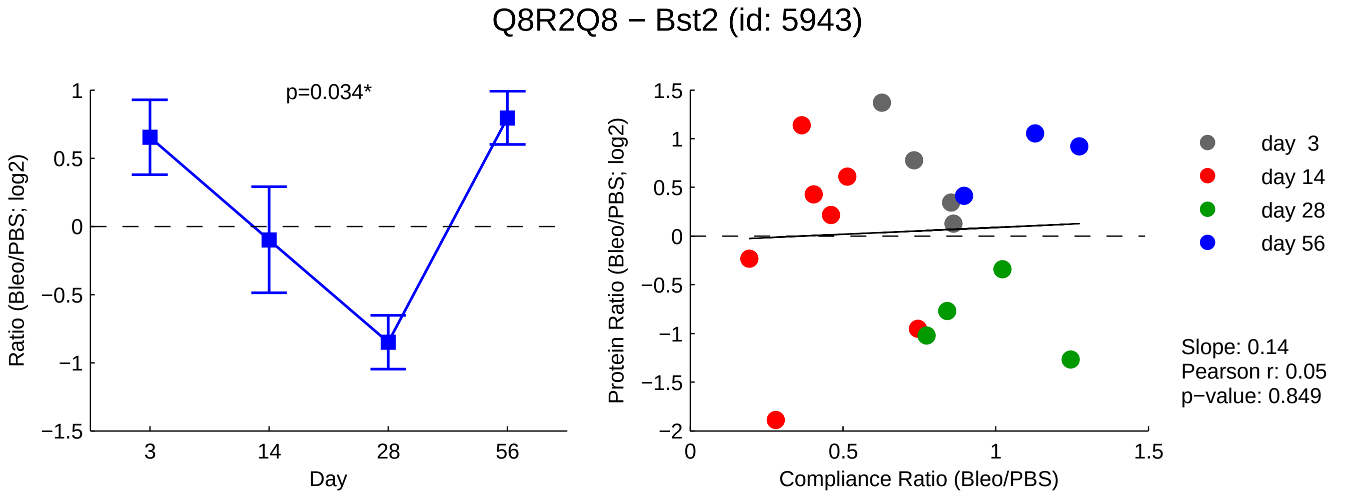
Q8R2Q8 − Bst2 (id: 5943)
p=0.034*
Ratio (Bleo/PBS; log2)
Day
Protein Ratio (Bleo/PBS; log2)
Compliance Ratio (Bleo/PBS)
day 3
day 14
day 28
day 56
Slope: 0.14
Pearson r: 0.05
p−value: 0.849

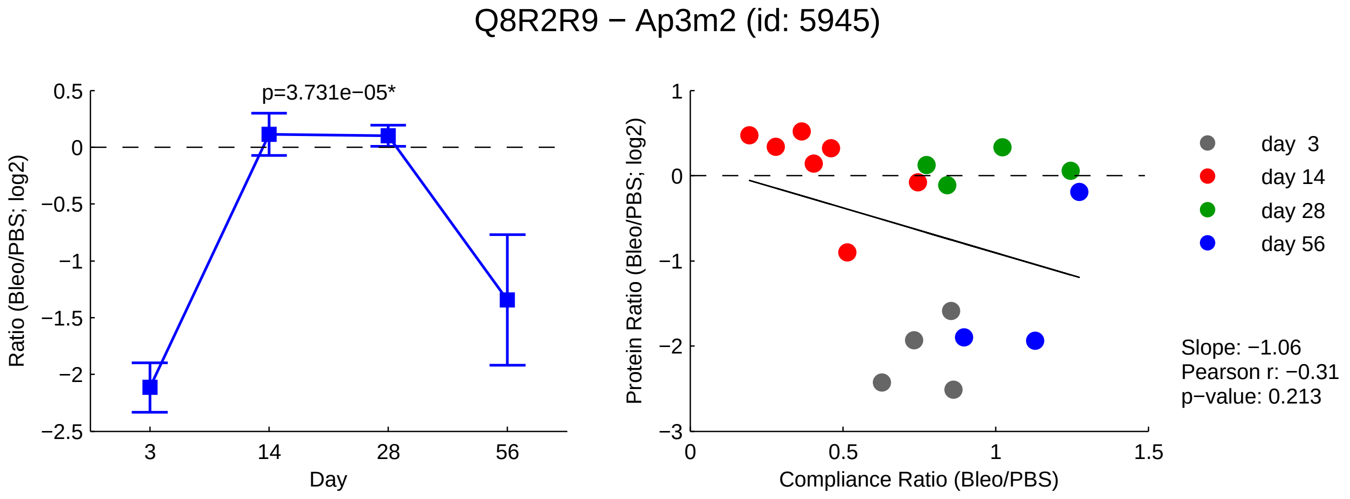
Q8R2R9 − Ap3m2 (id: 5945)
p=3.731e−05*
Ratio (Bleo/PBS; log2)
Day
Protein Ratio (Bleo/PBS; log2)
Compliance Ratio (Bleo/PBS)
day 3
day 14
day 28
day 56
Slope: −1.06
Pearson r: −0.31
p−value: 0.213

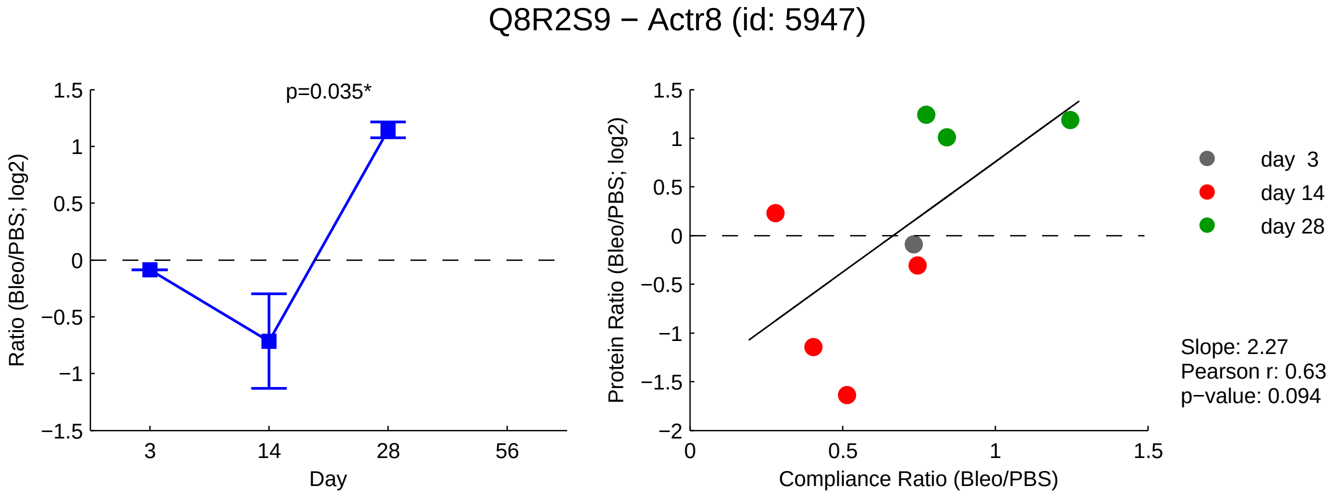
Q8R2S9 − Actr8 (id: 5947)
p=0.035*
Ratio (Bleo/PBS; log2)
Day
Protein Ratio (Bleo/PBS; log2)
Compliance Ratio (Bleo/PBS)
day 3
day 14
day 28
Slope: 2.27
Pearson r: 0.63
p−value: 0.094

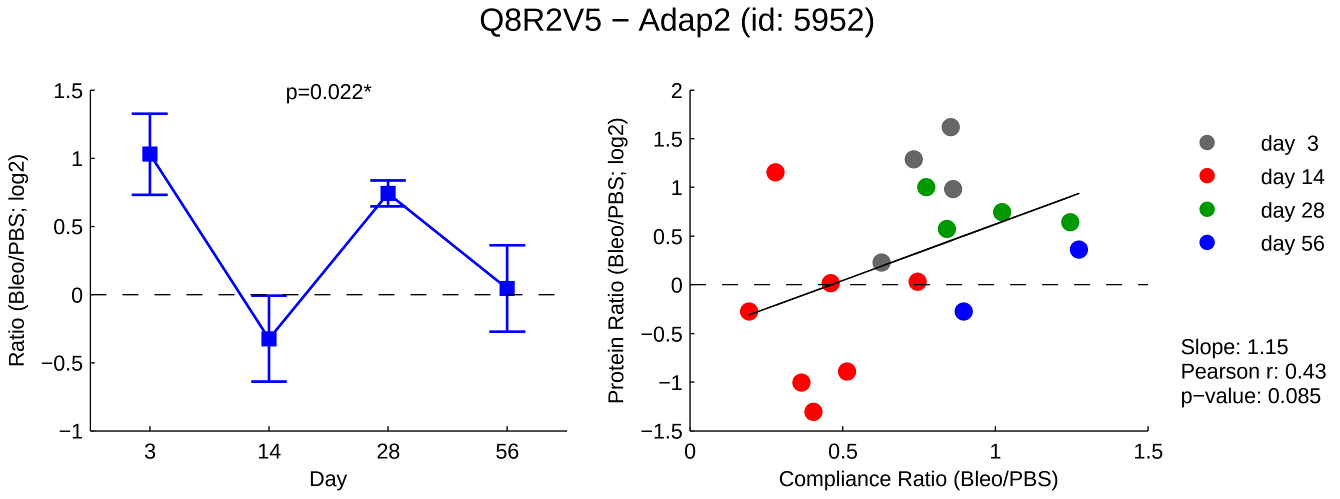
Q8R2V5 − Adap2 (id: 5952)
p=0.022*
Ratio (Bleo/PBS; log2)
Day
Protein Ratio (Bleo/PBS; log2)
Compliance Ratio (Bleo/PBS)
day 3
day 14
day 28
day 56
Slope: 1.15
Pearson r: 0.43
p−value: 0.085

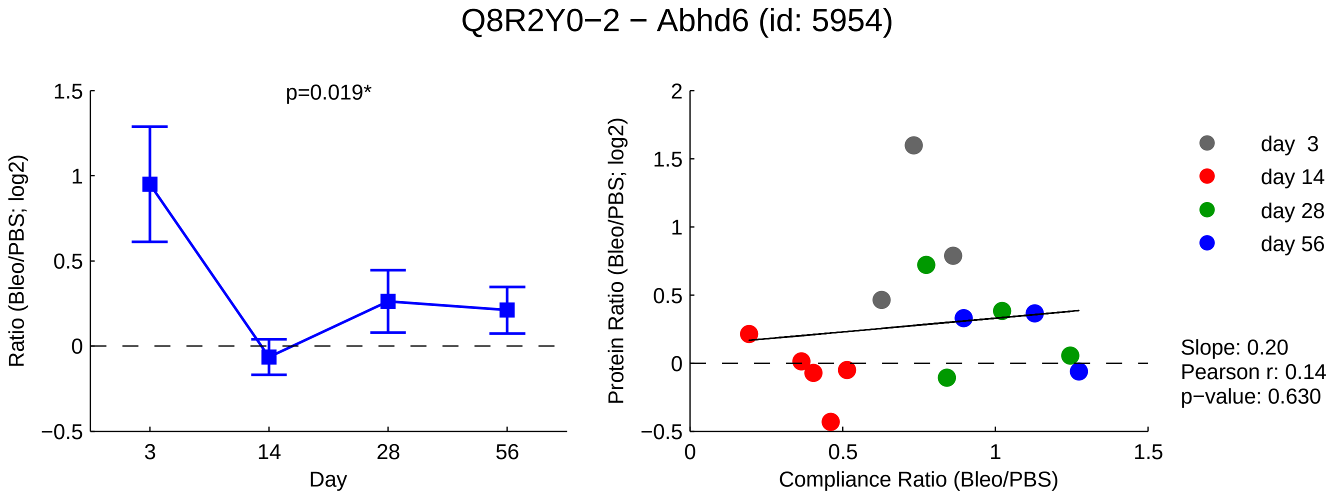
Q8R2Y0−2 − Abhd6 (id: 5954)
p=0.019*
Ratio (Bleo/PBS; log2)
Day
Protein Ratio (Bleo/PBS; log2)
Compliance Ratio (Bleo/PBS)
day 3
day 14
day 28
day 56
Slope: 0.20
Pearson r: 0.14
p−value: 0.630

Q8R2Y2 − Mcam (id: 5955)

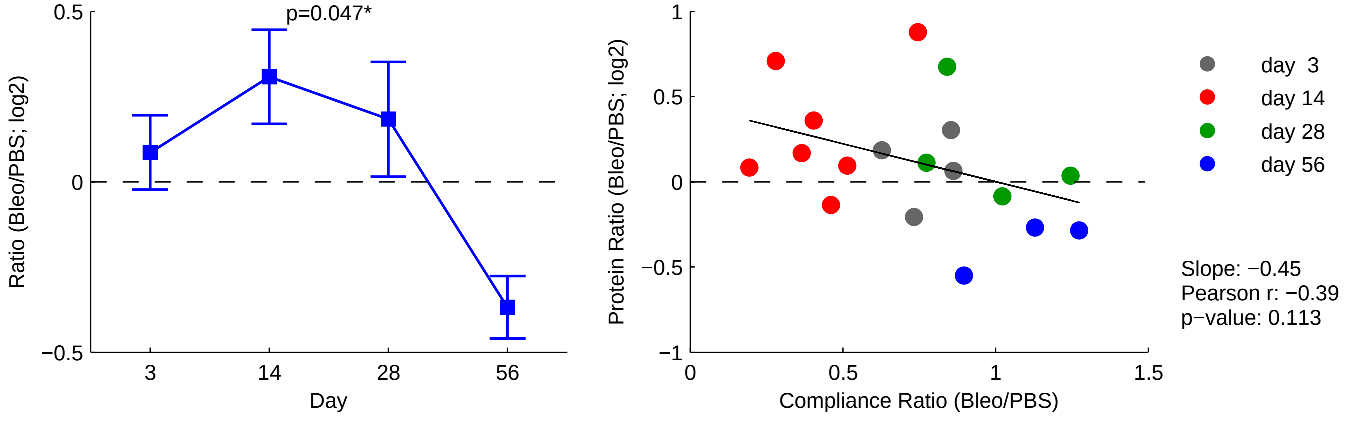

Q8R2Y8 − Ptrh2 (id: 5957)

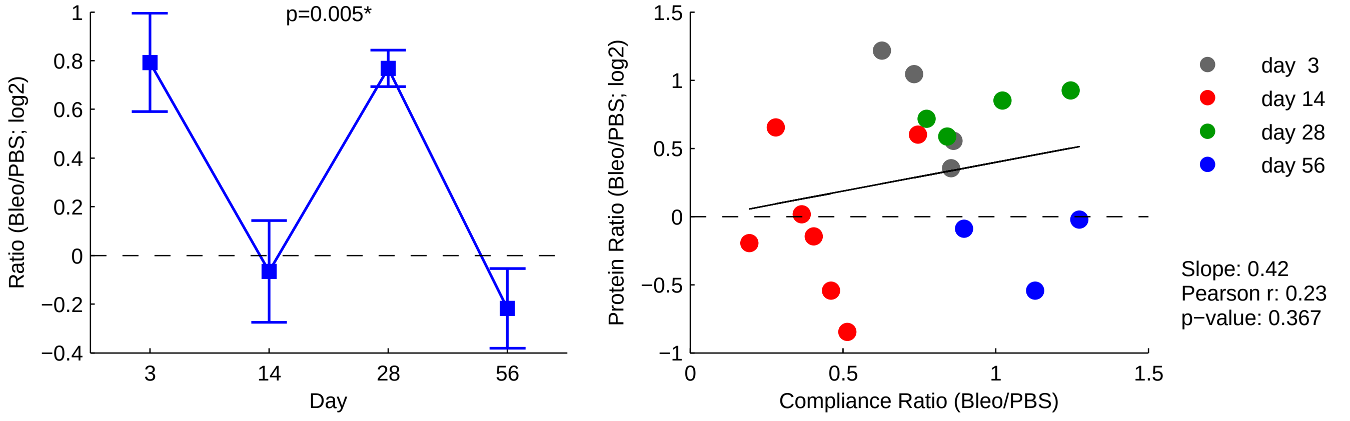

Q8R2Z0 − Fam132a (id: 5958)

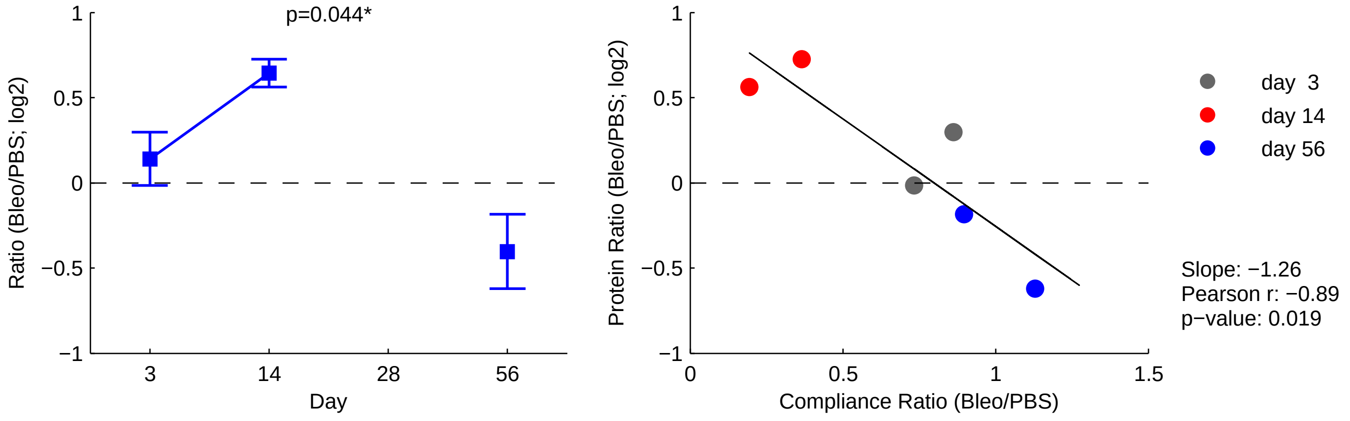

Q8R310−2 − Tmcc3 (id: 5961)

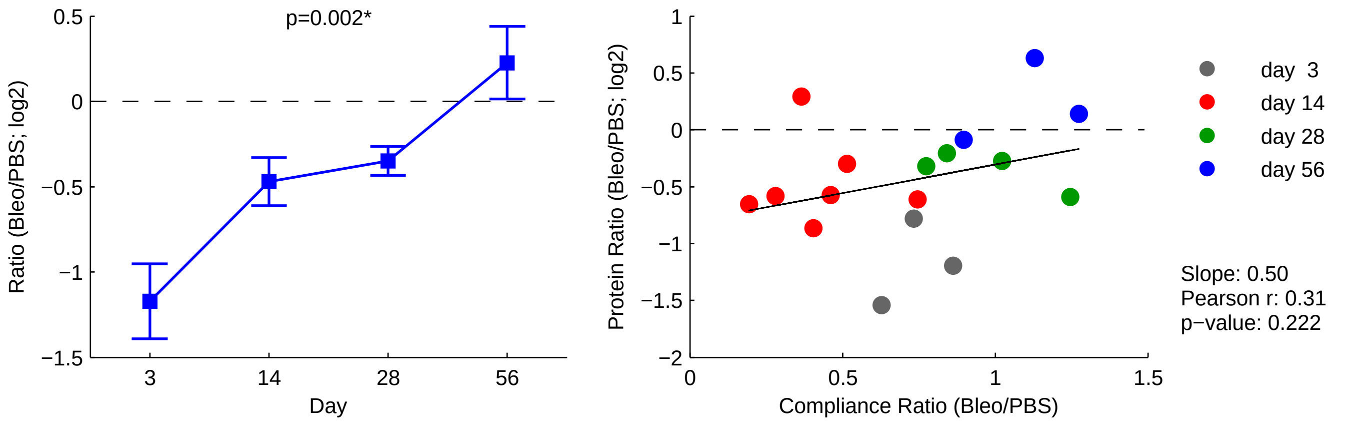

Q8R317−2 − Ubqln1 (id: 5962)

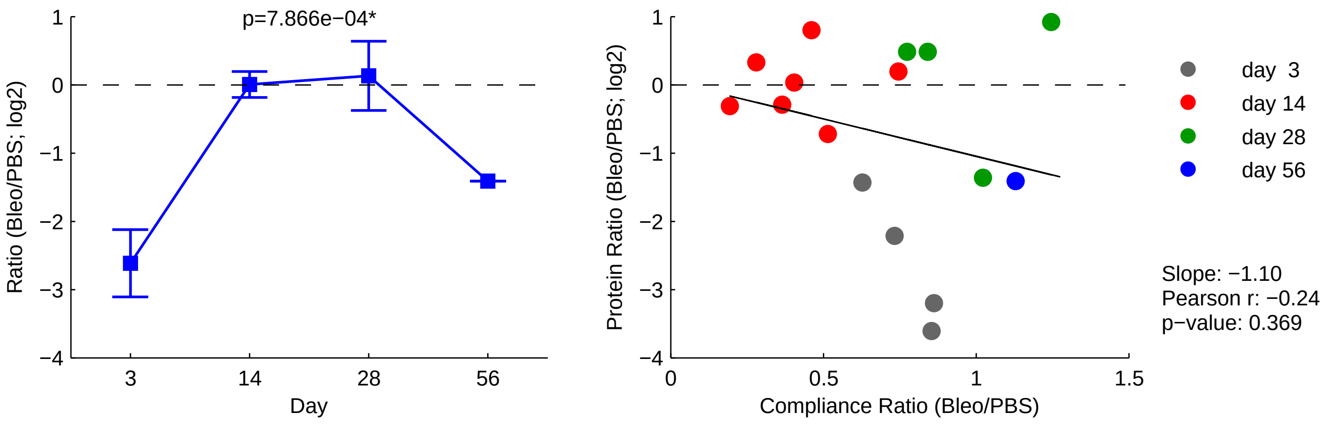

Q8R322 − Gle1 (id: 5963)

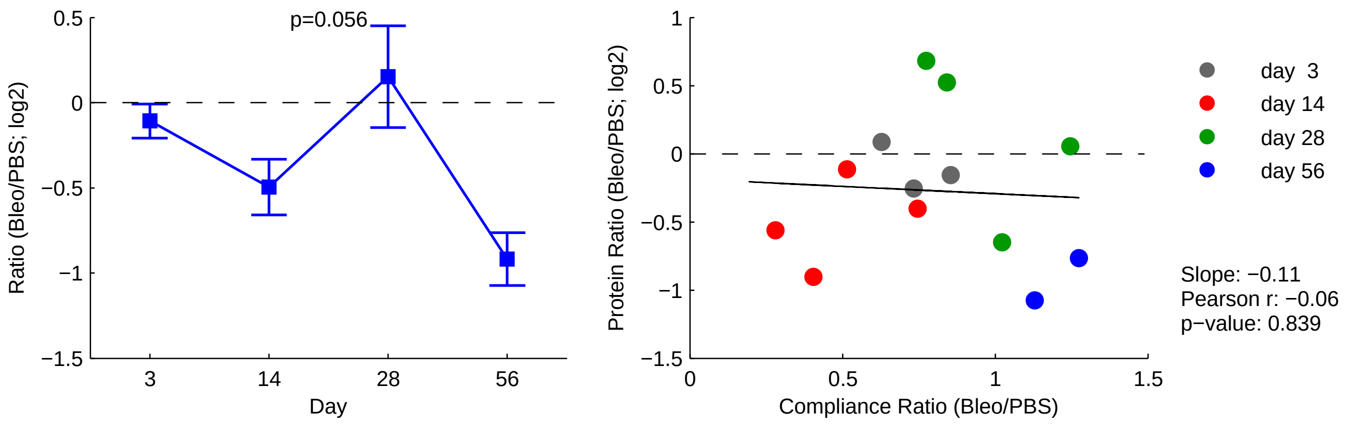

Q8R323 − Rfc3 (id: 5964)

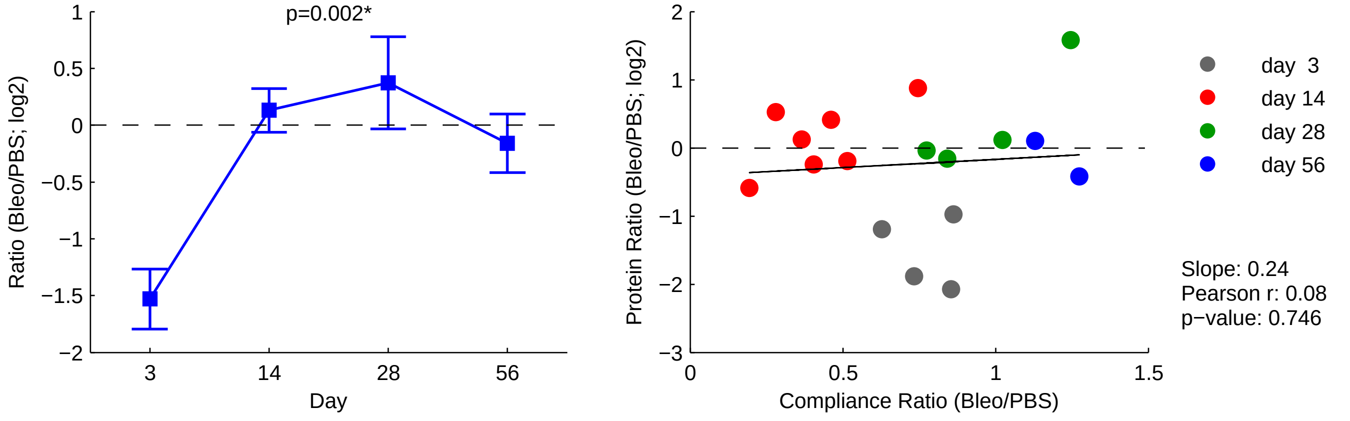

Q8R326−2 − Pspc1 (id: 5965)

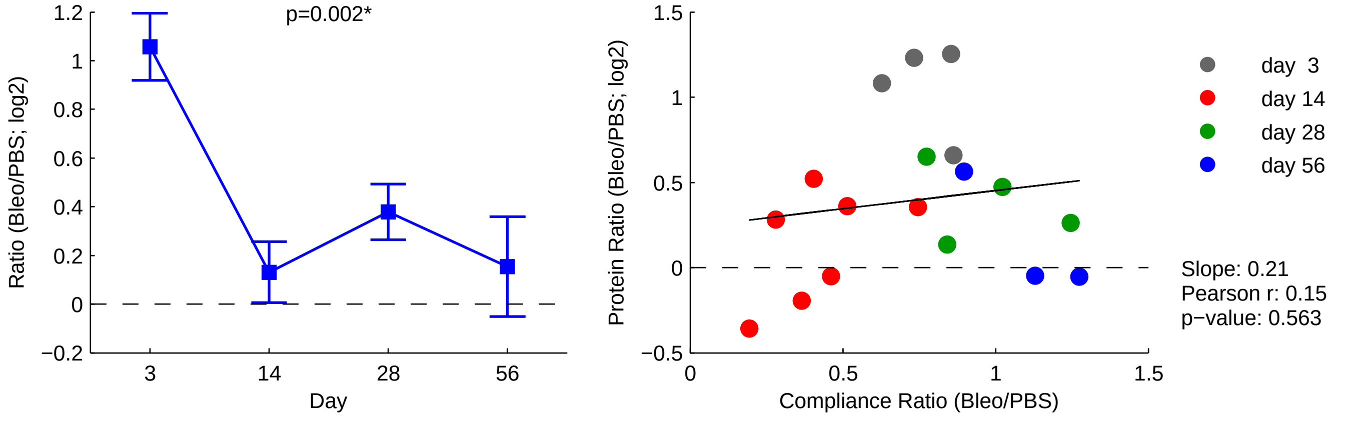

Q8R361 − Rab11fip5 (id: 5969)

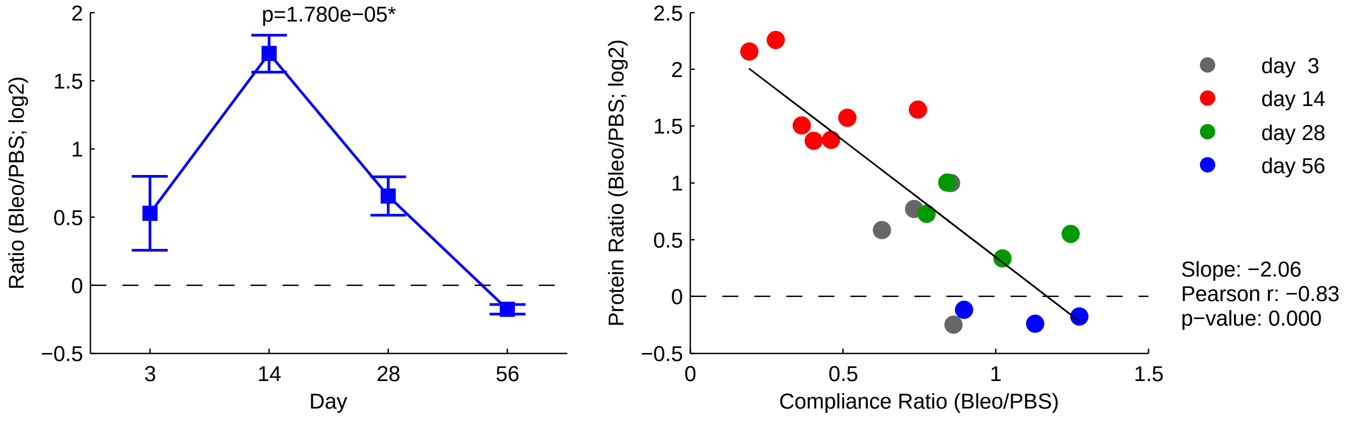

Q8R370 − Ushbp1 (id: 5970)

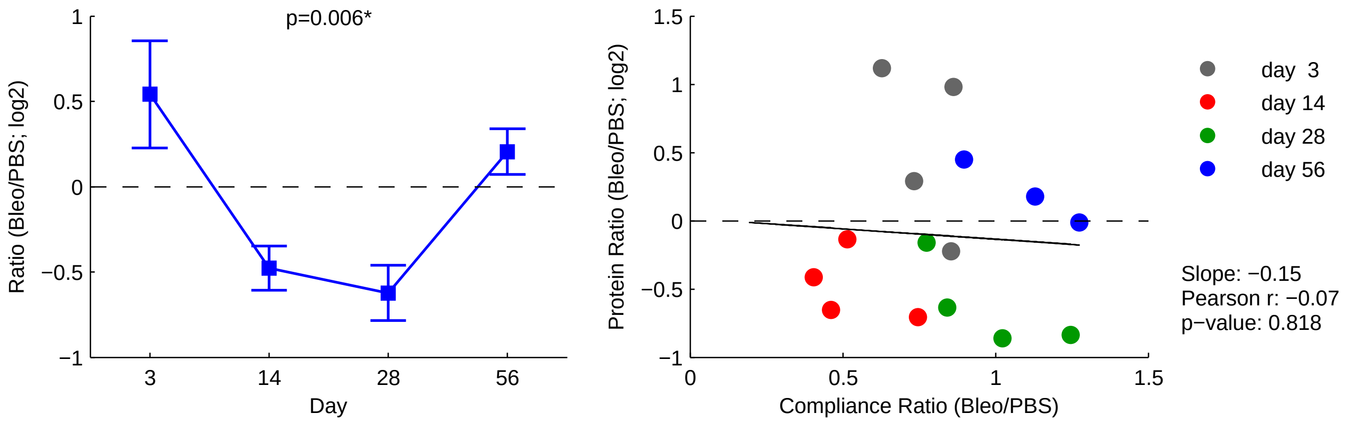

Q8R395 − Commd5 (id: 5972)

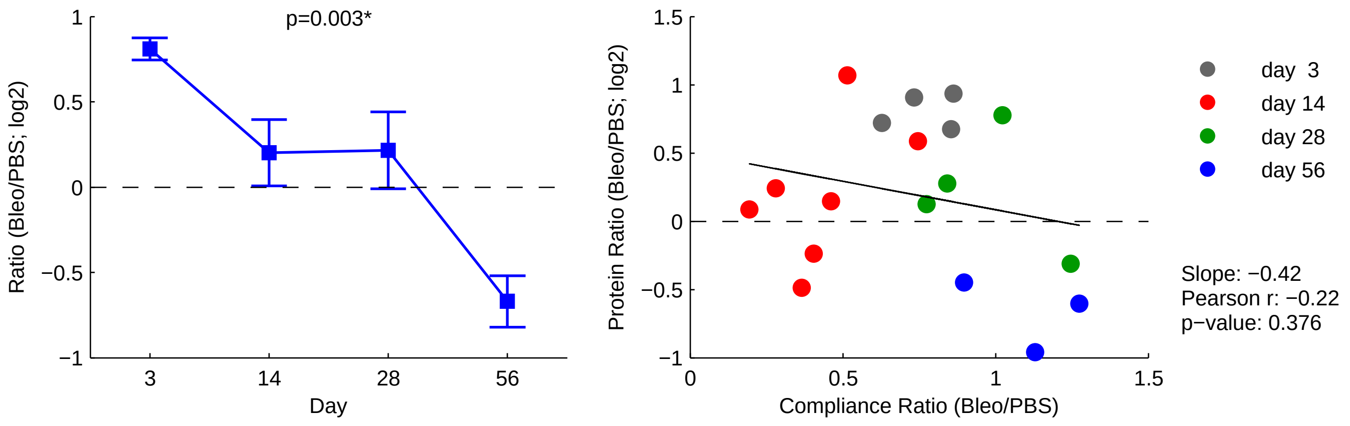

Q8R3G9 − Tspan8 (id: 5979)

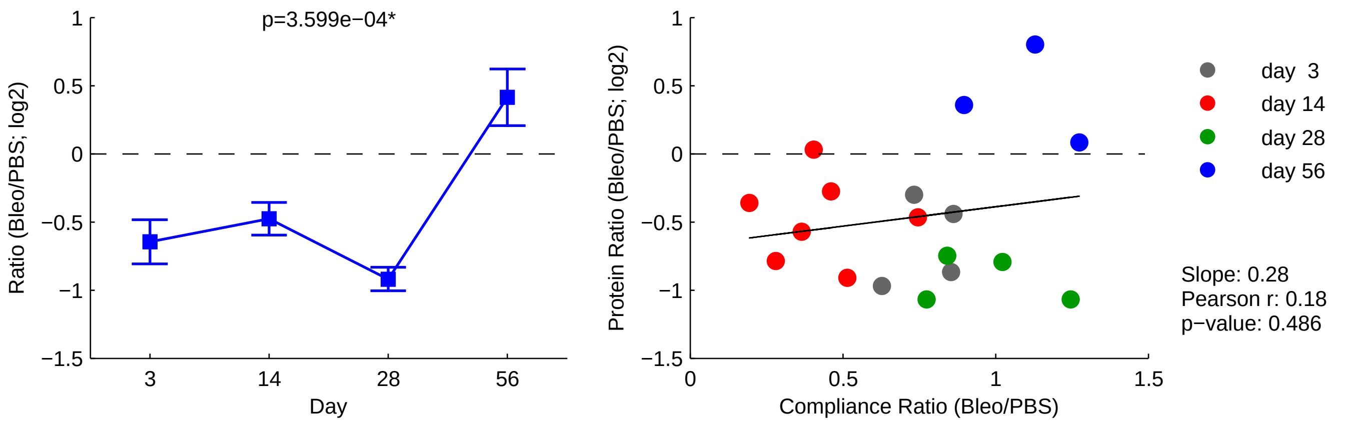

Q8R3H7 − Hs2st1 (id: 5980)

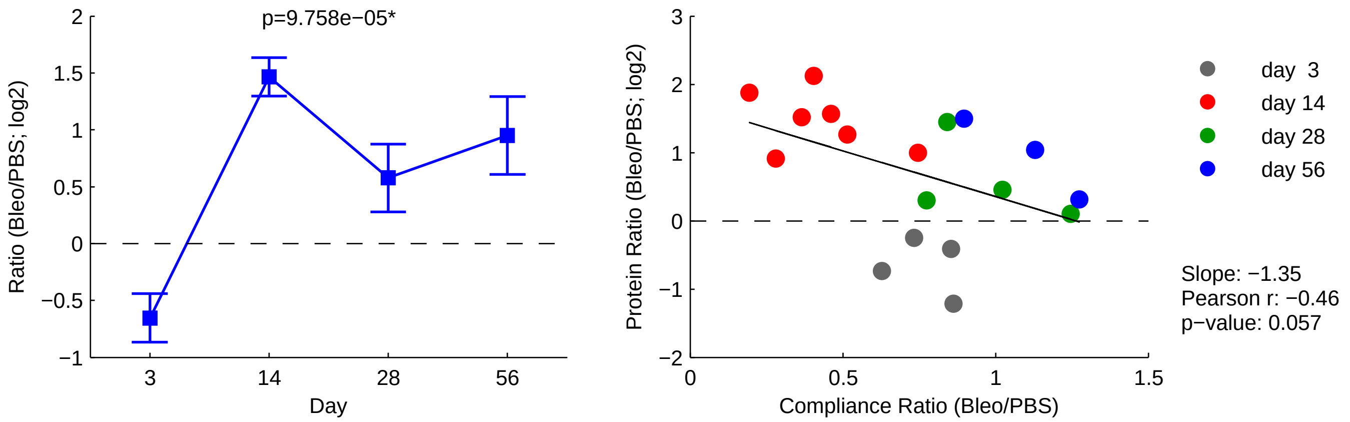

Q8R3L2−4 − Tcf25 (id: 5983)

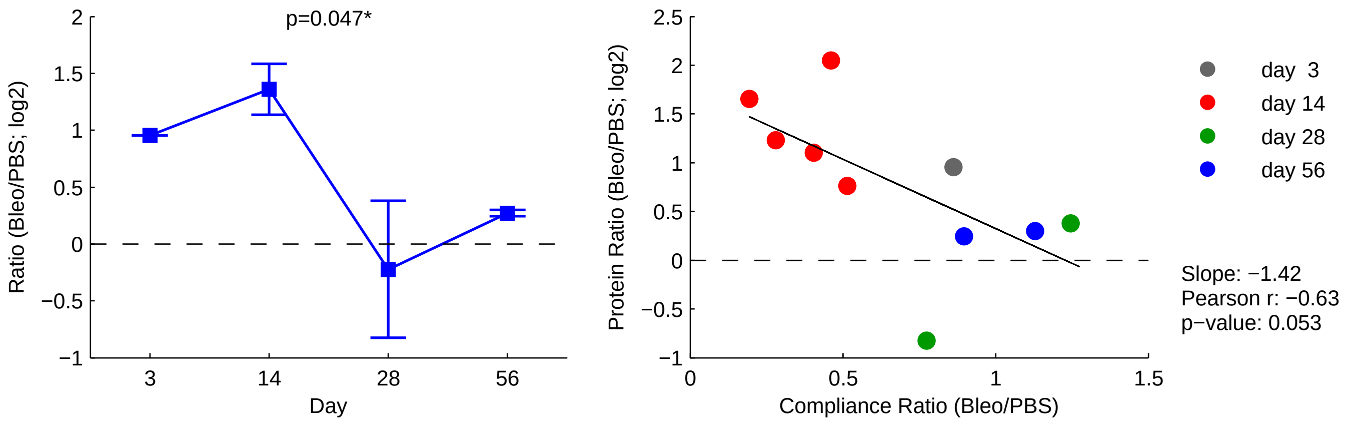

Q8R3L5−2 − Slco3a1 (id: 5984)

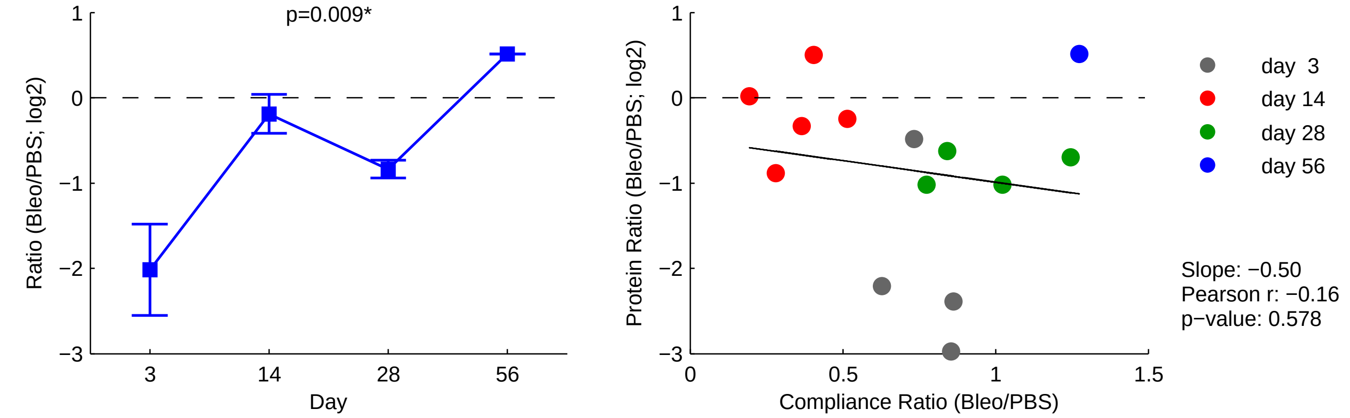

Q8R3Q0 − Tmem66 (id: 5988)

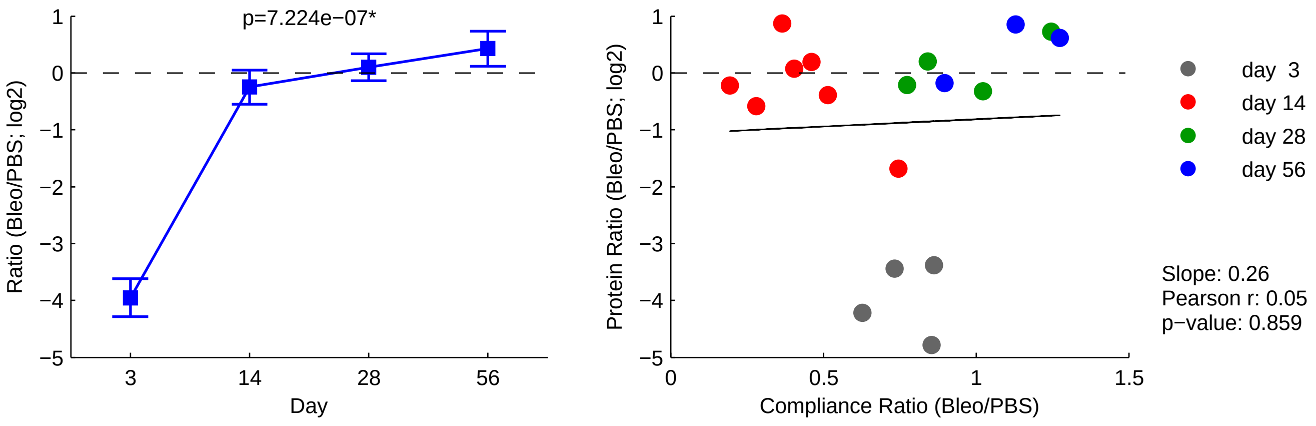

Q8R3V5−1 − Sh3glb2 (id: 5989)

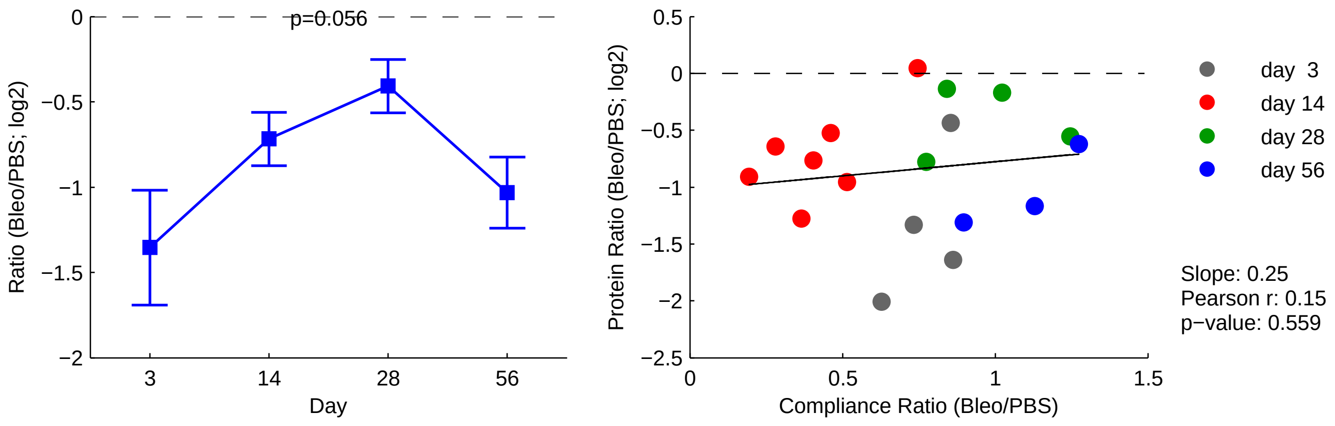

Q8R3W7 − Agr3 (id: 5990)

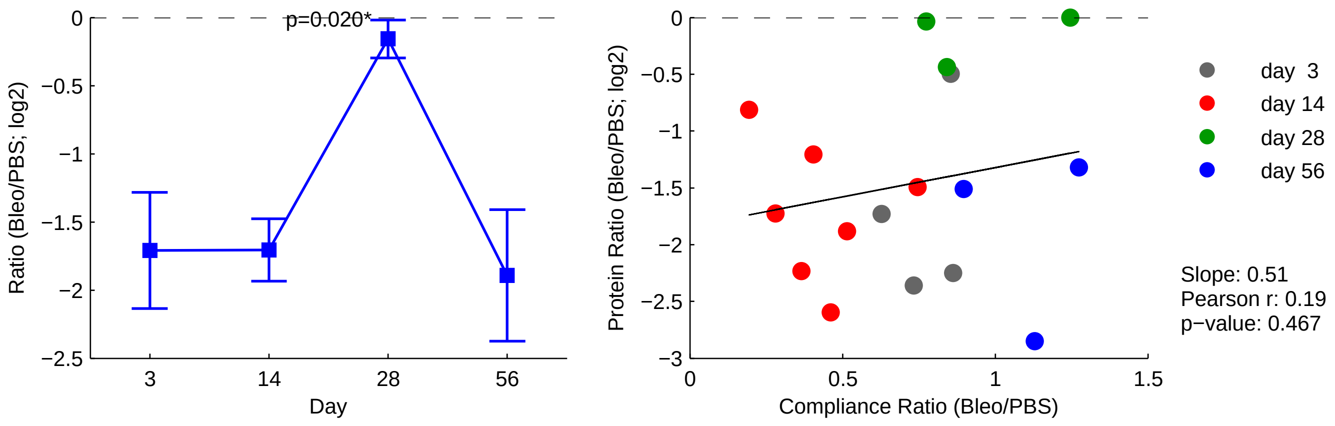

Q8R3Y8−2 − Irf2bp1 (id: 5991)

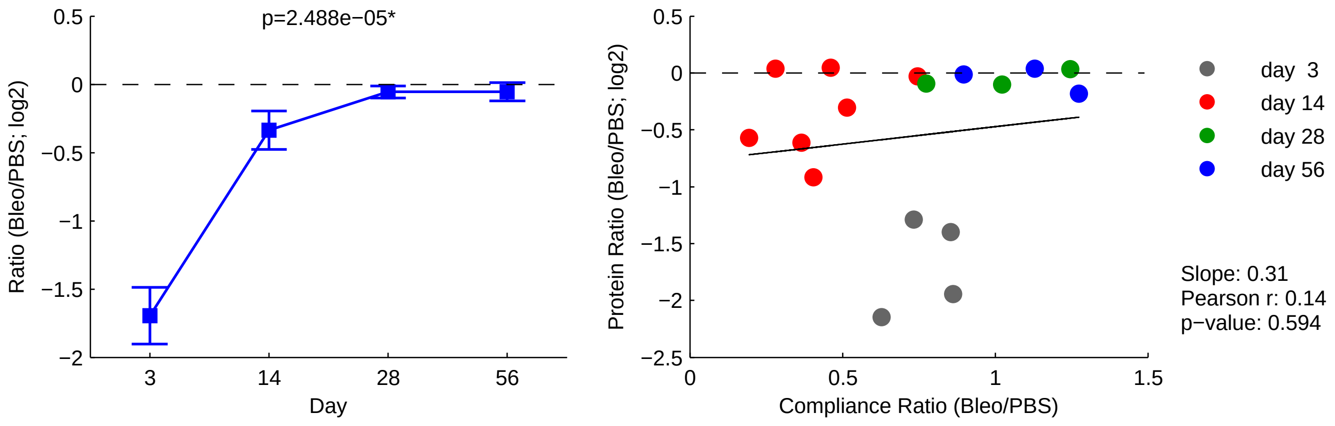

Q8R404 − Qil1 (id: 5992)

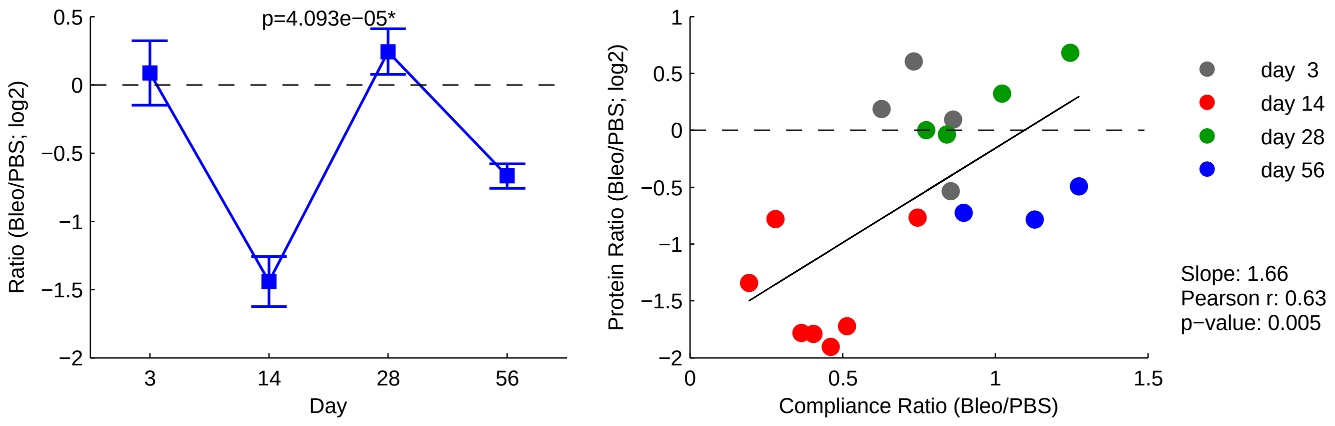

Q8R411 − Myct1 (id: 5994)

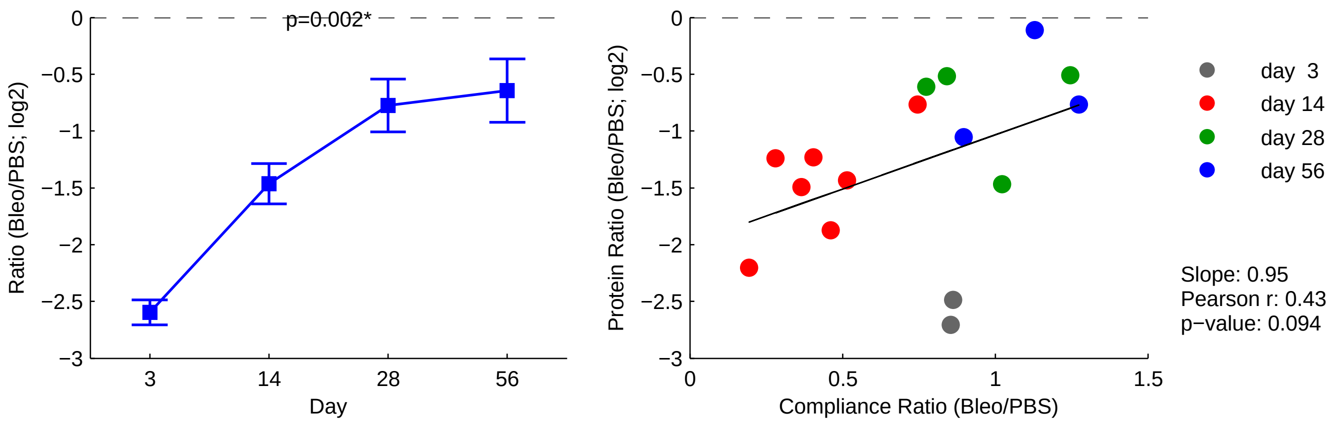

Q8R420 − Abca3 (id: 5995)

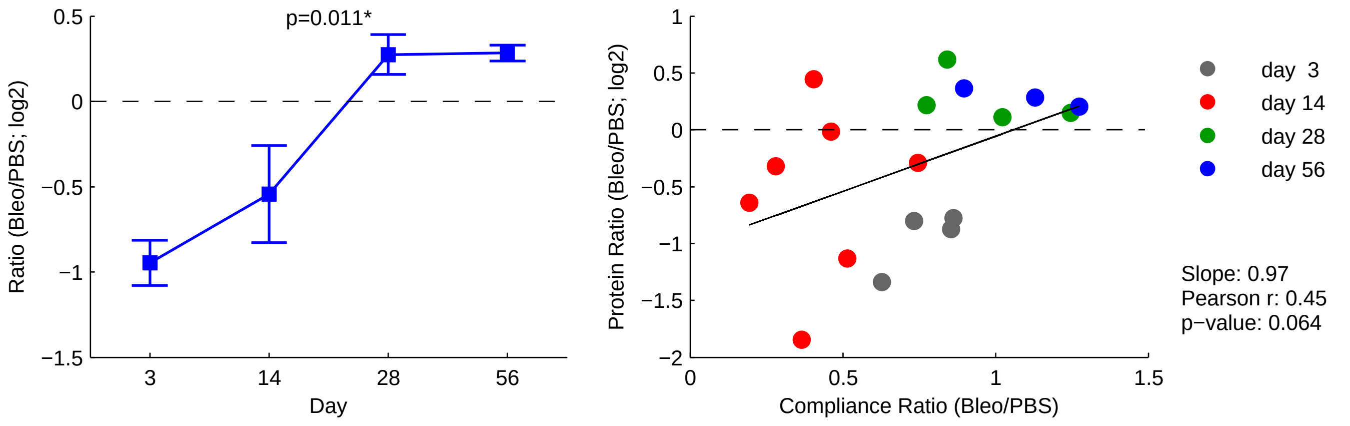

Q8R429 − Atp2a1 (id: 5997)

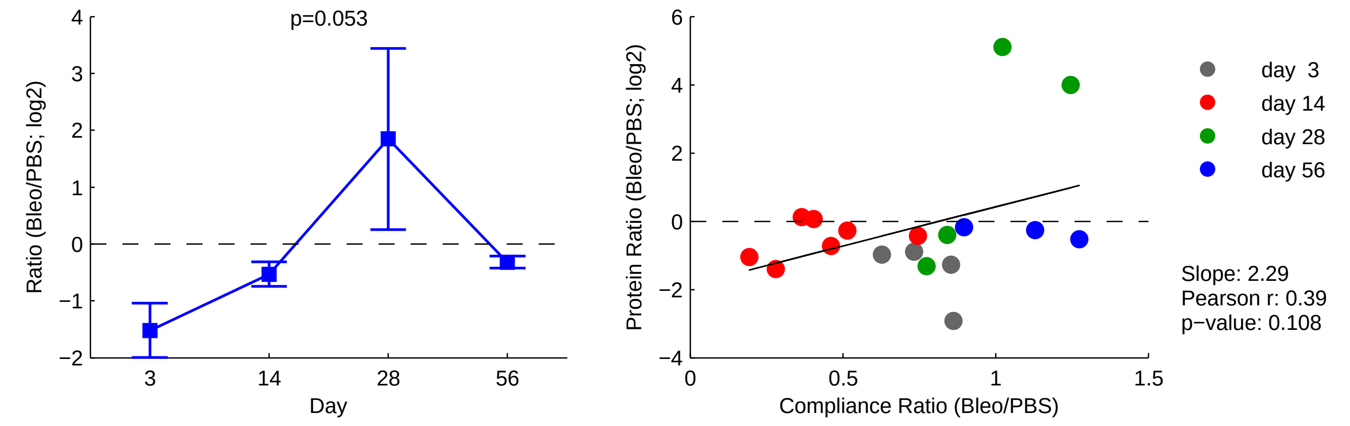

Q8R4N0 − Clybl (id: 6003)

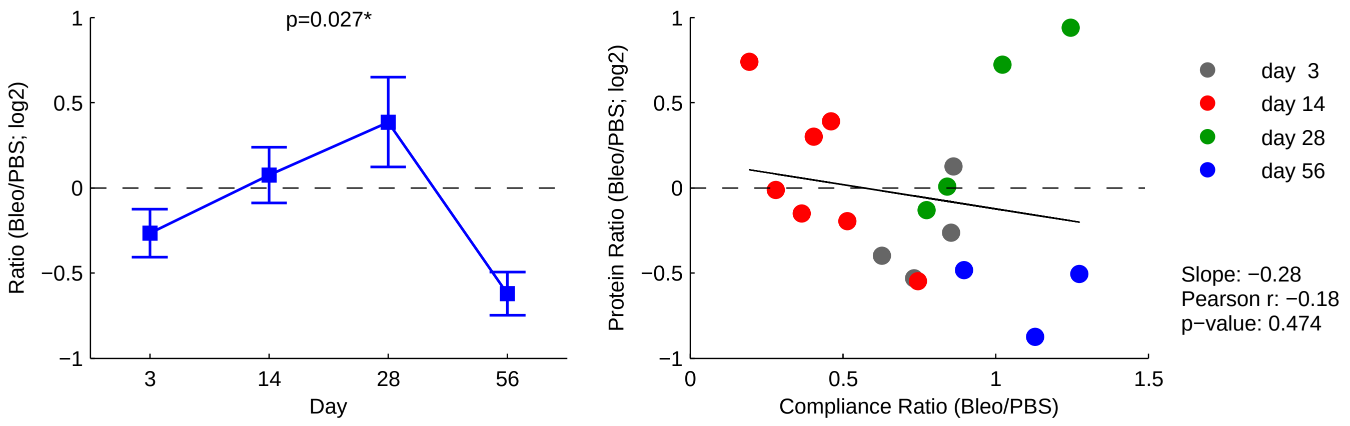

Q8R4R6 − Nup35 (id: 6004)

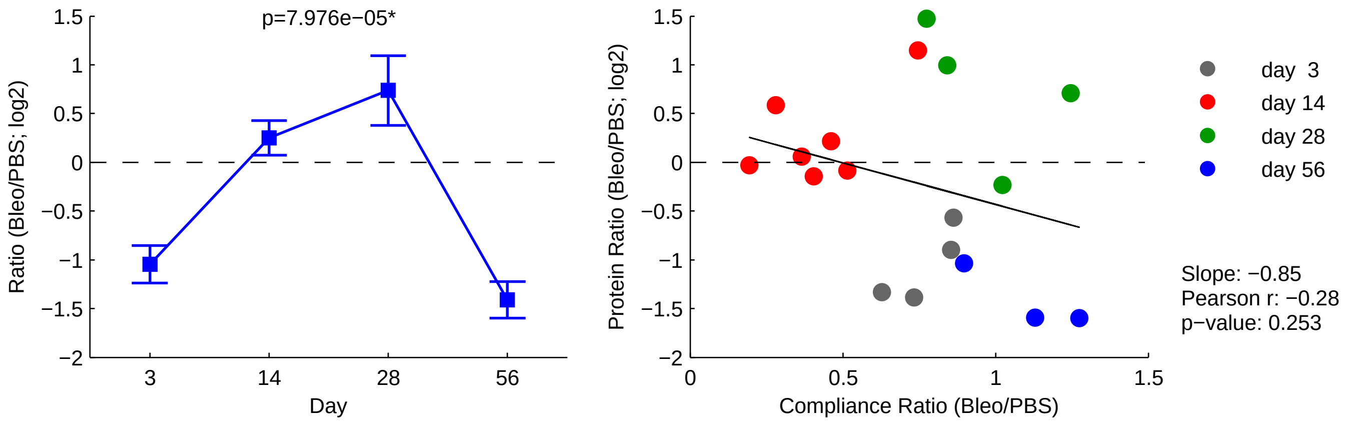

Q8R4U7 − Luzp1 (id: 6006)

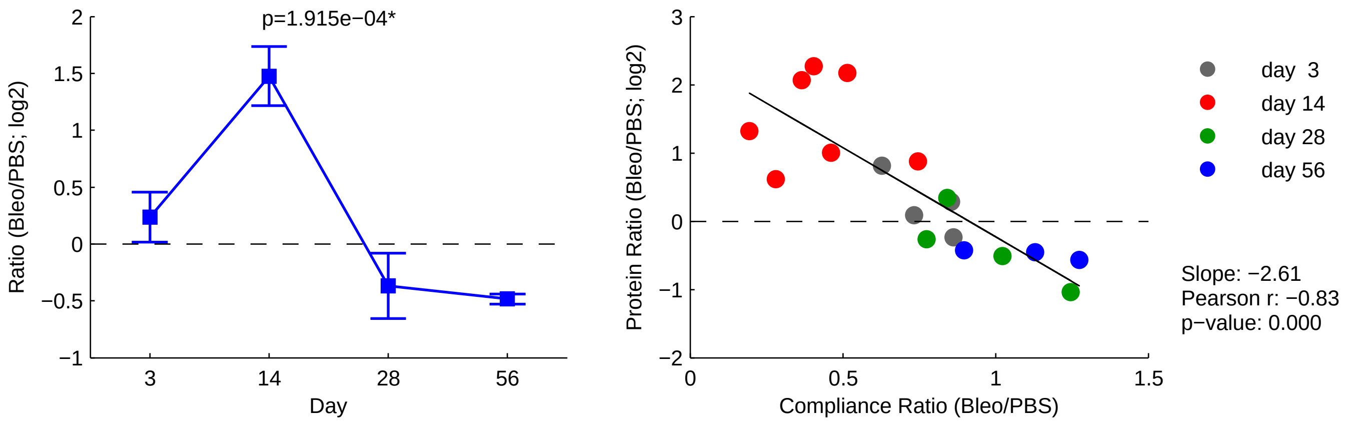

Q8R4W6 − Pcolce2 (id: 6008)

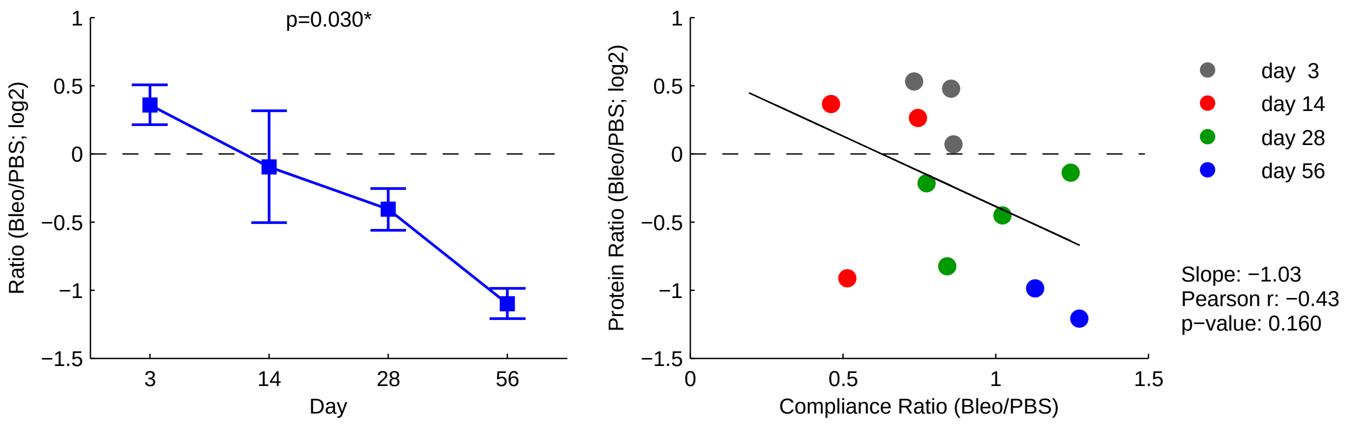

Q8R502 − Lrrc8c (id: 6010)

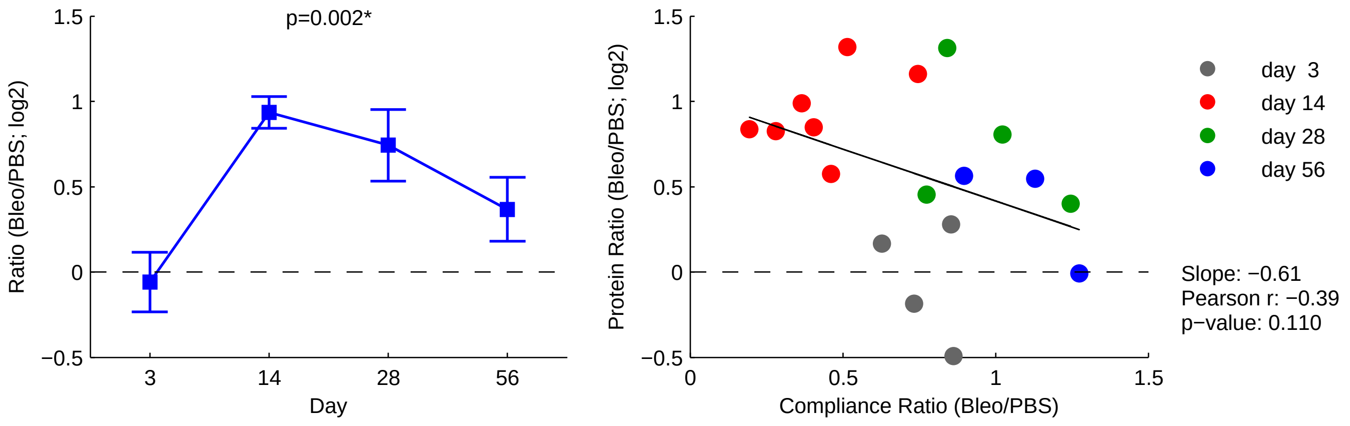

Q8R550−2 − Sh3kbp1 (id: 6013)

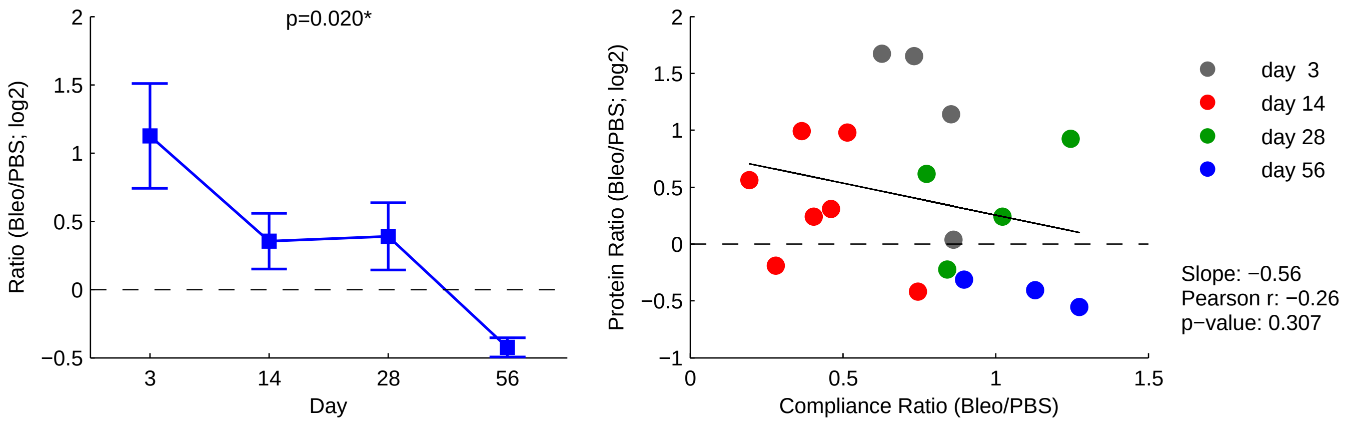

Q8R570 − Snap47 (id: 6015)

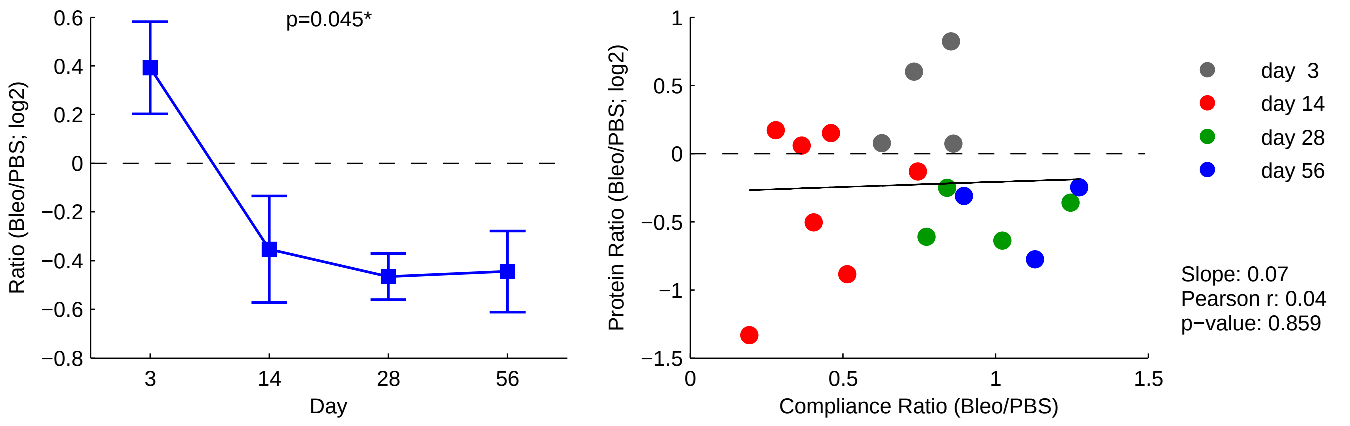

Q8R574 − Prpsap2 (id: 6016)

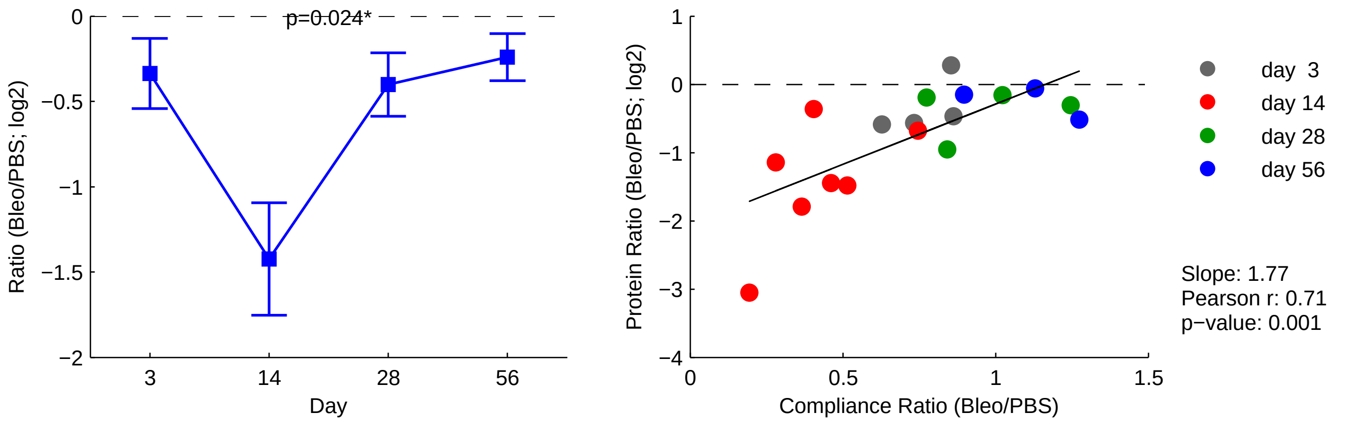

Q8R5F7−2 − Ifih1 (id: 6022)

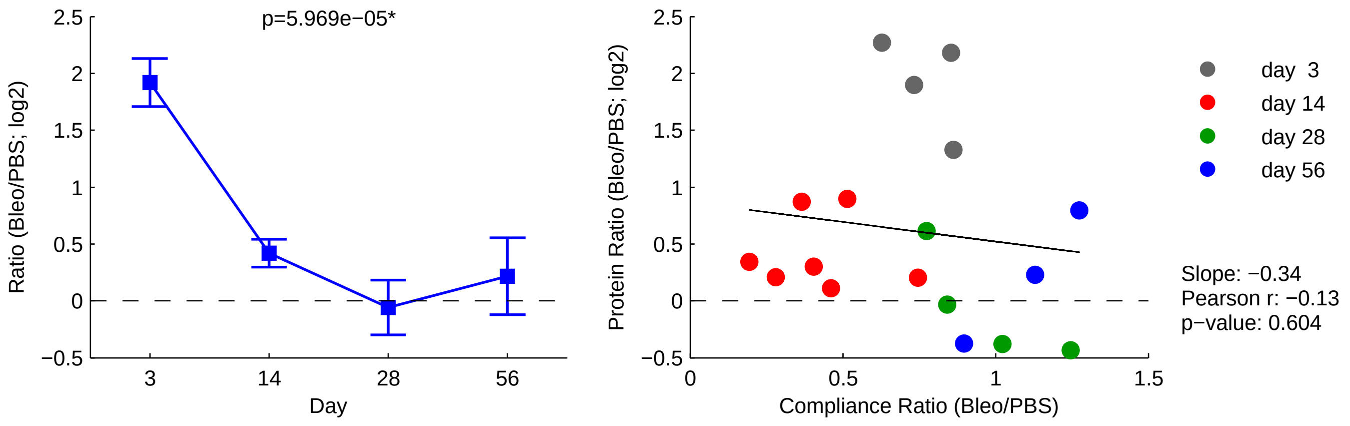

Q8R5F8 − Eps8l1 (id: 6023)

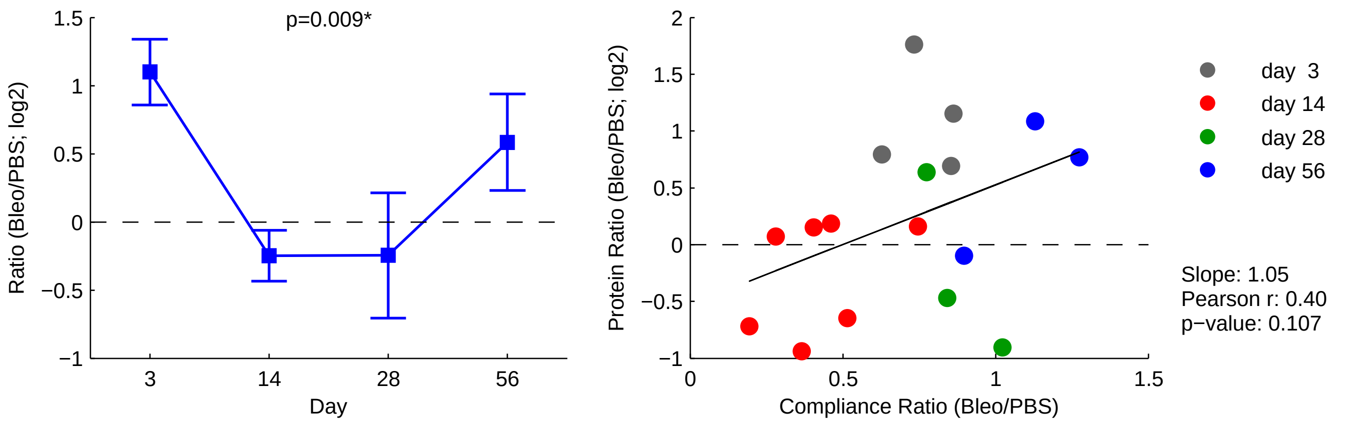

Q8R5G7−2 − Arap3 (id: 6024)

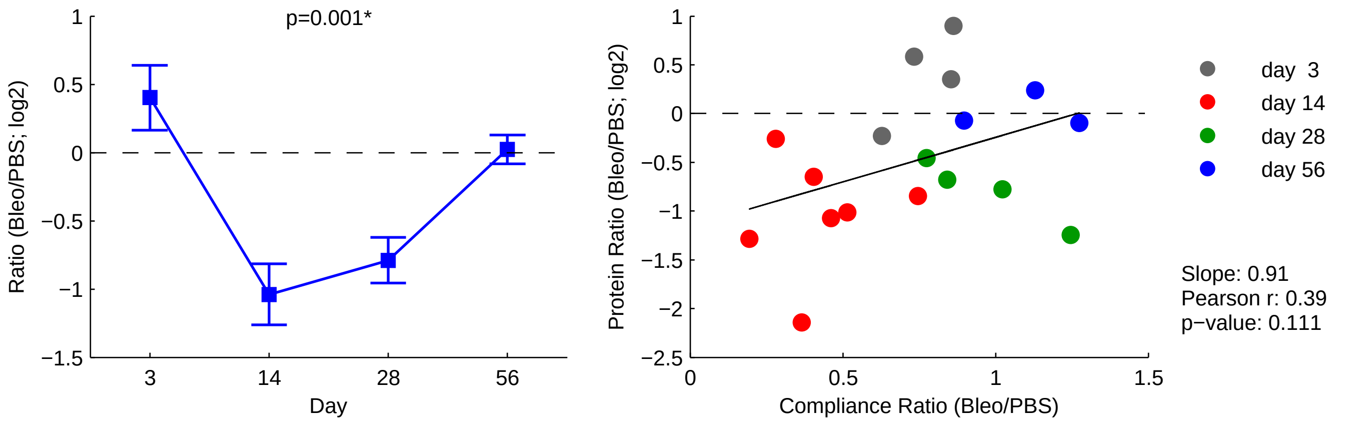

Q8R5J9 − Arl6ip5 (id: 6026)

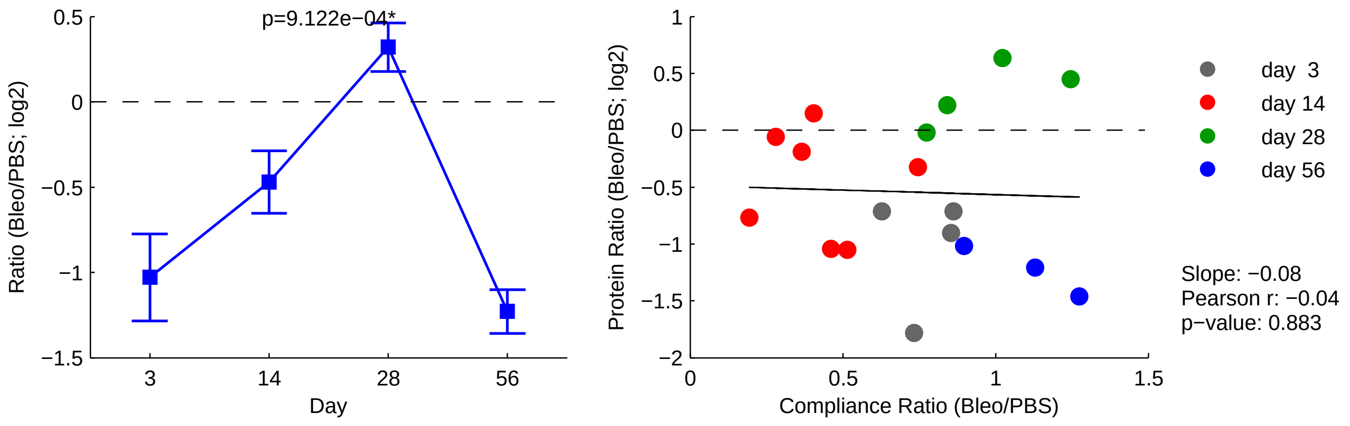

Q8R5K2−2 − Usp33 (id: 6027)

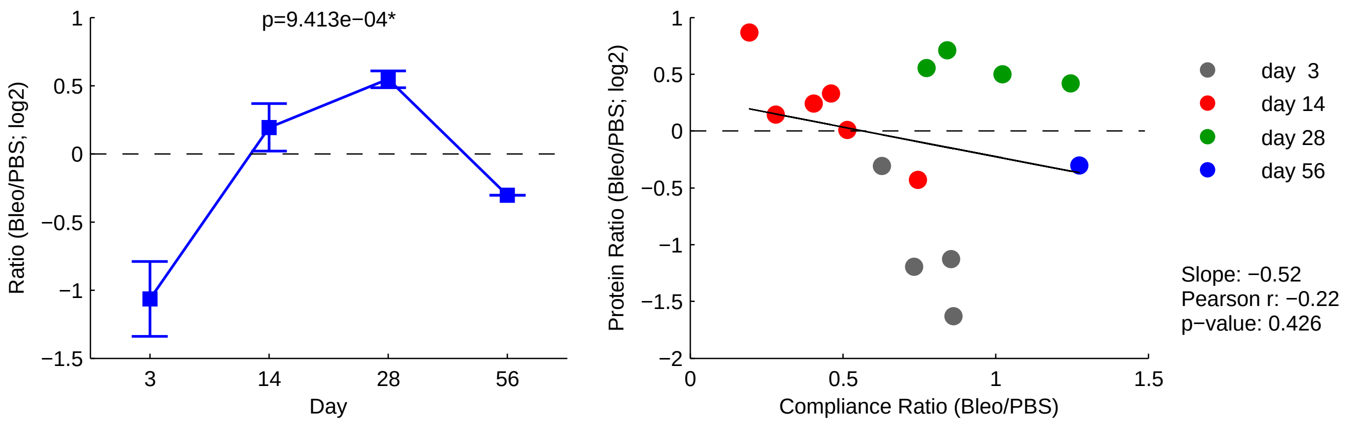

Q8R5L1 − C1qbp (id: 6029)

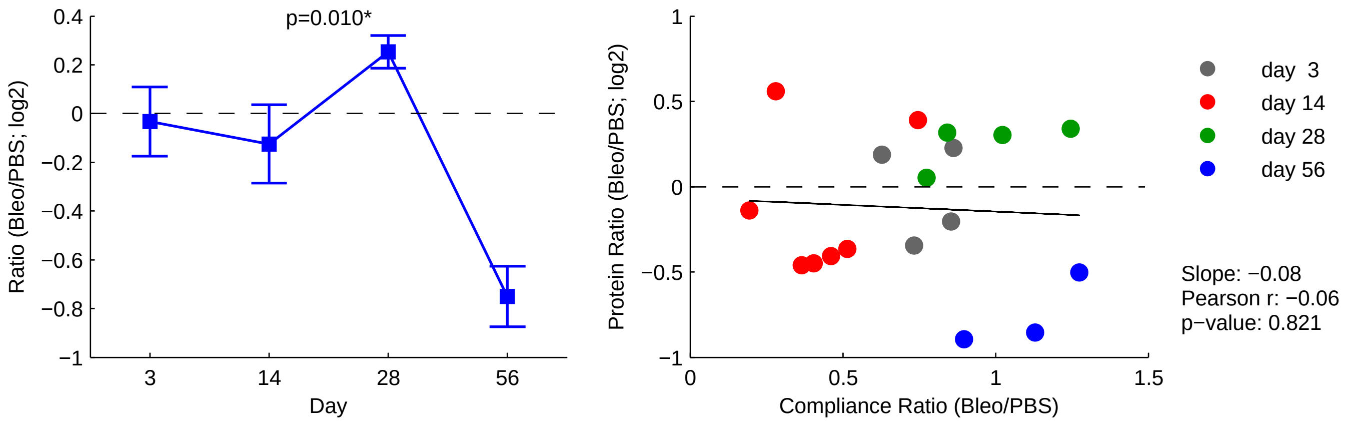

Q8R5L3−2 − Vps39 (id: 6030)

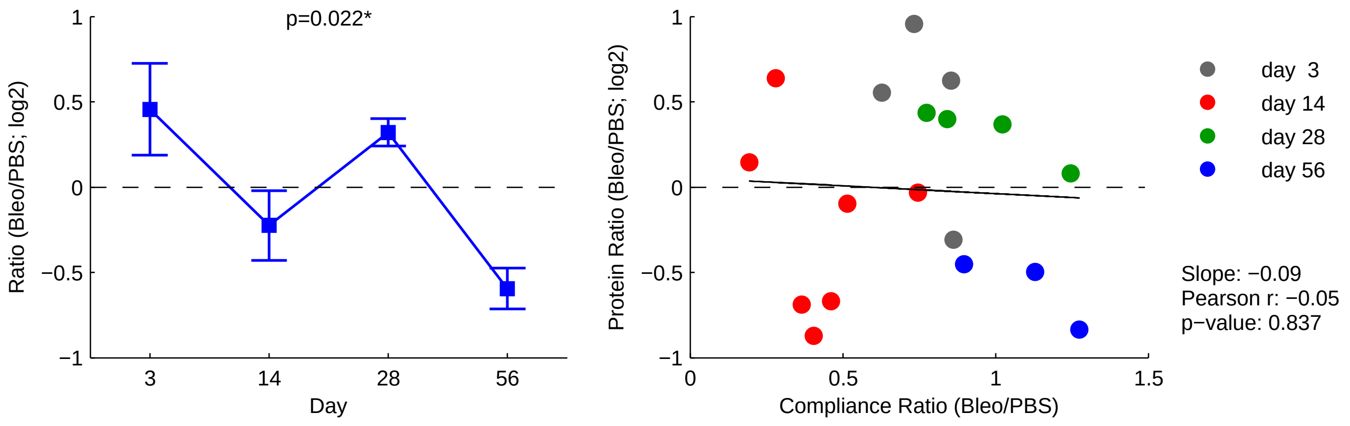

Q8VBV7 − Cops8 (id: 6034)

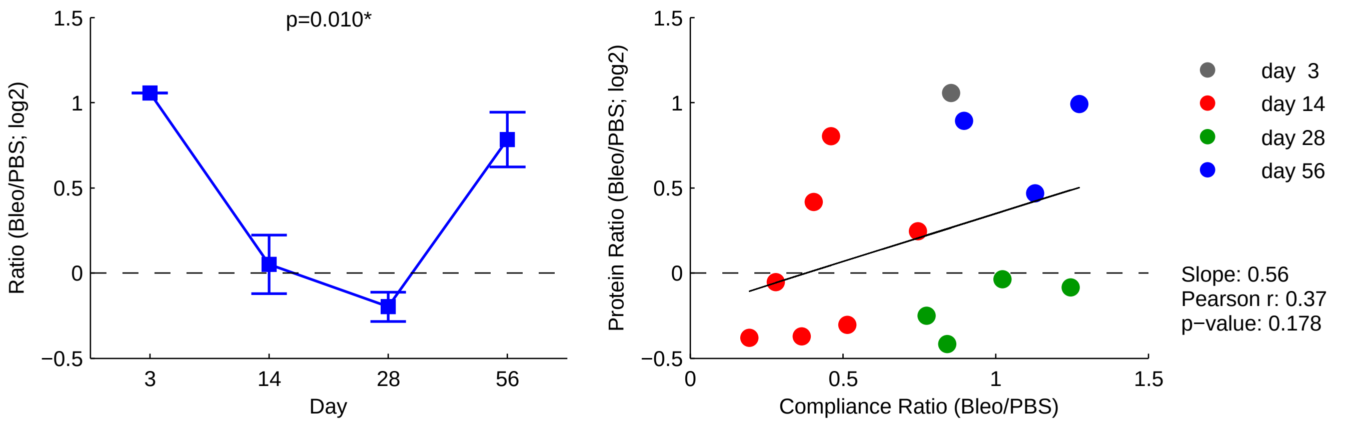

Q8VBZ3 − Clptm1 (id: 6036)

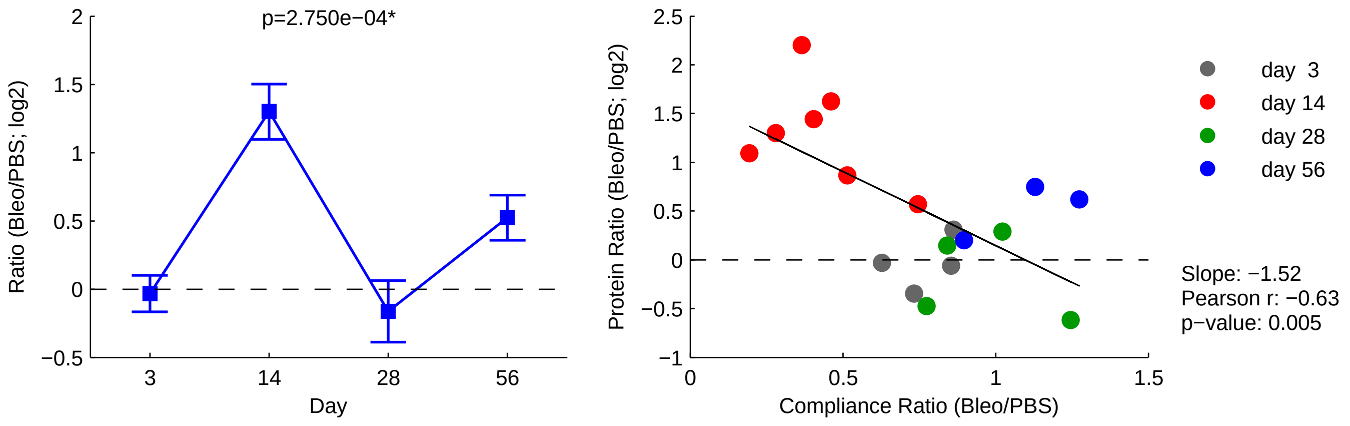

Q8VC42 − Mic1 (id: 6039)

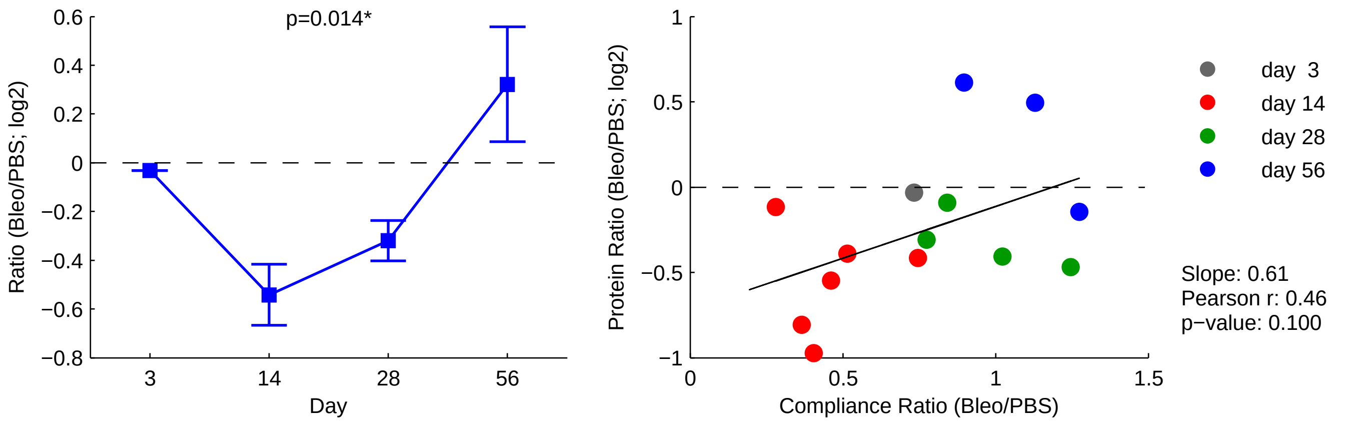

Q8VC88 − Gca (id: 6045)

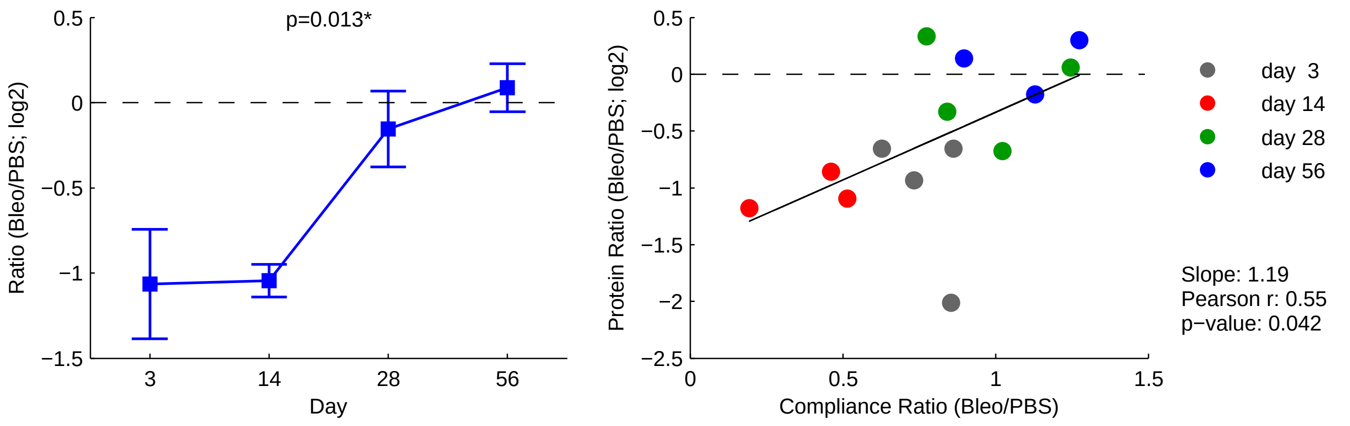

Q8VCB1 − Tmem48 (id: 6046)

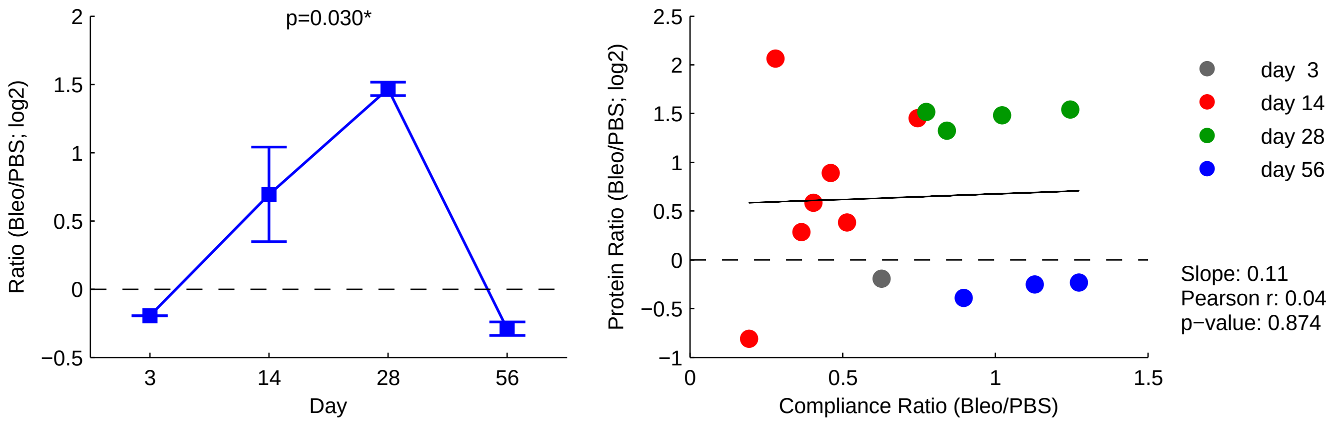

Q8VCC1 − Hpgd (id: 6048)

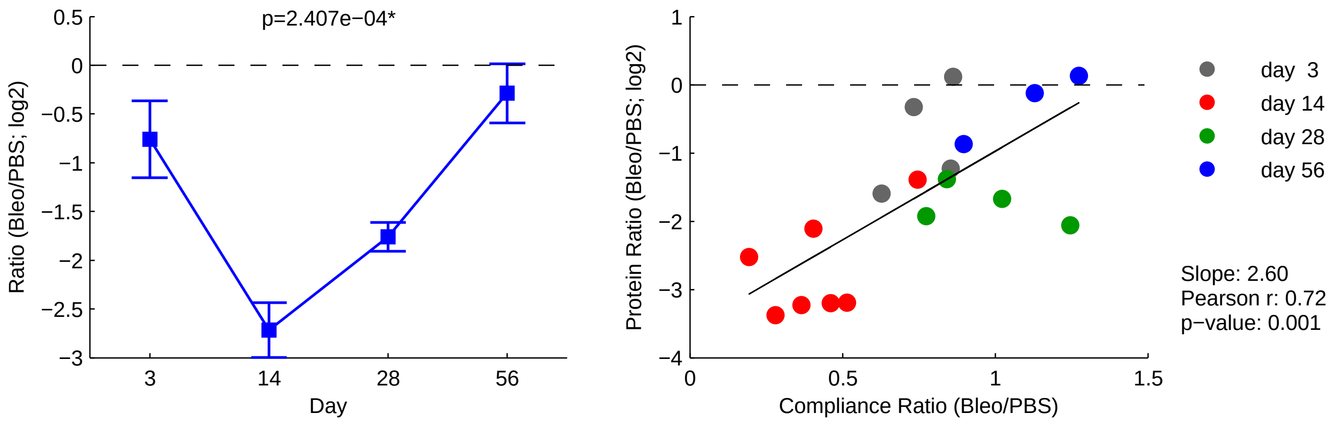

Q8VCC9 − Spon1 (id: 6050)

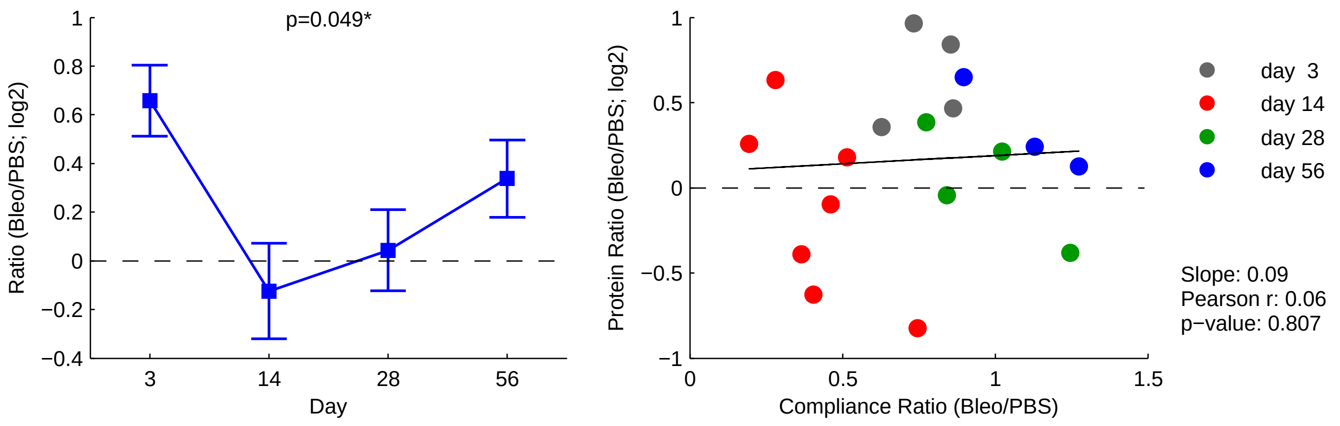

Q8VCF1 − Cant1 (id: 6053)

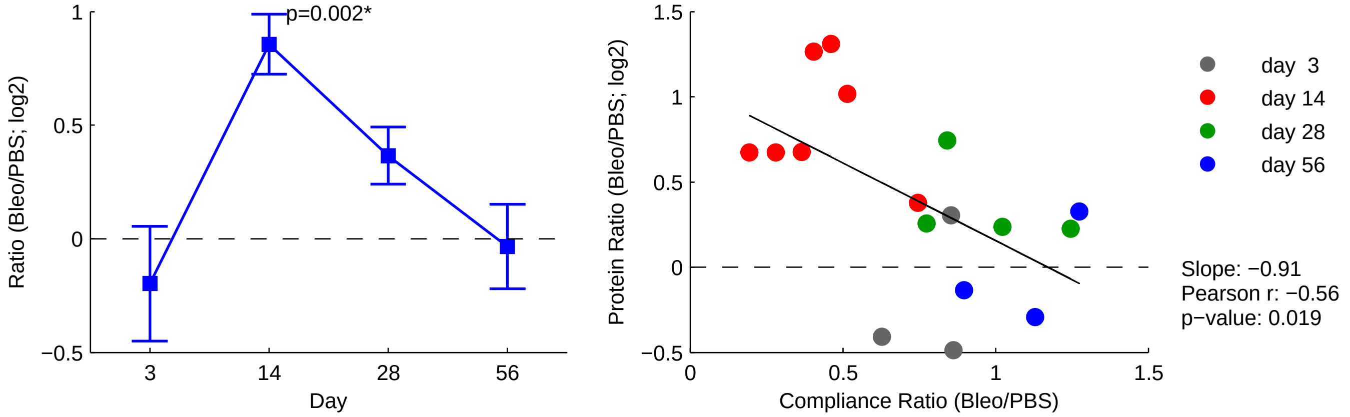

Q9CQ43 − Dut (id: 6054)

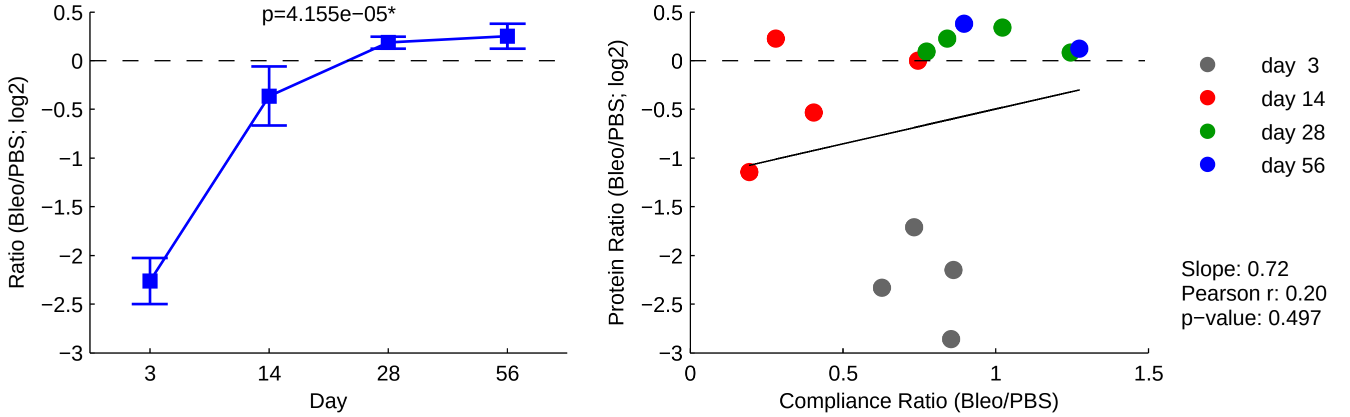

Q8VCH6 − Dhcr24 (id: 6057)

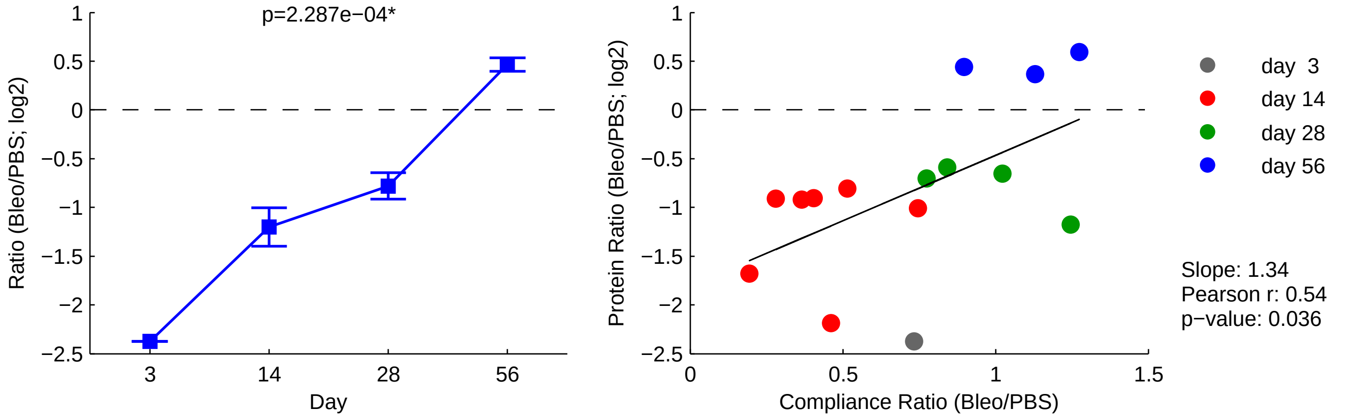

Q8VCH7 − Rdh10 (id: 6058)

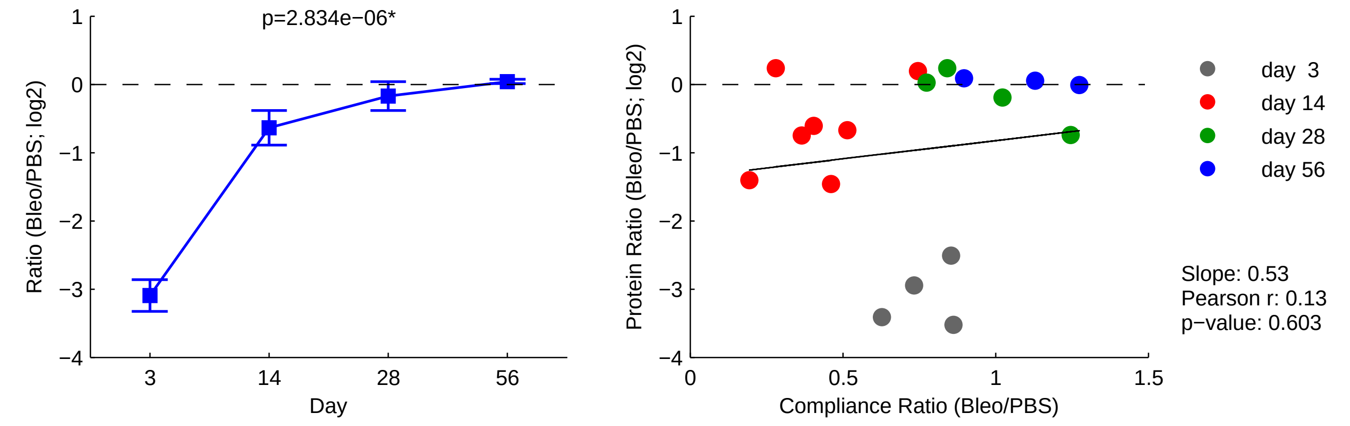

Q8VCI0 − Plbd1 (id: 6060)

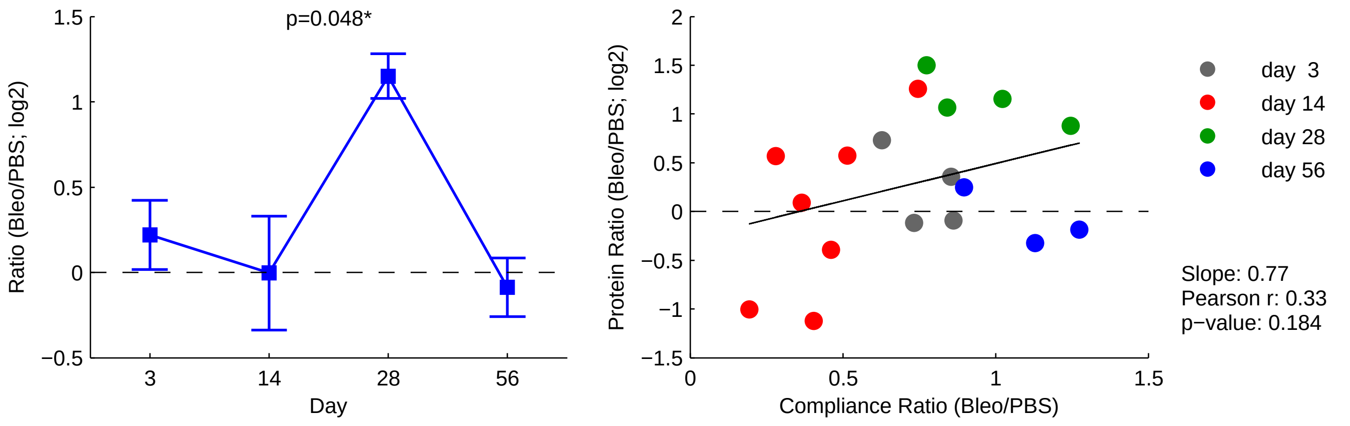

Q8VCI5 − Pex19 (id: 6061)

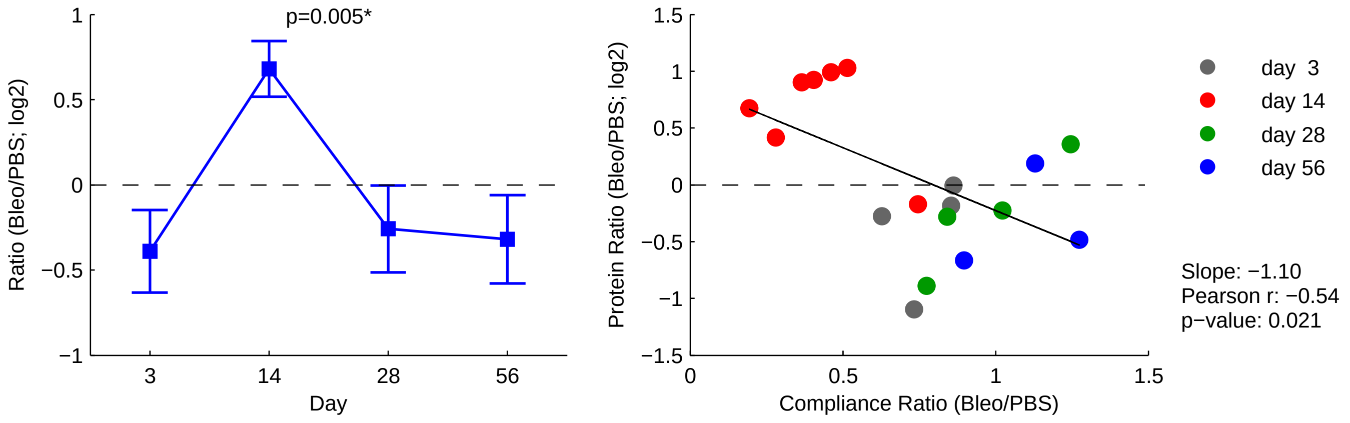

Q8VCL2 − Sco2 (id: 6063)

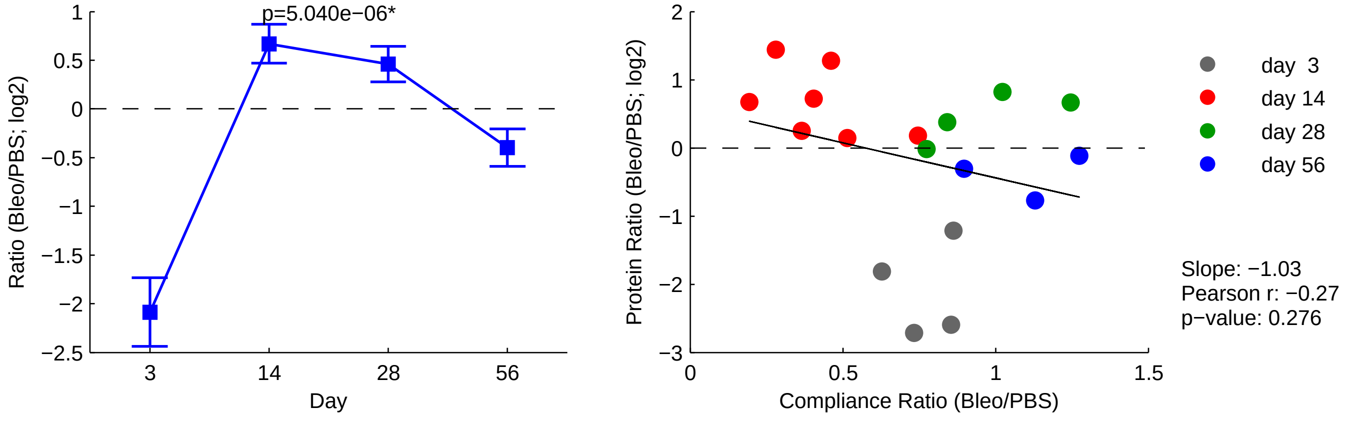

Q8VCM7 − Fgg (id: 6067)

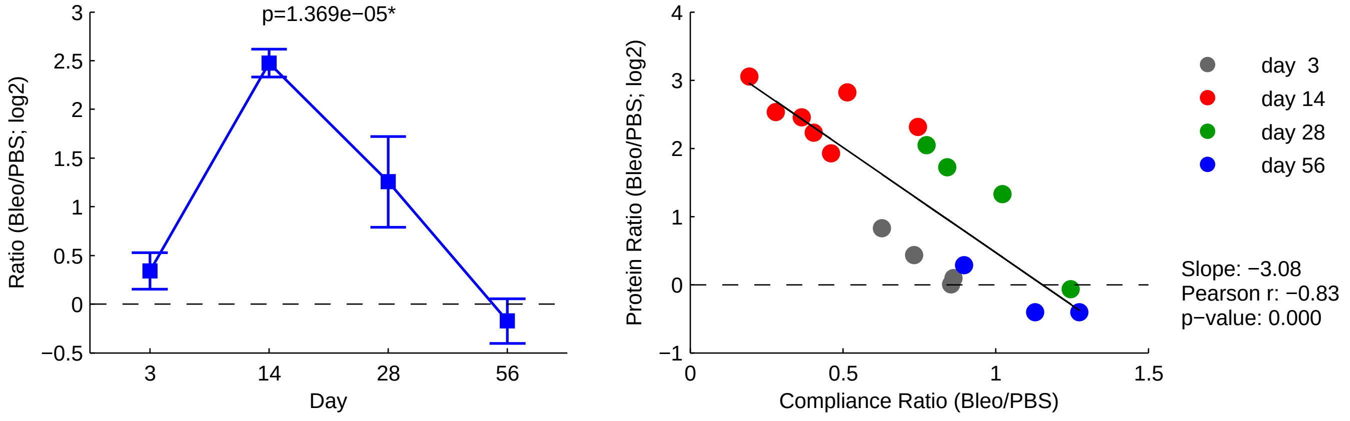

Q8VCM8 − Ncln (id: 6068)

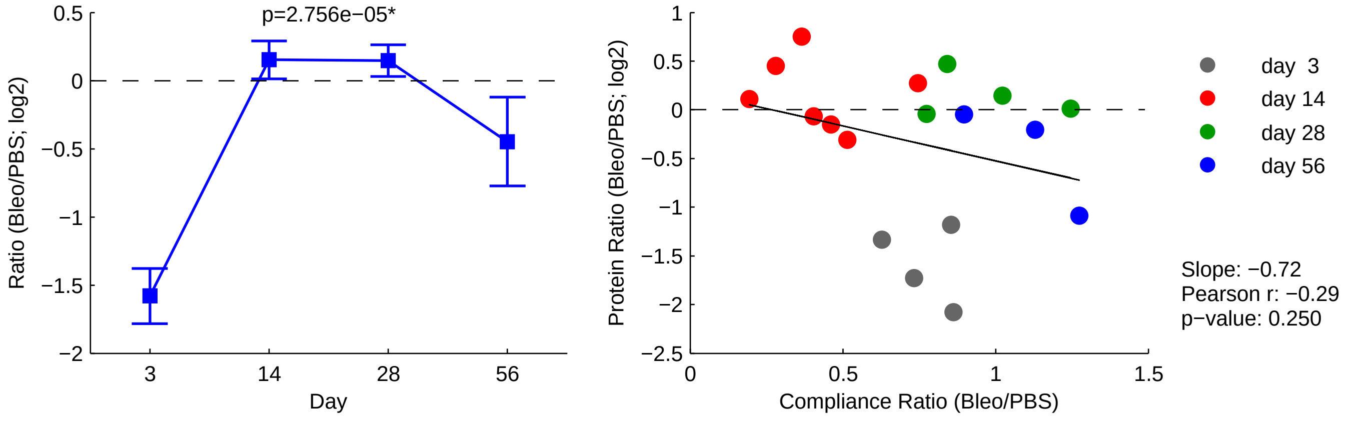

Q8VCN6 − Cd99 (id: 6070)

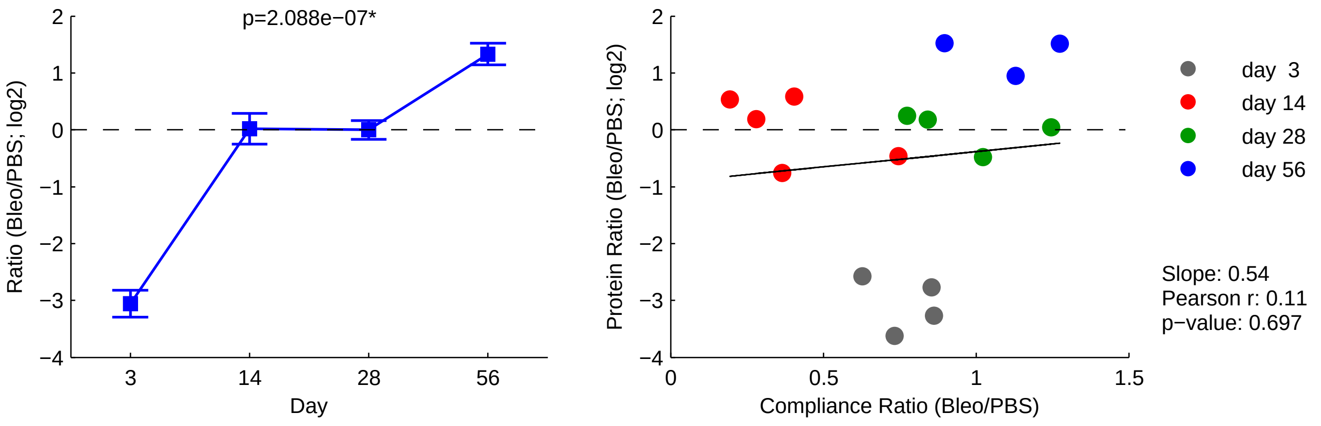

Q8VCP9 − Clec14a (id: 6071)

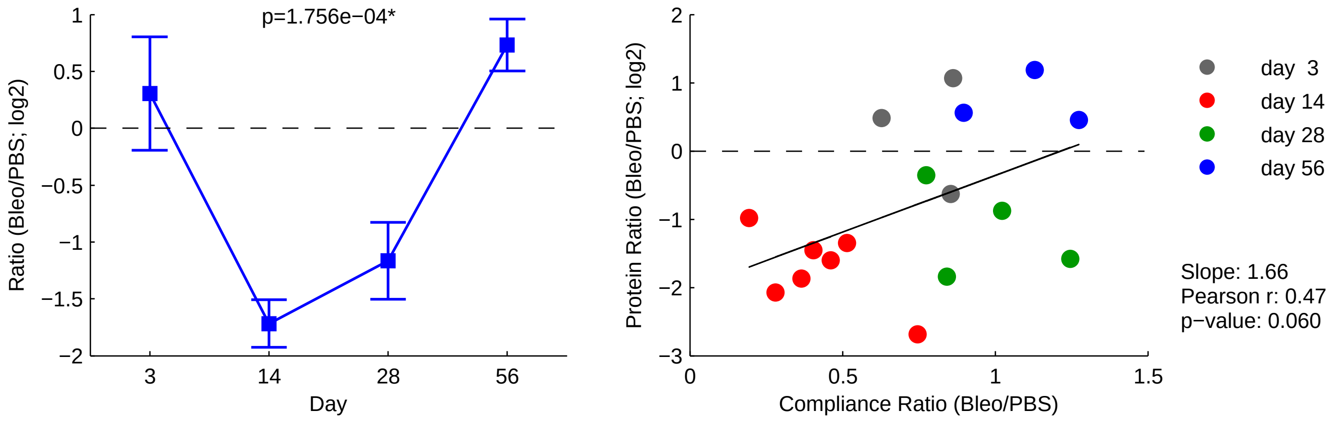

Q8VCS3 − Fam20b (id: 6074)

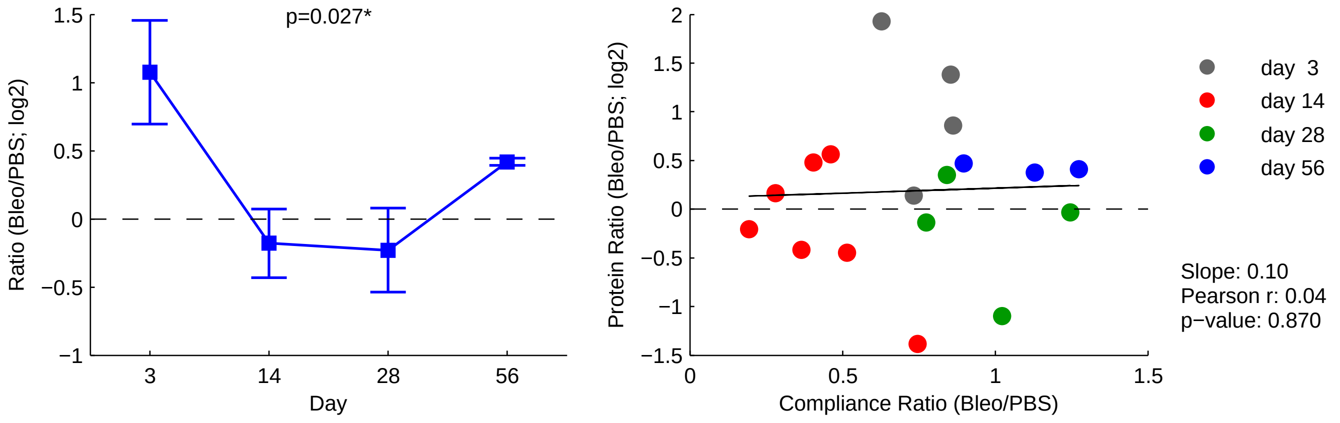

Q8VCT3 − Rnpep (id: 6075)

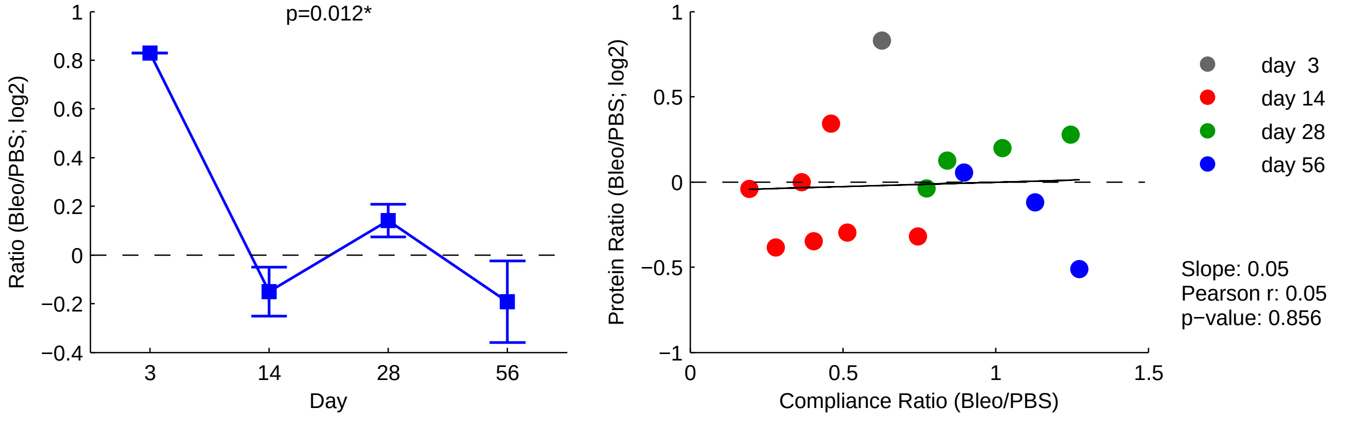

Q8VCT4 − Ces1d (id: 6076)

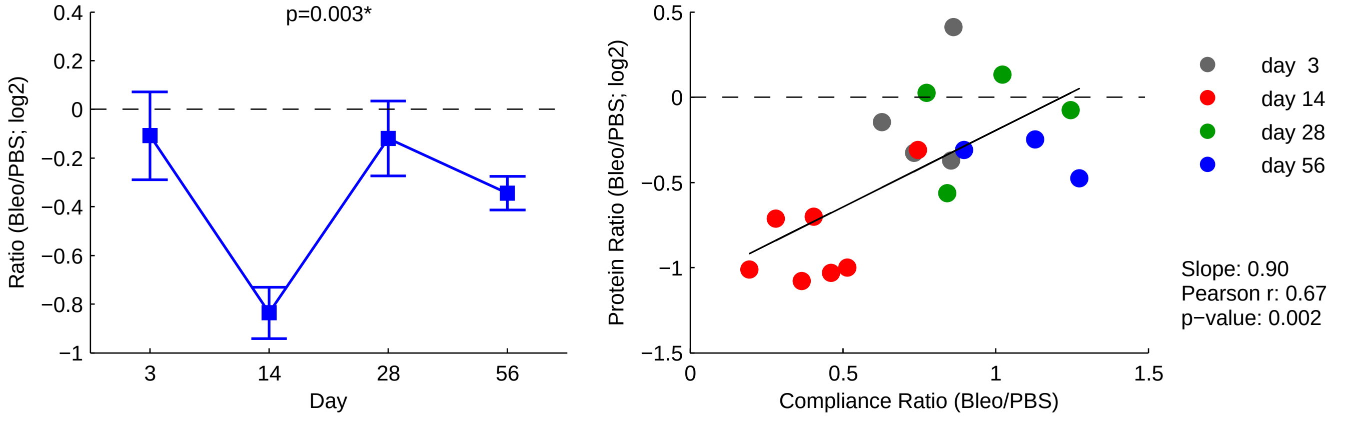

Q8VCU2 − Gpld1 (id: 6077)

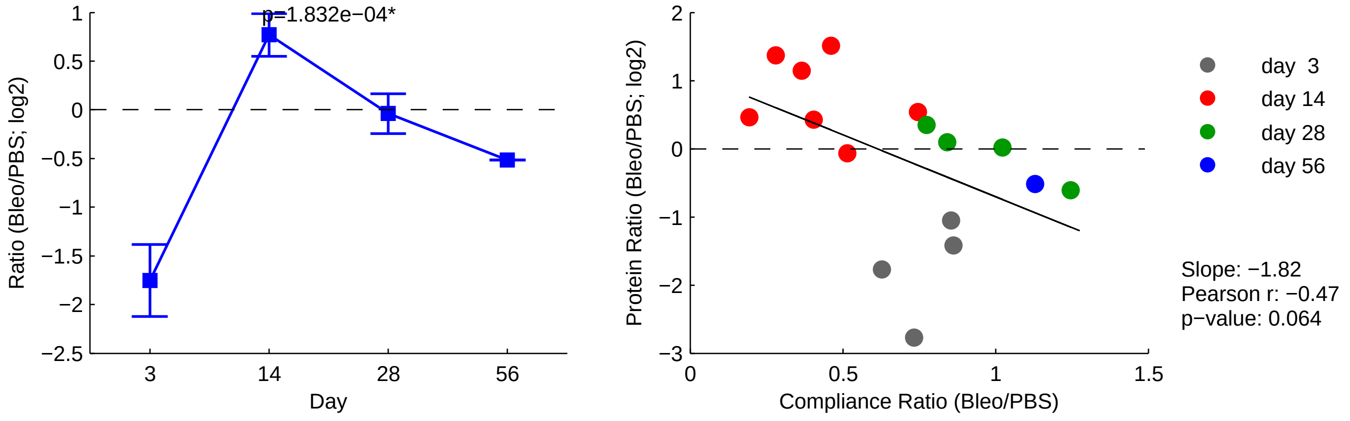

Q8VCV1 − Abhd17c (id: 6078)

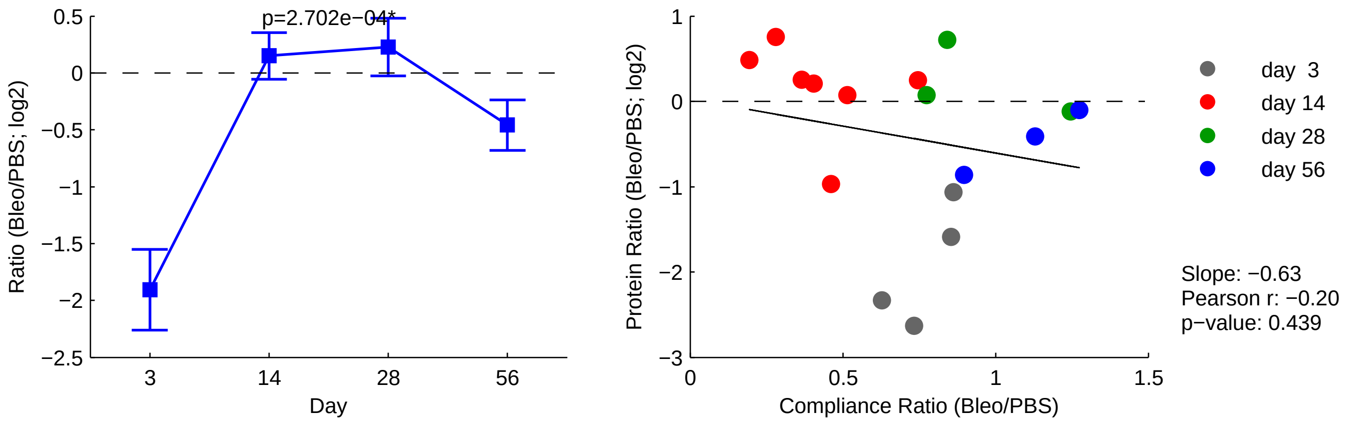

Q8VCW8 − Acsf2 (id: 6080)

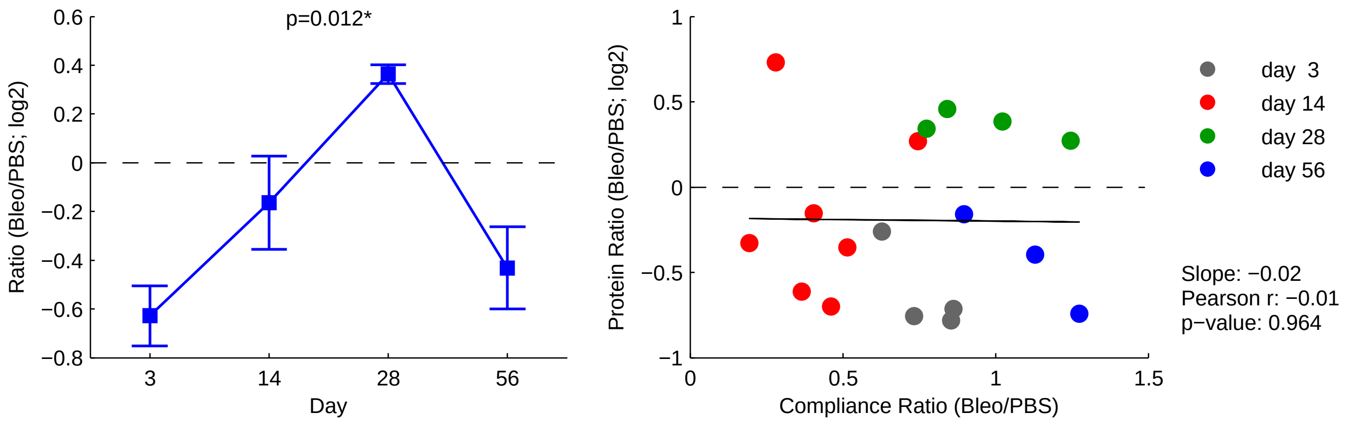

Q8VCX5 − Micu1 (id: 6081)

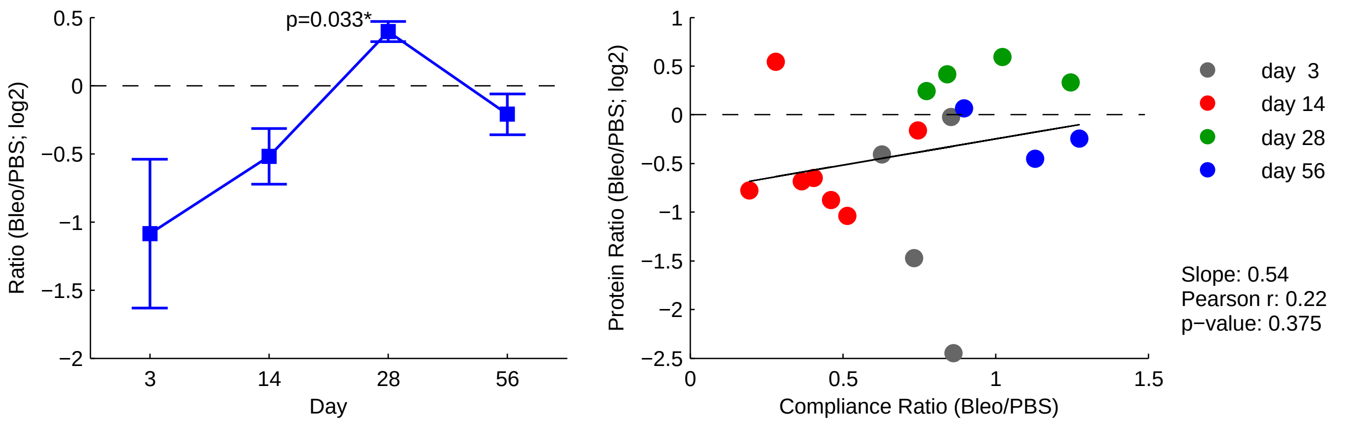

Q8VD58 − Evi2b (id: 6084)

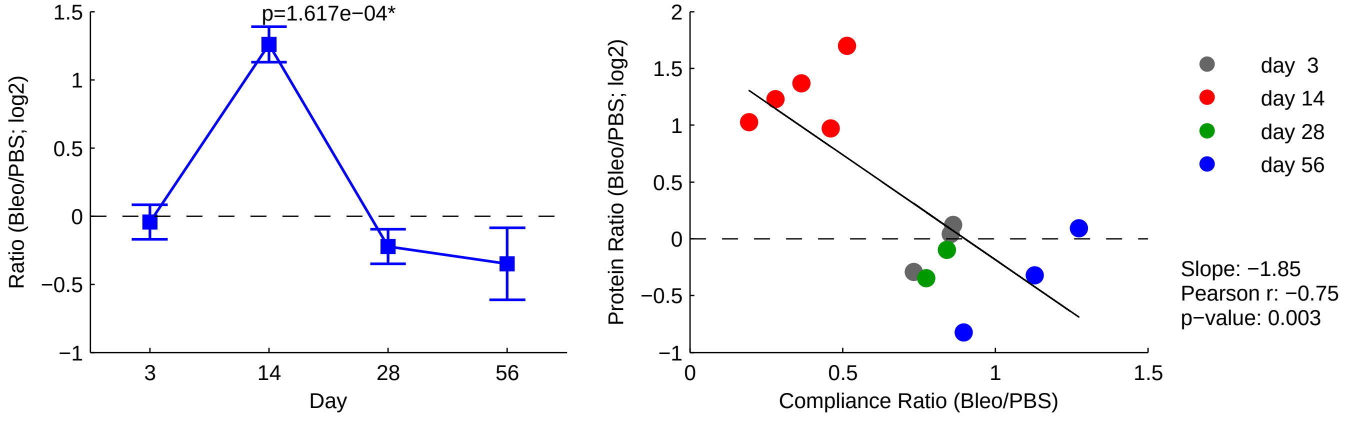

Q8VD65 − Pik3r4 (id: 6085)

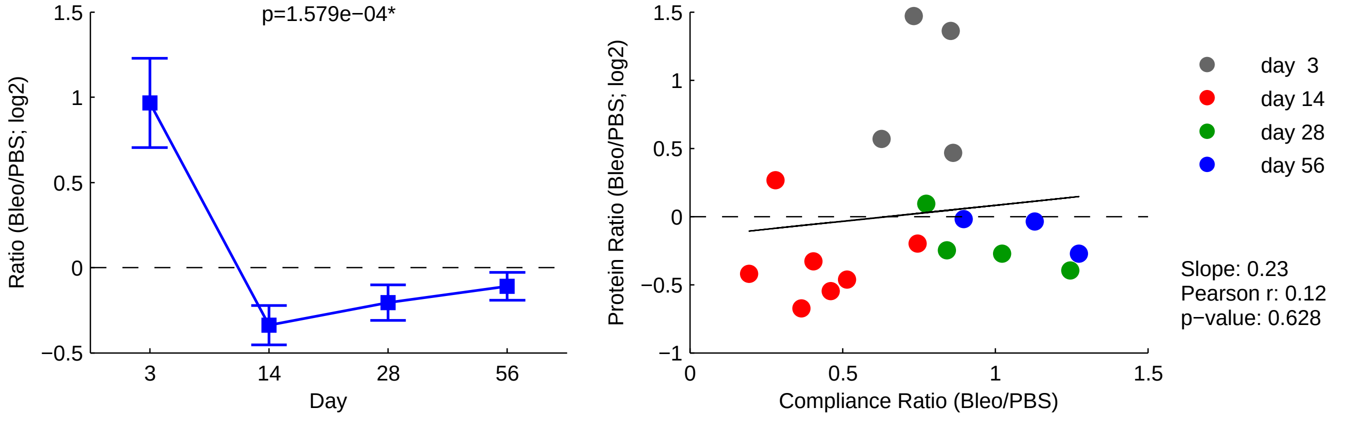

Q8VD72−2 − Ttc8 (id: 6086)

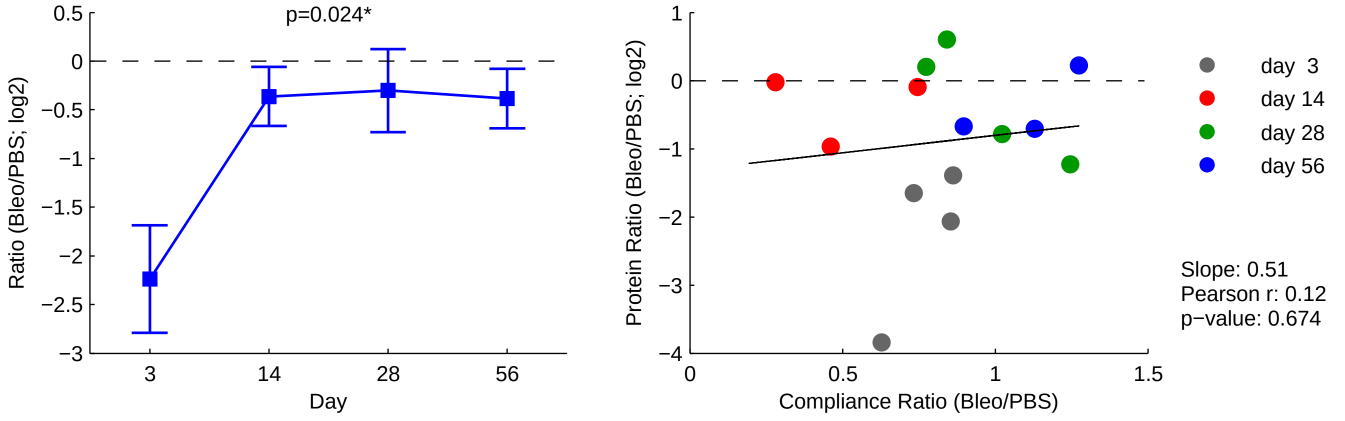

Q8VD75 − Hip1 (id: 6087)

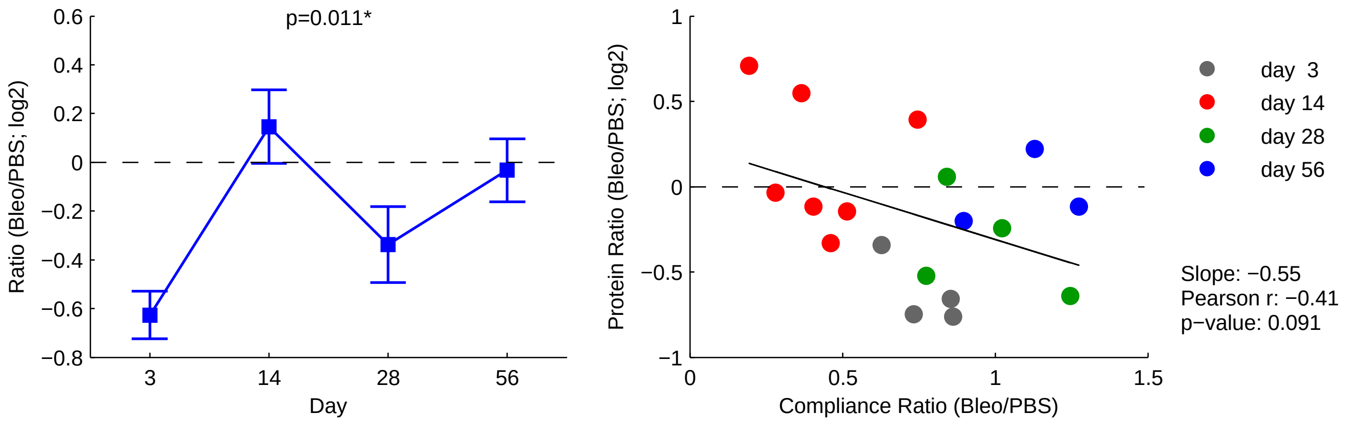

Q8VDC0 − Lars2 (id: 6089)

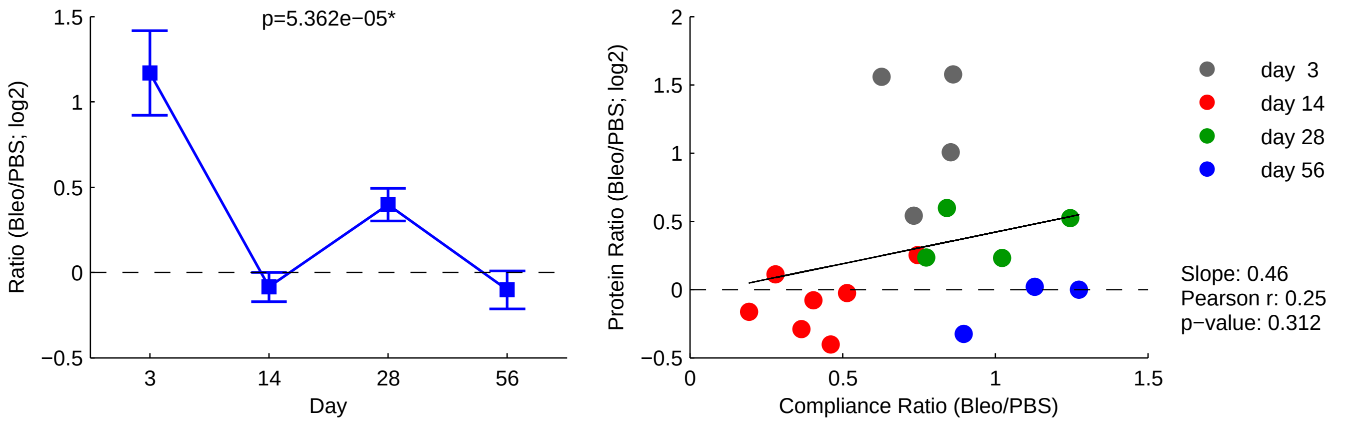

Q8VDD5 − Myh9 (id: 6091)

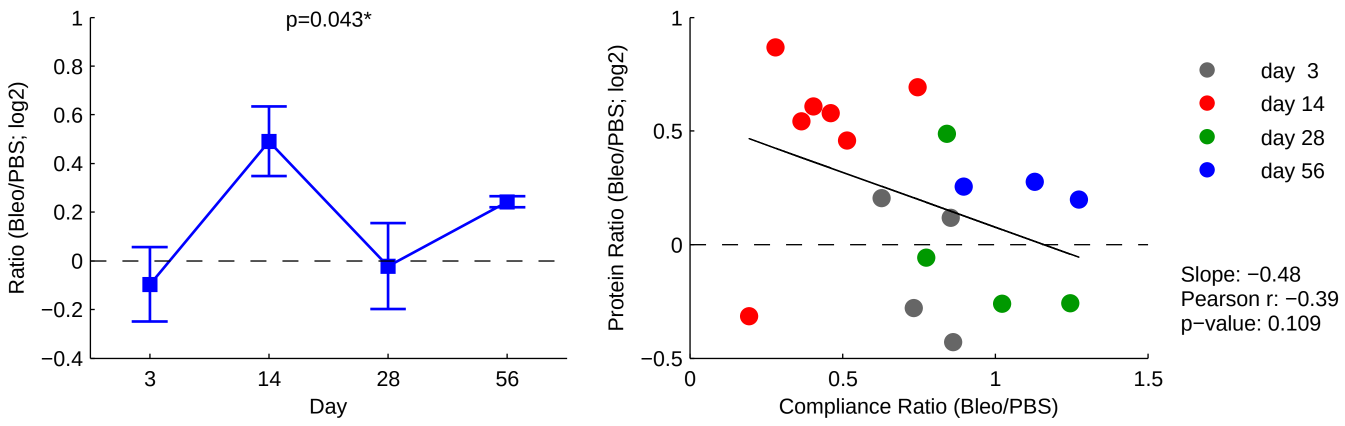

Q8VDD8 − Wash1 (id: 6092)

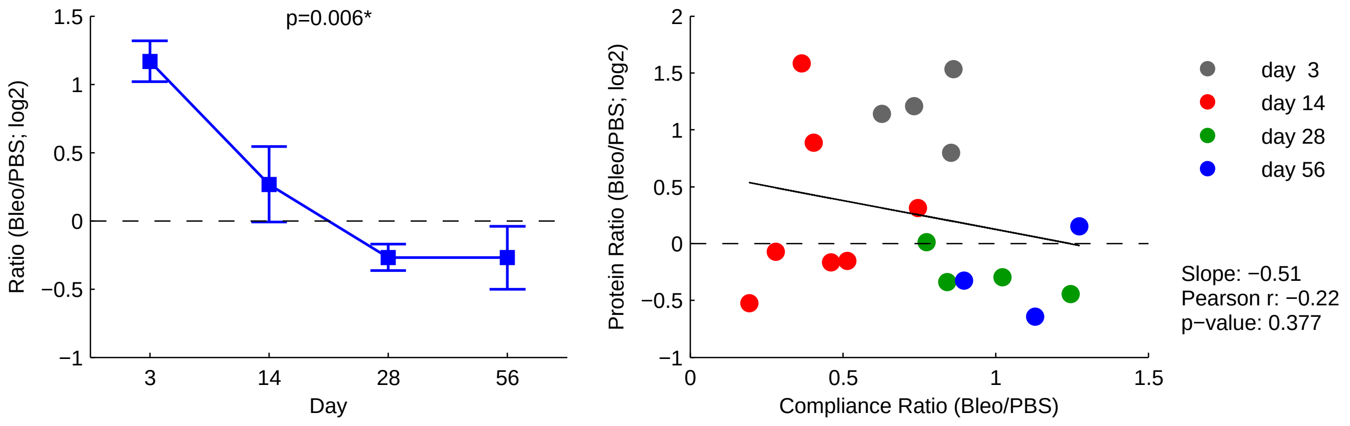

Q8VDF3−2 − (id: 6094)

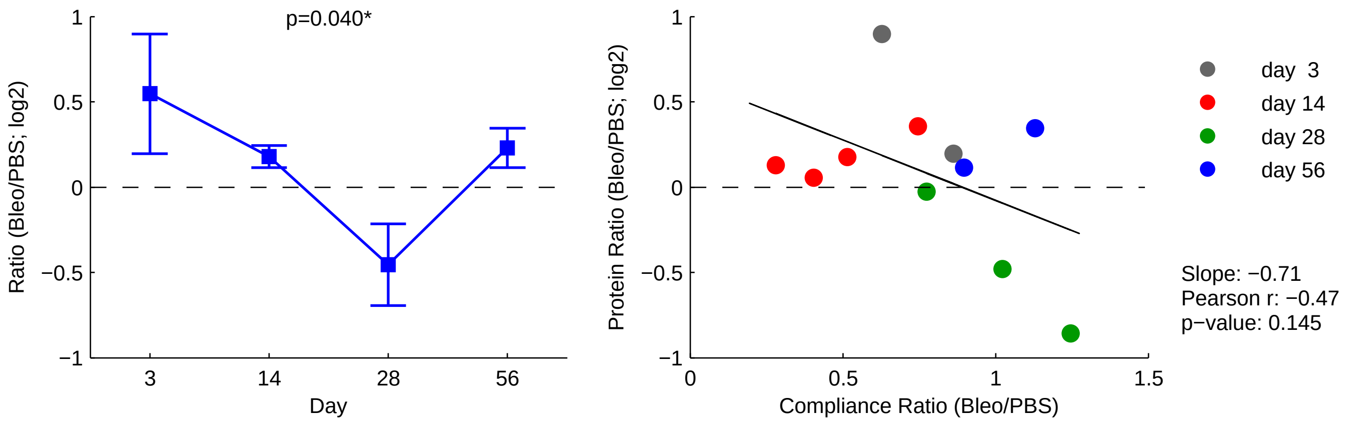

Q8VDI9 − Alg9 (id: 6096)

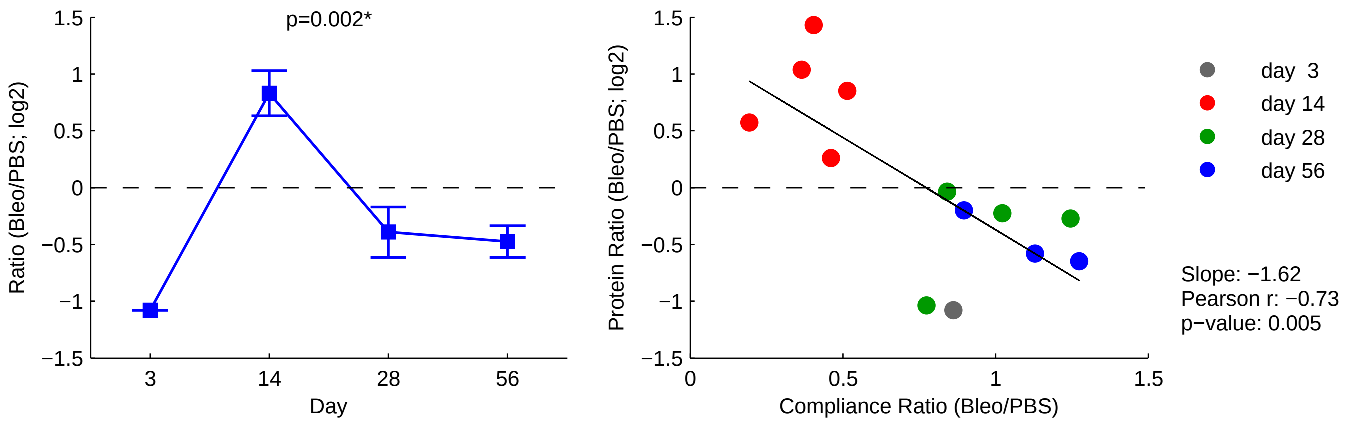

Q8VDJ3 − Hdlbp (id: 6097)

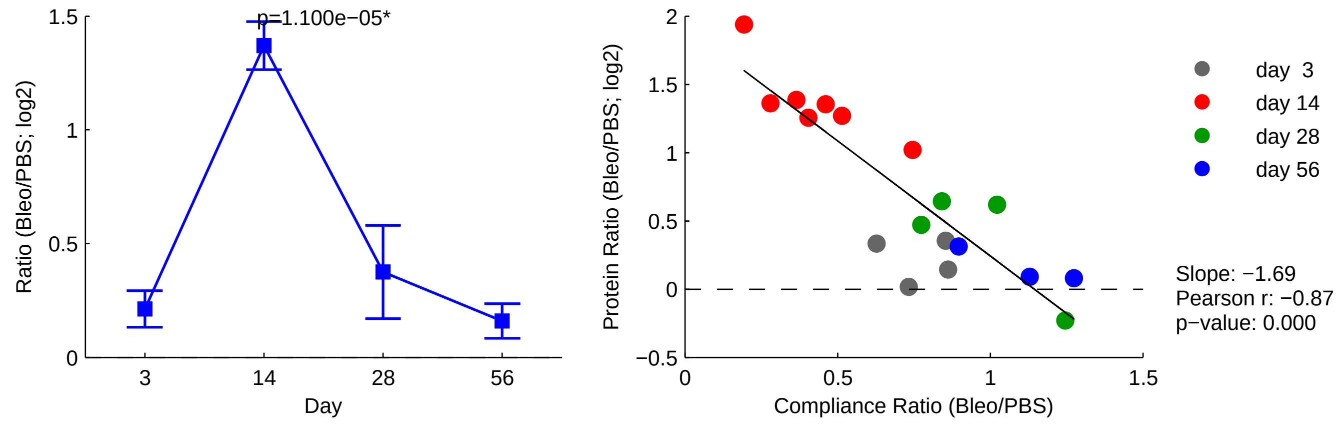

Q8VDK1−2 − Nit1 (id: 6098)

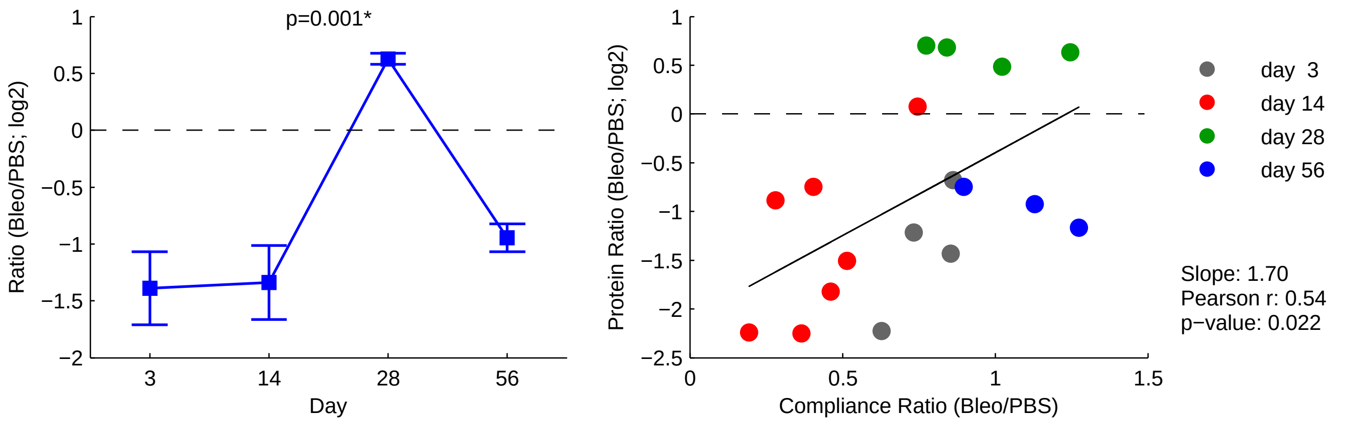

Q8VDL4−3 − Adpgk (id: 6099)

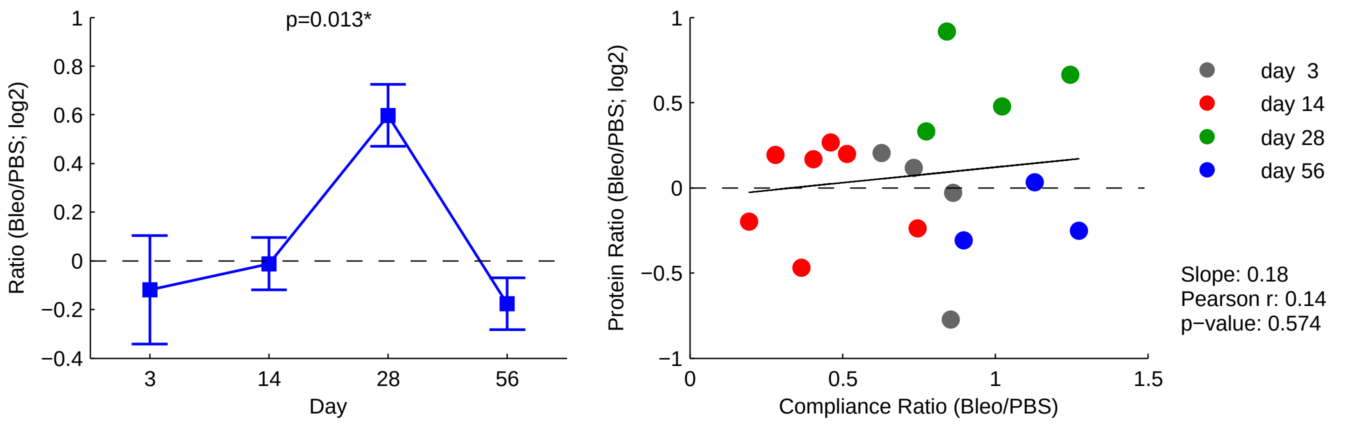

Q8VDM4 − Psmd2 (id: 6100)

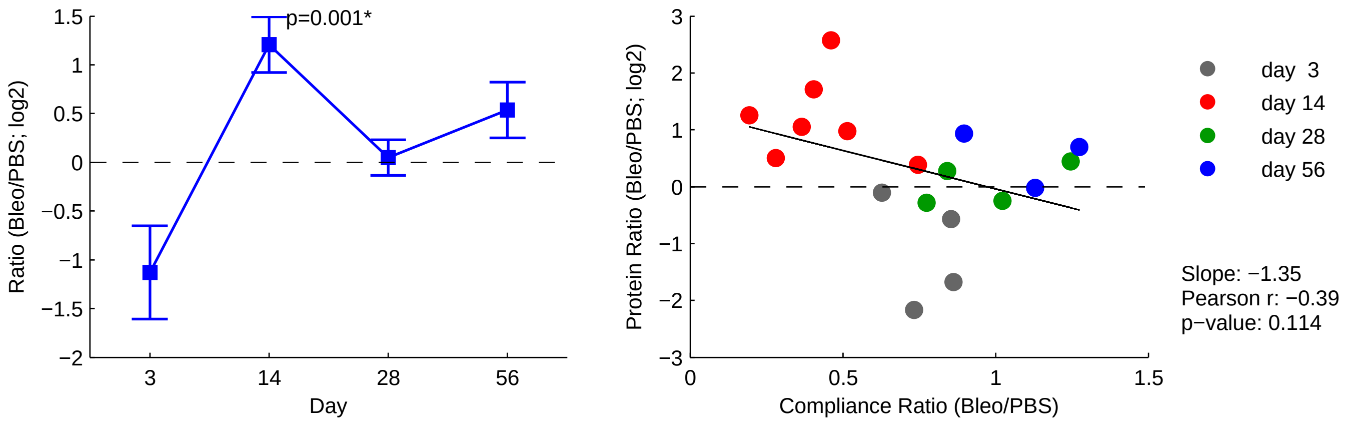

Q8VDN2 − Atp1a1 (id: 6102)

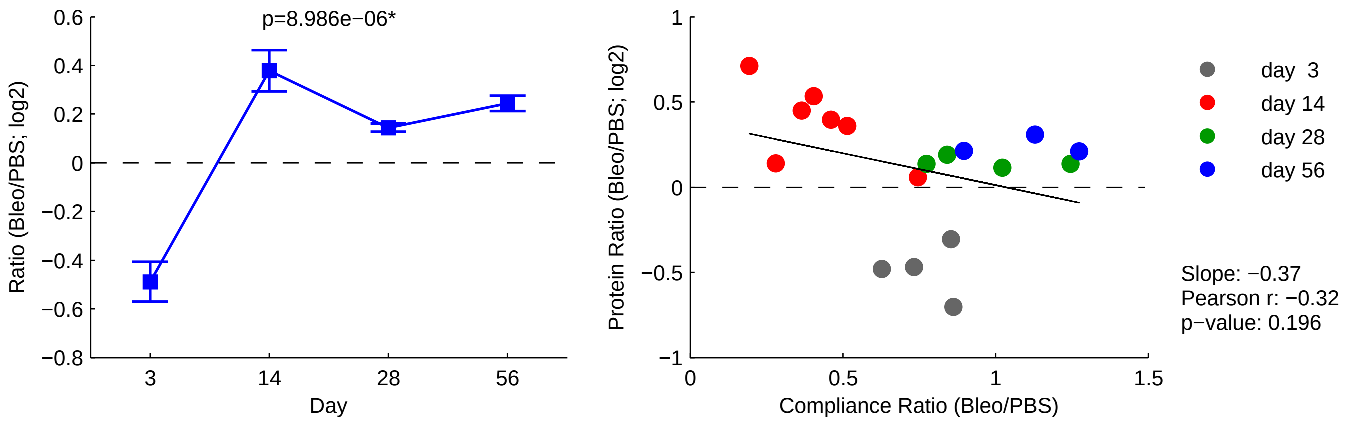

Q8VDP2 − (id: 6103)

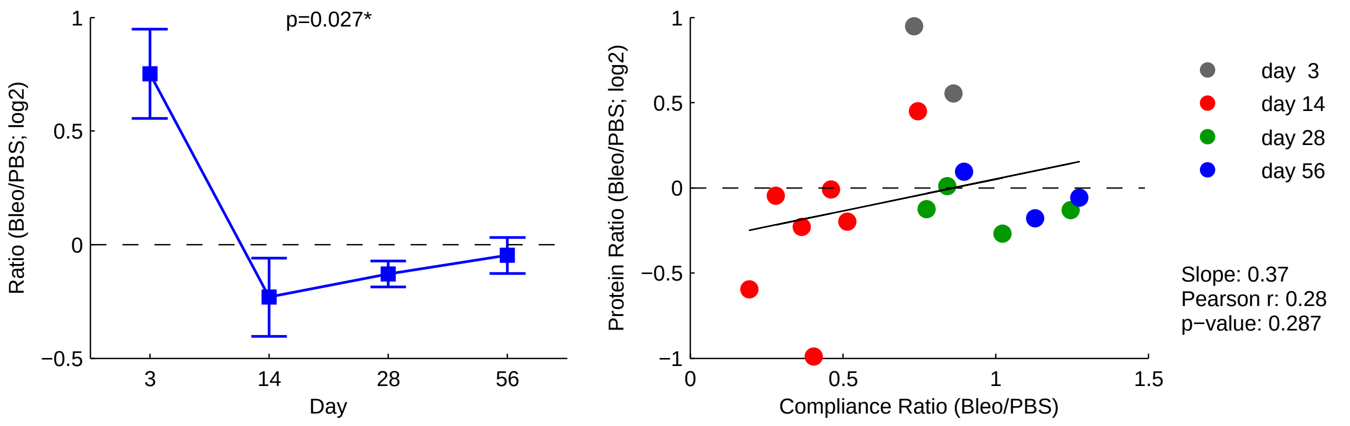

Q8VDP3−3 − Mical1 (id: 6104)

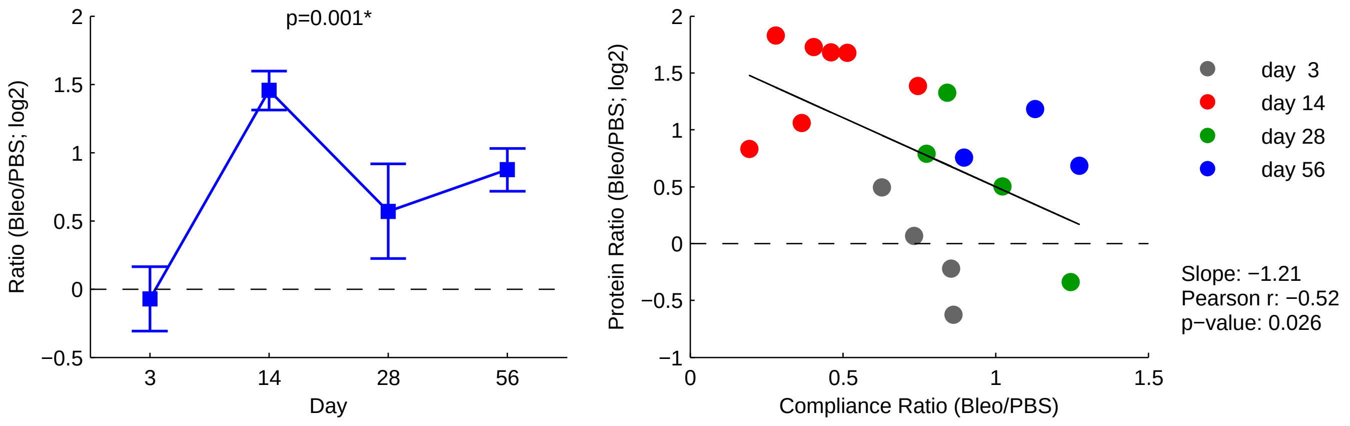

Q8VDP4 − (id: 6105)

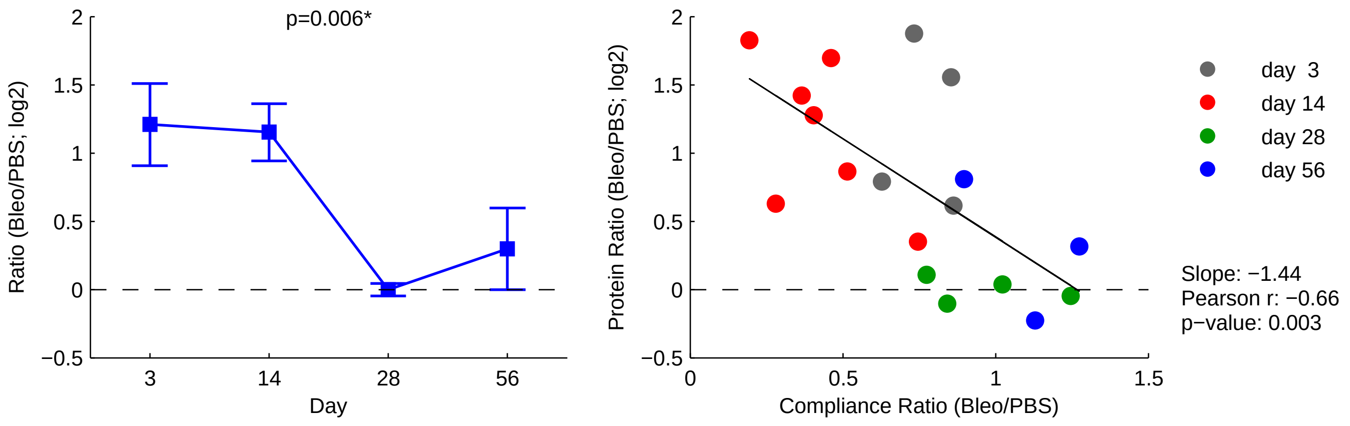

Q8VDP6 − Cdipt (id: 6106)

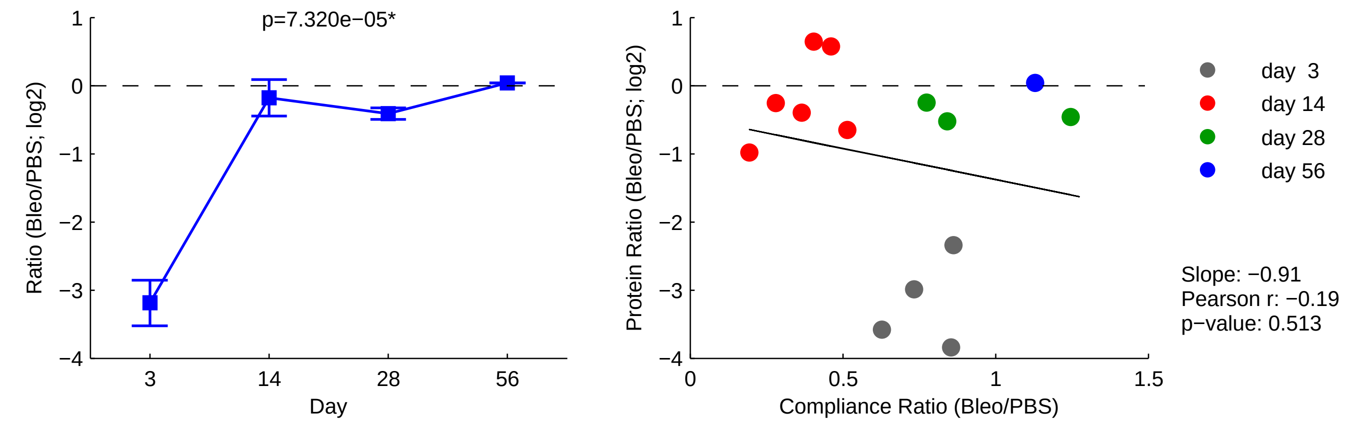

Q8VDQ8−2 − Sirt2 (id: 6108)

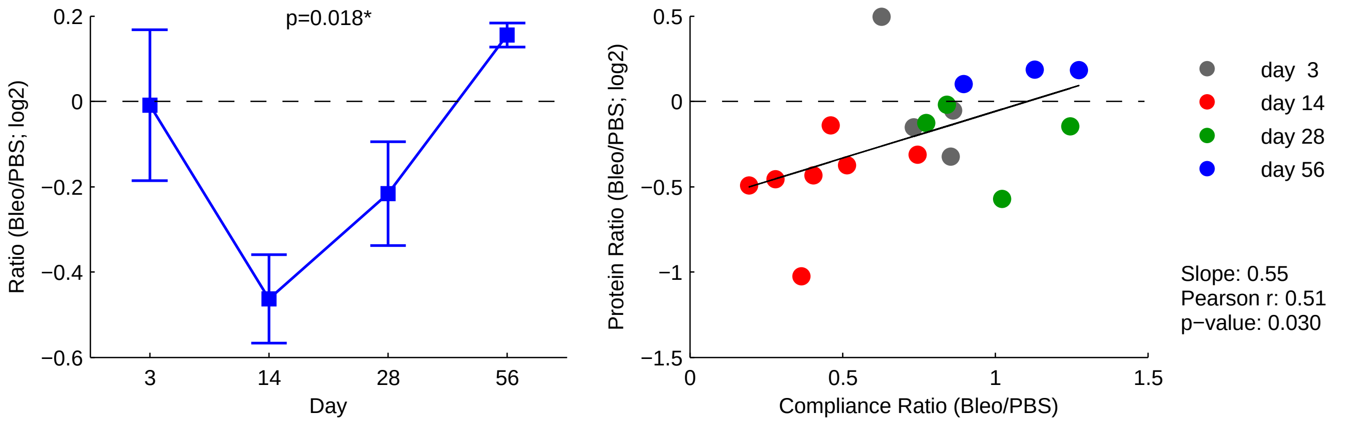

Q8VDT9 − Mrpl50 (id: 6111)

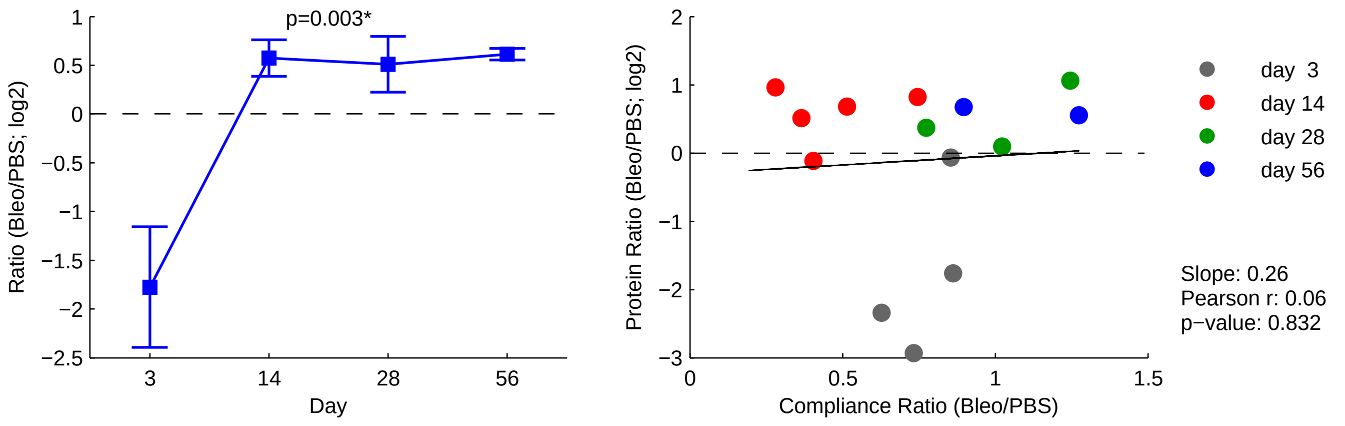

Q8VDW0 − Ddx39a (id: 6114)

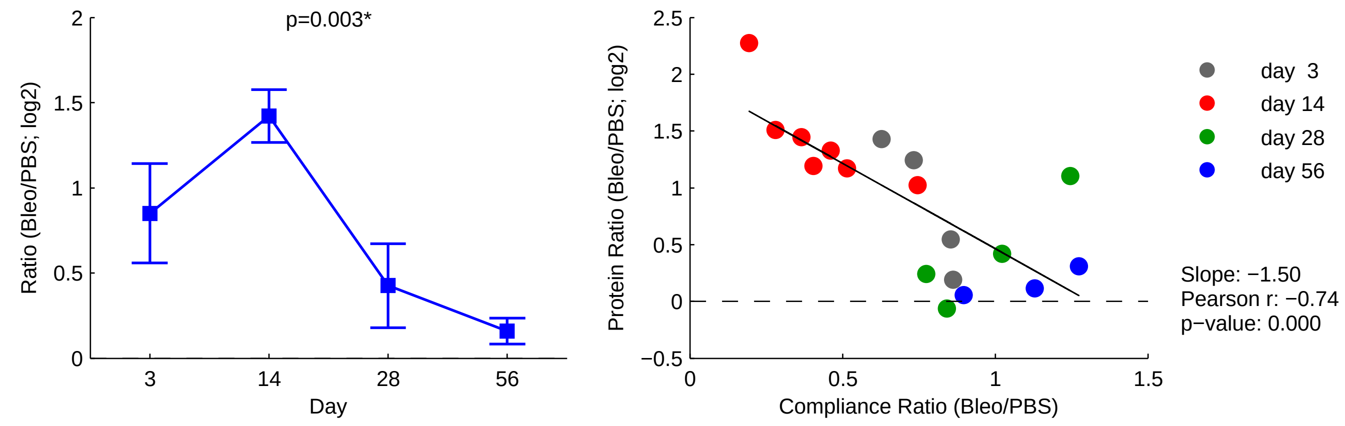

Q8VDZ4 − Zdhhc5 (id: 6115)

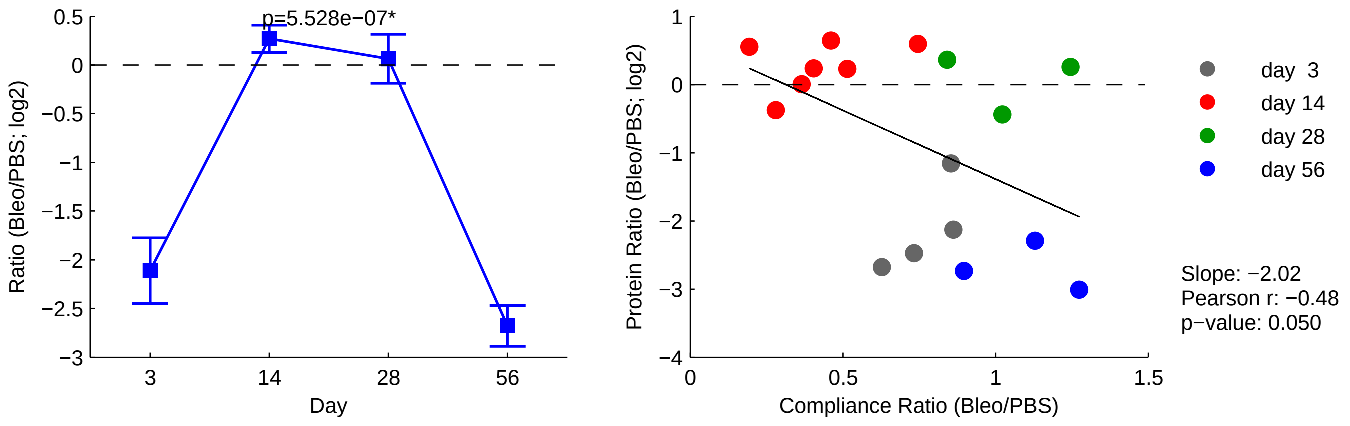

Q8VE11 − Mtmr6 (id: 6117)

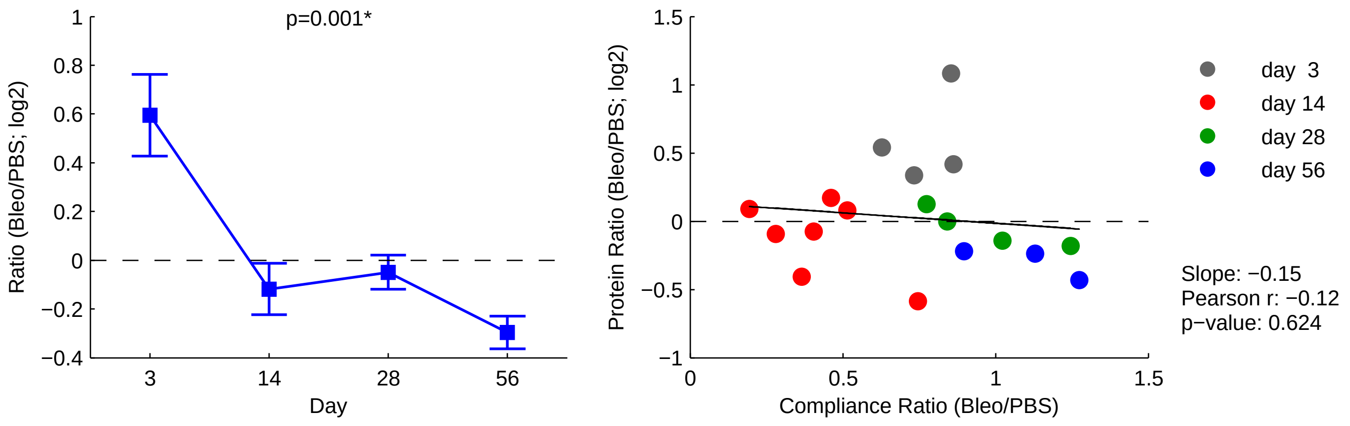

Q8VE22 − Mrps23 (id: 6119)

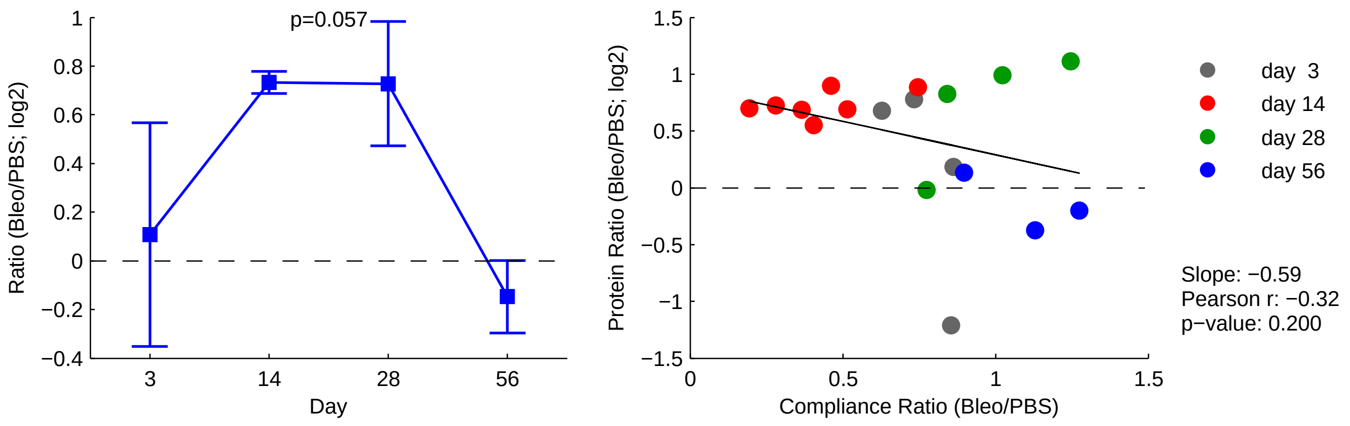

Q8VE37 − Rcc1 (id: 6120)

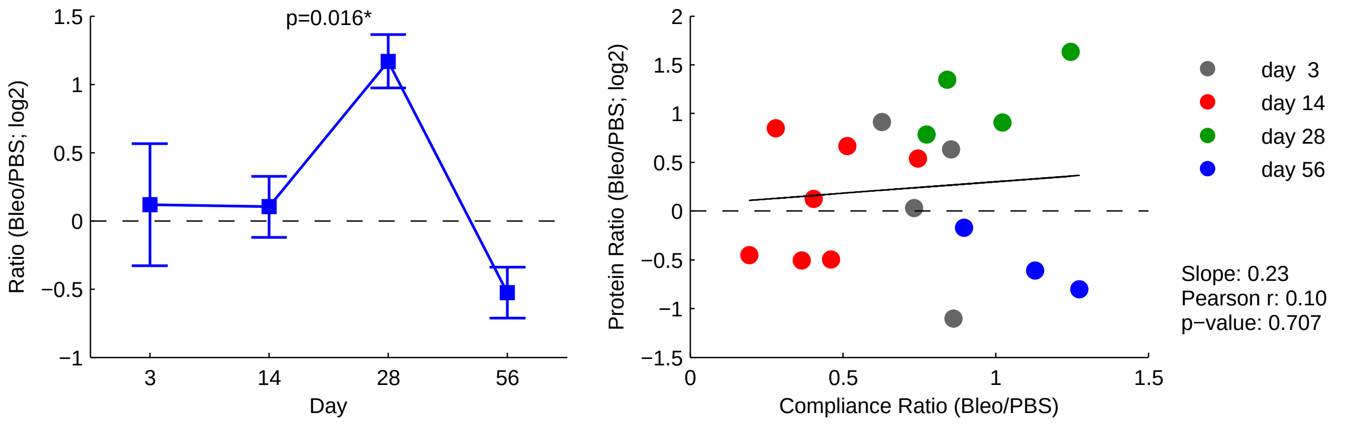

Q8VE73 − Cul7 (id: 6124)

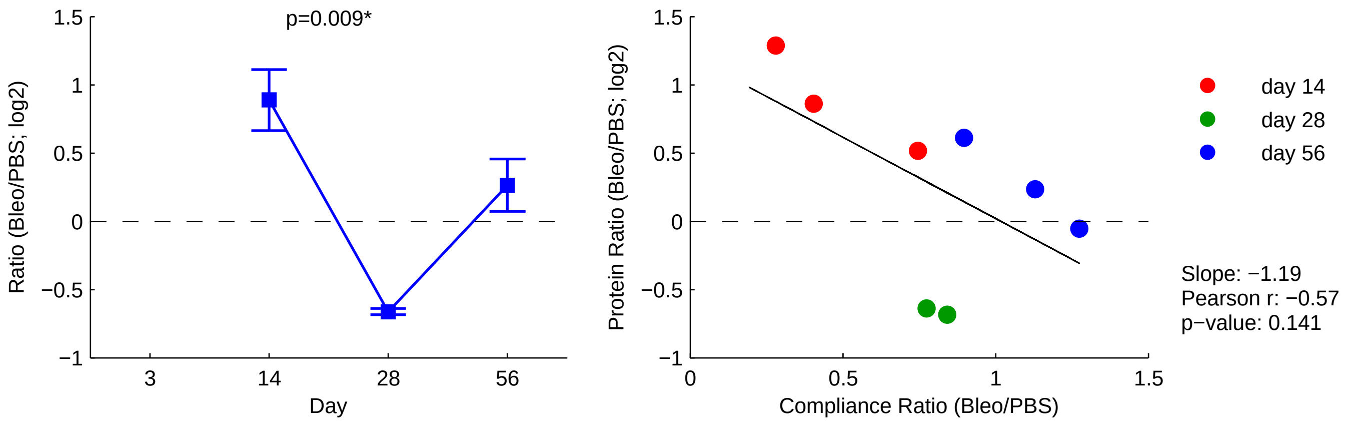

Q8VE99 − Ccdc115 (id: 6128)

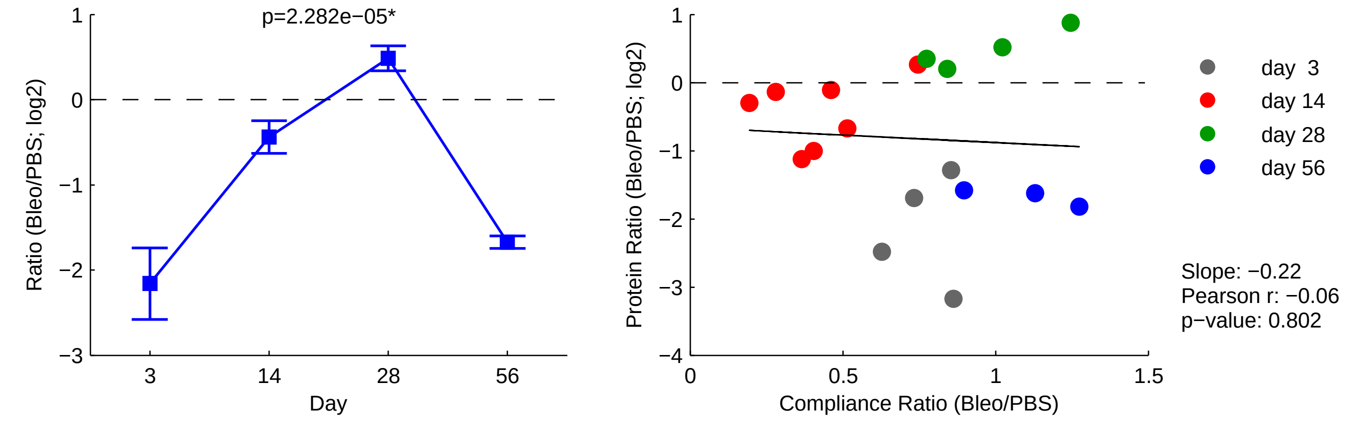

Q8VEE1 − Lmcd1 (id: 6135)

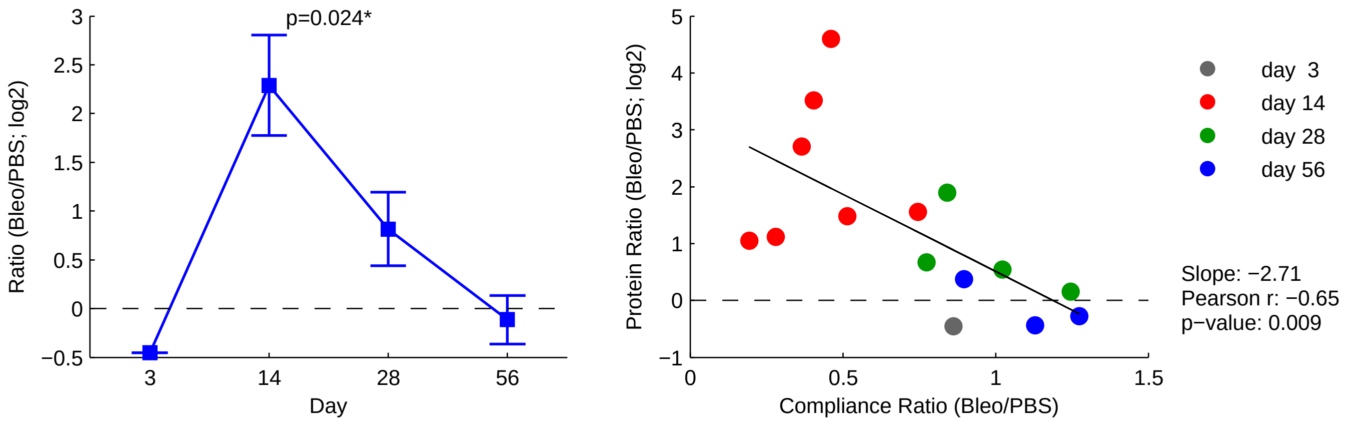

Q8VEH3 − Arl8a (id: 6137)

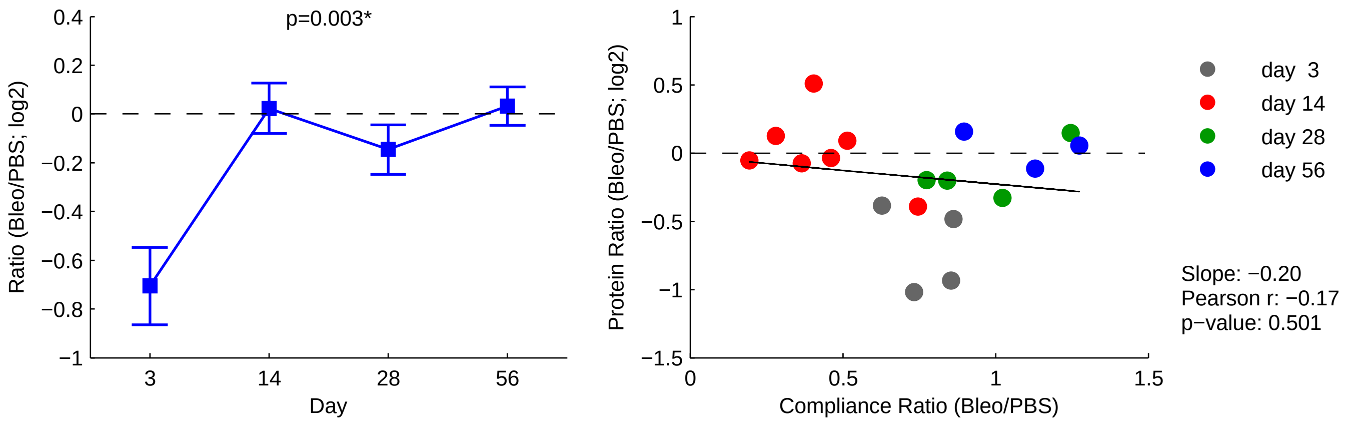

Q8VEH8 − Erlec1 (id: 6138)

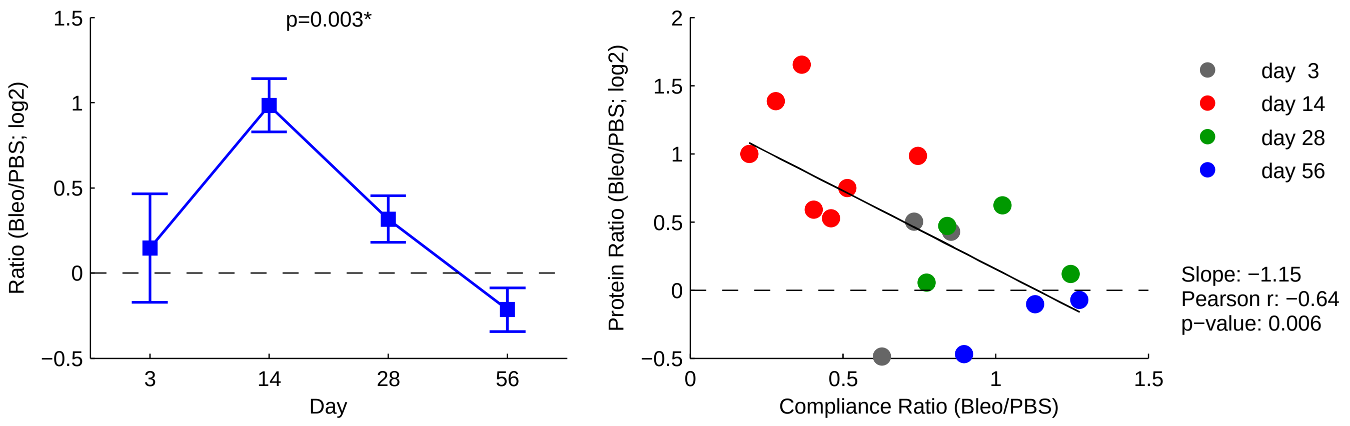

Q8VEJ9 − Vps4a (id: 6140)

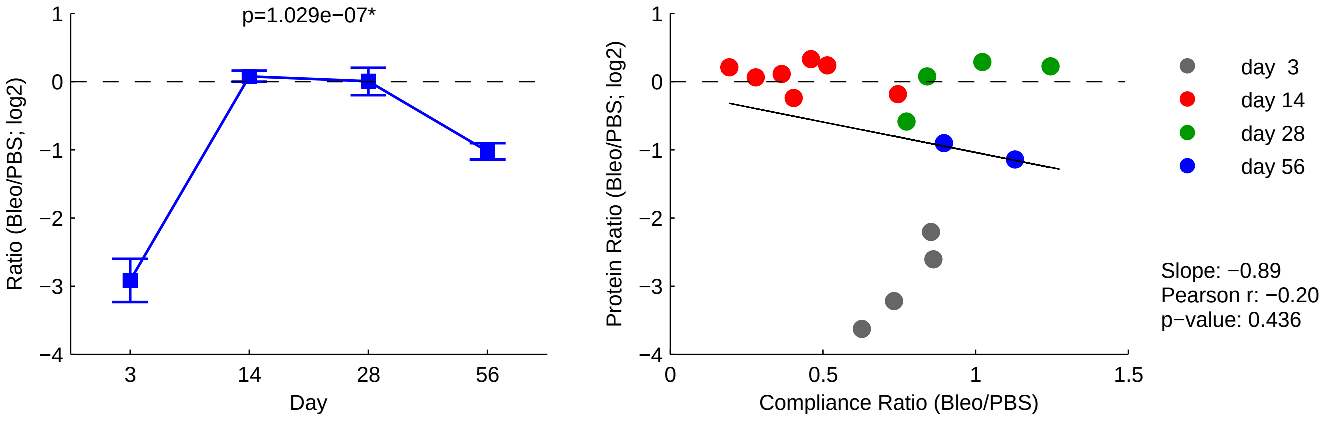

Q8VEK3 − Hnrnpu (id: 6141)

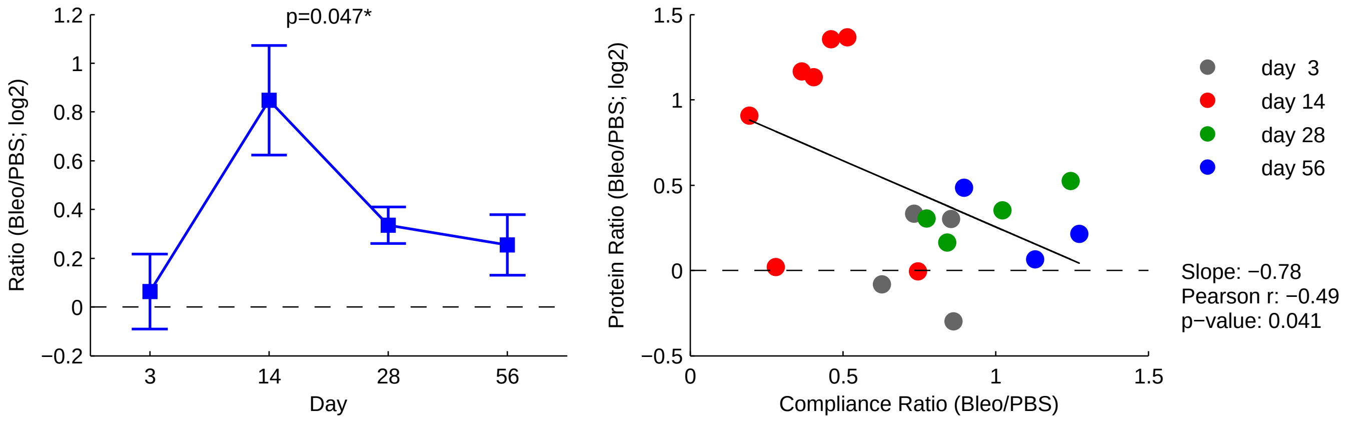

Q8VEM8 − Slc25a3 (id: 6144)

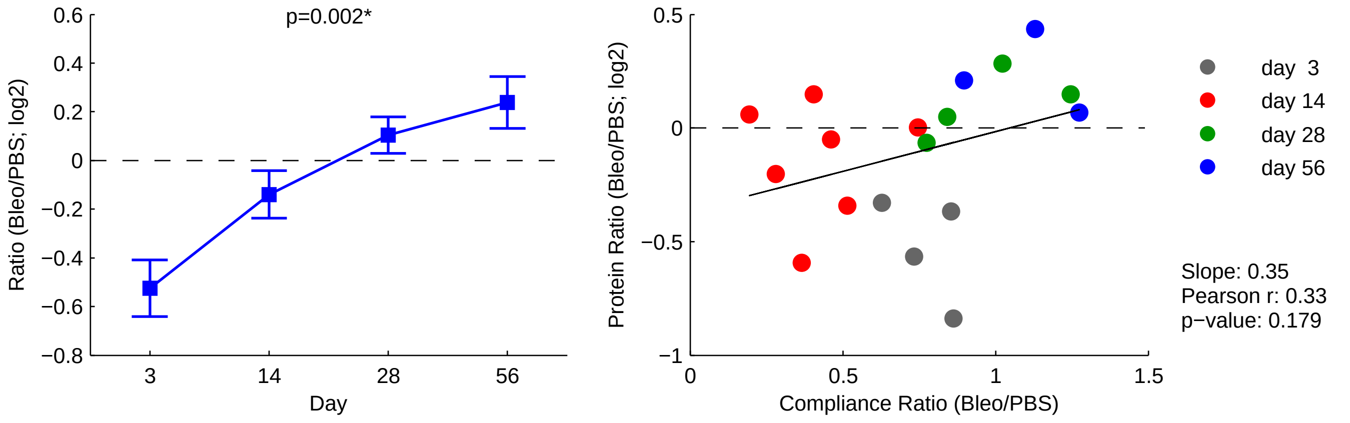

Q8VH51−2 − Rbm39 (id: 6147)

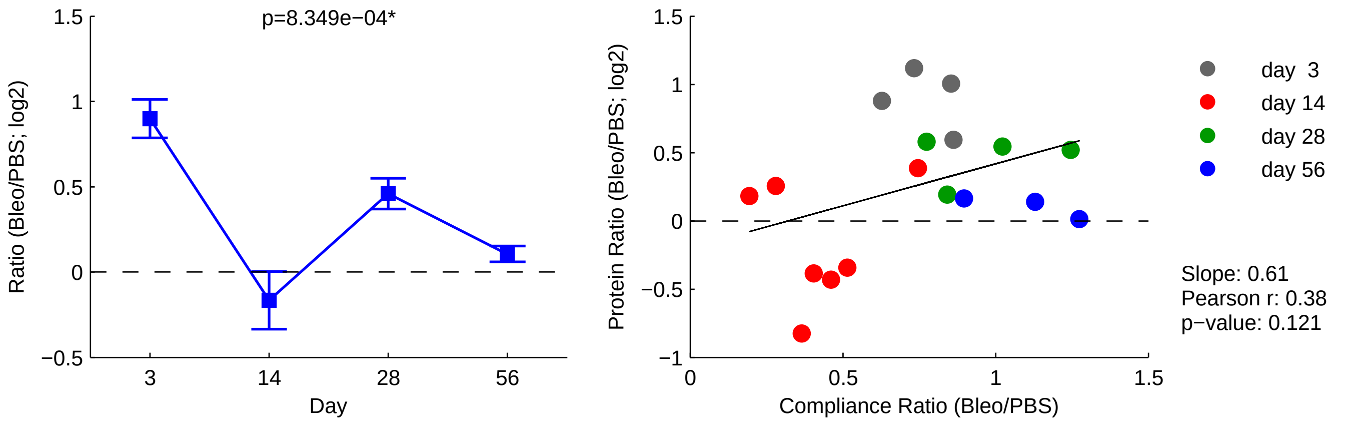

Q8VHE0 − Sec63 (id: 6149)

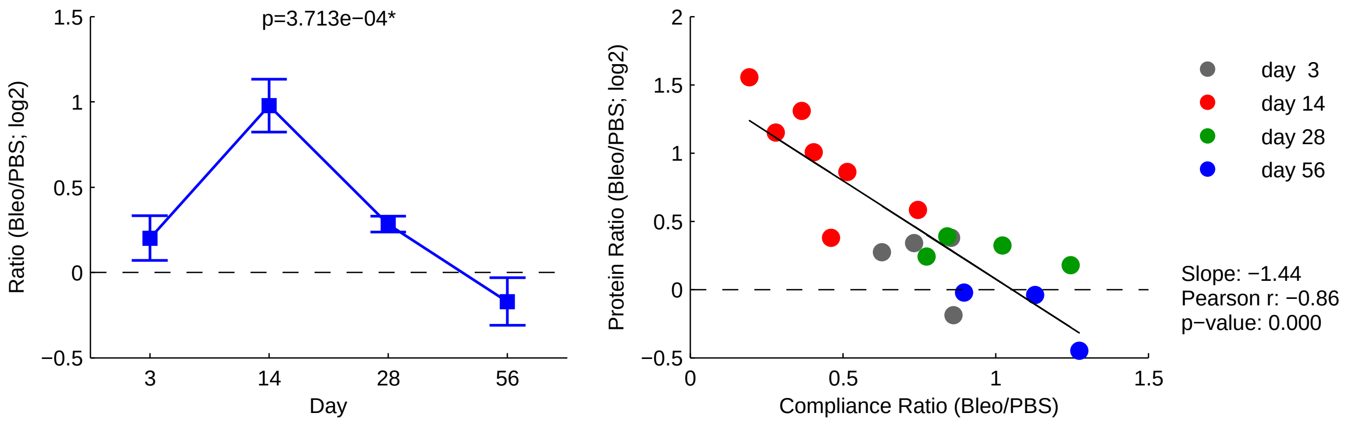

Q8VHE6 − Dnah5 (id: 6150)

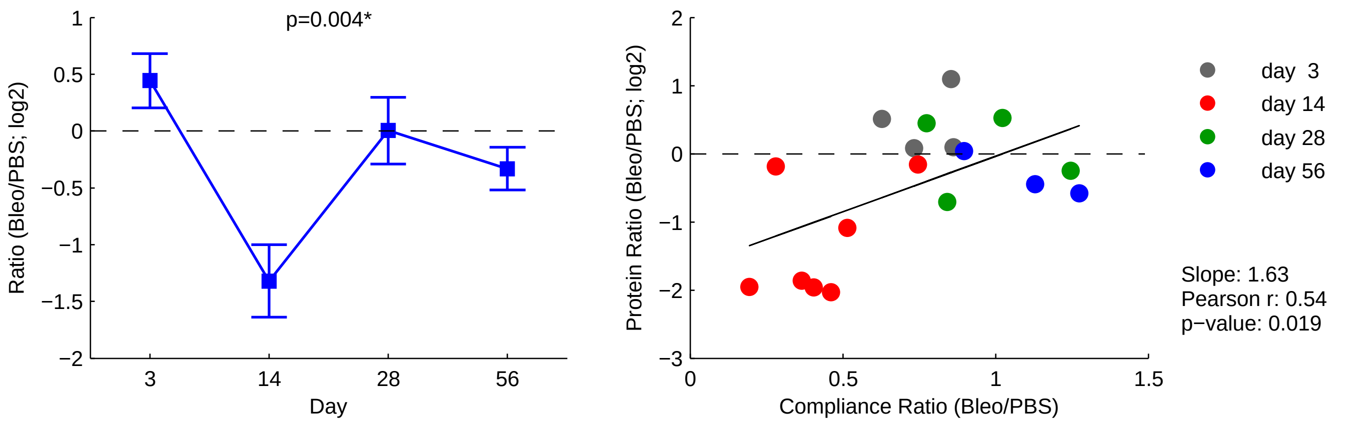

Q8VHI3 − Pofut2 (id: 6152)

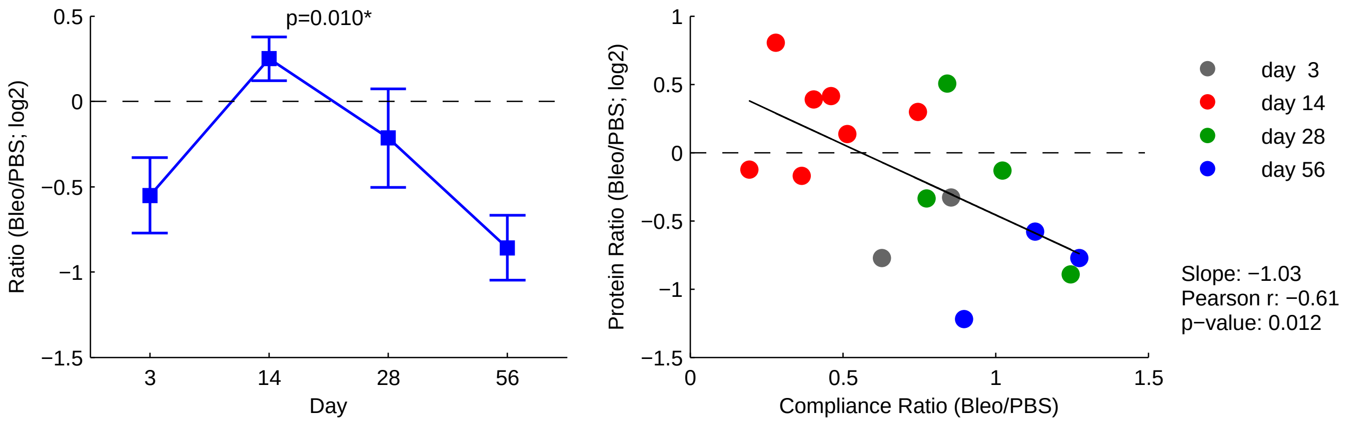

Q8VHK1 − Caskin2 (id: 6156)

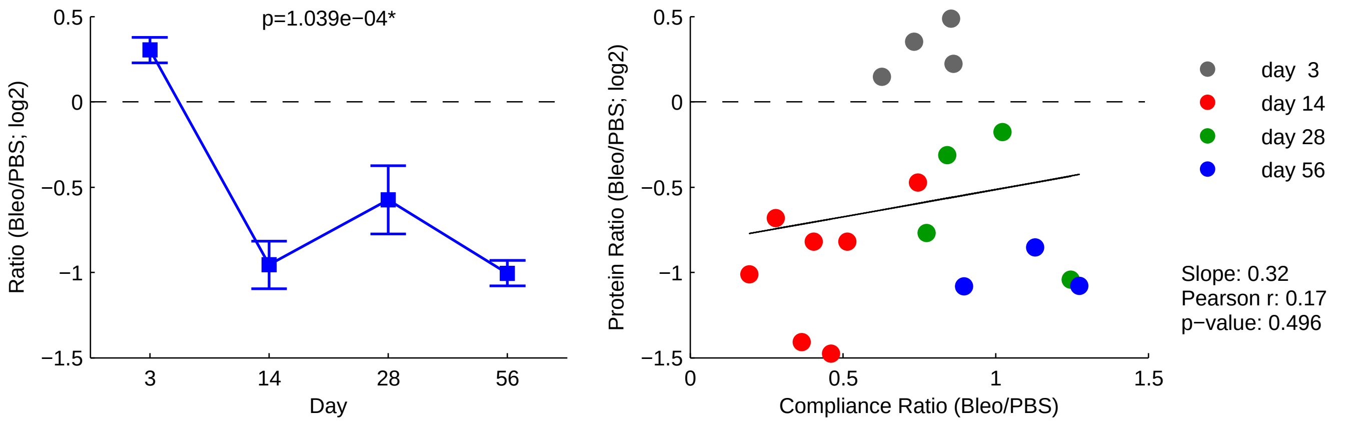

Q8VHM5 − Hnrnpr (id: 6159)

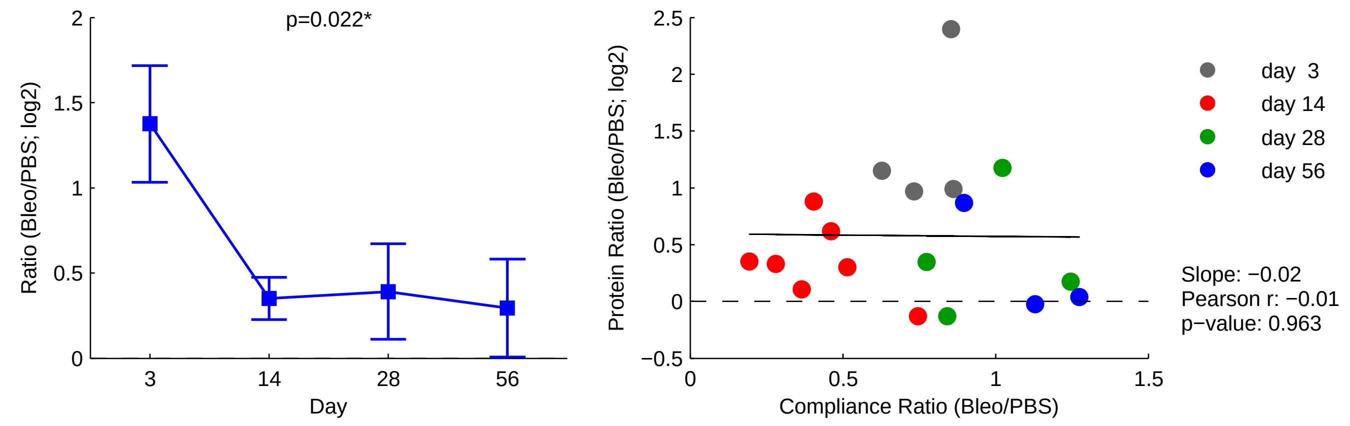

Q8VHY0 − Cspg4 (id: 6164)

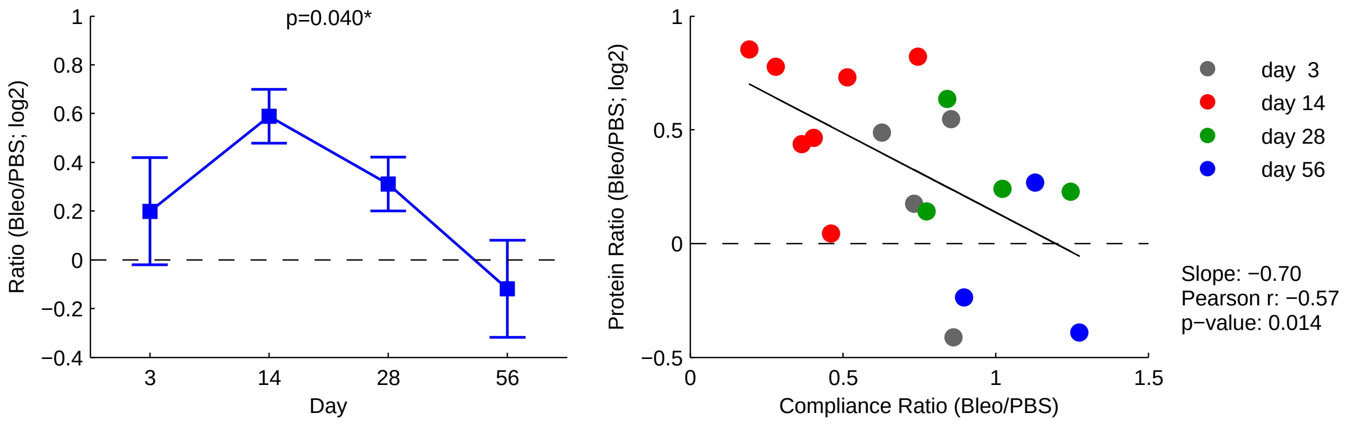

Q8VI84 − Noc3l (id: 6168)

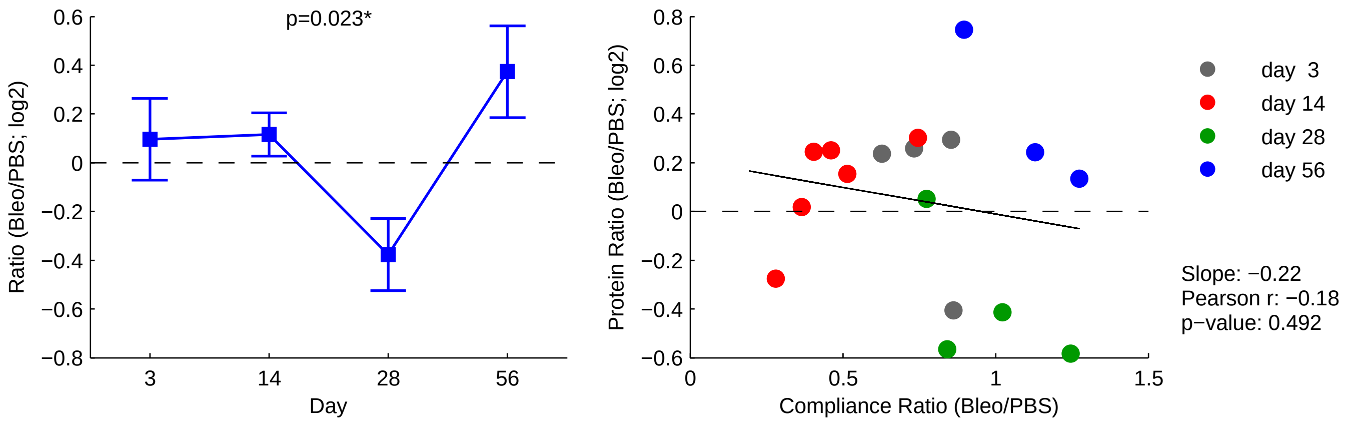

Q8VIB3 − St3gal6 (id: 6170)

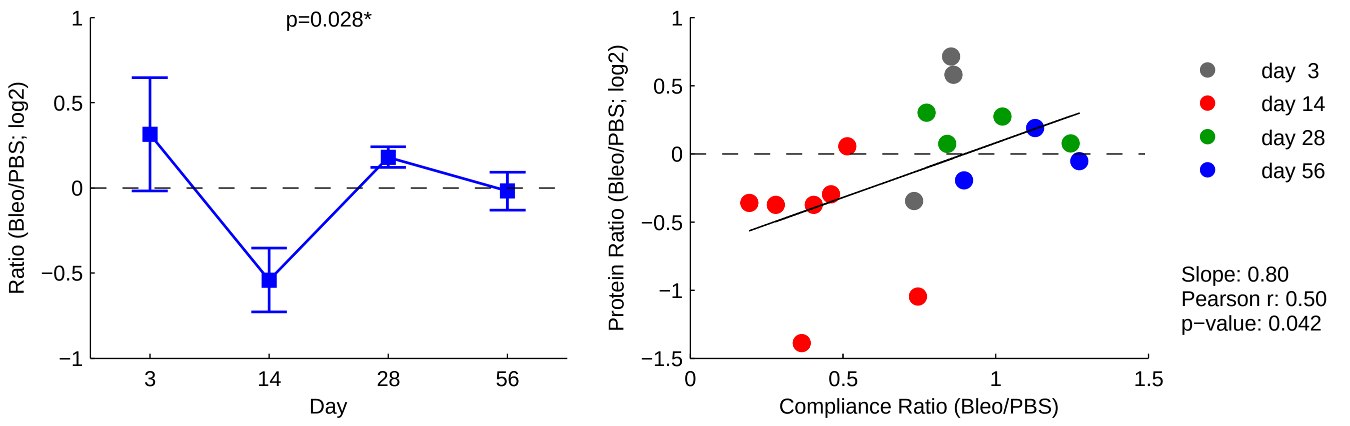

Q91UZ1 − Plcb4 (id: 6174)

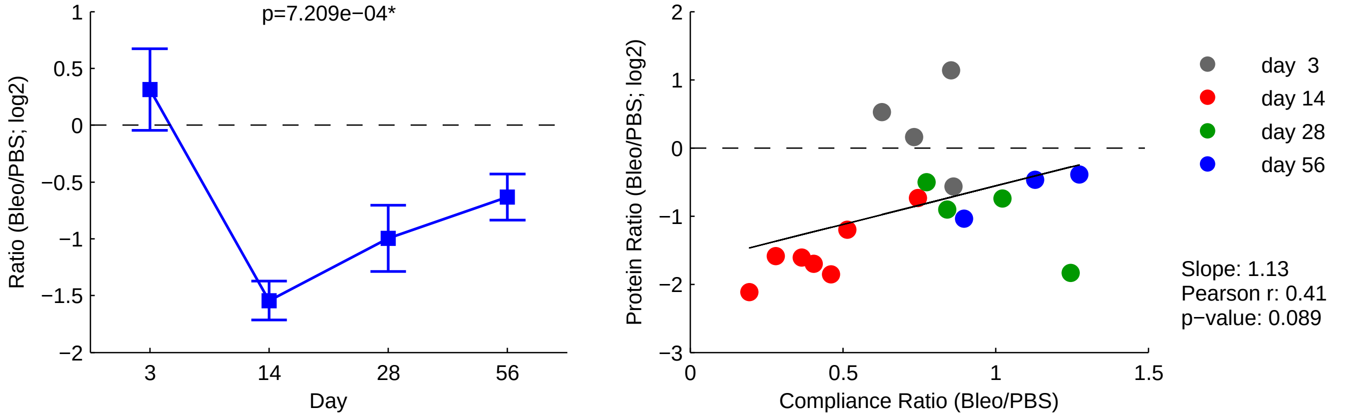

Q91V09 − Wdr13 (id: 6178)

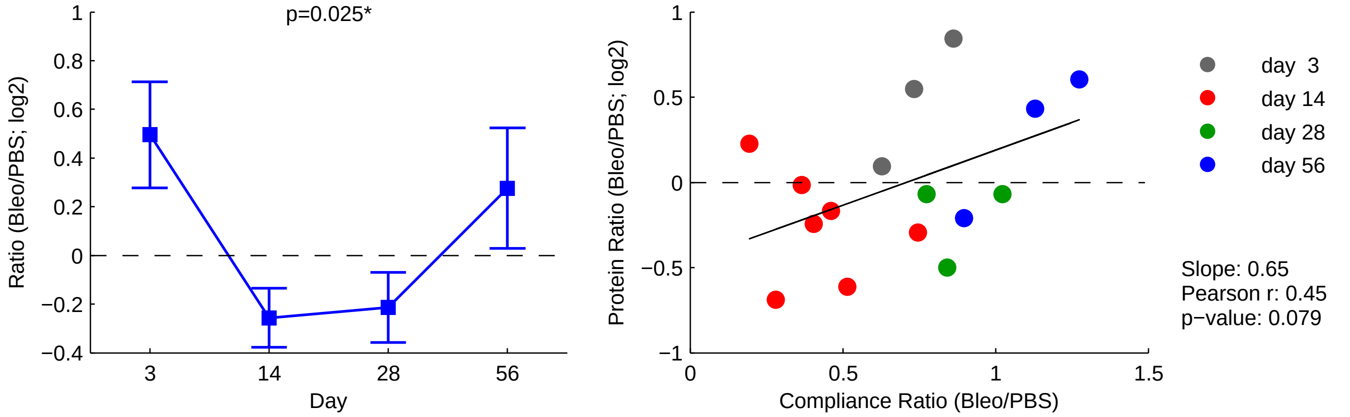

Q91V16 − Lyrm5 (id: 6180)

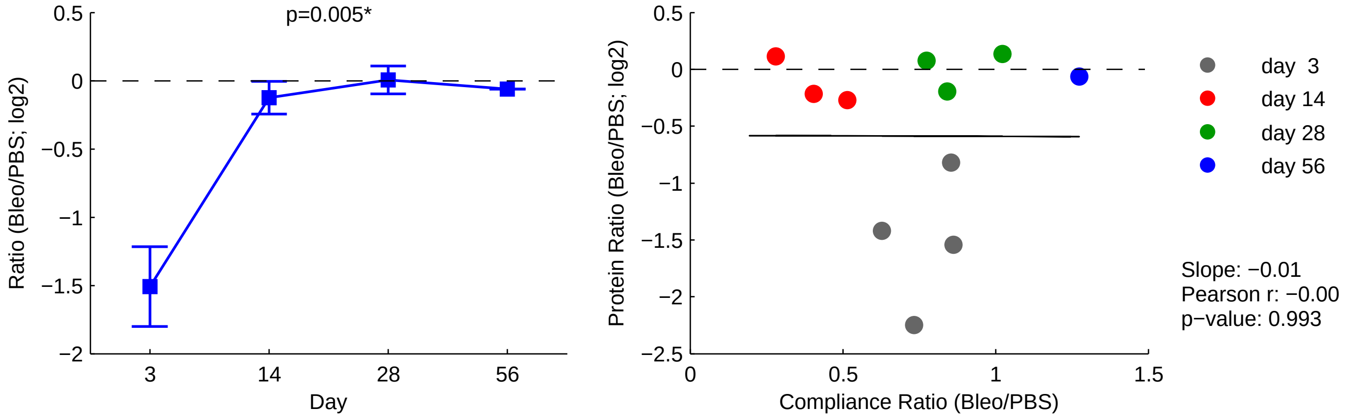

Q91V41 − Rab14 (id: 6182)

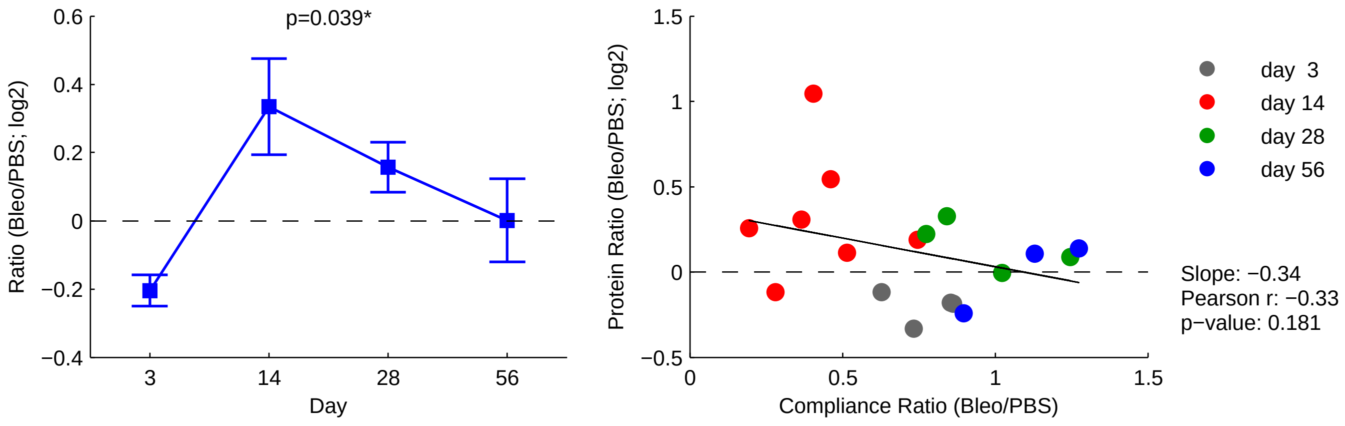

Q91V55 − Rps5 (id: 6183)

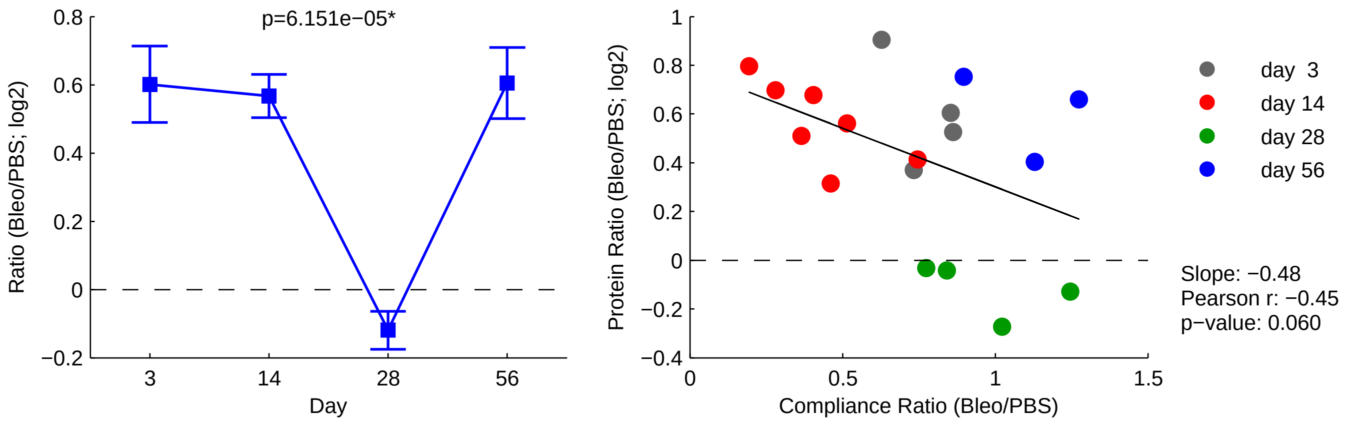

Q91V61 − Sfxn3 (id: 6184)

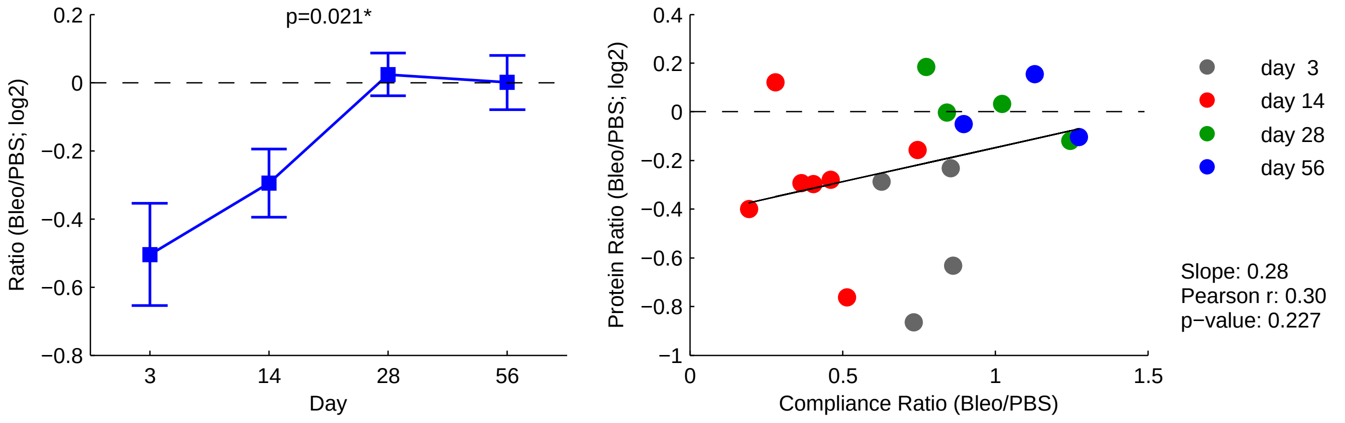

Q91V64 − Isoc1 (id: 6185)

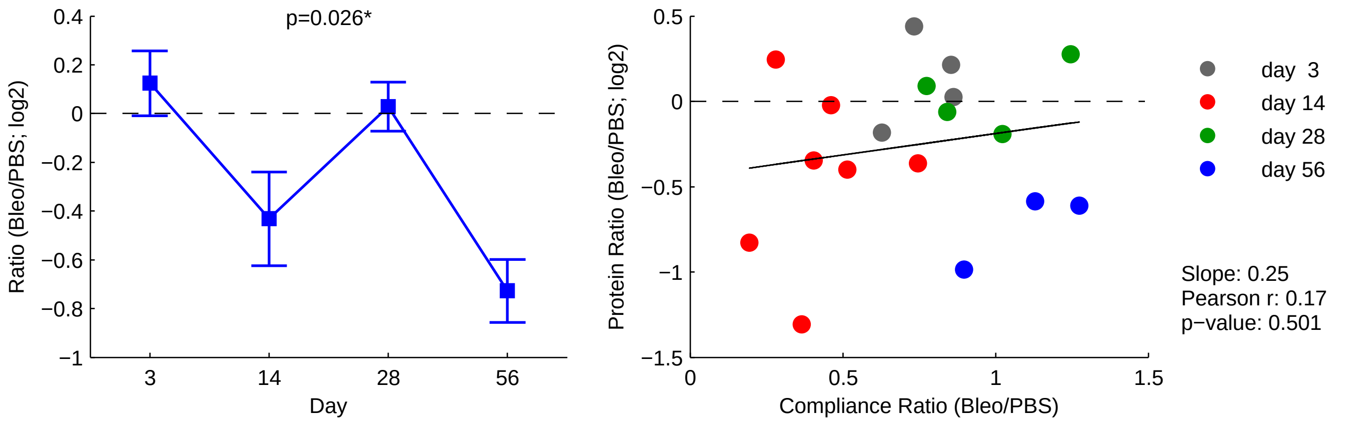

Q91V76 − (id: 6186)

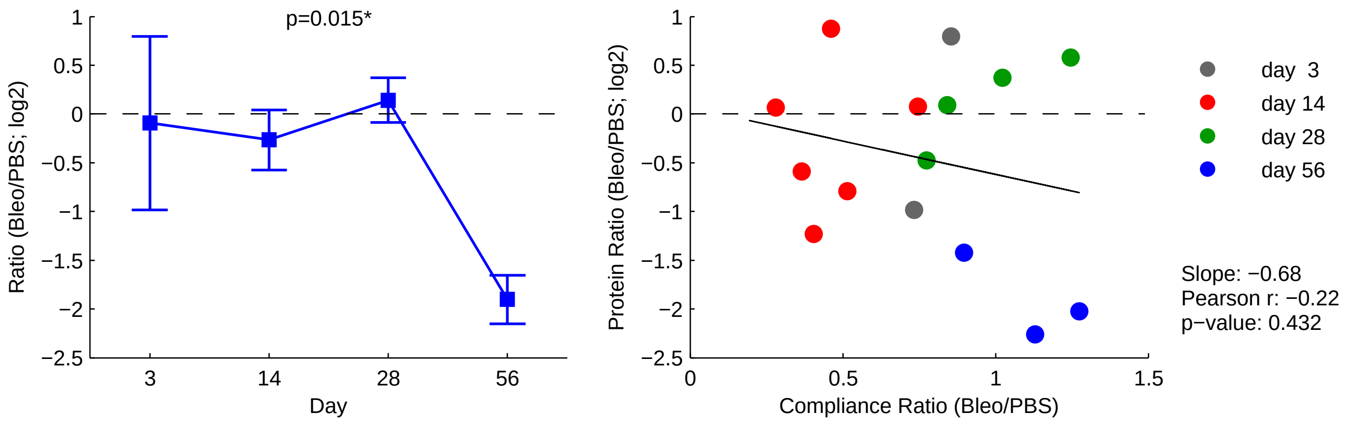

Q91V77 − S100a1 (id: 6187)

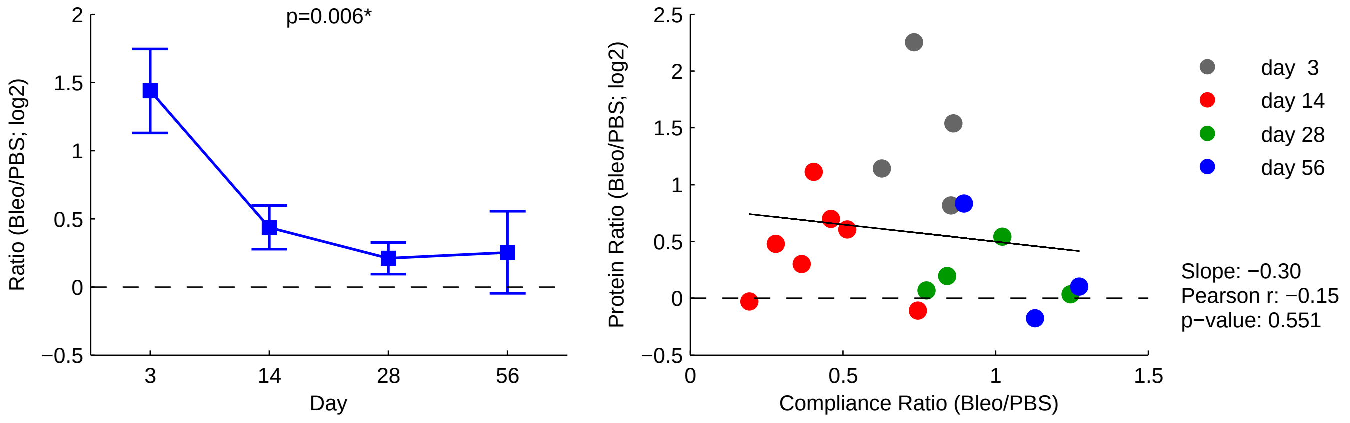

Q91V80 − Apof (id: 6188)

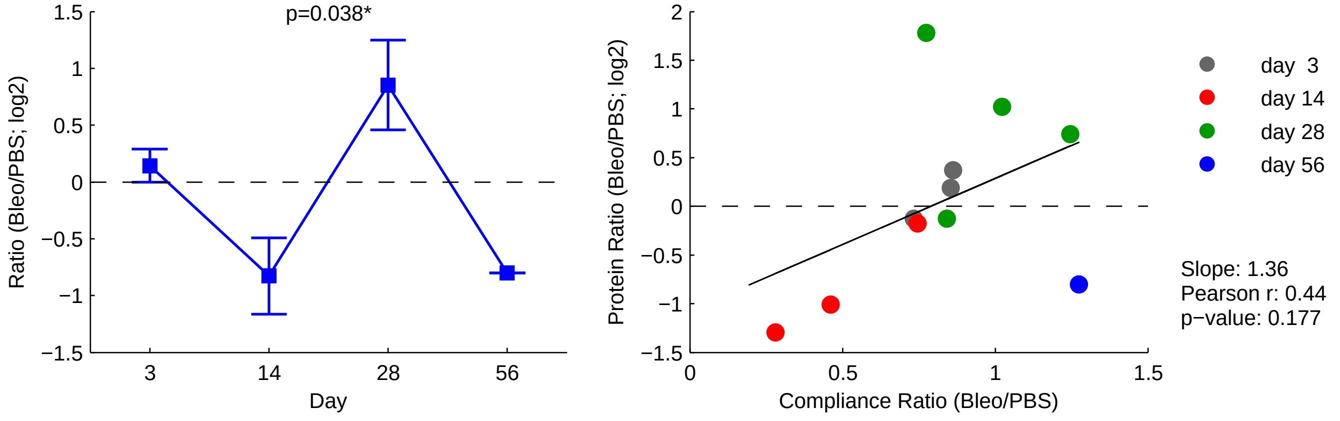

Q91V88−2 − Npnt (id: 6189)

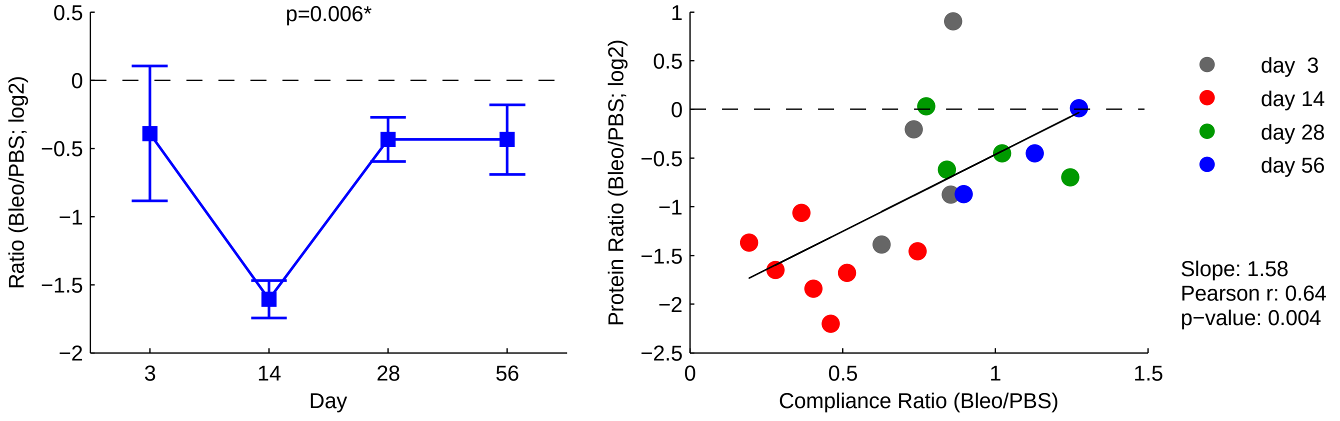

Q91VA0 − Acsm1 (id: 6191)

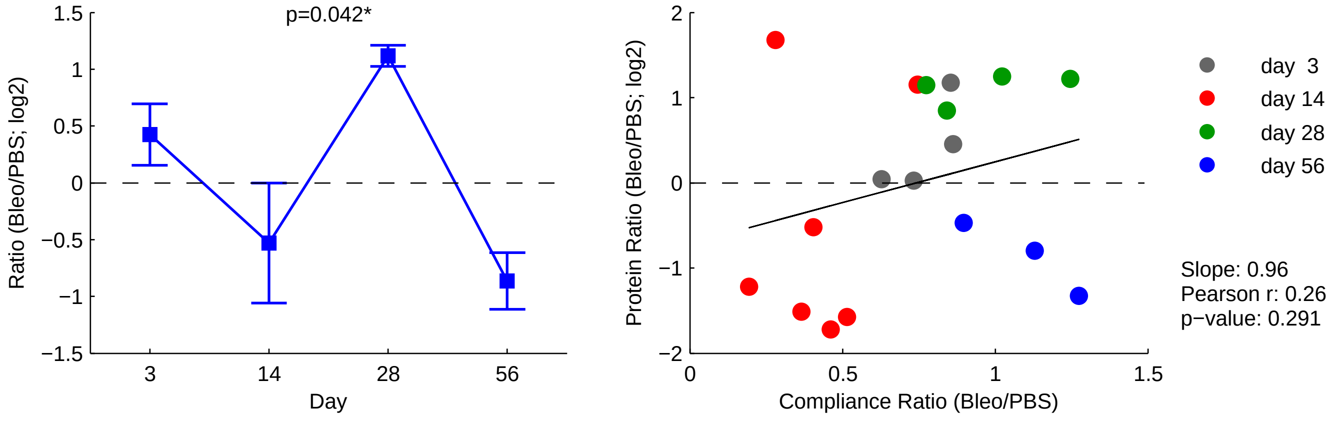

Q91VA7 − Idh3b (id: 6192)

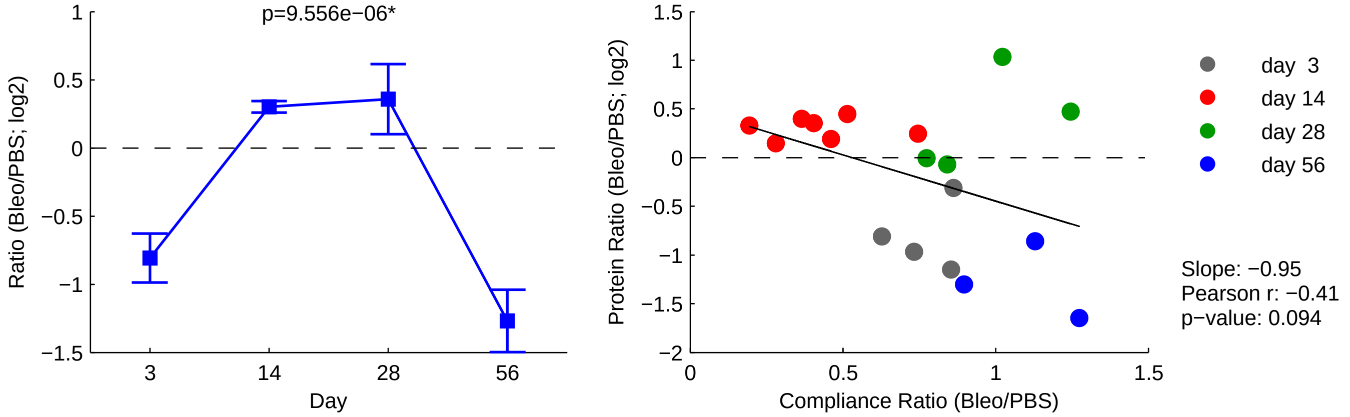

Q91VB8 − Hba−a1 (id: 6193)

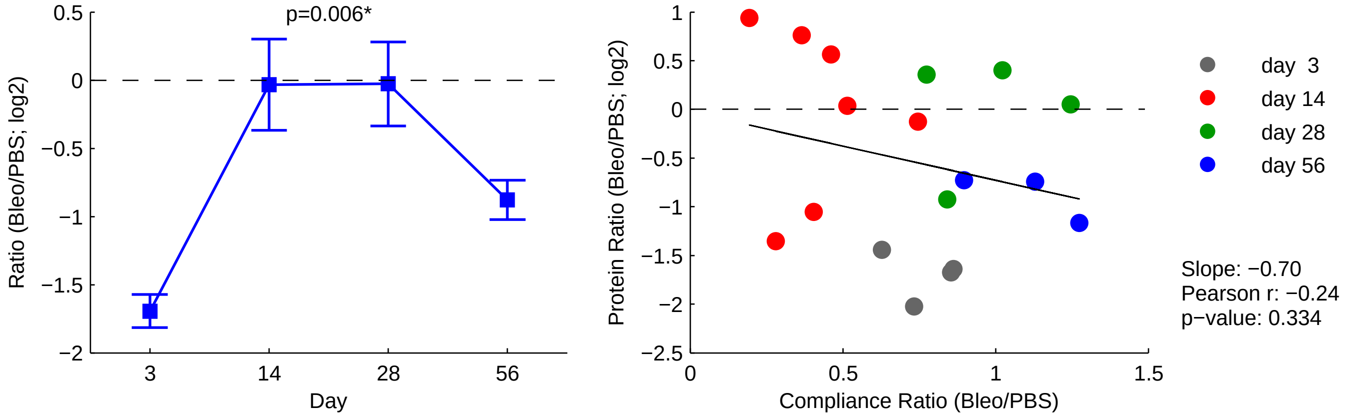

Q91VC7 − Ppp1r14a (id: 6195)

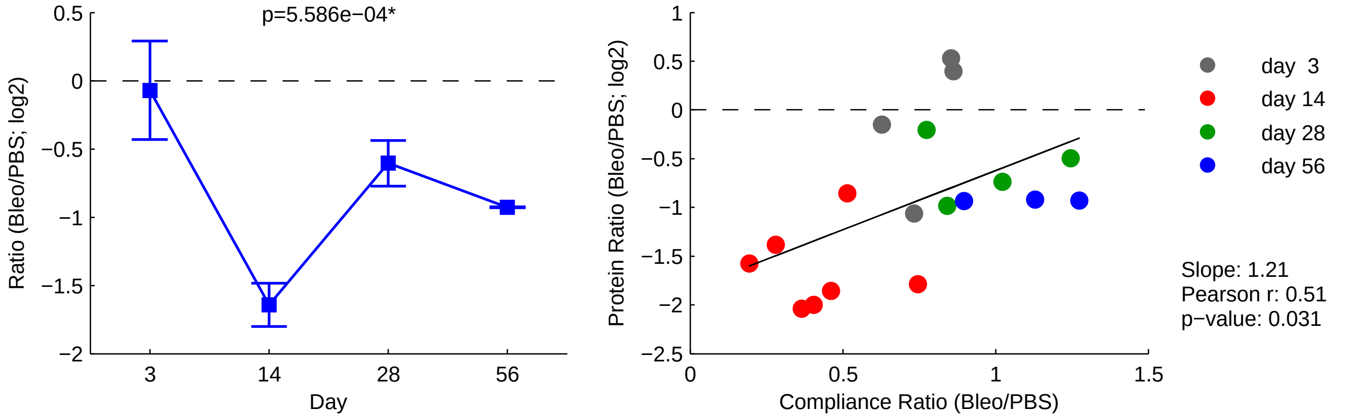

Q91VD9 − Ndufs1 (id: 6197)

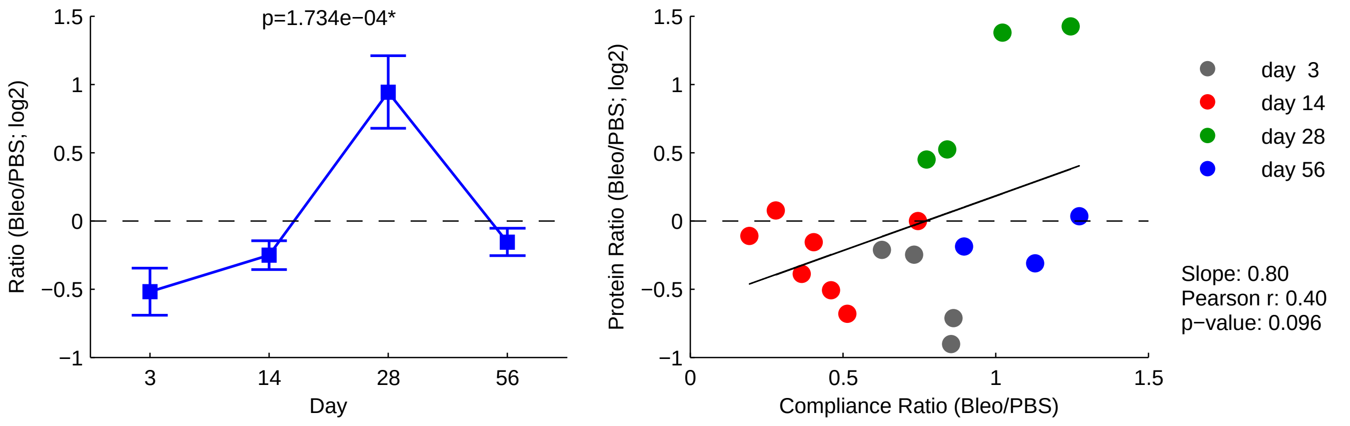

Q91VF5−2 − Emid1 (id: 6200)

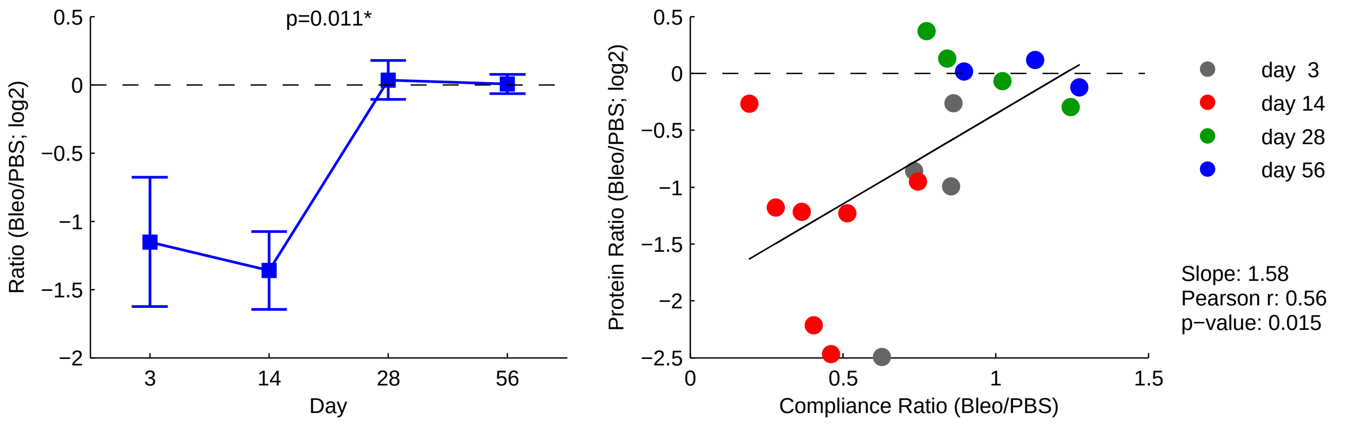

Q91VH6 − Memo1 (id: 6202)

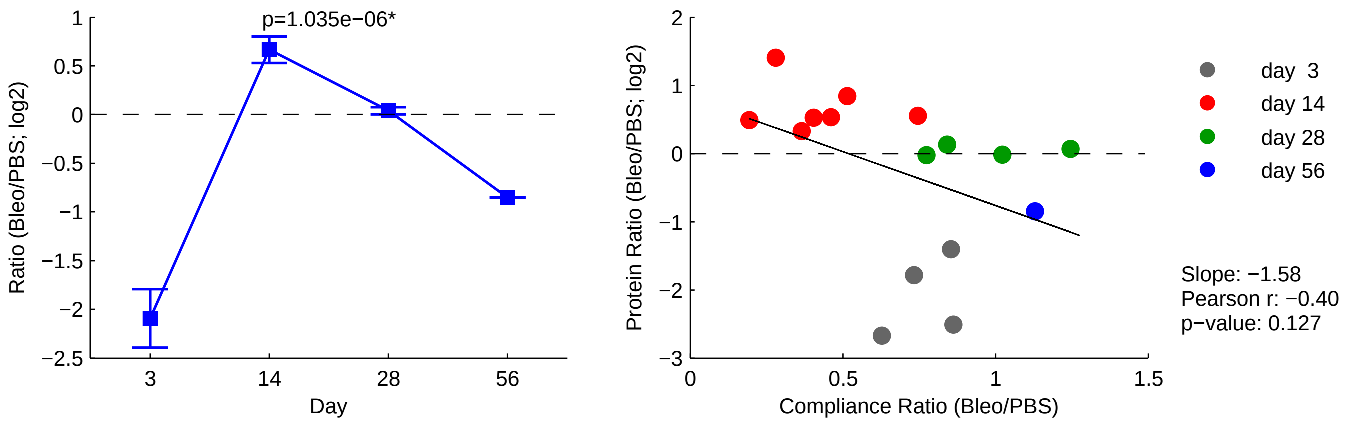

Q91VI7 − Rnh1 (id: 6203)

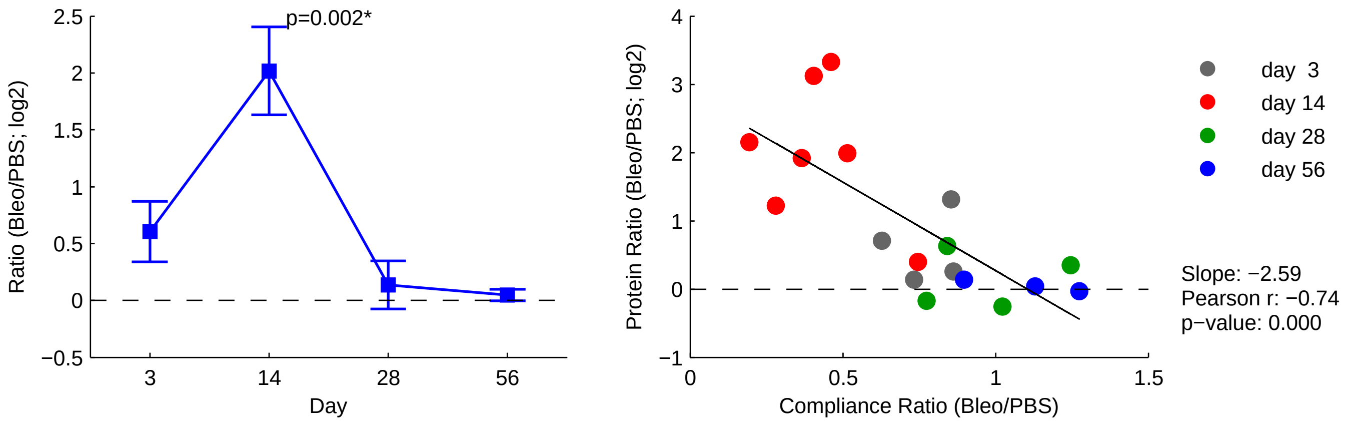

Q91VK1 − Bzw2 (id: 6206)

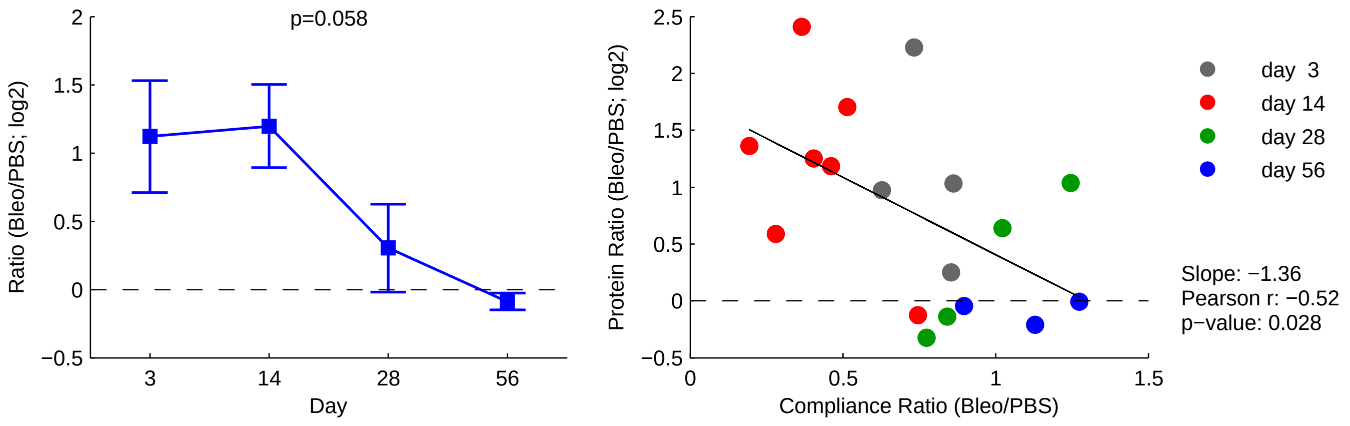

Q91VL8 − Terf2ip (id: 6209)

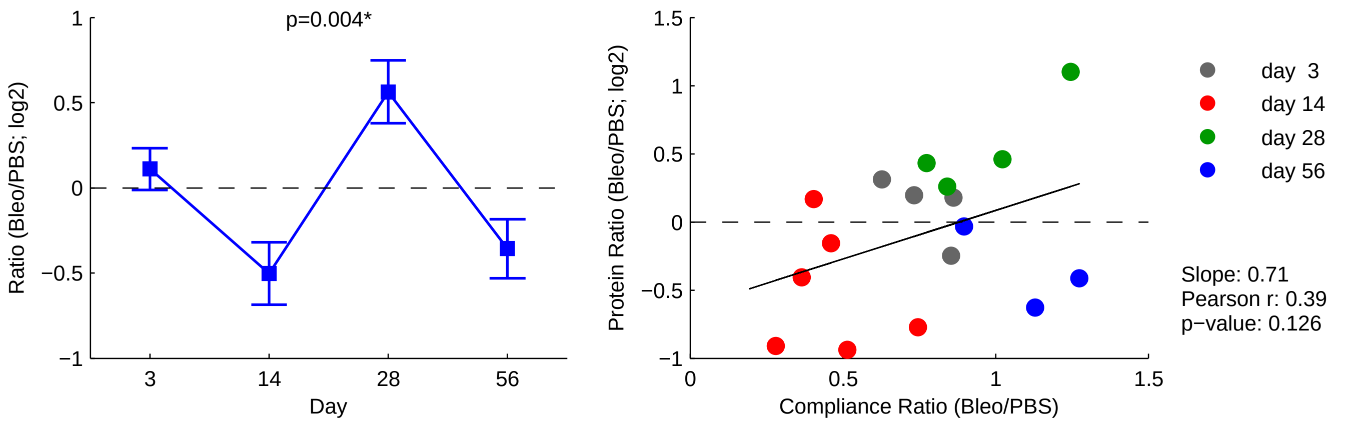

Q91VM9 − Ppa2 (id: 6213)

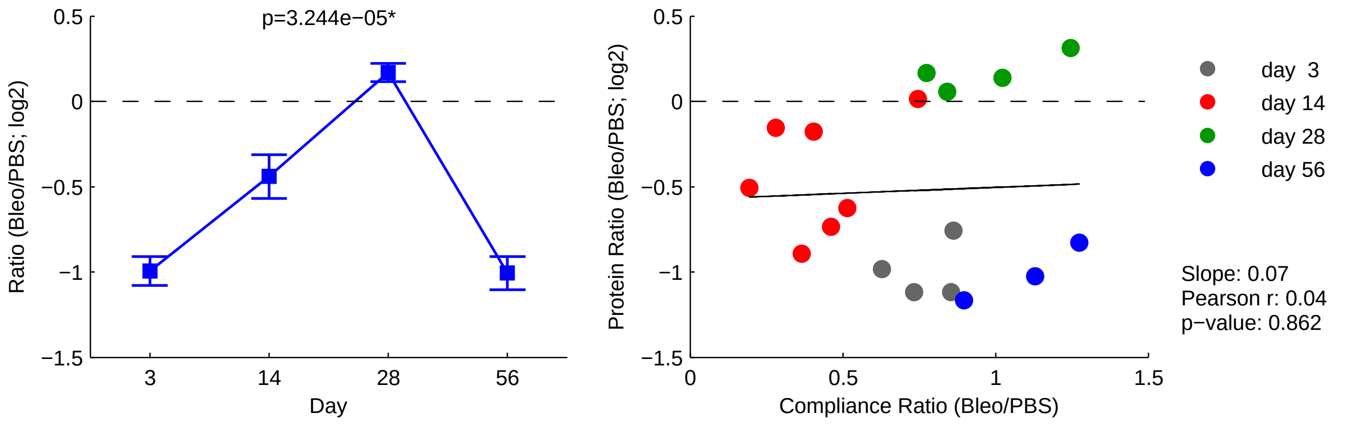

Q91VN4 − Chchd6 (id: 6215)

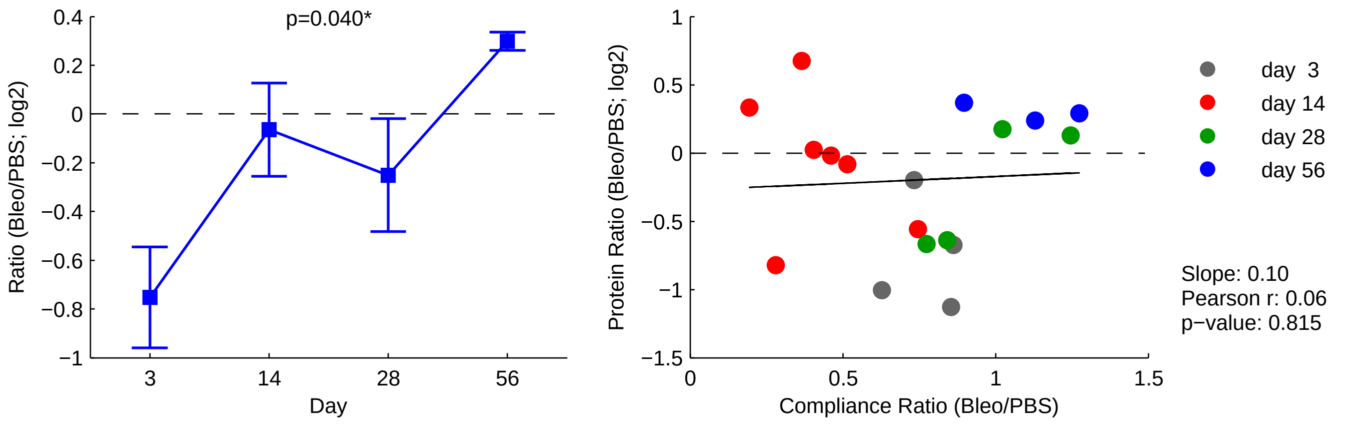

Q91VR5 − Ddx1 (id: 6217)

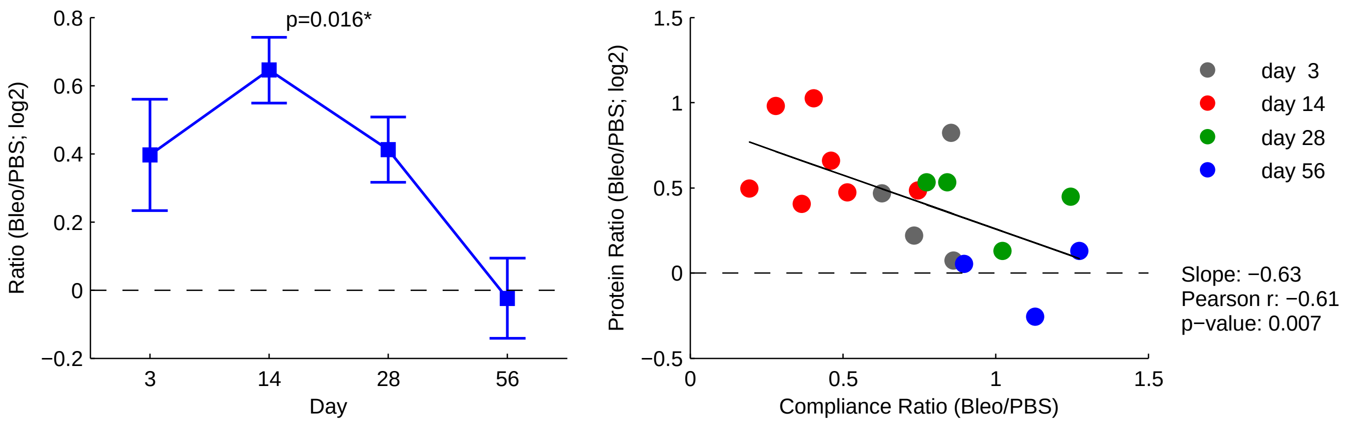

Q91VR8 − Brk1 (id: 6219)

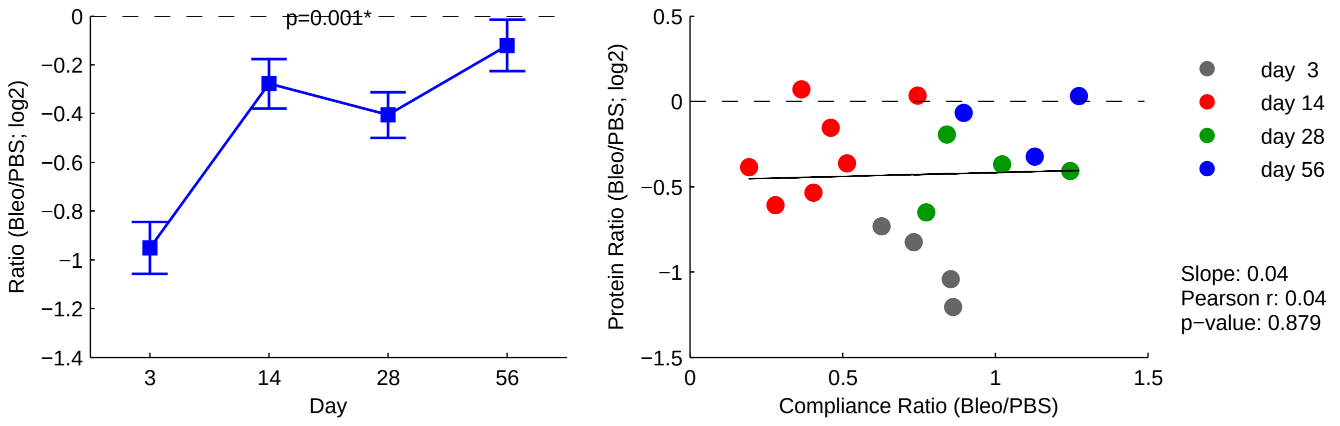

Q91VS8 − Farp2 (id: 6220)

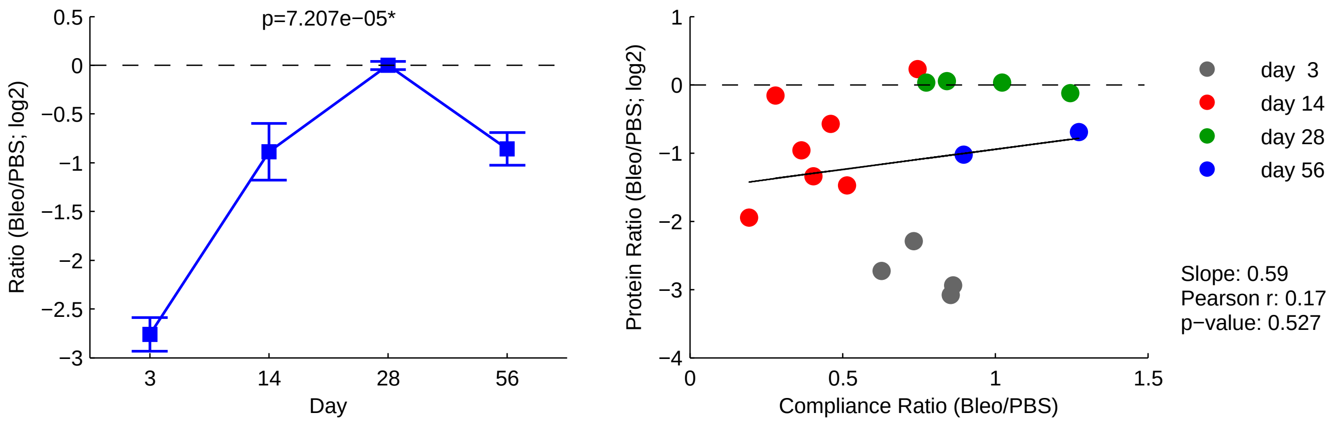

Q91VT4 − Cbr4 (id: 6222)

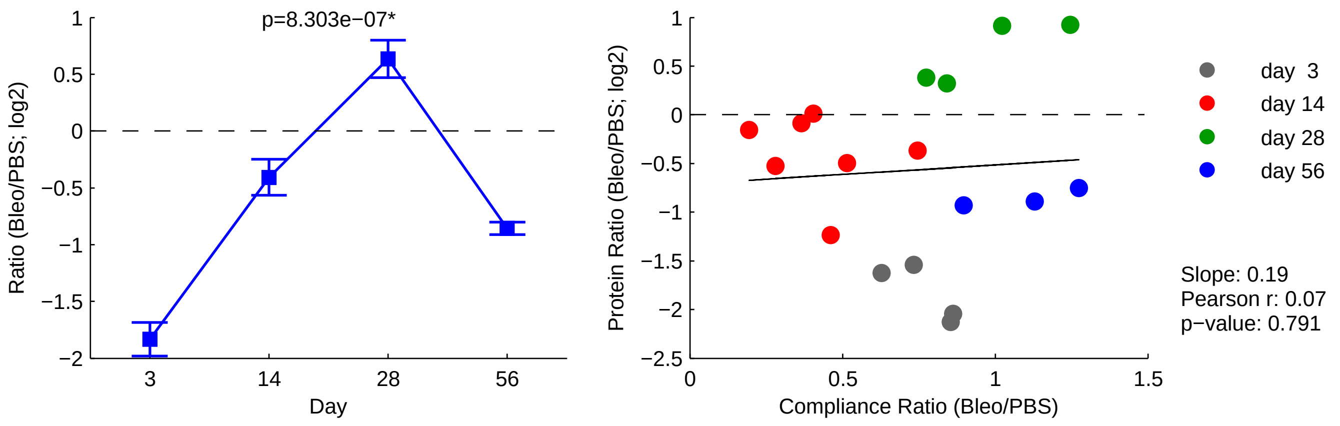

Q91VV4−2 − Dennd2d (id: 6223)

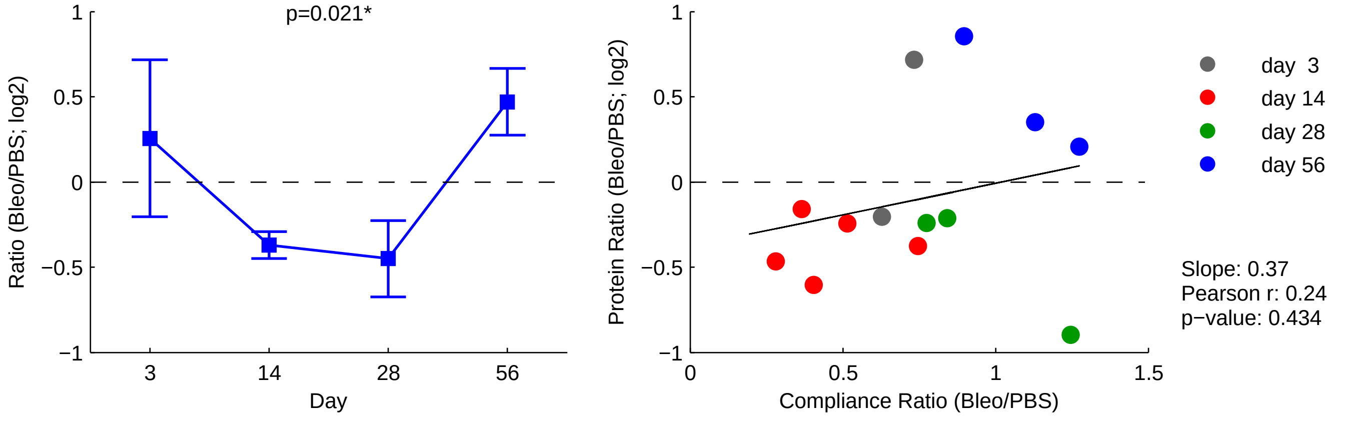

Q91VW3 − Sh3bgrl3 (id: 6224)

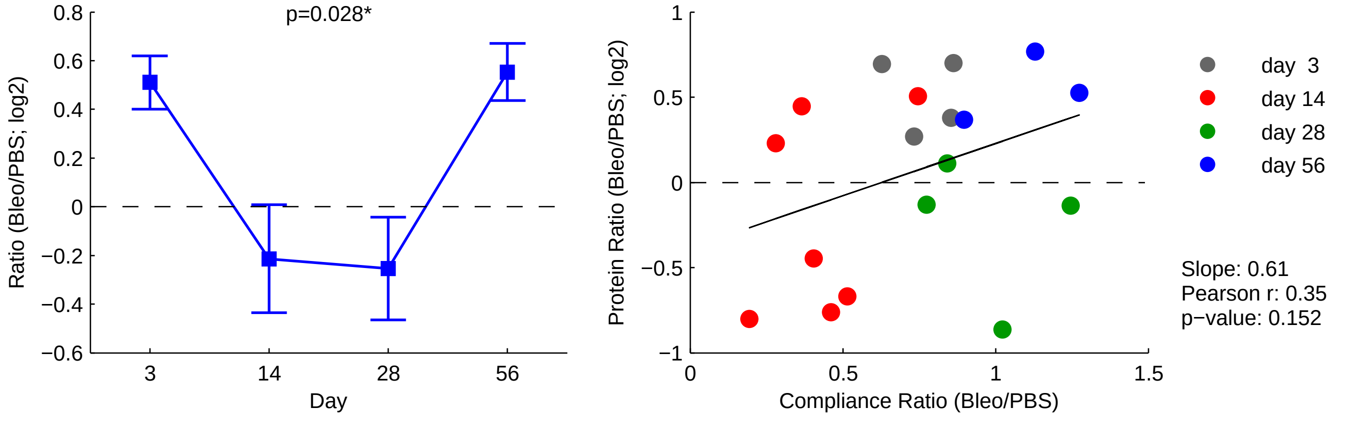

Q91VW5 − Golga4 (id: 6225)

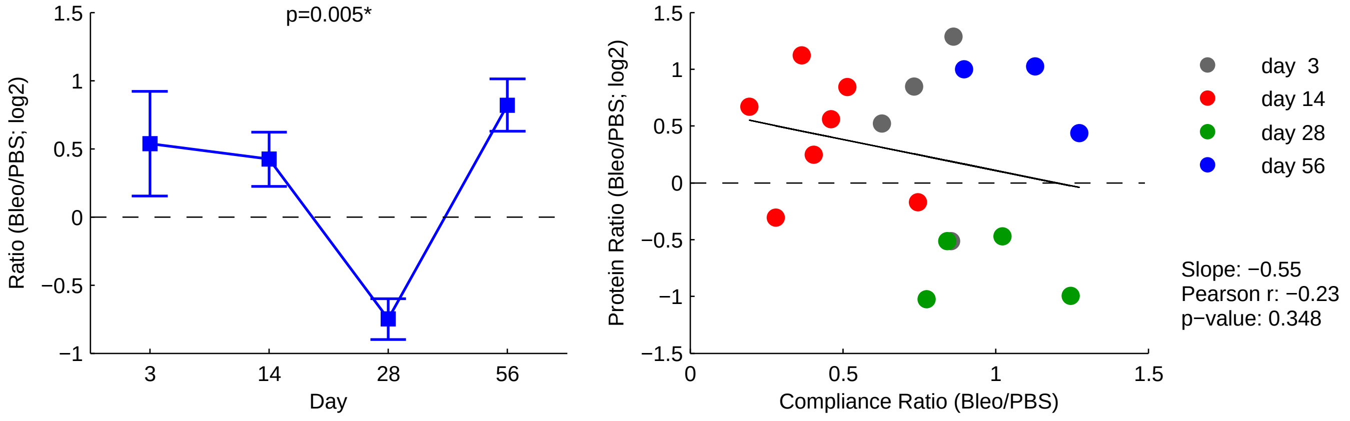

Q91VX9 − Tmem168 (id: 6226)

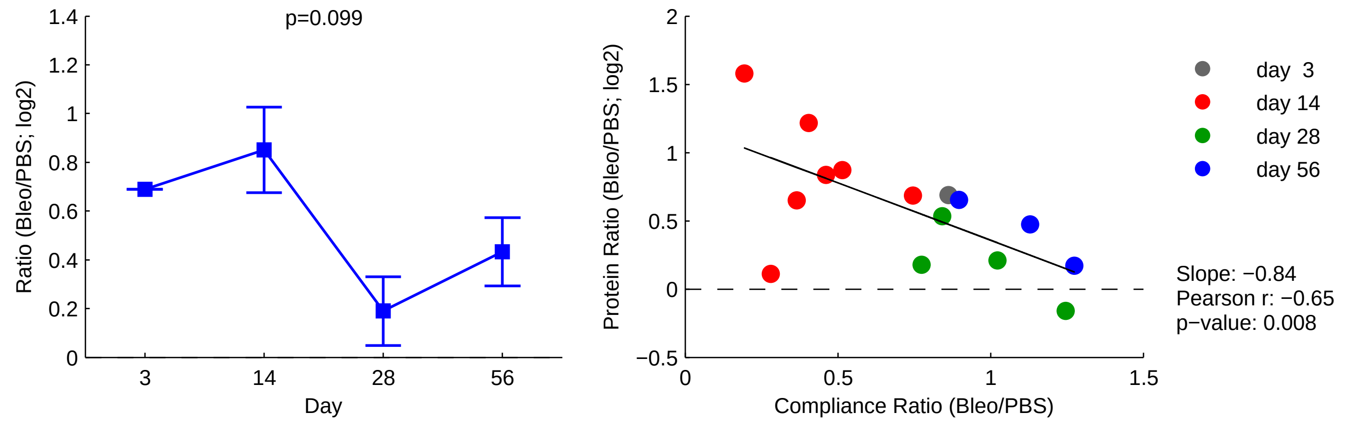

Q91VZ6 − Smap1 (id: 6228)

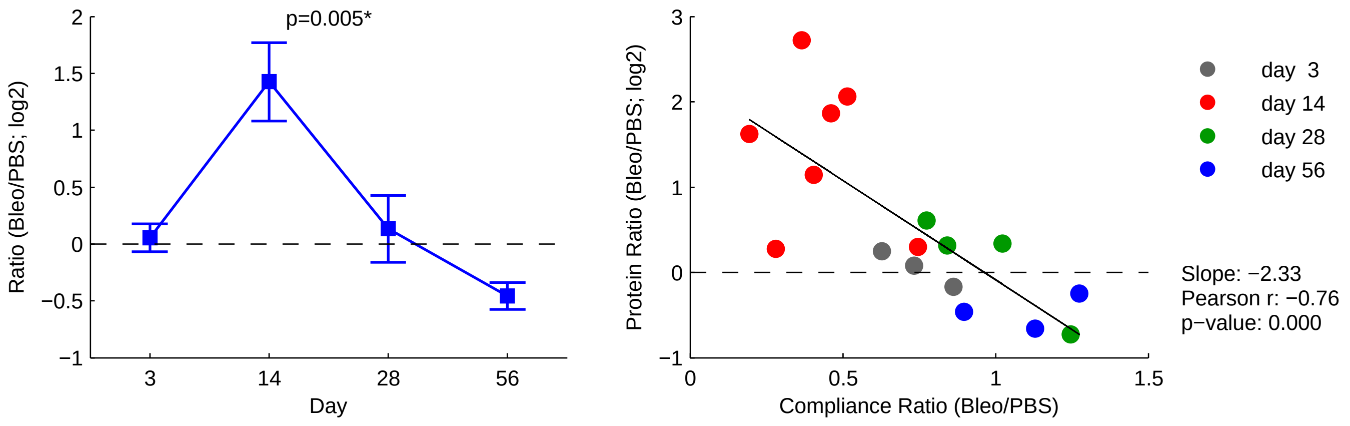

Q91W10−2 − Slc39a8 (id: 6229)

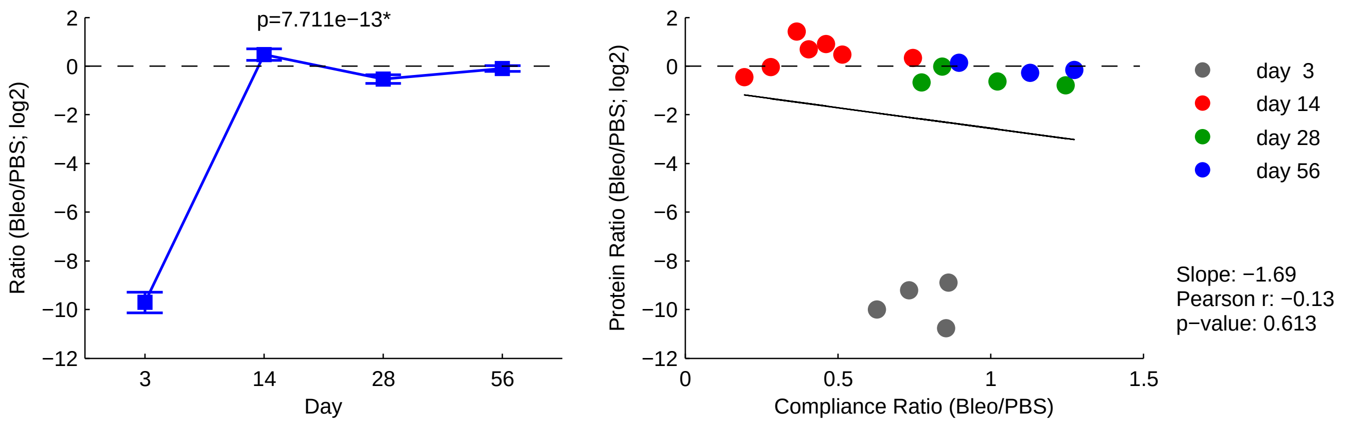

Q91W18−2 − Tdrd3 (id: 6230)

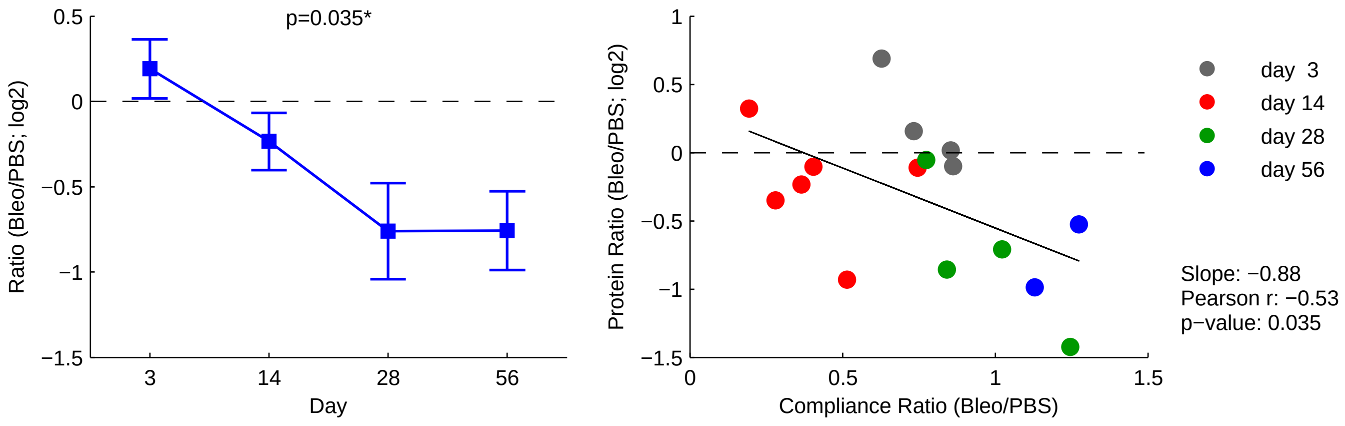

Q91W29 − Cox4i2 (id: 6231)

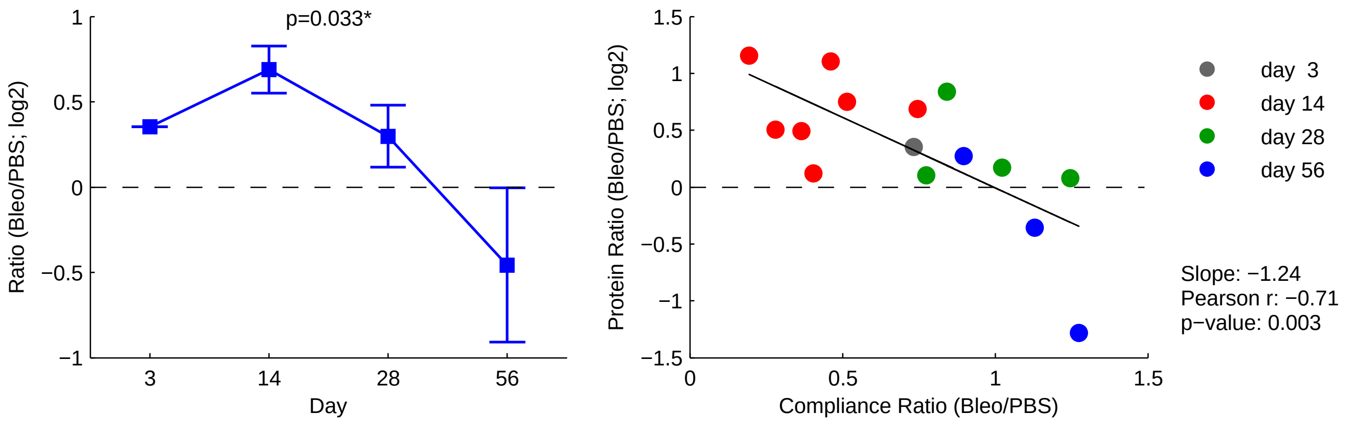

Q91W34 − (id: 6232)

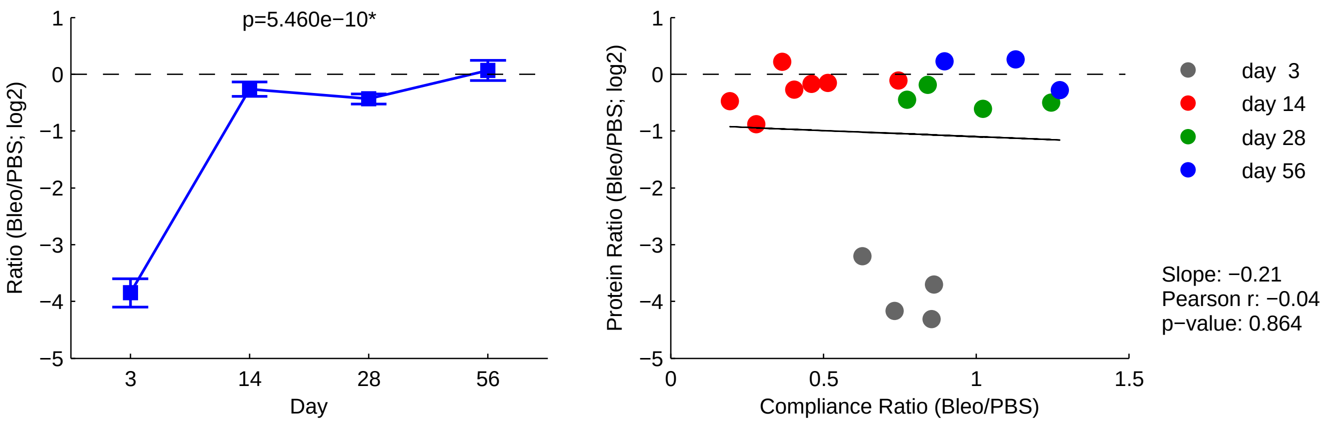

Q91W39 − Ncoa5 (id: 6233)

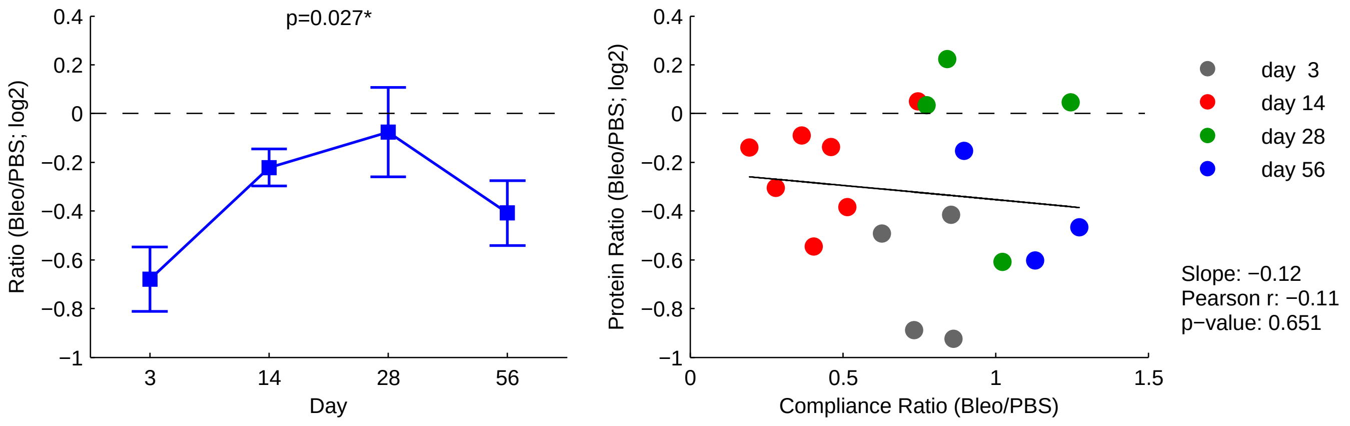

Q91W50 − Csde1 (id: 6234)

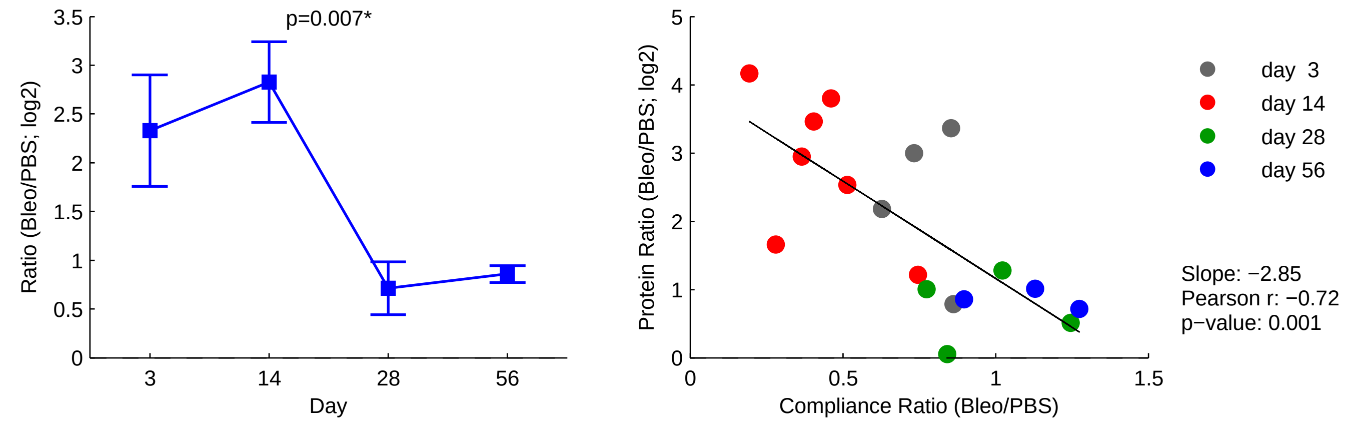

Q91W92 − Cdc42ep1 (id: 6239)

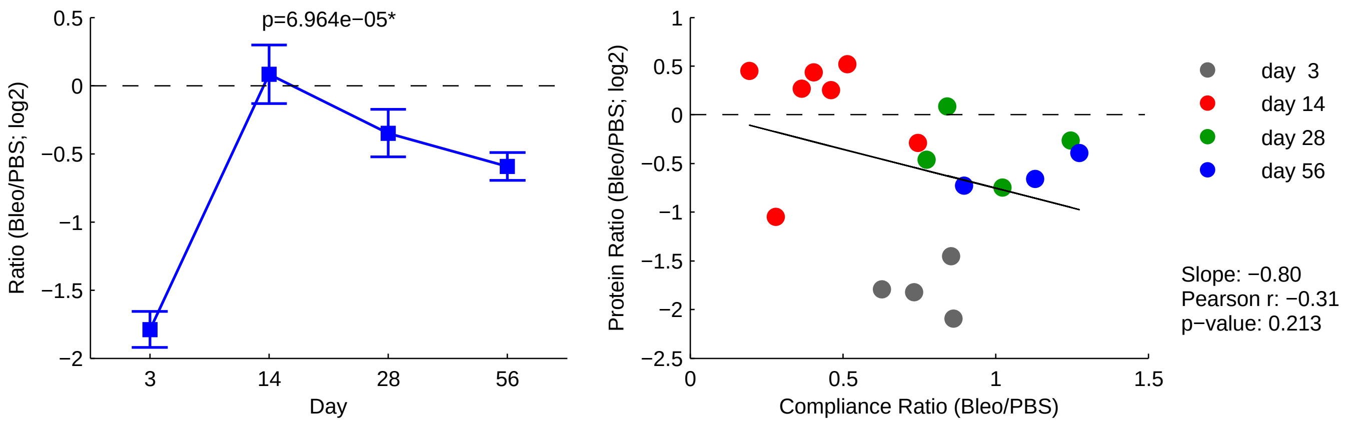

Q91W98 − Slc15a4 (id: 6242)

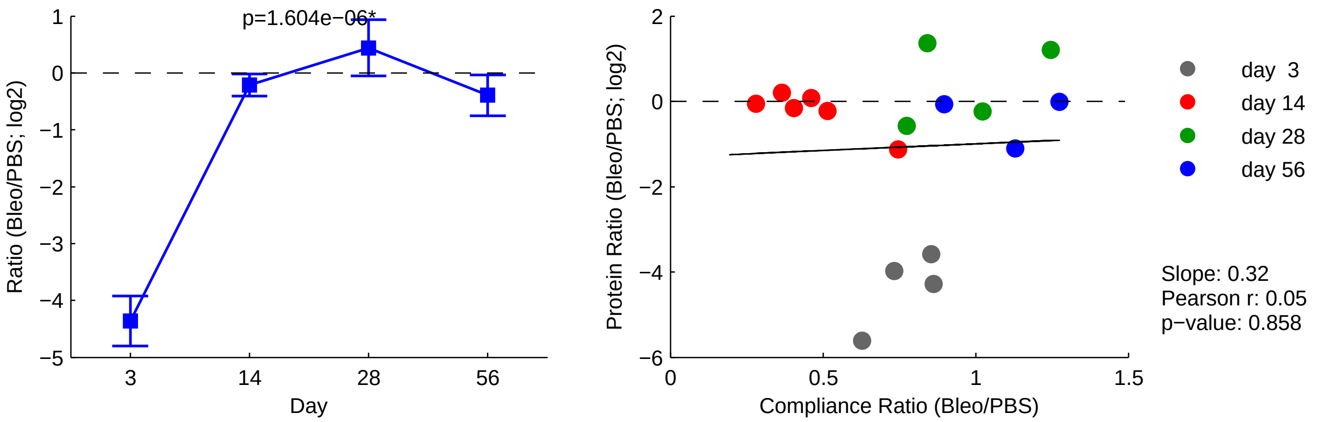

Q91WC9 − Daglb (id: 6244)

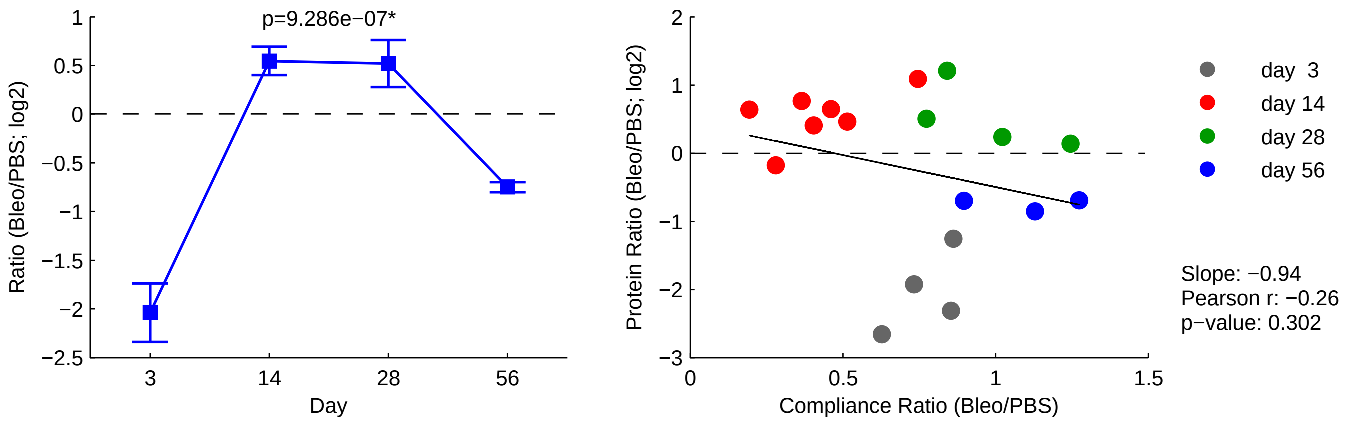

Q91WD5 − Ndufs2 (id: 6246)

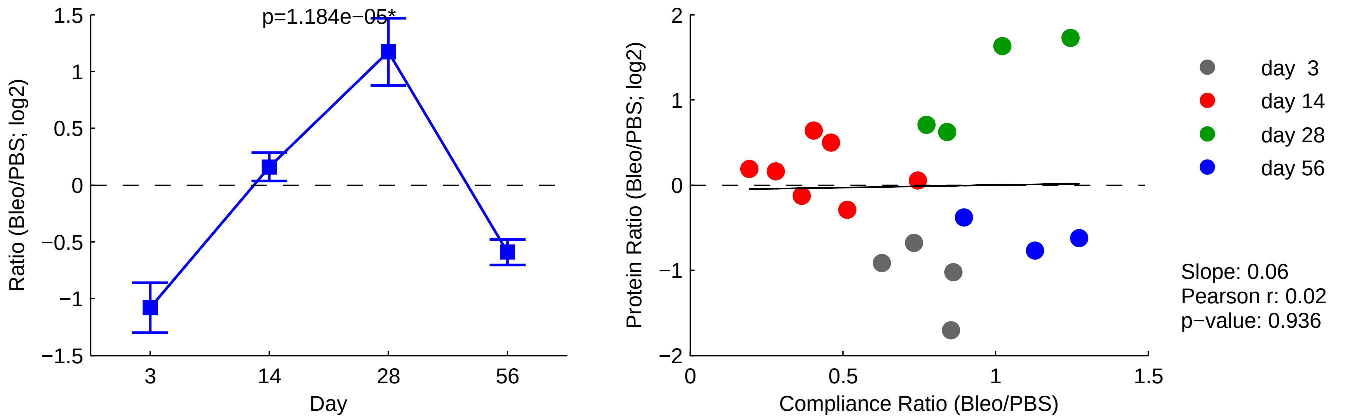

Q91WE6 − Cdkal1 (id: 6248)

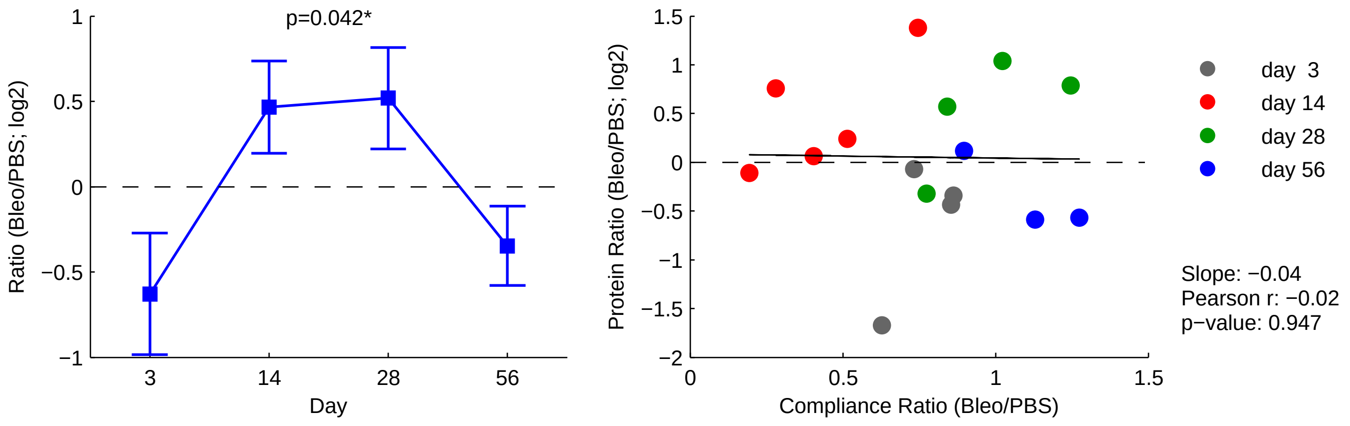

Q91WF3 − Adcy4 (id: 6249)

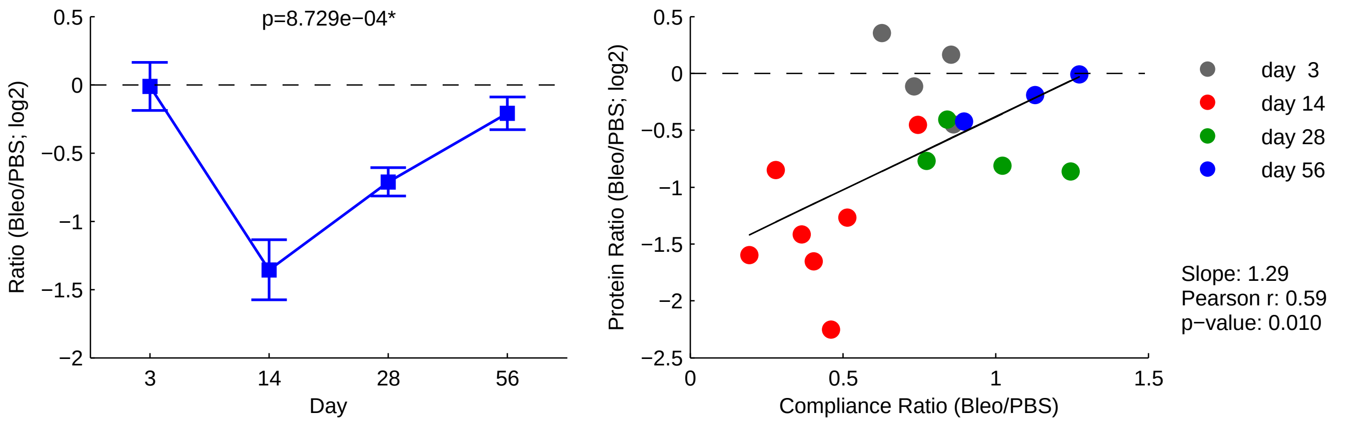

Q91WG4−2 − Elp2 (id: 6252)

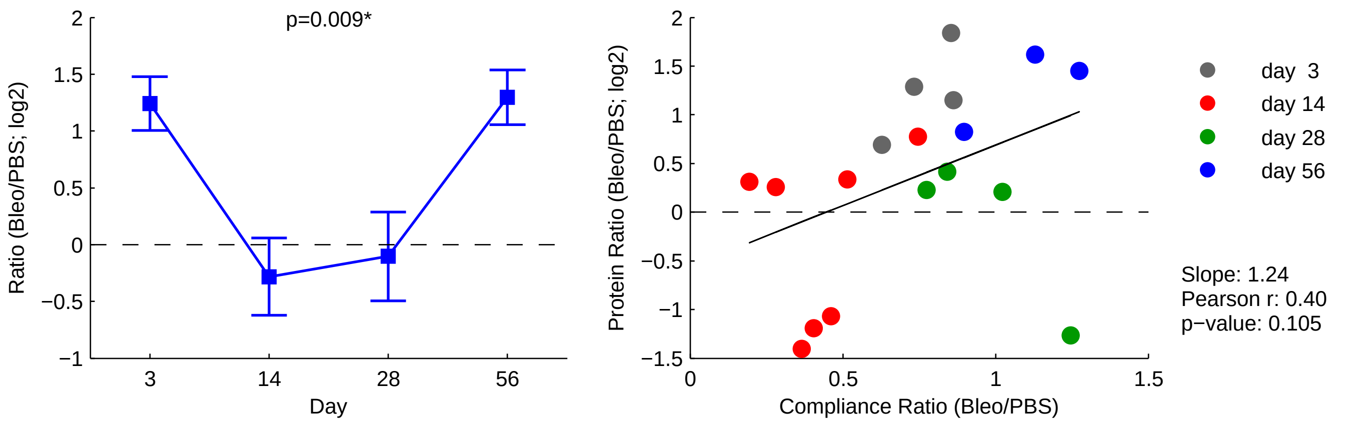

Q91WK1 − Spryd4 (id: 6253)

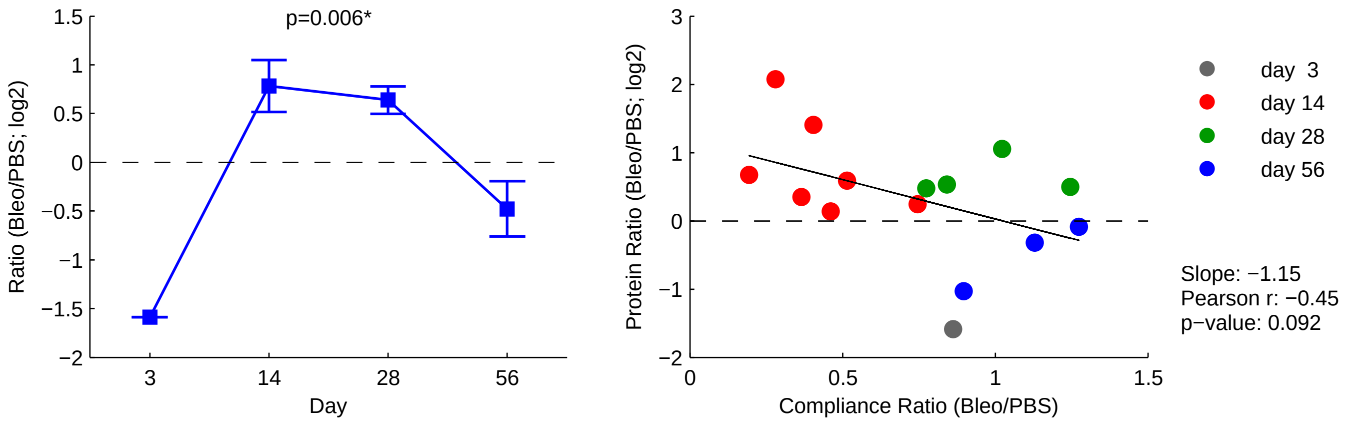

Q91WK2 − Eif3h (id: 6254)

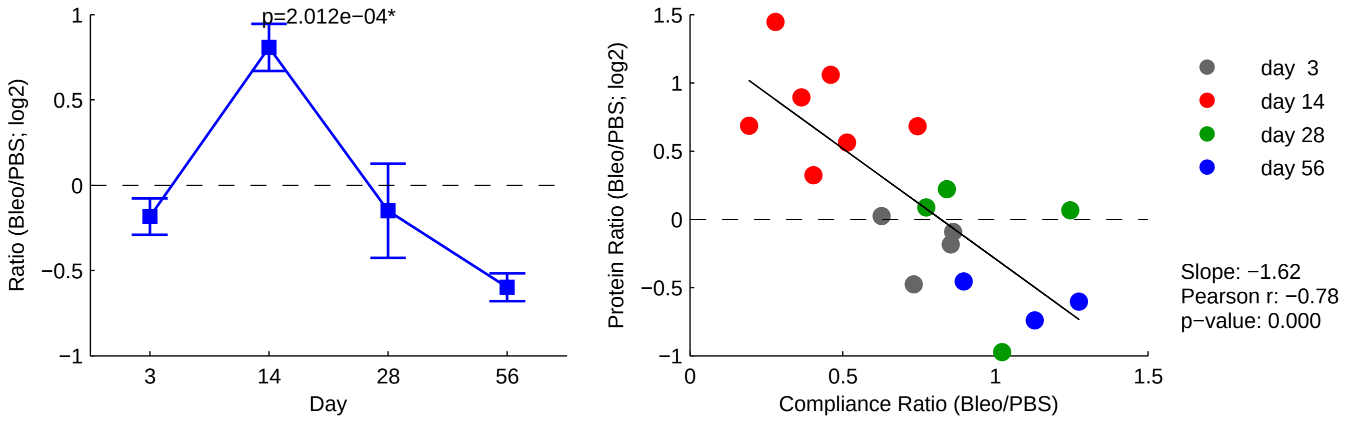

Q91WK5 − Gcsh (id: 6255)

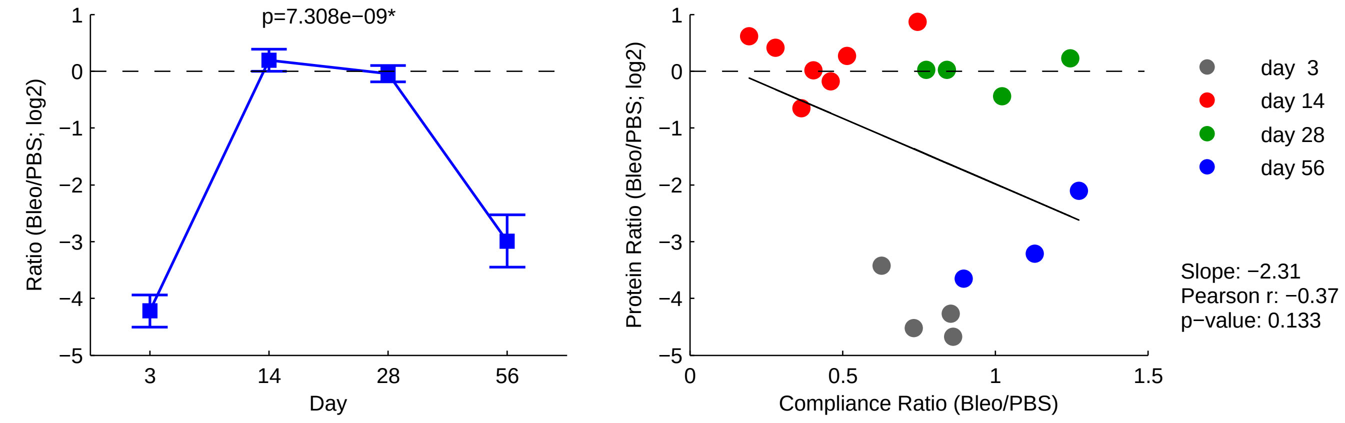

Q91WL8 − Wwox (id: 6256)

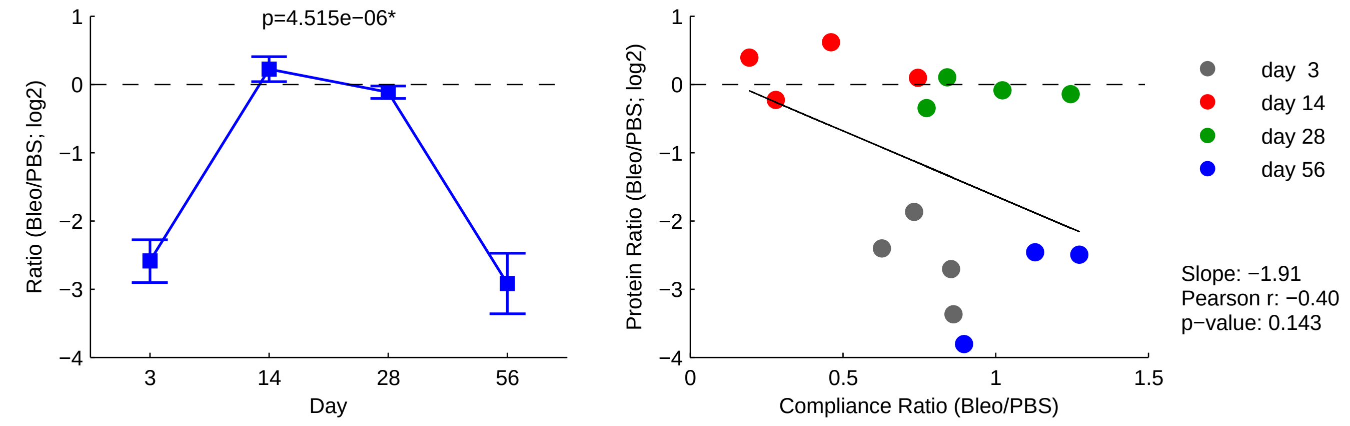

Q91WM2 − Cecr5 (id: 6257)

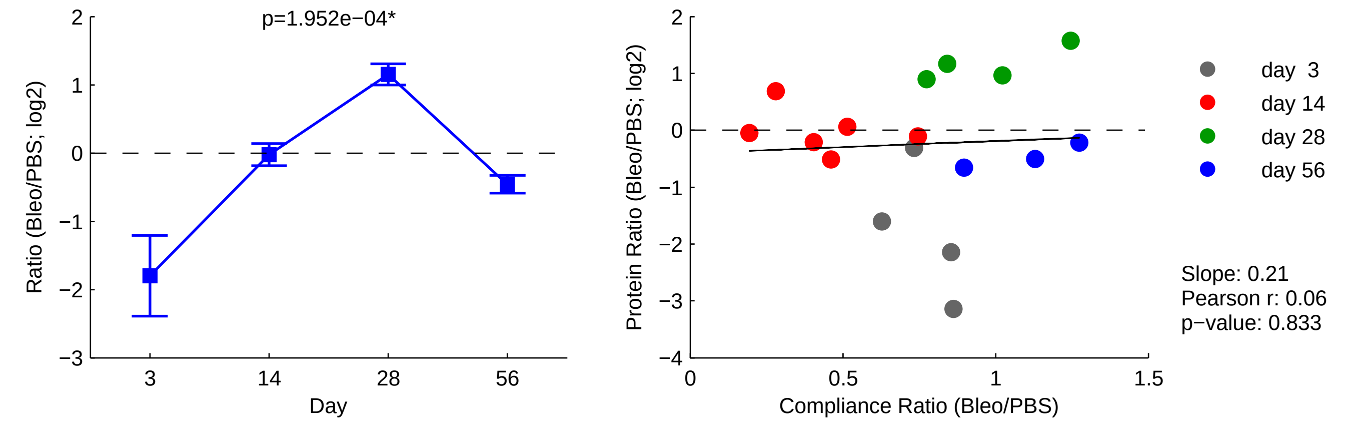

Q91WN1 − Dnajc9 (id: 6259)

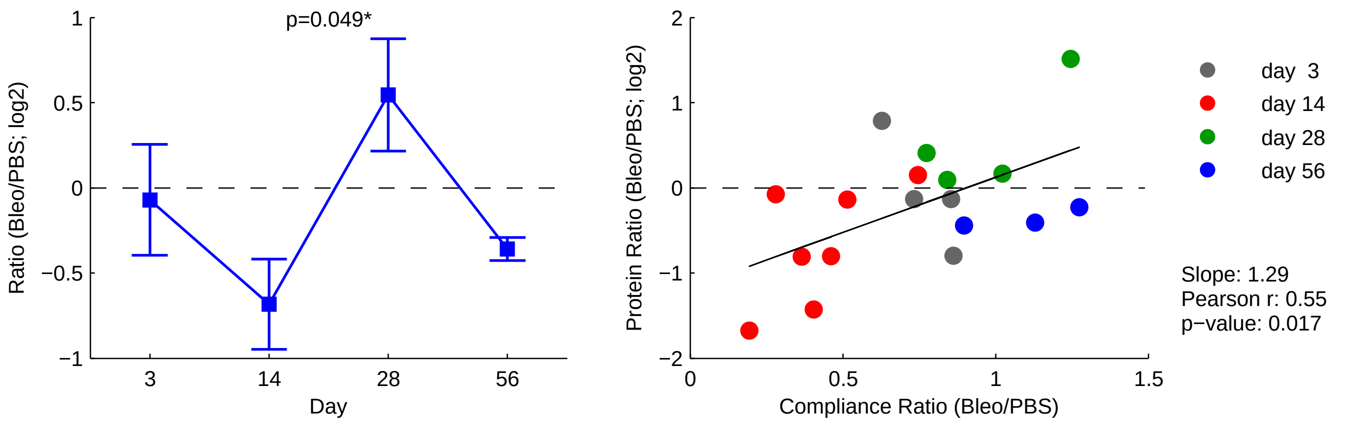

Q91WS0 − Cisd1 (id: 6264)

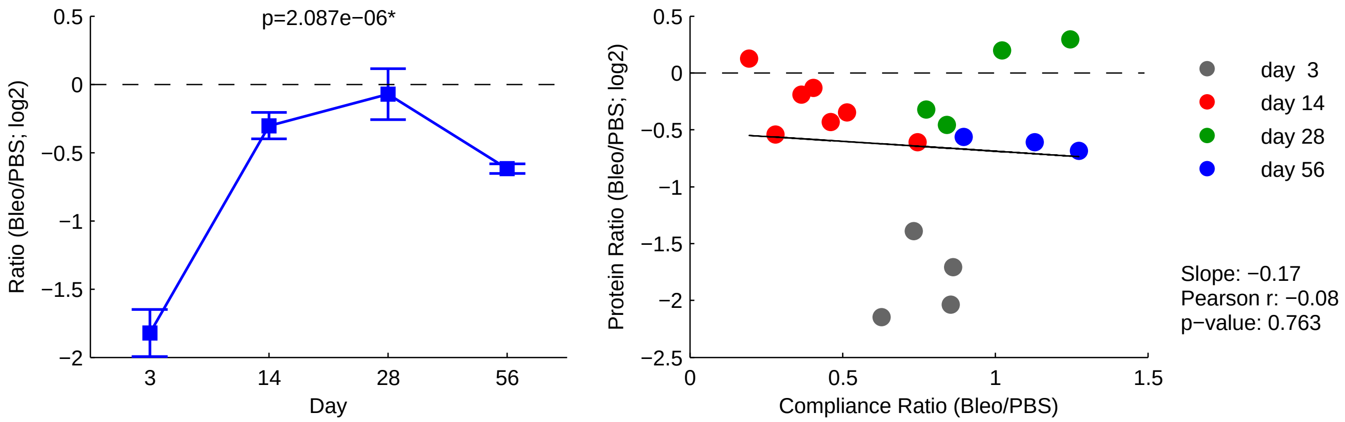

Q91WT7 − Akr1c14 (id: 6266)

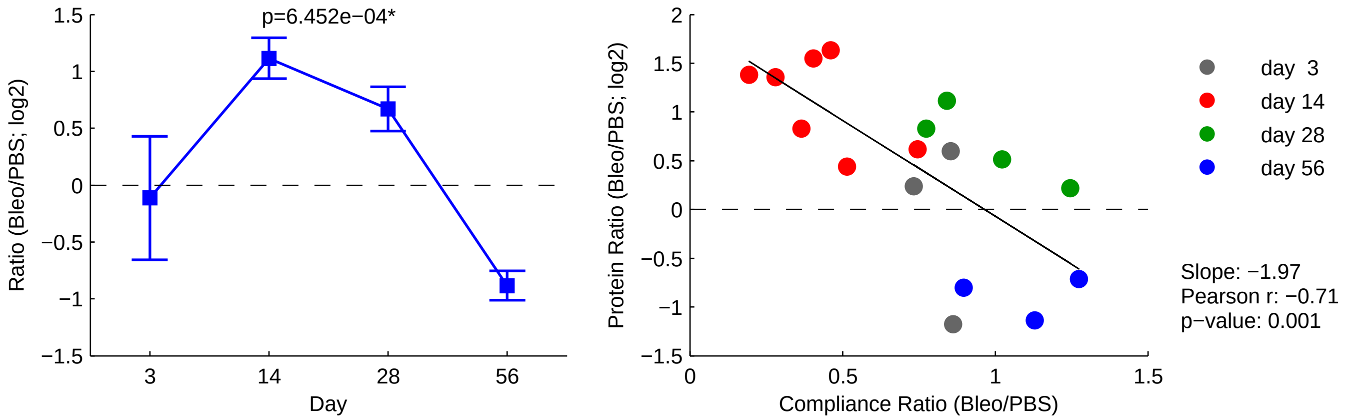

Q91WU0 − Ces1f (id: 6268)

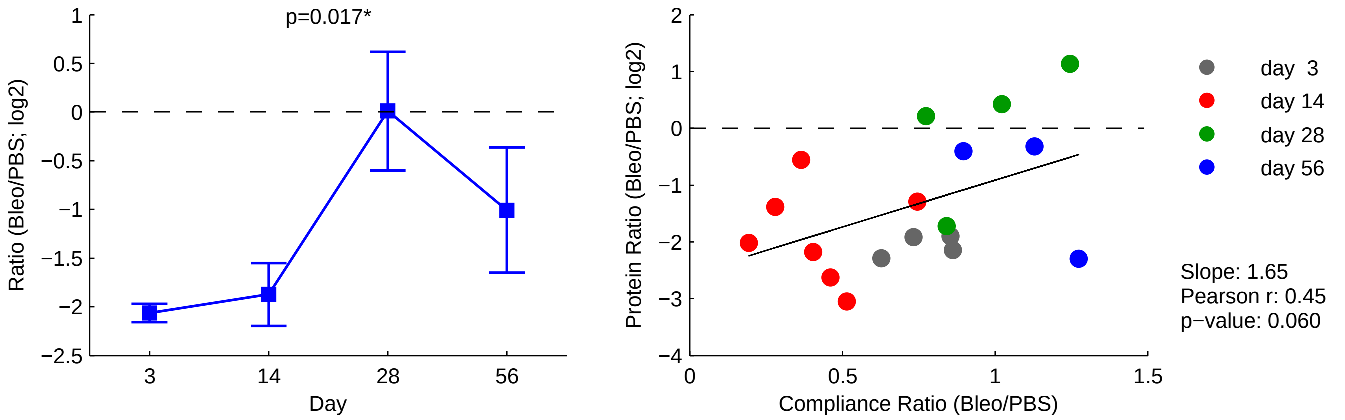

Q91X21 − Kiaa2013 (id: 6271)

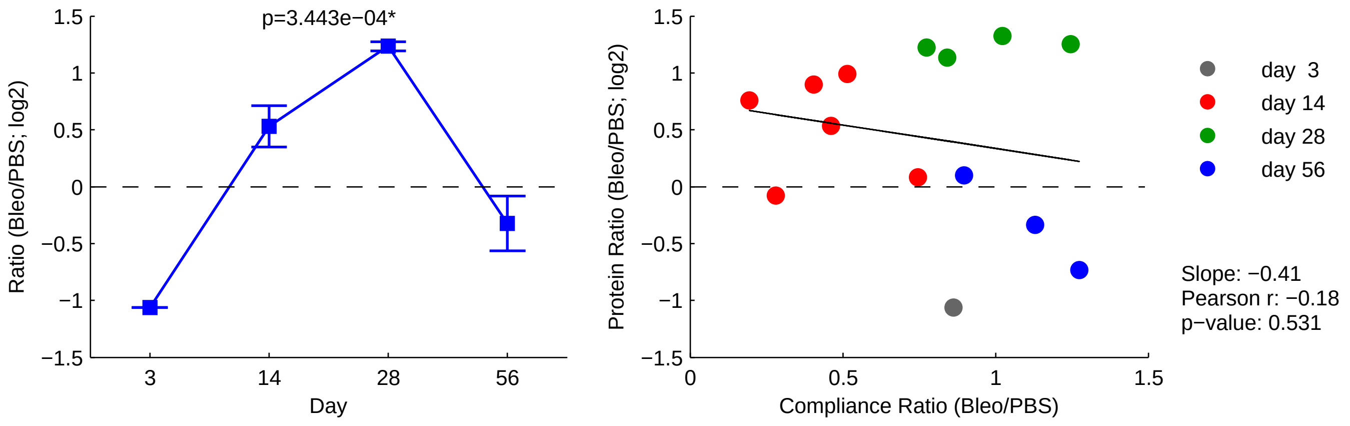

Q91X51 − Gorasp1 (id: 6274)

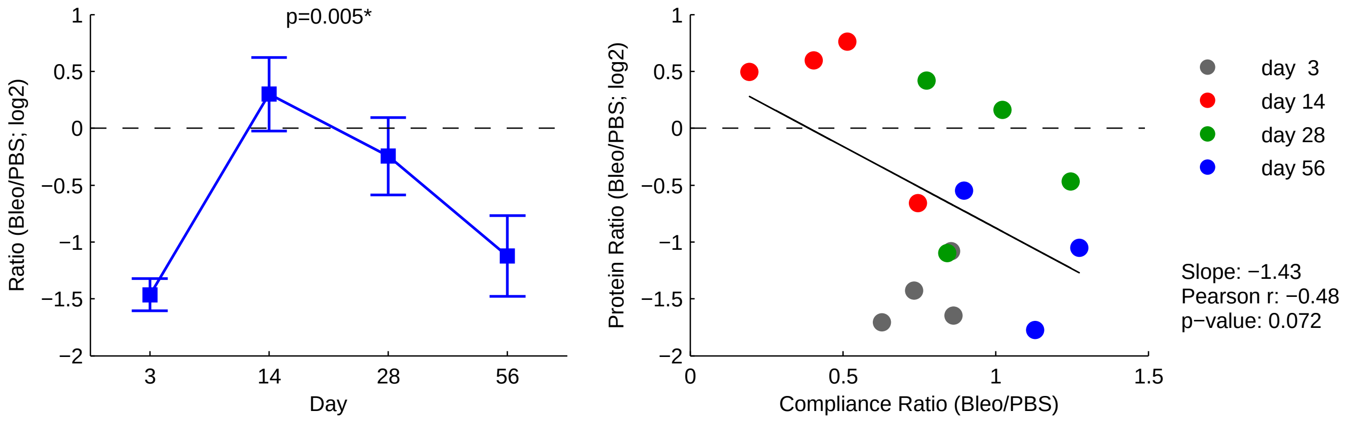

Q91X72 − Hpx (id: 6276)

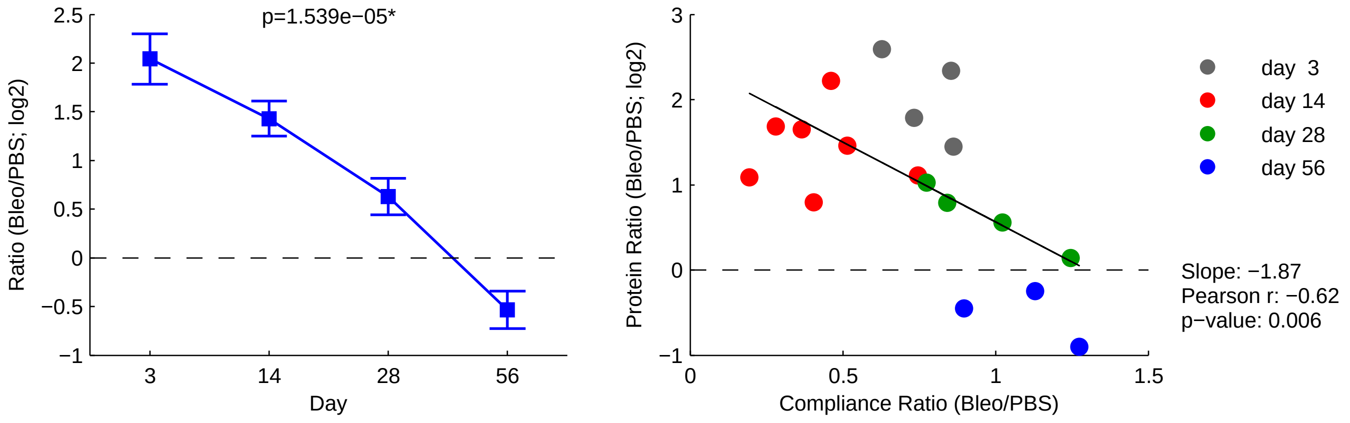

Q91X75 − Cyp2a5 (id: 6277)

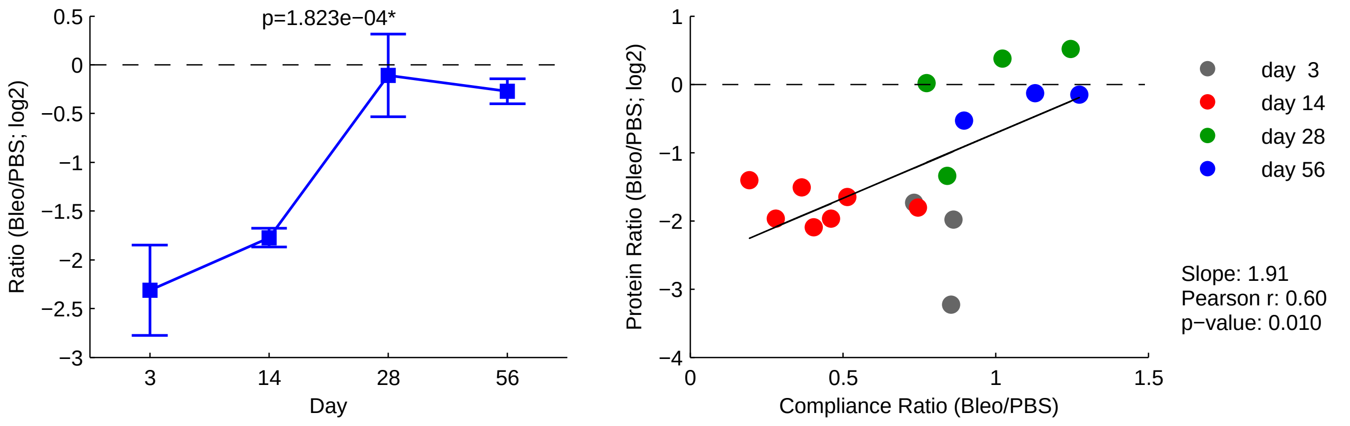

Q91X76 − Nt5dc2 (id: 6278)

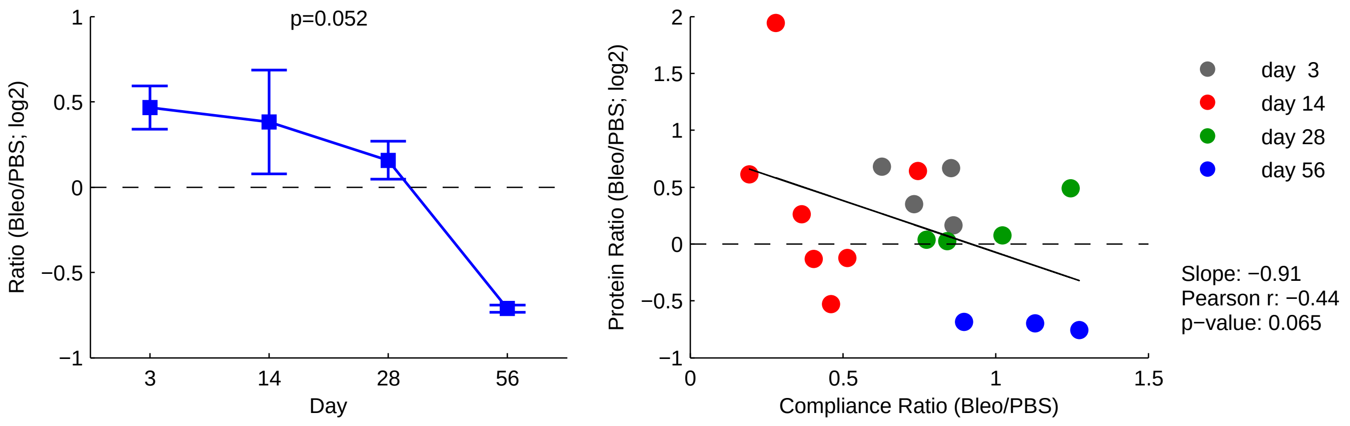

Q91X78 − Erlin1 (id: 6279)

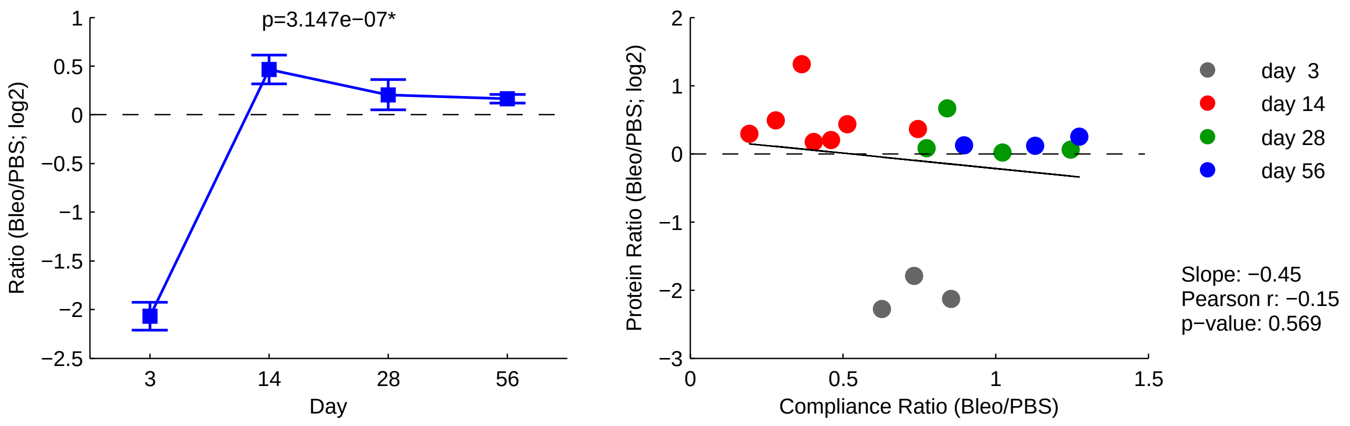

Q91X88−2 − Pomgnt1 (id: 6283)

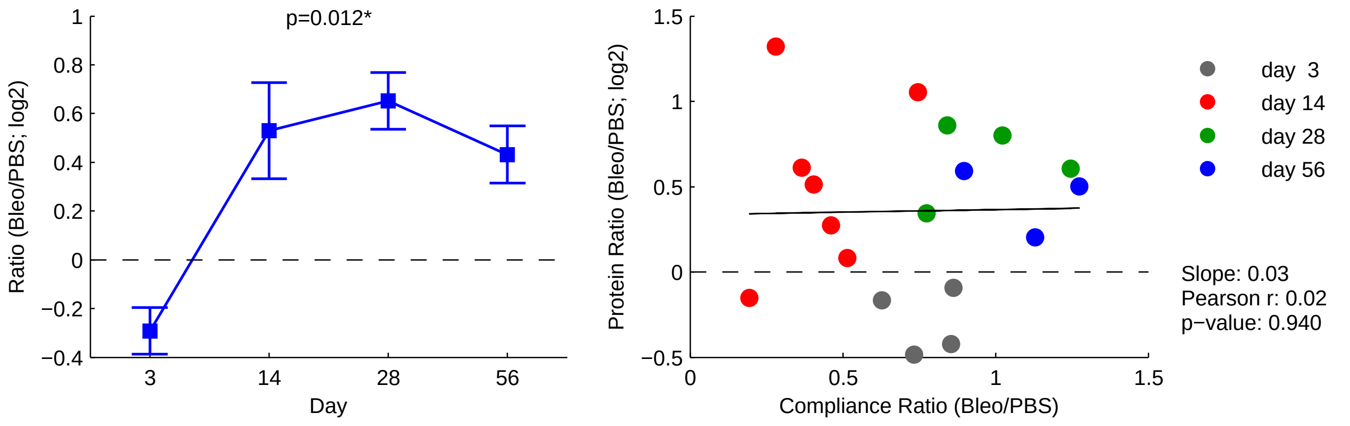

Q91X97 − Ncald (id: 6284)

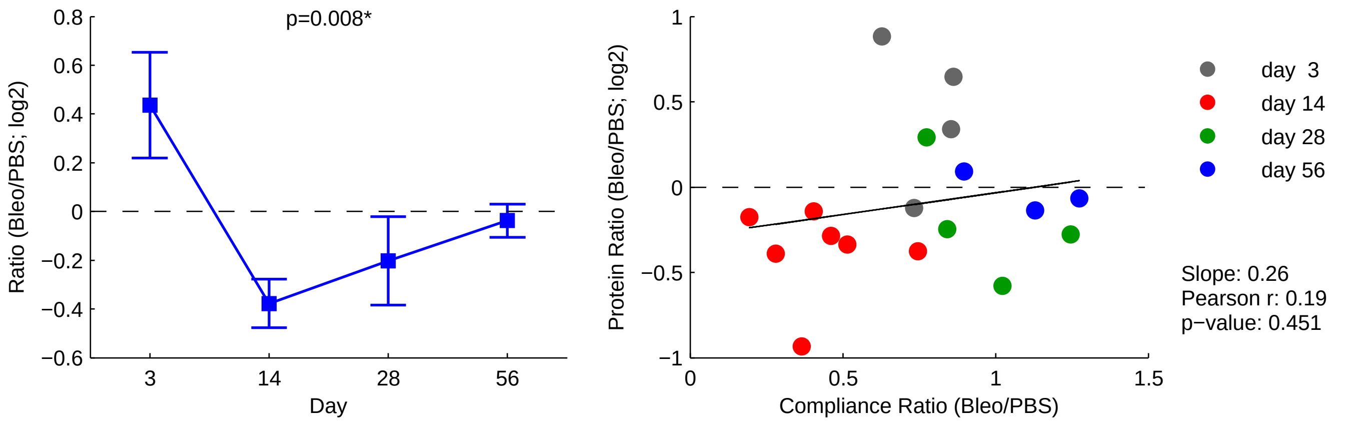

Q91XA2 − Golm1 (id: 6285)

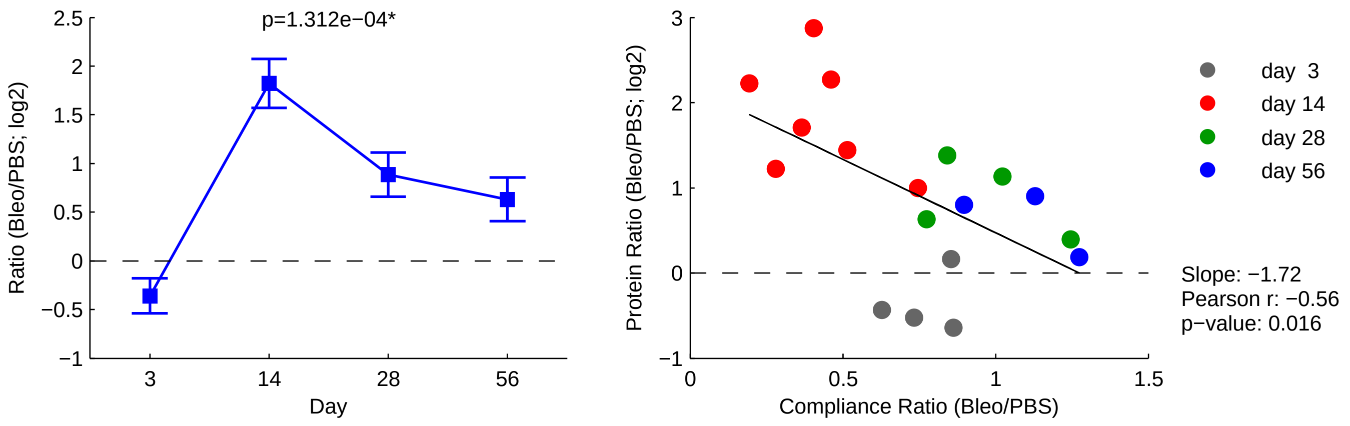

Q91XB0 − Trex1 (id: 6287)

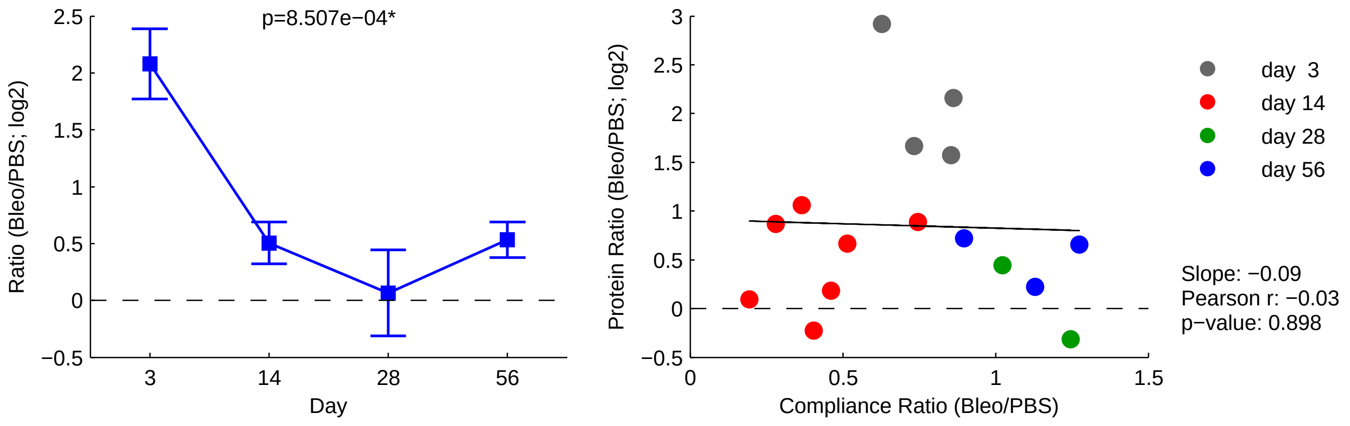

Q91XB7 − Yif1a (id: 6288)

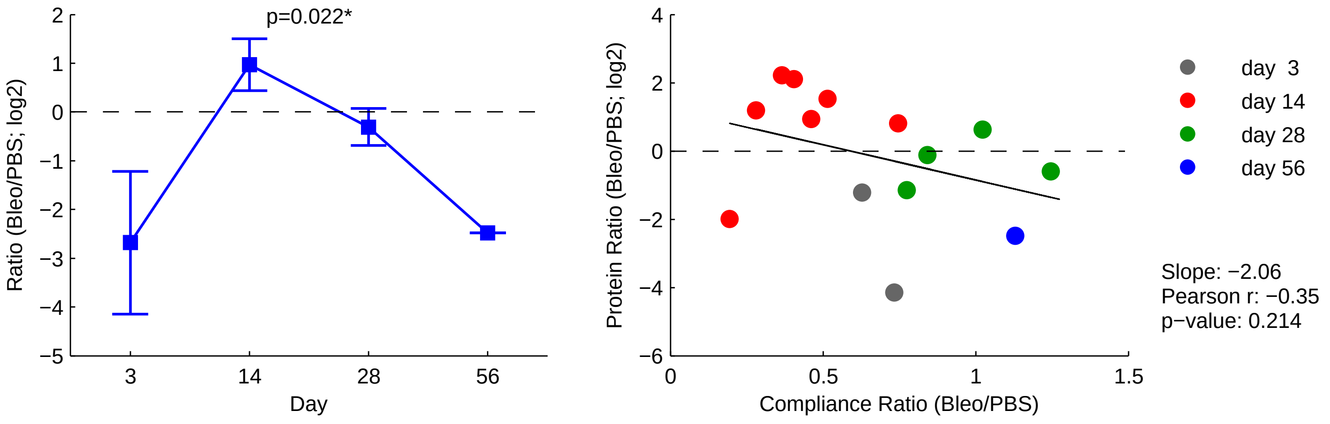

Q91XC9 − Pex16 (id: 6290)

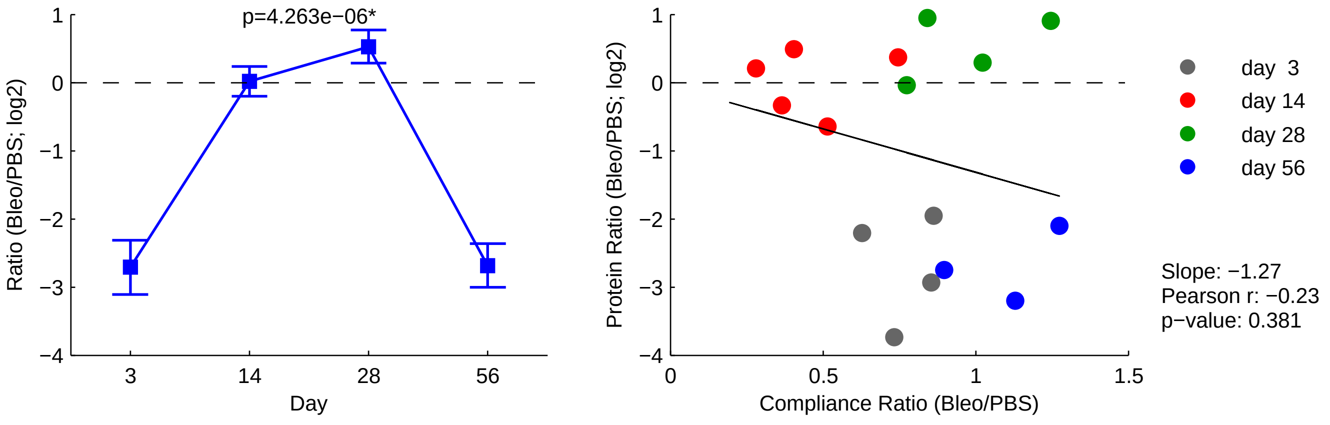

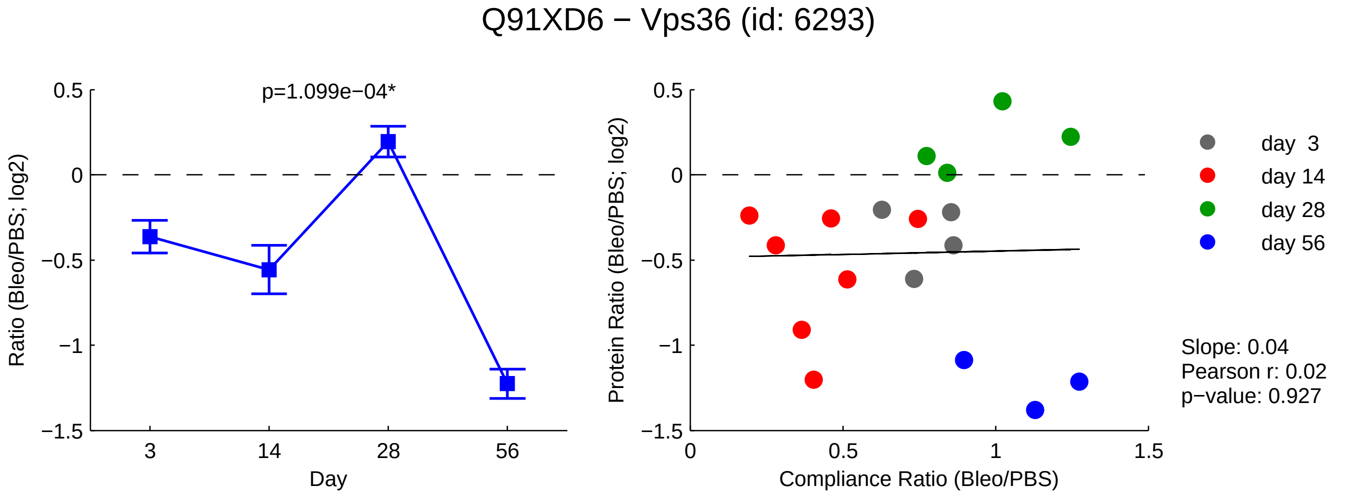
Q91XD6 − Vps36 (id: 6293)
p=1.099e−04*
Ratio (Bleo/PBS; log2)
Day
Protein Ratio (Bleo/PBS; log2)
Compliance Ratio (Bleo/PBS)
day 3
day 14
day 28
day 56
Slope: 0.04
Pearson r: 0.02
p−value: 0.927

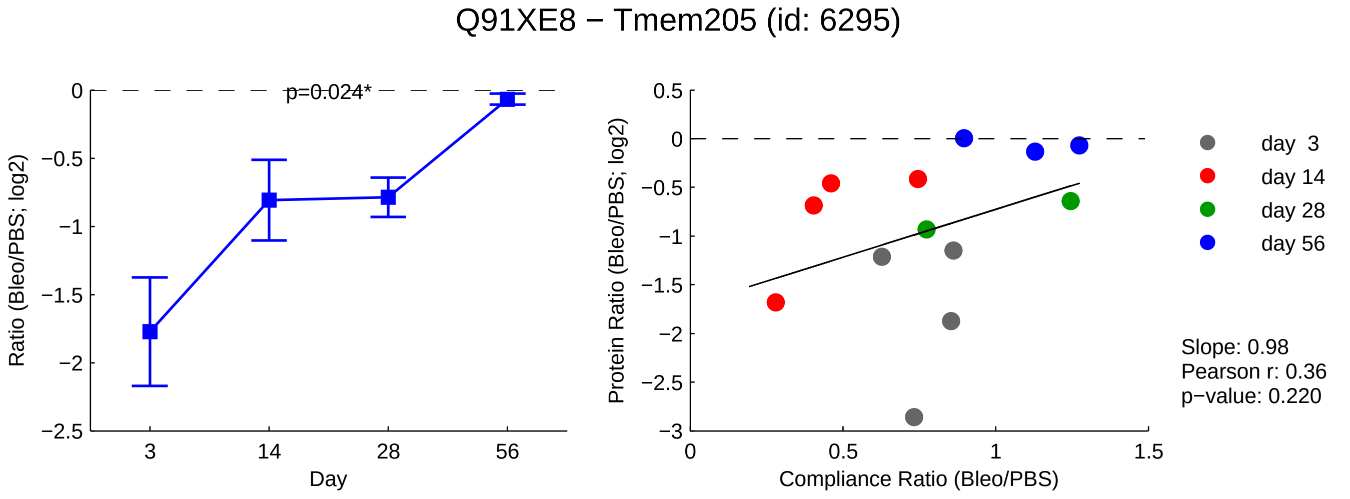
Q91XE8 − Tmem205 (id: 6295)
p=0.024*
Ratio (Bleo/PBS; log2)
Day
Protein Ratio (Bleo/PBS; log2)
Compliance Ratio (Bleo/PBS)
day 3
day 14
day 28
day 56
Slope: 0.98
Pearson r: 0.36
p−value: 0.220

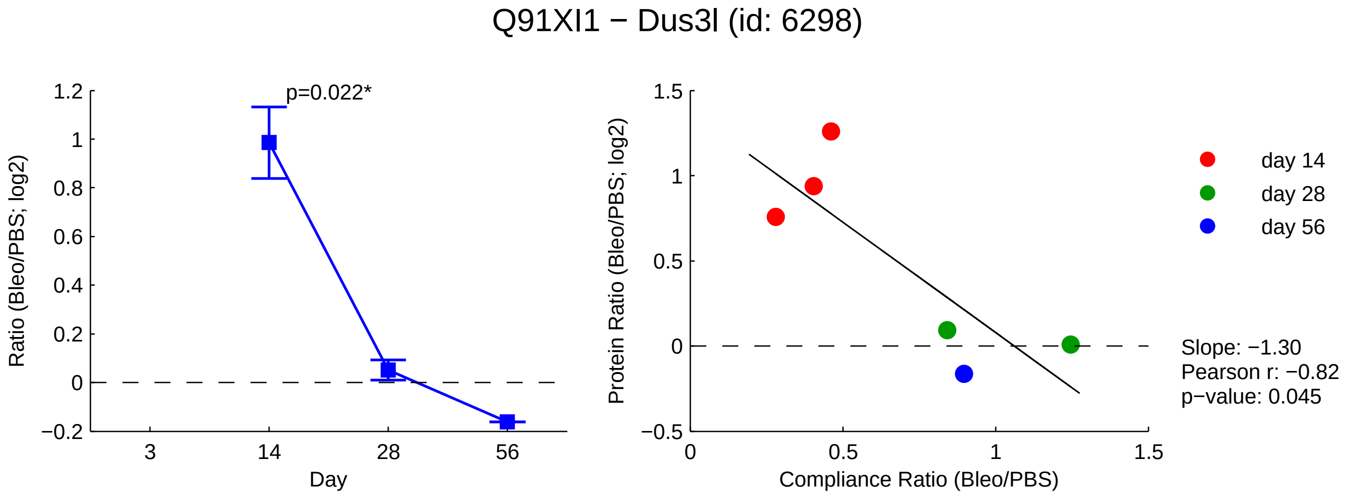
Q91XI1 − Dus3l (id: 6298)
p=0.022*
Ratio (Bleo/PBS; log2)
Day
Protein Ratio (Bleo/PBS; log2)
Compliance Ratio (Bleo/PBS)
day 14
day 28
day 56
Slope: −1.30
Pearson r: −0.82
p−value: 0.045

Q91XU0 − Wrnip1 (id: 6302)

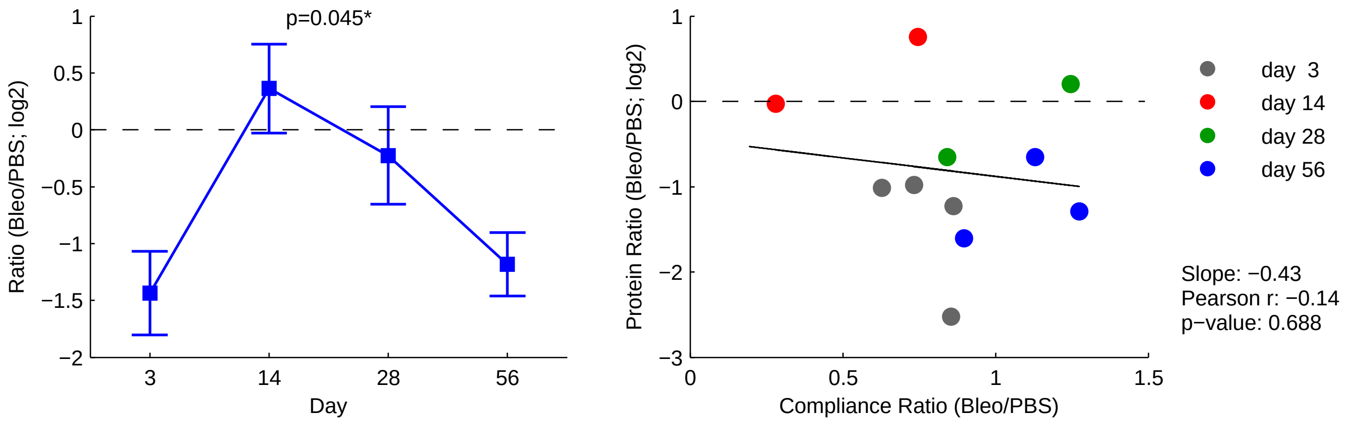

Q91XU3 − Pip4k2c (id: 6303)

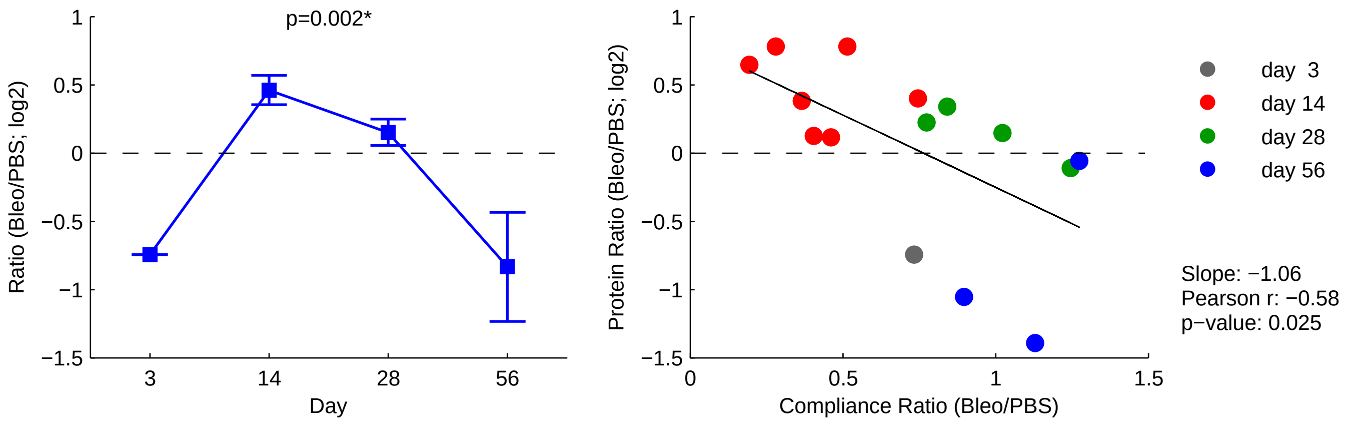

Q91XV3 − Basp1 (id: 6304)

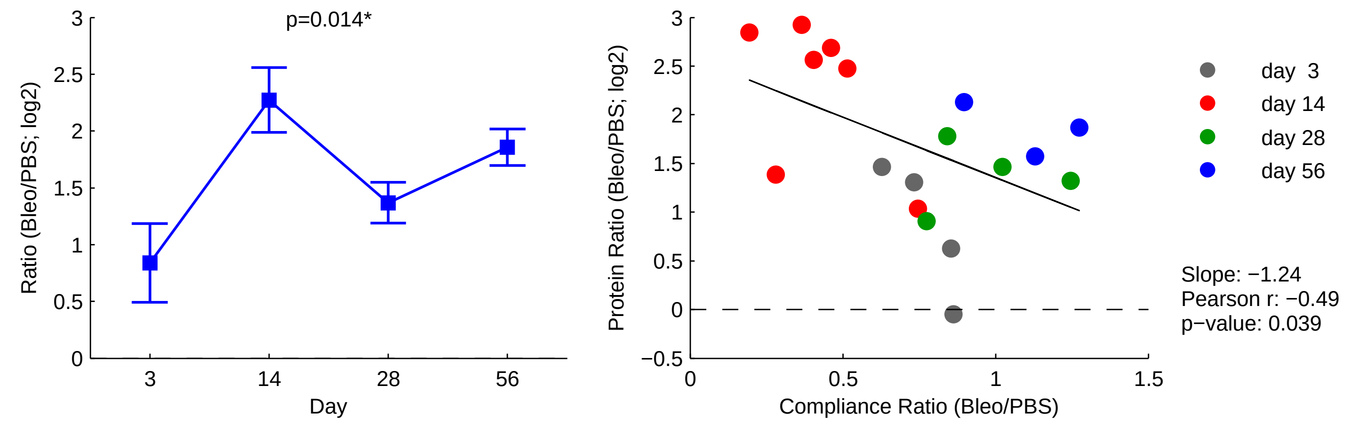

Q91Y74 − St3gal4 (id: 6306)

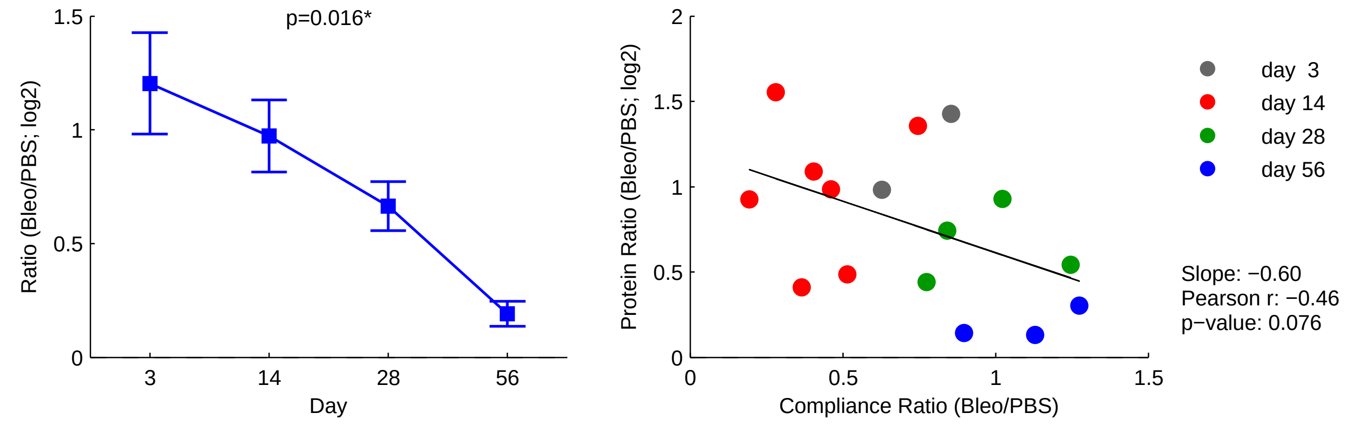

Q91YD3 − Dcp1a (id: 6308)

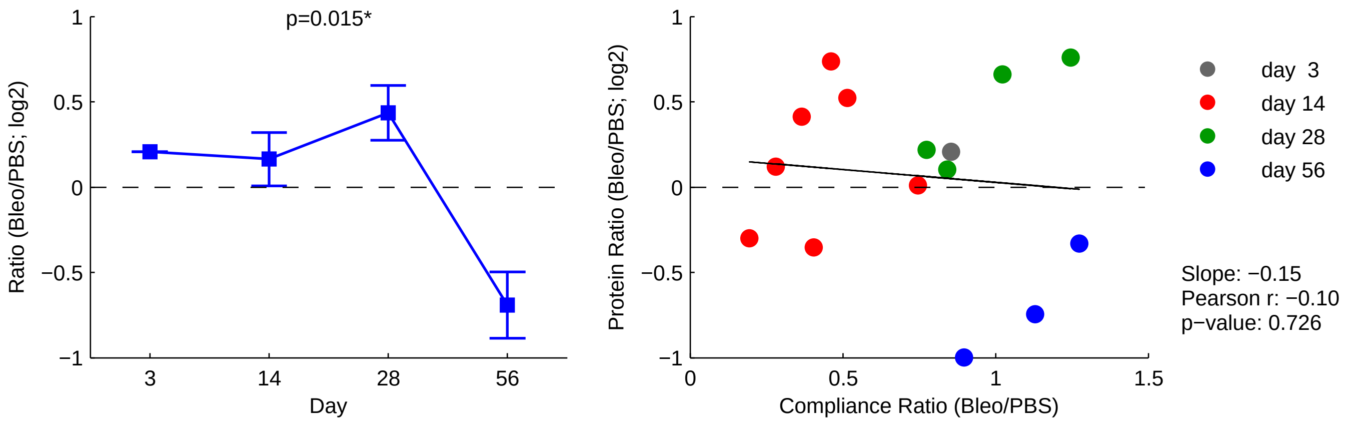

Q91YE7−2 − Rbm5 (id: 6310)

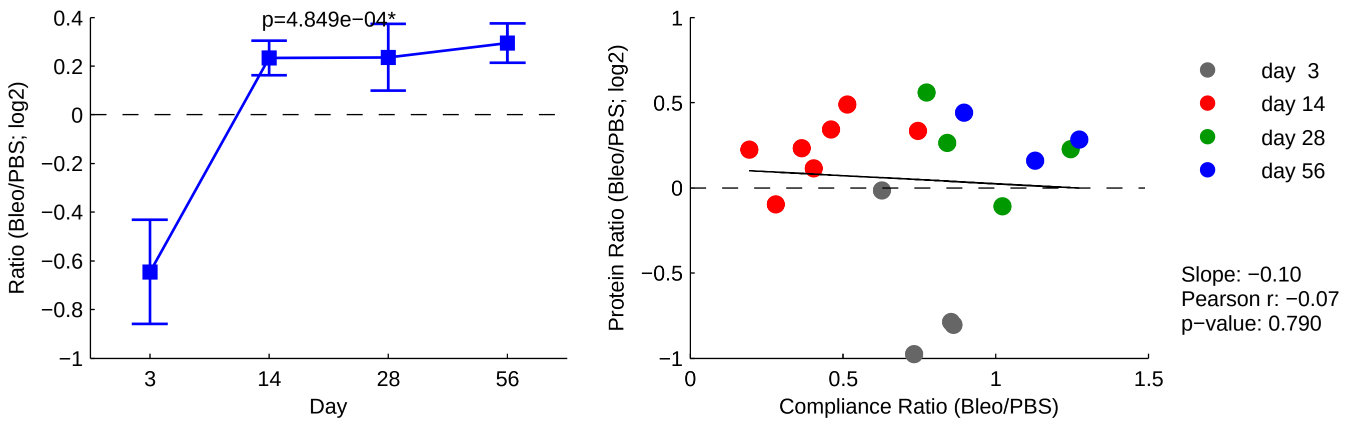

Q91YH5 − Atl3 (id: 6312)

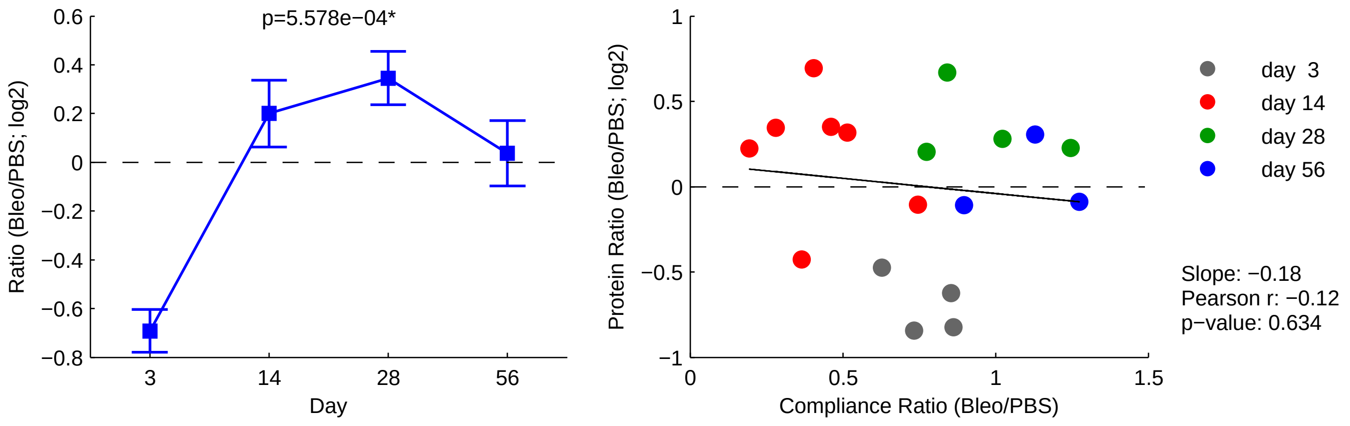

Q91YI4 − Arrb2 (id: 6313)

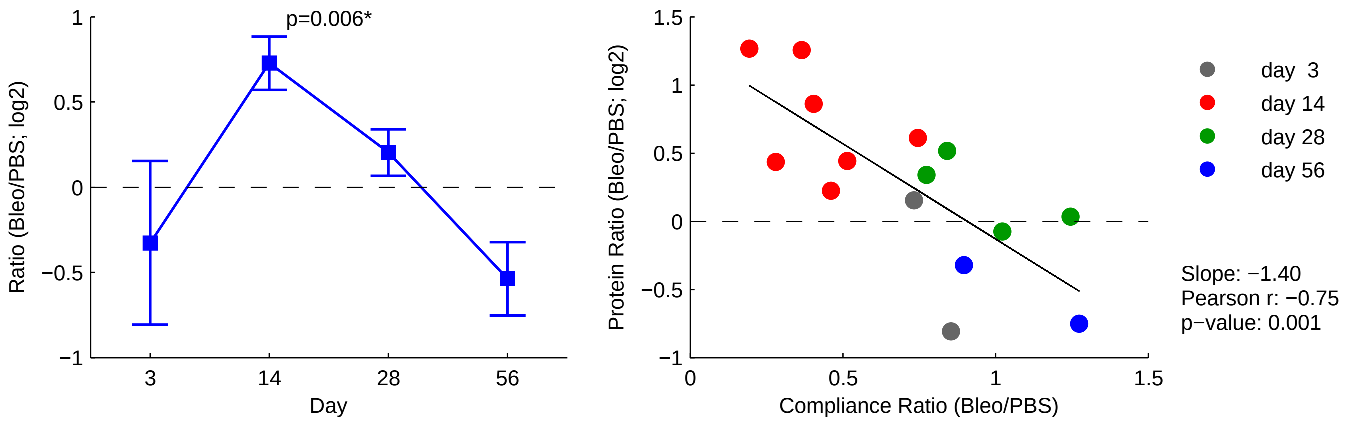

Q91YJ2 − Snx4 (id: 6315)

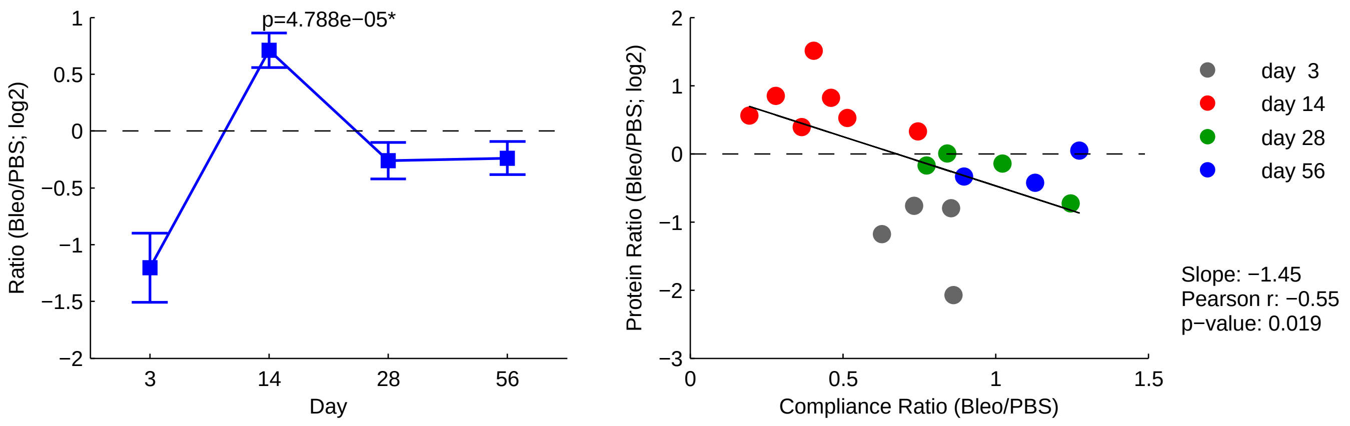

Q91YJ3 − Thyn1 (id: 6316)

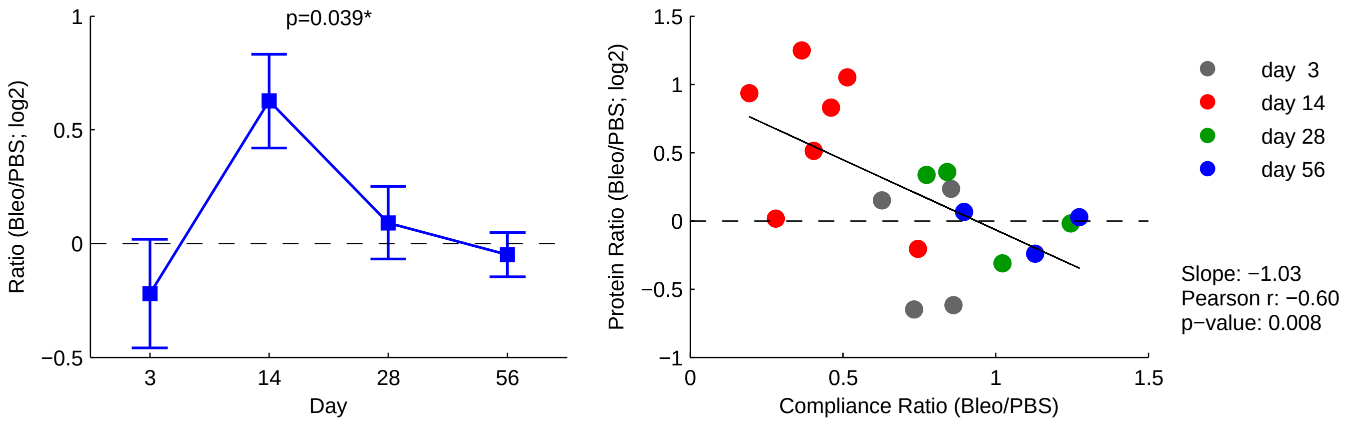

Q91YJ5 − Mtif2 (id: 6317)

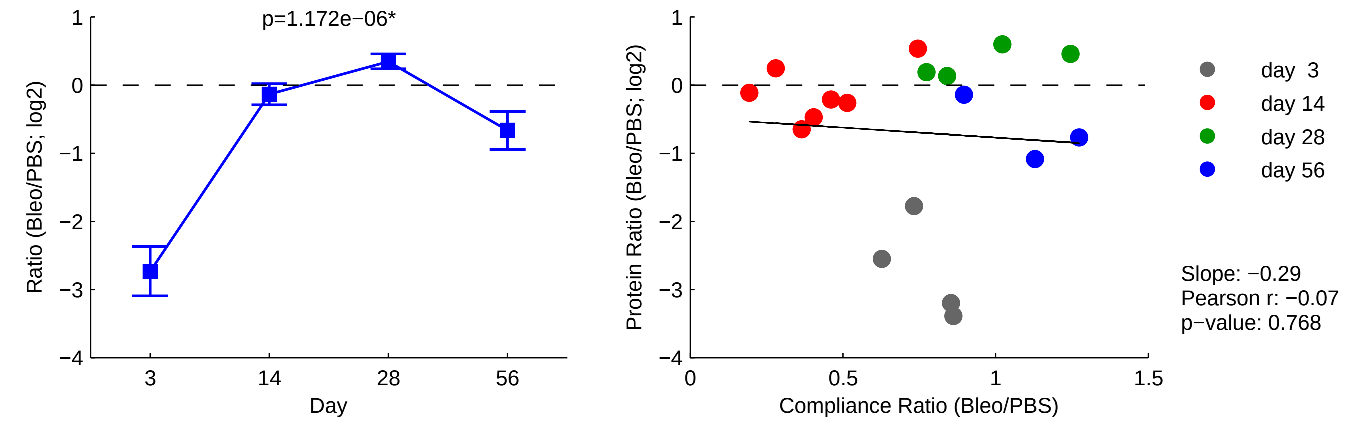

Q91YN0 − D6Wsu163e (id: 6321)

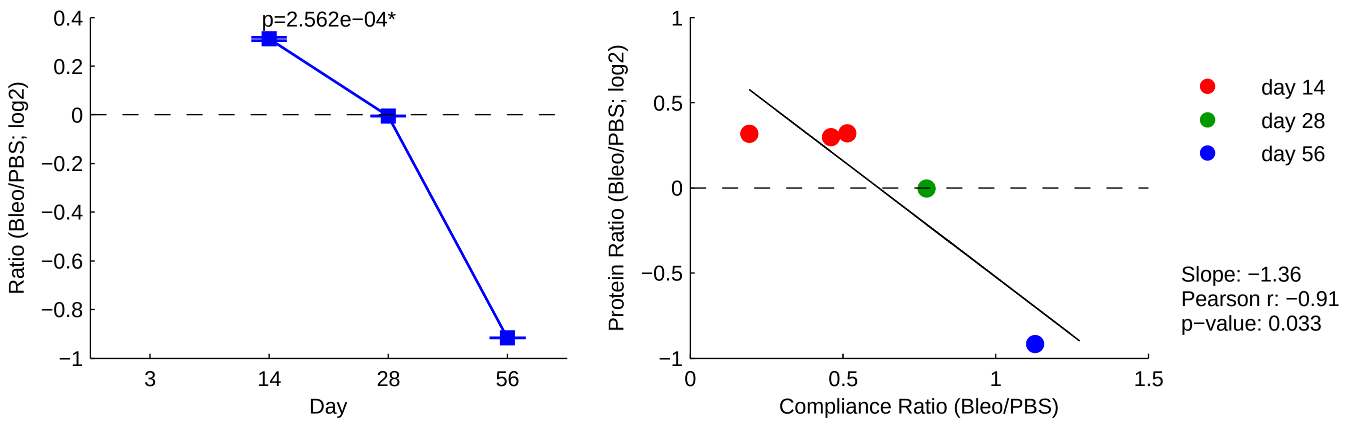

Q91YP0 − L2hgdh (id: 6323)

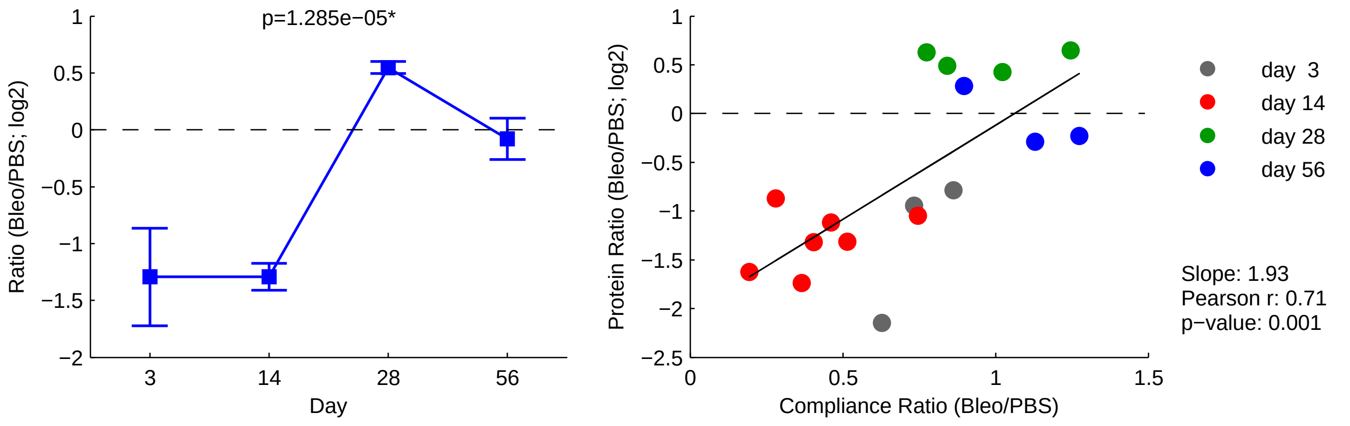

Q91YP2 − Nln (id: 6324)

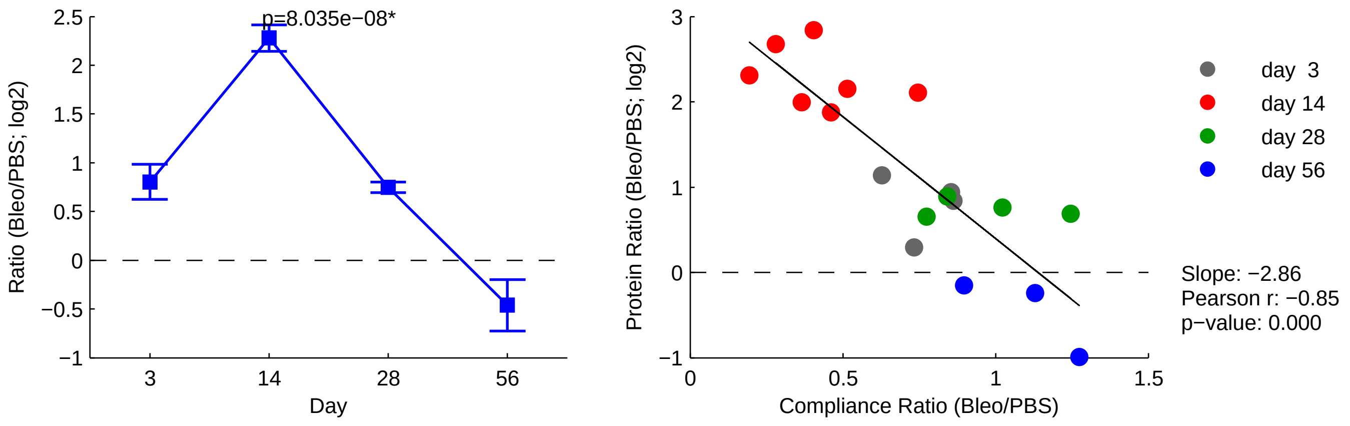

Q91YQ5 − Rpn1 (id: 6326)

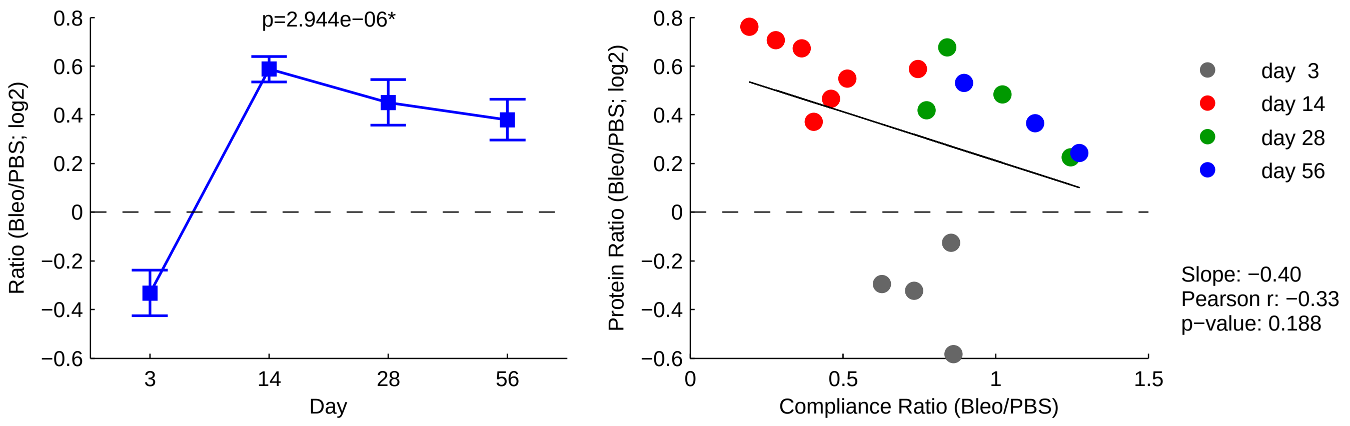

Q91YR9 − Ptgr1 (id: 6330)

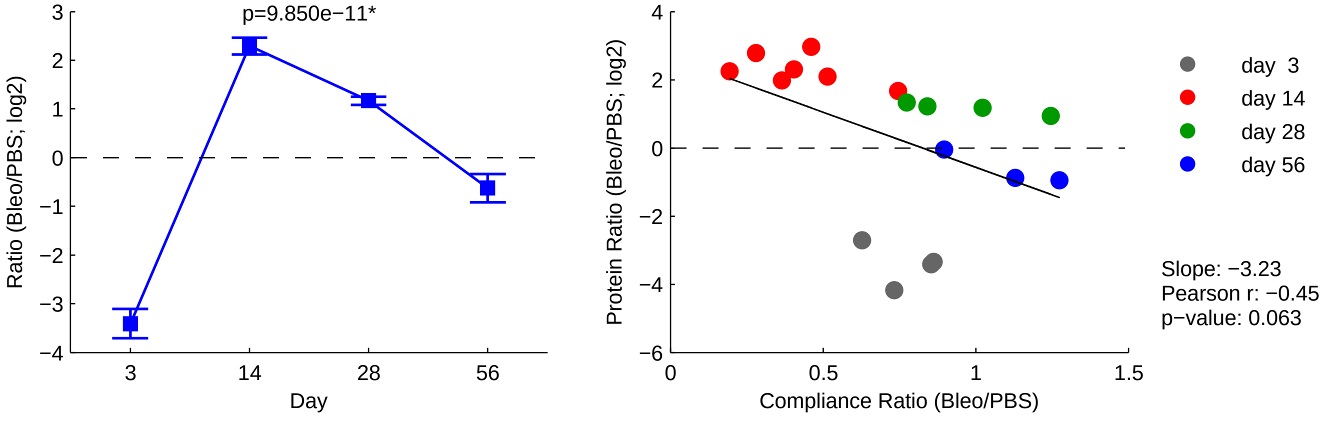

Q91YT7 − Ythdf2 (id: 6332)

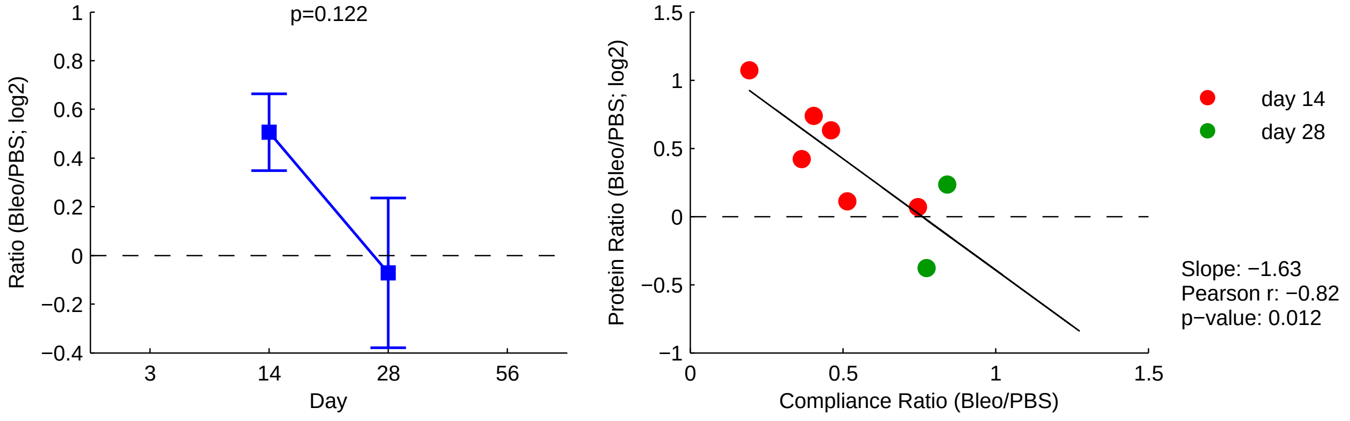

Q91YU6 − Lzts2 (id: 6334)

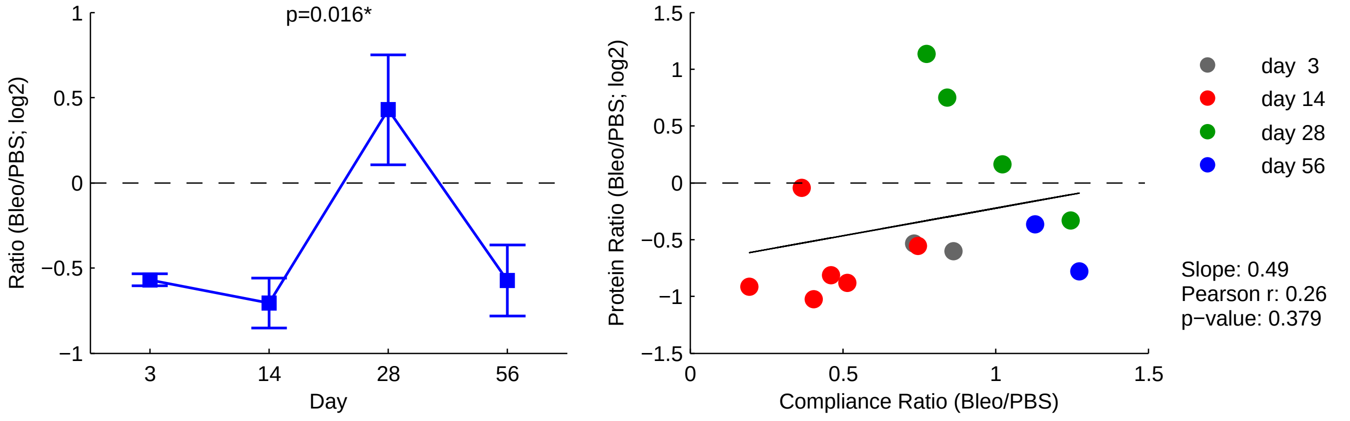

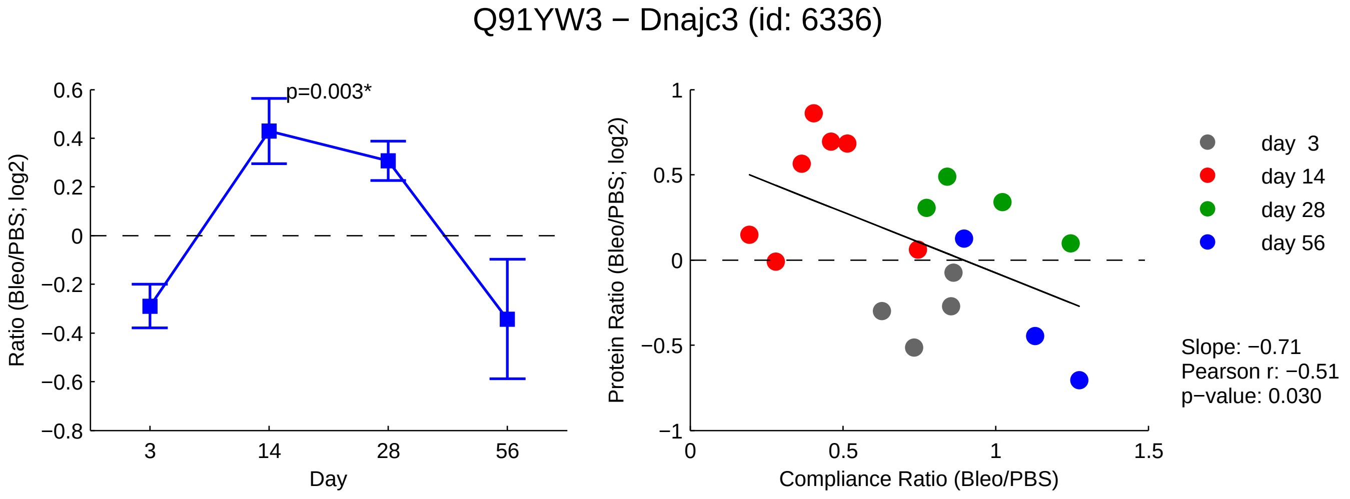
Q91YW3 − Dnajc3 (id: 6336)
p=0.003*
Ratio (Bleo/PBS; log2)
Day
Protein Ratio (Bleo/PBS; log2)
Compliance Ratio (Bleo/PBS)
day 3
day 14
day 28
day 56
Slope: −0.71
Pearson r: −0.51
p−value: 0.030

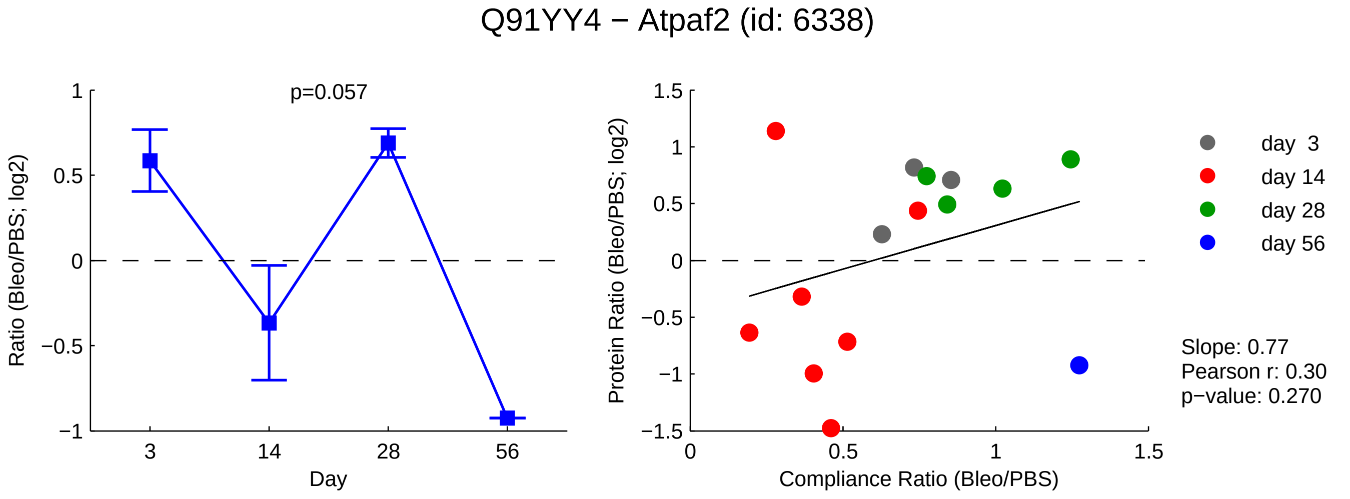
Q91YY4 − Atpaf2 (id: 6338)
p=0.057
Ratio (Bleo/PBS; log2)
Day
Protein Ratio (Bleo/PBS; log2)
Compliance Ratio (Bleo/PBS)
day 3
day 14
day 28
day 56
Slope: 0.77
Pearson r: 0.30
p−value: 0.270

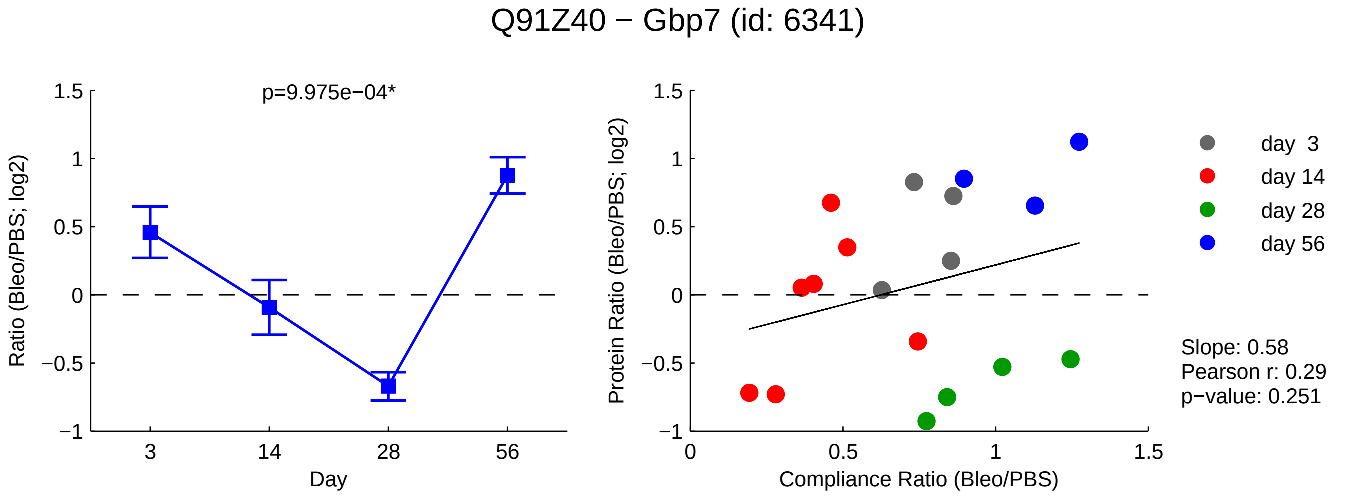
Q91Z40 − Gbp7 (id: 6341)
p=9.975e−04*
Ratio (Bleo/PBS; log2)
Day
Protein Ratio (Bleo/PBS; log2)
Compliance Ratio (Bleo/PBS)
day 3
day 14
day 28
day 56
Slope: 0.58
Pearson r: 0.29
p−value: 0.251

Q91Z49 − Fyttd1 (id: 6342)

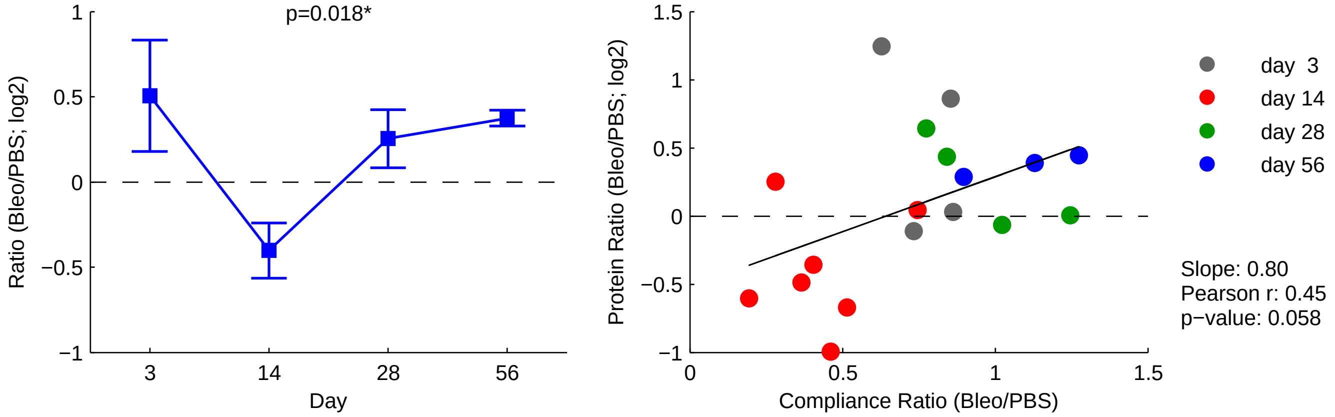

Q91Z58 − (id: 6345)

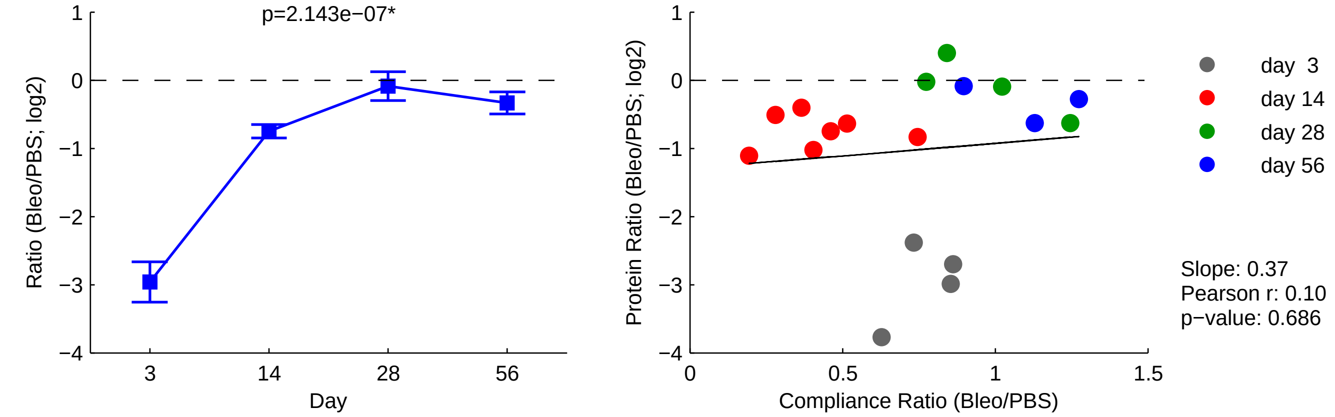

Q91Z67 − Srgap2 (id: 6346)

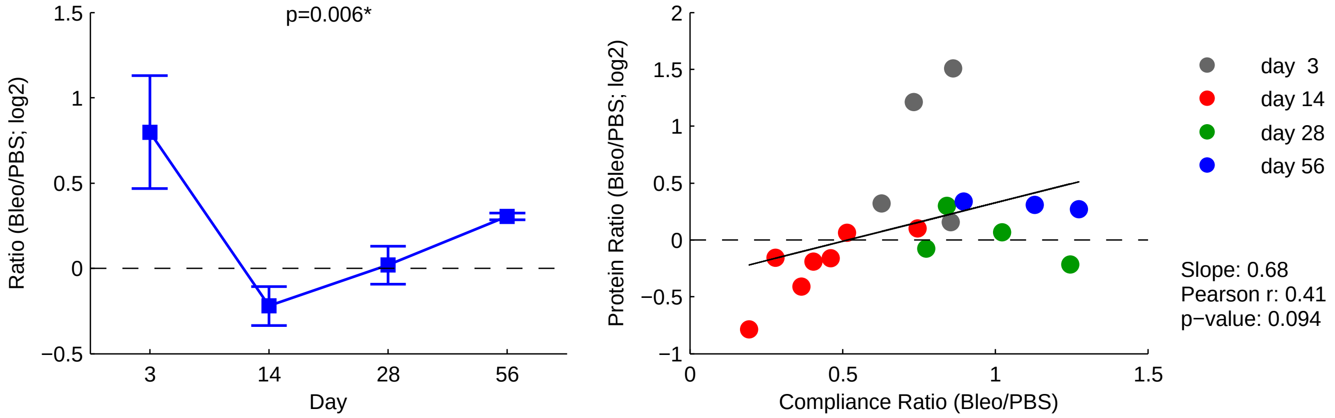

Q91Z96 − Bmp2k (id: 6349)

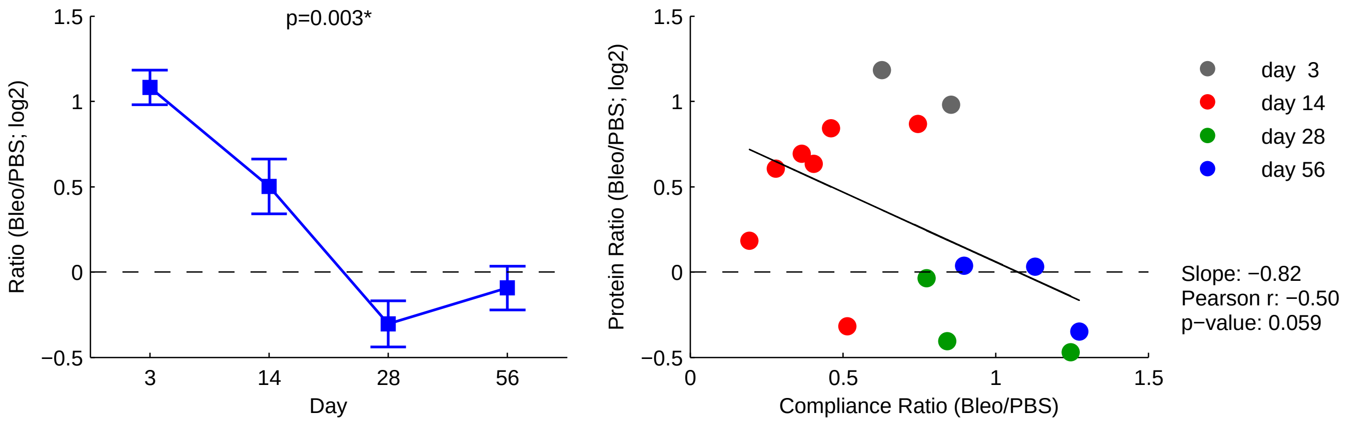

Q91ZA3 − Pcca (id: 6351)

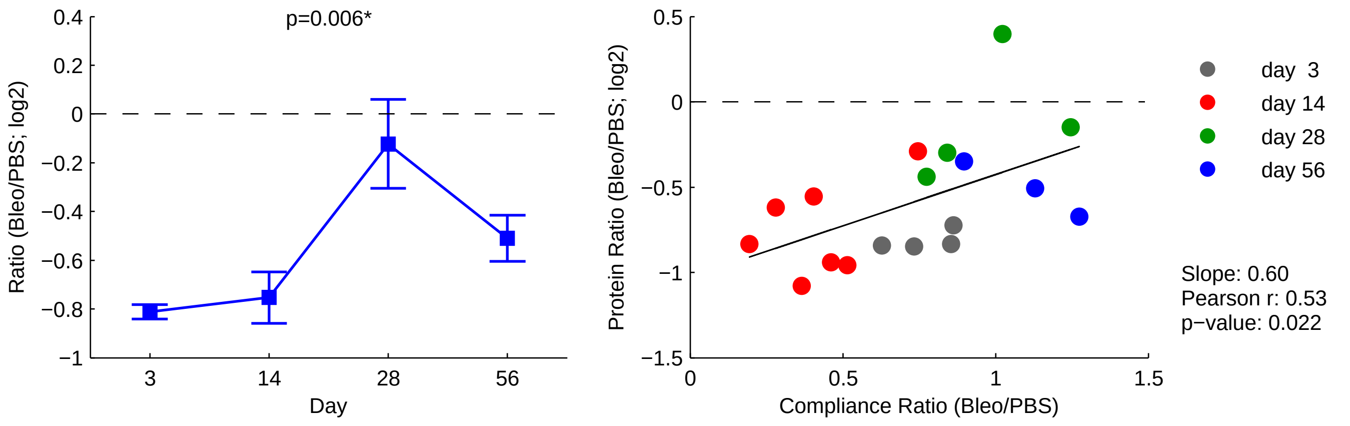

Q91ZE0 − Tmlhe (id: 6352)

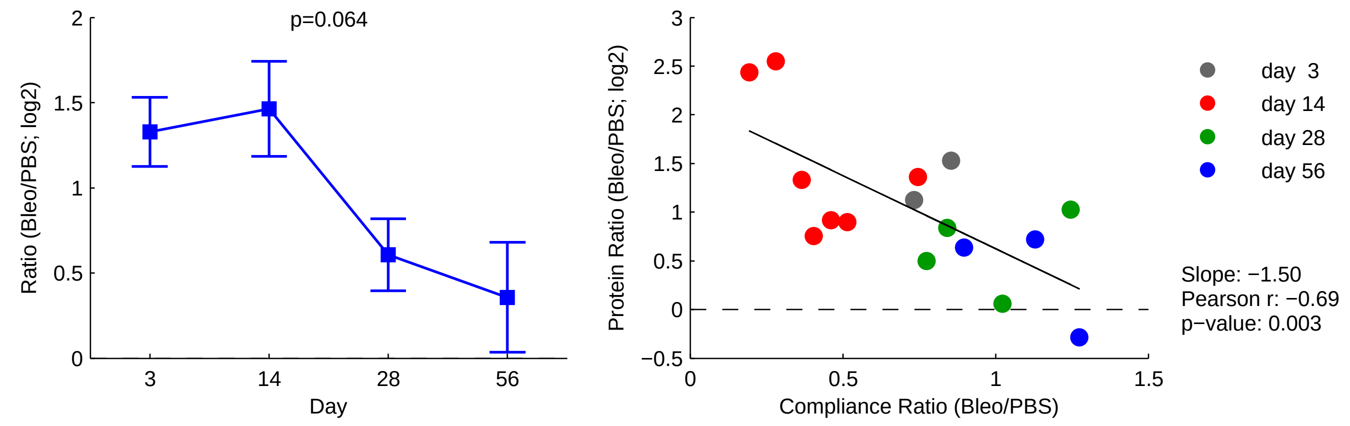

Q91ZX7 − Lrp1 (id: 6361)

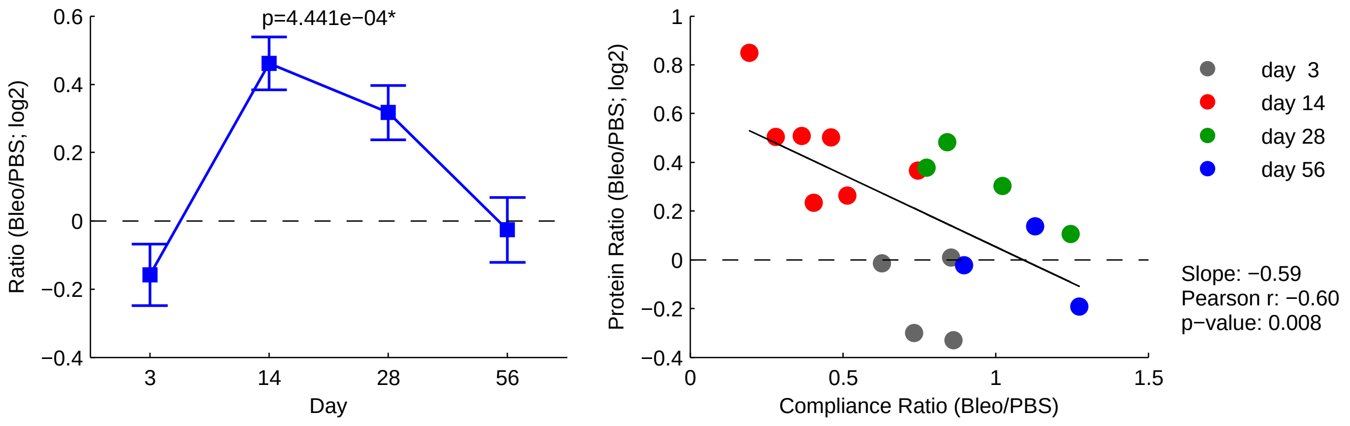

Q920A5 − Scpep1 (id: 6362)

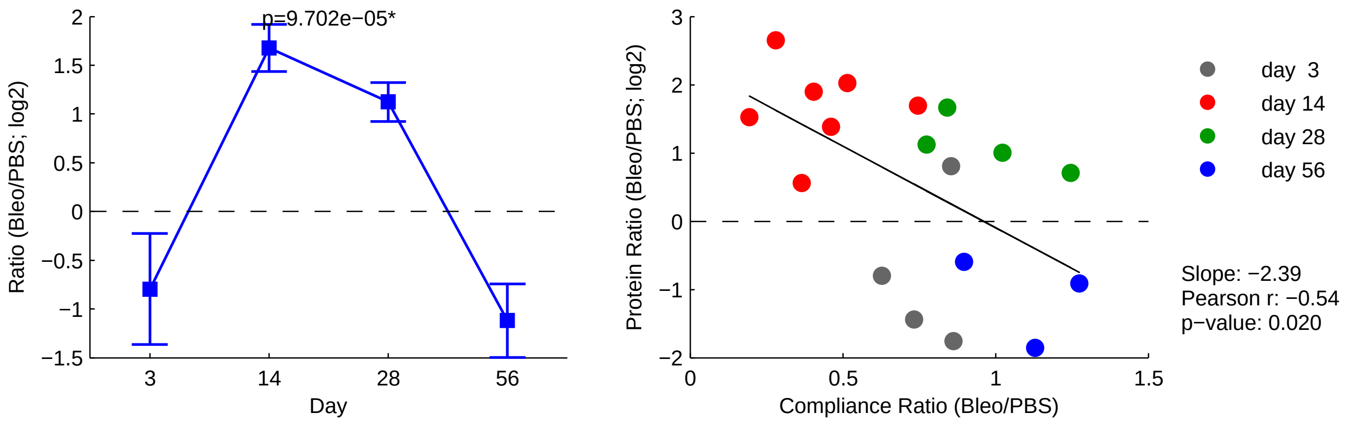

Q920A7 − Afg3l1 (id: 6363)

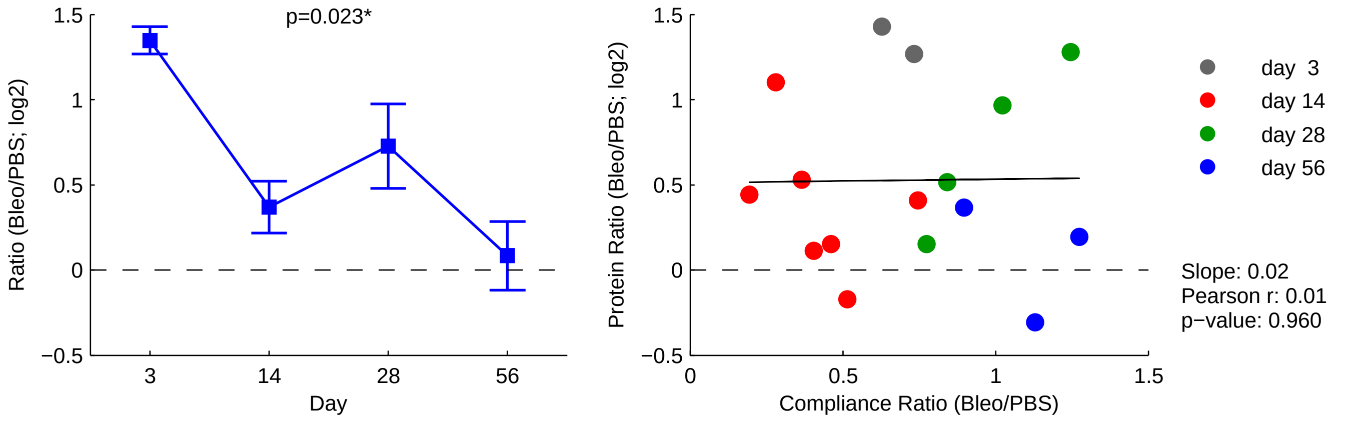

Q920L1 − Fads1 (id: 6369)

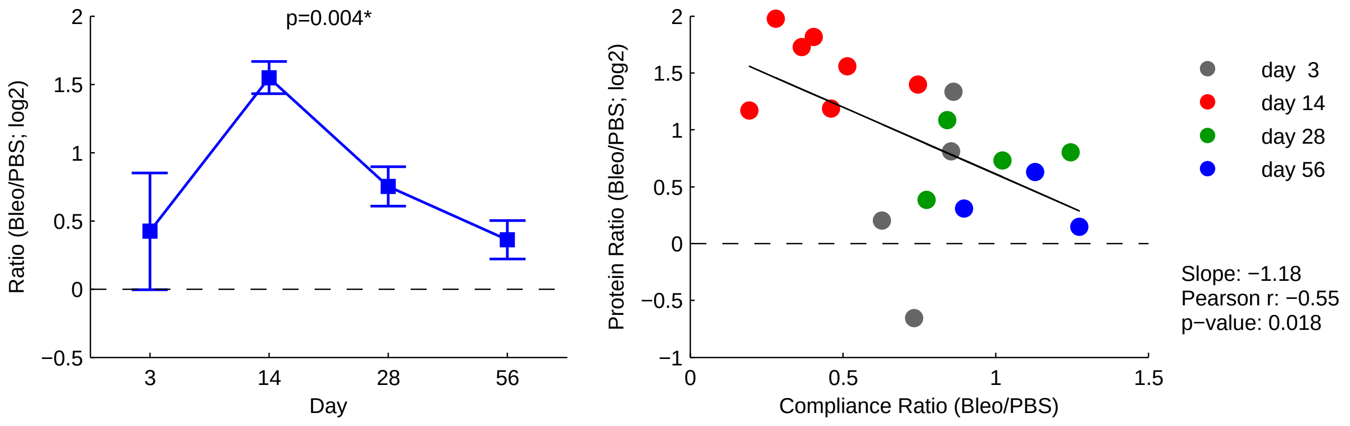

Q921C5 − Bicd2 (id: 6373)

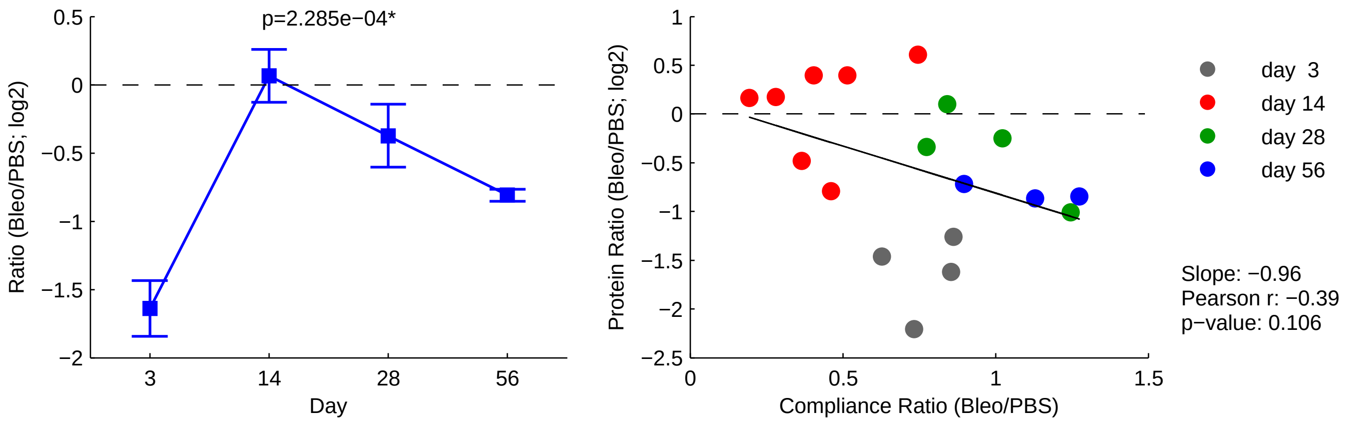

Q921G8 − Tubgcp2 (id: 6378)

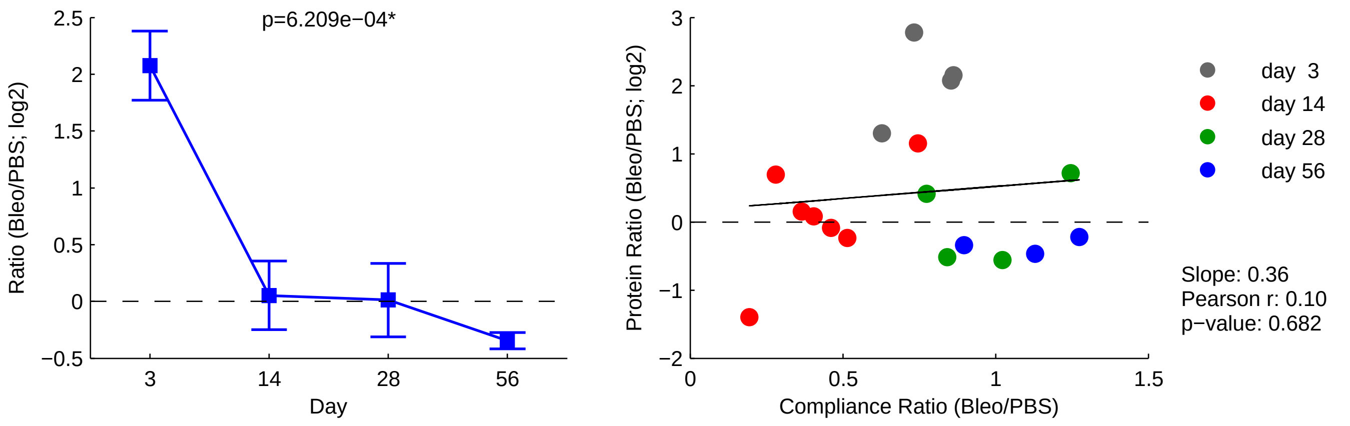

Q921H8 − Acaa1a (id: 6380)

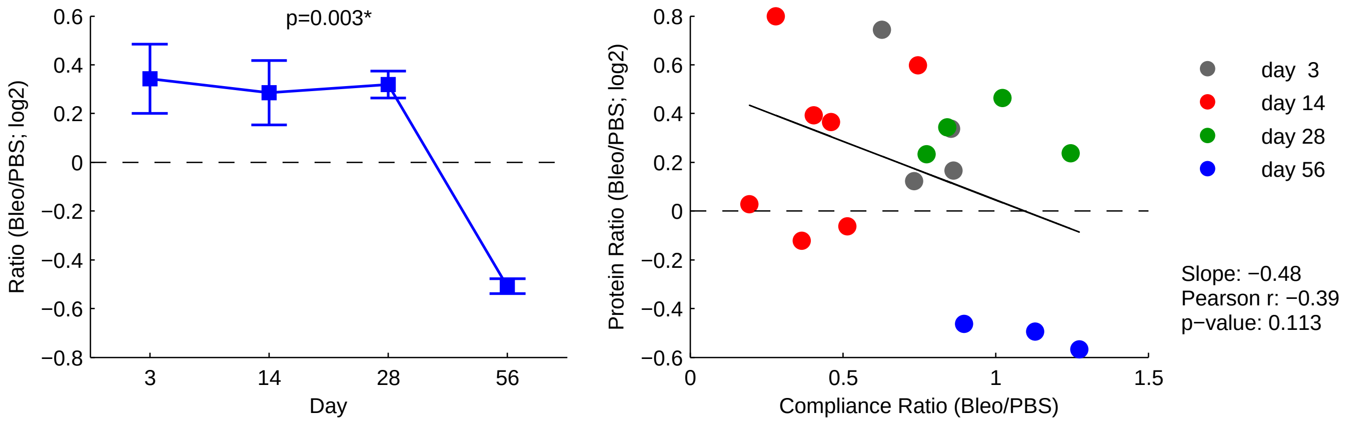

Q921H9 − Selrc1 (id: 6381)

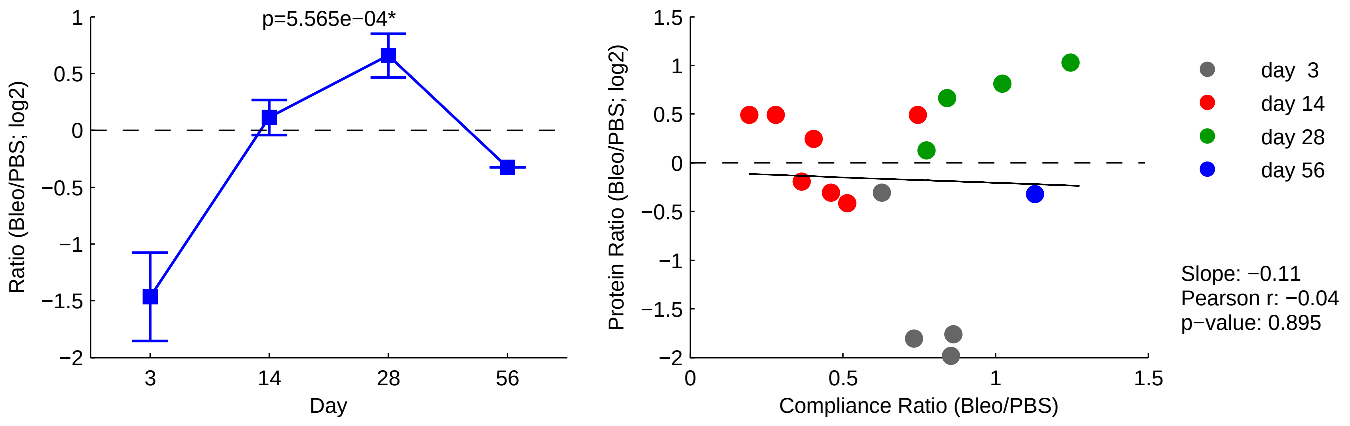

Q921I1 − Tf (id: 6383)

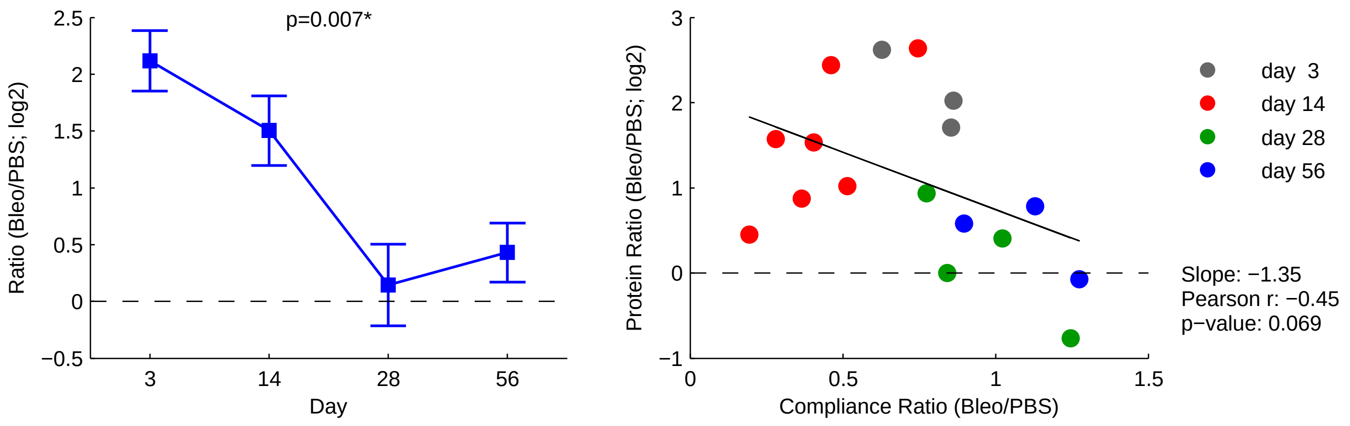

Q921I6 − Sh3bp4 (id: 6384)

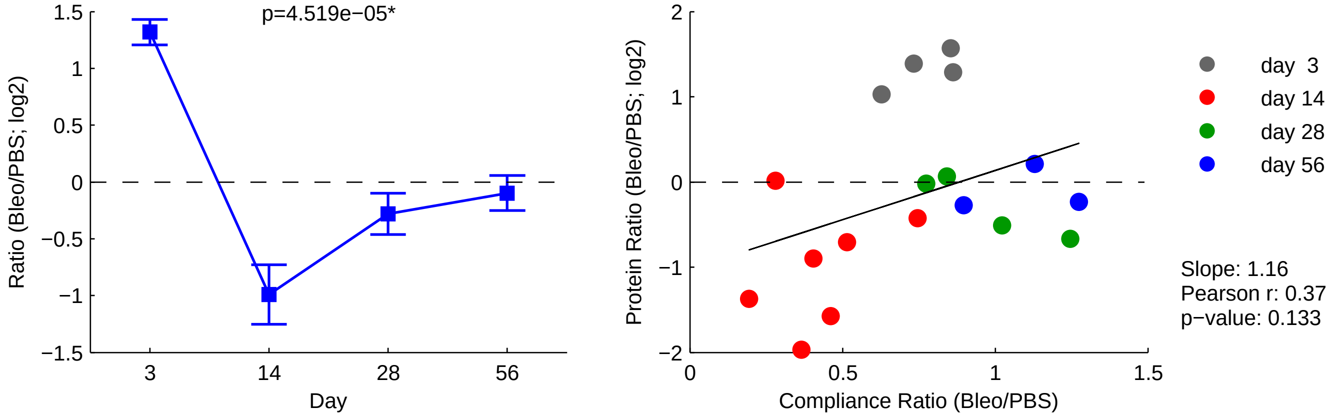

Q921I9 − Exosc4 (id: 6385)

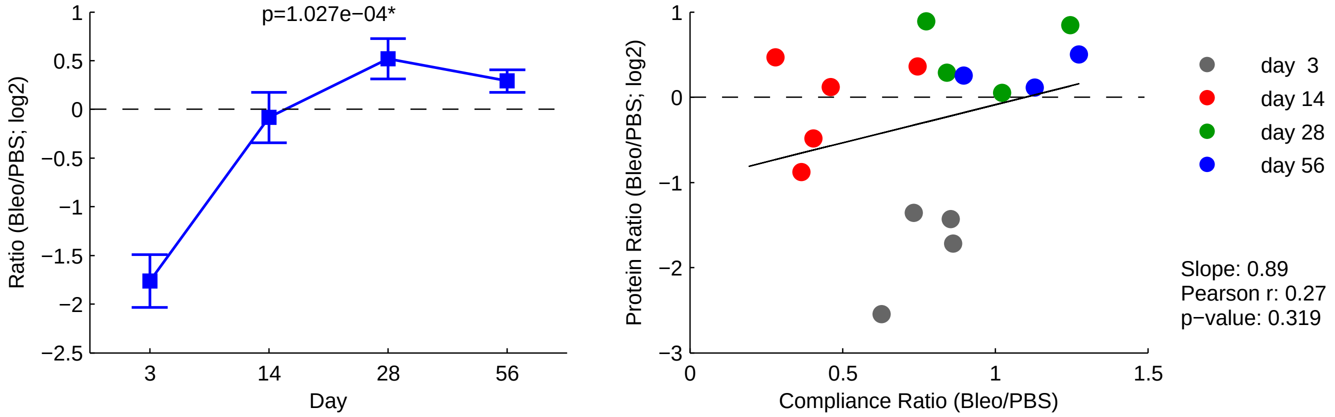

Q921L3 − Tmco1 (id: 6389)

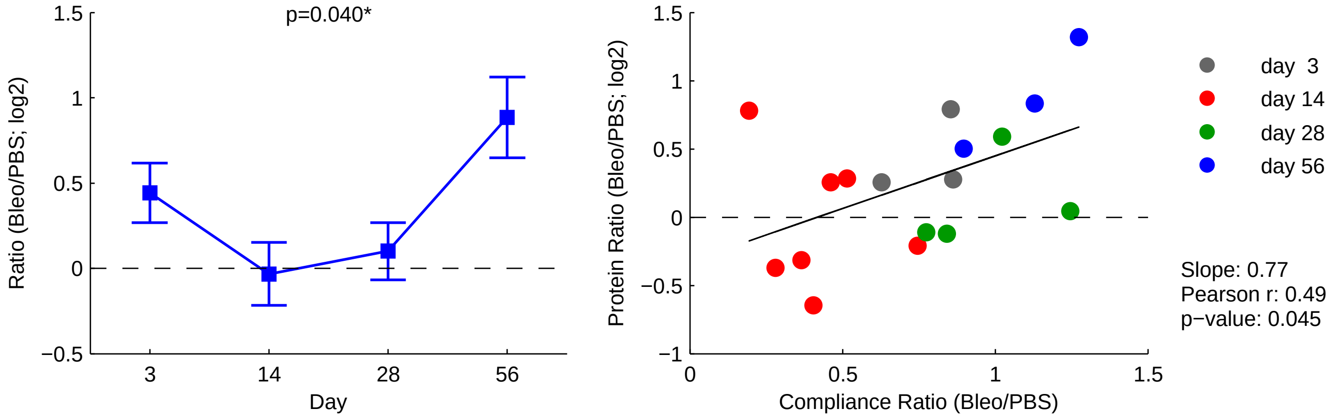

Q921N6 − Ddx27 (id: 6393)

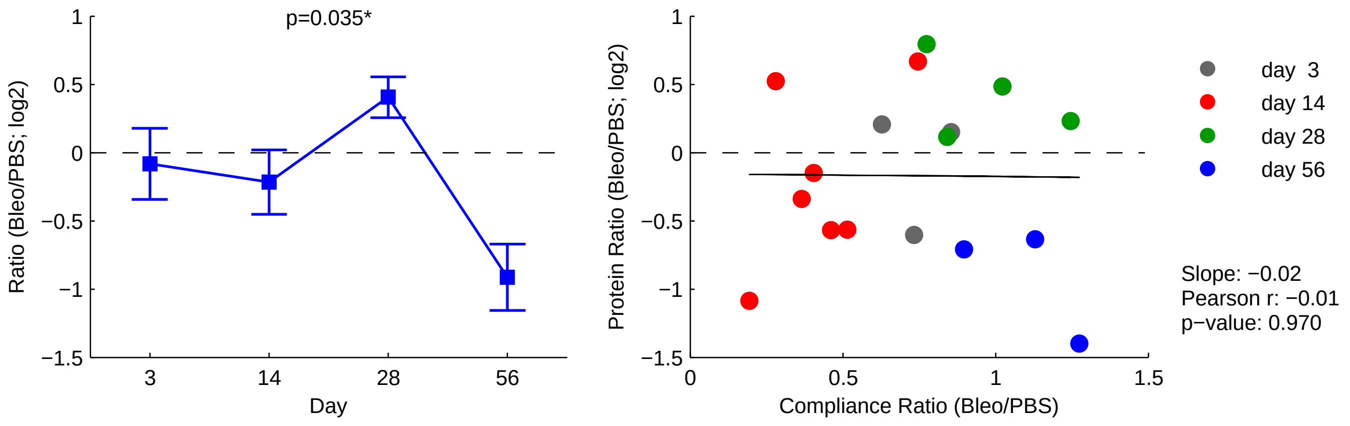

Q921Q3−2 − Alg1 (id: 6395)

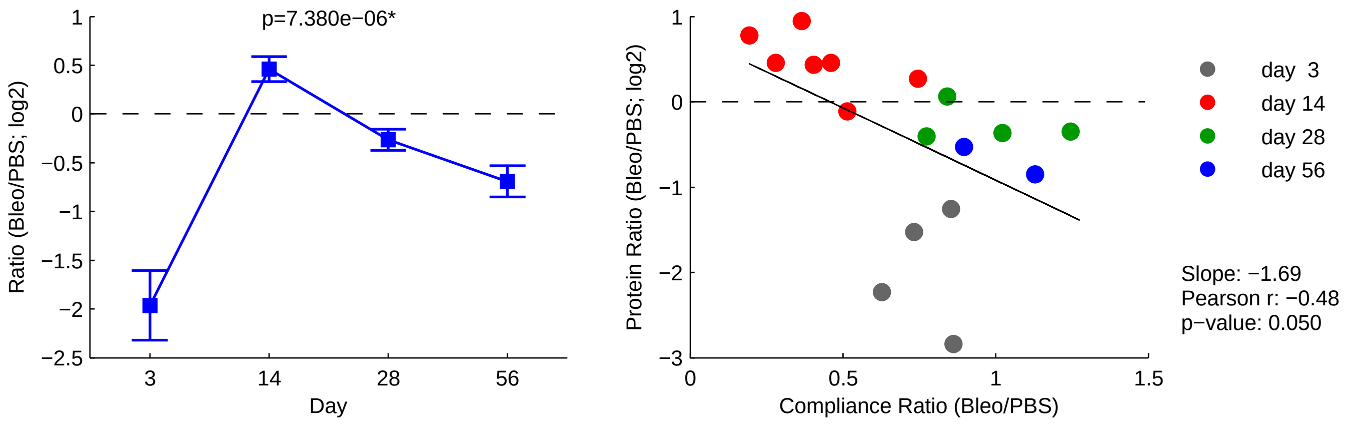

Q921S7 − Mrpl37 (id: 6397)

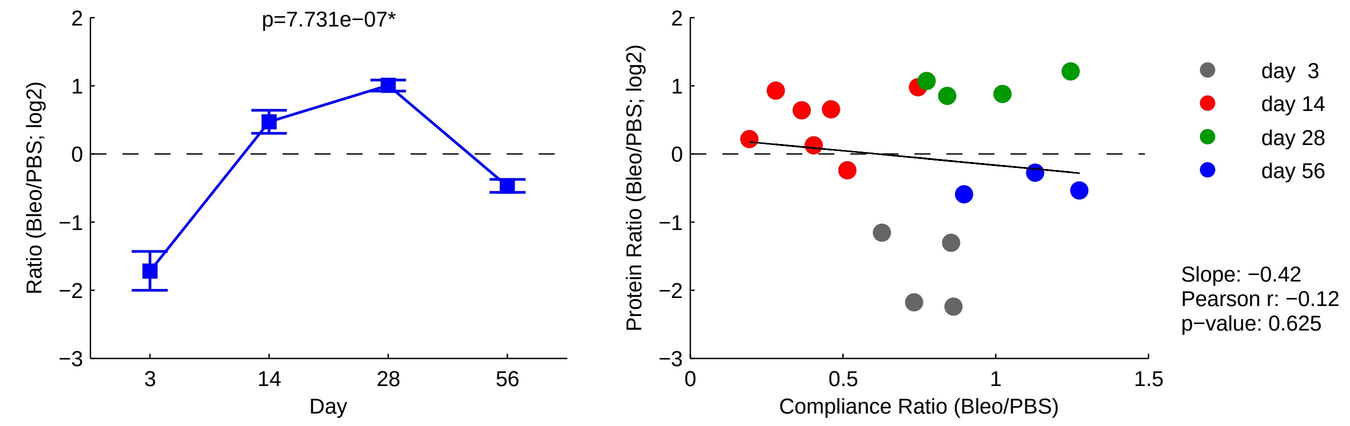

Q921T2−2 − Tor1aip1 (id: 6398)

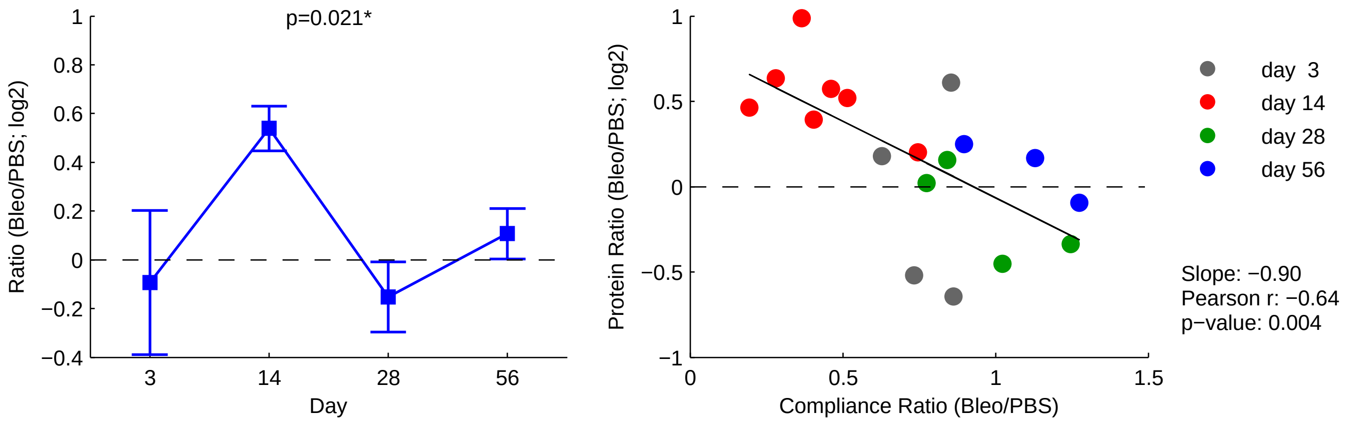

Q921W0 − Chmp1a (id: 6400)

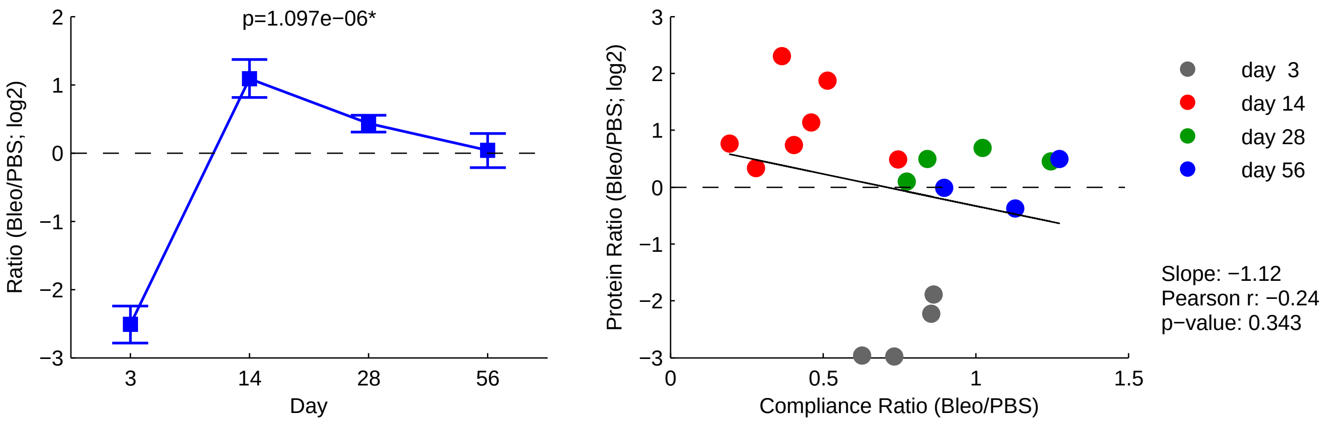

Q921W4 − Cryzl1 (id: 6401)

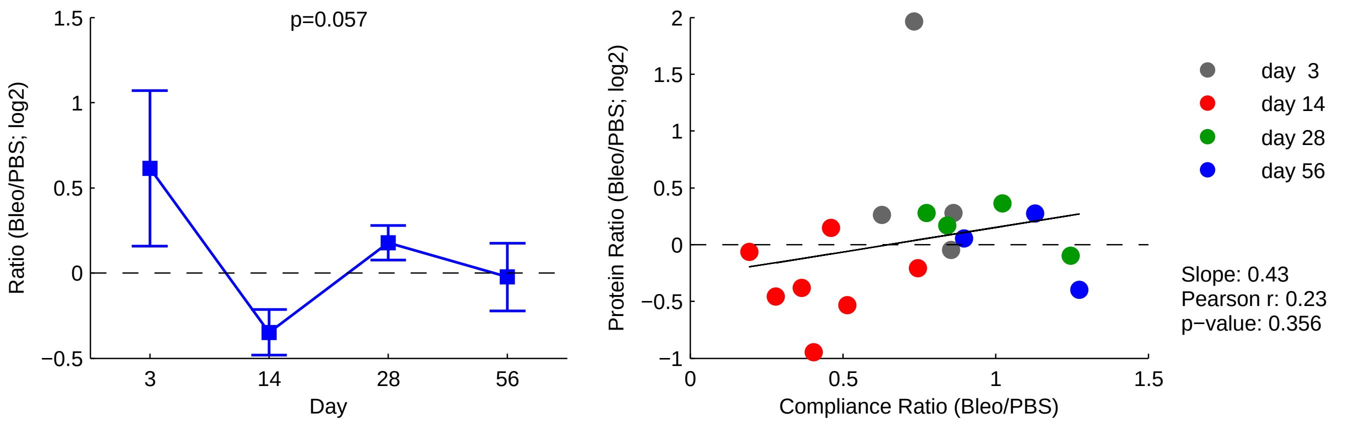

Q921W7 − Tes (id: 6402)

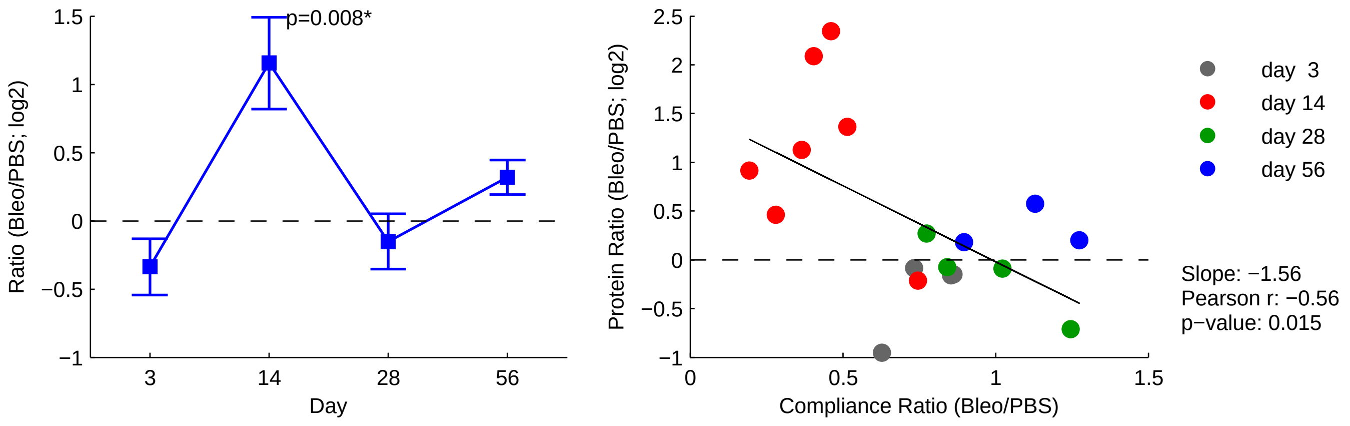

Q921X9 − Pdia5 (id: 6403)

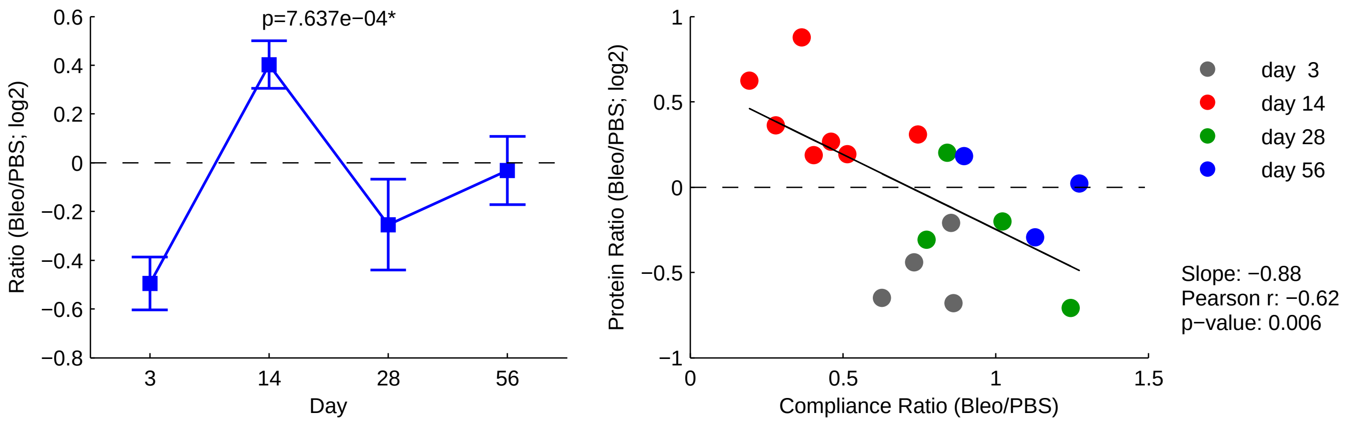

Q921Y2 − Imp3 (id: 6405)

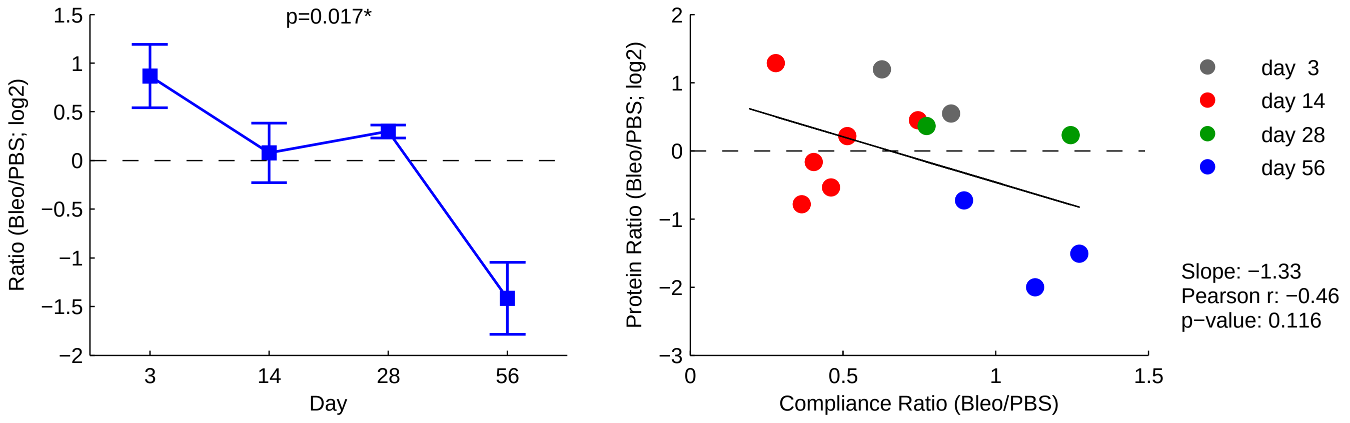

Q922D4−2 − Ppp6r3 (id: 6408)

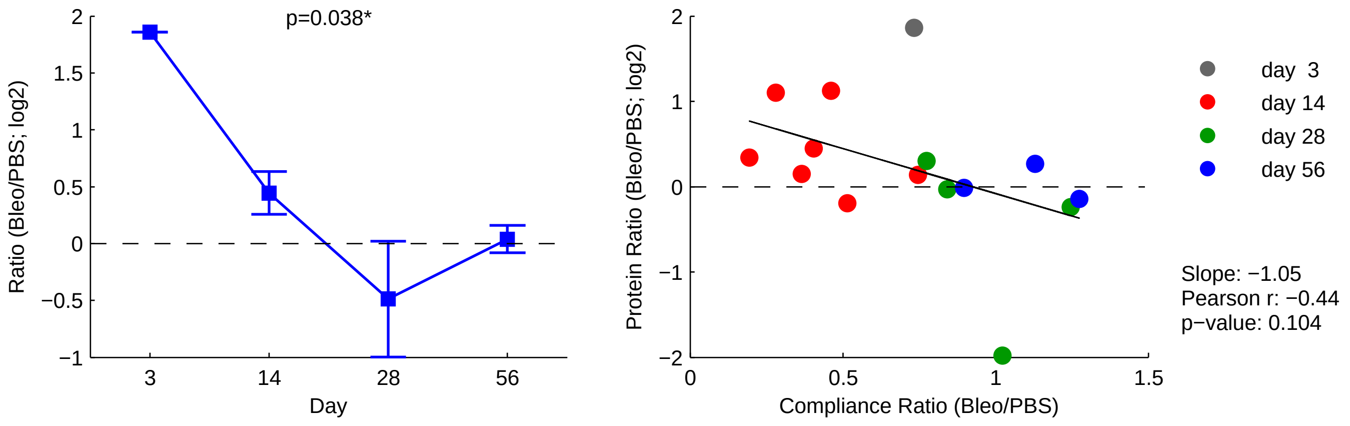

Q922F4 − Tubb6 (id: 6410)

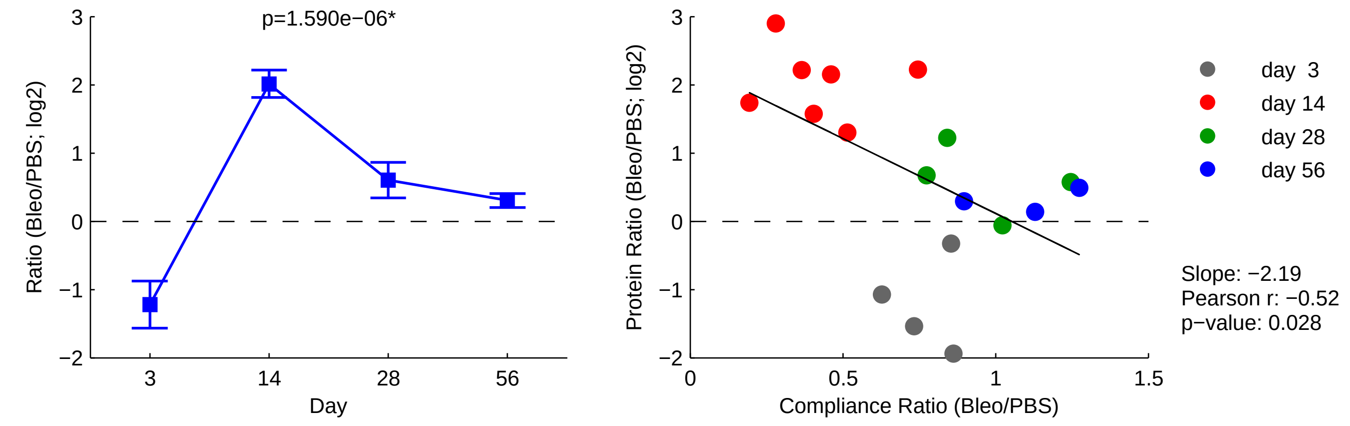

Q922G0 − Slc25a36 (id: 6411)

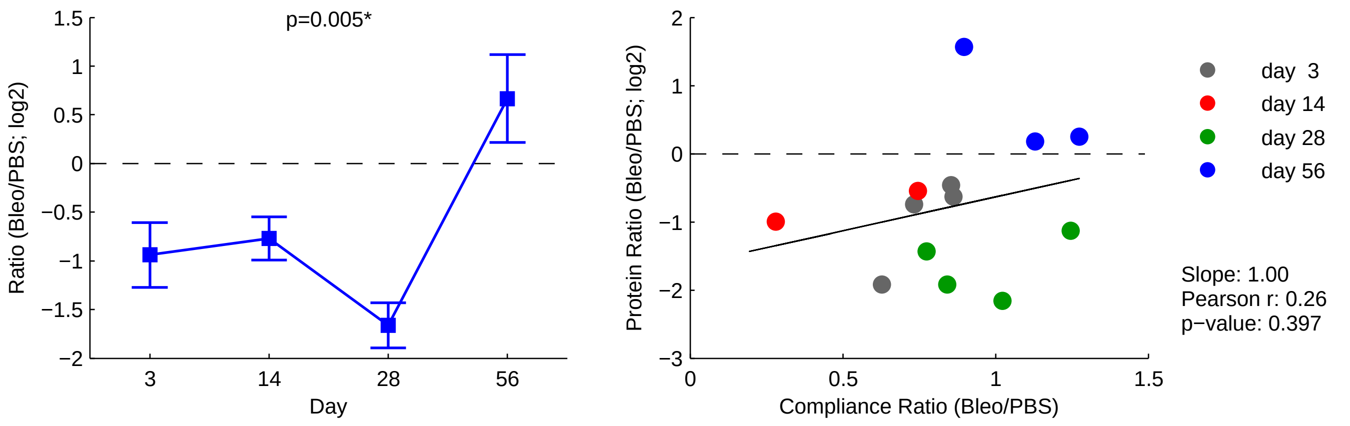

Q922J9 − Far1 (id: 6417)

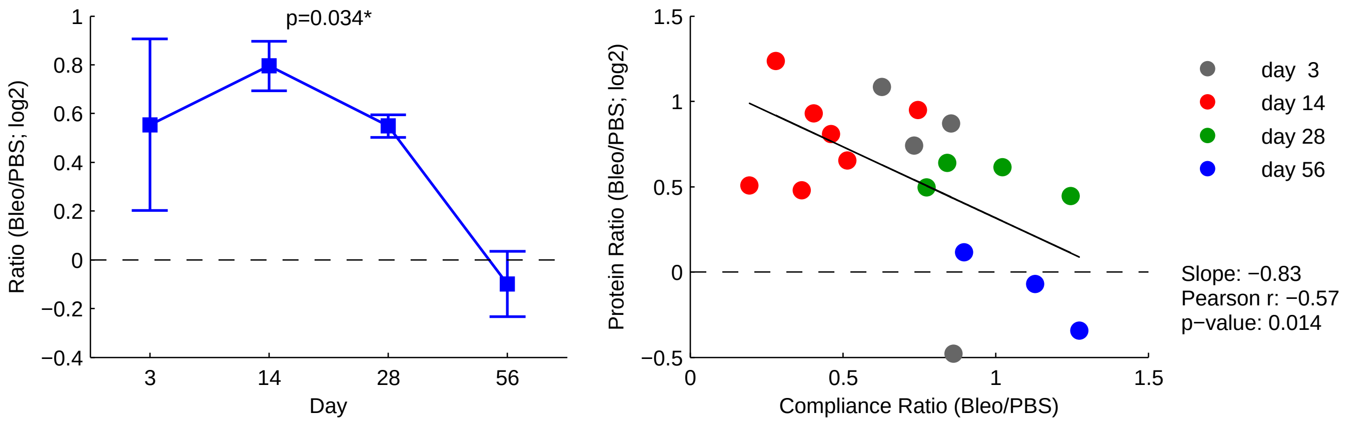

Q922M3 − Kctd10 (id: 6418)

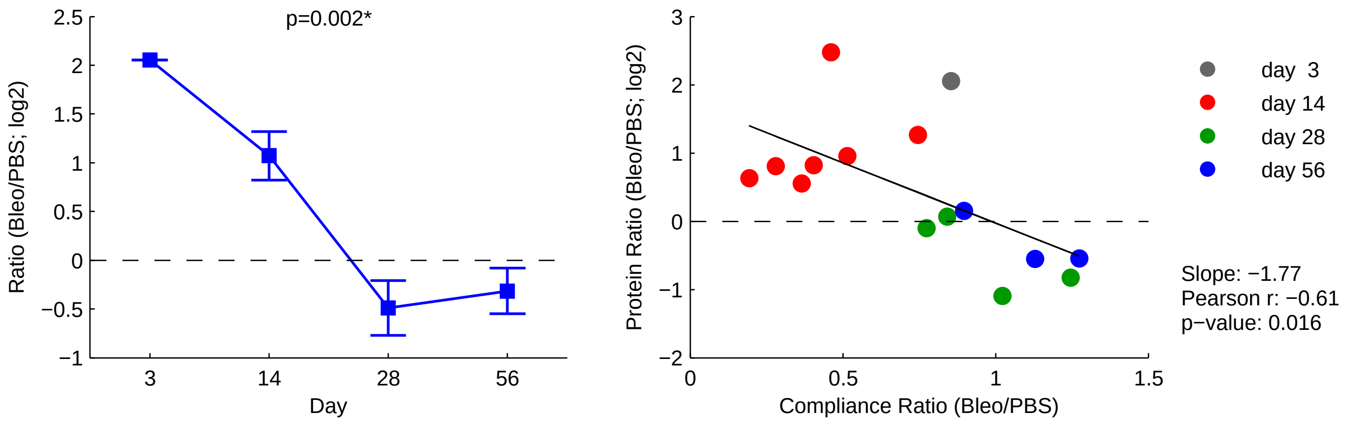

Q922Q1 − Marc2 (id: 6420)

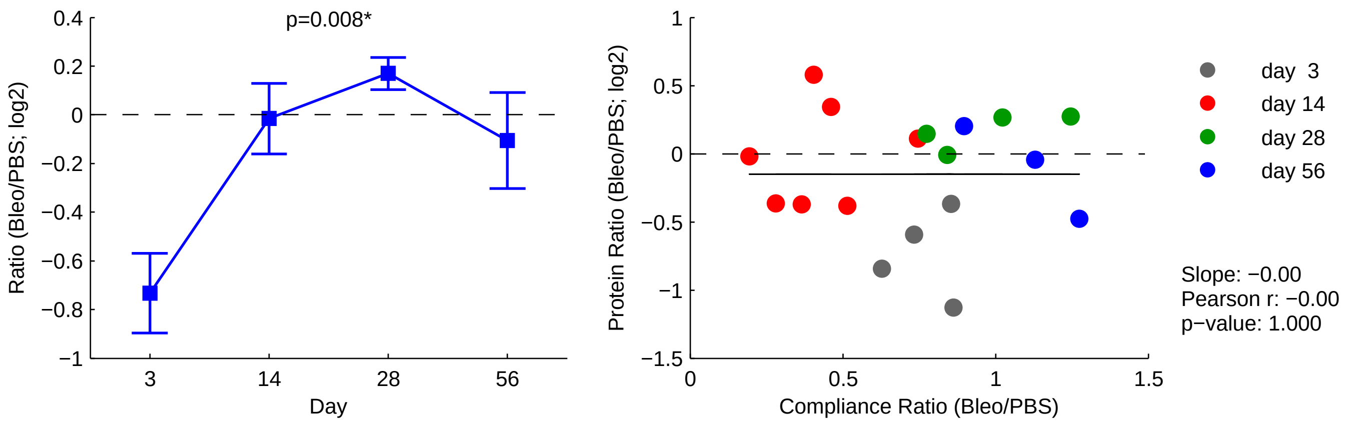

Q922Q4 − Pycr2 (id: 6421)

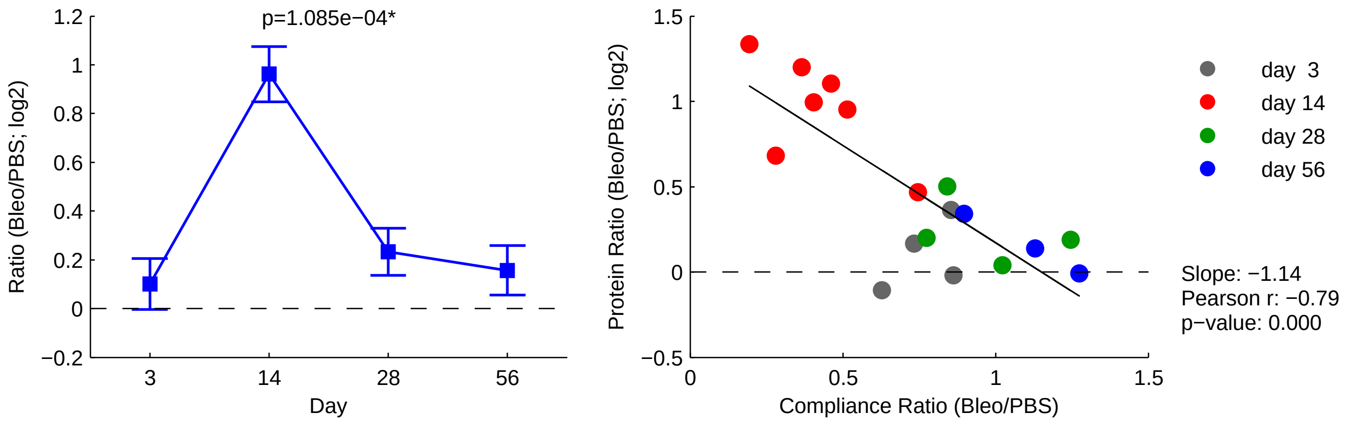

Q922Q8 − Lrrc59 (id: 6422)

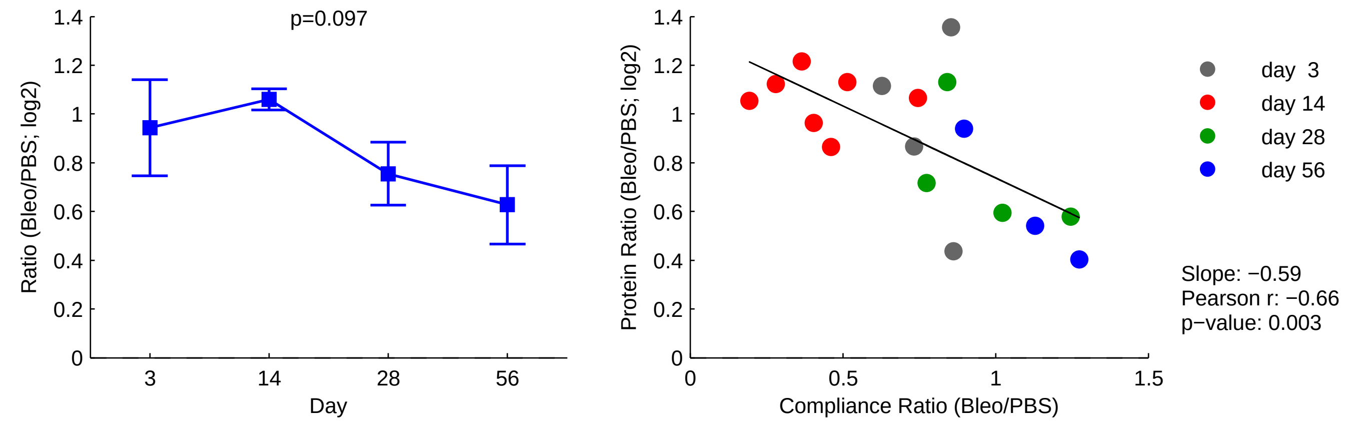

Q922Q9 − Chid1 (id: 6423)

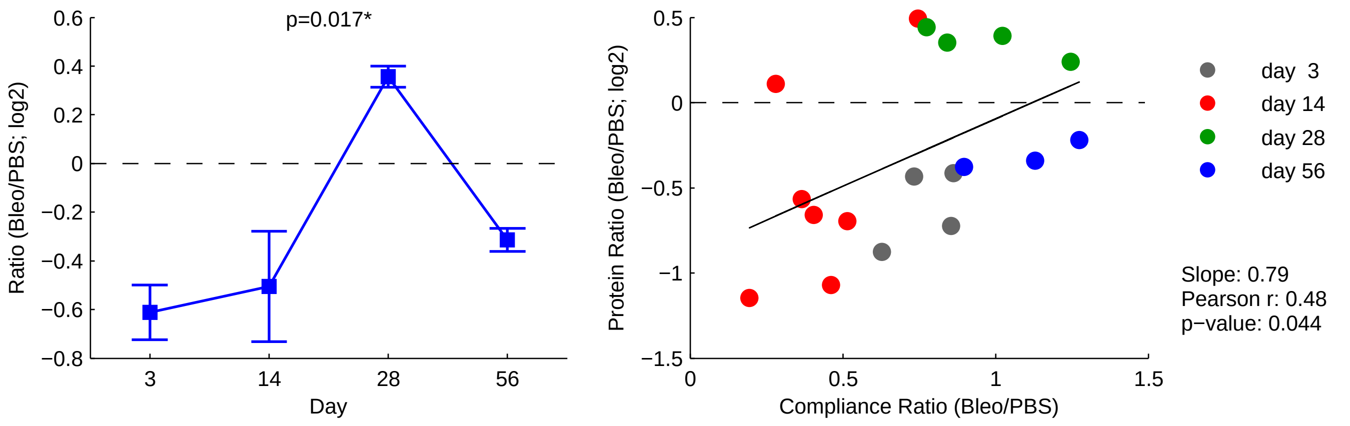

Q922U1 − Prpf3 (id: 6425)

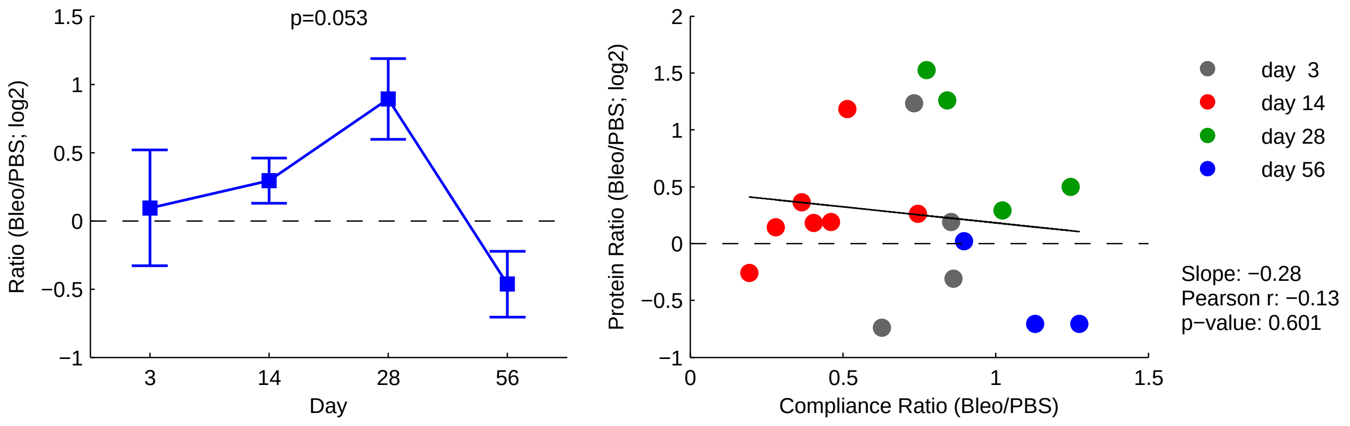

Q922W5 − Pycr1 (id: 6427)

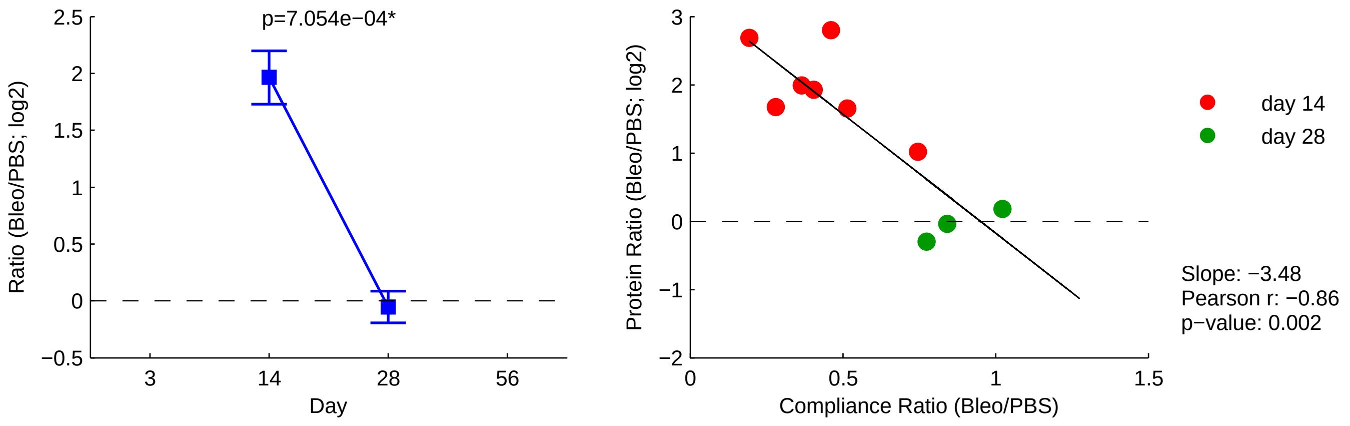

Q923B1 − Dbr1 (id: 6431)

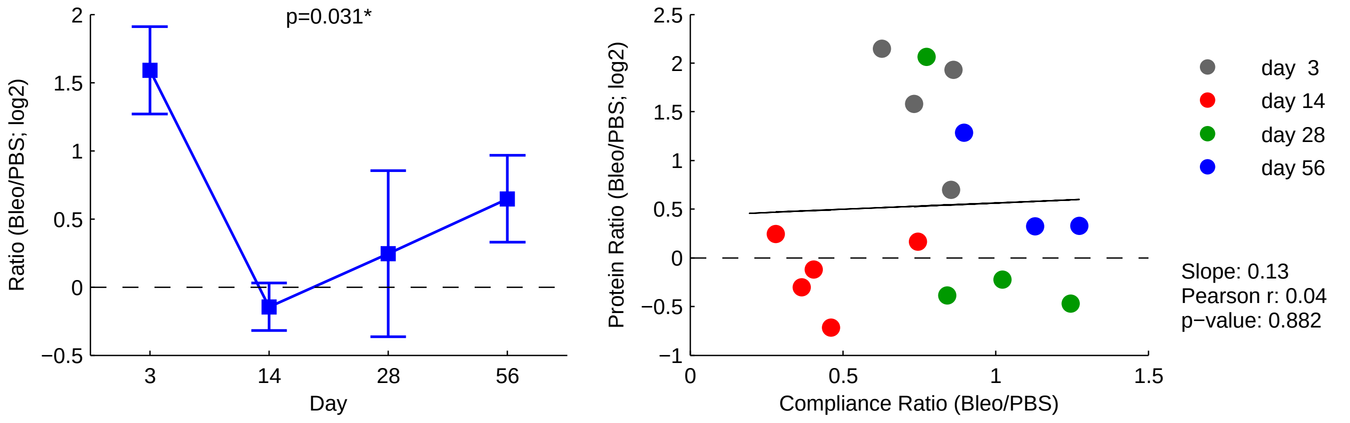

Q923B6 − Steap4 (id: 6432)

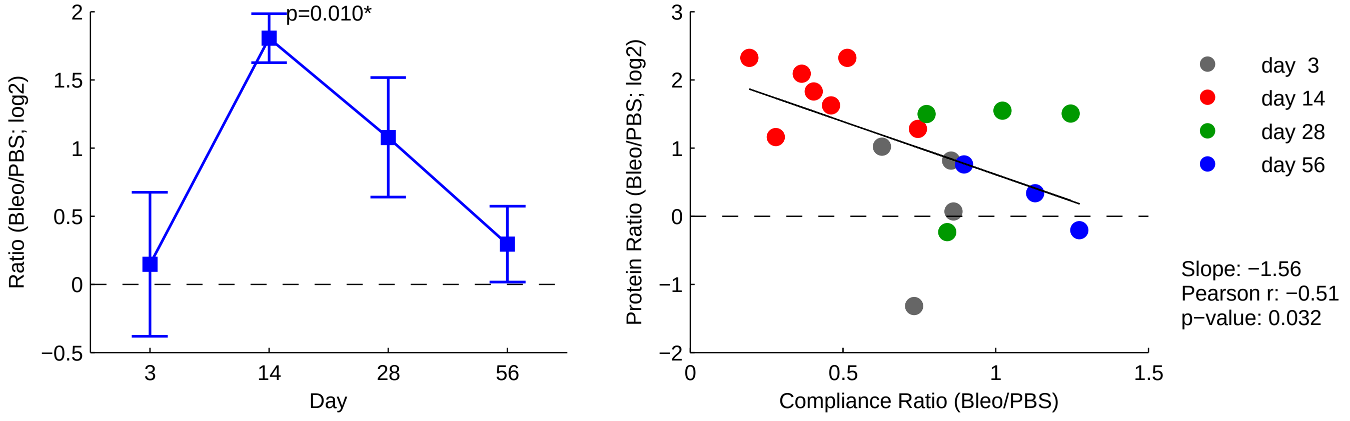

Q923D2 − Blvrb (id: 6433)

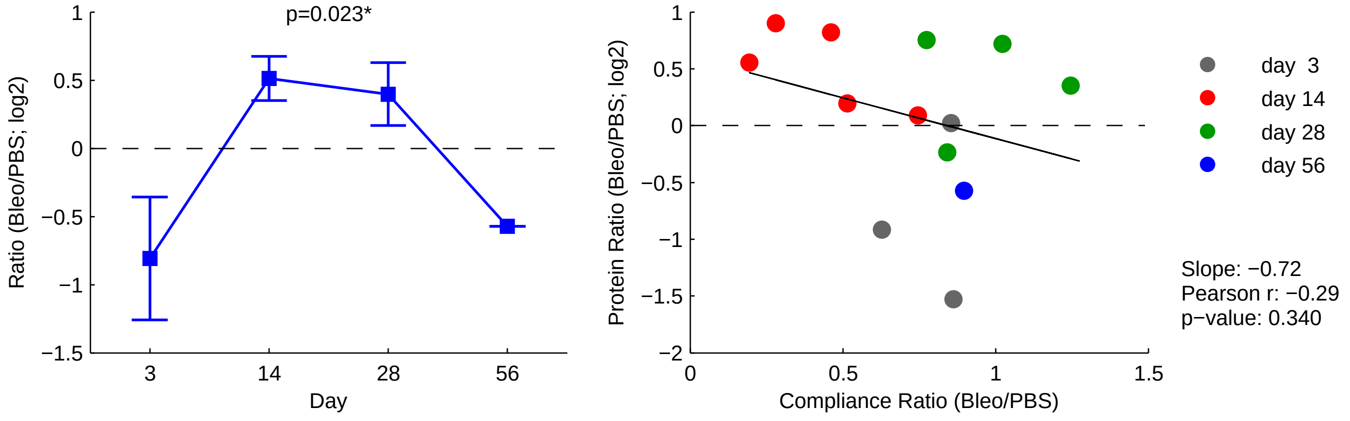

Q923D4 − Sf3b5 (id: 6434)

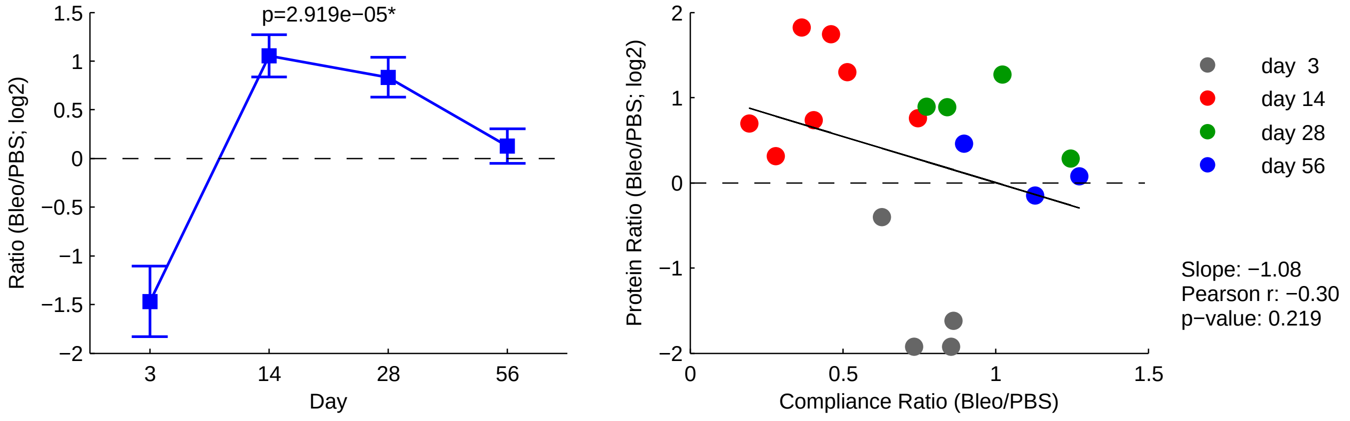

Q923D5 − Wbp11 (id: 6435)

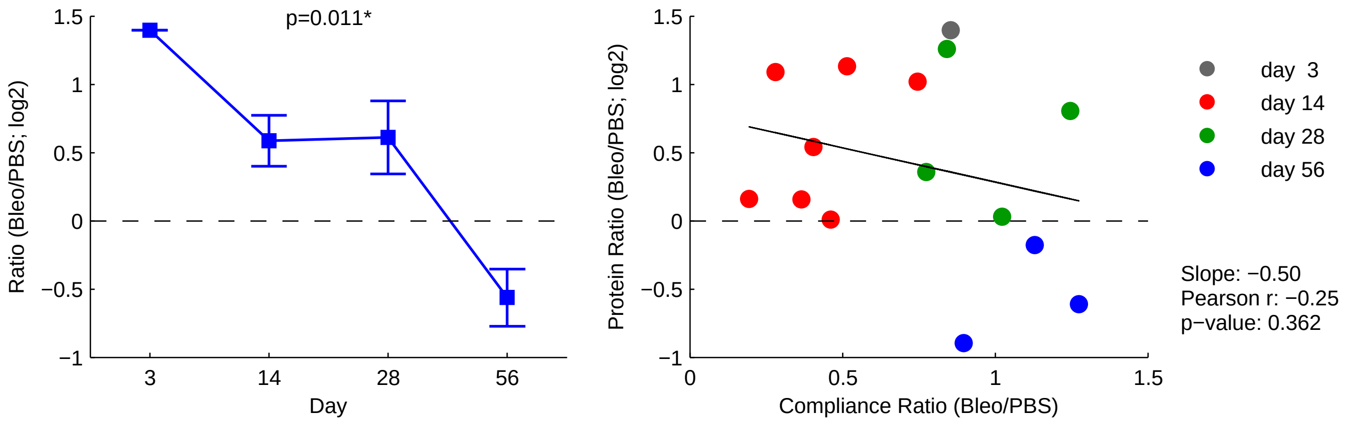

Q923Q2 − Stard13 (id: 6440)

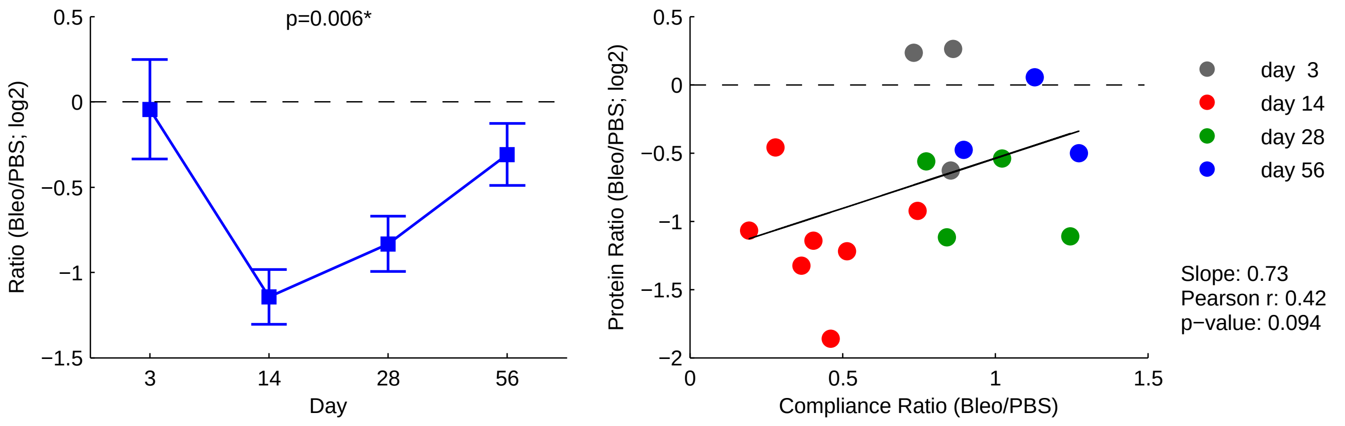

Q923T9−3 − Camk2g (id: 6441)

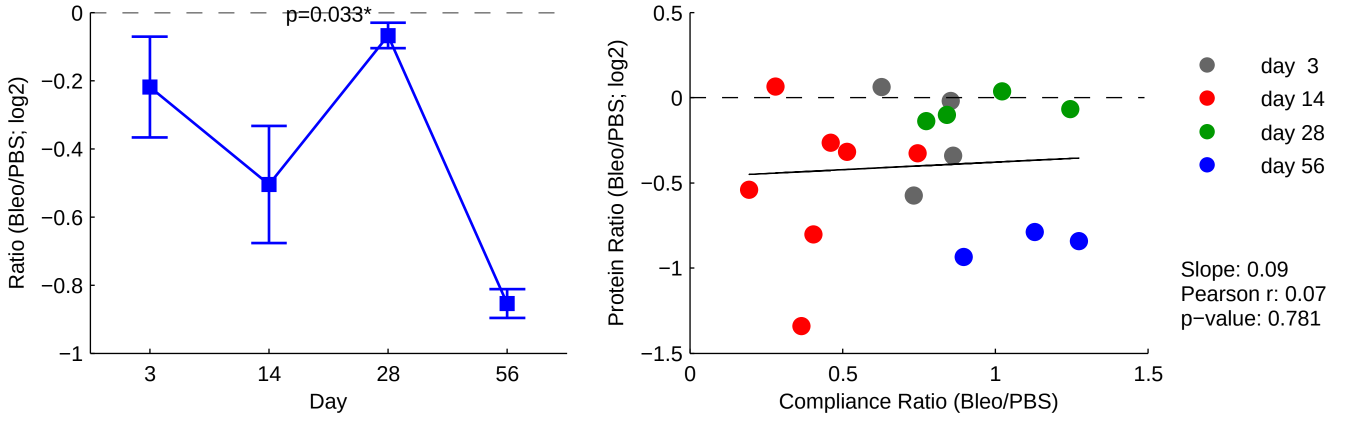

Q923U0 − Tom1l1 (id: 6442)

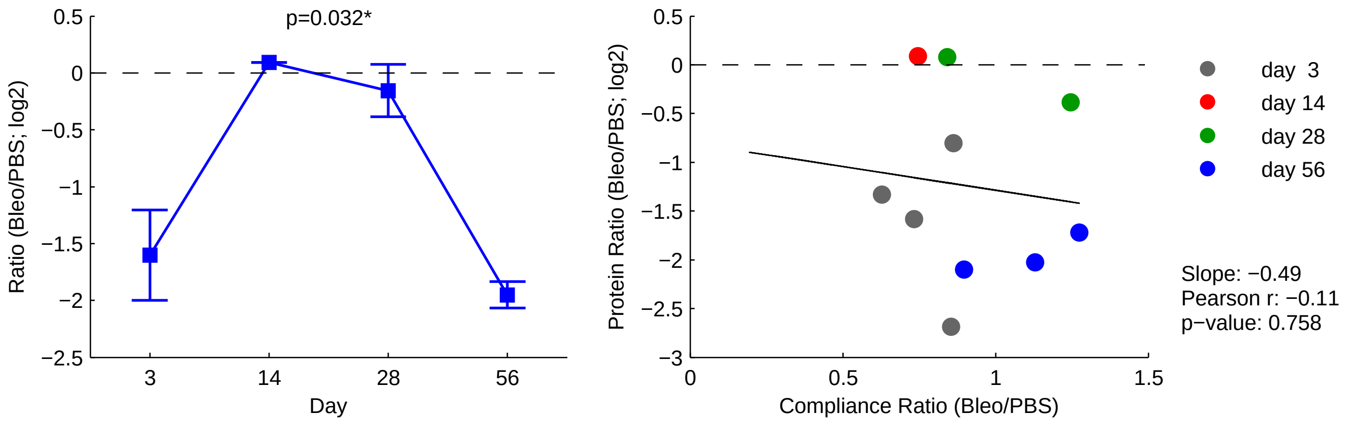

Q923X1 − Eltd1 (id: 6443)

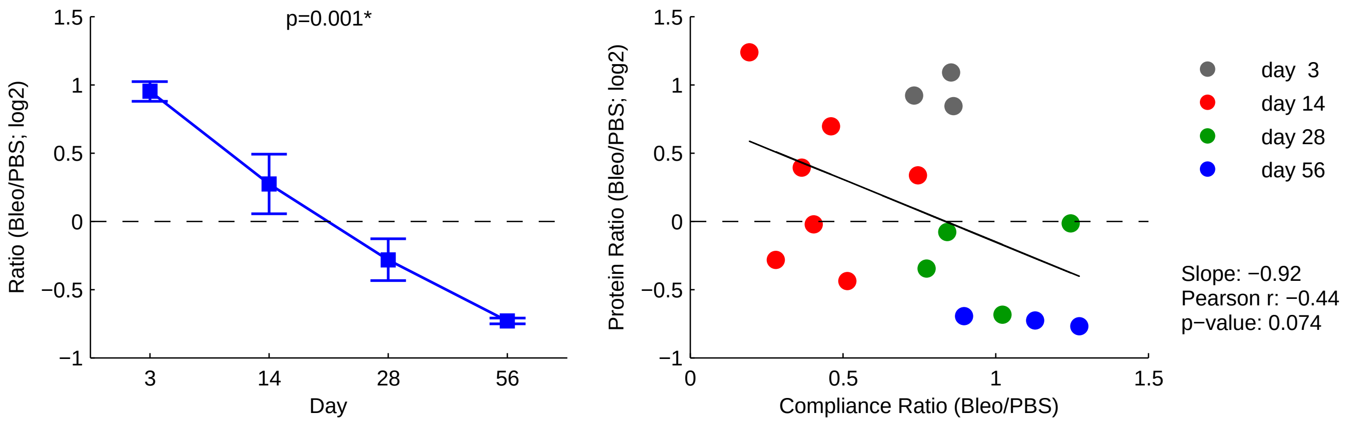

Q924D0 − Rtn4ip1 (id: 6447)

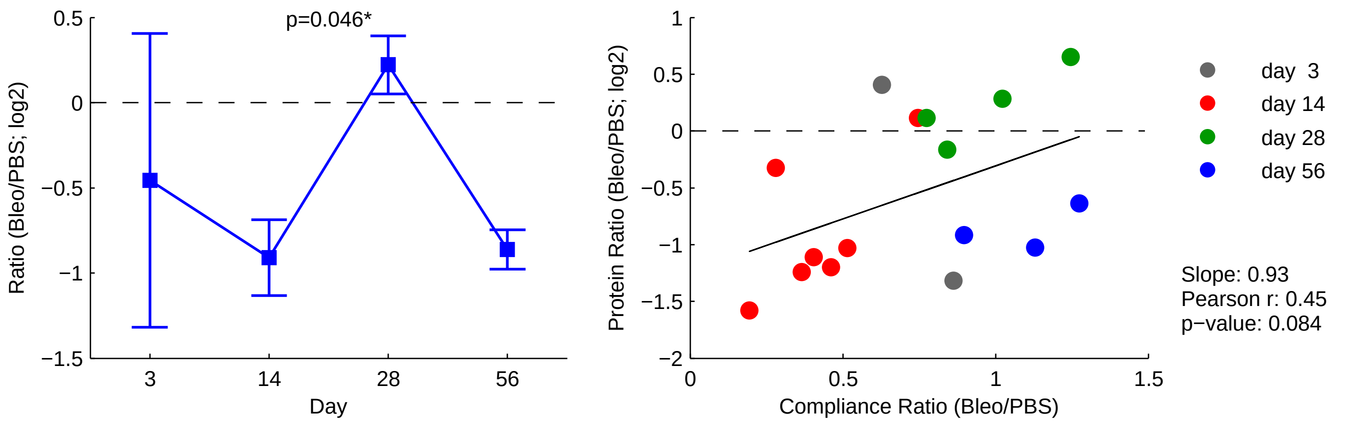

Q924L1 − Letmd1 (id: 6451)

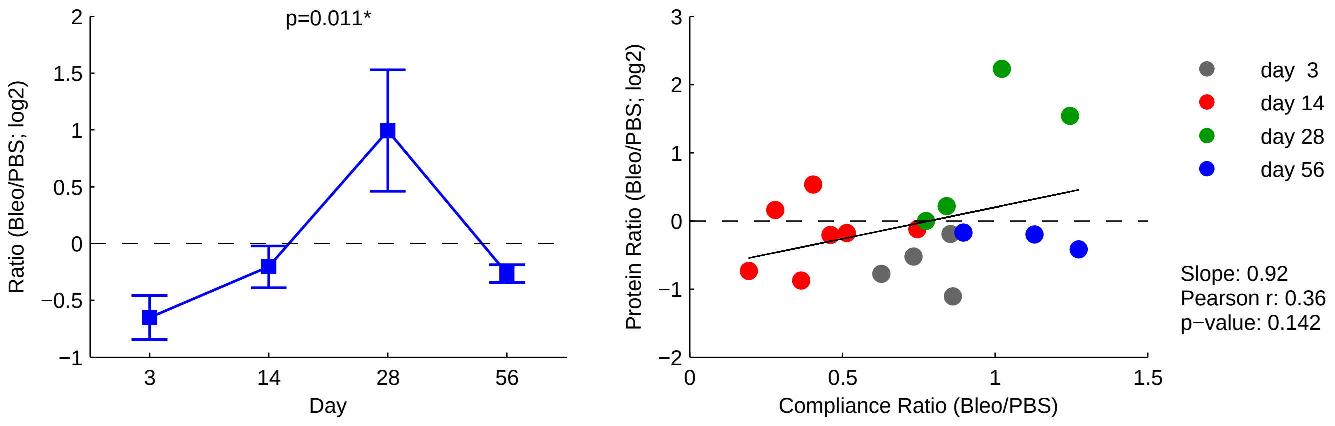

Q924W7 − St5 (id: 6456)

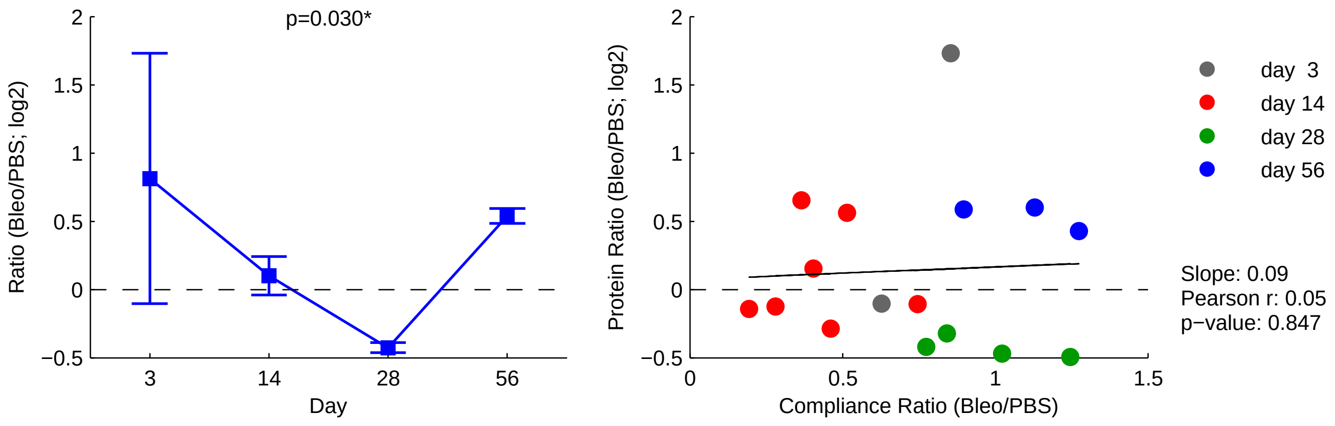

Q924X2 − Cpt1b (id: 6457)

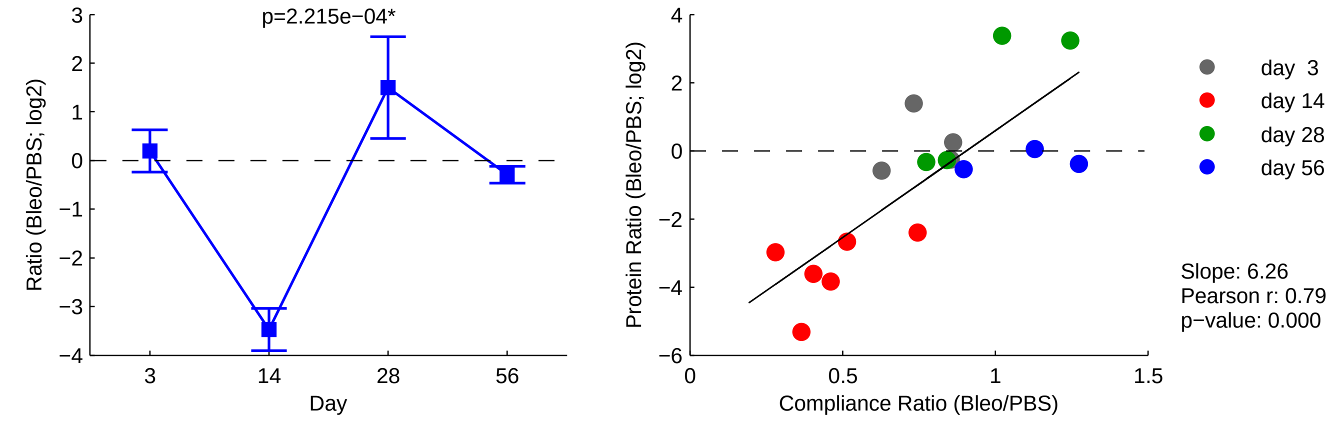

Q924Z5 − Tram2 (id: 6458)

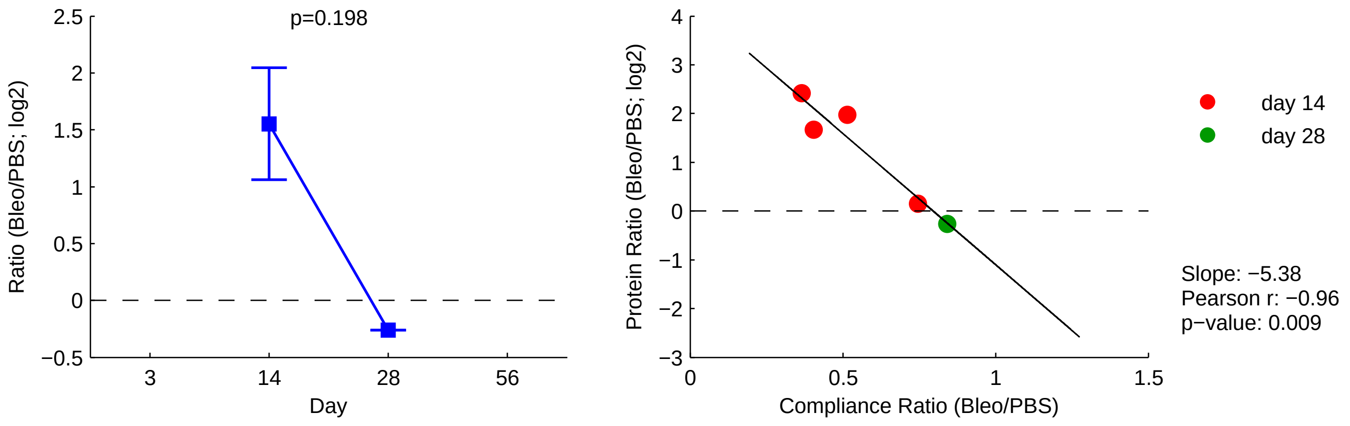

Q925B0 − Pawr (id: 6459)

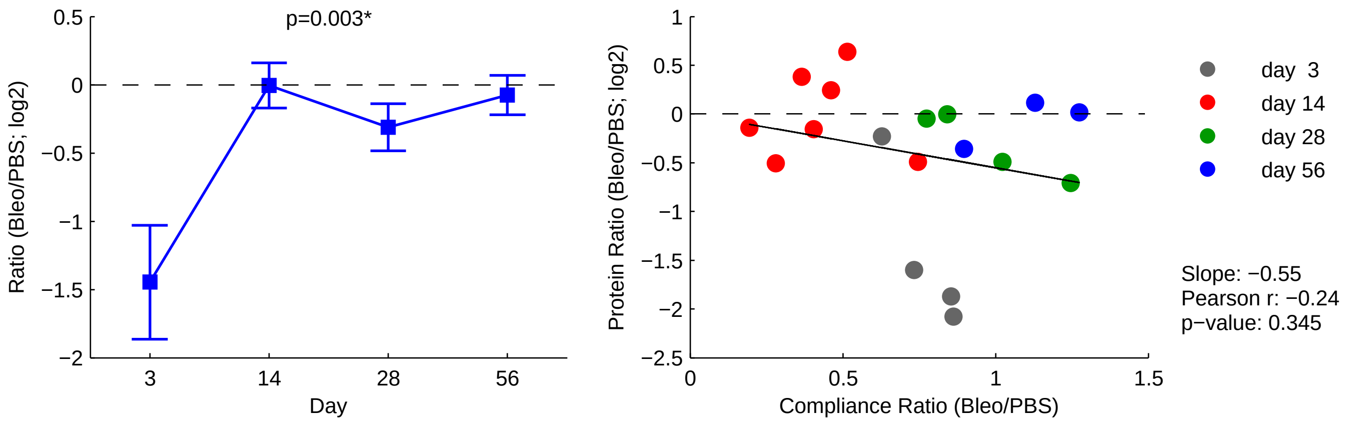

Q925F2 − Esam (id: 6460)

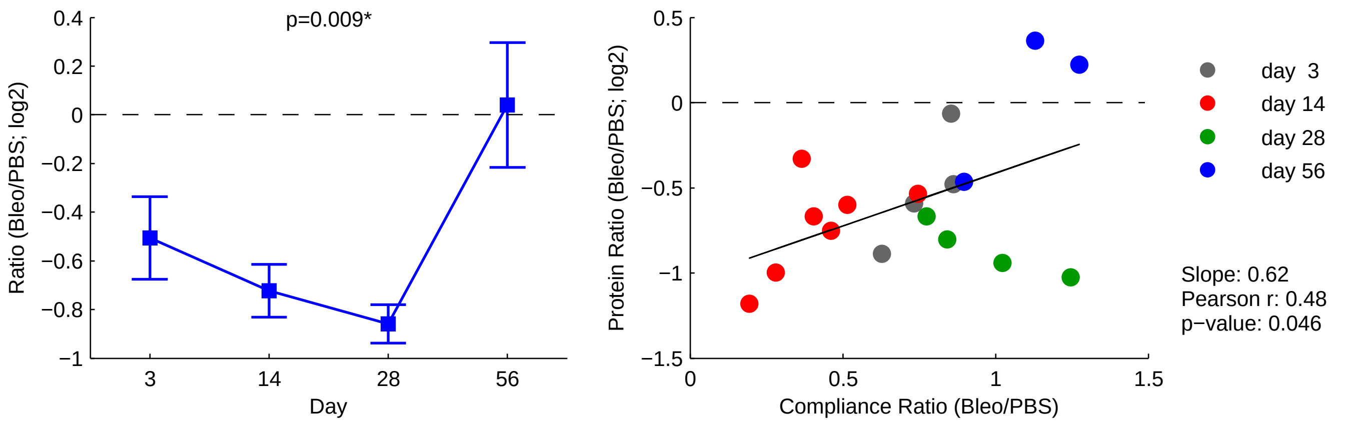

Q925I1 − Atad3 (id: 6461)

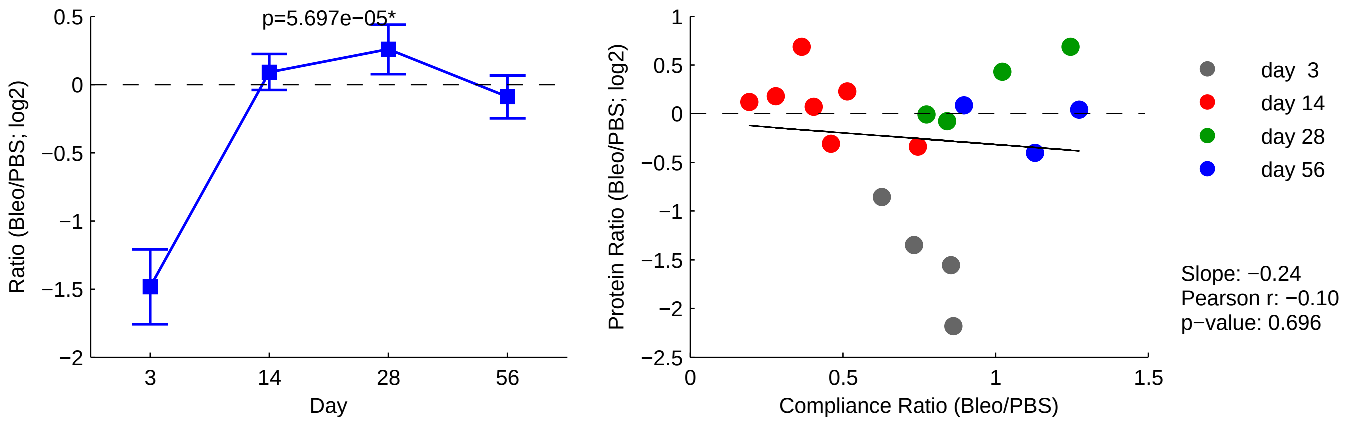

Q925N0 − Sfxn5 (id: 6464)

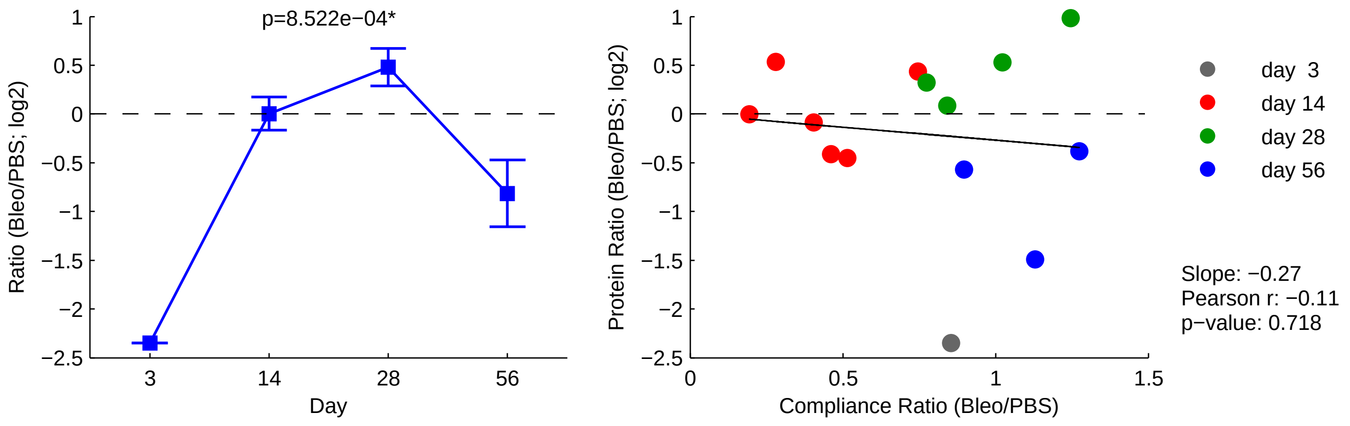

Q925N2 − Sfxn2 (id: 6465)

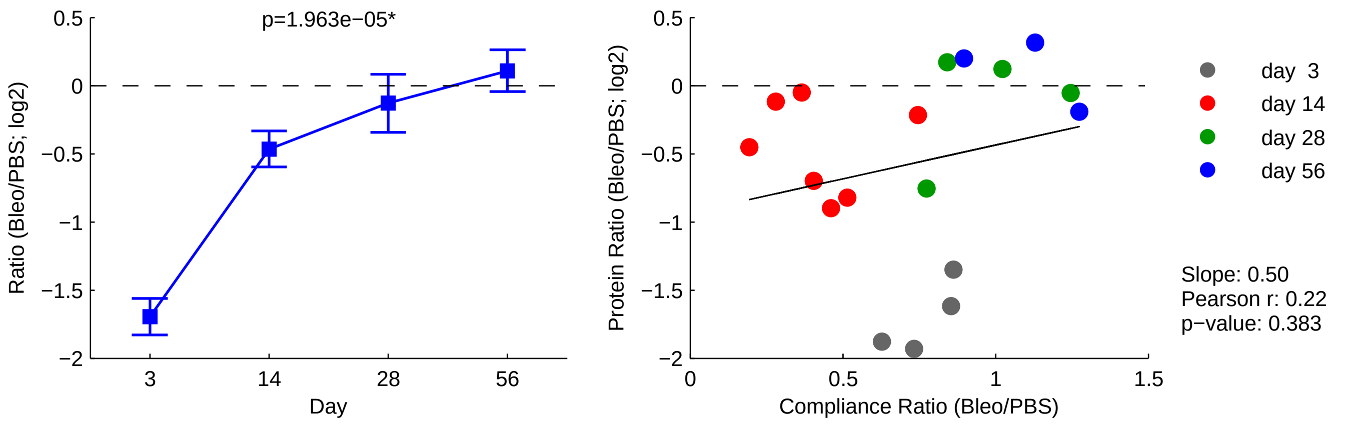

Q99J39−2 − Mlycd (id: 6474)

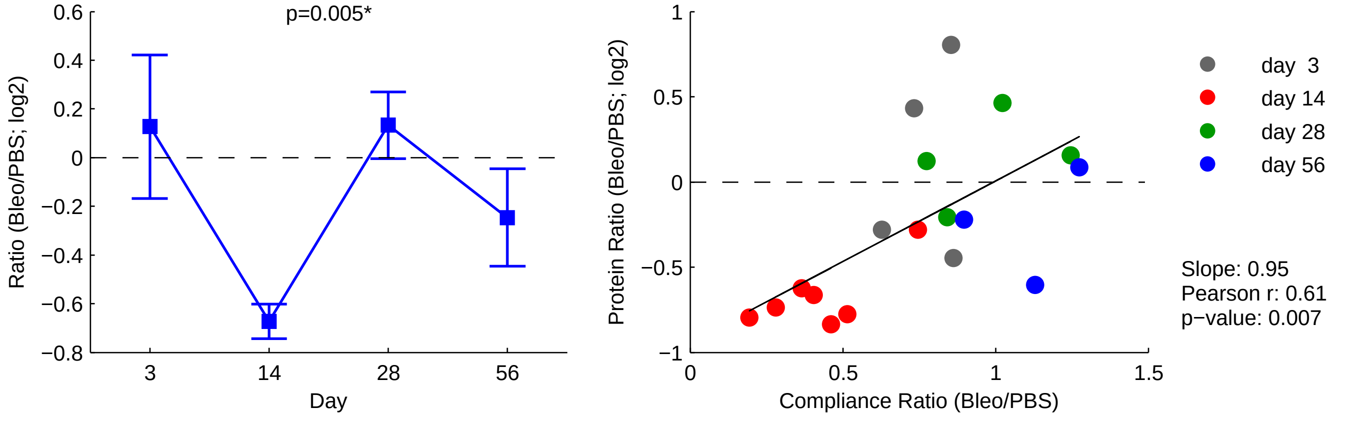

Q99J47−2 − Dhrs7b (id: 6476)

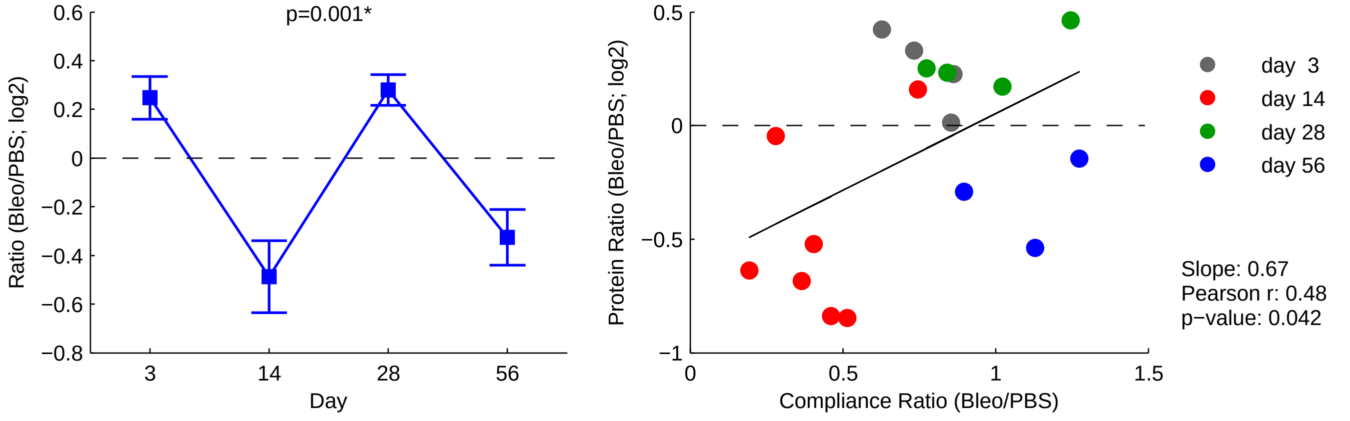

Q99J77 − Nans (id: 6478)

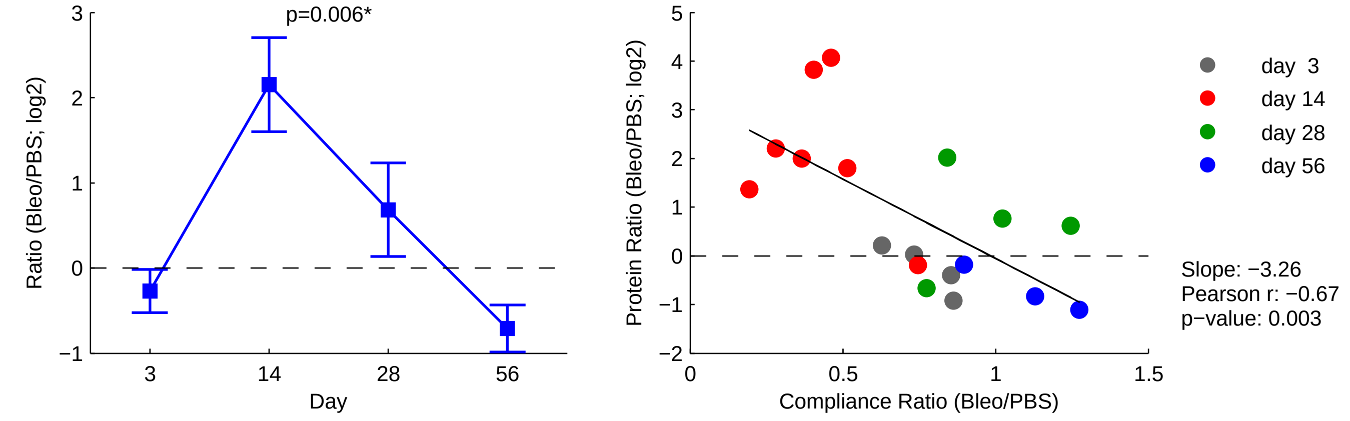

Q99J95 − Cdk9 (id: 6481)

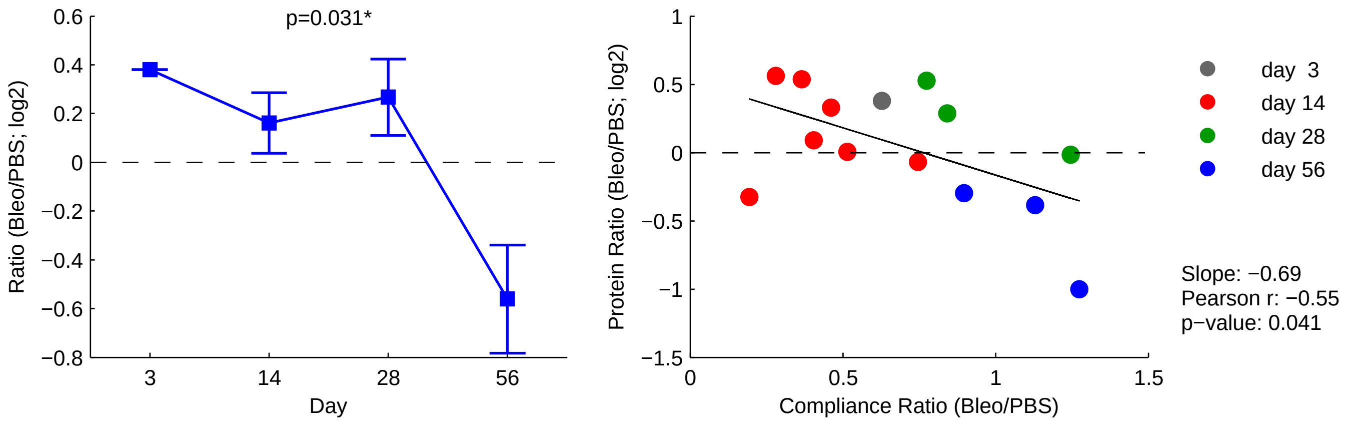

Q99JB2 − Stoml2 (id: 6482)

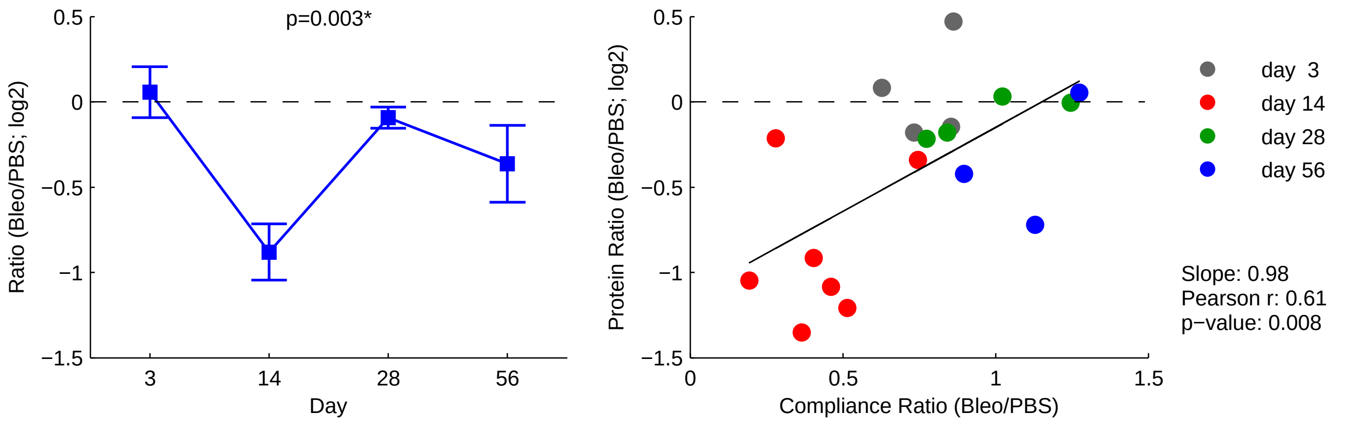

Q99JB8 − Pacsin3 (id: 6483)

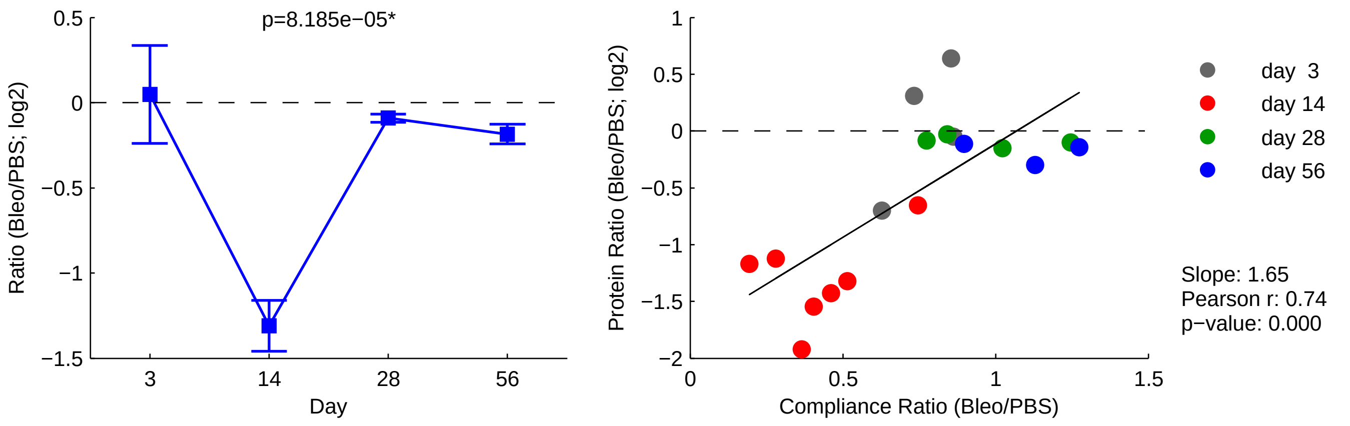

Q99JF8 − Psip1 (id: 6484)

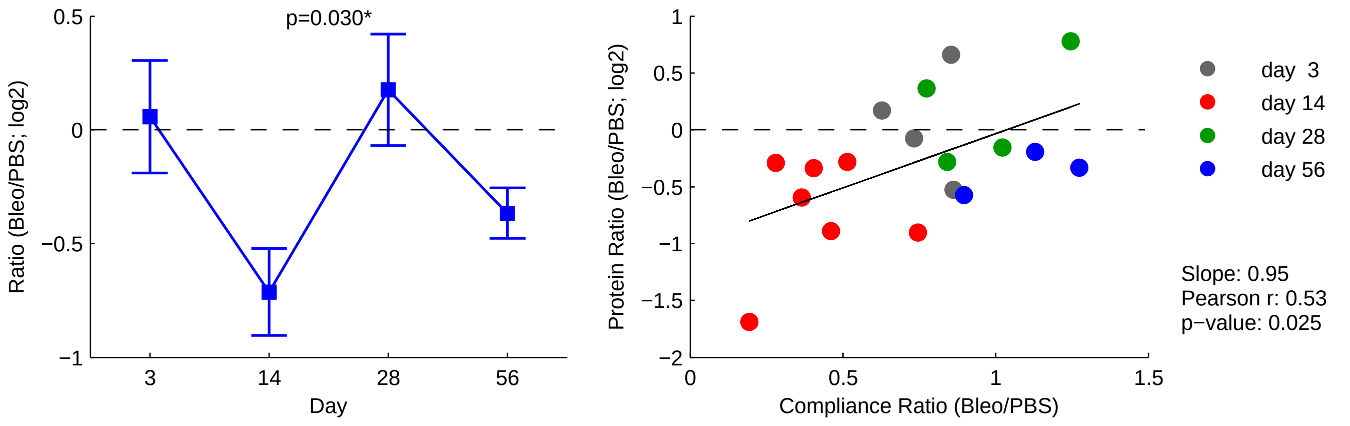

Q99JI4 − Psmd6 (id: 6486)

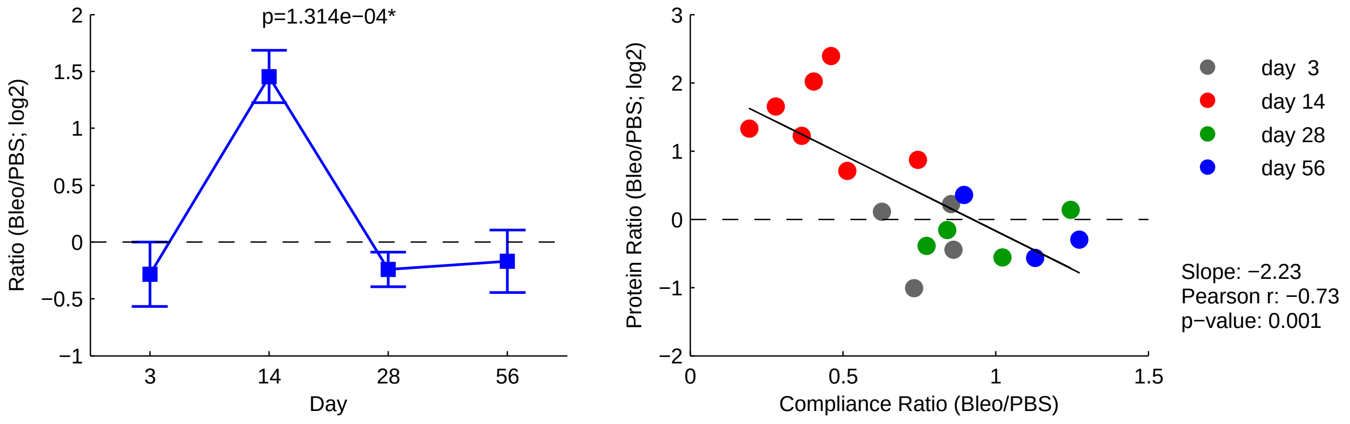

Q99JI6 − Rap1b (id: 6487)

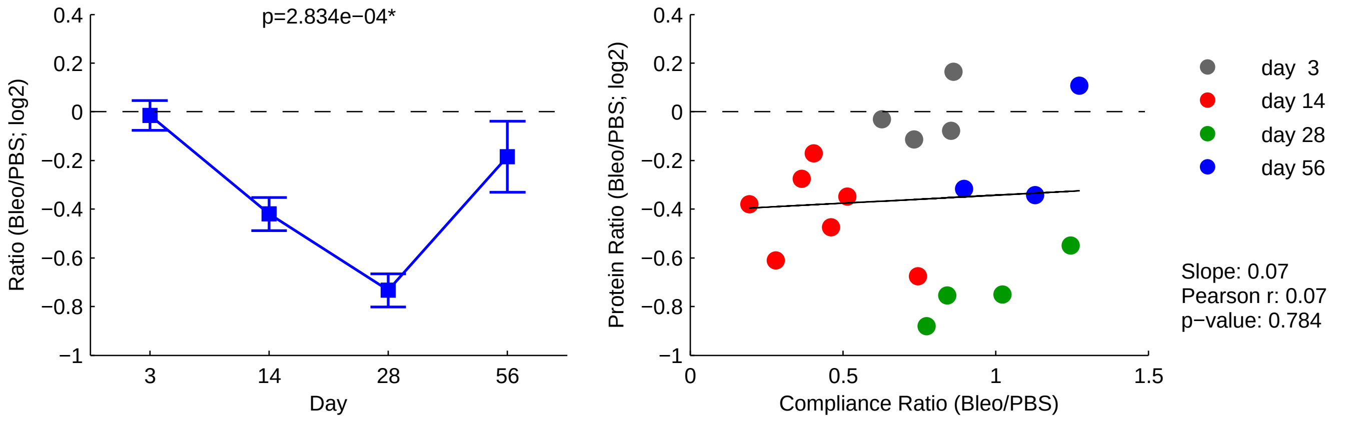

Q99JP6−2 − Homer3 (id: 6489)

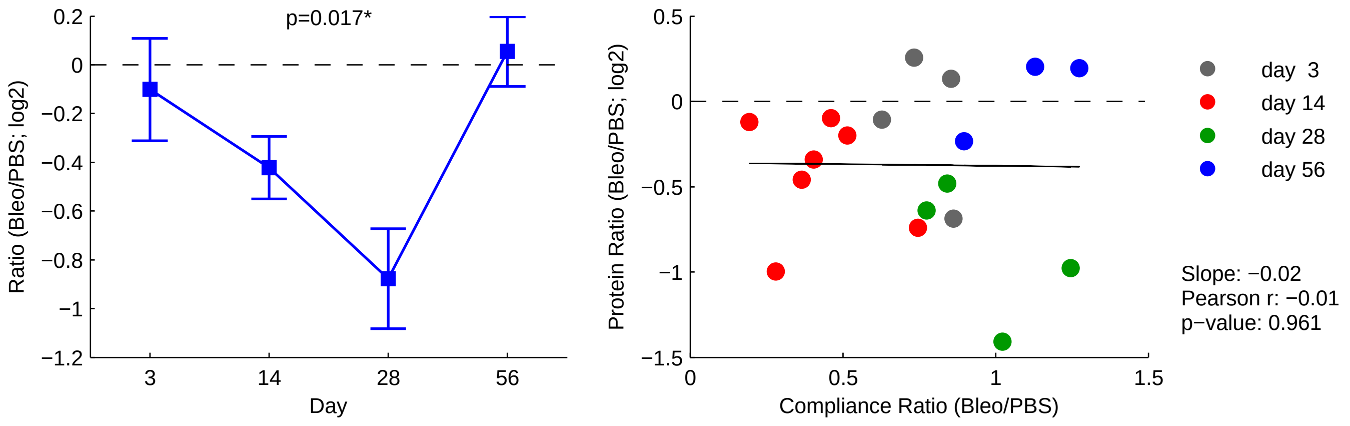

Q99JR1 − Sfxn1 (id: 6490)

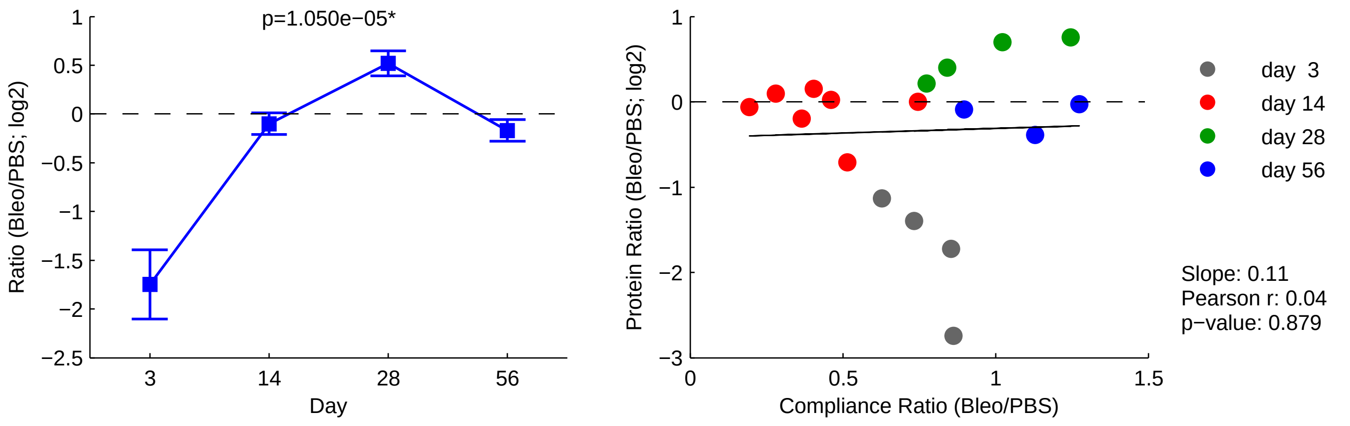

Q99JW5 − Epcam (id: 6495)

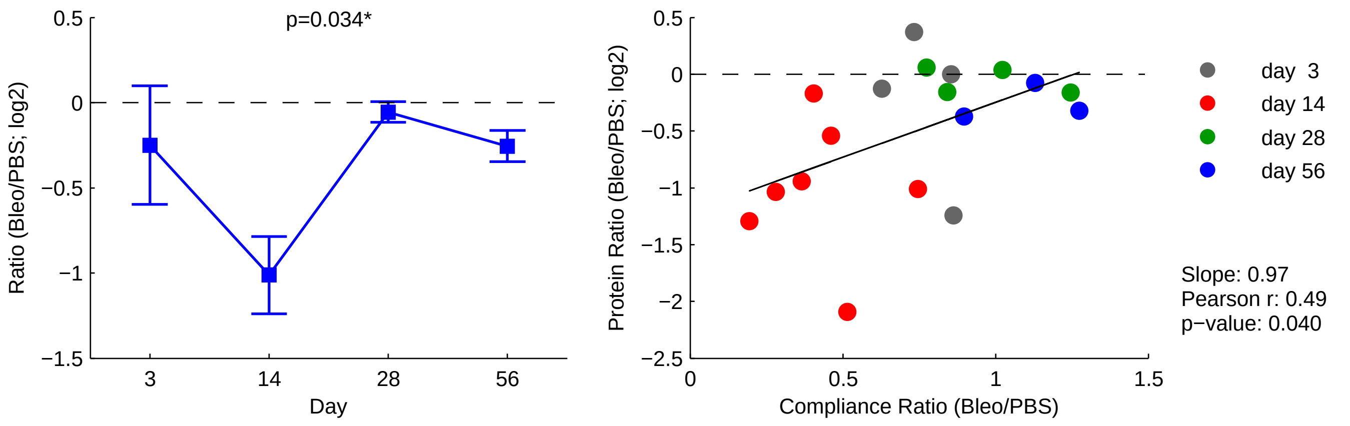

Q99JX7 − Nxf1 (id: 6497)

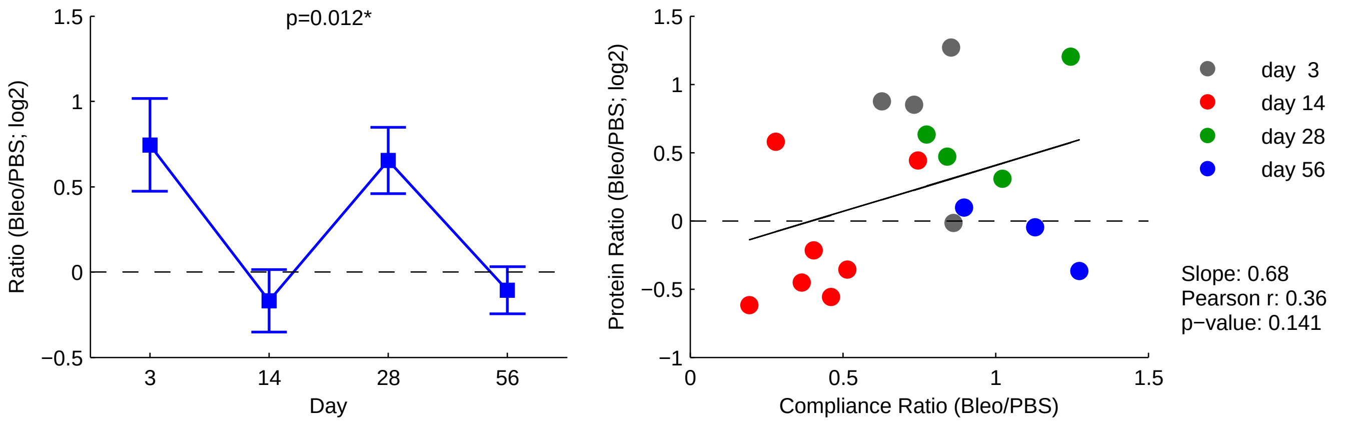

Q99JY0 − Hadhb (id: 6498)

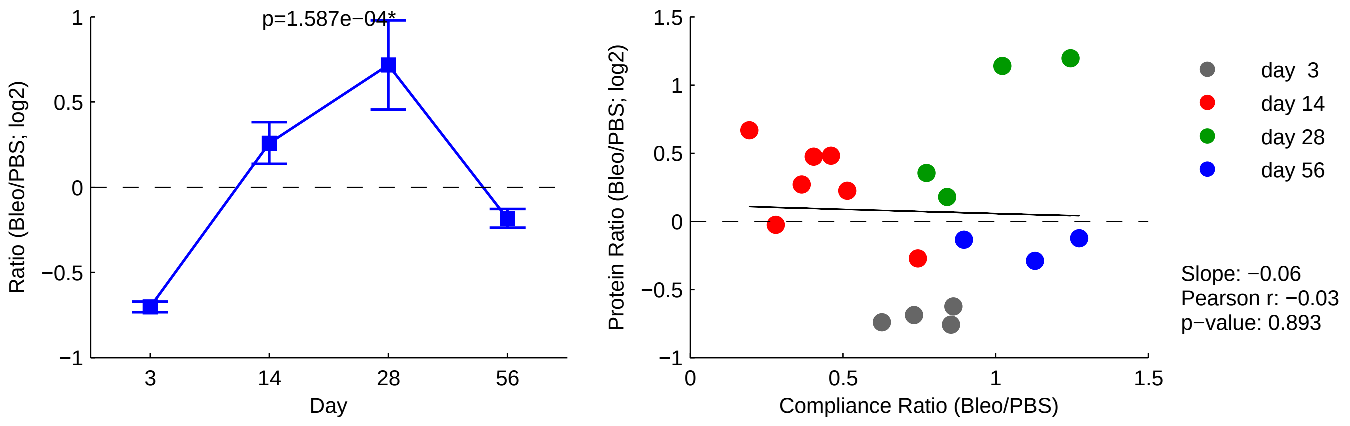

Q99JY4 − Trabd (id: 6499)

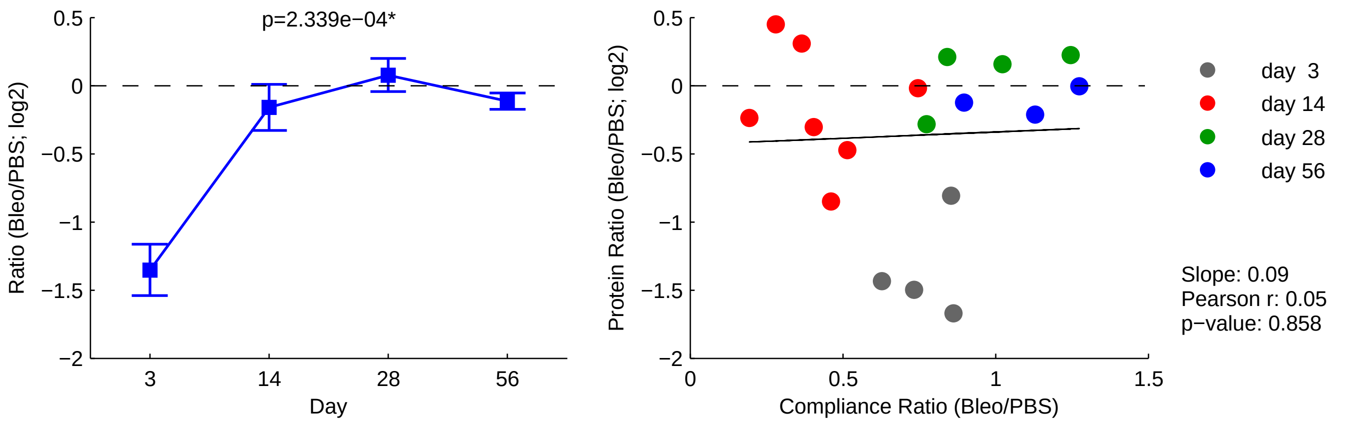

Q99JY6 − Higd1b (id: 6500)

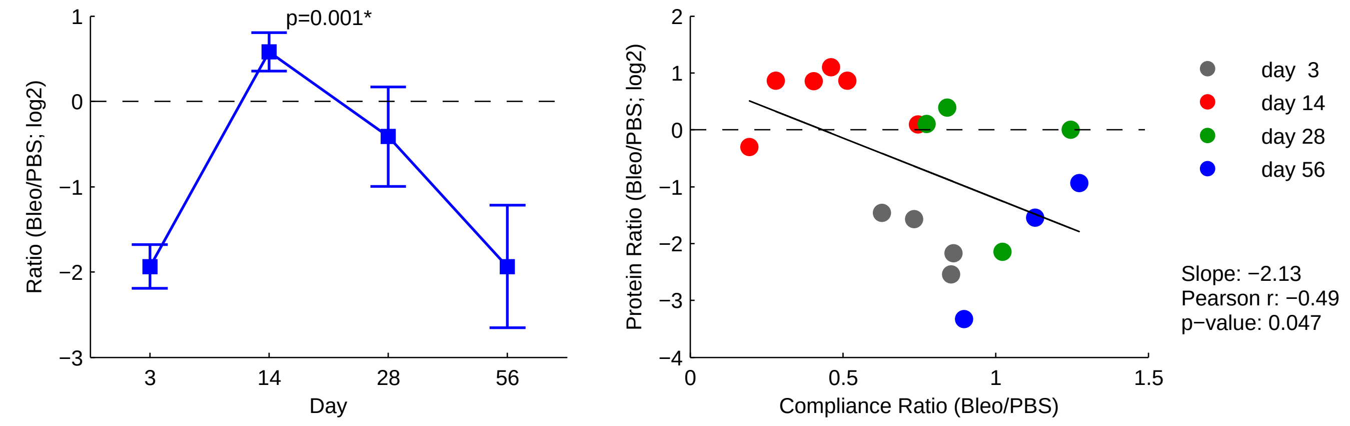

Q99JY8 − Ppap2b (id: 6501)

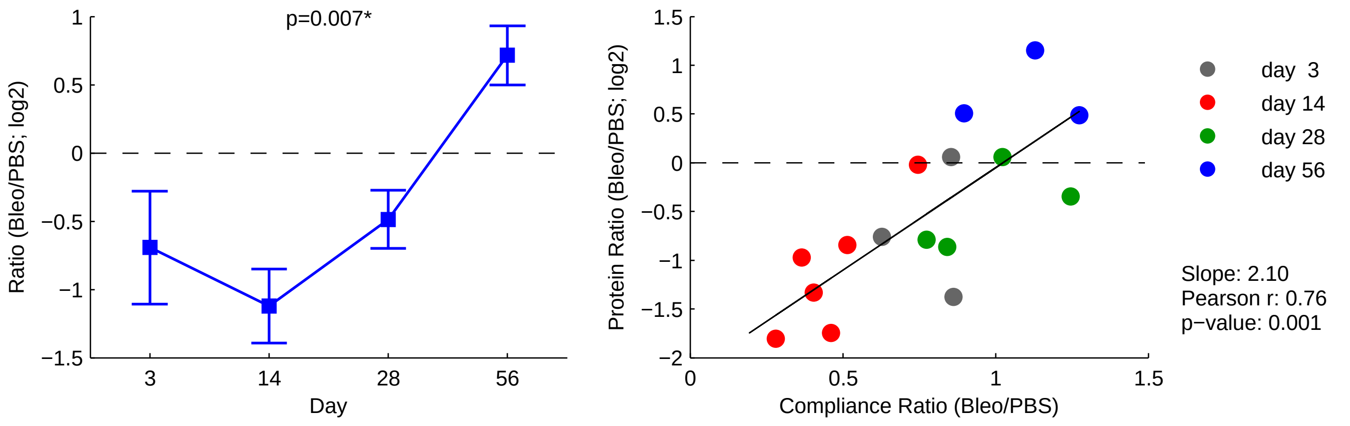

Q99K01−2 − Pdxdc1 (id: 6504)

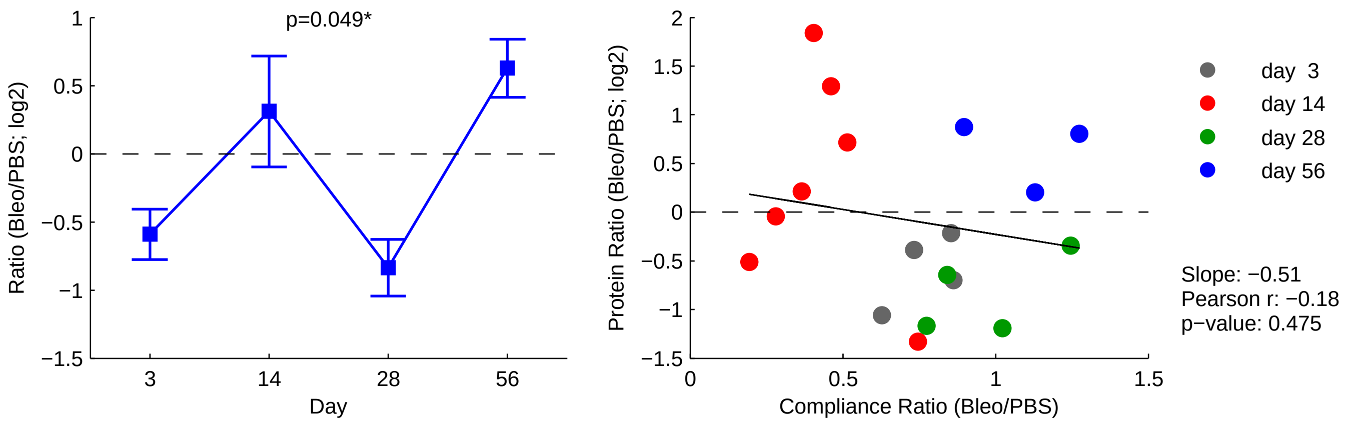

Q99K28 − Arfgap2 (id: 6506)

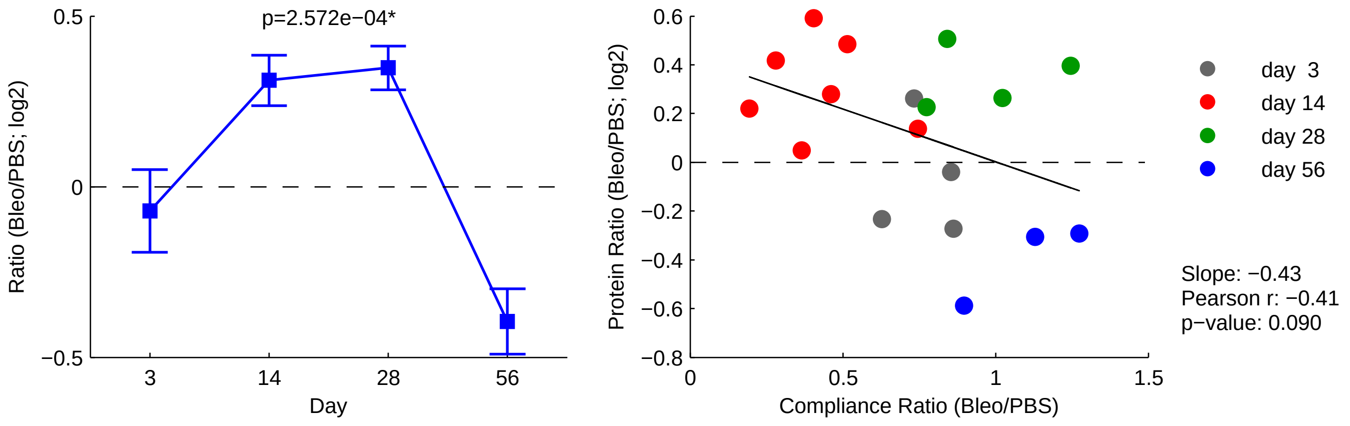

Q99K30 − Eps8l2 (id: 6507)

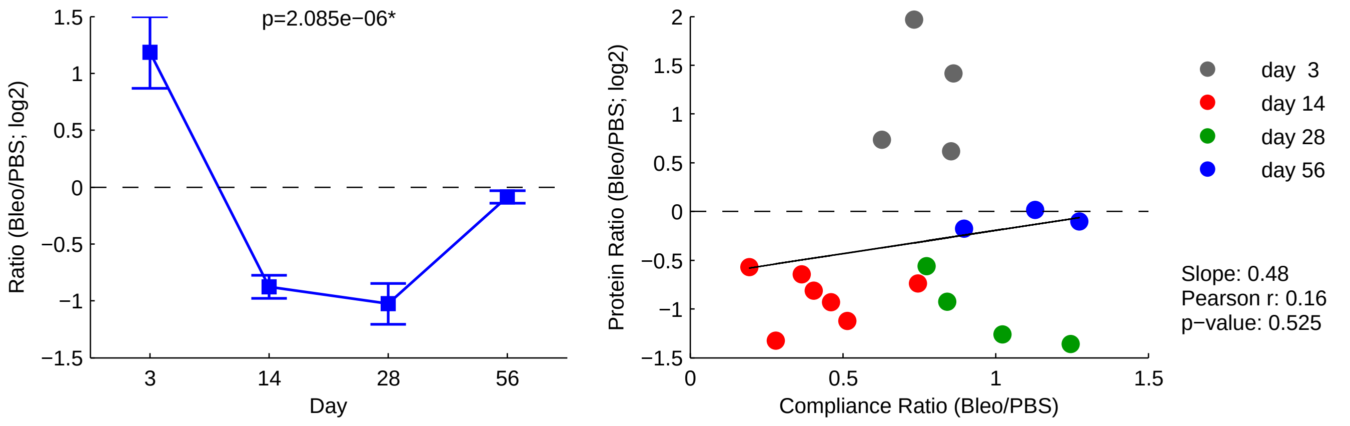

Q99K41 − Emilin1 (id: 6508)

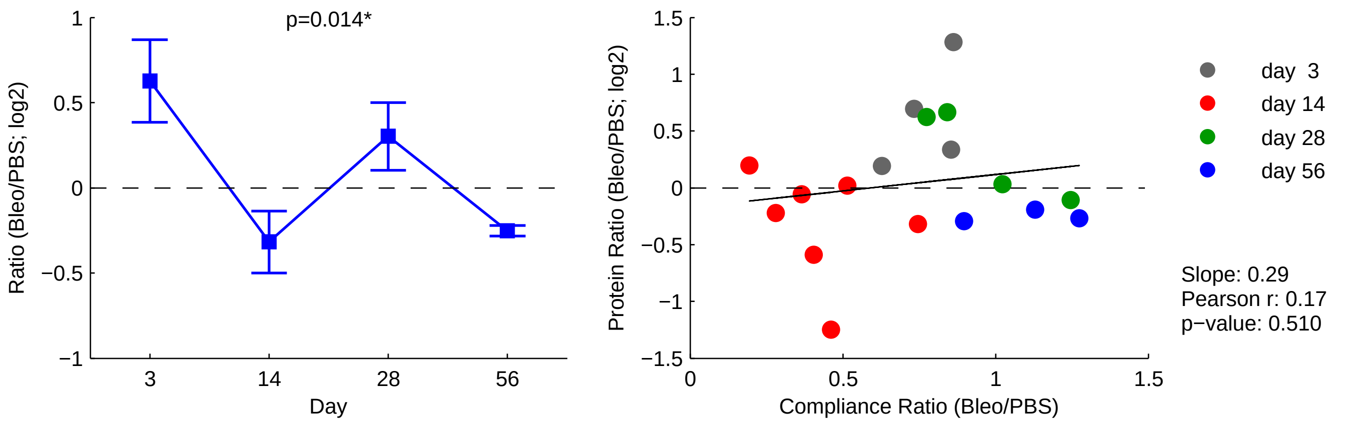

Q99K48 − Nono (id: 6509)

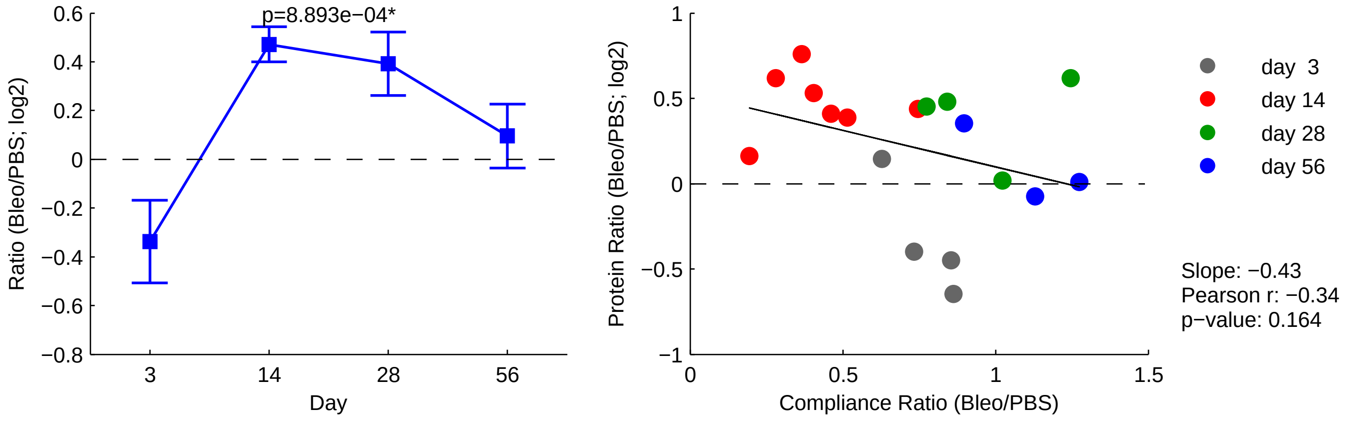

Q99KC8 − Vwa5a (id: 6516)

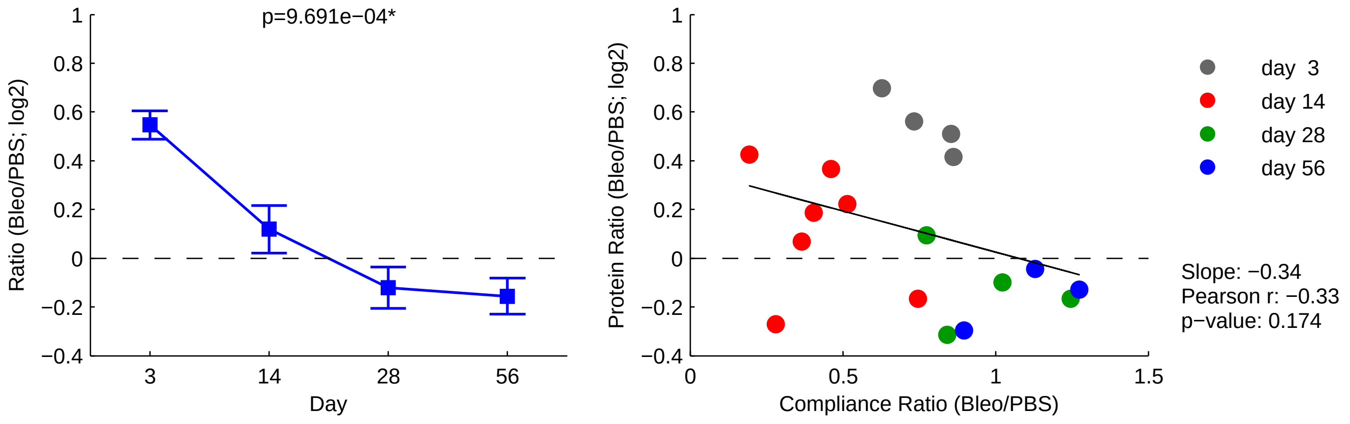

Q99KE1 − Me2 (id: 6518)

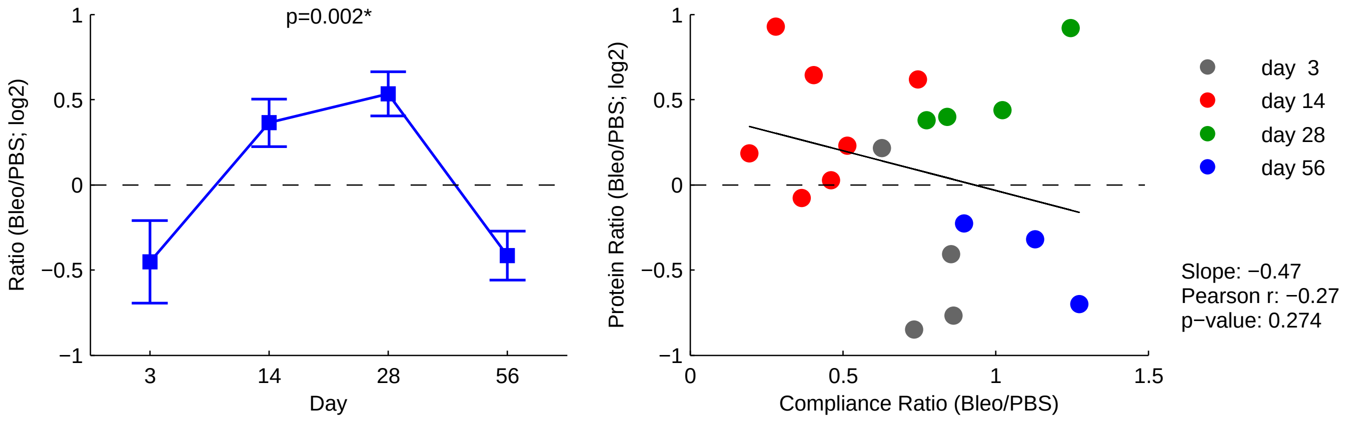

Q99KF1 − Tmed9 (id: 6519)

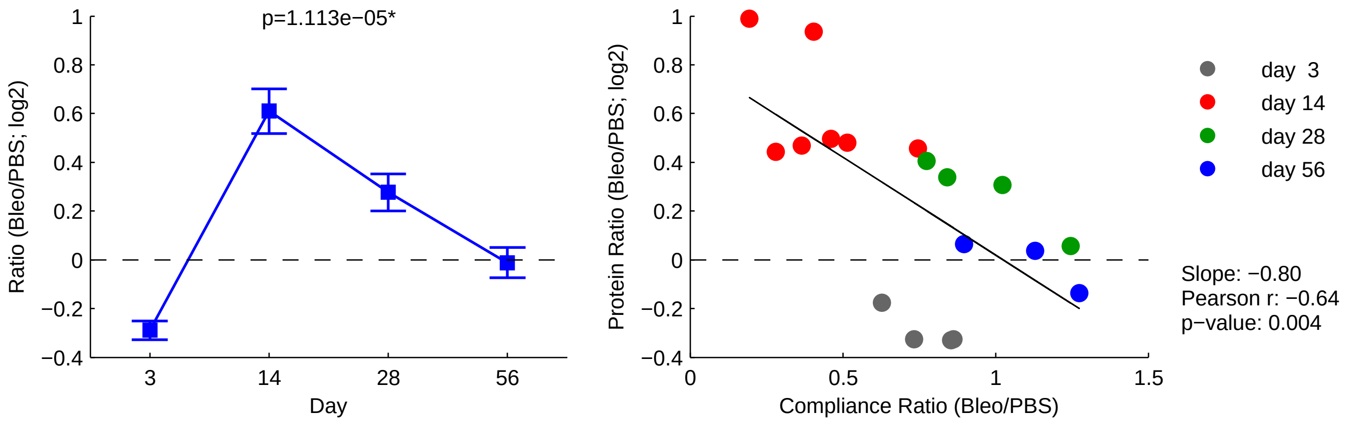

Q99KG3−3 − (id: 6521)

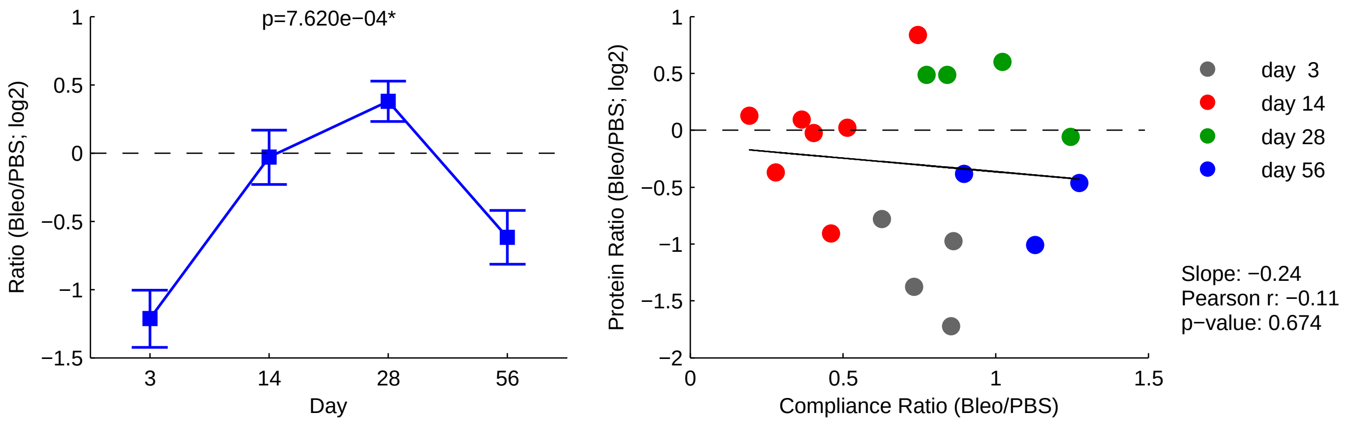

Q99KH8 − Stk24 (id: 6522)

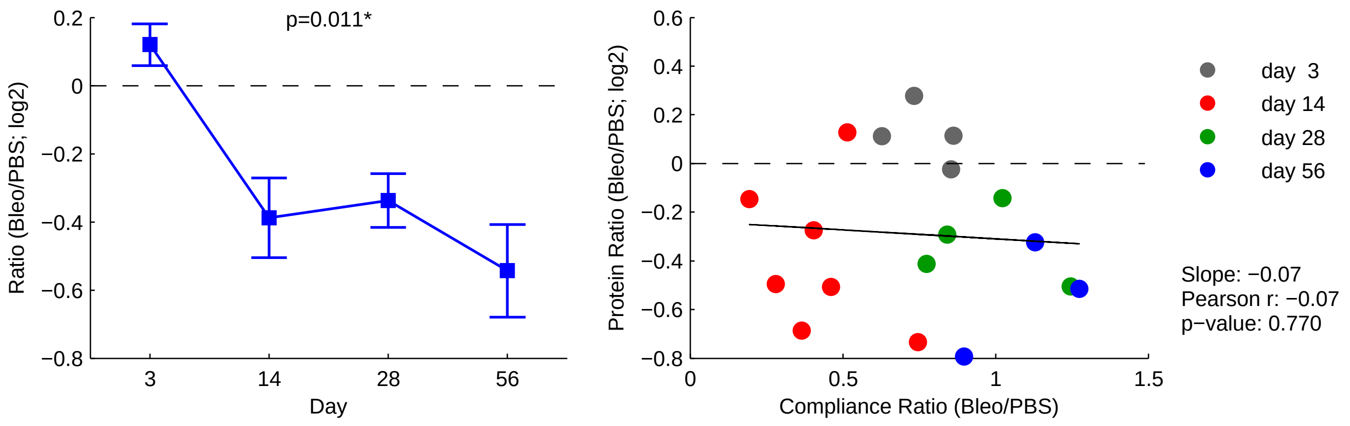

Q99KI0 − Aco2 (id: 6523)

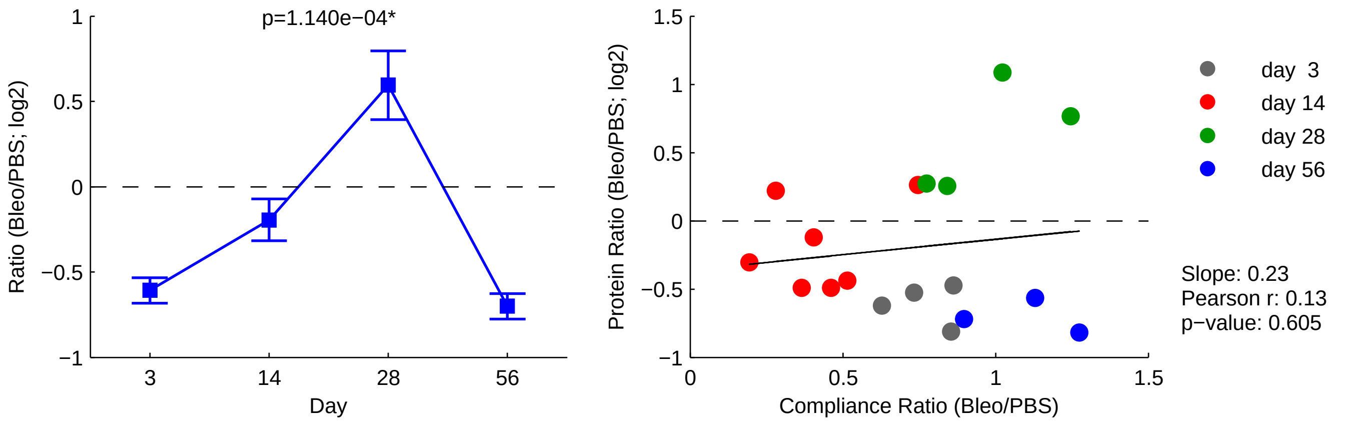

Q99KJ8 − Dctn2 (id: 6525)

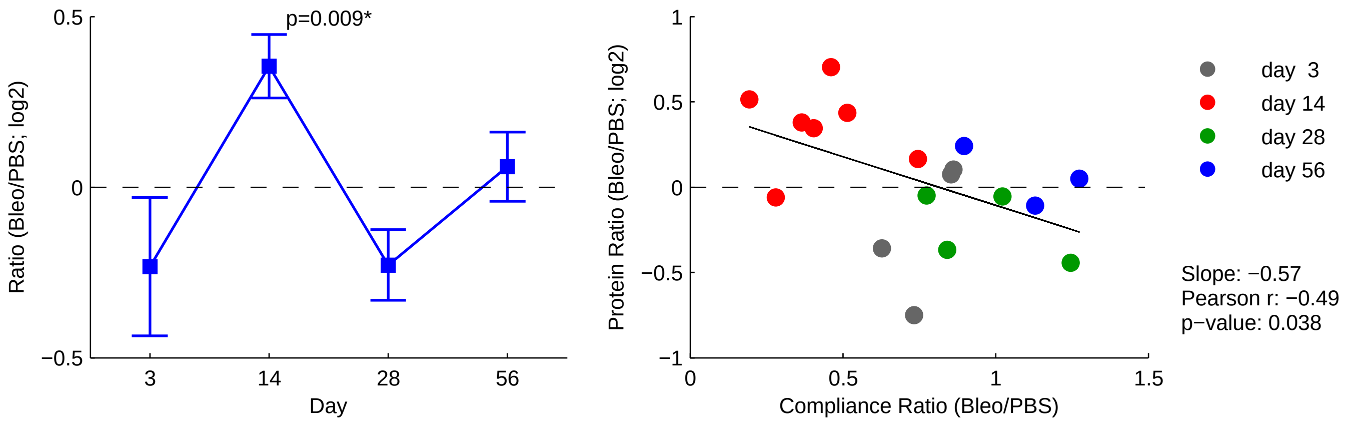

Q99KK9 − Hars2 (id: 6529)

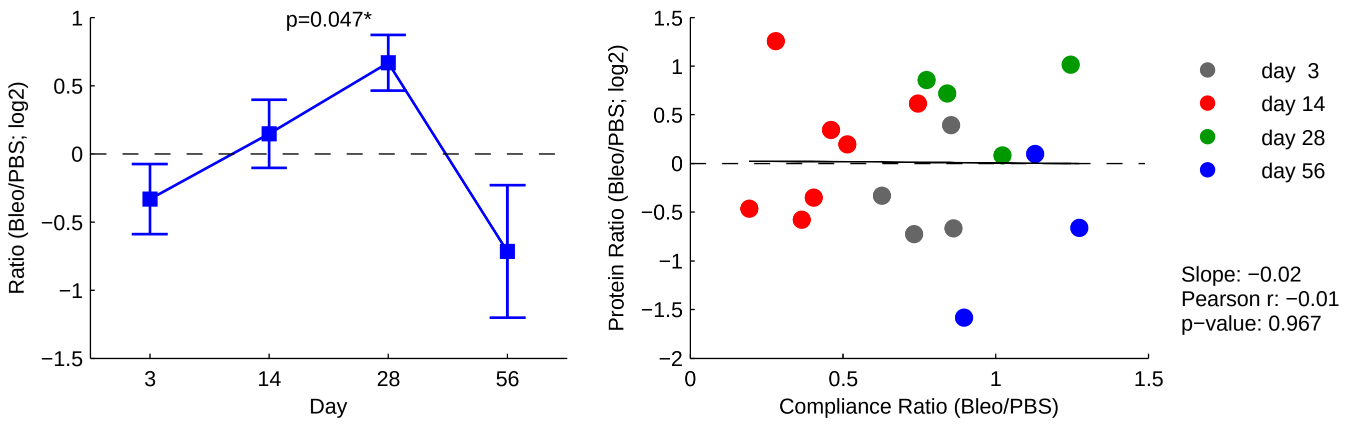

Q99KR3 − Lactb2 (id: 6533)

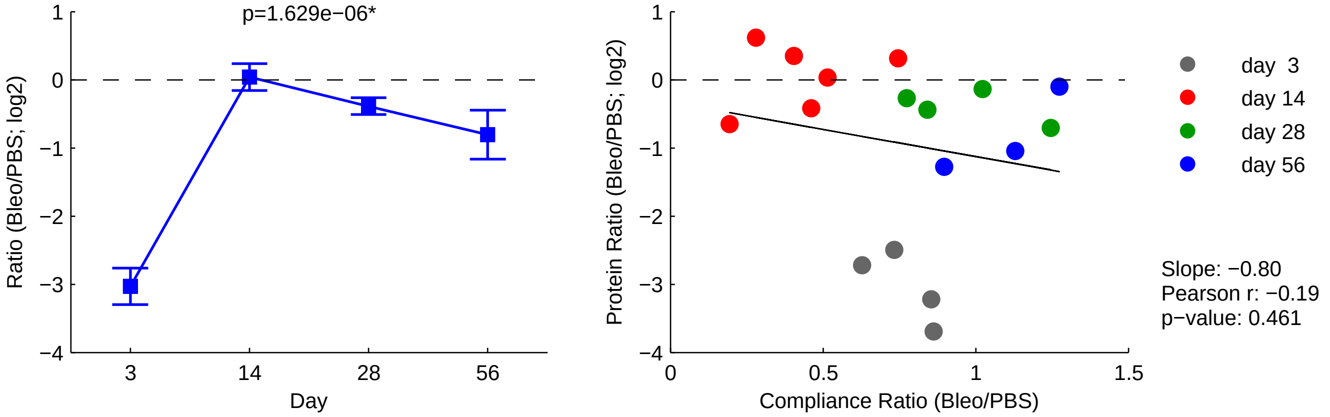

Q99KR7 − Ppif (id: 6534)

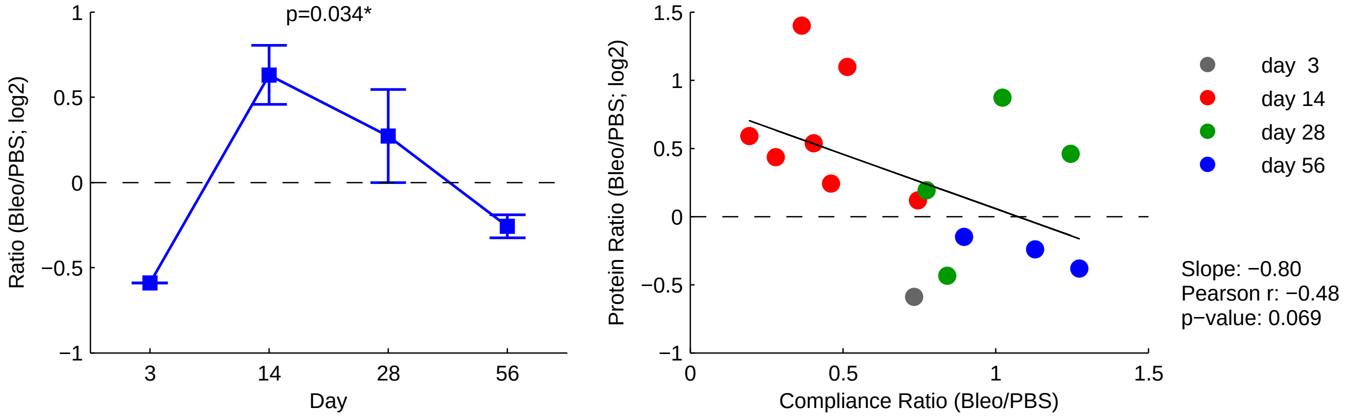

Q99KU0 − Vmp1 (id: 6535)

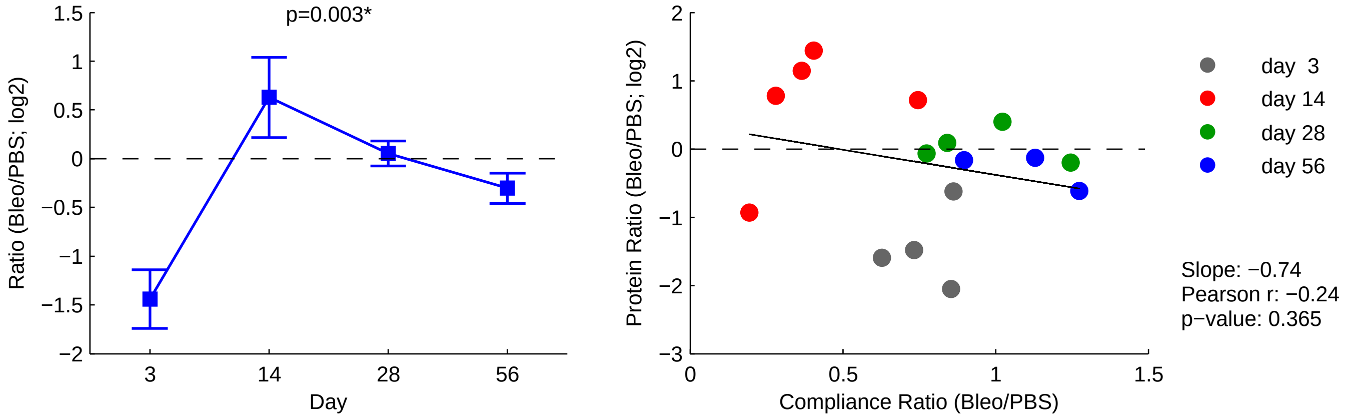

Q99KV1 − Dnajb11 (id: 6537)

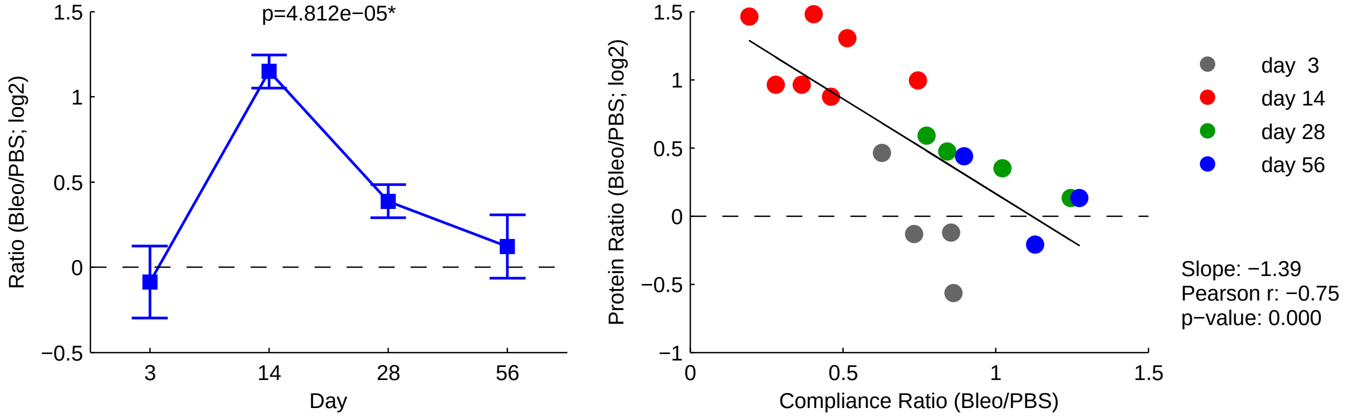

Q99KW9 − Itfg1 (id: 6540)

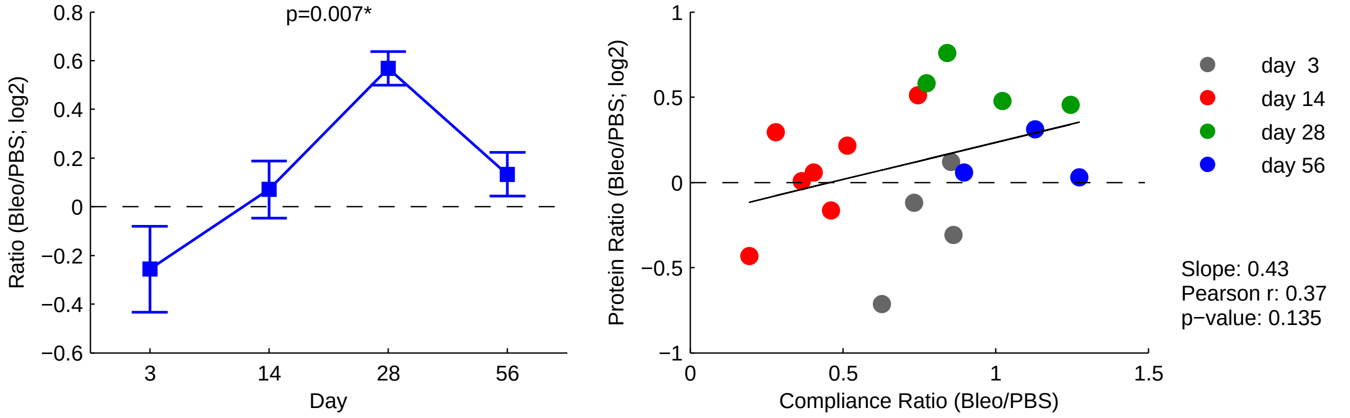

Q99KX1 − Mlf2 (id: 6541)

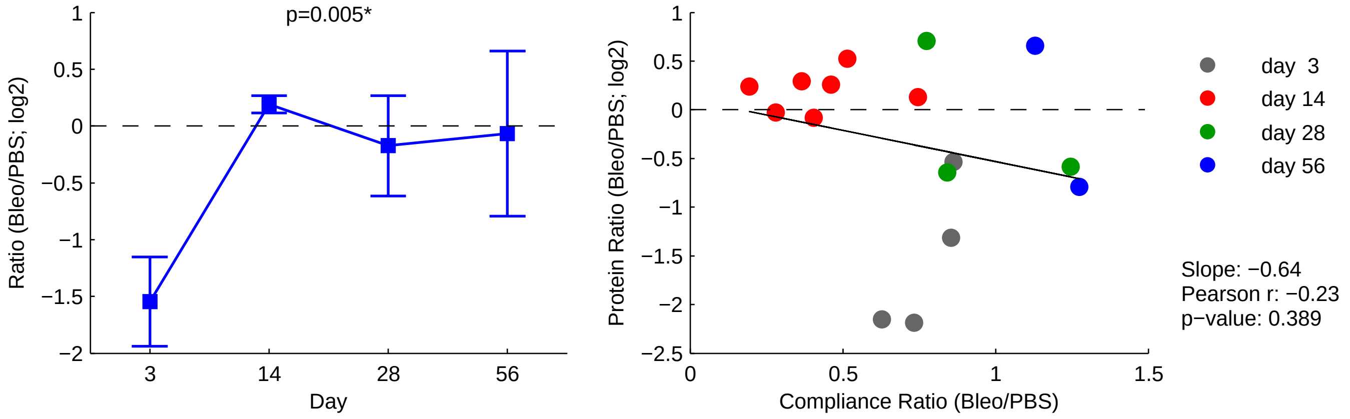

Q99KY4−2 − Gak (id: 6542)

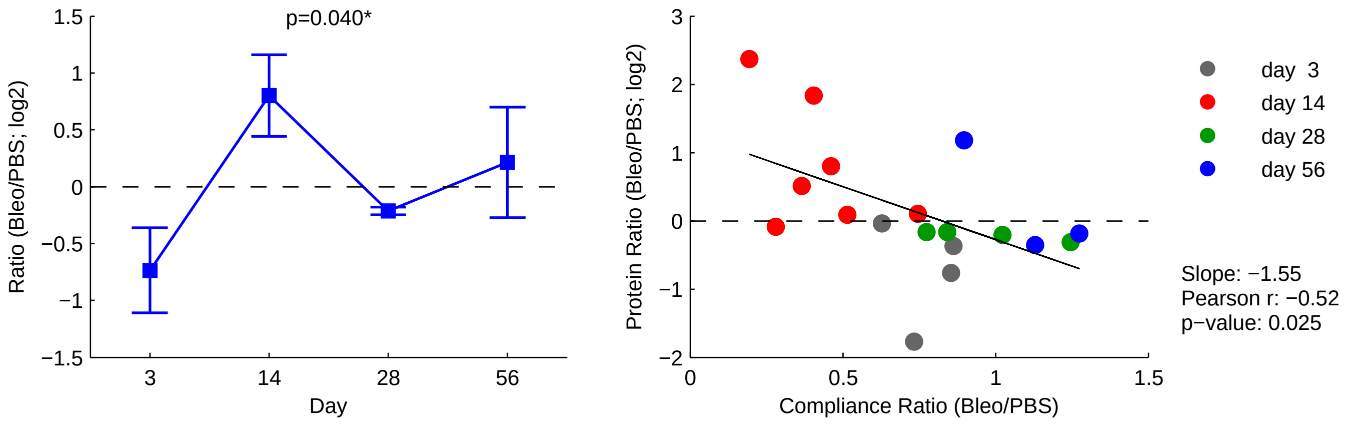

Q99L04 − Dhrs1 (id: 6543)

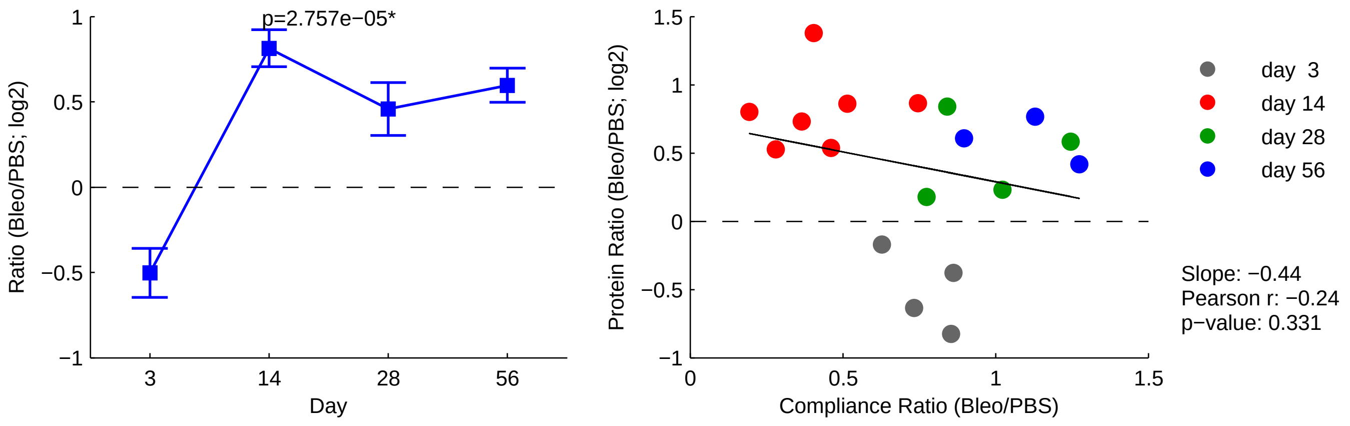

Q99L13 − Hibadh (id: 6544)

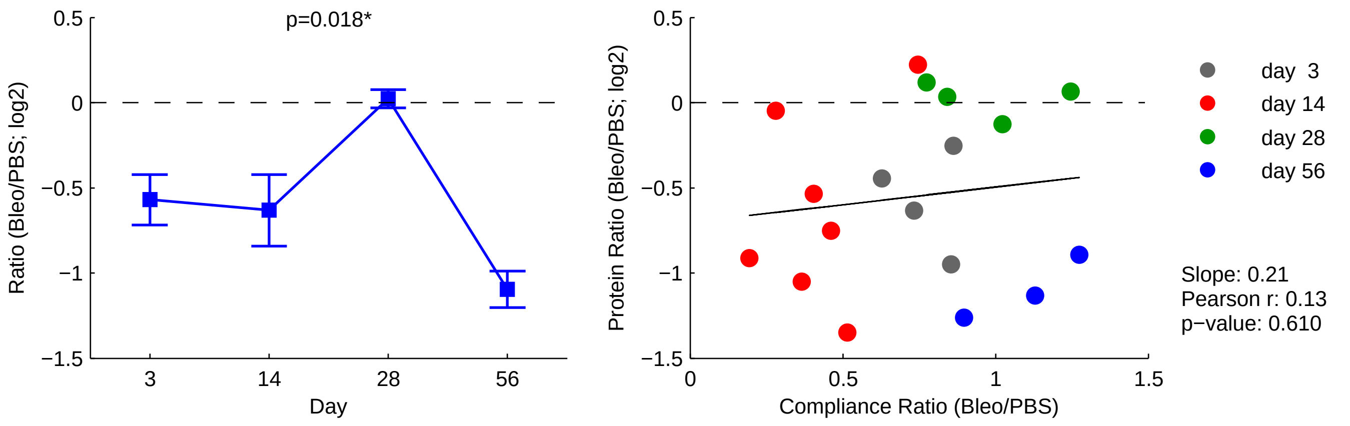

Q99L20 − Gstt3 (id: 6545)

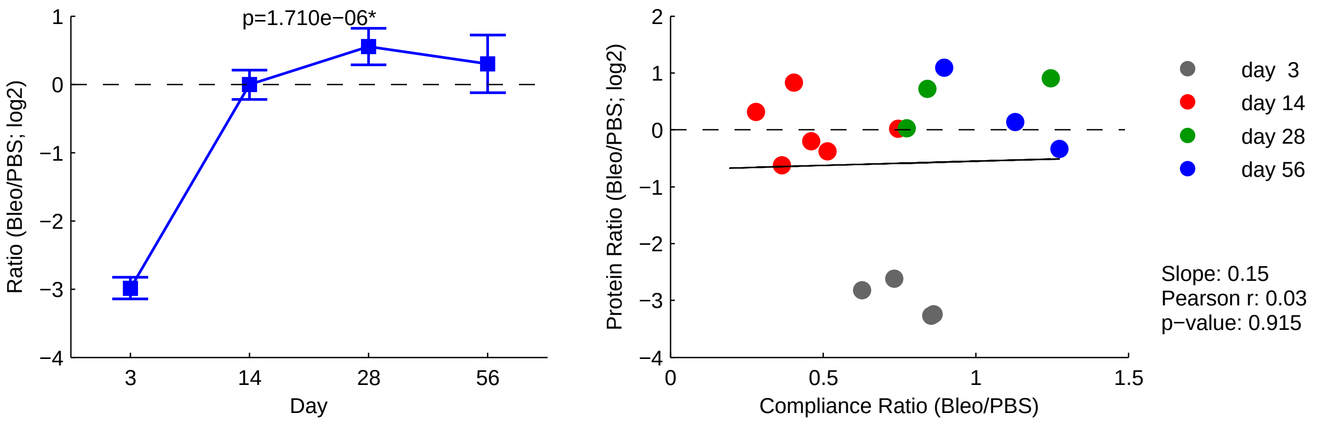

Q99L27 − Gmpr2 (id: 6546)

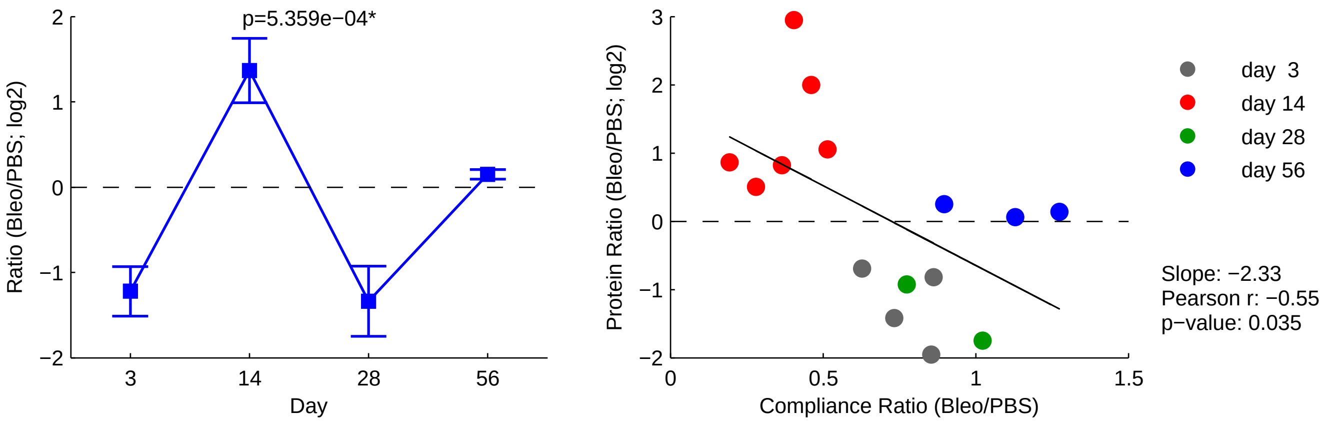

Q99L43 − Cds2 (id: 6547)

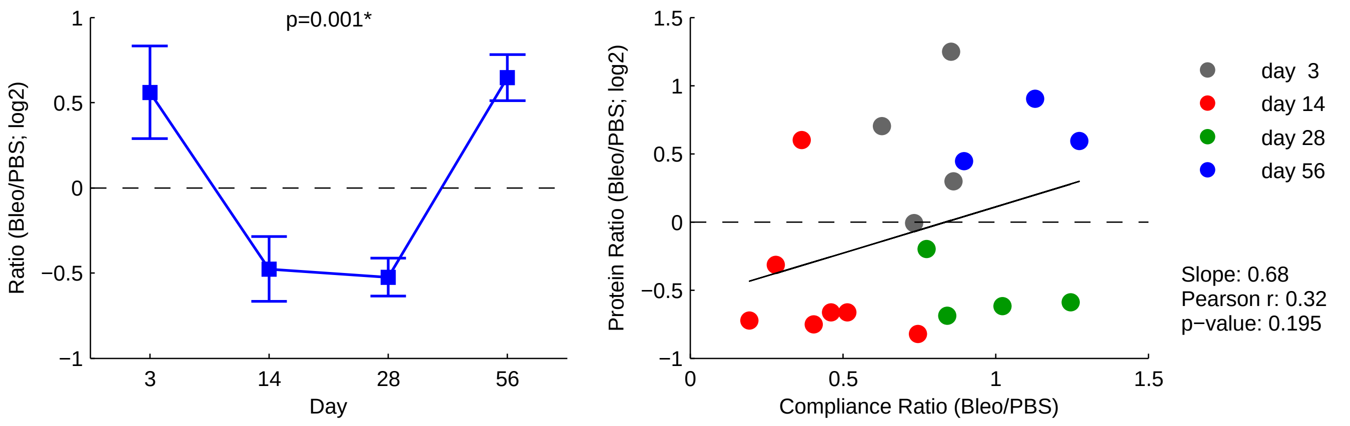

Q99L45 − Eif2s2 (id: 6548)

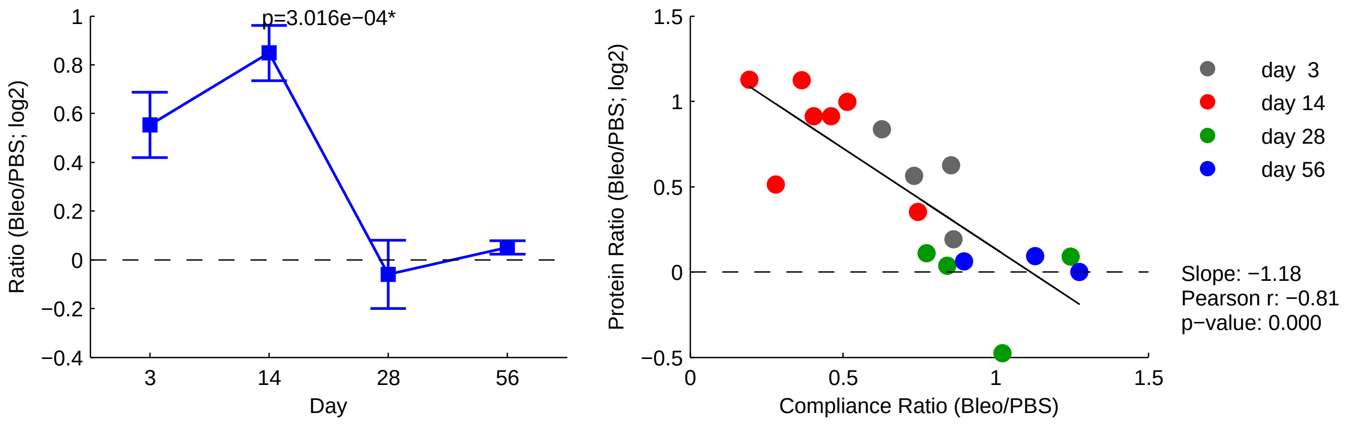

Q99L60−2 − Atp6v1c2 (id: 6550)

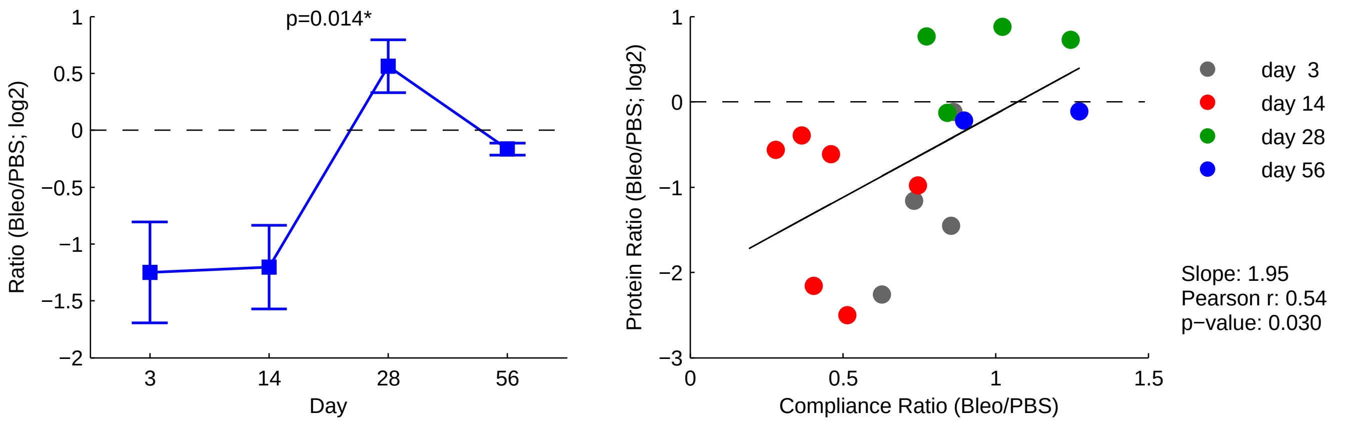

Q99L88 − Sntb1 (id: 6552)

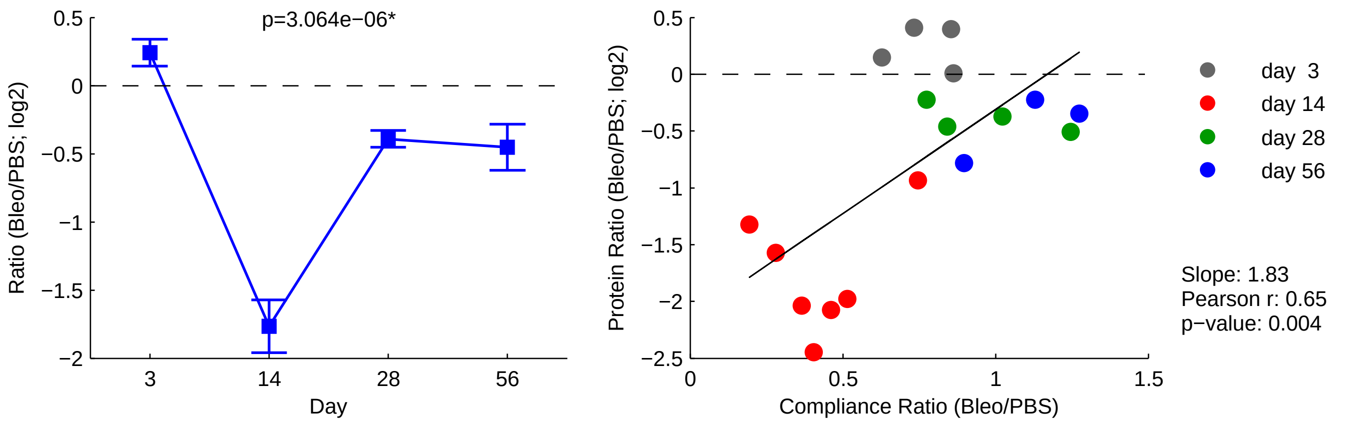

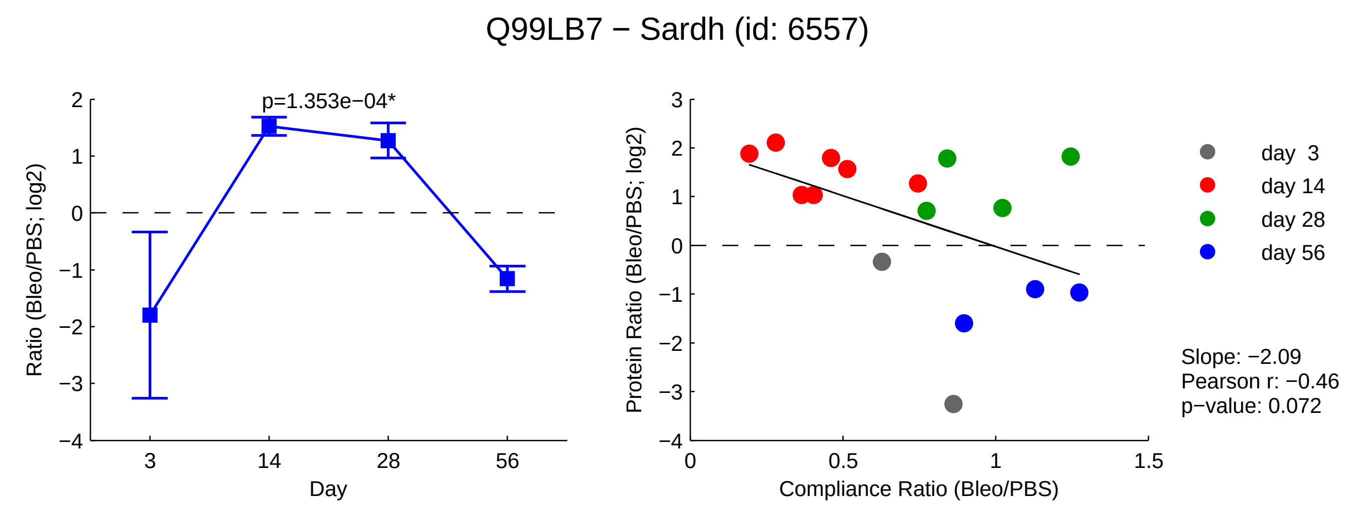

Q99LB7 − Sardh (id: 6557)
p=1.353e−04*
Ratio (Bleo/PBS; log2)
Day
Protein Ratio (Bleo/PBS; log2)
Compliance Ratio (Bleo/PBS)
day 3
day 14
day 28
day 56
Slope: −2.09
Pearson r: −0.46
p−value: 0.072

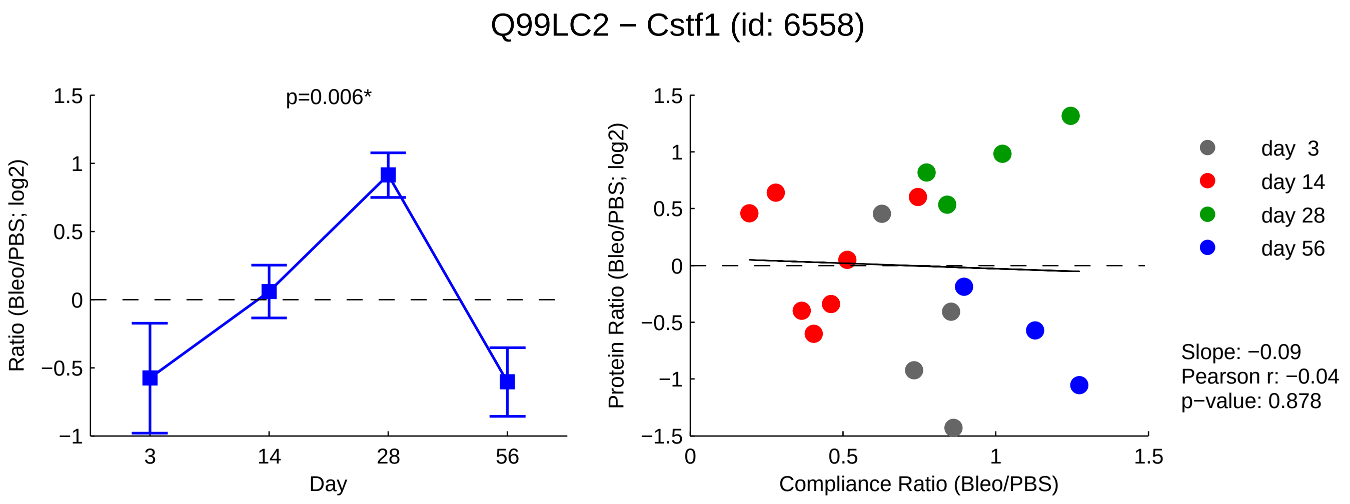

Q99LC2 − Cstf1 (id: 6558)
p=0.006*
Ratio (Bleo/PBS; log2)
Day
Protein Ratio (Bleo/PBS; log2)
Compliance Ratio (Bleo/PBS)
day 3
day 14
day 28
day 56
Slope: −0.09
Pearson r: −0.04
p−value: 0.878

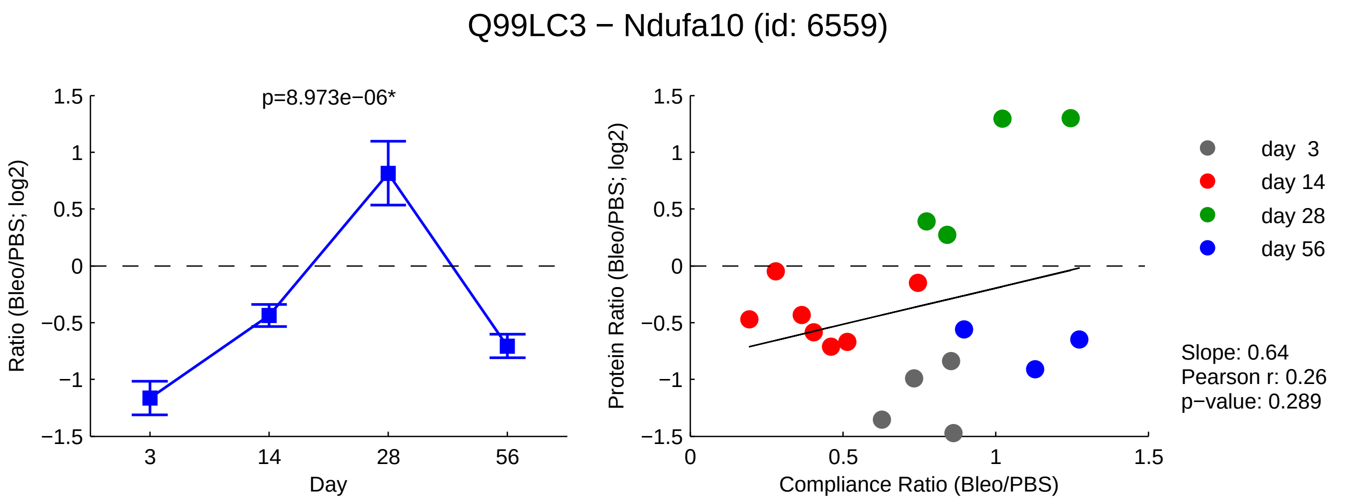

Q99LC3 − Ndufa10 (id: 6559)
p=8.973e−06*
Ratio (Bleo/PBS; log2)
Day
Protein Ratio (Bleo/PBS; log2)
Compliance Ratio (Bleo/PBS)
day 3
day 14
day 28
day 56
Slope: 0.64
Pearson r: 0.26
p−value: 0.289

Q99LC5 − Etfa (id: 6560)

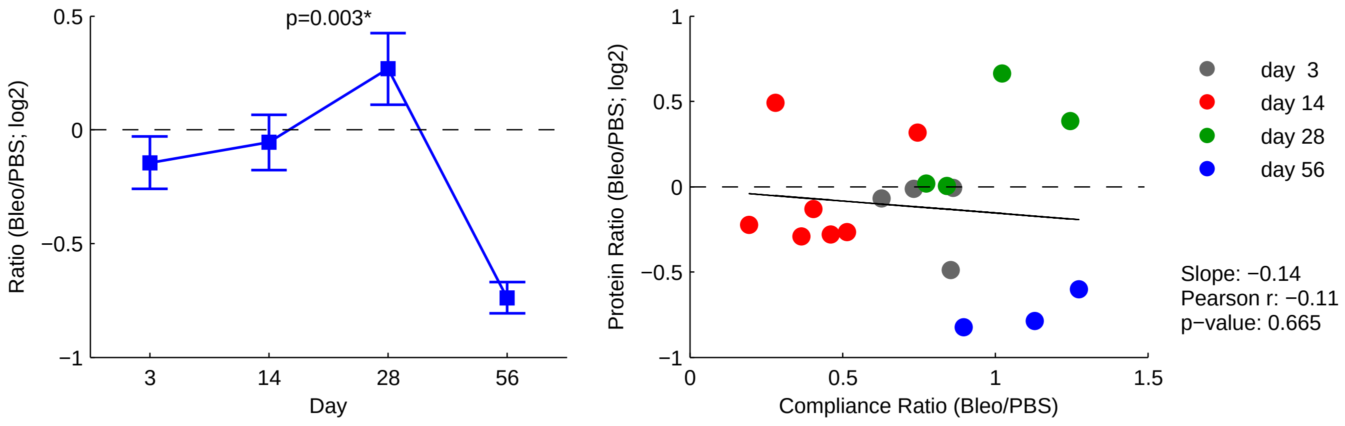

Q99LC8 − Eif2b1 (id: 6561)

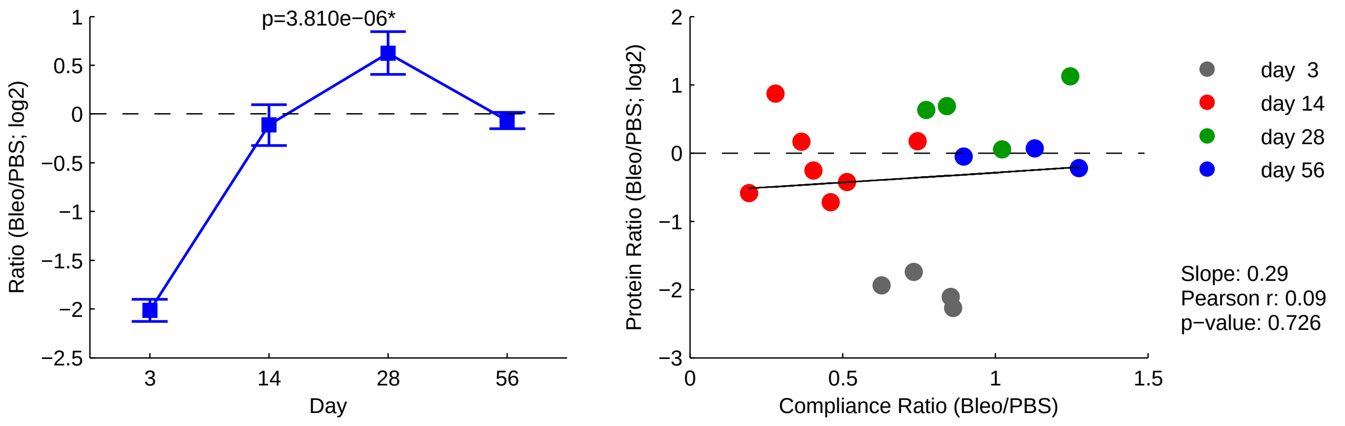

Q99LD8 − Ddah2 (id: 6563)

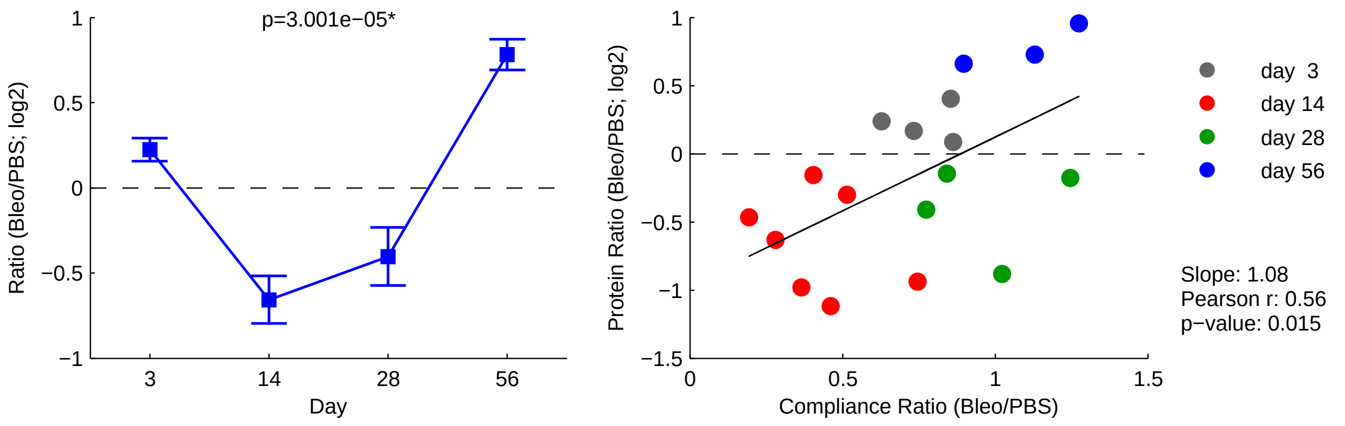

Q99LD9 − Eif2b2 (id: 6564)

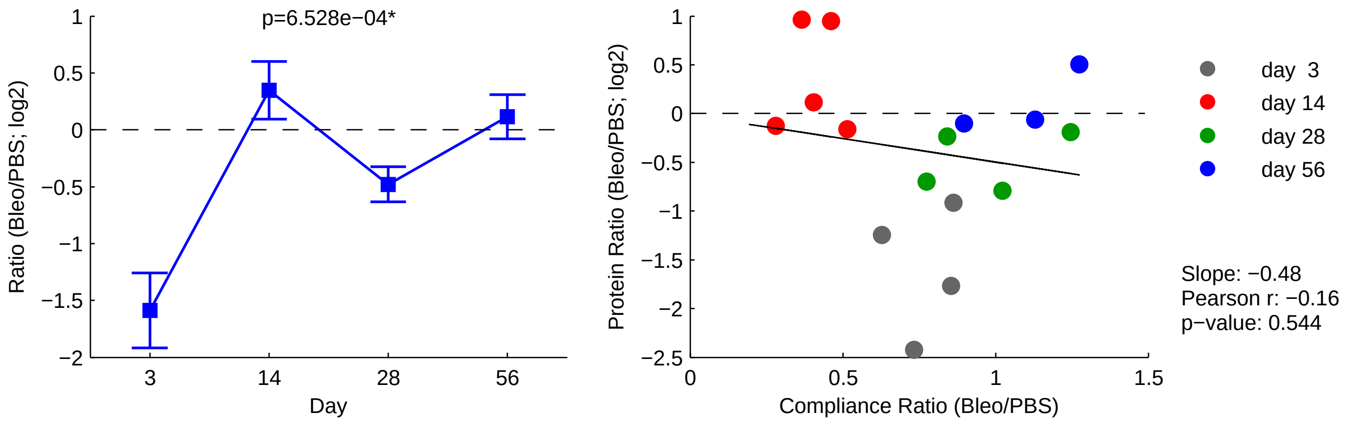

Q99LE6 − Abcf2 (id: 6565)

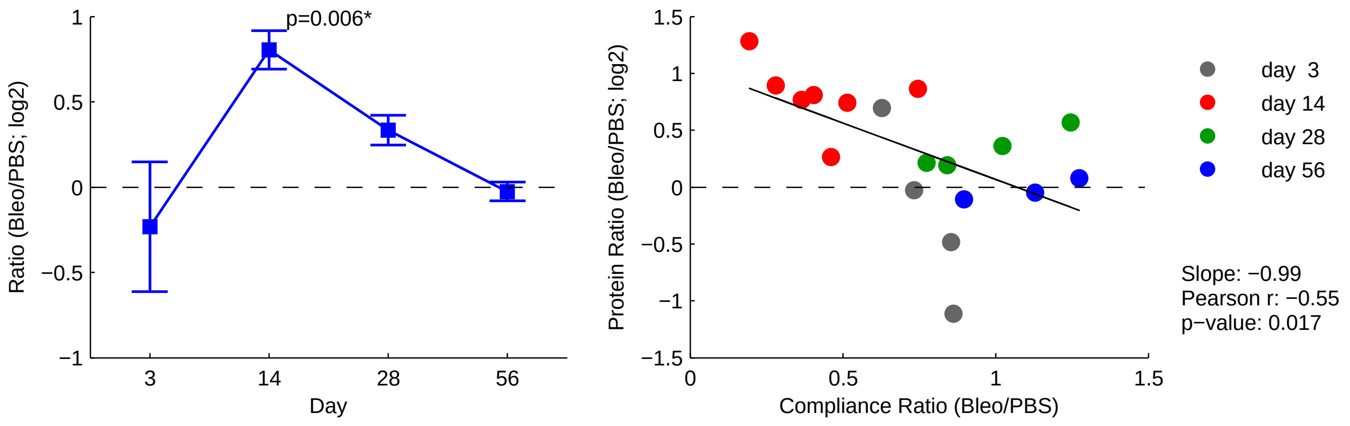

Q99LF4 − D10Wsu52e (id: 6566)

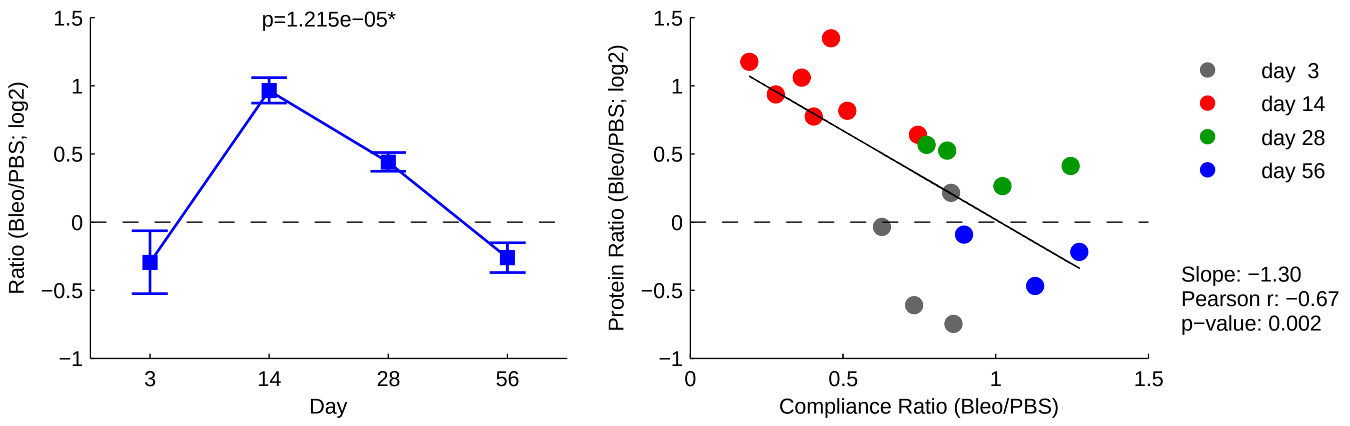

Q99LH2 − Ptdss1 (id: 6569)

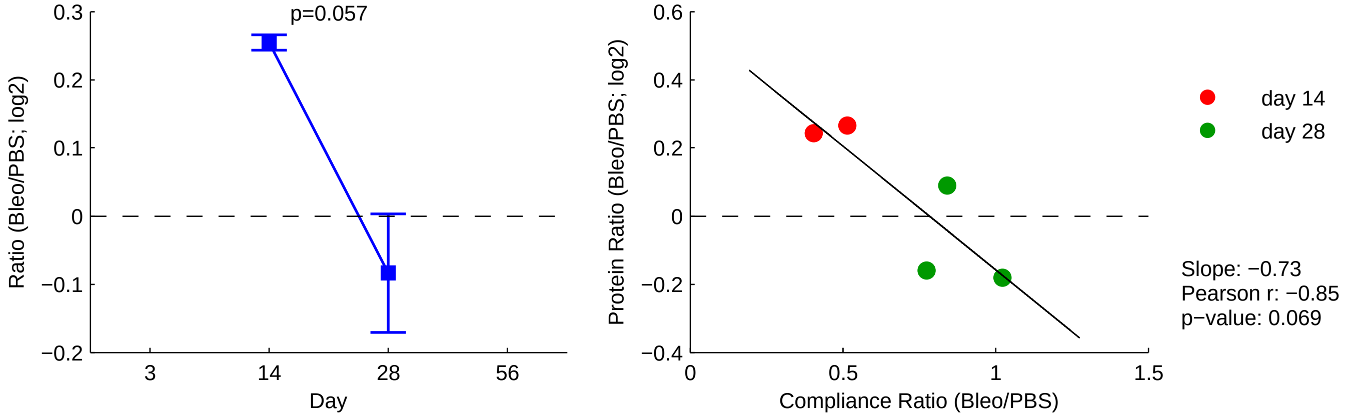

Q99LI7 − Cstf3 (id: 6571)

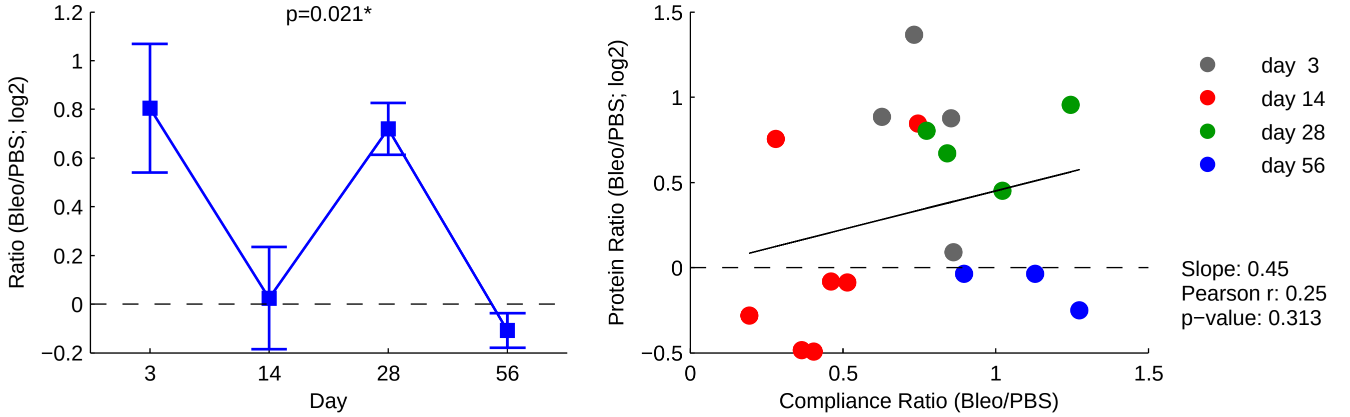

Q99LJ0 − Cttnbp2nl (id: 6572)

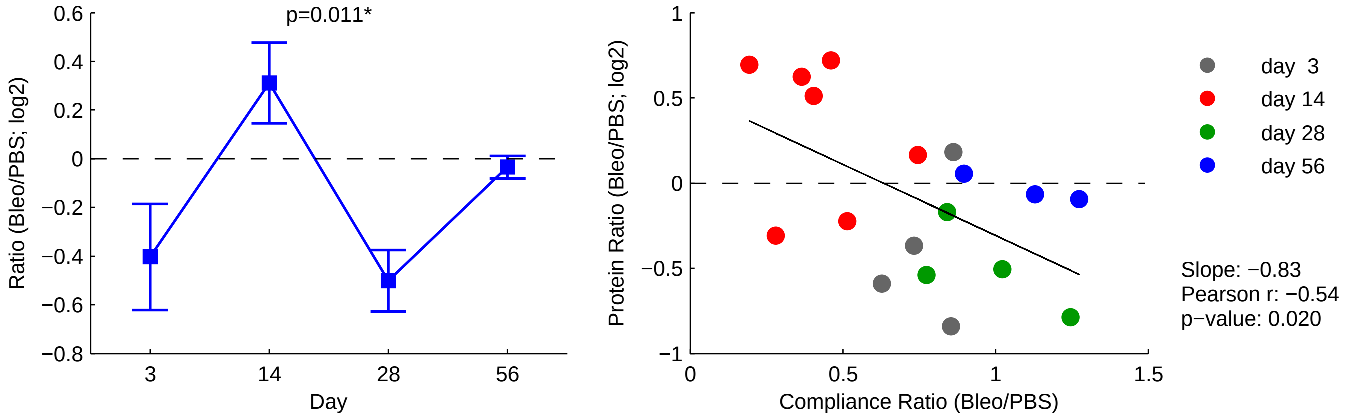

Q99LJ6 − Gpx7 (id: 6575)

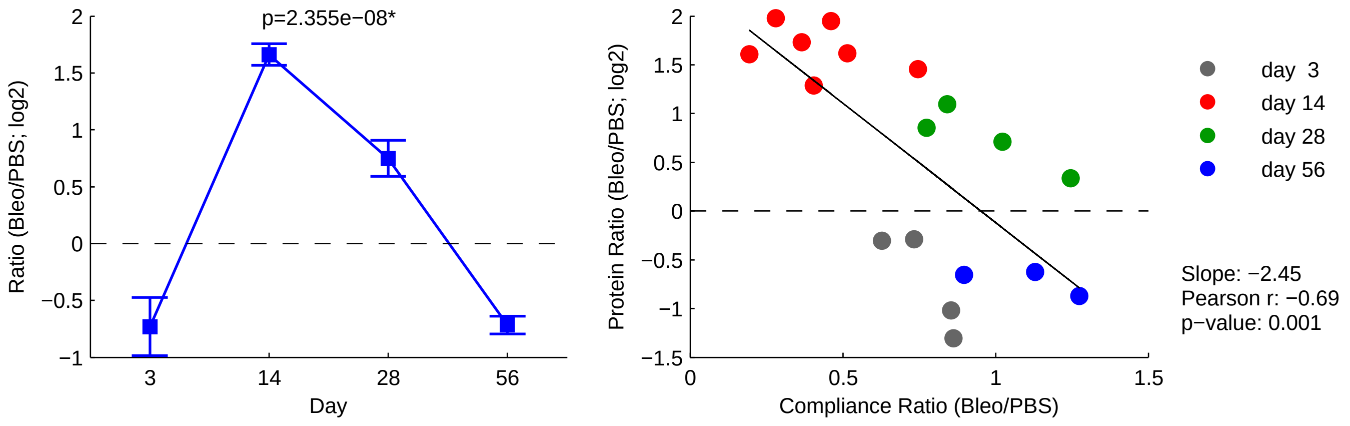

Q99LM2 − Cdk5rap3 (id: 6578)

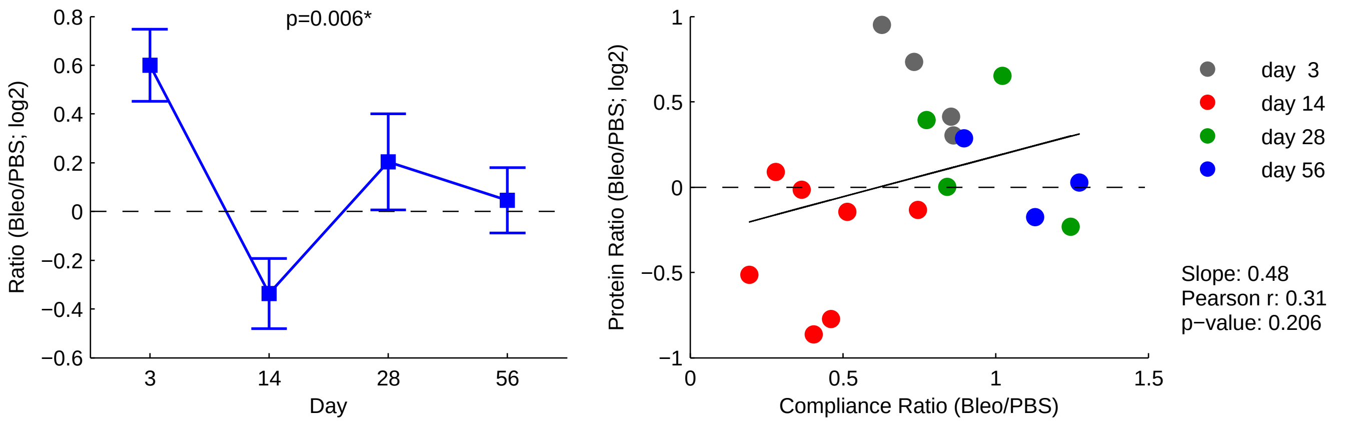

Q99LM9 − Tada1 (id: 6579)

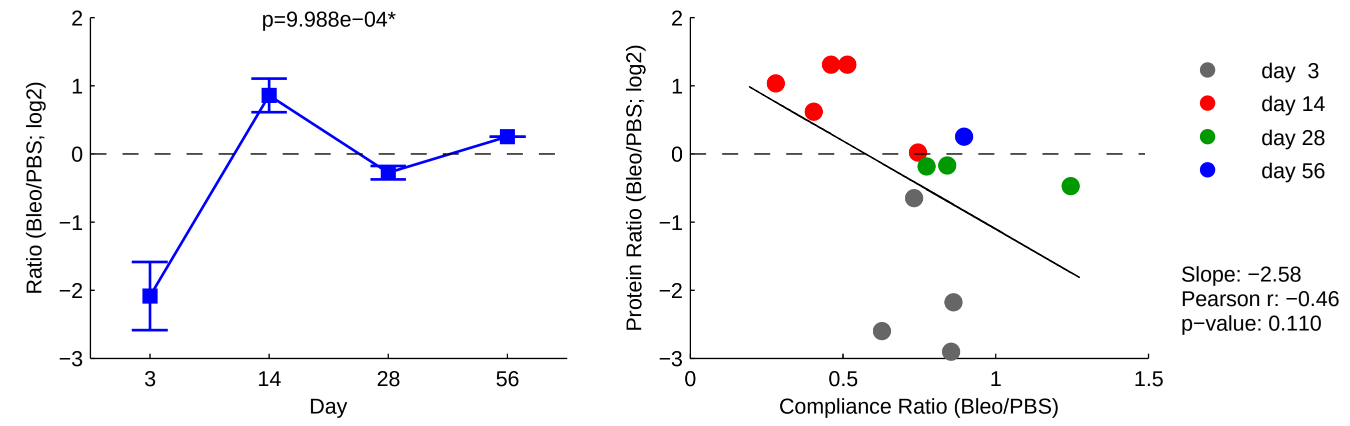

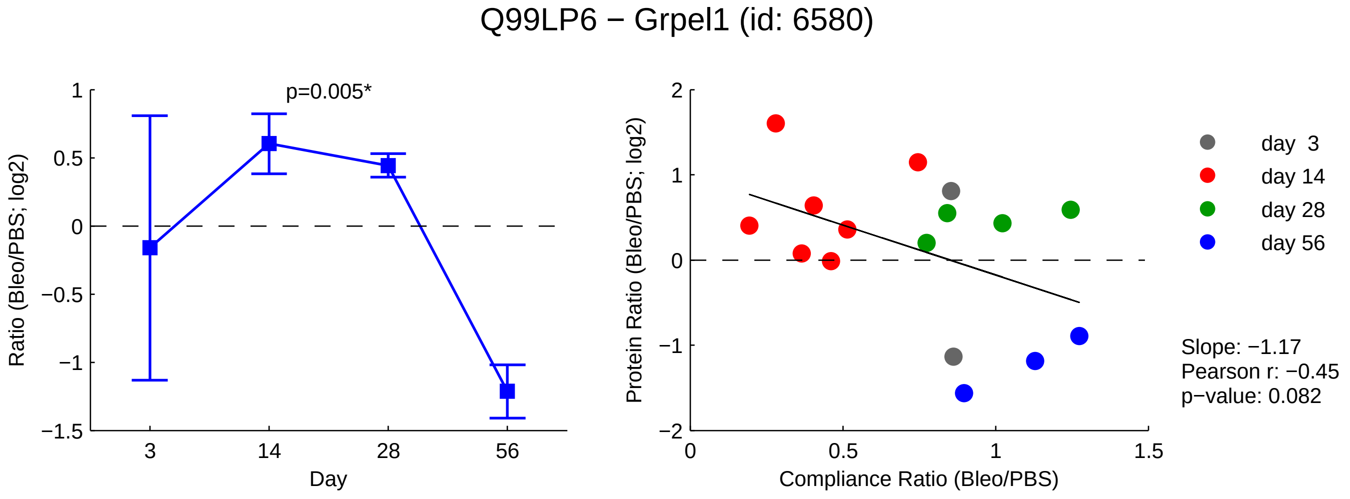
Q99LP6 − Grpel1 (id: 6580)
p=0.005*
Ratio (Bleo/PBS; log2)
Day
Protein Ratio (Bleo/PBS; log2)
Compliance Ratio (Bleo/PBS)
day 3
day 14
day 28
day 56
Slope: −1.17
Pearson r: −0.45
p−value: 0.082

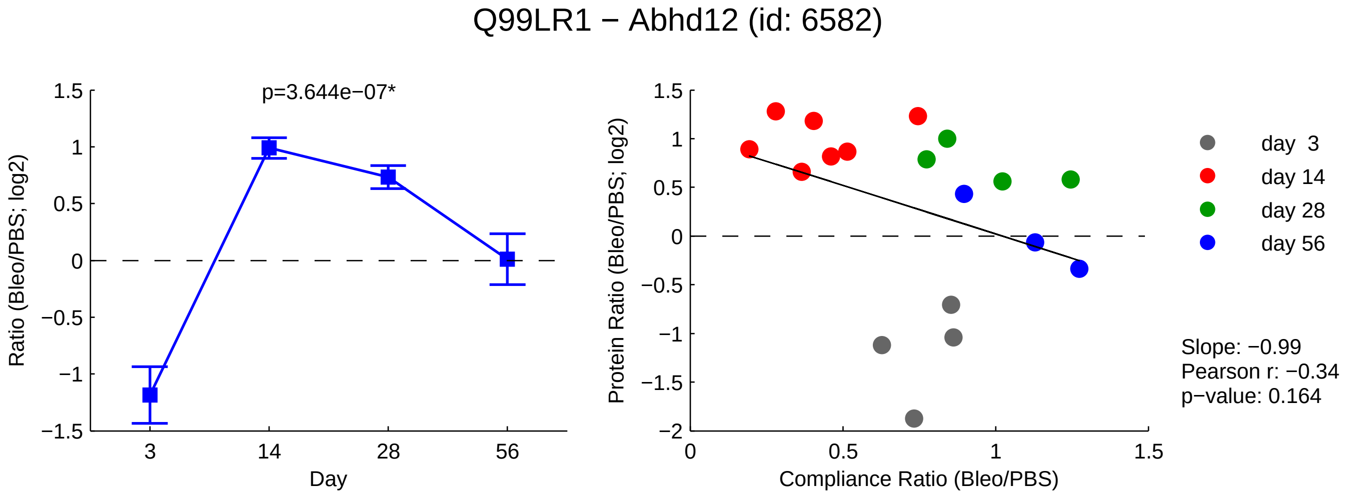
Q99LR1 − Abhd12 (id: 6582)
p=3.644e−07*
Ratio (Bleo/PBS; log2)
Day
Protein Ratio (Bleo/PBS; log2)
Compliance Ratio (Bleo/PBS)
day 3
day 14
day 28
day 56
Slope: −0.99
Pearson r: −0.34
p−value: 0.164

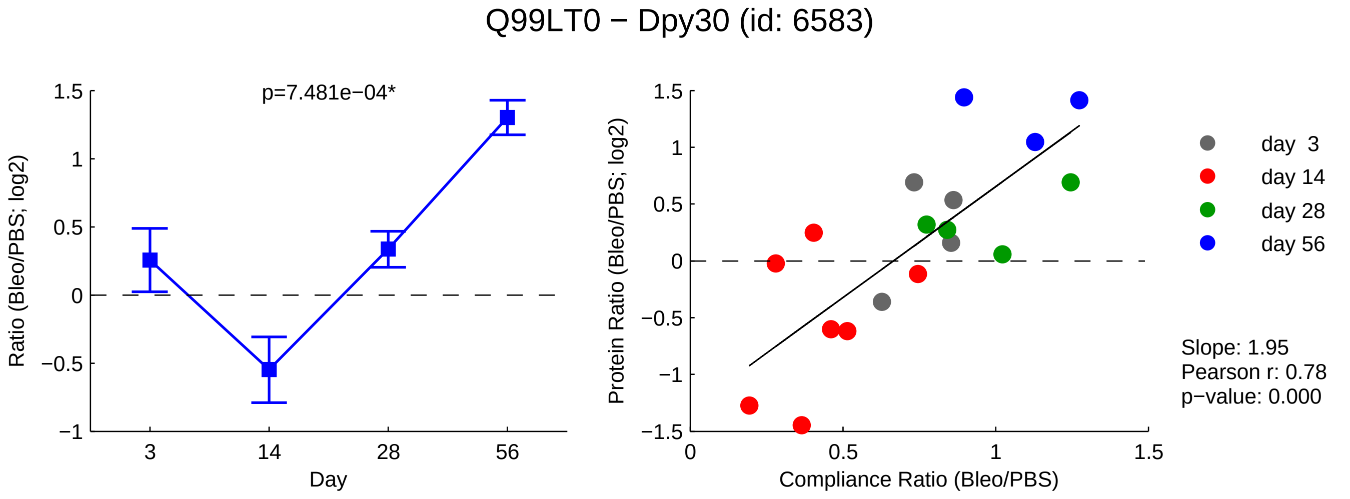
Q99LT0 − Dpy30 (id: 6583)
p=7.481e−04*
Ratio (Bleo/PBS; log2)
Day
Protein Ratio (Bleo/PBS; log2)
Compliance Ratio (Bleo/PBS)
day 3
day 14
day 28
day 56
Slope: 1.95
Pearson r: 0.78
p−value: 0.000

Q99LX0 − Park7 (id: 6585)

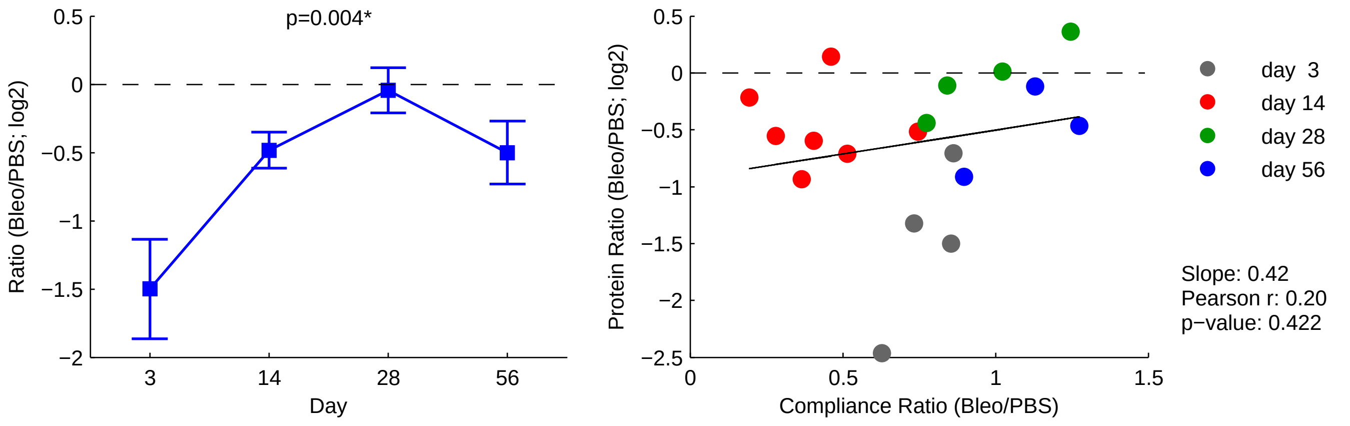

Q99M04 − Lias (id: 6588)

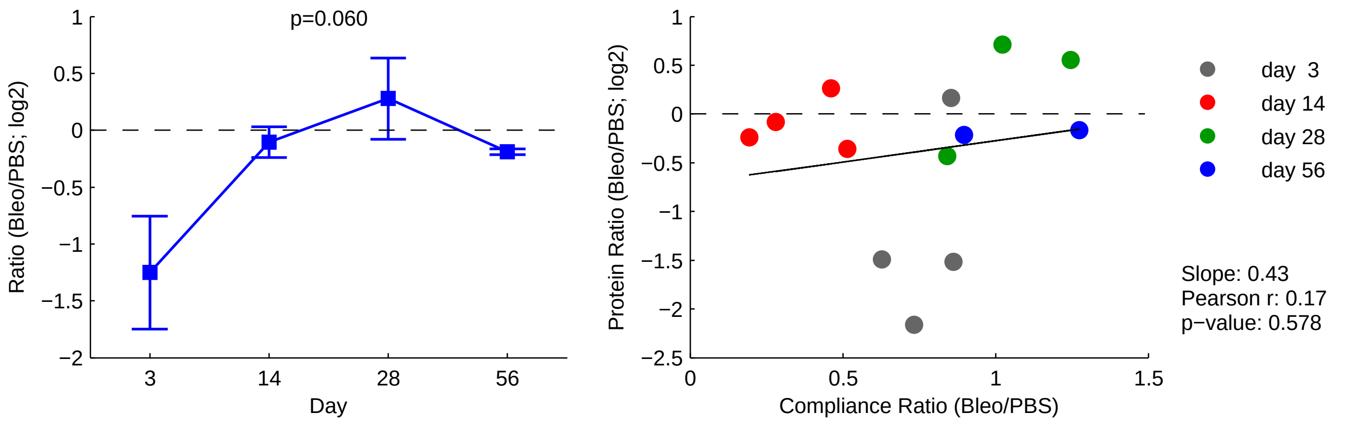

Q99M28 − Rnps1 (id: 6593)

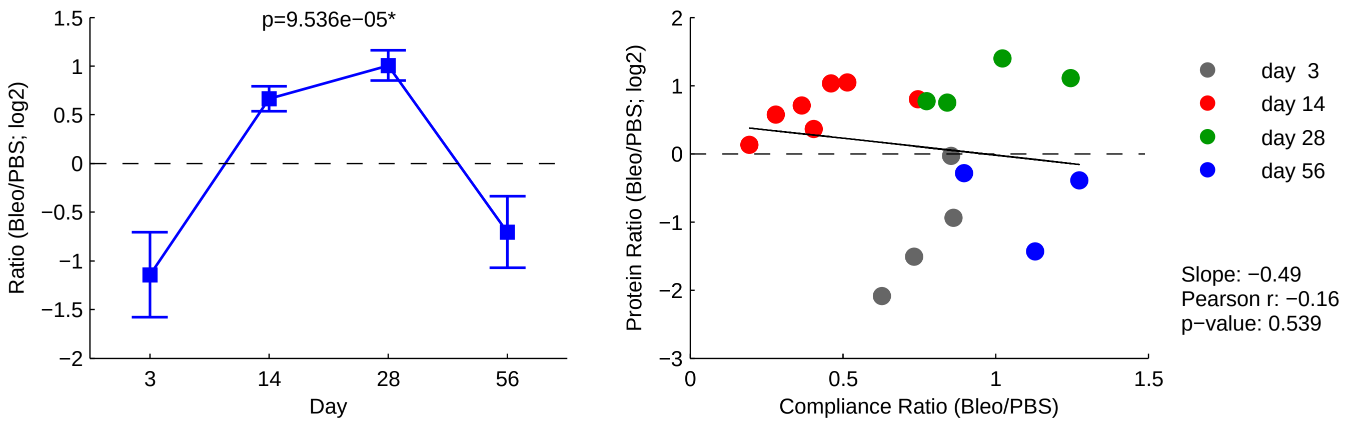

Q99M51 − Nck1 (id: 6596)

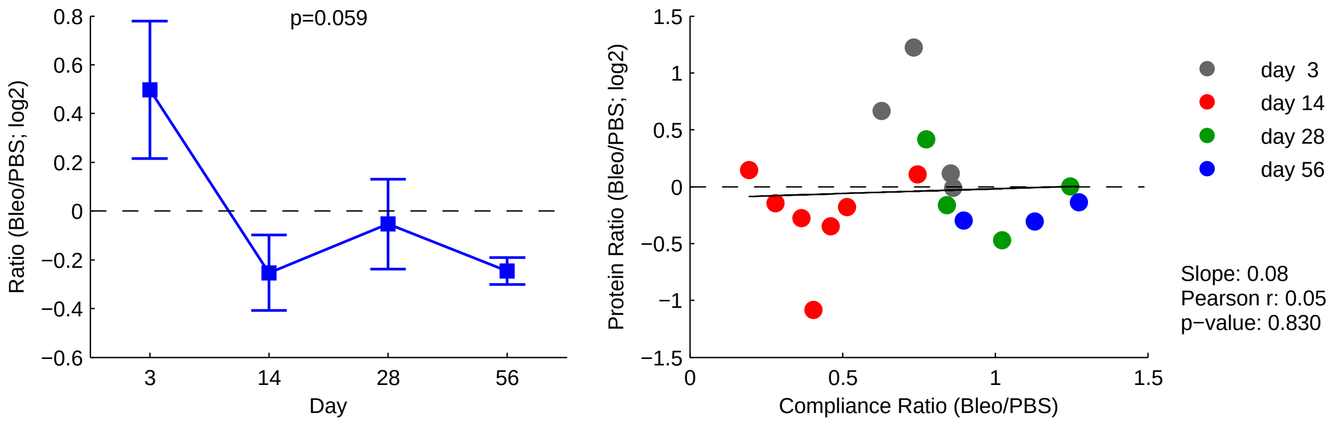

Q99M87 − Dnaja3 (id: 6599)

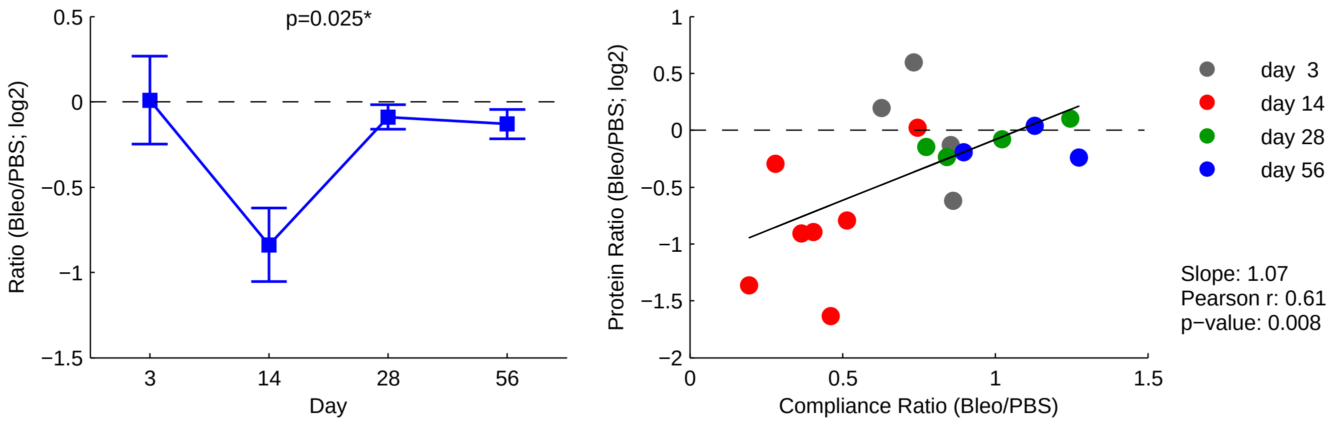

Q99MB1 − Tlr3 (id: 6601)

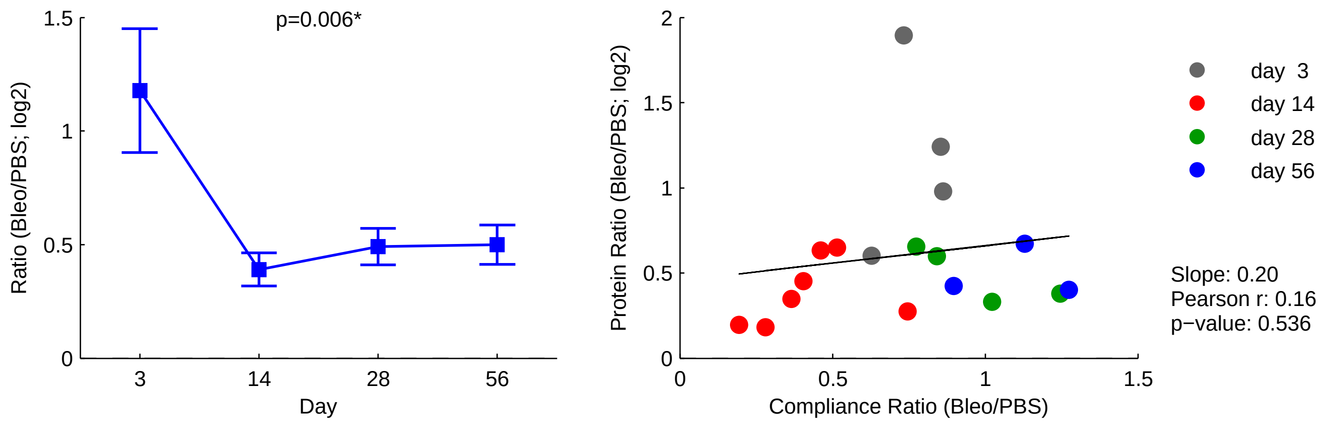

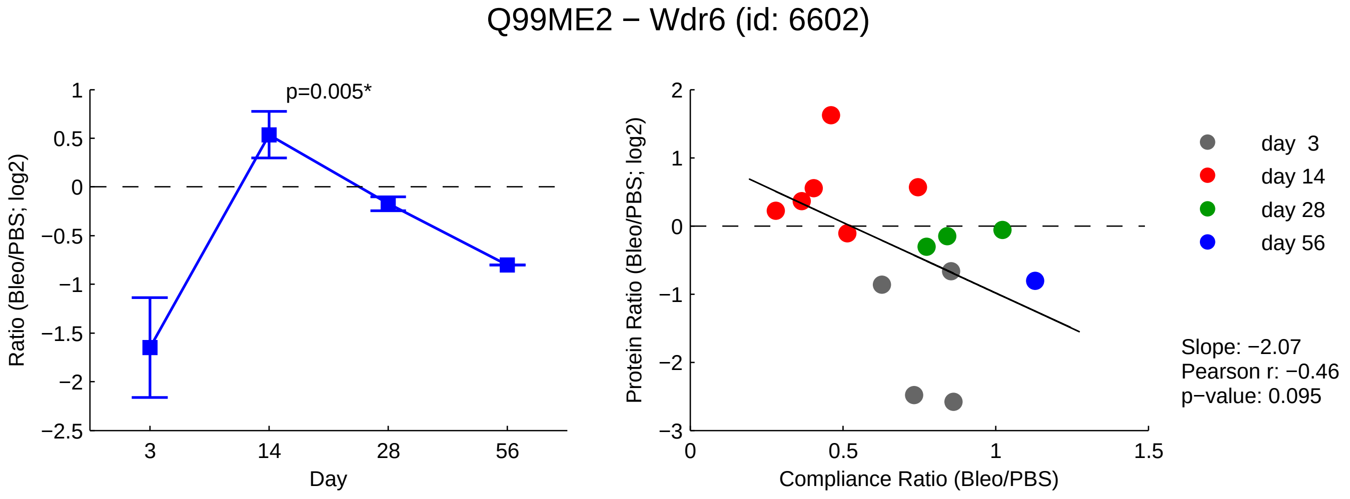
Q99ME2 − Wdr6 (id: 6602)
p=0.005*
Ratio (Bleo/PBS; log2)
Day
Protein Ratio (Bleo/PBS; log2)
Compliance Ratio (Bleo/PBS)
day 3
day 14
day 28
day 56
Slope: −2.07
Pearson r: −0.46
p−value: 0.095

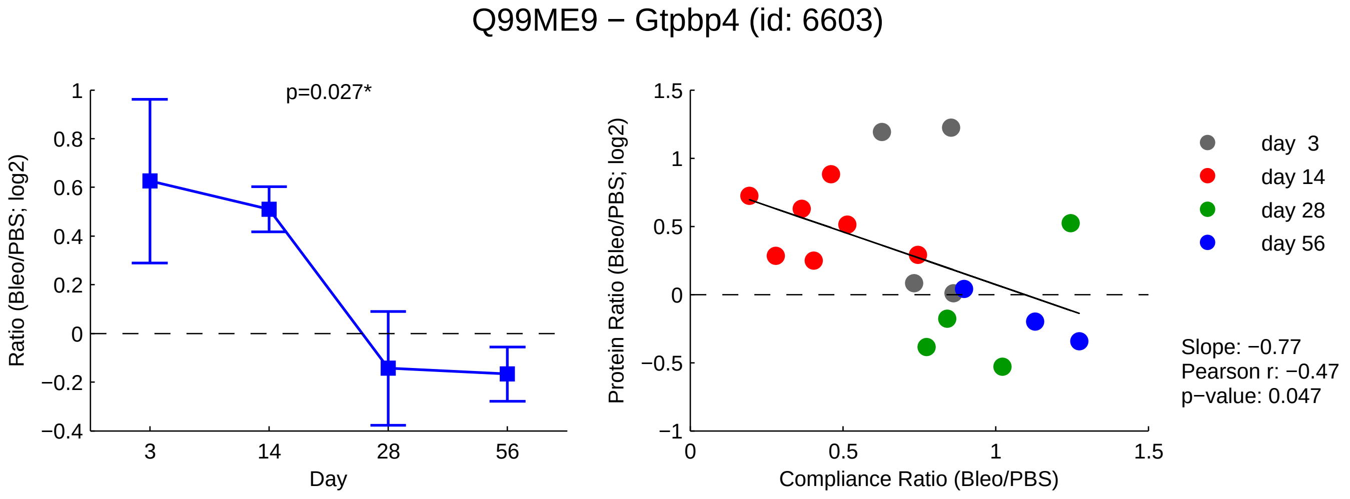
Q99ME9 − Gtpbp4 (id: 6603)
p=0.027*
Ratio (Bleo/PBS; log2)
Day
Protein Ratio (Bleo/PBS; log2)
Compliance Ratio (Bleo/PBS)
day 3
day 14
day 28
day 56
Slope: −0.77
Pearson r: −0.47
p−value: 0.047

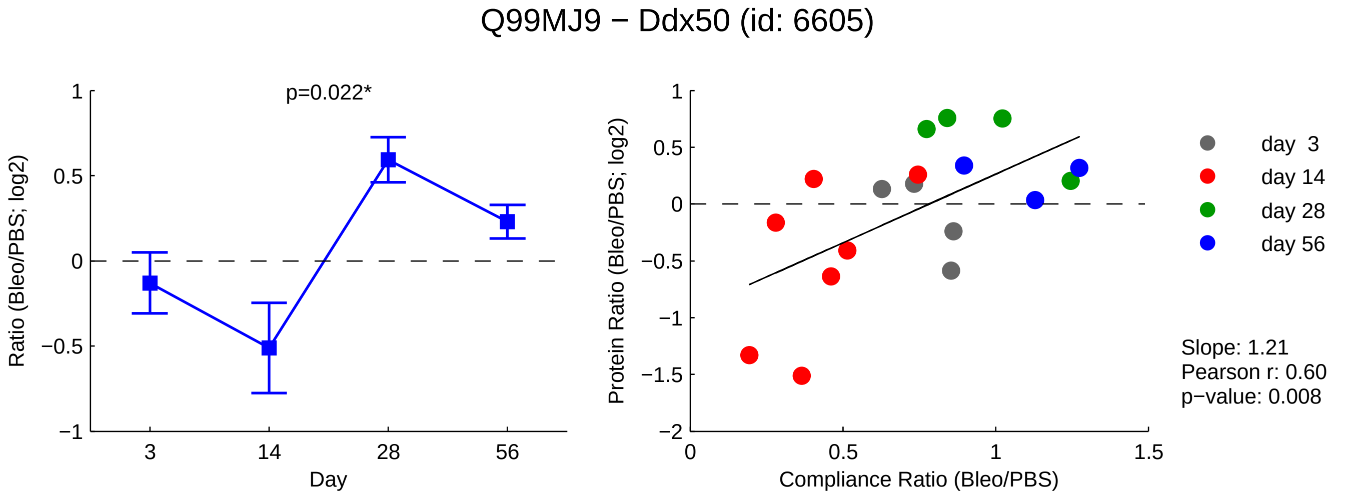
Q99MJ9 − Ddx50 (id: 6605)
p=0.022*
Ratio (Bleo/PBS; log2)
Day
Protein Ratio (Bleo/PBS; log2)
Compliance Ratio (Bleo/PBS)
day 3
day 14
day 28
day 56
Slope: 1.21
Pearson r: 0.60
p−value: 0.008

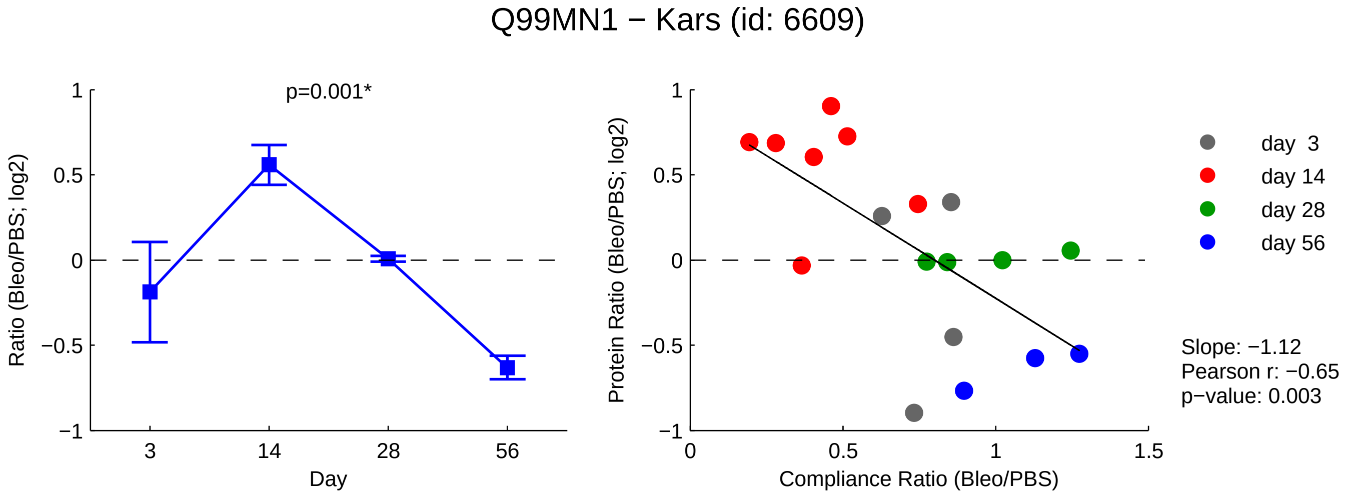
Q99MN1 − Kars (id: 6609)
p=0.001*
Ratio (Bleo/PBS; log2)
Day
Protein Ratio (Bleo/PBS; log2)
Compliance Ratio (Bleo/PBS)
day 3
day 14
day 28
day 56
Slope: −1.12
Pearson r: −0.65
p−value: 0.003

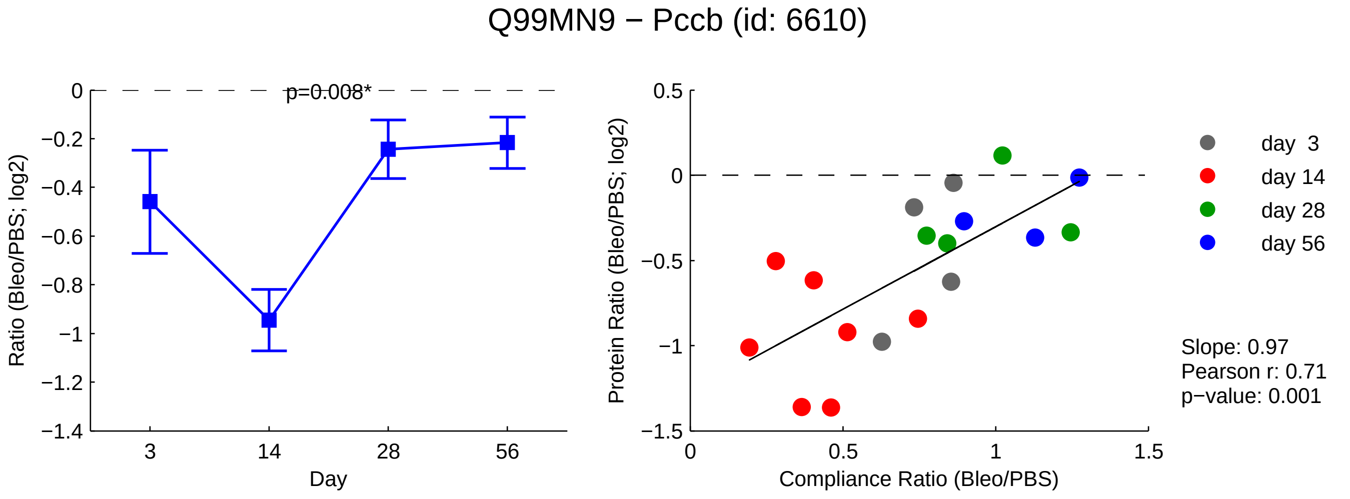
Q99MN9 − Pccb (id: 6610)
p=0.008*
Ratio (Bleo/PBS; log2)
Day
Protein Ratio (Bleo/PBS; log2)
Compliance Ratio (Bleo/PBS)
day 3
day 14
day 28
day 56
Slope: 0.97
Pearson r: 0.71
p−value: 0.001

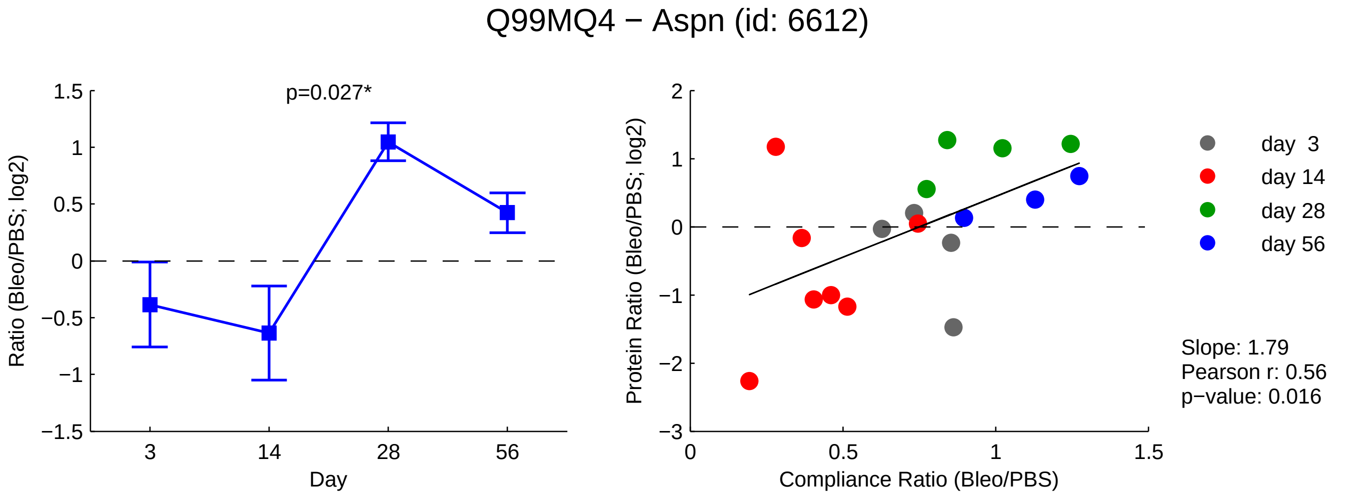
Q99MQ4 − Aspn (id: 6612)
p=0.027*
Ratio (Bleo/PBS; log2)
Day
Protein Ratio (Bleo/PBS; log2)
Compliance Ratio (Bleo/PBS)
day 3
day 14
day 28
day 56
Slope: 1.79
Pearson r: 0.56
p−value: 0.016

Q99MR1 − Gigyf1 (id: 6613)

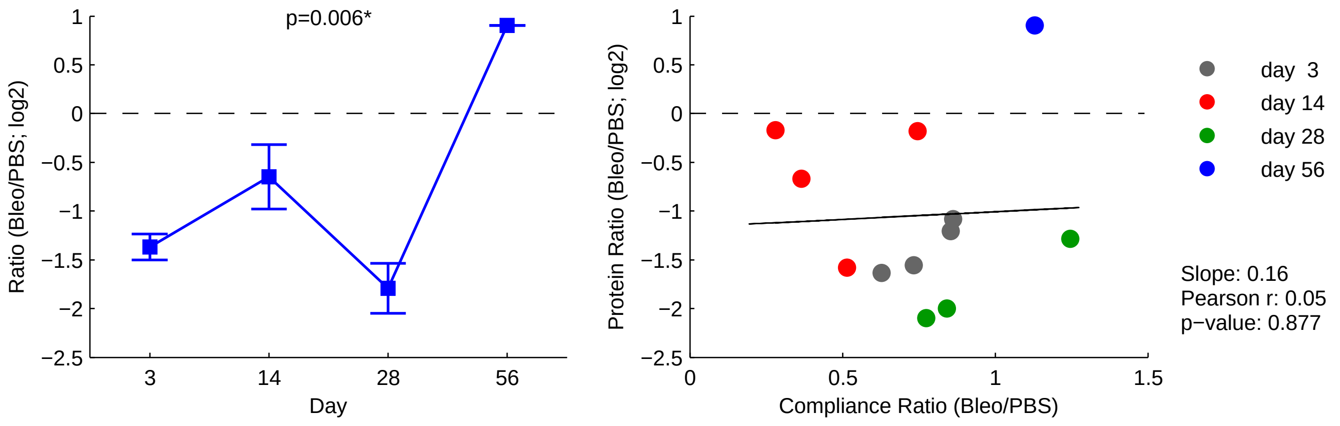

Q99MR3 − Slc12a9 (id: 6614)

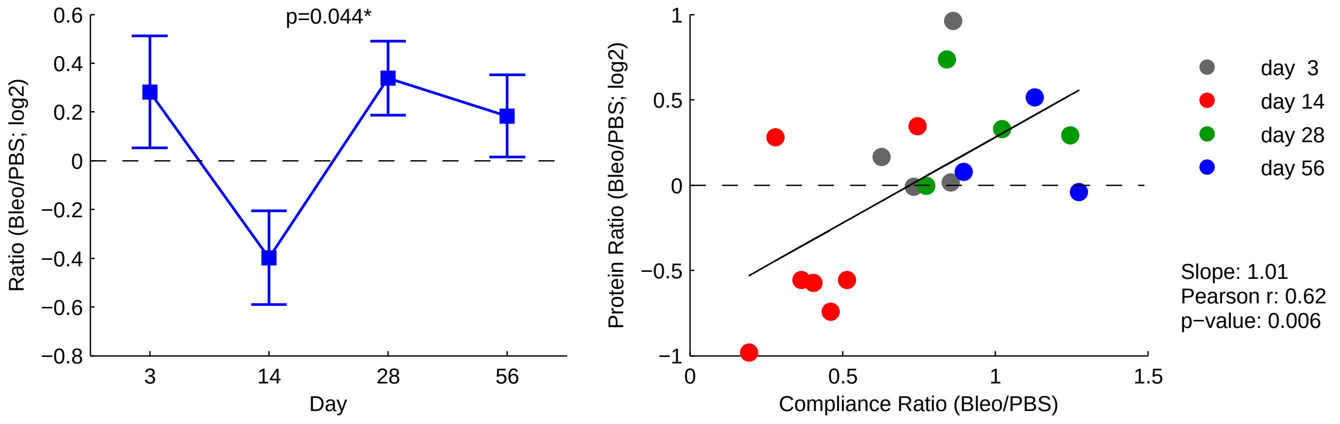

Q99MR8 − Mccc1 (id: 6616)

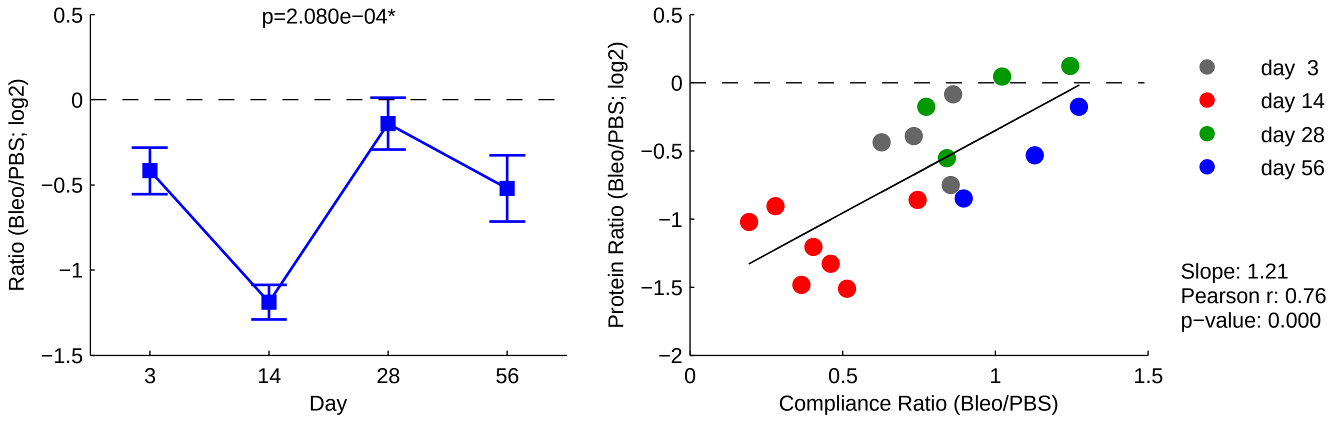

Q99N15 − Hsd17b10 (id: 6620)

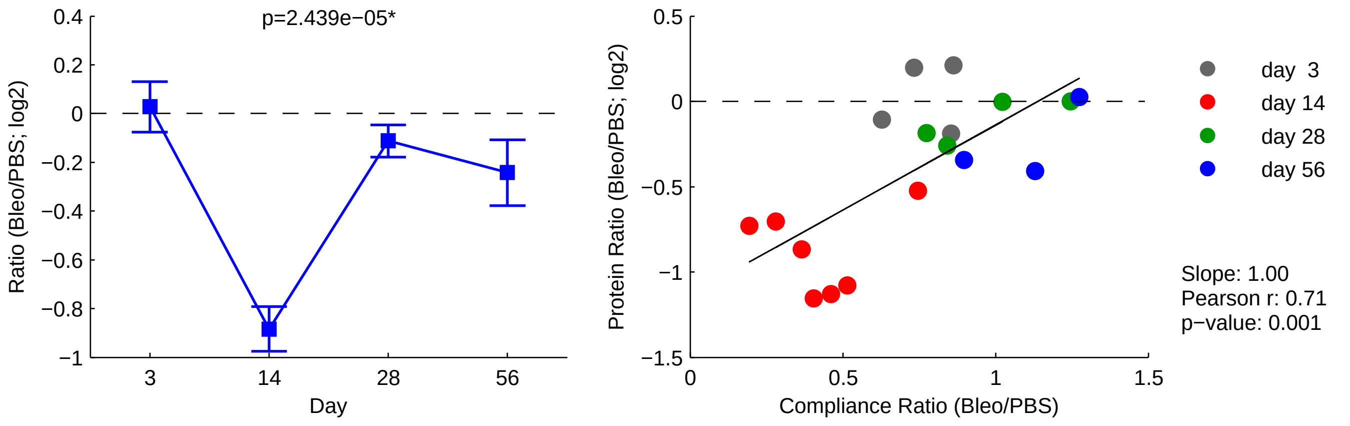

Q99N16 − Cyp4f3 (id: 6621)

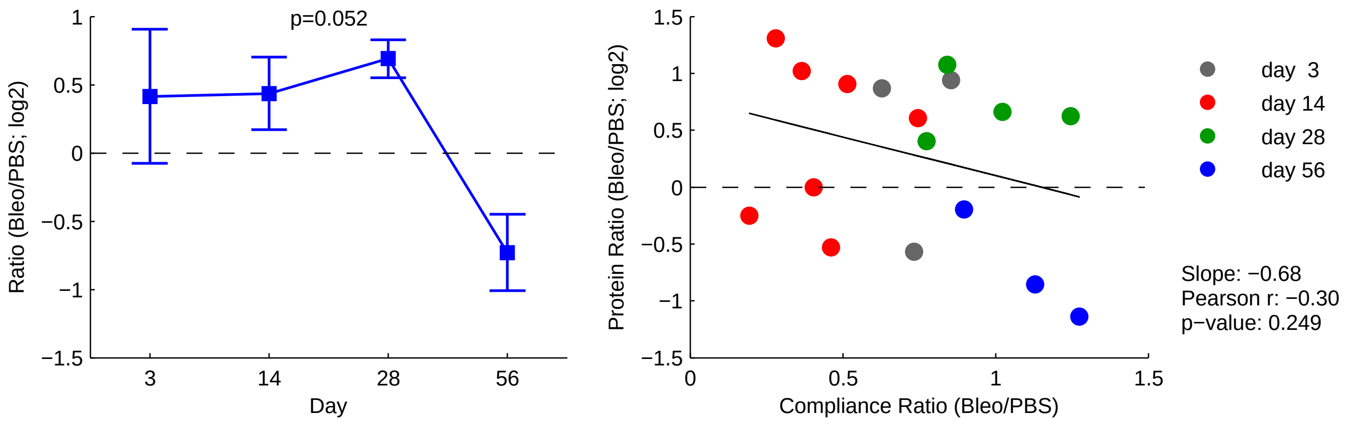

Q99N57 − Raf1 (id: 6625)

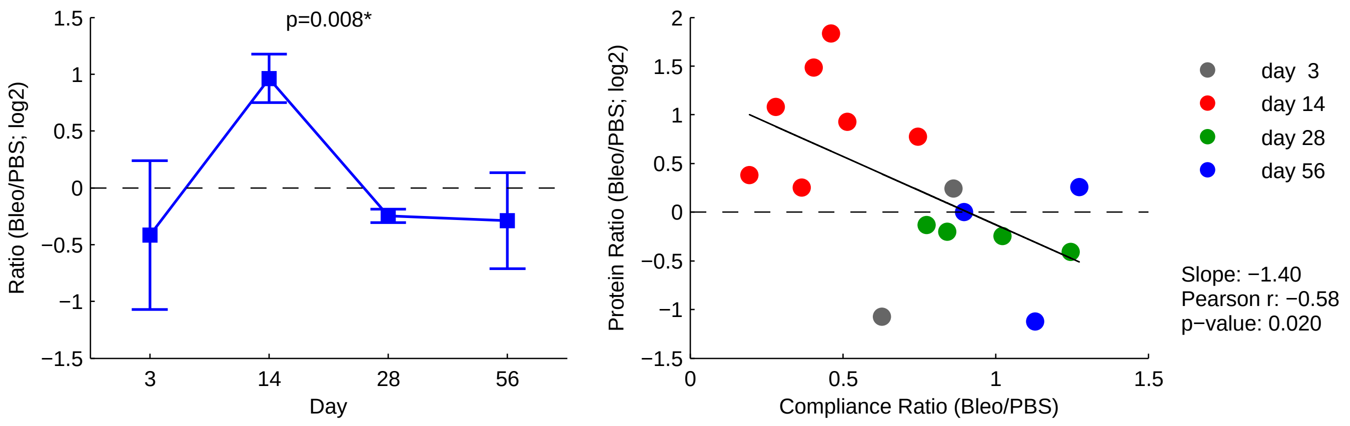

Q99N84 − Mrps18b (id: 6627)

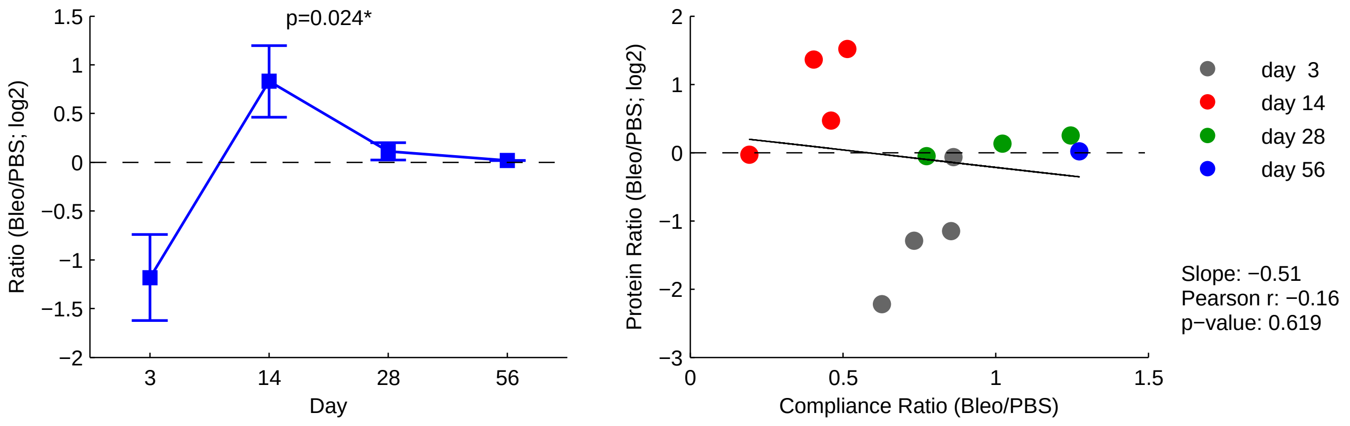

Q99N92 − Mrpl27 (id: 6629)

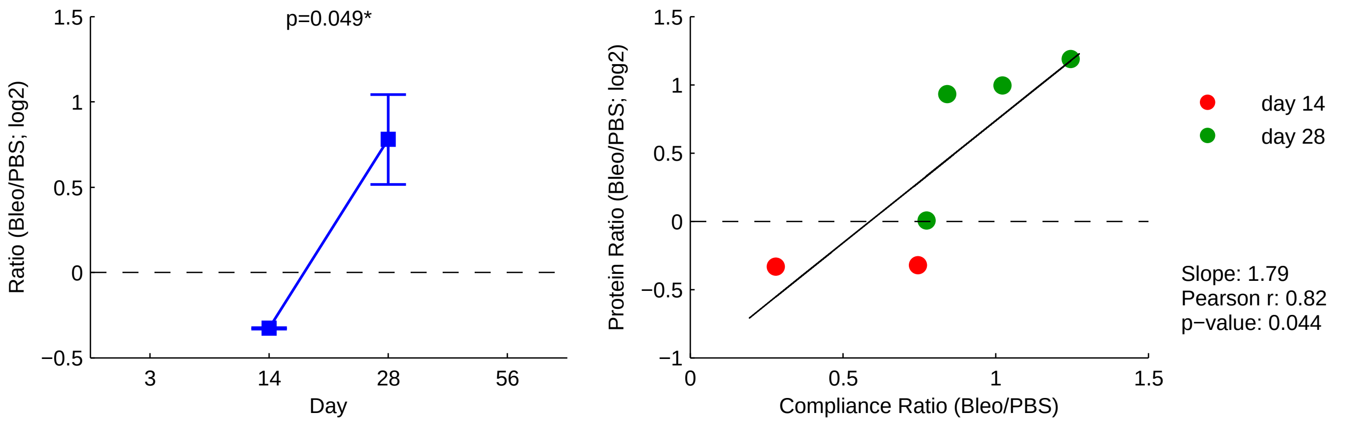

Q99N93 − Mrpl16 (id: 6630)

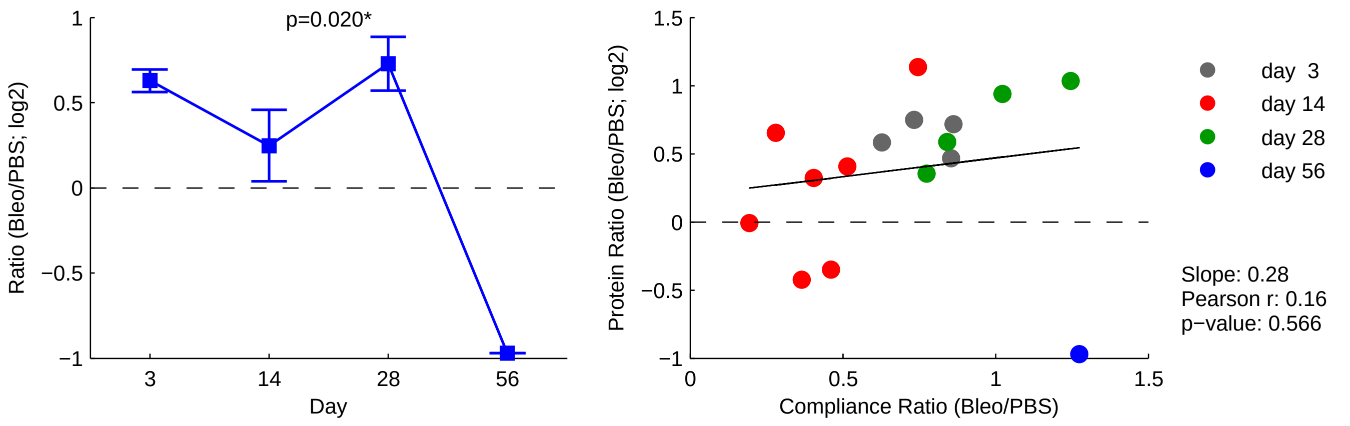

Q99N94 − Mrpl9 (id: 6631)

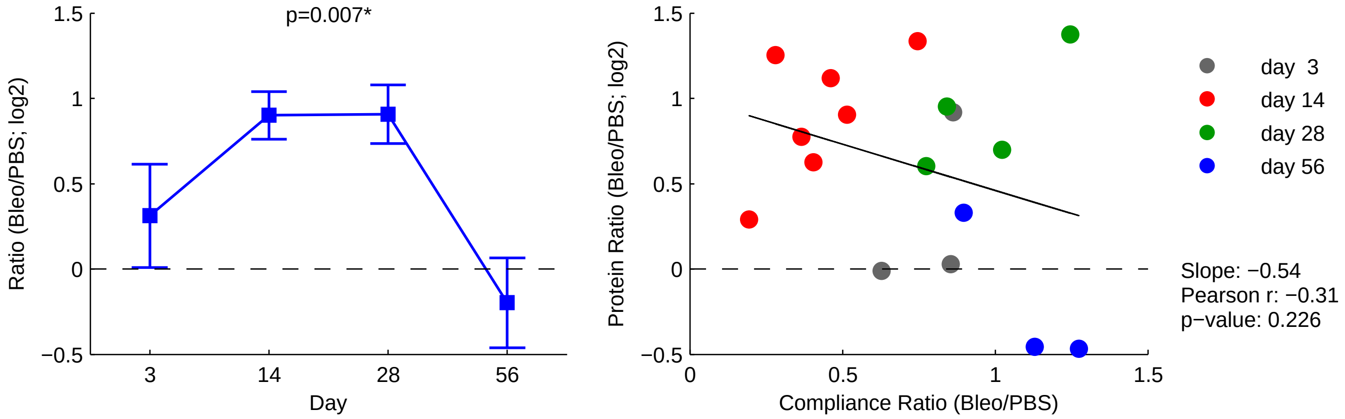

Q99N95 − Mrpl3 (id: 6632)

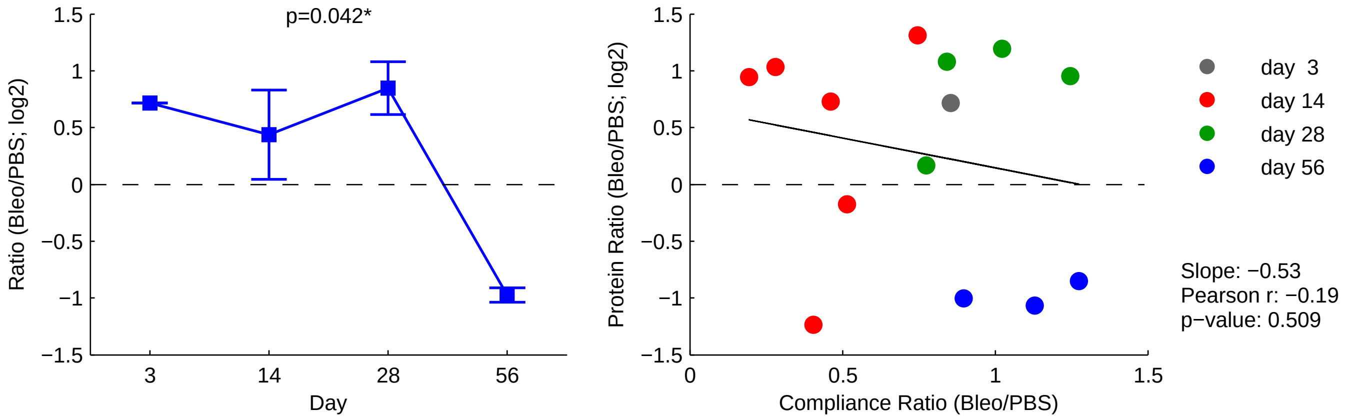

Q99N96 − Mrpl1 (id: 6633)

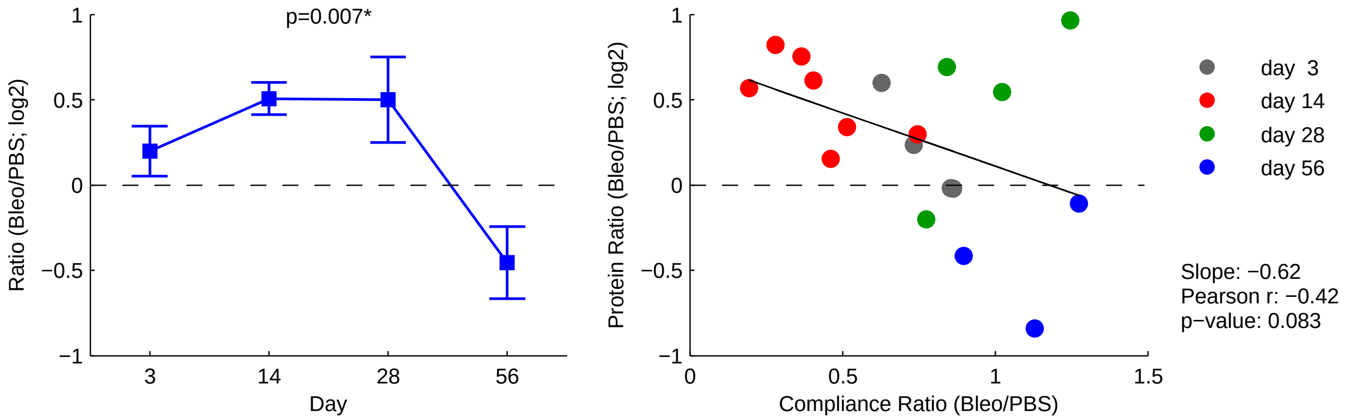

Q99NB1 − Acss1 (id: 6634)

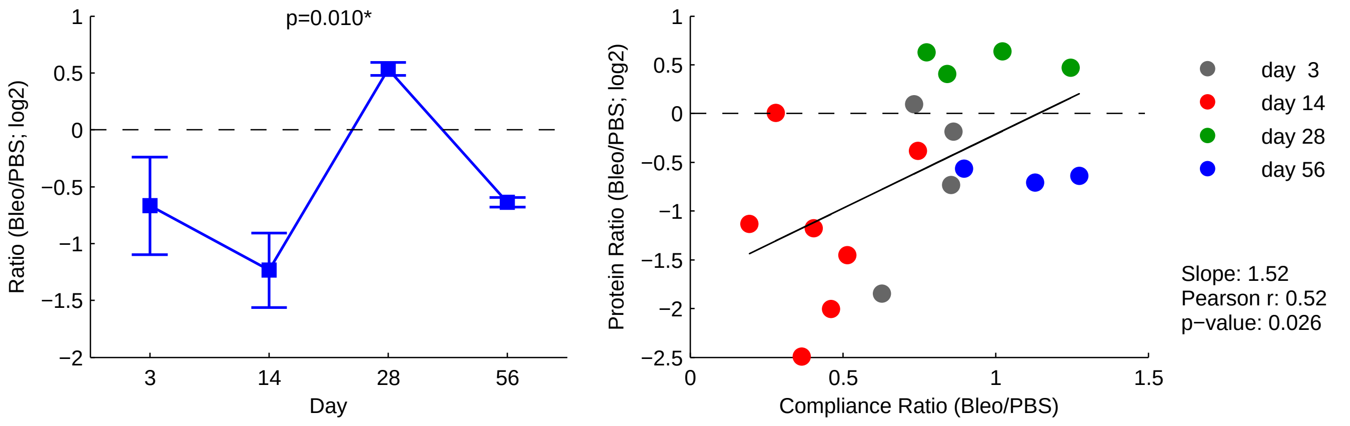

Q99P72 − Rtn4 (id: 6638)

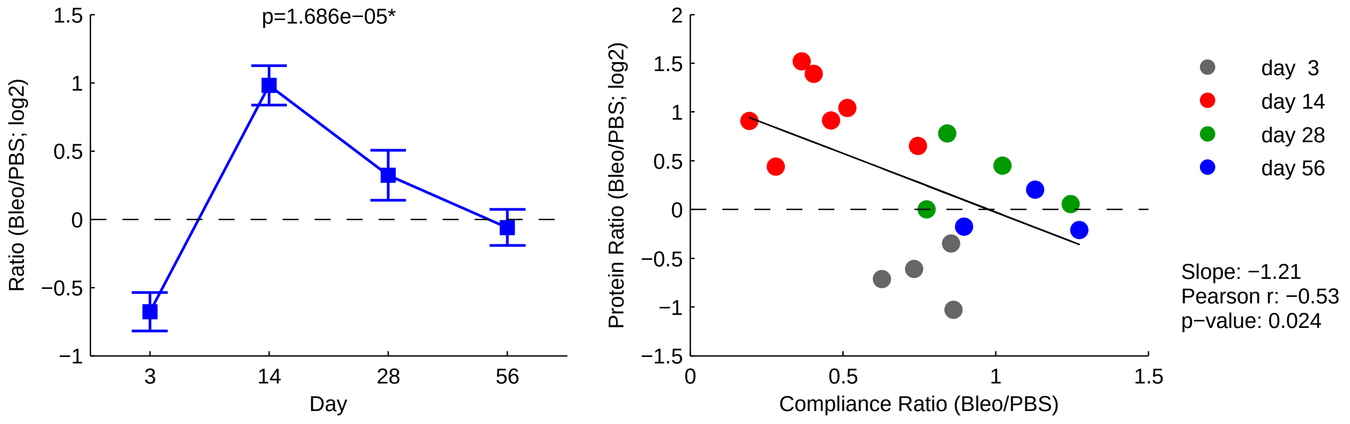

Q99PT1 − Arhgdia (id: 6645)

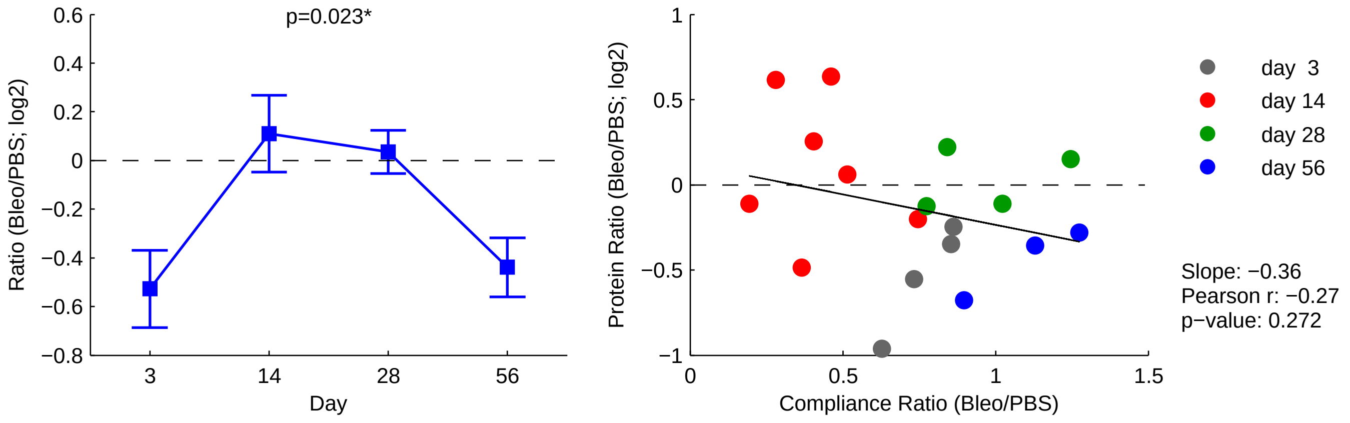

Q99PV0 − Prpf8 (id: 6647)

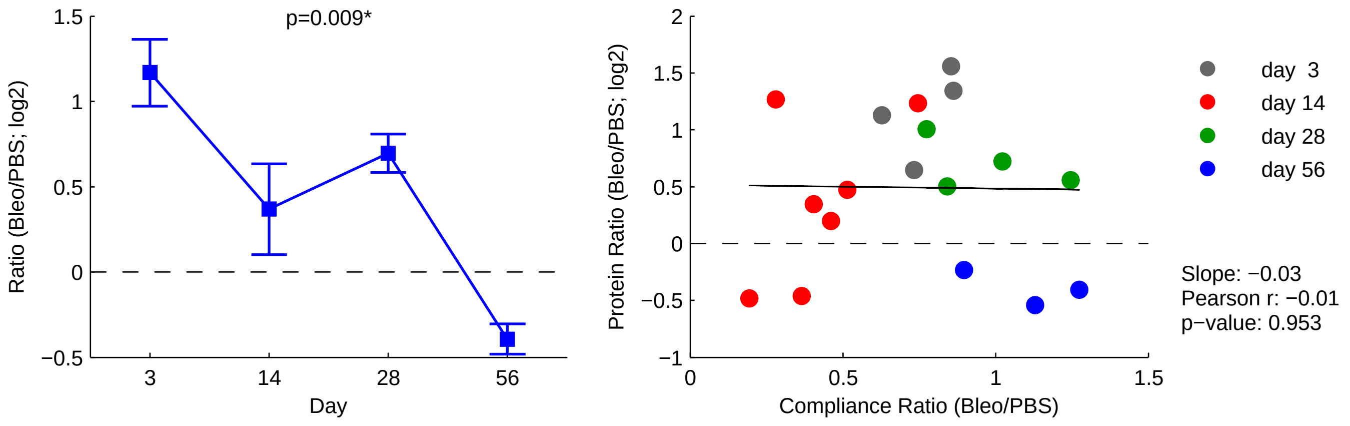

Q9BCZ4 − Vimp (id: 6649)

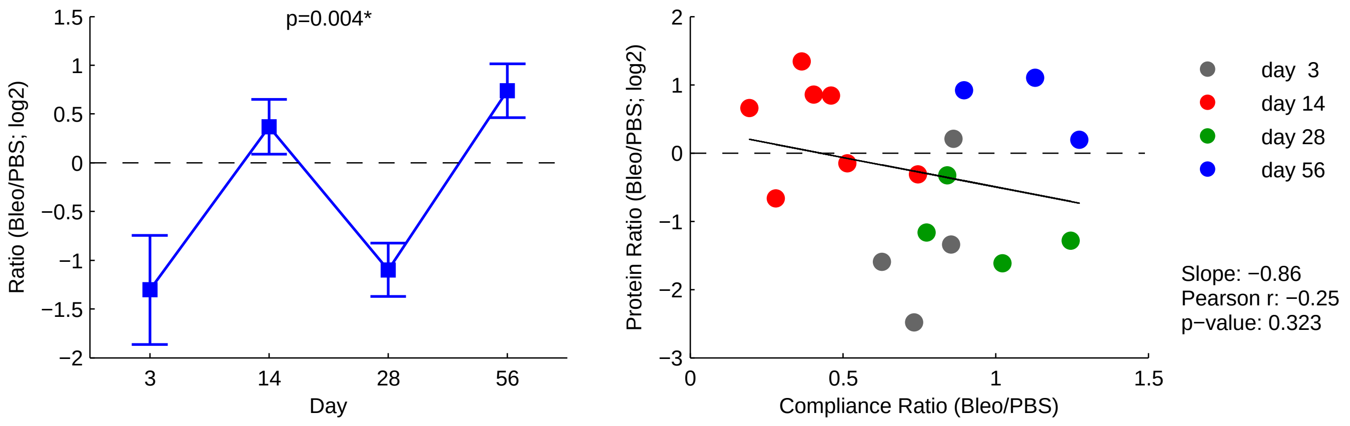

Q9CPP6 − Ndufa5 (id: 6653)

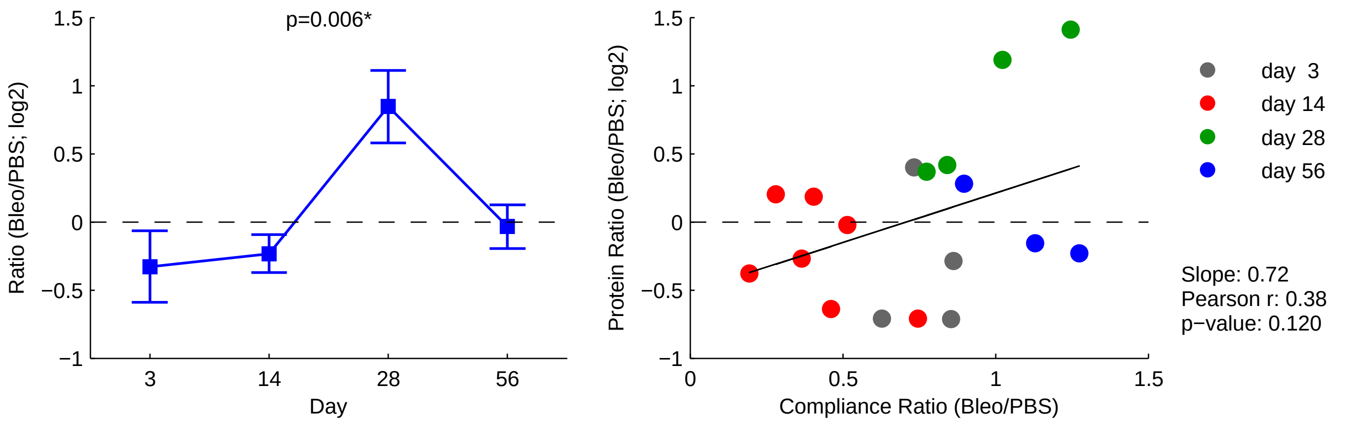

Q9CPQ1 − Cox6c (id: 6654)

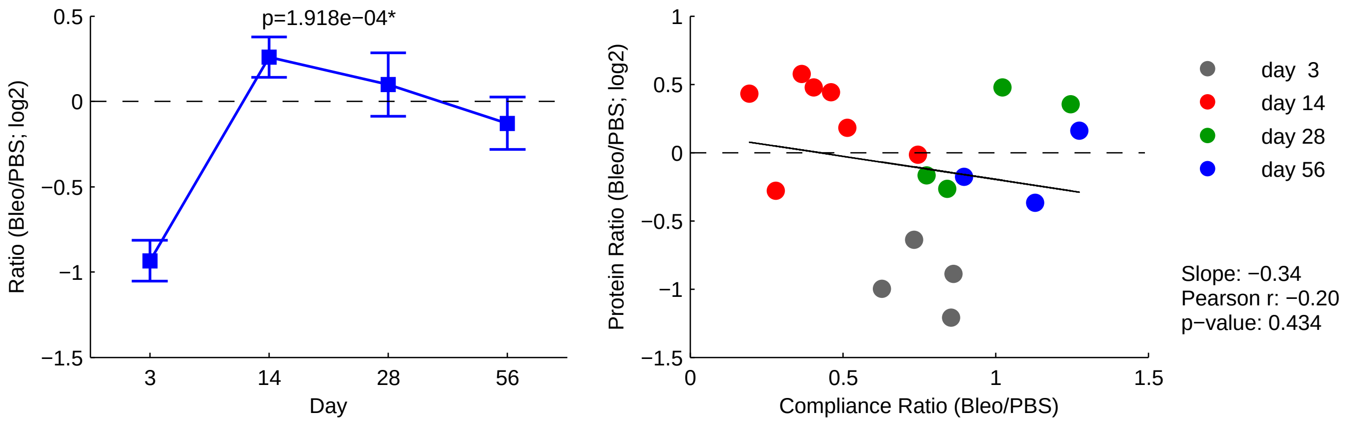

Q9CPQ3 − Tomm22 (id: 6655)

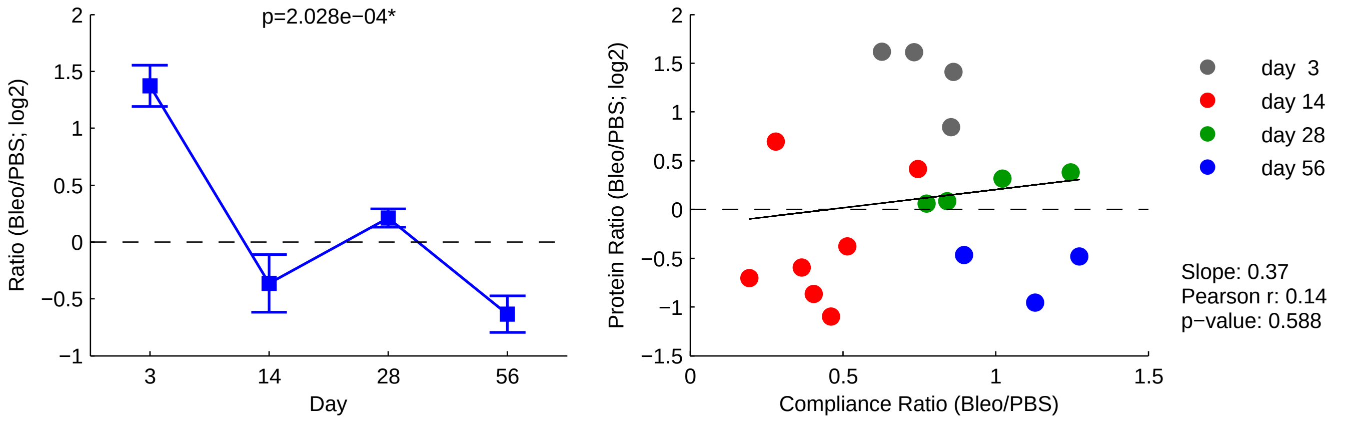

Q9CPQ8 − Atp5l (id: 6657)

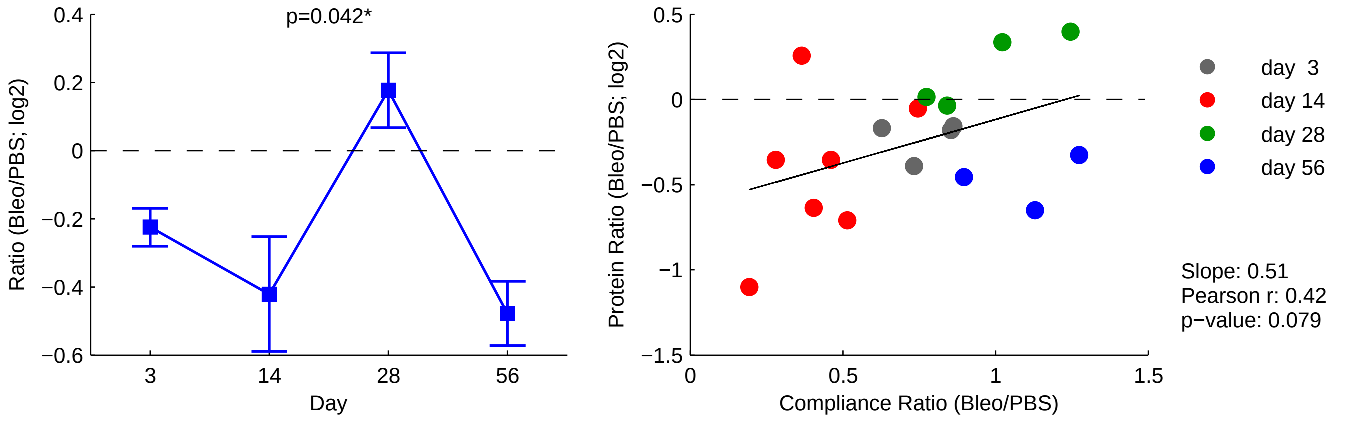

Q9CPR5 − Mrpl15 (id: 6658)

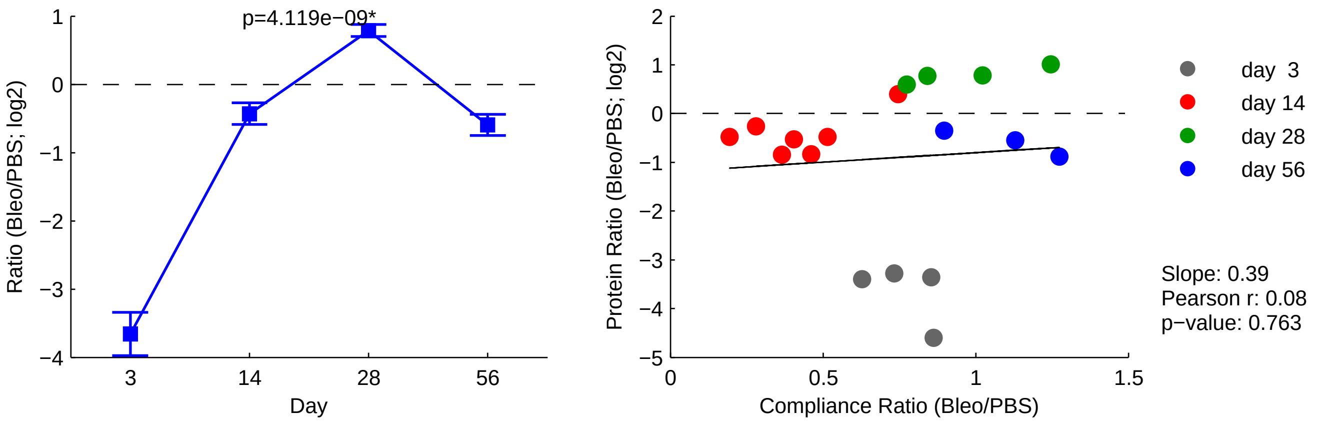

Q9CPS6 − Hint3 (id: 6660)

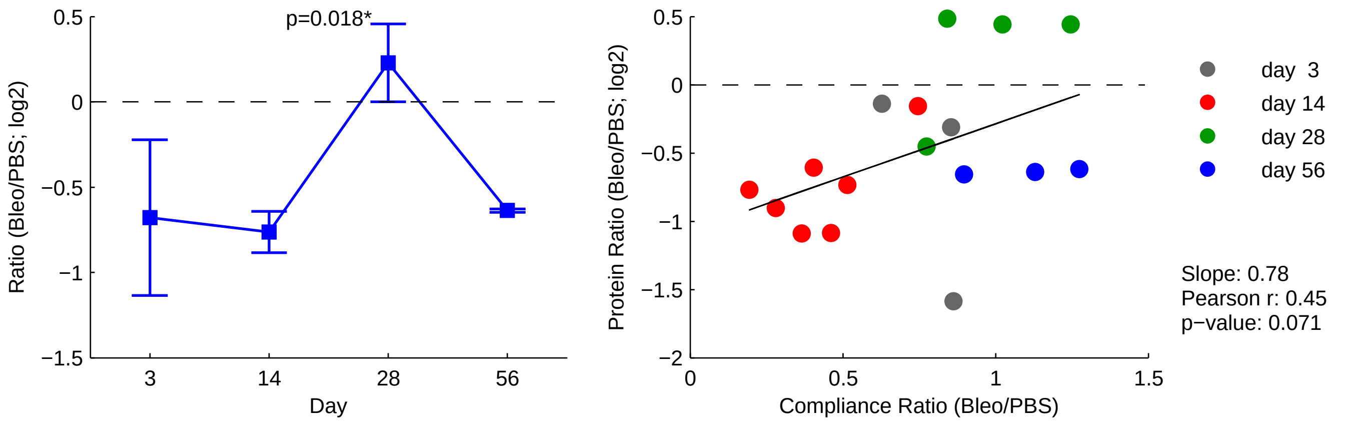

Q9CPT4 − D17Wsu104e (id: 6662)

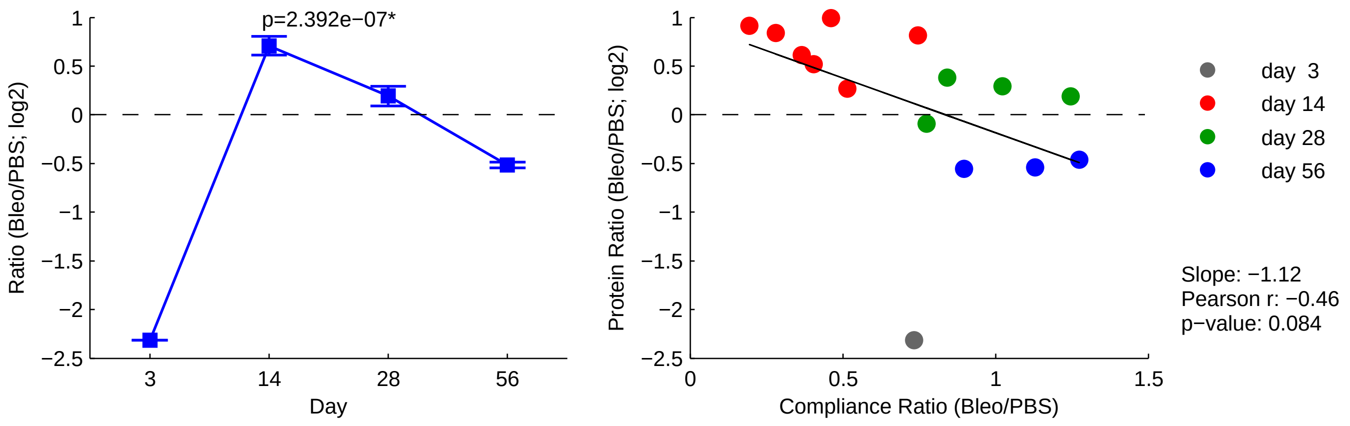

Q9CPU0 − Glo1 (id: 6663)

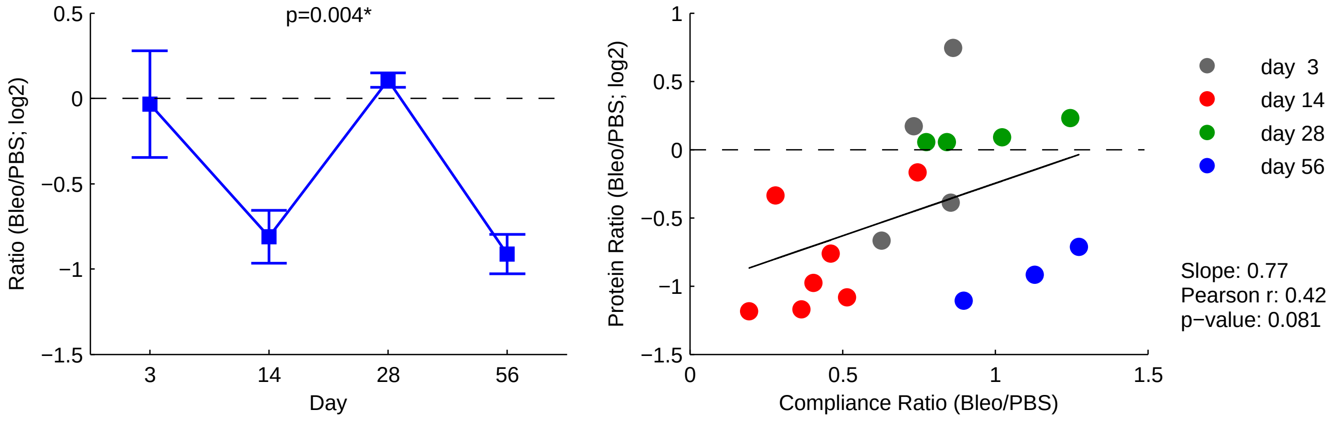

Q9CPV4 − Glod4 (id: 6667)

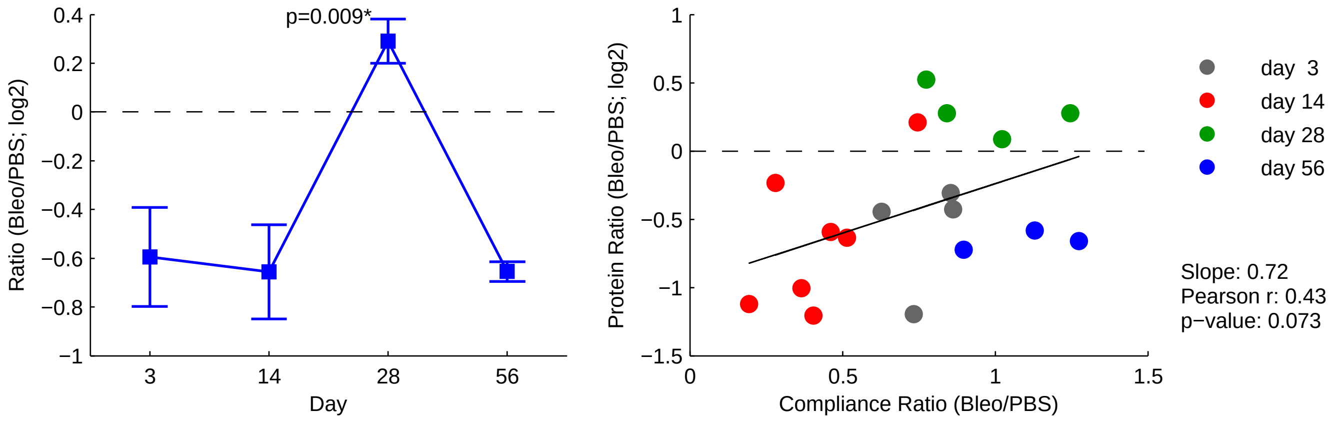

Q9CPW2 − Fdx1l (id: 6668)

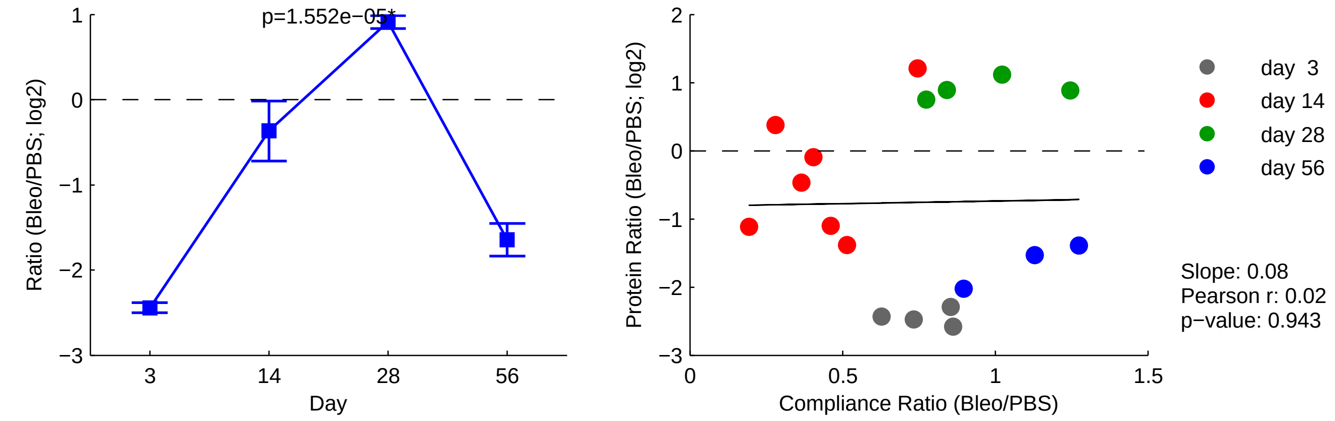

Q9CPW3 − Mrpl54 (id: 6669)

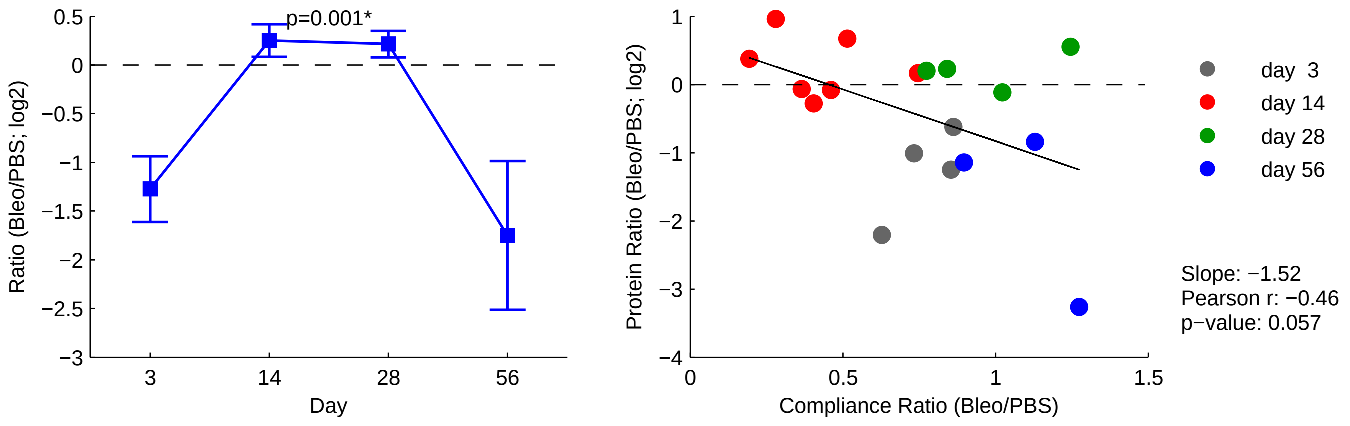

Q9CPW4 − Arpc5 (id: 6670)

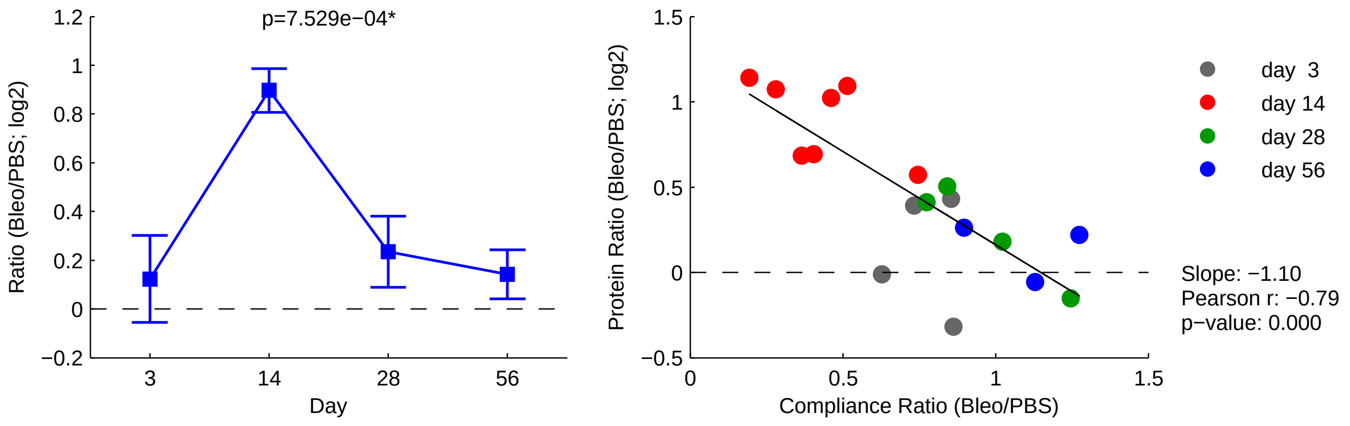

Q9CPX4 − Ftl1 (id: 6672)

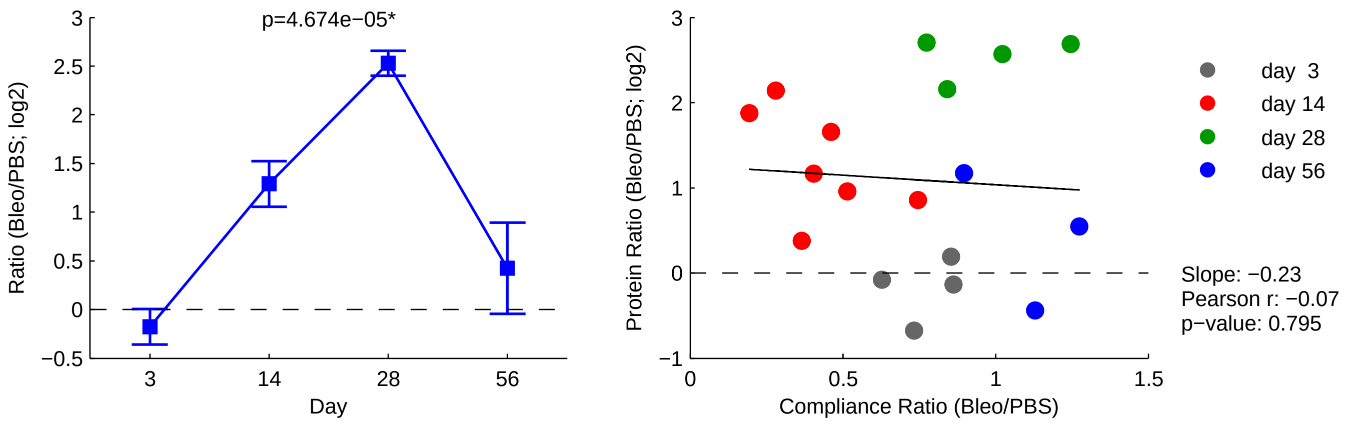

Q9CPX8 − Uqcr11 (id: 6675)

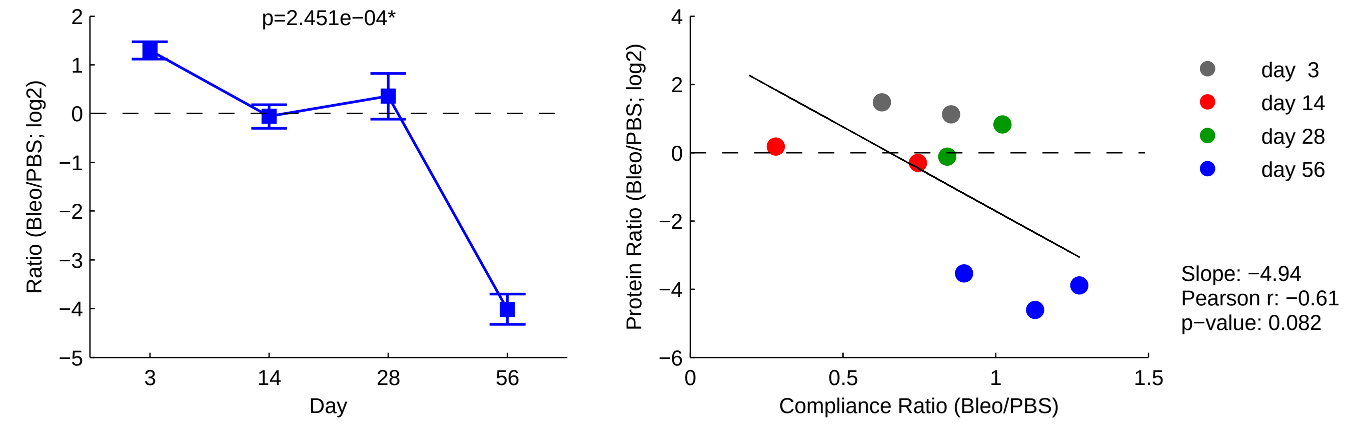

Q9CPY7−2 − Lap3 (id: 6678)

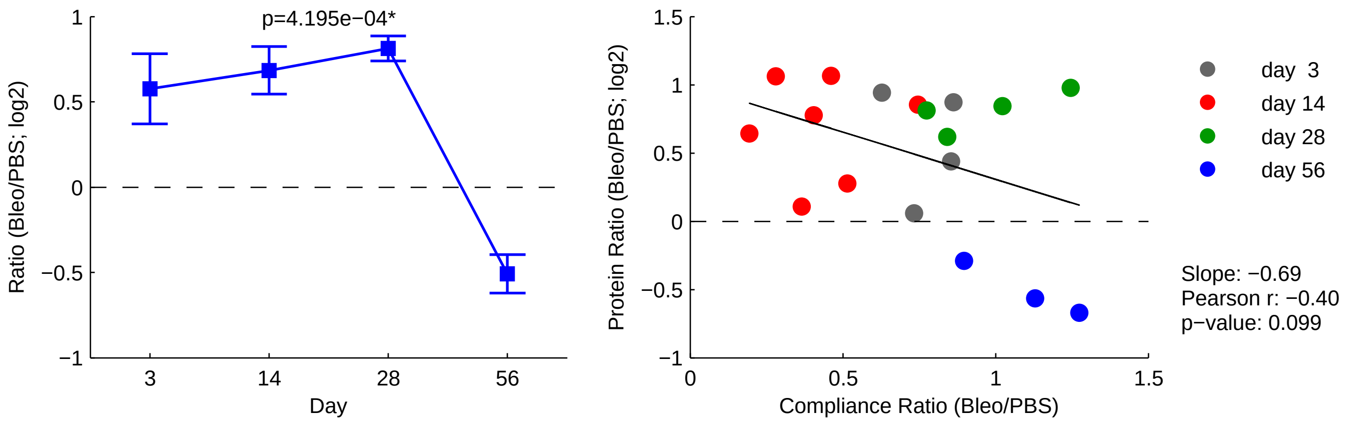

Q9CQ00 − (id: 6681)

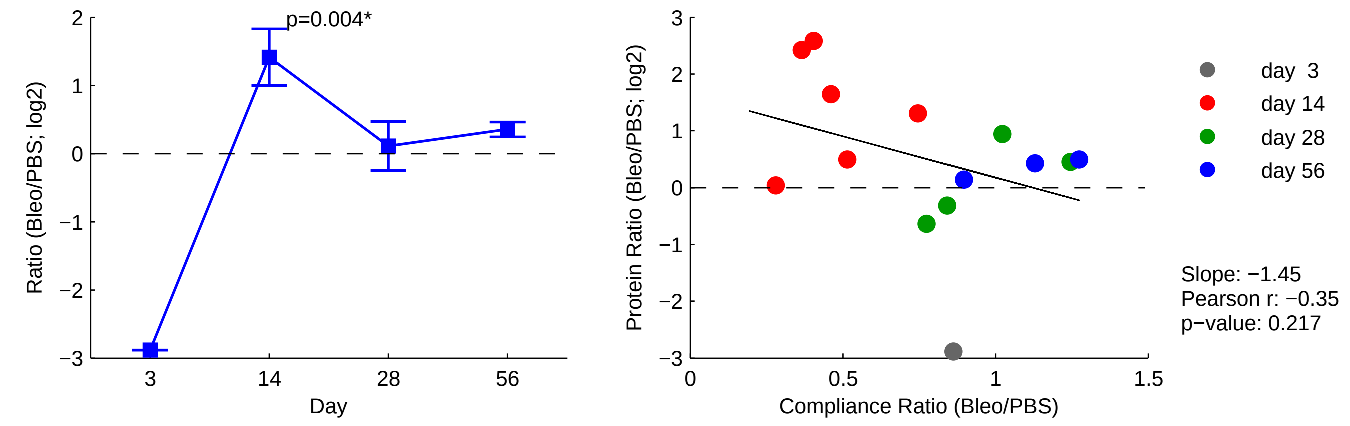

Q9CQ01 − Rnaset2 (id: 6682)

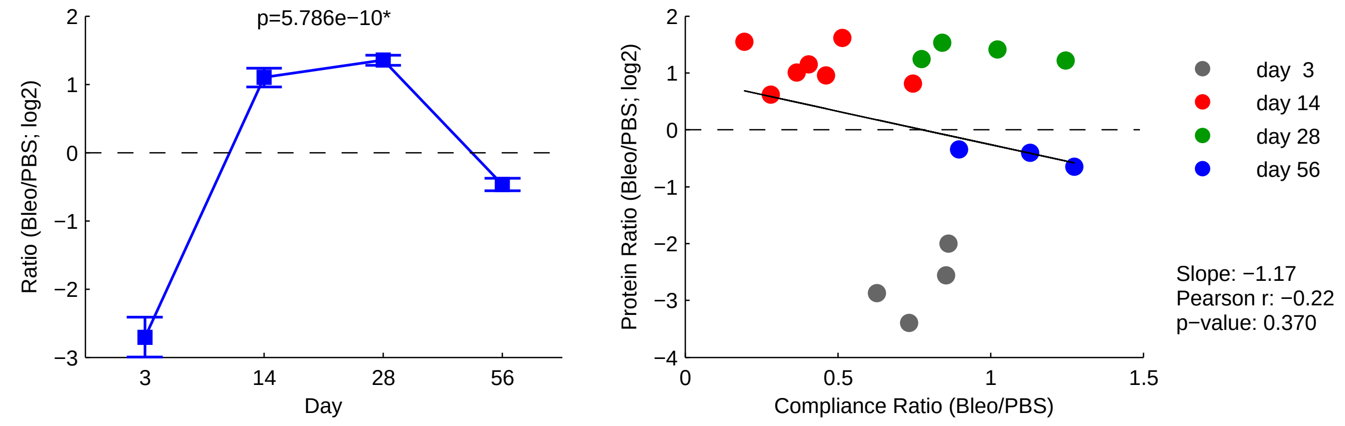

Q9CQ02 − Commd4 (id: 6683)

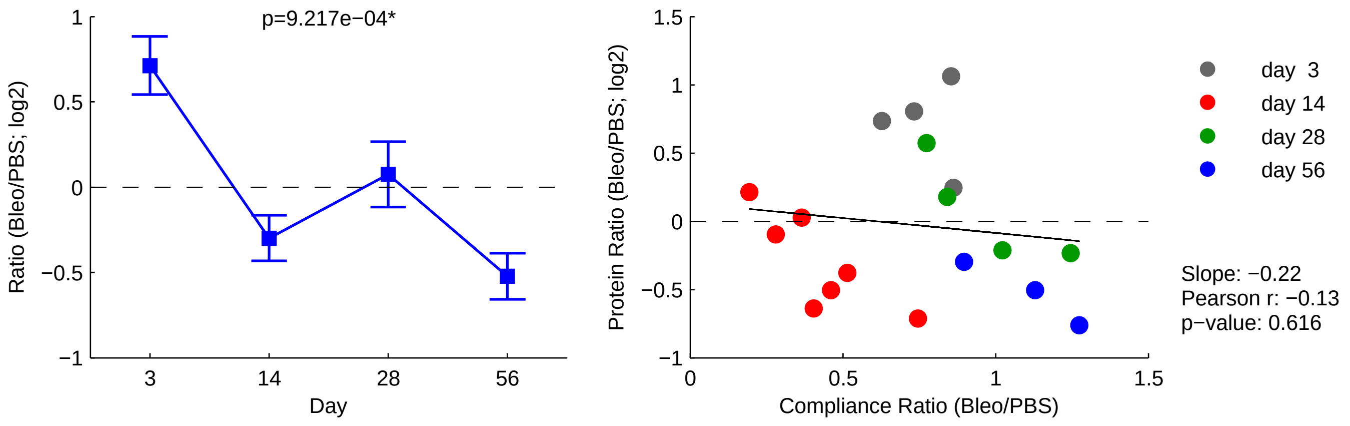

Q9CQ06 − Mrpl24 (id: 6684)

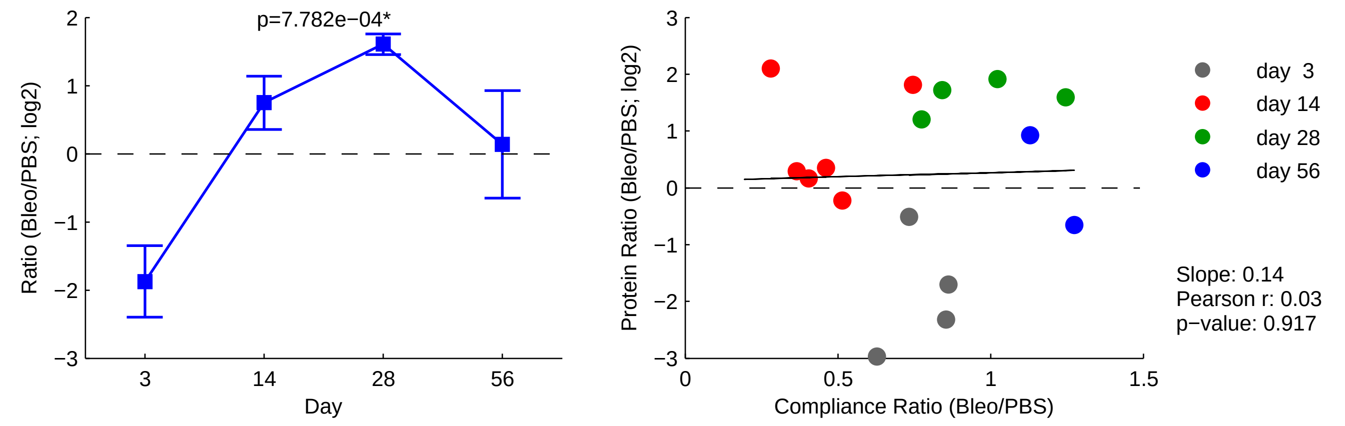

Q9CQ22 − Lamtor1 (id: 6689)

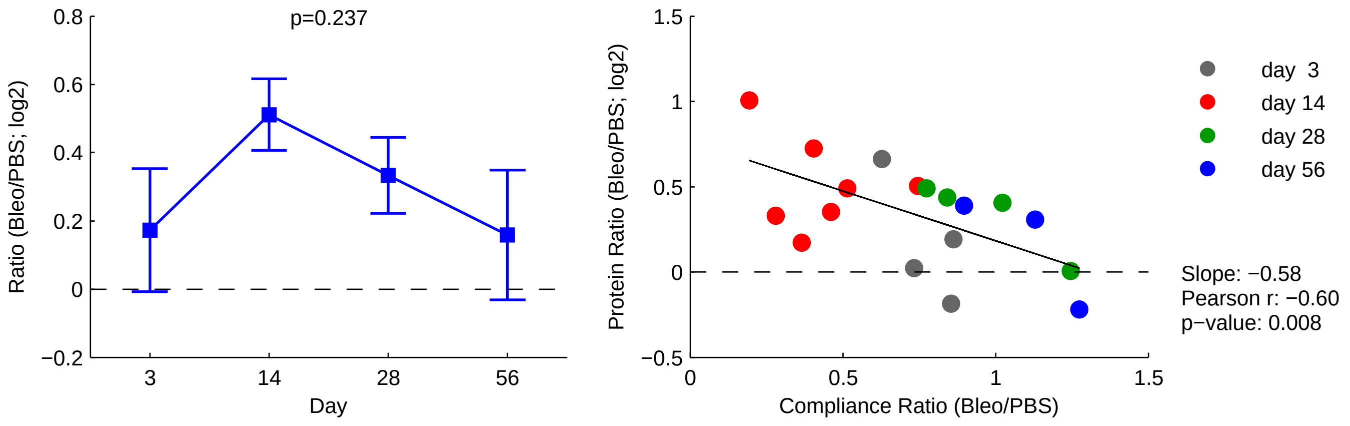

Q9CQ26 − Stambp (id: 6691)

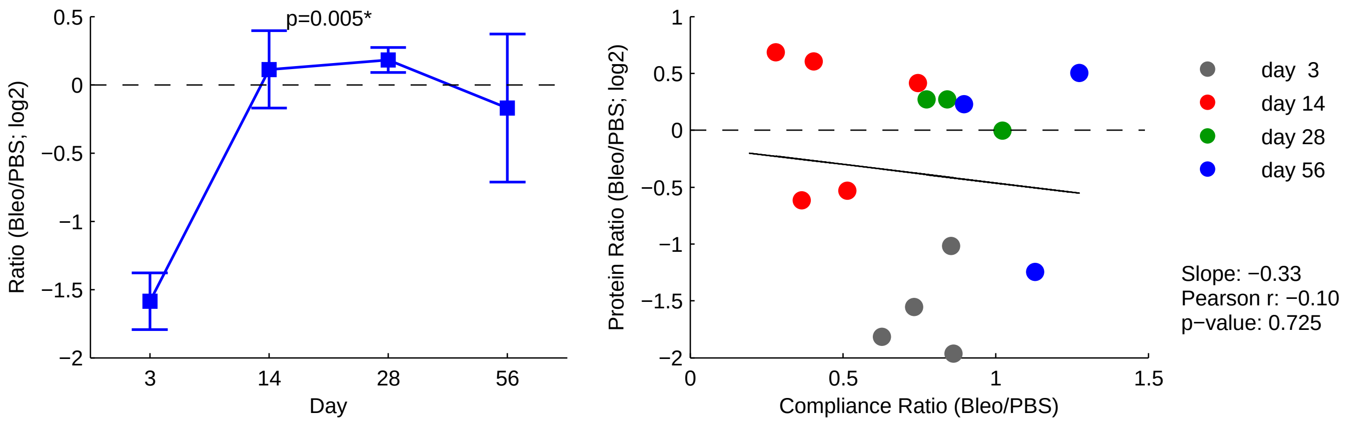

Q9CQ40 − Mrpl49 (id: 6693)

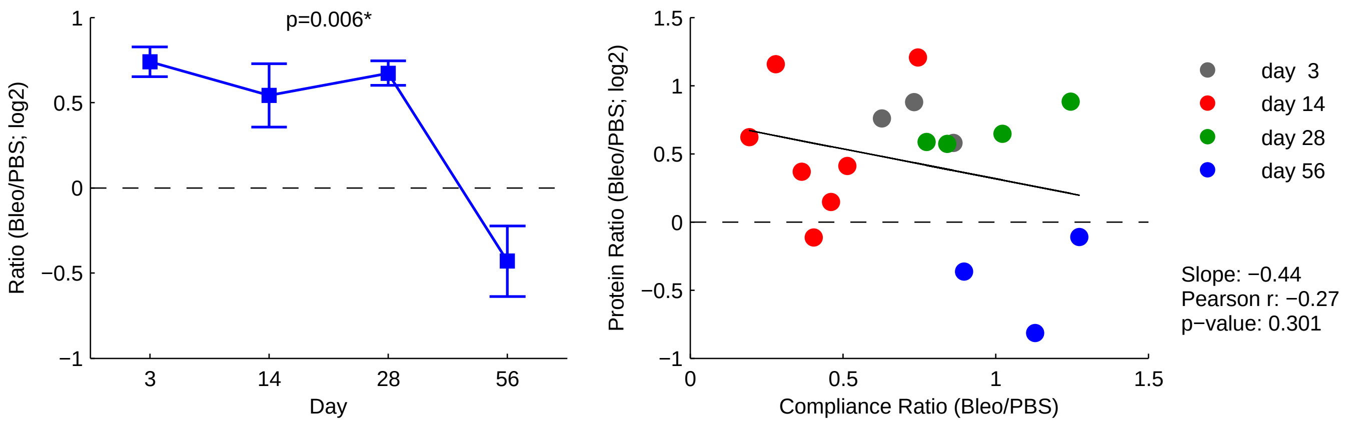

Q9CQ45 − Nenf (id: 6694)

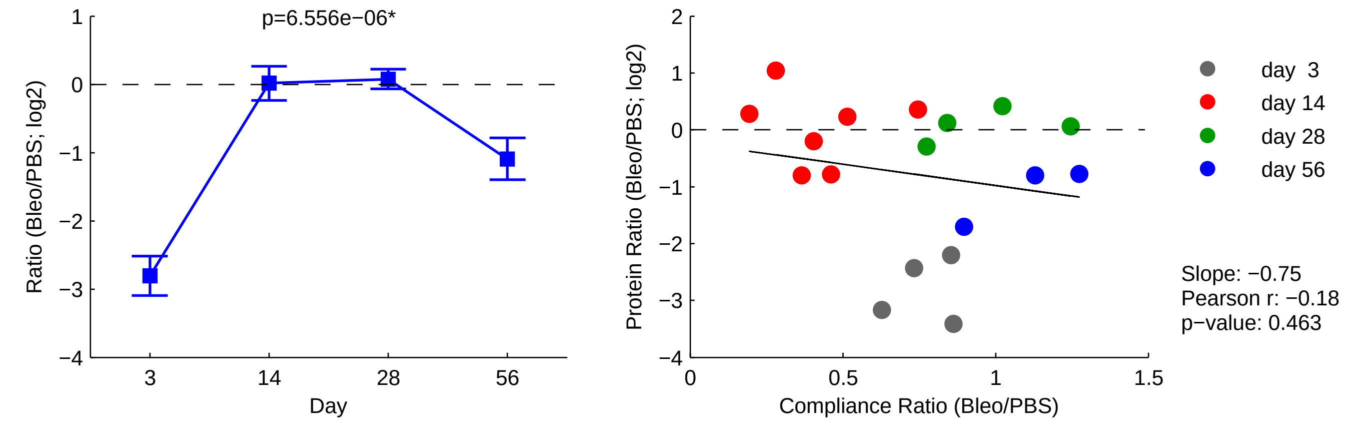

Q9CQ54 − Ndufc2 (id: 6696)

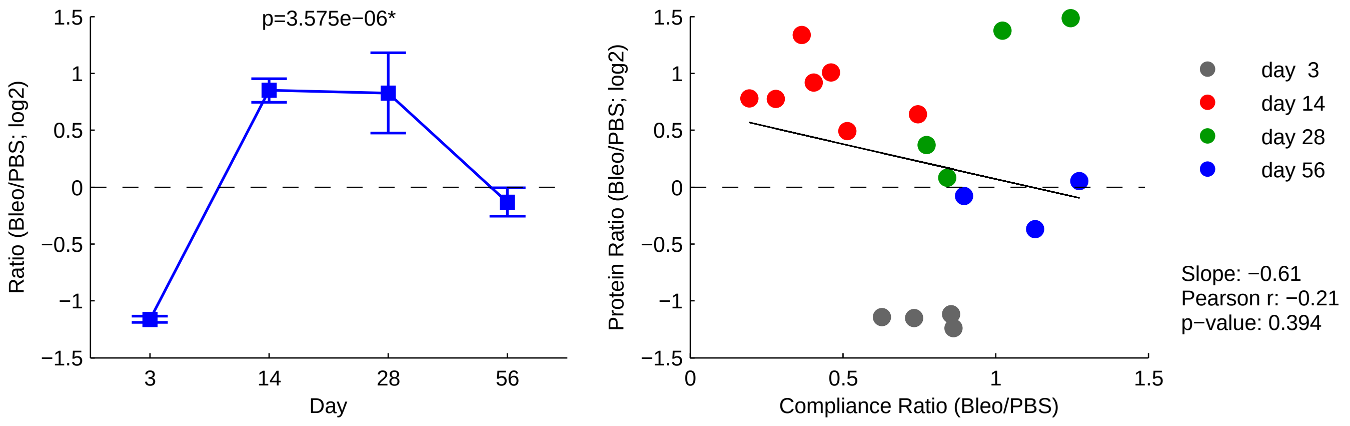

Q9CQ62 − Decr1 (id: 6698)

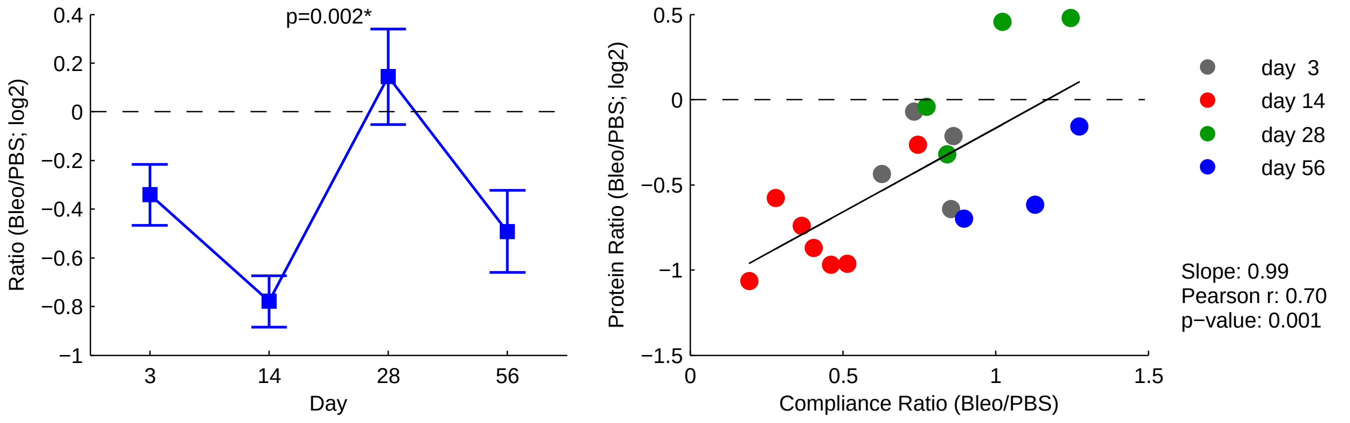

Q9CQ73 − Pkp2 (id: 6702)

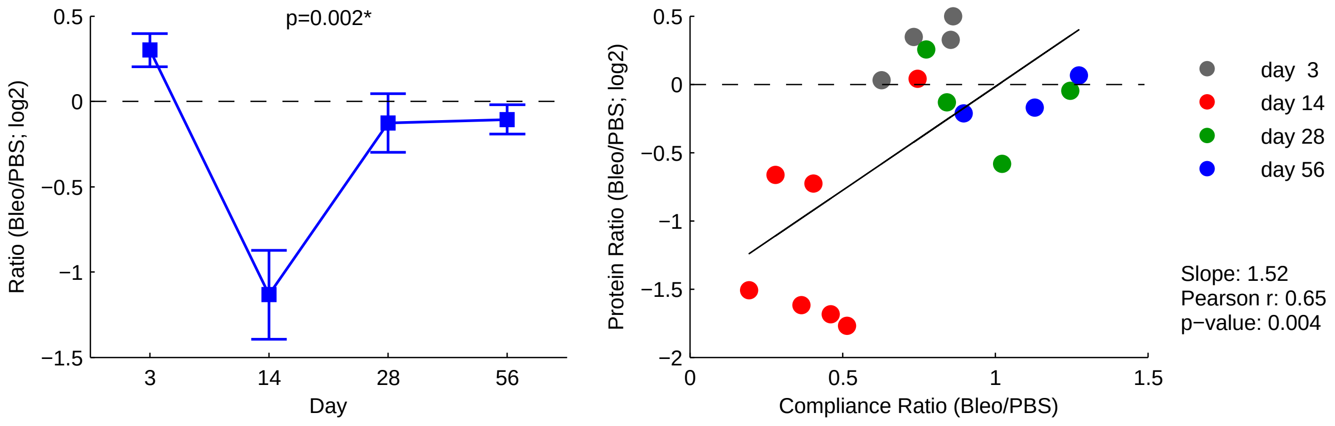

Q9CQ75 − Ndufa2 (id: 6703)

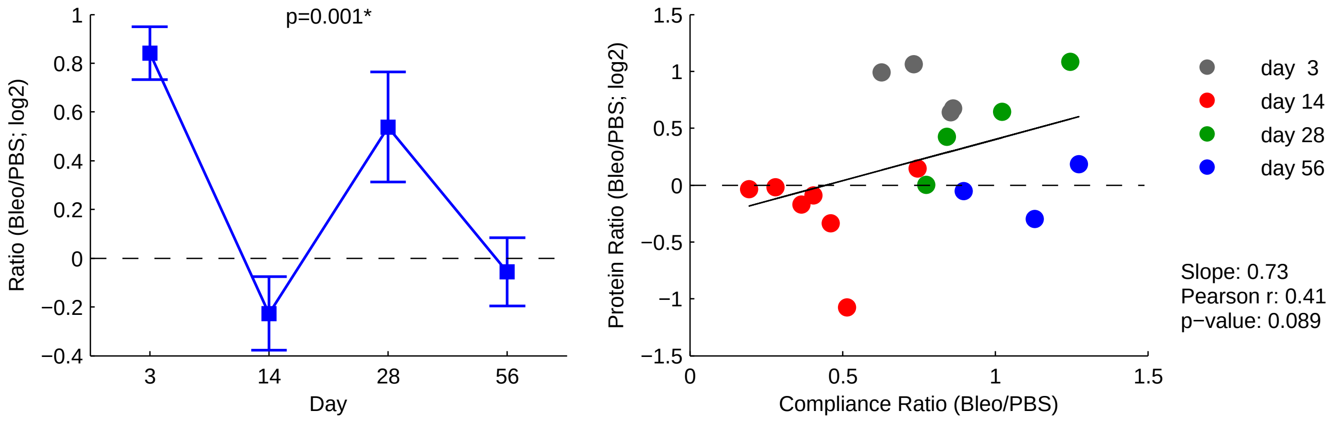

Q9CQ79 − Txndc9 (id: 6704)

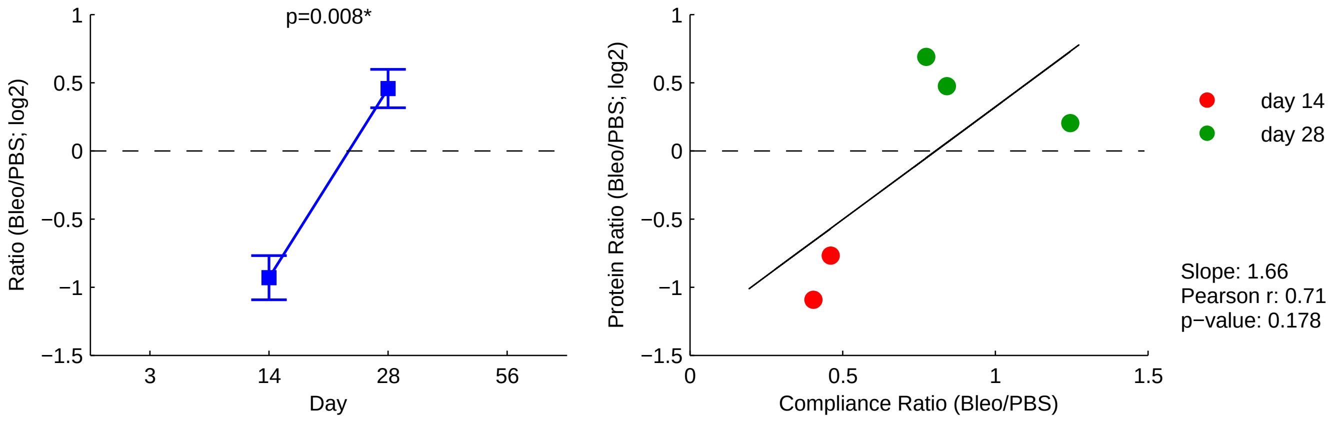

Q9CQ91 − Ndufa3 (id: 6706)

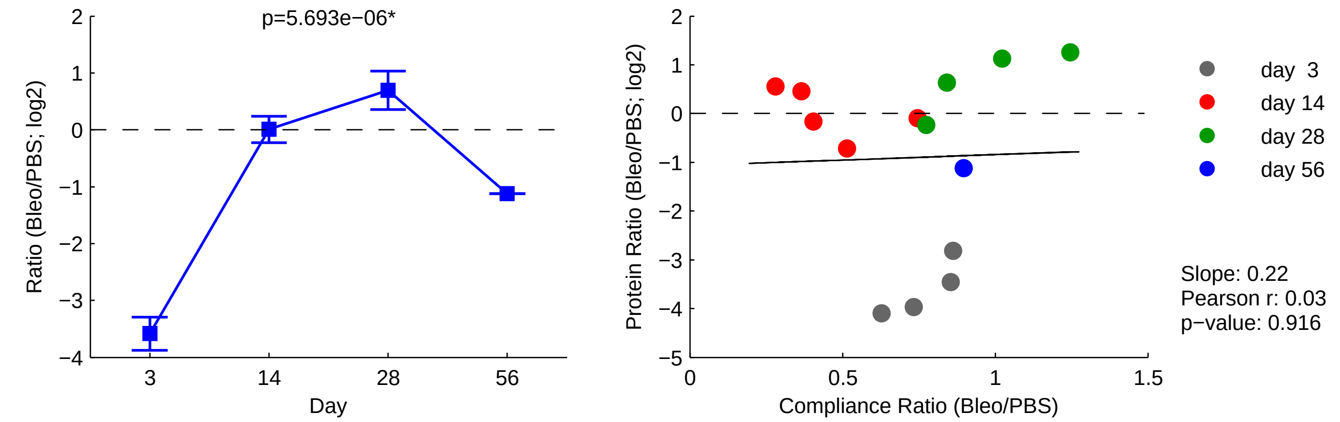

Q9CQ92 − Fis1 (id: 6707)

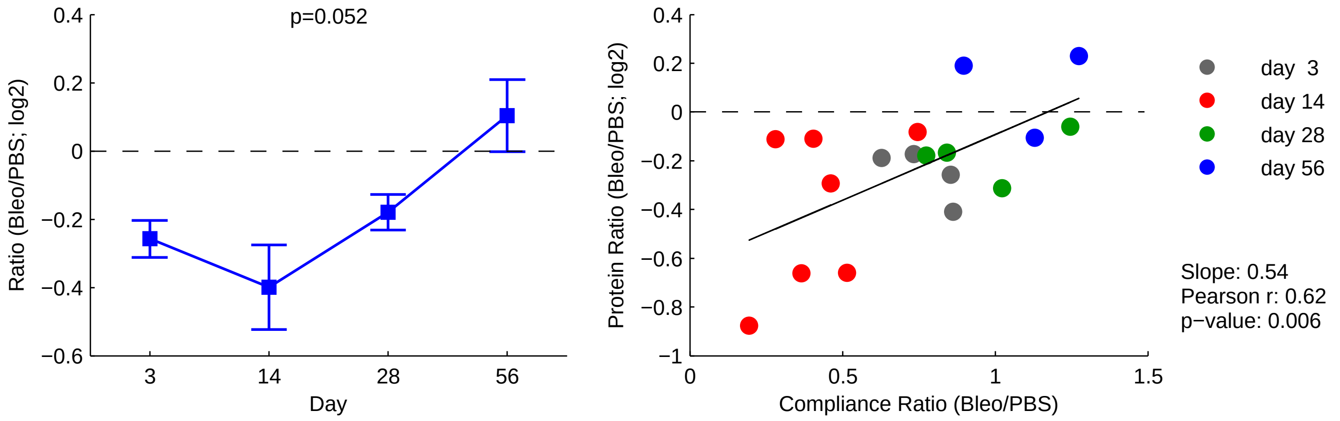

Q9CQA1 − Trappc5 (id: 6708)

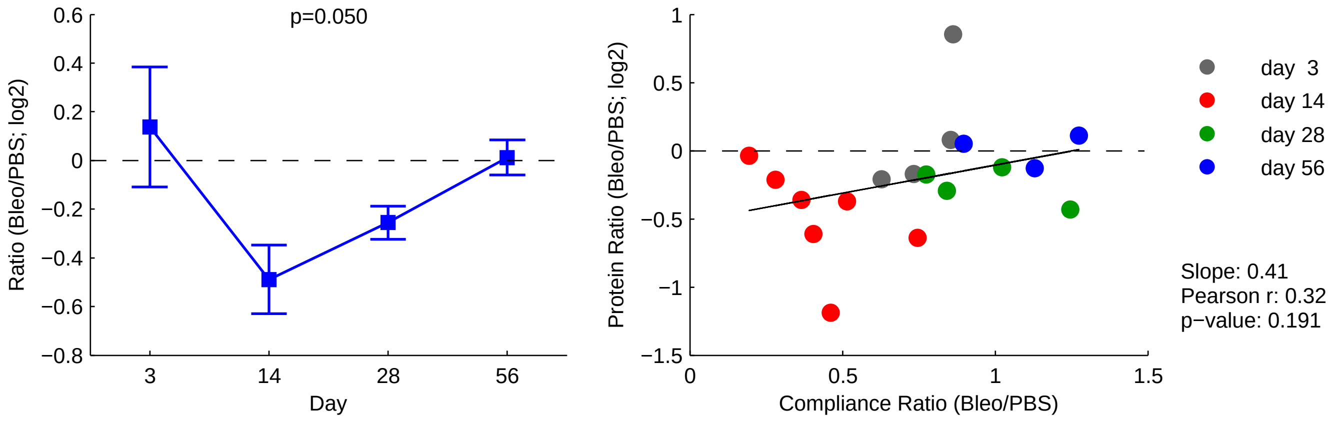

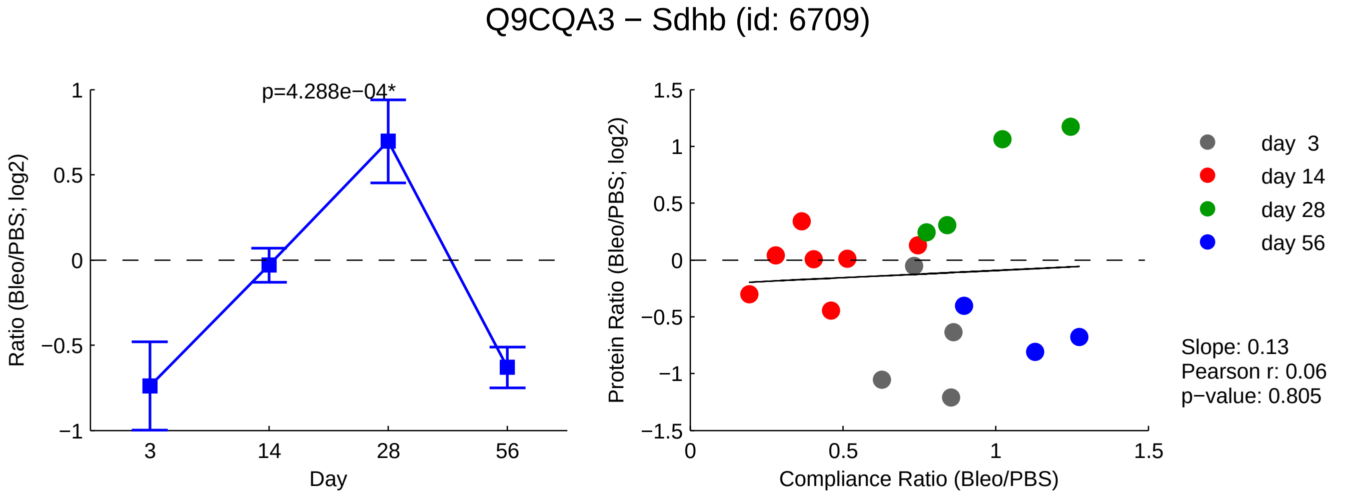

Q9CQA3 − Sdhb (id: 6709)
p=4.288e−04*
Ratio (Bleo/PBS; log2)
Day
Protein Ratio (Bleo/PBS; log2)
Compliance Ratio (Bleo/PBS)
day 3
day 14
day 28
day 56
Slope: 0.13
Pearson r: 0.06
p−value: 0.805

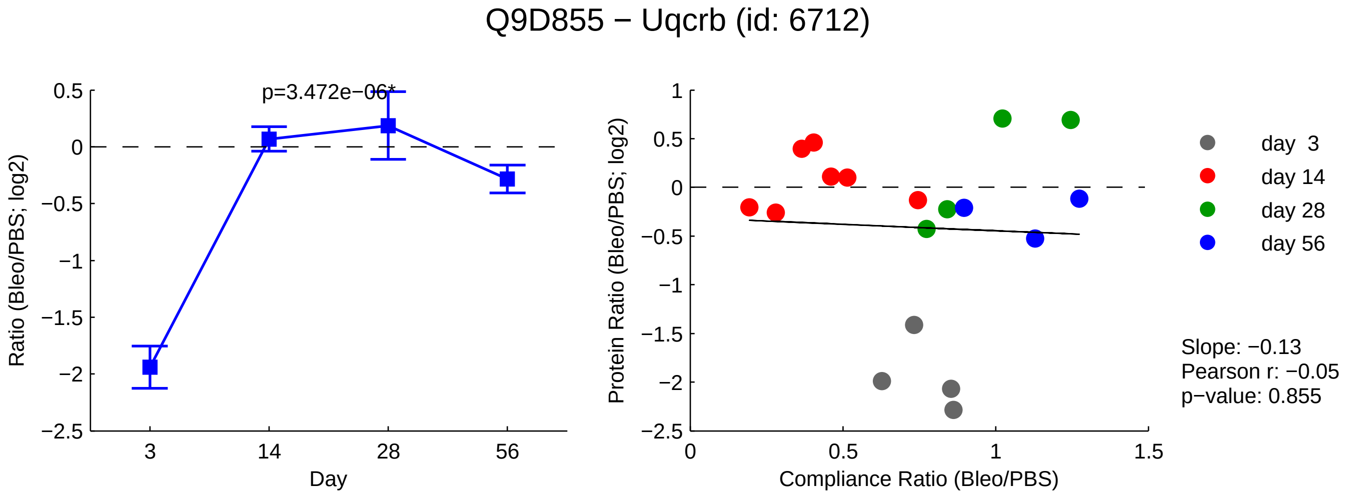

Q9D855 − Uqcrb (id: 6712)
p=3.472e−06*
Ratio (Bleo/PBS; log2)
Day
Protein Ratio (Bleo/PBS; log2)
Compliance Ratio (Bleo/PBS)
day 3
day 14
day 28
day 56
Slope: −0.13
Pearson r: −0.05
p−value: 0.855

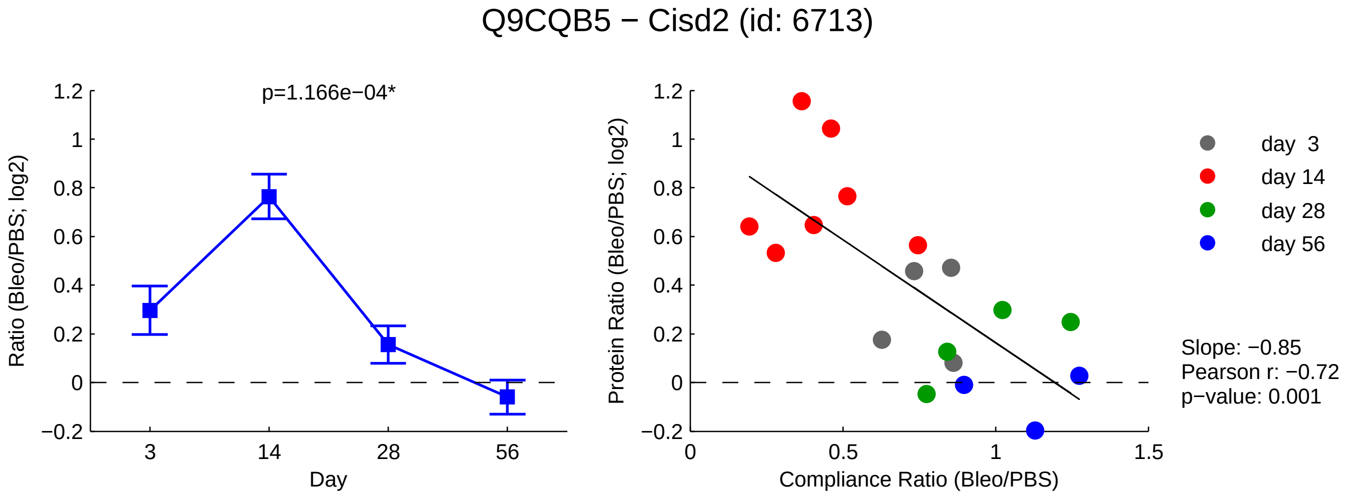

Q9CQB5 − Cisd2 (id: 6713)
p=1.166e−04*
Ratio (Bleo/PBS; log2)
Day
Protein Ratio (Bleo/PBS; log2)
Compliance Ratio (Bleo/PBS)
day 3
day 14
day 28
day 56
Slope: −0.85
Pearson r: −0.72
p−value: 0.001

Q9CQC6 − Bzw1 (id: 6714)

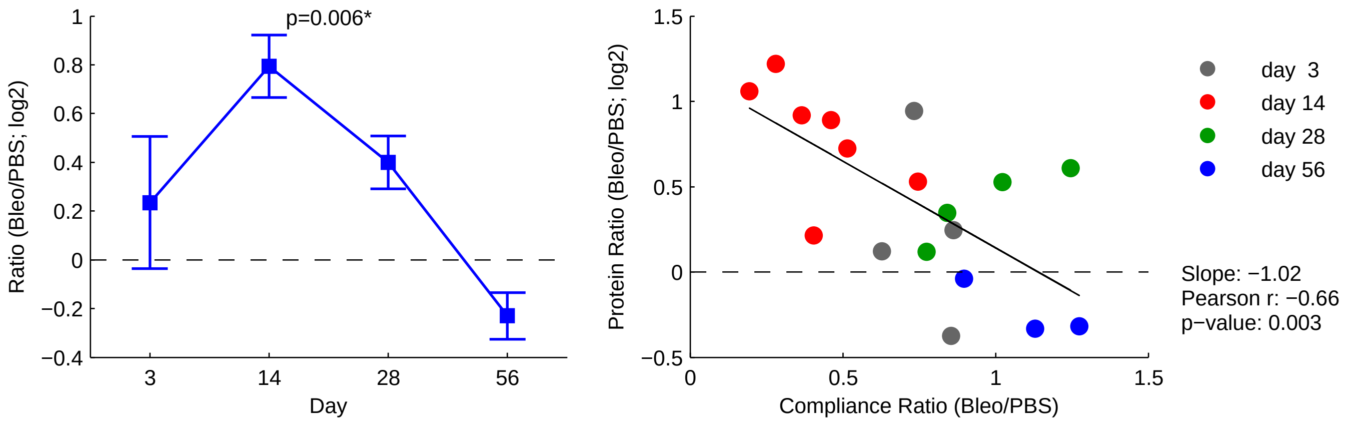

Q9CQC7 − Ndufb4 (id: 6715)

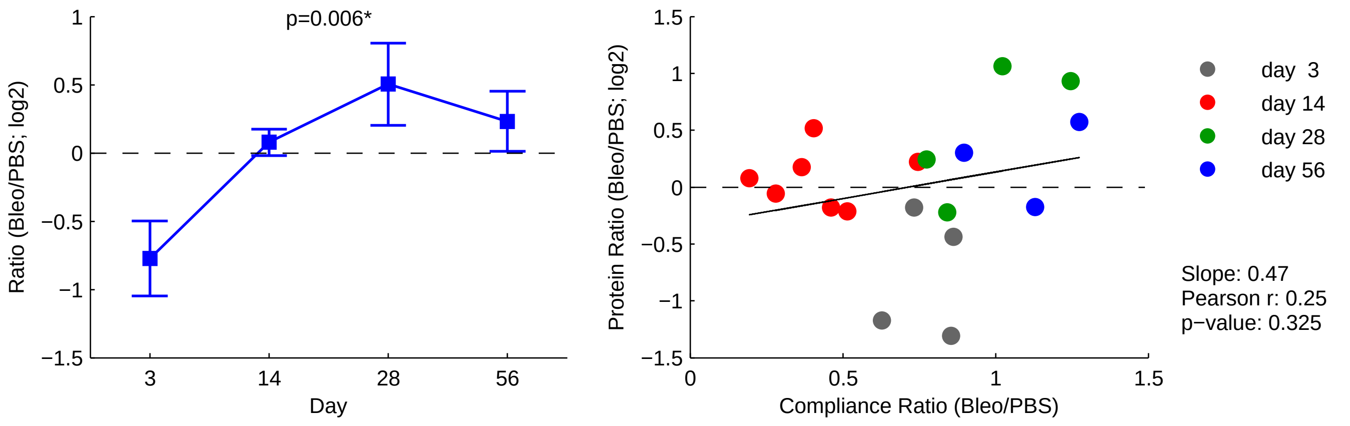

Q9CQC8 − Spg21 (id: 6716)

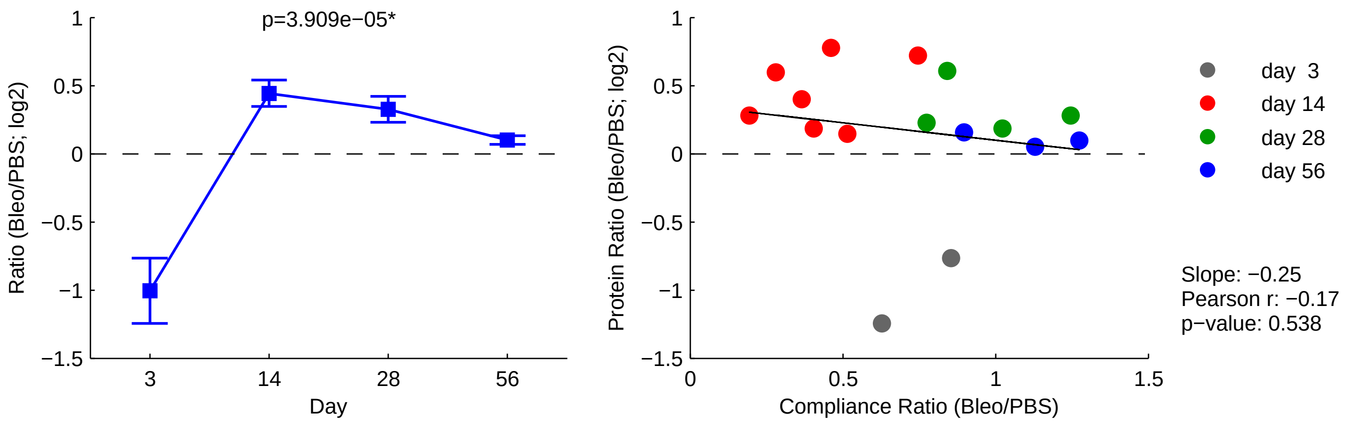

Q9CQC9 − Sar1b (id: 6717)

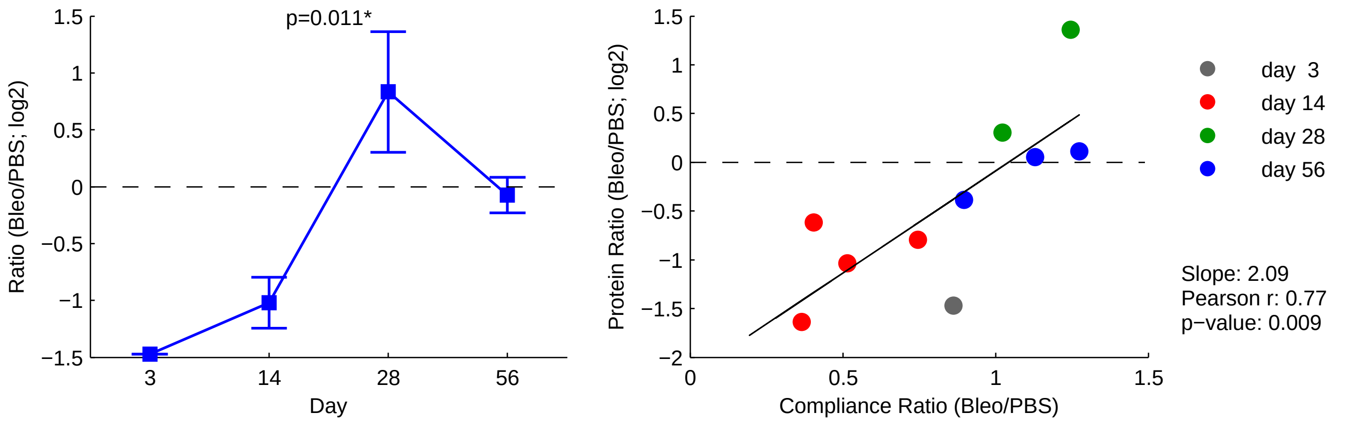

Q9CQD1 − Rab5a (id: 6718)

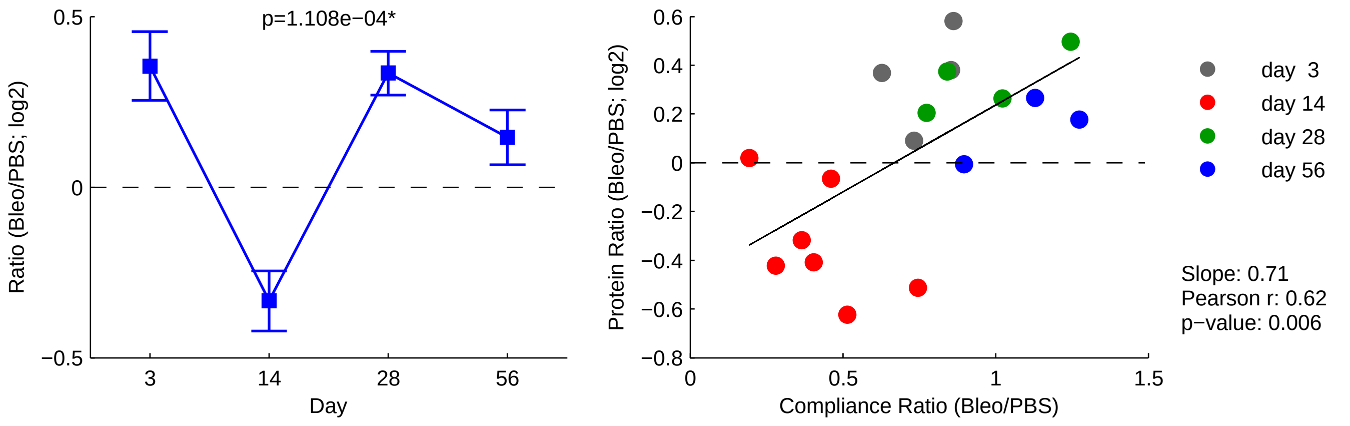

Q9CQD4 − Chmp1b2 (id: 6719)

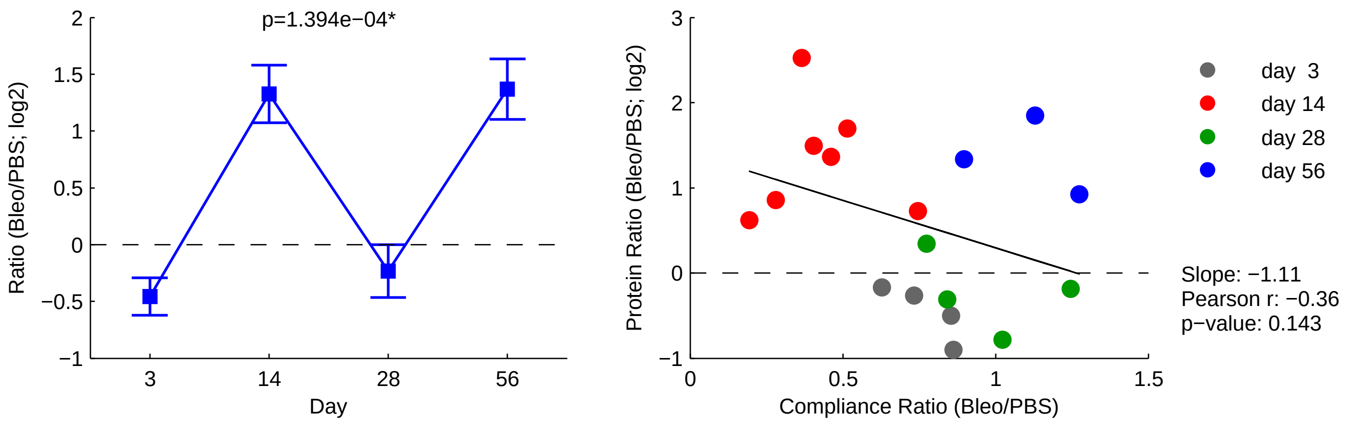

Q9CQE1 − Nipsnap3b (id: 6720)

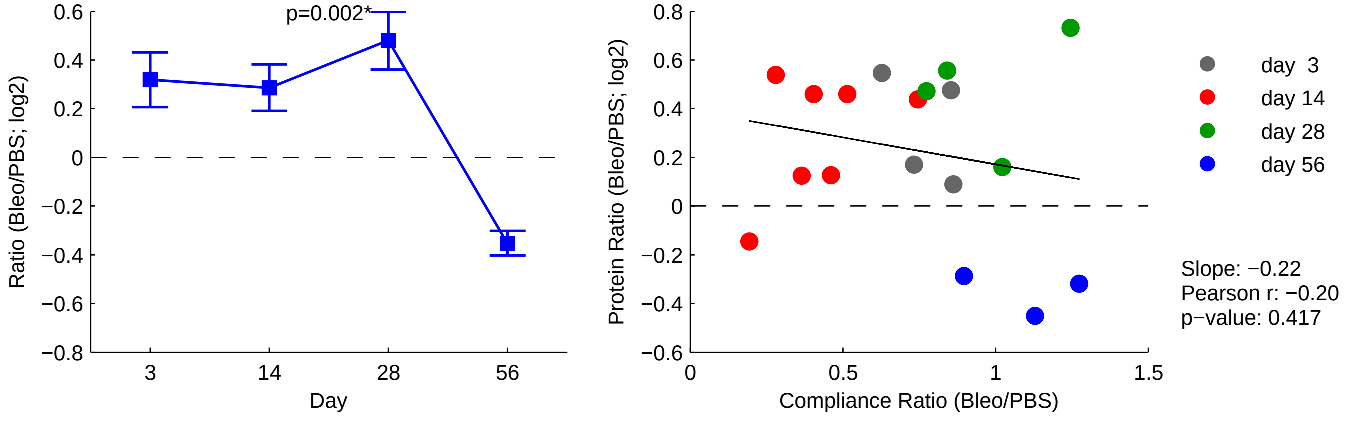

Q9CQE5 − Rgs10 (id: 6723)

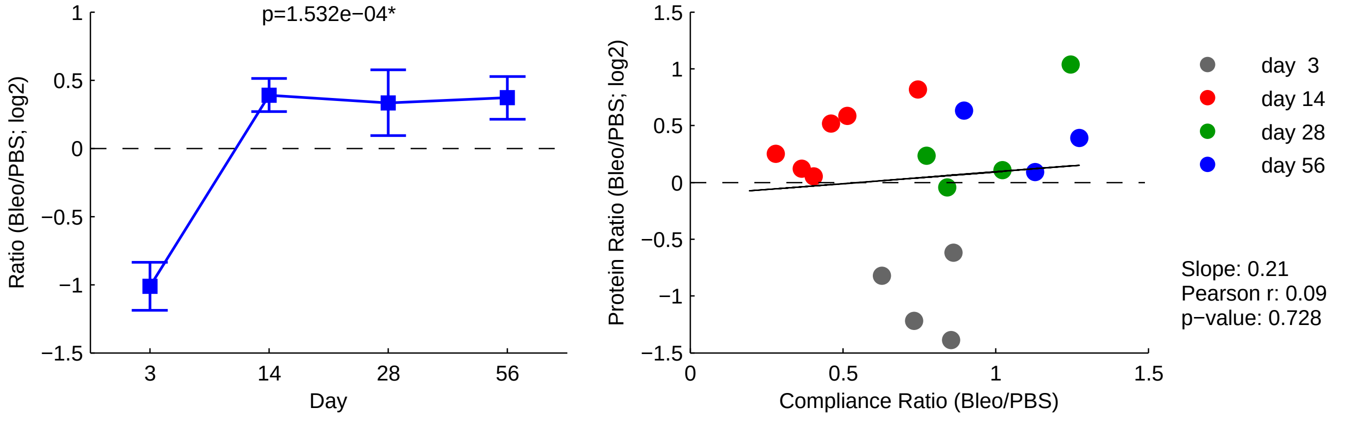

Q9CQE7 − Ergic3 (id: 6725)

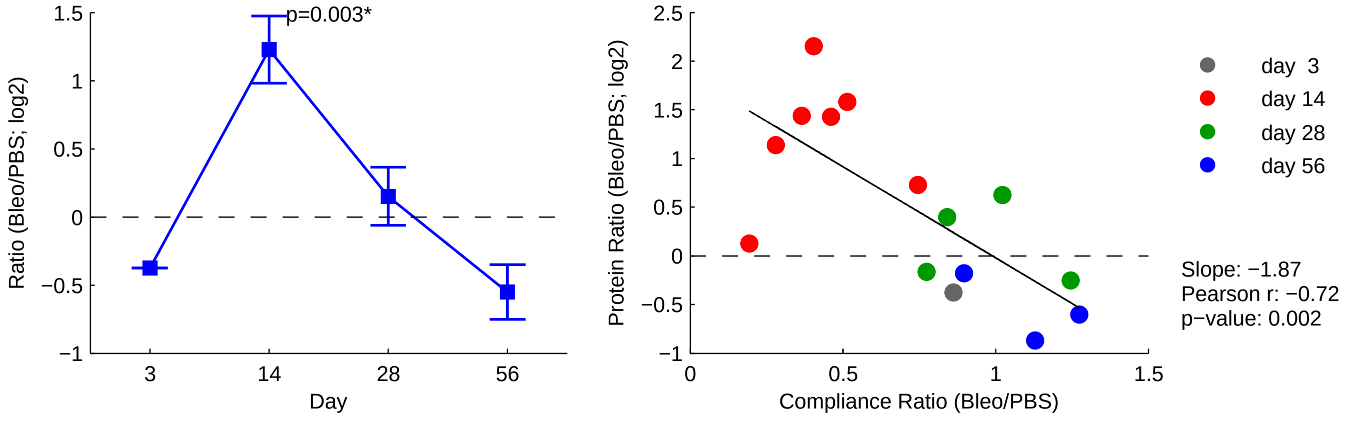

Q9CQE8 − Gm10704 (id: 6726)

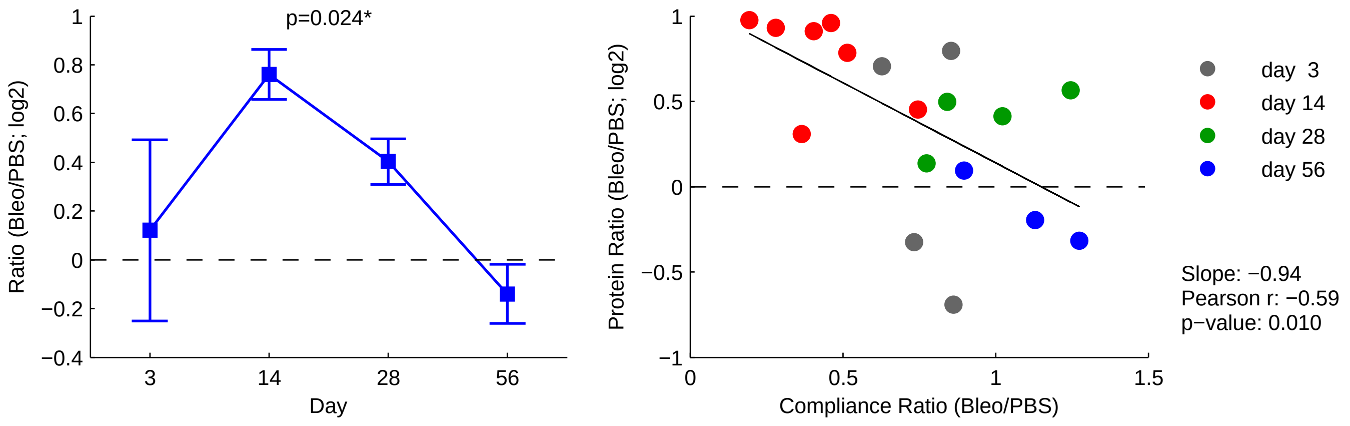

Q9CQF0 − Mrpl11 (id: 6727)

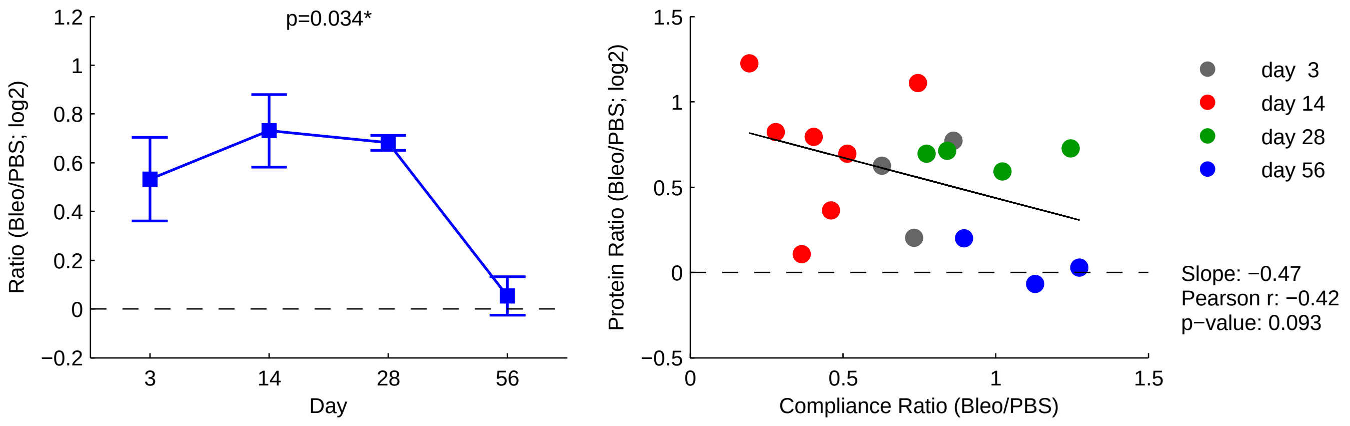

Q9CWM4 − Pfdn1 (id: 6730)

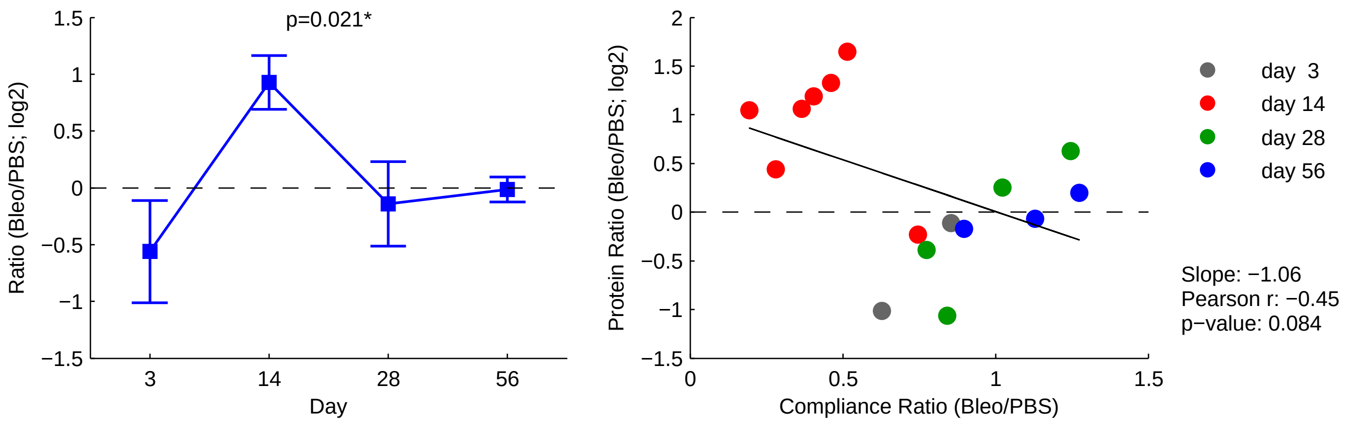

Q9CQF9 − Pcyox1 (id: 6732)

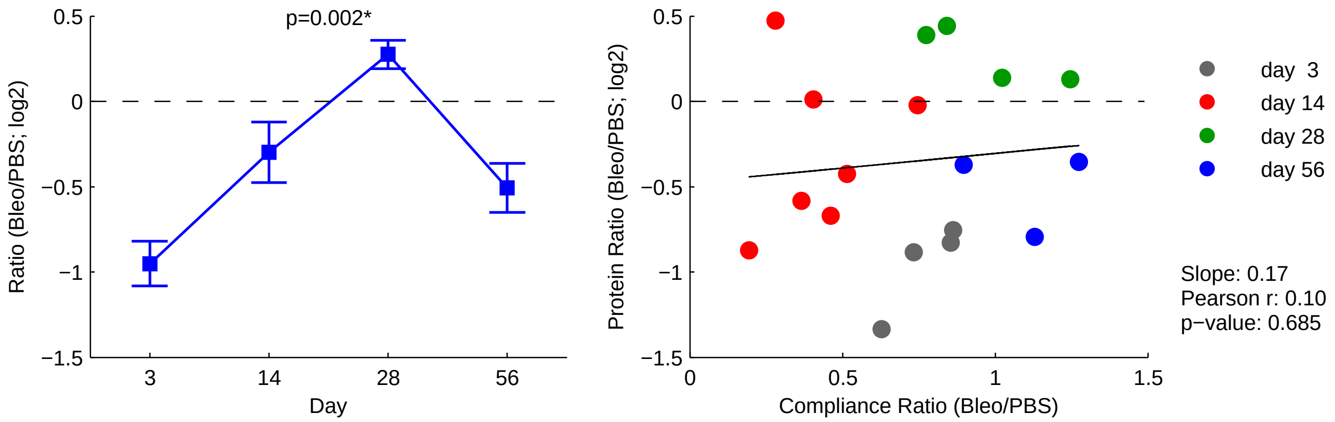

Q9CQG9 − Tmem100 (id: 6733)

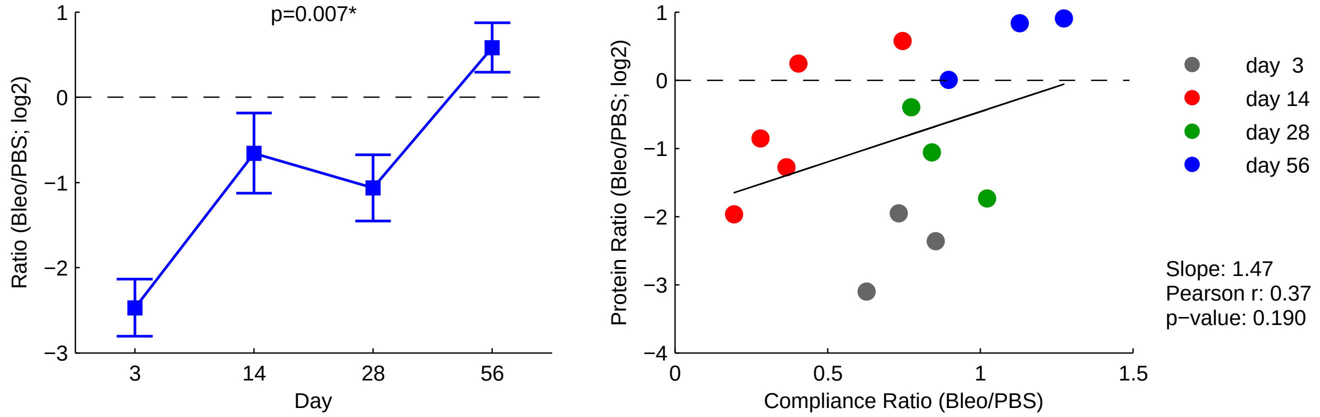

Q9CQH3 − Ndufb5 (id: 6734)

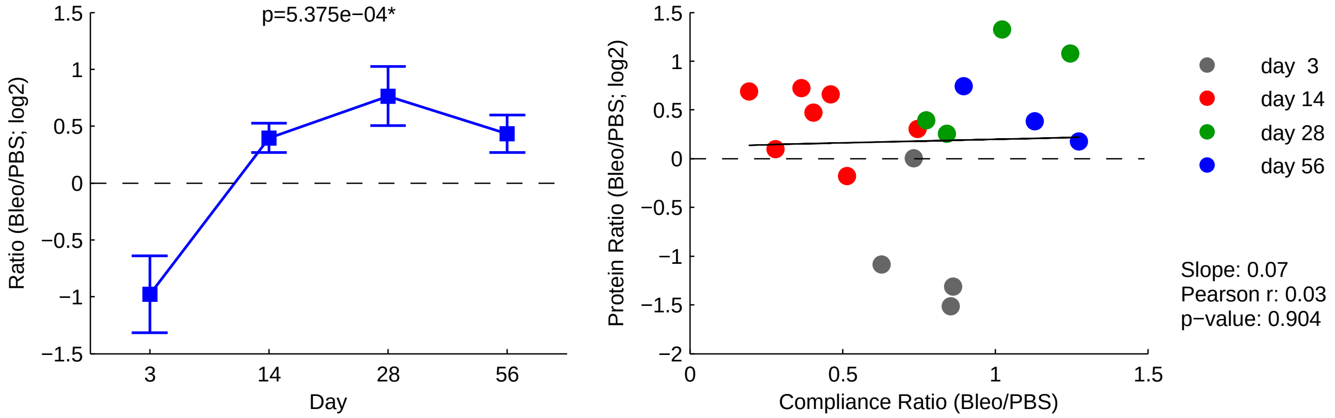

Q9CQH7 − Btf3l4 (id: 6735)

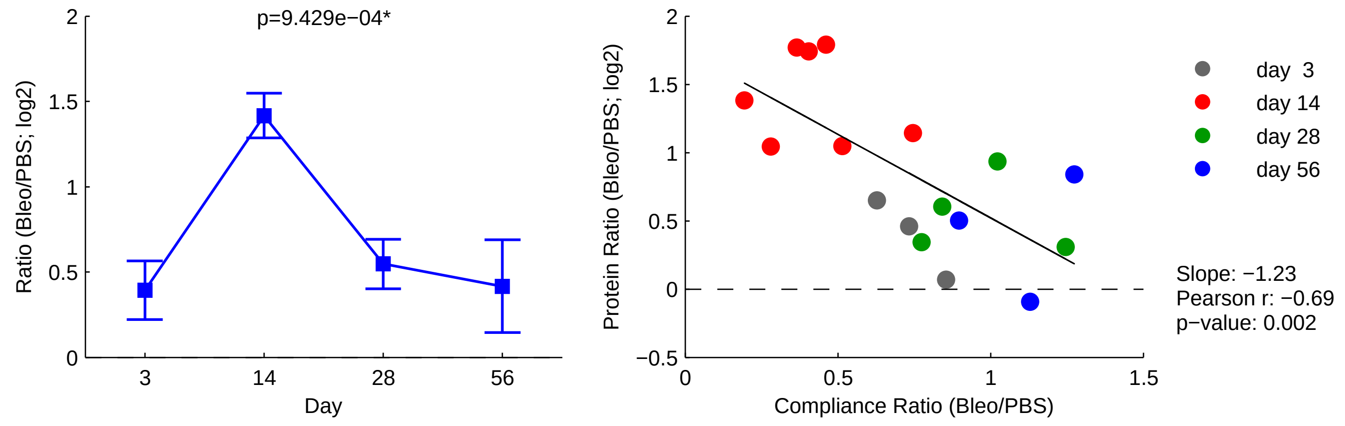

Q9CQI1 − Sftpa1 (id: 6736)

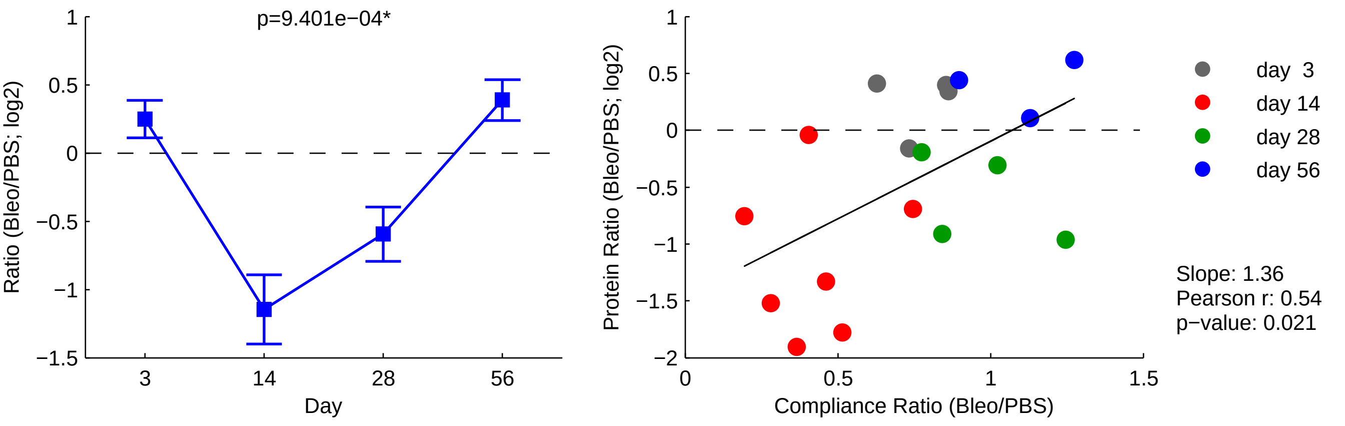

Q9CQI6 − Cotl1 (id: 6738)

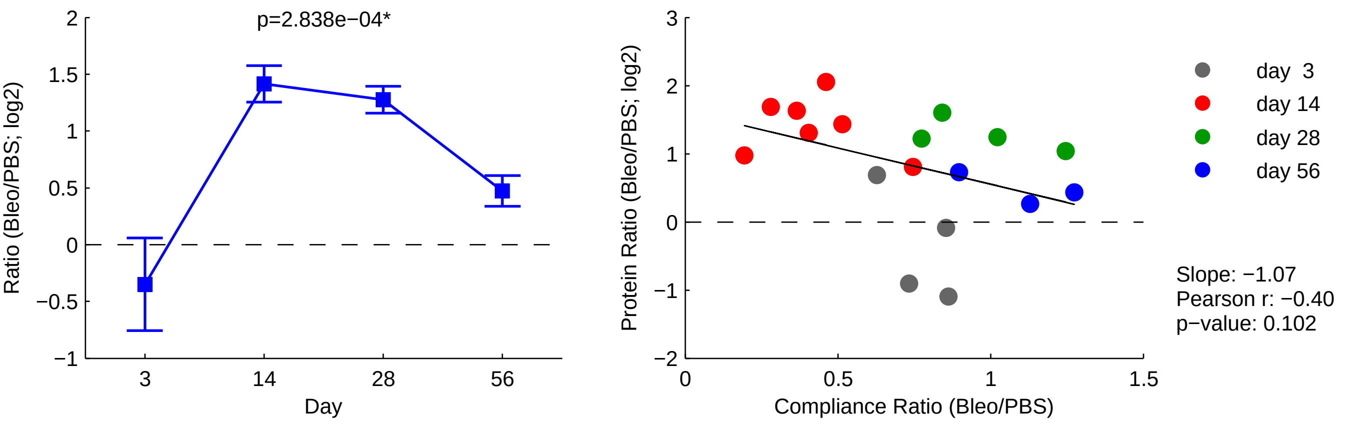

Q9CQI7 − Snrpb2 (id: 6739)

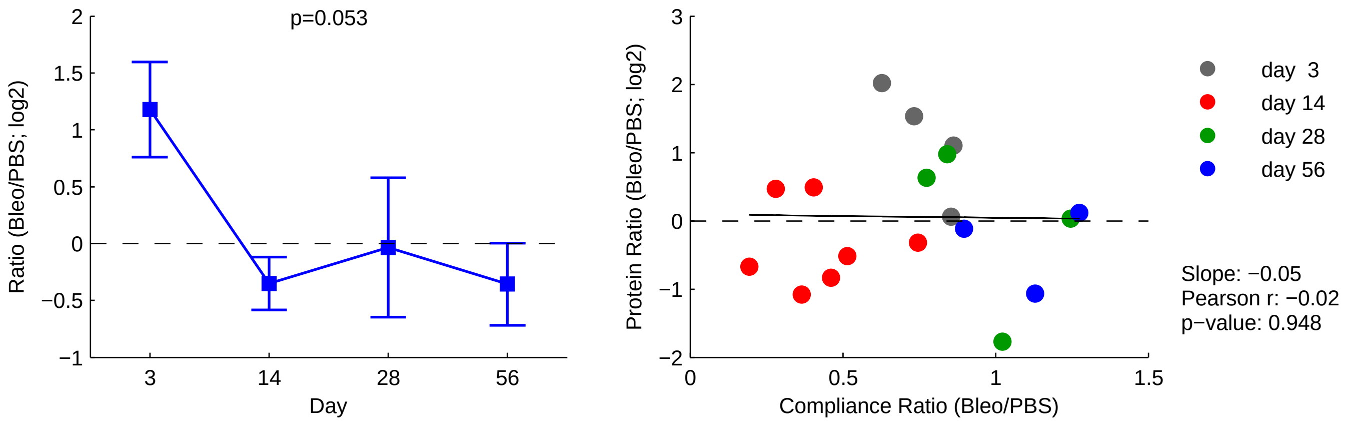

Q9CQJ1 − Higd2a (id: 6742)

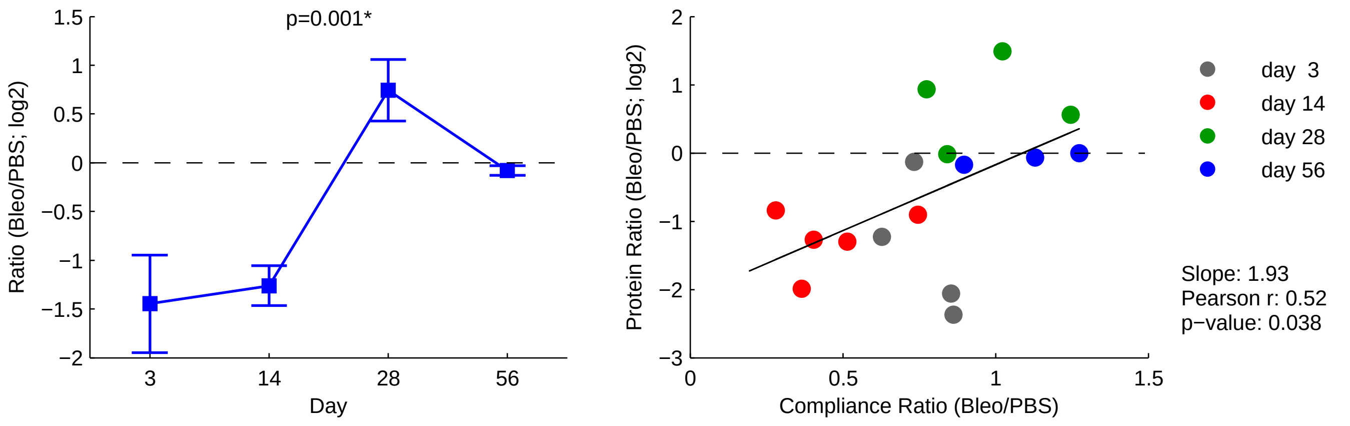

Q9CQJ6 − Denr (id: 6744)

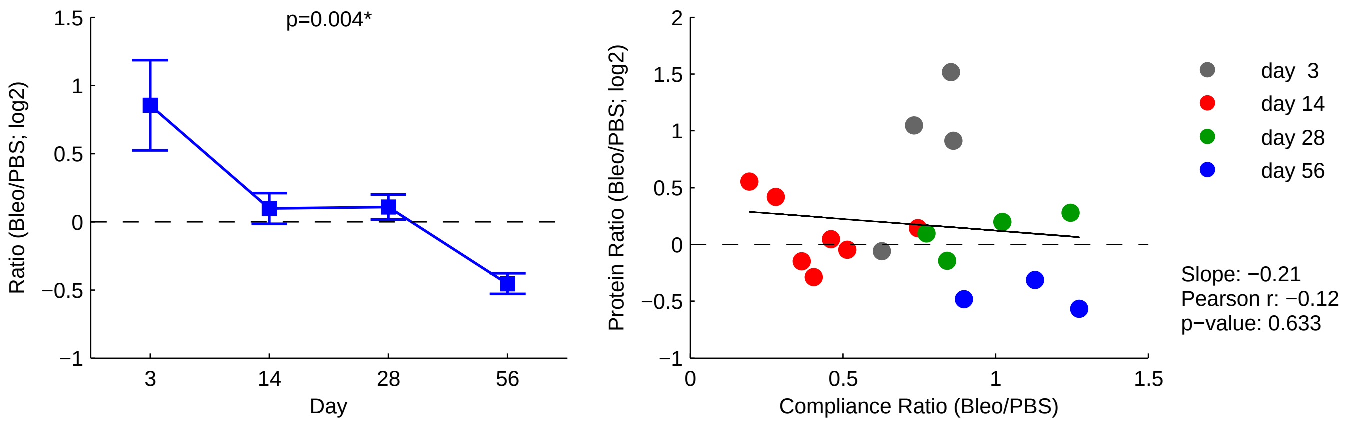

Q9CQJ8 − Ndufb9 (id: 6745)

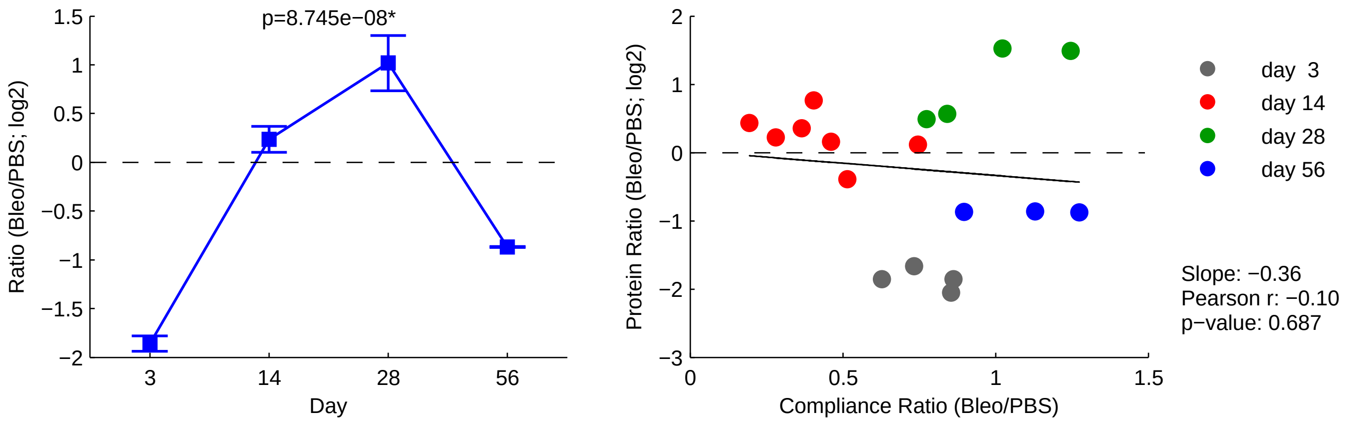

Q9CQL5 − Mrpl18 (id: 6748)

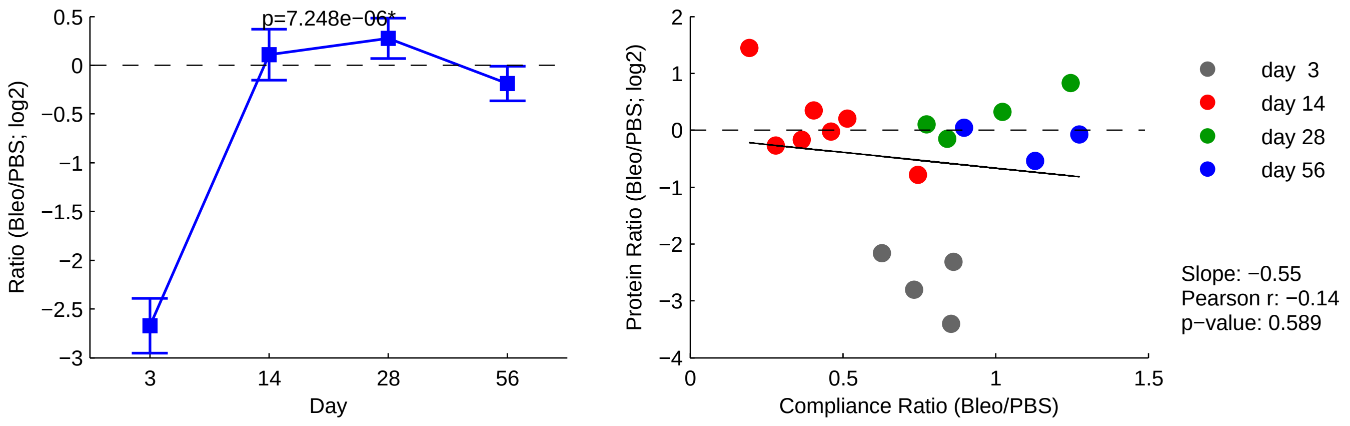

Q9CQN1 − Trap1 (id: 6751)

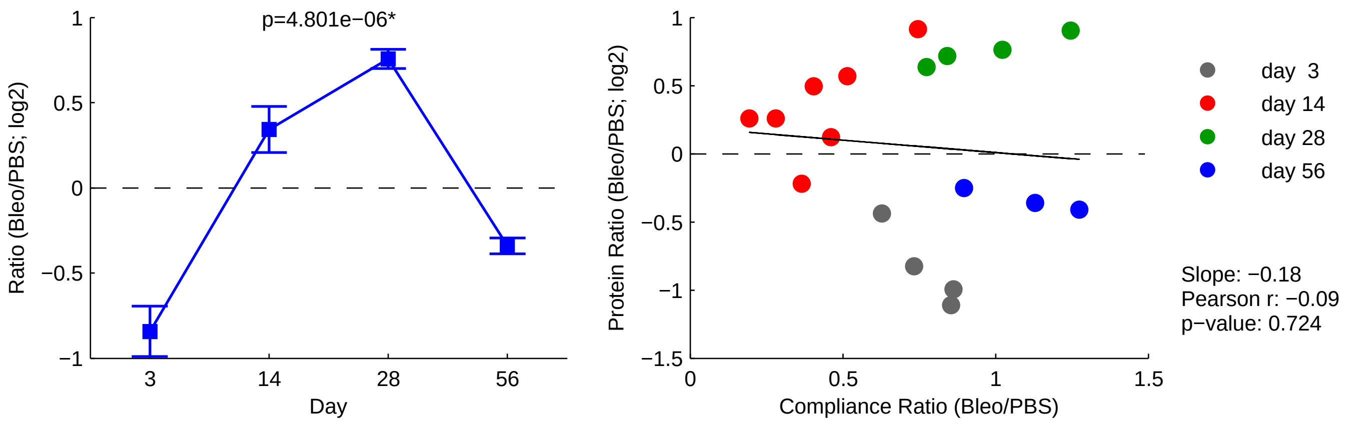

Q9CQN7 − Mrpl41 (id: 6754)

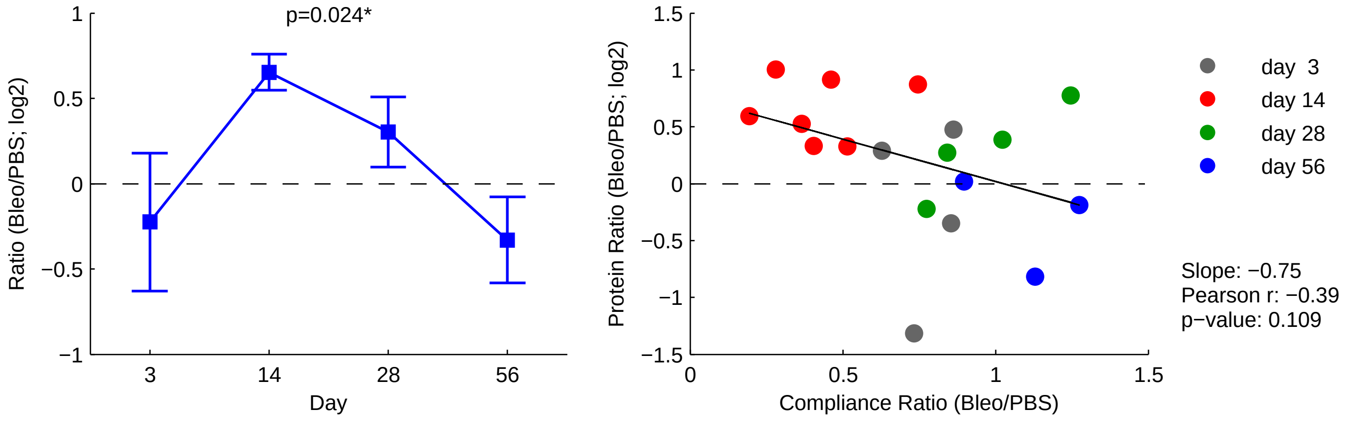

Q9CQP0 − Mrpl33 (id: 6755)

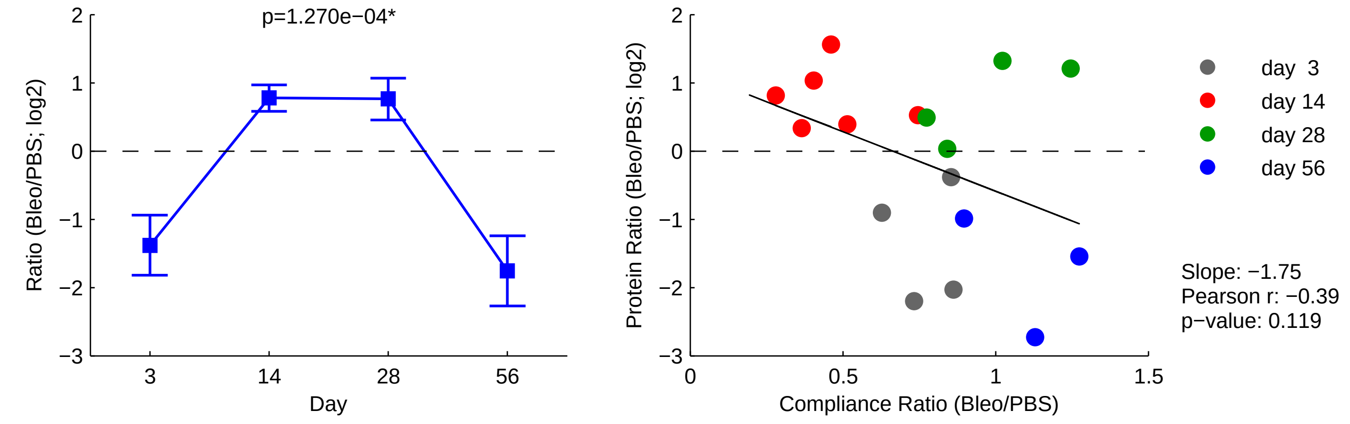

Q9CQP2 − Trappc2 (id: 6756)

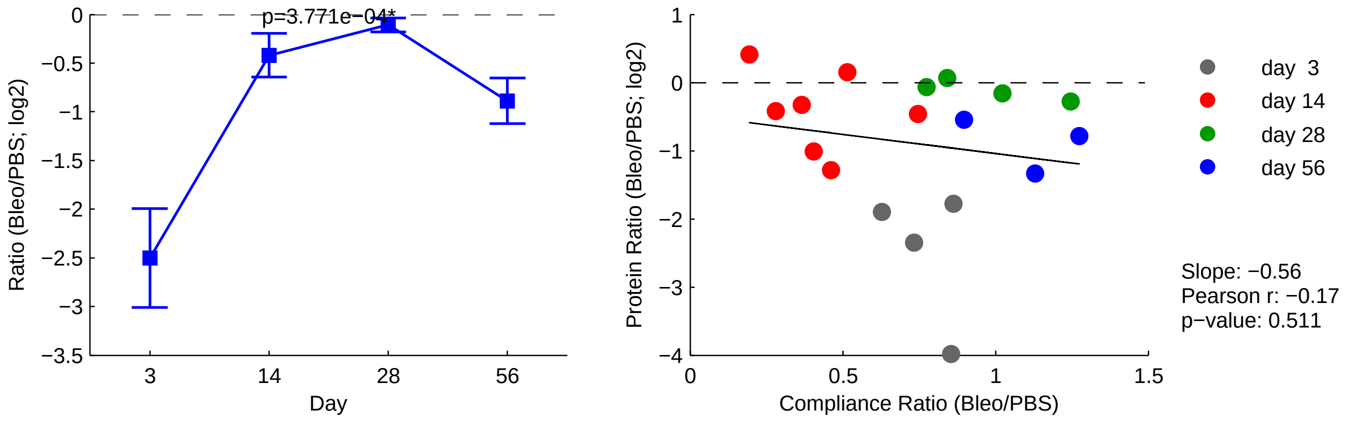

Q9CQQ7 − Atp5f1 (id: 6759)

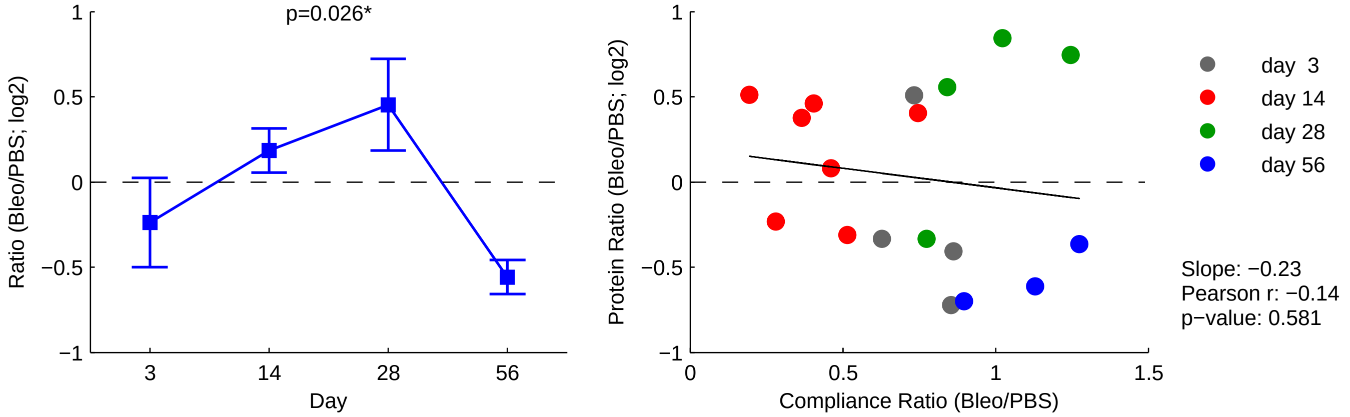

Q9CQR2 − Rps21 (id: 6761)

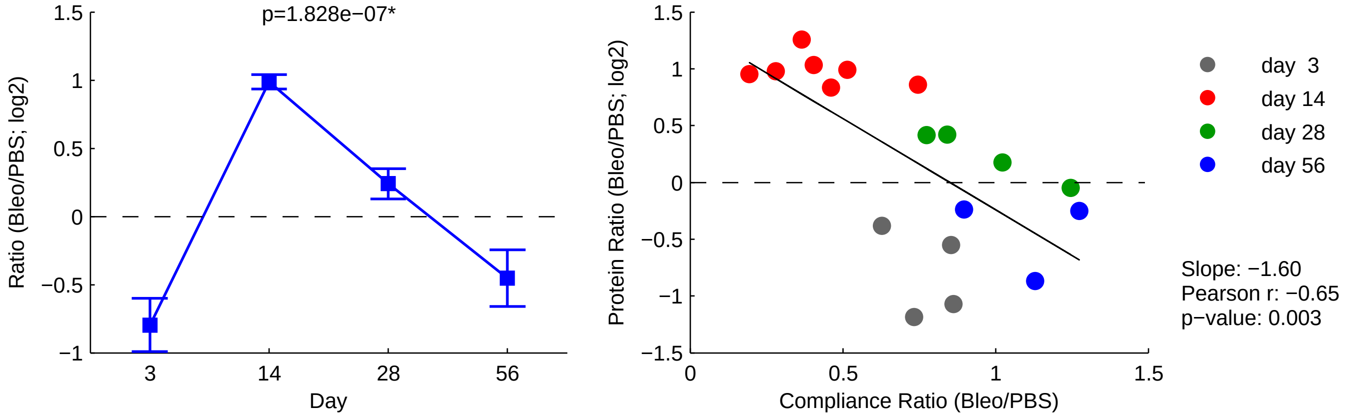

Q9CQR4 − Acot13 (id: 6762)

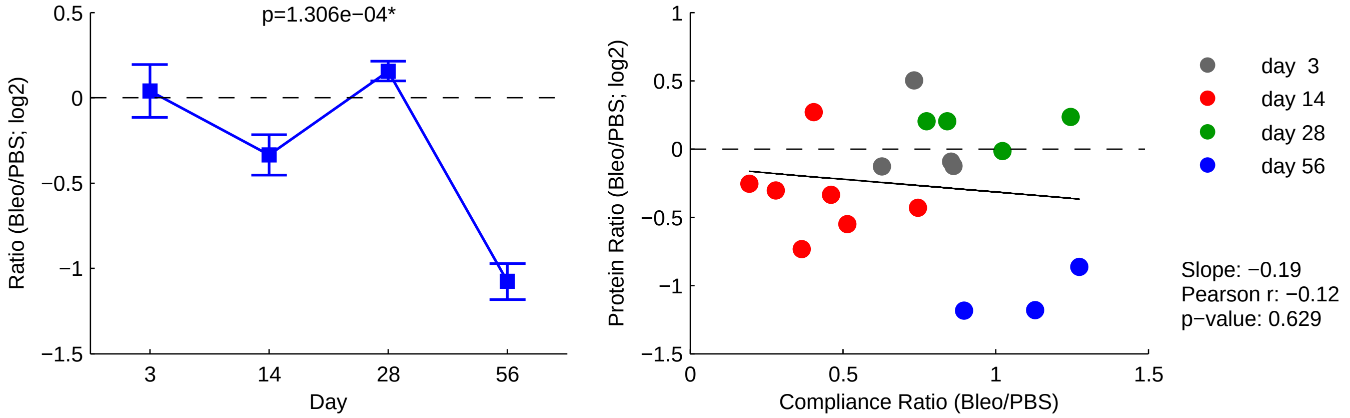

Q9CQR6 − Ppp6c (id: 6763)

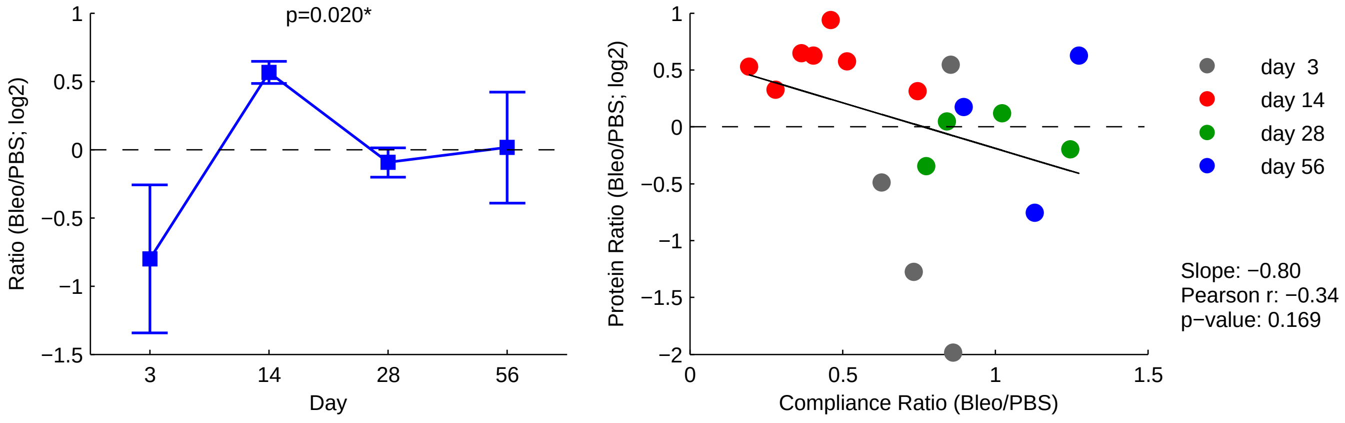

Q9CQS4 − Slc25a46 (id: 6765)

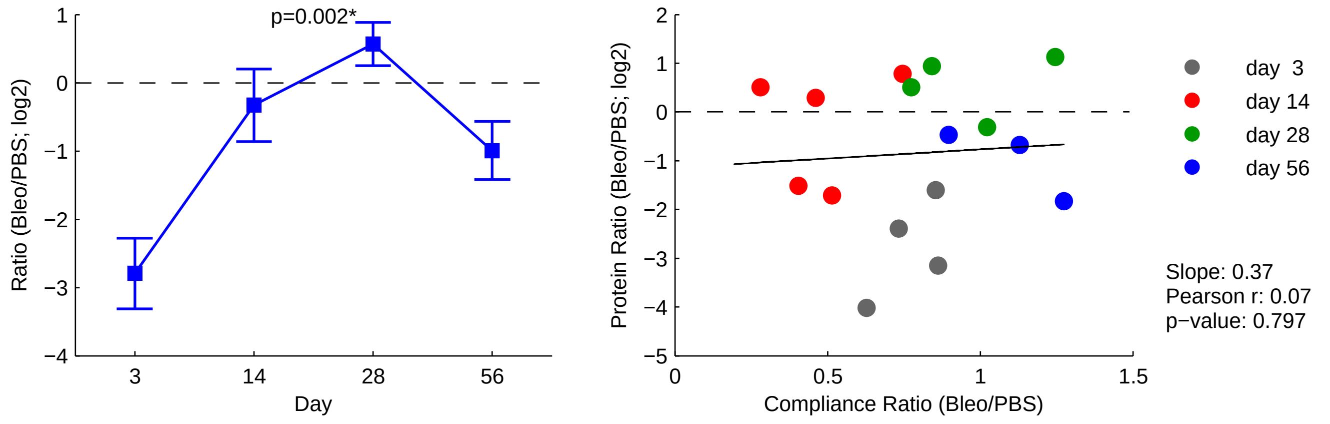

Q9CQS8 − Sec61b (id: 6766)

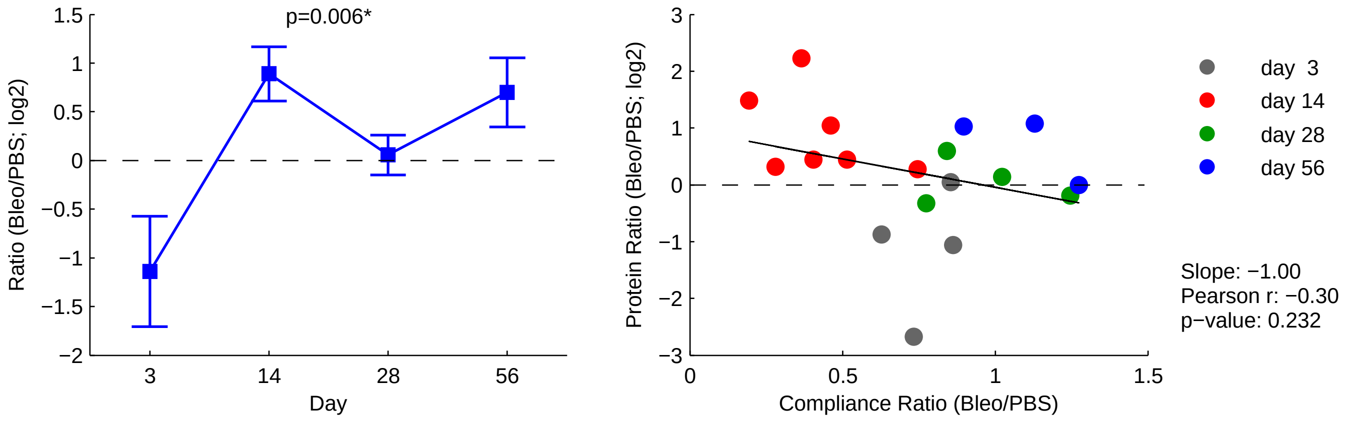

Q9CQT1 − Mri1 (id: 6768)

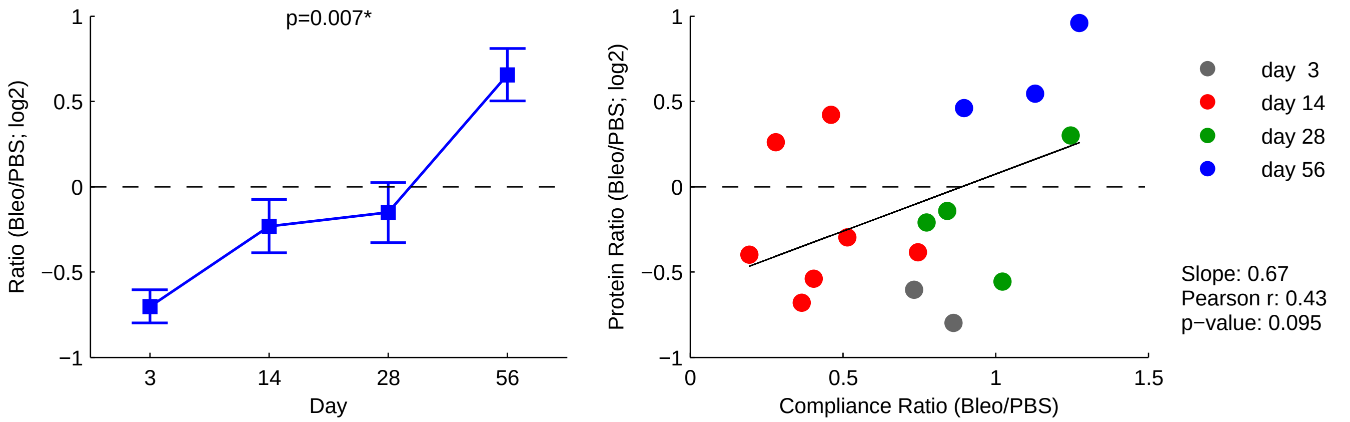

Q9CQU0 − Txndc12 (id: 6771)

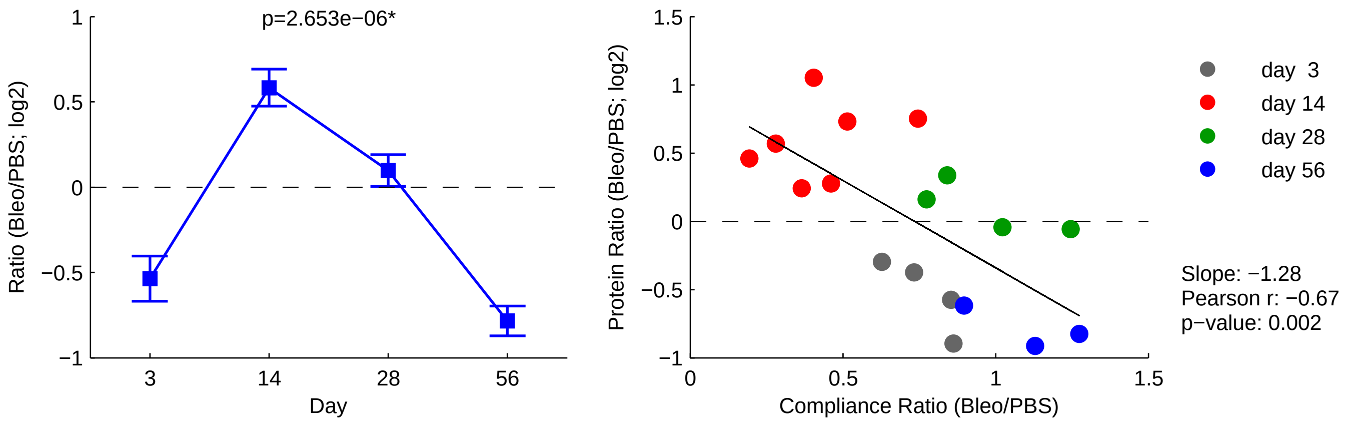

Q9CQV5 − Mrps24 (id: 6776)

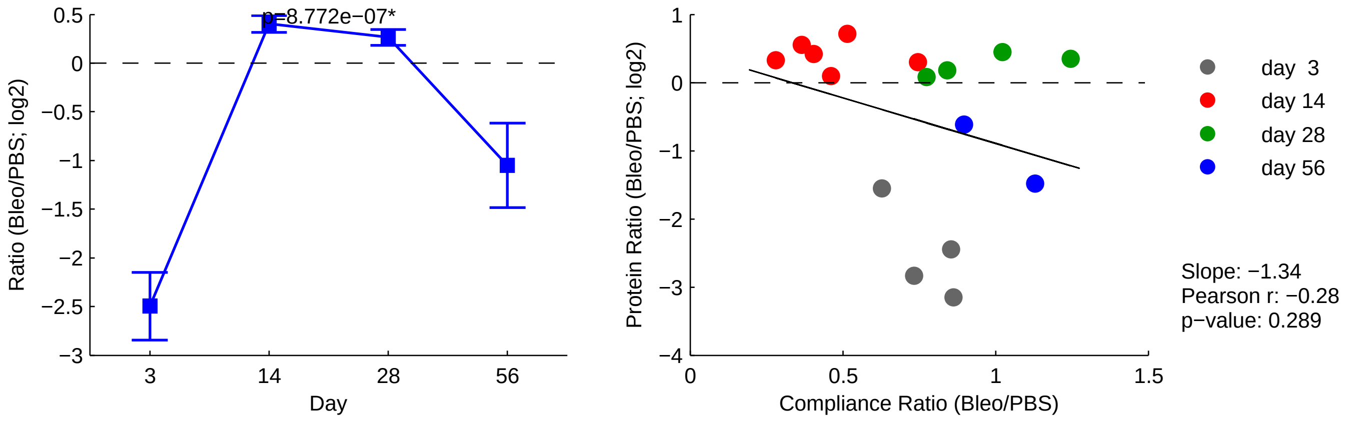

Q9CQV6 − Map1lc3b (id: 6777)

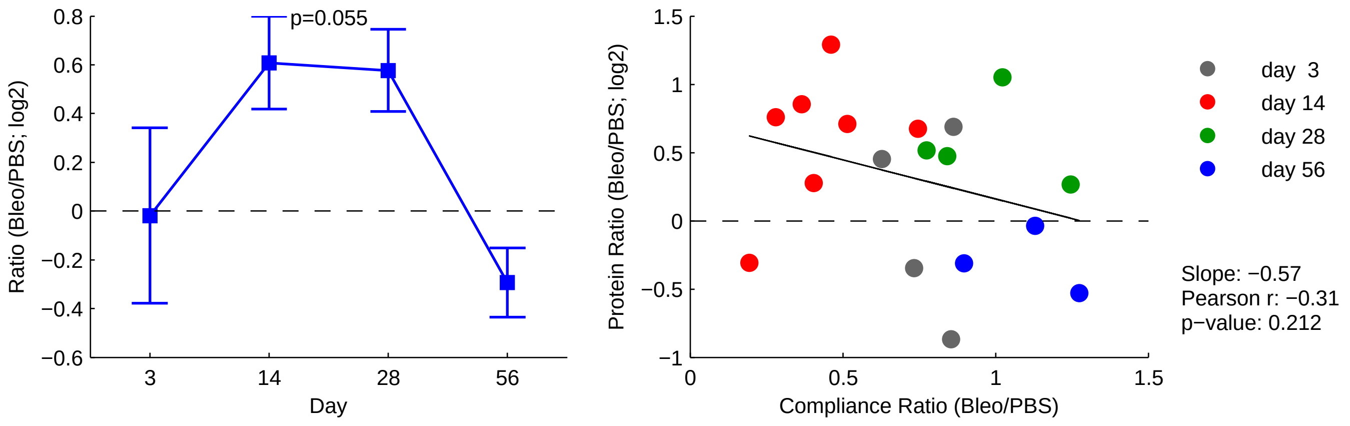

Q9CQV8 − Ywhab (id: 6779)

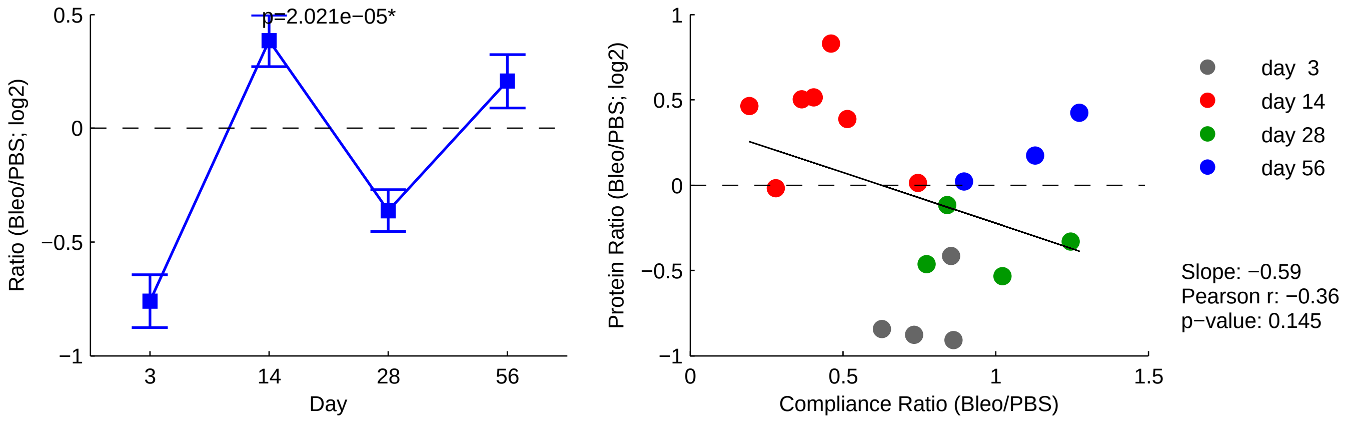

Q9CQW1 − Ykt6 (id: 6780)

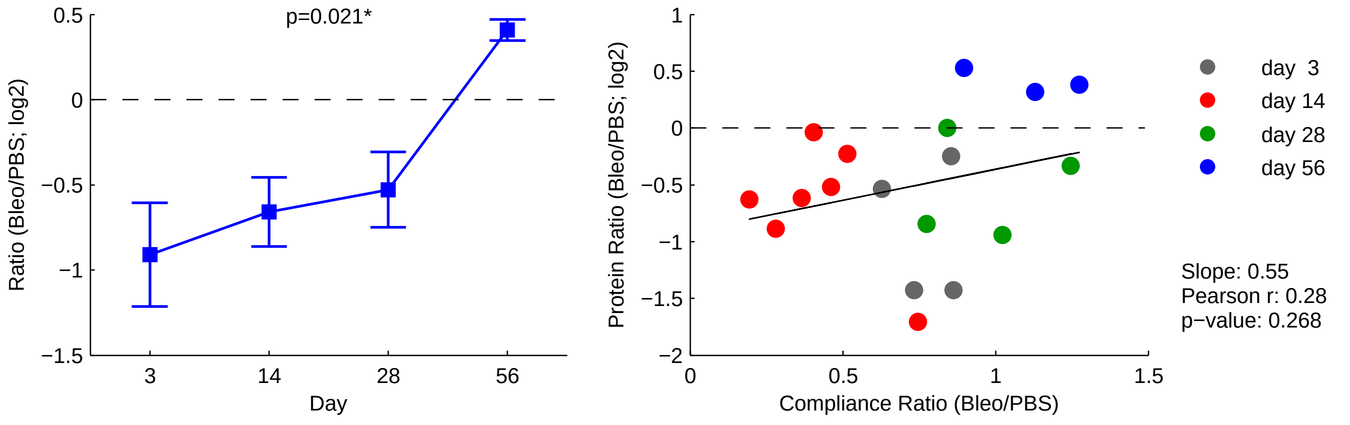

Q9CQW2 − Arl8b (id: 6781)

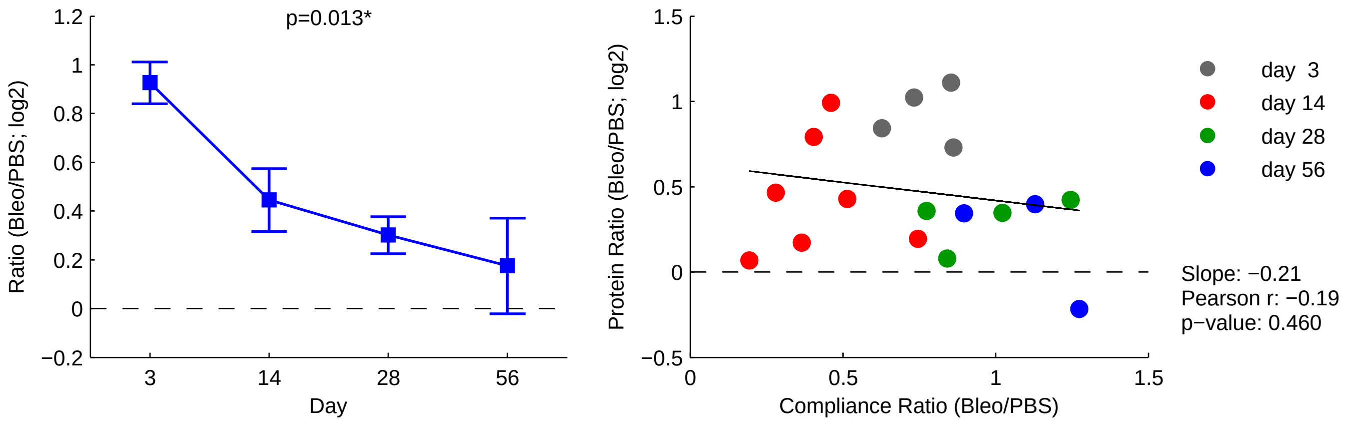

Q9CQW9 − Ifitm3 (id: 6782)

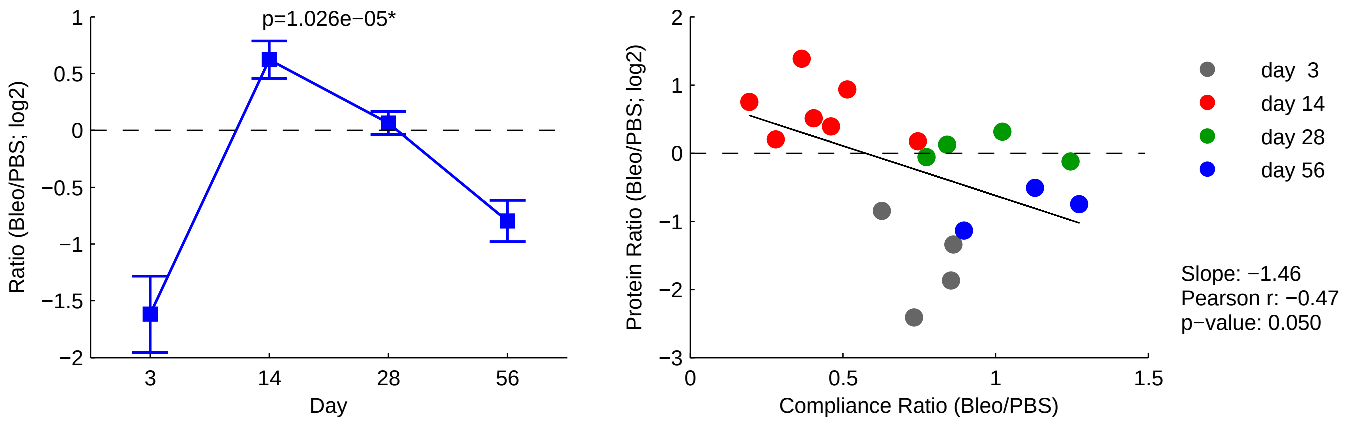

Q9CQX2 − Cyb5b (id: 6783)

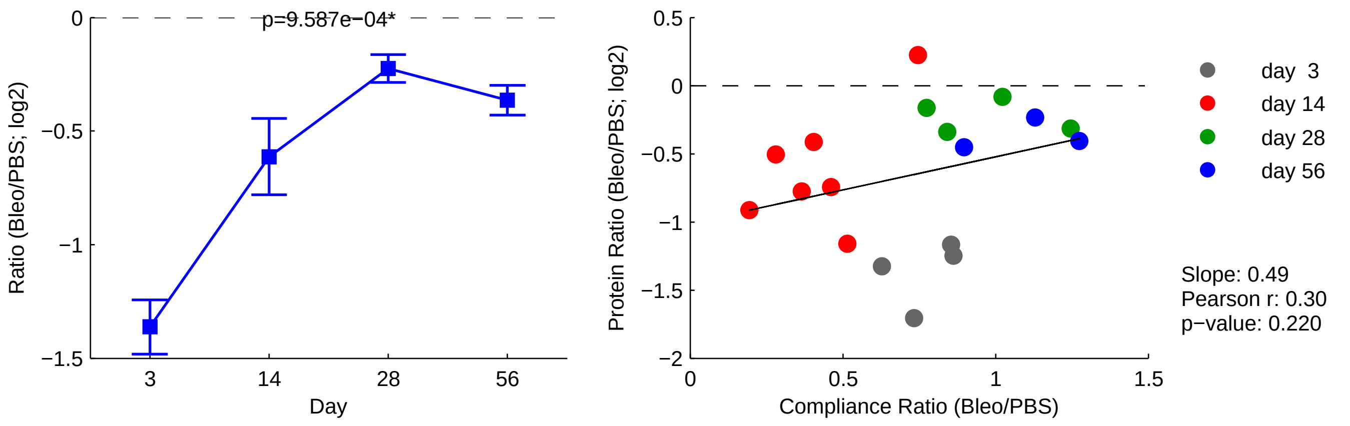

Q9CQX8 − Mrps36 (id: 6785)

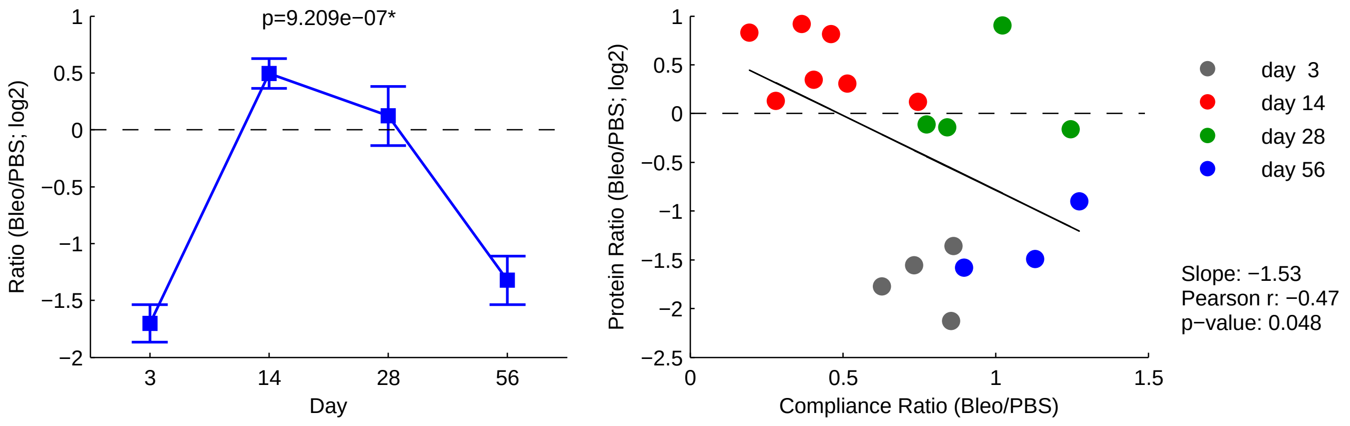

Q9CQZ1 − Hsbp1 (id: 6786)

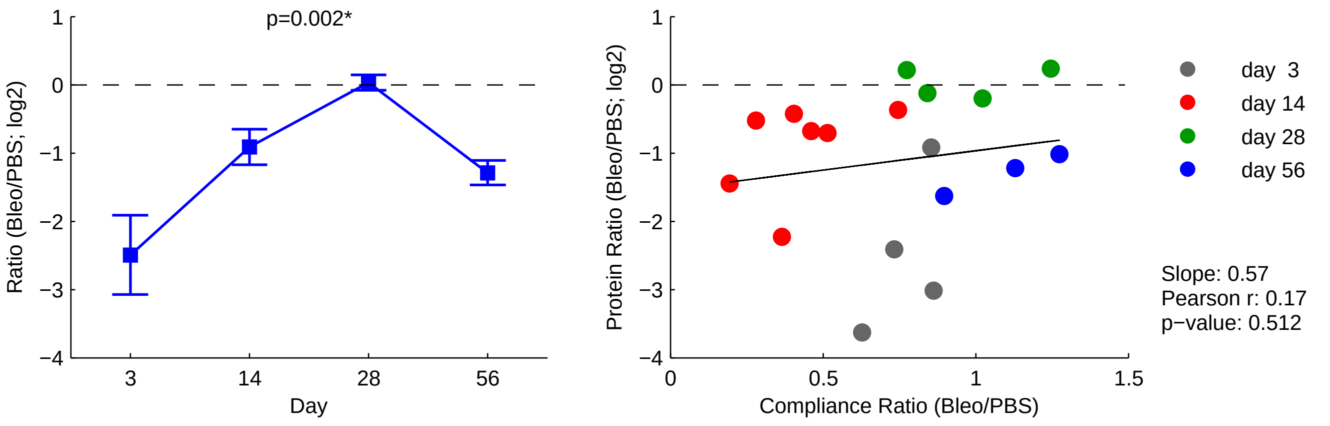

Q9CQZ5 − Ndufa6 (id: 6787)

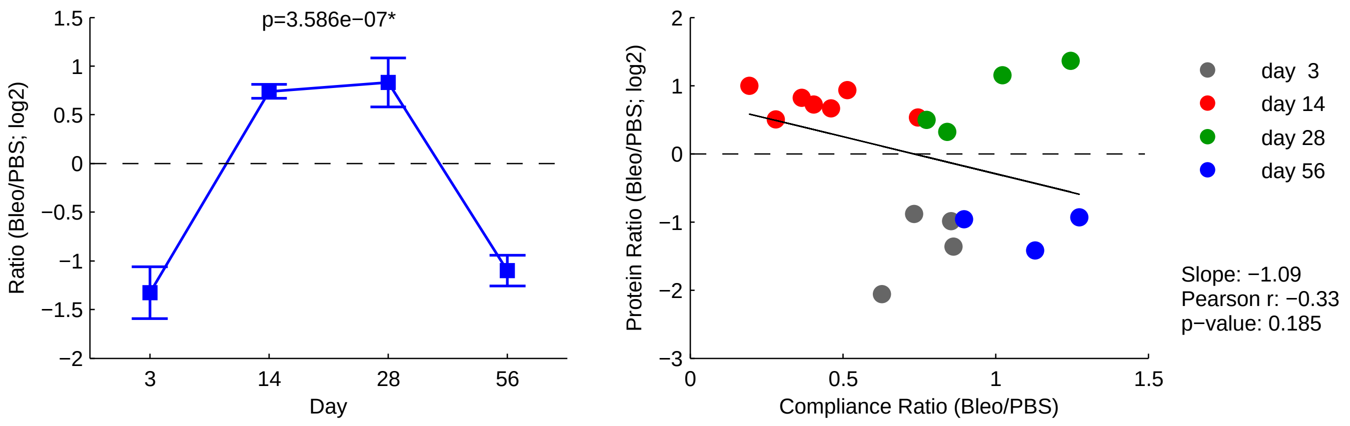

Q9CR00 − Psmd9 (id: 6789)

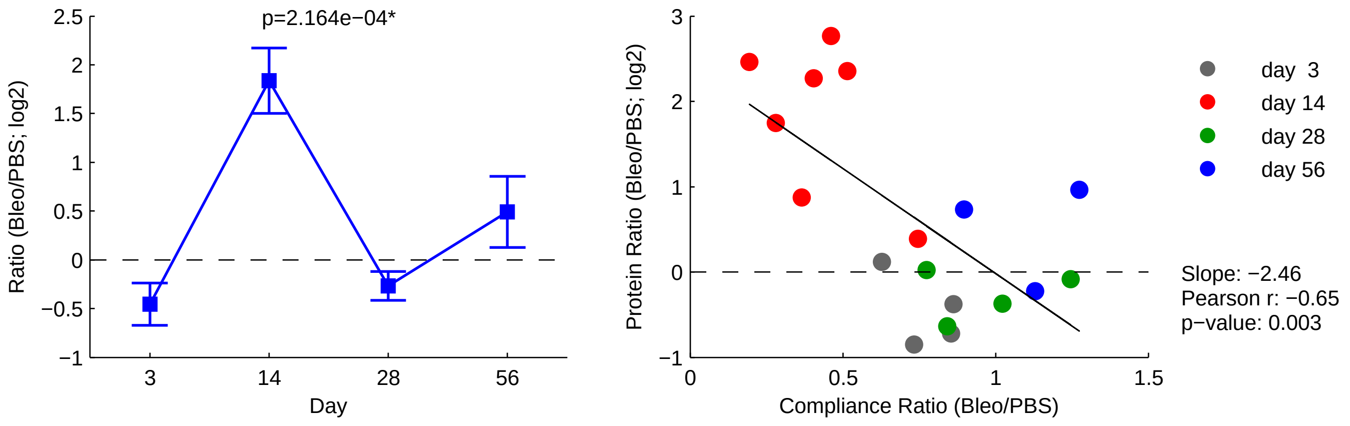

Q9CR09 − Ufc1 (id: 6791)

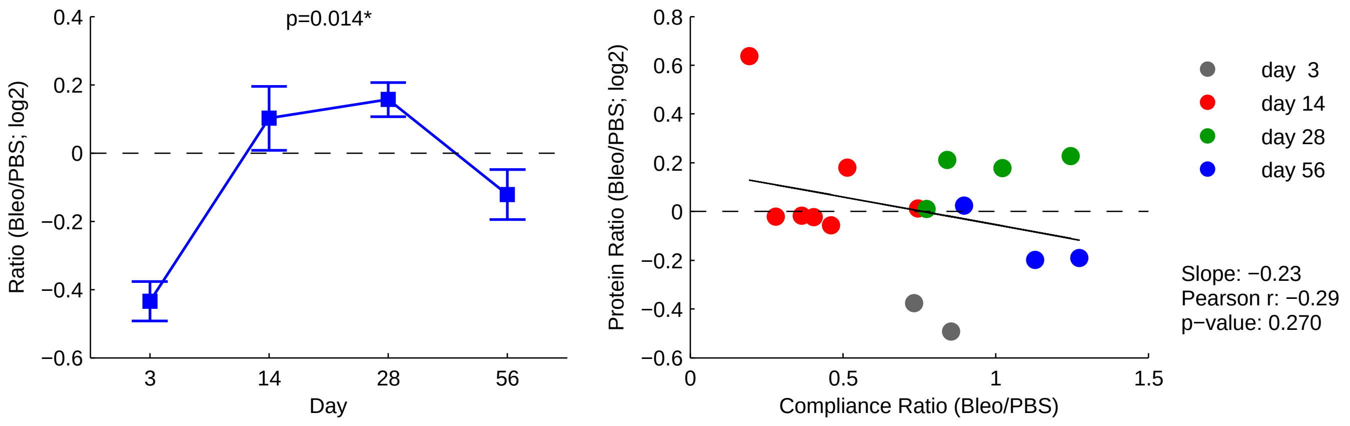

Q9CR11 − Yeats4 (id: 6793)

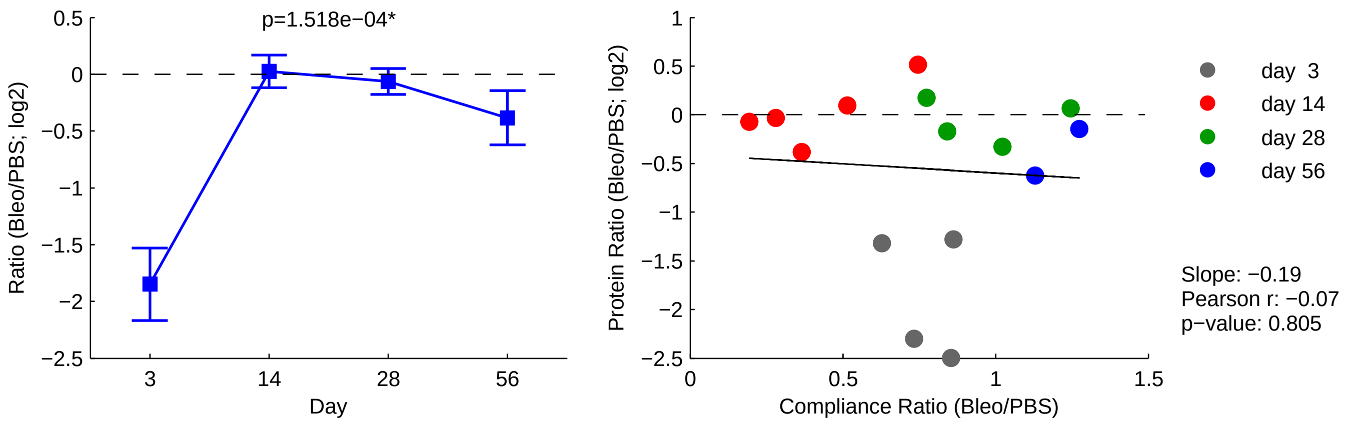

Q9CR24 − Nudt8 (id: 6795)

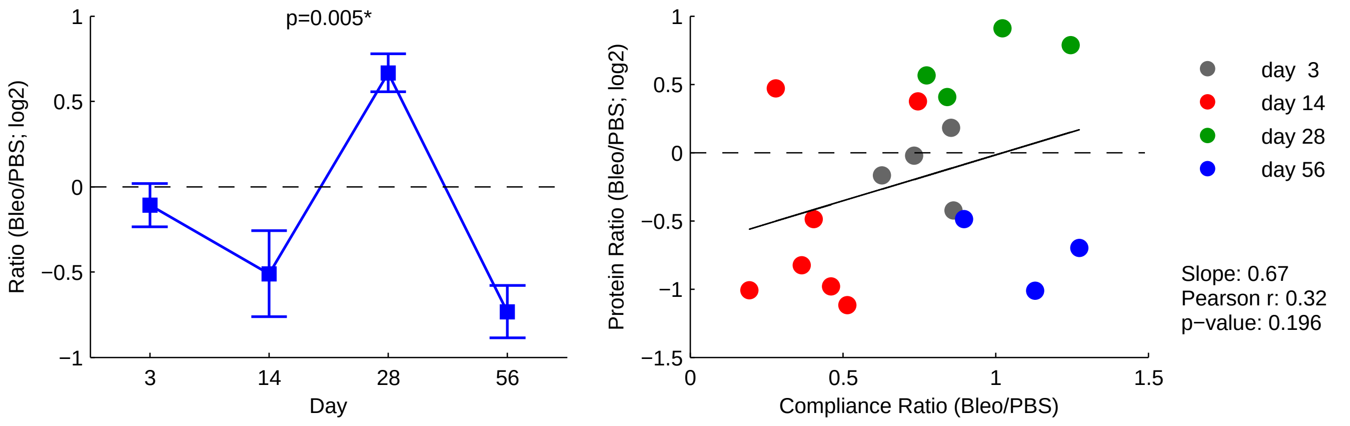

Q9CR39 − Wdr45b (id: 6799)

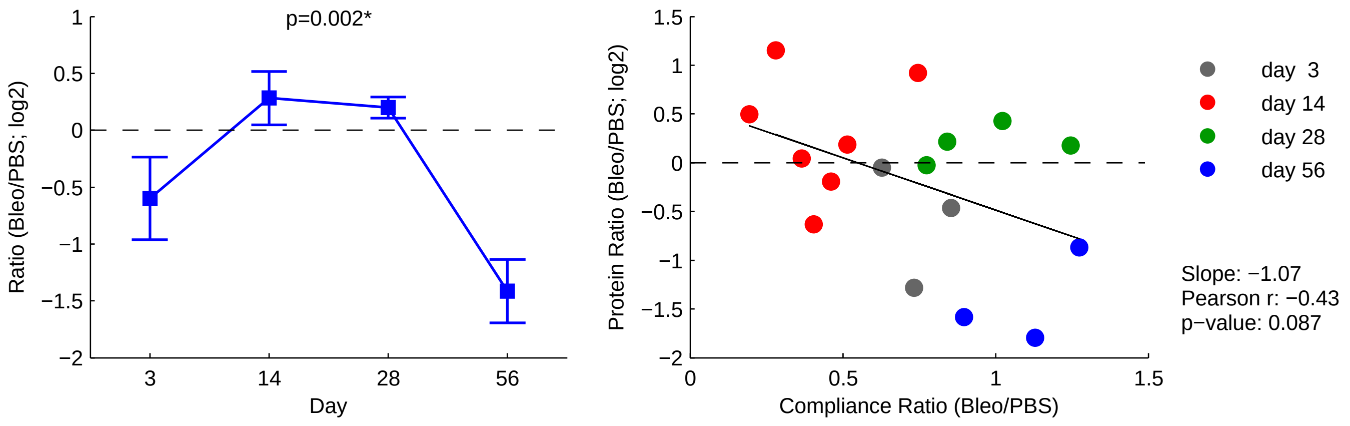

Q9CR41−2 − Hypk (id: 6800)

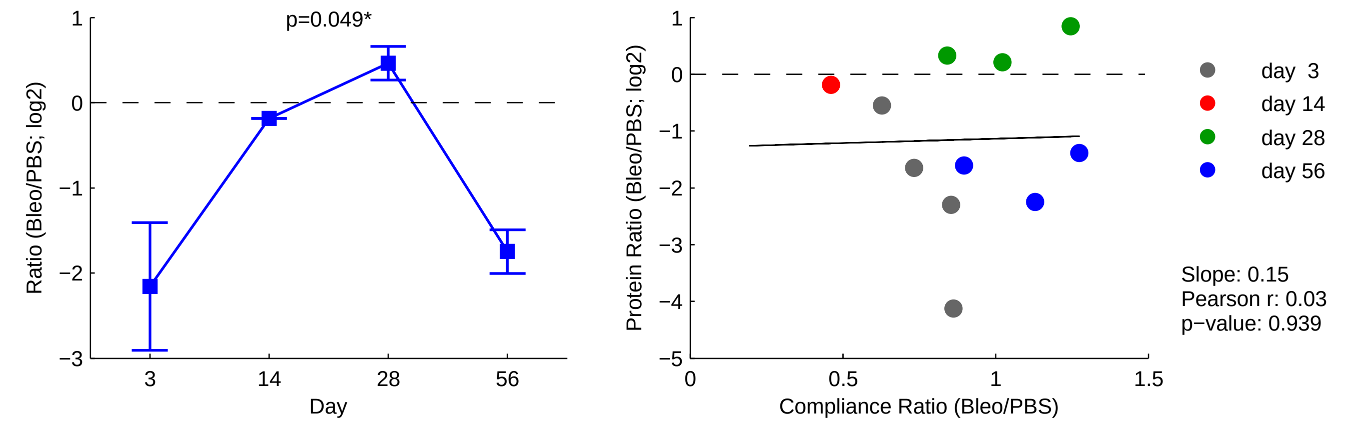

Q9CR51 − Atp6v1g1 (id: 6802)

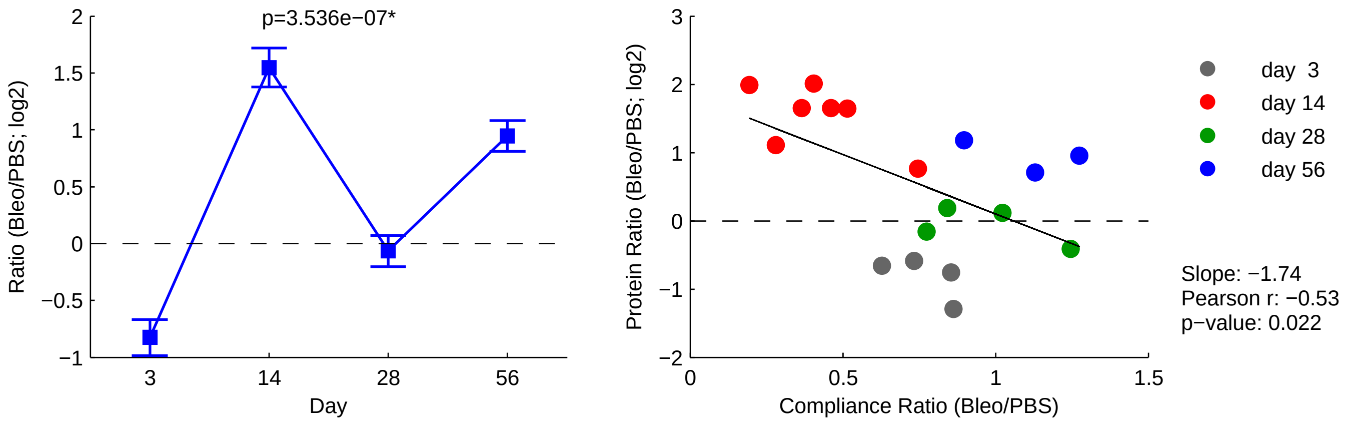

Q9CR57 − Rpl14 (id: 6804)

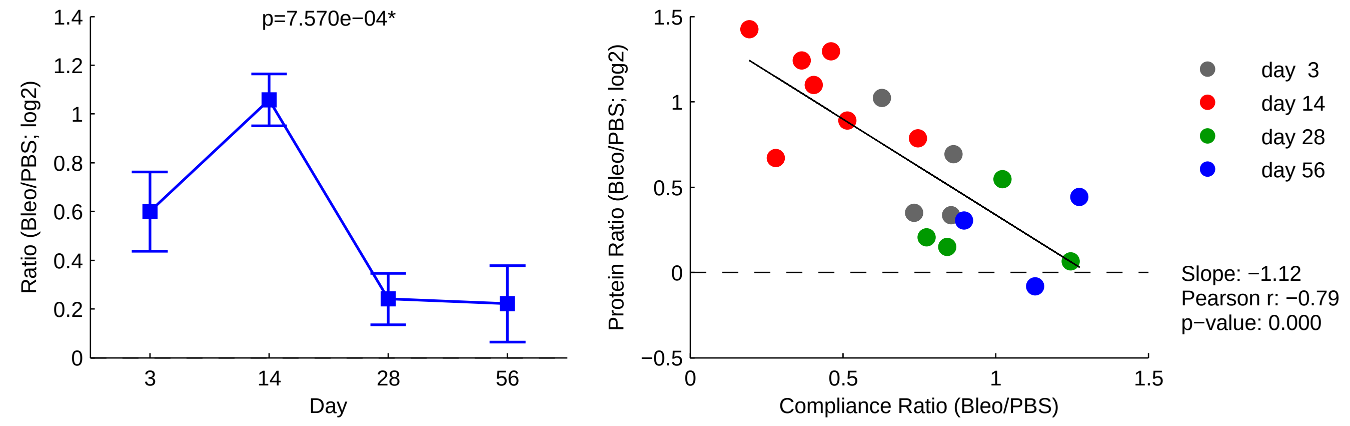

Q9CR61 − Ndufb7 (id: 6806)

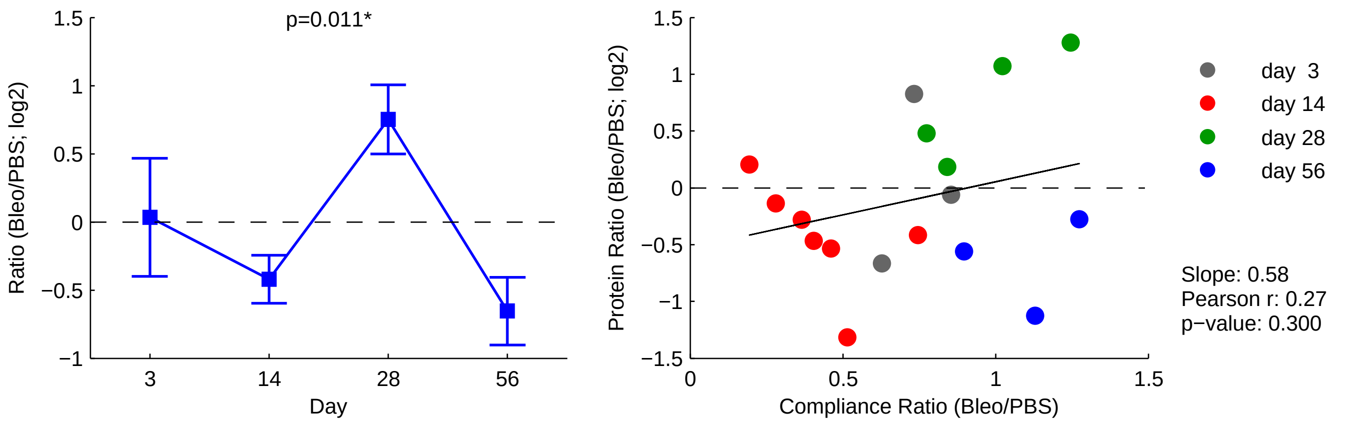

Q9CR62 − Slc25a11 (id: 6807)

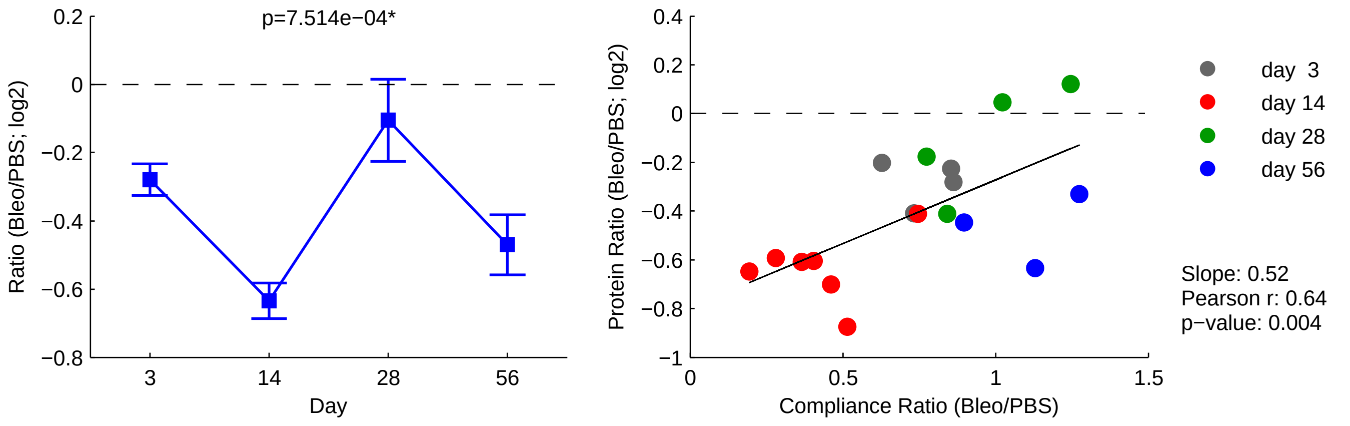

Q9CR68 − Uqcrfs1 (id: 6809)

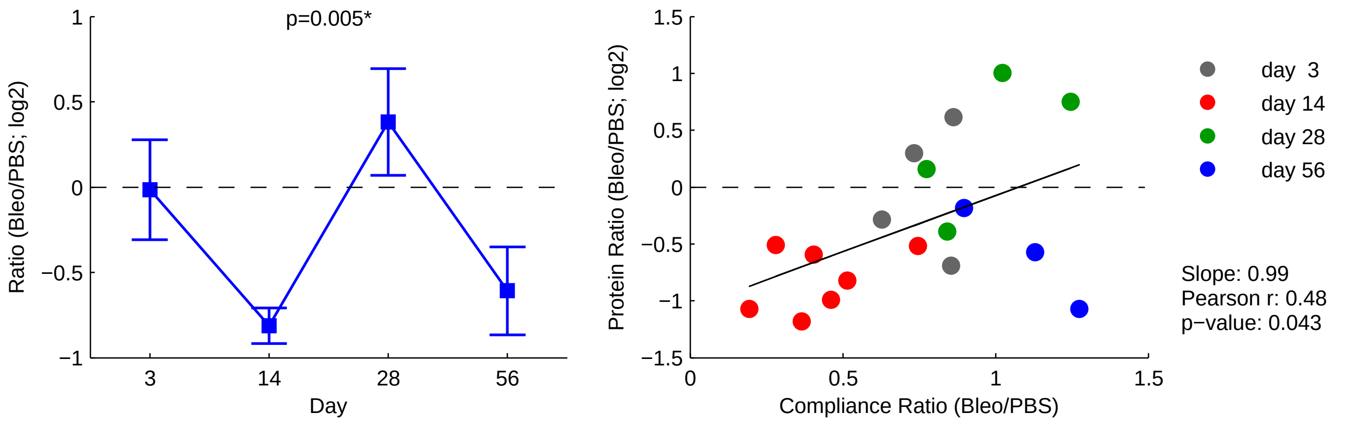

Q9CR89 − Ergic2 (id: 6811)

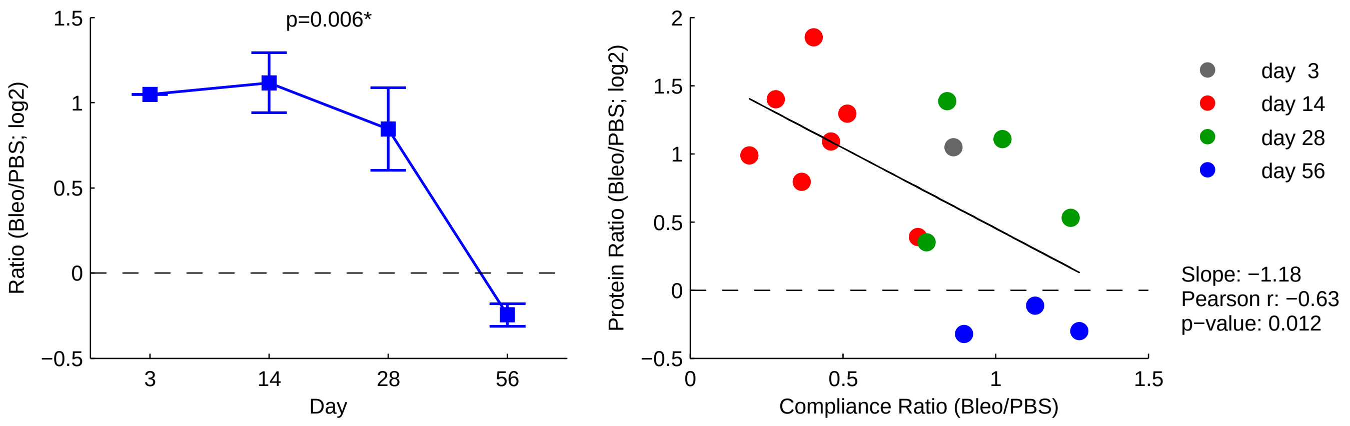

Q9CR95 − Necap1 (id: 6812)

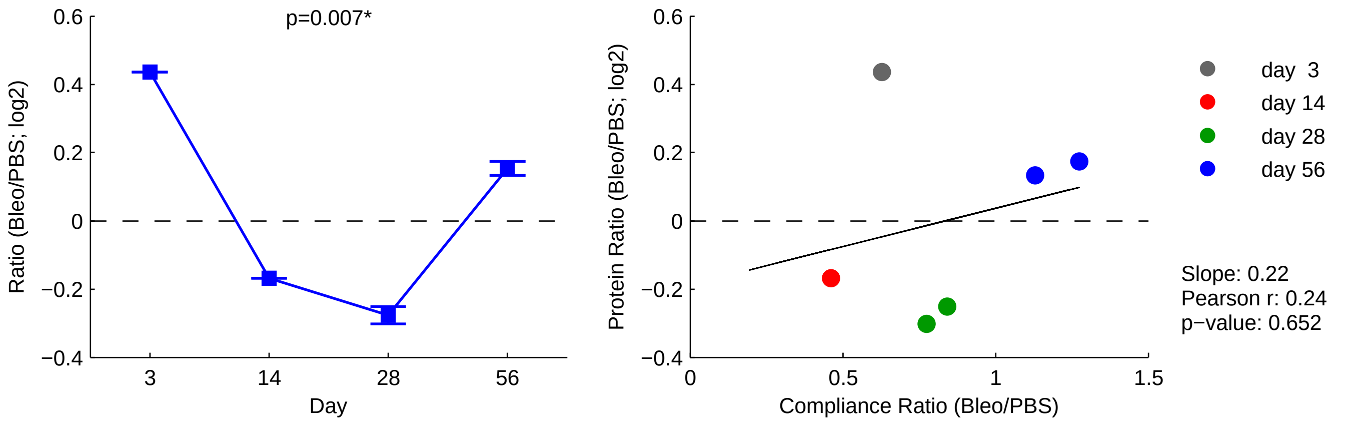

Q9CRA4 − Msmo1 (id: 6816)

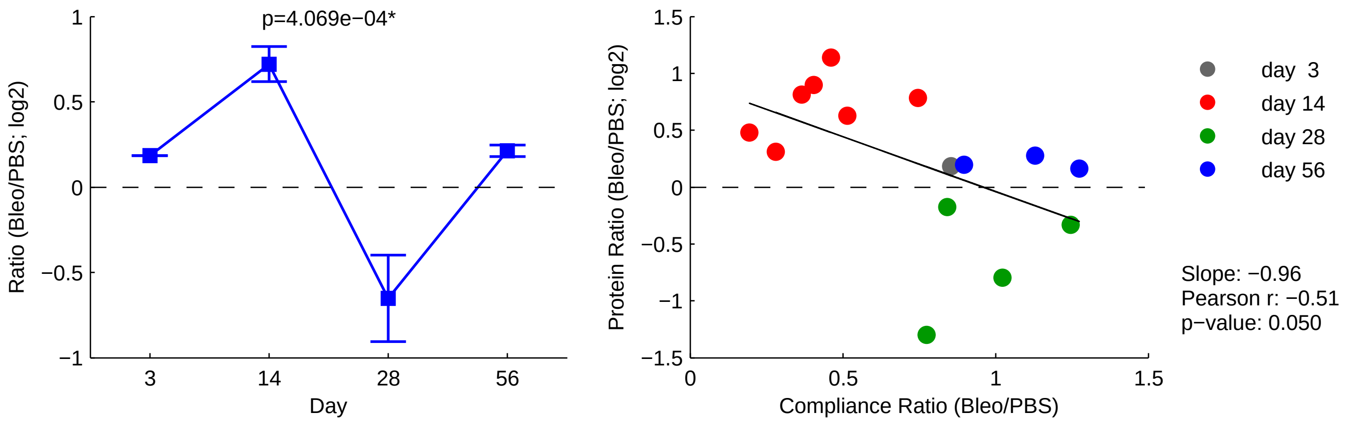

Q9CRA5 − Golph3 (id: 6817)

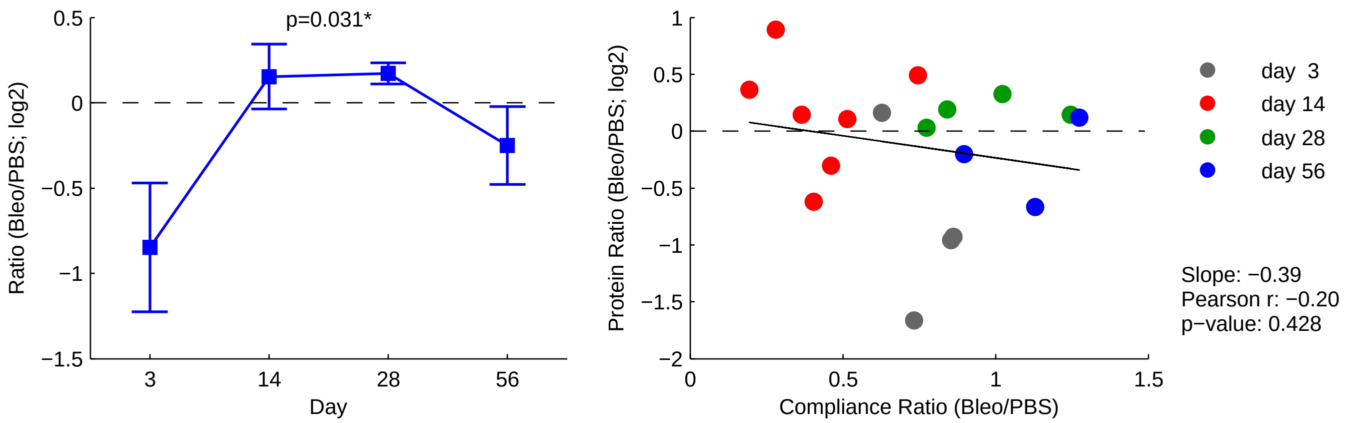

Q9CRB2 − Nhp2 (id: 6822)

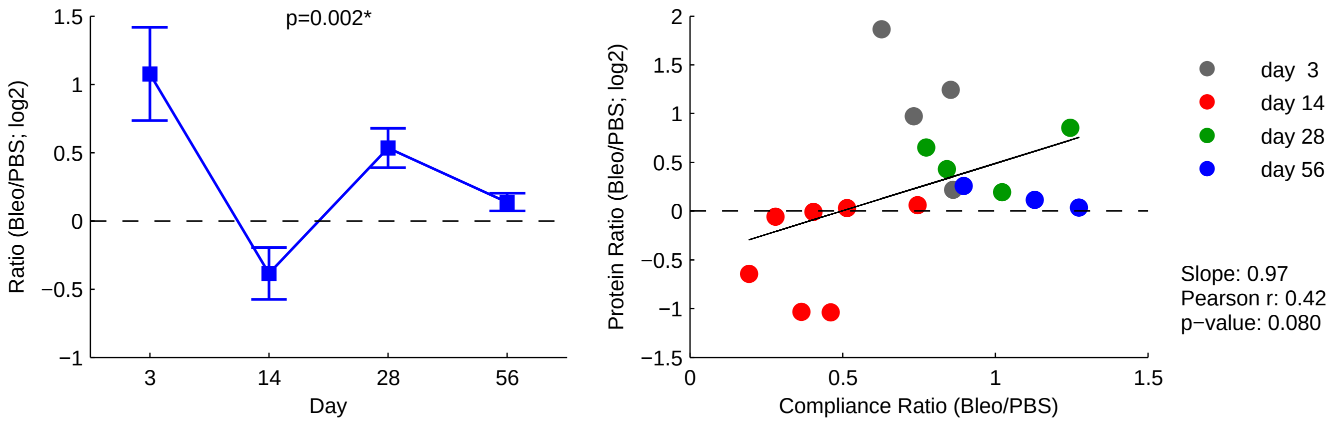

Q9CRB6 − Tppp3 (id: 6823)

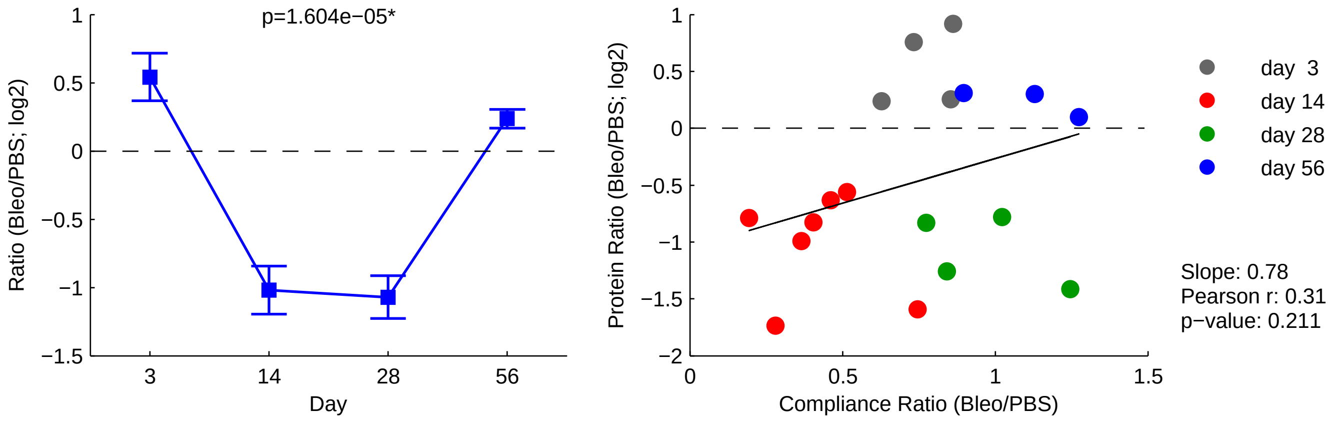

Q9CRB9 − Chchd3 (id: 6825)

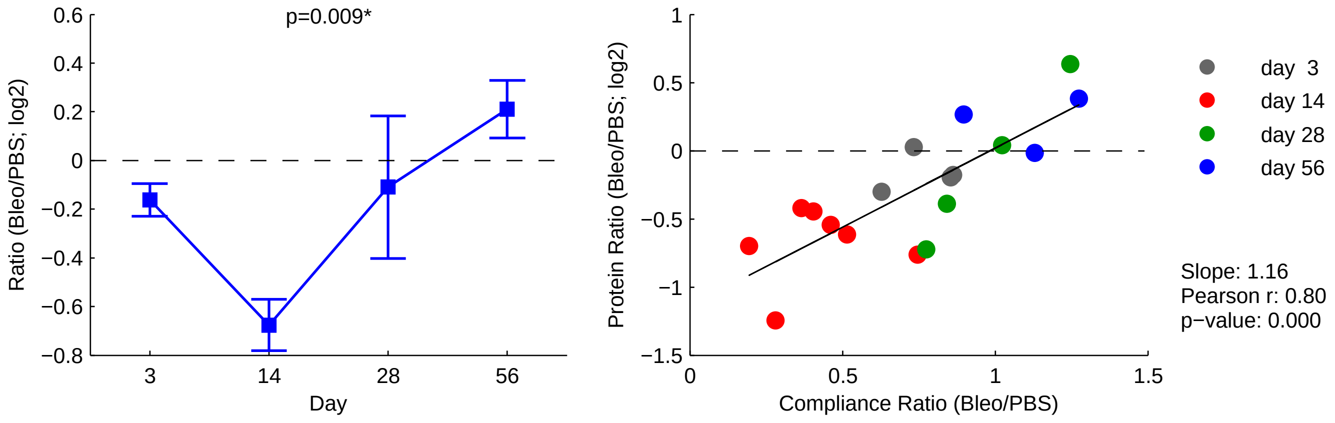

Q9CRC6 − (id: 6828)

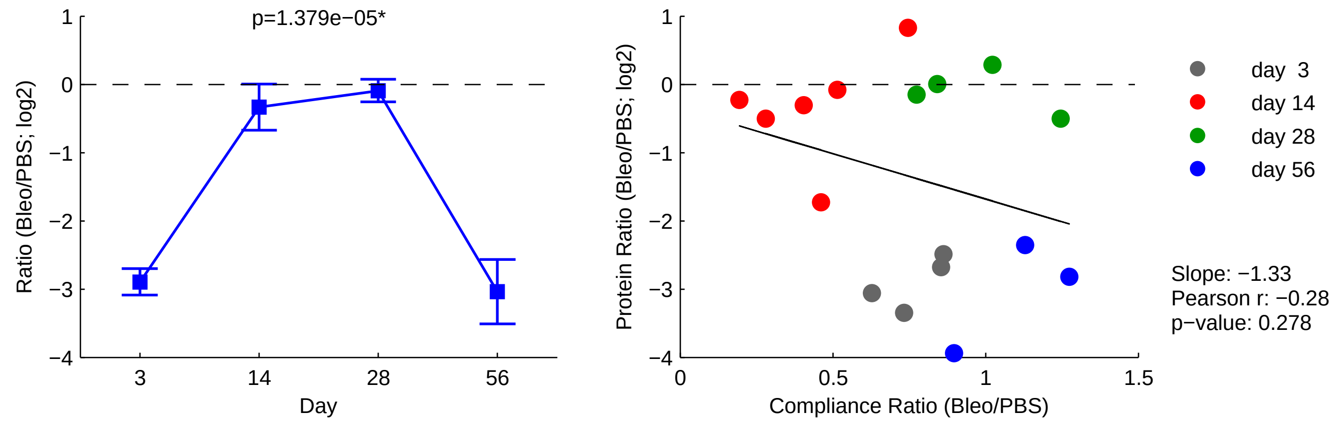

Q9CRC9 − Gnpda2 (id: 6830)

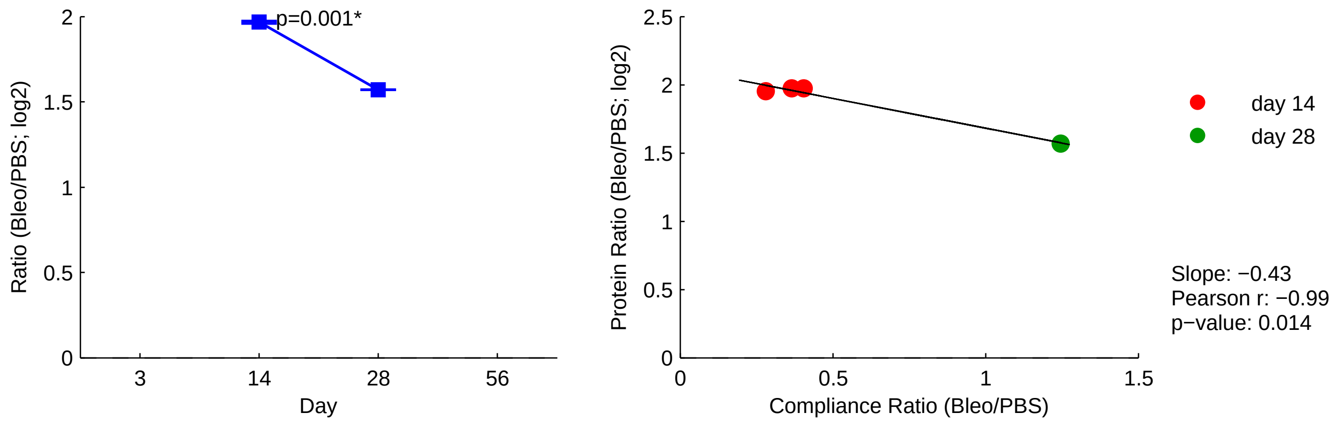

Q9CRD0 − Ociad1 (id: 6831)

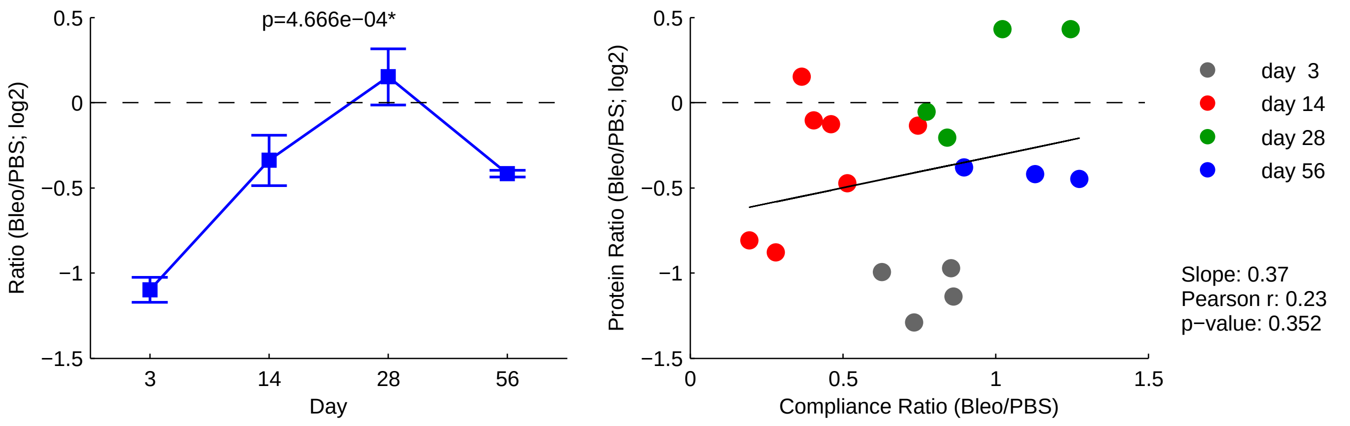

Q9CRD2 − Emc2 (id: 6833)

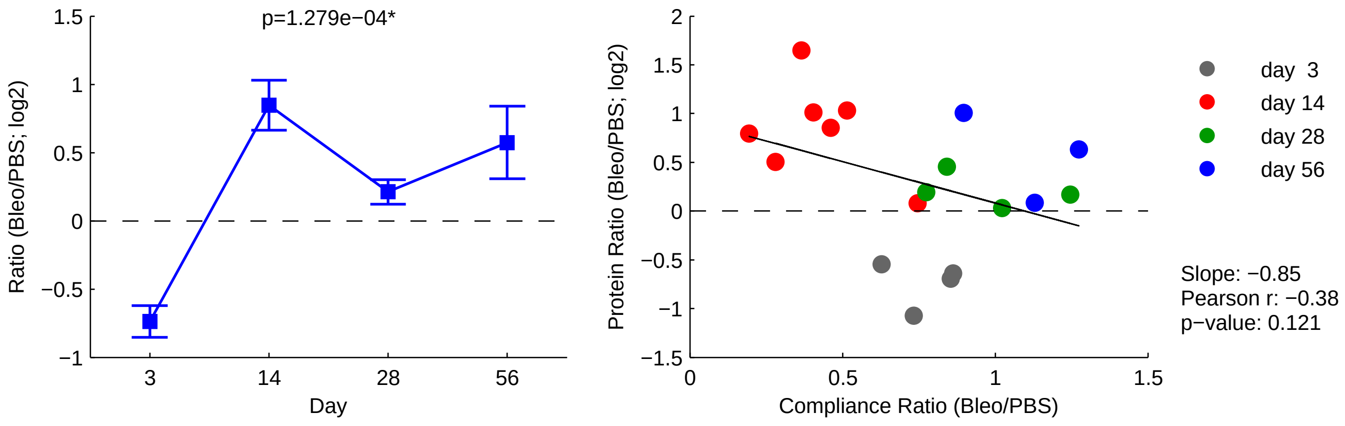

Q9CRY7 − Gdpd1 (id: 6835)

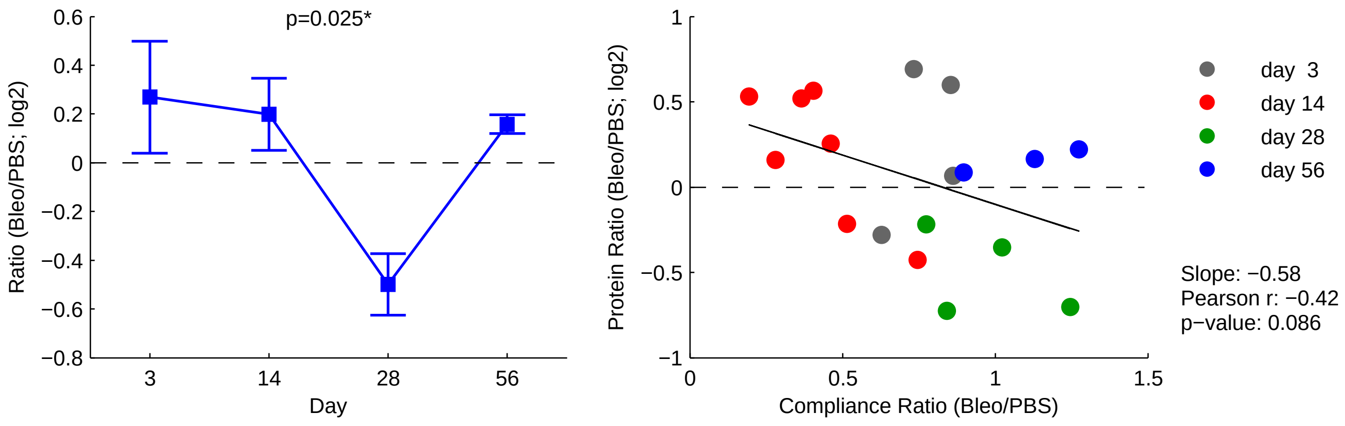

Q9CS42 − Prps2 (id: 6837)

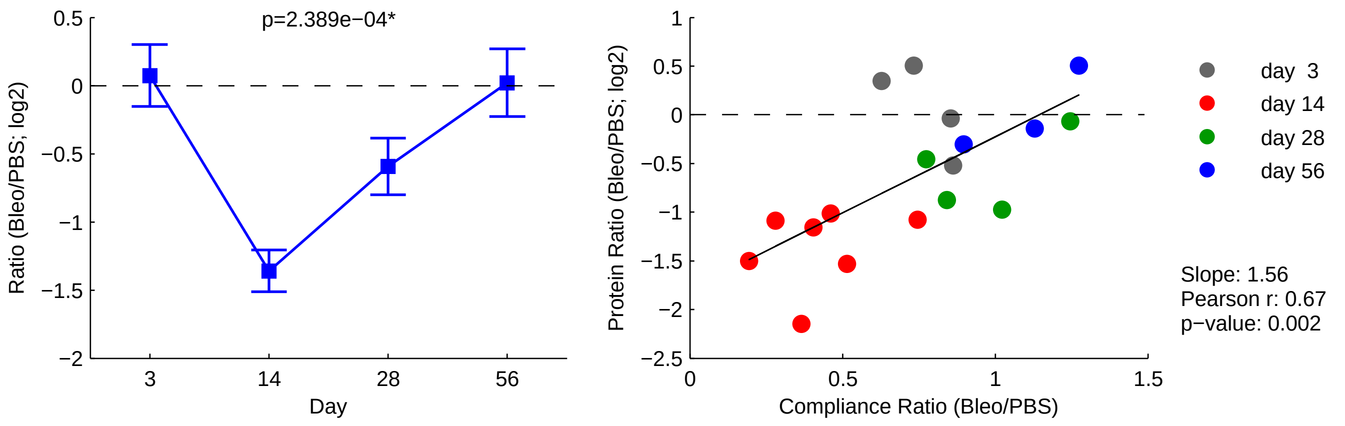

Q9CSB4 − Pard3b (id: 6839)

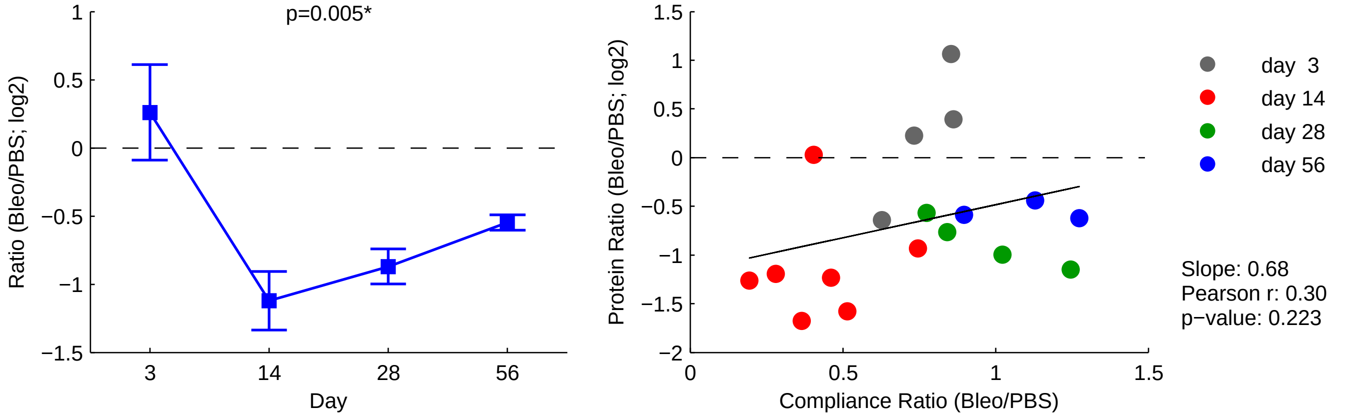

Q9CSU0 − Rprd1b (id: 6842)

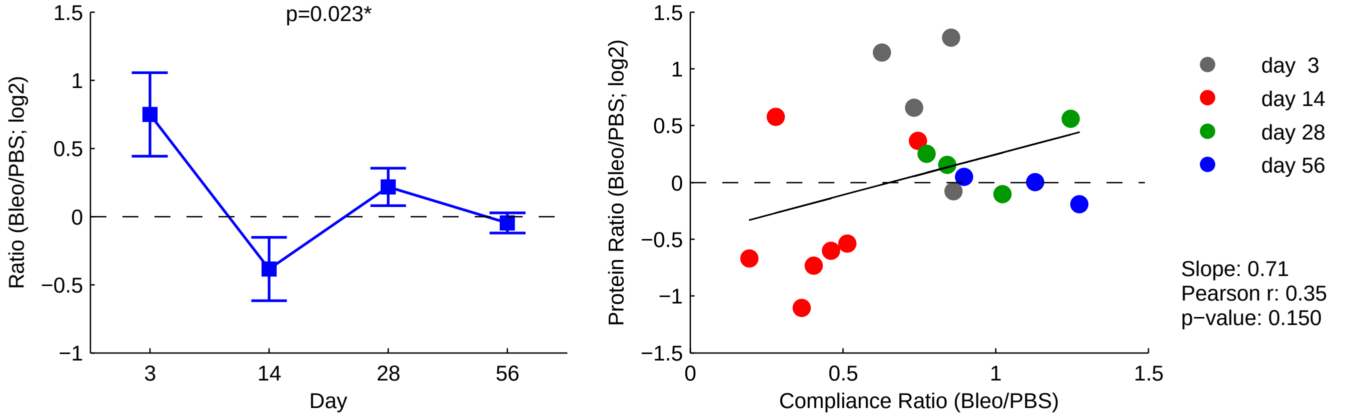

Q9CVB6 − Arpc2 (id: 6846)

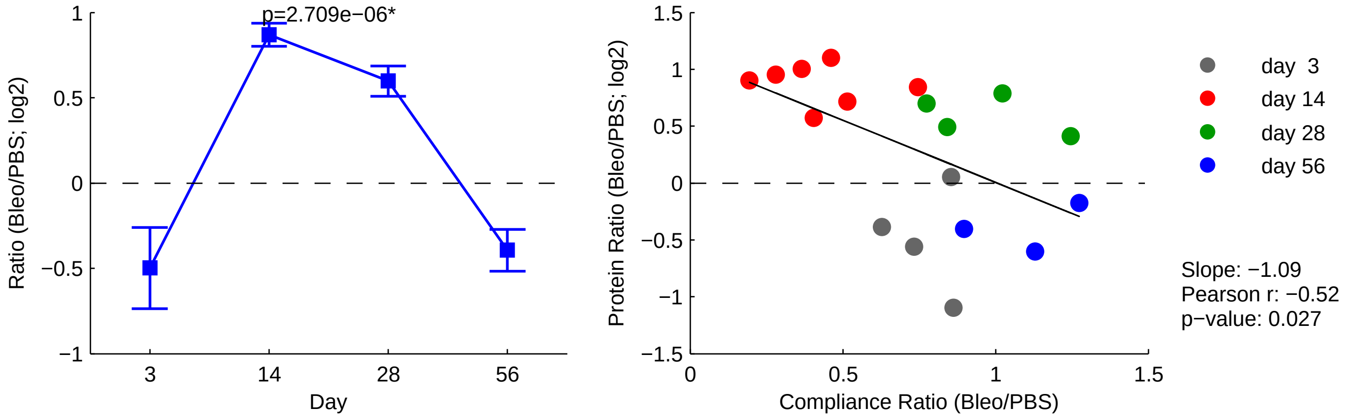

Q9CWD8 − Nubpl (id: 6850)

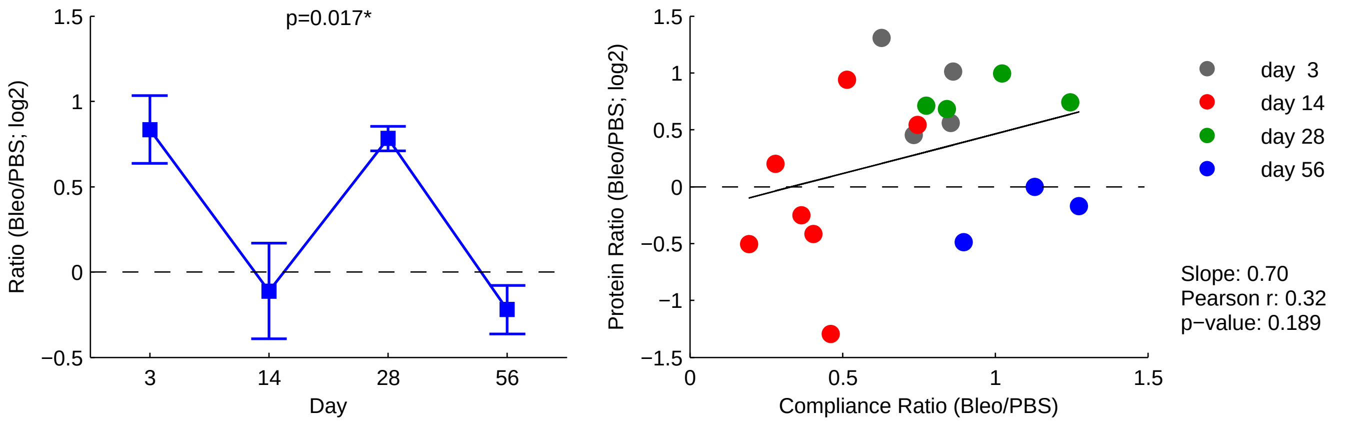

Q9CWE0 − Mtfr1l (id: 6851)

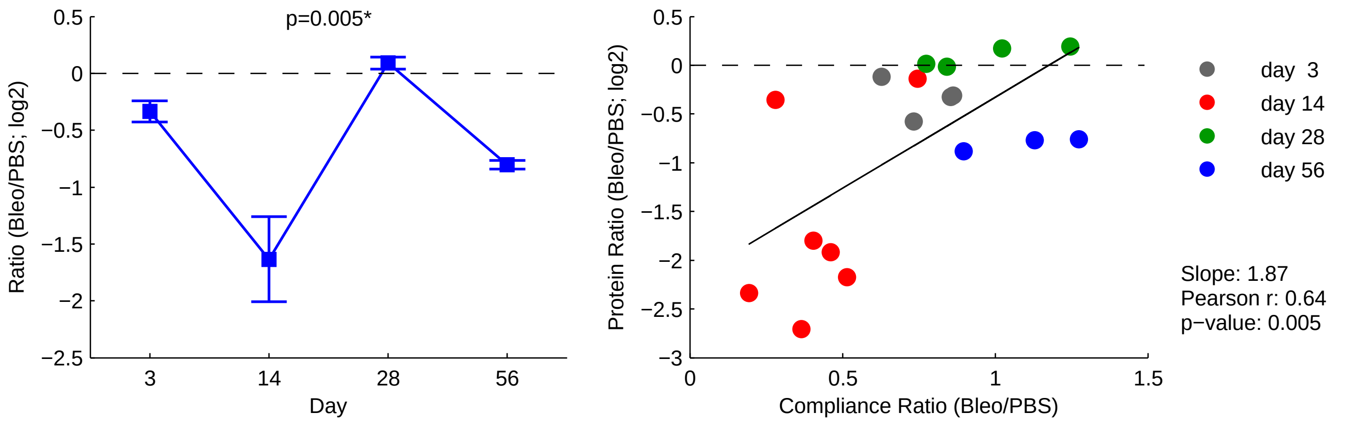

Q9CWF2 − Tubb2b (id: 6852)

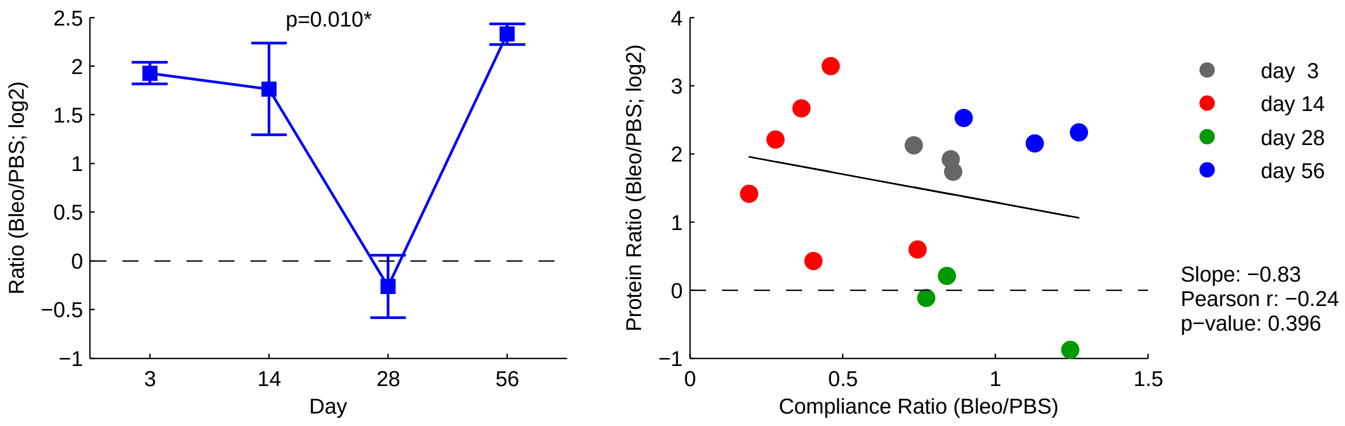

Q9CWG8 − Ndufaf7 (id: 6853)

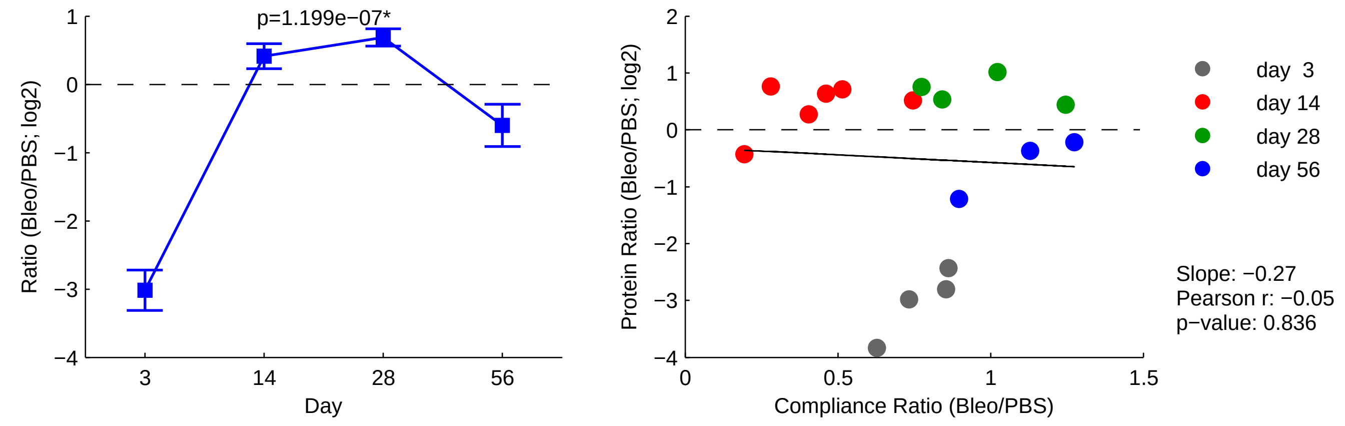

Q9CWJ9 − Atic (id: 6856)

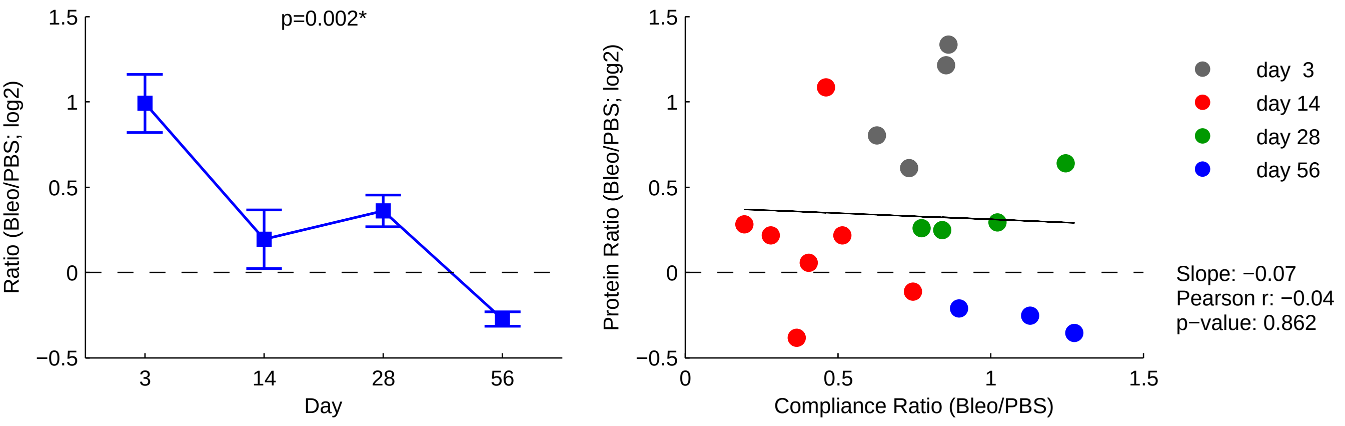

Q9CWK8 − Snx2 (id: 6858)

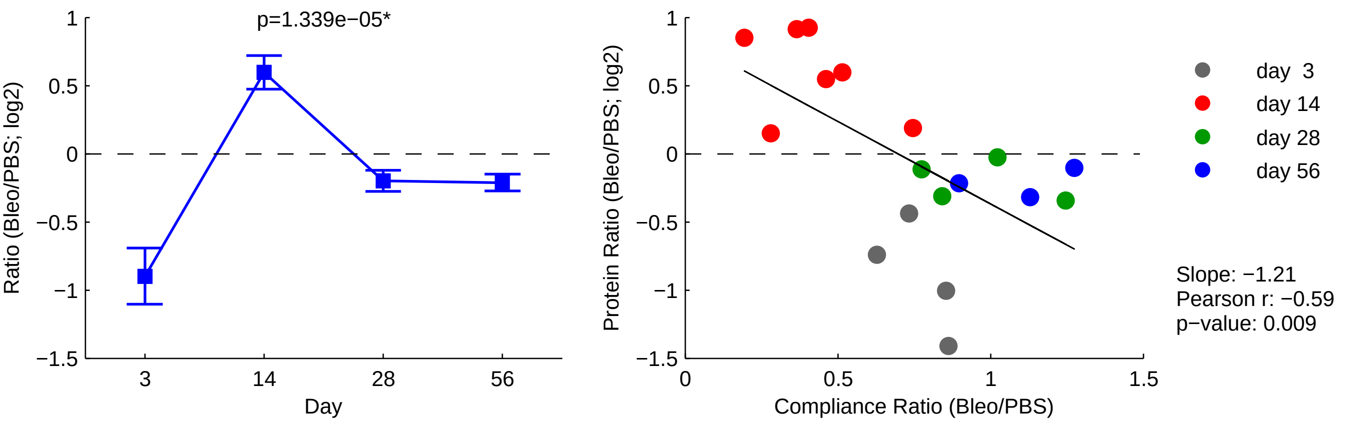

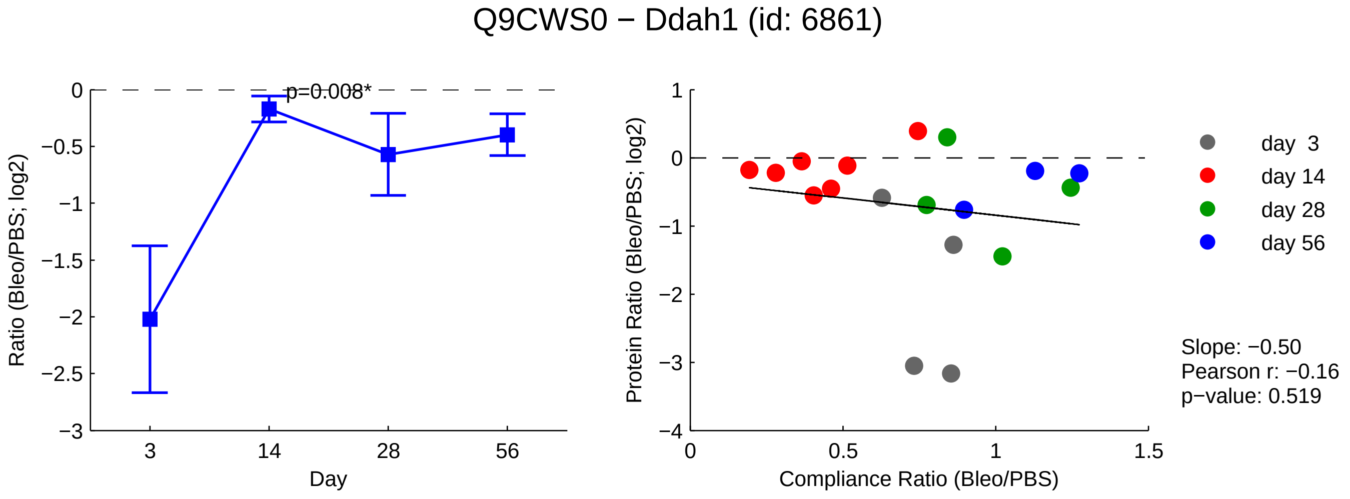
Q9CWS0 − Ddah1 (id: 6861)
p=0.008*
Ratio (Bleo/PBS; log2)
Day
Protein Ratio (Bleo/PBS; log2)
Compliance Ratio (Bleo/PBS)
day 3
day 14
day 28
day 56
Slope: −0.50
Pearson r: −0.16
p−value: 0.519

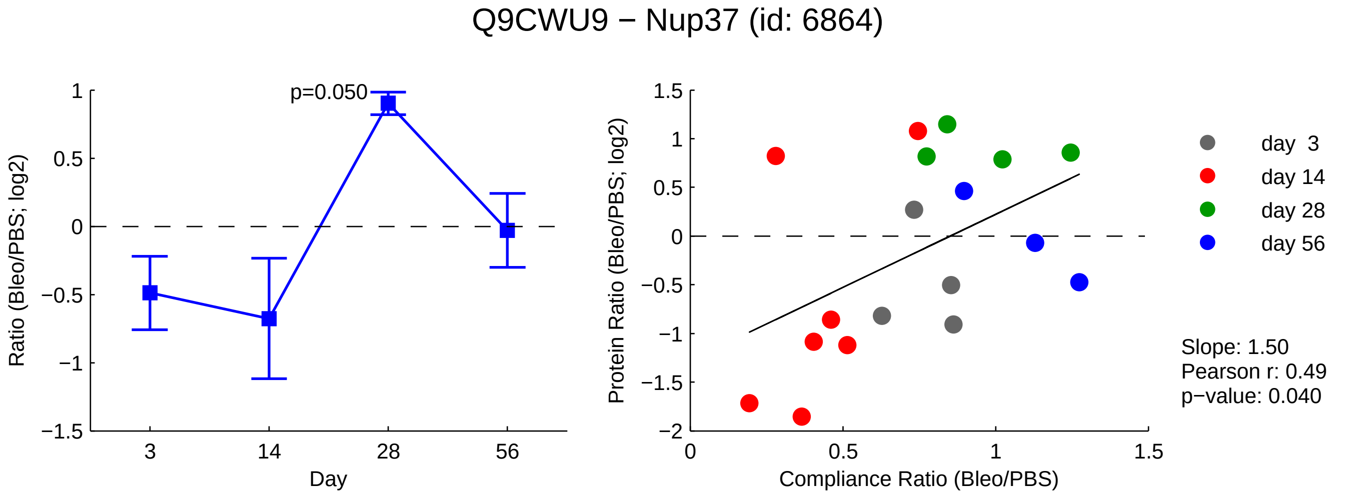
Q9CWU9 − Nup37 (id: 6864)
p=0.050
Ratio (Bleo/PBS; log2)
Day
Protein Ratio (Bleo/PBS; log2)
Compliance Ratio (Bleo/PBS)
day 3
day 14
day 28
day 56
Slope: 1.50
Pearson r: 0.49
p−value: 0.040

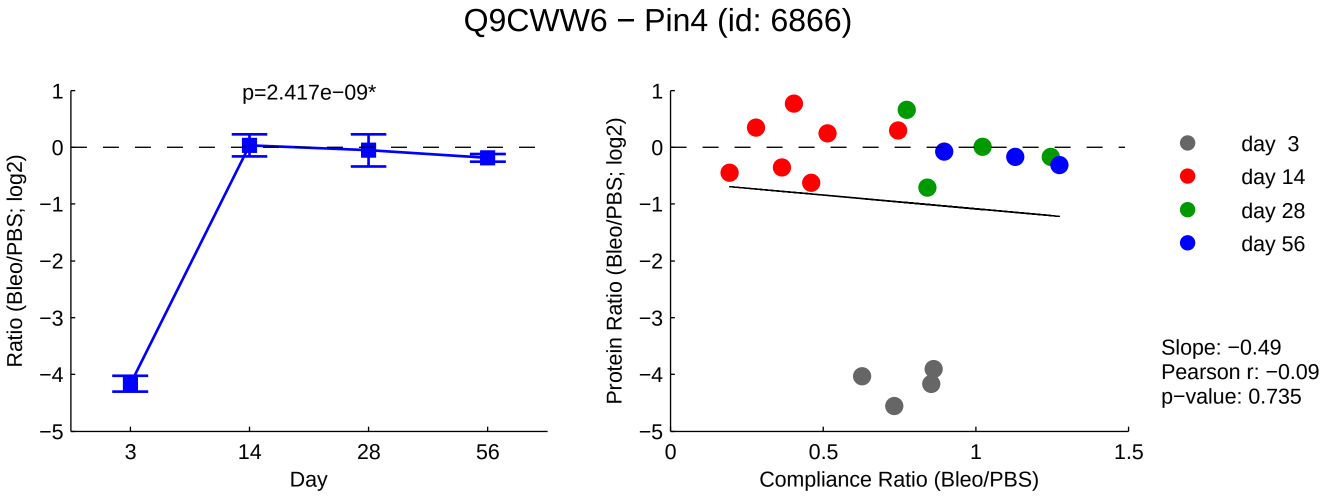
Q9CWW6 − Pin4 (id: 6866)
p=2.417e−09*
Ratio (Bleo/PBS; log2)
Day
Protein Ratio (Bleo/PBS; log2)
Compliance Ratio (Bleo/PBS)
day 3
day 14
day 28
day 56
Slope: −0.49
Pearson r: −0.09
p−value: 0.735

Q9CWX9 − Ddx47 (id: 6867)

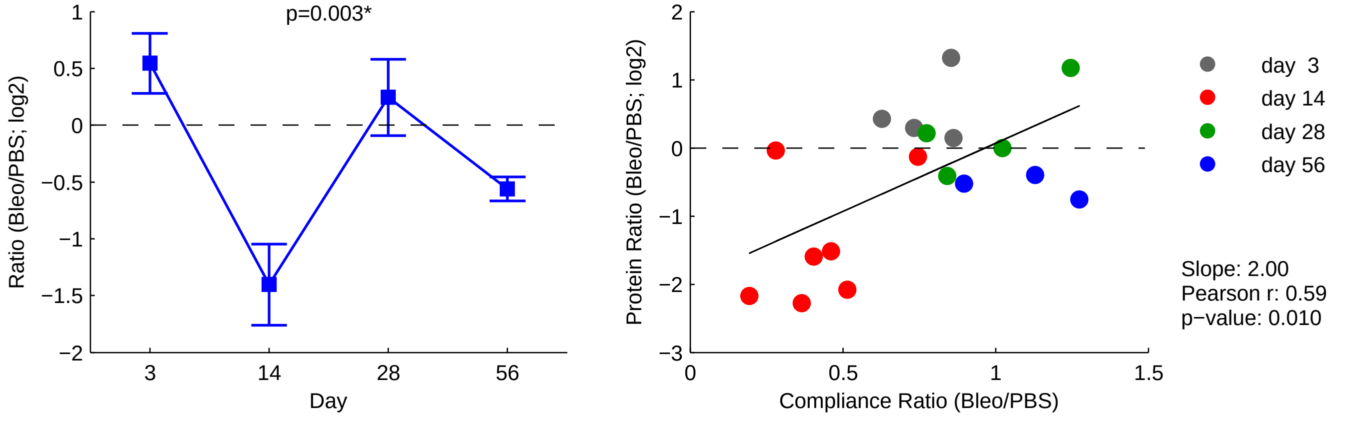

Q9CWZ3−2 − Rbm8a (id: 6868)

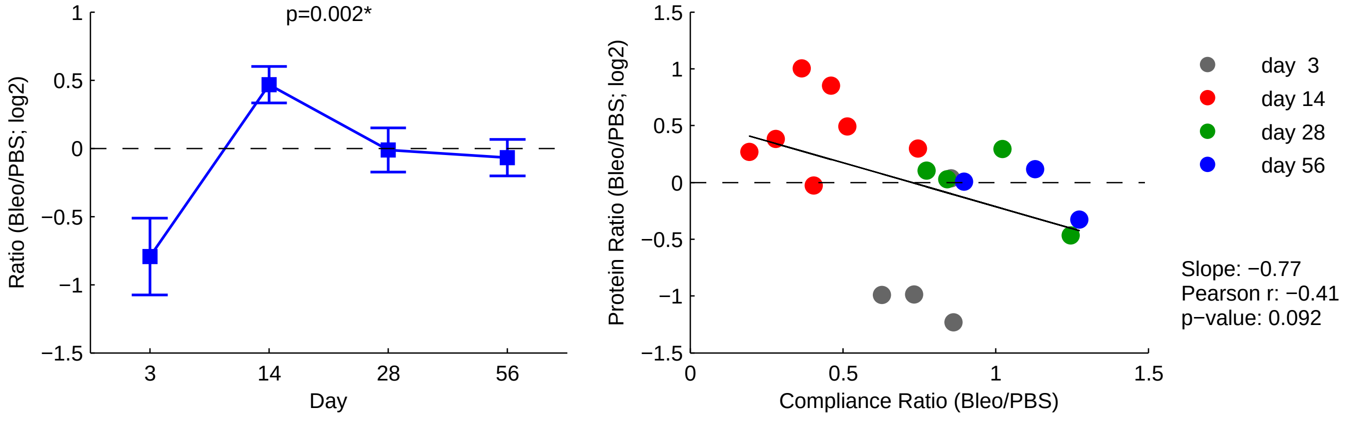

Q9CX56 − Psmd8 (id: 6875)

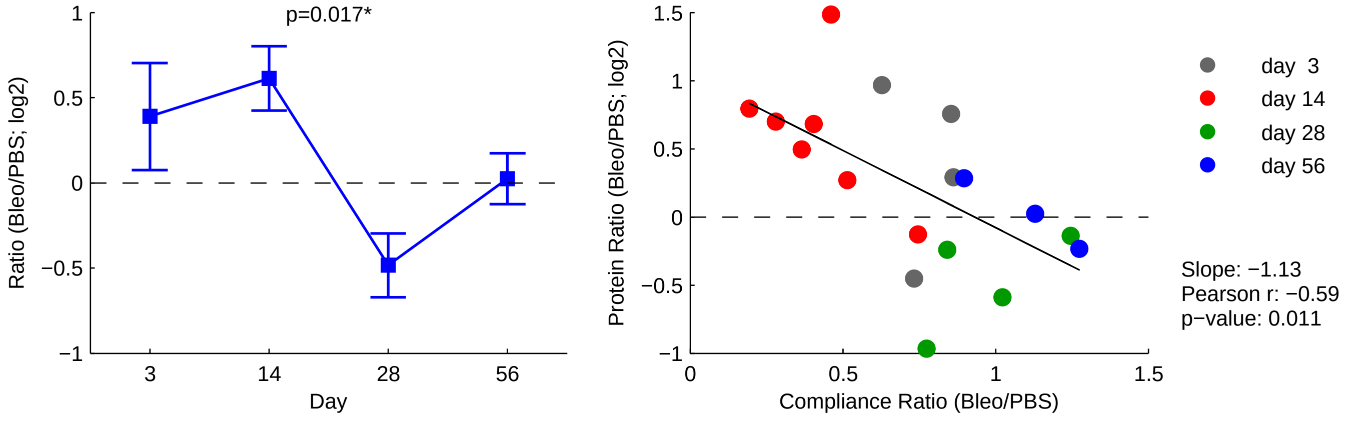

Q9CX80 − Cygb (id: 6876)

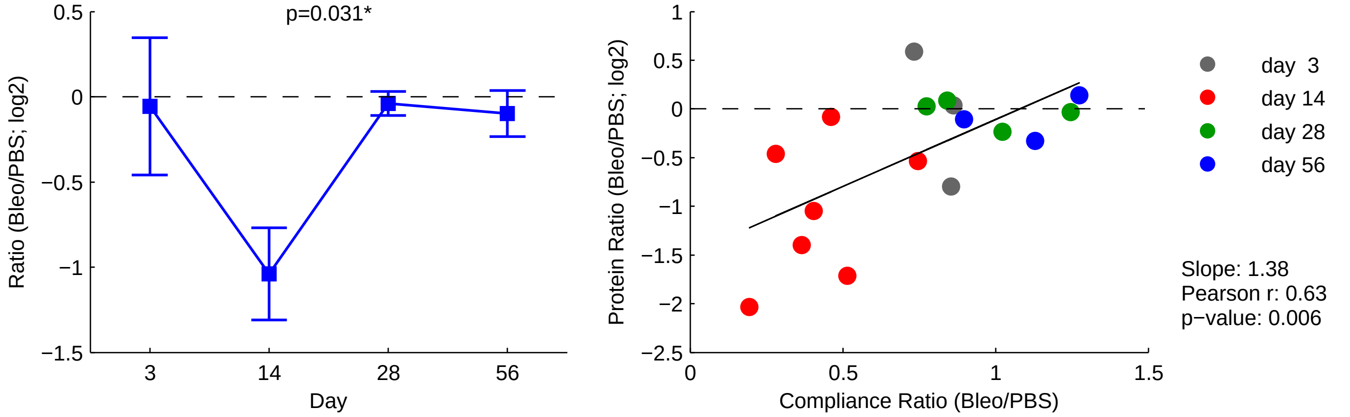

Q9CX86 − Hnrnpa0 (id: 6877)

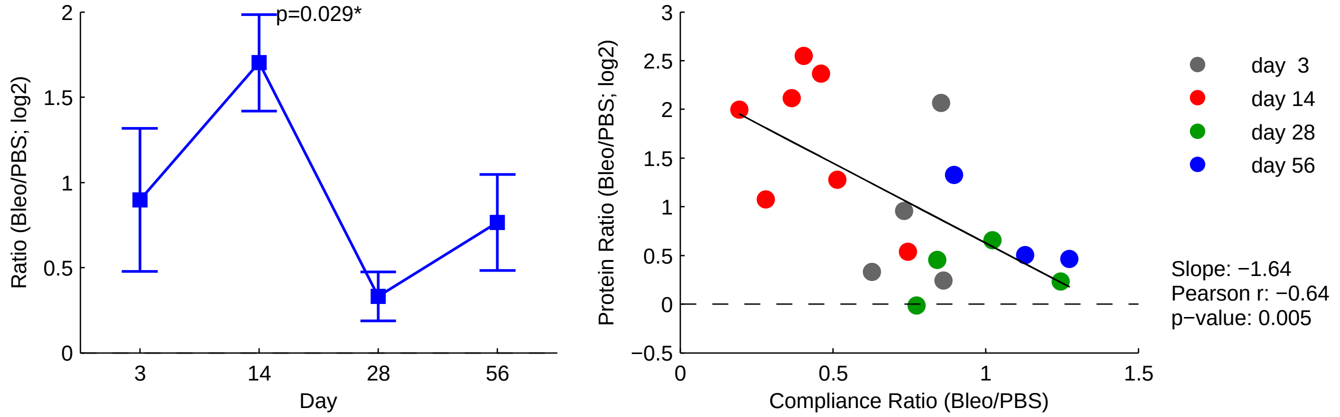

Q9CXD6 − Mcur1 (id: 6880)

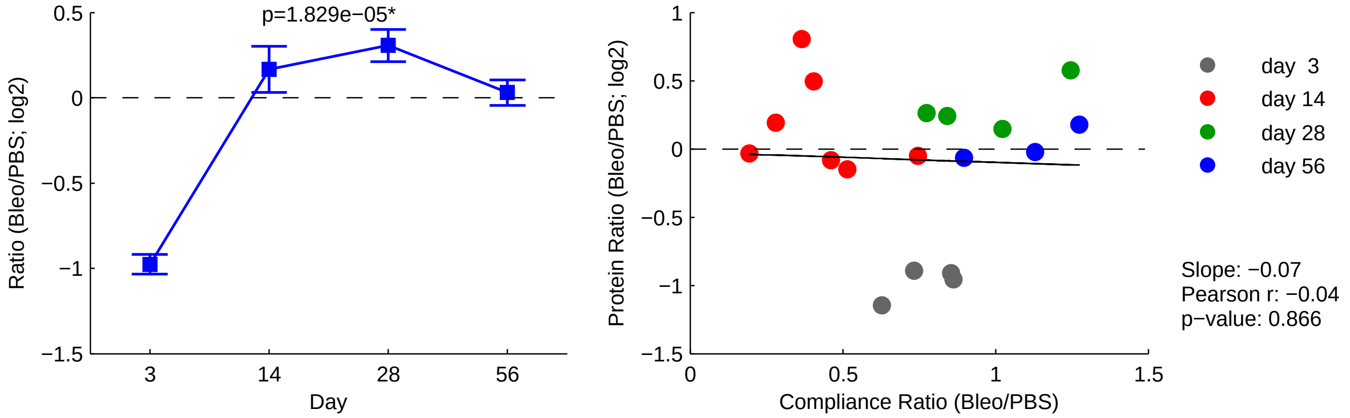

Q9CXD9 − Lrrc17 (id: 6881)

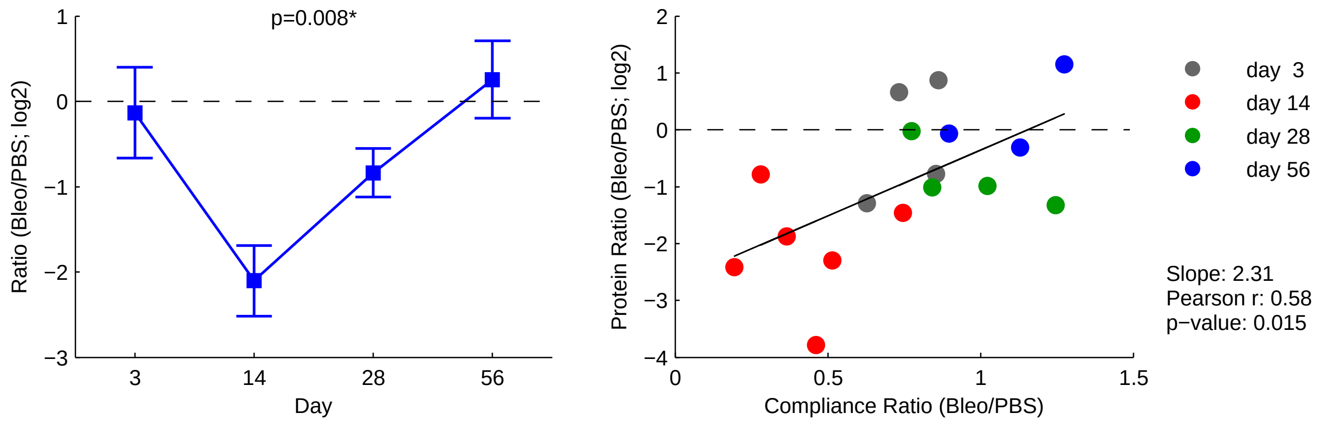

Q9CXE7 − Tmed5 (id: 6882)

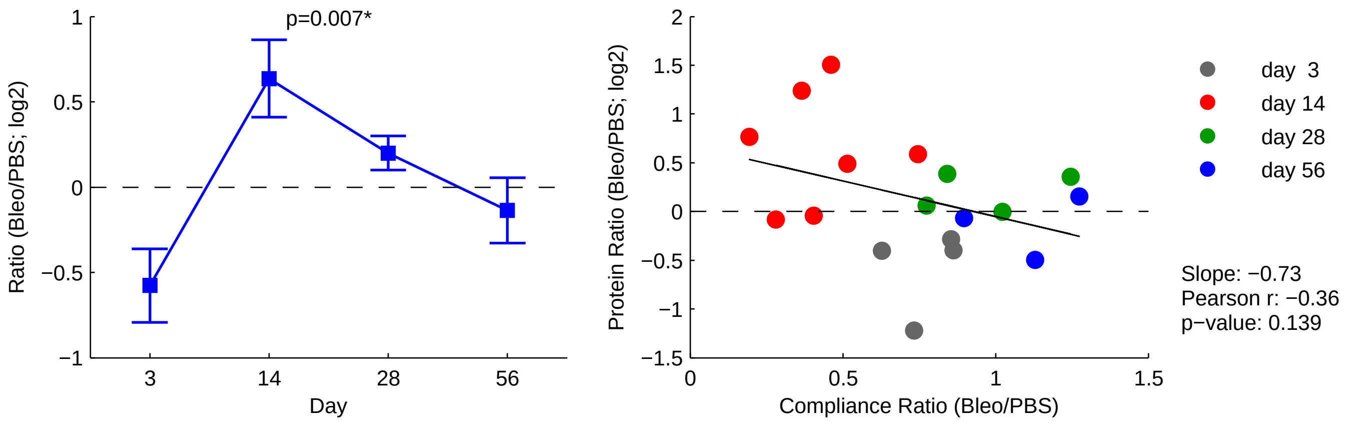

Q9CXG3 − Ppil4 (id: 6884)

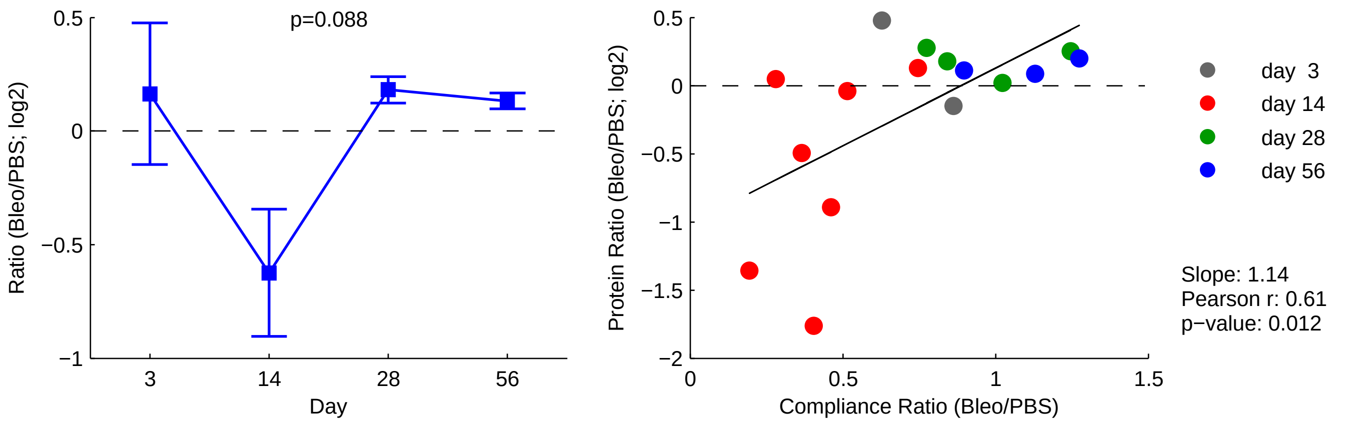

Q9CXI0 − Coq5 (id: 6885)

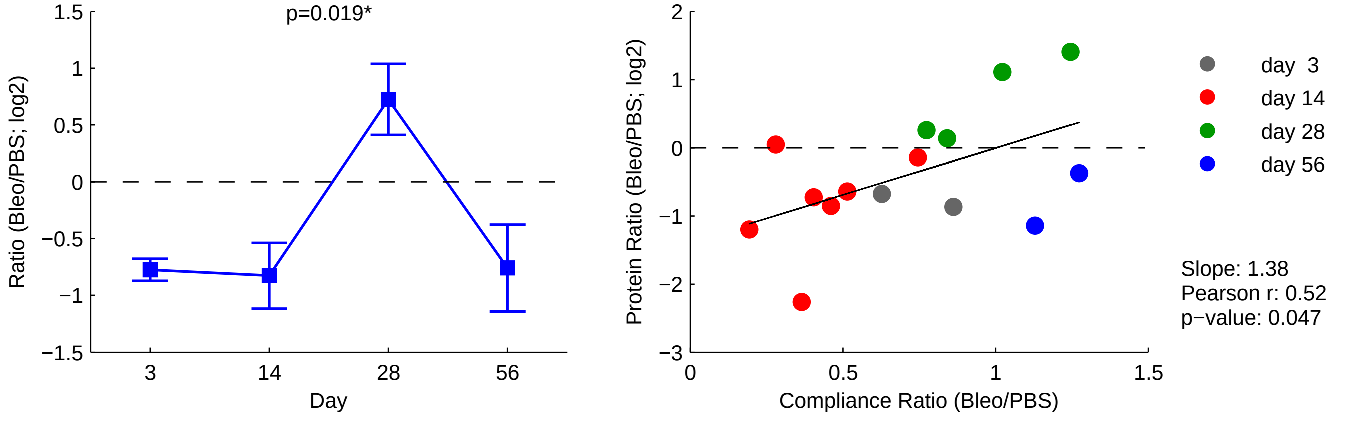

Q9CXJ4 − Abcb8 (id: 6887)

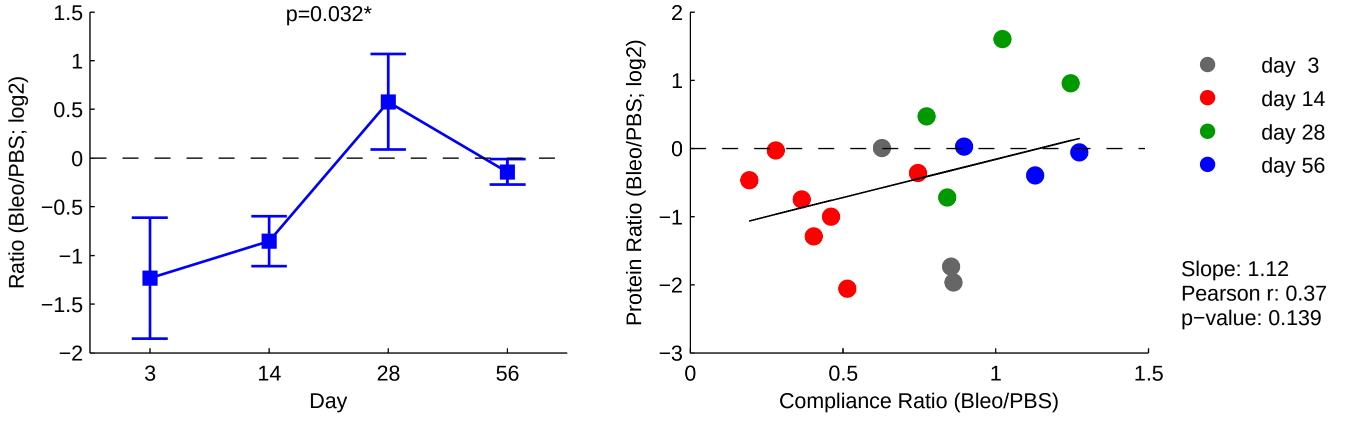

Q9CXK8 − Nip7 (id: 6888)

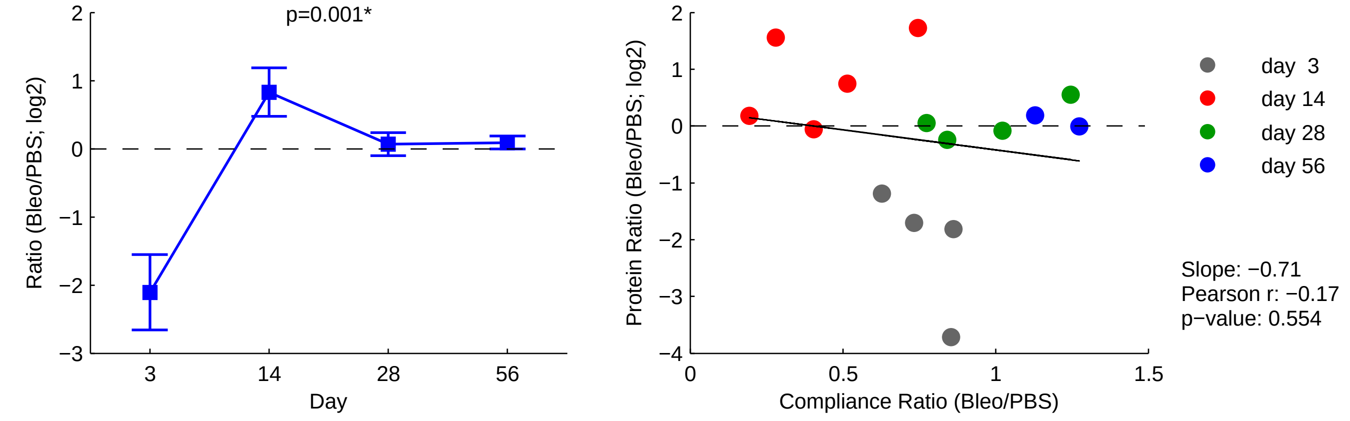

Q9CXR1 − Dhrs7 (id: 6892)

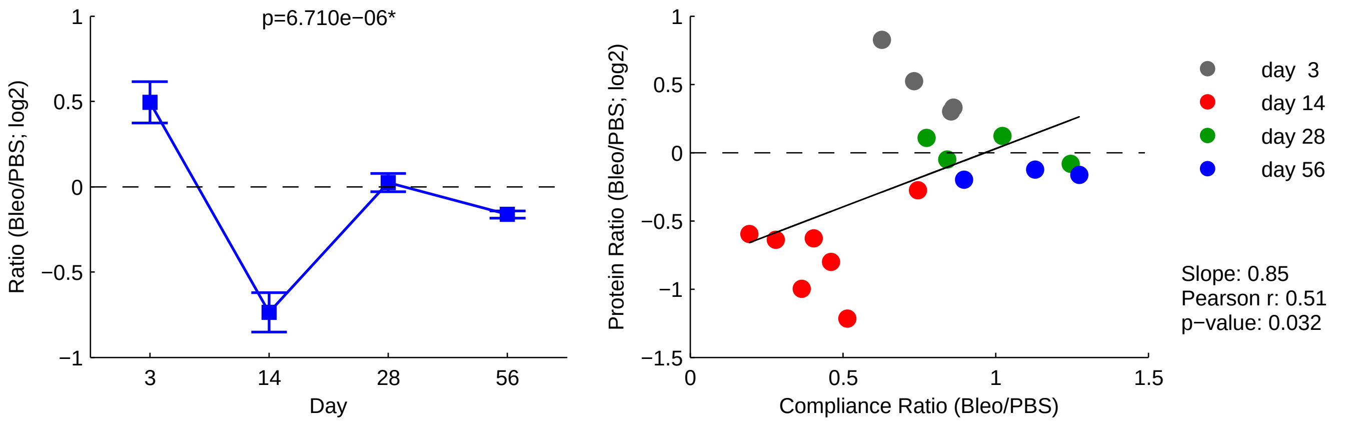

Q9CXS4 − Cenpv (id: 6893)

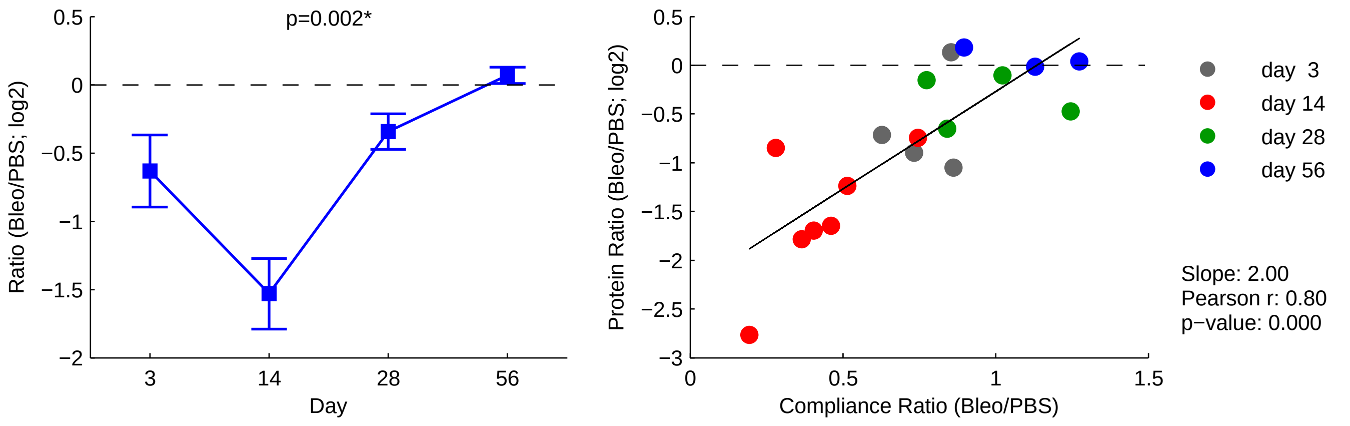

Q9CXT8 − Pmpcb (id: 6895)

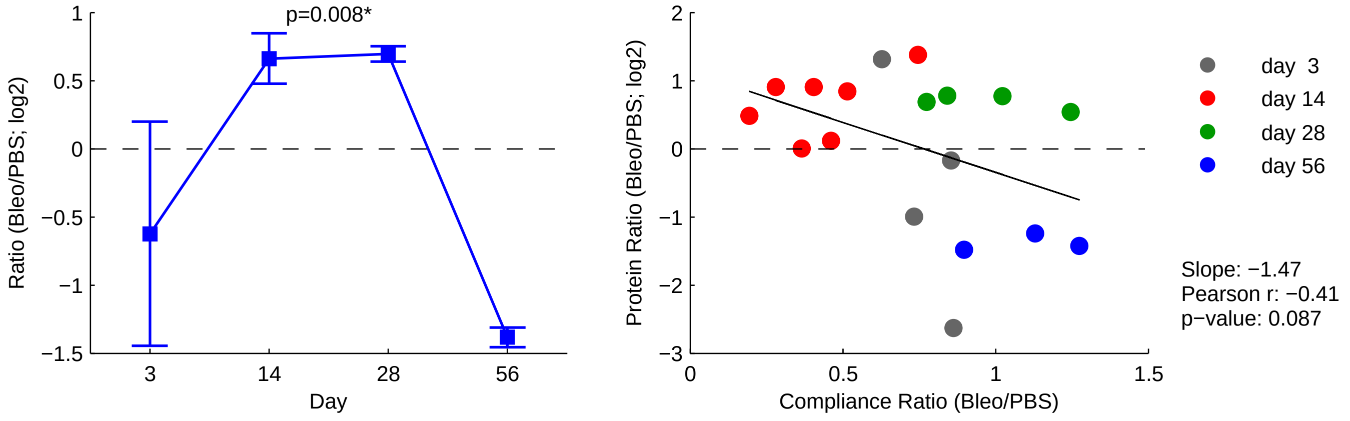

Q9CXU9 − Eif1b (id: 6899)

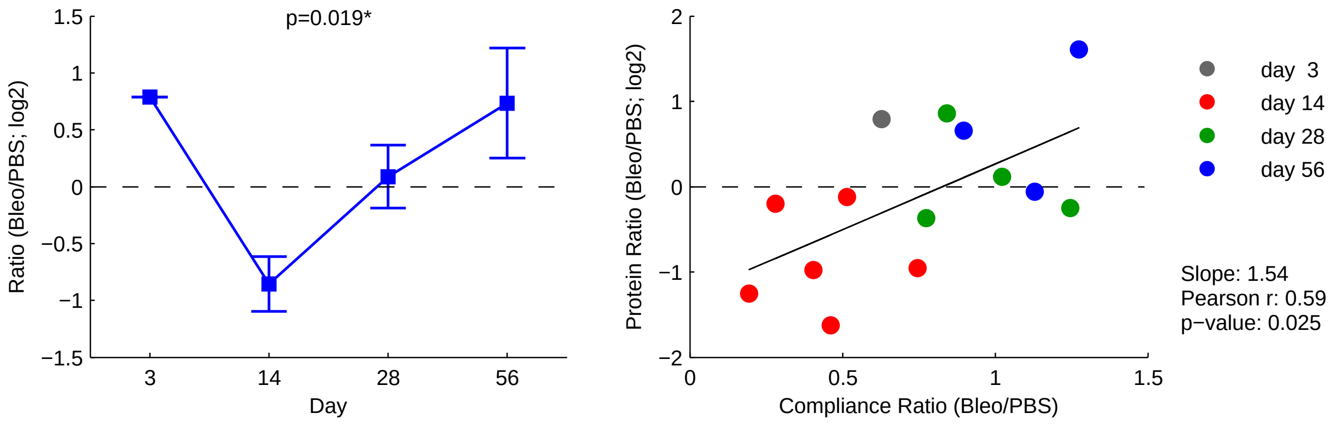

Q9CXY6 − Ilf2 (id: 6904)

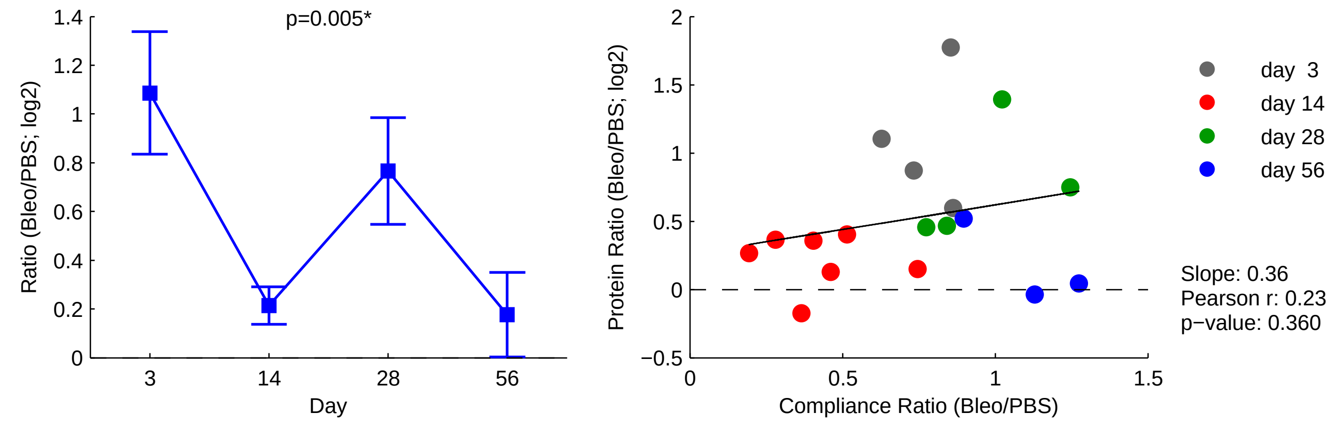

Q9CY16 − Mrps28 (id: 6906)

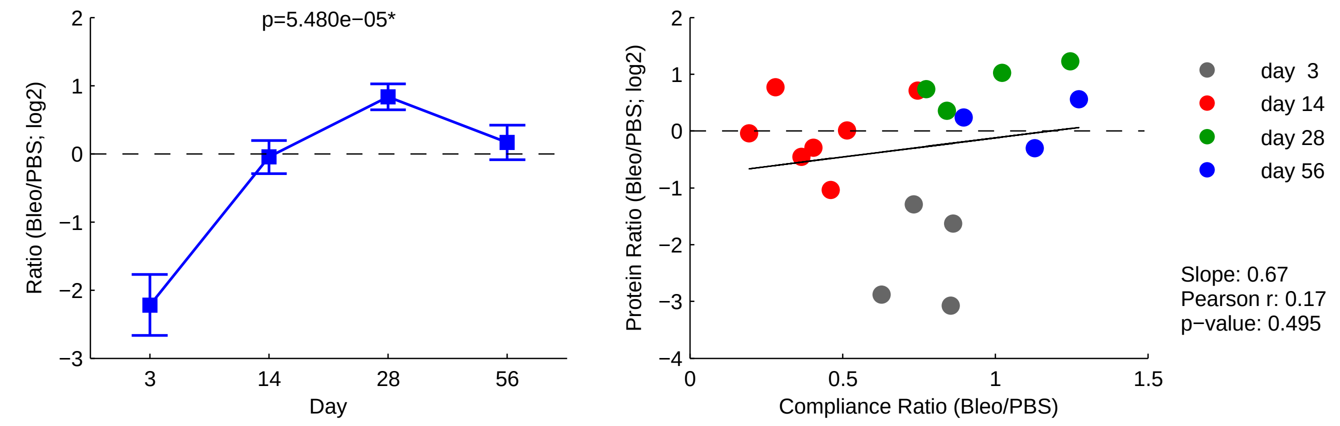

Q9CY50 − Ssr1 (id: 6911)

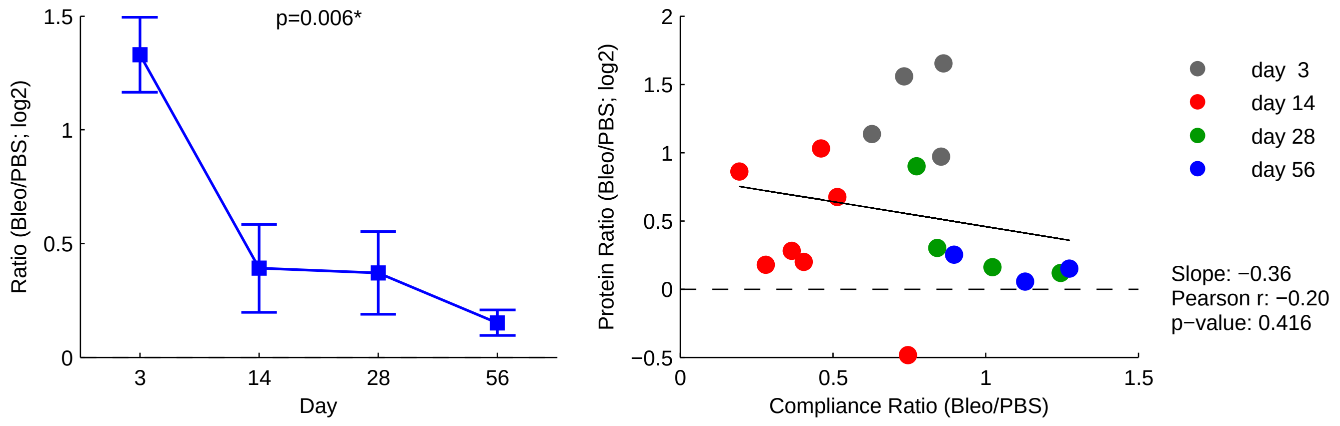

Q9CY58−2 − (id: 6913)

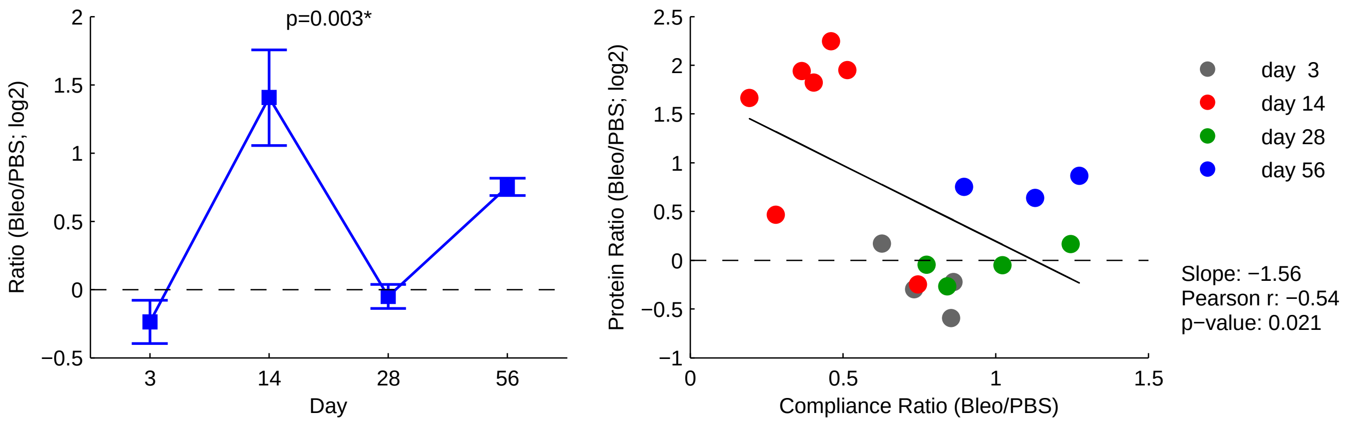

Q9CY73 − Mrpl44 (id: 6915)

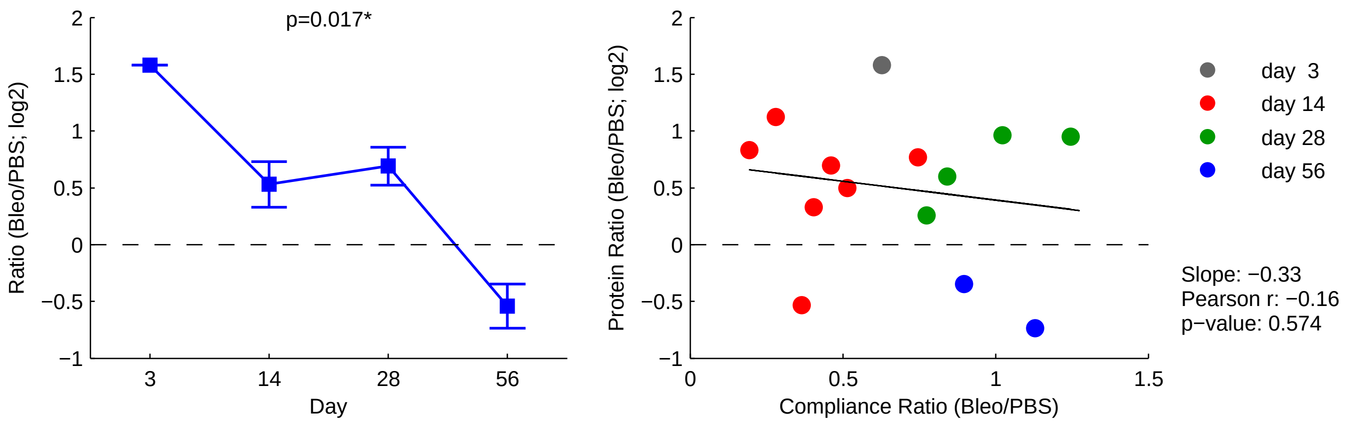

Q9CYD3 − Crtap (id: 6921)

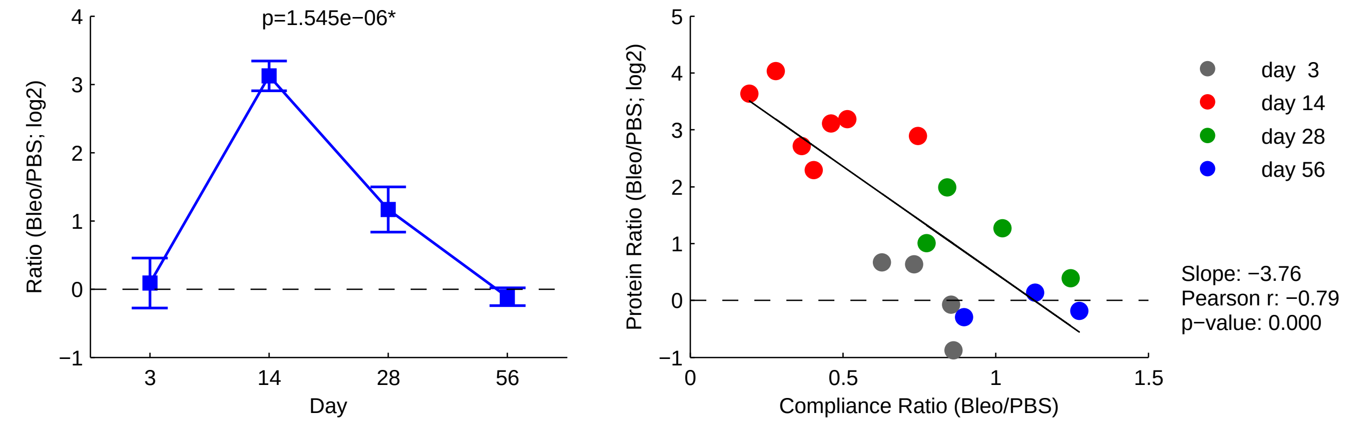

Q9CYI4 − Luc7l (id: 6927)

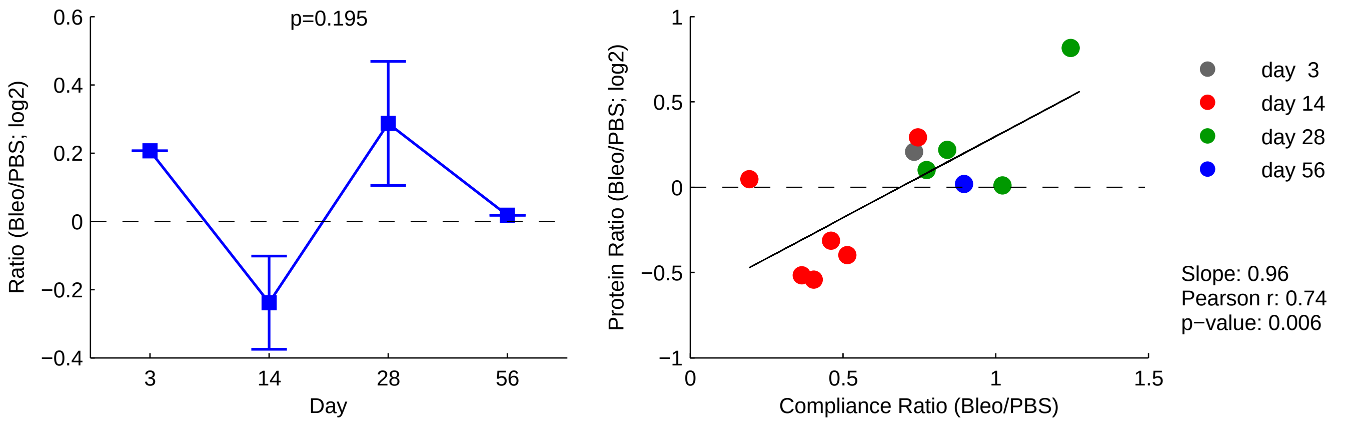

Q9CYL5 − Glipr2 (id: 6930)

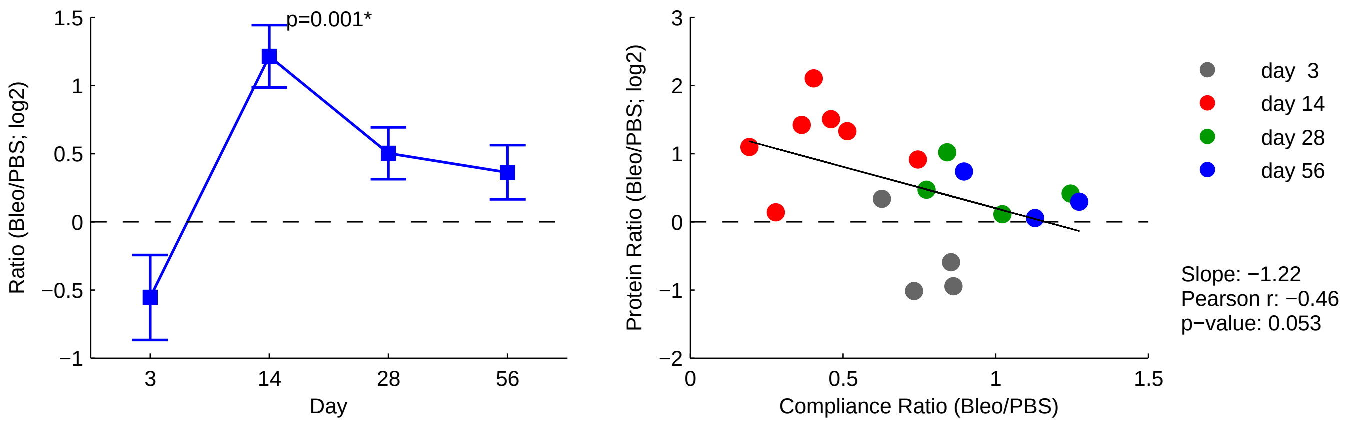

Q9CYN2 − Spcs2 (id: 6931)

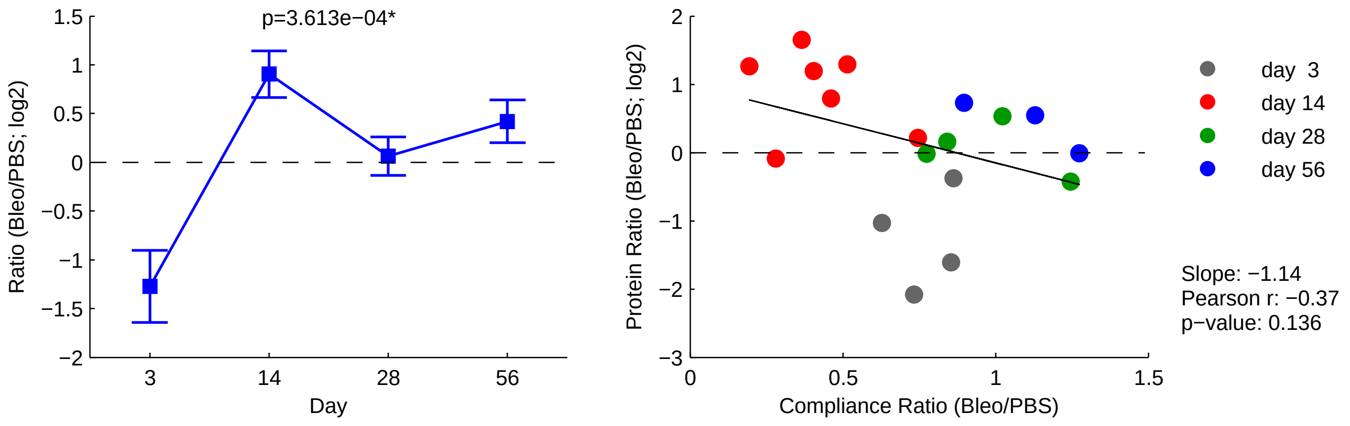

Q9CYW4 − Hdhd3 (id: 6935)

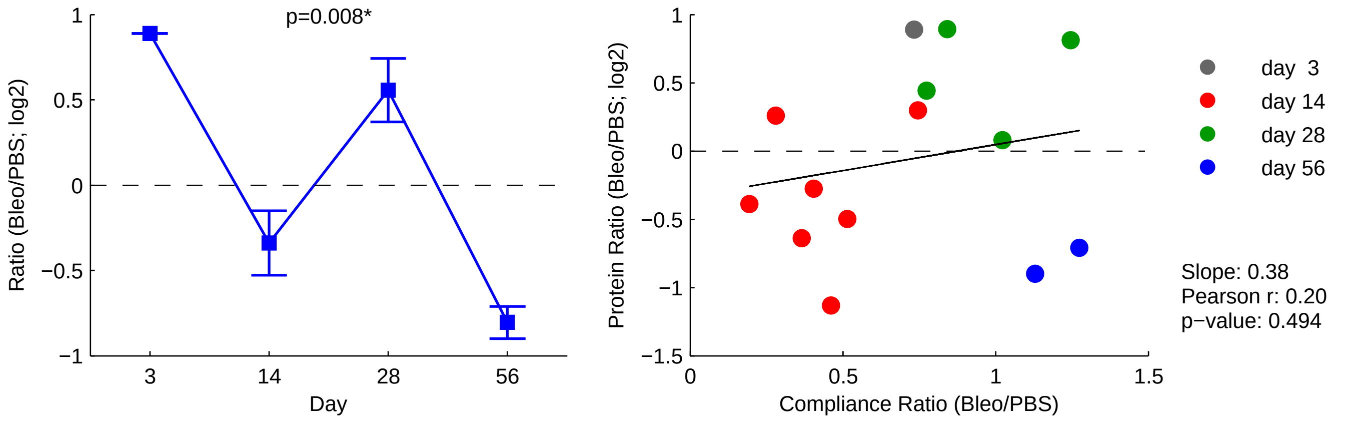

Q9CYX7 − Rrp15 (id: 6936)

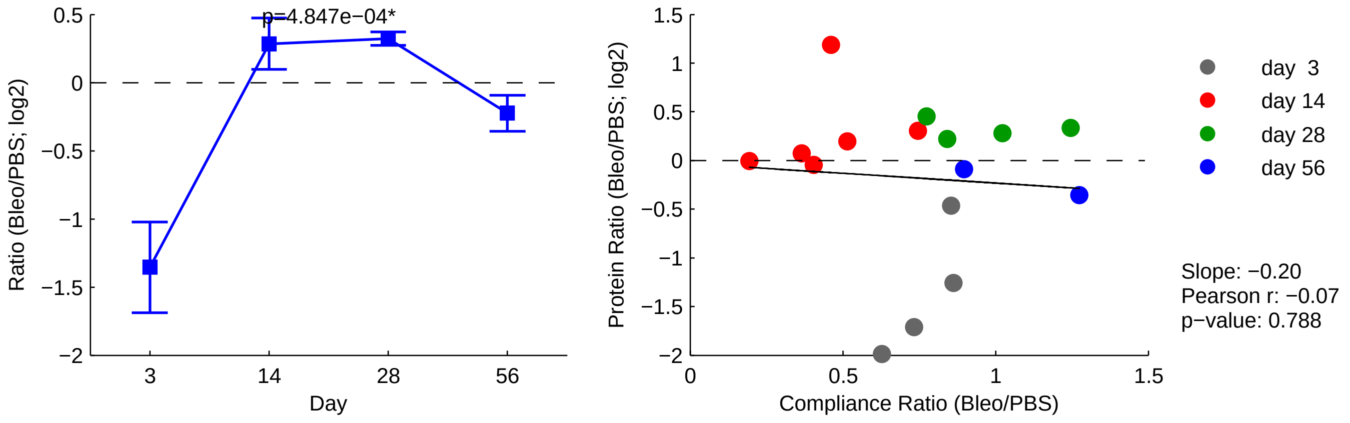

Q9CZ13 − Uqcrc1 (id: 6937)

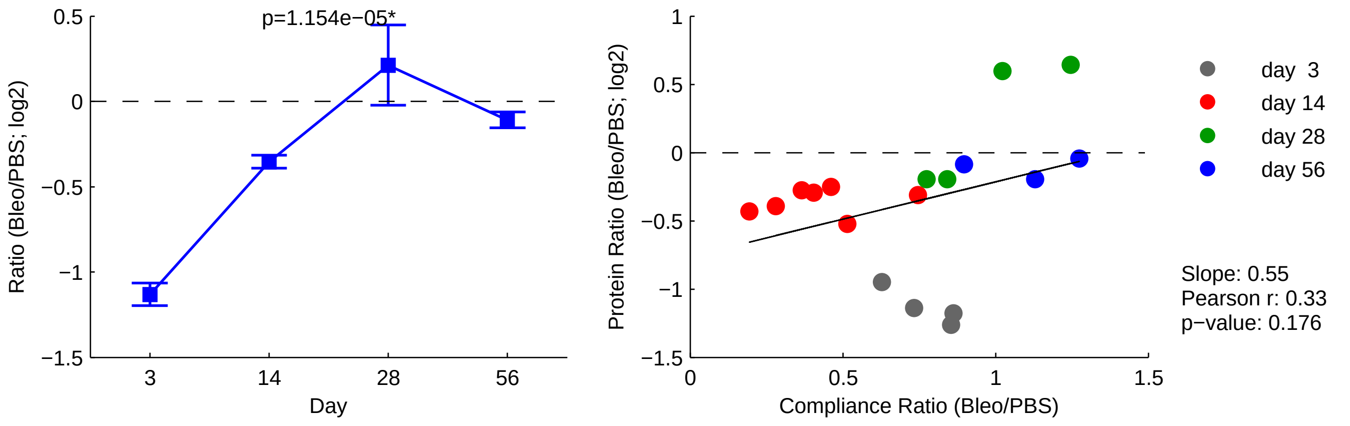

Q9CZ28 − Snf8 (id: 6938)

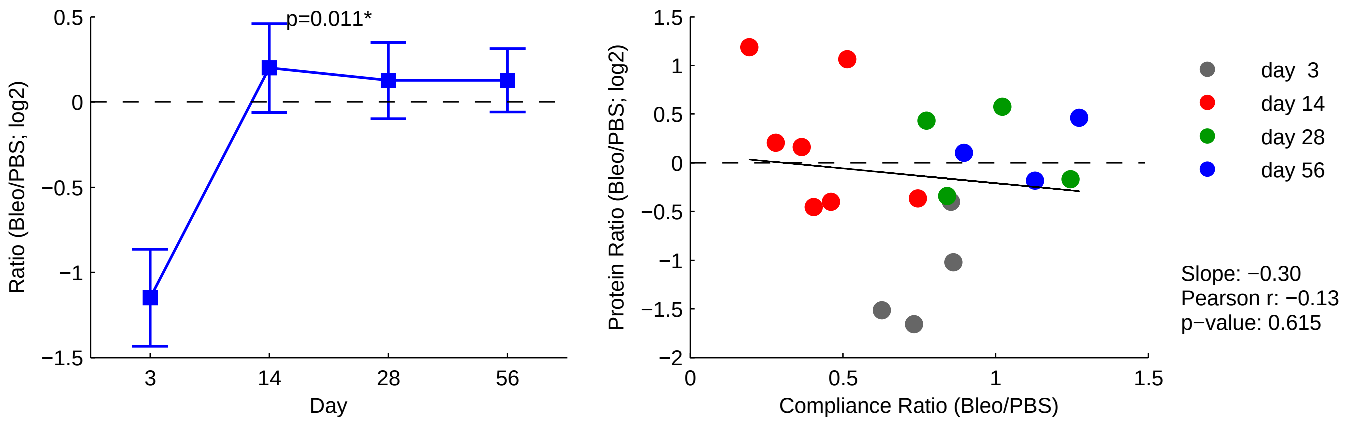

Q9CZ30 − Ola1 (id: 6939)

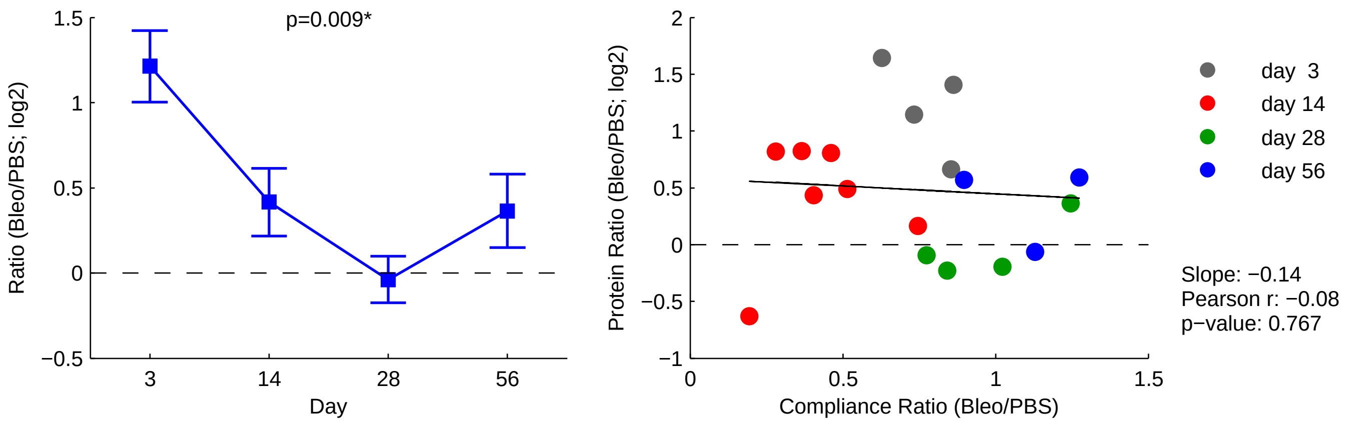

Q9CZ42−2 − (id: 6940)

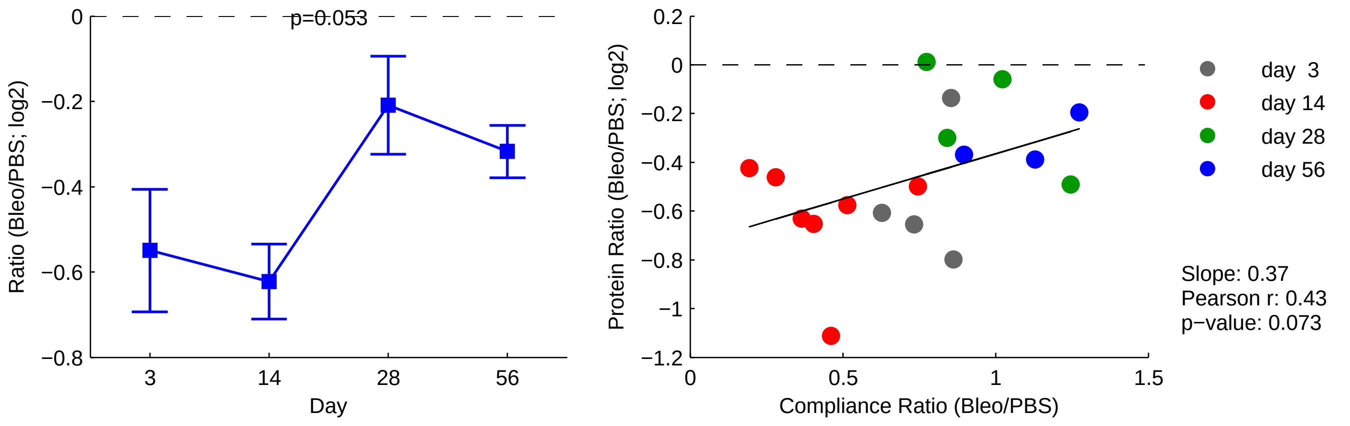

Q9CZ44 − Nsfl1c (id: 6941)

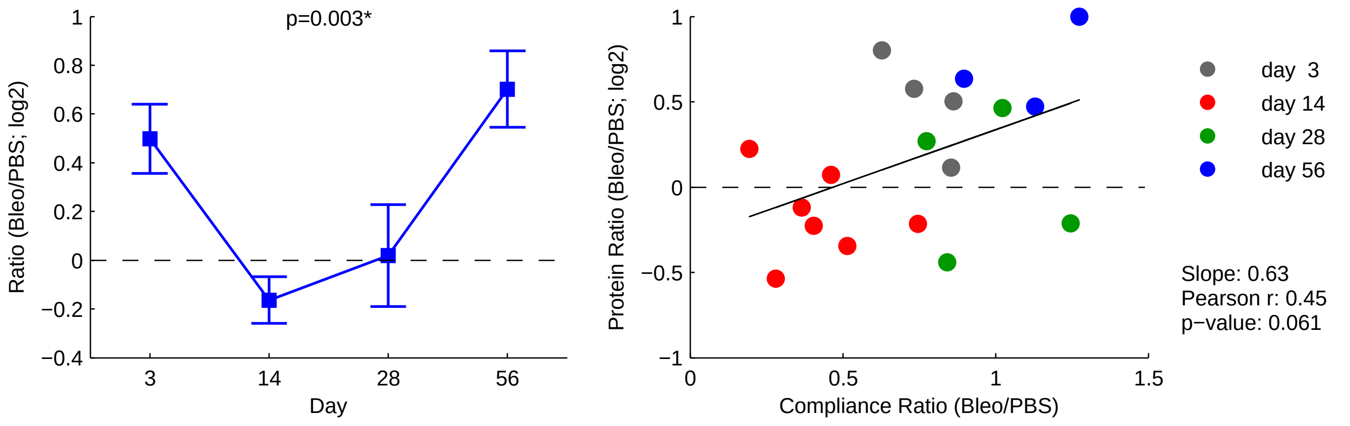

Q9CZ52−2 − Antxr1 (id: 6942)

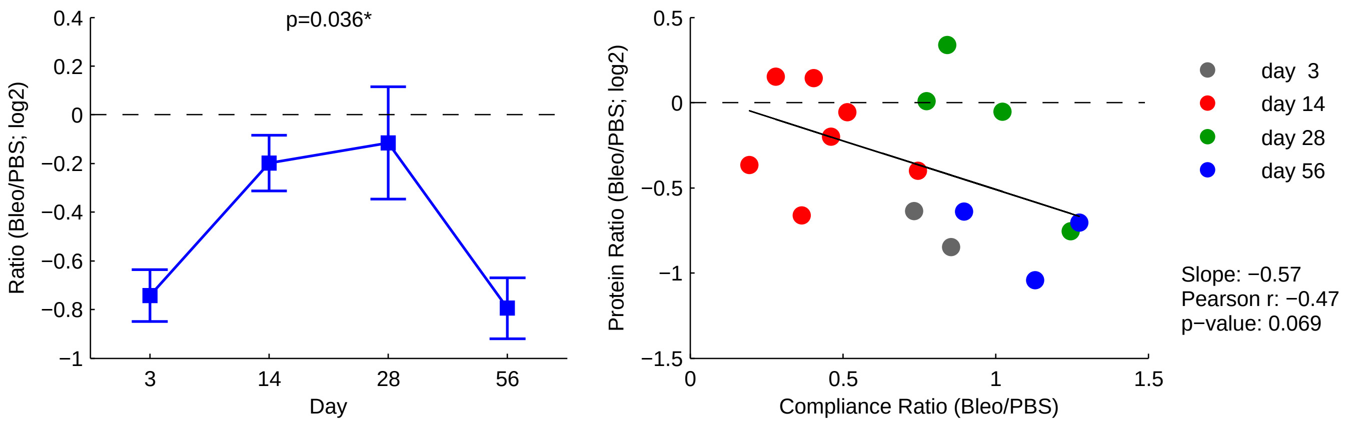

Q9CZA6−4 − Nde1 (id: 6944)

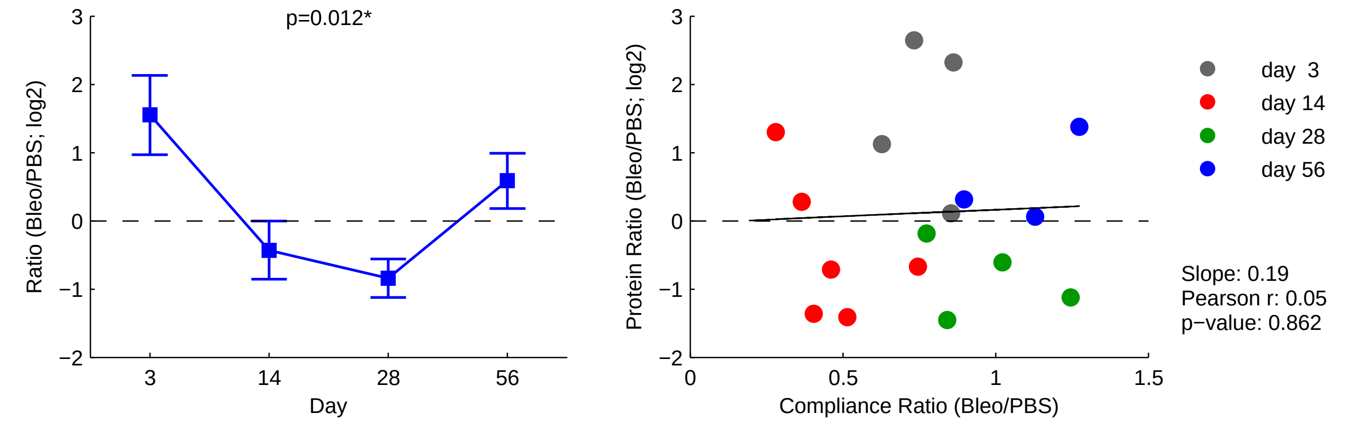

Q9CZC8 − Scrn1 (id: 6945)

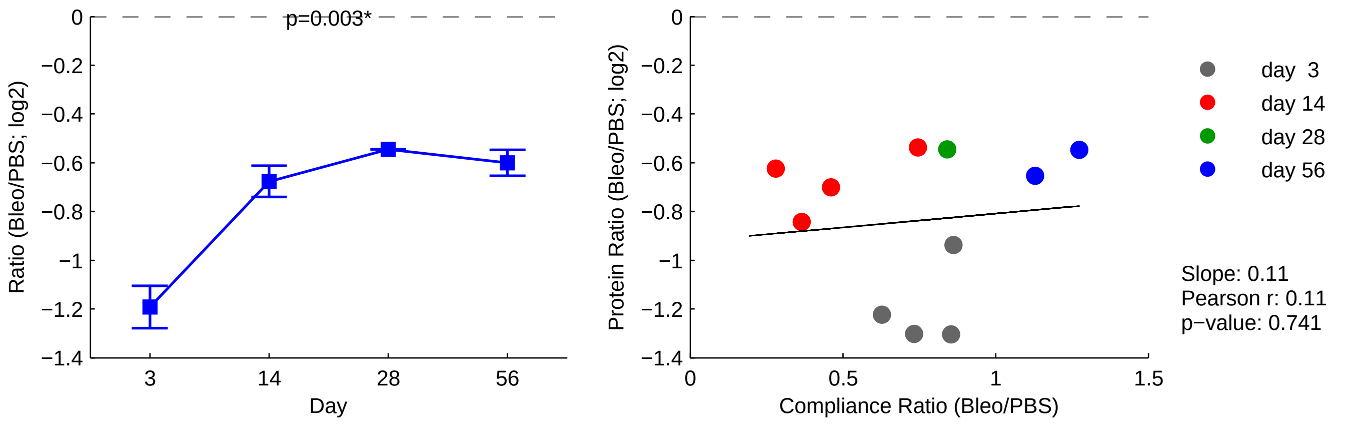

Q9CZD3 − Gars (id: 6946)

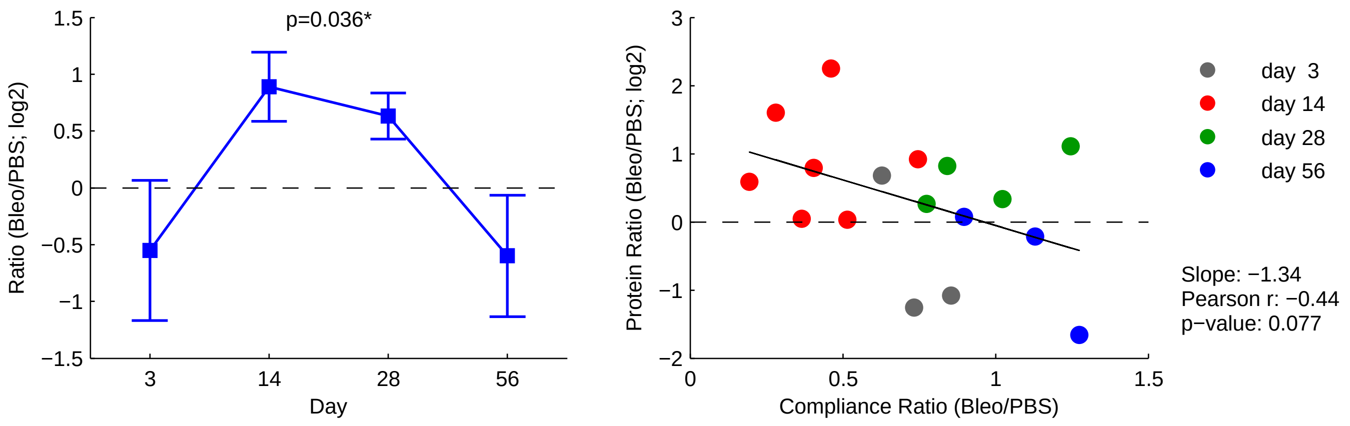

Q9CZE3 − Rab32 (id: 6948)

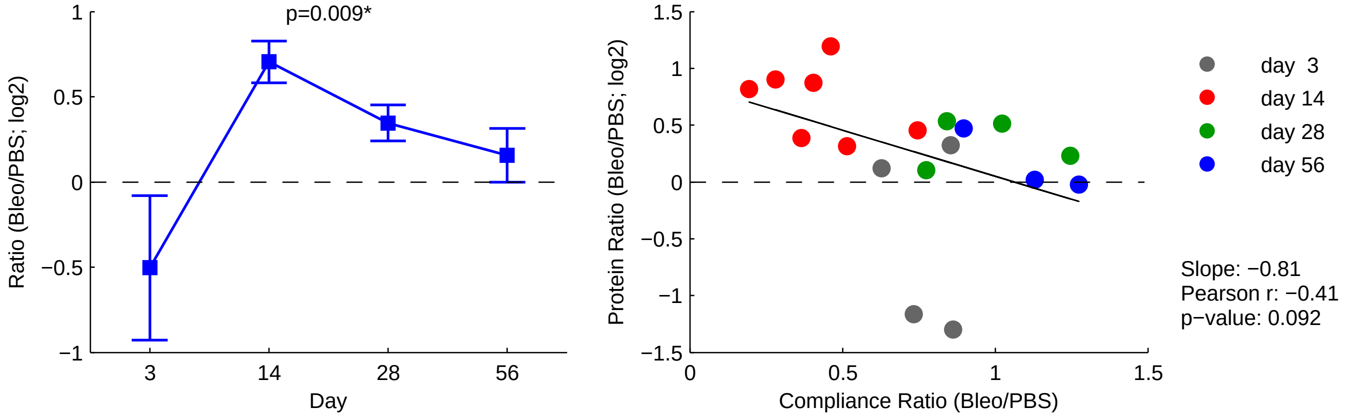

Q9CZG3 − Commd8 (id: 6949)

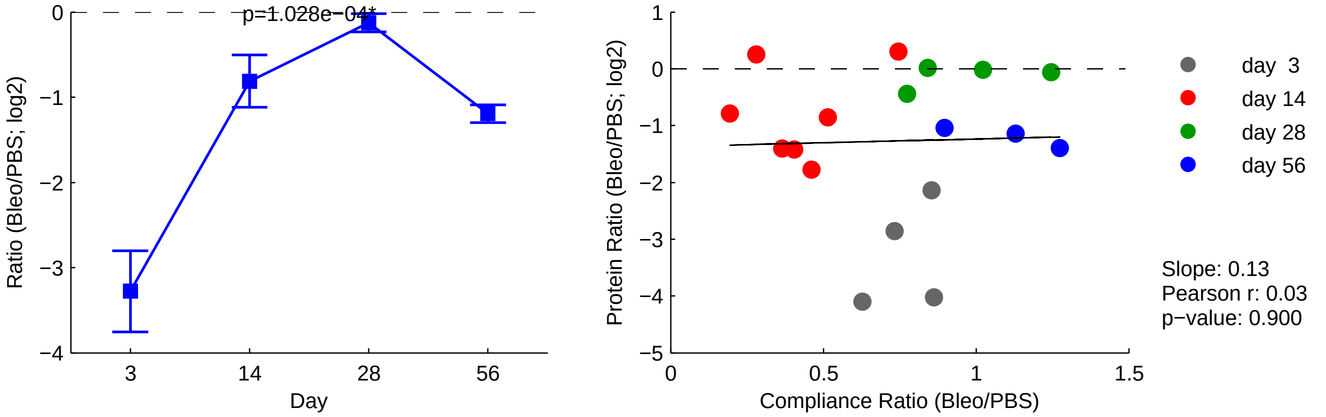

Q9CZG9 − Pdzd11 (id: 6950)

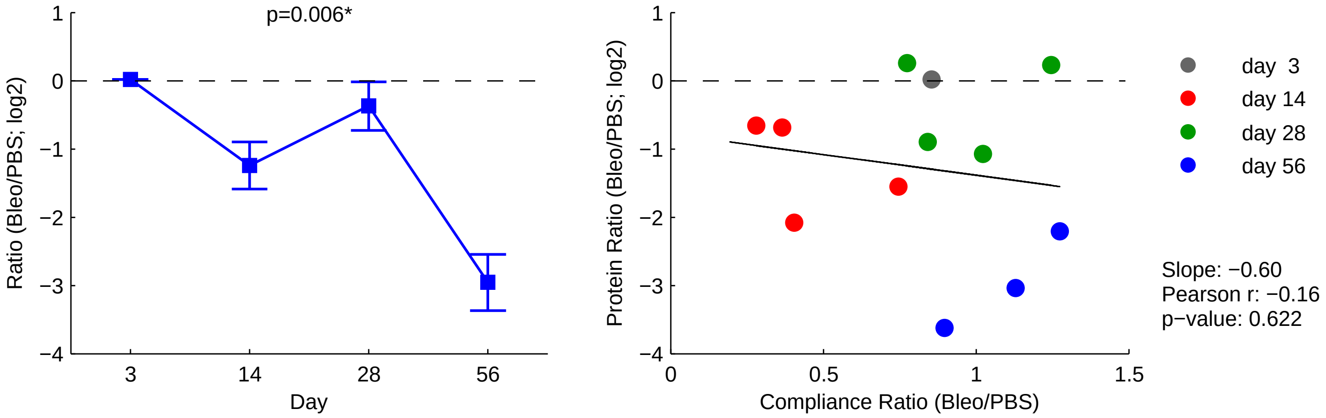

Q9CZH7 − Mxra7 (id: 6951)

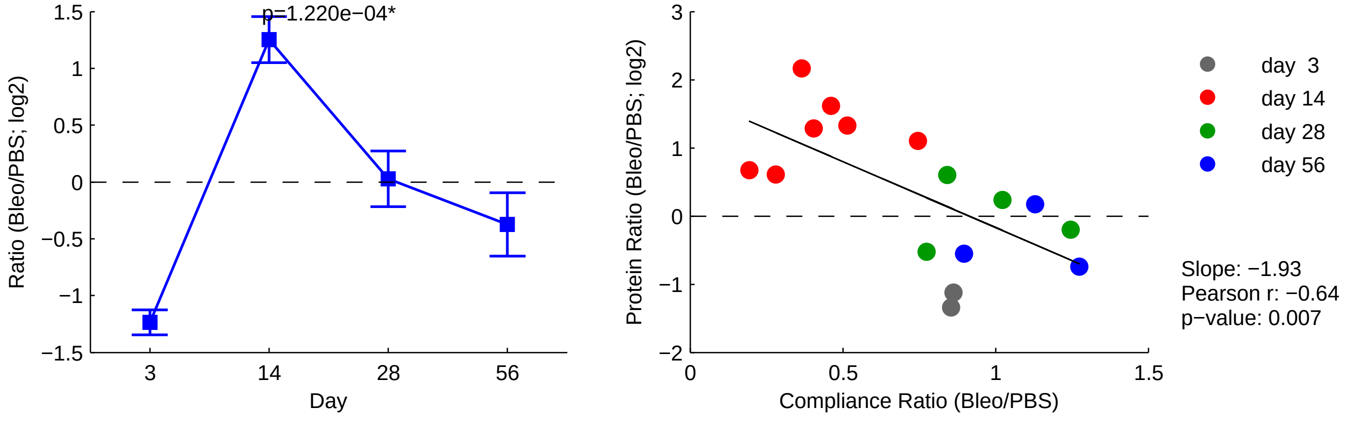

Q9CZJ2 − Hspa12b (id: 6953)

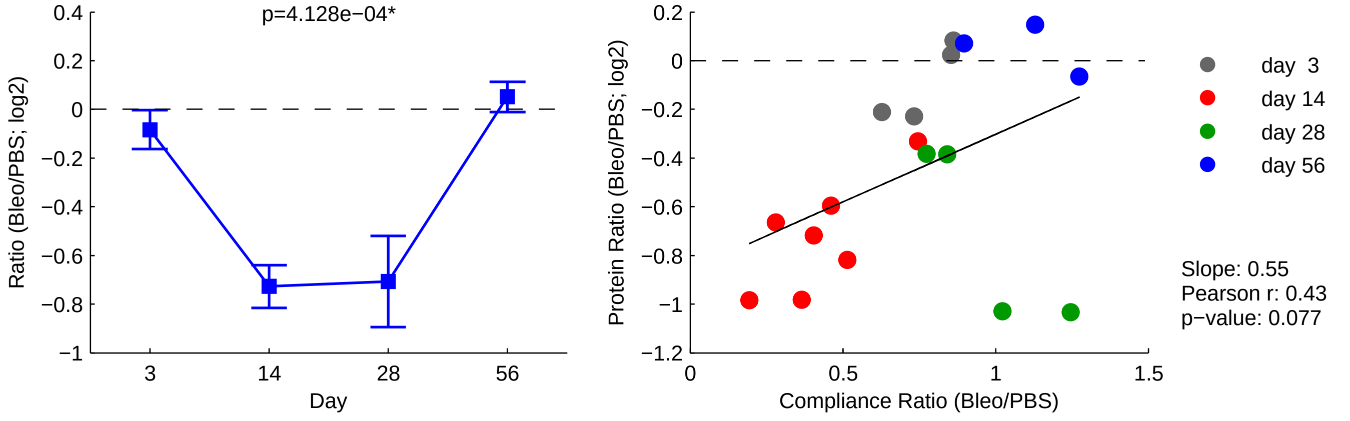

Q9CZP5 − Bcs1l (id: 6957)

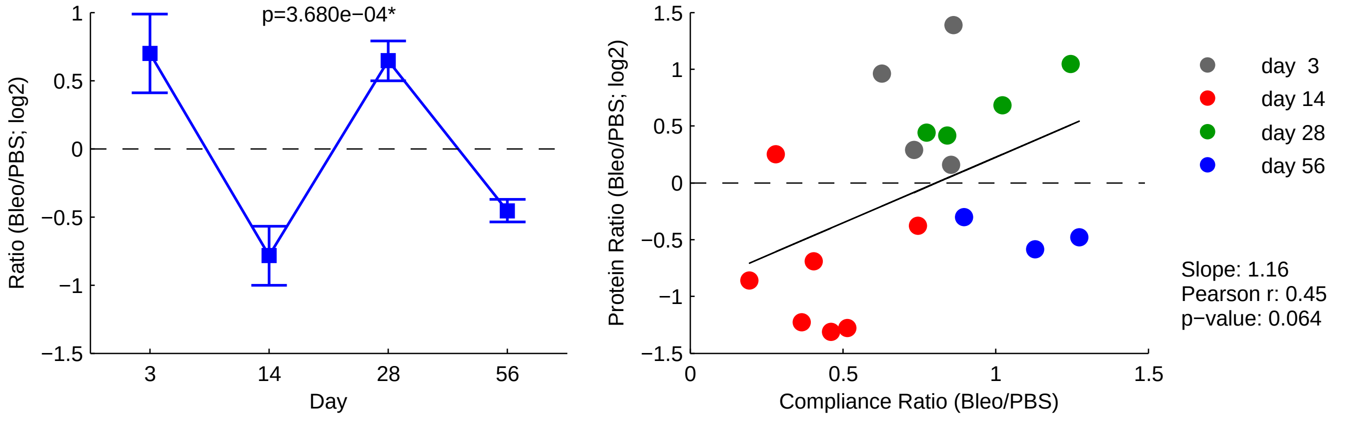

Q9CZR3 − Tomm40l (id: 6958)

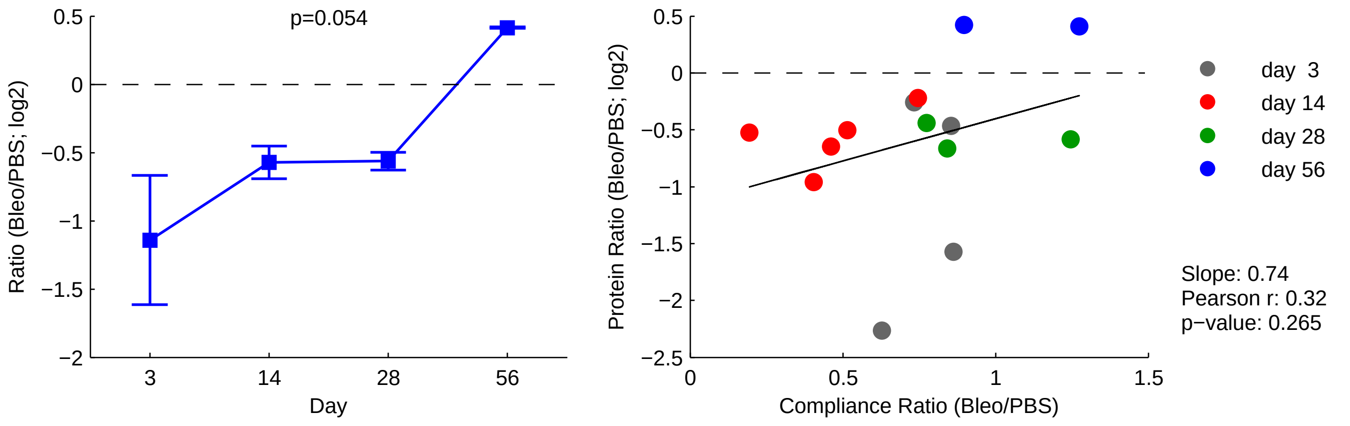

Q9CZR8 − Tsfm (id: 6959)

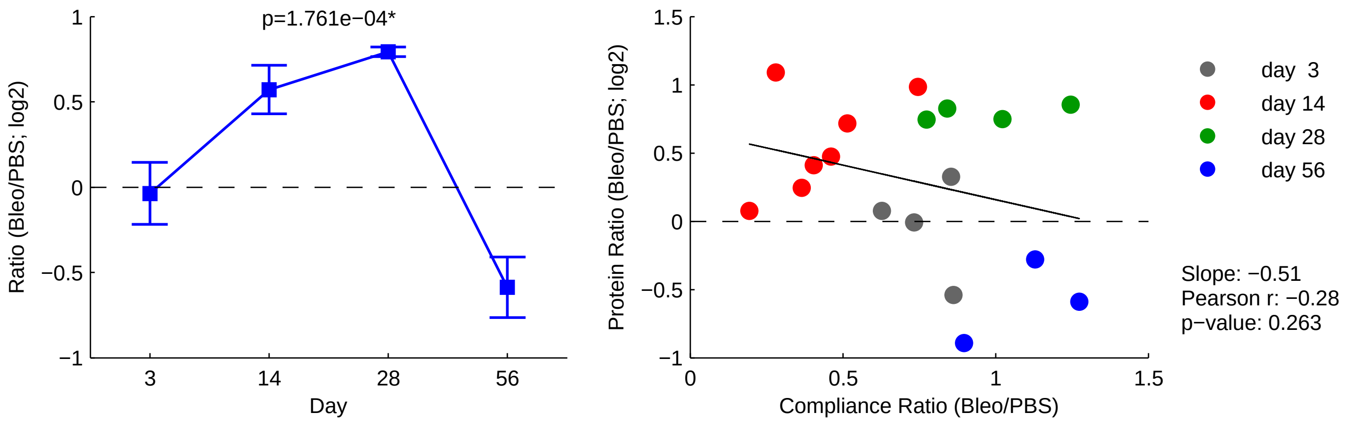

Q9CZS1 − Aldh1b1 (id: 6960)

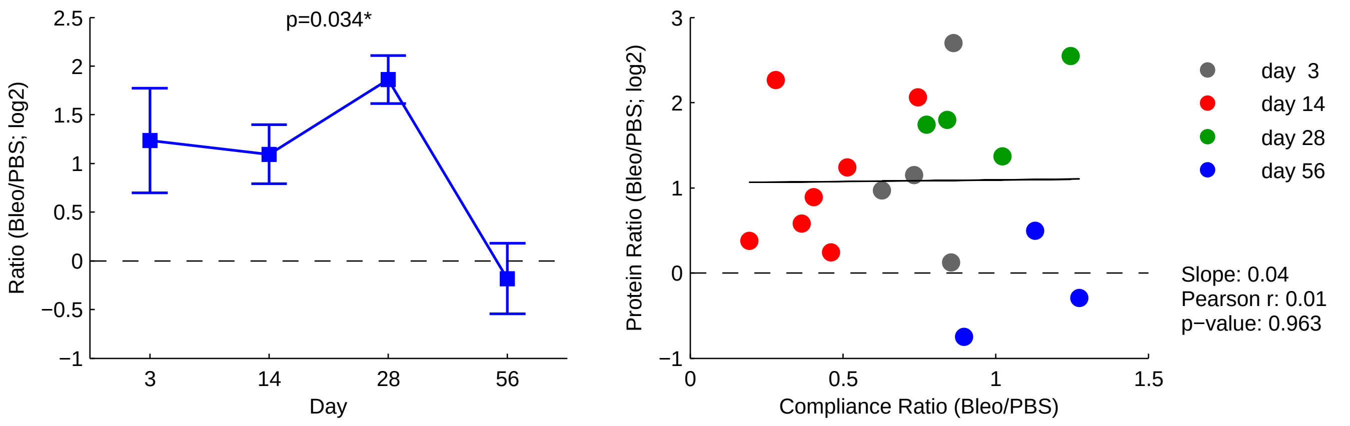

Q9CZV8 − Fbxl20 (id: 6967)

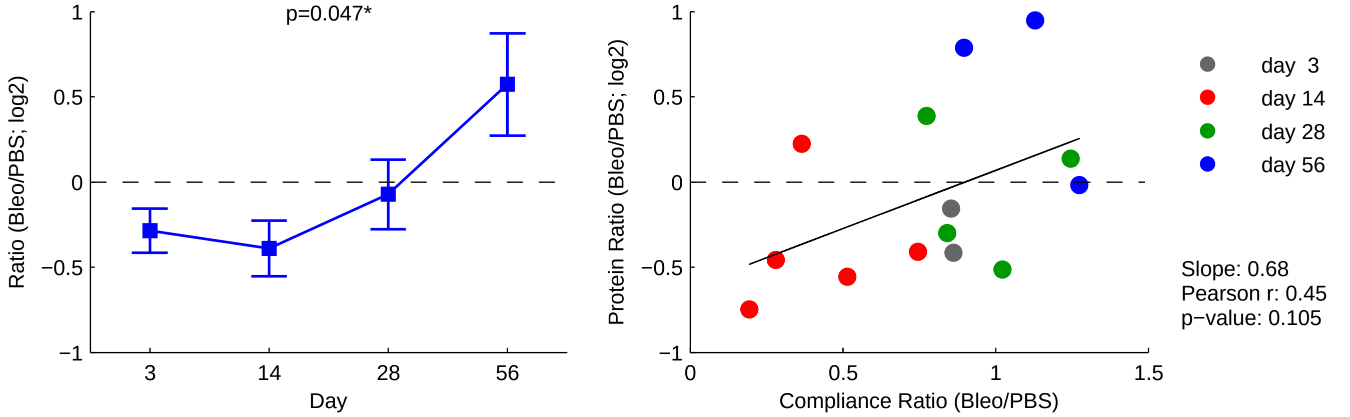

Q9CZW5 − Tomm70a (id: 6969)

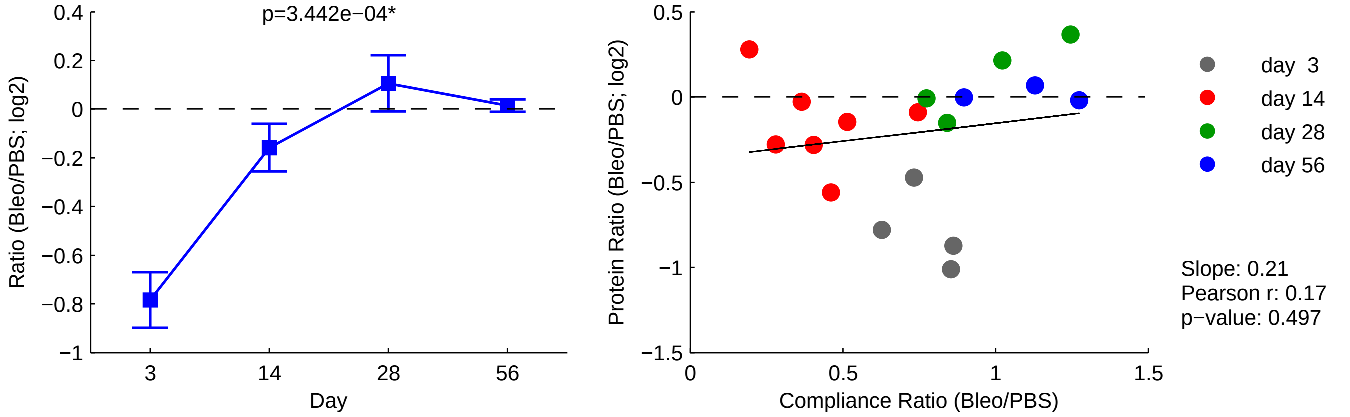

Q9CZX0−2 − Elp3 (id: 6970)

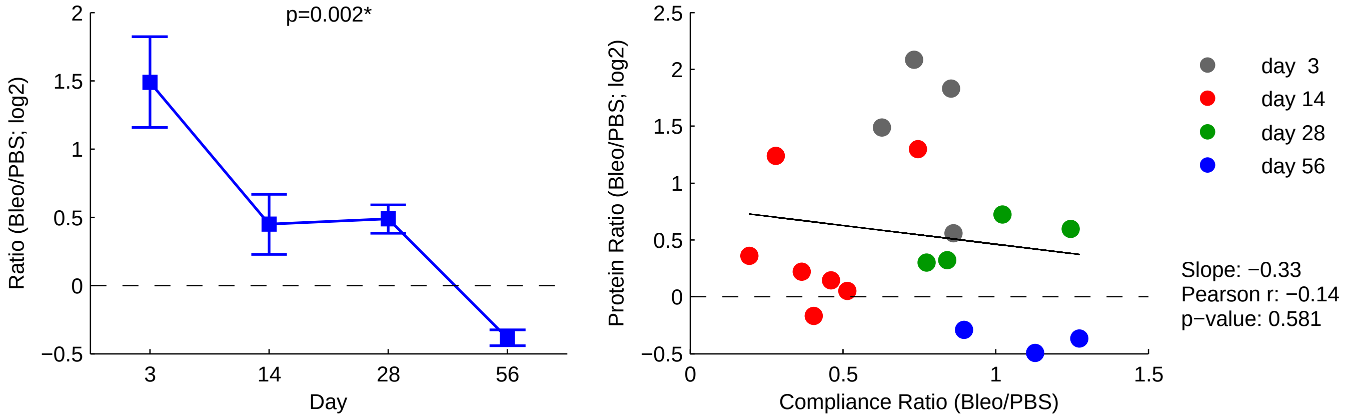

Q9CZX8 − Rps19 (id: 6972)

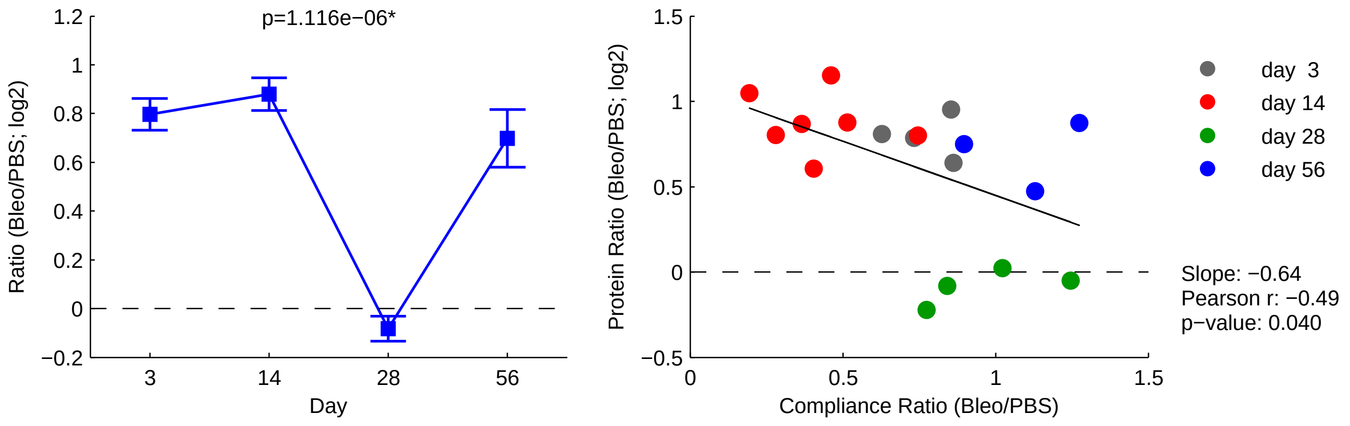

Q9D020 − Nt5c3 (id: 6976)

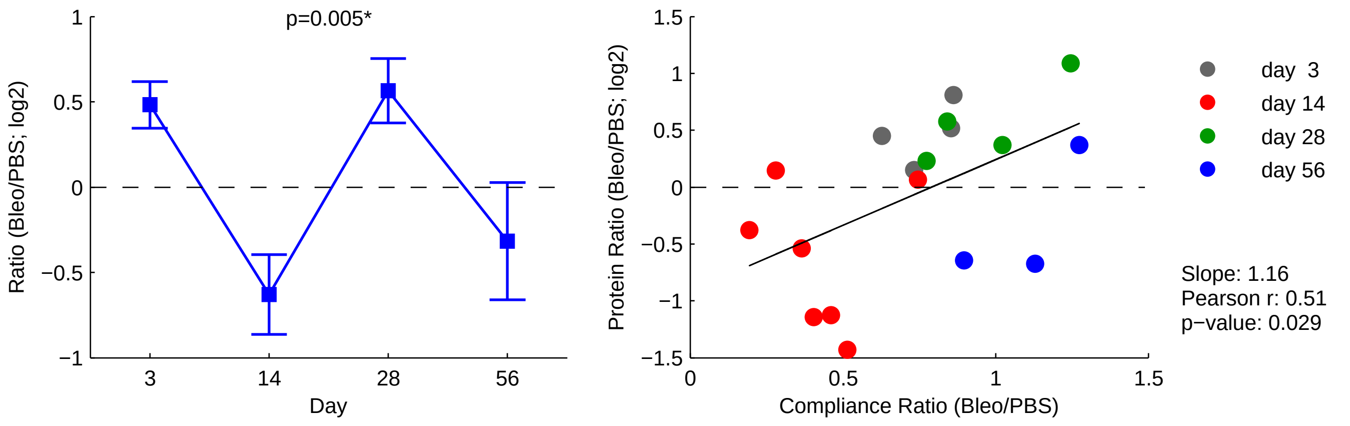

Q9D023 − Mpc2 (id: 6977)

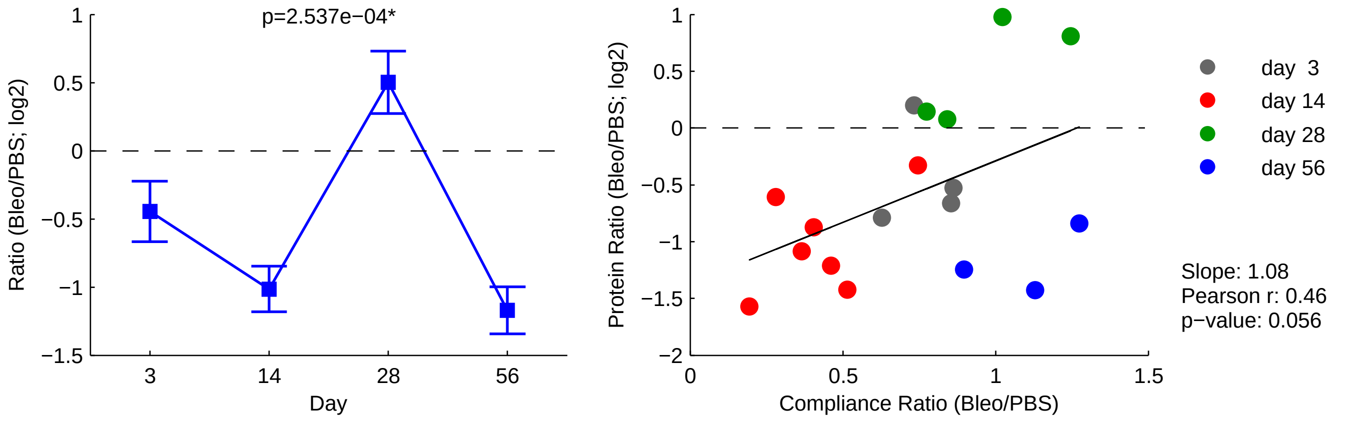

Q9D024 − Ccdc47 (id: 6978)

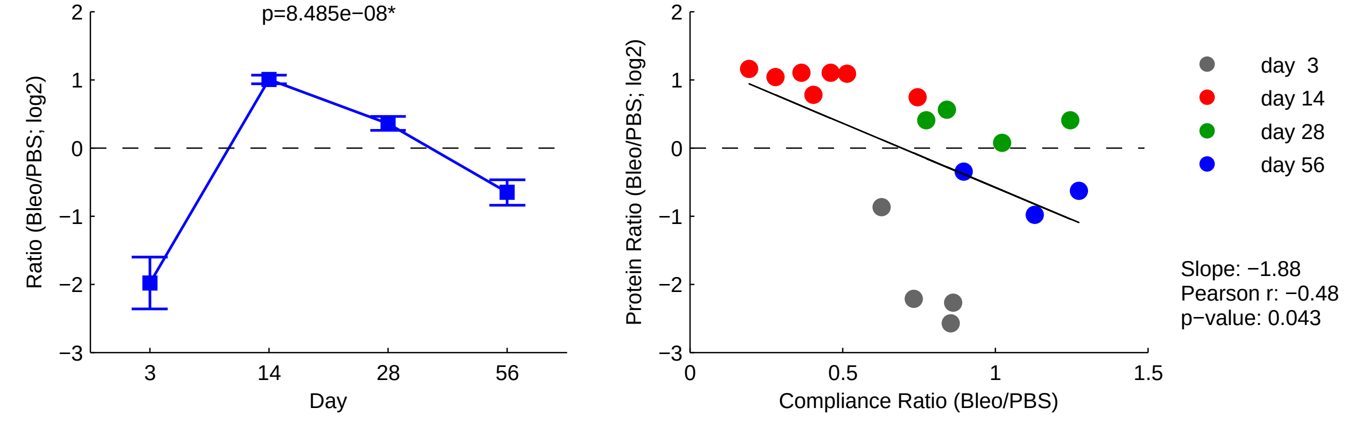

Q9D031 − Rsu1 (id: 6979)

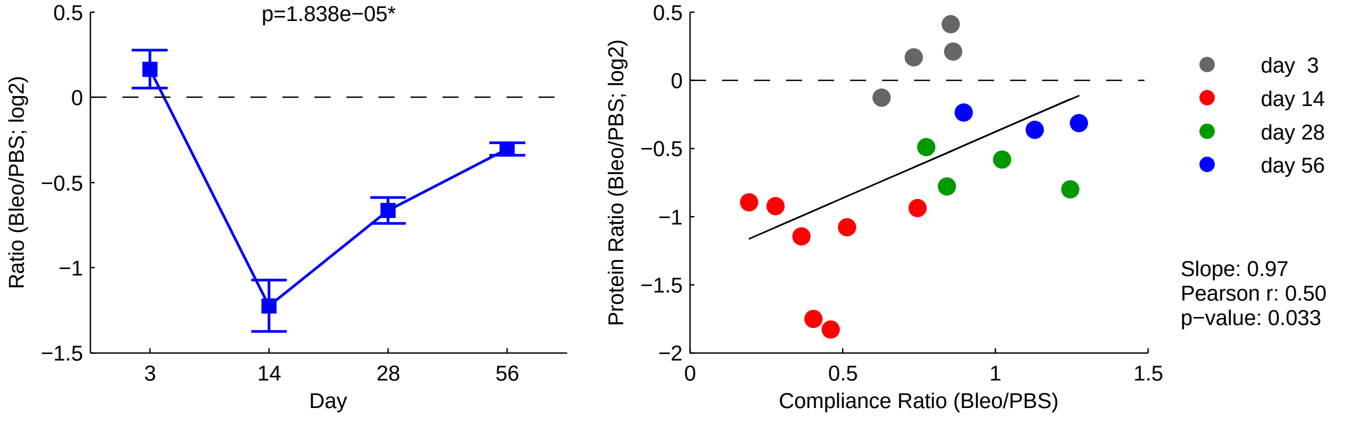

Q9D051 − Pdhb (id: 6980)

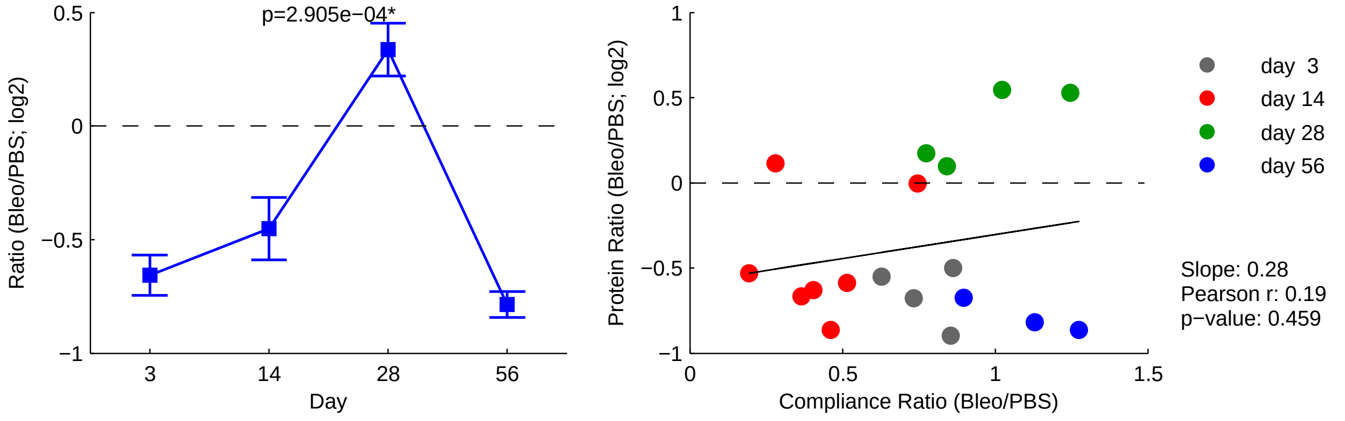

Q9D071−2 − Mms19 (id: 6981)

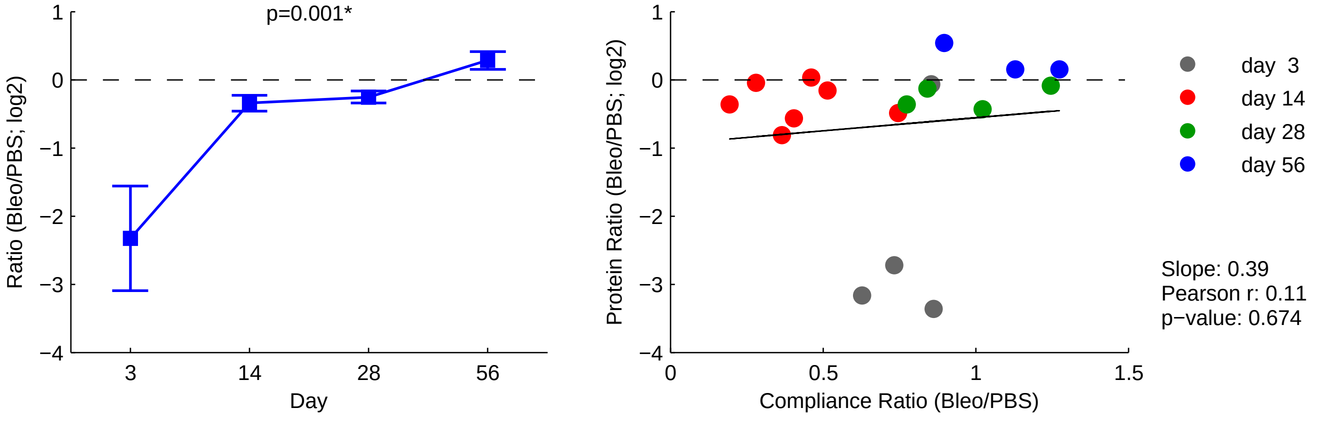

Q9D074 − Mgrn1 (id: 6982)

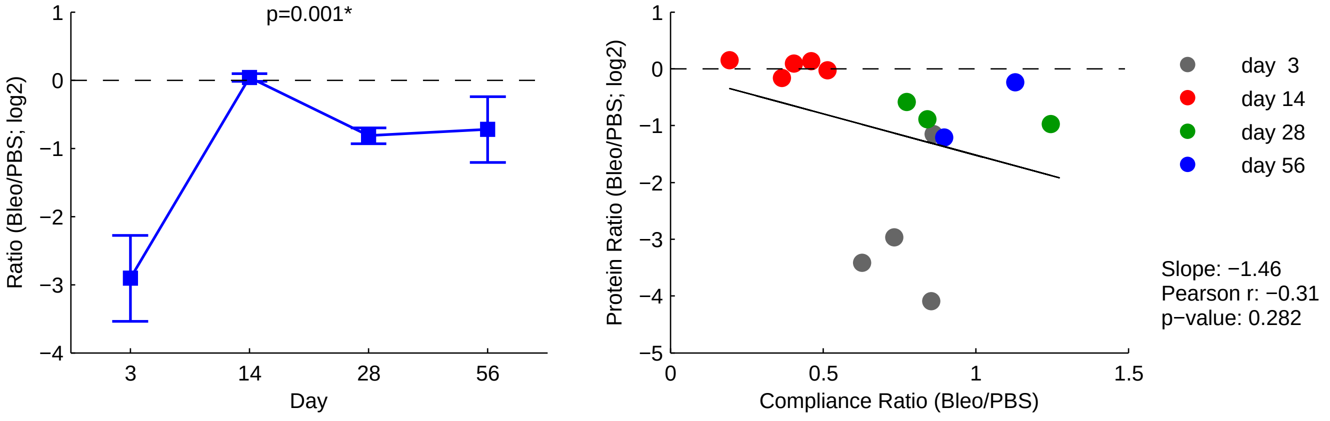

Q9D081 − Alg14 (id: 6983)

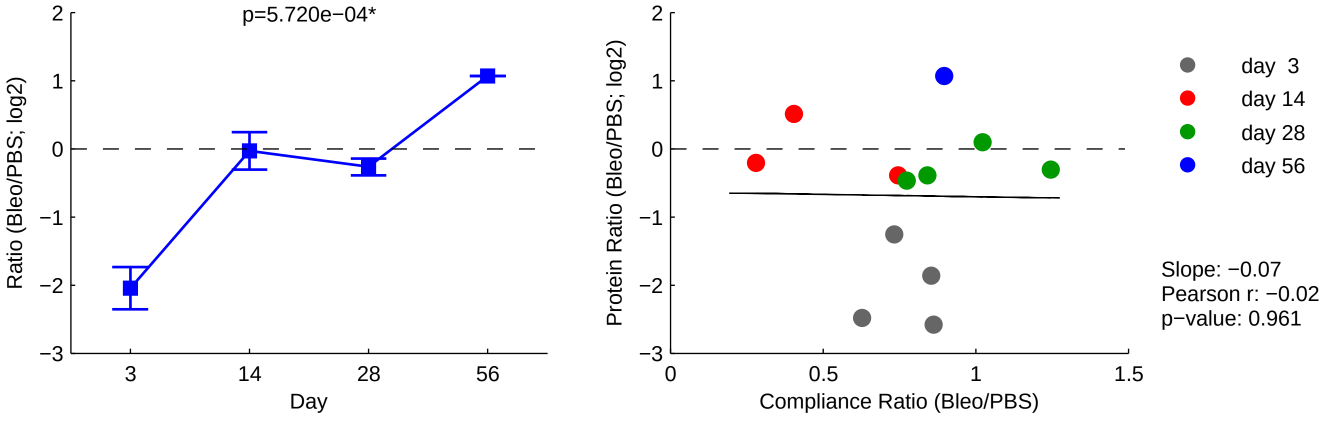

Q9D0B6 − Pbdc1 (id: 6987)

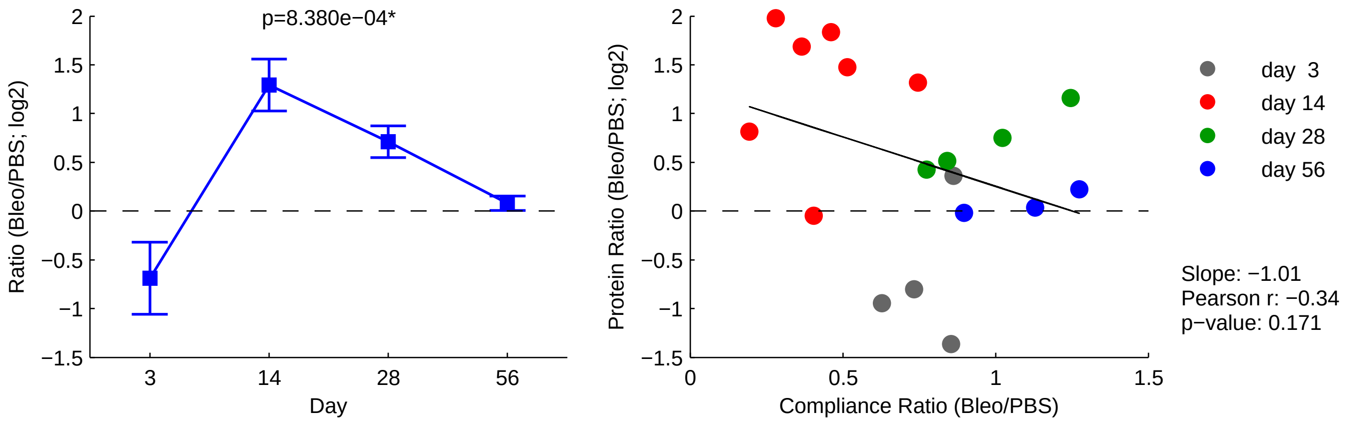

Q9D0D4 − Dimt1 (id: 6989)

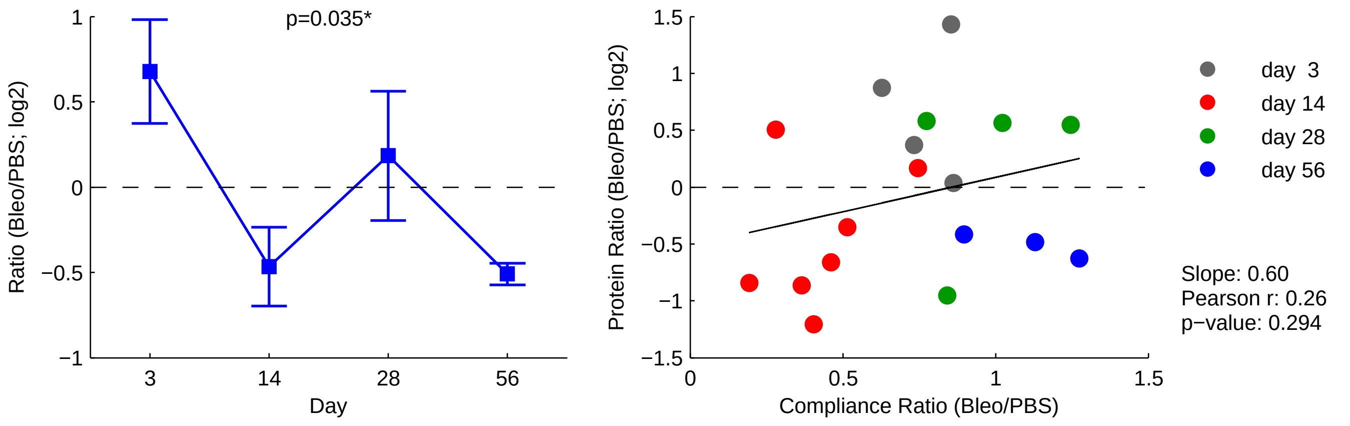

Q9D0E1−2 − Hnrnpm (id: 6991)

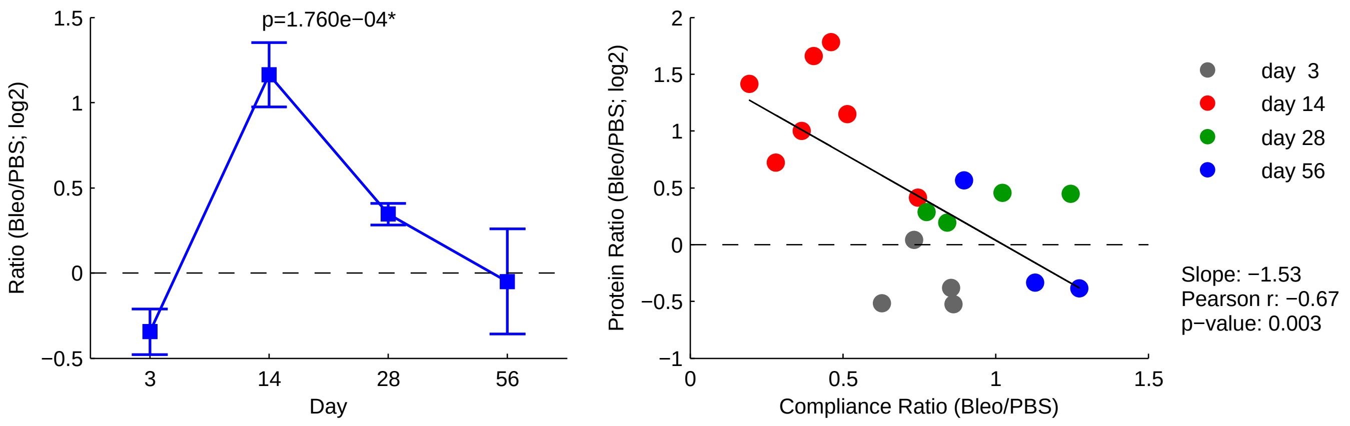

Q9D0F3 − Lman1 (id: 6992)

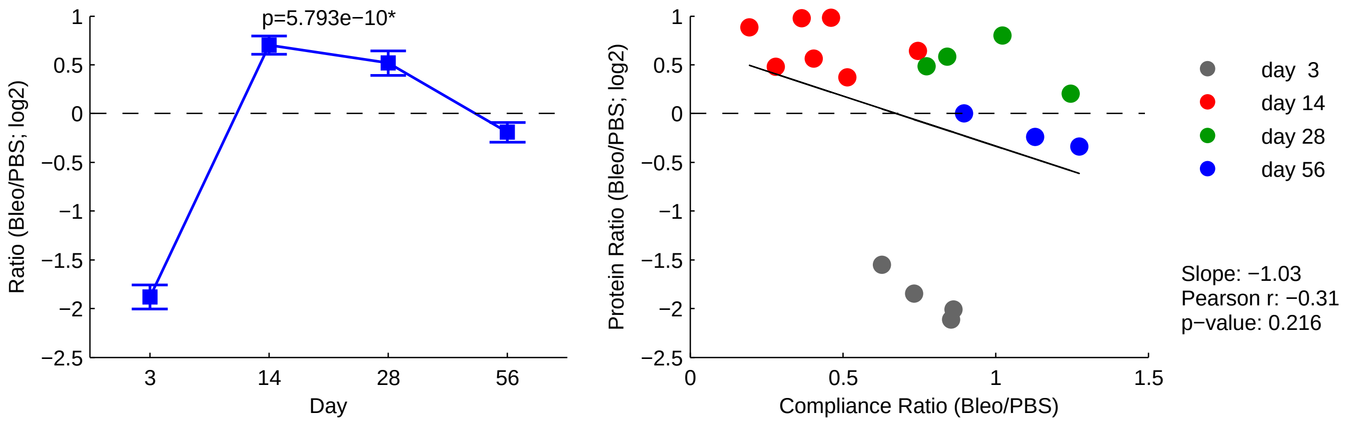

Q9D0F6 − Rfc5 (id: 6994)

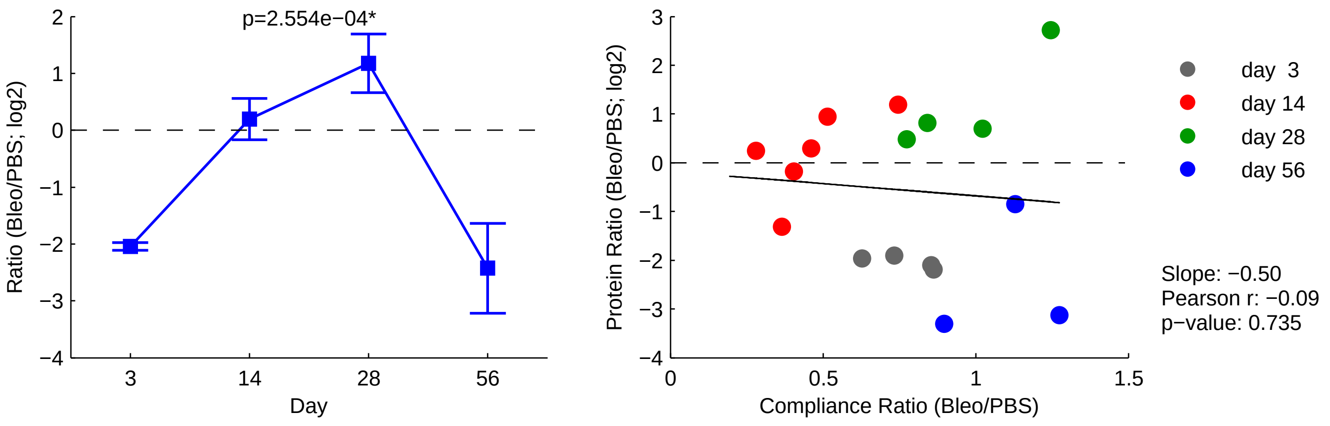

Q9D0F9 − Pgm1 (id: 6995)

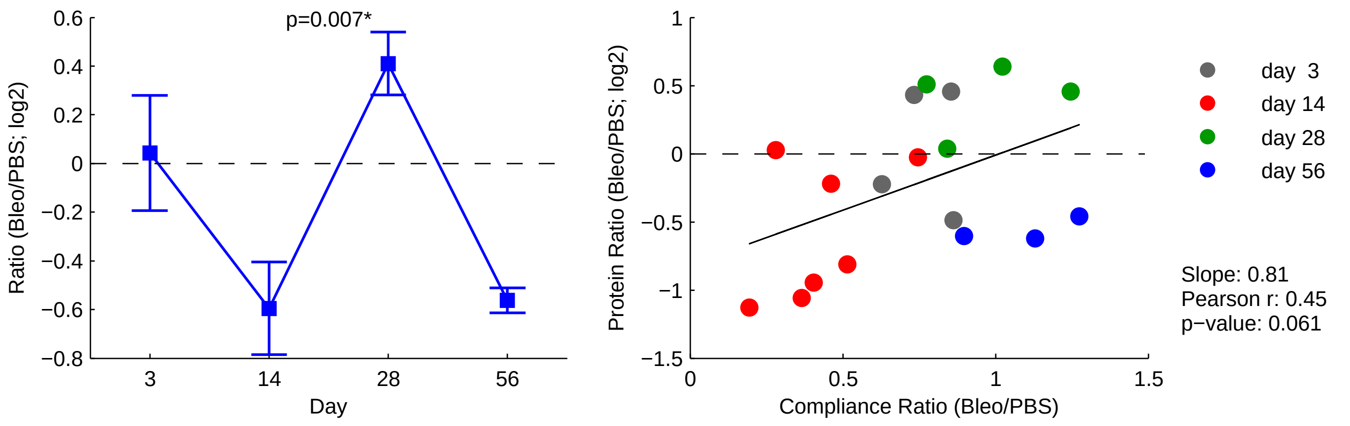

Q9D0G0 − Mrps30 (id: 6996)

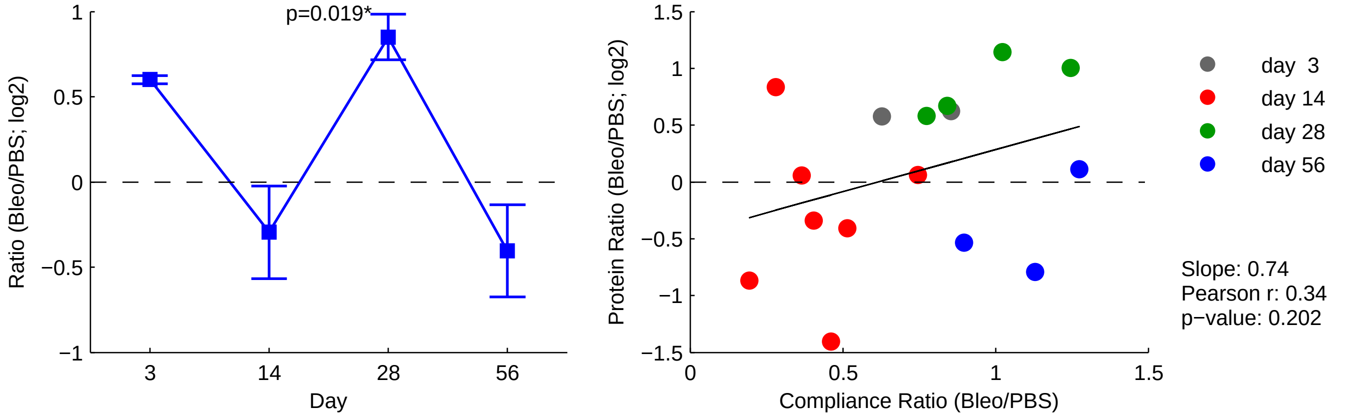

Q9D0I9 − Rars (id: 6999)

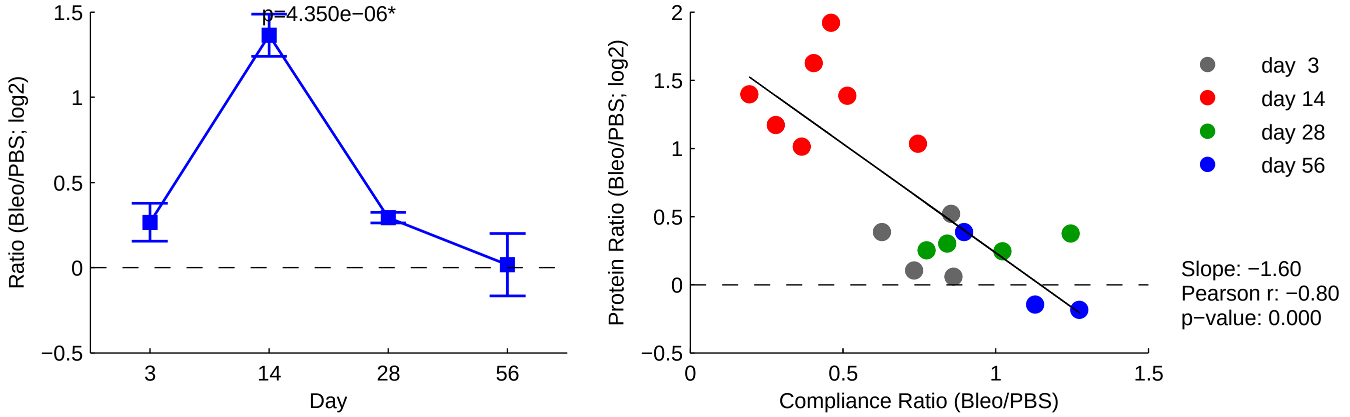

Q9D0K2 − Oxct1 (id: 7003)

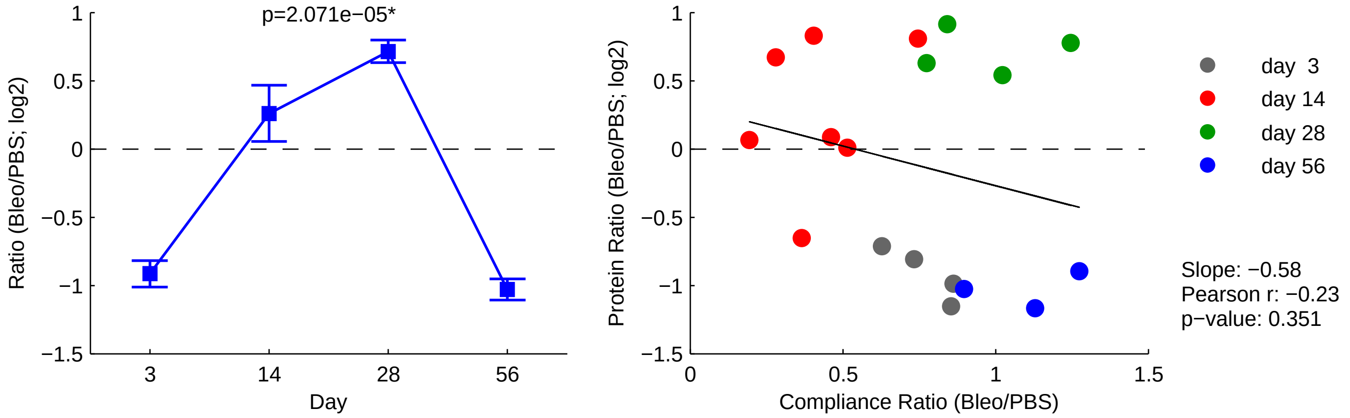

Q9D0L4 − Adck1 (id: 7004)

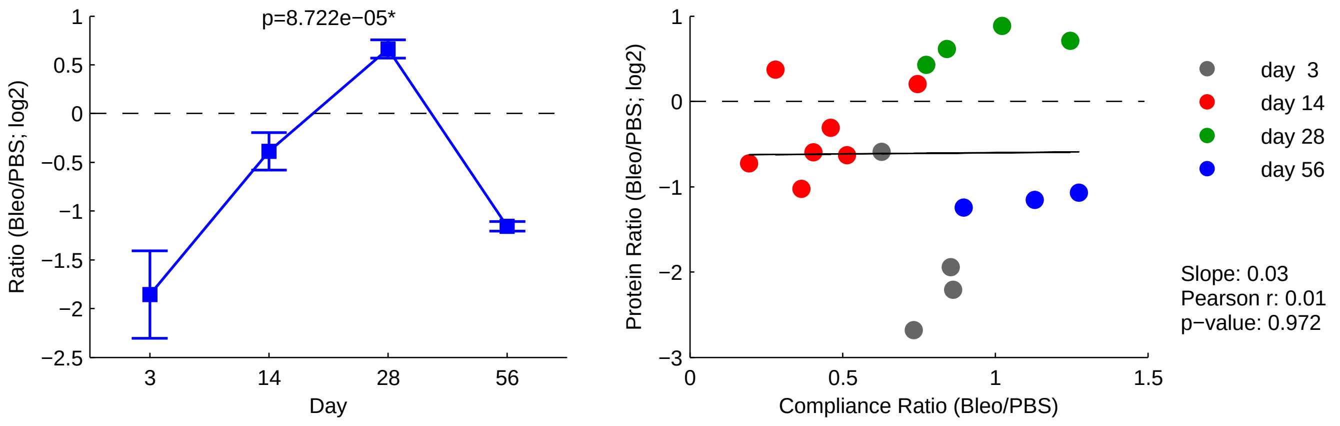

Q9D0L7 − Armc10 (id: 7005)

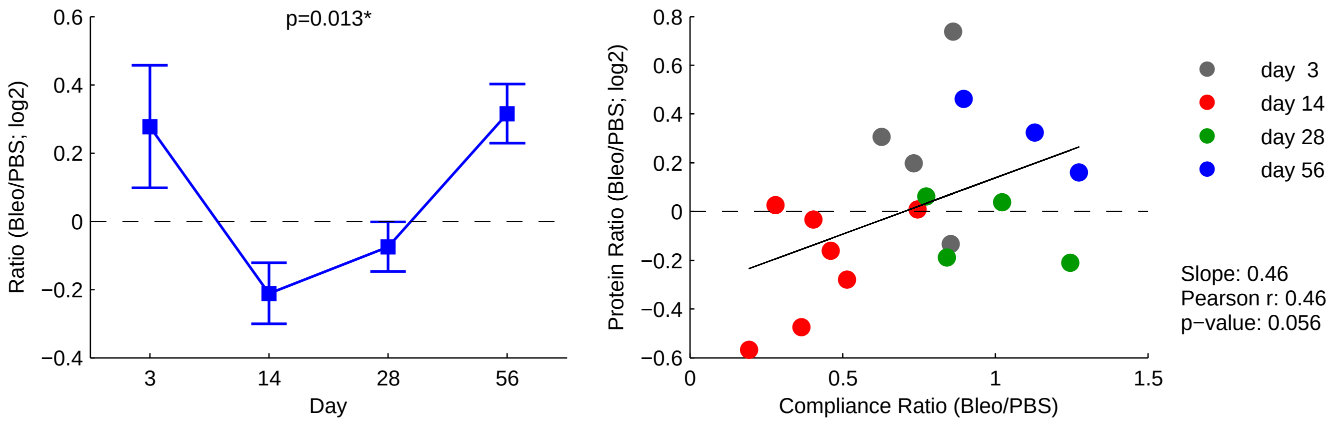

Q9D0M3−2 − Cyc1 (id: 7008)

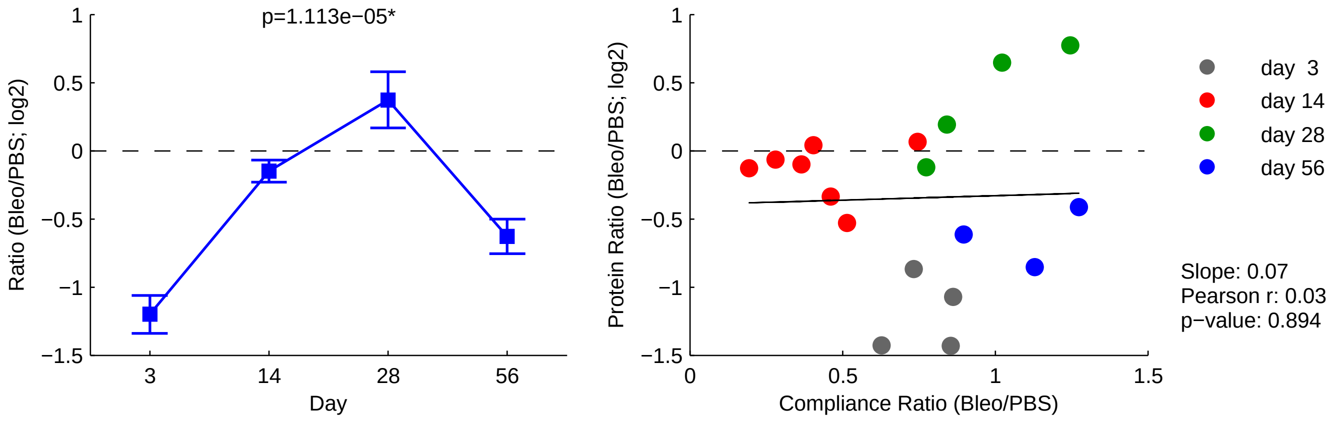

Q9D0M5 − Dynll2 (id: 7009)

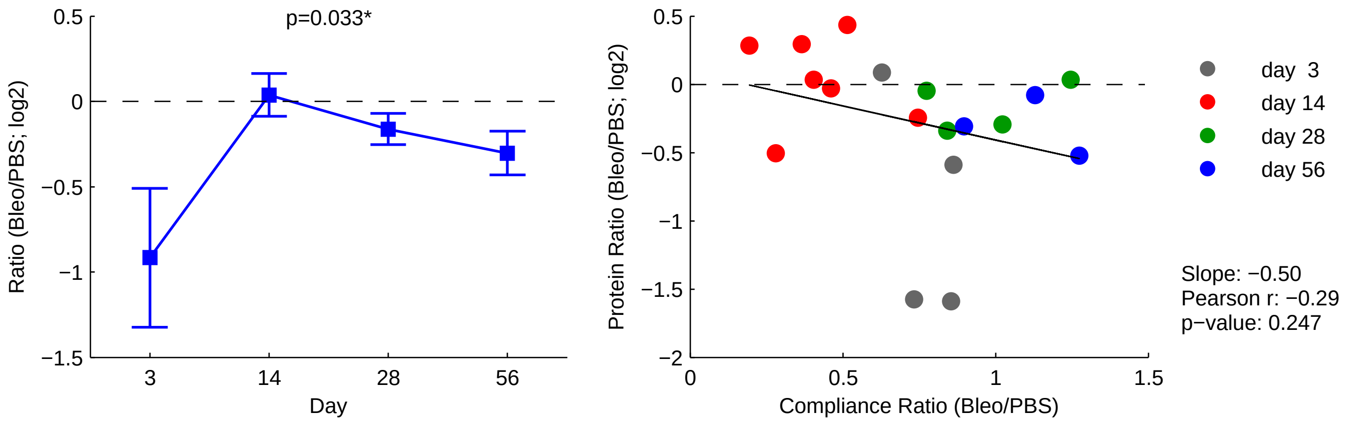

Q9D0Q7 − Mrpl45 (id: 7010)

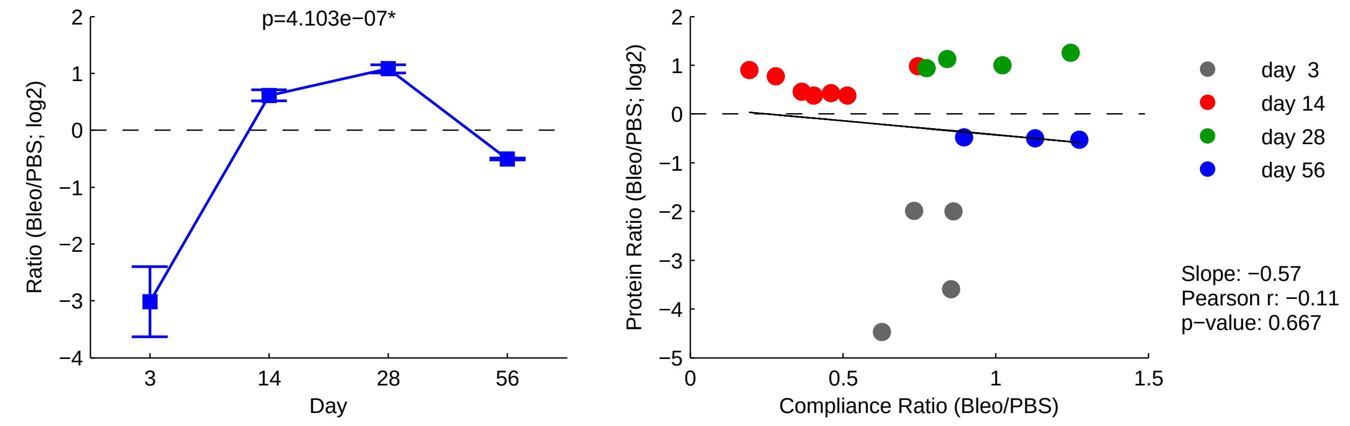

Q9D0R8 − Lsm12 (id: 7013)

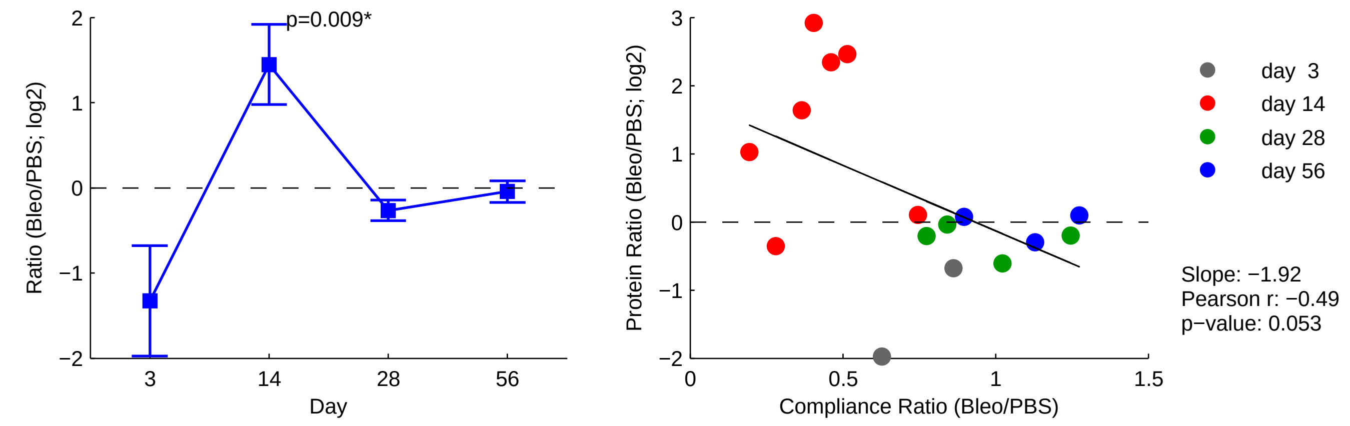

Q9D0S9 − Hint2 (id: 7014)

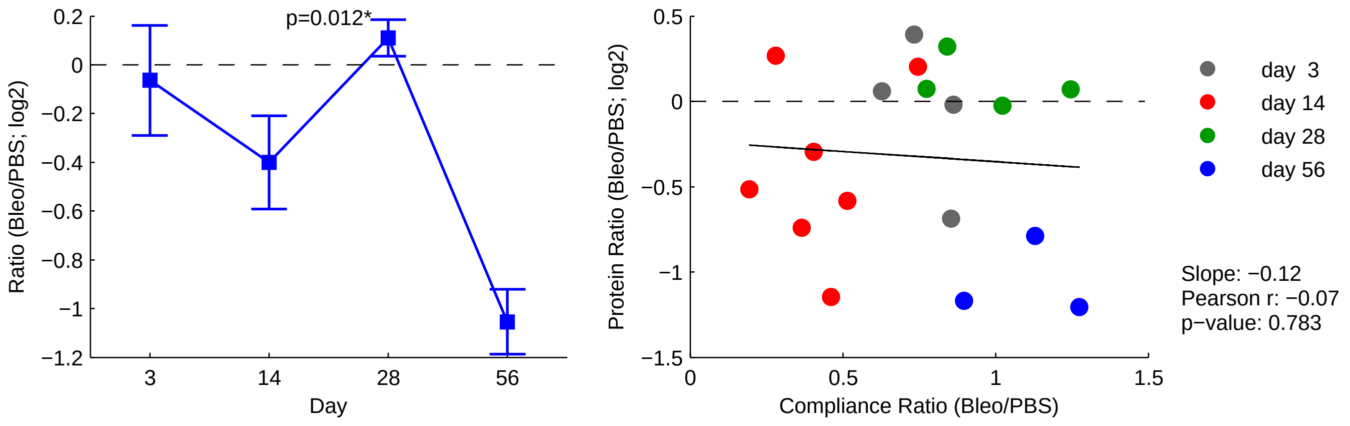

Q9D0W5 − Ppil1 (id: 7017)

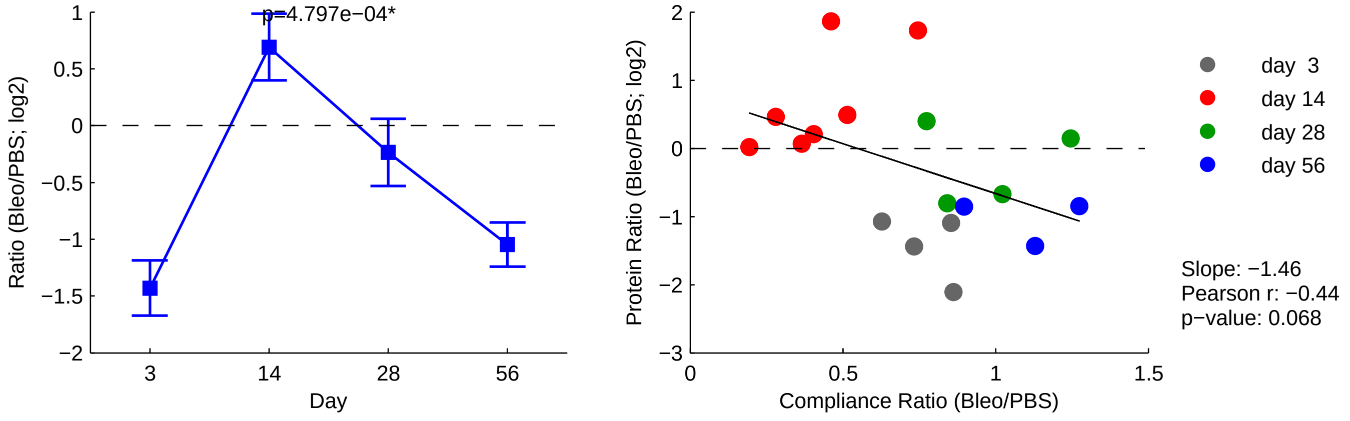

Q9D104 − Srp19 (id: 7019)

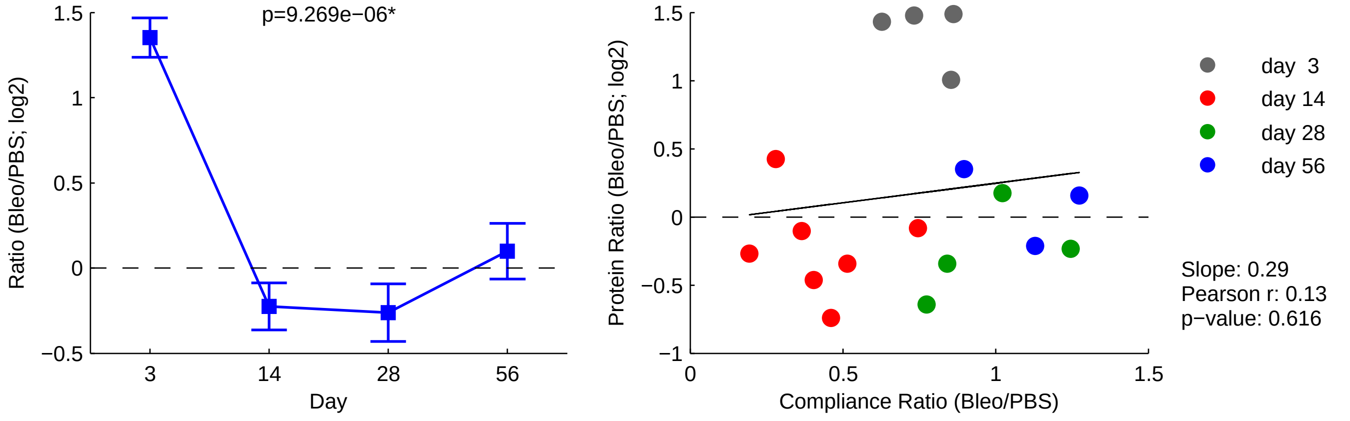

Q9D115 − Znf706 (id: 7020)

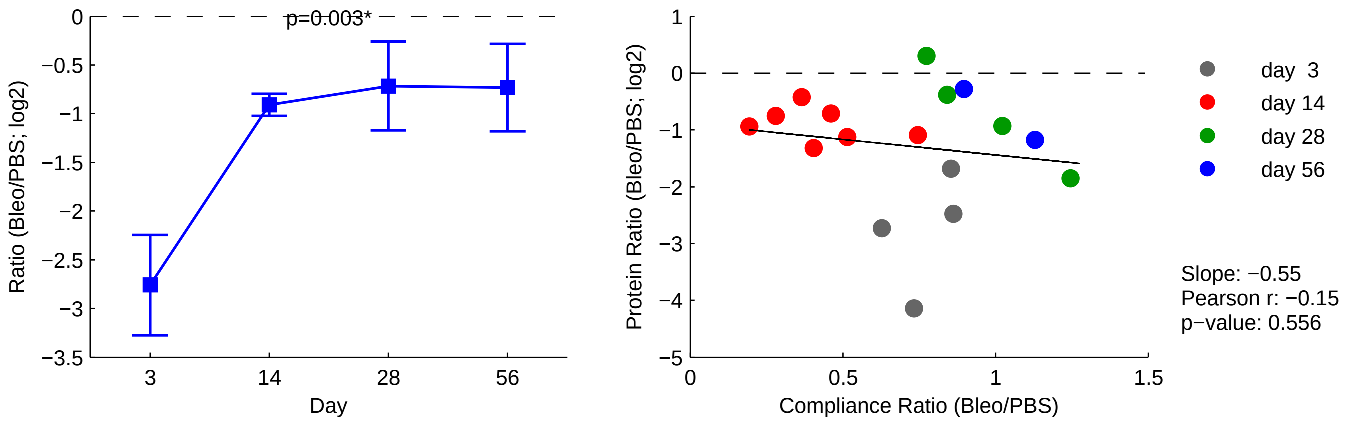

Q9D125 − Mrps25 (id: 7021)

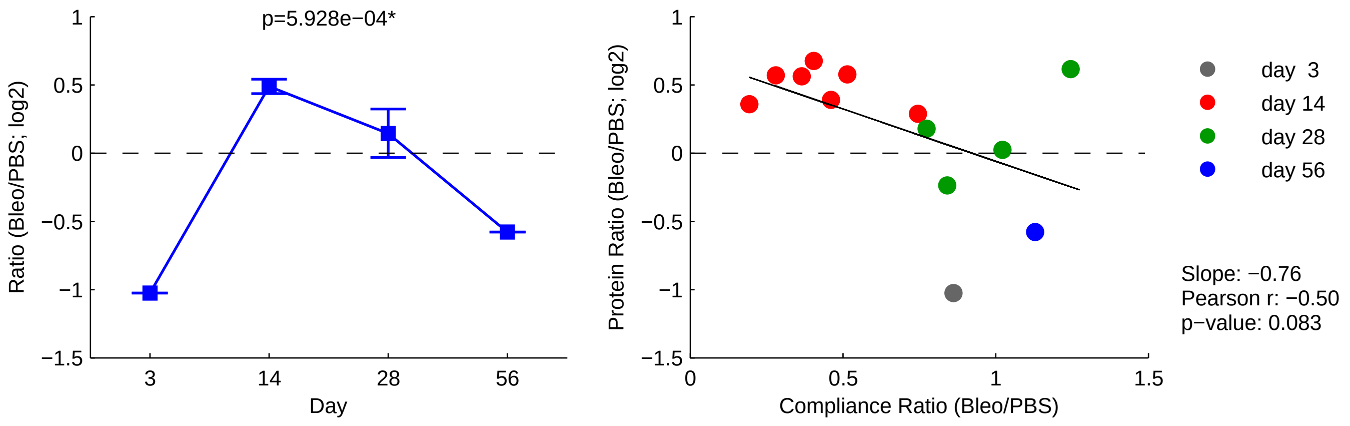

Q9D136 − Ogfod3 (id: 7022)

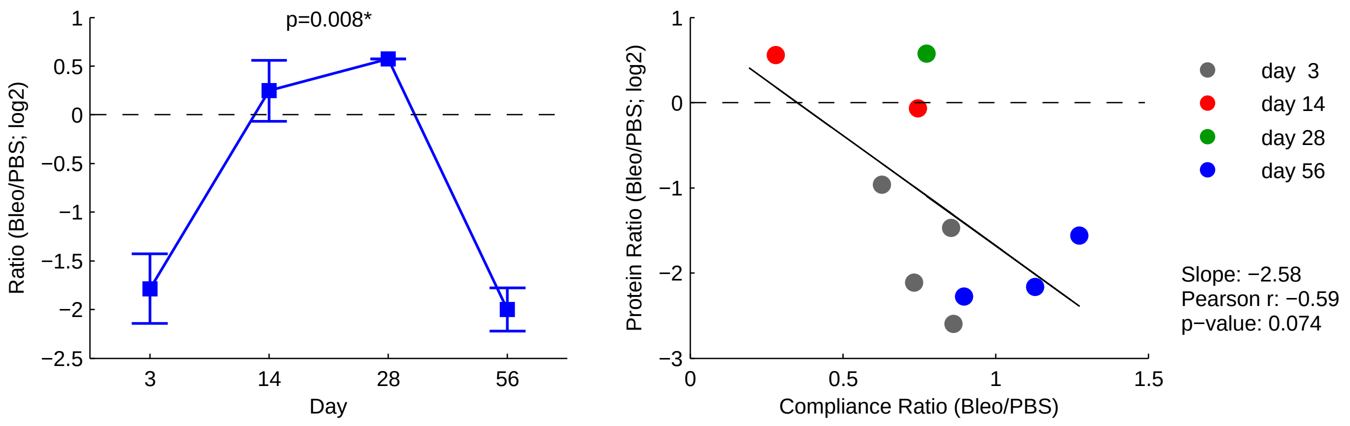

Q9D154 − Serpinb1a (id: 7023)

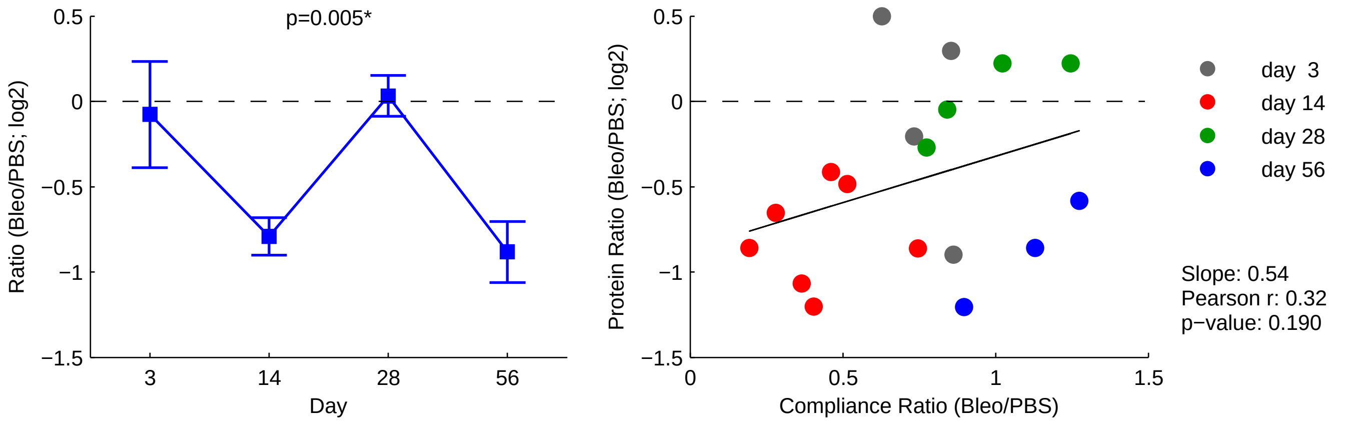

Q9D173 − Tomm7 (id: 7027)

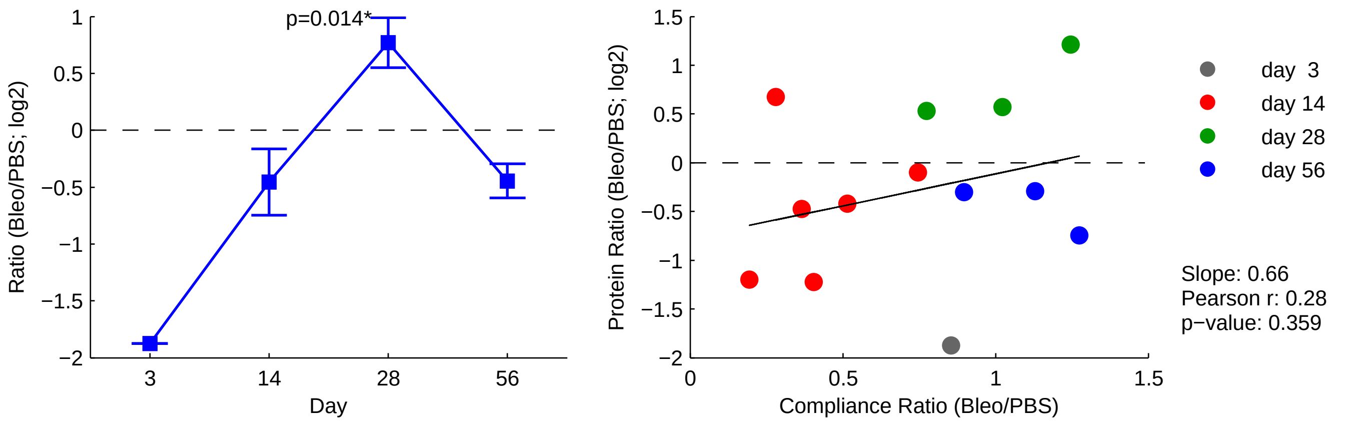

Q9D1A2 − Cndp2 (id: 7031)

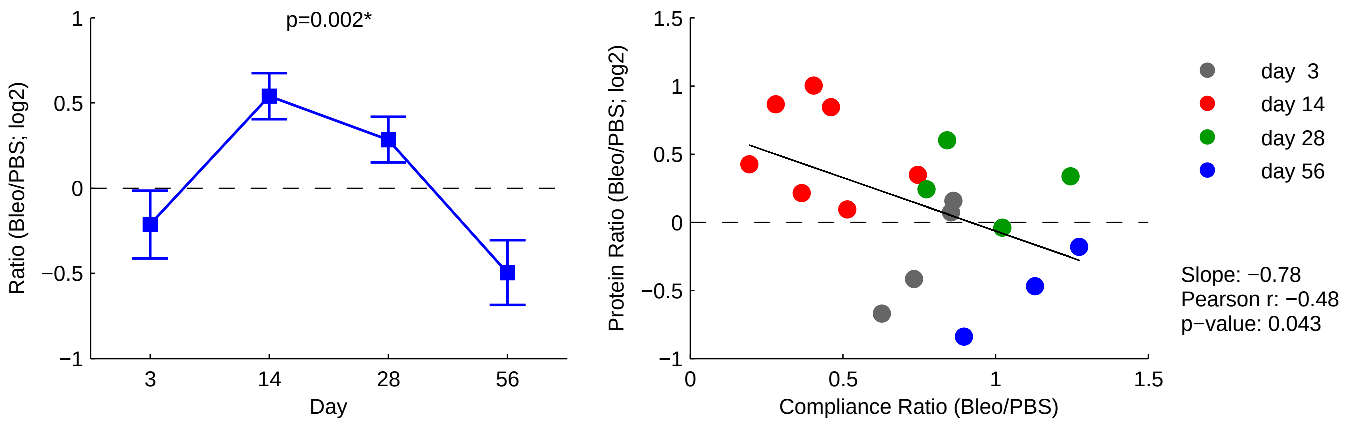

Q9D1B9 − Mrpl28 (id: 7032)

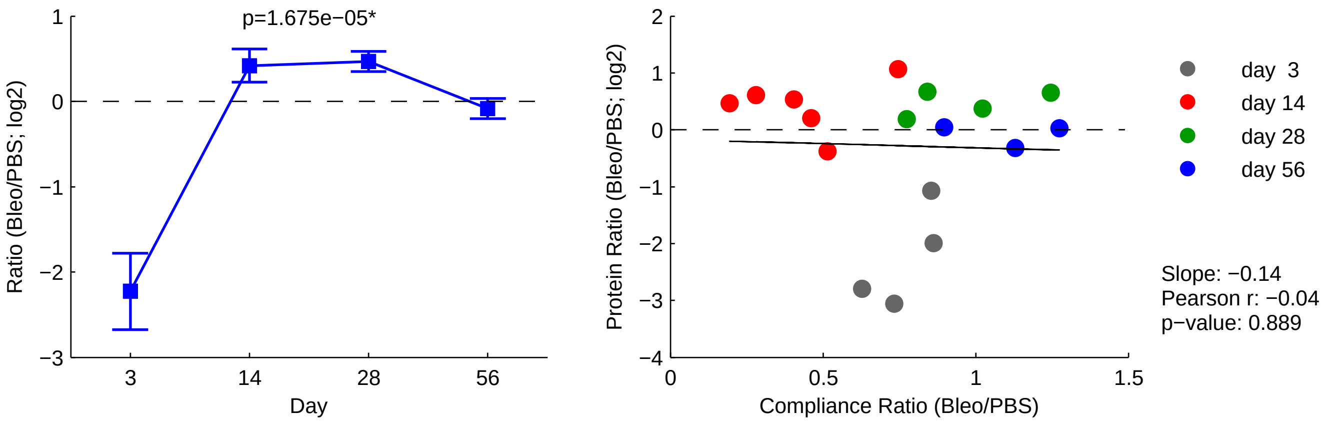

Q9D1C8 − Vps28 (id: 7035)

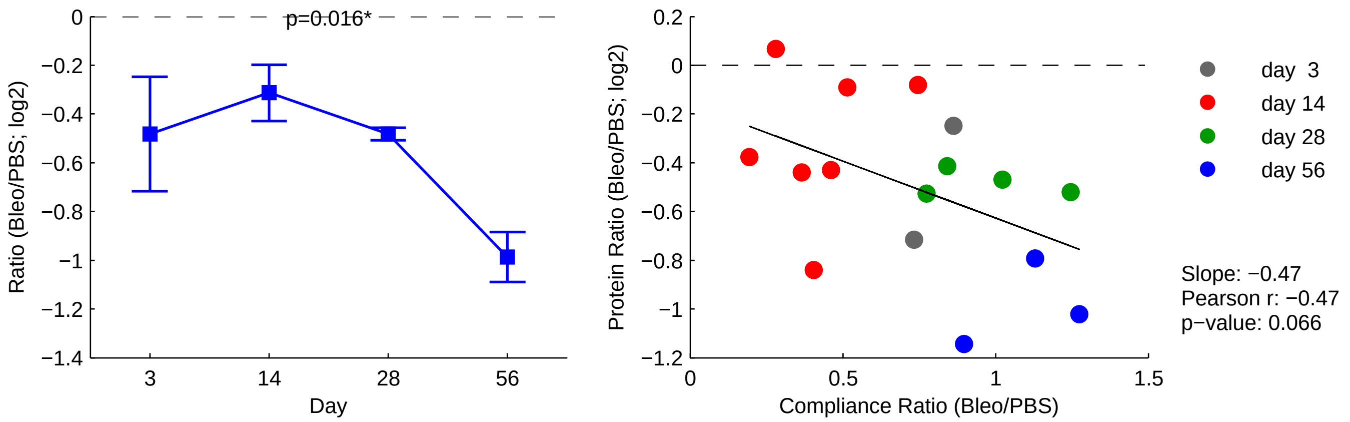

Q9D1D4 − Tmed10 (id: 7037)

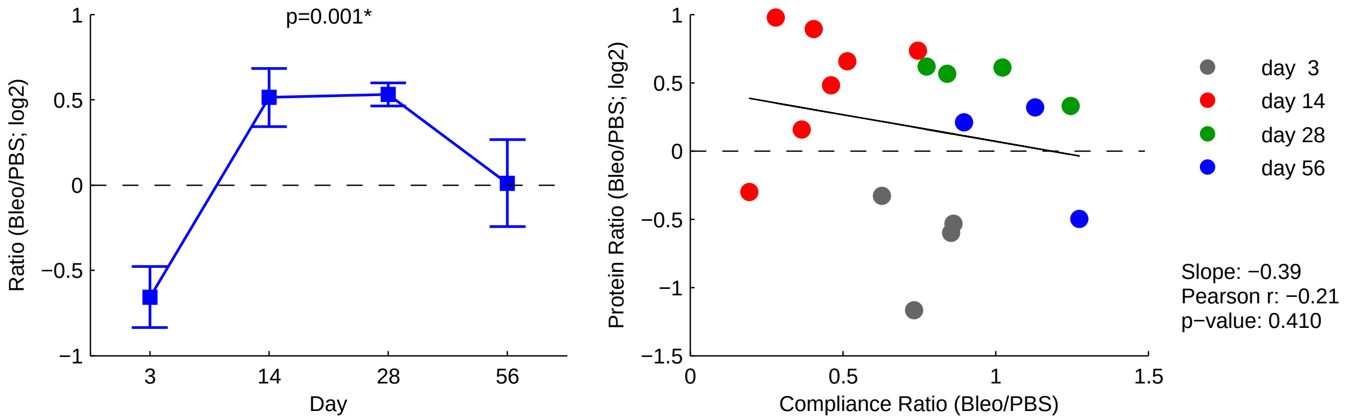

Q9D1I5 − Mcee (id: 7046)

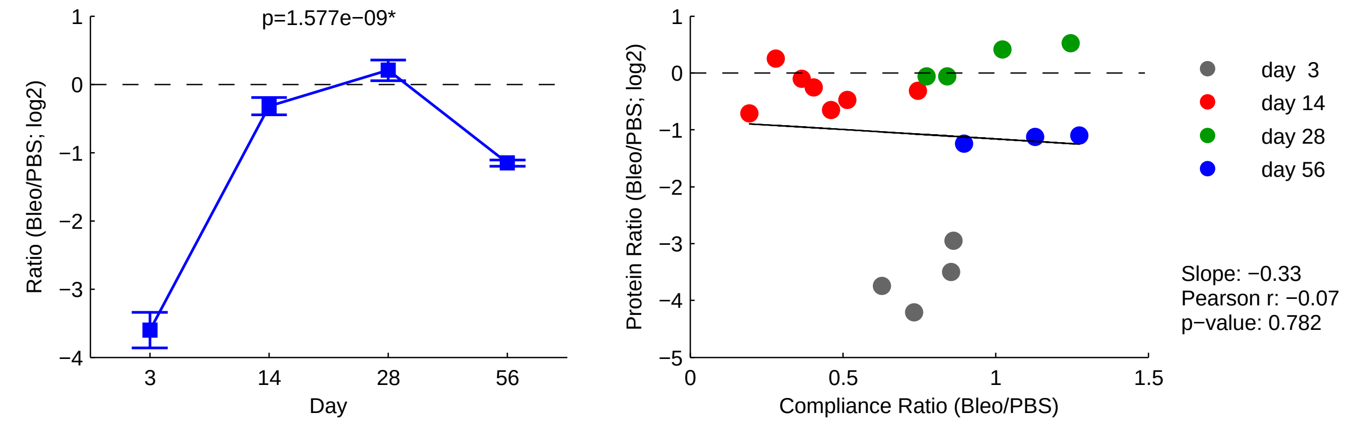

Q9D1J1 − Necap2 (id: 7048)

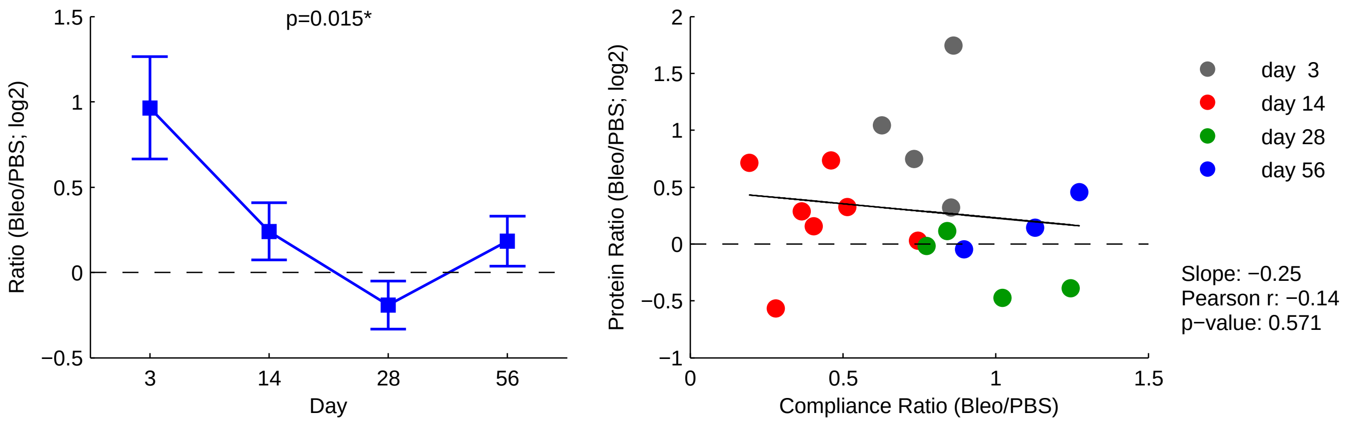

Q9D1J3 − Sarnp (id: 7049)

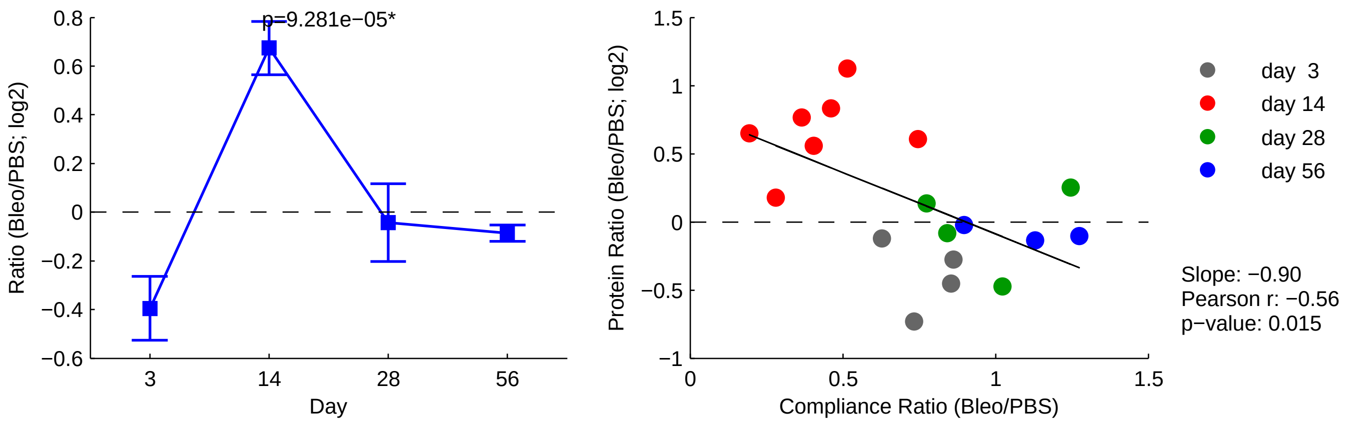

Q9D1K2 − Atp6v1f (id: 7050)

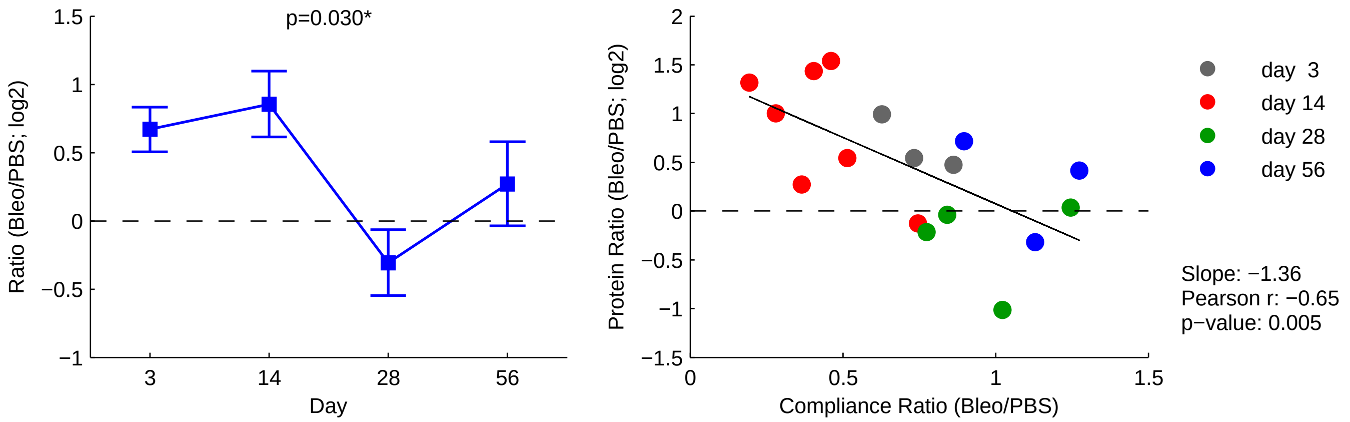

Q9D1L9 − Lamtor5 (id: 7051)

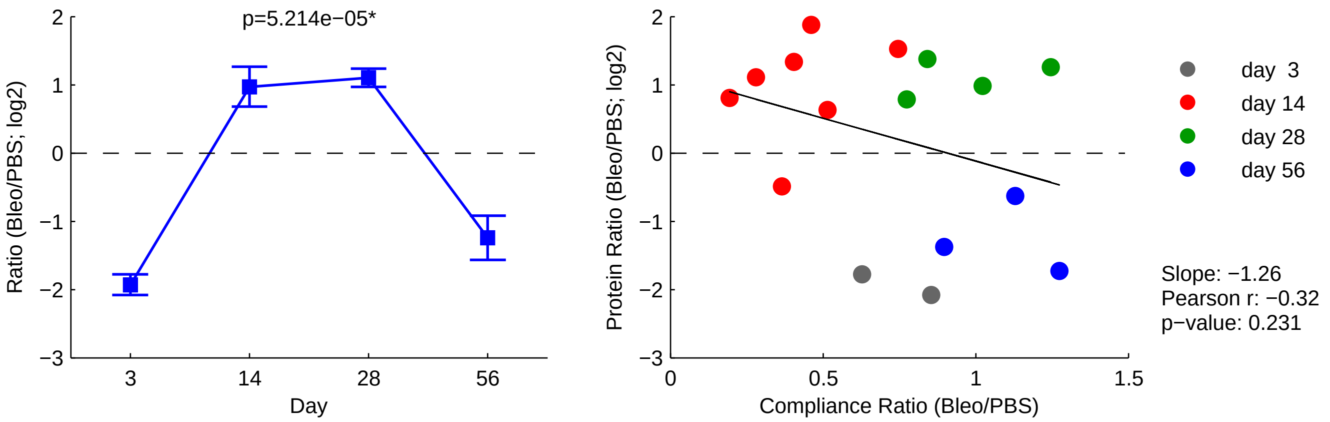

Q9D1M0 − Sec13 (id: 7052)

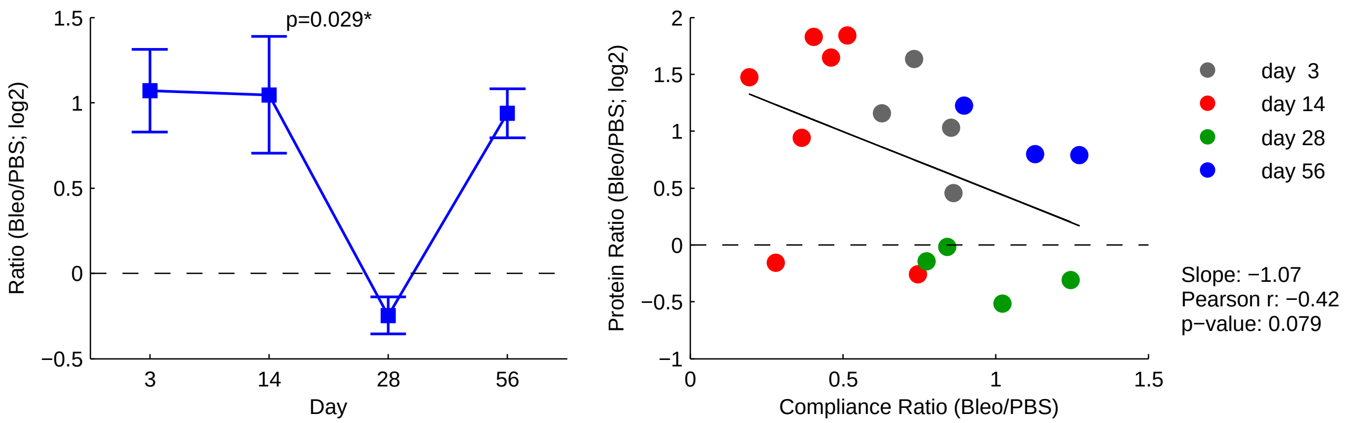

Q9D1M4 − Eef1e1 (id: 7053)

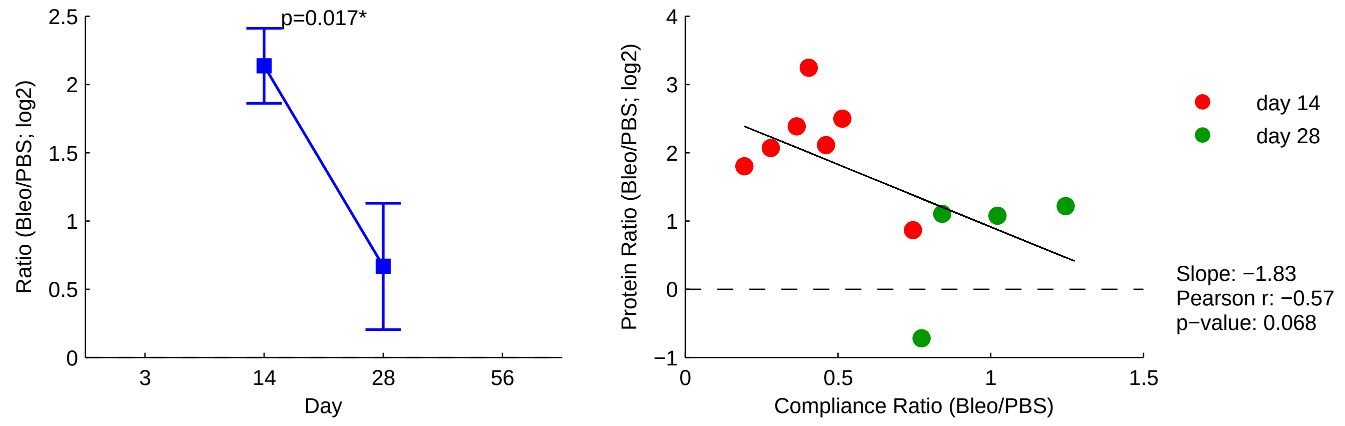

Q9D1N2 − Gpihbp1 (id: 7055)

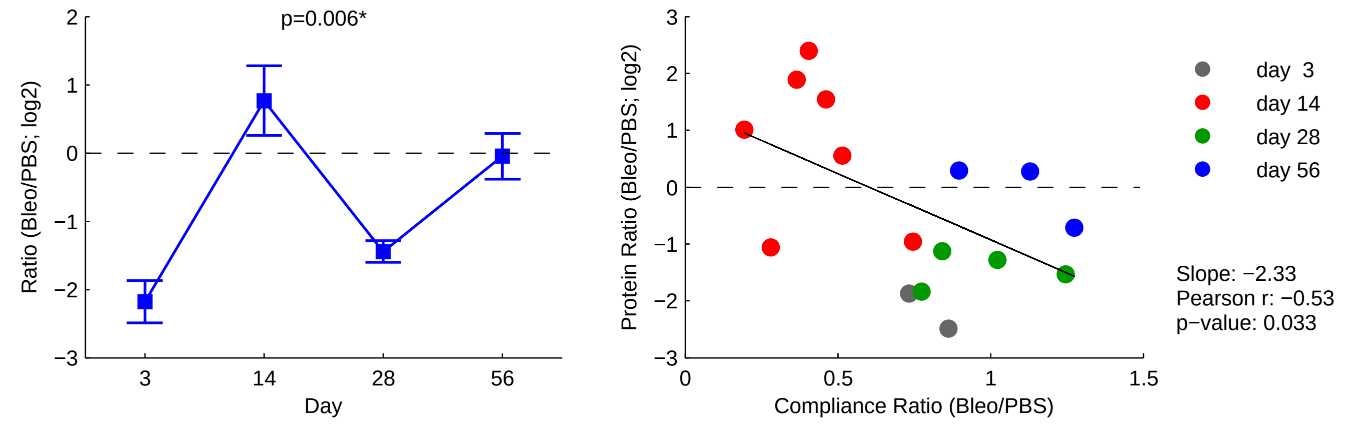

Q9D1P0 − Mrpl13 (id: 7057)

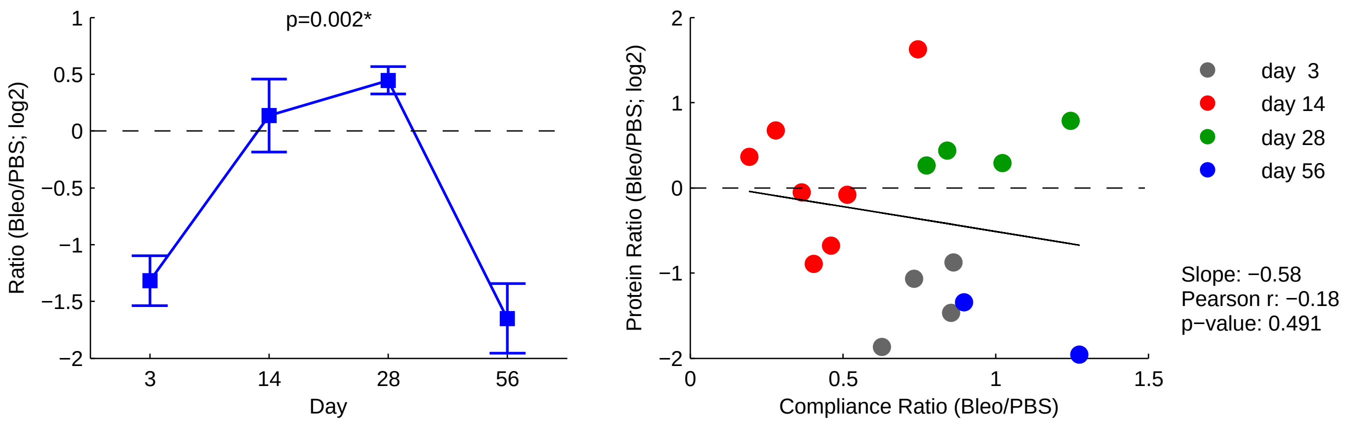

Q9D1P4 − Chordc1 (id: 7059)

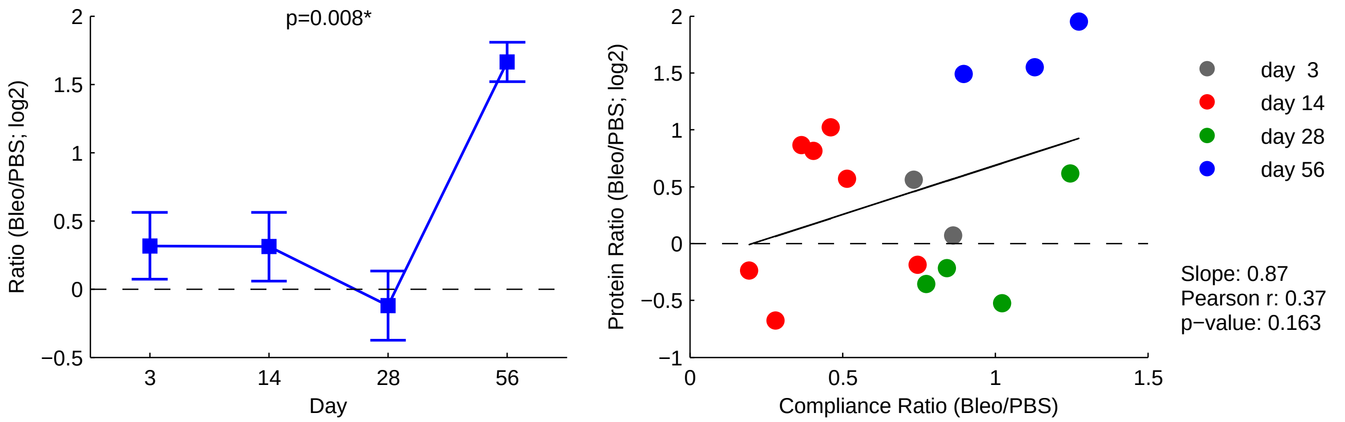

Q9D1Q6 − Erp44 (id: 7061)

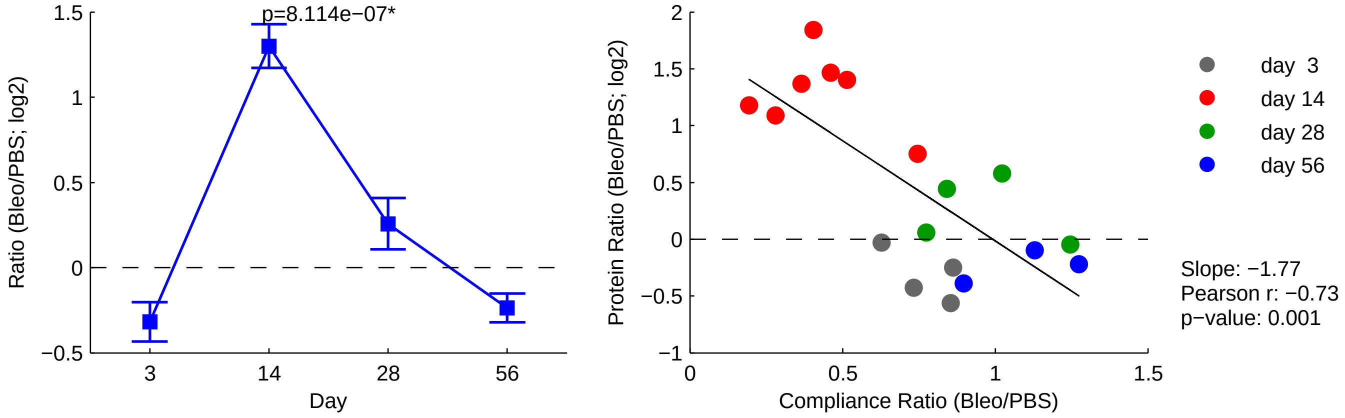

Q9D287 − Bcas2 (id: 7070)

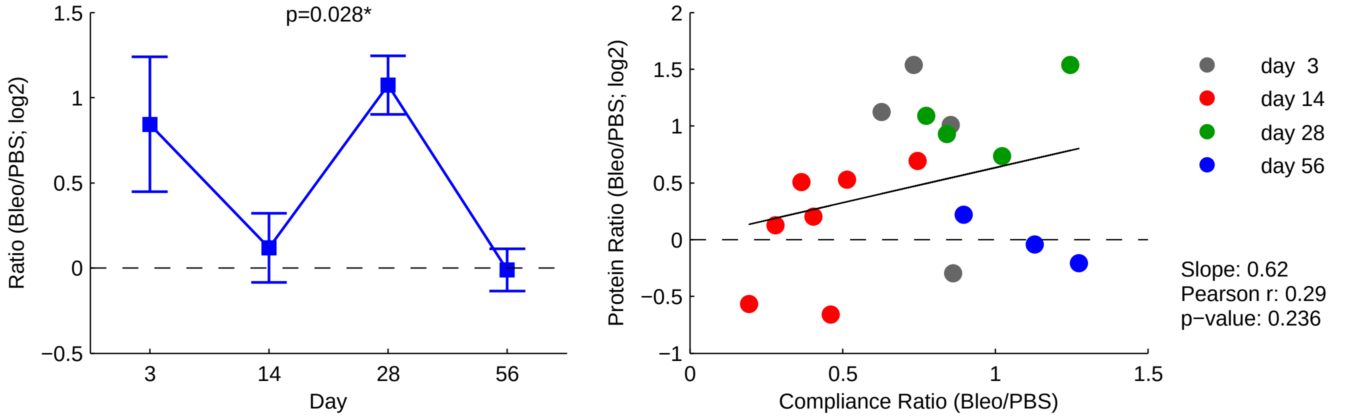

Q9D289 − Trappc6b (id: 7071)

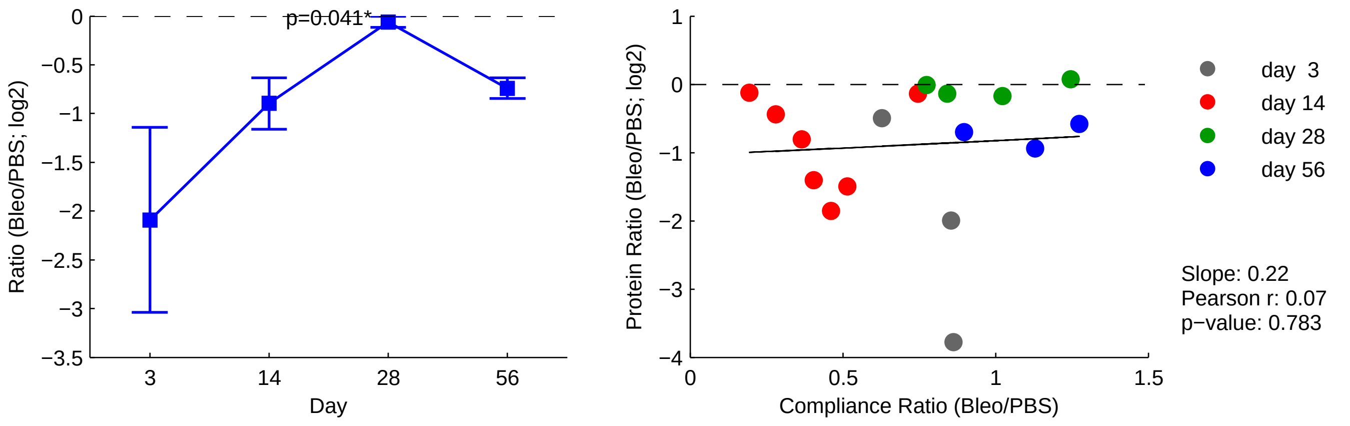

Q9D2E2 − Toe1 (id: 7073)

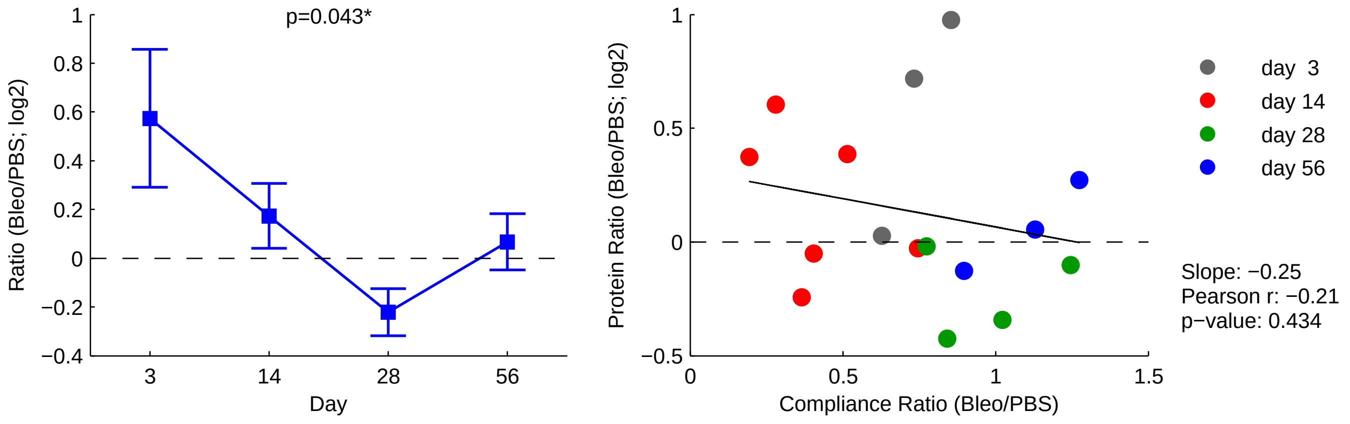

Q9D2G2 − Dlst (id: 7074)

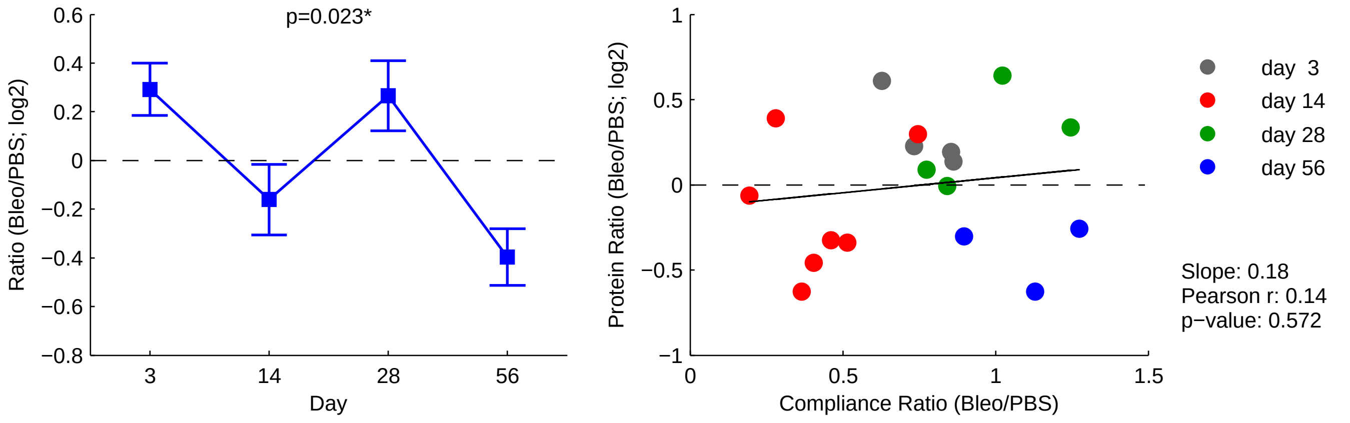

Q9D2M8 − Ube2v2 (id: 7076)

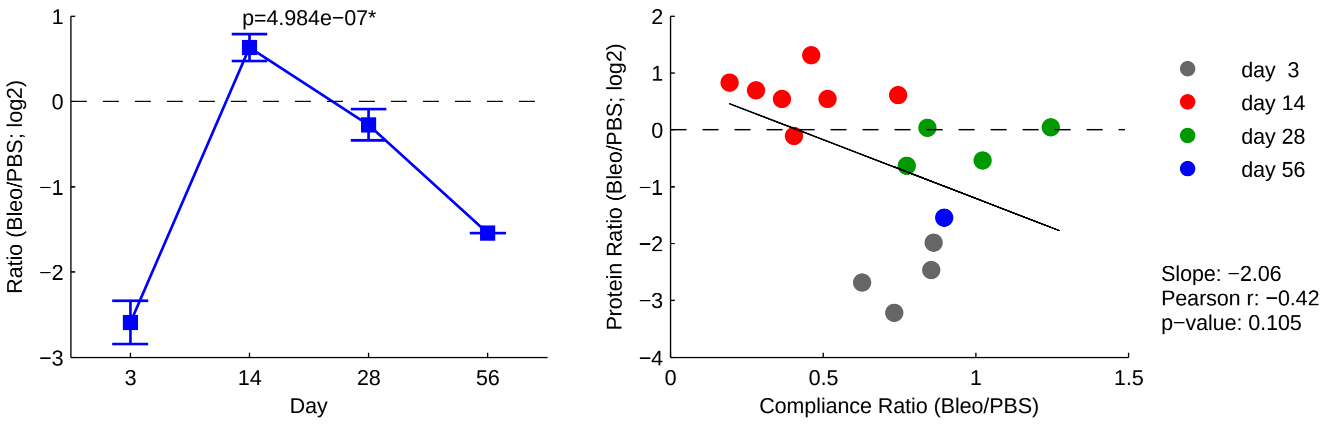

Q9D2Q3 − (id: 7079)

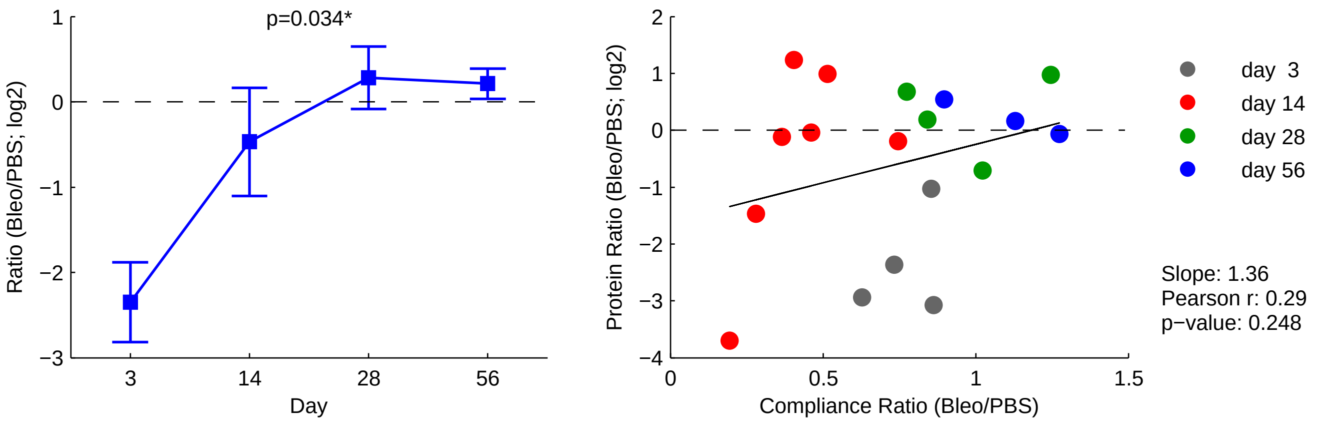

Q9D2U9 − Hist3h2ba (id: 7083)

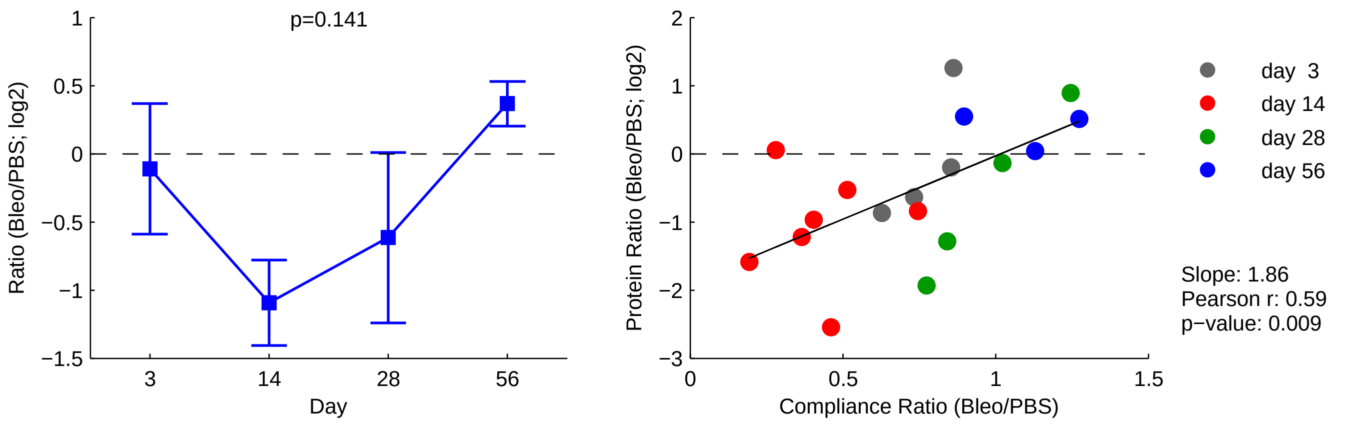

Q9D2Y4−2 − Mlkl (id: 7086)

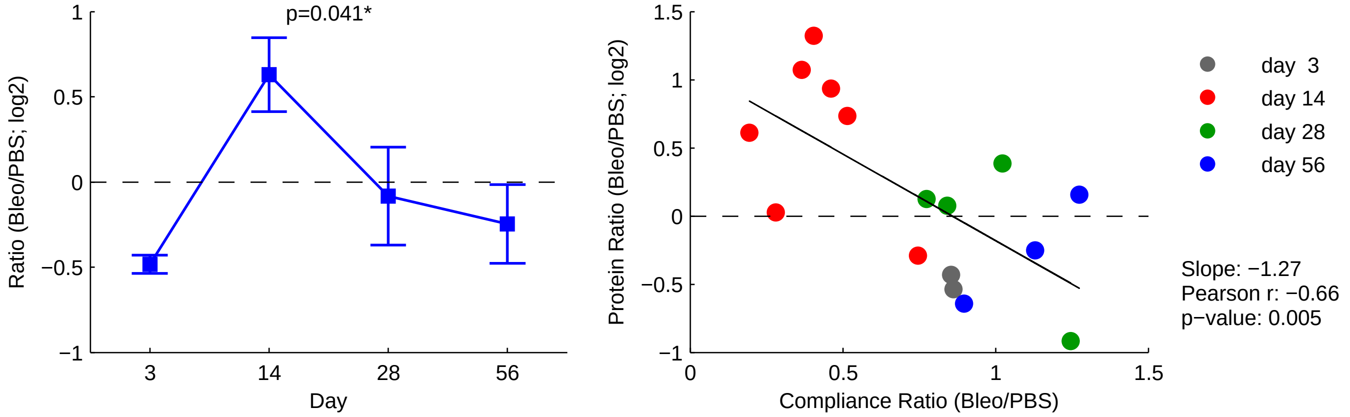

Q9D338 − Mrpl19 (id: 7088)

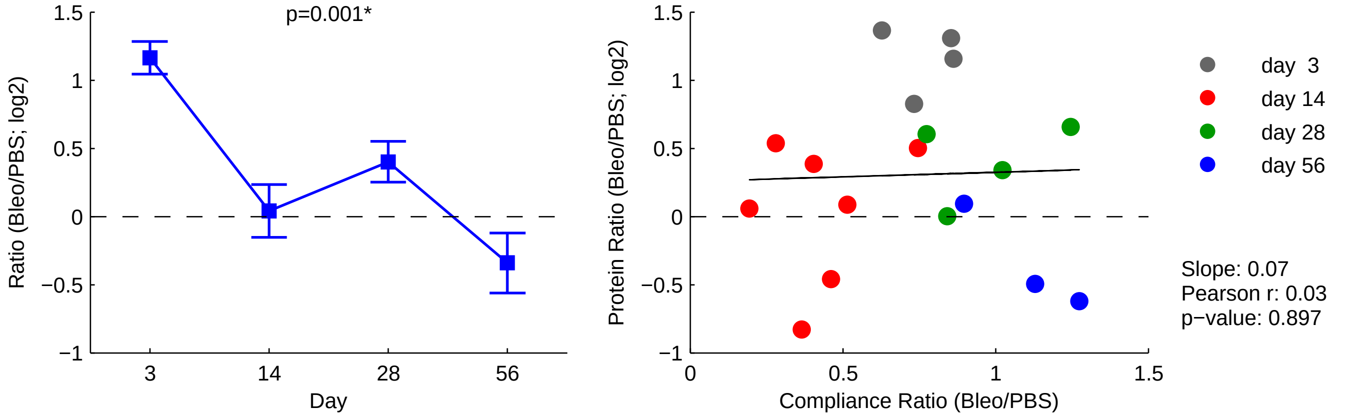

Q9D365 − Spcs3 (id: 7089)

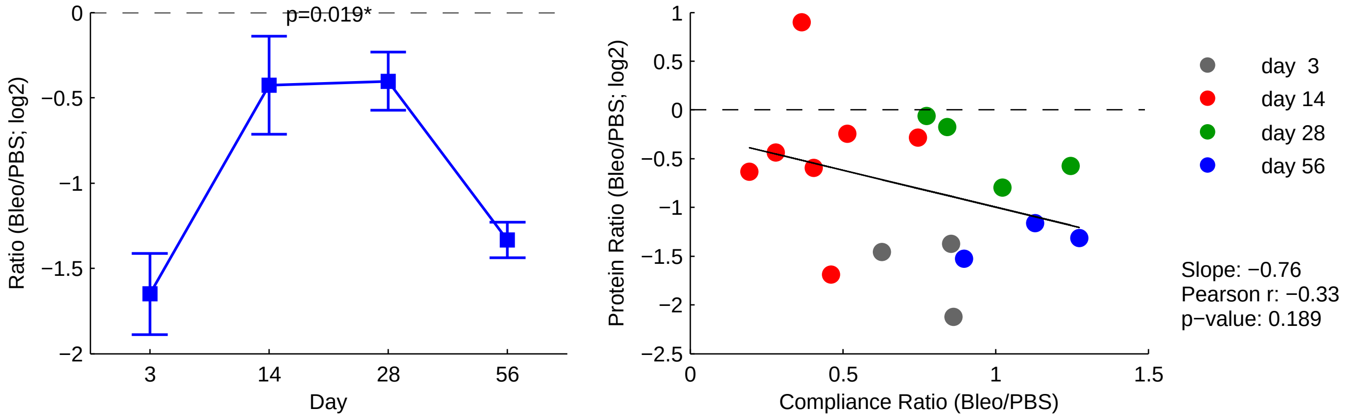

Q9D379 − Ephx1 (id: 7090)

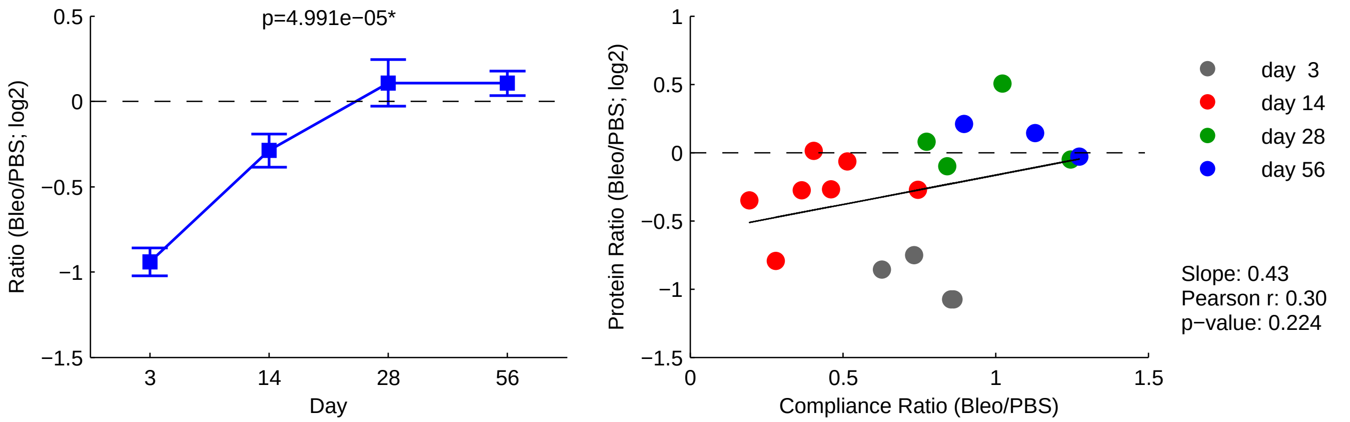

Q9D394−2 − Rufy3 (id: 7092)

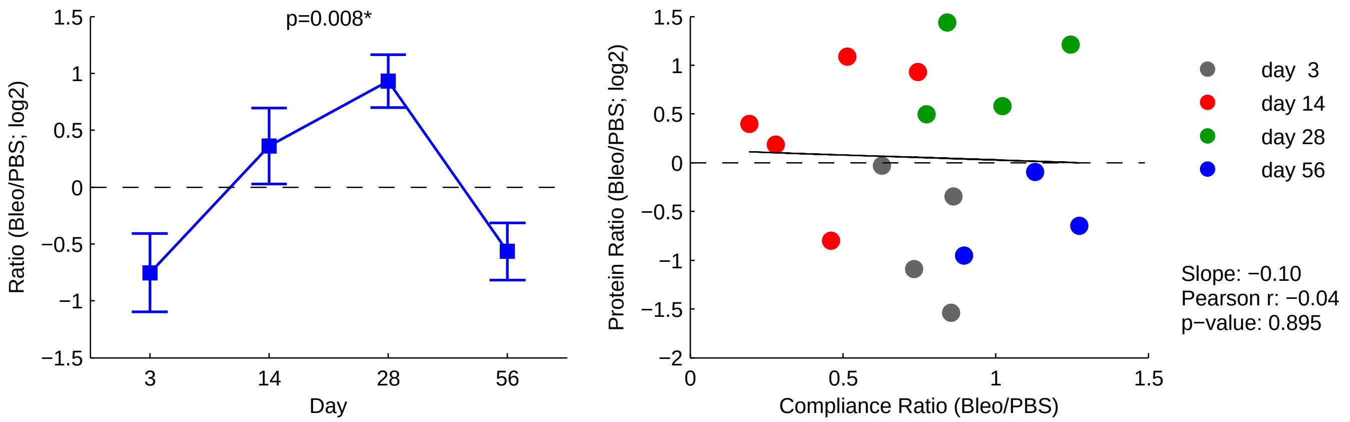

Q9D3D9 − Atp5d (id: 7094)

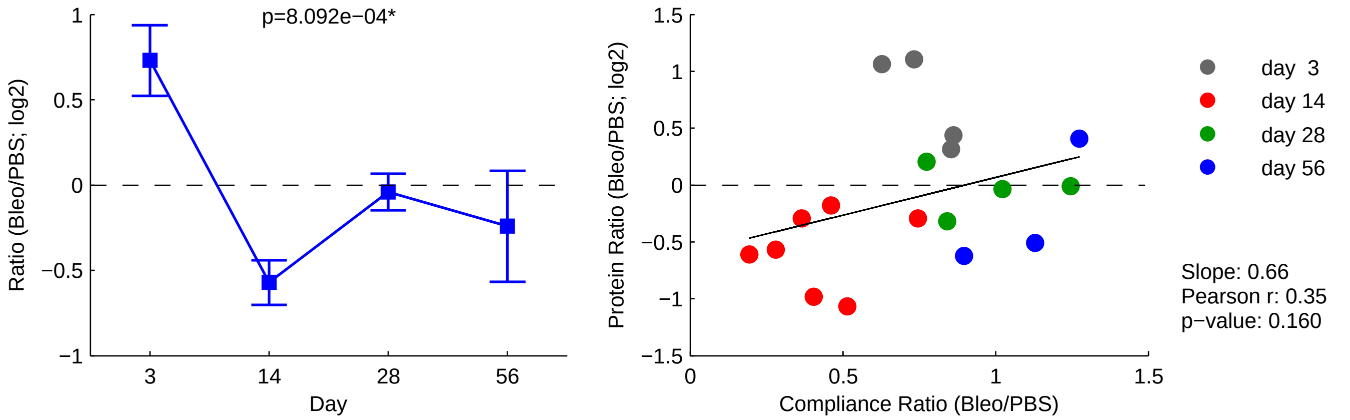

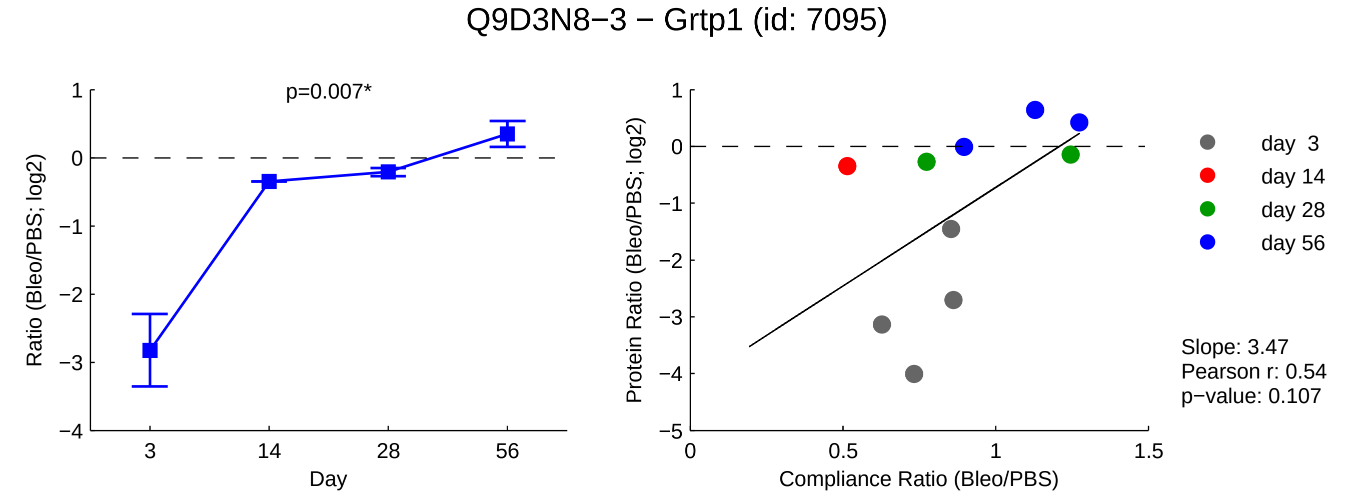
Q9D3N8−3 − Grtp1 (id: 7095)
p=0.007*
Ratio (Bleo/PBS; log2)
Day
Protein Ratio (Bleo/PBS; log2)
Compliance Ratio (Bleo/PBS)
day 3
day 14
day 28
day 56
Slope: 3.47
Pearson r: 0.54
p−value: 0.107

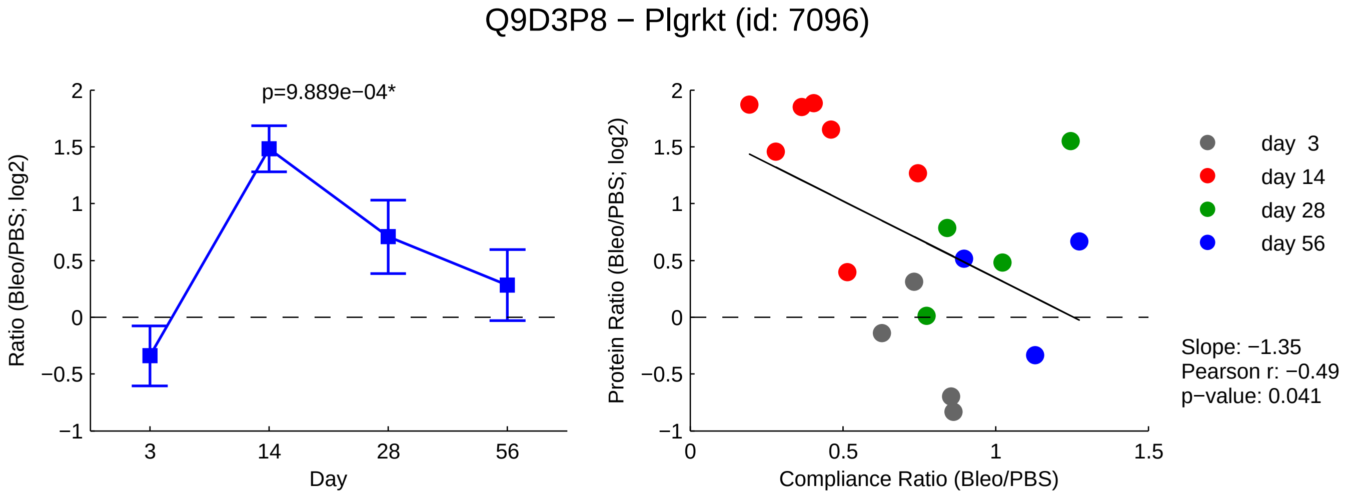
Q9D3P8 − Plgrkt (id: 7096)
p=9.889e−04*
Ratio (Bleo/PBS; log2)
Day
Protein Ratio (Bleo/PBS; log2)
Compliance Ratio (Bleo/PBS)
day 3
day 14
day 28
day 56
Slope: −1.35
Pearson r: −0.49
p−value: 0.041

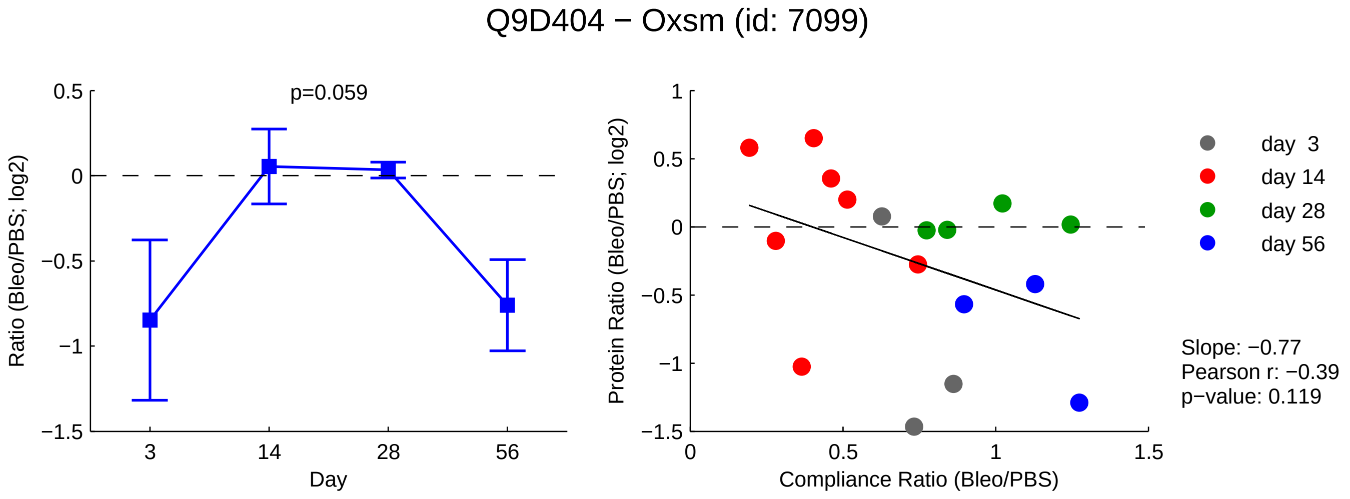
Q9D404 − Oxsm (id: 7099)
p=0.059
Ratio (Bleo/PBS; log2)
Day
Protein Ratio (Bleo/PBS; log2)
Compliance Ratio (Bleo/PBS)
day 3
day 14
day 28
day 56
Slope: −0.77
Pearson r: −0.39
p−value: 0.119

Q9D4H1 − Exoc2 (id: 7104)

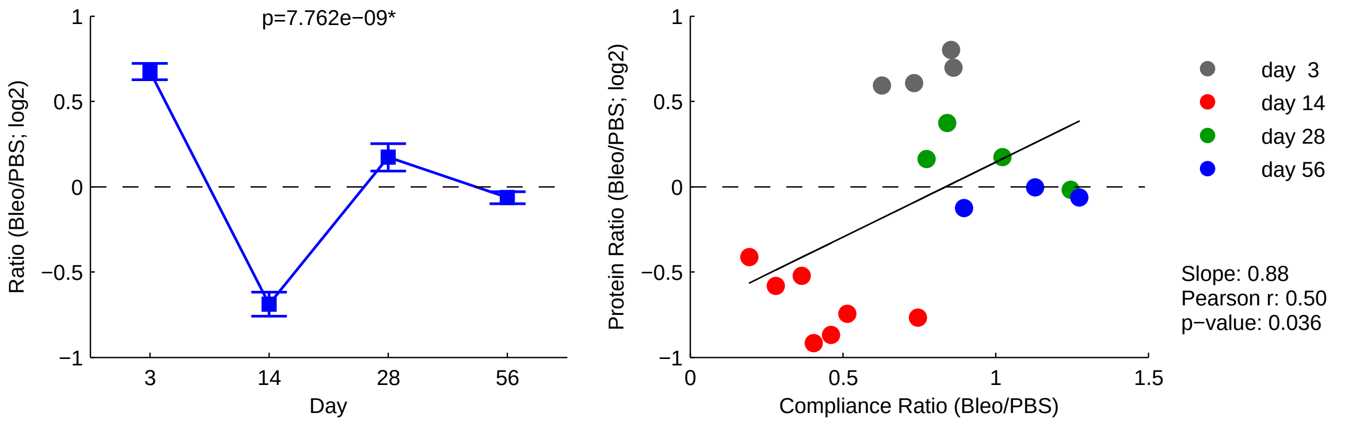

Q9D4H8 − Cul2 (id: 7105)

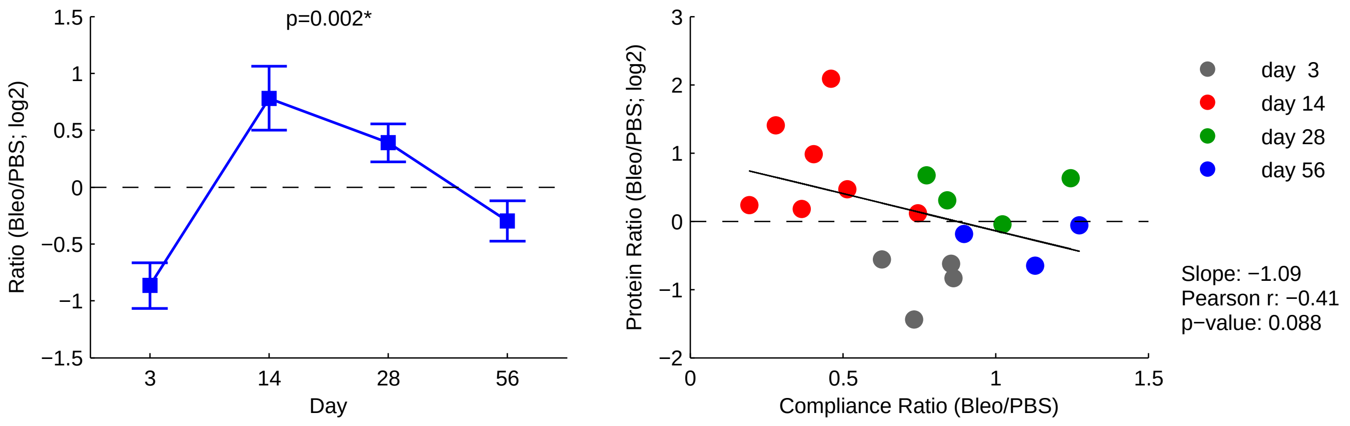

Q9D5R3 − Ccdc41 (id: 7114)

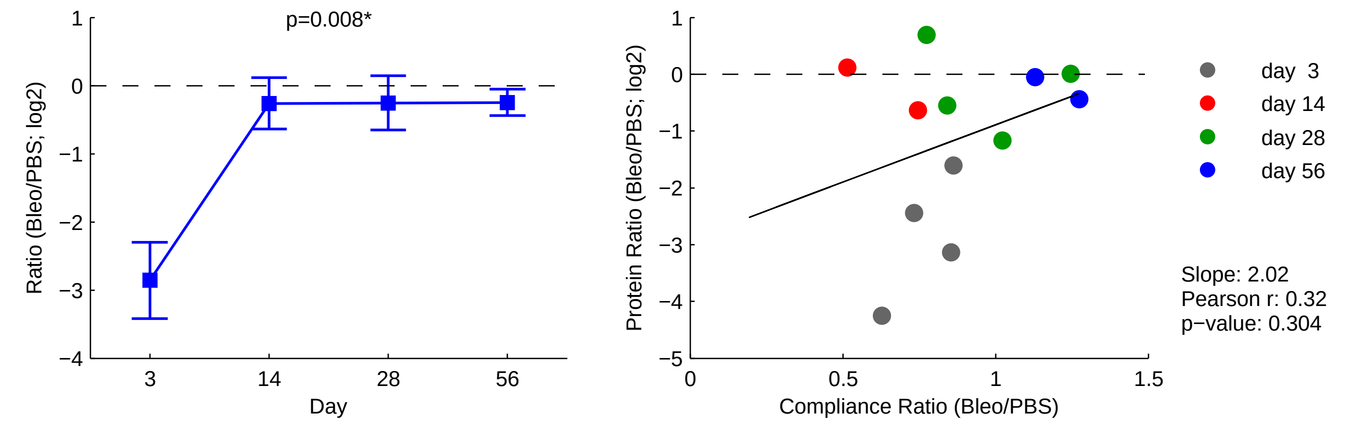

Q9D5T0 − Atad1 (id: 7115)

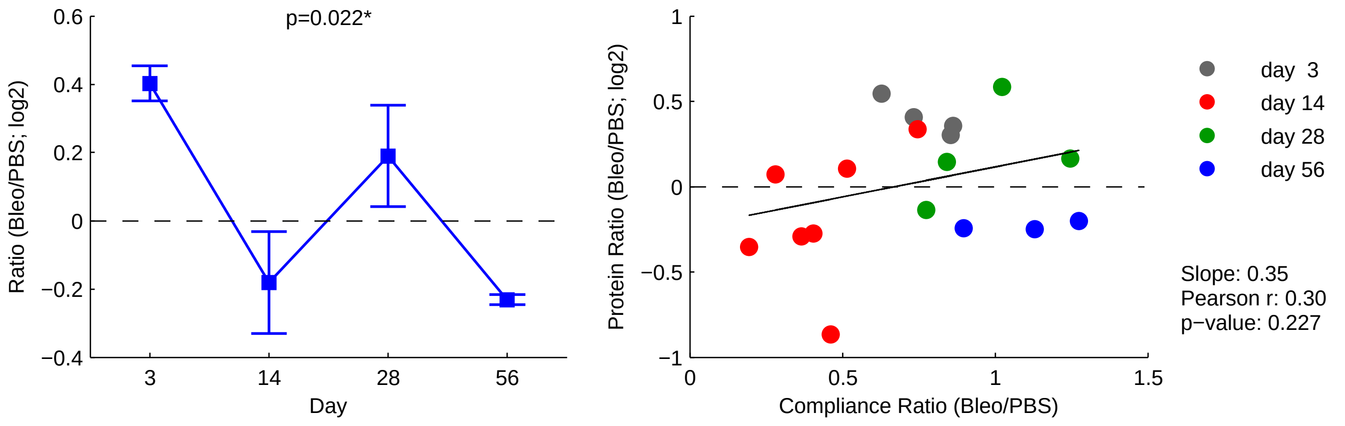

Q9D5V5 − Cul5 (id: 7116)

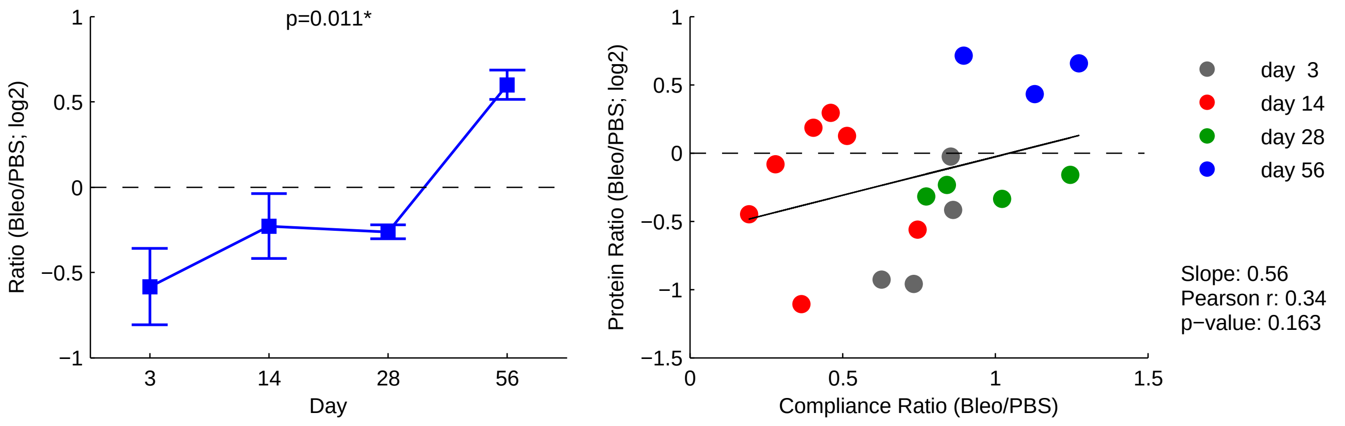

Q9D5V6 − Syap1 (id: 7117)

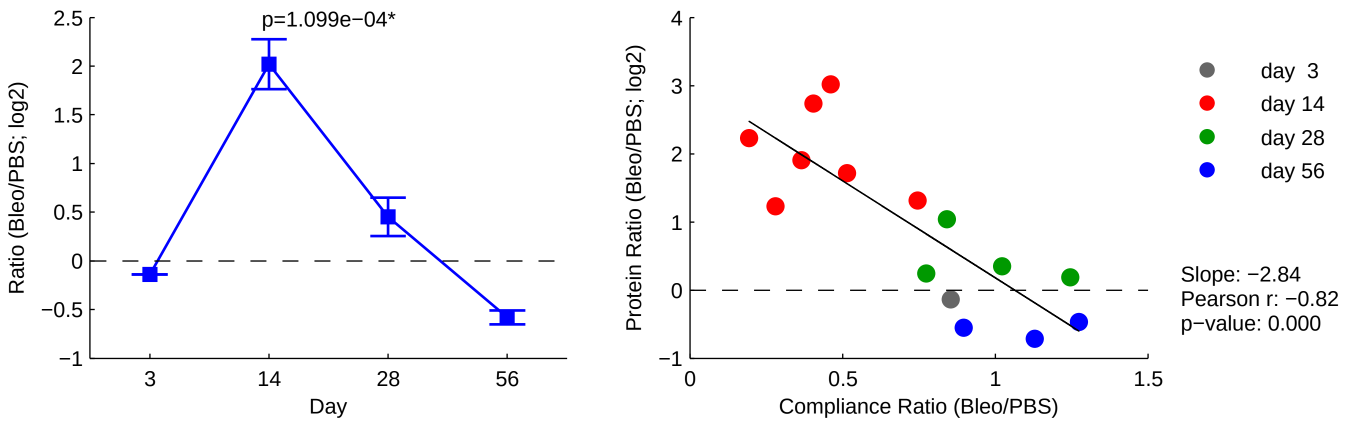

Q9D5Y1 − Ccdc39 (id: 7118)

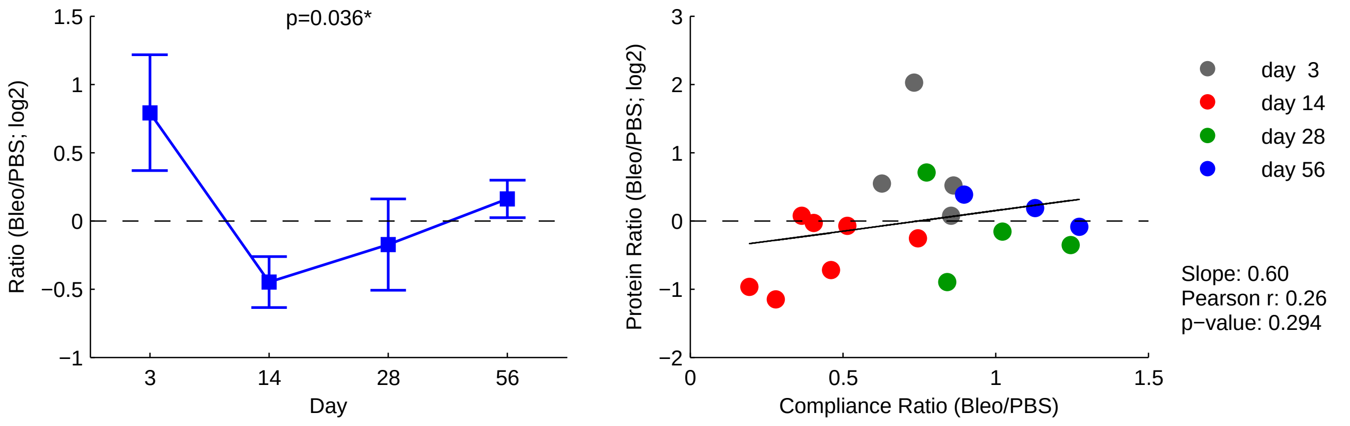

Q9D659 − 4632428N05Rik (id: 7120)

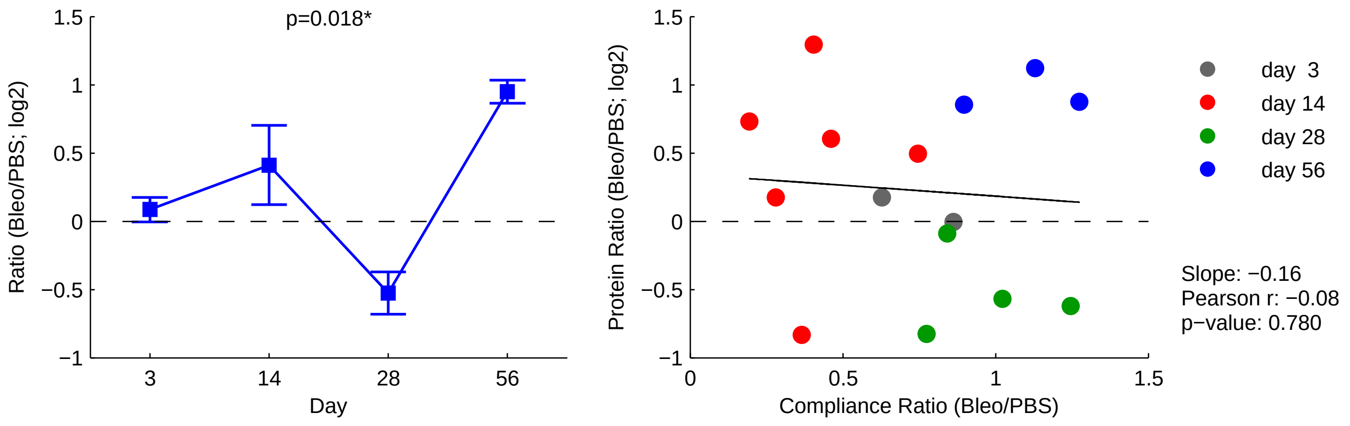

Q9D662 − Sec23b (id: 7121)

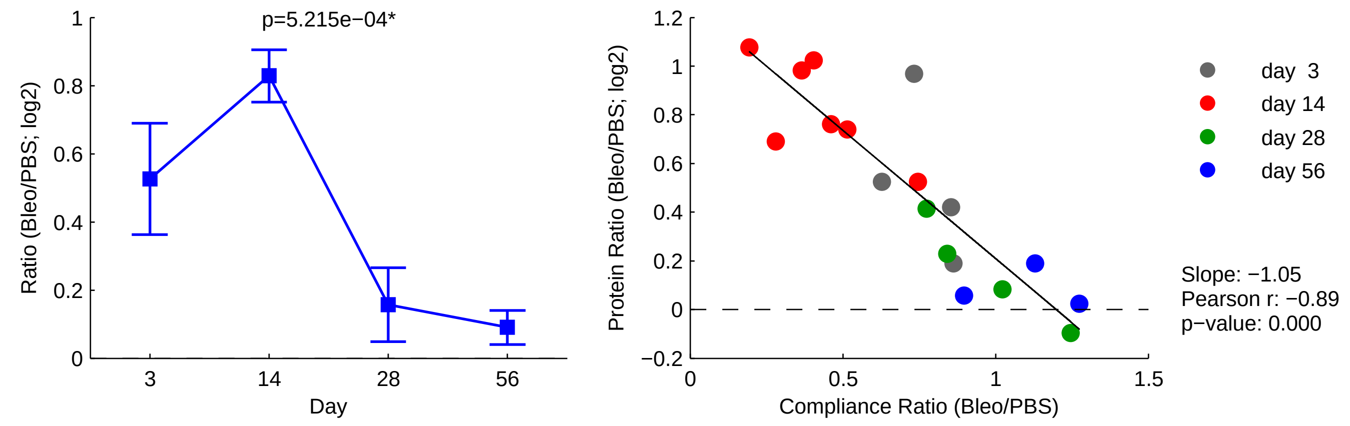

Q9D6I7 − Fam69a (id: 7124)

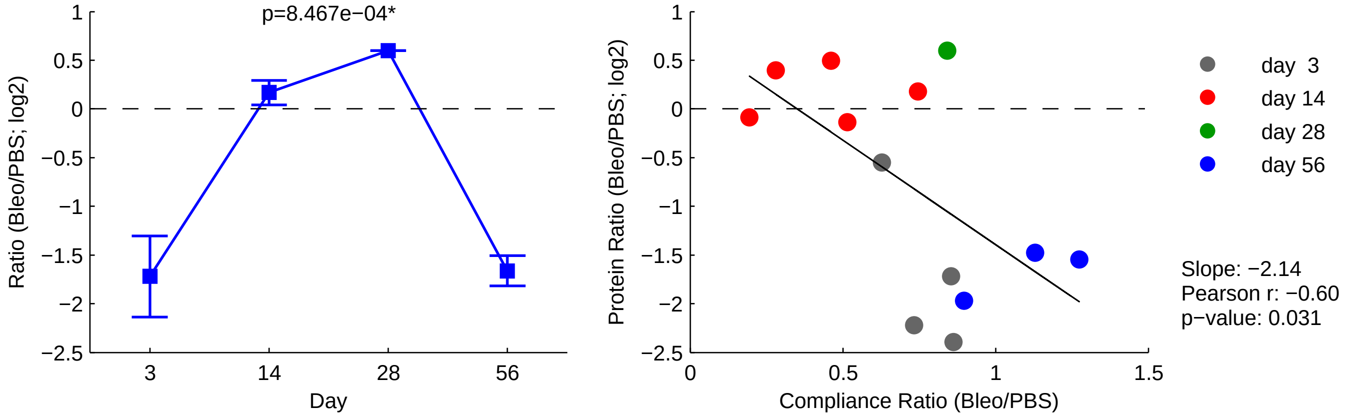

Q9D6J5 − Ndufb8 (id: 7127)

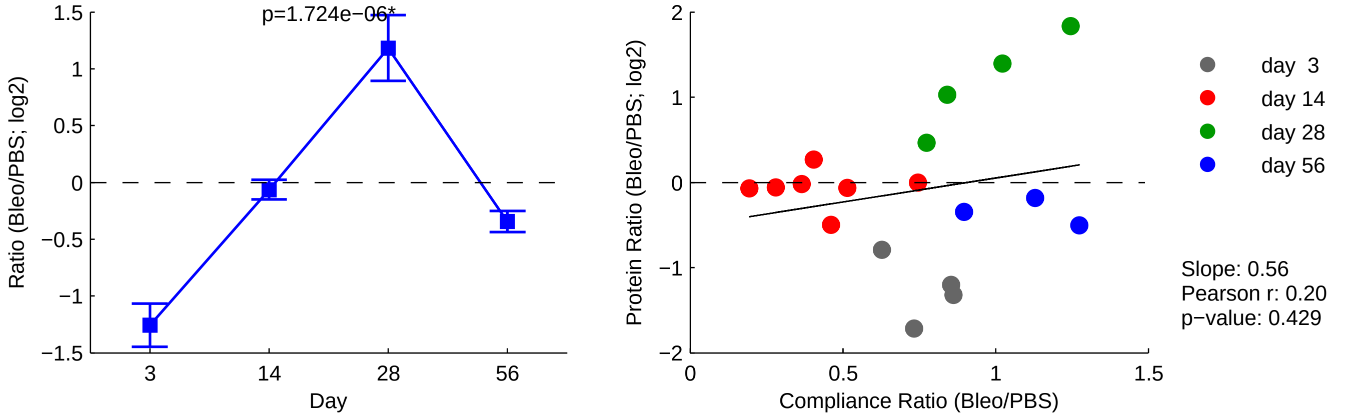

Q9D6J6 − Ndufv2 (id: 7128)

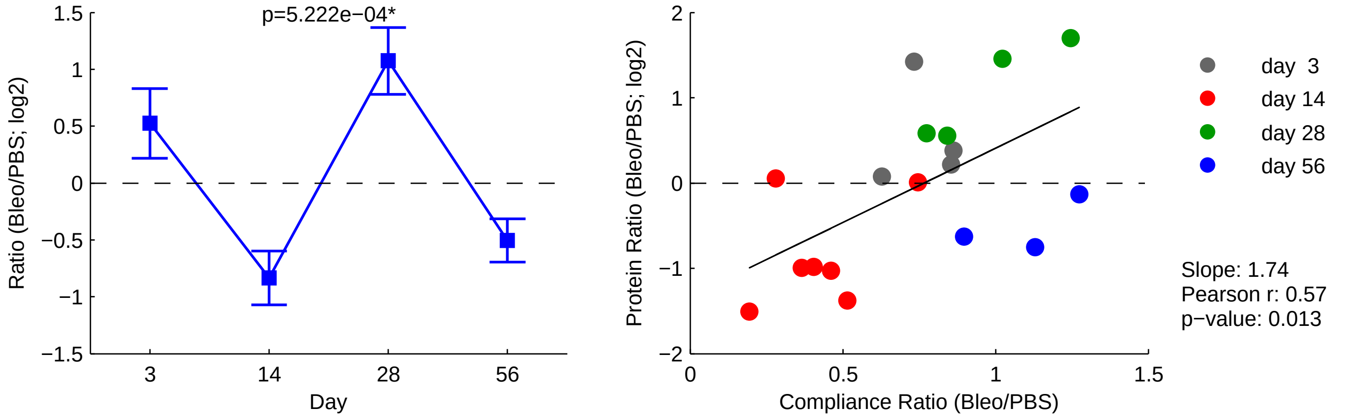

Q9D6K8 − Fundc2 (id: 7129)

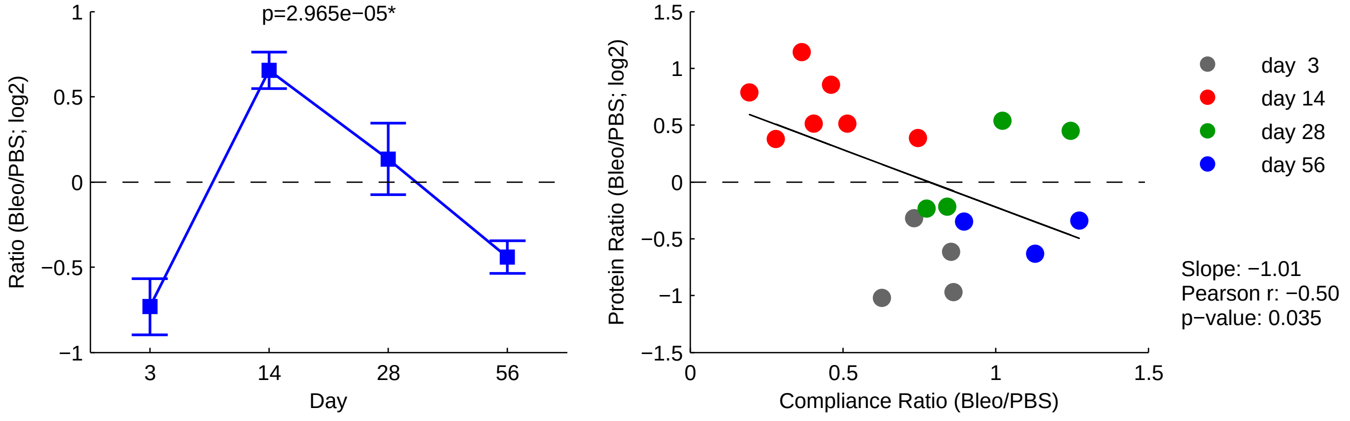

Q9D6K9 − Cers5 (id: 7130)

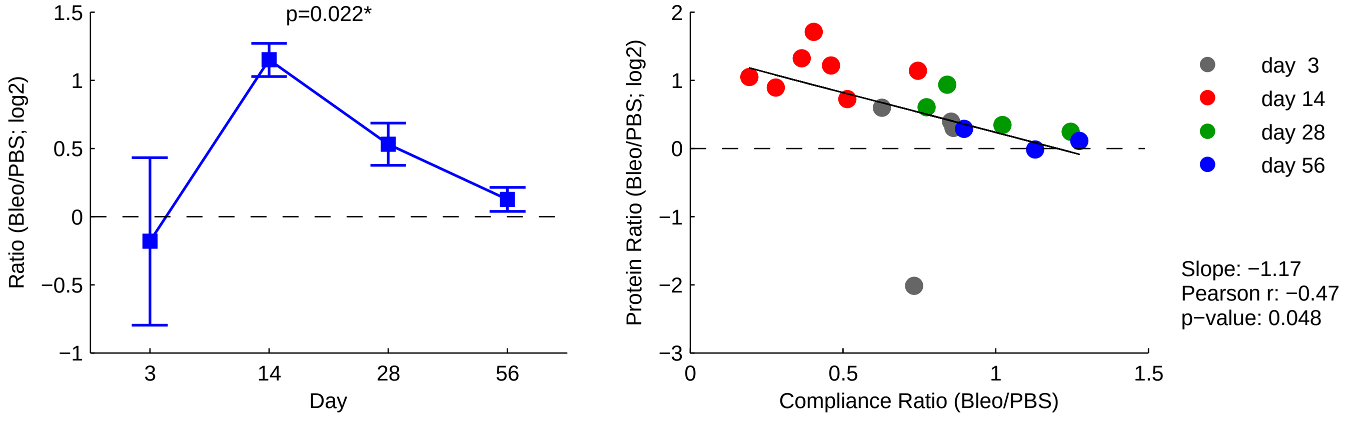

Q9D6L8 − Ppil3 (id: 7131)

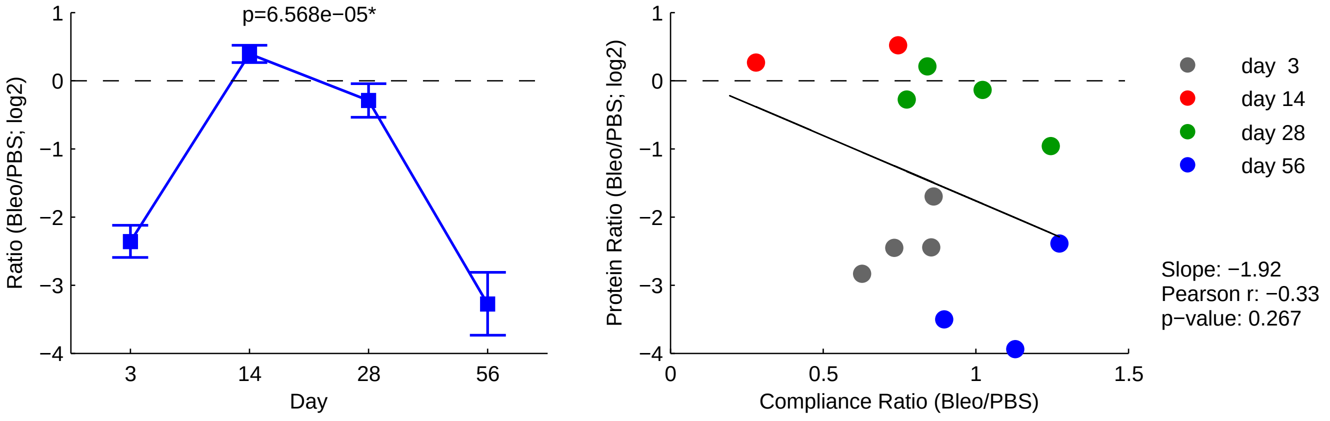

Q9D6P8 − Calml3 (id: 7133)

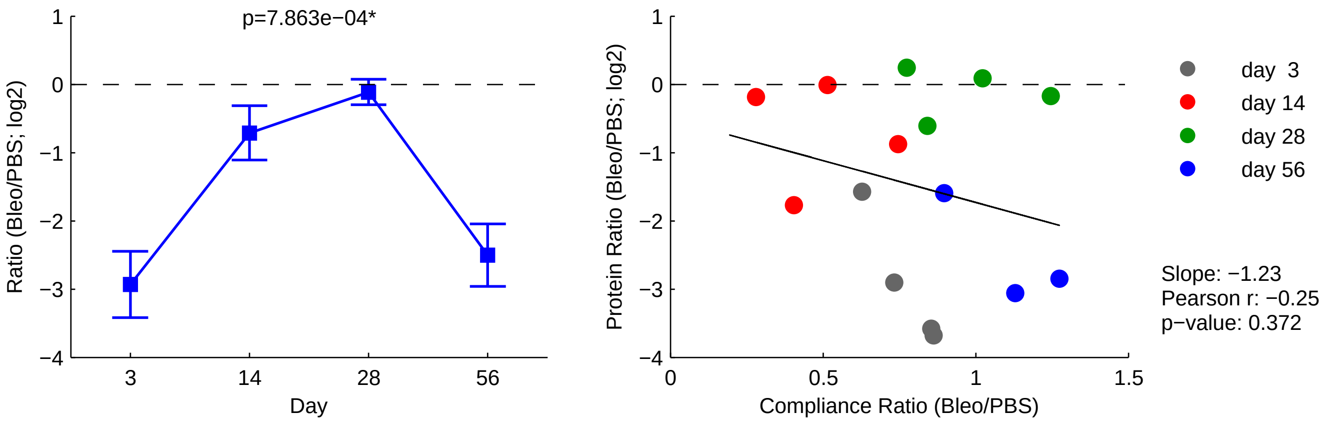

Q9D6R2 − Idh3a (id: 7134)

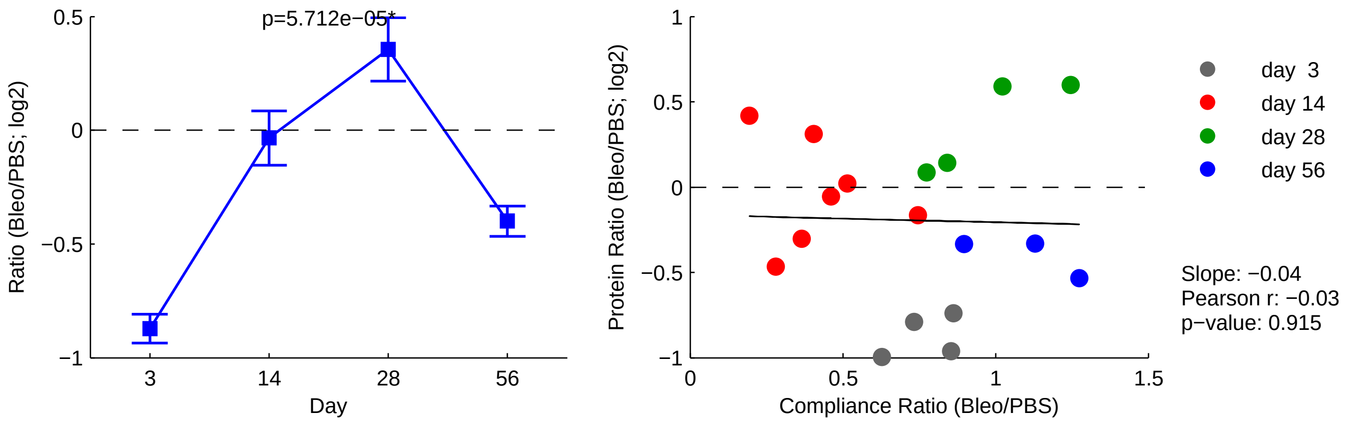

Q9D6S7 − Mrrf (id: 7135)

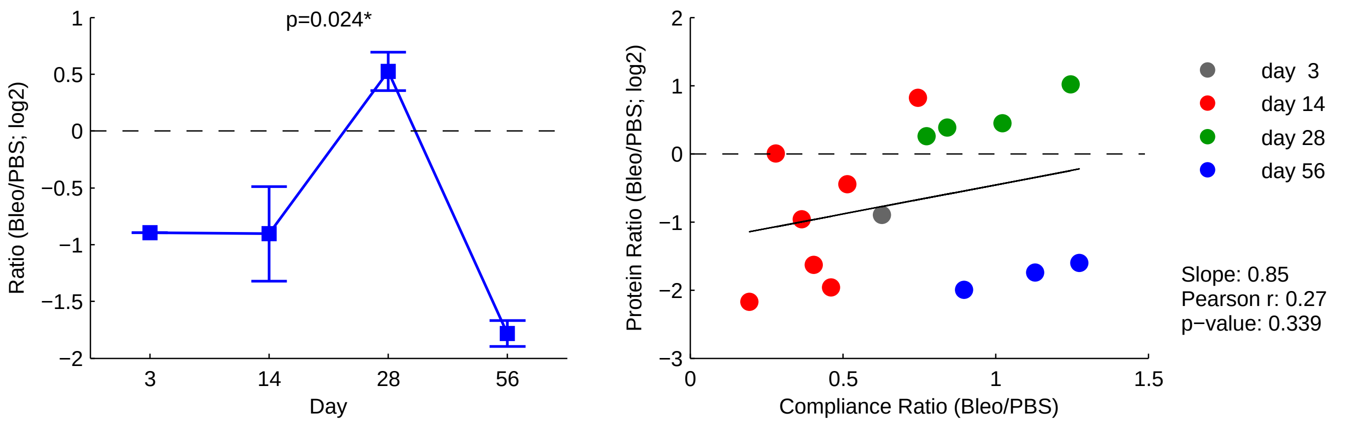

Q9D6T9 − Mrps36 (id: 7137)

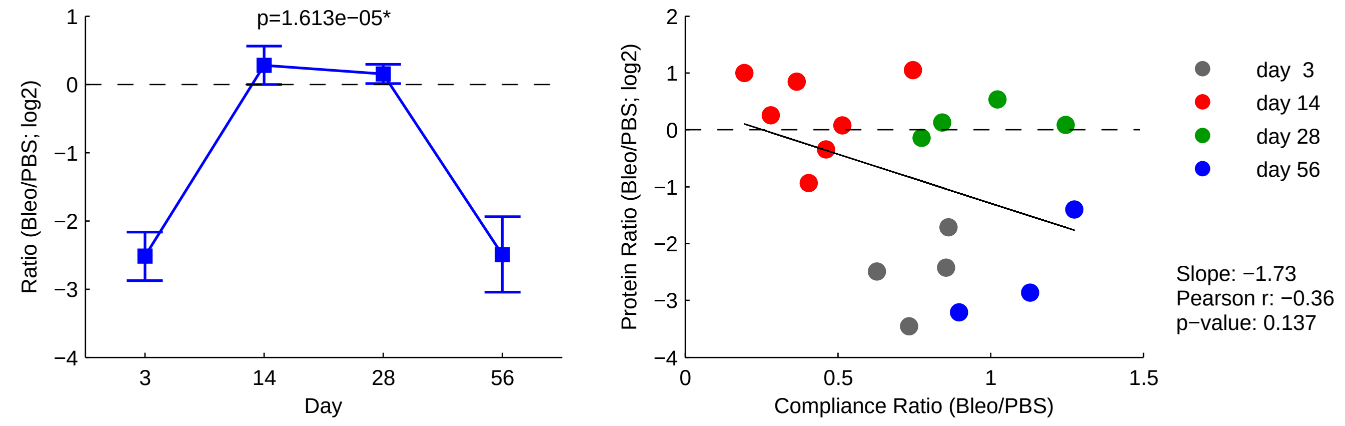

Q9D6U8 − Fam162a (id: 7138)

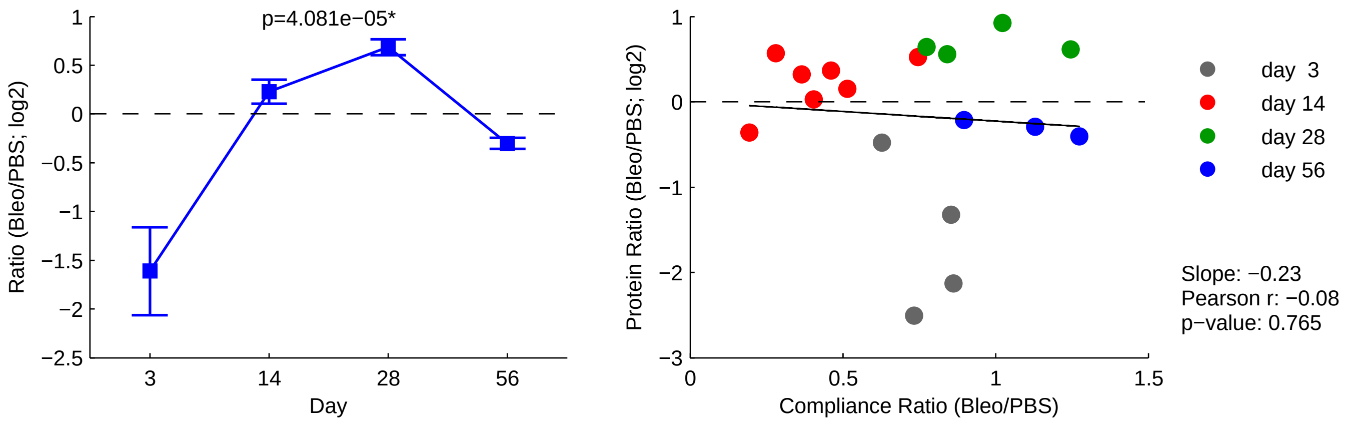

Q9D6W8 − (id: 7139)

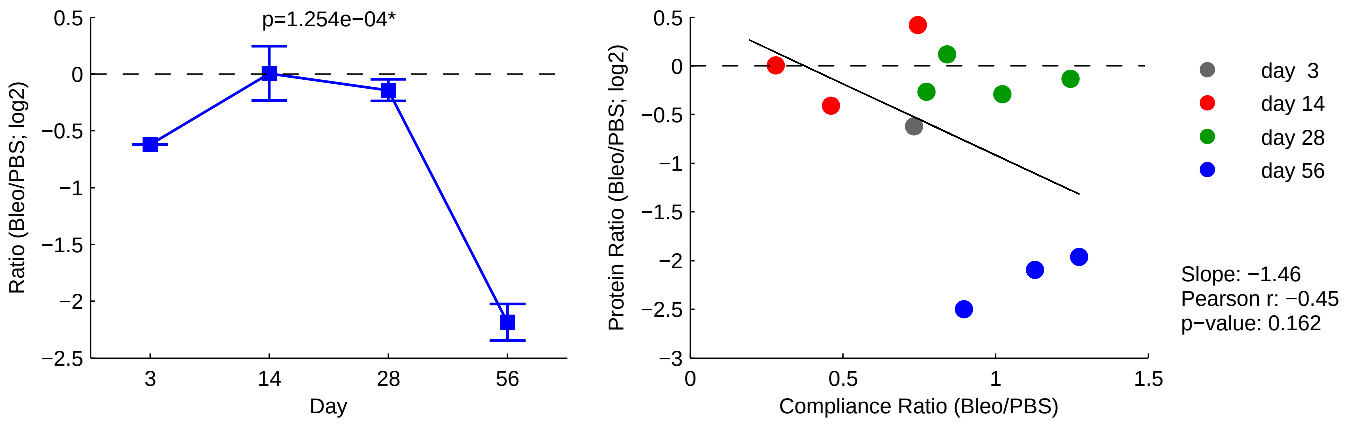

Q9D6Y7−3 − Msra (id: 7141)

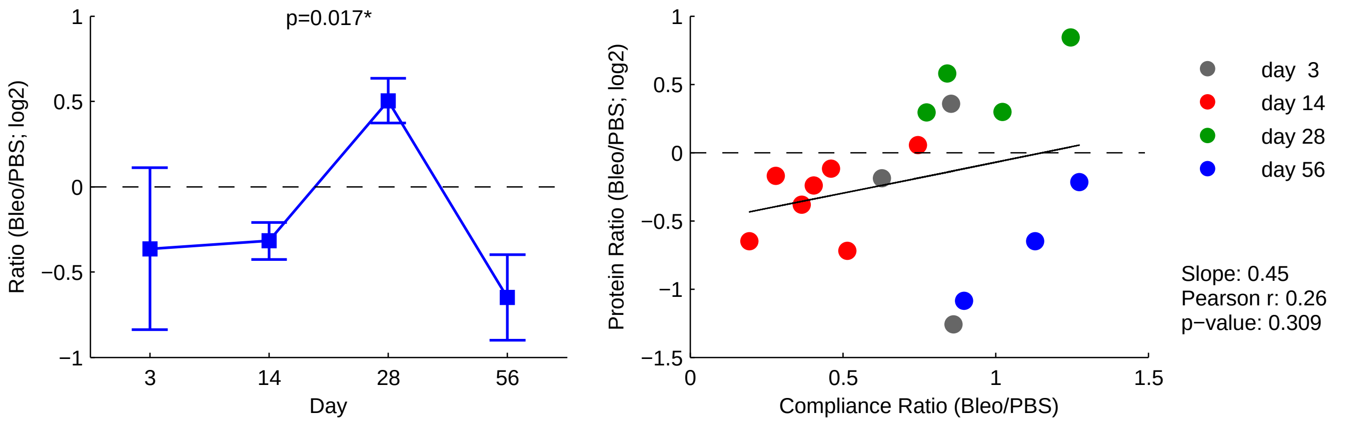

Q9D708 − S100a16 (id: 7146)

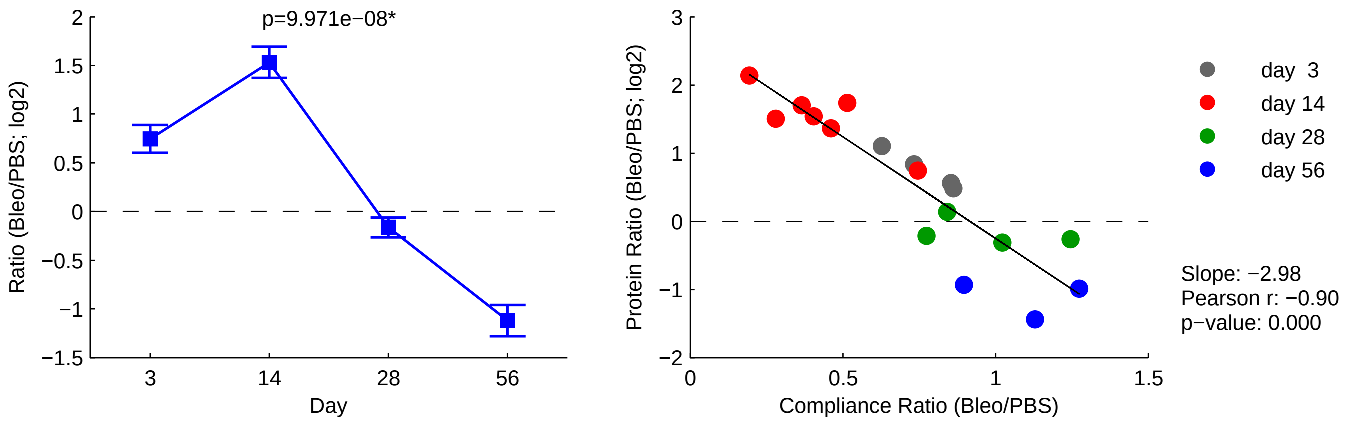

Q9D735 − (id: 7148)

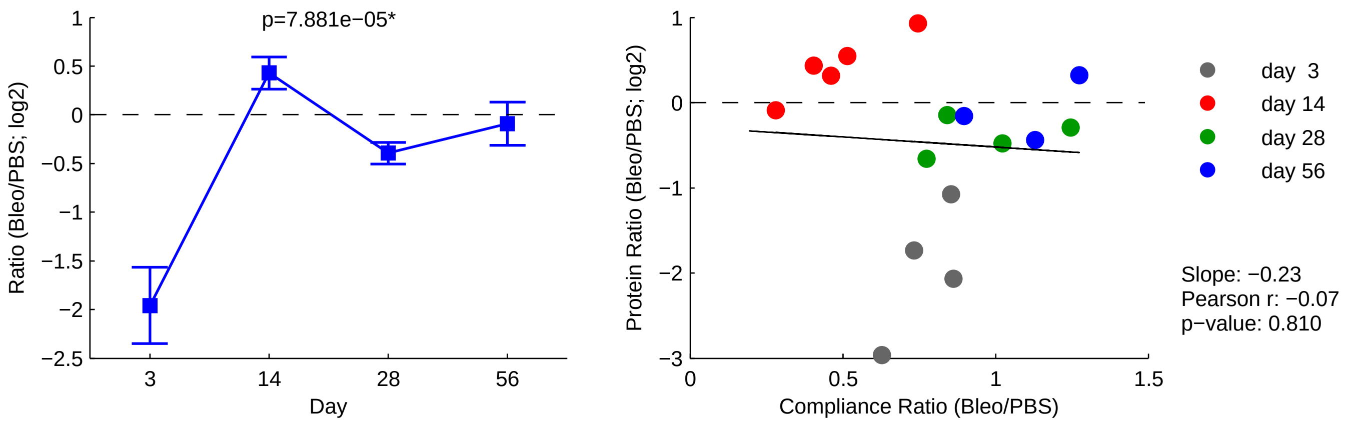

Q9D7B6 − Acad8 (id: 7156)

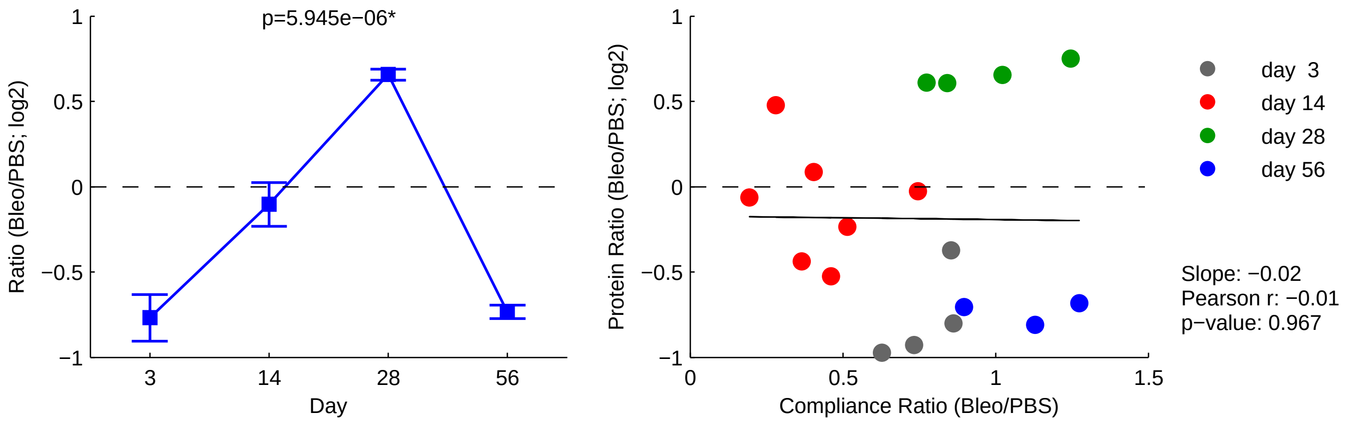

Q9D7B7 − Gpx8 (id: 7157)

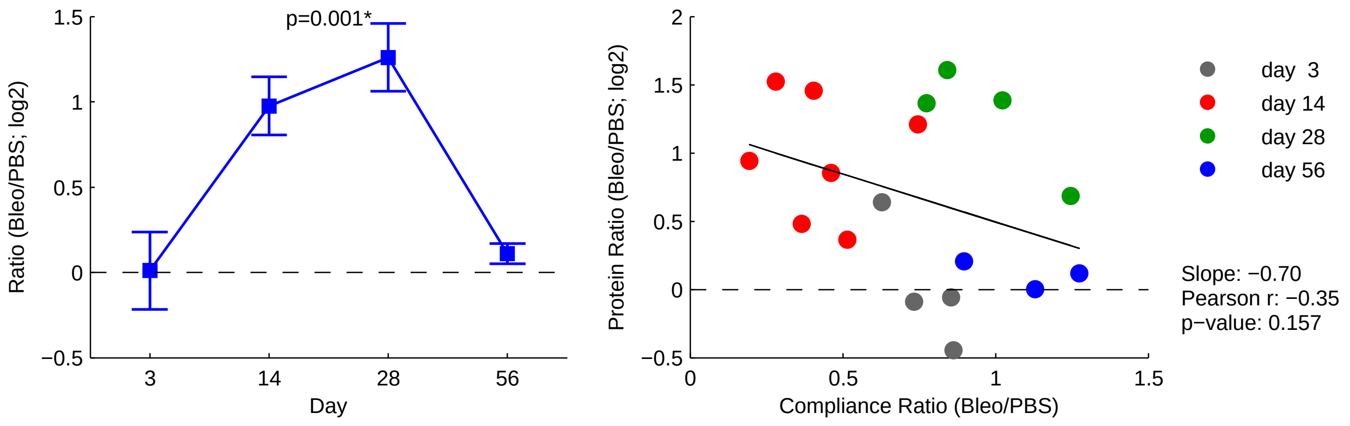

Q9D7H3 − RtcA (id: 7159)

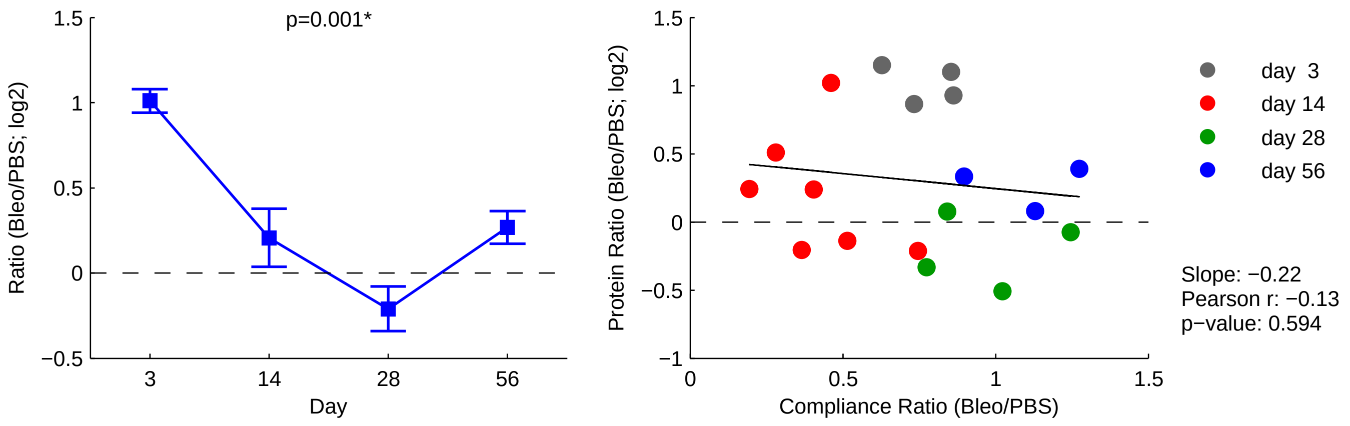

Q9D7J4 − Cox20 (id: 7161)

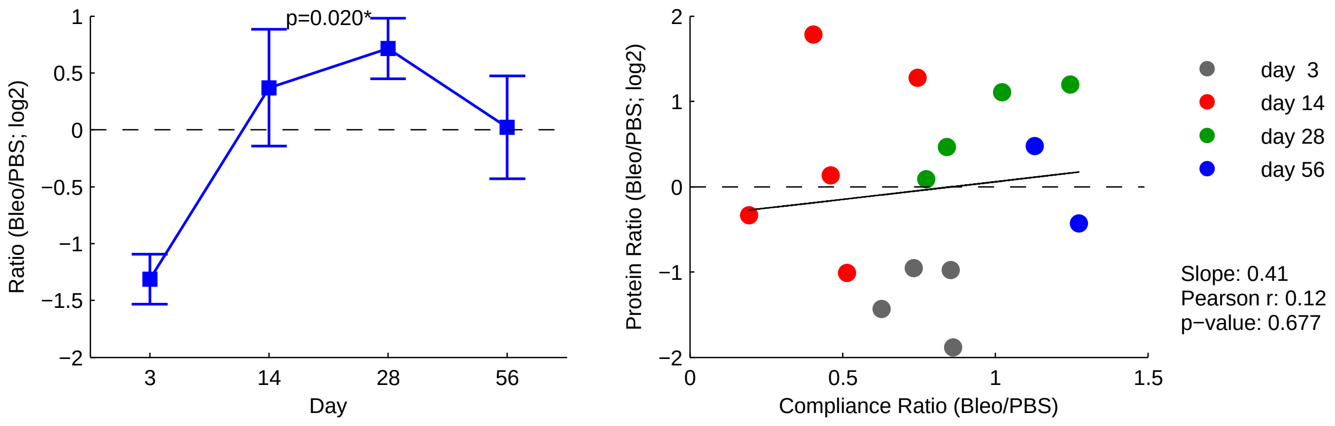

Q9D7J6 − Dnase1l1 (id: 7162)

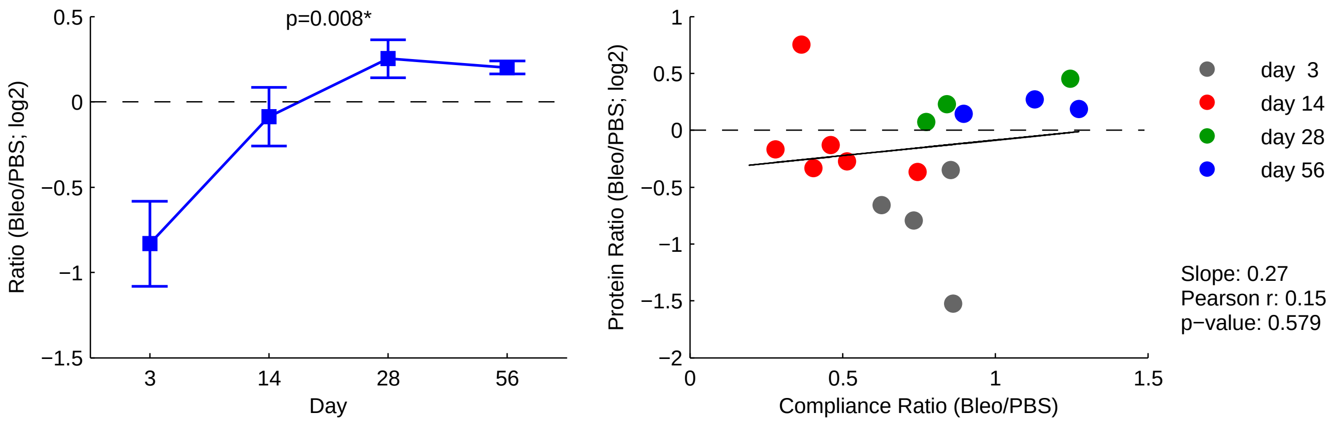

Q9D7J9 − Echdc3 (id: 7163)

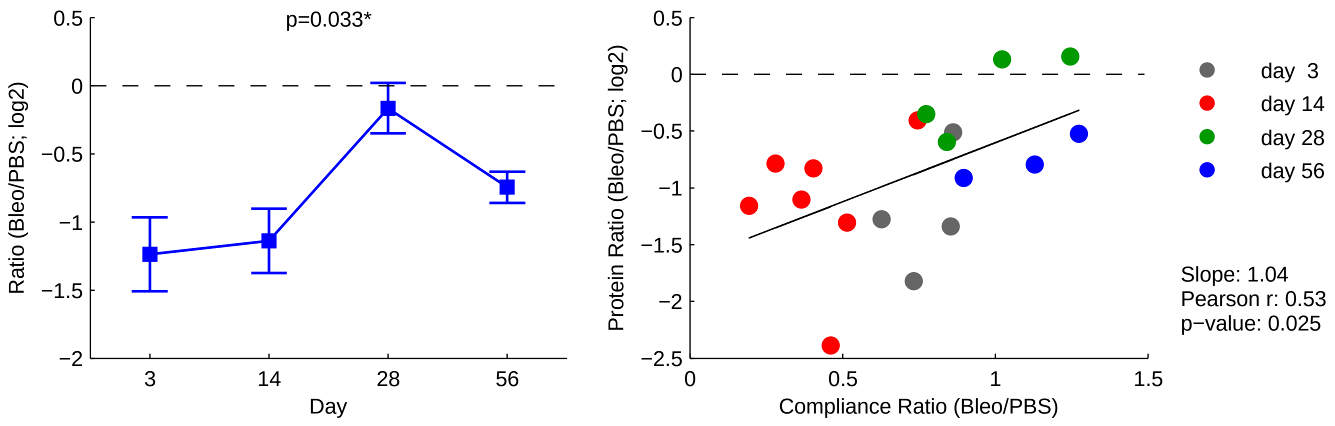

Q9D7M1 − Gid8 (id: 7164)

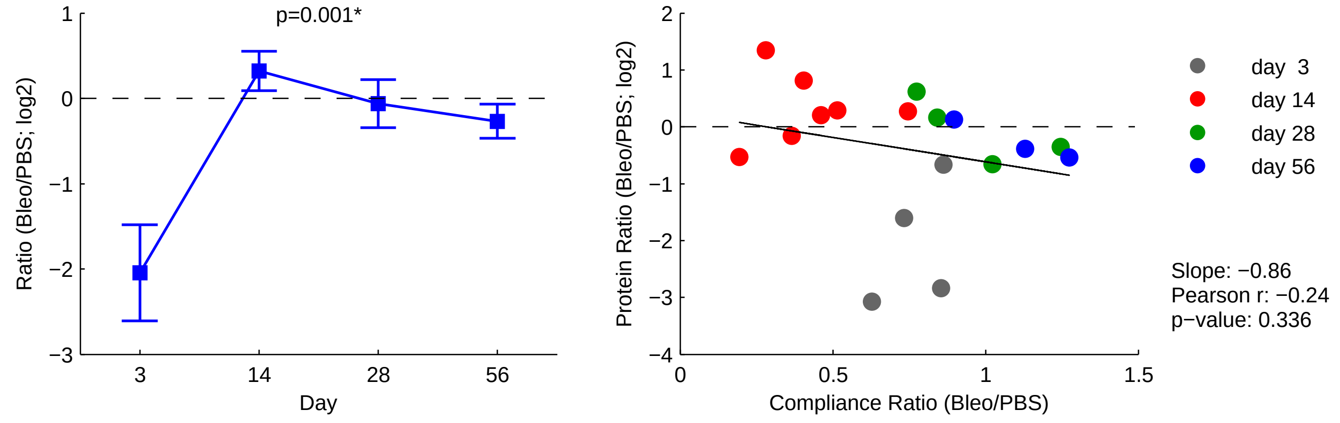

Q9D7M8 − Polr2d (id: 7165)

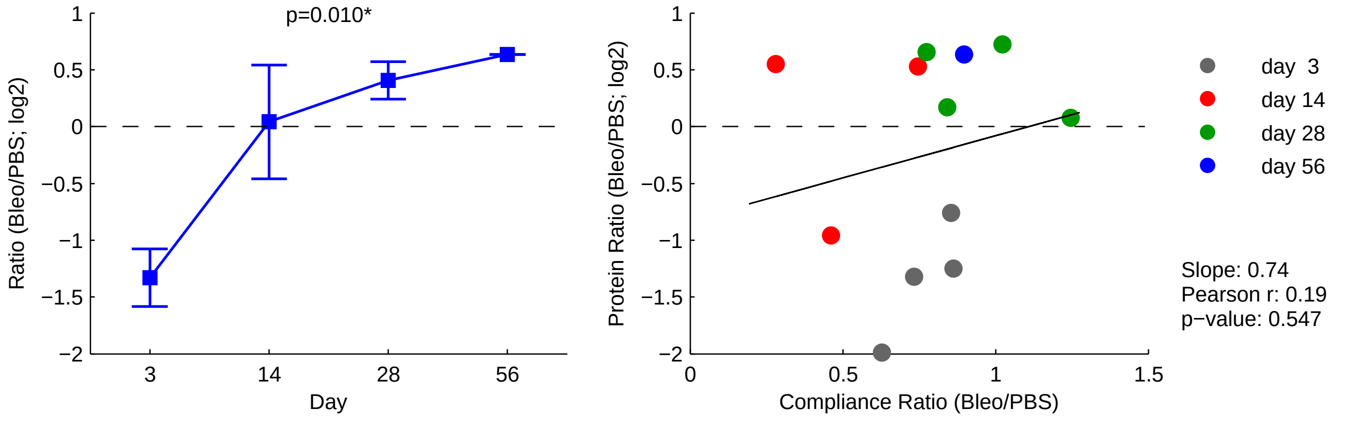

Q9D7N6 − Mrpl30 (id: 7167)

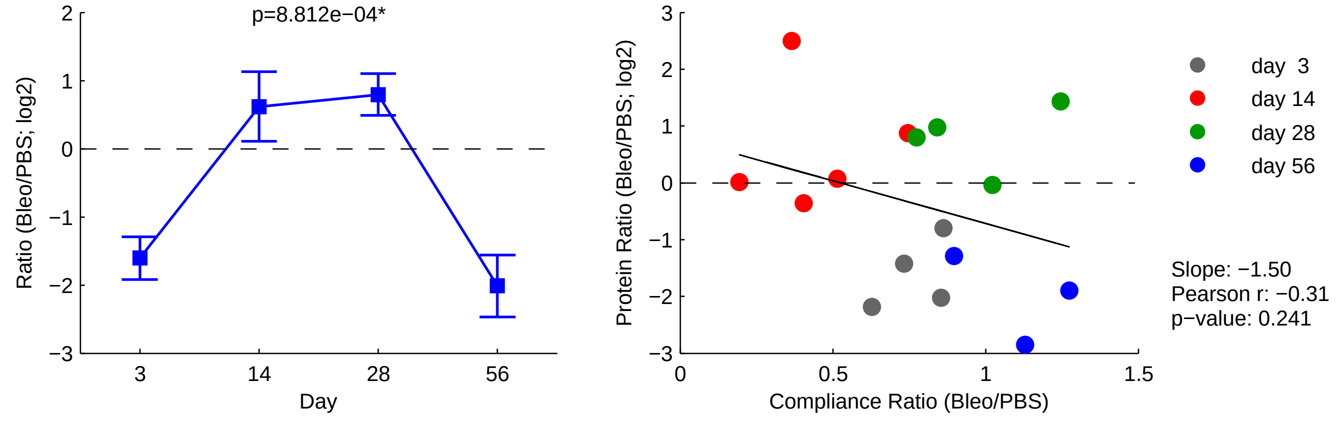

Q9D7N9 − Apmap (id: 7168)

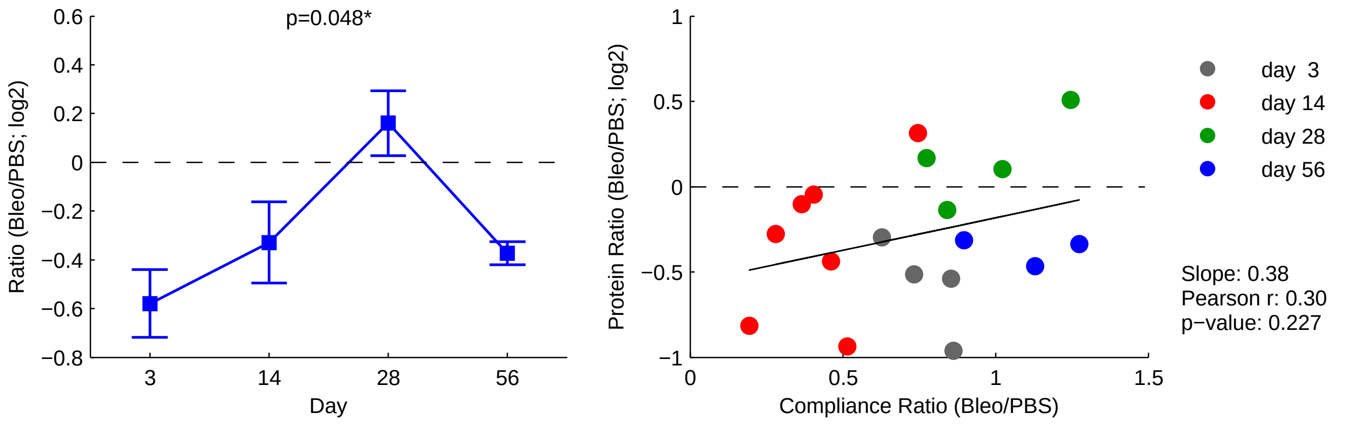

Q9D7P6 − Iscu (id: 7169)

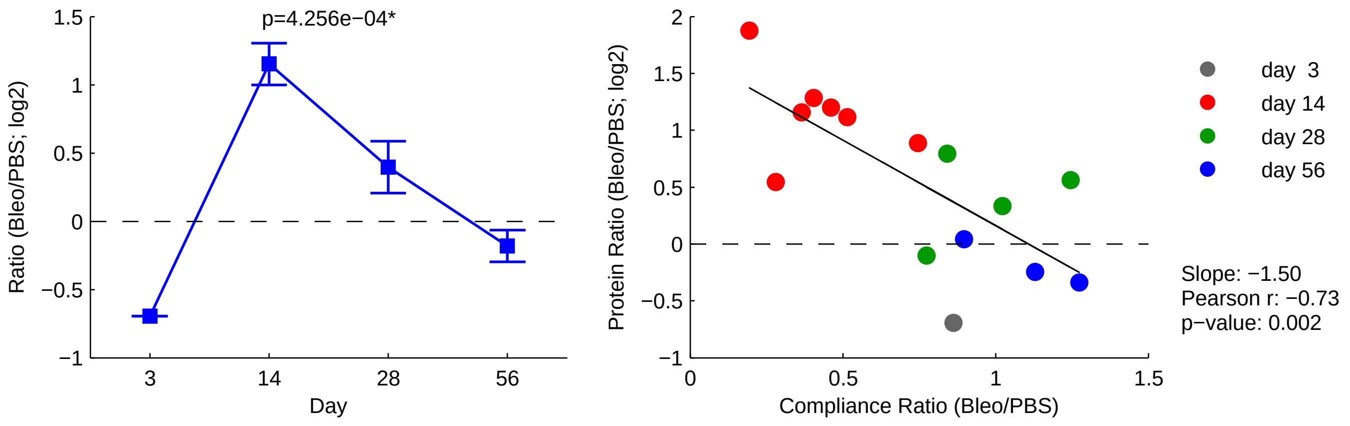

Q9D7P9 − Serpinb12 (id: 7171)

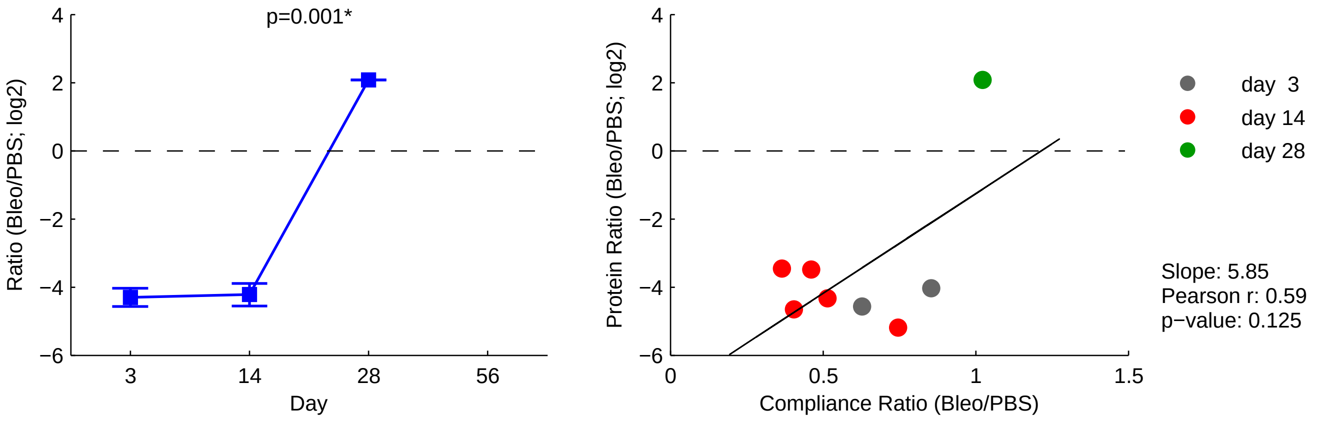

Q9D7S7−2 − Rpl22l1 (id: 7172)

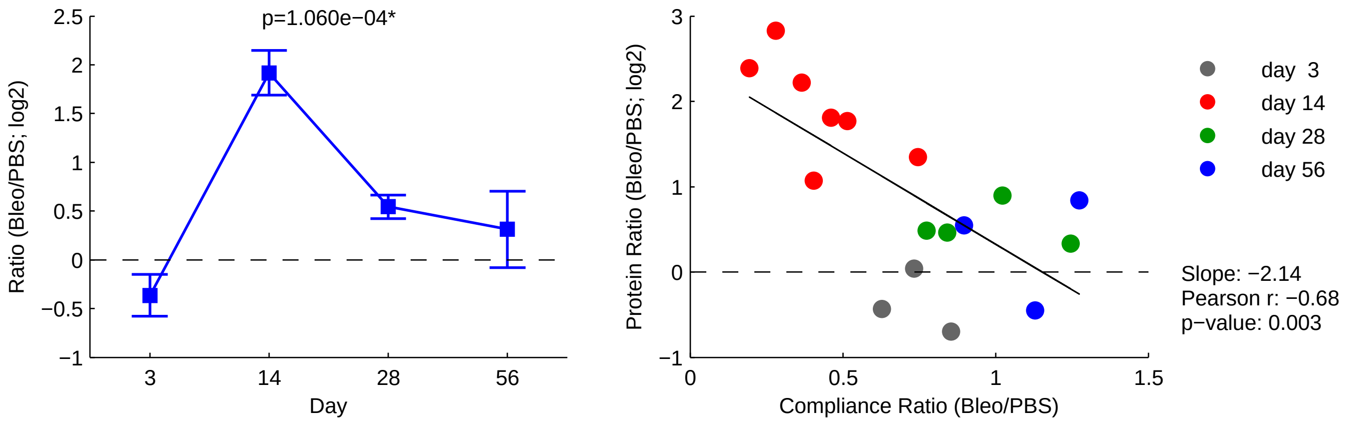

Q9D7S9 − Chmp5 (id: 7173)

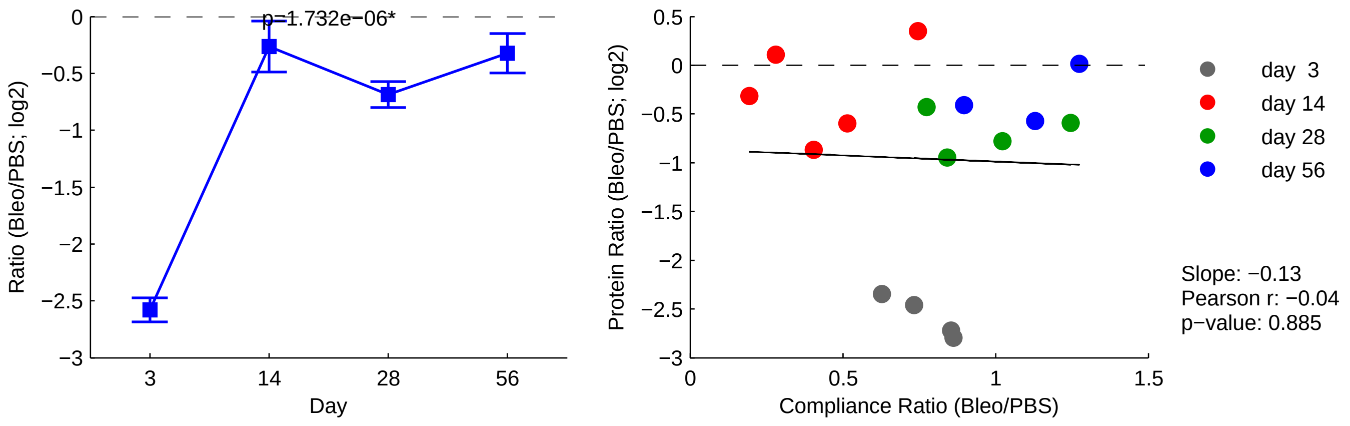

Q9D7Z3 − Nol7 (id: 7176)

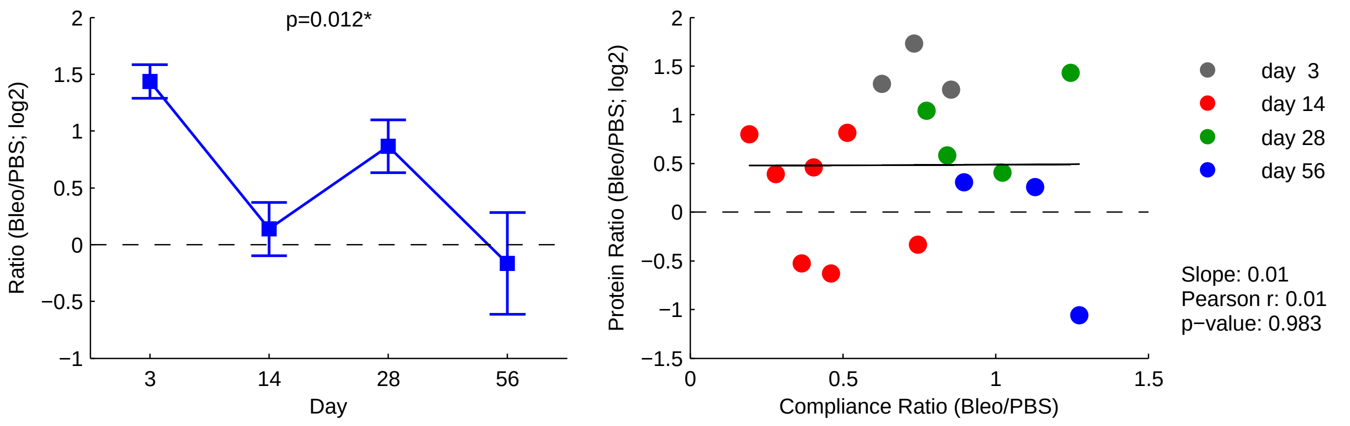

Q9D819 − Ppa1 (id: 7179)

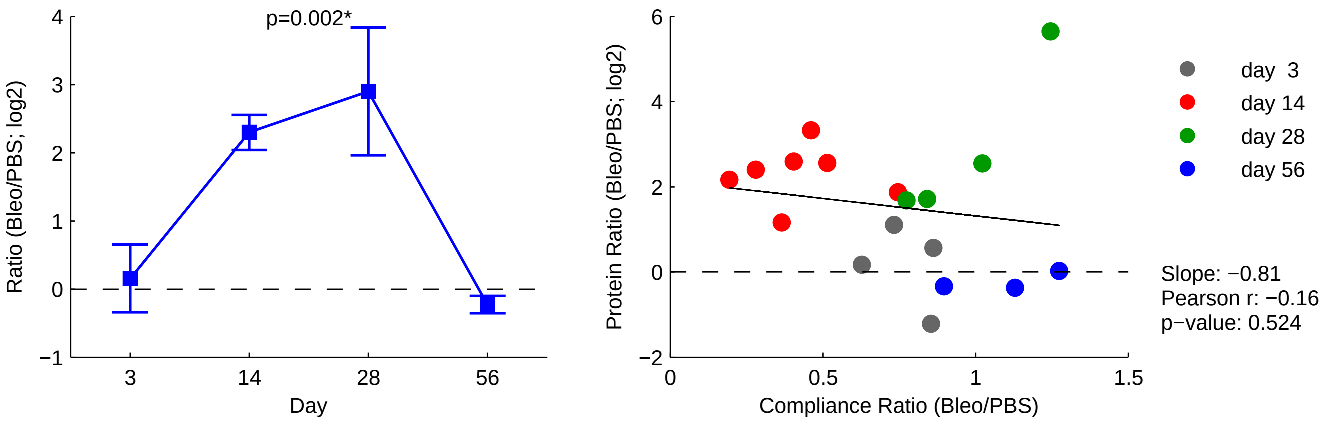

Q9D832 − Dnajb4 (id: 7182)

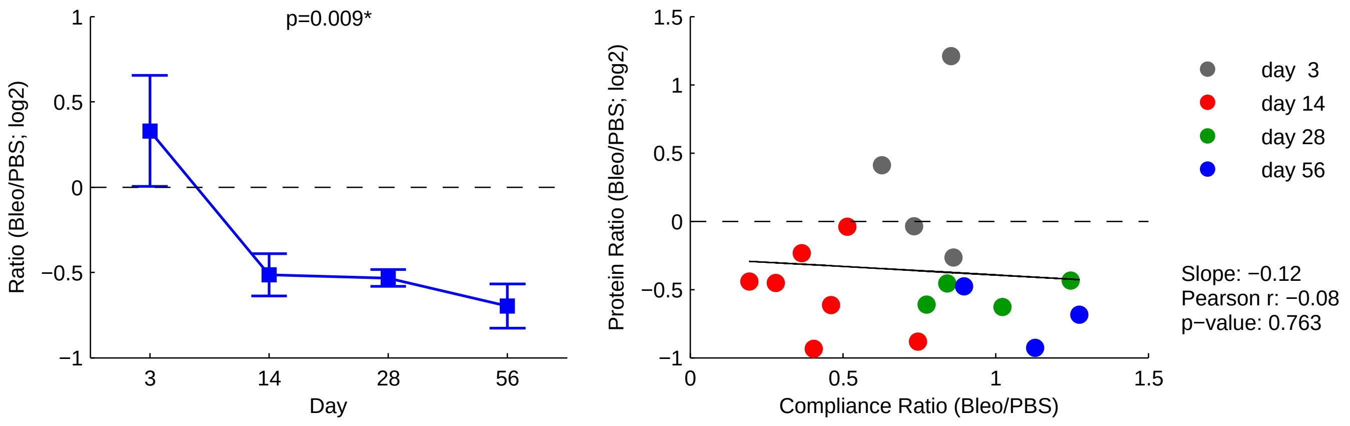

Q9D868−2 − Ppih (id: 7184)

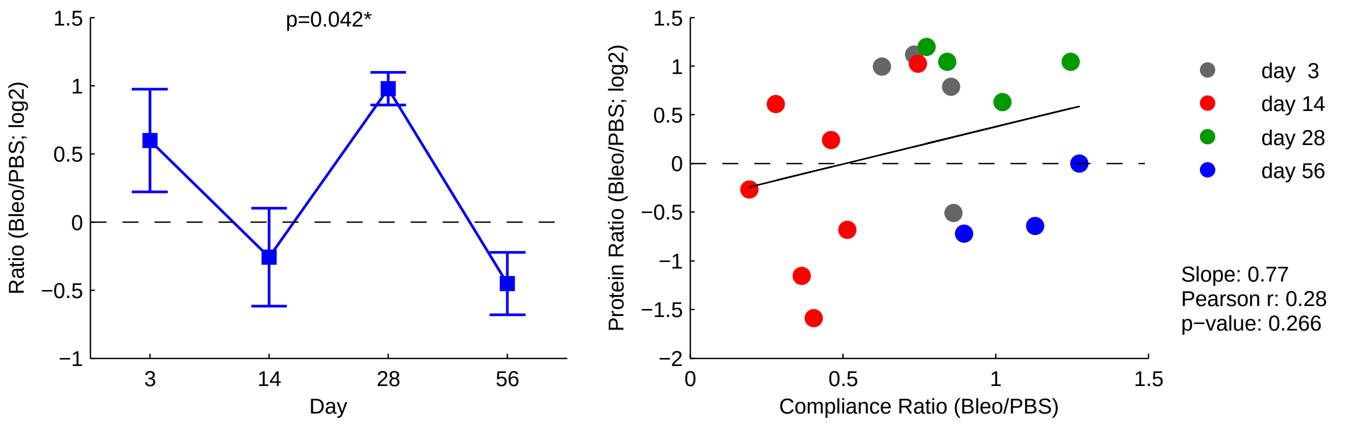

Q9D880 − Timm50 (id: 7185)

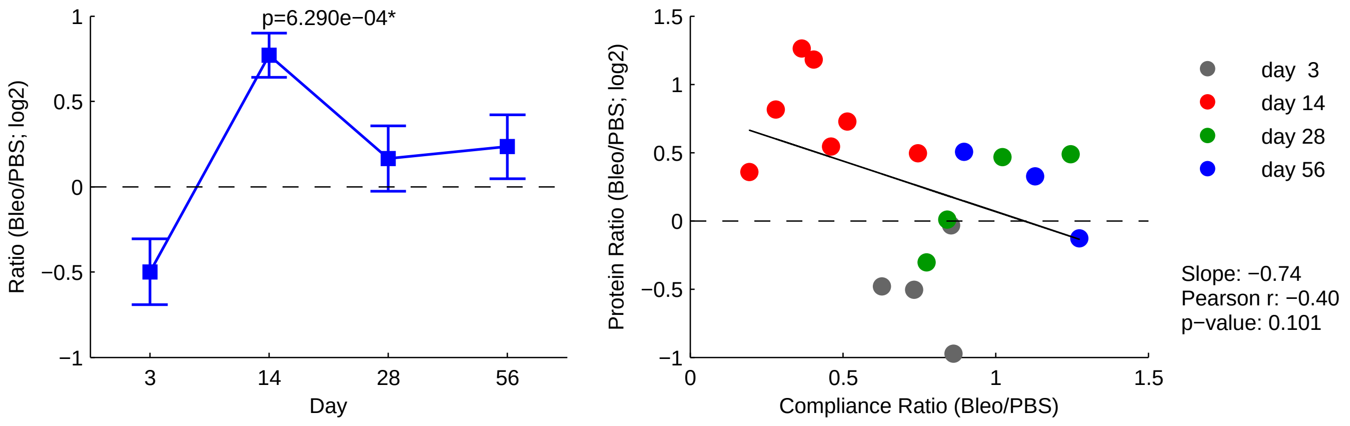

Q9D883 − U2af1 (id: 7186)

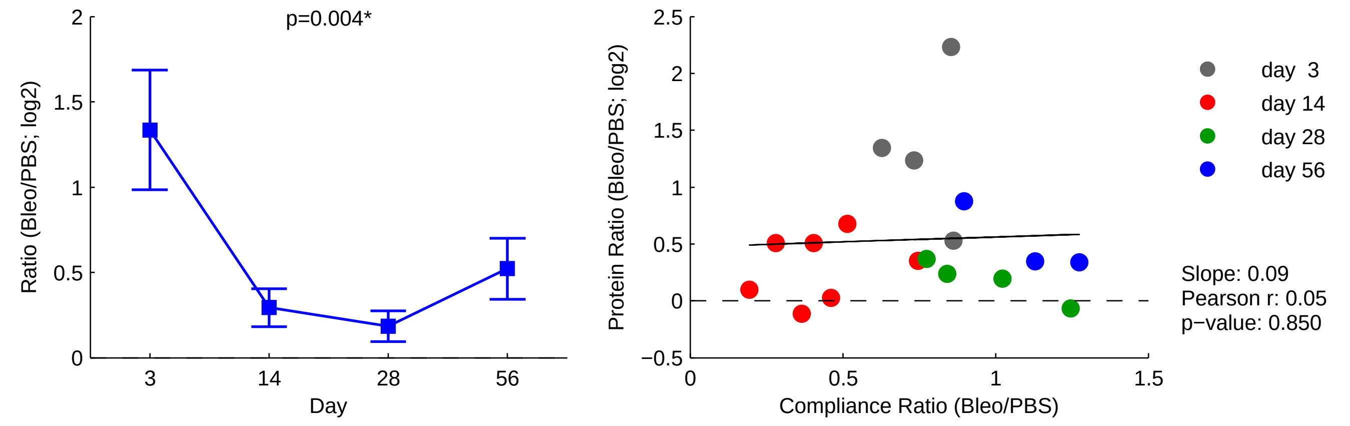

Q9D8B3 − Chmp4b (id: 7188)

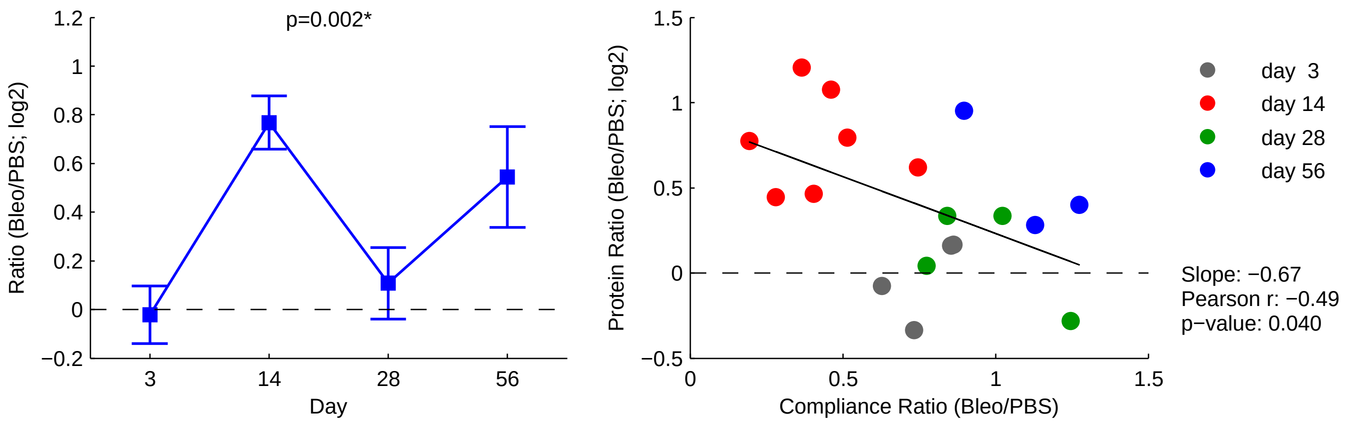

Q9D8B6 − Fam210b (id: 7189)

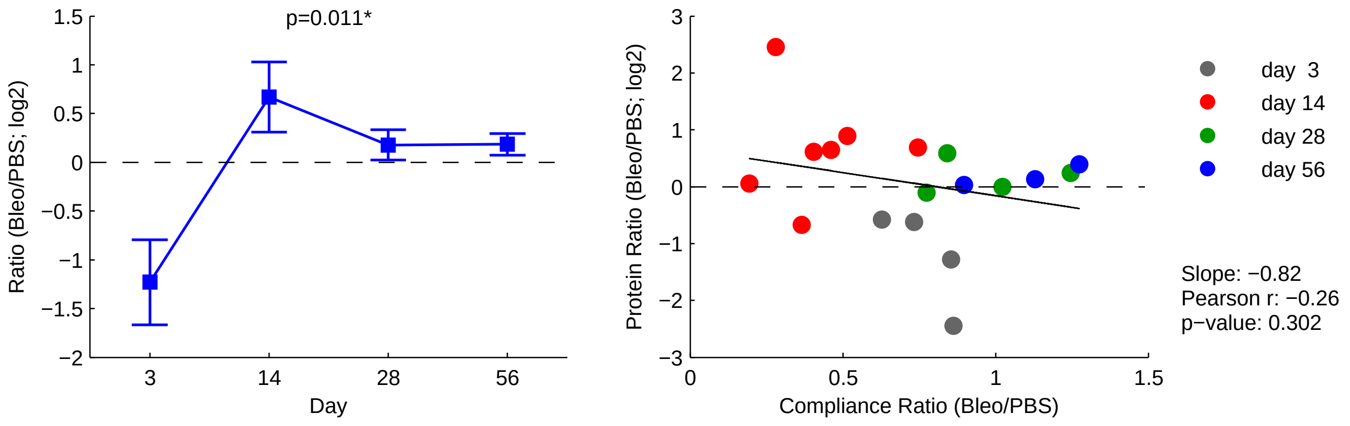

Q9D8B7 − Jam3 (id: 7190)

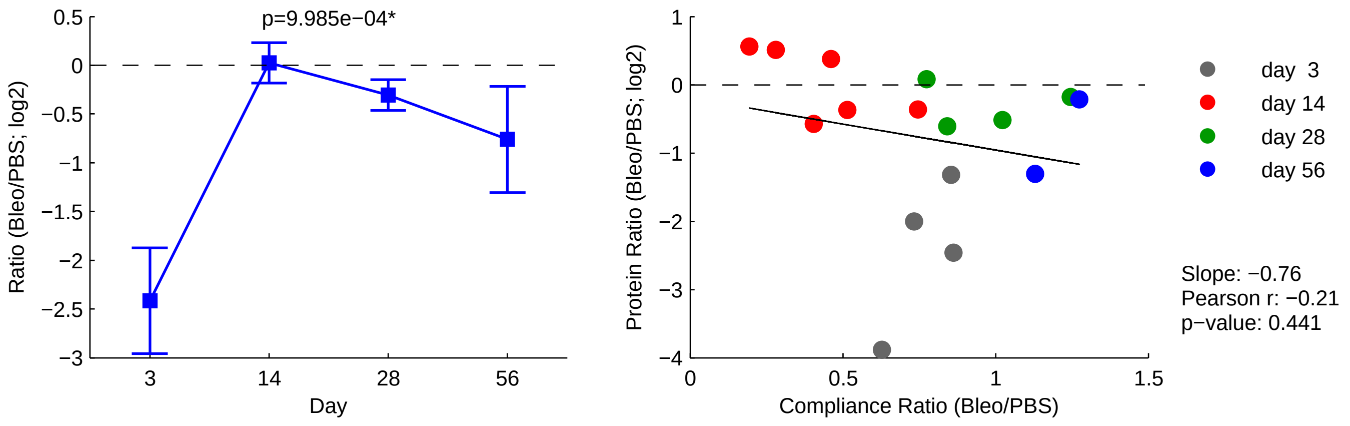

Q9D8C4 − Ifi35 (id: 7193)

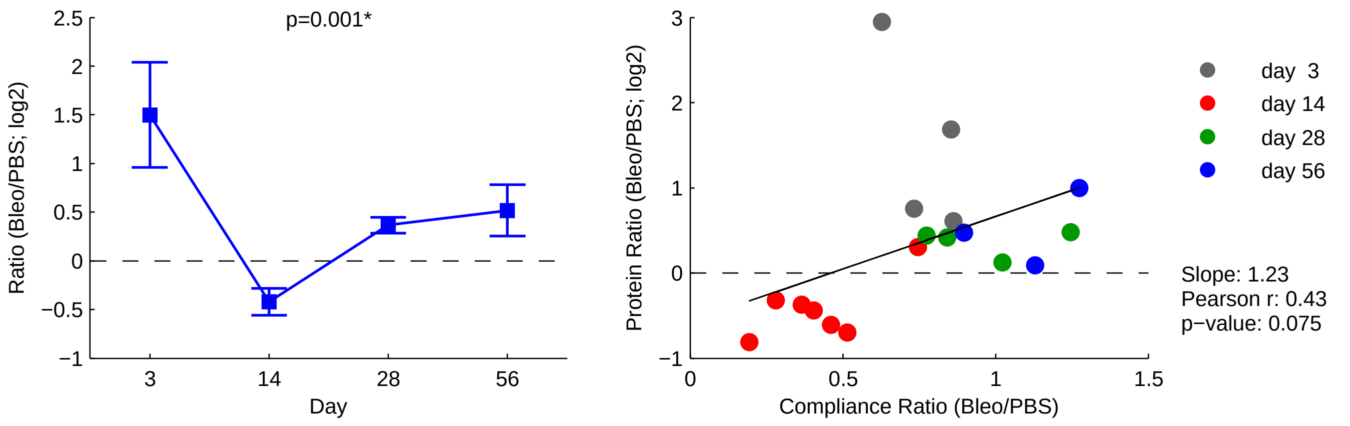

Q9D8E6 − Rpl4 (id: 7195)

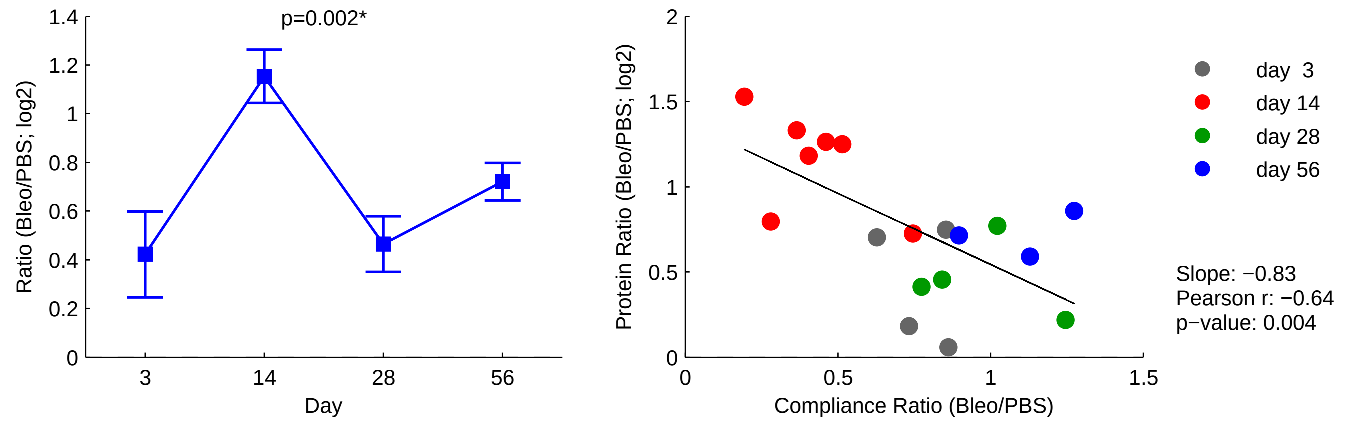

Q9D8N0 − Eef1g (id: 7199)

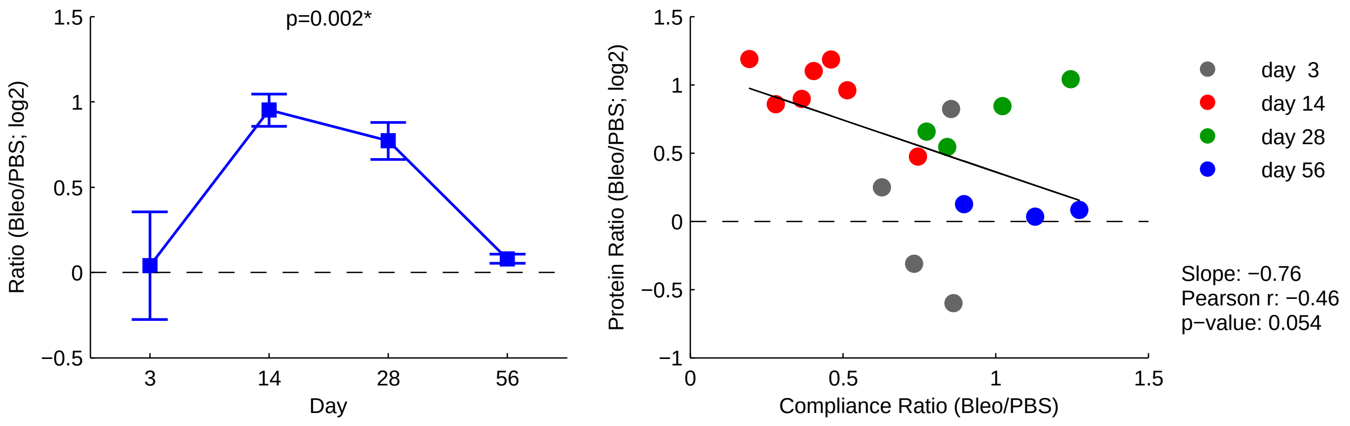

Q9D8N2 − Fam45a (id: 7200)

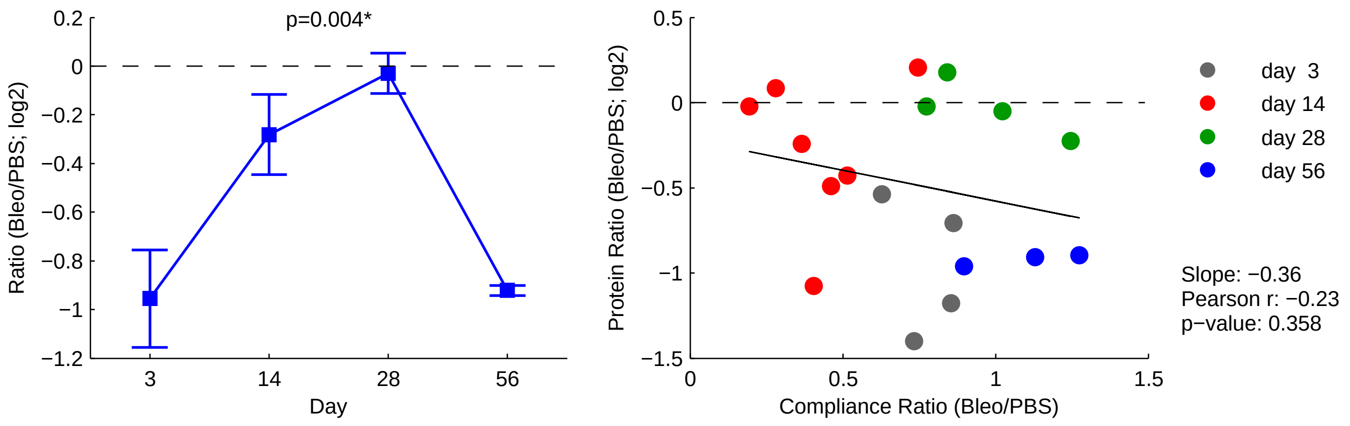

Q9D8S3 − Arfgap3 (id: 7202)

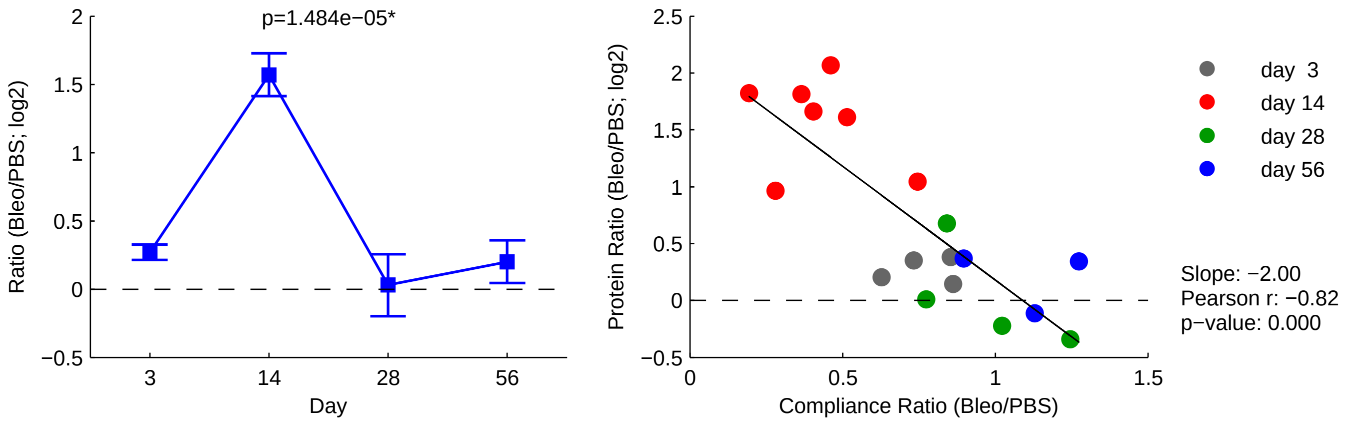

Q9D8S4 − Rexo2 (id: 7203)

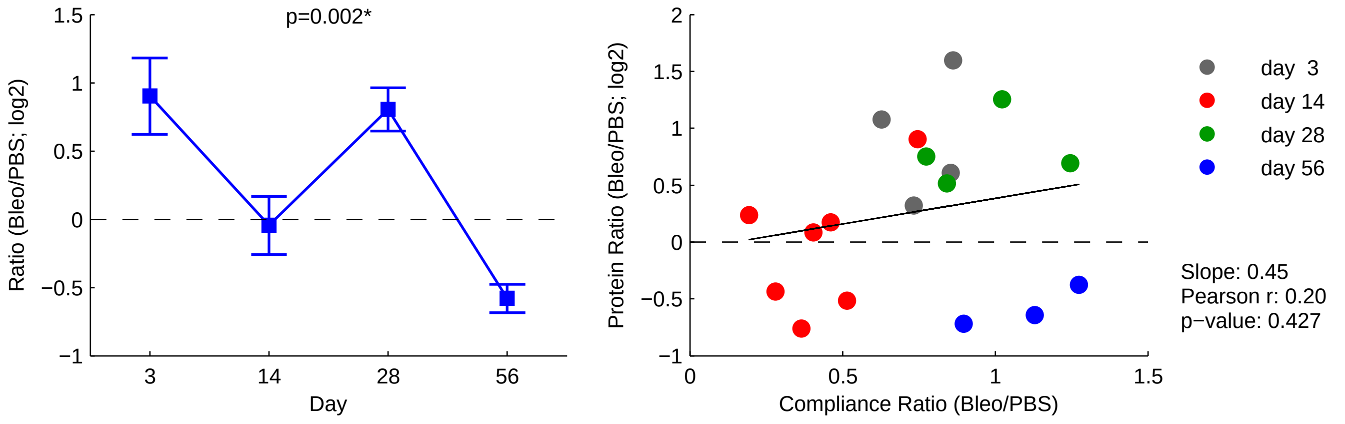

Q9D8T0 − Fam3a (id: 7204)

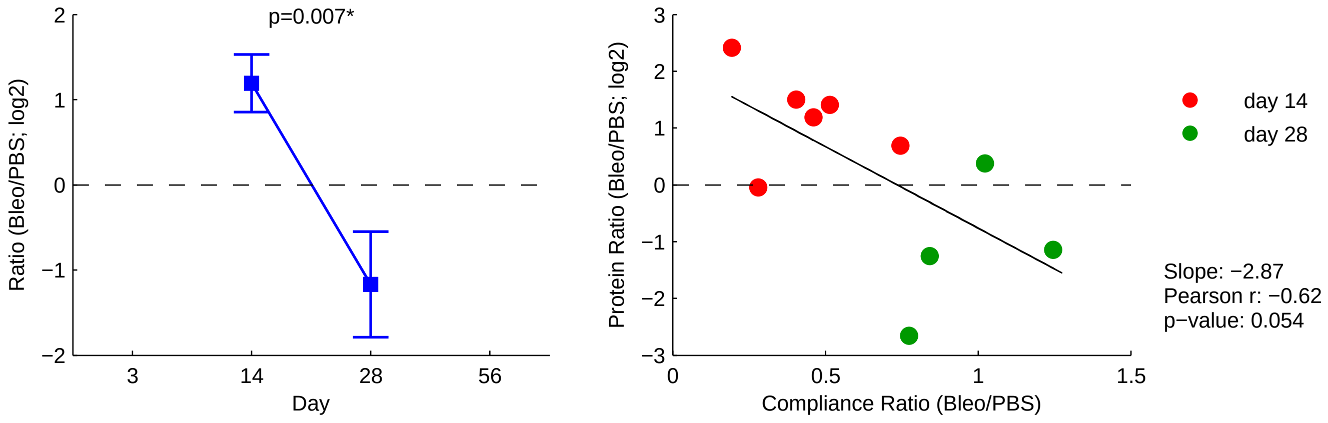

Q9D8T4 − Tvp23b (id: 7206)

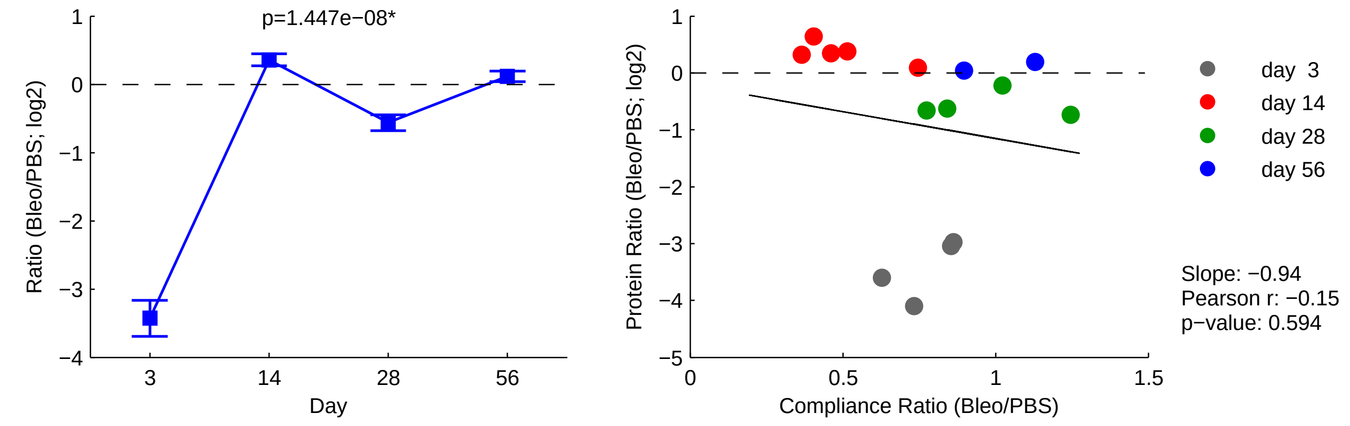

Q9D8T7 − Slirp (id: 7207)

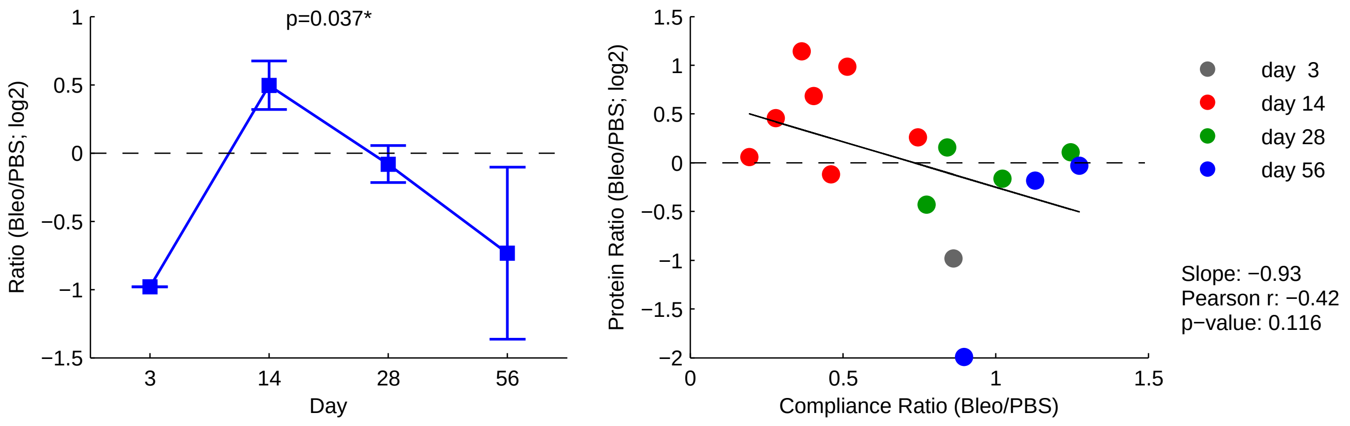

Q9D8U6 − Mcemp1 (id: 7208)

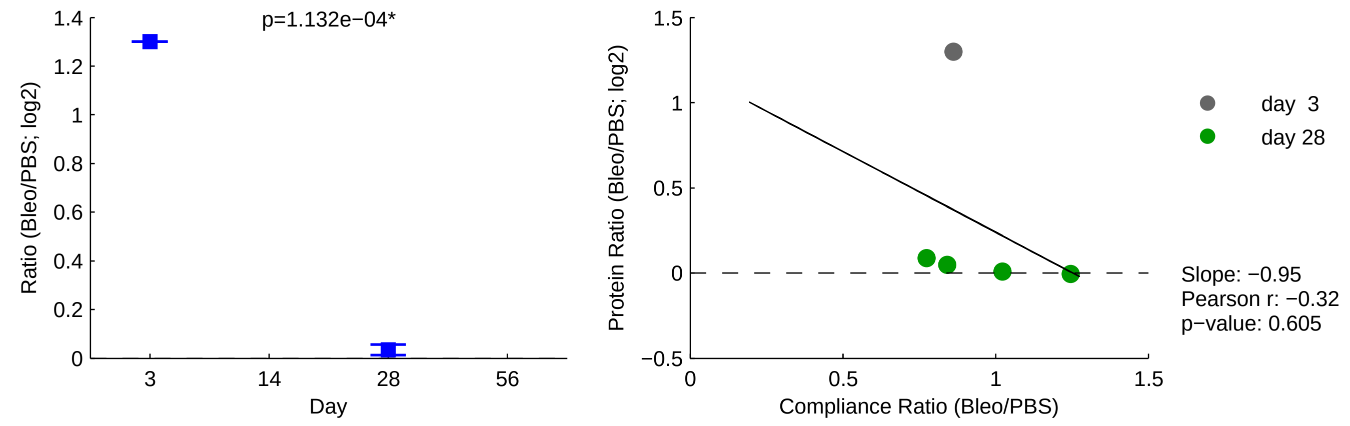

Q9D8U8 − Snx5 (id: 7209)

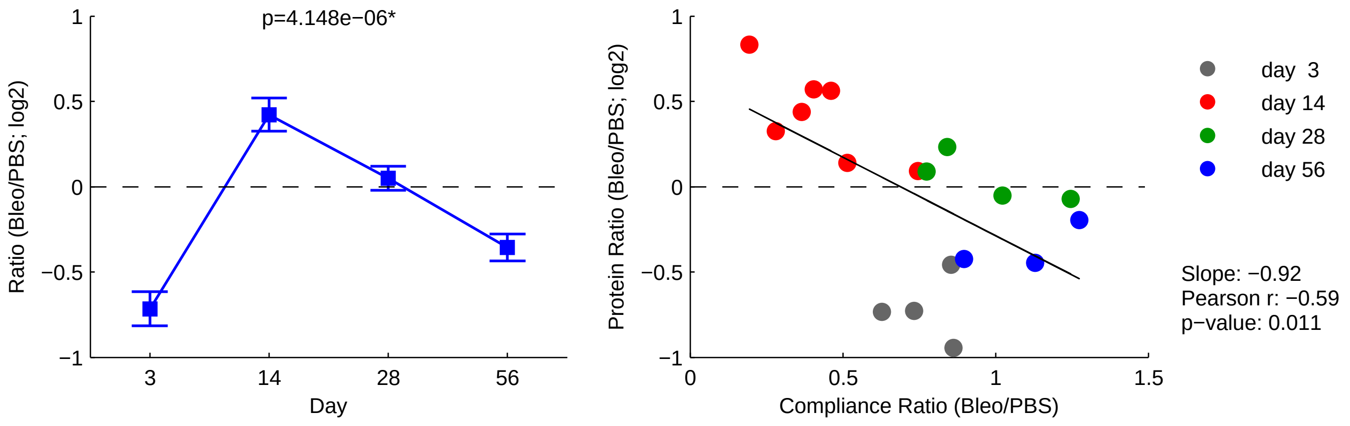

Q9D8V0−3 − Hm13 (id: 7210)

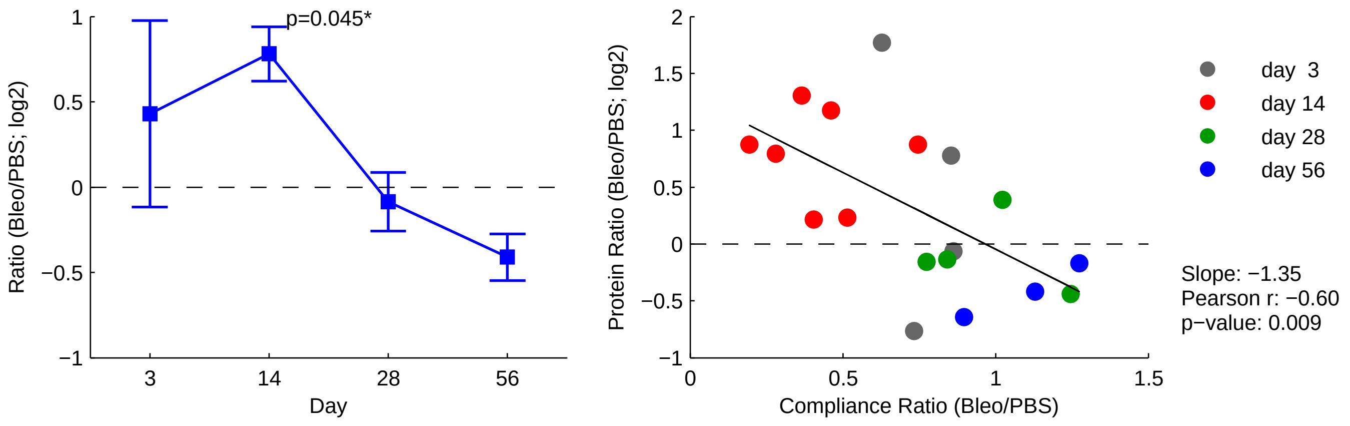

Q9D8W5 − Psmd12 (id: 7212)

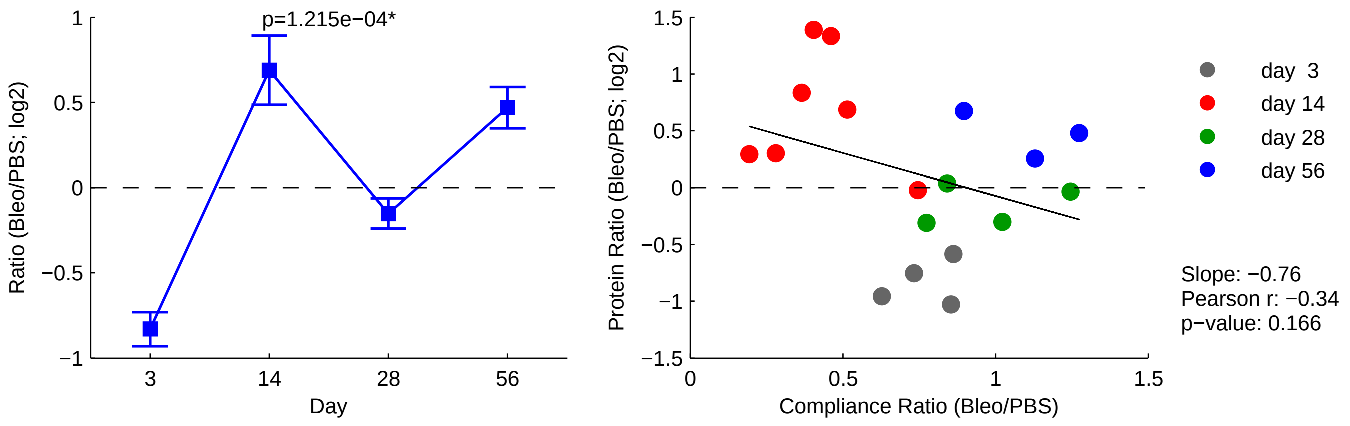

Q9D8W7 − Ociad2 (id: 7213)

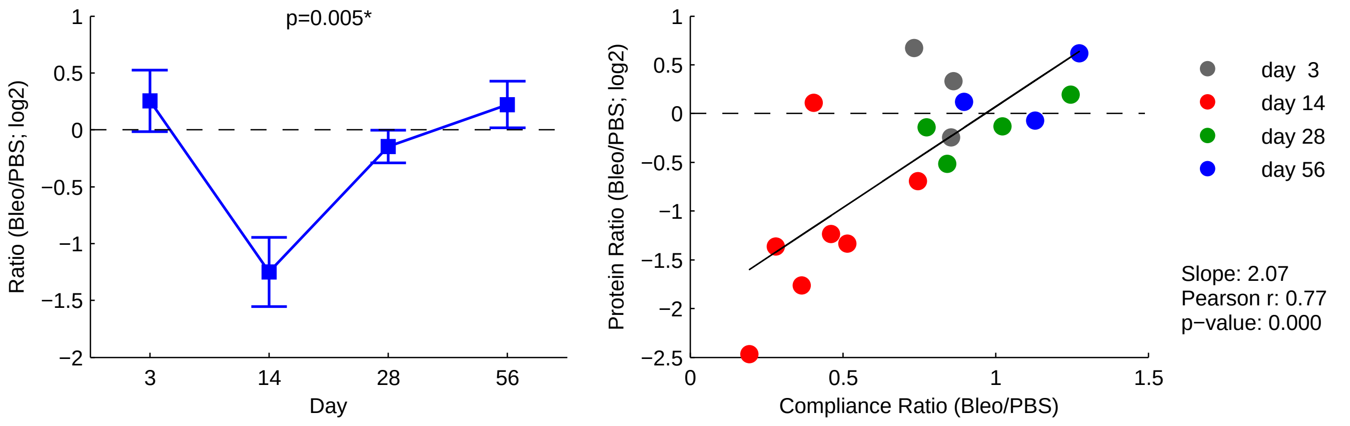

Q9D903 − Ebna1bp2 (id: 7221)

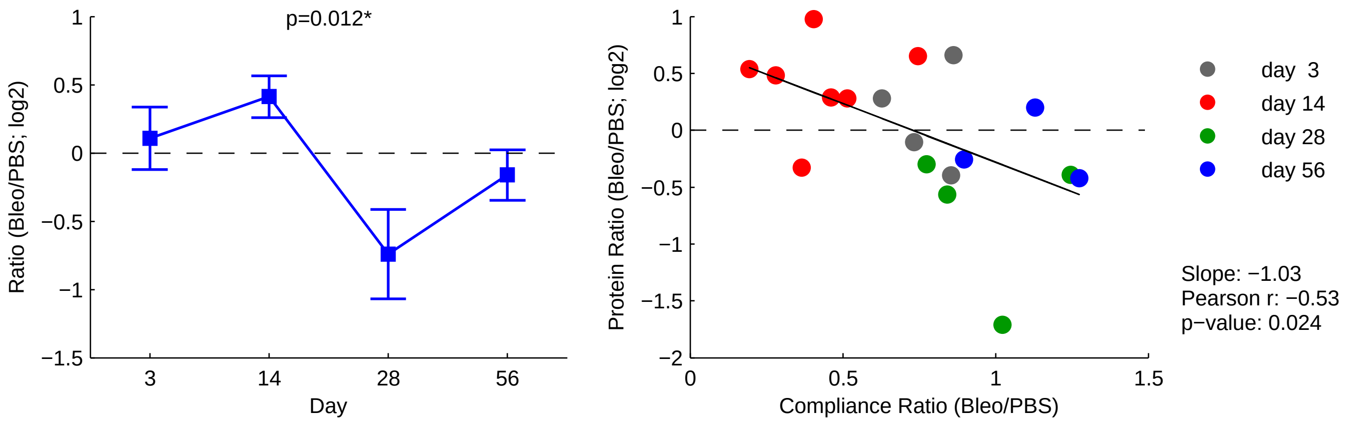

Q9D906 − Atg7 (id: 7222)

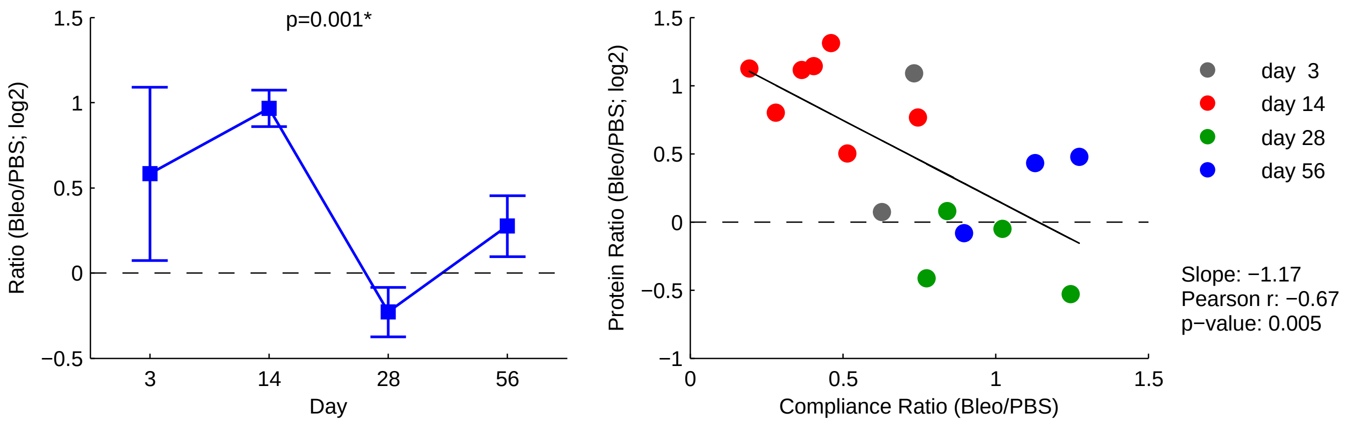

Q9D938 − Tmem160 (id: 7224)

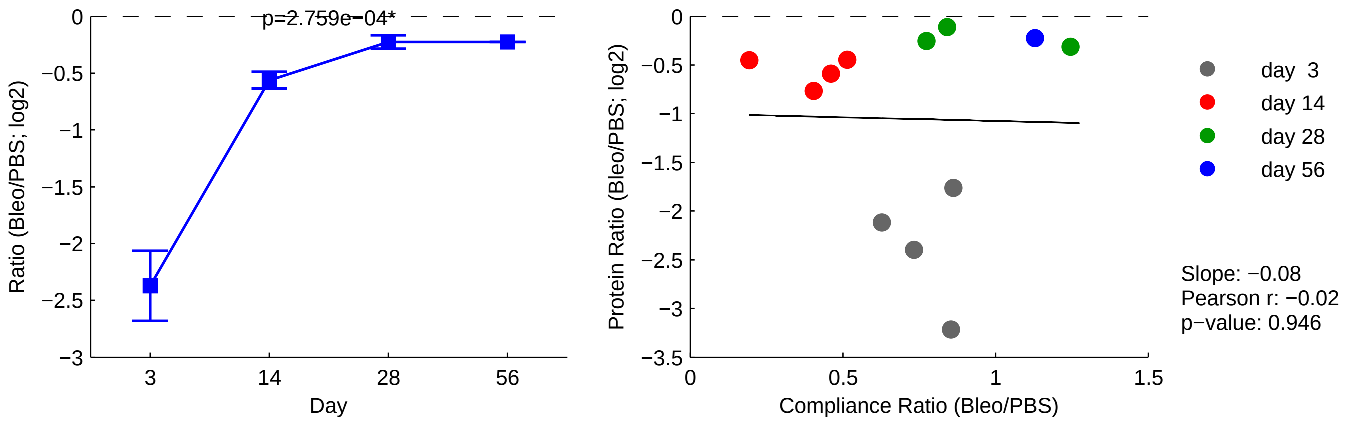

Q9D952 − Evpl (id: 7225)

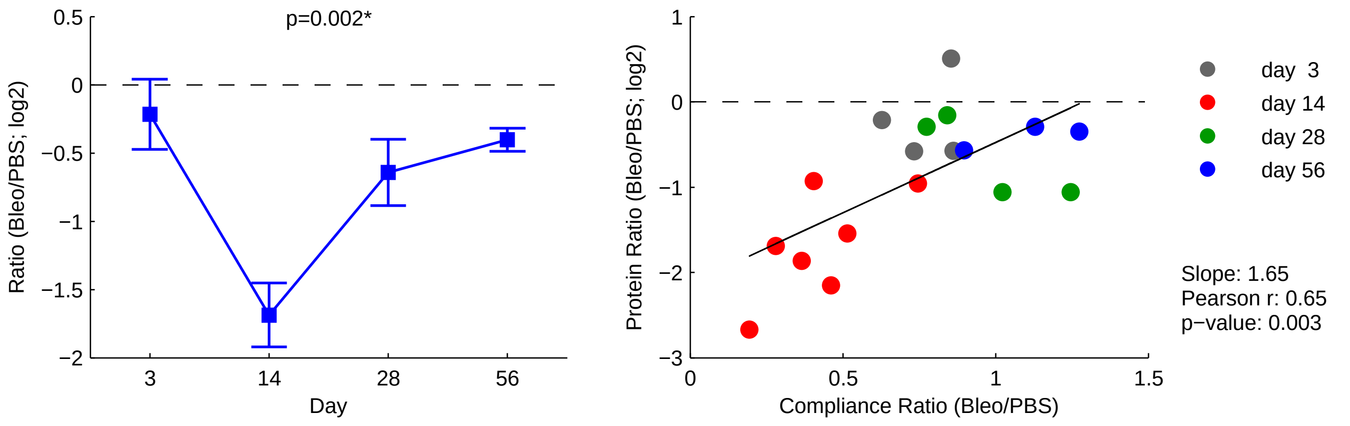

Q9D958 − Spcs1 (id: 7226)

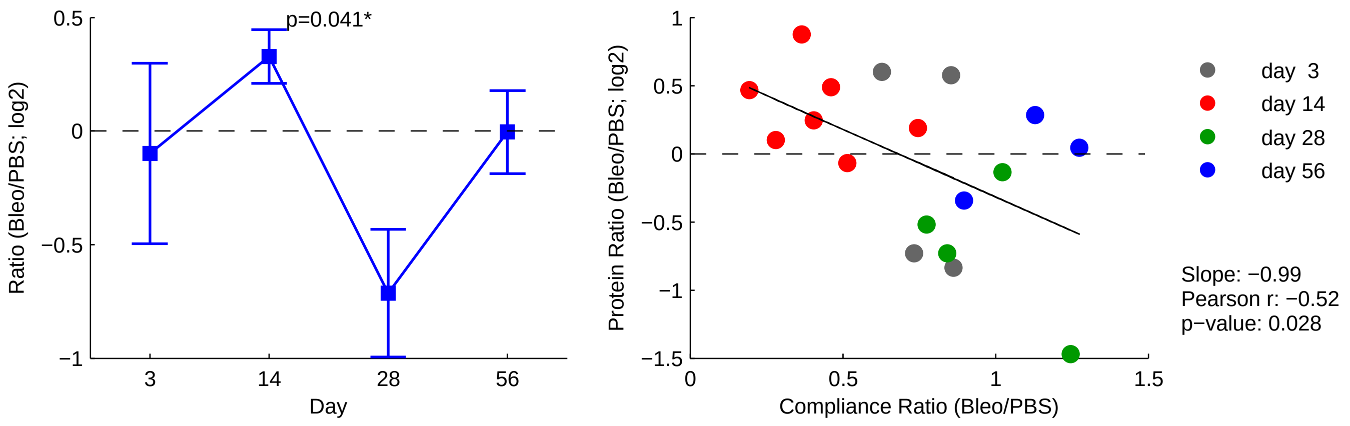

Q9D964 − Gatm (id: 7227)

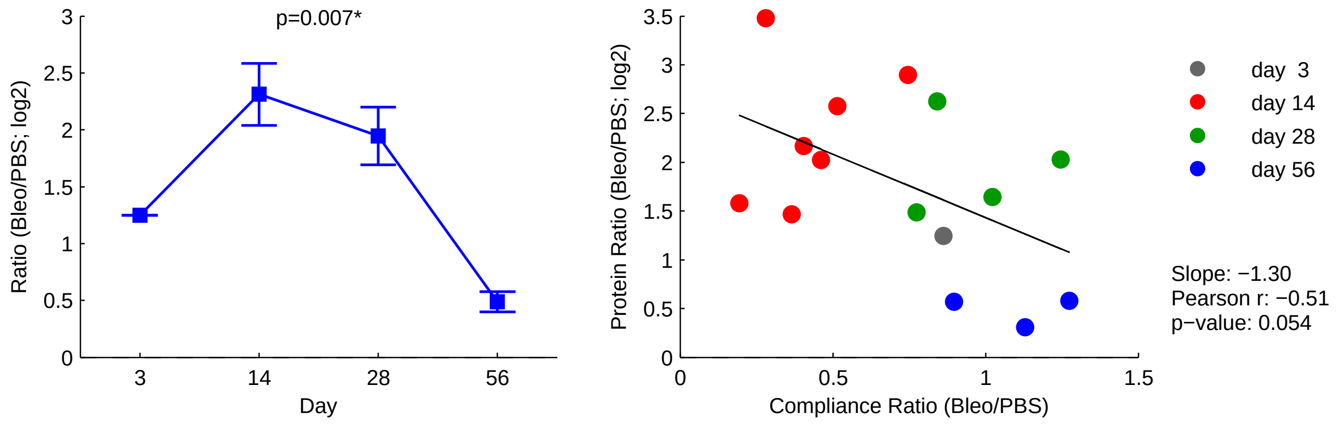

Q9D967 − Mdp1 (id: 7228)

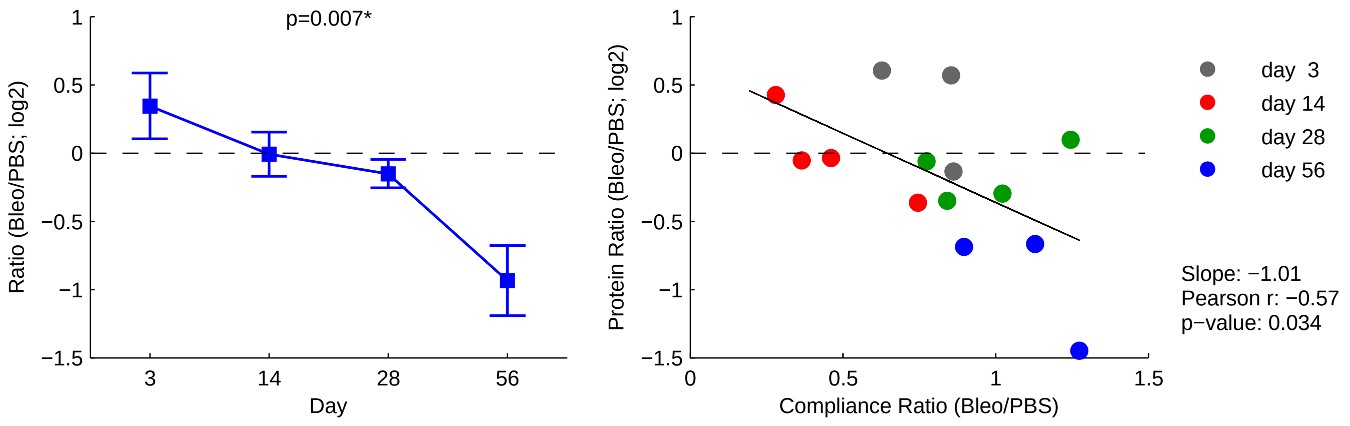

Q9D9V3−2 − Echdc1 (id: 7232)

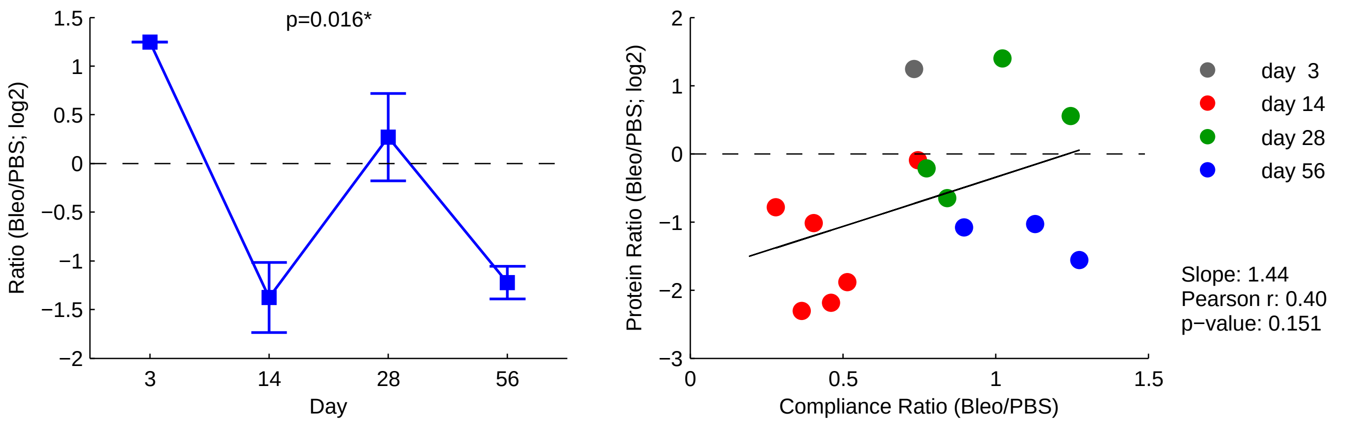

Q9DAI2 − Rabl5 (id: 7239)

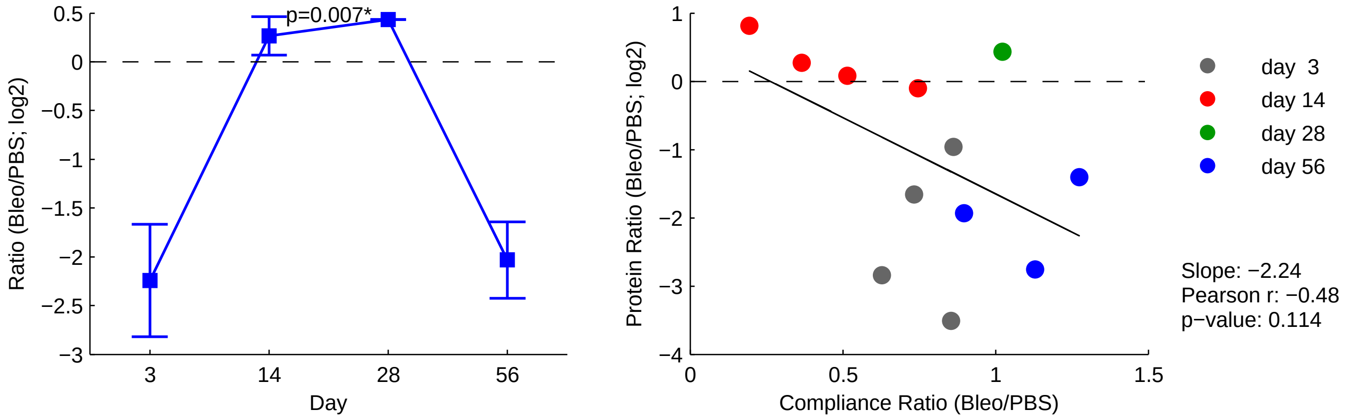

Q9DAJ2 − Tekt1 (id: 7240)

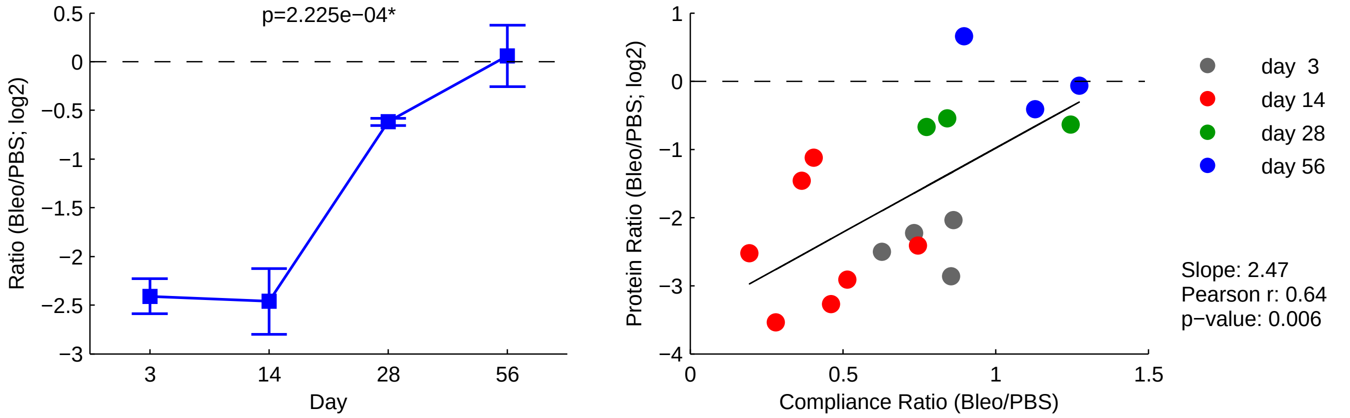

Q9DAK2 − Pacrg (id: 7242)

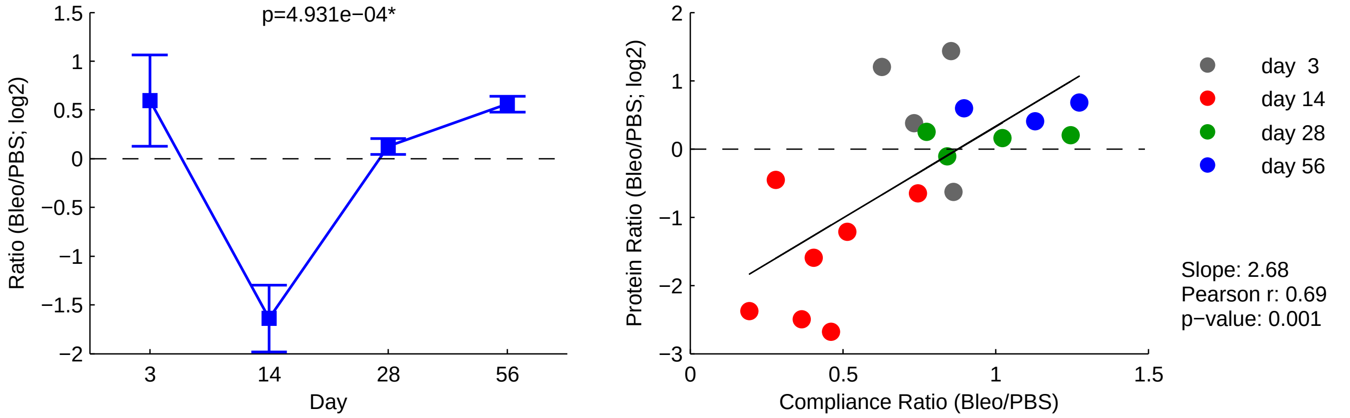

Q9DAM7 − (id: 7245)

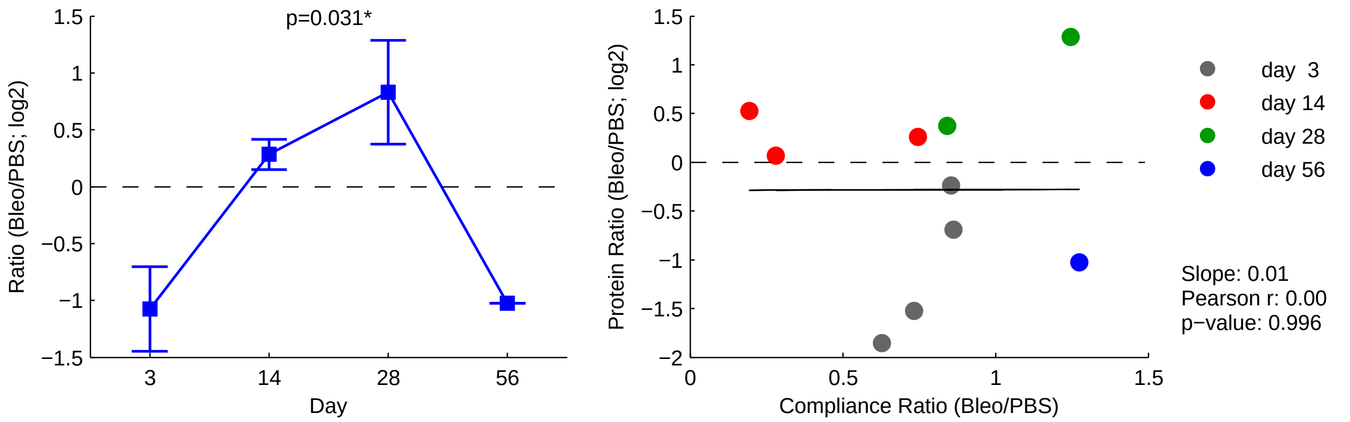

Q9DAR7 − Dcps (id: 7246)

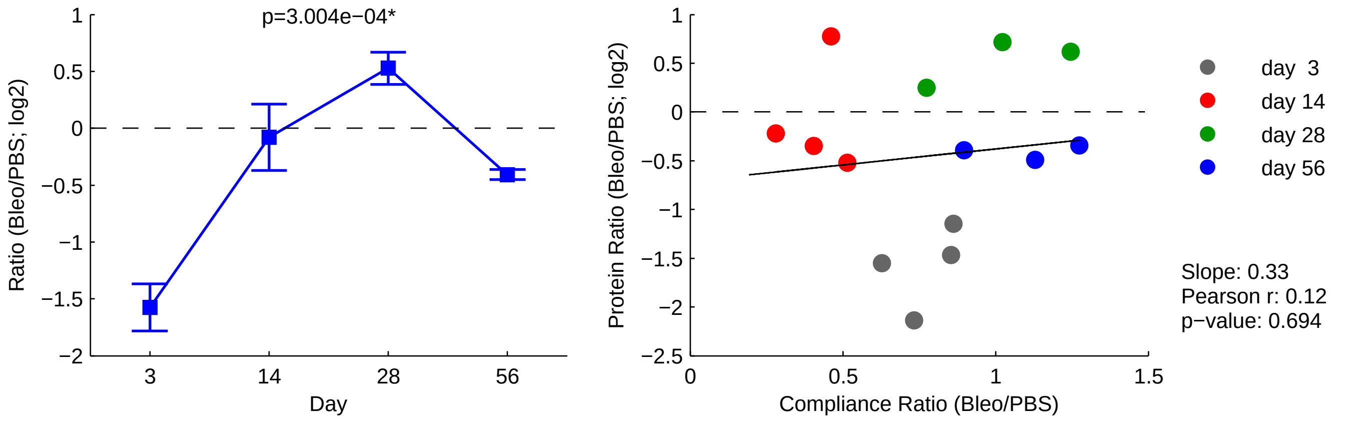

Q9DAT5 − Trmu (id: 7249)

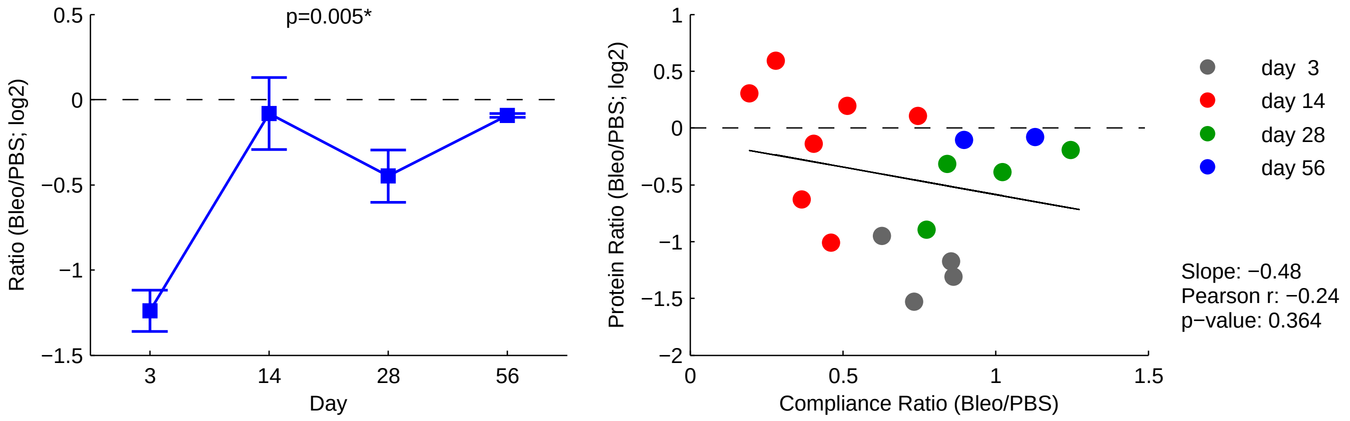

Q9DAU1 − Cnpy3 (id: 7250)

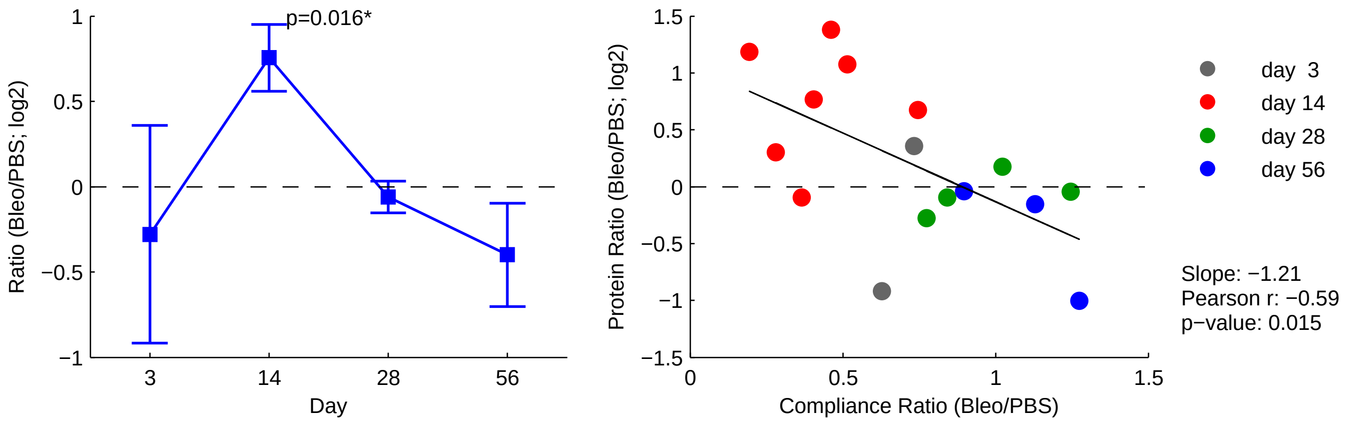

Q9DAW6 − Prpf4 (id: 7253)

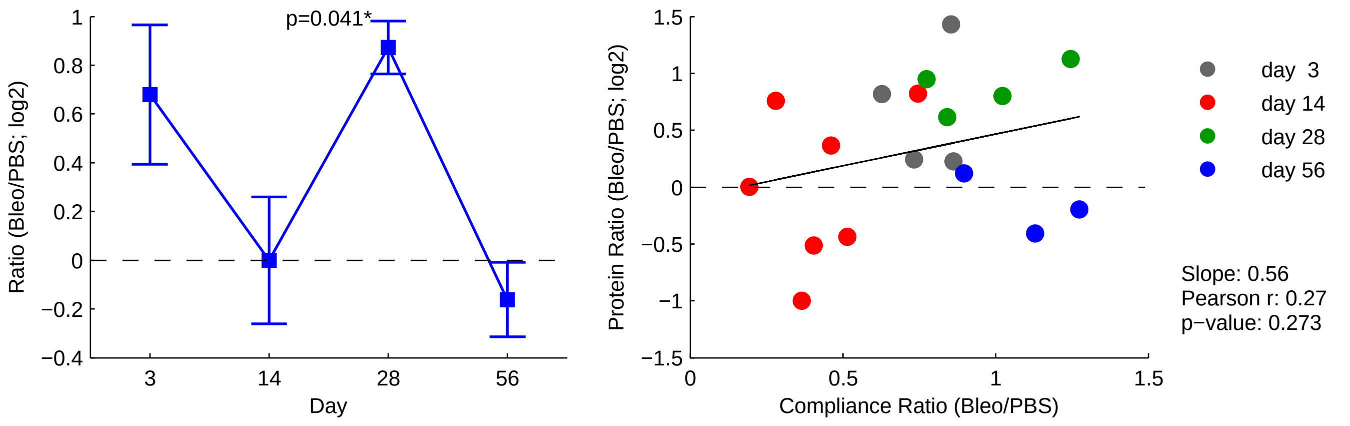

Q9DAW9 − Cnn3 (id: 7254)

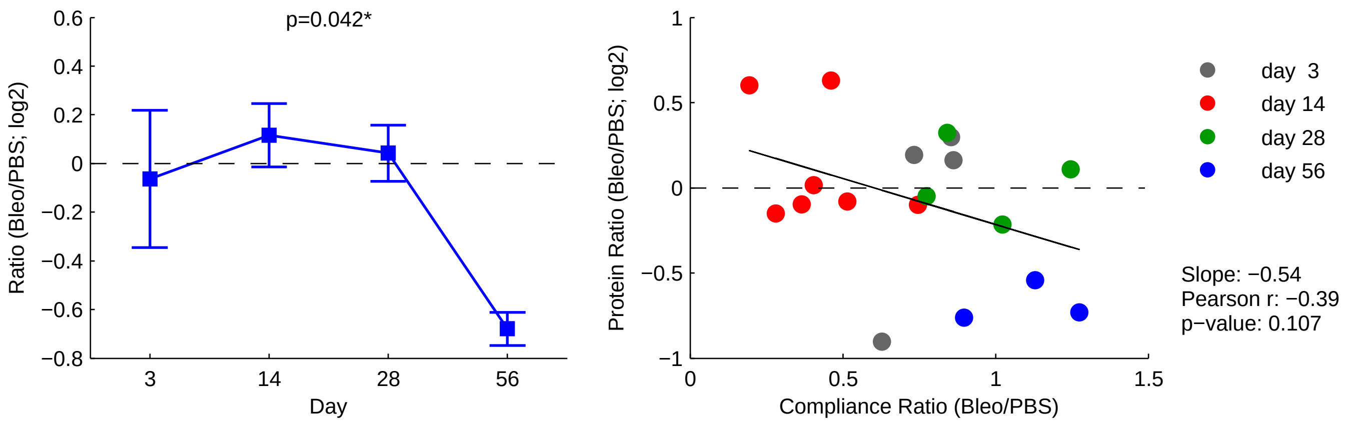

Q9DB05 − Napa (id: 7256)

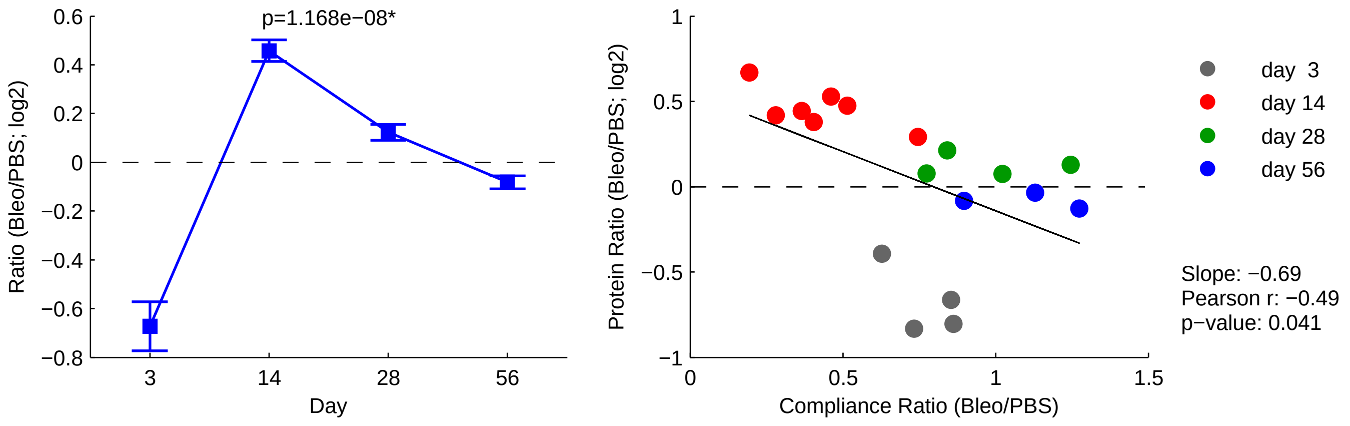

Q9DB07 − Ift46 (id: 7257)

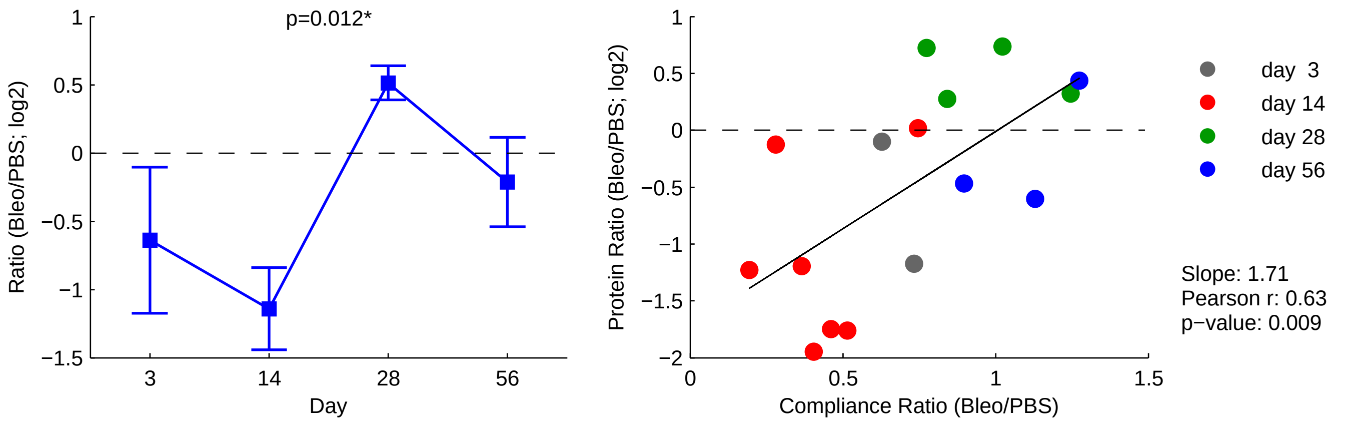

Q9DB10 − Smdt1 (id: 7258)

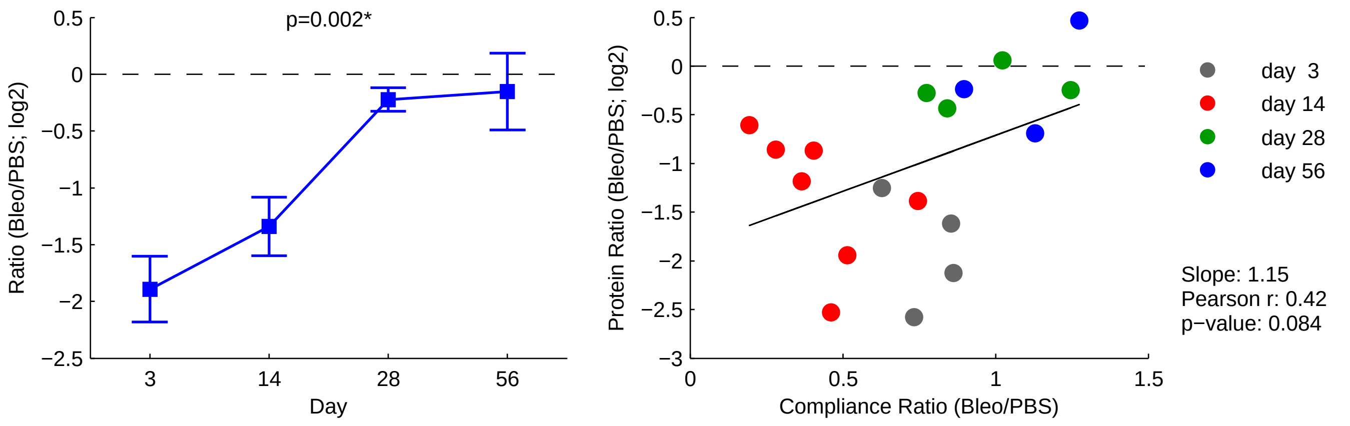

Q9DB15 − Mrpl12 (id: 7259)

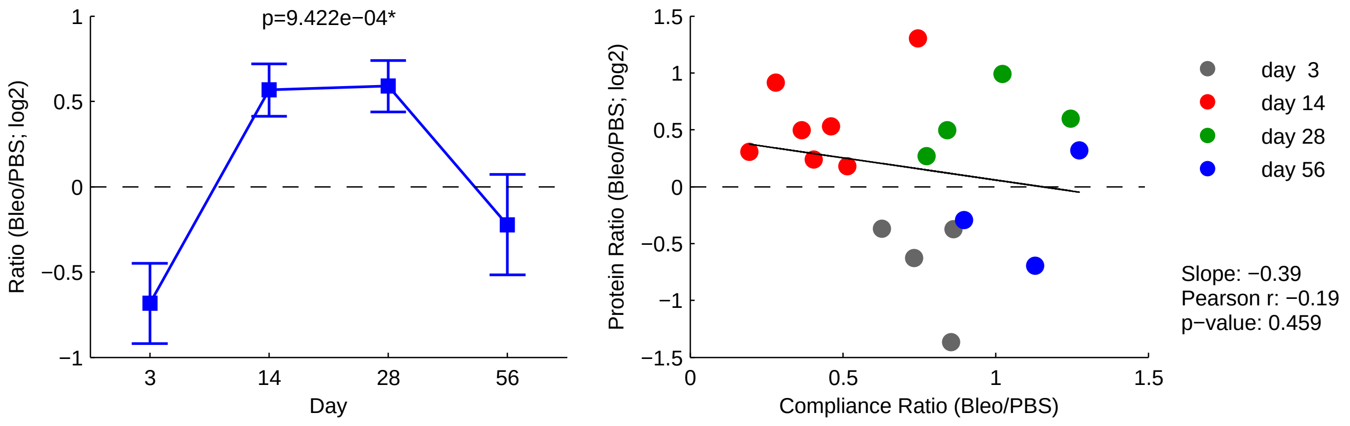

Q9DB16 − Cab39l (id: 7260)

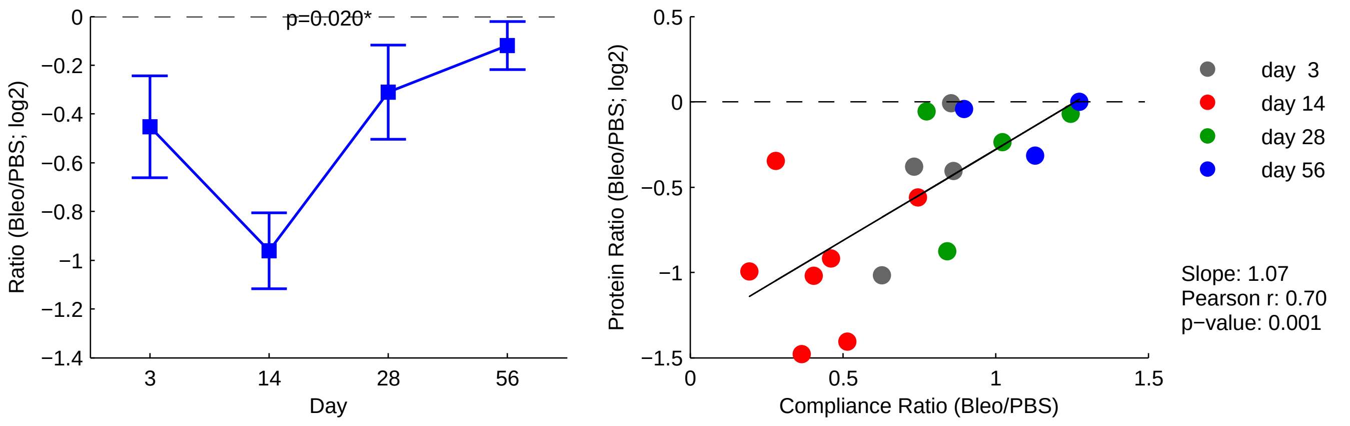

Q9DB20 − Atp5o (id: 7261)

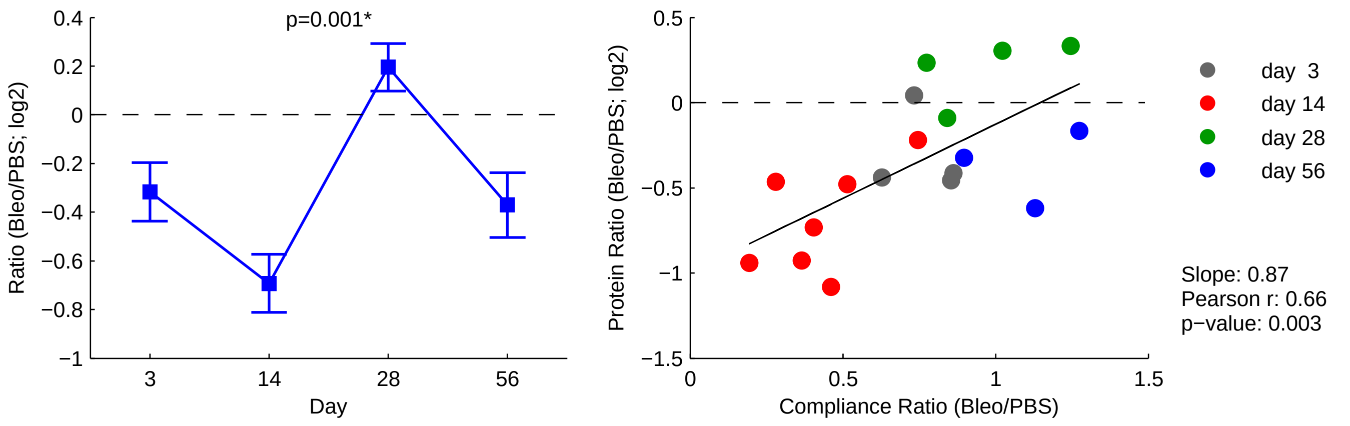

Q9DB25 − Alg5 (id: 7262)

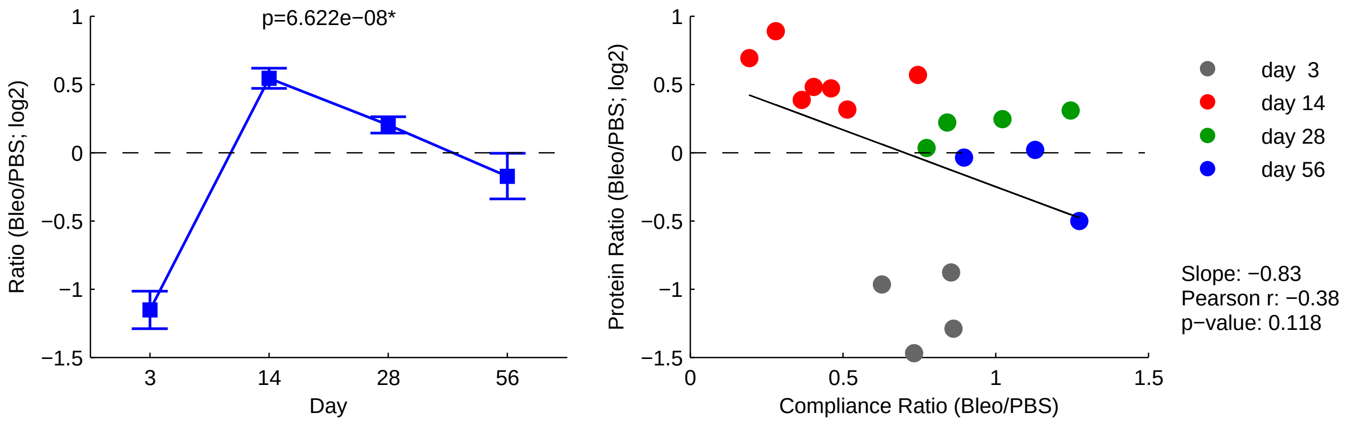

Q9DB34 − Chmp2a (id: 7265)

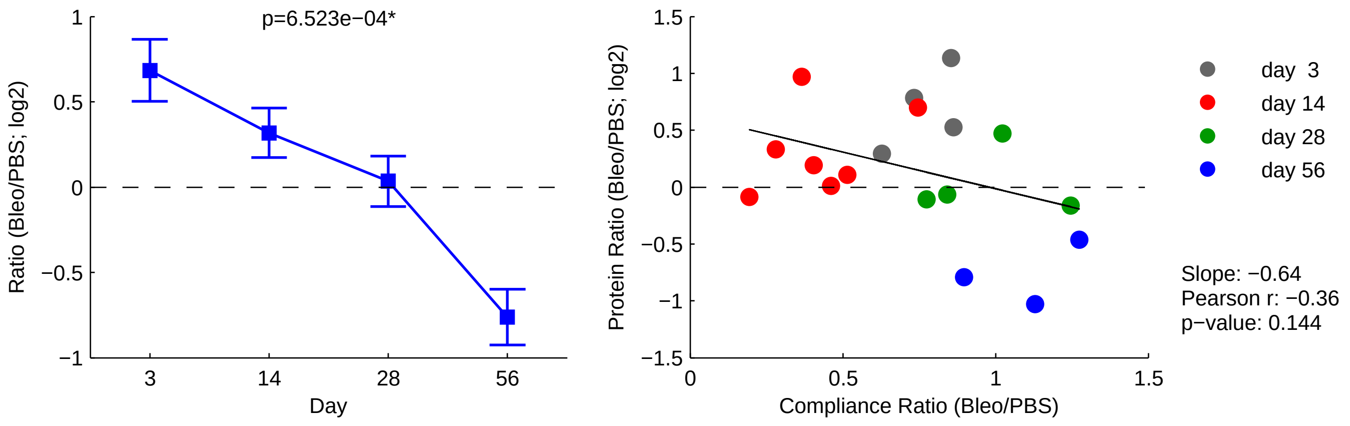

Q9DB60 − Fam213b (id: 7267)

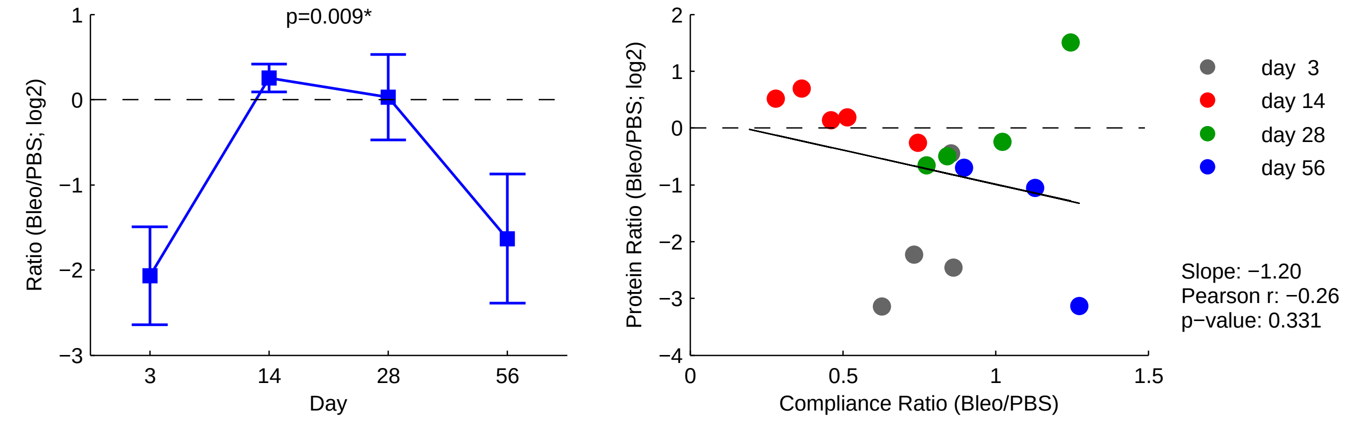

Q9DB70 − Fundc1 (id: 7268)

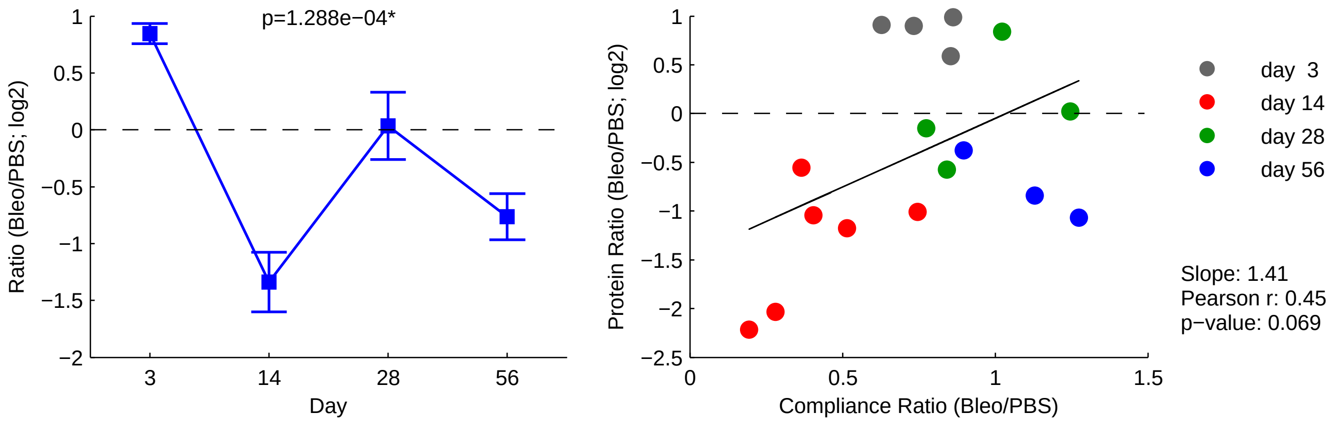

Q9DB73 − Cyb5r1 (id: 7269)

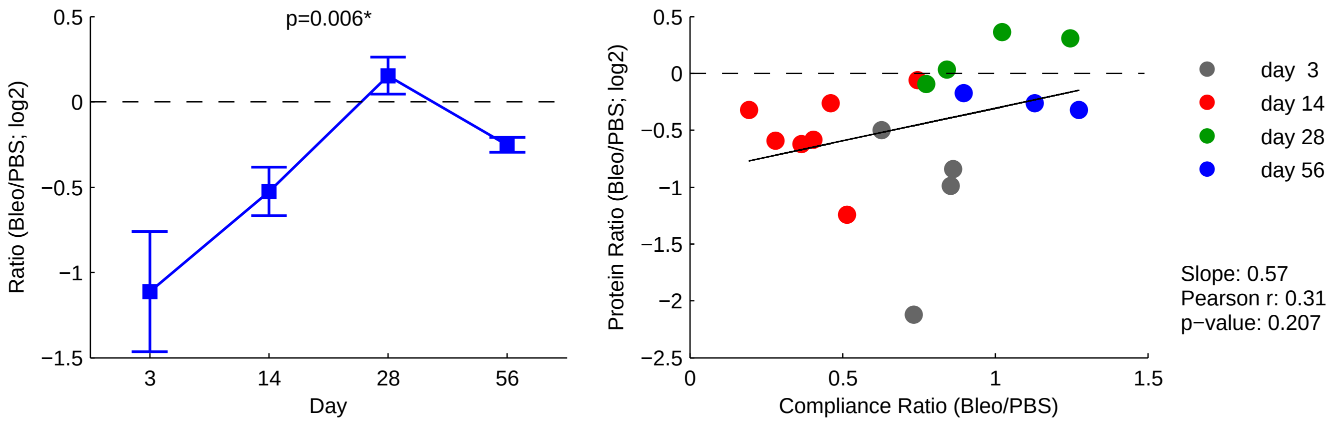

Q9DB77 − Uqcrc2 (id: 7270)

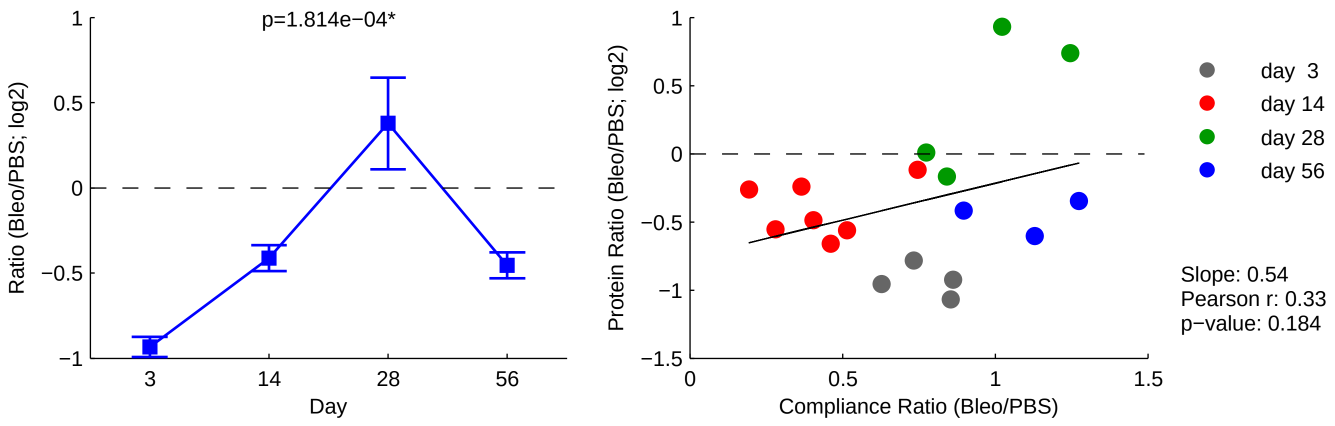

Q9DBB8 − Dhdh (id: 7276)

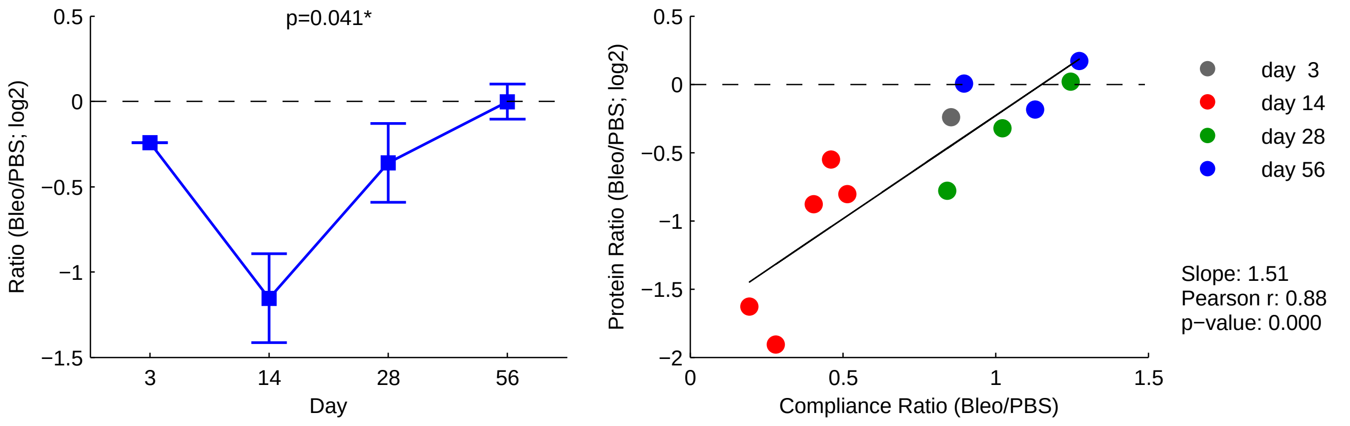

Q9DBC3 − Ftsjd2 (id: 7279)

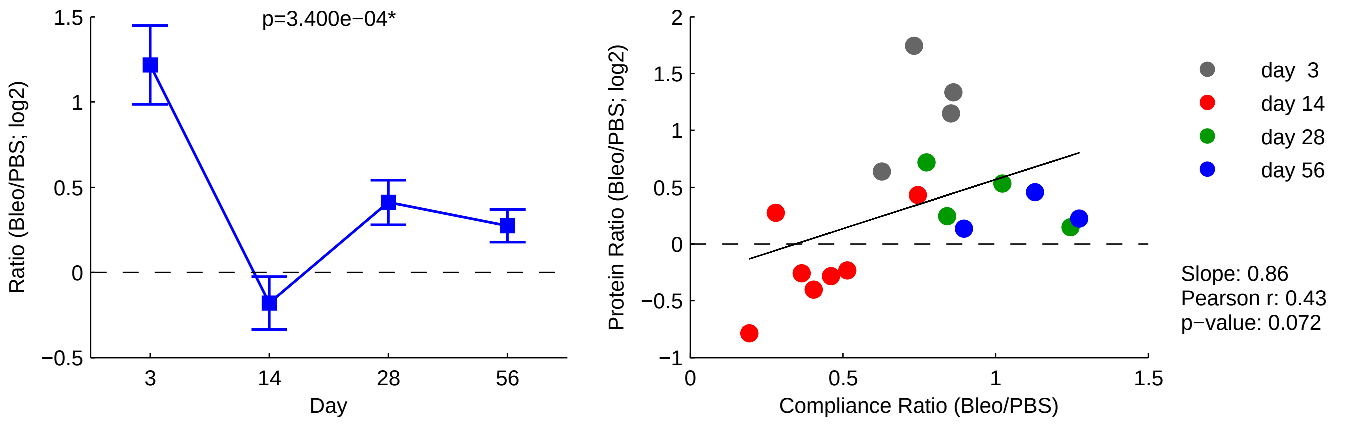

Q9DBC7 − Prkar1a (id: 7280)

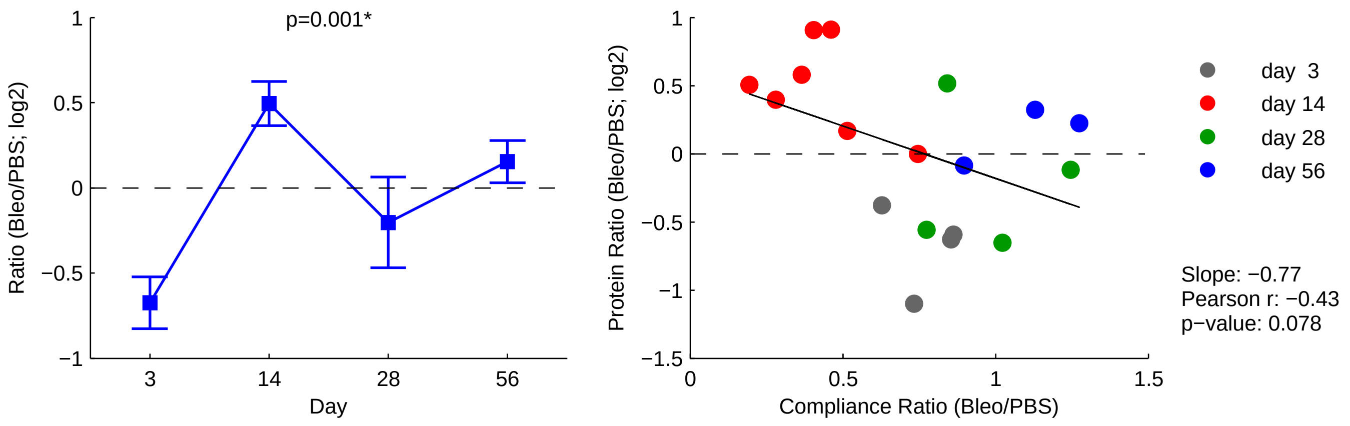

Q9DBD5 − Pelp1 (id: 7281)

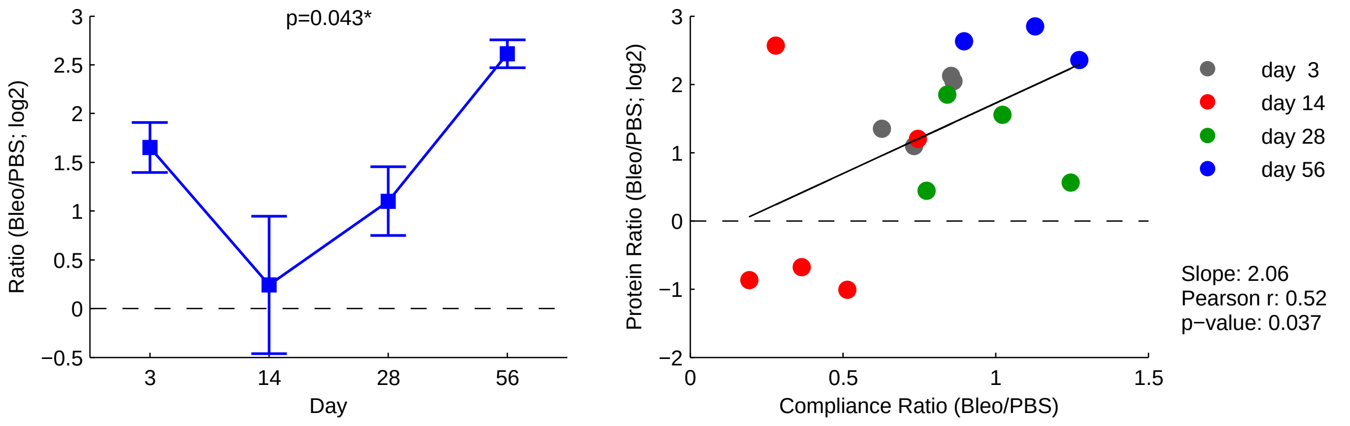

Q9DBE8 − Alg2 (id: 7283)

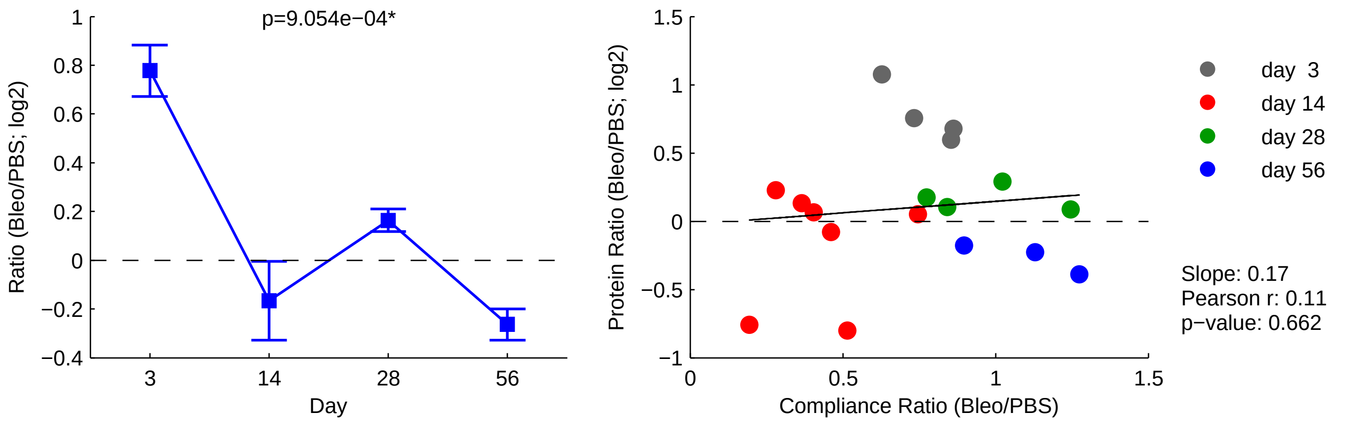

Q9DBF1−2 − Aldh7a1 (id: 7285)

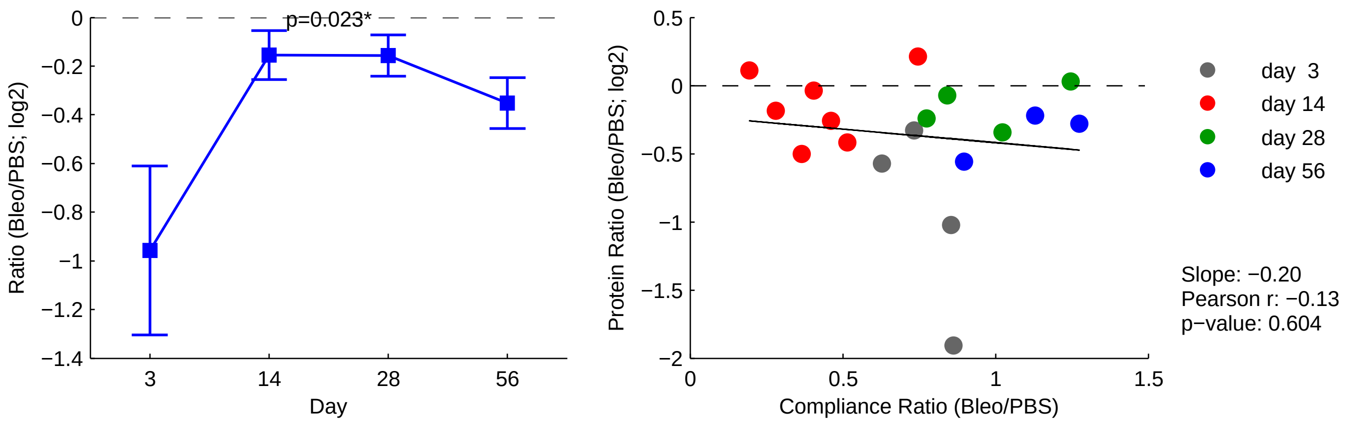

Q9DBG1 − Cyp27a1 (id: 7286)

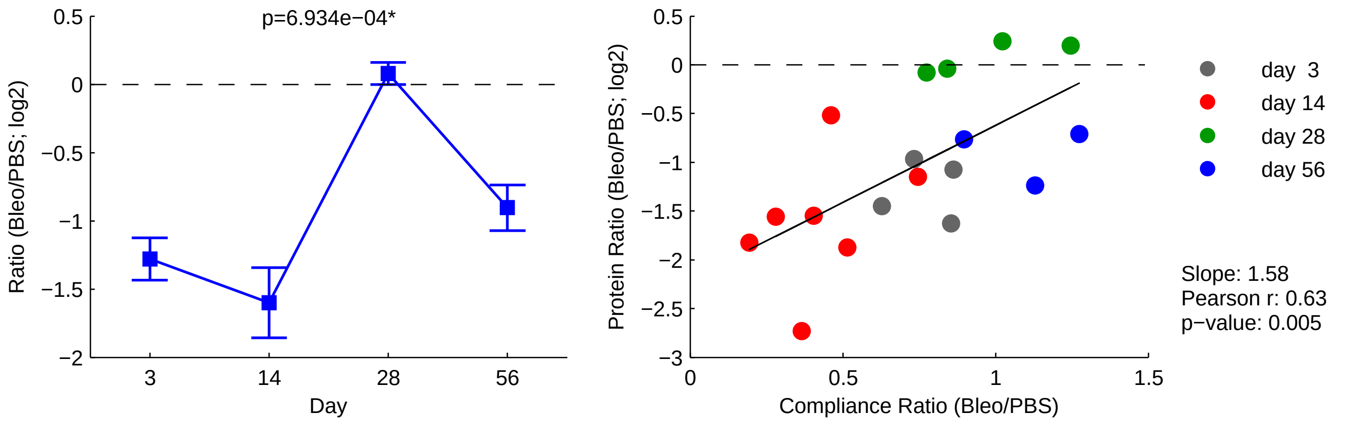

Q9DBG7 − Srpr (id: 7290)

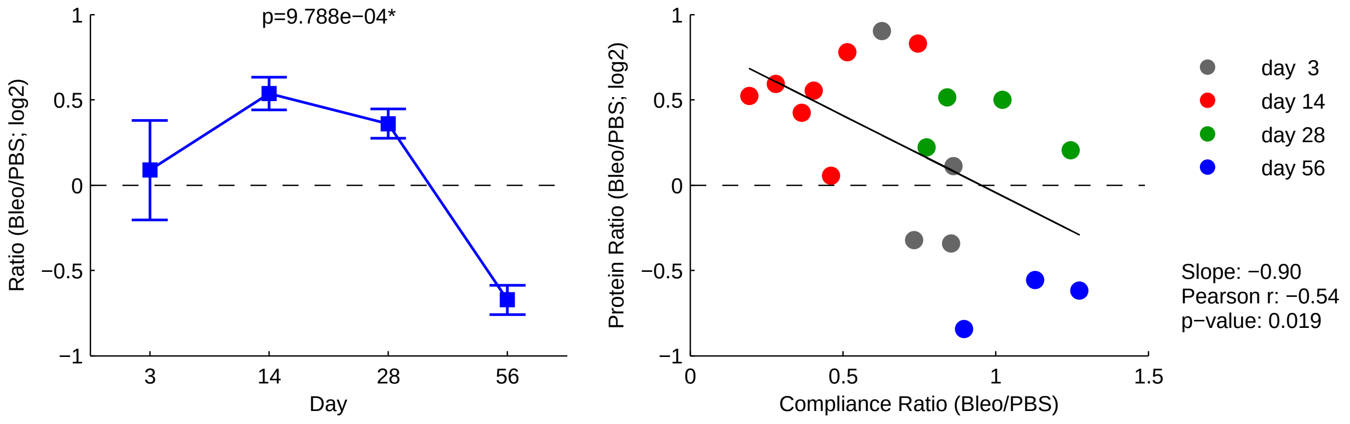

Q9DBG9 − Tax1bp3 (id: 7291)

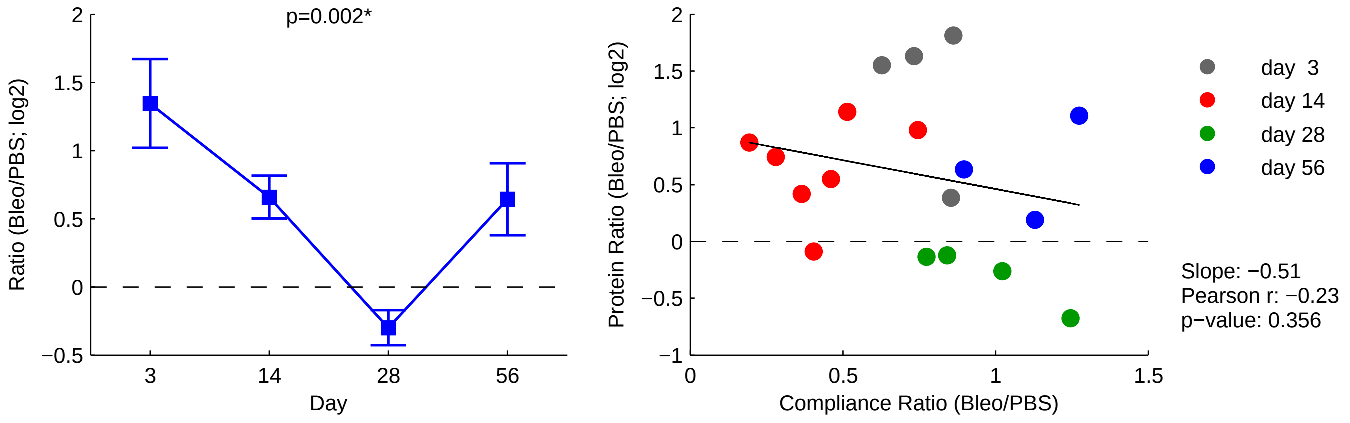

Q9DBH0 − Wwp2 (id: 7292)

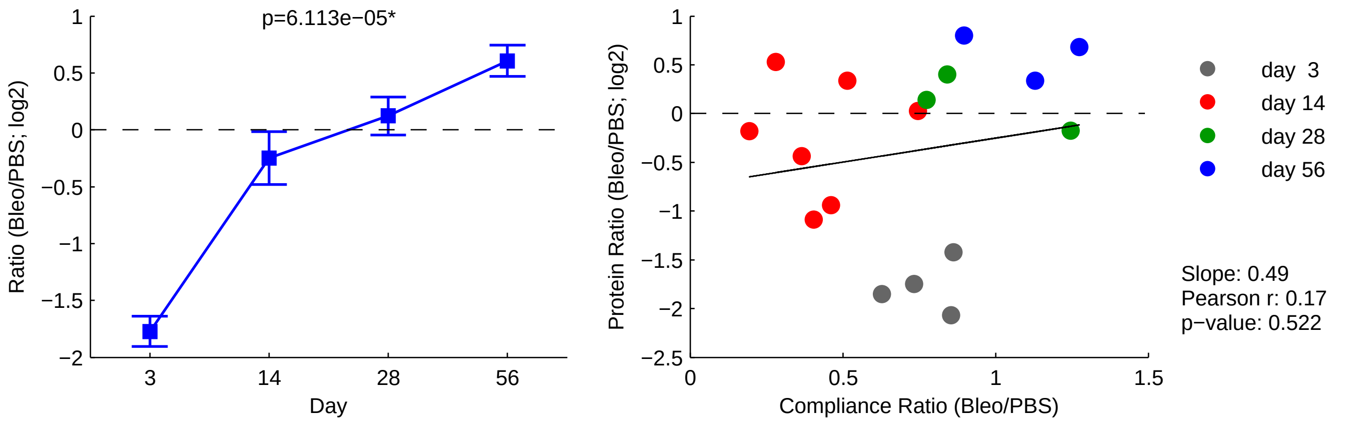

Q9DBH5 − Lman2 (id: 7293)

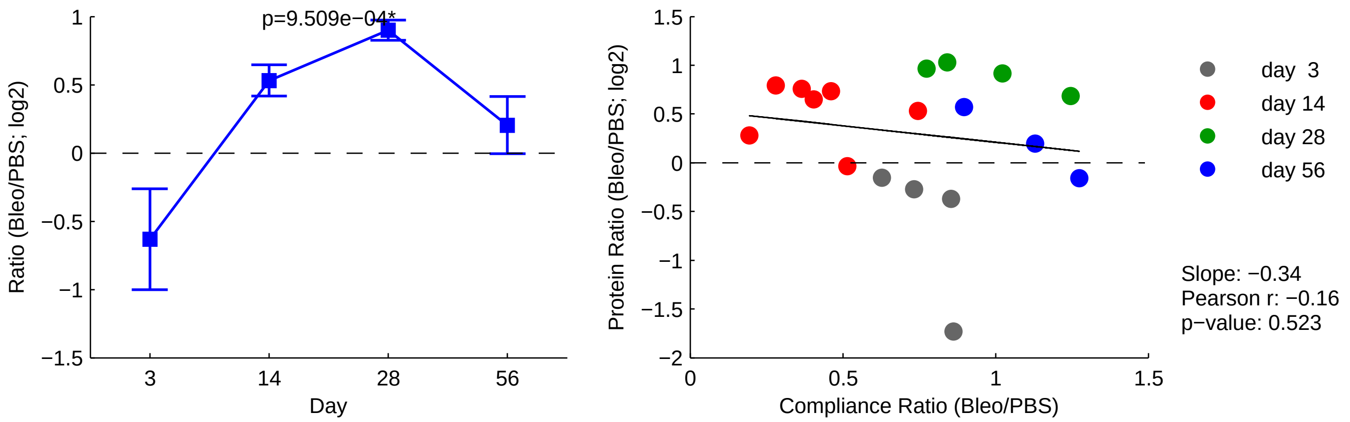

Q9DBJ1 − Pgam1 (id: 7294)

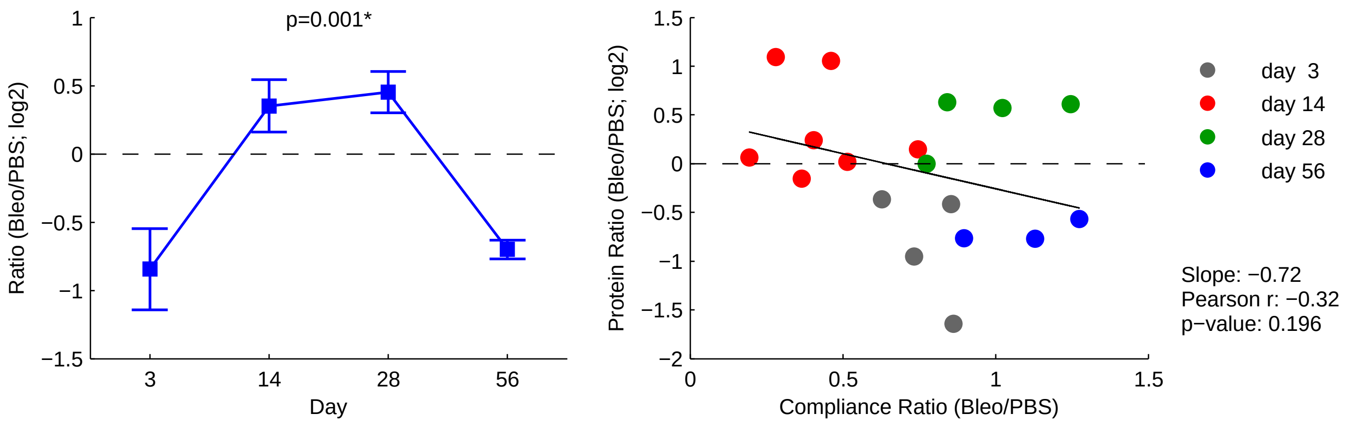

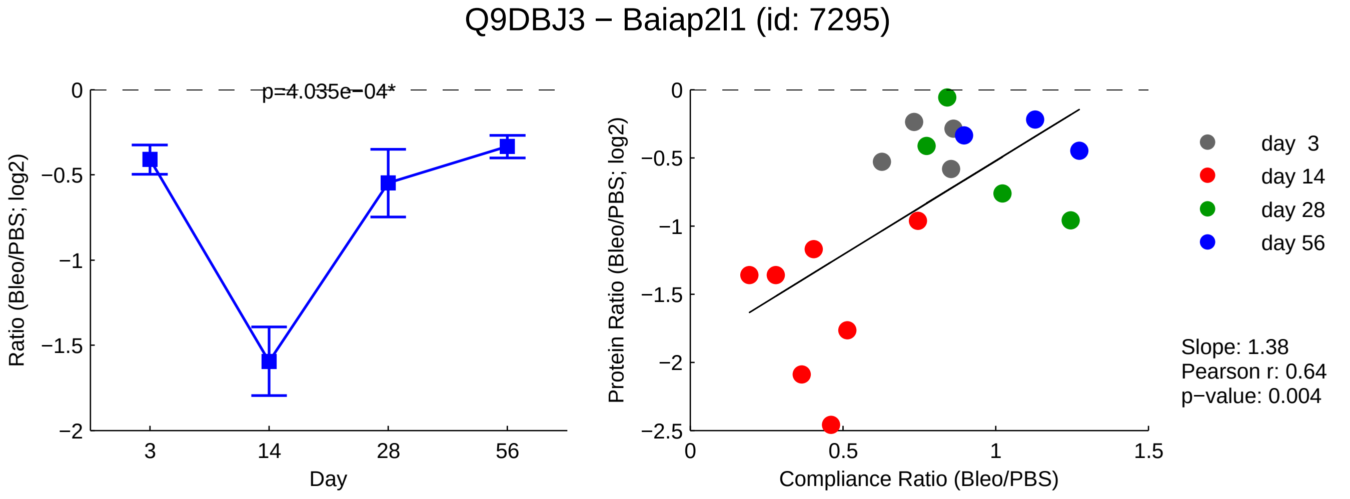
Q9DBJ3 − Baiap2l1 (id: 7295)
p=4.035e−04*
Ratio (Bleo/PBS; log2)
0
−0.5
−1
−1.5
−2
3
14
28
56
Day
Protein Ratio (Bleo/PBS; log2)
0
−0.5
−1
−1.5
−2
−2.5
0
0.5
1
1.5
Compliance Ratio (Bleo/PBS)
day 3
day 14
day 28
day 56
Slope: 1.38
Pearson r: 0.64
p−value: 0.004

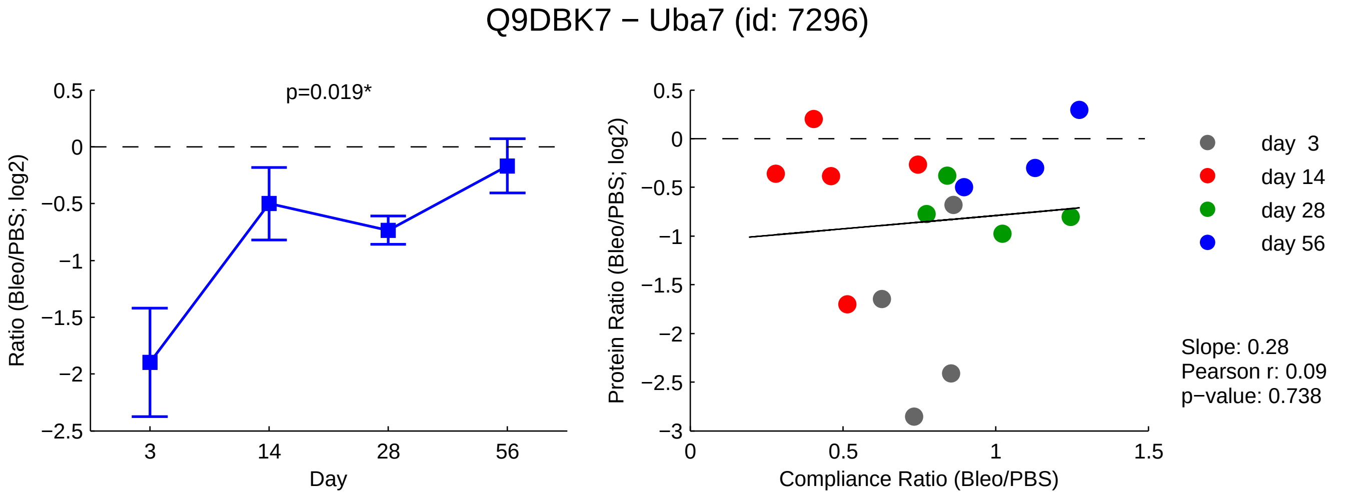
Q9DBK7 − Uba7 (id: 7296)
p=0.019*
Ratio (Bleo/PBS; log2)
0.5
0
−0.5
−1
−1.5
−2
−2.5
3
14
28
56
Day
Protein Ratio (Bleo/PBS; log2)
0.5
0
−0.5
−1
−1.5
−2
−2.5
−3
0
0.5
1
1.5
Compliance Ratio (Bleo/PBS)
day 3
day 14
day 28
day 56
Slope: 0.28
Pearson r: 0.09
p−value: 0.738

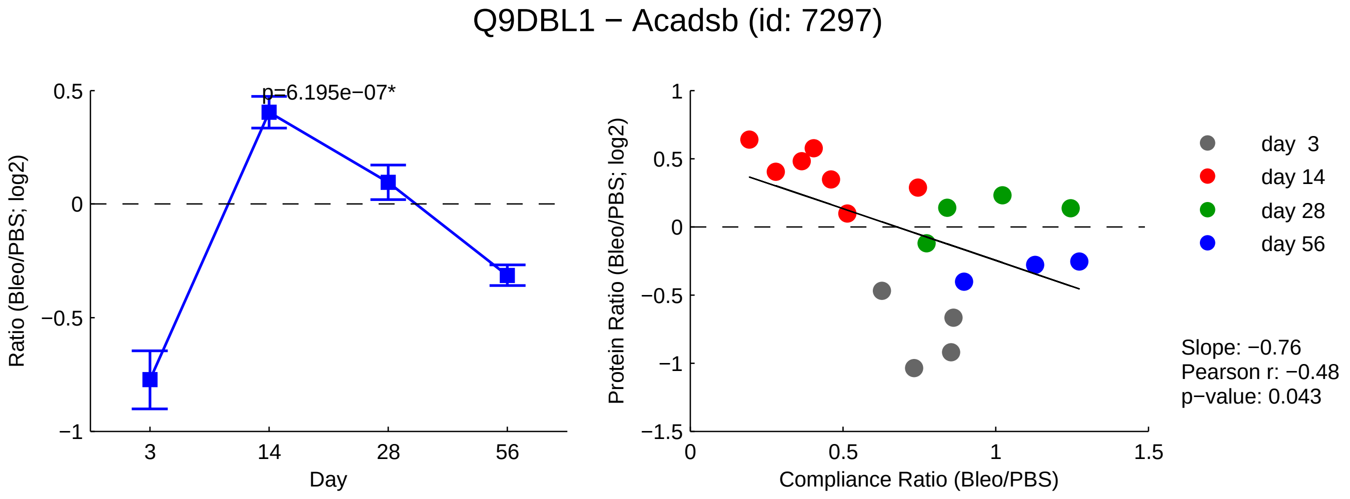
Q9DBL1 − Acadsb (id: 7297)
p=6.195e−07*
Ratio (Bleo/PBS; log2)
0.5
0
−0.5
−1
3
14
28
56
Day
Protein Ratio (Bleo/PBS; log2)
1
0.5
0
−0.5
−1
−1.5
0
0.5
1
1.5
Compliance Ratio (Bleo/PBS)
day 3
day 14
day 28
day 56
Slope: −0.76
Pearson r: −0.48
p−value: 0.043

Q9DBL7 − Coasy (id: 7299)

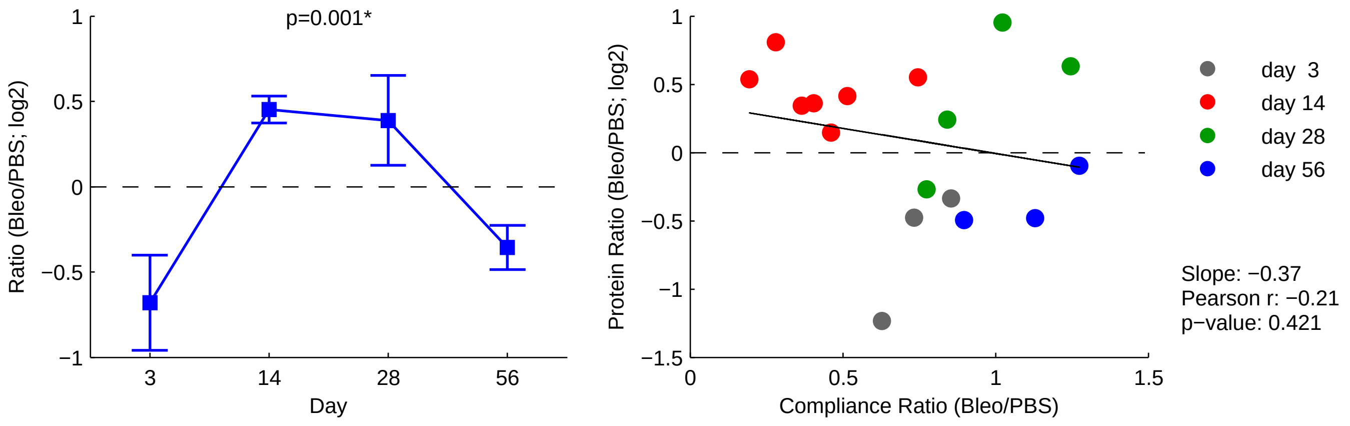

Q9DBL9−2 − Abhd5 (id: 7300)

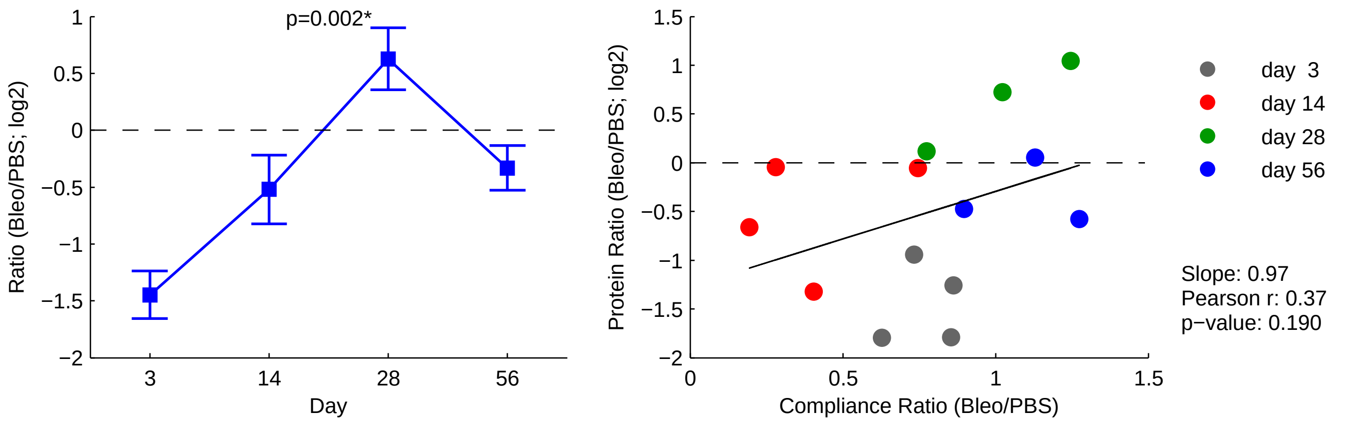

Q9DBM2 − Ehhadh (id: 7301)

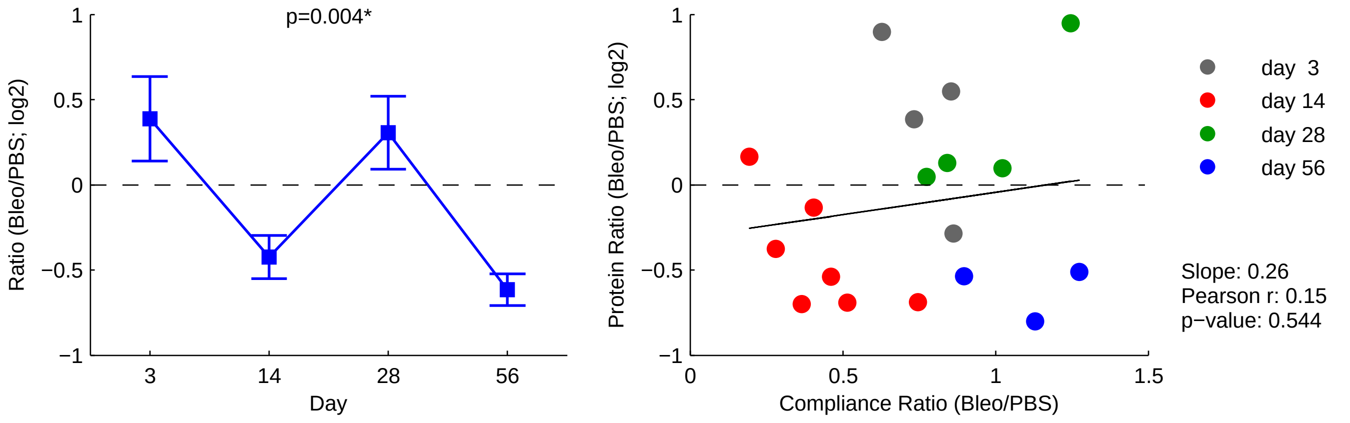

Q9DBN5 − Lonp2 (id: 7302)

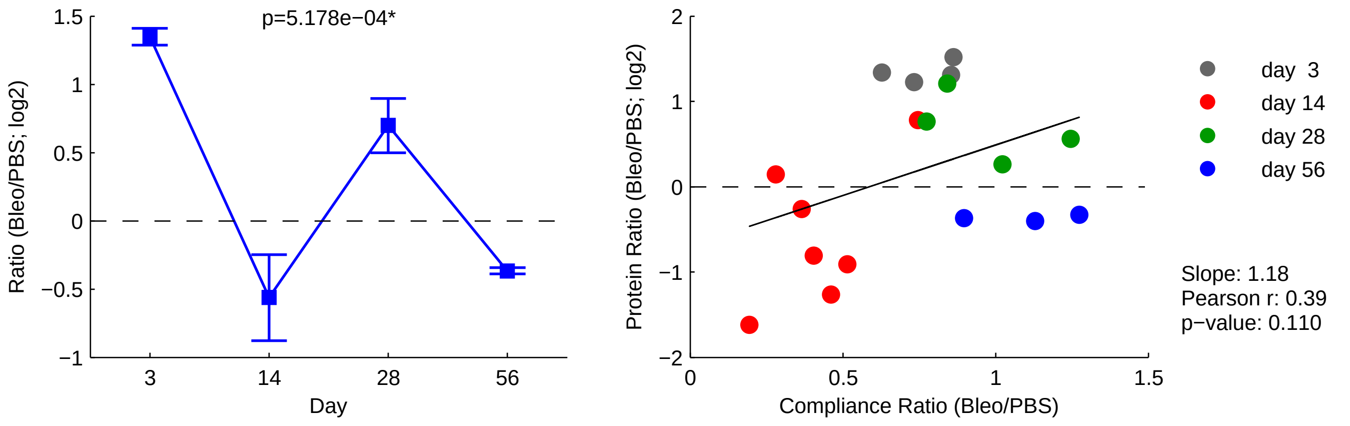

Q9DBP5 − Cmpk1 (id: 7304)

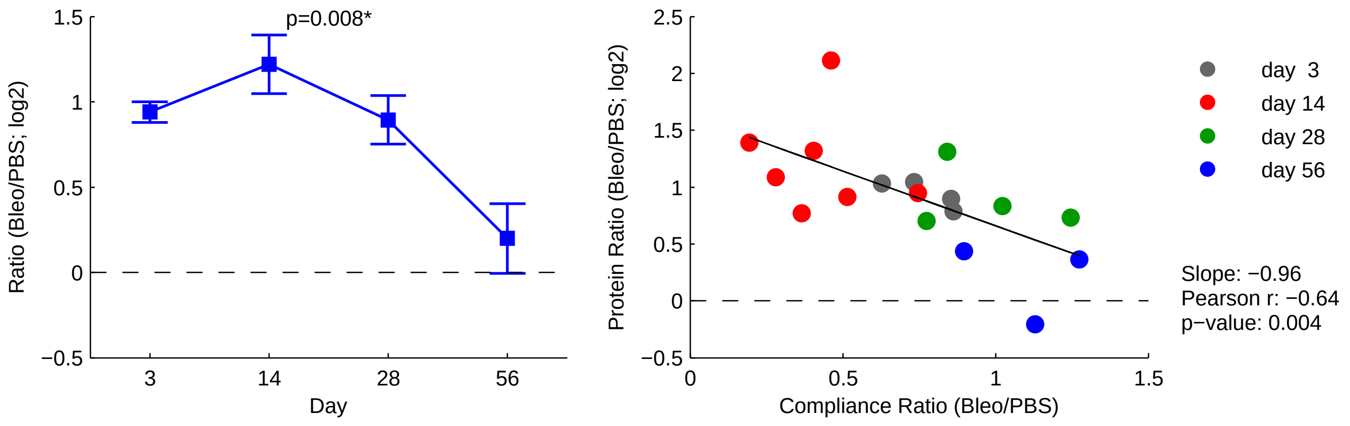

Q9DBR1−2 − Xrn2 (id: 7306)

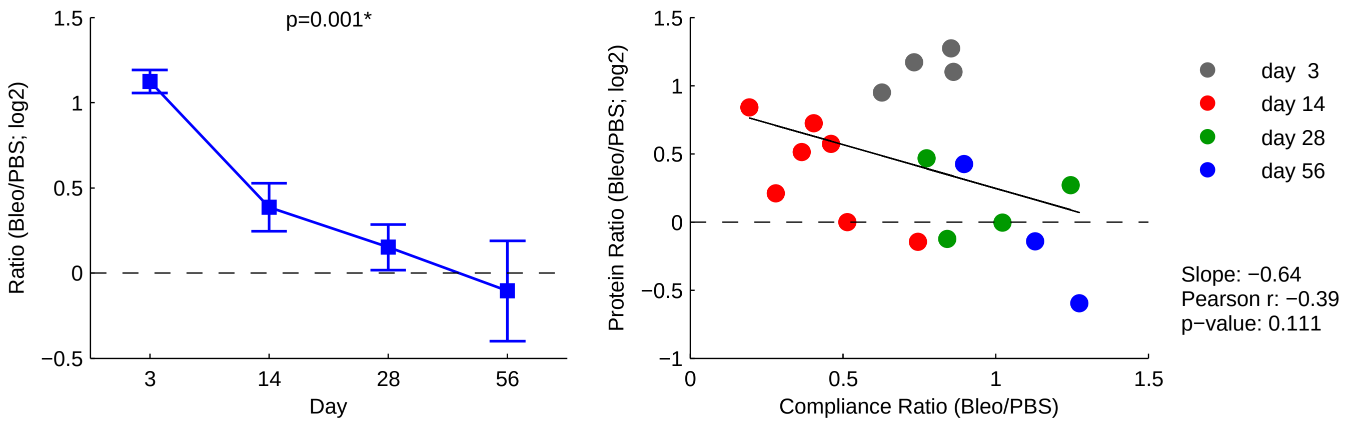

Q9DBR7 − Ppp1r12a (id: 7307)

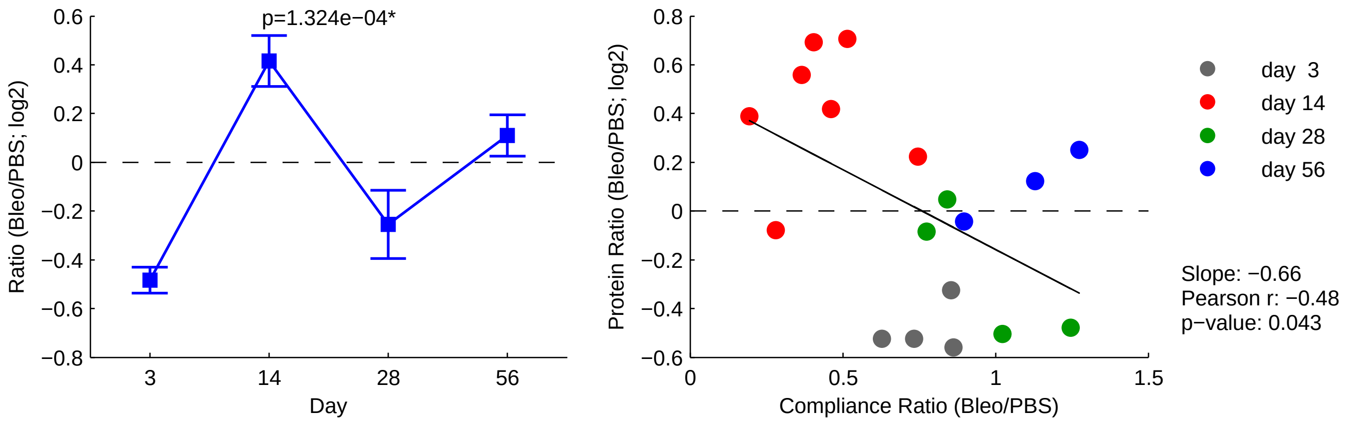

Q9DBS1 − Tmem43 (id: 7308)

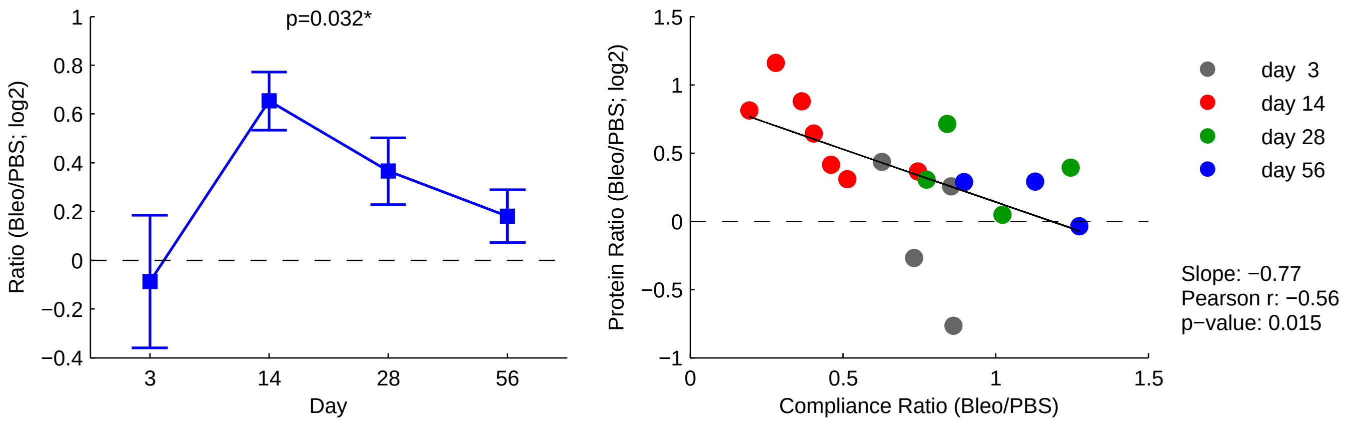

Q9DBS8 − Poc5 (id: 7312)

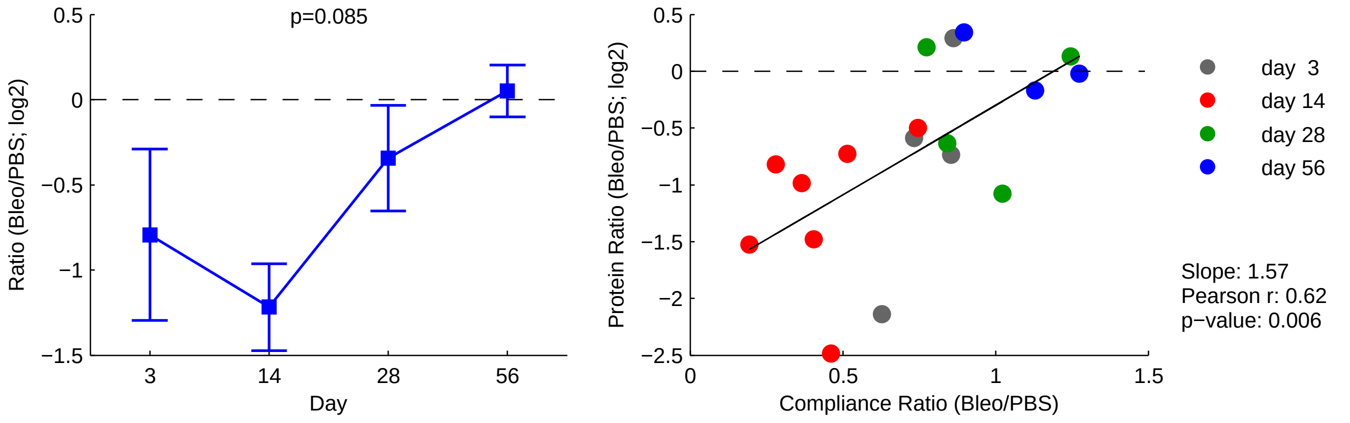

Q9DBV4 − Mxra8 (id: 7313)

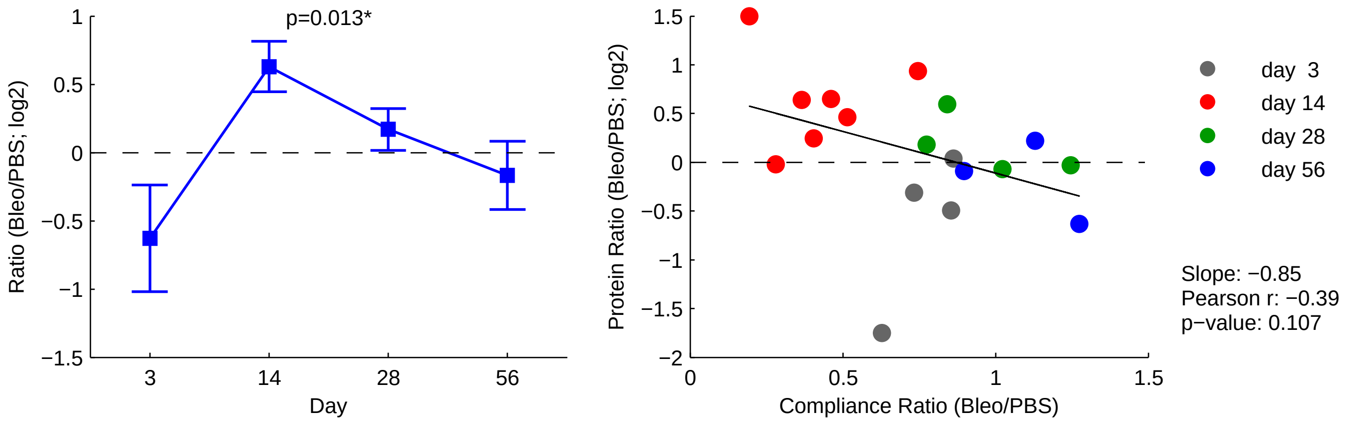

Q9DBW0 − Cyp4v2 (id: 7314)

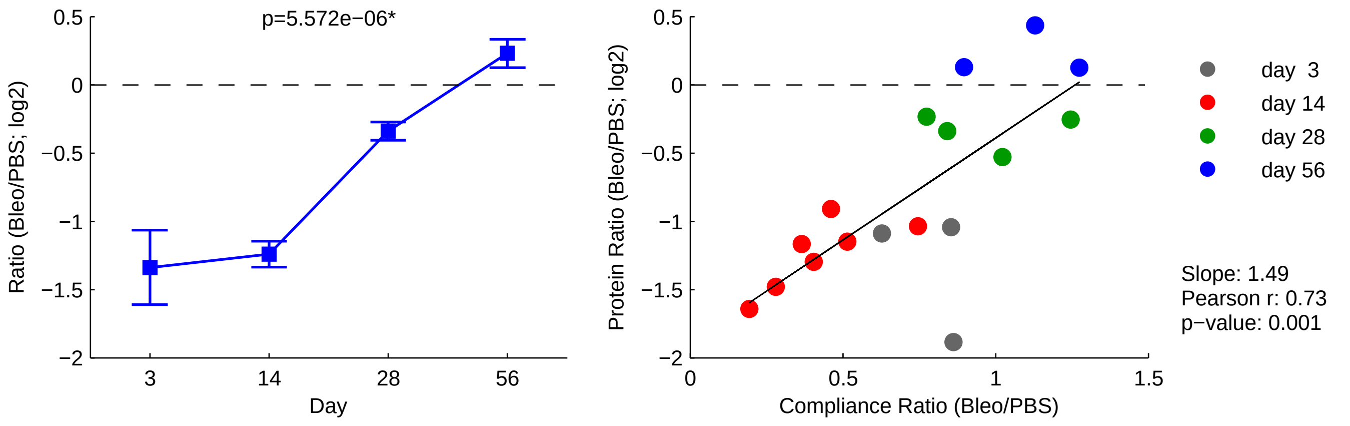

Q9DBX2 − Pdcl (id: 7316)

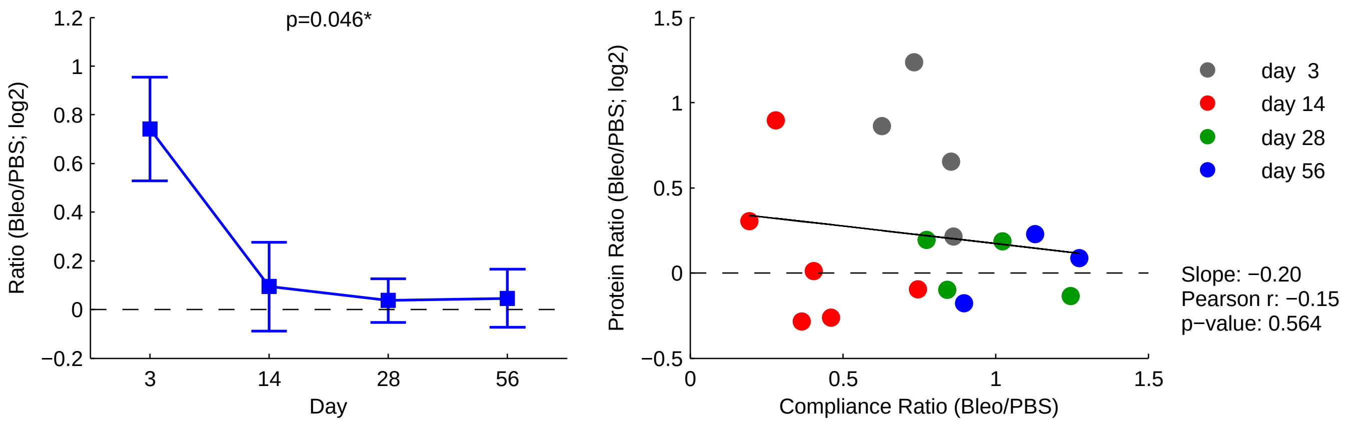

Q9DBX3 − Susd2 (id: 7317)

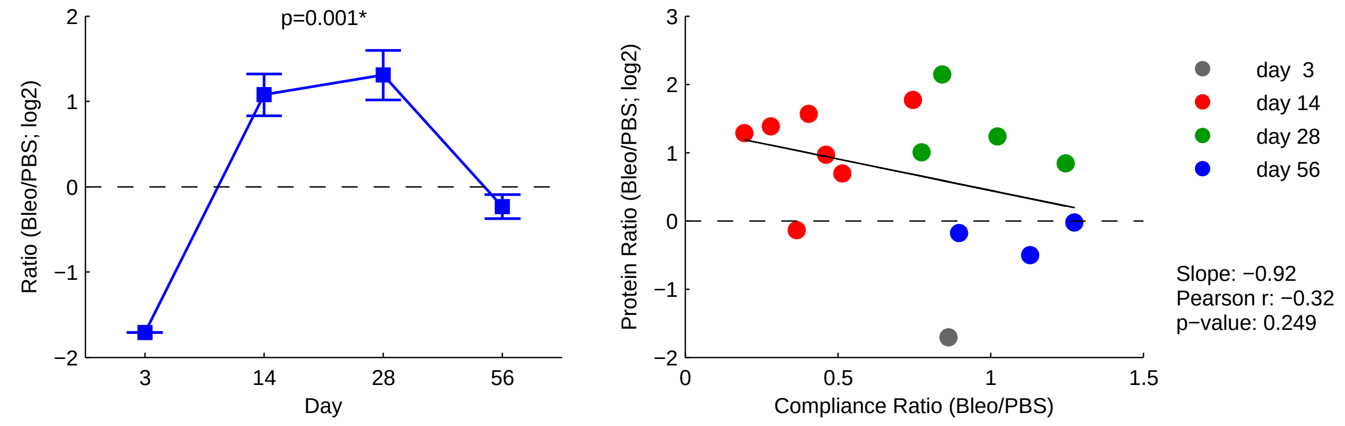

Q9DBX6 − Cyp2s1 (id: 7318)

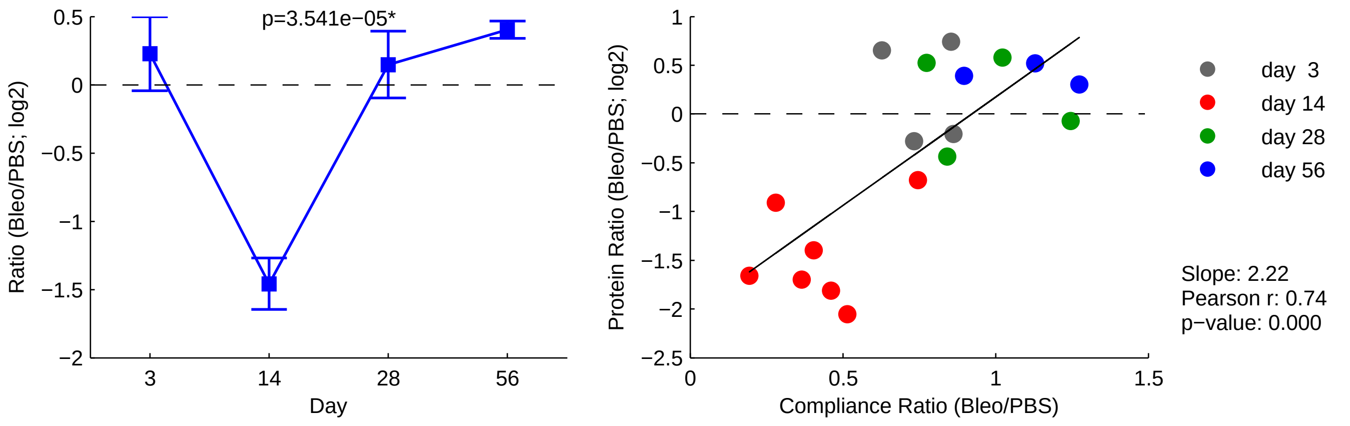

Q9DBZ1−2 − (id: 7321)

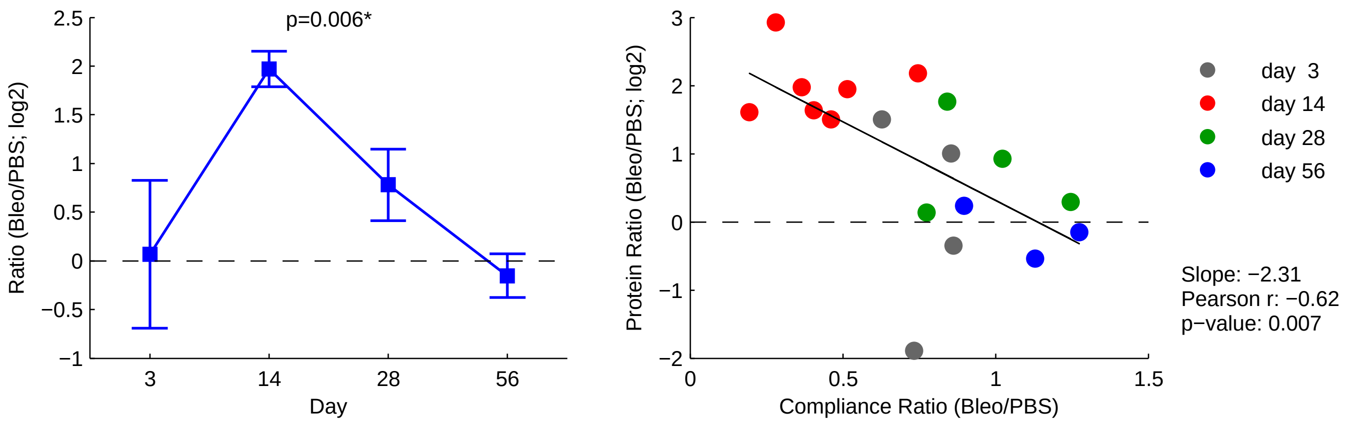

Q9DBZ5 − Eif3k (id: 7322)

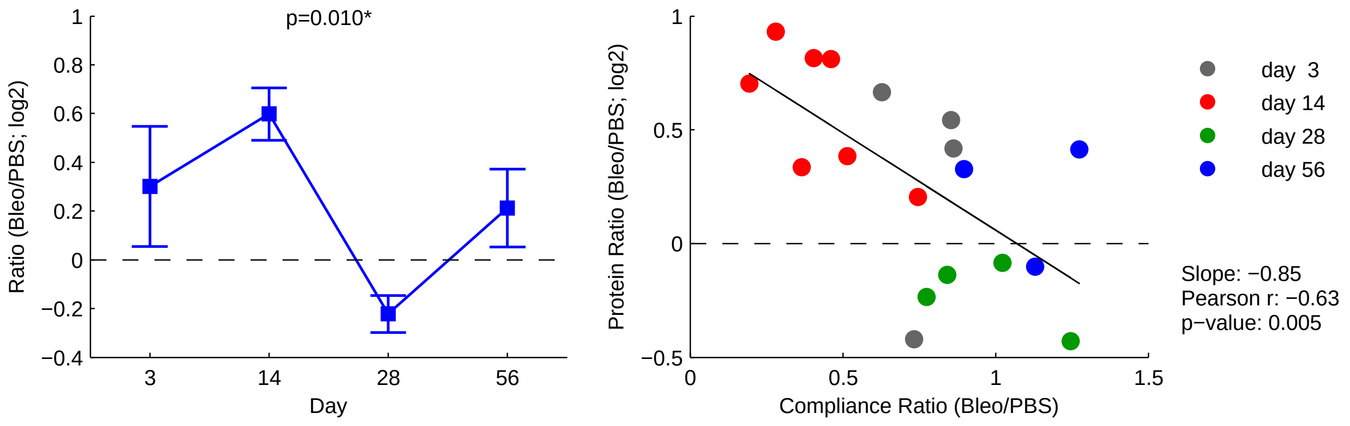

Q9DC07 − Nebl (id: 7324)

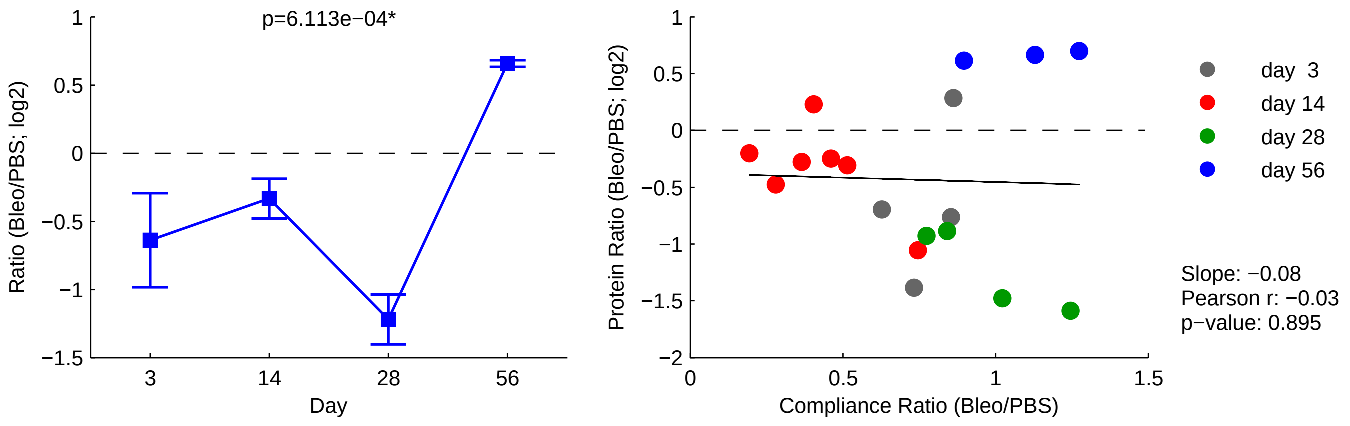

Q9DC23 − Dnajc10 (id: 7326)

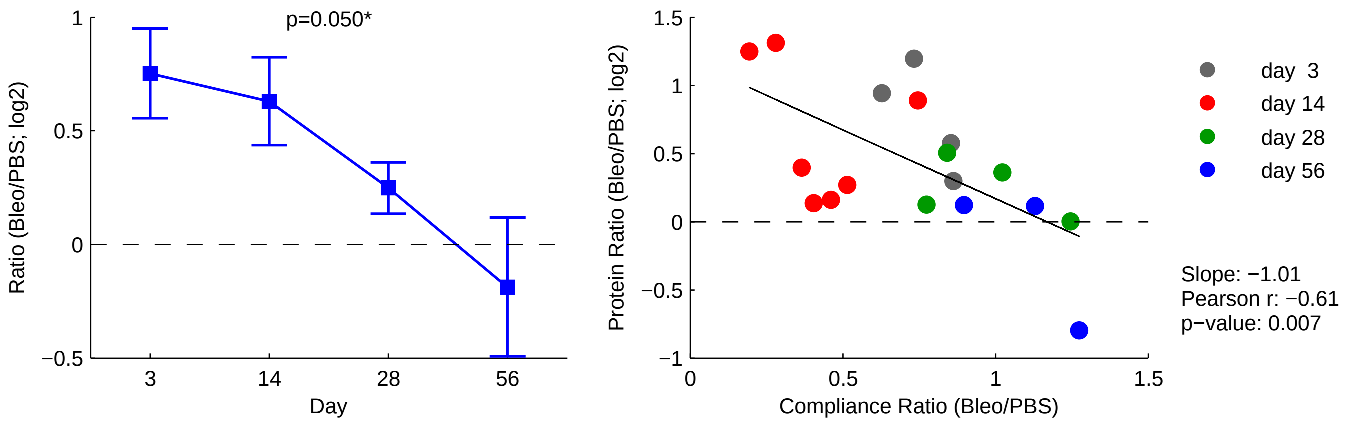

Q9DC50 − Crot (id: 7332)

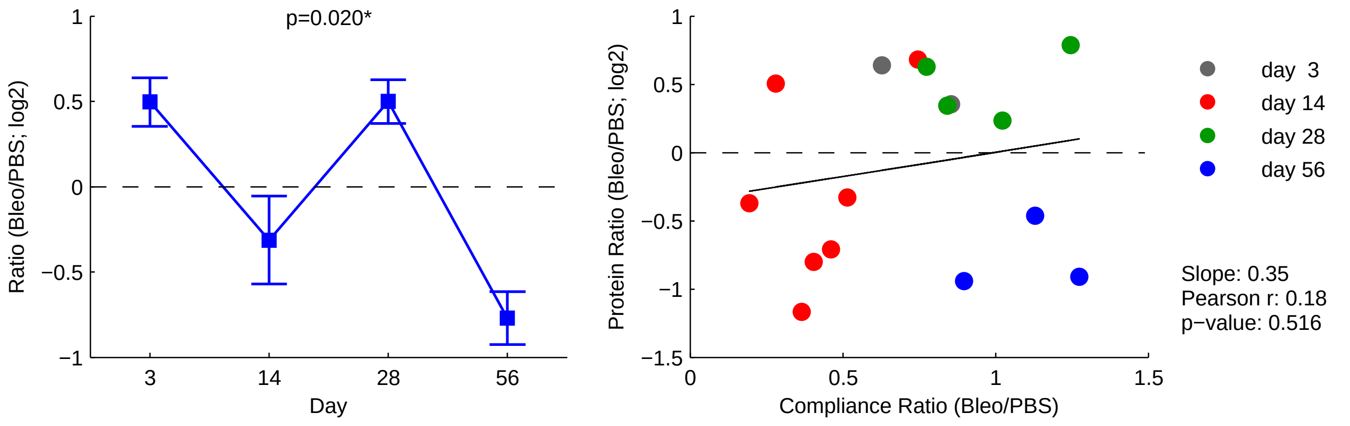

Q9DC61 − Pmpca (id: 7334)

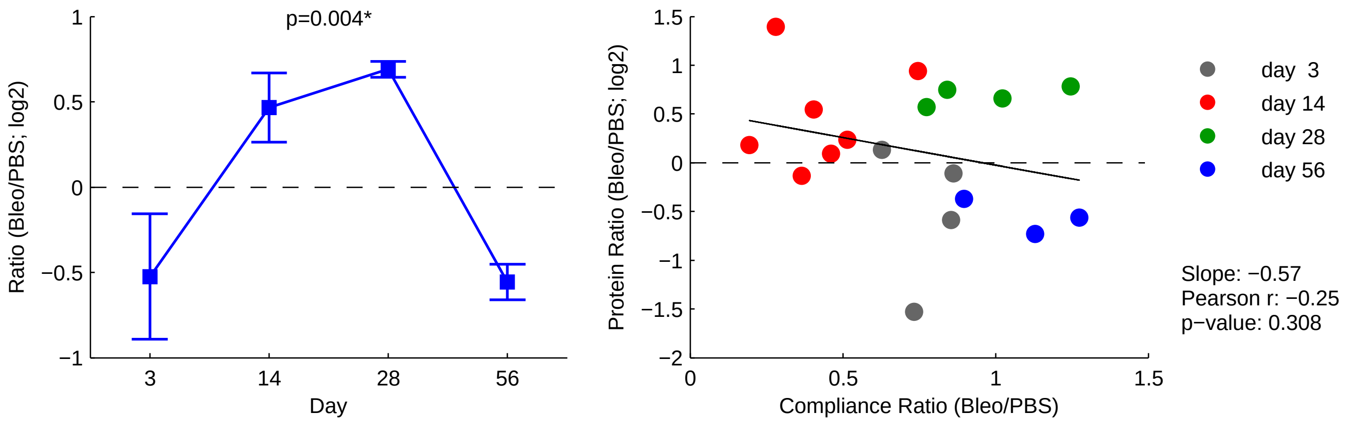

Q9DC69 − Ndufa9 (id: 7335)

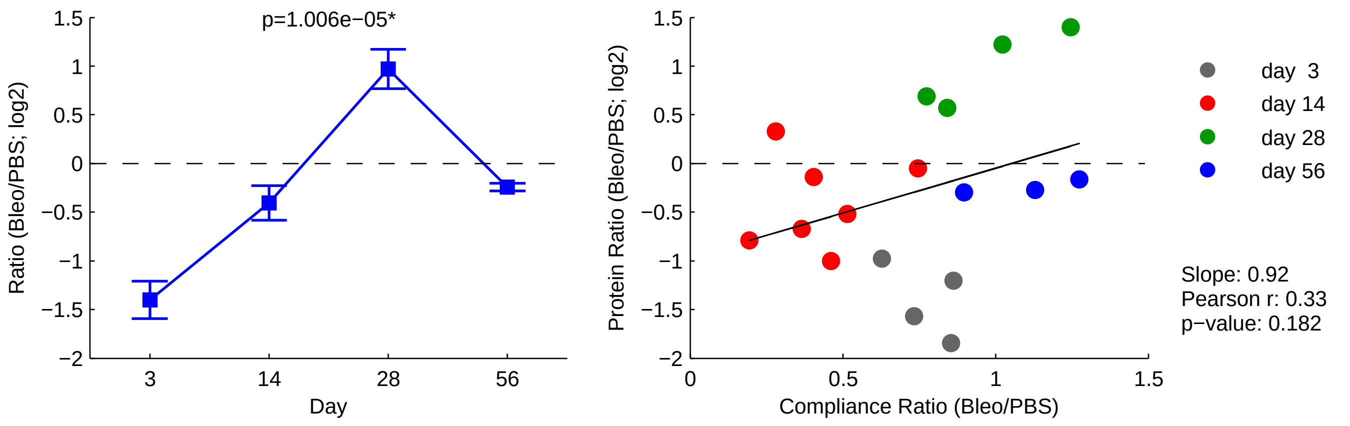

Q9DC70 − Ndufs7 (id: 7336)

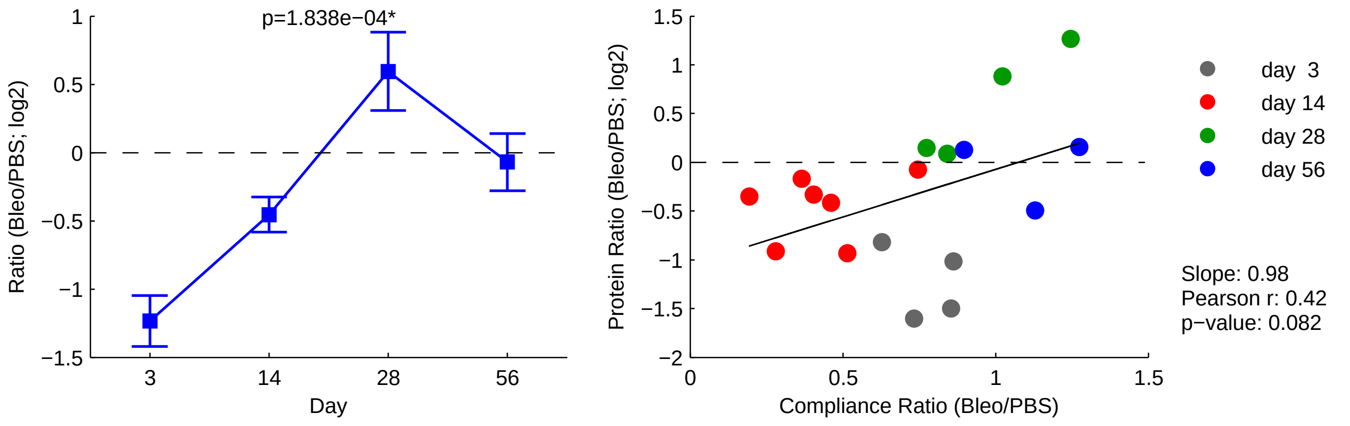

Q9DCB8 − Isca2 (id: 7341)

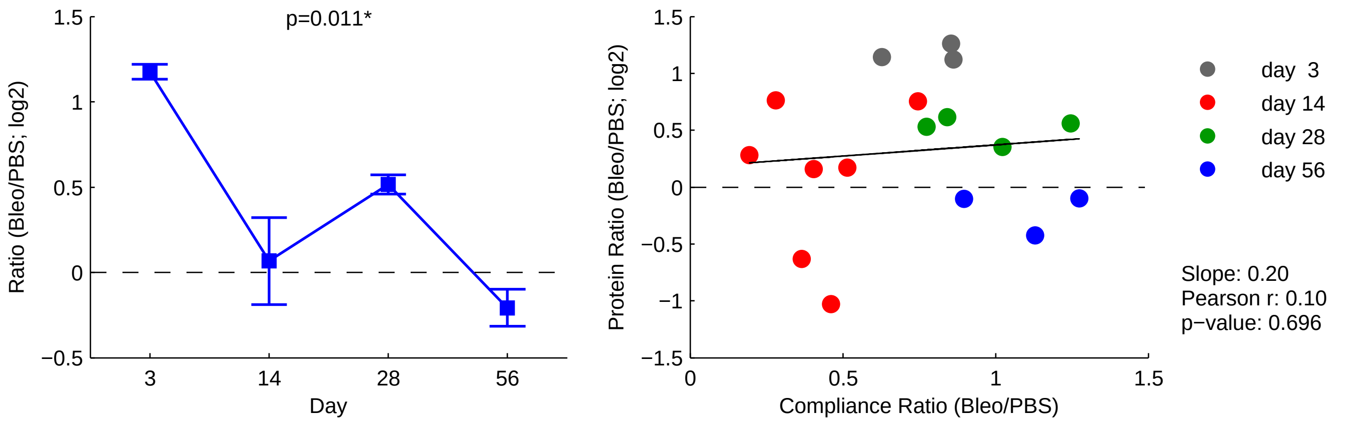

Q9DCC4 − Pycrl (id: 7342)

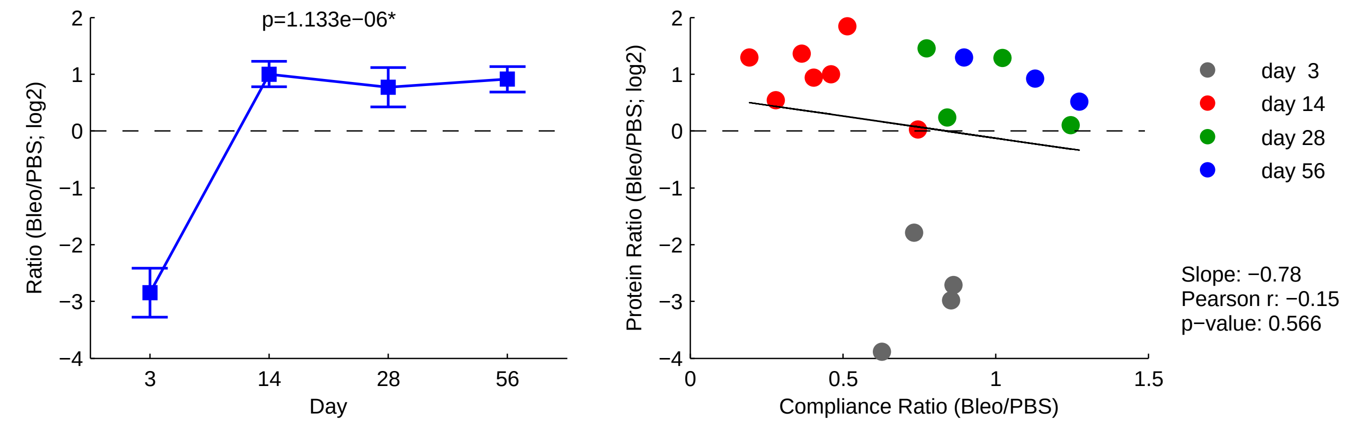

Q9DCC7 − Isoc2b (id: 7344)

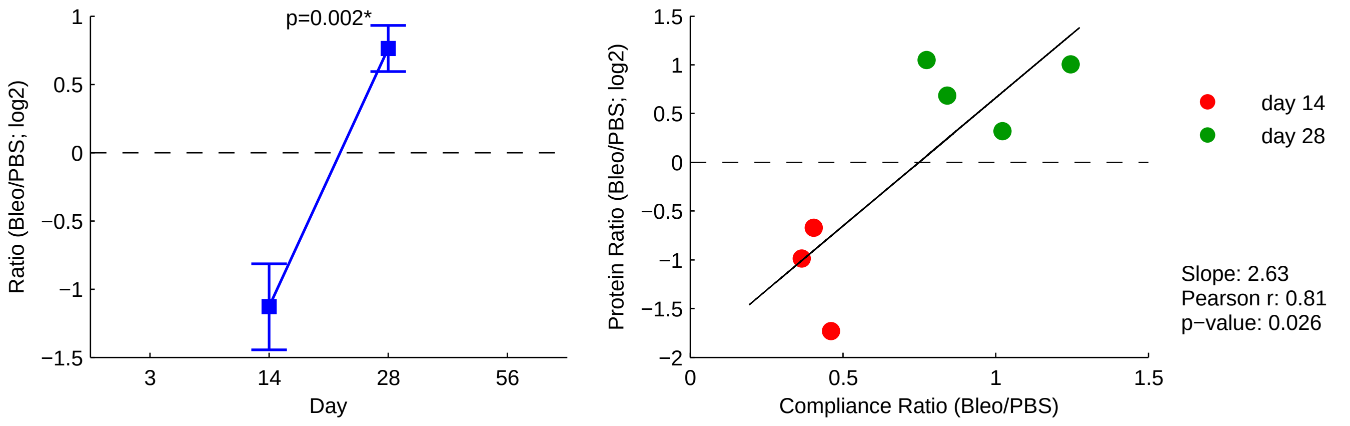

Q9DCC8 − Tomm20 (id: 7345)

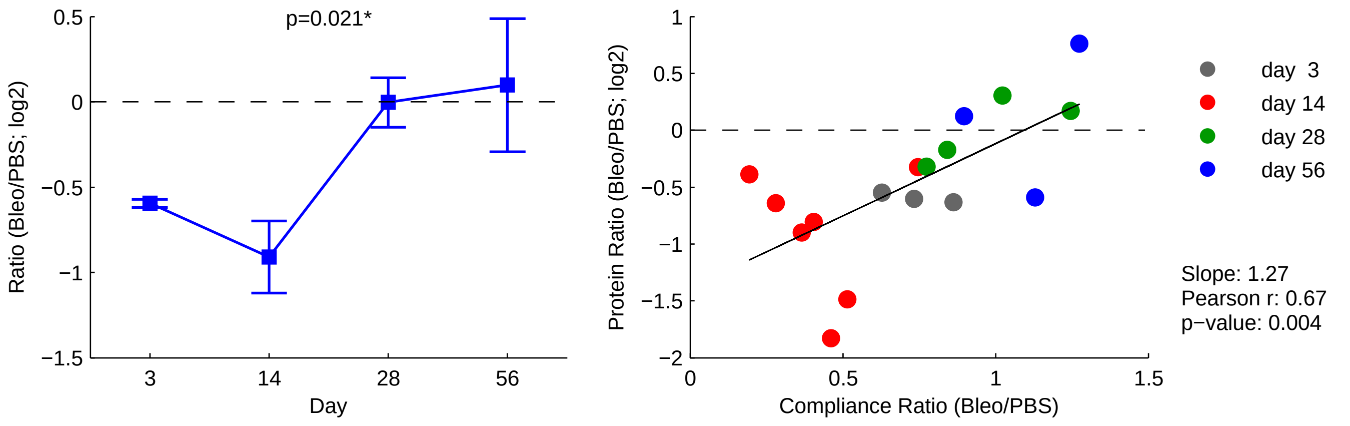

Q9DCD0 − Pgd (id: 7346)

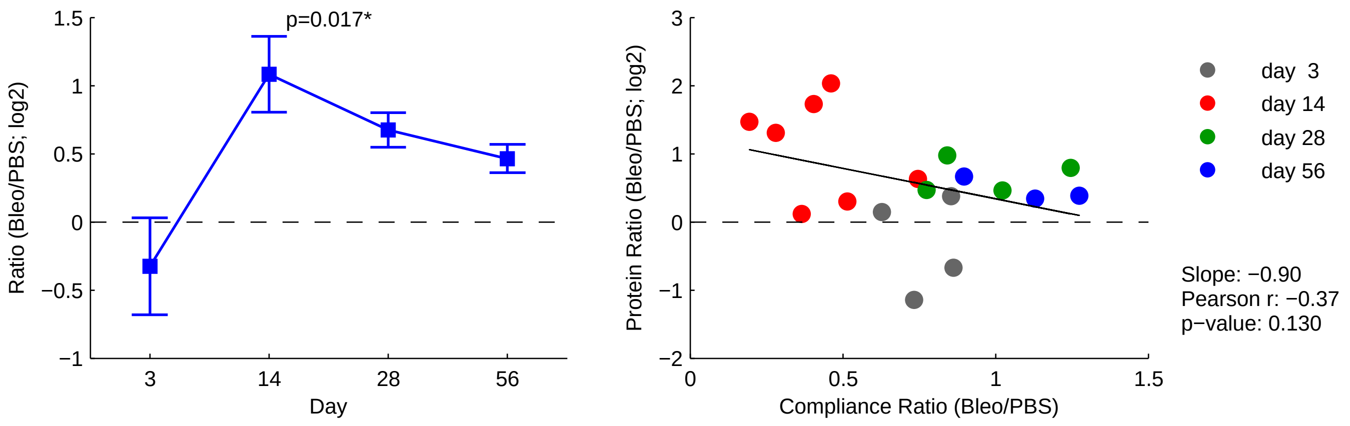

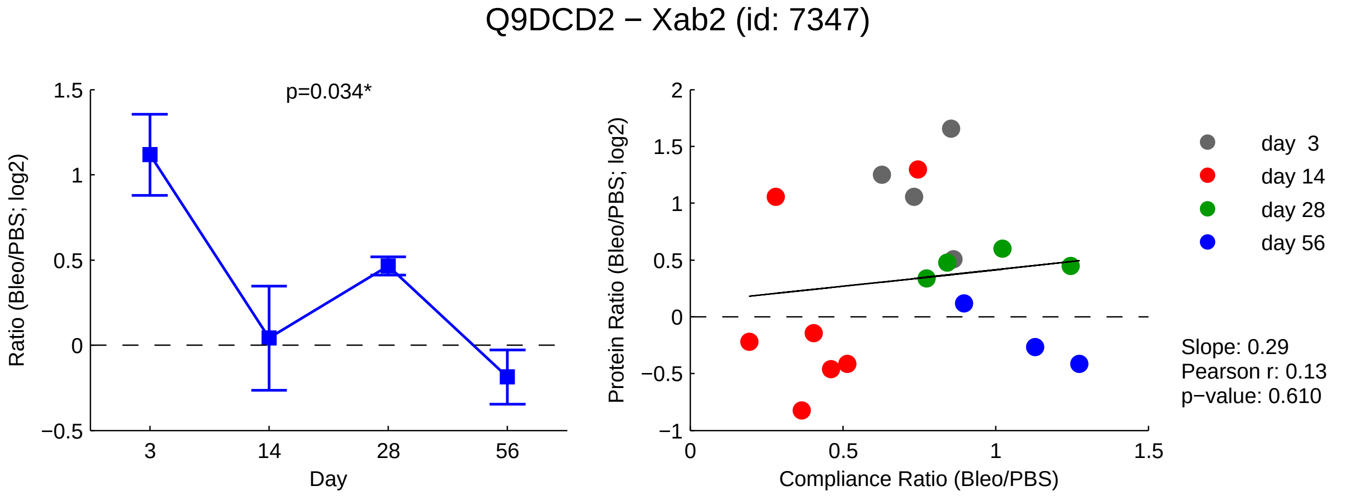

Q9DCD2 − Xab2 (id: 7347)
p=0.034*
Ratio (Bleo/PBS; log2)
Day
Protein Ratio (Bleo/PBS; log2)
Compliance Ratio (Bleo/PBS)
day 3
day 14
day 28
day 56
Slope: 0.29
Pearson r: 0.13
p−value: 0.610

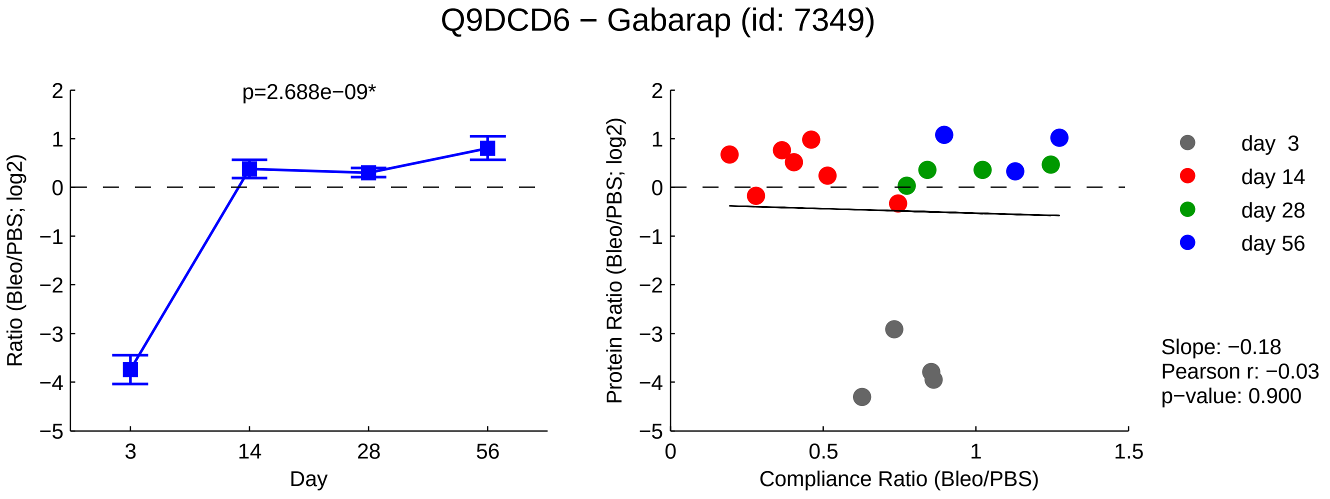

Q9DCD6 − Gabarap (id: 7349)
p=2.688e−09*
Ratio (Bleo/PBS; log2)
Day
Protein Ratio (Bleo/PBS; log2)
Compliance Ratio (Bleo/PBS)
day 3
day 14
day 28
day 56
Slope: −0.18
Pearson r: −0.03
p−value: 0.900

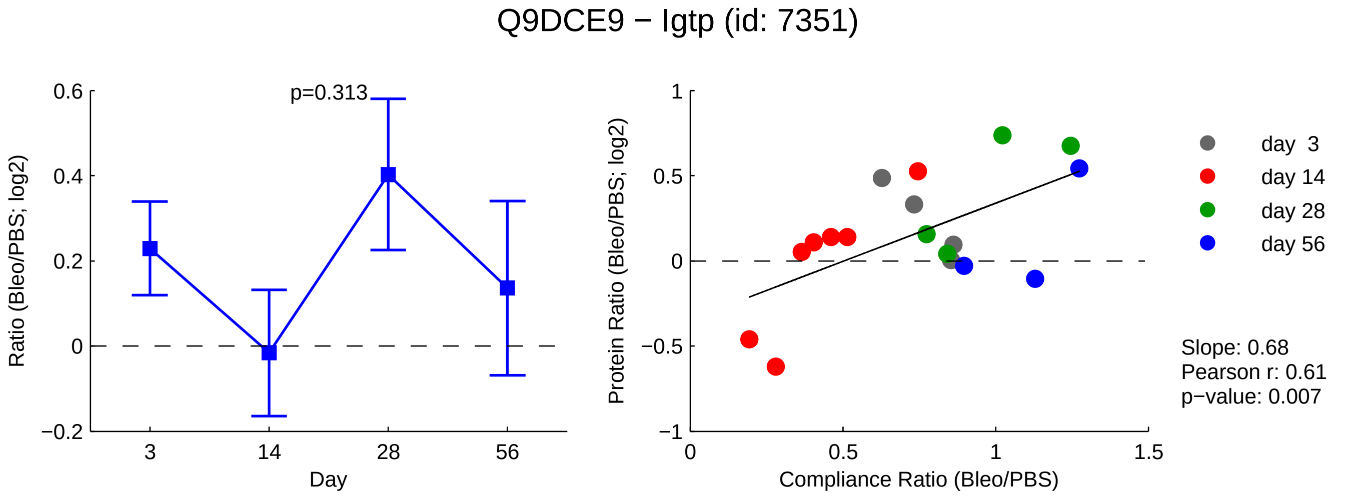

Q9DCE9 − Igtp (id: 7351)
p=0.313
Ratio (Bleo/PBS; log2)
Day
Protein Ratio (Bleo/PBS; log2)
Compliance Ratio (Bleo/PBS)
day 3
day 14
day 28
day 56
Slope: 0.68
Pearson r: 0.61
p−value: 0.007

Q9DCG9 − Trmt112 (id: 7354)

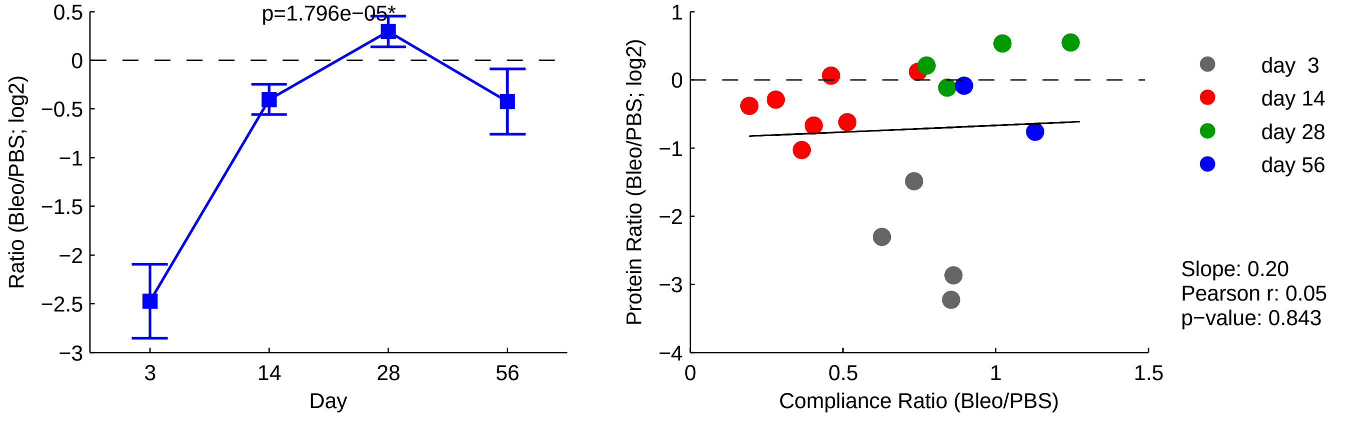

Q9DCH4 − Eif3f (id: 7355)

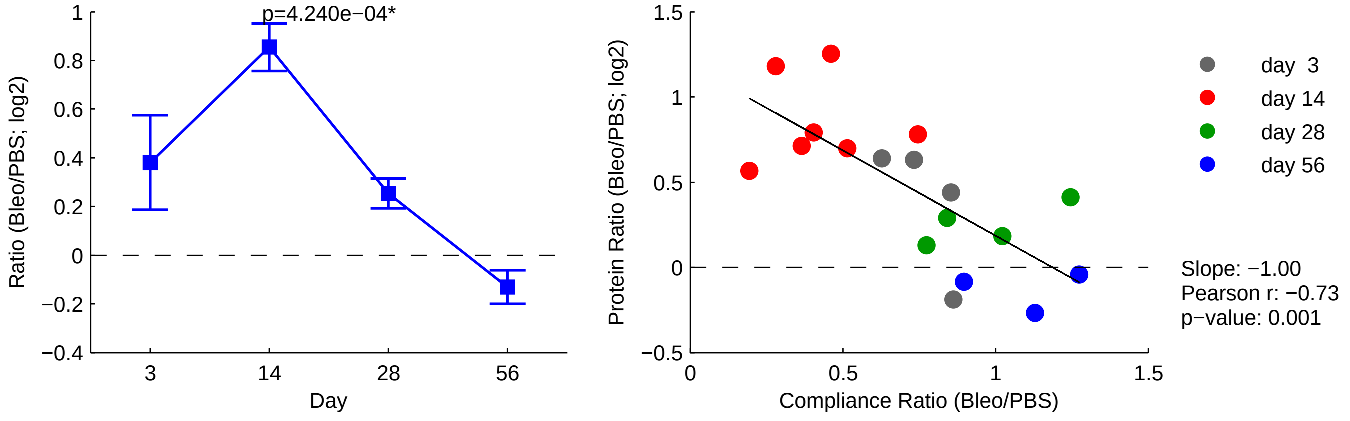

Q9DCJ1 − Mlst8 (id: 7357)

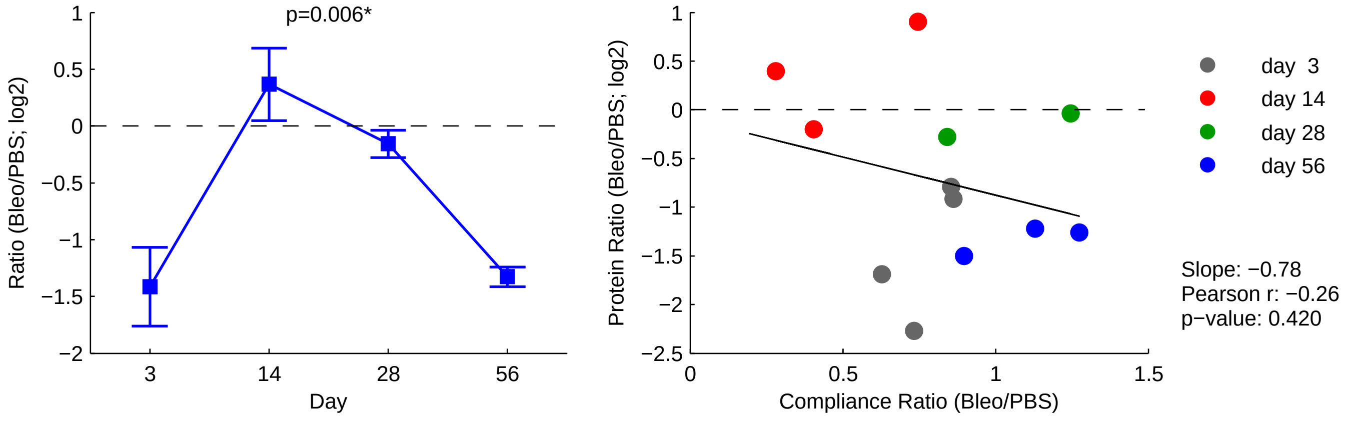

Q9DCM0 − Ethe1 (id: 7362)

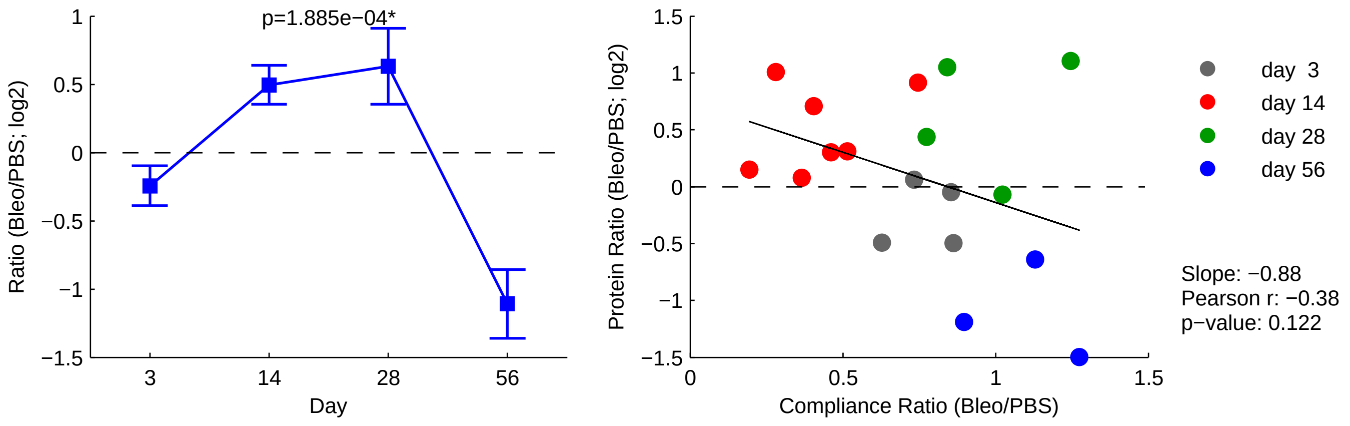

Q9DCM2 − Gstk1 (id: 7363)

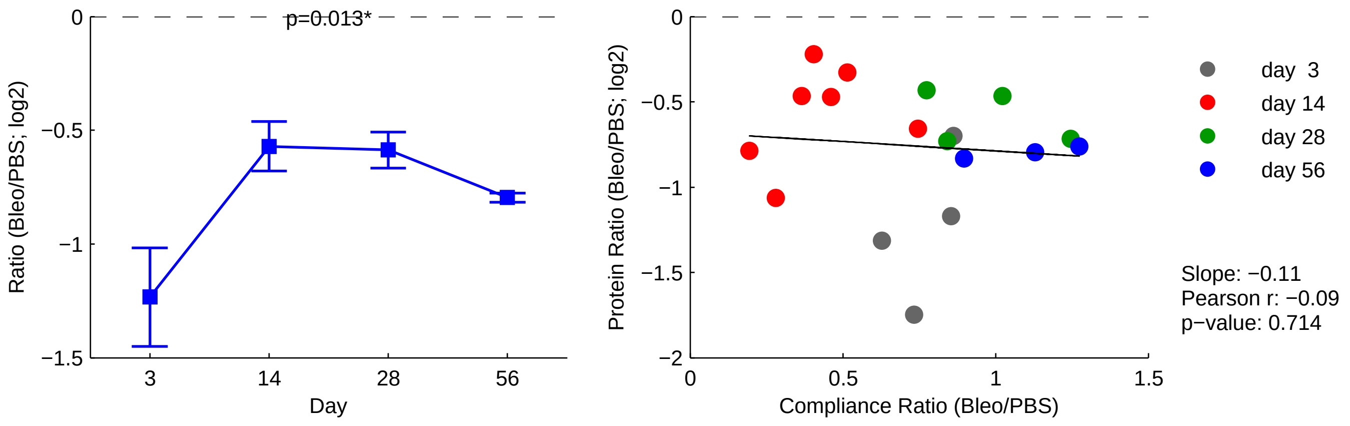

Q9DCN2−2 − Cyb5r3 (id: 7365)

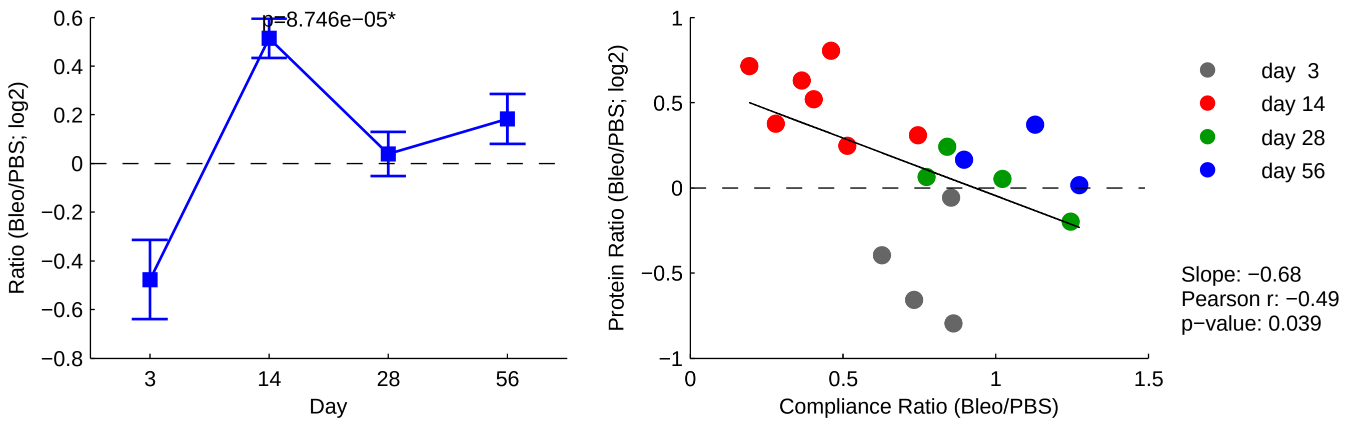

Q9DCR2 − Ap3s1 (id: 7367)

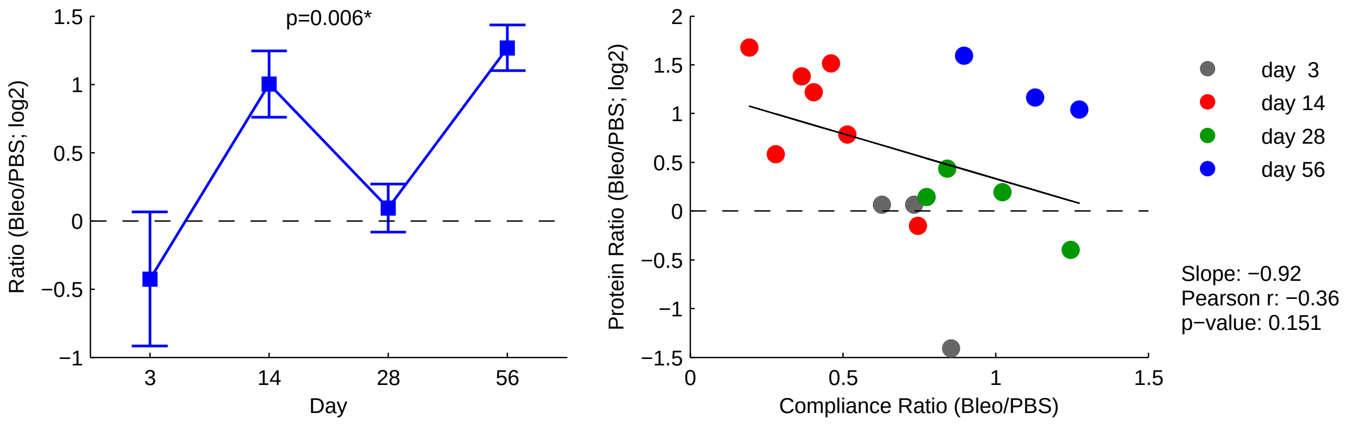

Q9DCS3 − Mecr (id: 7368)

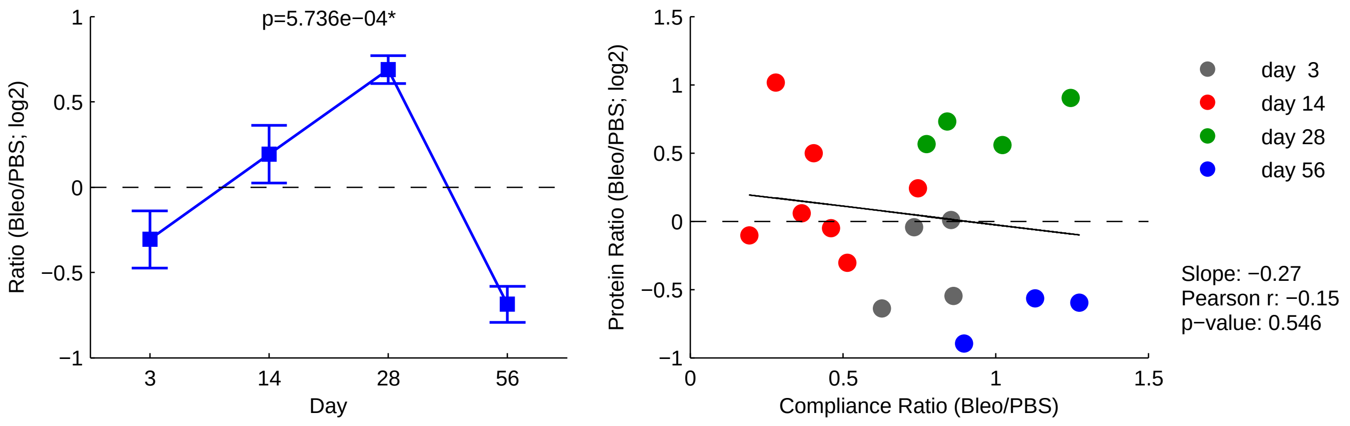

Q9DCS9 − Ndufb10 (id: 7369)

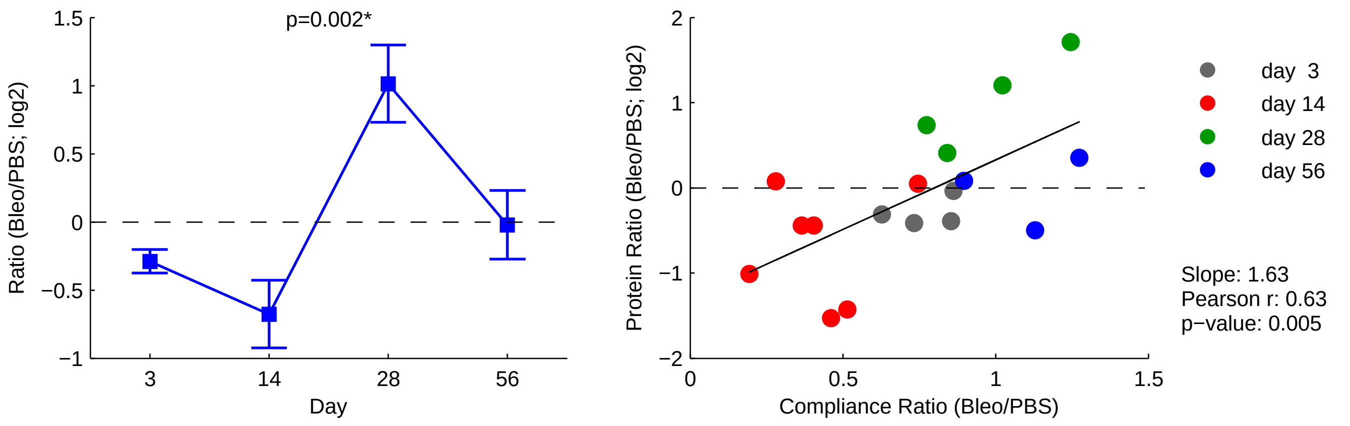

Q9DCT1 − Akr1e2 (id: 7370)

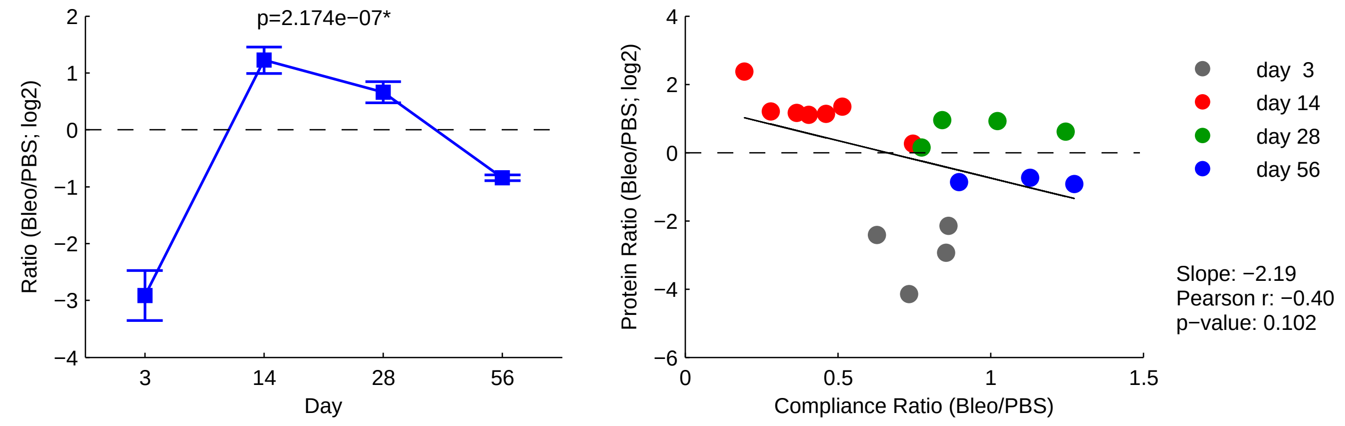

Q9DCT2 − Ndufs3 (id: 7371)

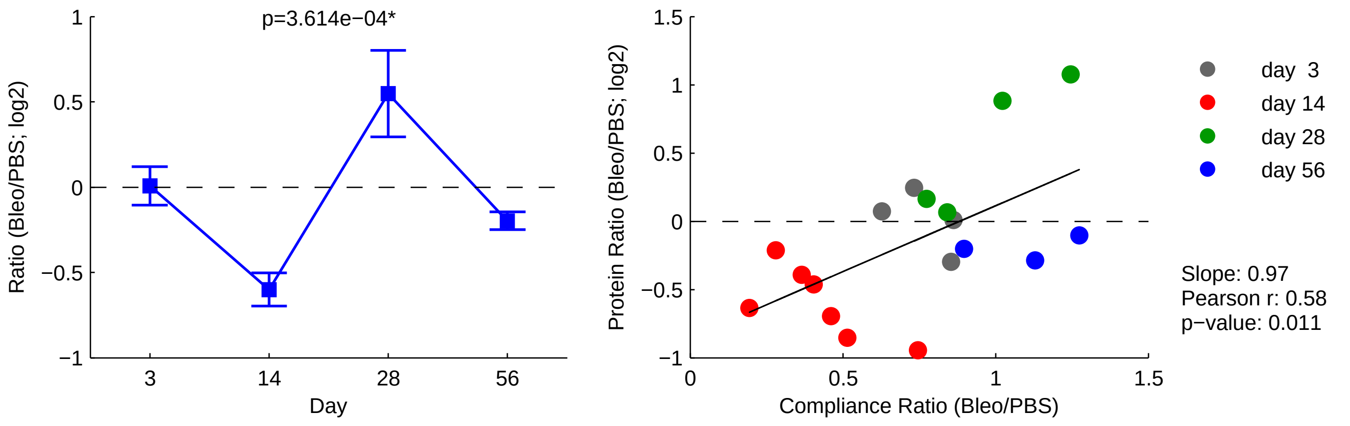

Q9DCT5 − Sdf2 (id: 7372)

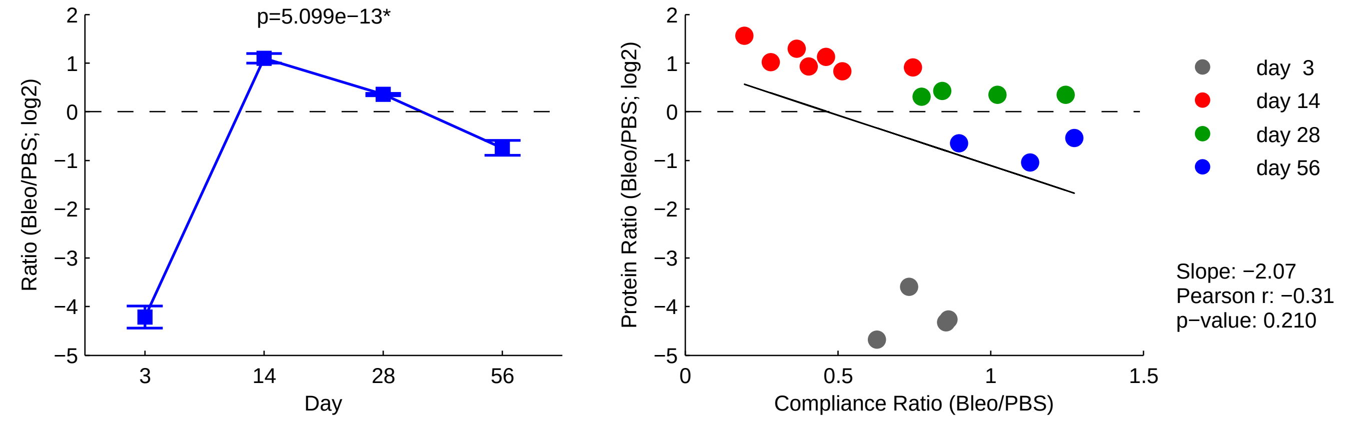

Q9DCT8 − Crip2 (id: 7373)

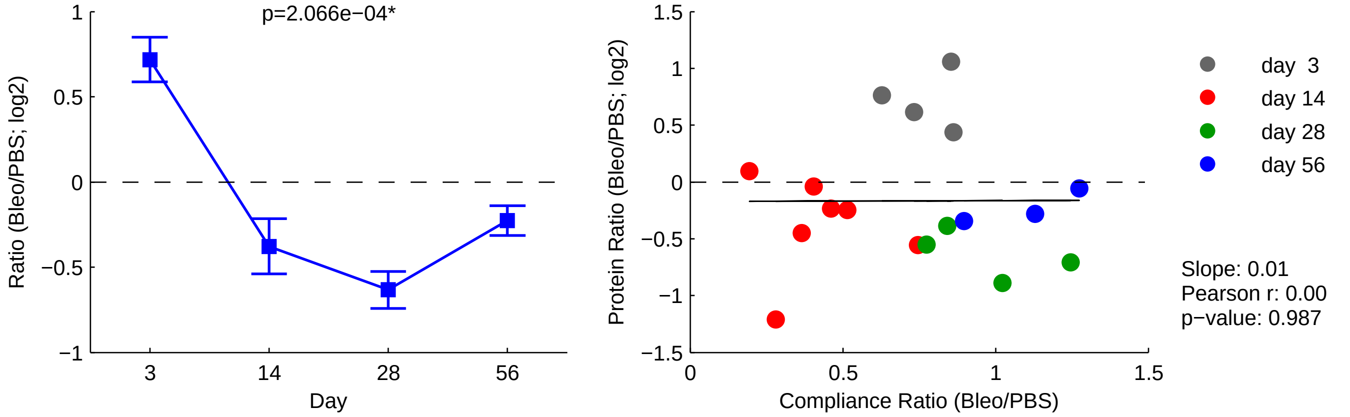

Q9DCU6 − Mrpl4 (id: 7374)

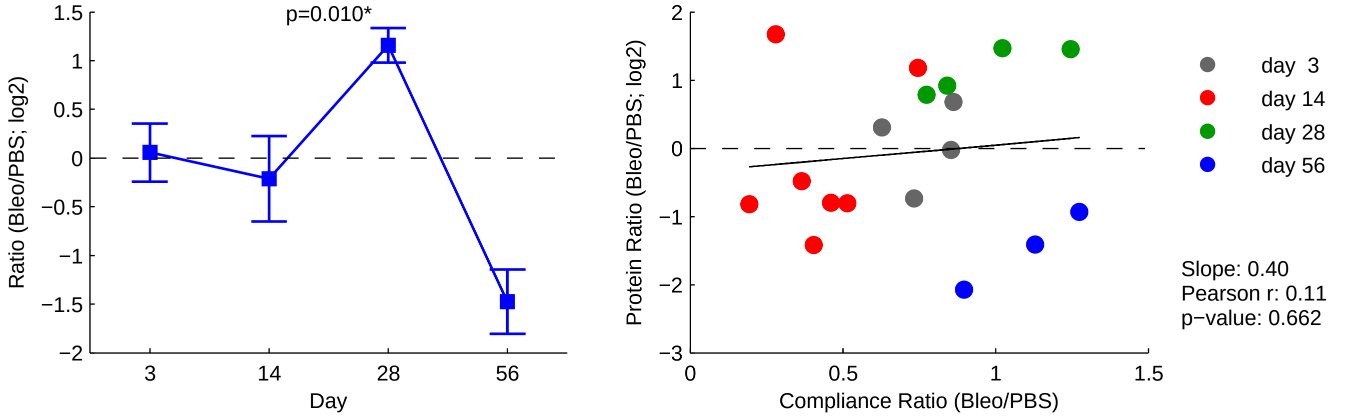

Q9DCV4 − Rmdn1 (id: 7376)

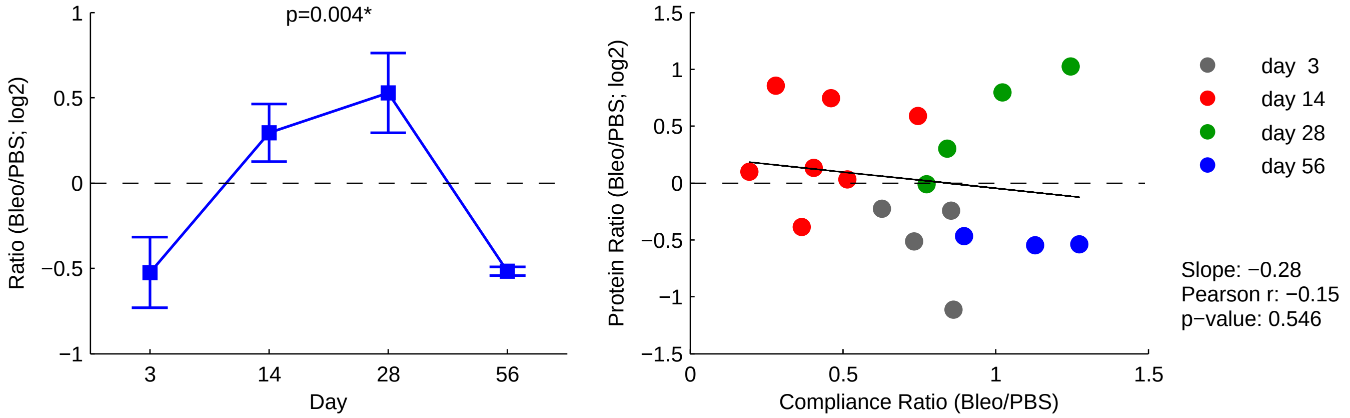

Q9DCW4 − Etfb (id: 7377)

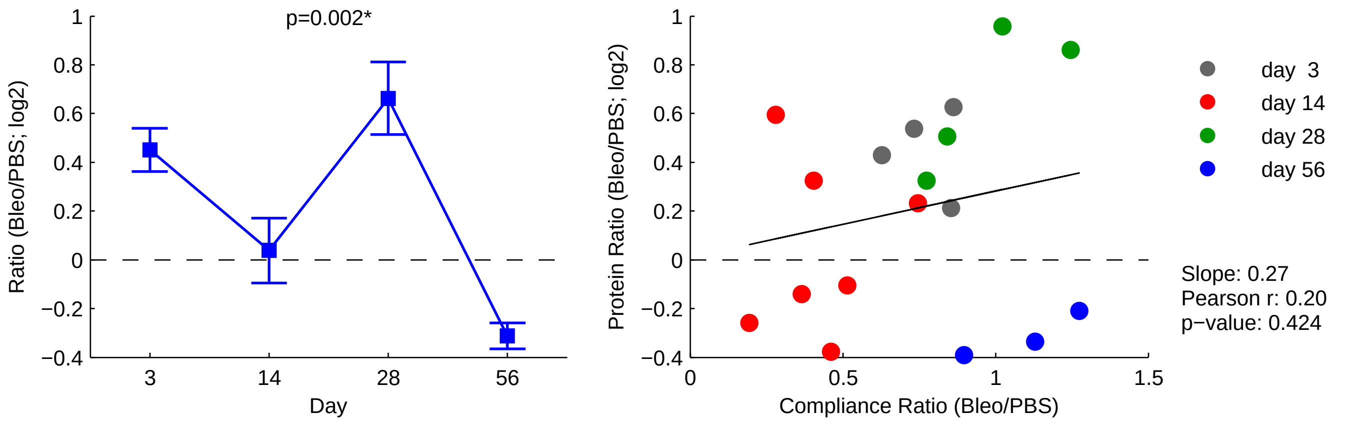

Q9DCX2 − Atp5h (id: 7379)

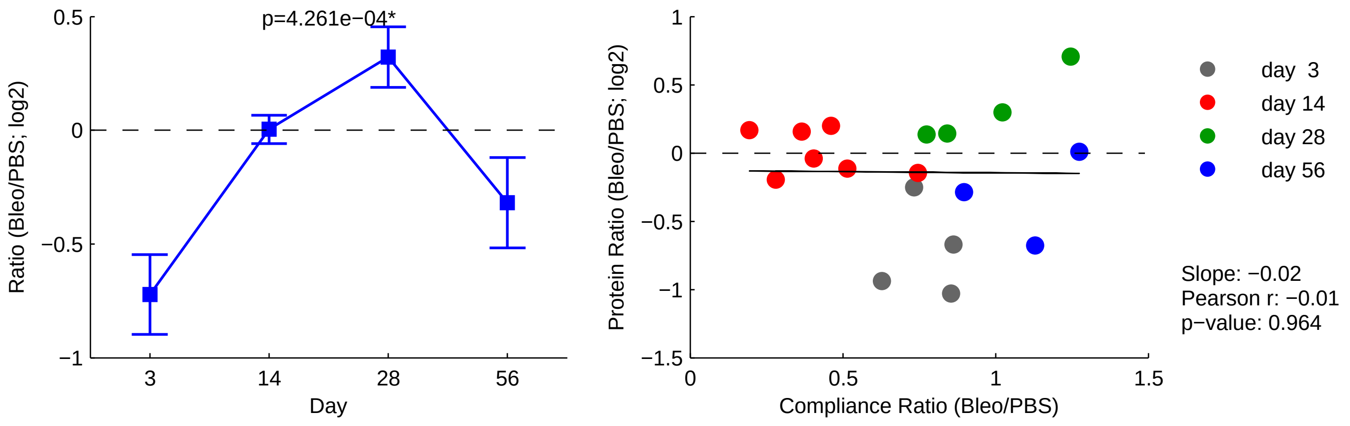

Q9DCX8 − Iyd (id: 7380)

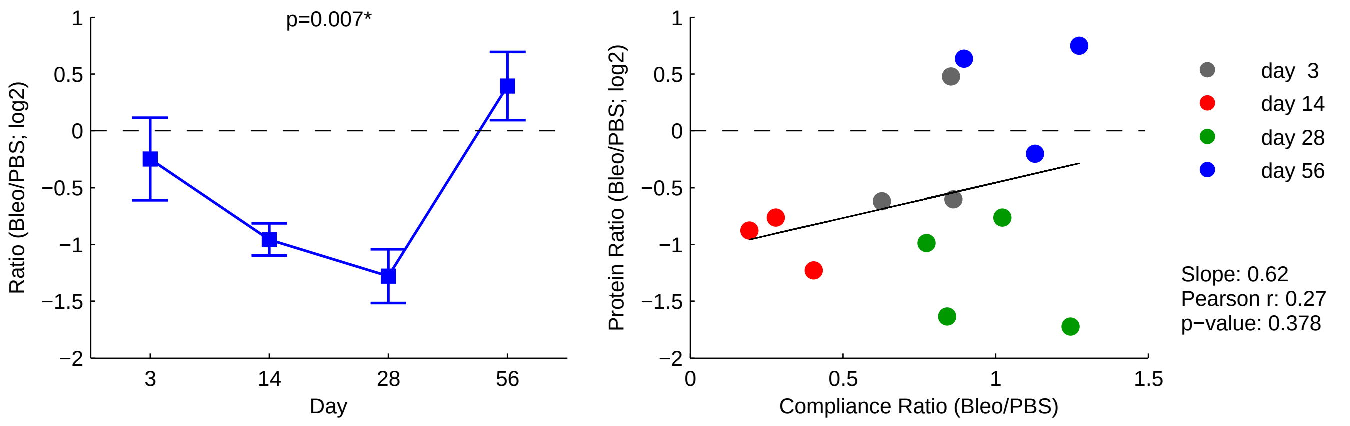

Q9DD03 − Rab13 (id: 7383)

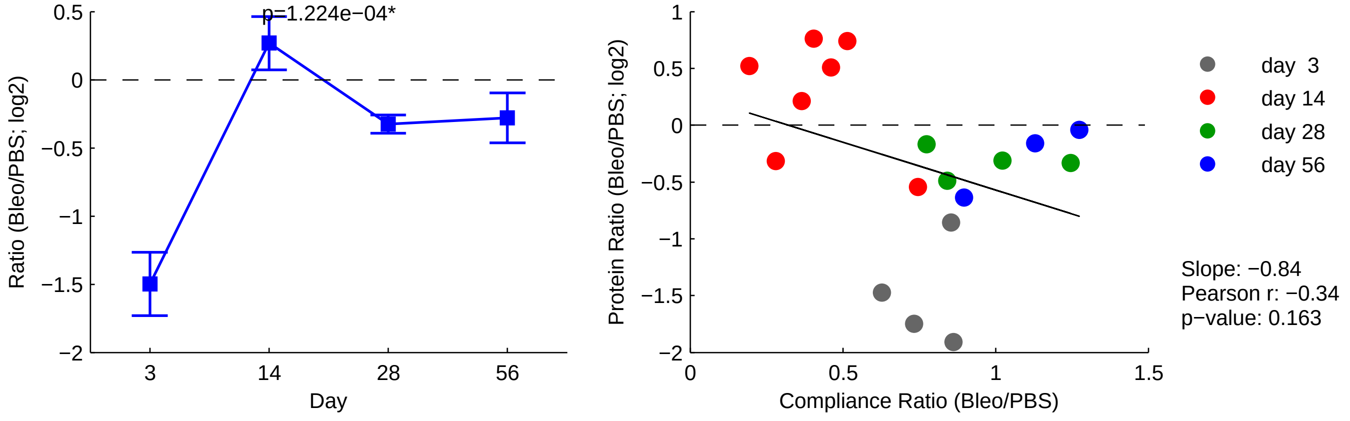

Q9DD06 − Rarres2 (id: 7384)

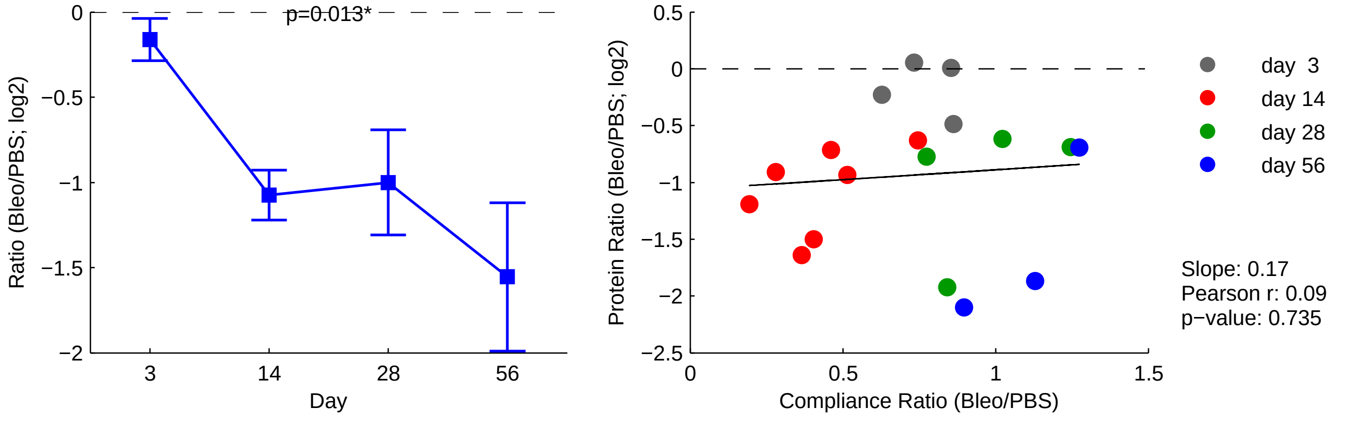

Q9DD23 − Lypd2 (id: 7387)

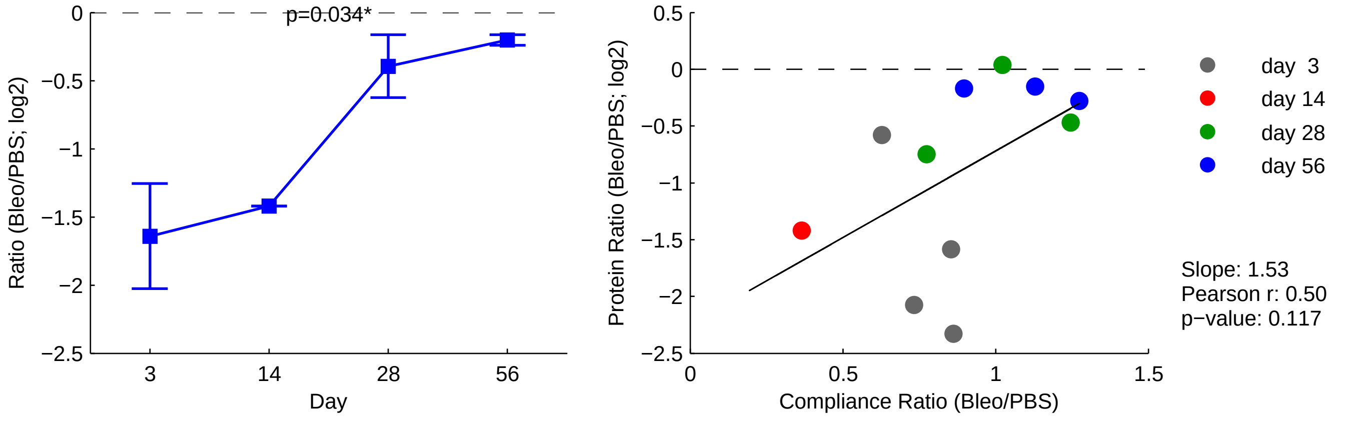

Q9EP69 − Sacm1l (id: 7390)

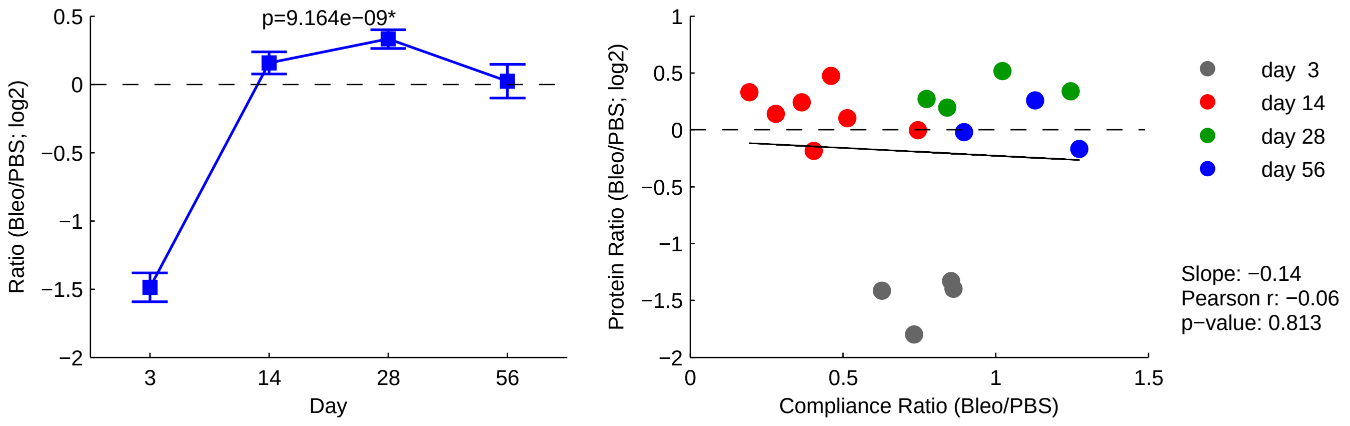

Q9EP72 − Emc7 (id: 7392)

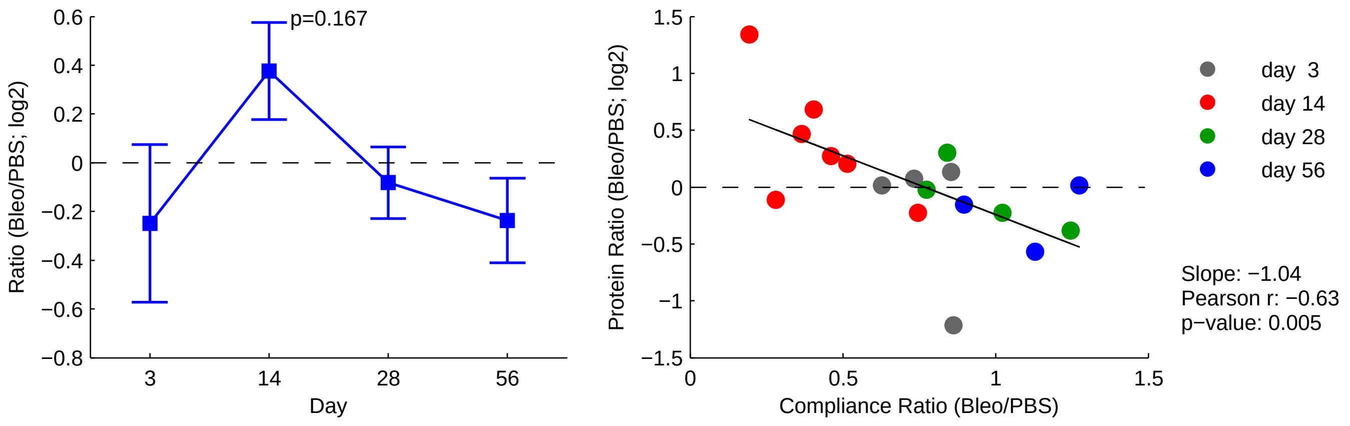

Q9EP89 − Lactb (id: 7395)

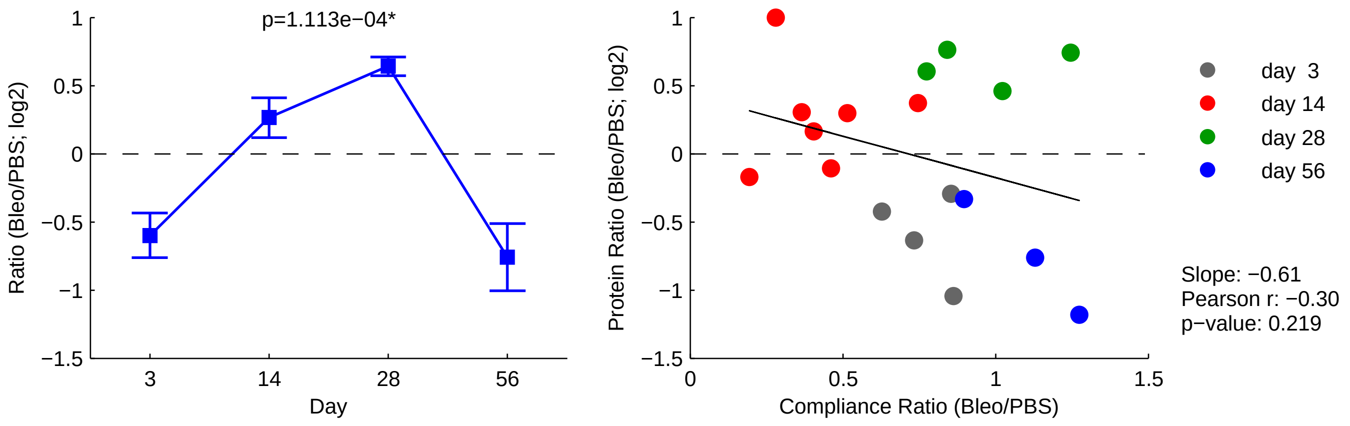

Q9EPC1 − Parva (id: 7399)

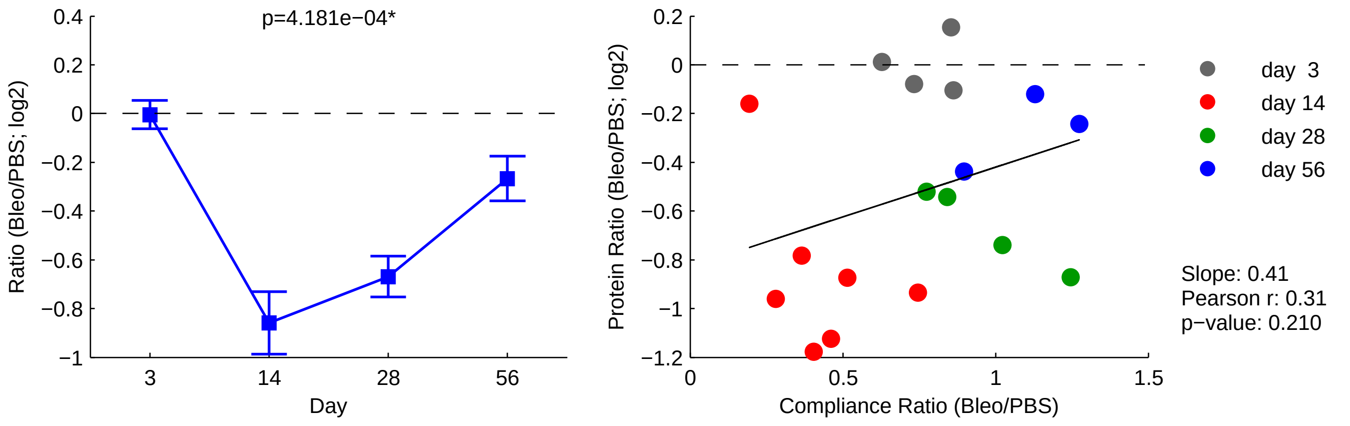

Q9EPJ9 − Arfgap1 (id: 7401)

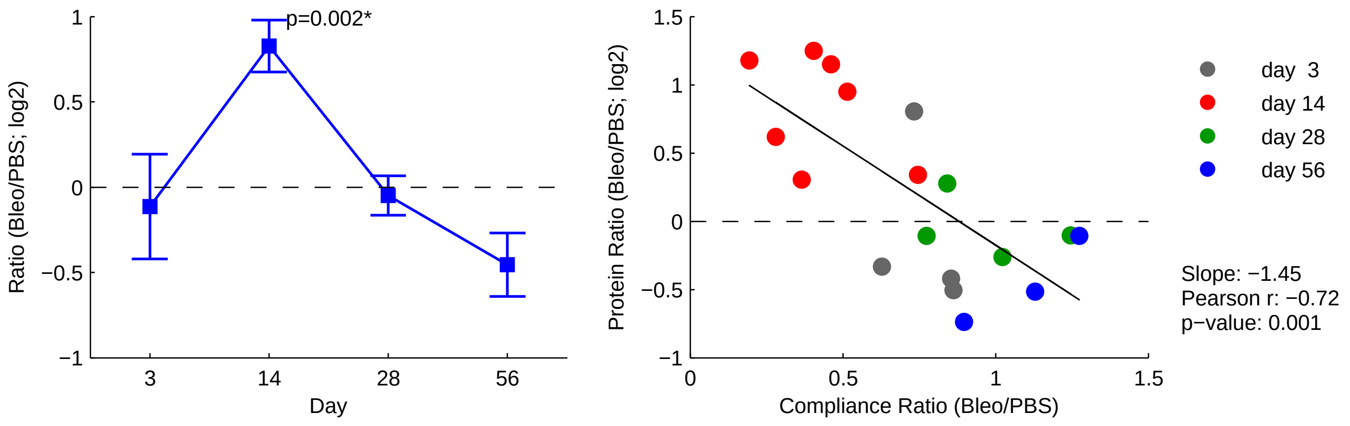

Q9EPK6 − Sil1 (id: 7404)

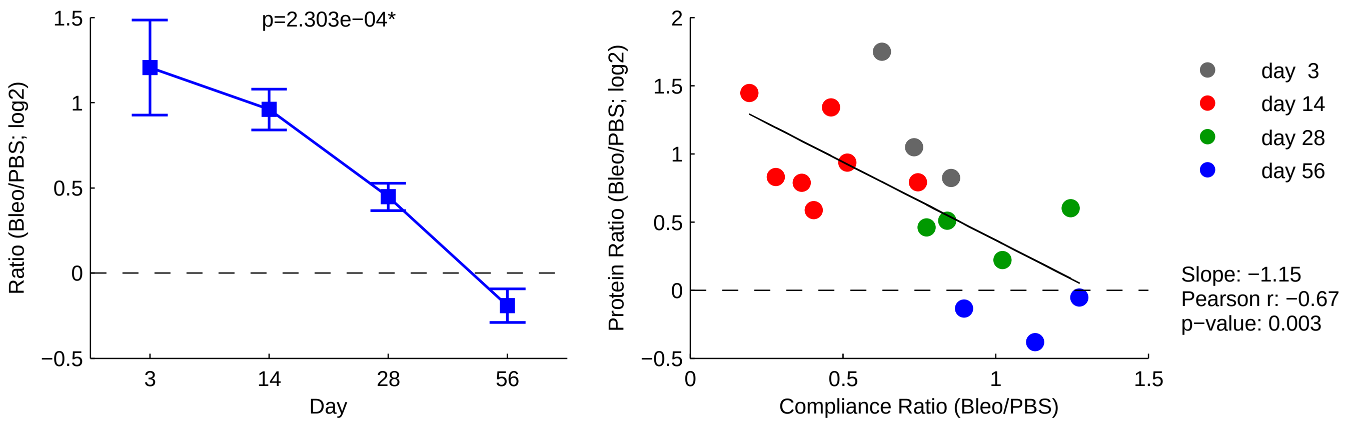

Q9EPL8 − Ipo7 (id: 7408)

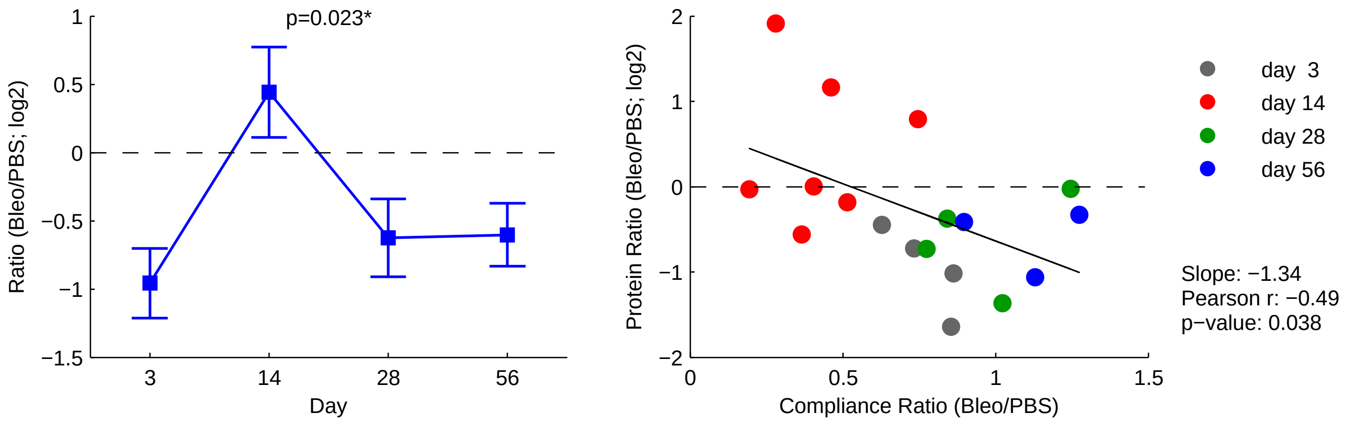

Q9EPL9 − Acox3 (id: 7409)

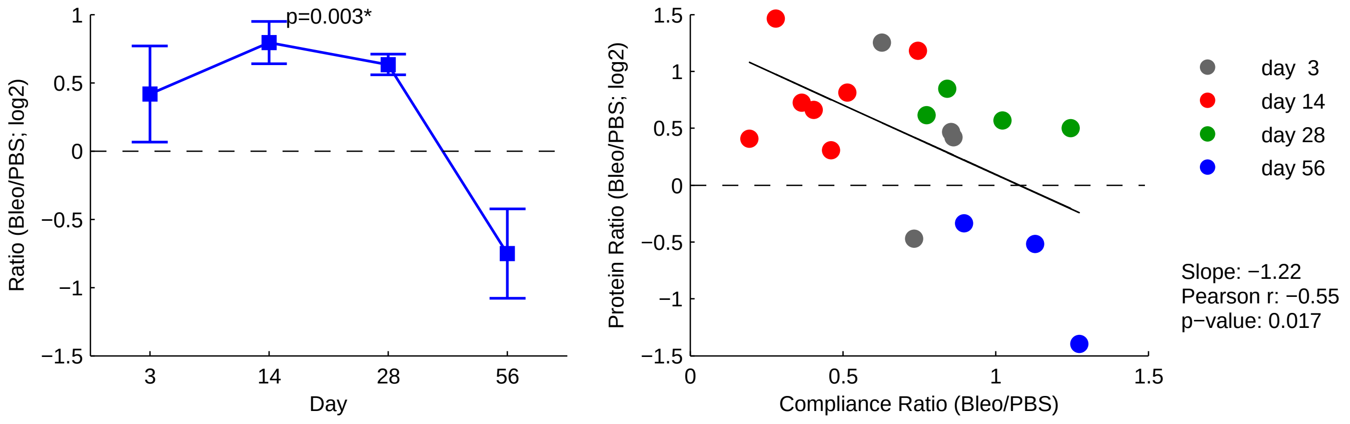

Q9EPN1−3 − Nbea (id: 7410)

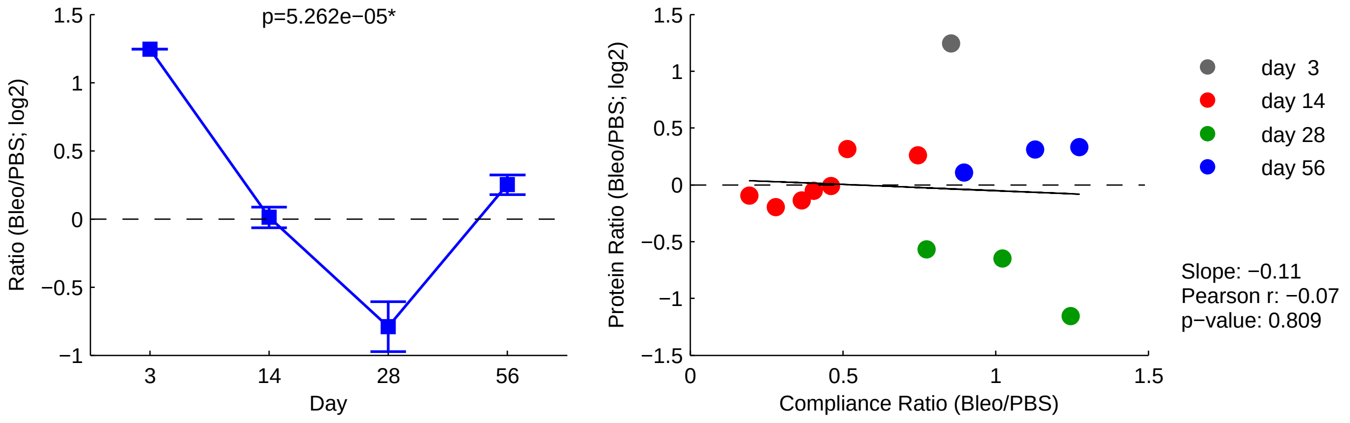

Q9EPR4 − Slc23a2 (id: 7411)

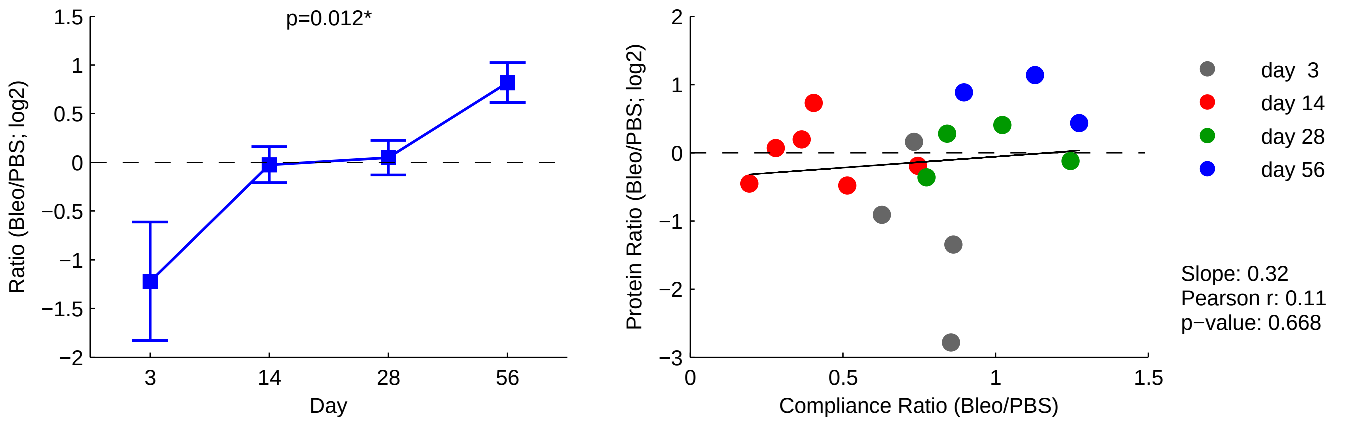

Q9EPR5 − Sorcs2 (id: 7412)

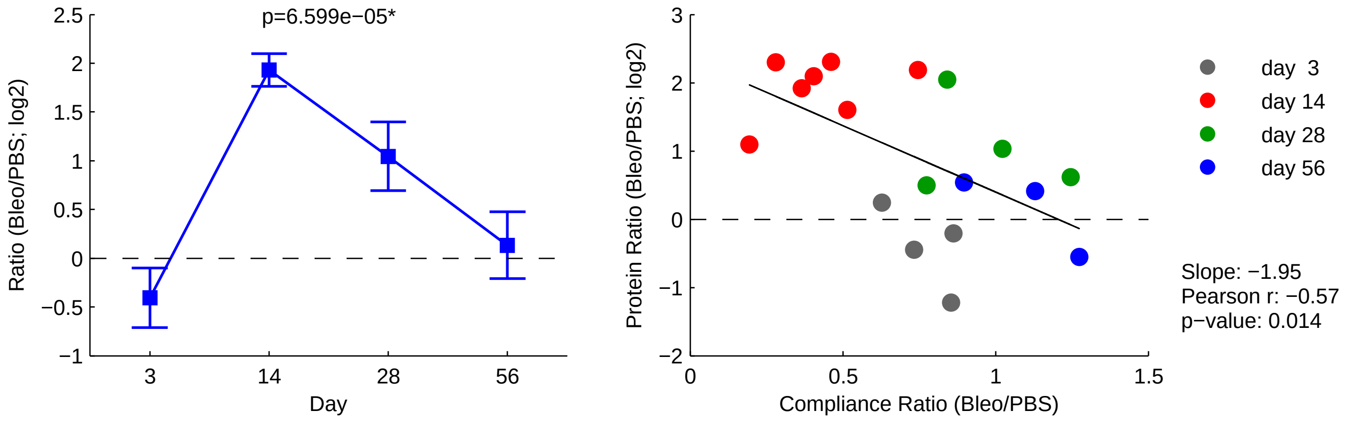

Q9EPT5 − Slco2a1 (id: 7414)

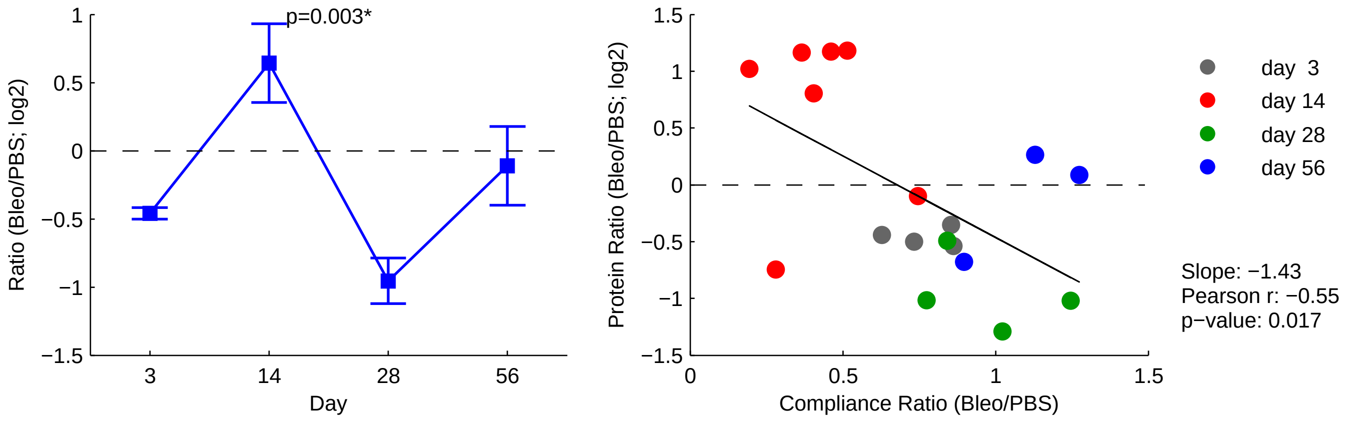

Q9EPU0−2 − Upf1 (id: 7415)

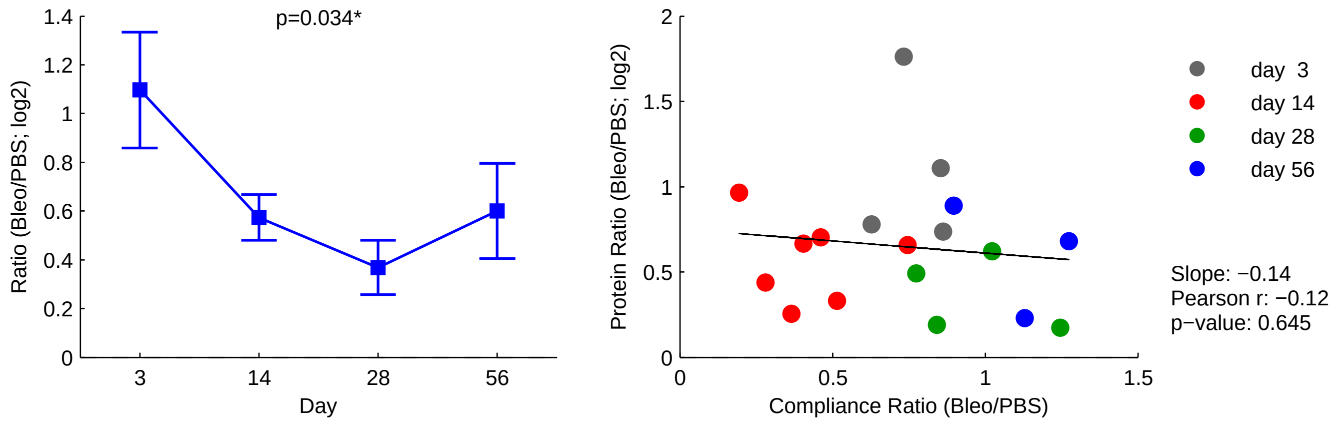

Q9EPU4 − Cpsf1 (id: 7416)

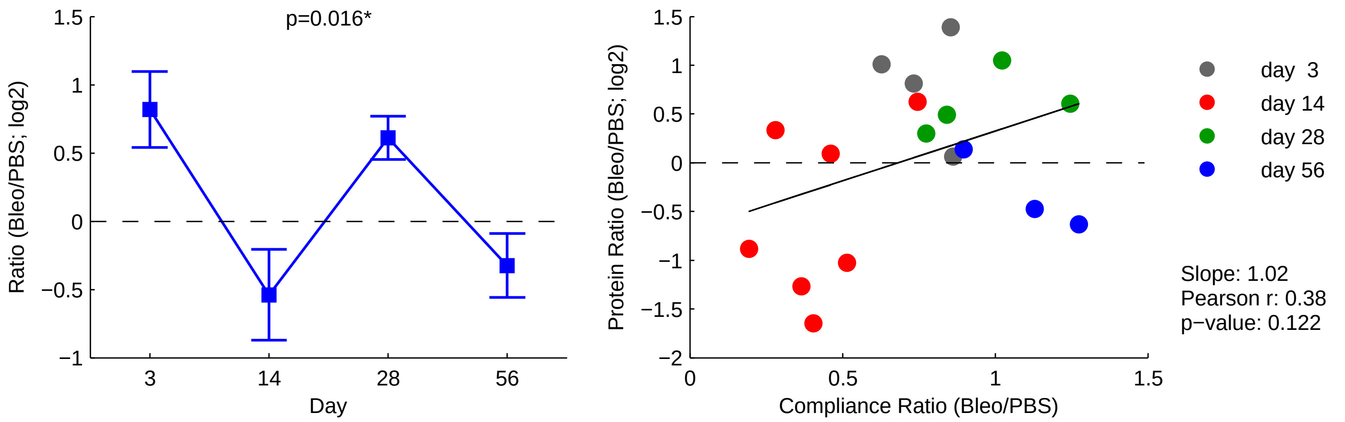

Q9EQ06 − Hsd17b11 (id: 7419)

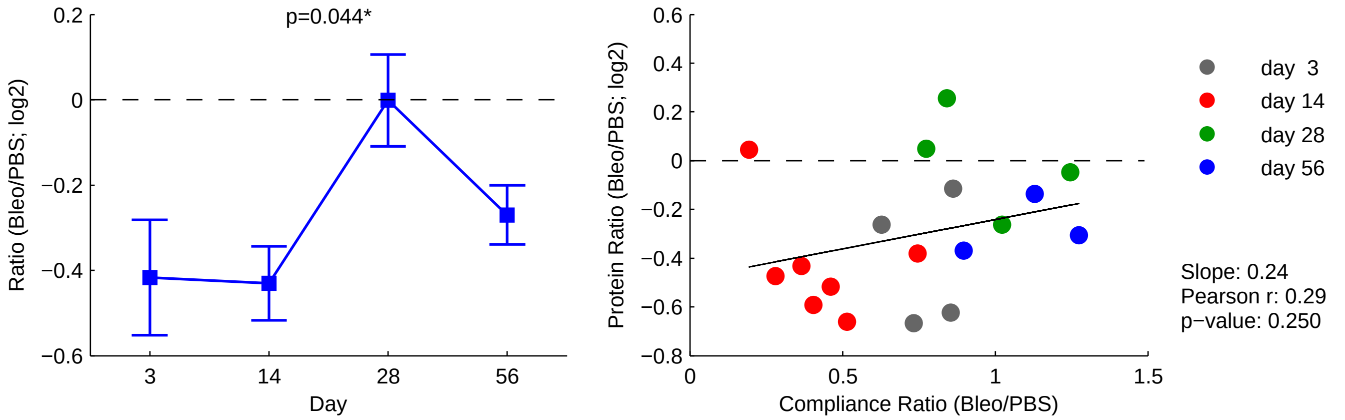

Q9EQ08 − Sgsh (id: 7420)

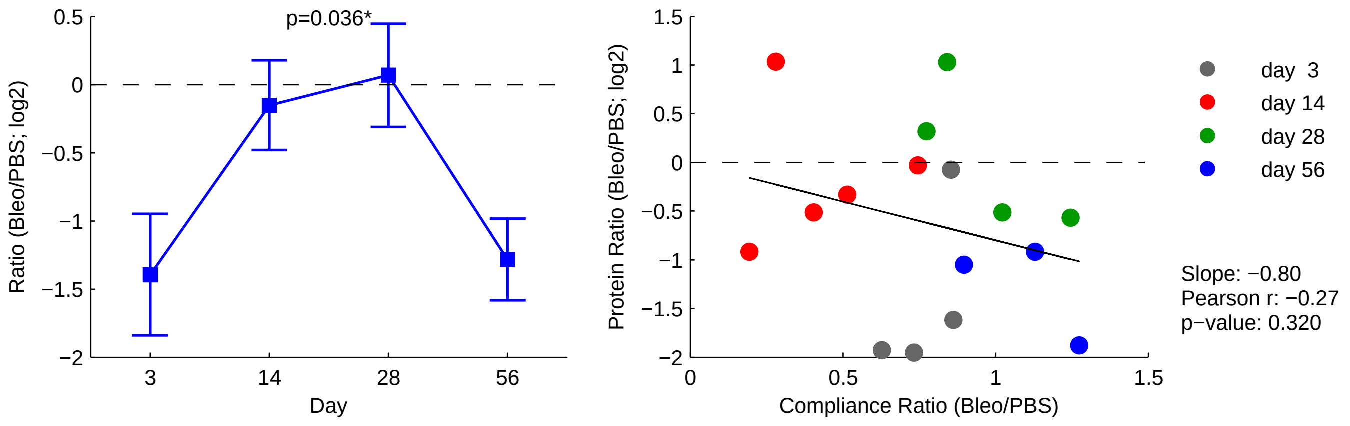

Q9EQ20 − Aldh6a1 (id: 7421)

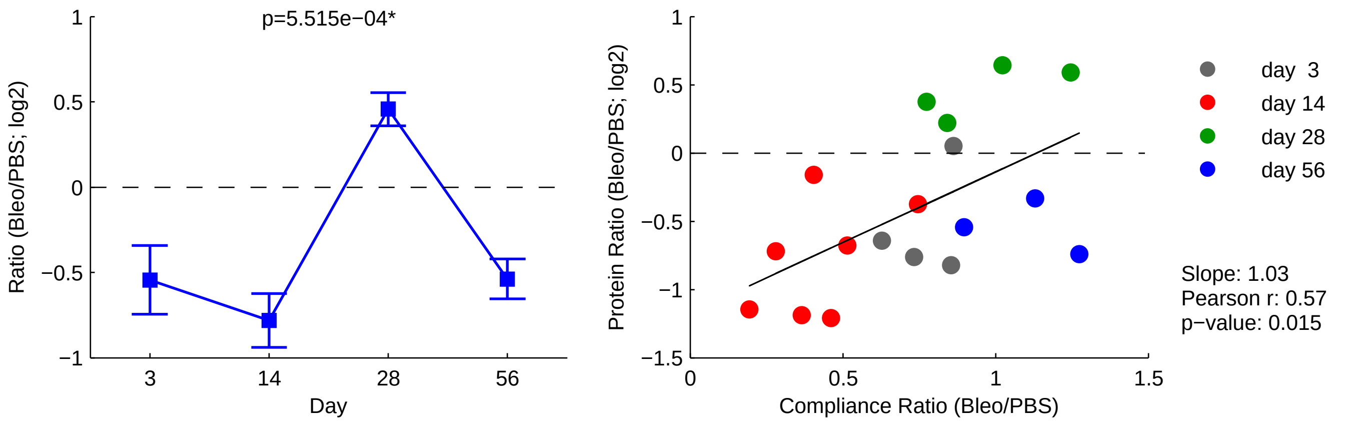

Q9EQC5 − Scyl1 (id: 7424)

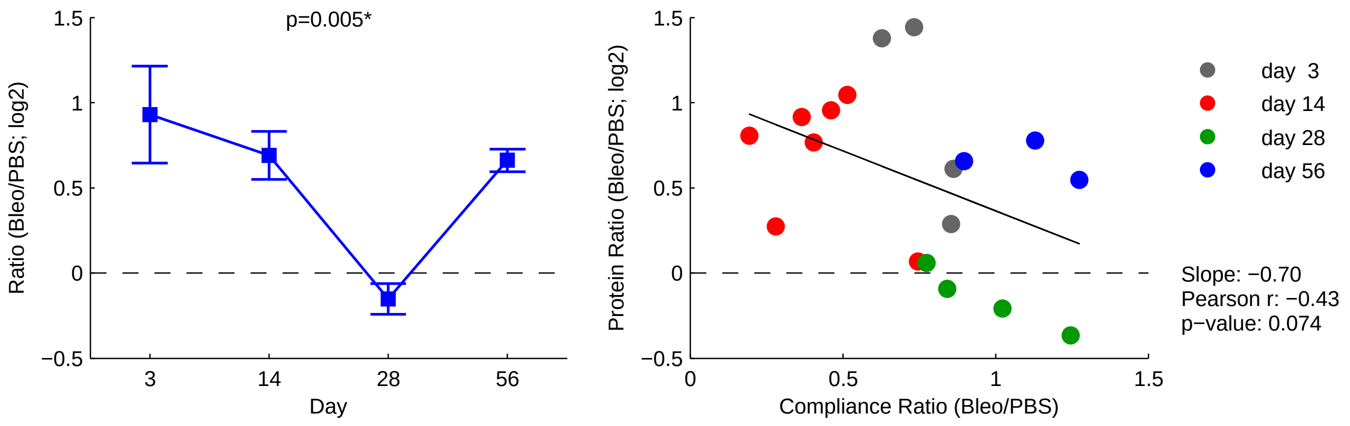

Q9EQF6 − Dpysl5 (id: 7428)

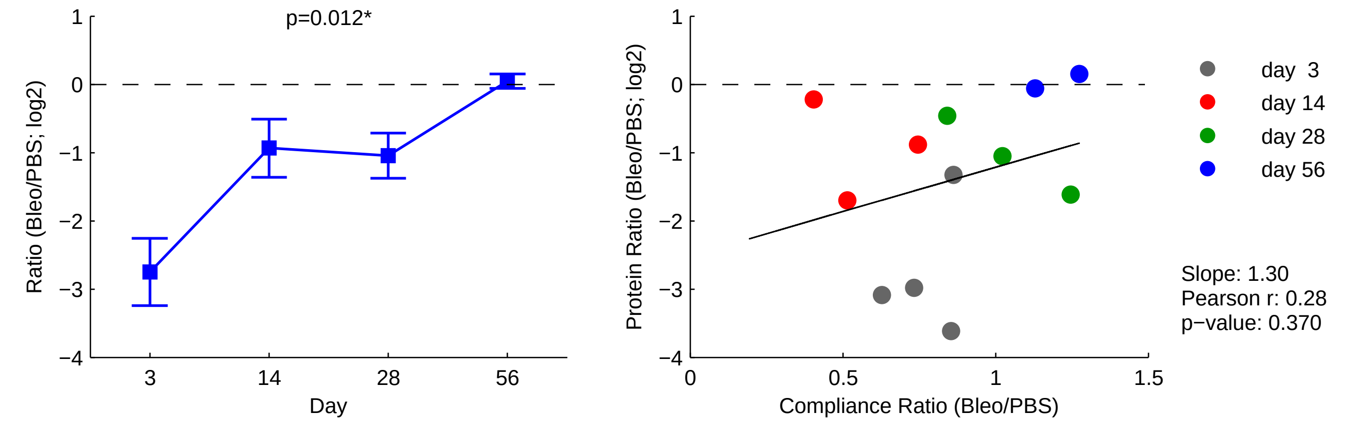

Q9EQG3 − Scel (id: 7429)

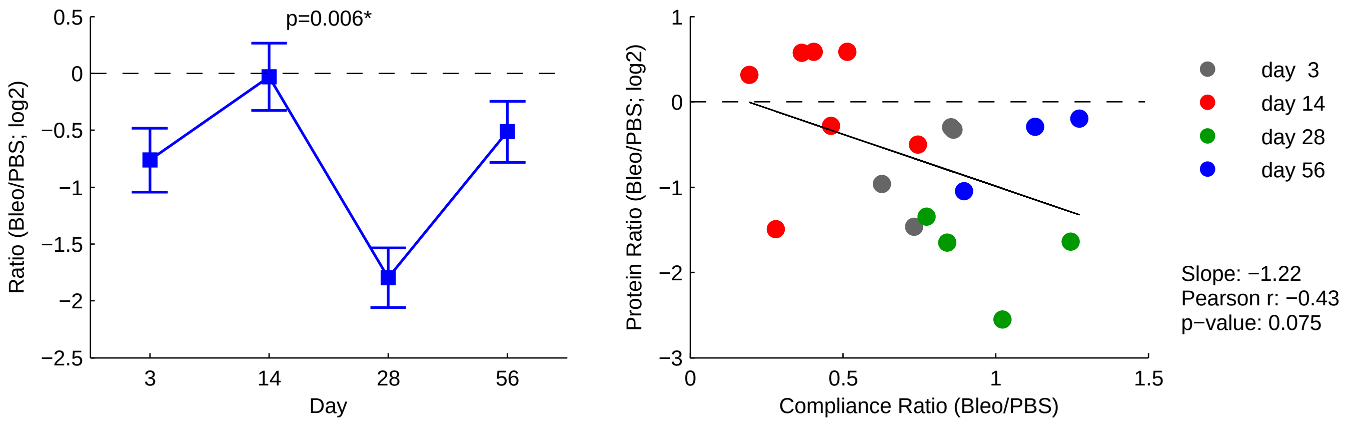

Q9EQG7 − Enpp5 (id: 7430)

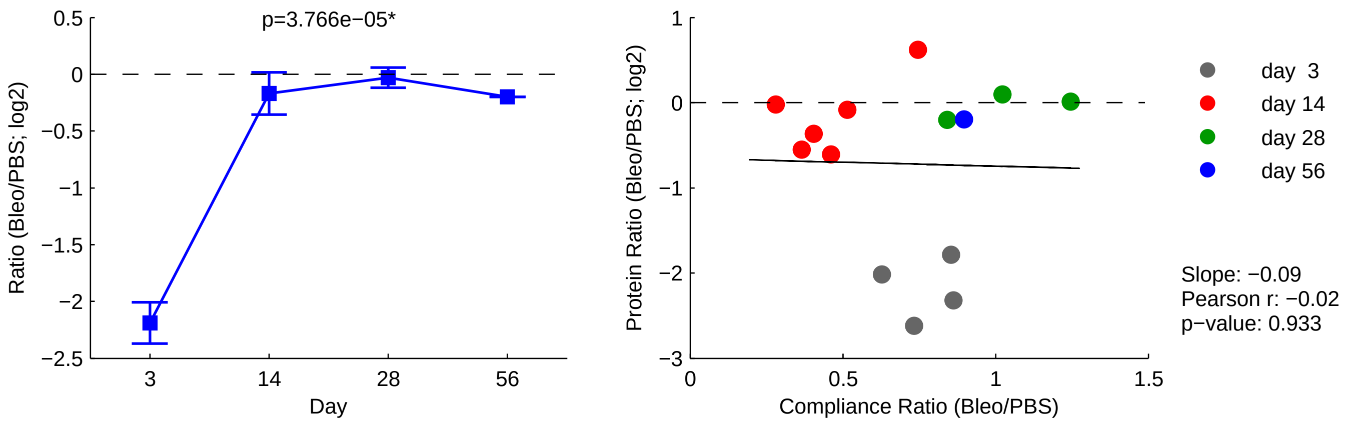

Q9EQG9−2 − Col4a3bp (id: 7431)

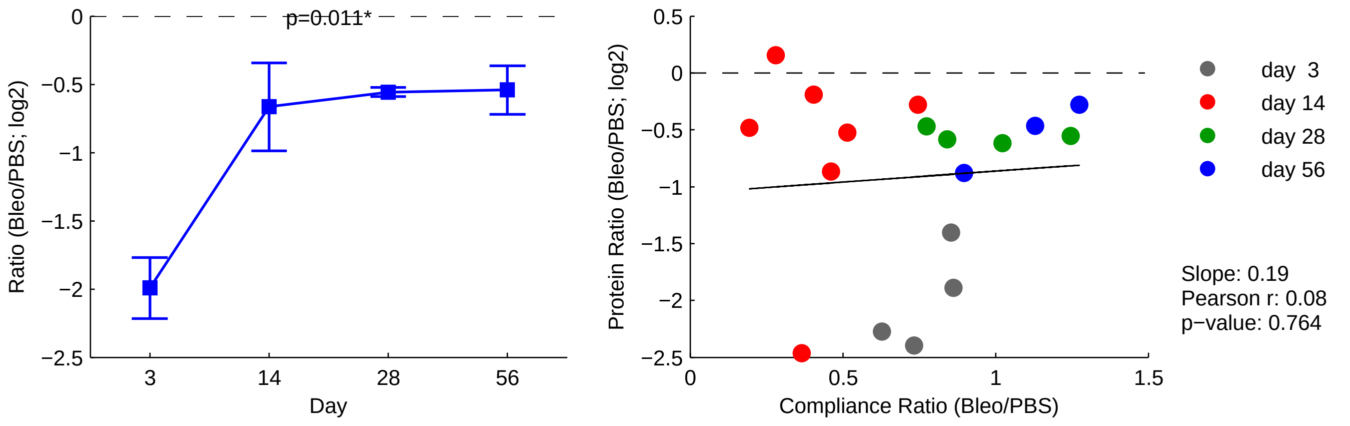

Q9EQH2 − Erap1 (id: 7432)

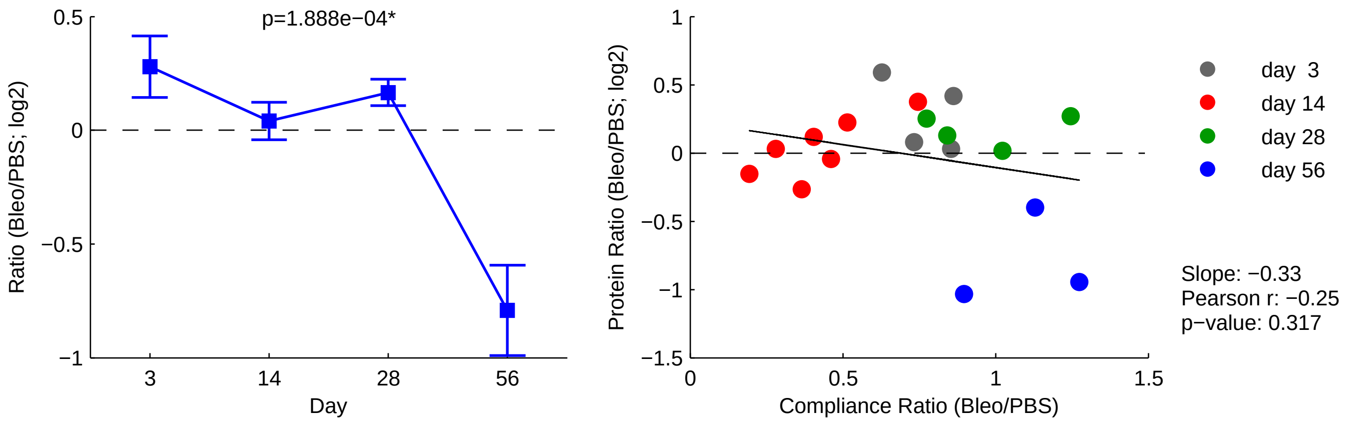

Q9EQH3 − Vps35 (id: 7433)

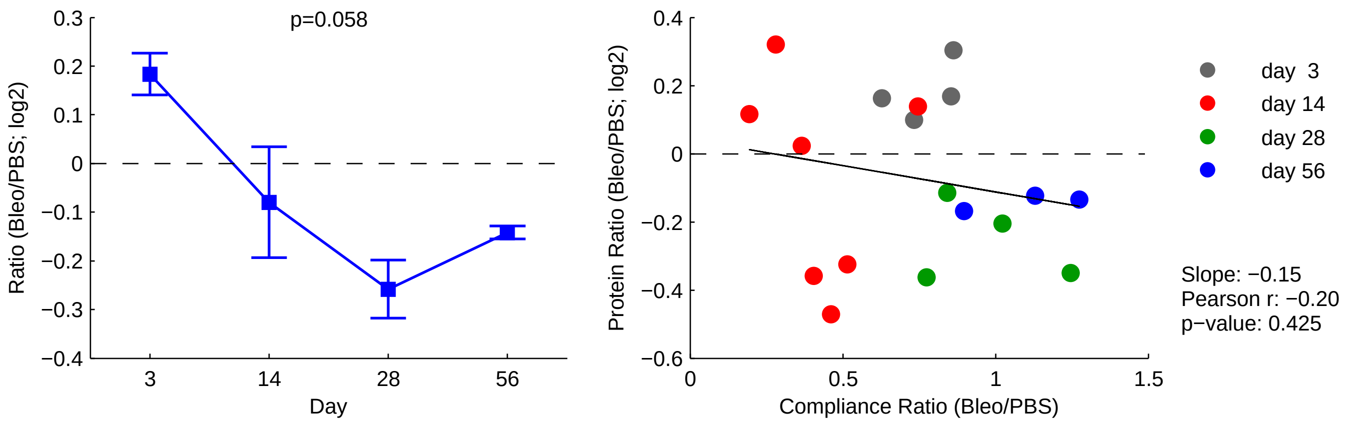

Q9EQI8 − Mrpl46 (id: 7436)

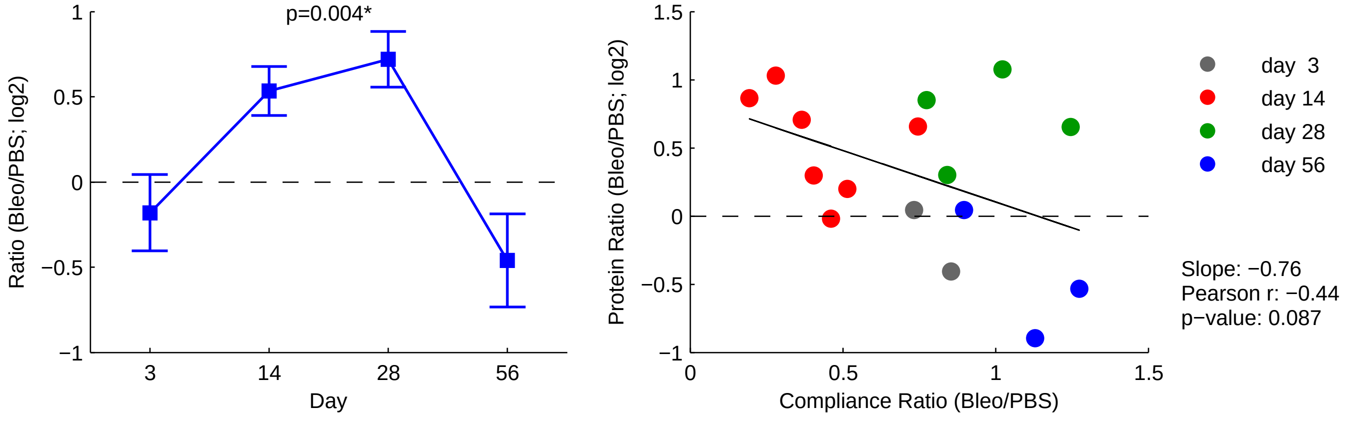

Q9EQJ0 − Tpcn1 (id: 7437)

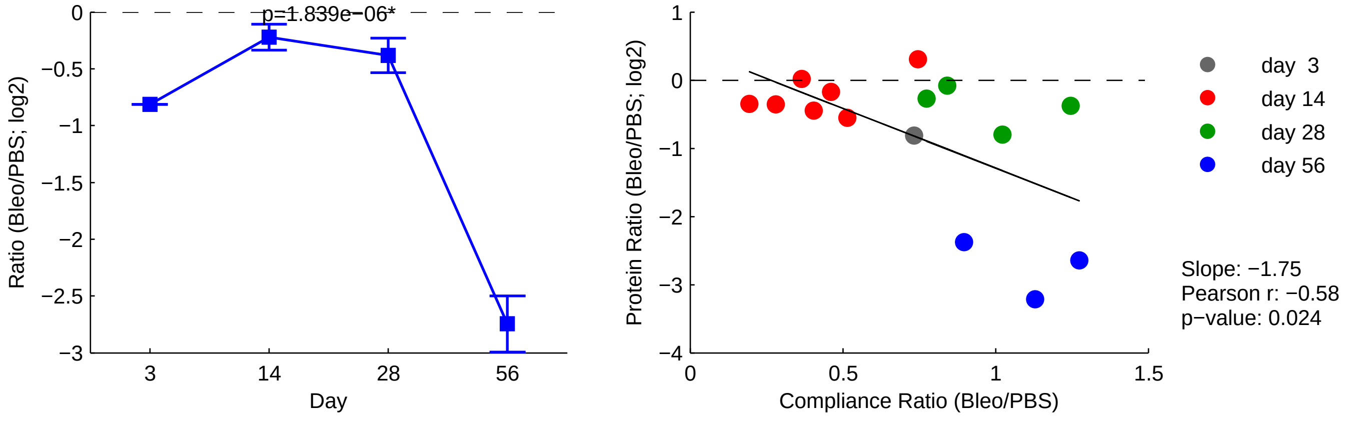

Q9EQP2 − Ehd4 (id: 7440)

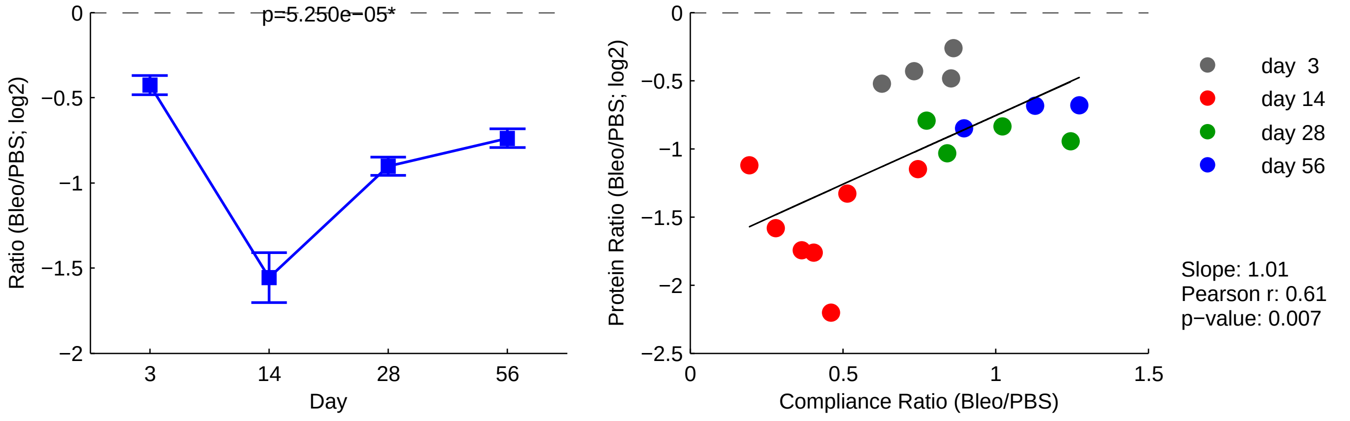

Q9EQU5−2 − Set (id: 7445)

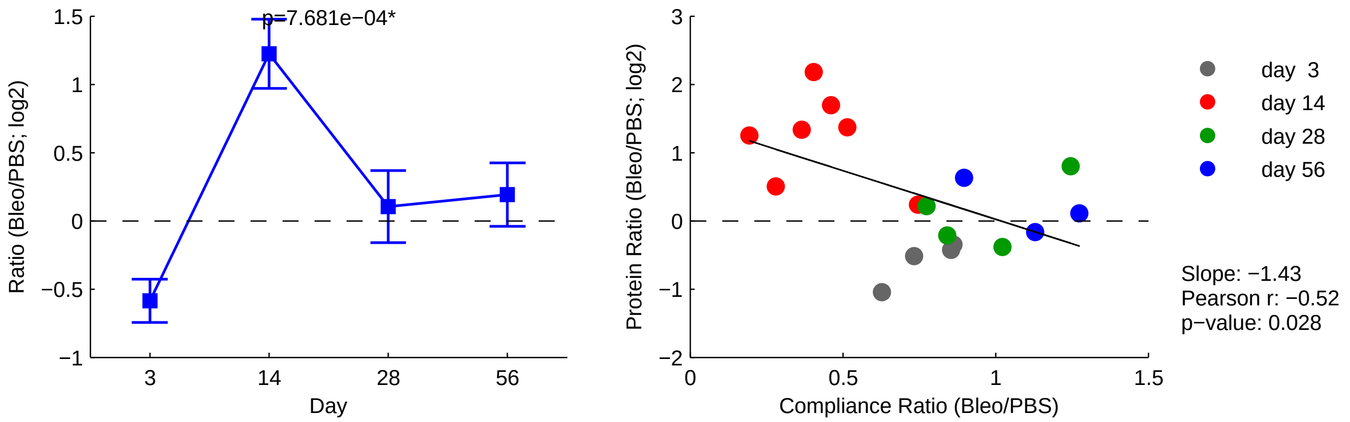

Q9ER38 − Tor3a (id: 7449)

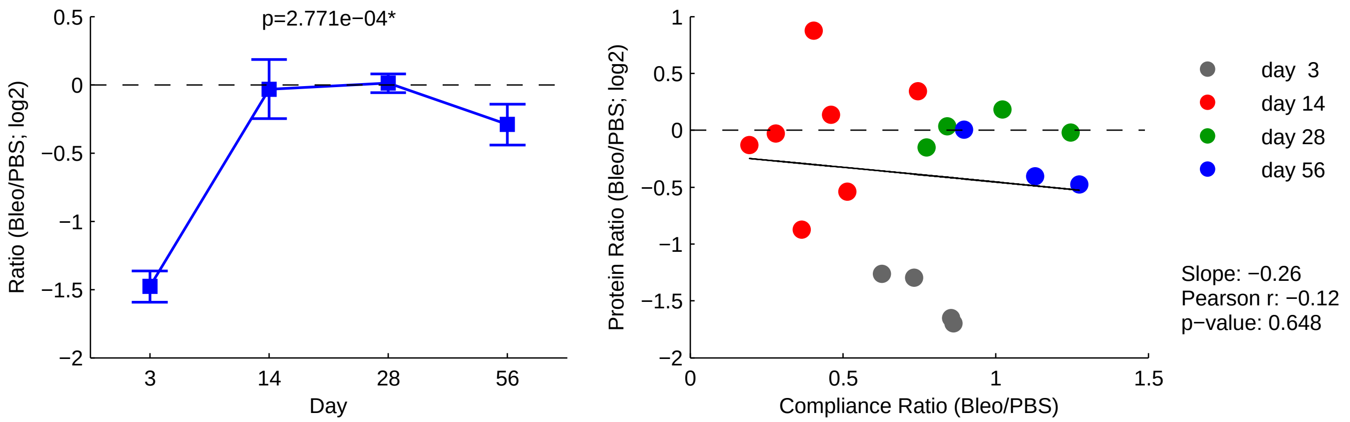

Q9ER41 − Tor1b (id: 7451)

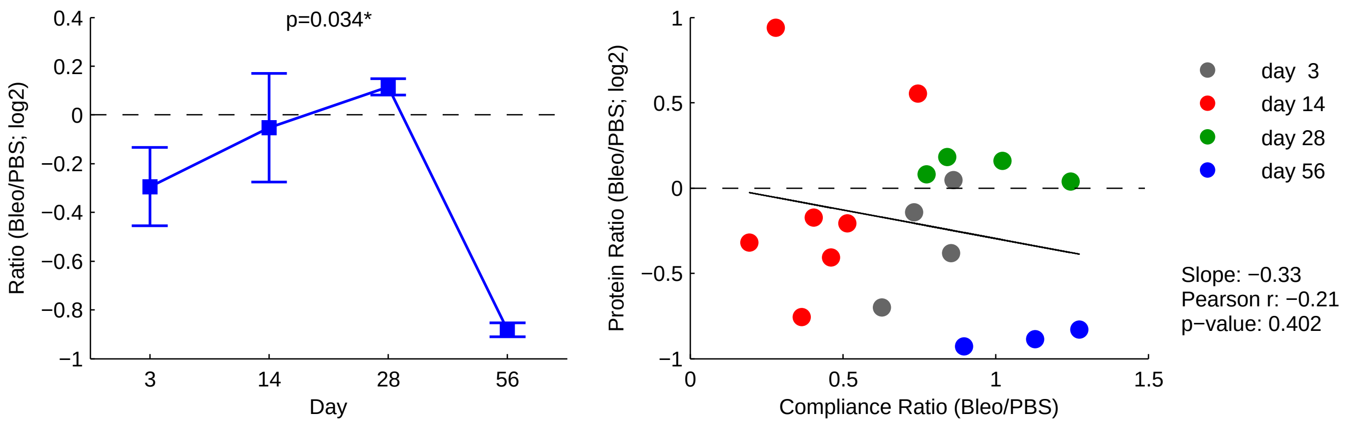

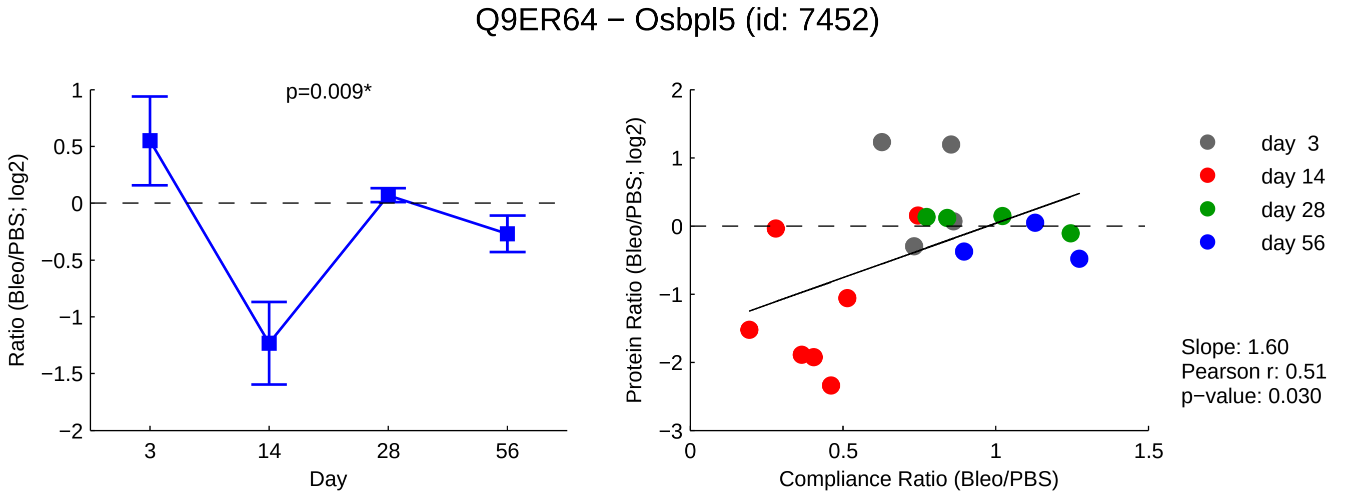
Q9ER64 − Osbpl5 (id: 7452)
p=0.009*
Ratio (Bleo/PBS; log2)
Day
Protein Ratio (Bleo/PBS; log2)
Compliance Ratio (Bleo/PBS)
day 3
day 14
day 28
day 56
Slope: 1.60
Pearson r: 0.51
p−value: 0.030

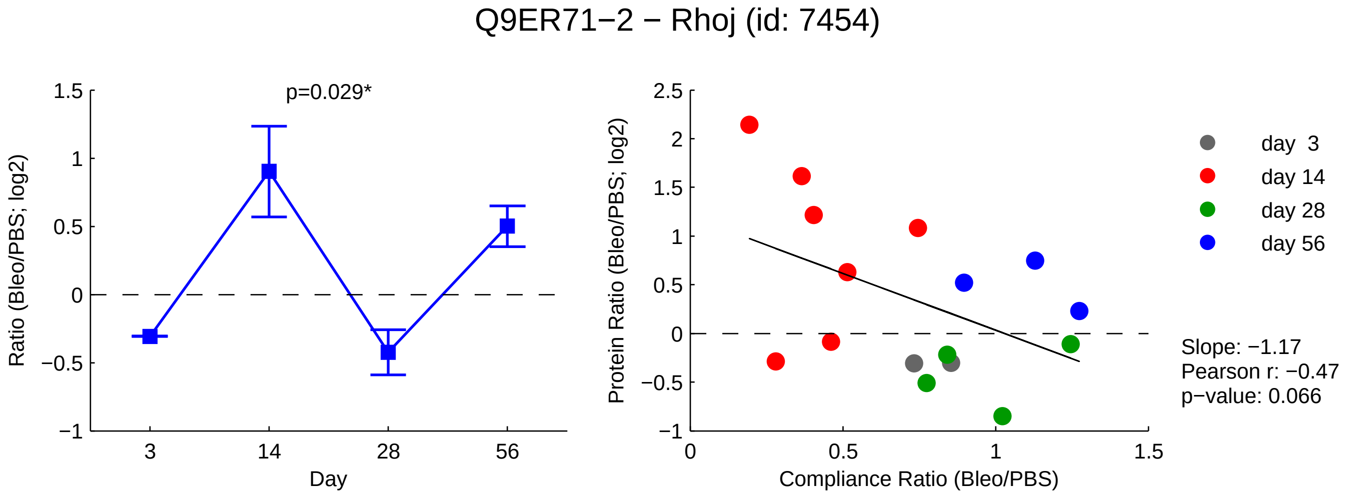
Q9ER71−2 − Rhoj (id: 7454)
p=0.029*
Ratio (Bleo/PBS; log2)
Day
Protein Ratio (Bleo/PBS; log2)
Compliance Ratio (Bleo/PBS)
day 3
day 14
day 28
day 56
Slope: −1.17
Pearson r: −0.47
p−value: 0.066

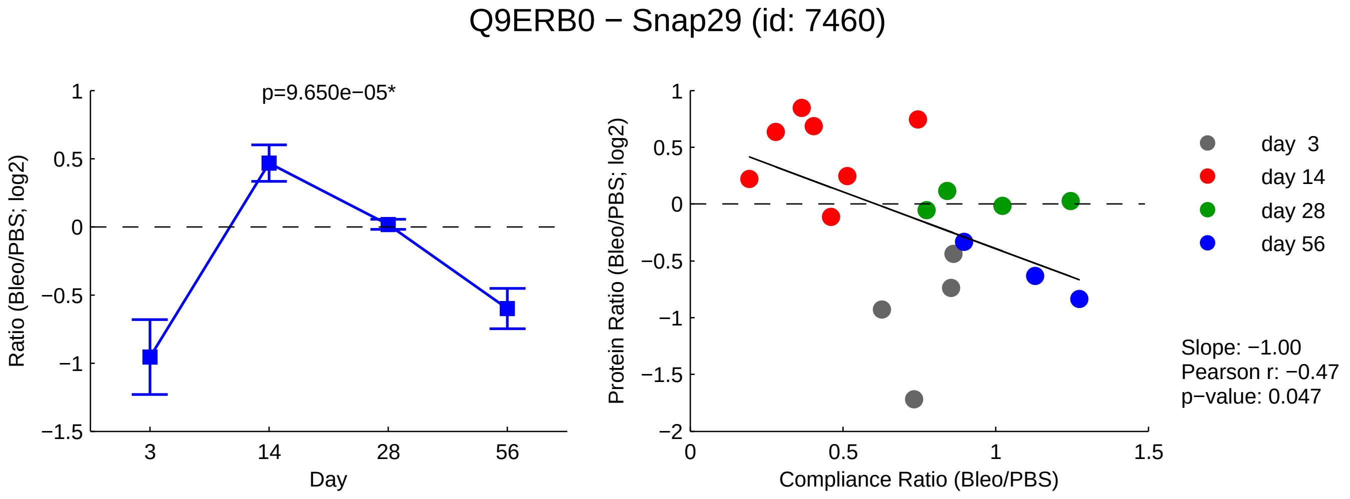
Q9ERB0 − Snap29 (id: 7460)
p=9.650e−05*
Ratio (Bleo/PBS; log2)
Day
Protein Ratio (Bleo/PBS; log2)
Compliance Ratio (Bleo/PBS)
day 3
day 14
day 28
day 56
Slope: −1.00
Pearson r: −0.47
p−value: 0.047

Q9ERD7 − Tubb3 (id: 7461)

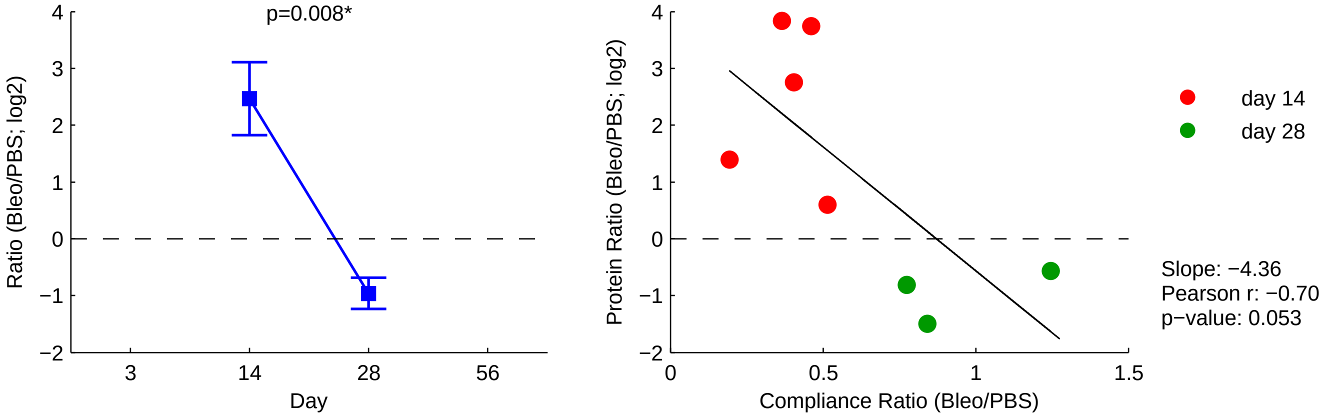

Q9ERE7 − Mesdc2 (id: 7462)

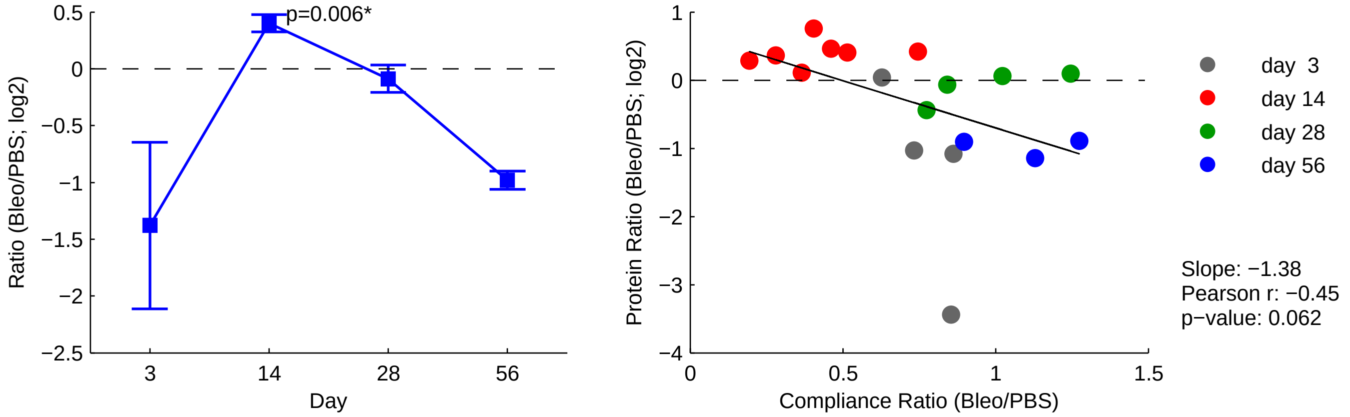

Q9ERG0 − Lima1 (id: 7465)

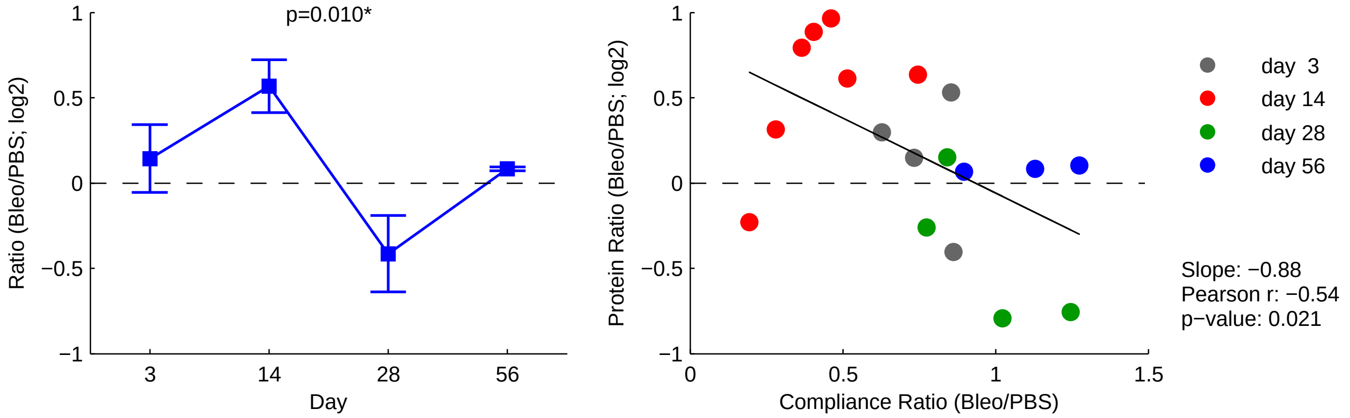

Q9ERI6 − Rdh14 (id: 7467)

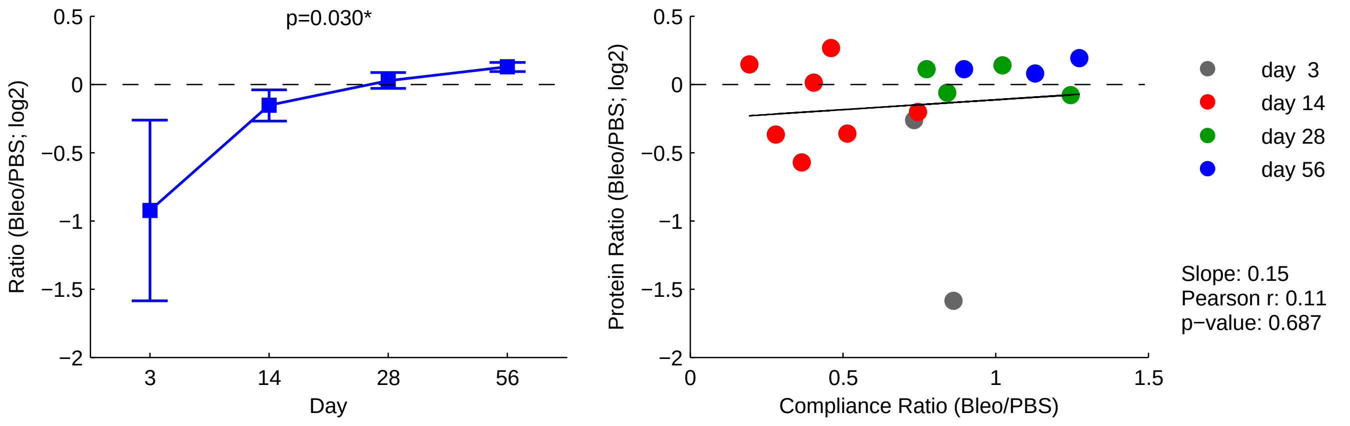

Q9ERK4 − Cse1l (id: 7468)

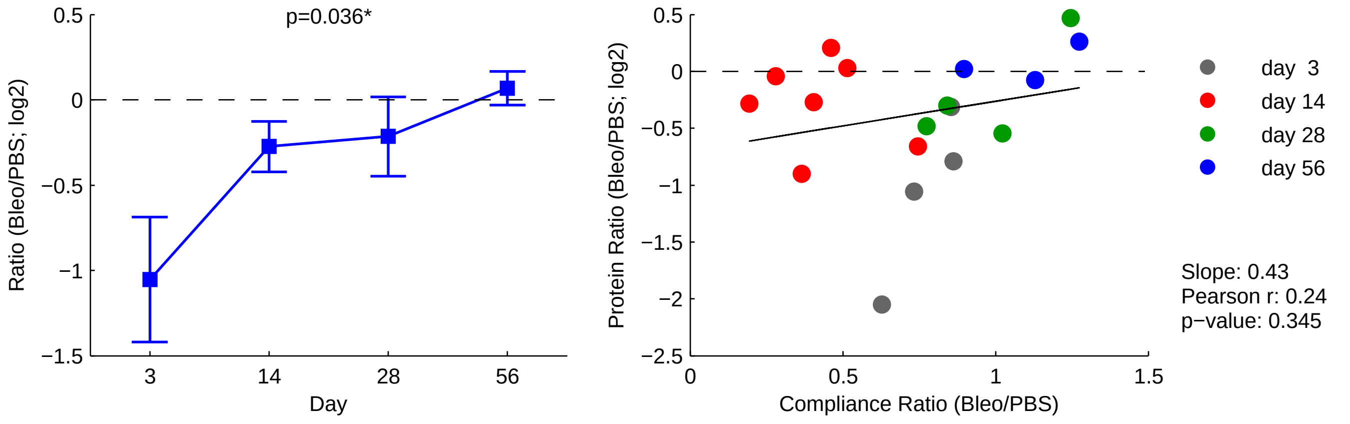

Q9ERL7 − Gmfg (id: 7469)

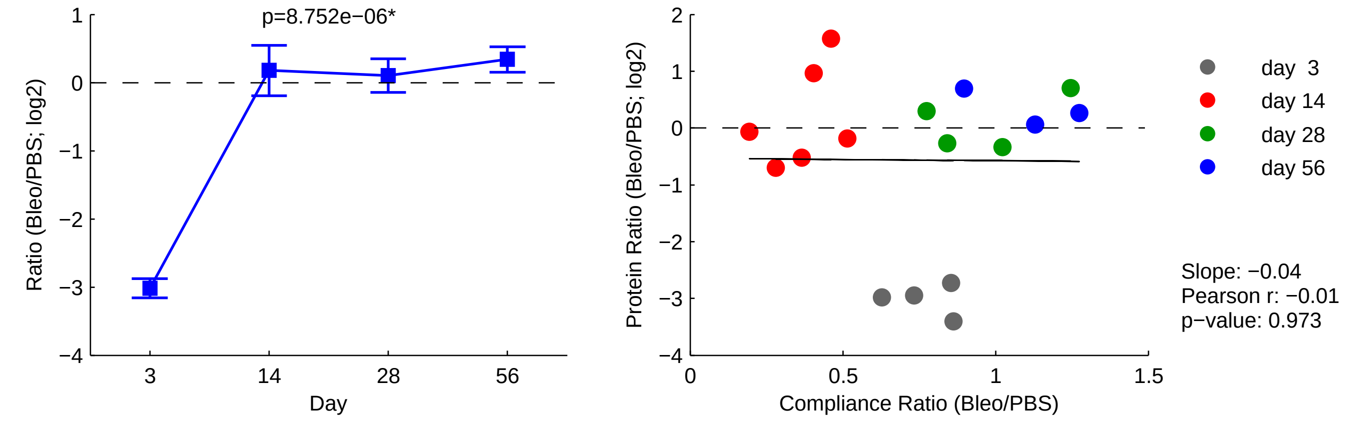

Q9ERN0 − Scamp2 (id: 7471)

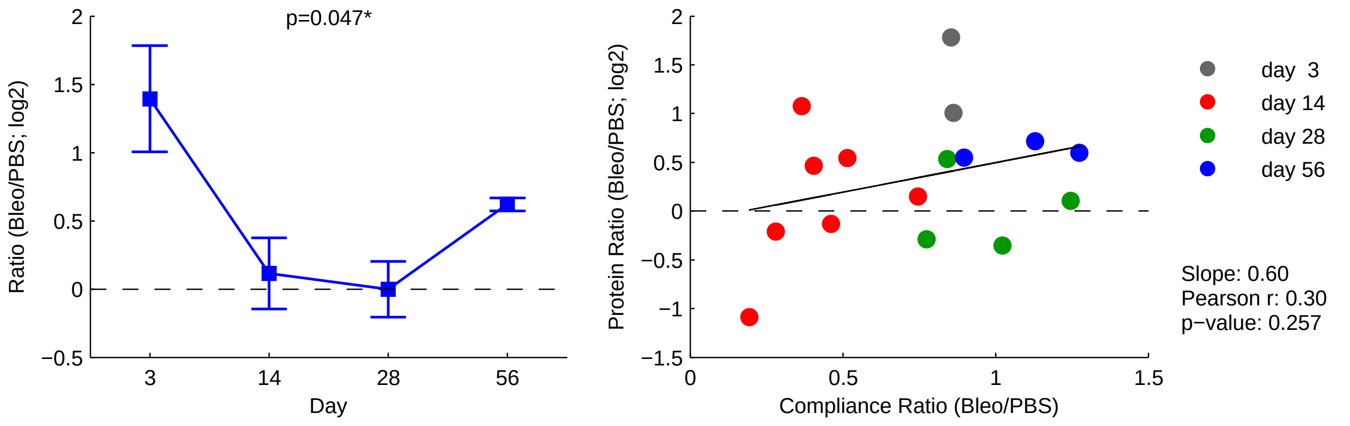

Q9ERR7 − Sep15 (id: 7473)

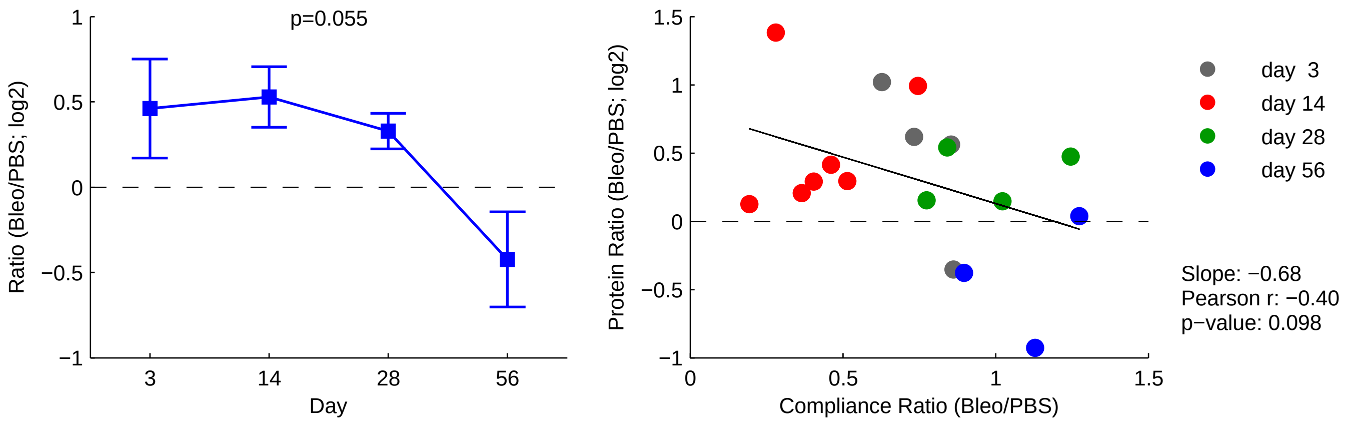

Q9ERS2 − Ndufa13 (id: 7474)

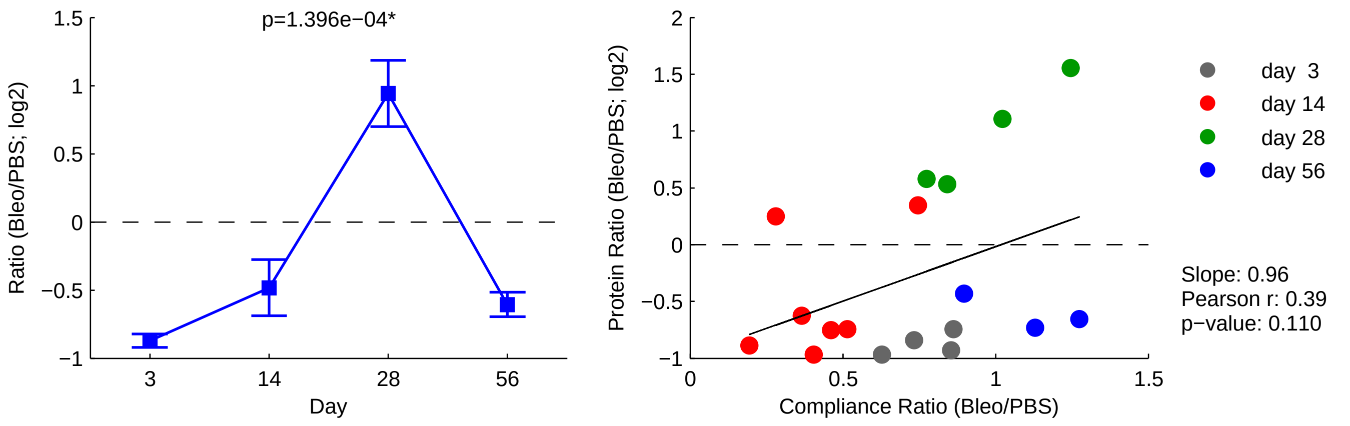

Q9ERU3 − Znf22 (id: 7475)

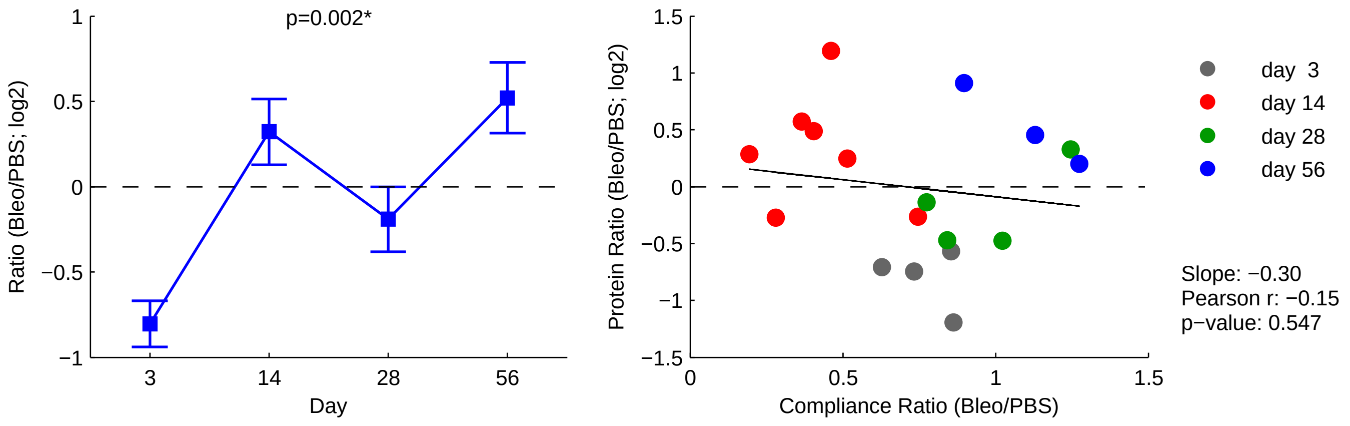

Q9ERU9 − Ranbp2 (id: 7476)

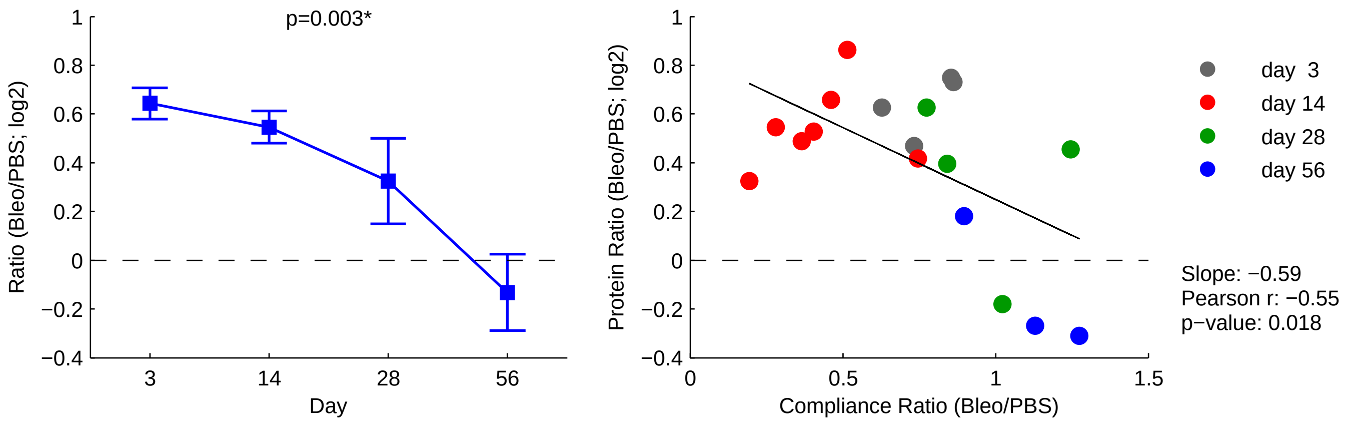

Q9ES28−3 − (id: 7480)

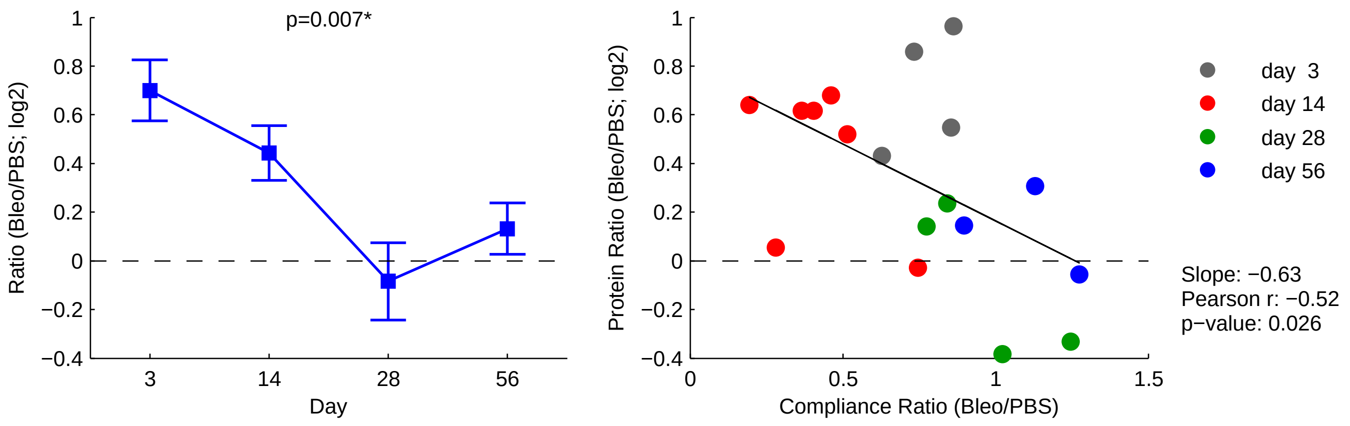

Q9ES74 − Nek7 (id: 7485)

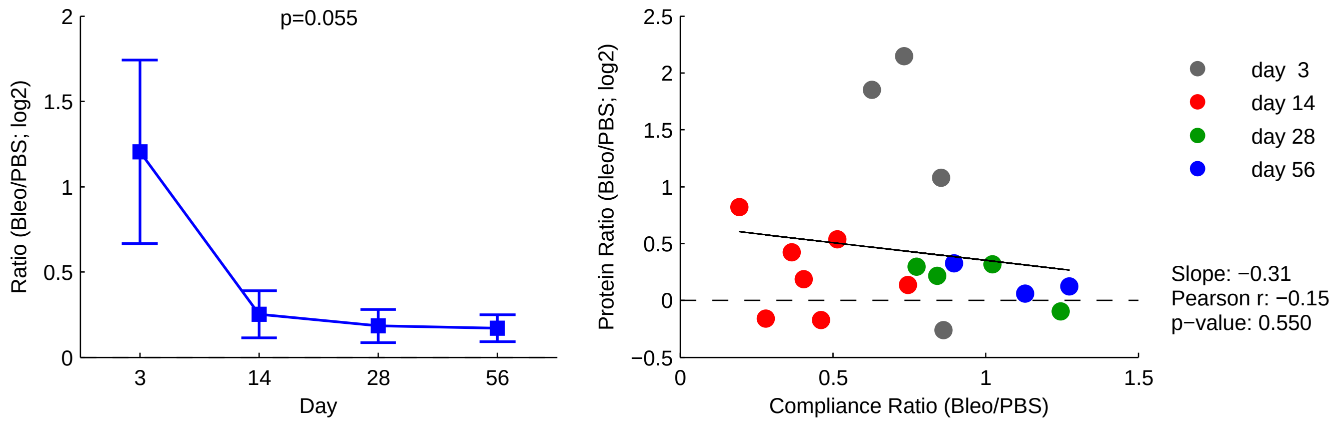

Q9ES97−3 − Rtn3 (id: 7486)

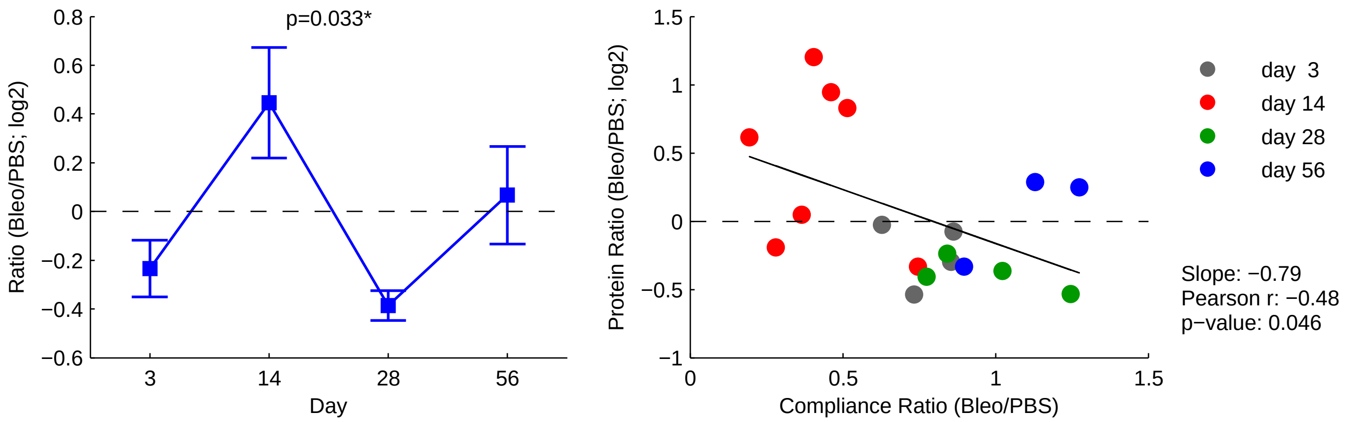

Q9ESB3 − Hrg (id: 7487)

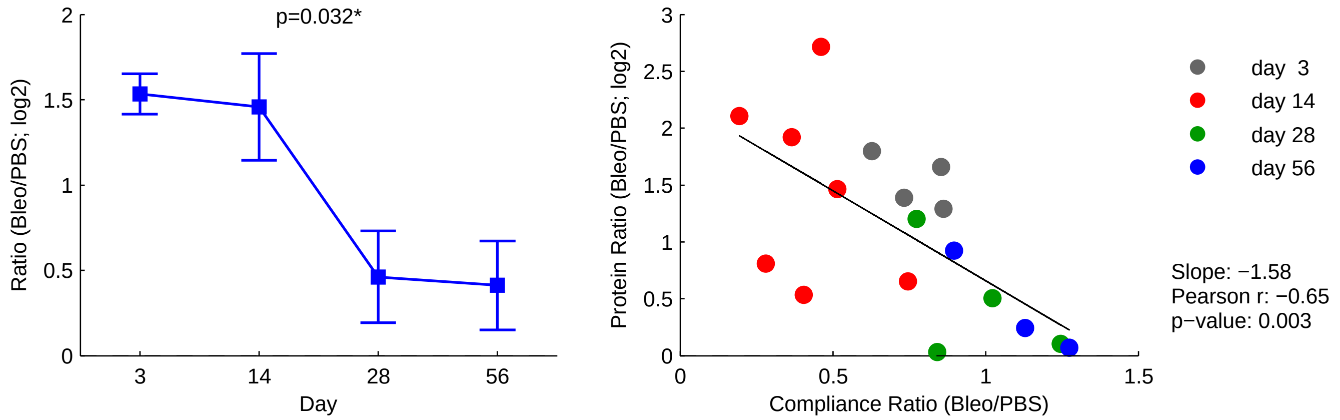

Q9ESD7−2 − Dysf (id: 7490)

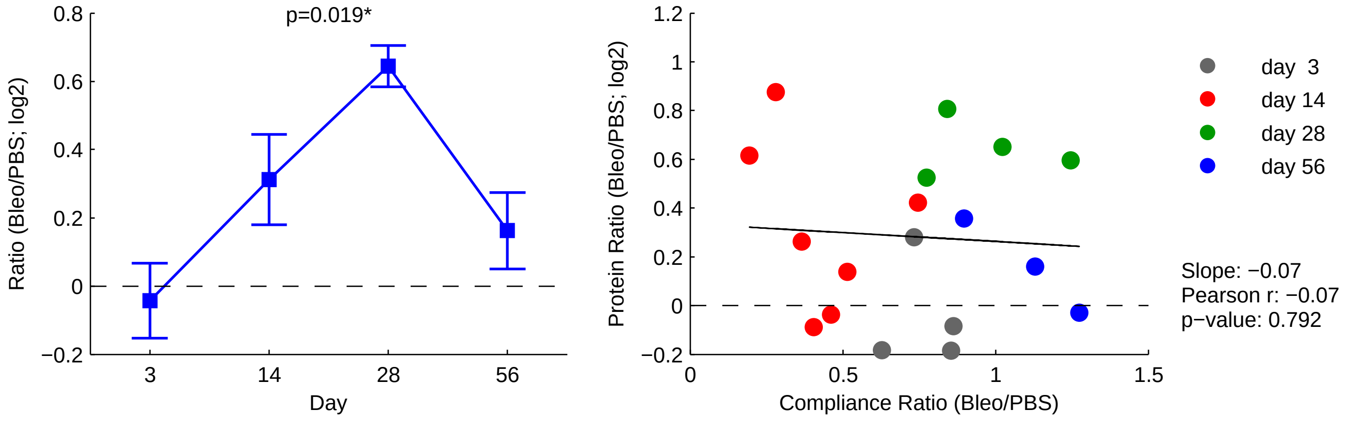

Q9ESN6 − Trim2 (id: 7495)

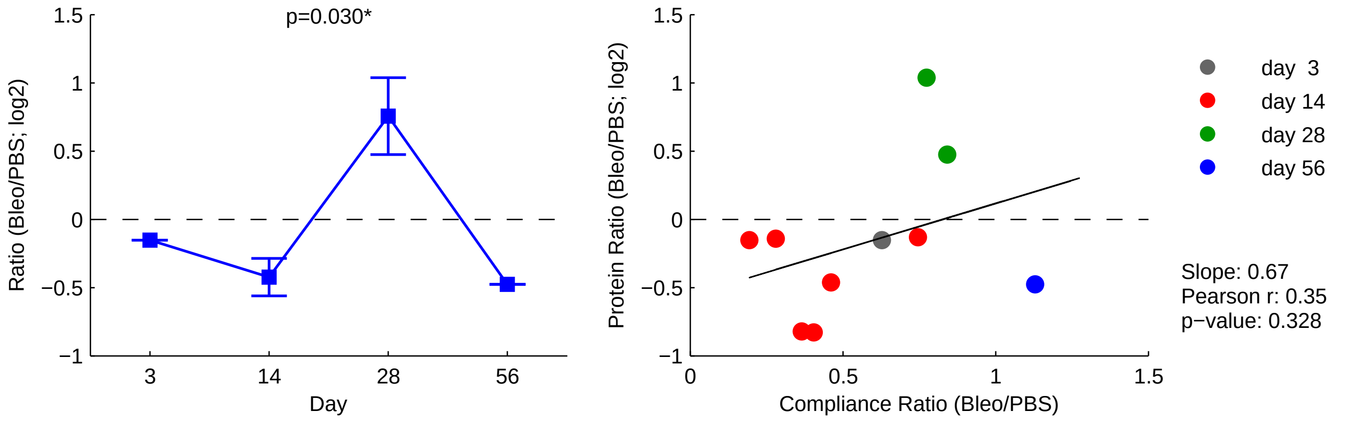

Q9ESP1 − Sdf2l1 (id: 7496)

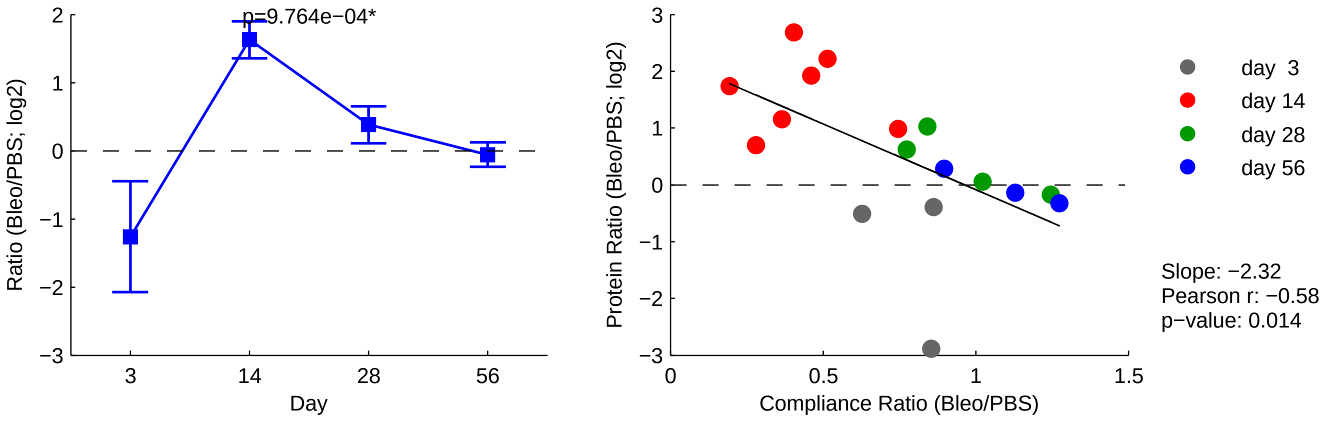

Q9ESW4 − Agk (id: 7500)

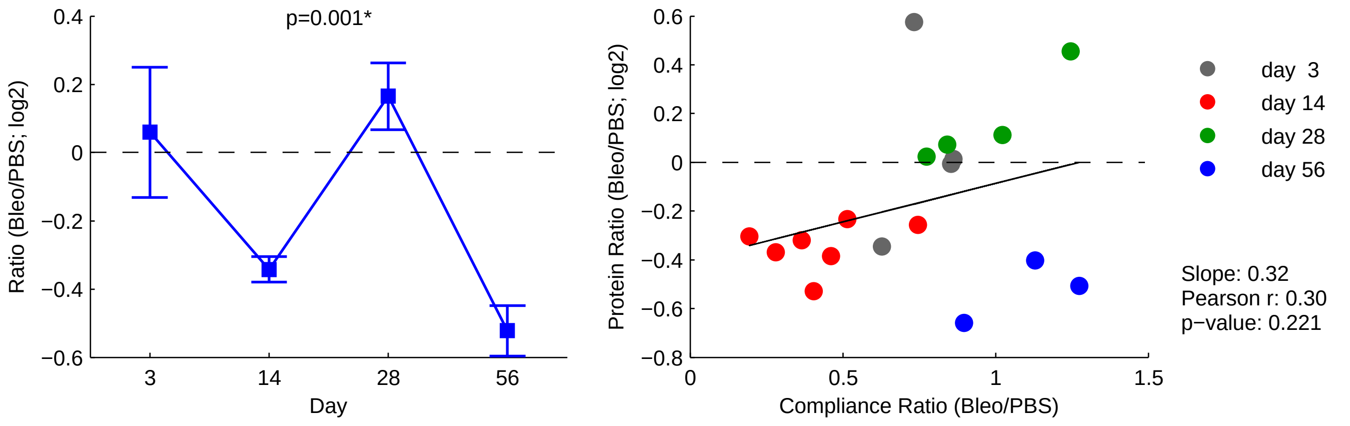

Q9ET22 − Dpp7 (id: 7504)

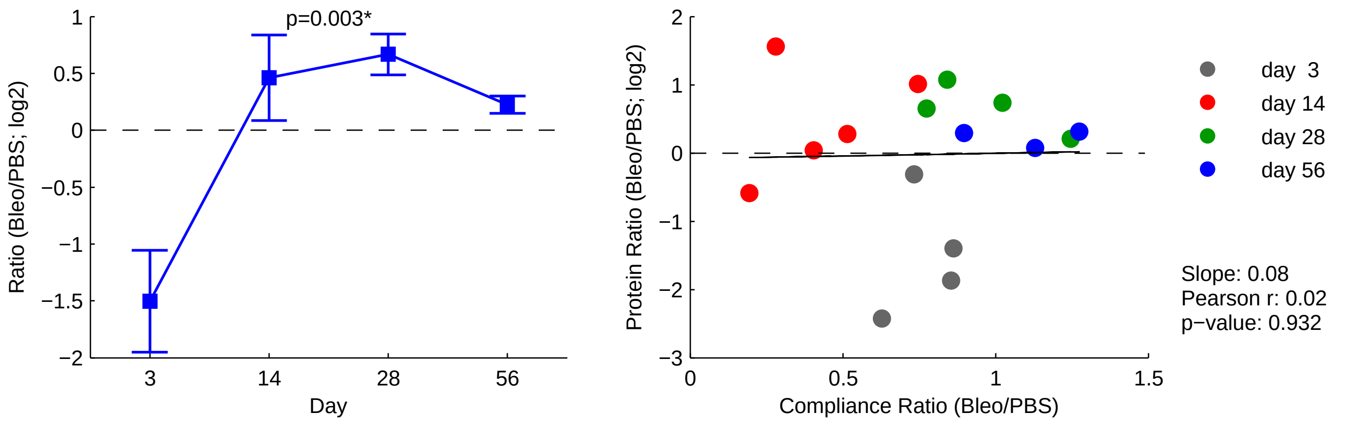

Q9ET30 − Tm9sf3 (id: 7506)

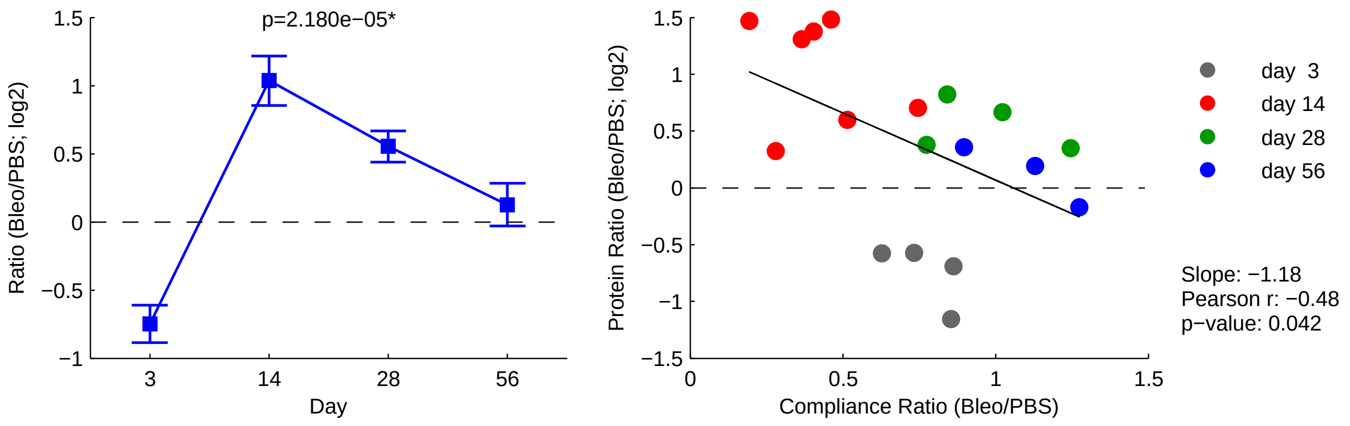

Q9ET54−3 − Palld (id: 7507)

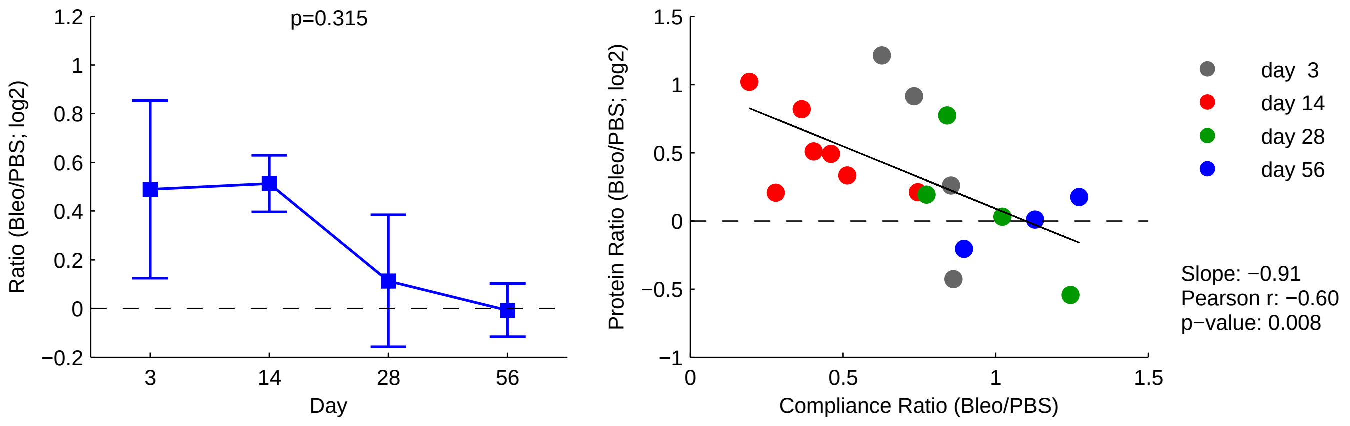

Q9ET78 − Jph2 (id: 7508)

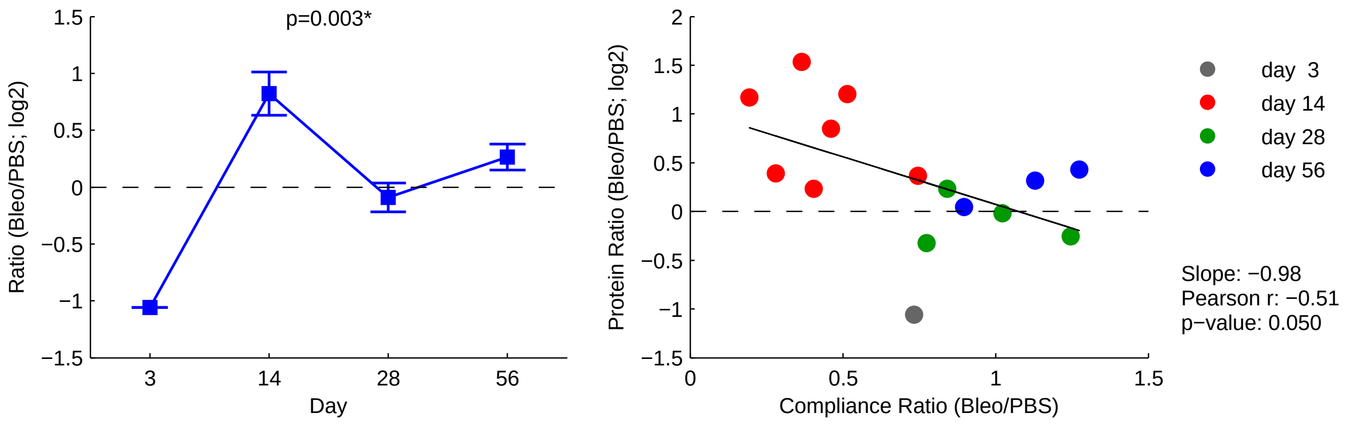

Q9JHC9−3 − Elf2 (id: 7511)

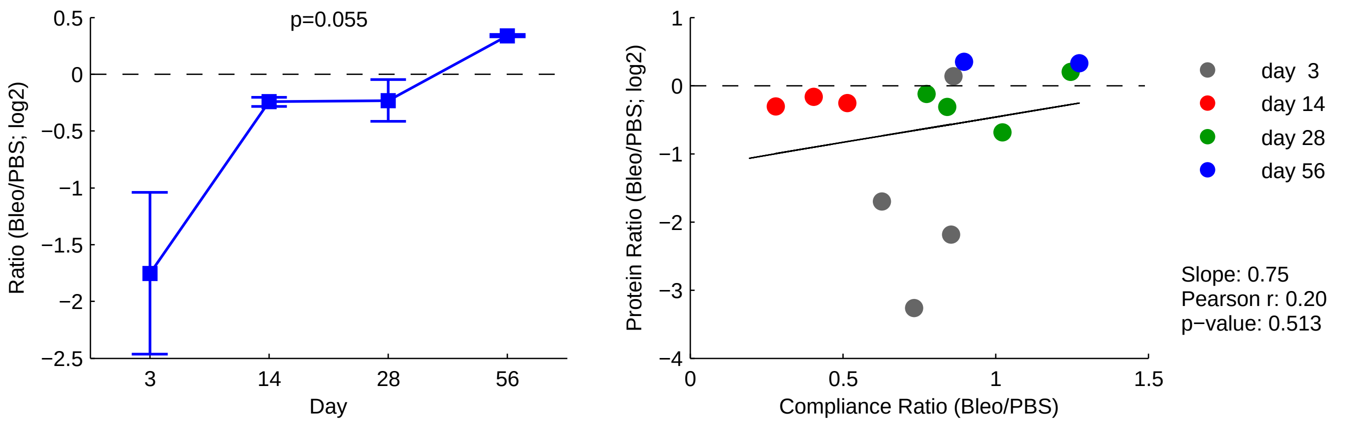

Q9JHH9 − Copz2 (id: 7516)

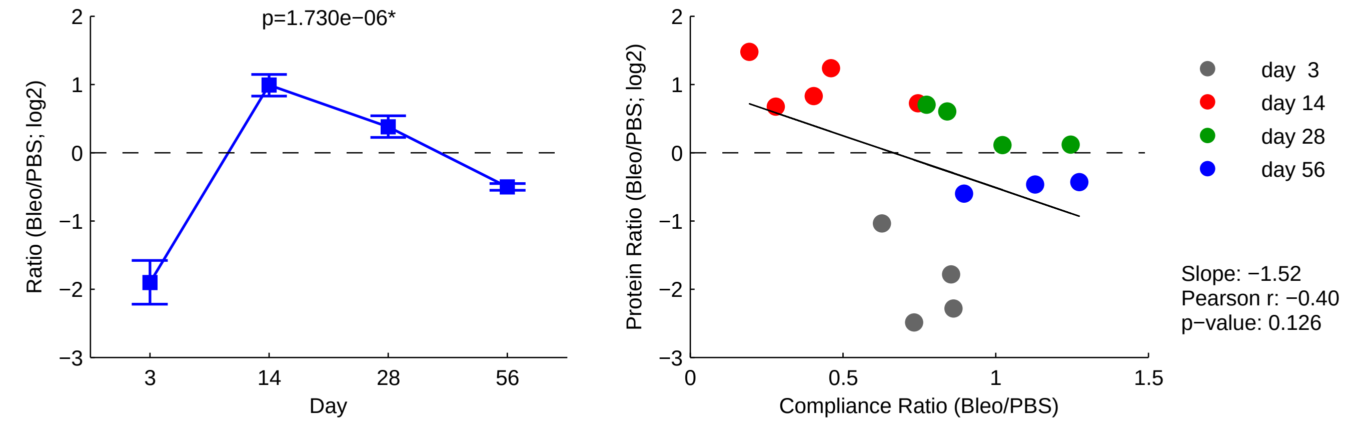

Q9JHI5 − Ivd (id: 7518)

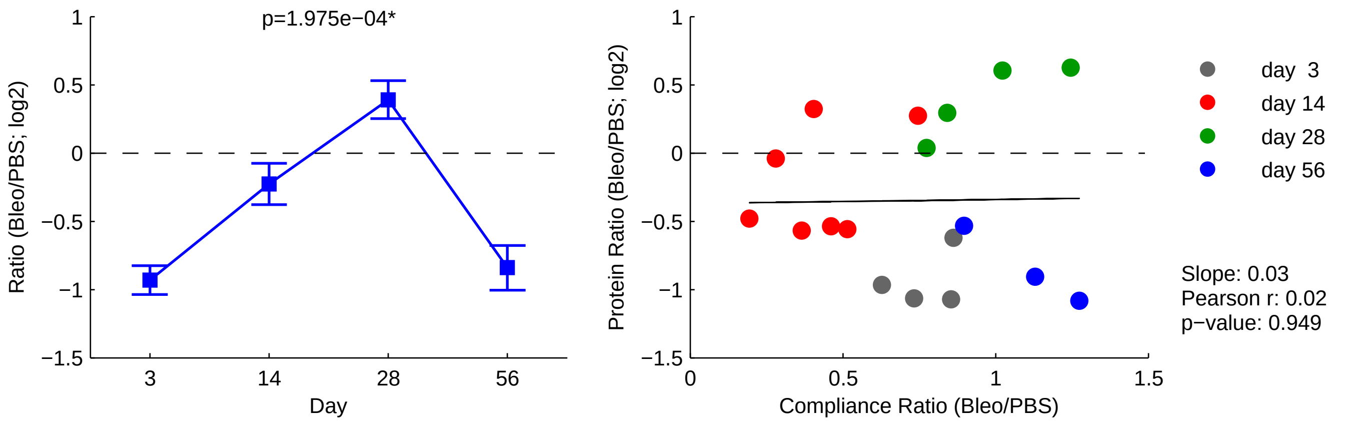

Q9JHL1 − Slc9a3r2 (id: 7521)

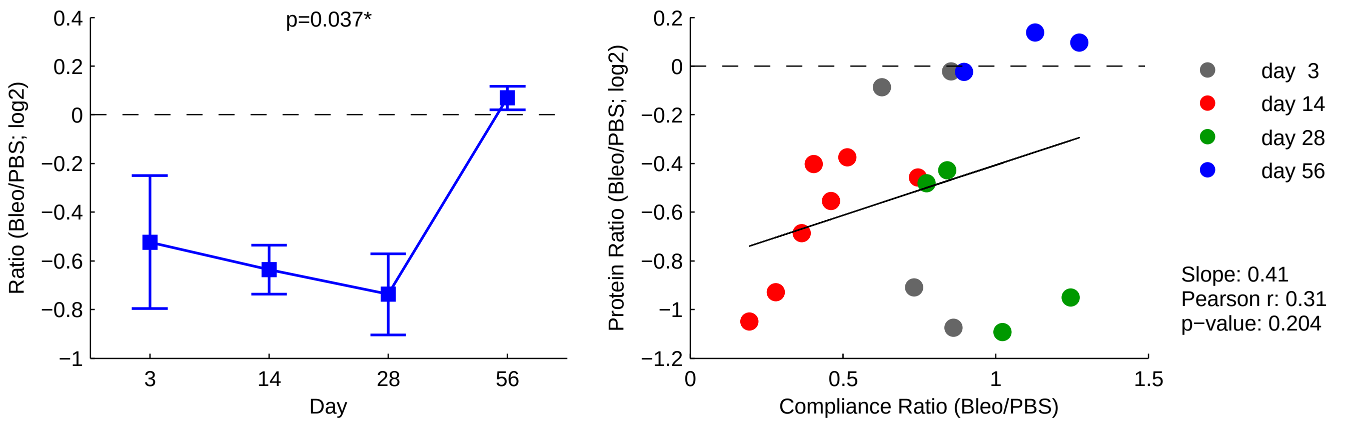

Q9JHP7 − Kdelc1 (id: 7524)

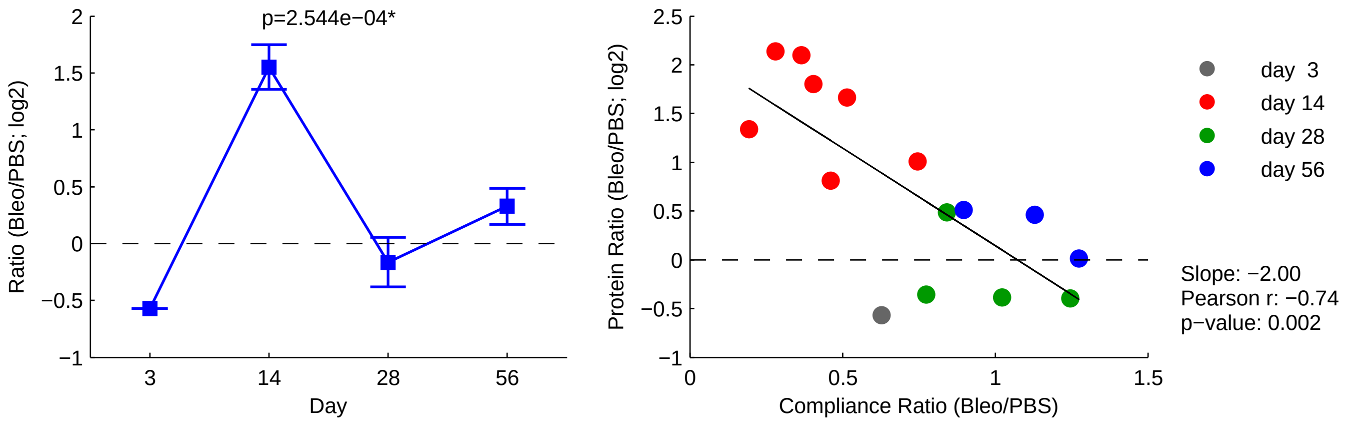

Q9JHQ0 − Anxa9 (id: 7525)

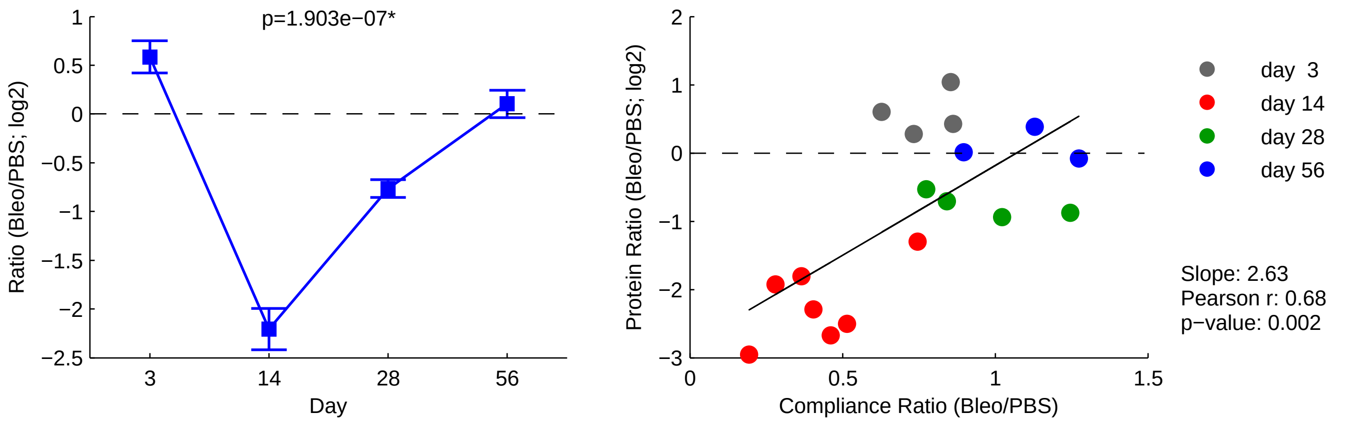

Q9JHQ5 − Lztfl1 (id: 7526)

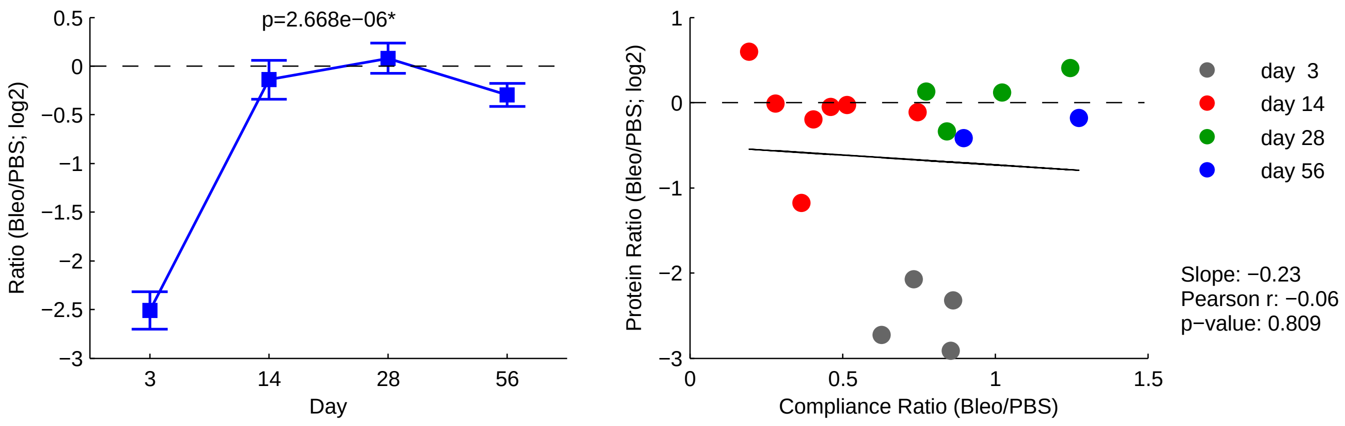

Q9JHS3 − Lamtor2 (id: 7527)

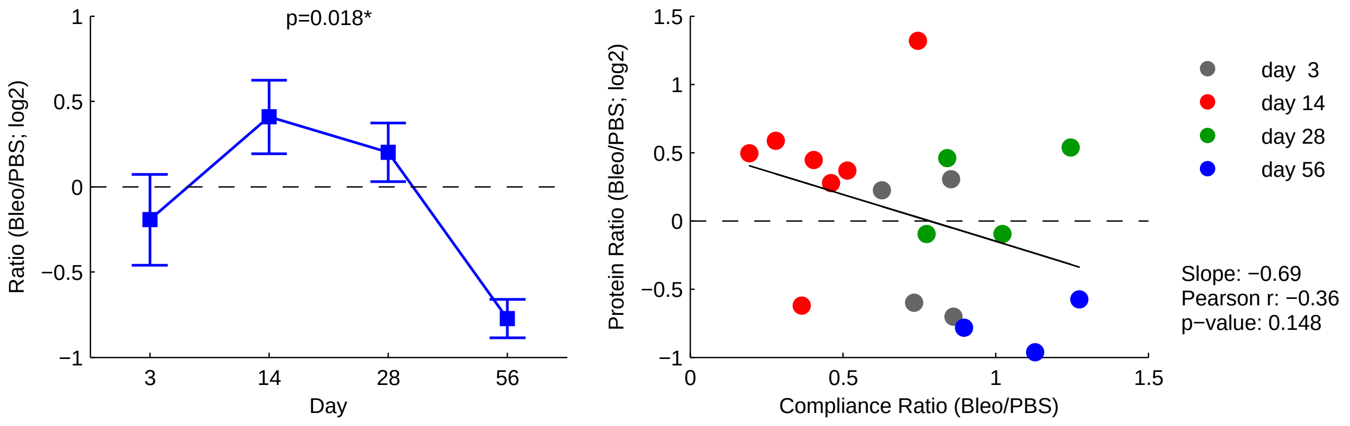

Q9JHS9 − Cwc15 (id: 7528)

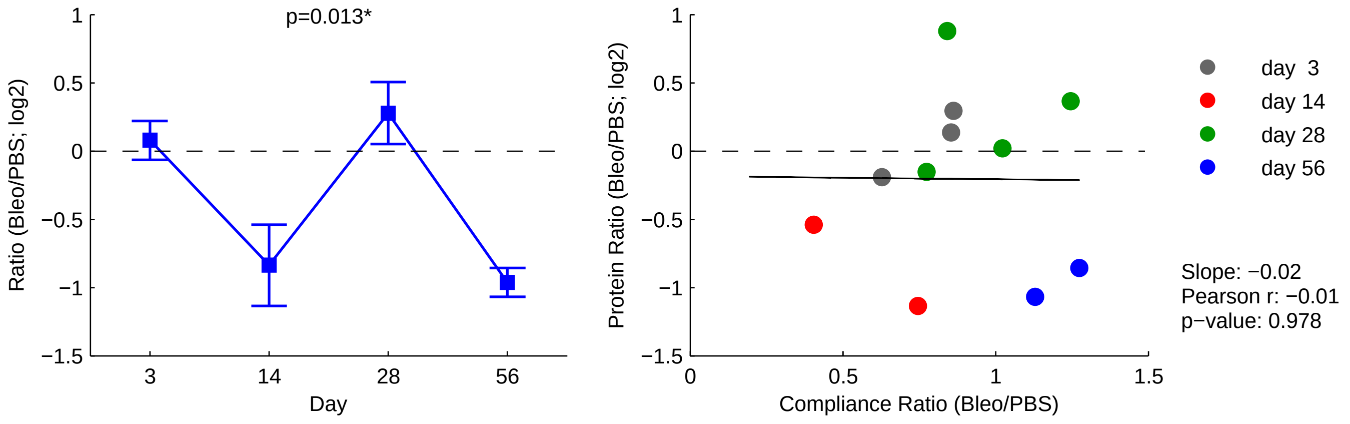

Q9JHU2 − Palmd (id: 7529)

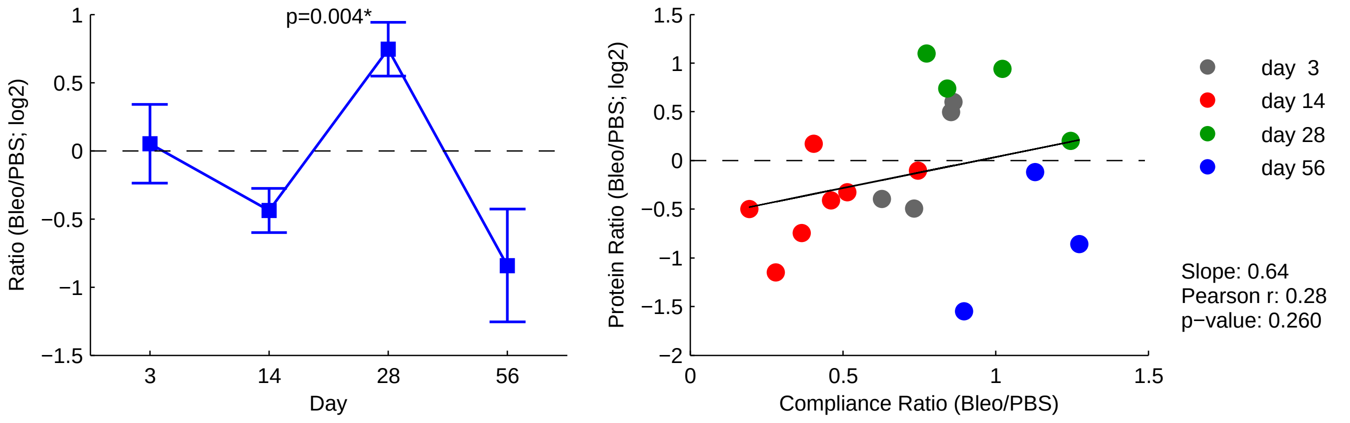

Q9JHW2 − Nit2 (id: 7532)

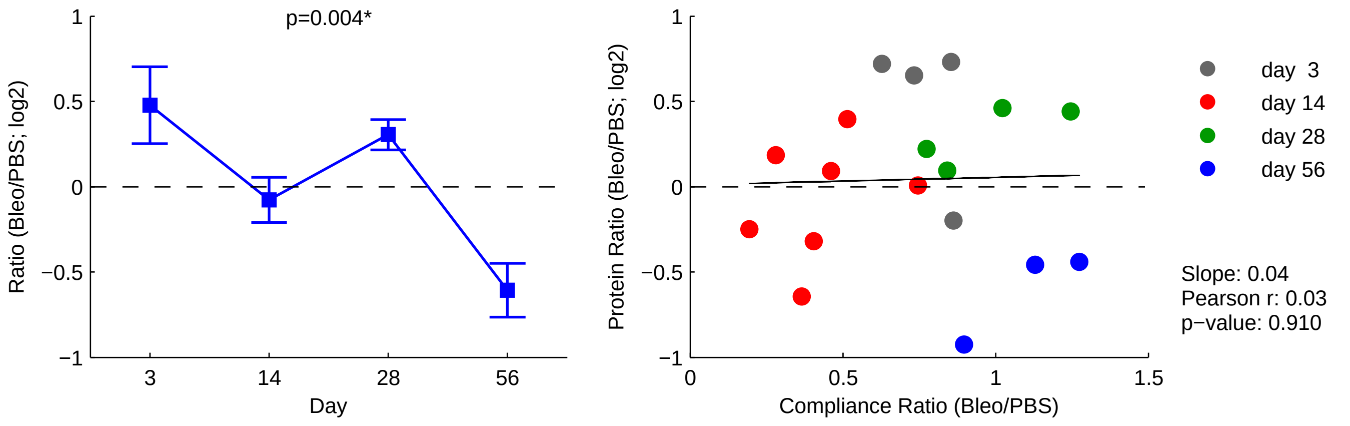

Q9JI10 − Stk3 (id: 7536)

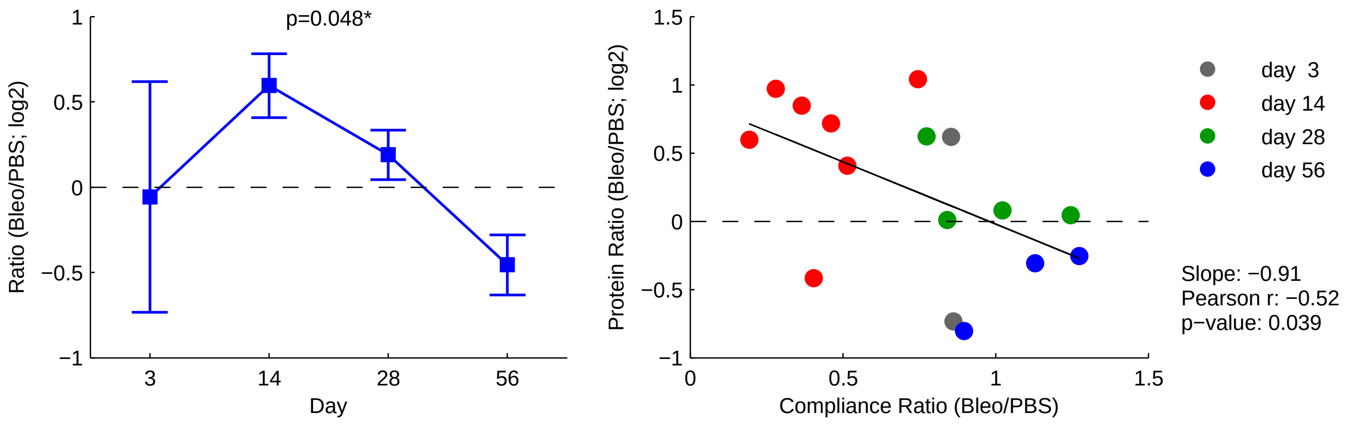

Q9JI11 − Stk4 (id: 7537)

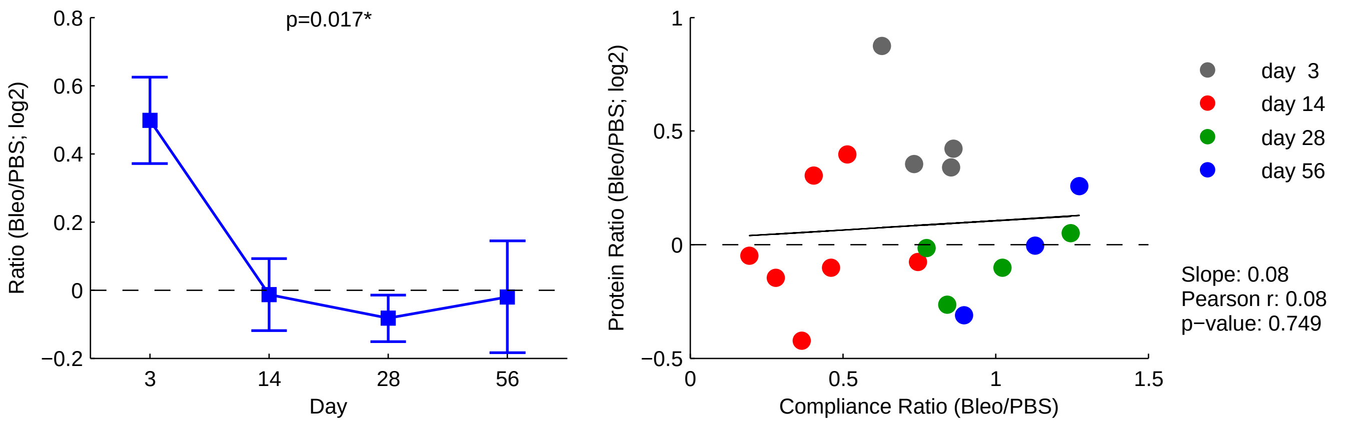

Q9JI44 − Dmap1 (id: 7541)

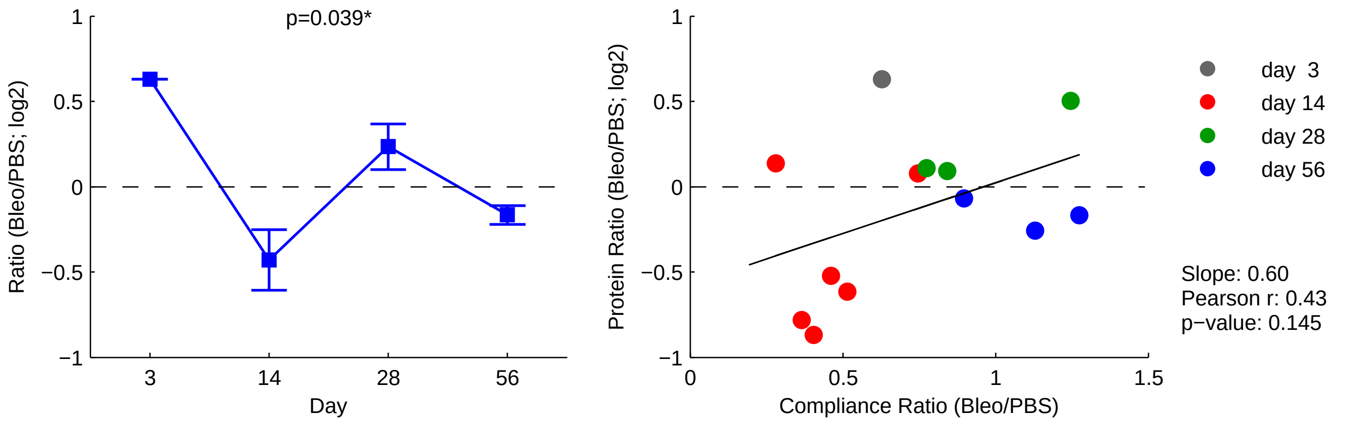

Q9JI59 − Jam2 (id: 7543)

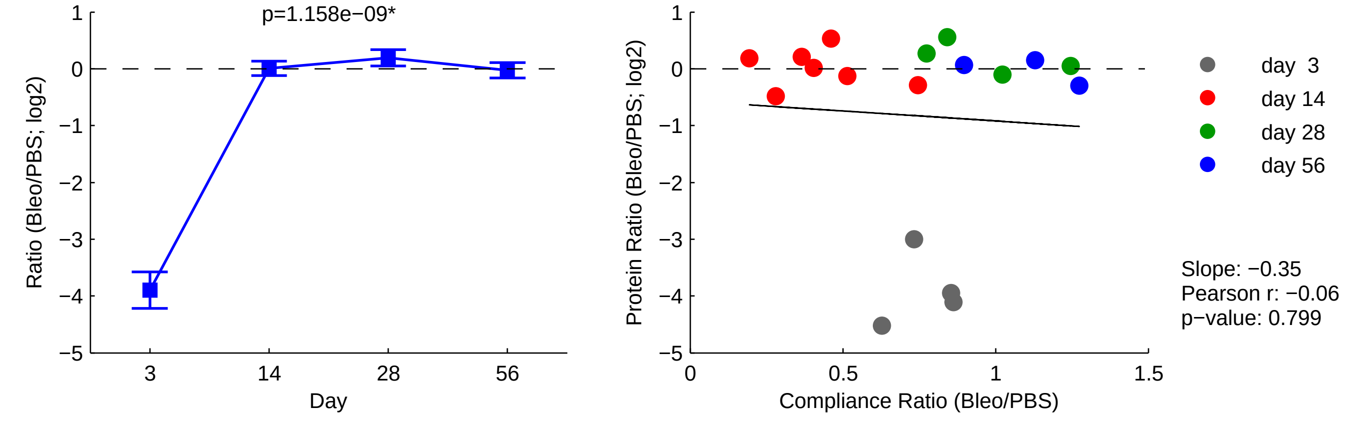

Q9JI60 − Lrat (id: 7544)

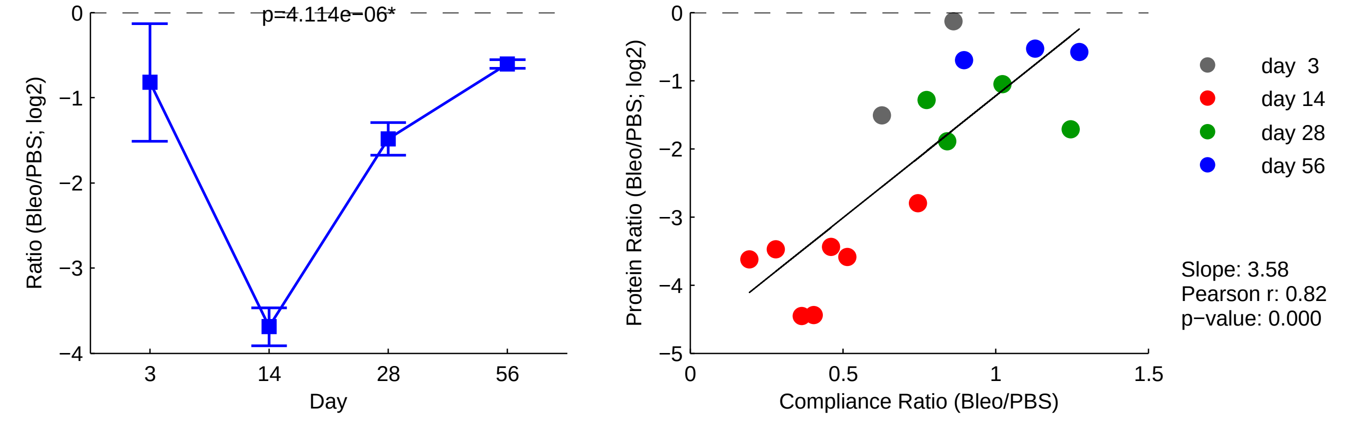

Q9JI75 − Nqo2 (id: 7545)

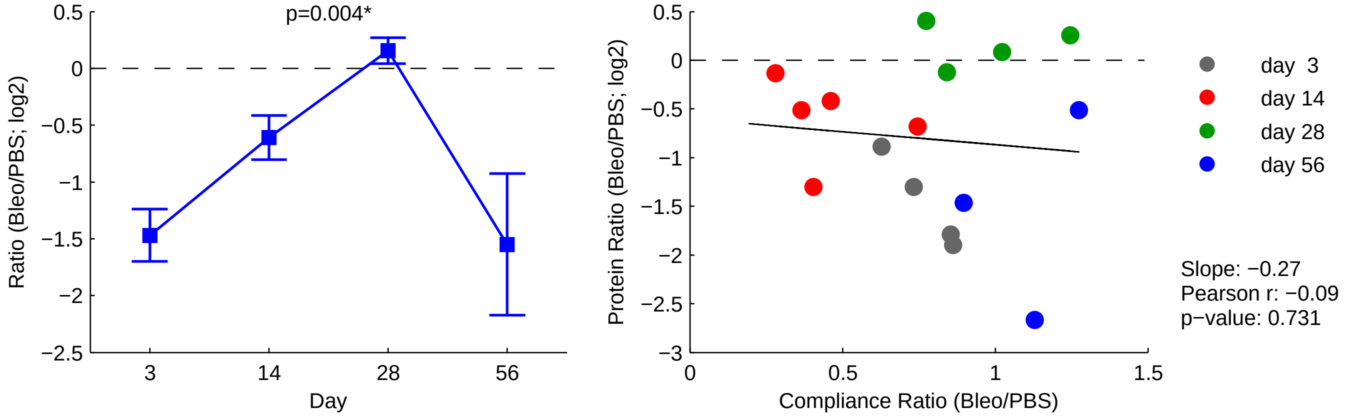

Q9JI91 − Actn2 (id: 7548)

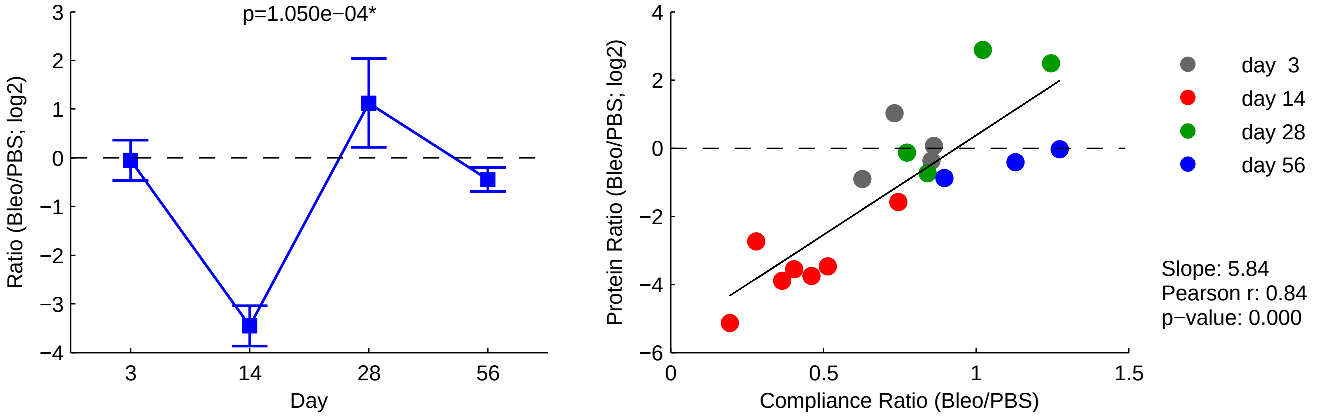

Q9JIF7 − Copb1 (id: 7552)

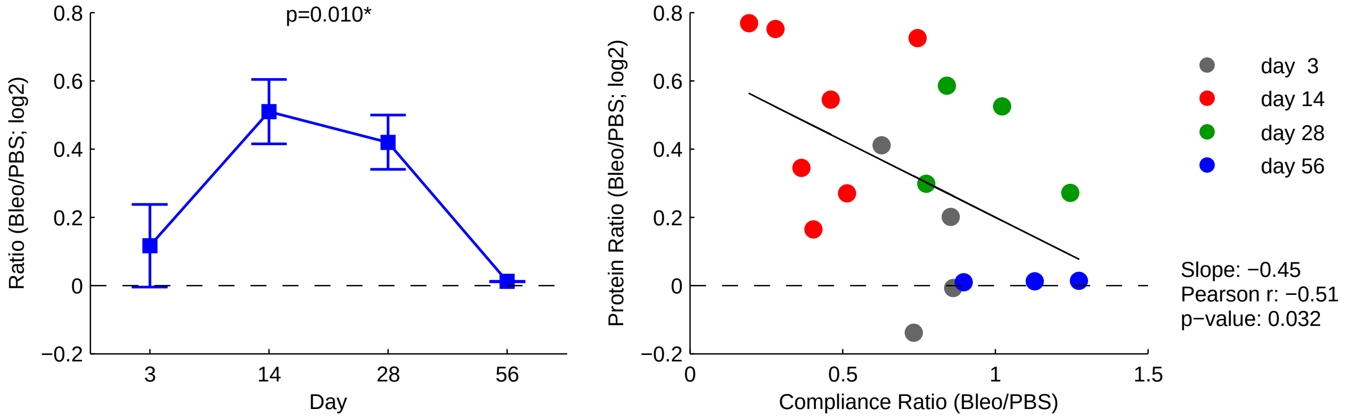

Q9JIF9 − Myot (id: 7553)

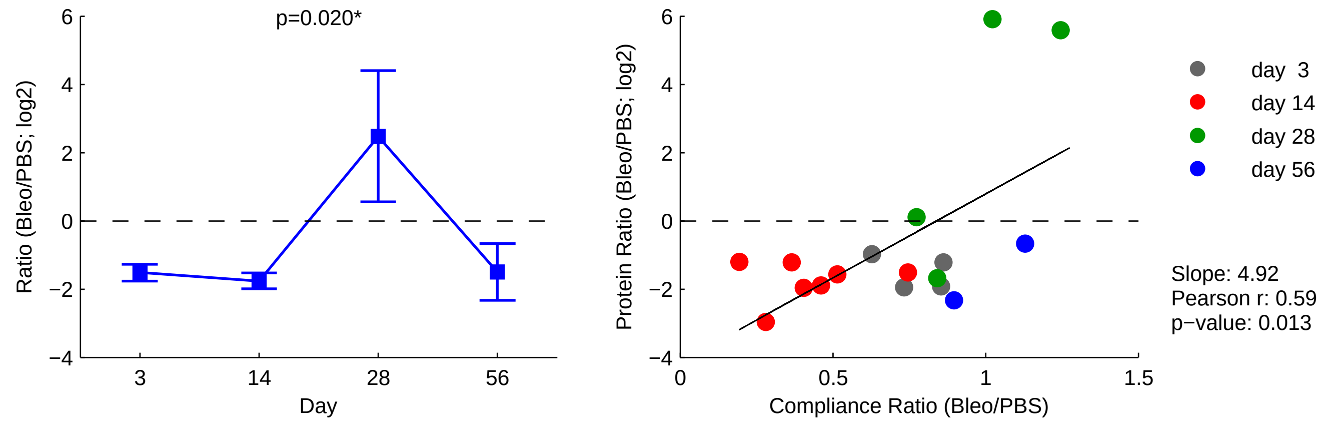

Q9JIG7 − Ccdc22 (id: 7554)

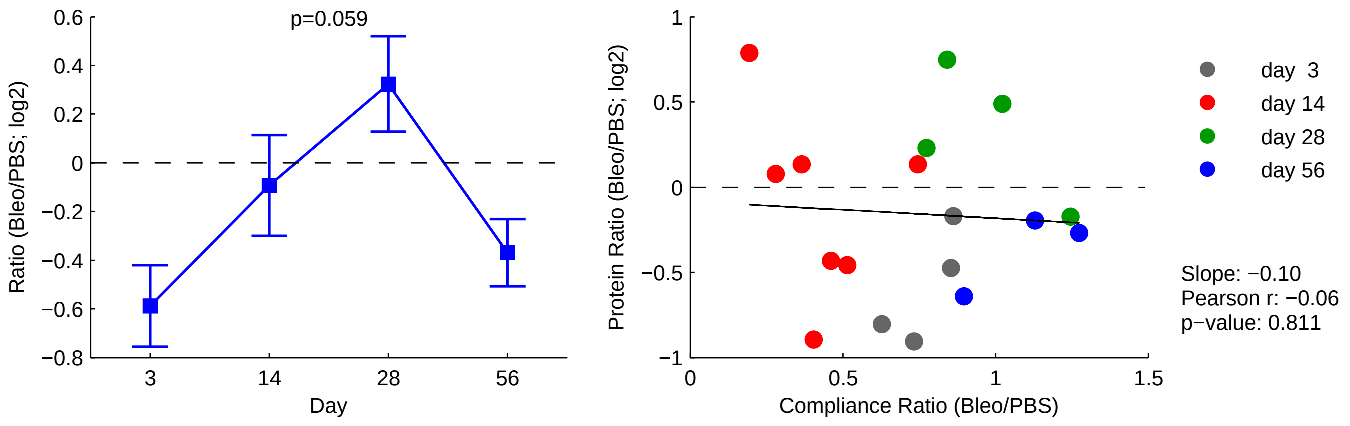

Q9JIG8 − Praf2 (id: 7555)

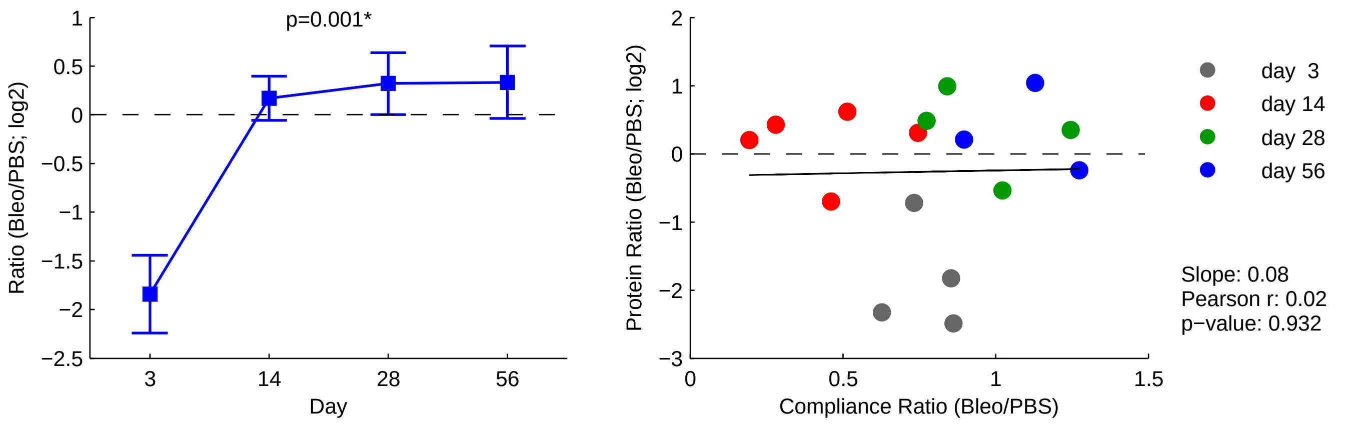

Q9JII6 − Akr1a1 (id: 7557)

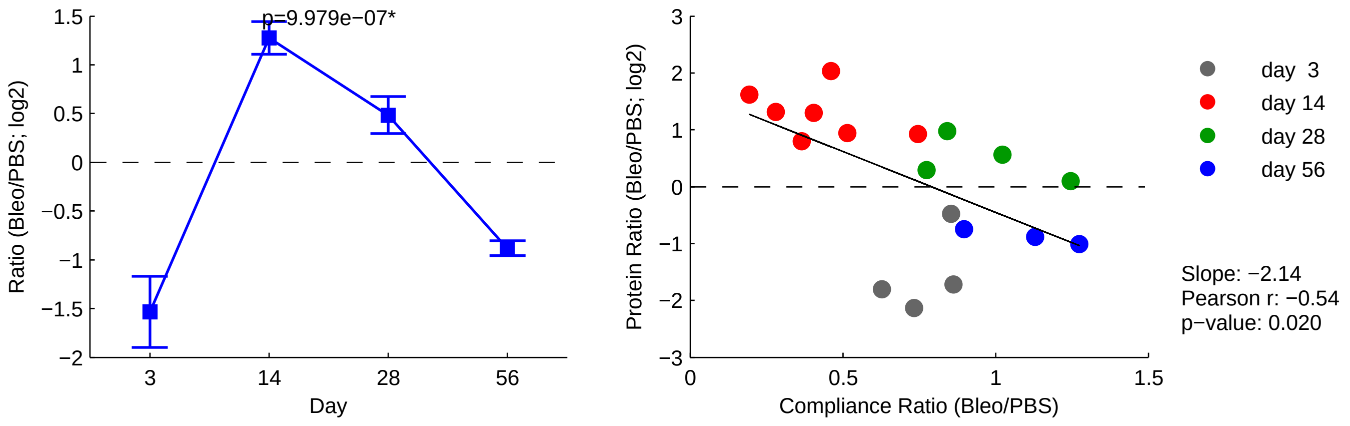

Q9JIQ3 − Diablo (id: 7560)

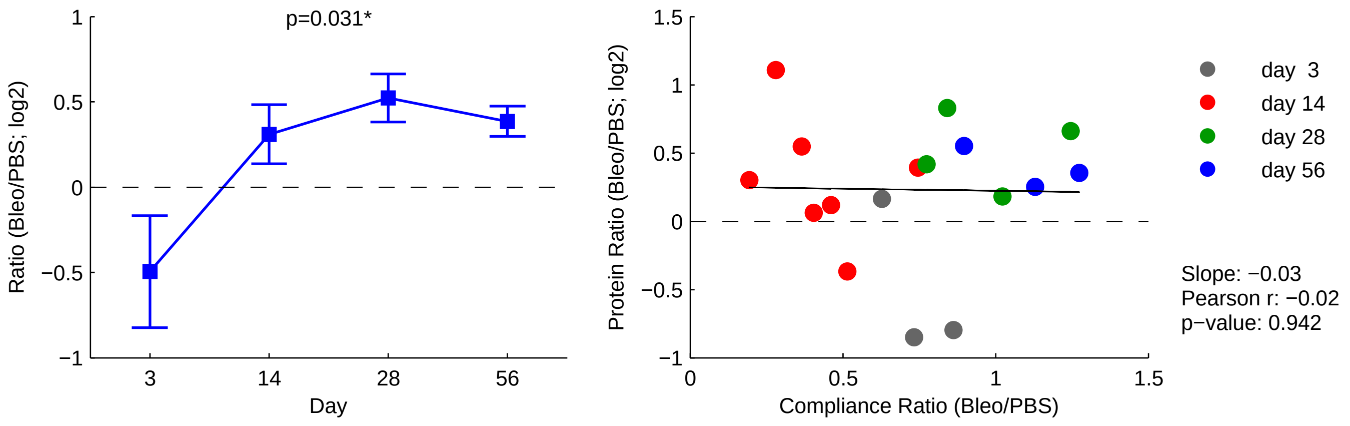

Q9JIW9 − Ralb (id: 7562)

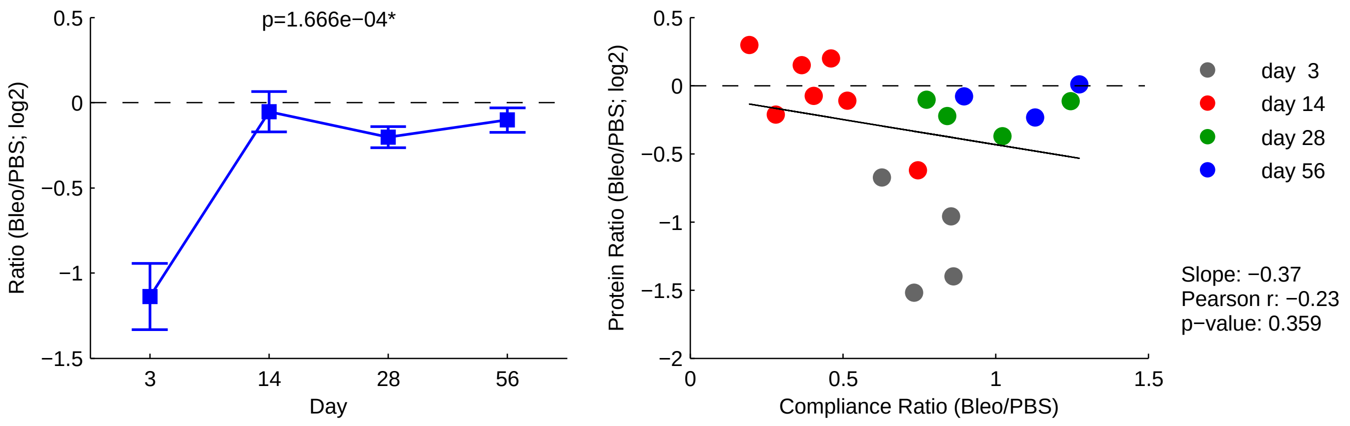

Q9JIY0 − Plekho1 (id: 7565)

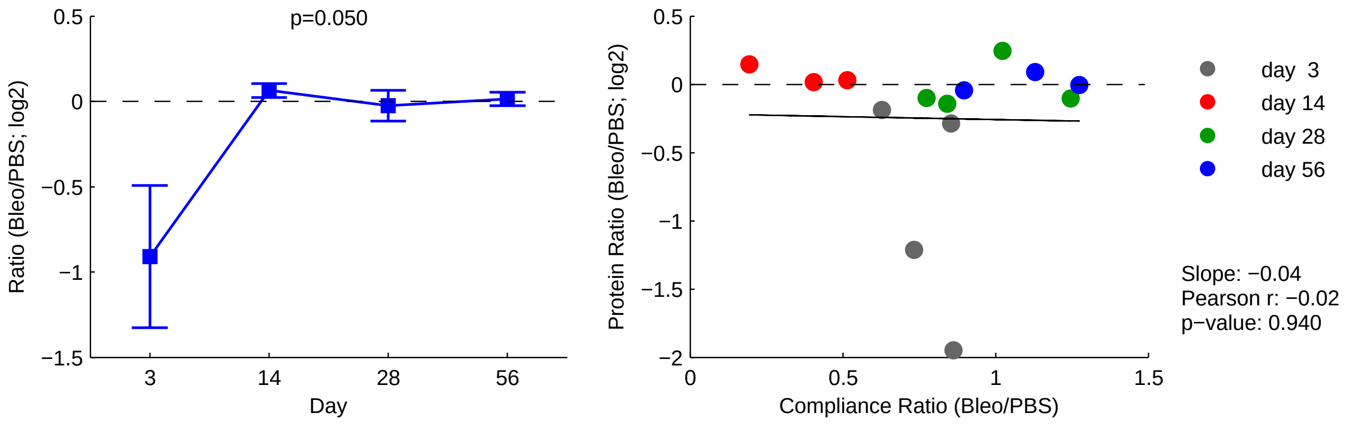

Q9JJ00 − Plscr1 (id: 7569)

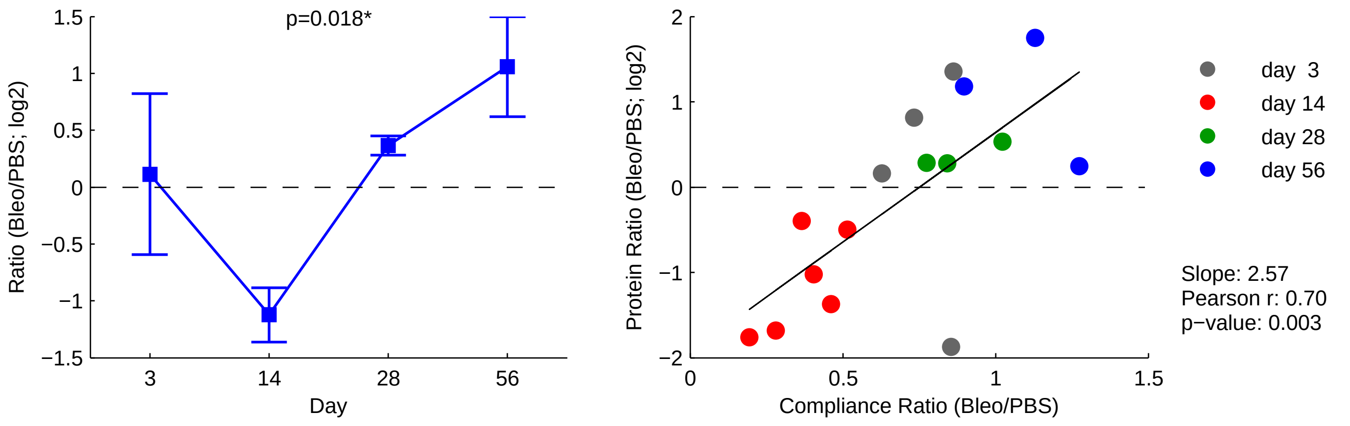

Q9JJ06 − C1galt1 (id: 7570)

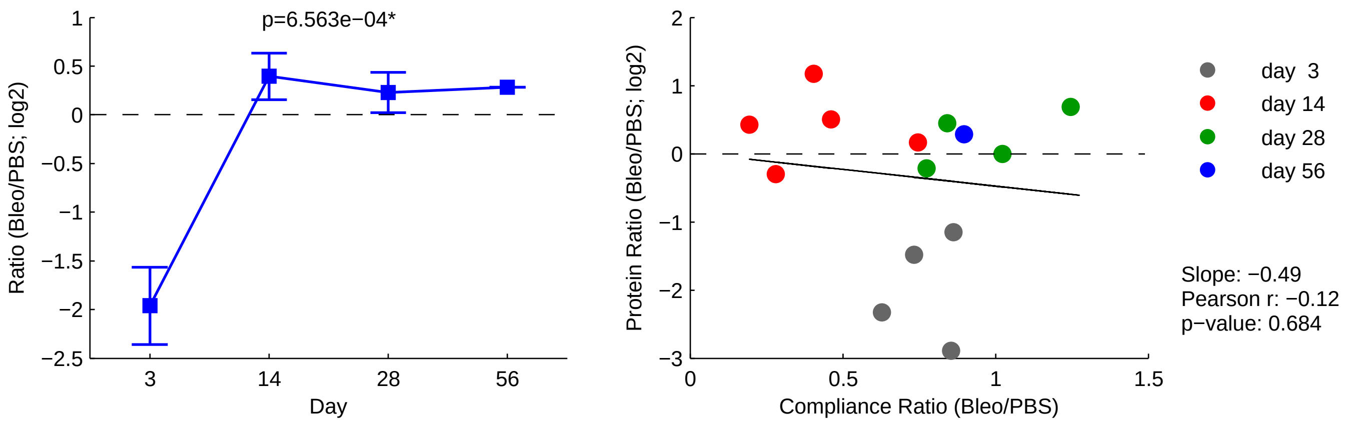

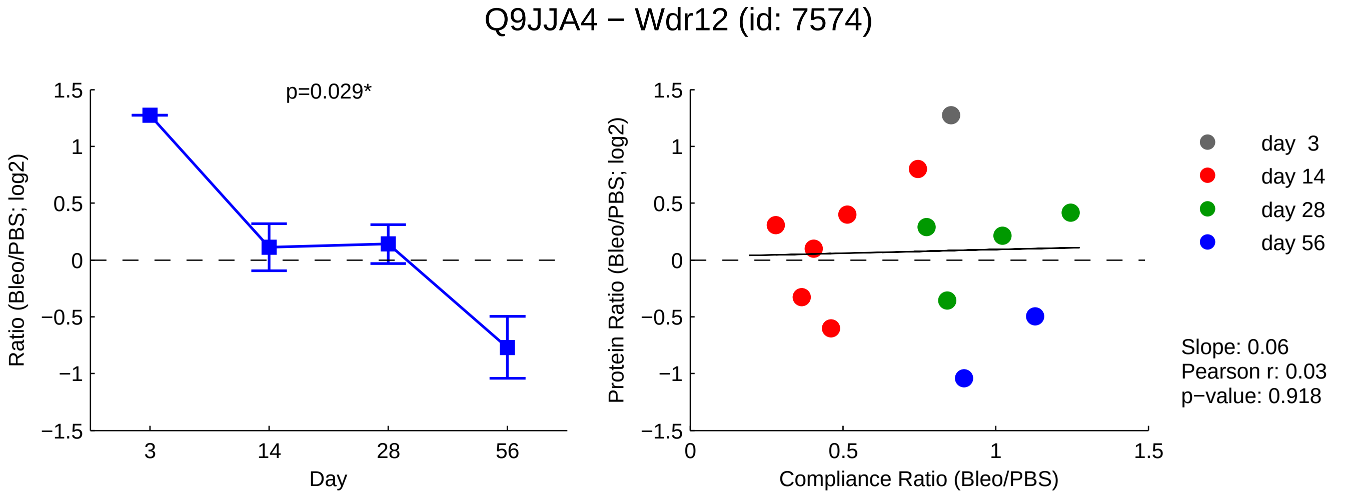
Q9JJA4 − Wdr12 (id: 7574)
p=0.029*
Ratio (Bleo/PBS; log2)
Day
Protein Ratio (Bleo/PBS; log2)
Compliance Ratio (Bleo/PBS)
day 3
day 14
day 28
day 56
Slope: 0.06
Pearson r: 0.03
p−value: 0.918

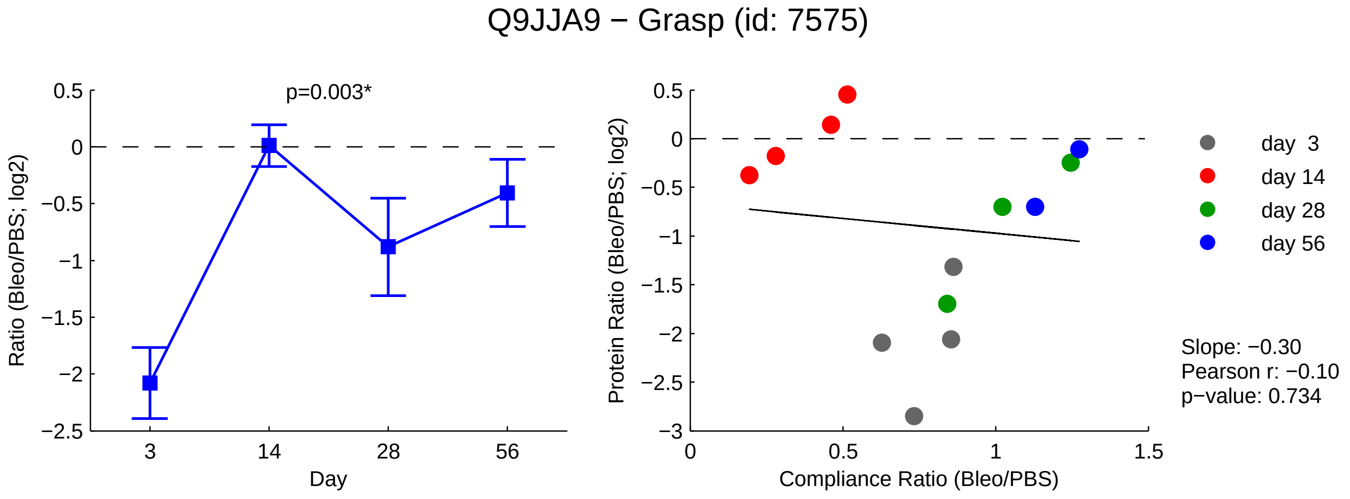
Q9JJA9 − Grasp (id: 7575)
p=0.003*
Ratio (Bleo/PBS; log2)
Day
Protein Ratio (Bleo/PBS; log2)
Compliance Ratio (Bleo/PBS)
day 3
day 14
day 28
day 56
Slope: −0.30
Pearson r: −0.10
p−value: 0.734

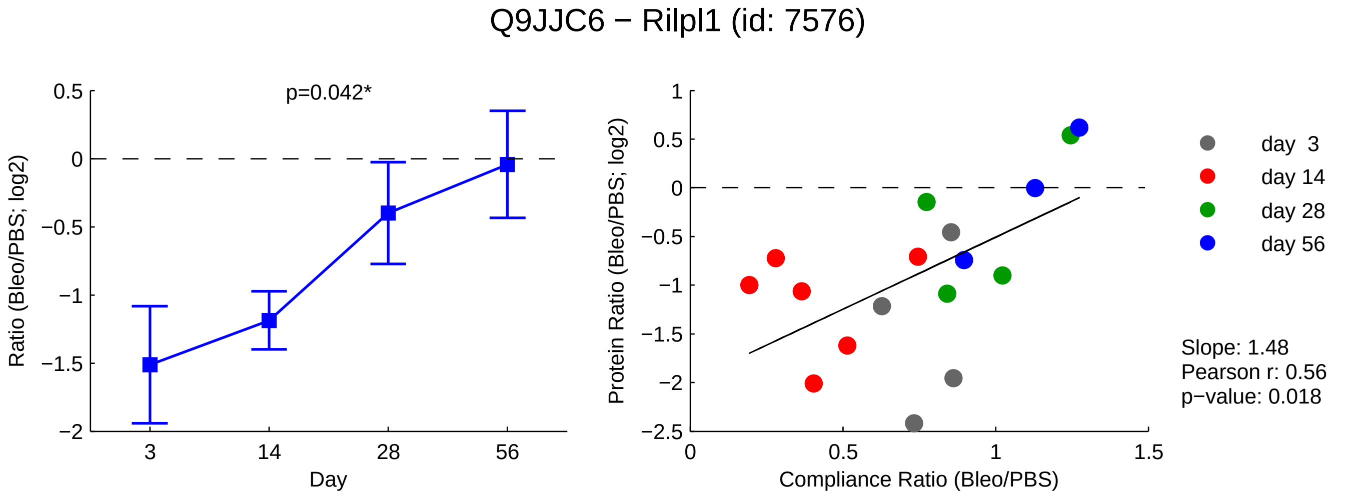
Q9JJC6 − Rilpl1 (id: 7576)
p=0.042*
Ratio (Bleo/PBS; log2)
Day
Protein Ratio (Bleo/PBS; log2)
Compliance Ratio (Bleo/PBS)
day 3
day 14
day 28
day 56
Slope: 1.48
Pearson r: 0.56
p−value: 0.018

Q9JJE7 − Fads3 (id: 7579)

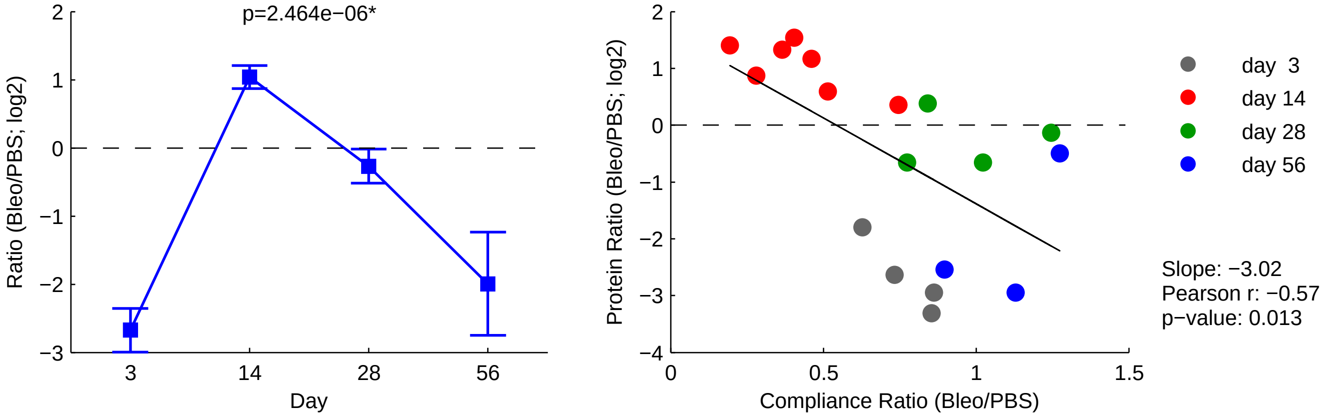

Q9JJI8 − Rpl38 (id: 7582)

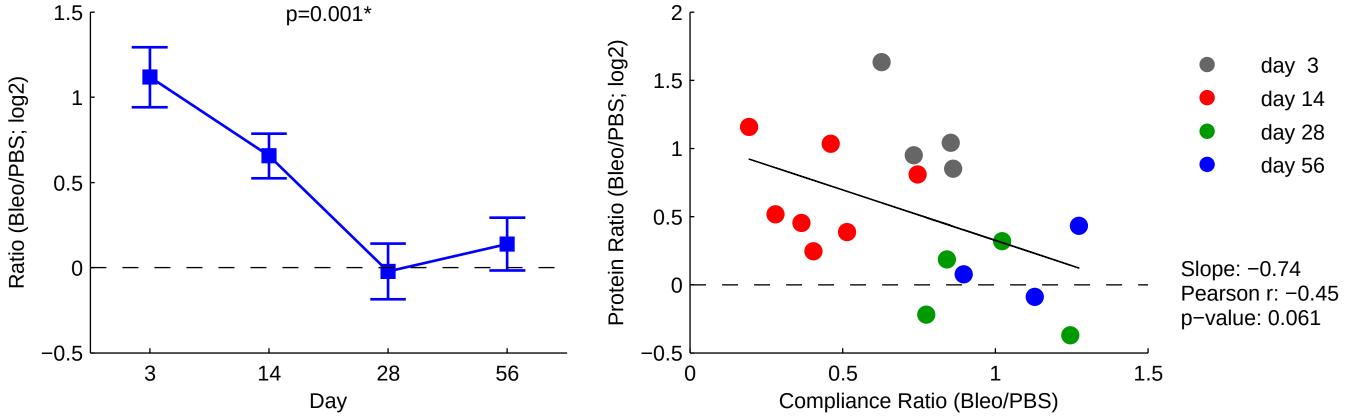

Q9JJN5 − Cpn1 (id: 7585)

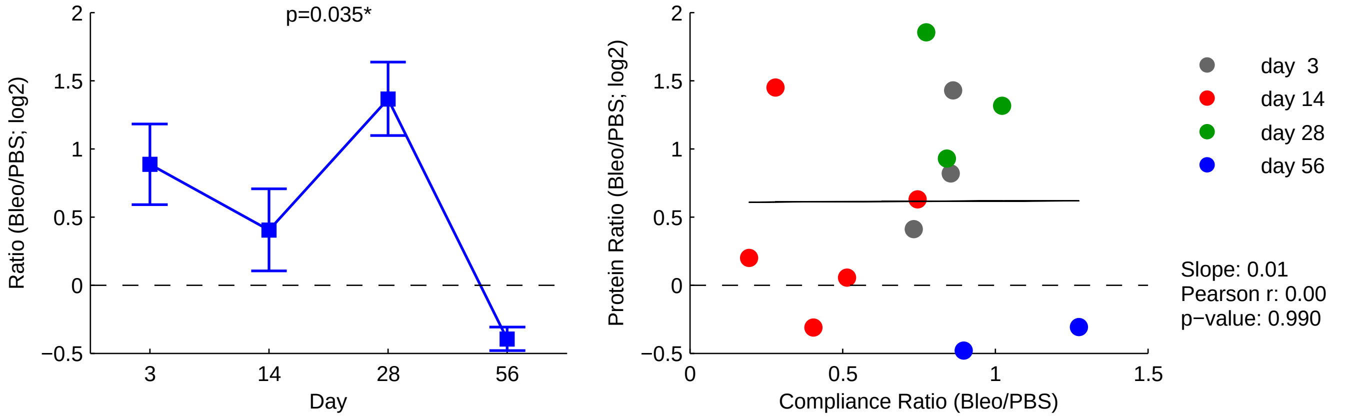

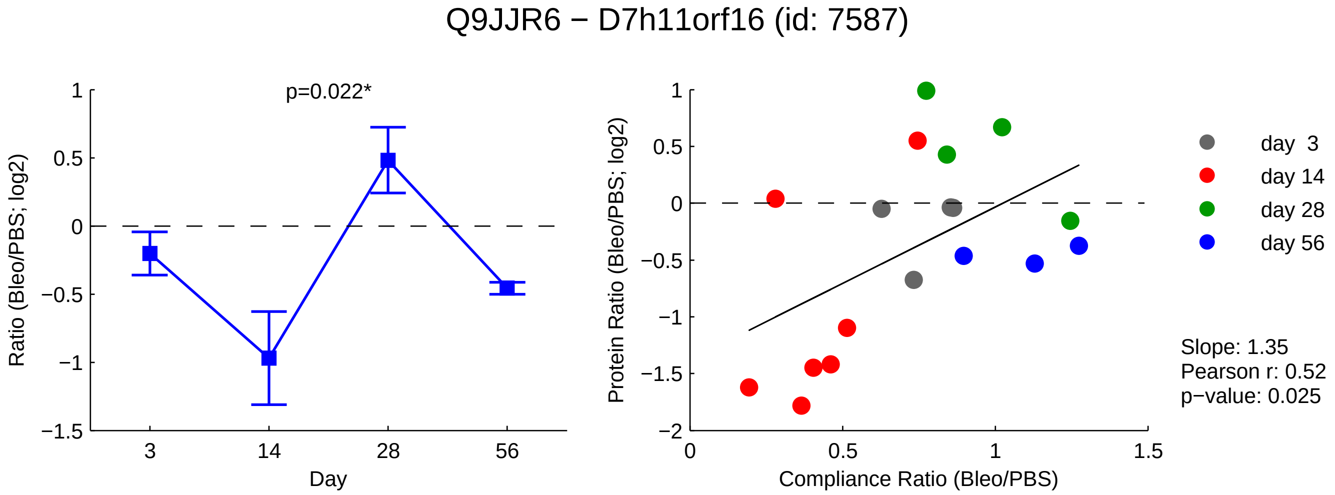
Q9JJR6 − D7h11orf16 (id: 7587)
p=0.022*
Ratio (Bleo/PBS; log2)
Day
Protein Ratio (Bleo/PBS; log2)
Compliance Ratio (Bleo/PBS)
day 3
day 14
day 28
day 56
Slope: 1.35
Pearson r: 0.52
p−value: 0.025

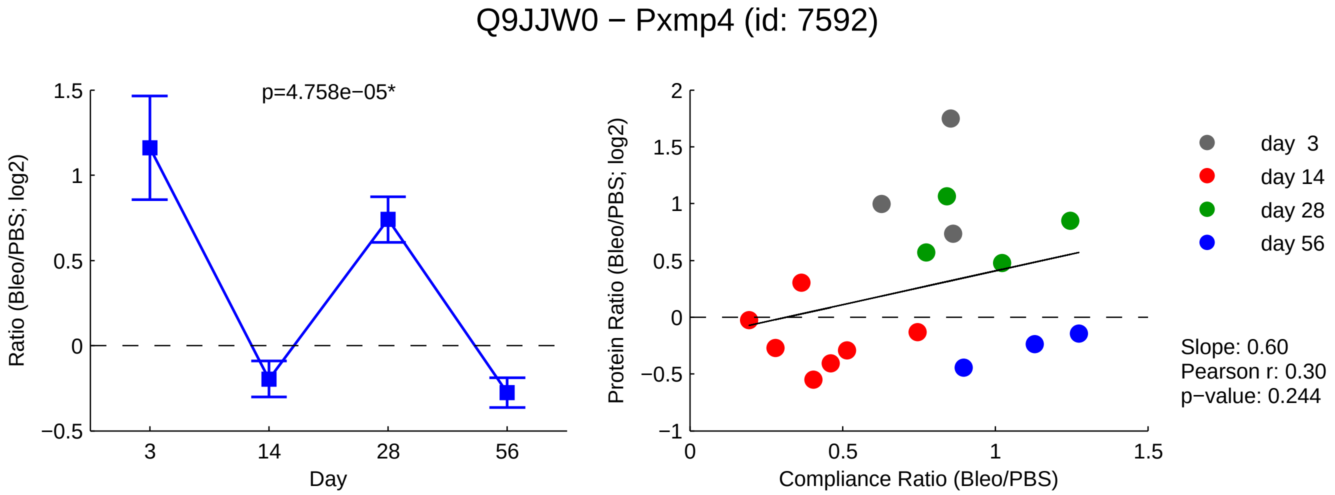
Q9JJW0 − Pxmp4 (id: 7592)
p=4.758e−05*
Ratio (Bleo/PBS; log2)
Day
Protein Ratio (Bleo/PBS; log2)
Compliance Ratio (Bleo/PBS)
day 3
day 14
day 28
day 56
Slope: 0.60
Pearson r: 0.30
p−value: 0.244

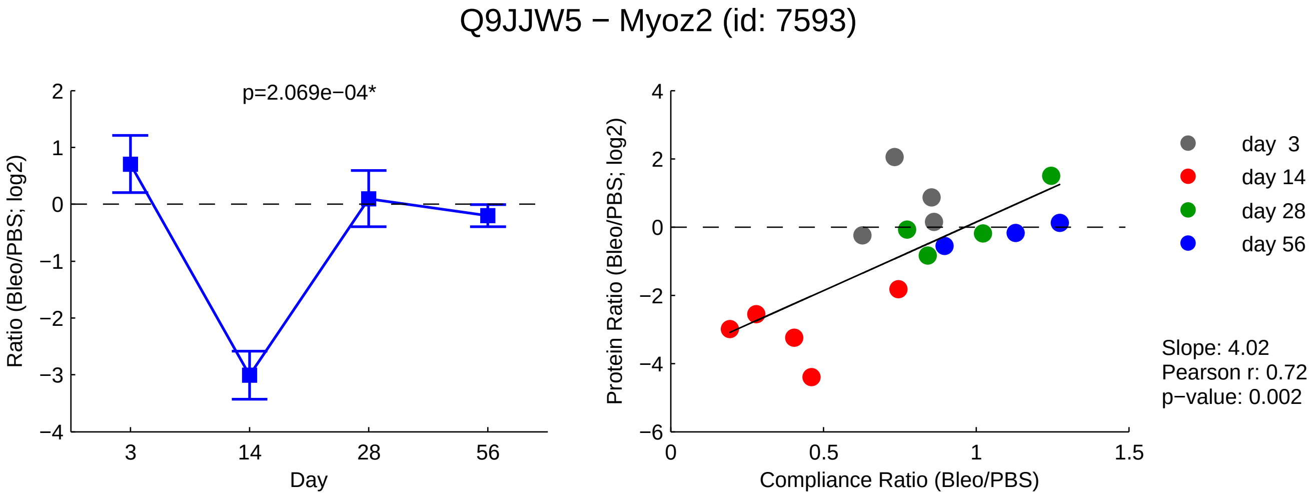
Q9JJW5 − Myoz2 (id: 7593)
p=2.069e−04*
Ratio (Bleo/PBS; log2)
Day
Protein Ratio (Bleo/PBS; log2)
Compliance Ratio (Bleo/PBS)
day 3
day 14
day 28
day 56
Slope: 4.02
Pearson r: 0.72
p−value: 0.002

Q9JJZ2 − Tuba8 (id: 7595)

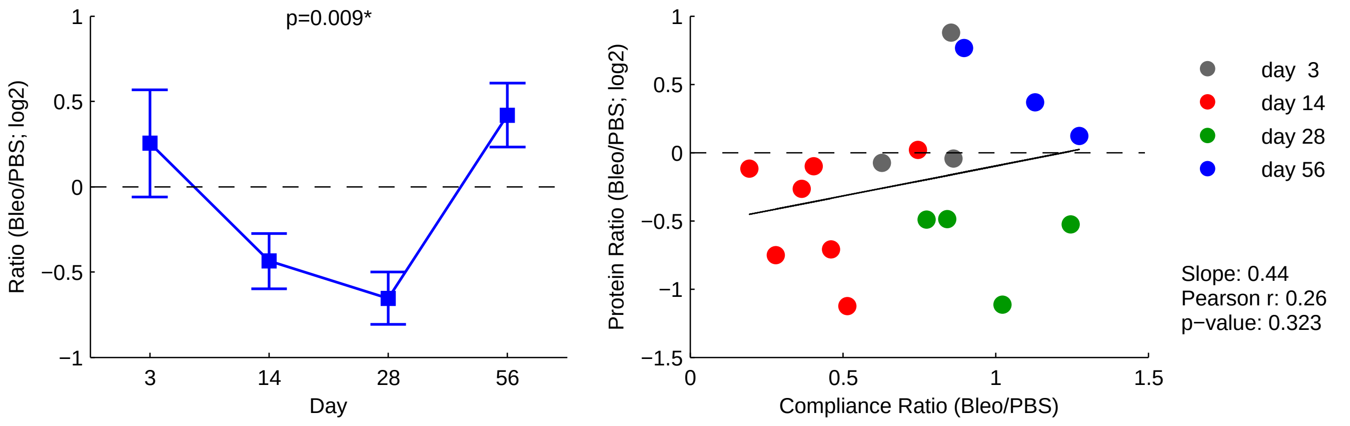

Q9JJZ4 − Ube2j1 (id: 7597)

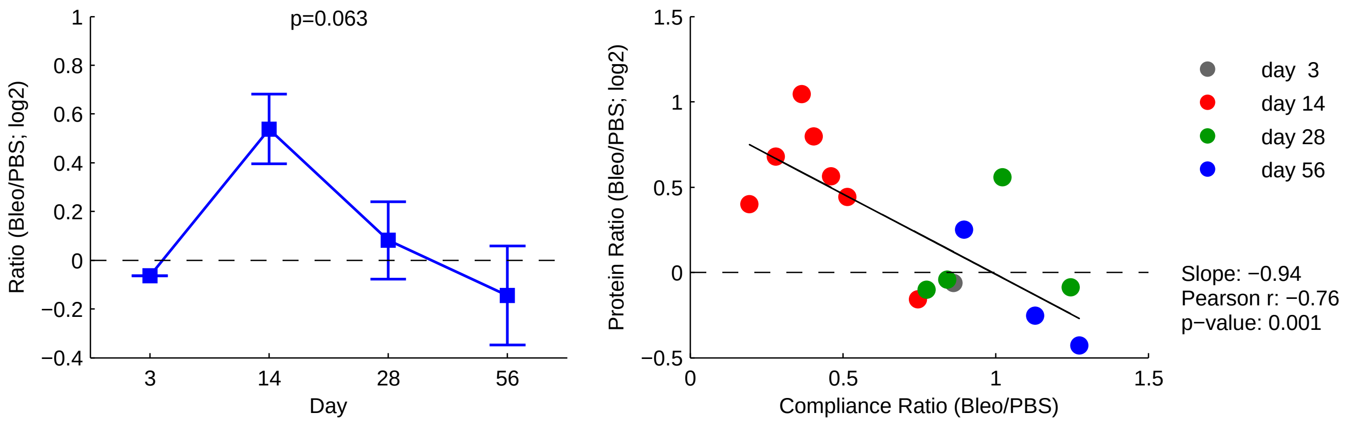

Q9JK38 − Gnpnat1 (id: 7600)

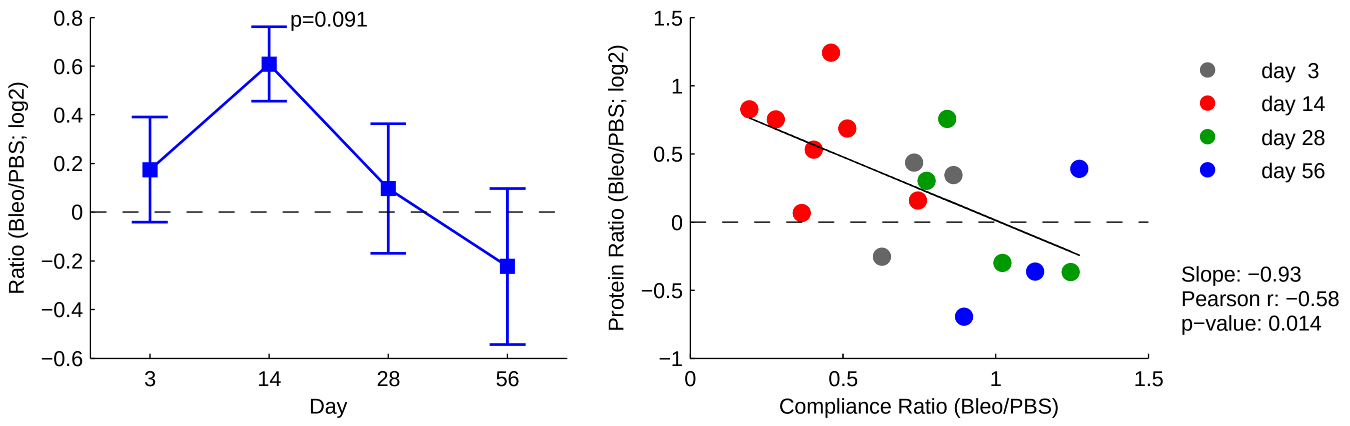

Q9JK42 − Pdk2 (id: 7601)

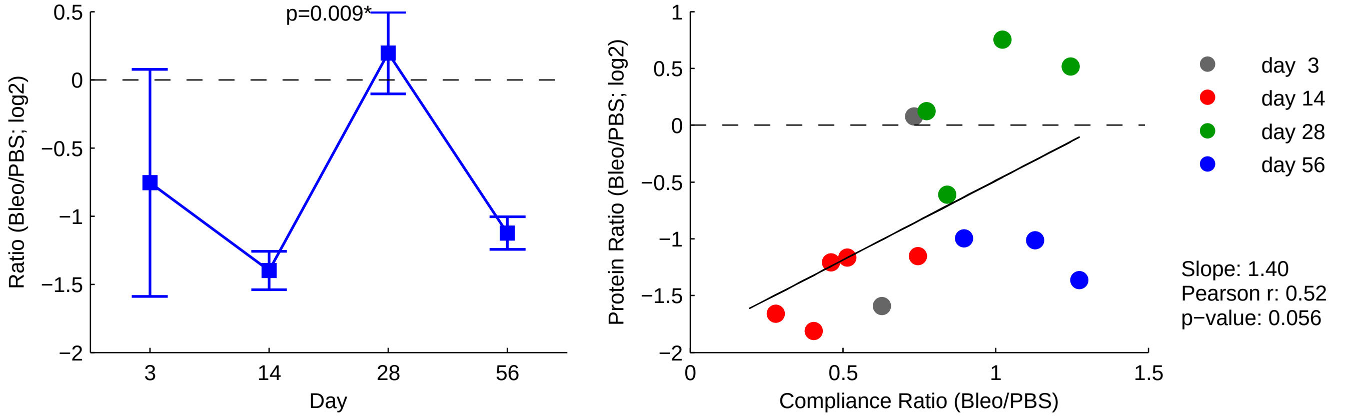

Q9JK48 − Sh3glb1 (id: 7602)

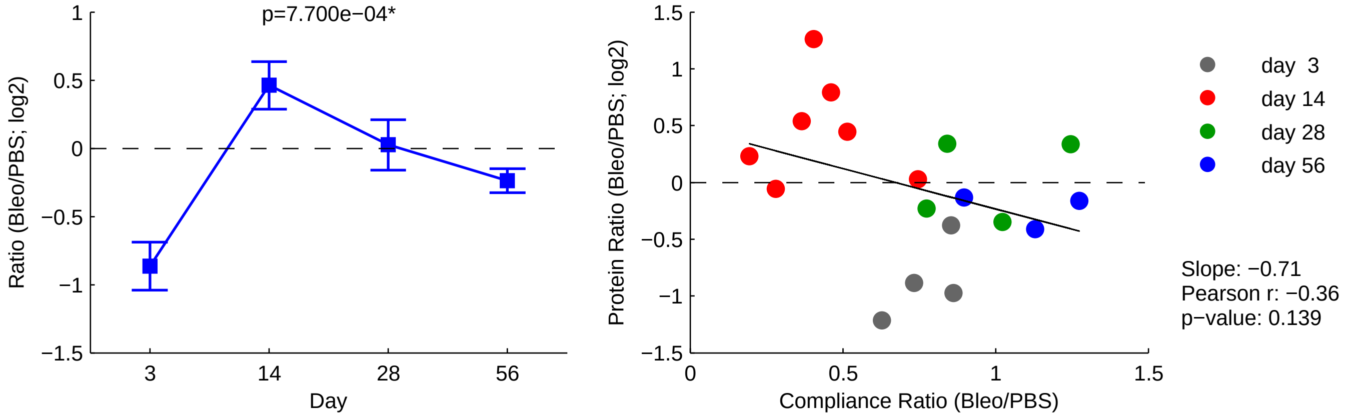

Q9JK53 − Prelp (id: 7603)

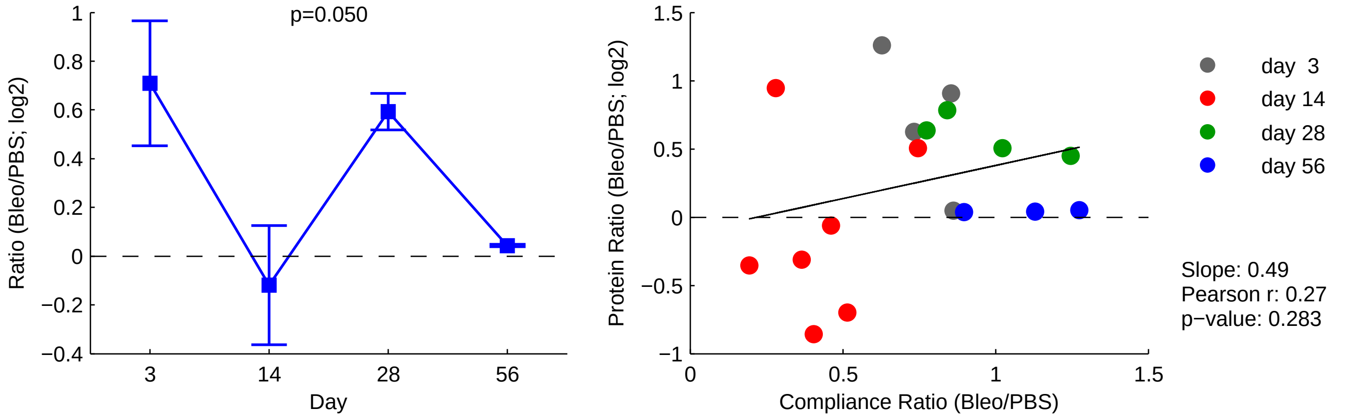

Q9JK83 − Pard6b (id: 7604)

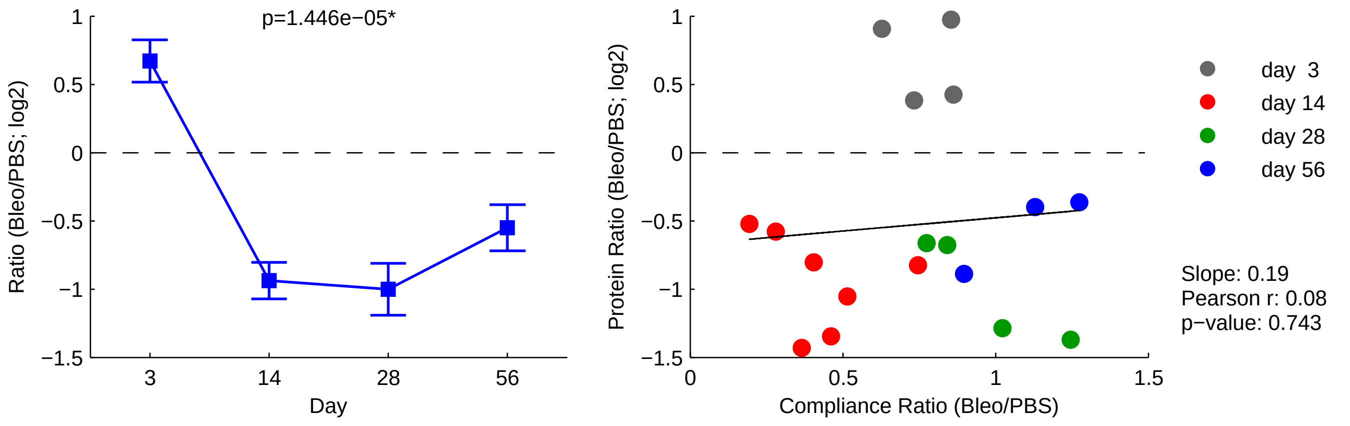

Q9JKF6 − Pvrl1 (id: 7610)

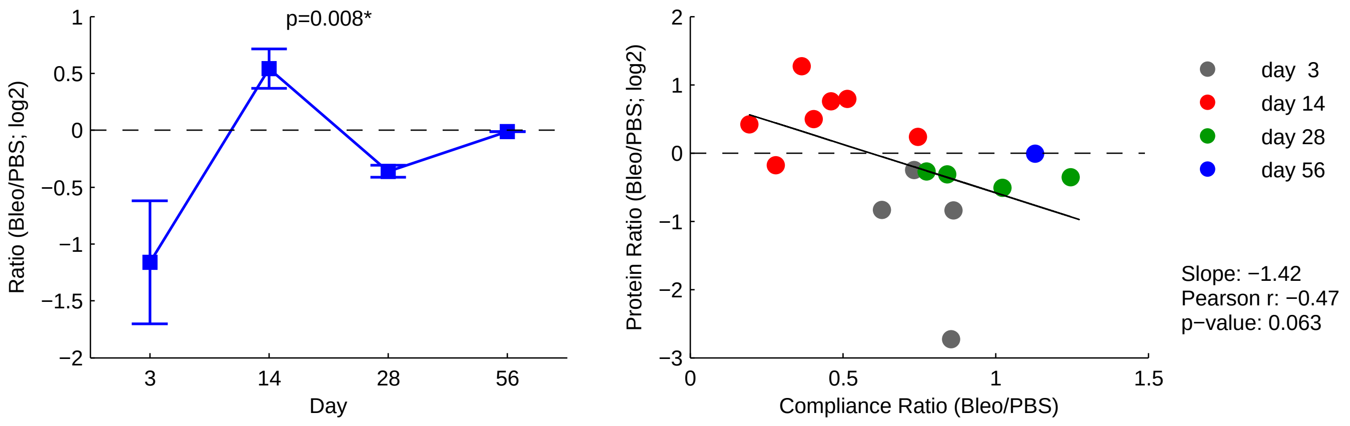

Q9JKF7 − Mrpl39 (id: 7611)

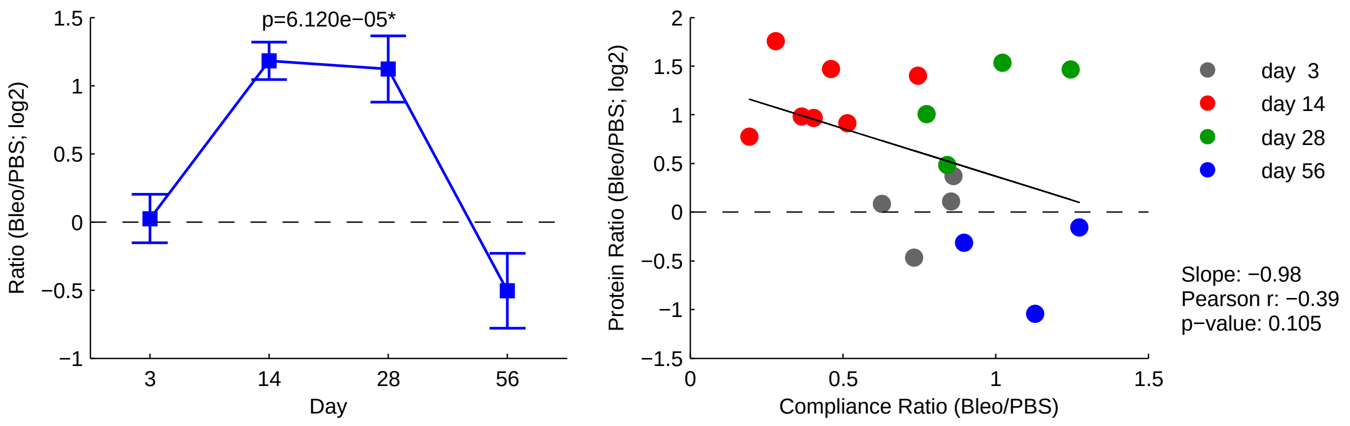

Q9JKJ9 − Cyp39a1 (id: 7612)

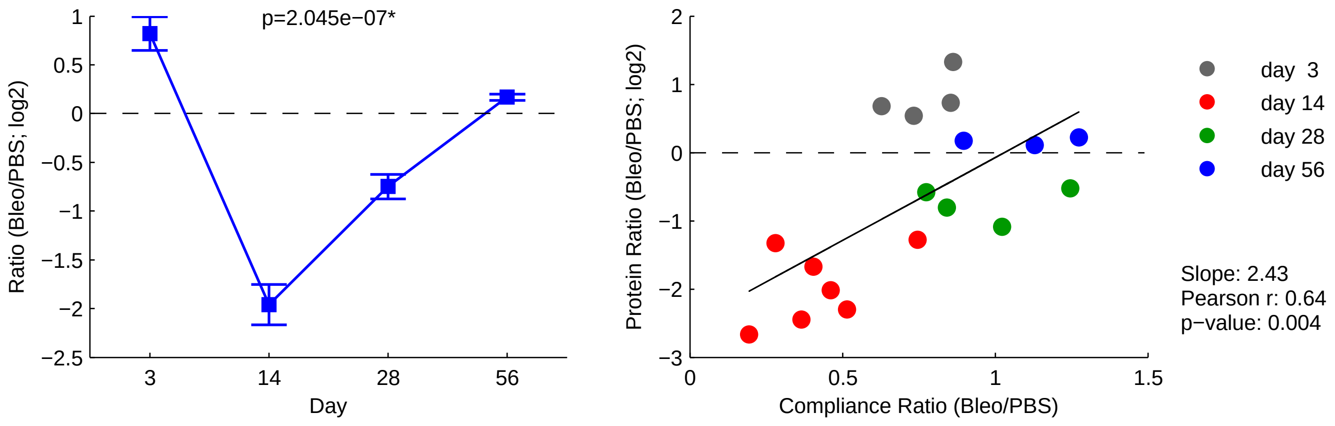

Q9JKK1−2 − Stx6 (id: 7613)

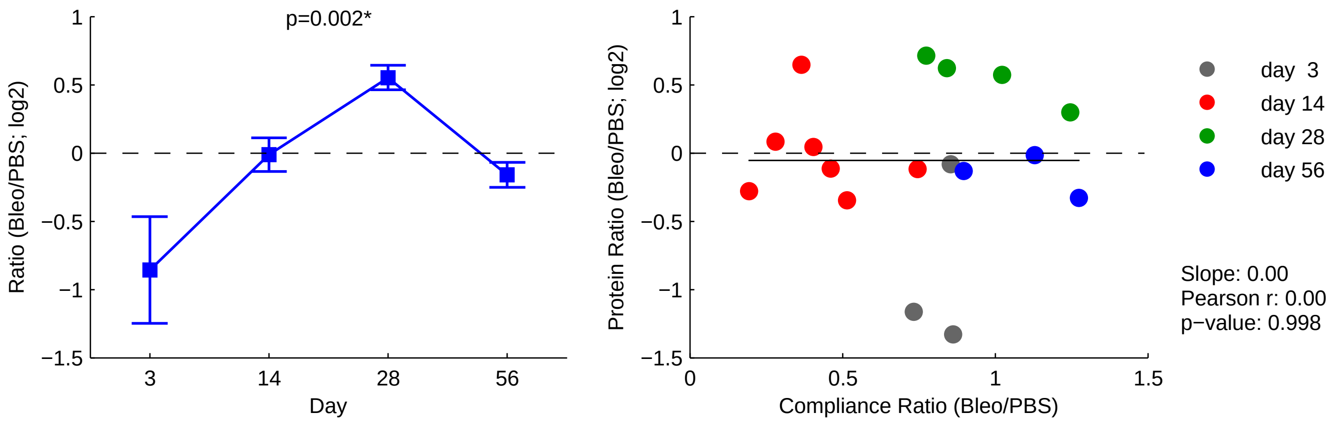

Q9JKL4 − Ndufaf3 (id: 7615)

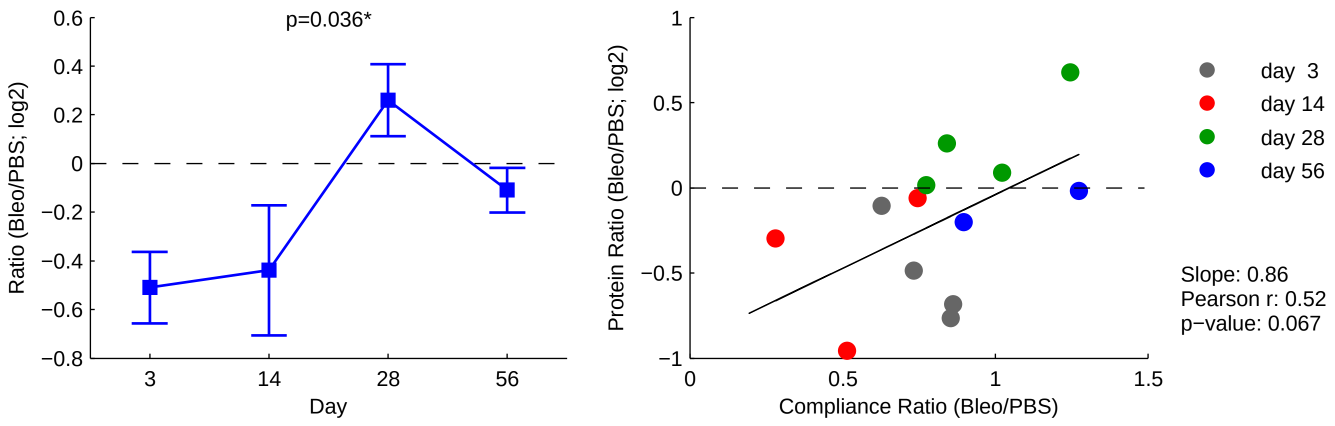

Q9JKR6 − Hyou1 (id: 7619)

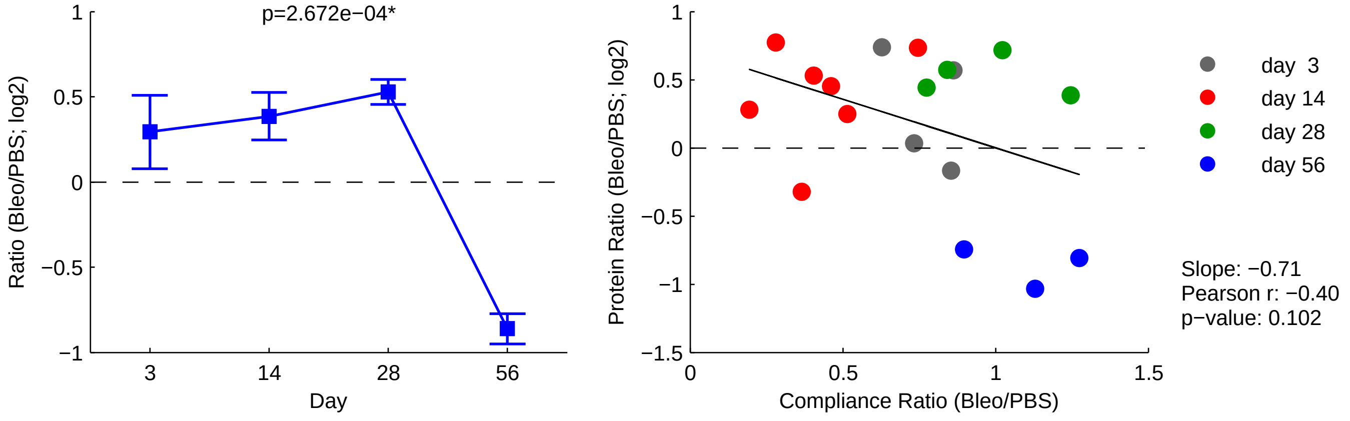

Q9JKS4−3 − Ldb3 (id: 7620)

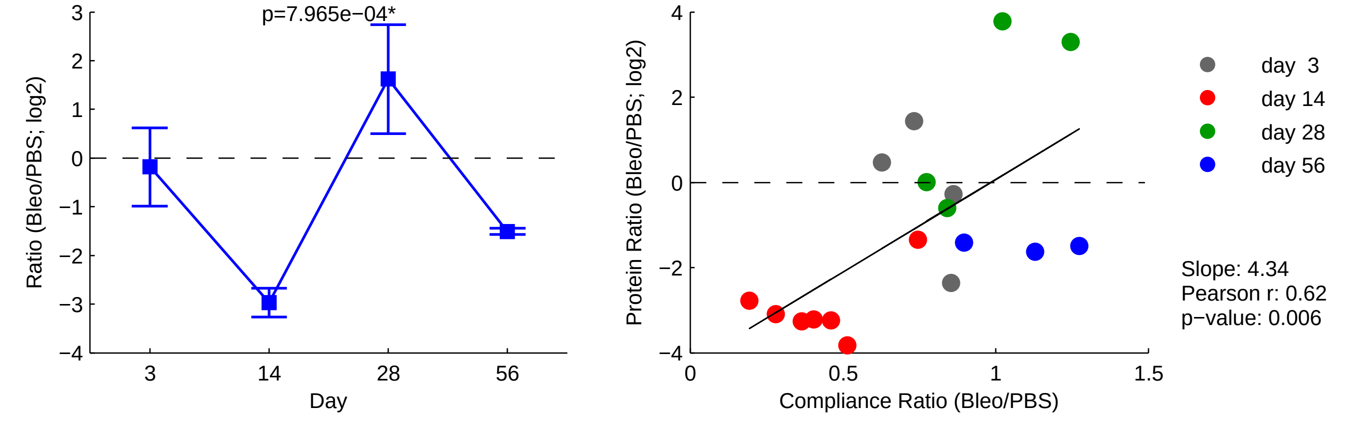

Q9JKV1 − Adrm1 (id: 7622)

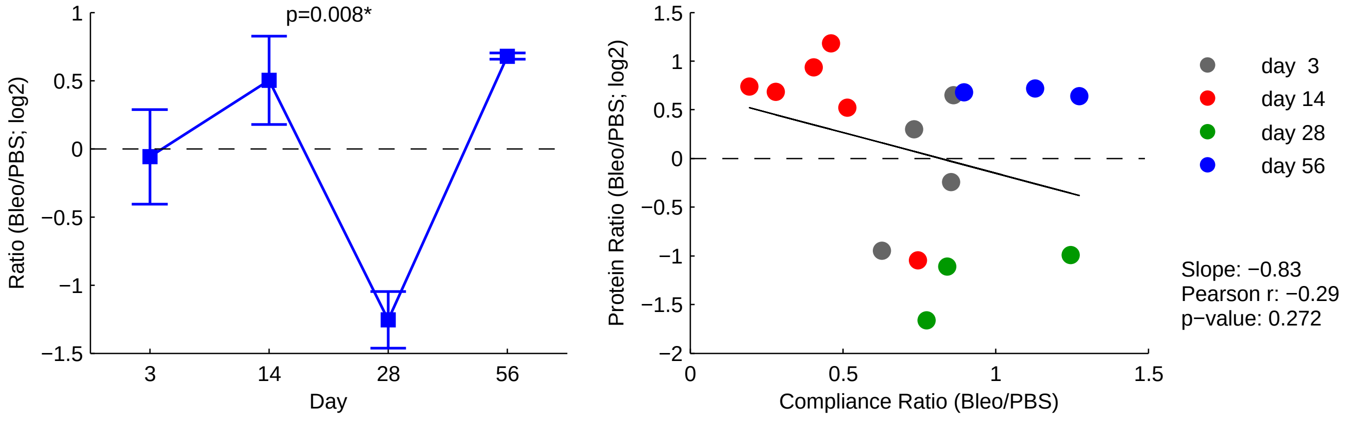

Q9JKW0 − Arl6ip1 (id: 7624)

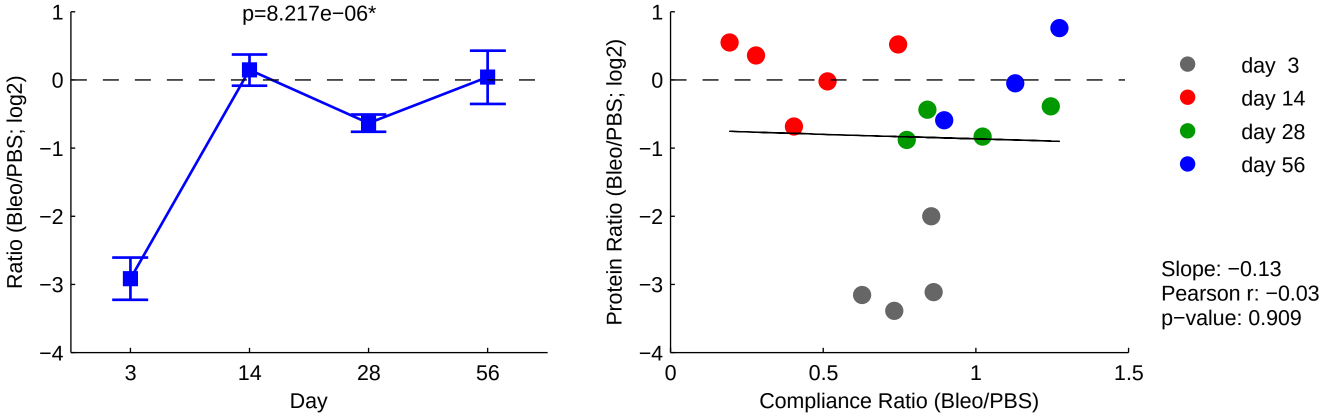

Q9JKY7 − Cyp2d22 (id: 7629)

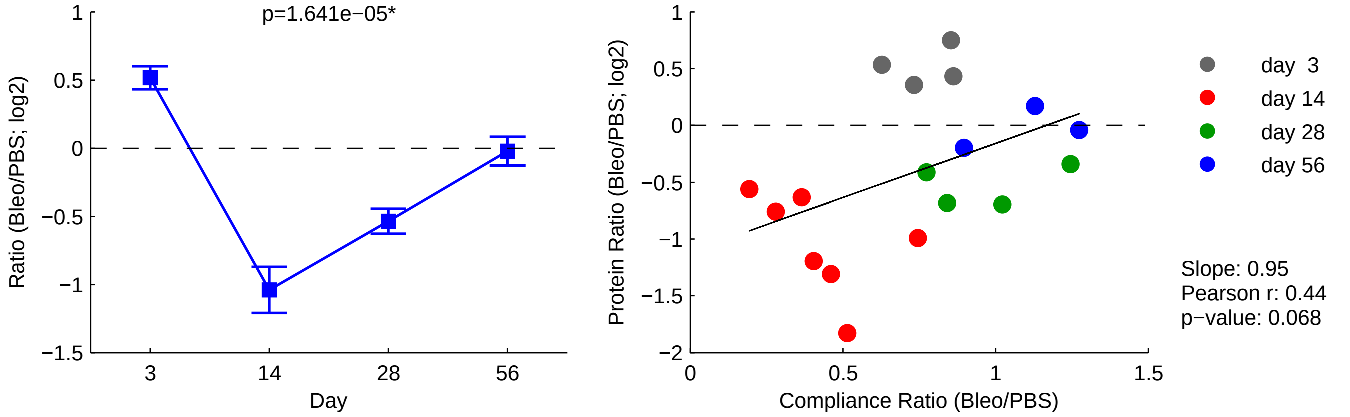

Q9JL62 − Gltp (id: 7635)

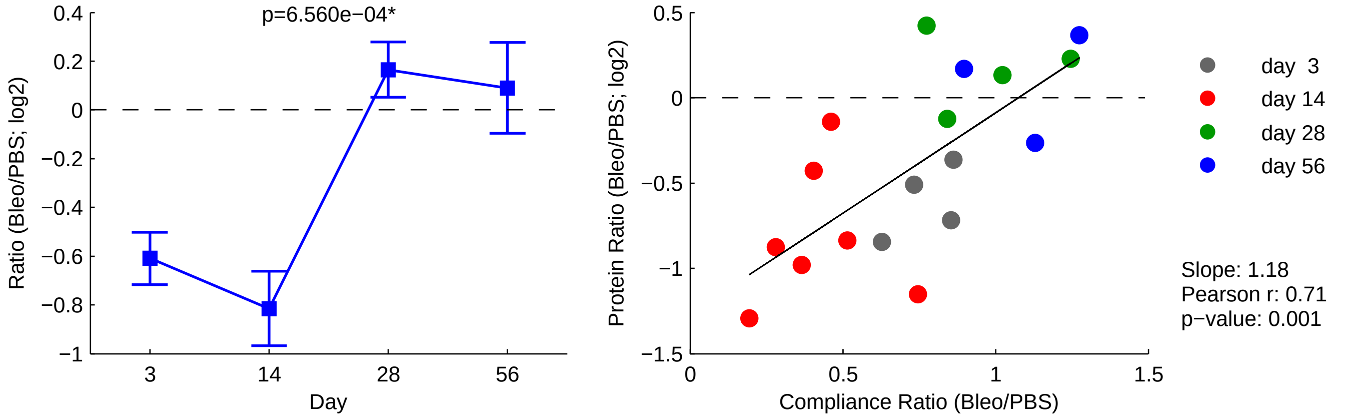

Q9JL95 − Prg3 (id: 7636)

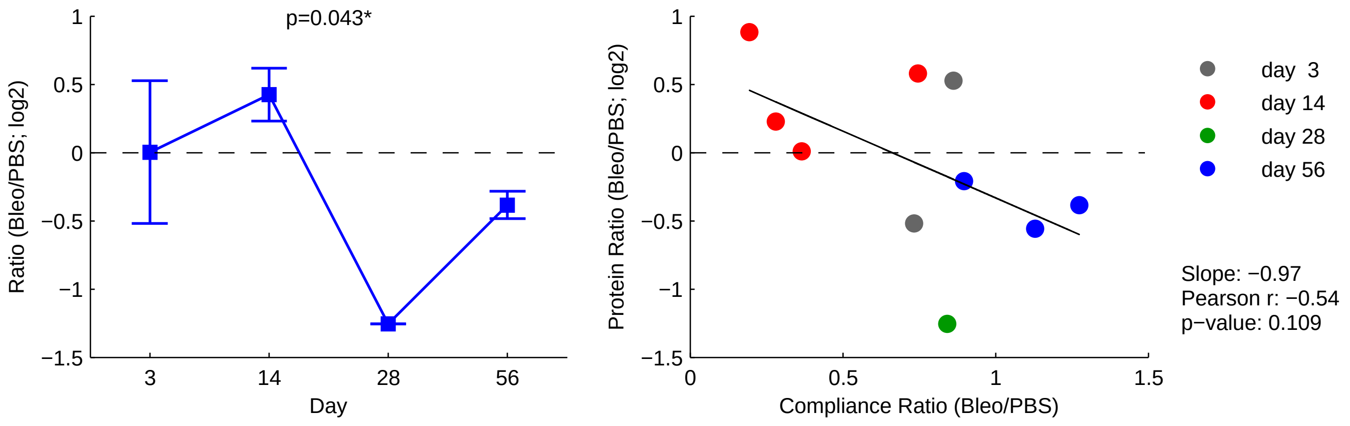

Q9JLB0−2 − Mpp6 (id: 7638)

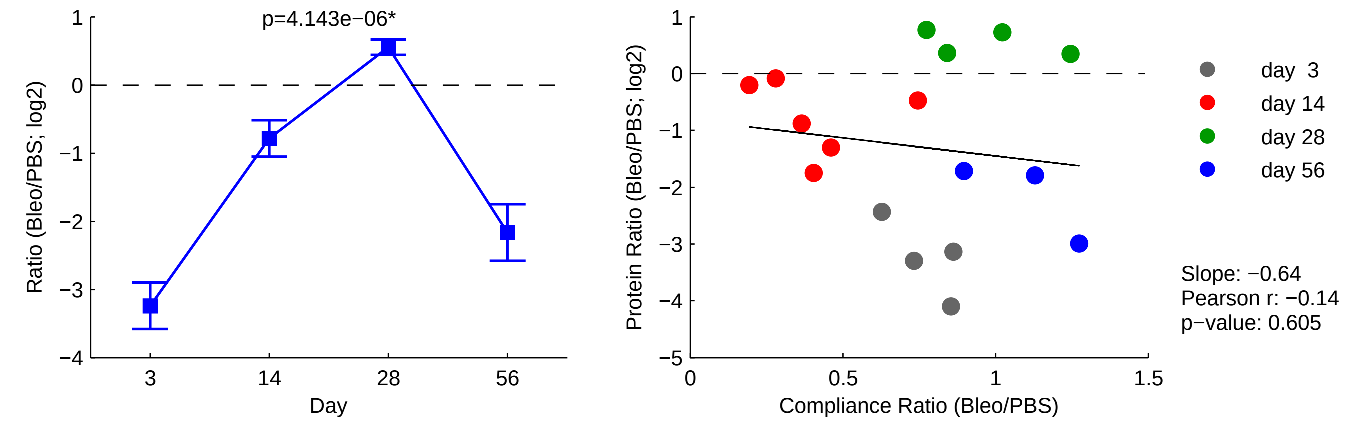

Q9JLB9 − Pvrl3 (id: 7640)

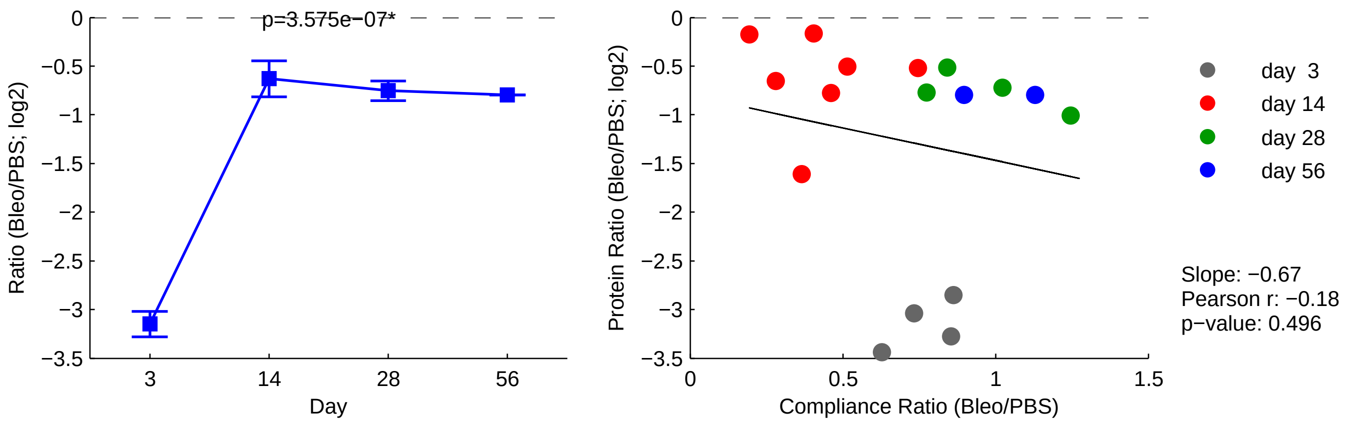

Q9JLF6 − Tgm1 (id: 7642)

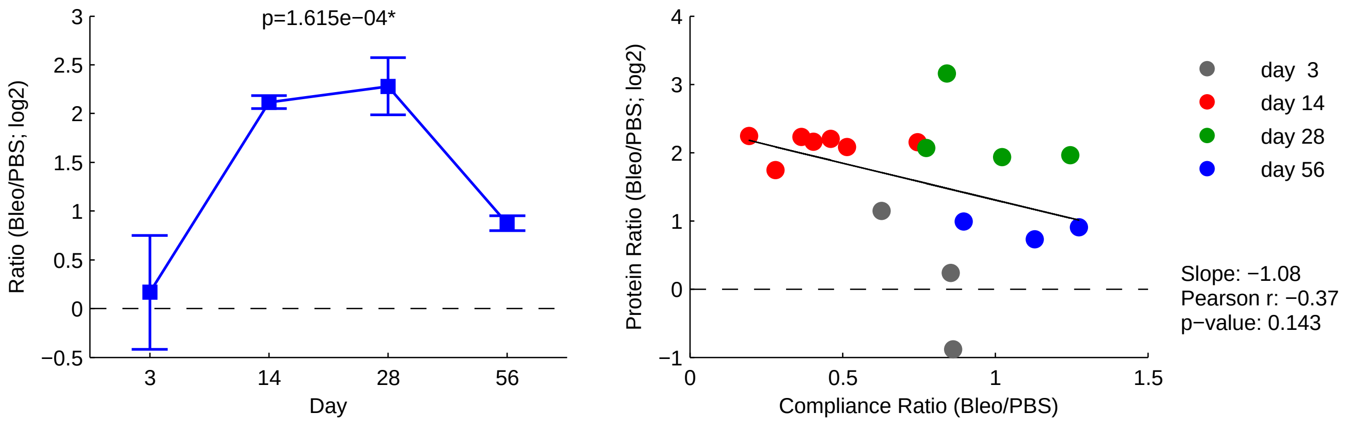

Q9JLH8 − Tmod4 (id: 7643)

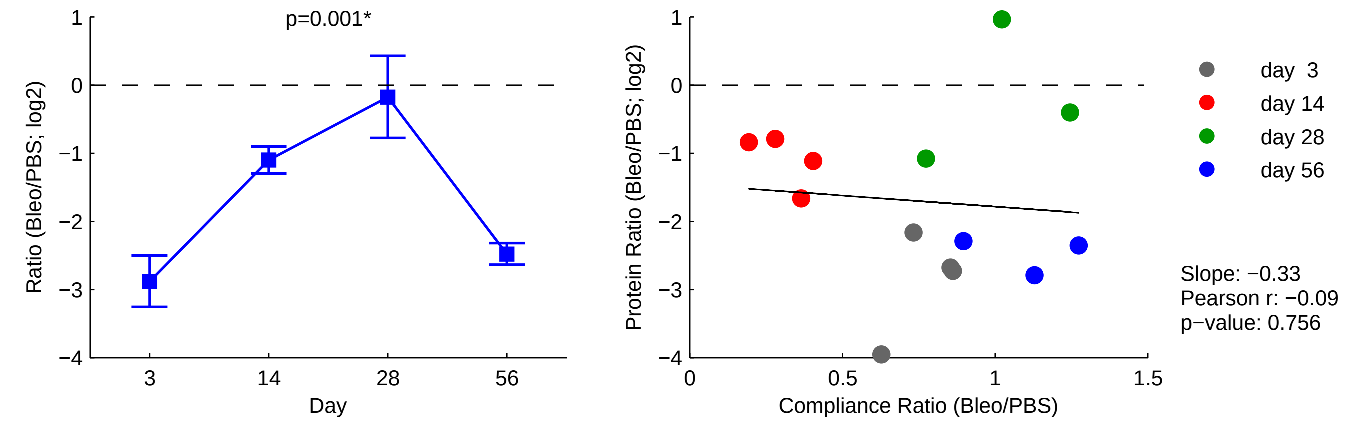

Q9JLJ5 − Elovl1 (id: 7647)

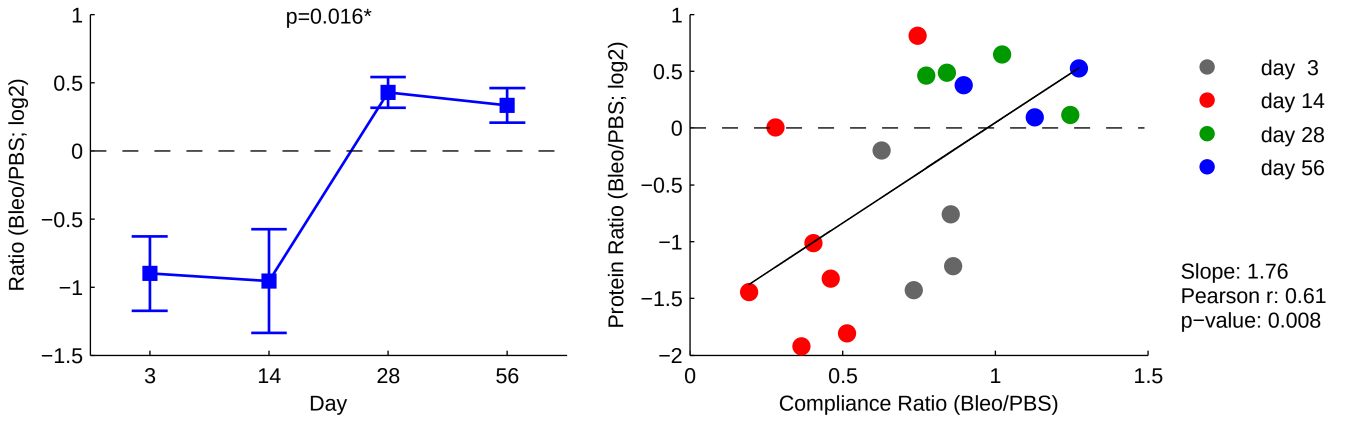

Q9JLM9 − Grb14 (id: 7648)

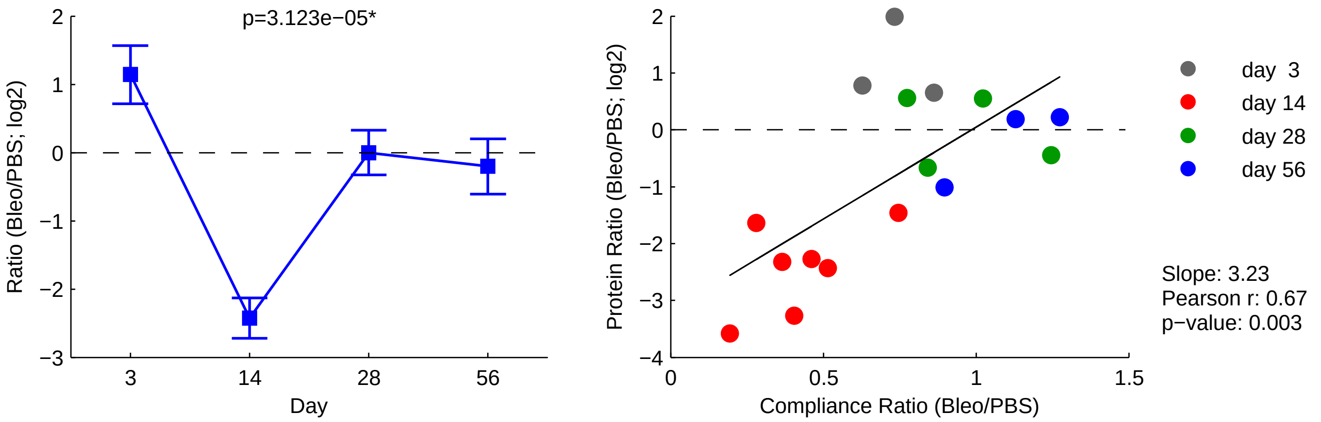

Q9JLN9 − Mtor (id: 7649)

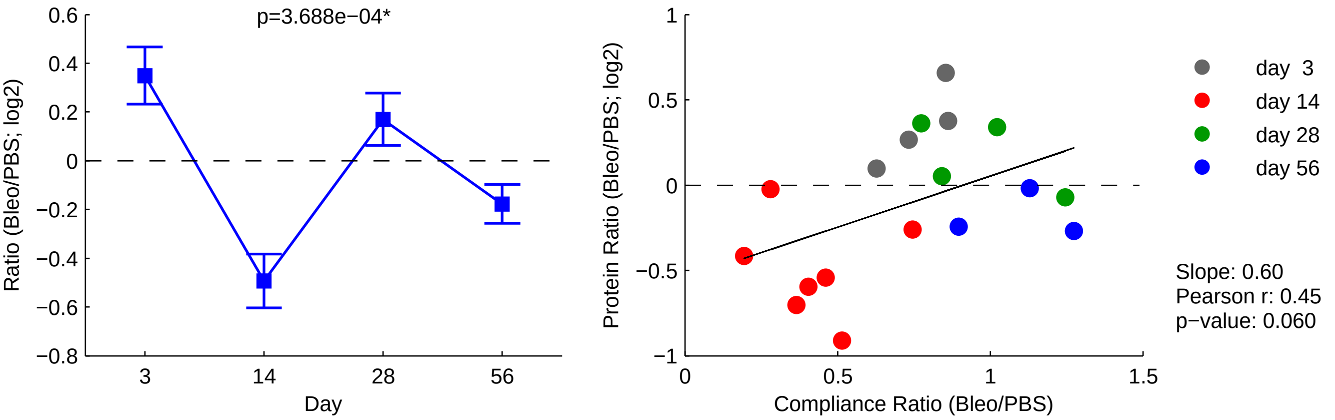

Q9JLQ0 − Cd2ap (id: 7650)

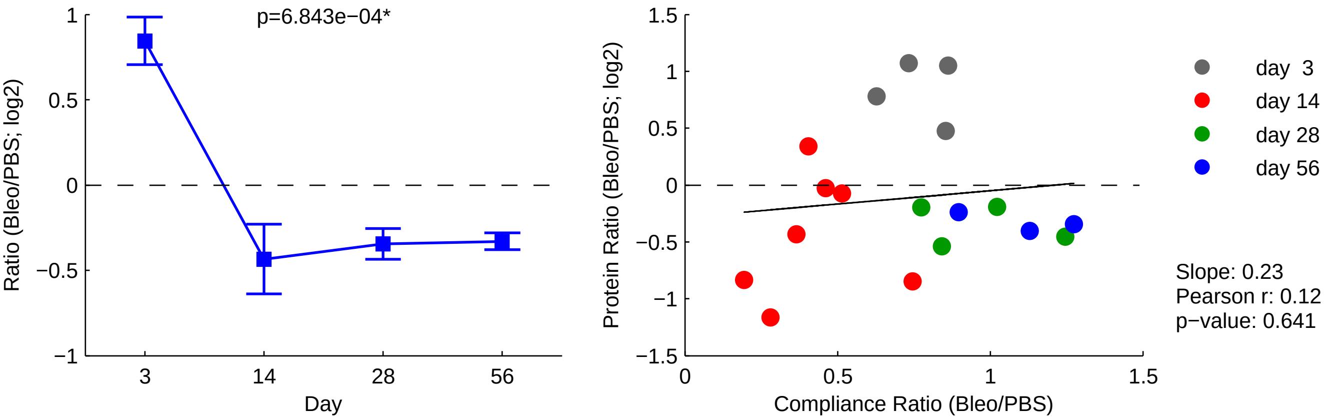

Q9JLQ2 − Git2 (id: 7651)

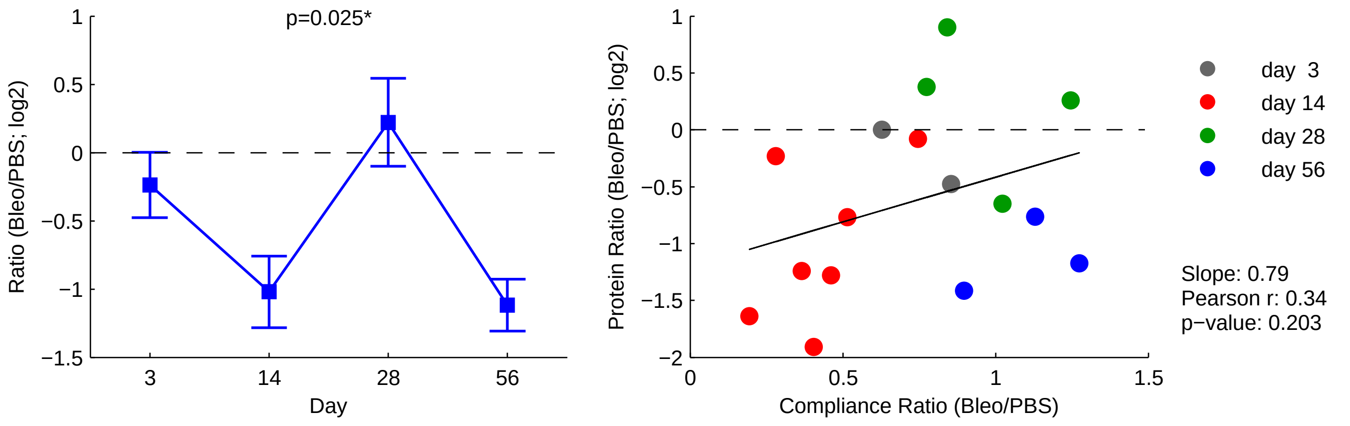

Q9JLR9 − Higd1a (id: 7652)

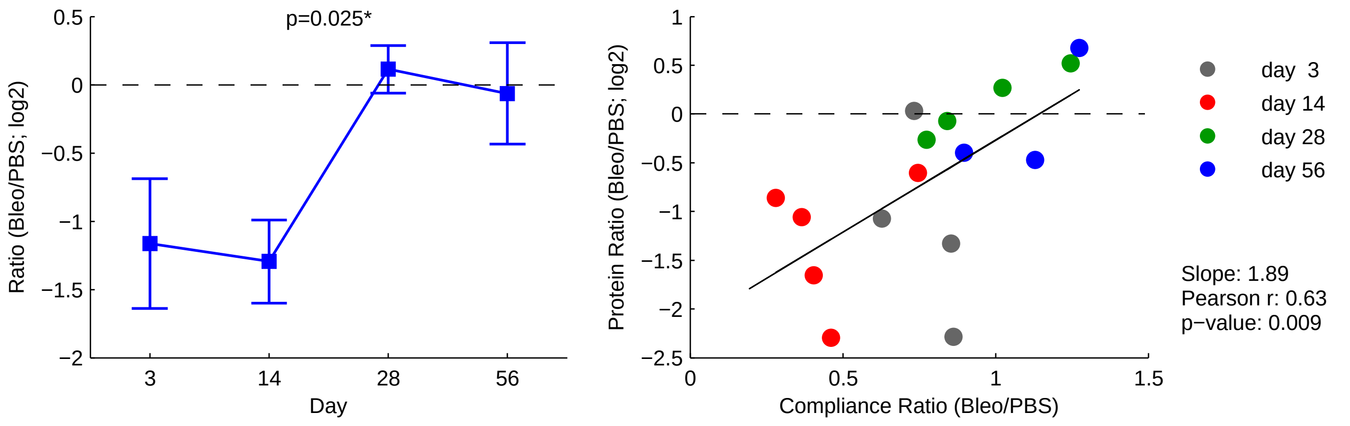

Q9JLV1 − Bag3 (id: 7653)

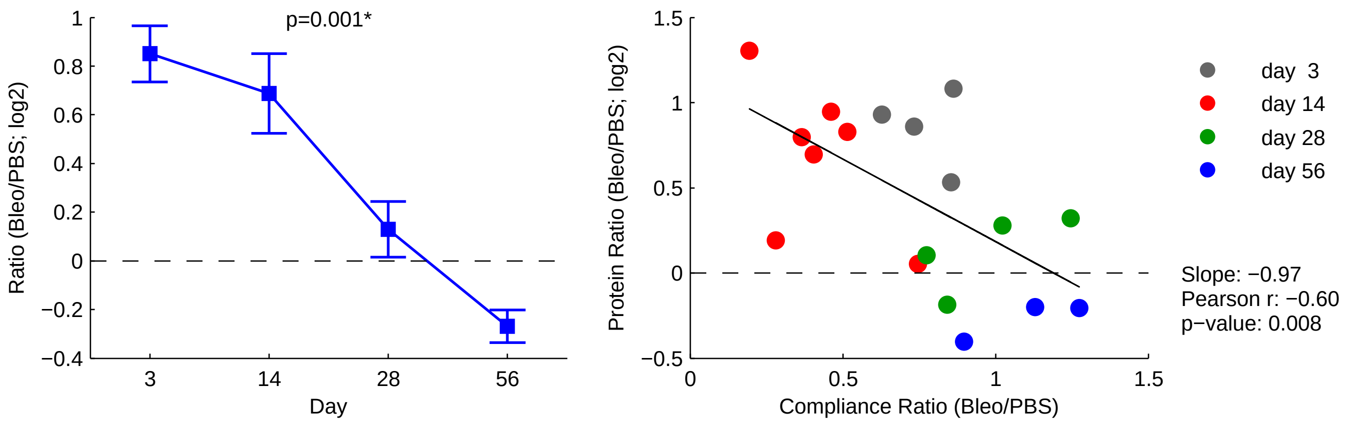

Q9JLY7 − Dusp14 (id: 7655)

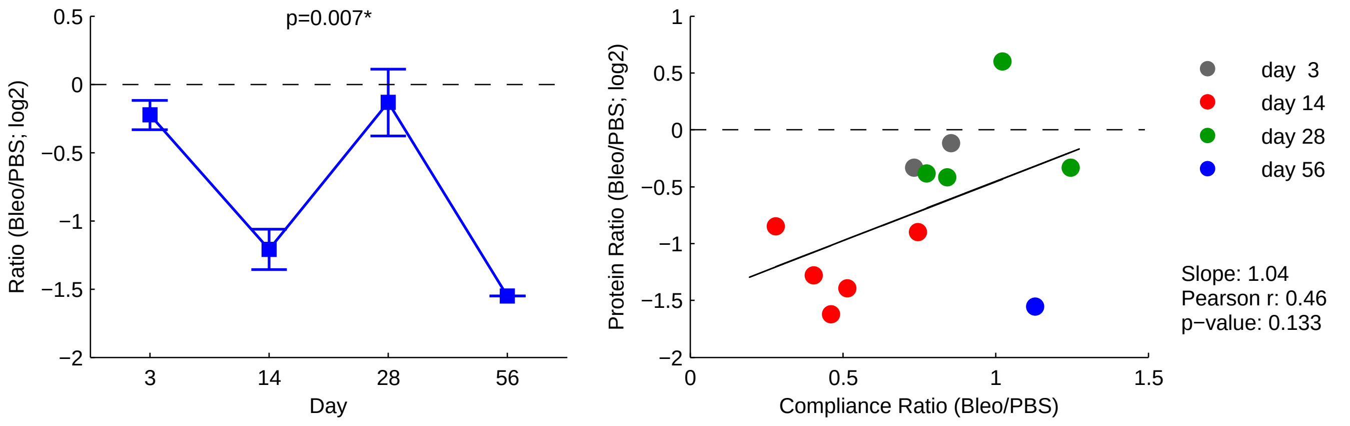

Q9JM13 − Rabgef1 (id: 7658)

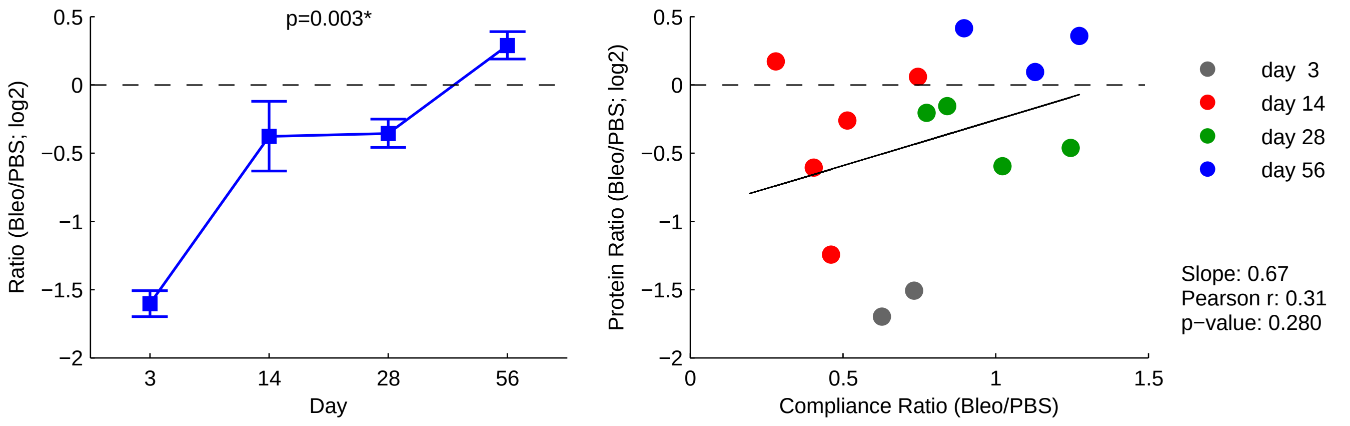

Q9JM51 − Ptges (id: 7660)

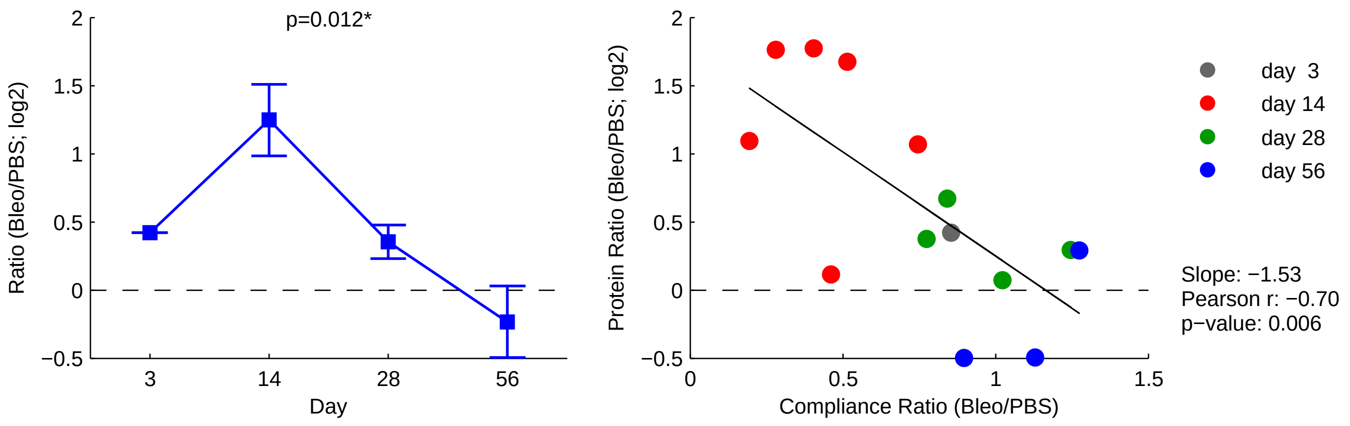

Q9JM58 − Crlf1 (id: 7661)

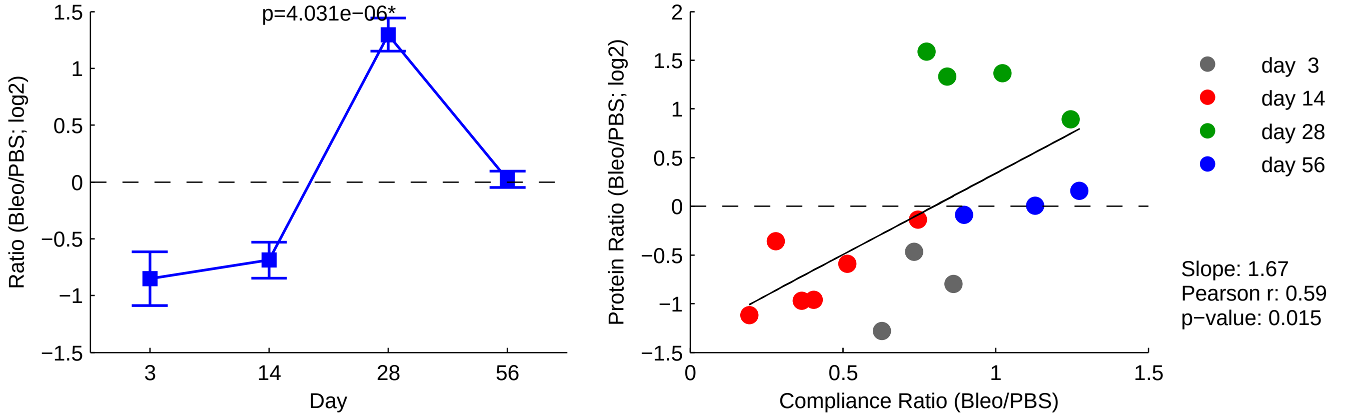

Q9JM62−2 − Reep6 (id: 7662)

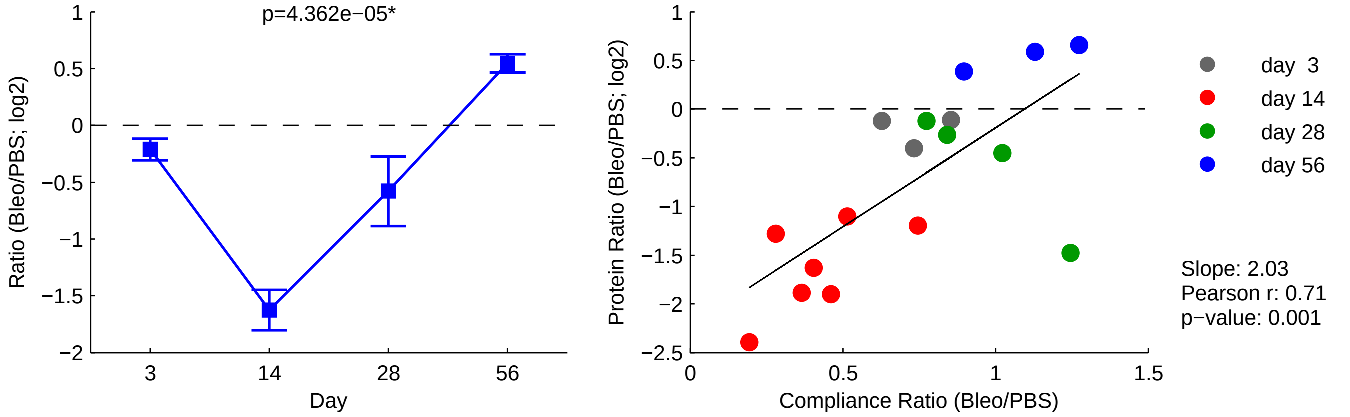

Q9JM76 − Arpc3 (id: 7663)

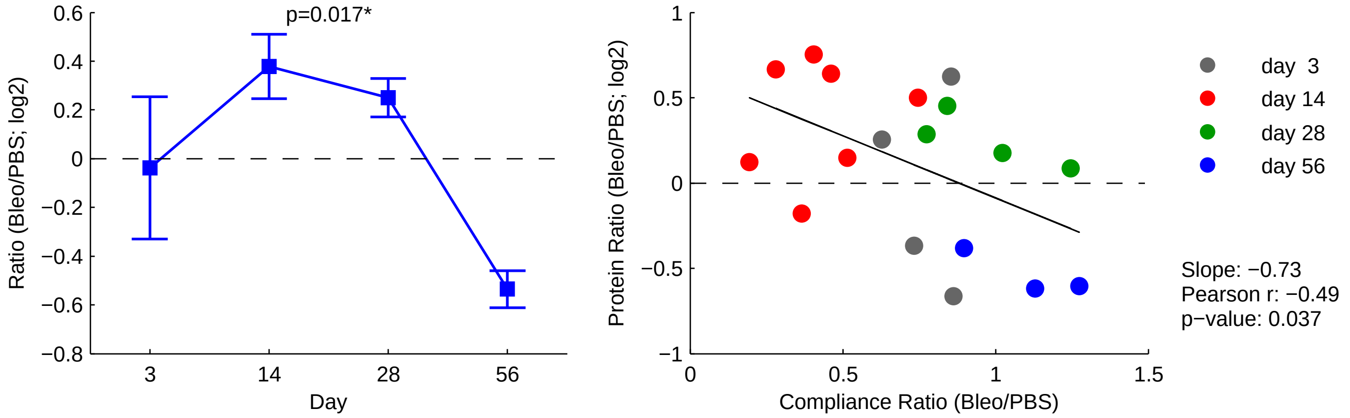

Q9JM90 − Stap1 (id: 7665)

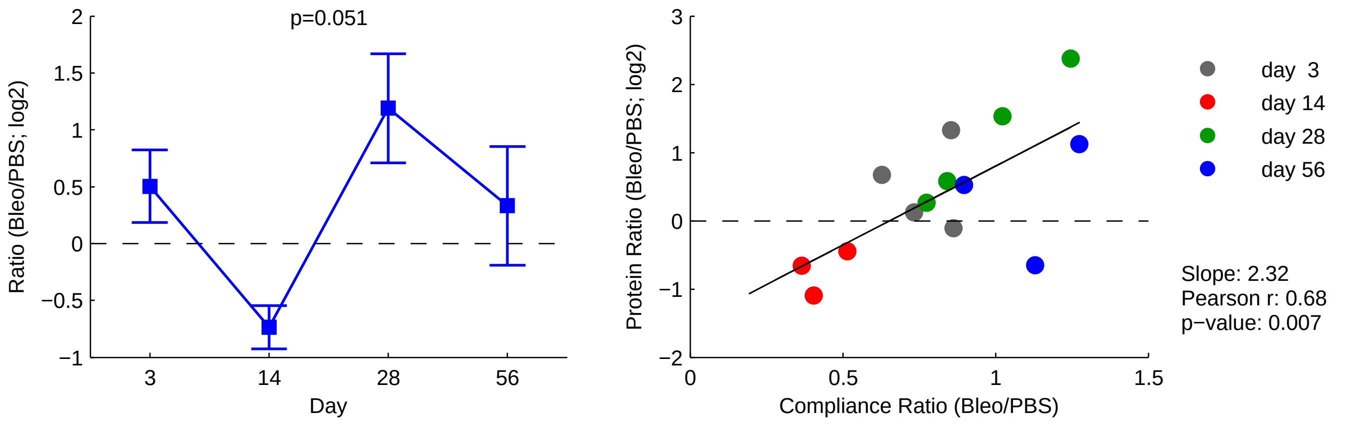

Q9JM96 − Cdc42ep4 (id: 7667)

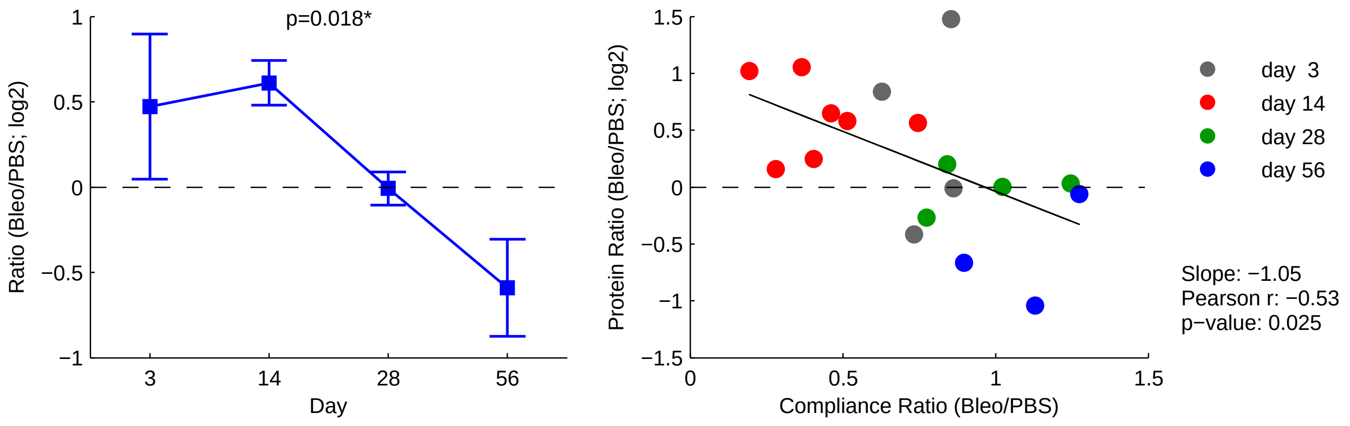

Q9JMA9 − Slc6a14 (id: 7669)

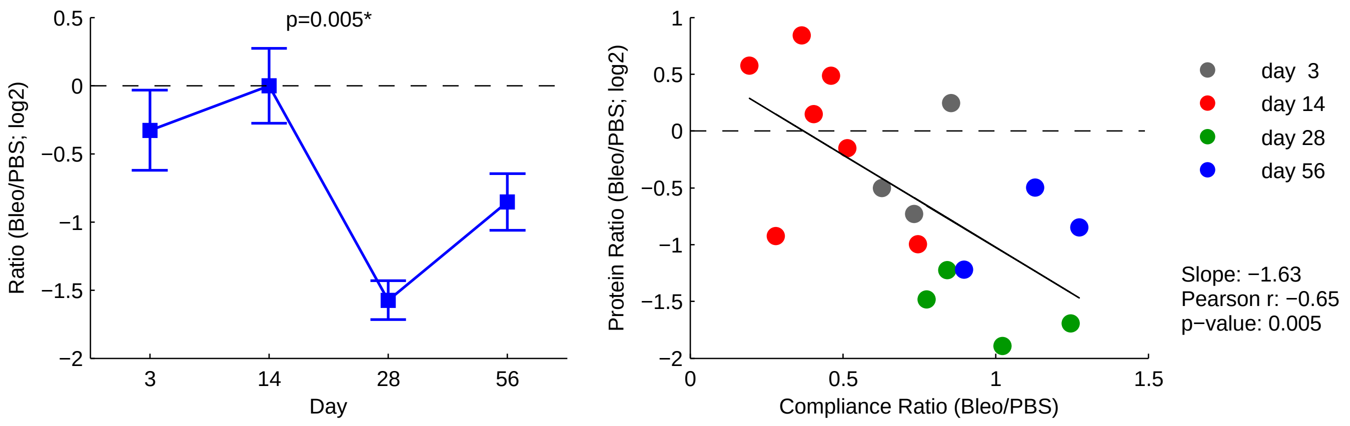

Q9JMC8 − Epb41l4b (id: 7671)

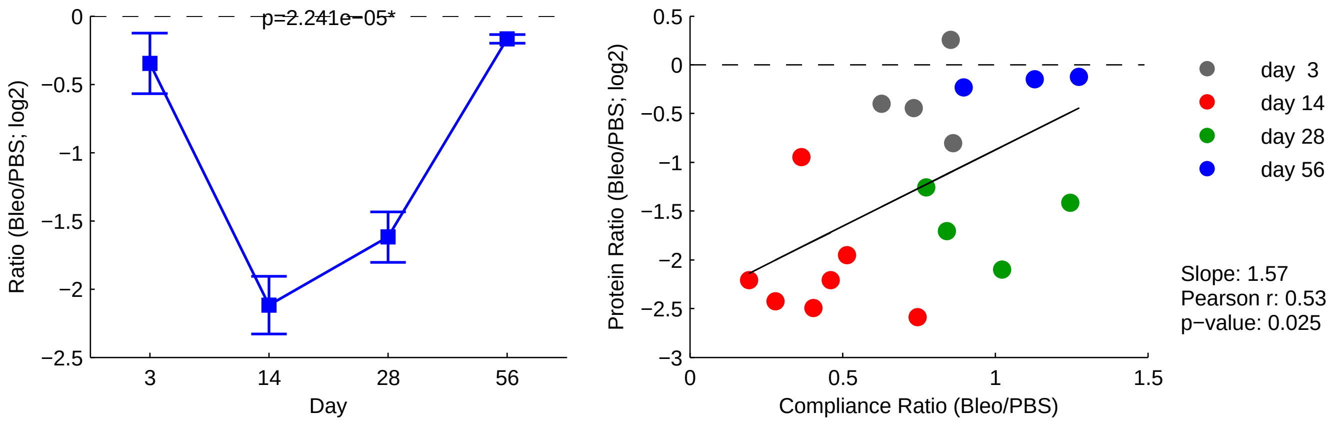

Q9JMG7 − Hdgfrp3 (id: 7675)

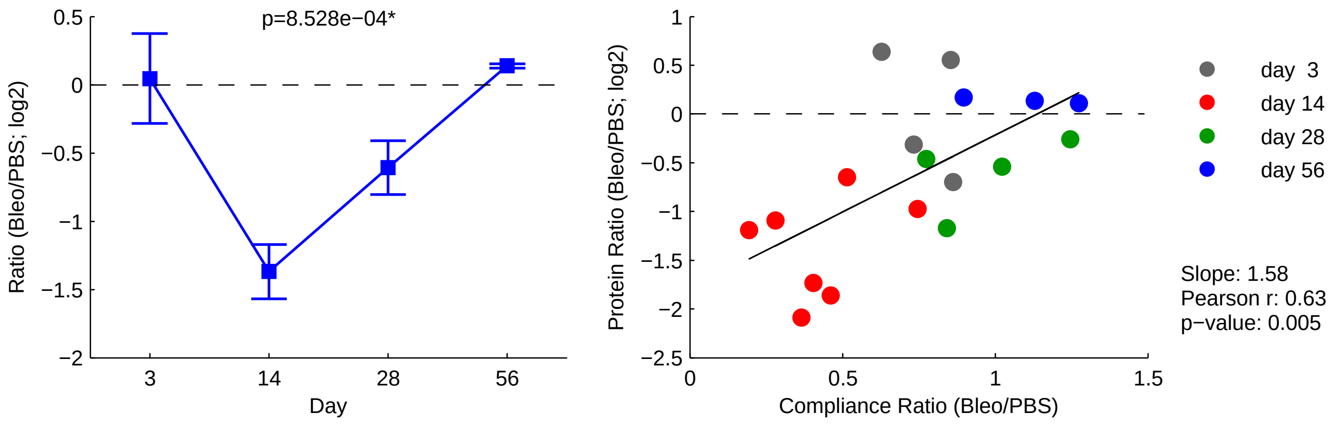

Q9QUG9 − Rasgrp2 (id: 7679)

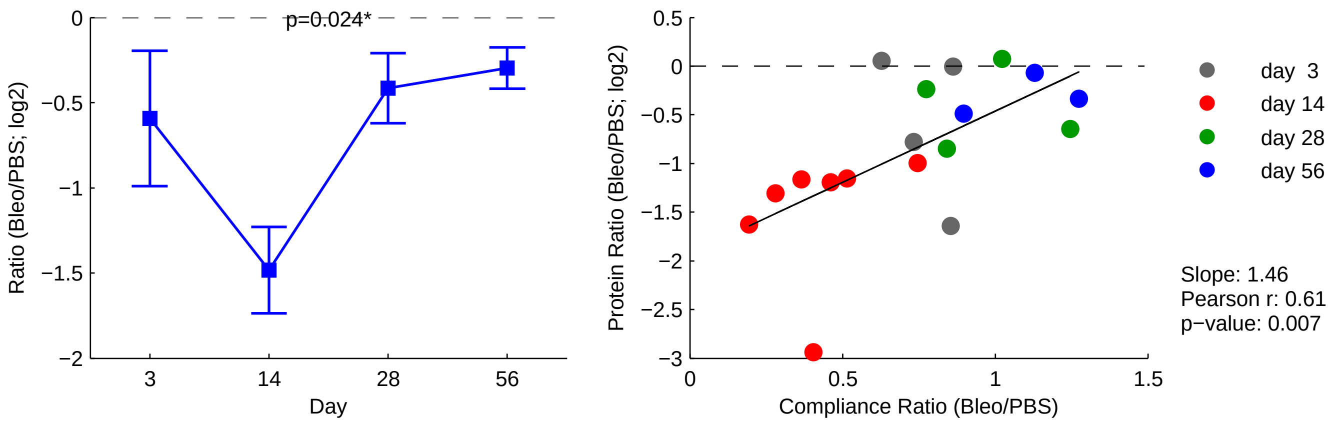

Q9QUH0 − Glrx (id: 7680)

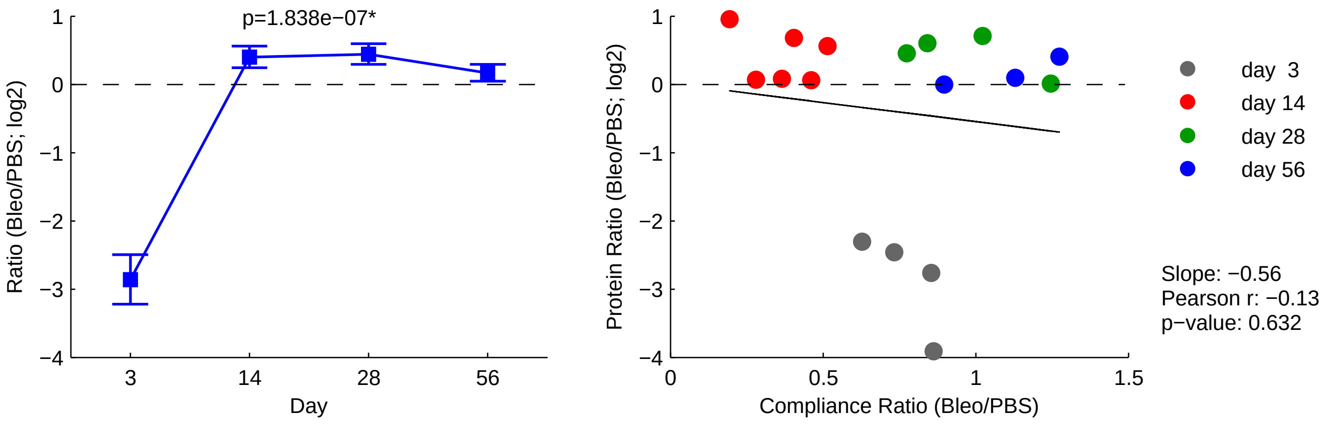

Q9QUI0 − Rhoa (id: 7681)

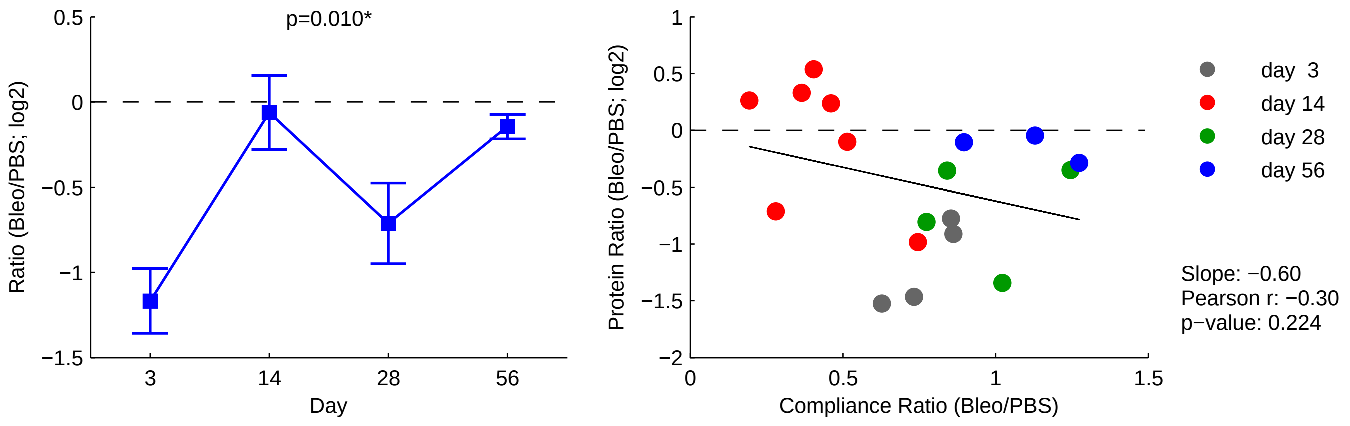

Q9QUJ7−2 − Acsl4 (id: 7682)

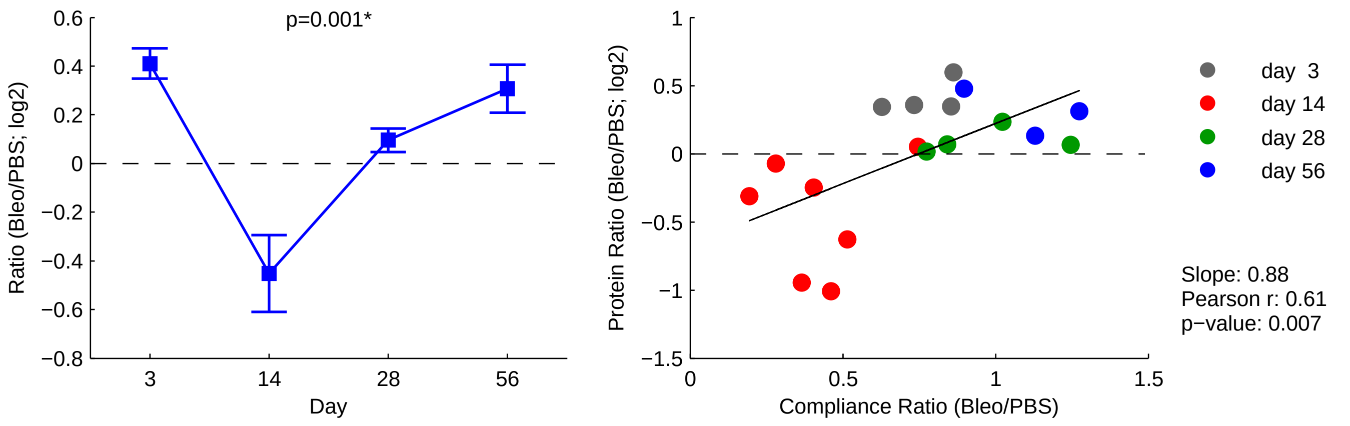

Q9QUM0 − Itga2b (id: 7685)

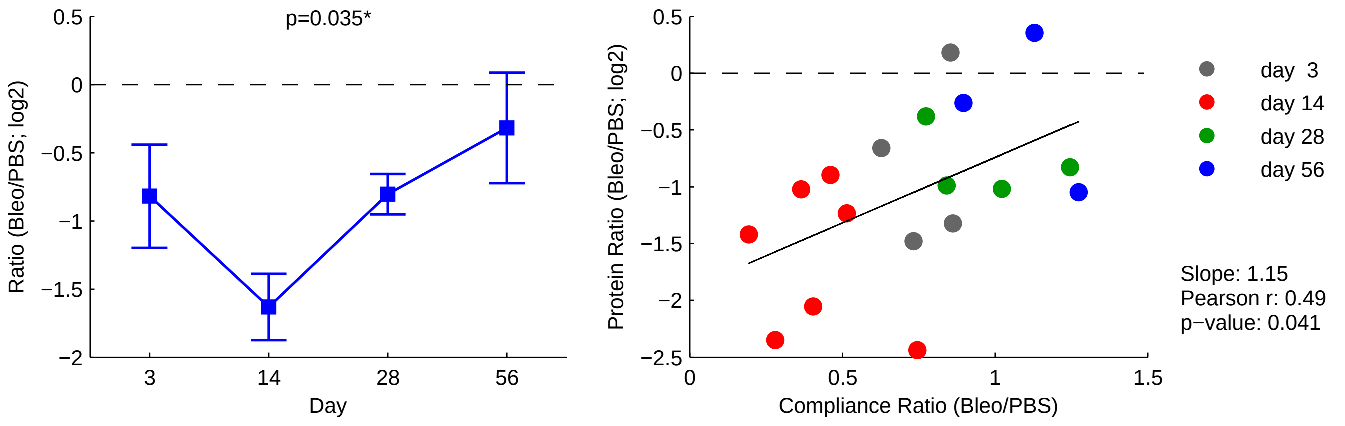

Q9QUM9 − Psma6 (id: 7687)

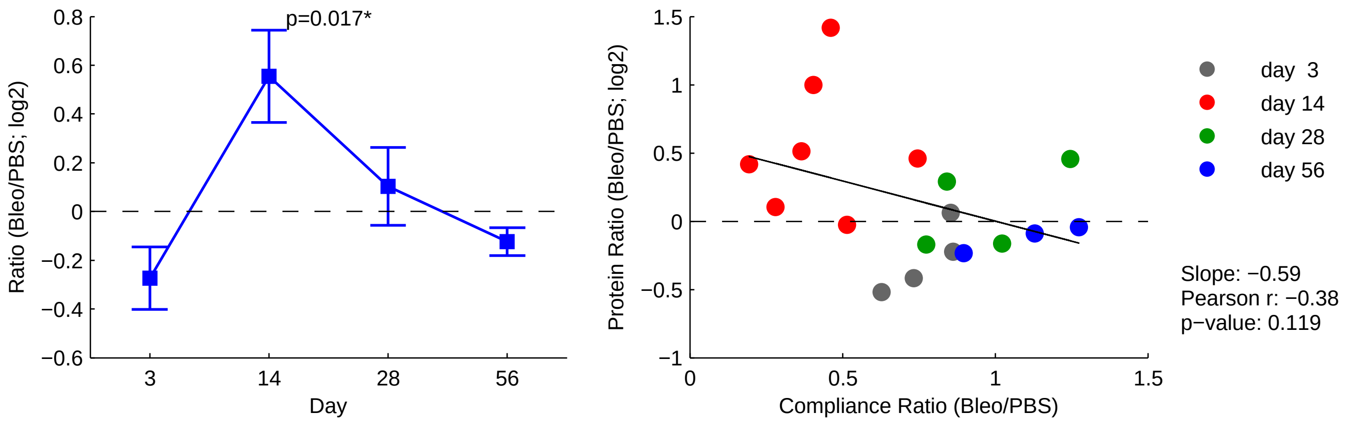

Q9QUR6 − Prep (id: 7690)

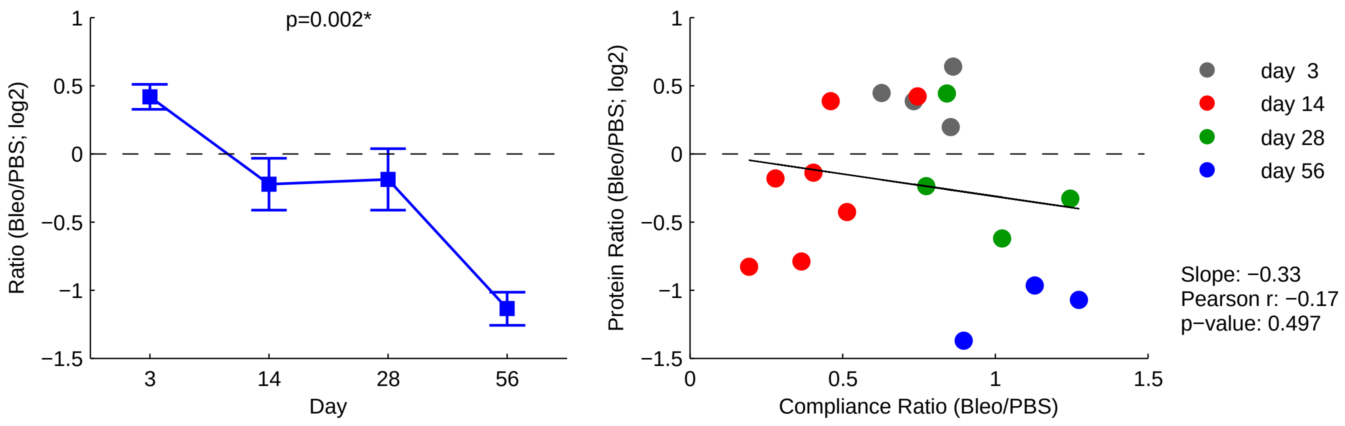

Q9QUR7 − Pin1 (id: 7691)

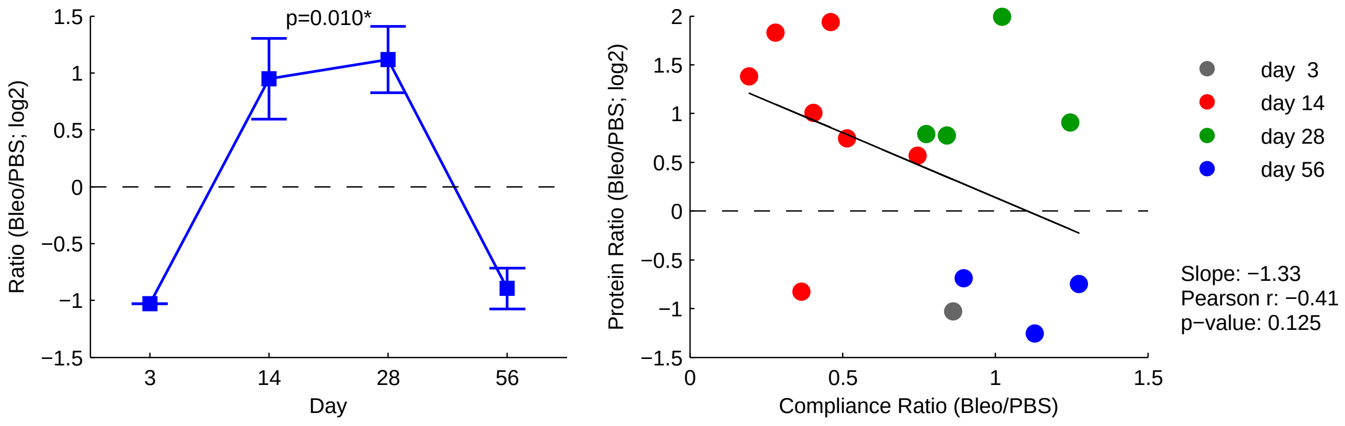

Q9QUR8 − Sema7a (id: 7692)

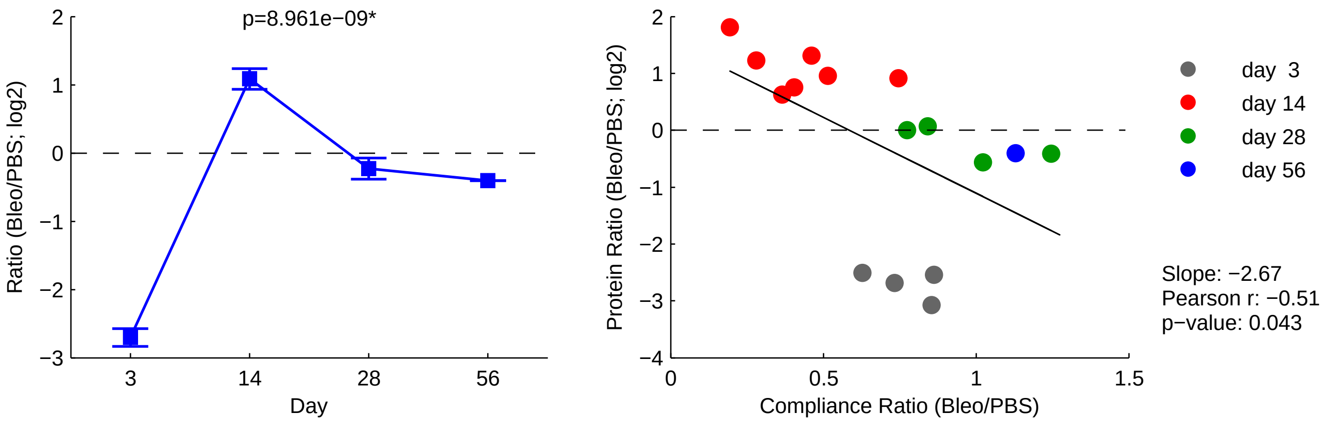

Q9QVP4 − Myl7 (id: 7693)

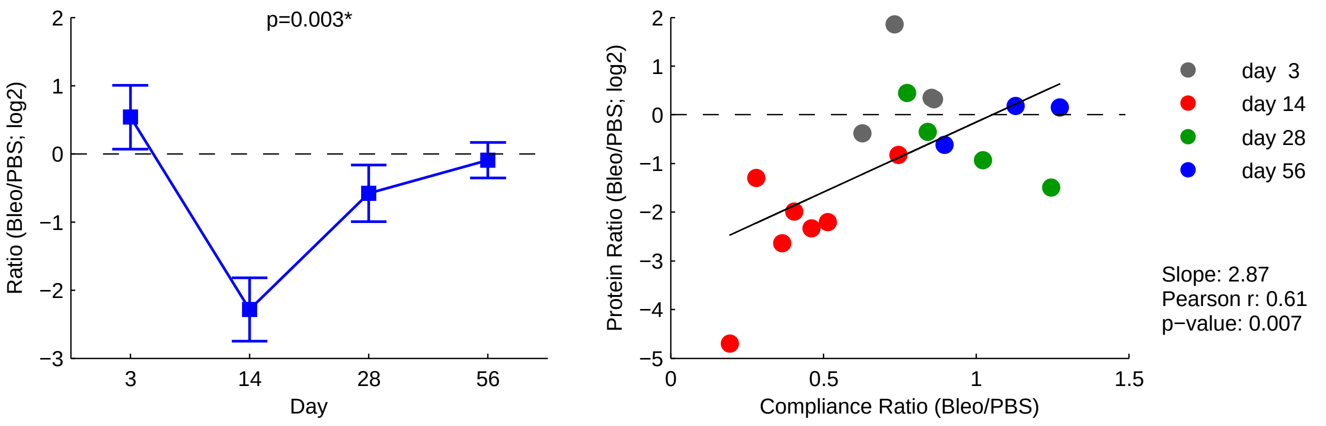

Q9QWR8 − Naga (id: 7697)

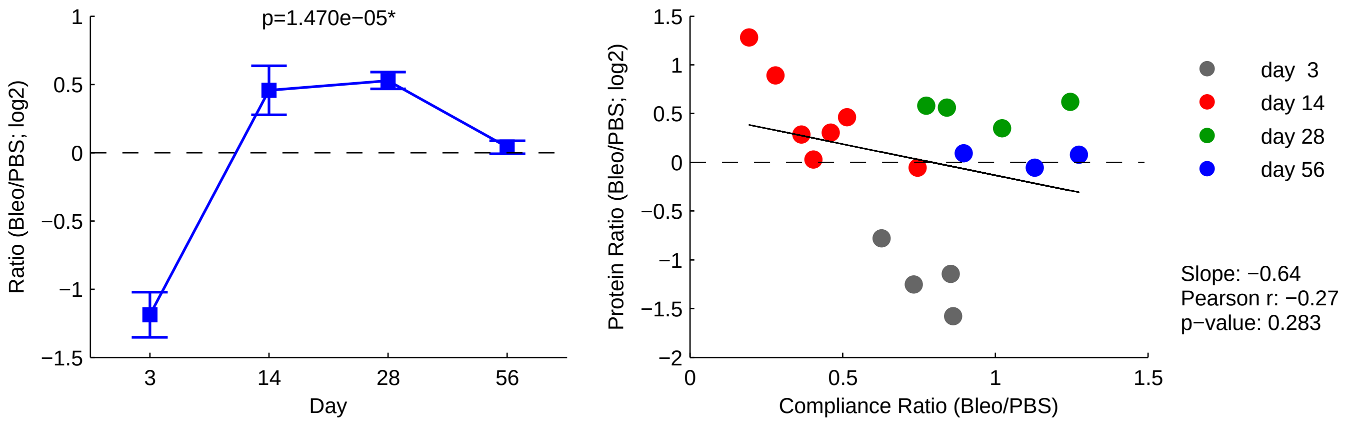

Q9QWV4 − Mlf1 (id: 7698)

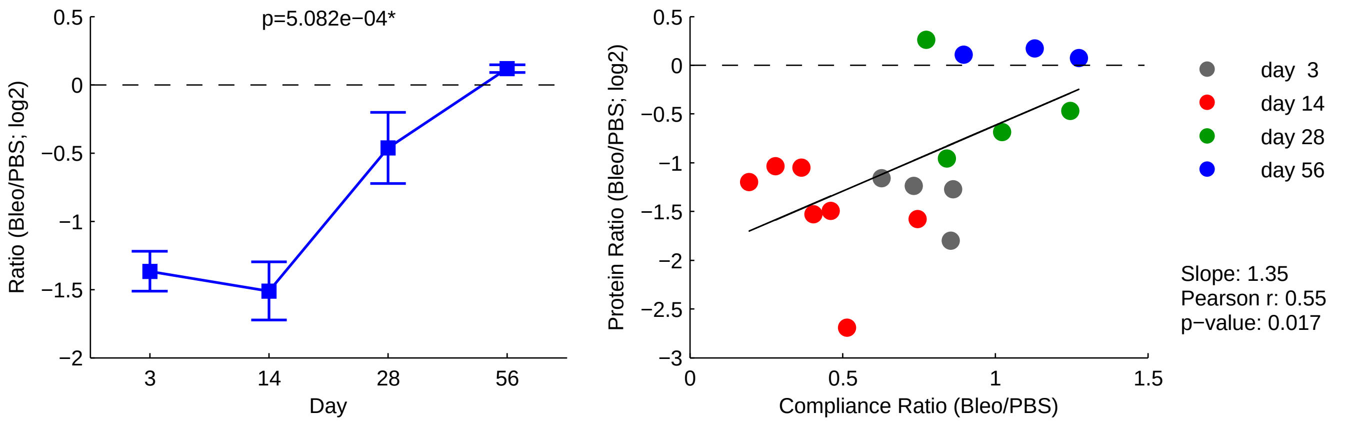

Q9QX60−2 − Dguok (id: 7702)

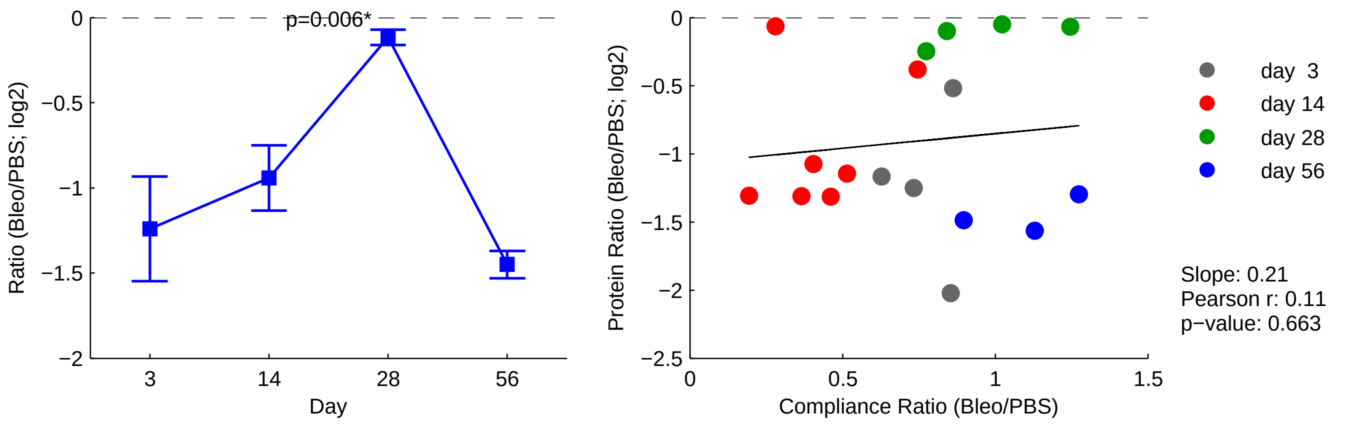

Q9QXH4 − Itgax (id: 7710)

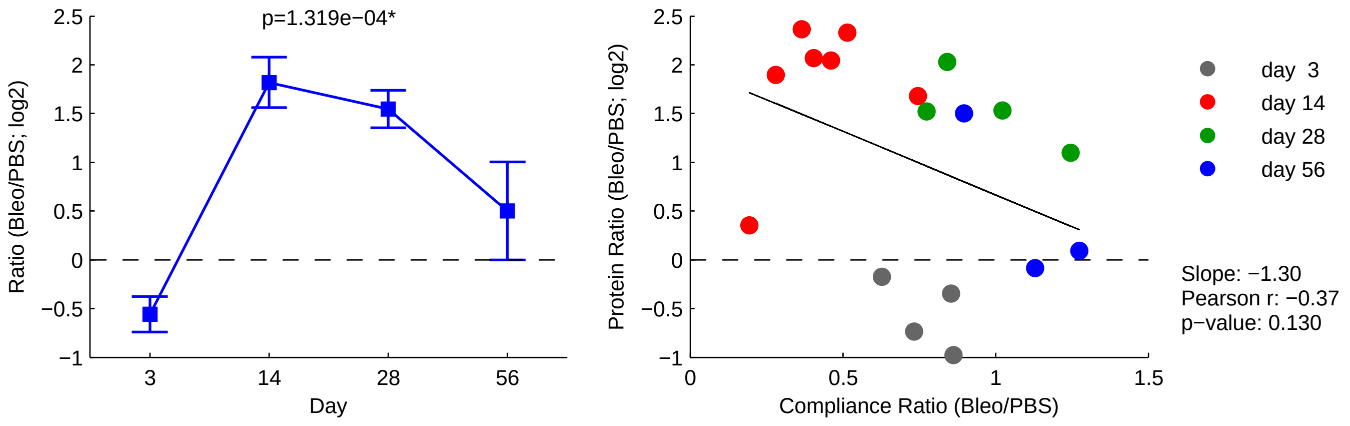

Q9QXK3 − Copg2 (id: 7712)

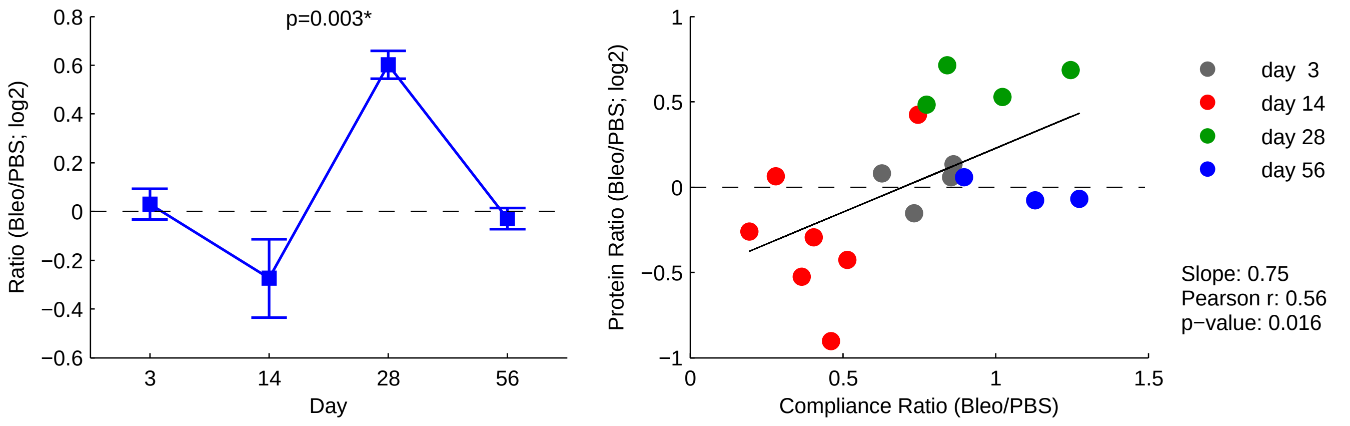

Q9QXS6−3 − Dbn1 (id: 7719)

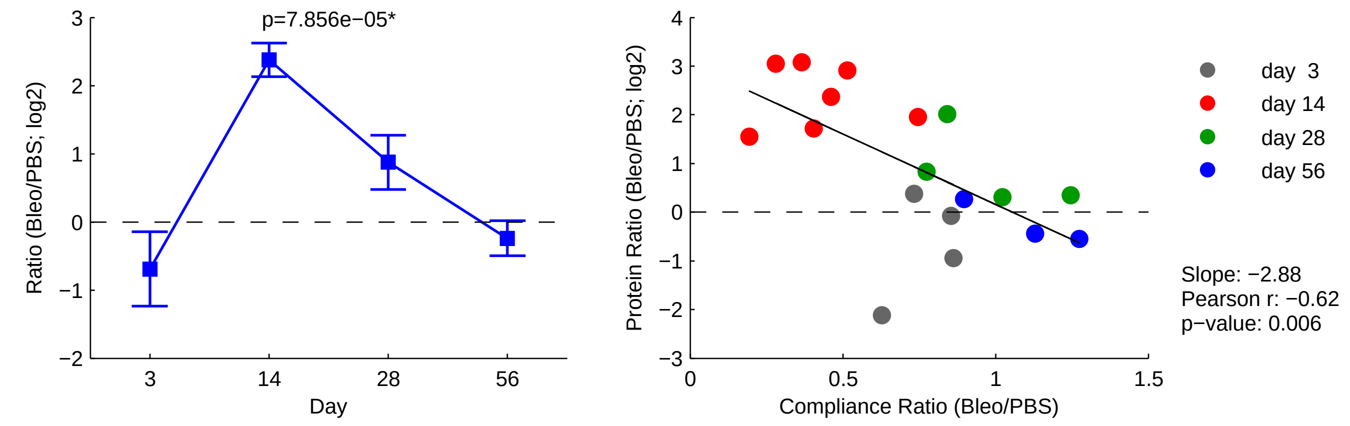

Q9QXT0 − Cnpy2 (id: 7720)

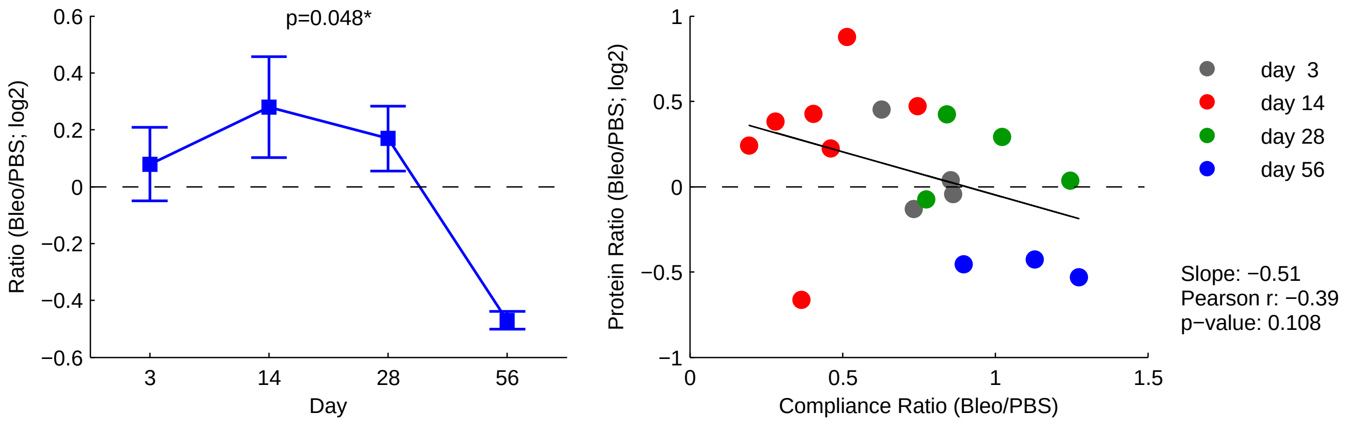

Q9QXV1 − Cbx8 (id: 7721)

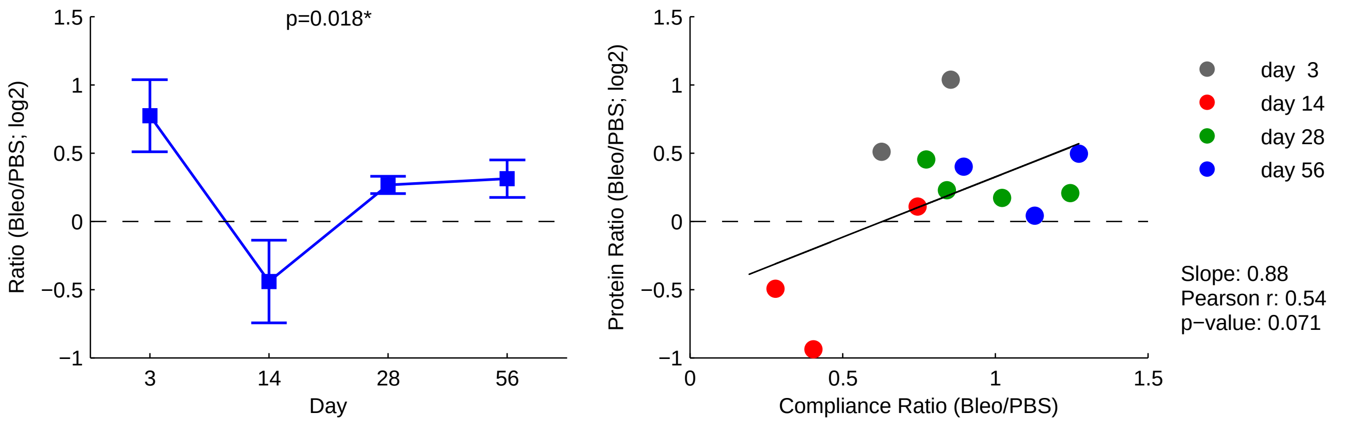

Q9QXX4 − Slc25a13 (id: 7725)

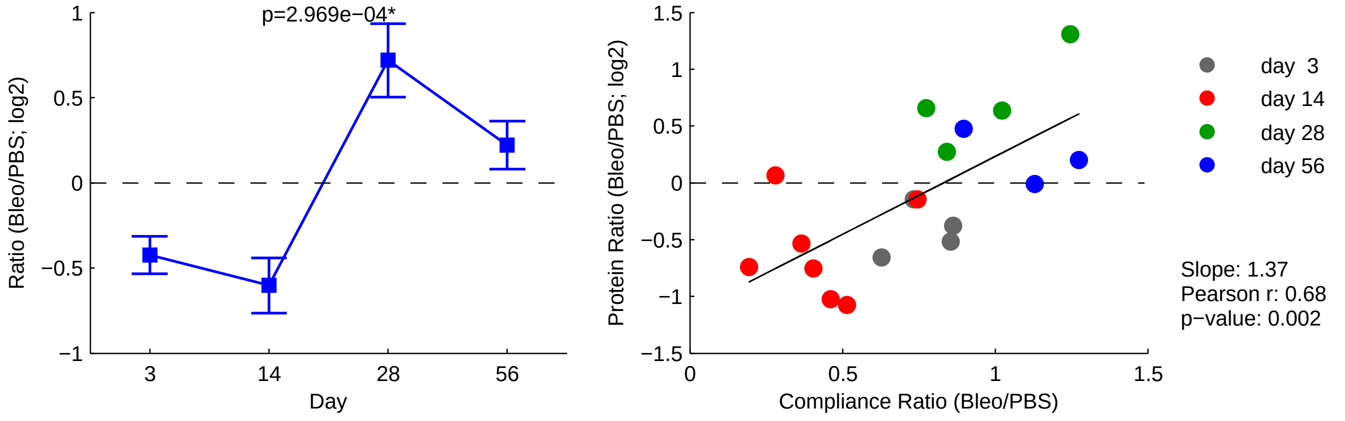

Q9QXY9 − Pex3 (id: 7727)

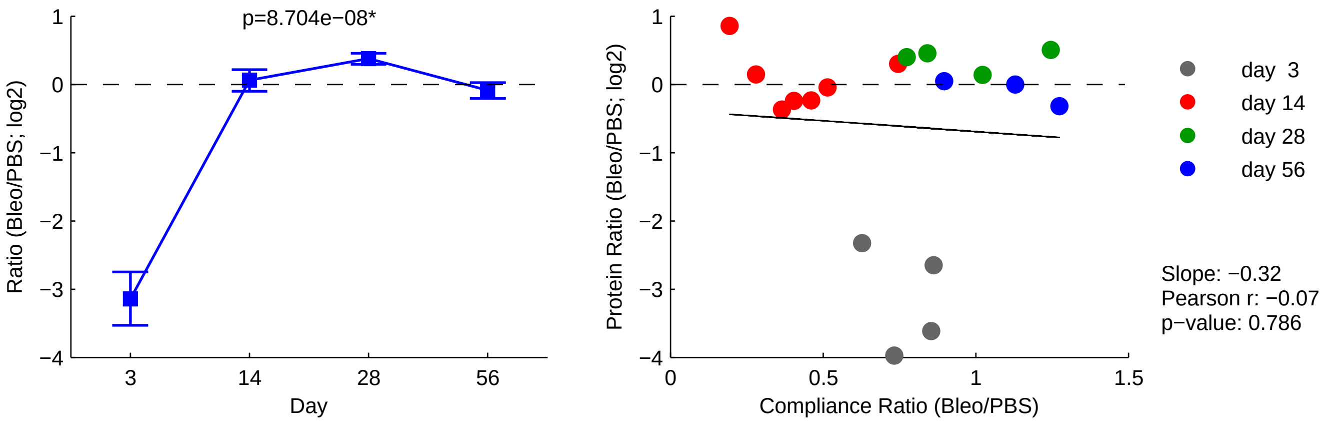

Q9QY81 − Nup210 (id: 7730)

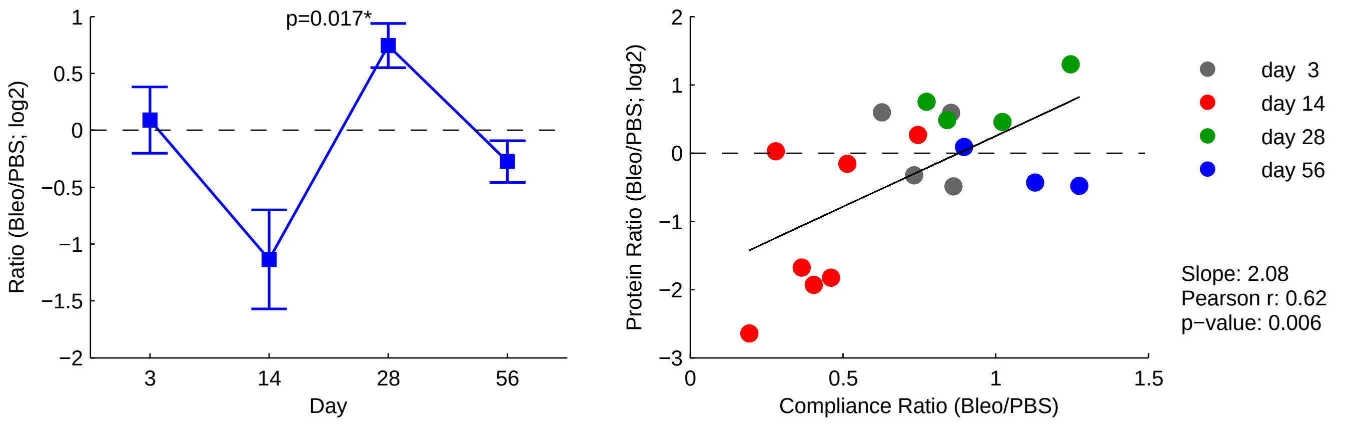

Q9QYC7 − Ggcx (id: 7738)

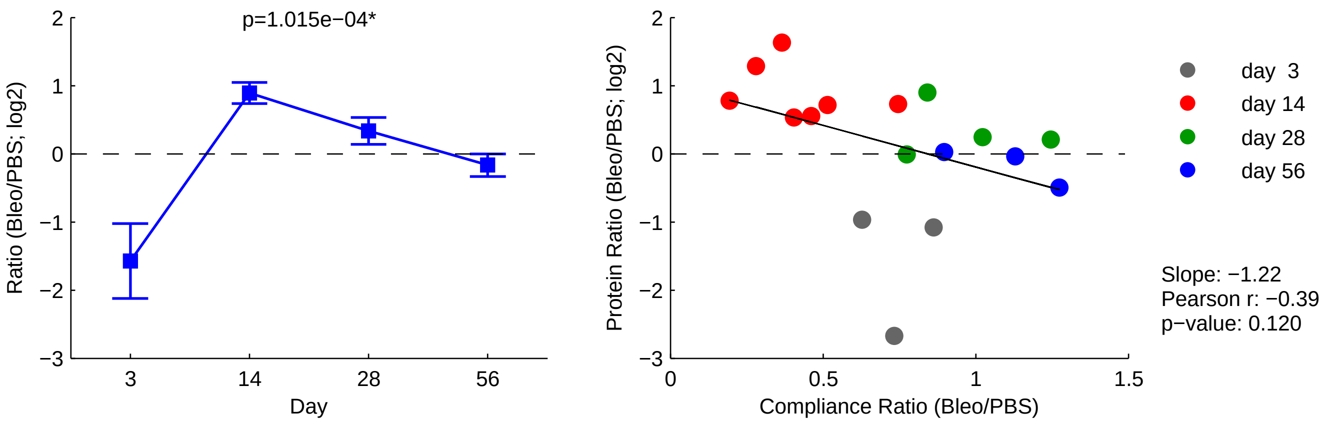

Q9QYE6 − Golga5 (id: 7739)

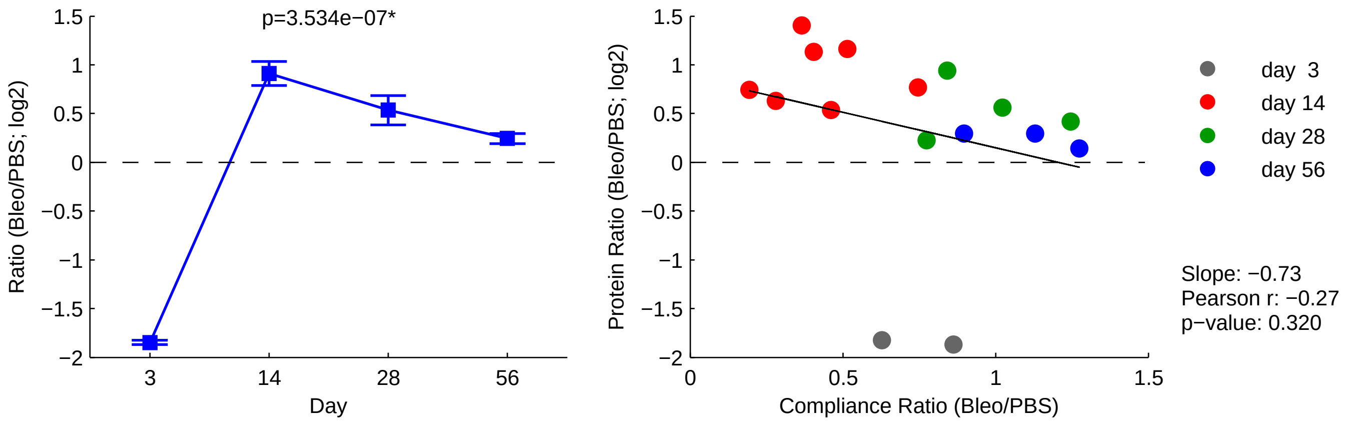

Q9R1R8 − Rdh11 (id: 7740)

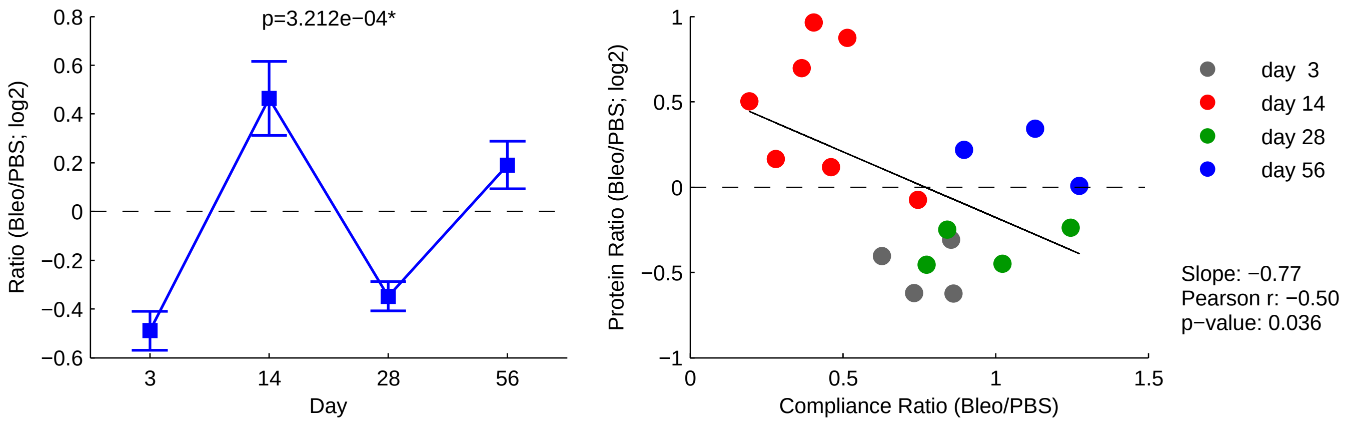

Q9QYI5 − Dnajb2 (id: 7744)

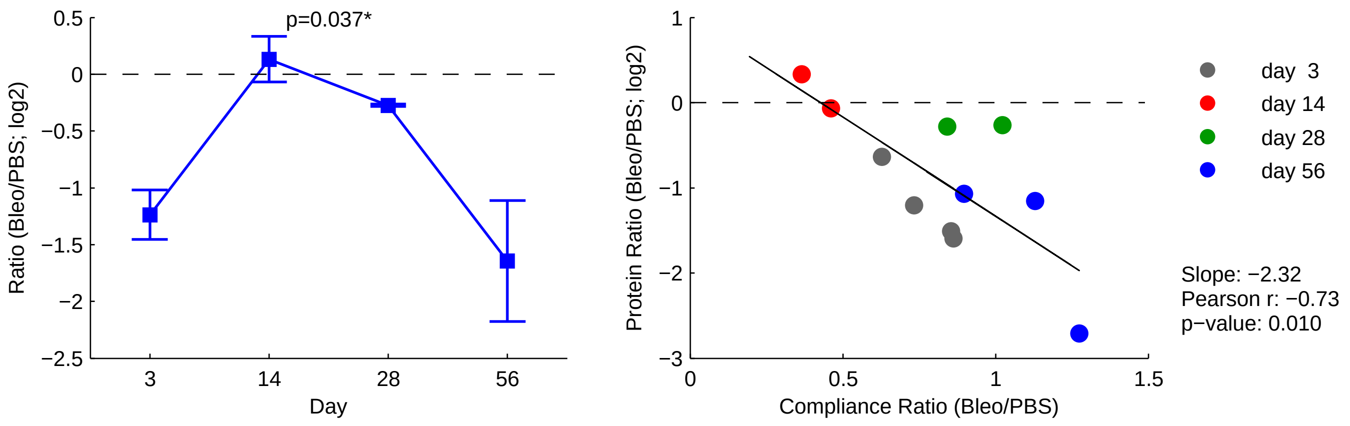

Q9QYJ3 − Dnajb1 (id: 7746)

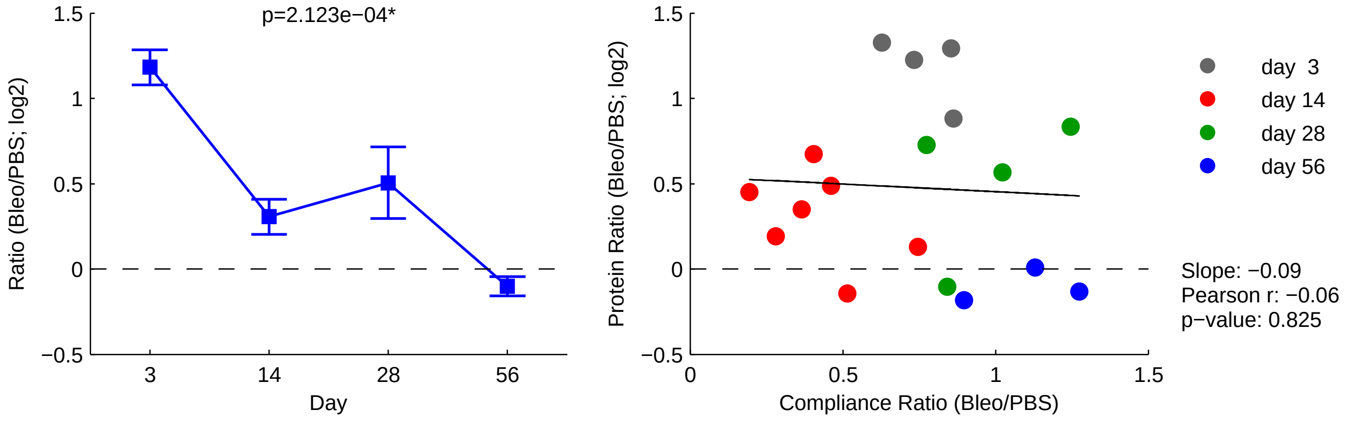

Q9QYR9 − Acot2 (id: 7748)

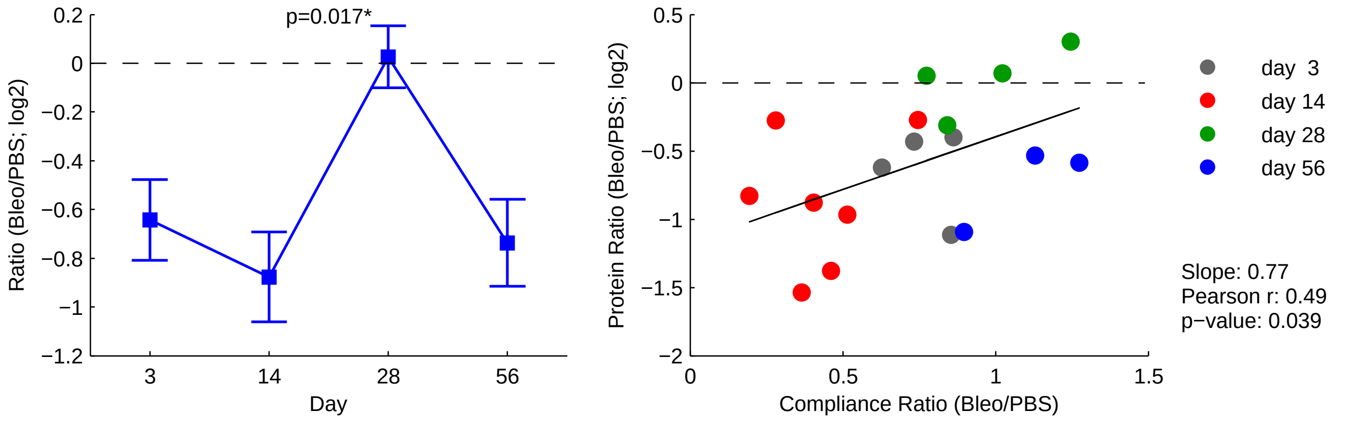

Q9QZ23 − Nfu1 (id: 7753)

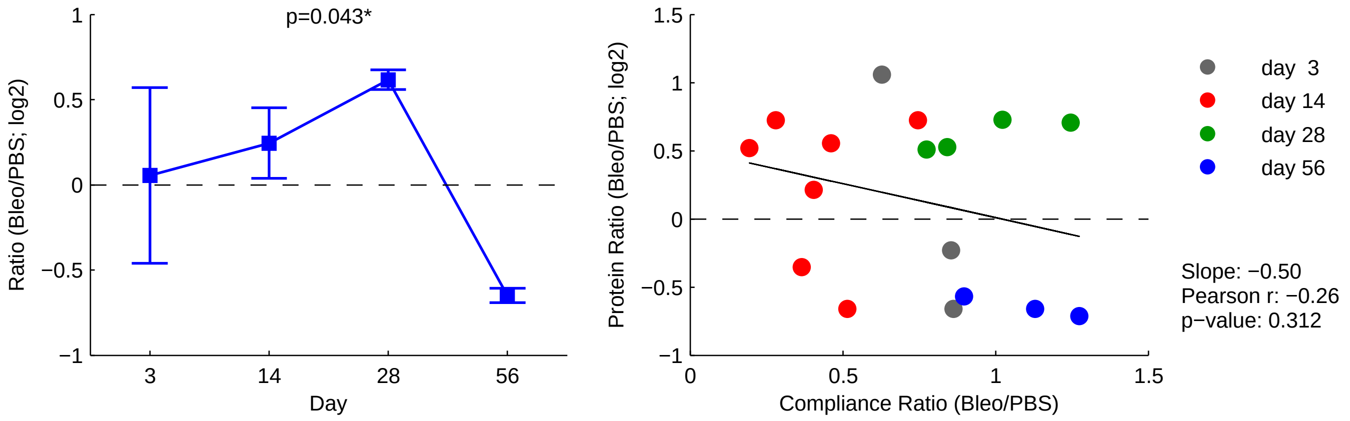

Q9QZ73 − Dcun1d1 (id: 7755)

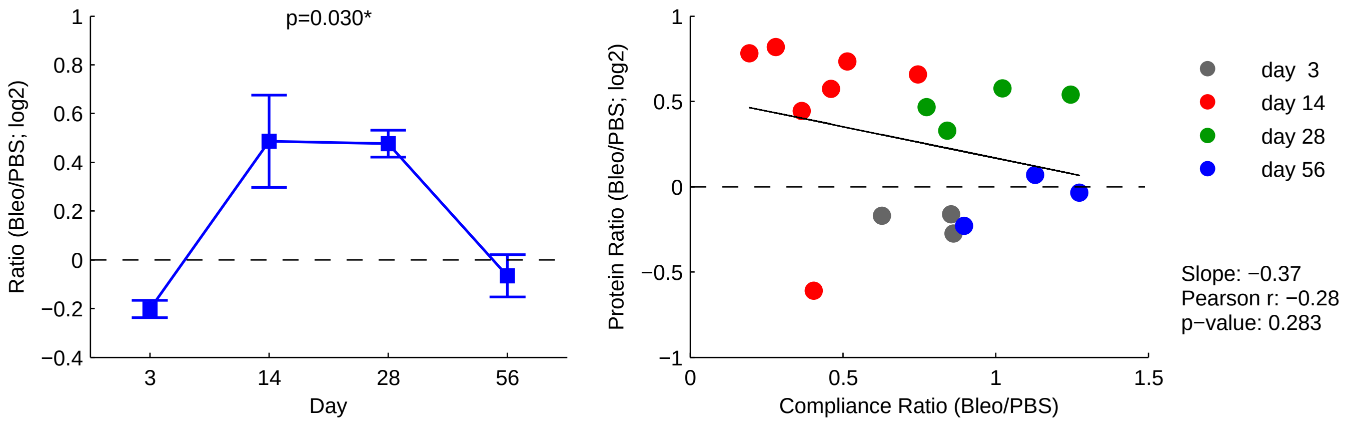

Q9QZ88 − Vps29 (id: 7757)

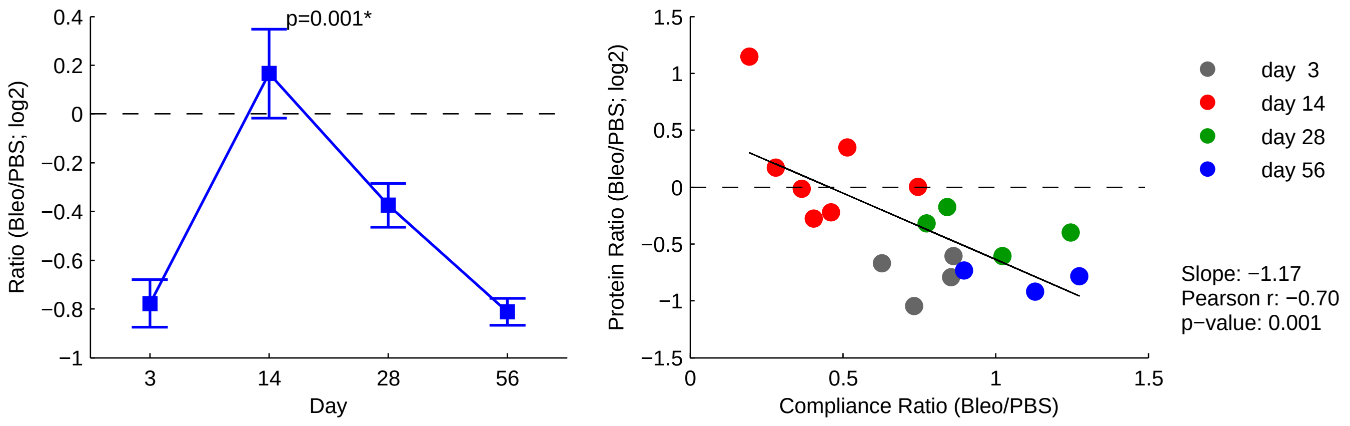

Q9QZB7 − Actr10 (id: 7759)

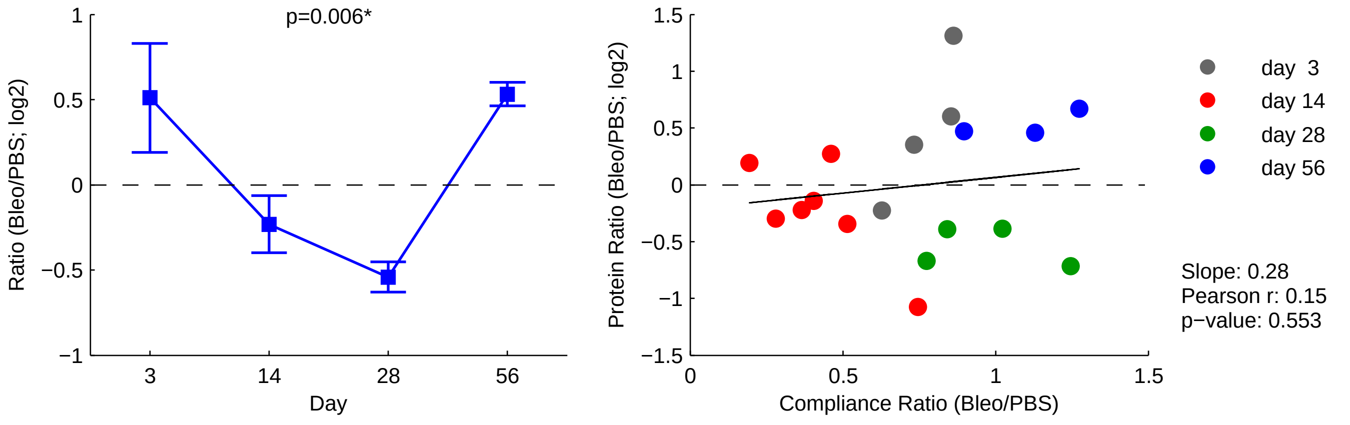

Q9QZB9 − Dctn5 (id: 7760)

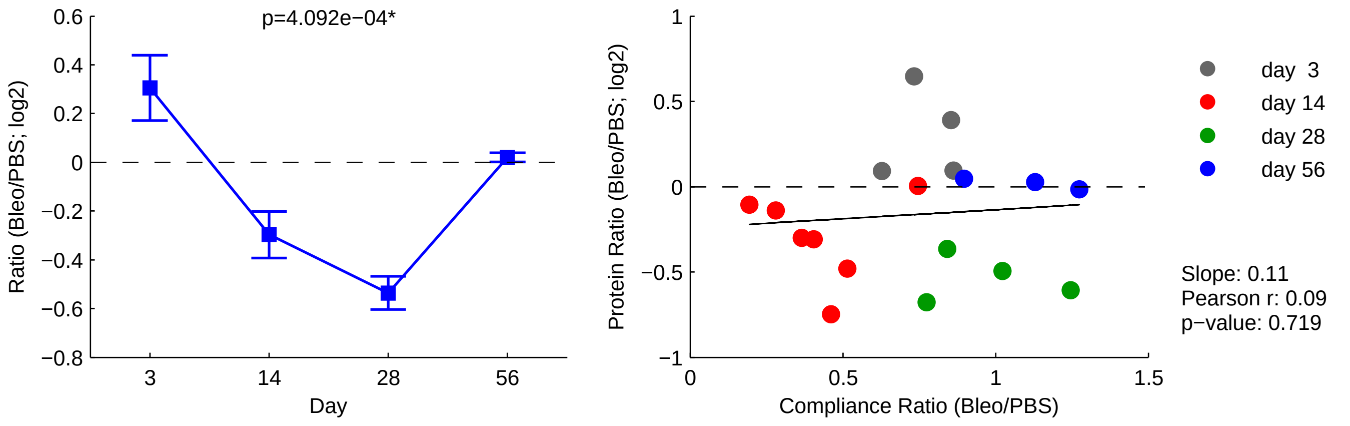

Q9QZD8 − Slc25a10 (id: 7763)

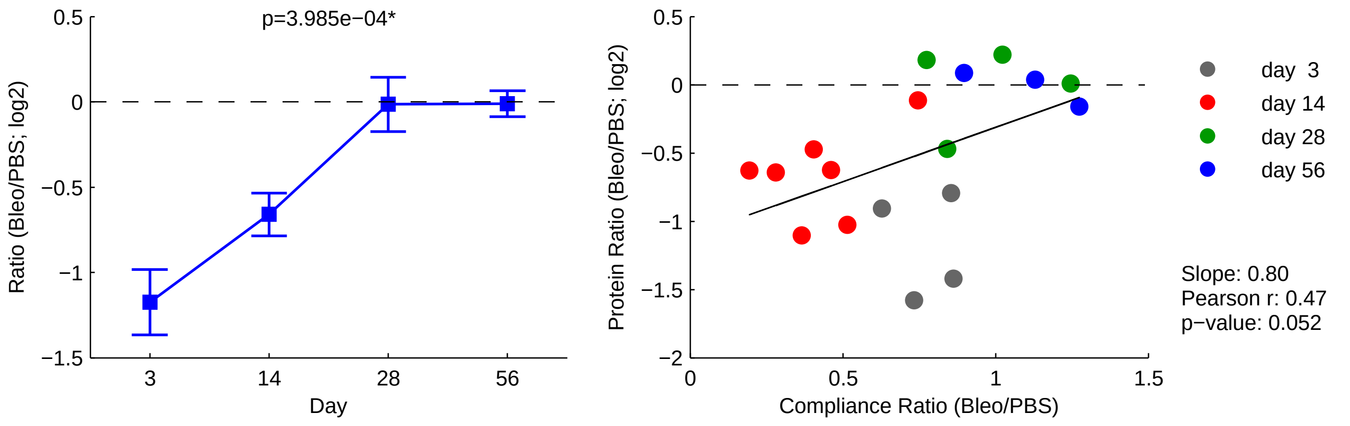

Q9QZD9 − Eif3i (id: 7764)

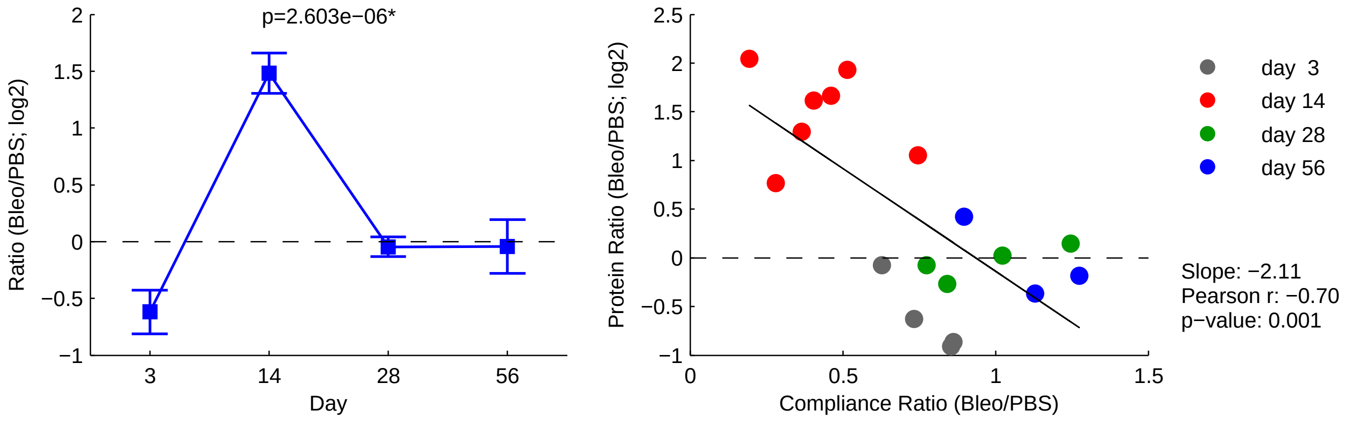

Q9QZE5 − Copg1 (id: 7765)

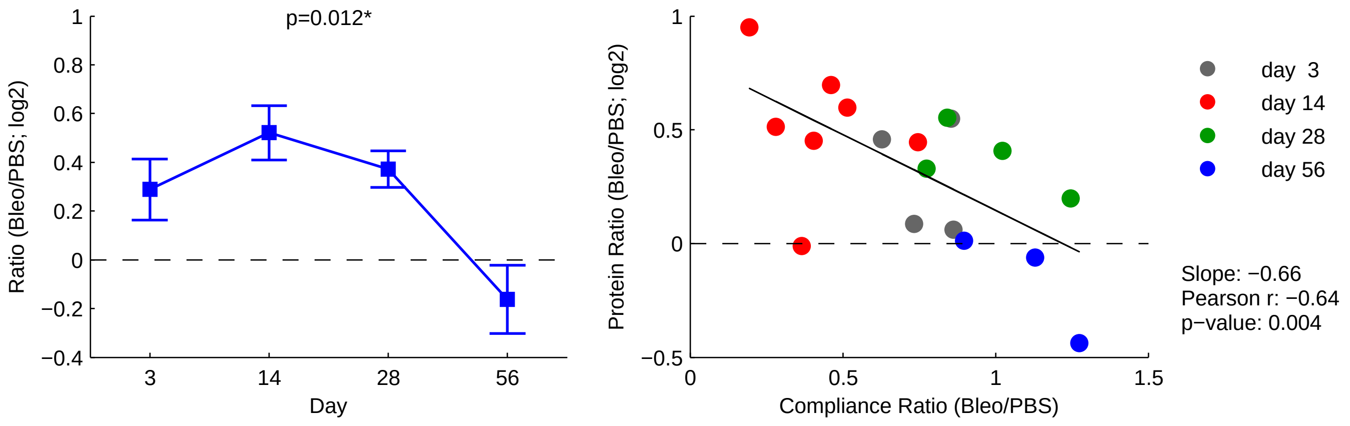

Q9QZH6 − Ecsit (id: 7769)

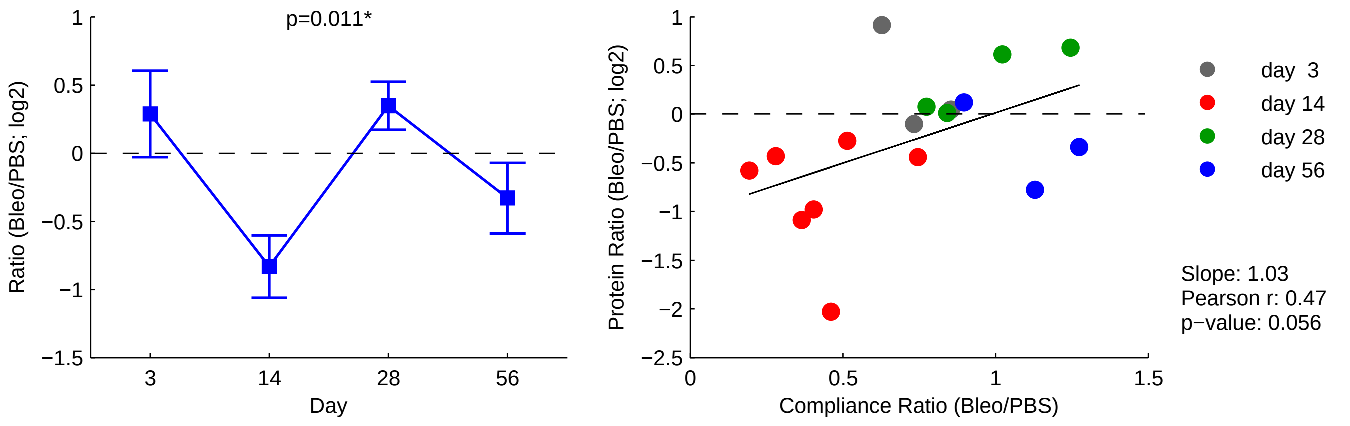

Q9QZM0 − Ubqln2 (id: 7771)

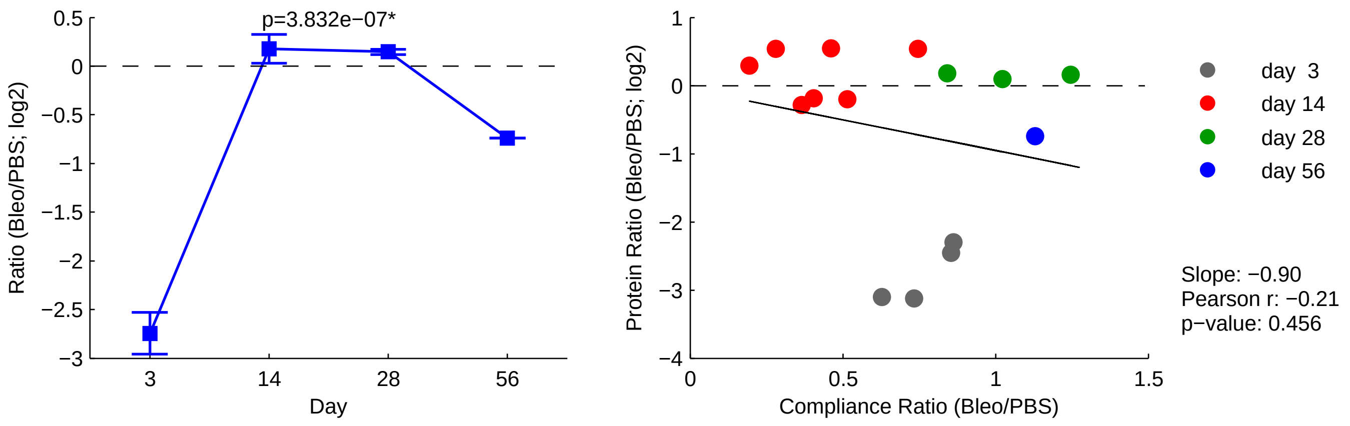

Q9QZQ8 − H2afy (id: 7773)

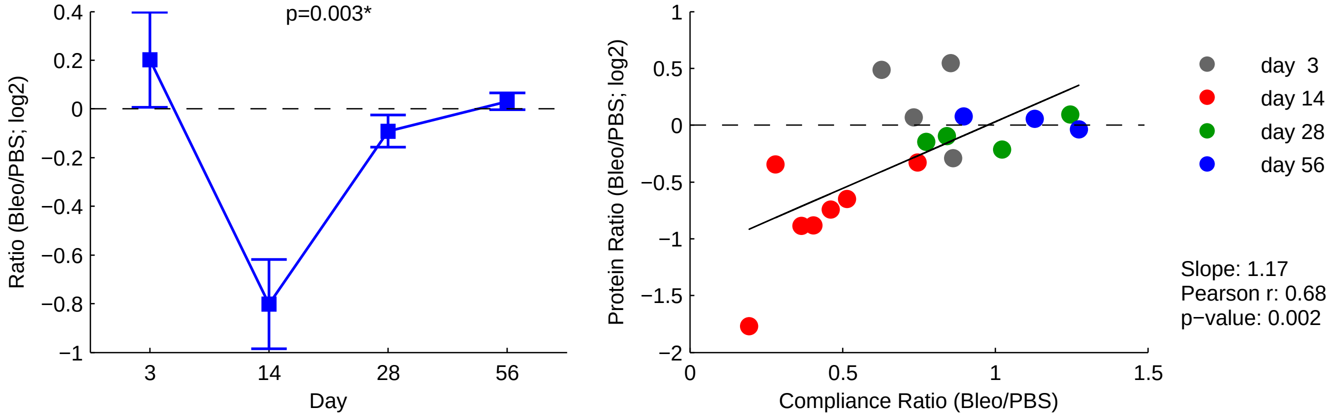

Q9QZQ8−2 − (id: 7774)

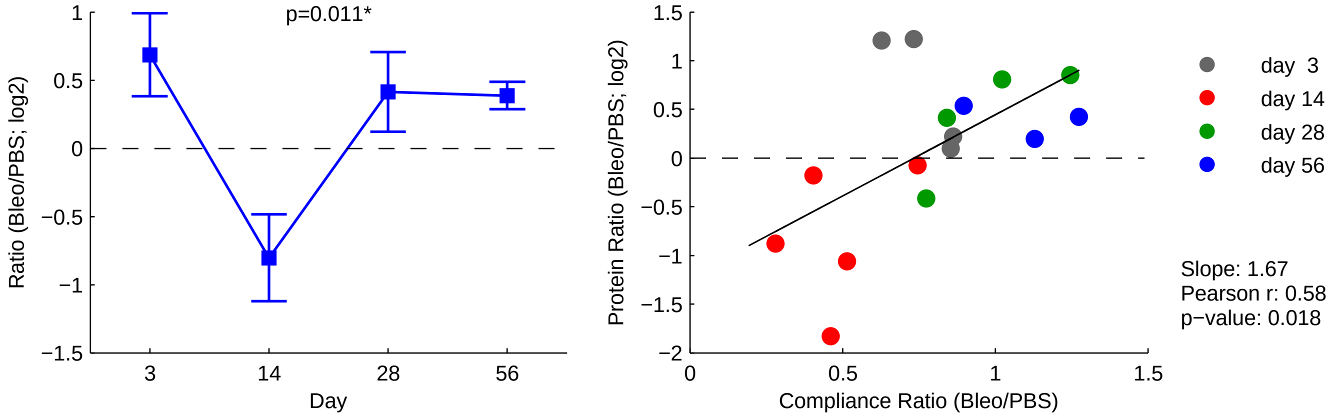

Q9QZR9 − Col4a4 (id: 7775)

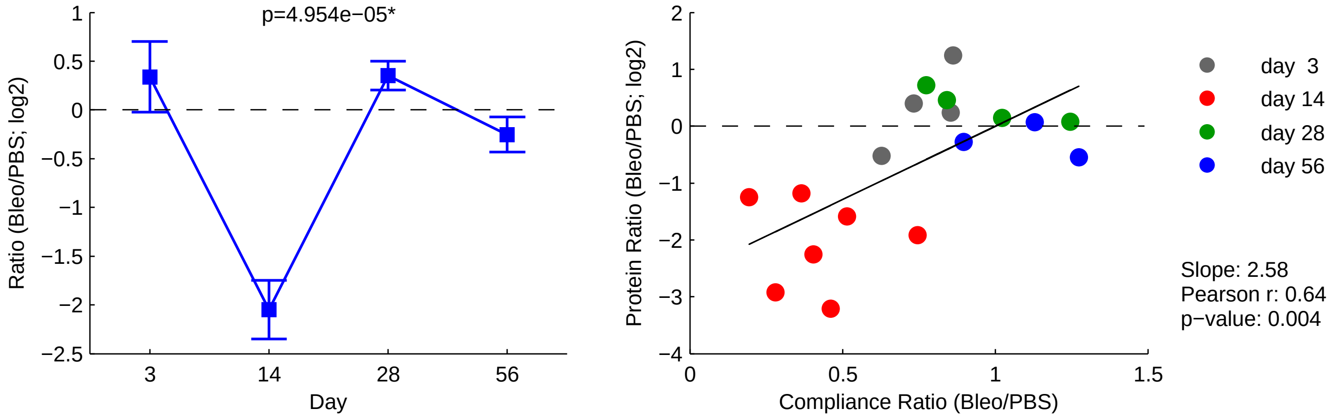

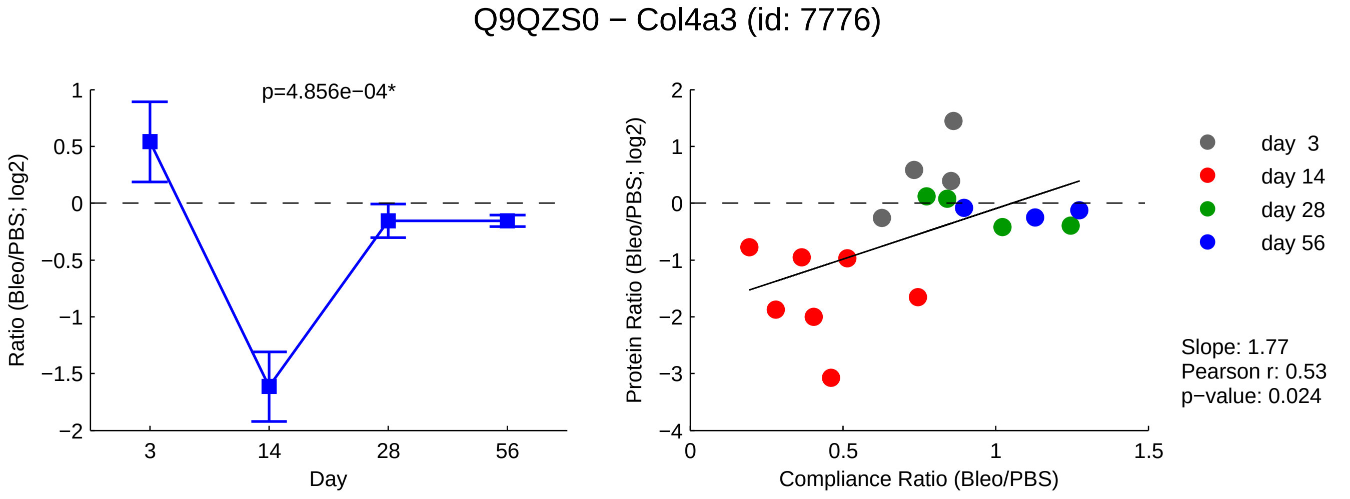

Q9QZS0 − Col4a3 (id: 7776)
p=4.856e−04*
Ratio (Bleo/PBS; log2)
Day
Protein Ratio (Bleo/PBS; log2)
Compliance Ratio (Bleo/PBS)
day 3
day 14
day 28
day 56
Slope: 1.77
Pearson r: 0.53
p−value: 0.024

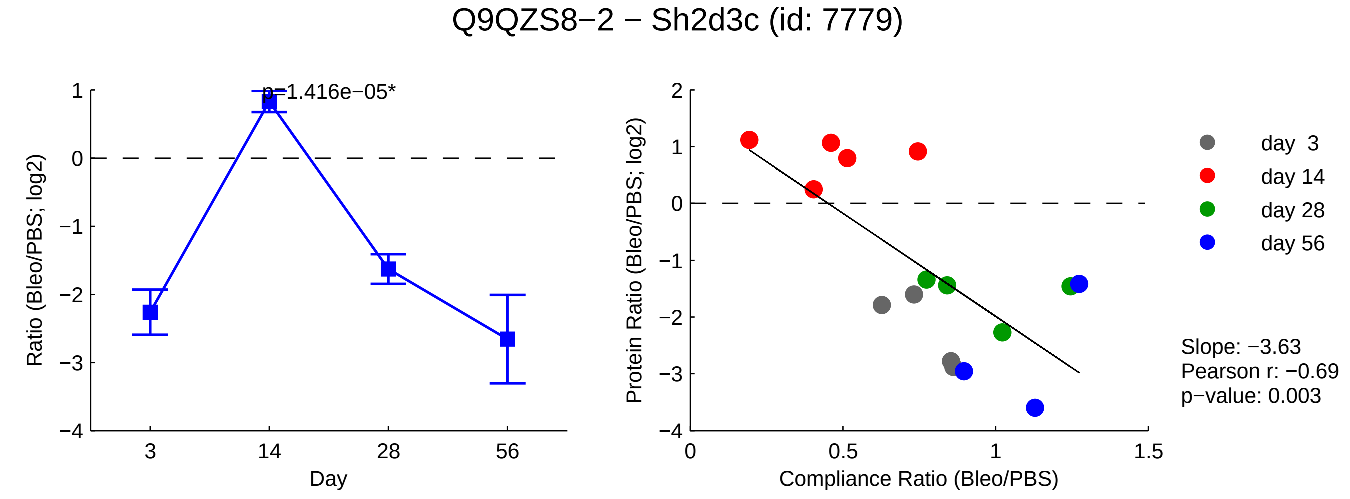

Q9QZS8−2 − Sh2d3c (id: 7779)
p=1.416e−05*
Ratio (Bleo/PBS; log2)
Day
Protein Ratio (Bleo/PBS; log2)
Compliance Ratio (Bleo/PBS)
day 3
day 14
day 28
day 56
Slope: −3.63
Pearson r: −0.69
p−value: 0.003

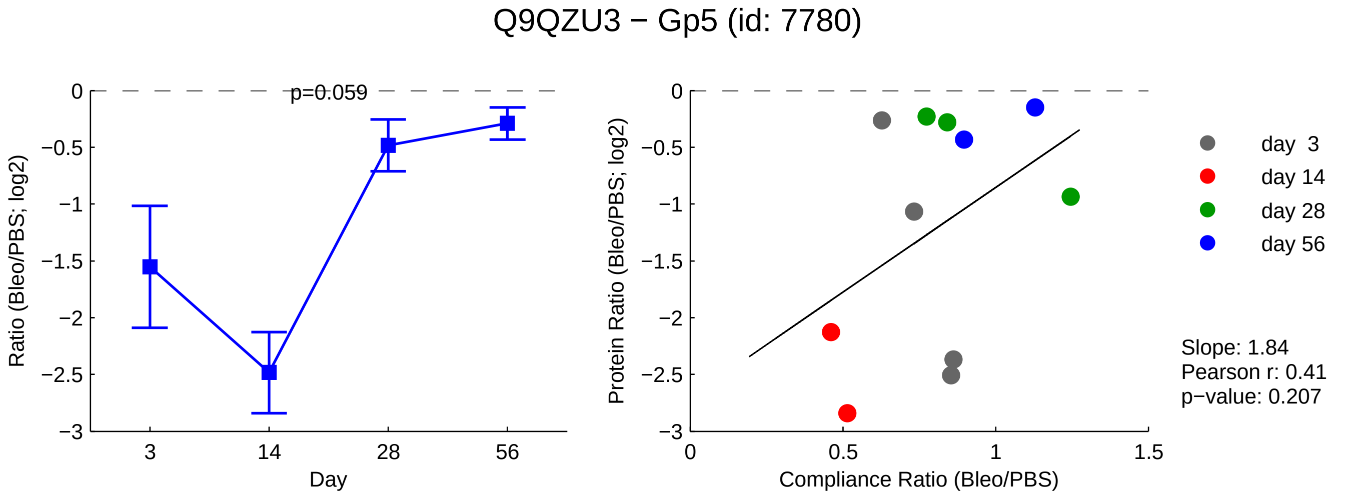

Q9QZU3 − Gp5 (id: 7780)
p=0.059
Ratio (Bleo/PBS; log2)
Day
Protein Ratio (Bleo/PBS; log2)
Compliance Ratio (Bleo/PBS)
day 3
day 14
day 28
day 56
Slope: 1.84
Pearson r: 0.41
p−value: 0.207

Q9R045 − Angptl2 (id: 7786)

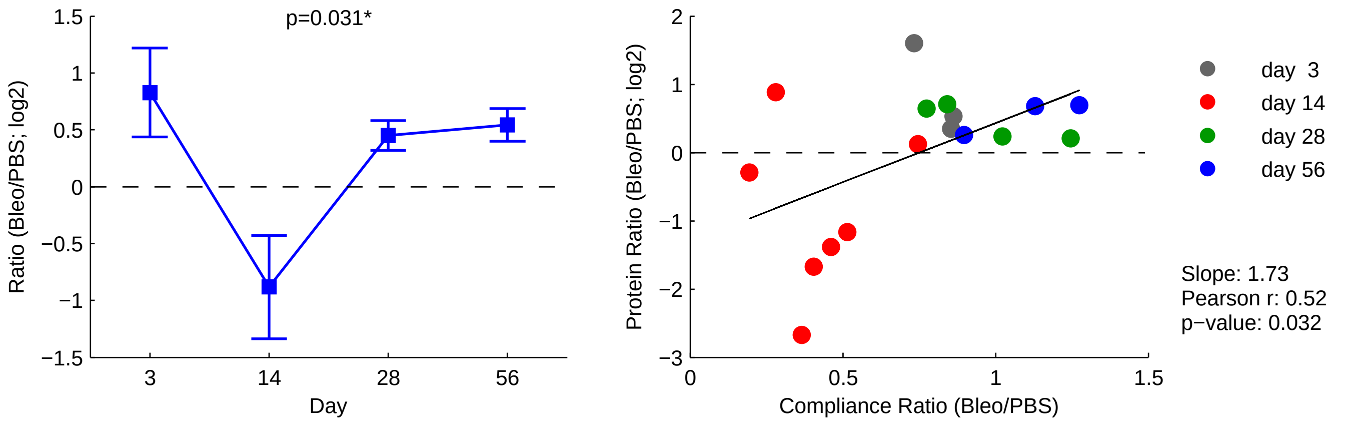

Q9R062 − Gyg1 (id: 7790)

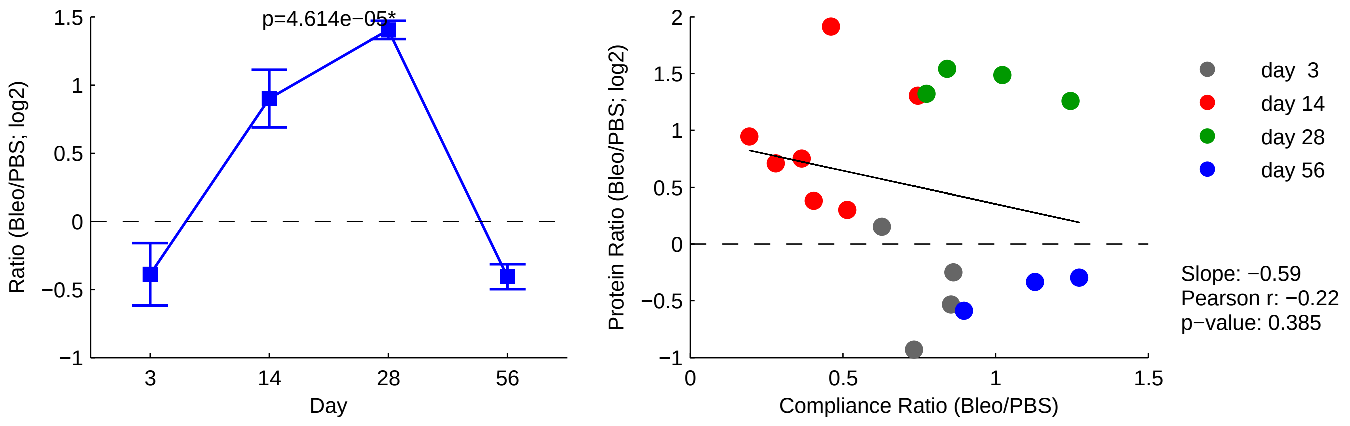

Q9R069 − Bcam (id: 7791)

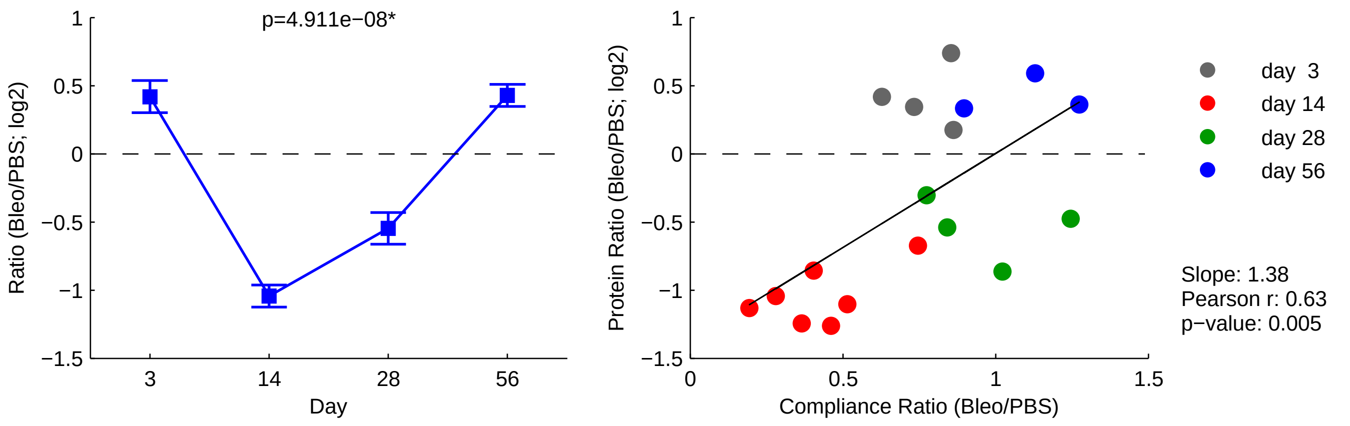

Q9R078 − Prkab1 (id: 7792)

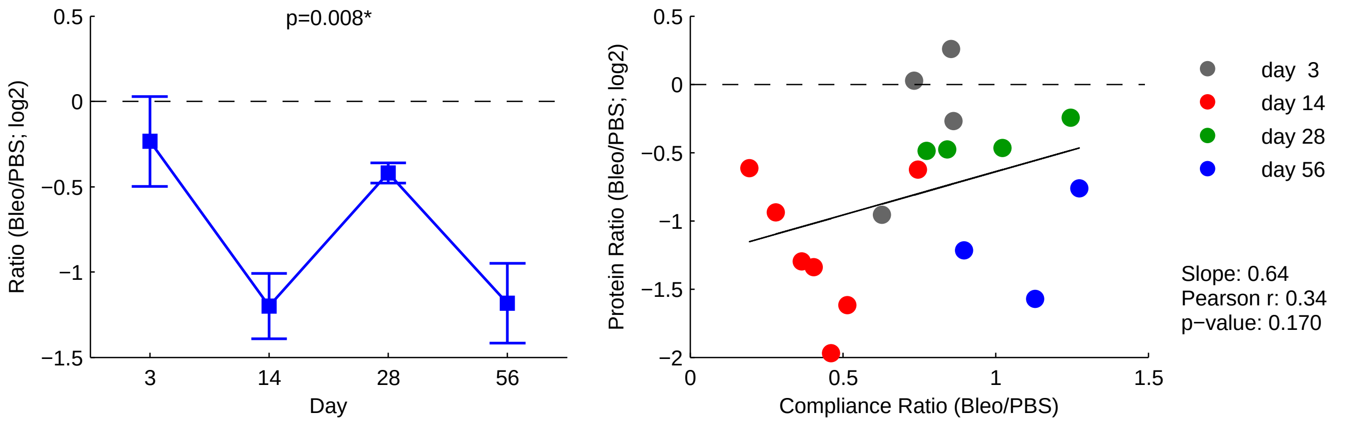

Q9R097 − Spint1 (id: 7793)

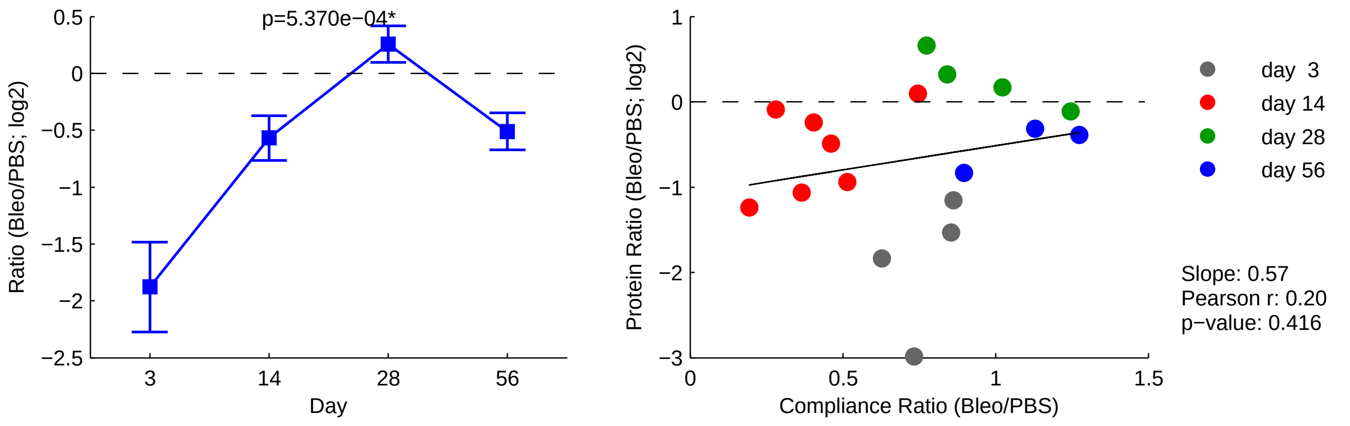

Q9R099 − Tbl2 (id: 7795)

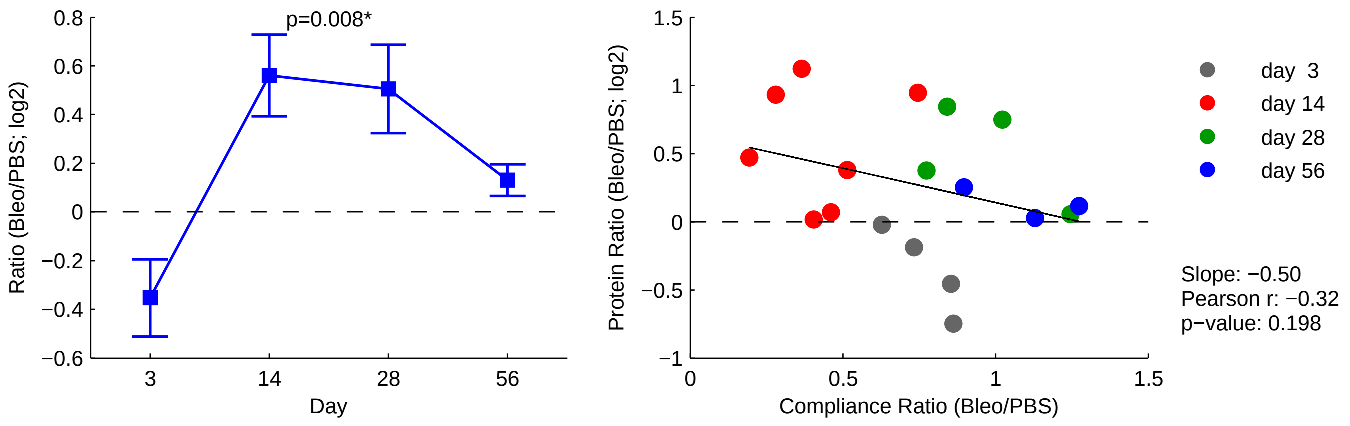

Q9R0A0 − Pex14 (id: 7796)

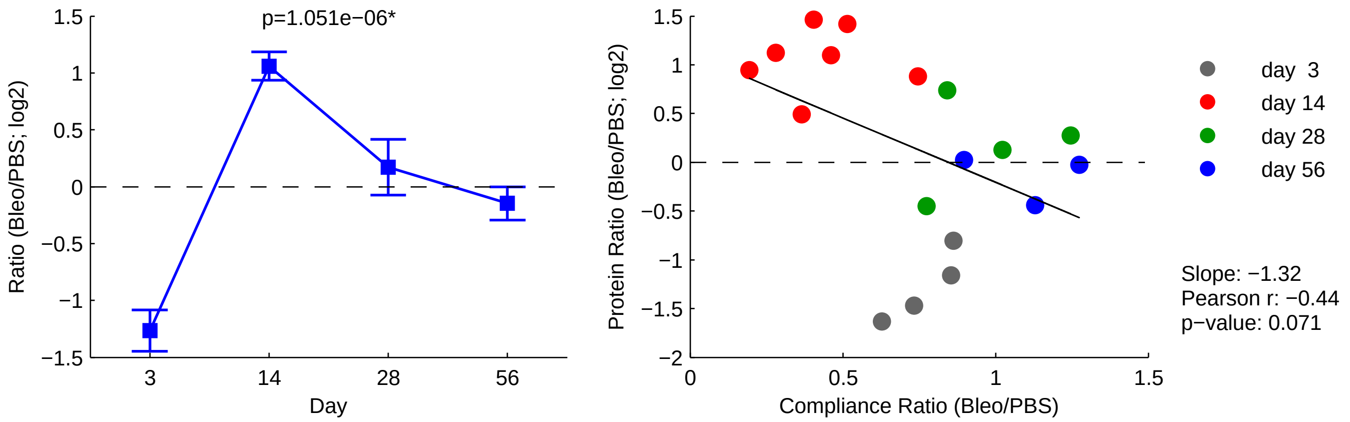

Q9R0E1 − Plod3 (id: 7800)

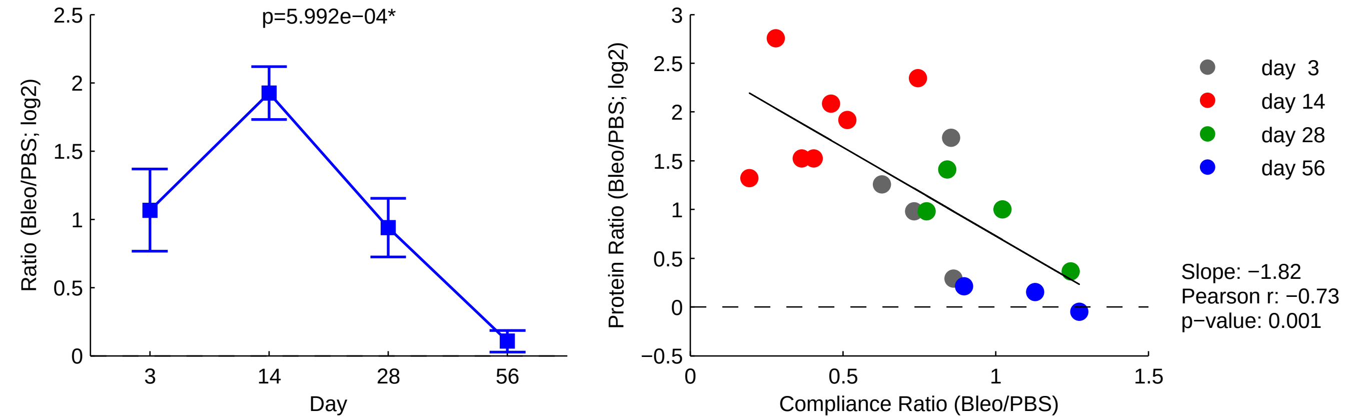

Q9R0E2 − Plod1 (id: 7801)

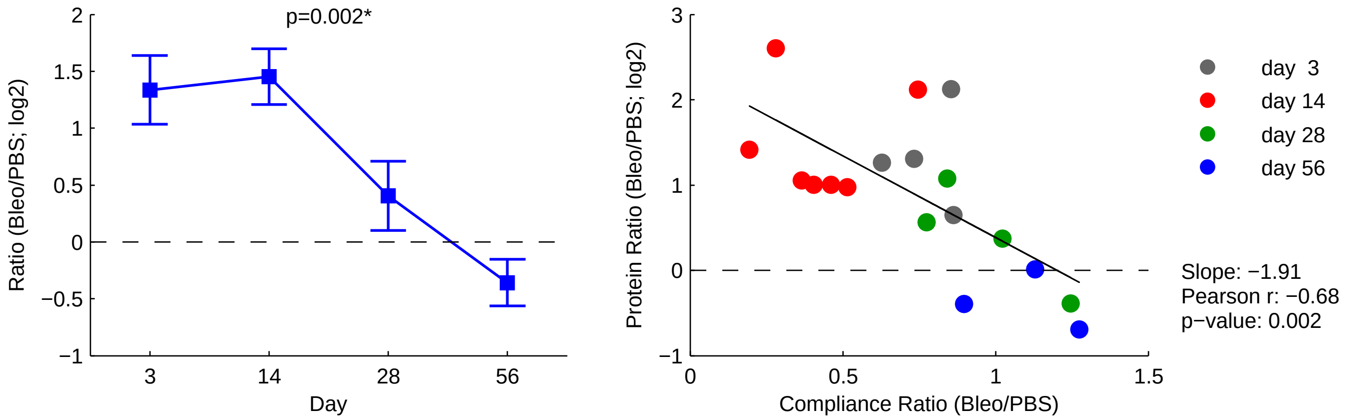

Q9R0H0−2 − Acox1 (id: 7803)

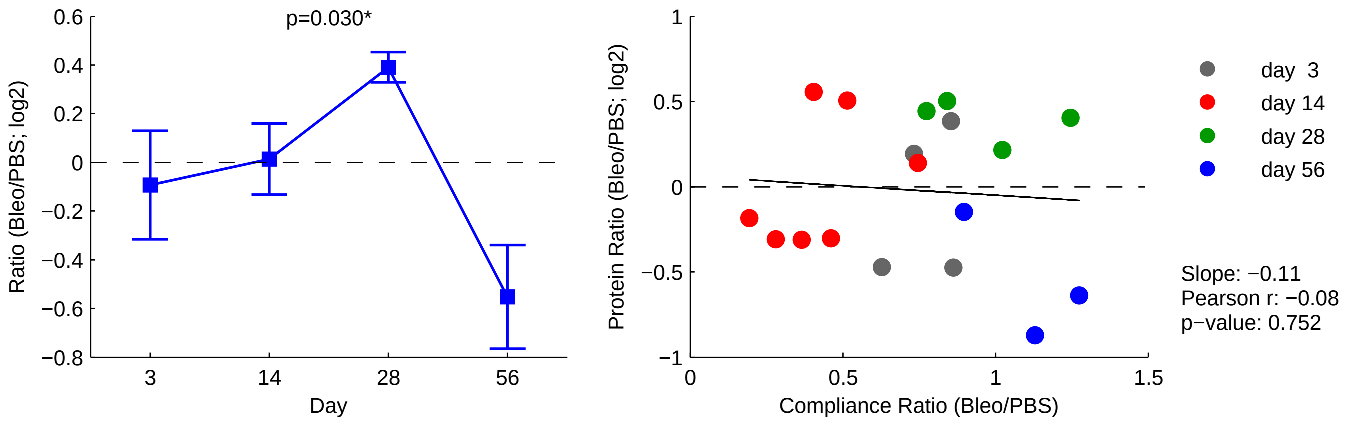

Q9R0H2−4 − Emcn (id: 7804)

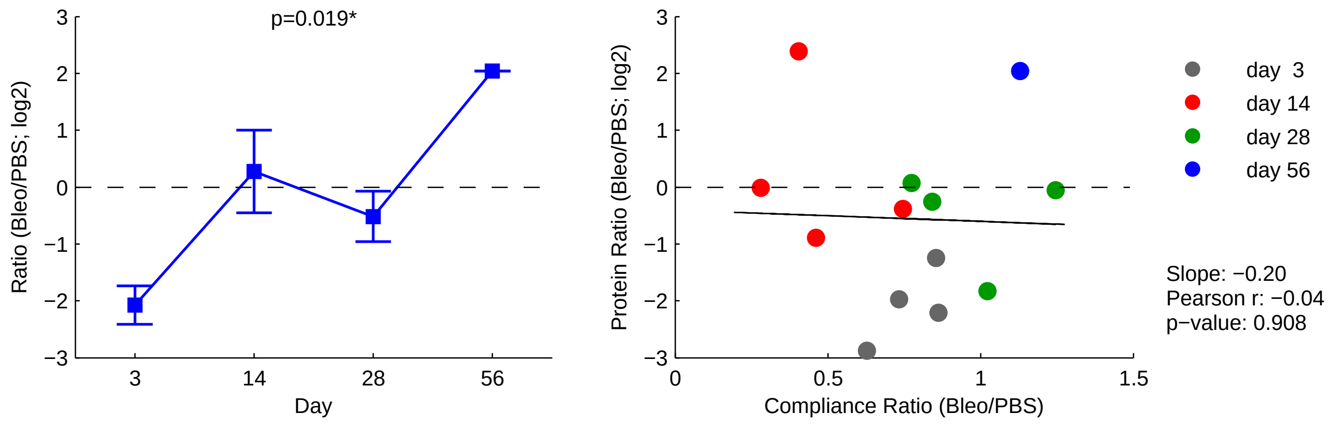

Q9R0L6 − Pcm1 (id: 7805)

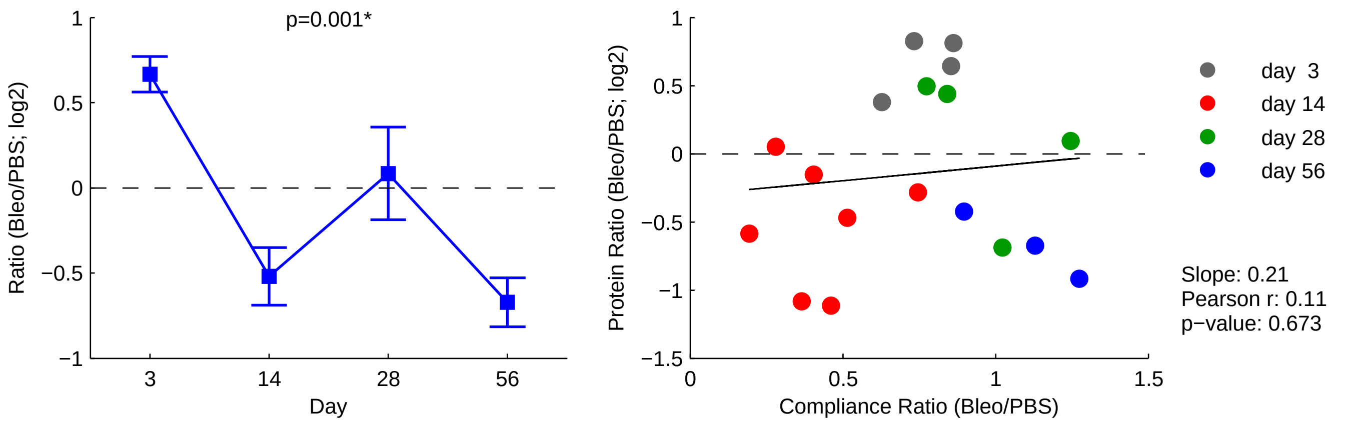

Q9R0M4 − Podxl (id: 7806)

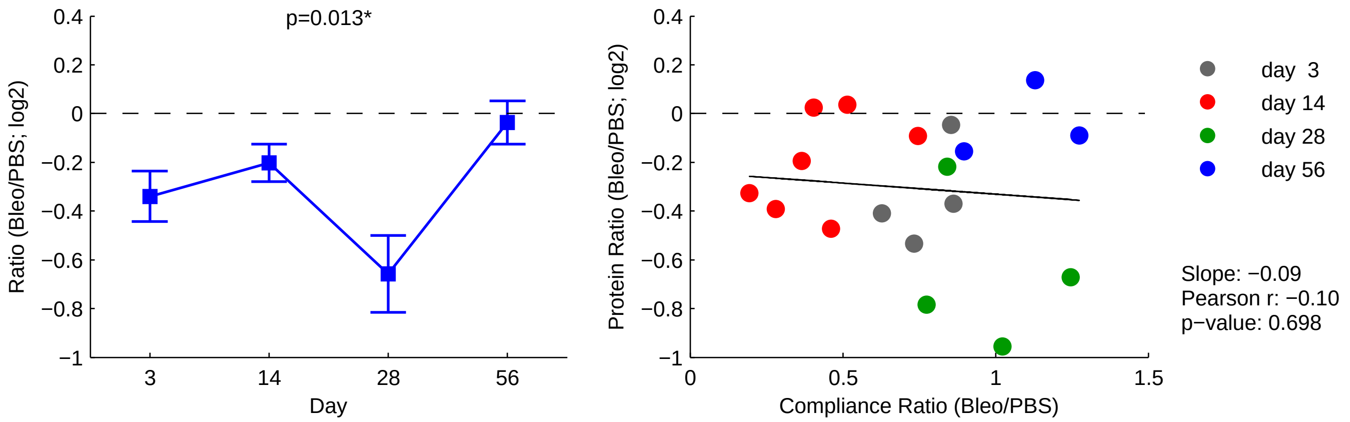

Q9R0P4 − Smap (id: 7807)

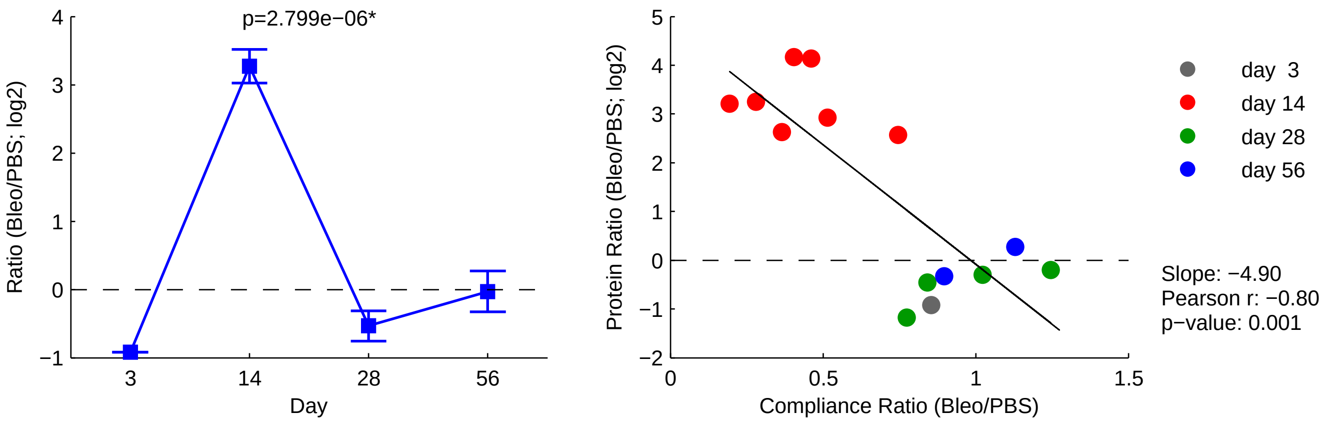

Q9R0P5 − Dstn (id: 7808)

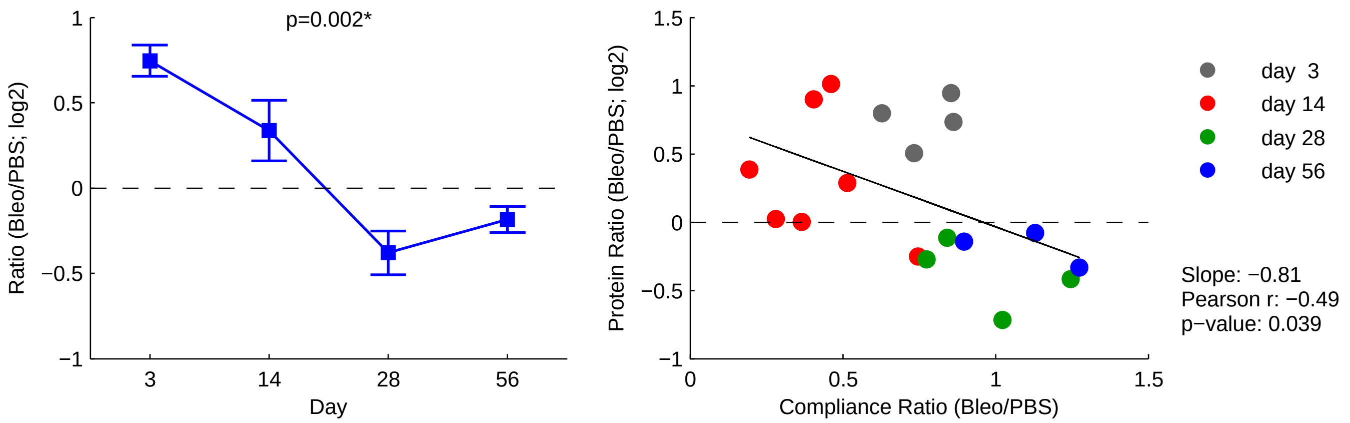

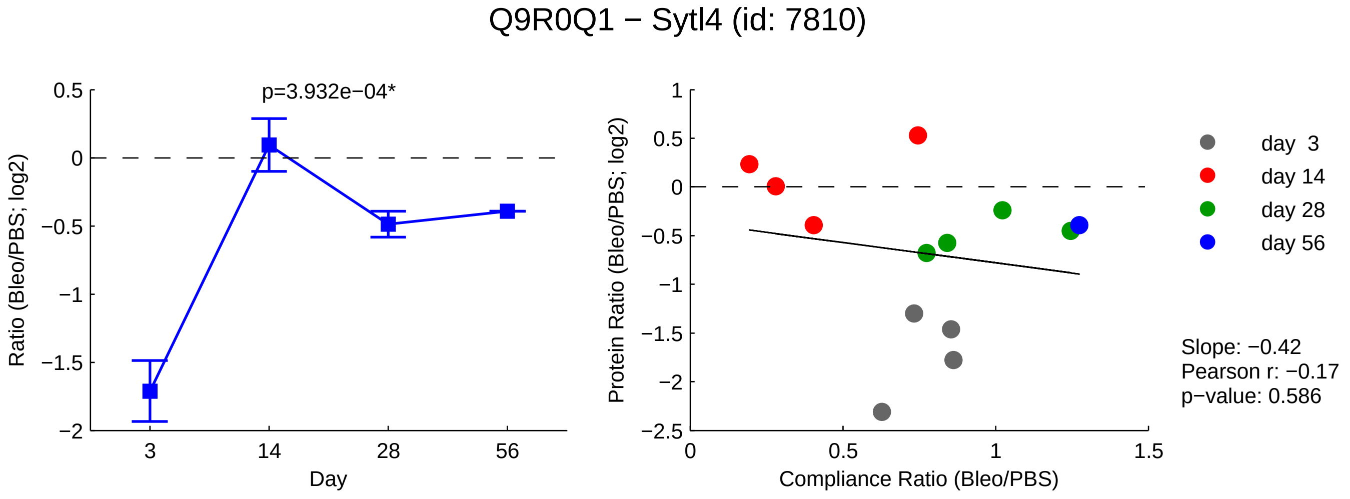
Q9R0Q1 − Sytl4 (id: 7810)
p=3.932e−04*
Ratio (Bleo/PBS; log2)
Day
Protein Ratio (Bleo/PBS; log2)
Compliance Ratio (Bleo/PBS)
day 3
day 14
day 28
day 56
Slope: −0.42
Pearson r: −0.17
p−value: 0.586

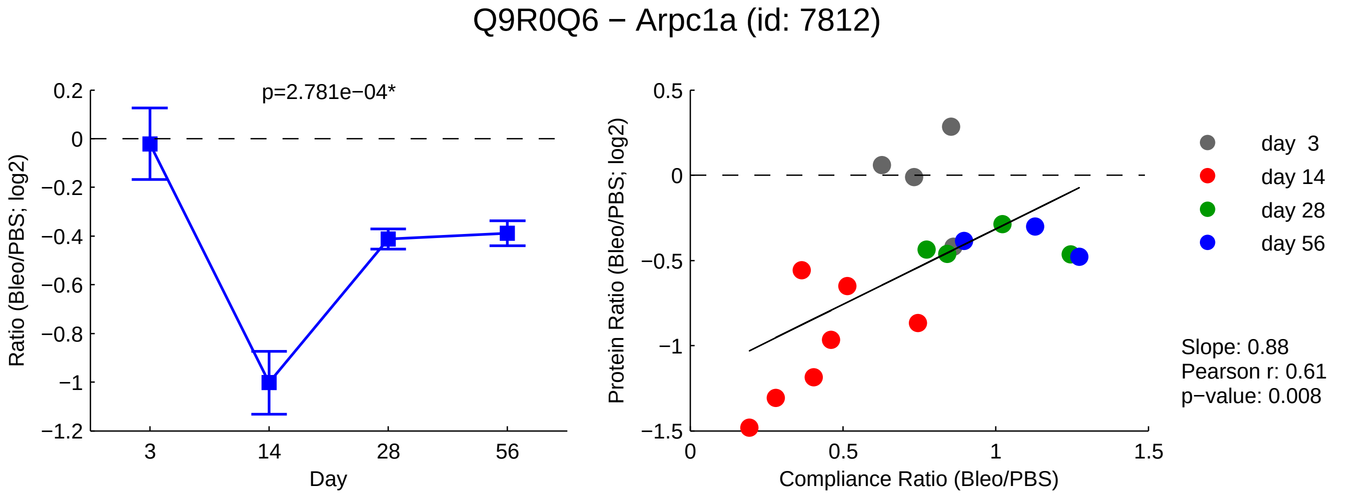
Q9R0Q6 − Arpc1a (id: 7812)
p=2.781e−04*
Ratio (Bleo/PBS; log2)
Day
Protein Ratio (Bleo/PBS; log2)
Compliance Ratio (Bleo/PBS)
day 3
day 14
day 28
day 56
Slope: 0.88
Pearson r: 0.61
p−value: 0.008

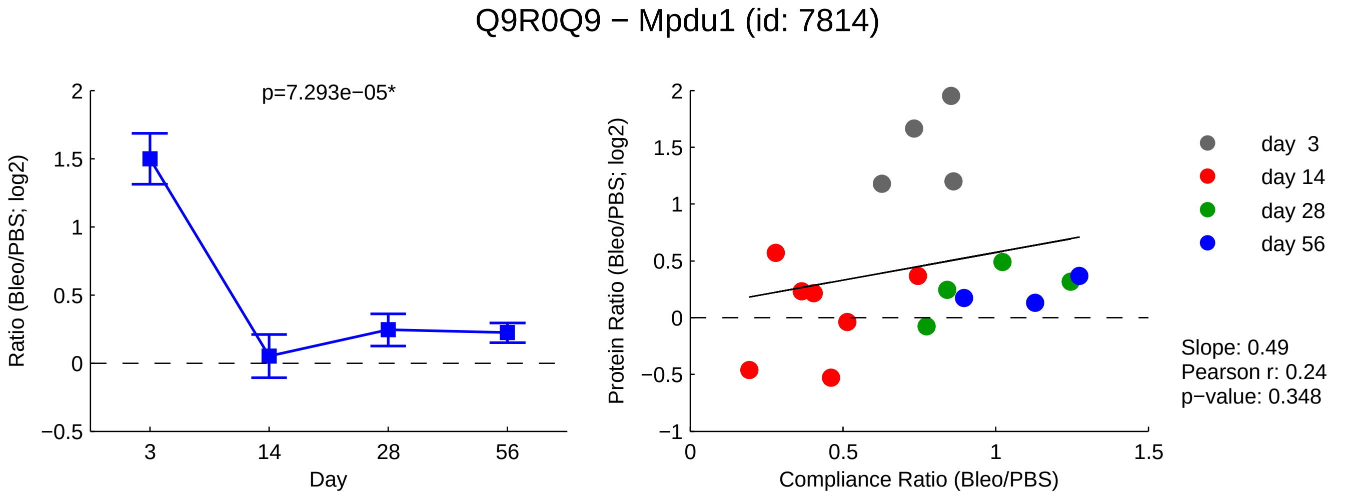
Q9R0Q9 − Mpdu1 (id: 7814)
p=7.293e−05*
Ratio (Bleo/PBS; log2)
Day
Protein Ratio (Bleo/PBS; log2)
Compliance Ratio (Bleo/PBS)
day 3
day 14
day 28
day 56
Slope: 0.49
Pearson r: 0.24
p−value: 0.348

Q9R0X4 − Acot9 (id: 7817)

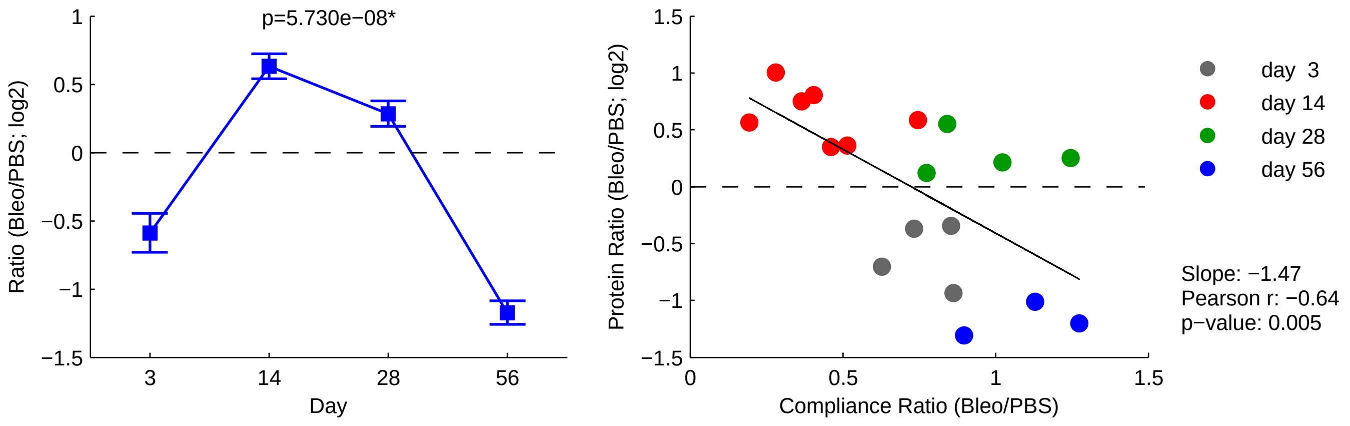

Q9R0Y5 − Ak1 (id: 7818)

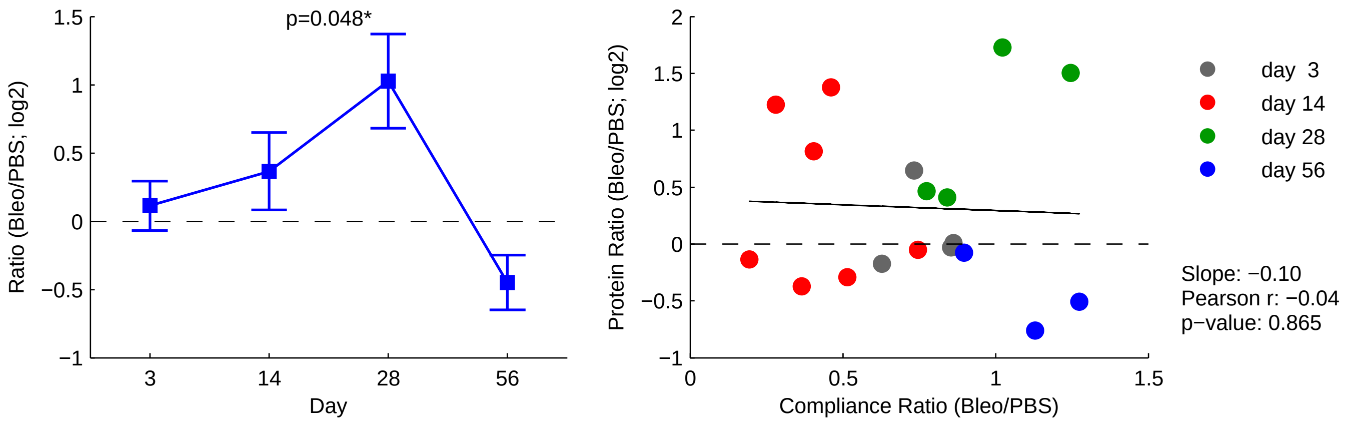

Q9R111 − Gda (id: 7819)

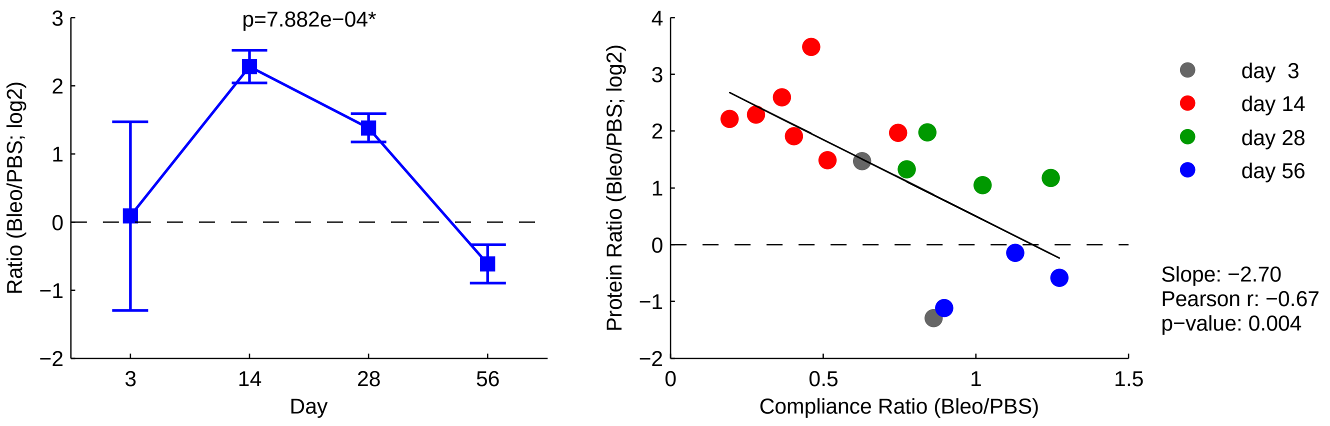

Q9R112 − Sqrdl (id: 7820)

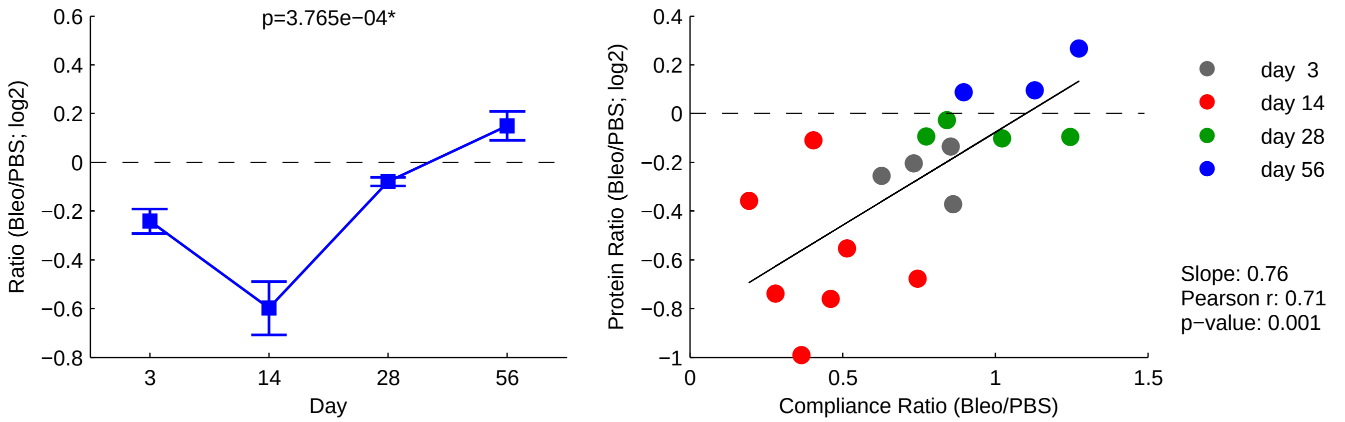

Q9R118 − Htra1 (id: 7821)

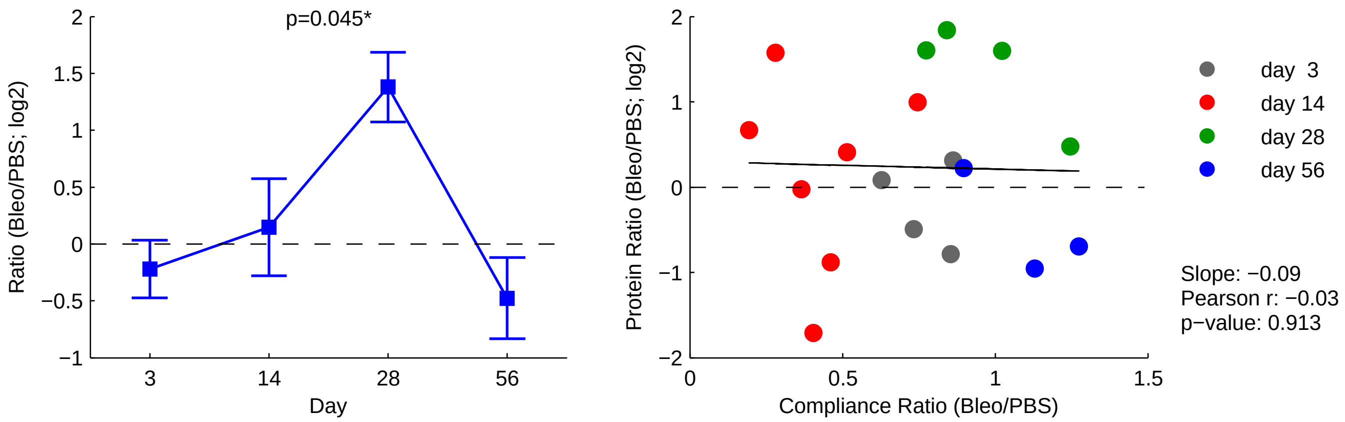

Q9R1C6 − Dgke (id: 7824)

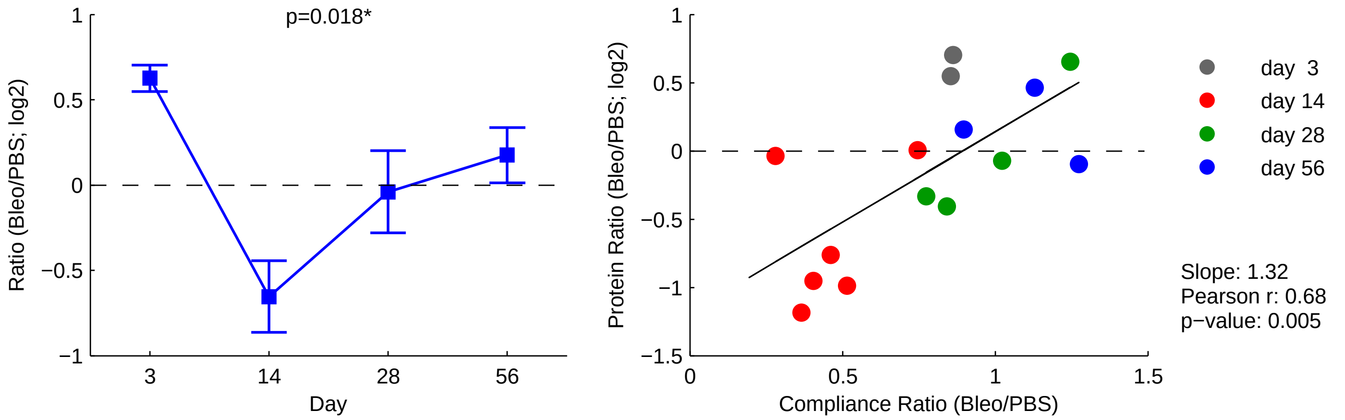

Q9R1J0 − Nsdhl (id: 7826)

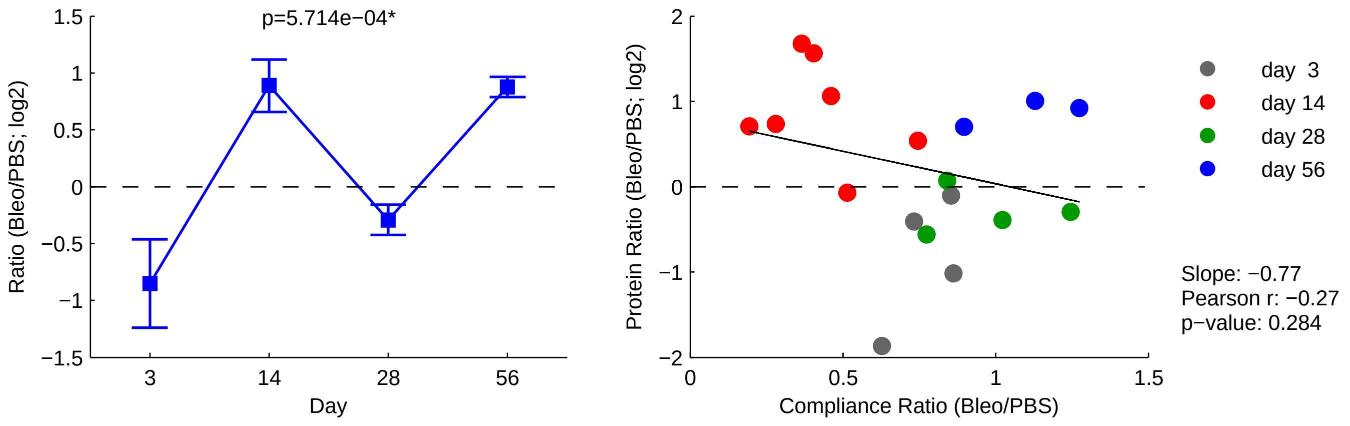

Q9R1P0 − Psma4 (id: 7828)

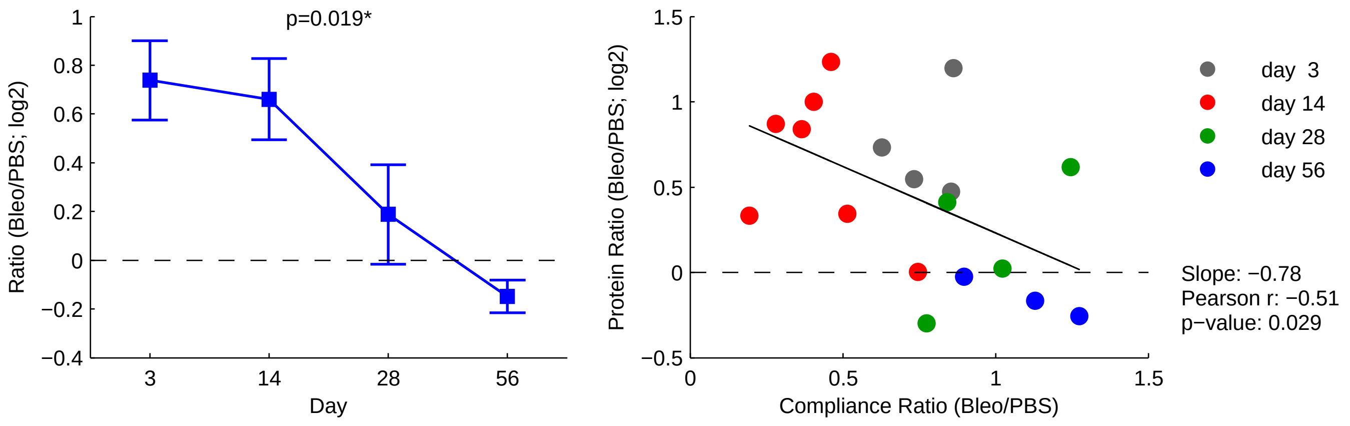

Q9R1P3 − Psmb2 (id: 7830)

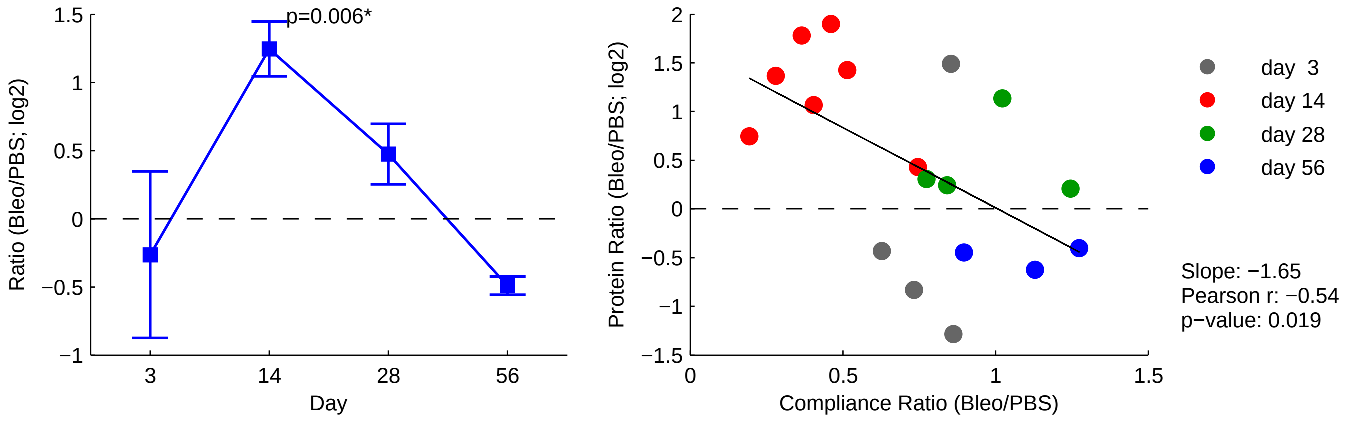

Q9R1P4 − Psma1 (id: 7831)

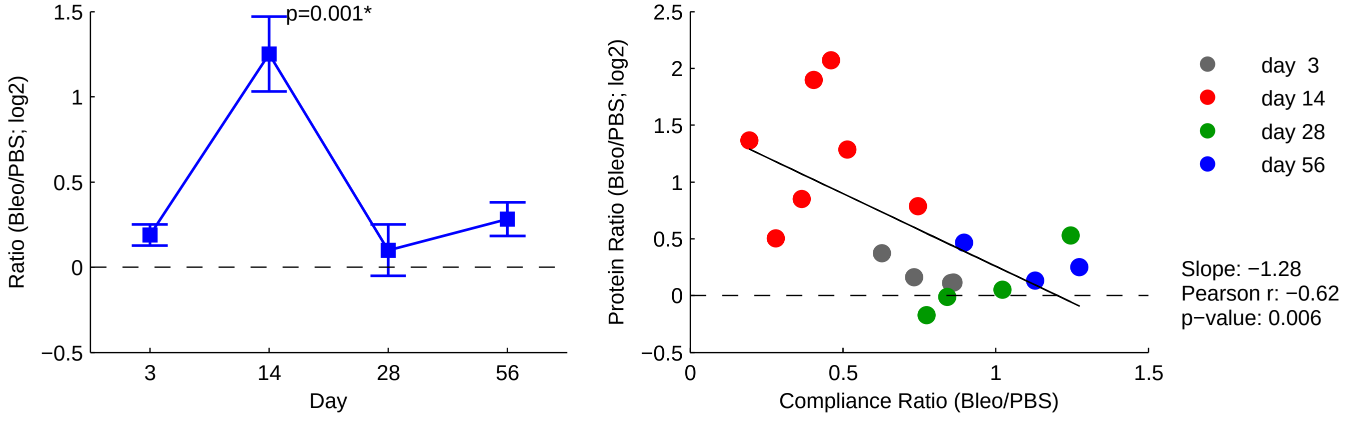

Q9R1Q6 − Tmem176b (id: 7832)

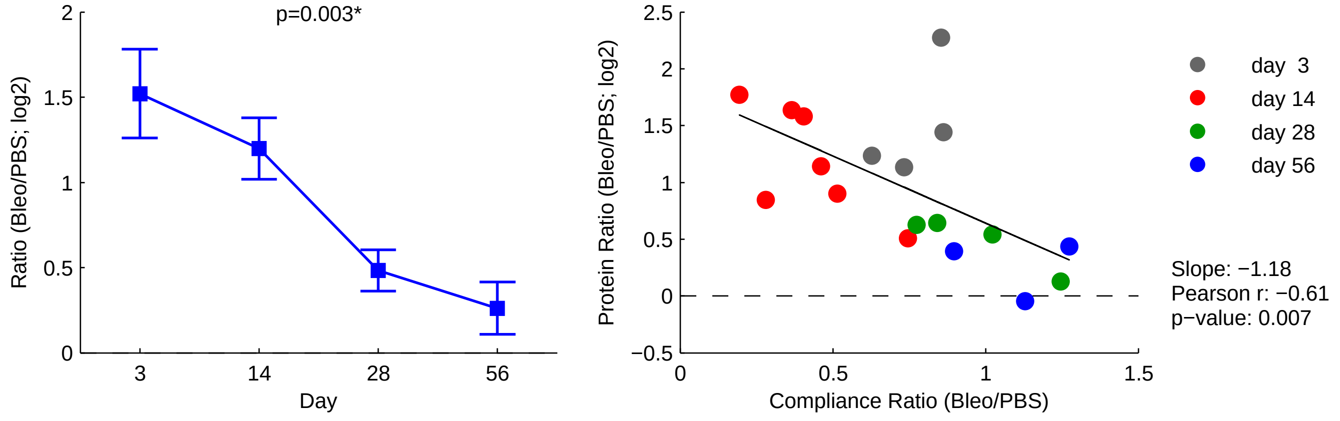

Q9R1Q7 − Plp2 (id: 7833)

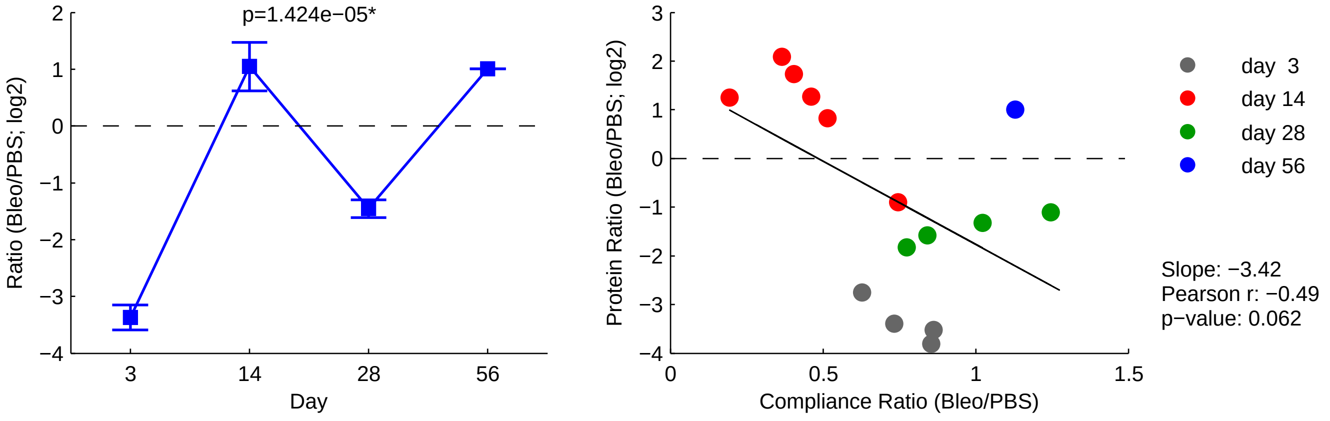

Q9R1W5 − Calcrl (id: 7838)

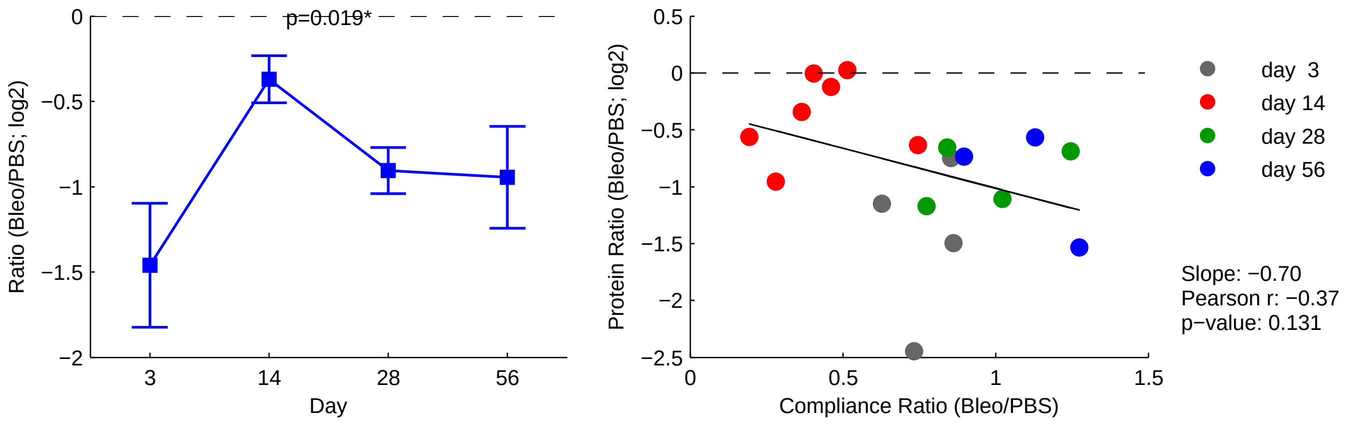

Q9R1Z8 − Sorbs3 (id: 7842)

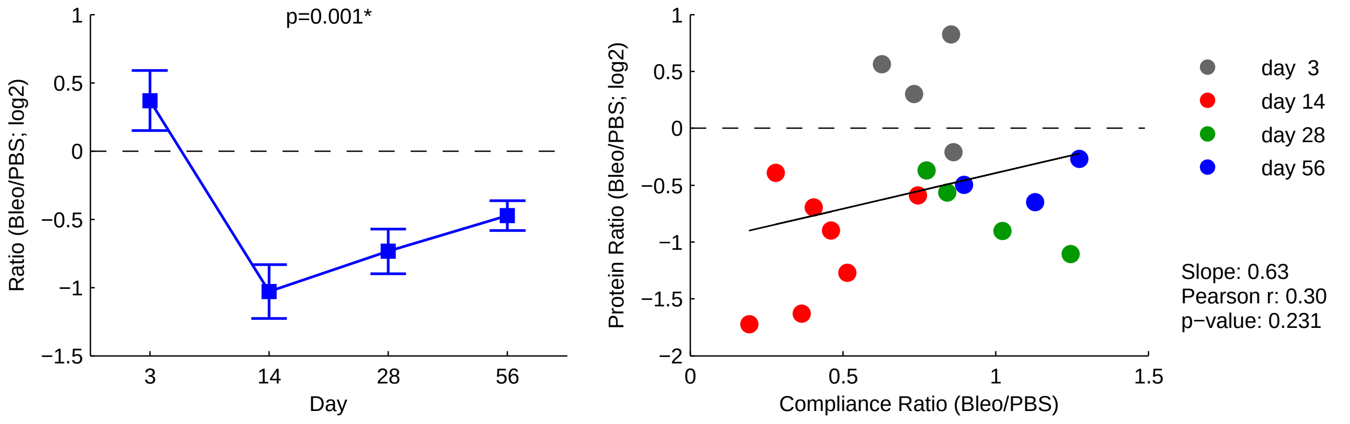

Q9R226 − Khdrbs3 (id: 7845)

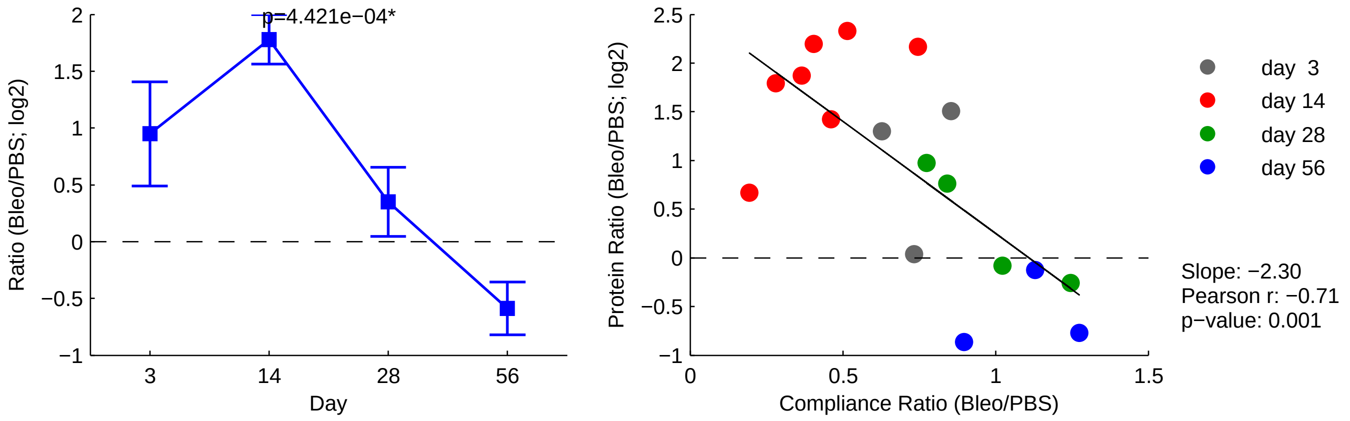

Q9R257 − Hebp1 (id: 7846)

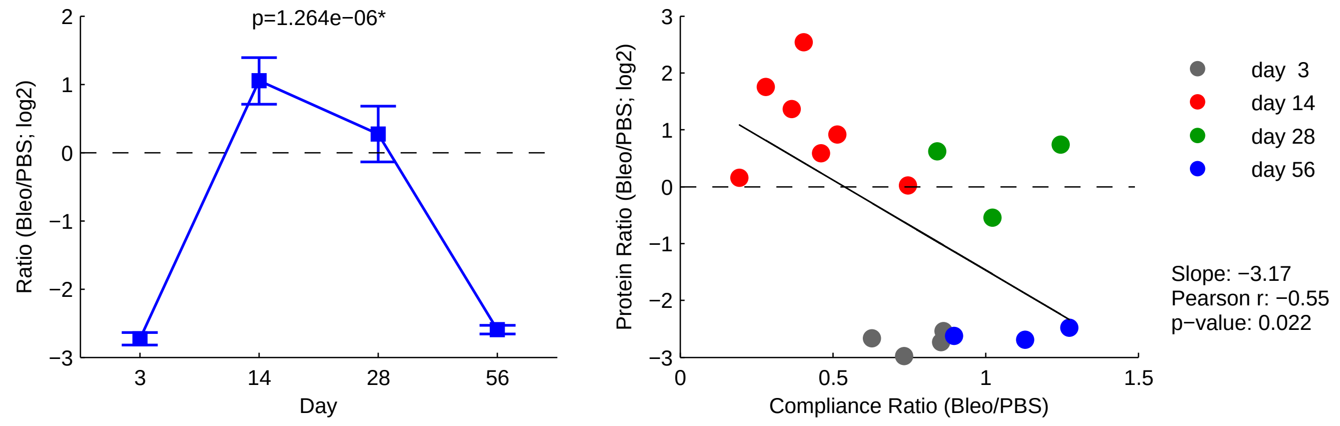

Q9WTI7−3 − (id: 7849)

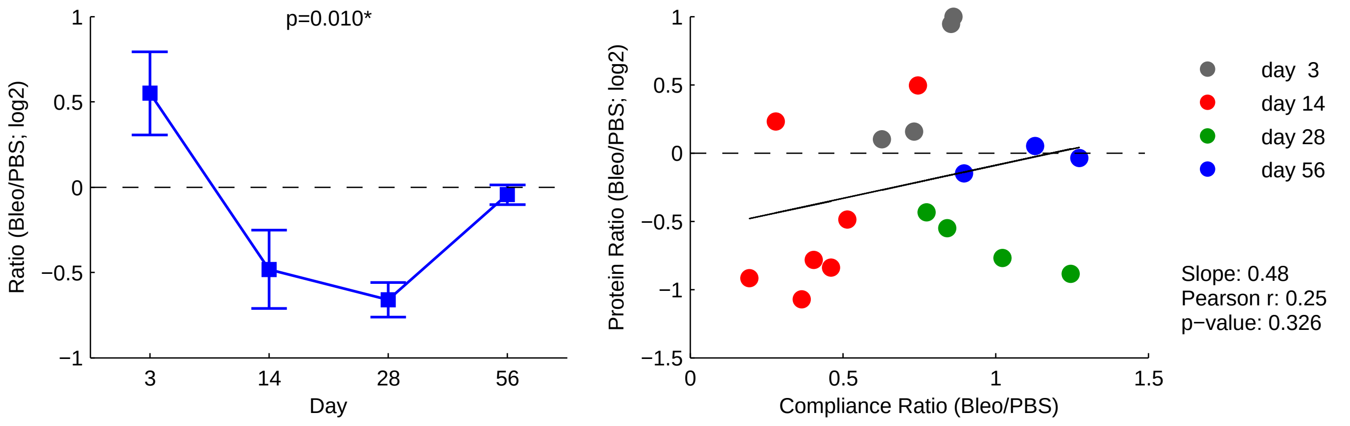

Q9WTK5 − Nfkb2 (id: 7851)

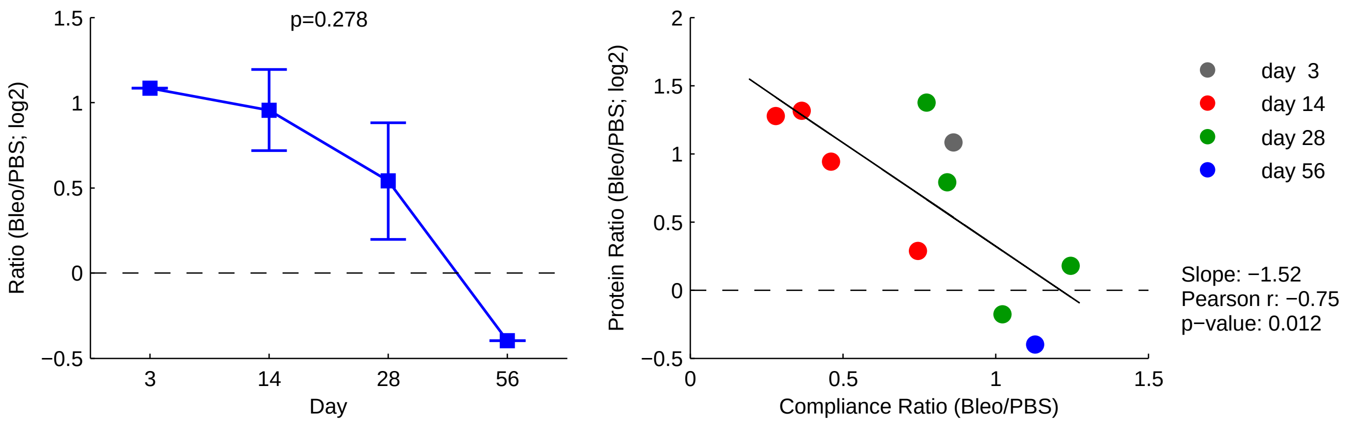

Q9WTL7 − Lypla2 (id: 7853)

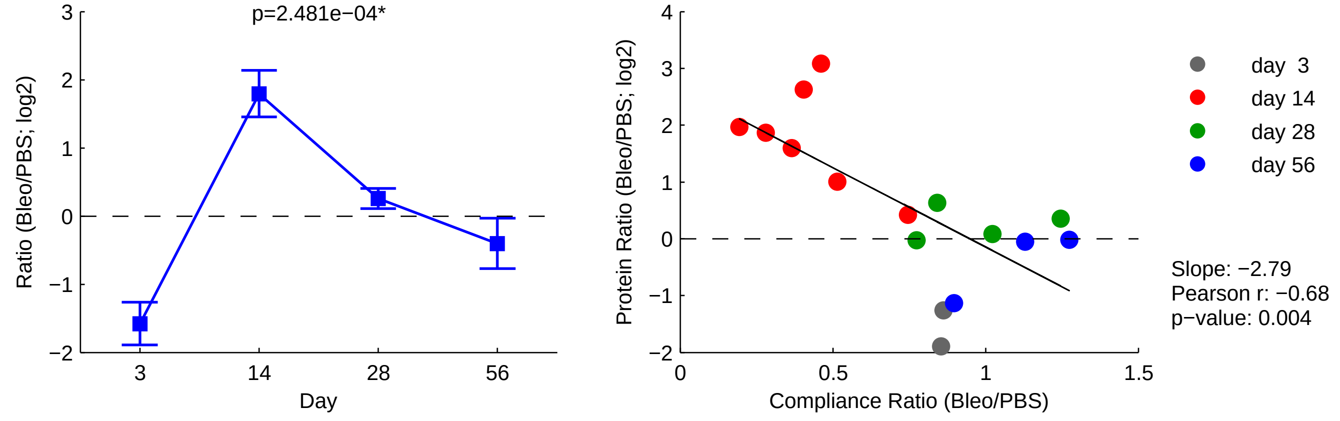

Q9WTP6−2 − Ak2 (id: 7857)

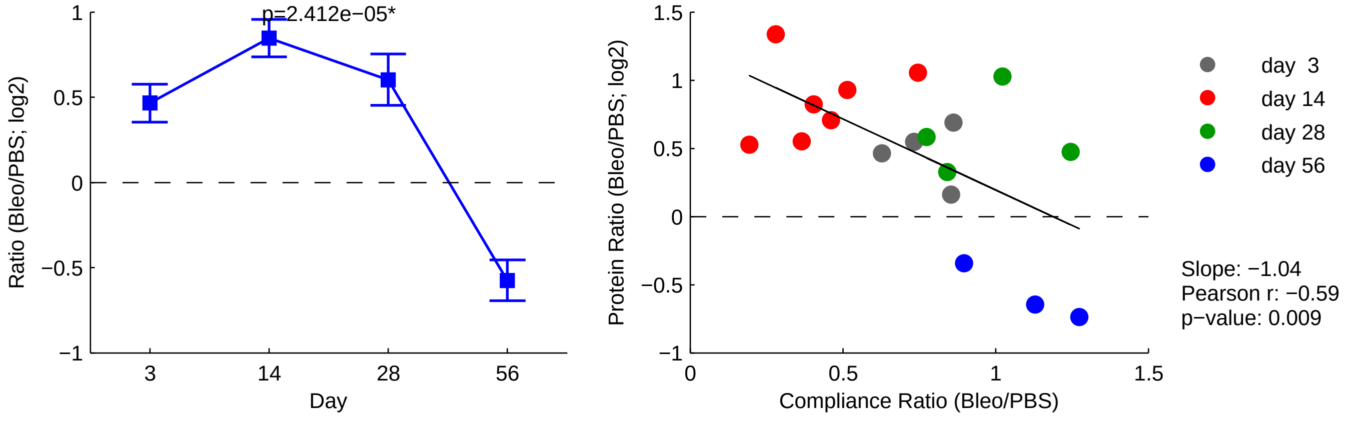

Q9WTP7 − Ak3 (id: 7858)

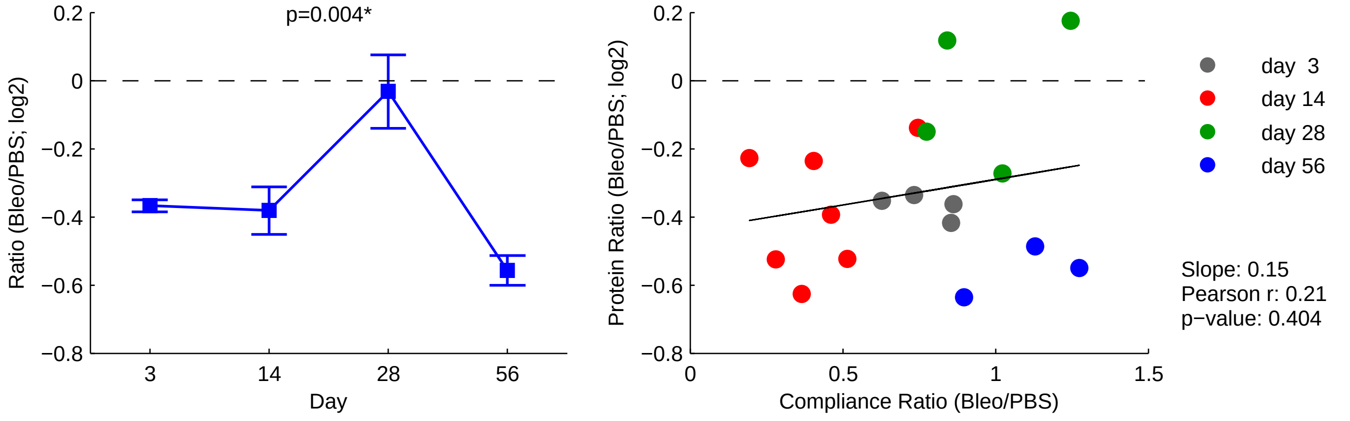

Q9WTR1 − Trpv2 (id: 7860)

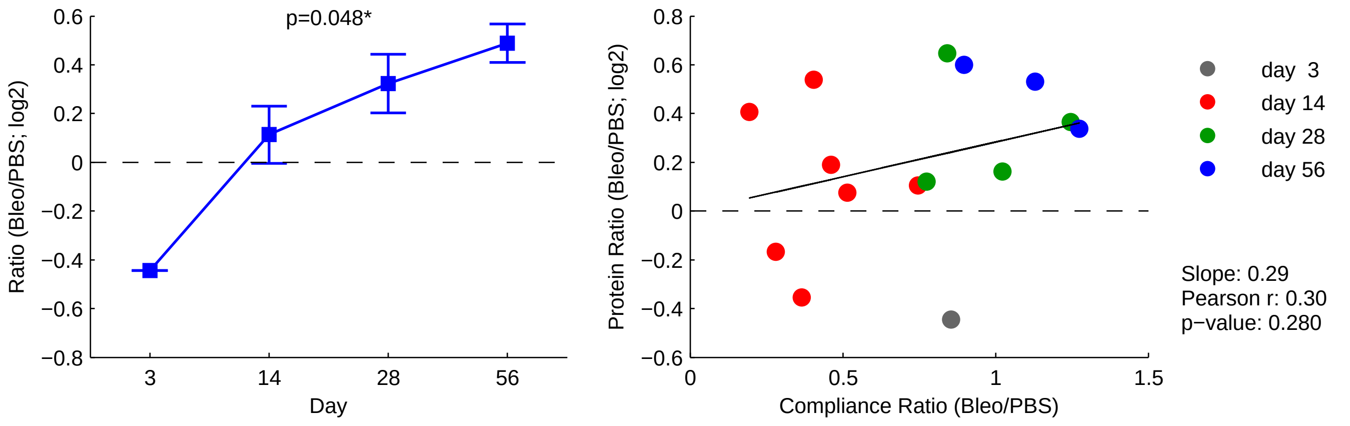

Q9WTR5 − Cdh13 (id: 7862)

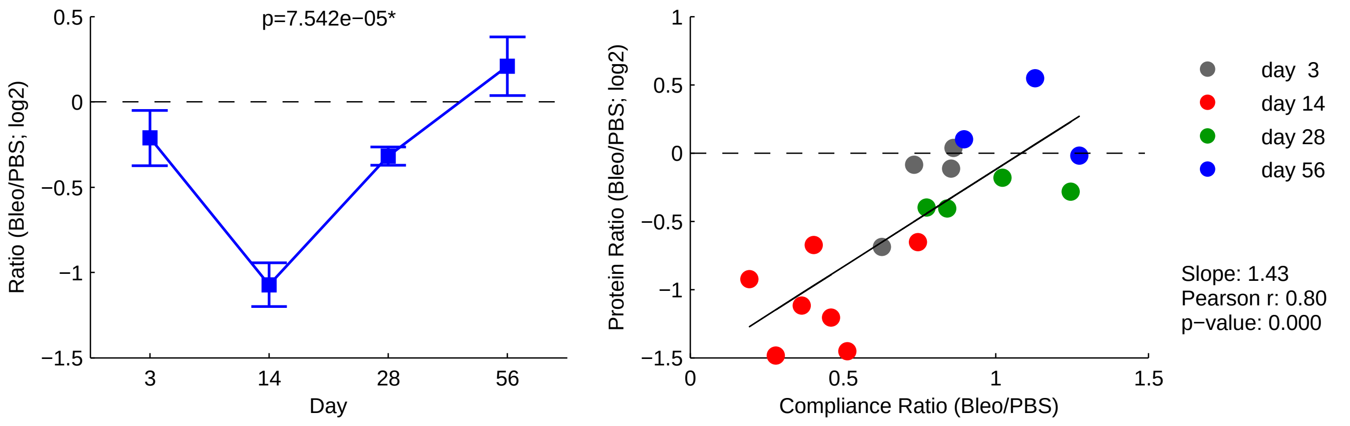

Q9WTS2 − Fut8 (id: 7863)

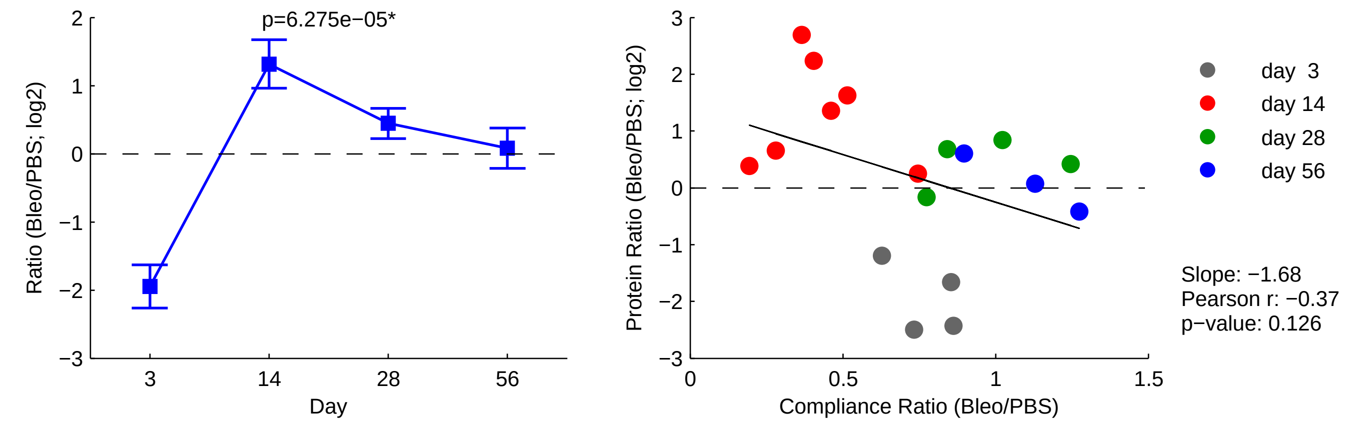

Q9WTU0 − Phf2 (id: 7864)

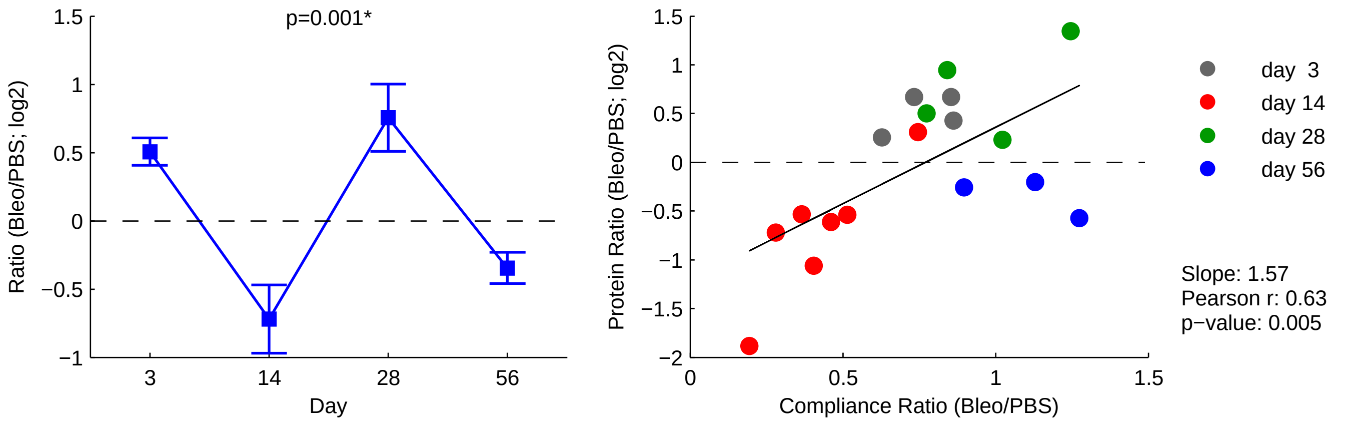

Q9WTU6−3 − Mapk9 (id: 7865)

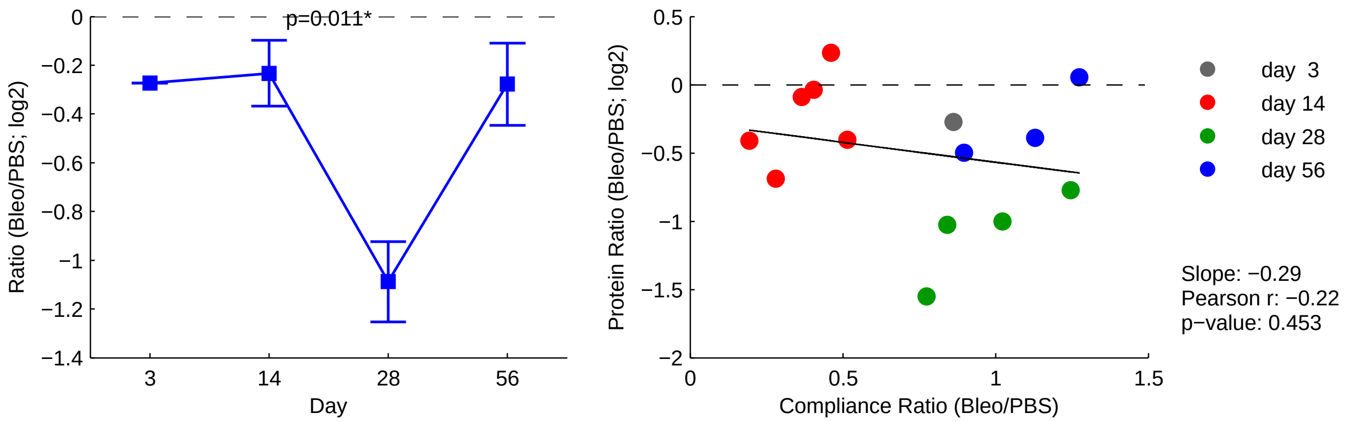

Q9WTV6 − Usp18 (id: 7866)

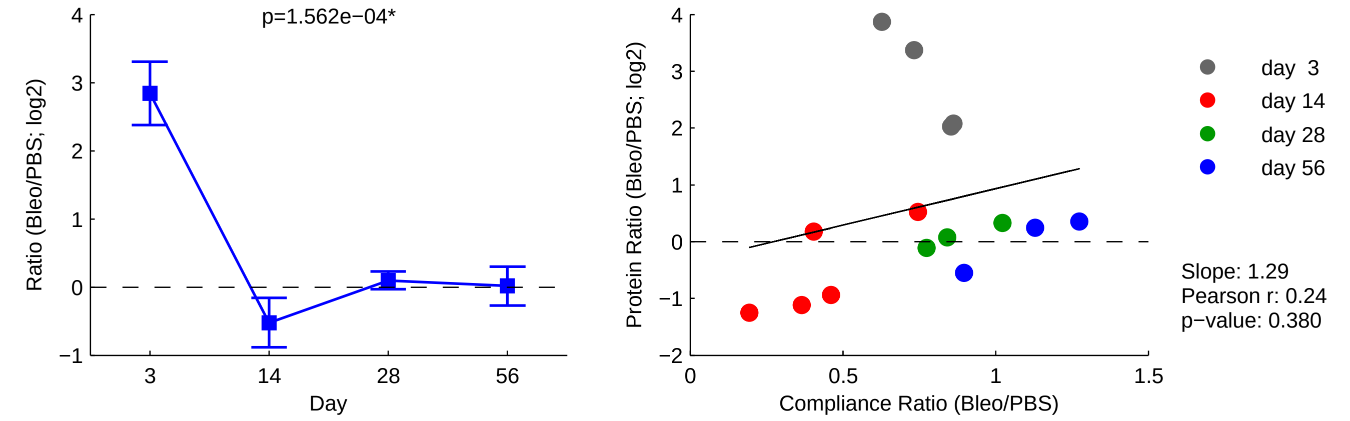

Q9WTX5 − Skp1 (id: 7869)

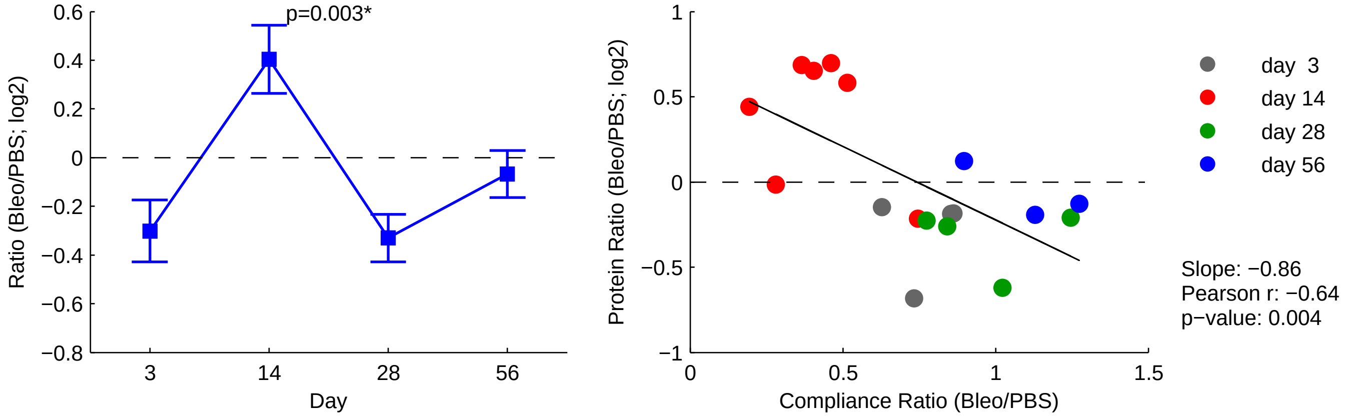

Q9WTX8 − Mad1l1 (id: 7871)

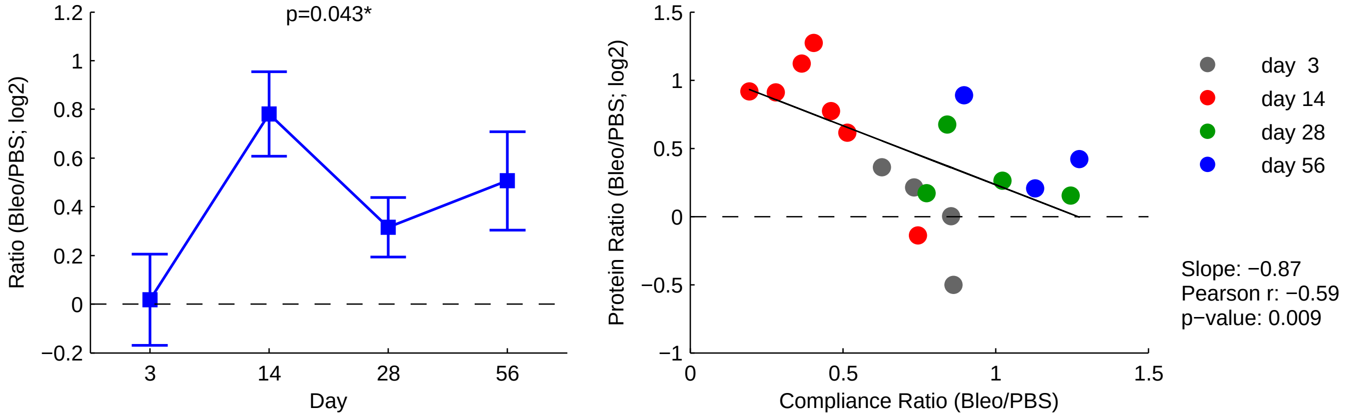

Q9WU28 − Pfdn5 (id: 7873)

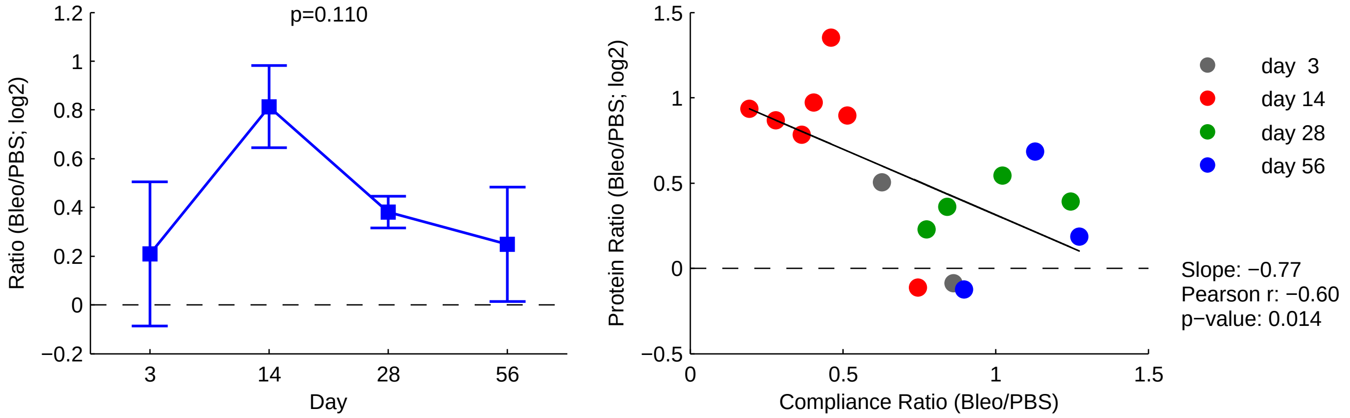

Q9WU38 − Scnn1b (id: 7874)

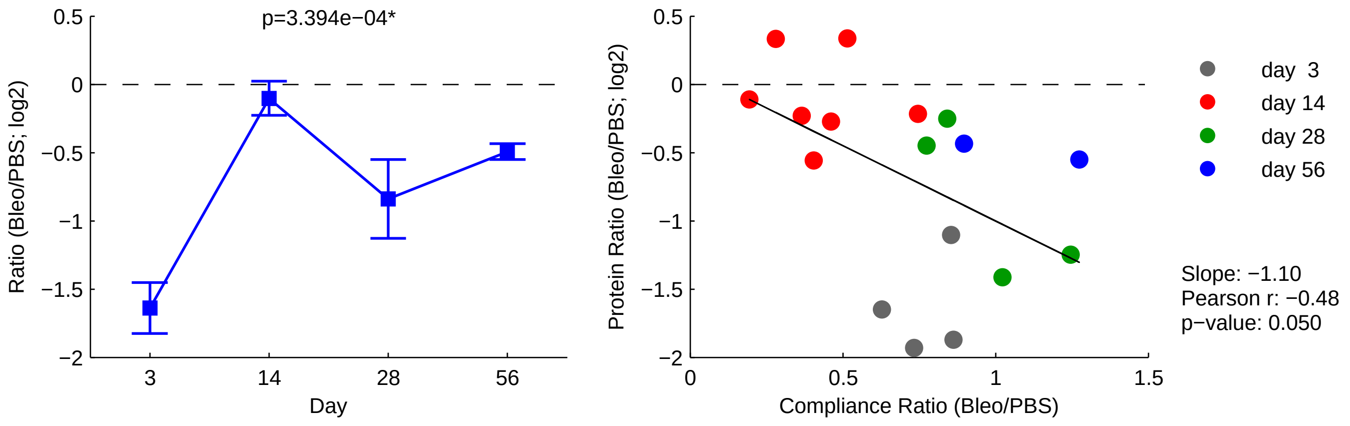

Q9WU79 − Prodh (id: 7878)

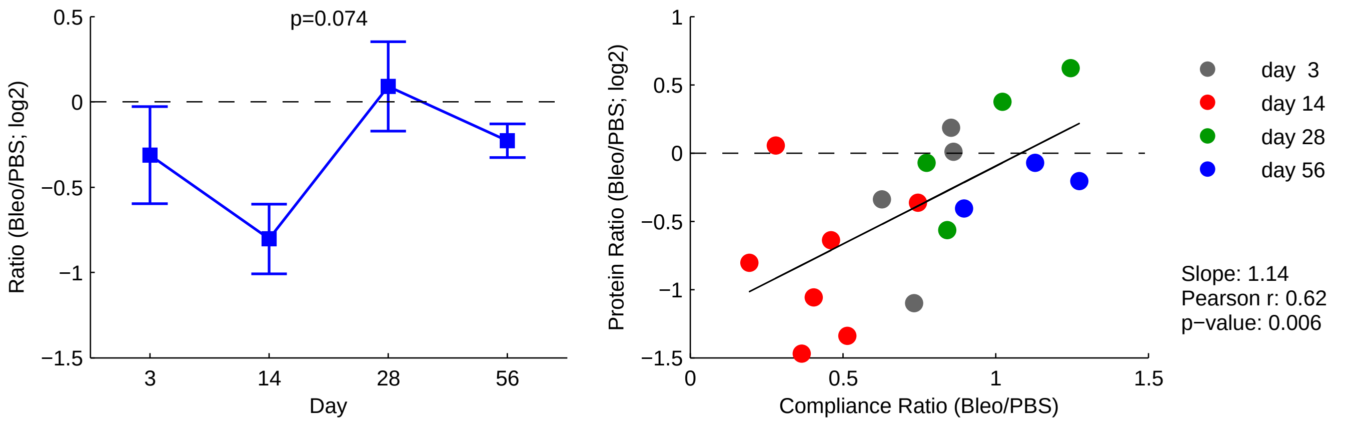

Q9WUB3 − Pygm (id: 7881)

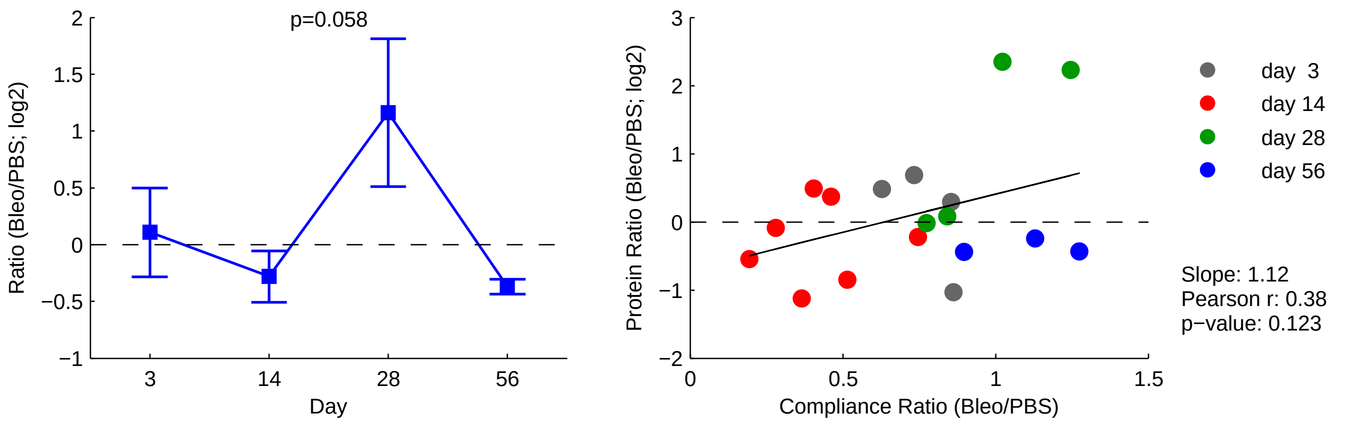

Q9WUD0 − Cyp2b10 (id: 7882)

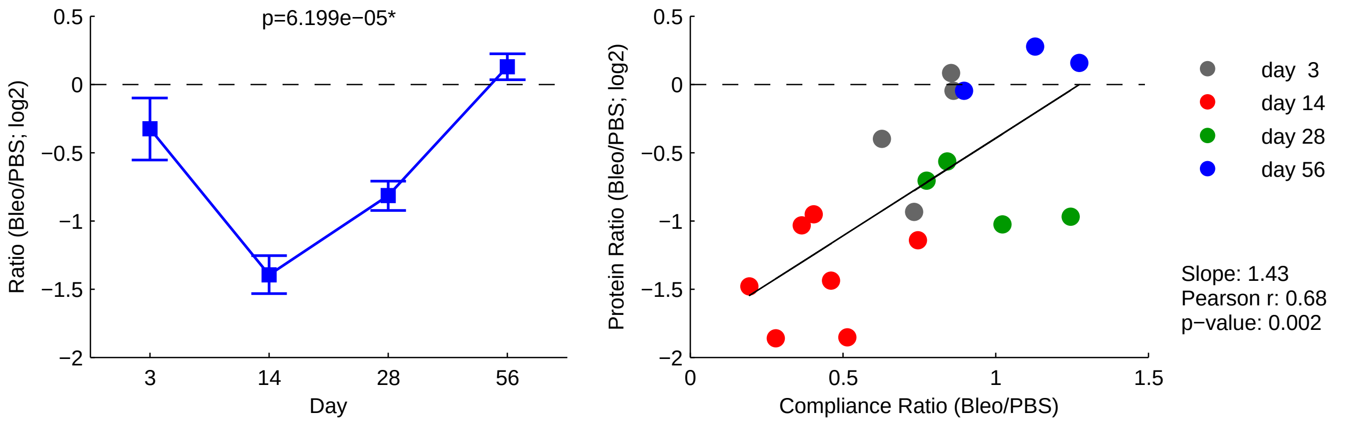

Q9WUD1 − Stub1 (id: 7883)

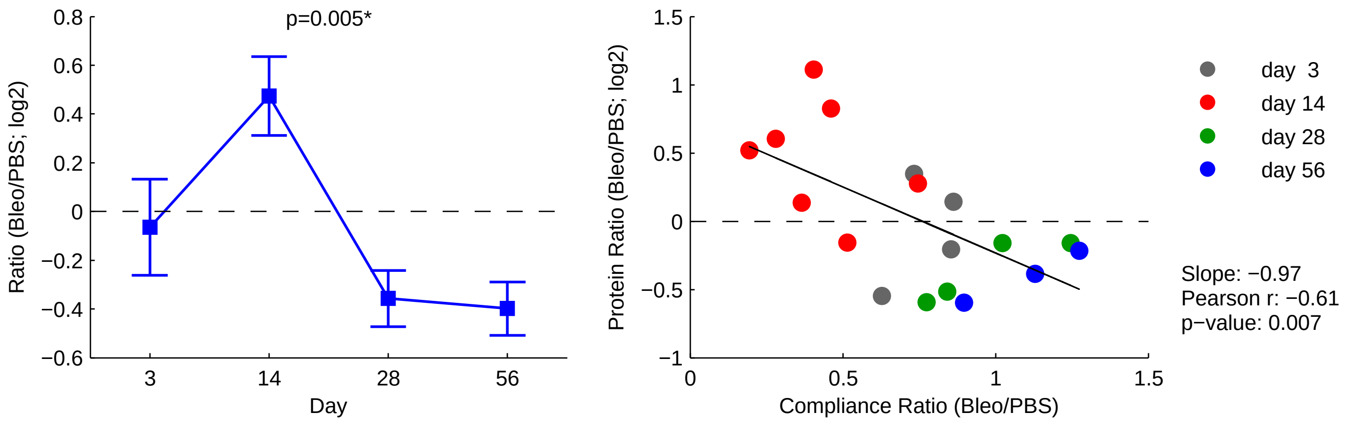

Q9WUH1 − Tmem115 (id: 7885)

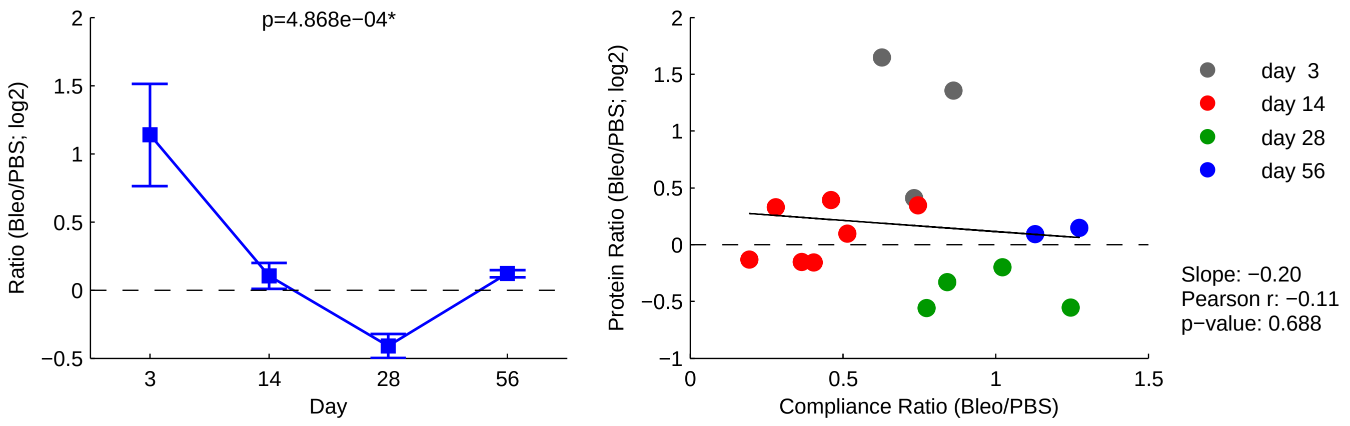

Q9WUK2 − Eif4h (id: 7886)

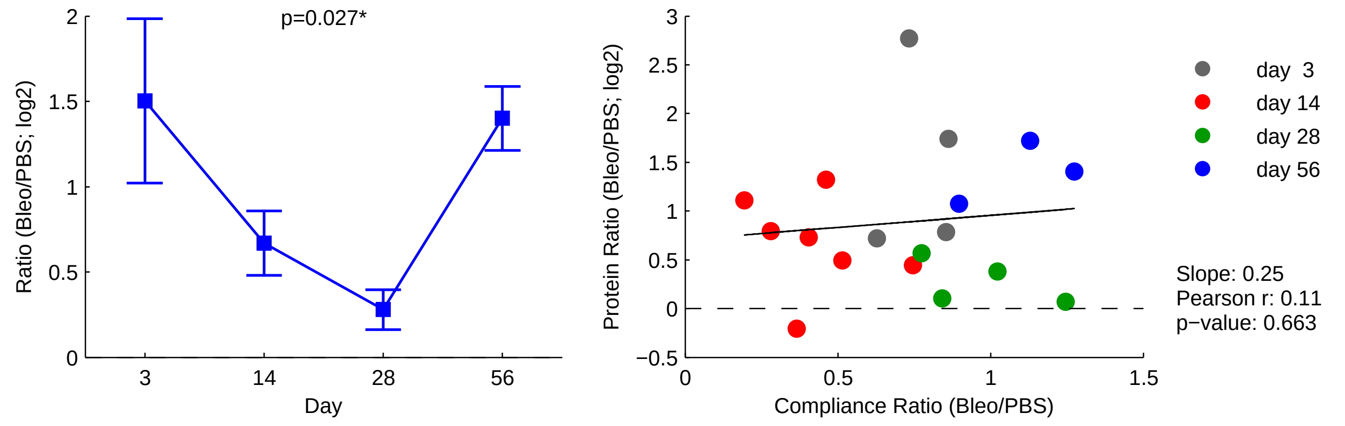

Q9WUM3 − Coro1b (id: 7889)

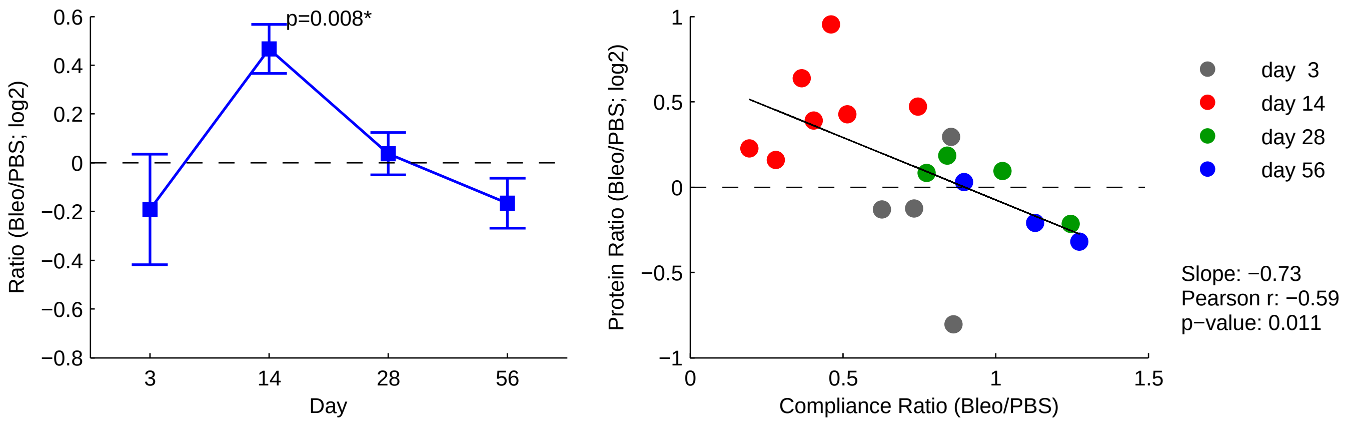

Q9WUM4 − Coro1c (id: 7890)

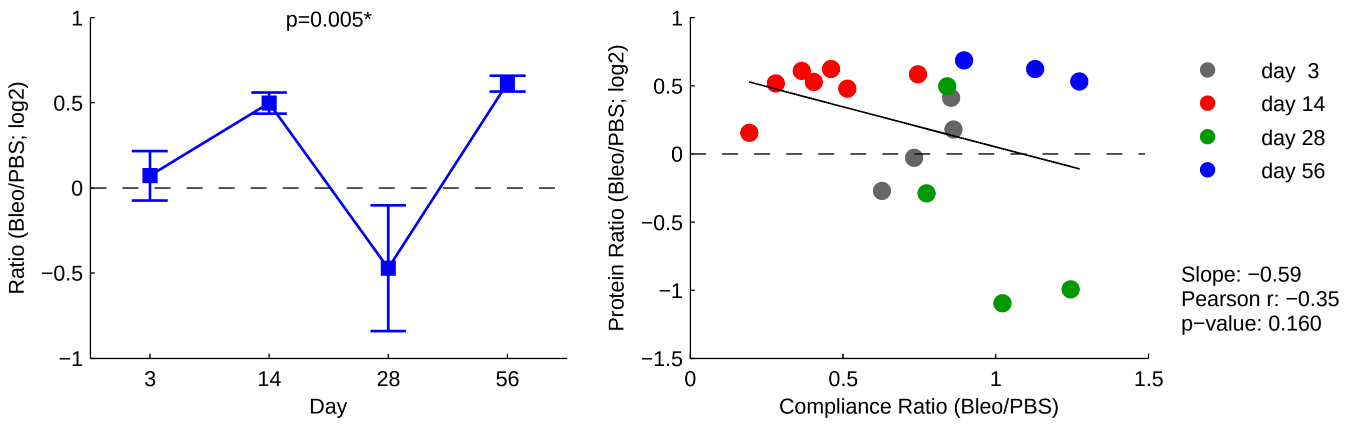

Q9WUM5 − Suclg1 (id: 7891)

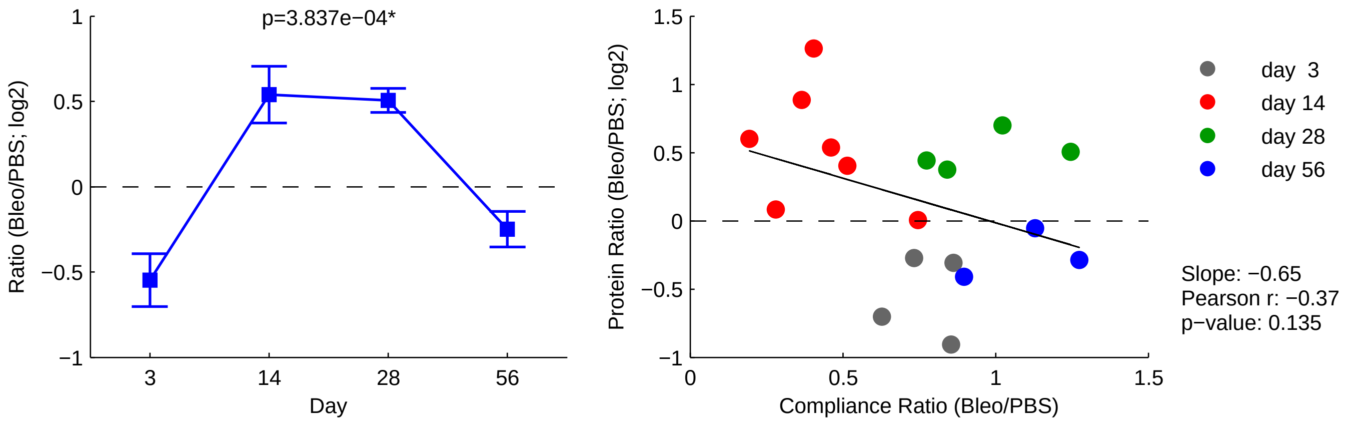

Q9WUN2 − Tbk1 (id: 7892)

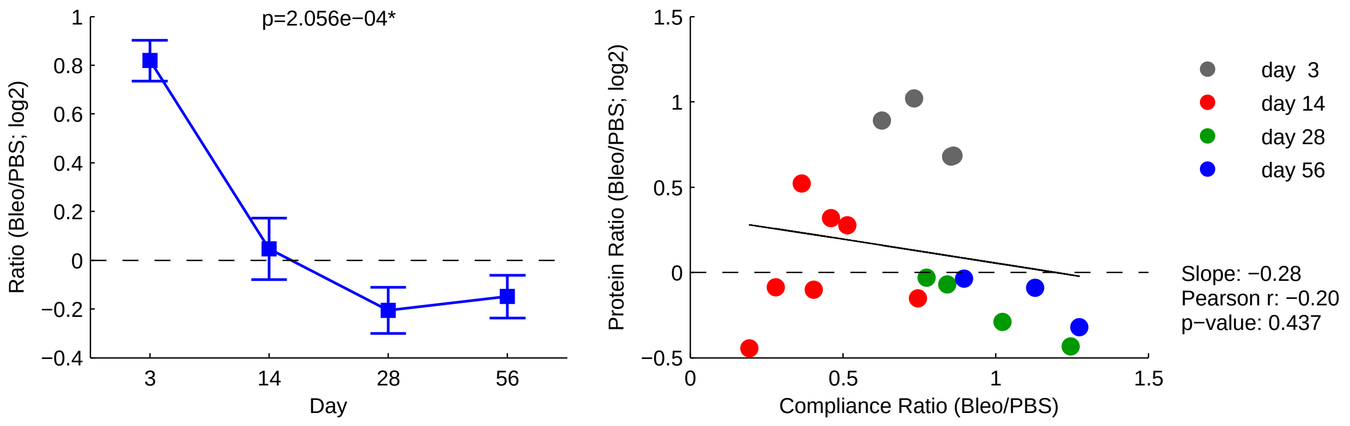

Q9WUP0 − Ramp2 (id: 7893)

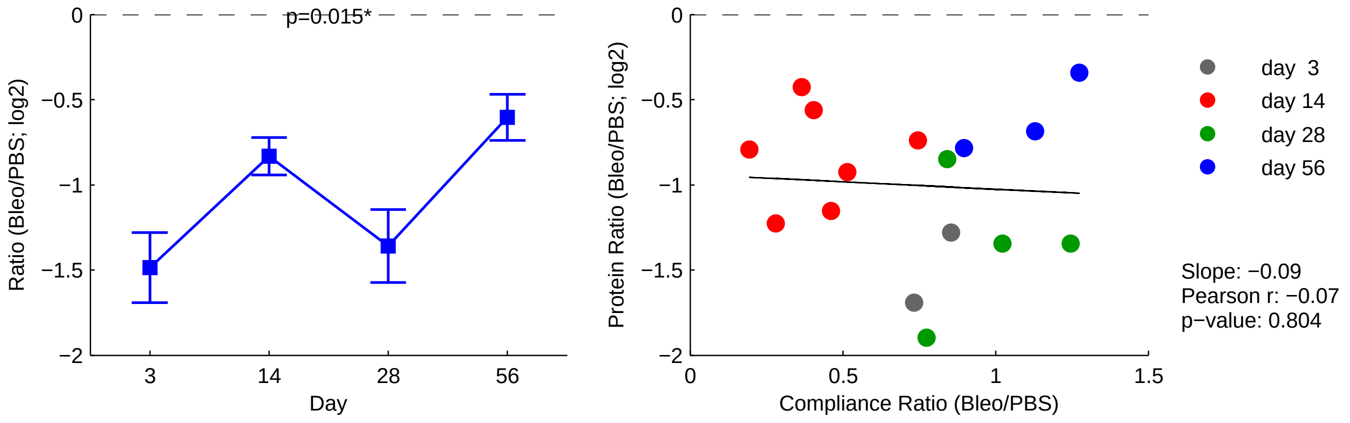

Q9WUR0 − Tinag (id: 7896)

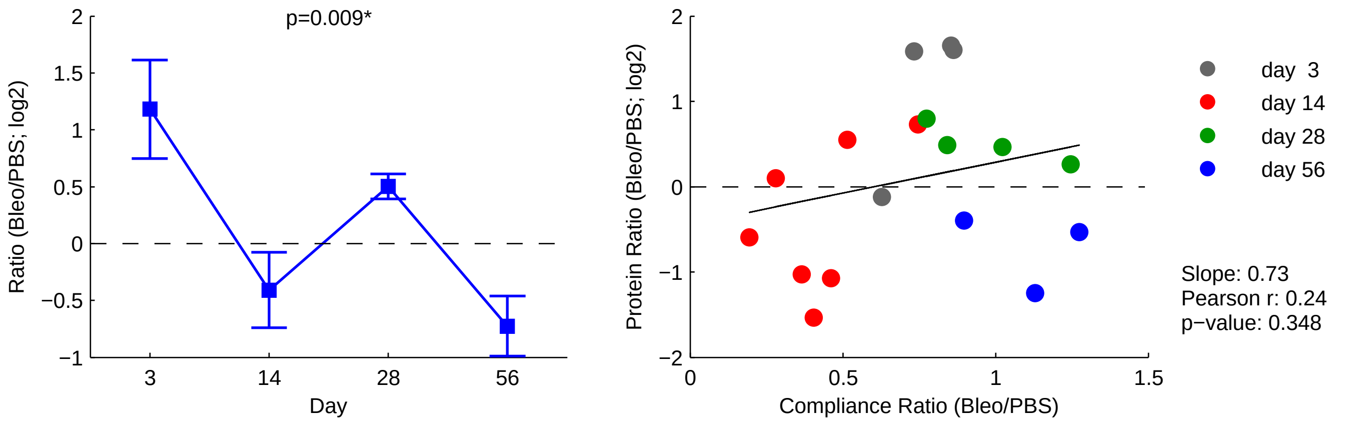

Q9WUR2−2 − Eci2 (id: 7897)

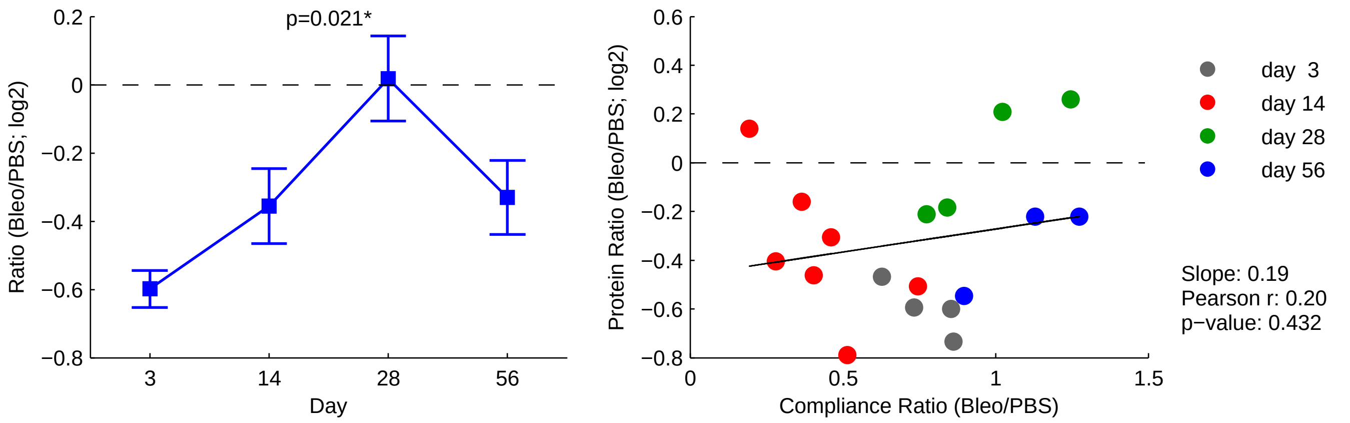

Q9WUU7 − Ctsz (id: 7899)

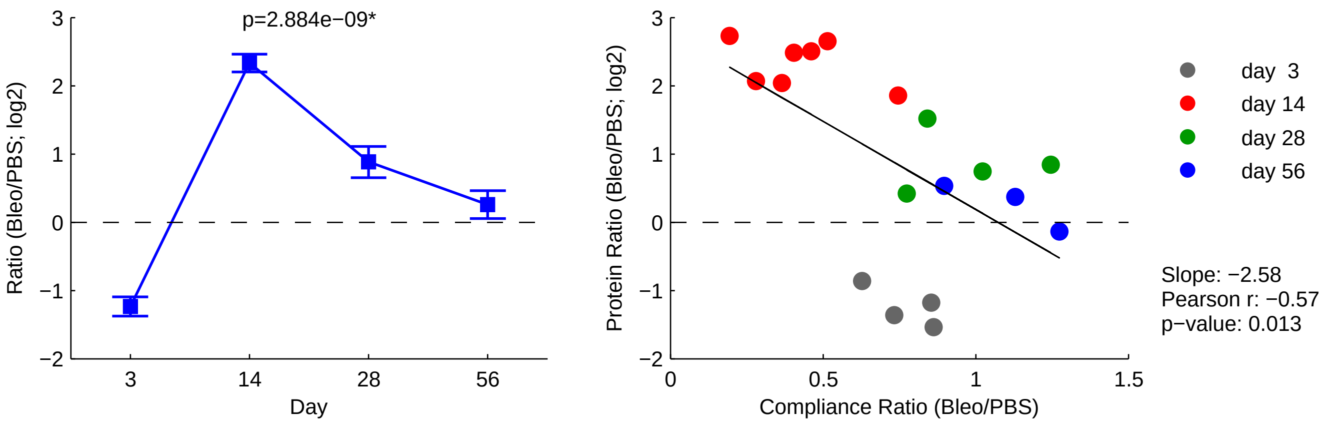

Q9WUU9 − Mcm3ap (id: 7900)

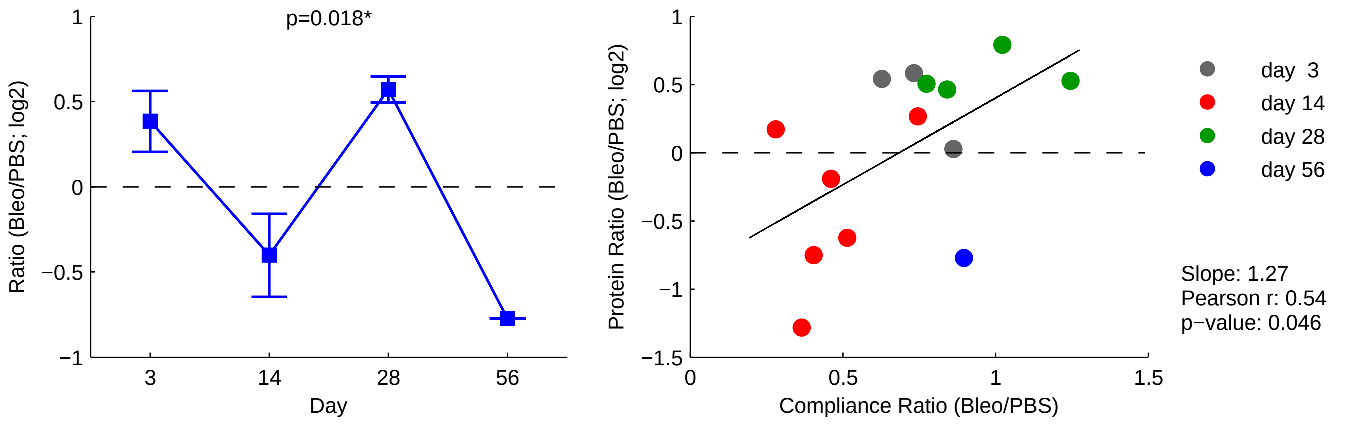

Q9WV27 − Atp1a4 (id: 7905)

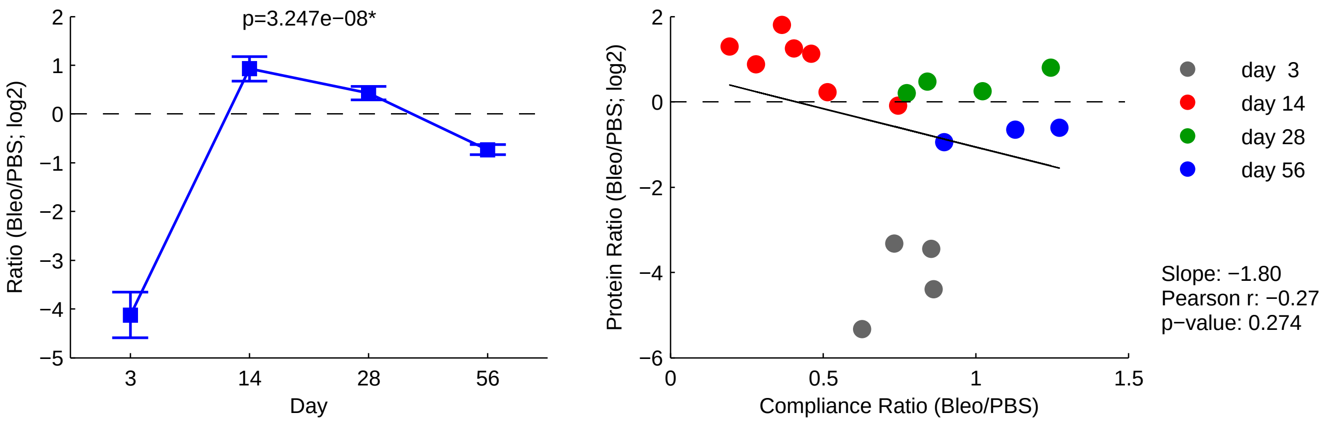

Q9WV32 − Arpc1b (id: 7906)

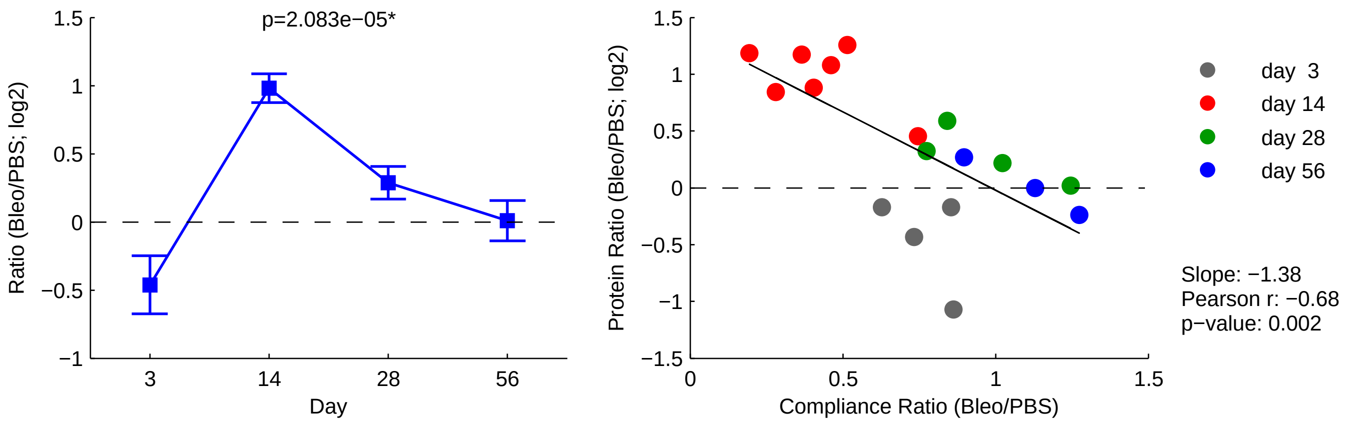

Q9WV34 − Mpp2 (id: 7907)

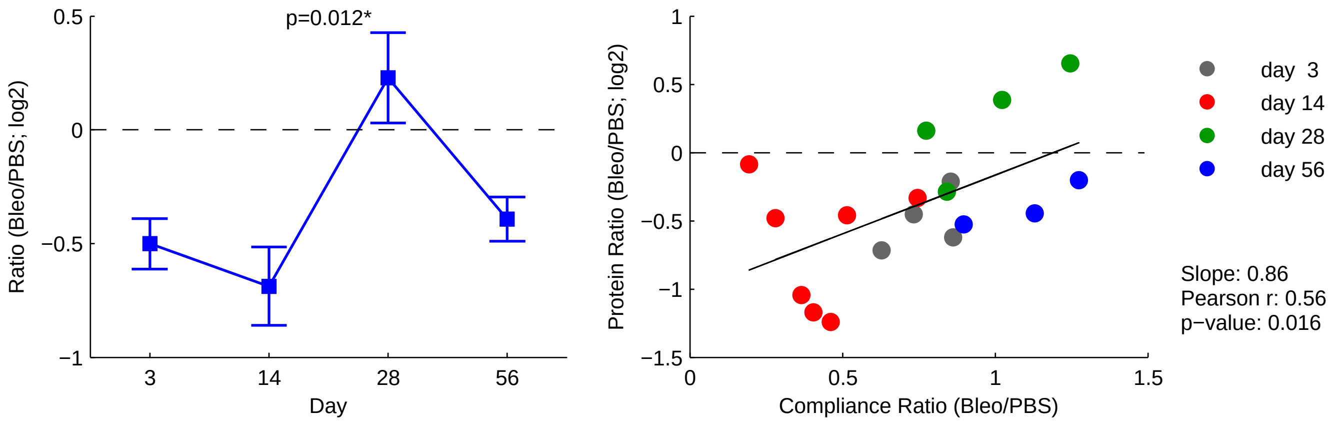

Q9WV35 − Apobec2 (id: 7908)

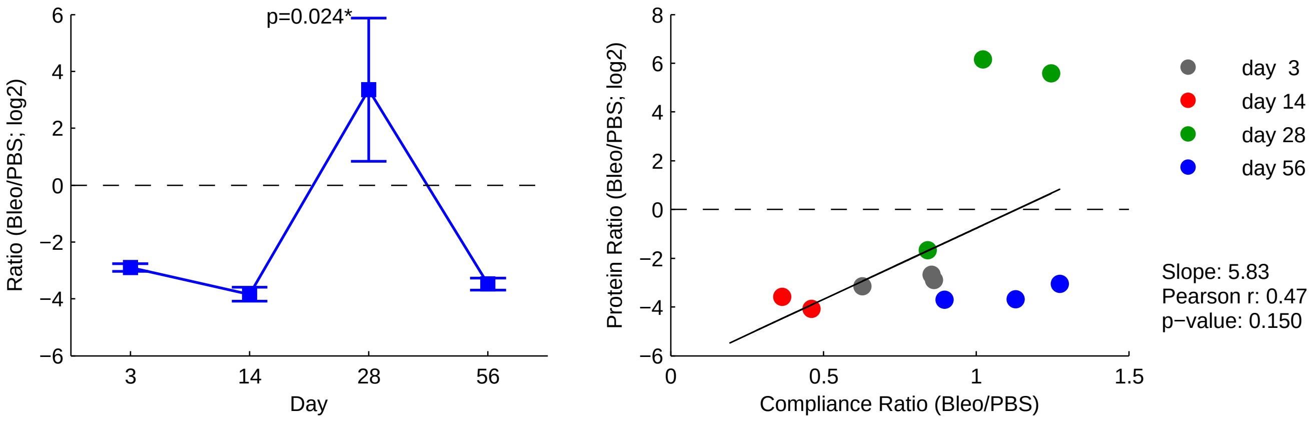

Q9WV54 − Asah1 (id: 7909)

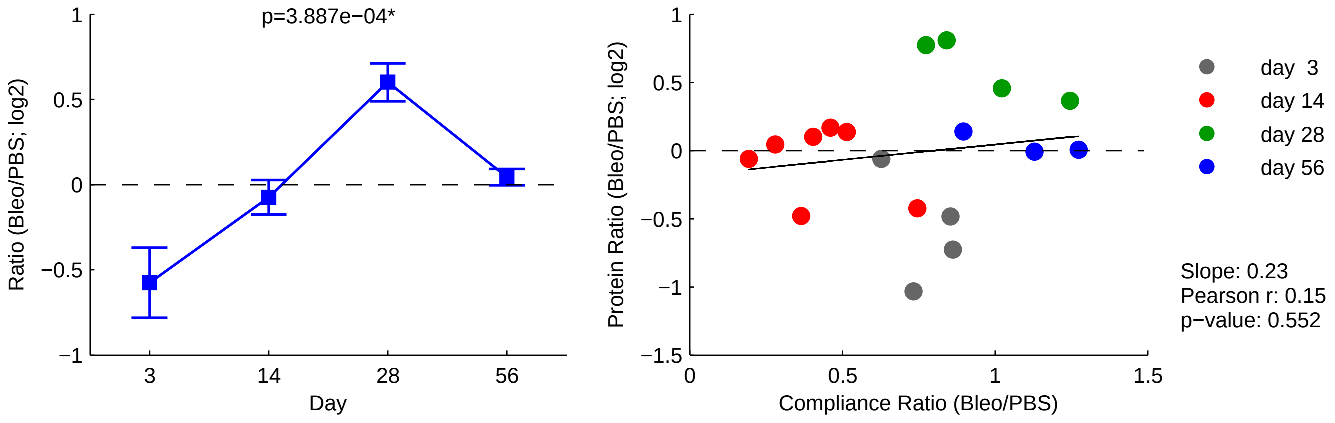

Q9WV55 − Vapa (id: 7910)

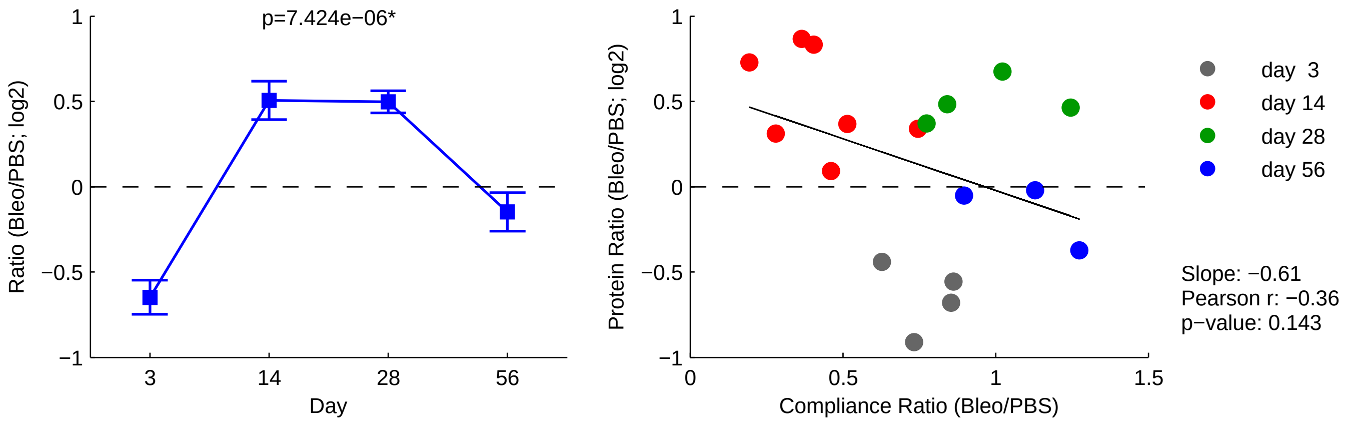

Q9WV68 − Decr2 (id: 7911)

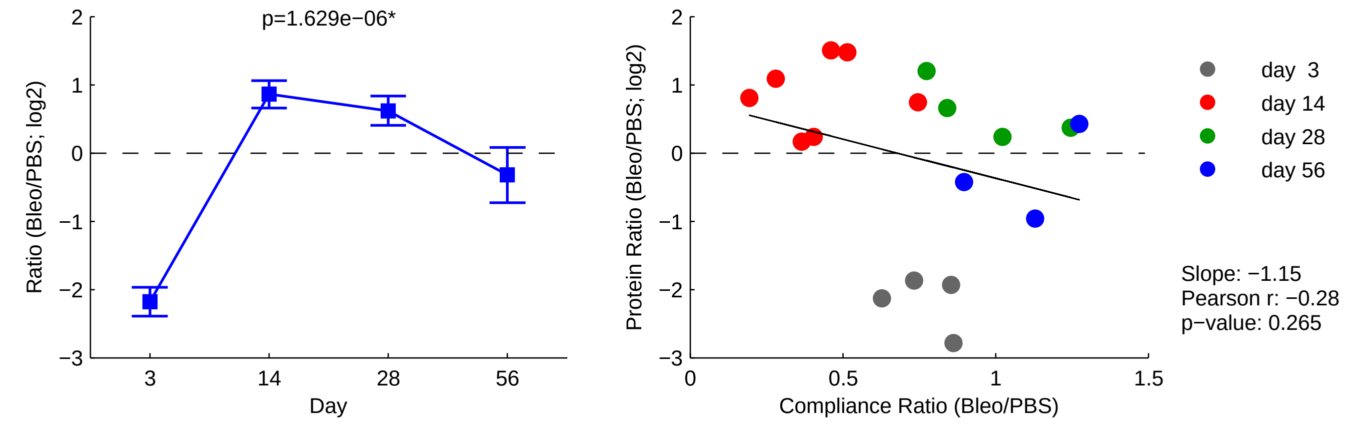

Q9WV85 − Nme3 (id: 7912)

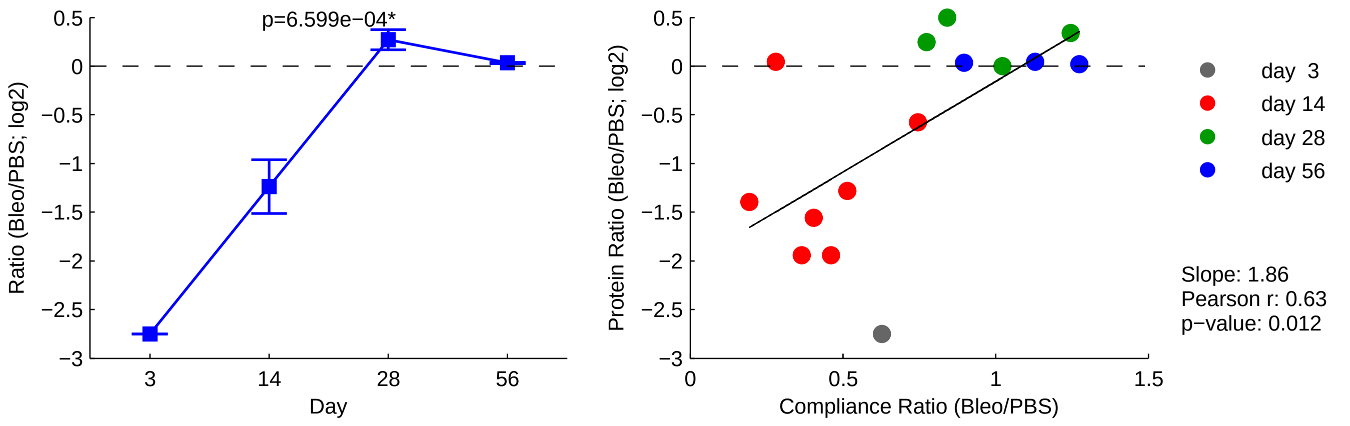

Q9WV91 − Ptgfrn (id: 7914)

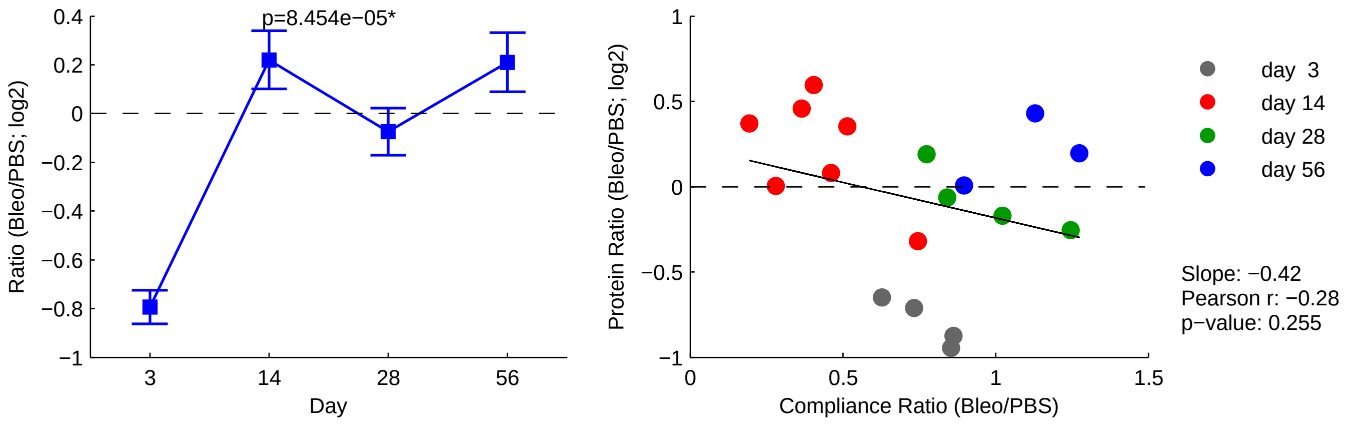

Q9WV95 − Phlda3 (id: 7917)

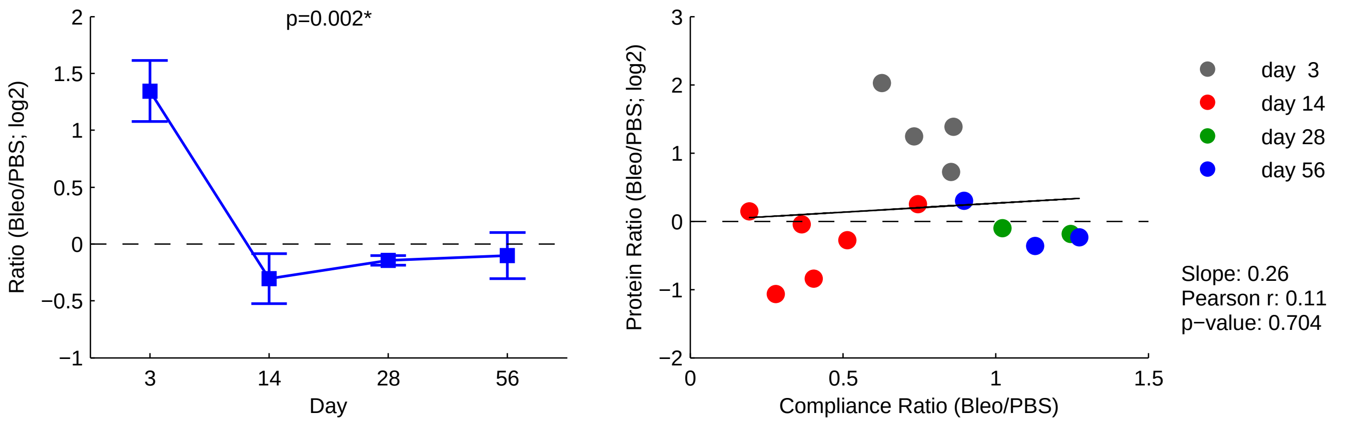

Q9WV98 − Timm9 (id: 7918)

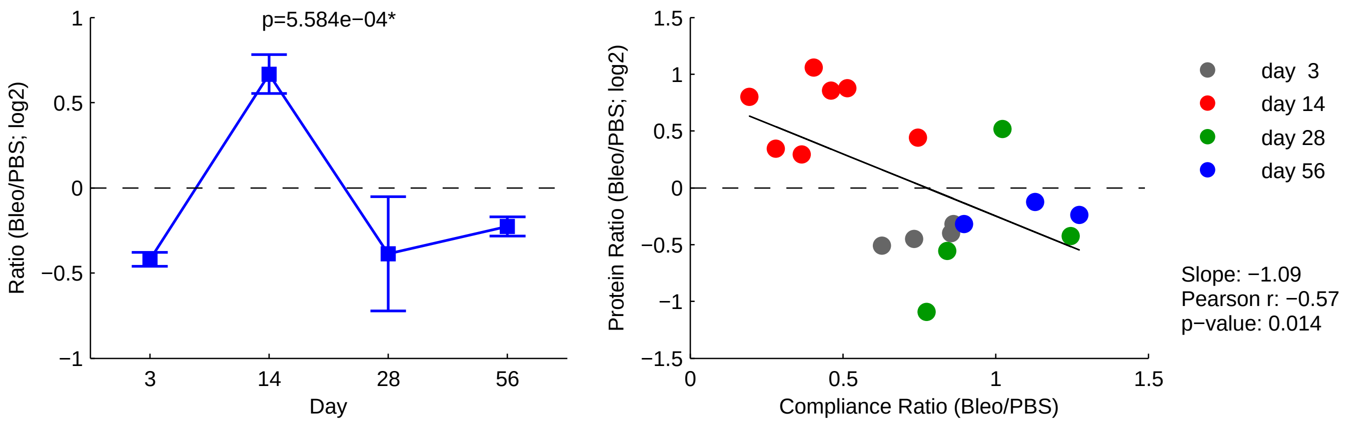

Q9WVB0 − Rbpms (id: 7922)

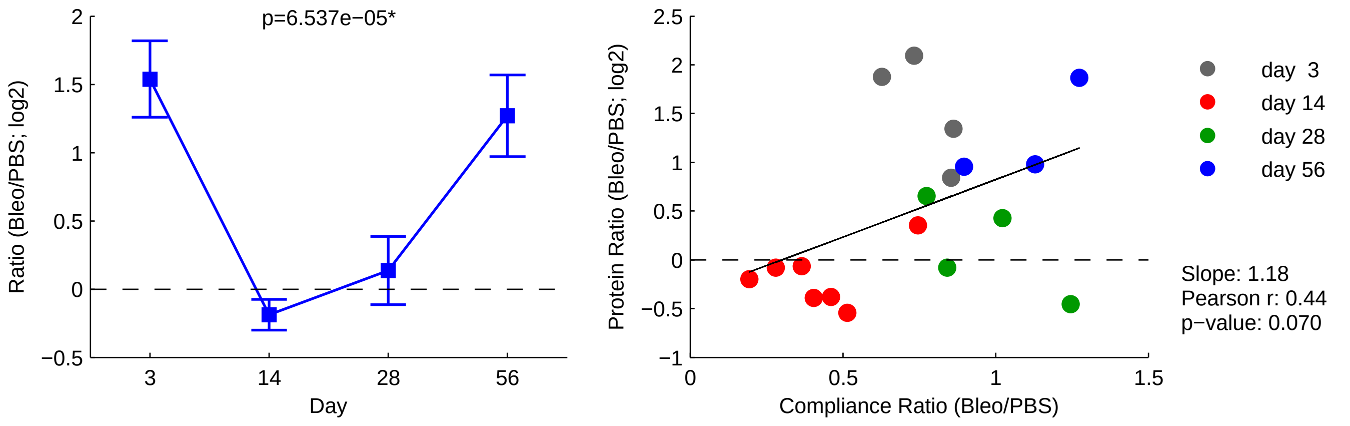

Q9WVB4 − Slit3 (id: 7923)

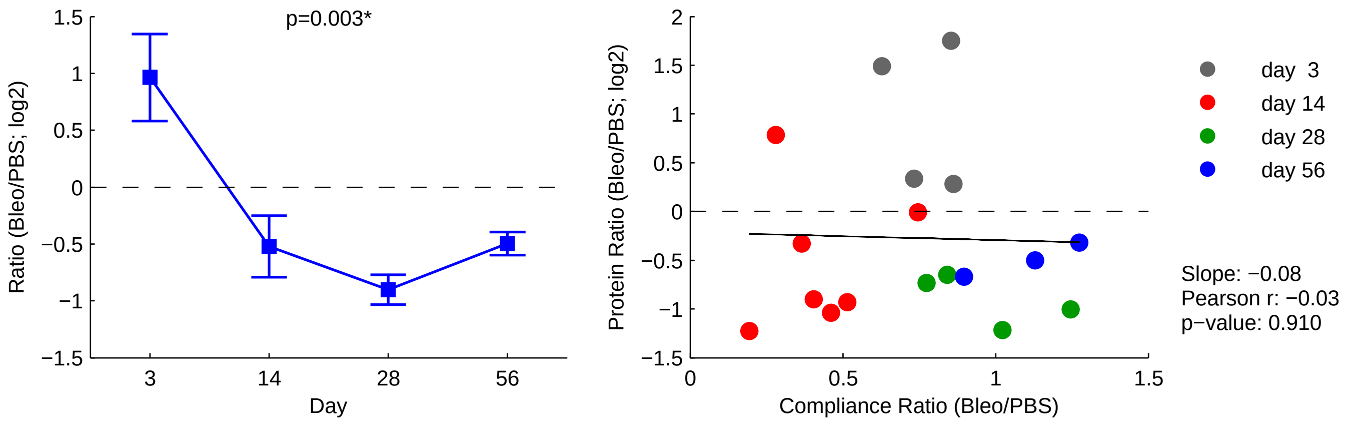

Q9WVC3 − Cav2 (id: 7924)

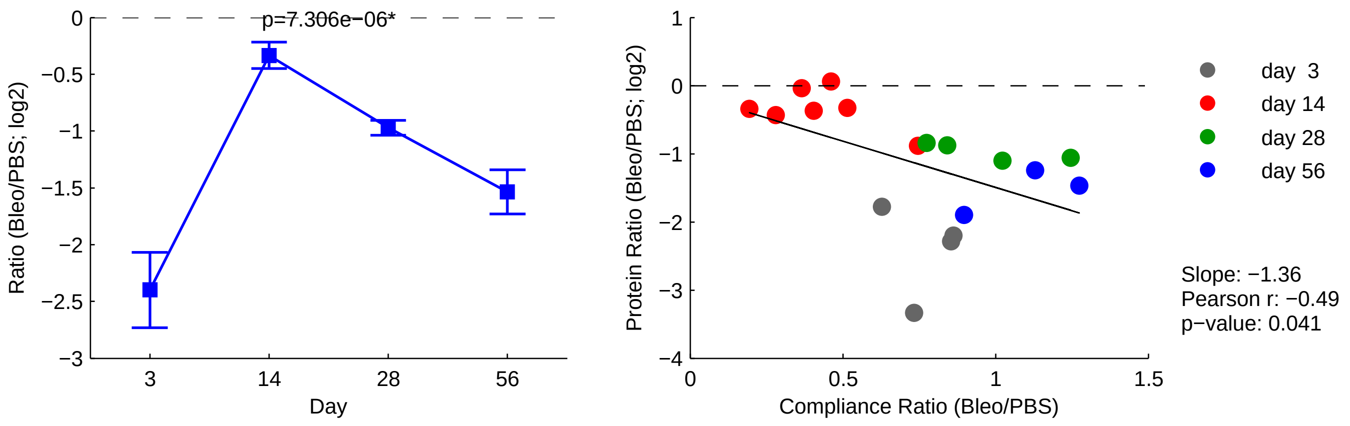

Q9WVD5 − Slc25a15 (id: 7925)

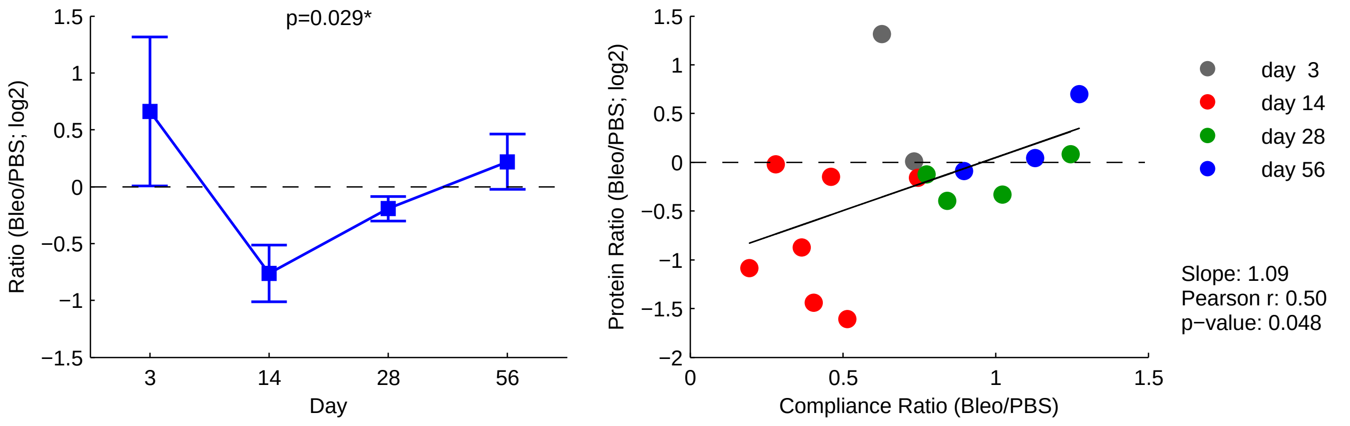

Q9WVH9 − Fbln5 (id: 7929)

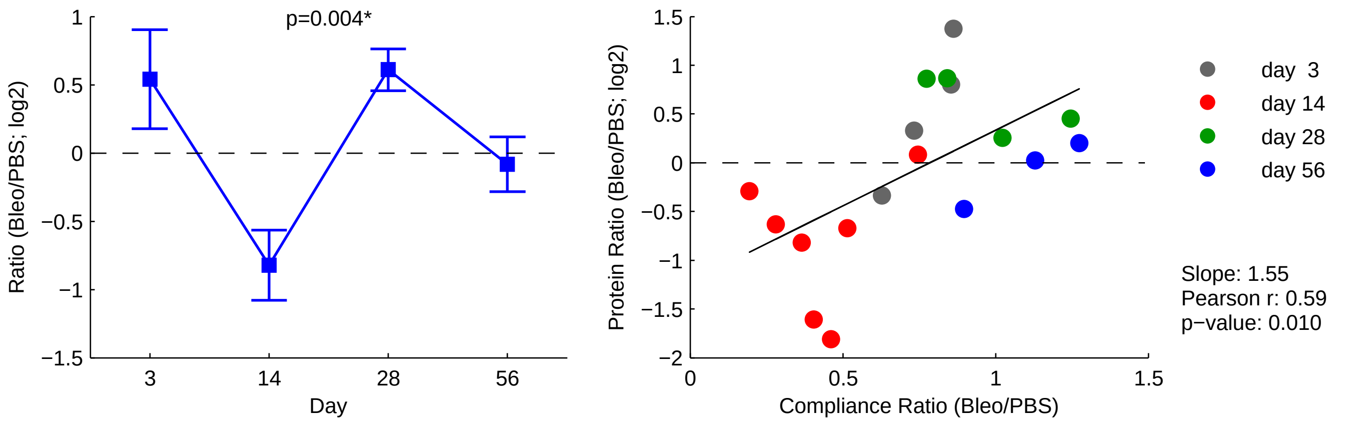

Q9WVJ2 − Psmd13 (id: 7930)

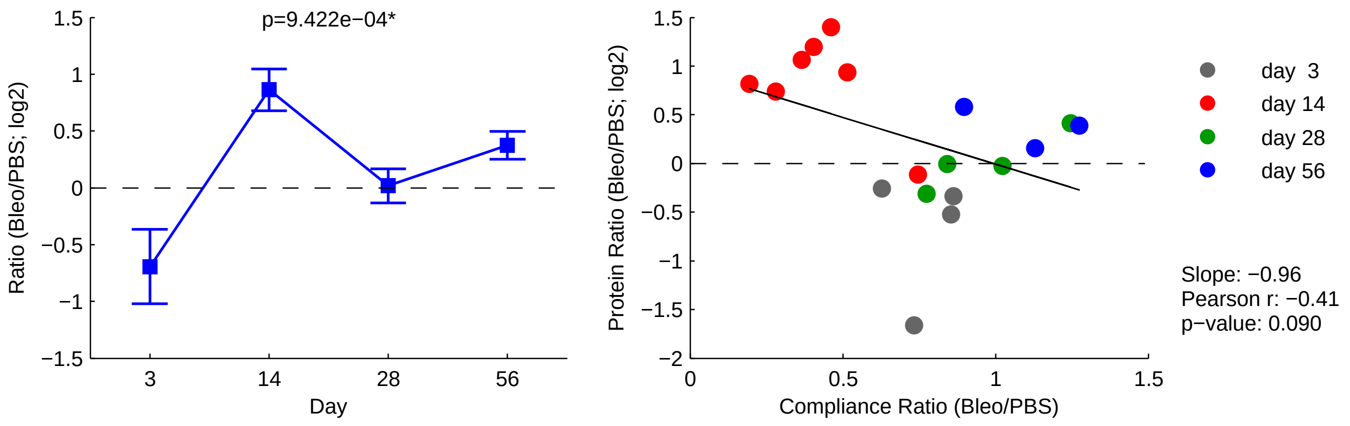

Q9WVJ3−2 − Cpq (id: 7931)

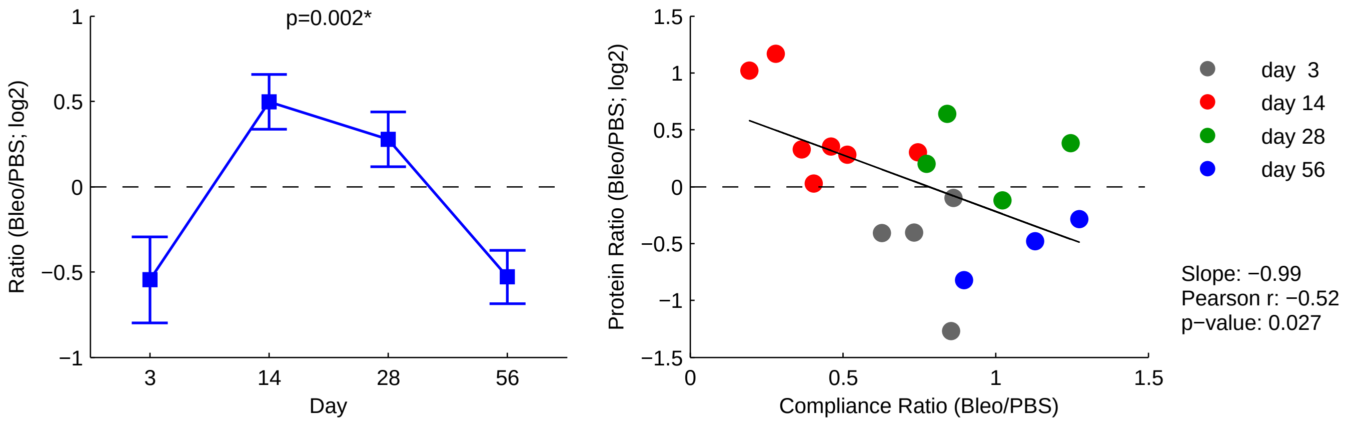

Q9WVL0 − Gstz1 (id: 7934)

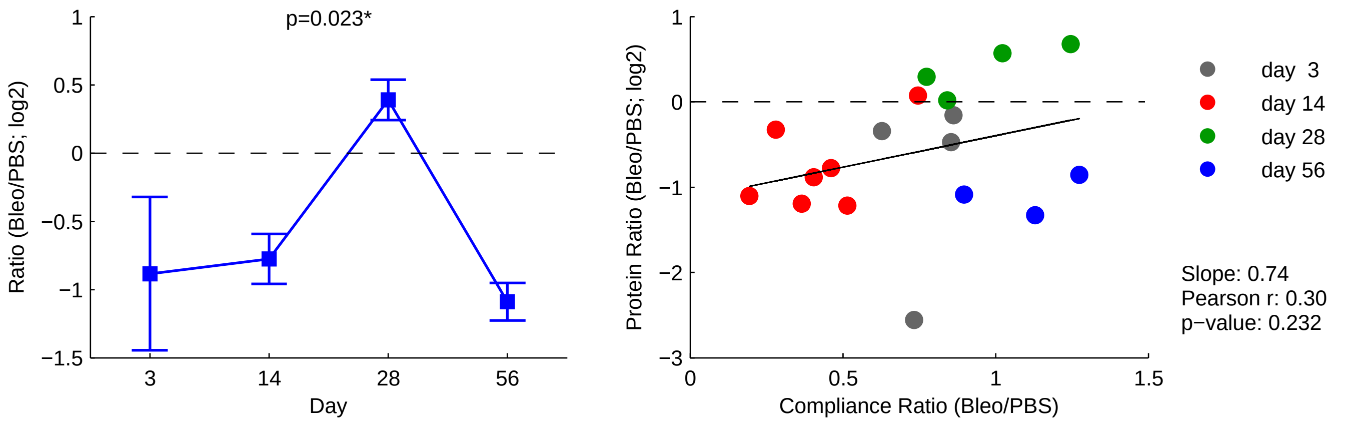

Q9WVL3 − Slc12a7 (id: 7935)

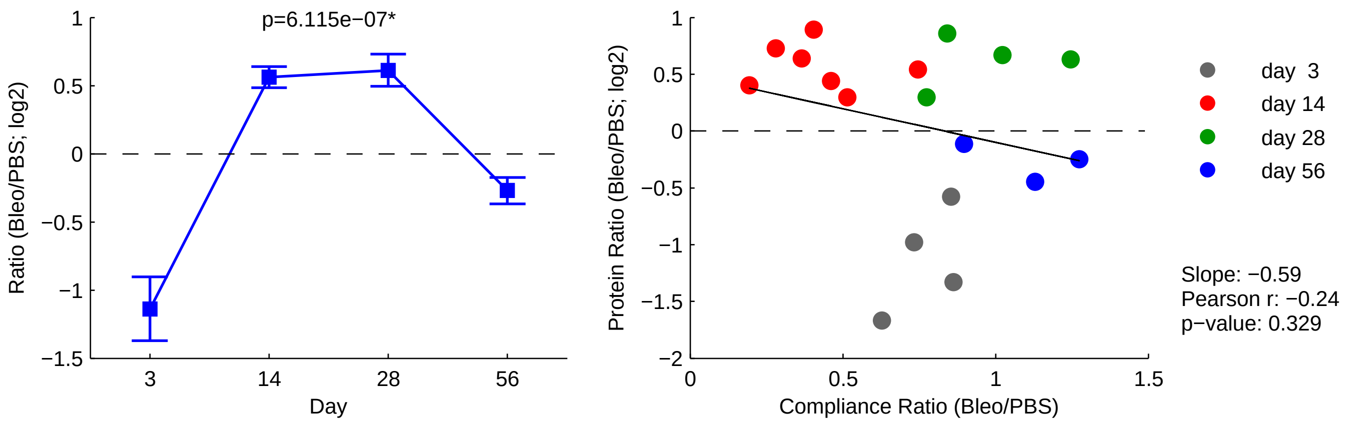

Q9WVP1 − Ap1m2 (id: 7938)

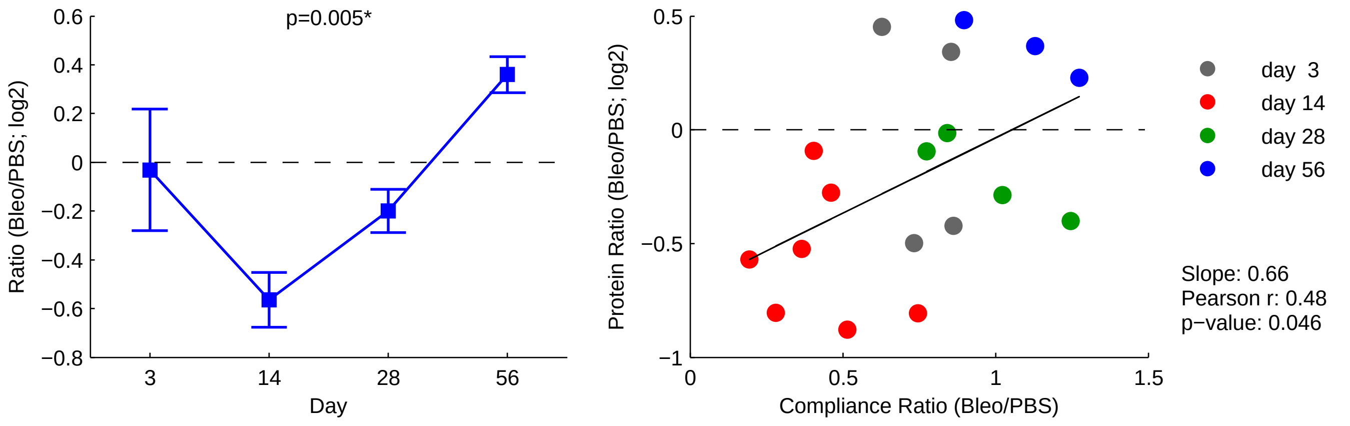

Q9WVQ5 − Apip (id: 7939)

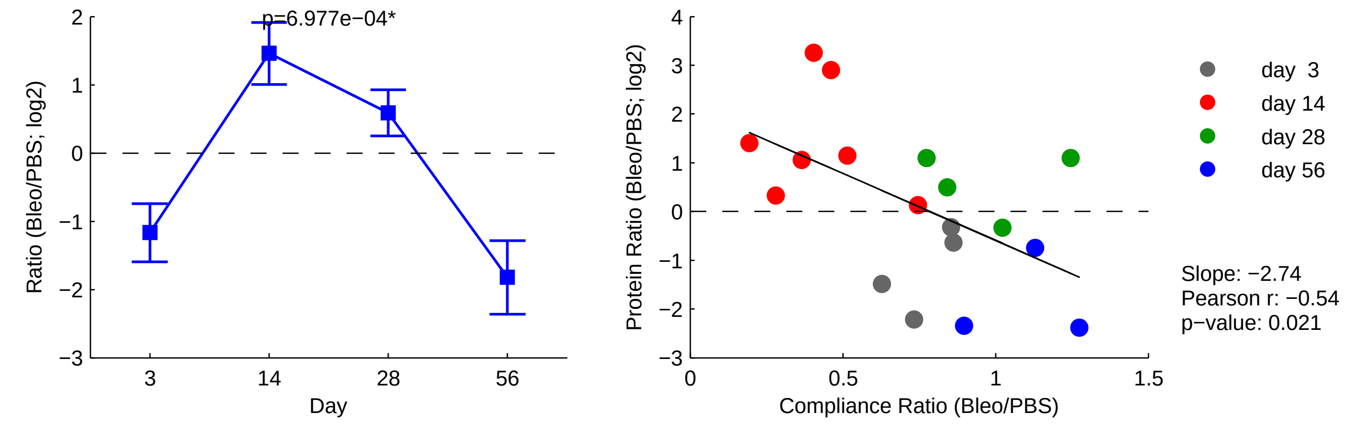

Q9WVT6 − Ca14 (id: 7940)

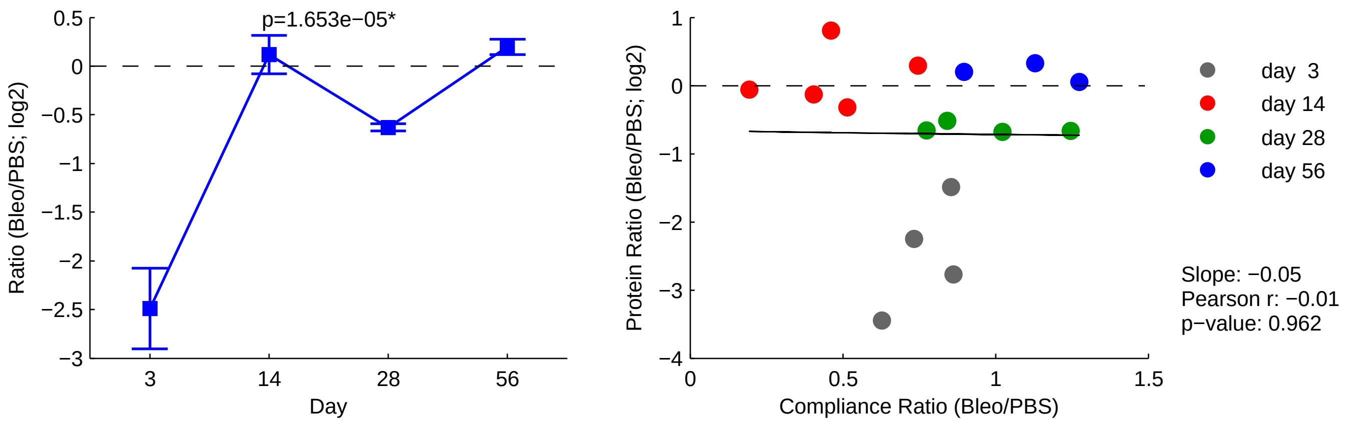

Q9Z0E6 − Gbp2 (id: 7941)

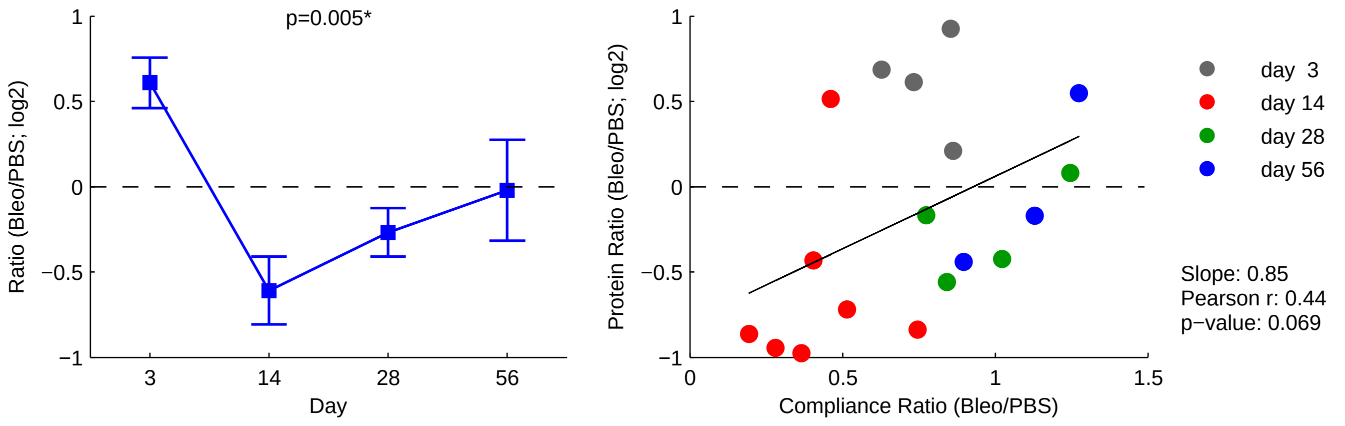

Q9Z0F4 − Cib1 (id: 7942)

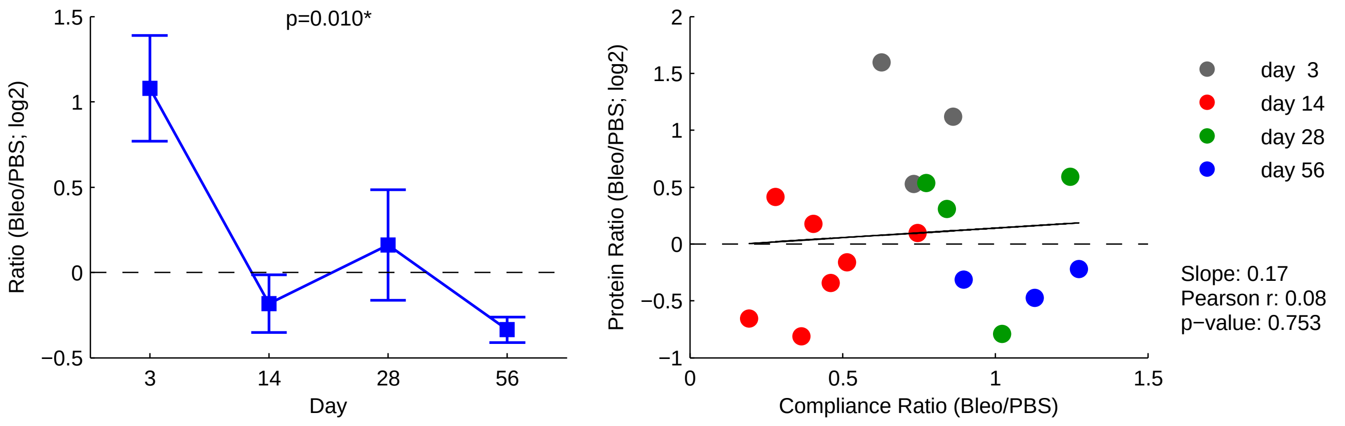

Q9Z0G9−2 − Cldn3 (id: 7946)

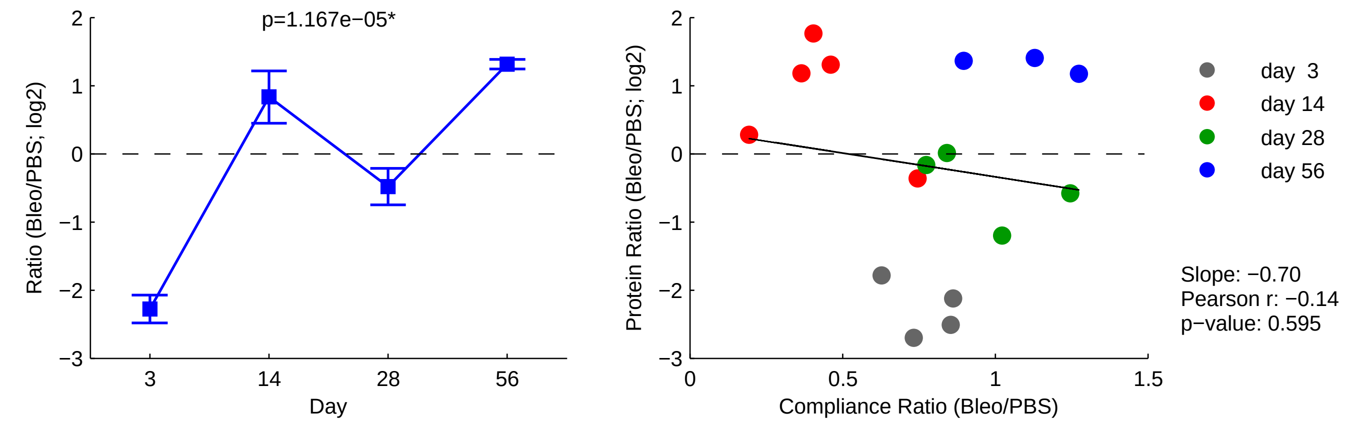

Q9Z0H3 − Smarcb1 (id: 7948)

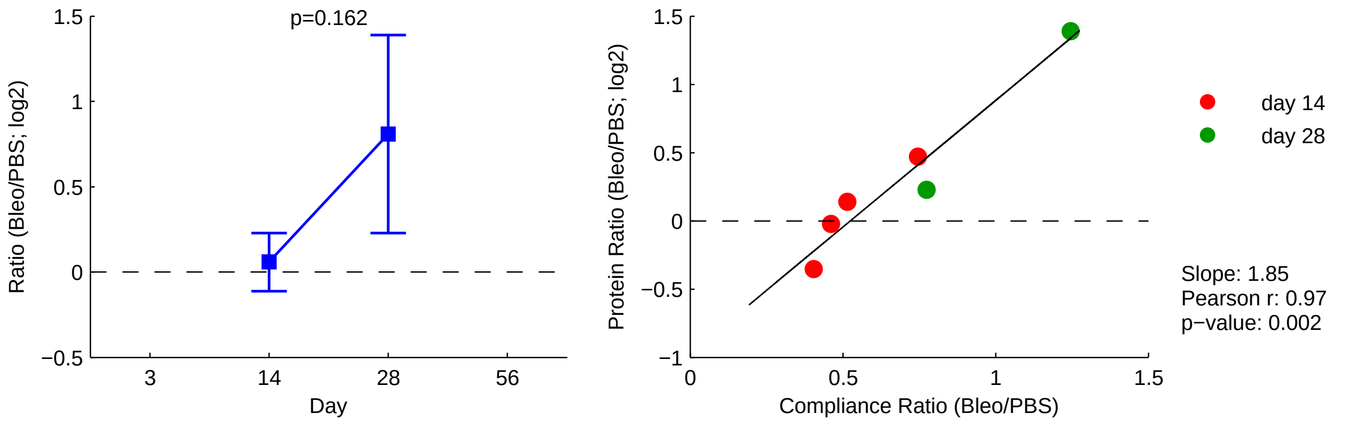

Q9Z0H3−2 − (id: 7949)

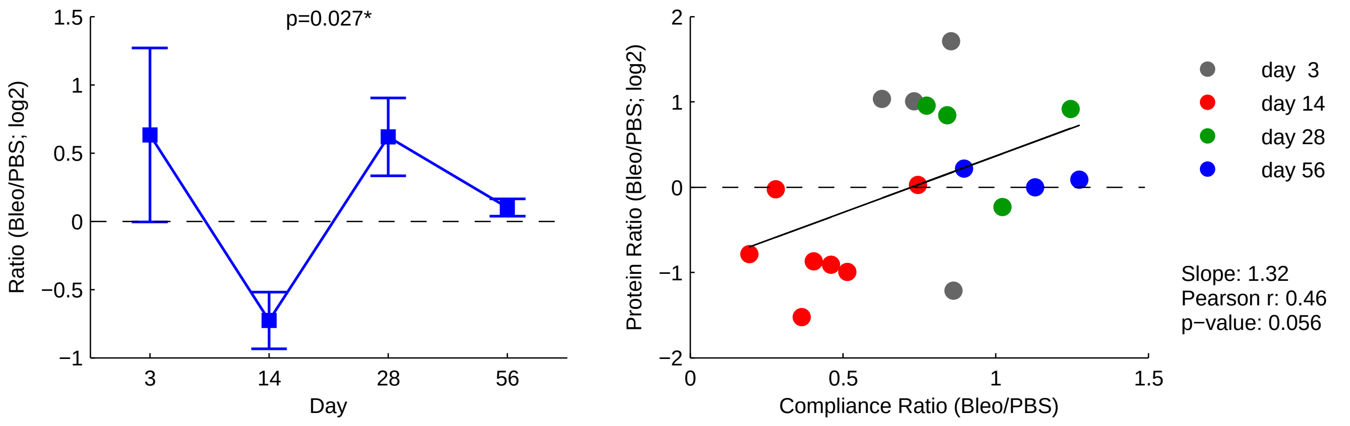

Q9Z0H8−2 − Clip2 (id: 7951)

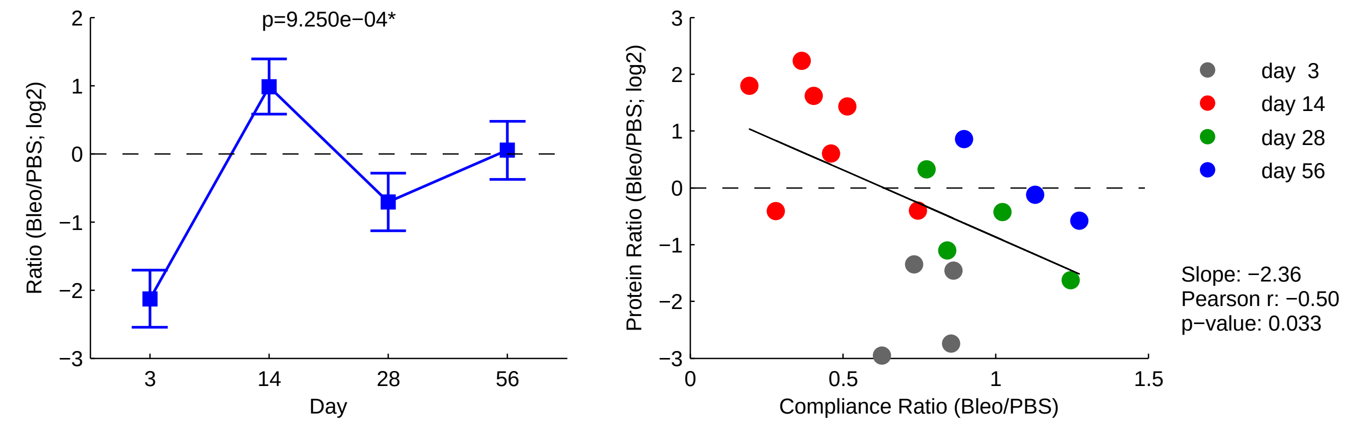

Q9Z0J0 − Npc2 (id: 7952)

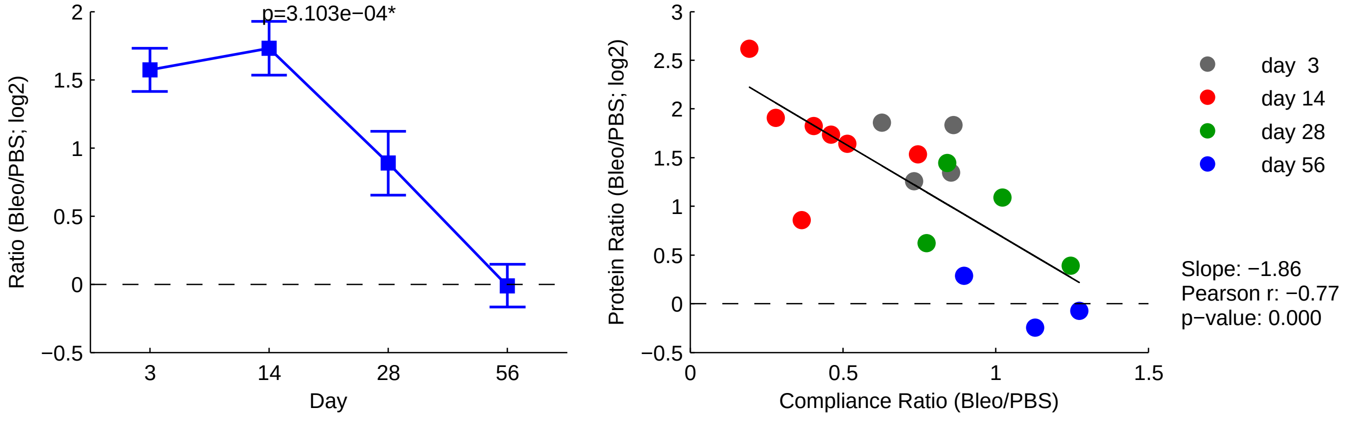

Q9Z0K8 − Vnn1 (id: 7954)

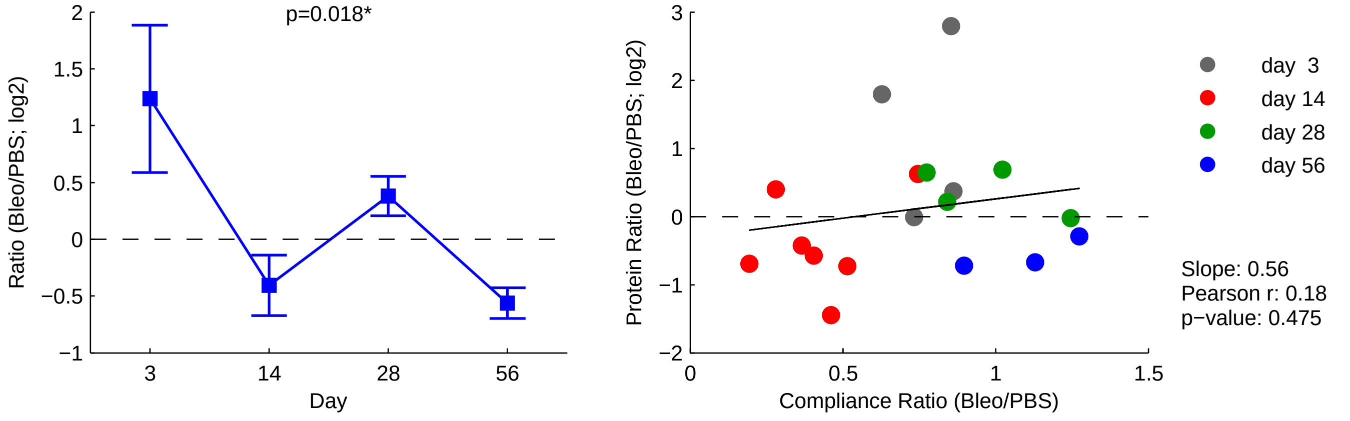

Q9Z0M5 − Lipa (id: 7956)

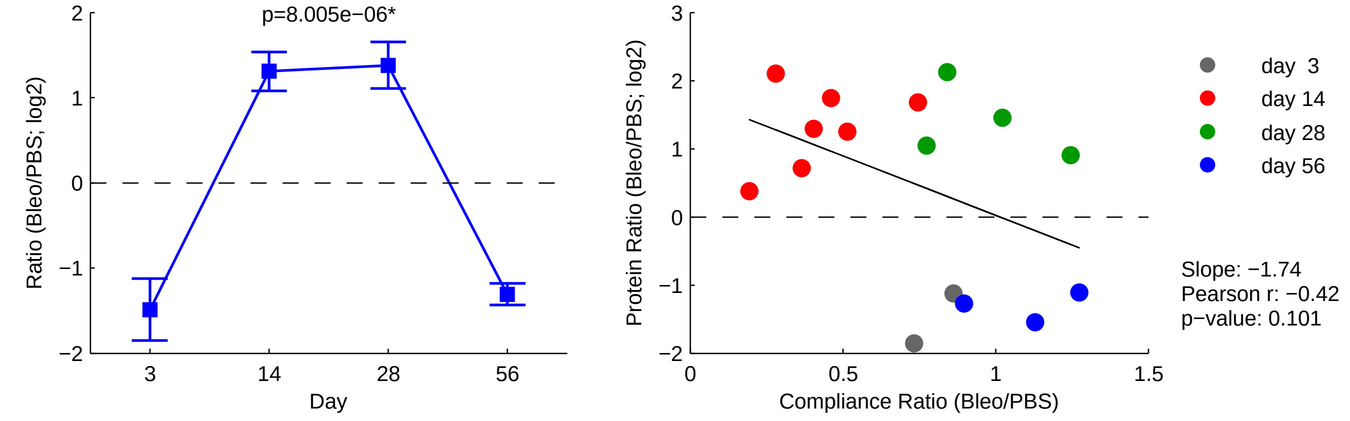

Q9Z0N1 − Eif2s3x (id: 7957)

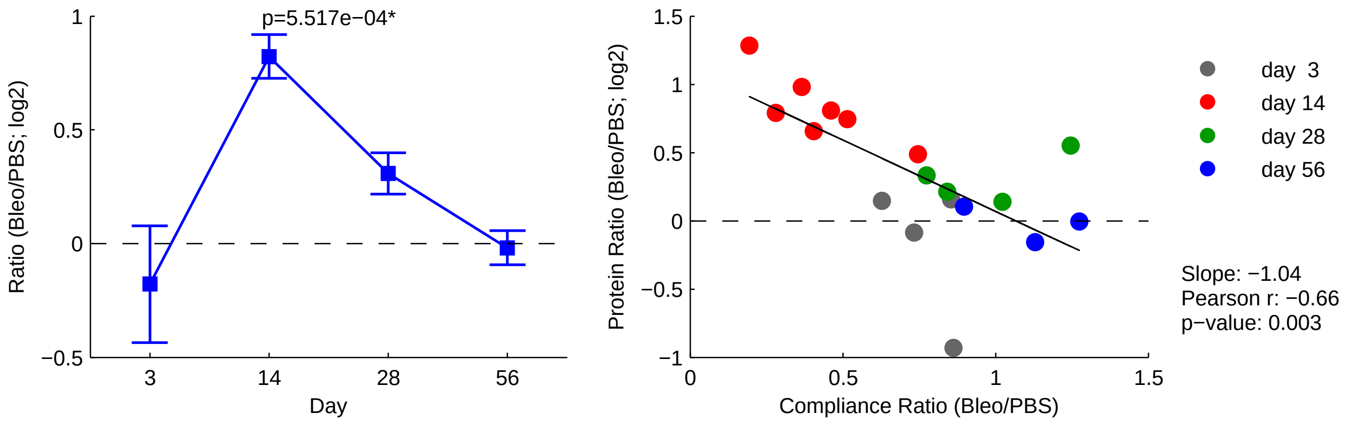

Q9Z0R4 − Itsn1 (id: 7961)

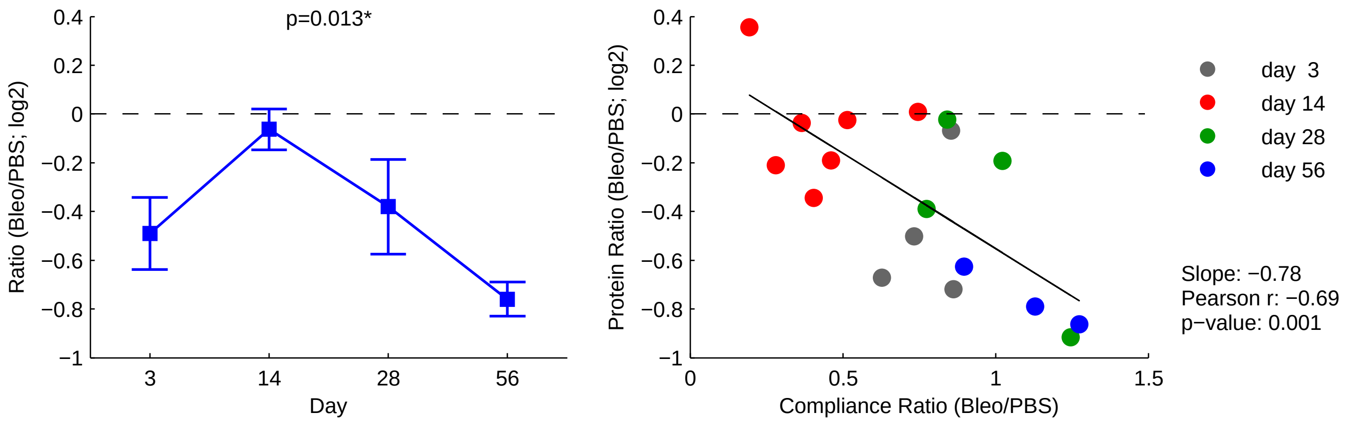

Q9Z0R9 − Fads2 (id: 7962)

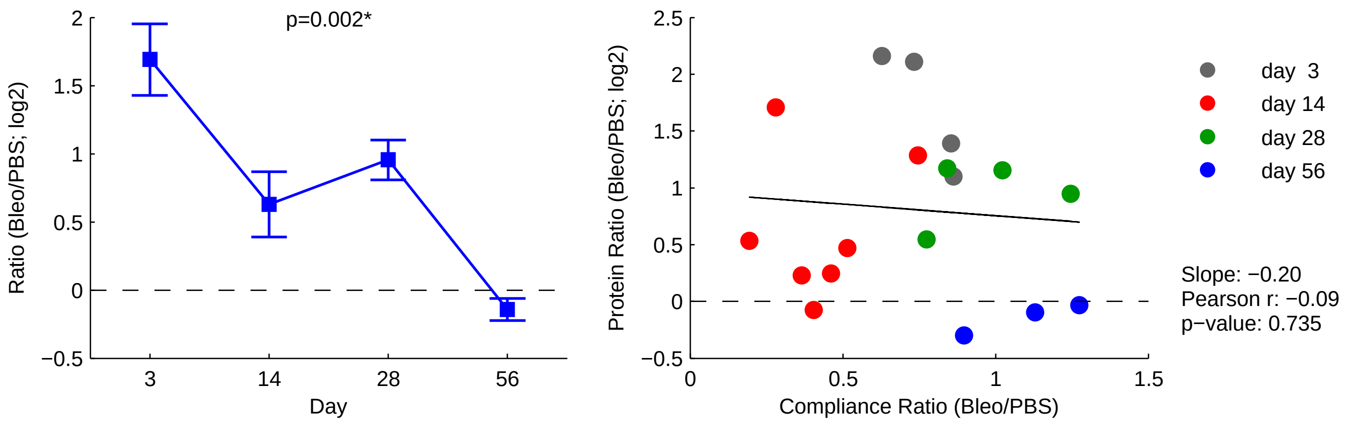

Q9Z0S1 − Bpnt1 (id: 7963)

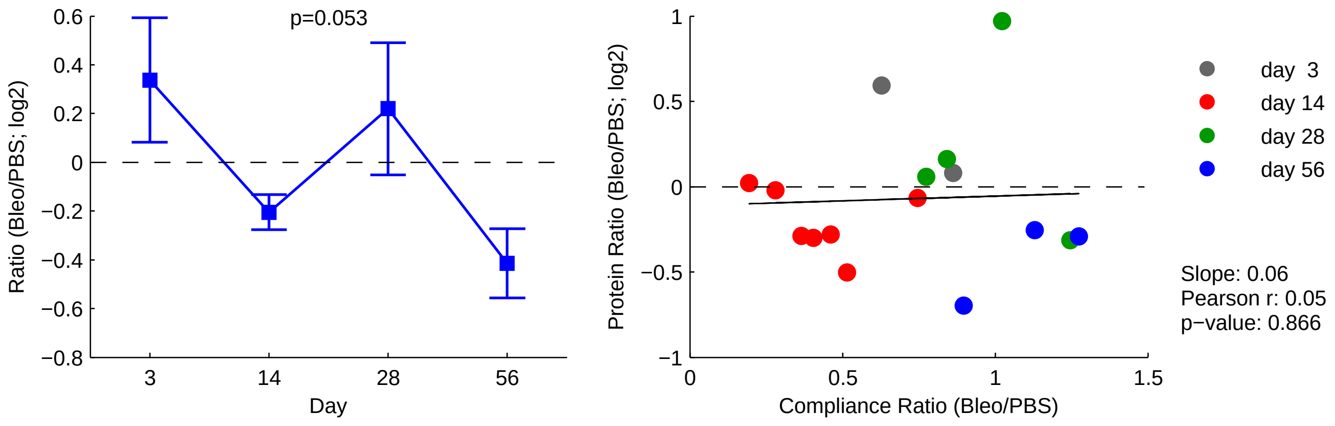

Q9Z0S2 − Lhx2 (id: 7964)

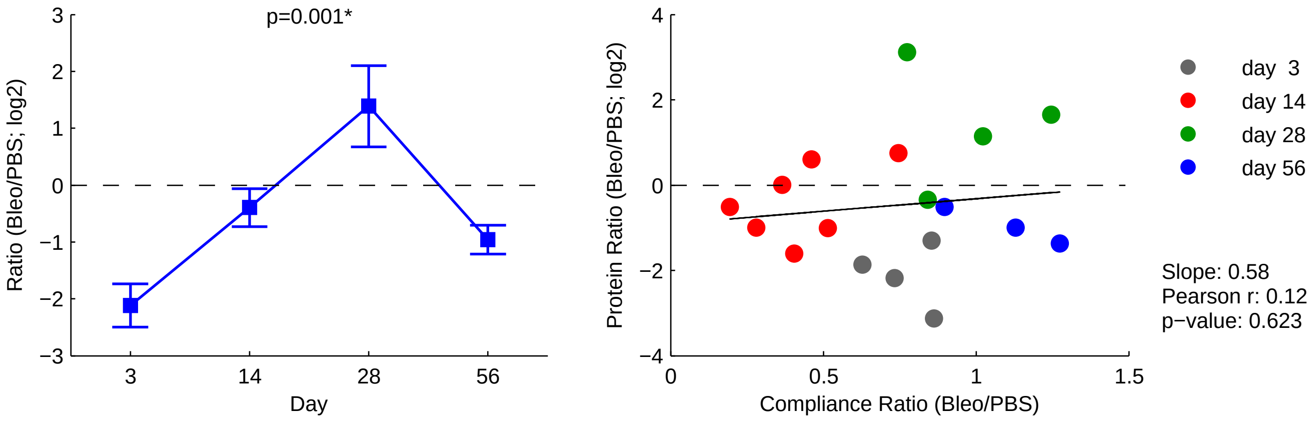

Q9Z0U1 − Tjp2 (id: 7966)

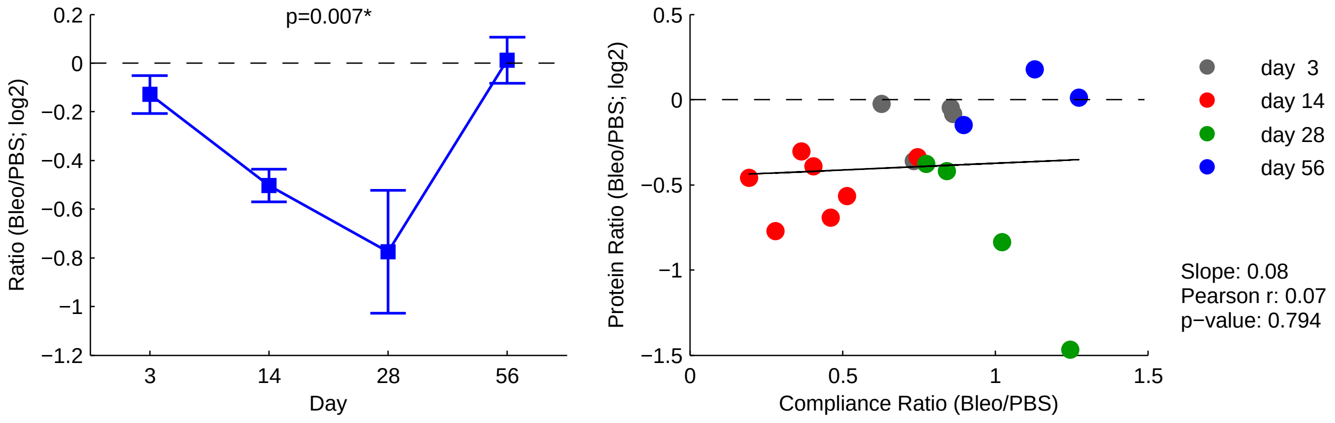

Q9Z0X1 − Aifm1 (id: 7970)

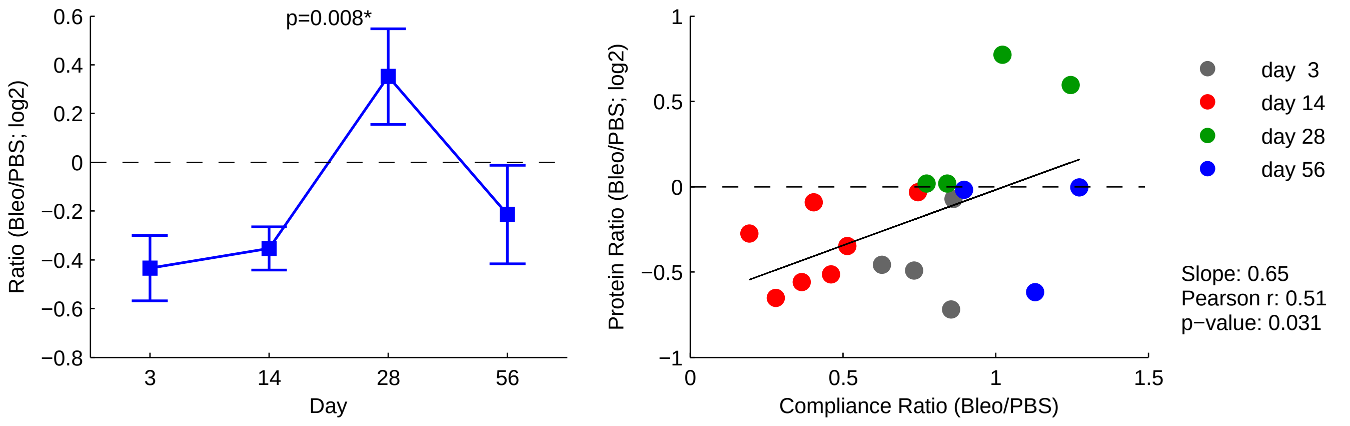

Q9Z0Z4−2 − Heph (id: 7973)

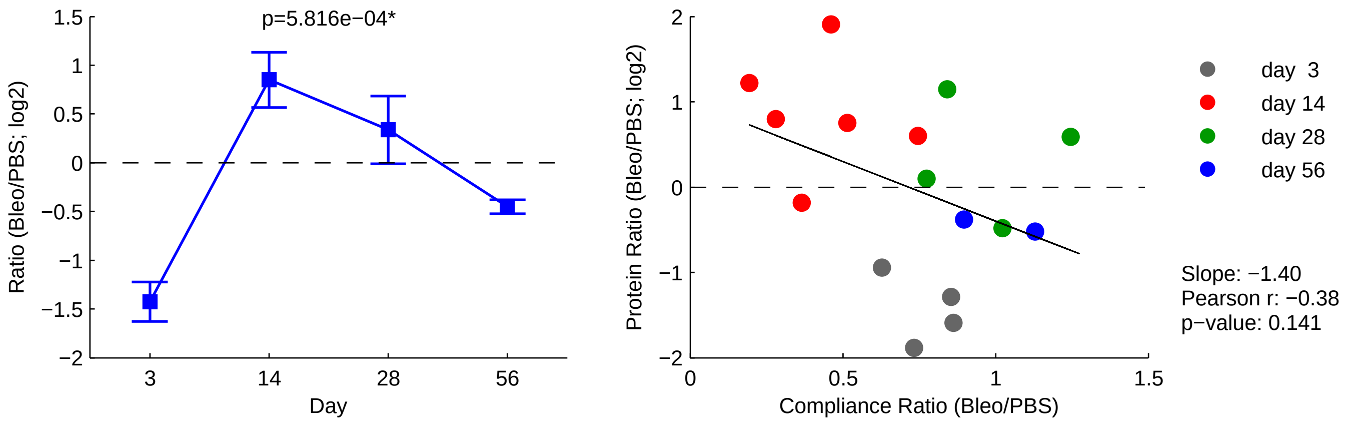

Q9Z110 − Aldh18a1 (id: 7976)

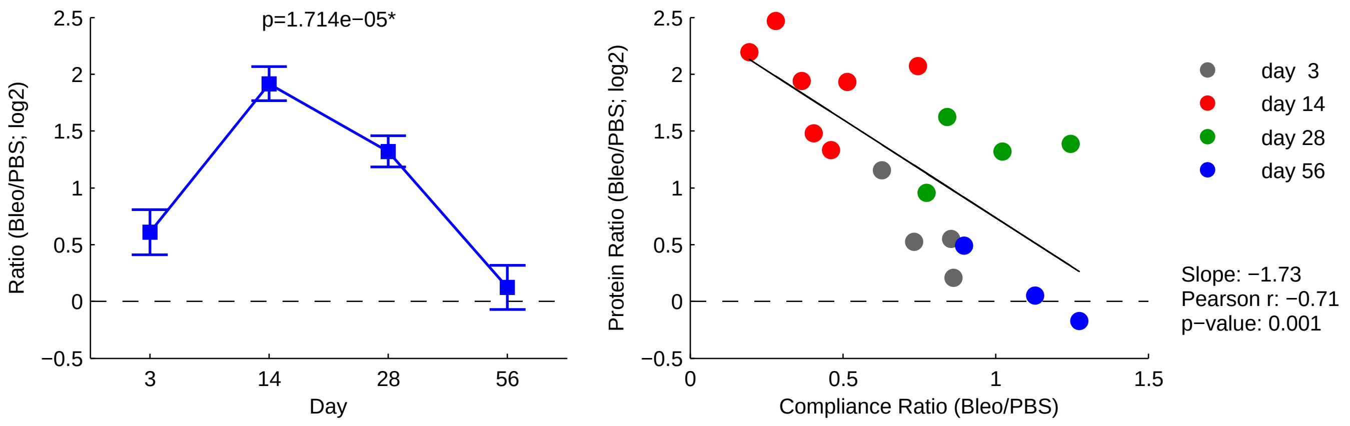

Q9Z130 − Hnrpdl (id: 7979)

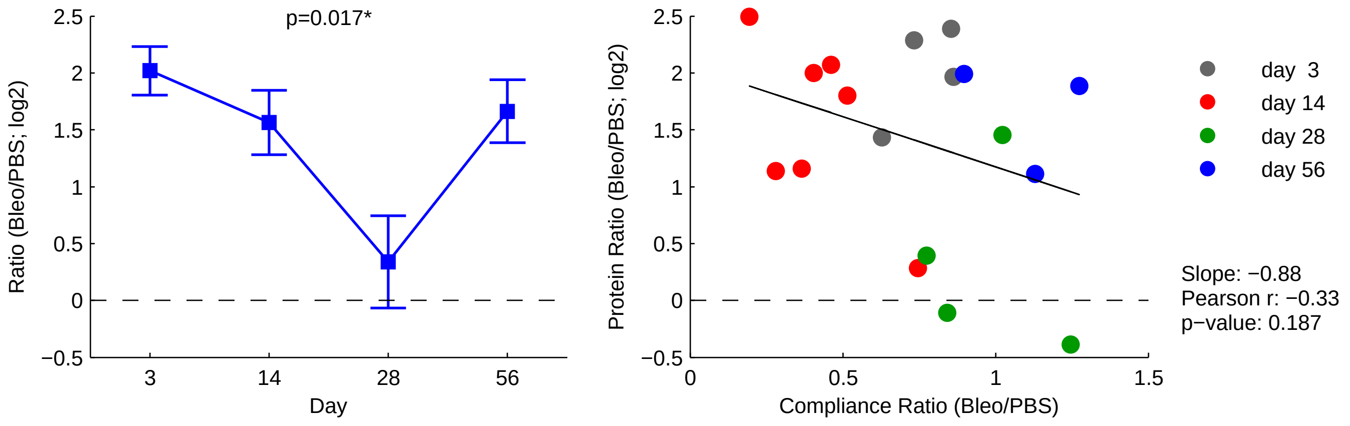

Q9Z148−2 − Ehmt2 (id: 7980)

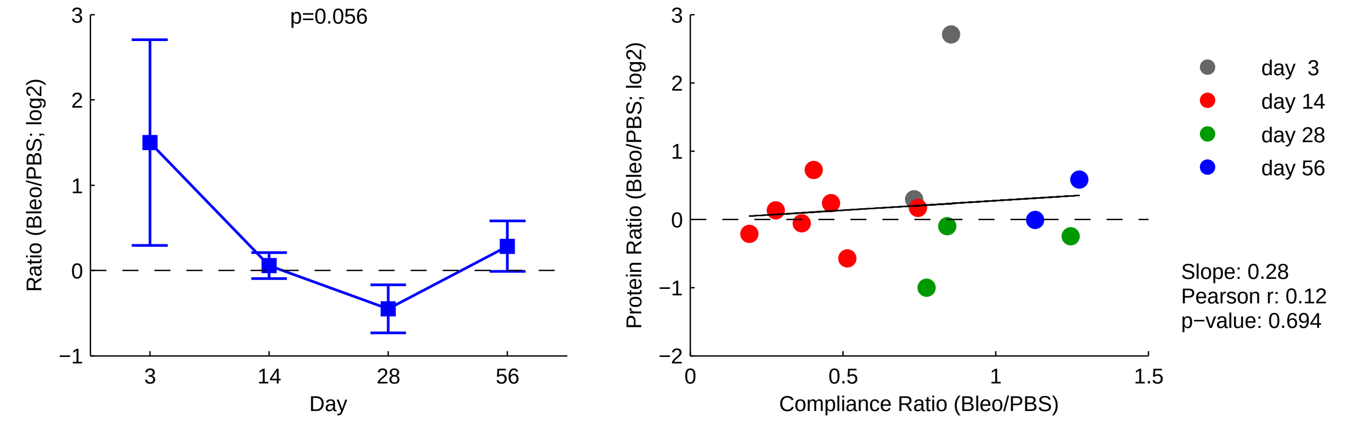

Q9Z1A1 − Tfg (id: 7984)

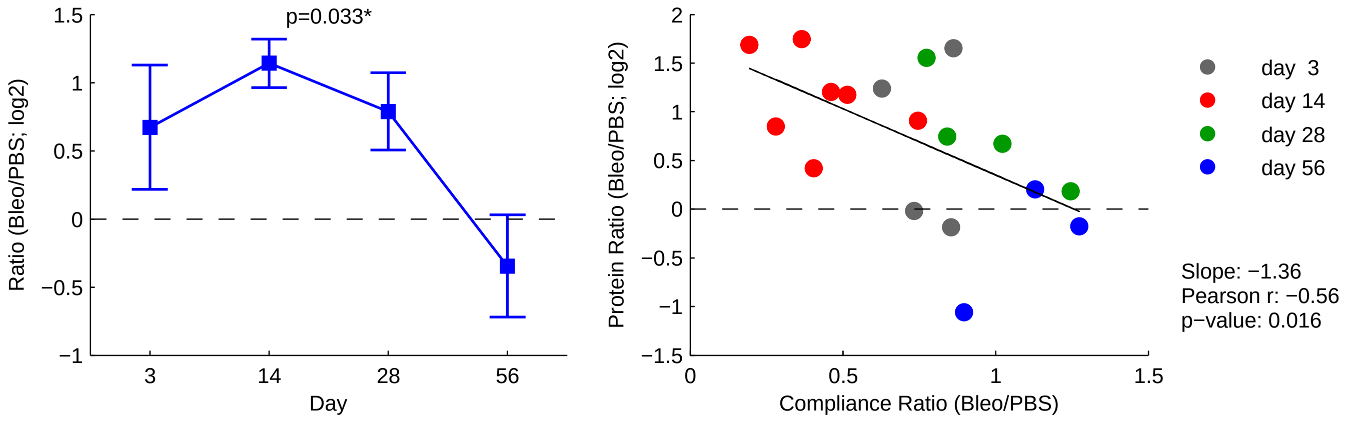

Q9Z1B3 − Plcb1 (id: 7986)

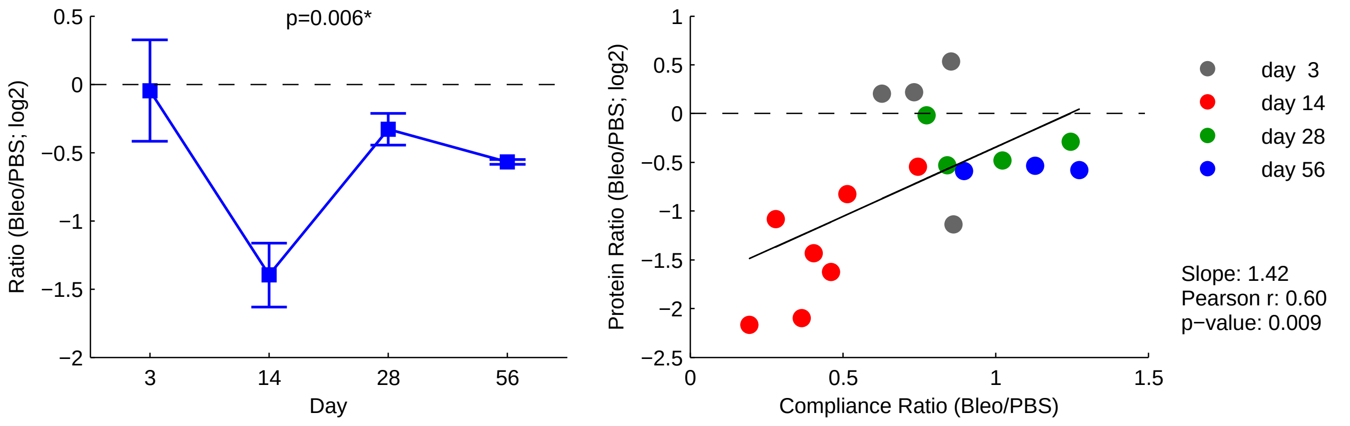

Q9Z1D1 − Eif3g (id: 7990)

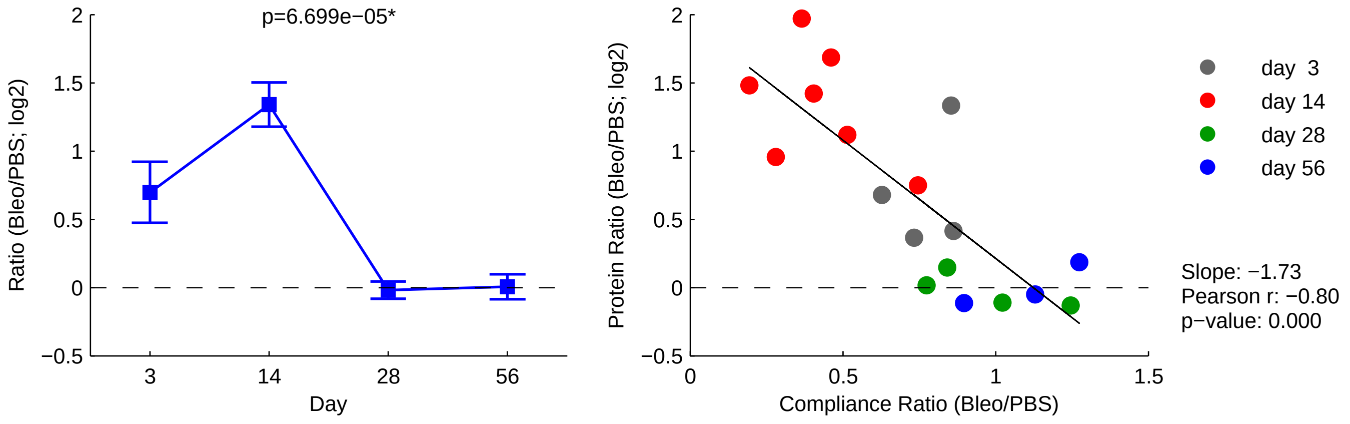

Q9Z1G3 − Atp6v1c1 (id: 7992)

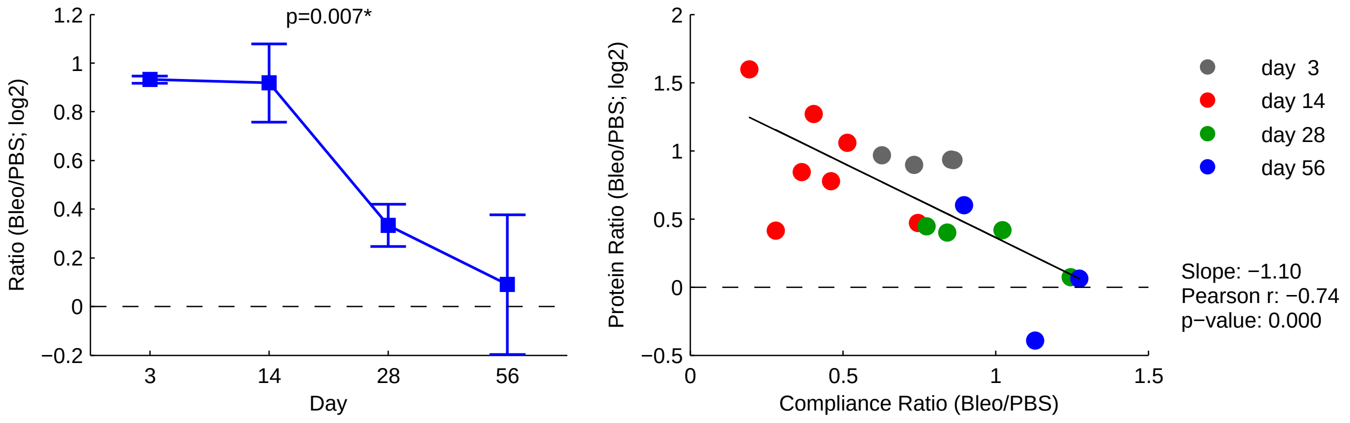

Q9Z1M0 − P2rx7 (id: 7993)

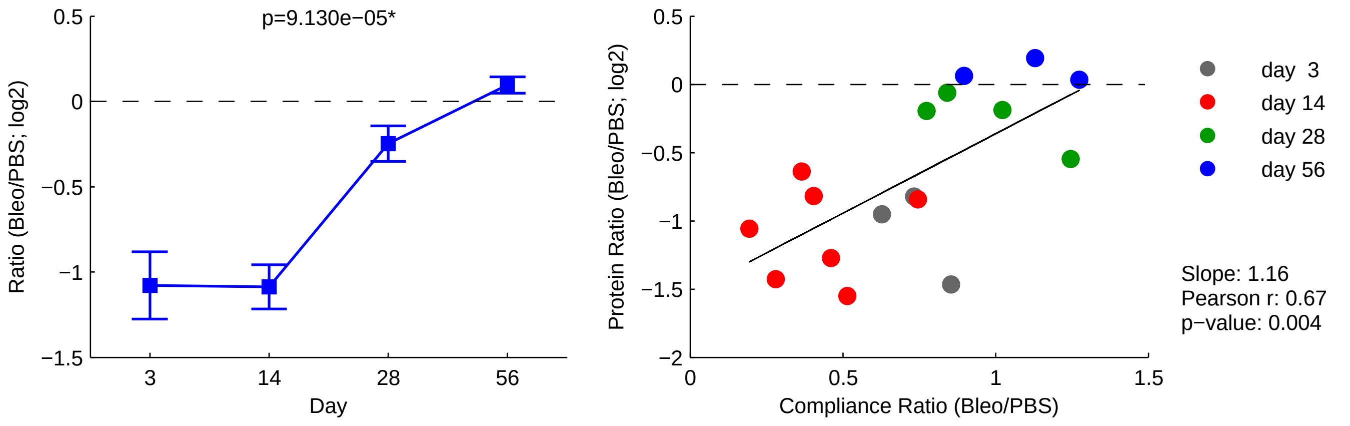

Q9Z1N5 − Ddx39b (id: 7996)

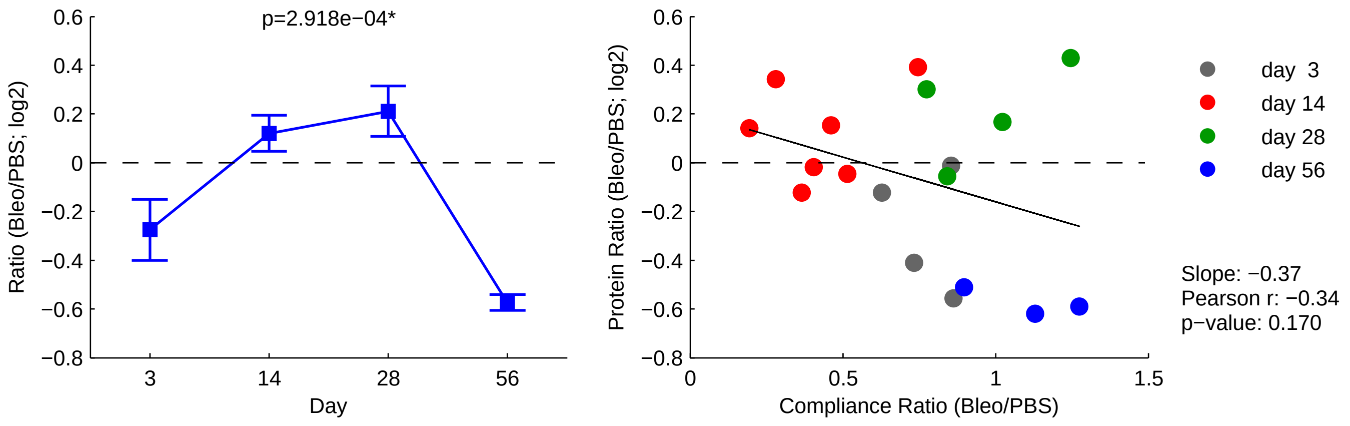

Q9Z1P6 − Ndufa7 (id: 7997)

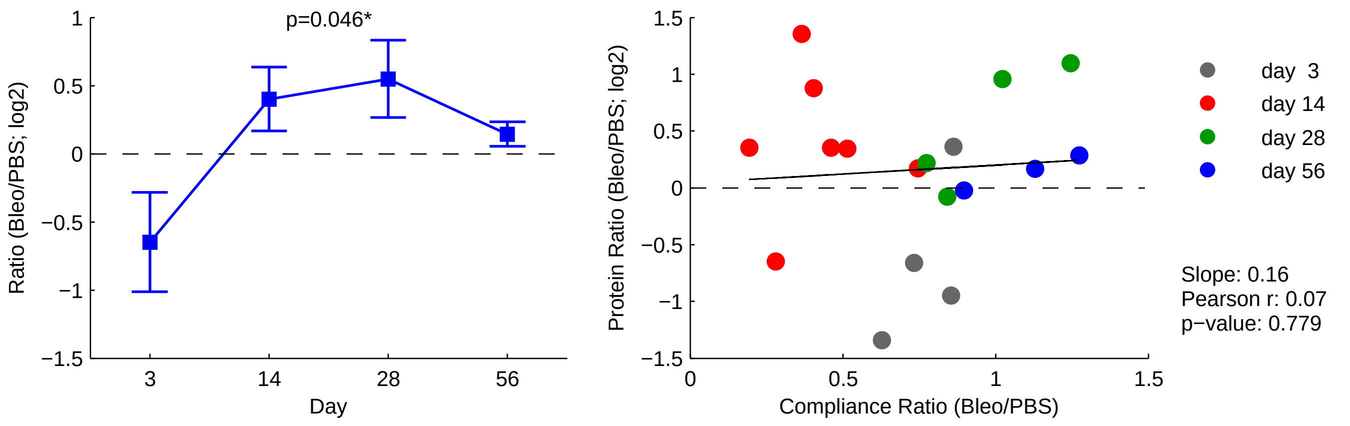

Q9Z1P7 − Kank3 (id: 7998)

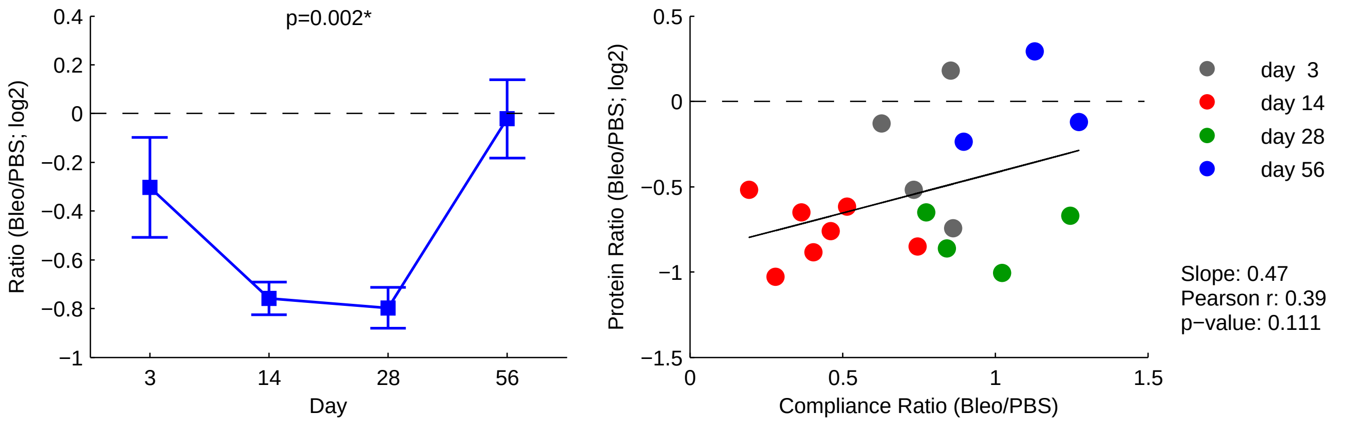

Q9Z1Q2 − Abhd16a (id: 7999)

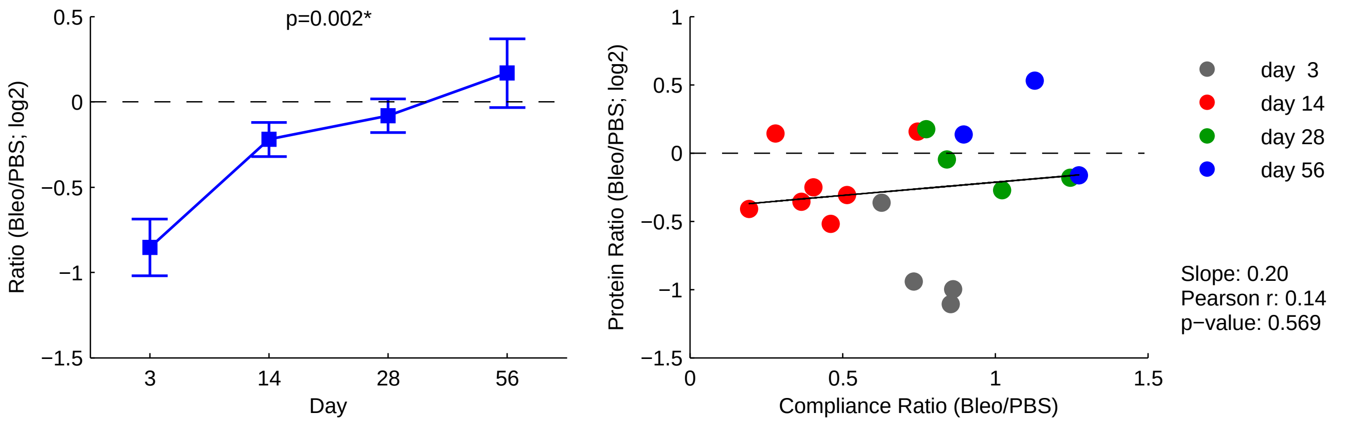

Q9Z1Q9 − Vars (id: 8001)

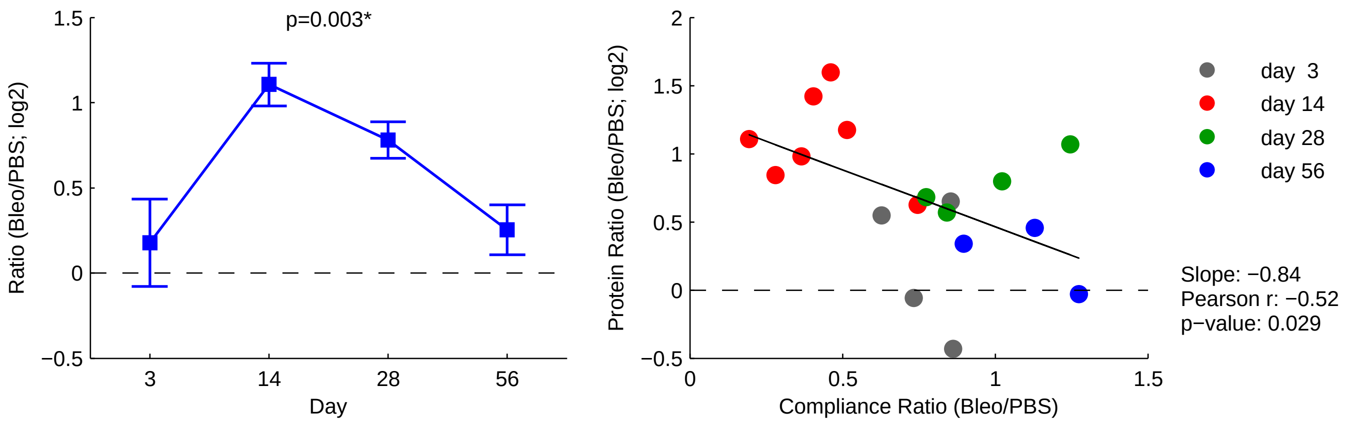

Q9Z1R4 − D17h6s53e (id: 8002)

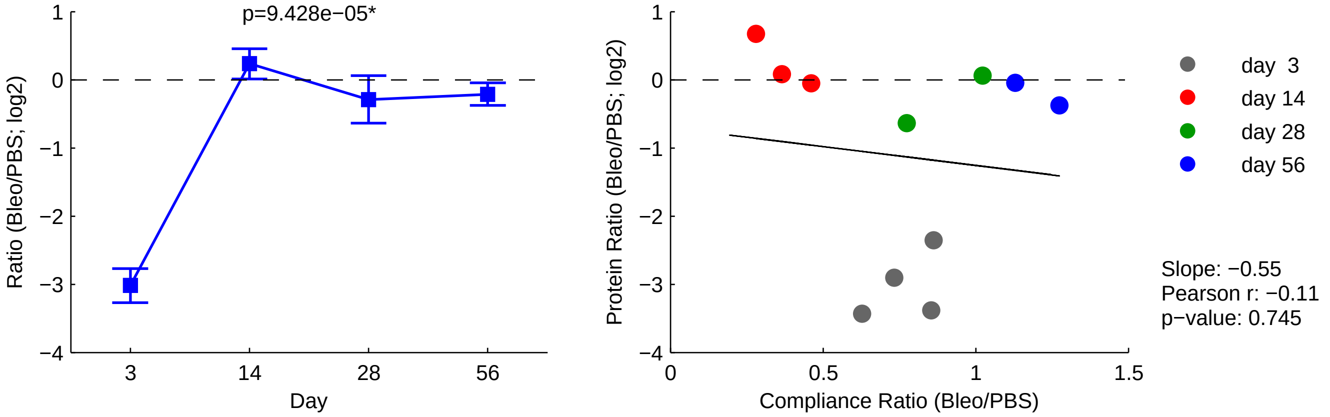

Q9Z1T1 − Ap3b1 (id: 8003)

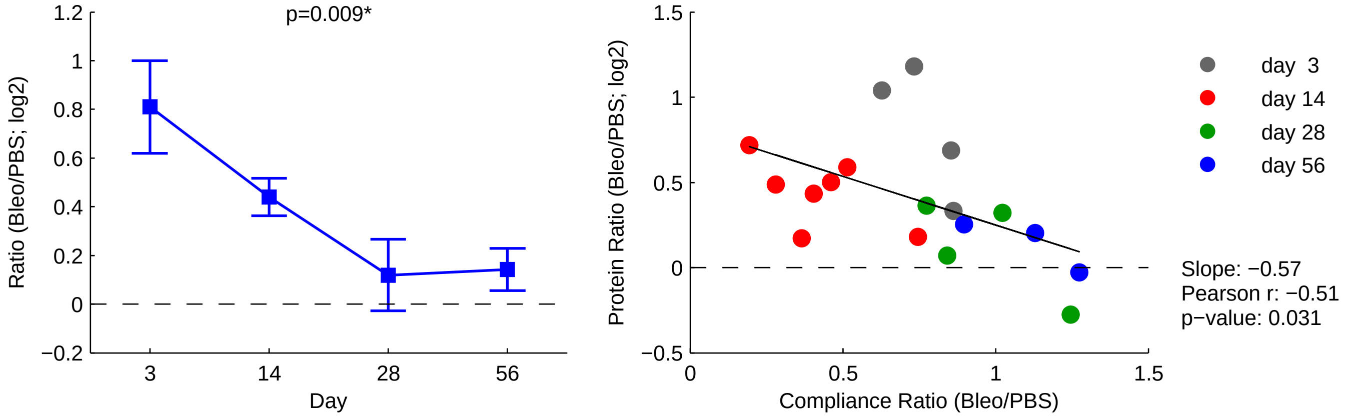

Q9Z1X4 − Ilf3 (id: 8009)

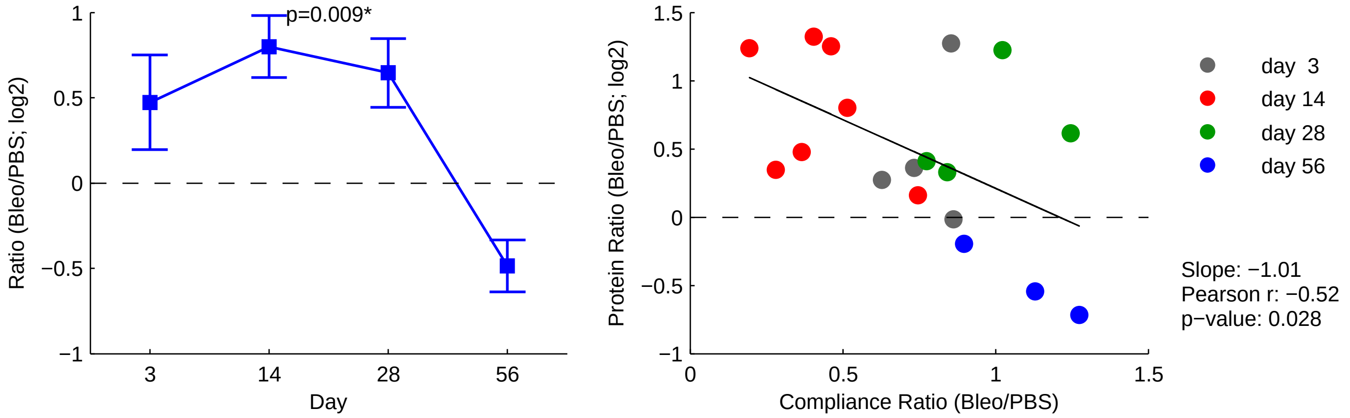

Q9Z1Y4 − Trip6 (id: 8010)

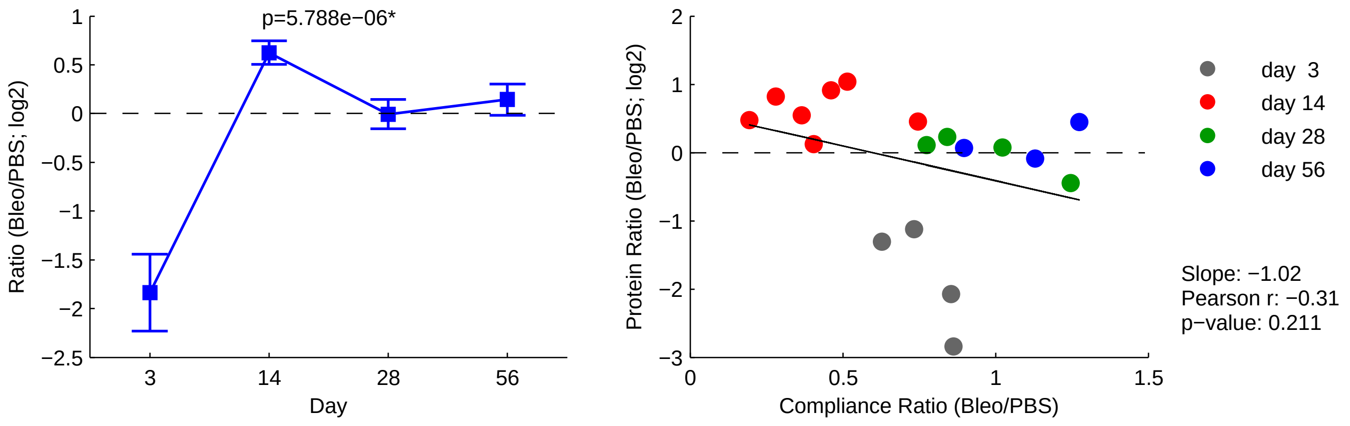

Q9Z1Z0 − Uso1 (id: 8011)

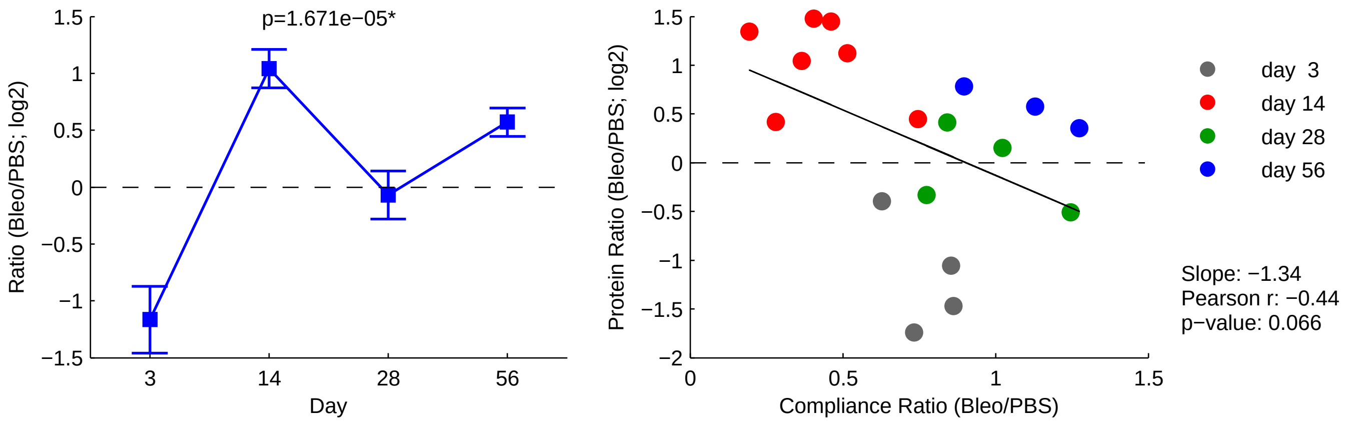

Q9Z1Z2 − Strap (id: 8012)

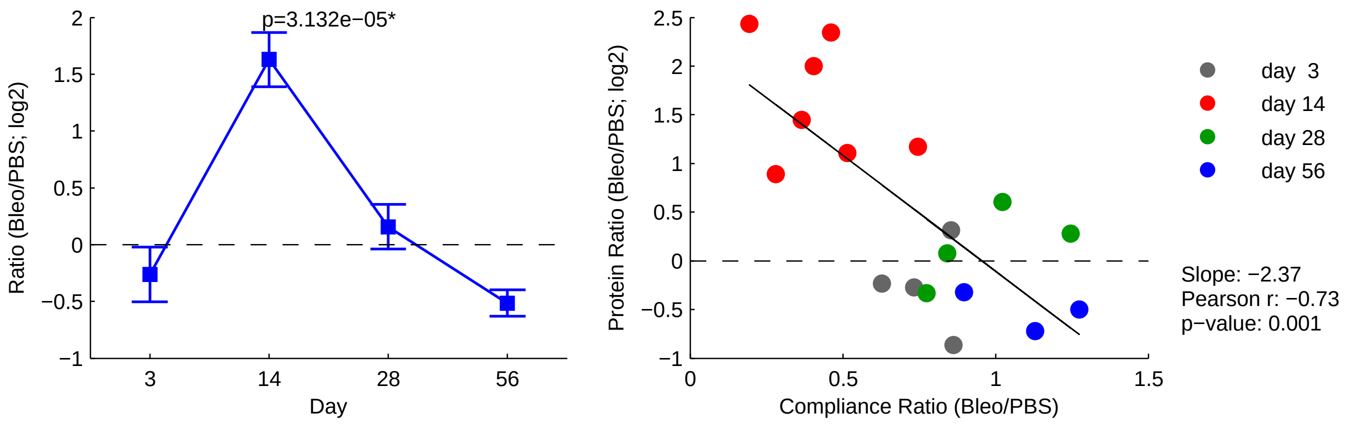

Q9Z247 − Fkbp9 (id: 8015)

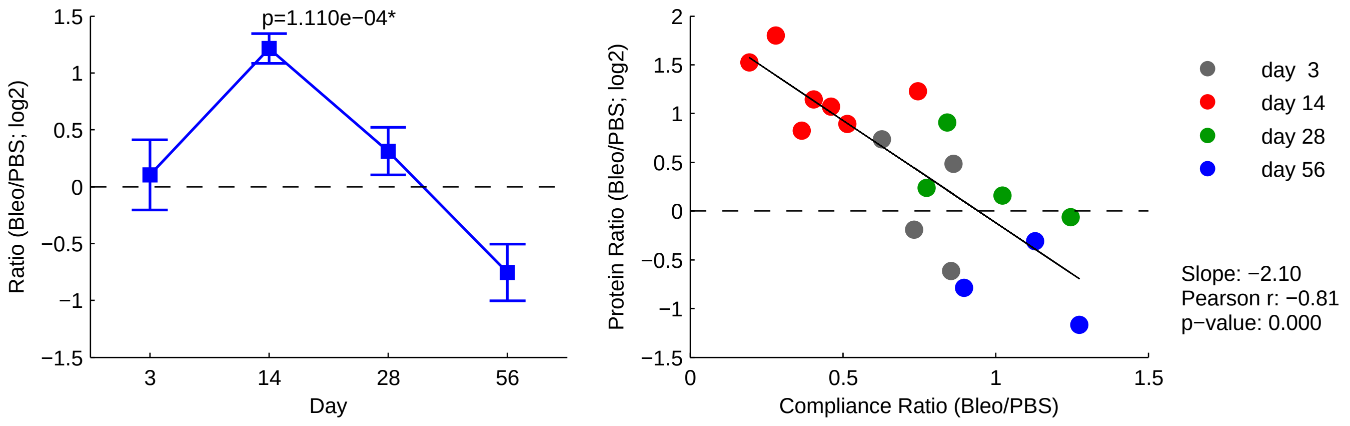

Q9Z257 − P2rx4 (id: 8016)

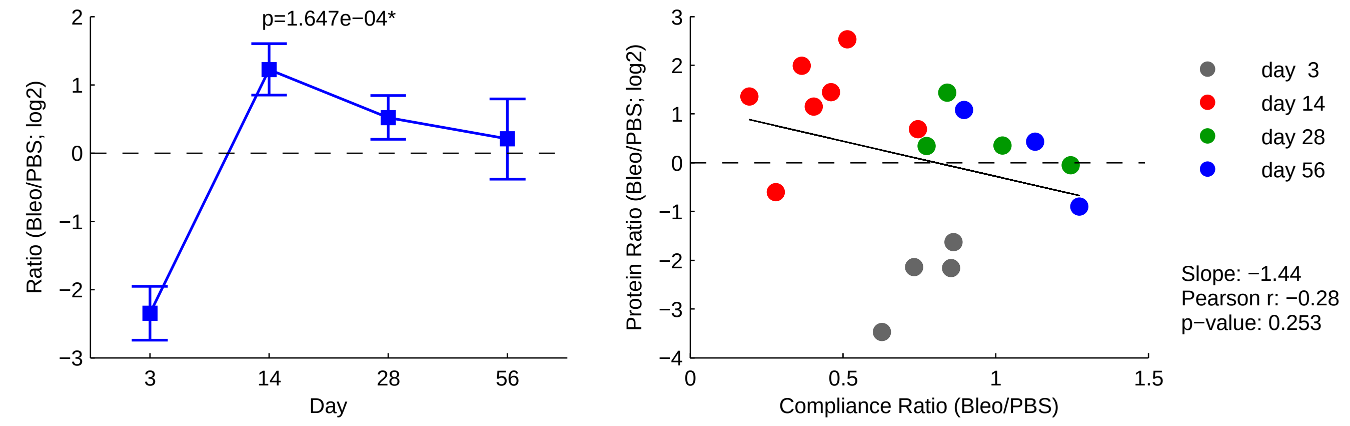

Q9Z266 − Snapin (id: 8017)

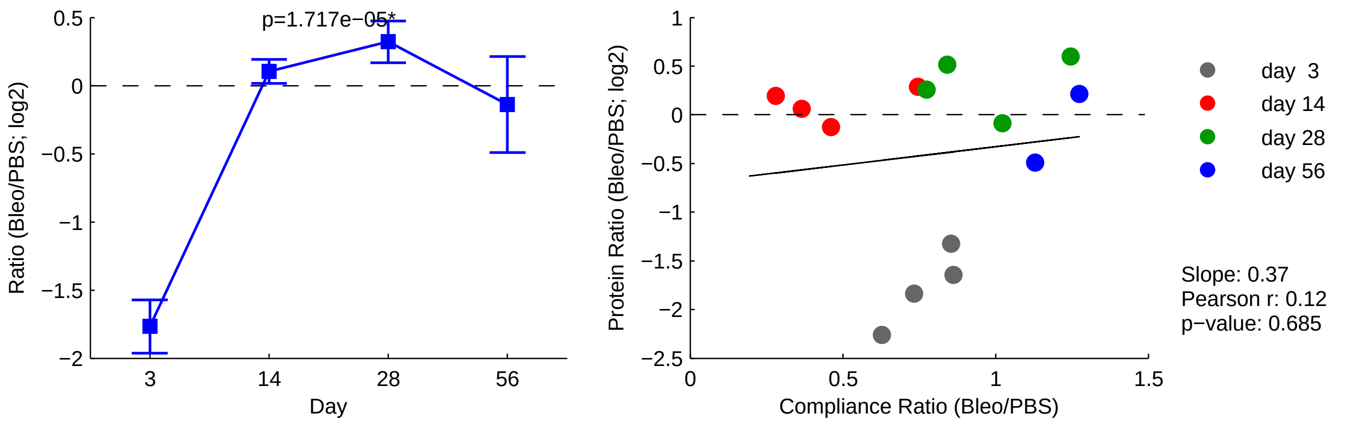

Q9Z2B9 − Rps6ka4 (id: 8021)

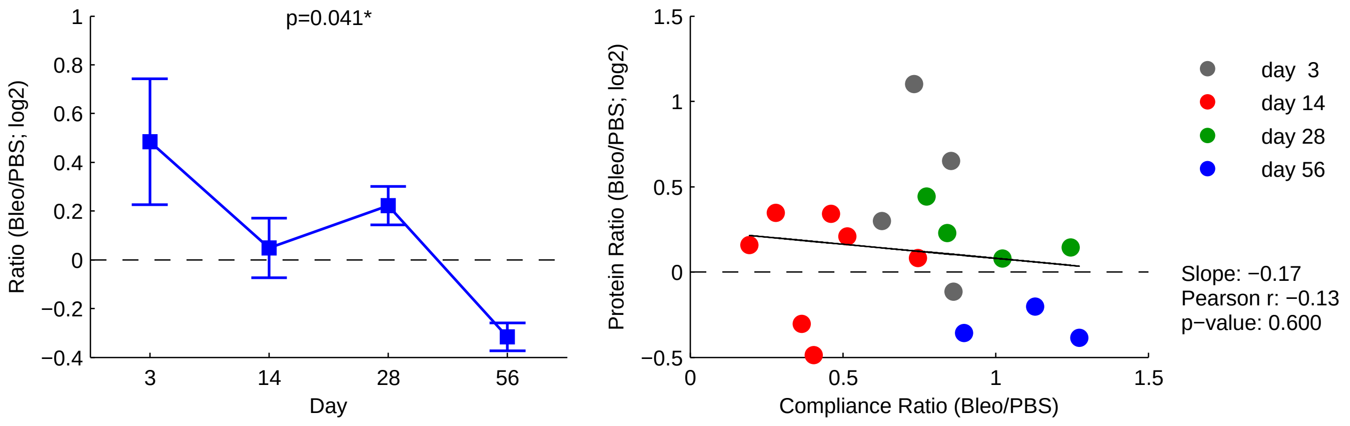

Q9Z2D0 − Mtmr9 (id: 8023)

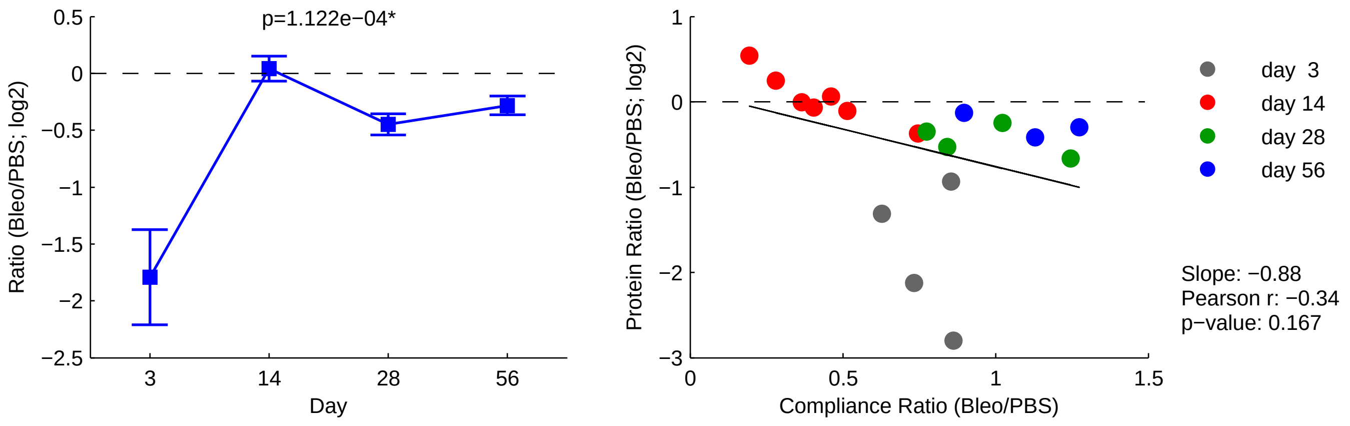

Q9Z2D1 − Mtmr2 (id: 8024)

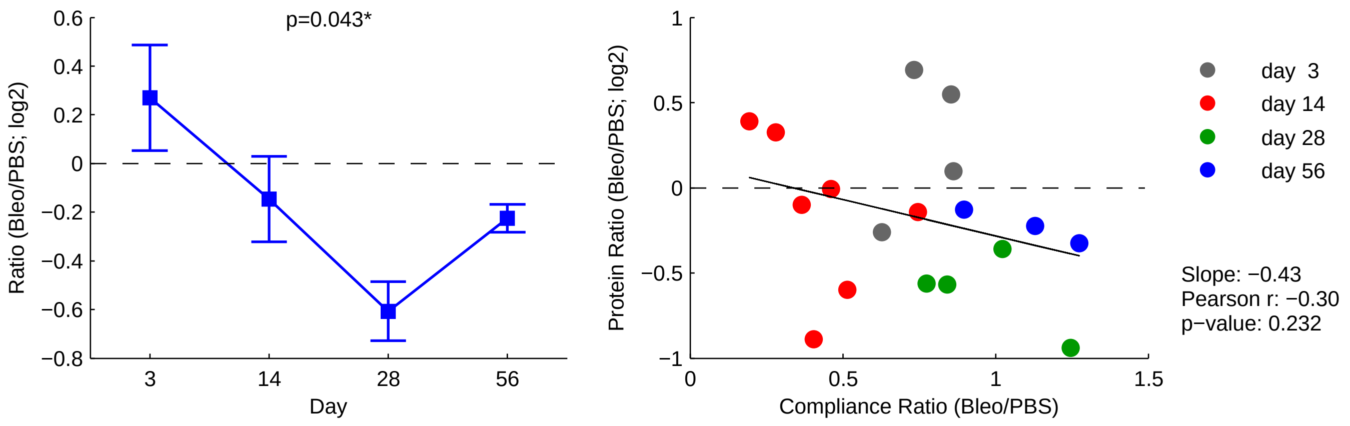

Q9Z2D6−2 − Mecp2 (id: 8025)

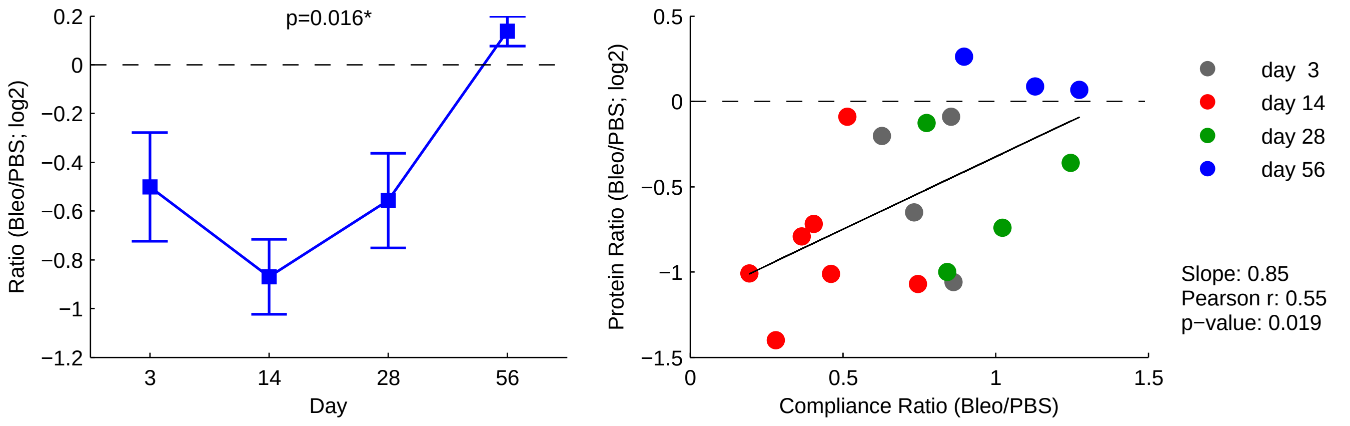

Q9Z2E2−5 − Mbd1 (id: 8027)

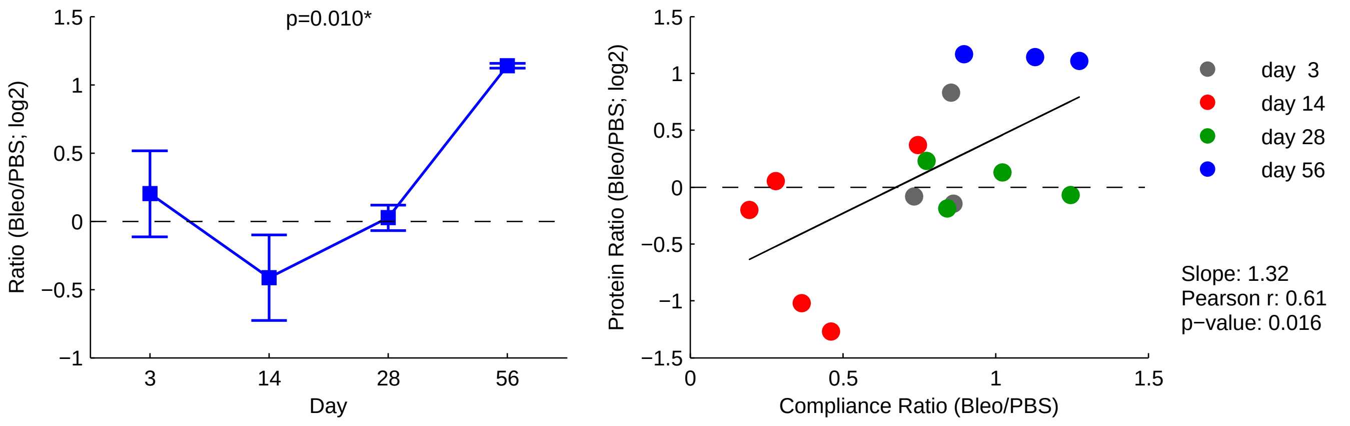

Q9Z2G6 − Sel1l (id: 8030)

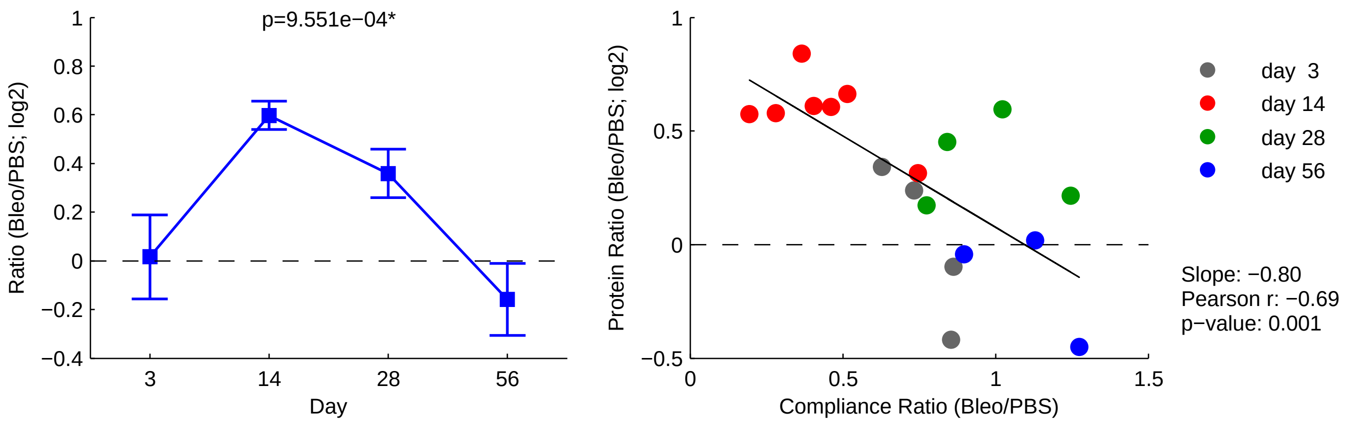

Q9Z2G9 − Htatip2 (id: 8031)

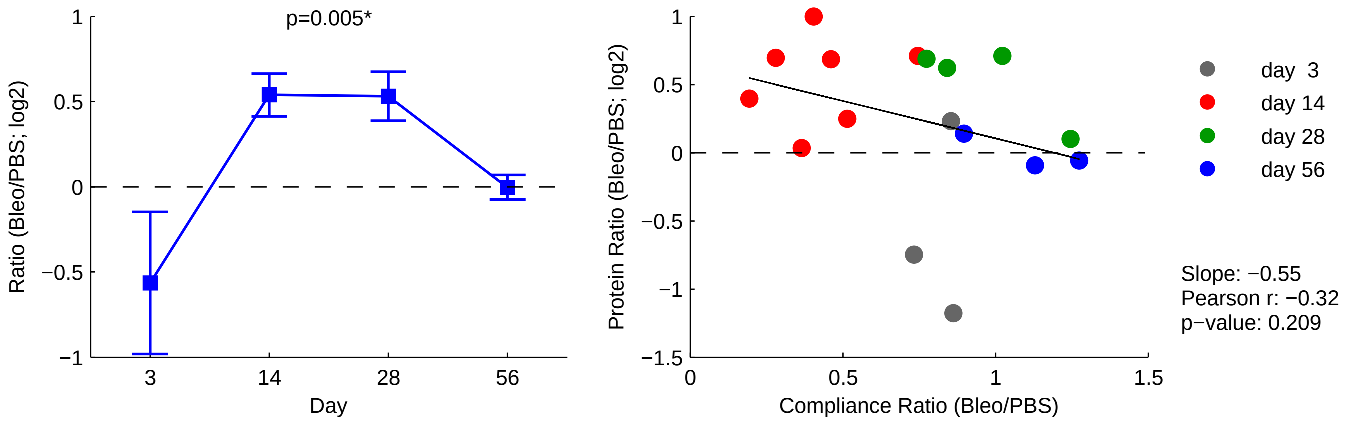

Q9Z2H7 − Gipc2 (id: 8032)

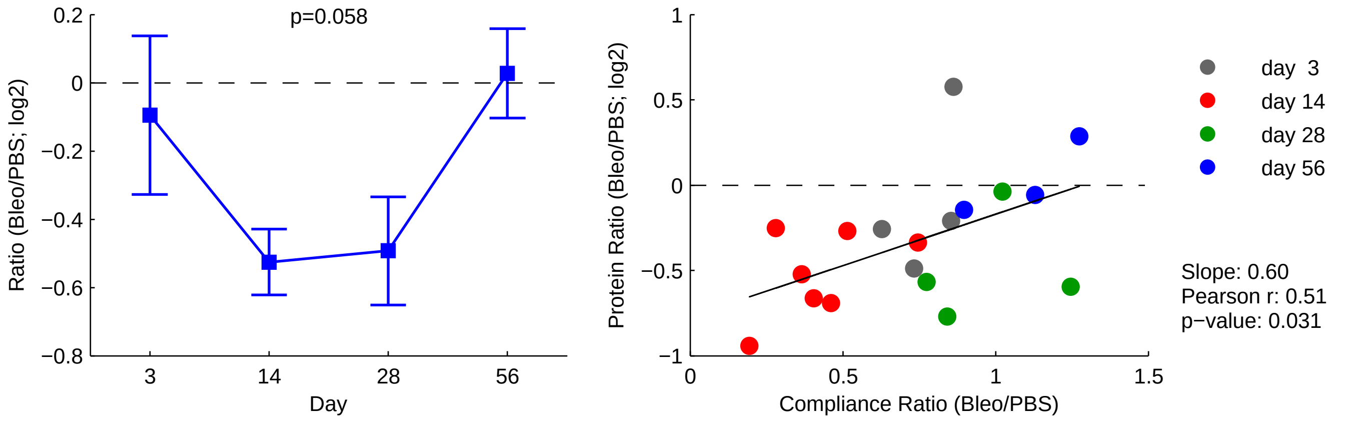

Q9Z2I0 − Letm1 (id: 8033)

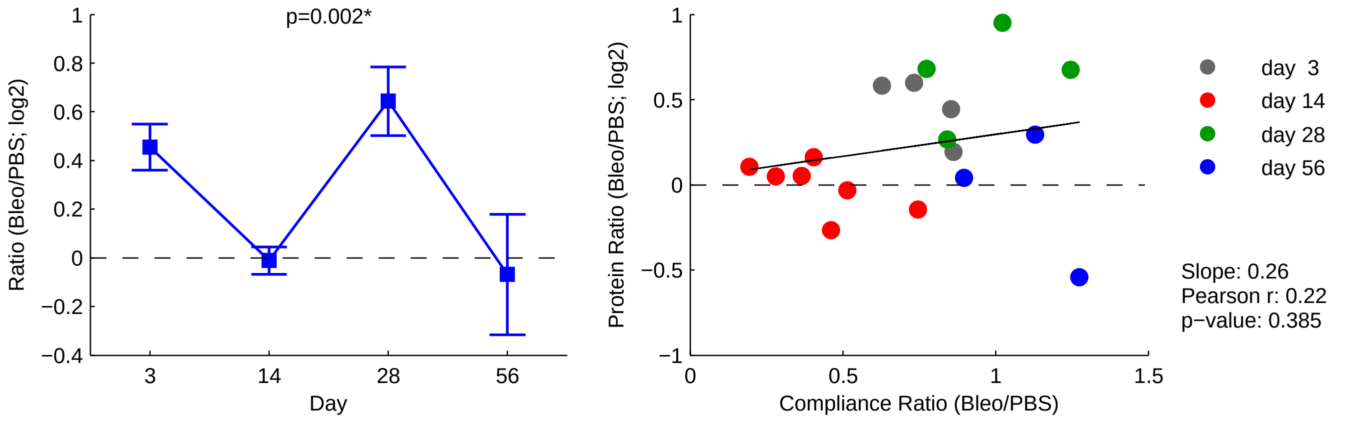

Q9Z2I8 − Suclg2 (id: 8034)

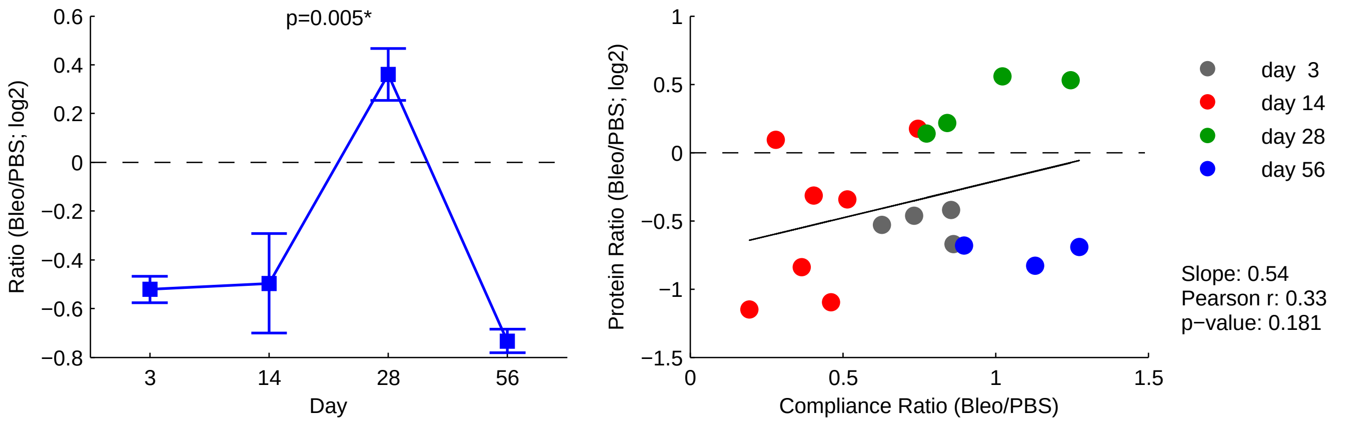

Q9Z2I9 − Sucla2 (id: 8035)

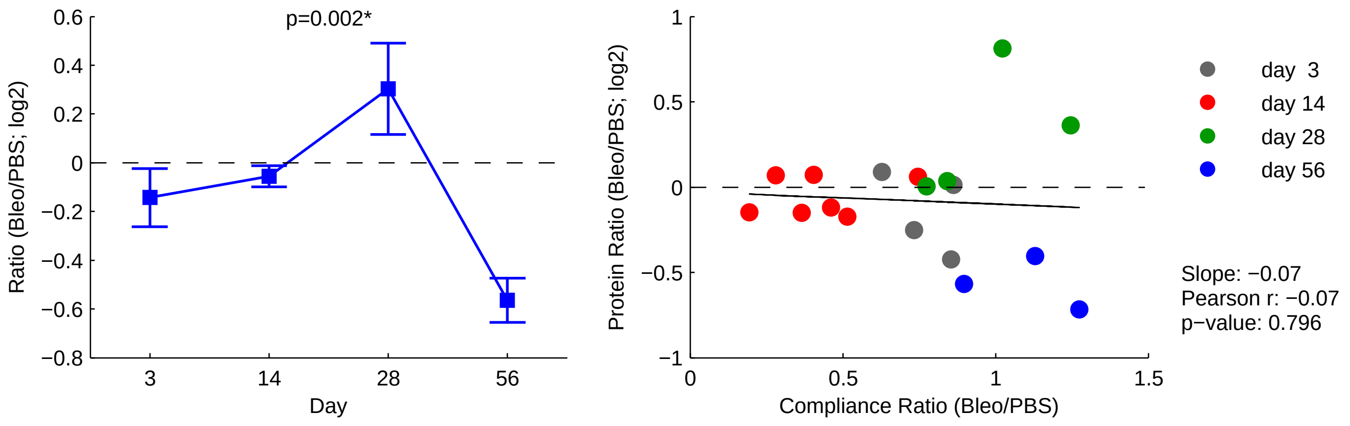

Q9Z2M6 − Ubl3 (id: 8037)

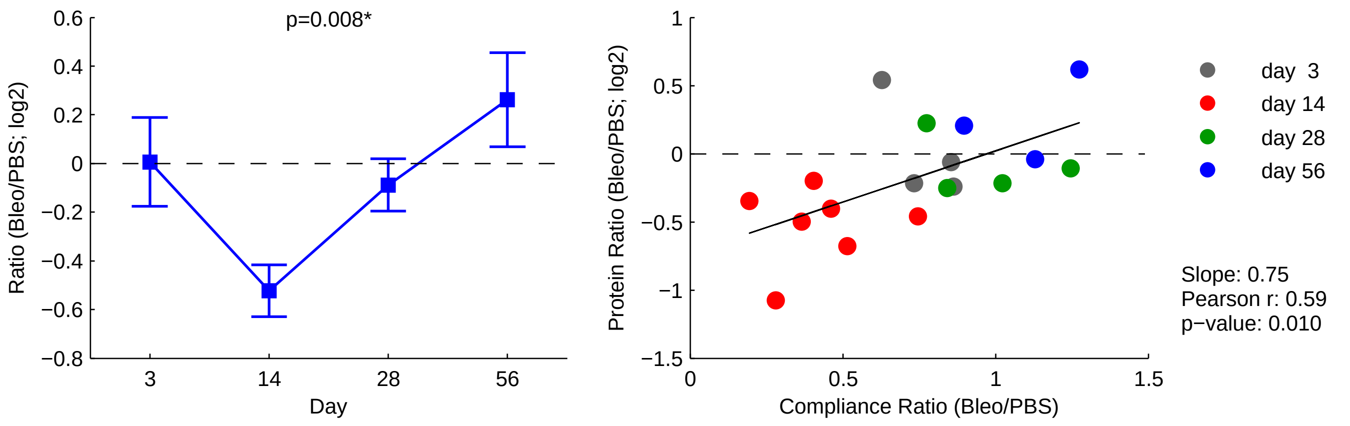

Q9Z2M7 − Pmm2 (id: 8038)

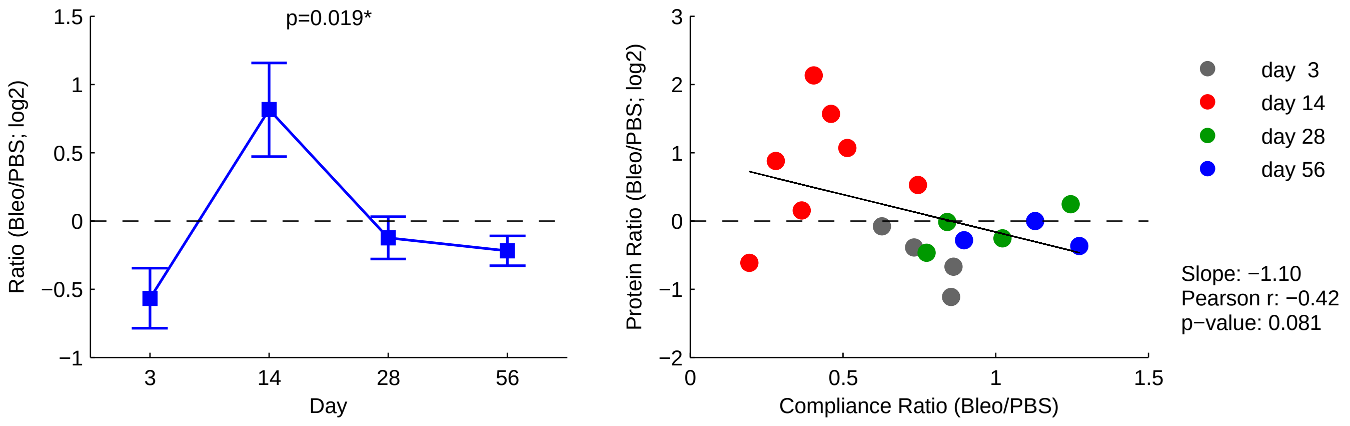

Q9Z2N8 − Actl6a (id: 8039)

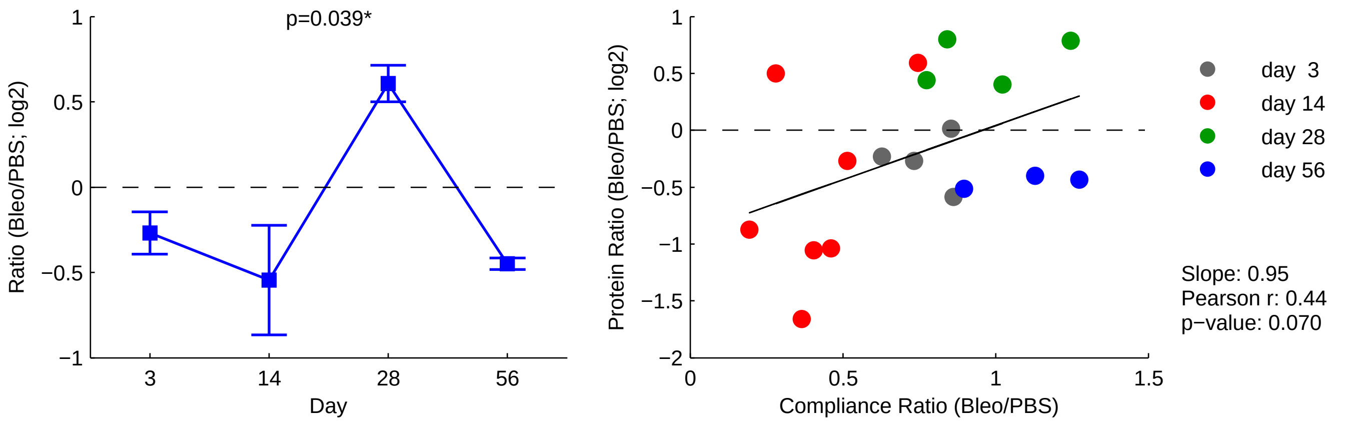

Q9Z2P8 − Vamp5 (id: 8040)

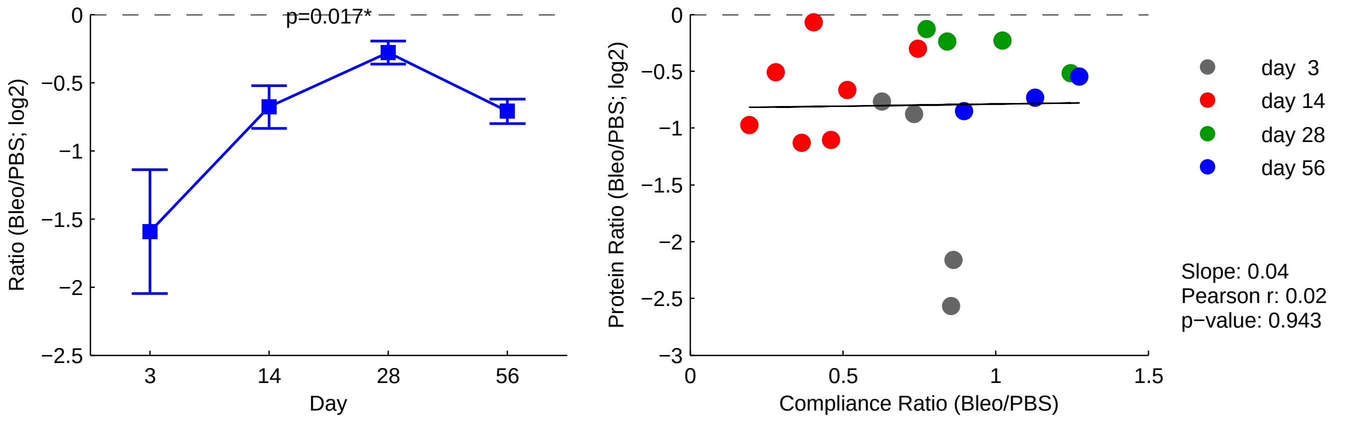

Q9Z2Q6 − Sept5 (id: 8041)

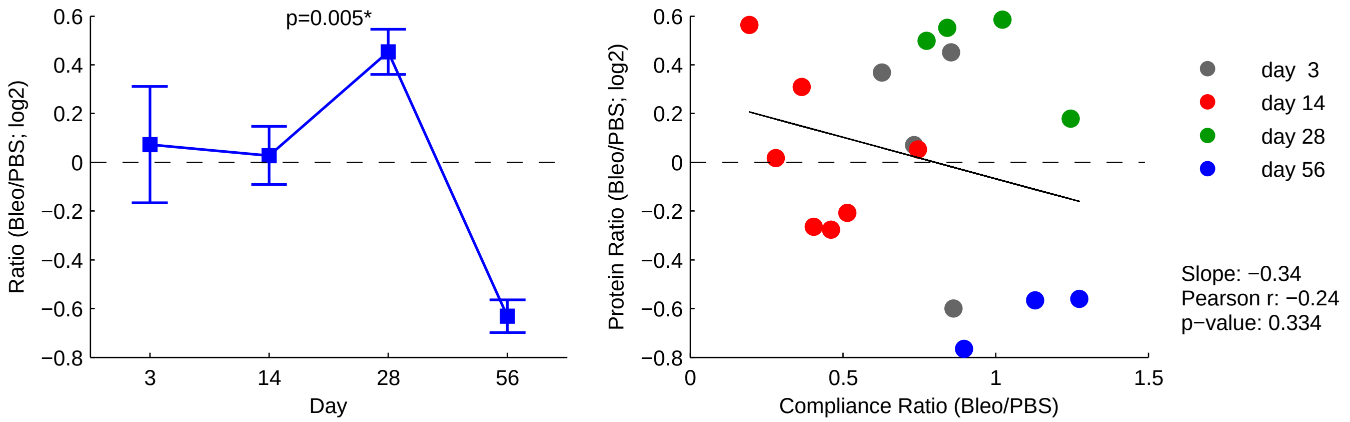

Q9Z2U0 − Psma7 (id: 8042)

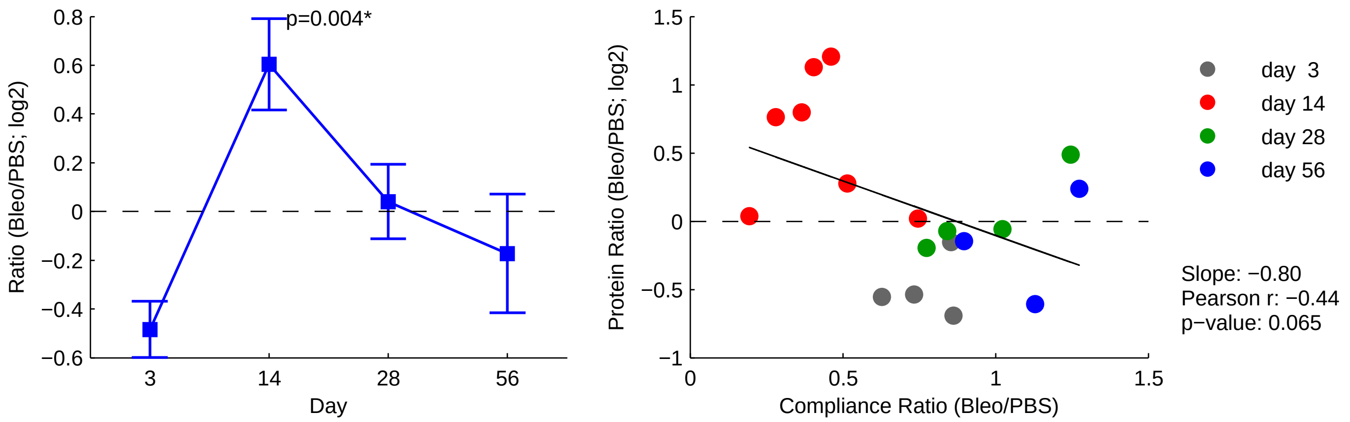

Q9Z2U1 − Psma5 (id: 8043)

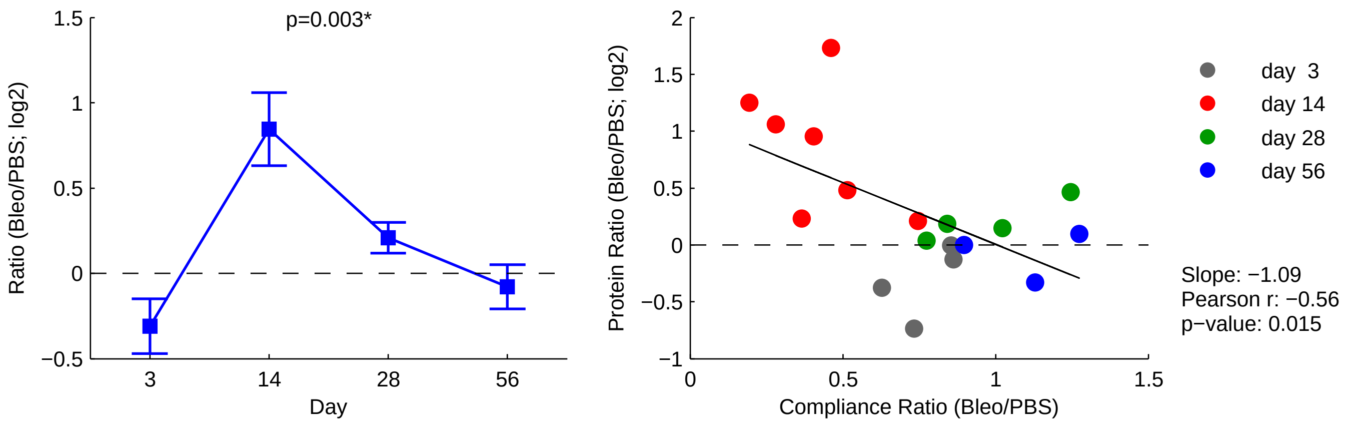

Q9Z2W1 − Stk25 (id: 8046)

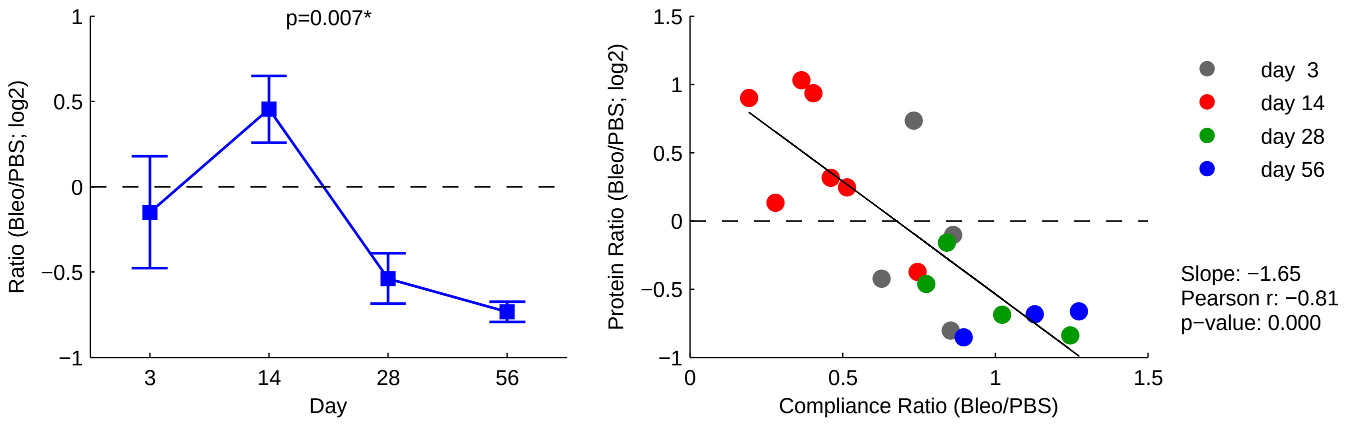

Q9Z2X1 − Hnrnpf (id: 8047)

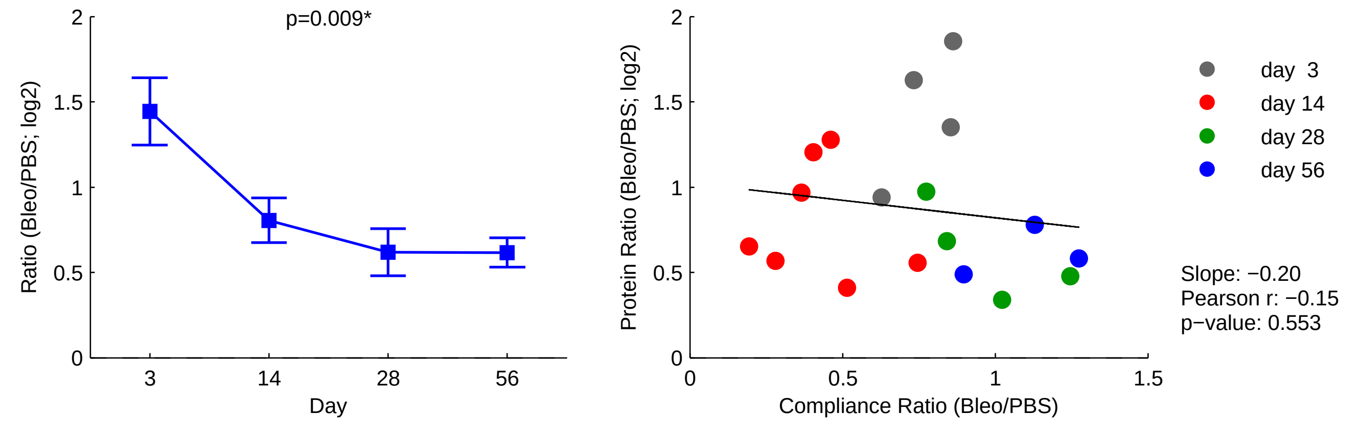

Q9Z2X2 − Psmd10 (id: 8048)

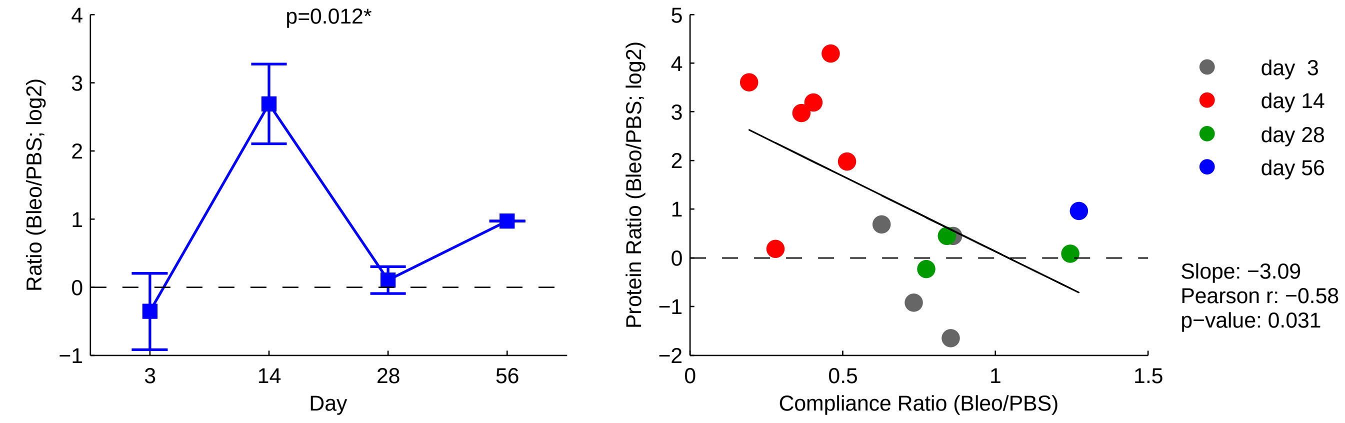

Q9Z2X8 − Keap1 (id: 8049)

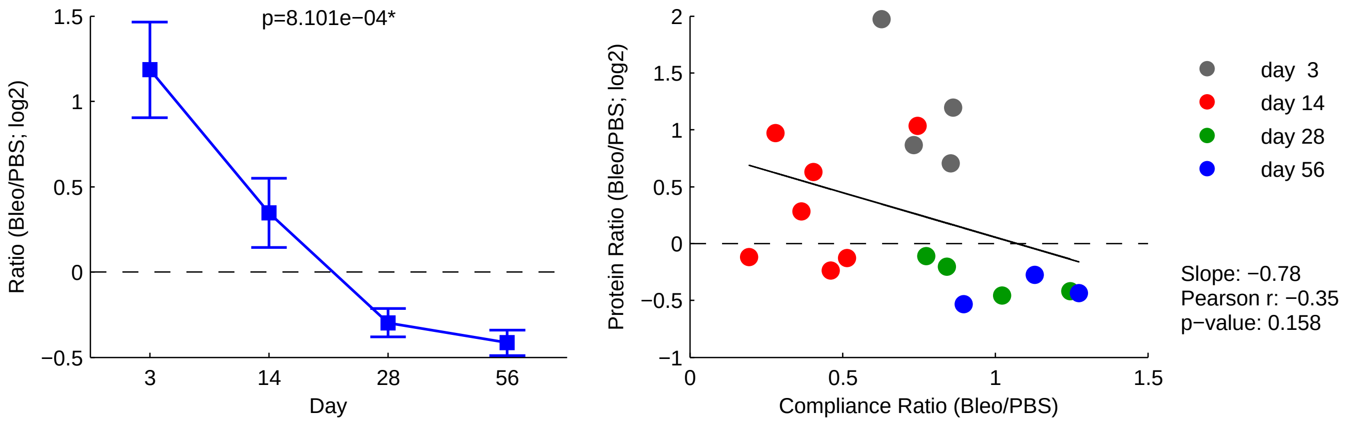

Q9Z2Z6 − Slc25a20 (id: 8052)

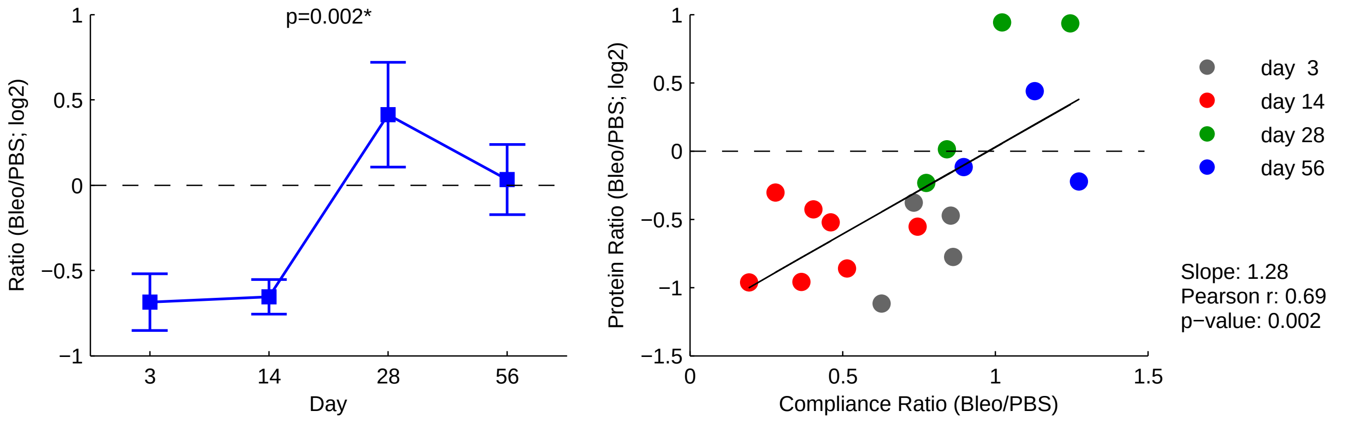

Q9Z315 − Sart1 (id: 8054)

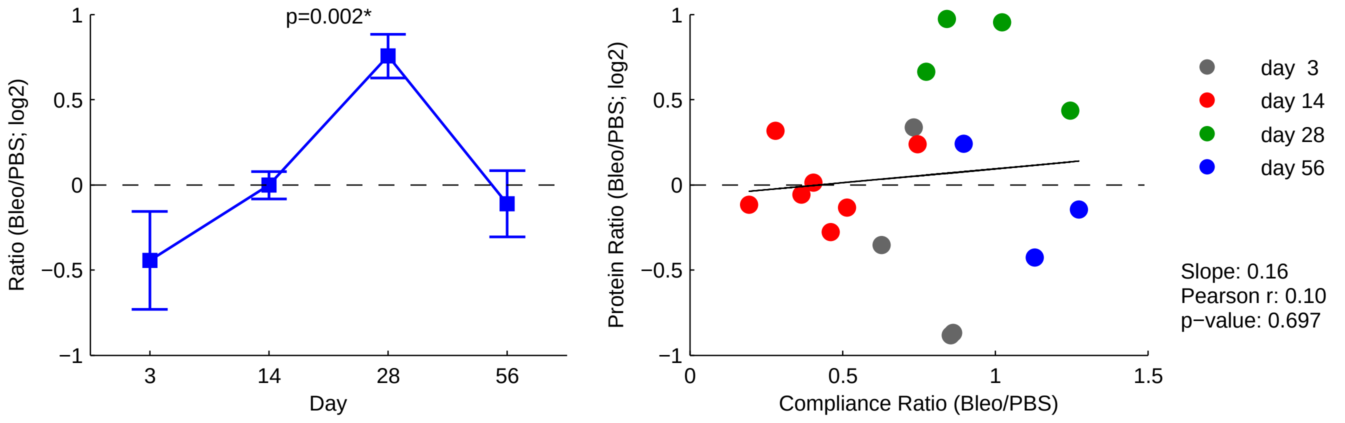

Q9Z329 − Itpr2 (id: 8057)

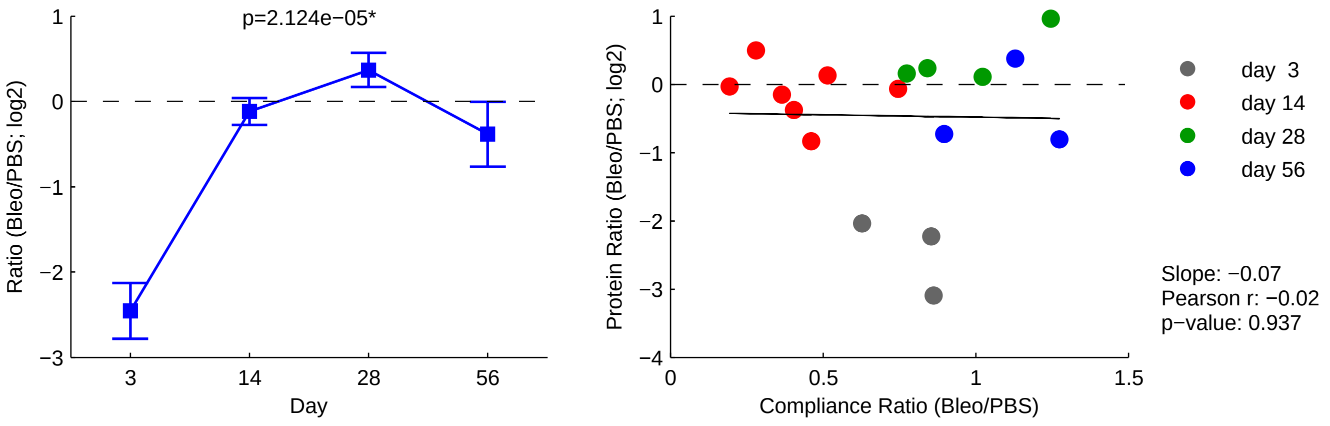

Supplement: Supplementary file 7 [file TissuePlots_Table EV6.pdf › Table EV6/TissuePlots_Table EV6.pdf]
