# Supplementary material for: Time- and compartment-resolved proteome profiling of the extracellular niche in lung injury and repair
Source: Mol Syst Biol. 2015 Jul 14;11(7):819. doi: 10.15252/msb.20156123 (PMC4547847; doi:10.15252/msb.20156123)
Supplement: Supplementary file 13 [file msb0011-0819-sd13.pdf]

Time- and compartment-resolved proteome profiling of the extracellular niche in lung injury and repair

Herbert B. Schiller, Isis E. Fernandez, Gerald Burgstaller, Christoph Schaab, Richard Scheltema, Thomas Schwarzmayr, Tim M. Strom, Oliver Eickelberg and Matthias Mann

Corresponding author: Matthias Mann, Max-Planck Institute of Biochemistry

Review timeline:

Submission date:	26 February 2015
Editorial Decision:	03 May 2015
Revision received:	11 May 2015
Accepted:	18 May 2015

Editor: Thomas Lemberger

Transaction Report:

(Note: With the exception of the correction of typographical or spelling errors that could be a source of ambiguity, letters and reports are not edited. The original formatting of letters and referee reports may not be reflected in this compilation.)

1st Editorial Decision 03 May 2015

Thank you again for submitting your work to Molecular Systems Biology. We have now finally heard back from the two referees who accepted to evaluate the study. As you will see, the referees find the topic of your study of potential interest and are supportive. They raise however a series of concerns and make suggestions for modifications, which we would ask you to carefully address in a revision of the present work. The recommendations provided by the reviewers are very clear in this regard and there is no need for further experimentation.

On an editorial level and in view of the comments from reviewer #1, we would encourage you to limit supplementary figures to data that are pertinent to the conclusions of this study.

Reviewer #1:

This manuscript describes application of mass-spectrometric analyses to the progression of changes occurring in a mouse model of lung fibrosis. State-of-the-art methods are applied to determine the proteins of the extracellular matrix and alveolar fluid that are up- or down-regulated during the inititation, progression and resolution of lung inflammation.
RNAseq data are also obtained and correlated with the proteomic data showing differential regulation at the RNA and protein levels - not surprising but useful nonetheless.
These data will be of value for future work on lung fibrosis.
Some of the presentation of results needs to be clarified.
Some of the bioinformatics (data-mining) analyses may be overkill and go too far beyond the actual data but some interesting inferences and hypotheses are offered by the authors.

© European Molecular Biology Organization

Fig. 1 Aspects of this figure and QDSP protocol need better explanation for clarity.
What exactly comprises Fr1? Is it [1] the PBS extract or [2] that plus the Buffer 1 extract or [3] the Buffer 1 + Buffer 2 extract - the Methods are unclear? Option 3 makes the most sense - the first two detergent extracts - but that is nowhere specified..
Related question - page 14 - what precisely were the two soluble fractions?

What is the definition of "matrisome" and "matrisome-associated" in Fig.1 panels C and D and Fig. 2?

Fig. 2 - some "matrisome-associated" proteins are enriched in the insoluble fraction, others are not. Can the authors comment on the fact that certain subsets (e.g. crosslinking enzymes) are enriched and, thus, presumably ECM-bound.

Fig. 4. Apart from TGFb, the relevance of the inferred regulators depicted in 4B is unclear. How were these inferred and what if any data validate these inferences?
The Methods are insufficiently clear for the reader to understand fully the basis for these conclusions.

Please provide the list of proteins whose expression correlates significantly with lung compliance and the associated values of Pearson correlation and fit slope value. Although reference is made to Table S7, that table does not seem to contain these data.

Figure 6 G-L needs some indication of what exactly are the structures shown - which are vessels, which are airways etc?

Figure S4: In addition to providing H&E staining of lung sections at the different experimental time points it would help to show ECM deposition (Masson's Trichrome staining or IHC using an anti-collagen I antibody) in the time course of the experiments.

The authors note (page 5, second paragraph) that all the components of the basement membrane (type 4 collagens, laminins, nidogen and perlecan), become "significantly more soluble" in bleomycin-treated lungs as compared to control lungs. Please discuss the possible biological relevance or implications of this observation.

Not actually clear that Fig. S3A is necessary - it duplicates Fig 1A plus well established RNASeq methods.

Fig S5 I was unconvinced of the significance of this figure.

A paper by Decaris et al (MCP, PMID 24741116) also used quantitative proteomics to study changes in the lung ECM during bleomycin-induced fibrosis. Although not as thorough as this paper, the Decaris study has some merits and should be cited and discussed.

Several references are incomplete (no volume or page numbers).

Reviewer #2:

The manuscript by Schiller et al. entitled "Time- and compartment-resolved proteome profiling of the extracellular niche in lung injury repair" describes an impressive set of proteomics (and transcriptomics) experiments to study in particular the ECM and ECM-associated proteins in the context of lung injury and repair using the bleomycin injury model.

The manuscripts comprises
a) Proteomics of lung tissue homogenate with and without bleomycin-induced injury (n = 4 each), 14 days post injury, using a novel 'quantitative detergent solubility profiling'.
b) Comparative proteomics/transcriptomics using the same samples as described above also for RNA-seq experiments.
c) Time-resolved proteomics experiments using lung tissue homogenate taken on four different days

post bleomycin-induced injury (and respective PBS-controls).
d) Bronchioalveolar lavage fluid proteomes taken on 6 different days post bleomycin-induced lung injury and appropriate PBS controls (n = 4 for each time point and treatment).

Each of these experiments could have been published individually. Instead, the Mann Group combined all these data into one impressive (tour de force) proteomics manuscript, extracting highly relevant information about the composition of the matrisome and proteins involved in the fibrogenesis and/or remodeling.

The manuscript itself is well written and is enjoyable to read. The manuscript is almost ready for being accepted in Molecular Systems Biology once the following issues have been addressed:

1) Time course experiments (page 6): the authors report first 8019 proteins and later on 'after filtering' 6236. Unfortunately, no details about the filtering are provided, as such it is not clear why 8019 proteins are reported in the first place (even 6300 proteins is impressive!). Please clarify and expand.

2) Time course experiments (page 6): the number of biological repeats/animals for the control experiments is not completely clear. There are 4 time points, but 34 controls (how are those 34 animals distributed over the 4 time points?). However, for the subsequent analysis the median of 16 PBS controls samples is used. How was this subset of 16 control samples selected? Please clarify the numbers and the selection process.

3) Page 6/last paragraph: Please introduce the concept of lung compliance. Not all readers of MSB are fully trained pulmonary specialists, i.e. the authors should not expect that the readers know/understand what the authors are talking about.

4) Page 8/bottom paragraph: the authors refer to a t-test based enrichment score, which was constructed to generate figure 7C. While the main message of this figure is an intensity vs. intensity plot, it is not completely clear how this t-test based enrichment score was constructed. Since such score can be very simple, but can also be quite sophisticated it would be very helpful if the authors provided some additional details about this enrichment score (e.g.: is there a statistical reason as to why this score goes from -3 to +3).

5) Throughout the text: The authors quantified the proteins using label free quantification methods. In the figures, sometimes iBAQ values (e.g. Figure 1 G to I) are reported and sometimes the MaxLFQ values (e.g. Figure 7C). Please clarify the motivation for using sometimes the former, and sometimes the latter. For the iBAQ-based dynamic range curves shown in Figures 6B, 6E, 7H and 7I, the use of iBAQ is obviously the appropriate value; however, it is less clear in e.g. Figure 1G and I, where also the MaxLFQ values could have been used.

1st Revision - authors' response 11 May 2015

Point by point reply to the reviewers

We thank both reviewers for their time and the constructive comments they made to improve our manuscript. Please find the detailed point by point reply to your questions/comments below:

Reviewer #1:

This manuscript describes application of mass-spectrometric analyses to the progression of changes occurring in a mouse model of lung fibrosis. State-of-the-art methods are applied to determine the proteins of the extracellular matrix and alveolar fluid that are up- or down-regulated during the inititation, progression and resolution of lung inflammation. RNAseq data are also obtained and correlated with the proteomic data showing differential regulation at the RNA and protein levels - not surprising but useful nonetheless. These data will be of value for future work on lung fibrosis. Some of the presentation of results needs to be clarified. Some of the bioinformatics (data-mining) analyses may be overkill and go too far beyond the actual data but some interesting inferences and hypotheses are offered by the authors.

Specific comments:

1. Fig. 1 Aspects of this figure and QDSP protocol need better explanation for clarity. What exactly comprises Fr1? Is it [1] the PBS extract or [2] that plus the Buffer 1 extract or [3] the Buffer 1 + Buffer 2 extract - the Methods are unclear? Option 3 makes the most sense - the first two detergent extracts - but that is nowhere specified.. Related question - page 14 - what precisely were the two soluble fractions?

Indeed the two combined fractions represent the initial tissue homogenate in PBS plus the proteins solubilized in buffer1 (1% NP40 non-ionic detergent). To make the methods description more clear on that point we changed the text to the following sentence (page 13): "The PBS from the tissue homogenate and the NP40 soluble fraction (*buffer 1*) was pooled, which together with the two fractions derived from ionic detergent extraction (*buffer 2 & 3*) resulted in a total of three soluble fractions and one insoluble pellet that were subjected to LC-MS/MS analysis."

We also thank the reviewer for pointing out that we did not carefully explain the buffers used for the timecourse experiment, which was done independently of the QDSP experiment in Figure 1. We therefore extended the description of these experiments in the methods part (page 14) to the following paragraph: "For tissue proteome time course analysis a similar sequential extraction procedure as described above was used. However, in these experiments we employed slightly different buffers for extraction following a commercially available protein extraction kit (Compartment Protein Extraction Kit, Millipore). We collected three protein fractions for LC-MS analysis (two soluble and one insoluble fraction). The first fraction measured was derived from proteins soluble in buffer M of the extraction kit [HEPES (pH7.9), $MgCl_2$, KCl, EDTA, Sucrose, Glycerol, Sodium deoxycholate, NP-40, Sodium OrthoVanadate]; the second fraction

was derived from proteins soluble in buffer CS [Buffer CS [Pipes (pH6.8), MgCl2, NaCl, EDTA, Sucrose, SDS, Sodium OrthoVanadate], and finally we also analyzed the proteins insoluble in buffer CS as described above for QDSP. To perform relative quantification of full proteomes in the various conditions, we summed up the peptide intensities of the three protein fractions in MaxQuant (Cox & Mann, 2008)."

2. What is the definition of "matrisome" and "matrisome-associated" in Fig.1 panels C and D and Fig. 2?

These definitions are introduced in our introduction section with the following sentence: "Bioinformatic analysis of protein domain architecture, together with literature mining, has defined an ECM component list (the 'matrisome') by classifying secreted proteins into structural constituents of the ECM ('core matrisome') and ECM interacting proteins ('matrisome-associated') (Cromar et al, 2012; Naba et al, 2011; Naba et al, 2012)" . To remind the reader where these terms are coming from we cite the work of Naba et al now also in the results text and the figure caption of the respective panels.

3. Fig. 2 - some "matrisome-associated" proteins are enriched in the insoluble fraction, others are not. Can the authors comment on the fact that certain subsets (e.g. crosslinking enzymes) are enriched and, thus, presumably ECM-bound.

As mentioned in the introduction section of the manuscript, the association of secreted proteins with the ECM can alter their function. A large body of literature has characterized ECM protein interactions based on surface plasmon resonance studies or ELISA based assay with purified ECM proteins or fragments thereof (Launay et al, 2014; Salza et al, 2014). We believe that in this study we provide for the first time an unbiased assessment of the strength of matrisome association of secreted proteins *in vivo*, which can be used for future annotation of these secreted proteins. However, in order to explore the molecular identity and the structural details of these extracellular interactions *in situ*, future progress in this field requires interaction studies exploiting both targeted approaches, such as antibody based proximity ligation assays (Soderberg et al, 2006), and unbiased methods, such as chemical crosslinking *in situ* combined with mass spectrometry analysis of crosslinked peptides (Walzthoeni et al, 2013).

4. Fig. 4. Apart from TGFb, the relevance of the inferred regulators depicted in 4B is unclear. How were these inferred and what if any data validate these inferences? The Methods are insufficiently clear for the reader to understand fully the basis for these conclusions.

To predict the activity of downstream biological processes and upstream transcriptional regulators and growth factors based on the observed protein abundance ratios, we used the Ingenuity® Pathway Analysis platform (IPA®, QIAGEN Redwood City, www.qiagen.com/ingenuity). We used a suite of algorithms and tools embedded in IPA for

inferring and scoring regulator networks upstream of gene-expression data based on a large-scale causal network derived from the Ingenuity Knowledge Base. The analytics tool ‘Upstream Regulator Analysis’ (Kramer et al, 2014) was used to compare the known effect (transcriptional activation or repression) of a transcriptional regulator on its target genes to the observed changes in protein abundance to assign an activation *Z*-score. Since it is *a priori* unknown which causal edges in the master network are applicable to the experimental context, the ‘Upstream Regulator Analysis’ tool uses a statistical approach to determine and score those regulators whose network connections to dataset genes as well as associated regulation directions are unlikely to occur in a random model (Kramer et al, 2014). In particular, the tool defines an overlap P-value measuring enrichment of network-regulated genes in the proteomic dataset, as well as an activation Z-score which can be used to find likely regulating molecules based on a statistically significant pattern match of up- and down-regulation, and also to predict the activation state (either activated or inhibited) of a putative regulator. In our analysis we restricted the ‘Upstream Regulator Analysis’ to the categories “transcriptional regulator” and “growth factor” in the IPA filter settings. We considered proteins with an overlap P-value of >3 (log10) that had an activation *Z*-score >2.5 as activated and those with an activation *Z*-score <−2.5 as inhibited. Using the ‘Downstream Effects Analysis’ (Kramer et al, 2014) embedded in IPA we aimed at identifying those biological processes and functions that are likely to be causally affected by up- and down-regulated proteins in the proteomic dataset. The approach is very similar to that of ‘Upstream Regulator Analysis’, except that the direction of edges connecting the dataset genes with the predicted entity (here, the biological process) is reversed. We confined our analysis to “Molecular and Cellular Functions & Physiological System Development and Function” and accepted an overlap P-value of >3 (log10). Biological processes with an activation *Z*-score >2 were considered as activated and those with an activation *Z*-score <−2 as inhibited.

The IPA analysis is now described in detail in the methods section on page 17. In addition, we added a data table with the activation Z-scores of the ‘Upstream Regulator Analysis’ and the ‘Downstream Effects Analysis’, which is now referenced in the results section as Table S7 on page 6 with the following sentence: “We assigned biological processes, and their upstream transcriptional regulators and growth factors to the consecutive phases of tissue repair (Fig. 4B; Table S7) (see Ingenuity Pathway Analysis in Methods).”

The IPA analysis was performed to identify unexpected transcriptional regulators in the consecutive phases of lung repair. The expectedly high activation Z-score of TGF-β at day14 validates the accuracy of this analysis. We also observed increased activity of transcriptional regulators of ER-stress response (Xbp1, Atf4) (Lenna & Trojanowska, 2012), autophagy (Tfeb) (Settembre et al, 2013), and hypoxia response (Hif1a), at day14 and day28 after injury, pointing to a massive increase in proteotoxic stress during these phases. Indeed, oxidative damage often appears together with ER-stress in inflammation (Chaudhari et al, 2014), which may explain the concerted upregulation of these factors. Furthermore, we found that the activity of Vegf was highest at day56, thus indicating the pro-resolution function of this master regulator of

angiogenesis in lung injury repair. Indeed, the activity of Vegf has previously been shown to promote fibrosis resolution and repair in mice (Yang et al, 2014). In addition to TGF-β activity, there is literature supporting possible roles of Hnf4a and Nrf2, which we also identified in the 'Upstream Regulator Analysis' to have significant activation Z-scores, in regulation of lung fibrogenesis. We discuss this literature in the discussion section on page 11. We are therefore confident that we identified both expected transcriptional regulators of fibrogenesis as well as novel factors (e.g. Tbx5; discussed on page 11) whose putative role in lung repair may be validated with additional experiments in the future.

5. Please provide the list of proteins whose expression correlates significantly with lung compliance and the associated values of Pearson correlation and fit slope value. Although reference is made to Table S7, that table does not seem to contain these data.

The referencing of Tables in the results section on page 6 may have been misleading. The information the reviewer is asking for is in Table S6. In Table S7 (now Table S8 due to the additional Table for the IPA results) we show the result of 1D annotation enrichment analysis (Cox & Mann, 2012) over the fit slope of the correlation analysis. To improve the readability of this part of the text we changed the results section on page 6 to the following: "For each protein, we determined how their temporal abundance profiles correlated with lung compliance changes (Supplementary Table S6). …. A statistical test (1D annotation enrichment) revealed gene categories that were enriched with negative and positive slopes, respectively (Fig. 4D; Supplementary Table S8)."

6. Figure 6 G-L needs some indication of what exactly are the structures shown - which are vessels, which are airways etc?

We modified Figure 6 according to this suggestions and labeled vessels (V) and airways (A).

7. Figure S4: In addition to providing H&E staining of lung sections at the different experimental time points it would help to show ECM deposition (Masson's Trichrome staining or IHC using an anti-collagen I antibody) in the time course of the experiments.

As suggested, we performed IHC using an anti-collagen I antibody for all time points of bleomycin treatment and included this data in Figure S4. To reference the new experiment in the results section we changed the text to the following on page 6: "The bleomycin mediated injury of the alveolar epithelium causes an inflammatory response, which leads to maximal fibrogenesis two weeks after injury. Subsequently, the provisional ECM becomes remodeled and repair is resolved within 8 weeks after injury (Bakowska & Adamson, 1998; Hecker et al, 2014). We characterized the dynamics of tissue repair upon bleomycin treatment using H&E (Supplementary Fig. S4A) and collagen type-I stainings (Supplementary Fig. S4B) at different time points after bleomycin injury and confirmed the almost complete resolution of tissue repair within eight weeks post bleomycin treatment."

8. The authors note (page 5, second paragraph) that all the components of the basement membrane (type 4 collagens, laminins, nidogen and perlecan), become "significantly more soluble" in bleomycin-treated lungs as compared to control lungs. Please discuss the possible biological relevance or implications of this observation.

The alveolar basement membrane is very important both in securing the structural integrity of alveoli and instructing the epithelial layer via active signal transduction. Any change in structural integrity and composition of the basement membrane (as observed in our study) is therefore likely to have important consequences. However, the exact mechanistic details and consequences of the phenomena observed here cannot be known at this point and require difficult functional studies. For instance, in the discussion section on page 10 we speculate about the role of basement membrane associated Netrins and their possible release due to structural disintegration of BM upon injury with the following paragraph: “QDSP uncovered increased solubility of basement membrane proteins, which could be due to proteolytic processing, as previously shown in lung injury and repair (Davey et al, 2011), and branching morphogenesis of the developing lung (Harunaga et al, 2014). Proteolytic processing might also explain the observed release of basement membrane associated Netrins from the insoluble compartment. Netrins inhibit the outgrowth of ectopic epithelial buds during branching morphogenesis in lung development (Liu et al, 2004), and it will be thus interesting to determine the functional relevance of their release upon injury.”

9. Not actually clear that Fig. S3A is necessary - it duplicates Fig 1A plus well established RNASeq methods.

We removed the panel A from Figure S3 as suggested.

10. Fig S5 I was unconvinced of the significance of this figure.

We removed Figure S5 as suggested.

11. A paper by Decaris et al (MCP, PMID 24741116) also used quantitative proteomics to study changes in the lung ECM during bleomycin-induced fibrosis. Although not as thorough as this paper, the Decaris study has some merits and should be cited and discussed.

We thank the reviewer for pointing this out. We included the Decaris study in our discussion with the following paragraph: “Proteomic studies of the ECM typically analyze the insoluble material left after application of different decellularization protocols (Barallobre-Barreiro et al, 2012; Didangelos et al, 2010; Naba et al, 2011; Naba et al, 2014; Rashid et al, 2012; Zanivan et al, 2013). These workflows, however, do not distinguish true ECM constituents from contaminant non-ECM proteins. Some recent studies have explored the possibility of measuring and comparing the protein pools after differential extraction from tissue (Barallobre-

Barreiro et al, 2012; Decaris et al, 2014). The latter study, for instance, quantified the turnover of ECM proteins and compared the insoluble compartment with the Guadinium hydrochloride soluble compartment (Decaris et al, 2014). We further explored the idea of a quantitative comparison of sequential protein extracts to characterize ECM proteins based on their solubility profiles, which we also used to directly measure the association of secreted proteins with ECM filaments in vivo."

12. Several references are incomplete (no volume or page numbers).

We thank the reviewer for noticing this – we corrected the incomplete references.

<u>Reviewer #2:</u>

The manuscript by Schiller et al. entitled "Time- and compartment-resolved proteome profiling of the extracellular niche in lung injury repair" describes an impressive set of proteomics (and transcriptomics) experiments to study in particular the ECM and ECM-associated proteins in the context of lung injury and repair using the bleomycin injury model.

The manuscripts comprises:

a) Proteomics of lung tissue homogenate with and without bleomycin-induced injury (n = 4 each), 14 days post injury, using a novel 'quantitative detergent solubility profiling'.
b) Comparative proteomics/transcriptomics using the same samples as described above also for RNA-seq experiments.
c) Time-resolved proteomics experiments using lung tissue homogenate taken on four different days post bleomycin-induced injury (and respective PBS-controls).
d) Bronchioalveolar lavage fluid proteomes taken on 6 different days post bleomycin-induced lung injury and appropriate PBS controls (n = 4 for each time point and treatment).

Each of these experiments could have been published individually. Instead, the Mann Group combined all these data into one impressive (tour de force) proteomics manuscript, extracting highly relevant information about the composition of the matrisome and proteins involved in the fibrogenesis and/or remodeling. The manuscript itself is well written and is enjoyable to read. The manuscript is almost ready for being accepted in Molecular Systems Biology once the following issues have been addressed.

Specific comments:

1. Time course experiments (page 6): the authors report first 8019 proteins and later on 'after filtering' 6236. Unfortunately, no details about the filtering are provided, as such it is not clear why 8019 proteins are reported in the first place (even 6300 proteins is impressive!). Please clarify and expand.

The applied filter is now explained in detail in the results section at page 6 of the manuscript with the following paragraph: “We quantified 8019 protein groups with a median number of 5020 identified proteins per single replicate sample (n=34), and generated abundance ratios (Bleo/PBS) by dividing individual replicates by the median value of all PBS control samples (n=16). In case of only missing intensity values (protein not identified) in one of the experimental conditions we used data imputation (see supplementary Methods) at the low end of the intensity dynamic range if we obtained at least 50% valid intensity values in the other condition. In this way, the MS-intensities of a total number of 6236 protein groups (with protein quantification for at least 3 replicates in one of the experimental conditions) were finally used for the ratiometric time course analysis (Supplementary Table S6).”

2. Time course experiments (page 6): the number of biological repeats/animals for the control experiments is not completely clear. There are 4 time points, but 34 controls (how are those 34 animals distributed over the 4 time points?). However, for the subsequent analysis the median of 16 PBS controls samples is used. How was this subset of 16 control samples selected? Please clarify the numbers and the selection process.

We used a total of 18 bleomycin treated mice and 16 control saline treated mice (n=34 for all experimental conditions). To express this more clearly in the results section we changed the text on page 6 to the following: “To characterize the proteome changes after injury associated with inflammation (day 3), fibrogenesis (day 14), remodeling (day 28), and resolution (day 54), we homogenized total lung lobes from 8 mice for each time point after a single intratracheal instillation of bleomycin (n=18; Bleo 3U/kg) or control saline (n=16; PBS) (Fig. 4A).”

3. Page 6/last paragraph: Please introduce the concept of lung compliance. Not all readers of MSB are fully trained pulmonary specialists, i.e. the authors should not expect that the readers know/understand what the authors are talking about.

We now explain the concept of lung compliance and its changes in human pathophysiology in the results section at page 6 with the following sentence: “In clinical practice, measurement of pulmonary compliance captures the lung's ability to stretch and expand, which is reduced in fibrosis and increased in lung emphysema. In the bleomycin model, we observed that the median lung compliance was slightly reduced at day 3 and severely reduced at day 14 after injury, after which it returned back to the level of PBS instilled control mice at days 28 and day 56.”

4. Page 8/bottom paragraph: the authors refer to a t-test based enrichment score, which was constructed to generate figure 7C. While the main message of this figure is an intensity vs. intensity plot, it is not completely clear how this t-test based enrichment score was constructed. Since such score can be very simple, but can also be quite sophisticated it would be very helpful if the authors provided some additional details about this enrichment score (e.g.: is there a statistical reason as to why this score goes from -3 to +3).

The t-test based enrichment score was calculated as in formula one in the paper by Tusher et al `Significance analysis of microarrays applied to the ionizing radiation response´ (Tusher et al, 2001). The statistic is based on the ratio of change in gene expression to standard deviation in the data for that gene. The ‘‘gene-specific scatter’’ s(i) is the standard deviation of the replicates. We explain this and cite the paper on page 17 in the methods part of the revised manuscript. There is no statistical reason for setting the color code threshold for the score from -3 to +3. This was done arbitrarily to visualize the score differences onto the scatter plot.

5. Throughout the text: The authors quantified the proteins using label free quantification methods. In the figures, sometimes iBAQ values (e.g. Figure 1 G to I) are reported and sometimes the MaxLFQ values (e.g. Figure 7C). Please clarify the motivation for using sometimes the former, and sometimes the latter. For the iBAQ-based dynamic range curves shown in Figures 6B, 6E, 7H and 7I, the use of iBAQ is obviously the appropriate value; however, it is less clear in e.g. Figure 1G and I, where also the MaxLFQ values could have been used.

We used iBAQ values (calculated from MaxLFQ values by dividing by the theoretical number of tryptic peptides for every protein) by default in most figures to keep the additional information on protein stoichiometry. Figure 7C is the only exception in which we kept the MaxLFQ value from the MaxQuant output. The information we want to convey in this panel is that we observe significantly enriched outliers over the whole range of MS-intensity. In this case it is more correct to use the MaxLFQ values not normalized for protein size. We will thus keep the original version of this panel.

References

Bakowska J, Adamson IY (1998) Collagenase and gelatinase activities in bronchoalveolar lavage fluids during bleomycin-induced lung injury. *The Journal of pathology* **185:** 319-323

Chaudhari N, Talwar P, Parimisetty A, Lefebvre d'Hellencourt C, Ravanan P (2014) A molecular web: endoplasmic reticulum stress, inflammation, and oxidative stress. *Frontiers in cellular neuroscience* **8:** 213

Cox J, Mann M (2008) MaxQuant enables high peptide identification rates, individualized p.p.b.-range mass accuracies and proteome-wide protein quantification. *Nature biotechnology* **26:** 1367-1372

Cox J, Mann M (2012) 1D and 2D annotation enrichment: a statistical method integrating quantitative proteomics with complementary high-throughput data. *BMC bioinformatics* **13 Suppl 16:** S12

Davey A, McAuley DF, O'Kane CM (2011) Matrix metalloproteinases in acute lung injury: mediators of injury and drivers of repair. *The European respiratory journal : official journal of the European Society for Clinical Respiratory Physiology* **38:** 959-970

Harunaga JS, Doyle AD, Yamada KM (2014) Local and global dynamics of the basement membrane during branching morphogenesis require protease activity and actomyosin contractility. *Developmental biology*

Hecker L, Logsdon NJ, Kurundkar D, Kurundkar A, Bernard K, Hock T, Meldrum E, Sanders YY, Thannickal VJ (2014) Reversal of persistent fibrosis in aging by targeting Nox4-Nrf2 redox imbalance. *Science translational medicine* **6:** 231ra247

Kramer A, Green J, Pollard J, Jr., Tugendreich S (2014) Causal analysis approaches in Ingenuity Pathway Analysis. *Bioinformatics* **30:** 523-530

Launay G, Salza R, Multedo D, Thierry-Mieg N, Ricard-Blum S (2014) MatrixDB, the extracellular matrix interaction database: updated content, a new navigator and expanded functionalities. *Nucleic acids research*

Lenna S, Trojanowska M (2012) The role of endoplasmic reticulum stress and the unfolded protein response in fibrosis. *Current opinion in rheumatology* **24:** 663-668

Liu Y, Stein E, Oliver T, Li Y, Brunken WJ, Koch M, Tessier-Lavigne M, Hogan BL (2004) Novel role for Netrins in regulating epithelial behavior during lung branching morphogenesis. *Current biology : CB* **14:** 897-905

Salza R, Peysselon F, Chautard E, Faye C, Moschcovich L, Weiss T, Perrin-Cocon L, Lotteau V, Kessler E, Ricard-Blum S (2014) Extended interaction network of procollagen C-proteinase enhancer-1 in the extracellular matrix. *The Biochemical journal* **457:** 137-149

Settembre C, Fraldi A, Medina DL, Ballabio A (2013) Signals from the lysosome: a control centre for cellular clearance and energy metabolism. *Nature reviews Molecular cell biology* **14:** 283-296

Soderberg O, Gullberg M, Jarvius M, Ridderstrale K, Leuchowius KJ, Jarvius J, Wester K, Hydbring P, Bahram F, Larsson LG, Landegren U (2006) Direct observation of individual endogenous protein complexes in situ by proximity ligation. *Nature methods* **3:** 995-1000

Tusher VG, Tibshirani R, Chu G (2001) Significance analysis of microarrays applied to the ionizing radiation response. *Proceedings of the National Academy of Sciences of the United States of America* **98:** 5116-5121

Walzthoeni T, Leitner A, Stengel F, Aebersold R (2013) Mass spectrometry supported determination of protein complex structure. *Current opinion in structural biology* **23:** 252-260

Yang L, Kwon J, Popov Y, Gajdos GB, Ordog T, Brekken RA, Mukhopadhyay D, Schuppan D, Bi Y, Simonetto D, Shah VH (2014) Vascular endothelial growth factor promotes fibrosis resolution and repair in mice. *Gastroenterology* **146:** 1339-1350 e1331
